# Causal Association Between Plasma Proteins and Pericarditis: A Mendelian Randomization Study With Therapeutic Target Identification

**DOI:** 10.1155/mi/4659271

**Published:** 2026-02-09

**Authors:** Zhexuan Chen, Zongqiang Chen, Lingfeng Peng, Huankai Zhang

**Affiliations:** ^1^ Department of Anesthesiology, Jieyang People’s Hospital, No. 107 Tianfu Road Rongcheng District, Jieyang, 522000, Guangdong, China; ^2^ Jinan University, No. 601 West Huangpu Avenue Tianhe District, Guangzhou, 510632, Guangdong, China, jnu.edu.cn; ^3^ Department of Intensive Care Unit, Jieyang People’s Hospital, No. 107 Tianfu Road Rongcheng District, Jieyang, 522000, Guangdong, China

**Keywords:** Mendelian randomization, network pharmacology, pericarditis, plasma proteins, single-cell rNA sequencing

## Abstract

**Background:**

Observational studies demonstrate that pro‐inflammatory cytokines play a critical role in pericarditis. However, the causal association between circulating plasma proteins and pericarditis remains unestablished.

**Objective:**

This research aimed to assess the causal association between plasma proteins and pericarditis and to investigate potential therapeutic targets.

**Methods:**

A genome‐wide association study (GWAS) involving 35,559 individuals of European ancestry was conducted using 4907 plasma proteins as instrumental variables (IVs). The causal relationship between plasma proteins and pericarditis was examined using inverse weighted median, variance weighting, and Mendelian randomization (MR)‐Egger methods. Horizontal pleiotropy was evaluated via MR‐Egger regression, and heterogeneity was quantified by Cochran’s Q statistic. In addition, enrichment analyses, construction of a protein–protein interaction (PPI) network, and single‐cell RNA sequencing (scRNA‐seq) analysis were performed. Molecular docking was used to predict potential drug targets.

**Results:**

MR analyses identified 67 plasma proteins with potential causal relationships with pericarditis, such as NEU1, GDNF, LAT, CASP8, ZFYVE27, and NAPA. Among them, elevated levels of NEU1, GDNF, and LAT increased the risk of pericarditis, whereas higher levels of CASP8, ZFYVE27, and NAPA decreased the risk of pericarditis. Pleiotropy tests and sensitivity analyses confirmed the stability of the findings. Moreover, scRNA‐seq analysis revealed that genes such as *extracellular matrix protein 1 (ECM1), CASP8, EPHA4*, and *CCL2* were specifically expressed in cardiac progenitor cells (CPCs). Molecular docking further identified compounds with anti‐inflammatory, antioxidant, or immunomodulatory potential, including phorbol 12‐myristate 13‐acetate (PMA), cerivastatin, and melatonin.

**Conclusion:**

This research examined the causal association between plasma proteins and pericarditis by MR analysis and identified several potential therapeutic targets. These findings provide theoretical evidence for targeted drug development and clinical prevention strategies for pericarditis.

## 1. Introduction

Pericarditis, defined as inflammation of the pericardial sac, is the most prevalent pericardial illness in clinical practice and accounts for 5% of emergency department visits for chest pain [1]. The annual incidence is ~27.7 cases per 100,000 people. It is characterized by fibrinous exudates and pericardial effusion and may progress to cardiac tamponade. About 15%–30% of individuals with acute pericarditis experience recurrence within 18 months [2], with half unable to achieve complete remission, significantly affecting their quality of life [3, 4]. Recurrence of pericarditis typically occurs within 4–6 weeks after the initial episode [5]. Current first‐line therapies, such as colchicine and nonsteroidal anti‐inflammatory drugs, along with second‐line corticosteroids, often yield limited efficacy [6]. Given that IL‐1 inhibitors offer new treatment options for colchicine‐resistant and corticosteroid‐dependent patients [7–9], newly proposed treatment algorithms recommend selecting a combination of IL‐1 inhibitors and colchicine or colchicine and corticosteroids based on whether inflammatory markers are elevated at initial presentation [10]. However, these recommendations are derived from expert opinion and lack support from prospective studies.

The onset of pericarditis is closely linked to autoinflammatory and autoimmune responses [11]. Evidence suggests that pro‐inflammatory cytokines released both systemically and locally, along with their role in inflammasome activation, are major drivers in the pathogenesis of pericarditis [11]. Existing studies have confirmed the critical roles of IL‐1β and IL‐6 in aseptic pericarditis models in rats [12]. Moreover, individuals with recurrent pericarditis often exhibit fever, elevated C‐reactive protein (CRP) levels, and a notable therapeutic response to IL‐1 antagonists, indicating that IL‐1–mediated inflammatory responses are central to disease development [13, 14]. Conversely, elevated serum concentrations of circulating TGF‐β and IL‐6 among individuals with viral or malignant pericarditis suggest a profibrotic mechanism mediated by TGF‐β [15, 16]. However, whether these plasma proteins are causally related to pericarditis remains unclear. Given that observational research is susceptible to residual confounders and selection bias, further research is needed to assess the causal impacts of genetically predicted (GP) circulating plasma proteins on pericarditis.

Mendelian randomization (MR) offers an effective approach for causal inference in observational research [17]. Owing to ethical constraints that hinder the implementation of randomized controlled trials examining the impacts of circulating plasma proteins on the risk of pericarditis, and given the limitations of observational studies in accounting for confounders, MR offers a valuable alternative for prospectively assessing the pathogenic potential of specific plasma proteins. Conceptually, MR is analogous to randomized controlled trials, as genetic variants are allocated randomly at conception, which helps minimize confounding and reduces reverse causation bias [18]. Prior MR research has demonstrated causal associations between GP plasma protein levels and the risk of stroke [19]. Another MR study provided important biological insights into therapeutic target discovery for lung adenocarcinoma based on the causal impacts of plasma proteins [20].

To date, no MR studies have identified a causal association between circulating plasma proteins and pericarditis. Therefore, this research aimed to examine whether GP plasma proteins are significantly associated with pericarditis risk through two‐sample MR (TSMR) analysis and single‐cell RNA sequencing (scRNA‐seq). By integrating observational evidence, MR analysis, and scRNA‐seq data, this research aimed to uncover the potential causal role of specific circulating plasma proteins in the pathogenesis of pericarditis. Furthermore, identifying cell‐specific expression patterns of key proteins and their potential binding affinity to candidate therapeutic compounds may facilitate the development of future targeted treatment strategies for pericarditis.

## 2. Materials and Methods

### 2.1. Study Design

**Figure 1:**
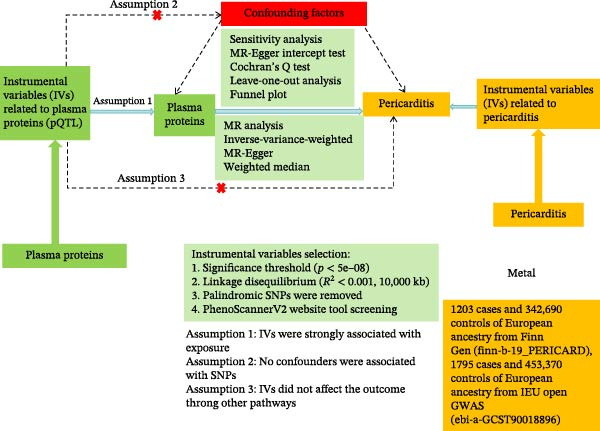
Study design and flowchart illustrating TSMR for plasma proteins and pericarditis. SNP, single nucleotide polymorphism; pQTL, protein quantitative trait loci.

Figure 1 illustrates the study design. This research applied a TSMR framework to examine the causal associations between 4907 plasma proteins and pericarditis using publicly available genome‐wide association study (GWAS) summary statistics (as detailed in Supporting Information 1: Table S1). Three core assumptions must be satisfied during TSMR analysis to ensure the stability of the MR results: (i) the genetic variants were strongly related to plasma proteins; (ii) the selected single nucleotide polymorphisms (SNPs) were independent of confounding variables; (iii) the instrumental variables (IVs) influenced the outcome exclusively through the exposure, without accounting for potential horizontal pleiotropy. Prior to MR analysis, relevant assumptions and independence assumptions were tested. R packages “TwoSampleMR,” “MR‐PRESSO” (version 4.2.3), and “fdrtool” were utilized to carry out MR and sensitivity analyses. Subsequently, the functions of the identified pericarditis‐related candidate proteins were annotated using Gene Ontology (GO) and Kyoto Encyclopedia of Genes and Genomes (KEGG) enrichment analyses, protein–protein interaction (PPI) network, and scRNA‐seq data. Potential pathogenic mechanisms and therapeutic pathways were inferred. Finally, molecular docking was applied to screen for high‐affinity active compounds, providing theoretical support for drug development.

### 2.2. Data Sources

#### 2.2.1. Plasma Protein Data Source

The 4907 plasma proteins were derived from the largest plasma proteomics GWAS conducted by Ferkingstad et al. [21], which analyzed data from 35,559 Icelandic individuals (mean age 55 years, 57% female, and 43% male).

#### 2.2.2. Pericarditis Data Source

A fixed‐effects meta‐analysis was performed using METAL software based on summary statistics from the FinnGen consortium and the IEU OpenGWAS database (ebi‐a‐GCST90018896), with effect sizes weighted by the inverse of their standard errors [22]. The FinnGen consortium dataset comprised 1203 European individuals diagnosed with pericarditis and 342,690 healthy controls. The IEU OpenGWAS dataset (ebi‐a‐GCST90018896) included 1795 European pericarditis cases and 453,370 healthy controls.

### 2.3. IV Selection Criteria

The criteria for selecting IVs included: (i) SNPs were significantly related to plasma protein levels (*p*  < 5  × 10^−8^) [23, 24]; (ii) linkage disequilibrium (LD) clumping was carried out to ensure independence among SNPs (*r* ^2^ < 0.001, kb = 10,000 kb); and (iii) SNPs directly related to pericarditis (*p*  < 0.05) were excluded. Furthermore, we calculated the F‐statistic for each SNP to validate the instrument strength. The formula *F* = *R* ^2^(N‐2)/(1‐*R* ^2^) was used, where *R* ^2^ was estimated by 2β^2^ × MAF × (1‐MAF). The results showed that the F‐statistics for all IVs were greater than 10 (mean: 202.296, range: 29.749–999.885), which were far higher than the threshold for weak IVs (typically *F* > 10), effectively avoiding weak instrumental bias. Detailed information is listed in Supporting Information 2: Table S2.

#### 2.3.1. Screening for Confounding Factors

Although we assessed the heterogeneity and horizontal pleiotropy of MR results using various sensitivity analyses to explore any SNPs that violated MR Hypotheses 2 and 3 (Figure 1), a small number of residual confounding IVs may still exist. Therefore, to satisfy the independence assumption of MR, the association between SNPs and phenotypes was analyzed using FastTraitR (version 1.0.1, https://www.ebi.ac.uk/gwas/), based on data from the NHGRI‐EBI Catalog database. SNPs associated with confounding factors were excluded, including tumor invasion of the pericardium, uremia, pericarditis after acute myocardial infarction, aortic dissection, postcardiac surgery, chest wall trauma, and autoimmune diseases (systemic lupus erythematosus and rheumatoid arthritis [RA]) [25, 26]. If any SNP was found to be associated with these confounding factors (*p*  < 5  × 10^−8^), the MR analysis should be repeated after excluding the SNP to ensure the robustness of the results.

### 2.4. Statistical Analysis

Three MR analysis methods, including inverse variance weighted (IVW), MR‐Egger regression, and weighted median, were used to assess the potential causal relationship between plasma protein levels and pericarditis. In addition, MR‐Egger regression and MR‐PRESSO (MR Pleiotropy RESidual Sum and Outlier) were utilized to detect pleiotropy. The IVW method was chosen as our primary analysis method because it can integrate the effect estimates of individual SNPs to obtain a comprehensive causal effect estimate. The IVW is particularly suitable when there is no horizontal pleiotropy because it uses a random effects meta‐analysis framework to effectively handle heterogeneity among different SNP effects. The weighted median method can produce effective estimates even if up to 50% of the IVs are invalid due to pleiotropy or other biases [27]. In addition, we performed MR‐Egger regression, which not only provided MR estimates but also detected directional pleiotropy through its intercept term. This method provides insights into the average pleiotropy of genetic variants as IVs [28]. However, due to its large standard error, the MR‐Egger method is generally considered inferior to IVW if there is no pleiotropy. The MR‐PRESSO method was also used because it can detect and adjust for outliers that may affect the results due to pleiotropy not considered by other methods. Finally, a Cochran Q analysis was performed to assess the heterogeneity among the individual causal effects estimated for each SNP. A *p*‐value > 0.05 indicated that the heterogeneity was not statistically significant, supporting the stability of the findings across different genetic variants.

### 2.5. Sensitivity Analyses

Multiple sensitivity analyses were carried out to test the robustness of MR results. Firstly, horizontal pleiotropy was assessed according to the intercept from the MR‐Egger regression. A significant horizontal pleiotropy was indicated if the intercept *p* value was less than 0.05. Secondly, heterogeneity among SNPs was examined by Cochran’s Q statistic. A *p* value greater than 0.05 suggested no significant heterogeneity. Thirdly, identification of outlier SNPs was conducted via leave‐one‐out analysis, where each SNP was excluded one by one, and the MR analysis was repeated to determine whether the results were driven by any single outlier. It should be noted that when the number of IVs was small (e.g., < 5 SNPs), the statistical power of MR‐Egger and weighted median methods may be insufficient, and the results of the sensitivity analysis should be interpreted with caution.

### 2.6. Candidate Protein Selection

The following steps were used to select plasma proteins significantly associated with pericarditis risk: (i) directional consistency: effect directions from MR‐Egger, IVW, and weighted median methods must be consistent (all odds ratios (ORs) > 1 or all < 1); (ii) exclusion of pleiotropic proteins: proteins with MR‐Egger intercept test P less than 0.05 were excluded; (iii) statistical significance: proteins with *p*  < 0.05 by the IVW method were retained.

### 2.7. PPI Network Construction and Visualization

Significant plasma proteins were used to generate a PPI network using the STRING database (https://string-db.org/), and key proteins were ranked based on STRING’s algorithm. The network was visualized via Cytoscape software (v3.10.3), and hub proteins were identified using the cytoHubba plugin. The top 10‐ranked proteins were defined as key core proteins to explore their potential as drug targets for pericarditis.

### 2.8. scRNA‐seq Analysis

scRNA‐seq data of cardiac progenitor cells (CPCs) were derived from the PanglaoDB database (https://panglaodb.se/, sample ID: SRA701836). The dataset was retrieved using the “Samples” module by filtering for “Human” and “Tissue” [29]. scRNA‐seq data were analyzed via the “Seurat” in the R package [30]. After quality control, t‐distributed stochastic neighbor embedding (t‐SNE) was employed to visualize the scRNA‐seq data. All publicly available datasets had obtained ethical approvals.

### 2.9. Molecular Docking and Prediction of Potential Therapeutic Compounds

To predict potential therapeutic drugs, drug enrichment analysis was carried out based on the selected key hub proteins using the DSigDB database (https://dsigdb.tanlab.org/), yielding candidate drugs and their target proteins. Subsequently, three‐dimensional structures of the target proteins were collected from the RCSB Protein Data Bank (http://www.rcsb.org), and molecular structures of drug compounds were retrieved from the PubChem database (https://pubchem.ncbi.nlm.nih.gov/). Molecular docking was then performed via the CB‐Dock2 (https://cadd.labshare.cn/), and the results were visualized by PyMOL v2.5.

### 2.10. GO and KEGG Enrichment Analyses

The functional characteristics and biological relevance of target proteins identified as being causally associated with pericarditis in MR were investigated through GO and KEGG enrichment analyses.

## 3. Results

### 3.1. MR Results and Sensitivity Analyses

The positive results were interpreted primarily based on the statistical significance (*p*  < 0.05) of IVW, and the consistency of the causal effect directions estimated by MR‐Egger regression and the weighted median method. As mentioned earlier, the IVW method integrates the effects of all effective IVs (SNPs), providing a single, comprehensive estimate of the causal effect, and exhibits high statistical power in the absence of level pleiotropy. If the effect directions obtained by MR‐Egger and the weighted median method were consistent with the IVW results, it would enhance the robustness of the results. Since pleiotropy in the same direction for all IVs was acceptable in the MR‐Egger regression method, and up to 50% of ineffective IVs were acceptable in the weighted median method, the consistent directions of their results can mitigate the risk of bias caused by pleiotropy in some IVs to some extent. Because the MR‐Egger method has low statistical power (larger standard error) in the absence of pleiotropy, we did not rely solely on its *p*‐value as the primary criterion, instead using its direction to complementarily verify the IVW results.

The validity of IVW results is highly dependent on the premise of no pleiotropy. Although the MR‐Egger intercept test (*p*  > 0.05) and MR‐PRESSO provided supporting evidence, residual undetected pleiotropy may still introduce bias. While the consistency of direction of MR‐Egger, weighted median, and IVW results enhances the robustness of the conclusions, due to the low statistical power of MR‐Egger, its statistical significance was not used as the primary criterion and was only used to complementarily verify the results. Therefore, this study used IVW results as the core finding and employed multiple methods to control for potential bias to the greatest extent. However, the conclusions still need to be interpreted with careful consideration.

**Figure 2:**
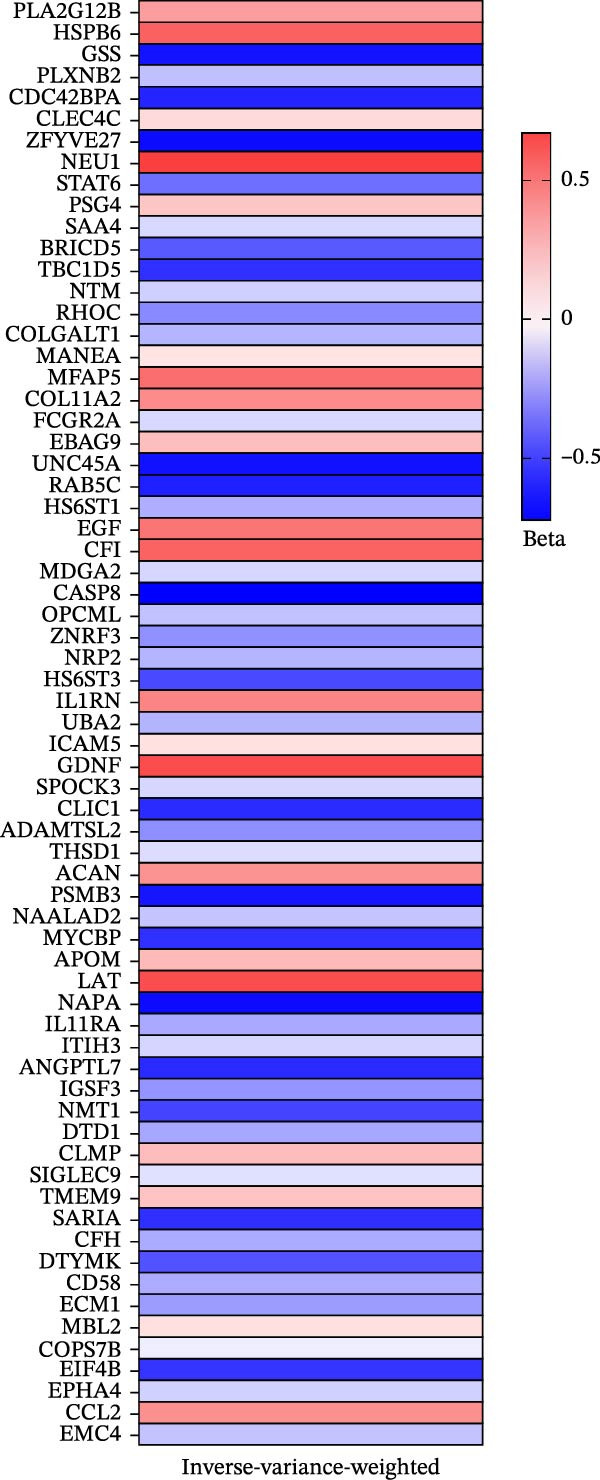
Heatmaps of the IVW analysis. Each colored block represents a different beta value.

As illustrated in Figure 2, a heatmap presents the MR estimates derived from the IVW method for the associations between 67 plasma proteins and pericarditis risk. Detailed estimates from the three MR methods (MR‐Egger, IVW, and weighted median), as well as sensitivity analysis results, are detailed in Supporting Information 3: Table S3. MR‐Egger regression demonstrated no significant horizontal pleiotropy (MR‐Egger intercept test, *p*  > 0.05). Supporting Information 4: Table S4 lists the detailed information on the IVs. Scatter plots of various tests assessing the associations between 67 plasma proteins and pericarditis risk are presented in Supporting Information 5: Figure S1. Among the significantly associated proteins (Figure 3), 21 (such as HSPB6, NEU1, and CFI) were correlated with an elevated risk of pericarditis (OR > 1), while 46 (such as CASP8, ZFYVE27, and GSS) were linked to a lower risk (OR < 1). Notably, the sensitivity analysis indicated that the results were inconsistent. For example, the IVW results for ANGPTL7 protein were significant (*p* = 0.038), but the MR‐Egger and weighted median results were not (*p* = 0.698 and *p* = 0.059, respectively). This inconsistency may be attributable to the use of a very small number of SNPs (many proteins use only 3–5 SNPs) as IVs. Although the F‐statistic was > 10, an insufficient number of IVs significantly reduced the statistical power and reliability of the results of MR‐Egger regression (used to detect pleiotropy) and the weighted median method. Therefore, this finding needs to be further validated by increasing the number of IVs in future studies.

Figure 3Forest plot of the MR estimates for the associations between 67 plasma proteins and pericarditis risk. (A) The first part of the analyzed plasma proteins; (B) The remaining plasma proteins. CI, confidence interval; OR, odds ratio.

**Figure:**
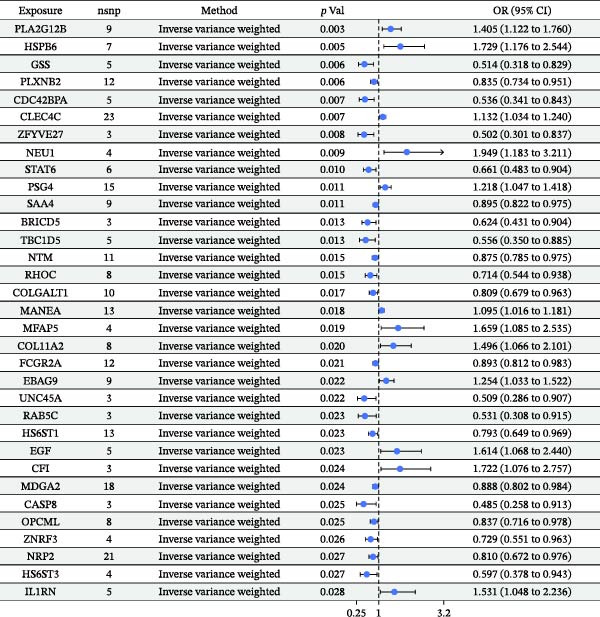
(A)

**Figure:**
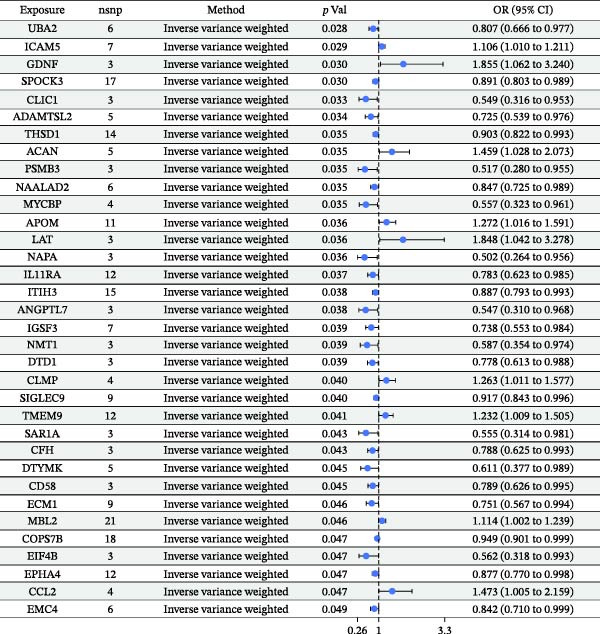
(B)

The leave‐one‐out analysis demonstrated that no single SNP substantially impacted the overall stability of the results (Supporting Information 6: Figure S2), and funnel plots showed a symmetric distribution of SNPs, suggesting no apparent bias (Supporting Information 7: Figure S3). Sensitivity analysis results are summarized in Supporting Information 8: Table S5, indicating the robustness and reliability of the findings (*p*‐values all > 0.05).

For these 67 candidate inflammatory proteins, we further manually examined the protein quantity trait loci (pQTLs) associated with the second common trait (tumor invasion of the pericardium, uremia, pericarditis after acute myocardial infarction, aortic dissection, postcardiac surgery, chest wall trauma, and autoimmune diseases [systemic lupus erythematosus, RA]). Using the NHGRI‐EBI GWAS Catalog database (https://www.ebi.ac.uk/gwas/) and the FastTraitR software package (v1.0.1), we found that the 67 pQTLs were not associated with any confounding factors. Supporting Information 3: Table S3 shows the corresponding gene information for the IVs of the 67 inflammatory proteins associated with the risk of pericarditis.

### 3.2. Statistical Visualization and PPI Network

**Figure 4:**
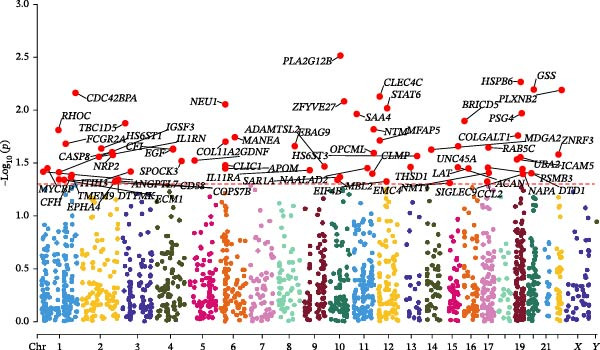
Manhattan plot displaying genomic loci associated with the 67 candidate plasma proteins. Significant genes were labeled.

**Figure 5:**
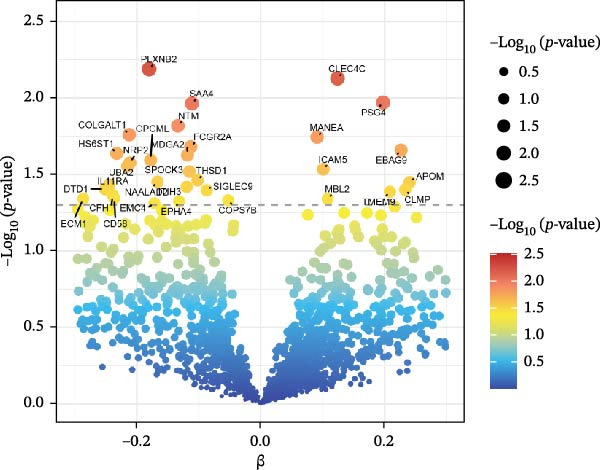
Volcano plot of MR analysis for plasma proteins and pericarditis risk. Beta indicates the effect size of protein on pericarditis risk.

The Manhattan plot (Figure 4) illustrates the genomic loci associated with the 67 plasma proteins. Several significant loci were annotated, including PLA2G12B, HSPB6, GSS, CLEC4C, STAT6, and PLXNB2. The volcano plot (Figure 5) highlights proteins with stronger statistical significance. Elevated levels of EBAG9, CLEC4C, PSG4, and MANEA correlated with a greater risk of pericarditis (positive effect), whereas higher levels of NTM, COLGALT1, and SAA4 were related to a reduced risk (negative effect).

Utilizing the STRING database, a PPI network was generated and analyzed with Cytoscape v3.10.3 to evaluate topological features (degree and betweenness centrality). Figure 6A presents the PPI network of the significantly associated proteins, and Figure 6B highlights the top 10 key hub proteins identified from the network (Table 1), among which epidermal growth factor (EGF), CCL2, and interleukin‐1 receptor antagonist (IL1RN) had the highest degree values.

Figure 6(A) The PPI network of potential targets. (B) The PPI network of core targets.

**Figure:**
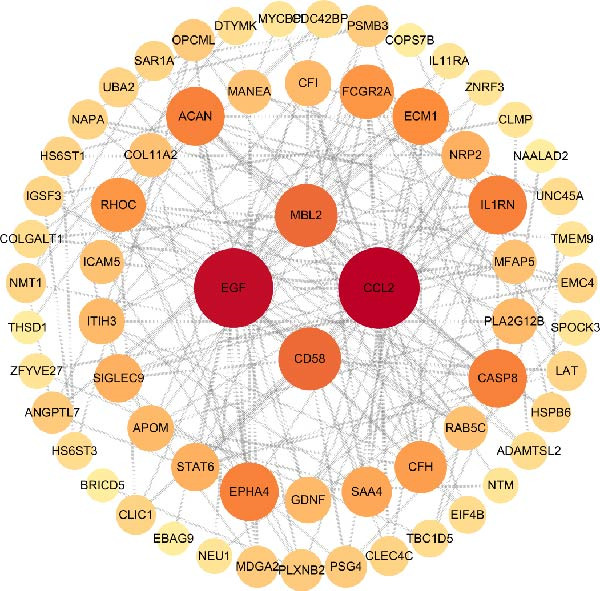
(A)

**Figure:**
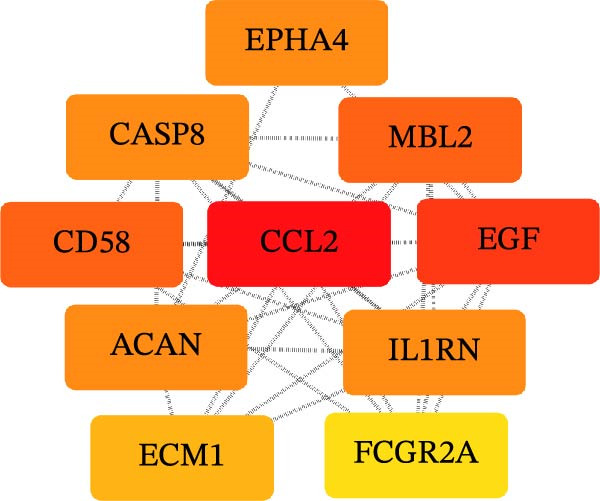
(B)

**Table 1:** Core targets screened from the PPI network.

Gene	Degree	Closeness centrality	Betweenness centrality	Topological coefficient
*EGF*	9	1.000	0.185	0.654
*CCL2*	8	0.899	0.046	0.722
*1LIRN*	8	0.8999	0.046	0.722
*CASP8*	7	0.818	0.024	0.761
*ACAN*	6	0.750	0.061	0.722
*FCGR2A*	6	0.750	0.005	0.796
*MBL2*	6	0.750	0.012	0.796
*CD58*	5	0.692	0.000	0.844
*ECM1*	5	0.692	0.005	0.822
*EPHA4*	2	0.562	0.000	0.833

The volcano and Manhattan plots provide different perspectives on statistically significant genes. Among these genes, the top 10 key hub proteins represented the most interconnected and central nodes within the PPI network.

### 3.3. scRNA‐seq Results

scRNA‐seq data from CPCs sample SRA701836 in the PanglaoDB database were analyzed. After quality control, dimensionality reduction and visualization were performed using t‐SNE. The analysis revealed that CPCs were divided into six cell clusters, including fibroblasts, cardiomyocytes, NK cells, endothelial cells, pericytes, and T cells. Focusing on the cell type‐specific expression of key hub proteins, extracellular matrix protein 1 (ECM1) was primarily enriched in the fibroblast and endothelial cell clusters, while CASP8 was enriched in fibroblast, T cell, and endothelial cell clusters (Figure 7). Similarly, CCL2 and EPHA4 also exhibited enrichment patterns in specific cell clusters.

Figure 7scRNA‐seq localization analysis of ECM1, CASP8, CCL2, and EPHA4. (A) CPCs were clustered into six cell types. (B) ECM1 was enriched in fibroblast and endothelial cell clusters. (C) CASP8 was enriched in fibroblast, endothelial cell, and T cell clusters. (D) CCL2 was enriched in fibroblast clusters. (E) EPHA4 was enriched in fibroblast and endothelial cell clusters.

**Figure:**
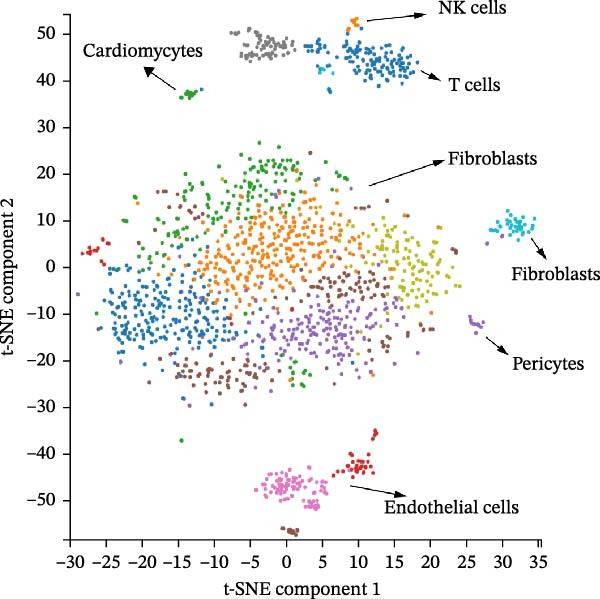
(A)

**Figure:**
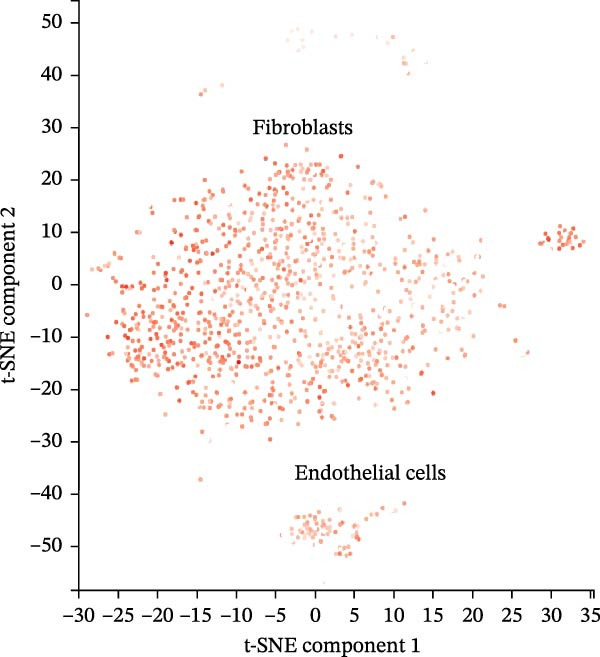
(B)

**Figure:**
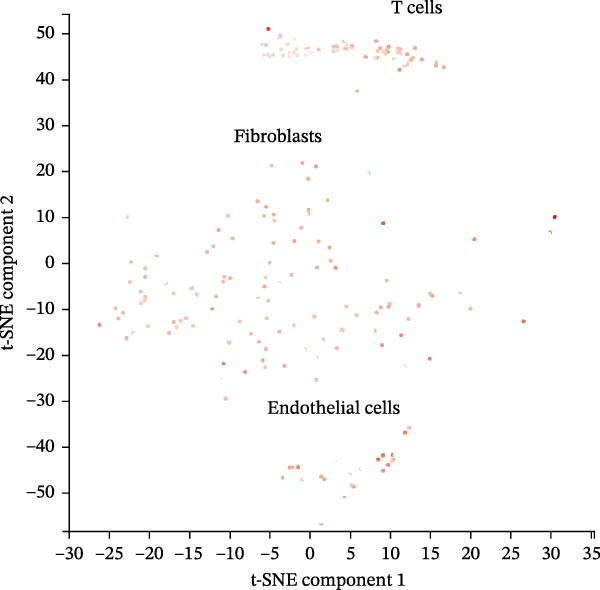
(C)

**Figure:**
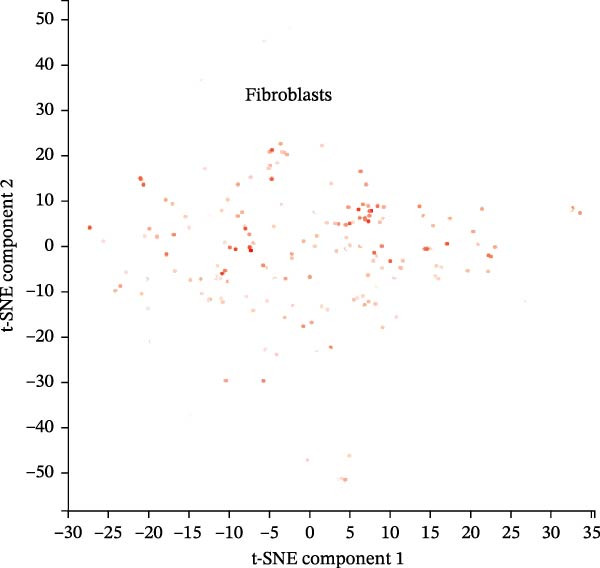
(D)

**Figure:**
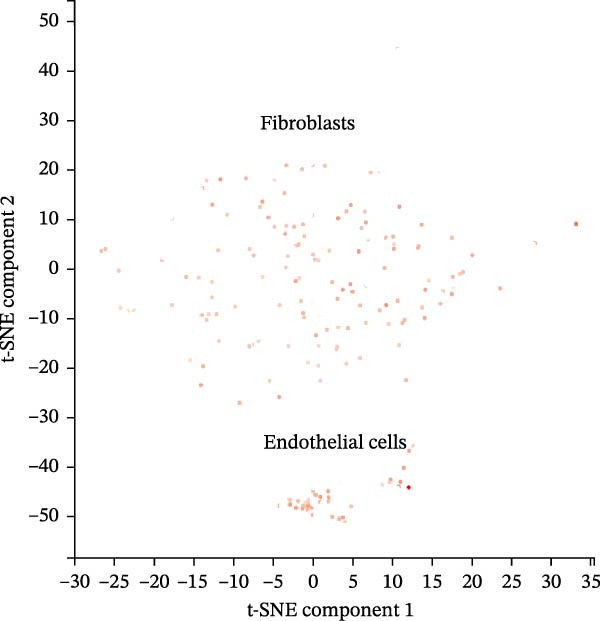
(E)

### 3.4. GO and KEGG Enrichment Analysis of Plasma Proteins

Significant enrichment of biological processes (BP, Figure 8) included leukocyte‐mediated immunity, positive regulation of endocytosis, regulation of endocytosis, regulation of peptidase activity, lymphocyte‐mediated immunity, adaptive immune response based on somatic recombination of immune receptors built from immunoglobulin superfamily domains, axonogenesis, positive regulation of cell development, epithelial cell proliferation, and epithelial morphogenesis. Significant enrichment of cellular components (CCs) encompassed secretory granule membrane, collagen‐containing ECM, lysosomal membrane, lytic vacuole membrane, vacuolar membrane, secretory granule lumen, cytoplasmic vesicle lumen, vesicle lumen, external side of plasma membrane, and endopeptidase complex. Significant enrichment of molecular functions (MFs) included glycosaminoglycan (GAG) binding, growth factor receptor binding, ECM structural constituent, cytokine‐receptor binding, sulfur compound binding, carbohydrate binding, protein tyrosine kinase binding, growth factor binding, cytokine binding, and heparin binding. KEGG pathway enrichment analysis demonstrated that plasma proteins were significantly enriched in pathways such as *Staphylococcus aureus* infection, GAG biosynthesis‐heparan sulfate/heparin, complement and coagulation cascades, Ras signaling pathway, legionellosis, phagosome, Yersinia infection, tuberculosis, JAK‐STAT signaling pathway, and PD‐L1 expression and PD‐1 checkpoint pathway in cancer (Figure 9).

Figure 8GO enrichment analysis of plasma proteins. (A) Bar chart displaying the top 10 enriched pathways. (B) Bubble plot showing the top 10 enriched pathways; bubble size corresponds to the number of genes involved, and bubble color intensity (redder) indicates greater statistical significance (smaller *p*‐value). (C) Circlize plot for top pathways.

**Figure:**
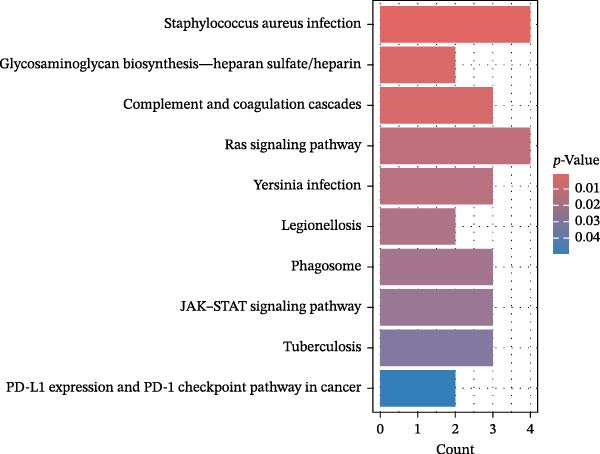
(A)

**Figure:**
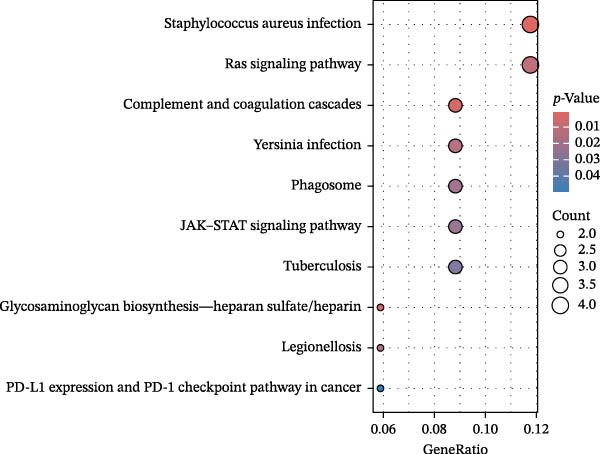
(B)

**Figure:**
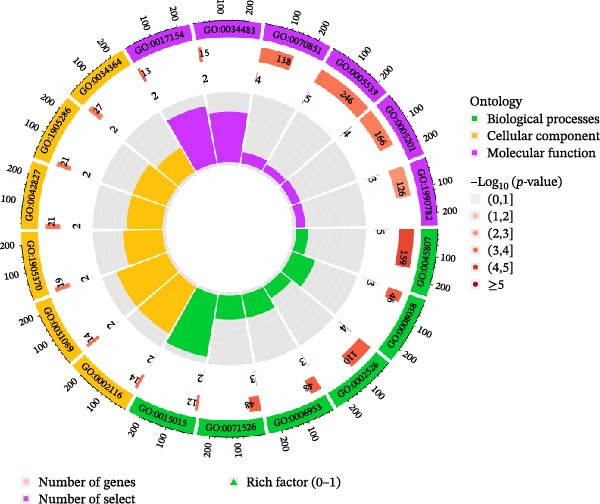
(C)

Figure 9KEGG pathway enrichment analysis. (A) Circos plot visualization of the top enriched KEGG pathways. The plot is divided into four layers from outside to inside: 1st layer (outermost): Coordinate axis representing the scale of gene numbers for each pathway ID. 2nd layer: Bars represent the number of background genes associated with the pathway, colored by enrichment significance (−log_10_ P‐value). Redder colors indicate higher significance. 3rd layer: Purple bars represent the number of differentially expressed genes (enriched genes) involved in each pathway. 4th layer (innermost): Bars represent the Rich Factor (ratio of enriched genes to background genes) for each pathway, colored by the pathway category (e.g., Human Diseases, Metabolism, etc.). (B) The corresponding descriptions and full names for the KEGG pathway IDs shown in panel (A).

**Figure:**
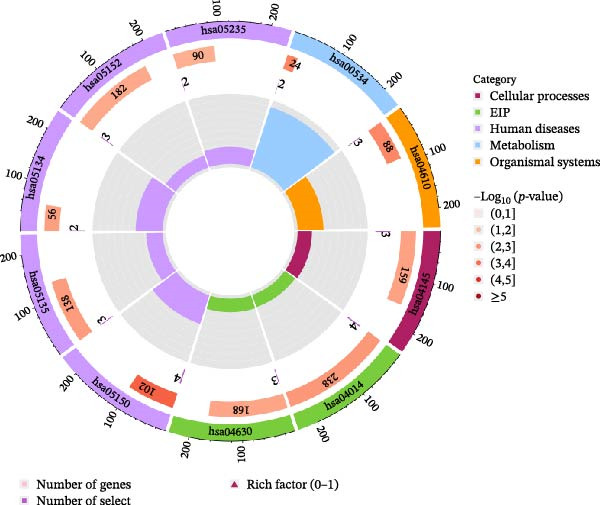
(A)

**Figure:**
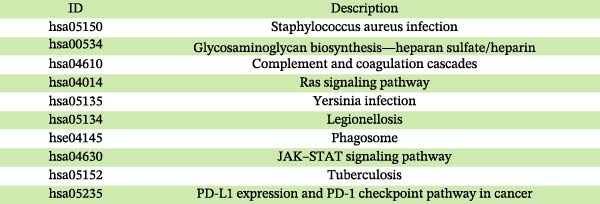
(B)

### 3.5. Drug Enrichment Analysis

**Figure 10:**
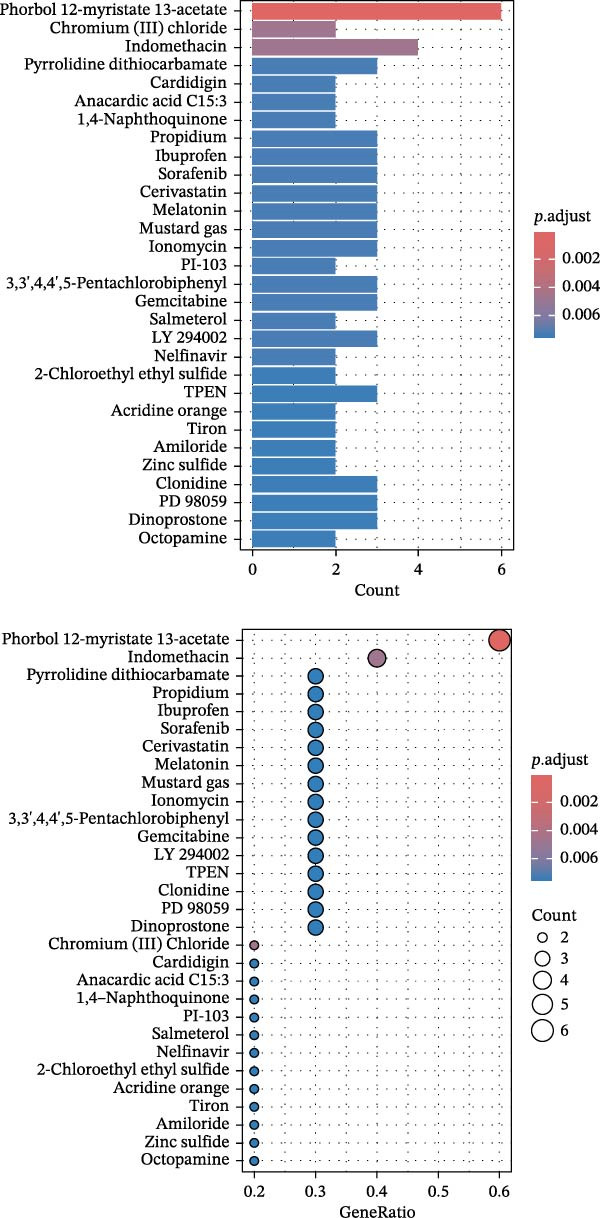
Drug enrichment analysis for core genes associated with pericarditis.

As presented in Figure 10, drug enrichment analysis identified compounds significantly related to the key hub plasma proteins involved in pericarditis, such as pyrrolidine dithiocarbamate, propidium, ibuprofen, phorbol 12‐myristate 13‐acetate (PMA), sorafenib, cerivastatin, and melatonin (Supporting Information 9: Table S6). In addition to established first‐line drugs for pericarditis (such as indomethacin, ibuprofen, and aspirin), the key hub proteins (IL1RN, MBL2, CD58, CCL2, CASP8, and EGF) were also significantly enriched in antioxidant agents, including glutathione (GSH) and N‐acetyl‐L‐cysteine (NAC), as well as immunomodulators such as PMA. Notably, cerivastatin showed strong associations with the CASP8/EGF/CCL2 target combination, and melatonin was significantly associated with the aggrecan (ACAN)/CASP8/EGF target combination.

### 3.6. Molecular Docking Results

Currently, treatment strategies for pericarditis primarily focus on inflammation suppression, immunoregulation, and fibrosis prevention. Based on the ranking of p‐adjust values from the drug enrichment analysis, compounds with relatively high fold enrichment and a gene count ≥ 3 were selected. These compounds, including PMA, cerivastatin, pyrrolidine dithiocarbamate, NAC, GSH, and melatonin, are known for their anti‐inflammatory, antioxidant, or immunomodulatory properties. These compounds were considered key active candidates and subsequently docked with five core target proteins: CD58, CASP8, EGF, CCL2, and ACAN. A binding energy (*ΔG*) threshold of ≤ –5.0 kcal/mol was applied to define effective binding. All valid docking results are illustrated in Figure 11, among which the PMA–CD58 complex (–94.5 kcal/mol) and cerivastatin‐CCL2 complex (–8.3 kcal/mol) showed the lowest binding energies, suggesting the strongest binding affinities. Notably, PMA, cerivastatin, and melatonin were predicted to bind with two or more key hub proteins. In particular, cerivastatin and melatonin exhibited strong binding affinities (ΔG ≤ –5.0 kcal/mol) with nearly all key hub proteins (Figure 11), indicating that these anti‐inflammatory, antioxidant, or immunoregulatory compounds have strong binding potential with the key hub proteins.

Figure 11(A) Molecular docking results for potential therapeutic compounds. (B) Docking results showing compounds with high binding affinity to candidate targets (binding energy < –5.0 kcal/mol).

**Figure:**
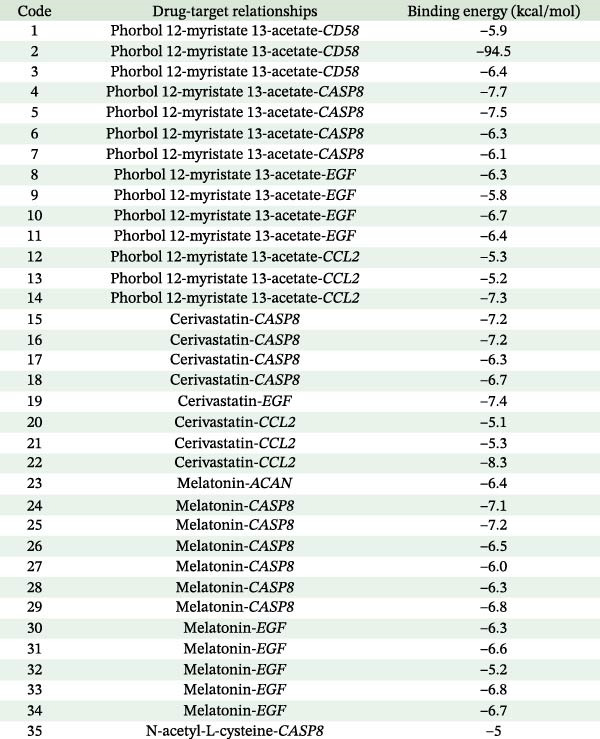
(A)

**Figure:**
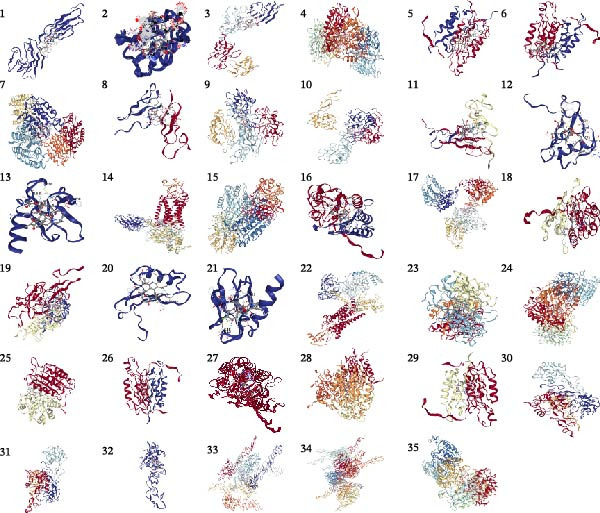
(B)

## 4. Discussion

This study systematically identified 67 plasma proteins causally associated with pericarditis, such as the pathogenic factor NEU1 and the protective factor CASP8, through TSMR analysis. KEGG and GO enrichment analyses underscored the role of key hub proteins (such as EGF, CD58, ECM1, and CASP8) within the pathological axis of “inflammation‐immunity‐fibrosis‐thromboinflammation.” PPI network analysis identified *EGF, CD58*, *EPHA4*, *ECM1*, *IL1RN, FCGR2A, ACAN, CASP8*, *CCL2*, and *MBL2* as hub genes. The scRNA‐seq, network pharmacology, and molecular docking collectively identified potential therapeutic compounds (such as cerivastatin and melatonin), providing a theoretical foundation for targeted interventions in pericarditis.

EGF, a pivotal growth factor, maintains tissue homeostasis by regulating cell differentiation and proliferation. However, its aberrant expression may drive pathological processes [31, 32]. Song et al. [33] reported that KLK8 can regulate macrophage function via paracrine EGF derived from cardiac fibroblasts, which might contribute to cardiac injury and fibrotic remodeling after myocardial infarction. In the present study, GP higher circulating EGF levels were significantly correlated with an elevated pericarditis risk (OR = 1.614, 95% CI: 1.068–2.440, PIVW = 0.023). The underlying mechanism may involve paracrine activation of macrophages by cardiac fibroblast‐derived EGF, thereby promoting pathological remodeling.

ACAN is a key proteoglycan responsible for maintaining cartilage elasticity [34]. In osteoarthritis, pro‐inflammatory factors, including IL‐1β and TNF‐α, reduce ACAN synthesis by suppressing the expression of Sox‐9 [35–37], while simultaneously promoting its degradation by activating matrix metalloproteinase‐13 (MMP‐13) and aggrecanases ADAMTS‐4 and ADAMTS‐5 [37, 38]. Notably, ACAN degradation fragments can activate macrophages via toll‐like receptor 2/4 (TLR‐2/4), thereby promoting the release of IL‐6 and TNF‐α, amplifying inflammation, and ultimately contributing to tissue damage [38–40]. Our MR analysis revealed that GP higher circulating ACAN levels correlated with a greater risk of pericarditis (OR = 1.459, 95% CI: 1.028–2.073, PIVW = 0.035). This finding may reflect a dual pathological role of ACAN in pericarditis. In camptodactyly‐arthropathy–coxa vara–pericarditis syndrome caused by PRG4 mutations [41], mechanical stress on the pericardium leads to extensive enzymatic degradation of ACAN (such as by MMP‐13 and ADAMTS‐4/5). The resulting ACAN fragments activate macrophages via TLR‐2/4, ultimately triggering the secretion of IL‐6 and TNF‐α and accelerating pericardial tissue injury. Genetically elevated baseline levels of ACAN may lead to increased generation of pro‐inflammatory fragments in inflammatory conditions, resulting in a vicious cycle of ACAN degradation, fragment accumulation, and inflammation amplification.

IL1RN, as a natural antagonist of IL‐1, blocks its pro‐inflammatory signaling by competitively binding to the IL‐1 receptor [42–44]. The local balance between IL‐1 and IL1RN is critical for inflammation regulation, and disruption of this balance may increase the risk of several diseases, including RA, atherosclerosis, coronary artery disease, and vasculitis [45]. The synthetic form of IL1RN, anakinra, effectively reduces pericarditis recurrence by inhibiting the binding of IL‐1α and IL‐1β to IL‐1R1 [7]. However, in this study, GP higher circulating IL1RN levels correlated with an increased risk of pericarditis (OR = 1.531, 95% CI: 1.048–2.236, PIVW = 0.028). This seemingly contradictory result suggests that elevated circulating IL1RN levels are more likely a compensatory response to overactive IL‐1 signaling rather than a direct pathogenic factor. This finding further supports that endogenous IL1RN compensation may be insufficient to completely antagonize the overactive IL‐1 signaling pathway in recurrent pericarditis. Thus, these results confirm that supplementation with the exogenous IL1RN (anakinra) is reasonable, providing human genetic evidence for its clinical application.

This study found that GP higher plasma CD58 levels correlated with a lower pericarditis risk (OR = 0.789, 95% CI: 0.626–0.995, PIVW = 0.045). CD58, known as lymphocyte function‐associated antigen‐3 (LFA‐3), is a ligand for the T‐cell costimulatory molecule CD2. The CD2–CD58 interaction promotes T‐cell adhesion to antigen‐presenting cells (including macrophages and B cells) and facilitates full activation of T cells [46, 47]. Studies on chronic heart failure have confirmed that CD58 on monocytes is a critical costimulatory signal driving activation of CD4+CD28^null^ T‐cells. Its activation leads to substantial secretion of pro‐inflammatory cytokines such as TNF‐α, ultimately aggravating cardiovascular inflammatory injury [48]. CD58 exists as a membrane‐bound form (mCD58) and can be cleaved by proteases and released into body fluids as soluble CD58 (sCD58). In individuals with RA, reduced serum sCD58 levels may lead to overactivation of the CD2 signaling pathway and exacerbation of inflammation. IL‐10 can significantly reduce the proliferation and TNF secretion of CD4+CD28^null^ T cells by inhibiting CD58 expression on monocytes. This suggests that decreased sCD58 may relieve the suppression of T cells, resulting in elevated production of pro‐inflammatory cytokines (including TNF‐α and IL‐2) and ultimately aggravating synovial inflammation and joint destruction [48, 49]. Given that pericarditis is one of the cardiovascular complications secondary to RA [50] and that the CD58–CD2 pathway directly modulates cardiovascular inflammation, the observed genetically higher plasma CD58 levels in this study, mainly reflecting sCD58, may competitively bind to CD2 and block the CD58–CD2 interaction. This could downregulate T‐cell activation and the release of pro‐inflammatory cytokines (such as IL‐2 and TNF‐α), thereby possibly reducing pericardial immune damage.

ECM1 is a soluble protein that regulates ECM homeostasis. It mediates various BPs by enhancing interactions with ECM components, including inhibiting the proteolytic activity of MMP‐9 (MMP9) [51], regulating endothelial cell proliferation [52], and facilitating Th2 lymphocyte migration [53]. In tuberculous pericarditis, neutrophils secrete MMP9 to promote ECM remodeling [54]. In IgG4‐related disease (IgG4‐RD), aberrant Th2‐type immune responses, such as activation of CD4+ cytotoxic T cells and involvement of cytokines (including IL‐4 and IL‐13), may contribute to pericardial fibrosis [55]. Collectively, the present study found that GP higher circulating ECM1 levels correlated with a lower pericarditis risk (OR = 0.751, 95% CI: 0.567–0.994, *p* = 0.046). The protective mechanisms may involve: (i) ECM1 may inhibit pathological remodeling by directly binding to and inhibiting MMP9 activity; (ii) it may help stabilize pericardial microvascular endothelial junctions and reduce inflammatory effusion exudate; and (iii) it may inhibit the migration of Th2 lymphocytes into the pericardial cavity and alleviate Th2‐type inflammatory fibrosis. These hypotheses need to be functionally validated in a pericarditis model.

CASP8, classified as apoptotic caspase‐8, is an initiator of extrinsic cell death. Recent research demonstrates that caspase‐8 exerts a crucial role in regulating inflammation. By cleaving pro‐necroptotic molecules such as CYLD, RIPK3, and RIPK1, caspase‐8 inhibits MLKL‐dependent activation of the NLRP3 inflammasome, thereby limiting the maturation and release of IL‐1β [56]. In dendritic cells deficient in caspase‐8, lipopolysaccharide (LPS) can directly activate the NLRP3 inflammasome without a secondary stimulus (such as ATP), resulting in elevated IL‐1β secretion [57]. However, a study by Lehle et al. [58] using caspase‐8‐deficient monocytes derived from individuals with very early‐onset inflammatory bowel disease observed reduced expression of the inflammasome sensor NLRP3 following LPS stimulation. This suggests that the regulation of inflammasomes by caspase‐8 may be dependent on cell types and stimulus conditions. This complexity suggests that the exact anti‐inflammatory mechanism of caspase‐8 in pericarditis needs to be further investigated. This study identified a causal association between GP circulating caspase‐8 levels and a reduced pericarditis risk (OR: 0.485, 95% CI: 0.258–0.913, PIVW = 0.024). This may be because caspase‐8 blocks NLRP3 inflammasome activation, thereby reducing IL‐1β activation and alleviating pericarditis. Additionally, caspase‐8‐activated inhibitors of apoptosis antagonists can eliminate host cells infected with pathogens such as Legionella and *Mycobacterium tuberculosis*, thereby restricting disease progression [59]. Our enrichment analysis further demonstrated the involvement of caspase‐8 in the pathogenesis of legionellosis and tuberculosis. Given that *Mycobacterium tuberculosis* is a major cause of tuberculous pericarditis, and Legionella infection can involve the pericardium, caspase‐8 may lower the risk of secondary pericarditis by suppressing excessive inflammation or enhancing pathogen clearance in these contexts.

EPHA4, a core member of the ephrin receptor tyrosine kinase family, mediates angiogenic and lymphangiogenic remodeling [60], axon guidance and cortical development [61], as well as promoting tumor angiogenesis [62]. Deficiency of EphA4 may cause the leakage of the blood–spinal cord barrier and disrupt astrocyte–vascular coupling [63]. In addition, it regulates monocyte–endothelial adhesion within atherosclerotic plaques [64]. In thrombosis, EphA4 constitutively interacts with platelet αIIbβ3 integrin and its surface expression is significantly upregulated upon platelet activation [65]. Chu et al. [66] found that the functional spectrum of EPHA4 lies at the intersection of arterial biology, vascular homeostasis, and immune regulation. Given its pivotal role in the vascular–immune axis, our MR analysis revealed that GP higher circulating EPHA4 levels correlated with a lower pericarditis risk (OR: 0.877; 95% CI: 0.770–0.998; PIVW = 0.0473). This protective effect may involve modulation of monocyte–endothelial adhesion, platelet activation, and vascular barrier integrity within the thromboinflammatory microenvironment of pericarditis.

FCGR2A (low‐affinity immunoglobulin gamma Fc region receptor II‐a/CD32) is a surface receptor expressed on neutrophils and macrophages. By binding to the Fc region of IgG, it initiates phagocytosis and anti‐pathogen immune responses, and also acts as a CRP receptor involved in inflammatory regulation [67, 68]. MR analysis has indicated that higher circulating FCGR2A levels are related to a lower risk of atrial fibrillation, suggesting its potential as a therapeutic target [69]. Notably, Szpakowicz et al. [68] identified FCGR2A as an independent biomarker for carotid atherosclerotic plaques, implying a tissue‐specific role in cardiovascular diseases. The present study revealed that lower GP circulating FCGR2A levels correlated with an elevated risk of pericarditis (OR = 0.893, 95% CI: 0.812–0.983, *p* = 0.021), which aligns with its reported protective role in atrial fibrillation [69]. A possible explanation is that reduced FCGR2A expression may impair phagocyte‐mediated clearance of antigen–antibody complexes in the pericardial cavity, leading to persistent inflammation, amplification of CRP‐mediated proinflammatory signaling, and ultimately promoting pericardial tissue damage.

A bioinformatics study identified CCL2 as a shared hub gene in both COVID‐19 and pericarditis. It may promote the progression of pericarditis by promoting the infiltration of immune cells (including monocytes and macrophages) into pericardial tissue and activating inflammatory pathways, including IL‐17 and TNF signaling [70]. In line with this, this study demonstrated that GP higher circulating levels of CCL2 were related to a greater pericarditis risk (OR = 1.473, 95% CI: 1.004–2.159, PIVW = 0.047), further providing genetic evidence for its pro‐inflammatory role in pericarditis. Jakab et al. [71] observed that MBL, a liver‐derived serum protein involved in innate immune defense, may contribute to the risk of pericarditis among individuals with systemic lupus erythematosus via promoter polymorphisms in the MBL2 gene (such as XA/XA), which result in reduced serum MBL levels. This study offers genetic evidence that elevated circulating MBL2 levels are also linked to an increased risk of pericarditis (OR = 1.114, 95% CI: 1.002–1.239, PIVW = 0.046), reinforcing the notion that MBL imbalance represents an independent risk factor for the development of pericarditis.

GO and KEGG enrichment analyses jointly revealed the core pathological features of plasma protein profiles in pericarditis, particularly in recurrent and etiology‐specific subtypes (tuberculous, viral, autoimmune, and radiation‐induced). These features were prominently characterized by aberrant immune activation, especially the interplay between innate and adaptive immunity, along with inflammasome‐driven inflammatory storms, profibrotic signaling, and a unique thromboinflammatory signature. In terms of BP, the enriched pathways included “lymphocyte‐mediated immunity” and “adaptive immune responses based on immunoglobulin superfamily domain‐based immune receptor somatic cell remodeling,” suggesting that B cells and T cells may be deeply involved in the pathological process of pericarditis. The enriched pathways in KEGG analysis, including “phagosomes,” “PD‐L1 expression in cancer,” and the “PD‐1 checkpoint pathway,” are consistent with this hypothesis. In tuberculous pericarditis, CD4^+^ T cells exert a central role in antimicrobial defense through IFN‐γ secretion [54]. However, in idiopathic recurrent acute pericarditis (IRAP) or recurrent pericarditis, B cells generate diverse autoantibodies such as anti‐heart and anti‐intercalated disk antibodies through somatic recombination. These autoantibodies may drive sustained subacute inflammation and tissue injury via Fcγ receptor (such as FCGR2A)‐mediated antibody‐dependent cellular phagocytosis and complement activation [11, 14]. Significantly enriched pathways, including “leukocyte‐mediated immunity”, “inflammatory response,” and “regulation of peptidase activity” in the BP enrichment results, as well as “cytokine receptor binding” and “cytokine binding” in MF, suggest that the excessive activation of neutrophils and macrophages in innate immune cells may be the core mechanism. KEGG pathways “JAK‐STAT signaling” and “Ras signaling” collectively outlined the core signaling networks involved in inflammatory activation. Neutrophil infiltration, release of reactive oxygen species (ROS), neutrophil extracellular traps (NETs), and IL‐1β have been identified as critical drivers in both recurrent and tuberculous pericarditis. Mechanistically, macrophages and activated neutrophils cleave pro‐IL‐1β into active IL‐1β via NLRP3 inflammasome‐dependent caspase‐1 activation [72]. In MF, the enriched pathways such as “cytokine receptor binding” and “cytokine binding” are highly consistent with its mechanism. IL‐1β binding to its receptor IL‐1R1 is a pivotal step that amplifies downstream inflammatory cascades, providing a mechanistic rationale for the clinical efficacy of IL‐1R antagonists such as anakinra in recurrent pericarditis [73]. This persistent inflammatory state is reflected in elevated clinical biomarkers such as CRP and serum amyloid A (SAA) in IRAP [11]. The enriched pathways in MF, including “glycosaminoglycan binding” and “extracellular matrix structural components” and the presence of BP in “regulation of peptidase activity,” “epithelial cell proliferation” and “epithelial morphogenesis,” suggest that abnormal ECM metabolism may play an important role in the development of pericarditis fibrosis. The KEGG pathway “GAG biosynthesis‐heparan sulfate/heparin” was directly linked to this process. Chronic or etiology‐specific pericarditis (such as tuberculous, radiation‐induced, RA‐associated) often progresses to fibrotic and constrictive forms. TGF‐β serves as a central profibrotic mediator by activating pericardial fibroblasts and promoting excessive collagen deposition [54, 74]. MMPs (such as MMP‐9) are involved in the imbalance of ECM degradation and remodeling, a process that may lead to pericardial stiffening [54]. MF terms such as “GAG binding” and “heparin binding” further implied involvement of coagulation‐related processes. The KEGG pathway “complement and coagulation cascades” serves as the molecular basis of this phenomenon. GO and KEGG analyses together highlight the close interaction between the complement system (C3a, C5a) and the coagulation system, suggesting that the unique thrombotic inflammation microenvironment may be a characteristic feature of pericarditis [11]. NETs released by neutrophils act as a key hub in this process: heparin‐binding proteins within NETs, such as platelet factor 4, exhibit strong procoagulant activity; NETs themselves provide a procoagulant surface; and complement activation products (such as C5a) can further activate neutrophils and platelets, creating a vicious cycle. The elevated levels of D‐dimer, a product of fibrin degradation, in IRAP support the presence of this pathological process [11].

The protein–drug interaction network illustrated that several drugs, including PMA, cerivastatin, melatonin, and NAC, may exert therapeutic effects by targeting hub genes. Melatonin can inhibit mitochondrial oxidative stress and prevent the opening of the mitochondrial permeability transition pore by activating the JAK2/STAT3 signaling pathway, ultimately reducing cardiomyocyte apoptosis and alleviating ischemia–reperfusion injury (IRI) [75]. Currently, melatonin is mainly used to manage cardiovascular diseases (such as hypertension and atherosclerosis), liver and lung fibrosis, and autoimmune diseases (such as multiple sclerosis). It exerts its cardiovascular protective effect by relieving inflammation and improving endothelial function [76]. This MR analysis revealed that EGF, ACAN, and CASP8 were associated with the genetic susceptibility to pericarditis. Further molecular docking results suggest that melatonin may have a high binding affinity to these core proteins. Therefore, we speculate that melatonin may inhibit pericardial inflammatory response and fibrosis by targeting and regulating EGF, ACAN, and CASP8‐related pathways. However, it must be emphasized that the molecular docking results are only estimated and predicted by computer simulations, and these findings are preliminary and need to be validated in future experiments. Similarly, molecular docking predicts that cerivastatin has a high affinity for multiple targets. Previous research has shown that cerivastatin lowers the production of adhesion molecules, such as VCAM‐1 and ICAM‐1, and decreases leukocyte adhesion by inhibiting RhoA geranylgeranylation and downstream MAPK/NF‐κB signaling, thereby attenuating atherosclerosis and vascular inflammation [77]. Furthermore, by inhibiting the isoprenoid pathway, cerivastatin upregulates KLF2 expression and subsequently suppresses the inflammatory gene CCL2 and its receptor CCR2, thereby attenuating macrophage inflammation and exerting anti‐atherosclerotic effects [78]. It should be noted that cerivastatin was withdrawn from the global market in 2001 due to the risk of rhabdomyolysis [79]. Although it has a high affinity for multiple targets, its safety needs to be rigorously tested in subsequent clinical translation. NAC alleviates IRI in myocardial infarction by reducing ROS through its antioxidant activity and inhibiting NF‐κB/TNF‐α signaling. In cardiothoracic surgery, NAC lowers postoperative IL‐6 levels and atrial fibrillation risk. In atherosclerosis, NAC attenuates macrophage activation (such as via MMP‐9) and improves endothelial function. These effects are primarily attributed to its ability to enhance GSH‐mediated antioxidant capacity and modulate inflammatory pathways [80]. Molecular docking results indicate that NAC may have a high binding affinity to the core protein CASP8. NAC has been reported to have antioxidant and anti‐inflammatory effects. Hence, we propose that NAC may mediate its anti‐inflammatory effect in part by regulating the CASP8‐related pathway, thereby potentially playing a therapeutic role in pericarditis. Similarly, this hypothesis is entirely based on predictions by computer simulations, and CASP8 is only one of its potential targets. The current results are only preliminary, and its actual efficacy in pericarditis still needs to be verified by rigorous experiments. Molecular docking predicts that PMA can bind to multiple core targets of pericarditis with high affinity. This finding needs to be interpreted with caution. PMA is a potent protein kinase C (PKC) activator that usually induces pro‐inflammatory signals (such as NF‐κB activation and TNF‐α secretion) and promotes tissue damage in immune cells [81]. This evidence contradicts the prediction result that it may serve as a potential therapeutic agent for pericarditis. One possible explanation is that molecular docking may overestimate the static binding energy and ignore the protein conformational changes and downstream pro‐inflammatory cascades caused by PKC activated by PMA, leading to a false positive risk. In addition, no literature supports the protective effect of PMA against pericarditis under specific conditions, and PMA itself has significant pro‐tumor activity [82]. Therefore, we emphasize that the predicted results of PMA are more likely to reflect the limitations of computational methods than the actual therapeutic potential. Further exploration of PMA must be extremely cautious, and its actual binding affinity to the target must be verified through rigorous experiments. The above discussion on the mechanisms of action of melatonin, cerivastatin, and NAC is based on their known studies in myocardial ischemia‐reperfusion injury, atherosclerosis, atrial fibrillation, and other cardiovascular diseases. Given that the targets and pathways of these drugs may potentially overlap with the core pathological mechanisms of pericarditis found in this study, it is speculated that they may exert potential therapeutic effects in pericarditis through similar mechanisms. However, it must be clearly stated that the therapeutic potential of all compounds in this study (including melatonin, cerivastatin, NAC, and PMA) is entirely predicted based on computer simulations. These prediction results are preliminary and hypothetical and do not represent proven mechanisms of action or therapeutic effects. In particular, the prediction results for PMA are highly questionable. All prediction results must be validated through extensive follow‐up studies. Any conclusions regarding the use of these compounds in the treatment of pericarditis are premature.

This study conducted a large‐scale MR analysis based on data from 35,559 individuals of European ancestry and 4907 plasma proteins, identifying for the first time 67 plasma proteins with a potential causal relationship with pericarditis. Moreover, sensitivity analyses (including MR‐Egger and IVW) were applied to ensure the robustness of causal inference. Integrated analyses of scRNA‐seq, PPI networks, and pathway enrichment further revealed that key hub proteins such as ECM1 and CCL2 were specifically expressed in CPCs and may participate in the modulation of inflammatory and immune pathways. Last, molecular docking was employed to identify potential therapeutic compounds (such as cerivastatin and melatonin), providing a theoretical basis for targeted therapy of pericarditis. Nonetheless, several limitations must be noted. First, the GWAS data were derived exclusively from the FinnGen consortium and the IEU OpenGWAS database, with participants limited to individuals of European descent. Second, though multiple sensitivity analyses were conducted to validate the MR hypothesis, we were unable to completely eliminate the effects of horizontal pleiotropy and residual confounding. Due to the unavailability of the PhenoScanner platform, an alternative screening approach, GWAS catalog‐based screening, was employed to exclude SNPs associated with known confounding factors. No significant associations were found, but the possibility of pleiotropy (horizontal pleiotropy) caused by unscreened or unknown confounding factors could not be completely ruled out. Furthermore, the causal inferences for some plasma proteins were based on a limited number of IVs, which may reduce the reliability of sensitivity analysis methods (such as MR‐Egger regression and weighted median). Therefore, the causal effect estimates in this study should be interpreted with caution, and future research should further validate this finding using more IVs. Third, the effects of different plasma proteins may be context‐dependent and interact in complex ways during disease progression. Thus, more advanced algorithms to dissect the underlying plasma–protein interaction networks are required. Last, this study relied solely on publicly available databases and lacked validation through experimental or clinical datasets. Further laboratory and cohort studies are necessary to corroborate these findings.

## 5. Conclusion

This study used MR analysis to reveal a potential causal relationship between 67 specific plasma proteins (e.g., NEU1, GDNF, LAT, CASP8, ZFYVE27, and NAPA) and the risk of pericarditis. Molecular docking analysis predicted multiple candidate drug targets. Notably, PMA, cerivastatin, and melatonin all bound to two or more key protein targets, among which cerivastatin and melatonin showed high affinity for almost all key proteins. It should be emphasized that all docking results are hypothetical and generated only by computer simulations and have not yet been validated. Future research should validate the feasibility of these targets, and the efficacy and safety of candidate drugs need to be evaluated in clinical trials.

## Author Contributions

**Zongqiang Chen:** conceptualization, methodology, software, writing – original draft preparation. **Lingfeng Peng**: data curation, writing – original draft preparation, supervision. **Huankai Zhang**: visualization, investigation, reviewing and editing. **Zhexuan Chen**: software, validation, writing – reviewing and editing.

## Acknowledgments

The authors have nothing to report.

## Funding

There is no funding for this project.

## Ethics Statement

All publicly available datasets had obtained ethical approvals.

## Conflicts of Interest

The authors declare no conflicts of interest.

## Supporting Information

Additional supporting information can be found online in the Supporting Information section.

## Supporting information


**Supporting Information 1** Table S1. Brief characteristics description of 4,907 proteins and pericarditis GWAS cohorts involved in this study.

**Supporting Information 2** Table S2. MR analysis of the associations between 67 plasma proteins and pericarditis.

**Supporting Information 3** Table S3. Characteristics of instrumental variables used for 67 identified plasma proteins in this study.

**Supporting Information 4** Table S4. Horizontal pleiotropy and heterogeneity analysis for 67 identified plasma proteins.

**Supporting Information 5** Figure S1. Scatter plots results of MR analysis between plasmas proteins and Pericarditis

**Supporting Information 6** Figure S2.Leave‐one‐out results of MR analysis between plasmas proteins and Pericarditis

**Supporting Information 7** Figure S3. Funnel plot results of MR analysis between plasmas proteins and Pericarditis

**Supporting Information 8** Table S5. Drug enrichment analysis of pericarditis core genes.

**Supporting Information 9** Table S6. Instrumental variable characteristics and R and *F* values of 4907 plasma proteins

## Data Availability

All relevant data are within the manuscript and its Supporting Information files.
