# Supplementary material for: Causal Association Between Plasma Proteins and Pericarditis: A Mendelian Randomization Study With Therapeutic Target Identification
Source: Mediators Inflamm. 2026 Feb 9;2026:4659271. doi: 10.1155/mi/4659271 (PMC12887433; doi:10.1155/mi/4659271)
Supplement: Supplementary file 1 — Supporting Information 1 Table S1. Brief characteristics description of 4,907 proteins and pericarditis GWAS cohorts involved in this study. [file MI-2026-4659271-s008.docx]

**Table S1.** Brief characteristics description of 4,907 proteins and pericarditis GWAS cohorts involved in this study.

| **Exposure or outcome** | **Source** | **Gender** | **Sample size（total）** | **Ancestry** | **Access Link** |
| --- | --- | --- | --- | --- | --- |
| 4,907 proteins | deCODE genetics  /Amgen, Inc. | male and female | 35,559 participants | European | doi: 10.1038/s41588-021-00978-w |
| pericarditis | Finn Gen | male and female | 343,893 participants | European | <https://www.finngen.fi/en> |
| pericarditis | IEU Open GWAS | male and female | 455,165 participants | European | https://gwas.mrcieu.ac.uk/ |
