# Supplementary material for: Causal Association Between Plasma Proteins and Pericarditis: A Mendelian Randomization Study With Therapeutic Target Identification
Source: Mediators Inflamm. 2026 Feb 9;2026:4659271. doi: 10.1155/mi/4659271 (PMC12887433; doi:10.1155/mi/4659271)
Supplement: Supplementary file 2 — Supporting Information 2 Table S2. MR analysis of the associations between 67 plasma proteins and pericarditis. [file MI-2026-4659271-s001.doc]

**Table S2.** MR analysis of the associations between 67 plasma proteins and pericarditis.

| **Plasma proteins** | | **Number of SNPs** | **OR (95% CI)** | **P-value** |
| --- | --- | --- | --- | --- |
| ACAN | |  |  |  |
|  | Inverse variance weighted | 5 | 1.459(1.028-2.073) | 0.035 |
|  | MR Egger | 5 | 1.544(0.588-4.051) | 0.443 |
|  | Weighted median | 5 | 1.490(0.963-2.306) | 0.073 |
| ADAMTSL2 | |  |  |  |
|  | Inverse variance weighted | 5 | 0.725(0.539-0.976) | 0.034 |
|  | MR Egger | 5 | 0.635(0.248-1.624) | 0.413 |
|  | Weighted median | 5 | 0.714(0.503-1.013) | 0.059 |
| ANGPTL7 | |  |  |  |
|  | Inverse variance weighted | 3 | 0.547(0.310-0.968) | 0.038 |
|  | MR Egger | 3 | 0.372(0.008-16.419) | 0.699 |
|  | Weighted median | 3 | 0.538(0.282-1.025) | 0.060 |
| APOM | |  |  |  |
|  | Inverse variance weighted | 11 | 1.272(1.016-1.591) | 0.035 |
|  | MR Egger | 11 | 1.491(1.070-2.080) | 0.043 |
|  | Weighted median | 11 | 1.323(0.995-1.758) | 0.054 |
| BRICD5 | |  |  |  |
|  | Inverse variance weighted | 3 | 0.624(0.431-0.904) | 0.013 |
|  | MR Egger | 3 | 0.736(0.163-3.321) | 0.758 |
|  | Weighted median | 3 | 0.632(0.421-0.948) | 0.026 |
| CASP8 | |  |  |  |
|  | Inverse variance weighted | 3 | 0.485(0.258-0.913) | 0.025 |
|  | MR Egger | 3 | 0.870(0.012-62.922) | 0.960 |
|  | Weighted median | 3 | 0.485(0.246-0.959) | 0.037 |
| CCL2 | |  |  |  |
|  | Inverse variance weighted | 4 | 1.473(1.005-2.159) | 0.047 |
|  | MR Egger | 4 | 1.504(0.524-4.318) | 0.527 |
|  | Weighted median | 4 | 1.498(0.941-2.384) | 0.088 |
| CD58 | |  |  |  |
|  | Inverse variance weighted | 3 | 0.789(0.626-0.995) | 0.045 |
|  | MR Egger | 3 | 0.869(0.647-1.166) | 0.521 |
|  | Weighted median | 3 | 0.816(0.637-1.045) | 0.107 |
| CDC42BPA | |  |  |  |
|  | Inverse variance weighted | 5 | 0.536(0.341-0.843) | 0.007 |
|  | MR Egger | 5 | 0.573(0.231-1.422) | 0.316 |
|  | Weighted median | 5 | 0.496(0.289-0.852) | 0.011 |
| CFH | |  |  |  |
|  | Inverse variance weighted | 3 | 0.788(0.625-0.993) | 0.043 |
|  | MR Egger | 3 | 0.750(0.137-4.116) | 0.796 |
|  | Weighted median | 3 | 0.805(0.634-1.021) | 0.073 |
| CFI | |  |  |  |
|  | Inverse variance weighted | 3 | 1.722(1.076-2.757) | 0.024 |
|  | MR Egger | 3 | 1.845(0.271-12.541) | 0.644 |
|  | Weighted median | 3 | 1.717(1.020-2.891) | 0.042 |
| CLEC4C | |  |  |  |
|  | Inverse variance weighted | 23 | 1.132(1.034-1.240) | 0.007 |
|  | MR Egger | 23 | 1.100(0.962-1.257) | 0.177 |
|  | Weighted median | 23 | 1.136(1.012-1.275) | 0.031 |
| CLIC1 | |  |  |  |
|  | Inverse variance weighted | 3 | 0.549(0.316-0.953) | 0.033 |
|  | MR Egger | 3 | 0.160(0.002-12.229) | 0.560 |
|  | Weighted median | 3 | 0.482(0.249-0.932) | 0.030 |
| CLMP | |  |  |  |
|  | Inverse variance weighted | 4 | 1.263(1.011-1.577) | 0.040 |
|  | MR Egger | 4 | 1.087(0.699-1.689) | 0.747 |
|  | Weighted median | 4 | 1.229(0.969-1.559) | 0.089 |
| COL11A2 | |  |  |  |
|  | Inverse variance weighted | 8 | 1.496(1.066-2.101) | 0.020 |
|  | MR Egger | 8 | 1.663(0.393-7.031) | 0.515 |
|  | Weighted median | 8 | 1.498(0.972-2.307) | 0.067 |
| COLGALT1 | |  |  |  |
|  | Inverse variance weighted | 10 | 0.809(0.679-0.963) | 0.017 |
|  | MR Egger | 10 | 0.702(0.501-0.985) | 0.075 |
|  | Weighted median | 10 | 0.792(0.643-0.975) | 0.028 |
| COPS7B | |  |  |  |
|  | Inverse variance weighted | 18 | 0.949(0.901-0.999) | 0.047 |
|  | MR Egger | 18 | 0.956(0.897-1.019) | 0.184 |
|  | Weighted median | 18 | 0.956(0.906-1.009) | 0.101 |
| DTD1 | |  |  |  |
|  | Inverse variance weighted | 3 | 0.778(0.613-0.988) | 0.039 |
|  | MR Egger | 3 | 0.864(0.536-1.394) | 0.657 |
|  | Weighted median | 3 | 0.797(0.619-1.026) | 0.078 |
| DTYMK | |  |  |  |
|  | Inverse variance weighted | 5 | 0.611(0.377-0.989) | 0.045 |
|  | MR Egger | 5 | 0.591(0.199-1.757) | 0.414 |
|  | Weighted median | 5 | 0.492(0.271-0.893) | 0.020 |
| EBAG9 | |  |  |  |
|  | Inverse variance weighted | 9 | 1.254(1.033-1.522) | 0.022 |
|  | MR Egger | 9 | 1.336(0.993-1.798) | 0.097 |
|  | Weighted median | 9 | 1.213(0.945-1.557) | 0.130 |
| ECM1 | |  |  |  |
|  | Inverse variance weighted | 9 | 0.751(0.567-0.994) | 0.046 |
|  | MR Egger | 9 | 0.739(0.427-1.280) | 0.317 |
|  | Weighted median | 9 | 0.738(0.515-1.057) | 0.098 |
| EGF | |  |  |  |
|  | Inverse variance weighted | 5 | 1.614(1.068-2.440) | 0.023 |
|  | MR Egger | 5 | 1.534(0.812-2.899) | 0.279 |
|  | Weighted median | 5 | 1.872(1.146-3.057) | 0.012 |
| EIF4B | |  |  |  |
|  | Inverse variance weighted | 3 | 0.562(0.318-0.993) | 0.047 |
|  | MR Egger | 3 | 0.587(0.022-15.878) | 0.805 |
|  | Weighted median | 3 | 0.574(0.303-1.085) | 0.088 |
| EMC4 | |  |  |  |
|  | Inverse variance weighted | 6 | 0.842(0.710-0.999) | 0.049 |
|  | MR Egger | 6 | 0.807(0.543-1.200) | 0.349 |
|  | Weighted median | 6 | 0.839(0.695-1.013) | 0.068 |
| EPHA4 | |  |  |  |
|  | Inverse variance weighted | 12 | 0.877(0.770-0.998) | 0.047 |
|  | MR Egger | 12 | 0.825(0.693-0.981) | 0.055 |
|  | Weighted median | 12 | 0.865(0.747-1.001) | 0.053 |
| FCGR2A | |  |  |  |
|  | Inverse variance weighted | 12 | 0.893(0.812-0.983) | 0.021 |
|  | MR Egger | 12 | 0.959(0.818-1.124) | 0.613 |
|  | Weighted median | 12 | 0.906(0.805-1.020) | 0.101 |
| GDNF | |  |  |  |
|  | Inverse variance weighted | 3 | 1.855(1.062-3.240) | 0.030 |
|  | MR Egger | 3 | 2.486(0.787-7.853) | 0.364 |
|  | Weighted median | 3 | 1.947(1.049-3.616) | 0.035 |
| GSS | |  |  |  |
|  | Inverse variance weighted | 5 | 0.514(0.318-0.829) | 0.006 |
|  | MR Egger | 5 | 0.606(0.188-1.958) | 0.464 |
|  | Weighted median | 5 | 0.518(0.290-0.925) | 0.026 |
| HS6ST1 | |  |  |  |
|  | Inverse variance weighted | 13 | 0.793(0.649-0.969) | 0.023 |
|  | MR Egger | 13 | 0.722(0.458-1.138) | 0.189 |
|  | Weighted median | 13 | 0.790(0.608-1.027) | 0.078 |
| HS6ST3 | |  |  |  |
|  | Inverse variance weighted | 4 | 0.597(0.378-0.943) | 0.027 |
|  | MR Egger | 4 | 0.507(0.201-1.286) | 0.289 |
|  | Weighted median | 4 | 0.575(0.343-0.965) | 0.036 |
| HSPB6 | |  |  |  |
|  | Inverse variance weighted | 7 | 1.729(1.176-2.544) | 0.005 |
|  | MR Egger | 7 | 2.433(0.270-21.961) | 0.464 |
|  | Weighted median | 7 | 1.782(1.096-2.897) | 0.020 |
| ICAM5 | |  |  |  |
|  | Inverse variance weighted | 7 | 1.106(1.010-1.211) | 0.029 |
|  | MR Egger | 7 | 1.101(0.964-1.257) | 0.214 |
|  | Weighted median | 7 | 1.092(0.994-1.199) | 0.065 |
| IGSF3 | |  |  |  |
|  | Inverse variance weighted | 7 | 0.738(0.553-0.984) | 0.039 |
|  | MR Egger | 7 | 0.772(0.423-1.408) | 0.437 |
|  | Weighted median | 7 | 0.759(0.535-1.076) | 0.121 |
| IL11RA | |  |  |  |
|  | Inverse variance weighted | 12 | 0.783(0.623-0.985) | 0.0370 |
|  | MR Egger | 12 | 0.758(0.504-1.139) | 0.211 |
|  | Weighted median | 12 | 0.807(0.596-1.093) | 0.166 |
| IL1RN | |  |  |  |
|  | Inverse variance weighted | 5 | 1.531(1.048-2.236) | 0.028 |
|  | MR Egger | 5 | 1.256(0.589-2.675) | 0.597 |
|  | Weighted median | 5 | 1.538(1.009-2.346) | 0.045 |
| ITIH3 | |  |  |  |
|  | Inverse variance weighted | 15 | 0.887(0.793-0.993) | 0.038 |
|  | MR Egger | 15 | 0.947(0.818-1.095) | 0.474 |
|  | Weighted median | 15 | 0.917(0.802-1.048) | 0.203 |
| LAT | |  |  |  |
|  | Inverse variance weighted | 3 | 1.848(1.042-3.278) | 0.036 |
|  | MR Egger | 3 | 17.475(0.272-1122.429) | 0.407 |
|  | Weighted median | 3 | 2.014(1.031-3.934) | 0.041 |
| MANEA | |  |  |  |
|  | Inverse variance weighted | 13 | 1.095(1.016-1.181) | 0.018 |
|  | MR Egger | 13 | 1.049(0.934-1.179) | 0.437 |
|  | Weighted median | 13 | 1.085(0.993-1.186) | 0.070 |
| MBL2 | |  |  |  |
|  | Inverse variance weighted | 21 | 1.114(1.002-1.239) | 0.046 |
|  | MR Egger | 21 | 1.120(0.919-1.364) | 0.276 |
|  | Weighted median | 21 | 1.148(0.999-1.318) | 0.051 |
| MDGA2 | |  |  |  |
|  | Inverse variance weighted | 18 | 0.888(0.802-0.984) | 0.024 |
|  | MR Egger | 18 | 0.917(0.809-1.040) | 0.198 |
|  | Weighted median | 18 | 0.902(0.797-1.020) | 0.100 |
| MFAP5 | |  |  |  |
|  | Inverse variance weighted | 4 | 1.659(1.085-2.535) | 0.019 |
|  | MR Egger | 4 | 1.207(0.433-3.364) | 0.753 |
|  | Weighted median | 4 | 1.704(1.042-2.787) | 0.034 |
| MYCBP | |  |  |  |
|  | Inverse variance weighted | 4 | 0.557(0.323-0.961) | 0.035 |
|  | MR Egger | 4 | 0.051(1.763-145.874) | 0.539 |
|  | Weighted median | 4 | 0.503(0.259-0.977) | 0.042 |
| NAALAD2 | |  |  |  |
|  | Inverse variance weighted | 6 | 0.847(0.725-0.989) | 0.035 |
|  | MR Egger | 6 | 0.767(0.599-0.982) | 0.104 |
|  | Weighted median | 6 | 0.813(0.690-0.958) | 0.013 |
| NAPA | |  |  |  |
|  | Inverse variance weighted | 3 | 0.502(0.264-0.956) | 0.036 |
|  | MR Egger | 3 | 0.064(8.766-46.301) | 0.563 |
|  | Weighted median | 3 | 0.452(0.220-0.930) | 0.031 |
| NEU1 | |  |  |  |
|  | Inverse variance weighted | 4 | 1.949(1.183-3.211) | 0.009 |
|  | MR Egger | 4 | 1.036(0.095-11.298) | 0.979 |
|  | Weighted median | 4 | 2.003(1.107-3.626) | 0.021 |
| NMT1 | |  |  |  |
|  | Inverse variance weighted | 3 | 0.587(0.354-0.974) | 0.039 |
|  | MR Egger | 3 | 0.822(0.081-8.383) | 0.896 |
|  | Weighted median | 3 | 0.603(0.341-1.065) | 0.081 |
| NRP2 | |  |  |  |
|  | Inverse variance weighted | 21 | 0.810(0.672-0.976) | 0.027 |
|  | MR Egger | 21 | 0.796(0.528-1.200) | 0.289 |
|  | Weighted median | 21 | 0.916(0.698-1.201) | 0.525 |
| NTM | |  |  |  |
|  | Inverse variance weighted | 11 | 0.875(0.785-0.975) | 0.015 |
|  | MR Egger | 11 | 0.949(0.788-1.144) | 0.598 |
|  | Weighted median | 11 | 0.893(0.786-1.014) | 0.081 |
| OPCML | |  |  |  |
|  | Inverse variance weighted | 8 | 0.837(0.716-0.978) | 0.025 |
|  | MR Egger | 8 | 0.960(0.712-1.295) | 0.798 |
|  | Weighted median | 8 | 0.856(0.714-1.026) | 0.0927 |
| PLA2G12B | |  |  |  |
|  | Inverse variance weighted | 9 | 1.405(1.122-1.760) | 0.003 |
|  | MR Egger | 9 | 1.059(0.589-1.906) | 0.853 |
|  | Weighted median | 9 | 1.293(0.961-1.740) | 0.090 |
| PLXNB2 | |  |  |  |
|  | Inverse variance weighted | 12 | 0.835(0.734-0.951) | 0.006 |
|  | MR Egger | 12 | 0.794(0.634-0.995) | 0.072 |
|  | Weighted median | 12 | 0.836(0.710-0.983) | 0.030 |
| PSG4 | |  |  |  |
|  | Inverse variance weighted | 15 | 1.218(1.047-1.418) | 0.011 |
|  | MR Egger | 15 | 1.199(0.948-1.517) | 0.154 |
|  | Weighted median | 15 | 1.216(1.009-1.465) | 0.040 |
| PSMB3 | |  |  |  |
|  | Inverse variance weighted | 3 | 0.517(0.280-0.955) | 0.035 |
|  | MR Egger | 3 | 0.425(0.006-30.133) | 0.761 |
|  | Weighted median | 3 | 0.517(0.265-1.012) | 0.054 |
| RAB5C | |  |  |  |
|  | Inverse variance weighted | 3 | 0.531(0.308-0.915) | 0.023 |
|  | MR Egger | 3 | 0.627(0.014-28.630) | 0.851 |
|  | Weighted median | 3 | 0.546(0.291-1.026) | 0.060 |
| RHOC | |  |  |  |
|  | Inverse variance weighted | 8 | 0.714(0.544-0.938) | 0.015 |
|  | MR Egger | 8 | 0.798(0.320-1.991) | 0.646 |
|  | Weighted median | 8 | 0.739(0.519-1.052) | 0.093 |
| SAA4 | |  |  |  |
|  | Inverse variance weighted | 9 | 0.895(0.822-0.975) | 0.011 |
|  | MR Egger | 9 | 0.926(0.812-1.055) | 0.284 |
|  | Weighted median | 9 | 0.904(0.818-0.999) | 0.050 |
| SAR1A | |  |  |  |
|  | Inverse variance weighted | 3 | 0.555(0.314-0.981) | 0.043 |
|  | MR Egger | 3 | 0.420(0.061-2.857) | 0.540 |
|  | Weighted median | 3 | 0.567(0.291-1.104) | 0.095 |
| SIGLEC9 | |  |  |  |
|  | Inverse variance weighted | 9 | 0.917(0.843-0.996) | 0.040 |
|  | MR Egger | 9 | 0.967(0.852-1.096) | 0.613 |
|  | Weighted median | 9 | 0.927(0.846-1.016) | 0.107 |
| SPOCK3 | |  |  |  |
|  | Inverse variance weighted | 17 | 0.891(0.803-0.989) | 0.030 |
|  | MR Egger | 17 | 0.902(0.792-1.028) | 0.144 |
|  | Weighted median | 17 | 0.867(0.753-0.999) | 0.050 |
| STAT6 | |  |  |  |
|  | Inverse variance weighted | 6 | 0.616(0.483-0.904) | 0.010 |
|  | MR Egger | 6 | 0.796(0.480-1.319) | 0.426 |
|  | Weighted median | 6 | 0.633(0.419-0.956) | 0.030 |
| TBC1D5 | |  |  |  |
|  | Inverse variance weighted | 5 | 0.556(0.350-0.885) | 0.013 |
|  | MR Egger | 5 | 0.168(0.006-4.362) | 0.362 |
|  | Weighted median | 5 | 0.501(0.280-0.896) | 0.020 |
| THSD1 | |  |  |  |
|  | Inverse variance weighted | 14 | 0.903(0.822-0.993) | 0.035 |
|  | MR Egger | 14 | 0.902(0.719-1.132) | 0.391 |
|  | Weighted median | 14 | 0.907(0.810-1.015) | 0.086 |
| TMEM9 | |  |  |  |
|  | Inverse variance weighted | 12 | 1.232(1.009-1.505) | 0.041 |
|  | MR Egger | 12 | 1.045(0.750-1.457) | 0.799 |
|  | Weighted median | 12 | 1.132(0.861-1.490) | 0.372 |
| UBA2 | |  |  |  |
|  | Inverse variance weighted | 6 | 0.807(0.666-0.977) | 0.028 |
|  | MR Egger | 6 | 0.815(0.583-1.139) | 0.297 |
|  | Weighted median | 6 | 0.814(0.653-1.015) | 0.068 |
| UNC45A | |  |  |  |
|  | Inverse variance weighted | 3 | 0.509(0.286-0.907) | 0.022 |
|  | MR Egger | 3 | 0.322(0.001-195.481) | 0.787 |
|  | Weighted median | 3 | 0.502(0.247-1.021) | 0.057 |
| ZFYVE27 | |  |  |  |
|  | Inverse variance weighted | 3 | 0.502(0.301-0.837) | 0.008 |
|  | MR Egger | 3 | 0.545(0.225-1.320) | 0.407 |
|  | Weighted median | 3 | 0.524(0.294-0.933) | 0.028 |
| ZNRF3 | |  |  |  |
|  | Inverse variance weighted | 4 | 0.728(0.551-0.963) | 0.026 |
|  | MR Egger | 4 | 0.898(0.344-2.348) | 0.847 |
|  | Weighted median | 4 | 0.729(0.537-0.989) | 0.042 |
