# Supplementary material for: Causal Association Between Plasma Proteins and Pericarditis: A Mendelian Randomization Study With Therapeutic Target Identification
Source: Mediators Inflamm. 2026 Feb 9;2026:4659271. doi: 10.1155/mi/4659271 (PMC12887433; doi:10.1155/mi/4659271)
Supplement: Supplementary file 3 — Supporting Information 3 Table S3. Characteristics of instrumental variables used for 67 identified plasma proteins in this study. [file MI-2026-4659271-s002.doc]

**Table S3.** Characteristics of instrumental variables used for 67 identified plasma proteins in this study.

| plasma proteins | SNP | Chr | Position | effect allele | Beta | SE | *P*-value |
| --- | --- | --- | --- | --- | --- | --- | --- |
|
| ACAN | rs10922098 | chr1 | 196695521 | T | -0.0661 | 0.008191 | 7.013E-16 |
| ACAN | rs11144945 | chr9 | 76535950 | A | -0.0541 | 0.008963 | 1.579E-09 |
| ACAN | rs12153751 | chr5 | 83697010 | T | -0.0543 | 0.00856 | 2.245E-10 |
| ACAN | rs34949187 | chr15 | 88843421 | A | -0.1415 | 0.010752 | 1.491E-39 |
| ACAN | rs9272799 | chr6 | 32642799 | T | -0.0497 | 0.007939 | 3.847E-10 |
| ADAMTSL2 | rs11534419 | chr9 | 133523101 | T | 0.1118 | 0.008717 | 1.176E-37 |
| ADAMTSL2 | rs2027962 | chr9 | 133720519 | A | -0.0687 | 0.008612 | 1.491E-15 |
| ADAMTSL2 | rs217184 | chr16 | 72072066 | C | 0.0677 | 0.010951 | 6.328E-10 |
| ADAMTSL2 | rs61830291 | chr1 | 220827800 | C | 0.1328 | 0.013809 | 6.781E-22 |
| ADAMTSL2 | rs77262773 | chr17 | 69253570 | T | 0.2174 | 0.027953 | 7.402E-15 |
| ANGPTL7 | rs1354034 | chr3 | 56815721 | C | 0.0614 | 0.008099 | 3.431E-14 |
| ANGPTL7 | rs3917538 | chr7 | 95308581 | A | 0.0607 | 0.009498 | 1.65E-10 |
| ANGPTL7 | rs4980320 | chr11 | 198986 | C | 0.044 | 0.007901 | 2.563E-08 |
| APOM | rs12660700 | chr6 | 31475961 | A | -0.1947 | 0.035513 | 4.192E-08 |
| APOM | rs1362076 | chr6 | 29473392 | T | -0.4253 | 0.04025 | 4.265E-26 |
| APOM | rs1707652 | chr3 | 165761011 | T | 0.0566 | 0.008837 | 1.502E-10 |
| APOM | rs1800961 | chr20 | 44413724 | T | -0.2149 | 0.019723 | 1.209E-27 |
| APOM | rs364950 | chr6 | 33008119 | G | -0.1873 | 0.025807 | 3.933E-13 |
| APOM | rs369560730 | chr6 | 33389536 | T | -0.2155 | 0.035654 | 1.501E-09 |
| APOM | rs3782735 | chr12 | 6775910 | A | -0.0483 | 0.00846 | 1.135E-08 |
| APOM | rs41285751 | chr6 | 31932038 | T | 0.1434 | 0.014948 | 8.511E-22 |
| APOM | rs72786786 | chr16 | 56951602 | A | 0.0762 | 0.008864 | 8.243E-18 |
| APOM | rs780093 | chr2 | 27519736 | C | -0.0578 | 0.008647 | 2.313E-11 |
| APOM | rs805295 | chr6 | 31707520 | T | -0.4845 | 0.02098 | 5.356E-118 |
| BRICD5 | rs12710562 | chr22 | 37633716 | G | 0.1294 | 0.010303 | 3.552E-36 |
| BRICD5 | rs1354034 | chr3 | 56815721 | C | 0.0751 | 0.008211 | 5.869E-20 |
| BRICD5 | rs59502684 | chr22 | 37636704 | C | -0.1146 | 0.014801 | 9.717E-15 |
| CASP8 | rs1047286 | chr19 | 6713251 | A | -0.0523 | 0.009326 | 2.049E-08 |
| CASP8 | rs1706435 | chr3 | 165743688 | G | 0.0692 | 0.008251 | 5.009E-17 |
| CASP8 | rs7774739 | chr6 | 32041524 | G | 0.0967 | 0.011132 | 3.74E-18 |
| CCL2 | rs12075 | chr1 | 159205564 | A | 0.075 | 0.008094 | 1.934E-20 |
| CCL2 | rs1799864 | chr3 | 46357717 | A | 0.1472 | 0.014002 | 7.524E-26 |
| CCL2 | rs4490403 | chr3 | 46059765 | C | -0.0472 | 0.008054 | 4.61E-09 |
| CCL2 | rs6542680 | chr2 | 3592552 | T | -0.0673 | 0.010341 | 7.603E-11 |
| CD58 | rs116614403 | chr1 | 118591986 | T | 0.2215 | 0.031178 | 1.209E-12 |
| CD58 | rs1706435 | chr3 | 165743688 | G | 0.0744 | 0.00824 | 1.735E-19 |
| CD58 | rs61789227 | chr1 | 116537006 | G | 0.6656 | 0.027448 | 6.699E-130 |
| CDC42BPA | rs12437339 | chr14 | 94683813 | T | 0.1257 | 0.018477 | 1.025E-11 |
| CDC42BPA | rs1354034 | chr3 | 56815721 | C | 0.0516 | 0.008105 | 1.933E-10 |
| CDC42BPA | rs1355538 | chr3 | 165787389 | G | -0.0445 | 0.008041 | 3.123E-08 |
| CDC42BPA | rs147863506 | chr14 | 94860009 | T | 0.2185 | 0.034944 | 4.031E-10 |
| CDC42BPA | rs704 | chr17 | 28367840 | A | -0.0461 | 0.007891 | 5.154E-09 |
| CFH | rs70620 | chr1 | 196735867 | A | -0.2752 | 0.010319 | 1.088E-156 |
| CFH | rs77834171 | chr1 | 196170343 | G | -0.1777 | 0.026005 | 8.291E-12 |
| CFH | rs78265616 | chr1 | 195599663 | C | -0.2667 | 0.032887 | 5.083E-16 |
| CFI | rs4355391 | chr4 | 109777388 | C | 0.0835 | 0.009447 | 9.646E-19 |
| CFI | rs4665972 | chr2 | 27375230 | C | -0.065 | 0.008621 | 4.715E-14 |
| CFI | rs6855357 | chr4 | 110142163 | C | -0.1869 | 0.03268 | 1.071E-08 |
| CLEC4C | rs10971418 | chr9 | 33122026 | A | -0.069 | 0.010298 | 2.075E-11 |
| CLEC4C | rs11051491 | chr12 | 31625835 | A | -0.0718 | 0.009787 | 2.198E-13 |
| CLEC4C | rs11055473 | chr12 | 7728138 | T | -0.4129 | 0.006359 | 1E-200 |
| CLEC4C | rs11125837 | chr2 | 60355425 | T | -0.0439 | 0.007819 | 1.975E-08 |
| CLEC4C | rs117812534 | chr12 | 7946084 | G | 0.3106 | 0.019853 | 3.608E-55 |
| CLEC4C | rs141613637 | chr12 | 8317335 | A | -0.2636 | 0.032265 | 3.088E-16 |
| CLEC4C | rs17804845 | chr12 | 9376266 | A | 0.1683 | 0.013931 | 1.325E-33 |
| CLEC4C | rs1837369 | chr2 | 111116699 | T | 0.0715 | 0.007804 | 5.104E-20 |
| CLEC4C | rs2331413 | chr17 | 64039089 | A | 0.0676 | 0.010711 | 2.768E-10 |
| CLEC4C | rs2395906 | chr8 | 129598547 | A | 0.0477 | 0.007973 | 2.19E-09 |
| CLEC4C | rs2661797 | chr2 | 65406056 | A | 0.063 | 0.007922 | 1.83E-15 |
| CLEC4C | rs3184504 | chr12 | 111446804 | C | -0.0492 | 0.008966 | 4.077E-08 |
| CLEC4C | rs6796 | chr7 | 6462736 | C | -0.0708 | 0.009199 | 1.395E-14 |
| CLEC4C | rs6818511 | chr4 | 105028111 | C | 0.1099 | 0.008289 | 3.996E-40 |
| CLEC4C | rs7129402 | chr11 | 121341047 | A | -0.117 | 0.008682 | 2.142E-41 |
| CLEC4C | rs7312313 | chr12 | 7788964 | C | -0.3101 | 0.010918 | 1.85E-177 |
| CLEC4C | rs73267361 | chr12 | 6436345 | A | -0.107 | 0.016732 | 1.607E-10 |
| CLEC4C | rs76428106 | chr13 | 28029870 | C | 0.433 | 0.033945 | 2.892E-37 |
| CLEC4C | rs7684212 | chr4 | 83233280 | G | -0.0583 | 0.008389 | 3.668E-12 |
| CLEC4C | rs7762909 | chr6 | 31353801 | G | 0.0605 | 0.007864 | 1.438E-14 |
| CLEC4C | rs77852018 | chr2 | 144833059 | A | -0.0737 | 0.012725 | 6.975E-09 |
| CLEC4C | rs7873862 | chr9 | 113351116 | T | 0.0718 | 0.010069 | 9.967E-13 |
| CLEC4C | rs9508005 | chr13 | 28215657 | G | -0.073 | 0.012774 | 1.098E-08 |
| CLIC1 | rs1354034 | chr3 | 56815721 | C | 0.054 | 0.008265 | 6.431E-11 |
| CLIC1 | rs1619994 | chr3 | 165744047 | G | 0.0679 | 0.008941 | 3.096E-14 |
| CLIC1 | rs710449 | chr3 | 186735031 | G | 0.0506 | 0.009154 | 3.243E-08 |
| CLMP | rs7131073 | chr11 | 123145655 | G | 0.0731 | 0.008629 | 2.427E-17 |
| CLMP | rs77924615 | chr16 | 20381010 | A | -0.066 | 0.010382 | 2.055E-10 |
| CLMP | rs7946718 | chr11 | 123197533 | G | 0.2113 | 0.008322 | 3.116E-142 |
| CLMP | rs9276171 | chr6 | 32731141 | G | 0.067 | 0.009882 | 1.204E-11 |
| CLMP | rs10922098 | chr1 | 196695521 | T | -0.0803 | 0.007978 | 7.826E-24 |
| CLMP | rs1355538 | chr3 | 165787389 | G | -0.047 | 0.007971 | 3.712E-09 |
| COL11A2 | rs10174098 | chr2 | 186628015 | A | -0.0502 | 0.009085 | 3.281E-08 |
| COL11A2 | rs12975366 | chr19 | 54255498 | C | -0.0693 | 0.008283 | 5.91E-17 |
| COL11A2 | rs2147161 | chr13 | 42408166 | C | 0.0485 | 0.008277 | 4.647E-09 |
| COL11A2 | rs2450083 | chr8 | 119051303 | C | 0.0459 | 0.008187 | 2.064E-08 |
| COL11A2 | rs271173 | chr6 | 132991083 | T | 0.0496 | 0.008291 | 2.199E-09 |
| COL11A2 | rs61729512 | chr12 | 7485173 | A | 0.0703 | 0.012405 | 1.452E-08 |
| COL11A2 | rs8051661 | chr16 | 88409532 | G | 0.1043 | 0.013251 | 3.523E-15 |
| COL11A2 | rs9963868 | chr18 | 48073063 | C | -0.0585 | 0.008586 | 9.552E-12 |
| COLGALT1 | rs10426931 | chr19 | 17587742 | T | 0.0472 | 0.008264 | 1.121E-08 |
| COLGALT1 | rs13225119 | chr7 | 6579021 | A | 0.0466 | 0.008226 | 1.468E-08 |
| COLGALT1 | rs144577567 | chr19 | 16194091 | A | 0.1908 | 0.033479 | 1.205E-08 |
| COLGALT1 | rs2169387 | chr8 | 9323885 | G | -0.1091 | 0.015174 | 6.491E-13 |
| COLGALT1 | rs35745856 | chr19 | 18444394 | C | -0.2289 | 0.040395 | 1.457E-08 |
| COLGALT1 | rs3752617 | chr7 | 127580863 | T | -0.1134 | 0.016357 | 4.125E-12 |
| COLGALT1 | rs4340676 | chr3 | 57596361 | G | -0.1085 | 0.010645 | 2.13E-24 |
| COLGALT1 | rs4808666 | chr19 | 17557324 | T | -0.1275 | 0.008253 | 7.6E-54 |
| COLGALT1 | rs4847398 | chr1 | 93112845 | T | -0.0531 | 0.008362 | 2.152E-10 |
| COLGALT1 | rs6796 | chr7 | 6462736 | C | 0.226 | 0.009553 | 1.009E-123 |
| COPS7B | rs1049739 | chr19 | 45475117 | G | -0.1456 | 0.008508 | 1.173E-65 |
| COPS7B | rs111696008 | chr2 | 216622986 | A | -0.1935 | 0.023306 | 1.02E-16 |
| COPS7B | rs11720167 | chr3 | 165768357 | G | 0.2273 | 0.008896 | 5.298E-144 |
| COPS7B | rs144202860 | chr2 | 216625740 | A | -0.1947 | 0.031226 | 4.515E-10 |
| COPS7B | rs148077724 | chr3 | 165952844 | G | 0.1775 | 0.021889 | 5.095E-16 |
| COPS7B | rs182320411 | chr19 | 45317999 | A | -0.2487 | 0.035058 | 1.303E-12 |
| COPS7B | rs2571068 | chr19 | 44361300 | T | -0.0772 | 0.008564 | 1.985E-19 |
| COPS7B | rs28573154 | chr6 | 32008772 | A | 0.0698 | 0.009794 | 1.026E-12 |
| COPS7B | rs3863087 | chr3 | 165792099 | A | -0.1874 | 0.012138 | 8.903E-54 |
| COPS7B | rs394353 | chr19 | 44864216 | G | -0.0714 | 0.008457 | 3.1E-17 |
| COPS7B | rs4704826 | chr5 | 156965071 | A | 0.0461 | 0.007268 | 2.256E-10 |
| COPS7B | rs62119281 | chr19 | 44640467 | T | 0.2204 | 0.020831 | 3.674E-26 |
| COPS7B | rs62290472 | chr3 | 162193597 | A | 0.0728 | 0.012887 | 1.613E-08 |
| COPS7B | rs73045691 | chr19 | 44937272 | A | 0.9955 | 0.005177 | 1E-200 |
| COPS7B | rs73169103 | chr3 | 167190675 | T | 0.1307 | 0.018985 | 5.807E-12 |
| COPS7B | rs77660932 | chr19 | 45091584 | C | -0.2708 | 0.011004 | 9.788E-134 |
| COPS7B | rs78980904 | chr2 | 20921704 | C | -0.0482 | 0.00803 | 1.941E-09 |
| COPS7B | rs887829 | chr2 | 233759924 | T | 0.0451 | 0.007539 | 2.205E-09 |
| DTD1 | rs1354034 | chr3 | 56815721 | C | 0.0744 | 0.008204 | 1.21E-19 |
| DTD1 | rs4759076 | chr12 | 54336088 | C | 0.0583 | 0.007998 | 3.11E-13 |
| DTD1 | rs6081231 | chr20 | 18590320 | A | -0.2005 | 0.008314 | 1.68E-128 |
| DTYMK | rs116616655 | chr2 | 242020515 | A | -0.2636 | 0.038788 | 1.076E-11 |
| DTYMK | rs1354034 | chr3 | 56815721 | C | 0.057 | 0.008283 | 5.916E-12 |
| DTYMK | rs142623301 | chr2 | 241753305 | A | -0.1745 | 0.028058 | 4.993E-10 |
| DTYMK | rs35979828 | chr12 | 54292096 | T | -0.0971 | 0.014529 | 2.337E-11 |
| DTYMK | rs4980320 | chr11 | 198986 | C | 0.0462 | 0.008071 | 1.041E-08 |
| EBAG9 | rs112430397 | chr6 | 25548089 | C | -0.2643 | 0.033613 | 3.751E-15 |
| EBAG9 | rs114531342 | chr6 | 25149451 | C | 0.2056 | 0.027169 | 3.804E-14 |
| EBAG9 | rs143654430 | chr6 | 30519527 | C | -0.8901 | 0.029407 | 1E-200 |
| EBAG9 | rs146431191 | chr6 | 31503428 | T | 0.3533 | 0.037152 | 1.915E-21 |
| EBAG9 | rs150581397 | chr6 | 37589310 | A | -0.1291 | 0.020632 | 3.92E-10 |
| EBAG9 | rs181369017 | chr6 | 33851220 | A | -0.478 | 0.026222 | 3.047E-74 |
| EBAG9 | rs28664709 | chr1 | 196702087 | A | 0.071 | 0.008059 | 1.254E-18 |
| EBAG9 | rs3176349 | chr6 | 36683600 | T | -0.1758 | 0.02148 | 2.74E-16 |
| EBAG9 | rs744865 | chr6 | 23627455 | C | -0.1771 | 0.023077 | 1.663E-14 |
| ECM1 | rs11577274 | chr1 | 153083768 | G | -0.0944 | 0.010883 | 4.155E-18 |
| ECM1 | rs116567297 | chr1 | 119981708 | A | -0.1652 | 0.019693 | 4.904E-17 |
| ECM1 | rs13796 | chr1 | 154273441 | C | 0.075 | 0.012456 | 1.733E-09 |
| ECM1 | rs185236008 | chr1 | 151205579 | A | 0.3505 | 0.023743 | 2.558E-49 |
| ECM1 | rs3811409 | chr1 | 151403108 | C | 0.1431 | 0.017112 | 6.129E-17 |
| ECM1 | rs41285751 | chr6 | 31932038 | T | 0.0794 | 0.01359 | 5.143E-09 |
| ECM1 | rs4411121 | chr1 | 118214411 | T | 0.0557 | 0.008733 | 1.79E-10 |
| ECM1 | rs4606261 | chr1 | 120147656 | C | 0.1626 | 0.019855 | 2.628E-16 |
| ECM1 | rs77262773 | chr17 | 69253570 | T | 0.163 | 0.024774 | 4.724E-11 |
| EGF | rs115294968 | chr4 | 109886222 | A | -0.5066 | 0.036259 | 2.326E-44 |
| EGF | rs4355391 | chr4 | 109777388 | C | 0.0623 | 0.009312 | 2.226E-11 |
| EGF | rs7073753 | chr10 | 63303060 | T | 0.0507 | 0.008087 | 3.634E-10 |
| EGF | rs75890625 | chr4 | 110940201 | G | -0.3681 | 0.03955 | 1.313E-20 |
| EGF | rs77107029 | chr4 | 111275272 | A | -0.1515 | 0.025203 | 1.843E-09 |
| EIF4B | rs1354034 | chr3 | 56815721 | C | 0.0691 | 0.00833 | 1.087E-16 |
| EIF4B | rs4980320 | chr11 | 198986 | C | 0.0497 | 0.008125 | 9.519E-10 |
| EIF4B | rs7297265 | chr12 | 54325905 | G | 0.047 | 0.008132 | 7.484E-09 |
| EMC4 | rs112668737 | chr6 | 32521568 | C | -0.0516 | 0.00906 | 1.233E-08 |
| EMC4 | rs144585235 | chr6 | 31932880 | A | 0.2934 | 0.019186 | 8.543E-53 |
| EMC4 | rs147233090 | chr15 | 43735849 | T | 0.2691 | 0.025851 | 2.238E-25 |
| EMC4 | rs163915 | chr19 | 6734760 | C | -0.1391 | 0.011299 | 7.911E-35 |
| EMC4 | rs402756 | chr19 | 6708309 | G | -0.2545 | 0.010015 | 1.837E-142 |
| EMC4 | rs411530 | chr1 | 196760515 | G | -0.0519 | 0.008198 | 2.435E-10 |
| EPHA4 | rs112155714 | chr2 | 222529863 | G | -0.3285 | 0.03962 | 1.12E-16 |
| EPHA4 | rs114022440 | chr2 | 221420548 | G | 0.2539 | 0.029843 | 1.77E-17 |
| EPHA4 | rs118167234 | chr9 | 133639513 | C | -0.2116 | 0.028364 | 8.644E-14 |
| EPHA4 | rs1260326 | chr2 | 27508073 | C | 0.0577 | 0.008624 | 2.217E-11 |
| EPHA4 | rs13108218 | chr4 | 3442204 | G | 0.0486 | 0.008403 | 7.296E-09 |
| EPHA4 | rs190696118 | chr2 | 221001417 | C | -0.2114 | 0.030226 | 2.671E-12 |
| EPHA4 | rs4661359 | chr1 | 16186658 | T | -0.0677 | 0.008334 | 4.527E-16 |
| EPHA4 | rs4674589 | chr2 | 221392836 | C | 0.0882 | 0.008224 | 7.765E-27 |
| EPHA4 | rs62576557 | chr9 | 132413424 | G | -0.1326 | 0.024311 | 4.916E-08 |
| EPHA4 | rs7470777 | chr9 | 133239789 | G | 0.5414 | 0.017662 | 1E-200 |
| EPHA4 | rs75212266 | chr9 | 133207463 | A | -0.3341 | 0.039348 | 2.051E-17 |
| EPHA4 | rs77924615 | chr16 | 20381010 | A | -0.0605 | 0.010252 | 3.603E-09 |
| FCGR2A | rs114014204 | chr1 | 160910354 | T | -0.6047 | 0.041243 | 1.131E-48 |
| FCGR2A | rs114499445 | chr1 | 160116508 | C | 0.2218 | 0.029223 | 3.2E-14 |
| FCGR2A | rs114844178 | chr1 | 161382477 | A | 0.6114 | 0.008844 | 1E-200 |
| FCGR2A | rs138293152 | chr1 | 161474240 | T | -0.7329 | 0.02285 | 1E-200 |
| FCGR2A | rs145300976 | chr1 | 152321672 | T | 0.1637 | 0.029689 | 3.512E-08 |
| FCGR2A | rs150752639 | chr1 | 161260983 | G | 0.418 | 0.019008 | 3.576E-107 |
| FCGR2A | rs17452514 | chr1 | 161981287 | T | -0.4113 | 0.030452 | 1.432E-41 |
| FCGR2A | rs34307129 | chr1 | 161305939 | A | -0.6 | 0.031527 | 9.408E-81 |
| FCGR2A | rs34436026 | chr1 | 161549650 | T | 0.5469 | 0.027388 | 1.037E-88 |
| FCGR2A | rs34676060 | chr1 | 159191101 | C | -0.289 | 0.040031 | 5.221E-13 |
| FCGR2A | rs55714927 | chr17 | 7176997 | T | 0.0753 | 0.008574 | 1.607E-18 |
| FCGR2A | rs72702031 | chr1 | 162399487 | G | 0.2576 | 0.042363 | 1.196E-09 |
| GDNF | rs1846190 | chr6 | 32616036 | A | 0.0464 | 0.008273 | 2.042E-08 |
| GDNF | rs211161 | chr6 | 96614917 | G | 0.1785 | 0.031397 | 1.306E-08 |
| GDNF | rs6457457 | chr6 | 31910331 | T | 0.0917 | 0.016078 | 1.174E-08 |
| GSS | rs11046893 | chr12 | 7250980 | T | 0.0481 | 0.008241 | 5.316E-09 |
| GSS | rs4811529 | chr20 | 38159789 | G | -0.0819 | 0.013507 | 1.33E-09 |
| GSS | rs6113872 | chr20 | 22980331 | T | -0.0487 | 0.008764 | 2.743E-08 |
| GSS | rs62213218 | chr20 | 36320792 | A | -0.135 | 0.024134 | 2.222E-08 |
| GSS | rs73102628 | chr20 | 31926687 | A | -0.1538 | 0.023414 | 5.078E-11 |
| HS6ST1 | rs113688927 | chr6 | 31361670 | G | -0.1862 | 0.020166 | 2.618E-20 |
| HS6ST1 | rs141236281 | chr5 | 100095884 | G | 0.1594 | 0.026439 | 1.649E-09 |
| HS6ST1 | rs143867680 | chr6 | 32557709 | T | -0.1337 | 0.021997 | 1.217E-09 |
| HS6ST1 | rs3184504 | chr12 | 111446804 | C | -0.0467 | 0.008366 | 2.372E-08 |
| HS6ST1 | rs4947328 | chr6 | 31593970 | G | 0.1114 | 0.018732 | 2.731E-09 |
| HS6ST1 | rs66476363 | chr2 | 128461807 | G | 0.076 | 0.013528 | 1.934E-08 |
| HS6ST1 | rs6952090 | chr7 | 130282658 | C | -0.0531 | 0.008552 | 5.337E-10 |
| HS6ST1 | rs704 | chr17 | 28367840 | A | -0.1286 | 0.008088 | 6.273E-57 |
| HS6ST1 | rs77262773 | chr17 | 69253570 | T | 0.2383 | 0.026866 | 7.312E-19 |
| HS6ST1 | rs78029512 | chr2 | 128060884 | C | -0.1456 | 0.022898 | 2.035E-10 |
| HS6ST1 | rs78705678 | chr10 | 17794281 | G | 0.0577 | 0.008436 | 7.917E-12 |
| HS6ST1 | rs840870 | chr2 | 128308686 | T | -0.1533 | 0.008176 | 1.935E-78 |
| HS6ST1 | rs907866 | chr2 | 20171619 | A | -0.0663 | 0.008134 | 3.599E-16 |
| HS6ST3 | rs55714927 | chr17 | 7176997 | T | 0.0577 | 0.010037 | 8.978E-09 |
| HS6ST3 | rs73021415 | chr11 | 126378312 | T | -0.1391 | 0.017244 | 7.227E-16 |
| HS6ST3 | rs77262773 | chr17 | 69253570 | T | 0.2054 | 0.027014 | 2.882E-14 |
| HS6ST3 | rs907866 | chr2 | 20171619 | A | -0.048 | 0.008164 | 4.115E-09 |
| HSPB6 | rs12342201 | chr9 | 93132682 | A | -0.065 | 0.008265 | 3.707E-15 |
| HSPB6 | rs3734537 | chr6 | 26422399 | A | 0.0807 | 0.014138 | 1.143E-08 |
| HSPB6 | rs7095308 | chr10 | 119666800 | A | 0.0523 | 0.009167 | 1.162E-08 |
| HSPB6 | rs77303334 | chr14 | 103974806 | C | 0.0785 | 0.014102 | 2.597E-08 |
| HSPB6 | rs77924615 | chr16 | 20381010 | A | -0.0655 | 0.010479 | 4.087E-10 |
| HSPB6 | rs9271738 | chr6 | 32625914 | G | 0.06 | 0.008979 | 2.357E-11 |
| HSPB6 | rs9318186 | chr13 | 73532802 | G | 0.0467 | 0.008301 | 1.847E-08 |
| ICAM5 | rs1275922 | chr2 | 26710019 | G | 0.0517 | 0.00927 | 2.442E-08 |
| ICAM5 | rs2116877 | chr19 | 11247764 | T | -0.0733 | 0.013339 | 3.907E-08 |
| ICAM5 | rs3093035 | chr19 | 10272297 | A | -0.6241 | 0.018321 | 1E-200 |
| ICAM5 | rs72658879 | chr19 | 11133332 | A | -0.3086 | 0.025466 | 8.459E-34 |
| ICAM5 | rs75806286 | chr17 | 7077441 | A | 0.0654 | 0.011883 | 3.716E-08 |
| ICAM5 | rs8101473 | chr19 | 10312662 | T | 0.5588 | 0.008258 | 1E-200 |
| ICAM5 | rs9916644 | chr17 | 69106726 | T | 0.2195 | 0.018135 | 1.009E-33 |
| IGSF3 | rs1260326 | chr2 | 27508073 | C | 0.053 | 0.008729 | 1.263E-09 |
| IGSF3 | rs1411623 | chr1 | 117576078 | A | -0.0939 | 0.014821 | 2.363E-10 |
| IGSF3 | rs200250001 | chr10 | 17770605 | A | 0.093 | 0.008649 | 5.721E-27 |
| IGSF3 | rs41276576 | chr1 | 117102002 | T | 0.1364 | 0.023777 | 9.656E-09 |
| IGSF3 | rs4704040 | chr5 | 72814688 | T | 0.0466 | 0.008214 | 1.401E-08 |
| IGSF3 | rs55714927 | chr17 | 7176997 | T | 0.0738 | 0.010152 | 3.605E-13 |
| IGSF3 | rs78705678 | chr10 | 17794281 | G | 0.1833 | 0.008584 | 3.533E-101 |
| IL11RA | rs112001035 | chr17 | 68827664 | A | -0.2743 | 0.014885 | 7.784E-76 |
| IL11RA | rs12150028 | chr17 | 68814006 | T | 0.0844 | 0.014695 | 9.278E-09 |
| IL11RA | rs1260326 | chr2 | 27508073 | C | 0.053 | 0.008622 | 7.898E-10 |
| IL11RA | rs12720926 | chr16 | 56965006 | G | 0.0462 | 0.008208 | 1.815E-08 |
| IL11RA | rs139849225 | chr9 | 33587521 | C | 0.1474 | 0.026357 | 2.239E-08 |
| IL11RA | rs4805762 | chr19 | 32473048 | G | 0.0825 | 0.011156 | 1.411E-13 |
| IL11RA | rs55683663 | chr9 | 32830672 | T | 0.1788 | 0.025216 | 1.333E-12 |
| IL11RA | rs61729933 | chr9 | 36835058 | A | 0.1871 | 0.032806 | 1.176E-08 |
| IL11RA | rs7232942 | chr18 | 470267 | T | 0.0648 | 0.010147 | 1.701E-10 |
| IL11RA | rs7236292 | chr18 | 322109 | G | 0.0501 | 0.008623 | 6.252E-09 |
| IL11RA | rs72729437 | chr9 | 35963179 | G | 0.1708 | 0.02318 | 1.727E-13 |
| IL11RA | rs78705678 | chr10 | 17794281 | G | 0.0931 | 0.008481 | 4.937E-28 |
| IL1RN | rs139133059 | chr2 | 113308958 | G | -0.2542 | 0.03815 | 2.679E-11 |
| IL1RN | rs28455809 | chr6 | 32557482 | C | 0.0502 | 0.008717 | 8.484E-09 |
| IL1RN | rs4848312 | chr2 | 113037620 | G | -0.1256 | 0.008695 | 2.712E-47 |
| IL1RN | rs6489859 | chr12 | 112774128 | G | -0.0452 | 0.008031 | 1.822E-08 |
| IL1RN | rs8047587 | chr16 | 53764710 | T | 0.0444 | 0.008043 | 3.385E-08 |
| ITIH3 | rs1025647 | chr1 | 216980584 | C | -0.0557 | 0.010136 | 3.905E-08 |
| ITIH3 | rs1070073 | chr12 | 103606541 | G | 0.0504 | 0.008135 | 5.806E-10 |
| ITIH3 | rs10922098 | chr1 | 196695521 | T | -0.0431 | 0.007822 | 3.584E-08 |
| ITIH3 | rs111989250 | chr3 | 54733249 | A | -0.3358 | 0.026939 | 1.158E-35 |
| ITIH3 | rs112875651 | chr8 | 125494452 | A | 0.0606 | 0.007861 | 1.273E-14 |
| ITIH3 | rs142044921 | chr3 | 49134297 | T | 0.1352 | 0.01578 | 1.055E-17 |
| ITIH3 | rs142131136 | chr3 | 42431036 | T | -0.1893 | 0.027265 | 3.837E-12 |
| ITIH3 | rs1495741 | chr8 | 18415371 | A | 0.063 | 0.009897 | 1.949E-10 |
| ITIH3 | rs35142429 | chr3 | 44959134 | A | -0.2674 | 0.027559 | 2.931E-22 |
| ITIH3 | rs4981022 | chr12 | 103756096 | A | 0.0622 | 0.008189 | 3.055E-14 |
| ITIH3 | rs62246138 | chr3 | 46412462 | A | -0.2353 | 0.021166 | 1.04E-28 |
| ITIH3 | rs6802320 | chr3 | 52207819 | C | 0.2885 | 0.00815 | 1E-200 |
| ITIH3 | rs74320783 | chr3 | 52802517 | A | -1.2672 | 0.023293 | 1E-200 |
| ITIH3 | rs77825069 | chr1 | 161547895 | A | 0.0821 | 0.011813 | 3.655E-12 |
| ITIH3 | rs9841474 | chr3 | 55172180 | A | -0.0545 | 0.00952 | 1.035E-08 |
| LAT | rs12586321 | chr14 | 105952978 | G | 0.0632 | 0.007934 | 1.638E-15 |
| LAT | rs2093214 | chr14 | 105877986 | G | 0.0449 | 0.007932 | 1.511E-08 |
| LAT | rs9273429 | chr6 | 32659679 | A | 0.0677 | 0.007804 | 4.123E-18 |
| MANEA | rs12201004 | chr6 | 76867934 | G | -0.1521 | 0.026825 | 1.427E-08 |
| MANEA | rs12975366 | chr19 | 54255498 | C | -0.0511 | 0.006863 | 9.632E-14 |
| MANEA | rs1451341 | chr6 | 98360489 | G | 0.0962 | 0.008546 | 2.133E-29 |
| MANEA | rs2501992 | chr6 | 94642011 | G | -0.3633 | 0.010775 | 1E-200 |
| MANEA | rs34463787 | chr6 | 95560335 | T | -0.5502 | 0.005697 | 1E-200 |
| MANEA | rs4552704 | chr6 | 91190583 | G | 0.0777 | 0.008122 | 1.108E-21 |
| MANEA | rs6937033 | chr6 | 92257121 | C | -0.1464 | 0.00843 | 1.458E-67 |
| MANEA | rs72895471 | chr6 | 81131957 | G | -0.1859 | 0.028424 | 6.141E-11 |
| MANEA | rs74726105 | chr6 | 94088897 | A | -0.5926 | 0.032653 | 1.32E-73 |
| MANEA | rs76285575 | chr6 | 101322761 | T | -0.2151 | 0.017923 | 3.497E-33 |
| MANEA | rs76935248 | chr12 | 89730519 | A | 0.0754 | 0.013613 | 3.043E-08 |
| MANEA | rs9403054 | chr6 | 95819488 | G | -0.3315 | 0.005406 | 1E-200 |
| MANEA | rs9922160 | chr16 | 69991931 | C | -0.0441 | 0.008055 | 4.374E-08 |
| MBL2 | rs10437383 | chr10 | 52164008 | A | 0.1451 | 0.008699 | 1.82E-62 |
| MBL2 | rs10823734 | chr10 | 51860626 | G | 0.0742 | 0.008476 | 2.064E-18 |
| MBL2 | rs10825251 | chr10 | 54170131 | A | -0.086 | 0.009327 | 2.968E-20 |
| MBL2 | rs11002760 | chr10 | 52617801 | G | -0.5566 | 0.023489 | 3.924E-124 |
| MBL2 | rs113953295 | chr10 | 43845335 | GC | -0.108 | 0.019459 | 2.854E-08 |
| MBL2 | rs11519274 | chr12 | 21209885 | T | -0.0635 | 0.009055 | 2.336E-12 |
| MBL2 | rs11595864 | chr10 | 35729105 | G | 0.1764 | 0.030221 | 5.317E-09 |
| MBL2 | rs117573752 | chr10 | 55348008 | T | 0.2176 | 0.018002 | 1.23E-33 |
| MBL2 | rs117917923 | chr10 | 54862132 | T | -0.2555 | 0.02982 | 1.053E-17 |
| MBL2 | rs117944916 | chr10 | 36528941 | T | -0.1445 | 0.0247 | 4.913E-09 |
| MBL2 | rs1260326 | chr2 | 27508073 | C | 0.0688 | 0.007393 | 1.333E-20 |
| MBL2 | rs150524062 | chr10 | 52987008 | A | 0.149 | 0.015347 | 2.768E-22 |
| MBL2 | rs1916565 | chr10 | 49677255 | A | -0.1876 | 0.013737 | 1.85E-42 |
| MBL2 | rs2261695 | chr1 | 11026382 | T | 0.1226 | 0.009138 | 4.814E-41 |
| MBL2 | rs2578062 | chr10 | 63972695 | C | -0.0697 | 0.010873 | 1.45E-10 |
| MBL2 | rs34529023 | chr10 | 53812313 | C | -0.2677 | 0.024043 | 8.531E-29 |
| MBL2 | rs3942920 | chr10 | 53371848 | C | 0.0839 | 0.014639 | 9.958E-09 |
| MBL2 | rs576123 | chr9 | 133268896 | T | -0.2327 | 0.007972 | 2.563E-187 |
| MBL2 | rs61850494 | chr10 | 55688442 | T | 0.2104 | 0.028798 | 2.749E-13 |
| MBL2 | rs71503158 | chr9 | 132811227 | A | 0.1214 | 0.021779 | 2.486E-08 |
| MBL2 | rs7475006 | chr10 | 52798850 | C | -0.2644 | 0.00834 | 1E-200 |
| MDGA2 | rs10149770 | chr14 | 35918522 | A | 0.0876 | 0.014043 | 4.438E-10 |
| MDGA2 | rs111629268 | chr14 | 47107546 | T | -0.1235 | 0.013036 | 2.695E-21 |
| MDGA2 | rs112351914 | chr14 | 48805400 | A | 0.1872 | 0.020382 | 4.132E-20 |
| MDGA2 | rs116174082 | chr14 | 48391779 | A | 1.0801 | 0.020399 | 1E-200 |
| MDGA2 | rs12147146 | chr14 | 44697112 | C | 0.596 | 0.033776 | 1.1E-69 |
| MDGA2 | rs146048337 | chr14 | 68666849 | T | 0.1582 | 0.027517 | 8.965E-09 |
| MDGA2 | rs147722382 | chr14 | 43985091 | T | -0.2174 | 0.039342 | 3.279E-08 |
| MDGA2 | rs149817759 | chr14 | 47198429 | A | -0.369 | 0.033482 | 3.037E-28 |
| MDGA2 | rs34231016 | chr2 | 233361281 | T | 0.0616 | 0.011211 | 3.919E-08 |
| MDGA2 | rs34489253 | chr14 | 68802341 | G | 0.0874 | 0.008128 | 5.766E-27 |
| MDGA2 | rs4665972 | chr2 | 27375230 | C | 0.0485 | 0.00816 | 2.787E-09 |
| MDGA2 | rs4788706 | chr16 | 73157810 | G | -0.0425 | 0.007758 | 4.298E-08 |
| MDGA2 | rs4900811 | chr14 | 47840365 | T | -0.5405 | 0.009024 | 1E-200 |
| MDGA2 | rs57844210 | chr15 | 37011201 | G | -0.1191 | 0.016173 | 1.785E-13 |
| MDGA2 | rs62621812 | chr7 | 127375029 | A | 0.1446 | 0.024288 | 2.622E-09 |
| MDGA2 | rs75694823 | chr14 | 50941270 | C | 0.3634 | 0.030385 | 5.767E-33 |
| MDGA2 | rs77544995 | chr14 | 47416698 | C | -0.2403 | 0.022097 | 1.523E-27 |
| MDGA2 | rs849133 | chr7 | 28152661 | T | -0.043 | 0.00773 | 2.657E-08 |
| MFAP5 | rs117294446 | chr9 | 126754554 | T | -0.1944 | 0.023185 | 5.082E-17 |
| MFAP5 | rs12342201 | chr9 | 93132682 | A | -0.0687 | 0.008141 | 3.199E-17 |
| MFAP5 | rs149685171 | chr9 | 127511179 | A | 0.1069 | 0.016351 | 6.239E-11 |
| MFAP5 | rs62578121 | chr9 | 126595085 | T | -0.1882 | 0.02772 | 1.127E-11 |
| MYCBP | rs1354034 | chr3 | 56815721 | C | 0.0503 | 0.008303 | 1.377E-09 |
| MYCBP | rs1355538 | chr3 | 165787389 | G | -0.0578 | 0.008224 | 2.094E-12 |
| MYCBP | rs342200 | chr7 | 106676033 | A | -0.0477 | 0.008116 | 4.175E-09 |
| MYCBP | rs4980320 | chr11 | 198986 | C | 0.0507 | 0.008084 | 3.564E-10 |
| NAALAD2 | rs1105878 | chr3 | 133842178 | A | -0.1014 | 0.008789 | 8.556E-31 |
| NAALAD2 | rs144861591 | chr6 | 26072764 | T | -0.3883 | 0.016017 | 7.816E-130 |
| NAALAD2 | rs73219477 | chr3 | 133996379 | T | 0.1425 | 0.026109 | 4.819E-08 |
| NAALAD2 | rs74357662 | chr3 | 133720984 | C | -0.3828 | 0.011285 | 1E-200 |
| NAALAD2 | rs75858866 | chr3 | 133507659 | C | 0.2559 | 0.032819 | 6.326E-15 |
| NAALAD2 | rs855791 | chr22 | 37066896 | G | -0.0644 | 0.008217 | 4.599E-15 |
| NAPA | rs1354034 | chr3 | 56815721 | C | 0.0499 | 0.008281 | 1.683E-09 |
| NAPA | rs1984983 | chr1 | 117598574 | T | -0.059 | 0.008103 | 3.31E-13 |
| NAPA | rs342200 | chr7 | 106676033 | A | -0.0453 | 0.008116 | 2.387E-08 |
| NEU1 | rs10778161 | chr12 | 101973606 | G | -0.0645 | 0.009617 | 1.986E-11 |
| NEU1 | rs2073044 | chr6 | 32371209 | T | 0.0555 | 0.009192 | 1.563E-09 |
| NEU1 | rs576123 | chr9 | 133268896 | T | 0.0541 | 0.009281 | 5.566E-09 |
| NEU1 | rs9346805 | chr6 | 160001183 | T | -0.0928 | 0.013198 | 2.042E-12 |
| NMT1 | rs1053733 | chr17 | 45105660 | A | 0.0461 | 0.008173 | 1.693E-08 |
| NMT1 | rs1354034 | chr3 | 56815721 | C | 0.0772 | 0.008267 | 9.832E-21 |
| NMT1 | rs35340377 | chr1 | 247874908 | A | -0.0554 | 0.009353 | 3.156E-09 |
| NRP2 | rs11111630 | chr12 | 103535806 | T | 0.0976 | 0.015074 | 9.494E-11 |
| NRP2 | rs113213551 | chr2 | 205494023 | A | 0.2786 | 0.030517 | 6.895E-20 |
| NRP2 | rs113341849 | chr3 | 46342713 | A | -0.0815 | 0.012898 | 2.639E-10 |
| NRP2 | rs115214655 | chr2 | 197160664 | C | -0.1459 | 0.021101 | 4.701E-12 |
| NRP2 | rs115832719 | chr3 | 53168922 | T | 0.1861 | 0.027774 | 2.078E-11 |
| NRP2 | rs1364076 | chr5 | 72828783 | G | 0.0493 | 0.009003 | 4.351E-08 |
| NRP2 | rs139404143 | chr12 | 99589602 | C | 0.1421 | 0.025315 | 1.985E-08 |
| NRP2 | rs141893000 | chr2 | 205734339 | G | -0.2458 | 0.03001 | 2.597E-16 |
| NRP2 | rs143840518 | chr3 | 53540401 | A | 0.1609 | 0.028695 | 2.055E-08 |
| NRP2 | rs16837000 | chr2 | 205130682 | T | -0.1504 | 0.010445 | 5.207E-47 |
| NRP2 | rs17629251 | chr2 | 199983531 | T | -0.1813 | 0.030173 | 1.871E-09 |
| NRP2 | rs2844572 | chr6 | 31367798 | C | -0.0771 | 0.008955 | 7.322E-18 |
| NRP2 | rs3096688 | chr6 | 32193257 | T | -0.063 | 0.0086 | 2.384E-13 |
| NRP2 | rs333947 | chr1 | 109928142 | A | -0.0737 | 0.011766 | 3.755E-10 |
| NRP2 | rs4981022 | chr12 | 103756096 | A | 0.0716 | 0.008901 | 8.696E-16 |
| NRP2 | rs56199187 | chr1 | 161551141 | T | 0.0786 | 0.01226 | 1.447E-10 |
| NRP2 | rs56287412 | chr7 | 17939205 | C | 0.0539 | 0.008353 | 1.099E-10 |
| NRP2 | rs72915925 | chr2 | 193920553 | G | -0.1725 | 0.031108 | 2.936E-08 |
| NRP2 | rs72958127 | chr2 | 207593750 | T | -0.1363 | 0.024017 | 1.385E-08 |
| NRP2 | rs77262773 | chr17 | 69253570 | T | 0.1515 | 0.027599 | 4.035E-08 |
| NRP2 | rs78004438 | chr12 | 103706537 | T | 0.1637 | 0.02883 | 1.362E-08 |
| NTM | rs12289924 | chr11 | 131440207 | G | 0.1038 | 0.009304 | 6.628E-29 |
| NTM | rs1260326 | chr2 | 27508073 | C | 0.0994 | 0.008823 | 1.945E-29 |
| NTM | rs12800878 | chr11 | 131246794 | A | 0.1964 | 0.008718 | 2.142E-112 |
| NTM | rs138580446 | chr11 | 132379412 | A | -0.1257 | 0.019019 | 3.866E-11 |
| NTM | rs2477669 | chr10 | 17764505 | A | -0.0808 | 0.010975 | 1.805E-13 |
| NTM | rs2511504 | chr11 | 131326501 | C | 0.3616 | 0.006654 | 1E-200 |
| NTM | rs2686401 | chr3 | 165771077 | C | -0.0793 | 0.008995 | 1.187E-18 |
| NTM | rs4592429 | chr11 | 130818963 | G | -0.0515 | 0.008777 | 4.42E-09 |
| NTM | rs540 | chr16 | 370907 | C | -0.05 | 0.008583 | 5.691E-09 |
| NTM | rs7118465 | chr11 | 132100419 | T | -0.1376 | 0.009354 | 5.59E-49 |
| NTM | rs78705678 | chr10 | 17794281 | G | 0.1237 | 0.008683 | 4.762E-46 |
| OPCML | rs11222592 | chr11 | 131376856 | T | -0.1012 | 0.013127 | 1.263E-14 |
| OPCML | rs12800878 | chr11 | 131246794 | A | 0.1378 | 0.008441 | 6.61E-60 |
| OPCML | rs1544300 | chr10 | 17781866 | G | -0.0753 | 0.01285 | 4.632E-09 |
| OPCML | rs2511504 | chr11 | 131326501 | C | 0.2551 | 0.008261 | 1E-200 |
| OPCML | rs7118465 | chr11 | 132100419 | T | -0.1064 | 0.009042 | 5.764E-32 |
| OPCML | rs763144 | chr16 | 379951 | C | -0.0454 | 0.008308 | 4.638E-08 |
| OPCML | rs780093 | chr2 | 27519736 | C | 0.0533 | 0.00861 | 6.001E-10 |
| OPCML | rs78705678 | chr10 | 17794281 | G | 0.093 | 0.008498 | 7.087E-28 |
| PLA2G12B | rs112172017 | chr1 | 109260021 | A | -0.2408 | 0.02774 | 3.94E-18 |
| PLA2G12B | rs2642438 | chr1 | 220796686 | G | 0.0617 | 0.00905 | 9.251E-12 |
| PLA2G12B | rs2792703 | chr10 | 112171932 | T | -0.0713 | 0.009295 | 1.714E-14 |
| PLA2G12B | rs3829126 | chr10 | 72954419 | T | -0.1494 | 0.013719 | 1.282E-27 |
| PLA2G12B | rs4665972 | chr2 | 27375230 | C | -0.0789 | 0.008605 | 4.77E-20 |
| PLA2G12B | rs56228609 | chr16 | 56953853 | T | 0.0712 | 0.008719 | 3.198E-16 |
| PLA2G12B | rs583169 | chr1 | 107424340 | C | 0.066 | 0.01155 | 1.103E-08 |
| PLA2G12B | rs662 | chr7 | 95308134 | C | -0.1275 | 0.009025 | 2.579E-45 |
| PLA2G12B | rs9260557 | chr6 | 29954002 | T | 0.0863 | 0.010095 | 1.241E-17 |
| PLXNB2 | rs11703790 | chr22 | 50241704 | C | 0.3662 | 0.012076 | 1E-200 |
| PLXNB2 | rs1260326 | chr2 | 27508073 | C | 0.0726 | 0.008704 | 7.359E-17 |
| PLXNB2 | rs1265889 | chr6 | 32065839 | A | -0.0769 | 0.012073 | 1.893E-10 |
| PLXNB2 | rs1277443 | chr10 | 17479396 | C | -0.0507 | 0.00887 | 1.09E-08 |
| PLXNB2 | rs142982411 | chr22 | 48885579 | T | -0.1496 | 0.027015 | 3.066E-08 |
| PLXNB2 | rs2477669 | chr10 | 17764505 | A | -0.0806 | 0.010856 | 1.135E-13 |
| PLXNB2 | rs28553432 | chr22 | 50285778 | A | 0.3084 | 0.020709 | 3.729E-50 |
| PLXNB2 | rs34666101 | chr10 | 17751396 | C | -0.1584 | 0.013592 | 2.19E-31 |
| PLXNB2 | rs5771217 | chr22 | 50189275 | T | -0.1533 | 0.008821 | 1.173E-67 |
| PLXNB2 | rs62259782 | chr3 | 58443333 | A | 0.0494 | 0.008999 | 4.029E-08 |
| PLXNB2 | rs73183310 | chr22 | 50011102 | G | 0.1257 | 0.009299 | 1.228E-41 |
| PLXNB2 | rs78705678 | chr10 | 17794281 | G | 0.1751 | 0.00857 | 8.592E-93 |
| PSG4 | rs111403605 | chr19 | 41301940 | A | 0.2469 | 0.022412 | 3.177E-28 |
| PSG4 | rs112059502 | chr19 | 43348781 | G | -0.2431 | 0.018253 | 1.817E-40 |
| PSG4 | rs113797028 | chr19 | 42837867 | A | -0.187 | 0.01876 | 2.099E-23 |
| PSG4 | rs1329424 | chr1 | 196677046 | G | -0.0451 | 0.007883 | 1.056E-08 |
| PSG4 | rs138744737 | chr19 | 43086509 | C | -0.271 | 0.03341 | 5.002E-16 |
| PSG4 | rs139960875 | chr19 | 43472873 | A | 0.2779 | 0.034491 | 7.812E-16 |
| PSG4 | rs142376868 | chr19 | 43044322 | A | -0.2819 | 0.036984 | 2.492E-14 |
| PSG4 | rs145949601 | chr19 | 41849960 | T | 0.4479 | 0.030095 | 4.254E-50 |
| PSG4 | rs147075006 | chr19 | 43241054 | C | -0.2456 | 0.026992 | 9.119E-20 |
| PSG4 | rs148053437 | chr19 | 40712270 | T | 0.2628 | 0.03701 | 1.24E-12 |
| PSG4 | rs149958555 | chr19 | 39290612 | T | 0.1977 | 0.034751 | 1.278E-08 |
| PSG4 | rs151145534 | chr19 | 43152147 | A | -0.1953 | 0.027523 | 1.285E-12 |
| PSG4 | rs192292781 | chr19 | 43153432 | A | 0.4957 | 0.014869 | 1E-200 |
| PSG4 | rs62119458 | chr19 | 41603019 | C | 0.1969 | 0.014694 | 6.025E-41 |
| PSG4 | rs73033341 | chr19 | 39359047 | G | -0.1137 | 0.018511 | 8.139E-10 |
| PSMB3 | rs1706435 | chr3 | 165743688 | G | 0.0632 | 0.008305 | 2.747E-14 |
| PSMB3 | rs3917538 | chr7 | 95308581 | A | 0.0612 | 0.009704 | 2.856E-10 |
| PSMB3 | rs7774739 | chr6 | 32041524 | G | 0.1312 | 0.011203 | 1.118E-31 |
| RAB5C | rs1354034 | chr3 | 56815721 | C | 0.066 | 0.008232 | 1.083E-15 |
| RAB5C | rs1355538 | chr3 | 165787389 | G | -0.049 | 0.008157 | 1.888E-09 |
| RAB5C | rs4980320 | chr11 | 198986 | C | 0.0493 | 0.008034 | 8.447E-10 |
| RHOC | rs10745330 | chr1 | 112540817 | T | 0.0998 | 0.008177 | 2.922E-34 |
| RHOC | rs11082304 | chr18 | 23141009 | T | -0.0498 | 0.008201 | 1.26E-09 |
| RHOC | rs112595944 | chr19 | 18314948 | A | -0.0905 | 0.016343 | 3.066E-08 |
| RHOC | rs1354034 | chr3 | 56815721 | C | 0.0706 | 0.008395 | 4.111E-17 |
| RHOC | rs3811444 | chr1 | 247876149 | T | -0.0546 | 0.008689 | 3.308E-10 |
| RHOC | rs3863087 | chr3 | 165792099 | A | -0.0883 | 0.014106 | 3.857E-10 |
| RHOC | rs73165061 | chr3 | 165764157 | A | 0.1203 | 0.010362 | 3.687E-31 |
| RHOC | rs7412 | chr19 | 44908822 | T | 0.0944 | 0.01726 | 4.522E-08 |
| SAA4 | rs117147437 | chr11 | 17943896 | A | 1.1372 | 0.017098 | 1E-200 |
| SAA4 | rs12418493 | chr11 | 16952634 | C | 0.3133 | 0.026259 | 8.165E-33 |
| SAA4 | rs12720922 | chr16 | 56966973 | A | -0.0965 | 0.01117 | 5.662E-18 |
| SAA4 | rs1356979 | chr11 | 18197693 | C | -0.3806 | 0.006799 | 1E-200 |
| SAA4 | rs1648233 | chr11 | 19210963 | A | -0.2834 | 0.020568 | 3.435E-43 |
| SAA4 | rs2702652 | chr11 | 19444928 | T | 0.0822 | 0.011125 | 1.481E-13 |
| SAA4 | rs34068567 | chr11 | 18269355 | C | 0.2436 | 0.012911 | 2.134E-79 |
| SAA4 | rs58810621 | chr11 | 19906548 | T | 0.0738 | 0.013056 | 1.582E-08 |
| SAA4 | rs73438377 | chr11 | 19162627 | T | -0.1752 | 0.025552 | 7.056E-12 |
| SAR1A | rs118085384 | chr10 | 70302388 | C | -0.1252 | 0.0215 | 5.774E-09 |
| SAR1A | rs1354034 | chr3 | 56815721 | C | 0.07 | 0.00835 | 5.134E-17 |
| SAR1A | rs4980320 | chr11 | 198986 | C | 0.0452 | 0.008143 | 2.849E-08 |
| SIGLEC9 | rs11084067 | chr19 | 51277740 | C | -0.2151 | 0.014974 | 8.588E-47 |
| SIGLEC9 | rs12493830 | chr3 | 98687950 | C | -0.1325 | 0.008638 | 4.222E-53 |
| SIGLEC9 | rs12984853 | chr19 | 51092115 | A | -0.5461 | 0.006213 | 1E-200 |
| SIGLEC9 | rs55714927 | chr17 | 7176997 | T | 0.0584 | 0.010558 | 3.18E-08 |
| SIGLEC9 | rs6905297 | chr6 | 32478323 | T | -0.0557 | 0.009788 | 1.264E-08 |
| SIGLEC9 | rs7258020 | chr19 | 51376905 | T | 0.0874 | 0.011484 | 2.724E-14 |
| SIGLEC9 | rs78410648 | chr19 | 51896148 | A | 0.1904 | 0.020006 | 1.776E-21 |
| SIGLEC9 | rs8101887 | chr19 | 51526223 | A | 0.1307 | 0.012121 | 4.142E-27 |
| SIGLEC9 | rs9272359 | chr6 | 32636767 | T | -0.0542 | 0.009563 | 1.449E-08 |
| SPOCK3 | rs1023875 | chr4 | 166817793 | G | -0.1932 | 0.025942 | 9.511E-14 |
| SPOCK3 | rs116614155 | chr4 | 167641203 | A | -0.354 | 0.028804 | 1.024E-34 |
| SPOCK3 | rs11668974 | chr19 | 44156638 | A | 0.1228 | 0.018981 | 9.833E-11 |
| SPOCK3 | rs13143783 | chr4 | 167209986 | T | -0.404 | 0.014183 | 1.778E-178 |
| SPOCK3 | rs138620872 | chr16 | 72672741 | G | 0.2799 | 0.029125 | 7.239E-22 |
| SPOCK3 | rs1434282 | chr1 | 199041592 | T | 0.0608 | 0.009109 | 2.476E-11 |
| SPOCK3 | rs17504449 | chr4 | 165724362 | T | 0.2635 | 0.024004 | 4.923E-28 |
| SPOCK3 | rs17599599 | chr4 | 167040587 | A | 0.9313 | 0.017419 | 1E-200 |
| SPOCK3 | rs181329862 | chr4 | 162031690 | A | 0.2677 | 0.039764 | 1.671E-11 |
| SPOCK3 | rs192358371 | chr4 | 167944432 | G | 0.7037 | 0.037077 | 2.518E-80 |
| SPOCK3 | rs342239 | chr7 | 106696456 | C | -0.0546 | 0.007991 | 8.318E-12 |
| SPOCK3 | rs67647638 | chr7 | 149731157 | A | -0.0823 | 0.014889 | 3.249E-08 |
| SPOCK3 | rs6902010 | chr6 | 107125336 | G | -0.0489 | 0.008013 | 1.046E-09 |
| SPOCK3 | rs7030887 | chr9 | 97926229 | T | -0.0504 | 0.00828 | 1.149E-09 |
| SPOCK3 | rs704 | chr17 | 28367840 | A | -0.0771 | 0.00795 | 3.086E-22 |
| SPOCK3 | rs75897620 | chr4 | 166735672 | A | 0.3922 | 0.019733 | 6.592E-88 |
| SPOCK3 | rs77904897 | chr4 | 167187687 | C | -0.2083 | 0.032449 | 1.369E-10 |
| STAT6 | rs12423621 | chr12 | 59160442 | A | -0.4831 | 0.036843 | 2.793E-39 |
| STAT6 | rs12810354 | chr12 | 60598617 | A | -0.3915 | 0.035099 | 6.824E-29 |
| STAT6 | rs1354034 | chr3 | 56815721 | C | 0.0723 | 0.008284 | 2.608E-18 |
| STAT6 | rs1398617 | chr3 | 165761968 | T | 0.0689 | 0.00926 | 1.002E-13 |
| STAT6 | rs4980320 | chr11 | 198986 | C | 0.0552 | 0.008079 | 8.361E-12 |
| STAT6 | rs78409925 | chr12 | 54421943 | C | -0.0948 | 0.017374 | 4.854E-08 |
| TBC1D5 | rs10876550 | chr12 | 54318524 | A | 0.0454 | 0.008083 | 1.946E-08 |
| TBC1D5 | rs12465887 | chr2 | 239575191 | C | -0.0634 | 0.011401 | 2.683E-08 |
| TBC1D5 | rs1354034 | chr3 | 56815721 | C | 0.0481 | 0.008232 | 5.12E-09 |
| TBC1D5 | rs1619994 | chr3 | 165744047 | G | 0.063 | 0.008901 | 1.465E-12 |
| TBC1D5 | rs4980320 | chr11 | 198986 | C | 0.0482 | 0.008022 | 1.872E-09 |
| THSD1 | rs11603123 | chr11 | 126435600 | A | 0.2303 | 0.02099 | 5.221E-28 |
| THSD1 | rs117541350 | chr9 | 133829389 | T | -0.2195 | 0.030643 | 7.885E-13 |
| THSD1 | rs117608058 | chr9 | 133540282 | A | -0.2355 | 0.026431 | 5.101E-19 |
| THSD1 | rs139562936 | chr9 | 134215558 | T | -0.1766 | 0.028875 | 9.593E-10 |
| THSD1 | rs140147078 | chr13 | 49511404 | A | 0.4609 | 0.032971 | 2.098E-44 |
| THSD1 | rs144876910 | chr13 | 53107811 | C | 0.2656 | 0.040041 | 3.284E-11 |
| THSD1 | rs146184955 | chr13 | 50029270 | T | 0.2307 | 0.025391 | 1.029E-19 |
| THSD1 | rs3205136 | chr9 | 133251244 | A | 0.1781 | 0.013907 | 1.502E-37 |
| THSD1 | rs600038 | chr9 | 133276354 | T | 0.4796 | 0.008971 | 1E-200 |
| THSD1 | rs61957359 | chr13 | 52290800 | T | -0.1824 | 0.022151 | 1.806E-16 |
| THSD1 | rs73199769 | chr13 | 51799262 | C | -0.227 | 0.023199 | 1.308E-22 |
| THSD1 | rs73485835 | chr13 | 51107561 | A | 0.3721 | 0.028564 | 8.591E-39 |
| THSD1 | rs74543965 | chr13 | 51887303 | G | 0.7992 | 0.016107 | 1E-200 |
| THSD1 | rs76518888 | chr9 | 132657117 | C | -0.2204 | 0.031787 | 4.102E-12 |
| TMEM9 | rs112164771 | chr9 | 114328722 | T | -0.2329 | 0.014034 | 7.492E-62 |
| TMEM9 | rs115667089 | chr3 | 98443972 | A | -0.3119 | 0.033806 | 2.805E-20 |
| TMEM9 | rs138498518 | chr3 | 74133329 | T | -0.1912 | 0.034709 | 3.617E-08 |
| TMEM9 | rs140666611 | chr3 | 75842392 | T | -0.1862 | 0.03353 | 2.803E-08 |
| TMEM9 | rs146473837 | chr3 | 87987857 | G | -0.5338 | 0.039397 | 8.019E-42 |
| TMEM9 | rs149389924 | chr3 | 97300531 | G | -0.1682 | 0.022551 | 8.738E-14 |
| TMEM9 | rs2842700 | chr1 | 207108804 | A | 0.1935 | 0.020056 | 5.012E-22 |
| TMEM9 | rs3131643 | chr6 | 31475005 | A | 0.1027 | 0.01243 | 1.426E-16 |
| TMEM9 | rs62259021 | chr3 | 81219047 | G | -0.1552 | 0.024105 | 1.207E-10 |
| TMEM9 | rs77539534 | chr3 | 86345875 | T | -0.1335 | 0.020642 | 9.969E-11 |
| TMEM9 | rs77608513 | chr3 | 87982377 | G | -0.3698 | 0.030199 | 1.777E-34 |
| TMEM9 | rs780093 | chr2 | 27519736 | C | -0.0524 | 0.008698 | 1.698E-09 |
| TMEM9 | rs4915486 | chr1 | 201141835 | T | 0.0656 | 0.008296 | 2.62E-15 |
| UBA2 | rs12146727 | chr12 | 7063032 | A | -0.0819 | 0.012541 | 6.543E-11 |
| UBA2 | rs12289048 | chr11 | 65159920 | T | -0.053 | 0.009223 | 9.105E-09 |
| UBA2 | rs1354034 | chr3 | 56815721 | C | 0.0484 | 0.008199 | 3.572E-09 |
| UBA2 | rs141622900 | chr19 | 44923535 | A | 0.4037 | 0.020019 | 1.971E-90 |
| UBA2 | rs186012201 | chr19 | 45175376 | A | -0.0742 | 0.012571 | 3.585E-09 |
| UBA2 | rs5167 | chr19 | 44945208 | G | 0.1905 | 0.008505 | 4.106E-111 |
| UNC45A | rs1354034 | chr3 | 56815721 | C | 0.0585 | 0.00838 | 2.93E-12 |
| UNC45A | rs1355538 | chr3 | 165787389 | G | -0.0497 | 0.008304 | 2.164E-09 |
| UNC45A | rs4980320 | chr11 | 198986 | C | 0.0476 | 0.008153 | 5.282E-09 |
| ZFYVE27 | rs1354034 | chr3 | 56815721 | C | 0.0466 | 0.008296 | 1.938E-08 |
| ZFYVE27 | rs17736427 | chr14 | 92729029 | T | 0.0471 | 0.008518 | 3.215E-08 |
| ZFYVE27 | rs72701845 | chr14 | 92750678 | A | -0.2065 | 0.026068 | 2.343E-15 |
| ZNRF3 | rs10512472 | chr17 | 35557785 | C | 0.0842 | 0.011188 | 5.234E-14 |
| ZNRF3 | rs1354034 | chr3 | 56815721 | C | 0.1451 | 0.008252 | 3.35E-69 |
| ZNRF3 | rs1619994 | chr3 | 165744047 | G | 0.0781 | 0.008963 | 2.956E-18 |
| ZNRF3 | rs56312618 | chr11 | 237209 | A | 0.0772 | 0.009387 | 1.972E-16 |
