# Supplementary material for: Causal Association Between Plasma Proteins and Pericarditis: A Mendelian Randomization Study With Therapeutic Target Identification
Source: Mediators Inflamm. 2026 Feb 9;2026:4659271. doi: 10.1155/mi/4659271 (PMC12887433; doi:10.1155/mi/4659271)
Supplement: Supplementary file 8 — Supporting Information 8 Table S5. Drug enrichment analysis of pericarditis core genes. [file MI-2026-4659271-s007.docx]

**Table S5.** Drug enrichment analysis of pericarditis core genes

| ID | Description | RichFactor | FoldEnrichment | zScore | pvalue | p.adjust | qvalue | geneID | Count |
| --- | --- | --- | --- | --- | --- | --- | --- | --- | --- |
| Phorbol 12-myristate 13-acetate | Phorbol 12-myristate 13-acetate | 0.0124223602484472 | 24.4062111801242 | 11.7533346844088 | 4.13424097030517e-08 | 2.87329747436209e-05 | 8.57311022263283e-06 | IL1RN/MBL2/CD58/CASP8/EGF/CCL2 | 6 |
| Chromium (Iii) Chloride | Chromium (Iii) Chloride | 0.181818181818182 | 357.218181818182 | 26.6676909381585 | 1.27930327026851e-05 | 0.00399408517687157 | 0.00119172249881666 | CASP8/CCL2 | 2 |
| indomethacin | indomethacin | 0.0117302052785924 | 23.0463343108504 | 9.26759245119588 | 1.72406554397334e-05 | 0.00399408517687157 | 0.00119172249881666 | IL1RN/CASP8/EGF/CCL2 | 4 |
| Pyrrolidine dithiocarbamate | Pyrrolidine dithiocarbamate | 0.0188679245283019 | 37.0698113207547 | 10.3052444244634 | 5.98648032506239e-05 | 0.00721048479575463 | 0.00215140553542395 | CASP8/EGF/CCL2 | 3 |
| Cardidigin | Cardidigin | 0.0833333333333333 | 163.725 | 18.0001738207357 | 6.39715446716926e-05 | 0.00721048479575463 | 0.00215140553542395 | CASP8/CCL2 | 2 |
| Anacardic acid C15:3 | Anacardic acid C15:3 | 0.0769230769230769 | 151.130769230769 | 17.2860320825896 | 7.52879120049538e-05 | 0.00721048479575463 | 0.00215140553542395 | CASP8/EGF | 2 |
| 1,4-NAPHTHOQUINONE | 1,4-NAPHTHOQUINONE | 0.0740740740740741 | 145.533333333333 | 16.9589866834231 | 8.12888680094381e-05 | 0.00721048479575463 | 0.00215140553542395 | CASP8/CCL2 | 2 |
| PROPIDIUM | PROPIDIUM | 0.0159574468085106 | 31.3515957446808 | 9.43625556649543 | 9.84805846722835e-05 | 0.00721048479575463 | 0.00215140553542395 | CASP8/EGF/CCL2 | 3 |
| ibuprofen | ibuprofen | 0.0158730158730159 | 31.1857142857143 | 9.40985127585109 | 0.000100042124317263 | 0.00721048479575463 | 0.00215140553542395 | IL1RN/EGF/CCL2 | 3 |
| Sorafenib | Sorafenib | 0.0144927536231884 | 28.4739130434783 | 8.96720424139959 | 0.000130982810443129 | 0.00721048479575463 | 0.00215140553542395 | EPHA4/CASP8/EGF | 3 |
| cerivastatin | cerivastatin | 0.0142857142857143 | 28.0671428571429 | 8.89891267234564 | 0.000136679038889158 | 0.00721048479575463 | 0.00215140553542395 | CASP8/EGF/CCL2 | 3 |
| Melatonin | Melatonin | 0.0136363636363636 | 26.7913636363636 | 8.68125023428687 | 0.000156830259490233 | 0.00721048479575463 | 0.00215140553542395 | ACAN/CASP8/EGF | 3 |
| Mustard gas | Mustard gas | 0.0129310344827586 | 25.4056034482759 | 8.43848232767662 | 0.000183457010214117 | 0.00721048479575463 | 0.00215140553542395 | CASP8/EGF/CCL2 | 3 |
| ionomycin | ionomycin | 0.0128755364806867 | 25.2965665236052 | 8.41908420363083 | 0.000185799999188058 | 0.00721048479575463 | 0.00215140553542395 | CASP8/EGF/CCL2 | 3 |
| PI-103 | PI-103 | 0.0487804878048781 | 95.8390243902439 | 13.7177854706234 | 0.000189185003031282 | 0.00721048479575463 | 0.00215140553542395 | CASP8/EGF | 2 |
| 3,3',4,4',5-Pentachlorobiphenyl | 3,3',4,4',5-Pentachlorobiphenyl | 0.0126582278481013 | 24.8696202531646 | 8.34269592881488 | 0.000195367019823287 | 0.00721048479575463 | 0.00215140553542395 | IL1RN/MBL2/CCL2 | 3 |
| gemcitabine | gemcitabine | 0.0126582278481013 | 24.8696202531646 | 8.34269592881488 | 0.000195367019823287 | 0.00721048479575463 | 0.00215140553542395 | CASP8/EGF/CCL2 | 3 |
| Salmeterol | Salmeterol | 0.0476190476190476 | 93.5571428571428 | 13.5503546364965 | 0.000198590315444453 | 0.00721048479575463 | 0.00215140553542395 | CASP8/CCL2 | 2 |
| LY 294002 | LY 294002 | 0.012448132780083 | 24.456846473029 | 8.26817448246156 | 0.0002052496391431 | 0.00721048479575463 | 0.00215140553542395 | CASP8/EGF/CCL2 | 3 |
| nelfinavir | nelfinavir | 0.0454545454545455 | 89.3045454545455 | 13.2326744289165 | 0.000218077153487861 | 0.00721048479575463 | 0.00215140553542395 | CASP8/CCL2 | 2 |
| 2-CHLOROETHYL ETHYL SULFIDE | 2-CHLOROETHYL ETHYL SULFIDE | 0.0444444444444444 | 87.32 | 13.0817846605503 | 0.000228158307660828 | 0.00721048479575463 | 0.00215140553542395 | IL1RN/CASP8 | 2 |
| TPEN | TPEN | 0.0118577075098814 | 23.296837944664 | 8.0550704312184 | 0.000236837396501 | 0.00721048479575463 | 0.00215140553542395 | CD58/CASP8/CCL2 | 3 |
| Acridine orange | Acridine orange | 0.0425531914893617 | 83.6042553191489 | 12.7944851172229 | 0.000248995158414549 | 0.00721048479575463 | 0.00215140553542395 | CASP8/EGF | 2 |
| TIRON | TIRON | 0.0425531914893617 | 83.6042553191489 | 12.7944851172229 | 0.000248995158414549 | 0.00721048479575463 | 0.00215140553542395 | CASP8/CCL2 | 2 |
| AMILORIDE | AMILORIDE | 0.04 | 78.588 | 12.3960769291255 | 0.000281934009871894 | 0.00743211139580087 | 0.00221753266940215 | CASP8/CCL2 | 2 |
| ZINC SULFIDE | ZINC SULFIDE | 0.0392156862745098 | 77.0470588235294 | 12.2710943267843 | 0.000293361839624269 | 0.00743211139580087 | 0.00221753266940215 | IL1RN/CCL2 | 2 |
| clonidine | clonidine | 0.0109489051094891 | 21.5113138686131 | 7.71560209445853 | 0.000299423192924352 | 0.00743211139580087 | 0.00221753266940215 | IL1RN/FCGR2A/CCL2 | 3 |
| PD 98059 | PD 98059 | 0.0109489051094891 | 21.5113138686131 | 7.71560209445853 | 0.000299423192924352 | 0.00743211139580087 | 0.00221753266940215 | CASP8/EGF/CCL2 | 3 |
| Dinoprostone | Dinoprostone | 0.0106382978723404 | 20.9010638297872 | 7.59611613513489 | 0.000325823435472818 | 0.0076129663116766 | 0.002271494681409 | IL1RN/EGF/CCL2 | 3 |
| octopamine | octopamine | 0.037037037037037 | 72.7666666666667 | 11.9170479163228 | 0.00032898737694549 | 0.0076129663116766 | 0.002271494681409 | IL1RN/CCL2 | 2 |
| glutathione | glutathione | 0.0104895104895105 | 20.6087412587413 | 7.53821187365173 | 0.000339571159225863 | 0.0076129663116766 | 0.002271494681409 | CASP8/EGF/CCL2 | 3 |
| Puerarin | Puerarin | 0.0344827586206897 | 67.748275862069 | 11.4880790581605 | 0.00037961264310577 | 0.00824471209245344 | 0.00245998982538936 | CASP8/EGF | 2 |
| enoxaparin | enoxaparin | 0.03125 | 61.396875 | 10.9210486852367 | 0.000462222039257728 | 0.00973467628133701 | 0.00290455316535159 | FCGR2A/EGF | 2 |
| beclomethasone | beclomethasone | 0.0294117647058824 | 57.7852941176471 | 10.5850906708369 | 0.000521725467038513 | 0.0106646823409343 | 0.00318204077419774 | IL1RN/CCL2 | 2 |
| letrozole | letrozole | 0.027027027027027 | 53.1 | 10.1326813412439 | 0.000617597003254809 | 0.0117053948756994 | 0.00349256007650554 | EGF/CCL2 | 2 |
| ns-398 | ns-398 | 0.027027027027027 | 53.1 | 10.1326813412439 | 0.000617597003254809 | 0.0117053948756994 | 0.00349256007650554 | CASP8/CCL2 | 2 |
| folic acid | folic acid | 0.00852272727272727 | 16.7446022727273 | 6.7263604184914 | 0.000623164907051623 | 0.0117053948756994 | 0.00349256007650554 | CASP8/EGF/CCL2 | 3 |
| mitomycin C | mitomycin C | 0.00826446280991736 | 16.2371900826446 | 6.6124002902306 | 0.000681585575492723 | 0.0124658414465116 | 0.00371945591058356 | CASP8/EGF/CCL2 | 3 |
| Bathocuproine disulfonate | Bathocuproine disulfonate | 0.025 | 49.1175 | 9.73162635261017 | 0.000721373096062771 | 0.0128552385067596 | 0.00383564102359962 | CASP8/CCL2 | 2 |
| bay 11-7082 | bay 11-7082 | 0.0240963855421687 | 47.3421686746988 | 9.54742097848309 | 0.000776213037021573 | 0.0131577575787803 | 0.00392590419238126 | CASP8/CCL2 | 2 |
| nimesulide | nimesulide | 0.0240963855421687 | 47.3421686746988 | 9.54742097848309 | 0.000776213037021573 | 0.0131577575787803 | 0.00392590419238126 | CASP8/CCL2 | 2 |
| chitosamine | chitosamine | 0.0232558139534884 | 45.6906976744186 | 9.37282122422754 | 0.000833014356067835 | 0.0137729254190532 | 0.00410945294593485 | ACAN/CCL2 | 2 |
| cycloheximide | cycloheximide | 0.00763358778625954 | 14.9977099236641 | 6.32546242971811 | 0.000858501982664587 | 0.0137729254190532 | 0.00410945294593485 | CASP8/EGF/CCL2 | 3 |
| pentoxifylline | pentoxifylline | 0.0227272727272727 | 44.6522727272727 | 9.26135160530022 | 0.000871969134651602 | 0.0137729254190532 | 0.00410945294593485 | CASP8/CCL2 | 2 |
| gedunin | gedunin | 0.0224719101123595 | 44.1505617977528 | 9.20701258095881 | 0.000891772149435101 | 0.0137729254190532 | 0.00410945294593485 | IL1RN/CASP8 | 2 |
| GNF-Pf-4325 | GNF-Pf-4325 | 0.021978021978022 | 43.1802197802198 | 9.10099802164152 | 0.0009320285238418 | 0.0140817353058707 | 0.00420159311663237 | CASP8/CCL2 | 2 |
| 7,8-Benzoflavone | 7,8-Benzoflavone | 0.0217391304347826 | 42.7108695652174 | 9.04927418591284 | 0.000952481521301446 | 0.0140845671766916 | 0.00420243806710828 | IL1RN/CCL2 | 2 |
| Prestwick-983 | Prestwick-983 | 0.00705882352941176 | 13.8684705882353 | 6.052316245 | 0.00107704116405521 | 0.0150312799378083 | 0.00448491048504086 | IL1RN/CD58/CCL2 | 3 |
| 33258 Hoechst | 33258 Hoechst | 0.0202020202020202 | 39.6909090909091 | 8.70913186074532 | 0.00110169875245148 | 0.0150312799378083 | 0.00448491048504086 | CASP8/EGF | 2 |
| nicotinamide | nicotinamide | 0.0202020202020202 | 39.6909090909091 | 8.70913186074532 | 0.00110169875245148 | 0.0150312799378083 | 0.00448491048504086 | EGF/CCL2 | 2 |
| ACMC-20mvek | ACMC-20mvek | 0.02 | 39.294 | 8.66343609599937 | 0.00112387708307955 | 0.0150312799378083 | 0.00448491048504086 | CASP8/CCL2 | 2 |
| Phenethyl isothiocyanate | Phenethyl isothiocyanate | 0.0198019801980198 | 38.9049504950495 | 8.61841044631037 | 0.00114627026863862 | 0.0150312799378083 | 0.00448491048504086 | CASP8/CCL2 | 2 |
| rofecoxib | rofecoxib | 0.0198019801980198 | 38.9049504950495 | 8.61841044631037 | 0.00114627026863862 | 0.0150312799378083 | 0.00448491048504086 | IL1RN/CCL2 | 2 |
| N-Acetyl-L-cysteine | N-Acetyl-L-cysteine | 0.00678733031674208 | 13.3350678733032 | 5.91895478281846 | 0.00120630760177392 | 0.0155256256154235 | 0.00463240930895636 | CASP8/EGF/CCL2 | 3 |
| gentamicin | gentamicin | 0.019047619047619 | 37.4228571428571 | 8.44468890763723 | 0.00123798796053024 | 0.0156436660467002 | 0.00466762924831495 | EGF/CCL2 | 2 |
| Imatinib | Imatinib | 0.0178571428571429 | 35.0839285714286 | 8.16303107638592 | 0.00140672430891283 | 0.017458453476686 | 0.00520911069277872 | CASP8/CCL2 | 2 |
| fluticasone | fluticasone | 0.0175438596491228 | 34.4684210526316 | 8.08728362801063 | 0.00145685168716318 | 0.0177633670627792 | 0.00530008831710337 | CASP8/CCL2 | 2 |
| carbachol | carbachol | 0.0169491525423729 | 33.3 | 7.94150882909693 | 0.00155965313084858 | 0.0186889469989614 | 0.00557625529541144 | EGF/CCL2 | 2 |
| IBMX | IBMX | 0.0165289256198347 | 32.4743801652893 | 7.83687155175231 | 0.00163897694135438 | 0.019026363186886 | 0.00567693078048701 | EGF/CCL2 | 2 |
| quinpirole | quinpirole | 0.00609756097560976 | 11.9798780487805 | 5.56593960458372 | 0.00164256372836426 | 0.019026363186886 | 0.00567693078048701 | IL1RN/CD58/CCL2 | 3 |
| ETHYLENE GLYCOL | ETHYLENE GLYCOL | 0.016 | 31.4352 | 7.70315279019762 | 0.00174769651087181 | 0.019591114113805 | 0.00584543654739807 | EGF/CCL2 | 2 |
| LITHOCHOLIC ACID | LITHOCHOLIC ACID | 0.016 | 31.4352 | 7.70315279019762 | 0.00174769651087181 | 0.019591114113805 | 0.00584543654739807 | CASP8/EGF | 2 |
| chloroquine | chloroquine | 0.015748031496063 | 30.940157480315 | 7.63863105106958 | 0.00180331930299899 | 0.0198937605648302 | 0.00593573772248624 | EGF/CCL2 | 2 |
| DEOXYNIVALENOL | DEOXYNIVALENOL | 0.0153846153846154 | 30.2261538461538 | 7.544601593 | 0.00188832825424803 | 0.0205060646359747 | 0.00611843200800761 | CASP8/CCL2 | 2 |
| Agarose | Agarose | 0.0150375939849624 | 29.5443609022556 | 7.45371003381259 | 0.00197522266554771 | 0.0211196885008563 | 0.00630152008279998 | CASP8/EGF | 2 |
| berberine | berberine | 0.0144927536231884 | 28.4739130434783 | 7.30873234859928 | 0.00212422361527407 | 0.0220348569047086 | 0.00657458055316561 | CASP8/CCL2 | 2 |
| ursodiol | ursodiol | 0.0144927536231884 | 28.4739130434783 | 7.30873234859928 | 0.00212422361527407 | 0.0220348569047086 | 0.00657458055316561 | CASP8/CCL2 | 2 |
| lorazepam | lorazepam | 0.0137931034482759 | 27.0993103448276 | 7.11824540492347 | 0.00234155941967413 | 0.0235852724155582 | 0.00703718086461942 | CASP8/EGF | 2 |
| TITANIUM DIOXIDE | TITANIUM DIOXIDE | 0.0137931034482759 | 27.0993103448276 | 7.11824540492347 | 0.00234155941967413 | 0.0235852724155582 | 0.00703718086461942 | IL1RN/CCL2 | 2 |
| Vandetanib | Vandetanib | 0.0133333333333333 | 26.196 | 6.99025201165884 | 0.00250300825486471 | 0.0248234093222674 | 0.00740660603784427 | EPHA4/EGF | 2 |
| butyric acid | butyric acid | 0.0132450331125828 | 26.0225165562914 | 6.96540223012175 | 0.00253591663579998 | 0.0248234093222674 | 0.00740660603784427 | EGF/CCL2 | 2 |
| lovastatin | lovastatin | 0.0127388535031847 | 25.028025477707 | 6.82121110169938 | 0.00273768250131078 | 0.0264262408112638 | 0.00788484580055883 | CASP8/EGF | 2 |
| Sorafenib tosylate | Sorafenib tosylate | 0.0121212121212121 | 23.8145454545455 | 6.64104652733858 | 0.00301816294399748 | 0.0287345650147705 | 0.00857358471474409 | CASP8/EGF | 2 |
| Coxistac | Coxistac | 0.0118343195266272 | 23.2508875739645 | 6.55568399097746 | 0.00316328919305836 | 0.0296698752944948 | 0.00885265495345017 | CASP8/CCL2 | 2 |
| bicalutamide | bicalutamide | 0.0113636363636364 | 22.3261363636364 | 6.4131867579553 | 0.0034250557817512 | 0.0296698752944948 | 0.00885265495345017 | EGF/CCL2 | 2 |
| actinomycin D | actinomycin D | 0.0112994350282486 | 22.2 | 6.39350513239689 | 0.00346325817530289 | 0.0296698752944948 | 0.00885265495345017 | CASP8/CCL2 | 2 |
| CALCIUM | CALCIUM | 0.0112359550561798 | 22.0752808988764 | 6.37398519152554 | 0.00350166184179581 | 0.0296698752944948 | 0.00885265495345017 | EGF/CCL2 | 2 |
| tobramycin | tobramycin | 0.0112359550561798 | 22.0752808988764 | 6.37398519152554 | 0.00350166184179581 | 0.0296698752944948 | 0.00885265495345017 | IL1RN/CCL2 | 2 |
| Velcade(Bortezomib) | Velcade(Bortezomib) | 0.0112359550561798 | 22.0752808988764 | 6.37398519152554 | 0.00350166184179581 | 0.0296698752944948 | 0.00885265495345017 | CASP8/EGF | 2 |
| dUTP | dUTP | 0.0111731843575419 | 21.9519553072626 | 6.35462468569497 | 0.0035402666085637 | 0.0296698752944948 | 0.00885265495345017 | CASP8/EGF | 2 |
| zardaverine | zardaverine | 0.0111731843575419 | 21.9519553072626 | 6.35462468569497 | 0.0035402666085637 | 0.0296698752944948 | 0.00885265495345017 | IL1RN/CCL2 | 2 |
| maltotriose | maltotriose | 0.010989010989011 | 21.5901098901099 | 6.29747793106974 | 0.00365728578527087 | 0.0296698752944948 | 0.00885265495345017 | CASP8/EGF | 2 |
| PhIP | PhIP | 0.0109289617486339 | 21.472131147541 | 6.27873352693074 | 0.00369669322836411 | 0.0296698752944948 | 0.00885265495345017 | CASP8/EGF | 2 |
| 1,9-Pyrazoloanthrone | 1,9-Pyrazoloanthrone | 0.0108695652173913 | 21.3554347826087 | 6.26013794392471 | 0.00373630090981672 | 0.0296698752944948 | 0.00885265495345017 | CASP8/EGF | 2 |
| GEMCITABINE HYDROCHLORIDE | GEMCITABINE HYDROCHLORIDE | 0.0108108108108108 | 21.24 | 6.24168917897334 | 0.00377610865752846 | 0.0296698752944948 | 0.00885265495345017 | CASP8/EGF | 2 |
| Erythorbic acid | Erythorbic acid | 0.0106951871657754 | 21.0128342245989 | 6.205224278 | 0.00385632366379919 | 0.0296698752944948 | 0.00885265495345017 | CASP8/EGF | 2 |
| Quercetin dihydrate | Quercetin dihydrate | 0.0106951871657754 | 21.0128342245989 | 6.205224278 | 0.00385632366379919 | 0.0296698752944948 | 0.00885265495345017 | CASP8/EGF | 2 |
| Imatinib mesylate | Imatinib mesylate | 0.0106382978723404 | 20.9010638297872 | 6.18720432107499 | 0.0038967305786285 | 0.0296698752944948 | 0.00885265495345017 | CASP8/EGF | 2 |
| Ethylparaben | Ethylparaben | 0.0105820105820106 | 20.7904761904762 | 6.16932353721993 | 0.0039373368722575 | 0.0296698752944948 | 0.00885265495345017 | CASP8/EGF | 2 |
| hydroquinone | hydroquinone | 0.0104712041884817 | 20.5727748691099 | 6.13397222854477 | 0.00401914690948989 | 0.0296698752944948 | 0.00885265495345017 | CASP8/CCL2 | 2 |
| Insulin | Insulin | 0.0104712041884817 | 20.5727748691099 | 6.13397222854477 | 0.00401914690948989 | 0.0296698752944948 | 0.00885265495345017 | CASP8/EGF | 2 |
| methanol | methanol | 0.0104166666666667 | 20.465625 | 6.11649815587197 | 0.00406035031011593 | 0.0296698752944948 | 0.00885265495345017 | CASP8/EGF | 2 |
| Acid red 87 | Acid red 87 | 0.0103626943005181 | 20.359585492228 | 6.09915615931035 | 0.00410175240358687 | 0.0296698752944948 | 0.00885265495345017 | CASP8/EGF | 2 |
| retinol | retinol | 0.0103626943005181 | 20.359585492228 | 6.09915615931035 | 0.00410175240358687 | 0.0296698752944948 | 0.00885265495345017 | MBL2/CCL2 | 2 |
| Aizen uranine | Aizen uranine | 0.0103092783505155 | 20.2546391752577 | 6.0819445441698 | 0.00414335301864881 | 0.0296698752944948 | 0.00885265495345017 | CASP8/EGF | 2 |
| thalidomide | thalidomide | 0.0103092783505155 | 20.2546391752577 | 6.0819445441698 | 0.00414335301864881 | 0.0296698752944948 | 0.00885265495345017 | CASP8/CCL2 | 2 |
| UNII-CXY7B3Q98Z | UNII-CXY7B3Q98Z | 0.0102564102564103 | 20.1507692307692 | 6.06486164614201 | 0.00418515198414175 | 0.0296698752944948 | 0.00885265495345017 | CASP8/EGF | 2 |
| 2-Butanone | 2-Butanone | 0.0101522842639594 | 19.946192893401 | 6.03107549193511 | 0.00426934428224939 | 0.0296698752944948 | 0.00885265495345017 | CASP8/EGF | 2 |
| Bisulfite | Bisulfite | 0.0101010101010101 | 19.8454545454545 | 6.01436905286784 | 0.00431173727301291 | 0.0296698752944948 | 0.00885265495345017 | CASP8/EGF | 2 |
| L-cysteine | L-cysteine | 0.0101010101010101 | 19.8454545454545 | 6.01436905286784 | 0.00431173727301291 | 0.0296698752944948 | 0.00885265495345017 | CASP8/EGF | 2 |
| streptozocin | streptozocin | 0.0101010101010101 | 19.8454545454545 | 6.01436905286784 | 0.00431173727301291 | 0.0296698752944948 | 0.00885265495345017 | CASP8/EGF | 2 |
| PLATINUM | PLATINUM | 0.01 | 19.647 | 5.98132170236116 | 0.00439711608403414 | 0.0299599086047268 | 0.00893919272265229 | CASP8/EGF | 2 |
| water | water | 0.00995024875621891 | 19.5492537313433 | 5.96497777243583 | 0.00444010156300267 | 0.0299599086047268 | 0.00893919272265229 | CASP8/EGF | 2 |
| Crizotinib | Crizotinib | 0.00980392156862745 | 19.2617647058824 | 5.91664739679443 | 0.00457024024797014 | 0.0300300844064862 | 0.00896013120496445 | EPHA4/EGF | 2 |
| chrysin | chrysin | 0.00975609756097561 | 19.1678048780488 | 5.90076634225082 | 0.00461401332458932 | 0.0300300844064862 | 0.00896013120496445 | CASP8/CCL2 | 2 |
| 2,2',4,4',5,5'-Hexachlorobiphenyl | 2,2',4,4',5,5'-Hexachlorobiphenyl | 0.00961538461538462 | 18.8913461538462 | 5.85379117685957 | 0.00474651071751265 | 0.0300300844064862 | 0.00896013120496445 | IL1RN/CASP8 | 2 |
| Sodium dichromate | Sodium dichromate | 0.00961538461538462 | 18.8913461538462 | 5.85379117685957 | 0.00474651071751265 | 0.0300300844064862 | 0.00896013120496445 | CASP8/CCL2 | 2 |
| Alitretinoin | Alitretinoin | 0.00943396226415094 | 18.5349056603774 | 5.79266737734289 | 0.0049259166132823 | 0.0300300844064862 | 0.00896013120496445 | CASP8/CCL2 | 2 |
| Atiprimod | Atiprimod | 0.1 | 196.47 | 13.9521791051994 | 0.00507935434884621 | 0.0300300844064862 | 0.00896013120496445 | CASP8 | 1 |
| Coralyne chloride | Coralyne chloride | 0.1 | 196.47 | 13.9521791051994 | 0.00507935434884621 | 0.0300300844064862 | 0.00896013120496445 | CCL2 | 1 |
| hydroxychloroquine | hydroxychloroquine | 0.1 | 196.47 | 13.9521791051994 | 0.00507935434884621 | 0.0300300844064862 | 0.00896013120496445 | CCL2 | 1 |
| picolinic acid | picolinic acid | 0.1 | 196.47 | 13.9521791051994 | 0.00507935434884621 | 0.0300300844064862 | 0.00896013120496445 | CCL2 | 1 |
| sulfadiazine | sulfadiazine | 0.1 | 196.47 | 13.9521791051994 | 0.00507935434884621 | 0.0300300844064862 | 0.00896013120496445 | EGF | 1 |
| tiopronin | tiopronin | 0.1 | 196.47 | 13.9521791051994 | 0.00507935434884621 | 0.0300300844064862 | 0.00896013120496445 | CCL2 | 1 |
| buflomedil | buflomedil | 0.00909090909090909 | 17.8609090909091 | 5.67530174284269 | 0.00529409128532498 | 0.0300300844064862 | 0.00896013120496445 | IL1RN/EGF | 2 |
| Aflodac | Aflodac | 0.00884955752212389 | 17.3867256637168 | 5.59126541246536 | 0.00557837221865932 | 0.0300300844064862 | 0.00896013120496445 | CASP8/EGF | 2 |
| 15(S)-HETE-d8 | 15(S)-HETE-d8 | 0.0909090909090909 | 178.609090909091 | 13.2964137877399 | 0.0055860104804607 | 0.0300300844064862 | 0.00896013120496445 | EGF | 1 |
| 5,6-Dichloro-1H-benzimidazole | 5,6-Dichloro-1H-benzimidazole | 0.0909090909090909 | 178.609090909091 | 13.2964137877399 | 0.0055860104804607 | 0.0300300844064862 | 0.00896013120496445 | CASP8 | 1 |
| aclarubicin | aclarubicin | 0.0909090909090909 | 178.609090909091 | 13.2964137877399 | 0.0055860104804607 | 0.0300300844064862 | 0.00896013120496445 | CASP8 | 1 |
| Alantolactone | Alantolactone | 0.0909090909090909 | 178.609090909091 | 13.2964137877399 | 0.0055860104804607 | 0.0300300844064862 | 0.00896013120496445 | CASP8 | 1 |
| Angelicin | Angelicin | 0.0909090909090909 | 178.609090909091 | 13.2964137877399 | 0.0055860104804607 | 0.0300300844064862 | 0.00896013120496445 | CASP8 | 1 |
| beta-Solamarine | beta-Solamarine | 0.0909090909090909 | 178.609090909091 | 13.2964137877399 | 0.0055860104804607 | 0.0300300844064862 | 0.00896013120496445 | CASP8 | 1 |
| Cadmium telluride | Cadmium telluride | 0.0909090909090909 | 178.609090909091 | 13.2964137877399 | 0.0055860104804607 | 0.0300300844064862 | 0.00896013120496445 | CASP8 | 1 |
| Saracatinib (AZD0530) | Saracatinib (AZD0530) | 0.0909090909090909 | 178.609090909091 | 13.2964137877399 | 0.0055860104804607 | 0.0300300844064862 | 0.00896013120496445 | EPHA4 | 1 |
| acetaldehyde | acetaldehyde | 0.0087719298245614 | 17.2342105263158 | 5.56396854422488 | 0.00567467770586641 | 0.0300300844064862 | 0.00896013120496445 | EGF/CCL2 | 2 |
| FENRETINIDE | FENRETINIDE | 0.00858369098712446 | 16.8643776824034 | 5.49721835867605 | 0.00591880875190853 | 0.0300300844064862 | 0.00896013120496445 | CD58/CASP8 | 2 |
| celecoxib | celecoxib | 0.00854700854700855 | 16.7923076923077 | 5.48411683193497 | 0.0059682108754451 | 0.0300300844064862 | 0.00896013120496445 | CASP8/EGF | 2 |
| docetaxel | docetaxel | 0.00854700854700855 | 16.7923076923077 | 5.48411683193497 | 0.0059682108754451 | 0.0300300844064862 | 0.00896013120496445 | CASP8/EGF | 2 |
| morphine | morphine | 0.00854700854700855 | 16.7923076923077 | 5.48411683193497 | 0.0059682108754451 | 0.0300300844064862 | 0.00896013120496445 | IL1RN/CASP8 | 2 |
| 4-Methylhistamine | 4-Methylhistamine | 0.0833333333333333 | 163.725 | 12.7241549817936 | 0.00609243439038065 | 0.0300300844064862 | 0.00896013120496445 | CCL2 | 1 |
| Aminolevulinic acid | Aminolevulinic acid | 0.0833333333333333 | 163.725 | 12.7241549817936 | 0.00609243439038065 | 0.0300300844064862 | 0.00896013120496445 | CASP8 | 1 |
| BISOPROLOL | BISOPROLOL | 0.0833333333333333 | 163.725 | 12.7241549817936 | 0.00609243439038065 | 0.0300300844064862 | 0.00896013120496445 | CCL2 | 1 |
| Canertinib dihydrochloride | Canertinib dihydrochloride | 0.0833333333333333 | 163.725 | 12.7241549817936 | 0.00609243439038065 | 0.0300300844064862 | 0.00896013120496445 | EGF | 1 |
| Cordycepin | Cordycepin | 0.0833333333333333 | 163.725 | 12.7241549817936 | 0.00609243439038065 | 0.0300300844064862 | 0.00896013120496445 | CASP8 | 1 |
| Eckol | Eckol | 0.0833333333333333 | 163.725 | 12.7241549817936 | 0.00609243439038065 | 0.0300300844064862 | 0.00896013120496445 | CASP8 | 1 |
| Enzacamene | Enzacamene | 0.0833333333333333 | 163.725 | 12.7241549817936 | 0.00609243439038065 | 0.0300300844064862 | 0.00896013120496445 | CCL2 | 1 |
| Ethanolamine | Ethanolamine | 0.0833333333333333 | 163.725 | 12.7241549817936 | 0.00609243439038065 | 0.0300300844064862 | 0.00896013120496445 | EGF | 1 |
| Modrasone | Modrasone | 0.0833333333333333 | 163.725 | 12.7241549817936 | 0.00609243439038065 | 0.0300300844064862 | 0.00896013120496445 | CCL2 | 1 |
| Phylloquinone | Phylloquinone | 0.0833333333333333 | 163.725 | 12.7241549817936 | 0.00609243439038065 | 0.0300300844064862 | 0.00896013120496445 | CASP8 | 1 |
| PP2 | PP2 | 0.0833333333333333 | 163.725 | 12.7241549817936 | 0.00609243439038065 | 0.0300300844064862 | 0.00896013120496445 | EPHA4 | 1 |
| Trovafloxacin | Trovafloxacin | 0.0833333333333333 | 163.725 | 12.7241549817936 | 0.00609243439038065 | 0.0300300844064862 | 0.00896013120496445 | CASP8 | 1 |
| 2-Aminoethanethiol | 2-Aminoethanethiol | 0.0769230769230769 | 151.130769230769 | 12.2190232855882 | 0.0065986261732226 | 0.0309867918269575 | 0.00924558574768742 | EGF | 1 |
| ACRYLONITRILE | ACRYLONITRILE | 0.0769230769230769 | 151.130769230769 | 12.2190232855882 | 0.0065986261732226 | 0.0309867918269575 | 0.00924558574768742 | CCL2 | 1 |
| Dehydroxymethylepoxyquinomicin | Dehydroxymethylepoxyquinomicin | 0.0769230769230769 | 151.130769230769 | 12.2190232855882 | 0.0065986261732226 | 0.0309867918269575 | 0.00924558574768742 | CASP8 | 1 |
| Diacerein | Diacerein | 0.0769230769230769 | 151.130769230769 | 12.2190232855882 | 0.0065986261732226 | 0.0309867918269575 | 0.00924558574768742 | ACAN | 1 |
| Palmatine | Palmatine | 0.0769230769230769 | 151.130769230769 | 12.2190232855882 | 0.0065986261732226 | 0.0309867918269575 | 0.00924558574768742 | CCL2 | 1 |
| ro 32-0432 | ro 32-0432 | 0.0769230769230769 | 151.130769230769 | 12.2190232855882 | 0.0065986261732226 | 0.0309867918269575 | 0.00924558574768742 | EGF | 1 |
| XCT790 | XCT790 | 0.0769230769230769 | 151.130769230769 | 12.2190232855882 | 0.0065986261732226 | 0.0309867918269575 | 0.00924558574768742 | CASP8 | 1 |
| HEMATOXYLIN | HEMATOXYLIN | 0.008 | 15.7176 | 5.28492972274918 | 0.00678458622951594 | 0.031054636584146 | 0.00926582871196784 | CASP8/EGF | 2 |
| oxyphenbutazone | oxyphenbutazone | 0.00796812749003984 | 15.6549800796813 | 5.27309390253288 | 0.00683722252582384 | 0.031054636584146 | 0.00926582871196784 | CASP8/EGF | 2 |
| Bendamustine | Bendamustine | 0.0714285714285714 | 140.335714285714 | 11.7688121987127 | 0.00710458592356722 | 0.031054636584146 | 0.00926582871196784 | CASP8 | 1 |
| deferiprone | deferiprone | 0.0714285714285714 | 140.335714285714 | 11.7688121987127 | 0.00710458592356722 | 0.031054636584146 | 0.00926582871196784 | CCL2 | 1 |
| GOLD | GOLD | 0.0714285714285714 | 140.335714285714 | 11.7688121987127 | 0.00710458592356722 | 0.031054636584146 | 0.00926582871196784 | CASP8 | 1 |
| Malondialdehyde | Malondialdehyde | 0.0714285714285714 | 140.335714285714 | 11.7688121987127 | 0.00710458592356722 | 0.031054636584146 | 0.00926582871196784 | IL1RN | 1 |
| meclocycline | meclocycline | 0.0714285714285714 | 140.335714285714 | 11.7688121987127 | 0.00710458592356722 | 0.031054636584146 | 0.00926582871196784 | CCL2 | 1 |
| NSC267099 | NSC267099 | 0.0714285714285714 | 140.335714285714 | 11.7688121987127 | 0.00710458592356722 | 0.031054636584146 | 0.00926582871196784 | CASP8 | 1 |
| safrole | safrole | 0.0714285714285714 | 140.335714285714 | 11.7688121987127 | 0.00710458592356722 | 0.031054636584146 | 0.00926582871196784 | CASP8 | 1 |
| Talmapimod | Talmapimod | 0.0714285714285714 | 140.335714285714 | 11.7688121987127 | 0.00710458592356722 | 0.031054636584146 | 0.00926582871196784 | CASP8 | 1 |
| THYMOL | THYMOL | 0.0714285714285714 | 140.335714285714 | 11.7688121987127 | 0.00710458592356722 | 0.031054636584146 | 0.00926582871196784 | CASP8 | 1 |
| 1-chloro-2,4-dinitrobenzene | 1-chloro-2,4-dinitrobenzene | 0.00763358778625954 | 14.9977099236641 | 5.14723779046261 | 0.00742864273805465 | 0.0311127532146718 | 0.00928316907730457 | EGF/CCL2 | 2 |
| ascorbic acid | ascorbic acid | 0.00763358778625954 | 14.9977099236641 | 5.14723779046261 | 0.00742864273805465 | 0.0311127532146718 | 0.00928316907730457 | CASP8/CCL2 | 2 |
| ellagic acid | ellagic acid | 0.00754716981132075 | 14.8279245283019 | 5.11422867184873 | 0.00759387392652817 | 0.0311127532146718 | 0.00928316907730457 | CASP8/CCL2 | 2 |
| suloctidil | suloctidil | 0.00754716981132075 | 14.8279245283019 | 5.11422867184873 | 0.00759387392652817 | 0.0311127532146718 | 0.00928316907730457 | IL1RN/CCL2 | 2 |
| Benzo[e]pyrene | Benzo[e]pyrene | 0.0666666666666667 | 130.98 | 11.36421363 | 0.00761031373596288 | 0.0311127532146718 | 0.00928316907730457 | CASP8 | 1 |
| Fostamatinib | Fostamatinib | 0.0666666666666667 | 130.98 | 11.36421363 | 0.00761031373596288 | 0.0311127532146718 | 0.00928316907730457 | EPHA4 | 1 |
| MELAMINE | MELAMINE | 0.0666666666666667 | 130.98 | 11.36421363 | 0.00761031373596288 | 0.0311127532146718 | 0.00928316907730457 | CCL2 | 1 |
| Methacholine chloride | Methacholine chloride | 0.0666666666666667 | 130.98 | 11.36421363 | 0.00761031373596288 | 0.0311127532146718 | 0.00928316907730457 | IL1RN | 1 |
| Oroxylin A | Oroxylin A | 0.0666666666666667 | 130.98 | 11.36421363 | 0.00761031373596288 | 0.0311127532146718 | 0.00928316907730457 | CASP8 | 1 |
| PCI-24781 | PCI-24781 | 0.0666666666666667 | 130.98 | 11.36421363 | 0.00761031373596288 | 0.0311127532146718 | 0.00928316907730457 | CASP8 | 1 |
| piperazine | piperazine | 0.0666666666666667 | 130.98 | 11.36421363 | 0.00761031373596288 | 0.0311127532146718 | 0.00928316907730457 | CASP8 | 1 |
| nitric oxide | nitric oxide | 0.00746268656716418 | 14.6619402985075 | 5.08175354289947 | 0.00776078303536871 | 0.0311629157178016 | 0.00929813615510324 | CASP8/EGF | 2 |
| metformin | metformin | 0.00740740740740741 | 14.5533333333333 | 5.06039283124976 | 0.00787298576009428 | 0.0311629157178016 | 0.00929813615510324 | CASP8/CCL2 | 2 |
| temozolomide | temozolomide | 0.00738007380073801 | 14.499631 | 5.04979769579829 | 0.00792936562541214 | 0.0311629157178016 | 0.00929813615510324 | CASP8/CCL2 | 2 |
| dihydroergocristine | dihydroergocristine | 0.0072992700729927 | 14.3408759124088 | 5.01834698601158 | 0.00809961726486247 | 0.0311629157178016 | 0.00929813615510324 | IL1RN/CCL2 | 2 |
| 1,3-Dimethylthiourea | 1,3-Dimethylthiourea | 0.0625 | 122.79375 | 10.9979890283993 | 0.00811580970492387 | 0.0311629157178016 | 0.00929813615510324 | CCL2 | 1 |
| 3-amino-1,2,4-triazole | 3-amino-1,2,4-triazole | 0.0625 | 122.79375 | 10.9979890283993 | 0.00811580970492387 | 0.0311629157178016 | 0.00929813615510324 | CCL2 | 1 |
| CHLOROBENZENE | CHLOROBENZENE | 0.0625 | 122.79375 | 10.9979890283993 | 0.00811580970492387 | 0.0311629157178016 | 0.00929813615510324 | CCL2 | 1 |
| Dracorhodin | Dracorhodin | 0.0625 | 122.79375 | 10.9979890283993 | 0.00811580970492387 | 0.0311629157178016 | 0.00929813615510324 | CASP8 | 1 |
| etofenamate | etofenamate | 0.0625 | 122.79375 | 10.9979890283993 | 0.00811580970492387 | 0.0311629157178016 | 0.00929813615510324 | CCL2 | 1 |
| Octinoxate | Octinoxate | 0.0625 | 122.79375 | 10.9979890283993 | 0.00811580970492387 | 0.0311629157178016 | 0.00929813615510324 | CCL2 | 1 |
| pd 168393 | pd 168393 | 0.0625 | 122.79375 | 10.9979890283993 | 0.00811580970492387 | 0.0311629157178016 | 0.00929813615510324 | CCL2 | 1 |
| hydrocortisone | hydrocortisone | 0.00709219858156028 | 13.9340425531915 | 4.93684609883841 | 0.00856174780954528 | 0.0322131525689599 | 0.00961149724511185 | EGF/CCL2 | 2 |
| amprenavir | amprenavir | 0.0588235294117647 | 115.570588235294 | 10.6644129493784 | 0.00862107392493028 | 0.0322131525689599 | 0.00961149724511185 | CCL2 | 1 |
| calphostin C | calphostin C | 0.0588235294117647 | 115.570588235294 | 10.6644129493784 | 0.00862107392493028 | 0.0322131525689599 | 0.00961149724511185 | CCL2 | 1 |
| Diethylcarbamodithioic acid | Diethylcarbamodithioic acid | 0.0588235294117647 | 115.570588235294 | 10.6644129493784 | 0.00862107392493028 | 0.0322131525689599 | 0.00961149724511185 | CASP8 | 1 |
| NVP-AEW541 | NVP-AEW541 | 0.0588235294117647 | 115.570588235294 | 10.6644129493784 | 0.00862107392493028 | 0.0322131525689599 | 0.00961149724511185 | EGF | 1 |
| ABT-737 | ABT-737 | 0.0555555555555556 | 109.15 | 10.3588892187694 | 0.00912610649042989 | 0.0326940412930349 | 0.00975498089318876 | CASP8 | 1 |
| allopurinol | allopurinol | 0.0555555555555556 | 109.15 | 10.3588892187694 | 0.00912610649042989 | 0.0326940412930349 | 0.00975498089318876 | CCL2 | 1 |
| isosorbide | isosorbide | 0.0555555555555556 | 109.15 | 10.3588892187694 | 0.00912610649042989 | 0.0326940412930349 | 0.00975498089318876 | CCL2 | 1 |
| METHYL METHACRYLATE | METHYL METHACRYLATE | 0.0555555555555556 | 109.15 | 10.3588892187694 | 0.00912610649042989 | 0.0326940412930349 | 0.00975498089318876 | CCL2 | 1 |
| Mollugin | Mollugin | 0.0555555555555556 | 109.15 | 10.3588892187694 | 0.00912610649042989 | 0.0326940412930349 | 0.00975498089318876 | CASP8 | 1 |
| napelline | napelline | 0.0555555555555556 | 109.15 | 10.3588892187694 | 0.00912610649042989 | 0.0326940412930349 | 0.00975498089318876 | IL1RN | 1 |
| Pentetic acid | Pentetic acid | 0.0555555555555556 | 109.15 | 10.3588892187694 | 0.00912610649042989 | 0.0326940412930349 | 0.00975498089318876 | EGF | 1 |
| Perifosine | Perifosine | 0.0555555555555556 | 109.15 | 10.3588892187694 | 0.00912610649042989 | 0.0326940412930349 | 0.00975498089318876 | CASP8 | 1 |
| 2-arachidonoylglycerol | 2-arachidonoylglycerol | 0.0526315789473684 | 103.405263157895 | 10.0776797592044 | 0.00963090749583495 | 0.0328571103504653 | 0.00980363610608354 | CCL2 | 1 |
| clobenpropit | clobenpropit | 0.0526315789473684 | 103.405263157895 | 10.0776797592044 | 0.00963090749583495 | 0.0328571103504653 | 0.00980363610608354 | CCL2 | 1 |
| dequalinium | dequalinium | 0.0526315789473684 | 103.405263157895 | 10.0776797592044 | 0.00963090749583495 | 0.0328571103504653 | 0.00980363610608354 | CCL2 | 1 |
| EIPA | EIPA | 0.0526315789473684 | 103.405263157895 | 10.0776797592044 | 0.00963090749583495 | 0.0328571103504653 | 0.00980363610608354 | CCL2 | 1 |
| GW440139A | GW440139A | 0.0526315789473684 | 103.405263157895 | 10.0776797592044 | 0.00963090749583495 | 0.0328571103504653 | 0.00980363610608354 | EPHA4 | 1 |
| Methoxycamptothecin | Methoxycamptothecin | 0.0526315789473684 | 103.405263157895 | 10.0776797592044 | 0.00963090749583495 | 0.0328571103504653 | 0.00980363610608354 | CASP8 | 1 |
| repaglinide | repaglinide | 0.0526315789473684 | 103.405263157895 | 10.0776797592044 | 0.00963090749583495 | 0.0328571103504653 | 0.00980363610608354 | CCL2 | 1 |
| Rubitecan | Rubitecan | 0.0526315789473684 | 103.405263157895 | 10.0776797592044 | 0.00963090749583495 | 0.0328571103504653 | 0.00980363610608354 | CASP8 | 1 |
| Tosyllysyl chloromethane | Tosyllysyl chloromethane | 0.0526315789473684 | 103.405263157895 | 10.0776797592044 | 0.00963090749583495 | 0.0328571103504653 | 0.00980363610608354 | CCL2 | 1 |
| terfenadine | terfenadine | 0.00666666666666667 | 13.098 | 4.76504373959731 | 0.00964438922517254 | 0.0328571103504653 | 0.00980363610608354 | IL1RN/CASP8 | 2 |
| Goniothalamin | Goniothalamin | 0.05 | 98.235 | 9.8177089908821 | 0.0101354770355251 | 0.0338661372100478 | 0.0101047012955387 | CASP8 | 1 |
| Nimbolide | Nimbolide | 0.05 | 98.235 | 9.8177089908821 | 0.0101354770355251 | 0.0338661372100478 | 0.0101047012955387 | CASP8 | 1 |
| PERINDOPRIL | PERINDOPRIL | 0.05 | 98.235 | 9.8177089908821 | 0.0101354770355251 | 0.0338661372100478 | 0.0101047012955387 | CCL2 | 1 |
| Trifluridine | Trifluridine | 0.05 | 98.235 | 9.8177089908821 | 0.0101354770355251 | 0.0338661372100478 | 0.0101047012955387 | CASP8 | 1 |
| geldanamycin | geldanamycin | 0.00638977635782748 | 12.5539936102236 | 4.64989273373946 | 0.0104627689596778 | 0.034554540031183 | 0.0103101013042682 | FCGR2A/EGF | 2 |
| AlphaRedisol | AlphaRedisol | 0.0476190476190476 | 93.5571428571428 | 9.57642010702049 | 0.0106398152038463 | 0.034554540031183 | 0.0103101013042682 | EGF | 1 |
| FLAVANONE | FLAVANONE | 0.0476190476190476 | 93.5571428571428 | 9.57642010702049 | 0.0106398152038463 | 0.034554540031183 | 0.0103101013042682 | CASP8 | 1 |
| Garcinol | Garcinol | 0.0476190476190476 | 93.5571428571428 | 9.57642010702049 | 0.0106398152038463 | 0.034554540031183 | 0.0103101013042682 | CCL2 | 1 |
| GW559768X | GW559768X | 0.0476190476190476 | 93.5571428571428 | 9.57642010702049 | 0.0106398152038463 | 0.034554540031183 | 0.0103101013042682 | EPHA4 | 1 |
| Polydatin | Polydatin | 0.0476190476190476 | 93.5571428571428 | 9.57642010702049 | 0.0106398152038463 | 0.034554540031183 | 0.0103101013042682 | CCL2 | 1 |
| bucladesine | bucladesine | 0.00626959247648903 | 12.317868338558 | 4.59902764667095 | 0.0108506866037734 | 0.0350754753005698 | 0.010465533713309 | IL1RN/EGF | 2 |
| Fulvestrant | Fulvestrant | 0.00625 | 12.279375 | 4.5906829661731 | 0.0109159623624628 | 0.0351231196384797 | 0.0104797494415456 | EGF/CCL2 | 2 |
| gambogic acid | gambogic acid | 0.0454545454545455 | 89.3045454545455 | 9.35166771166649 | 0.0111439220951113 | 0.0352046629822834 | 0.0105040796781671 | CASP8 | 1 |
| probucol | probucol | 0.0454545454545455 | 89.3045454545455 | 9.35166771166649 | 0.0111439220951113 | 0.0352046629822834 | 0.0105040796781671 | CCL2 | 1 |
| Roflumilast | Roflumilast | 0.0454545454545455 | 89.3045454545455 | 9.35166771166649 | 0.0111439220951113 | 0.0352046629822834 | 0.0105040796781671 | CCL2 | 1 |
| Sirtinol | Sirtinol | 0.0454545454545455 | 89.3045454545455 | 9.35166771166649 | 0.0111439220951113 | 0.0352046629822834 | 0.0105040796781671 | CCL2 | 1 |
| celastrol | celastrol | 0.00604229607250755 | 11.8712990936556 | 4.50128325210869 | 0.0116456771018075 | 0.0361393726495561 | 0.0107829707110376 | IL1RN/CASP8 | 2 |
| Chonsurid | Chonsurid | 0.0434782608695652 | 85.4217391304348 | 9.14163642437205 | 0.0116477978035979 | 0.0361393726495561 | 0.0107829707110376 | CCL2 | 1 |
| MLN-8054 | MLN-8054 | 0.0434782608695652 | 85.4217391304348 | 9.14163642437205 | 0.0116477978035979 | 0.0361393726495561 | 0.0107829707110376 | EPHA4 | 1 |
| vinorelbine | vinorelbine | 0.0434782608695652 | 85.4217391304348 | 9.14163642437205 | 0.0116477978035979 | 0.0361393726495561 | 0.0107829707110376 | EGF | 1 |
| R-atenolol | R-atenolol | 0.00591715976331361 | 11.6254437869822 | 4.4465690081113 | 0.0121211280167757 | 0.0368788318094678 | 0.0110036044929423 | IL1RN/CCL2 | 2 |
| L-sorbose | L-sorbose | 0.0416666666666667 | 81.8625 | 8.94477834124591 | 0.0121514424235513 | 0.0368788318094678 | 0.0110036044929423 | CCL2 | 1 |
| Perfluoroheptanoic acid | Perfluoroheptanoic acid | 0.0416666666666667 | 81.8625 | 8.94477834124591 | 0.0121514424235513 | 0.0368788318094678 | 0.0110036044929423 | MBL2 | 1 |
| Prolinedithiocarbamate | Prolinedithiocarbamate | 0.0416666666666667 | 81.8625 | 8.94477834124591 | 0.0121514424235513 | 0.0368788318094678 | 0.0110036044929423 | CCL2 | 1 |
| S-1,2-Dichlorovinyl-N-acetylcysteine | S-1,2-Dichlorovinyl-N-acetylcysteine | 0.0416666666666667 | 81.8625 | 8.94477834124591 | 0.0121514424235513 | 0.0368788318094678 | 0.0110036044929423 | CASP8 | 1 |
| IRON | IRON | 0.00588235294117647 | 11.5570588235294 | 4.43123213329442 | 0.0122585454804127 | 0.0369543065301754 | 0.011026124023392 | CD58/CCL2 | 2 |
| ALW-II-49-7 | ALW-II-49-7 | 0.04 | 78.588 | 8.7597643978831 | 0.0126548560491824 | 0.0369543065301754 | 0.011026124023392 | EPHA4 | 1 |
| apicidin | apicidin | 0.04 | 78.588 | 8.7597643978831 | 0.0126548560491824 | 0.0369543065301754 | 0.011026124023392 | CASP8 | 1 |
| betazole | betazole | 0.04 | 78.588 | 8.7597643978831 | 0.0126548560491824 | 0.0369543065301754 | 0.011026124023392 | CD58 | 1 |
| EMETINE | EMETINE | 0.04 | 78.588 | 8.7597643978831 | 0.0126548560491824 | 0.0369543065301754 | 0.011026124023392 | CCL2 | 1 |
| GW768505A | GW768505A | 0.04 | 78.588 | 8.7597643978831 | 0.0126548560491824 | 0.0369543065301754 | 0.011026124023392 | EPHA4 | 1 |
| ICN 1229 | ICN 1229 | 0.04 | 78.588 | 8.7597643978831 | 0.0126548560491824 | 0.0369543065301754 | 0.011026124023392 | CASP8 | 1 |
| indinavir | indinavir | 0.04 | 78.588 | 8.7597643978831 | 0.0126548560491824 | 0.0369543065301754 | 0.011026124023392 | CCL2 | 1 |
| Pomalidomide | Pomalidomide | 0.04 | 78.588 | 8.7597643978831 | 0.0126548560491824 | 0.0369543065301754 | 0.011026124023392 | CCL2 | 1 |
| clomipramine | clomipramine | 0.00576368876080692 | 11.3239193083573 | 4.37854869656307 | 0.0127449902814327 | 0.0370617918225762 | 0.0110581946066604 | IL1RN/CCL2 | 2 |
| Alloxan | Alloxan | 0.0384615384615385 | 75.5653846153846 | 8.58544612351191 | 0.0131580387746697 | 0.0377885824313861 | 0.011275048449804 | CCL2 | 1 |
| dimethoate | dimethoate | 0.0384615384615385 | 75.5653846153846 | 8.58544612351191 | 0.0131580387746697 | 0.0377885824313861 | 0.011275048449804 | CCL2 | 1 |
| lonidamine | lonidamine | 0.0384615384615385 | 75.5653846153846 | 8.58544612351191 | 0.0131580387746697 | 0.0377885824313861 | 0.011275048449804 | CASP8 | 1 |
| methylergometrine | methylergometrine | 0.00564971751412429 | 11.1 | 4.32735597533669 | 0.0132399241131368 | 0.0378262491332216 | 0.0112862871325174 | IL1RN/CD58 | 2 |
| simvastatin | simvastatin | 0.00558659217877095 | 10.9759776536313 | 4.29874441130851 | 0.0135265341274958 | 0.0378262491332216 | 0.0112862871325174 | CASP8/CCL2 | 2 |
| acetylcholine | acetylcholine | 0.037037037037037 | 72.7666666666667 | 8.42082526047275 | 0.0136609906941563 | 0.0378262491332216 | 0.0112862871325174 | IL1RN | 1 |
| Acitretin | Acitretin | 0.037037037037037 | 72.7666666666667 | 8.42082526047275 | 0.0136609906941563 | 0.0378262491332216 | 0.0112862871325174 | CASP8 | 1 |
| CP-55940 | CP-55940 | 0.037037037037037 | 72.7666666666667 | 8.42082526047275 | 0.0136609906941563 | 0.0378262491332216 | 0.0112862871325174 | CCL2 | 1 |
| Indoxyl sulfate | Indoxyl sulfate | 0.037037037037037 | 72.7666666666667 | 8.42082526047275 | 0.0136609906941563 | 0.0378262491332216 | 0.0112862871325174 | CCL2 | 1 |
| Lonafarnib | Lonafarnib | 0.037037037037037 | 72.7666666666667 | 8.42082526047275 | 0.0136609906941563 | 0.0378262491332216 | 0.0112862871325174 | CASP8 | 1 |
| Nebivolol | Nebivolol | 0.037037037037037 | 72.7666666666667 | 8.42082526047275 | 0.0136609906941563 | 0.0378262491332216 | 0.0112862871325174 | CCL2 | 1 |
| Perfluorohexanesulfonic acid | Perfluorohexanesulfonic acid | 0.037037037037037 | 72.7666666666667 | 8.42082526047275 | 0.0136609906941563 | 0.0378262491332216 | 0.0112862871325174 | MBL2 | 1 |
| 15442-64-5 | 15442-64-5 | 0.0357142857142857 | 70.1678571428571 | 8.26502940611232 | 0.0141637119017535 | 0.0380336221329077 | 0.0113481613936885 | CASP8 | 1 |
| ALW-II-38-3 | ALW-II-38-3 | 0.0357142857142857 | 70.1678571428571 | 8.26502940611232 | 0.0141637119017535 | 0.0380336221329077 | 0.0113481613936885 | EPHA4 | 1 |
| Digoxigenin | Digoxigenin | 0.0357142857142857 | 70.1678571428571 | 8.26502940611232 | 0.0141637119017535 | 0.0380336221329077 | 0.0113481613936885 | CCL2 | 1 |
| gsno | gsno | 0.0357142857142857 | 70.1678571428571 | 8.26502940611232 | 0.0141637119017535 | 0.0380336221329077 | 0.0113481613936885 | CASP8 | 1 |
| Norcantharidin | Norcantharidin | 0.0357142857142857 | 70.1678571428571 | 8.26502940611232 | 0.0141637119017535 | 0.0380336221329077 | 0.0113481613936885 | CASP8 | 1 |
| NSC321521 | NSC321521 | 0.0357142857142857 | 70.1678571428571 | 8.26502940611232 | 0.0141637119017535 | 0.0380336221329077 | 0.0113481613936885 | CASP8 | 1 |
| Pemetrexed disodium | Pemetrexed disodium | 0.0357142857142857 | 70.1678571428571 | 8.26502940611232 | 0.0141637119017535 | 0.0380336221329077 | 0.0113481613936885 | EGF | 1 |
| Capsaicin | Capsaicin | 0.00543478260869565 | 10.6777173913043 | 4.22915977815669 | 0.0142550438116335 | 0.0380336221329077 | 0.0113481613936885 | CASP8/EGF | 2 |
| dimethyl sulfoxide | dimethyl sulfoxide | 0.00540540540540541 | 10.62 | 4.2155642242922 | 0.0144027902852705 | 0.0380336221329077 | 0.0113481613936885 | EGF/CCL2 | 2 |
| Compound C (Dorsomorphin) | Compound C (Dorsomorphin) | 0.0344827586206897 | 67.748275862069 | 8.11729231512528 | 0.0146662024915385 | 0.0380336221329077 | 0.0113481613936885 | EPHA4 | 1 |
| Epirubicin | Epirubicin | 0.0344827586206897 | 67.748275862069 | 8.11729231512528 | 0.0146662024915385 | 0.0380336221329077 | 0.0113481613936885 | CASP8 | 1 |
| formononetin | formononetin | 0.0344827586206897 | 67.748275862069 | 8.11729231512528 | 0.0146662024915385 | 0.0380336221329077 | 0.0113481613936885 | CASP8 | 1 |
| Lopinavir | Lopinavir | 0.0344827586206897 | 67.748275862069 | 8.11729231512528 | 0.0146662024915385 | 0.0380336221329077 | 0.0113481613936885 | CCL2 | 1 |
| Mangostin | Mangostin | 0.0344827586206897 | 67.748275862069 | 8.11729231512528 | 0.0146662024915385 | 0.0380336221329077 | 0.0113481613936885 | CASP8 | 1 |
| NG-25 | NG-25 | 0.0344827586206897 | 67.748275862069 | 8.11729231512528 | 0.0146662024915385 | 0.0380336221329077 | 0.0113481613936885 | EPHA4 | 1 |
| phosphatidylcholines | phosphatidylcholines | 0.0344827586206897 | 67.748275862069 | 8.11729231512528 | 0.0146662024915385 | 0.0380336221329077 | 0.0113481613936885 | CASP8 | 1 |
| ZIRAM | ZIRAM | 0.0344827586206897 | 67.748275862069 | 8.11729231512528 | 0.0146662024915385 | 0.0380336221329077 | 0.0113481613936885 | CASP8 | 1 |
| monocrotaline | monocrotaline | 0.0333333333333333 | 65.49 | 7.97693784384898 | 0.0151684625575533 | 0.0388964002667762 | 0.0116055900833849 | EGF | 1 |
| Perfluorodecanoic acid | Perfluorodecanoic acid | 0.0333333333333333 | 65.49 | 7.97693784384898 | 0.0151684625575533 | 0.0388964002667762 | 0.0116055900833849 | MBL2 | 1 |
| sulfapyridine | sulfapyridine | 0.0333333333333333 | 65.49 | 7.97693784384898 | 0.0151684625575533 | 0.0388964002667762 | 0.0116055900833849 | CCL2 | 1 |
| (-)-isoprenaline | (-)-isoprenaline | 0.00523560209424084 | 10.286387434555 | 4.13612326692597 | 0.0153034765650321 | 0.0388964002667762 | 0.0116055900833849 | IL1RN/CCL2 | 2 |
| Dienogest | Dienogest | 0.032258064516129 | 63.3774193548387 | 7.8433667665083 | 0.0156704921938091 | 0.0388964002667762 | 0.0116055900833849 | CCL2 | 1 |
| Digitoxigenin | Digitoxigenin | 0.032258064516129 | 63.3774193548387 | 7.8433667665083 | 0.0156704921938091 | 0.0388964002667762 | 0.0116055900833849 | CCL2 | 1 |
| fenvalerate | fenvalerate | 0.032258064516129 | 63.3774193548387 | 7.8433667665083 | 0.0156704921938091 | 0.0388964002667762 | 0.0116055900833849 | CCL2 | 1 |
| Honokiol | Honokiol | 0.032258064516129 | 63.3774193548387 | 7.8433667665083 | 0.0156704921938091 | 0.0388964002667762 | 0.0116055900833849 | CASP8 | 1 |
| L 741211 | L 741211 | 0.032258064516129 | 63.3774193548387 | 7.8433667665083 | 0.0156704921938091 | 0.0388964002667762 | 0.0116055900833849 | CCL2 | 1 |
| MANEB | MANEB | 0.032258064516129 | 63.3774193548387 | 7.8433667665083 | 0.0156704921938091 | 0.0388964002667762 | 0.0116055900833849 | CASP8 | 1 |
| stavudine | stavudine | 0.032258064516129 | 63.3774193548387 | 7.8433667665083 | 0.0156704921938091 | 0.0388964002667762 | 0.0116055900833849 | CCL2 | 1 |
| TAE684 | TAE684 | 0.032258064516129 | 63.3774193548387 | 7.8433667665083 | 0.0156704921938091 | 0.0388964002667762 | 0.0116055900833849 | EPHA4 | 1 |
| 7-Hydroxystaurosporine | 7-Hydroxystaurosporine | 0.03125 | 61.396875 | 7.71604587534867 | 0.0161722914942809 | 0.0392985587343692 | 0.0117255828408493 | CASP8 | 1 |
| chelerythrine | chelerythrine | 0.03125 | 61.396875 | 7.71604587534867 | 0.0161722914942809 | 0.0392985587343692 | 0.0117255828408493 | EGF | 1 |
| DEXMEDETOMIDINE | DEXMEDETOMIDINE | 0.03125 | 61.396875 | 7.71604587534867 | 0.0161722914942809 | 0.0392985587343692 | 0.0117255828408493 | CASP8 | 1 |
| METHOXYACETIC ACID | METHOXYACETIC ACID | 0.03125 | 61.396875 | 7.71604587534867 | 0.0161722914942809 | 0.0392985587343692 | 0.0117255828408493 | EGF | 1 |
| Poloxalene | Poloxalene | 0.03125 | 61.396875 | 7.71604587534867 | 0.0161722914942809 | 0.0392985587343692 | 0.0117255828408493 | EGF | 1 |
| sodium fluoride | sodium fluoride | 0.03125 | 61.396875 | 7.71604587534867 | 0.0161722914942809 | 0.0392985587343692 | 0.0117255828408493 | CASP8 | 1 |
| rapamycin | rapamycin | 0.0050761421319797 | 9.97309644670051 | 4.06013749509625 | 0.0162283256931855 | 0.0392985587343692 | 0.0117255828408493 | CASP8/EGF | 2 |
| 6-Deoxy-D-galactose | 6-Deoxy-D-galactose | 0.0303030303030303 | 59.5363636363636 | 7.59449891130005 | 0.016673860552913 | 0.0395298893831332 | 0.0117946053895907 | EGF | 1 |
| CADMIUM SELENIDE | CADMIUM SELENIDE | 0.0303030303030303 | 59.5363636363636 | 7.59449891130005 | 0.016673860552913 | 0.0395298893831332 | 0.0117946053895907 | CCL2 | 1 |
| danthron | danthron | 0.0303030303030303 | 59.5363636363636 | 7.59449891130005 | 0.016673860552913 | 0.0395298893831332 | 0.0117946053895907 | CASP8 | 1 |
| juglone | juglone | 0.0303030303030303 | 59.5363636363636 | 7.59449891130005 | 0.016673860552913 | 0.0395298893831332 | 0.0117946053895907 | CASP8 | 1 |
| lithocholic acid | lithocholic acid | 0.0303030303030303 | 59.5363636363636 | 7.59449891130005 | 0.016673860552913 | 0.0395298893831332 | 0.0117946053895907 | CCL2 | 1 |
| rosavin | rosavin | 0.0303030303030303 | 59.5363636363636 | 7.59449891130005 | 0.016673860552913 | 0.0395298893831332 | 0.0117946053895907 | EGF | 1 |
| mifepristone | mifepristone | 0.00498753117206983 | 9.79900249376559 | 4.01730574805729 | 0.0167788739108263 | 0.0395298893831332 | 0.0117946053895907 | CASP8/EGF | 2 |
| NICKEL CHLORIDE | NICKEL CHLORIDE | 0.00498753117206983 | 9.79900249376559 | 4.01730574805729 | 0.0167788739108263 | 0.0395298893831332 | 0.0117946053895907 | EGF/CCL2 | 2 |
| BISPHENOL A DIGLYCIDYL ETHER | BISPHENOL A DIGLYCIDYL ETHER | 0.0294117647058824 | 57.7852941176471 | 7.47829897258258 | 0.0171751994636122 | 0.0396570220173106 | 0.0118325381861571 | CCL2 | 1 |
| MANCOZEB | MANCOZEB | 0.0294117647058824 | 57.7852941176471 | 7.47829897258258 | 0.0171751994636122 | 0.0396570220173106 | 0.0118325381861571 | CASP8 | 1 |
| Pemetrexed | Pemetrexed | 0.0294117647058824 | 57.7852941176471 | 7.47829897258258 | 0.0171751994636122 | 0.0396570220173106 | 0.0118325381861571 | CCL2 | 1 |
| POLY I-C | POLY I-C | 0.0294117647058824 | 57.7852941176471 | 7.47829897258258 | 0.0171751994636122 | 0.0396570220173106 | 0.0118325381861571 | CASP8 | 1 |
| riluzole | riluzole | 0.0294117647058824 | 57.7852941176471 | 7.47829897258258 | 0.0171751994636122 | 0.0396570220173106 | 0.0118325381861571 | CASP8 | 1 |
| Xanthohumol | Xanthohumol | 0.0294117647058824 | 57.7852941176471 | 7.47829897258258 | 0.0171751994636122 | 0.0396570220173106 | 0.0118325381861571 | CASP8 | 1 |
| amsacrine | amsacrine | 0.0285714285714286 | 56.1342857142857 | 7.36706212477822 | 0.0176763083202567 | 0.0398864749434363 | 0.0119010004753608 | EGF | 1 |
| fludroxycortide | fludroxycortide | 0.0285714285714286 | 56.1342857142857 | 7.36706212477822 | 0.0176763083202567 | 0.0398864749434363 | 0.0119010004753608 | CCL2 | 1 |
| Luronit | Luronit | 0.0285714285714286 | 56.1342857142857 | 7.36706212477822 | 0.0176763083202567 | 0.0398864749434363 | 0.0119010004753608 | CASP8 | 1 |
| Methylbenzethonium chloride | Methylbenzethonium chloride | 0.0285714285714286 | 56.1342857142857 | 7.36706212477822 | 0.0176763083202567 | 0.0398864749434363 | 0.0119010004753608 | CCL2 | 1 |
| oxozinc | oxozinc | 0.0285714285714286 | 56.1342857142857 | 7.36706212477822 | 0.0176763083202567 | 0.0398864749434363 | 0.0119010004753608 | CCL2 | 1 |
| Pregna-4,17(20)-diene-3,16-dione | Pregna-4,17(20)-diene-3,16-dione | 0.0285714285714286 | 56.1342857142857 | 7.36706212477822 | 0.0176763083202567 | 0.0398864749434363 | 0.0119010004753608 | CASP8 | 1 |
| pyrogallol | pyrogallol | 0.0285714285714286 | 56.1342857142857 | 7.36706212477822 | 0.0176763083202567 | 0.0398864749434363 | 0.0119010004753608 | CASP8 | 1 |
| Destruxin B | Destruxin B | 0.0277777777777778 | 54.575 | 7.26044199390408 | 0.0181771872166872 | 0.0404908497294794 | 0.0120813288855849 | CASP8 | 1 |
| TAXIFOLIN | TAXIFOLIN | 0.0277777777777778 | 54.575 | 7.26044199390408 | 0.0181771872166872 | 0.0404908497294794 | 0.0120813288855849 | EGF | 1 |
| trolox | trolox | 0.0277777777777778 | 54.575 | 7.26044199390408 | 0.0181771872166872 | 0.0404908497294794 | 0.0120813288855849 | CASP8 | 1 |
| uridine triphosphate | uridine triphosphate | 0.0277777777777778 | 54.575 | 7.26044199390408 | 0.0181771872166872 | 0.0404908497294794 | 0.0120813288855849 | EGF | 1 |
| 2,2',5,5'-TETRACHLOROBIPHENYL | 2,2',5,5'-TETRACHLOROBIPHENYL | 0.027027027027027 | 53.1 | 7.15812516861471 | 0.0186778362467128 | 0.0412098291792553 | 0.0122958520989221 | IL1RN | 1 |
| bupropion | bupropion | 0.027027027027027 | 53.1 | 7.15812516861471 | 0.0186778362467128 | 0.0412098291792553 | 0.0122958520989221 | CASP8 | 1 |
| dioxidanide | dioxidanide | 0.027027027027027 | 53.1 | 7.15812516861471 | 0.0186778362467128 | 0.0412098291792553 | 0.0122958520989221 | CCL2 | 1 |
| 2-Naphthoxyacetic acid | 2-Naphthoxyacetic acid | 0.0263157894736842 | 51.7026315789474 | 7.05982727220896 | 0.019178255504109 | 0.0418944049858861 | 0.0125001102343348 | CASP8 | 1 |
| adenosine | adenosine | 0.0263157894736842 | 51.7026315789474 | 7.05982727220896 | 0.019178255504109 | 0.0418944049858861 | 0.0125001102343348 | CASP8 | 1 |
| calcium folinate | calcium folinate | 0.0263157894736842 | 51.7026315789474 | 7.05982727220896 | 0.019178255504109 | 0.0418944049858861 | 0.0125001102343348 | EGF | 1 |
| 15-delta prostaglandin J2 | 15-delta prostaglandin J2 | 0.00464037122969838 | 9.1169373549884 | 3.8450220440967 | 0.0192292304899247 | 0.0418944049858861 | 0.0125001102343348 | EPHA4/CCL2 | 2 |
| Antimycin A | Antimycin A | 0.0256410256410256 | 50.3769230769231 | 6.96528959208597 | 0.0196784450826167 | 0.0423421651158472 | 0.0126337092431986 | CCL2 | 1 |
| C2-ceramide | C2-ceramide | 0.0256410256410256 | 50.3769230769231 | 6.96528959208597 | 0.0196784450826167 | 0.0423421651158472 | 0.0126337092431986 | EGF | 1 |
| Psoralen | Psoralen | 0.0256410256410256 | 50.3769230769231 | 6.96528959208597 | 0.0196784450826167 | 0.0423421651158472 | 0.0126337092431986 | CASP8 | 1 |
| ribavirin | ribavirin | 0.0256410256410256 | 50.3769230769231 | 6.96528959208597 | 0.0196784450826167 | 0.0423421651158472 | 0.0126337092431986 | CCL2 | 1 |
| 124020-07-1 | 124020-07-1 | 0.025 | 49.1175 | 6.87427617549601 | 0.0201784050759447 | 0.0428868242439803 | 0.012796220183361 | CCL2 | 1 |
| bisindolylmaleimide IX | bisindolylmaleimide IX | 0.025 | 49.1175 | 6.87427617549601 | 0.0201784050759447 | 0.0428868242439803 | 0.012796220183361 | CASP8 | 1 |
| DMA-4 herbicide | DMA-4 herbicide | 0.025 | 49.1175 | 6.87427617549601 | 0.0201784050759447 | 0.0428868242439803 | 0.012796220183361 | CASP8 | 1 |
| SB 415286 | SB 415286 | 0.025 | 49.1175 | 6.87427617549601 | 0.0201784050759447 | 0.0428868242439803 | 0.012796220183361 | CASP8 | 1 |
| 4,5,6,7-tetrabromobenzotriazole | 4,5,6,7-tetrabromobenzotriazole | 0.024390243902439 | 47.919512195122 | 6.78657131720972 | 0.0206781355777673 | 0.0428994156016367 | 0.0127999770897727 | CASP8 | 1 |
| asparagine | asparagine | 0.024390243902439 | 47.919512195122 | 6.78657131720972 | 0.0206781355777673 | 0.0428994156016367 | 0.0127999770897727 | EGF | 1 |
| Cladribine | Cladribine | 0.024390243902439 | 47.919512195122 | 6.78657131720972 | 0.0206781355777673 | 0.0428994156016367 | 0.0127999770897727 | CASP8 | 1 |
| Dacinostat | Dacinostat | 0.024390243902439 | 47.919512195122 | 6.78657131720972 | 0.0206781355777673 | 0.0428994156016367 | 0.0127999770897727 | CASP8 | 1 |
| Esculetin | Esculetin | 0.024390243902439 | 47.919512195122 | 6.78657131720972 | 0.0206781355777673 | 0.0428994156016367 | 0.0127999770897727 | CASP8 | 1 |
| MLN8054 | MLN8054 | 0.024390243902439 | 47.919512195122 | 6.78657131720972 | 0.0206781355777673 | 0.0428994156016367 | 0.0127999770897727 | EPHA4 | 1 |
| niflumic acid | niflumic acid | 0.024390243902439 | 47.919512195122 | 6.78657131720972 | 0.0206781355777673 | 0.0428994156016367 | 0.0127999770897727 | CASP8 | 1 |
| Regorafenib | Regorafenib | 0.024390243902439 | 47.919512195122 | 6.78657131720972 | 0.0206781355777673 | 0.0428994156016367 | 0.0127999770897727 | EPHA4 | 1 |
| cyclophosphamide | cyclophosphamide | 0.00441501103752759 | 8.67417218543046 | 3.7290430302071 | 0.0211181085130883 | 0.0432895808641178 | 0.012916391412694 | CASP8/EGF | 2 |
| Acteoside | Acteoside | 0.0238095238095238 | 46.7785714285714 | 6.70197737809121 | 0.0211776366817267 | 0.0432895808641178 | 0.012916391412694 | CCL2 | 1 |
| amlodipine | amlodipine | 0.0238095238095238 | 46.7785714285714 | 6.70197737809121 | 0.0211776366817267 | 0.0432895808641178 | 0.012916391412694 | CASP8 | 1 |
| Aziridine | Aziridine | 0.0238095238095238 | 46.7785714285714 | 6.70197737809121 | 0.0211776366817267 | 0.0432895808641178 | 0.012916391412694 | EGF | 1 |
| RUTIN | RUTIN | 0.0238095238095238 | 46.7785714285714 | 6.70197737809121 | 0.0211776366817267 | 0.0432895808641178 | 0.012916391412694 | CCL2 | 1 |
| 9,12-Octadecadienoic acid | 9,12-Octadecadienoic acid | 0.0232558139534884 | 45.6906976744186 | 6.62031288426516 | 0.0216769084814299 | 0.0436679750567935 | 0.0130292935799899 | CCL2 | 1 |
| Afatinib | Afatinib | 0.0232558139534884 | 45.6906976744186 | 6.62031288426516 | 0.0216769084814299 | 0.0436679750567935 | 0.0130292935799899 | EGF | 1 |
| Andrographolide | Andrographolide | 0.0232558139534884 | 45.6906976744186 | 6.62031288426516 | 0.0216769084814299 | 0.0436679750567935 | 0.0130292935799899 | CASP8 | 1 |
| Gadodiamide hydrate | Gadodiamide hydrate | 0.0232558139534884 | 45.6906976744186 | 6.62031288426516 | 0.0216769084814299 | 0.0436679750567935 | 0.0130292935799899 | CCL2 | 1 |
| halcinonide | halcinonide | 0.0232558139534884 | 45.6906976744186 | 6.62031288426516 | 0.0216769084814299 | 0.0436679750567935 | 0.0130292935799899 | CCL2 | 1 |
| Zoledronic acid | Zoledronic acid | 0.00432900432900433 | 8.5051948051948 | 3.68384675069089 | 0.0219128322353364 | 0.0438968282922613 | 0.0130975769383953 | CASP8/CCL2 | 2 |
| Acetovanillone | Acetovanillone | 0.0227272727272727 | 44.6522727272727 | 6.54141086519275 | 0.0221759510704521 | 0.0438968282922613 | 0.0130975769383953 | CCL2 | 1 |
| betamethasone | betamethasone | 0.0227272727272727 | 44.6522727272727 | 6.54141086519275 | 0.0221759510704521 | 0.0438968282922613 | 0.0130975769383953 | CCL2 | 1 |
| Rhein | Rhein | 0.0227272727272727 | 44.6522727272727 | 6.54141086519275 | 0.0221759510704521 | 0.0438968282922613 | 0.0130975769383953 | EGF | 1 |
| STYRENE | STYRENE | 0.0227272727272727 | 44.6522727272727 | 6.54141086519275 | 0.0221759510704521 | 0.0438968282922613 | 0.0130975769383953 | CCL2 | 1 |
| Vorinostat | Vorinostat | 0.00426439232409382 | 8.37825159914712 | 3.64953661034737 | 0.0225396775887956 | 0.0438968282922613 | 0.0130975769383953 | CASP8/EGF | 2 |
| 139890-68-9 | 139890-68-9 | 0.0222222222222222 | 43.66 | 6.46511739595777 | 0.0226747645423335 | 0.0438968282922613 | 0.0130975769383953 | EGF | 1 |
| 2,2',3,4,4',5,5'-HEPTACHLOROBIPHENYL | 2,2',3,4,4',5,5'-HEPTACHLOROBIPHENYL | 0.0222222222222222 | 43.66 | 6.46511739595777 | 0.0226747645423335 | 0.0438968282922613 | 0.0130975769383953 | IL1RN | 1 |
| 22-Hydroxycholesterol | 22-Hydroxycholesterol | 0.0222222222222222 | 43.66 | 6.46511739595777 | 0.0226747645423335 | 0.0438968282922613 | 0.0130975769383953 | CCL2 | 1 |
| alclometasone | alclometasone | 0.0222222222222222 | 43.66 | 6.46511739595777 | 0.0226747645423335 | 0.0438968282922613 | 0.0130975769383953 | CCL2 | 1 |
| Bromoacetate | Bromoacetate | 0.0222222222222222 | 43.66 | 6.46511739595777 | 0.0226747645423335 | 0.0438968282922613 | 0.0130975769383953 | CASP8 | 1 |
| Lysergide | Lysergide | 0.0222222222222222 | 43.66 | 6.46511739595777 | 0.0226747645423335 | 0.0438968282922613 | 0.0130975769383953 | EGF | 1 |
| methimazole | methimazole | 0.0222222222222222 | 43.66 | 6.46511739595777 | 0.0226747645423335 | 0.0438968282922613 | 0.0130975769383953 | CCL2 | 1 |
| ramipril | ramipril | 0.0222222222222222 | 43.66 | 6.46511739595777 | 0.0226747645423335 | 0.0438968282922613 | 0.0130975769383953 | CCL2 | 1 |
| 2-[6-fluoro-2-methyl-3-[(4-methylsulfonylphenyl)methylidene]inden-1-yl]acetic acid | 2-[6-fluoro-2-methyl-3-[(4-methylsulfonylphenyl)methylidene]inden-1-yl]acetic acid | 0.0217391304347826 | 42.7108695652174 | 6.39129031475235 | 0.0231733489905827 | 0.0442458174408103 | 0.0132017054688976 | CASP8 | 1 |
| EXEMESTANE | EXEMESTANE | 0.0217391304347826 | 42.7108695652174 | 6.39129031475235 | 0.0231733489905827 | 0.0442458174408103 | 0.0132017054688976 | EGF | 1 |
| methoxsalen | methoxsalen | 0.0217391304347826 | 42.7108695652174 | 6.39129031475235 | 0.0231733489905827 | 0.0442458174408103 | 0.0132017054688976 | CASP8 | 1 |
| pomiferin | pomiferin | 0.0217391304347826 | 42.7108695652174 | 6.39129031475235 | 0.0231733489905827 | 0.0442458174408103 | 0.0132017054688976 | EGF | 1 |
| Salicylate, sodium | Salicylate, sodium | 0.0217391304347826 | 42.7108695652174 | 6.39129031475235 | 0.0231733489905827 | 0.0442458174408103 | 0.0132017054688976 | EGF | 1 |
| 2-Mercaptobenzothiazole | 2-Mercaptobenzothiazole | 0.0212765957446809 | 41.8021276595745 | 6.31979809120605 | 0.0236717045086728 | 0.0445849177060369 | 0.0133028834351977 | CCL2 | 1 |
| chlorambucil | chlorambucil | 0.0212765957446809 | 41.8021276595745 | 6.31979809120605 | 0.0236717045086728 | 0.0445849177060369 | 0.0133028834351977 | CASP8 | 1 |
| loratadine | loratadine | 0.0212765957446809 | 41.8021276595745 | 6.31979809120605 | 0.0236717045086728 | 0.0445849177060369 | 0.0133028834351977 | CASP8 | 1 |
| T-2 TOXIN | T-2 TOXIN | 0.0212765957446809 | 41.8021276595745 | 6.31979809120605 | 0.0236717045086728 | 0.0445849177060369 | 0.0133028834351977 | ACAN | 1 |
| TOSYLPHENYLALANYL CHLOROMETHYL KETONE | TOSYLPHENYLALANYL CHLOROMETHYL KETONE | 0.0212765957446809 | 41.8021276595745 | 6.31979809120605 | 0.0236717045086728 | 0.0445849177060369 | 0.0133028834351977 | CCL2 | 1 |
| fluorometholone | fluorometholone | 0.0204081632653061 | 40.0959183673469 | 6.18333935758382 | 0.0246677291281094 | 0.0457175246507628 | 0.0136408214406668 | CCL2 | 1 |
| Osajin | Osajin | 0.0204081632653061 | 40.0959183673469 | 6.18333935758382 | 0.0246677291281094 | 0.0457175246507628 | 0.0136408214406668 | EGF | 1 |
| p-benzoquinone | p-benzoquinone | 0.0204081632653061 | 40.0959183673469 | 6.18333935758382 | 0.0246677291281094 | 0.0457175246507628 | 0.0136408214406668 | CCL2 | 1 |
| Platelet activating factor | Platelet activating factor | 0.0204081632653061 | 40.0959183673469 | 6.18333935758382 | 0.0246677291281094 | 0.0457175246507628 | 0.0136408214406668 | EGF | 1 |
| spironolactone | spironolactone | 0.0204081632653061 | 40.0959183673469 | 6.18333935758382 | 0.0246677291281094 | 0.0457175246507628 | 0.0136408214406668 | CCL2 | 1 |
| Ursolic acid | Ursolic acid | 0.0204081632653061 | 40.0959183673469 | 6.18333935758382 | 0.0246677291281094 | 0.0457175246507628 | 0.0136408214406668 | CASP8 | 1 |
| Cabozantinib | Cabozantinib | 0.02 | 39.294 | 6.11815448167157 | 0.0251653984162368 | 0.0463924453561925 | 0.0138421987658764 | EPHA4 | 1 |
| Leptomycin B | Leptomycin B | 0.02 | 39.294 | 6.11815448167157 | 0.0251653984162368 | 0.0463924453561925 | 0.0138421987658764 | CCL2 | 1 |
| DEHP | DEHP | 0.0196078431372549 | 38.5235294117647 | 6.05486623689331 | 0.025662839147769 | 0.0468127905713897 | 0.0139676179364843 | CASP8 | 1 |
| flumetasone | flumetasone | 0.0196078431372549 | 38.5235294117647 | 6.05486623689331 | 0.025662839147769 | 0.0468127905713897 | 0.0139676179364843 | CCL2 | 1 |
| OLEANOLIC ACID | OLEANOLIC ACID | 0.0196078431372549 | 38.5235294117647 | 6.05486623689331 | 0.025662839147769 | 0.0468127905713897 | 0.0139676179364843 | CASP8 | 1 |
| triamcinolone | triamcinolone | 0.0196078431372549 | 38.5235294117647 | 6.05486623689331 | 0.025662839147769 | 0.0468127905713897 | 0.0139676179364843 | CCL2 | 1 |
| choline | choline | 0.0192307692307692 | 37.7826923076923 | 5.99338327990092 | 0.0261600514160136 | 0.0469799372974922 | 0.0140174898108383 | CCL2 | 1 |
| dacarbazine | dacarbazine | 0.0192307692307692 | 37.7826923076923 | 5.99338327990092 | 0.0261600514160136 | 0.0469799372974922 | 0.0140174898108383 | CASP8 | 1 |
| fludrocortisone | fludrocortisone | 0.0192307692307692 | 37.7826923076923 | 5.99338327990092 | 0.0261600514160136 | 0.0469799372974922 | 0.0140174898108383 | CCL2 | 1 |
| harmaline | harmaline | 0.0192307692307692 | 37.7826923076923 | 5.99338327990092 | 0.0261600514160136 | 0.0469799372974922 | 0.0140174898108383 | CCL2 | 1 |
| nevirapine | nevirapine | 0.0192307692307692 | 37.7826923076923 | 5.99338327990092 | 0.0261600514160136 | 0.0469799372974922 | 0.0140174898108383 | EGF | 1 |
| Zebularine | Zebularine | 0.0192307692307692 | 37.7826923076923 | 5.99338327990092 | 0.0261600514160136 | 0.0469799372974922 | 0.0140174898108383 | CASP8 | 1 |
| busulfan | busulfan | 0.0188679245283019 | 37.0698113207547 | 5.93362032027531 | 0.026657035314245 | 0.0475042039574366 | 0.0141739162129724 | CASP8 | 1 |
| CHEBI:18224 | CHEBI:18224 | 0.0188679245283019 | 37.0698113207547 | 5.93362032027531 | 0.026657035314245 | 0.0475042039574366 | 0.0141739162129724 | CCL2 | 1 |
| O-Phospho-L-tyrosine | O-Phospho-L-tyrosine | 0.0188679245283019 | 37.0698113207547 | 5.93362032027531 | 0.026657035314245 | 0.0475042039574366 | 0.0141739162129724 | EGF | 1 |
| cadmium acetate | cadmium acetate | 0.0185185185185185 | 36.3833333333333 | 5.87549761410897 | 0.0271537909357032 | 0.0478981845185628 | 0.014291468913528 | CASP8 | 1 |
| HELENALIN | HELENALIN | 0.0185185185185185 | 36.3833333333333 | 5.87549761410897 | 0.0271537909357032 | 0.0478981845185628 | 0.014291468913528 | CASP8 | 1 |
| p,p'-DDE | p,p'-DDE | 0.0185185185185185 | 36.3833333333333 | 5.87549761410897 | 0.0271537909357032 | 0.0478981845185628 | 0.014291468913528 | CASP8 | 1 |
| suramin | suramin | 0.0185185185185185 | 36.3833333333333 | 5.87549761410897 | 0.0271537909357032 | 0.0478981845185628 | 0.014291468913528 | EGF | 1 |
| Chalcone | Chalcone | 0.0181818181818182 | 35.7218181818182 | 5.81894050844202 | 0.0276503183735965 | 0.0485277052263879 | 0.0144793001584224 | CASP8 | 1 |
| IN1152 | IN1152 | 0.0181818181818182 | 35.7218181818182 | 5.81894050844202 | 0.0276503183735965 | 0.0485277052263879 | 0.0144793001584224 | CASP8 | 1 |
| AZ-628 | AZ-628 | 0.0178571428571429 | 35.0839285714286 | 5.76387903062378 | 0.0281466177210988 | 0.0486614410849843 | 0.014519203171135 | EPHA4 | 1 |
| beclometasone | beclometasone | 0.0178571428571429 | 35.0839285714286 | 5.76387903062378 | 0.0281466177210988 | 0.0486614410849843 | 0.014519203171135 | CCL2 | 1 |
| isopropanol | isopropanol | 0.0178571428571429 | 35.0839285714286 | 5.76387903062378 | 0.0281466177210988 | 0.0486614410849843 | 0.014519203171135 | CCL2 | 1 |
| methoxychlor | methoxychlor | 0.0178571428571429 | 35.0839285714286 | 5.76387903062378 | 0.0281466177210988 | 0.0486614410849843 | 0.014519203171135 | CCL2 | 1 |
| montelukast | montelukast | 0.0178571428571429 | 35.0839285714286 | 5.76387903062378 | 0.0281466177210988 | 0.0486614410849843 | 0.014519203171135 | CCL2 | 1 |
| Tanespimycin | Tanespimycin | 0.0178571428571429 | 35.0839285714286 | 5.76387903062378 | 0.0281466177210988 | 0.0486614410849843 | 0.014519203171135 | CASP8 | 1 |
| 105156-22-7 | 105156-22-7 | 0.0175438596491228 | 34.4684210526316 | 5.71024751745572 | 0.0286426890713506 | 0.0492739329321501 | 0.0147019534837313 | CCL2 | 1 |
| Lenalidomide | Lenalidomide | 0.0175438596491228 | 34.4684210526316 | 5.71024751745572 | 0.0286426890713506 | 0.0492739329321501 | 0.0147019534837313 | CASP8 | 1 |
| 3-Butylidenephthalide | 3-Butylidenephthalide | 0.0172413793103448 | 33.8741379310345 | 5.65798427963904 | 0.0291385325174603 | 0.0493902469208366 | 0.0147366583012568 | CASP8 | 1 |
| alpha-Tocopherol | alpha-Tocopherol | 0.0172413793103448 | 33.8741379310345 | 5.65798427963904 | 0.0291385325174603 | 0.0493902469208366 | 0.0147366583012568 | CASP8 | 1 |
| Alvocidib | Alvocidib | 0.0172413793103448 | 33.8741379310345 | 5.65798427963904 | 0.0291385325174603 | 0.0493902469208366 | 0.0147366583012568 | CASP8 | 1 |
| Fisetin | Fisetin | 0.0172413793103448 | 33.8741379310345 | 5.65798427963904 | 0.0291385325174603 | 0.0493902469208366 | 0.0147366583012568 | CASP8 | 1 |
| Melitten | Melitten | 0.0172413793103448 | 33.8741379310345 | 5.65798427963904 | 0.0291385325174603 | 0.0493902469208366 | 0.0147366583012568 | CASP8 | 1 |
| TACROLIMUS MONOHYDRATE | TACROLIMUS MONOHYDRATE | 0.0172413793103448 | 33.8741379310345 | 5.65798427963904 | 0.0291385325174603 | 0.0493902469208366 | 0.0147366583012568 | CASP8 | 1 |
| Caffeic acid phenethyl ester | Caffeic acid phenethyl ester | 0.0169491525423729 | 33.3 | 5.60703129762205 | 0.029634148152502 | 0.0493902469208366 | 0.0147366583012568 | CCL2 | 1 |
| cilostazol | cilostazol | 0.0169491525423729 | 33.3 | 5.60703129762205 | 0.029634148152502 | 0.0493902469208366 | 0.0147366583012568 | CCL2 | 1 |
| DL-Homocysteine | DL-Homocysteine | 0.0169491525423729 | 33.3 | 5.60703129762205 | 0.029634148152502 | 0.0493902469208366 | 0.0147366583012568 | CCL2 | 1 |
| flunisolide | flunisolide | 0.0169491525423729 | 33.3 | 5.60703129762205 | 0.029634148152502 | 0.0493902469208366 | 0.0147366583012568 | CCL2 | 1 |
| INDIRUBIN-3'-MONOXIME | INDIRUBIN-3'-MONOXIME | 0.0169491525423729 | 33.3 | 5.60703129762205 | 0.029634148152502 | 0.0493902469208366 | 0.0147366583012568 | CASP8 | 1 |
| naloxone | naloxone | 0.0169491525423729 | 33.3 | 5.60703129762205 | 0.029634148152502 | 0.0493902469208366 | 0.0147366583012568 | CCL2 | 1 |
| Tyrphostin B42 | Tyrphostin B42 | 0.0169491525423729 | 33.3 | 5.60703129762205 | 0.029634148152502 | 0.0493902469208366 | 0.0147366583012568 | CCL2 | 1 |
| 2,4-Dinitrofluorobenzene | 2,4-Dinitrofluorobenzene | 0.0166666666666667 | 32.745 | 5.55733394543229 | 0.0301295360695158 | 0.0498572084959846 | 0.0148759864804376 | CCL2 | 1 |
| FERROUS SULFATE | FERROUS SULFATE | 0.0166666666666667 | 32.745 | 5.55733394543229 | 0.0301295360695158 | 0.0498572084959846 | 0.0148759864804376 | IL1RN | 1 |
| VERAPAMIL | VERAPAMIL | 0.0166666666666667 | 32.745 | 5.55733394543229 | 0.0301295360695158 | 0.0498572084959846 | 0.0148759864804376 | CASP8 | 1 |
