# Supplementary material for: Causal Association Between Plasma Proteins and Pericarditis: A Mendelian Randomization Study With Therapeutic Target Identification
Source: Mediators Inflamm. 2026 Feb 9;2026:4659271. doi: 10.1155/mi/4659271 (PMC12887433; doi:10.1155/mi/4659271)
Supplement: Supplementary file 5 — Supporting Information 5 Figure S1. Scatter plots results of MR analysis between plasmas proteins and Pericarditis [file MI-2026-4659271-s004.pdf]

Figure S1. Scatter plots results of MR analysis between plasmas proteins and Pericarditis

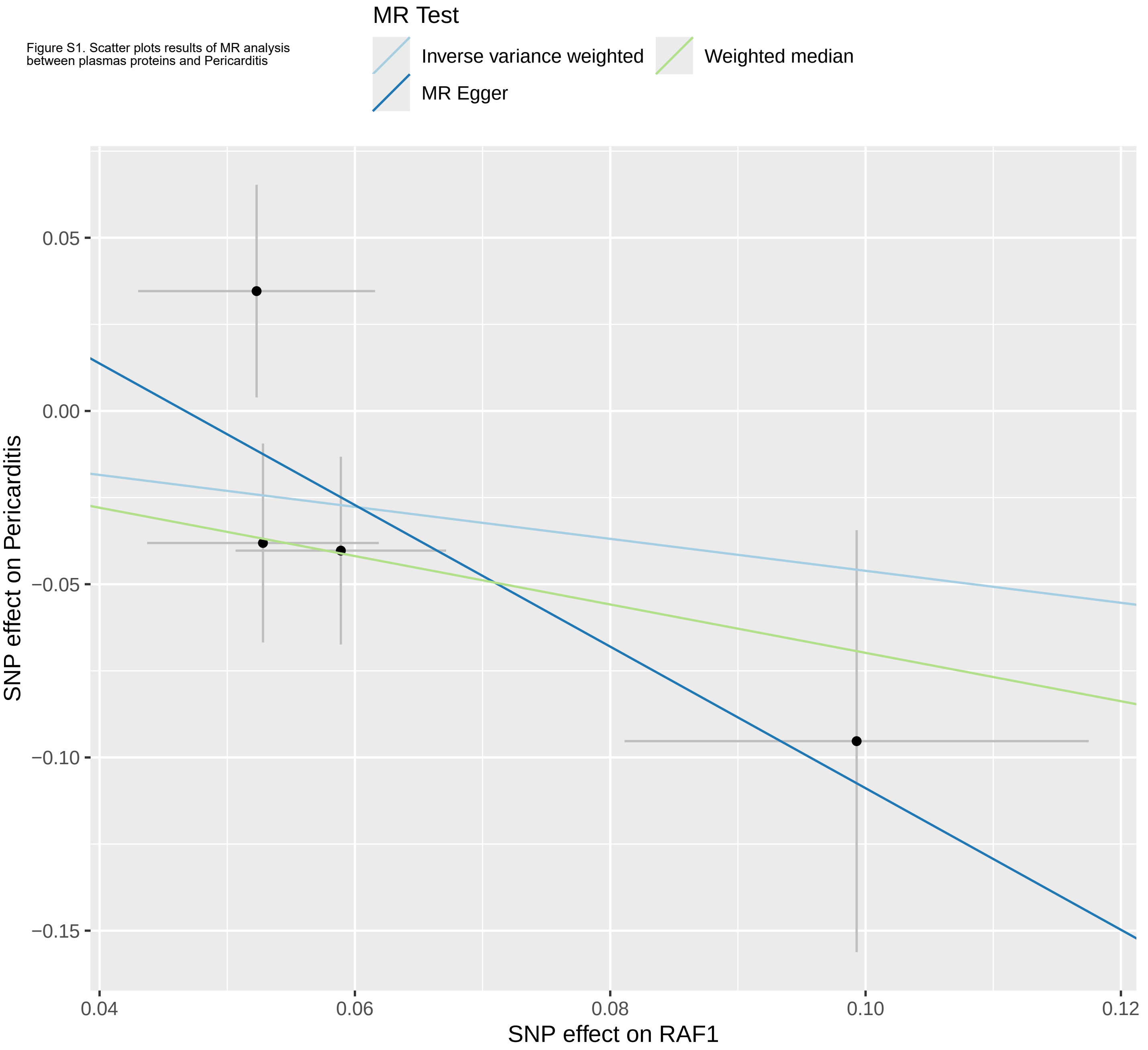

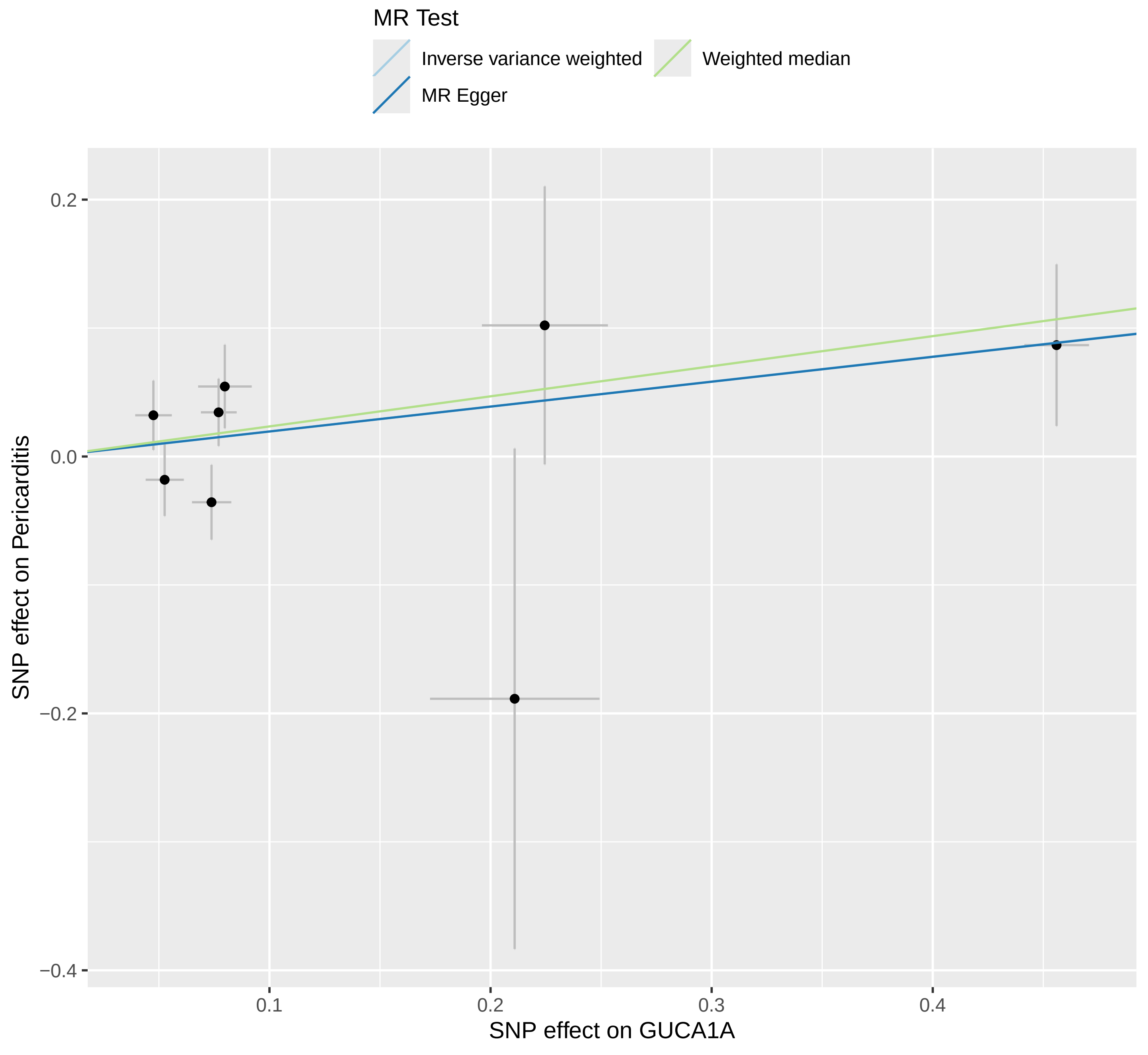
MR Test
Inverse variance weighted
Weighted median
MR Egger
0.2
0.0
−0.2
−0.4
0.1
0.2
0.3
0.4
SNP effect on Pericarditis
SNP effect on GUCA1A

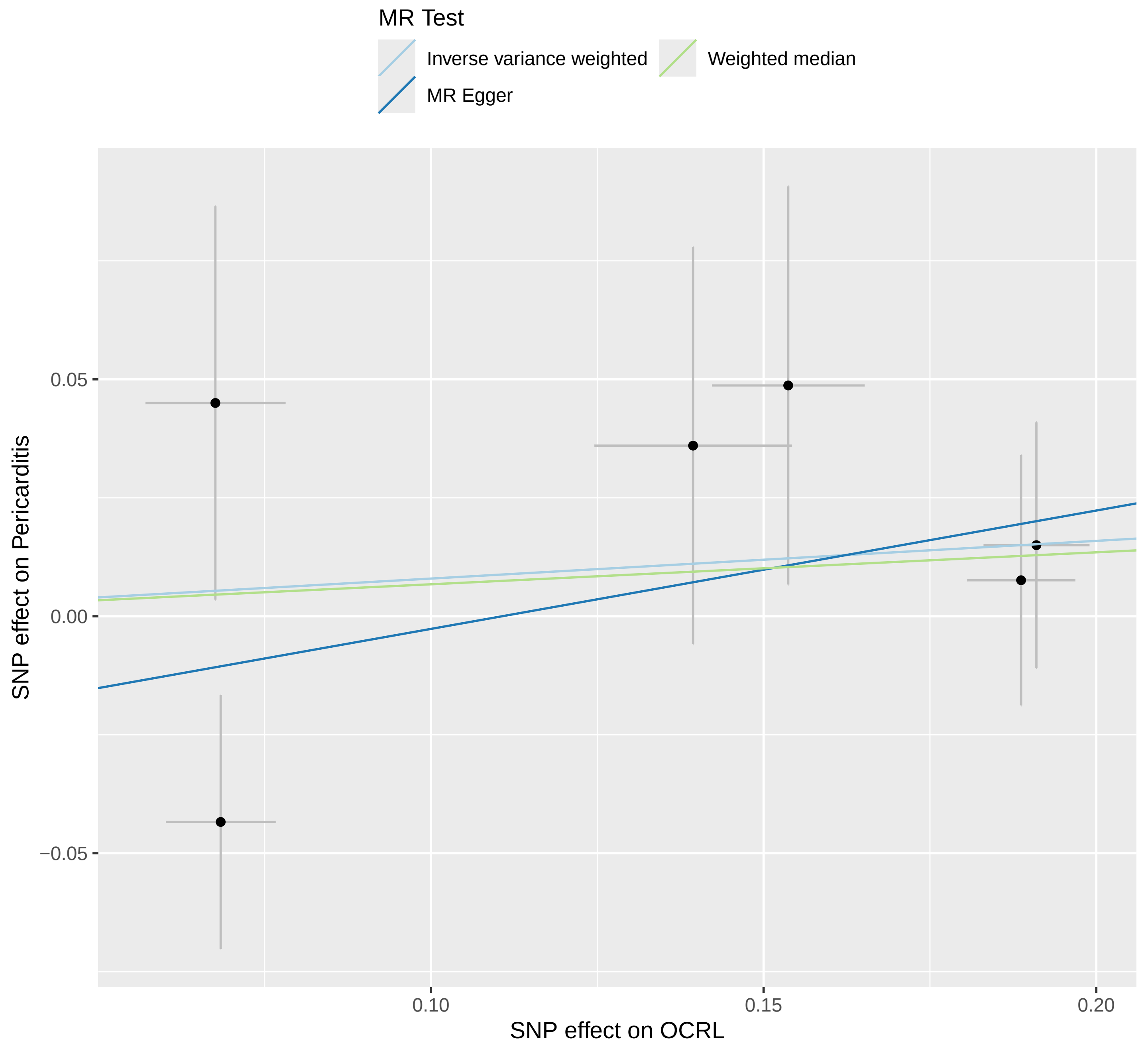

MR Test
Inverse variance weighted
MR Egger
Weighted median
SNP effect on Pericarditis
0.05
0.00
−0.05
0.10
0.15
0.20
SNP effect on OCRL

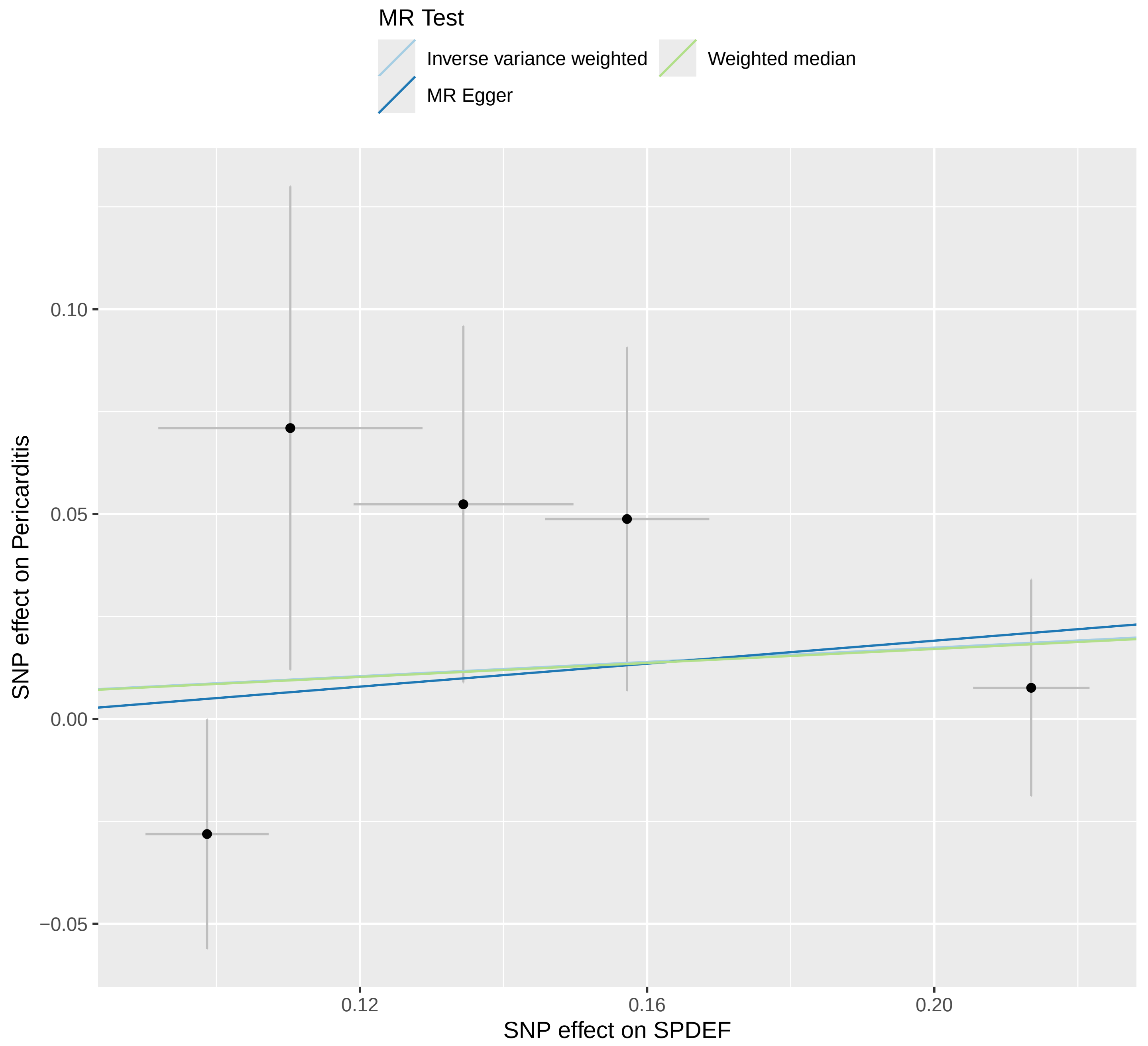

MR Test
Inverse variance weighted
Weighted median
MR Egger
0.10
0.05
0.00
−0.05
0.12
0.16
0.20
SNP effect on SPDEF
SNP effect on Pericarditis

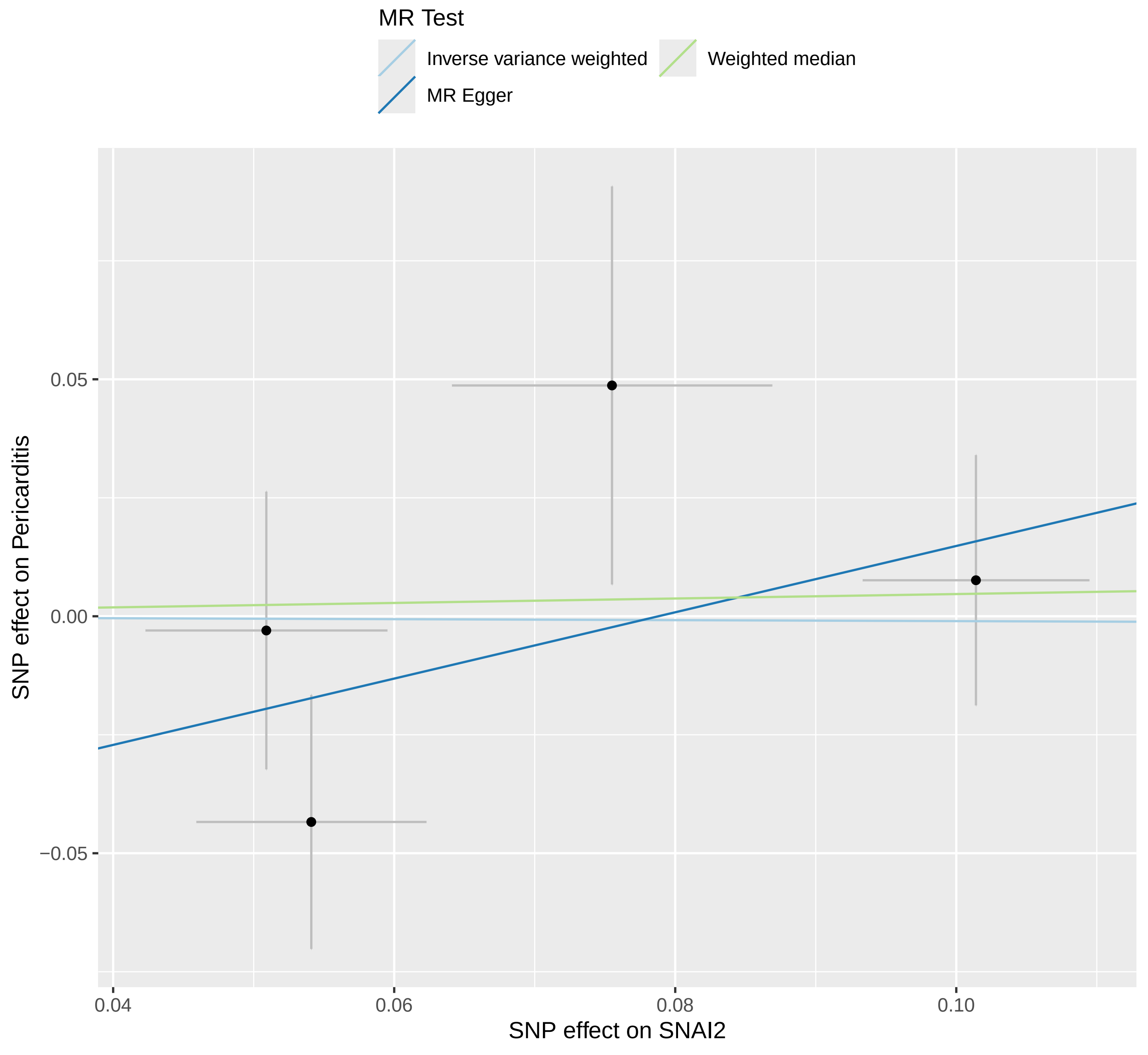
MR Test
Inverse variance weighted
Weighted median
MR Egger
0.05
0.00
−0.05
SNP effect on Pericarditis
0.04
0.06
0.08
0.10
SNP effect on SNAI2

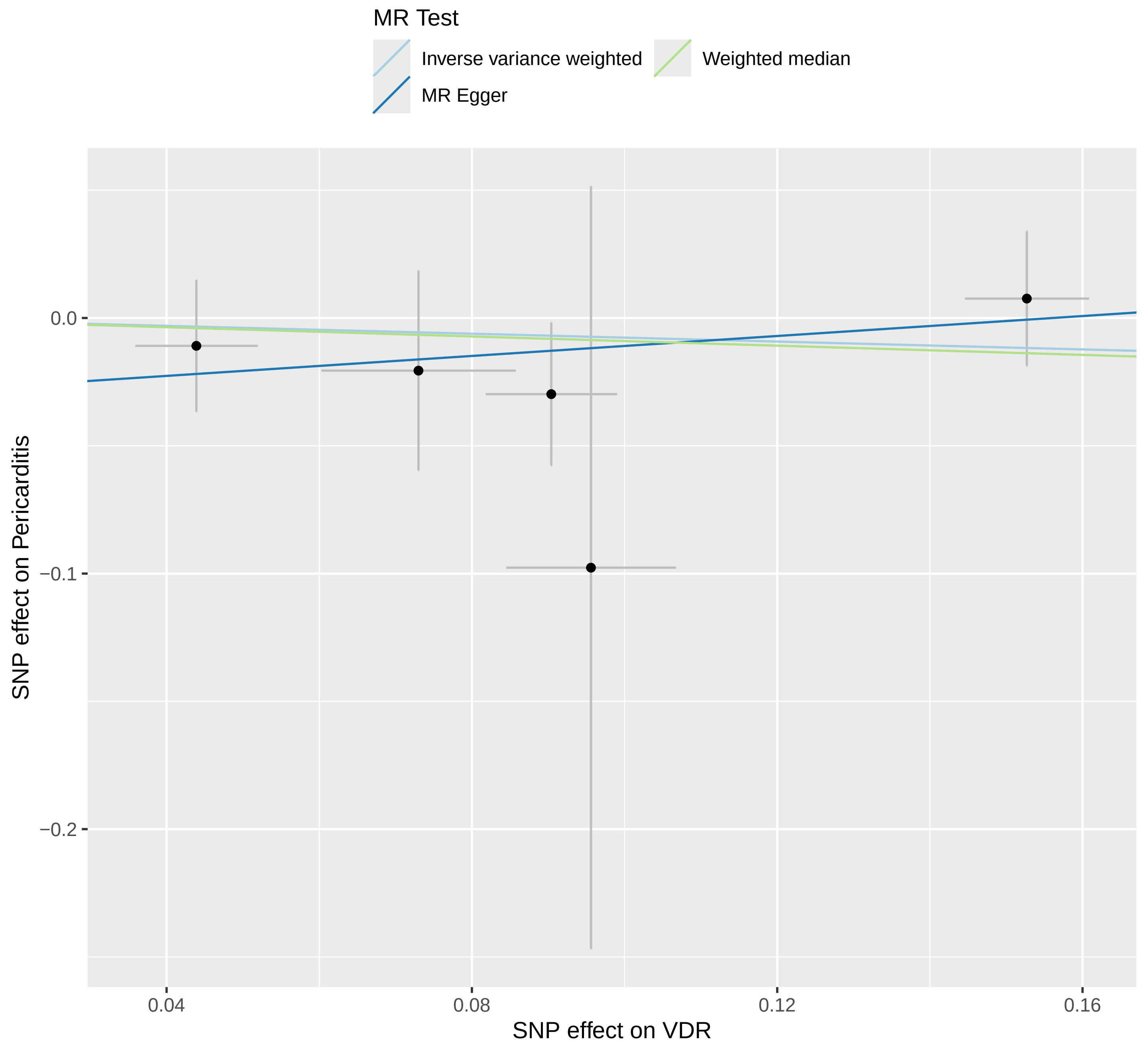
MR Test
Inverse variance weighted
Weighted median
MR Egger
0.0
−0.1
−0.2
SNP effect on Pericarditis
0.04
0.08
0.12
0.16
SNP effect on VDR

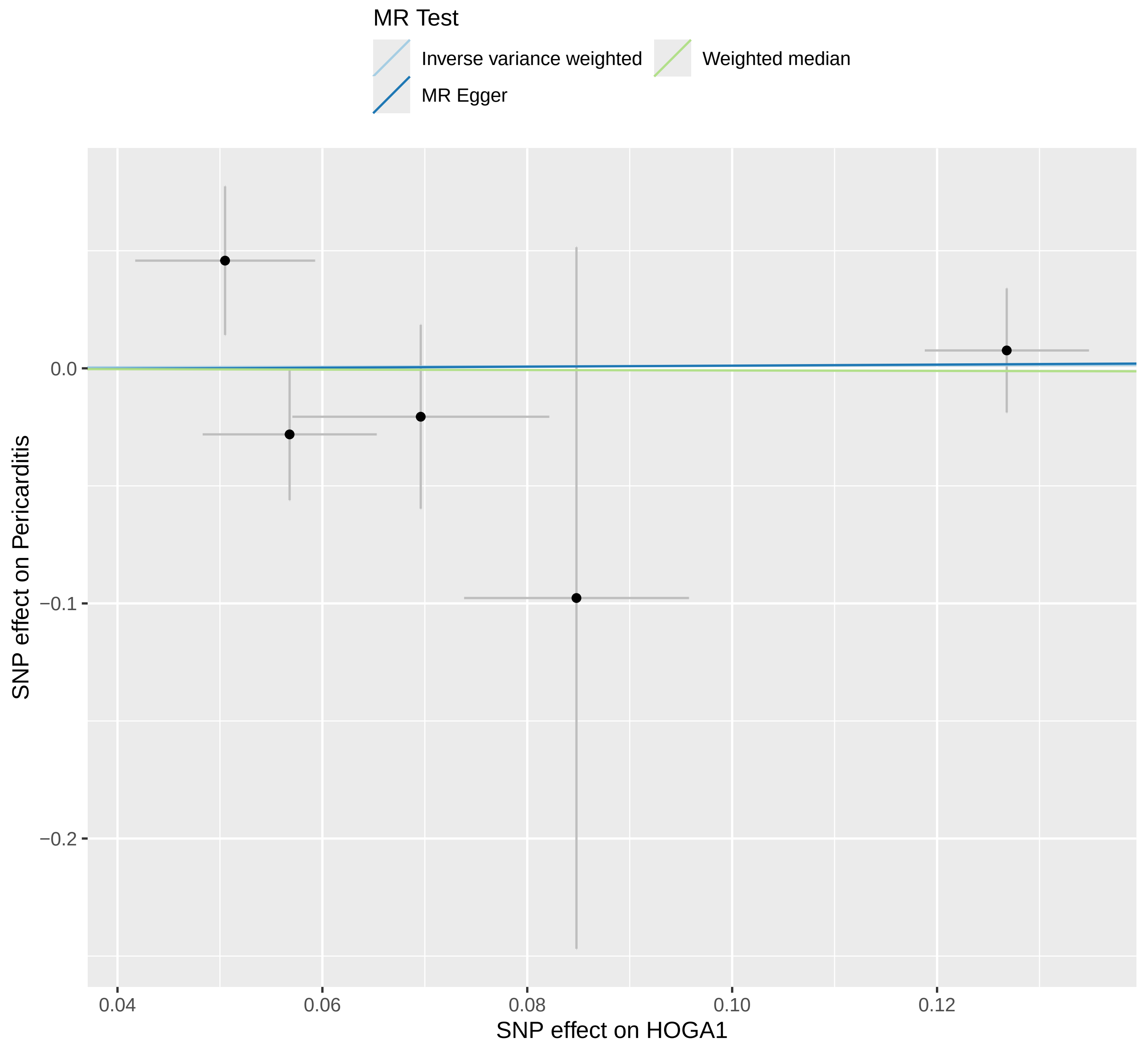

MR Test
Inverse variance weighted
Weighted median
MR Egger
0.0
−0.1
−0.2
SNP effect on Pericarditis
0.04
0.06
0.08
0.10
0.12
SNP effect on HOGA1

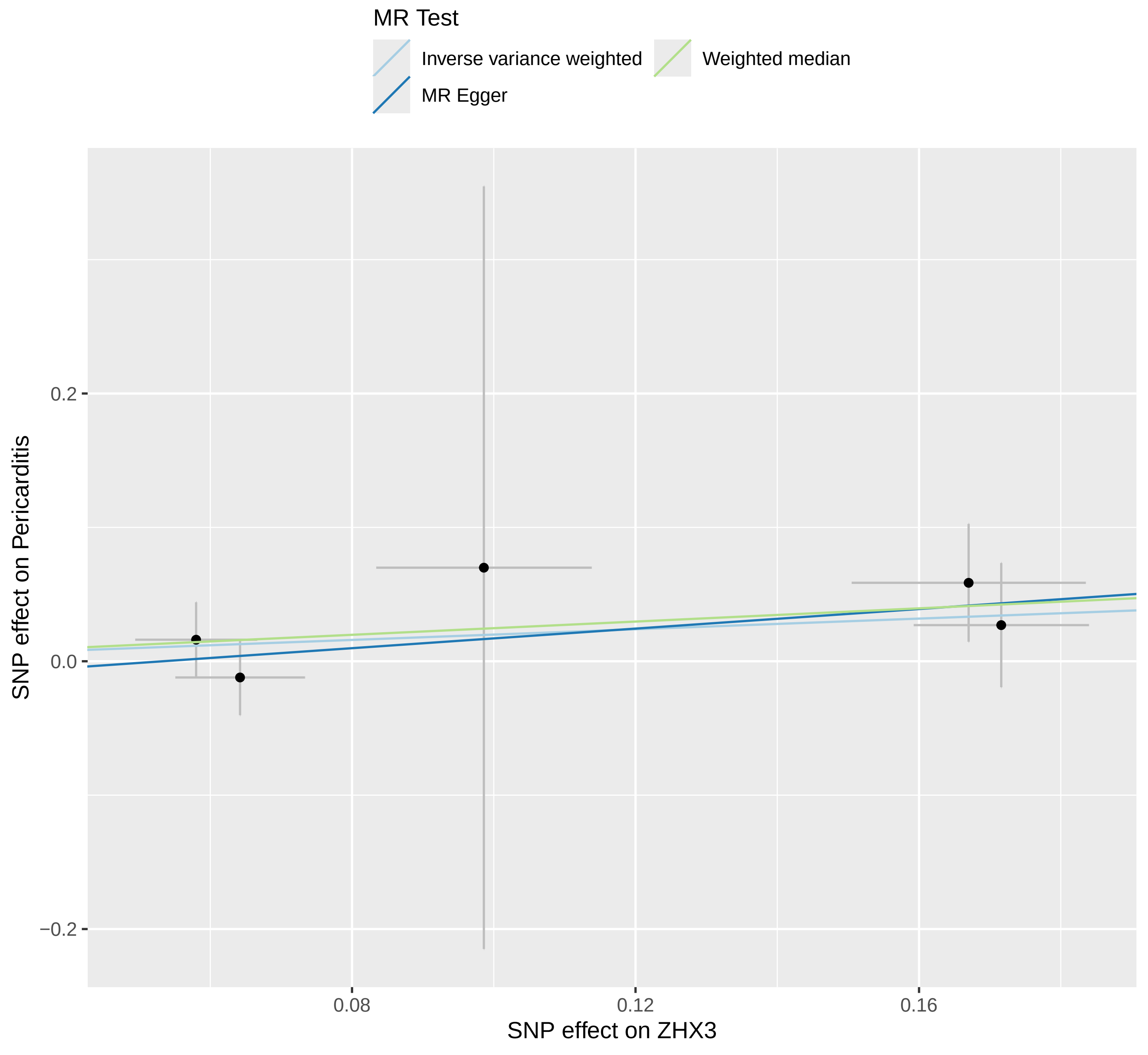
MR Test
Inverse variance weighted
Weighted median
MR Egger
0.2
0.0
−0.2
SNP effect on Pericarditis
0.08
0.12
0.16
SNP effect on ZHX3

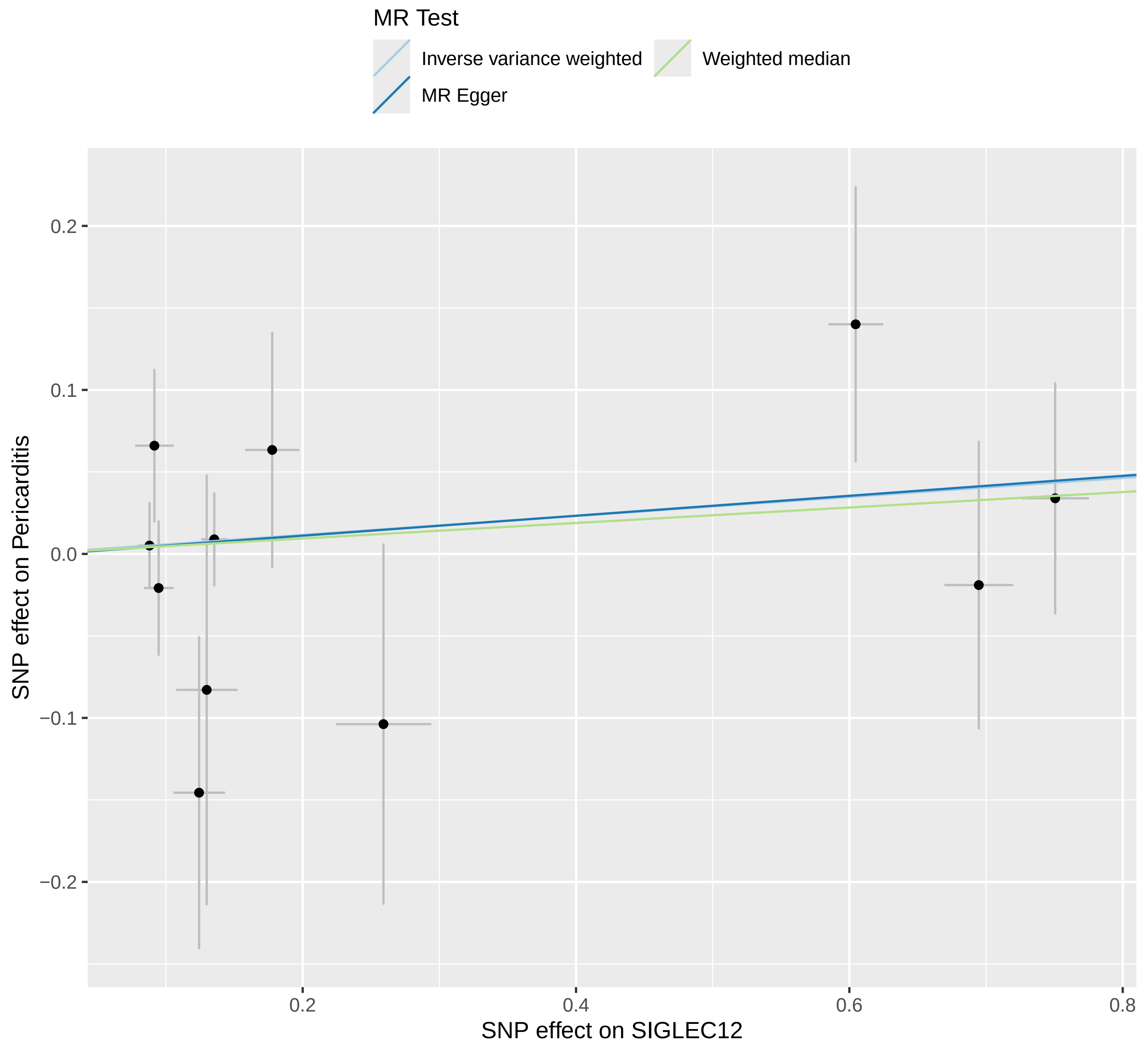
MR Test
Inverse variance weighted
Weighted median
MR Egger
0.2
0.1
0.0
−0.1
−0.2
SNP effect on Pericarditis
0.2
0.4
0.6
0.8
SNP effect on SIGLEC12

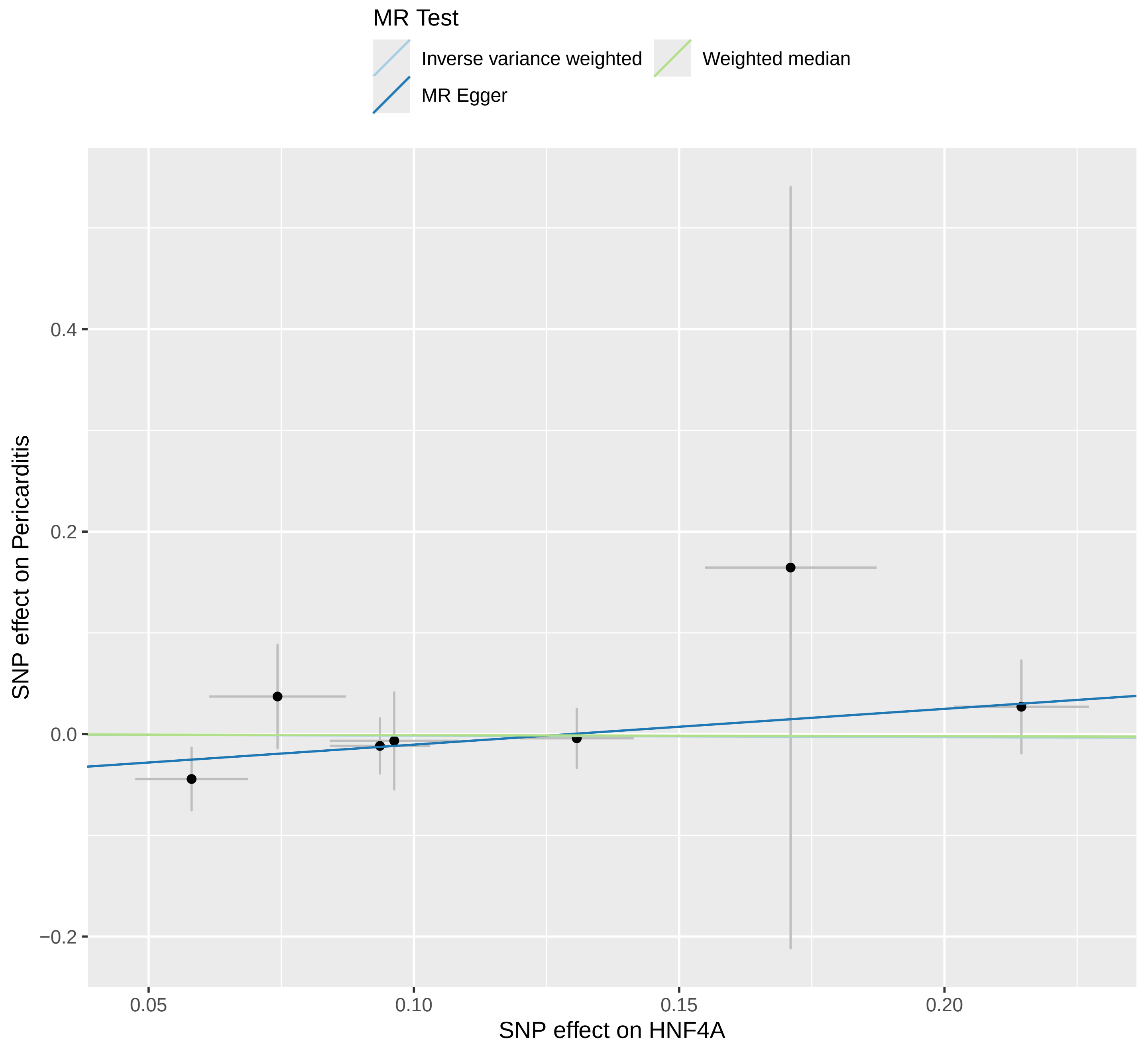

MR Test
Inverse variance weighted
Weighted median
MR Egger
SNP effect on Pericarditis
0.4
0.2
0.0
−0.2
0.05
0.10
0.15
0.20
SNP effect on HNF4A

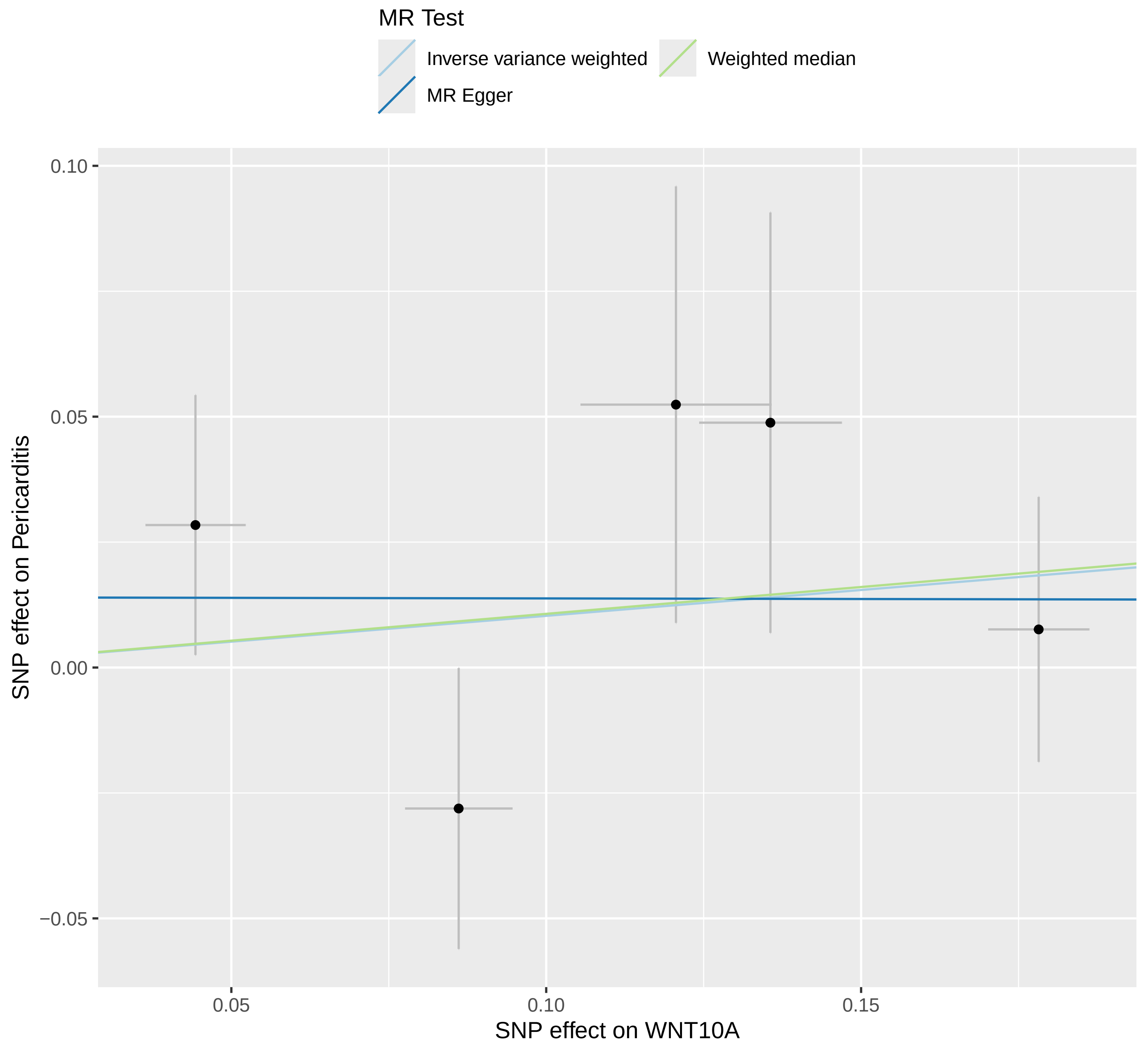
MR Test
Inverse variance weighted
MR Egger
Weighted median
SNP effect on Pericarditis
SNP effect on WNT10A
0.10
0.05
0.00
−0.05
0.05
0.10
0.15

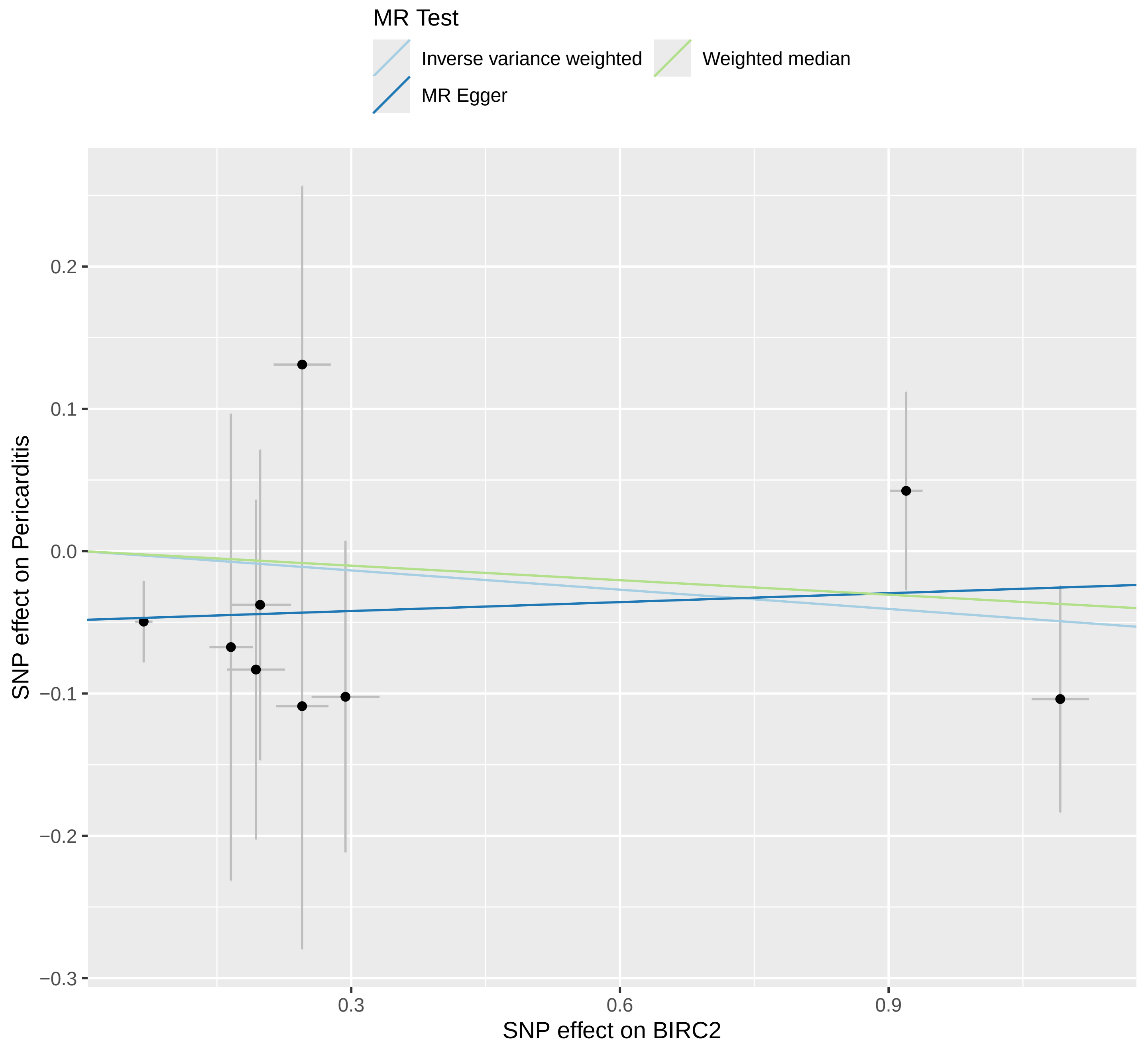

MR Test
Inverse variance weighted
Weighted median
MR Egger
0.2
0.1
0.0
−0.1
−0.2
−0.3
SNP effect on Pericarditis
0.3
0.6
0.9
SNP effect on BIRC2

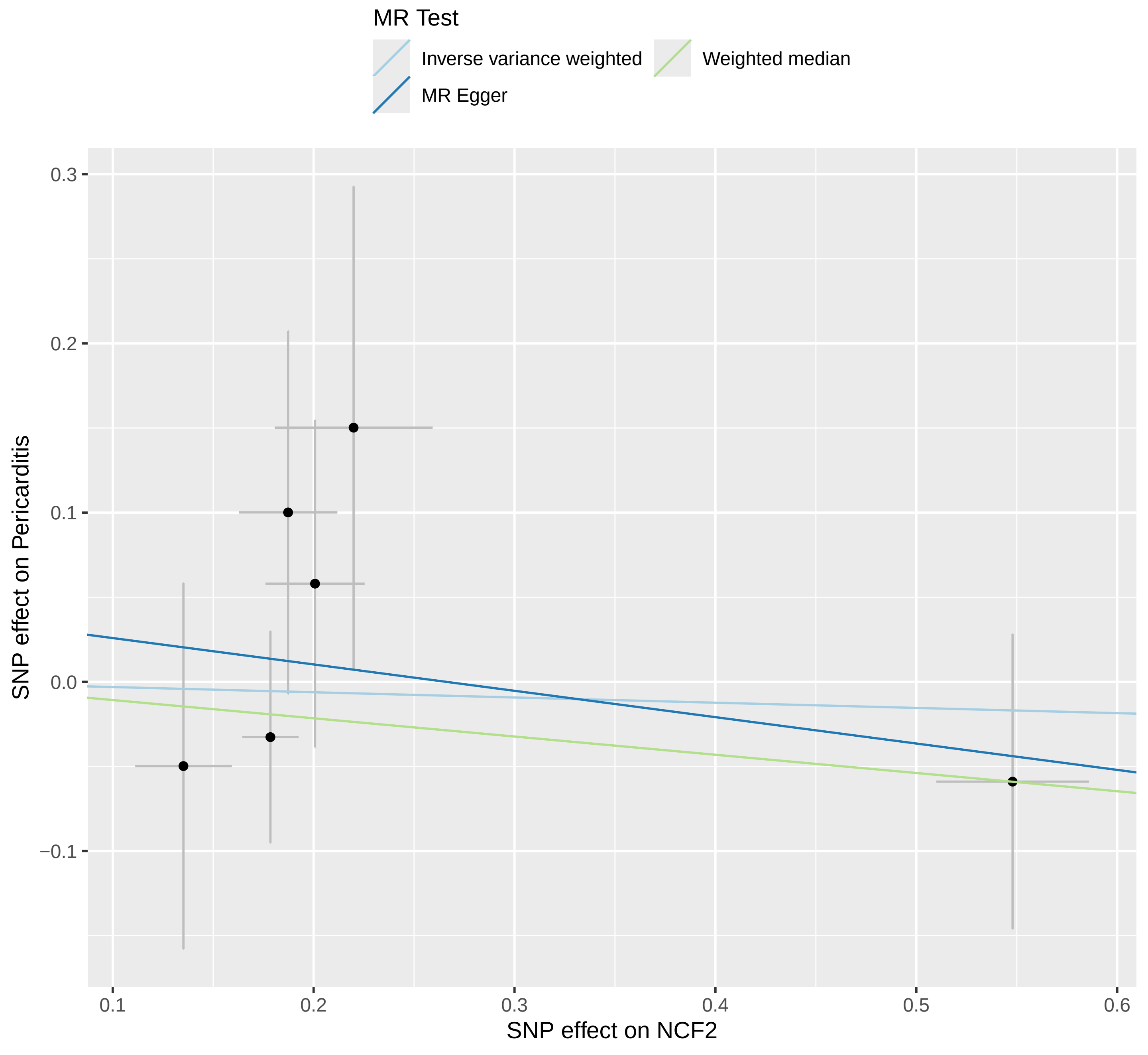
MR Test
Inverse variance weighted
Weighted median
MR Egger
SNP effect on Pericarditis
0.3
0.2
0.1
0.0
−0.1
0.1
0.2
0.3
0.4
0.5
0.6
SNP effect on NCF2

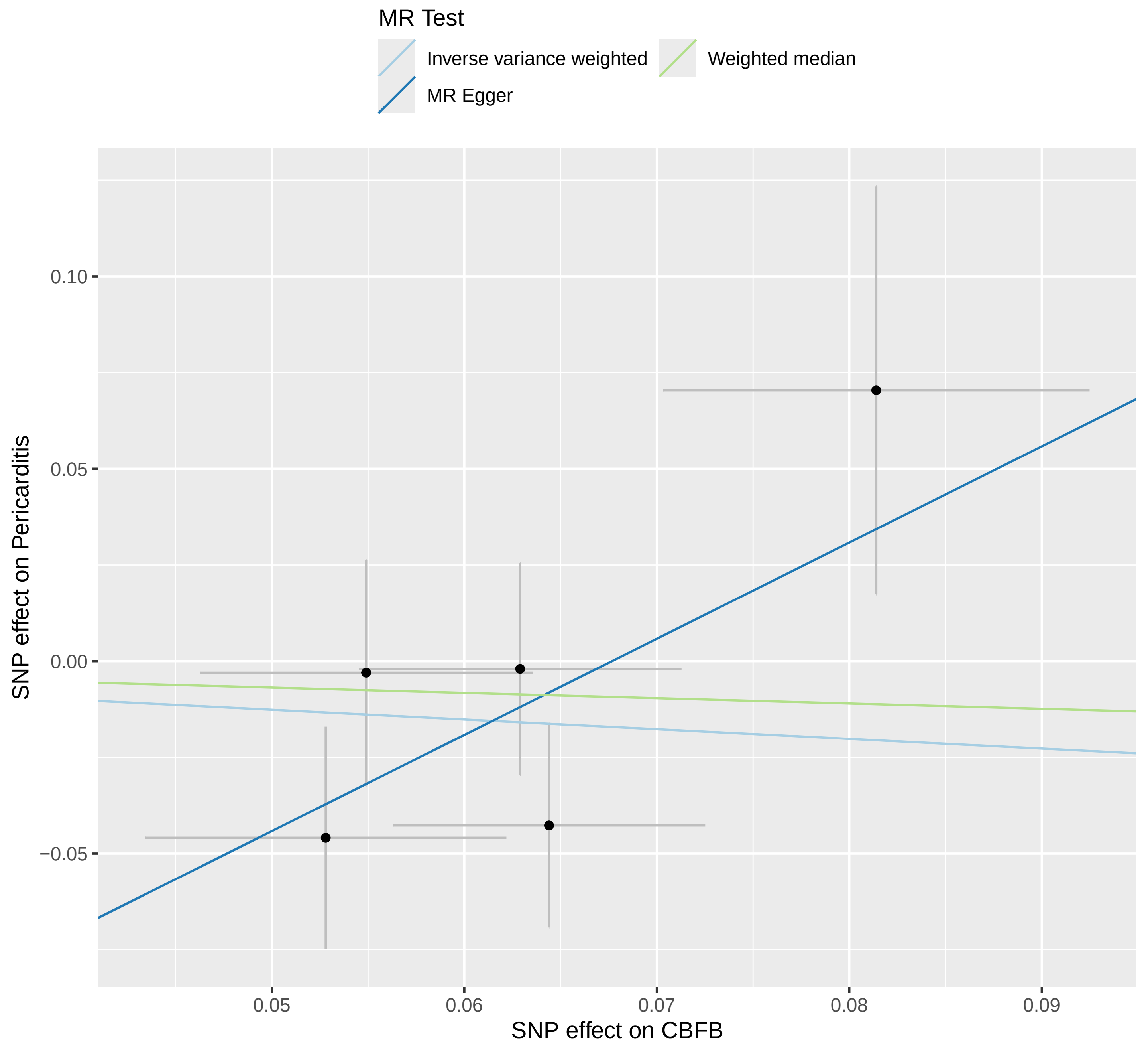
MR Test
Inverse variance weighted
Weighted median
MR Egger
SNP effect on Pericarditis
0.10
0.05
0.00
−0.05
0.05
0.06
0.07
0.08
0.09
SNP effect on CBFB

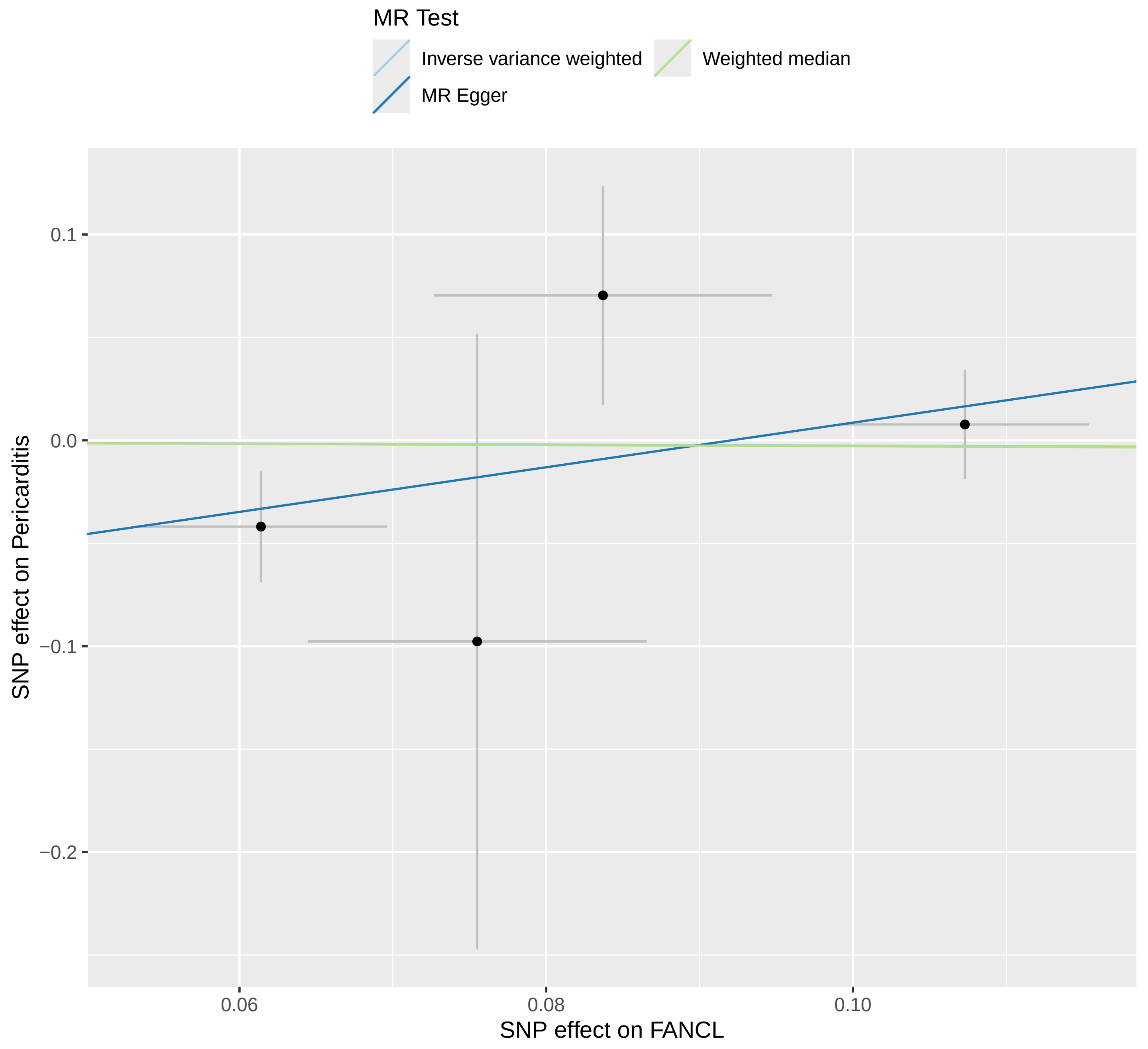
MR Test
Inverse variance weighted
Weighted median
MR Egger
0.1
0.0
−0.1
−0.2
SNP effect on Pericarditis
0.06
0.08
0.10
SNP effect on FANCL

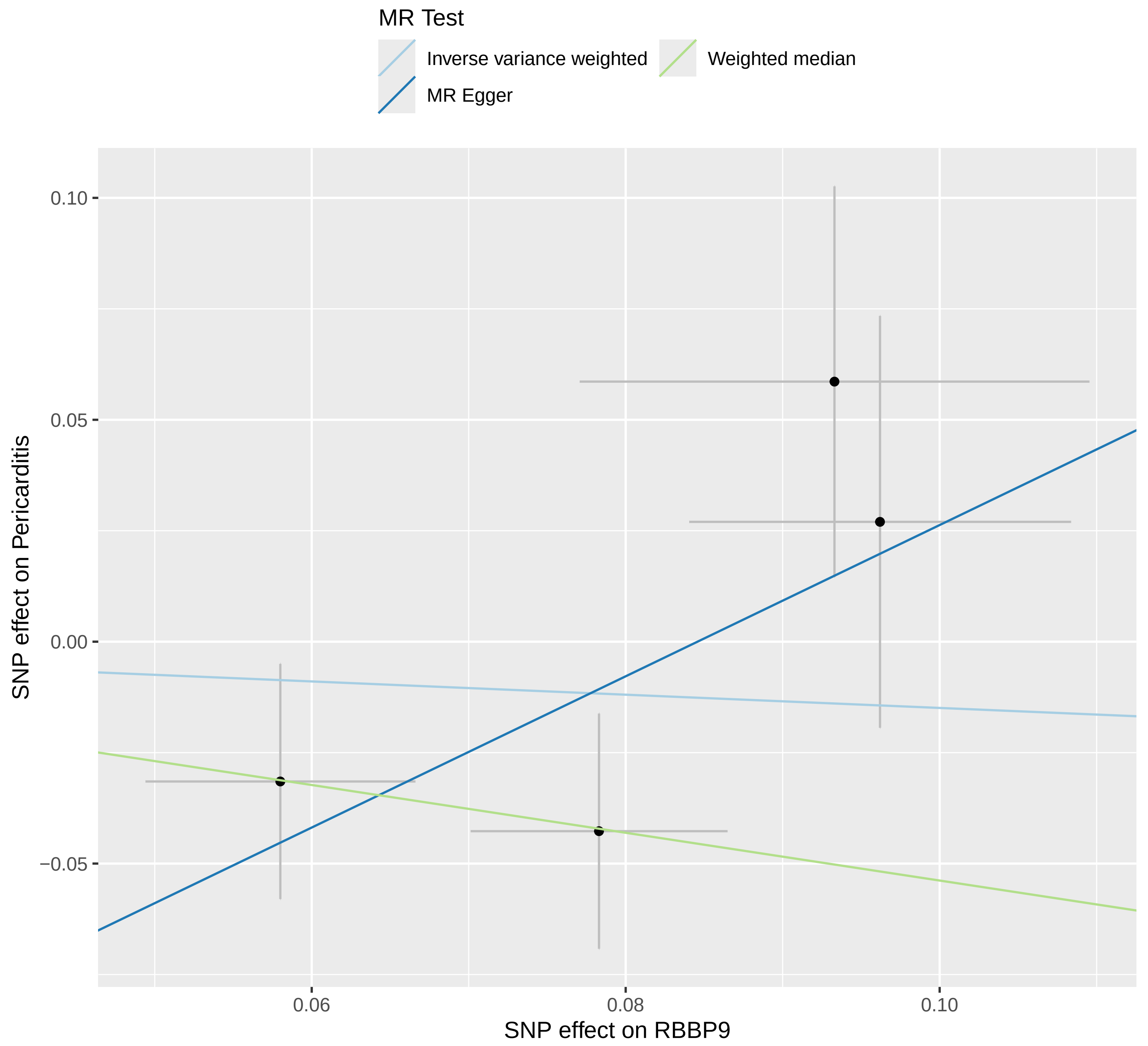
MR Test
Inverse variance weighted
Weighted median
MR Egger
SNP effect on Pericarditis
0.10
0.05
0.00
−0.05
0.06
0.08
0.10
SNP effect on RBBP9

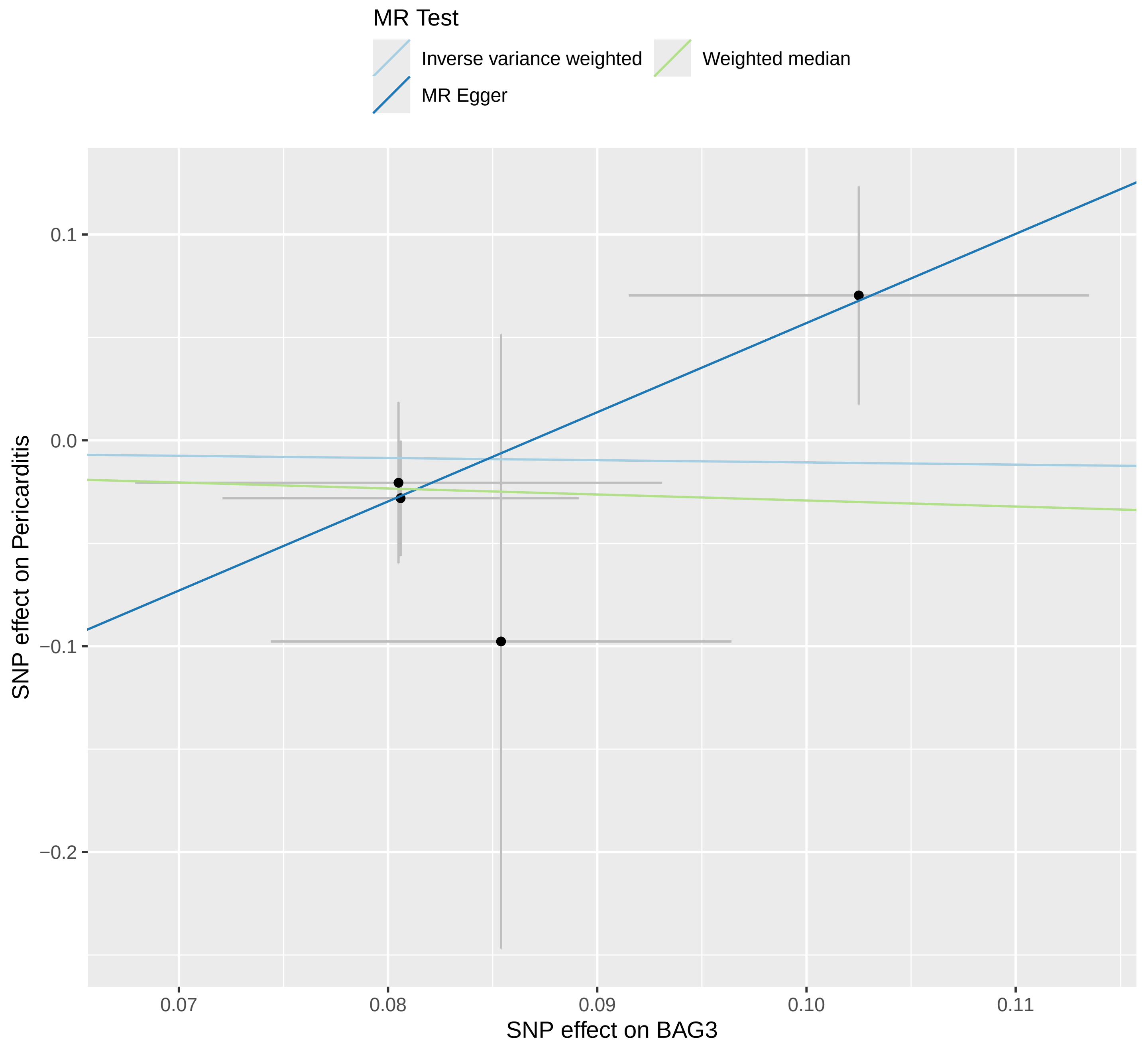
MR Test
Inverse variance weighted
Weighted median
MR Egger
0.1
0.0
−0.1
−0.2
SNP effect on Pericarditis
0.07
0.08
0.09
0.10
0.11
SNP effect on BAG3

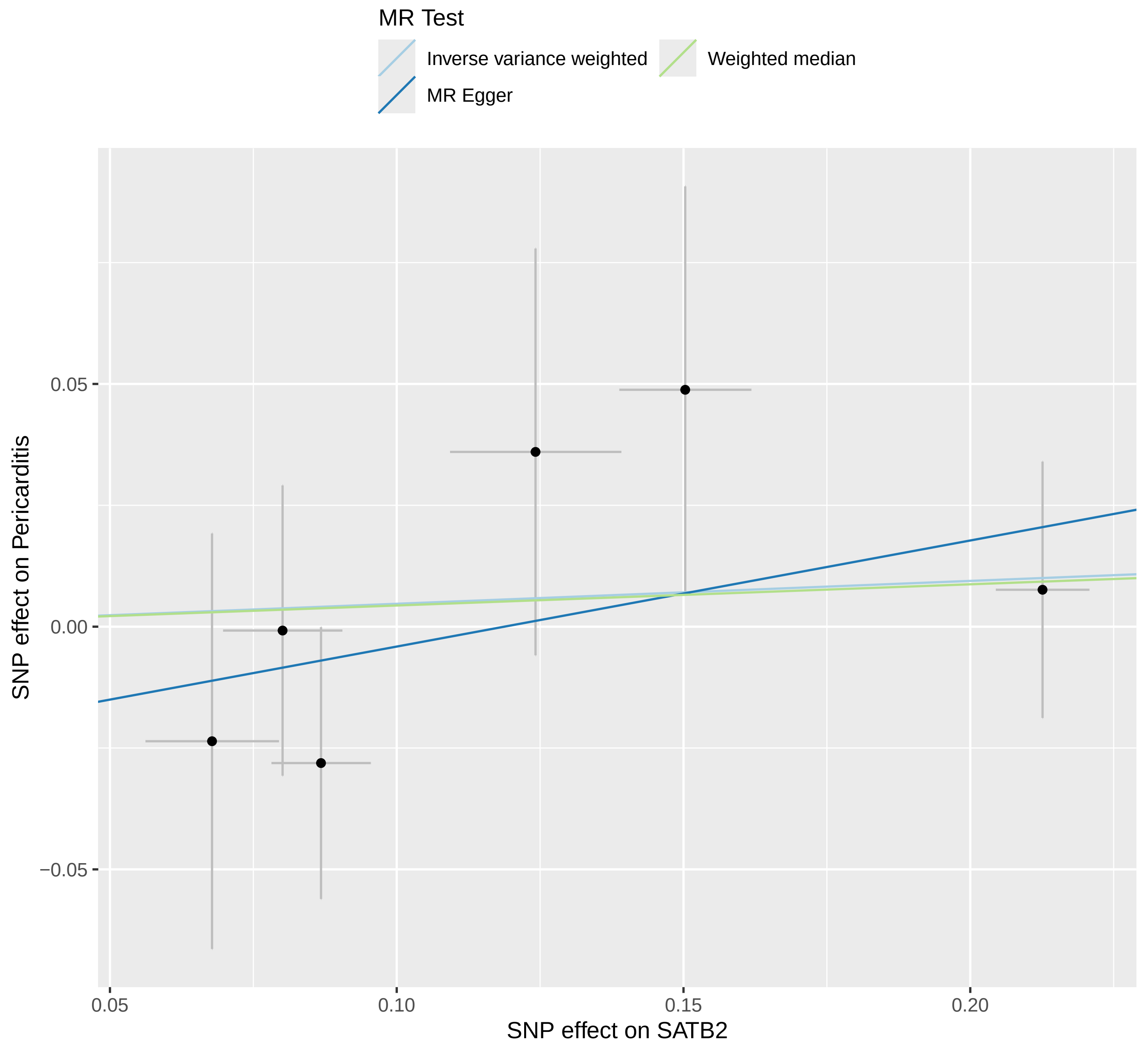
MR Test
Inverse variance weighted
MR Egger
Weighted median
SNP effect on Pericarditis
0.05
0.00
−0.05
0.05
0.10
0.15
0.20
SNP effect on SATB2

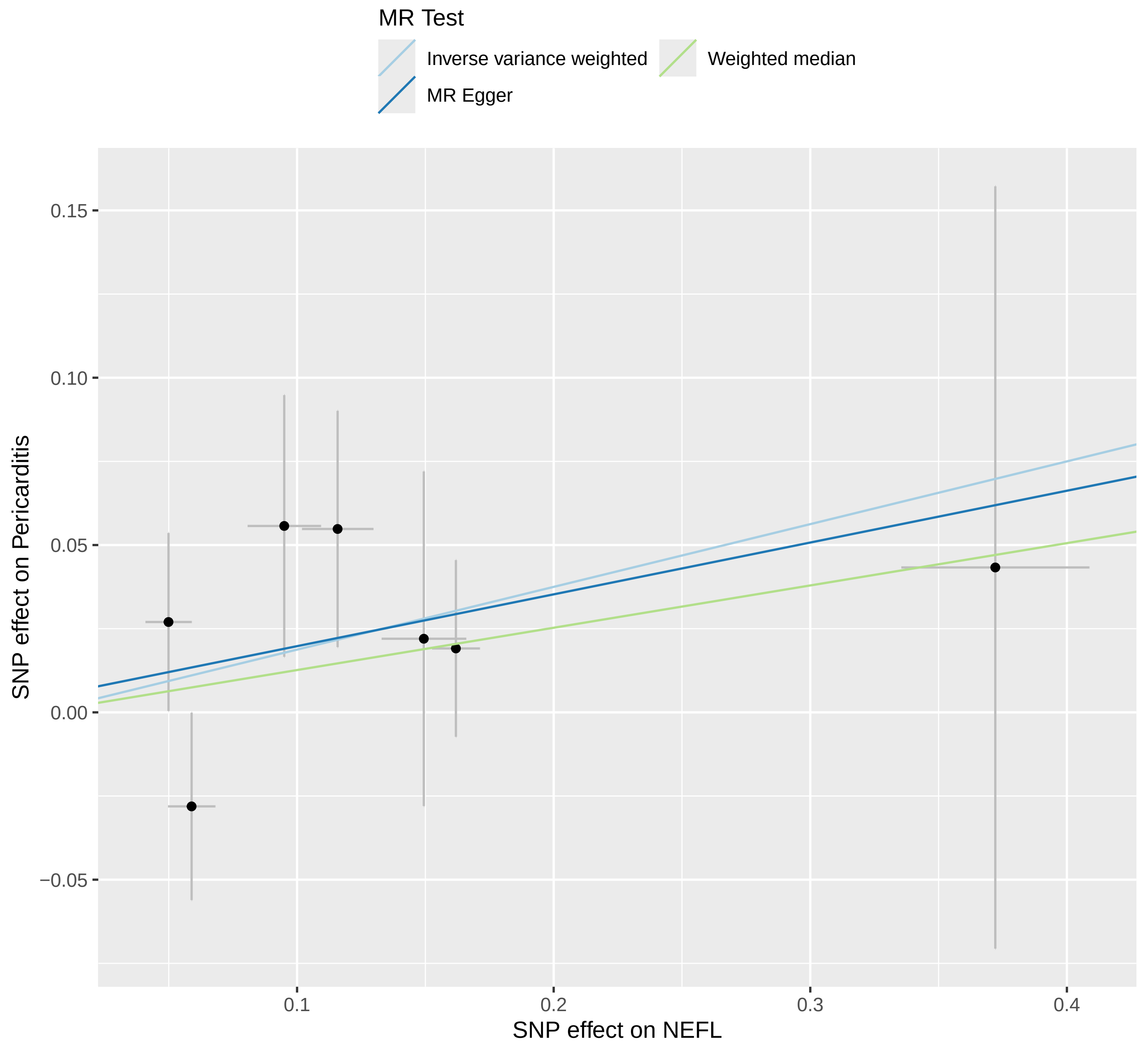
MR Test
Inverse variance weighted
MR Egger
Weighted median
0.15
0.10
0.05
0.00
−0.05
SNP effect on Pericarditis
0.1
0.2
0.3
0.4
SNP effect on NEFL

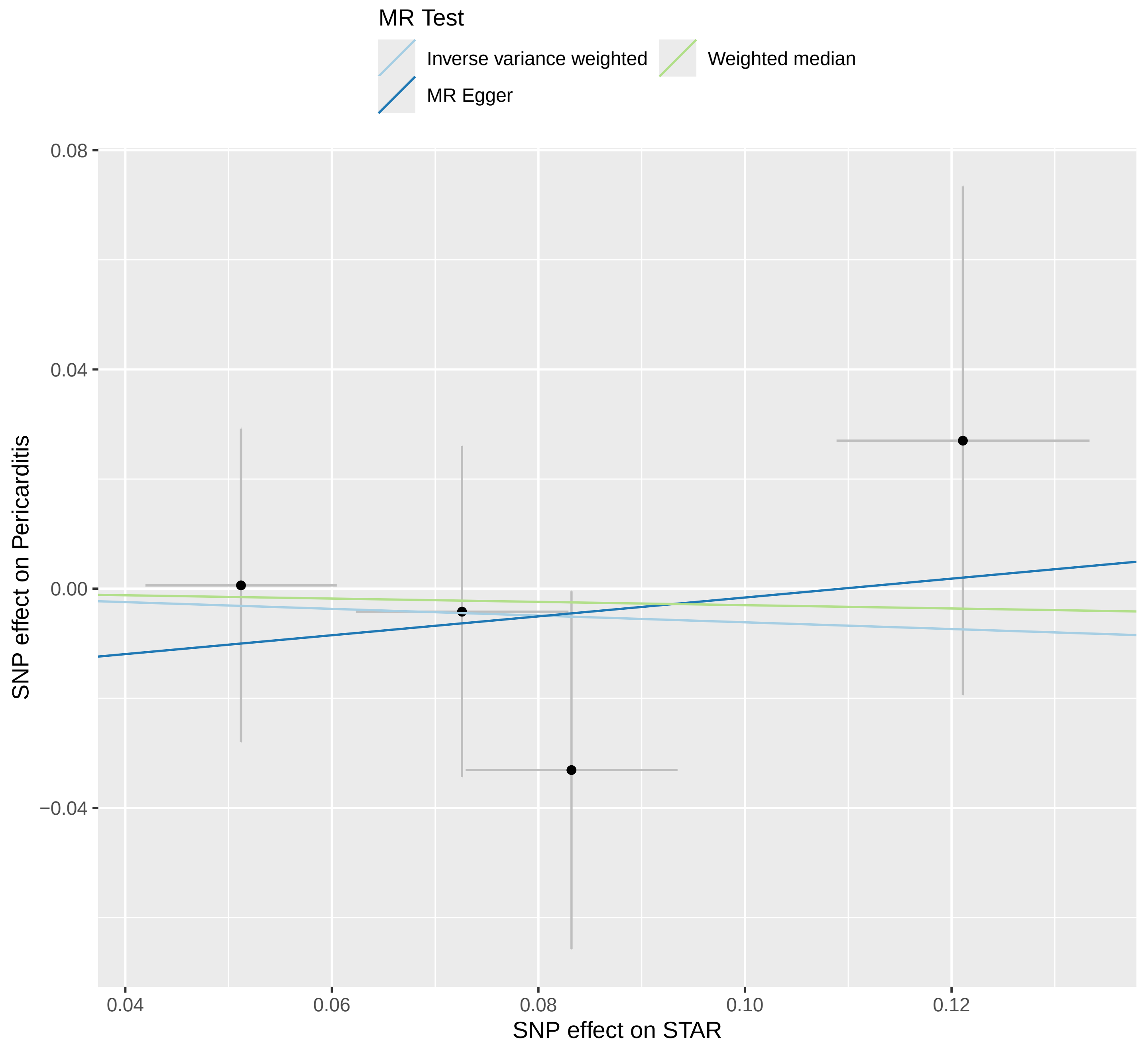
MR Test
Inverse variance weighted
MR Egger
Weighted median
0.08
0.04
0.00
−0.04
SNP effect on Pericarditis
0.04
0.06
0.08
0.10
0.12
SNP effect on STAR

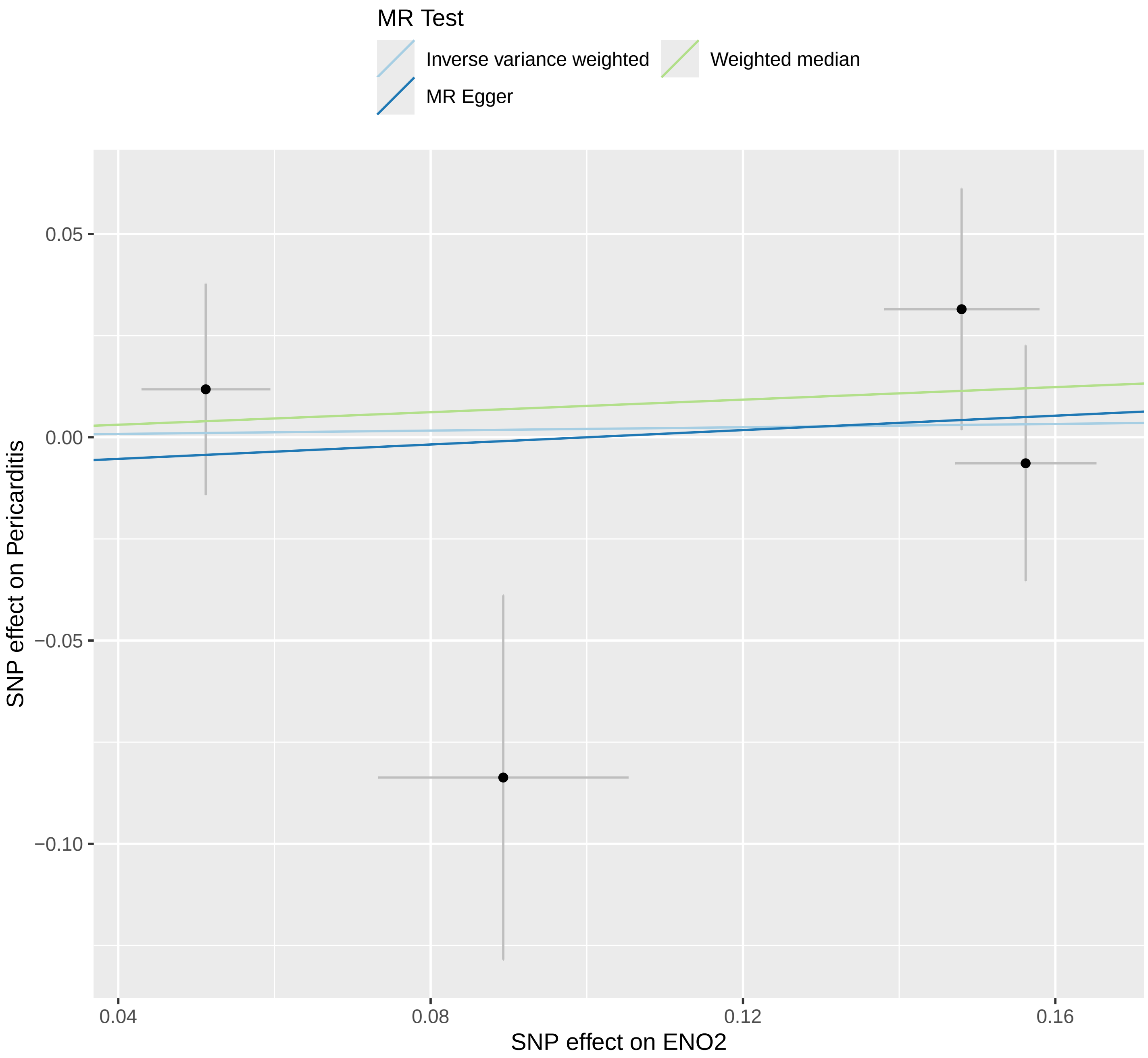
MR Test
Inverse variance weighted
MR Egger
Weighted median
0.05
0.00
−0.05
−0.10
SNP effect on Pericarditis
0.04
0.08
0.12
0.16
SNP effect on ENO2

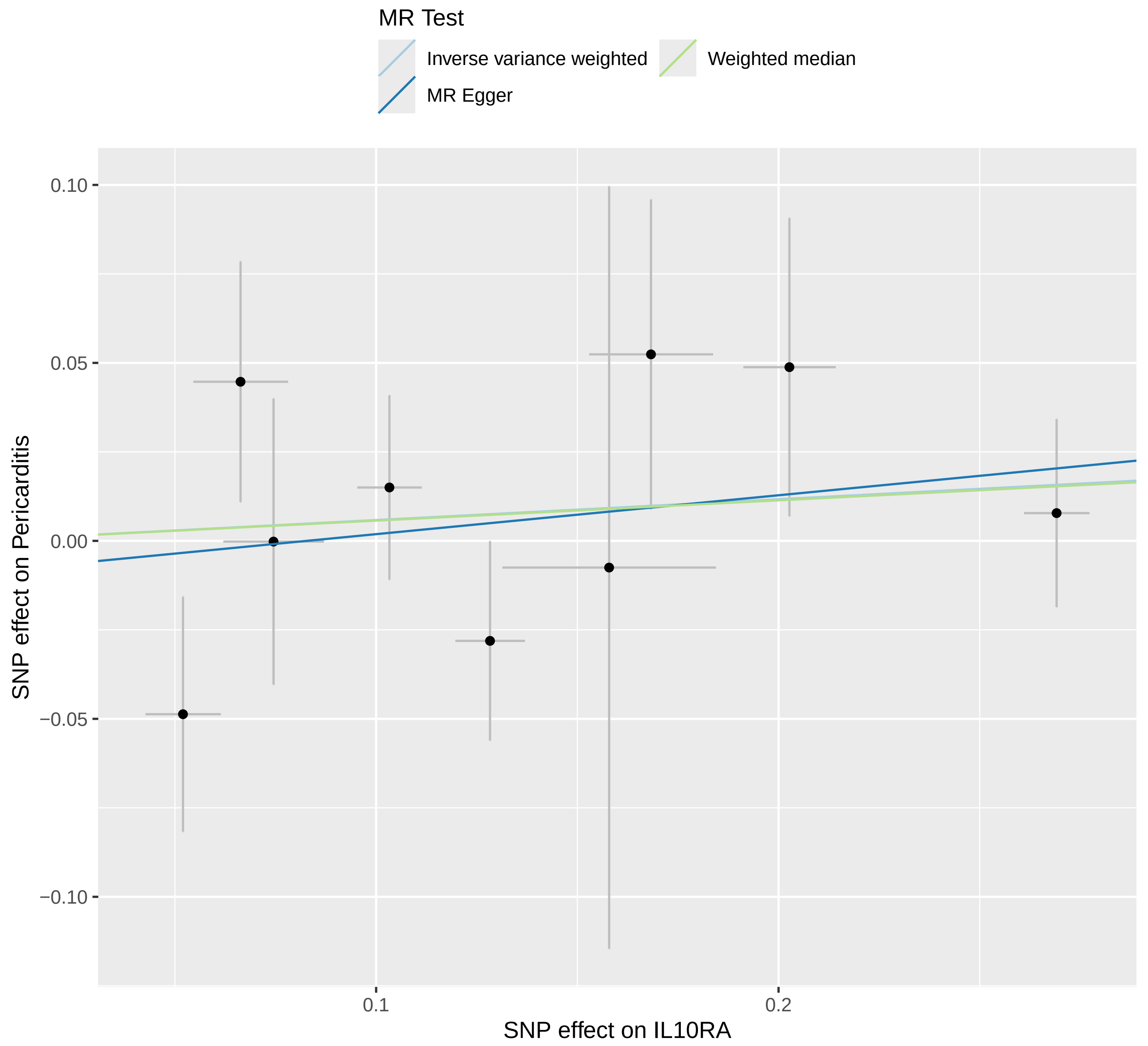

MR Test
Inverse variance weighted
Weighted median
MR Egger
0.10
0.05
0.00
−0.05
−0.10
0.1
0.2
SNP effect on Pericarditis
SNP effect on IL10RA

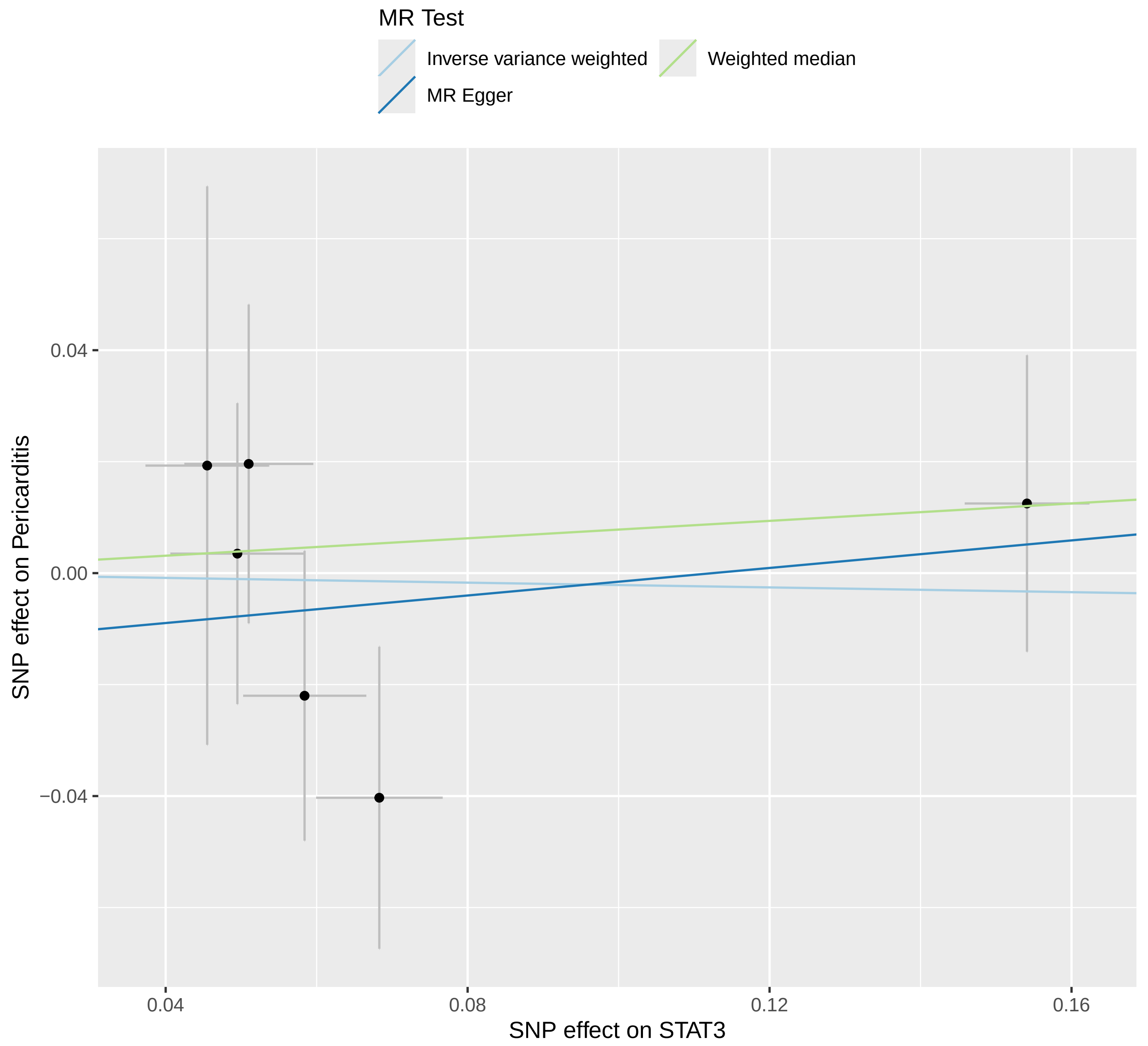
MR Test
Inverse variance weighted
Weighted median
MR Egger
0.04
0.00
−0.04
SNP effect on Pericarditis
0.04
0.08
0.12
0.16
SNP effect on STAT3

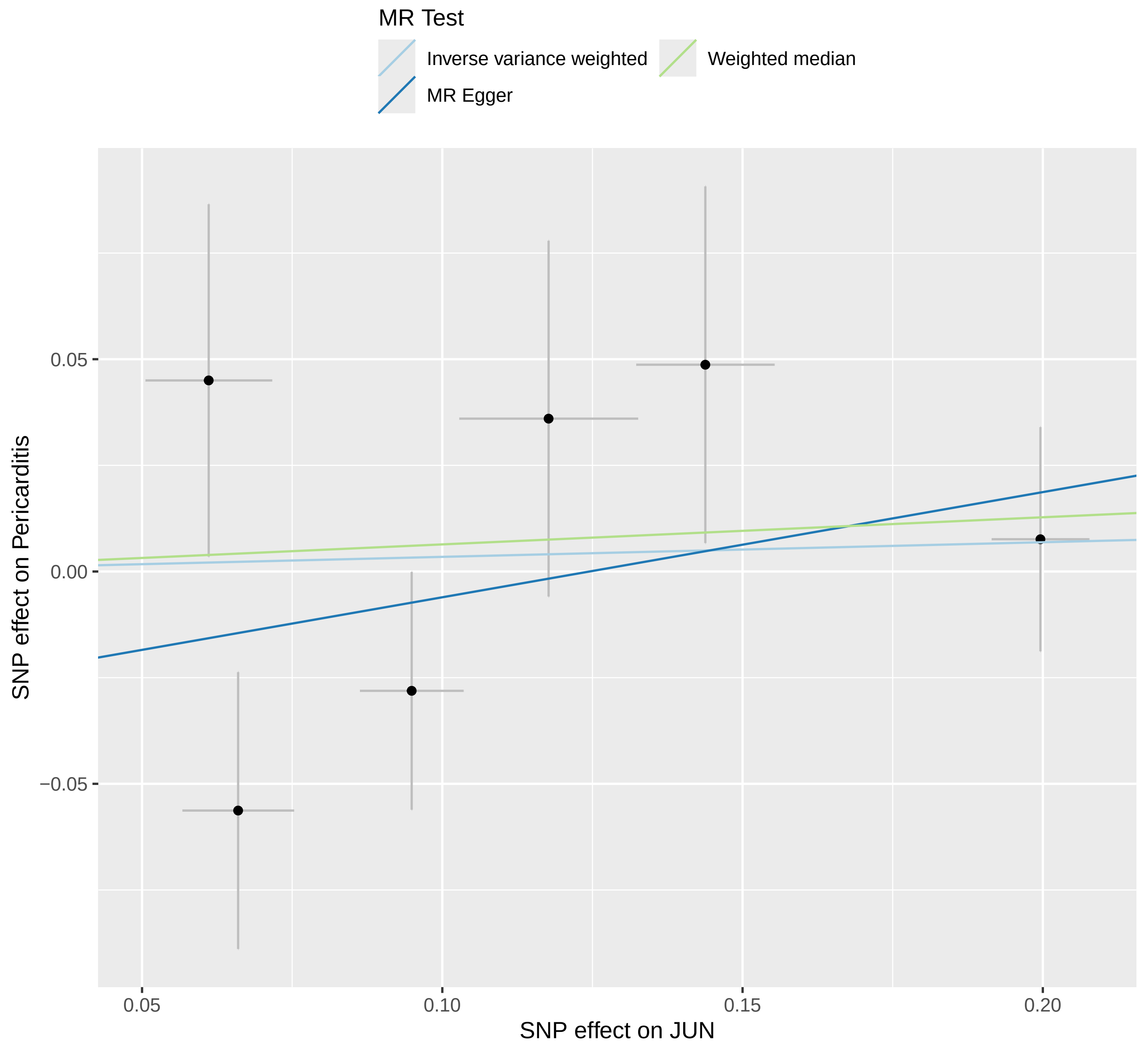
MR Test
Inverse variance weighted
Weighted median
MR Egger
0.05
0.00
−0.05
0.05
0.10
0.15
0.20
SNP effect on Pericarditis
SNP effect on JUN

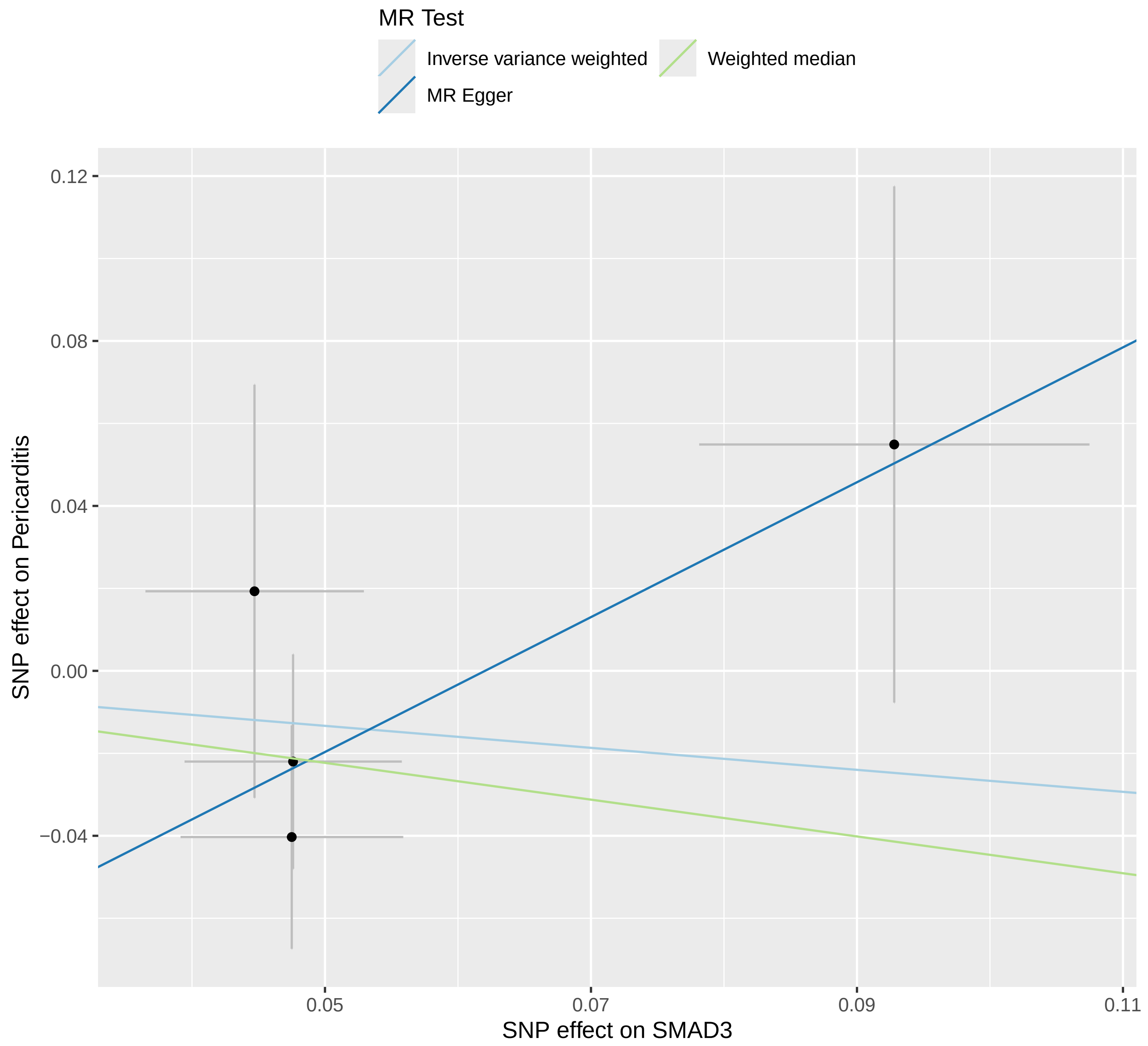
MR Test
Inverse variance weighted
Weighted median
MR Egger
0.12
0.08
0.04
0.00
−0.04
SNP effect on Pericarditis
0.05
0.07
0.09
0.11
SNP effect on SMAD3

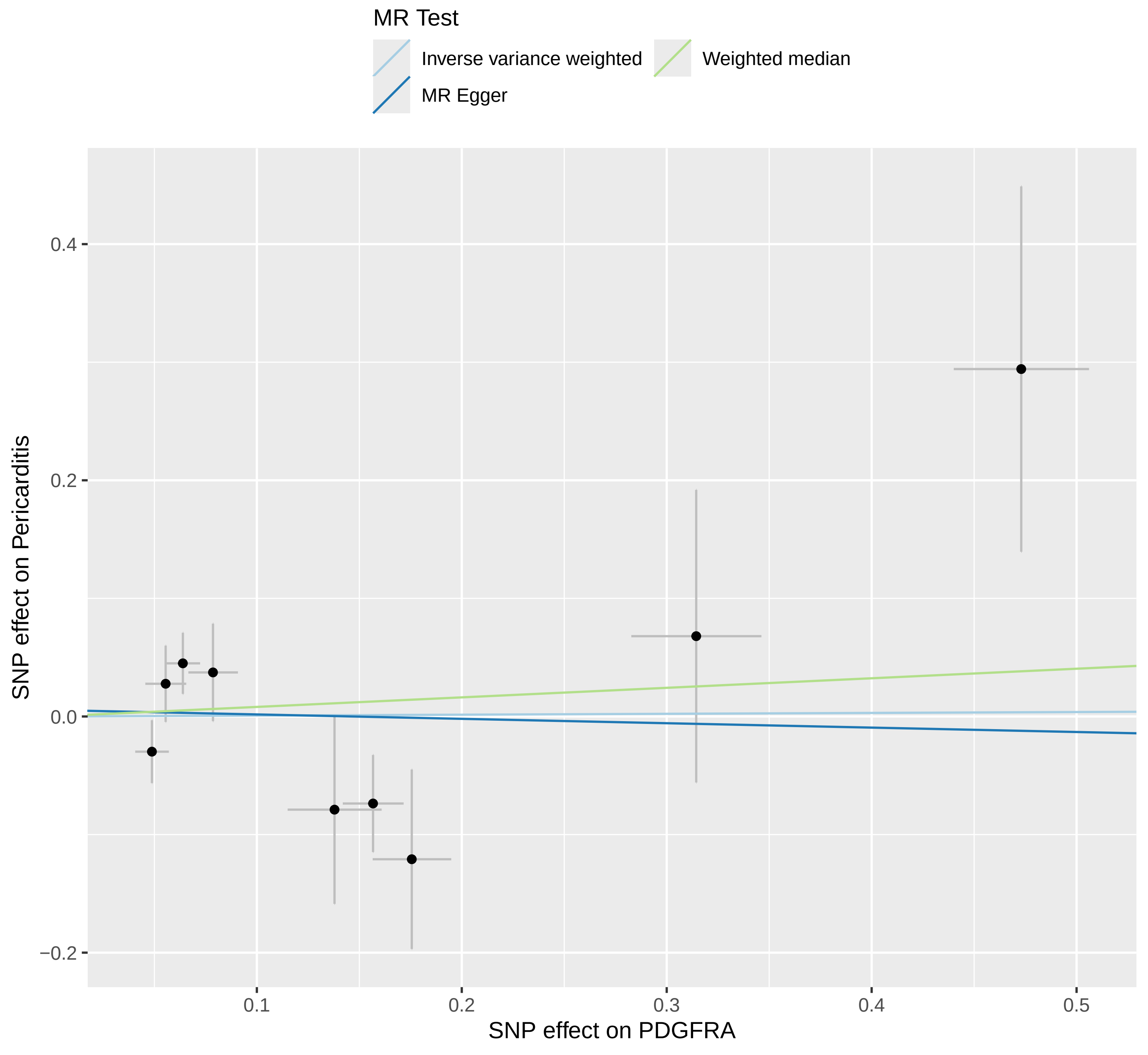

MR Test
Inverse variance weighted
Weighted median
MR Egger
SNP effect on Pericarditis
0.4
0.2
0.0
−0.2
0.1
0.2
0.3
0.4
0.5
SNP effect on PDGFRA

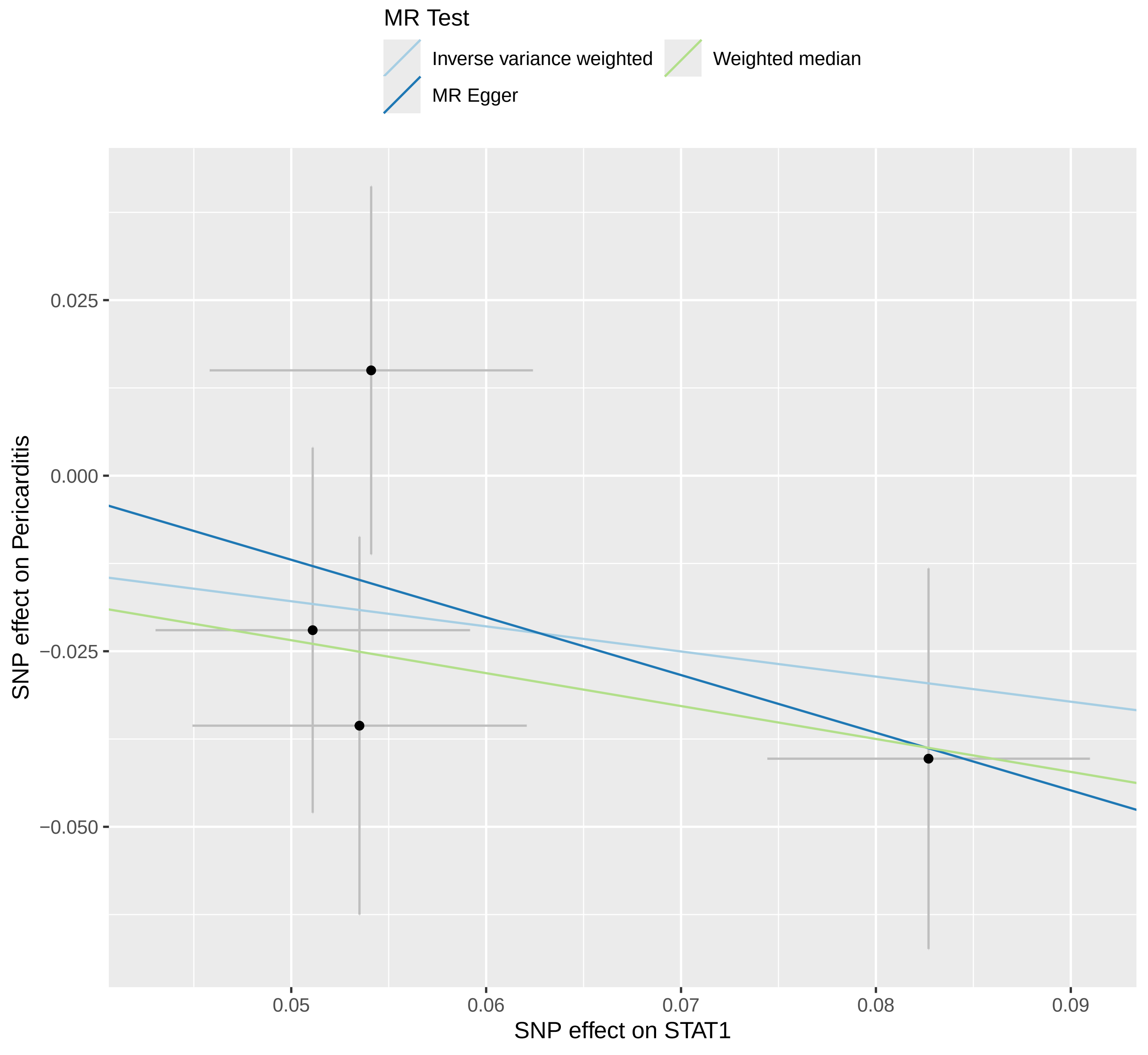

MR Test
Inverse variance weighted
MR Egger
Weighted median
0.025
0.000
−0.025
−0.050
SNP effect on Pericarditis
0.05
0.06
0.07
0.08
0.09
SNP effect on STAT1

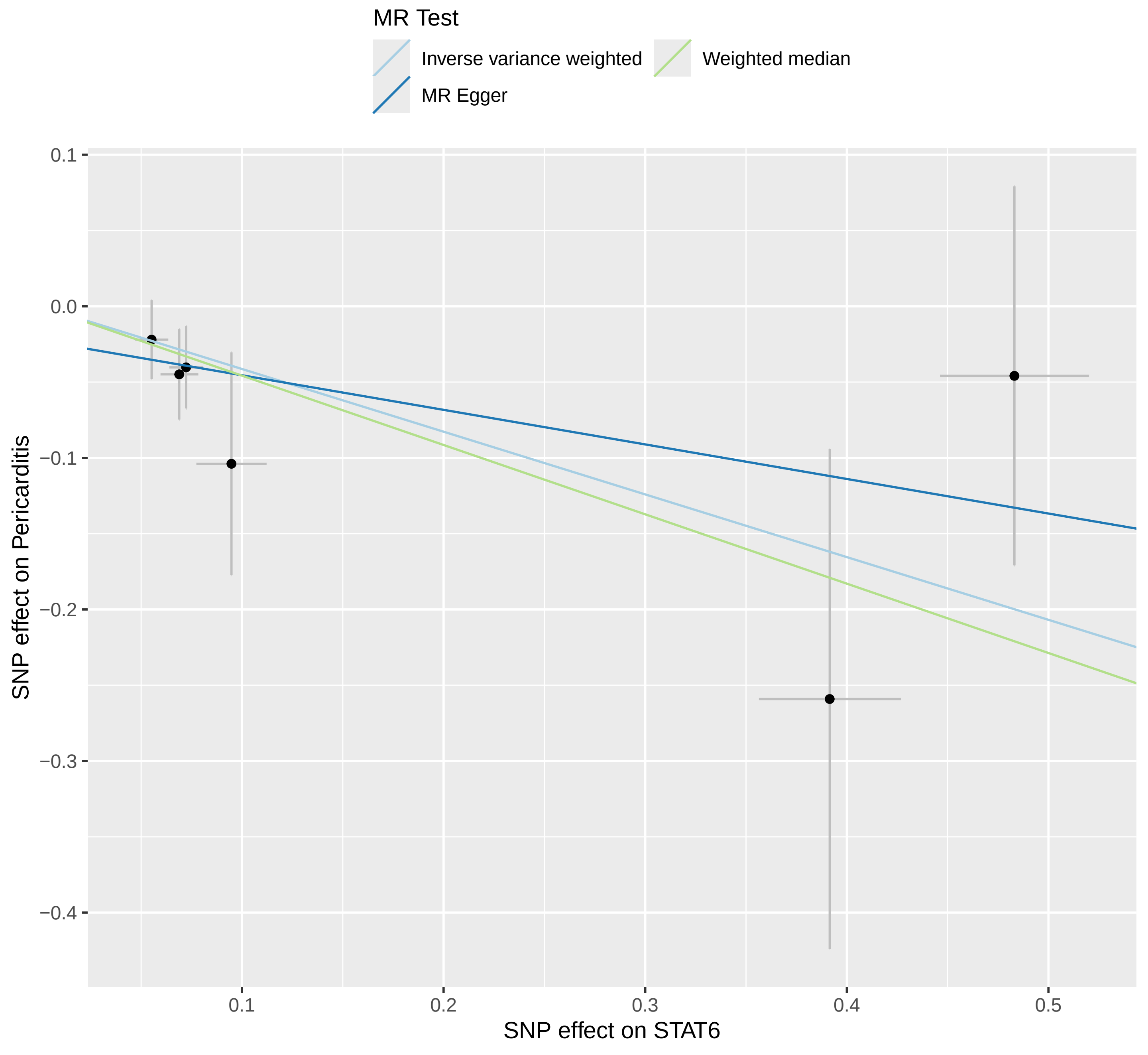
MR Test
Inverse variance weighted
Weighted median
MR Egger
0.1
0.0
−0.1
−0.2
−0.3
−0.4
SNP effect on Pericarditis
0.1
0.2
0.3
0.4
0.5
SNP effect on STAT6

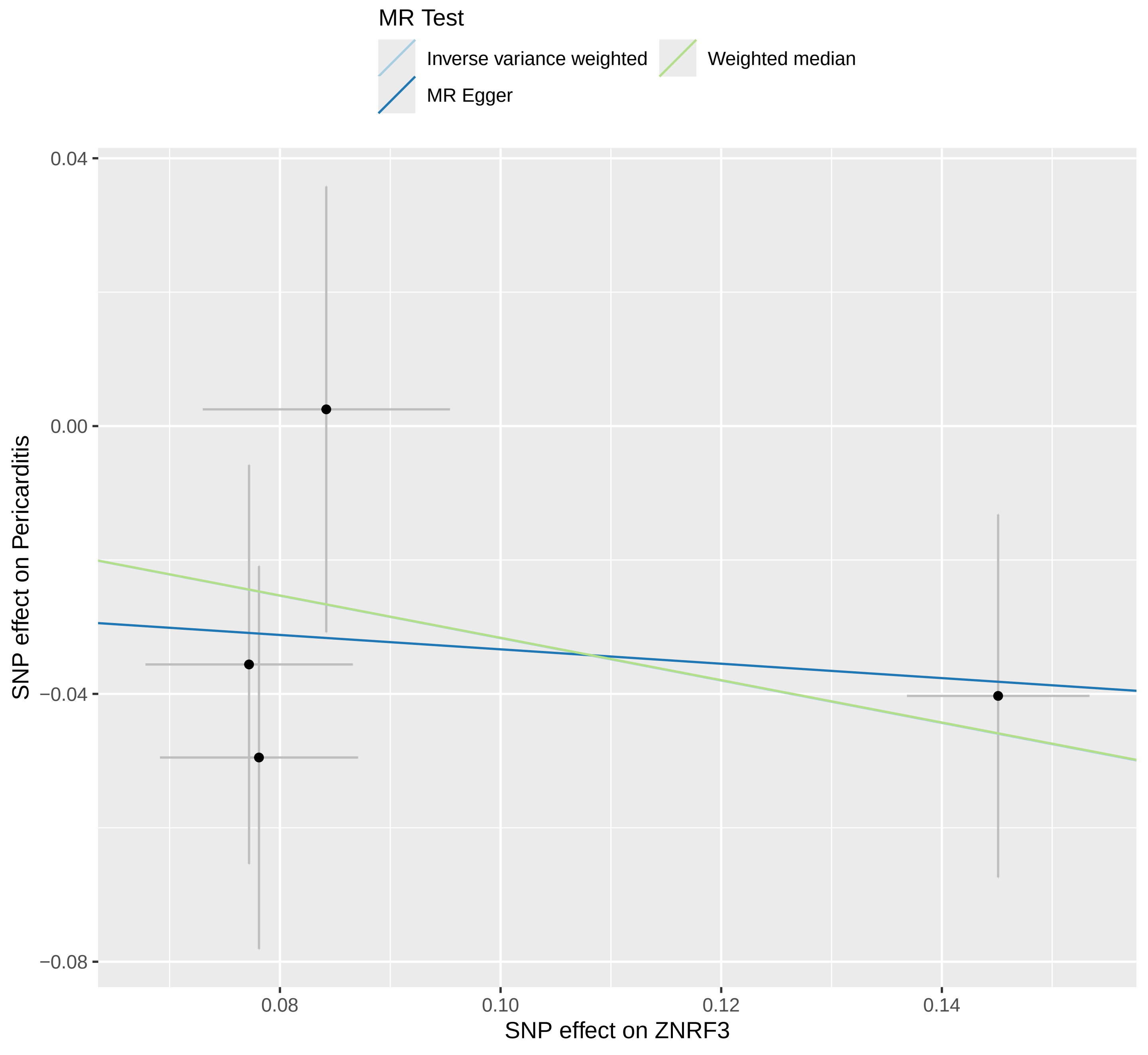
MR Test
Inverse variance weighted
MR Egger
Weighted median
0.04
0.00
−0.04
−0.08
SNP effect on Pericarditis
0.08
0.10
0.12
0.14
SNP effect on ZNRF3

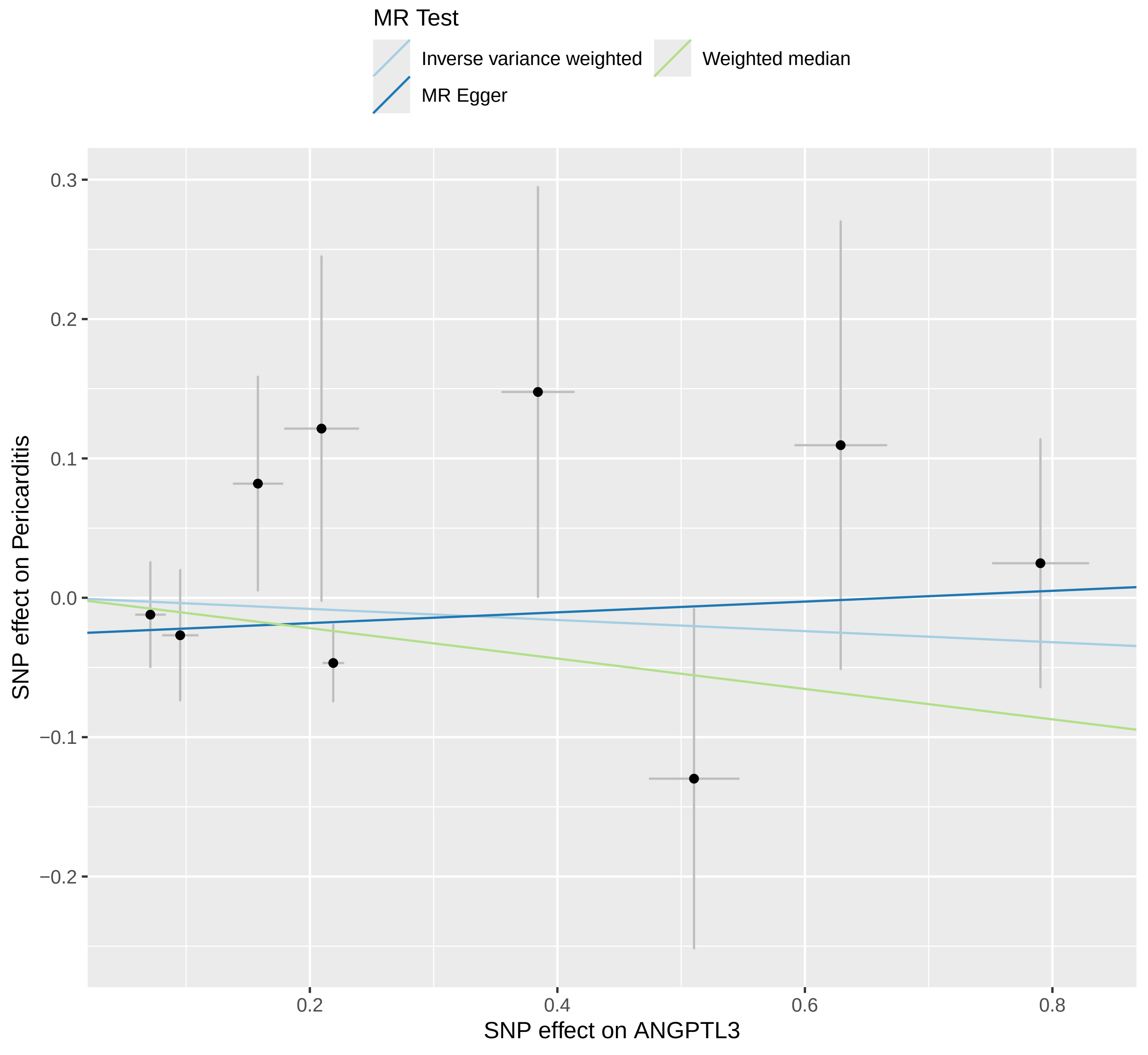

MR Test
Inverse variance weighted
Weighted median
MR Egger
0.3
0.2
0.1
0.0
−0.1
−0.2
SNP effect on Pericarditis
0.2
0.4
0.6
0.8
SNP effect on ANGPTL3

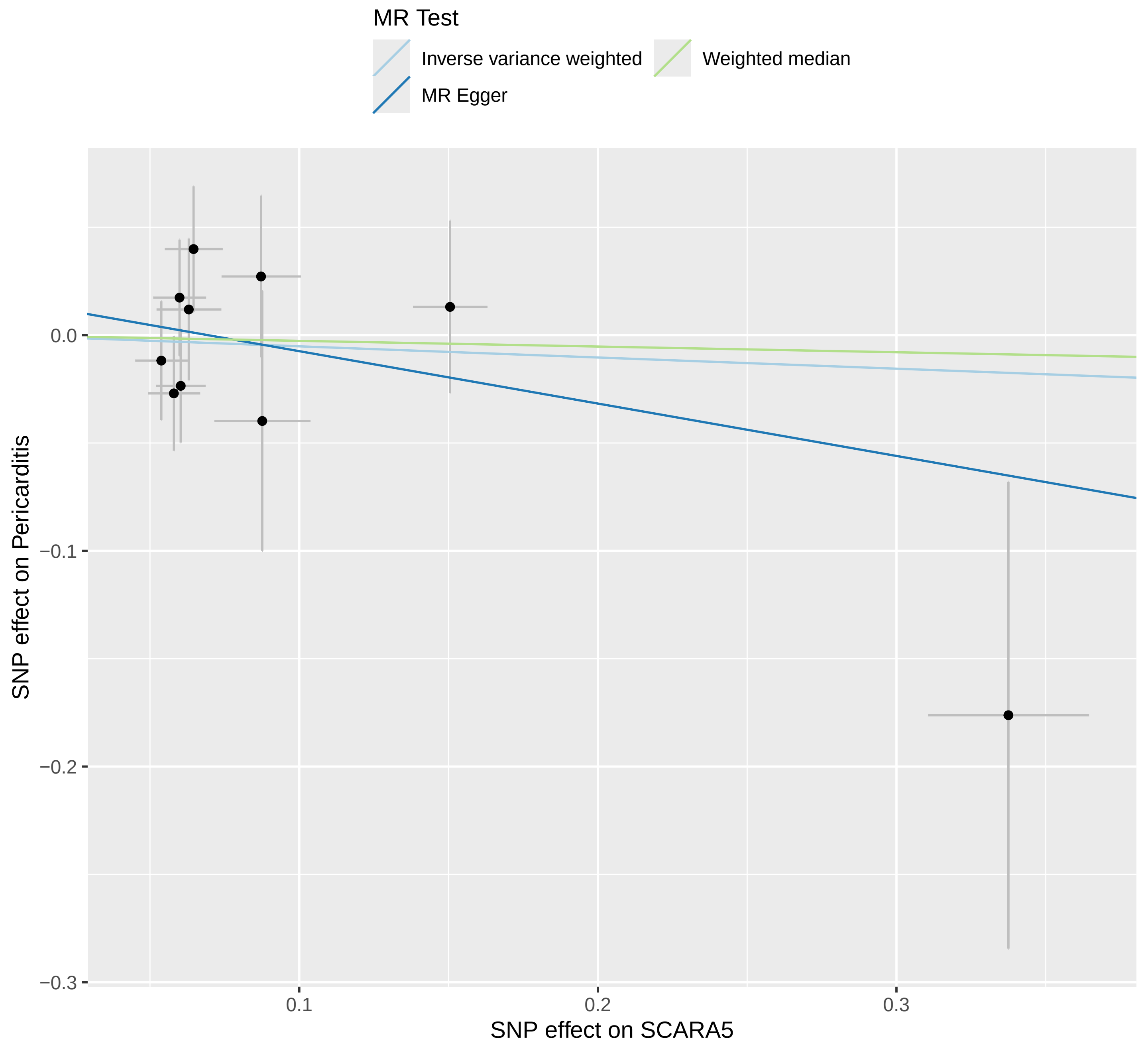

MR Test
Inverse variance weighted
Weighted median
MR Egger
SNP effect on Pericarditis
0.0
−0.1
−0.2
−0.3
0.1
0.2
0.3
SNP effect on SCARA5

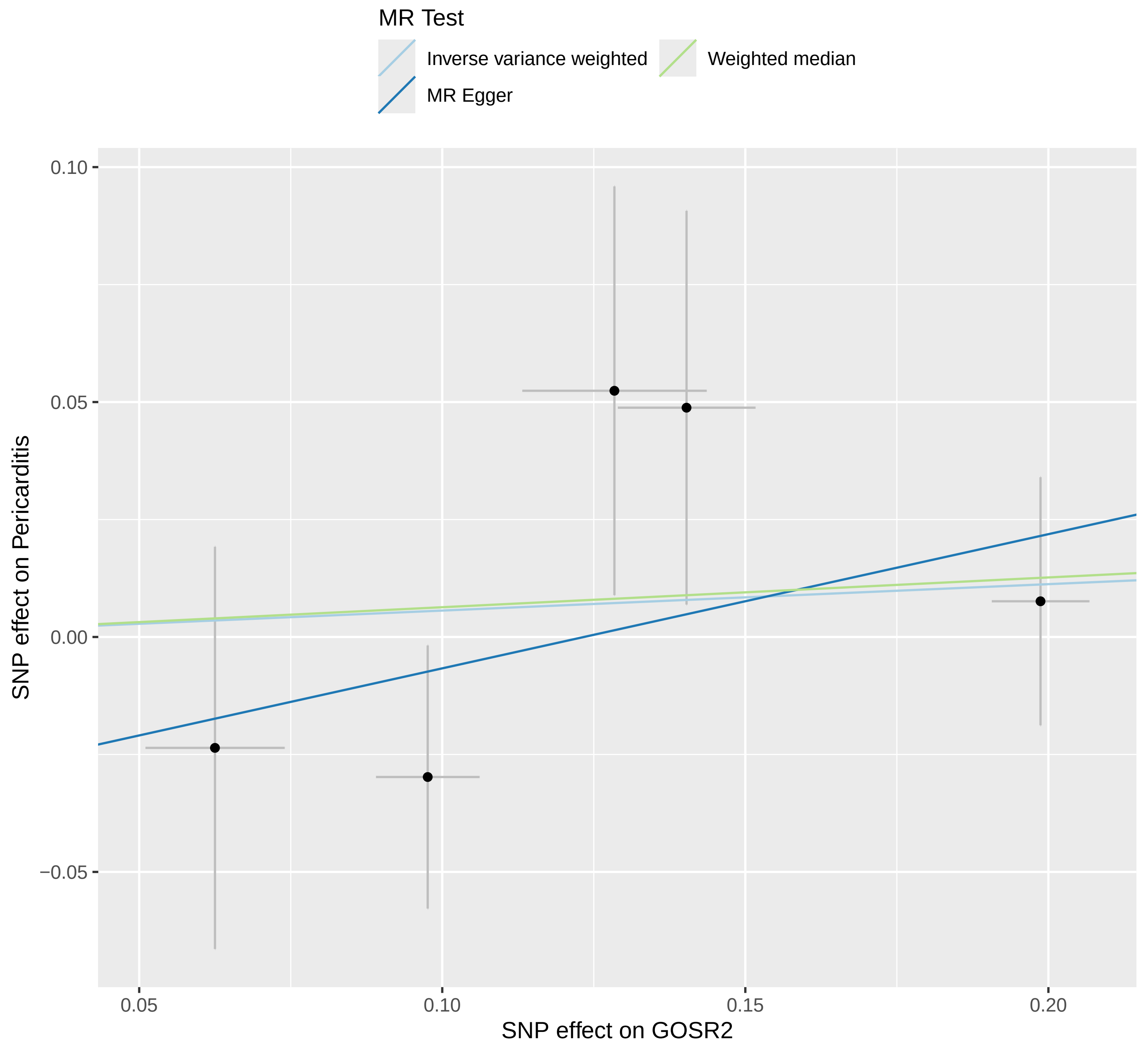
MR Test
Inverse variance weighted
MR Egger
Weighted median
0.10
0.05
0.00
−0.05
SNP effect on Pericarditis
0.05
0.10
0.15
0.20
SNP effect on GOSR2

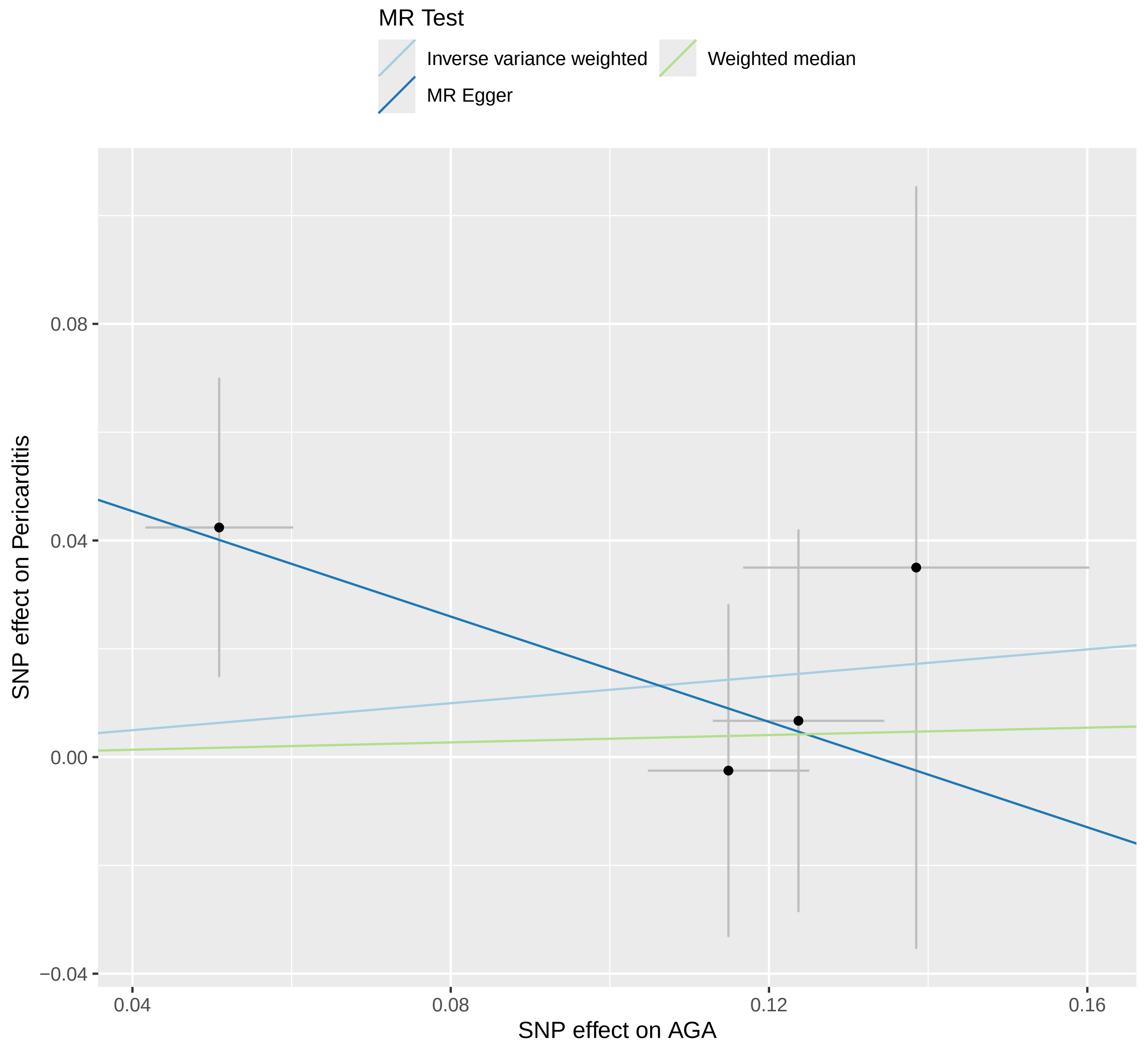
MR Test
Inverse variance weighted
MR Egger
Weighted median
SNP effect on Pericarditis
0.08
0.04
0.00
−0.04
0.04
0.08
0.12
0.16
SNP effect on AGA

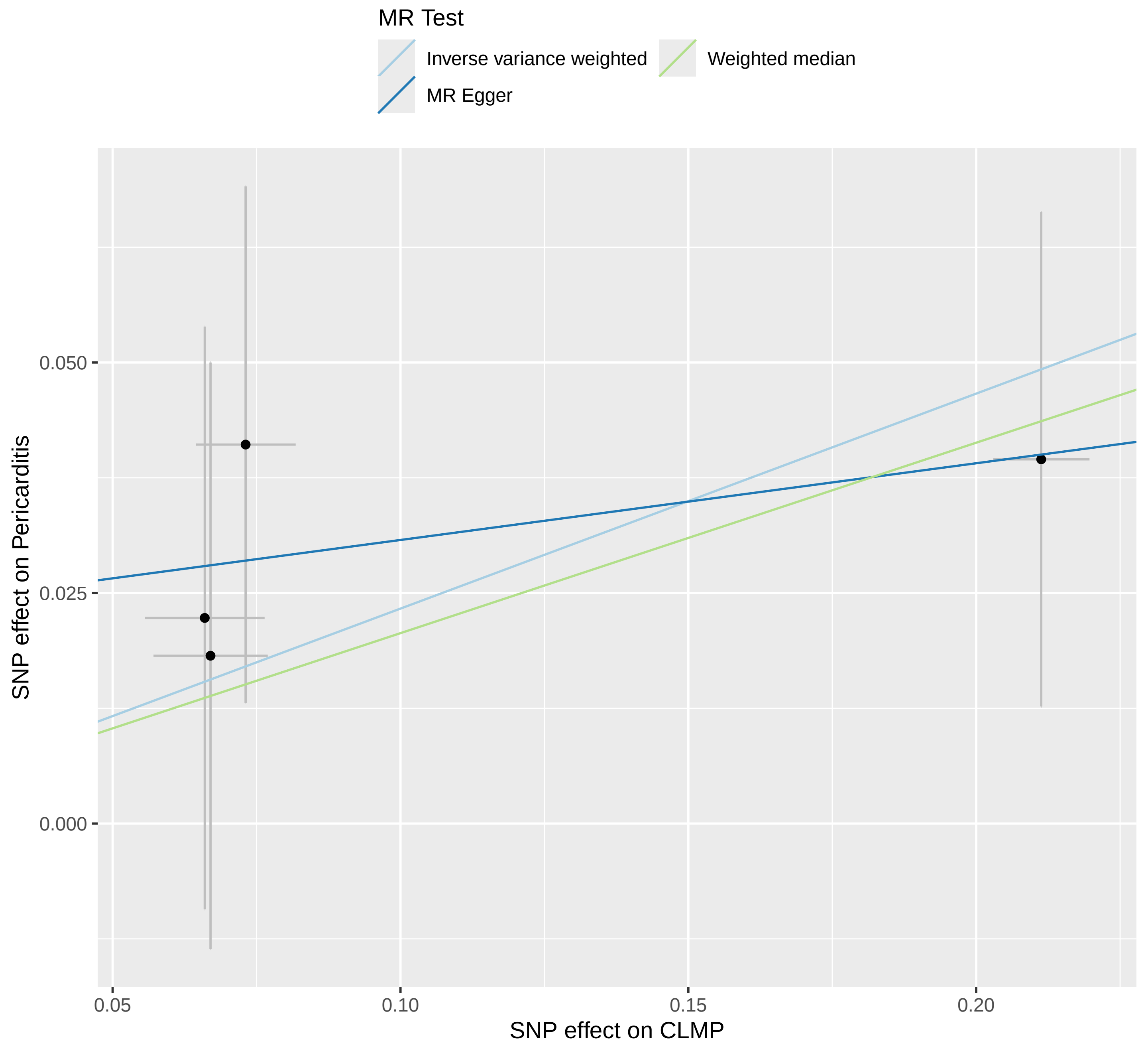
MR Test
Inverse variance weighted
MR Egger
Weighted median
SNP effect on Pericarditis
0.050
0.025
0.000
0.05
0.10
0.15
0.20
SNP effect on CLMP

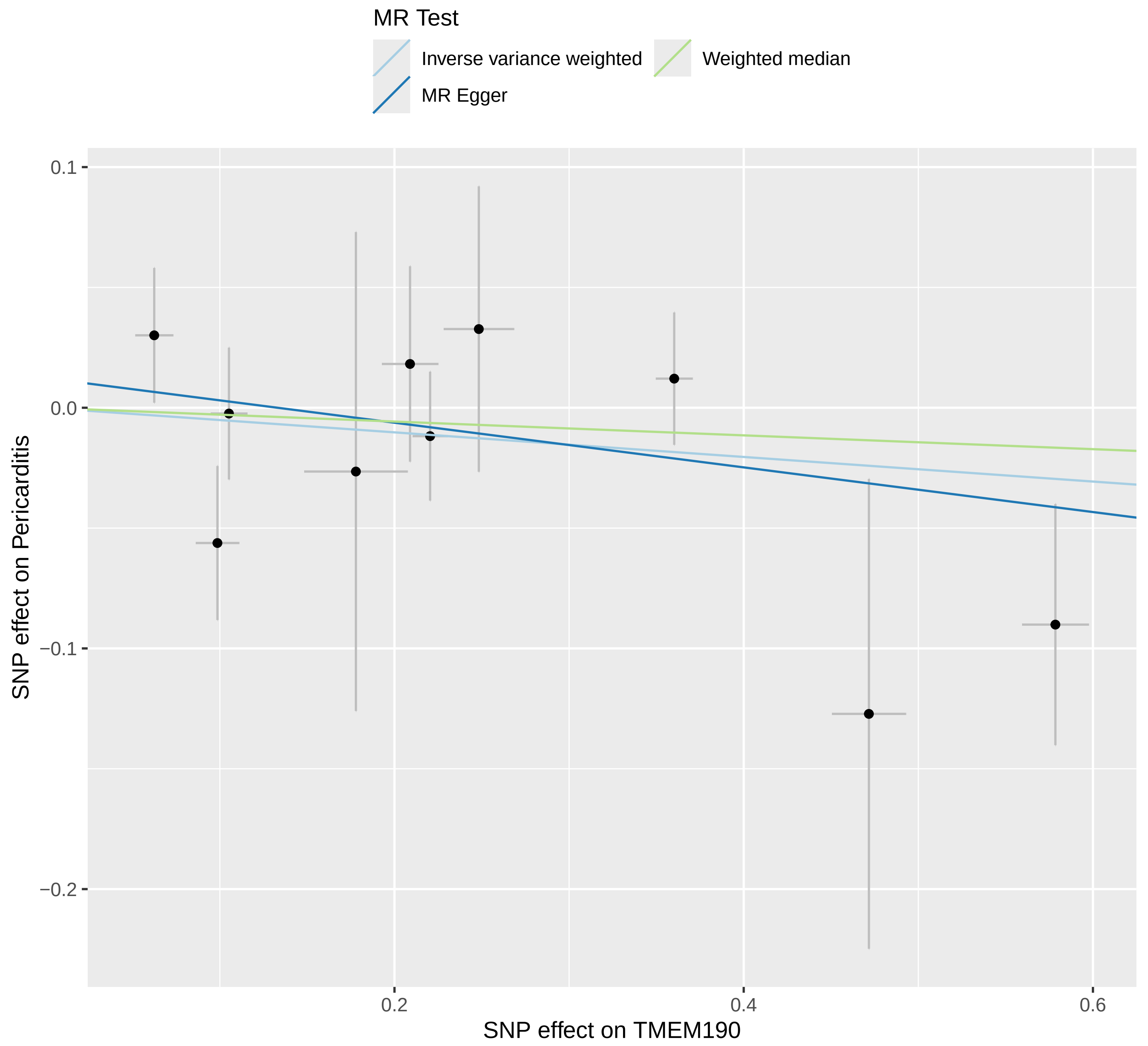

MR Test
Inverse variance weighted
MR Egger
Weighted median
0.1
0.0
−0.1
−0.2
SNP effect on Pericarditis
0.2
0.4
0.6
SNP effect on TMEM190

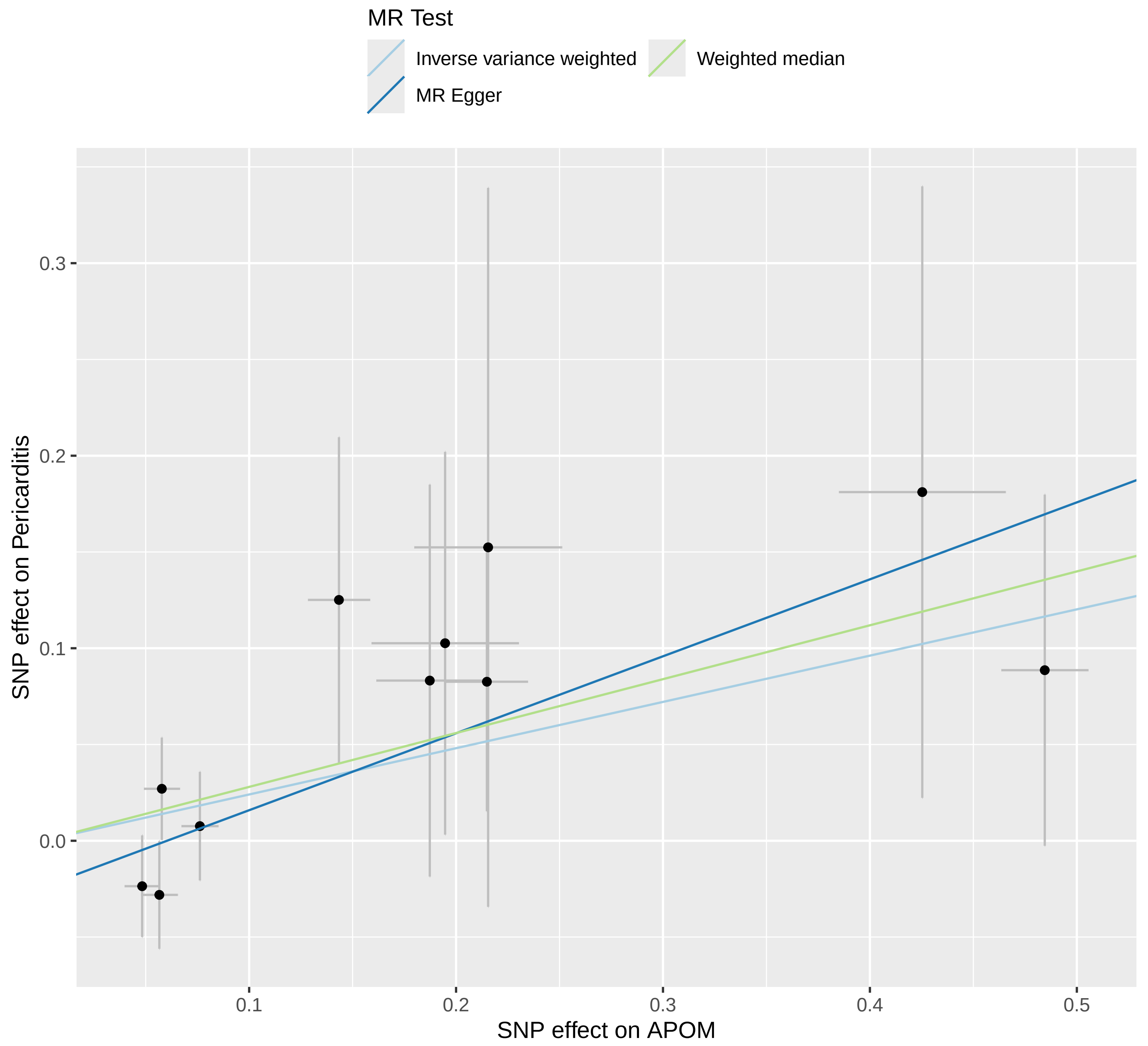
MR Test
Inverse variance weighted
MR Egger
Weighted median
0.3
0.2
0.1
0.0
SNP effect on Pericarditis
0.1
0.2
0.3
0.4
0.5
SNP effect on APOM

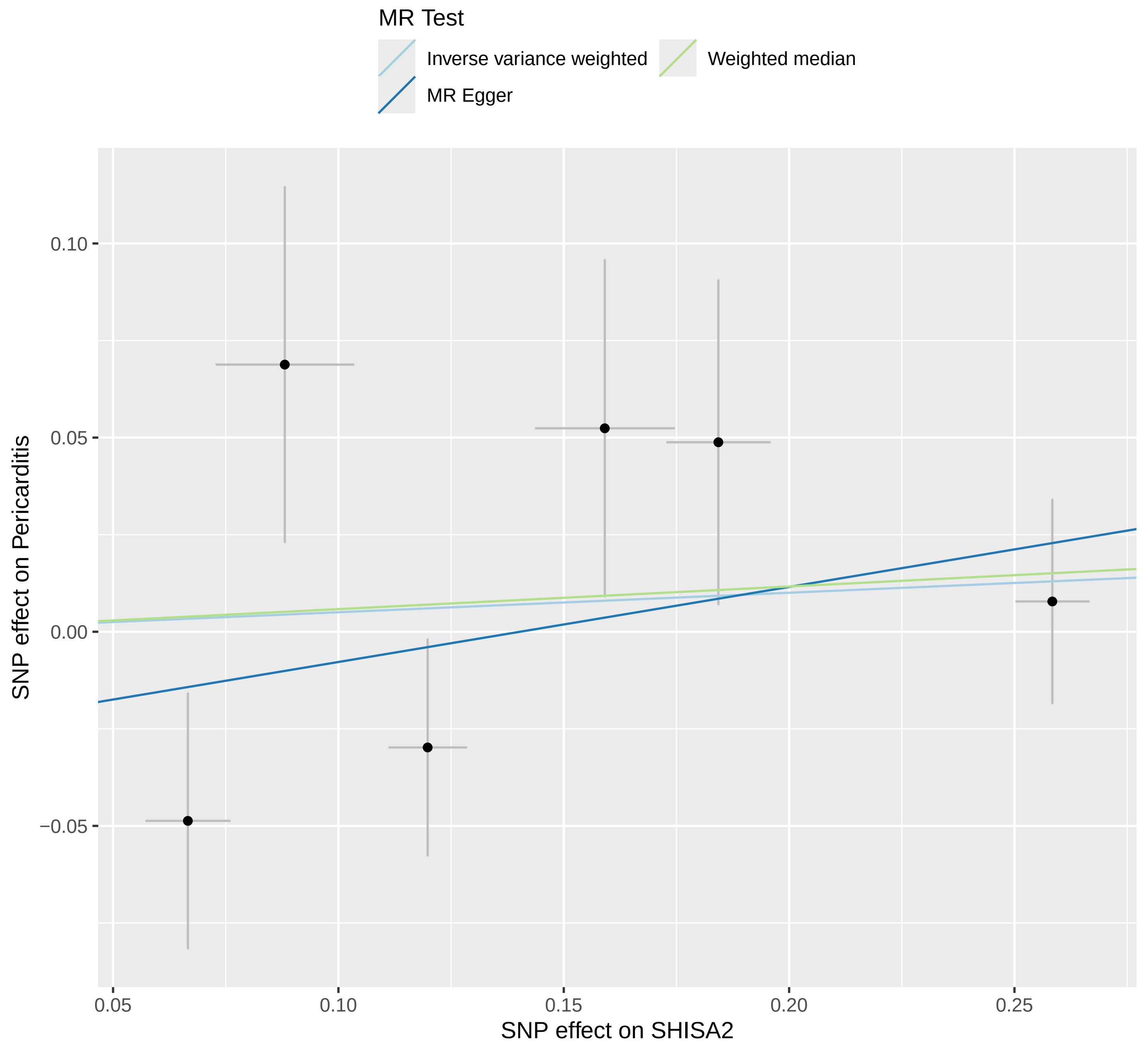

MR Test
Inverse variance weighted
MR Egger
Weighted median
SNP effect on Pericarditis
0.10
0.05
0.00
−0.05
0.05
0.10
0.15
0.20
0.25
SNP effect on SHISA2

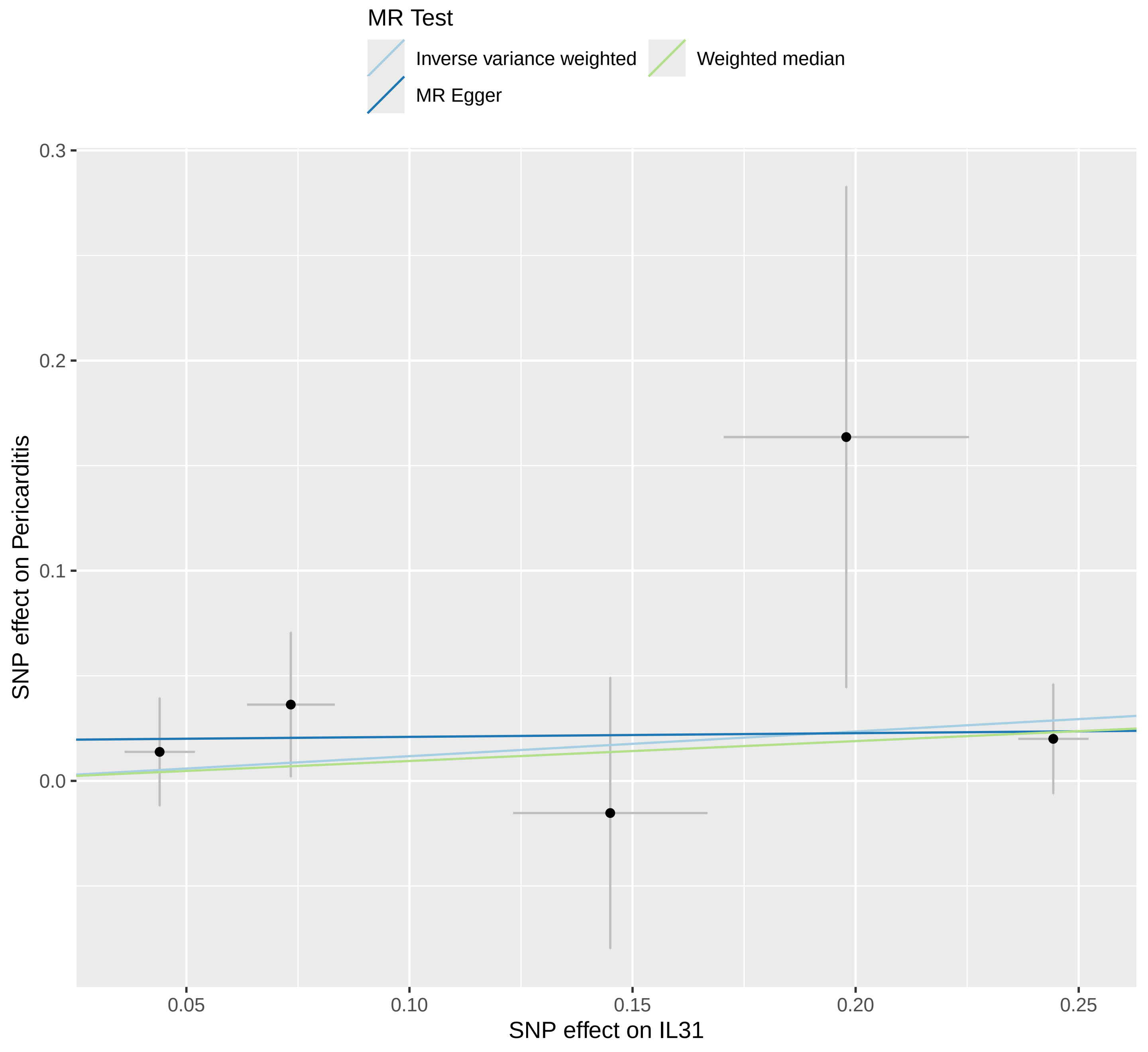

MR Test
Inverse variance weighted
MR Egger
Weighted median
0.3
0.2
0.1
0.0
0.05
0.10
0.15
0.20
0.25
SNP effect on Pericarditis
SNP effect on IL31

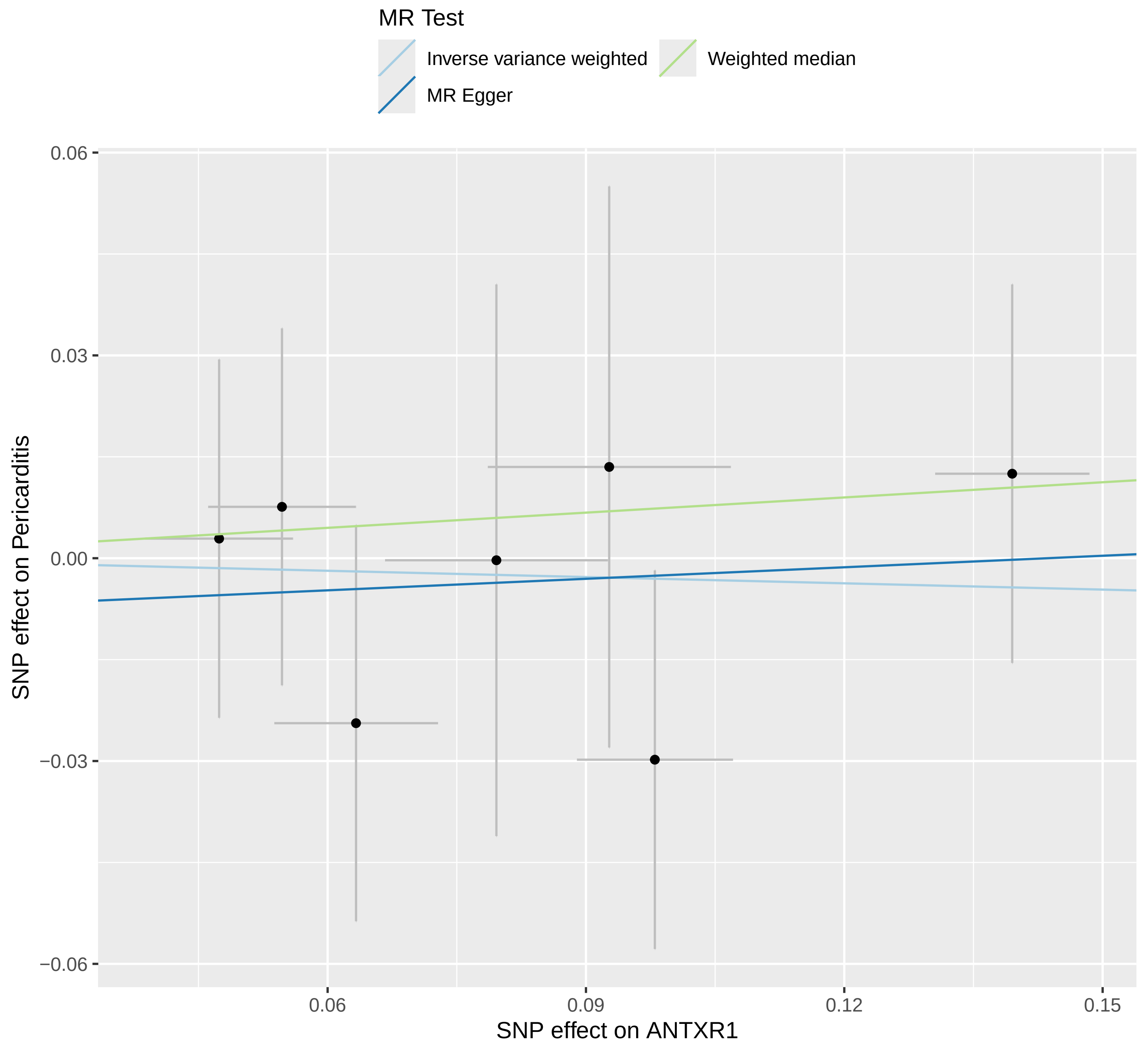

MR Test
Inverse variance weighted
Weighted median
MR Egger
0.06
0.03
0.00
−0.03
−0.06
SNP effect on Pericarditis
0.06
0.09
0.12
0.15
SNP effect on ANTXR1

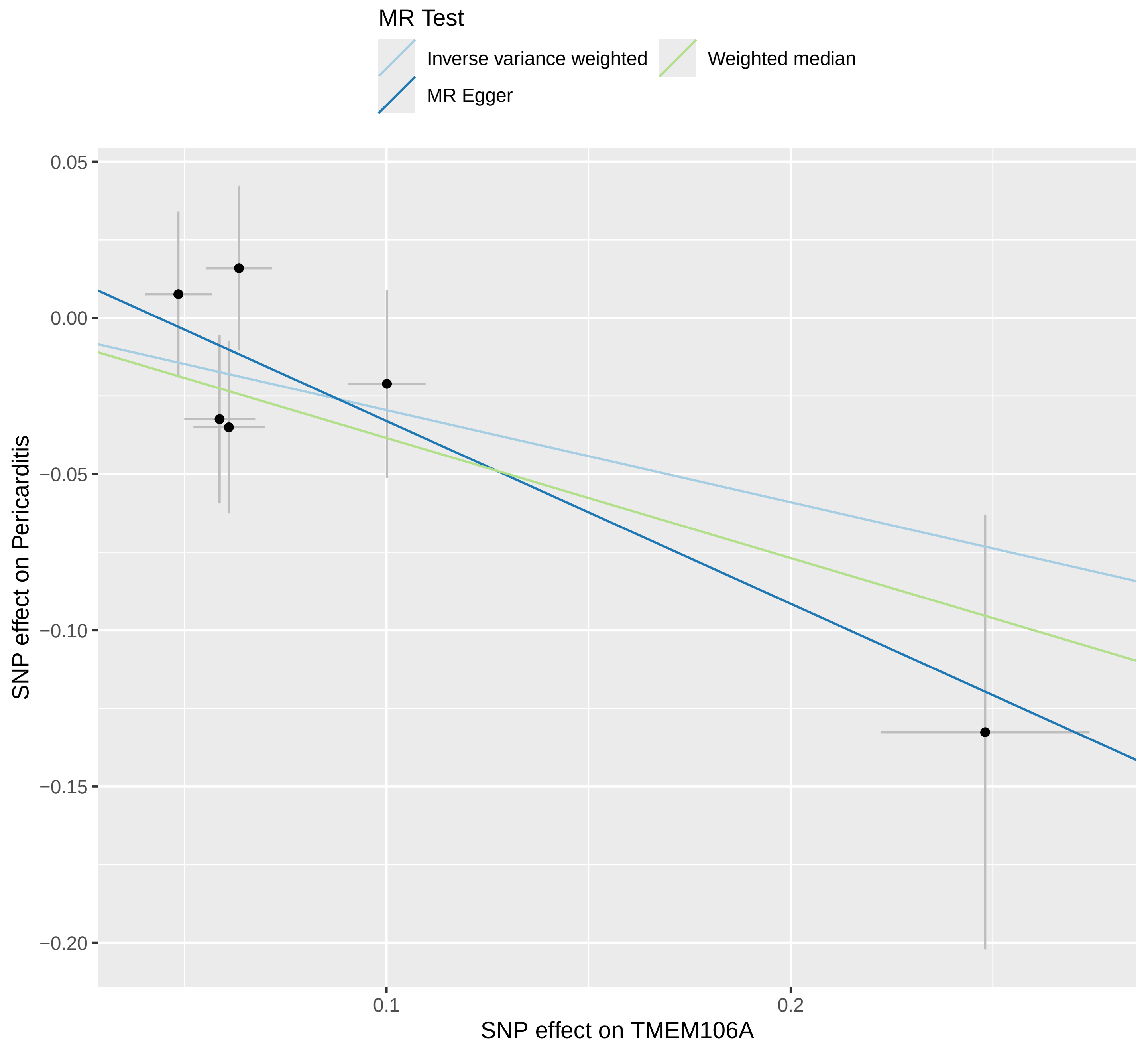
MR Test
Inverse variance weighted
Weighted median
MR Egger
0.05
0.00
−0.05
−0.10
−0.15
−0.20
0.1
0.2
SNP effect on Pericarditis
SNP effect on TMEM106A

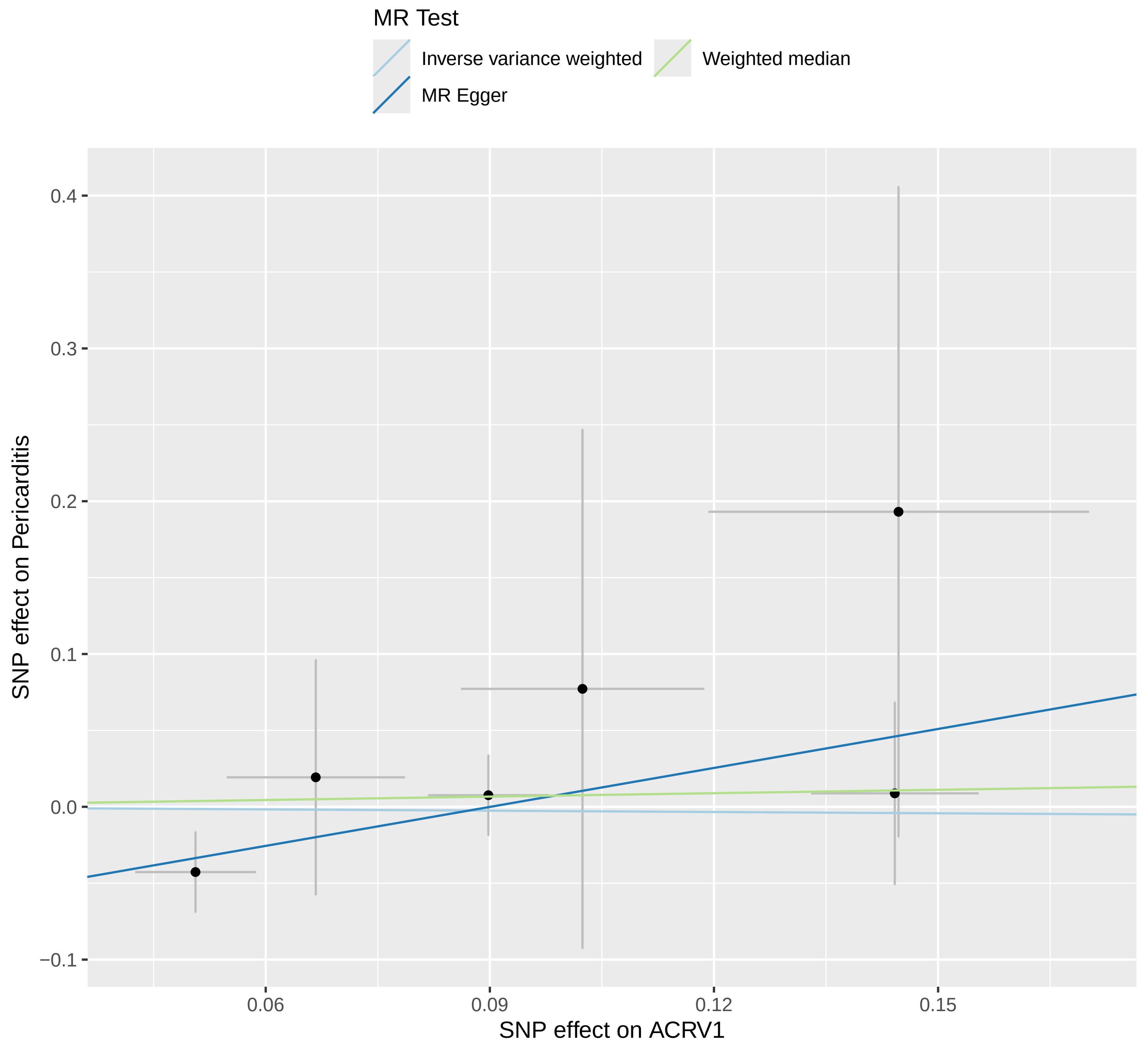
MR Test
Inverse variance weighted
Weighted median
MR Egger
SNP effect on Pericarditis
0.4
0.3
0.2
0.1
0.0
−0.1
0.06
0.09
0.12
0.15
SNP effect on ACRV1

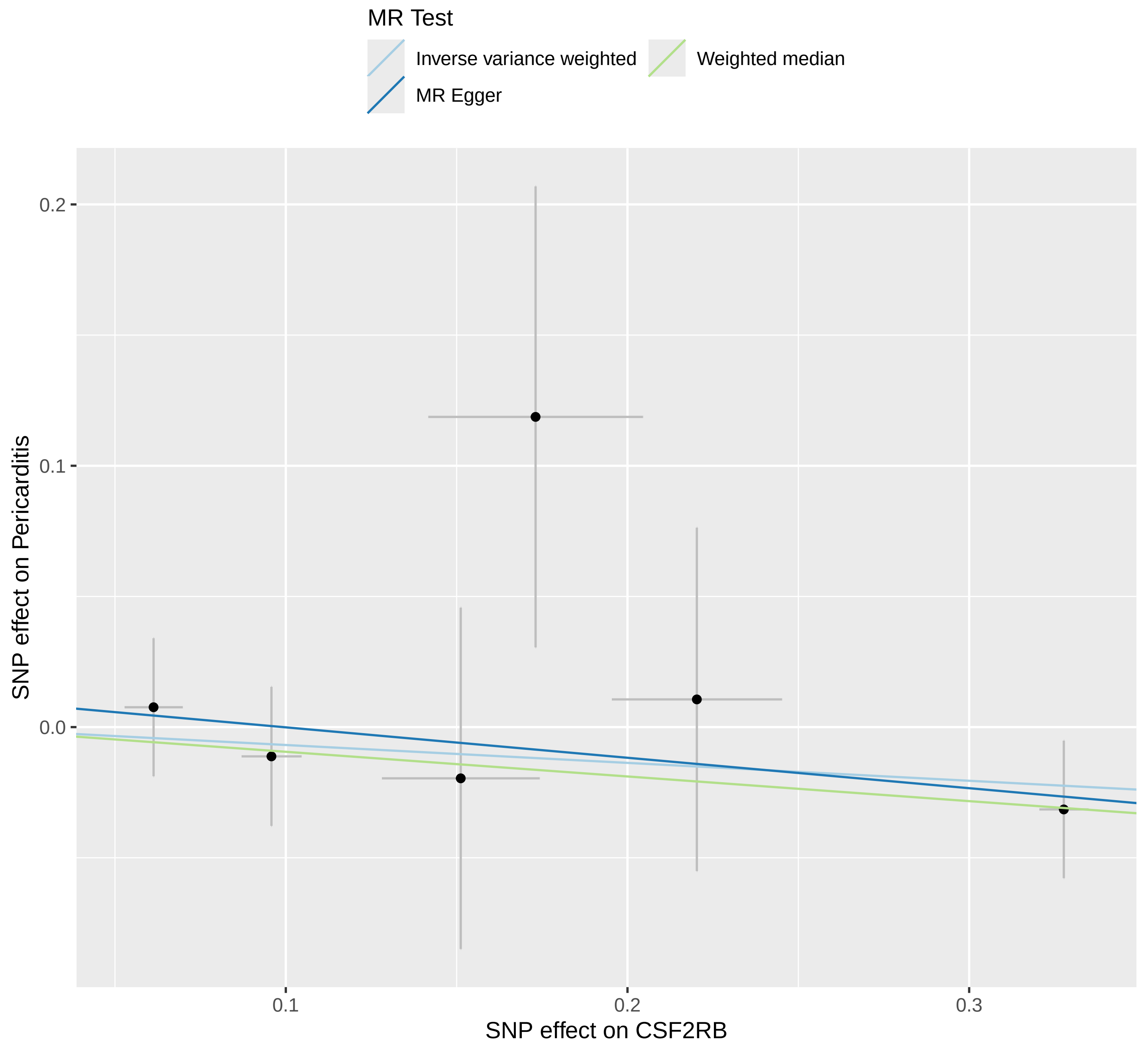
MR Test
Inverse variance weighted
Weighted median
MR Egger
0.2
0.1
0.0
0.1
0.2
0.3
SNP effect on Pericarditis
SNP effect on CSF2RB

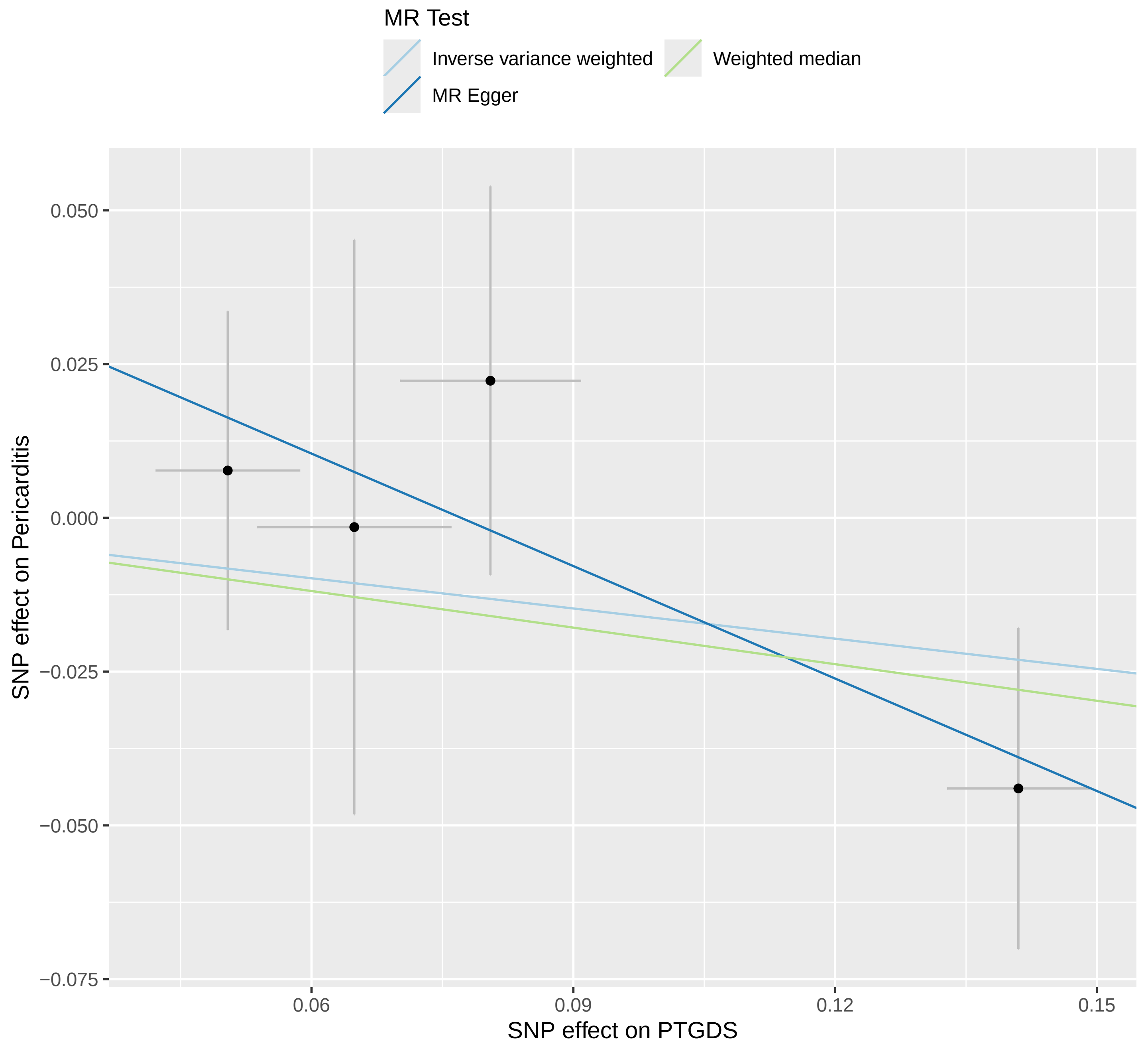
MR Test
Inverse variance weighted
MR Egger
Weighted median
0.050
0.025
0.000
−0.025
−0.050
−0.075
SNP effect on Pericarditis
0.06
0.09
0.12
0.15
SNP effect on PTGDS

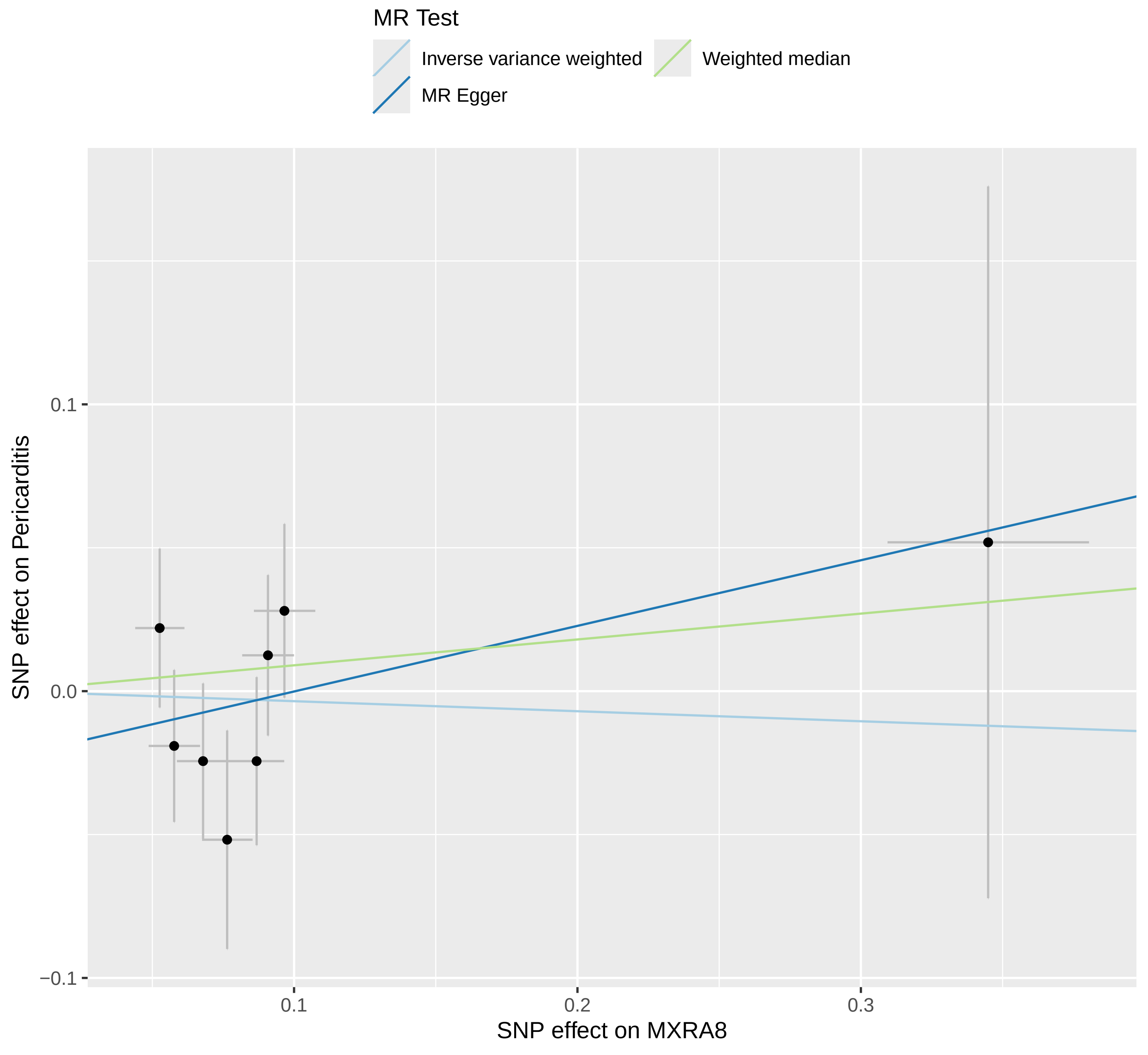
MR Test
Inverse variance weighted
Weighted median
MR Egger
SNP effect on Pericarditis
0.1
0.0
−0.1
0.1
0.2
0.3
SNP effect on MXRA8

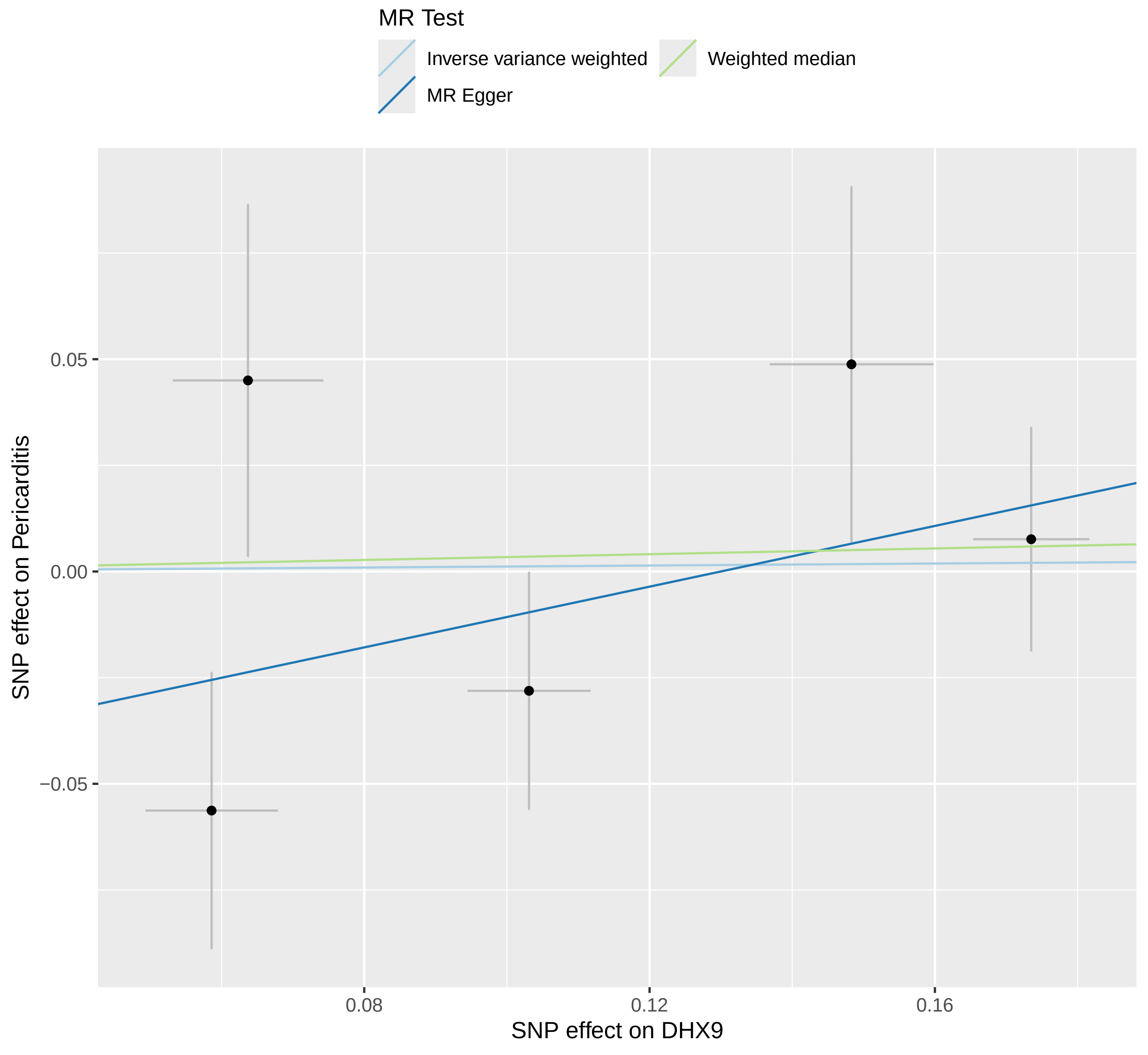

MR Test
Inverse variance weighted
MR Egger
Weighted median
0.05
0.00
−0.05
0.08
0.12
0.16
SNP effect on Pericarditis
SNP effect on DHX9

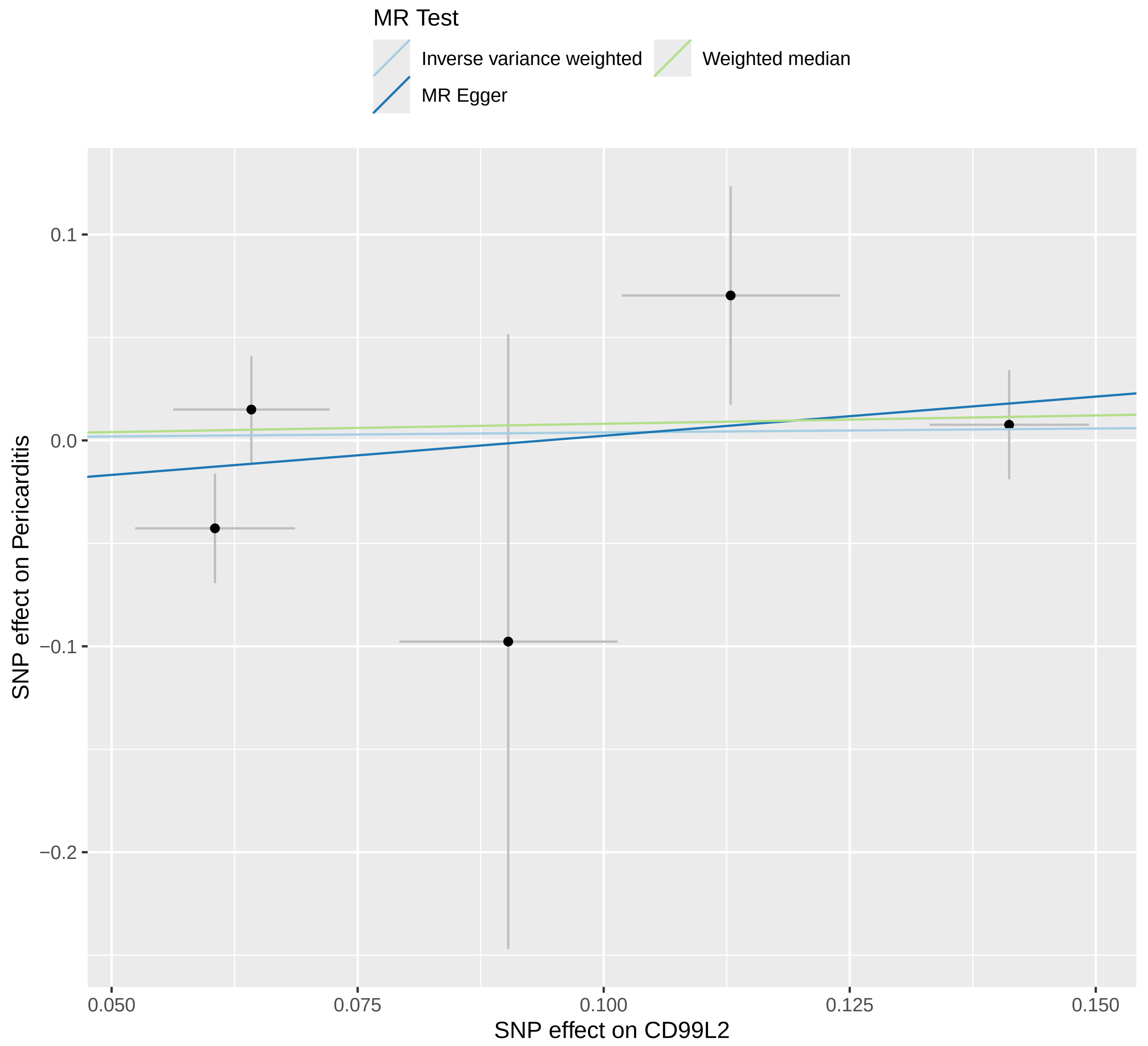
MR Test
Inverse variance weighted
Weighted median
MR Egger
0.1
0.0
−0.1
−0.2
SNP effect on Pericarditis
0.050
0.075
0.100
0.125
0.150
SNP effect on CD99L2

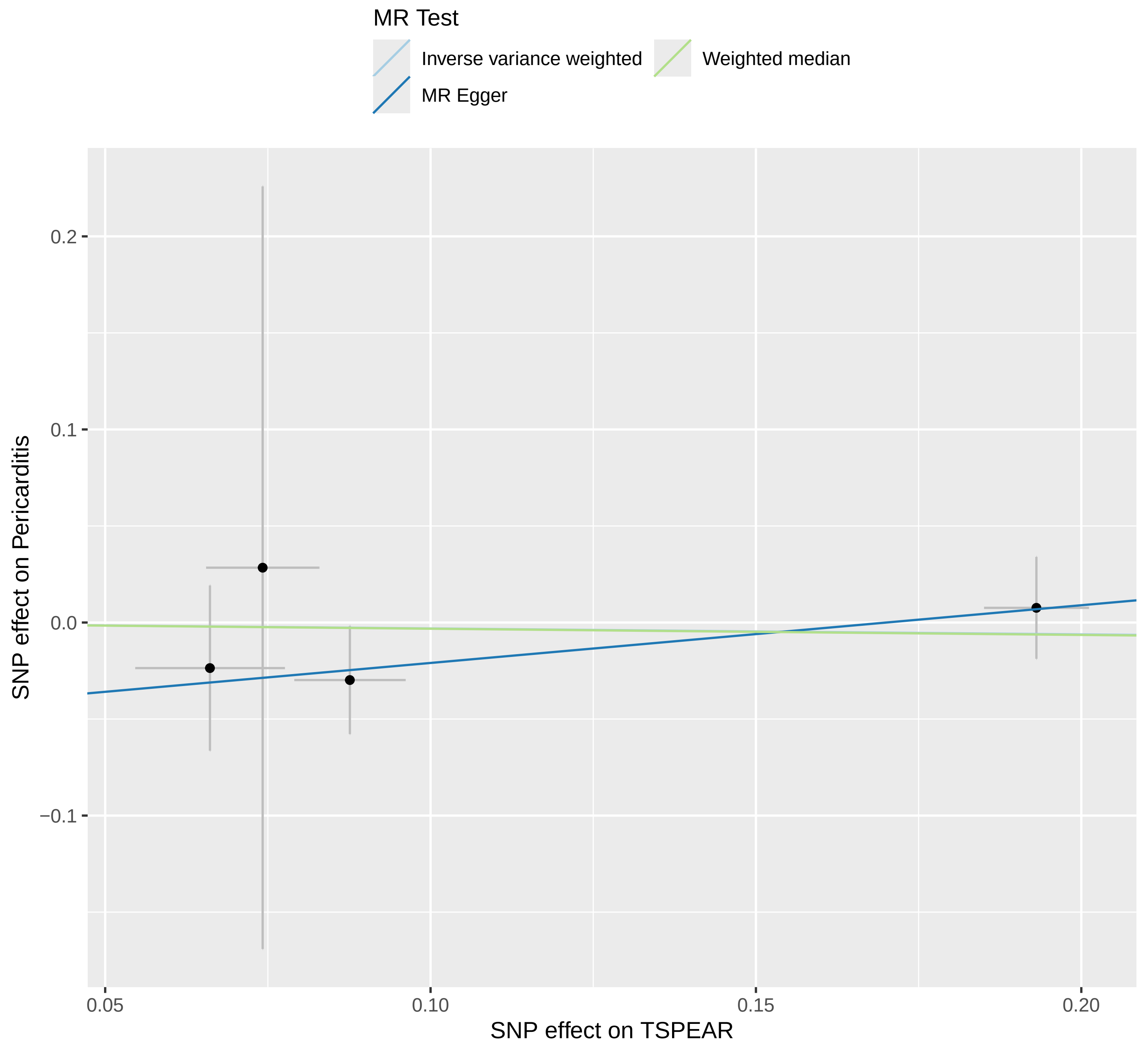
MR Test
Inverse variance weighted
Weighted median
MR Egger
SNP effect on Pericarditis
0.2
0.1
0.0
−0.1
0.05
0.10
0.15
0.20
SNP effect on TSPEAR

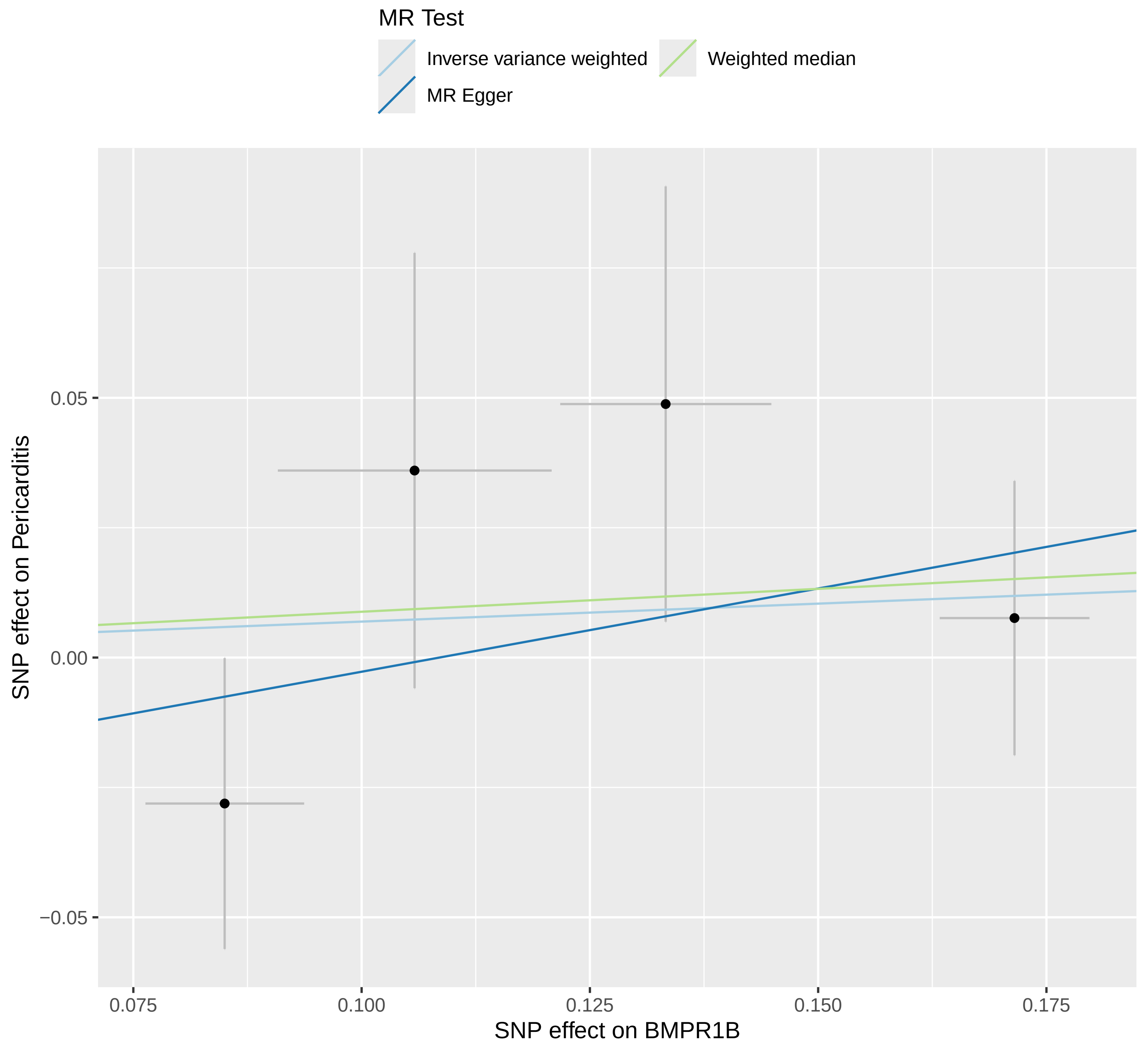

MR Test
Inverse variance weighted
MR Egger
Weighted median
SNP effect on Pericarditis
0.05
0.00
−0.05
0.075
0.100
0.125
0.150
0.175
SNP effect on BMPR1B

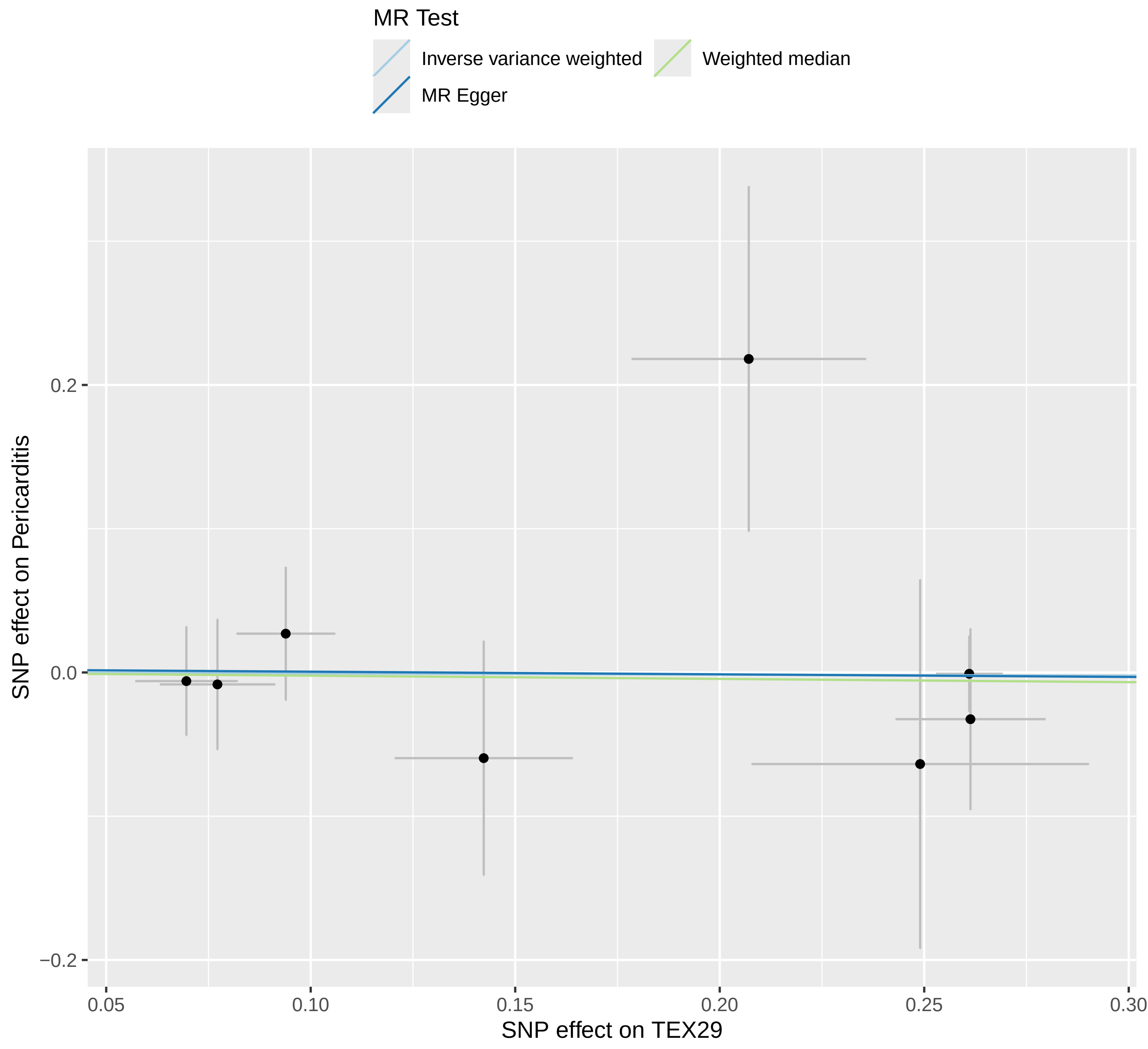

MR Test
Inverse variance weighted
MR Egger
Weighted median
SNP effect on Pericarditis
0.2
0.0
−0.2
0.05
0.10
0.15
0.20
0.25
0.30
SNP effect on TEX29

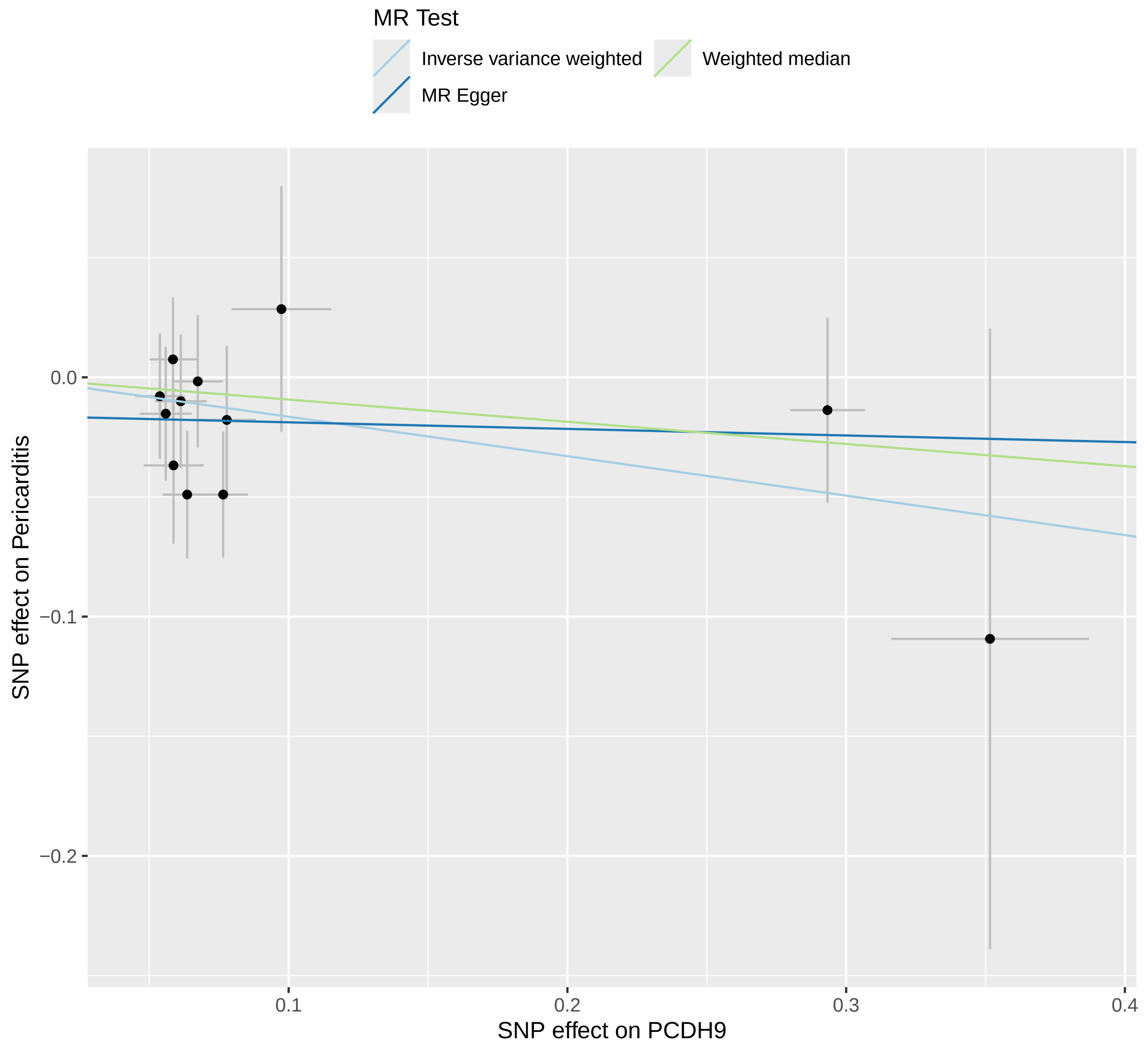

MR Test
Inverse variance weighted
Weighted median
MR Egger
0.0
−0.1
−0.2
SNP effect on Pericarditis
0.1
0.2
0.3
0.4
SNP effect on PCDH9

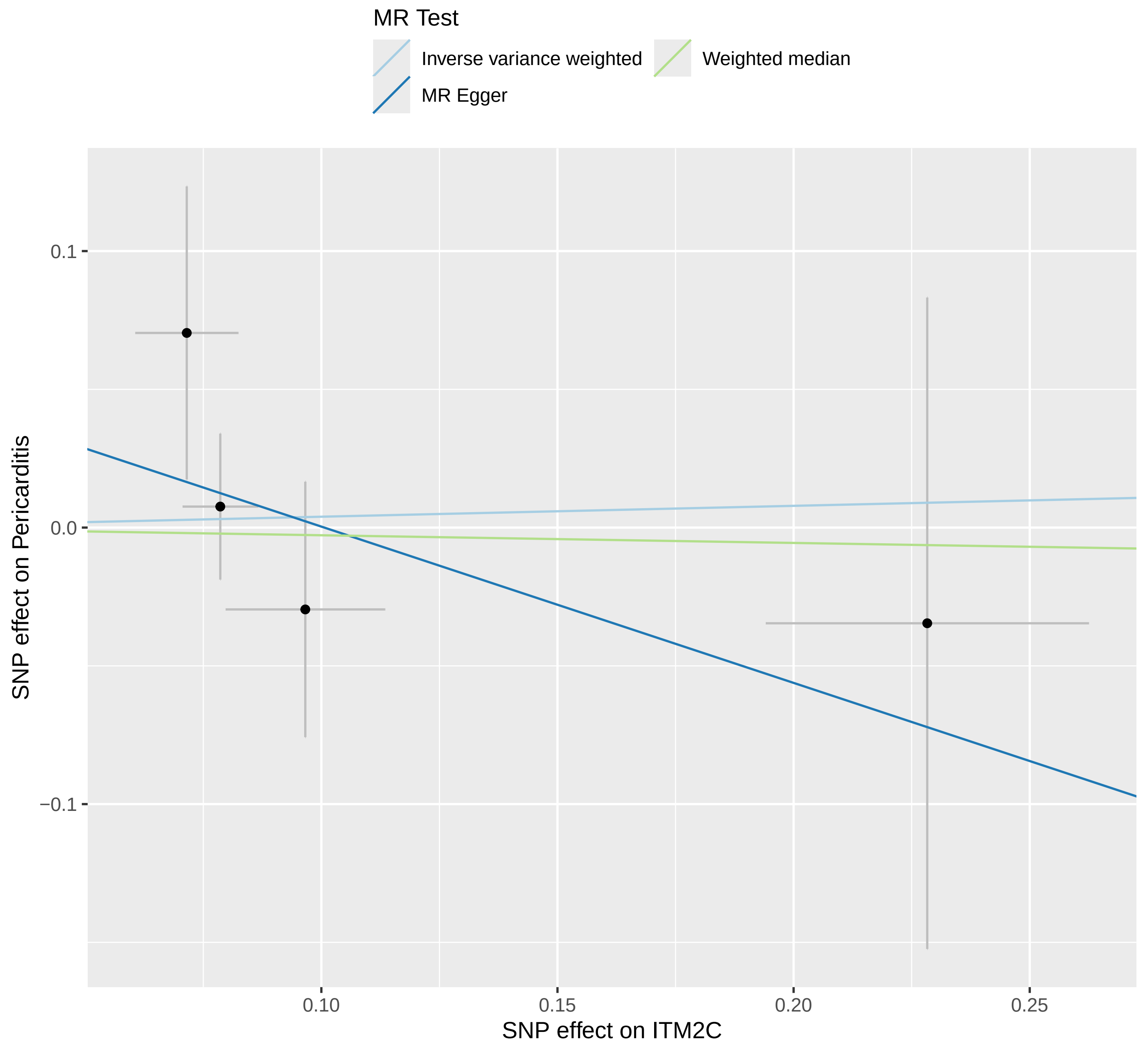

MR Test
Inverse variance weighted
Weighted median
MR Egger
0.1
0.0
−0.1
SNP effect on Pericarditis
0.10
0.15
0.20
0.25
SNP effect on ITM2C

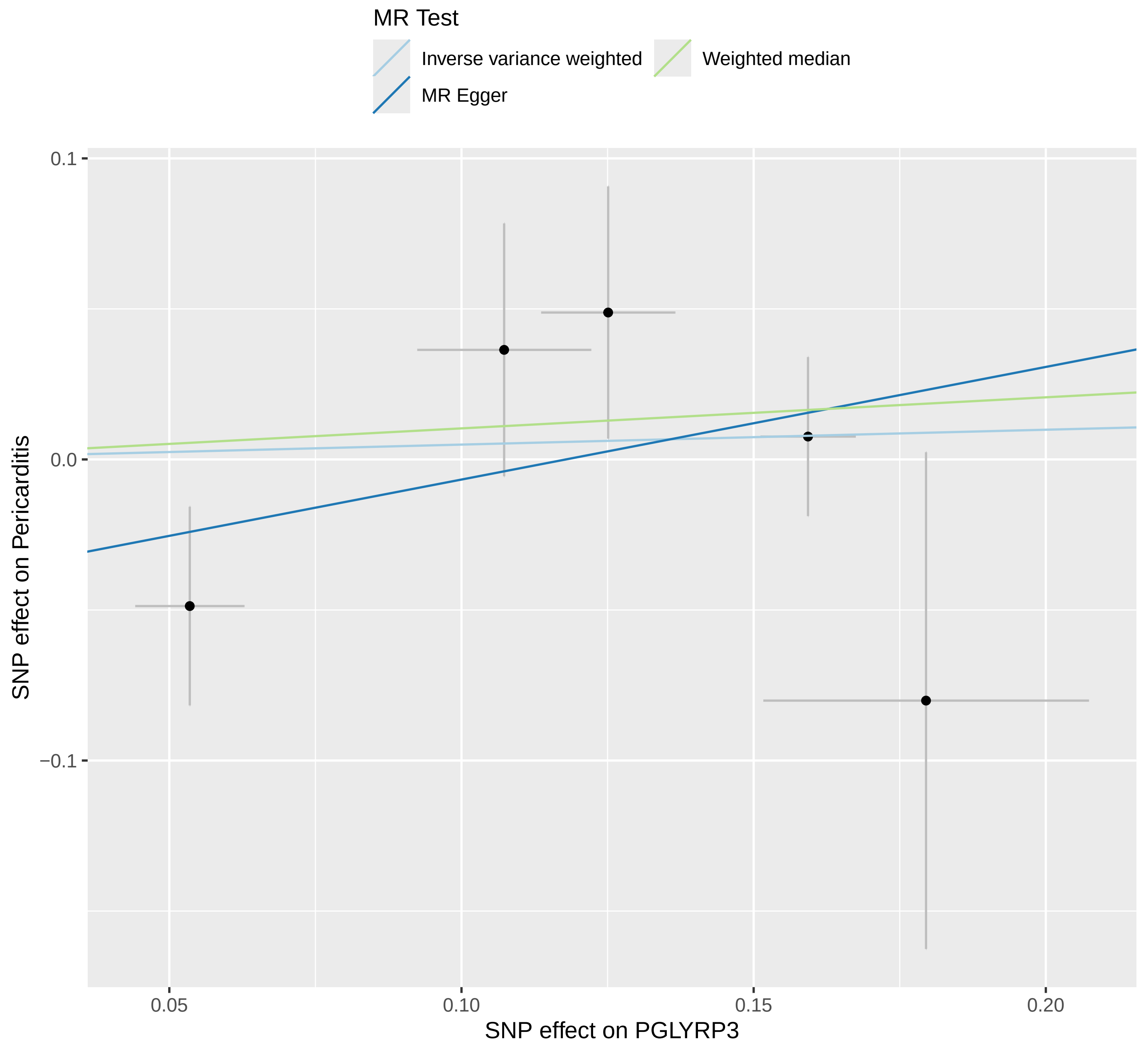
MR Test
Inverse variance weighted
Weighted median
MR Egger
0.1
0.0
−0.1
SNP effect on Pericarditis
0.05
0.10
0.15
0.20
SNP effect on PGLYRP3

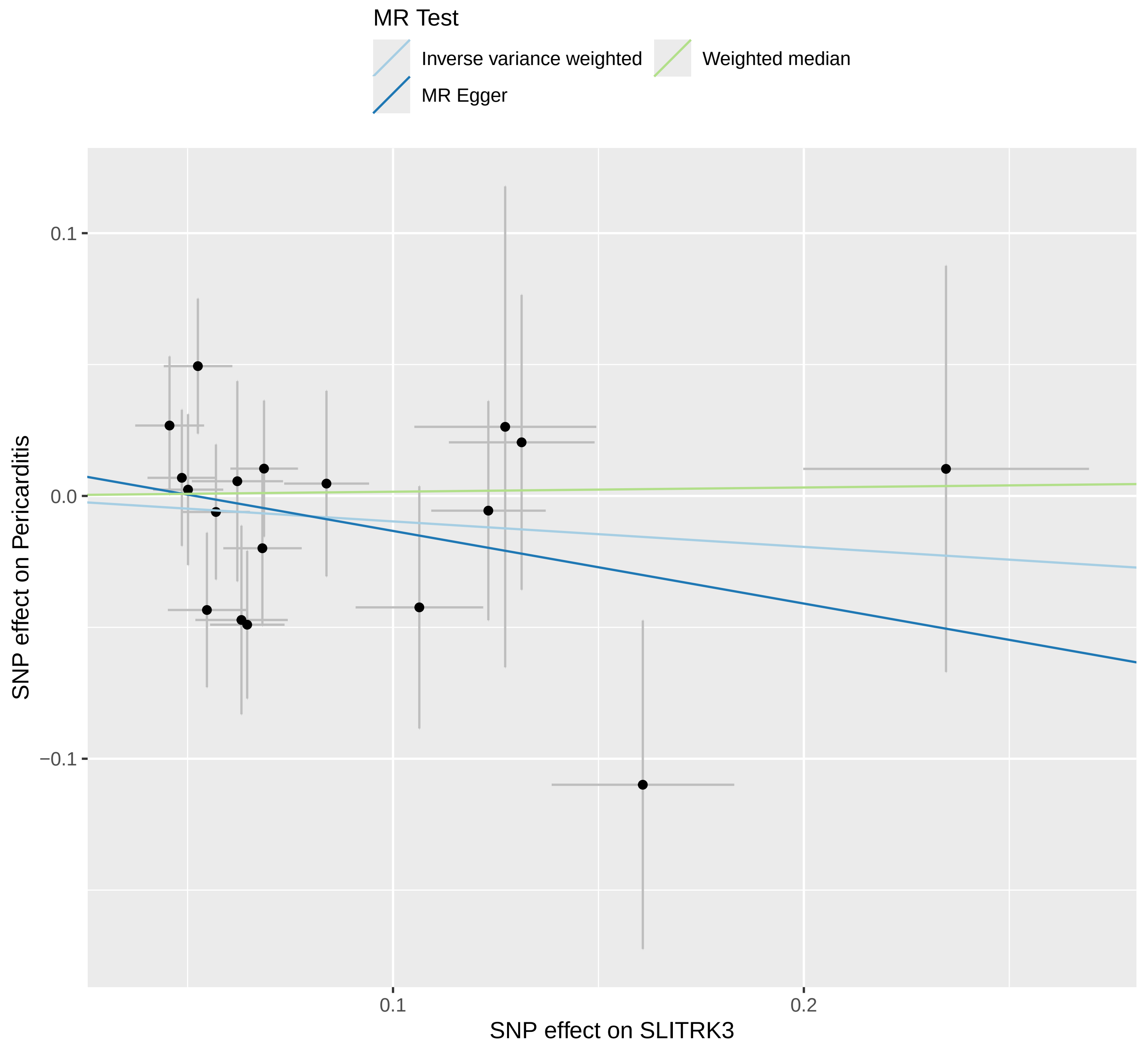
MR Test
Inverse variance weighted
Weighted median
MR Egger
0.1
0.0
−0.1
0.1
0.2
SNP effect on Pericarditis
SNP effect on SLITRK3

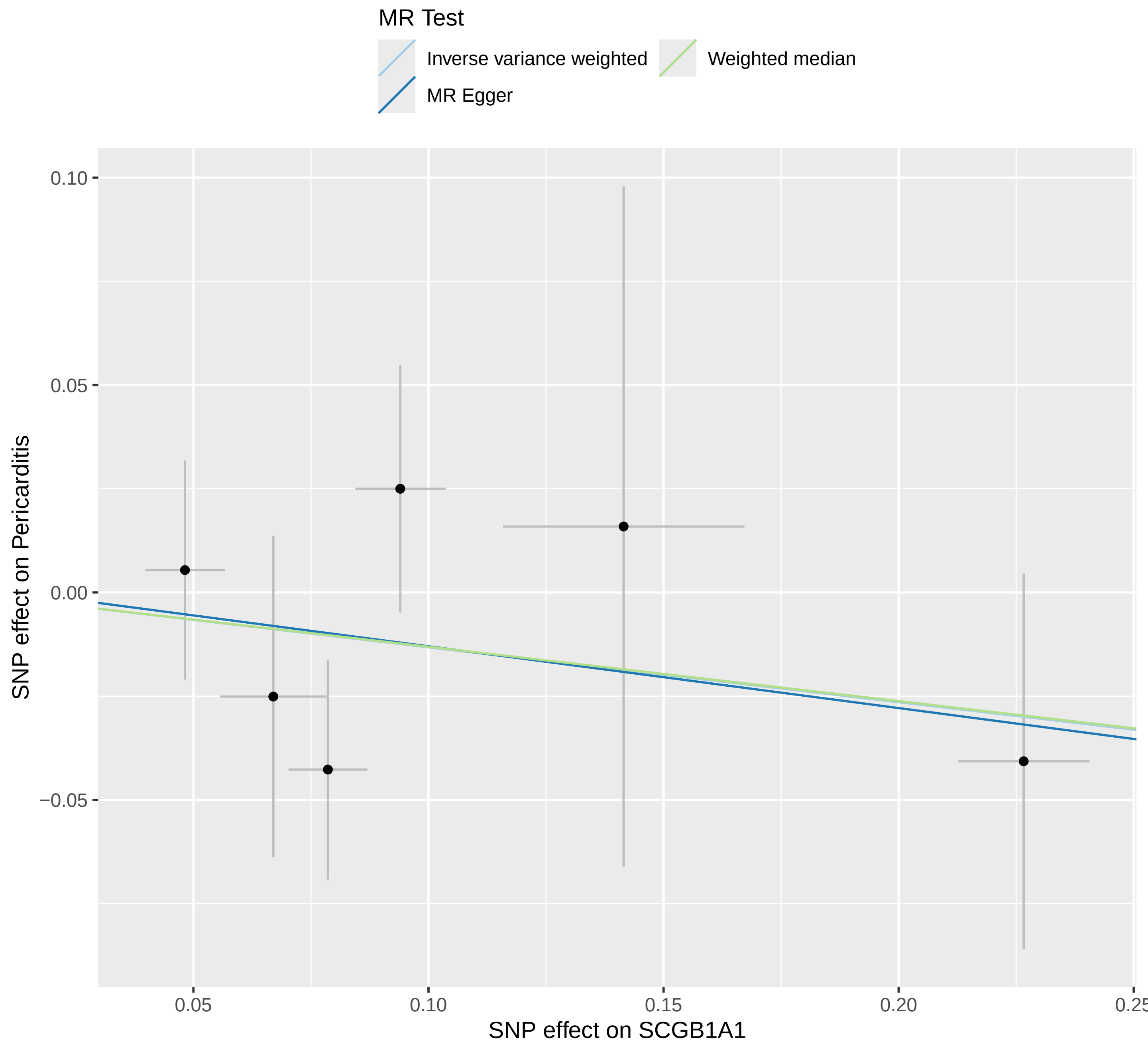
MR Test
Inverse variance weighted
Weighted median
MR Egger
0.10
0.05
0.00
−0.05
SNP effect on Pericarditis
0.05
0.10
0.15
0.20
0.25
SNP effect on SCGB1A1

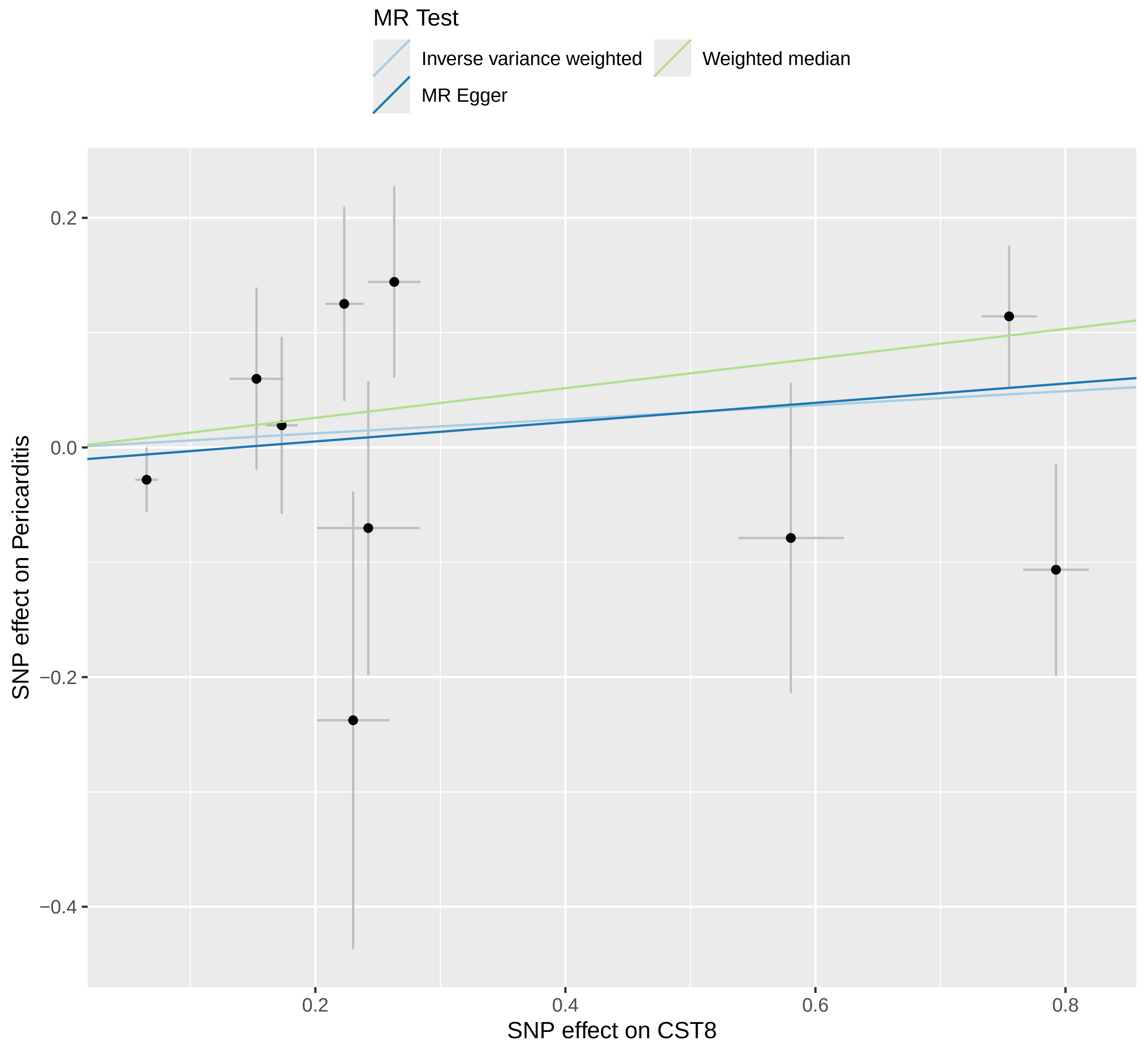

MR Test
Inverse variance weighted
Weighted median
MR Egger
SNP effect on Pericarditis
SNP effect on CST8
0.2
0.0
−0.2
−0.4
0.2
0.4
0.6
0.8

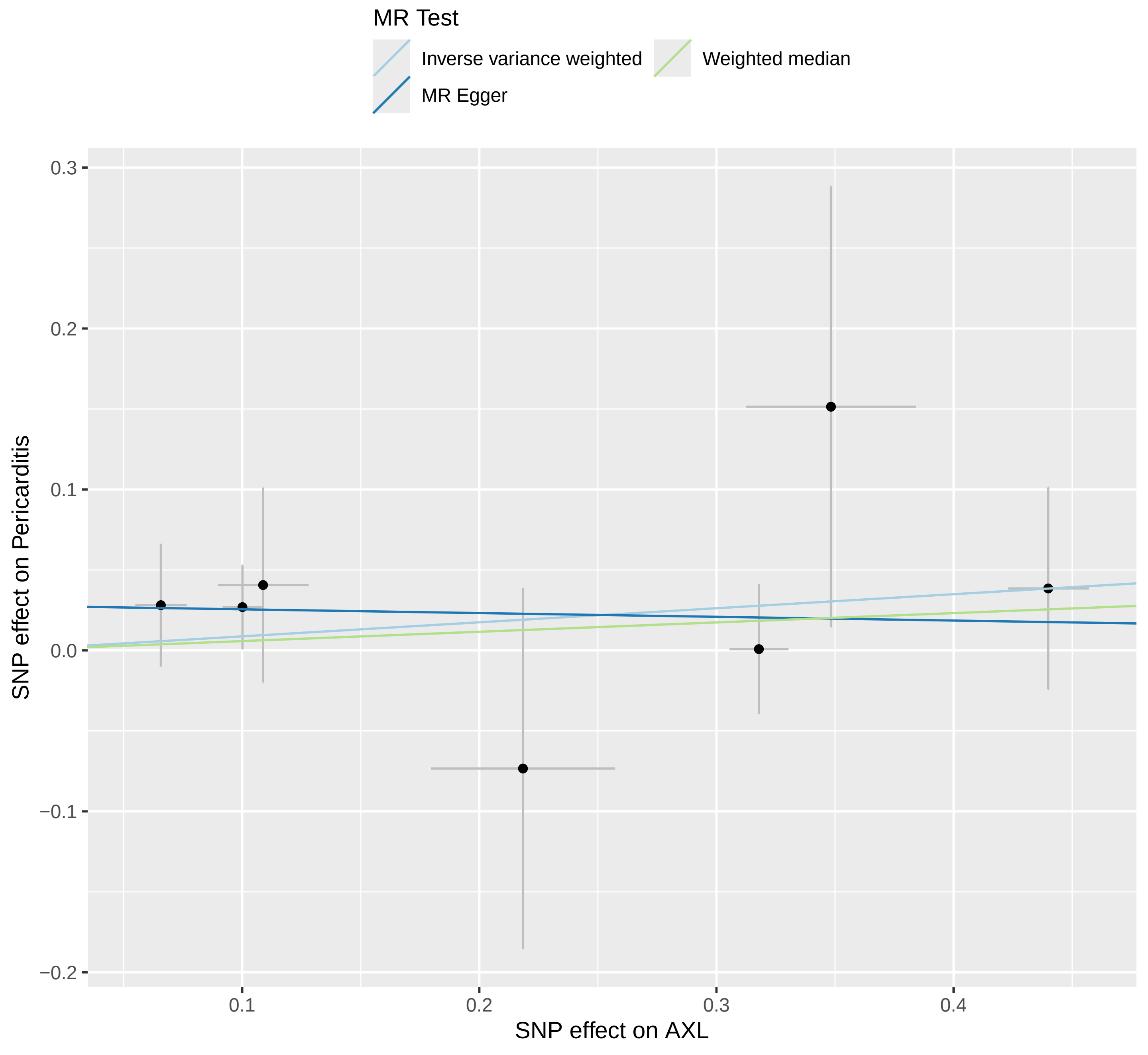
MR Test
Inverse variance weighted
Weighted median
MR Egger
0.3
0.2
0.1
0.0
−0.1
−0.2
SNP effect on Pericarditis
0.1
0.2
0.3
0.4
SNP effect on AXL

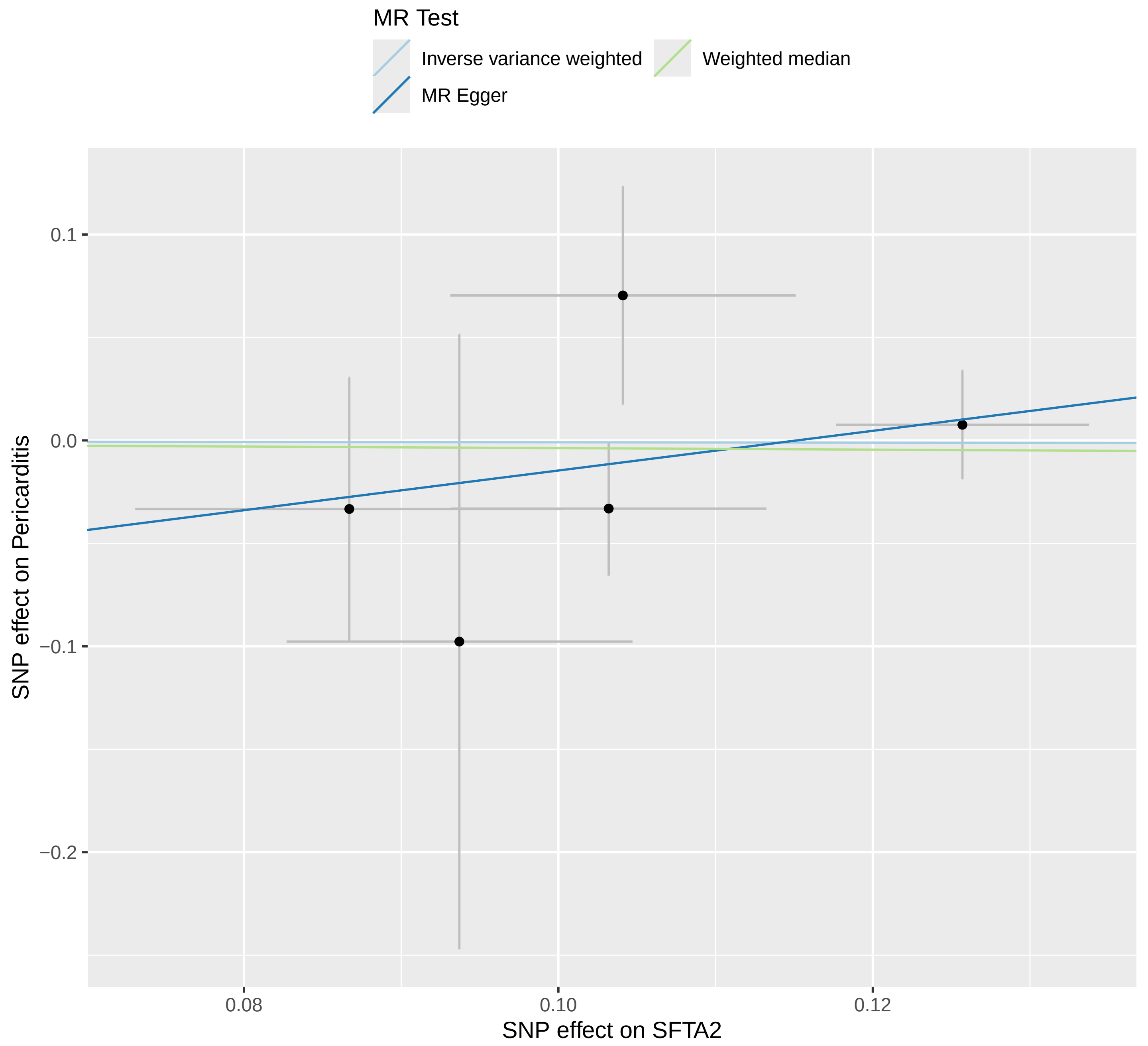
MR Test
Inverse variance weighted
Weighted median
MR Egger
0.1
0.0
−0.1
−0.2
SNP effect on Pericarditis
0.08
0.10
0.12
SNP effect on SFTA2

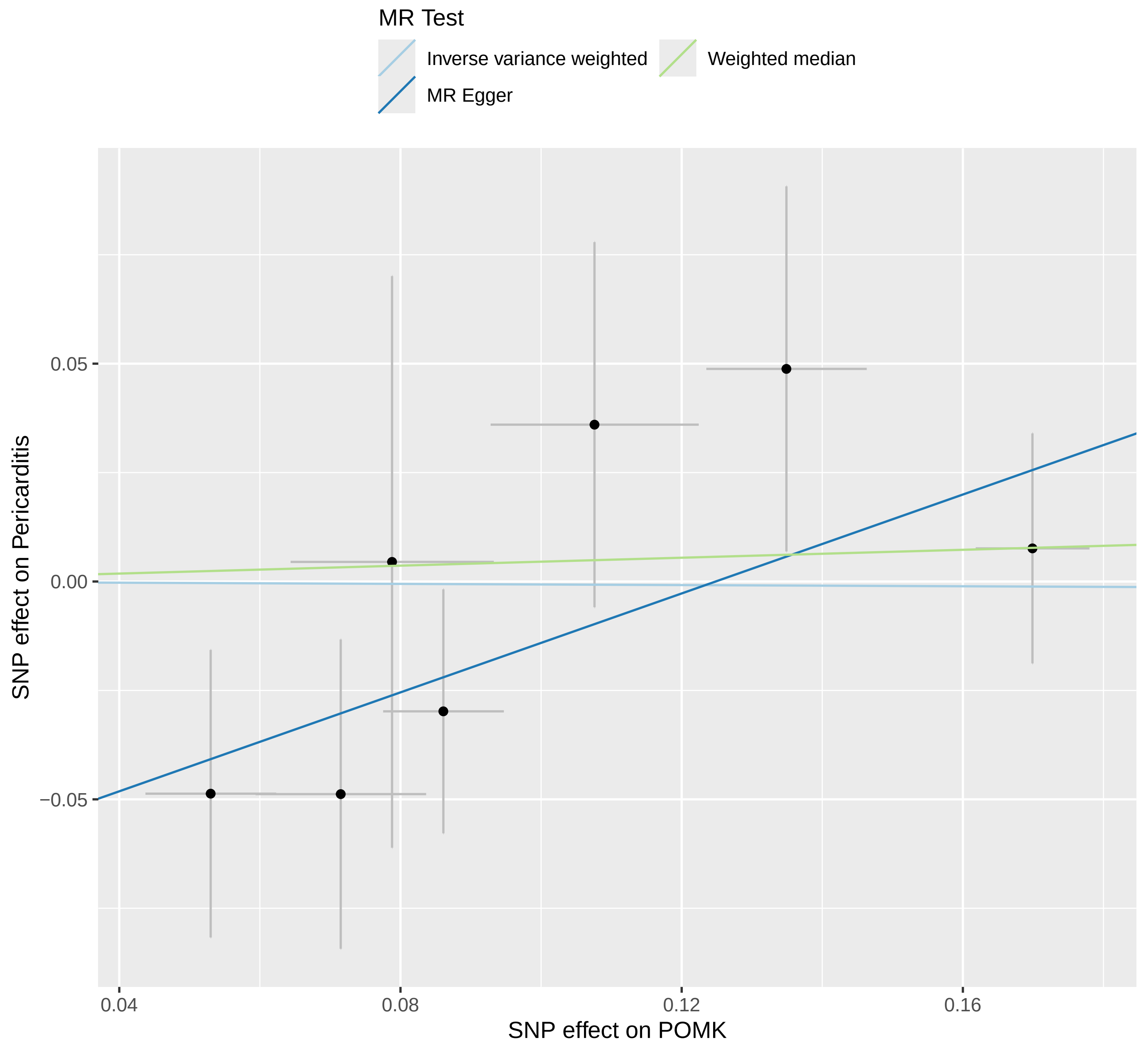
MR Test
Inverse variance weighted
MR Egger
Weighted median
0.05
0.00
−0.05
SNP effect on Pericarditis
0.04
0.08
0.12
0.16
SNP effect on POMK

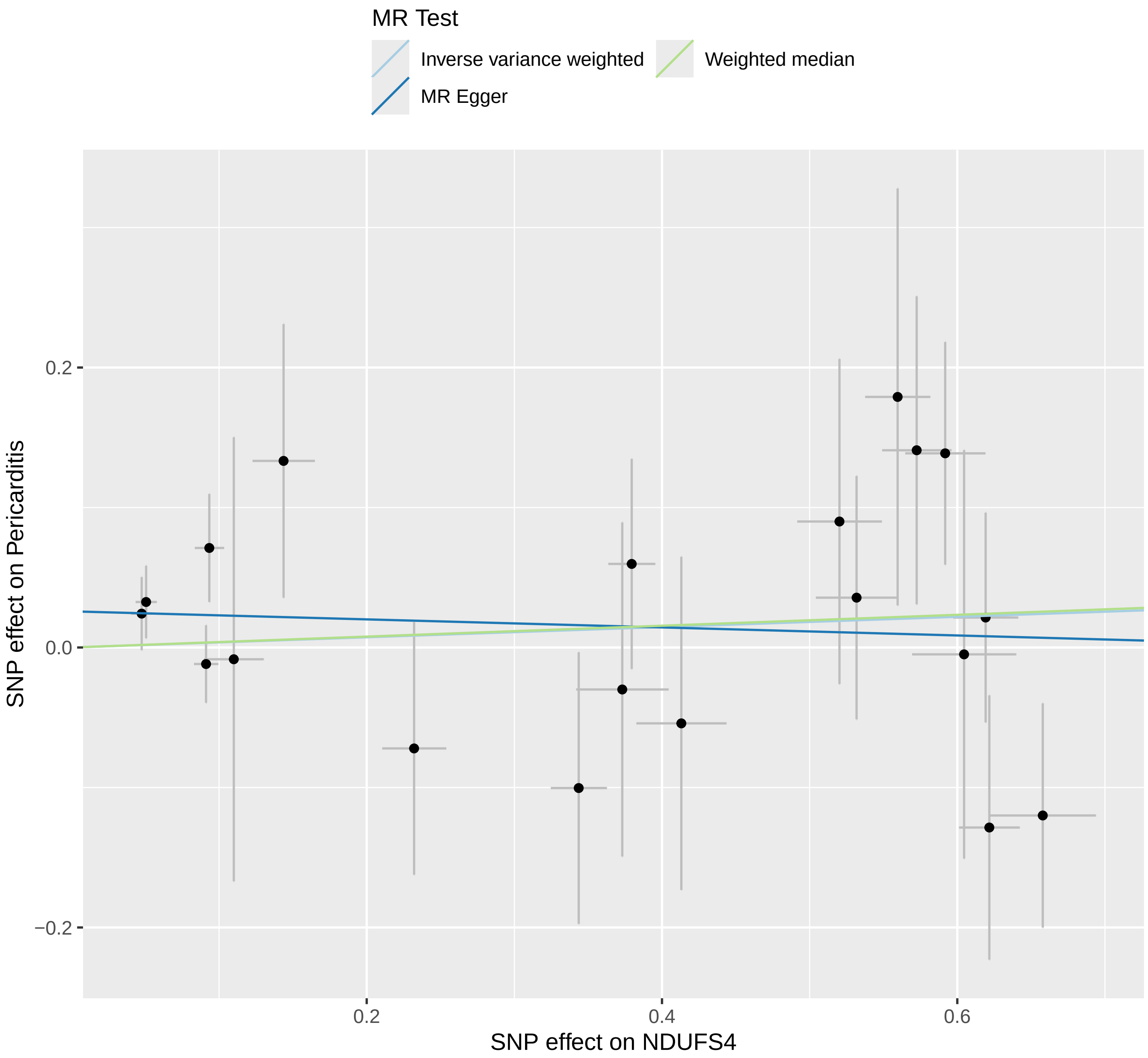
MR Test
Inverse variance weighted
Weighted median
MR Egger
0.2
0.0
−0.2
SNP effect on Pericarditis
0.2
0.4
0.6
SNP effect on NDUFS4

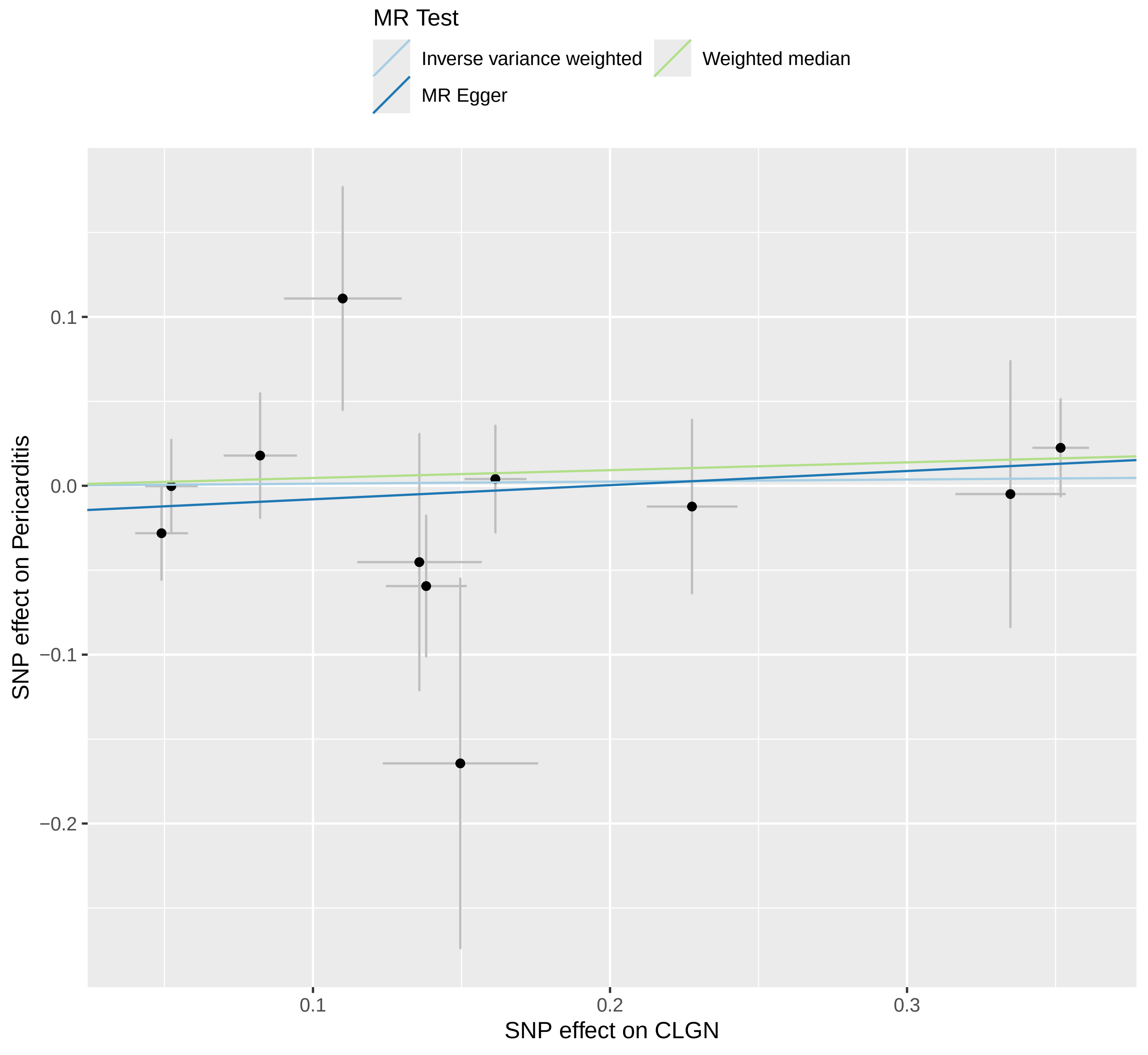

MR Test
Inverse variance weighted
Weighted median
MR Egger
0.1
0.0
−0.1
−0.2
0.1
0.2
0.3
SNP effect on Pericarditis
SNP effect on CLGN

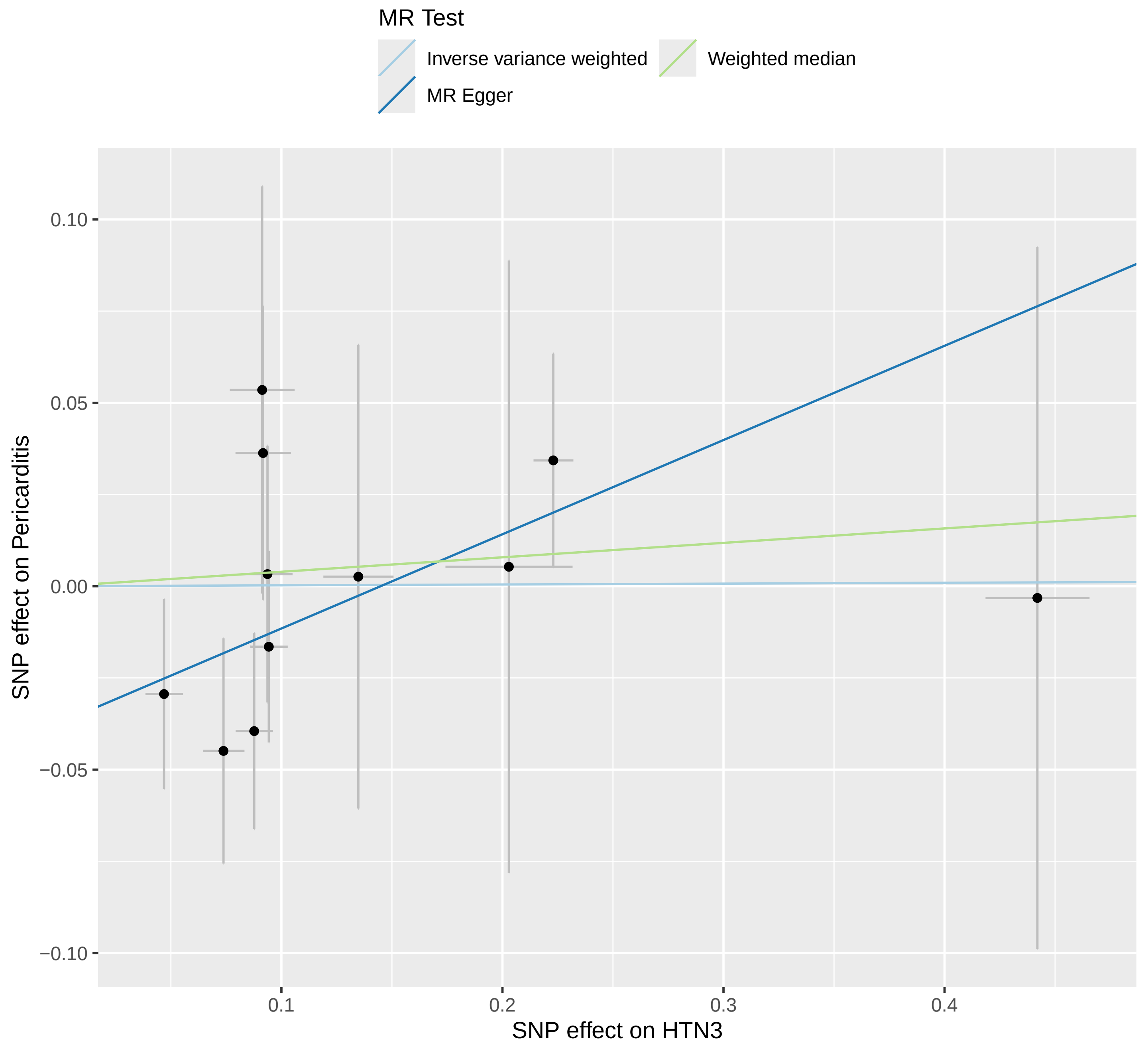
MR Test
Inverse variance weighted
MR Egger
Weighted median
0.10
0.05
0.00
−0.05
−0.10
SNP effect on Pericarditis
0.1
0.2
0.3
0.4
SNP effect on HTN3

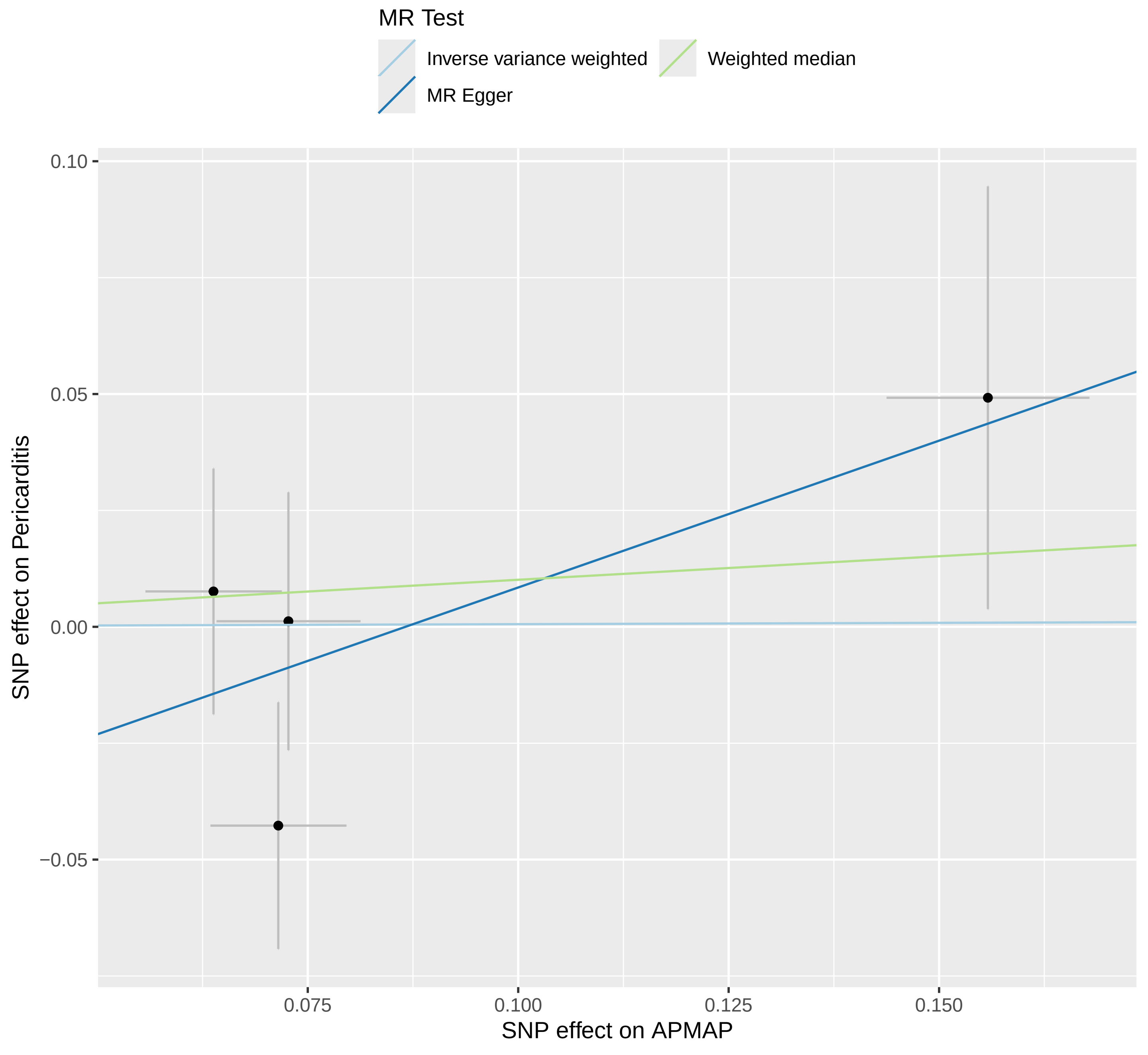

MR Test
Inverse variance weighted
MR Egger
Weighted median
0.10
0.05
0.00
−0.05
SNP effect on Pericarditis
0.075
0.100
0.125
0.150
SNP effect on APMAP

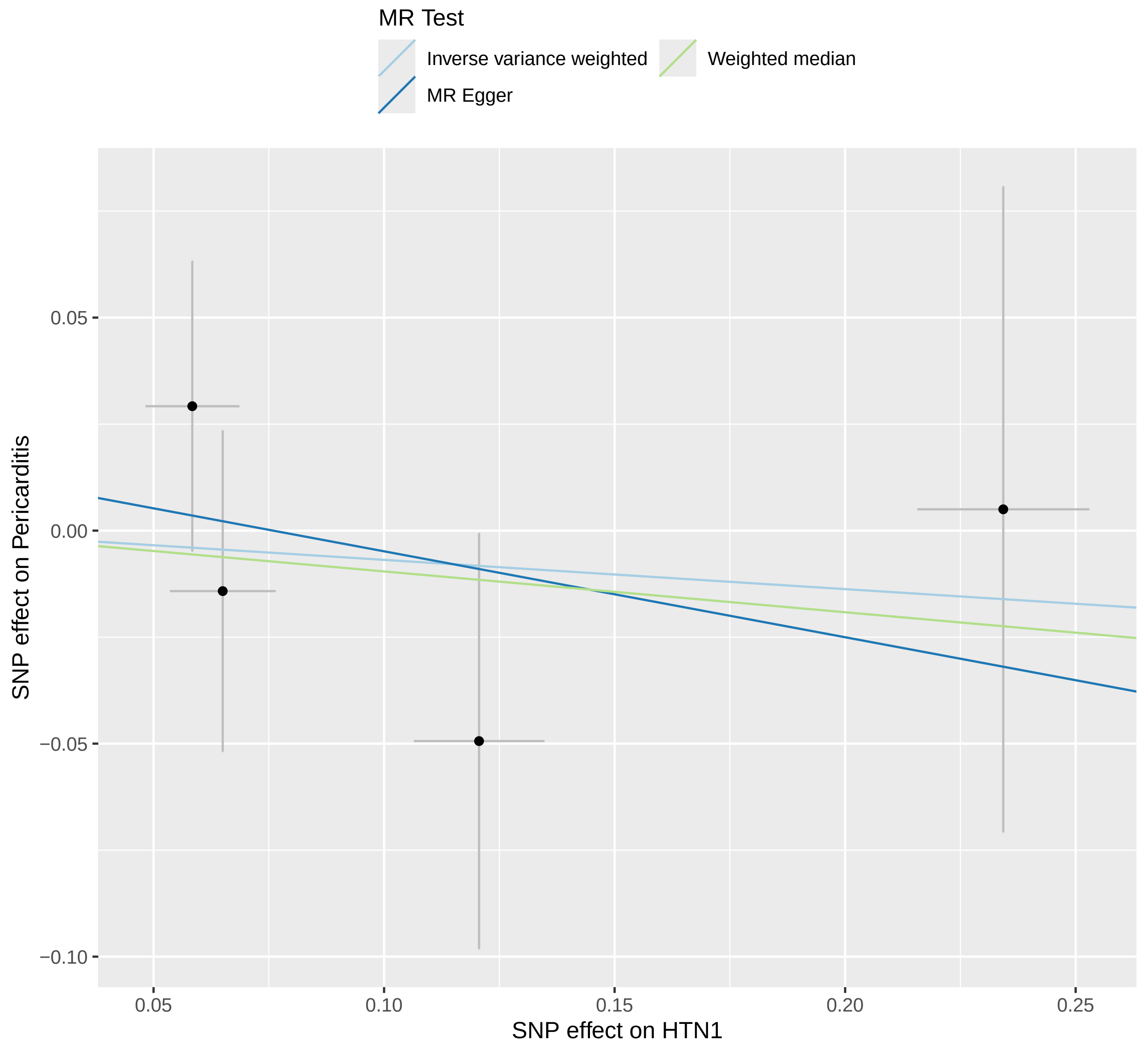

MR Test
Inverse variance weighted
Weighted median
MR Egger
0.05
0.00
−0.05
−0.10
SNP effect on Pericarditis
0.05
0.10
0.15
0.20
0.25
SNP effect on HTN1

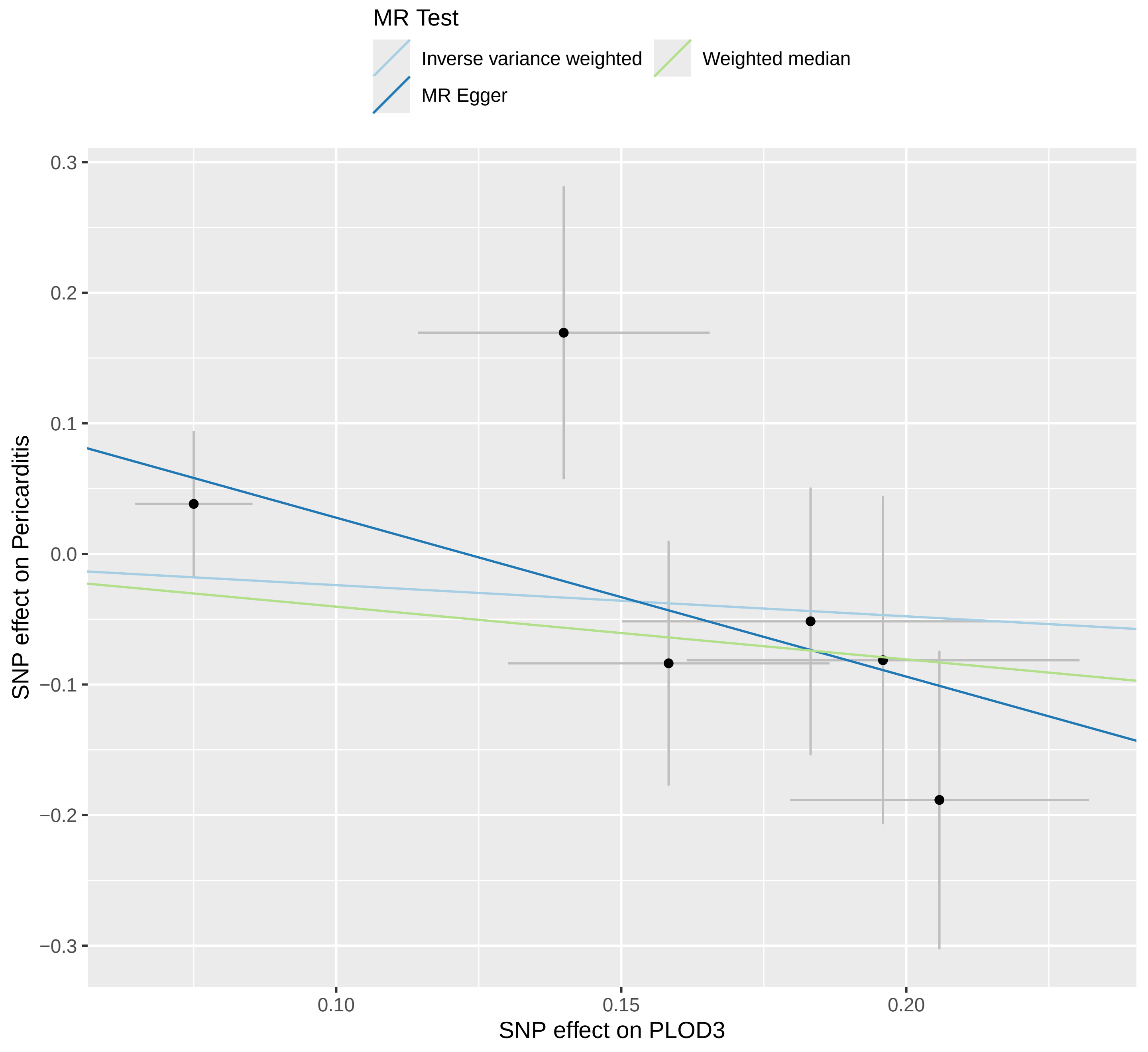

MR Test
Inverse variance weighted
Weighted median
MR Egger
0.3
0.2
0.1
0.0
−0.1
−0.2
−0.3
0.10
0.15
0.20
SNP effect on Pericarditis
SNP effect on PLOD3

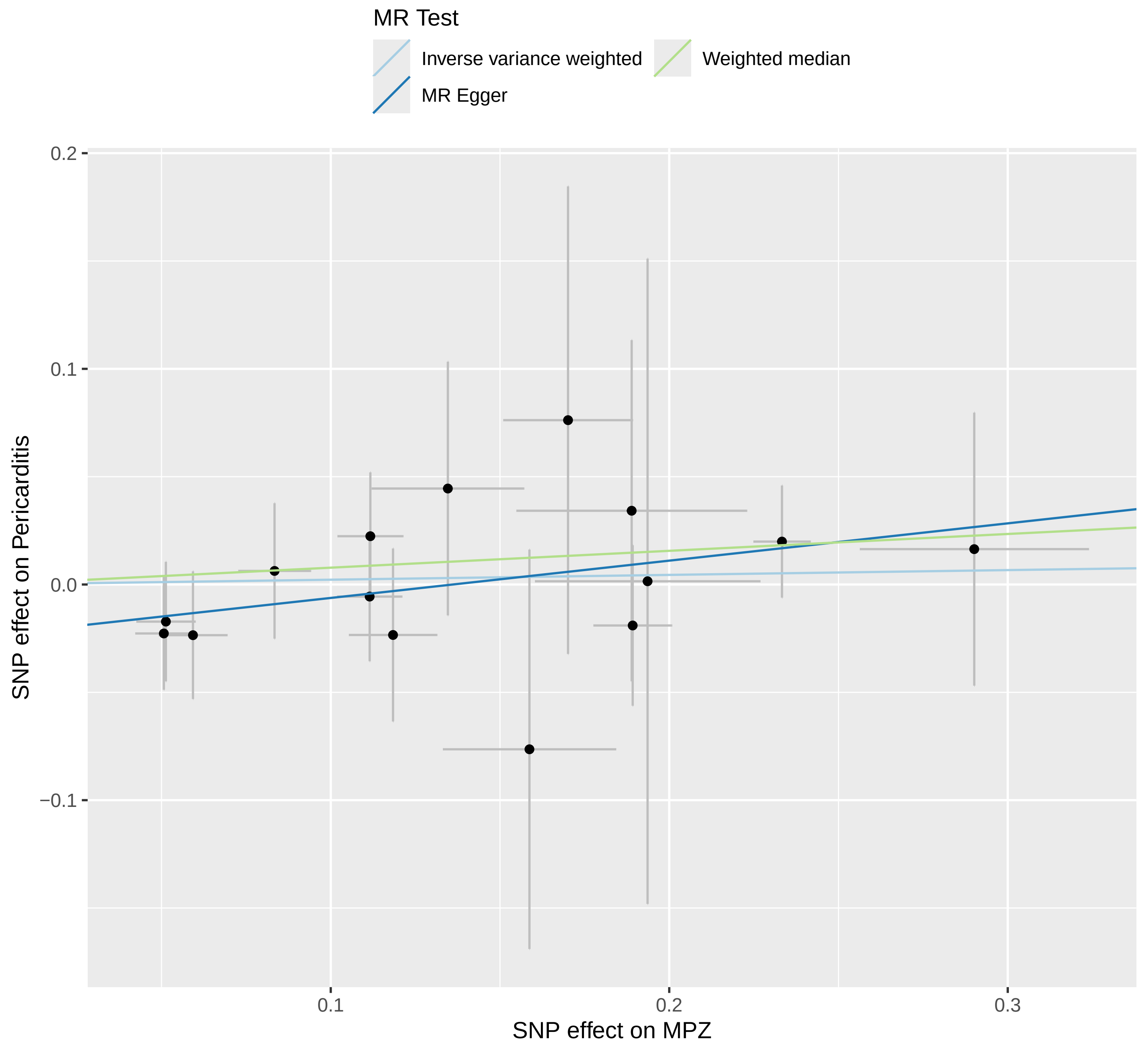
MR Test
Inverse variance weighted
Weighted median
MR Egger
0.2
0.1
0.0
−0.1
SNP effect on Pericarditis
0.1
0.2
0.3
SNP effect on MPZ

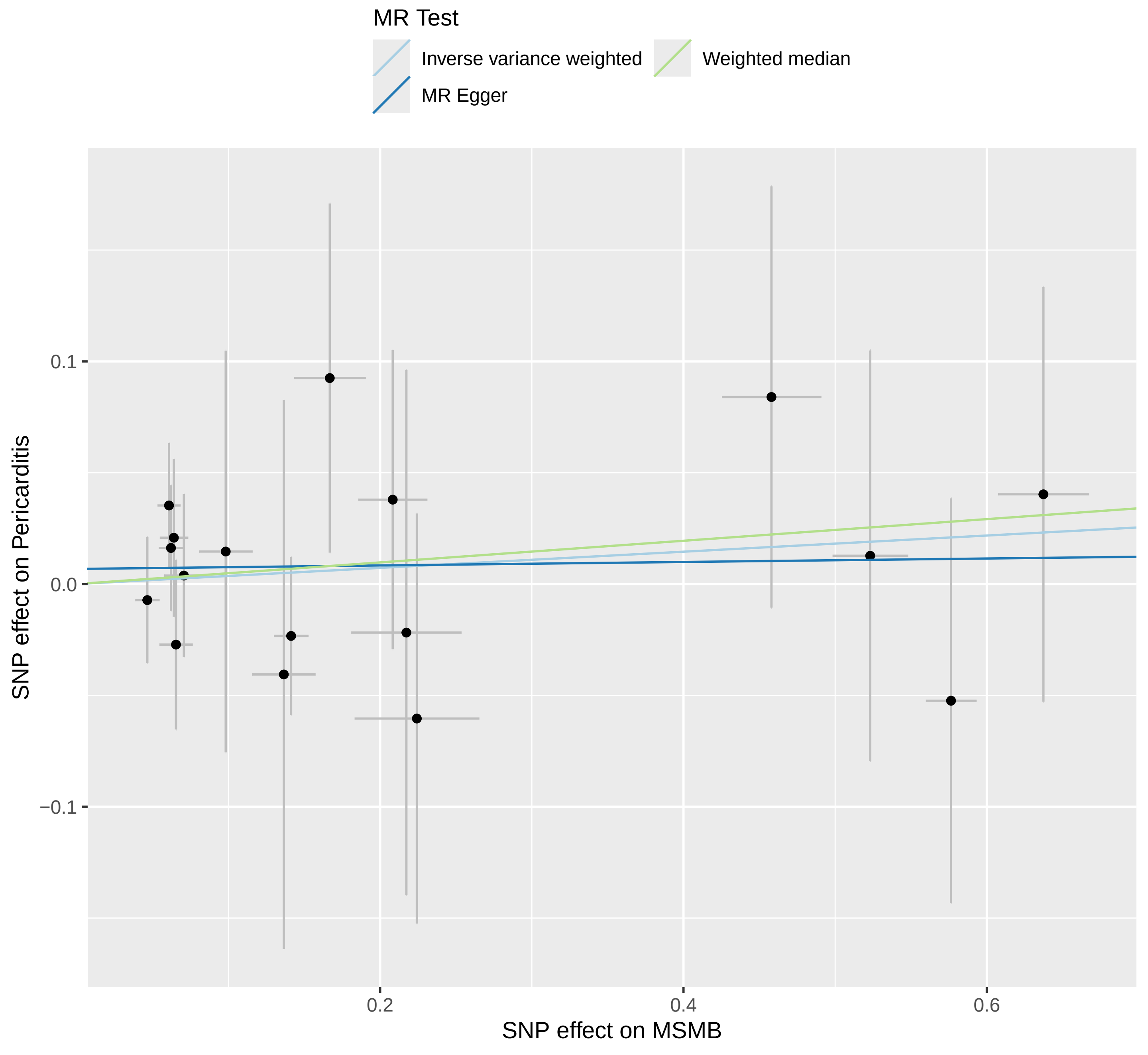
MR Test
Inverse variance weighted
Weighted median
MR Egger
SNP effect on Pericarditis
0.1
0.0
−0.1
0.2
0.4
0.6
SNP effect on MSMB

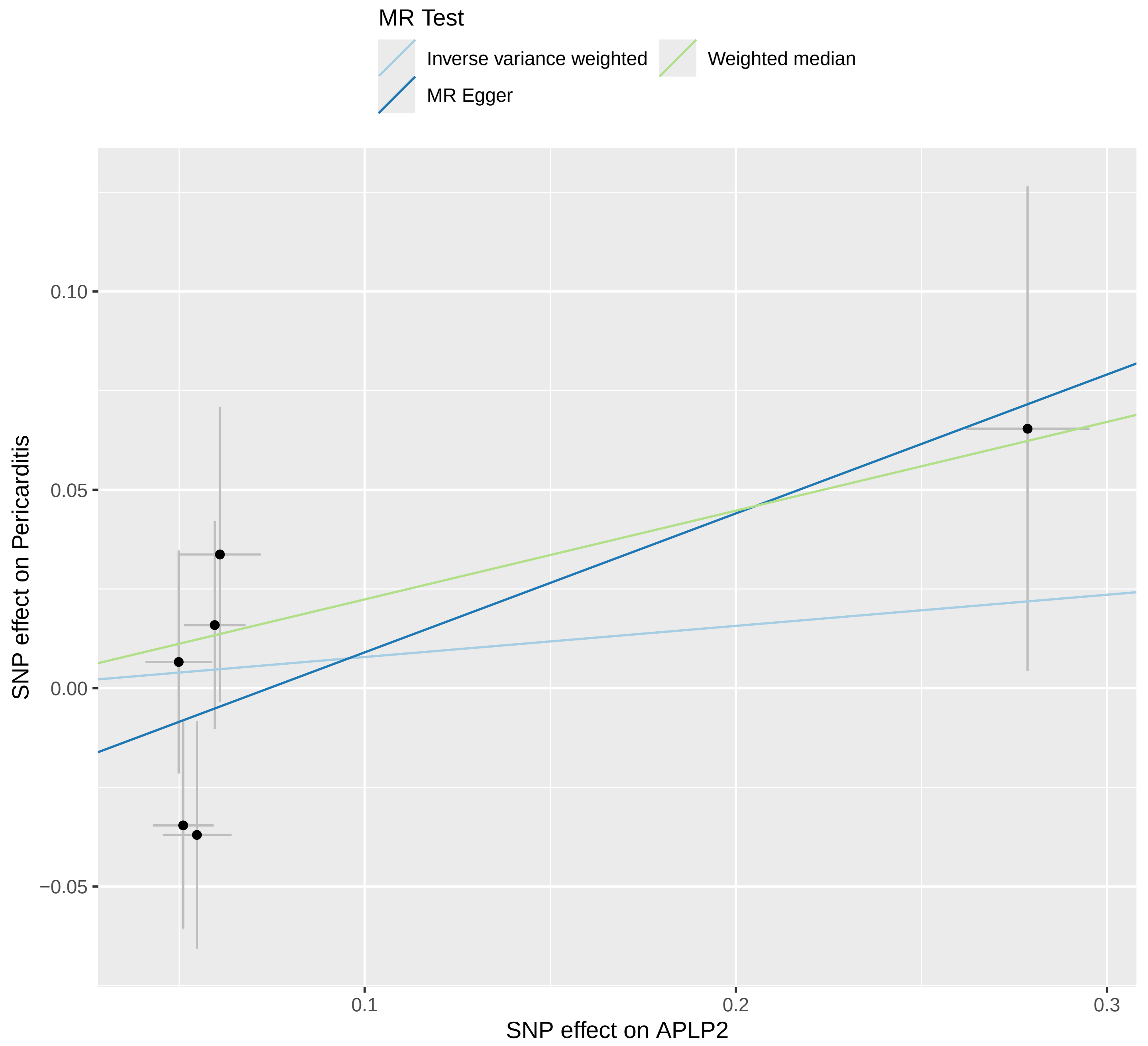
MR Test
Inverse variance weighted
MR Egger
Weighted median
0.10
0.05
0.00
−0.05
0.1
0.2
0.3
SNP effect on Pericarditis
SNP effect on APLP2

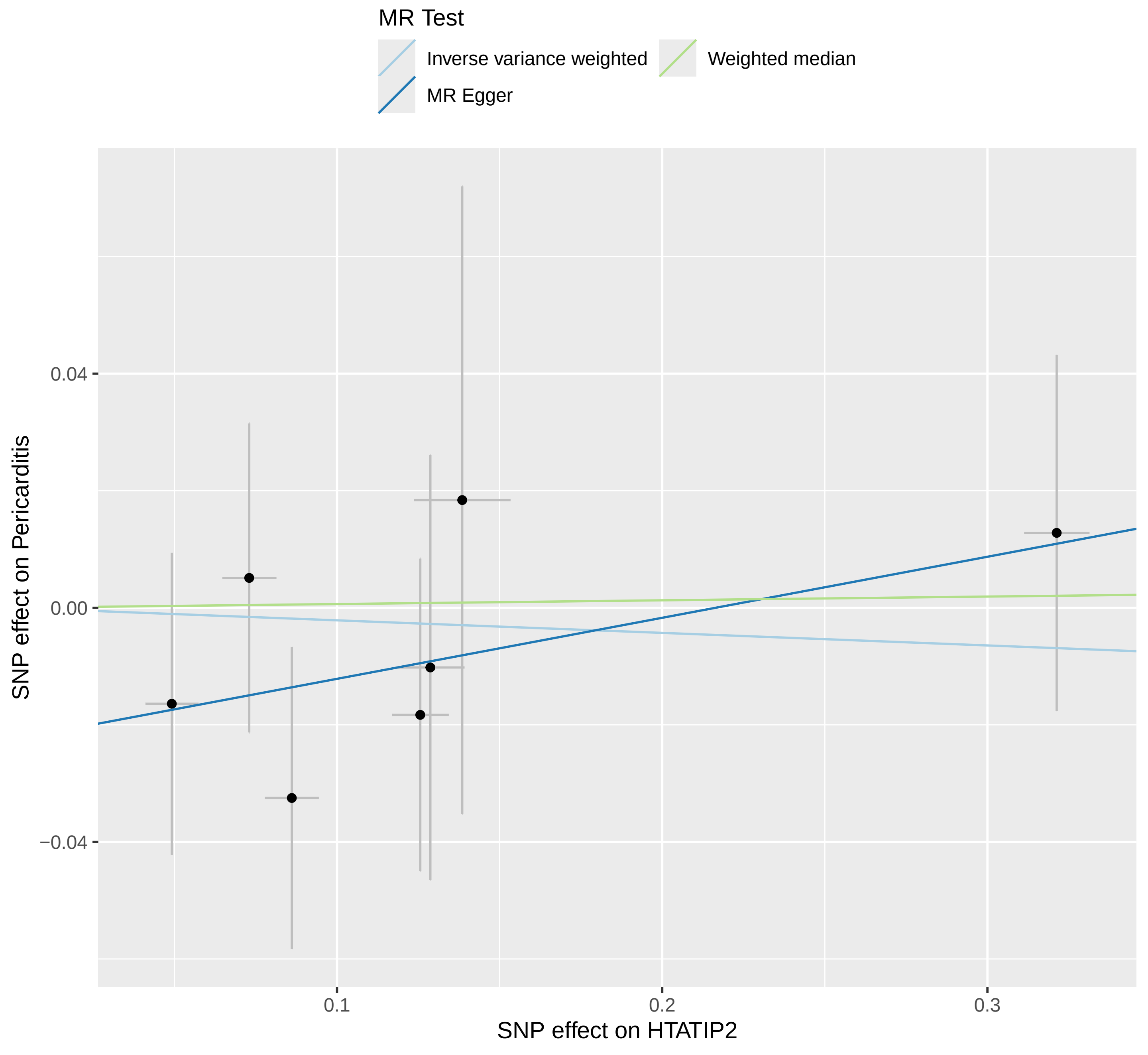

MR Test
Inverse variance weighted
Weighted median
MR Egger
0.04
0.00
−0.04
0.1
0.2
0.3
SNP effect on Pericarditis
SNP effect on HTATIP2

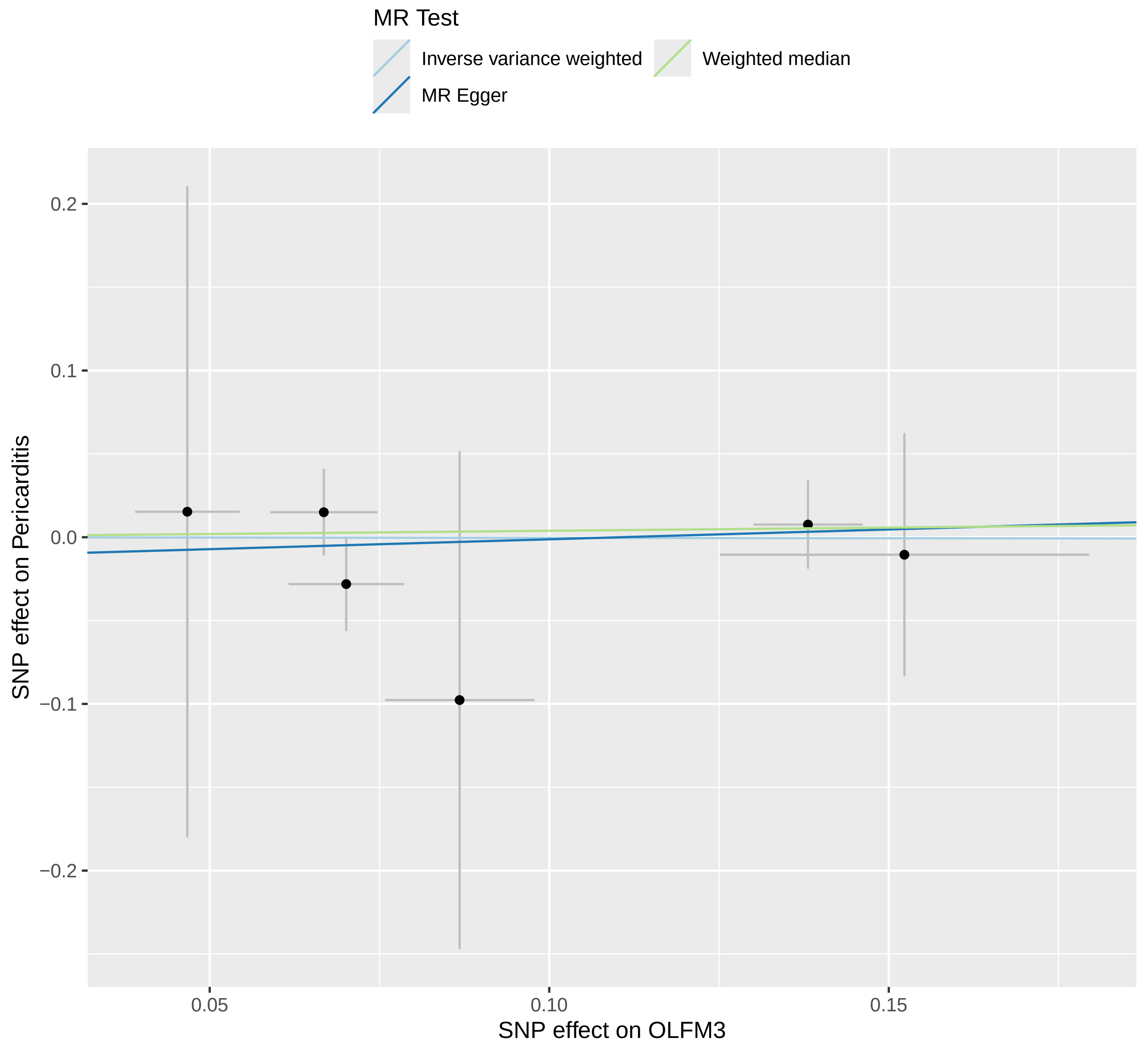

MR Test
Inverse variance weighted
Weighted median
MR Egger
0.2
0.1
0.0
−0.1
−0.2
SNP effect on Pericarditis
0.05
0.10
0.15
SNP effect on OLFM3

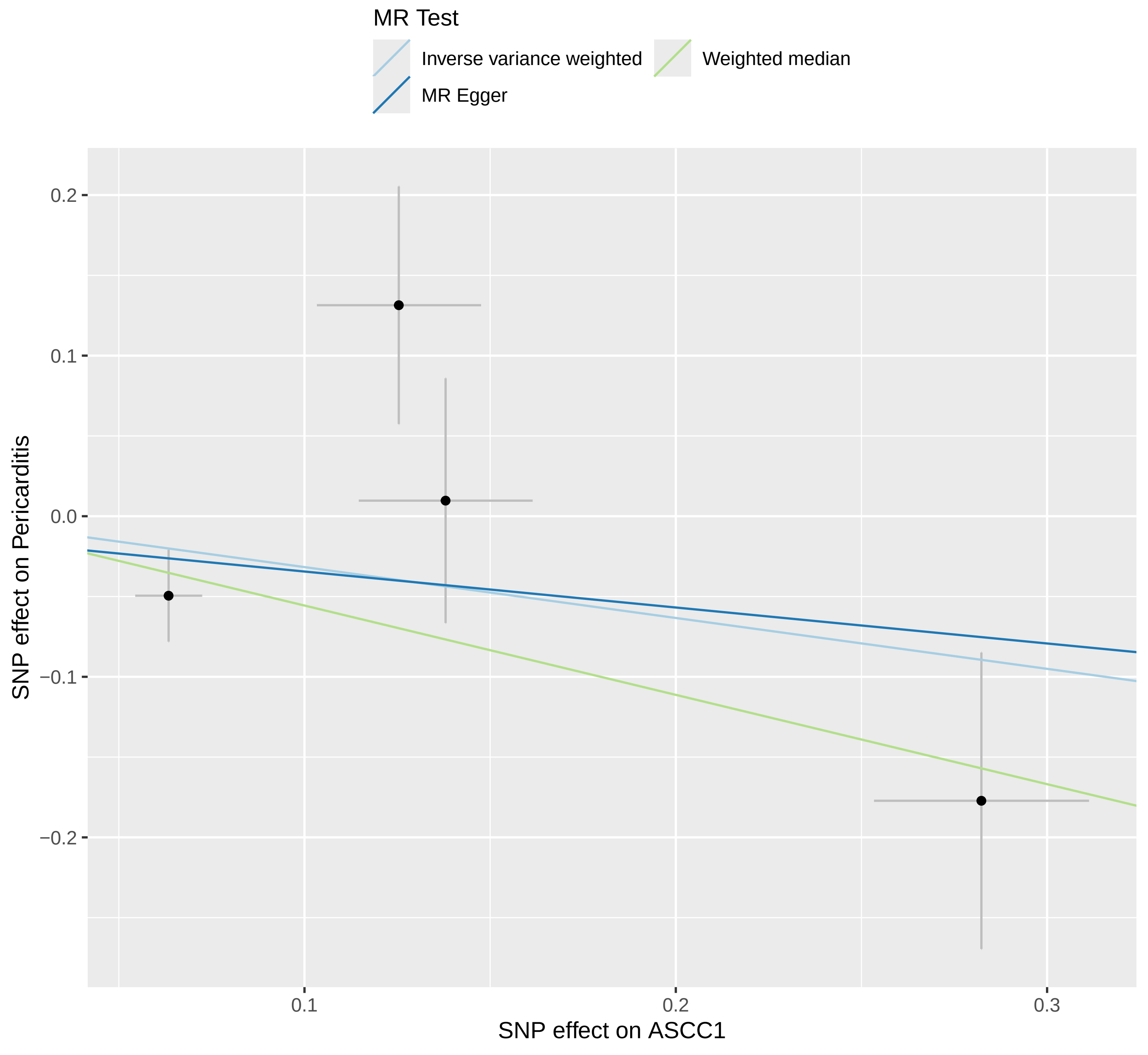
MR Test
Inverse variance weighted
MR Egger
Weighted median
SNP effect on Pericarditis
0.2
0.1
0.0
−0.1
−0.2
0.1
0.2
0.3
SNP effect on ASCC1

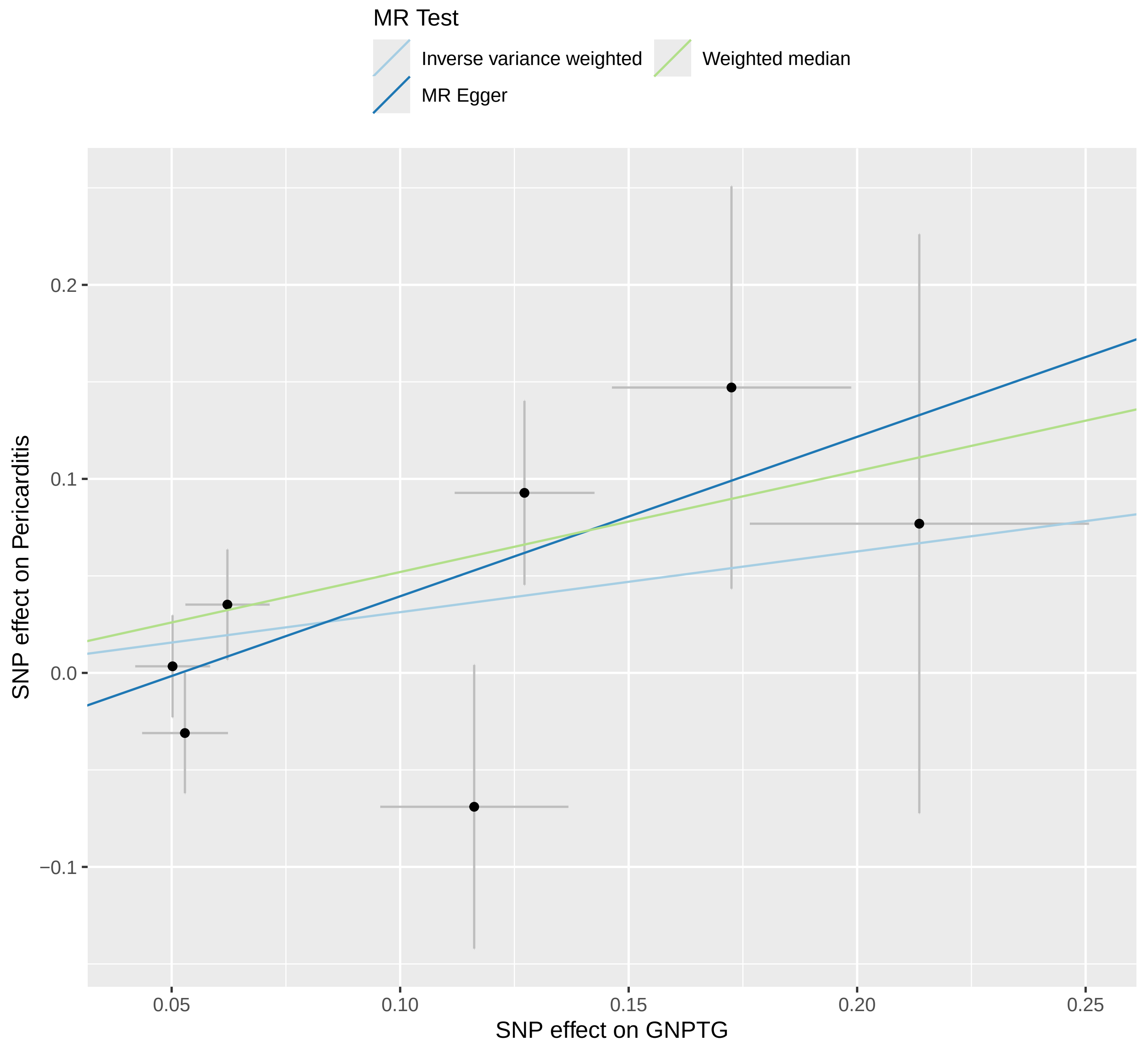

MR Test
Inverse variance weighted
Weighted median
MR Egger
0.2
0.1
0.0
−0.1
SNP effect on Pericarditis
0.05
0.10
0.15
0.20
0.25
SNP effect on GNPTG

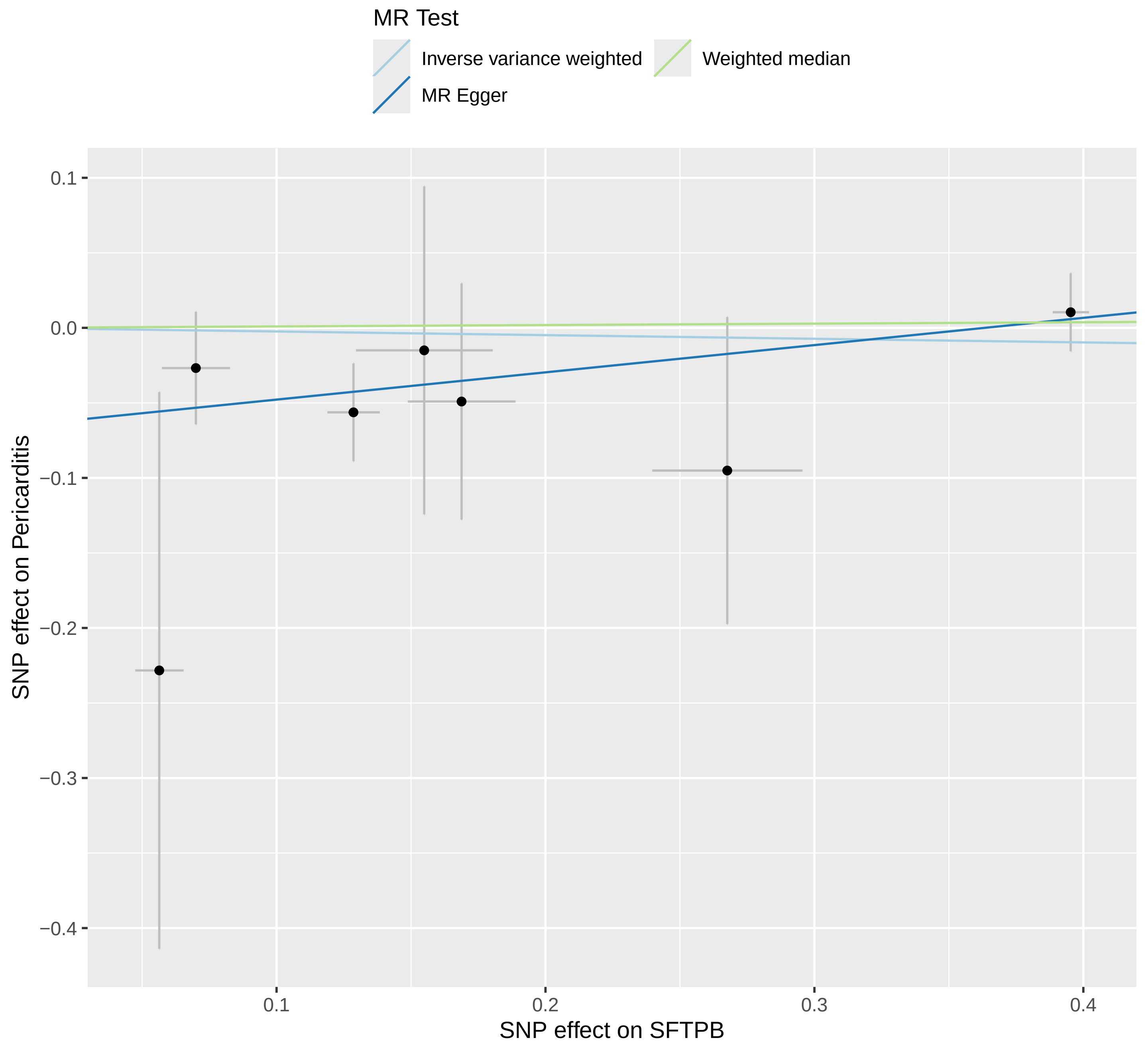
MR Test
Inverse variance weighted
Weighted median
MR Egger
0.1
0.0
−0.1
−0.2
−0.3
−0.4
SNP effect on Pericarditis
0.1
0.2
0.3
0.4
SNP effect on SFTPB

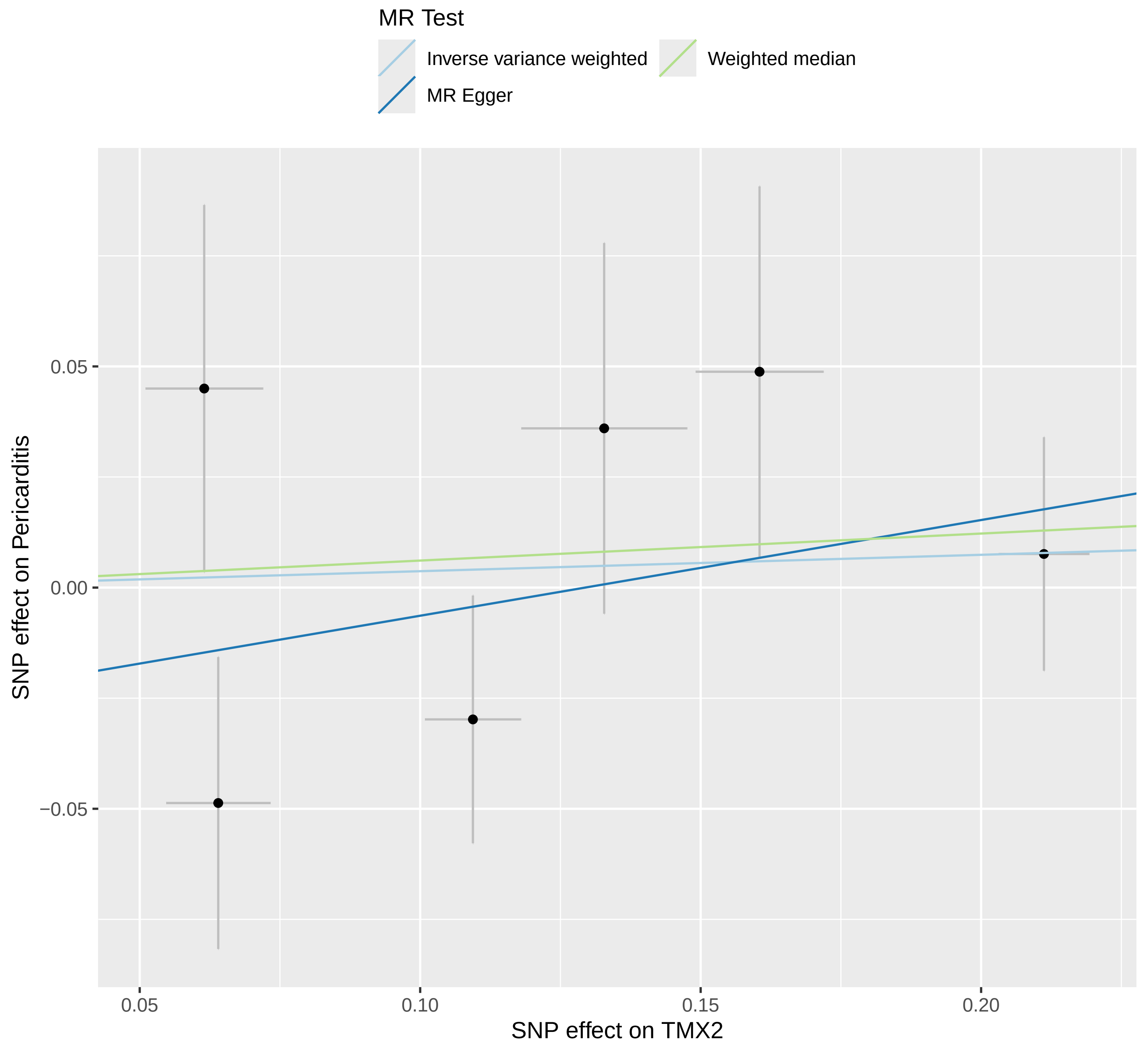
MR Test
Inverse variance weighted
MR Egger
Weighted median
SNP effect on Pericarditis
0.05
0.00
−0.05
0.05
0.10
0.15
0.20
SNP effect on TMX2

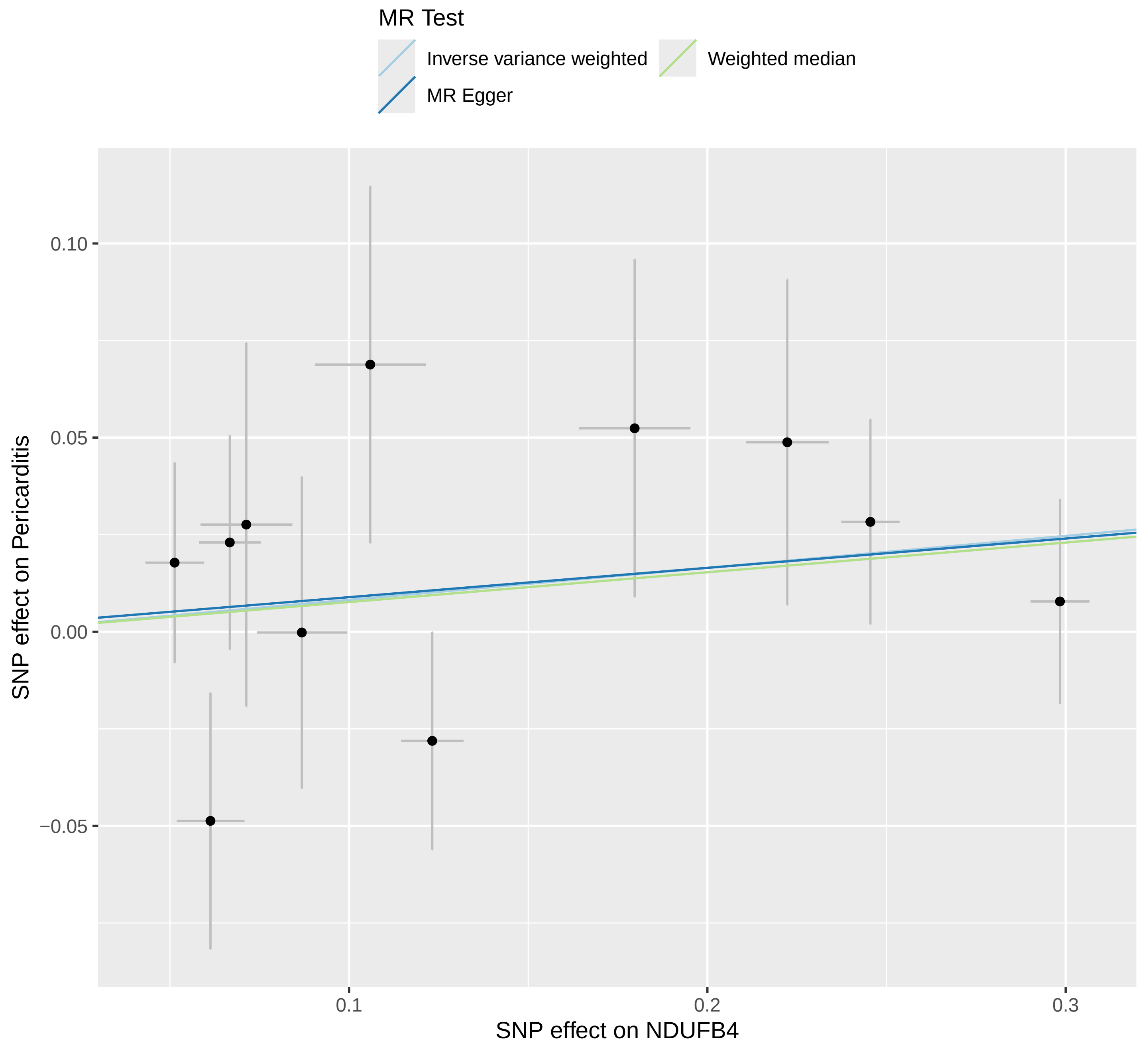
MR Test
Inverse variance weighted
Weighted median
MR Egger
0.10
0.05
0.00
−0.05
SNP effect on Pericarditis
0.1
0.2
0.3
SNP effect on NDUFB4

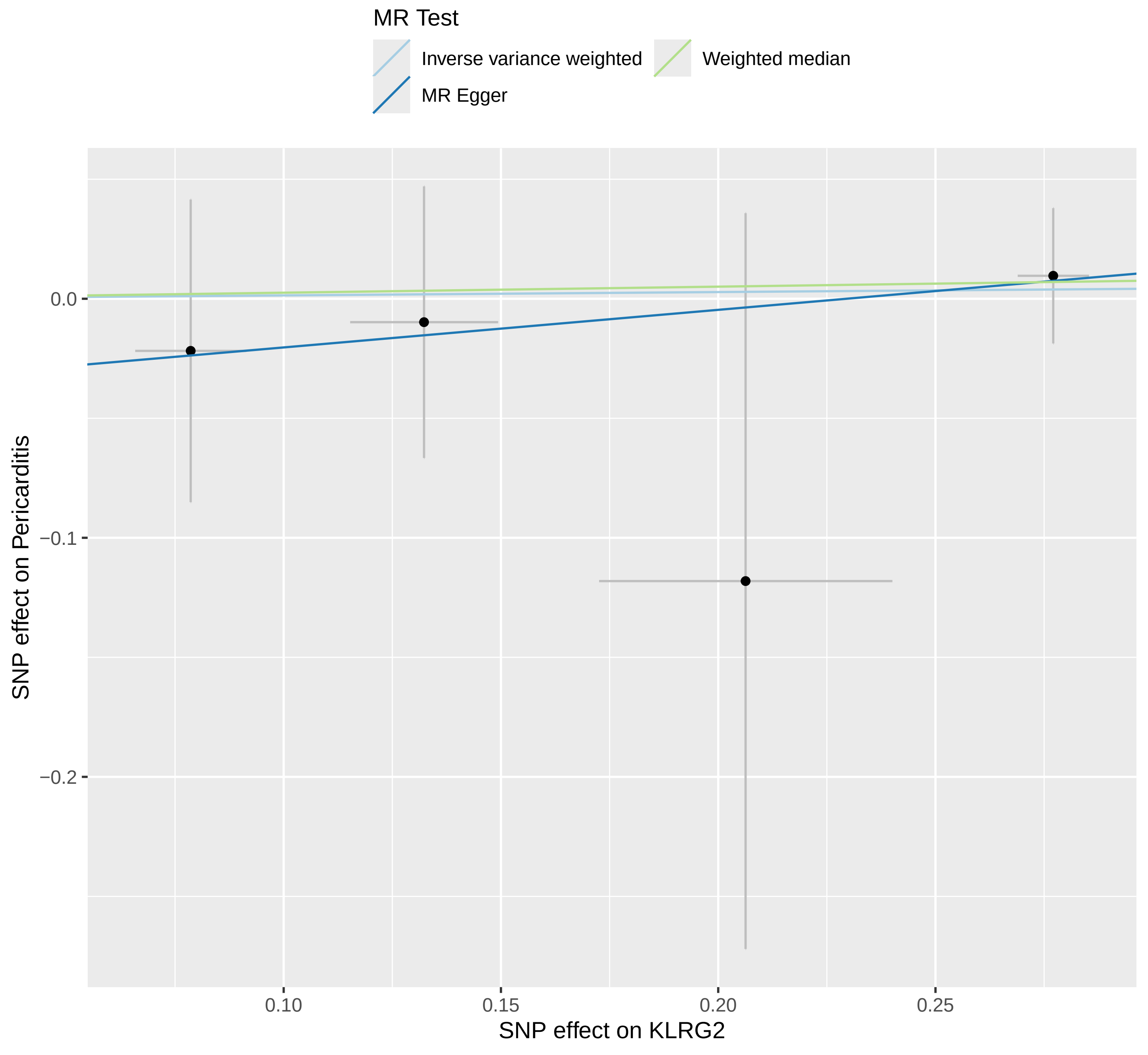

MR Test
Inverse variance weighted
Weighted median
MR Egger
0.0
−0.1
−0.2
SNP effect on Pericarditis
0.10
0.15
0.20
0.25
SNP effect on KLRG2

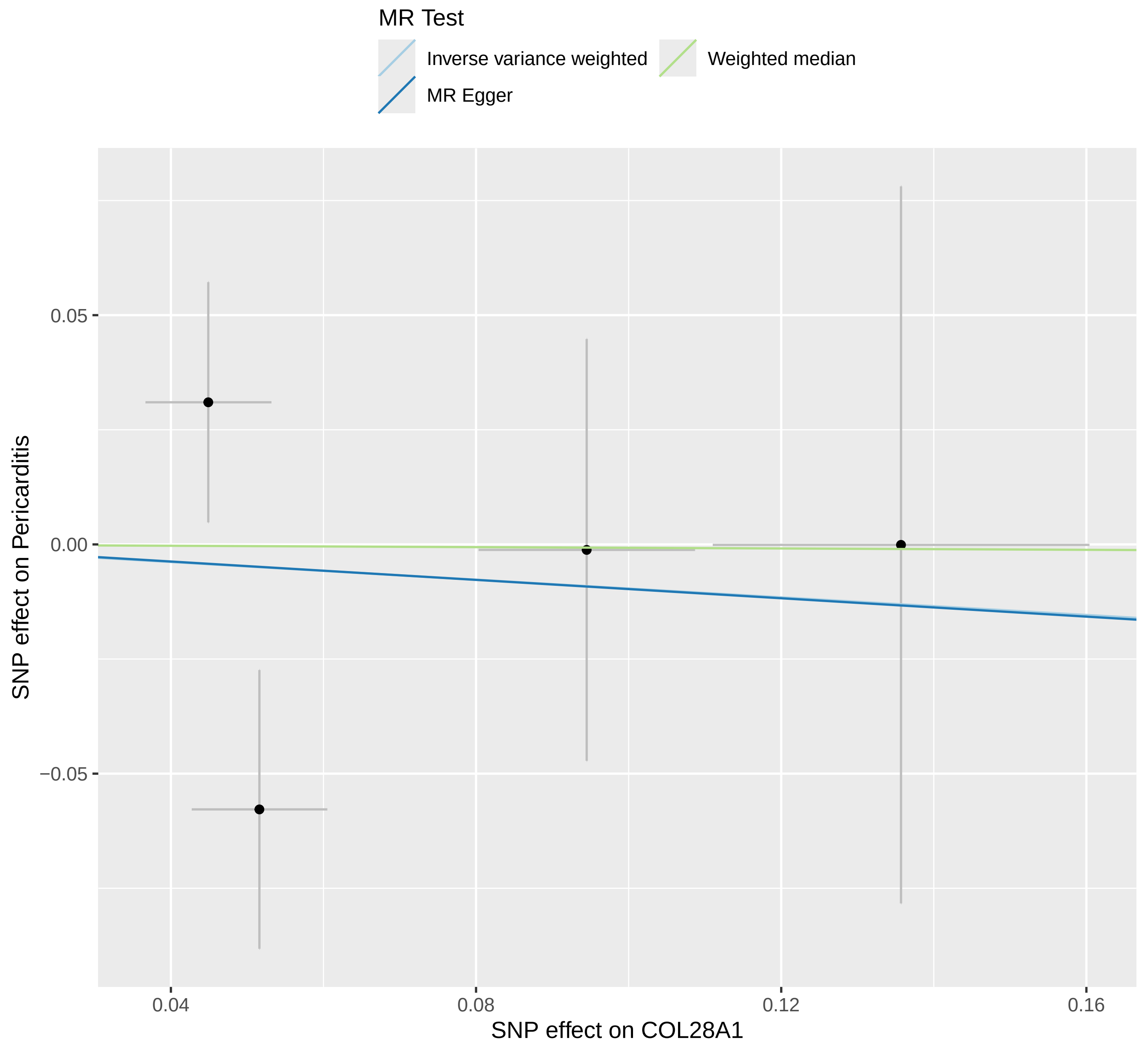
MR Test
Inverse variance weighted
Weighted median
MR Egger
0.05
0.00
−0.05
0.04
0.08
0.12
0.16
SNP effect on Pericarditis
SNP effect on COL28A1

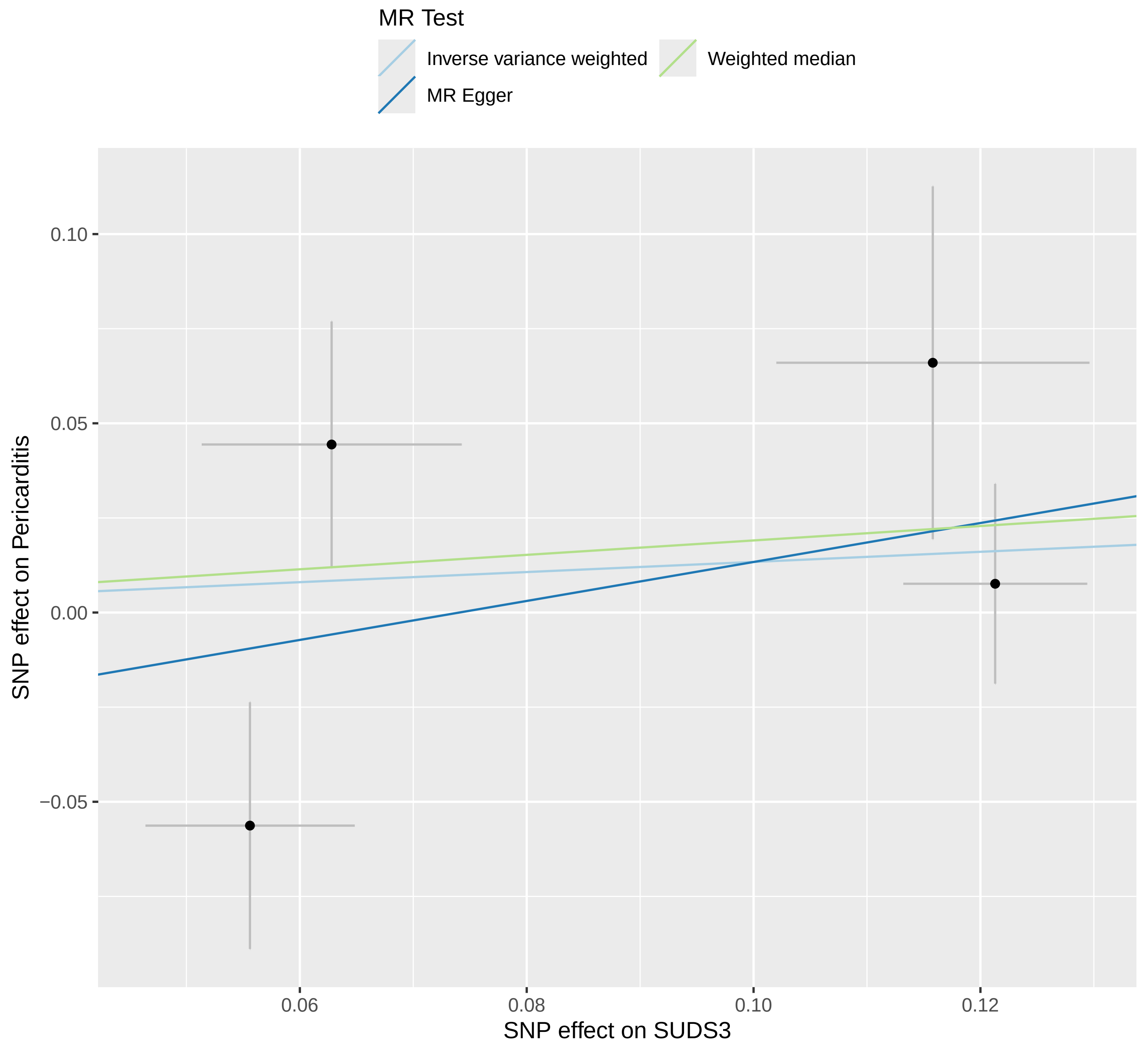

MR Test
Inverse variance weighted
Weighted median
MR Egger
0.10
0.05
0.00
−0.05
SNP effect on Pericarditis
0.06
0.08
0.10
0.12
SNP effect on SUDS3

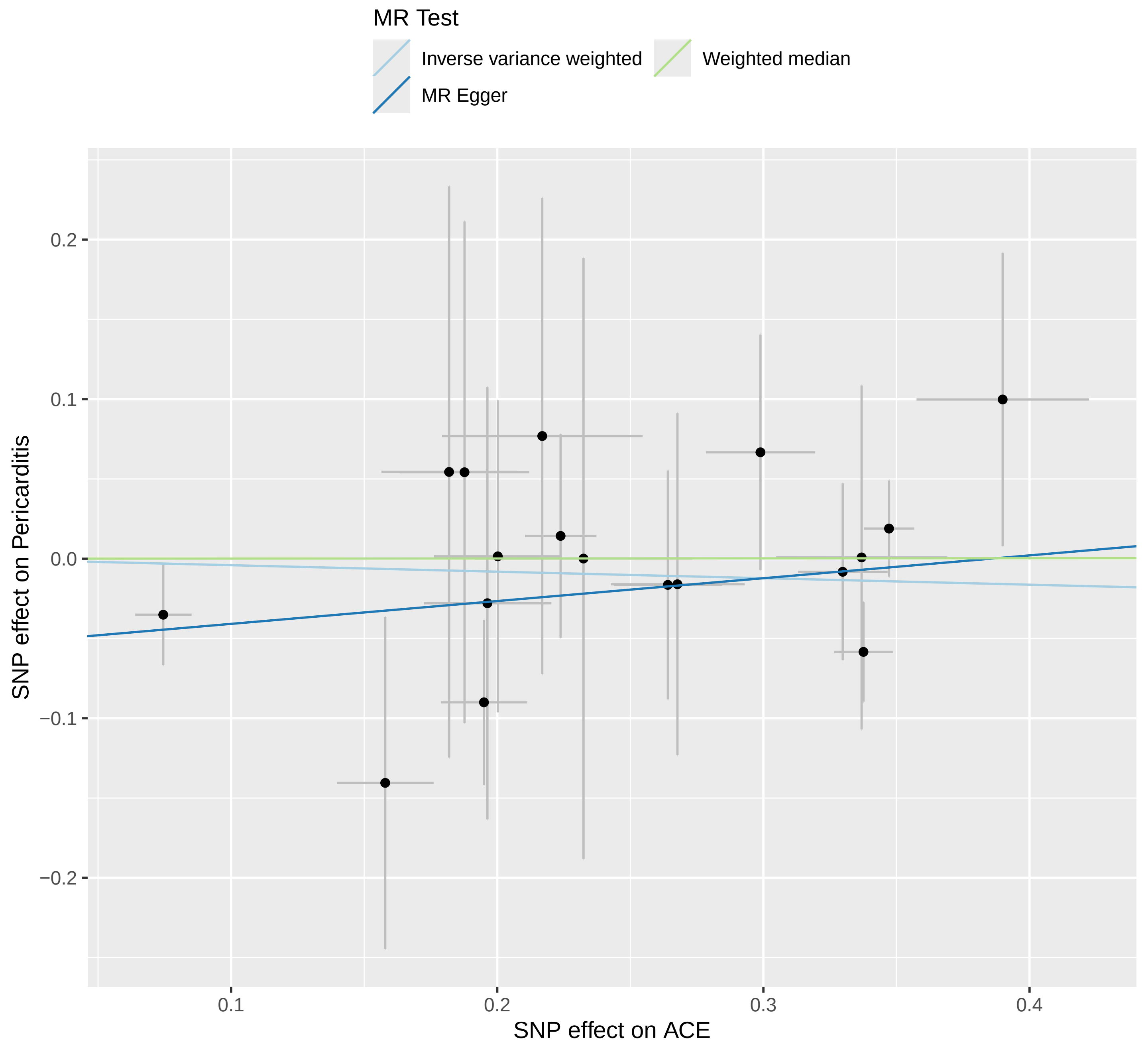

MR Test
Inverse variance weighted
Weighted median
MR Egger
SNP effect on Pericarditis
0.2
0.1
0.0
−0.1
−0.2
0.1
0.2
0.3
0.4
SNP effect on ACE

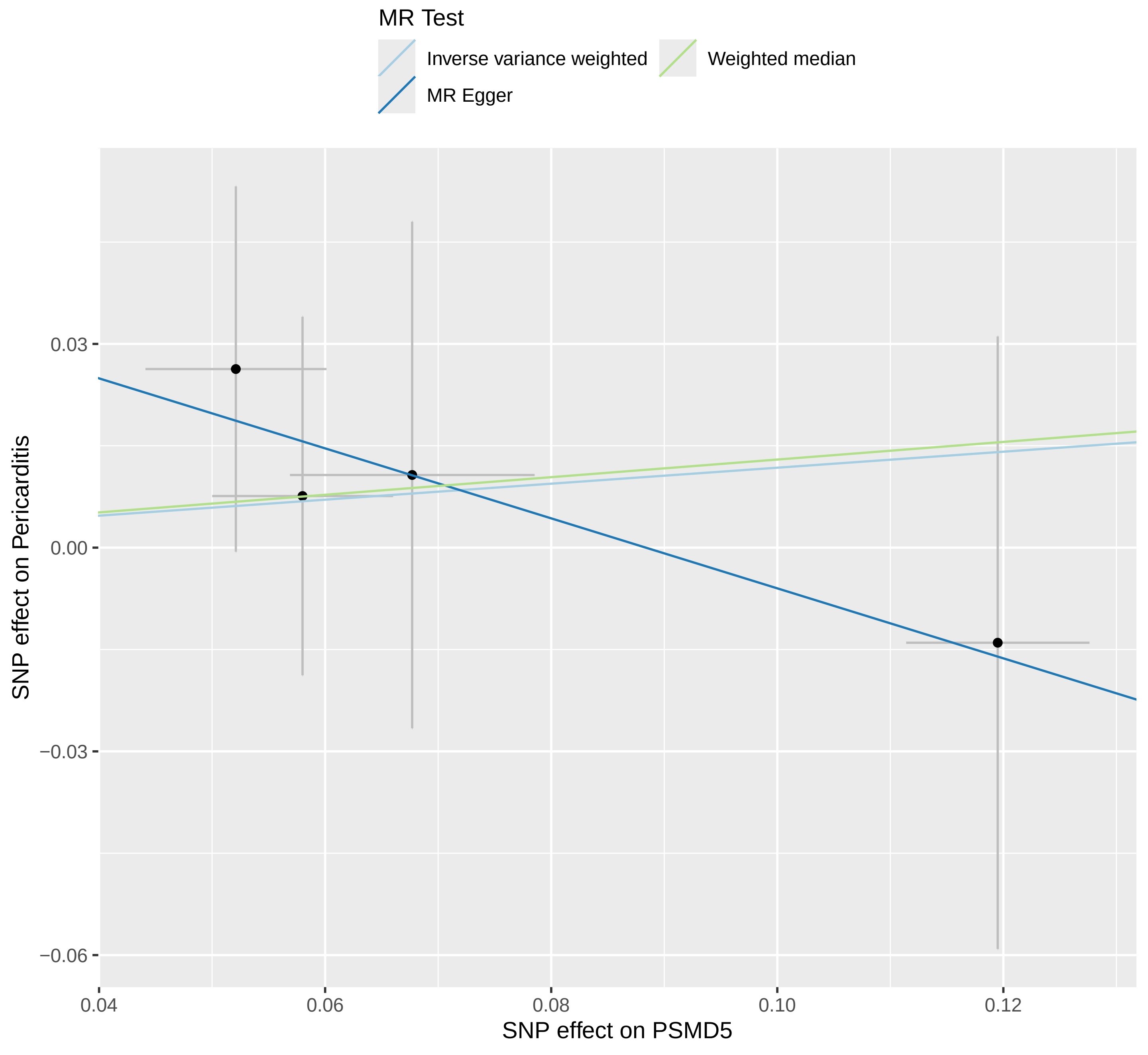
MR Test
Inverse variance weighted
Weighted median
MR Egger
SNP effect on Pericarditis
0.03
0.00
−0.03
−0.06
0.04
0.06
0.08
0.10
0.12
SNP effect on PSMD5

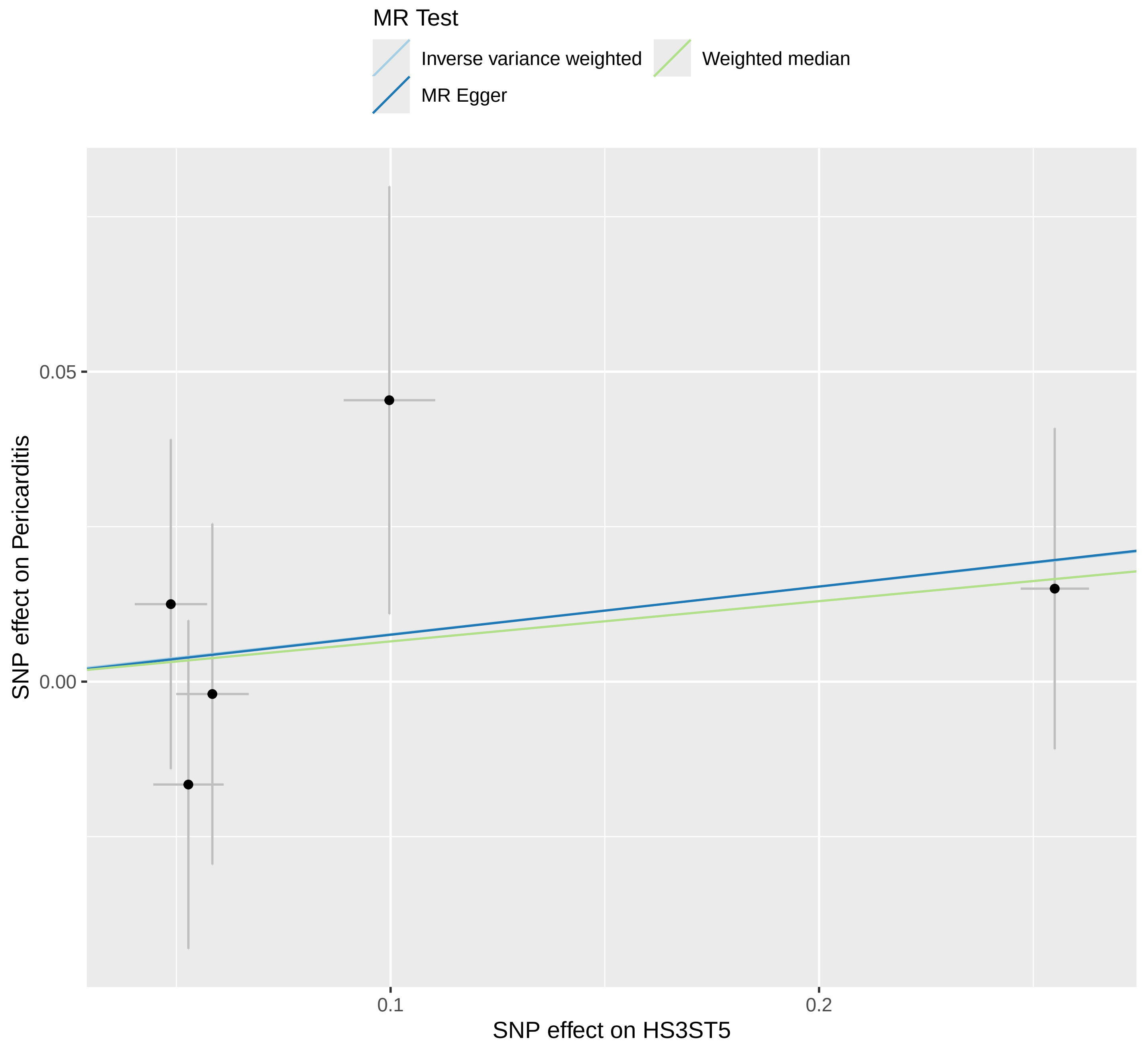

MR Test
Inverse variance weighted
Weighted median
MR Egger
0.05
0.00
0.1
0.2
SNP effect on Pericarditis
SNP effect on HS3ST5

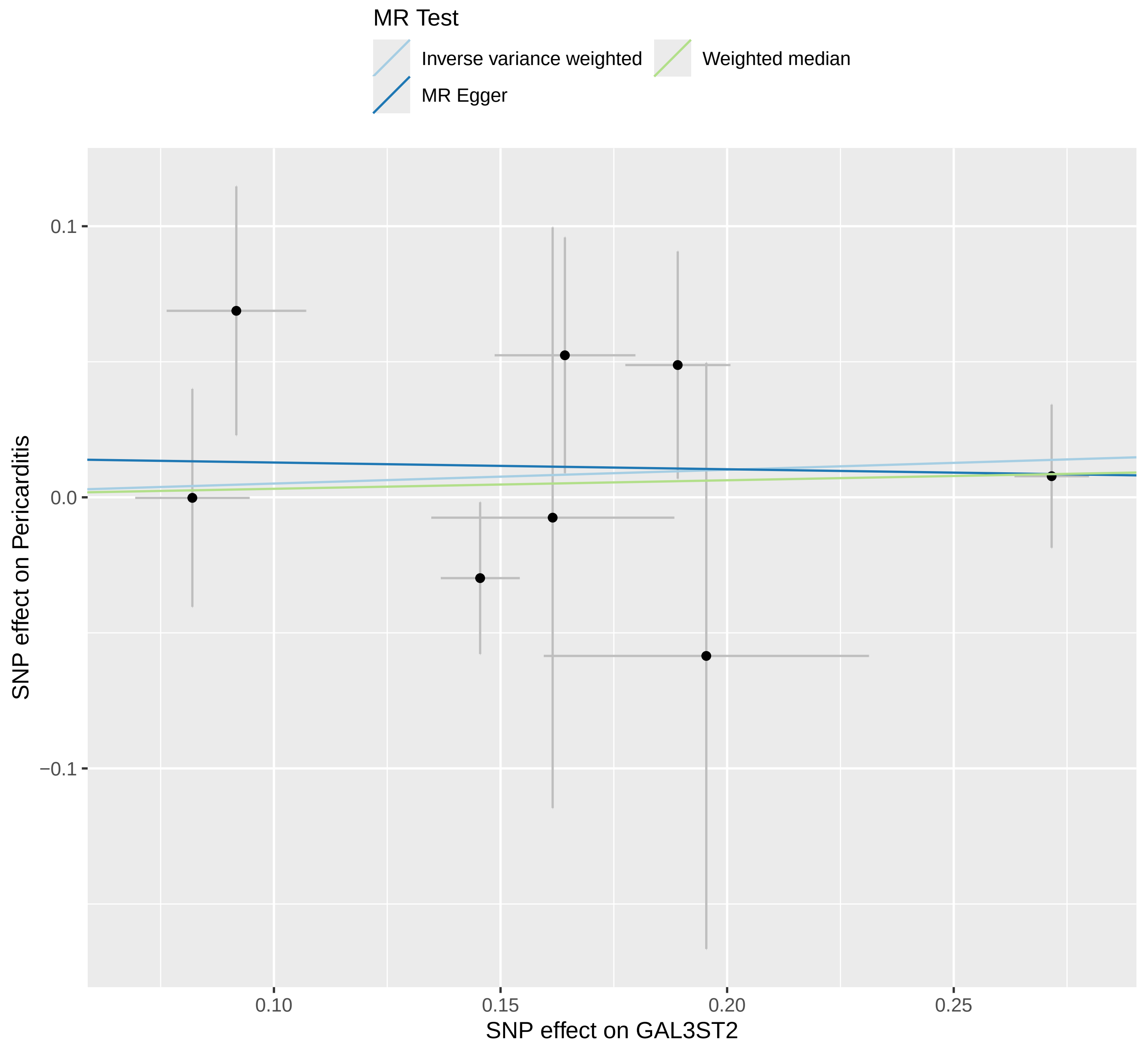
MR Test
Inverse variance weighted
Weighted median
MR Egger
0.1
0.0
−0.1
SNP effect on Pericarditis
0.10
0.15
0.20
0.25
SNP effect on GAL3ST2

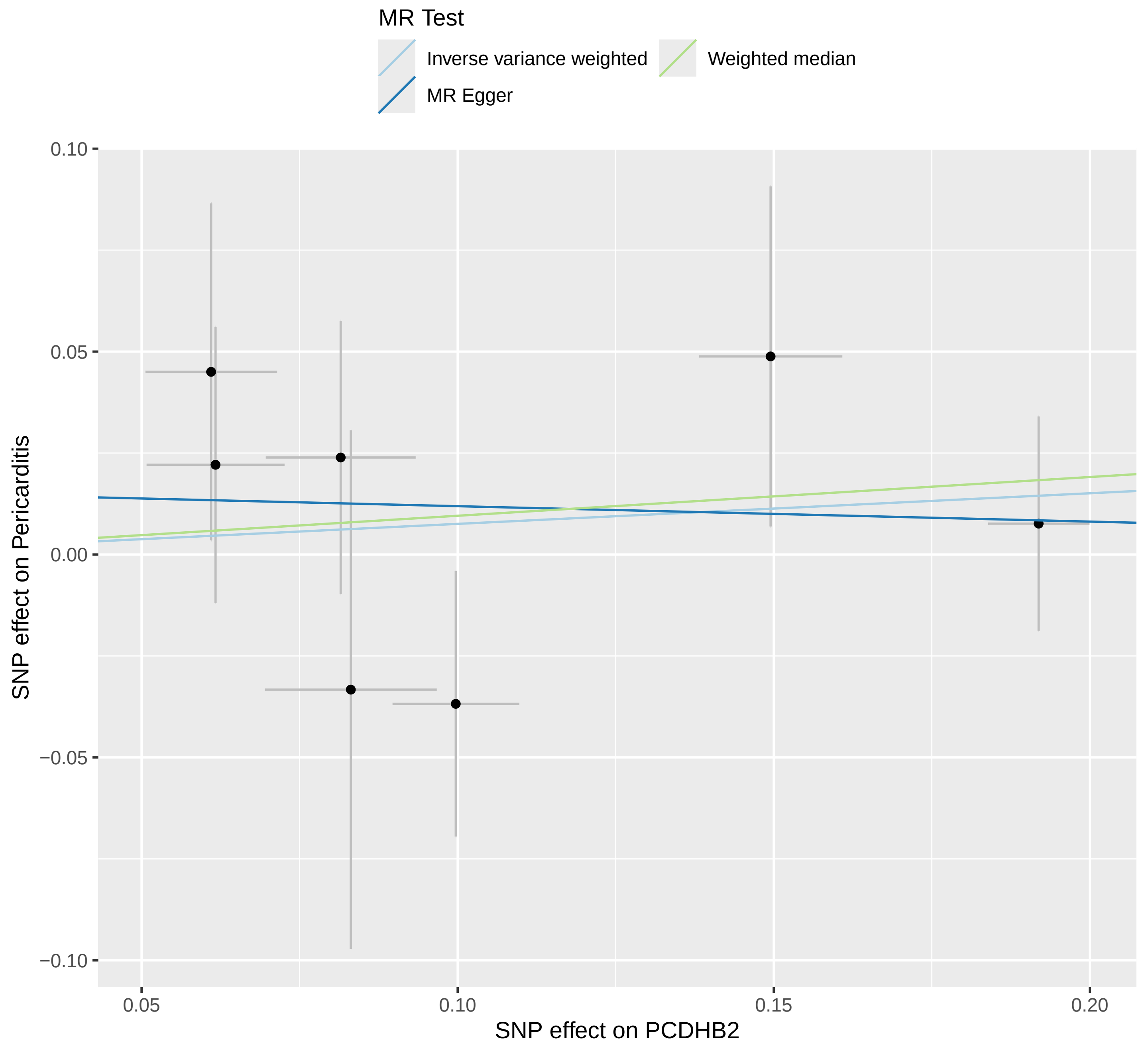

MR Test
Inverse variance weighted
MR Egger
Weighted median
0.10
0.05
0.00
−0.05
−0.10
SNP effect on Pericarditis
0.05
0.10
0.15
0.20
SNP effect on PCDHB2

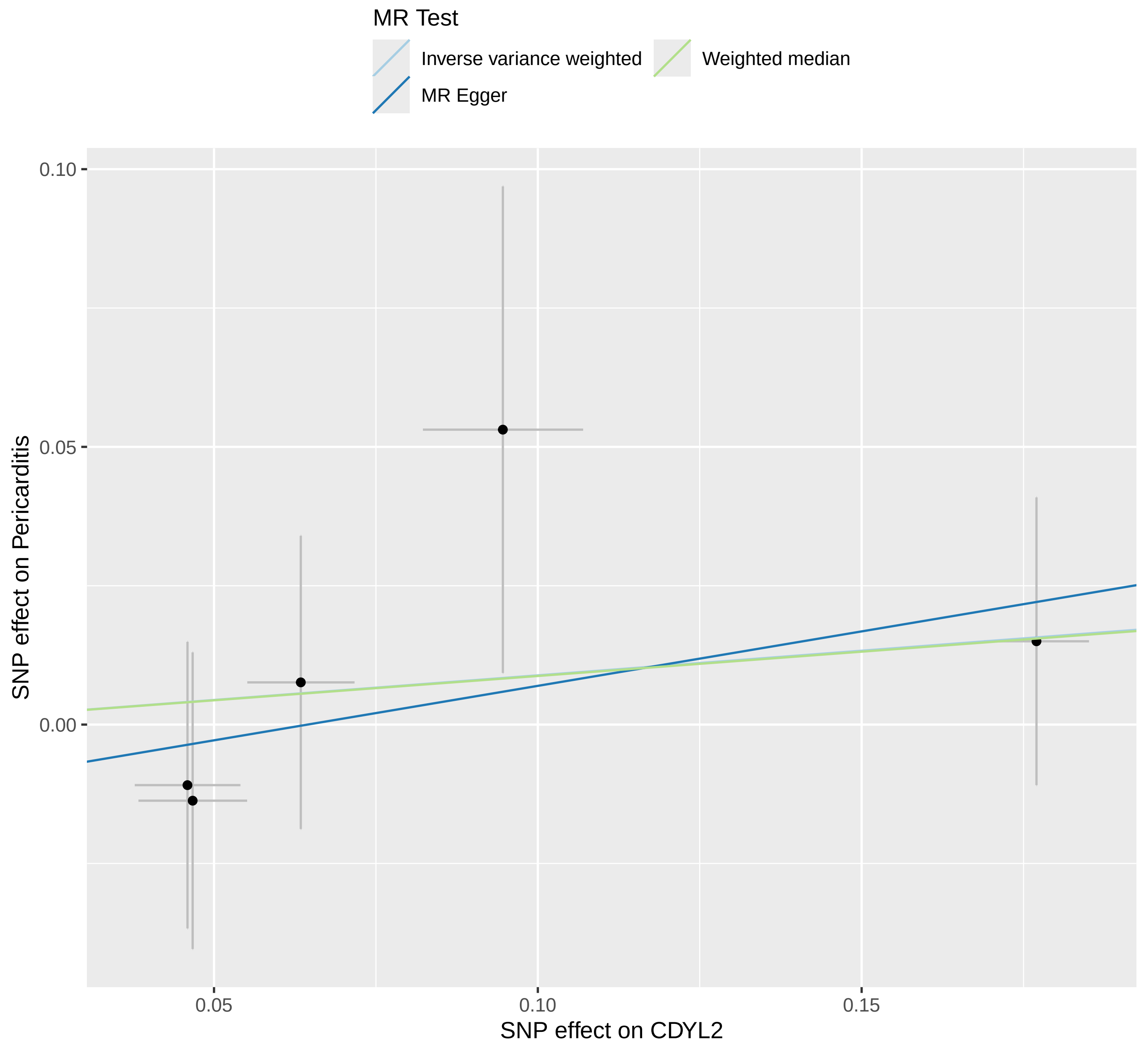
MR Test
Inverse variance weighted
Weighted median
MR Egger
0.10
0.05
0.00
SNP effect on Pericarditis
0.05
0.10
0.15
SNP effect on CDYL2

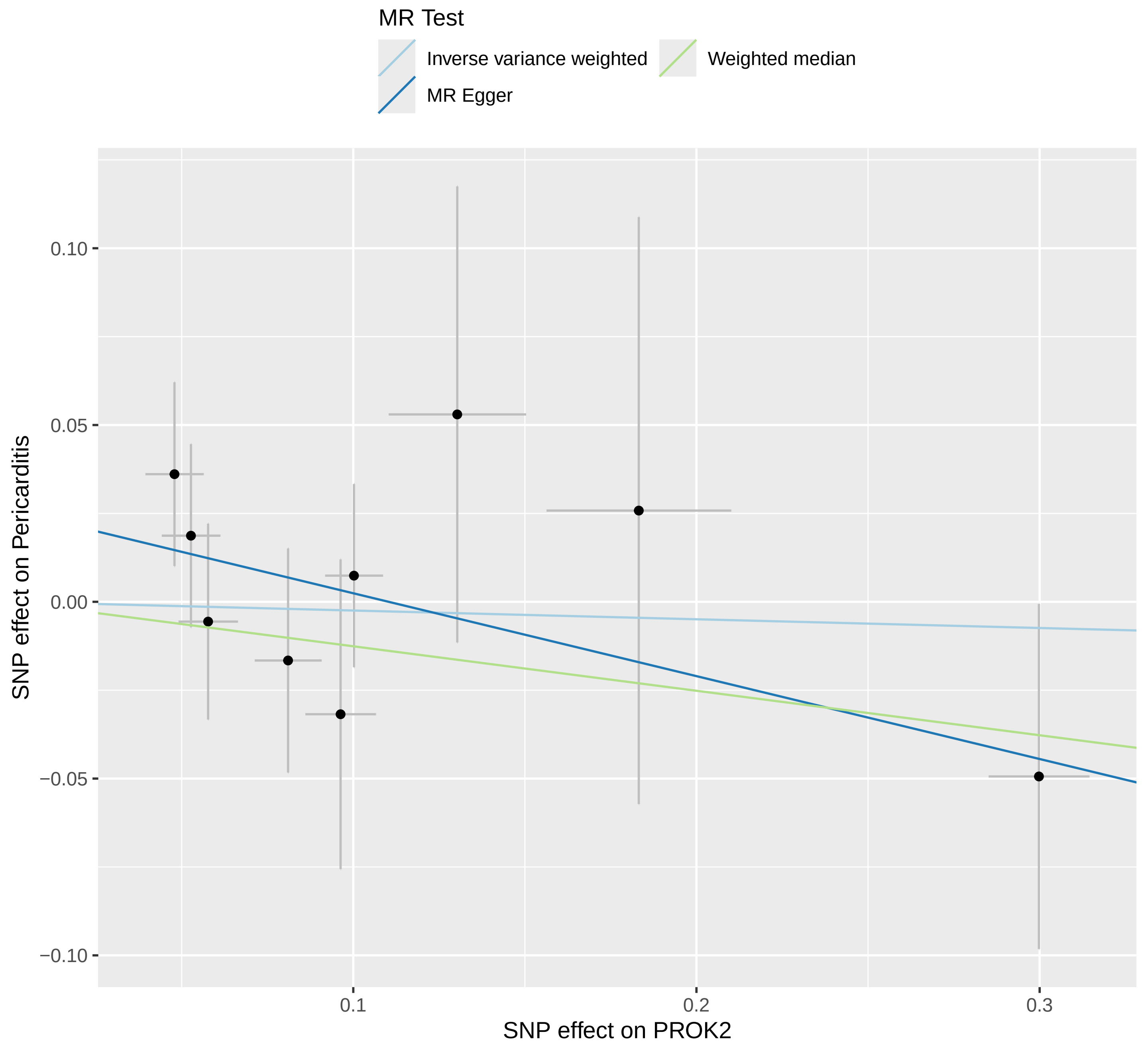

MR Test
Inverse variance weighted
Weighted median
MR Egger
SNP effect on Pericarditis
0.10
0.05
0.00
−0.05
−0.10
0.1
0.2
0.3
SNP effect on PROK2

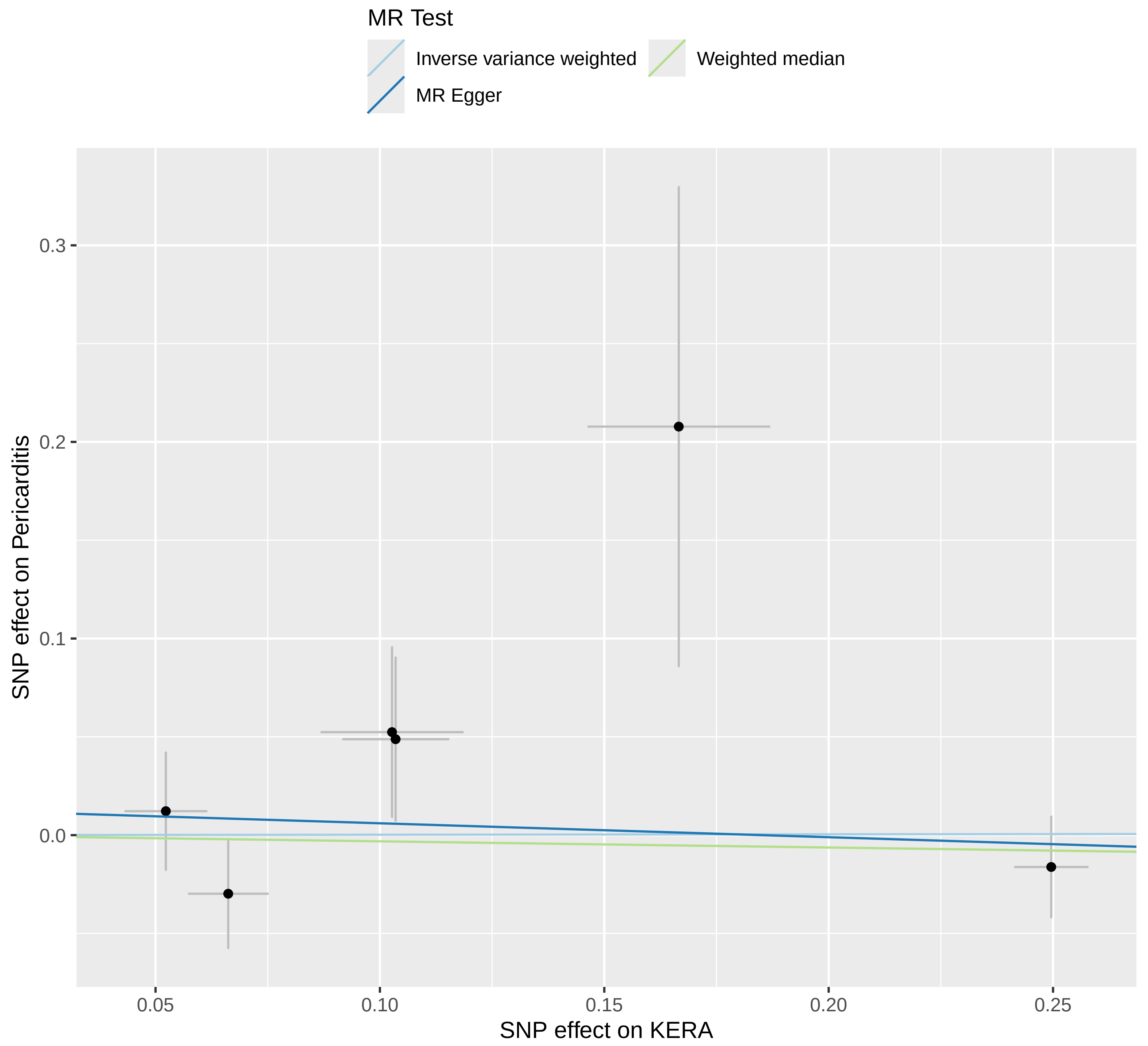

MR Test
Inverse variance weighted
MR Egger
Weighted median
SNP effect on Pericarditis
0.3
0.2
0.1
0.0
0.05
0.10
0.15
0.20
0.25
SNP effect on KERA

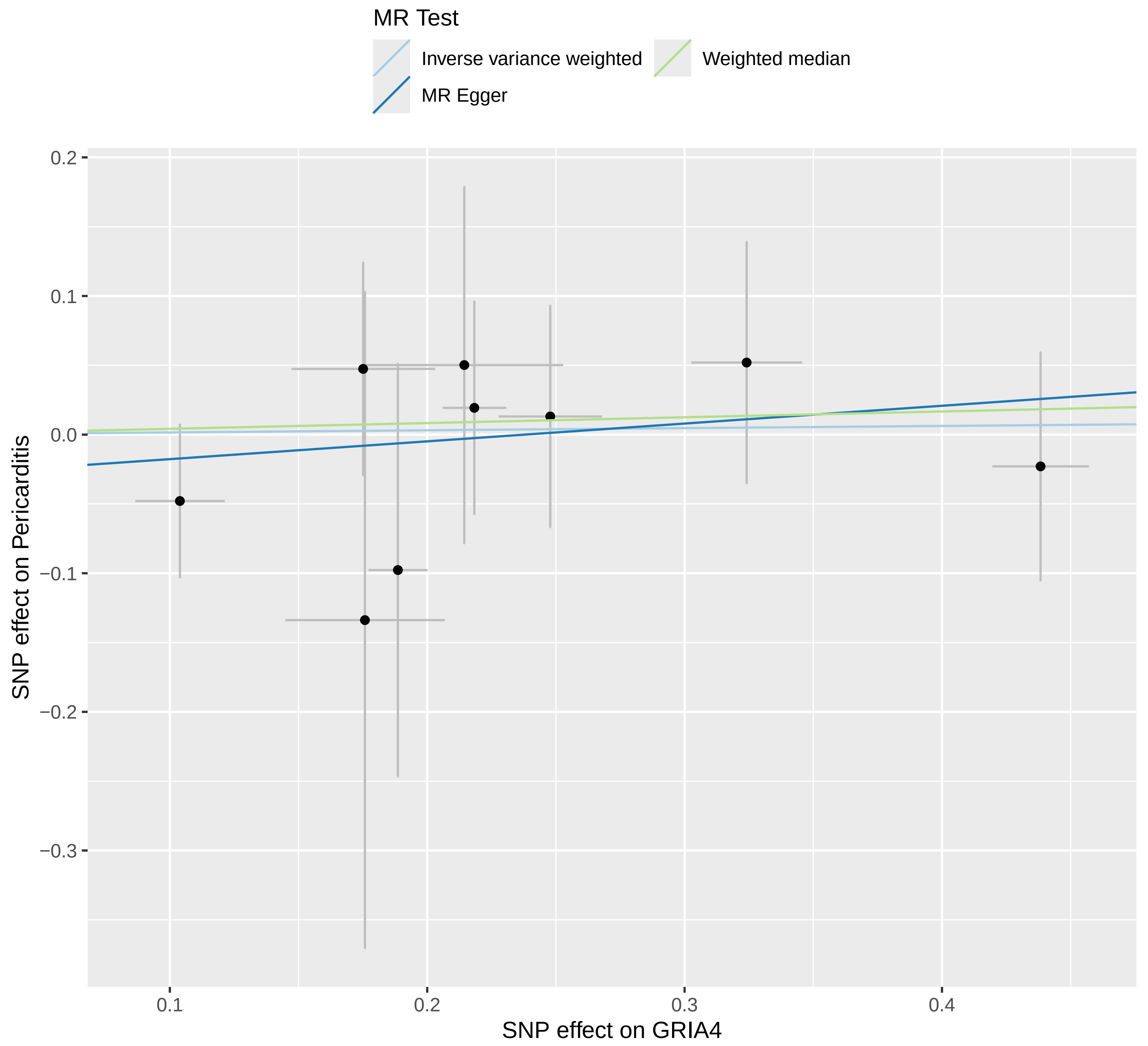
MR Test
Inverse variance weighted
Weighted median
MR Egger
SNP effect on Pericarditis
SNP effect on GRIA4
0.2
0.1
0.0
−0.1
−0.2
−0.3
0.1
0.2
0.3
0.4

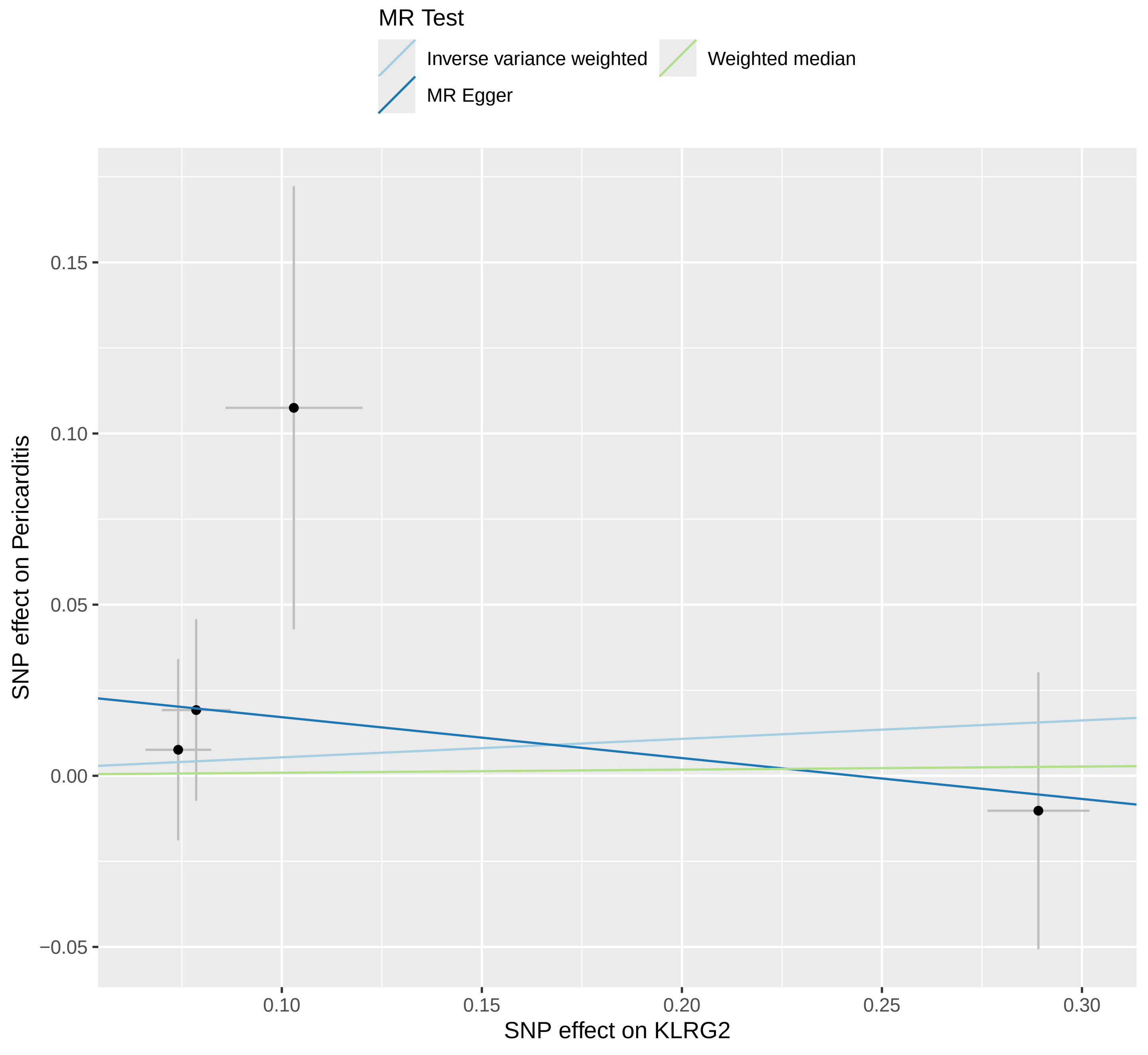

MR Test
Inverse variance weighted
Weighted median
MR Egger
0.15
0.10
0.05
0.00
−0.05
SNP effect on Pericarditis
0.10
0.15
0.20
0.25
0.30
SNP effect on KLRG2

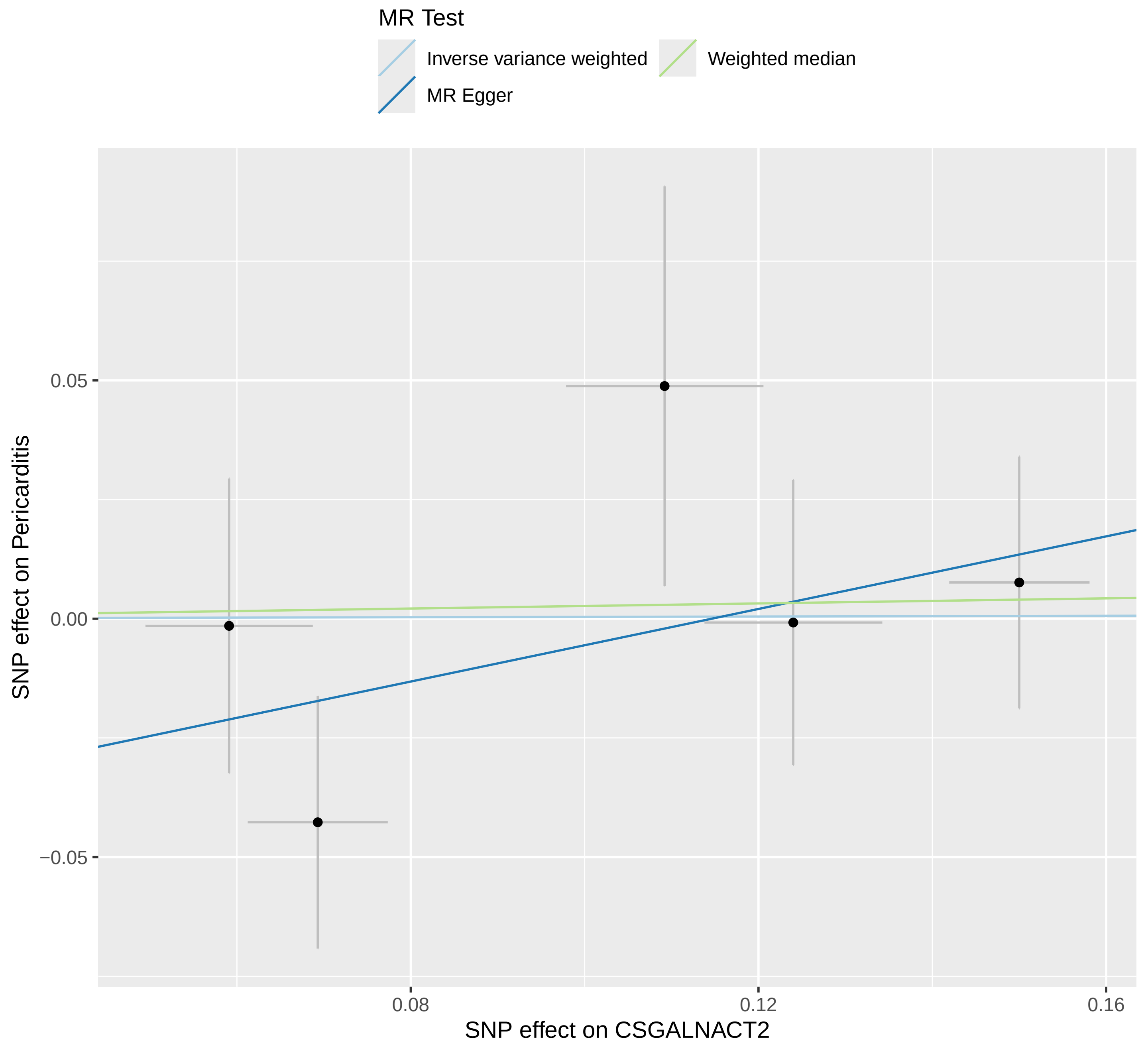

MR Test
Inverse variance weighted
Weighted median
MR Egger
0.05
0.00
−0.05
0.08
0.12
0.16
SNP effect on Pericarditis
SNP effect on CSGALNACT2

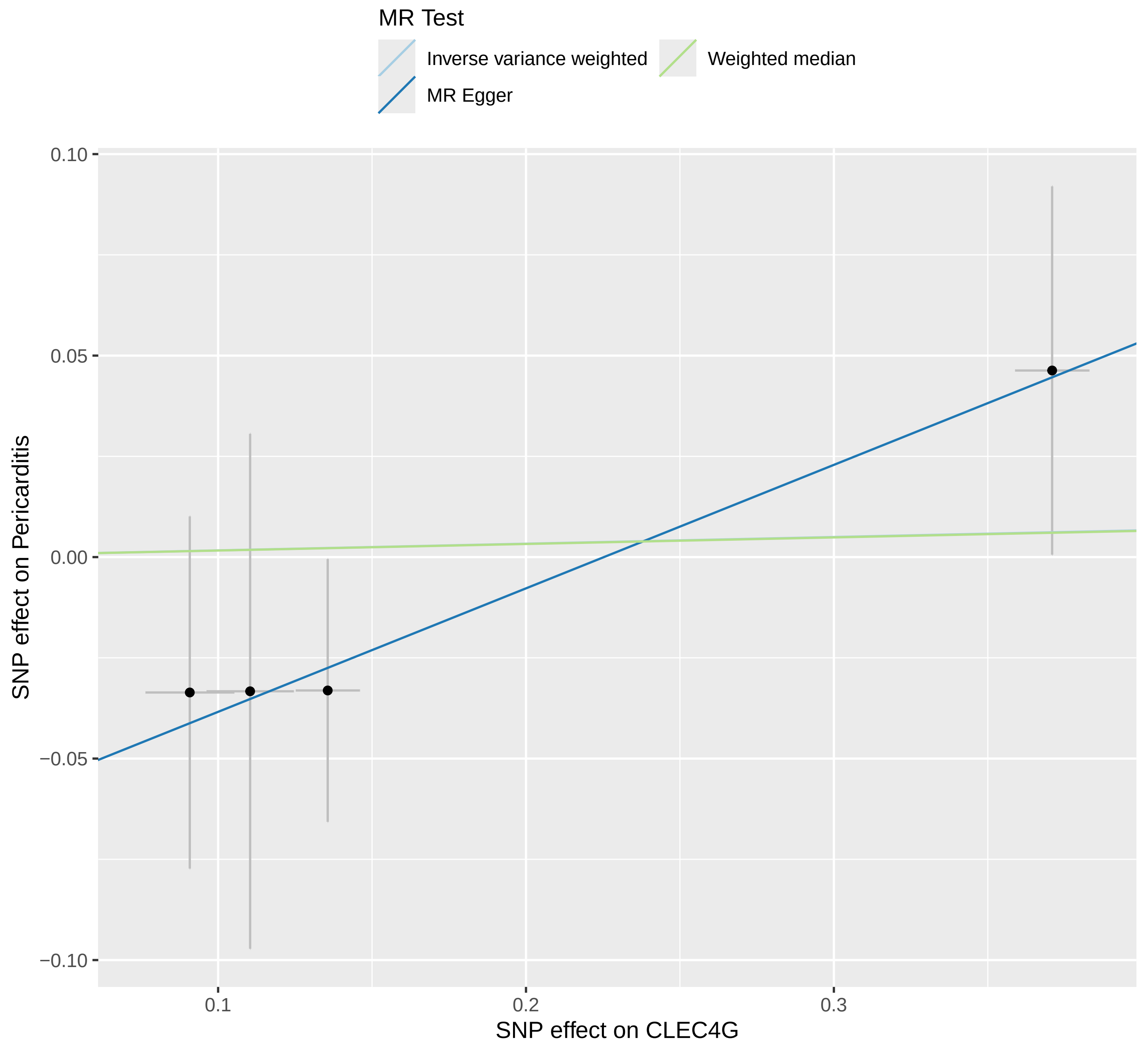
MR Test
Inverse variance weighted
Weighted median
MR Egger
0.10
0.05
0.00
−0.05
−0.10
SNP effect on Pericarditis
0.1
0.2
0.3
SNP effect on CLEC4G

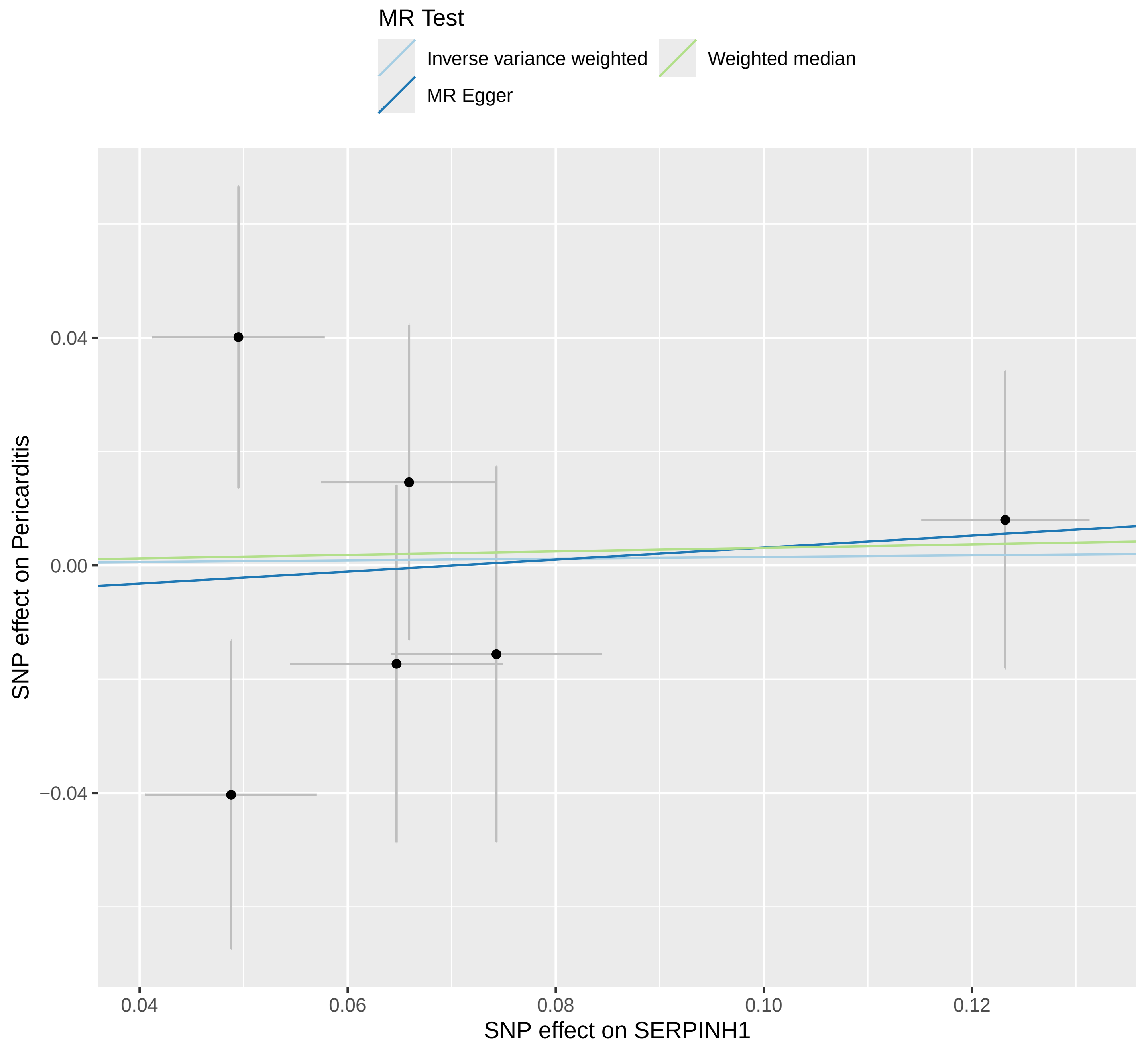
MR Test
Inverse variance weighted
Weighted median
MR Egger
0.04
0.00
−0.04
0.04
0.06
0.08
0.10
0.12
SNP effect on Pericarditis
SNP effect on SERPINH1

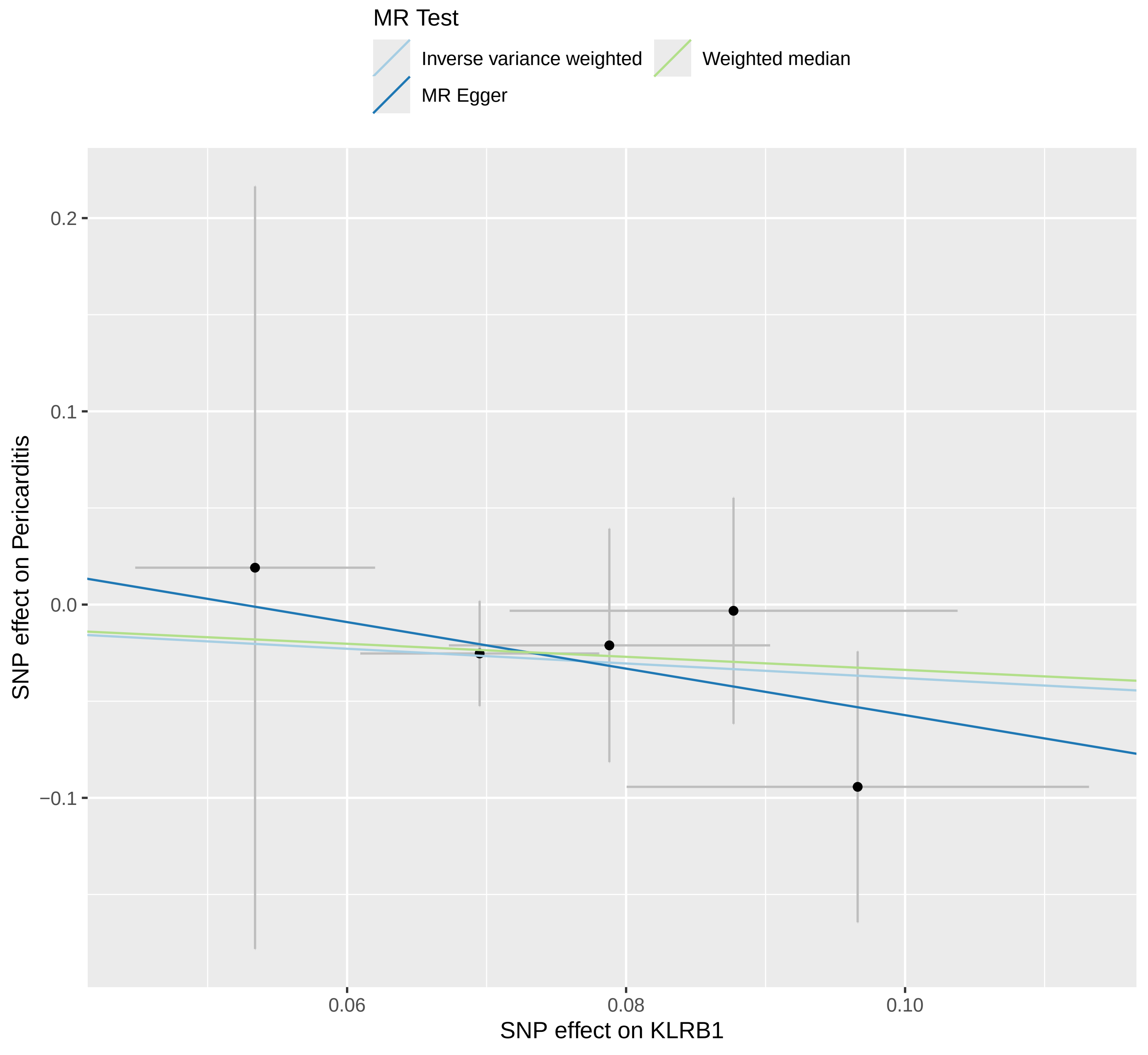
MR Test
Inverse variance weighted
Weighted median
MR Egger
0.2
0.1
0.0
−0.1
SNP effect on Pericarditis
0.06
0.08
0.10
SNP effect on KLRB1

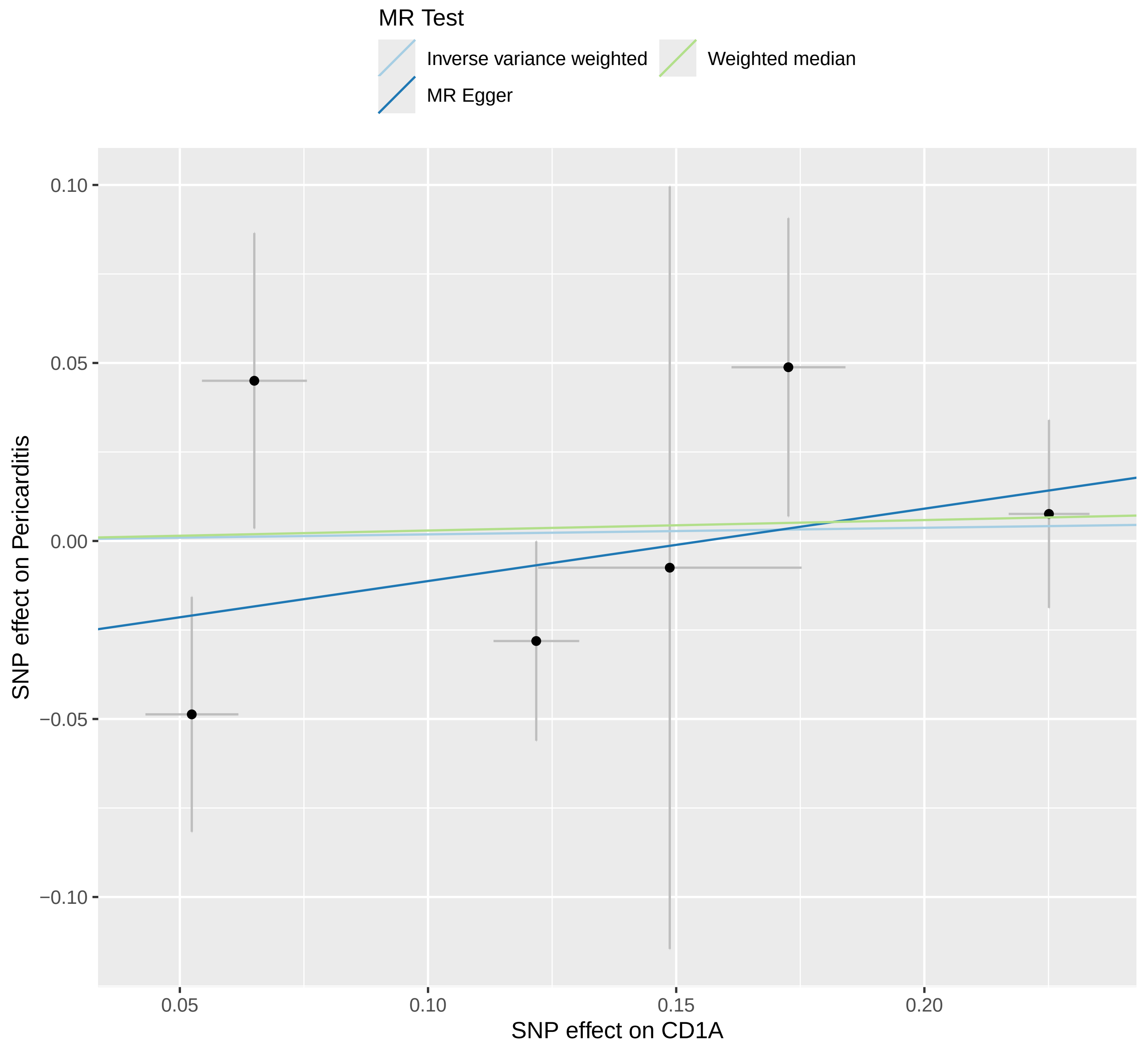
MR Test
Inverse variance weighted
Weighted median
MR Egger
SNP effect on Pericarditis
0.10
0.05
0.00
−0.05
−0.10
0.05
0.10
0.15
0.20
SNP effect on CD1A

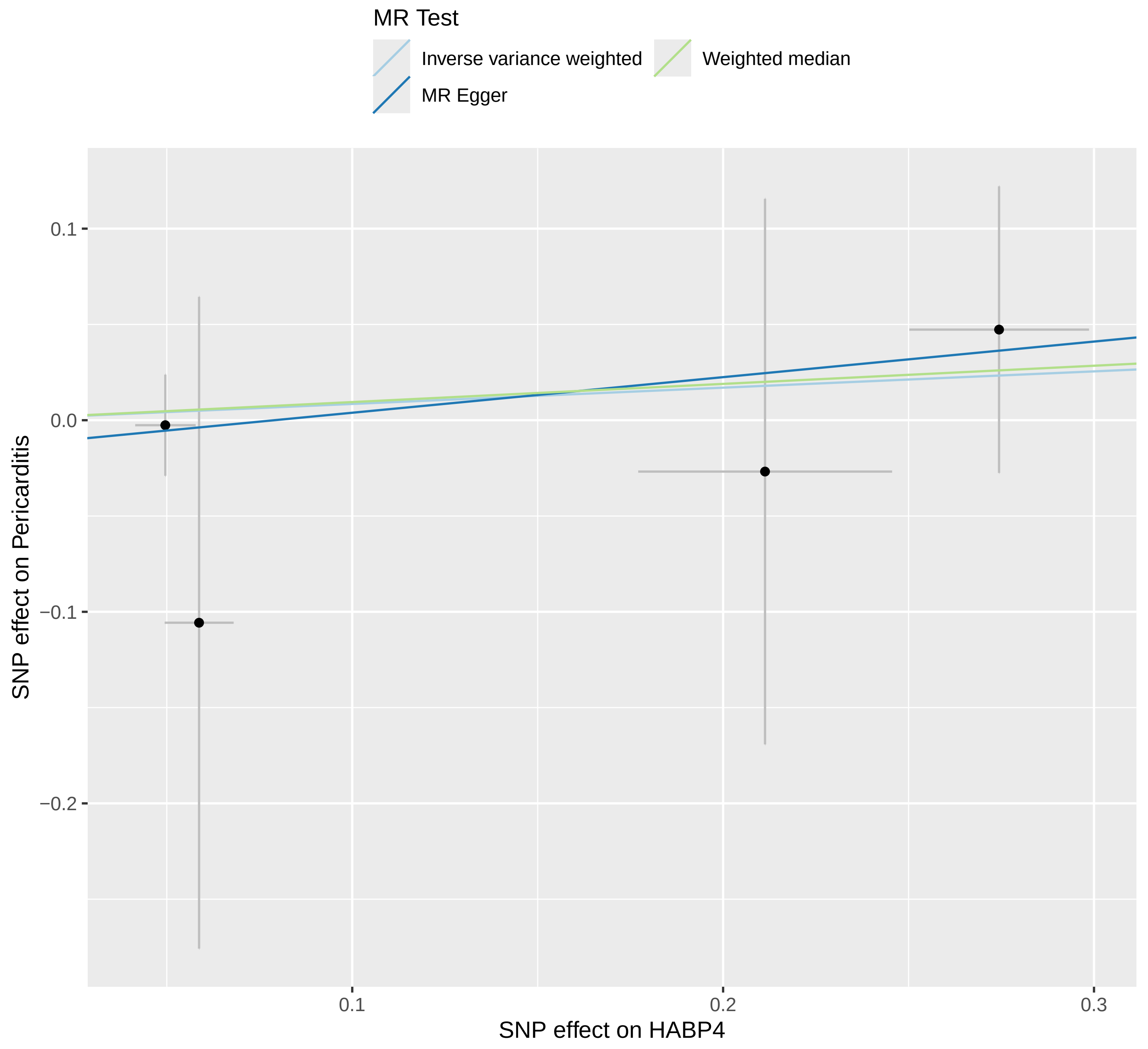

MR Test
Inverse variance weighted
Weighted median
MR Egger
0.1
0.0
−0.1
−0.2
SNP effect on Pericarditis
0.1
0.2
0.3
SNP effect on HABP4

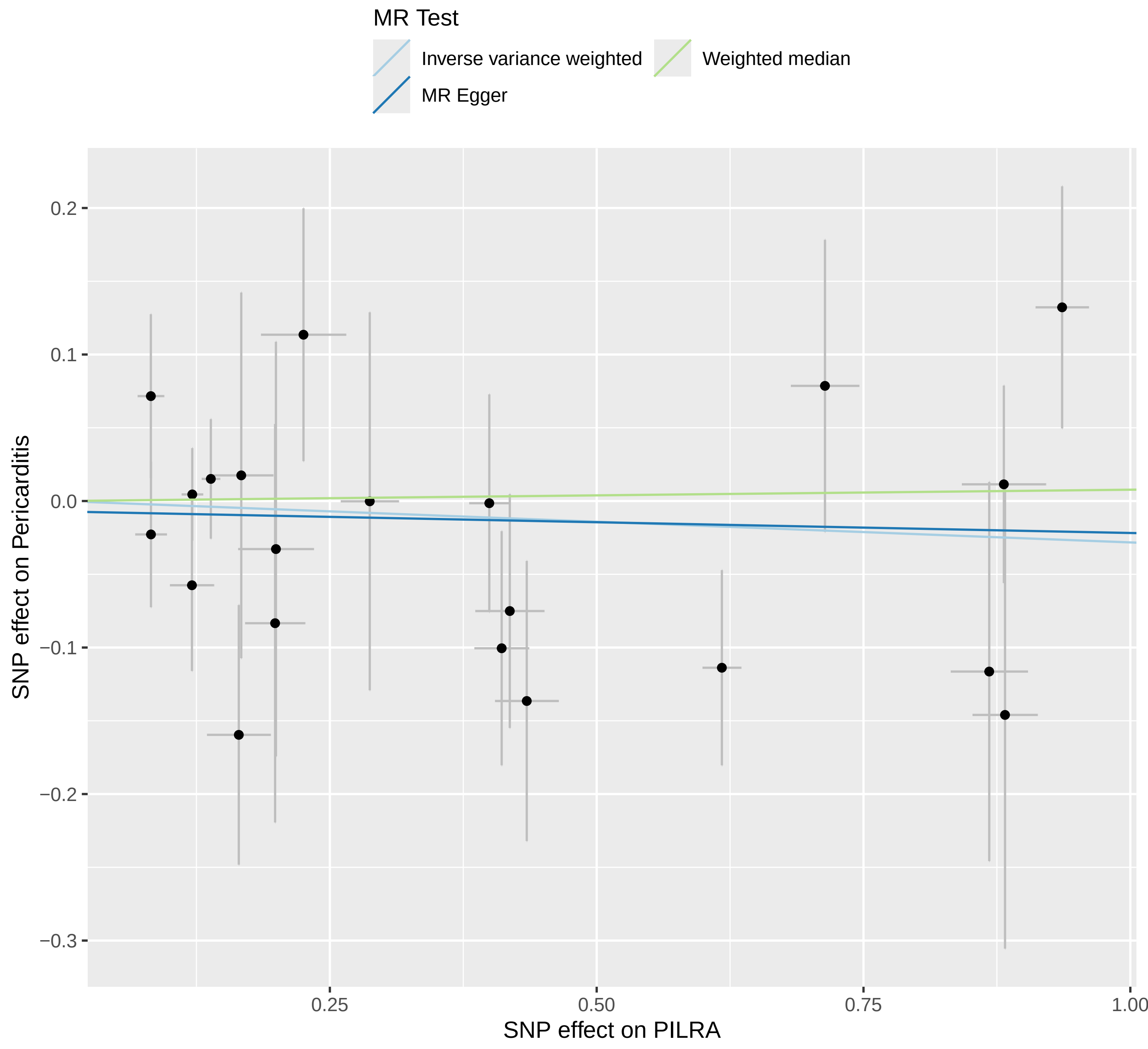

MR Test
Inverse variance weighted
Weighted median
MR Egger
0.2
0.1
0.0
−0.1
−0.2
−0.3
0.25
0.50
0.75
1.00
SNP effect on PILRA
SNP effect on Pericarditis

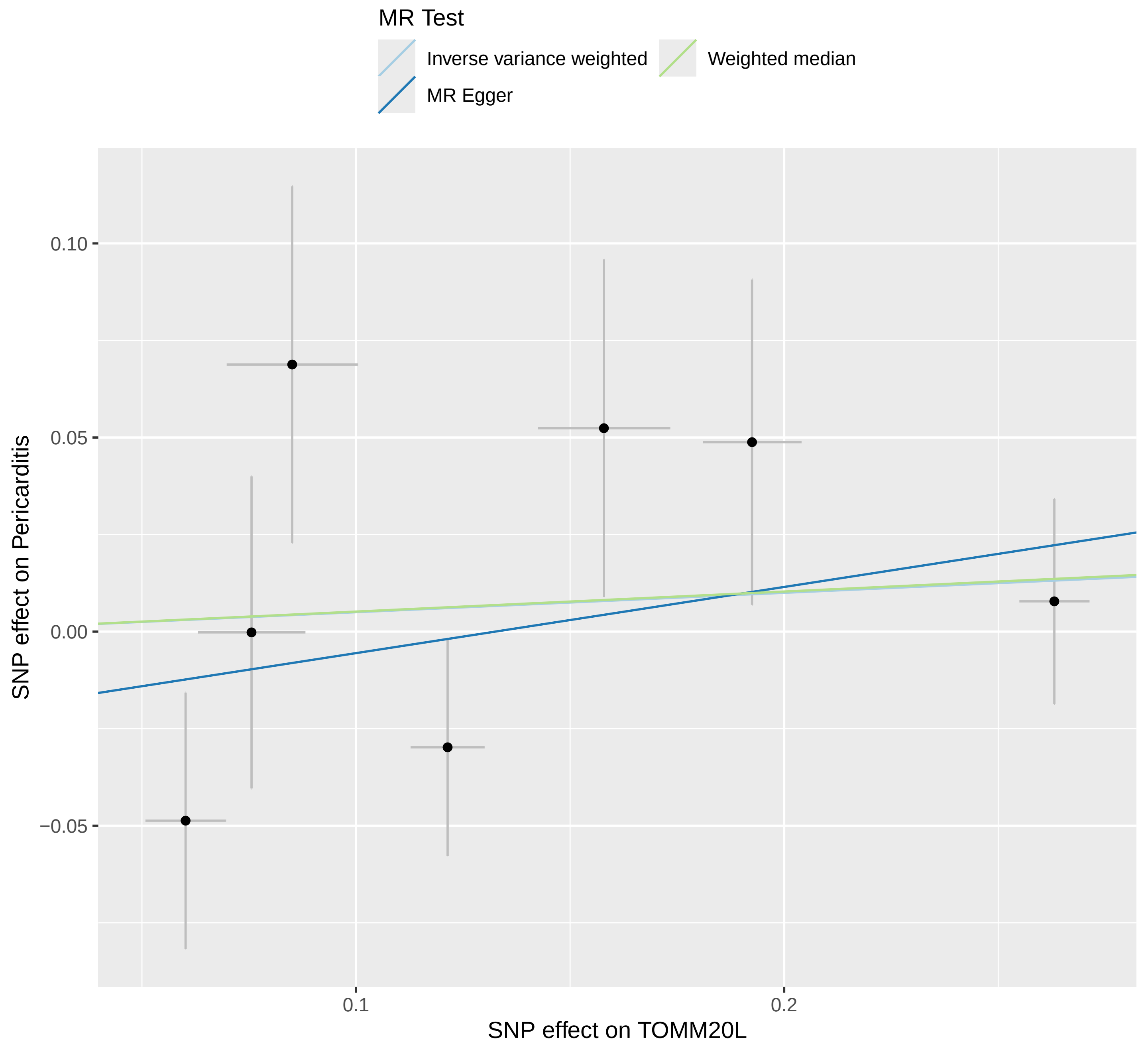

MR Test
Inverse variance weighted
Weighted median
MR Egger
0.10
0.05
0.00
−0.05
0.1
0.2
SNP effect on Pericarditis
SNP effect on TOMM20L

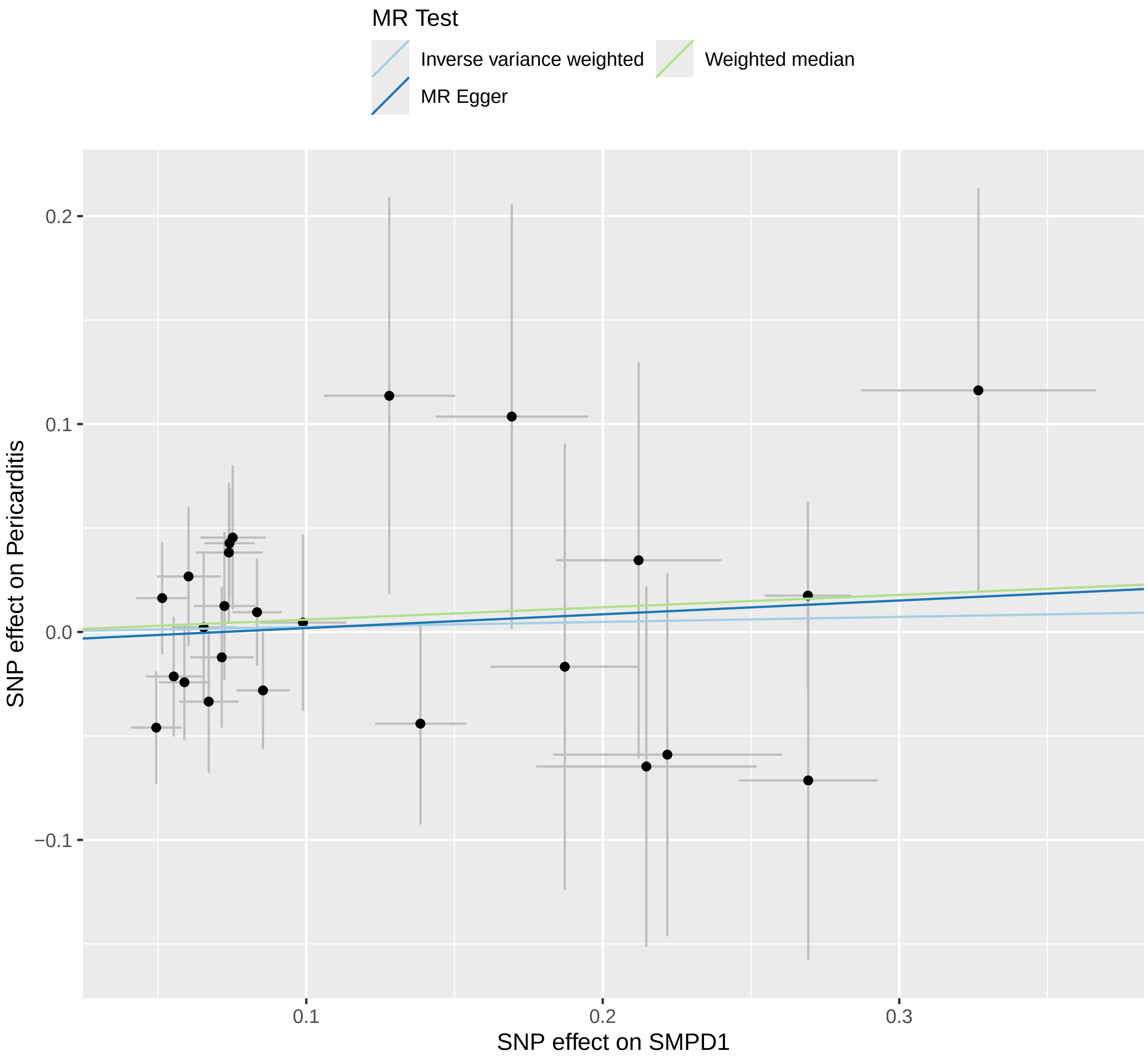
MR Test
Inverse variance weighted
Weighted median
MR Egger
0.2
0.1
0.0
−0.1
SNP effect on Pericarditis
0.1
0.2
0.3
SNP effect on SMPD1

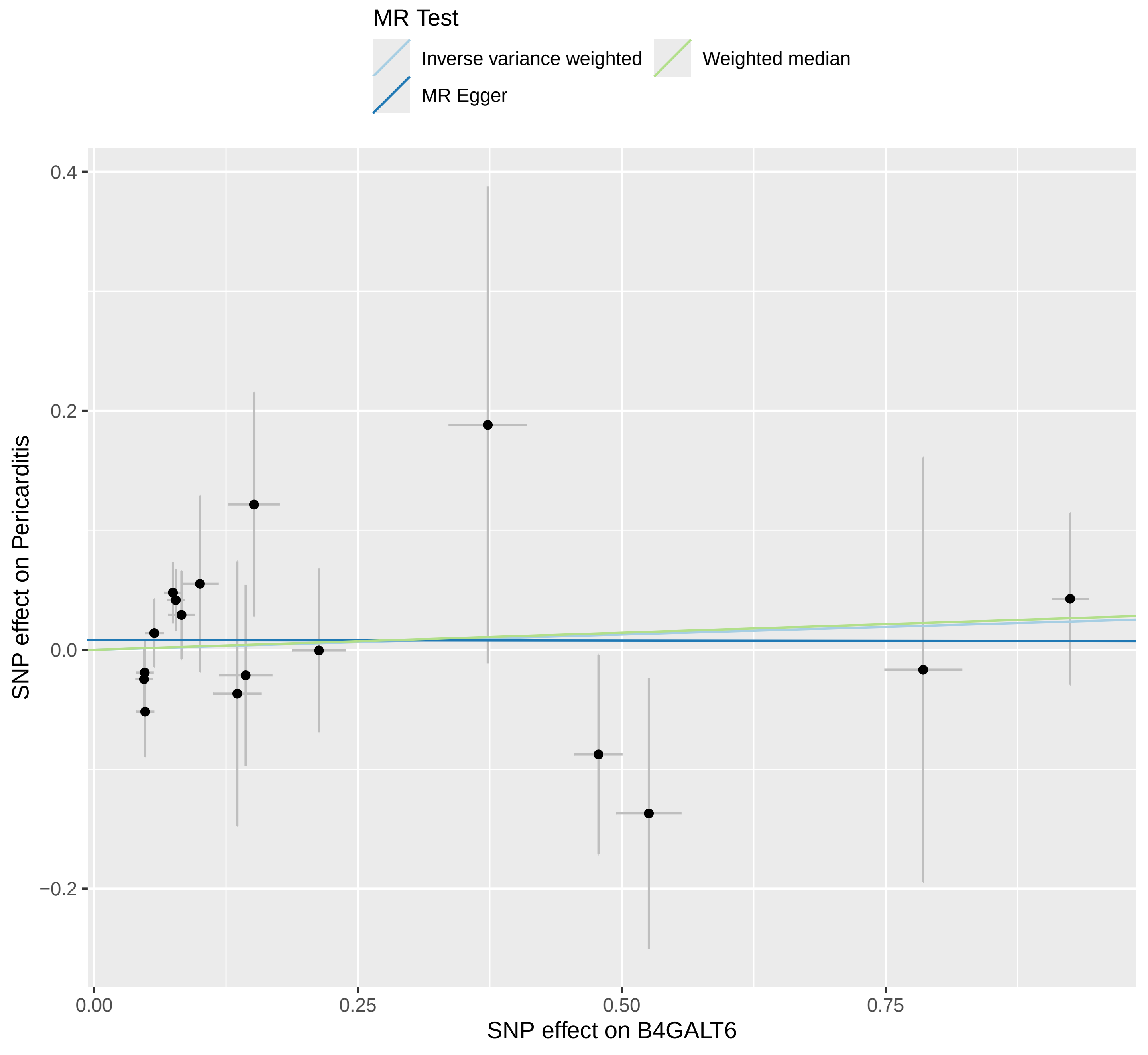

MR Test
Inverse variance weighted
Weighted median
MR Egger
0.4
0.2
0.0
−0.2
SNP effect on Pericarditis
0.00
0.25
0.50
0.75
SNP effect on B4GALT6

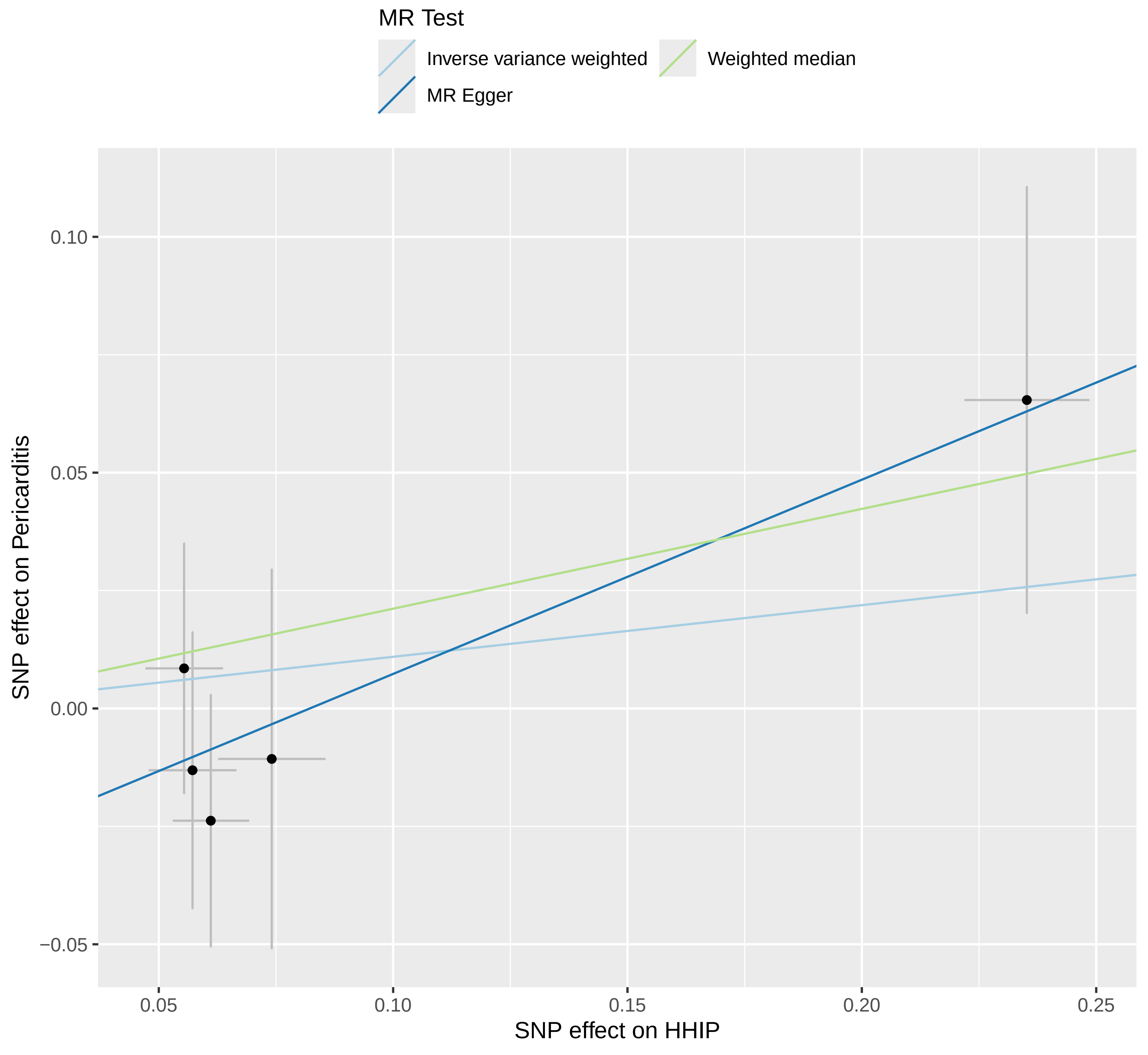
MR Test
Inverse variance weighted
Weighted median
MR Egger
0.10
0.05
0.00
−0.05
SNP effect on Pericarditis
0.05
0.10
0.15
0.20
0.25
SNP effect on HHIP

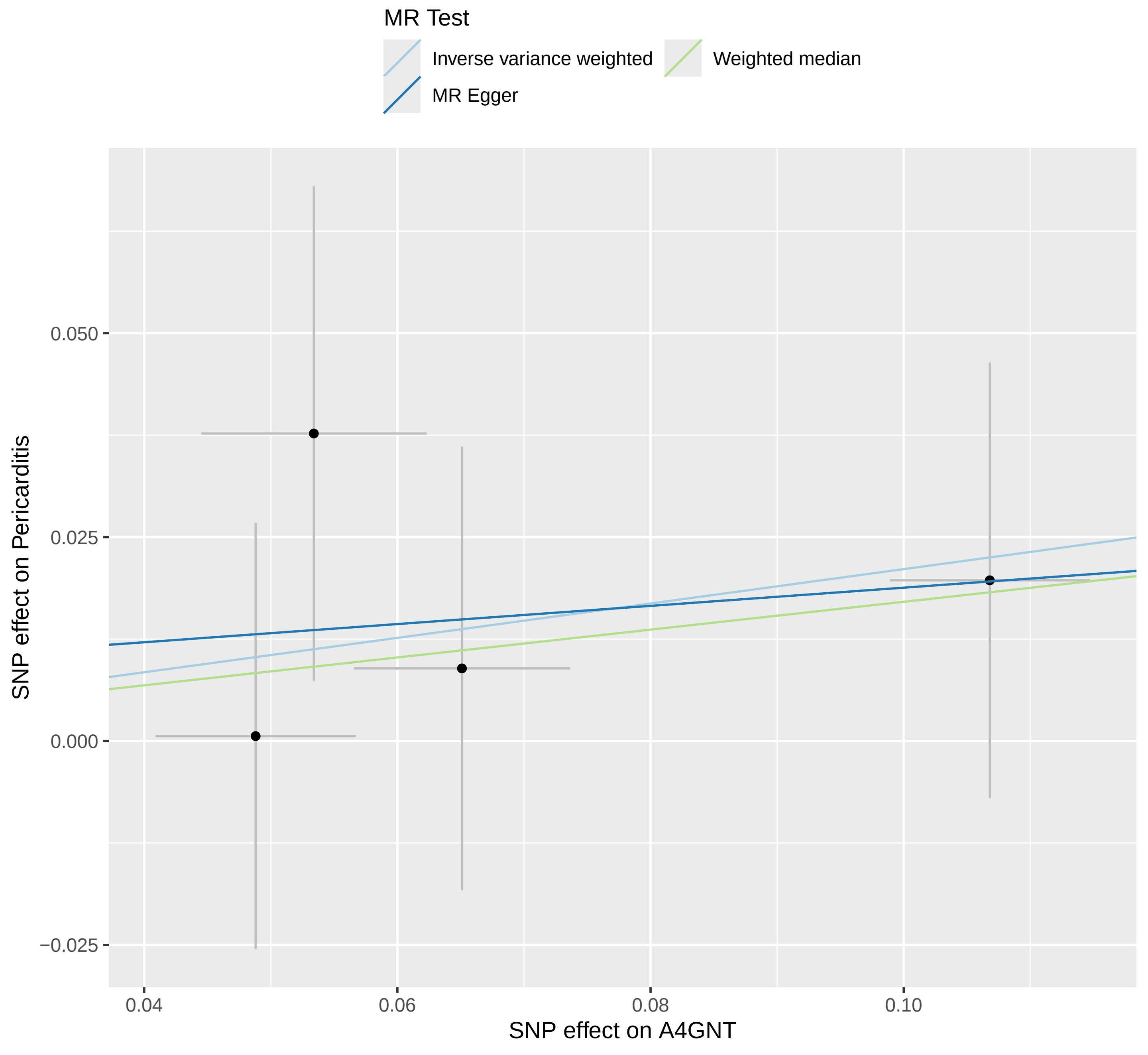
MR Test
Inverse variance weighted
MR Egger
Weighted median
0.050
0.025
0.000
−0.025
SNP effect on Pericarditis
0.04
0.06
0.08
0.10
SNP effect on A4GNT

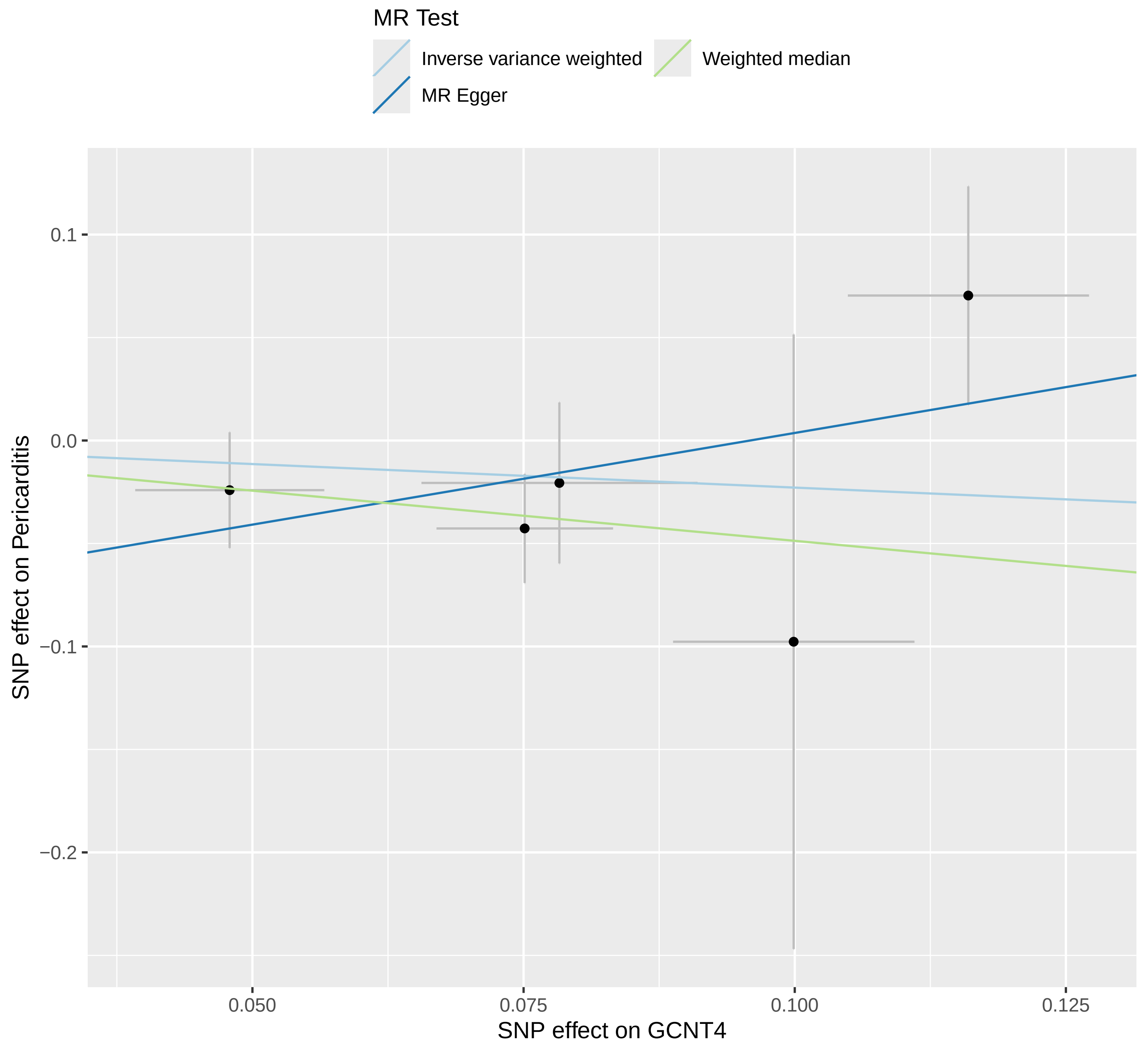
MR Test
Inverse variance weighted
Weighted median
MR Egger
0.1
0.0
−0.1
−0.2
SNP effect on Pericarditis
0.050
0.075
0.100
0.125
SNP effect on GCNT4

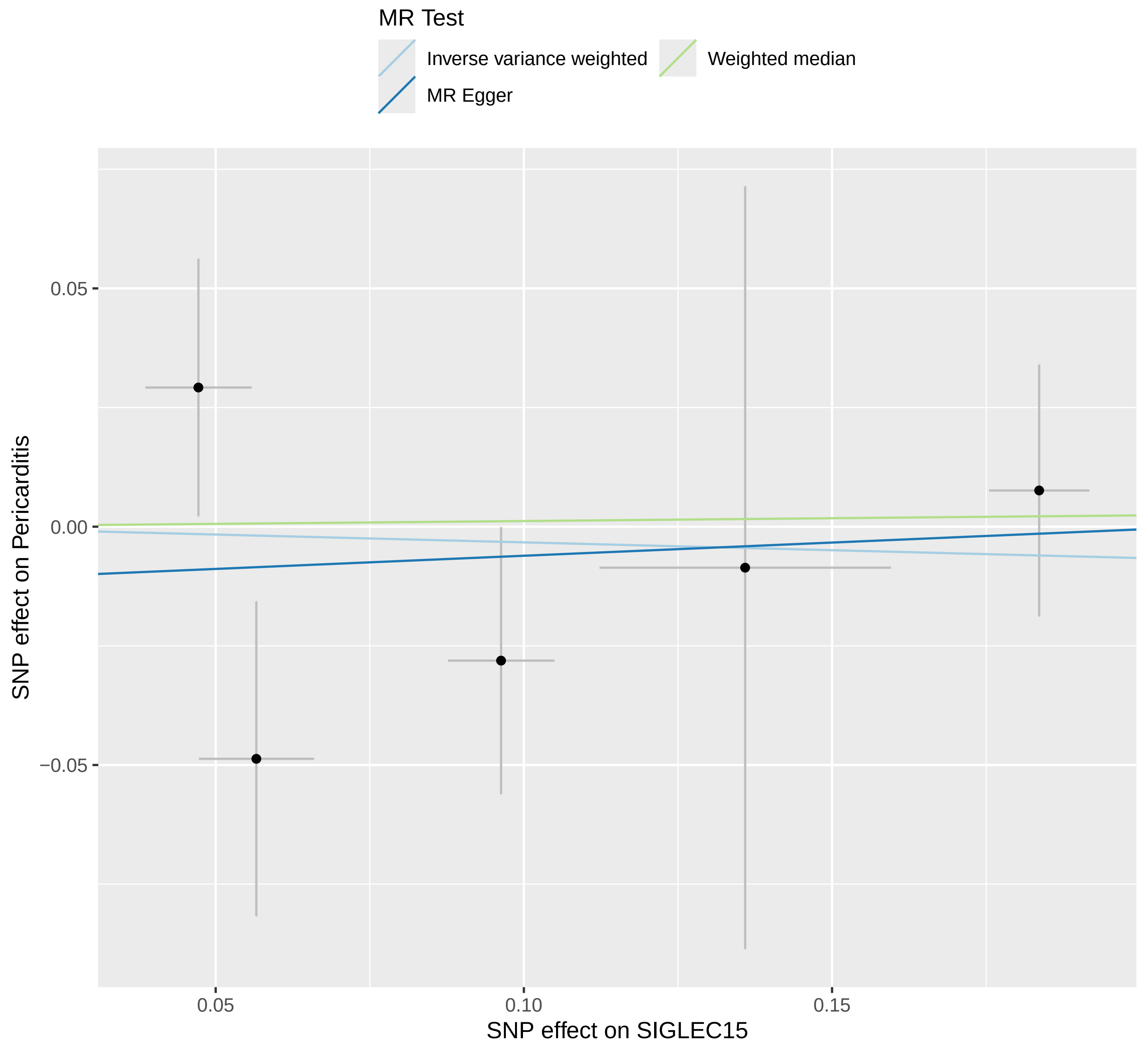
MR Test
Inverse variance weighted
MR Egger
Weighted median
0.05
0.00
−0.05
0.05
0.10
0.15
SNP effect on Pericarditis
SNP effect on SIGLEC15

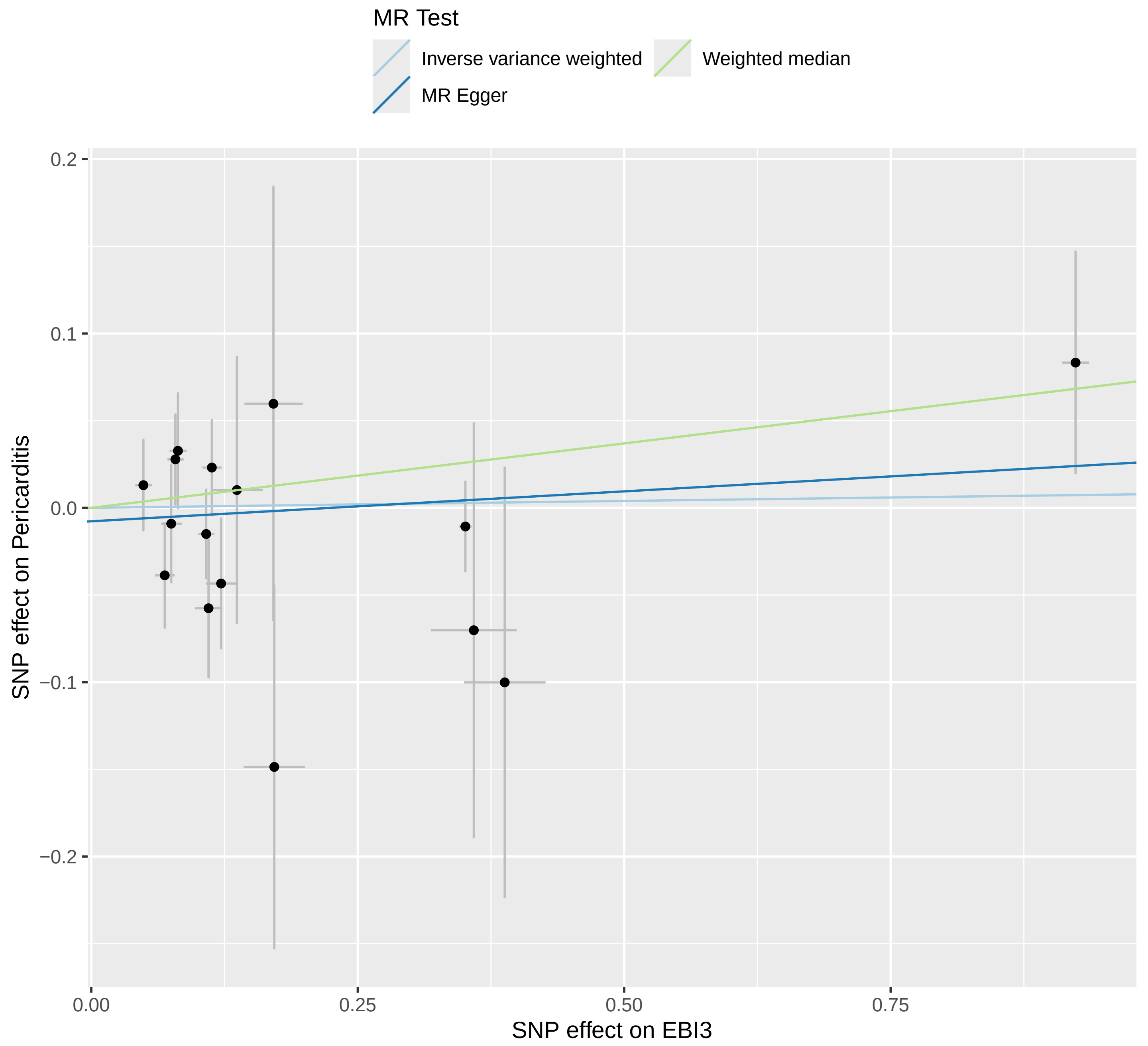

MR Test
Inverse variance weighted
Weighted median
MR Egger
0.2
0.1
0.0
−0.1
−0.2
SNP effect on Pericarditis
0.00
0.25
0.50
0.75
SNP effect on EBI3

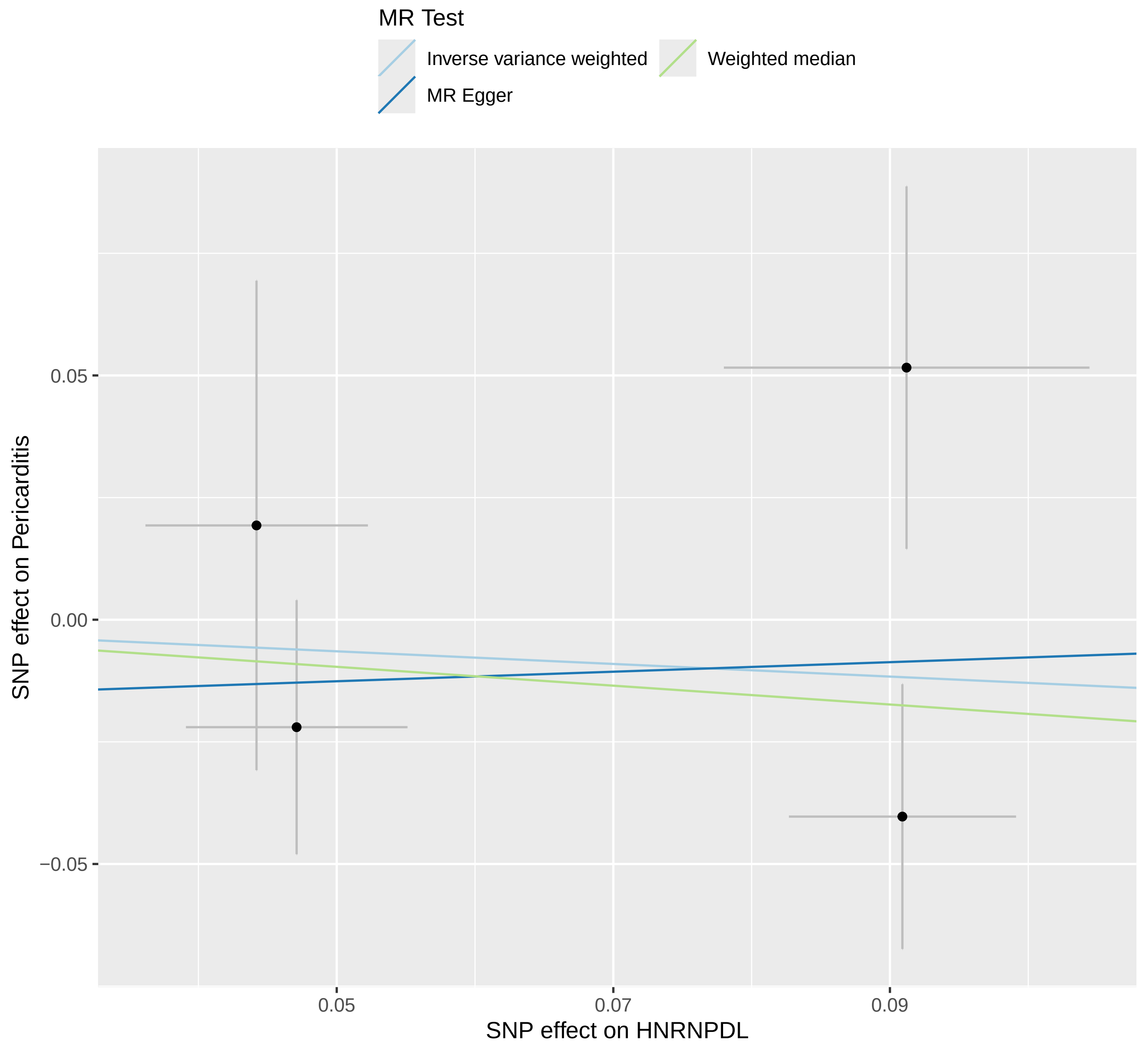

MR Test
Inverse variance weighted
MR Egger
Weighted median
0.05
0.00
−0.05
SNP effect on Pericarditis
0.05
0.07
0.09
SNP effect on HNRNPDL

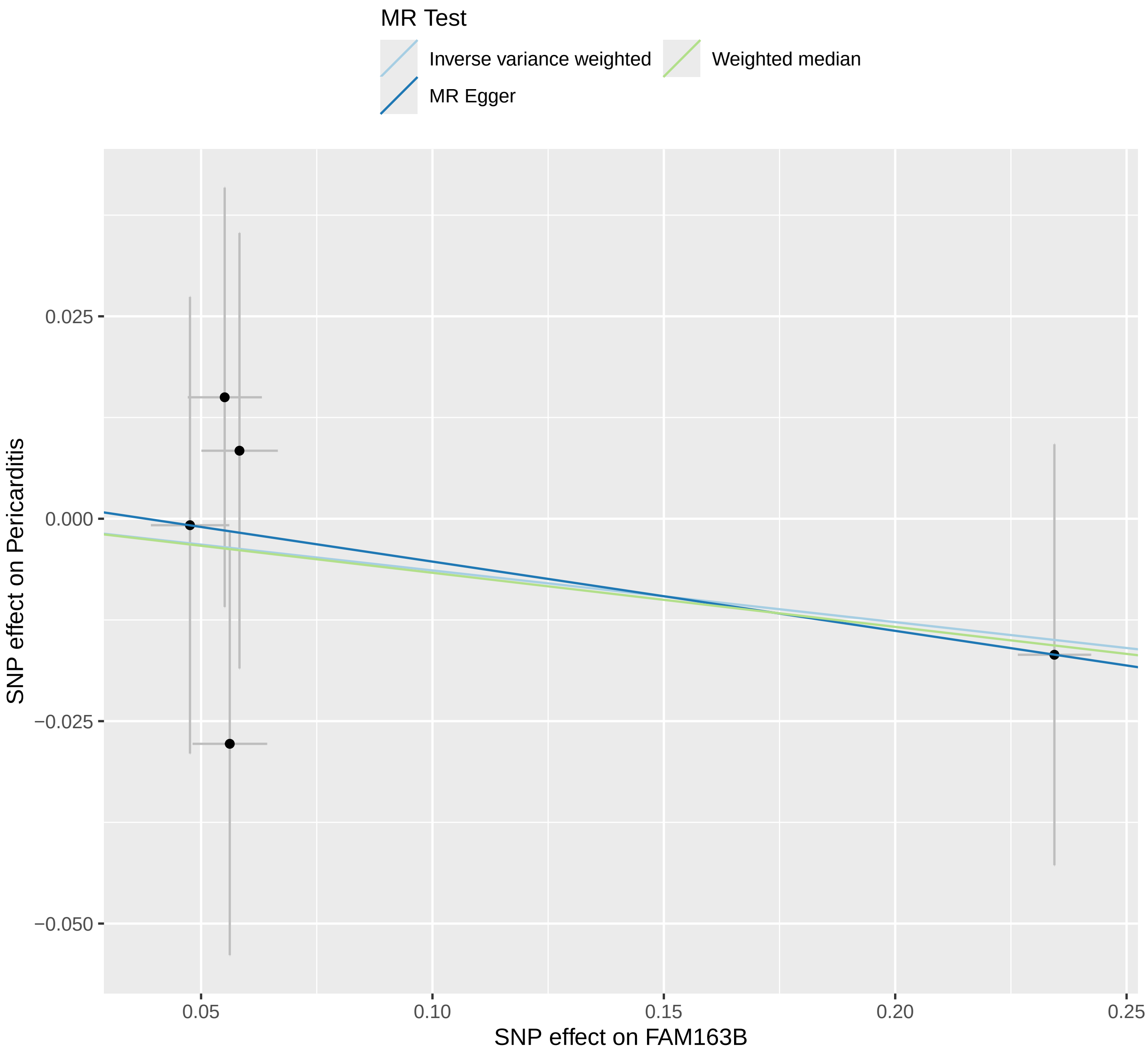
MR Test
Inverse variance weighted
Weighted median
MR Egger
0.025
0.000
−0.025
−0.050
SNP effect on Pericarditis
0.05
0.10
0.15
0.20
0.25
SNP effect on FAM163B

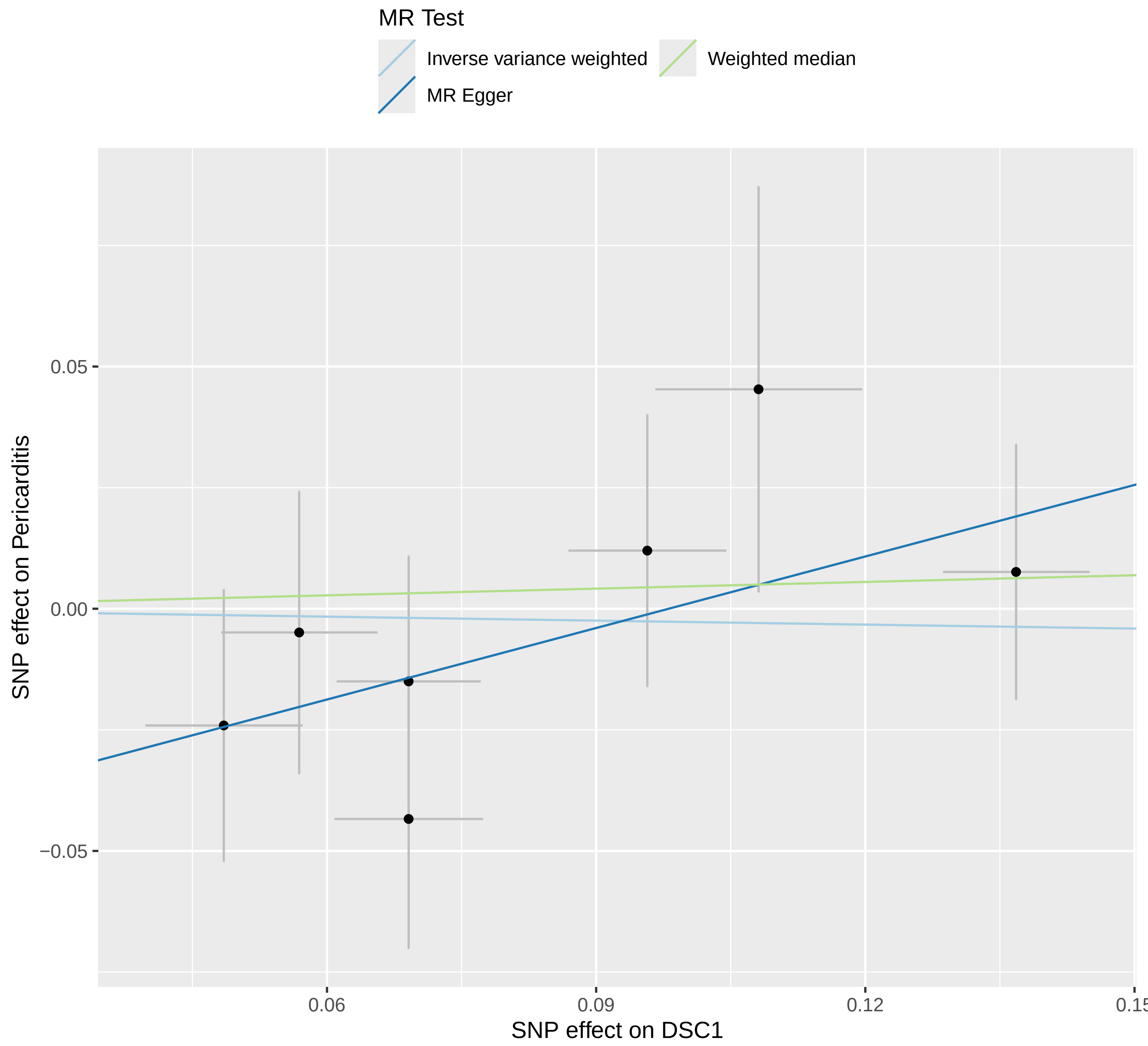
MR Test
Inverse variance weighted
MR Egger
Weighted median
0.05
0.00
−0.05
0.06
0.09
0.12
SNP effect on DSC1
SNP effect on Pericarditis

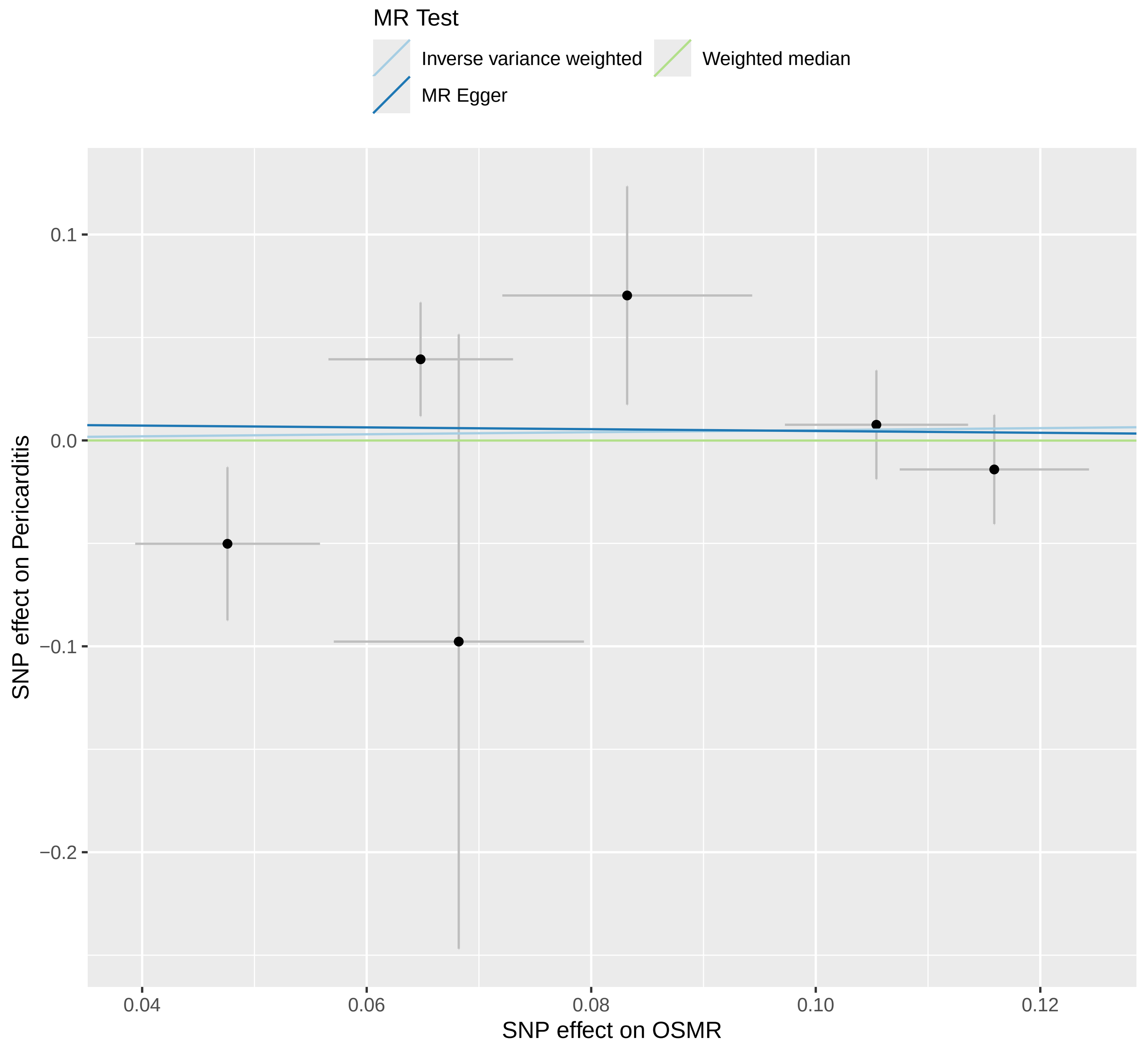
MR Test
Inverse variance weighted
Weighted median
MR Egger
0.1
0.0
−0.1
−0.2
SNP effect on Pericarditis
0.04
0.06
0.08
0.10
0.12
SNP effect on OSMR

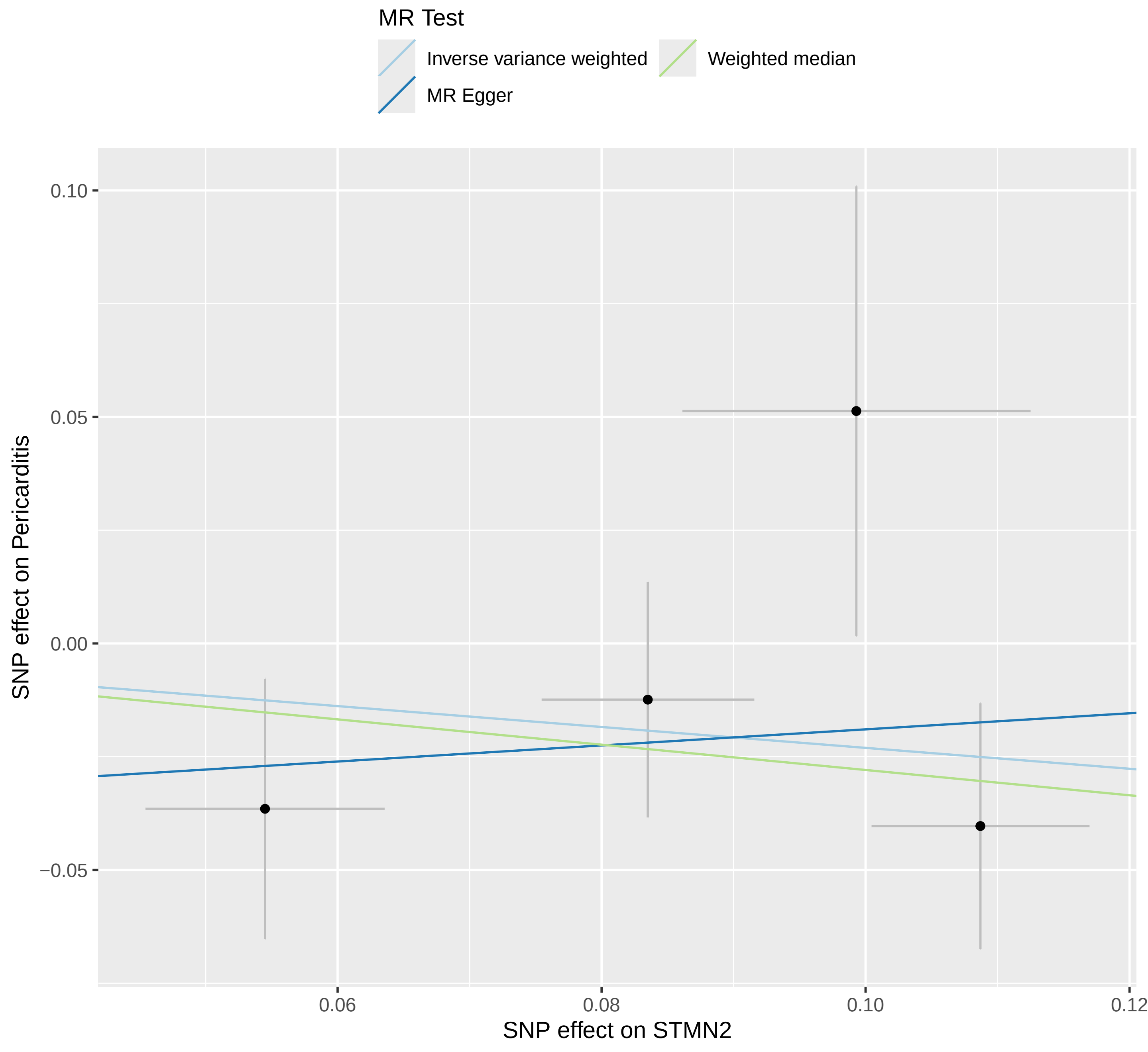

MR Test
Inverse variance weighted
Weighted median
MR Egger
0.10
0.05
0.00
−0.05
0.06
0.08
0.10
0.12
SNP effect on Pericarditis
SNP effect on STMN2

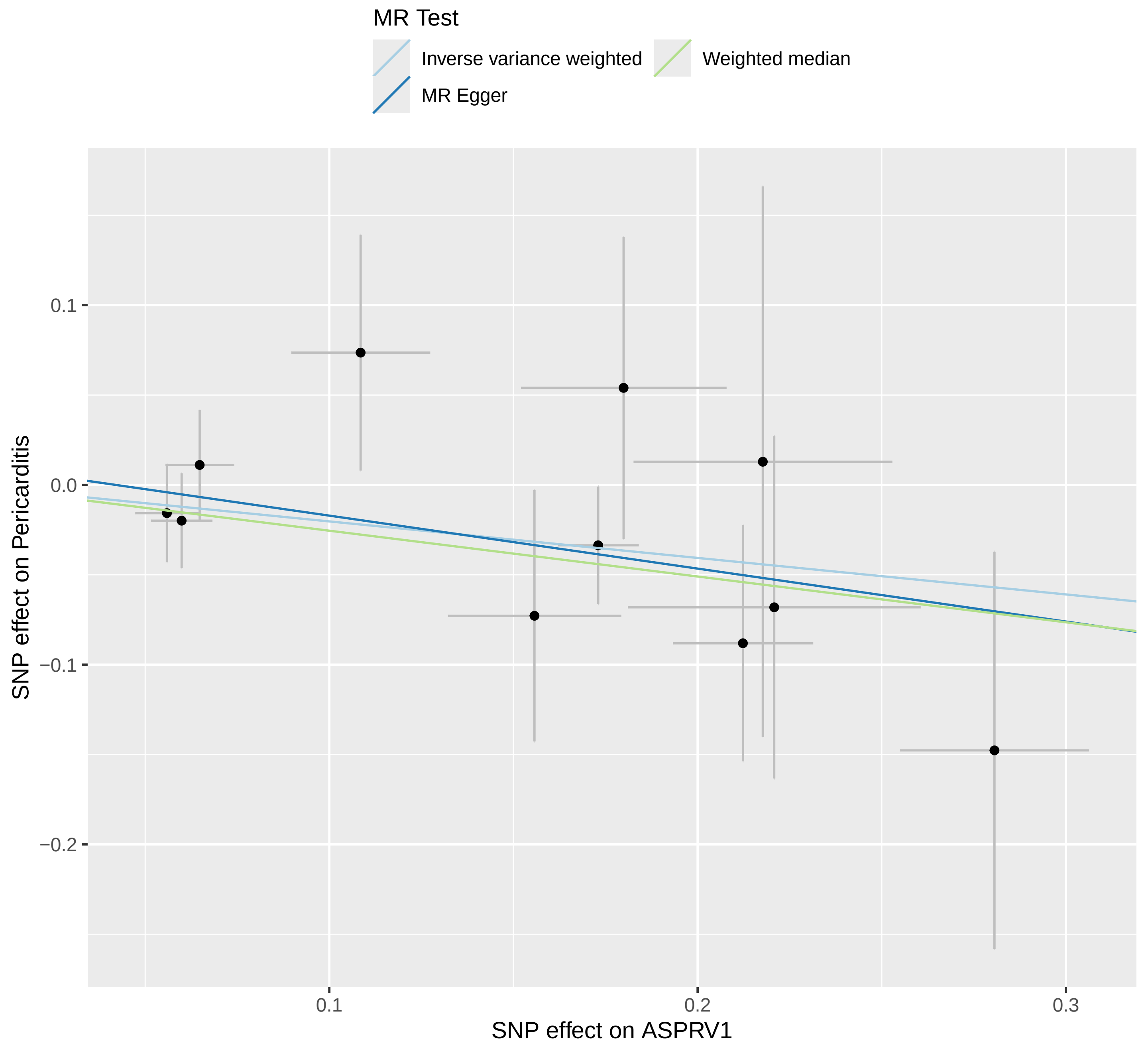
MR Test
Inverse variance weighted
Weighted median
MR Egger
SNP effect on Pericarditis
0.1
0.0
−0.1
−0.2
0.1
0.2
0.3
SNP effect on ASPRV1

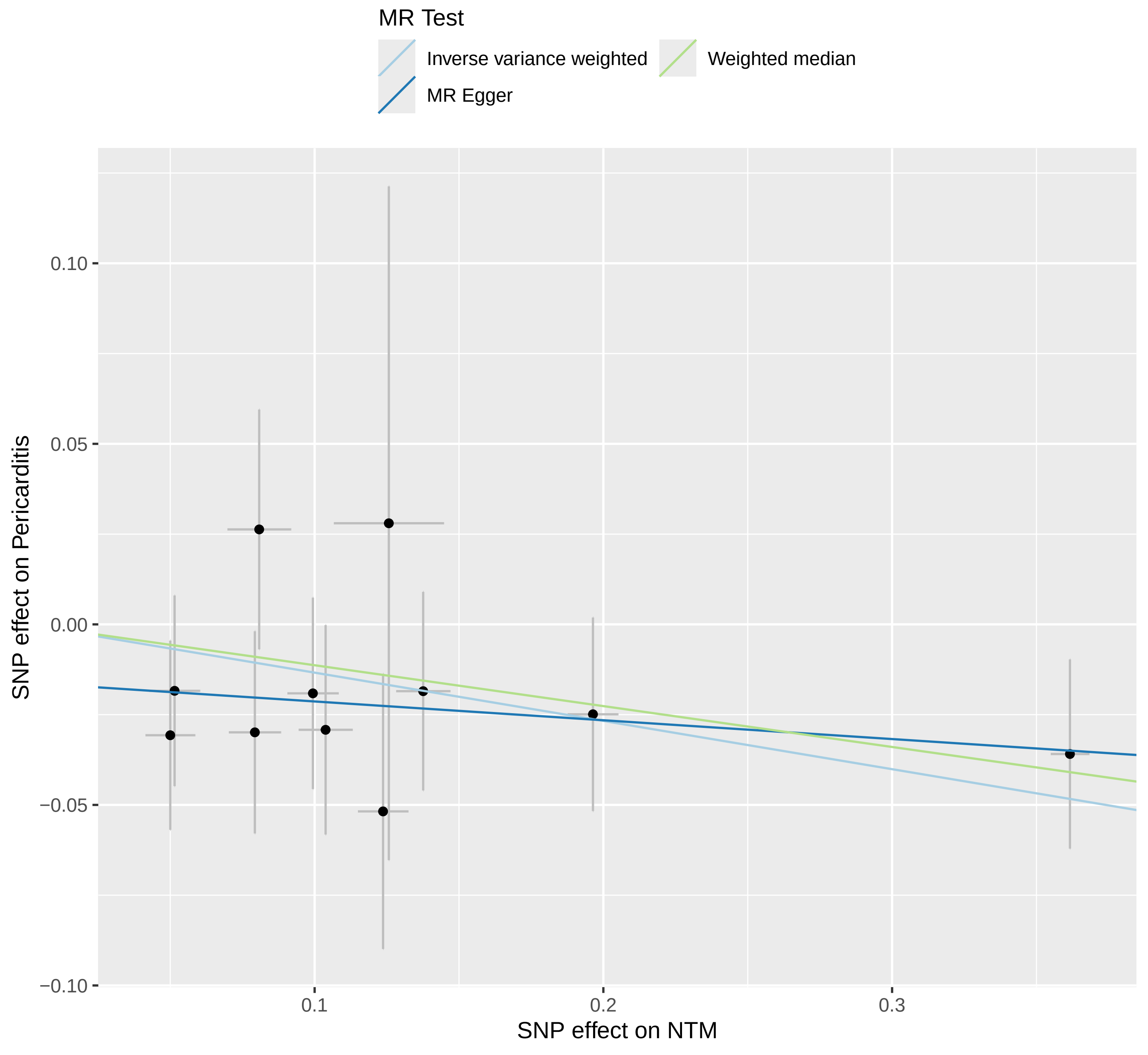

MR Test
Inverse variance weighted
Weighted median
MR Egger
SNP effect on Pericarditis
0.10
0.05
0.00
−0.05
−0.10
0.1
0.2
0.3
SNP effect on NTM

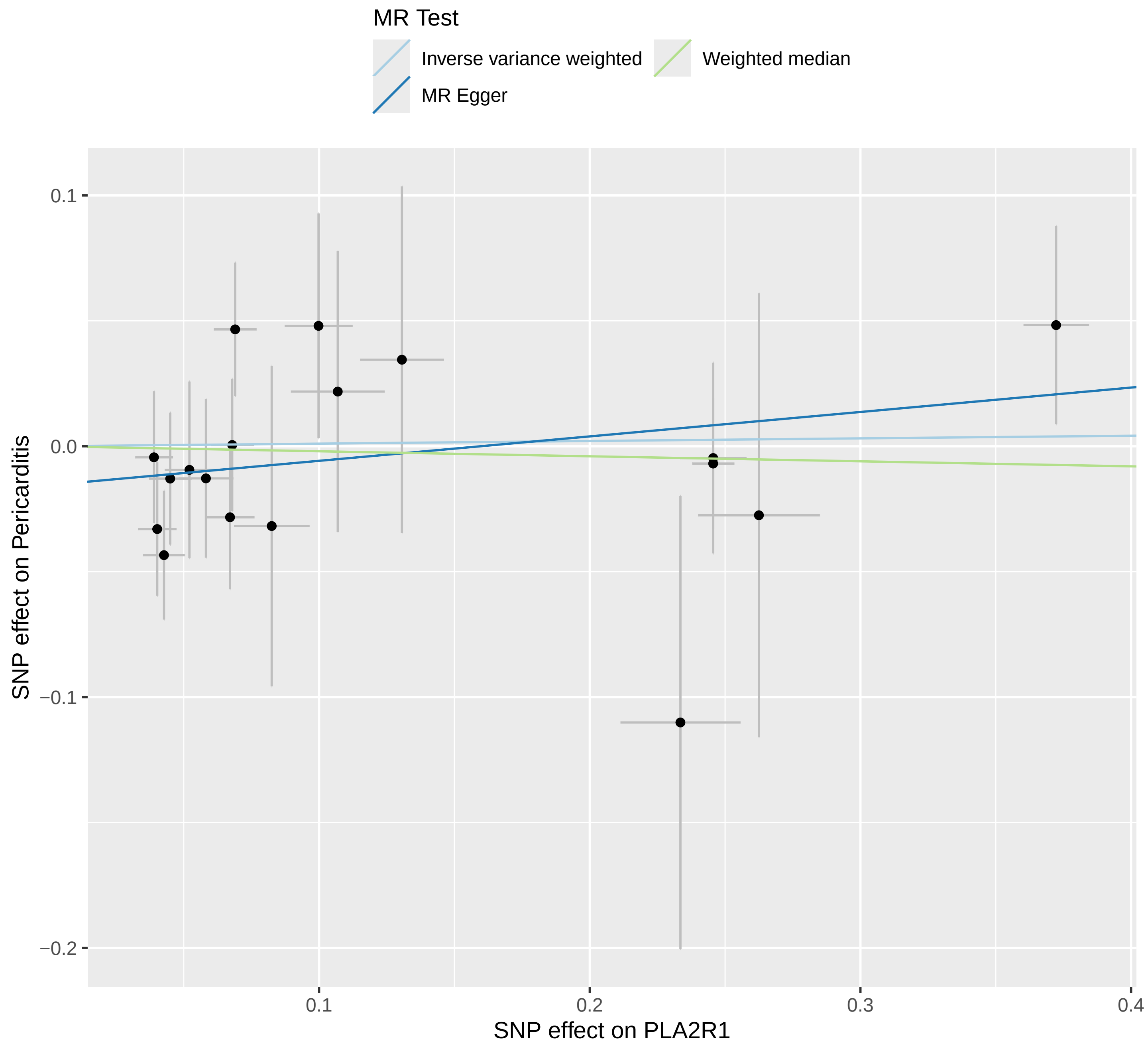

MR Test
Inverse variance weighted
Weighted median
MR Egger
SNP effect on Pericarditis
0.1
0.0
−0.1
−0.2
0.1
0.2
0.3
0.4
SNP effect on PLA2R1

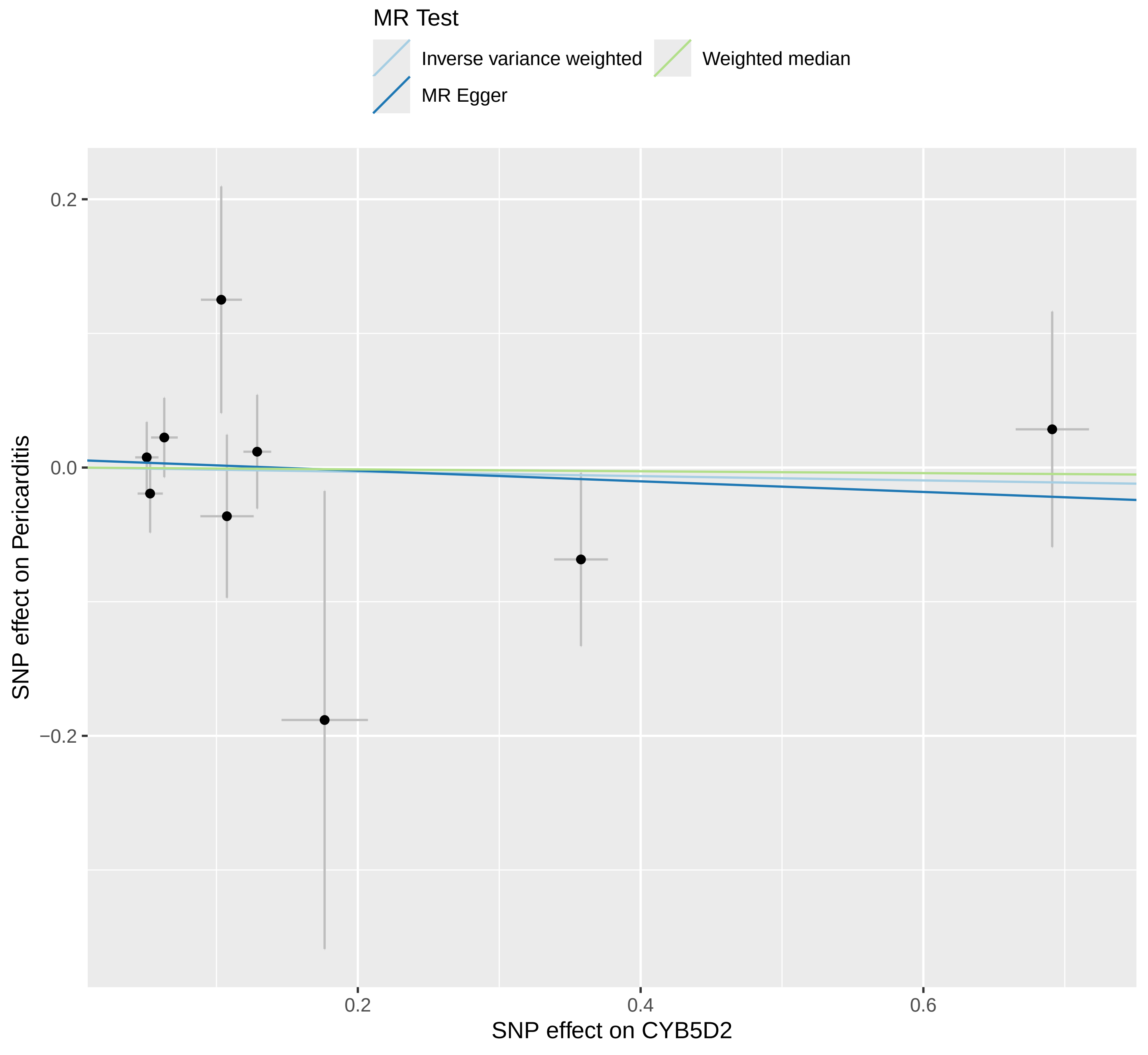
MR Test
Inverse variance weighted
Weighted median
MR Egger
0.2
0.0
−0.2
0.2
0.4
0.6
SNP effect on Pericarditis
SNP effect on CYB5D2

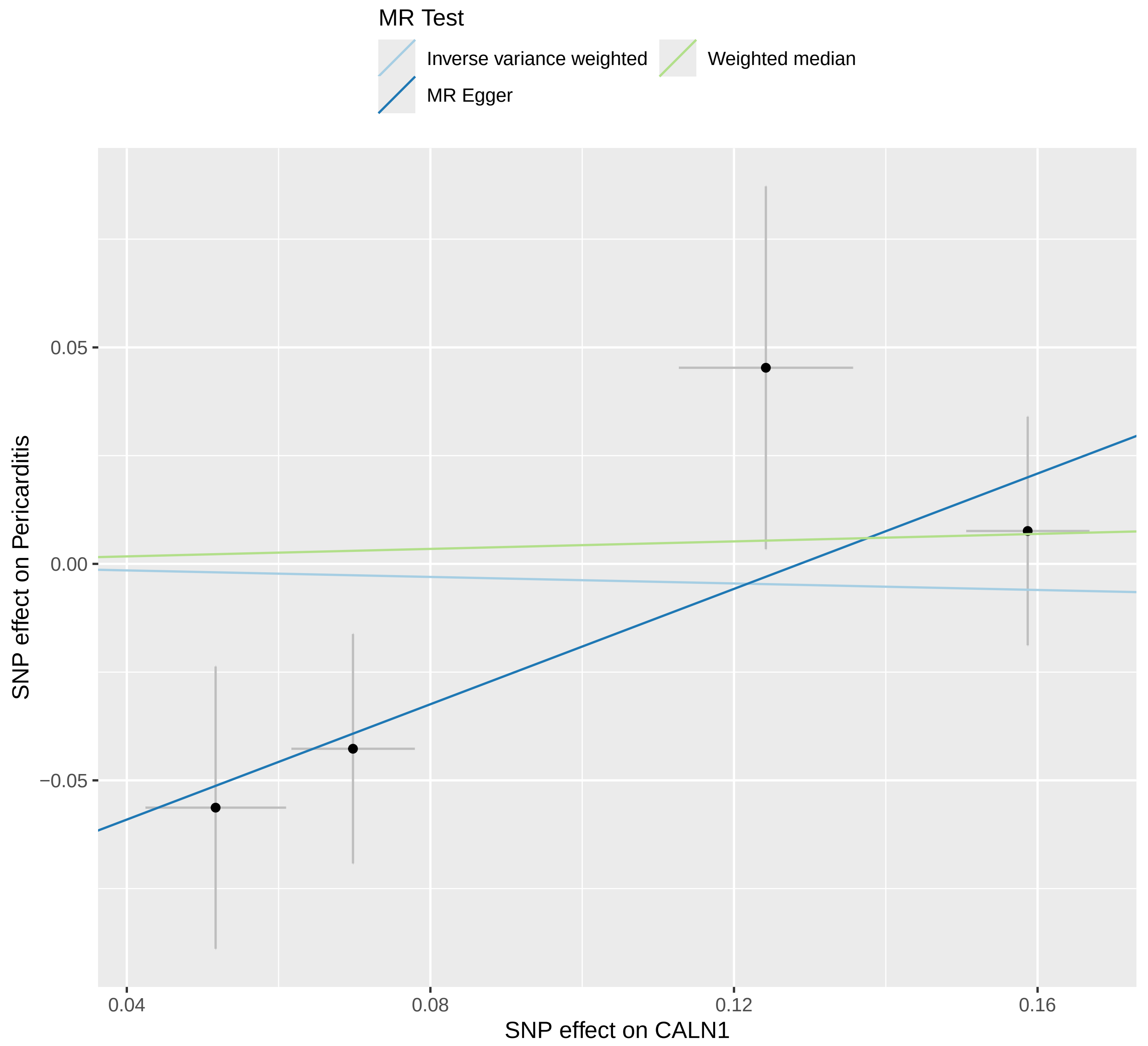
MR Test
Inverse variance weighted
MR Egger
Weighted median
0.05
0.00
−0.05
SNP effect on Pericarditis
0.04
0.08
0.12
0.16
SNP effect on CALN1

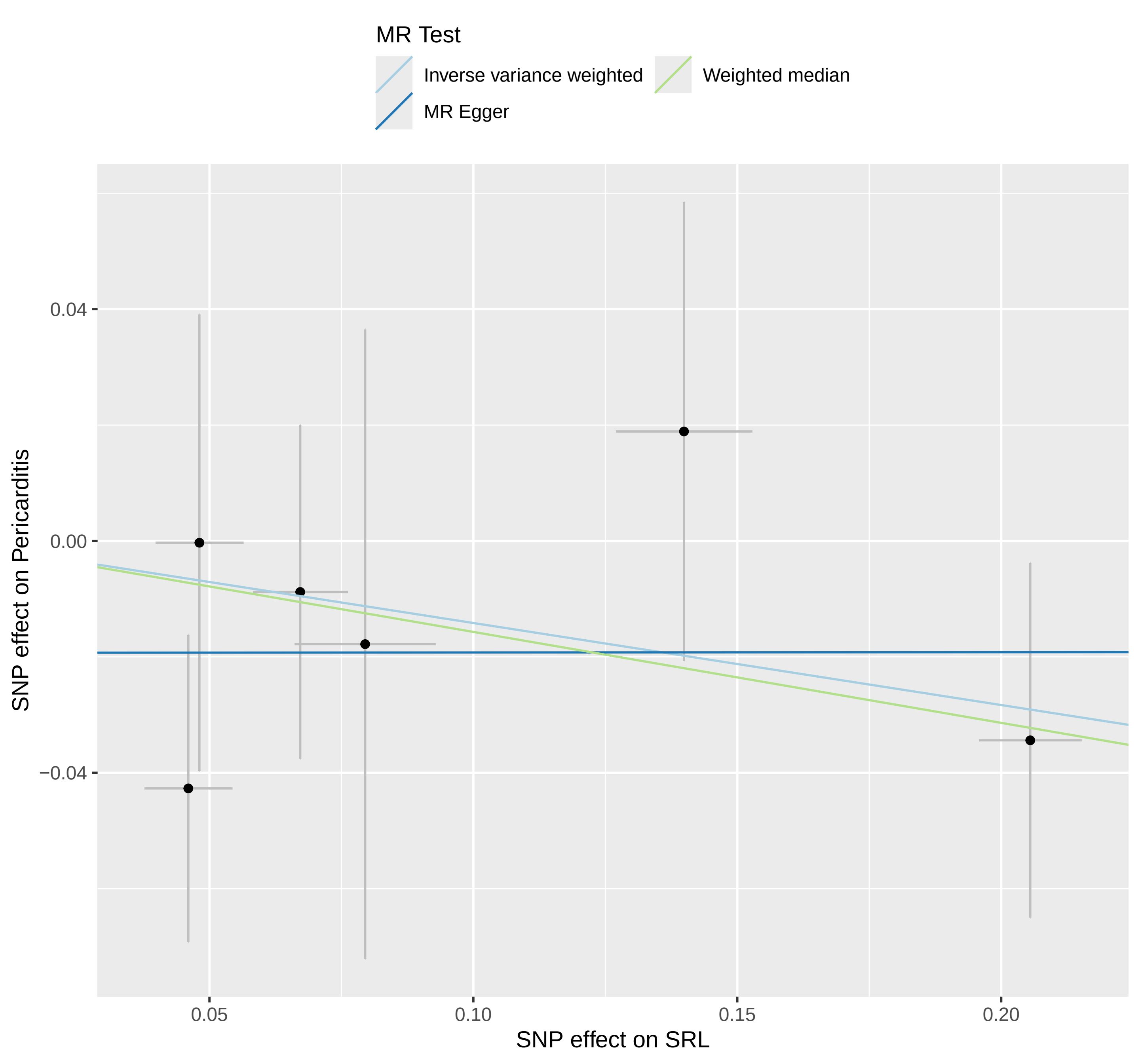
MR Test
Inverse variance weighted
Weighted median
MR Egger
0.04
0.00
−0.04
SNP effect on Pericarditis
0.05
0.10
0.15
0.20
SNP effect on SRL

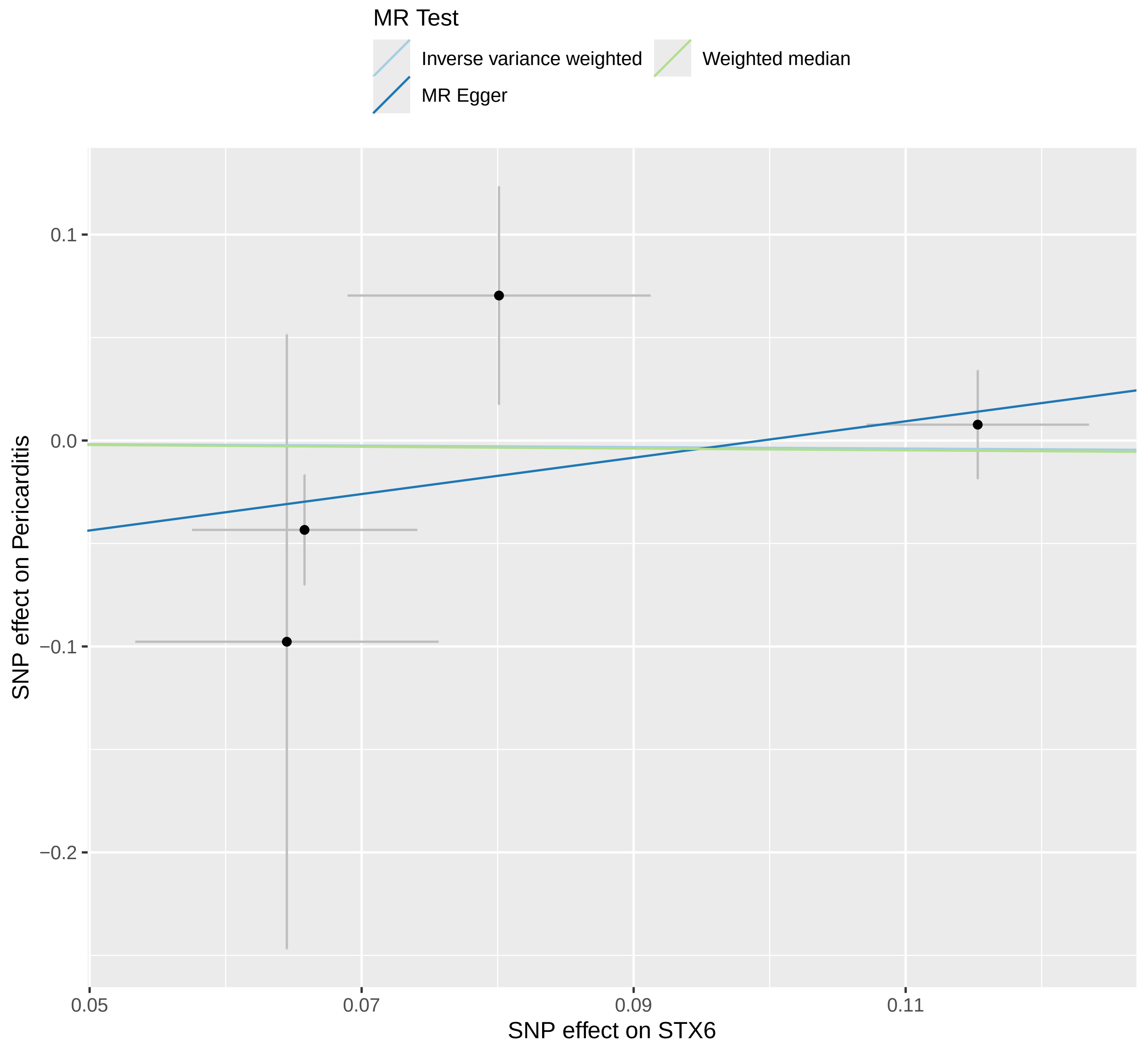

MR Test
Inverse variance weighted
Weighted median
MR Egger
0.1
0.0
−0.1
−0.2
SNP effect on Pericarditis
0.05
0.07
0.09
0.11
SNP effect on STX6

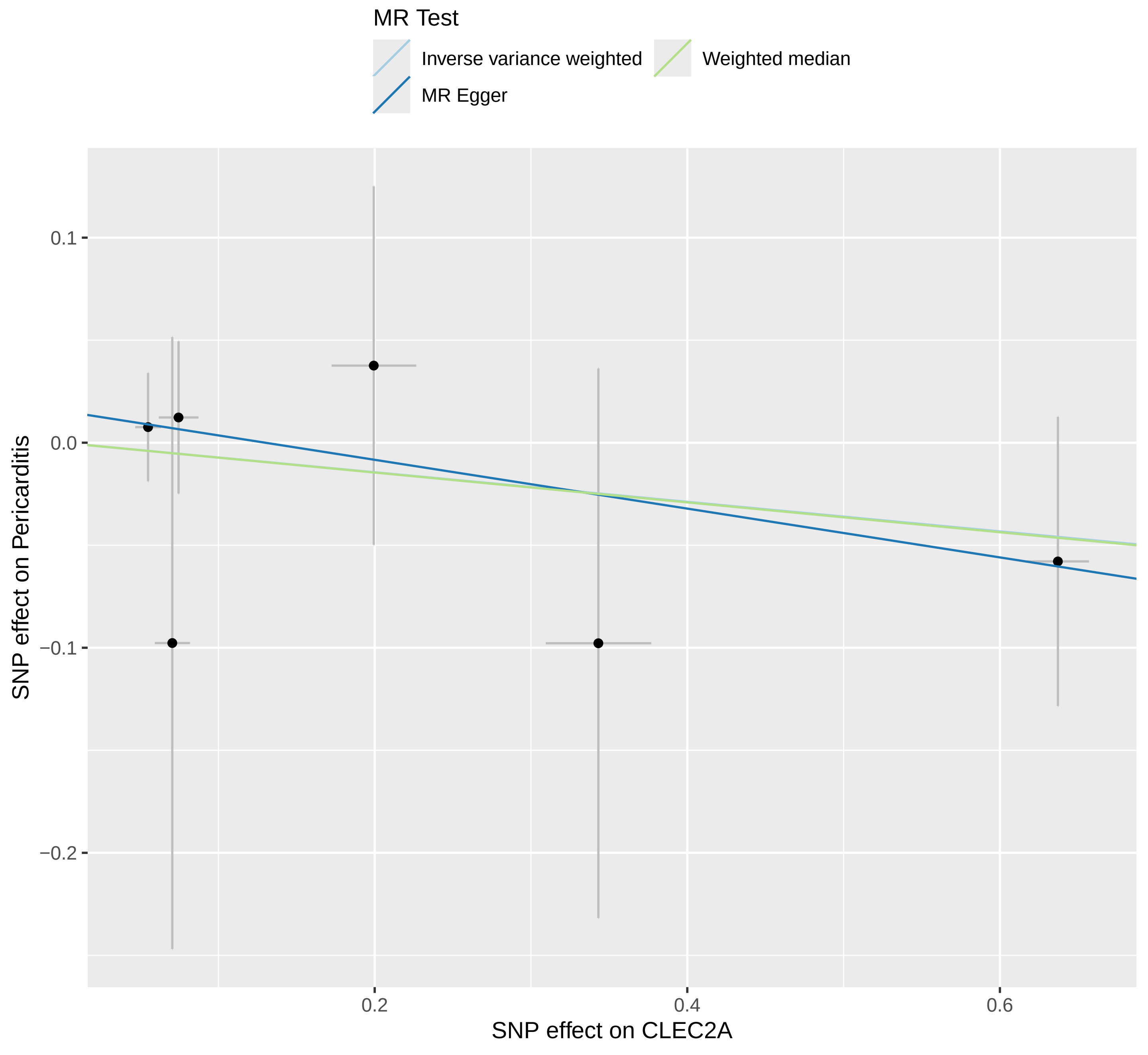

MR Test
Inverse variance weighted
Weighted median
MR Egger
0.1
0.0
−0.1
−0.2
0.2
0.4
0.6
SNP effect on Pericarditis
SNP effect on CLEC2A

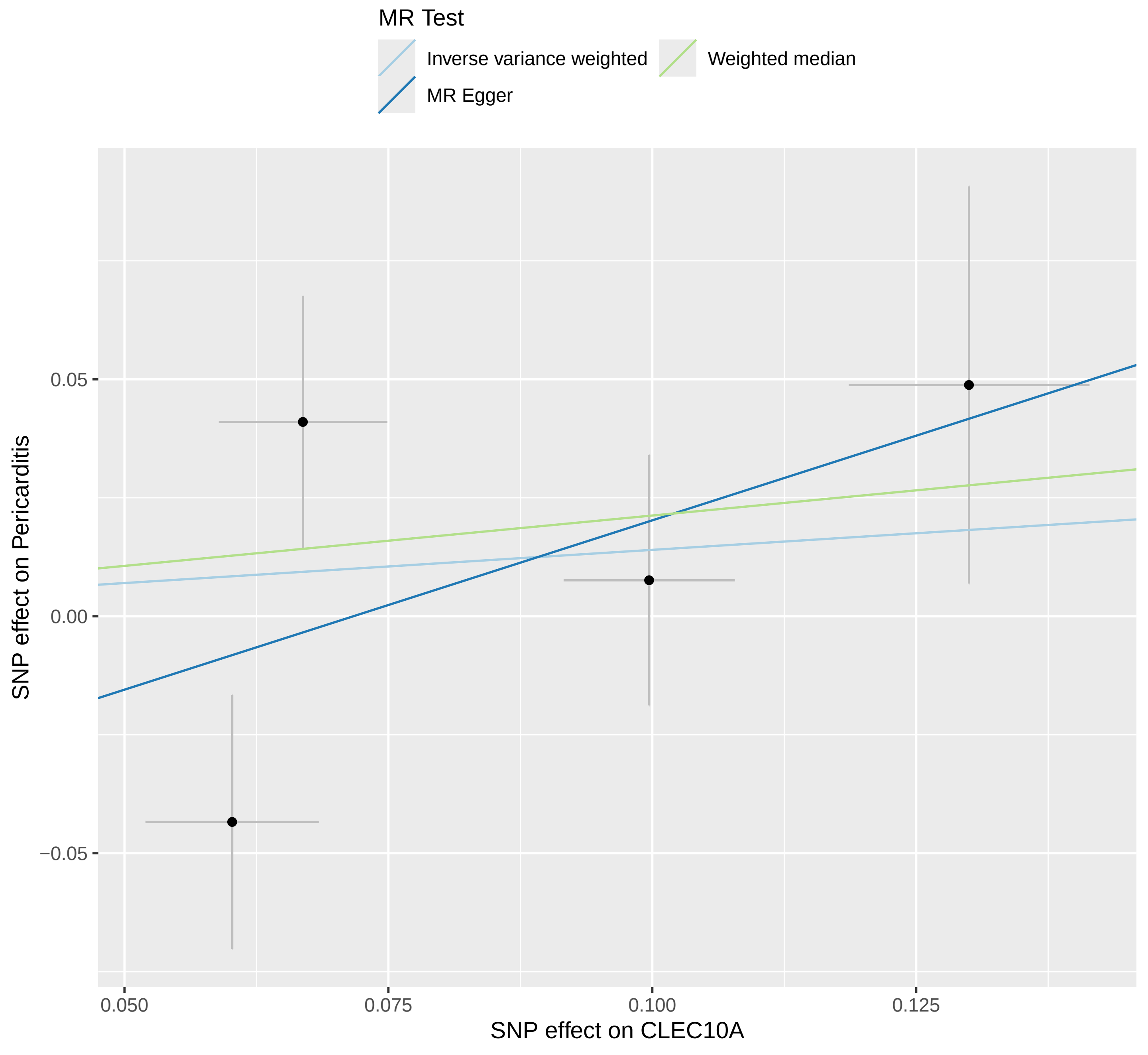
MR Test
Inverse variance weighted
MR Egger
Weighted median
SNP effect on Pericarditis
0.05
0.00
−0.05
0.050
0.075
0.100
0.125
SNP effect on CLEC10A

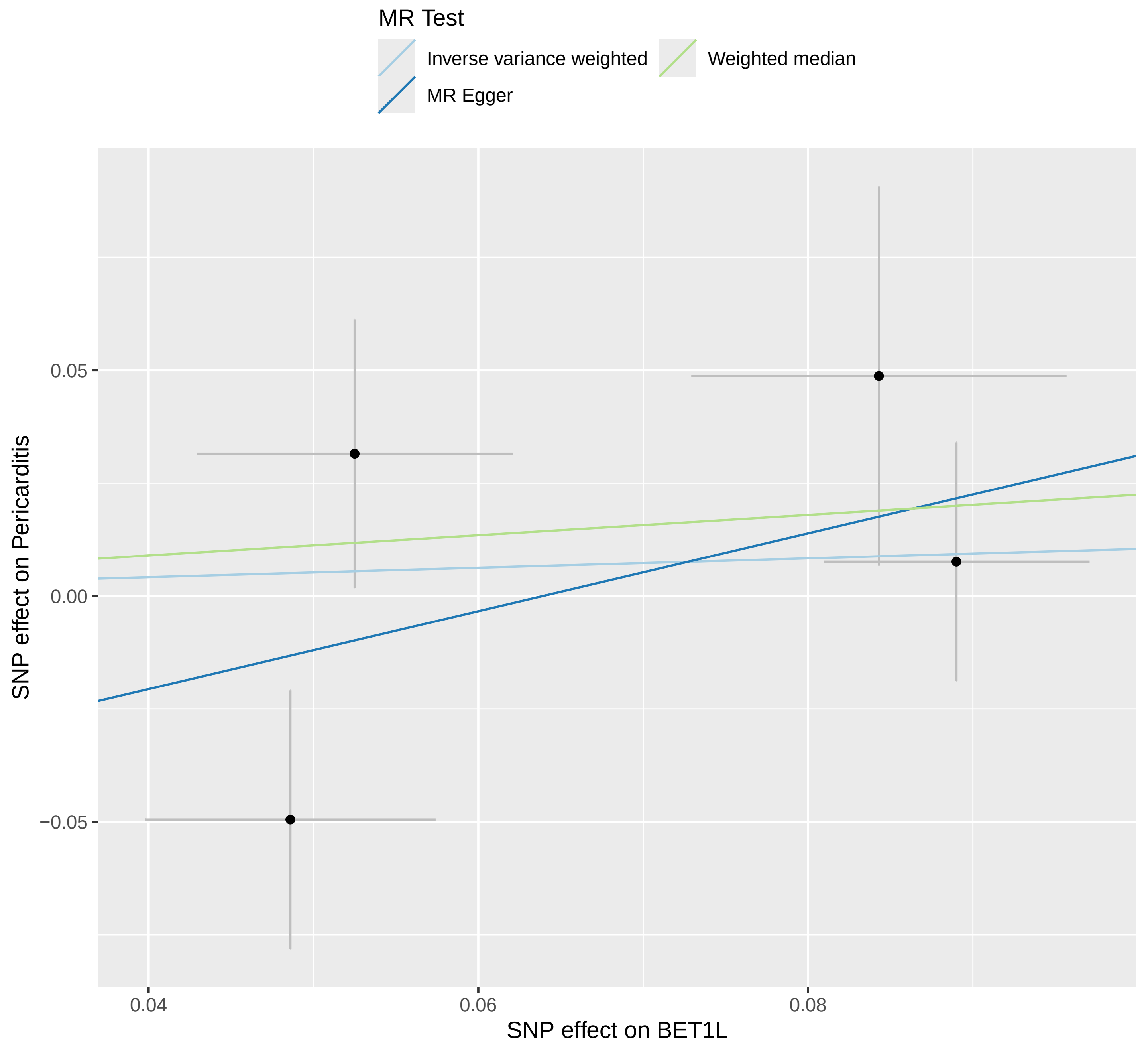

MR Test
Inverse variance weighted
MR Egger
Weighted median
0.05
0.00
−0.05
0.04
0.06
0.08
SNP effect on Pericarditis
SNP effect on BET1L

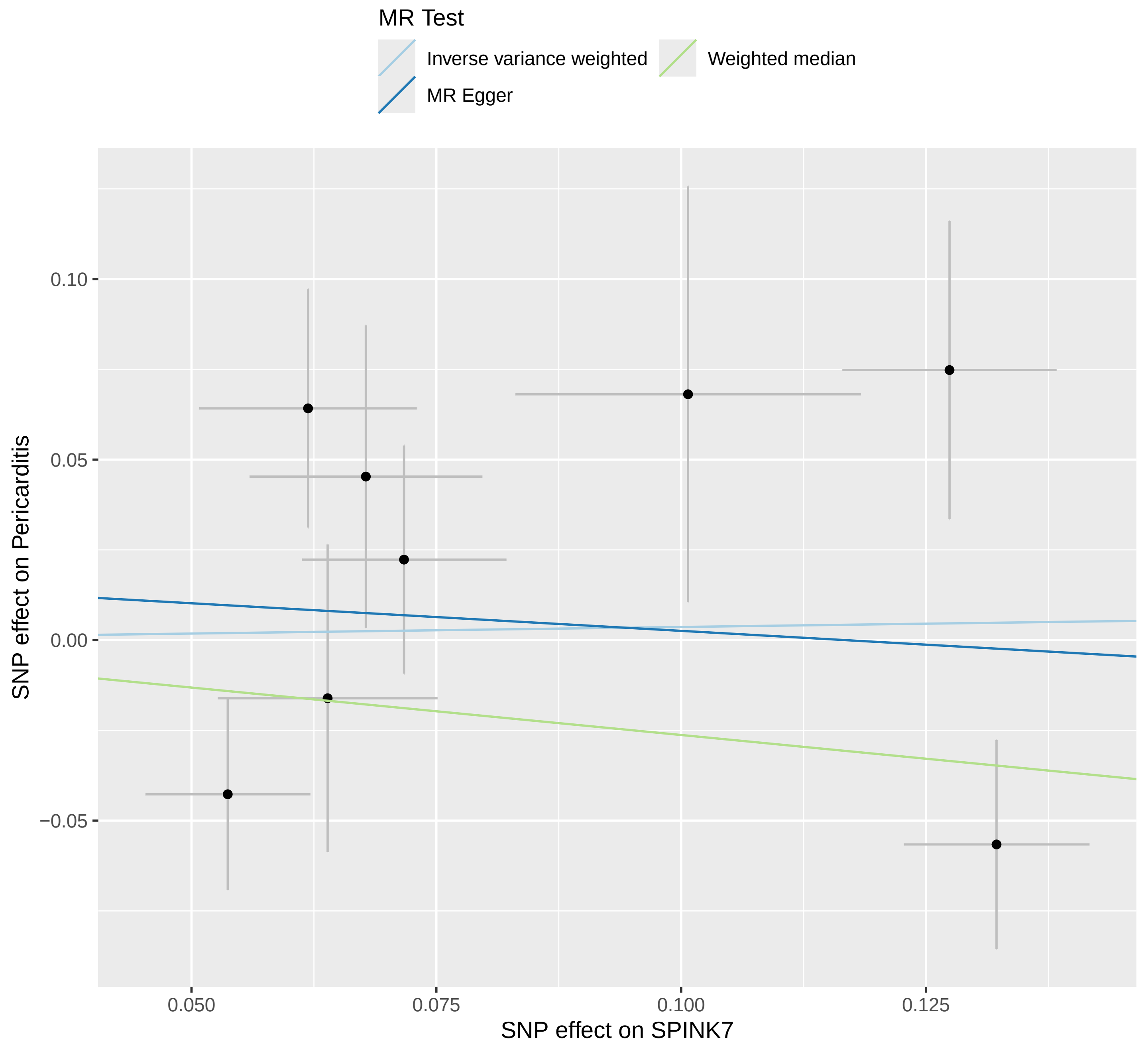
MR Test
Inverse variance weighted
Weighted median
MR Egger
0.10
0.05
0.00
−0.05
SNP effect on Pericarditis
0.050
0.075
0.100
0.125
SNP effect on SPINK7

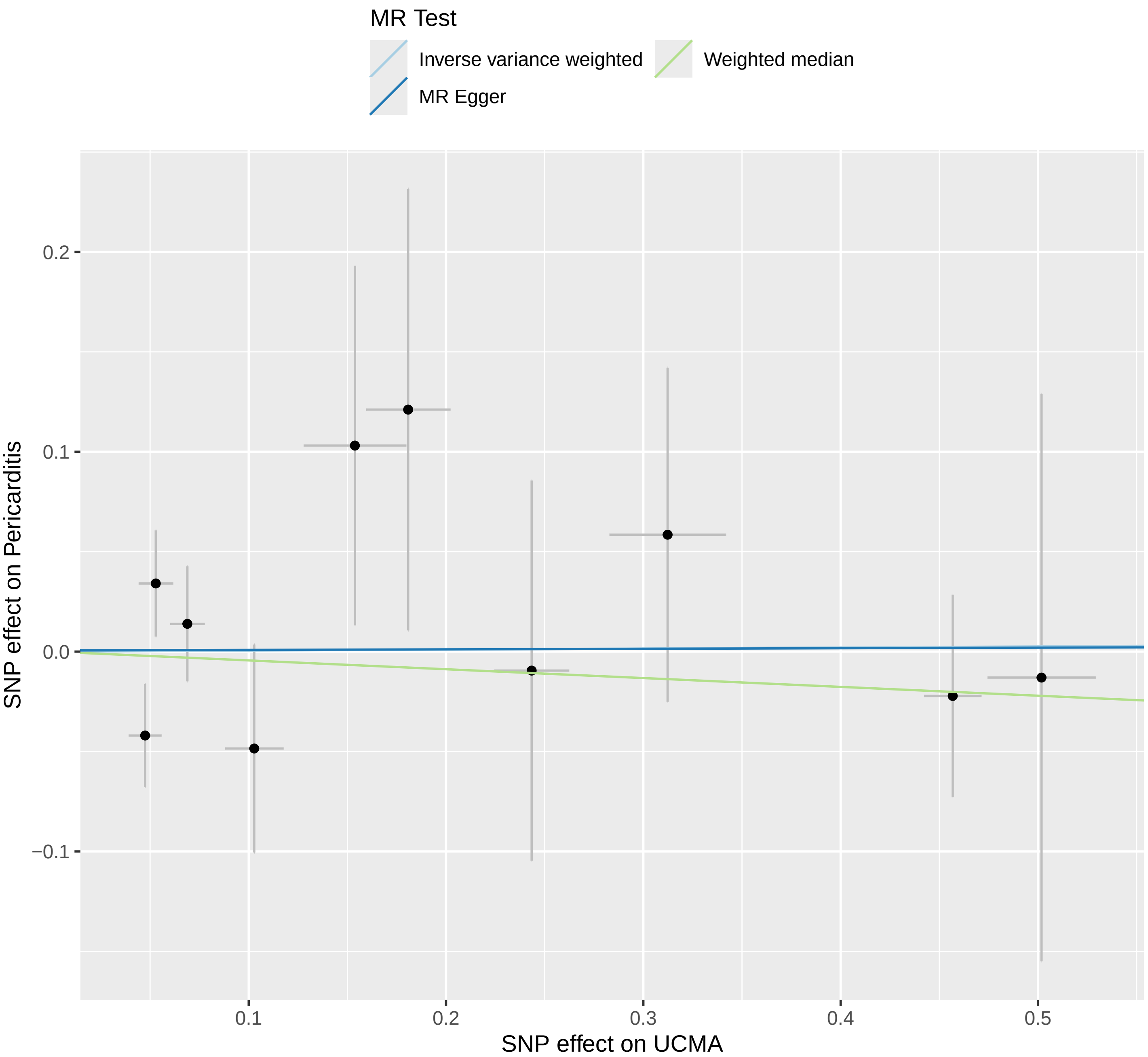

MR Test
Inverse variance weighted
MR Egger
Weighted median
0.2
0.1
0.0
−0.1
0.1
0.2
0.3
0.4
0.5
SNP effect on Pericarditis
SNP effect on UCMA

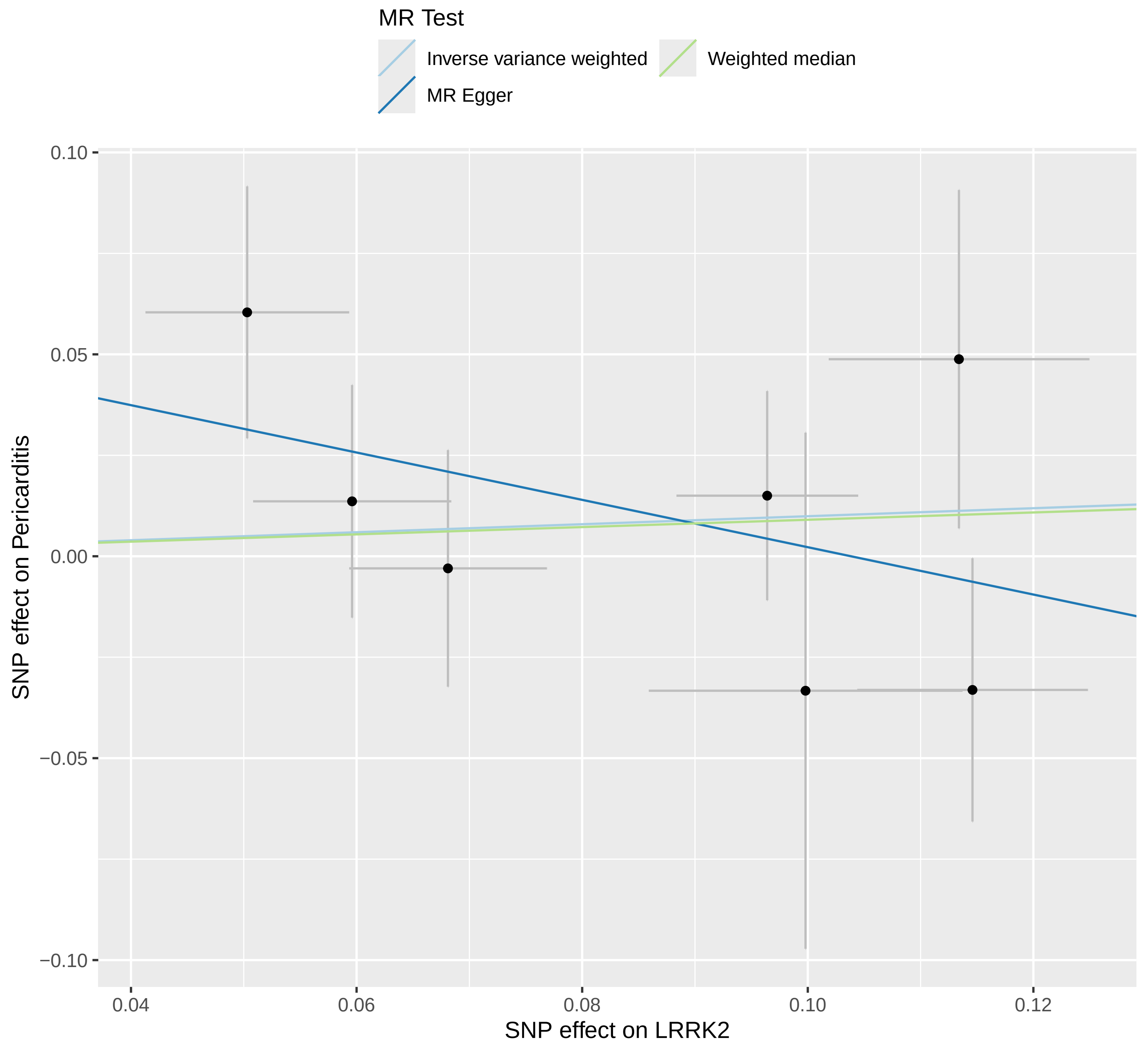
MR Test
Inverse variance weighted
Weighted median
MR Egger
0.10
0.05
0.00
−0.05
−0.10
SNP effect on Pericarditis
0.04
0.06
0.08
0.10
0.12
SNP effect on LRRK2

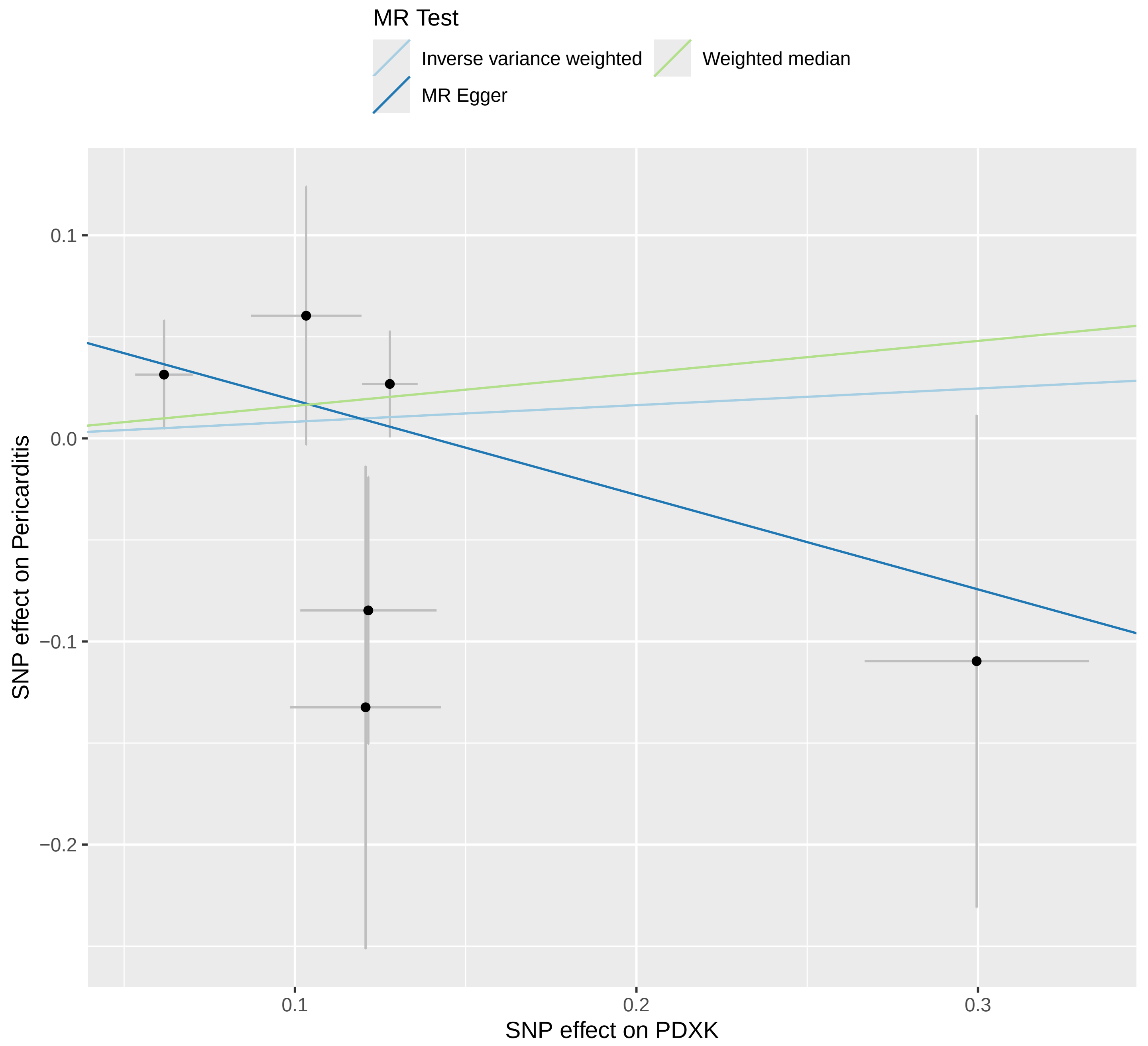

MR Test
Inverse variance weighted
Weighted median
MR Egger
0.1
0.0
−0.1
−0.2
SNP effect on Pericarditis
0.1
0.2
0.3
SNP effect on PDXK

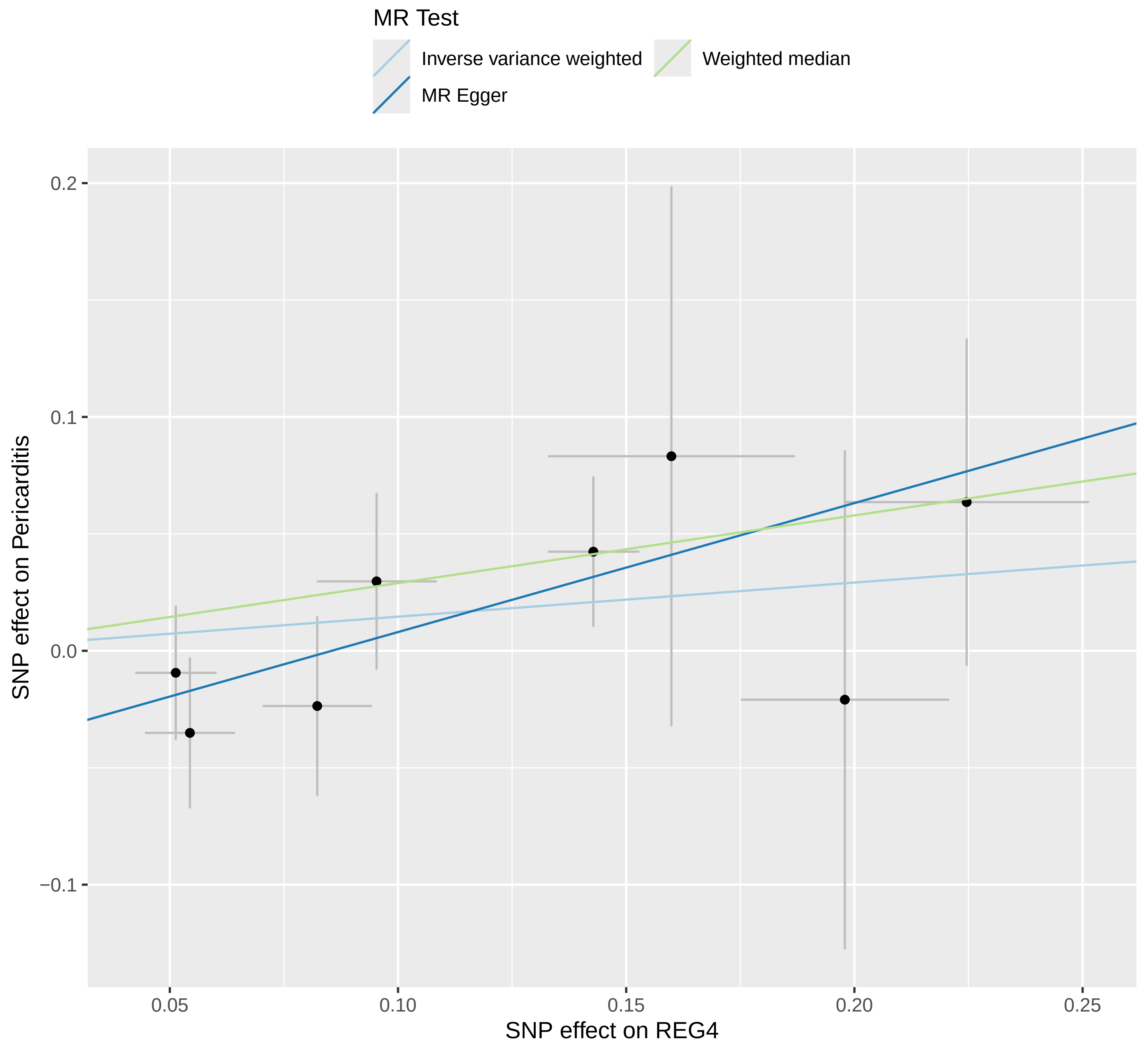

MR Test
Inverse variance weighted
Weighted median
MR Egger
0.2
0.1
0.0
−0.1
SNP effect on Pericarditis
0.05
0.10
0.15
0.20
0.25
SNP effect on REG4

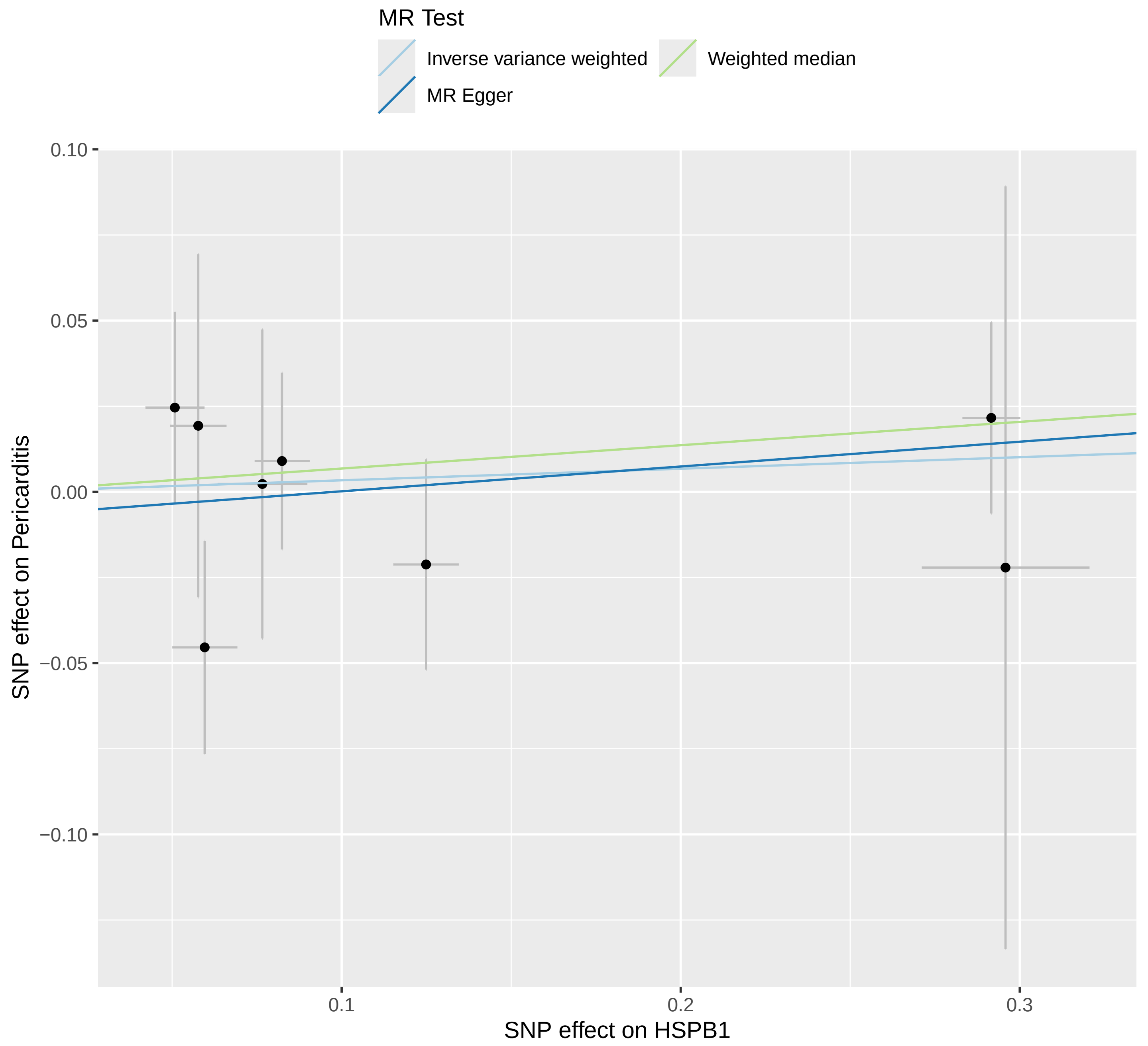
MR Test
Inverse variance weighted
Weighted median
MR Egger
0.10
0.05
0.00
−0.05
−0.10
SNP effect on Pericarditis
0.1
0.2
0.3
SNP effect on HSPB1

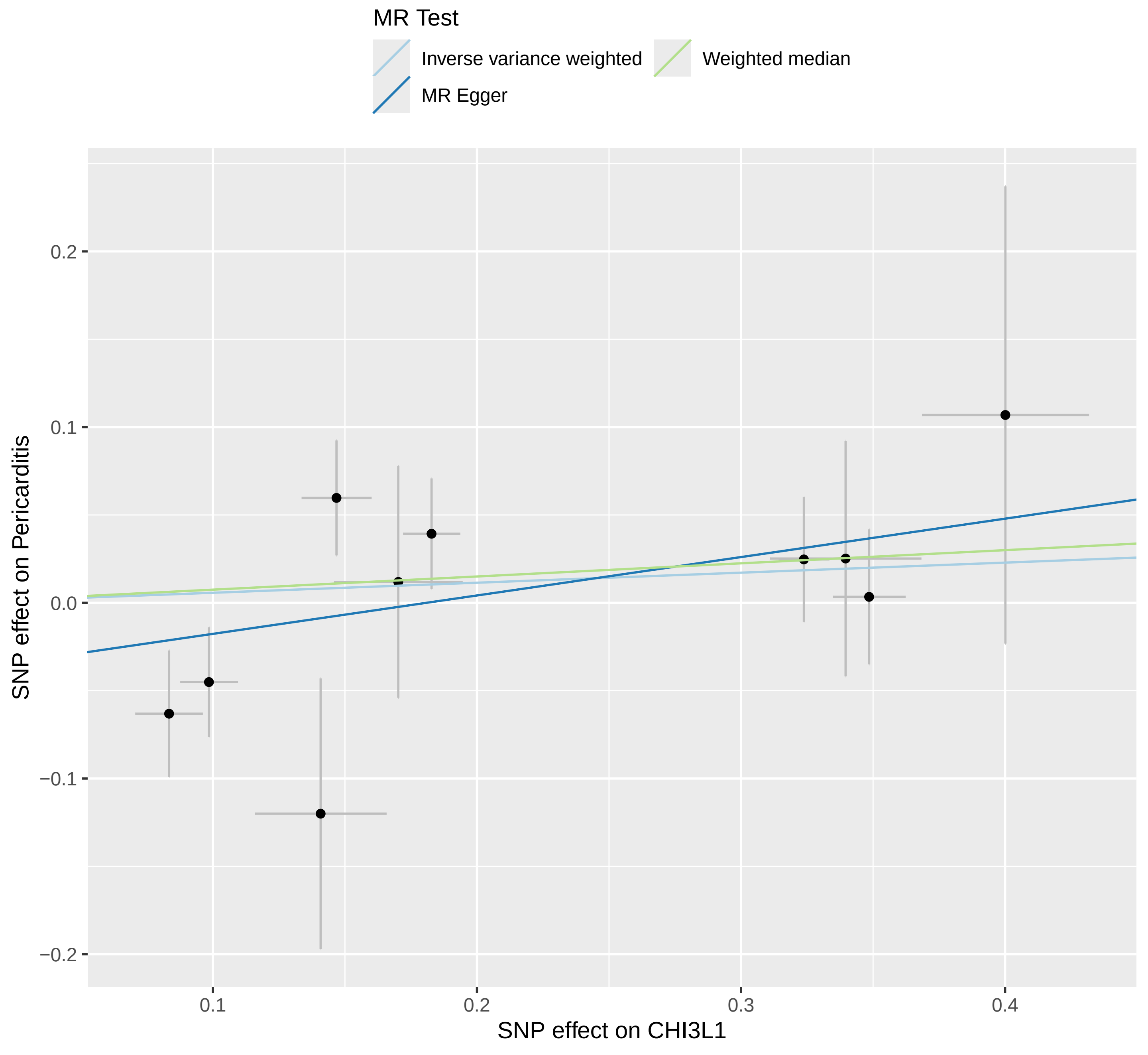
MR Test
Inverse variance weighted
Weighted median
MR Egger
0.2
0.1
0.0
−0.1
−0.2
SNP effect on Pericarditis
0.1
0.2
0.3
0.4
SNP effect on CHI3L1

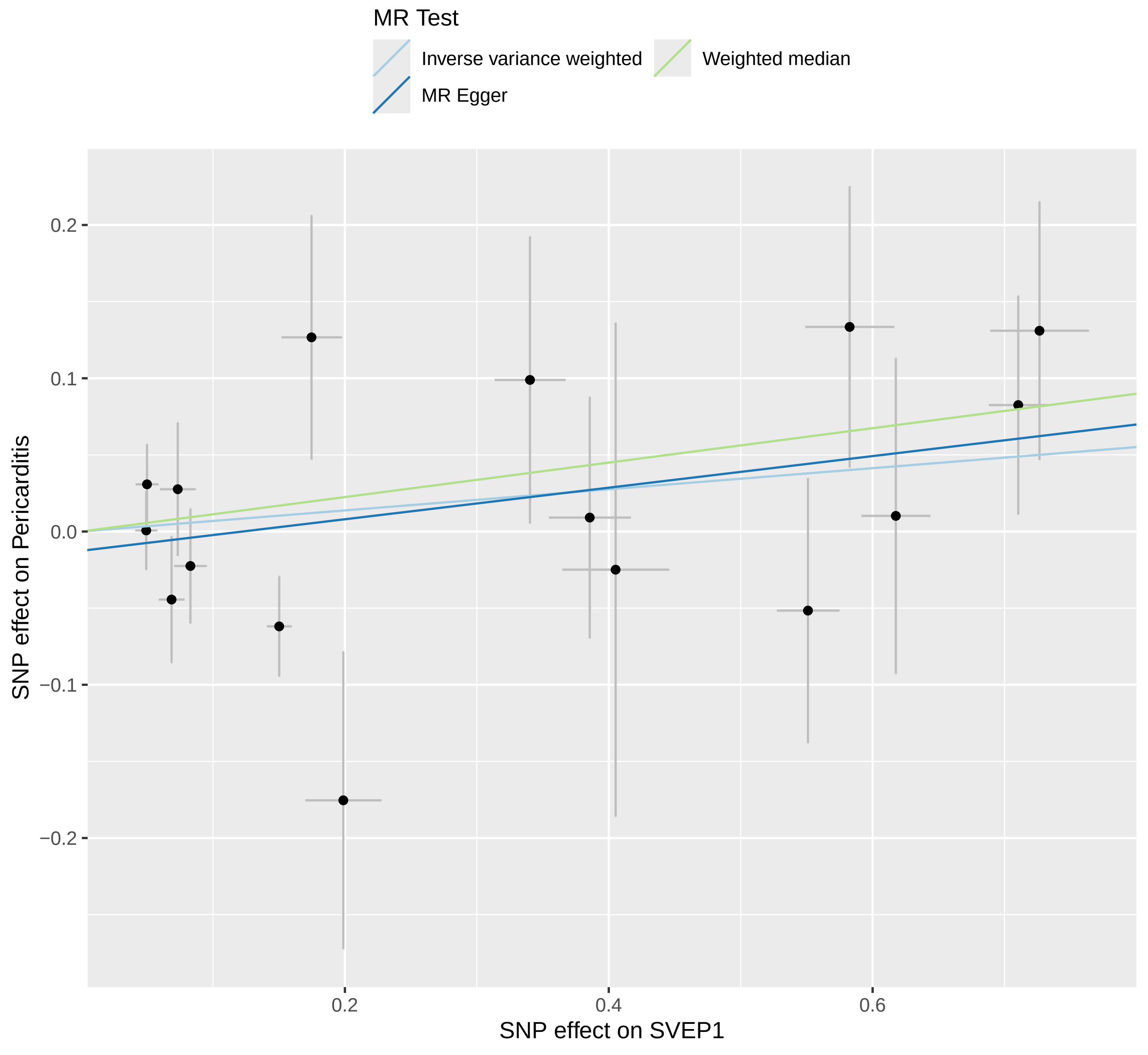
MR Test
Inverse variance weighted
Weighted median
MR Egger
0.2
0.1
0.0
−0.1
−0.2
SNP effect on Pericarditis
0.2
0.4
0.6
SNP effect on SVEP1

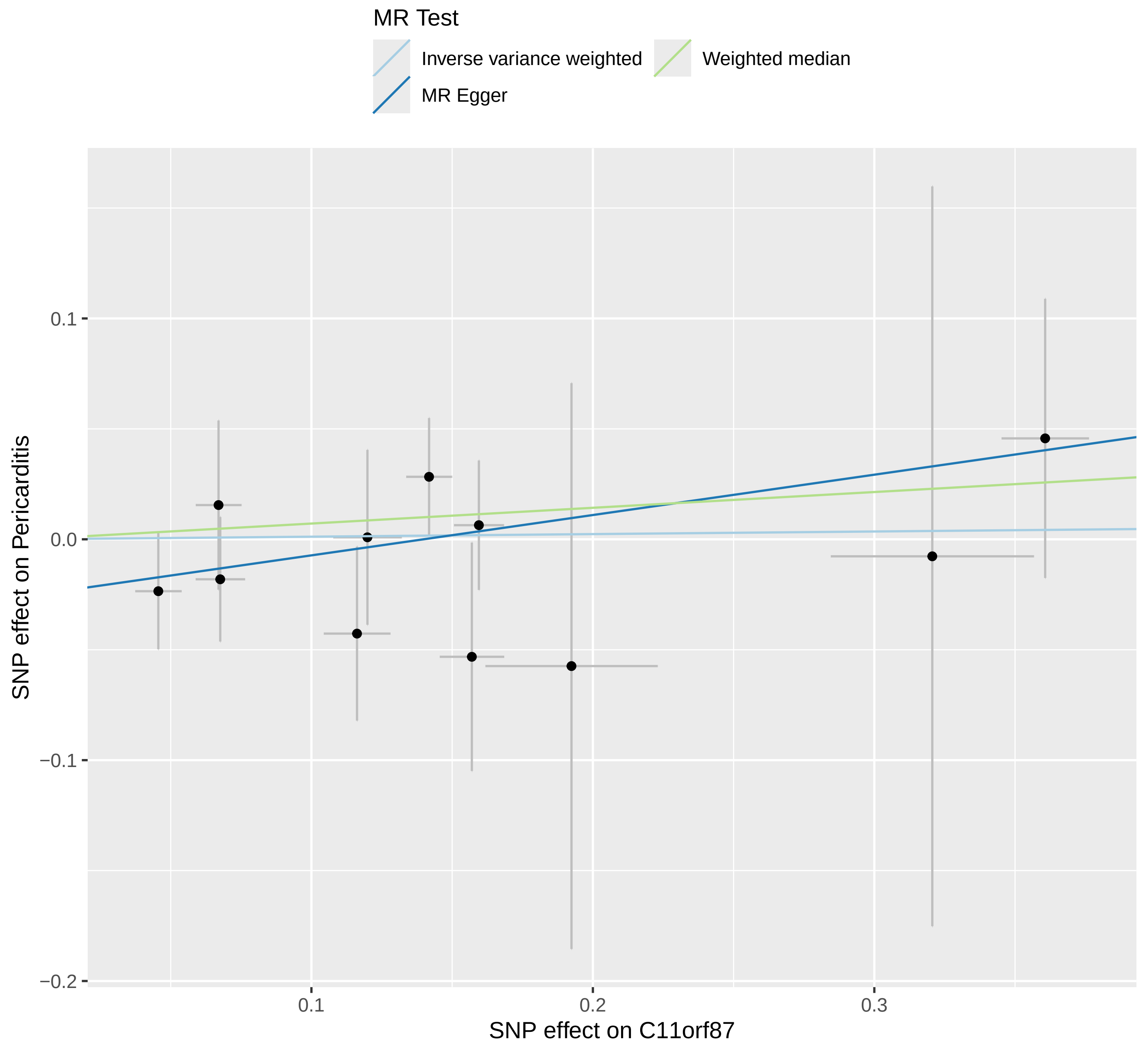
MR Test
Inverse variance weighted
Weighted median
MR Egger
0.1
0.0
−0.1
−0.2
0.1
0.2
0.3
SNP effect on Pericarditis
SNP effect on C11orf87

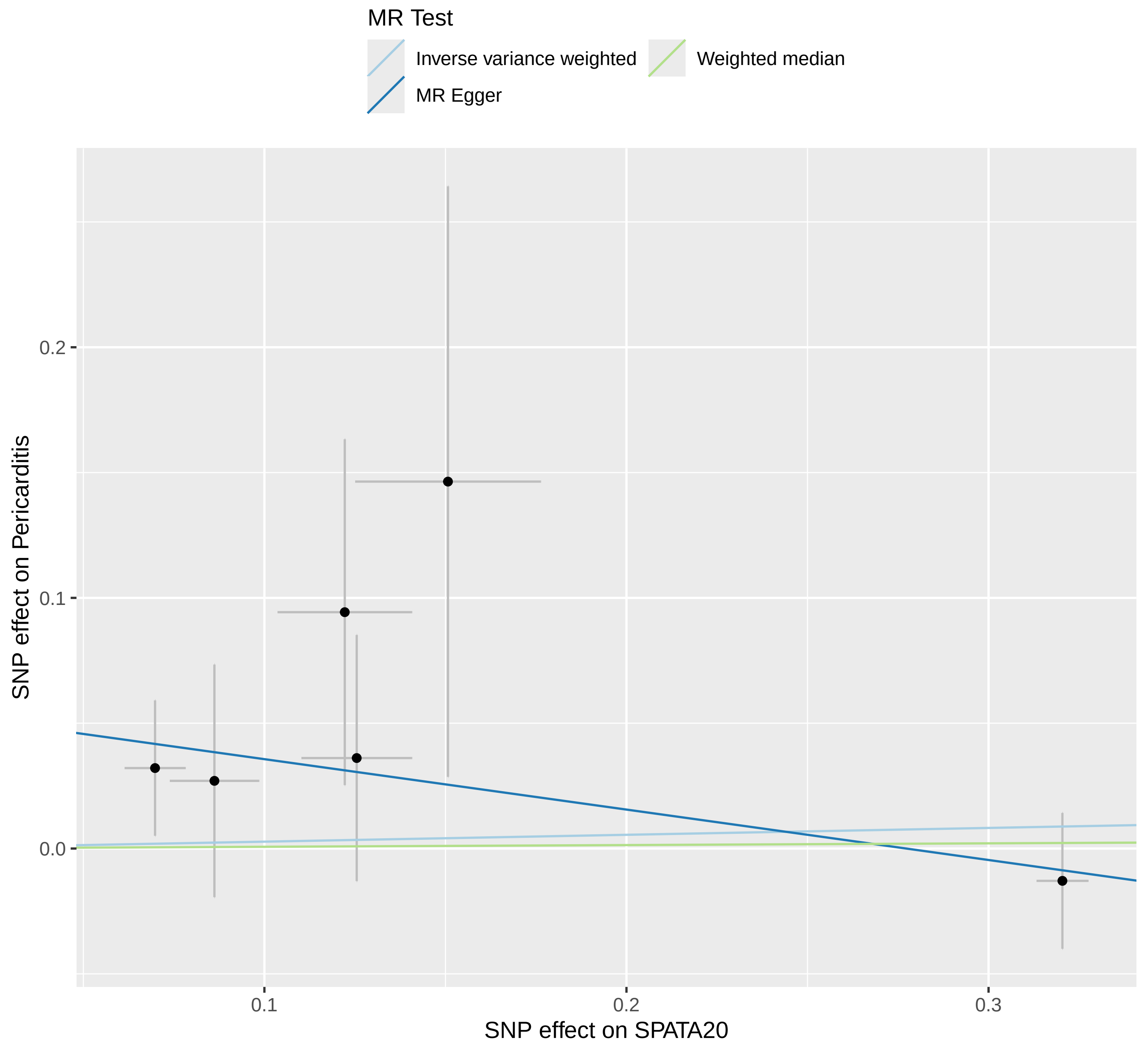

MR Test
Inverse variance weighted
MR Egger
Weighted median
0.2
0.1
0.0
SNP effect on Pericarditis
0.1
0.2
0.3
SNP effect on SPATA20

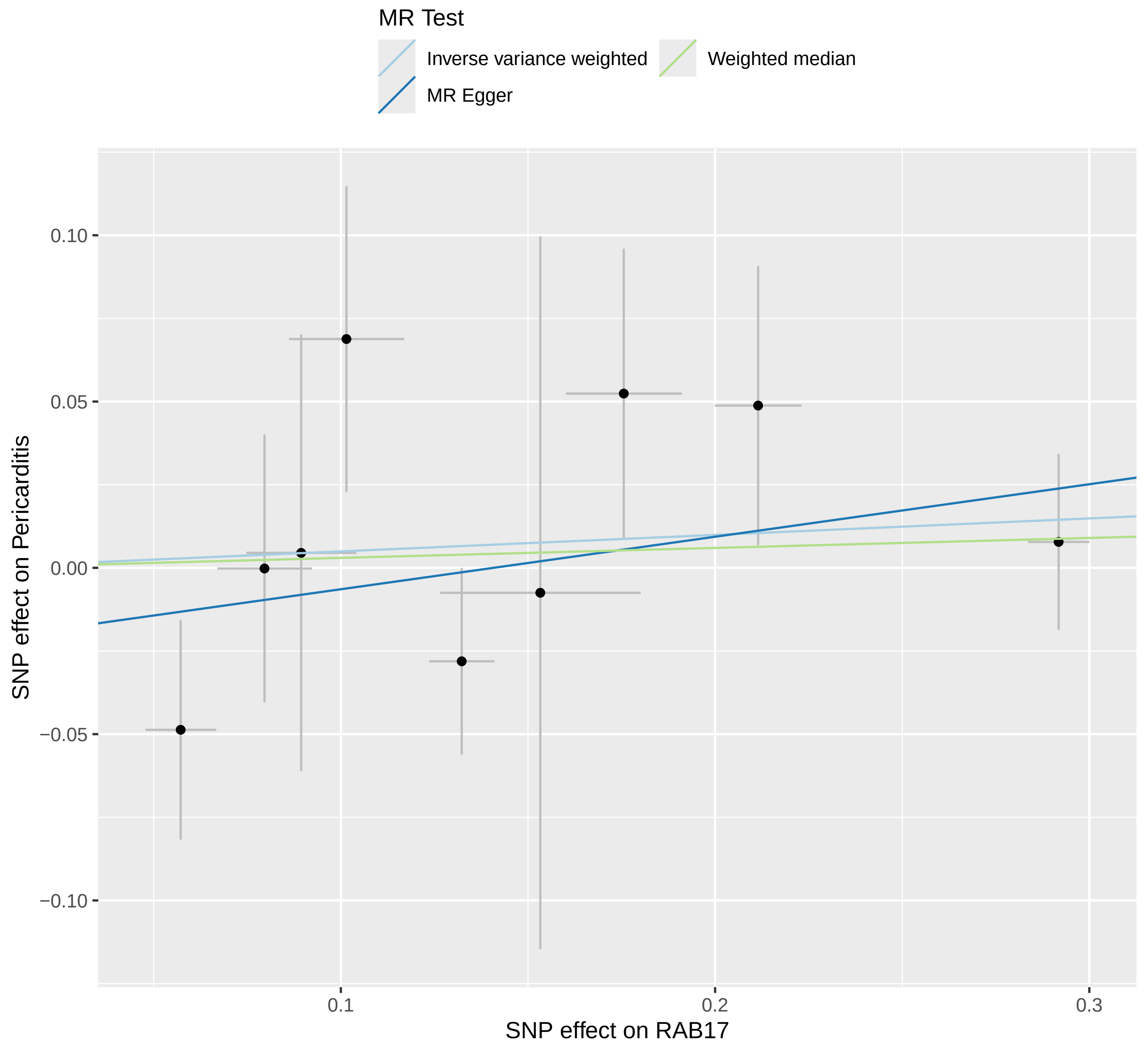

MR Test
Inverse variance weighted
MR Egger
Weighted median
0.10
0.05
0.00
−0.05
−0.10
0.1
0.2
0.3
SNP effect on Pericarditis
SNP effect on RAB17

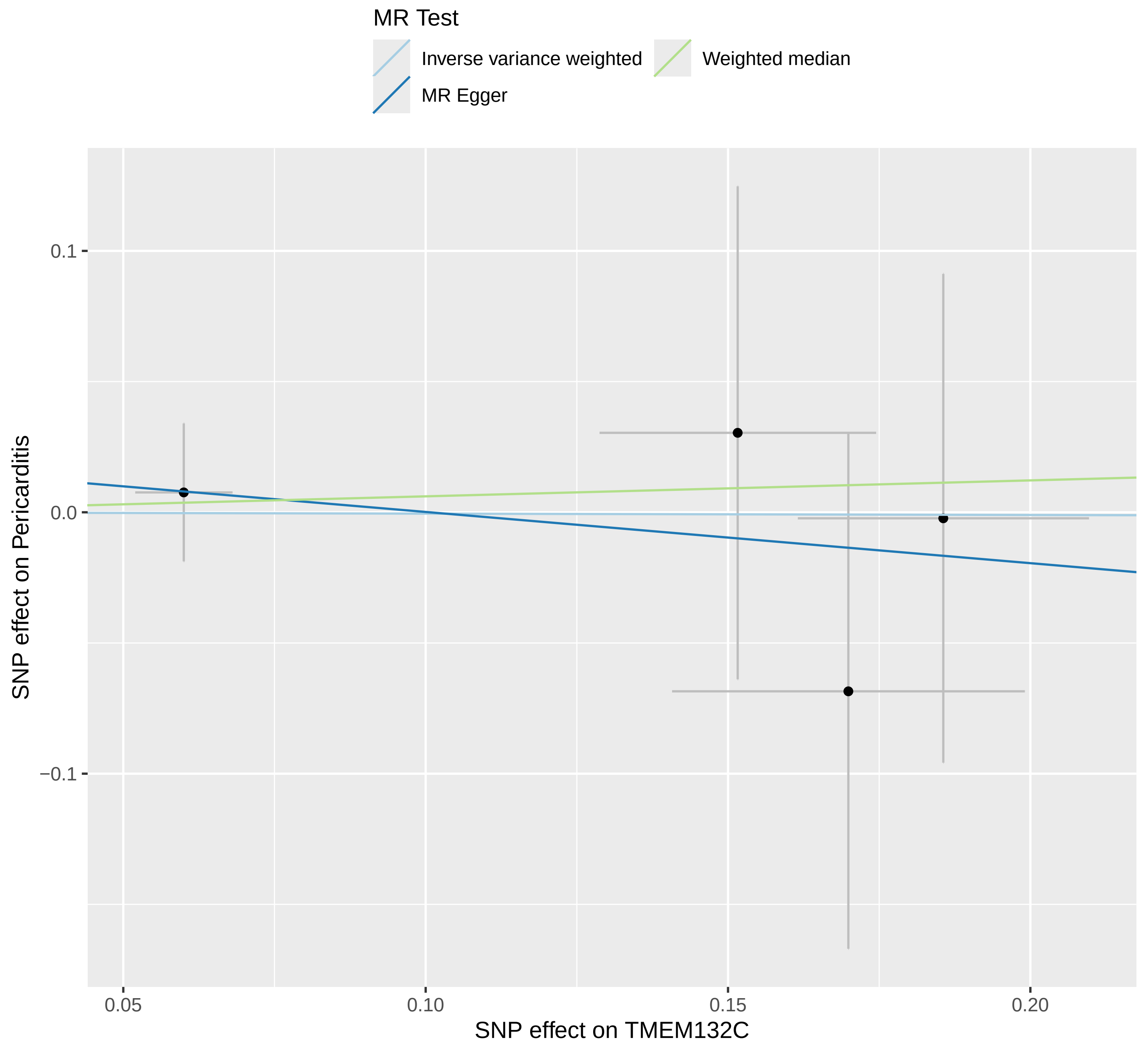
MR Test
Inverse variance weighted
Weighted median
MR Egger
0.1
0.0
−0.1
0.05
0.10
0.15
0.20
SNP effect on Pericarditis
SNP effect on TMEM132C

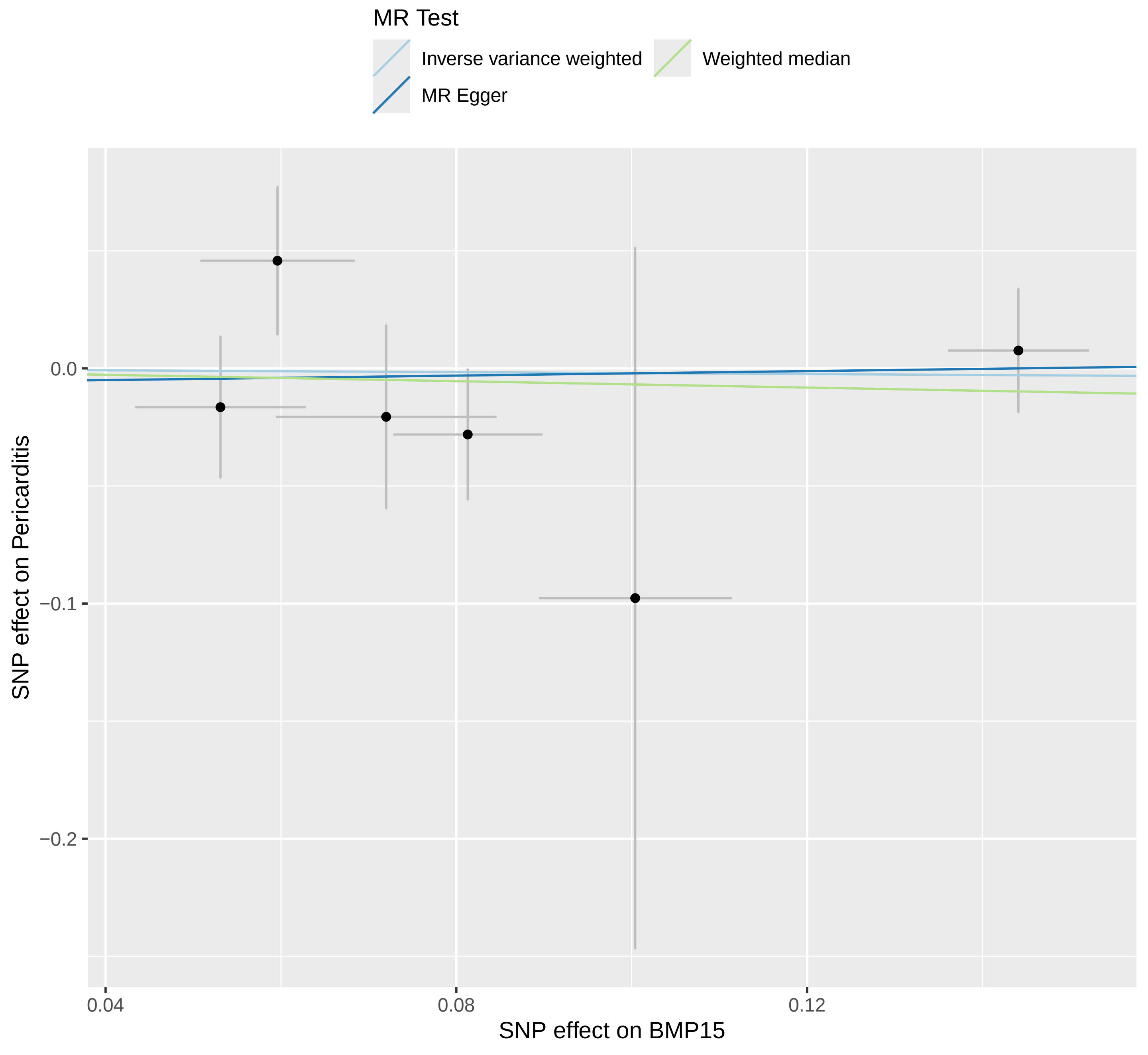

MR Test
Inverse variance weighted
Weighted median
MR Egger
0.0
−0.1
−0.2
0.04
0.08
0.12
SNP effect on Pericarditis
SNP effect on BMP15

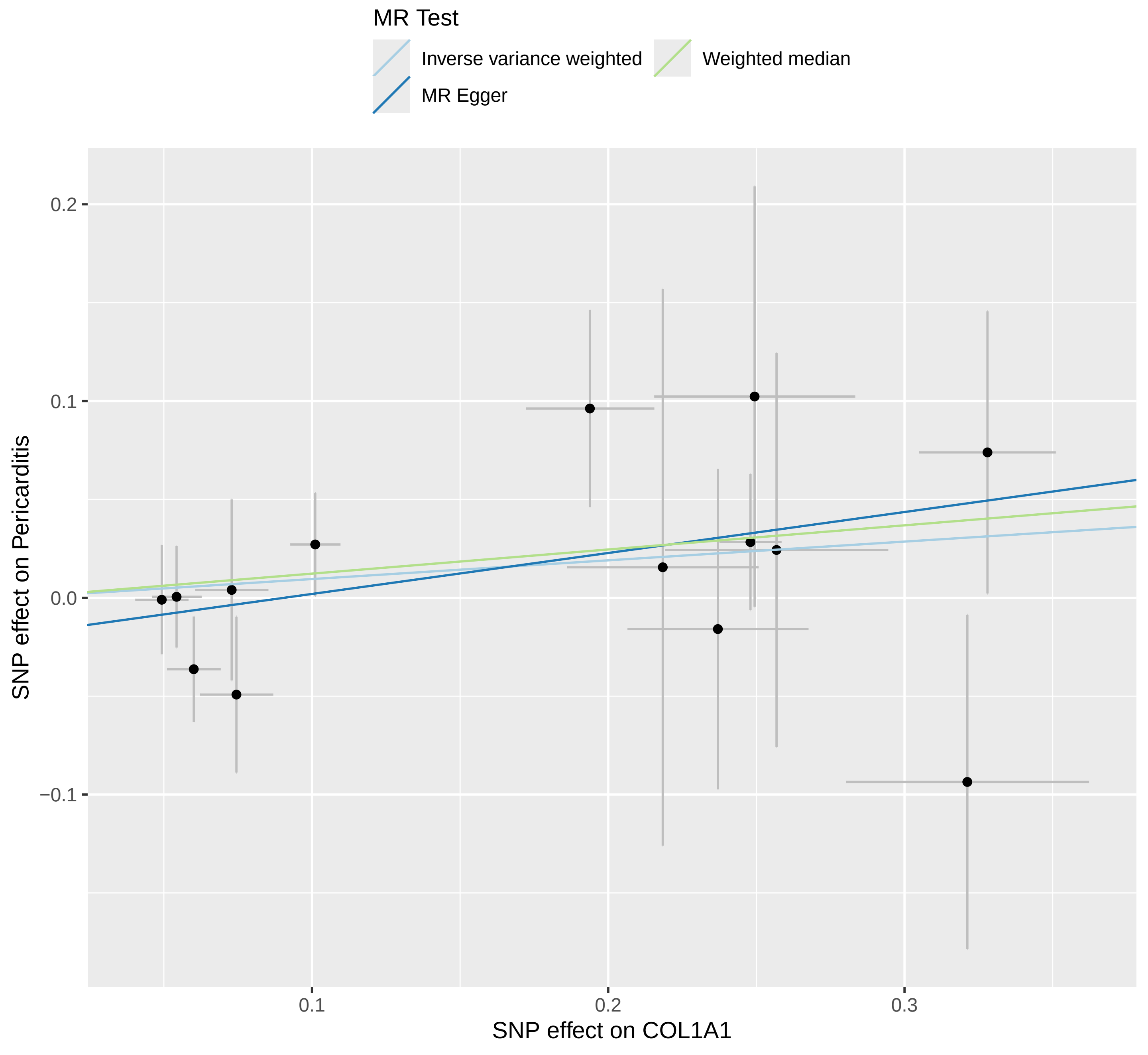
MR Test
Inverse variance weighted
Weighted median
MR Egger
SNP effect on Pericarditis
0.2
0.1
0.0
−0.1
0.1
0.2
0.3
SNP effect on COL1A1

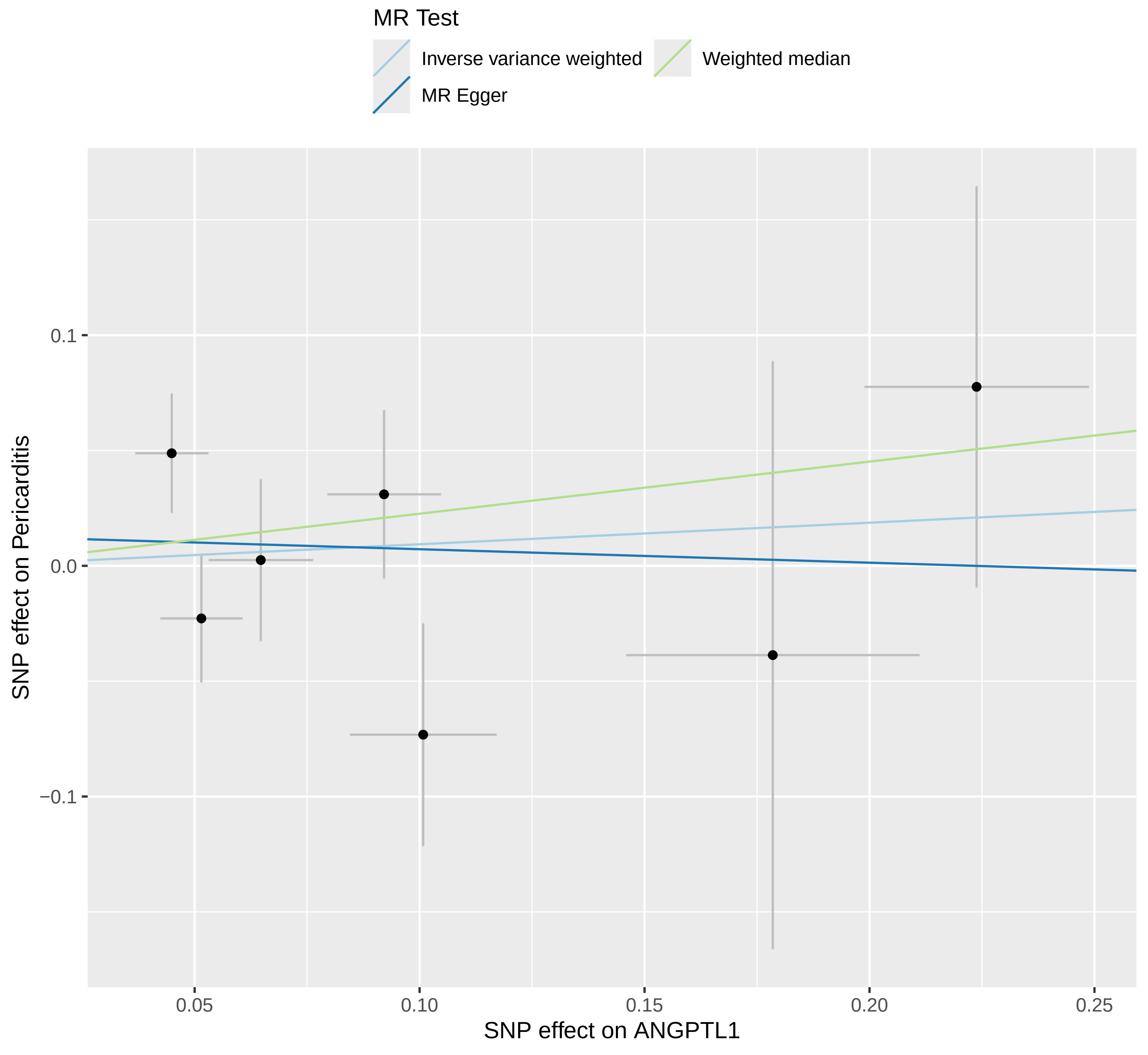
MR Test
Inverse variance weighted
MR Egger
Weighted median
0.1
0.0
−0.1
0.05
0.10
0.15
0.20
0.25
SNP effect on ANGPTL1
SNP effect on Pericarditis

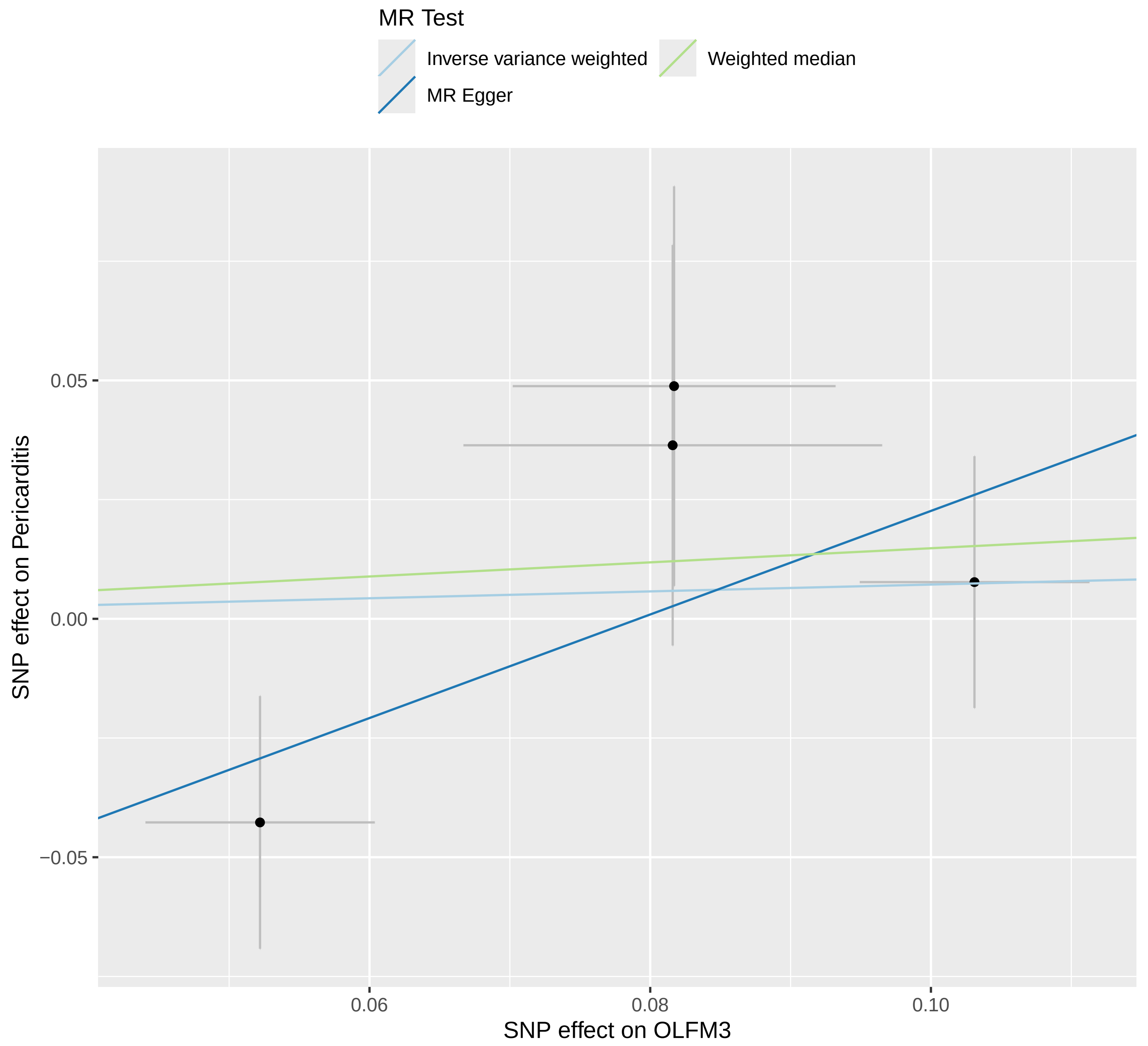
MR Test
Inverse variance weighted
MR Egger
Weighted median
0.05
0.00
−0.05
0.06
0.08
0.10
SNP effect on Pericarditis
SNP effect on OLFM3

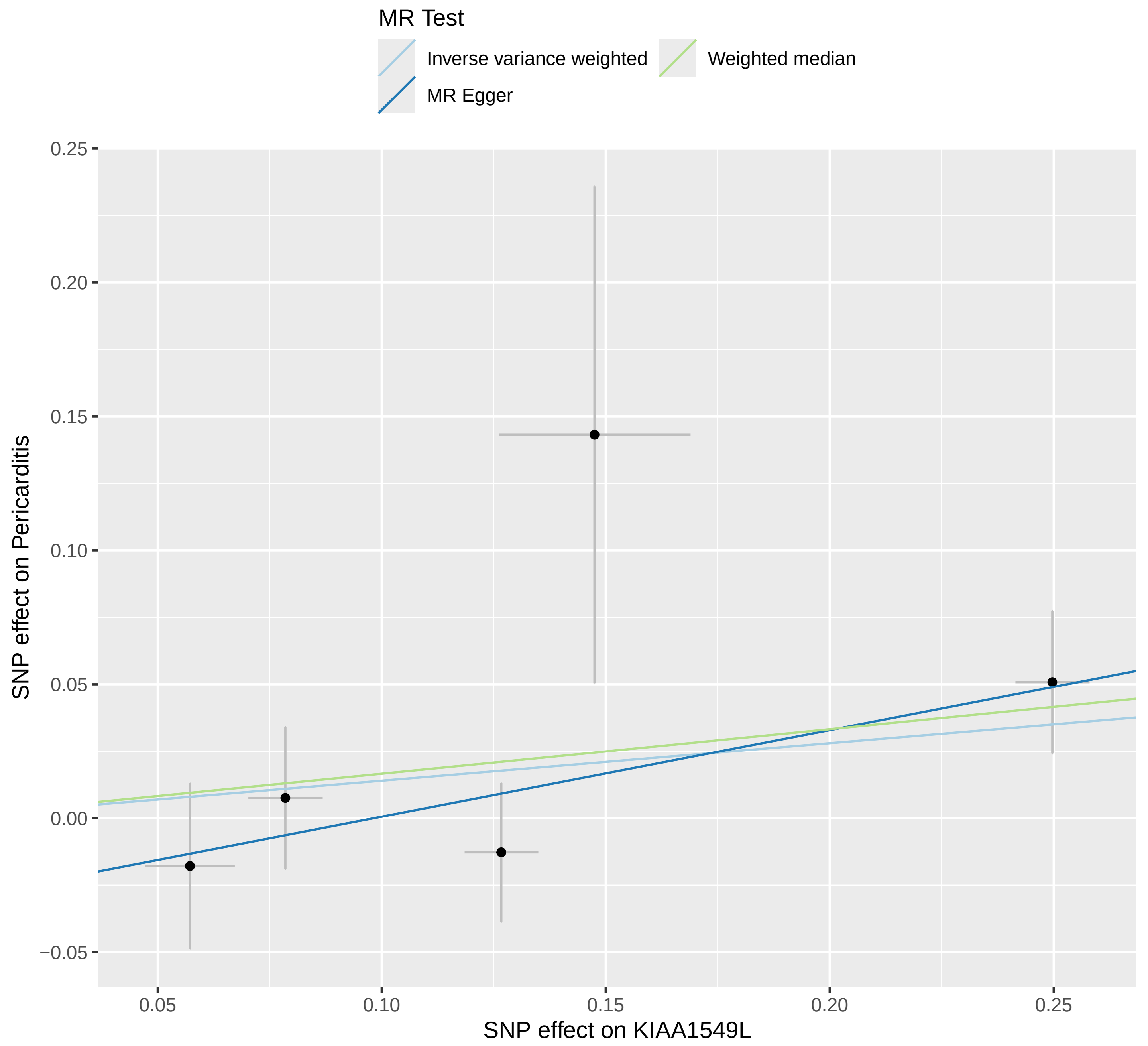
MR Test
Inverse variance weighted
Weighted median
MR Egger
0.25
0.20
0.15
0.10
0.05
0.00
−0.05
SNP effect on Pericarditis
0.05
0.10
0.15
0.20
0.25
SNP effect on KIAA1549L

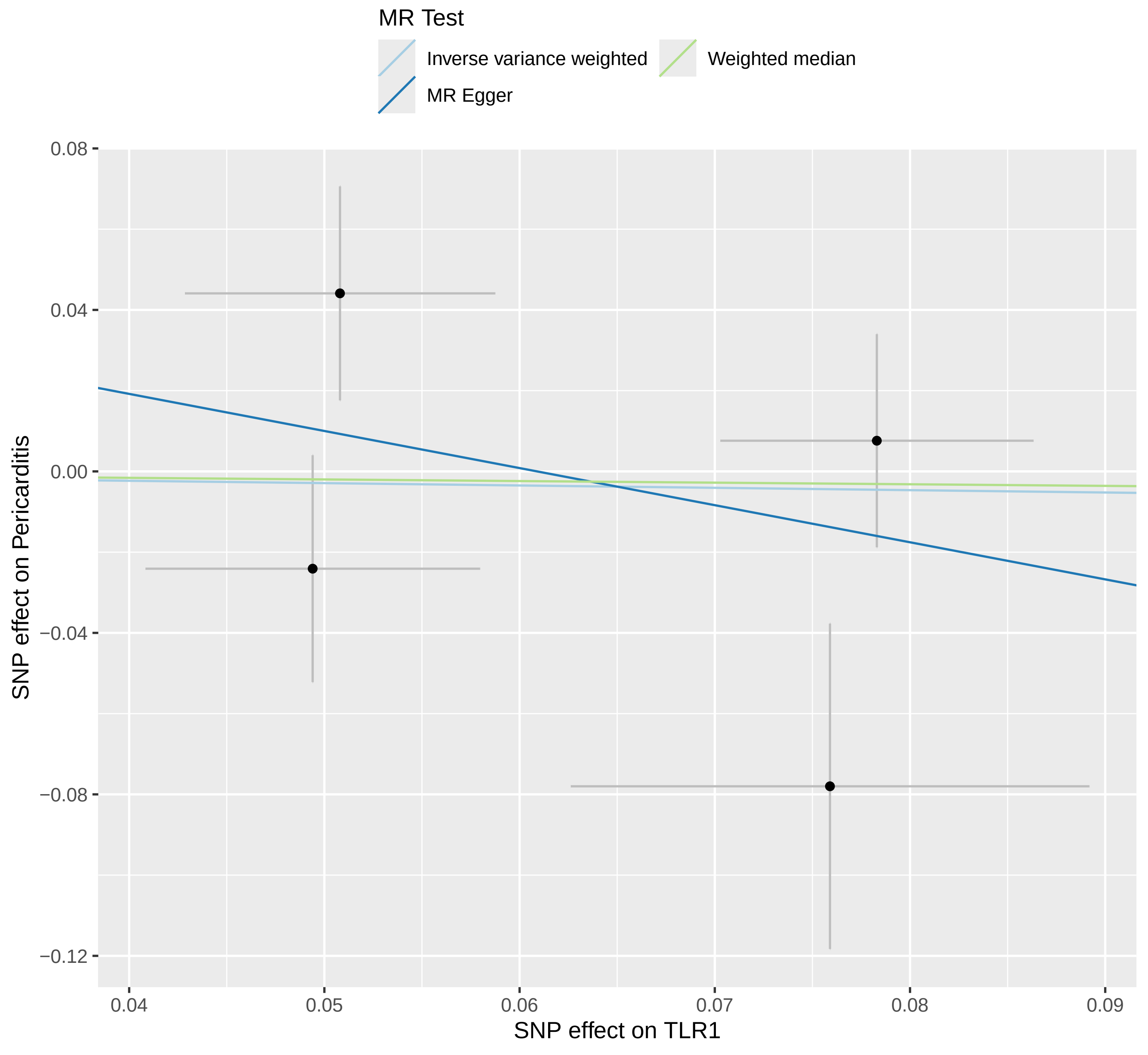
MR Test
Inverse variance weighted
Weighted median
MR Egger
0.08
0.04
0.00
−0.04
−0.08
−0.12
SNP effect on Pericarditis
0.04
0.05
0.06
0.07
0.08
0.09
SNP effect on TLR1

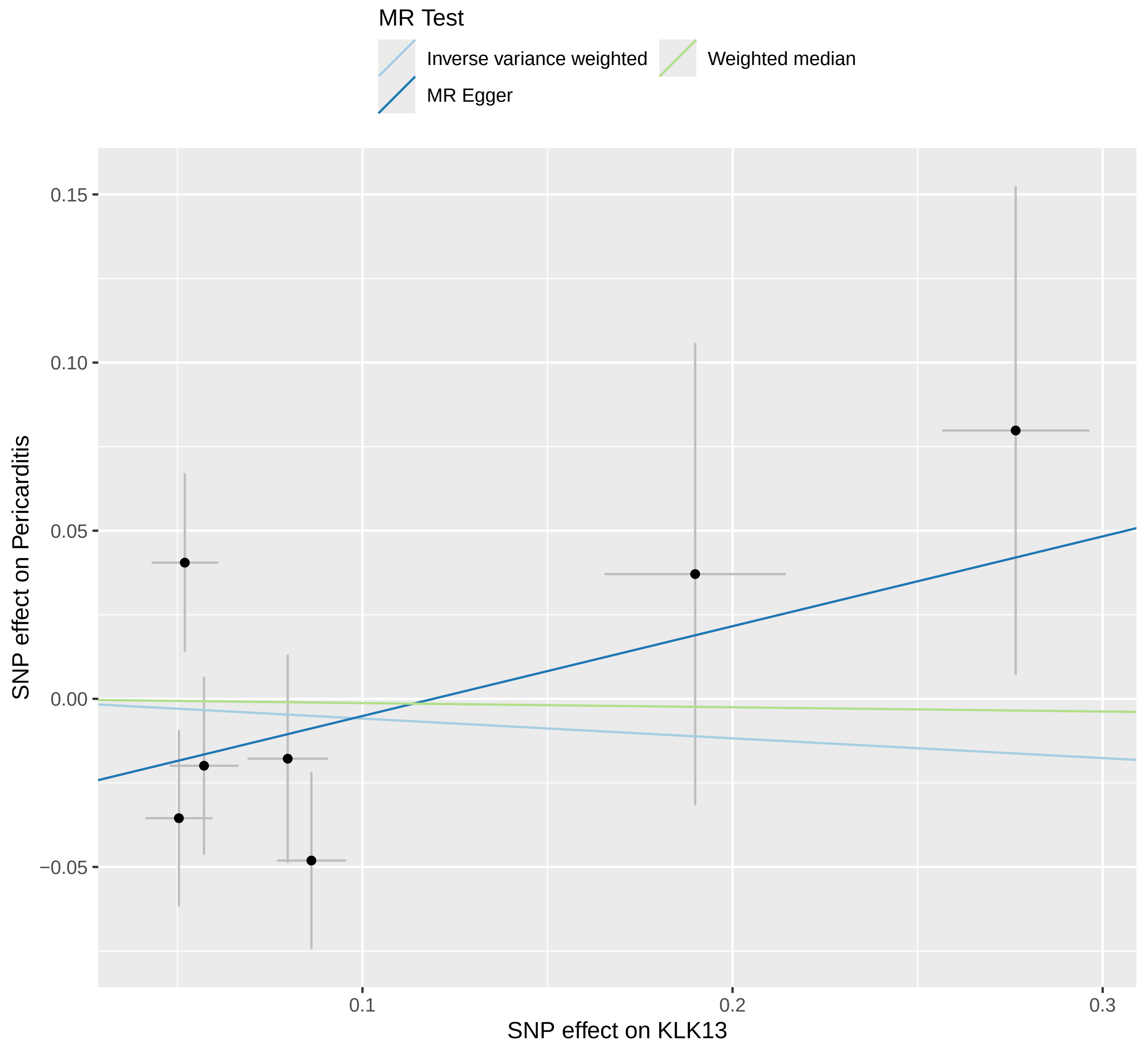
MR Test
Inverse variance weighted
Weighted median
MR Egger
0.15
0.10
0.05
0.00
−0.05
SNP effect on Pericarditis
0.1
0.2
0.3
SNP effect on KLK13

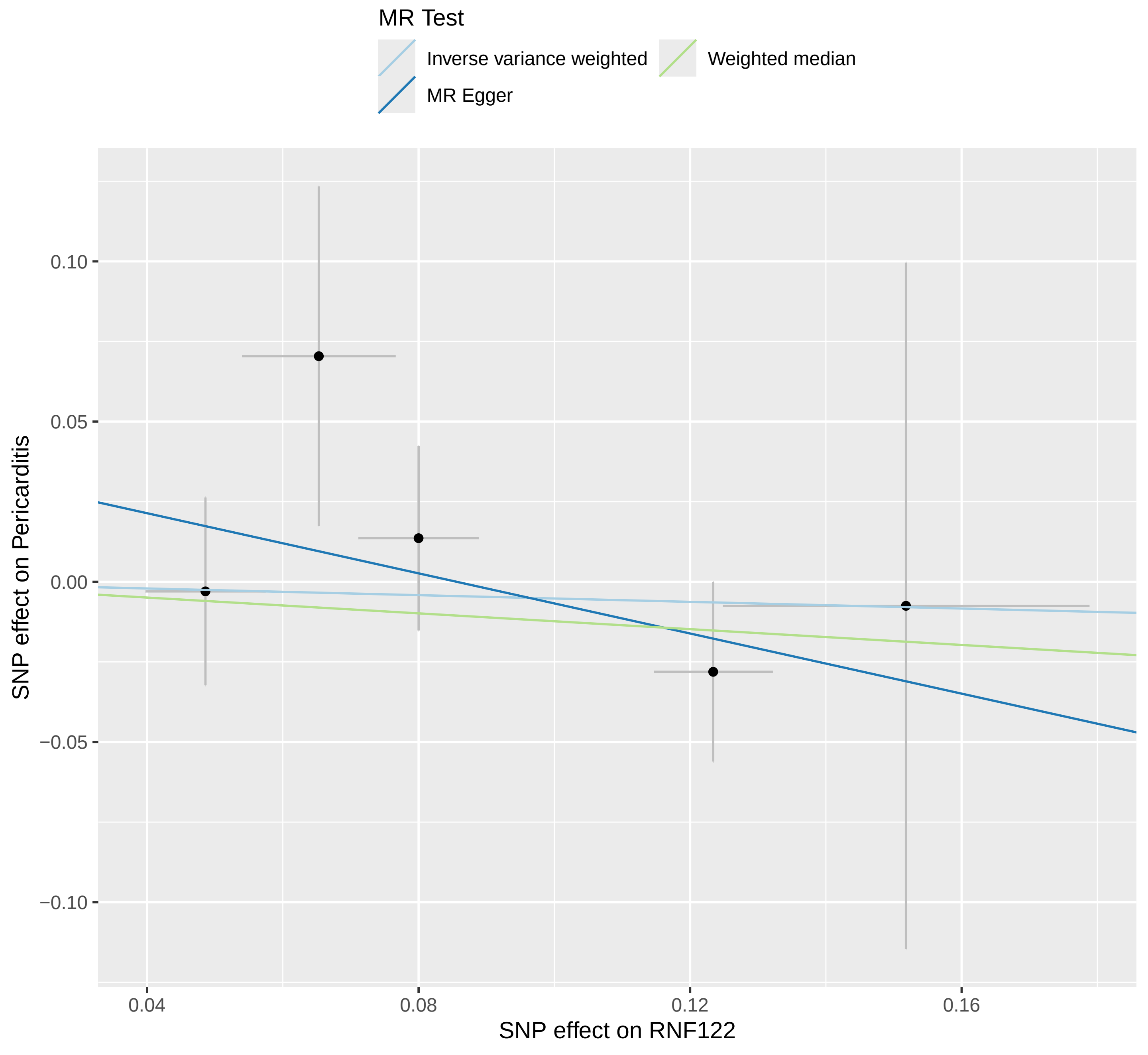
MR Test
Inverse variance weighted
Weighted median
MR Egger
SNP effect on Pericarditis
0.10
0.05
0.00
−0.05
−0.10
0.04
0.08
0.12
0.16
SNP effect on RNF122

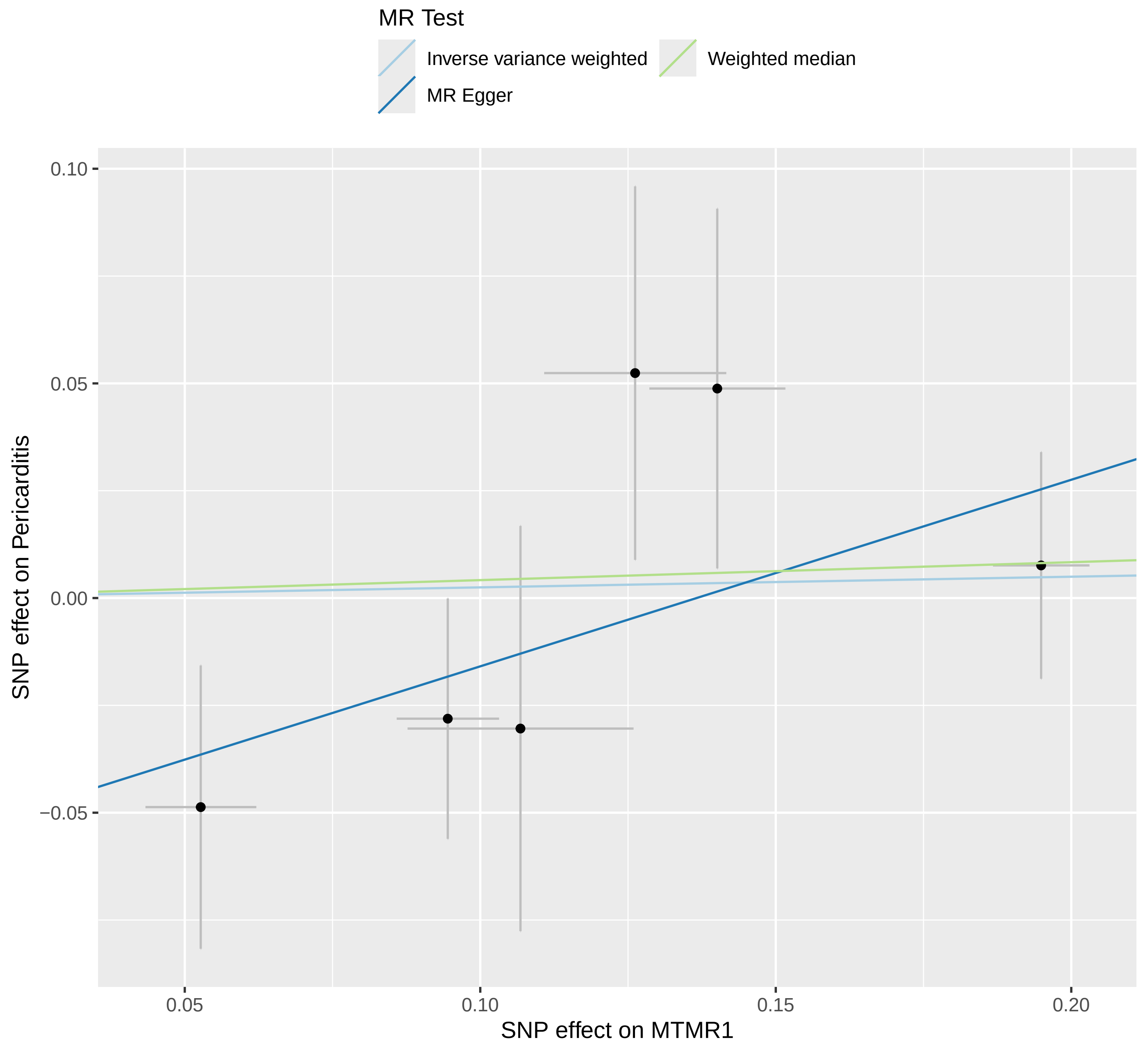
MR Test
Inverse variance weighted
Weighted median
MR Egger
0.10
0.05
0.00
−0.05
SNP effect on Pericarditis
0.05
0.10
0.15
0.20
SNP effect on MTMR1

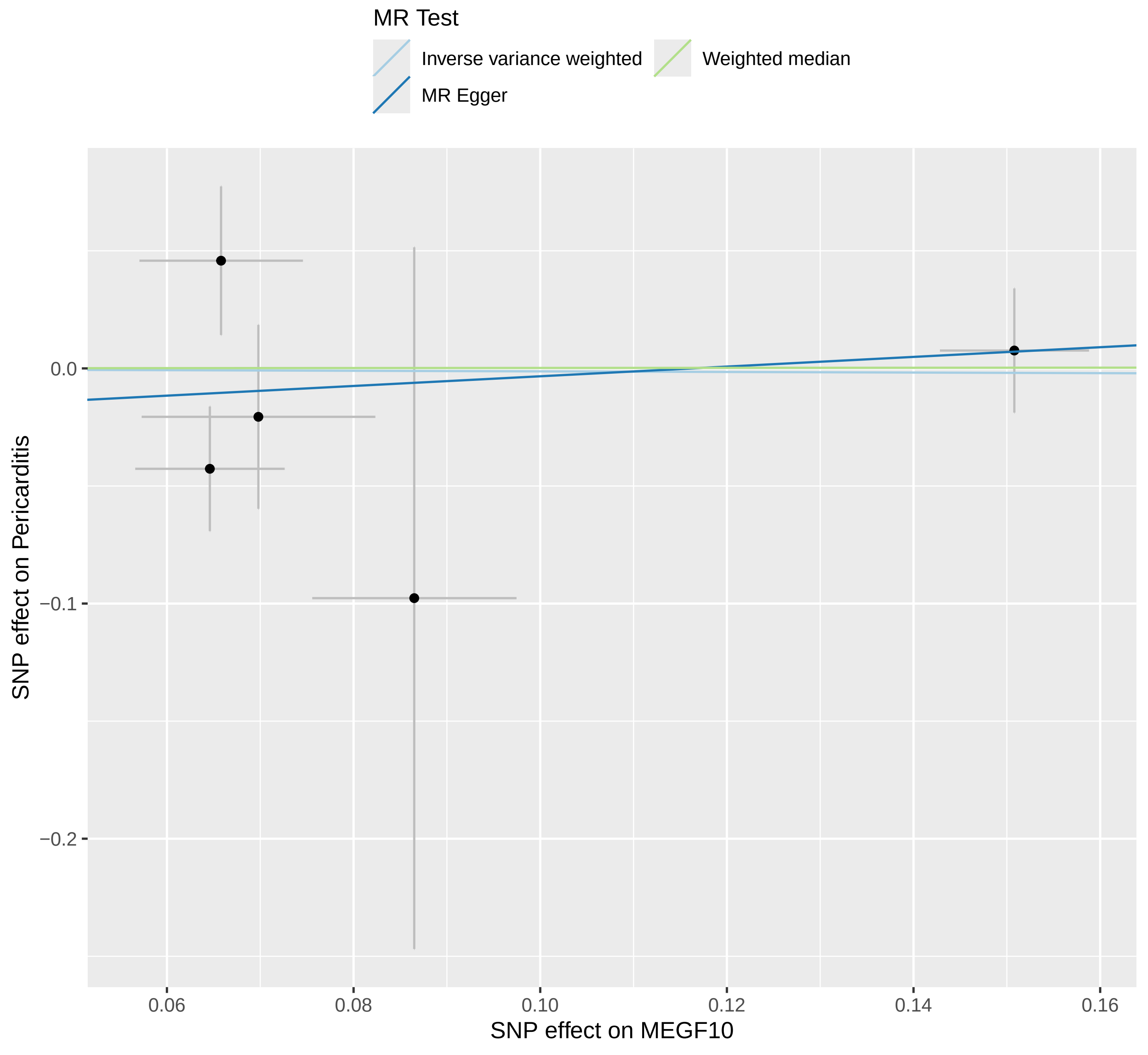
MR Test
Inverse variance weighted
Weighted median
MR Egger
SNP effect on Pericarditis
0.0
−0.1
−0.2
0.06
0.08
0.10
0.12
0.14
0.16
SNP effect on MEGF10

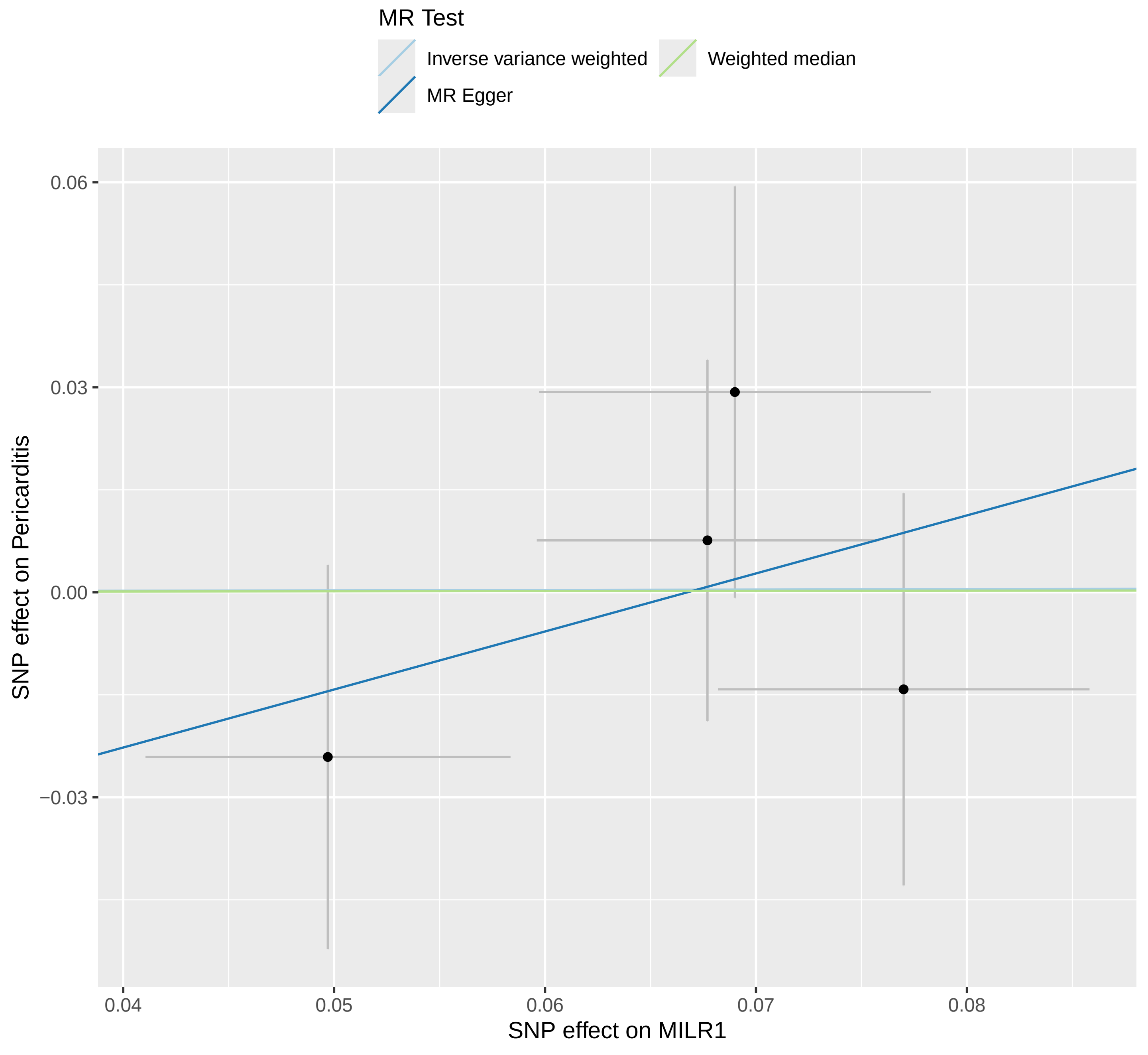
MR Test
Inverse variance weighted
MR Egger
Weighted median
0.06
0.03
0.00
−0.03
SNP effect on Pericarditis
0.04
0.05
0.06
0.07
0.08
SNP effect on MILR1

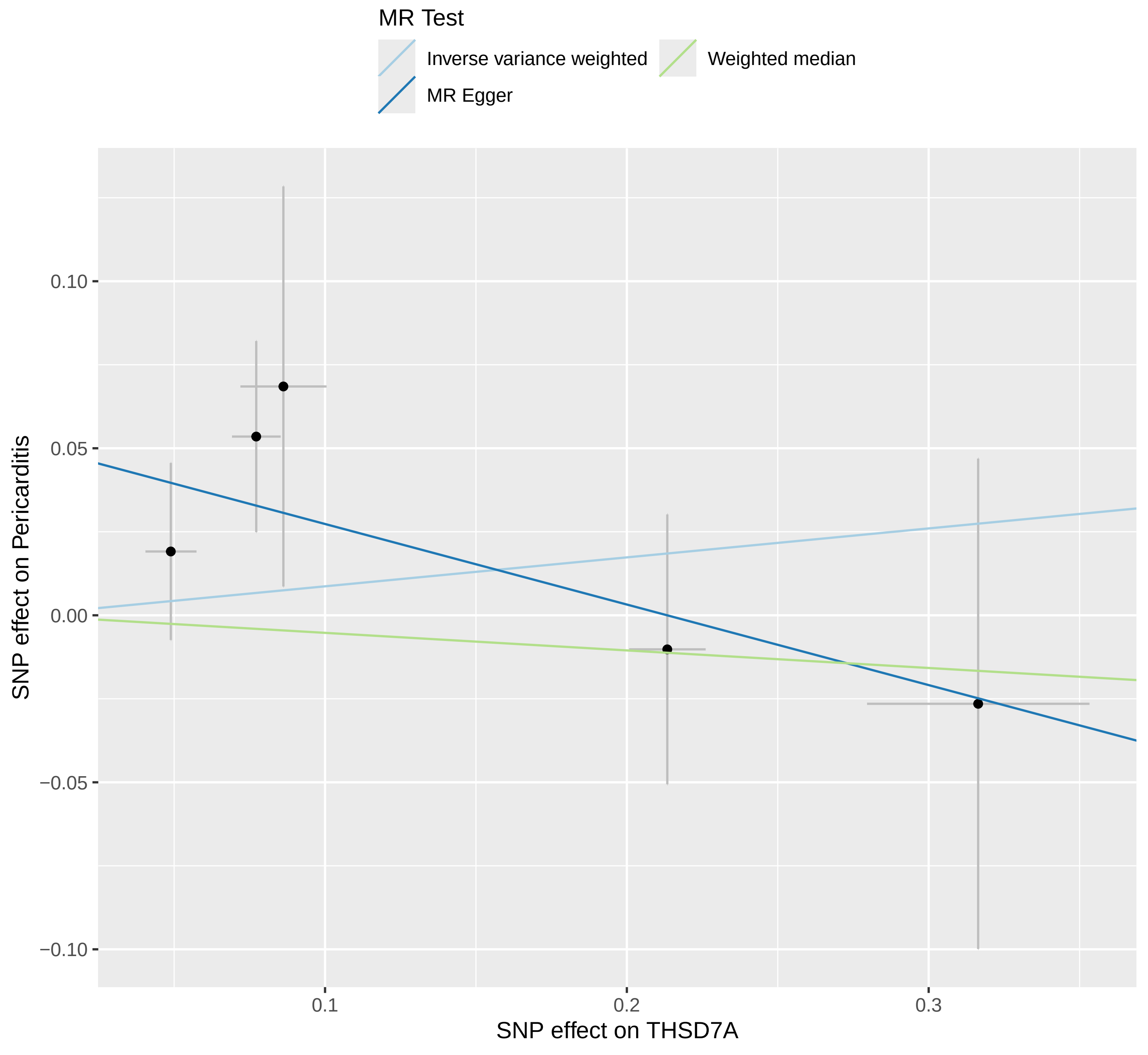

MR Test
Inverse variance weighted
MR Egger
Weighted median
0.10
0.05
0.00
−0.05
−0.10
SNP effect on Pericarditis
0.1
0.2
0.3
SNP effect on THSD7A

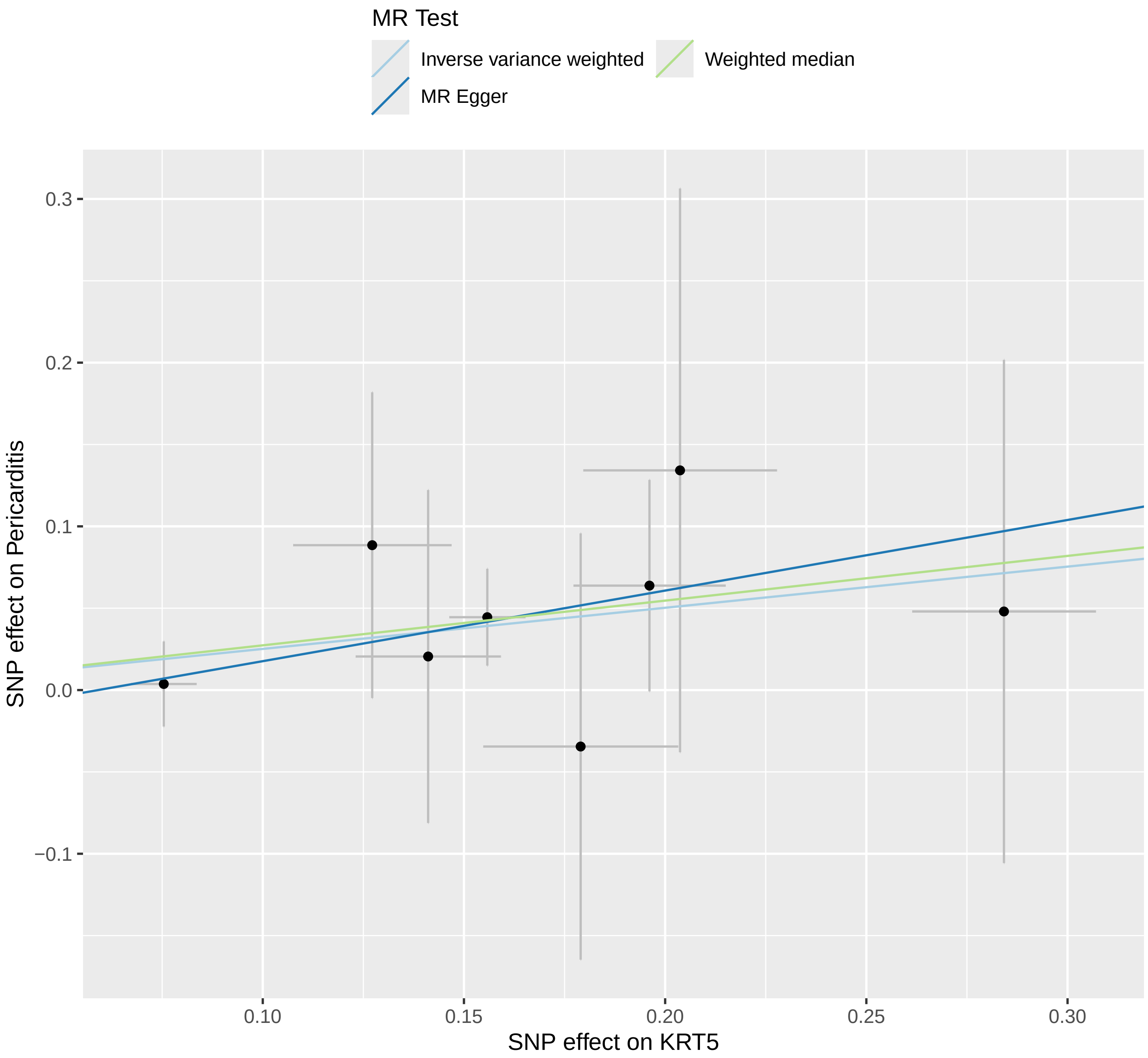

MR Test
Inverse variance weighted
Weighted median
MR Egger
SNP effect on Pericarditis
SNP effect on KRT5
0.3
0.2
0.1
0.0
−0.1
0.10
0.15
0.20
0.25
0.30

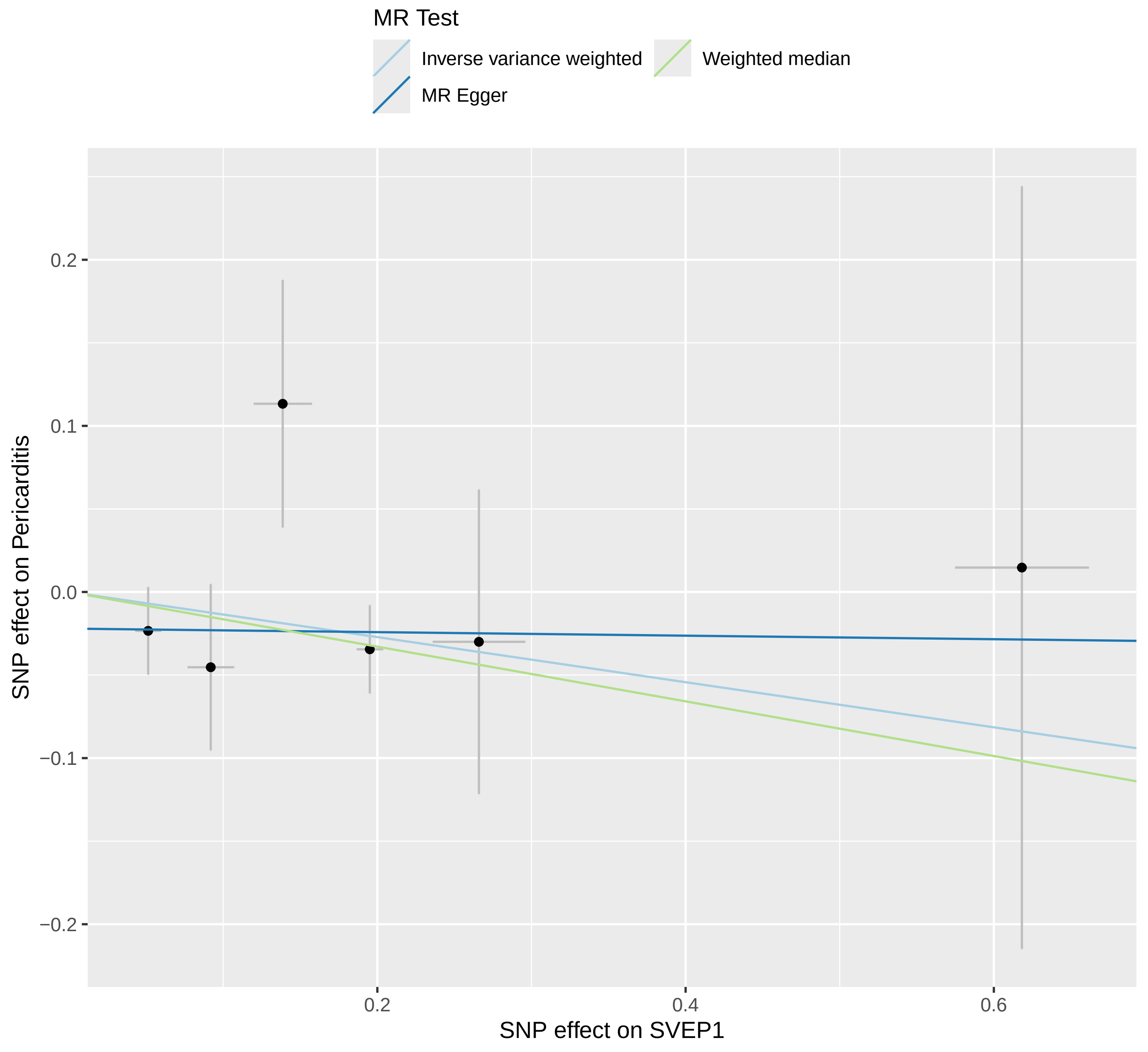
MR Test
Inverse variance weighted
MR Egger
Weighted median
SNP effect on Pericarditis
0.2
0.1
0.0
−0.1
−0.2
0.2
0.4
0.6
SNP effect on SVEP1

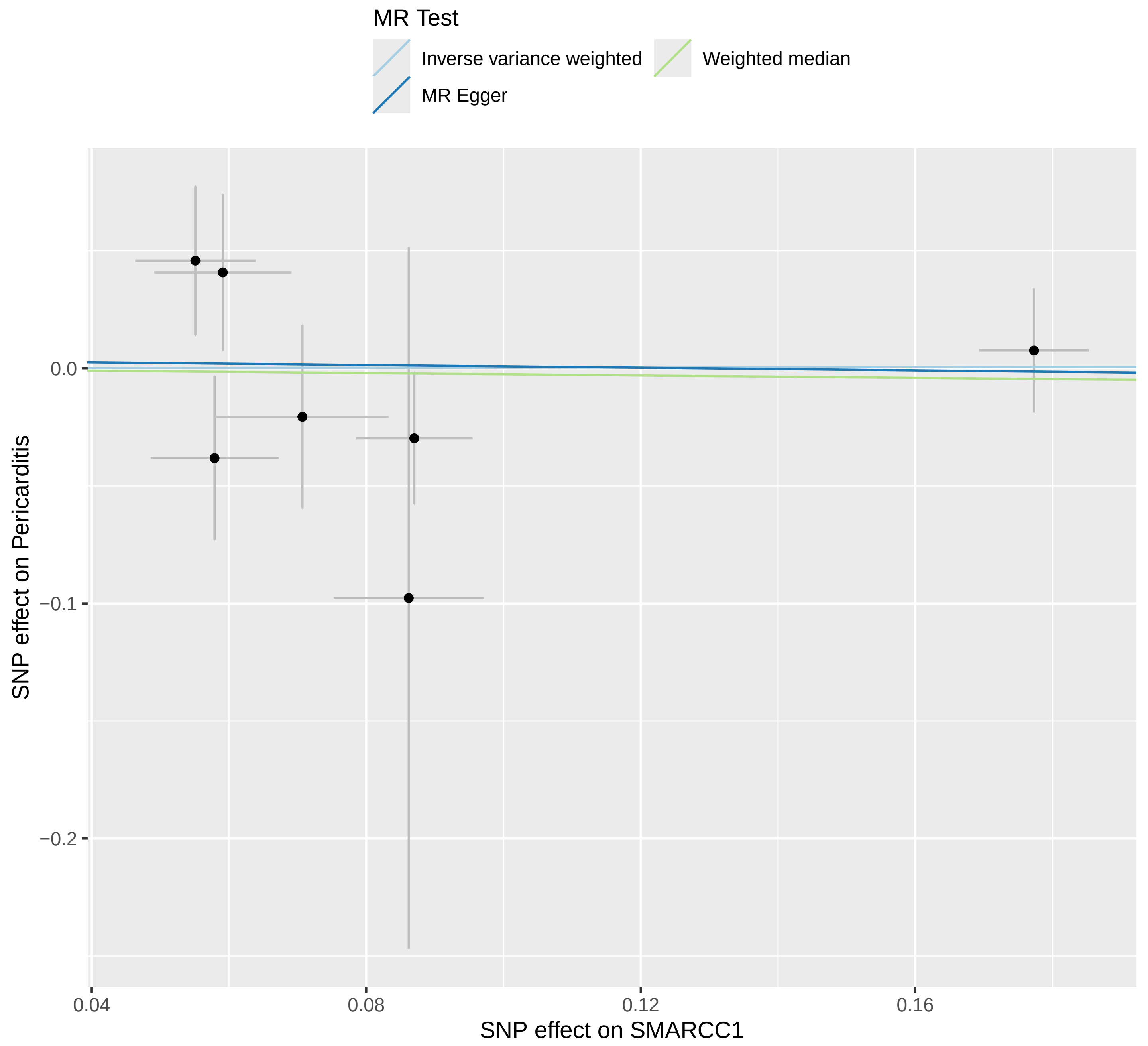

MR Test
Inverse variance weighted
MR Egger
Weighted median
0.0
−0.1
−0.2
SNP effect on Pericarditis
0.04
0.08
0.12
0.16
SNP effect on SMARCC1

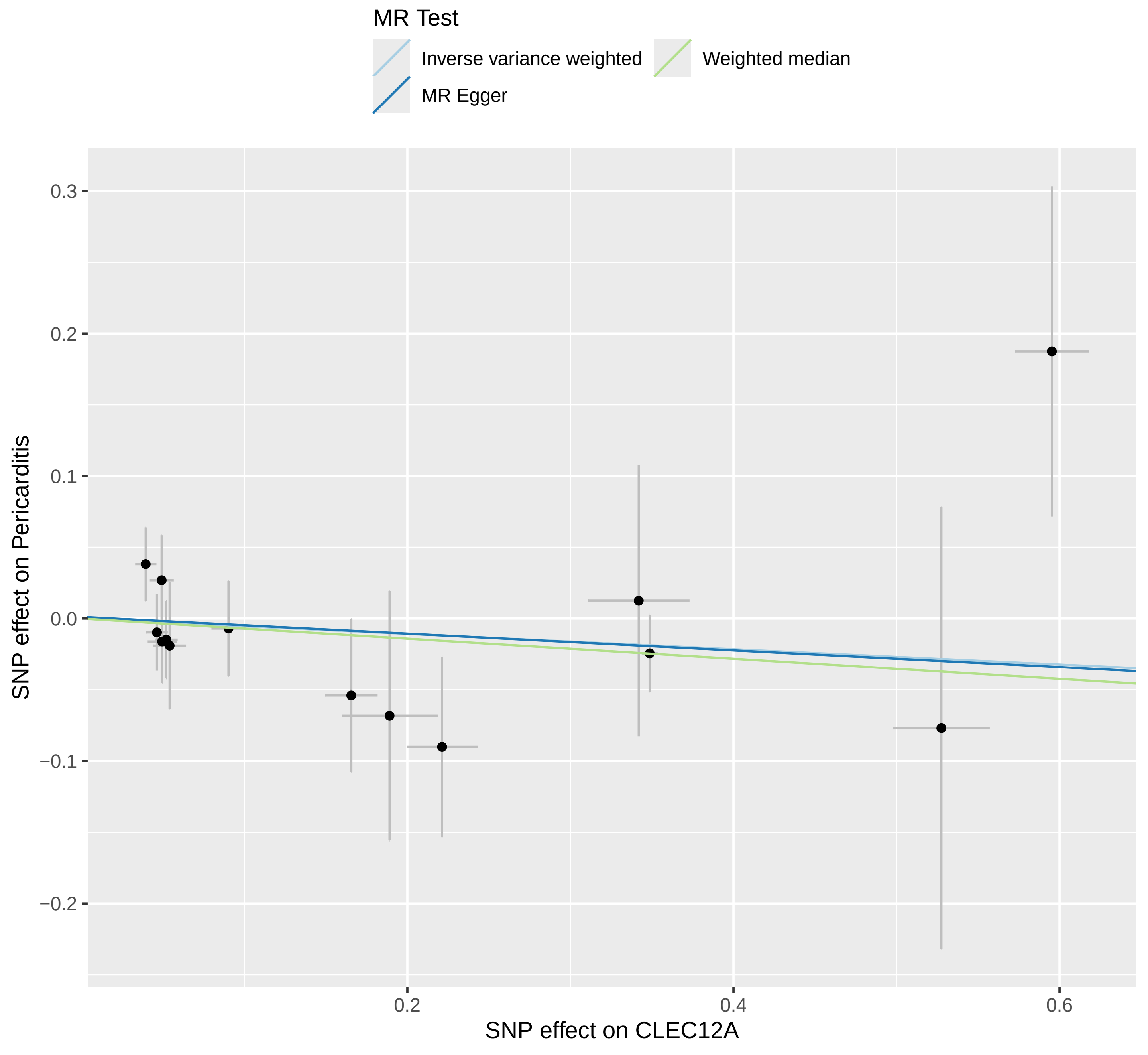
MR Test
Inverse variance weighted
Weighted median
MR Egger
SNP effect on Pericarditis
0.3
0.2
0.1
0.0
−0.1
−0.2
SNP effect on CLEC12A
0.2
0.4
0.6

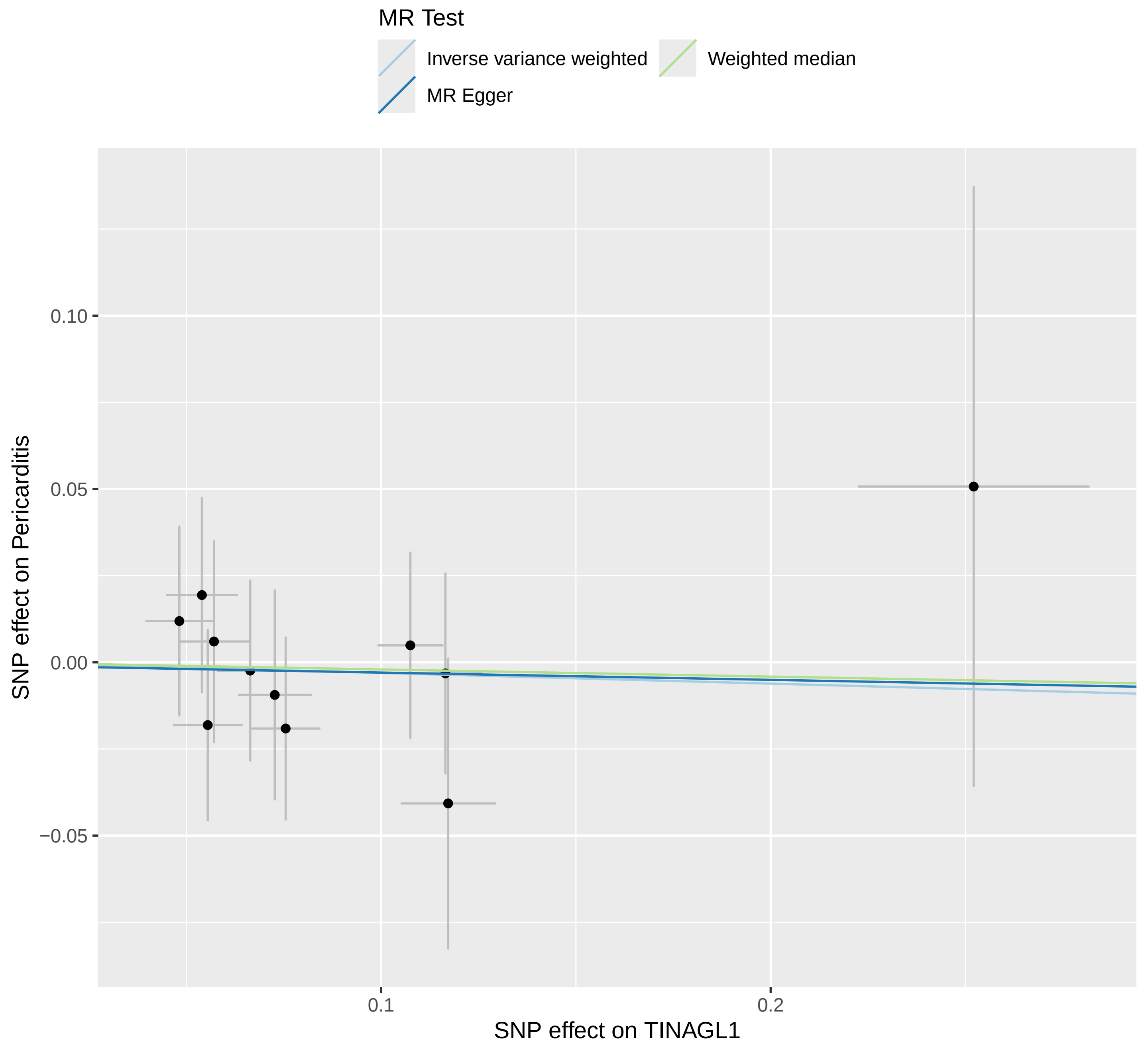
MR Test
Inverse variance weighted
Weighted median
MR Egger
0.10
0.05
0.00
−0.05
0.1
0.2
SNP effect on Pericarditis
SNP effect on TINAGL1

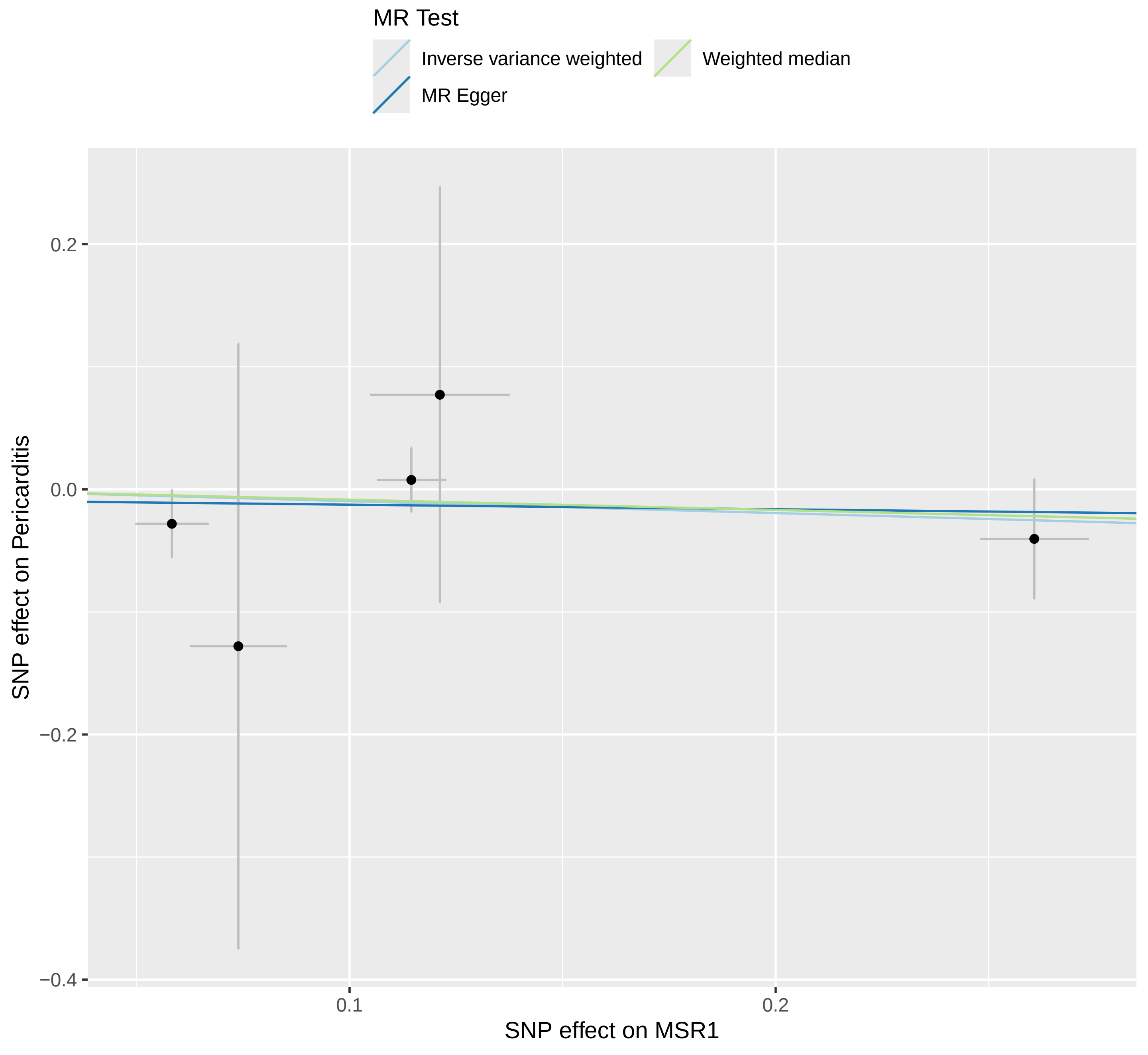
MR Test
Inverse variance weighted
Weighted median
MR Egger
0.2
0.0
−0.2
−0.4
SNP effect on Pericarditis
0.1
0.2
SNP effect on MSR1

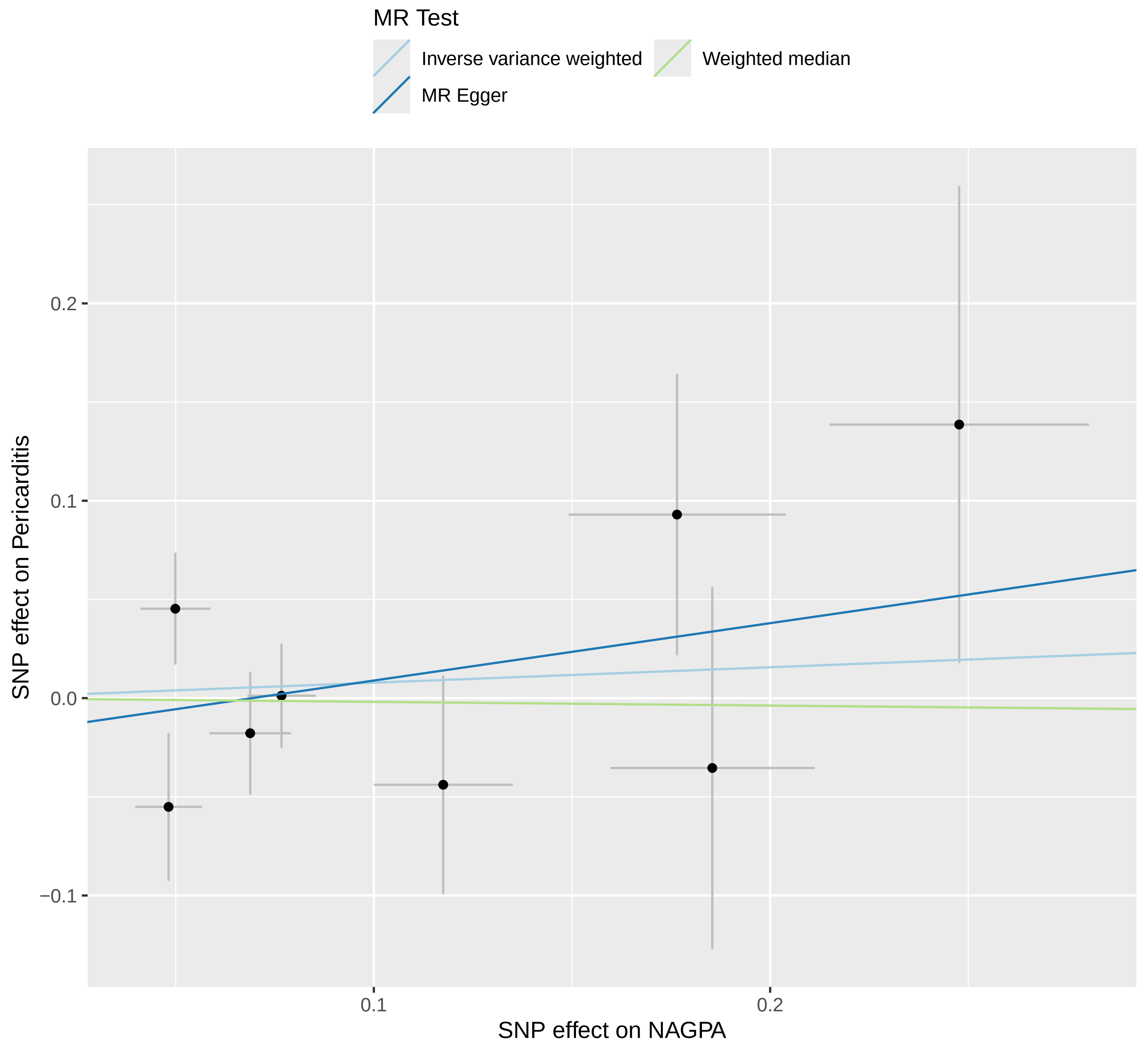

MR Test
Inverse variance weighted
MR Egger
Weighted median
SNP effect on Pericarditis
0.2
0.1
0.0
−0.1
0.1
0.2
SNP effect on NAGPA

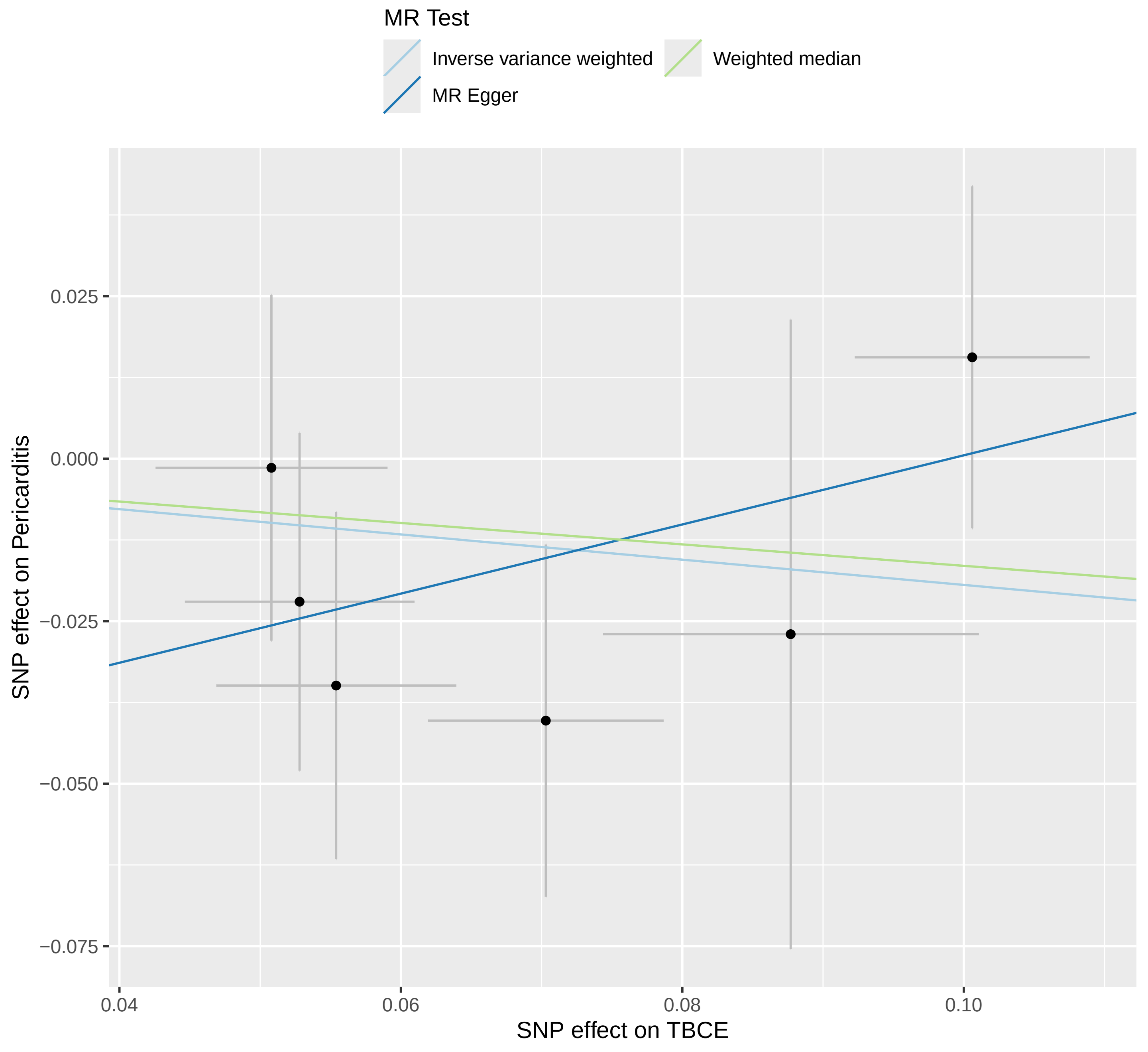
MR Test
Inverse variance weighted
MR Egger
Weighted median
SNP effect on Pericarditis
0.025
0.000
−0.025
−0.050
−0.075
0.04
0.06
0.08
0.10
SNP effect on TBCE

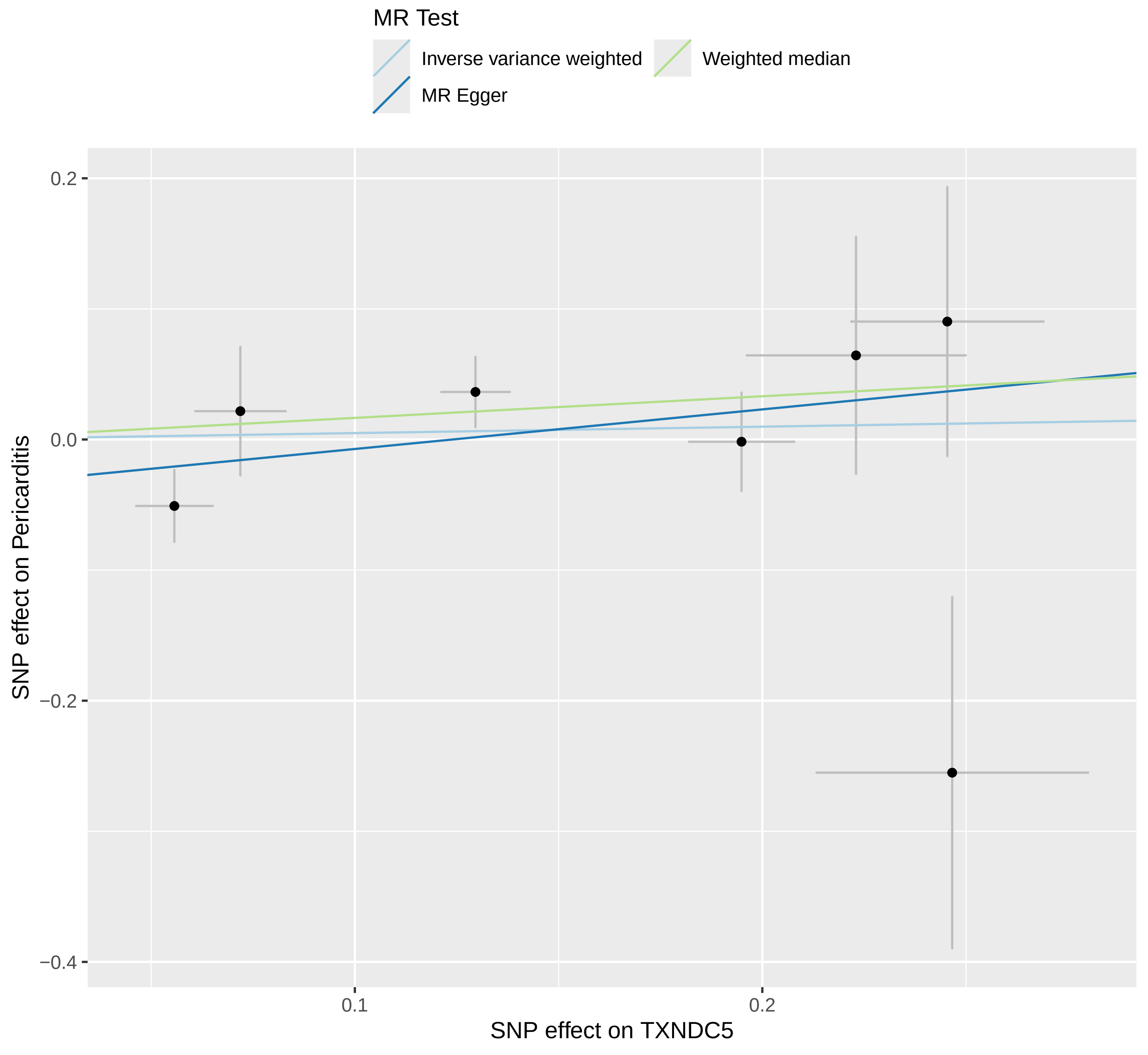
MR Test
Inverse variance weighted
MR Egger
Weighted median
0.2
0.0
−0.2
−0.4
SNP effect on Pericarditis
0.1
0.2
SNP effect on TXNDC5

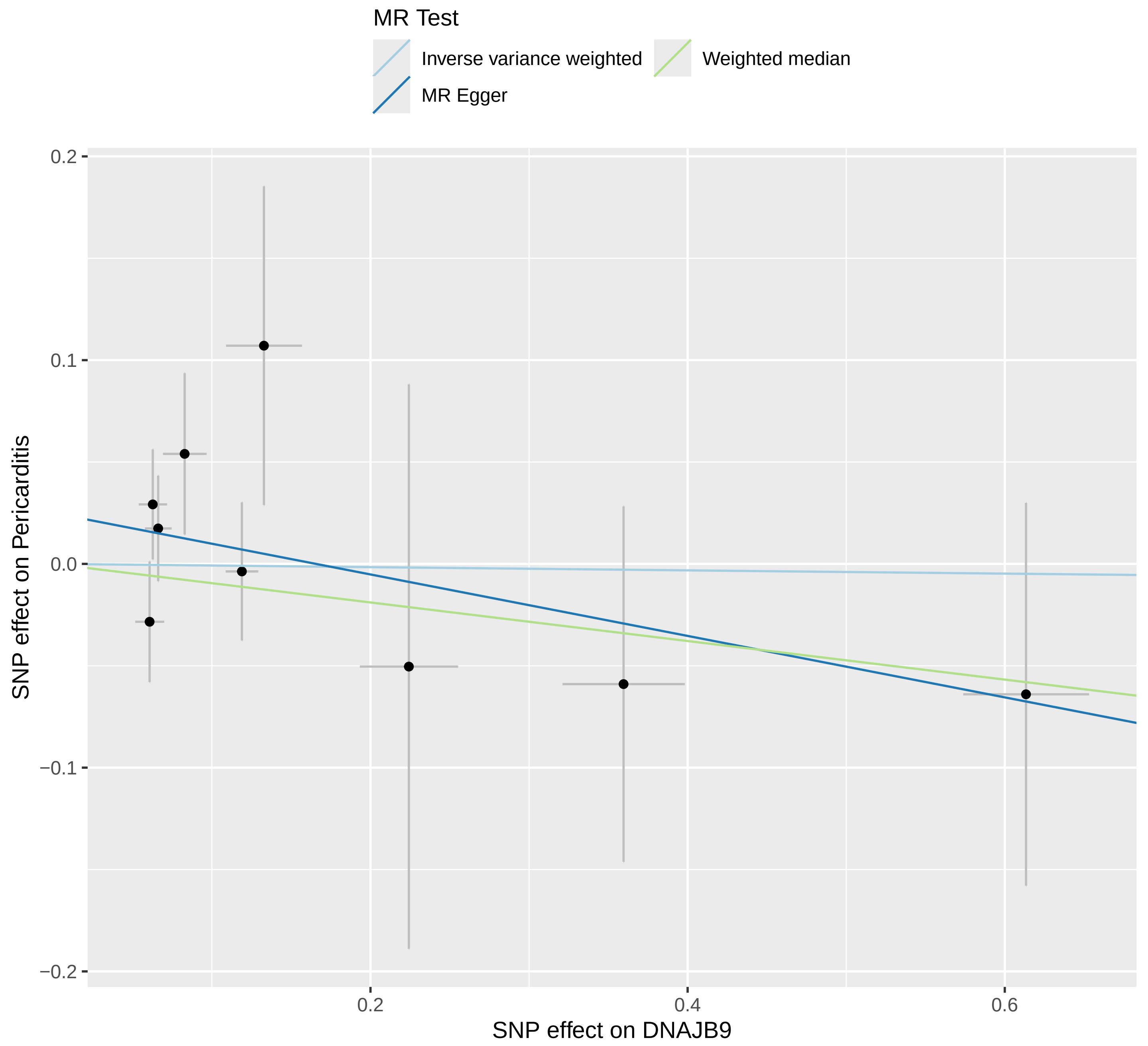
MR Test
Inverse variance weighted
Weighted median
MR Egger
0.2
0.1
0.0
−0.1
−0.2
SNP effect on Pericarditis
0.2
0.4
0.6
SNP effect on DNAJB9

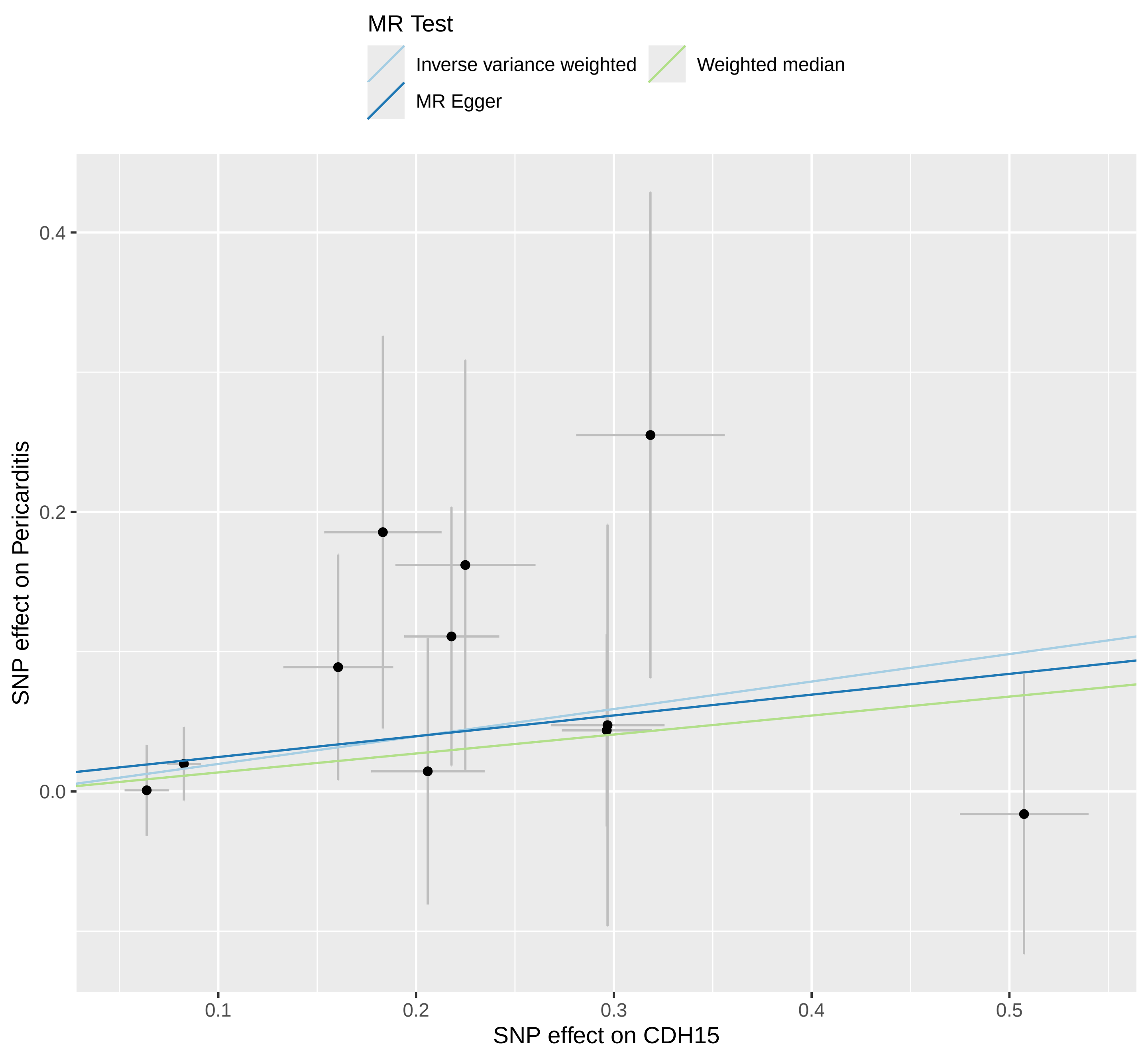

MR Test
Inverse variance weighted
Weighted median
MR Egger
SNP effect on Pericarditis
0.4
0.2
0.0
0.1
0.2
0.3
0.4
0.5
SNP effect on CDH15

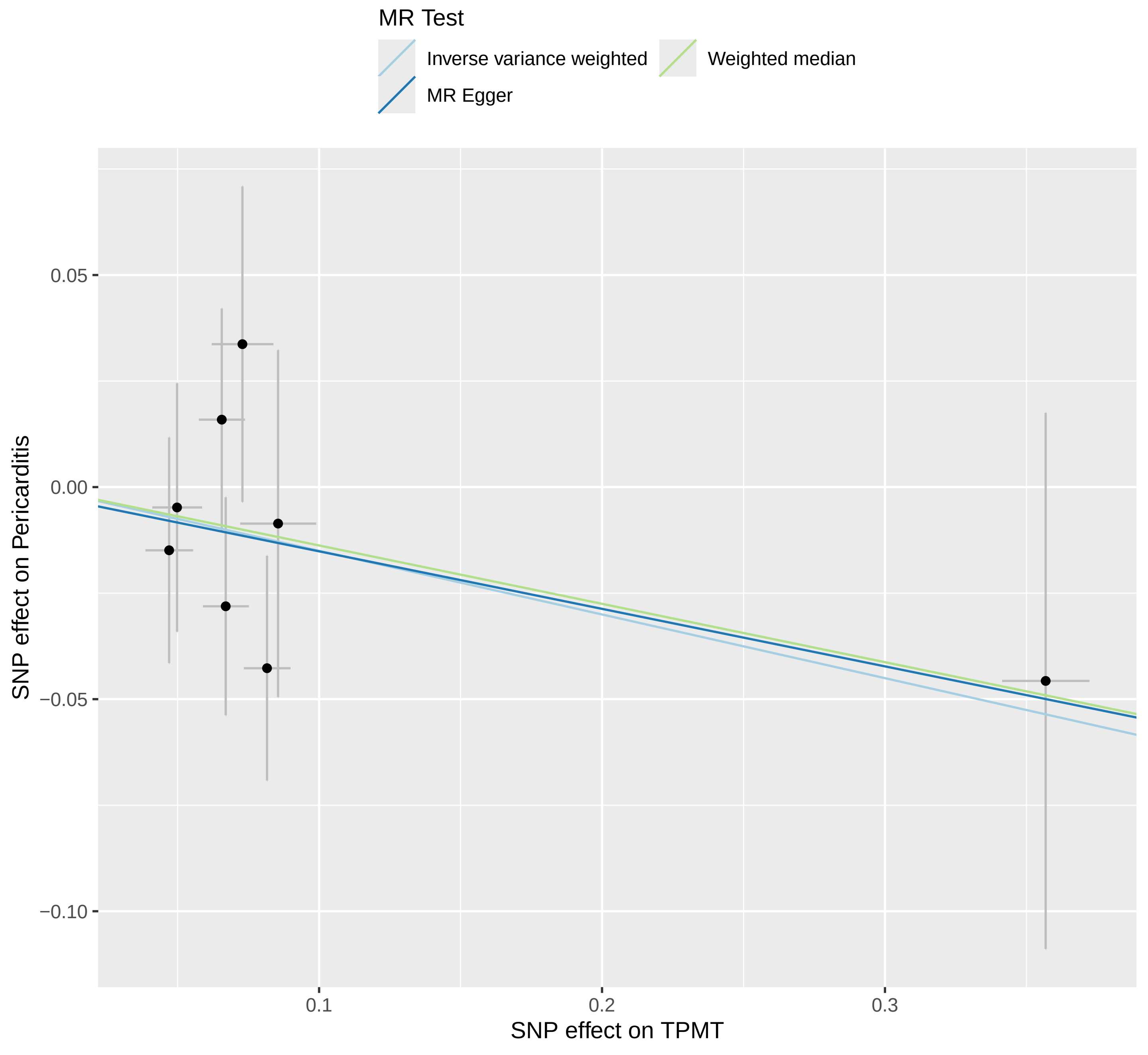

MR Test
Inverse variance weighted
MR Egger
Weighted median
0.05
0.00
−0.05
−0.10
SNP effect on Pericarditis
0.1
0.2
0.3
SNP effect on TPMT

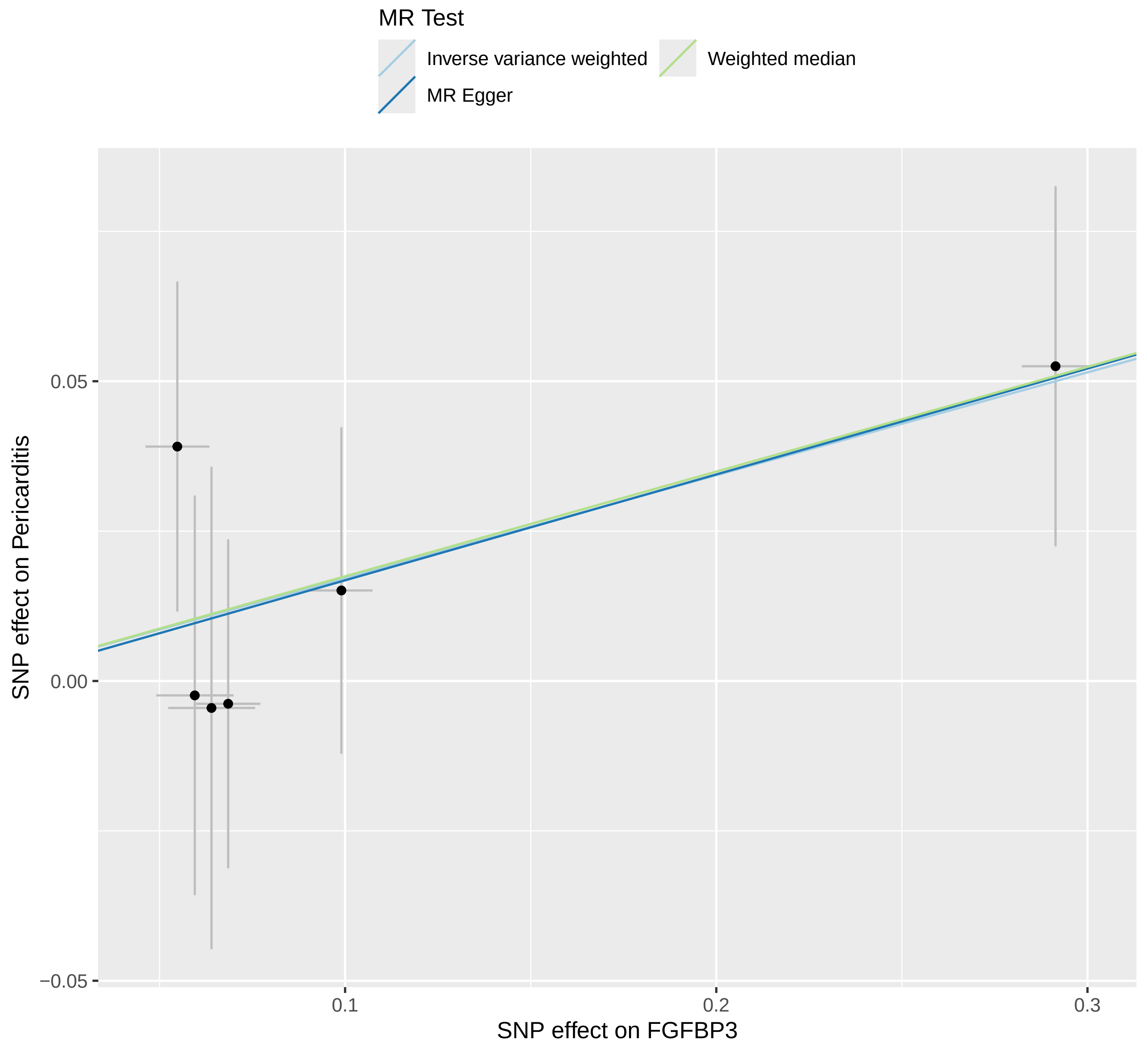
MR Test
Inverse variance weighted
Weighted median
MR Egger
SNP effect on Pericarditis
0.05
0.00
−0.05
0.1
0.2
0.3
SNP effect on FGFBP3

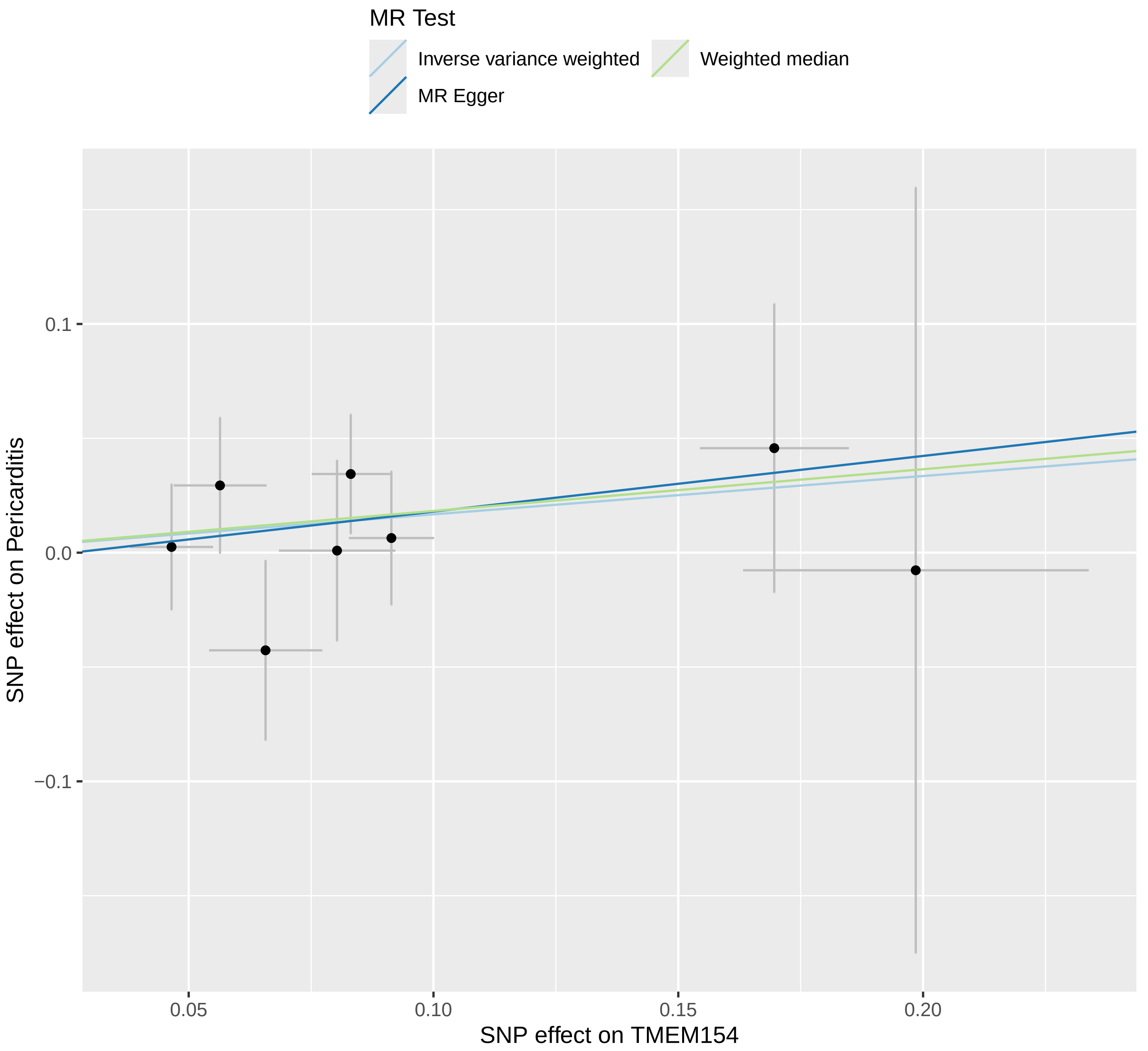
MR Test
Inverse variance weighted
Weighted median
MR Egger
0.1
0.0
−0.1
SNP effect on Pericarditis
0.05
0.10
0.15
0.20
SNP effect on TMEM154

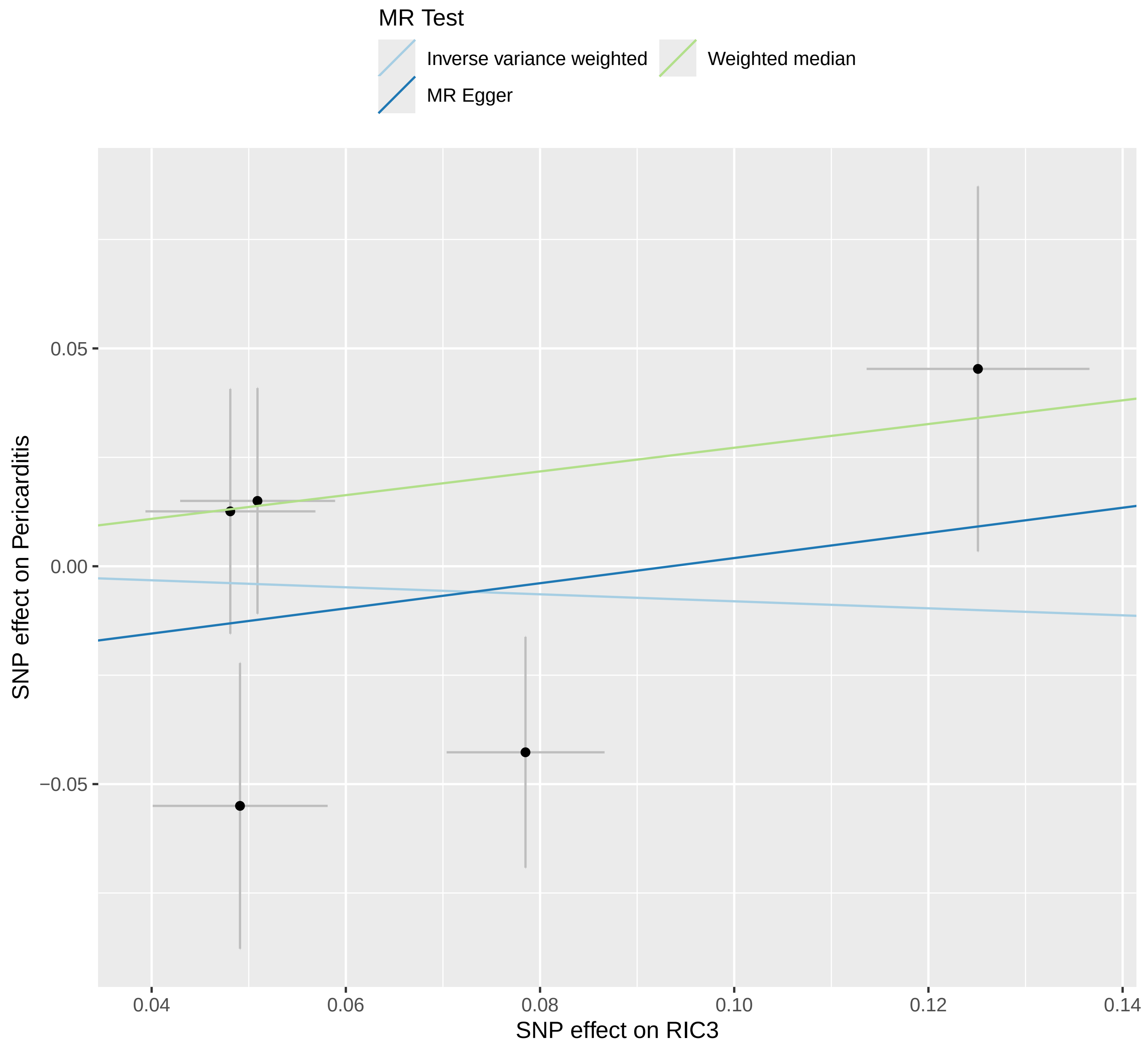
MR Test
Inverse variance weighted
MR Egger
Weighted median
0.05
0.00
−0.05
SNP effect on Pericarditis
0.04
0.06
0.08
0.10
0.12
0.14
SNP effect on RIC3

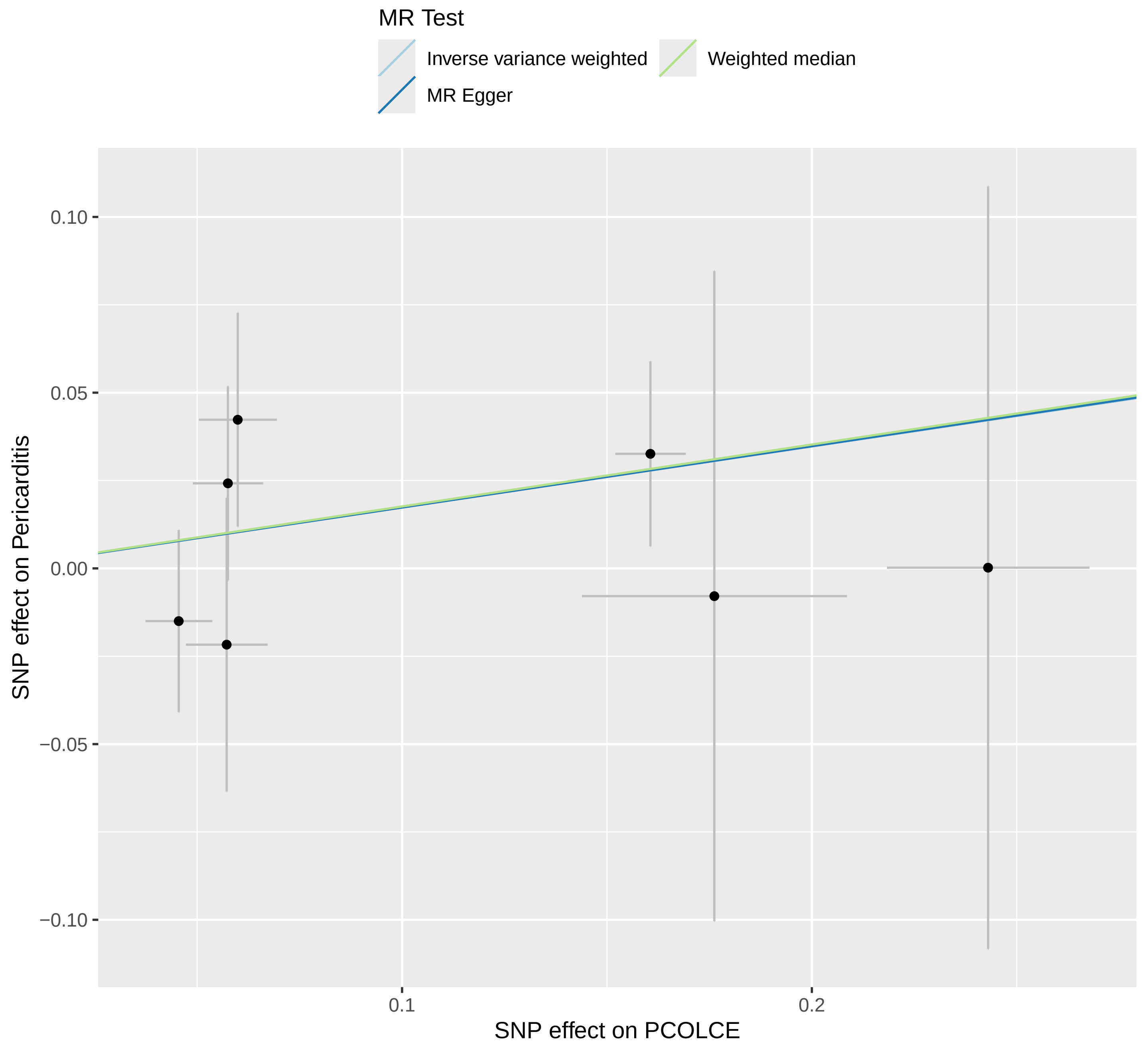

MR Test
Inverse variance weighted
Weighted median
MR Egger
0.10
0.05
0.00
−0.05
−0.10
SNP effect on Pericarditis
0.1
0.2
SNP effect on PCOLCE

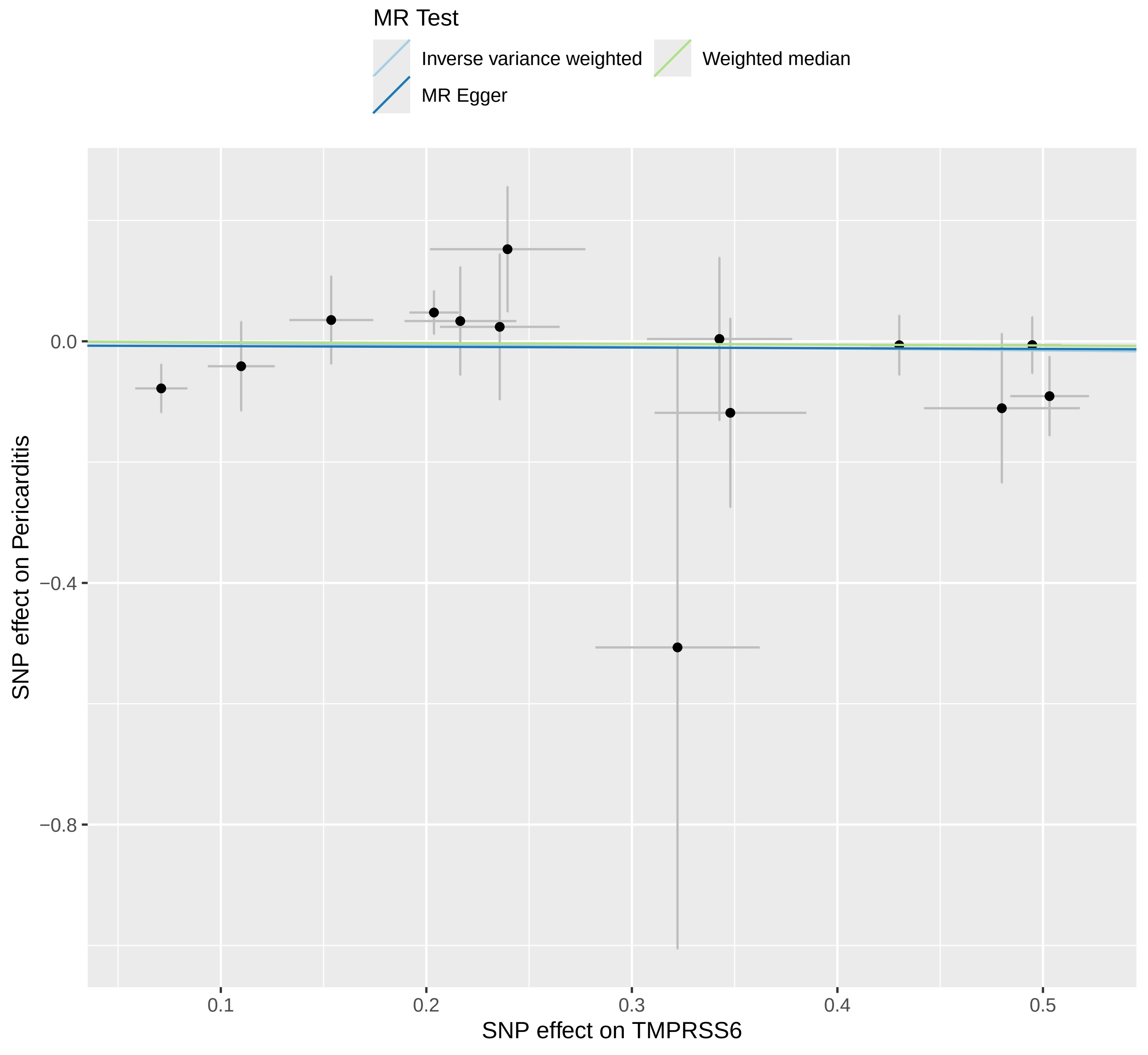
MR Test
Inverse variance weighted
Weighted median
MR Egger
SNP effect on Pericarditis
0.0
−0.4
−0.8
0.1
0.2
0.3
0.4
0.5
SNP effect on TMPRSS6

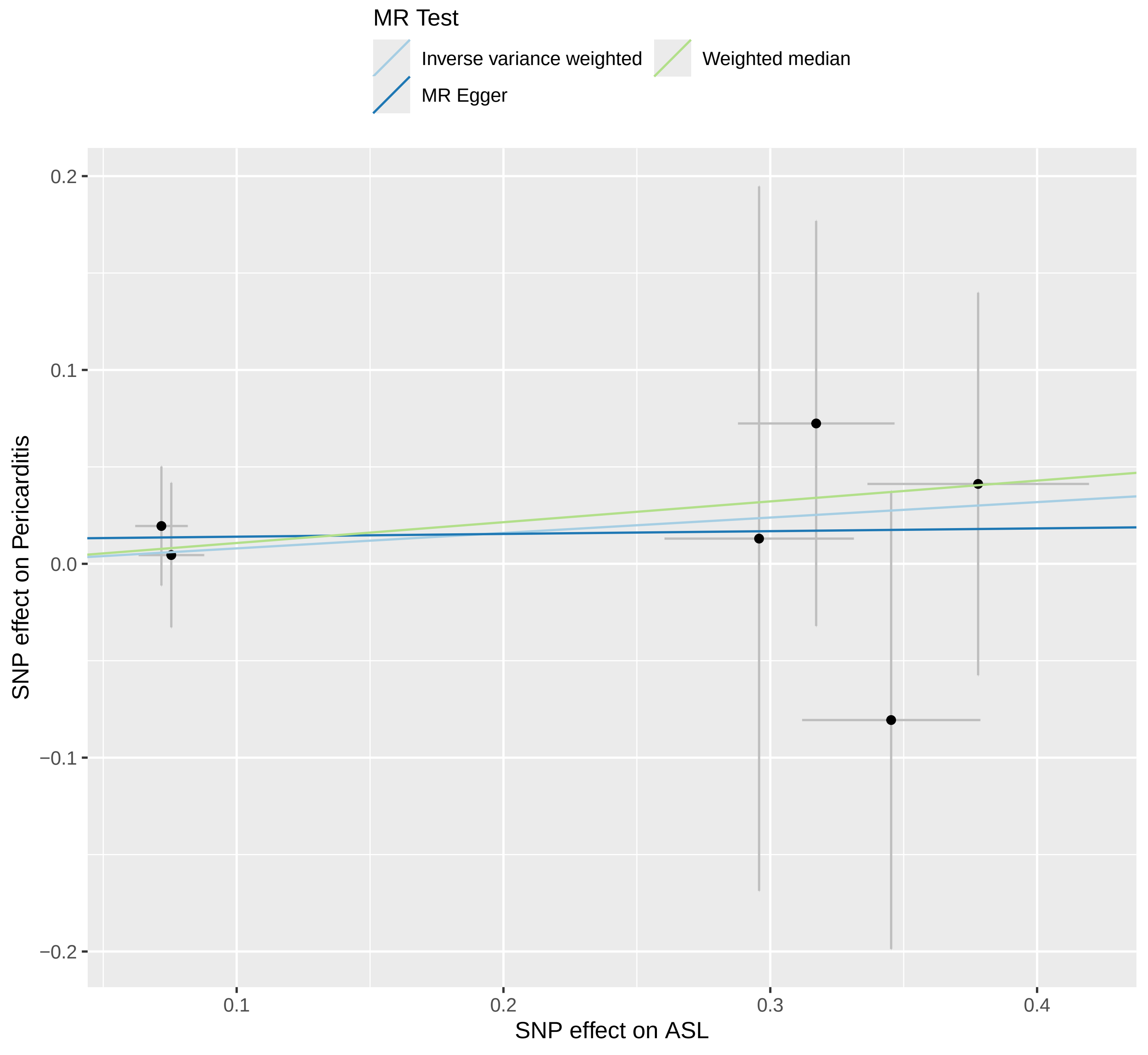

MR Test
Inverse variance weighted
Weighted median
MR Egger
0.2
0.1
0.0
−0.1
−0.2
SNP effect on Pericarditis
0.1
0.2
0.3
0.4
SNP effect on ASL

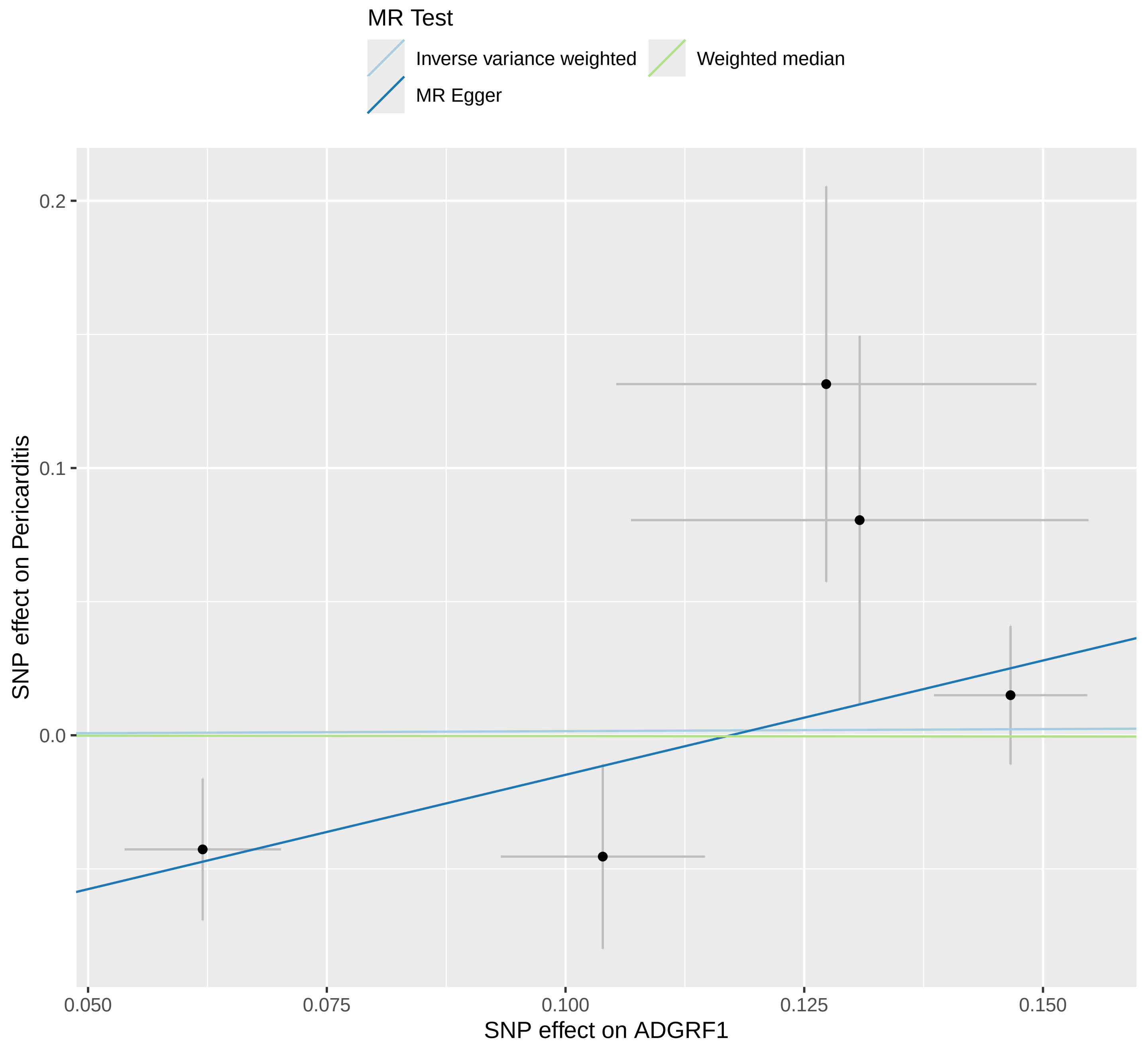
MR Test
Inverse variance weighted
MR Egger
Weighted median
0.2
0.1
0.0
SNP effect on Pericarditis
0.050
0.075
0.100
0.125
0.150
SNP effect on ADGRF1

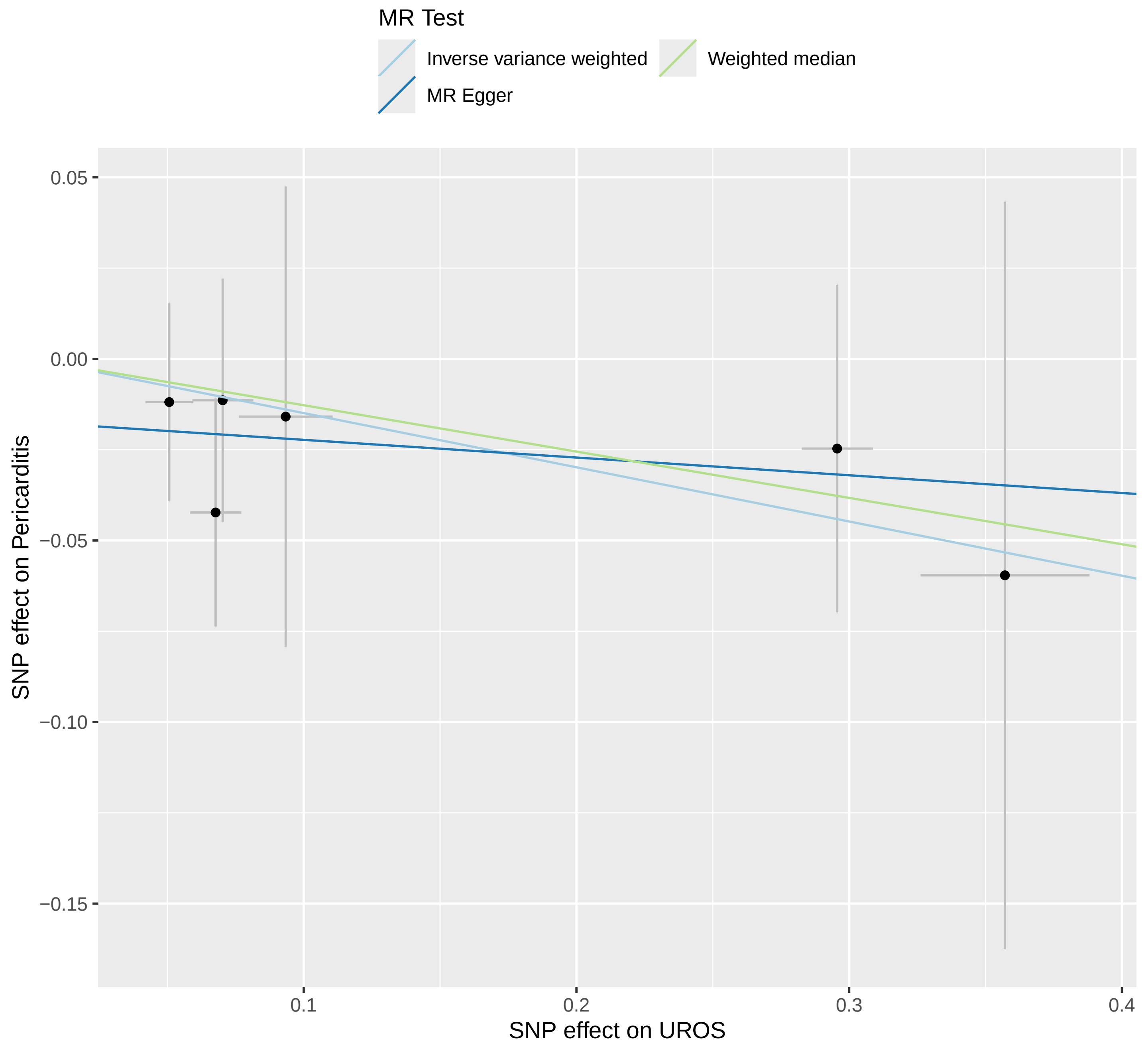
MR Test
Inverse variance weighted
Weighted median
MR Egger
0.05
0.00
−0.05
−0.10
−0.15
0.1
0.2
0.3
0.4
SNP effect on UROS
SNP effect on Pericarditis

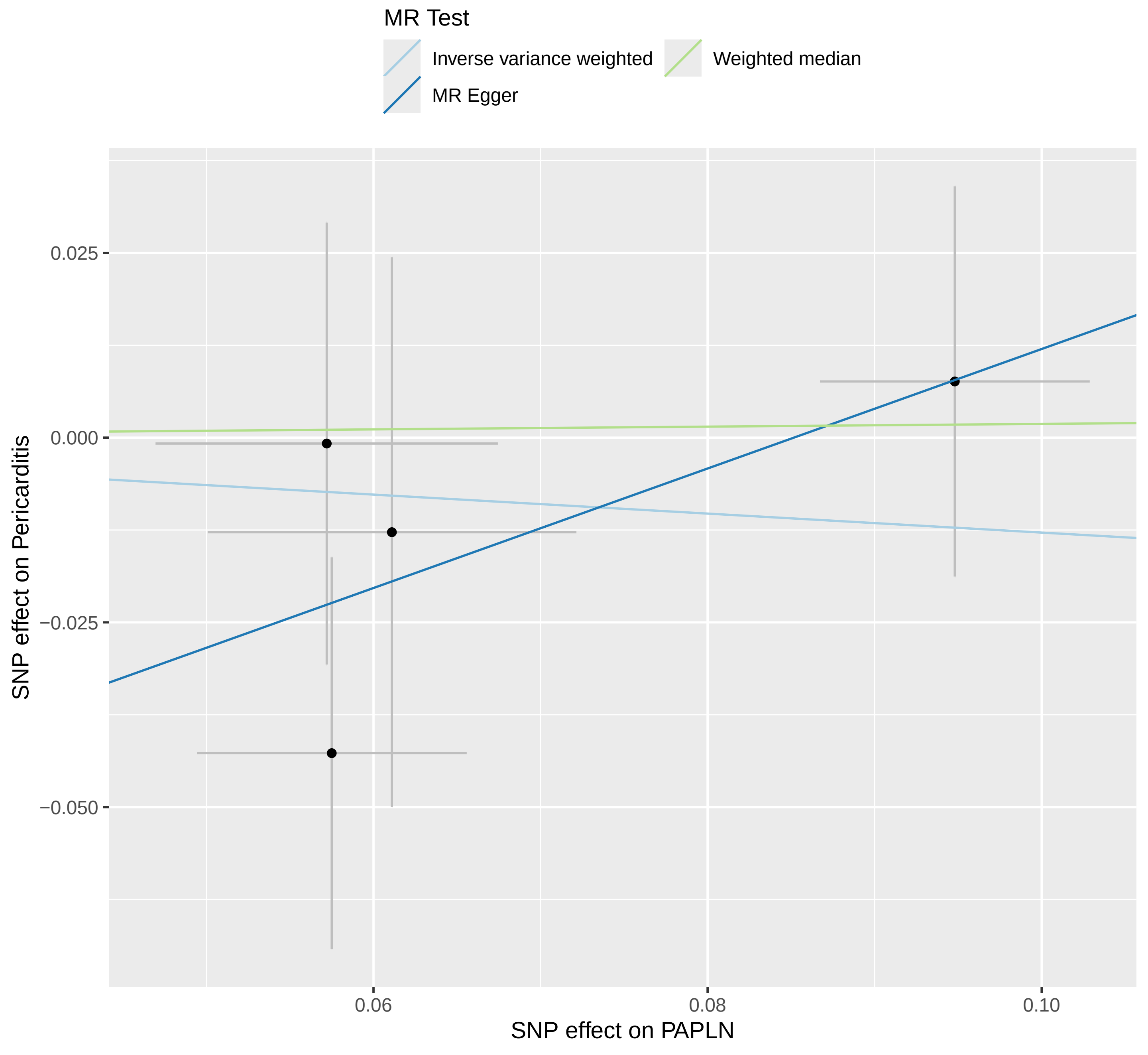

MR Test
Inverse variance weighted
MR Egger
Weighted median
0.025
0.000
−0.025
−0.050
SNP effect on Pericarditis
0.06
0.08
0.10
SNP effect on PAPLN

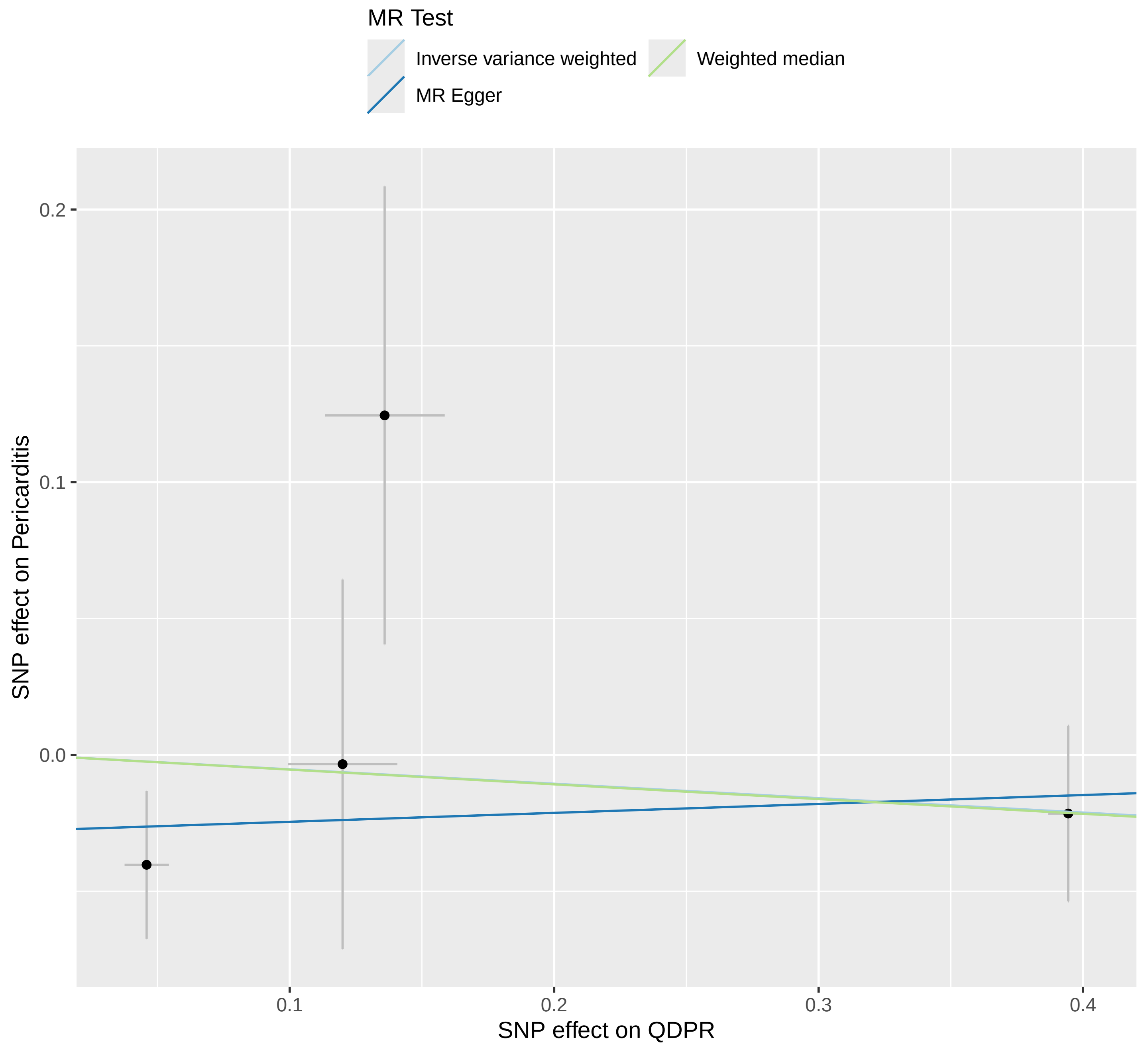
MR Test
Inverse variance weighted
Weighted median
MR Egger
0.2
0.1
0.0
SNP effect on Pericarditis
0.1
0.2
0.3
0.4
SNP effect on QDPR

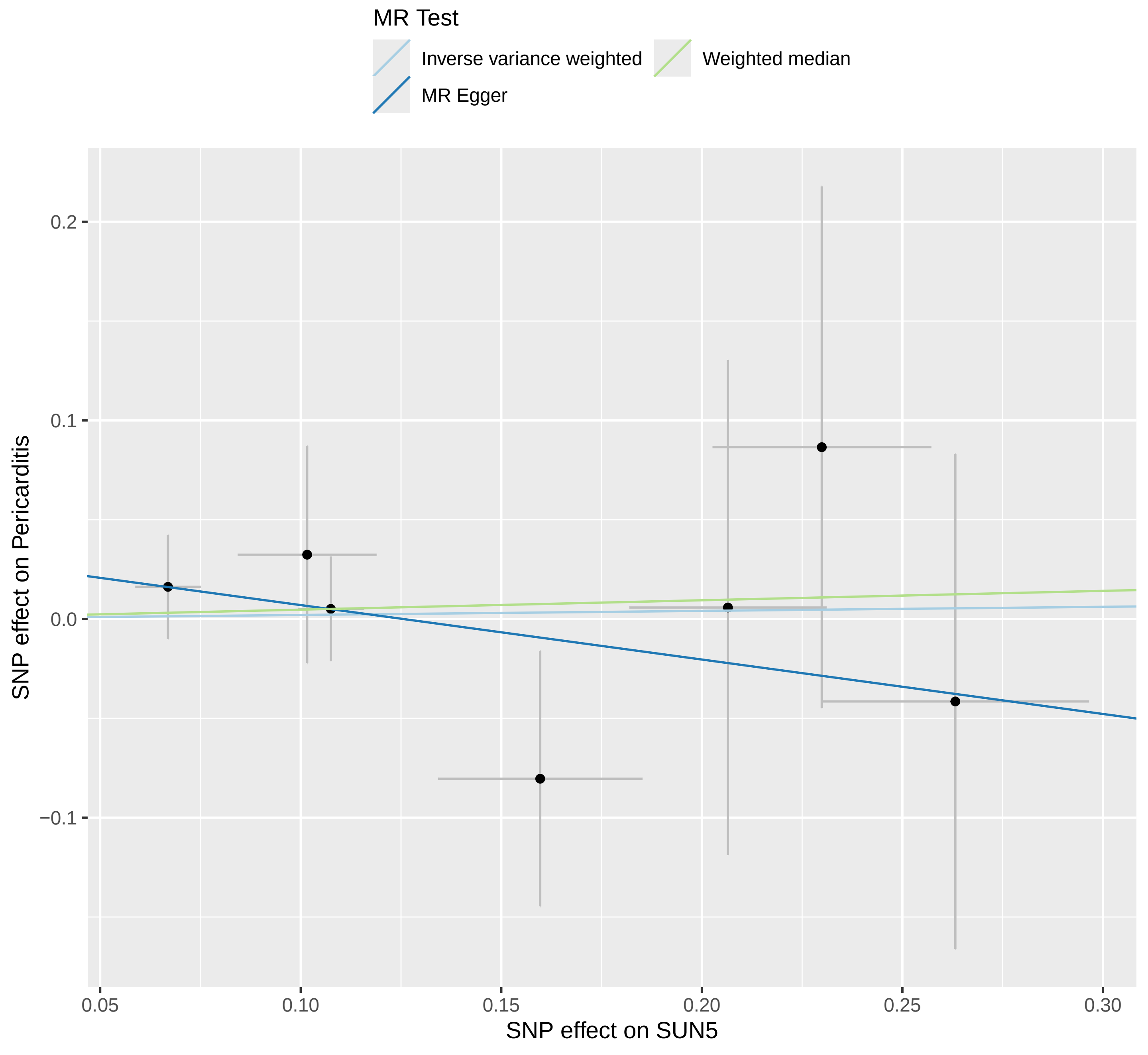

MR Test
Inverse variance weighted
Weighted median
MR Egger
0.2
0.1
0.0
−0.1
0.05
0.10
0.15
0.20
0.25
0.30
SNP effect on SUN5
SNP effect on Pericarditis

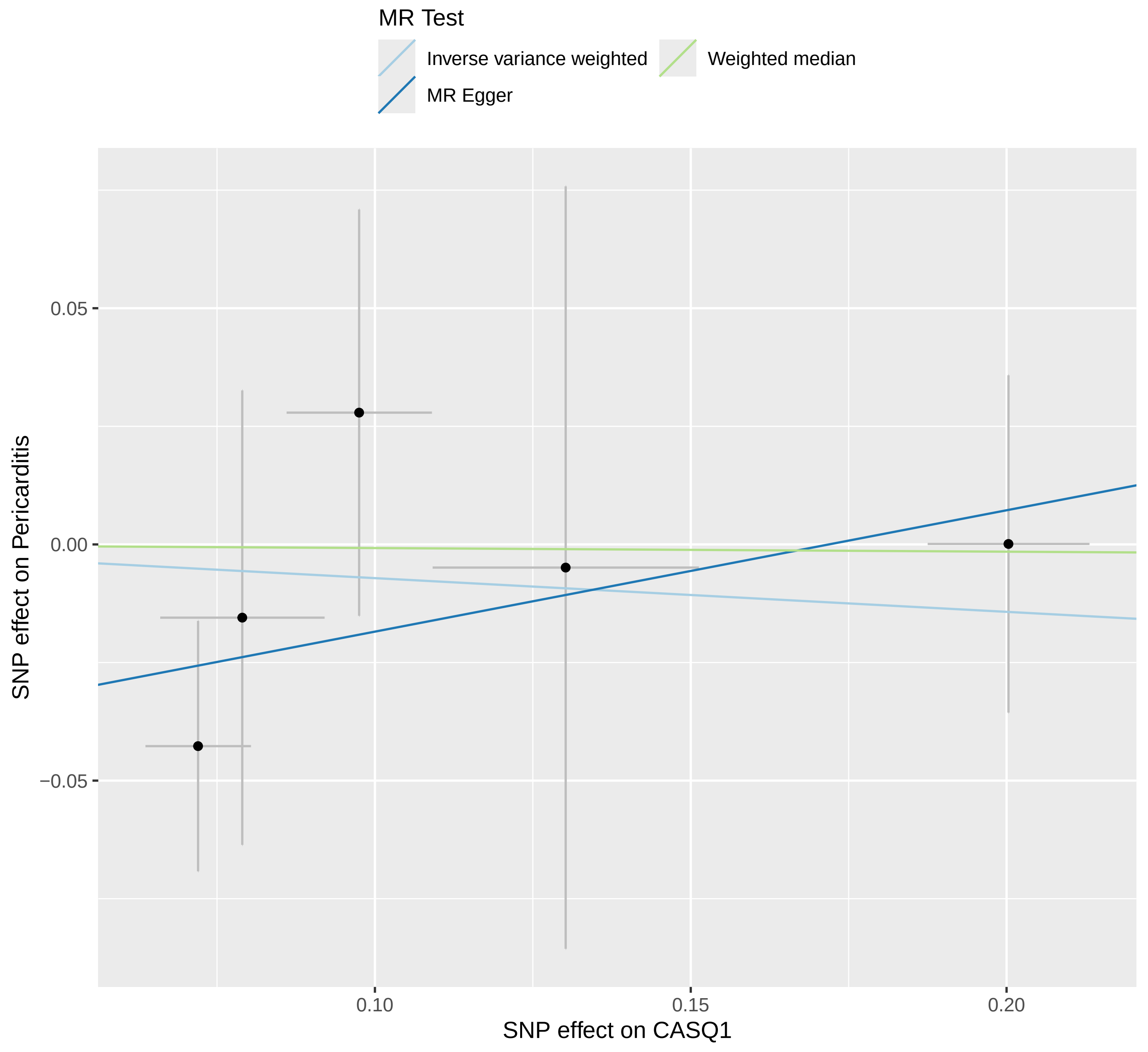
MR Test
Inverse variance weighted
MR Egger
Weighted median
SNP effect on Pericarditis
0.05
0.00
−0.05
0.10
0.15
0.20
SNP effect on CASQ1

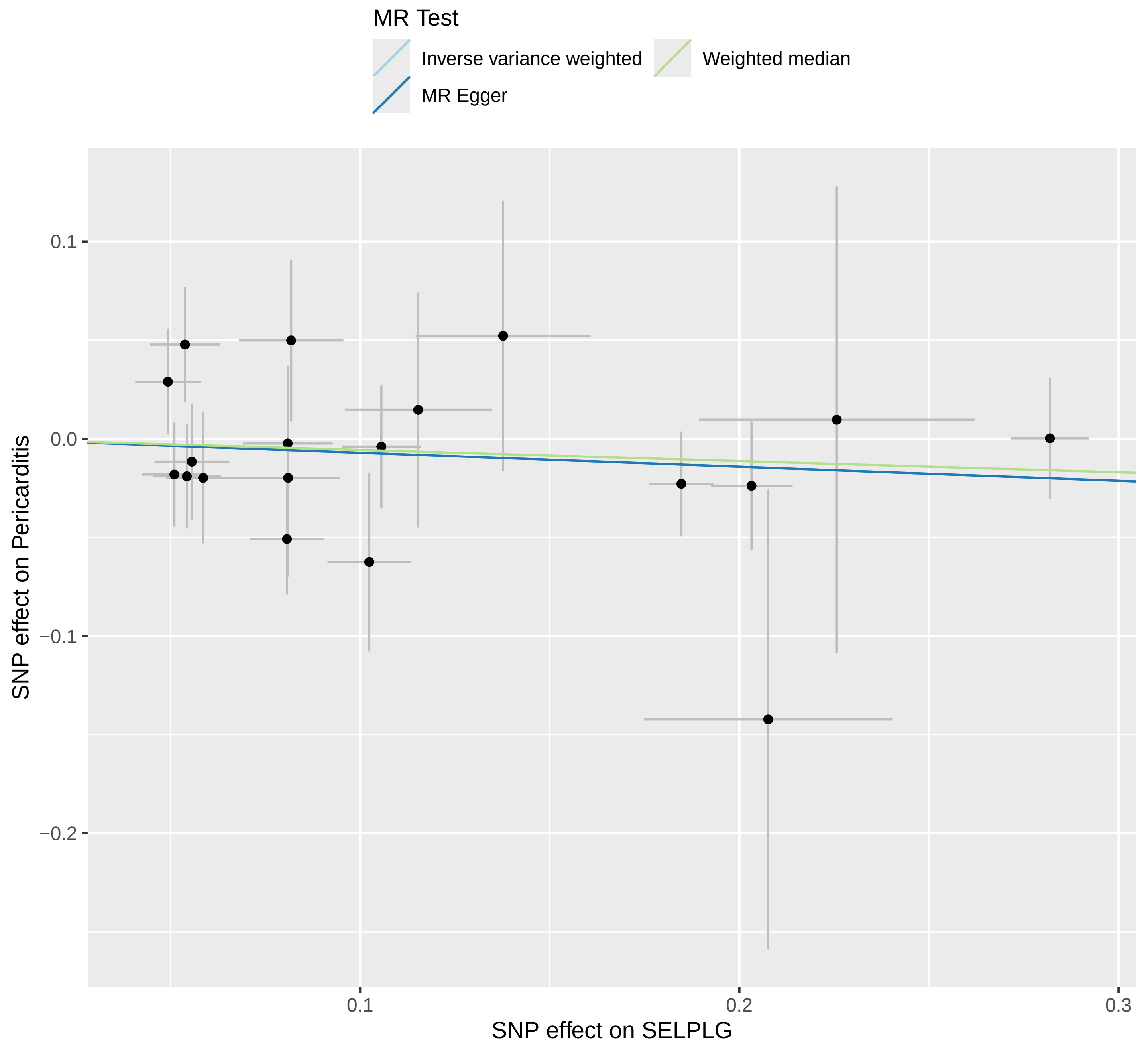
MR Test
Inverse variance weighted
Weighted median
MR Egger
0.1
0.0
−0.1
−0.2
SNP effect on Pericarditis
0.1
0.2
0.3
SNP effect on SELPLG

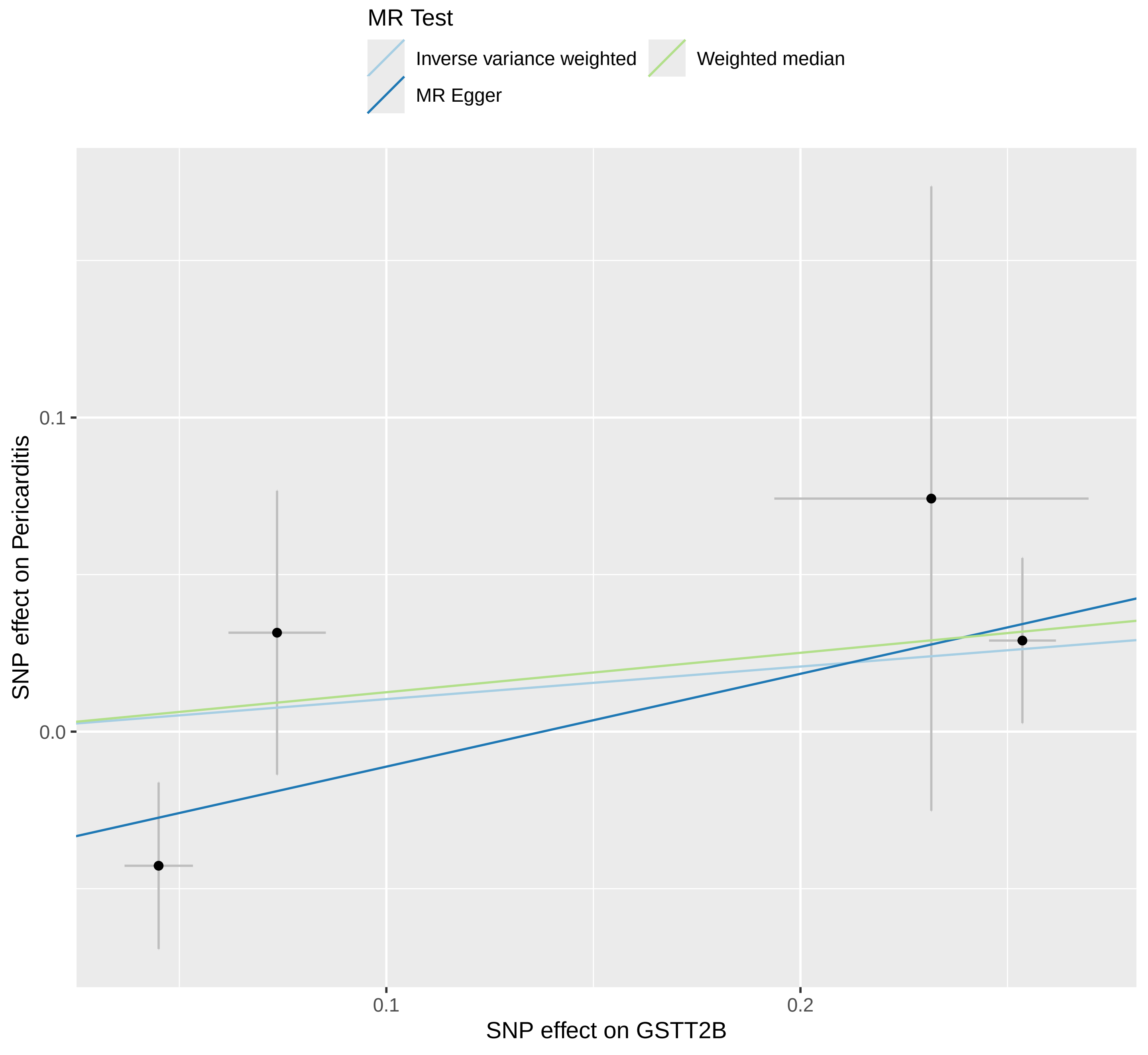
MR Test
Inverse variance weighted
MR Egger
Weighted median
0.1
0.0
0.1
0.2
SNP effect on Pericarditis
SNP effect on GSTT2B

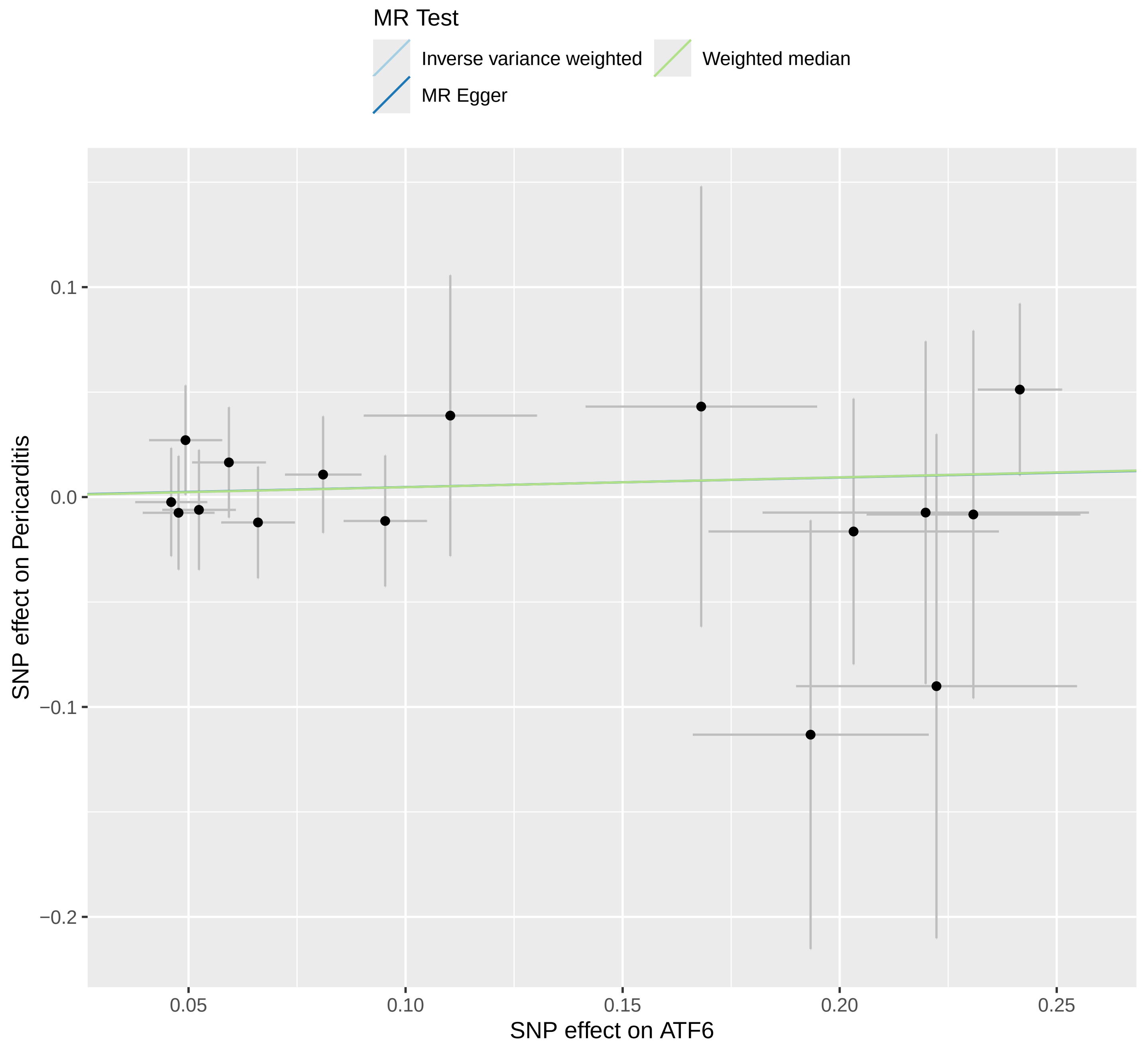

MR Test
Inverse variance weighted
Weighted median
MR Egger
0.1
0.0
−0.1
−0.2
SNP effect on Pericarditis
0.05
0.10
0.15
0.20
0.25
SNP effect on ATF6

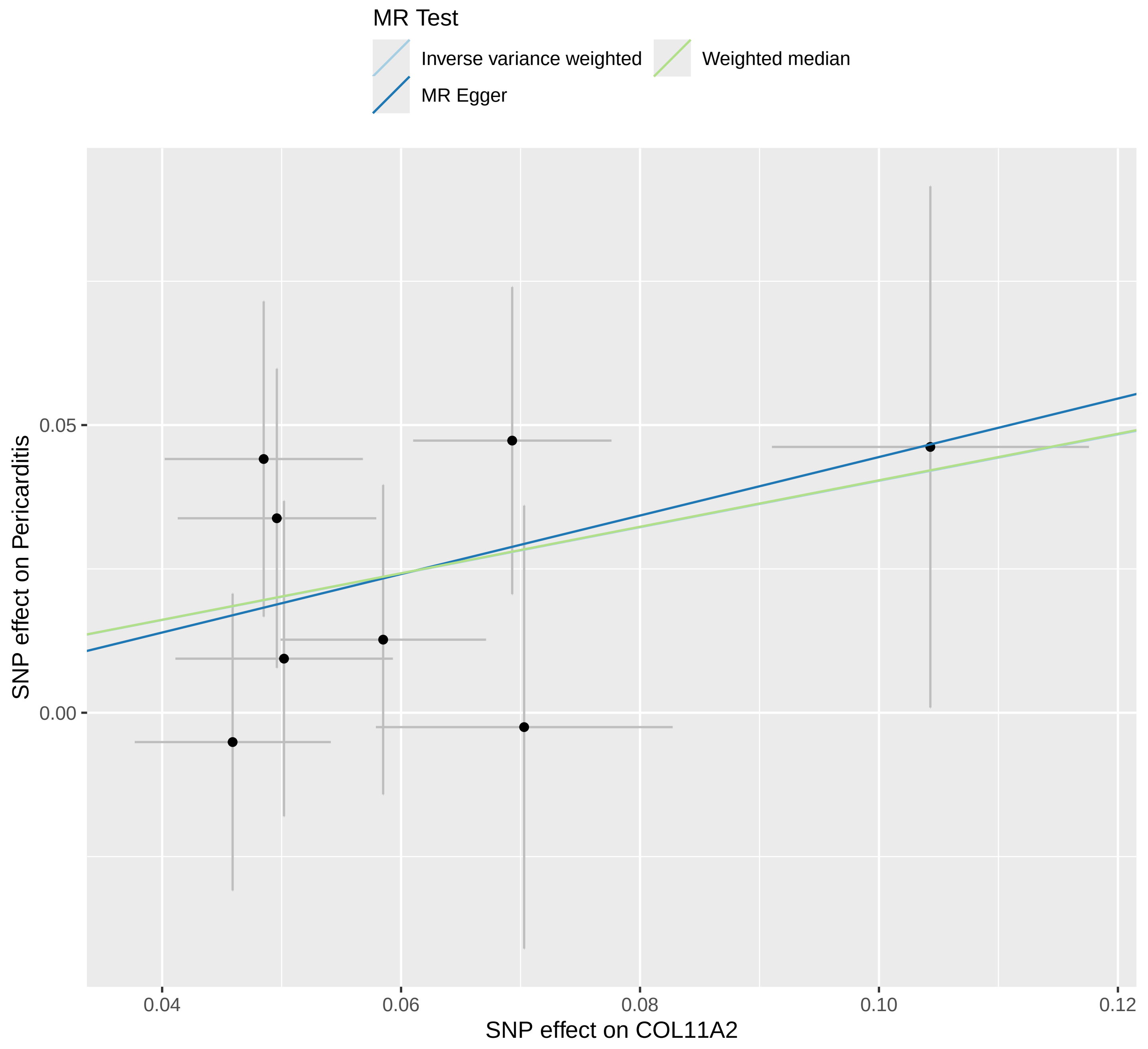

MR Test
Inverse variance weighted
Weighted median
MR Egger
SNP effect on Pericarditis
0.05
0.00
0.04
0.06
0.08
0.10
0.12
SNP effect on COL11A2

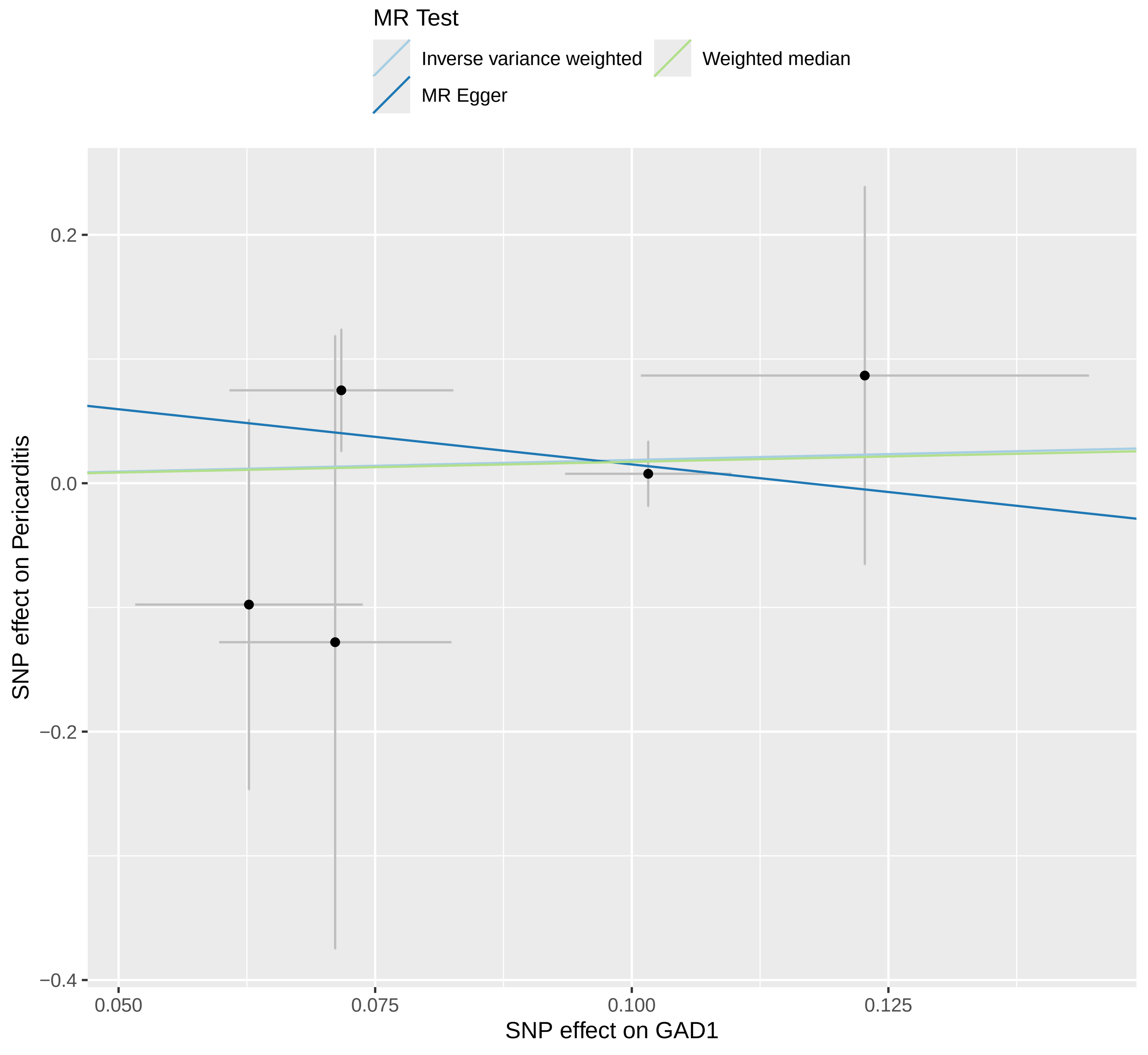

MR Test
Inverse variance weighted
Weighted median
MR Egger
0.2
0.0
−0.2
−0.4
SNP effect on Pericarditis
0.050
0.075
0.100
0.125
SNP effect on GAD1

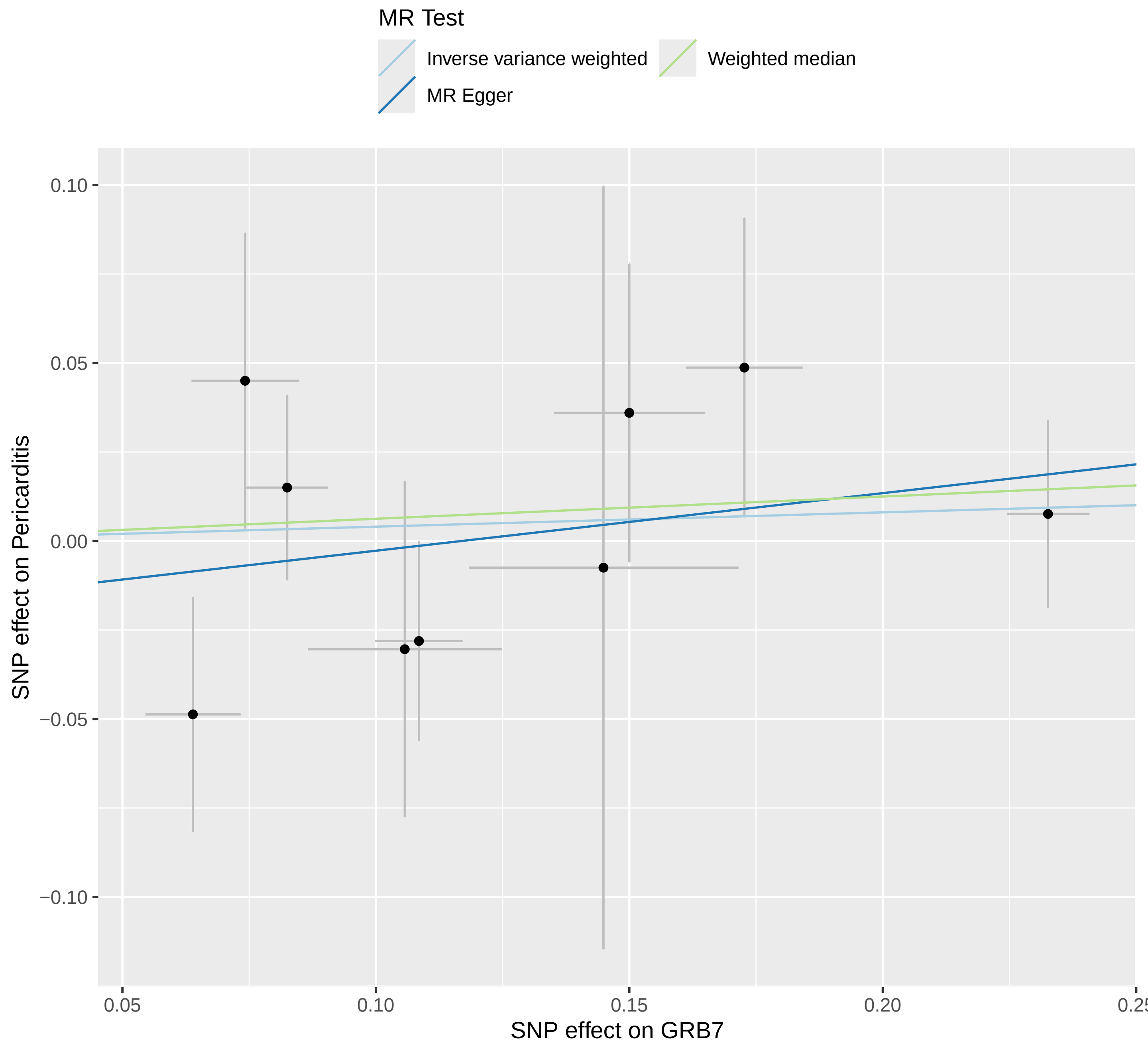

MR Test
Inverse variance weighted
Weighted median
MR Egger
SNP effect on Pericarditis
0.10
0.05
0.00
−0.05
−0.10
0.05
0.10
0.15
0.20
SNP effect on GRB7

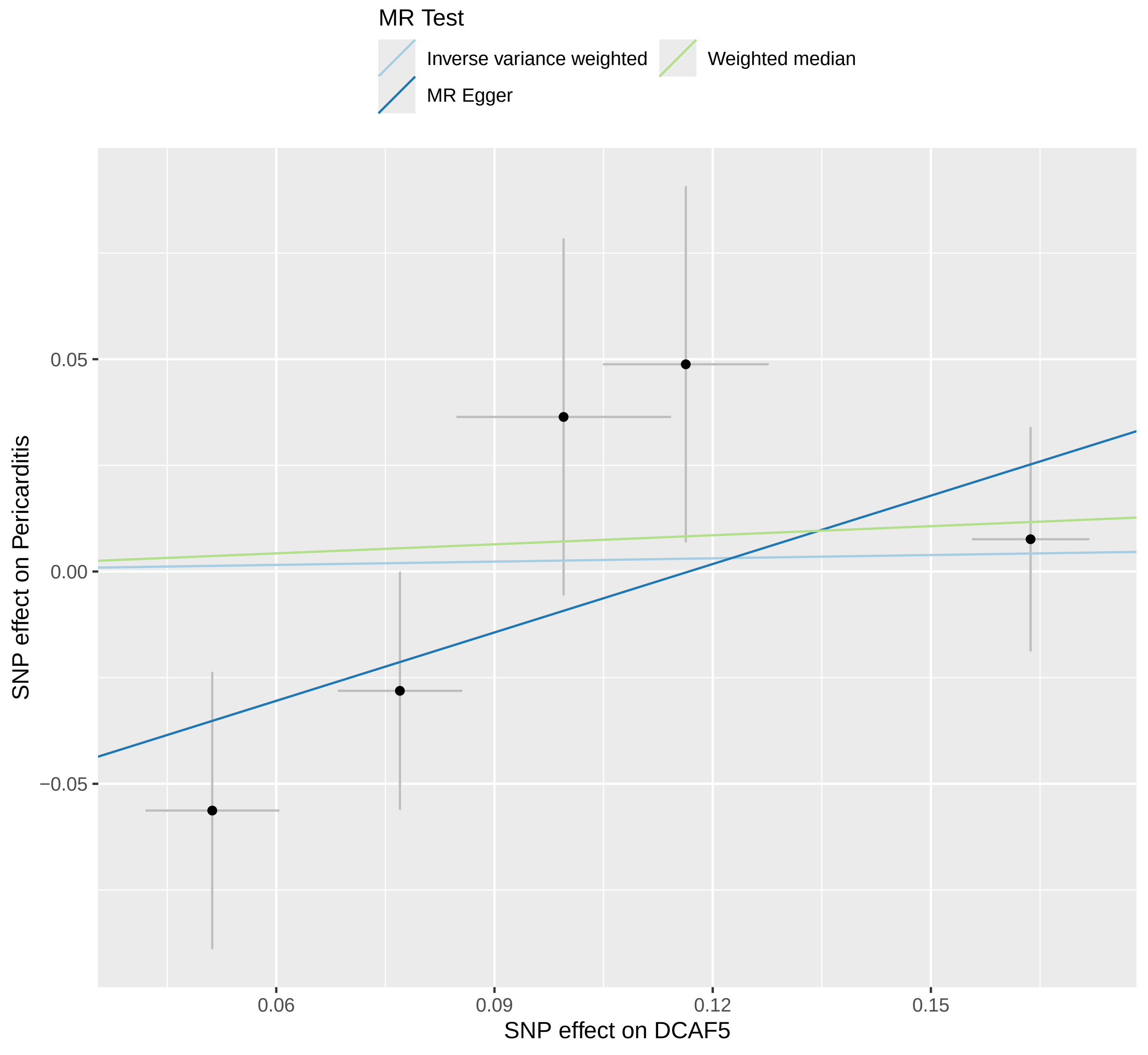
MR Test
Inverse variance weighted
Weighted median
MR Egger
0.05
0.00
−0.05
0.06
0.09
0.12
0.15
SNP effect on Pericarditis
SNP effect on DCAF5

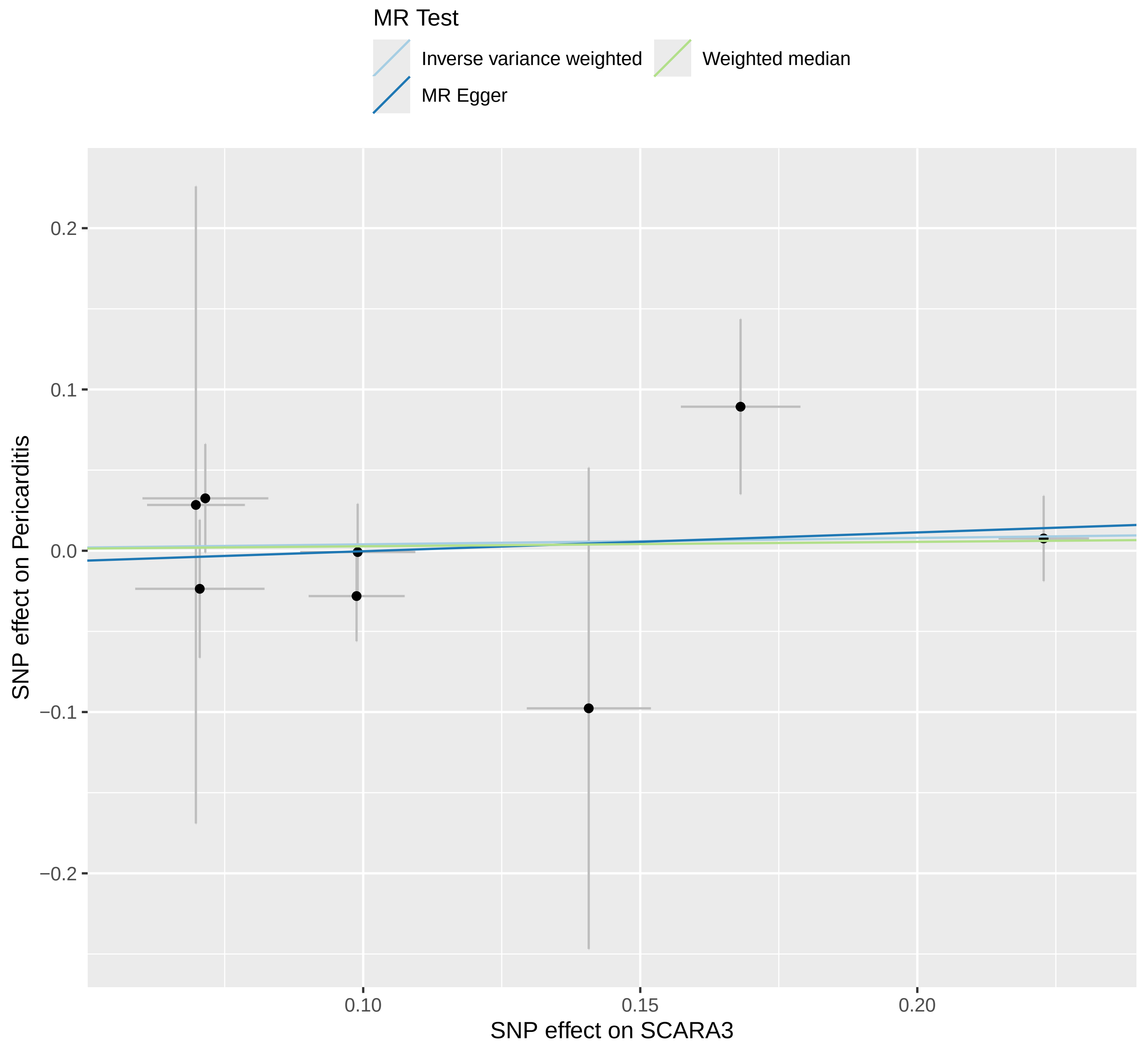

MR Test
Inverse variance weighted
Weighted median
MR Egger
0.2
0.1
0.0
−0.1
−0.2
SNP effect on Pericarditis
0.10
0.15
0.20
SNP effect on SCARA3

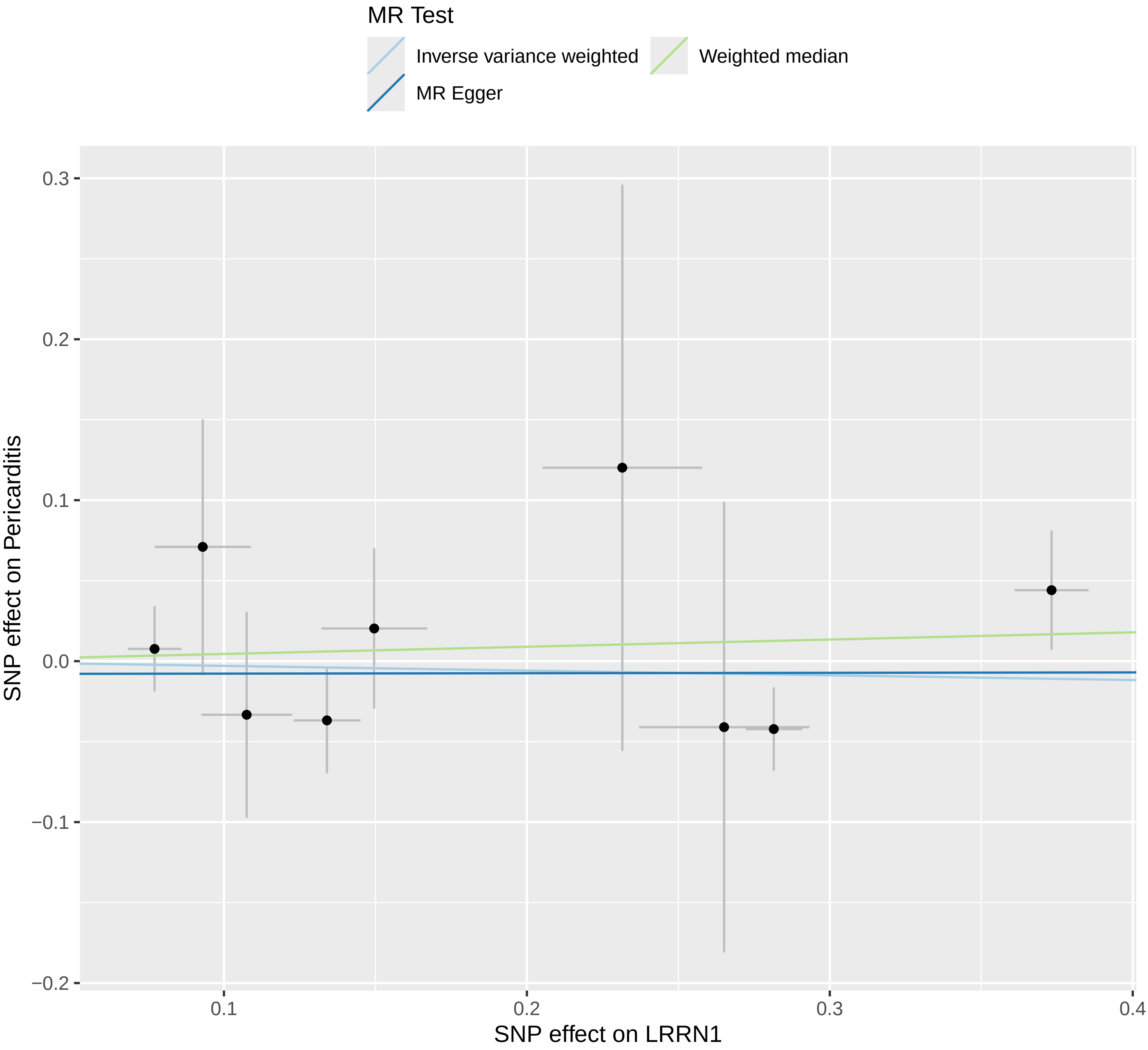

MR Test
Inverse variance weighted
Weighted median
MR Egger
0.3
0.2
0.1
0.0
−0.1
−0.2
SNP effect on Pericarditis
0.1
0.2
0.3
0.4
SNP effect on LRRN1

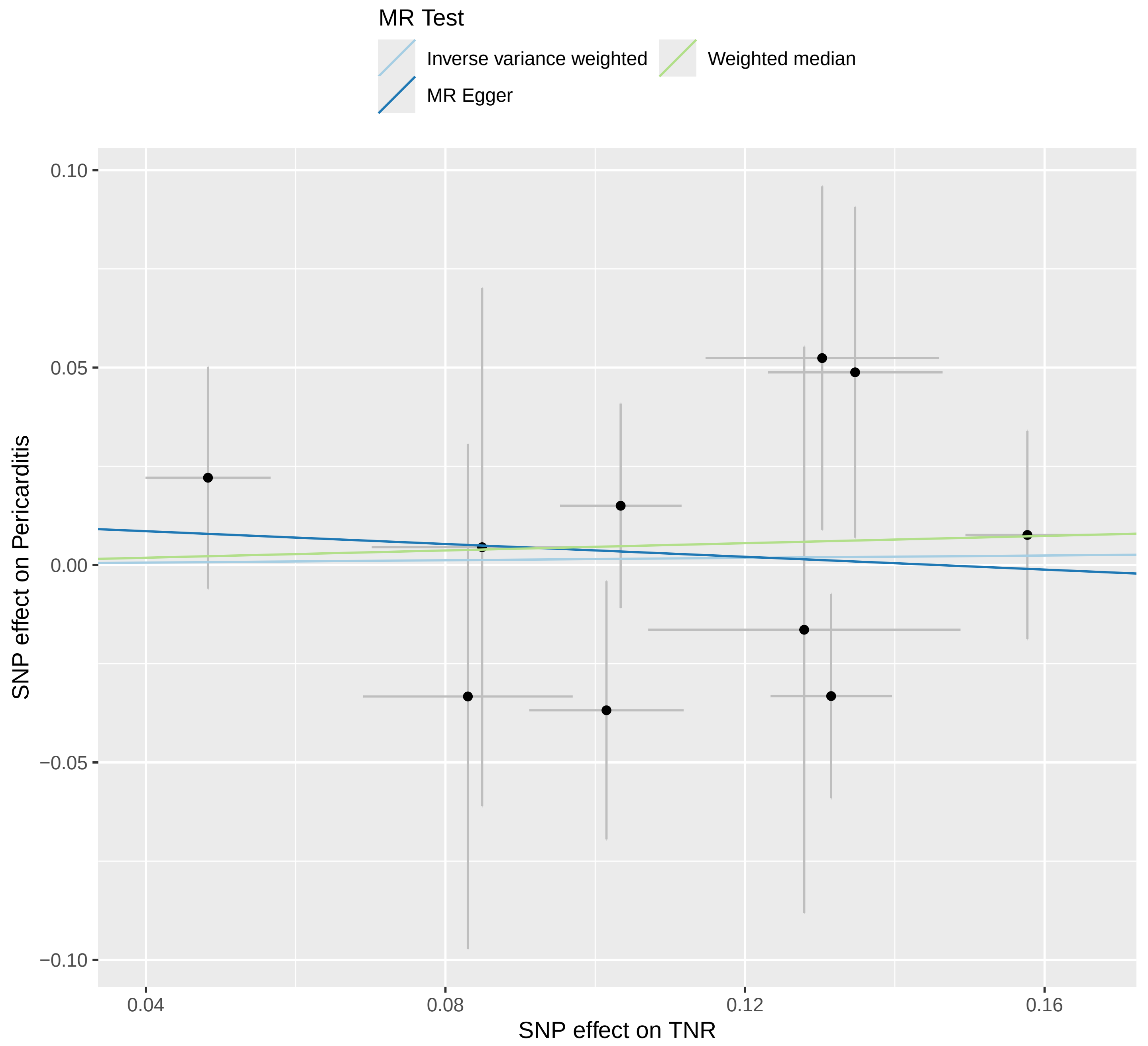
MR Test
Inverse variance weighted
MR Egger
Weighted median
0.10
0.05
0.00
−0.05
−0.10
SNP effect on Pericarditis
0.04
0.08
0.12
0.16
SNP effect on TNR

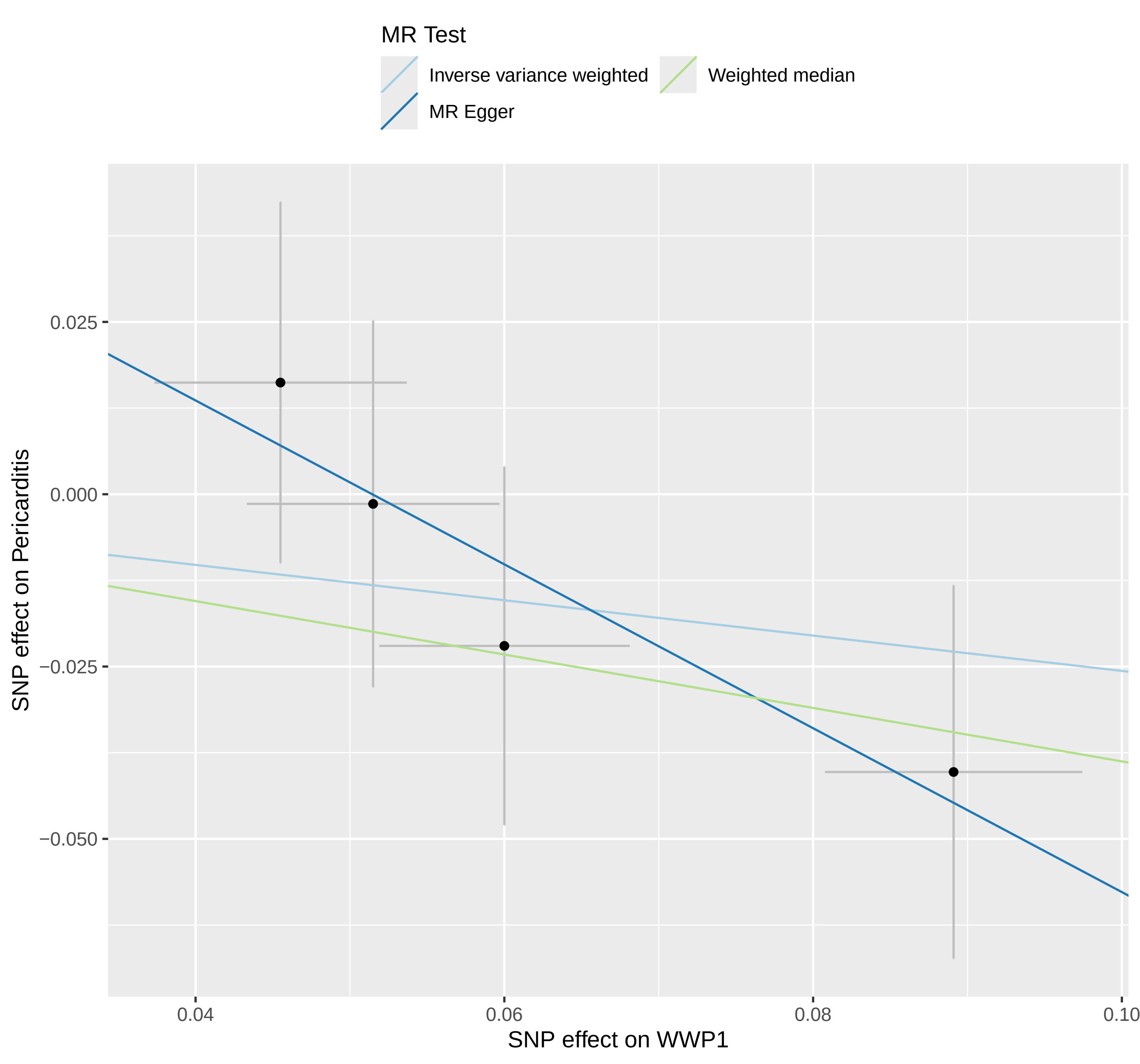
MR Test
Inverse variance weighted
Weighted median
MR Egger
0.025
0.000
−0.025
−0.050
SNP effect on Pericarditis
0.04
0.06
0.08
0.10
SNP effect on WWP1

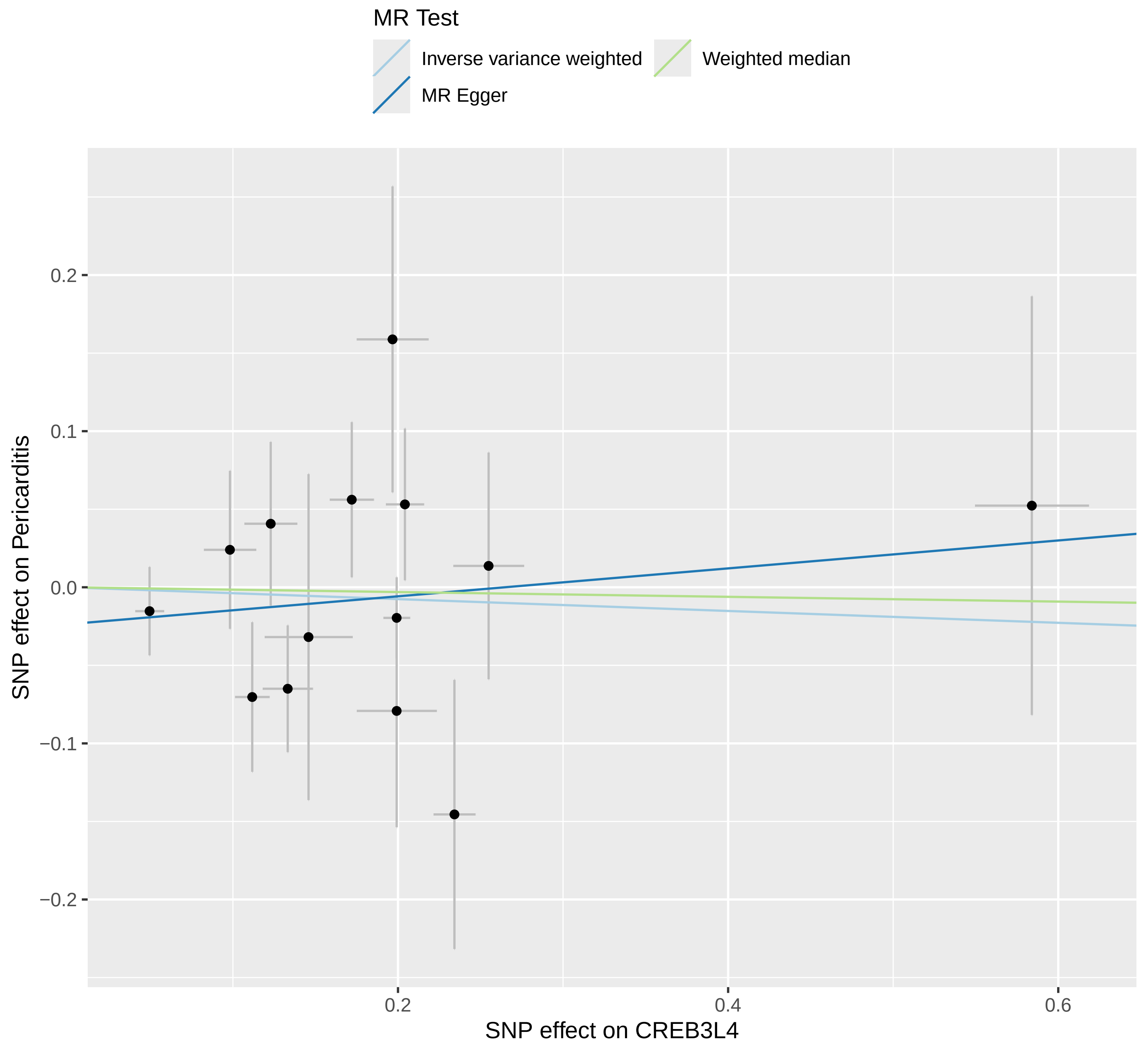
MR Test
Inverse variance weighted
Weighted median
MR Egger
0.2
0.1
0.0
−0.1
−0.2
SNP effect on Pericarditis
0.2
0.4
0.6
SNP effect on CREB3L4

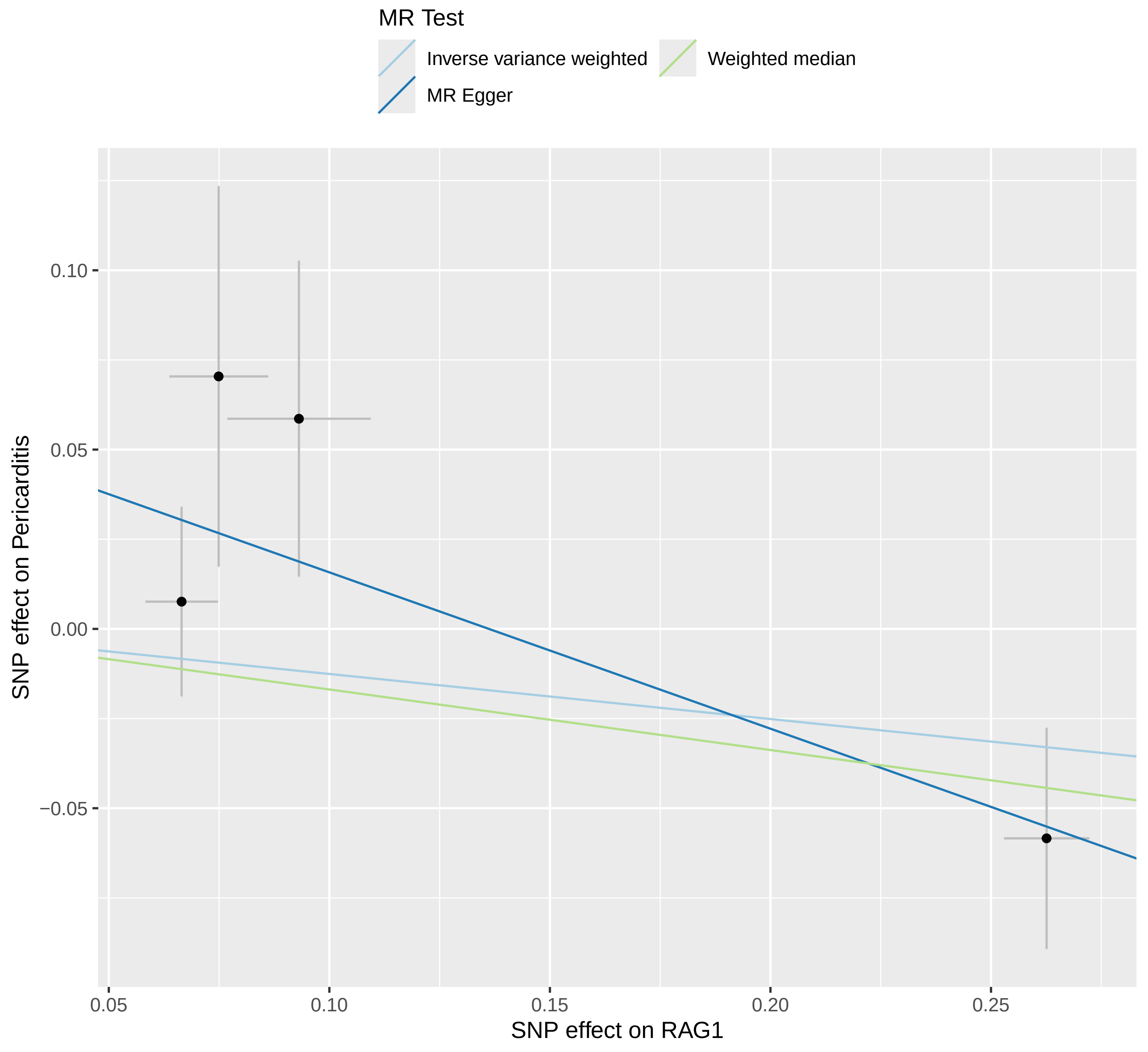
MR Test
Inverse variance weighted
Weighted median
MR Egger
SNP effect on Pericarditis
0.10
0.05
0.00
−0.05
0.05
0.10
0.15
0.20
0.25
SNP effect on RAG1

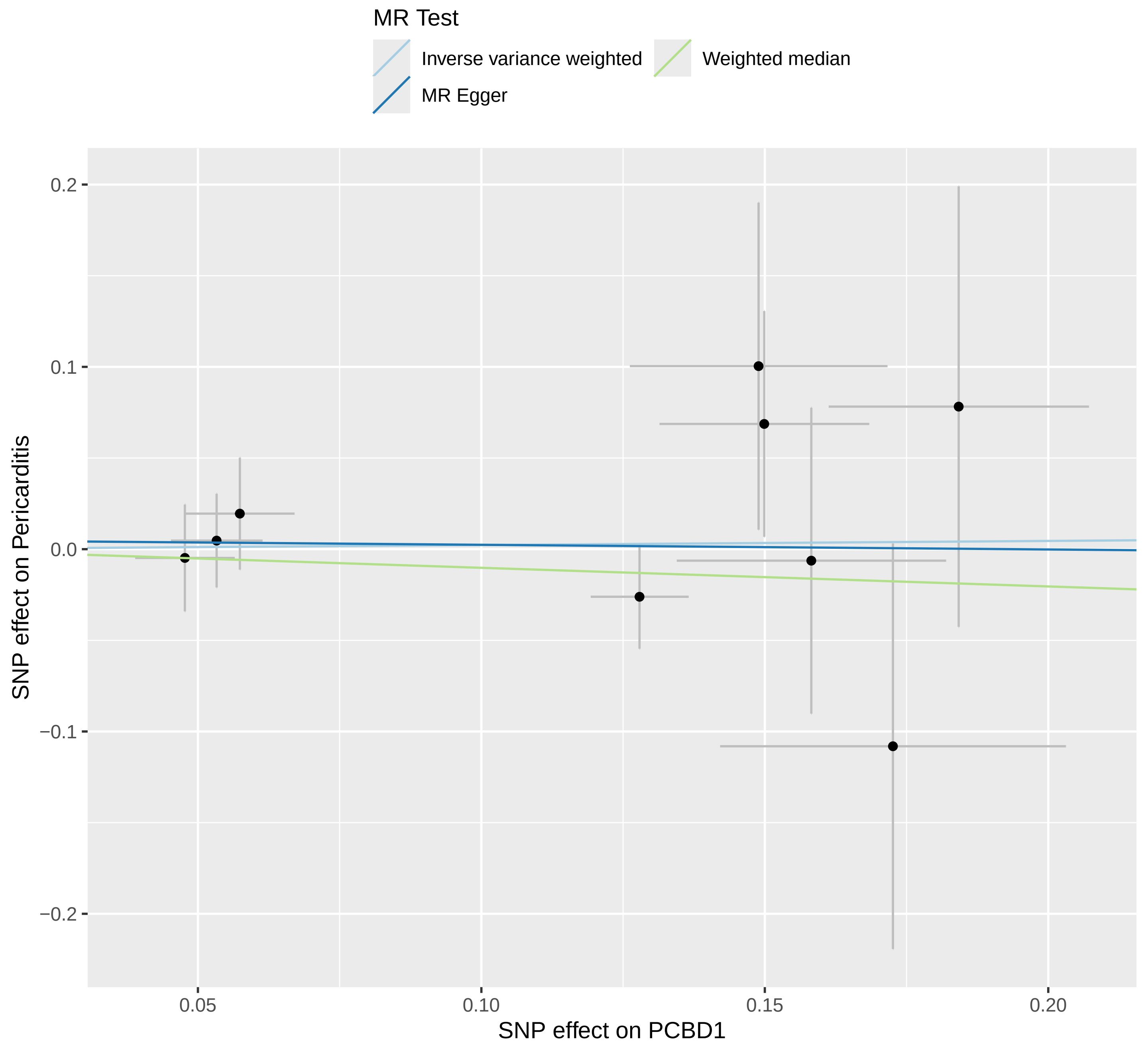

MR Test
Inverse variance weighted
MR Egger
Weighted median
0.2
0.1
0.0
−0.1
−0.2
SNP effect on Pericarditis
0.05
0.10
0.15
0.20
SNP effect on PCBD1

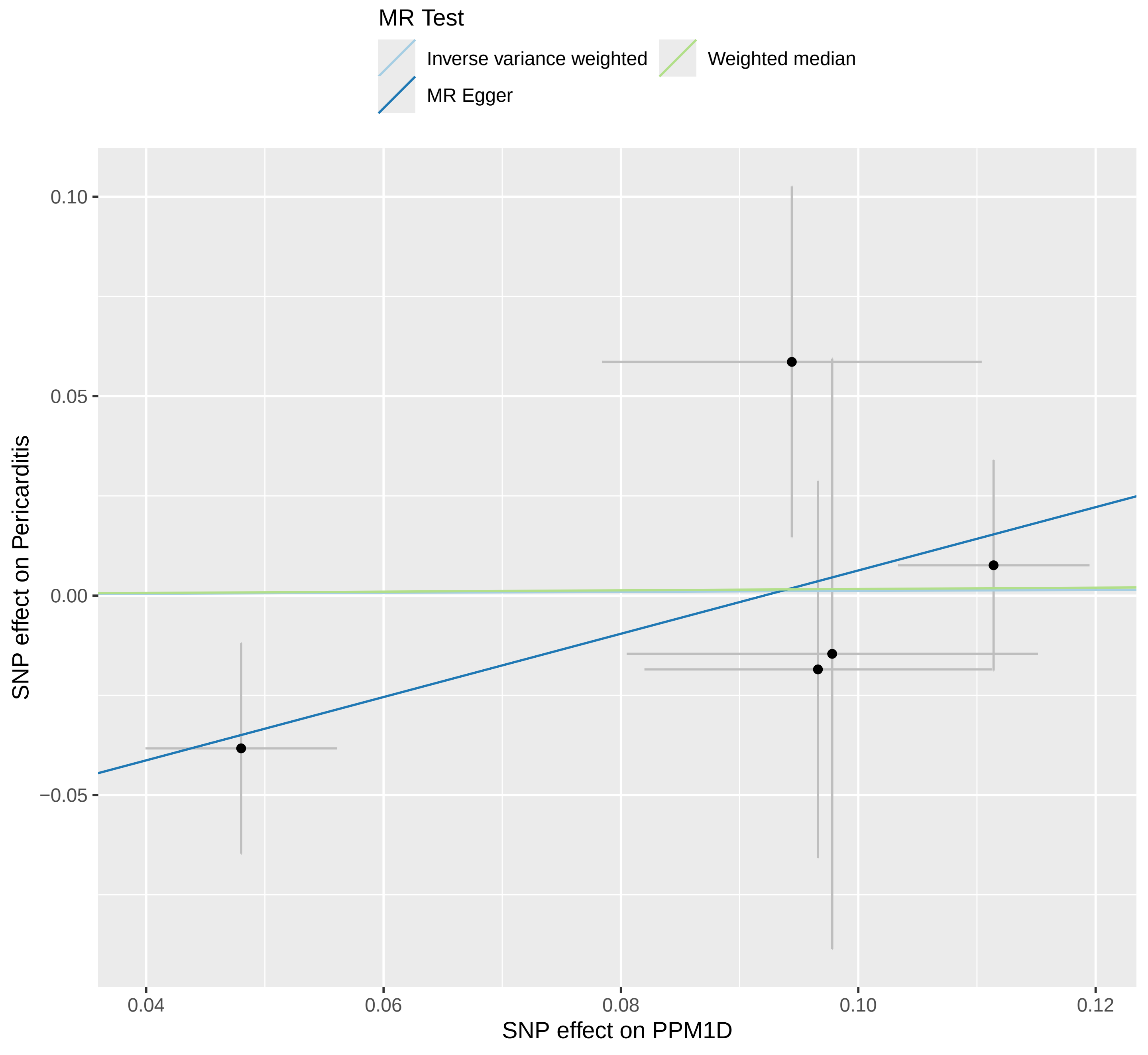
MR Test
Inverse variance weighted
Weighted median
MR Egger
SNP effect on Pericarditis
0.10
0.05
0.00
−0.05
0.04
0.06
0.08
0.10
0.12
SNP effect on PPM1D

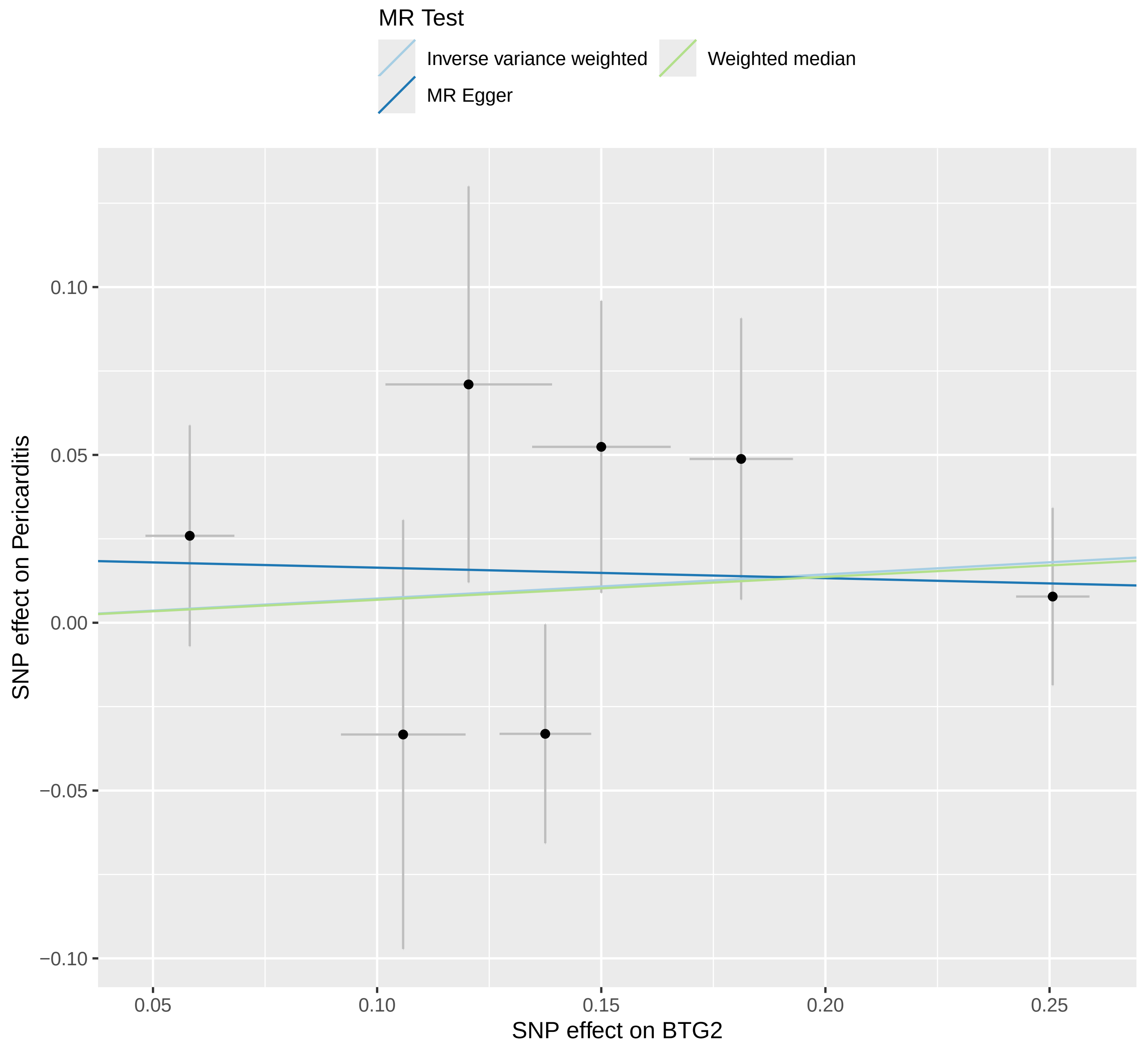

MR Test
Inverse variance weighted
MR Egger
Weighted median
SNP effect on Pericarditis
0.10
0.05
0.00
−0.05
−0.10
0.05
0.10
0.15
0.20
0.25
SNP effect on BTG2

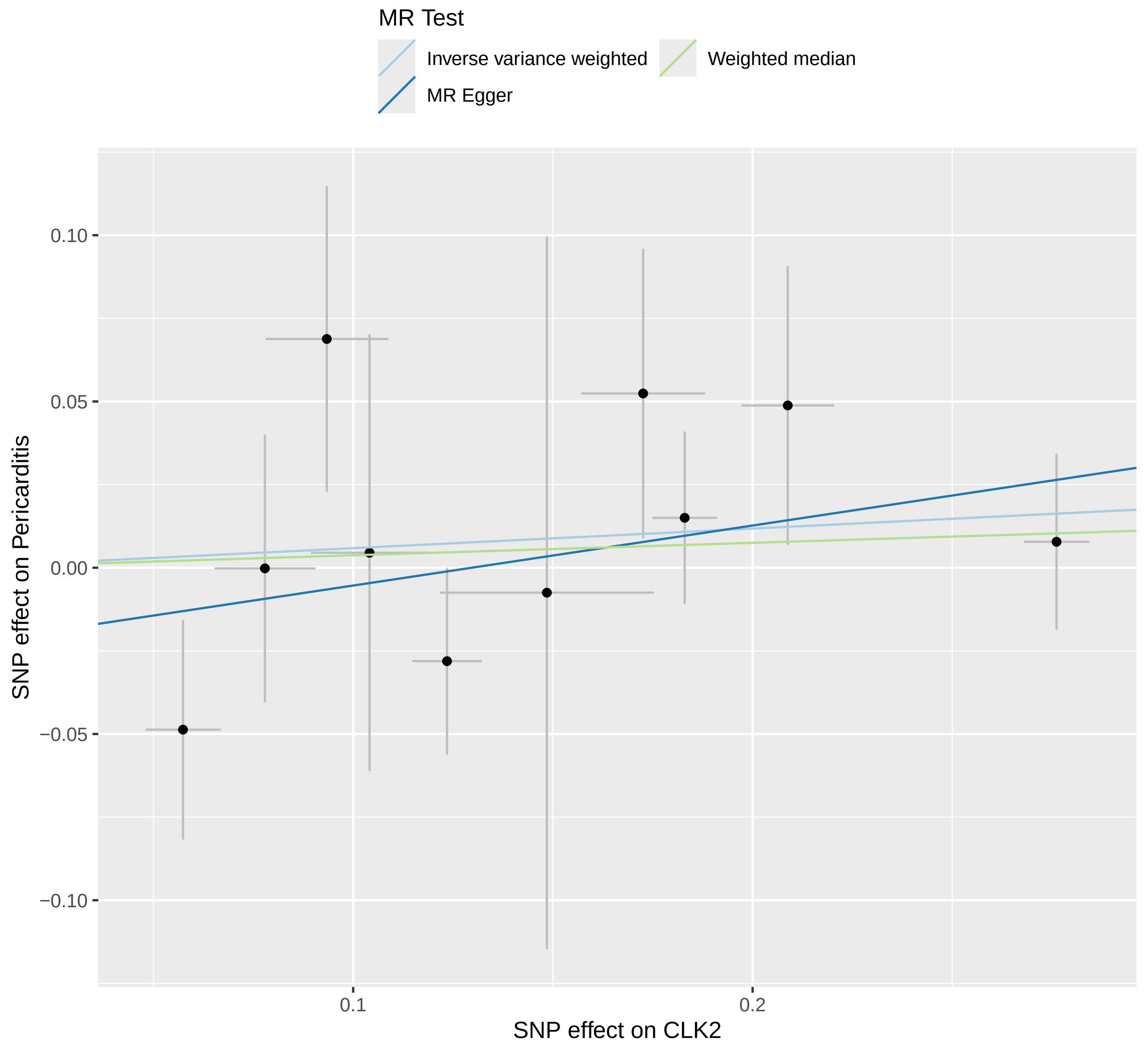
MR Test
Inverse variance weighted
Weighted median
MR Egger
0.10
0.05
0.00
−0.05
−0.10
SNP effect on Pericarditis
0.1
0.2
SNP effect on CLK2

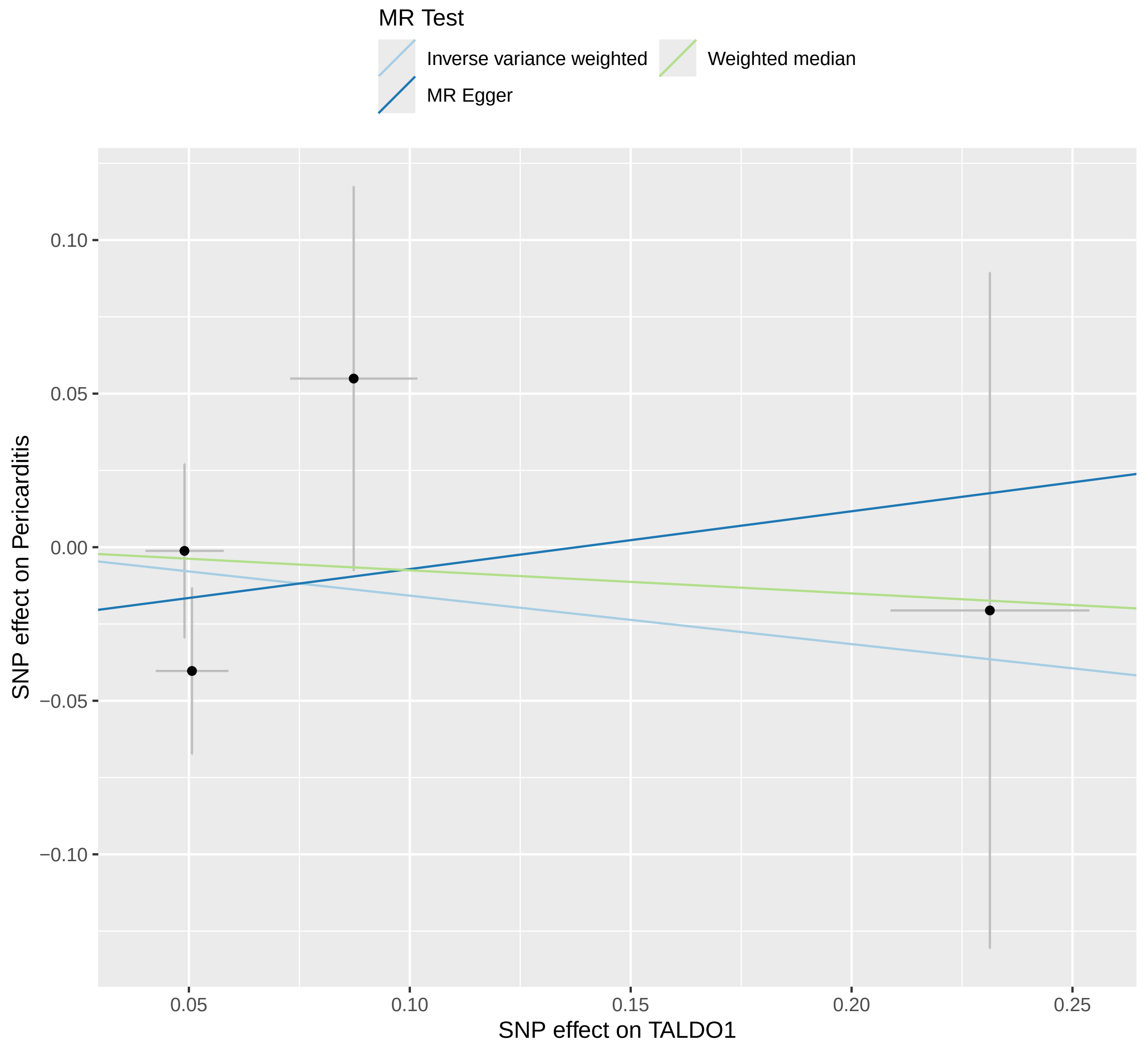
MR Test
Inverse variance weighted
MR Egger
Weighted median
0.10
0.05
0.00
−0.05
−0.10
SNP effect on Pericarditis
0.05
0.10
0.15
0.20
0.25
SNP effect on TALDO1

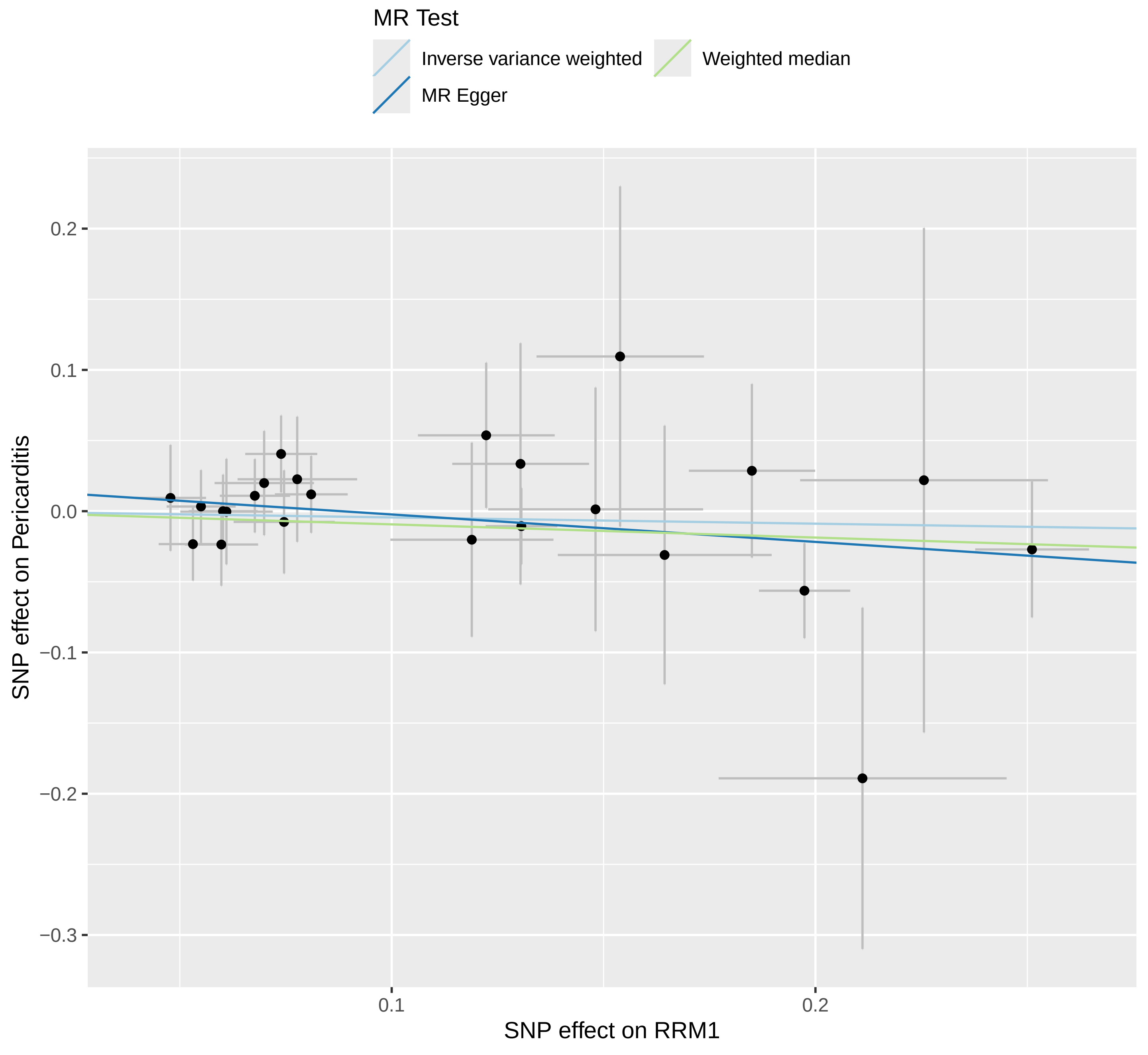
MR Test
Inverse variance weighted
Weighted median
MR Egger
0.2
0.1
0.0
−0.1
−0.2
−0.3
SNP effect on Pericarditis
0.1
0.2
SNP effect on RRM1

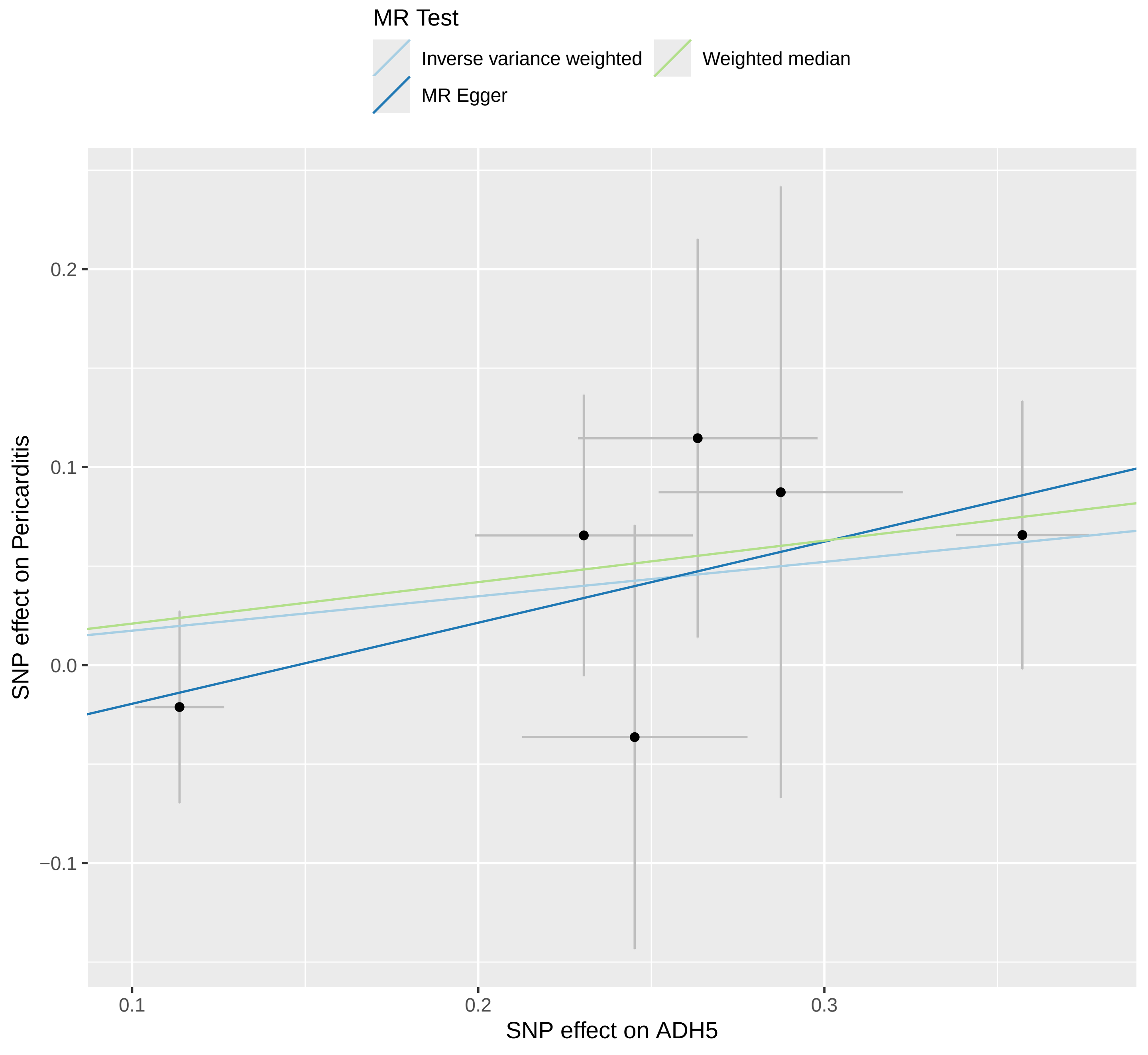
MR Test
Inverse variance weighted
Weighted median
MR Egger
SNP effect on Pericarditis
0.2
0.1
0.0
−0.1
SNP effect on ADH5
0.1
0.2
0.3

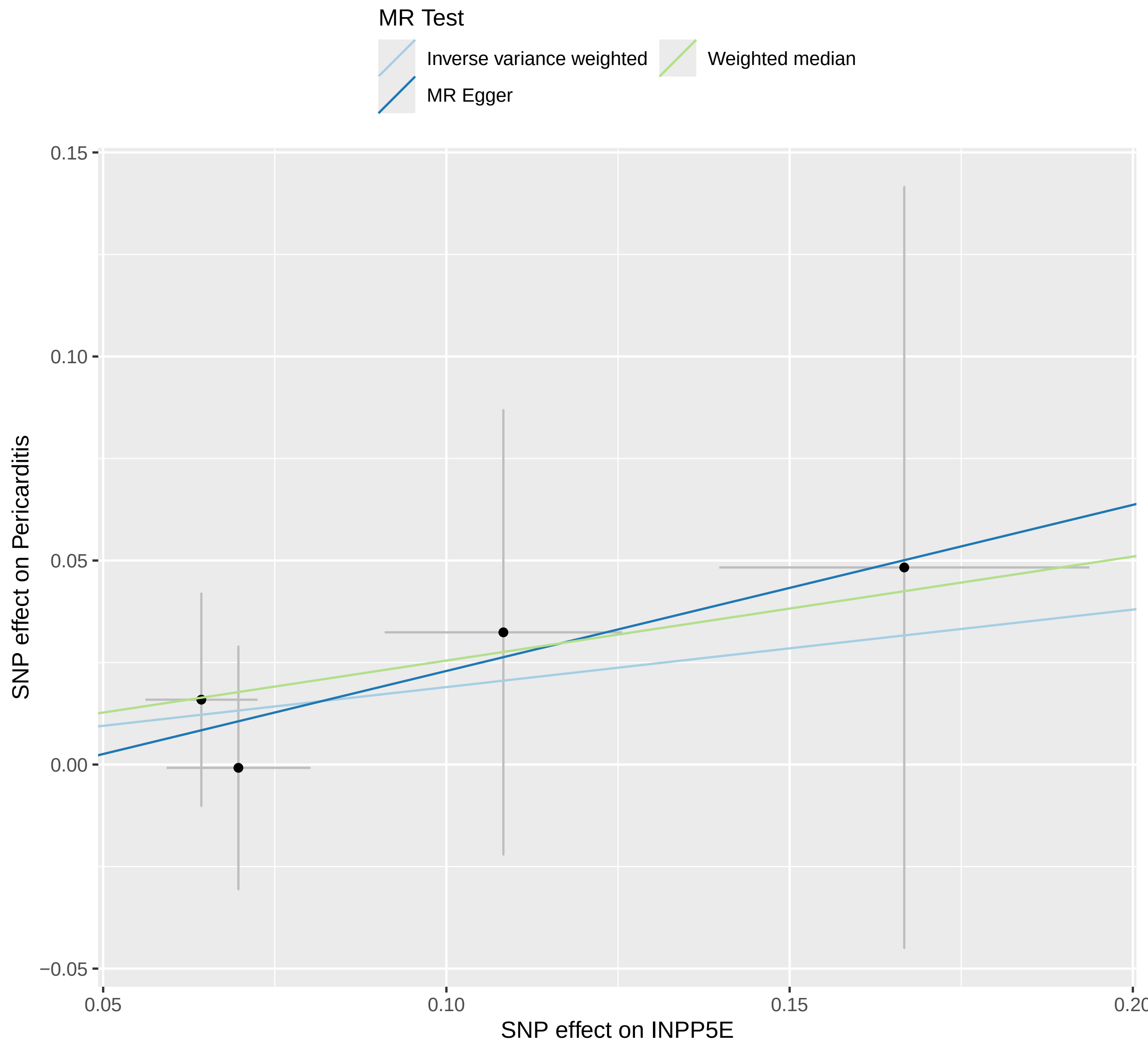

MR Test
Inverse variance weighted
Weighted median
MR Egger
0.15
0.10
0.05
0.00
−0.05
0.05
0.10
0.15
0.20
SNP effect on Pericarditis
SNP effect on INPP5E

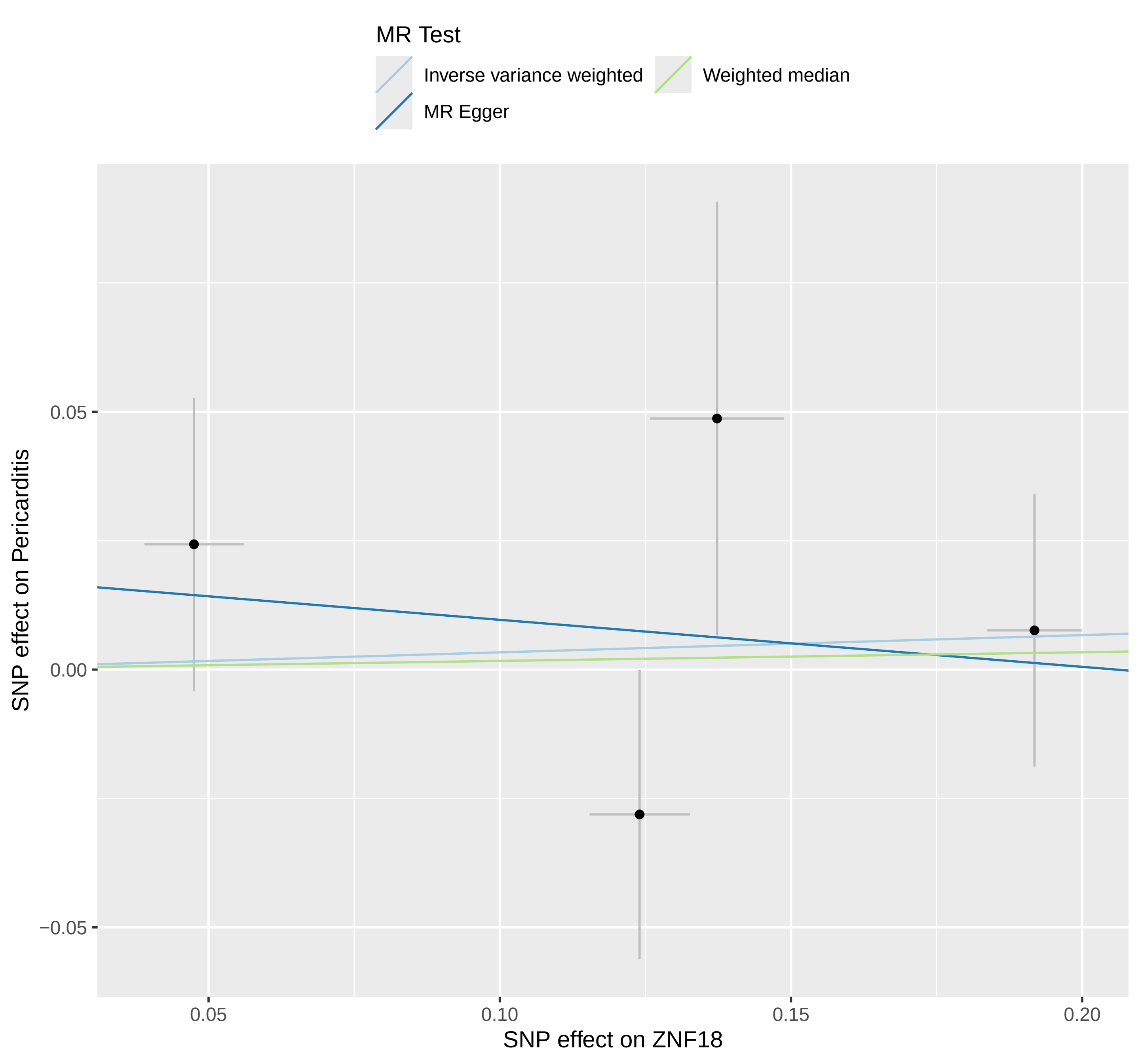

MR Test
Inverse variance weighted
Weighted median
MR Egger
SNP effect on Pericarditis
0.05
0.00
−0.05
0.05
0.10
0.15
0.20
SNP effect on ZNF18

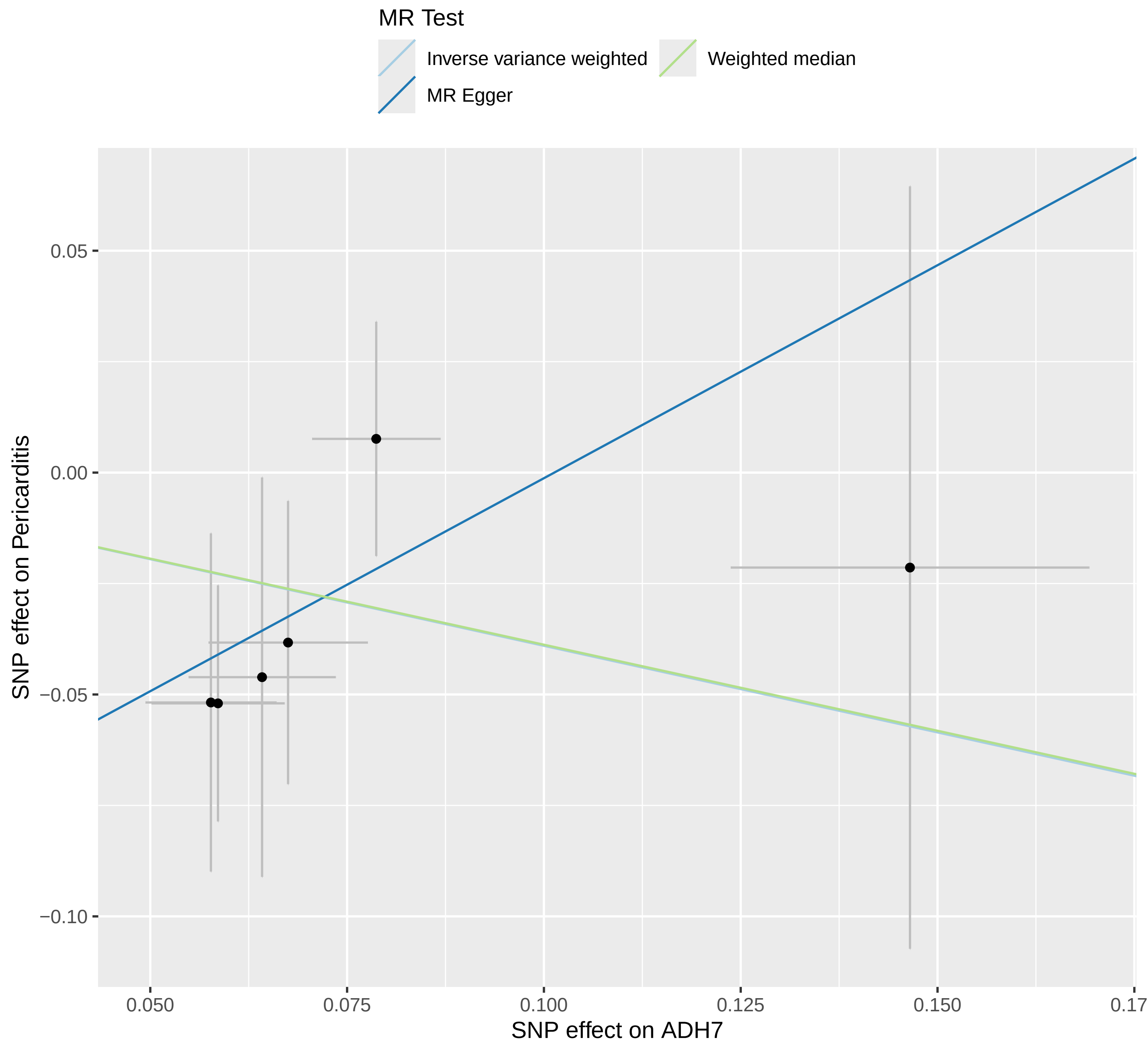

MR Test
Inverse variance weighted
Weighted median
MR Egger
0.05
0.00
−0.05
−0.10
SNP effect on Pericarditis
0.050
0.075
0.100
0.125
0.150
0.17
SNP effect on ADH7

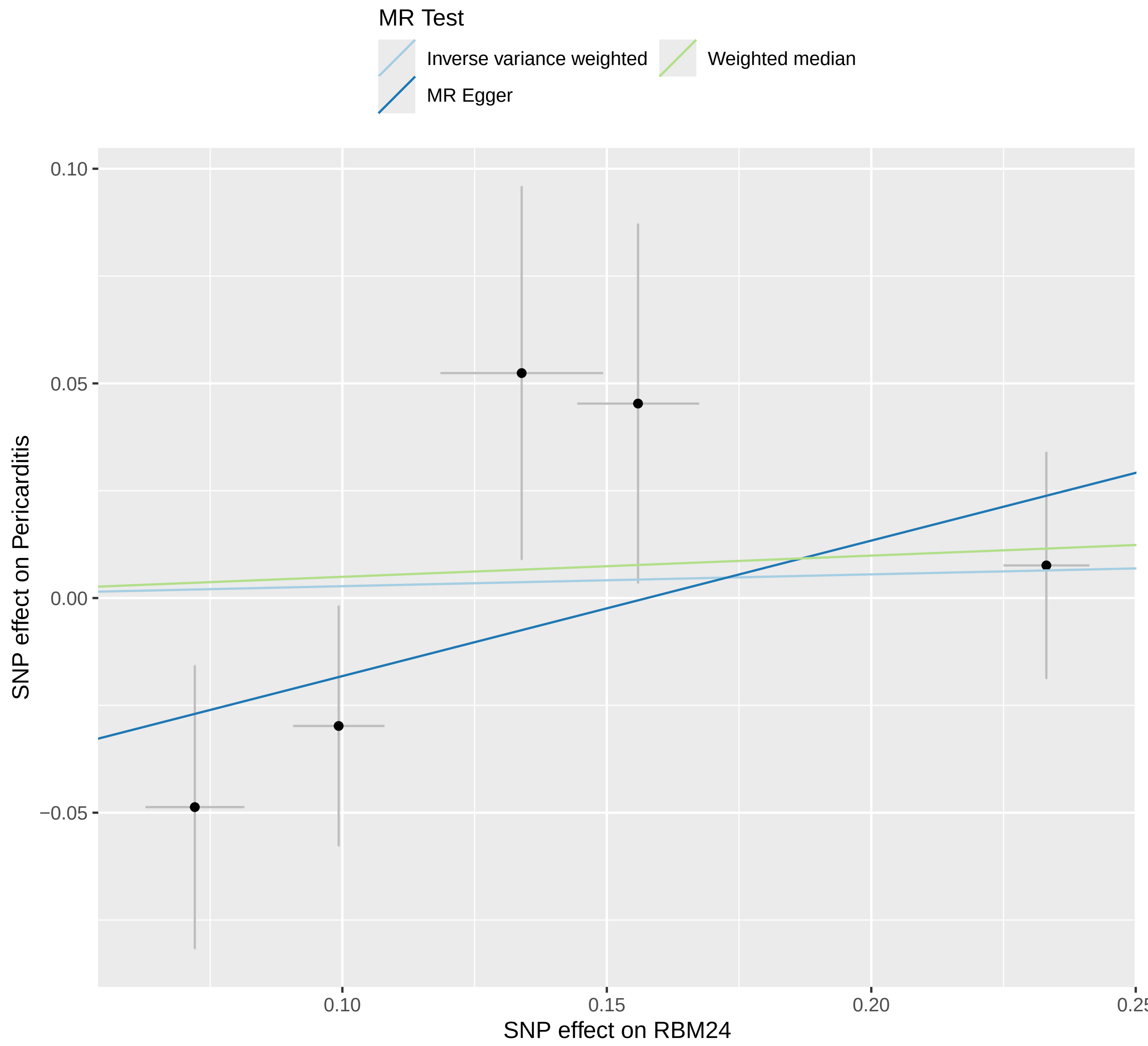

MR Test
Inverse variance weighted
Weighted median
MR Egger
0.10
0.05
0.00
−0.05
0.10
0.15
0.20
0.25
SNP effect on Pericarditis
SNP effect on RBM24

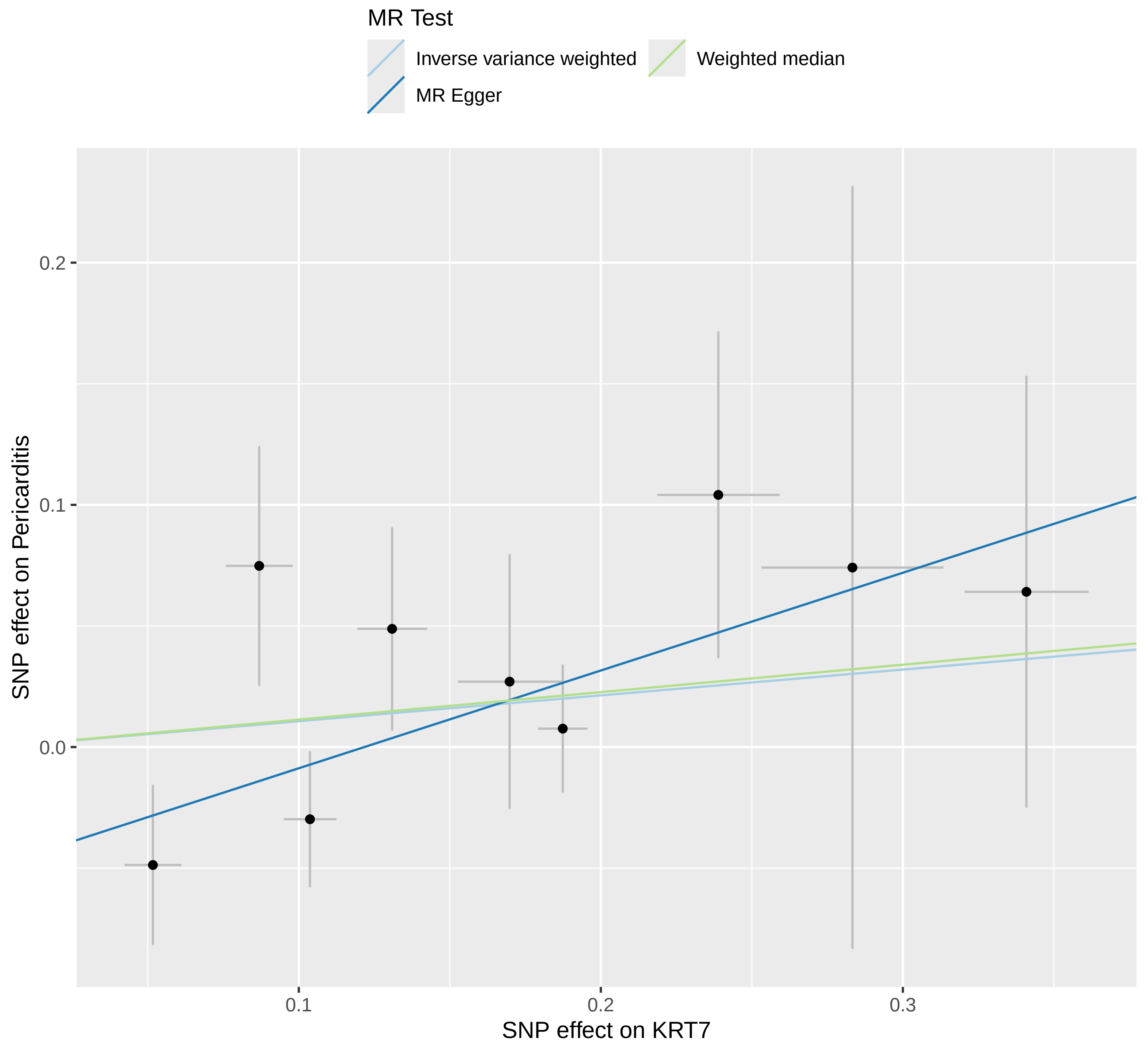
MR Test
Inverse variance weighted
Weighted median
MR Egger
0.2
0.1
0.0
SNP effect on Pericarditis
0.1
0.2
0.3
SNP effect on KRT7

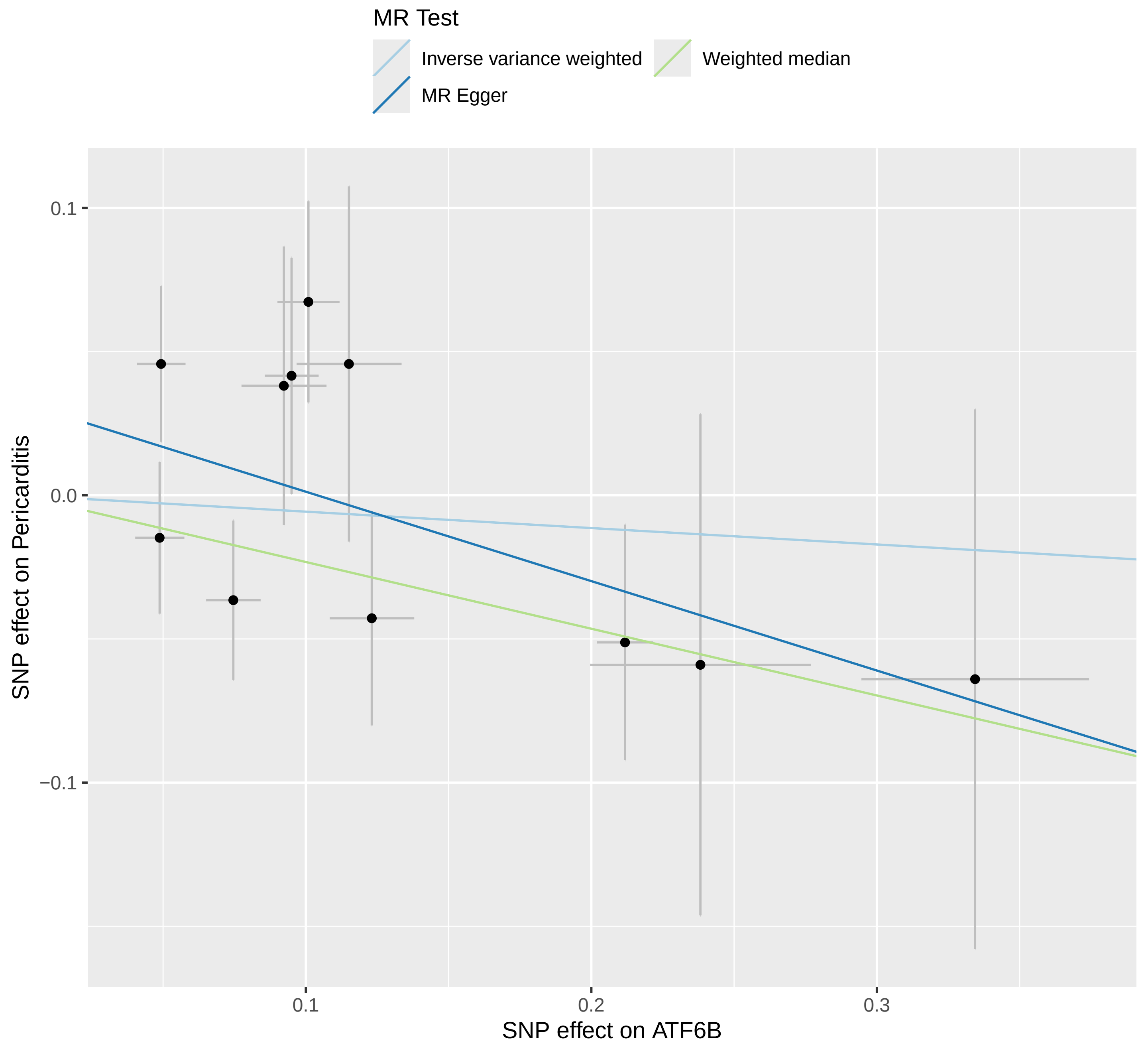
MR Test
Inverse variance weighted
Weighted median
MR Egger
0.1
0.0
−0.1
SNP effect on Pericarditis
0.1
0.2
0.3
SNP effect on ATF6B

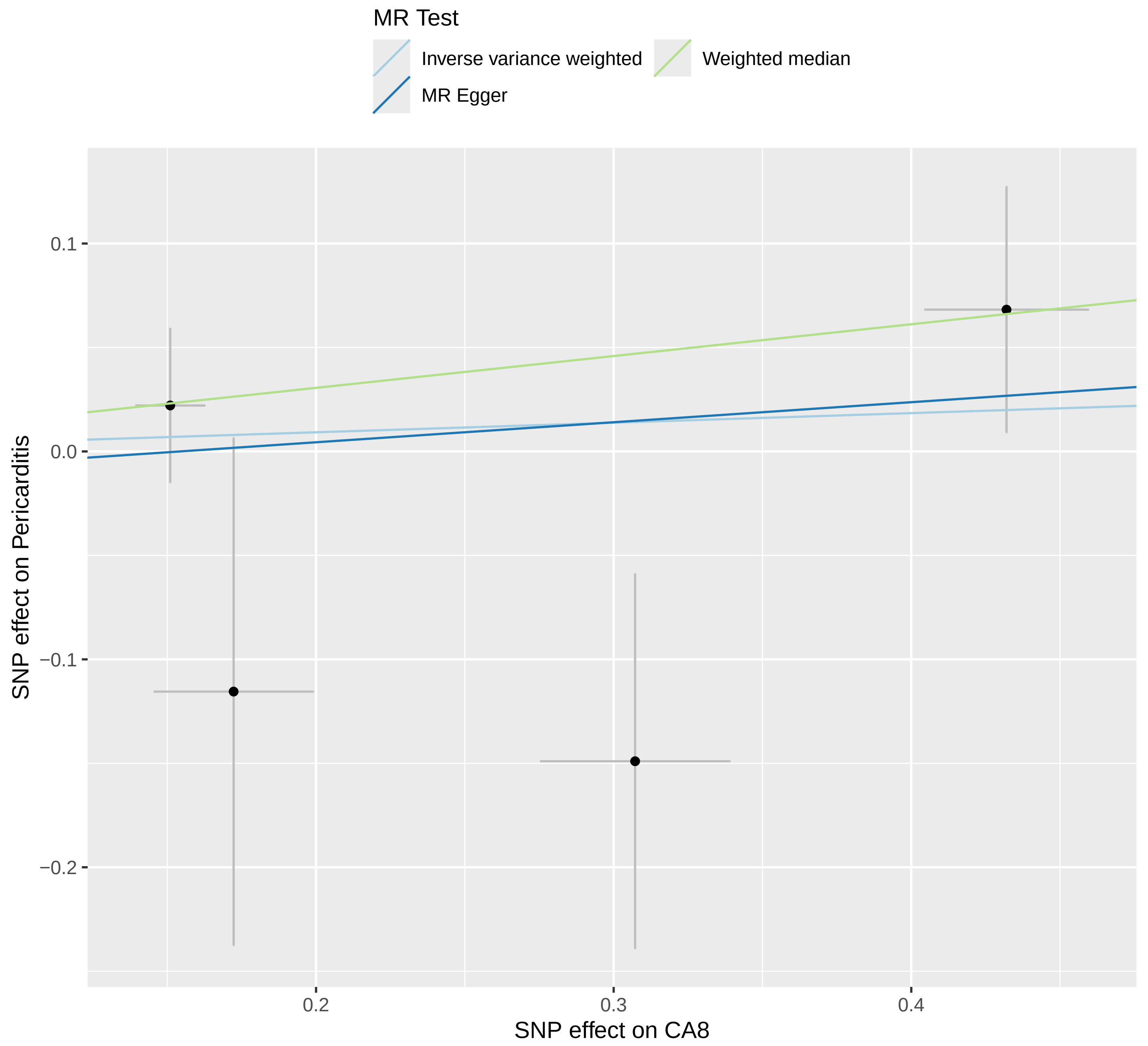
MR Test
Inverse variance weighted
MR Egger
Weighted median
0.1
0.0
−0.1
−0.2
SNP effect on Pericarditis
0.2
0.3
0.4
SNP effect on CA8

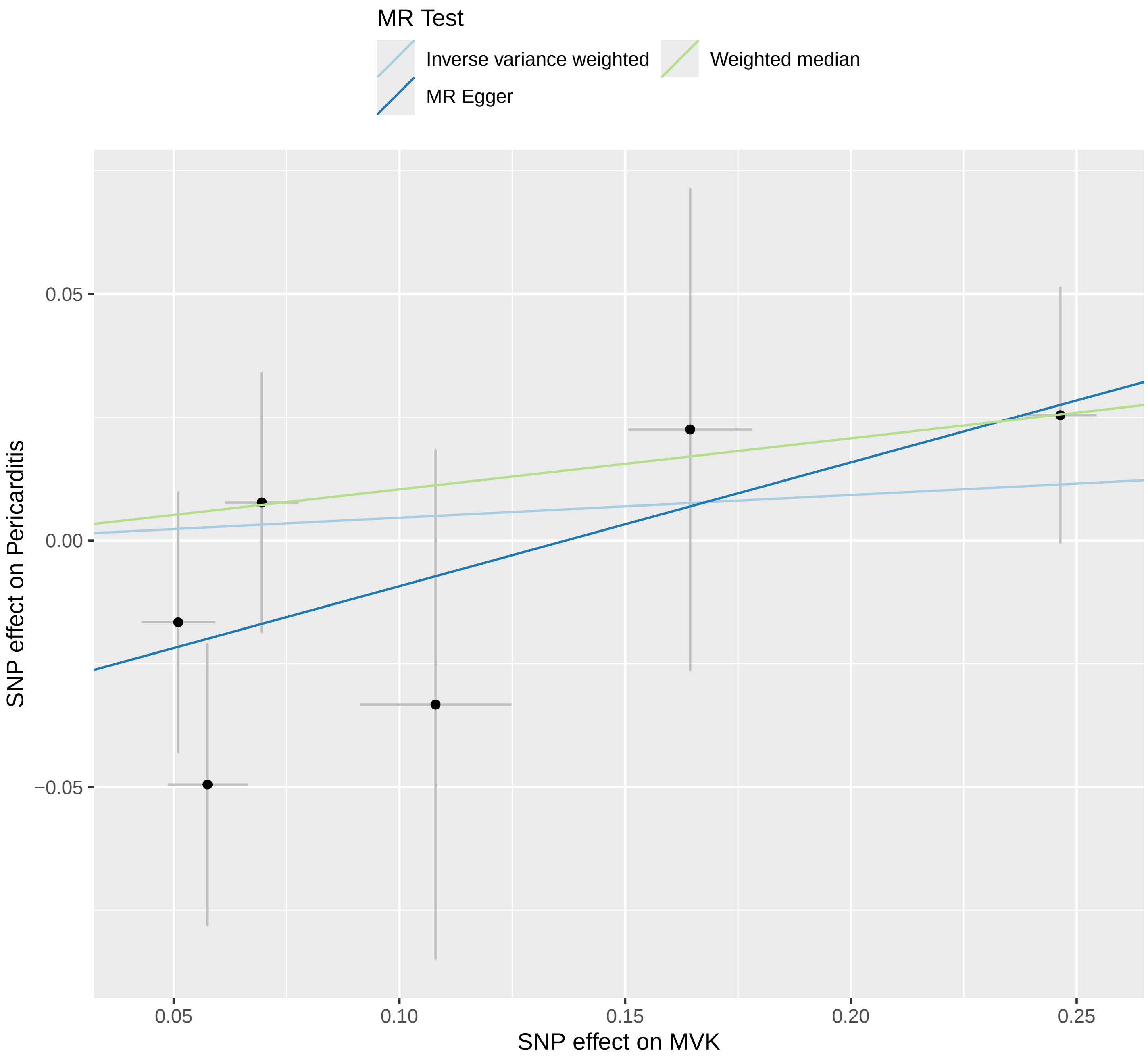
MR Test
Inverse variance weighted
MR Egger
Weighted median
SNP effect on Pericarditis
0.05
0.00
−0.05
0.05
0.10
0.15
0.20
0.25
SNP effect on MVK

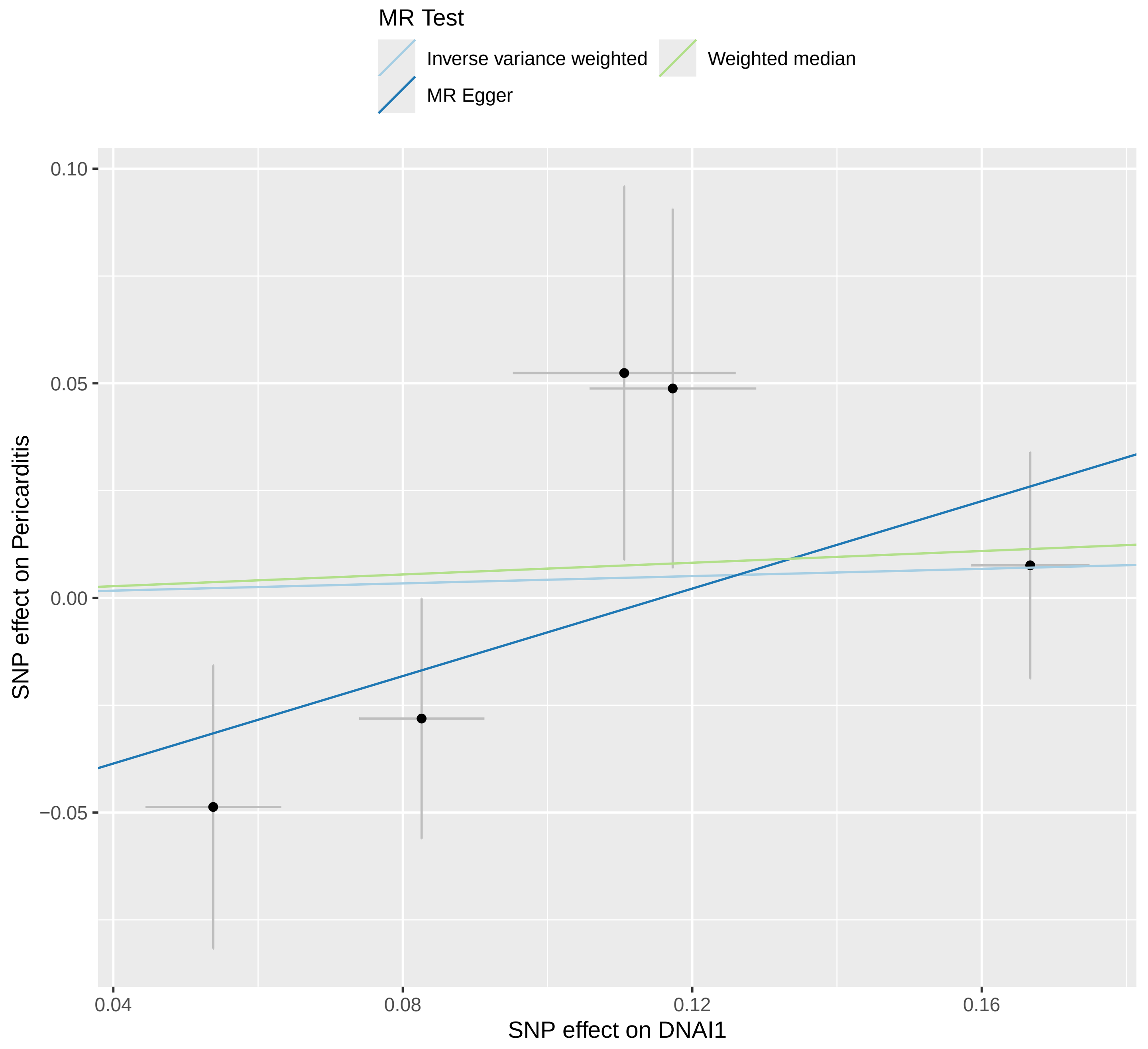
MR Test
Inverse variance weighted
MR Egger
Weighted median
0.10
0.05
0.00
−0.05
SNP effect on Pericarditis
0.04
0.08
0.12
0.16
SNP effect on DNAI1

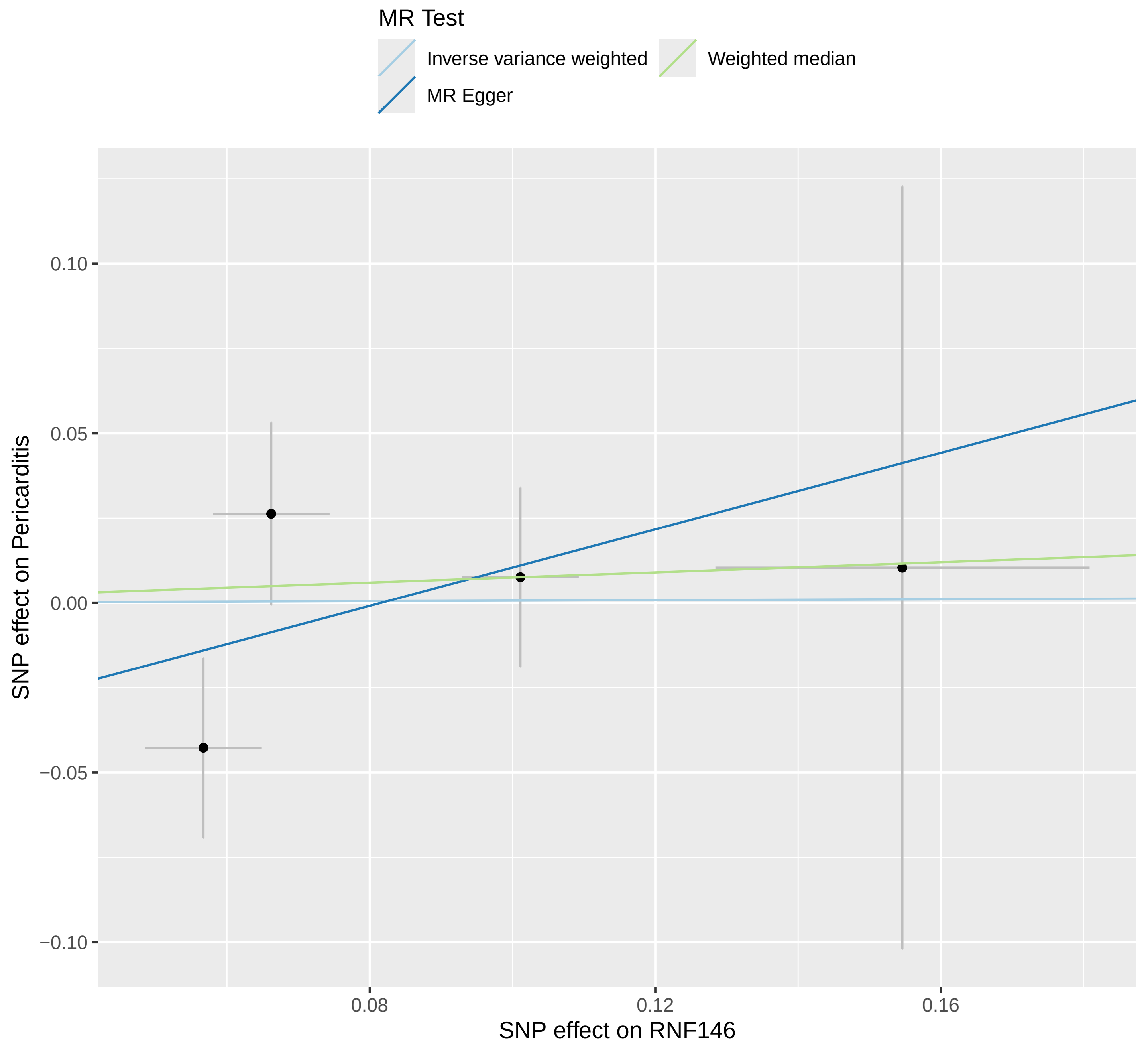

MR Test
Inverse variance weighted
MR Egger
Weighted median
0.10
0.05
0.00
−0.05
−0.10
SNP effect on Pericarditis
0.08
0.12
0.16
SNP effect on RNF146

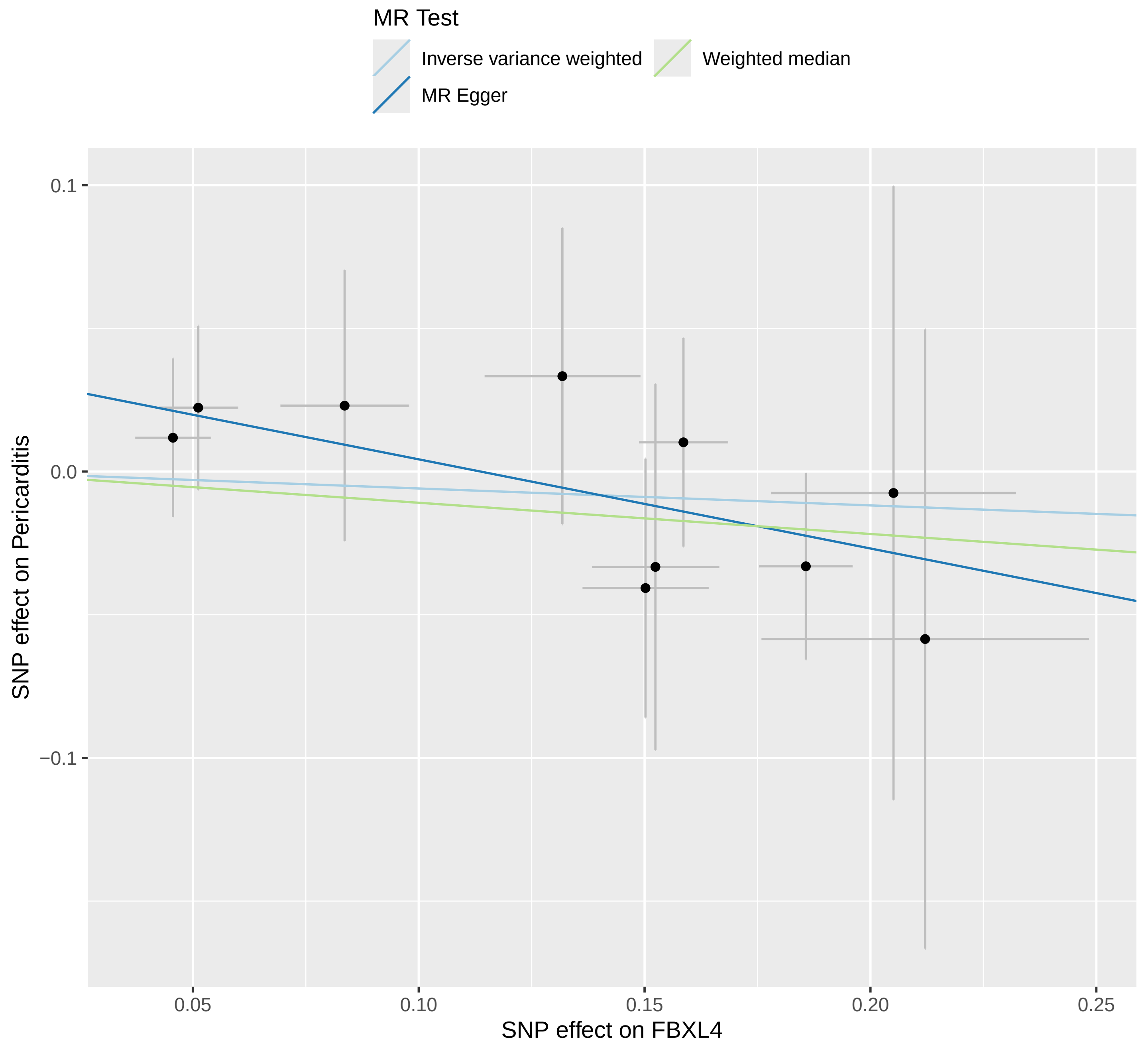

MR Test
Inverse variance weighted
Weighted median
MR Egger
0.1
0.0
−0.1
SNP effect on Pericarditis
0.05
0.10
0.15
0.20
0.25
SNP effect on FBXL4

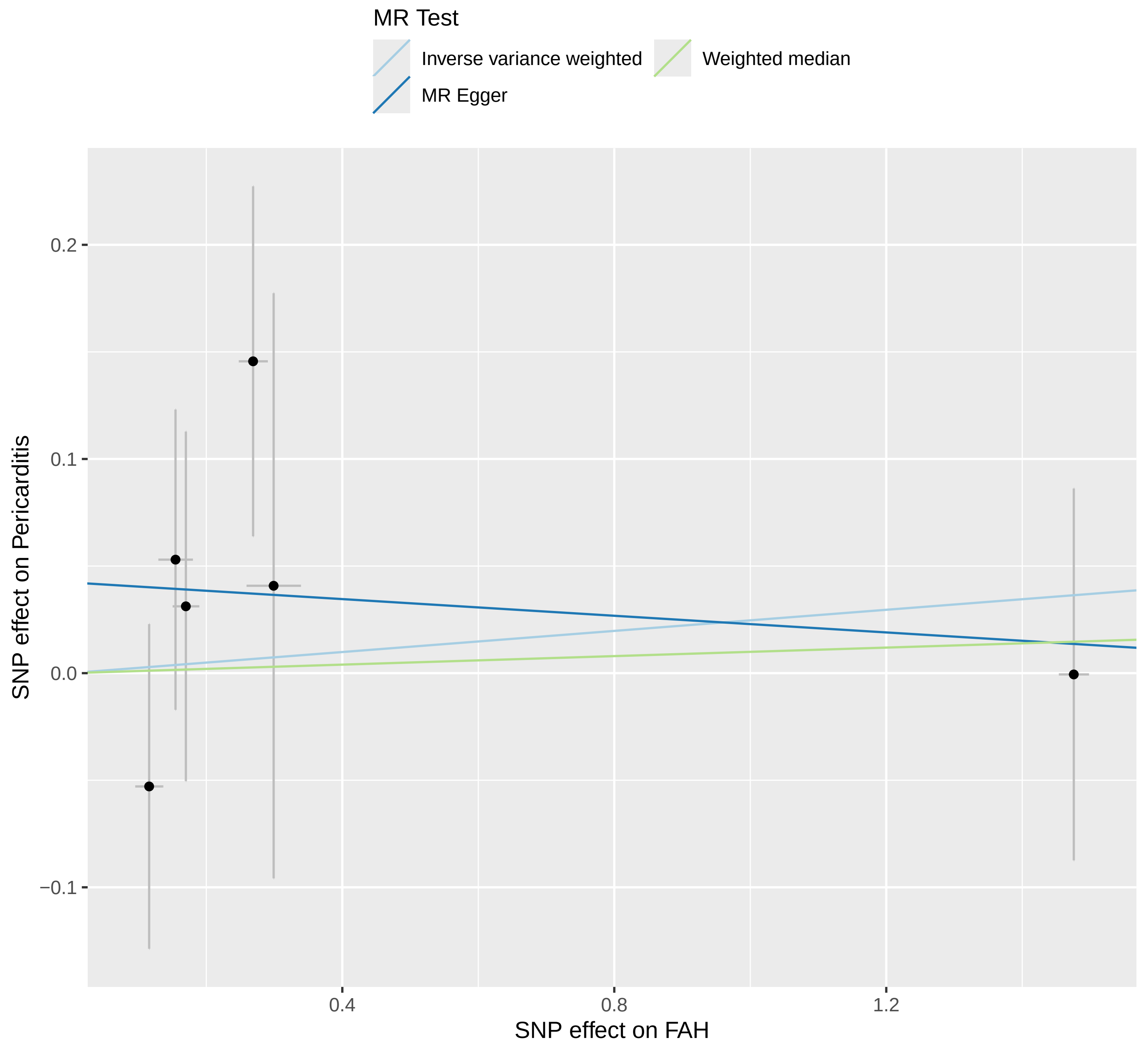

MR Test
Inverse variance weighted
Weighted median
MR Egger
0.2
0.1
0.0
−0.1
SNP effect on Pericarditis
0.4
0.8
1.2
SNP effect on FAH

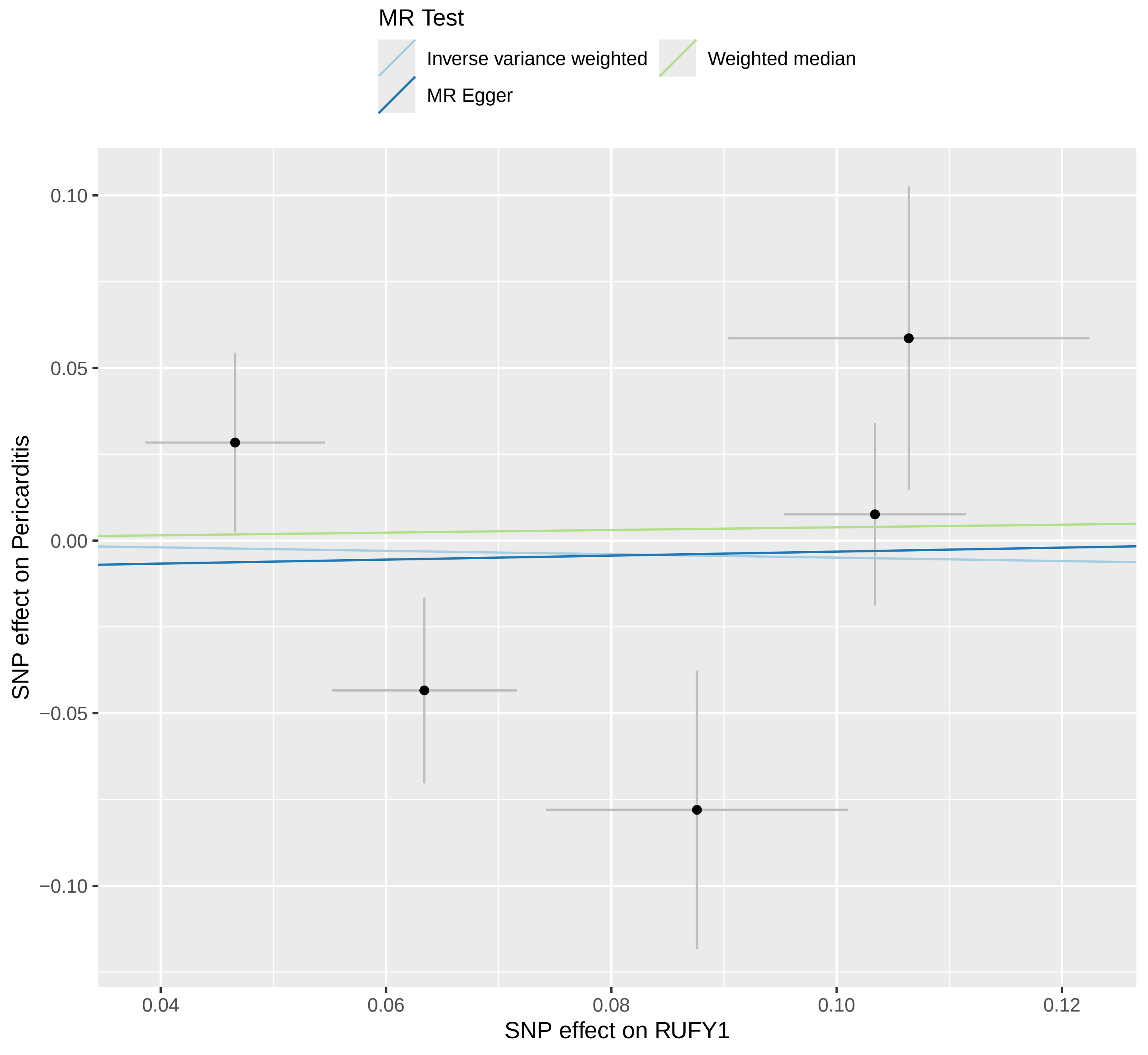

MR Test
Inverse variance weighted
Weighted median
MR Egger
0.10
0.05
0.00
−0.05
−0.10
SNP effect on Pericarditis
0.04
0.06
0.08
0.10
0.12
SNP effect on RUFY1

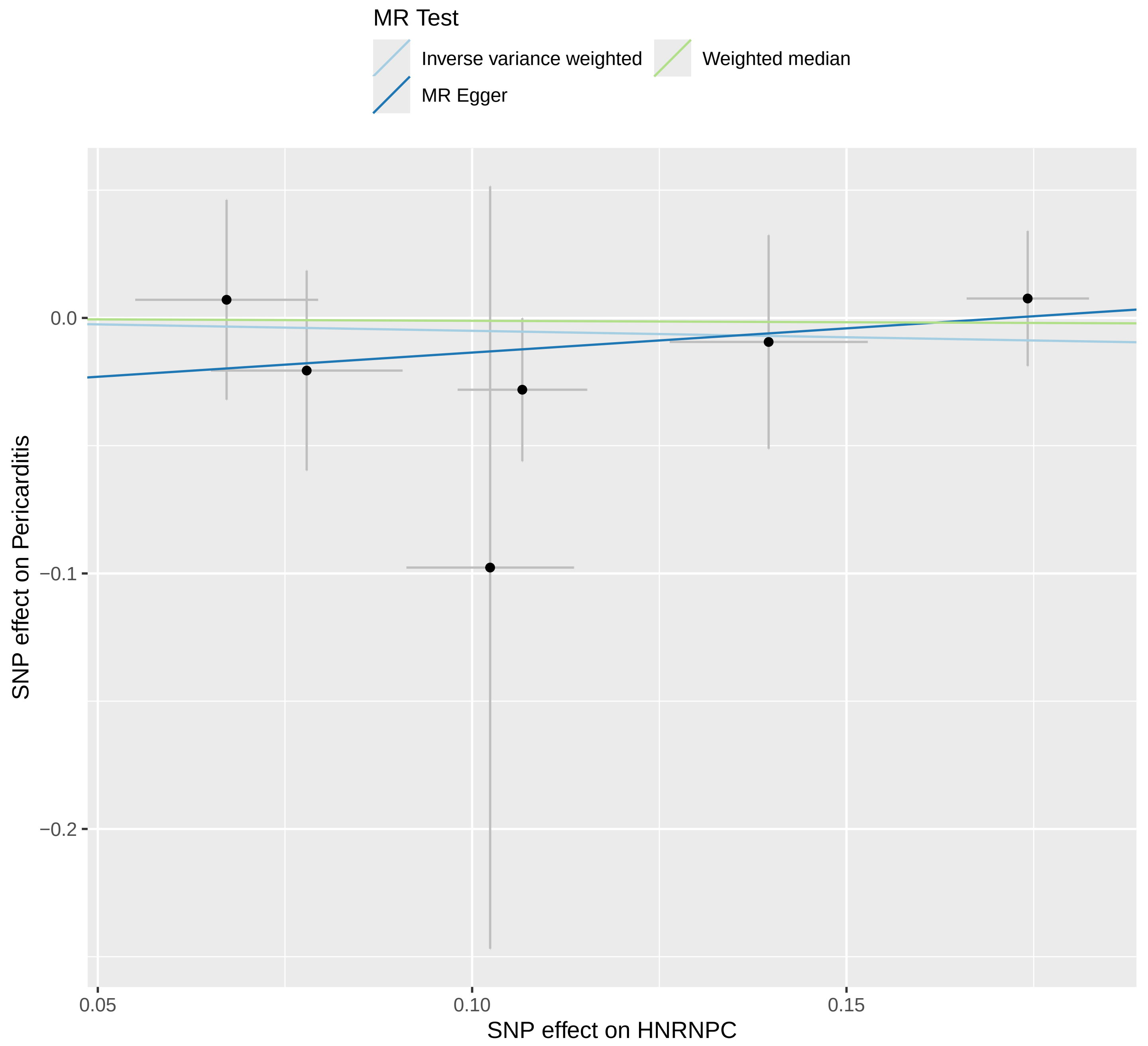

MR Test
Inverse variance weighted
Weighted median
MR Egger
SNP effect on Pericarditis
0.0
−0.1
−0.2
0.05
0.10
0.15
SNP effect on HNRNPC

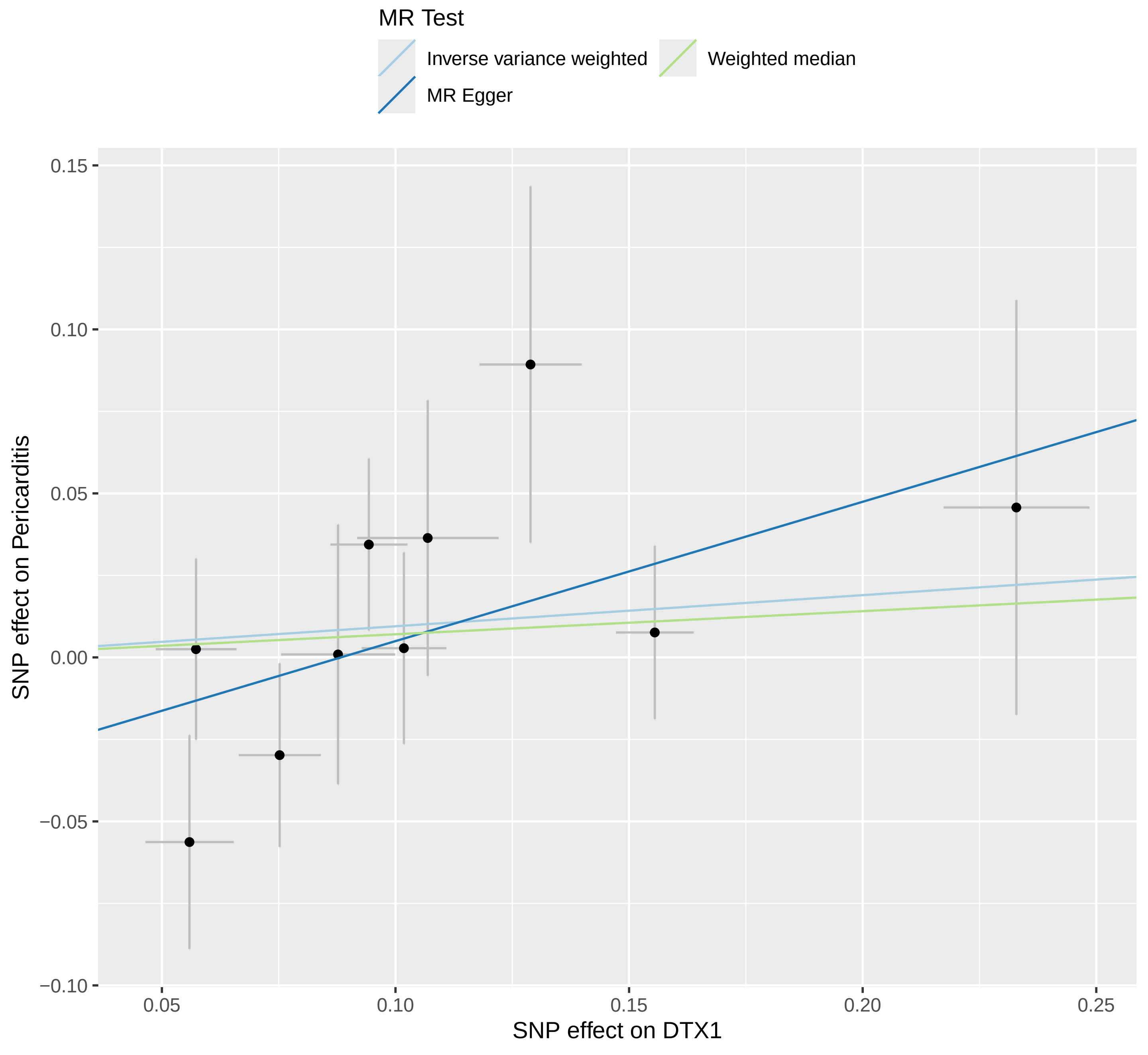

MR Test
Inverse variance weighted
MR Egger
Weighted median
SNP effect on Pericarditis
0.15
0.10
0.05
0.00
−0.05
−0.10
0.05
0.10
0.15
0.20
0.25
SNP effect on DTX1

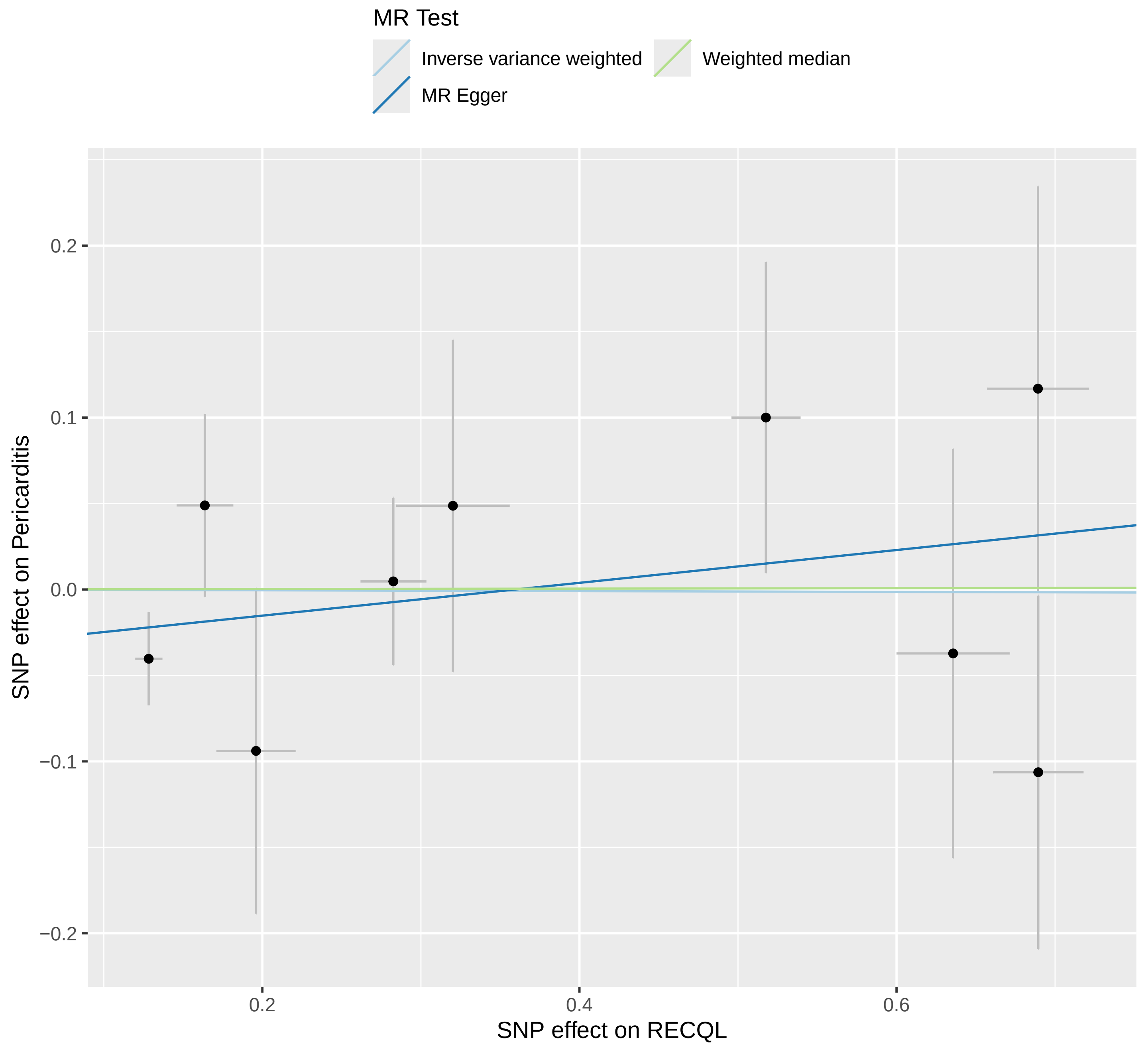
MR Test
Inverse variance weighted
Weighted median
MR Egger
0.2
0.1
0.0
−0.1
−0.2
SNP effect on Pericarditis
0.2
0.4
0.6
SNP effect on RECQL

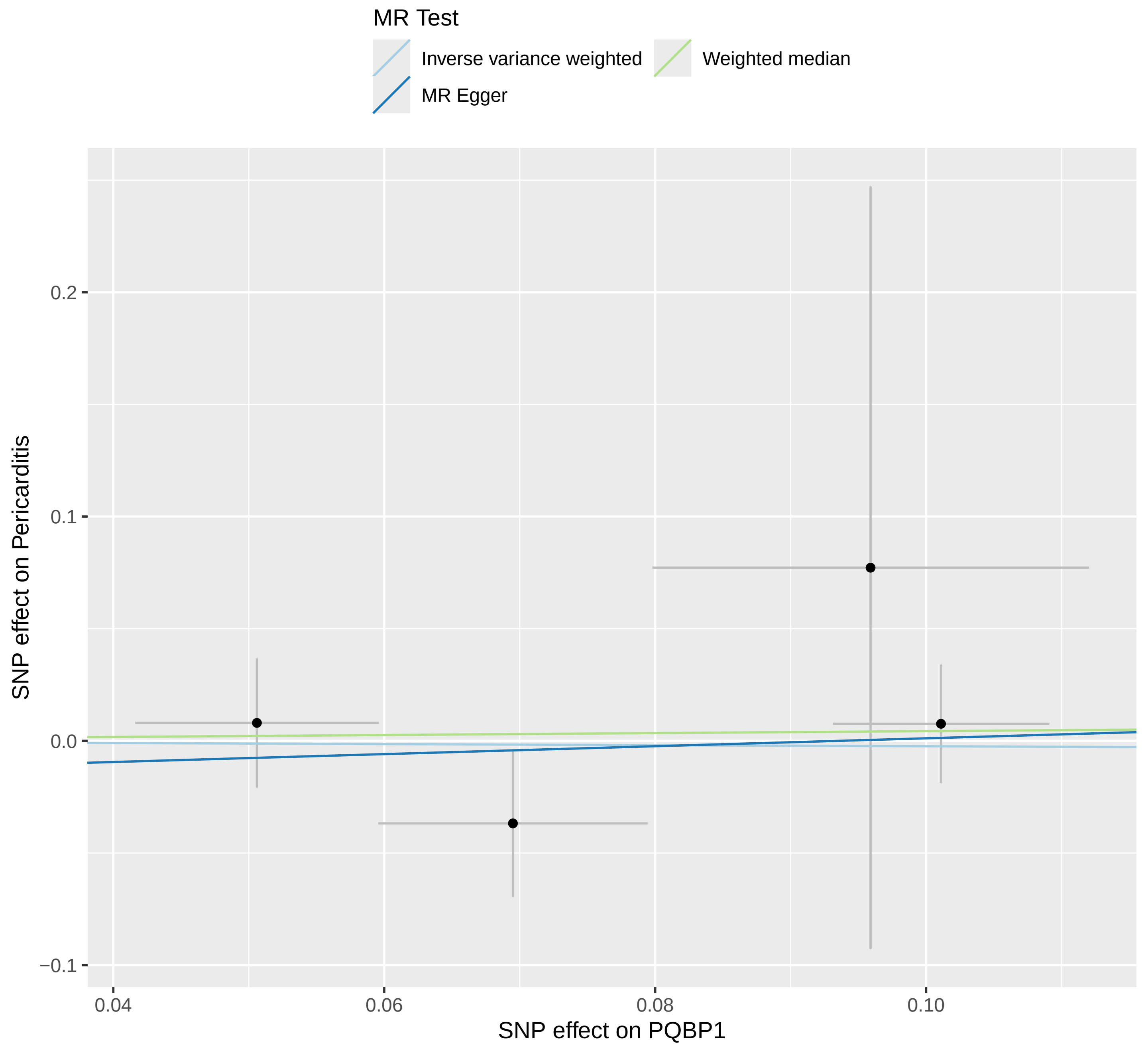
MR Test
Inverse variance weighted
Weighted median
MR Egger
SNP effect on Pericarditis
0.2
0.1
0.0
−0.1
0.04
0.06
0.08
0.10
SNP effect on PQBP1

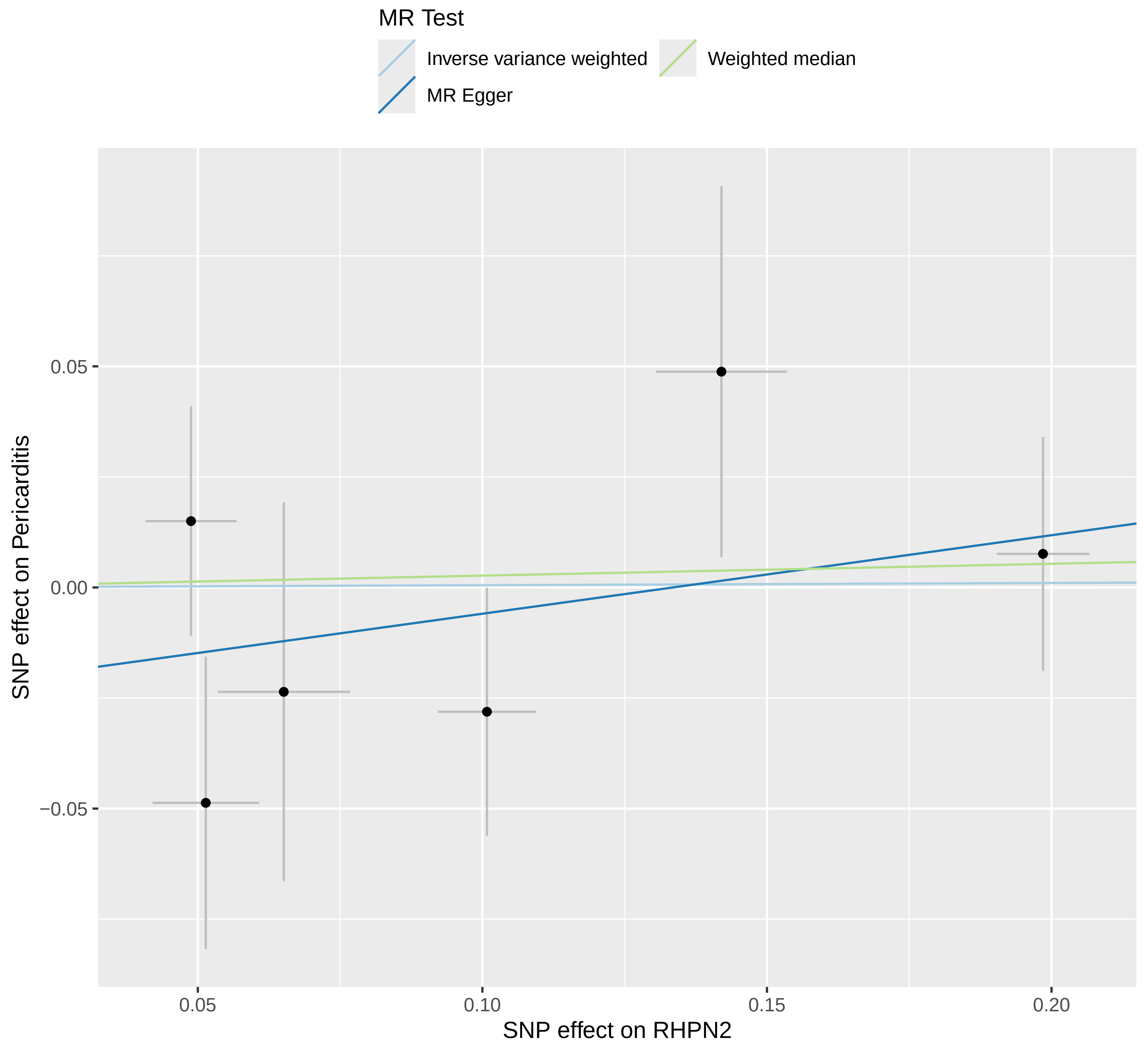
MR Test
Inverse variance weighted
Weighted median
MR Egger
0.05
0.00
−0.05
SNP effect on Pericarditis
0.05
0.10
0.15
0.20
SNP effect on RHPN2

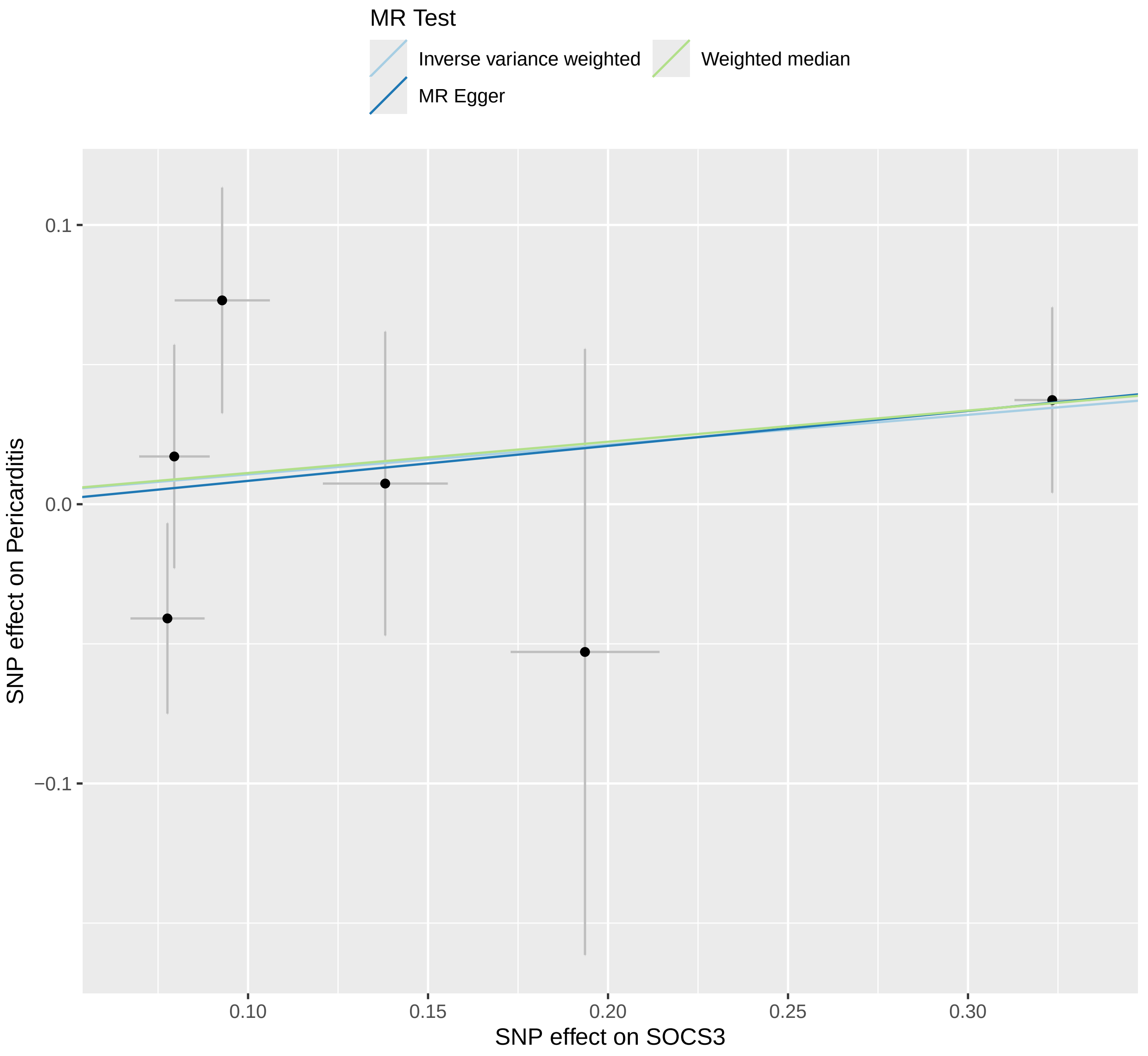
MR Test
Inverse variance weighted
Weighted median
MR Egger
0.1
0.0
−0.1
SNP effect on Pericarditis
0.10
0.15
0.20
0.25
0.30
SNP effect on SOCS3

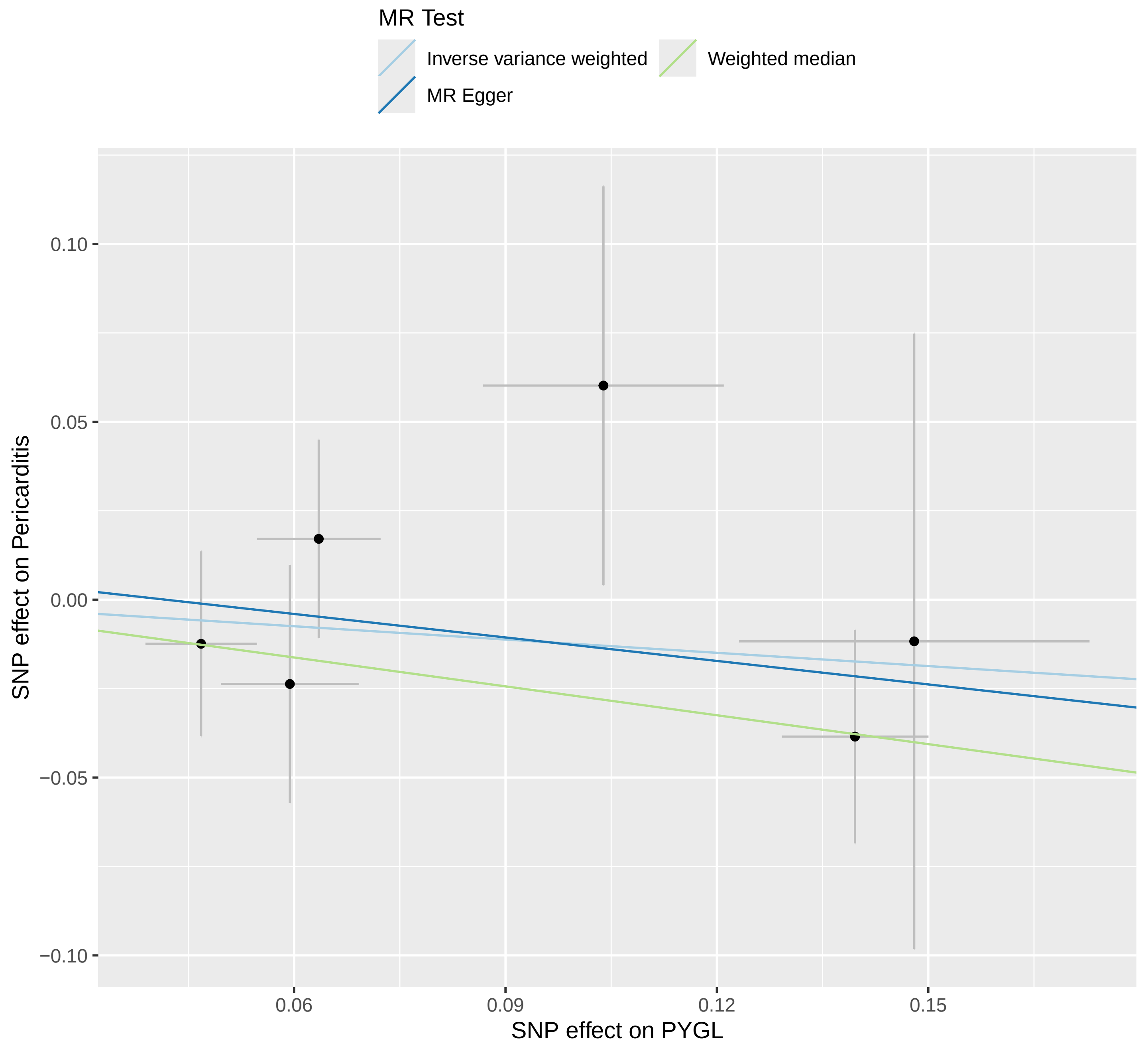
MR Test
Inverse variance weighted
MR Egger
Weighted median
SNP effect on Pericarditis
0.10
0.05
0.00
−0.05
−0.10
0.06
0.09
0.12
0.15
SNP effect on PYGL

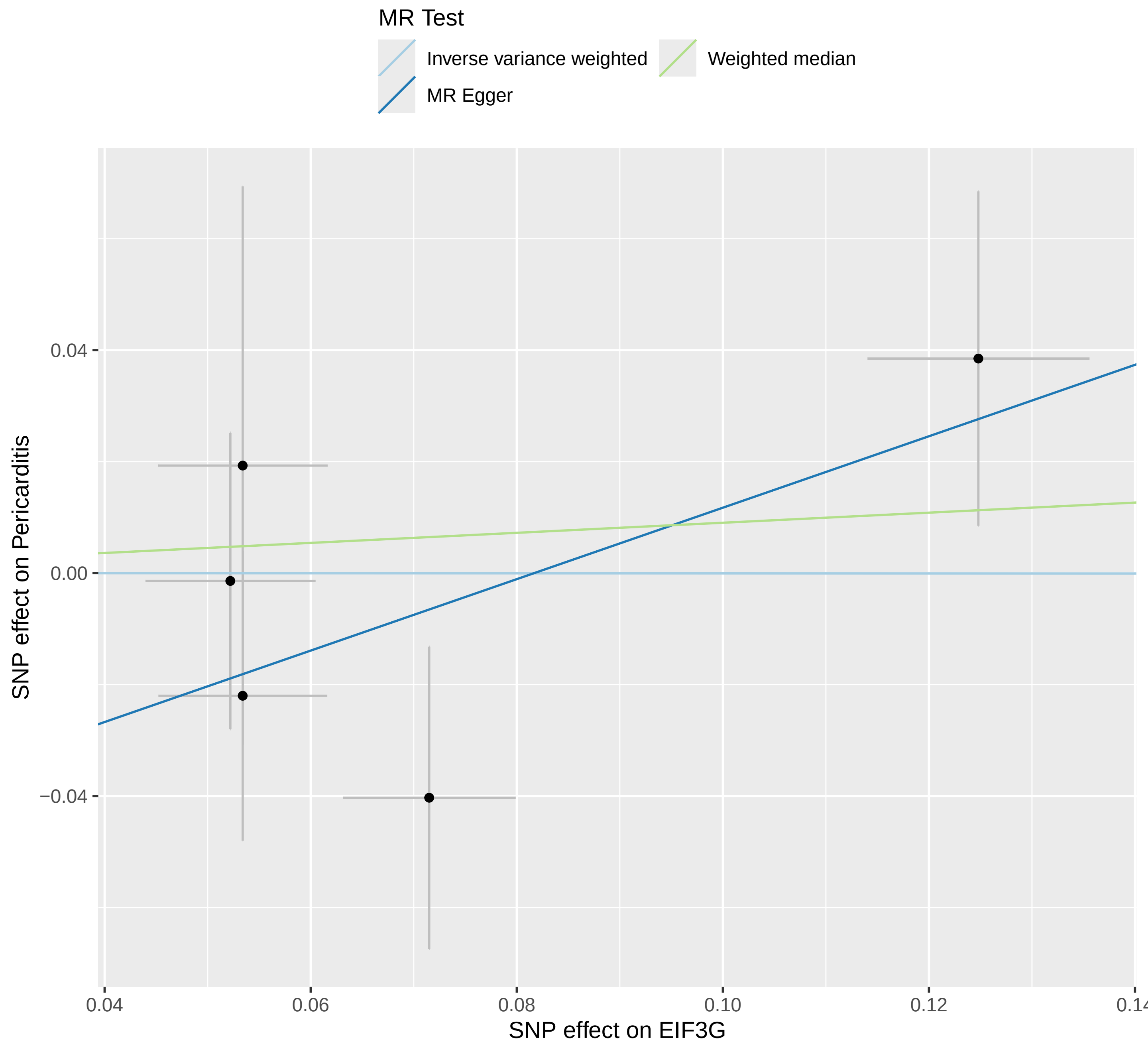
MR Test
Inverse variance weighted
Weighted median
MR Egger
0.04
0.00
−0.04
0.04
0.06
0.08
0.10
0.12
0.14
SNP effect on EIF3G
SNP effect on Pericarditis

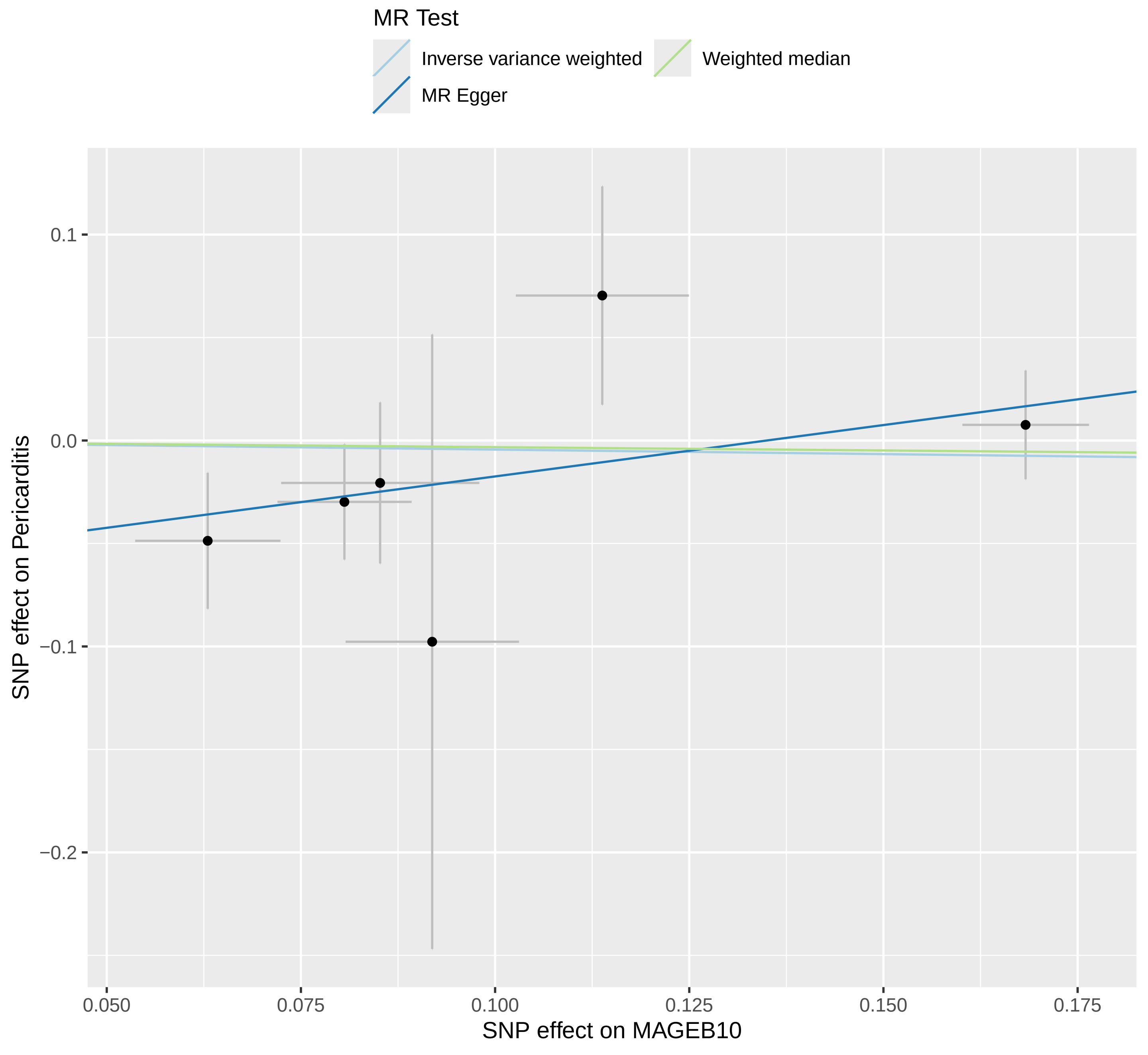
MR Test
Inverse variance weighted
Weighted median
MR Egger
SNP effect on Pericarditis
0.1
0.0
−0.1
−0.2
0.050
0.075
0.100
0.125
0.150
0.175
SNP effect on MAGEB10

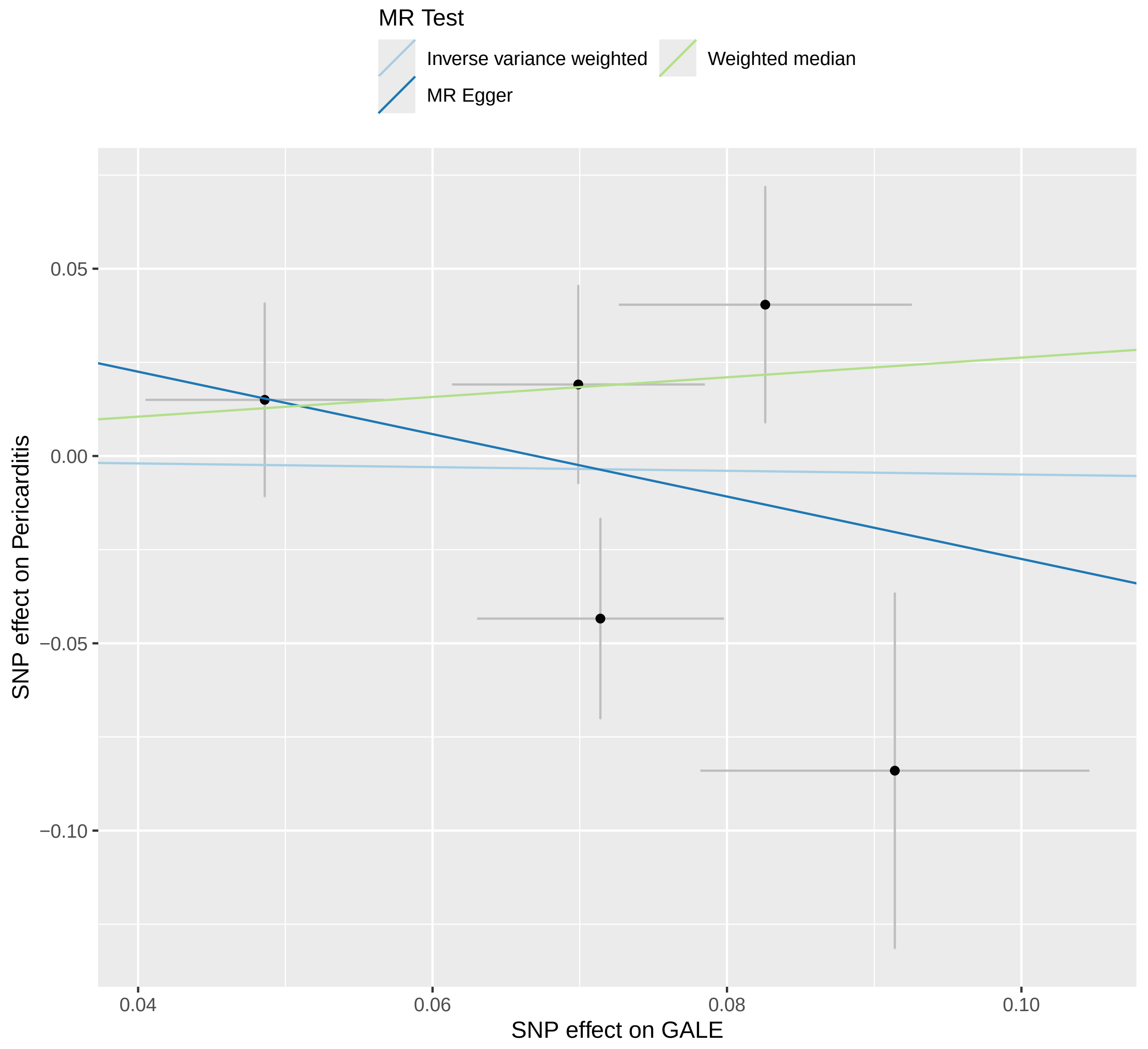

MR Test
Inverse variance weighted
Weighted median
MR Egger
0.05
0.00
−0.05
−0.10
SNP effect on Pericarditis
0.04
0.06
0.08
0.10
SNP effect on GALE

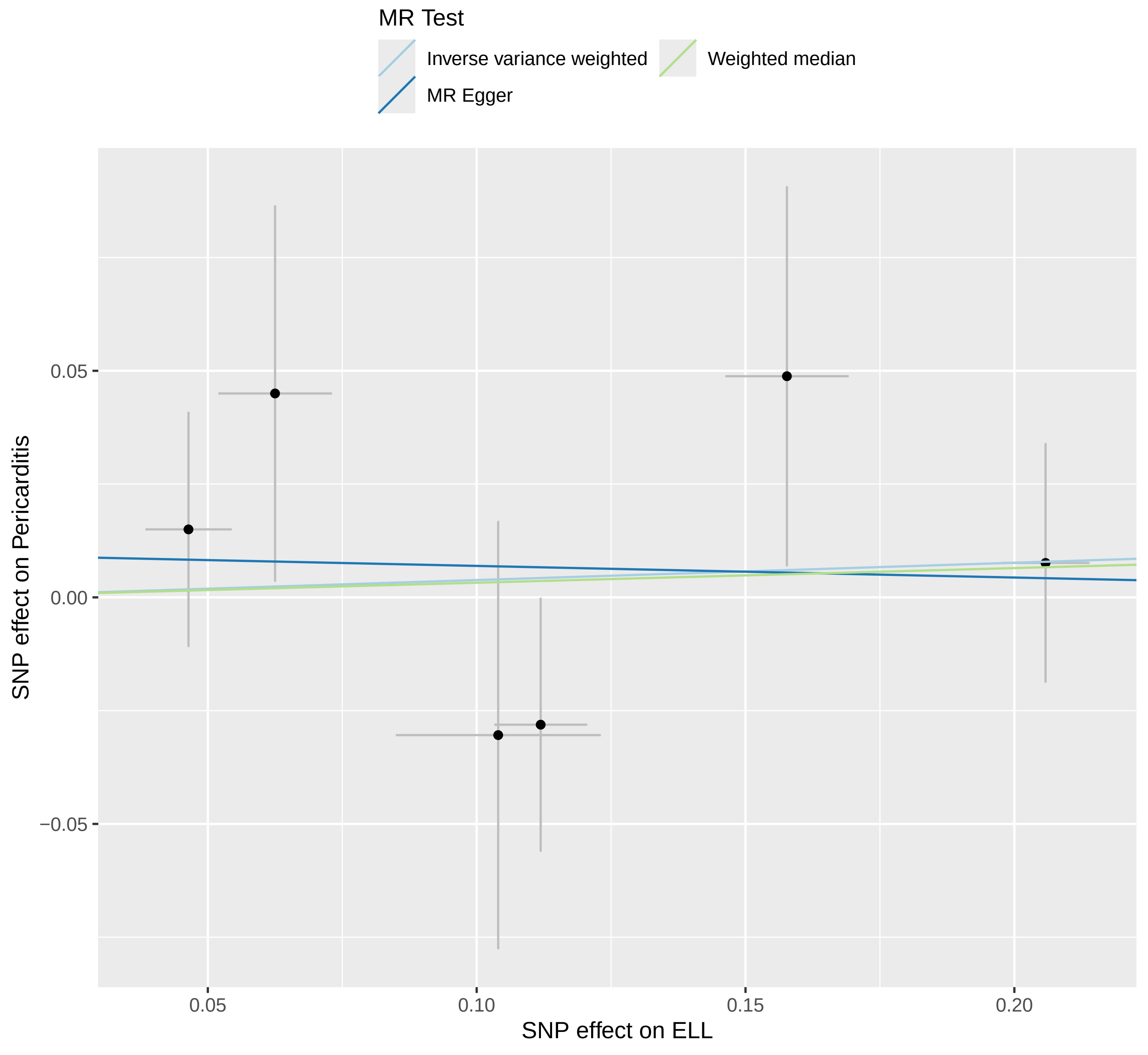
MR Test
Inverse variance weighted
Weighted median
MR Egger
0.05
0.00
−0.05
SNP effect on Pericarditis
0.05
0.10
0.15
0.20
SNP effect on ELL

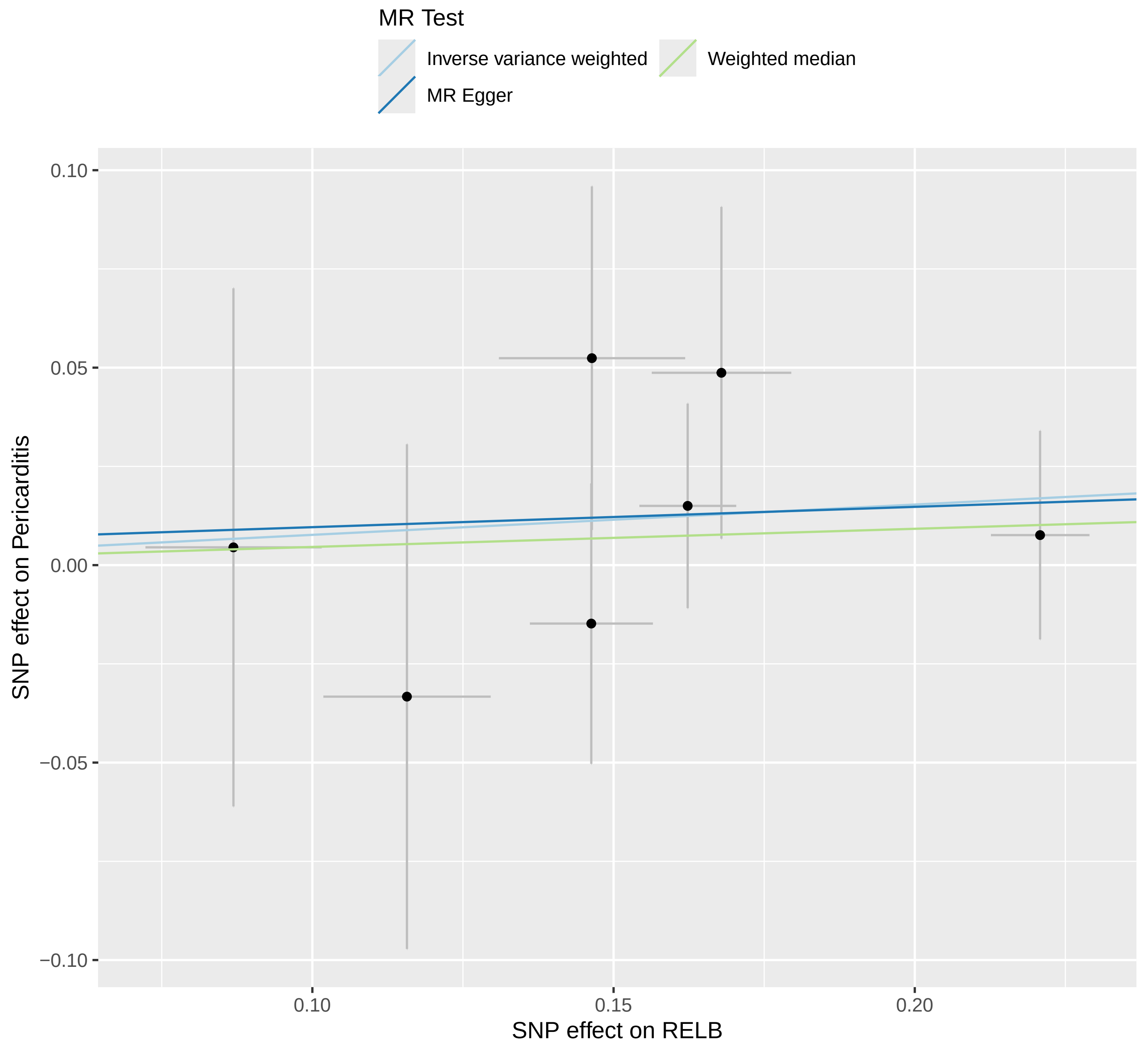
MR Test
Inverse variance weighted
MR Egger
Weighted median
0.10
0.05
0.00
−0.05
−0.10
SNP effect on Pericarditis
0.10
0.15
0.20
SNP effect on RELB

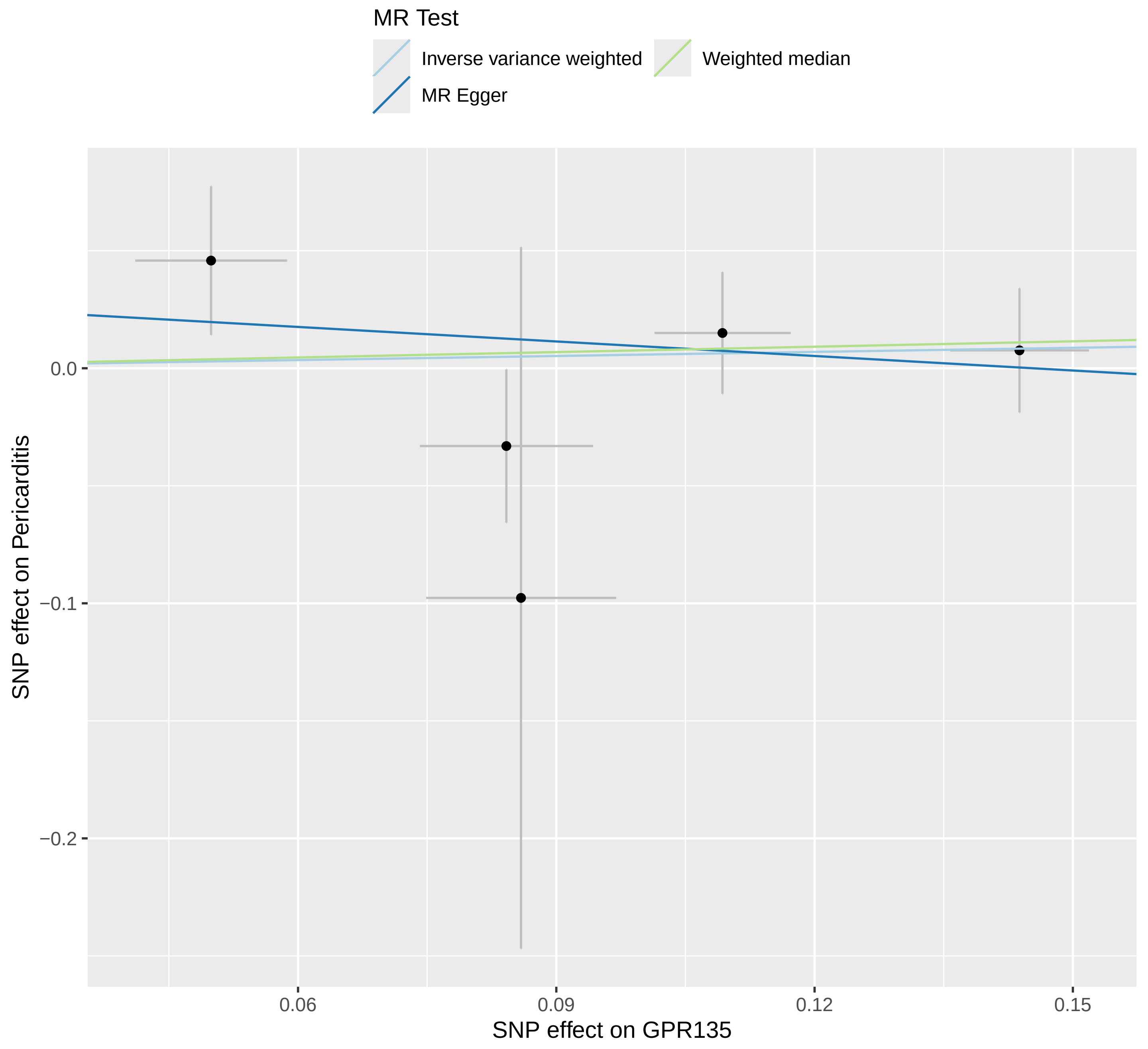
MR Test
Inverse variance weighted
Weighted median
MR Egger
0.0
−0.1
−0.2
0.06
0.09
0.12
0.15
SNP effect on Pericarditis
SNP effect on GPR135

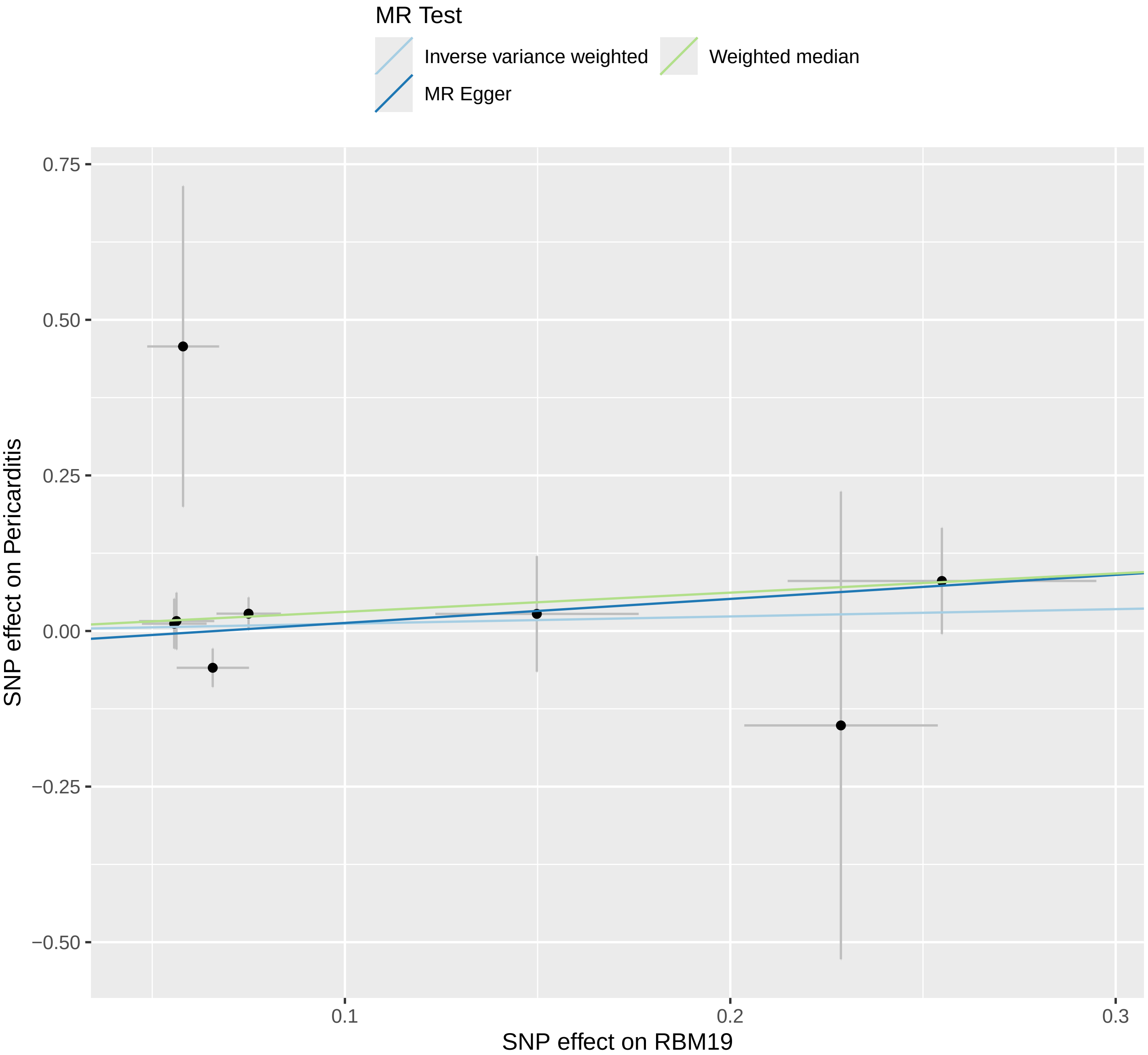
MR Test
Inverse variance weighted
MR Egger
Weighted median
0.75
0.50
0.25
0.00
−0.25
−0.50
SNP effect on Pericarditis
0.1
0.2
0.3
SNP effect on RBM19

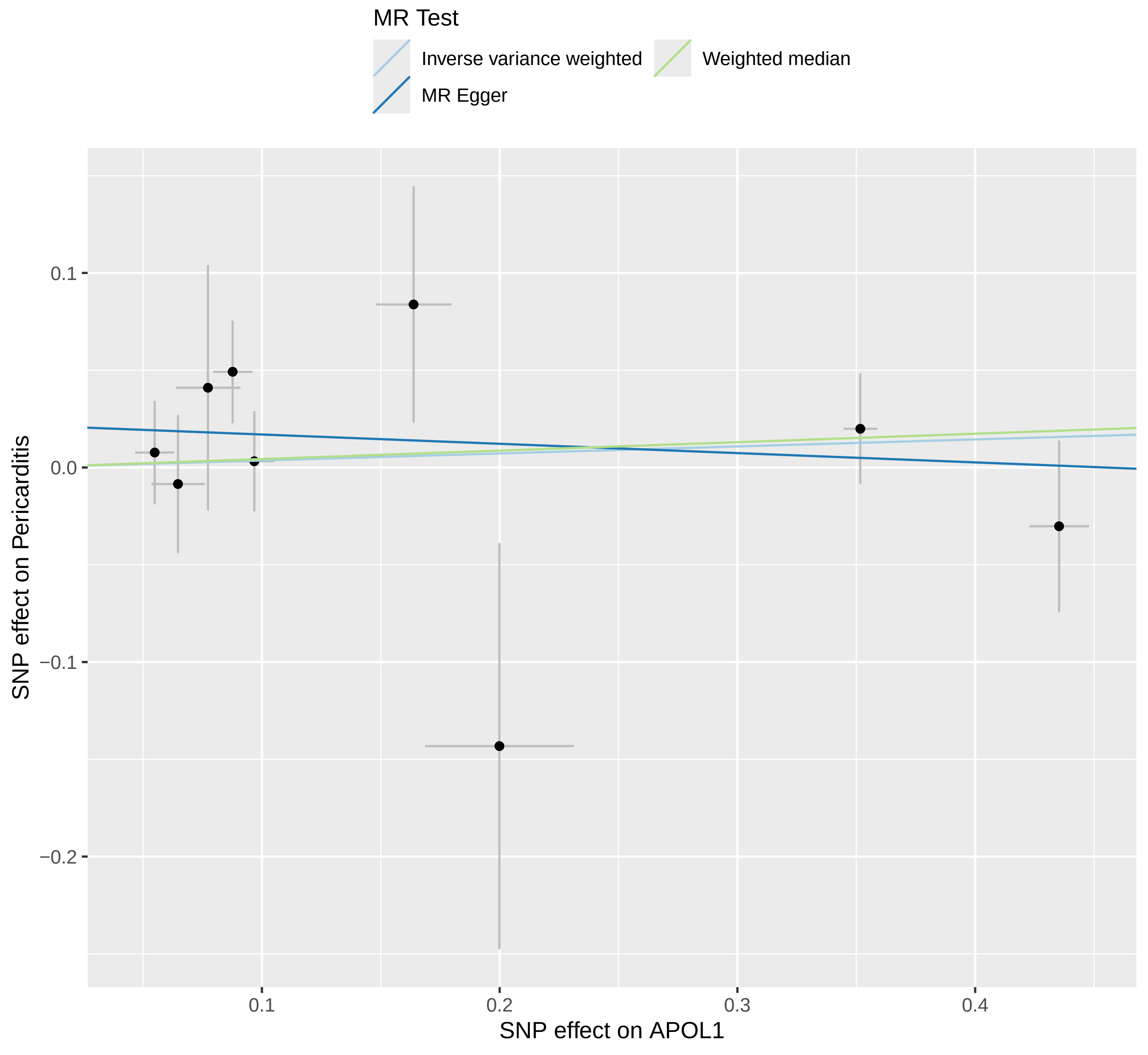

MR Test
Inverse variance weighted
Weighted median
MR Egger
0.1
0.0
−0.1
−0.2
SNP effect on Pericarditis
0.1
0.2
0.3
0.4
SNP effect on APOL1

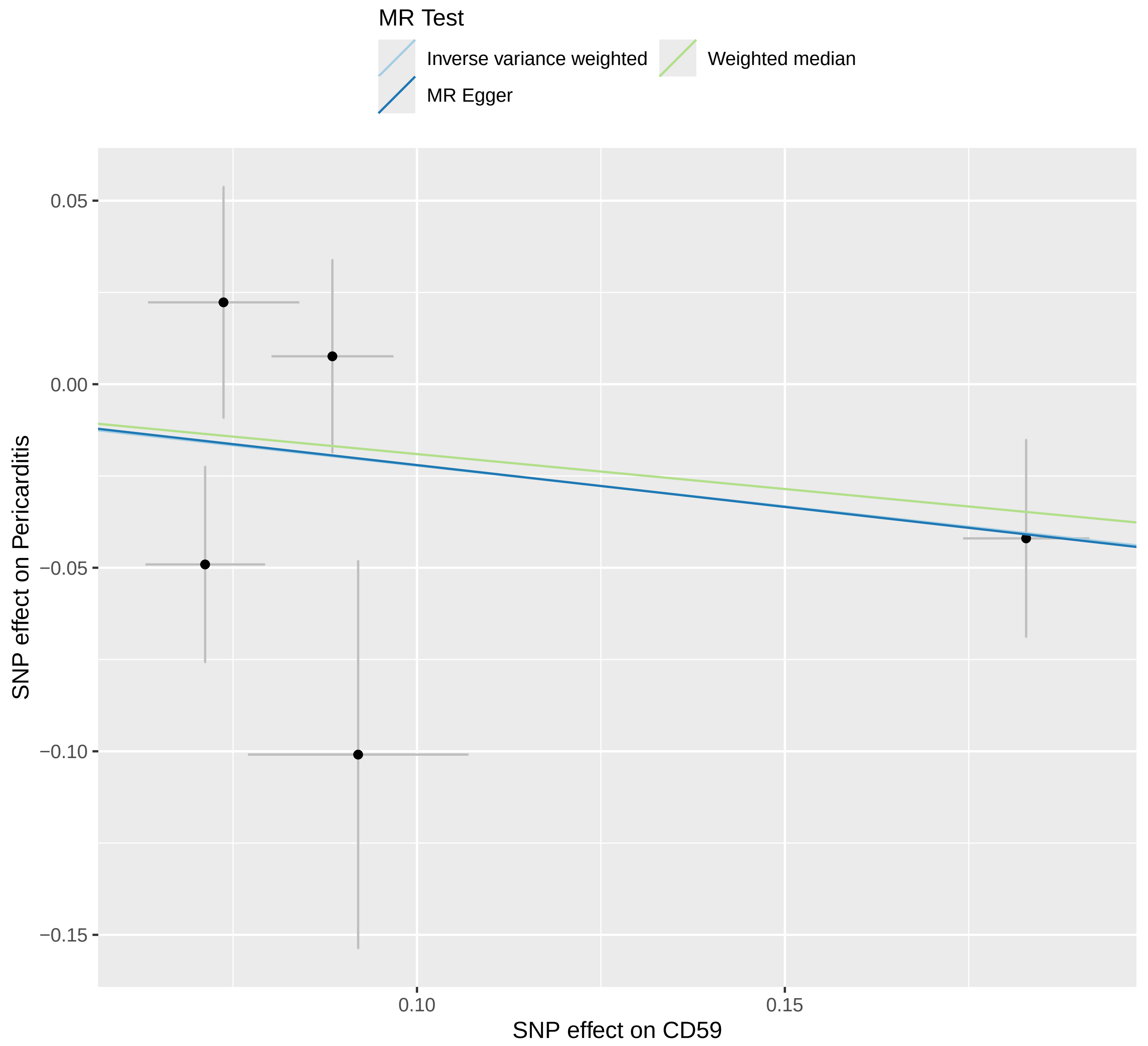
MR Test
Inverse variance weighted
Weighted median
MR Egger
0.05
0.00
−0.05
−0.10
−0.15
SNP effect on Pericarditis
0.10
0.15
SNP effect on CD59

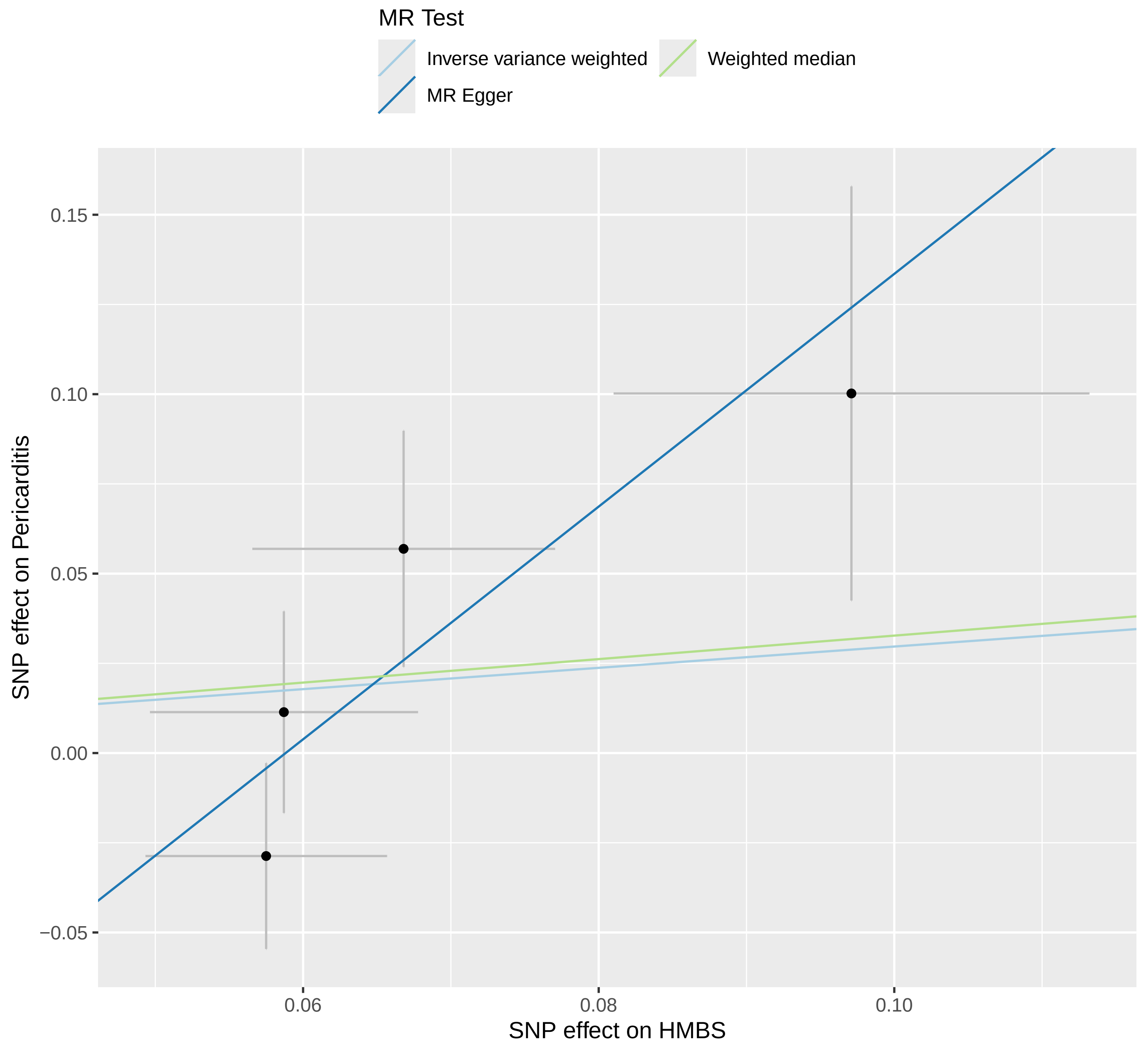
MR Test
Inverse variance weighted
Weighted median
MR Egger
0.15
0.10
0.05
0.00
−0.05
SNP effect on Pericarditis
0.06
0.08
0.10
SNP effect on HMBS

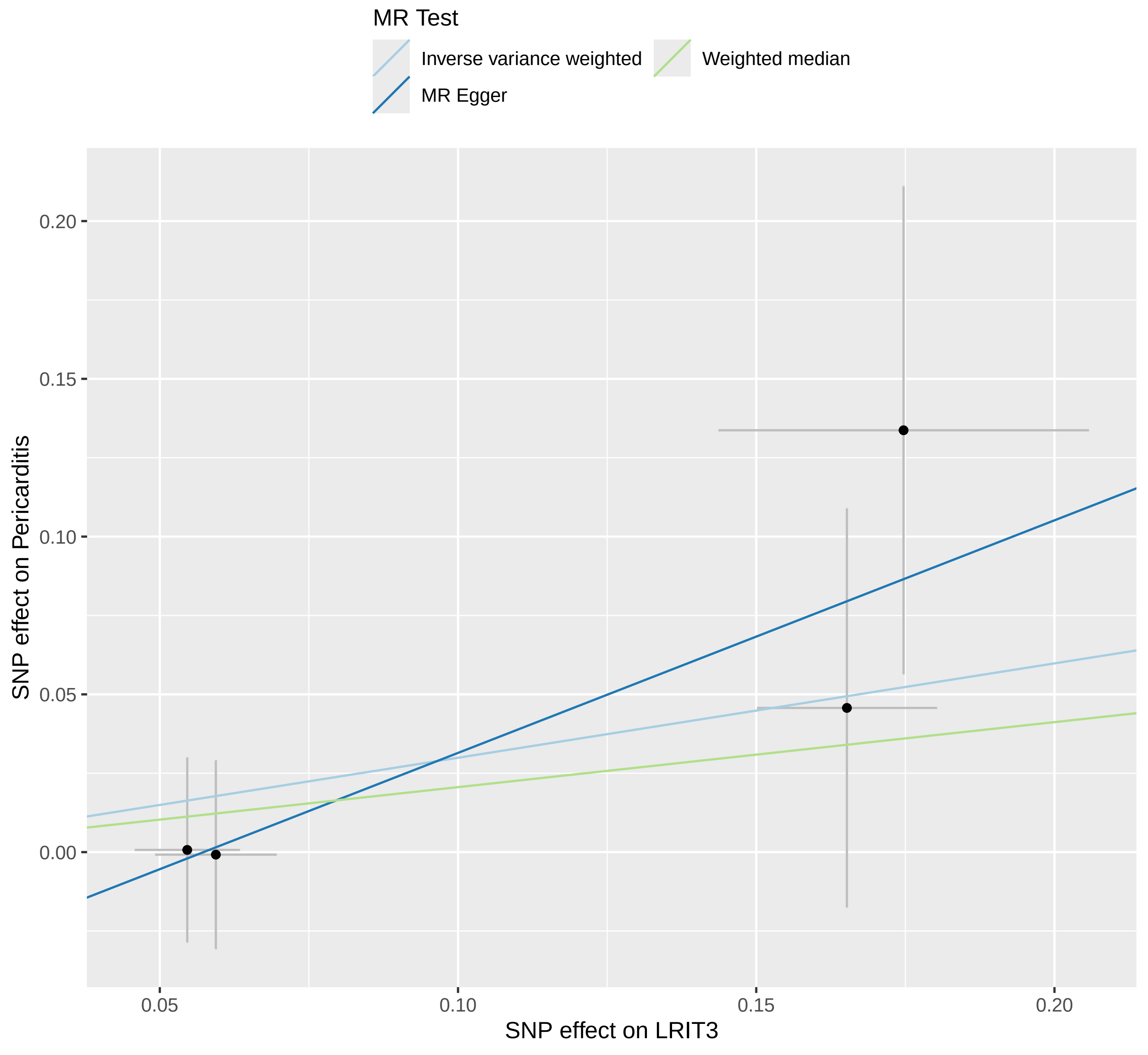
MR Test
Inverse variance weighted
Weighted median
MR Egger
SNP effect on Pericarditis
0.20
0.15
0.10
0.05
0.00
0.05
0.10
0.15
0.20
SNP effect on LRIT3

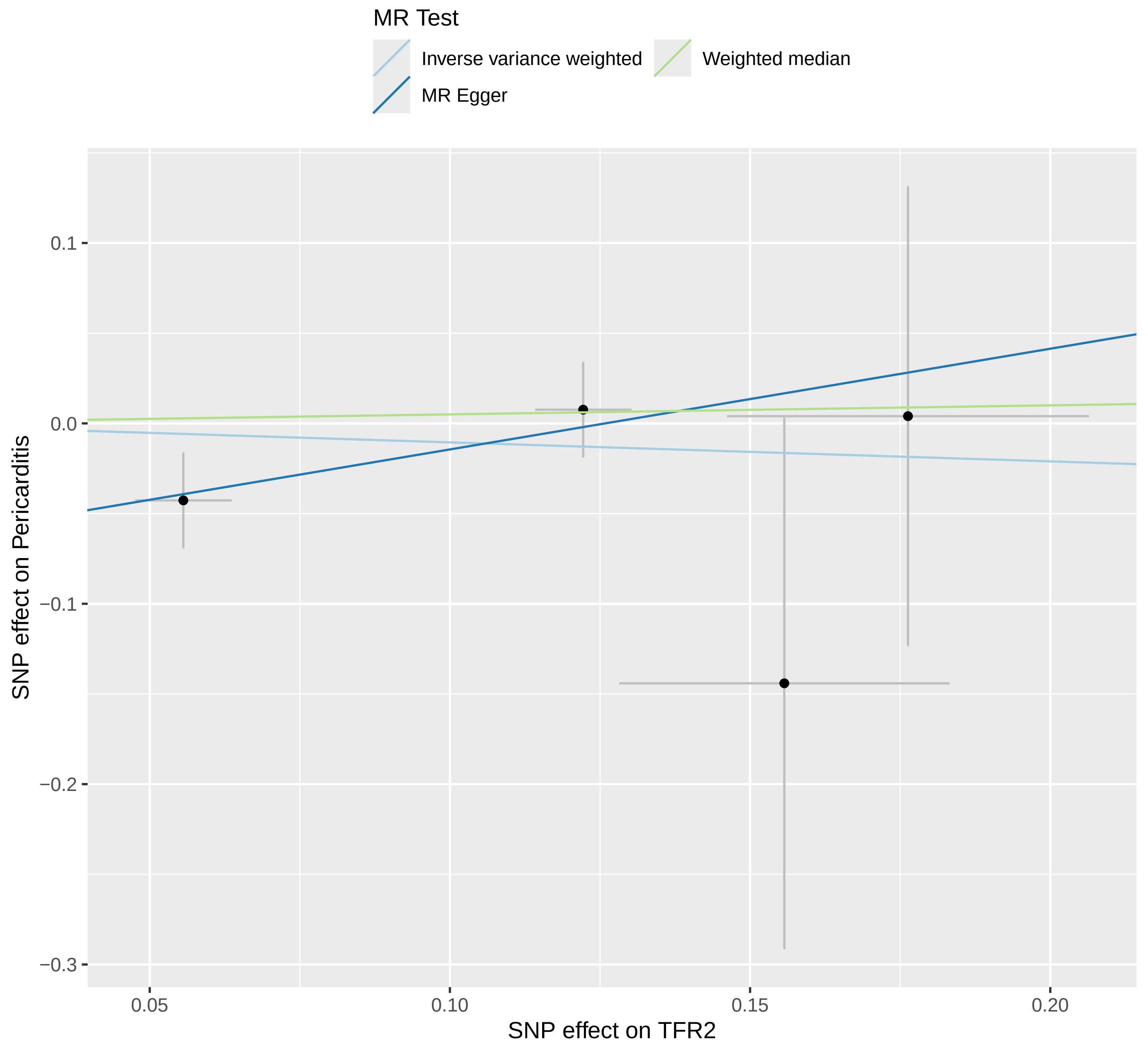
MR Test
Inverse variance weighted
Weighted median
MR Egger
0.1
0.0
−0.1
−0.2
−0.3
SNP effect on Pericarditis
0.05
0.10
0.15
0.20
SNP effect on TFR2

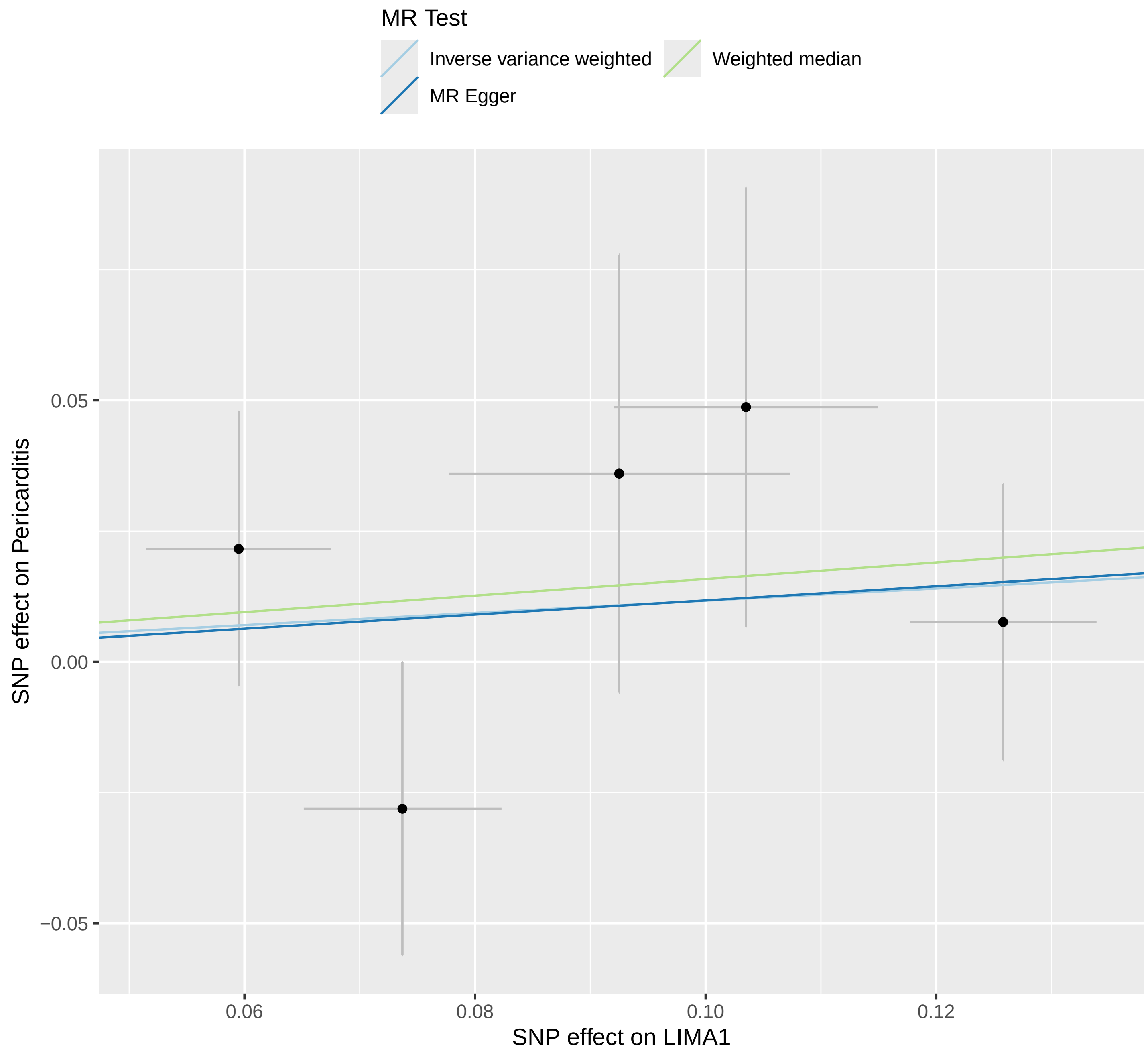
MR Test
Inverse variance weighted
MR Egger
Weighted median
0.05
0.00
−0.05
SNP effect on Pericarditis
0.06
0.08
0.10
0.12
SNP effect on LIMA1

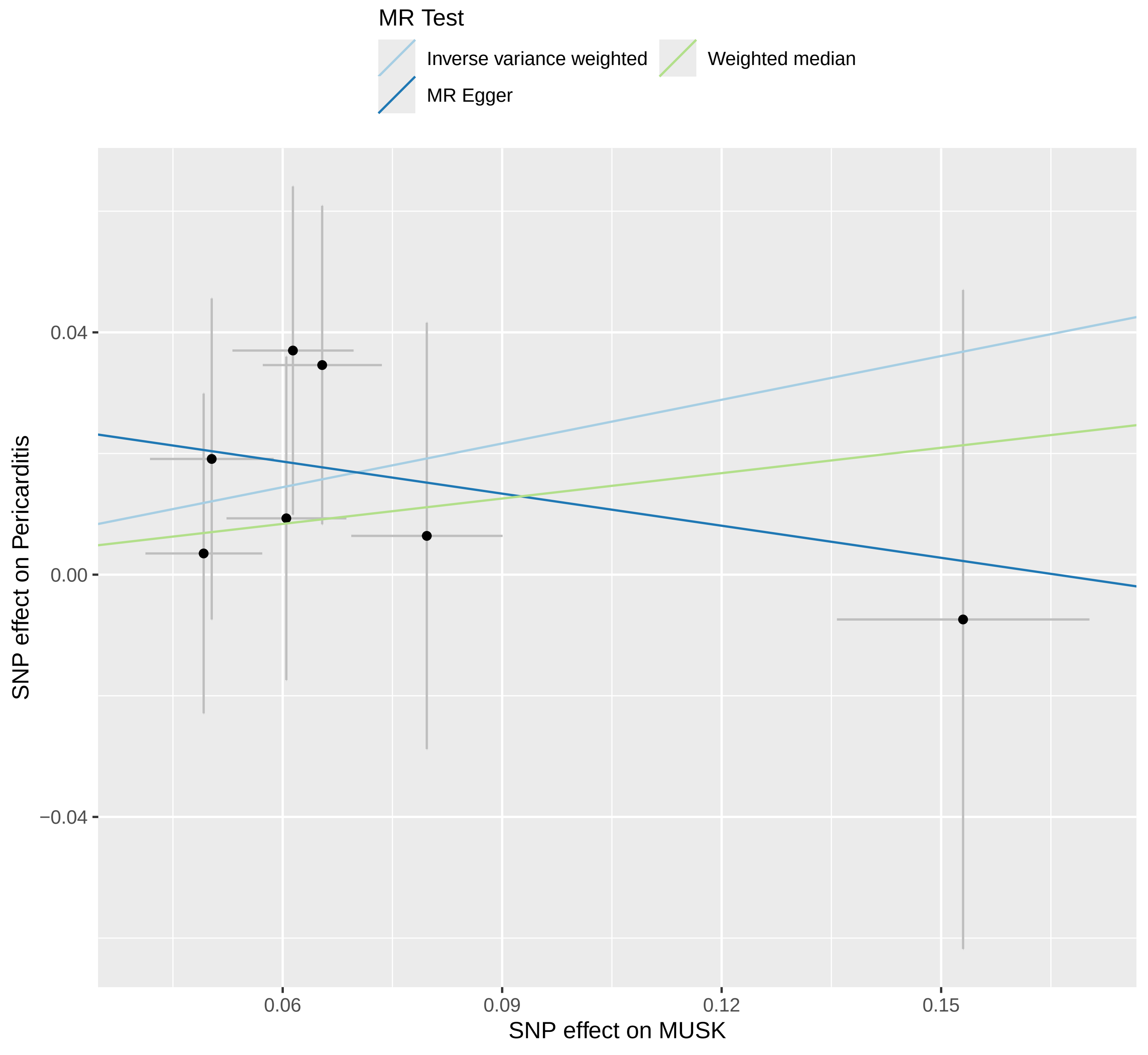

MR Test
Inverse variance weighted
Weighted median
MR Egger
0.04
0.00
−0.04
SNP effect on Pericarditis
0.06
0.09
0.12
0.15
SNP effect on MUSK

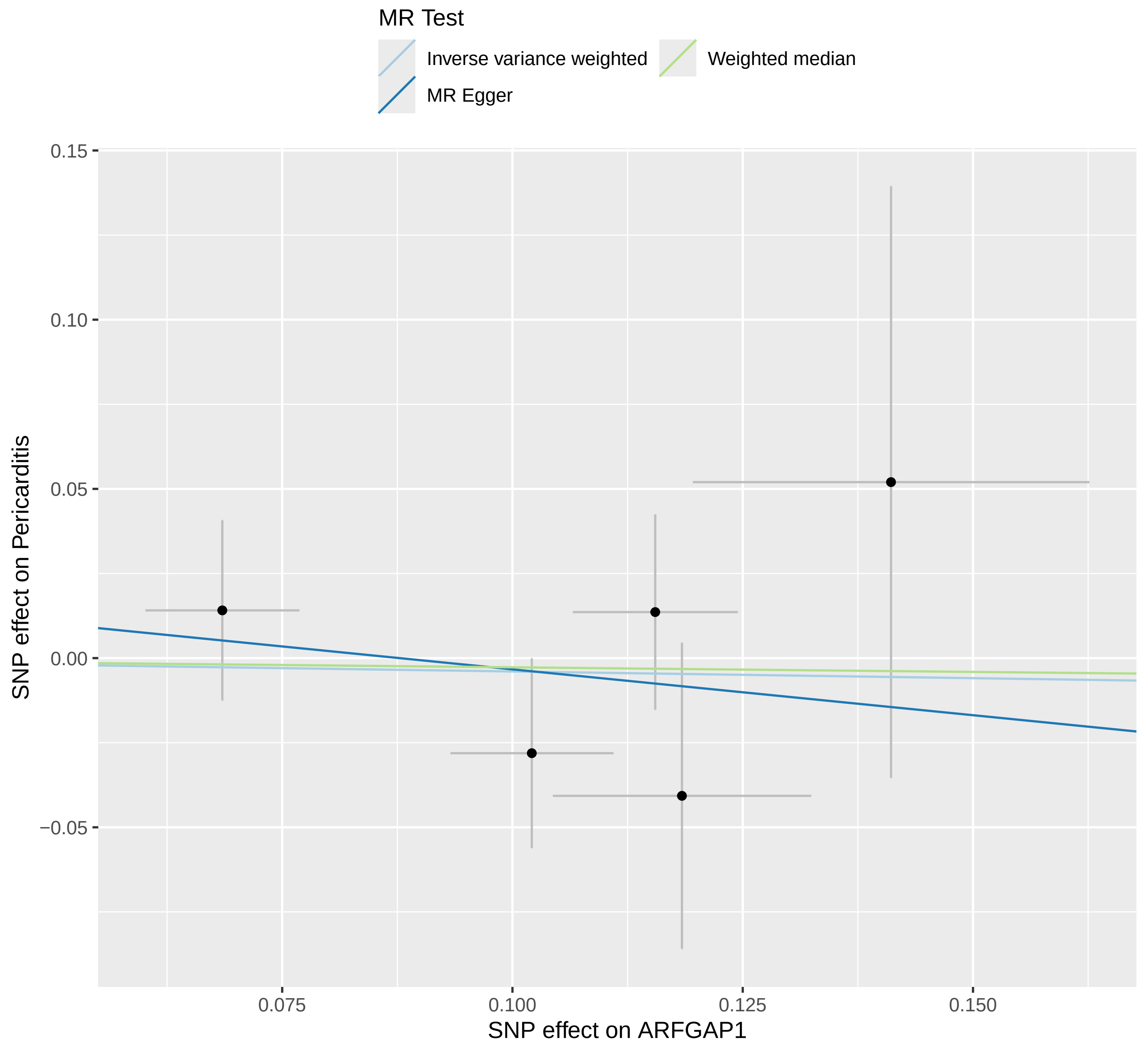
MR Test
Inverse variance weighted
Weighted median
MR Egger
0.15
0.10
0.05
0.00
−0.05
0.075
0.100
0.125
0.150
SNP effect on Pericarditis
SNP effect on ARFGAP1

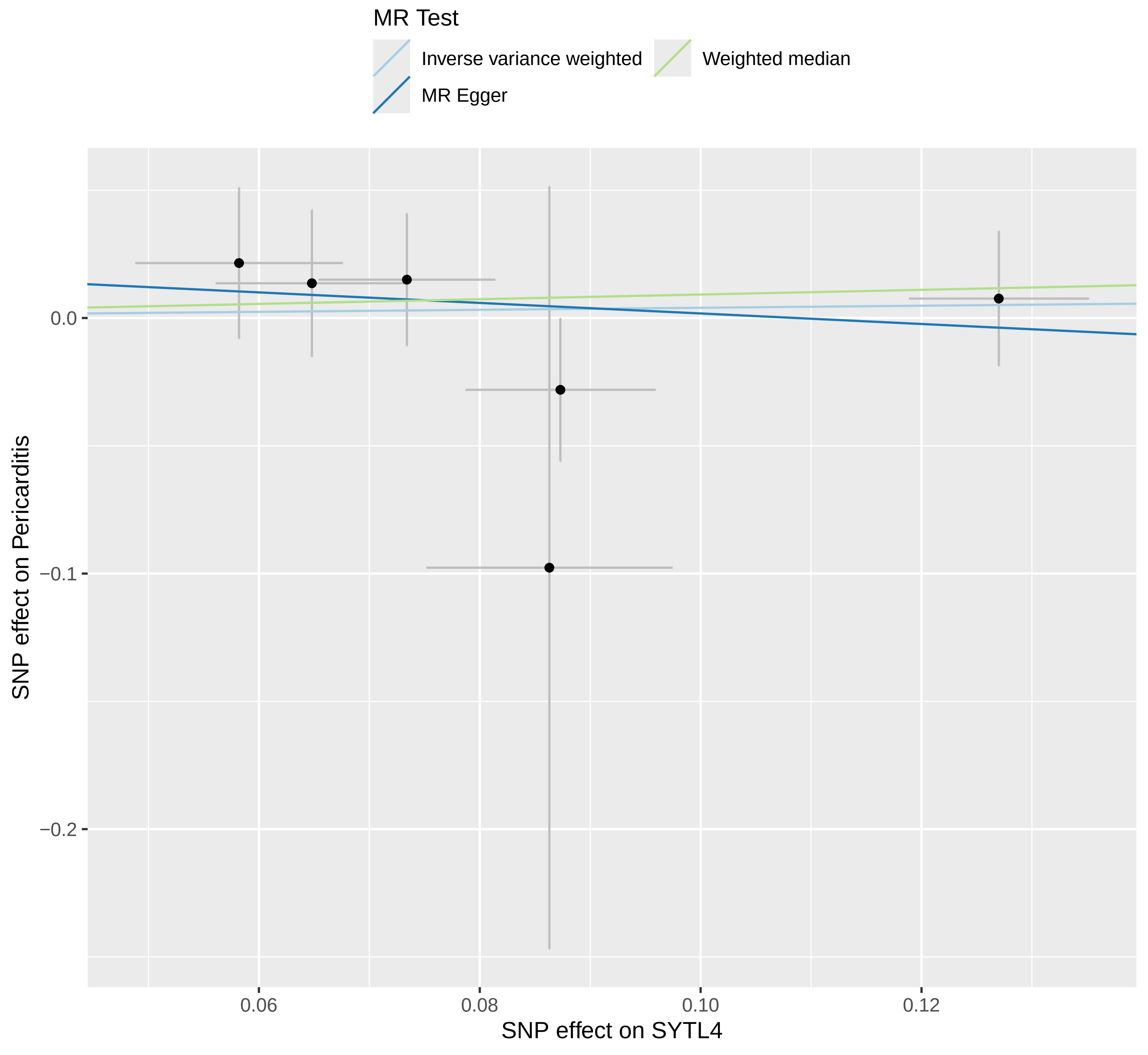
MR Test
Inverse variance weighted
Weighted median
MR Egger
0.0
−0.1
−0.2
0.06
0.08
0.10
0.12
SNP effect on SYTL4
SNP effect on Pericarditis

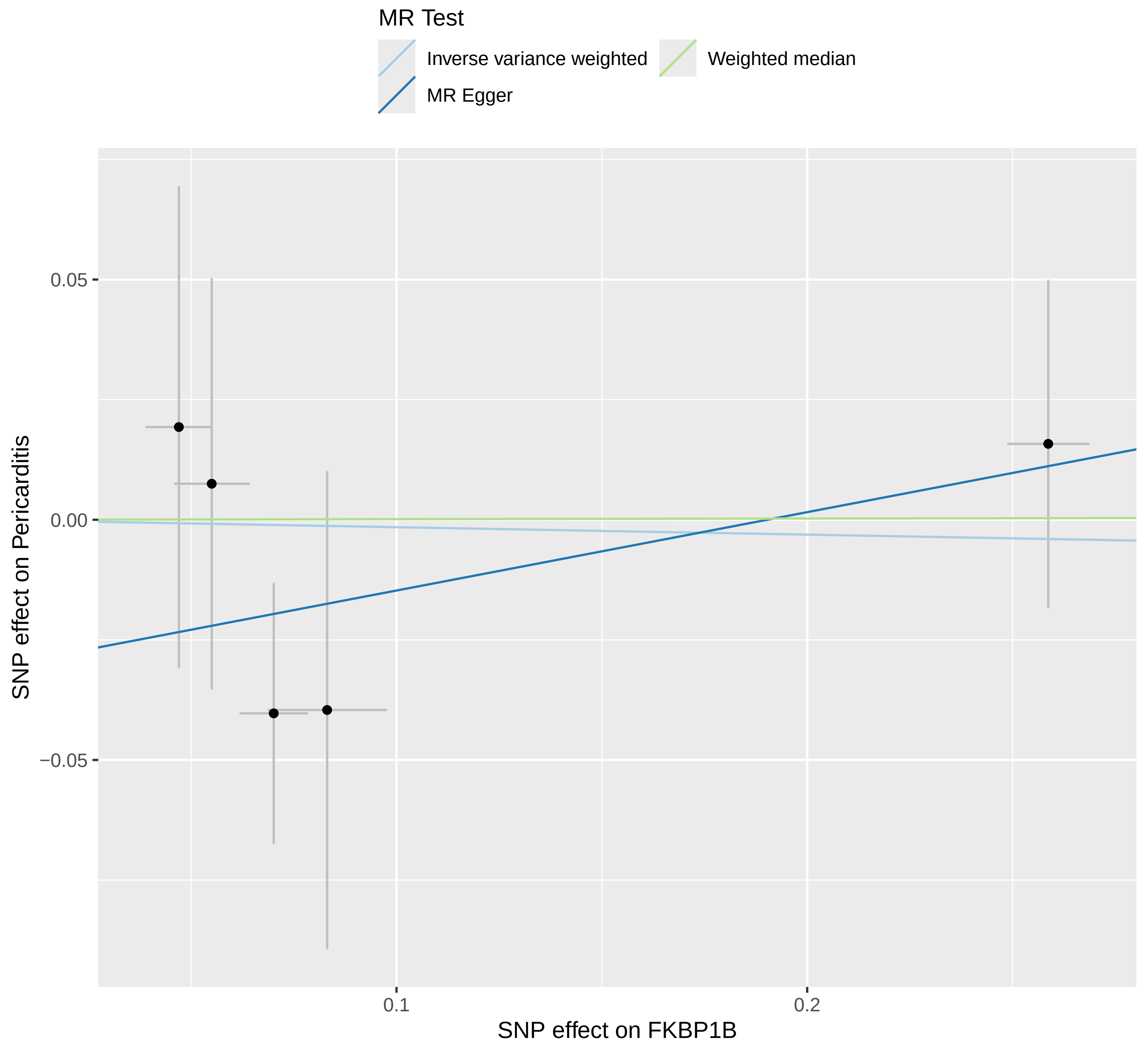
MR Test
Inverse variance weighted
Weighted median
MR Egger
0.05
0.00
−0.05
SNP effect on Pericarditis
0.1
0.2
SNP effect on FKBP1B

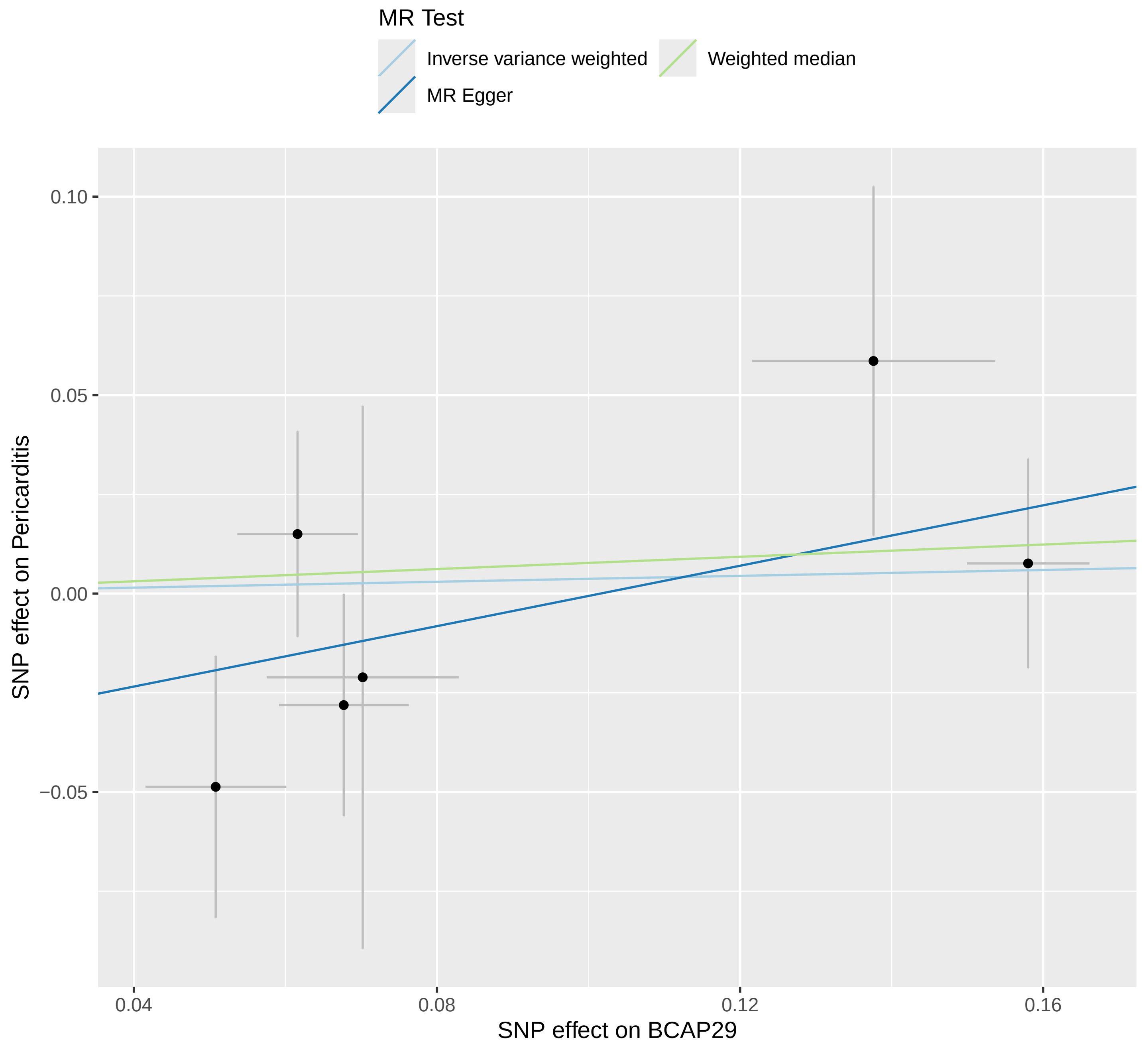

MR Test
Inverse variance weighted
Weighted median
MR Egger
0.10
0.05
0.00
−0.05
SNP effect on Pericarditis
0.04
0.08
0.12
0.16
SNP effect on BCAP29

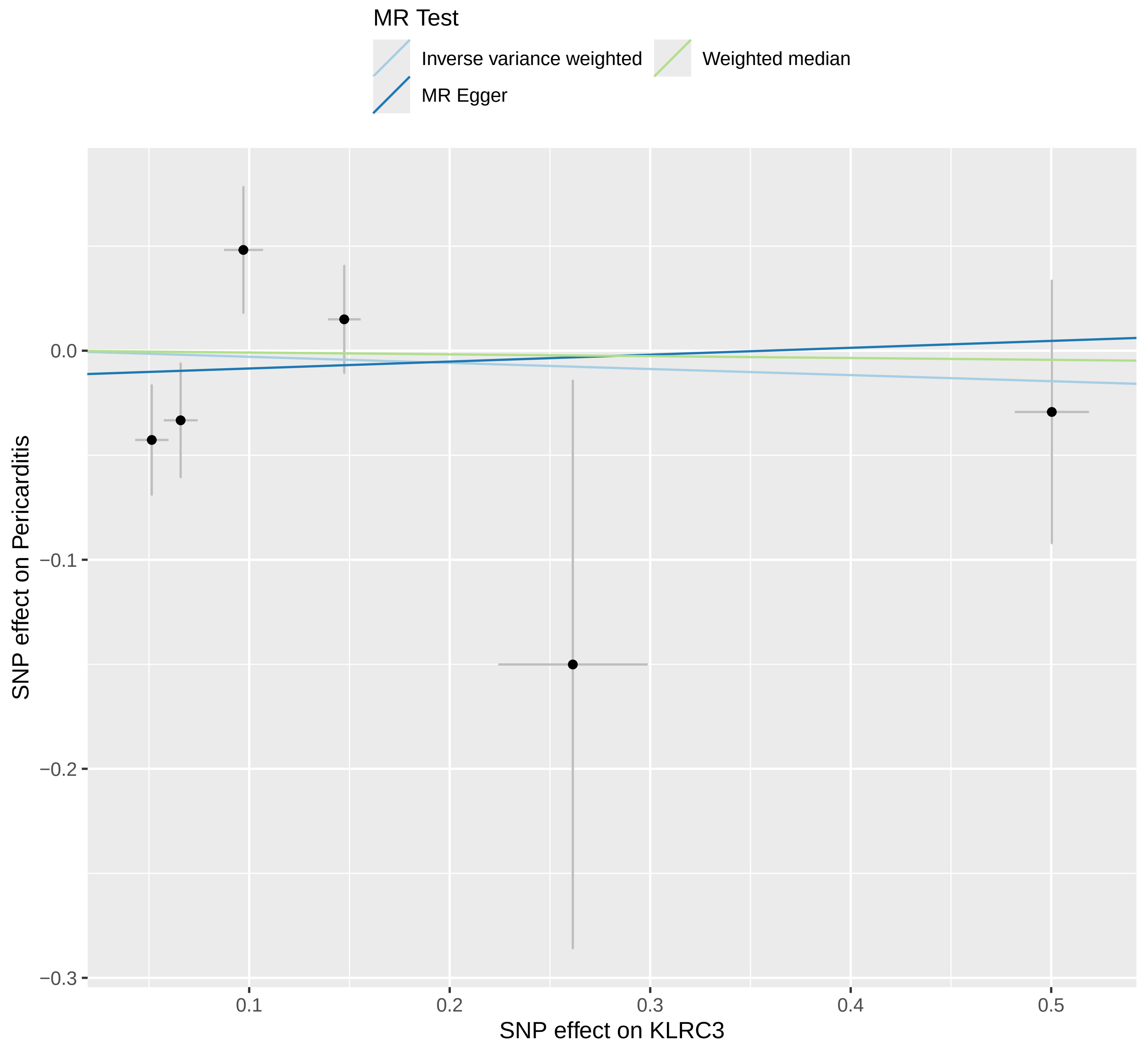
MR Test
Inverse variance weighted
Weighted median
MR Egger
0.0
−0.1
−0.2
−0.3
0.1
0.2
0.3
0.4
0.5
SNP effect on KLRC3
SNP effect on Pericarditis

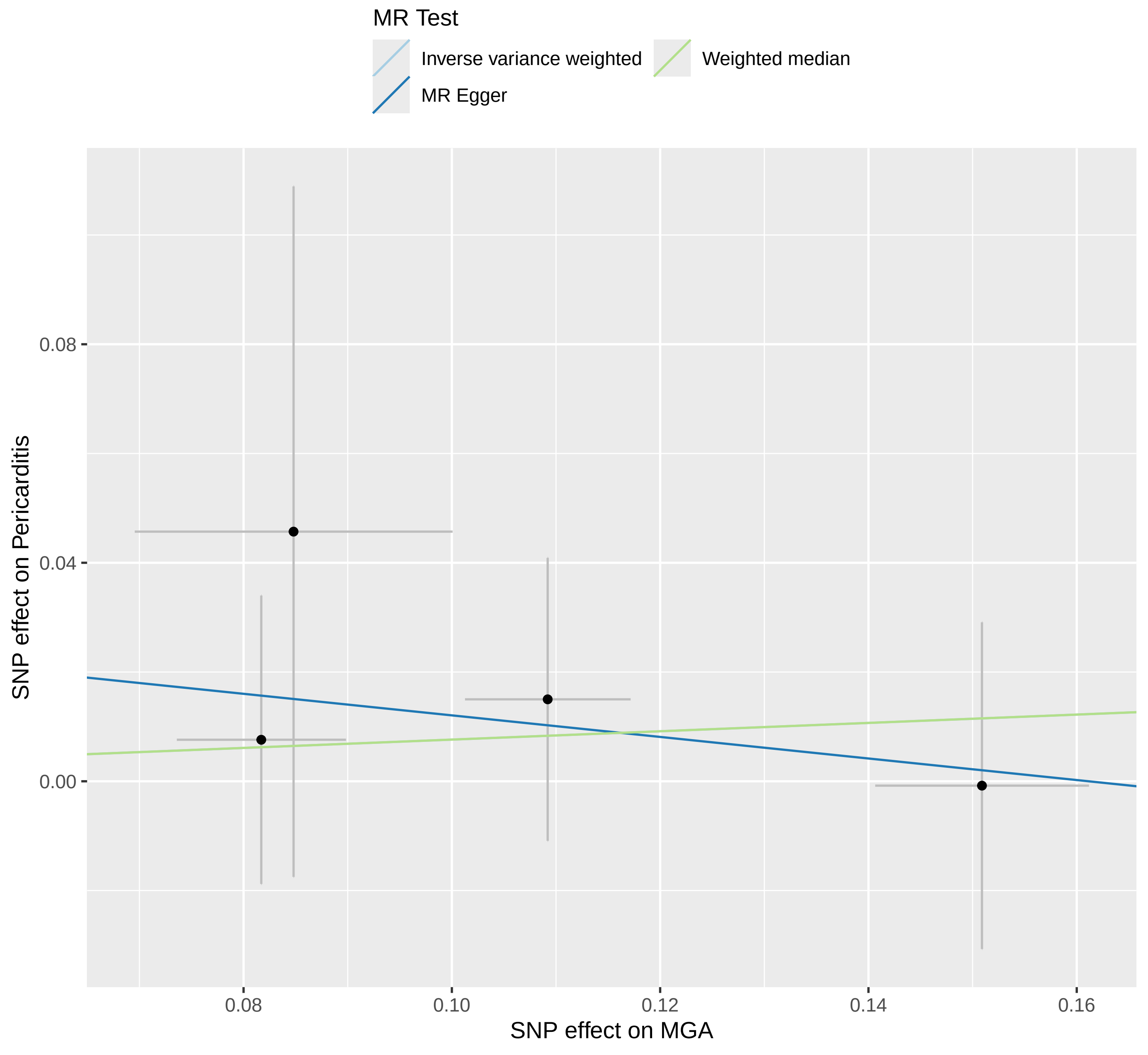
MR Test
Inverse variance weighted
Weighted median
MR Egger
SNP effect on Pericarditis
0.08
0.04
0.00
0.08
0.10
0.12
0.14
0.16
SNP effect on MGA

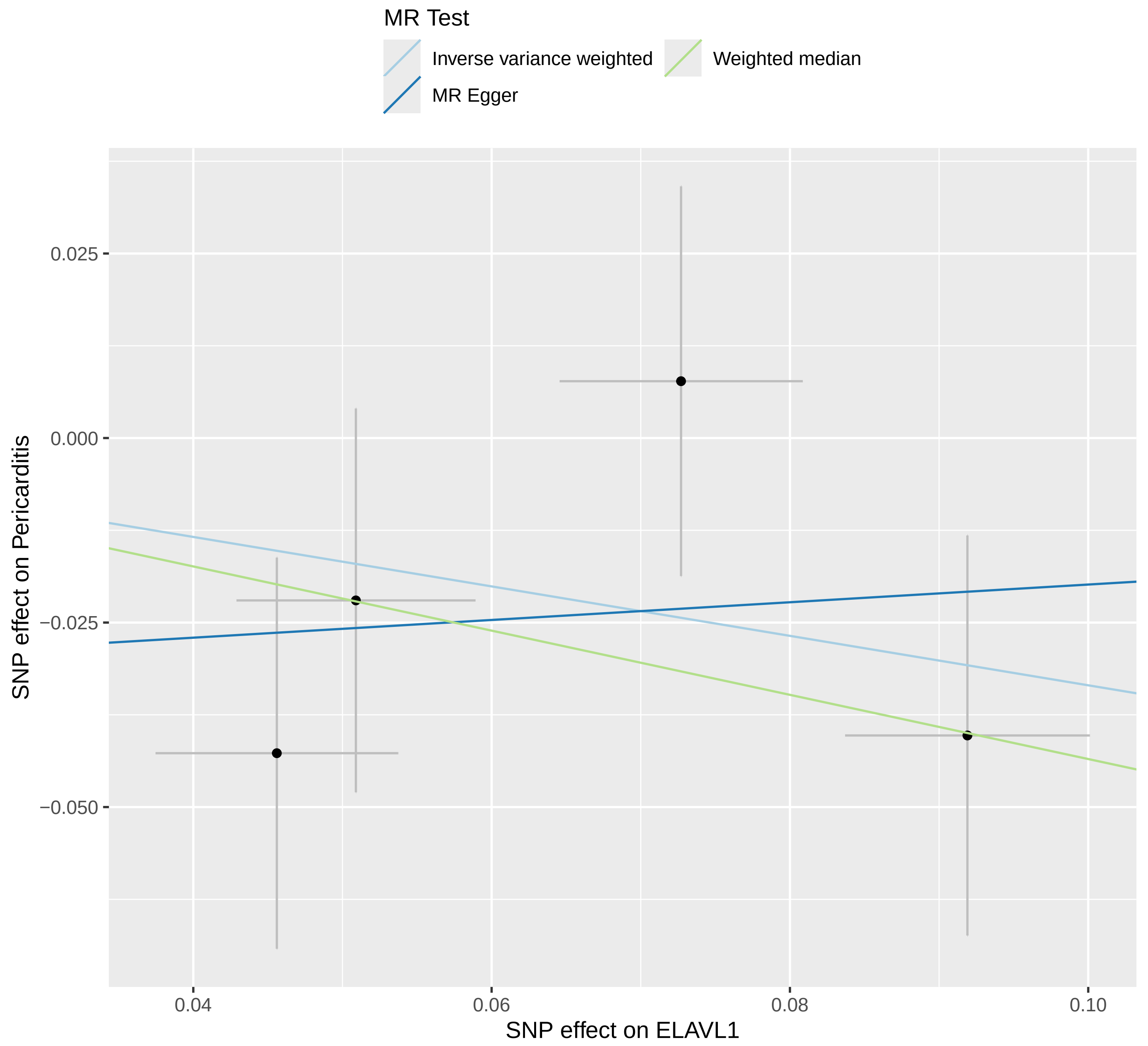

MR Test
Inverse variance weighted
MR Egger
Weighted median
0.025
0.000
−0.025
−0.050
SNP effect on Pericarditis
0.04
0.06
0.08
0.10
SNP effect on ELAVL1

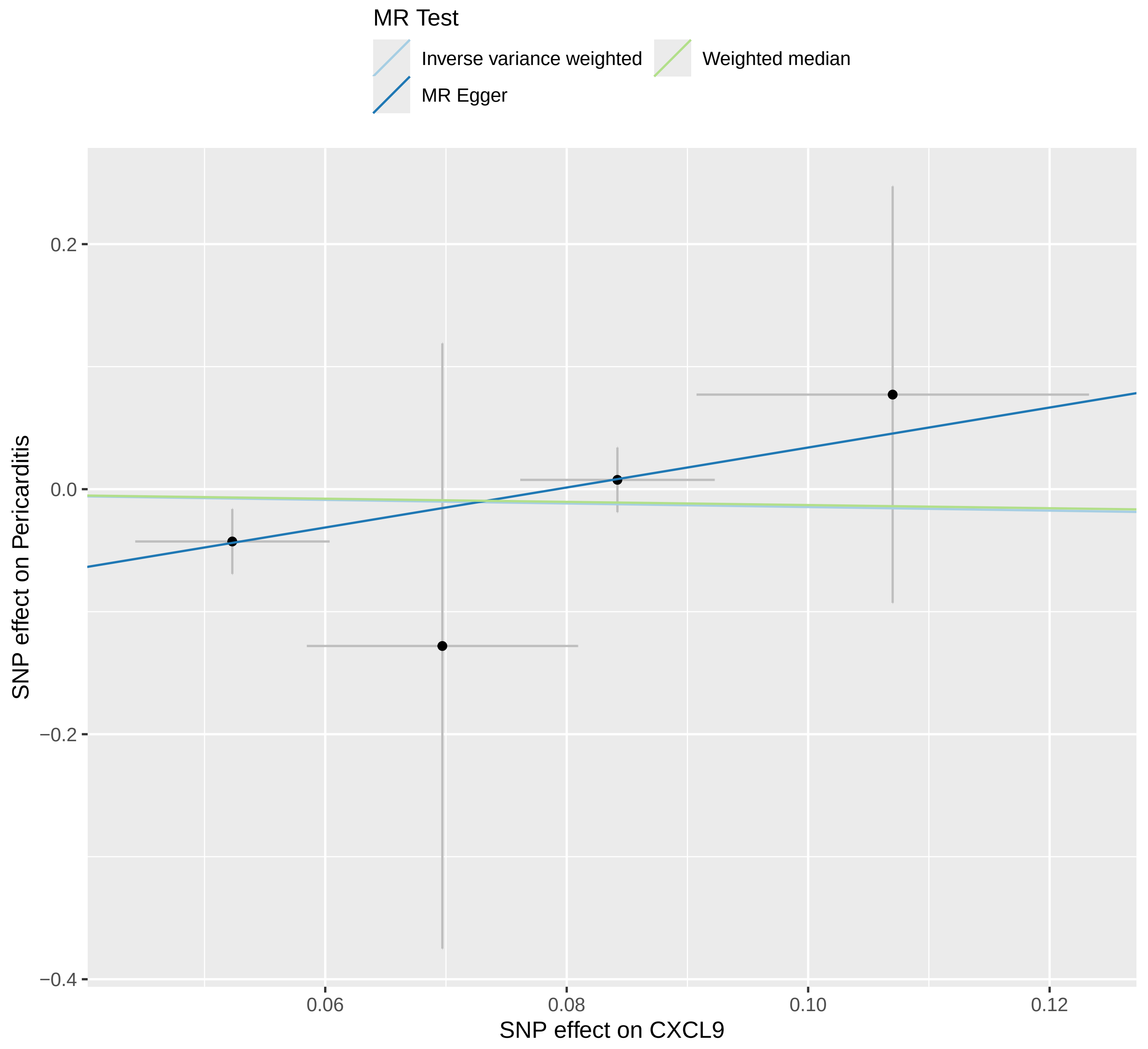

MR Test
Inverse variance weighted
Weighted median
MR Egger
0.2
0.0
−0.2
−0.4
0.06
0.08
0.10
0.12
SNP effect on Pericarditis
SNP effect on CXCL9

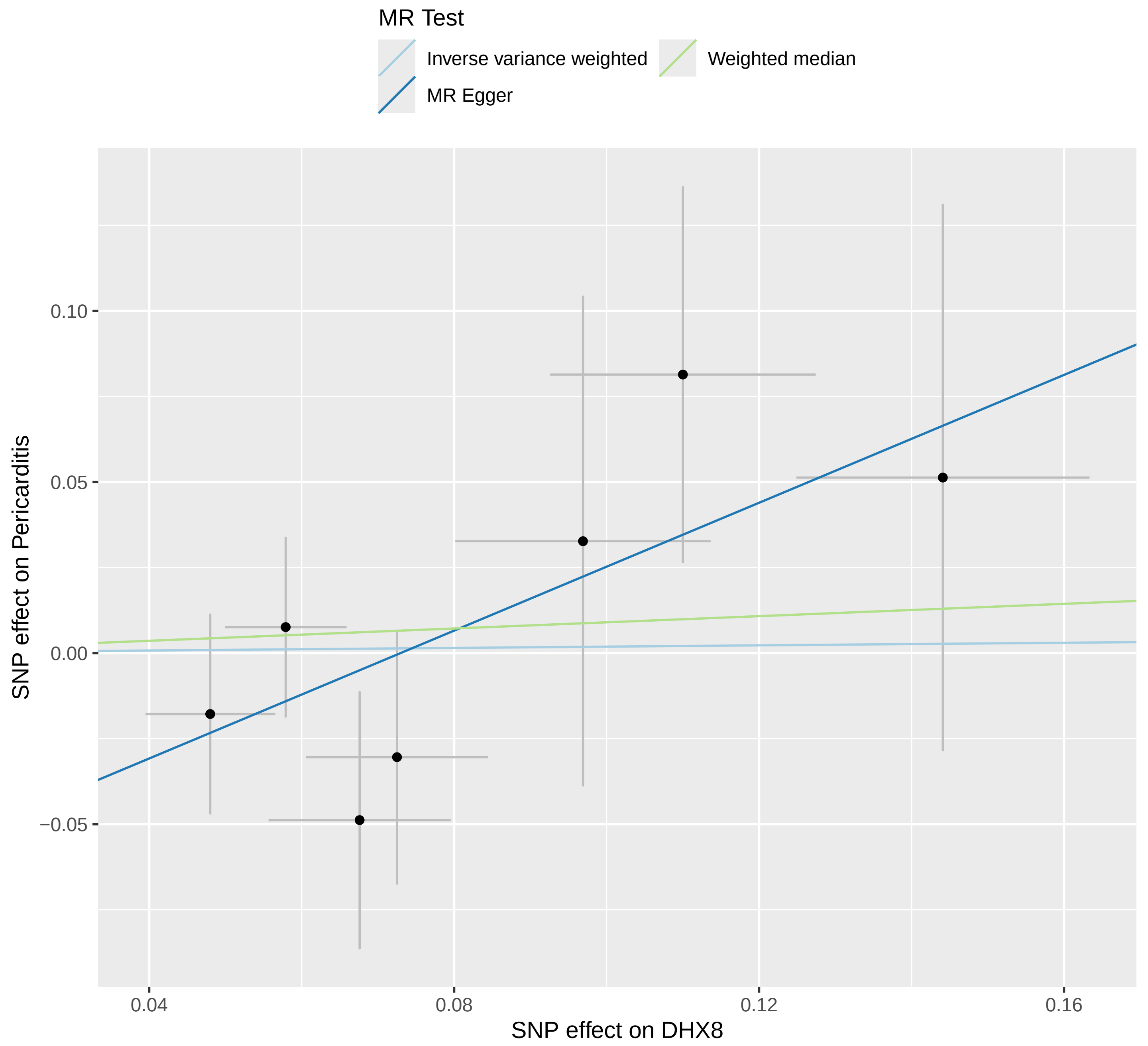

MR Test
Inverse variance weighted
Weighted median
MR Egger
0.10
0.05
0.00
−0.05
SNP effect on Pericarditis
0.04
0.08
0.12
0.16
SNP effect on DHX8

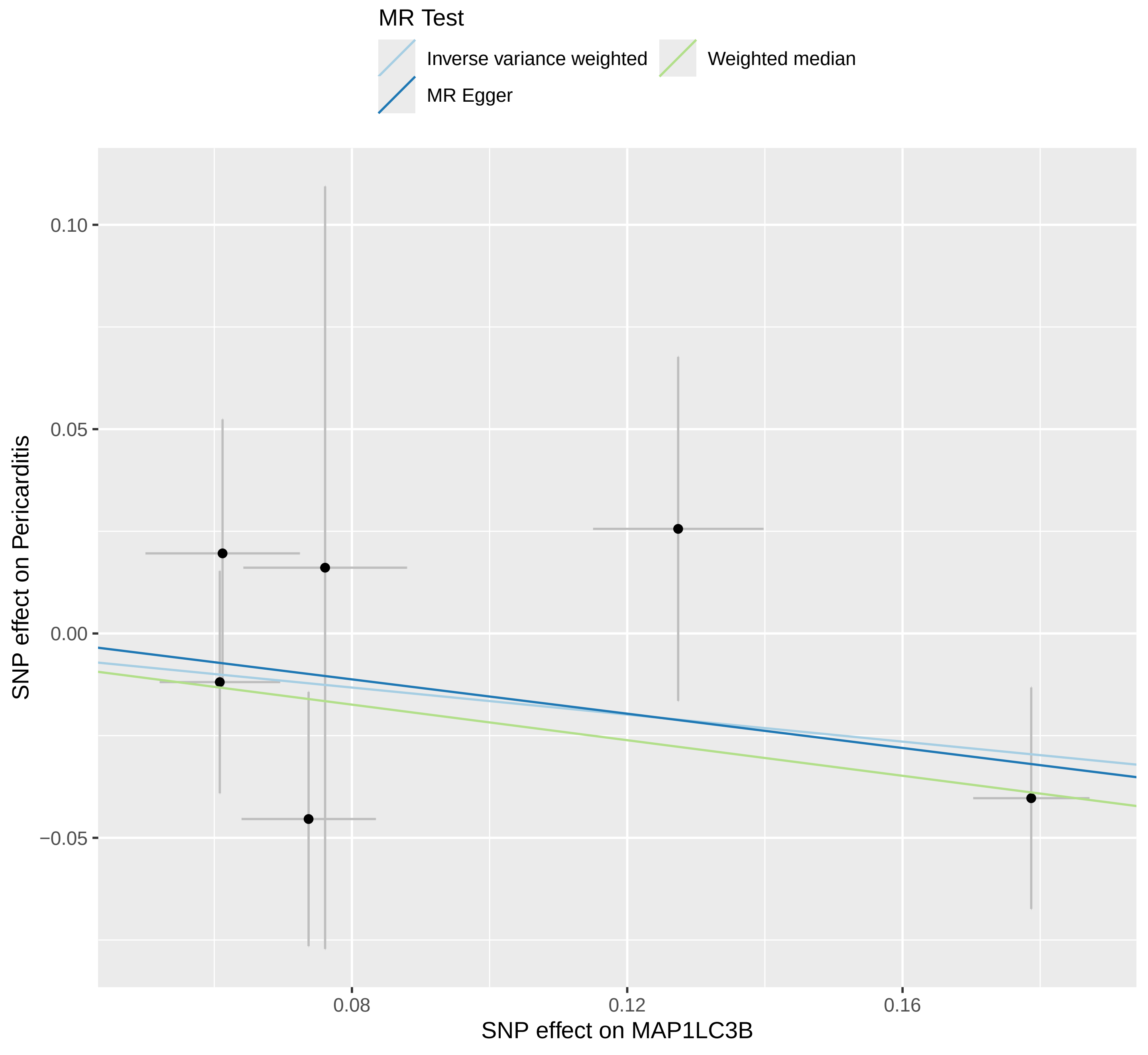
MR Test
Inverse variance weighted
MR Egger
Weighted median
0.10
0.05
0.00
−0.05
SNP effect on Pericarditis
0.08
0.12
0.16
SNP effect on MAP1LC3B

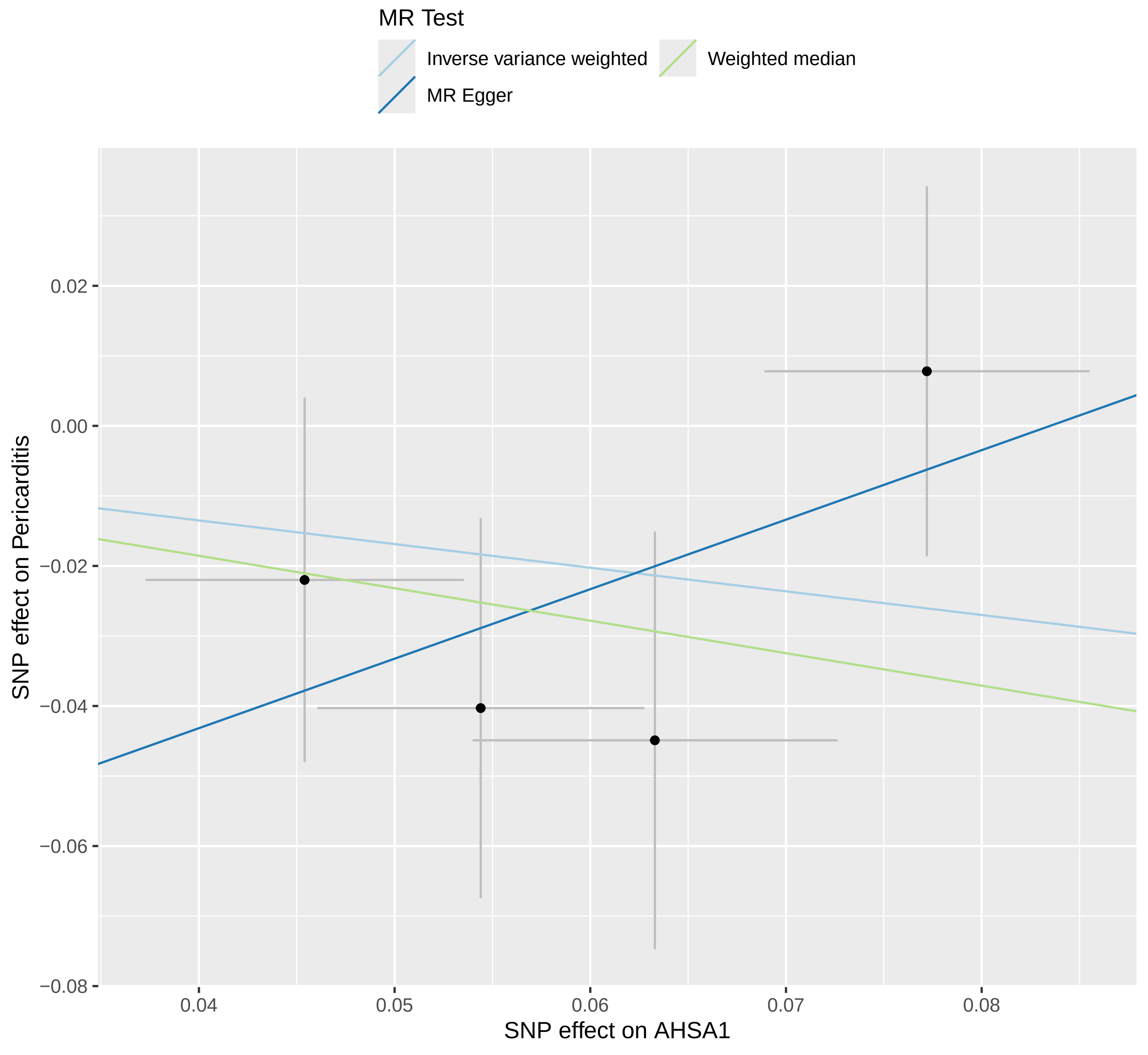

MR Test
Inverse variance weighted
Weighted median
MR Egger
0.02
0.00
−0.02
−0.04
−0.06
−0.08
0.04
0.05
0.06
0.07
0.08
SNP effect on Pericarditis
SNP effect on AHSA1

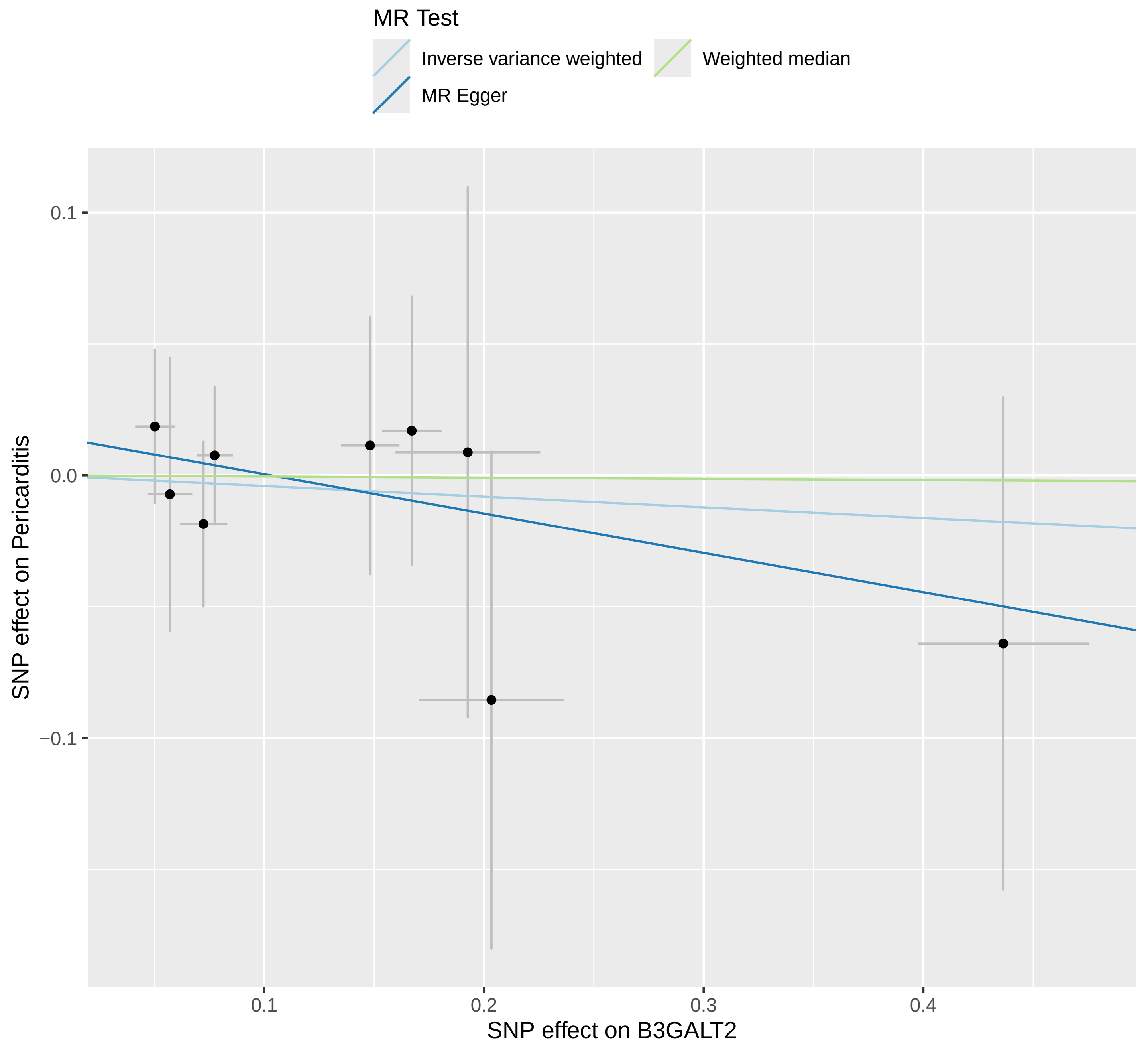
MR Test
Inverse variance weighted
MR Egger
Weighted median
SNP effect on Pericarditis
0.1
0.0
−0.1
0.1
0.2
0.3
0.4
SNP effect on B3GALT2

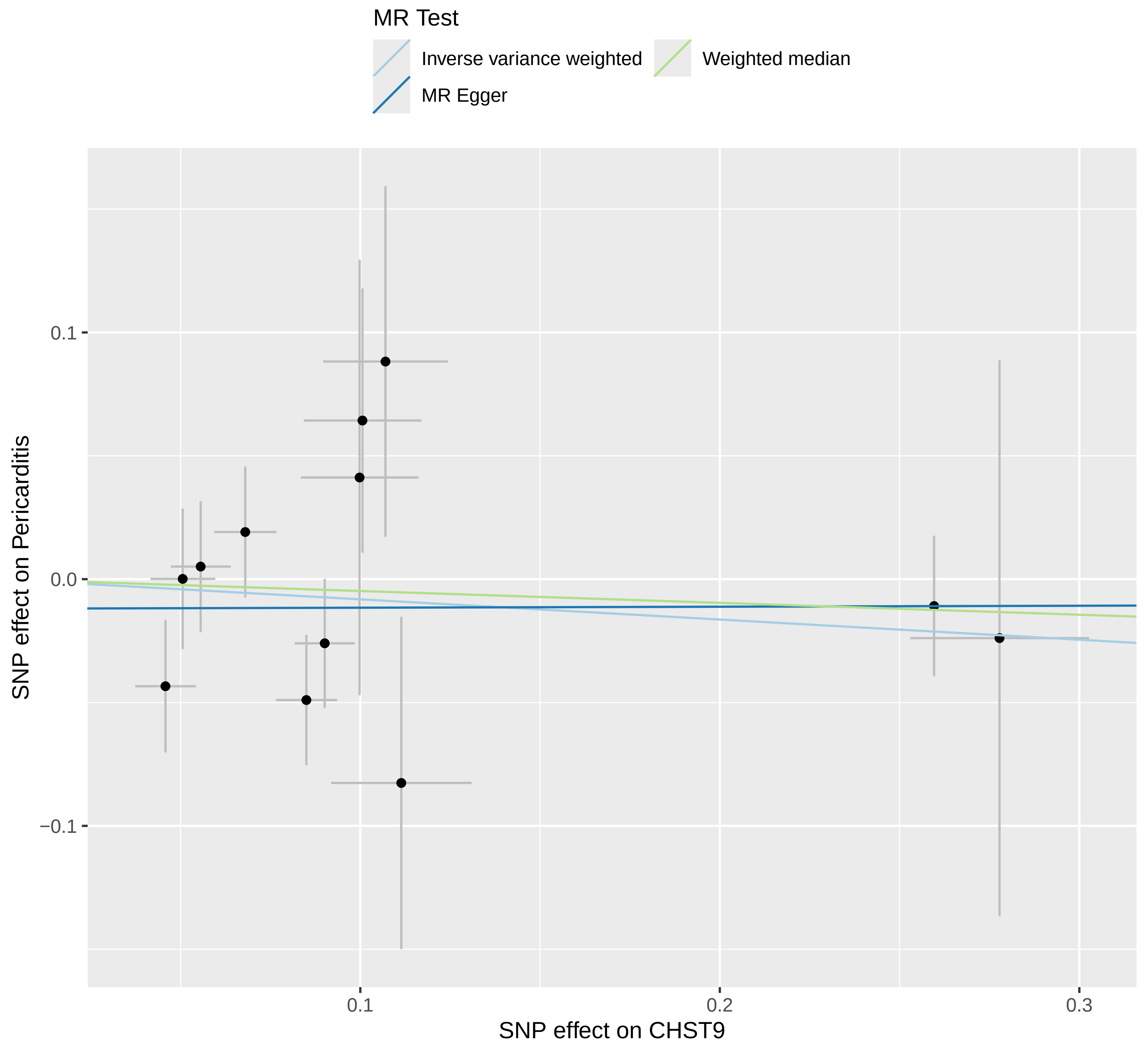

MR Test
Inverse variance weighted
Weighted median
MR Egger
0.1
0.0
−0.1
SNP effect on Pericarditis
0.1
0.2
0.3
SNP effect on CHST9

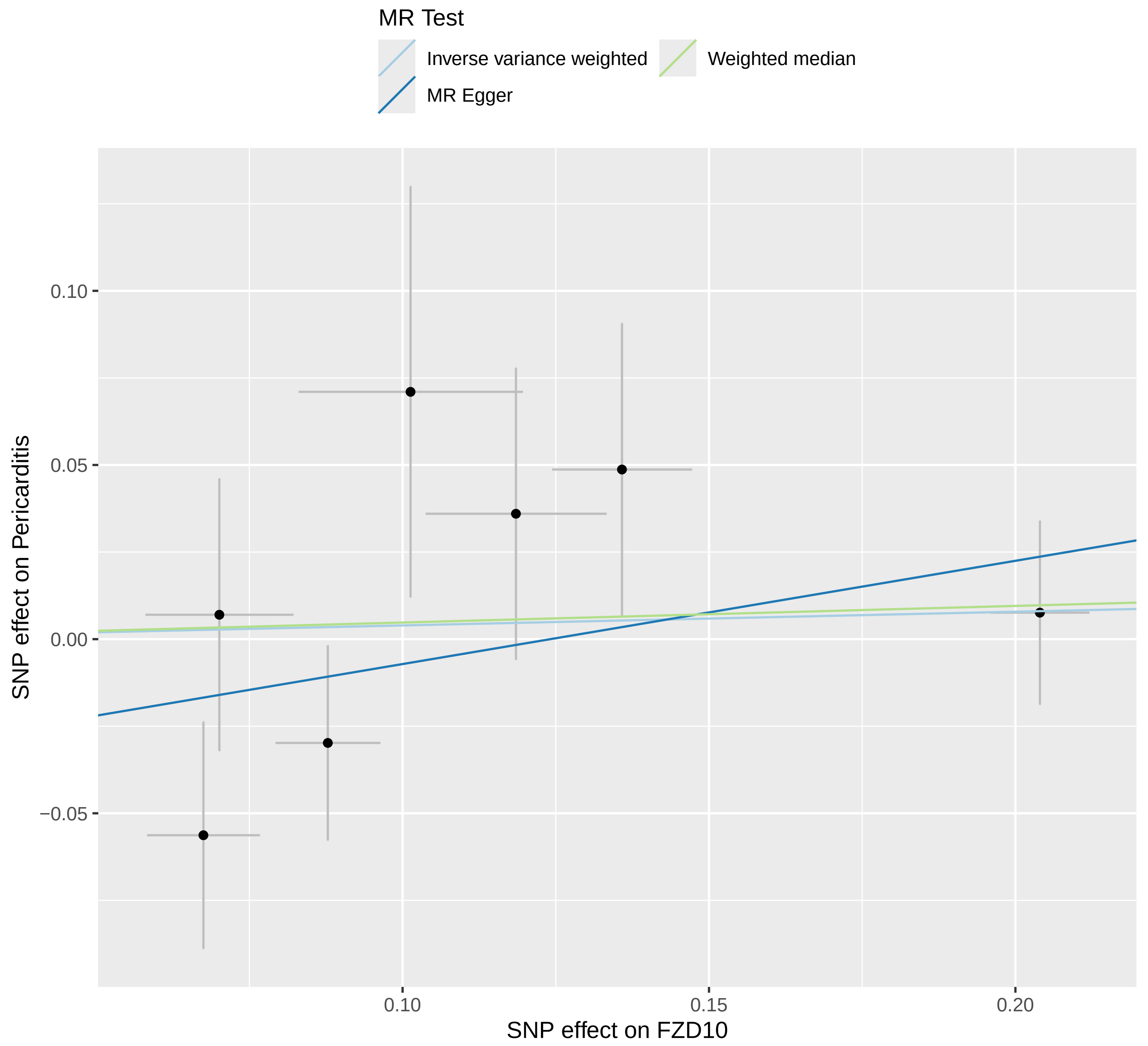
MR Test
Inverse variance weighted
Weighted median
MR Egger
SNP effect on Pericarditis
0.10
0.05
0.00
−0.05
0.10
0.15
0.20
SNP effect on FZD10

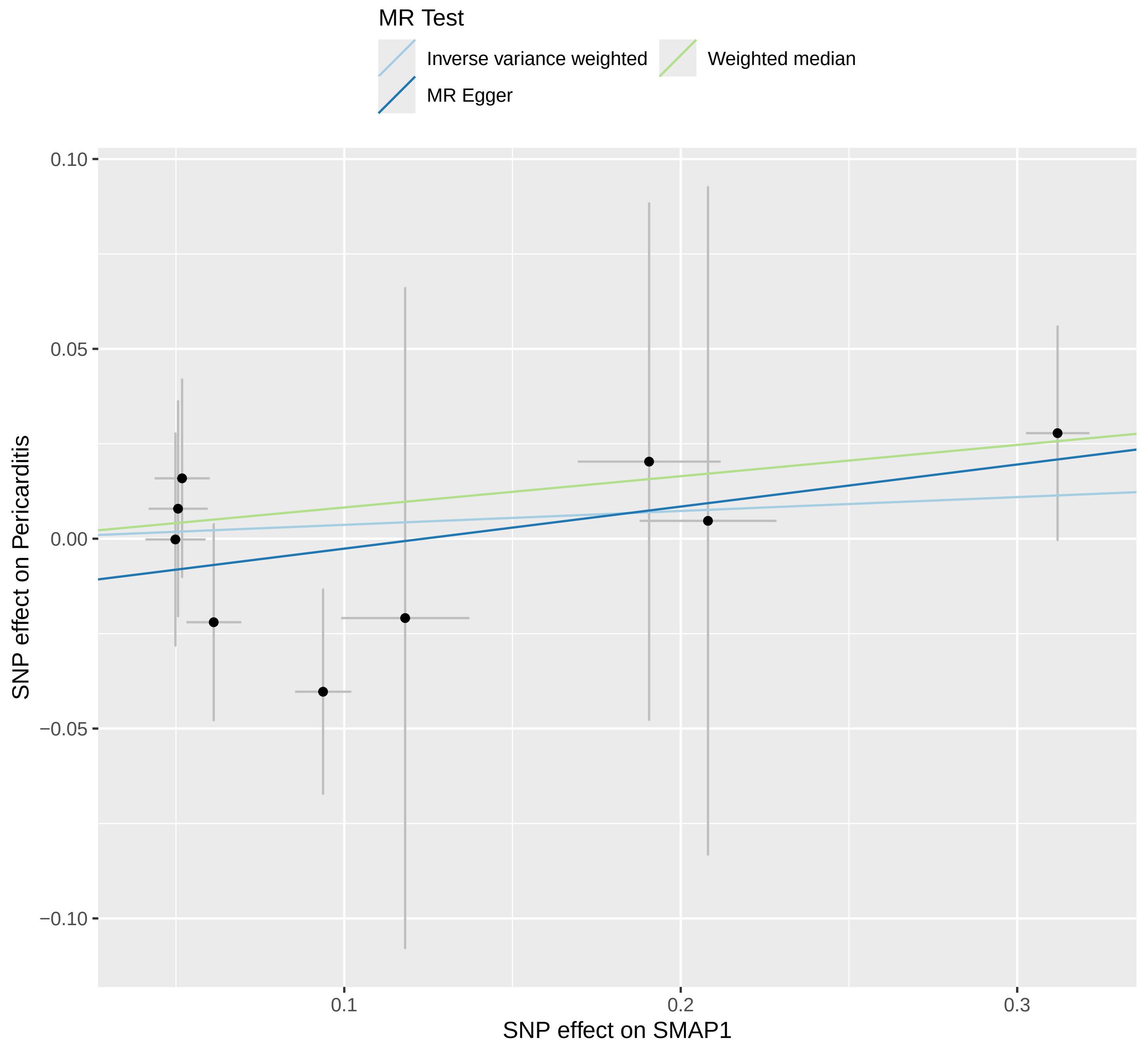
MR Test
Inverse variance weighted
Weighted median
MR Egger
0.10
0.05
0.00
−0.05
−0.10
0.1
0.2
0.3
SNP effect on Pericarditis
SNP effect on SMAP1

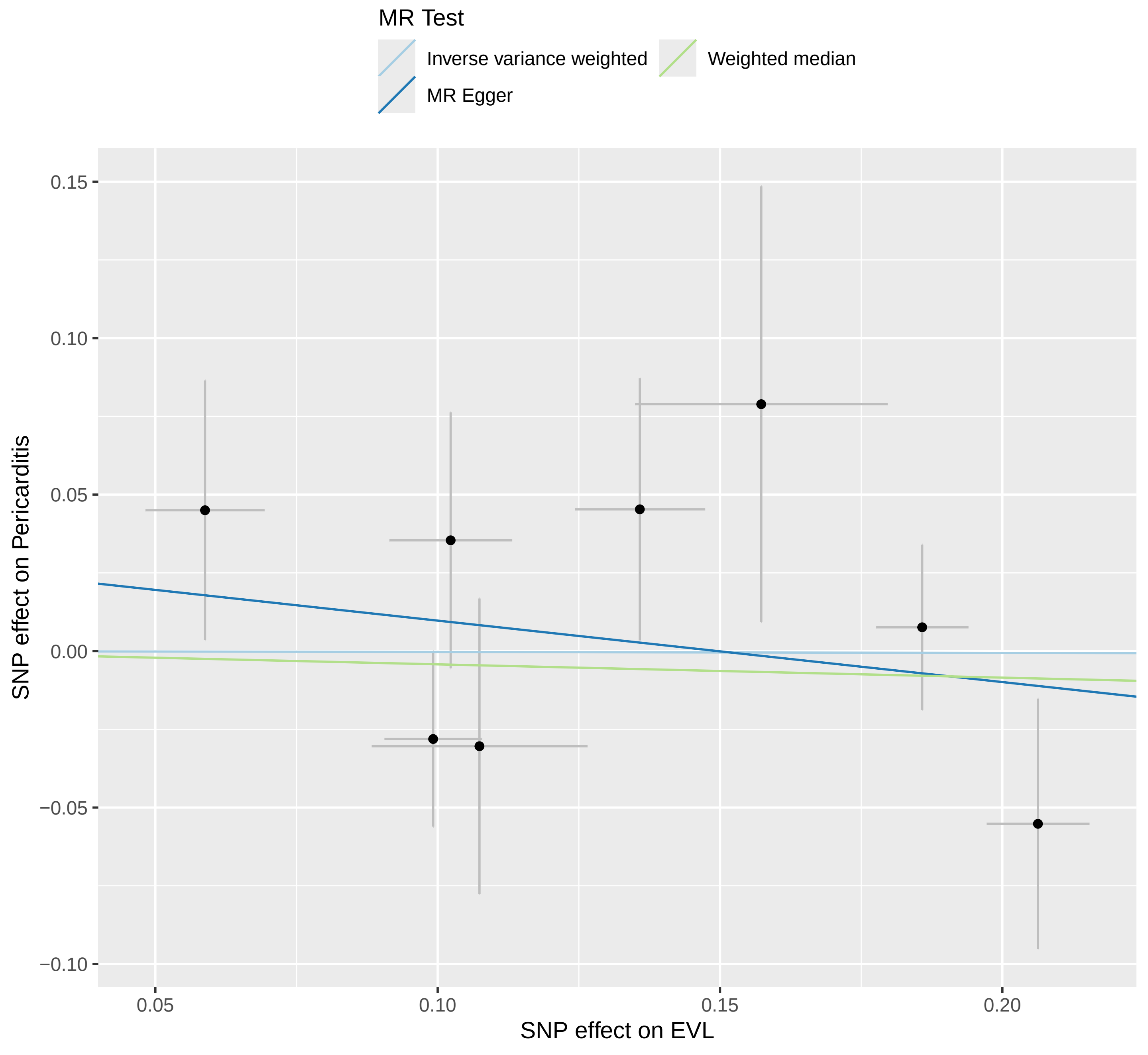
MR Test
Inverse variance weighted
Weighted median
MR Egger
SNP effect on Pericarditis
0.15
0.10
0.05
0.00
−0.05
−0.10
0.05
0.10
0.15
0.20
SNP effect on EVL

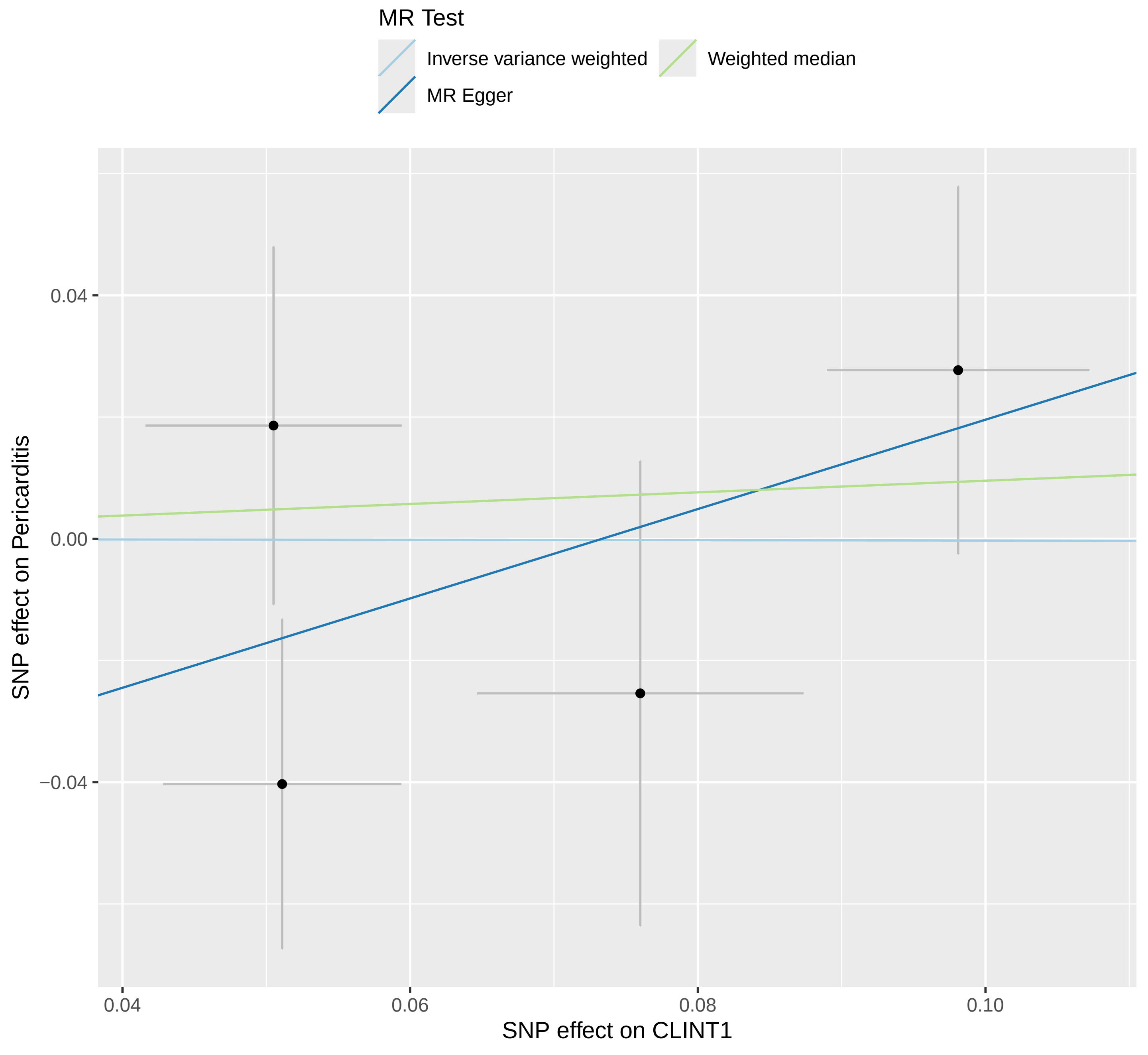
MR Test
Inverse variance weighted
Weighted median
MR Egger
SNP effect on Pericarditis
0.04
0.00
−0.04
0.04
0.06
0.08
0.10
SNP effect on CLINT1

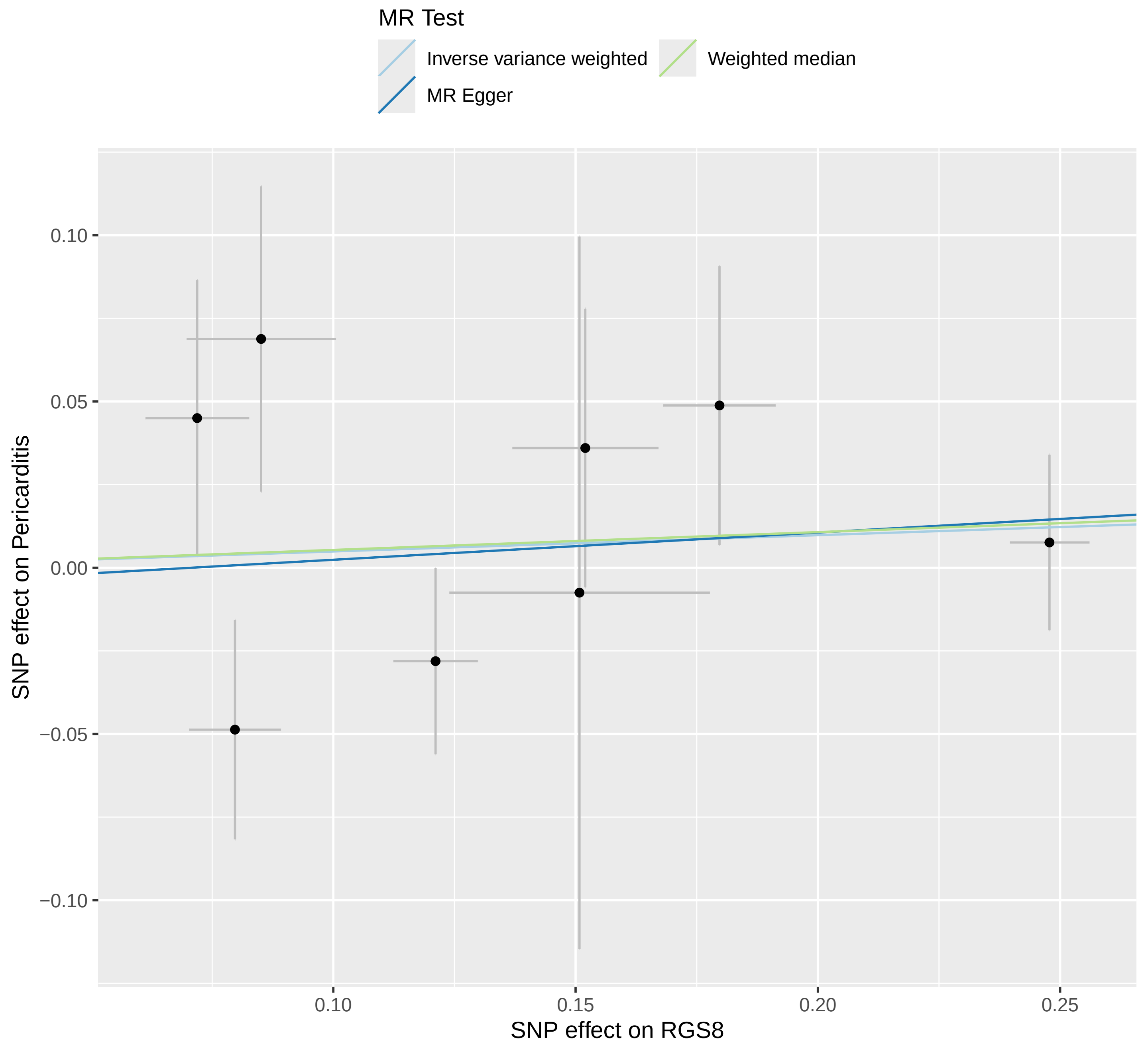
MR Test
Inverse variance weighted
Weighted median
MR Egger
0.10
0.05
0.00
−0.05
−0.10
SNP effect on Pericarditis
0.10
0.15
0.20
0.25
SNP effect on RGS8

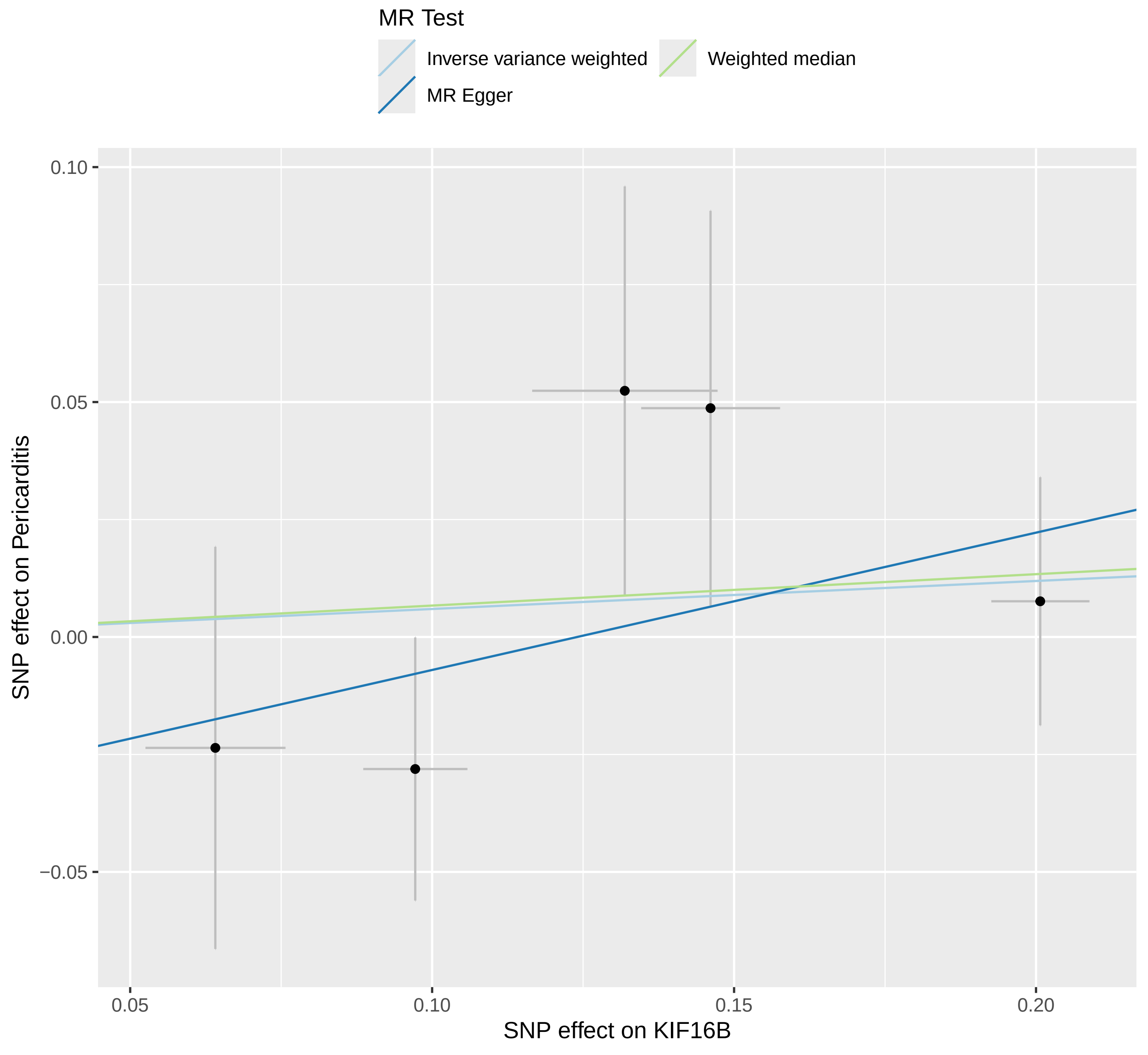

MR Test
Inverse variance weighted
Weighted median
MR Egger
0.10
0.05
0.00
−0.05
SNP effect on Pericarditis
0.05
0.10
0.15
0.20
SNP effect on KIF16B

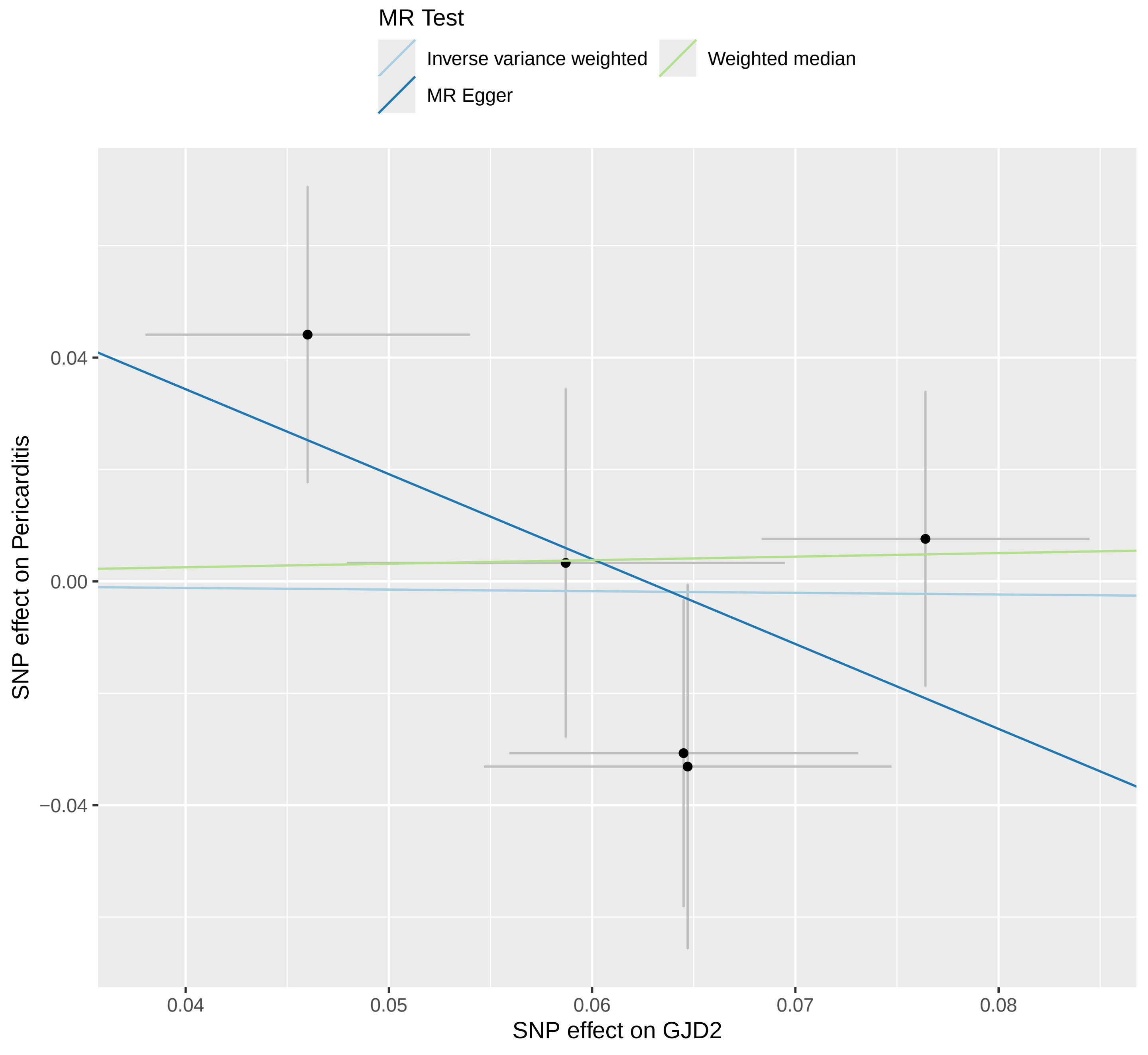
MR Test
Inverse variance weighted
MR Egger
Weighted median
SNP effect on Pericarditis
0.04
0.00
−0.04
0.04
0.05
0.06
0.07
0.08
SNP effect on GJD2

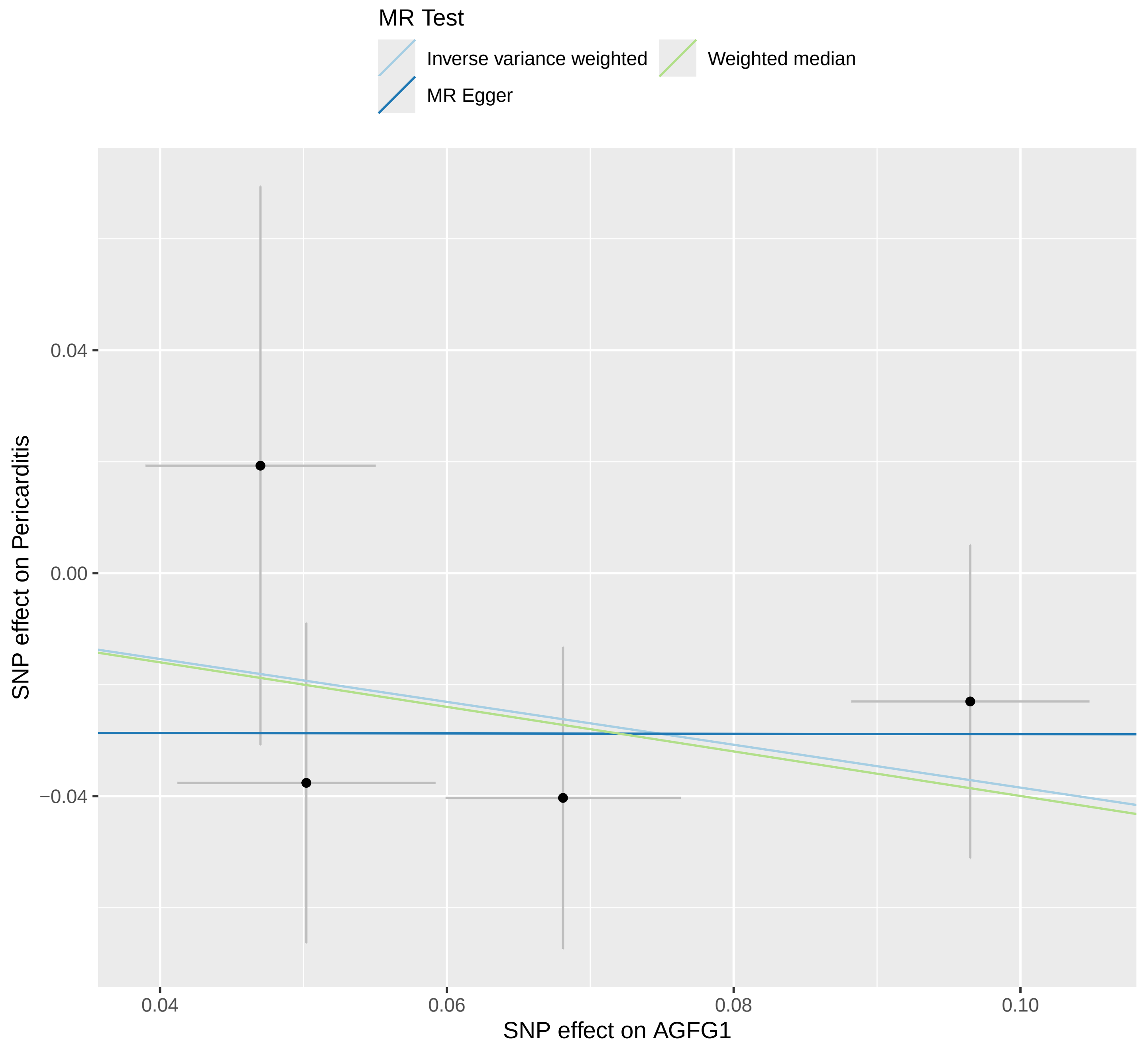
MR Test
Inverse variance weighted
Weighted median
MR Egger
0.04
0.00
−0.04
SNP effect on Pericarditis
0.04
0.06
0.08
0.10
SNP effect on AGFG1

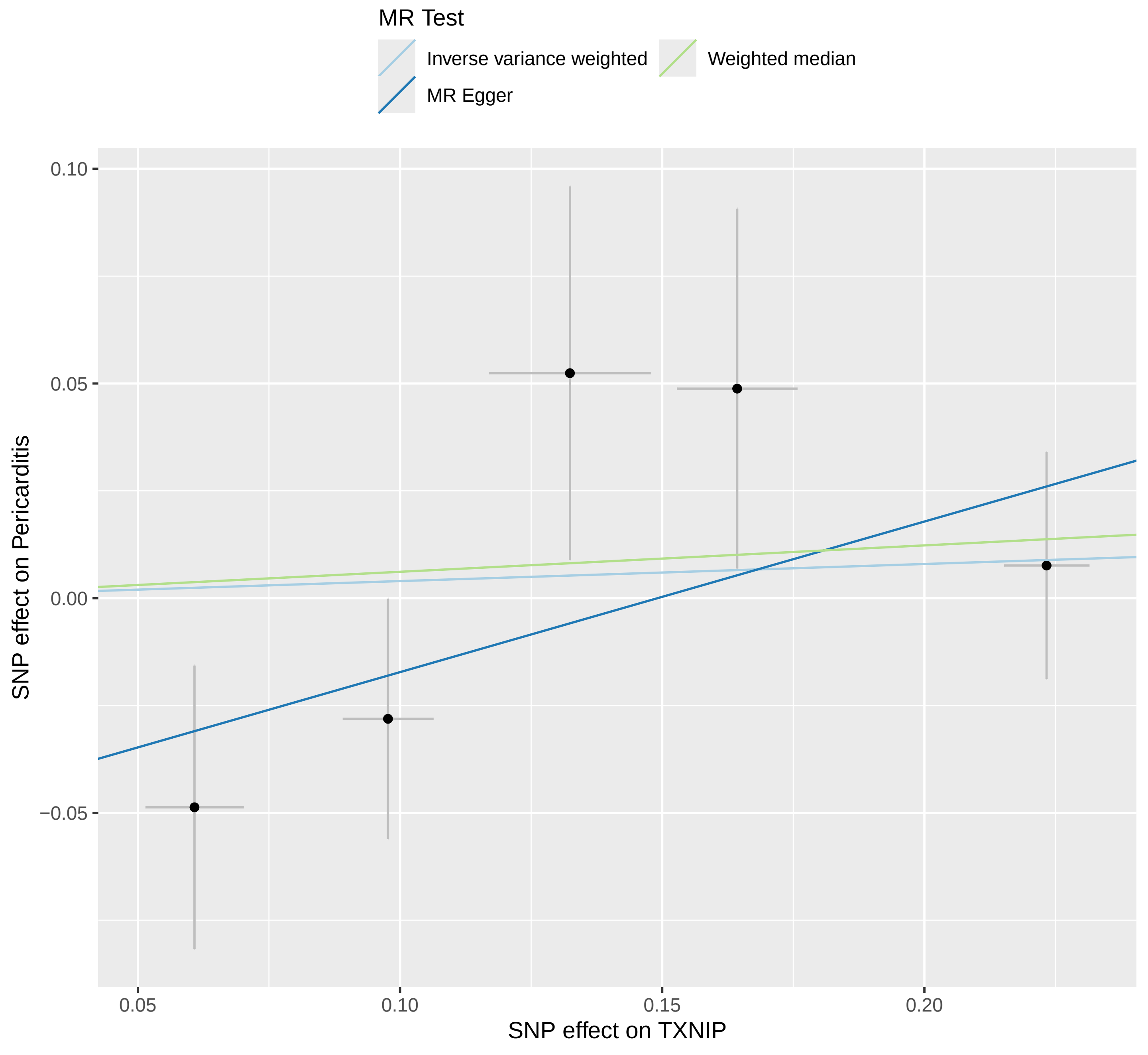
MR Test
Inverse variance weighted
Weighted median
MR Egger
0.10
0.05
0.00
−0.05
SNP effect on Pericarditis
0.05
0.10
0.15
0.20
SNP effect on TXNIP

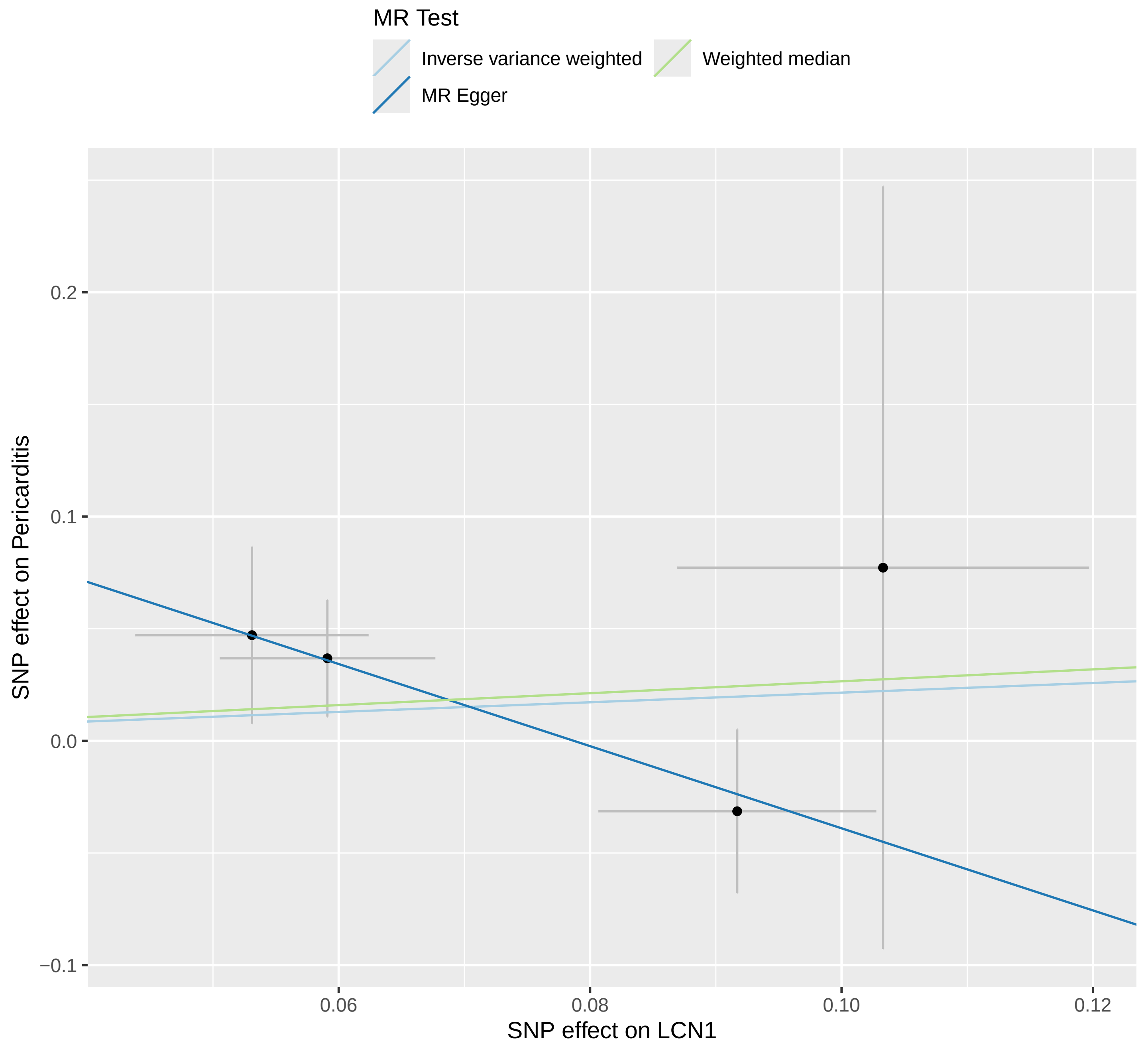

MR Test
Inverse variance weighted
Weighted median
MR Egger
0.2
0.1
0.0
−0.1
SNP effect on Pericarditis
0.06
0.08
0.10
0.12
SNP effect on LCN1

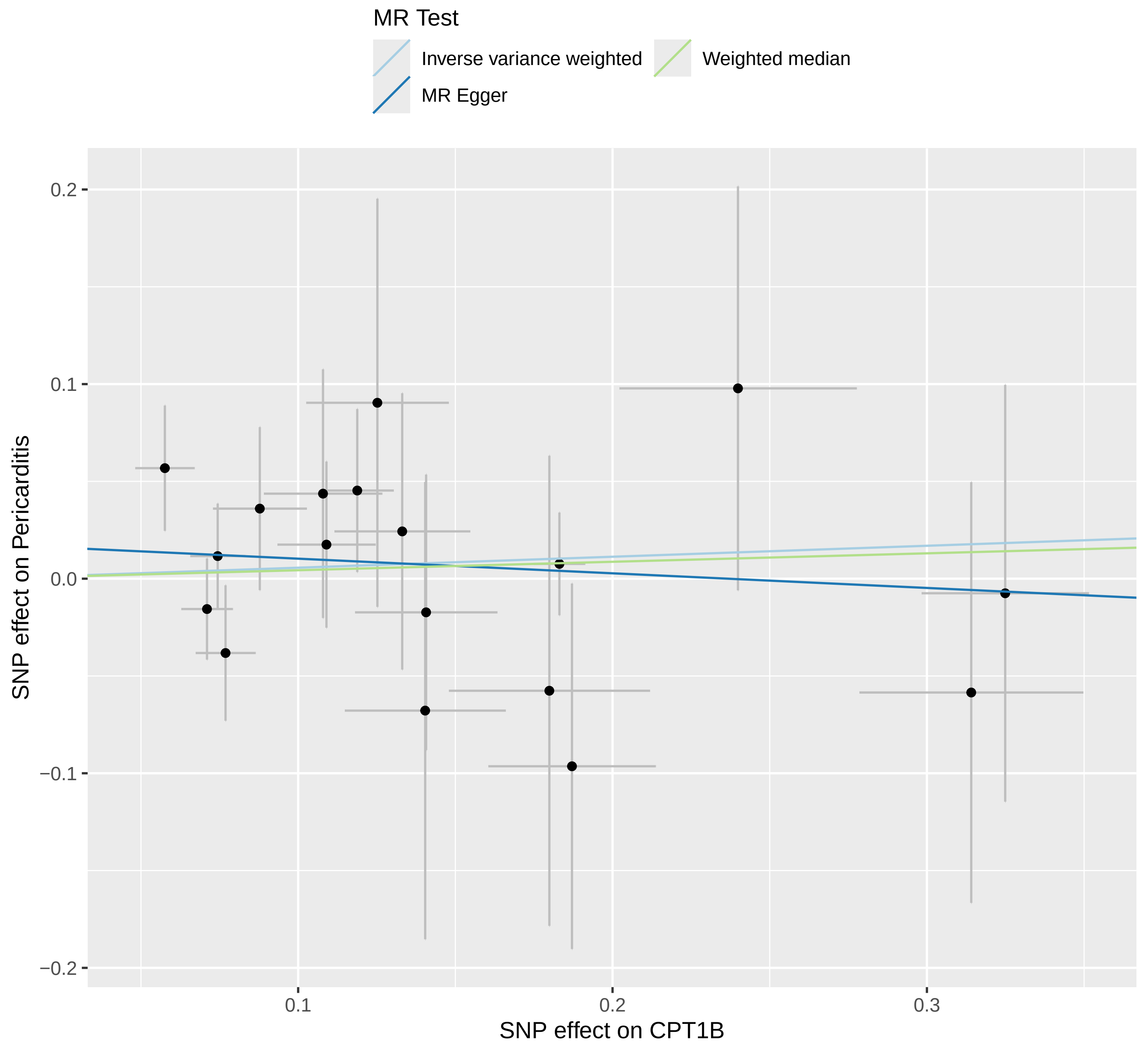
MR Test
Inverse variance weighted
Weighted median
MR Egger
0.2
0.1
0.0
−0.1
−0.2
0.1
0.2
0.3
SNP effect on Pericarditis
SNP effect on CPT1B

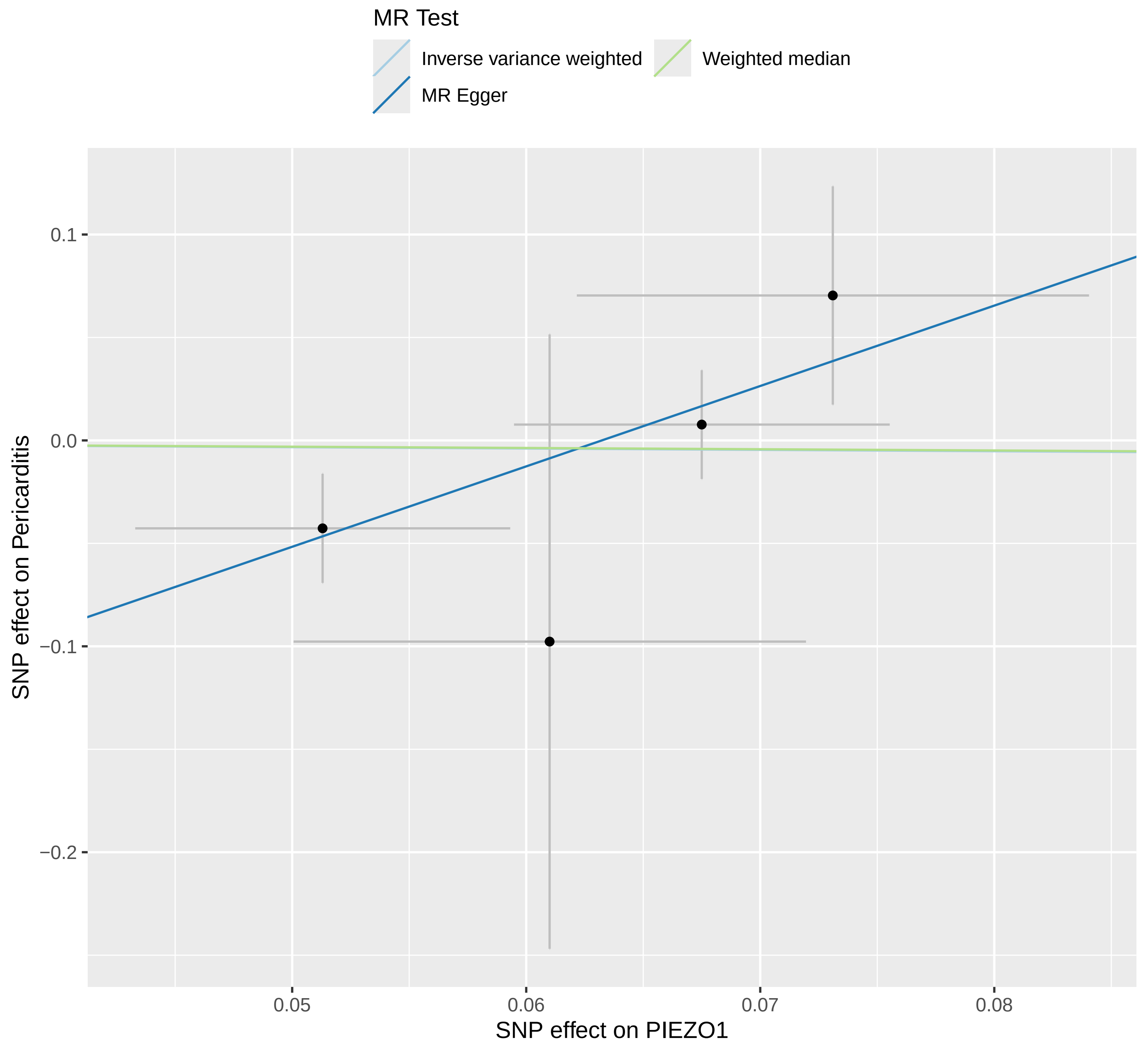
MR Test
Inverse variance weighted
MR Egger
Weighted median
0.1
0.0
−0.1
−0.2
SNP effect on Pericarditis
0.05
0.06
0.07
0.08
SNP effect on PIEZO1

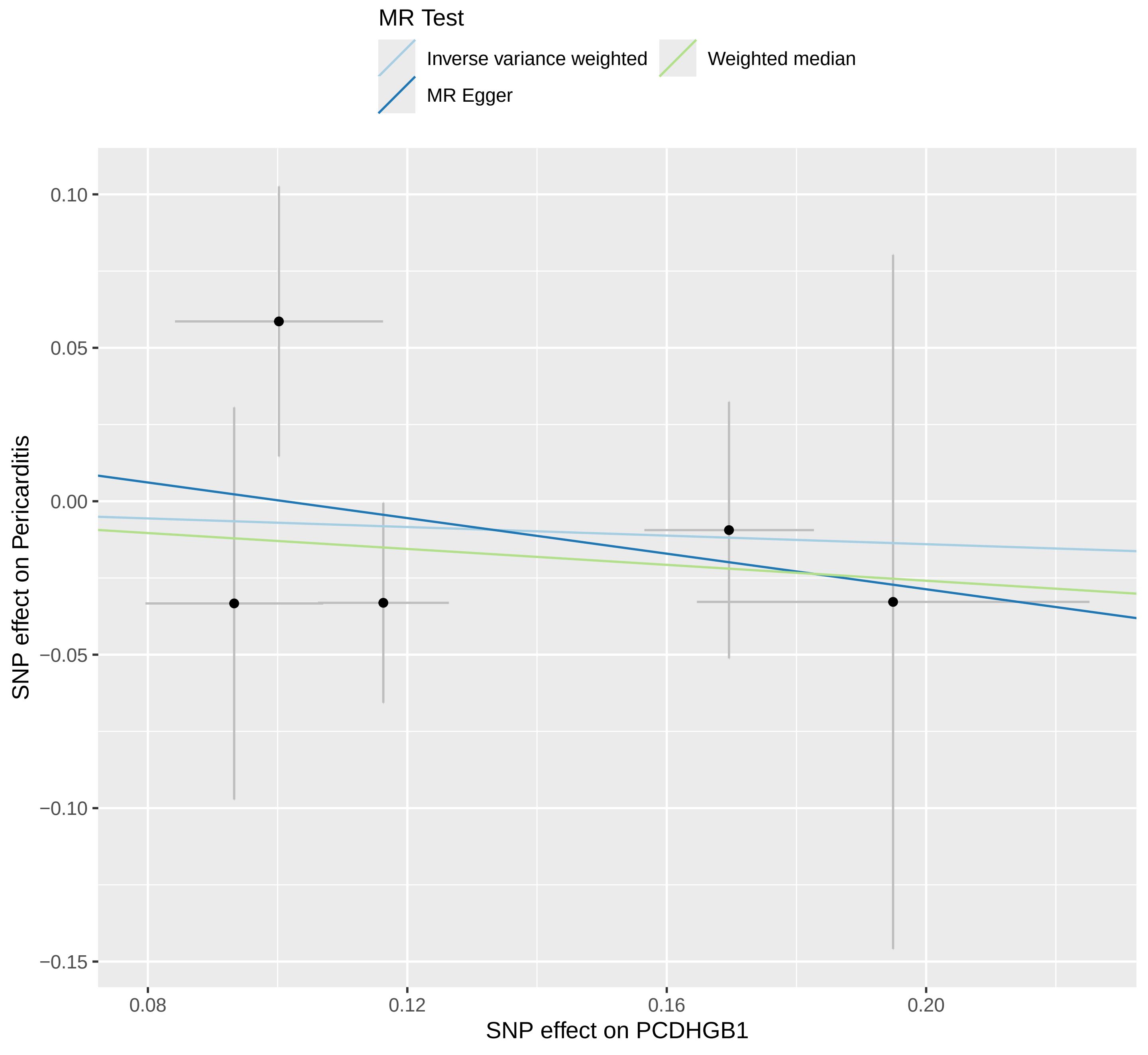

MR Test
Inverse variance weighted
Weighted median
MR Egger
0.10
0.05
0.00
−0.05
−0.10
−0.15
0.08
0.12
0.16
0.20
SNP effect on Pericarditis
SNP effect on PCDHGB1

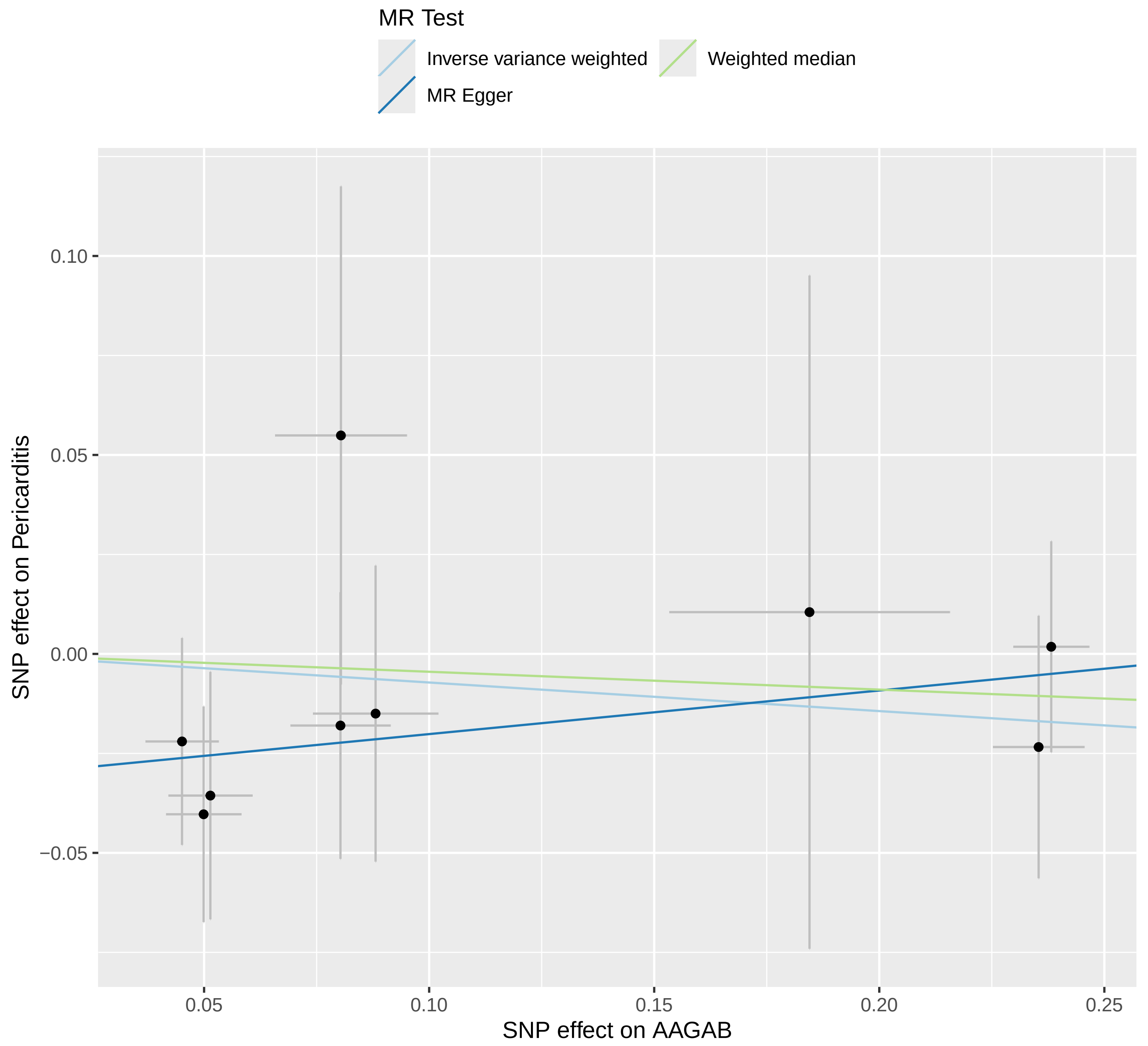

MR Test
Inverse variance weighted
Weighted median
MR Egger
0.10
0.05
0.00
−0.05
SNP effect on Pericarditis
0.05
0.10
0.15
0.20
0.25
SNP effect on AAGAB

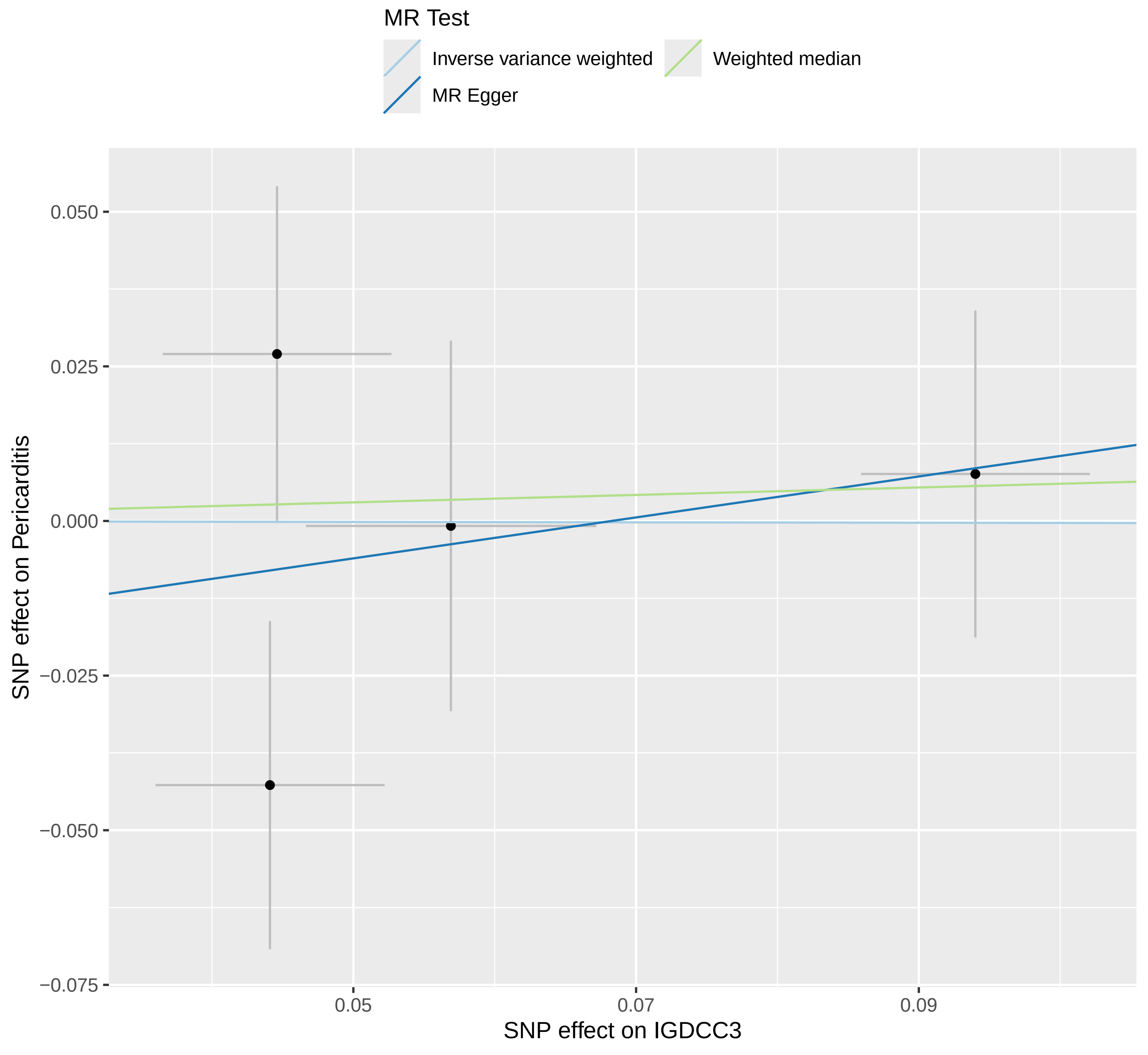
MR Test
Inverse variance weighted
Weighted median
MR Egger
SNP effect on Pericarditis
0.050
0.025
0.000
−0.025
−0.050
−0.075
0.05
0.07
0.09
SNP effect on IGDCC3

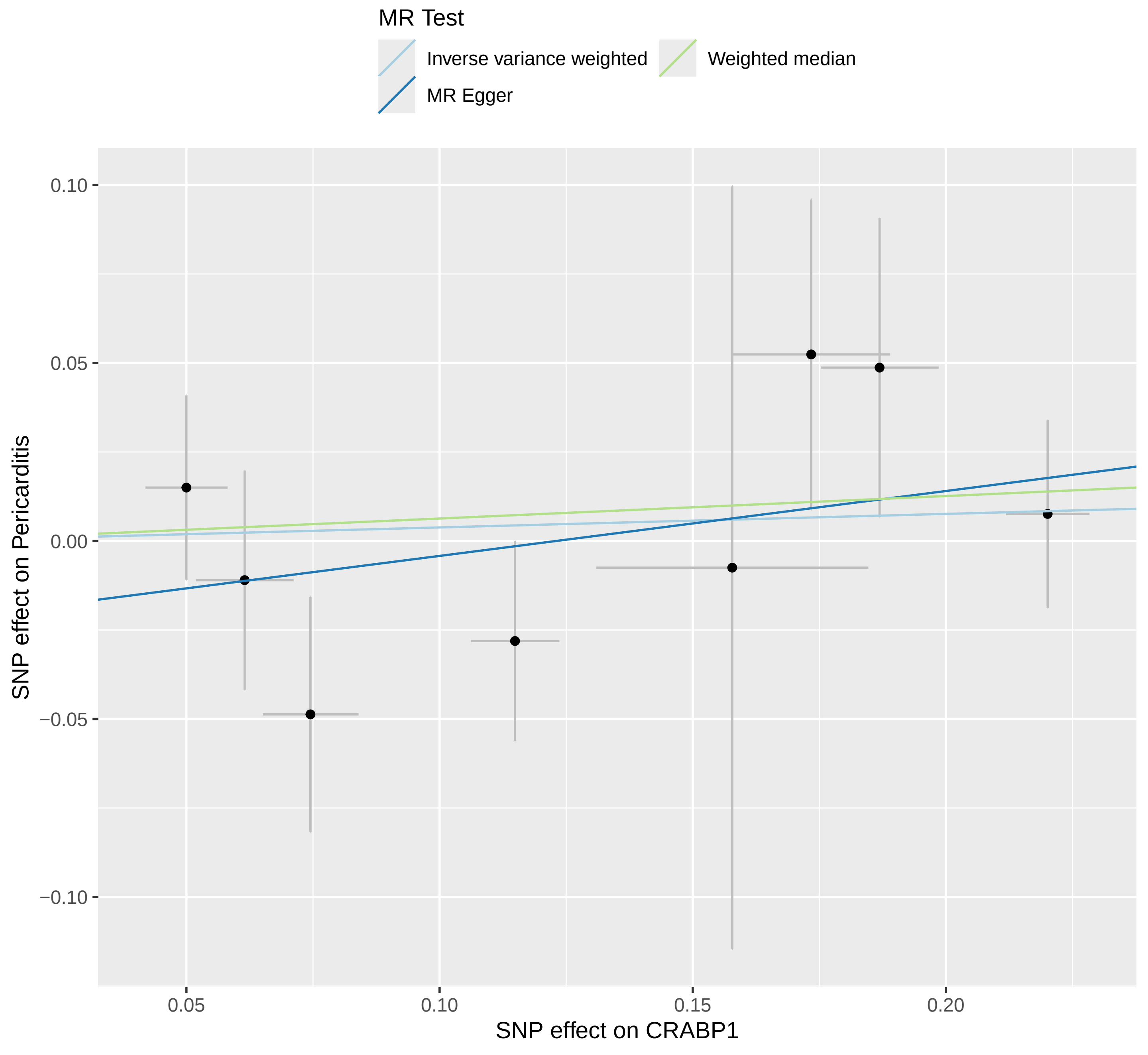
MR Test
Inverse variance weighted
Weighted median
MR Egger
0.10
0.05
0.00
−0.05
−0.10
SNP effect on Pericarditis
0.05
0.10
0.15
0.20
SNP effect on CRABP1

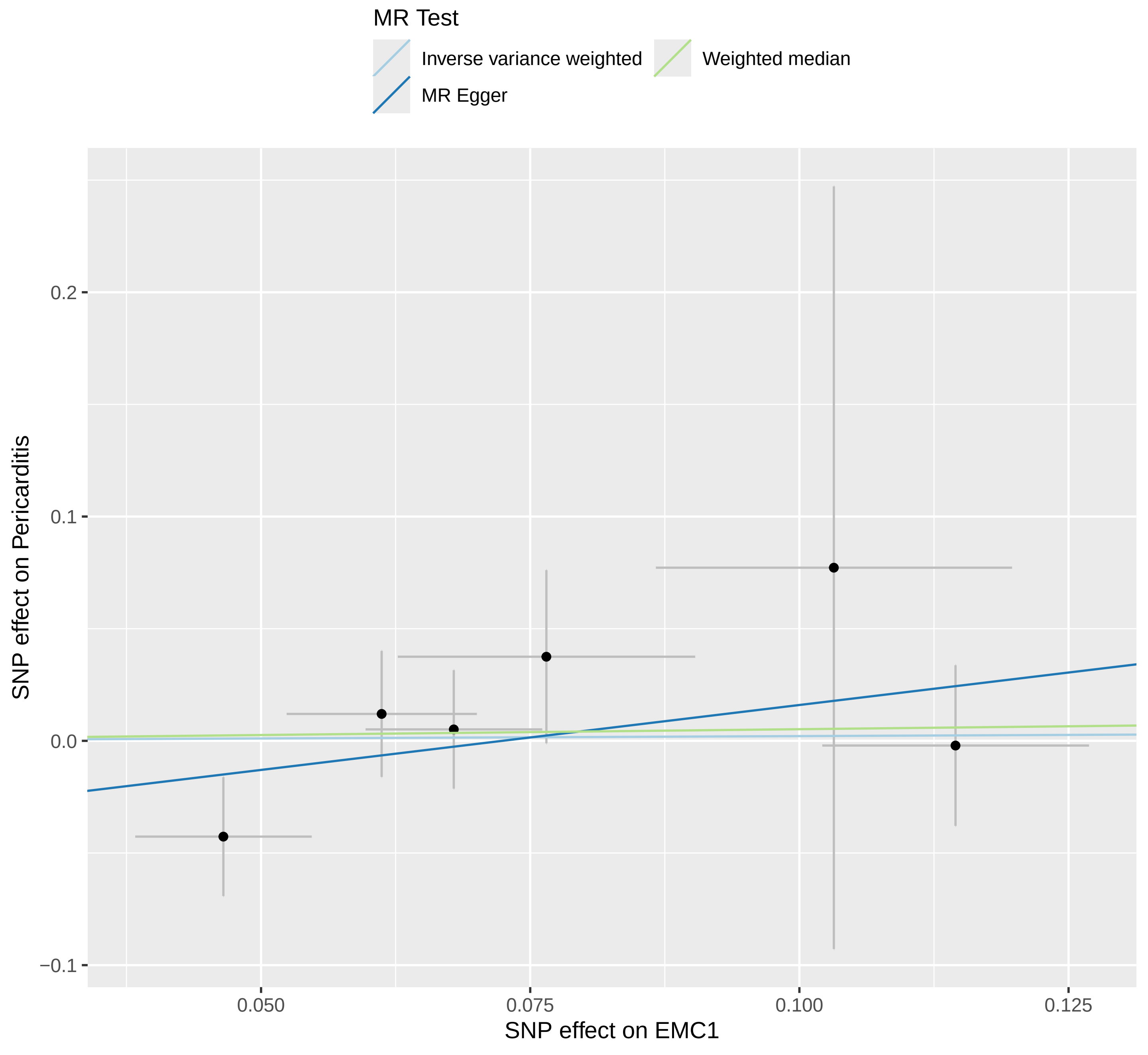

MR Test
Inverse variance weighted
Weighted median
MR Egger
0.2
0.1
0.0
−0.1
SNP effect on Pericarditis
0.050
0.075
0.100
0.125
SNP effect on EMC1

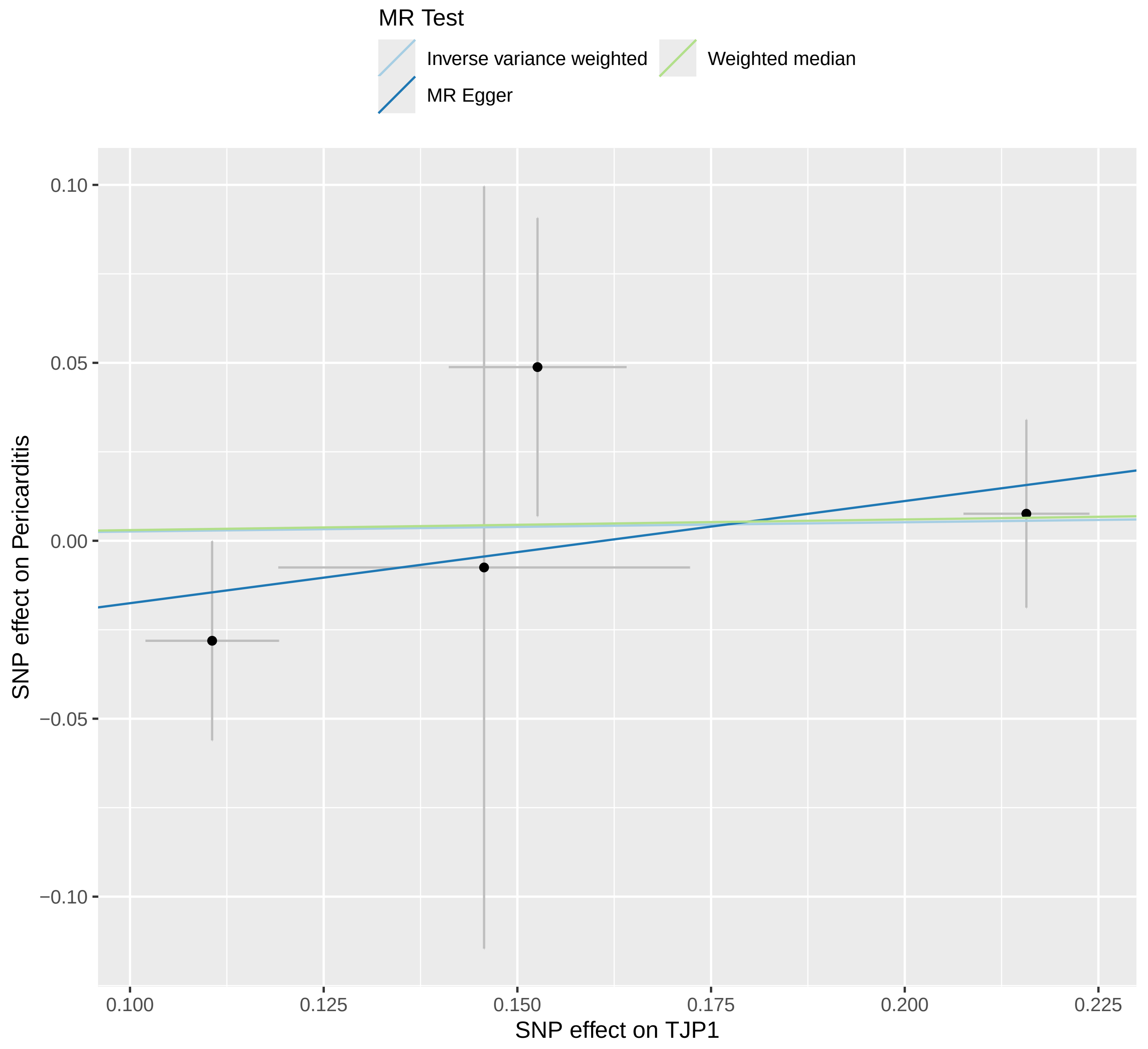
MR Test
Inverse variance weighted
Weighted median
MR Egger
0.10
0.05
0.00
−0.05
−0.10
SNP effect on Pericarditis
0.100
0.125
0.150
0.175
0.200
0.225
SNP effect on TJP1

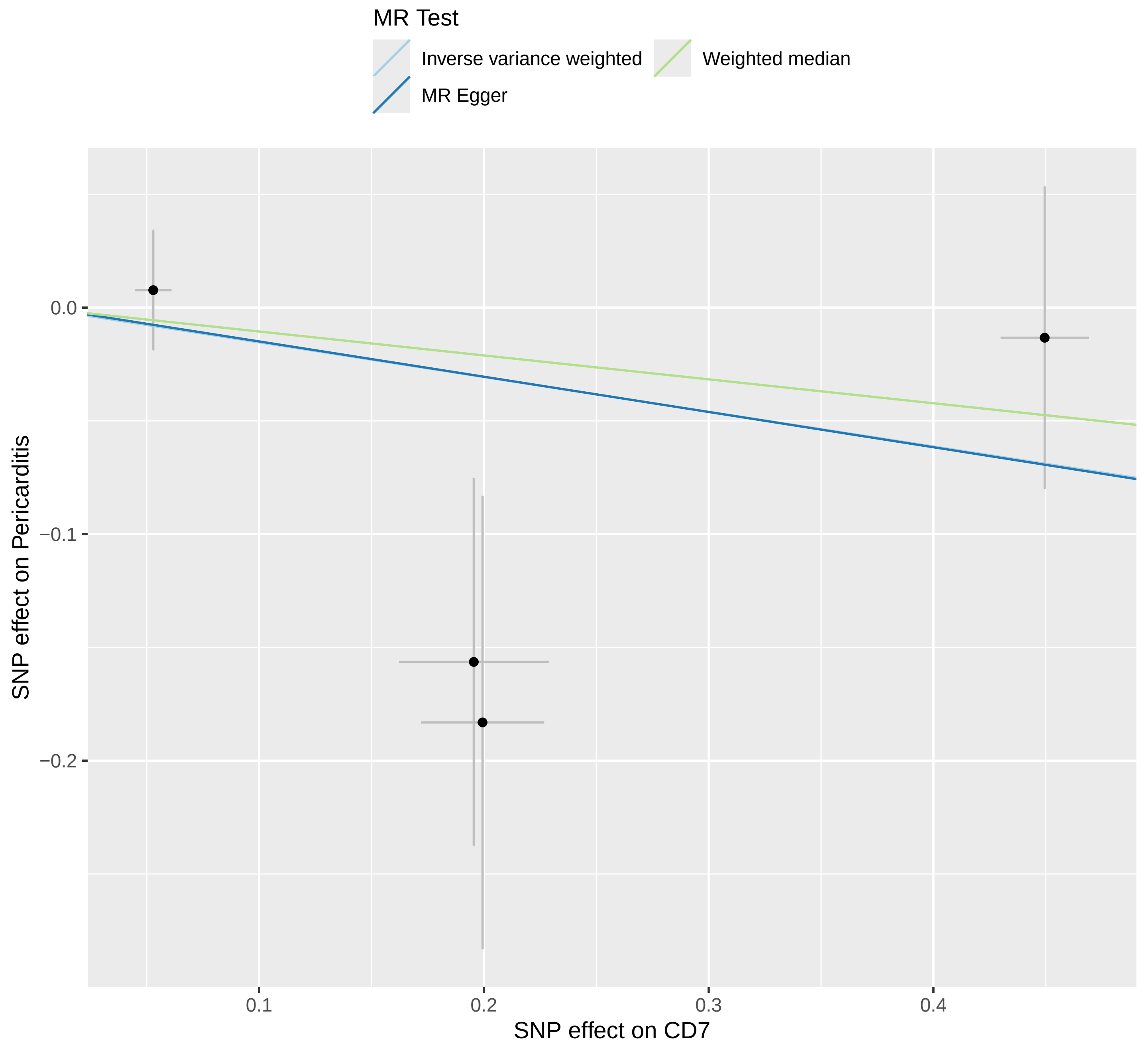
MR Test
Inverse variance weighted
Weighted median
MR Egger
0.0
−0.1
−0.2
SNP effect on Pericarditis
0.1
0.2
0.3
0.4
SNP effect on CD7

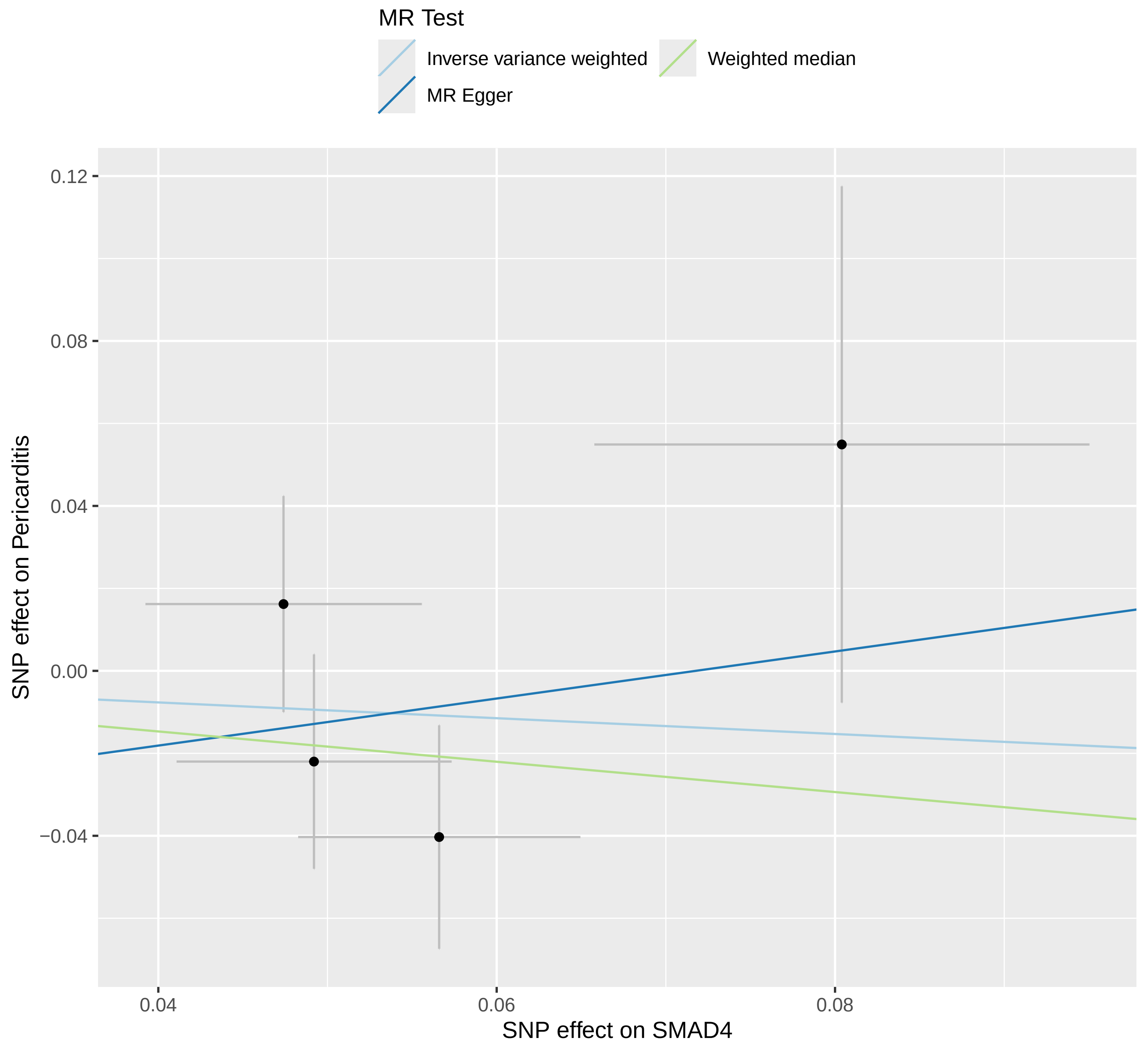
MR Test
Inverse variance weighted
Weighted median
MR Egger
0.12
0.08
0.04
0.00
−0.04
SNP effect on Pericarditis
0.04
0.06
0.08
SNP effect on SMAD4

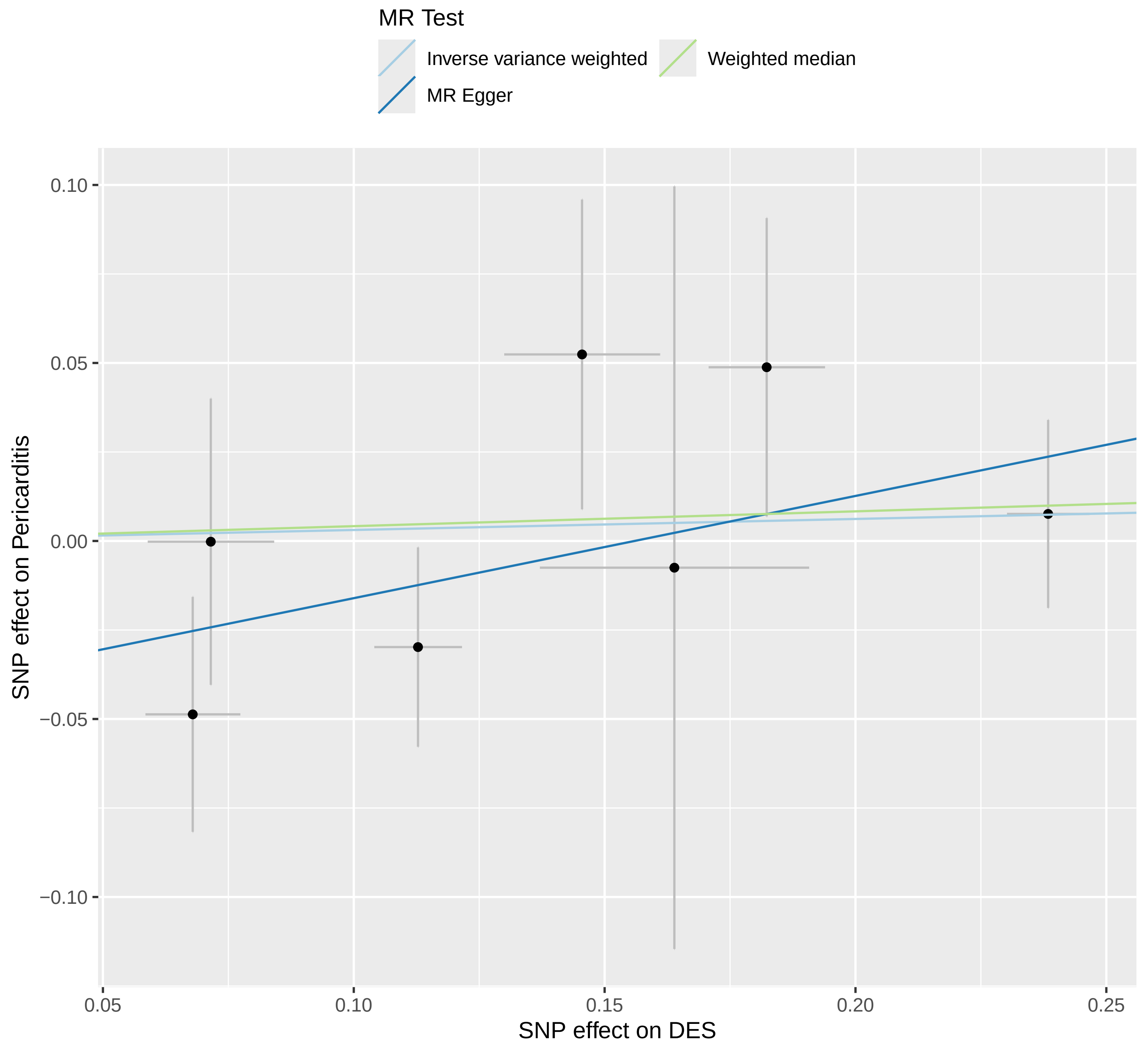
MR Test
Inverse variance weighted
MR Egger
Weighted median
SNP effect on Pericarditis
0.10
0.05
0.00
−0.05
−0.10
0.05
0.10
0.15
0.20
0.25
SNP effect on DES

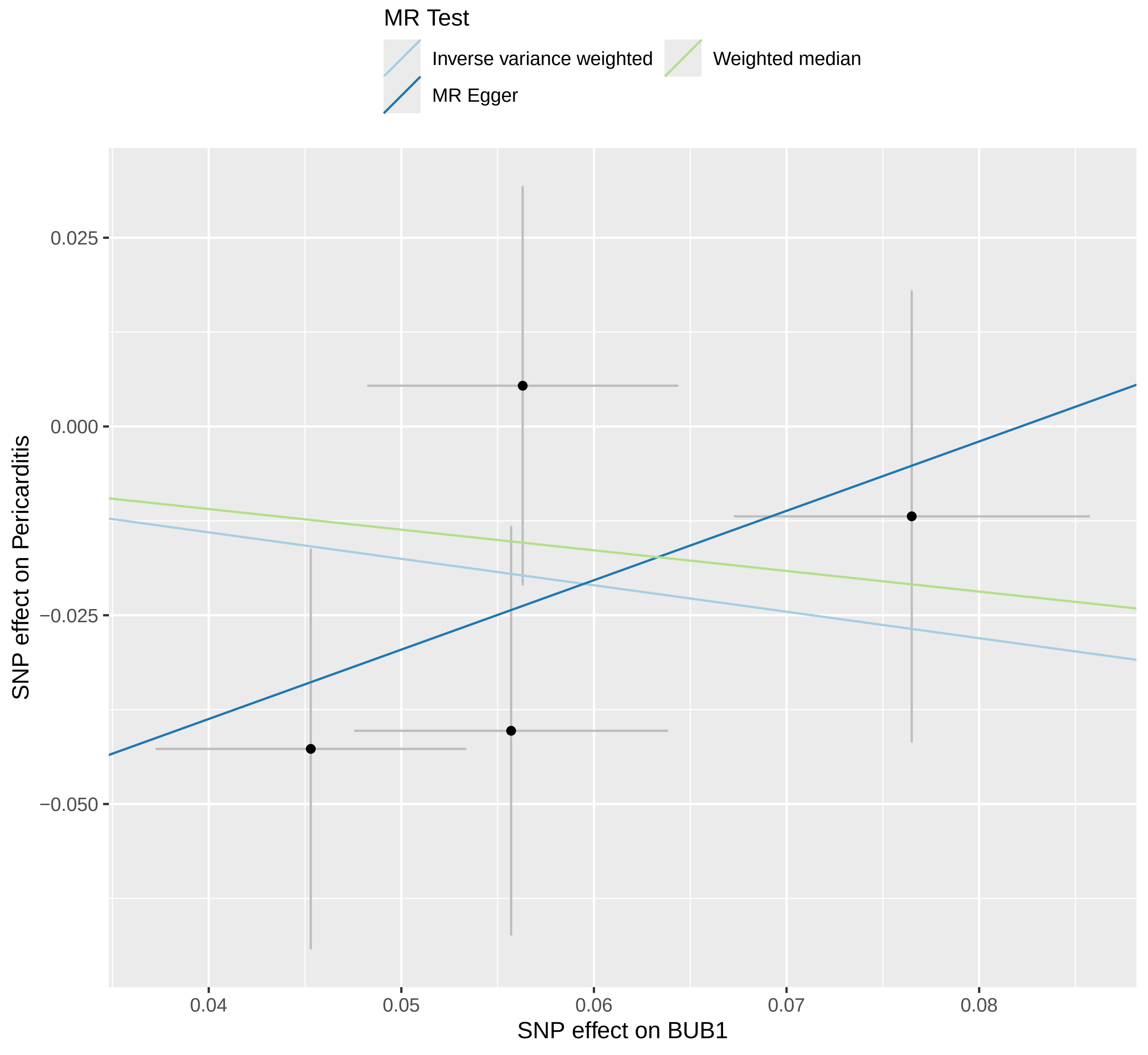

MR Test
Inverse variance weighted
MR Egger
Weighted median
0.025
0.000
−0.025
−0.050
SNP effect on Pericarditis
0.04
0.05
0.06
0.07
0.08
SNP effect on BUB1

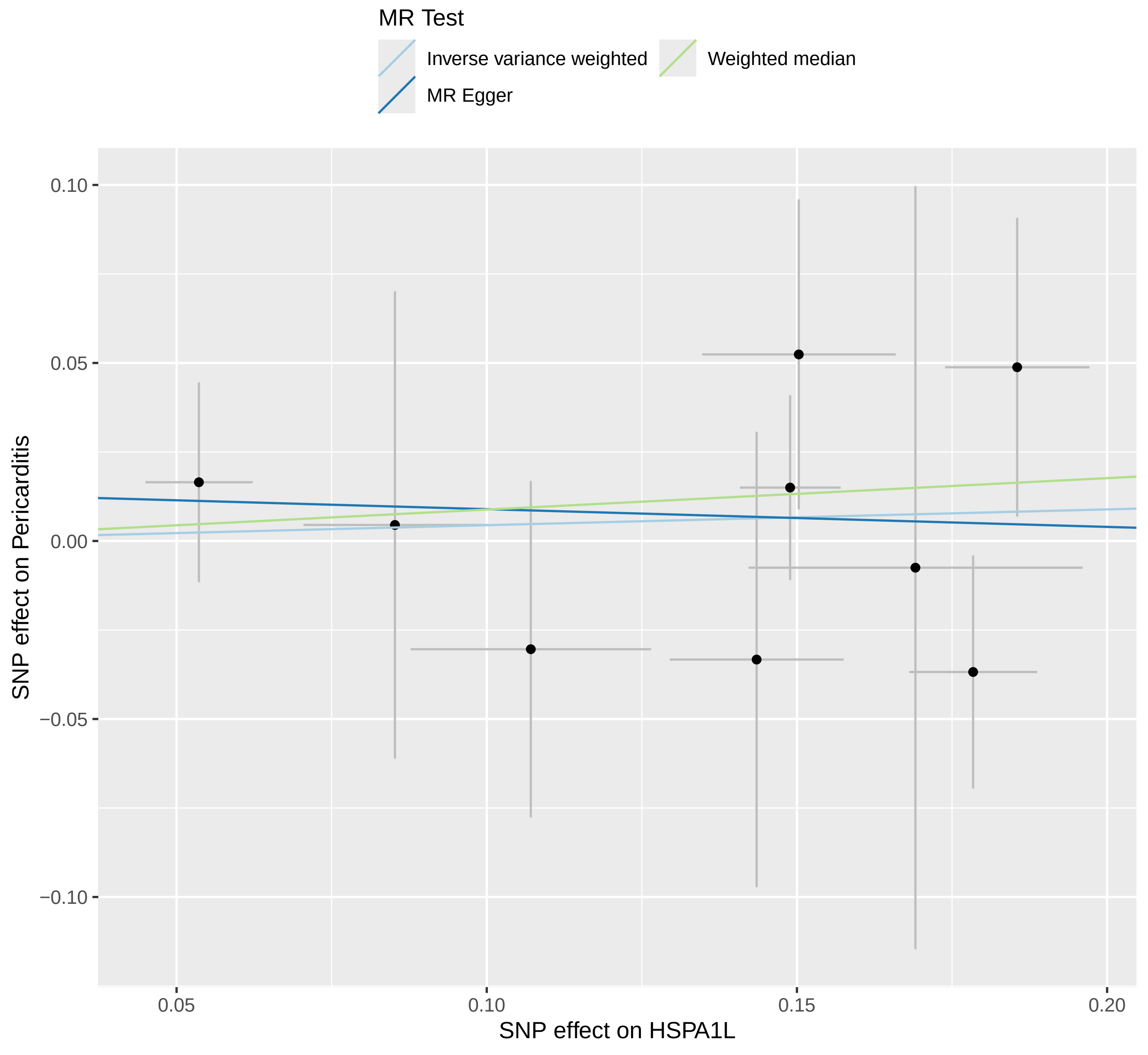
MR Test
Inverse variance weighted
Weighted median
MR Egger
0.10
0.05
0.00
−0.05
−0.10
SNP effect on Pericarditis
0.05
0.10
0.15
0.20
SNP effect on HSPA1L

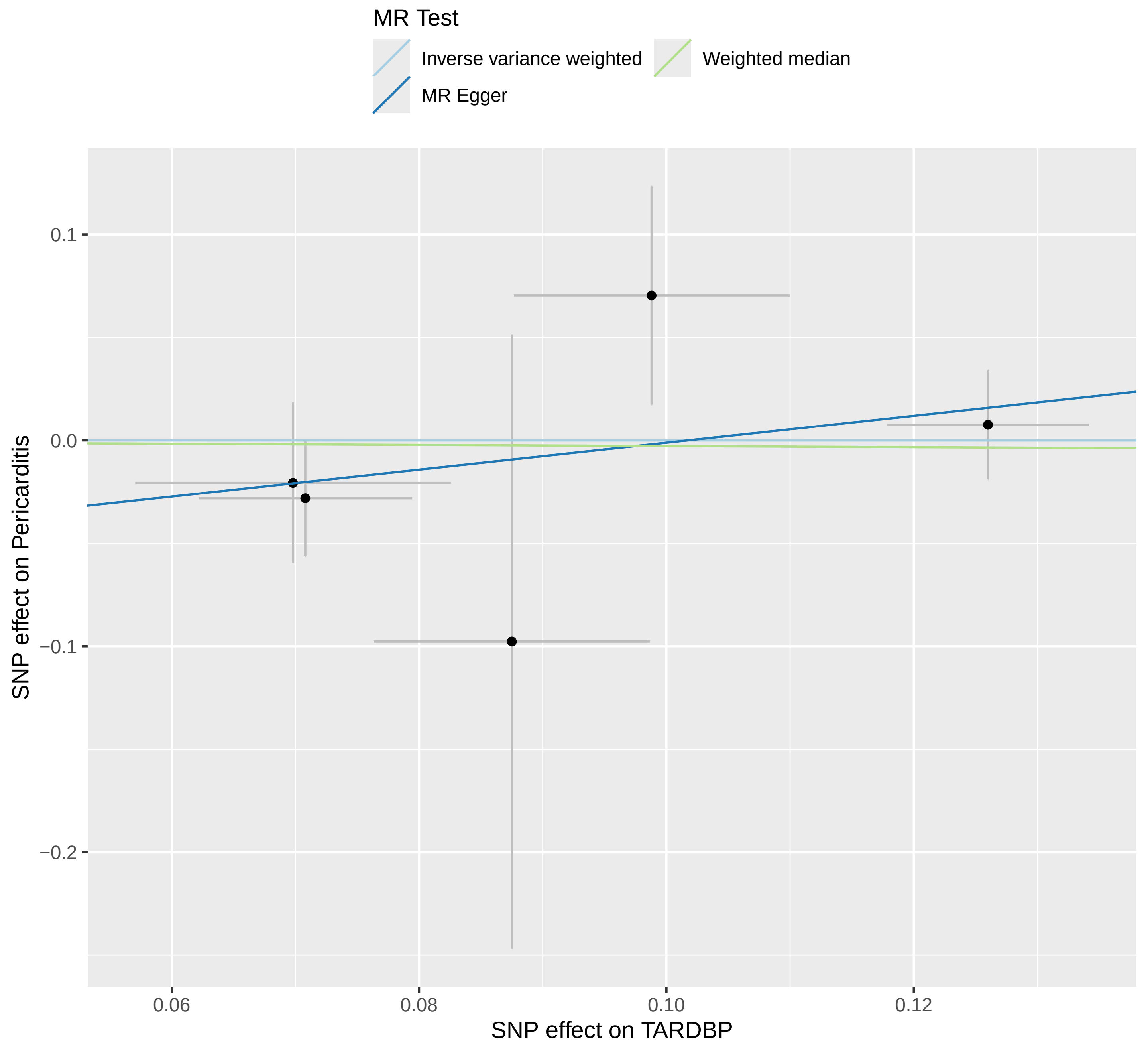
MR Test
Inverse variance weighted
Weighted median
MR Egger
0.1
0.0
−0.1
−0.2
SNP effect on Pericarditis
0.06
0.08
0.10
0.12
SNP effect on TARDBP

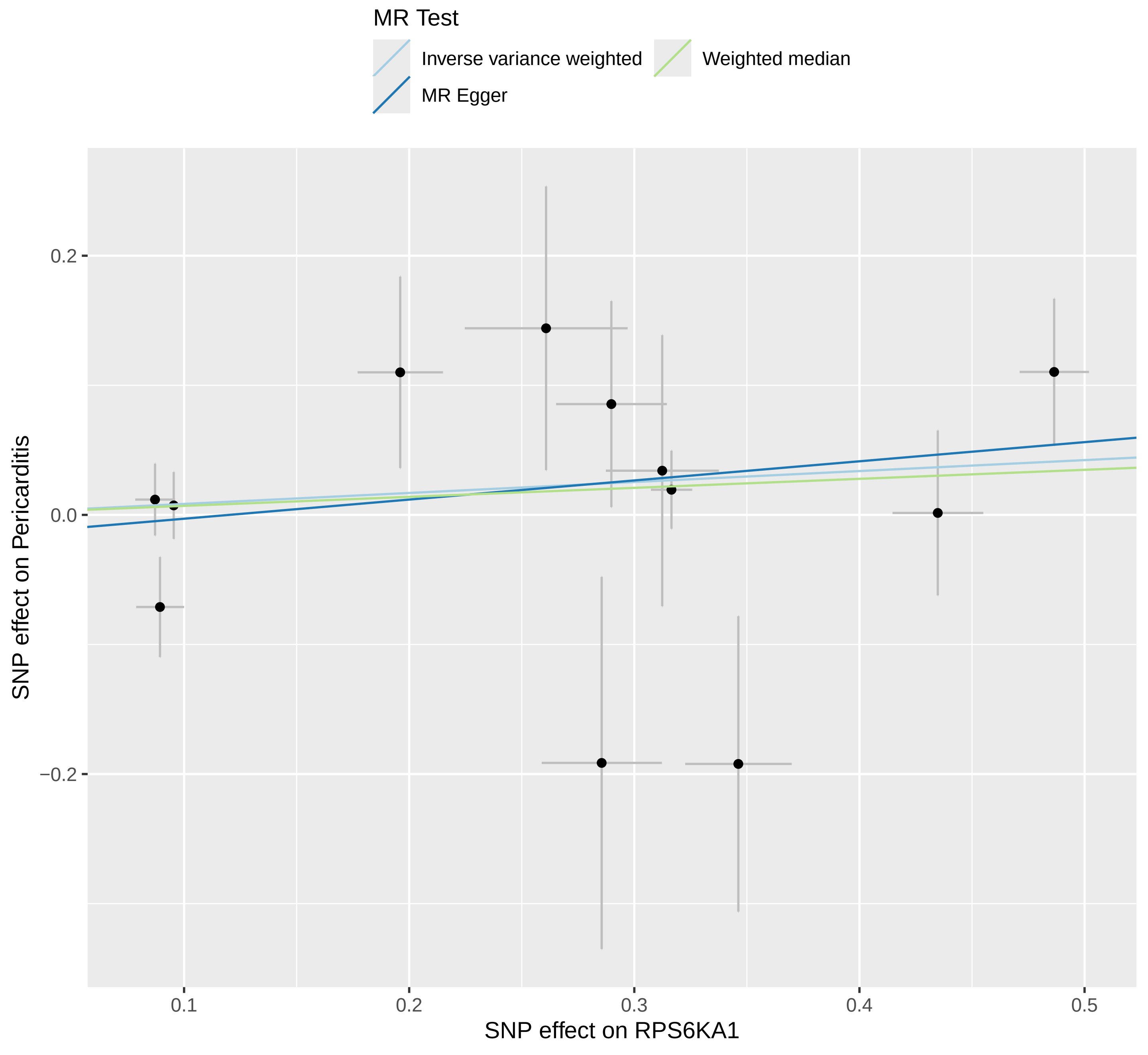

MR Test
Inverse variance weighted
Weighted median
MR Egger
0.2
0.0
−0.2
0.1
0.2
0.3
0.4
0.5
SNP effect on Pericarditis
SNP effect on RPS6KA1

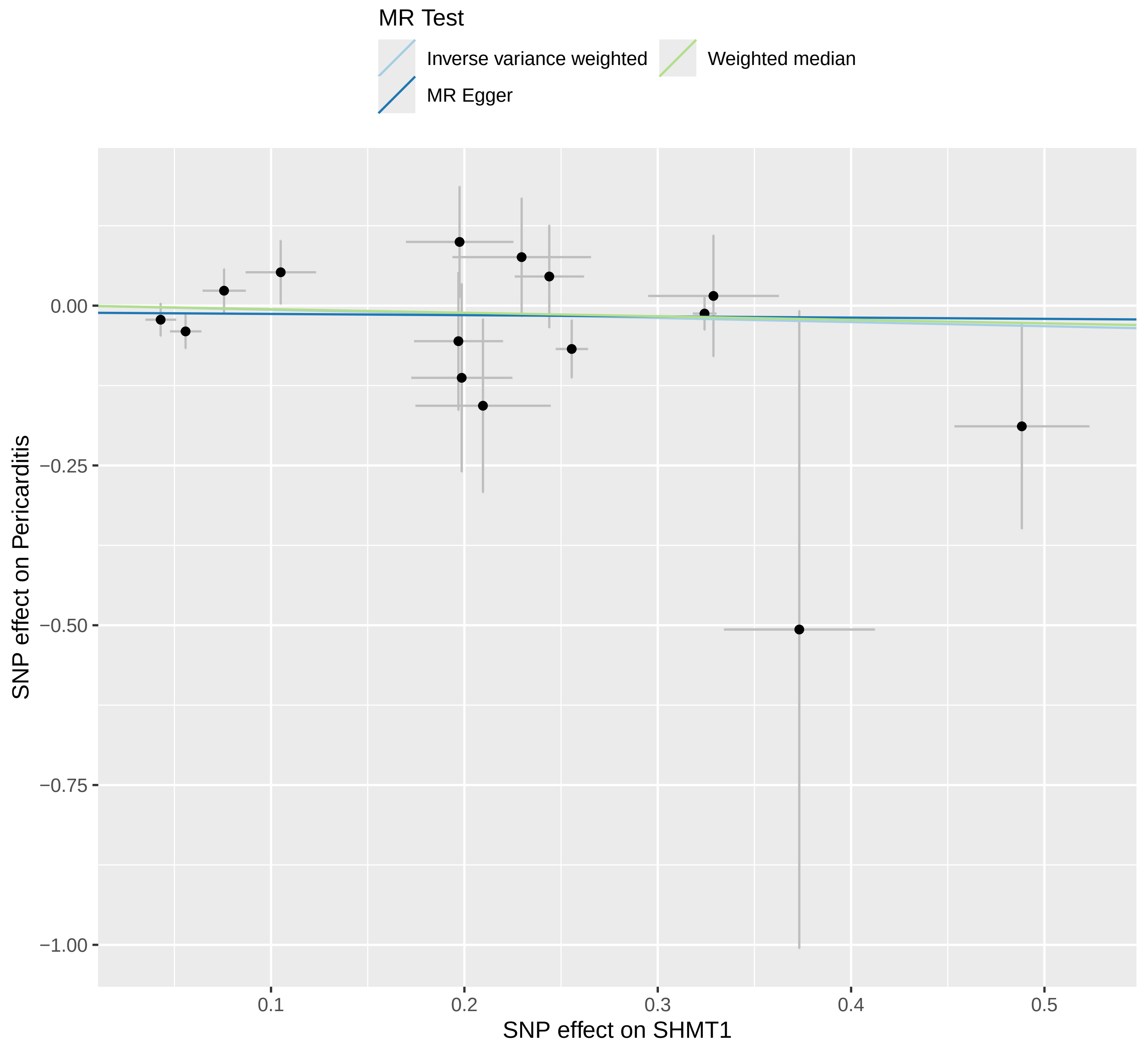

MR Test
Inverse variance weighted
Weighted median
MR Egger
SNP effect on Pericarditis
0.00
−0.25
−0.50
−0.75
−1.00
0.1
0.2
0.3
0.4
0.5
SNP effect on SHMT1

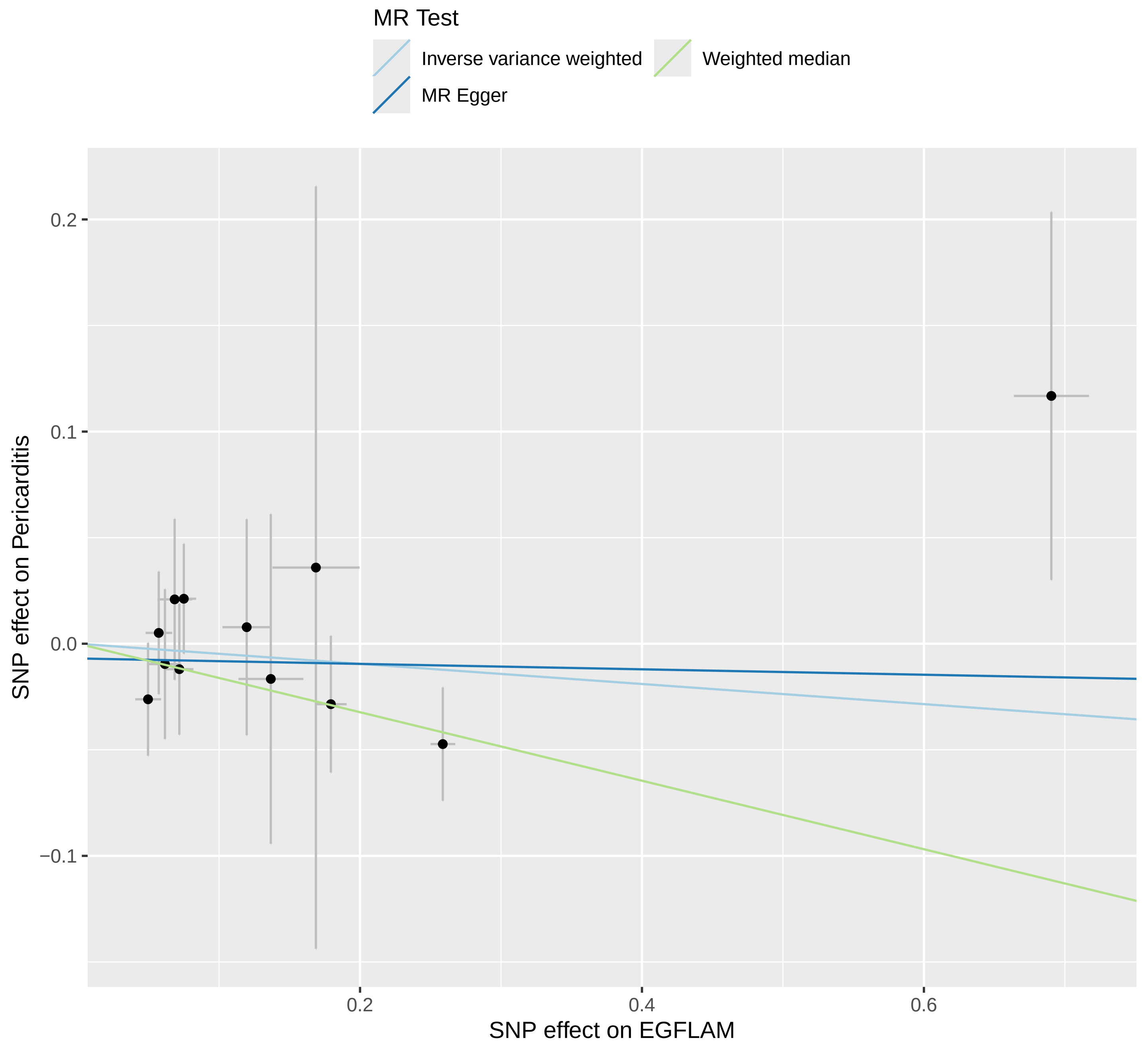
MR Test
Inverse variance weighted
Weighted median
MR Egger
0.2
0.1
0.0
−0.1
SNP effect on Pericarditis
0.2
0.4
0.6
SNP effect on EGFLAM

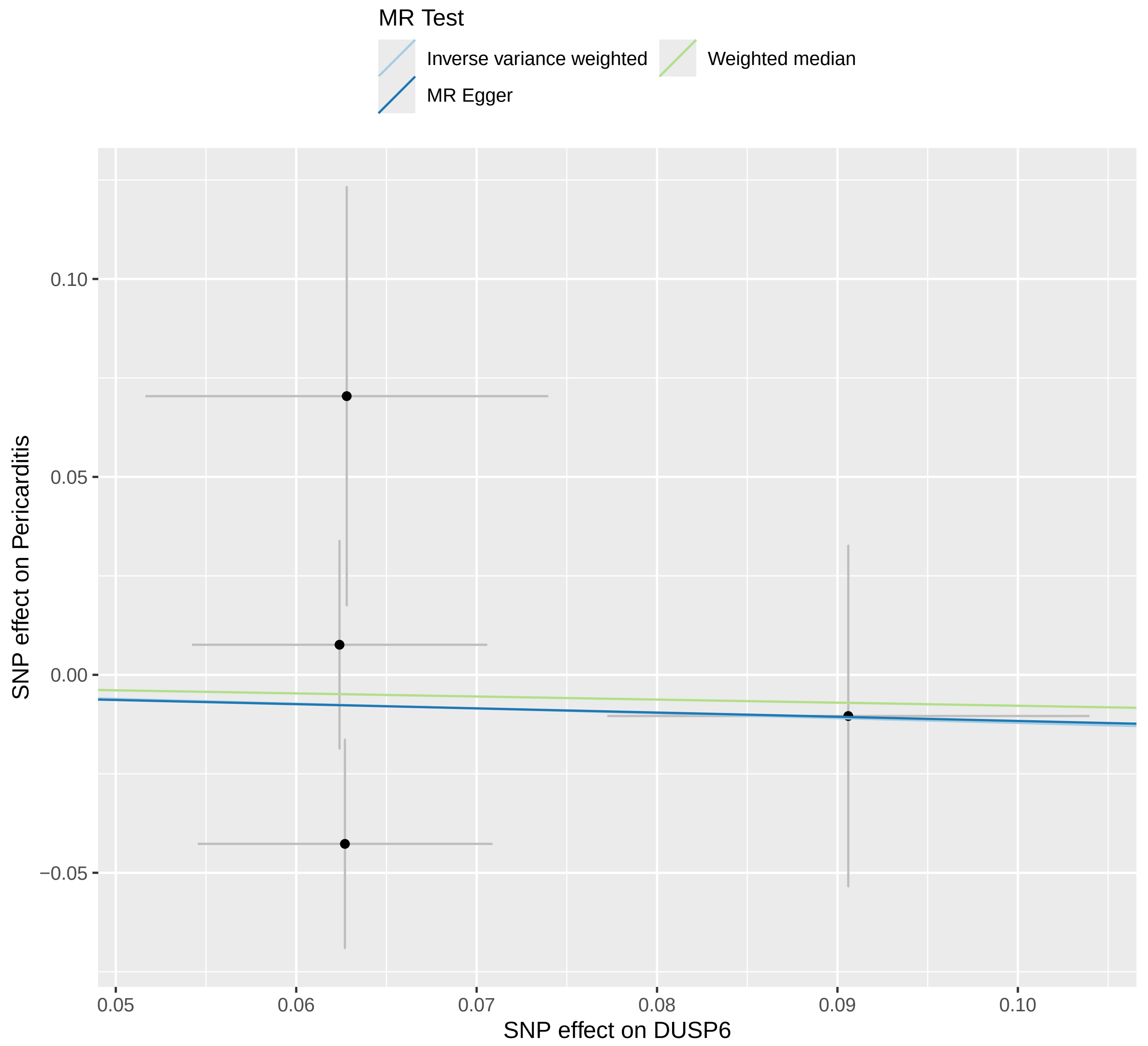

MR Test
Inverse variance weighted
Weighted median
MR Egger
SNP effect on Pericarditis
0.10
0.05
0.00
−0.05
0.05
0.06
0.07
0.08
0.09
0.10
SNP effect on DUSP6

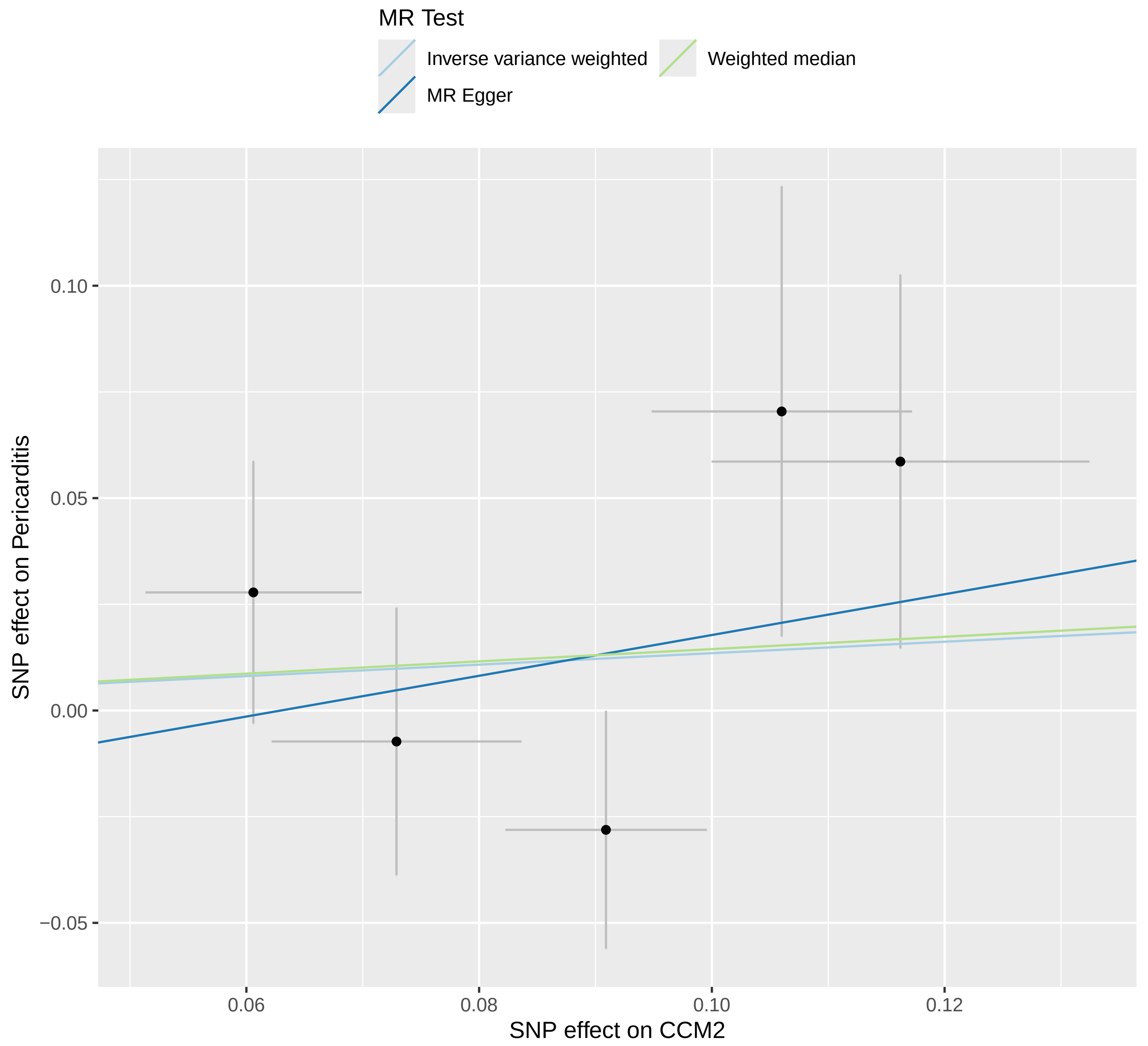
MR Test
Inverse variance weighted
MR Egger
Weighted median
0.10
0.05
0.00
−0.05
SNP effect on Pericarditis
0.06
0.08
0.10
0.12
SNP effect on CCM2

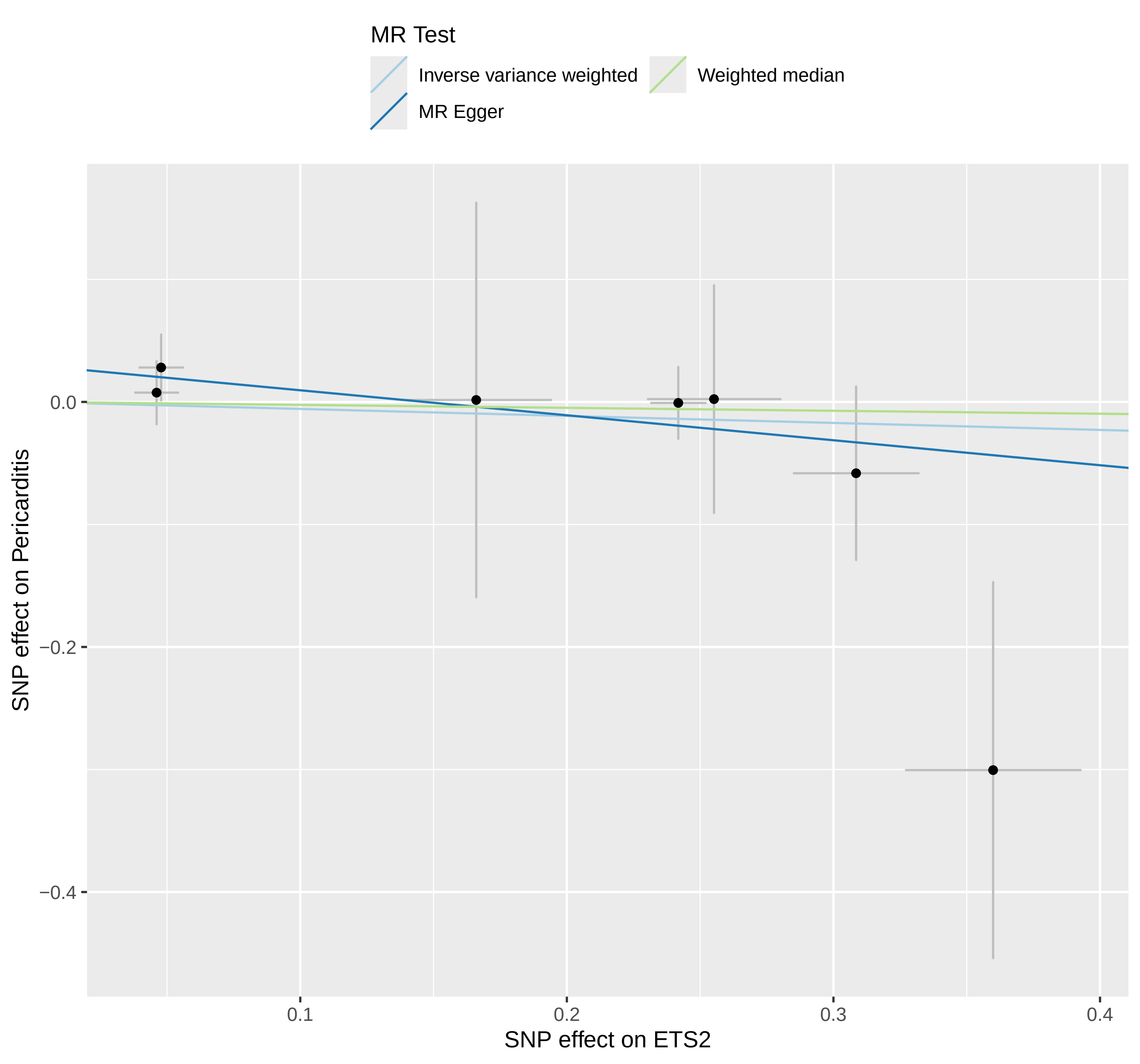

MR Test
Inverse variance weighted
Weighted median
MR Egger
0.0
−0.2
−0.4
0.1
0.2
0.3
0.4
SNP effect on Pericarditis
SNP effect on ETS2

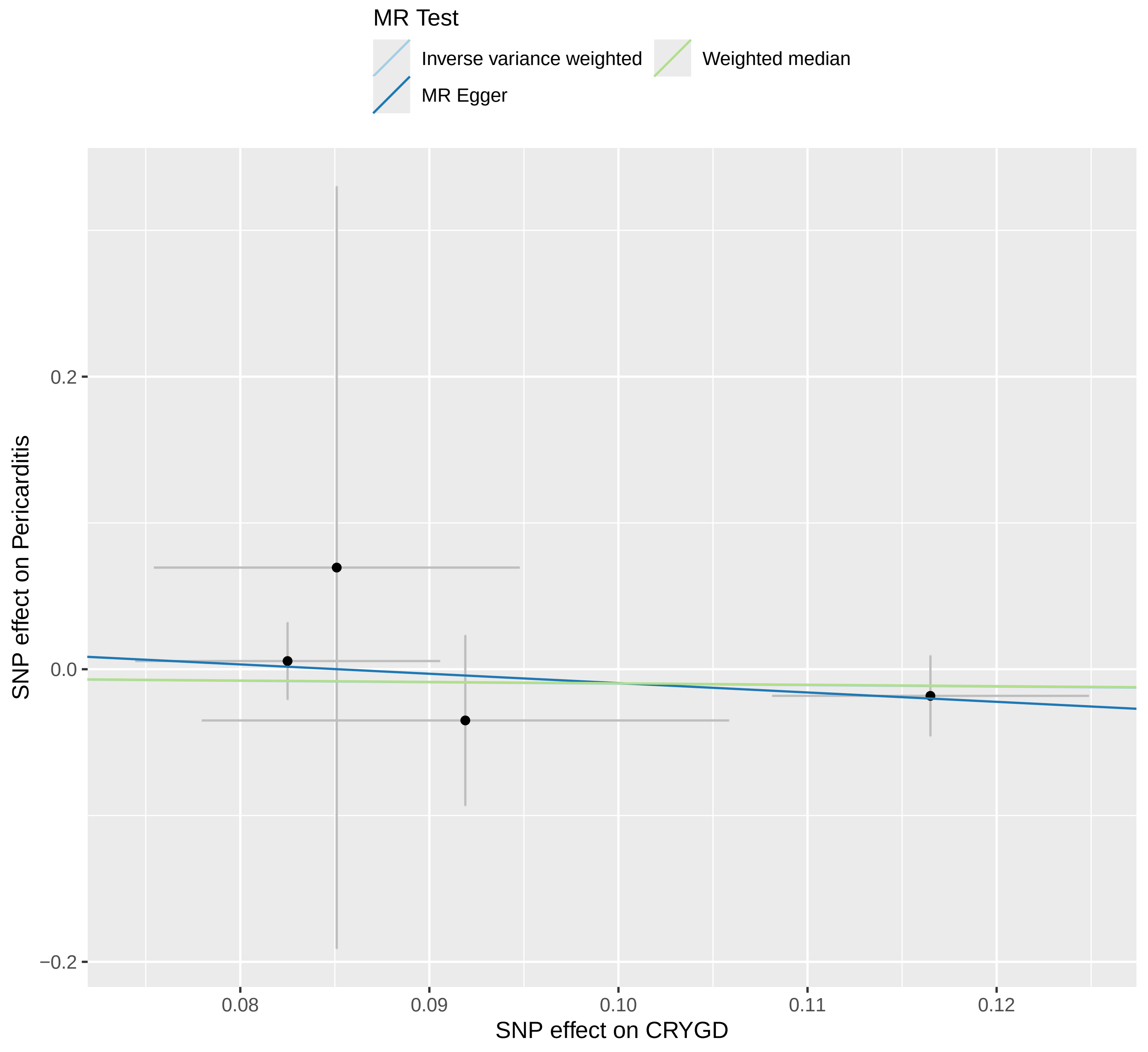
MR Test
Inverse variance weighted
MR Egger
Weighted median
0.2
0.0
−0.2
SNP effect on Pericarditis
0.08
0.09
0.10
0.11
0.12
SNP effect on CRYGD

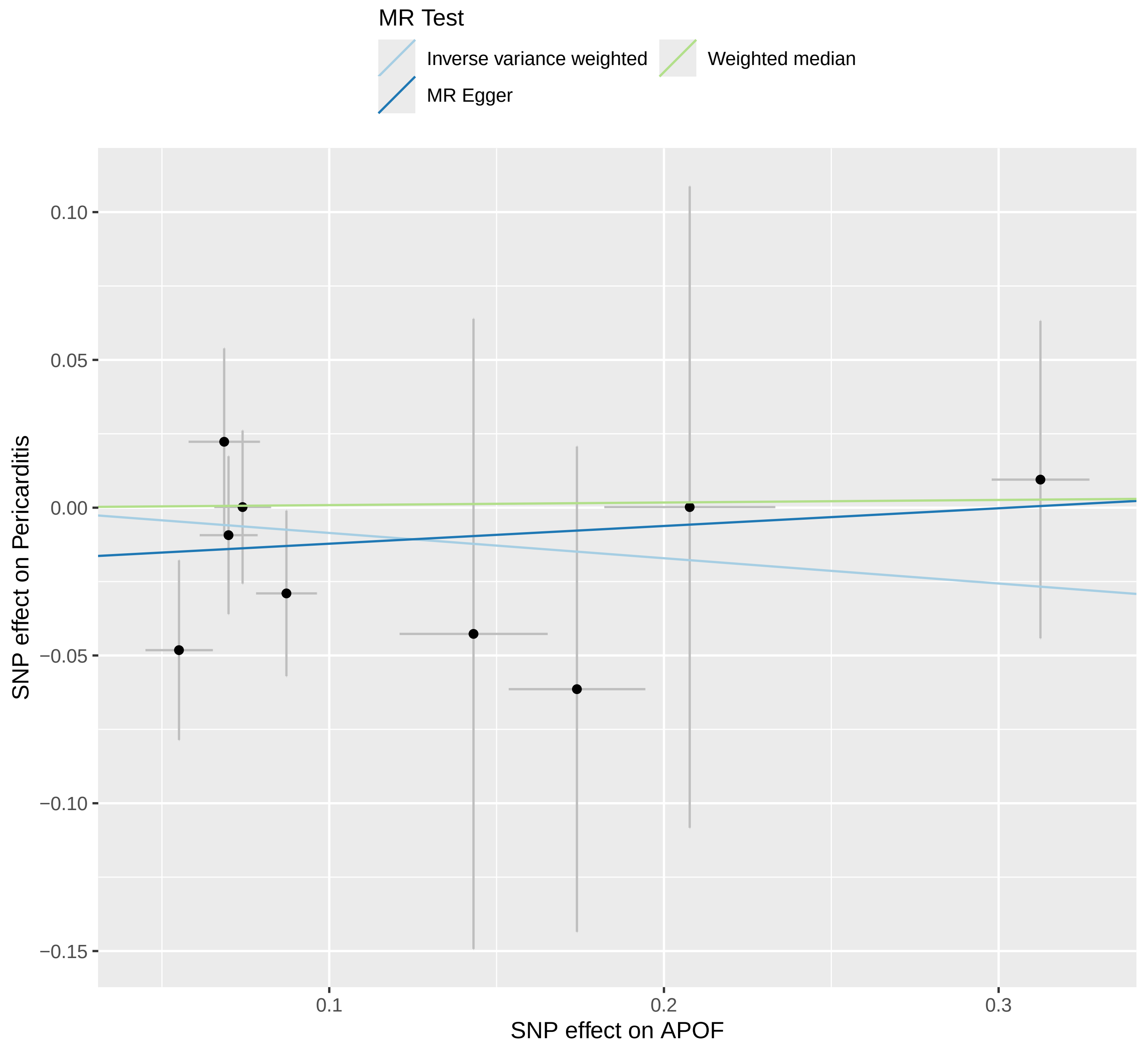
MR Test
Inverse variance weighted
MR Egger
Weighted median
0.10
0.05
0.00
−0.05
−0.10
−0.15
SNP effect on Pericarditis
0.1
0.2
0.3
SNP effect on APOF

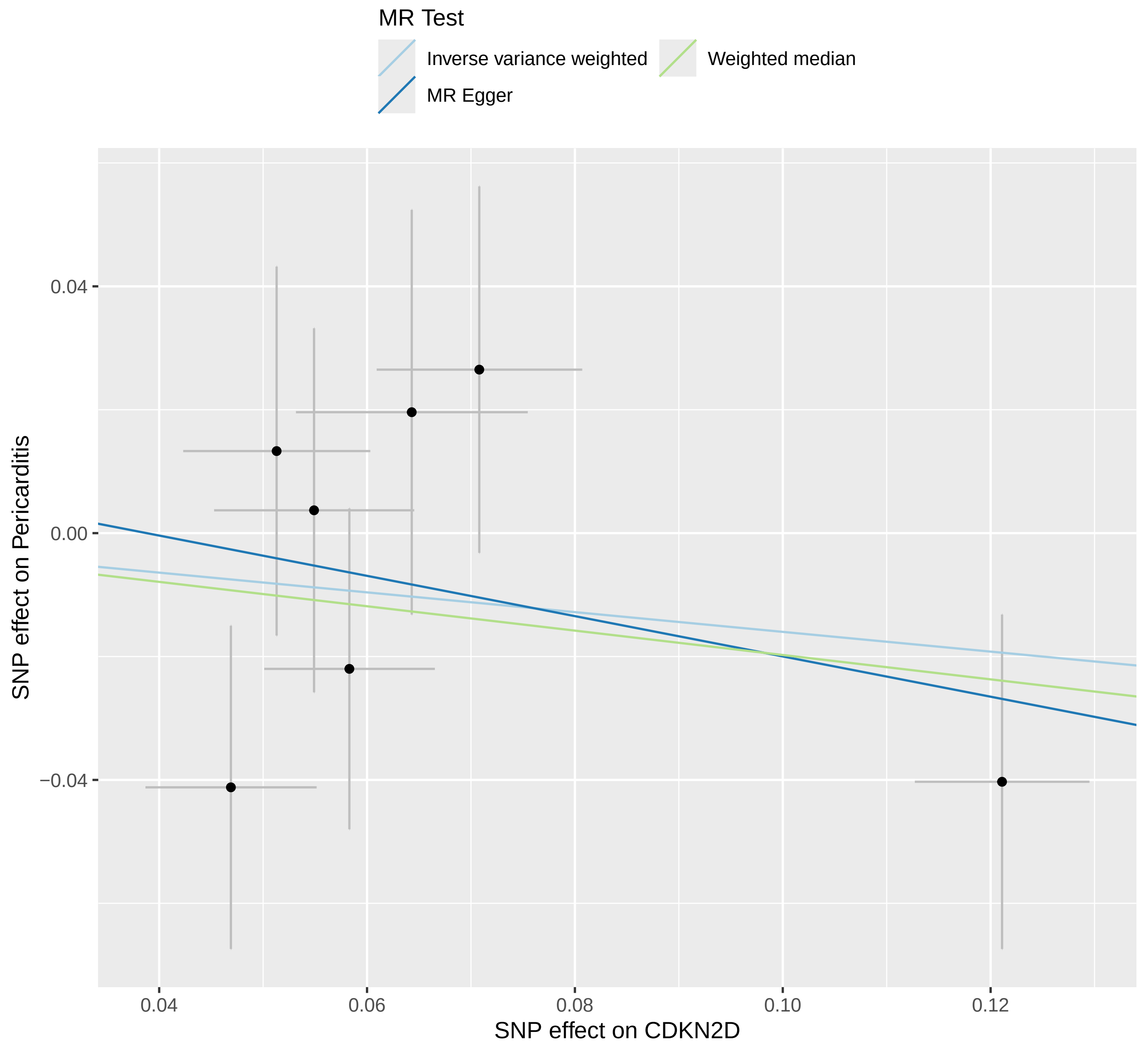
MR Test
Inverse variance weighted
MR Egger
Weighted median
SNP effect on Pericarditis
0.04
0.00
−0.04
0.04
0.06
0.08
0.10
0.12
SNP effect on CDKN2D

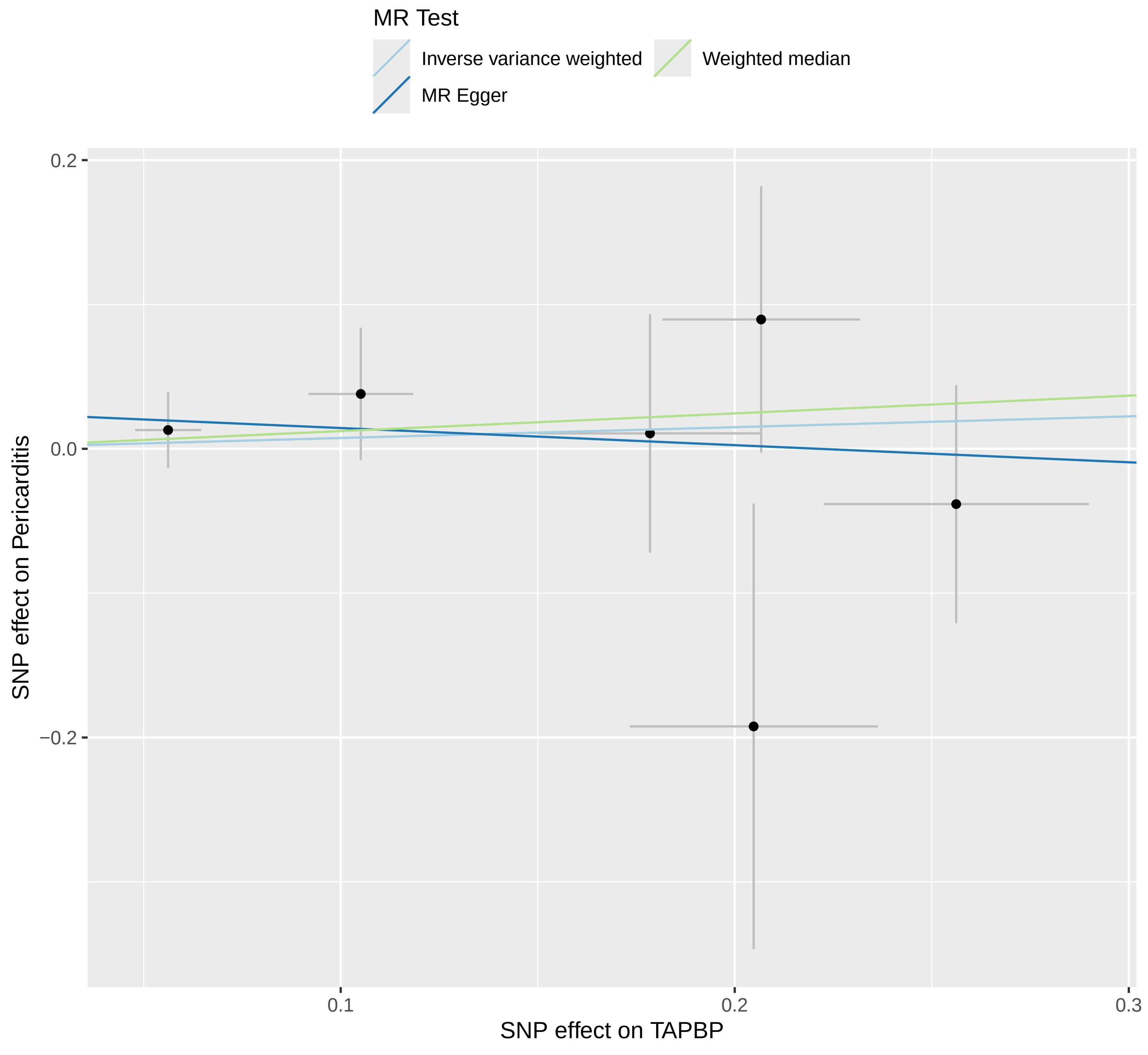

MR Test
Inverse variance weighted
Weighted median
MR Egger
0.2
0.0
−0.2
0.1
0.2
0.3
SNP effect on Pericarditis
SNP effect on TAPBP

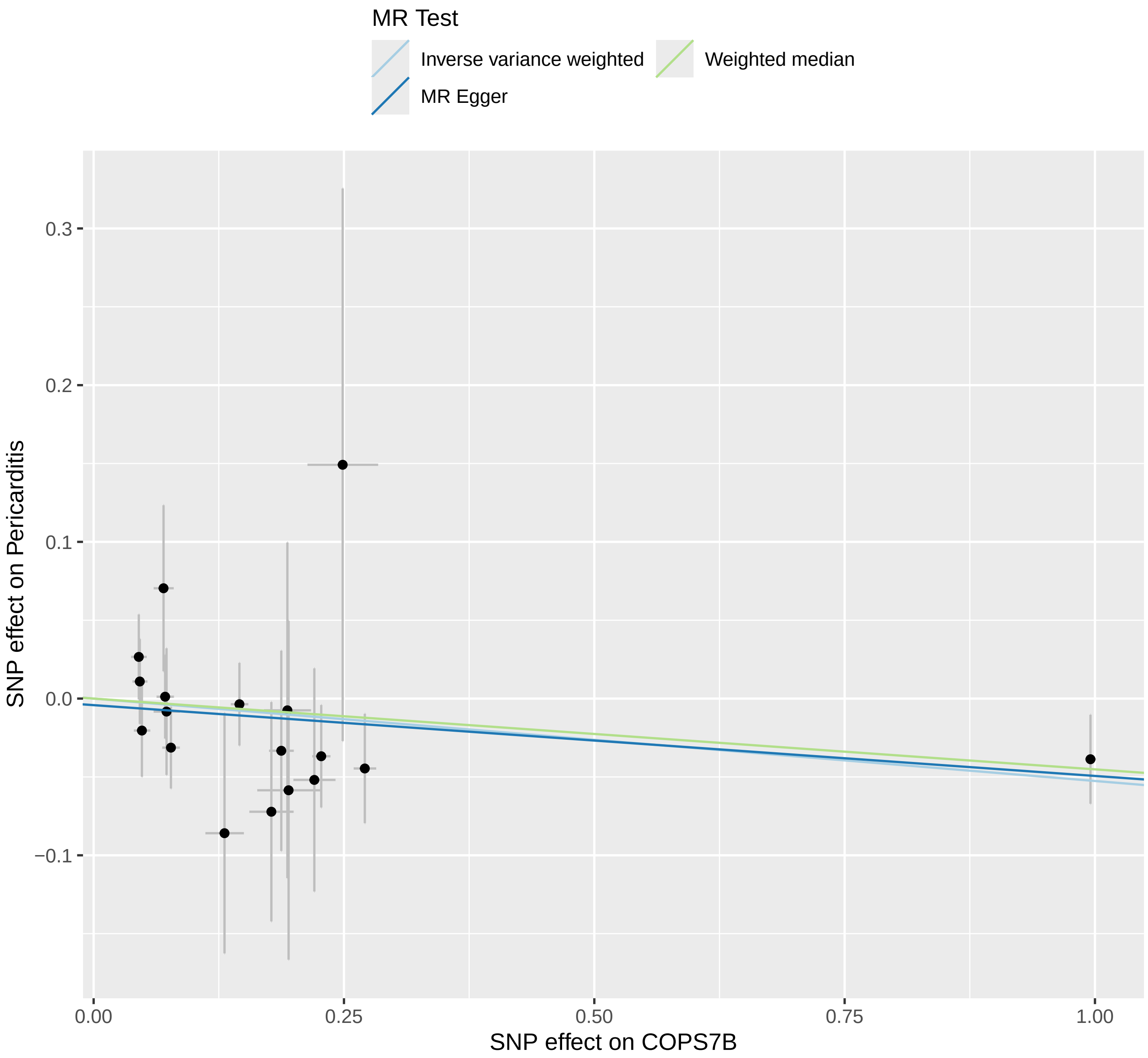
MR Test
Inverse variance weighted
Weighted median
MR Egger
0.3
0.2
0.1
0.0
−0.1
SNP effect on Pericarditis
0.00
0.25
0.50
0.75
1.00
SNP effect on COPS7B

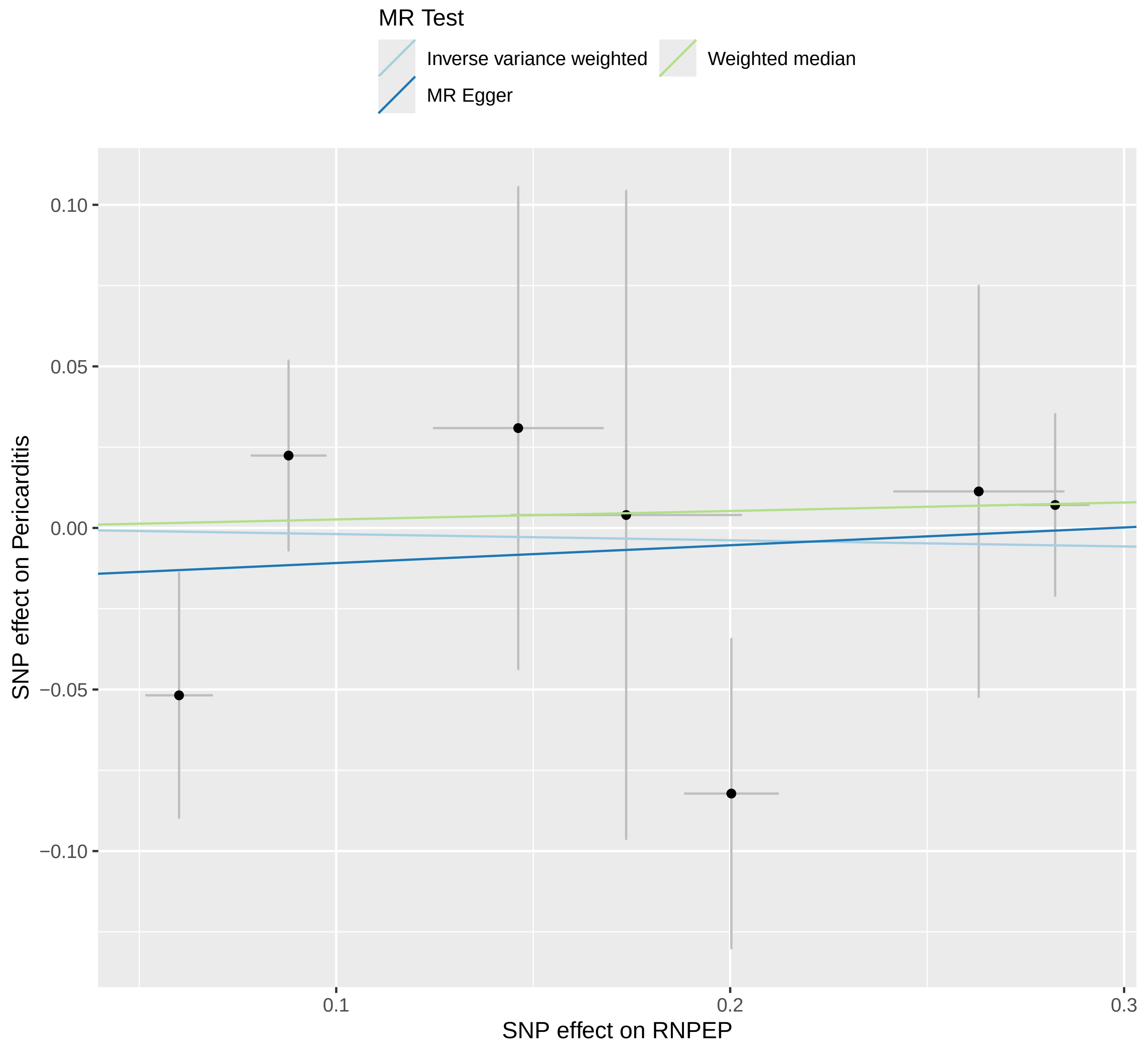

MR Test
Inverse variance weighted
Weighted median
MR Egger
0.10
0.05
0.00
−0.05
−0.10
SNP effect on Pericarditis
0.1
0.2
0.3
SNP effect on RNPEP

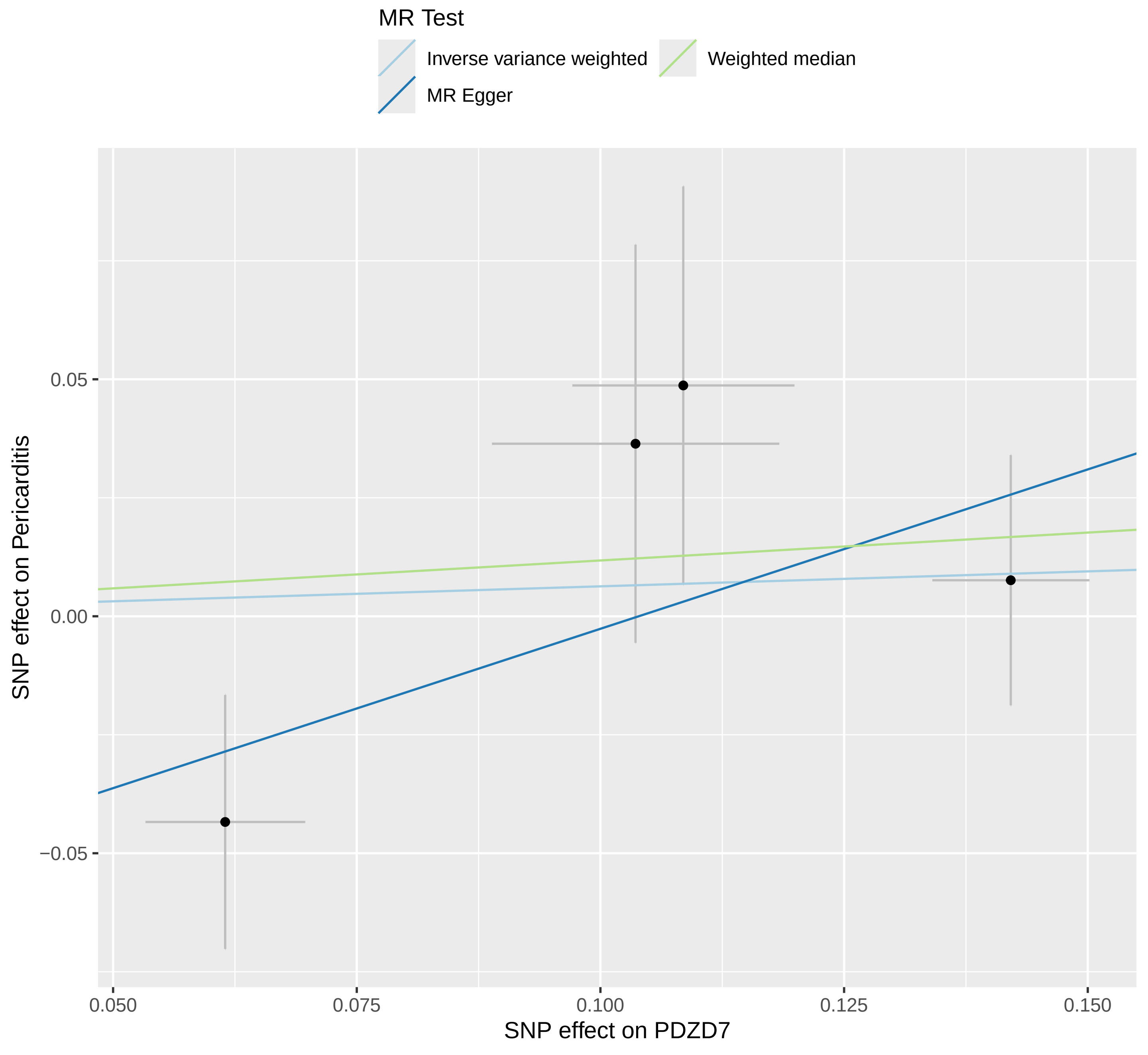
MR Test
Inverse variance weighted
Weighted median
MR Egger
0.05
0.00
−0.05
SNP effect on Pericarditis
0.050
0.075
0.100
0.125
0.150
SNP effect on PDZD7

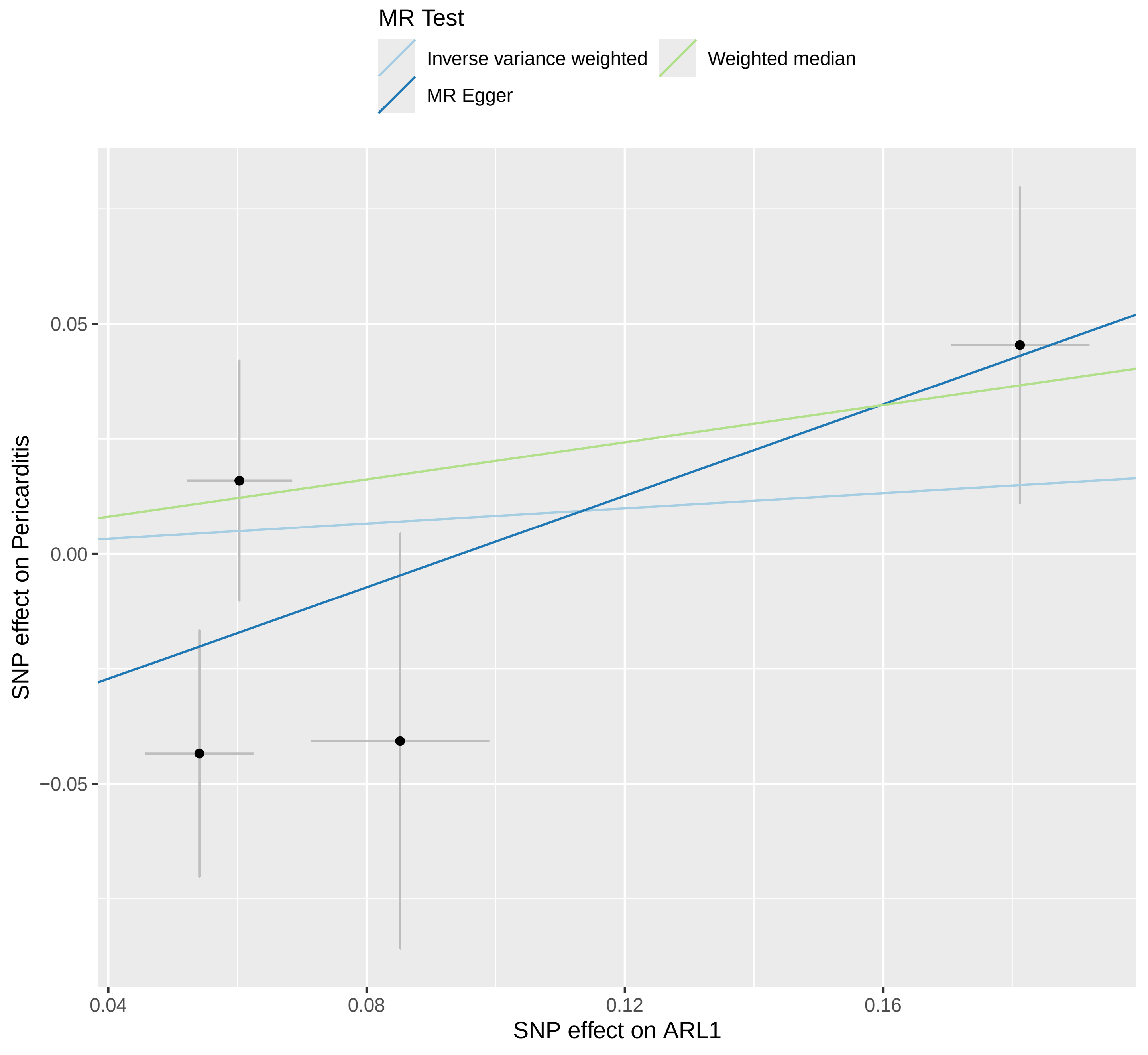
MR Test
Inverse variance weighted
MR Egger
Weighted median
SNP effect on Pericarditis
0.05
0.00
−0.05
0.04
0.08
0.12
0.16
SNP effect on ARL1

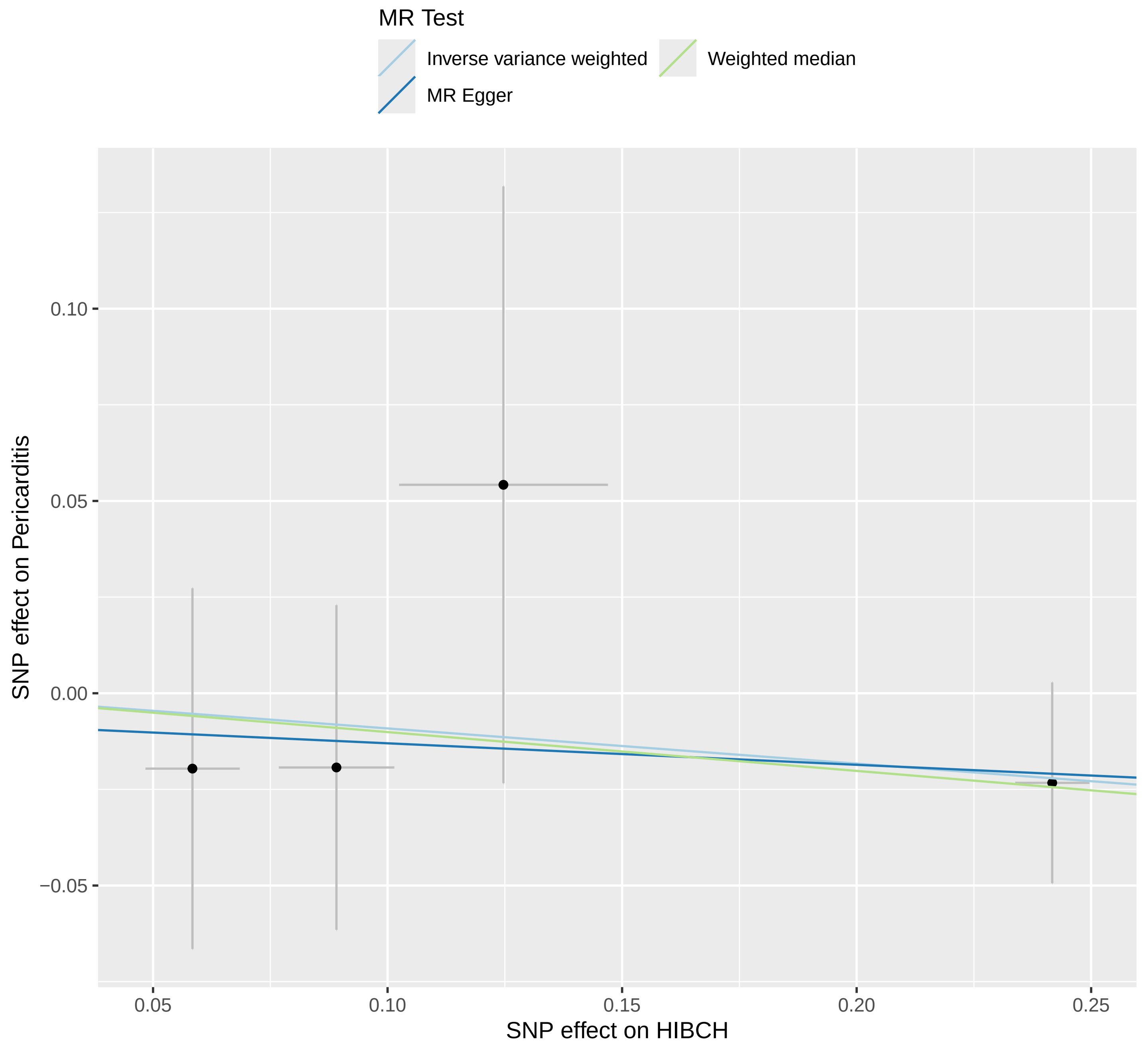

MR Test
Inverse variance weighted
Weighted median
MR Egger
SNP effect on Pericarditis
0.10
0.05
0.00
−0.05
0.05
0.10
0.15
0.20
0.25
SNP effect on HIBCH

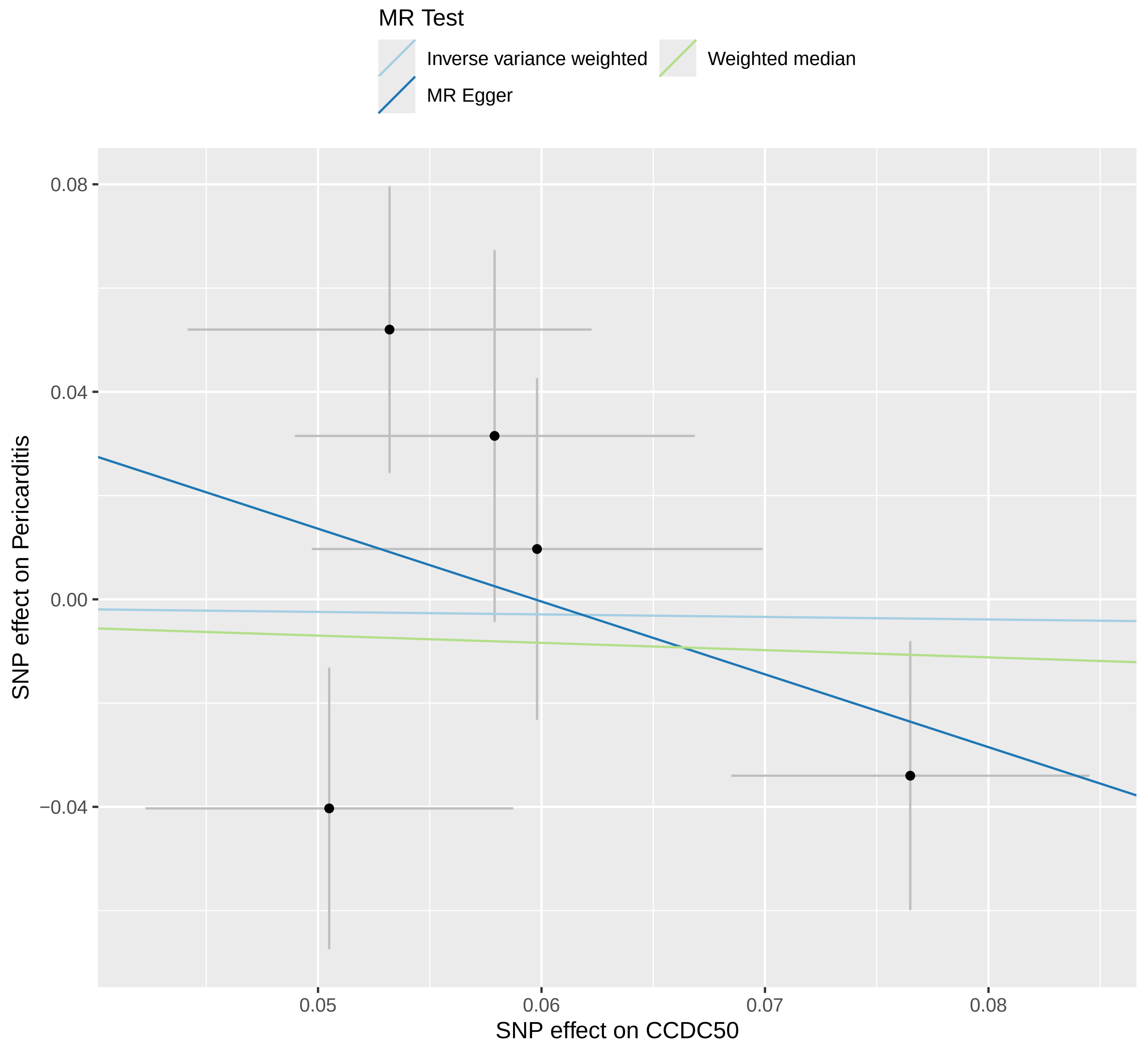

MR Test
Inverse variance weighted
Weighted median
MR Egger
0.08
0.04
0.00
−0.04
0.05
0.06
0.07
0.08
SNP effect on Pericarditis
SNP effect on CCDC50

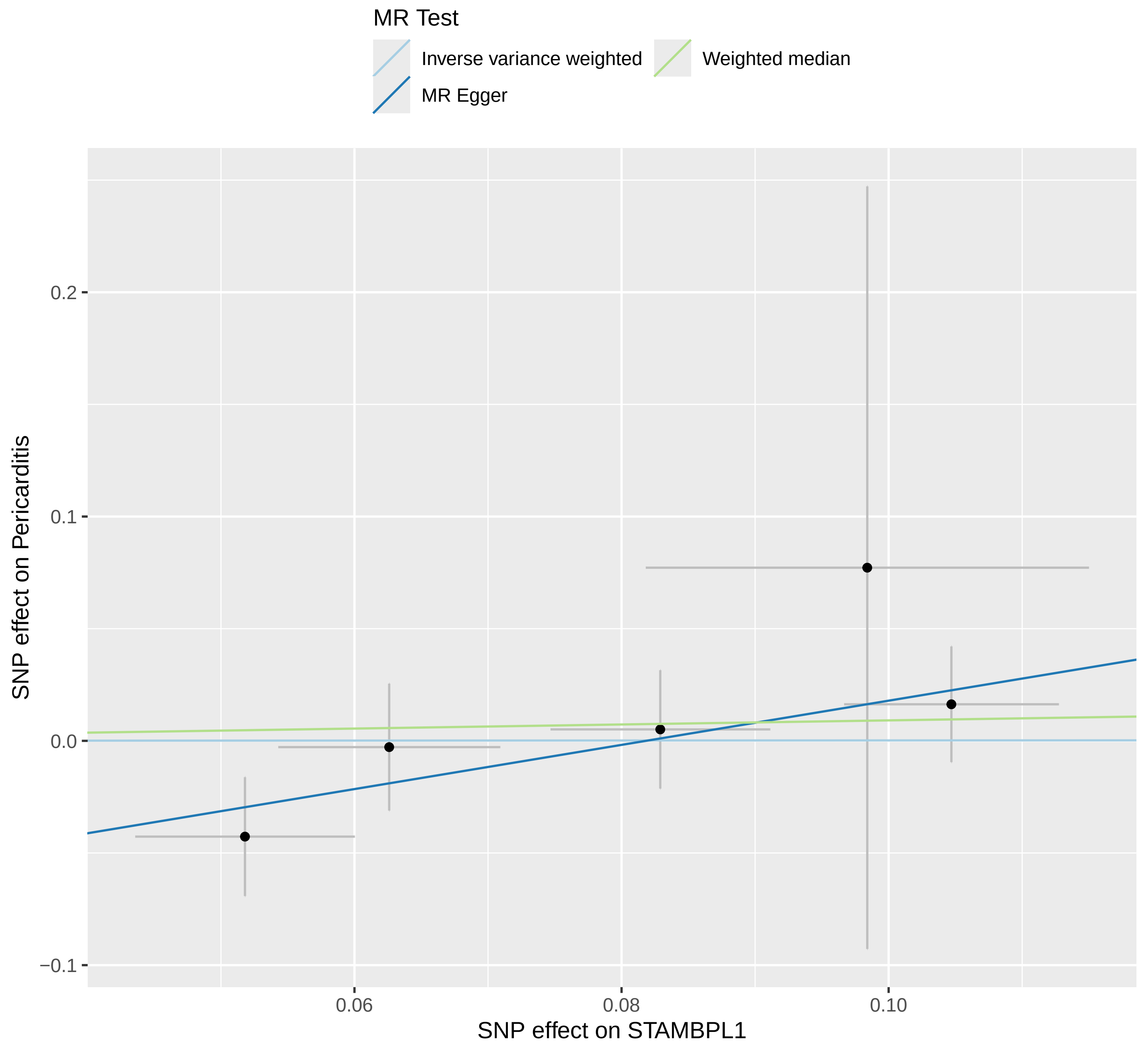
MR Test
Inverse variance weighted
Weighted median
MR Egger
0.2
0.1
0.0
−0.1
0.06
0.08
0.10
SNP effect on Pericarditis
SNP effect on STAMBPL1

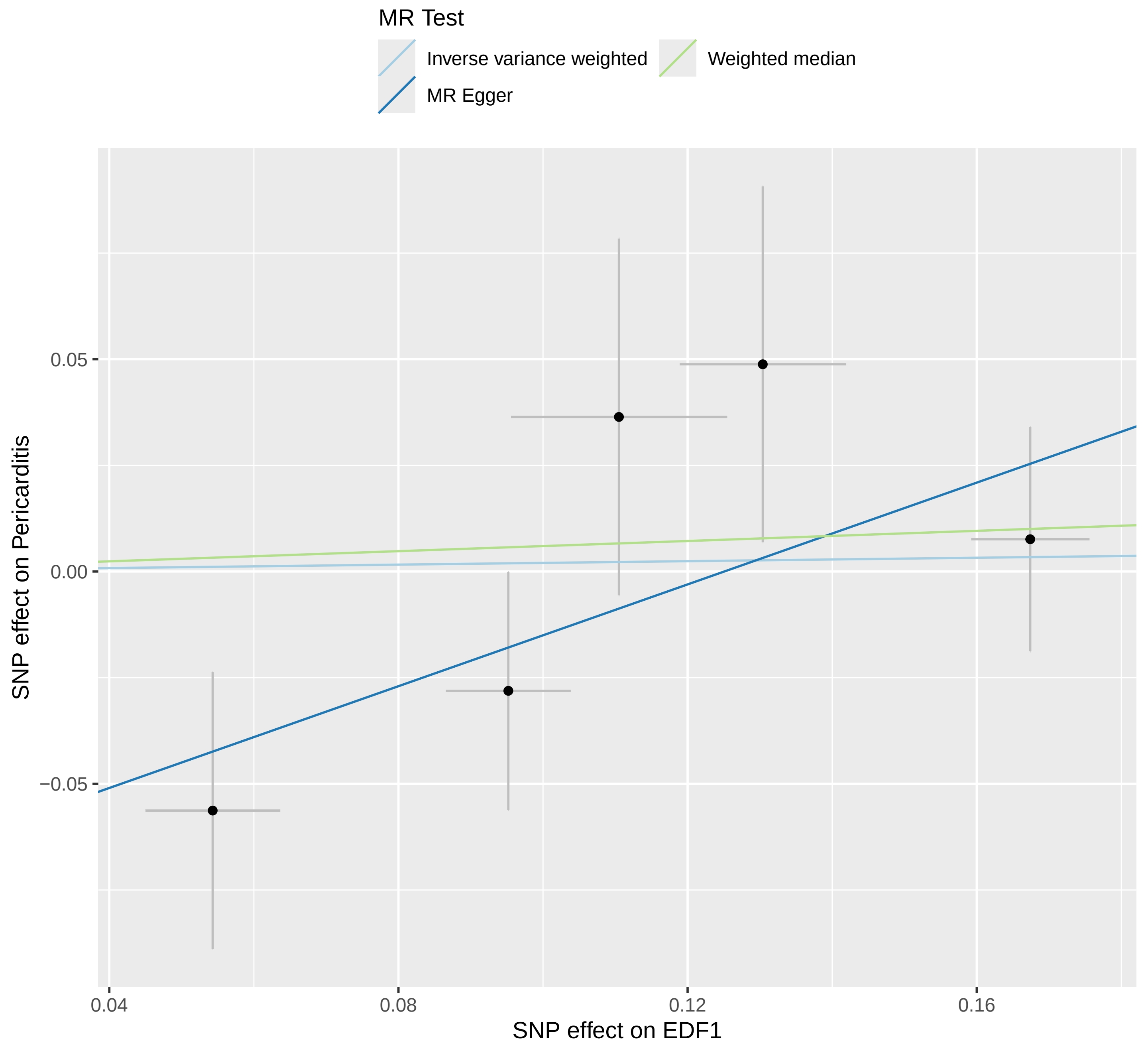
MR Test
Inverse variance weighted
MR Egger
Weighted median
SNP effect on Pericarditis
0.05
0.00
−0.05
0.04
0.08
0.12
0.16
SNP effect on EDF1

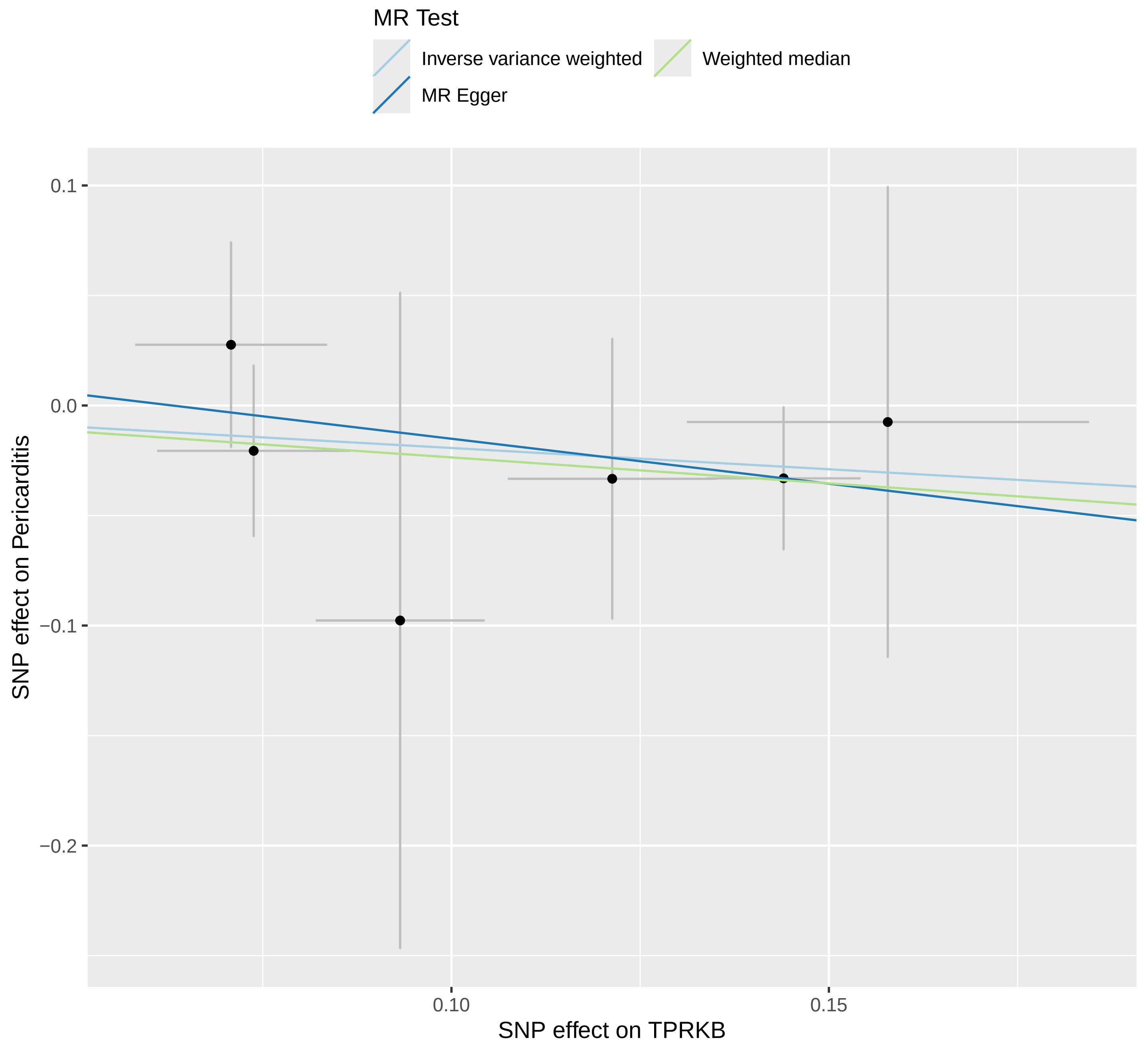

MR Test
Inverse variance weighted
MR Egger
Weighted median
0.1
0.0
−0.1
−0.2
SNP effect on Pericarditis
0.10
0.15
SNP effect on TPRKB

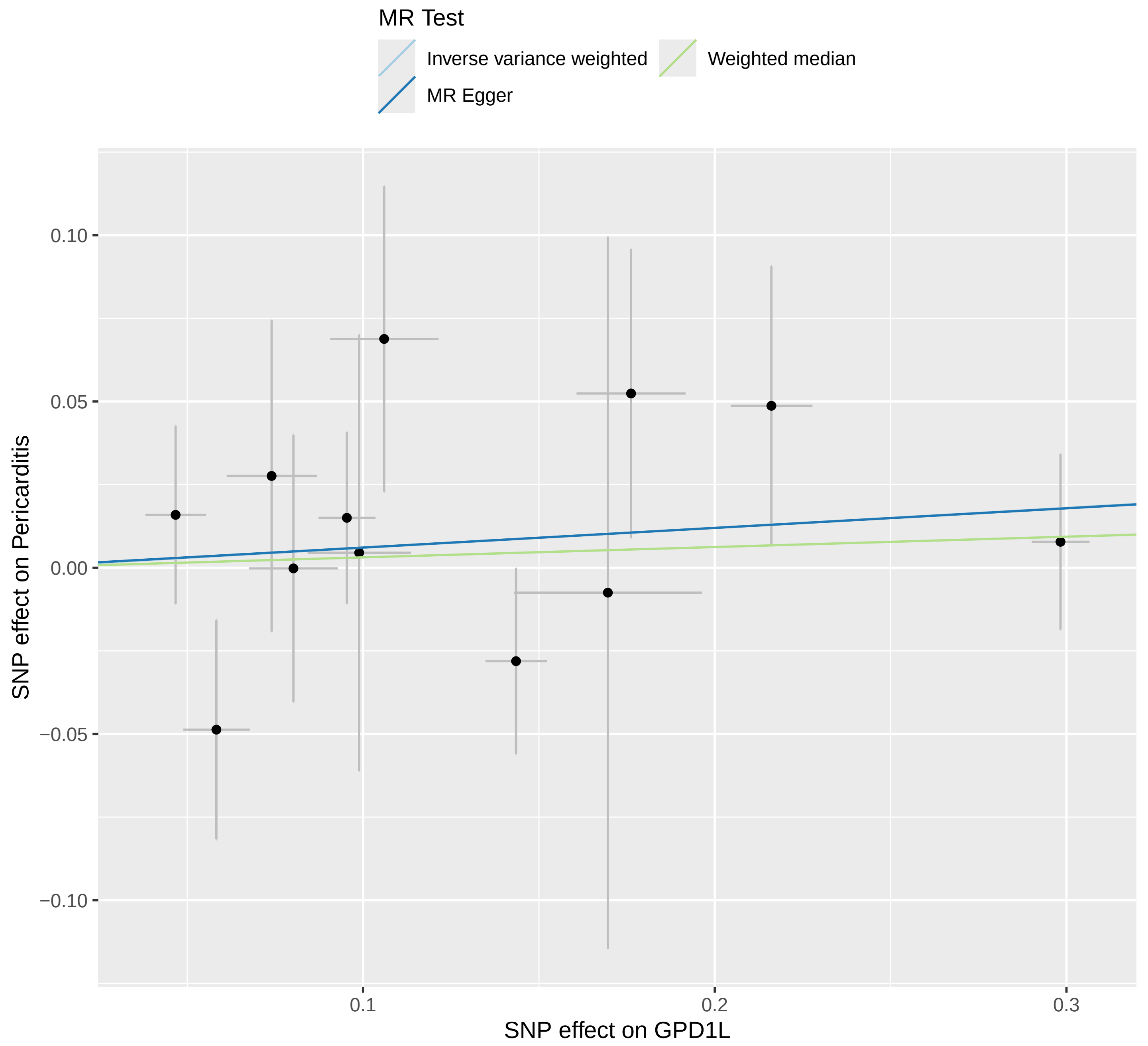

MR Test
Inverse variance weighted
MR Egger
Weighted median
0.10
0.05
0.00
−0.05
−0.10
SNP effect on Pericarditis
0.1
0.2
0.3
SNP effect on GPD1L

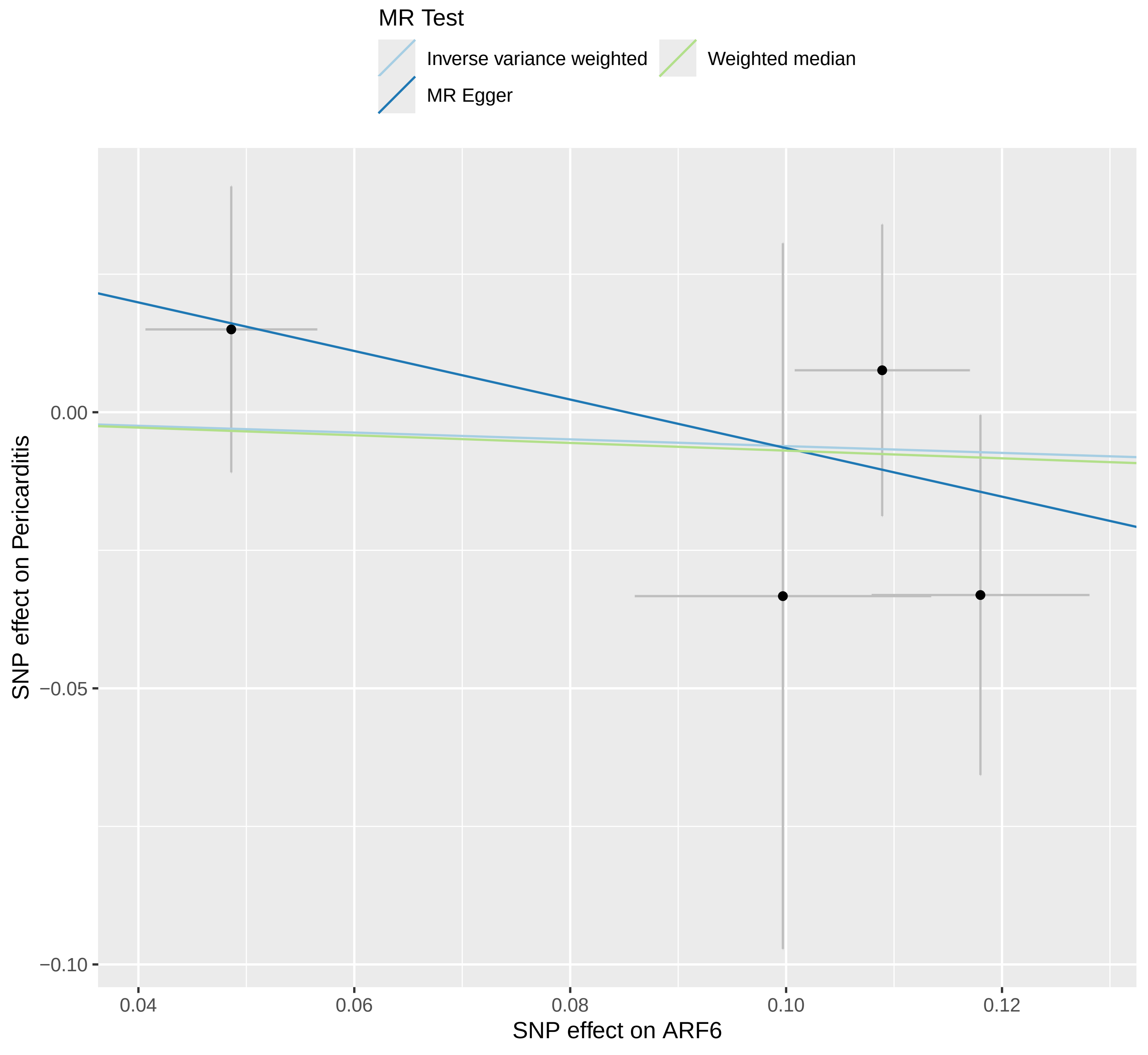
MR Test
Inverse variance weighted
MR Egger
Weighted median
0.00
−0.05
−0.10
SNP effect on Pericarditis
0.04
0.06
0.08
0.10
0.12
SNP effect on ARF6

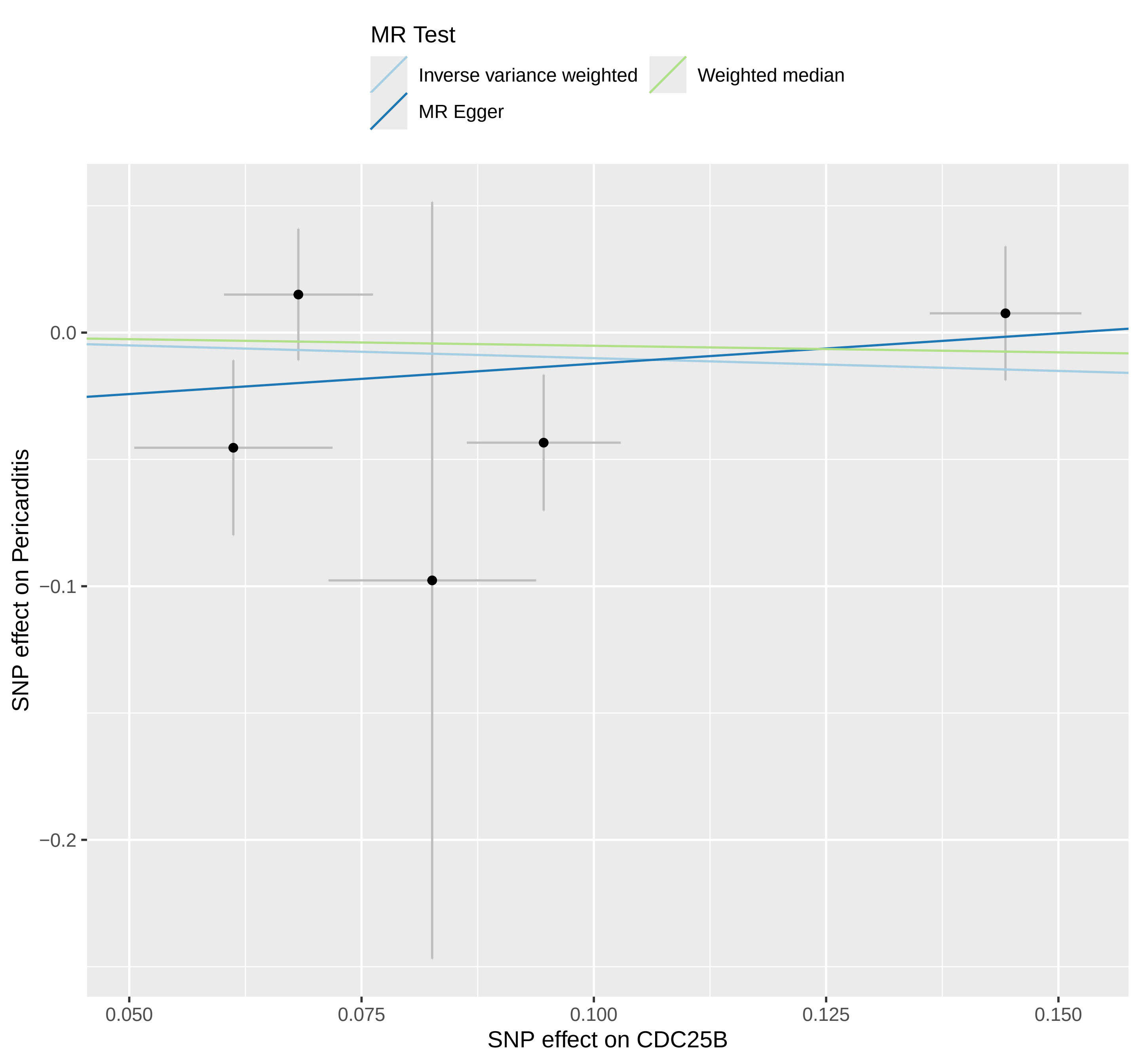
MR Test
Inverse variance weighted
Weighted median
MR Egger
0.0
−0.1
−0.2
0.050
0.075
0.100
0.125
0.150
SNP effect on Pericarditis
SNP effect on CDC25B

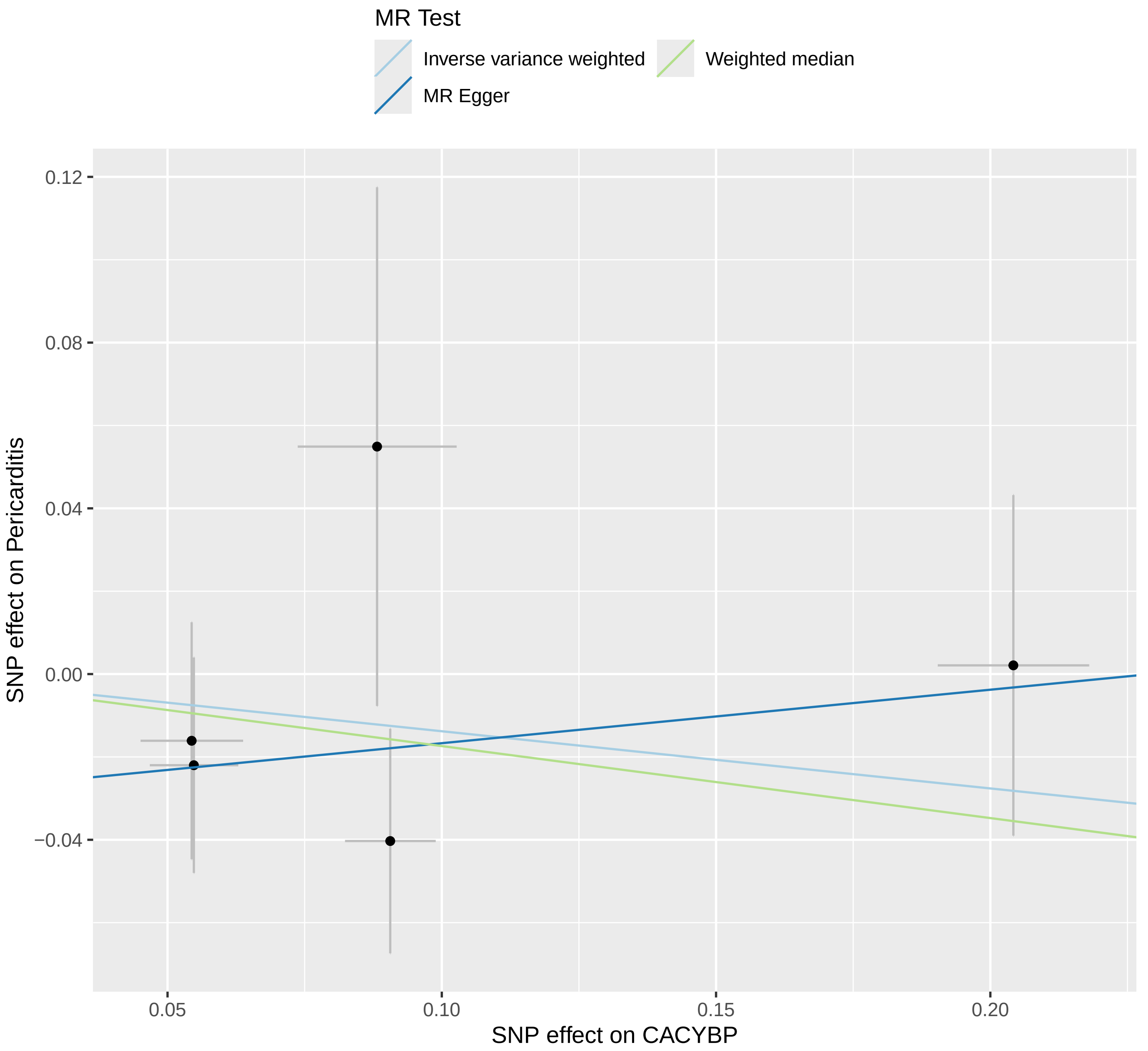

MR Test
Inverse variance weighted
Weighted median
MR Egger
0.12
0.08
0.04
0.00
−0.04
SNP effect on Pericarditis
0.05
0.10
0.15
0.20
SNP effect on CACYBP

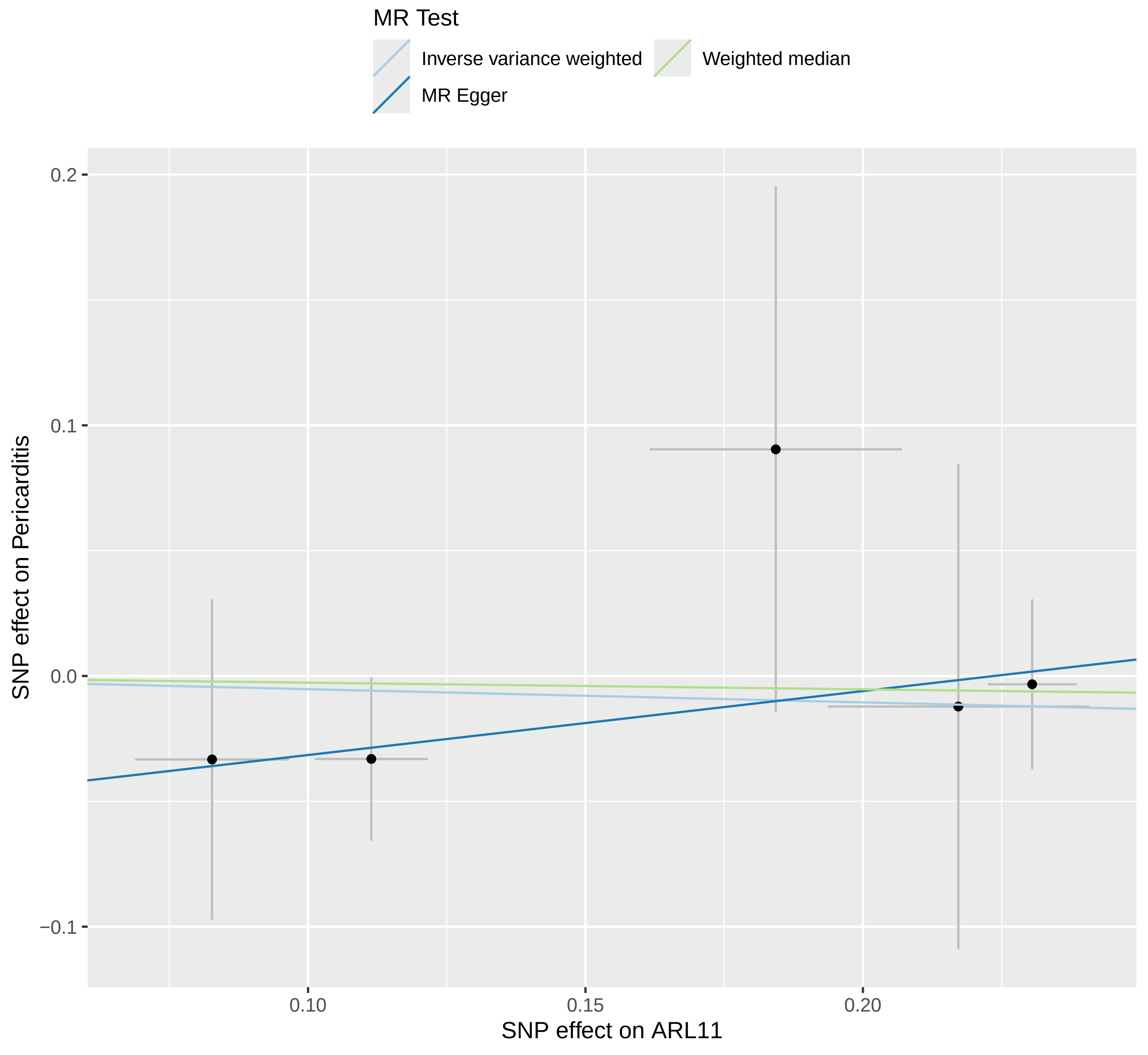
MR Test
Inverse variance weighted
Weighted median
MR Egger
0.2
0.1
0.0
−0.1
SNP effect on Pericarditis
0.10
0.15
0.20
SNP effect on ARL11

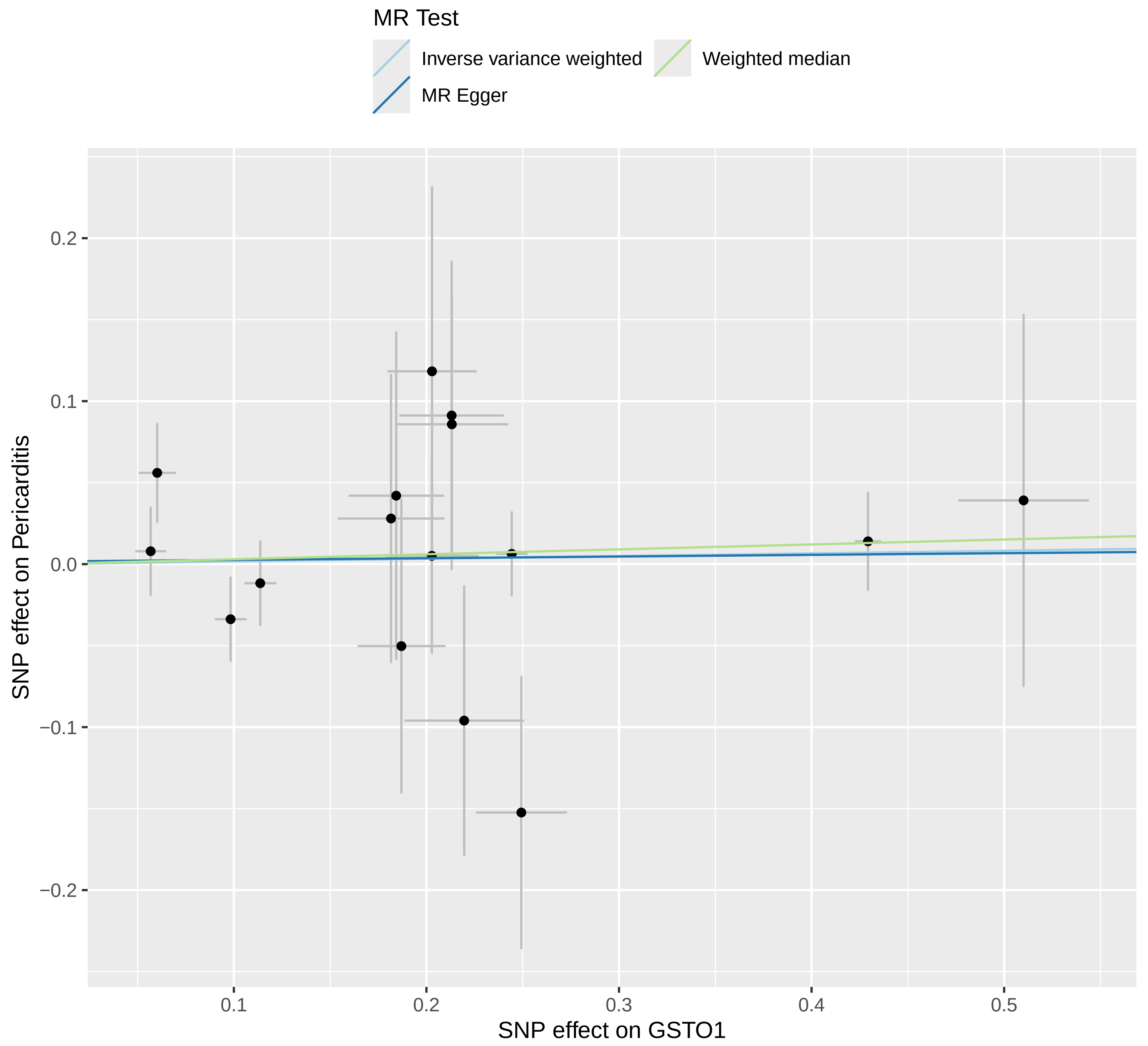

MR Test
Inverse variance weighted
Weighted median
MR Egger
0.2
0.1
0.0
−0.1
−0.2
0.1
0.2
0.3
0.4
0.5
SNP effect on Pericarditis
SNP effect on GSTO1

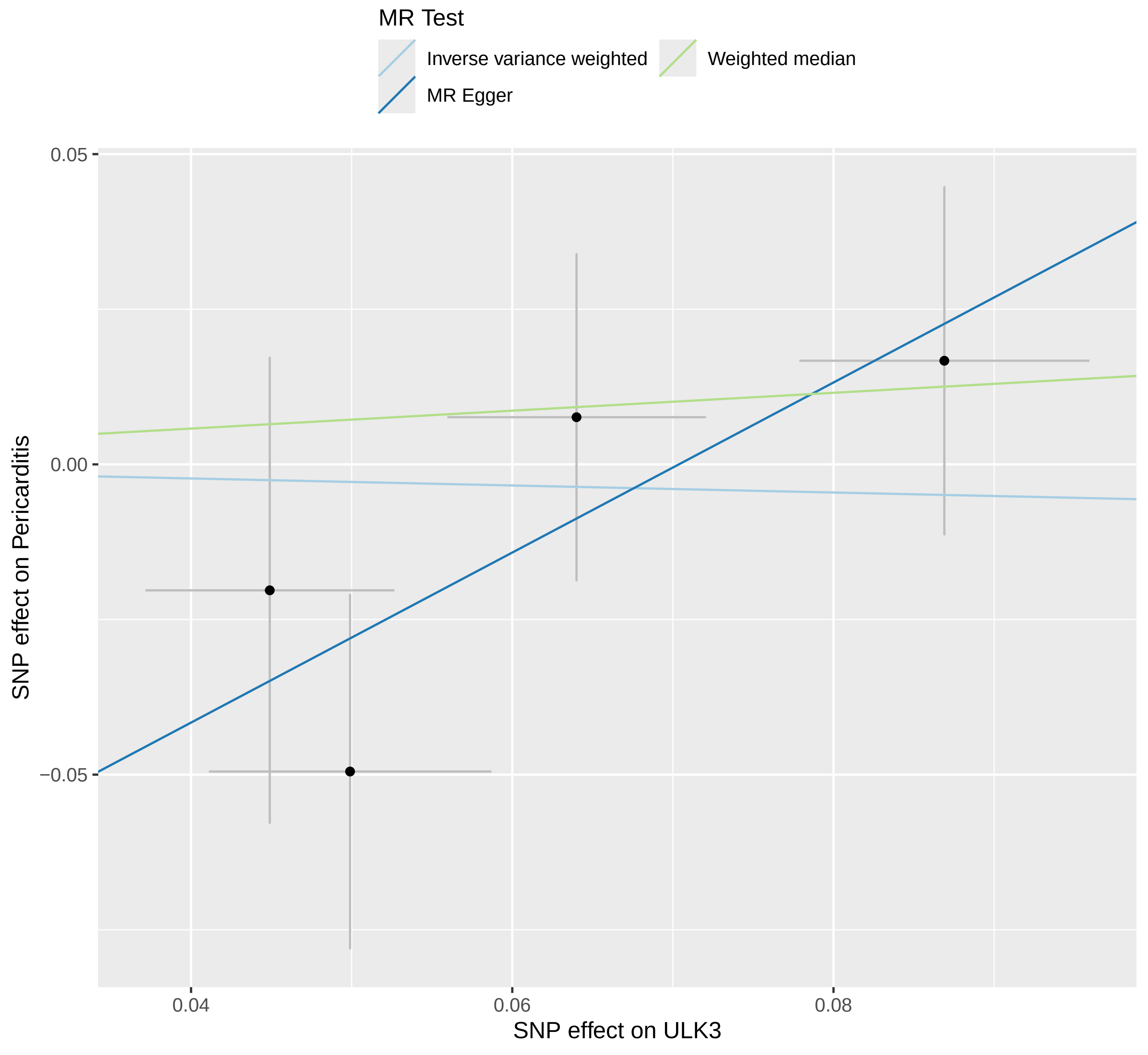
MR Test
Inverse variance weighted
MR Egger
Weighted median
0.05
0.00
−0.05
SNP effect on Pericarditis
0.04
0.06
0.08
SNP effect on ULK3

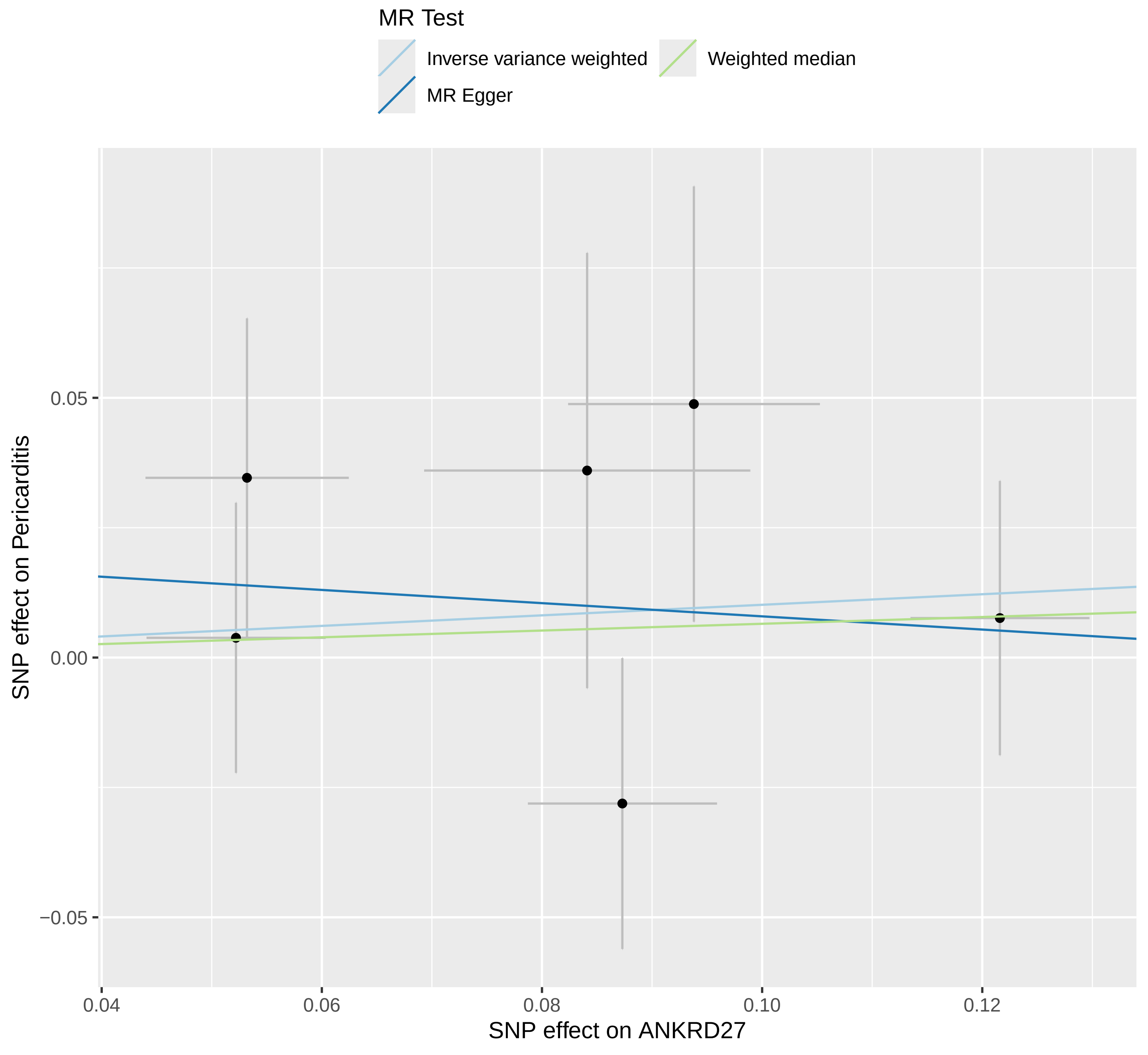
MR Test
Inverse variance weighted
Weighted median
MR Egger
SNP effect on Pericarditis
0.05
0.00
−0.05
0.04
0.06
0.08
0.10
0.12
SNP effect on ANKRD27

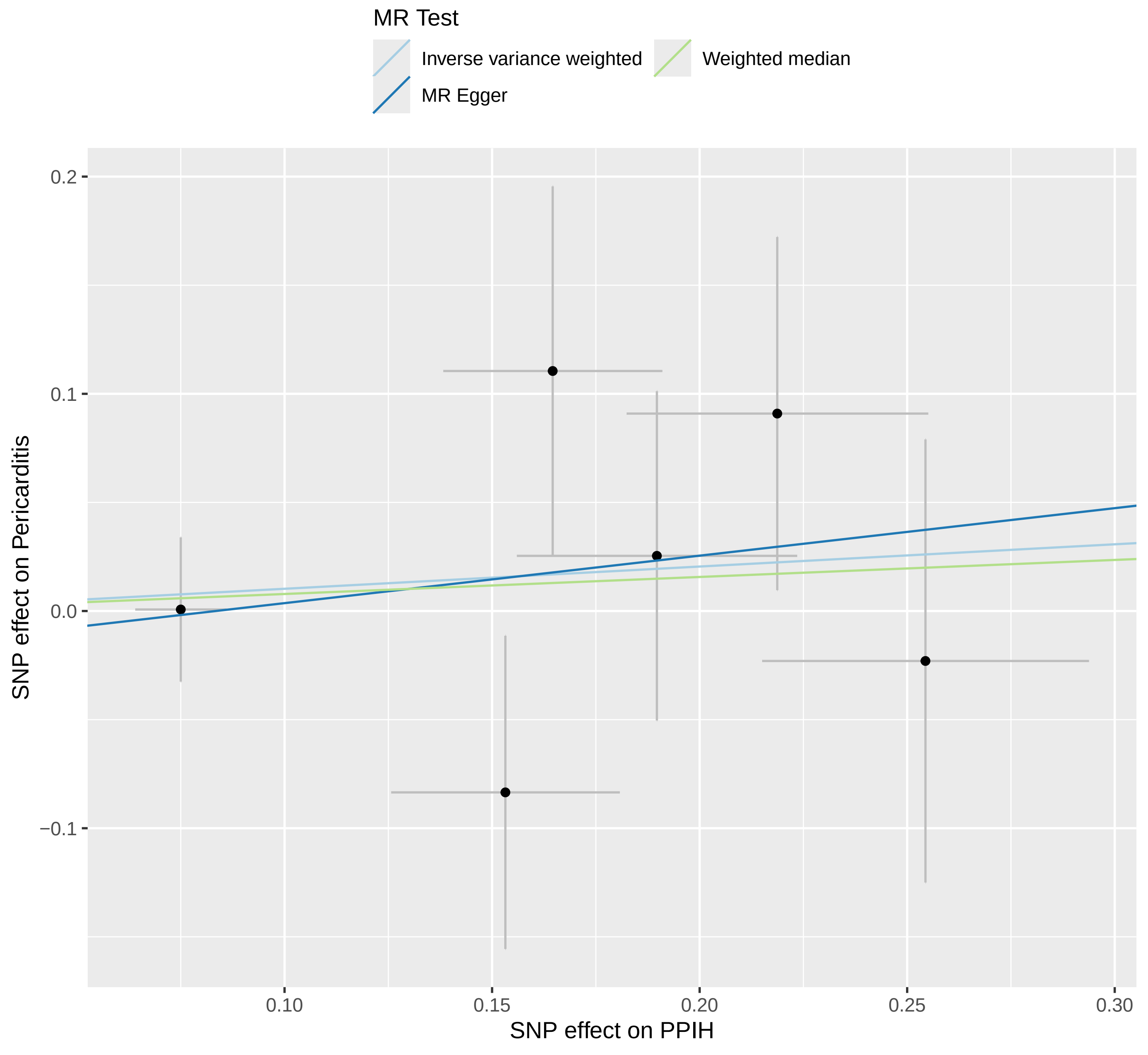
MR Test
Inverse variance weighted
Weighted median
MR Egger
SNP effect on Pericarditis
SNP effect on PPIH
0.2
0.1
0.0
−0.1
0.10
0.15
0.20
0.25
0.30

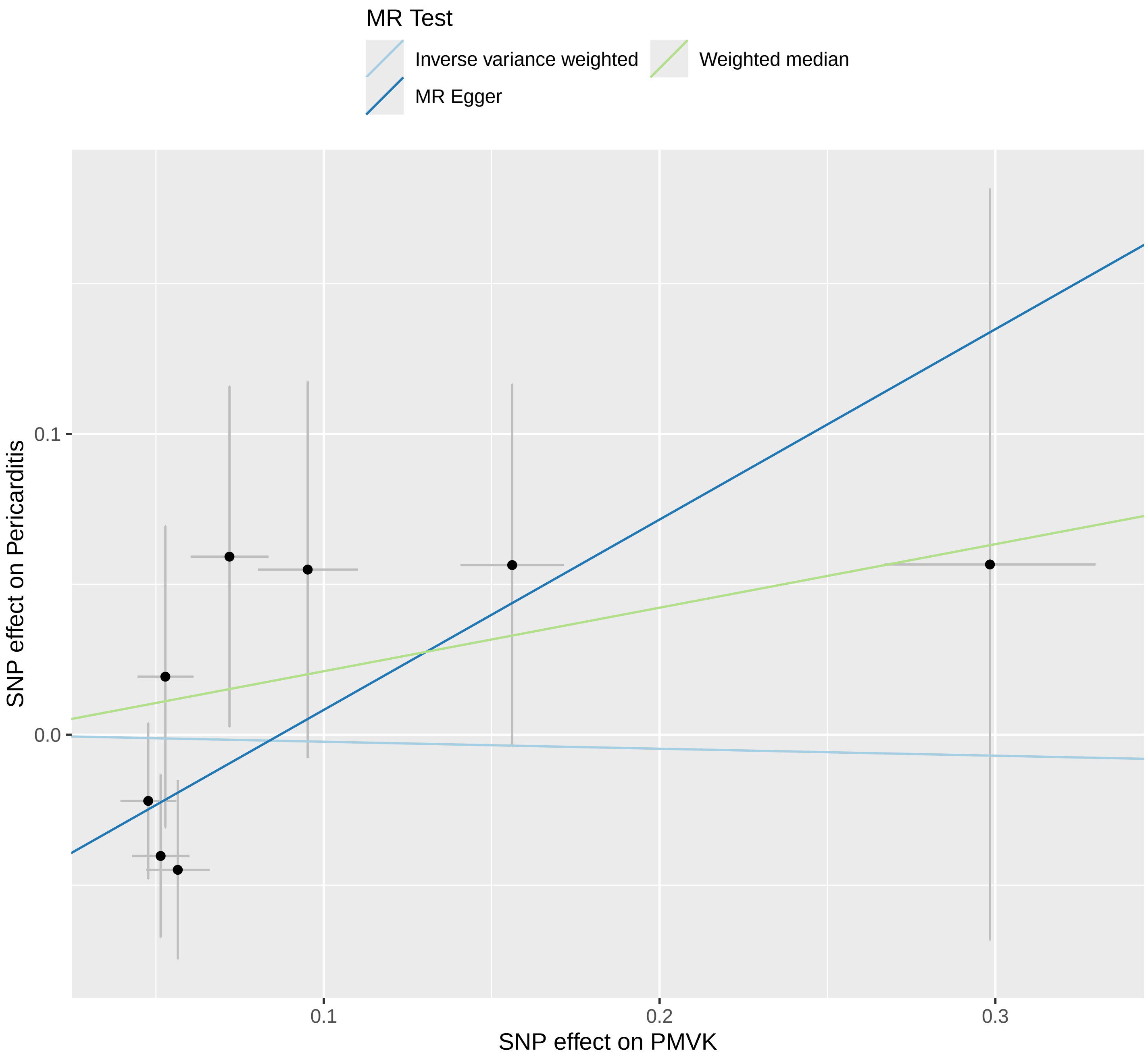

MR Test
Inverse variance weighted
Weighted median
MR Egger
0.1
0.0
SNP effect on Pericarditis
0.1
0.2
0.3
SNP effect on PMVK

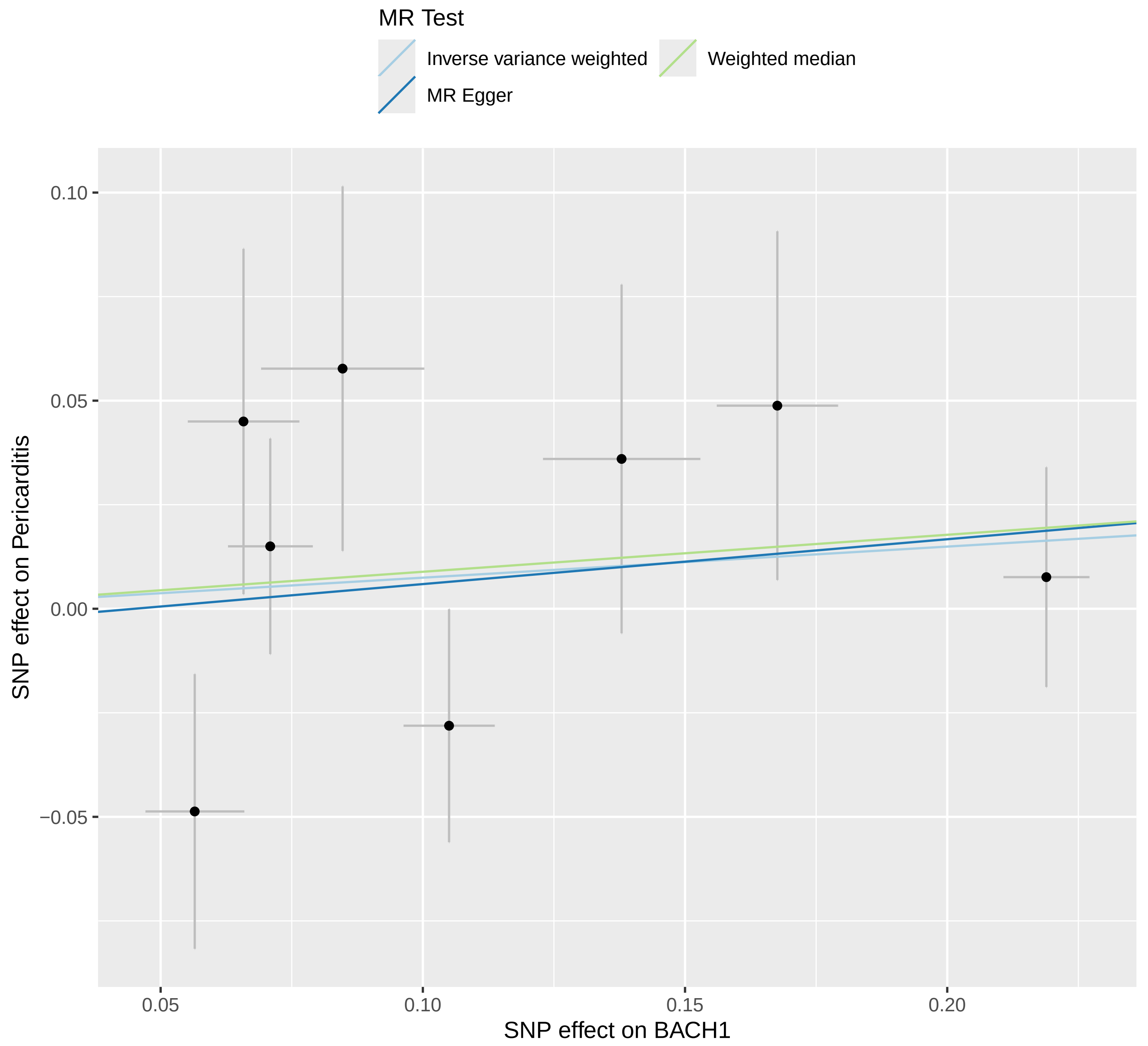
MR Test
Inverse variance weighted
Weighted median
MR Egger
0.10
0.05
0.00
−0.05
SNP effect on Pericarditis
0.05
0.10
0.15
0.20
SNP effect on BACH1

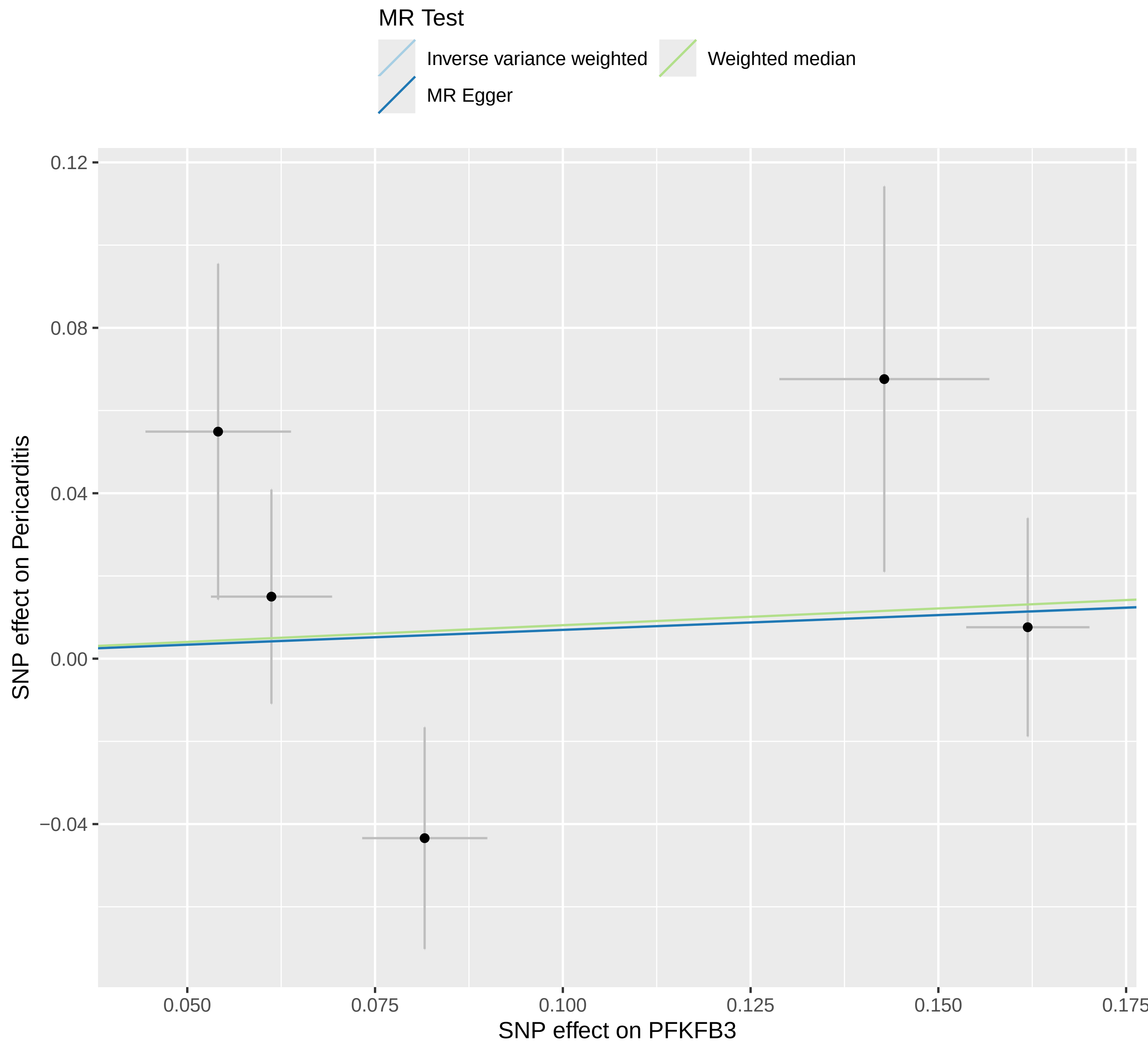
MR Test
Inverse variance weighted
MR Egger
Weighted median
0.12
0.08
0.04
0.00
−0.04
SNP effect on Pericarditis
0.050
0.075
0.100
0.125
0.150
0.175
SNP effect on PFKFB3

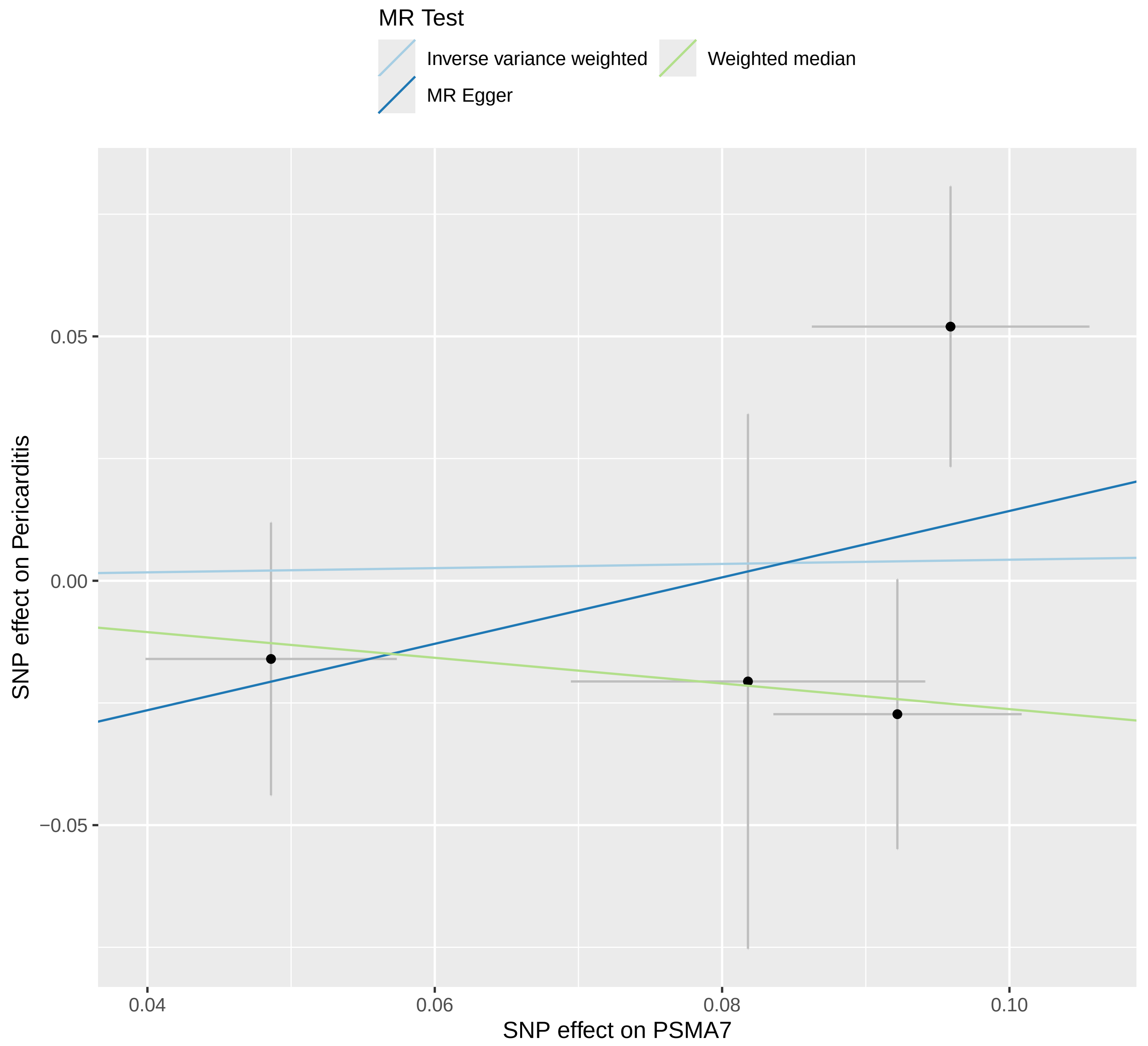

MR Test
Inverse variance weighted
MR Egger
Weighted median
0.05
0.00
−0.05
SNP effect on Pericarditis
0.04
0.06
0.08
0.10
SNP effect on PSMA7

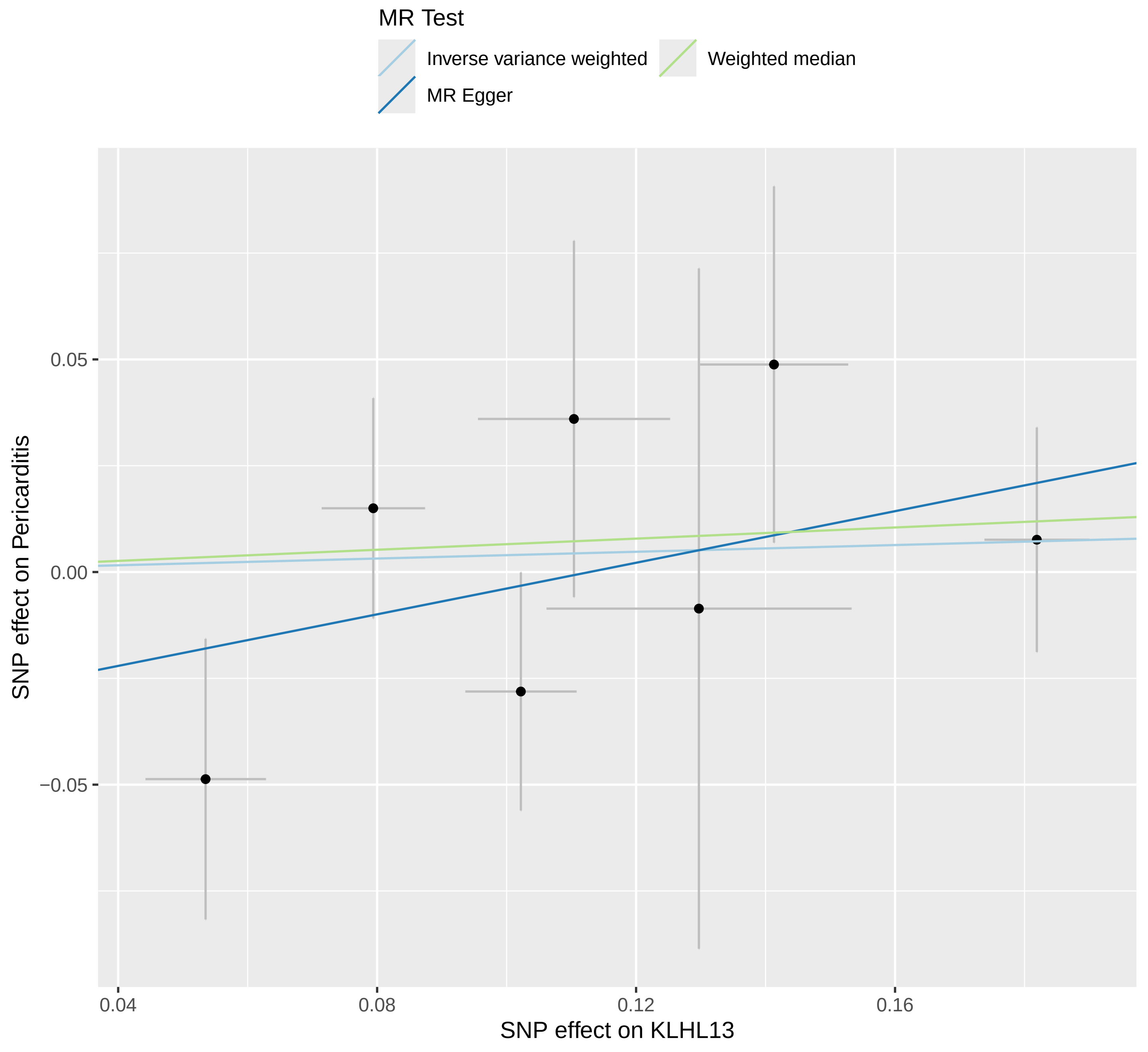

MR Test
Inverse variance weighted
MR Egger
Weighted median
0.05
0.00
−0.05
0.04
0.08
0.12
0.16
SNP effect on Pericarditis
SNP effect on KLHL13

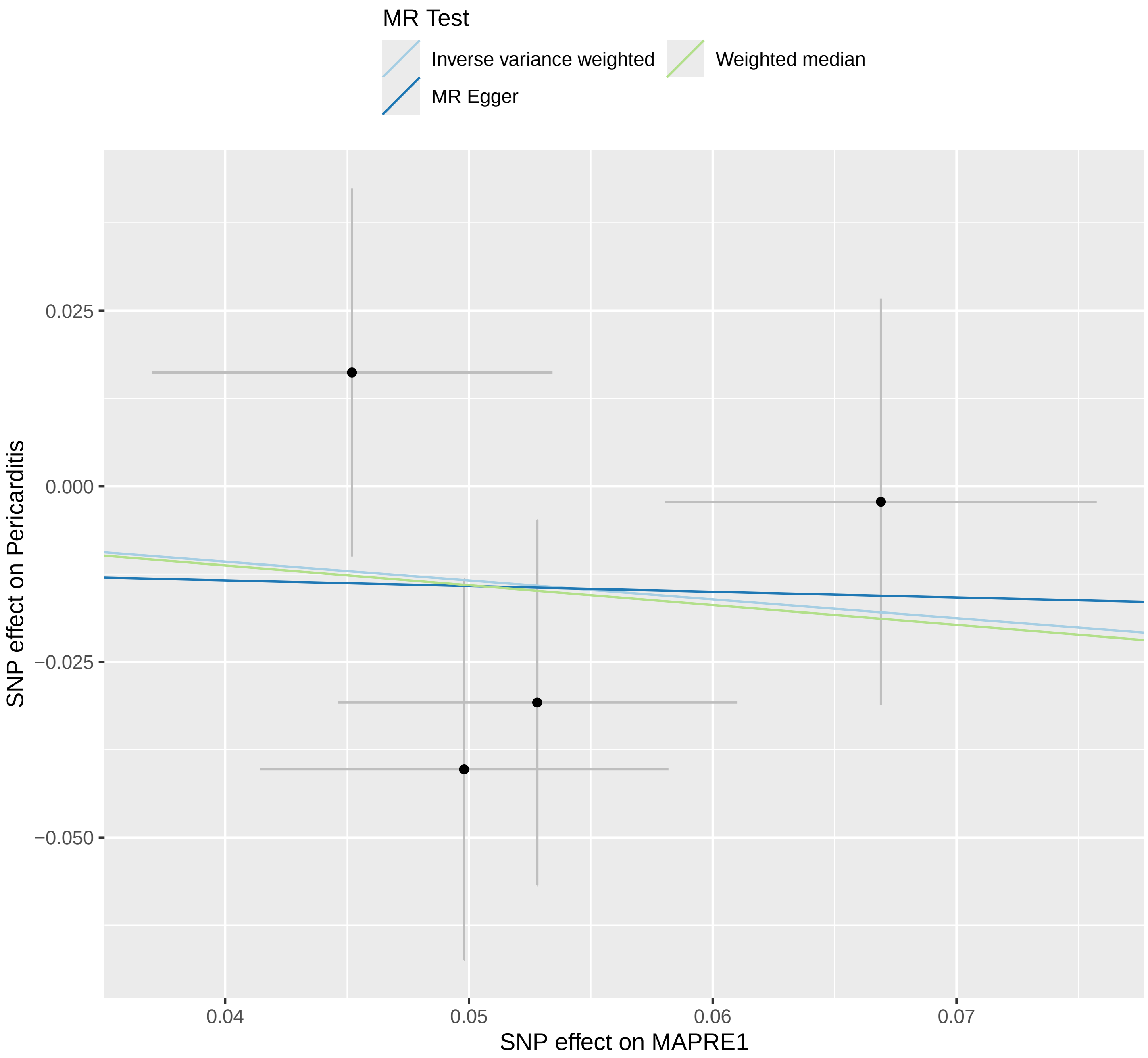
MR Test
Inverse variance weighted
Weighted median
MR Egger
SNP effect on Pericarditis
0.025
0.000
−0.025
−0.050
0.04
0.05
0.06
0.07
SNP effect on MAPRE1

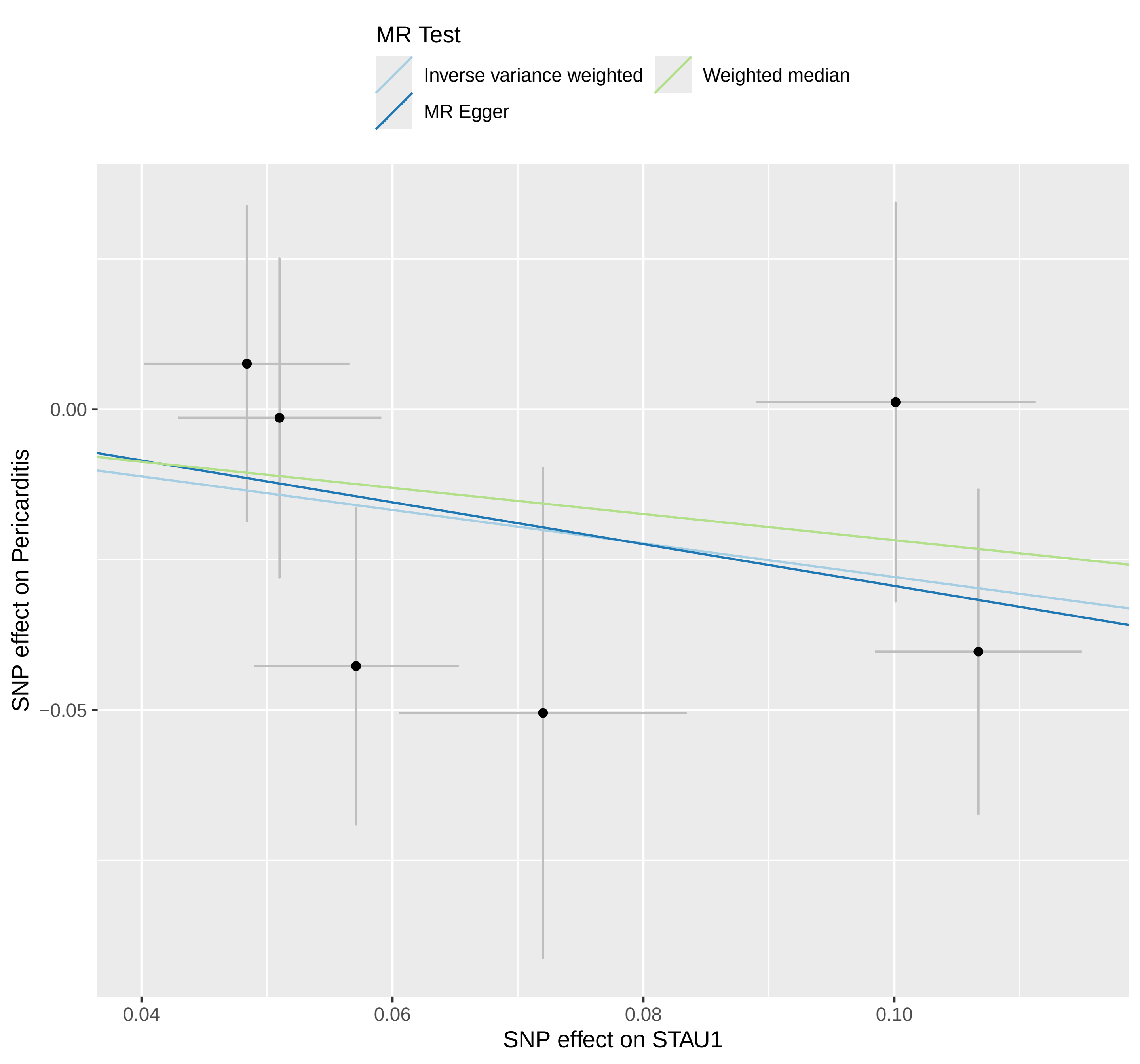

MR Test
Inverse variance weighted
MR Egger
Weighted median
0.00
−0.05
SNP effect on Pericarditis
0.04
0.06
0.08
0.10
SNP effect on STAU1

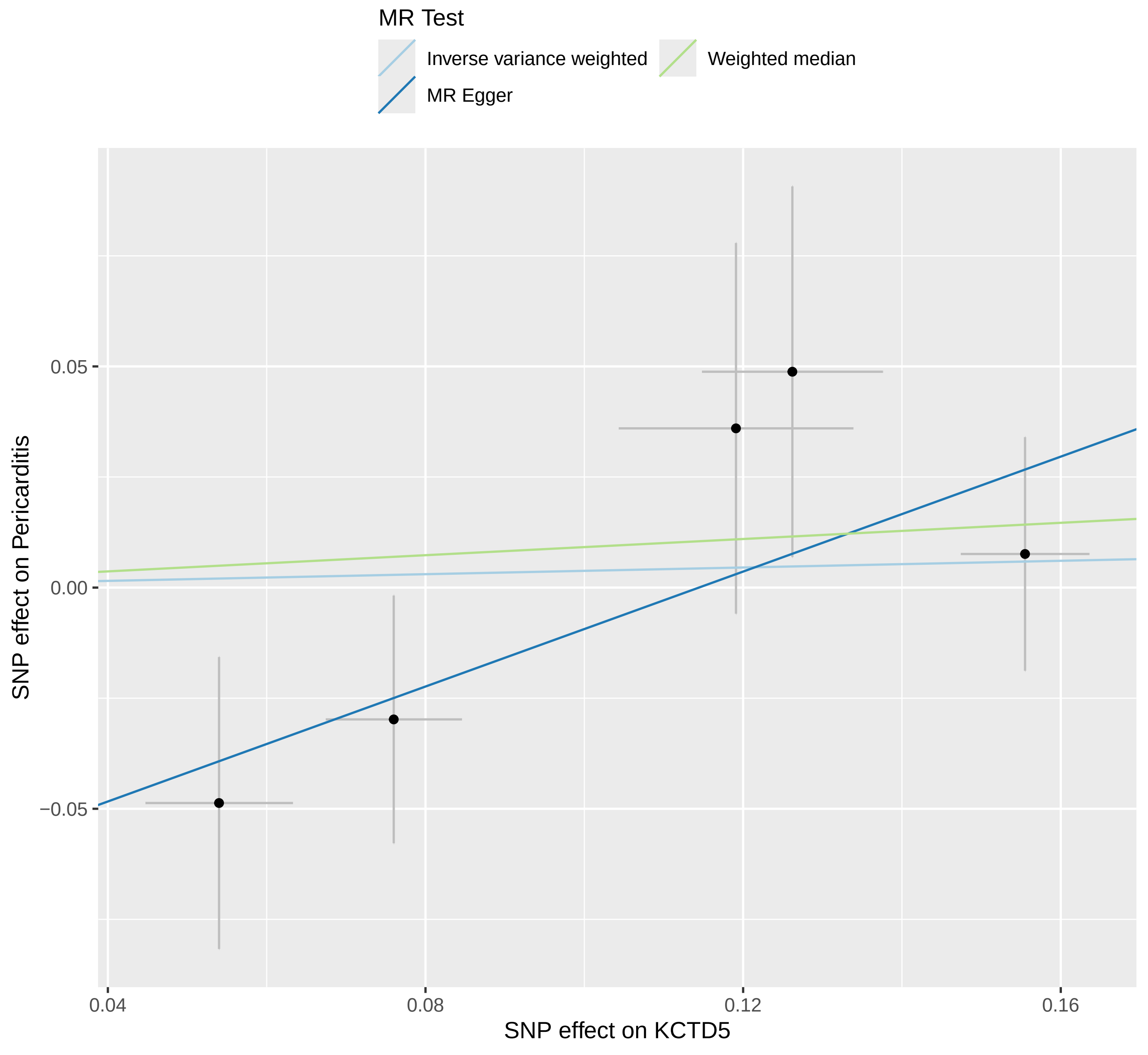
MR Test
Inverse variance weighted
Weighted median
MR Egger
0.05
0.00
−0.05
0.04
0.08
0.12
0.16
SNP effect on Pericarditis
SNP effect on KCTD5

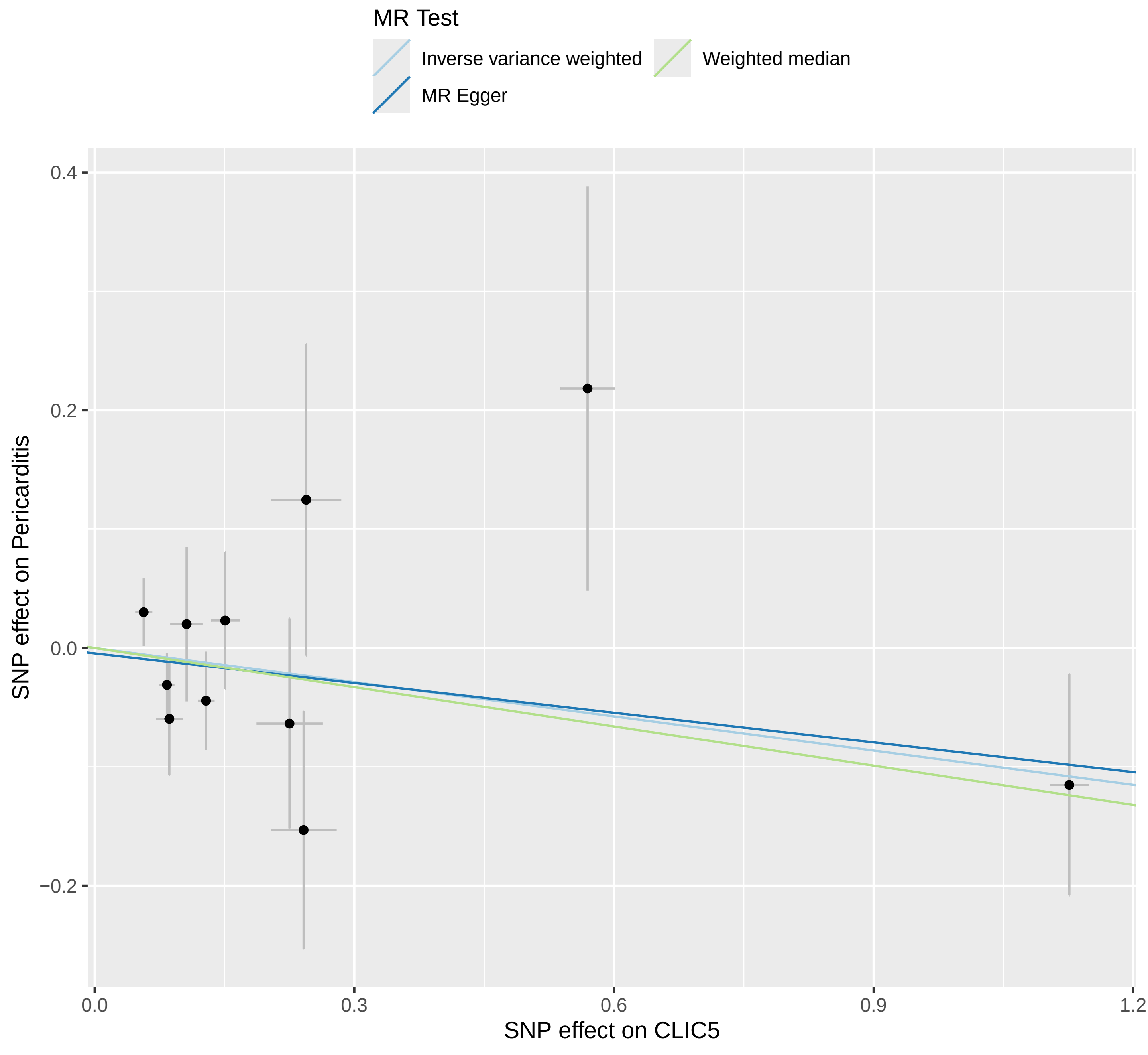
MR Test
Inverse variance weighted
MR Egger
Weighted median
0.4
0.2
0.0
−0.2
0.0
0.3
0.6
0.9
1.2
SNP effect on Pericarditis
SNP effect on CLIC5

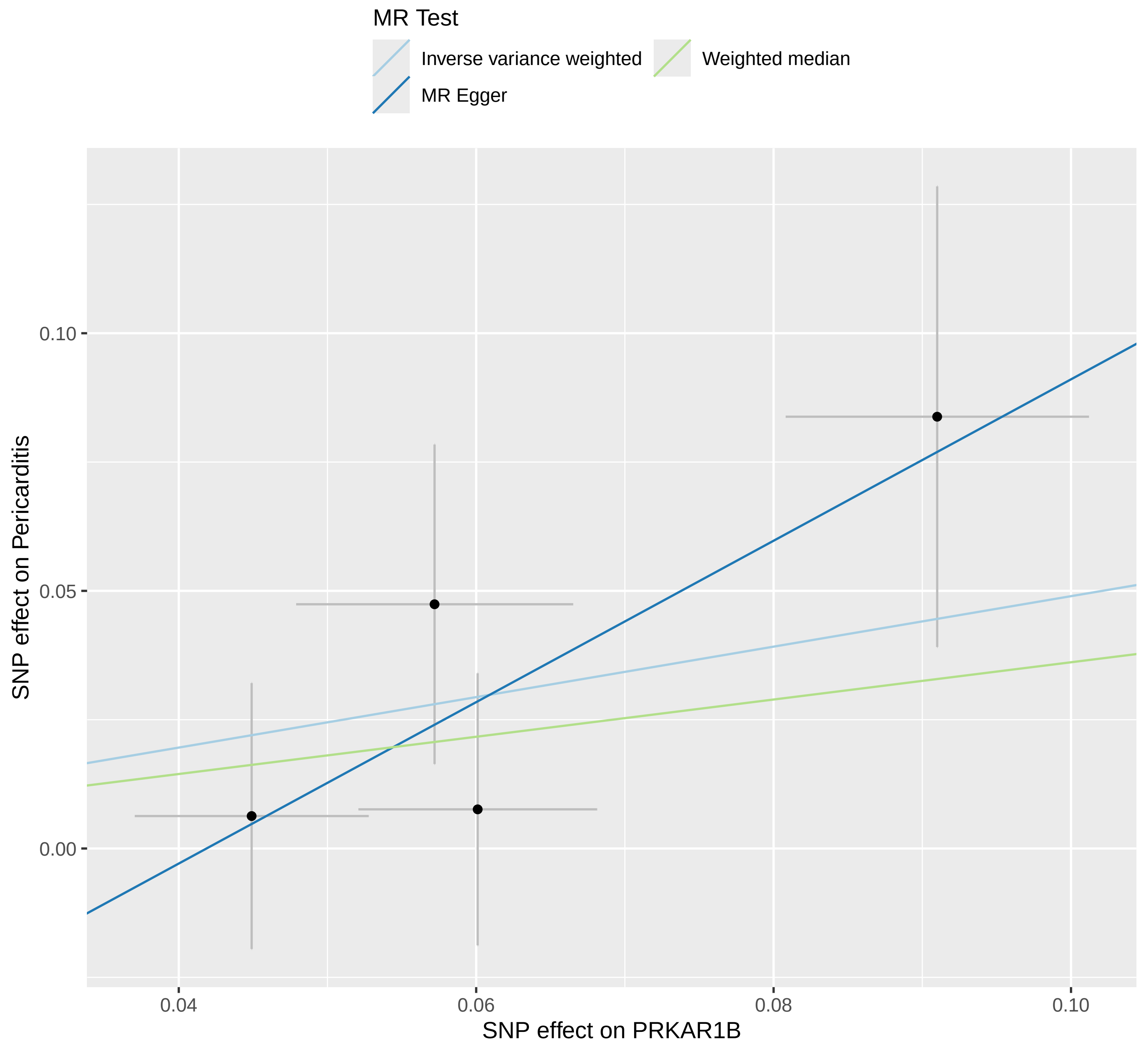

MR Test
Inverse variance weighted
Weighted median
MR Egger
0.10
0.05
0.00
SNP effect on Pericarditis
0.04
0.06
0.08
0.10
SNP effect on PRKAR1B

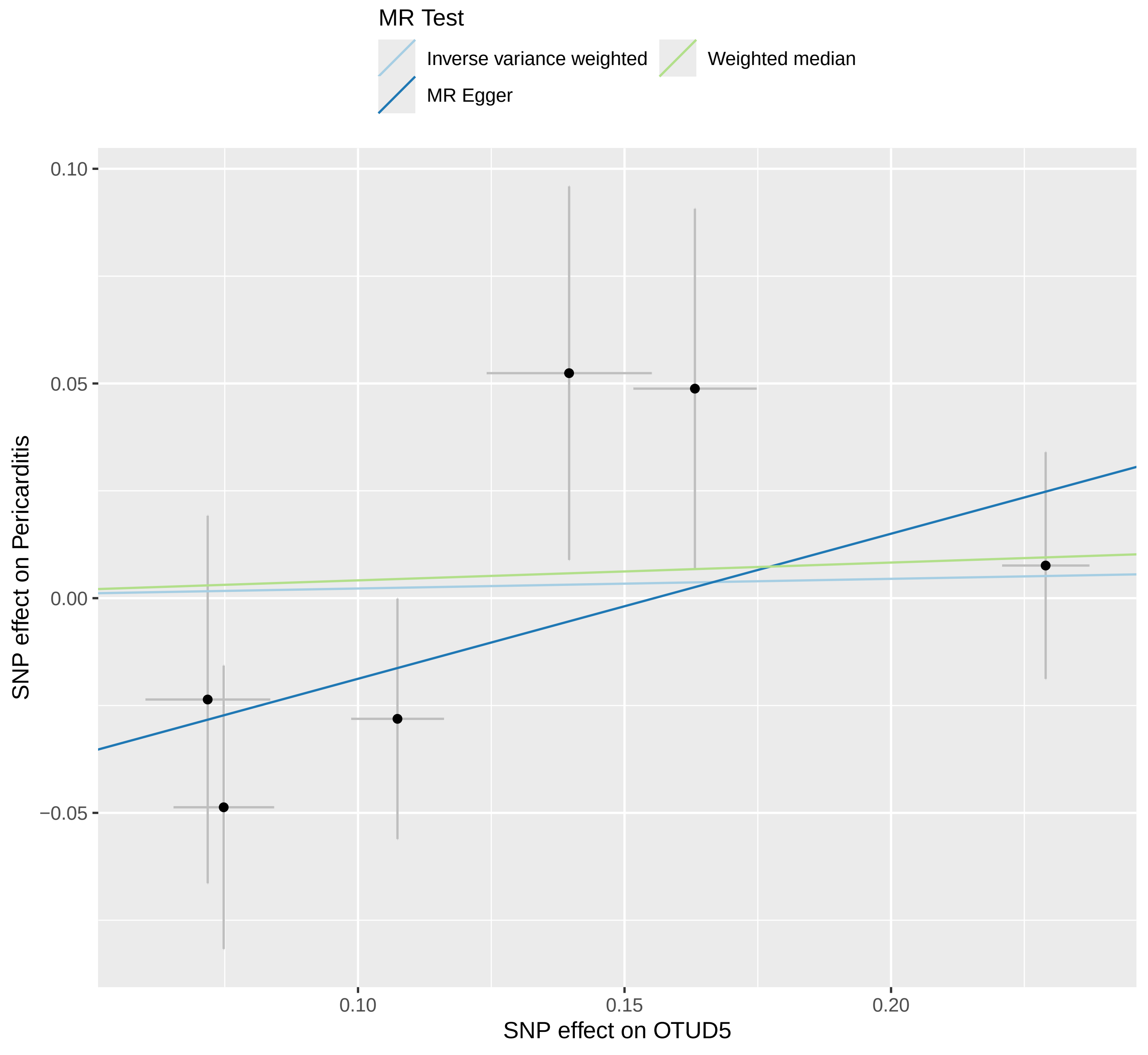
MR Test
Inverse variance weighted
MR Egger
Weighted median
SNP effect on Pericarditis
0.10
0.05
0.00
−0.05
0.10
0.15
0.20
SNP effect on OTUD5

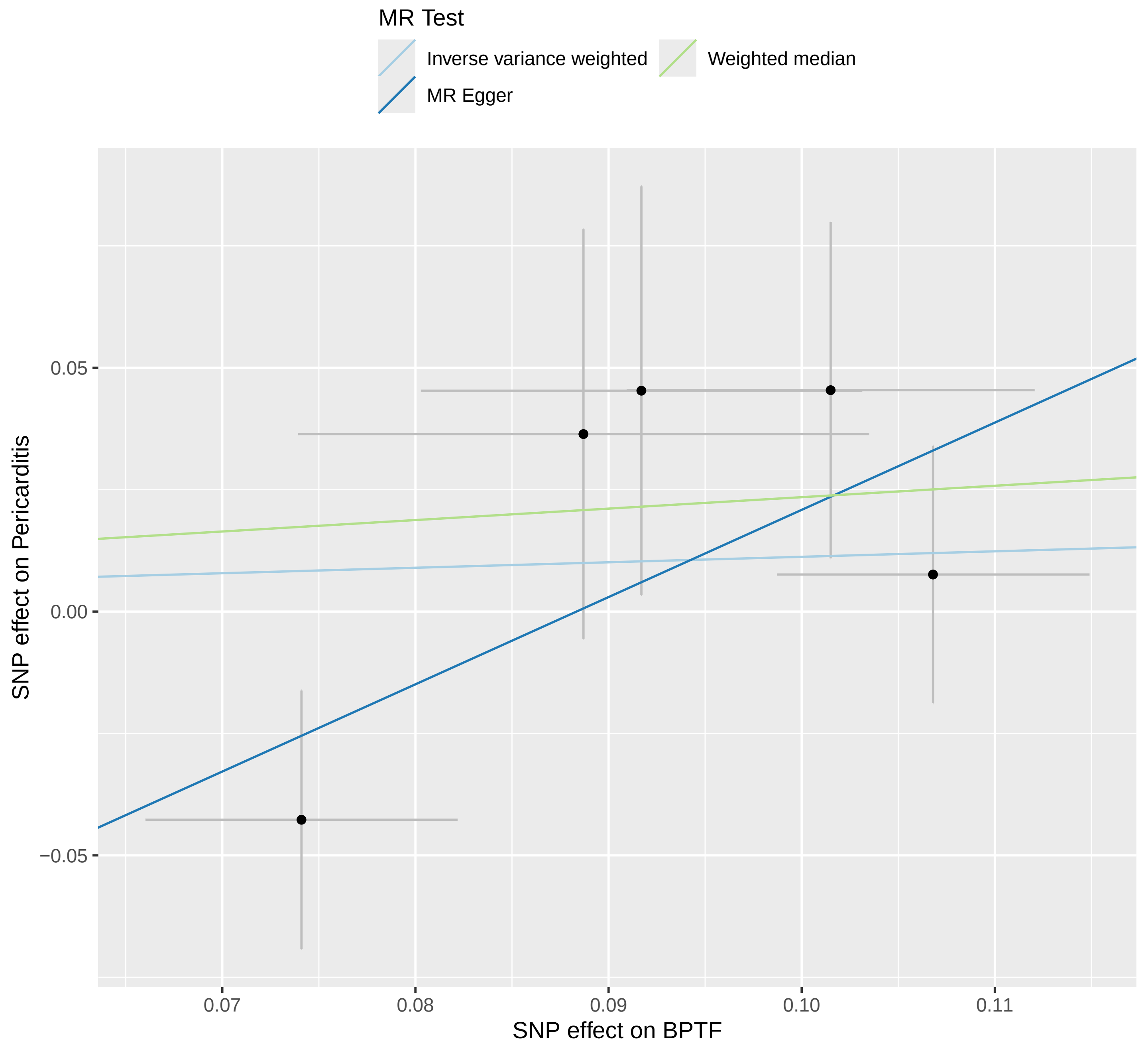
MR Test
Inverse variance weighted
Weighted median
MR Egger
SNP effect on Pericarditis
0.05
0.00
−0.05
0.07
0.08
0.09
0.10
0.11
SNP effect on BPTF

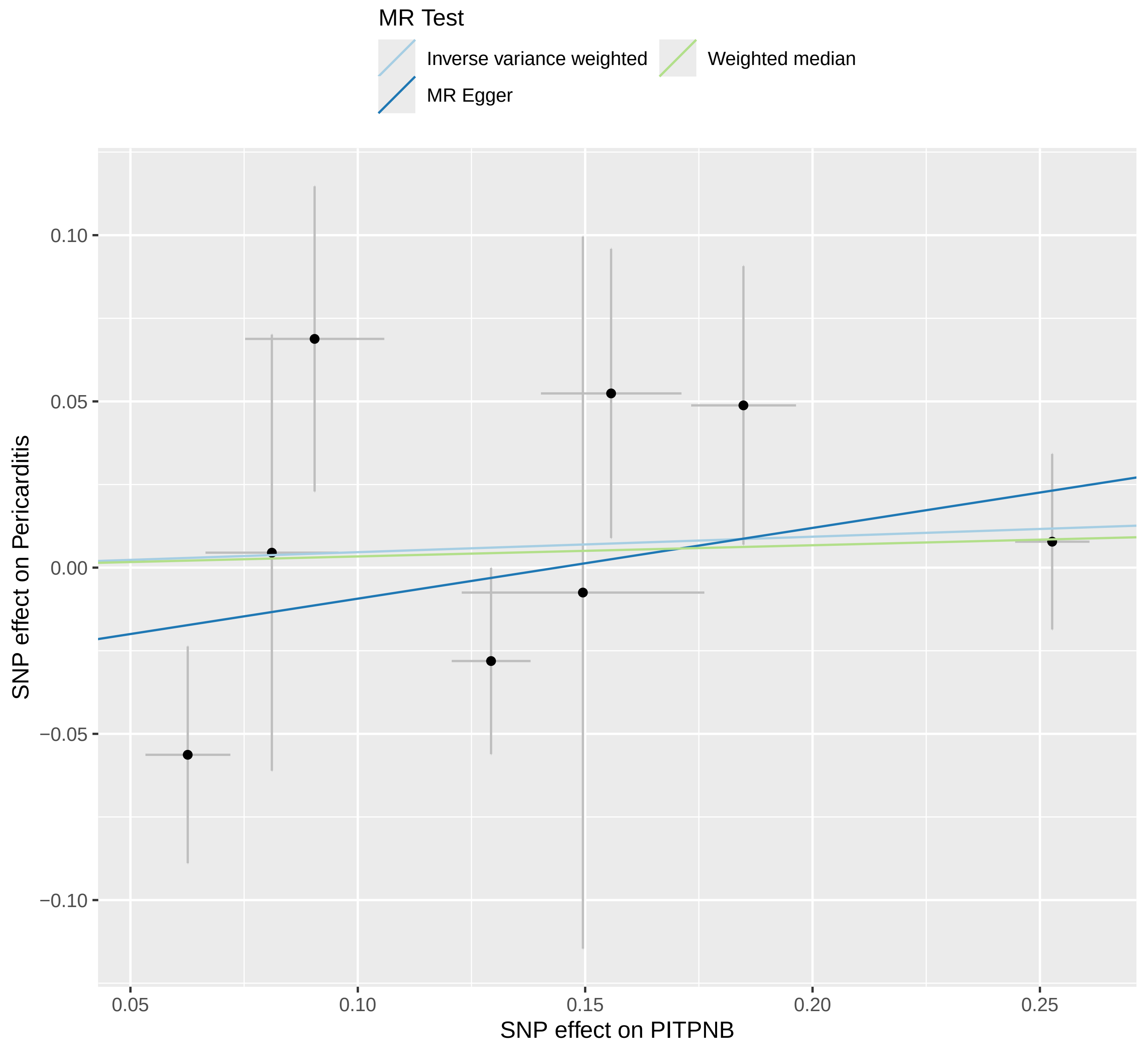

MR Test
Inverse variance weighted
MR Egger
Weighted median
0.10
0.05
0.00
−0.05
−0.10
SNP effect on Pericarditis
0.05
0.10
0.15
0.20
0.25
SNP effect on PITPNB

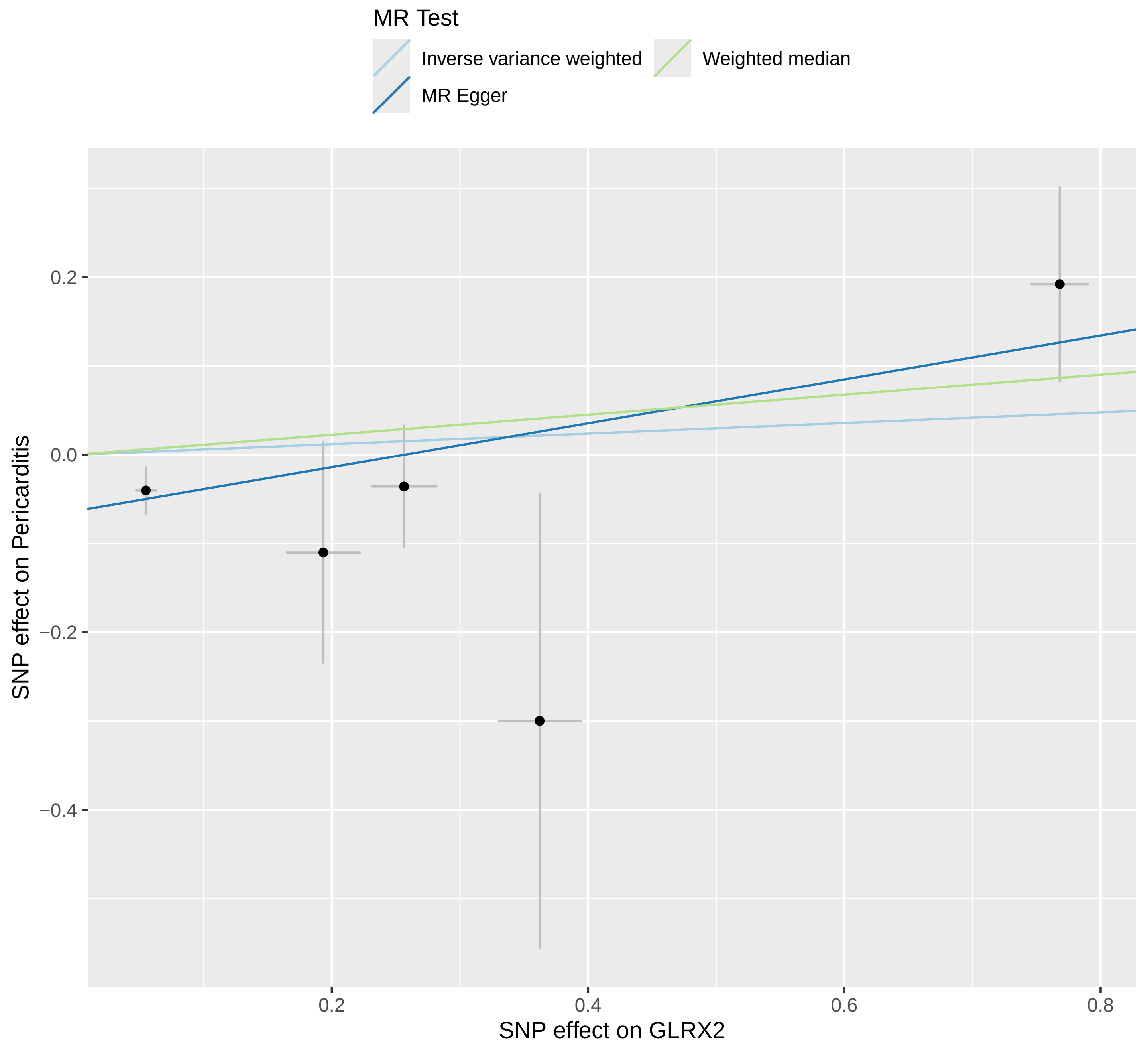

MR Test
Inverse variance weighted
Weighted median
MR Egger
0.2
0.0
−0.2
−0.4
SNP effect on Pericarditis
0.2
0.4
0.6
0.8
SNP effect on GLRX2

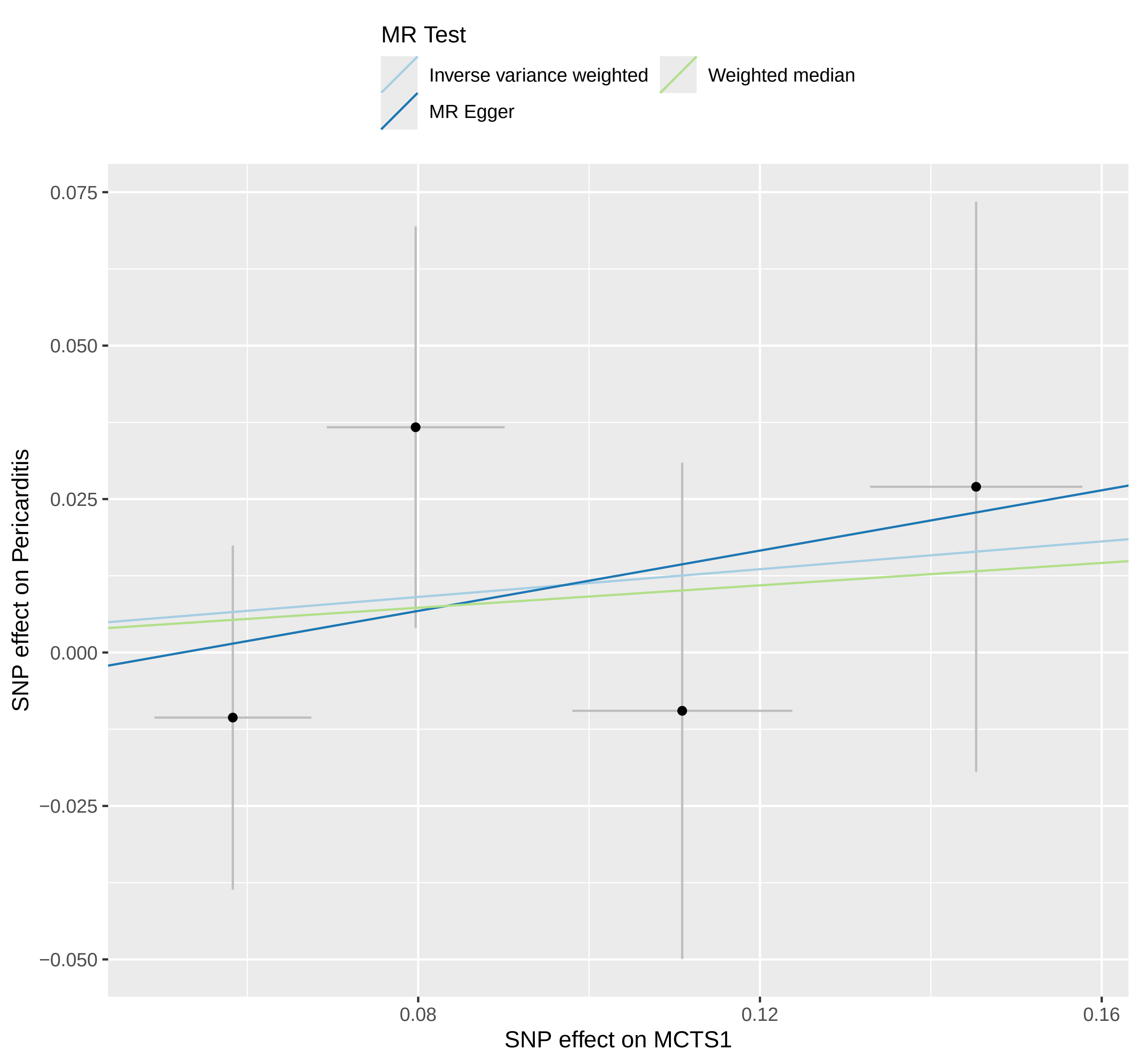

MR Test
Inverse variance weighted
Weighted median
MR Egger
0.075
0.050
0.025
0.000
−0.025
−0.050
SNP effect on Pericarditis
0.08
0.12
0.16
SNP effect on MCTS1

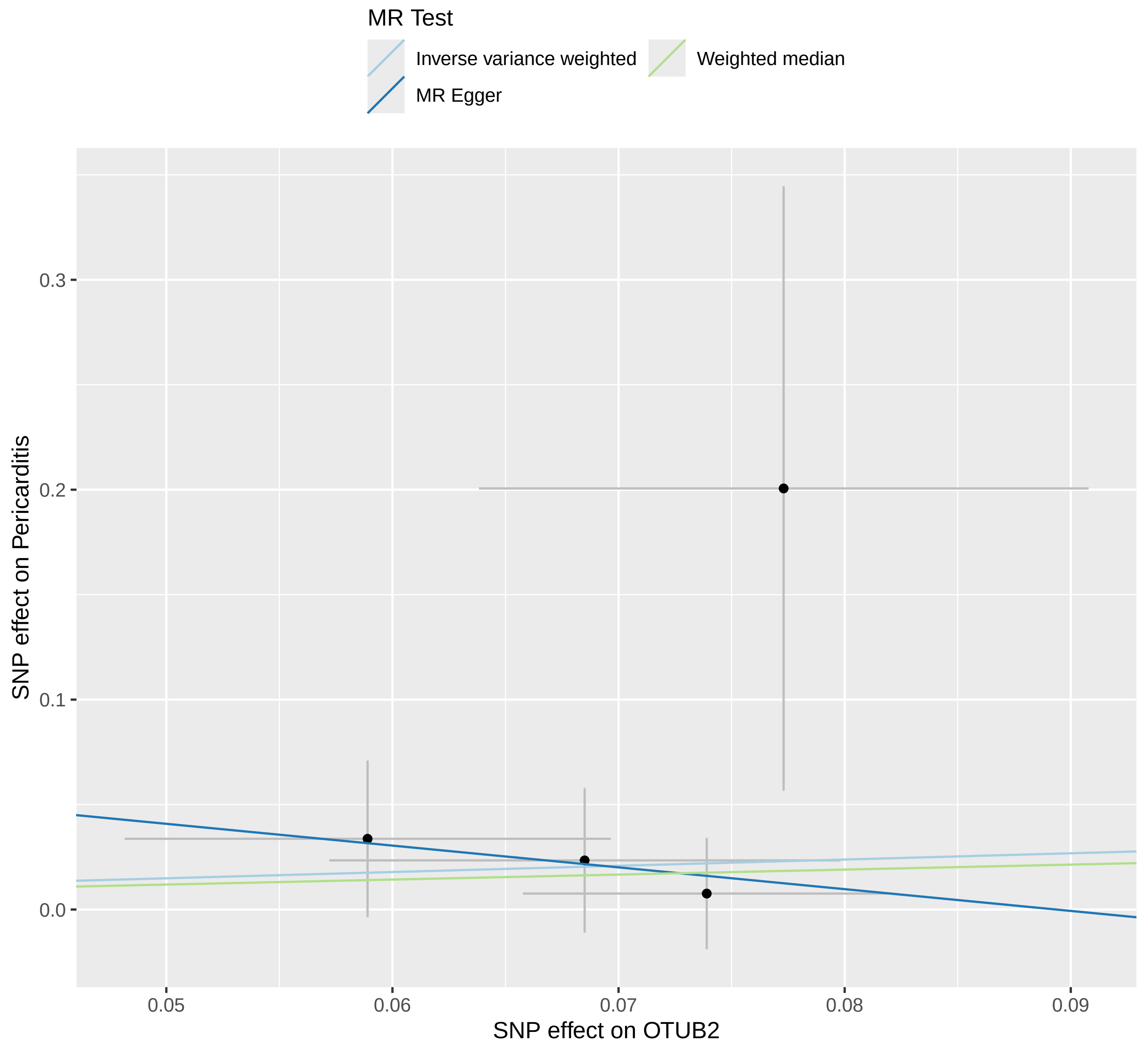

MR Test
Inverse variance weighted
Weighted median
MR Egger
SNP effect on Pericarditis
0.3
0.2
0.1
0.0
0.05
0.06
0.07
0.08
0.09
SNP effect on OTUB2

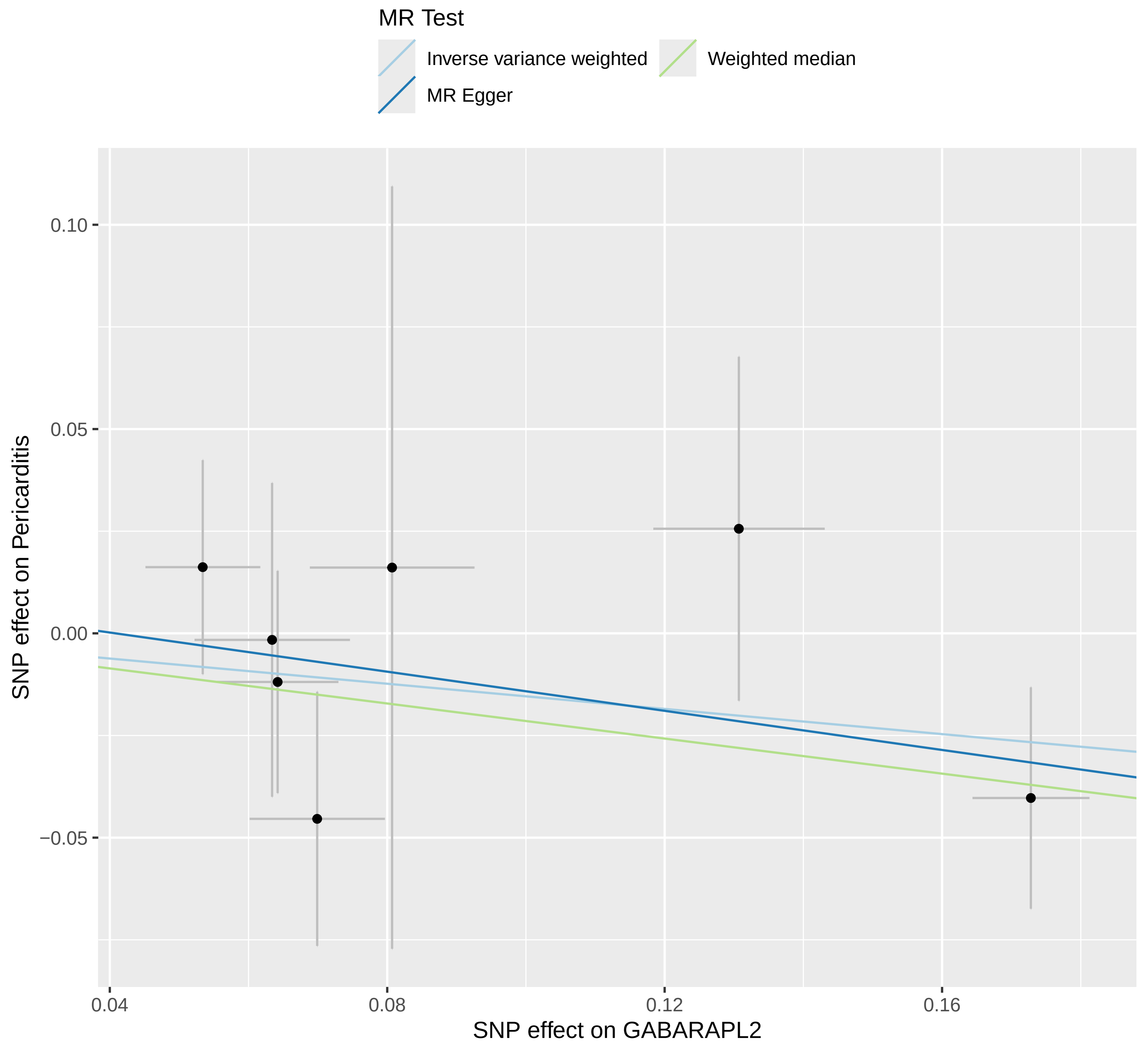
MR Test
Inverse variance weighted
MR Egger
Weighted median
0.10
0.05
0.00
−0.05
SNP effect on Pericarditis
0.04
0.08
0.12
0.16
SNP effect on GABARAPL2

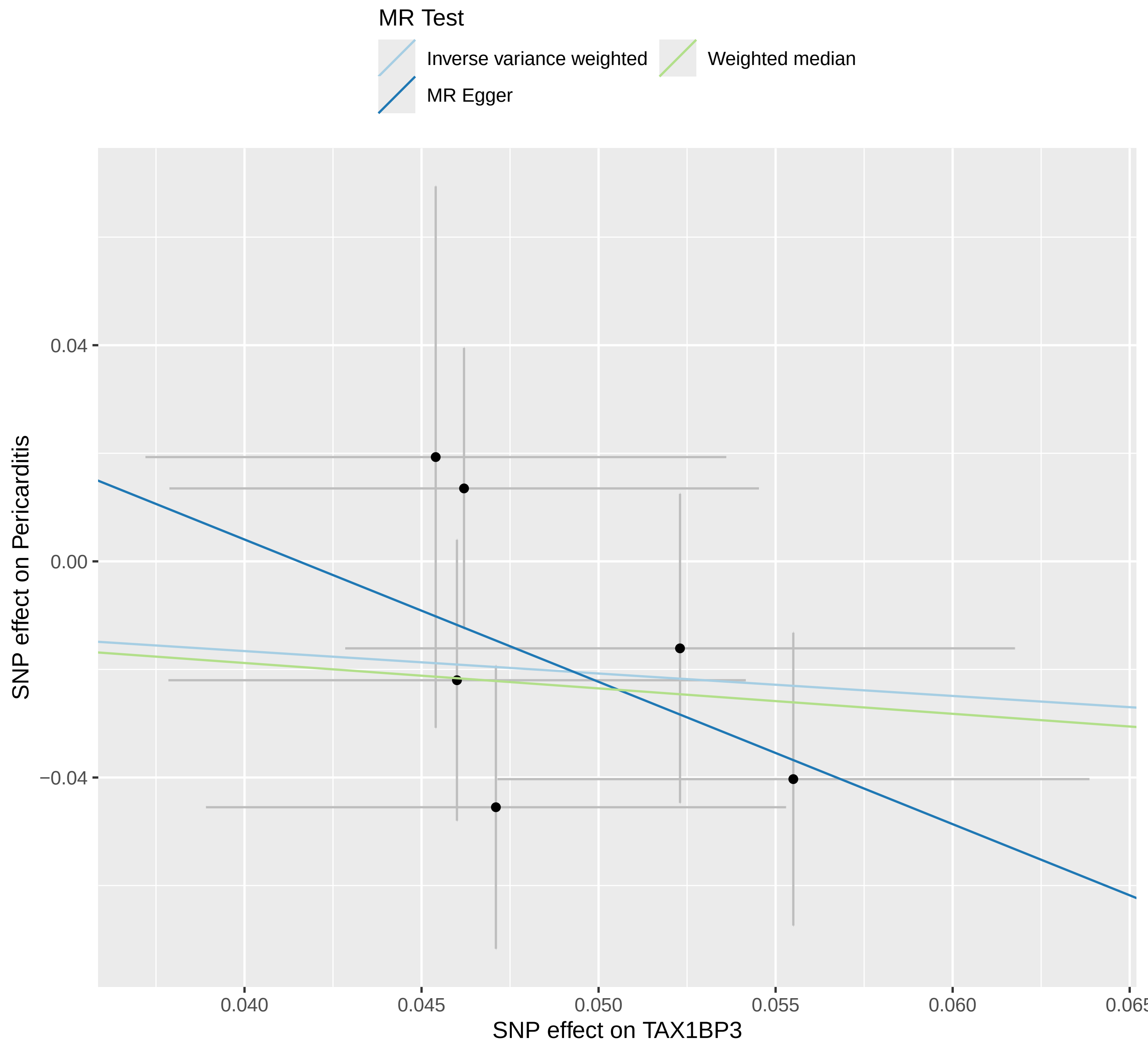

MR Test
Inverse variance weighted
Weighted median
MR Egger
SNP effect on Pericarditis
0.04
0.00
−0.04
0.040
0.045
0.050
0.055
0.060
0.065
SNP effect on TAX1BP3

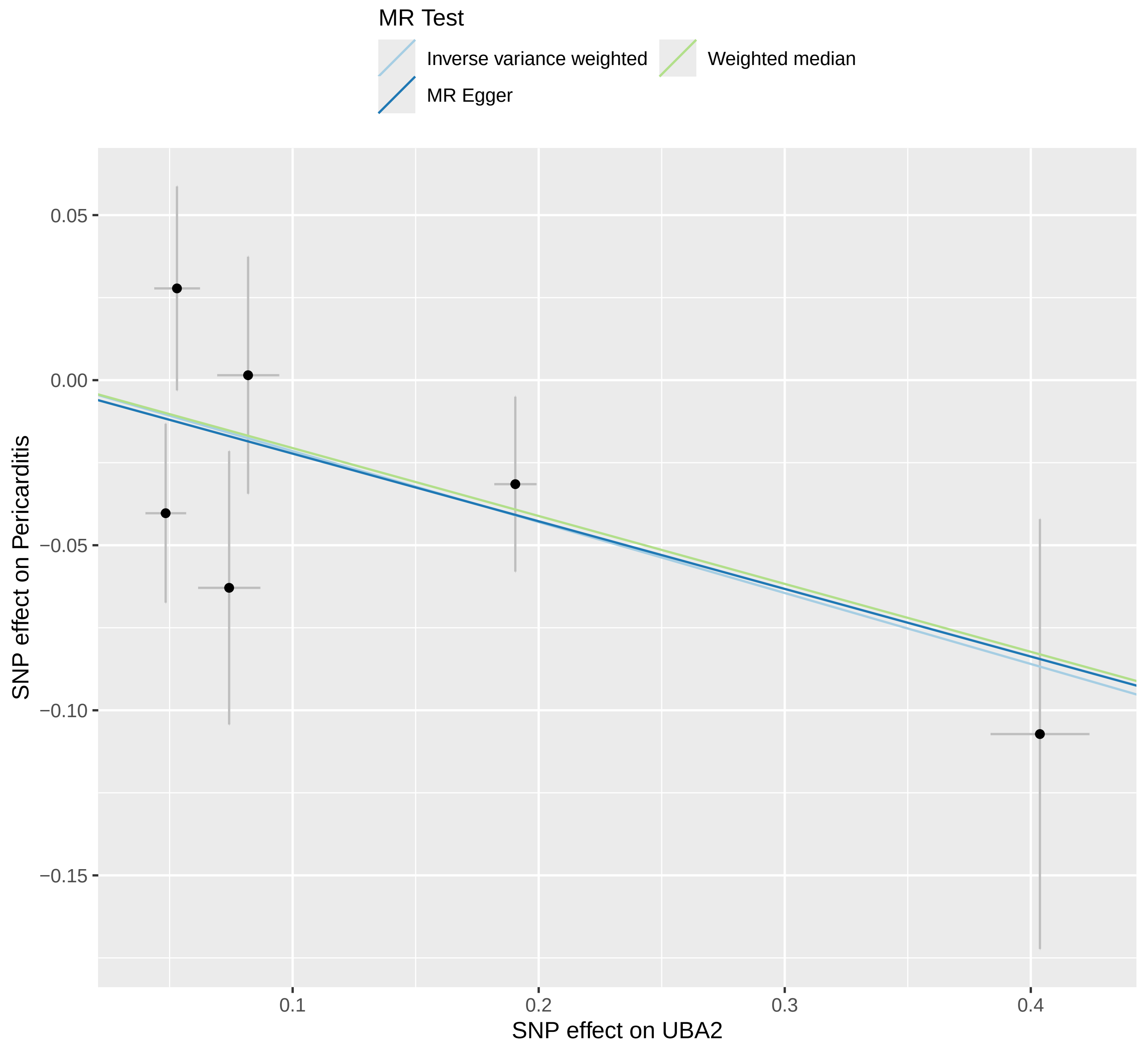
MR Test
Inverse variance weighted
Weighted median
MR Egger
0.05
0.00
−0.05
−0.10
−0.15
SNP effect on Pericarditis
0.1
0.2
0.3
0.4
SNP effect on UBA2

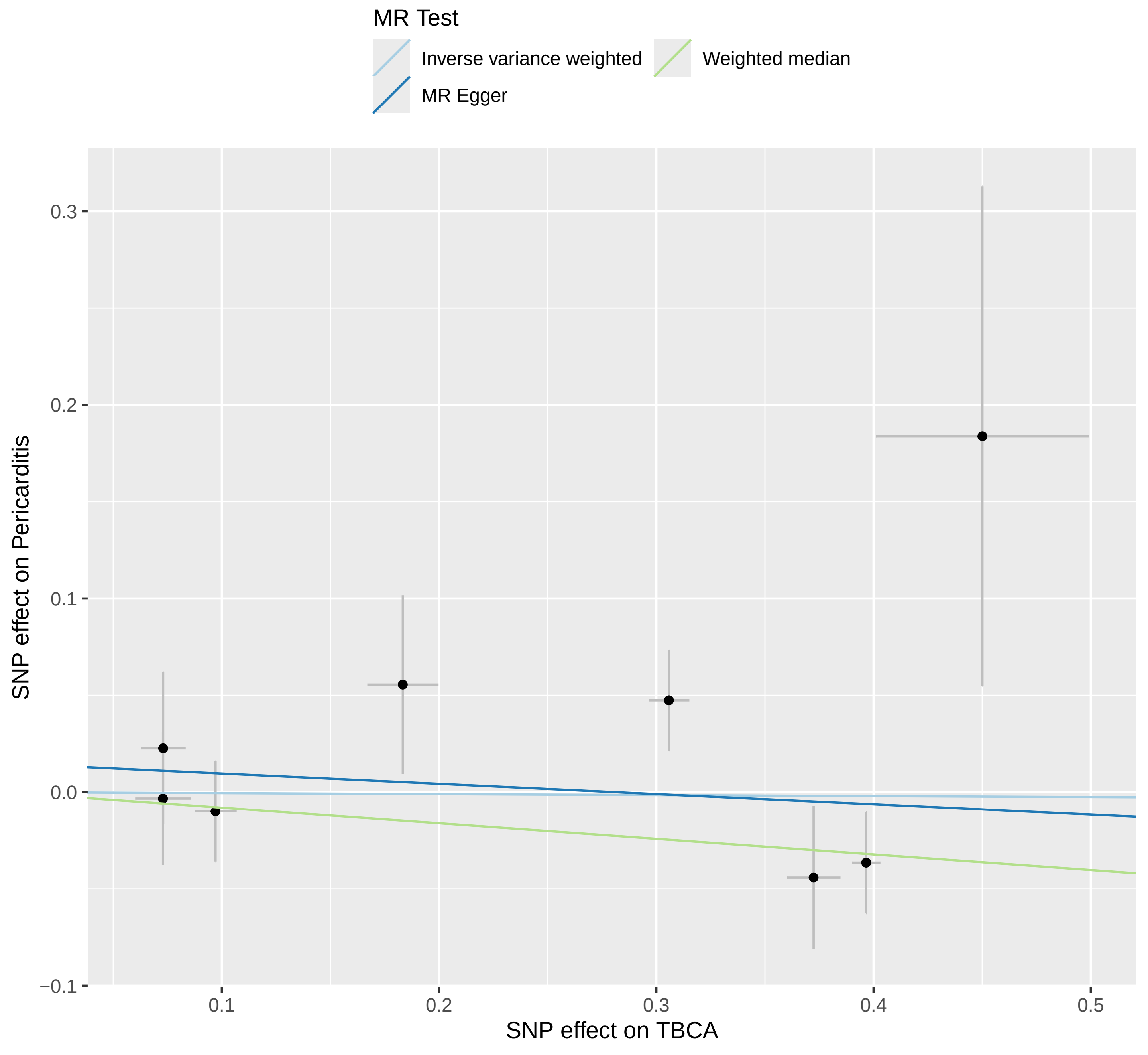
MR Test
Inverse variance weighted
MR Egger
Weighted median
0.3
0.2
0.1
0.0
−0.1
SNP effect on Pericarditis
0.1
0.2
0.3
0.4
0.5
SNP effect on TBCA

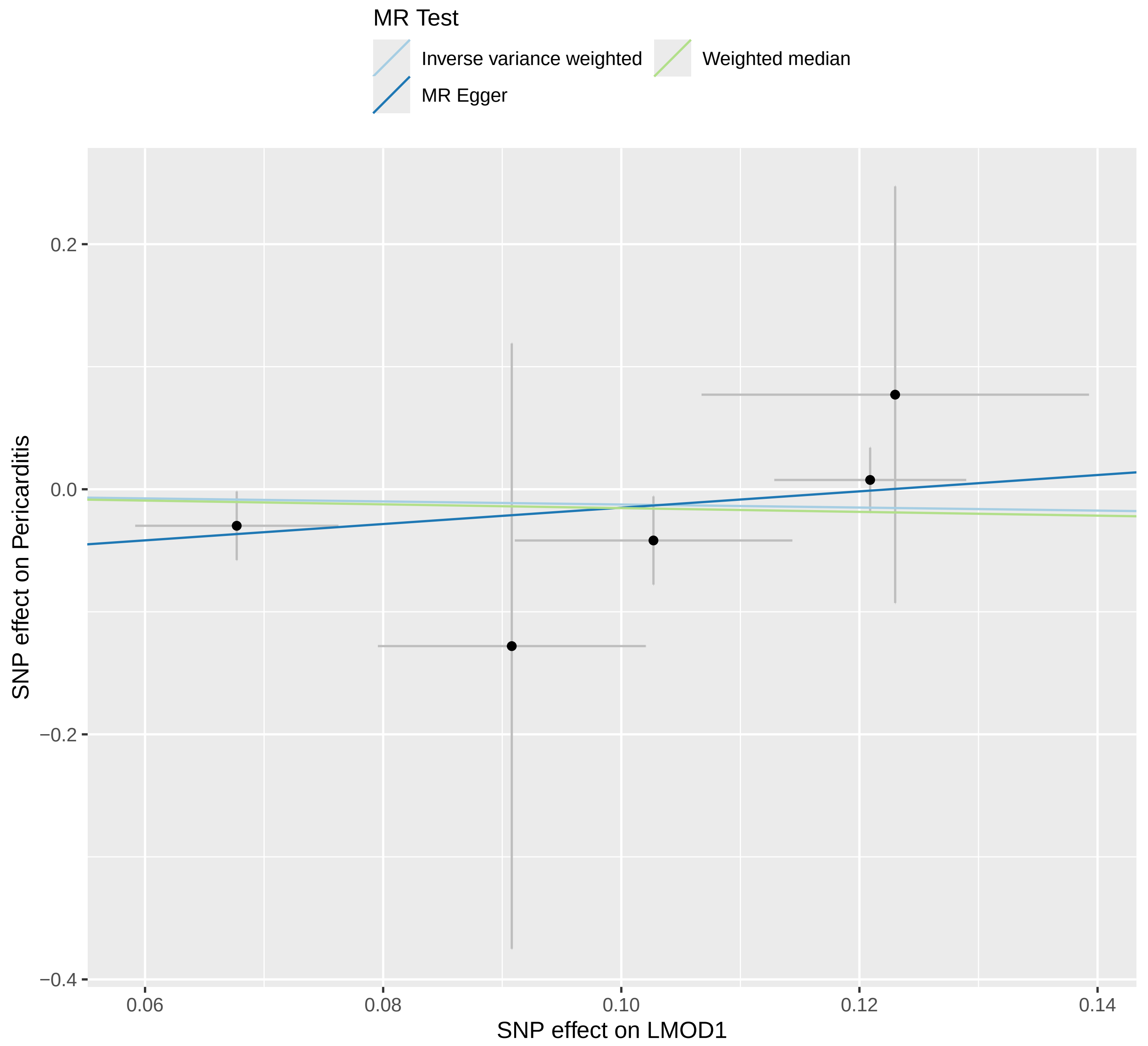
MR Test
Inverse variance weighted
Weighted median
MR Egger
0.2
0.0
−0.2
−0.4
SNP effect on Pericarditis
0.06
0.08
0.10
0.12
0.14
SNP effect on LMOD1

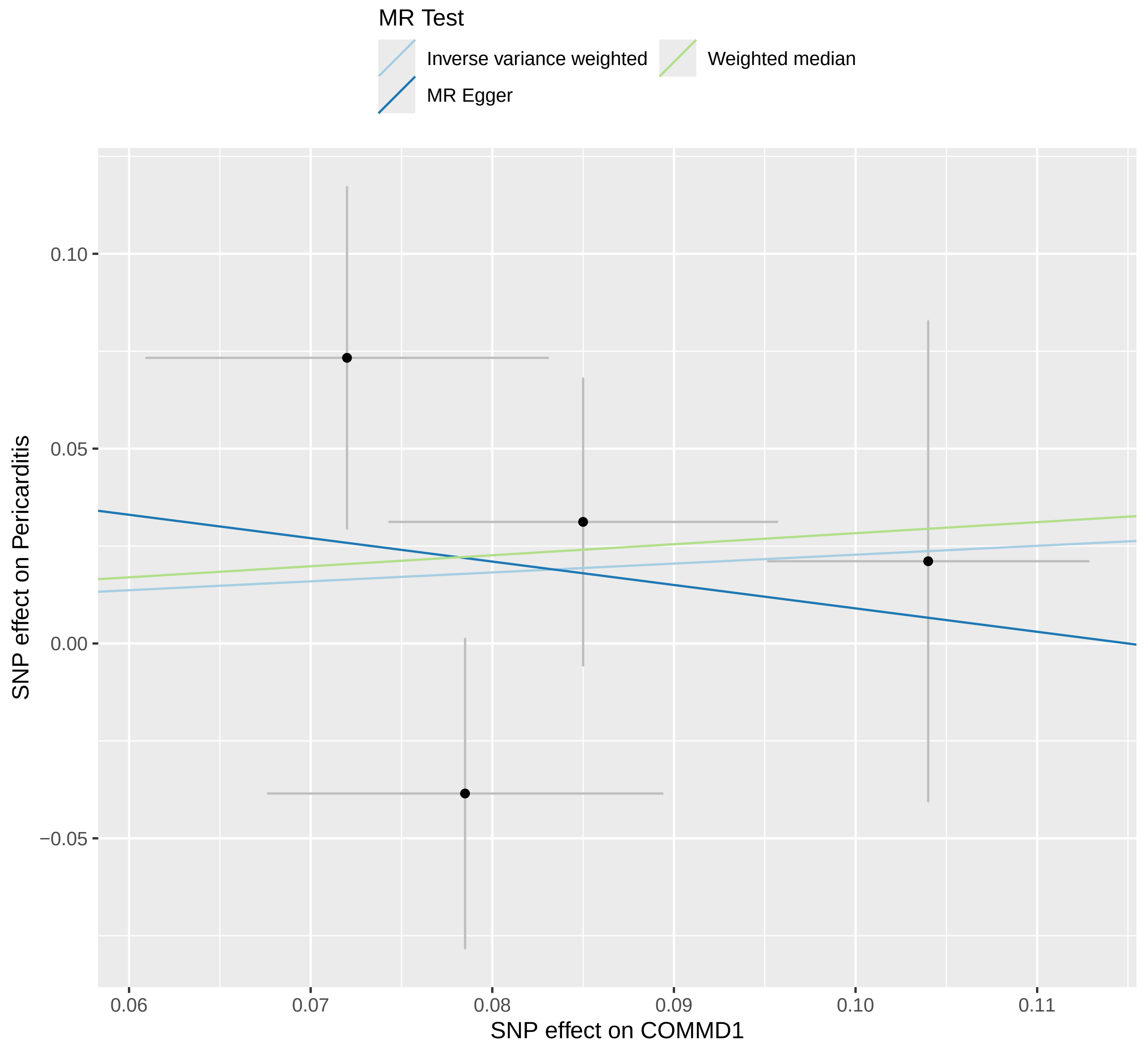
MR Test
Inverse variance weighted
Weighted median
MR Egger
0.10
0.05
0.00
−0.05
SNP effect on Pericarditis
0.06
0.07
0.08
0.09
0.10
0.11
SNP effect on COMMD1

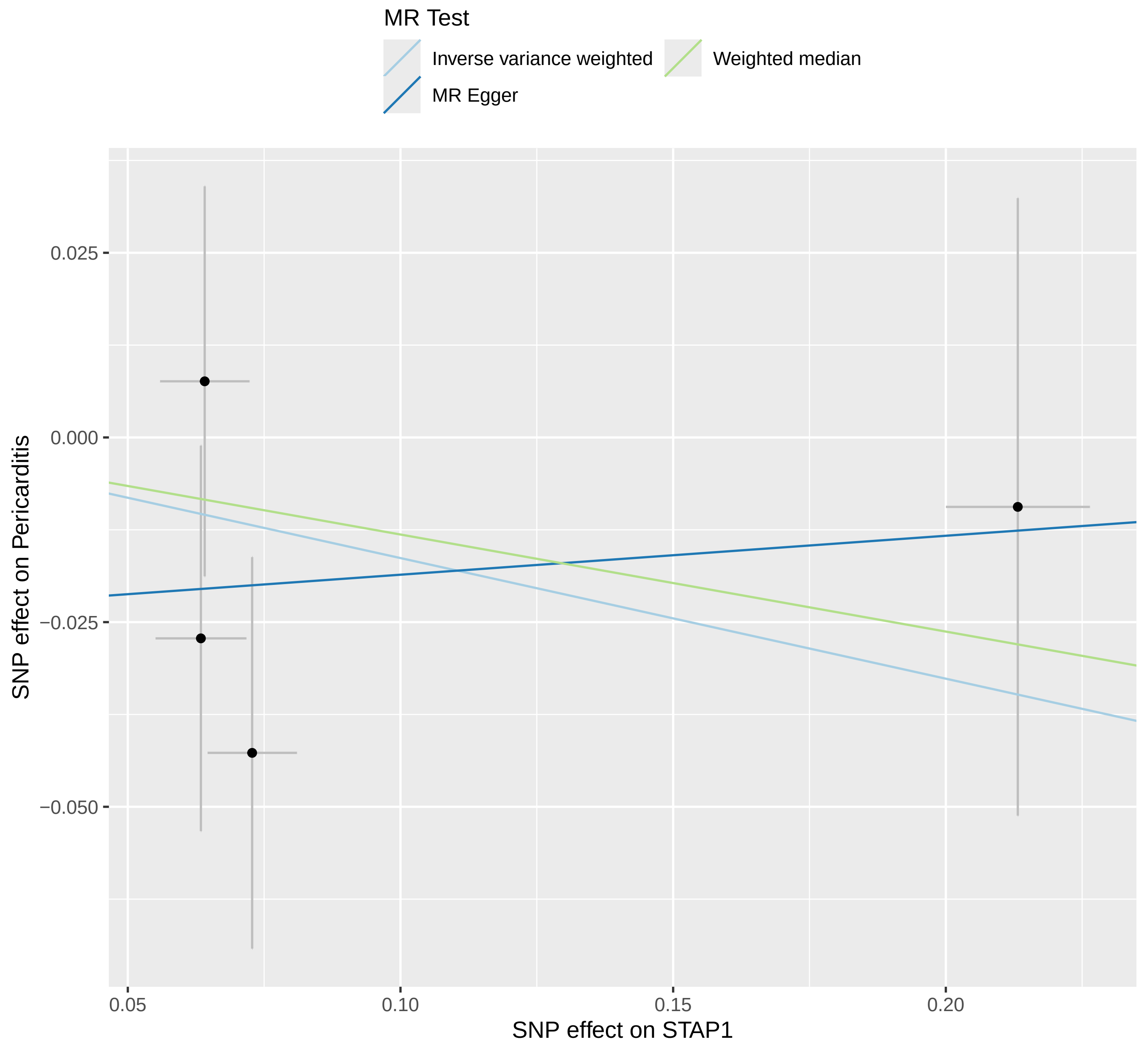
MR Test
Inverse variance weighted
Weighted median
MR Egger
0.025
0.000
−0.025
−0.050
SNP effect on Pericarditis
0.05
0.10
0.15
0.20
SNP effect on STAP1

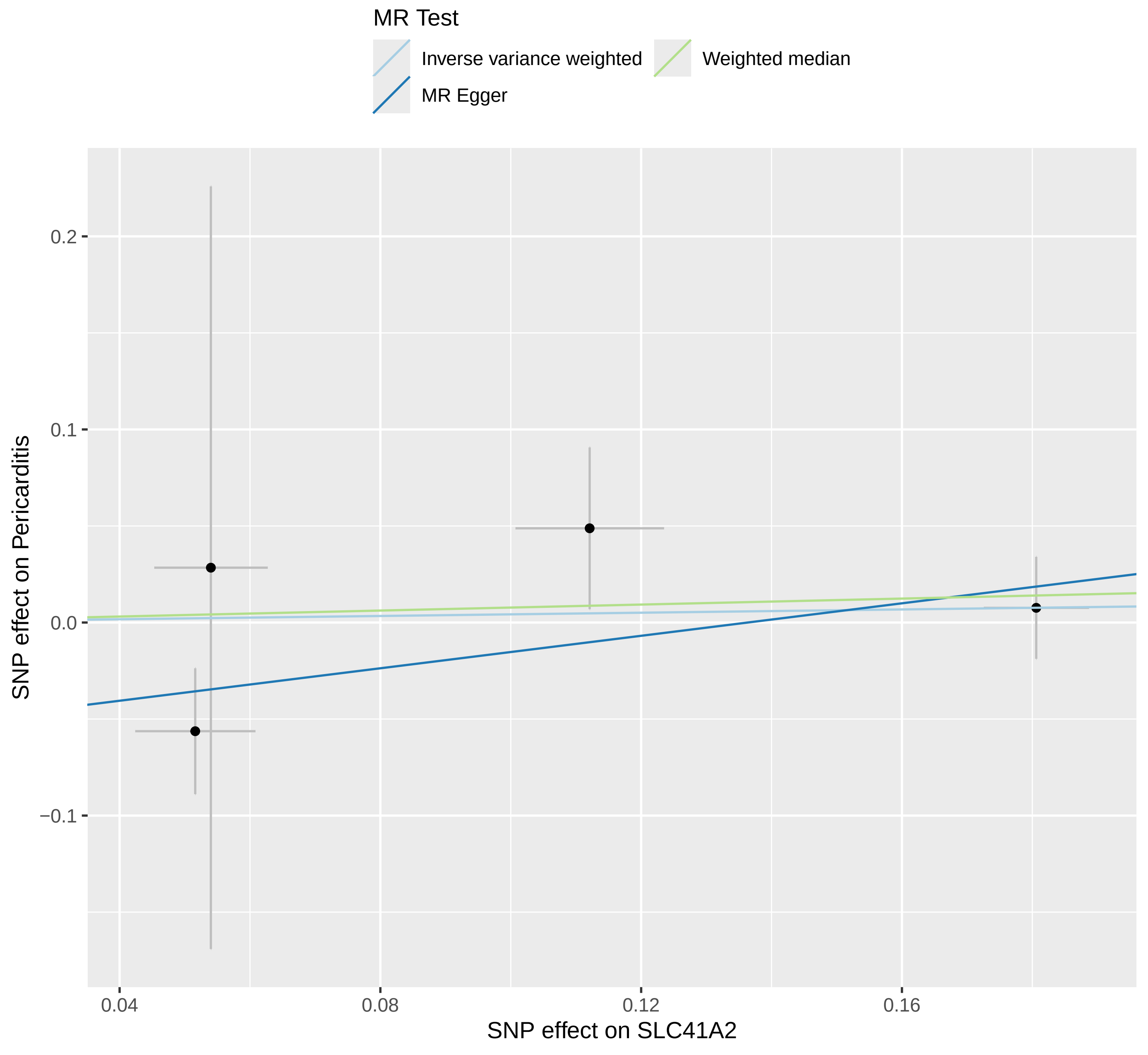
MR Test
Inverse variance weighted
Weighted median
MR Egger
0.2
0.1
0.0
−0.1
SNP effect on Pericarditis
0.04
0.08
0.12
0.16
SNP effect on SLC41A2

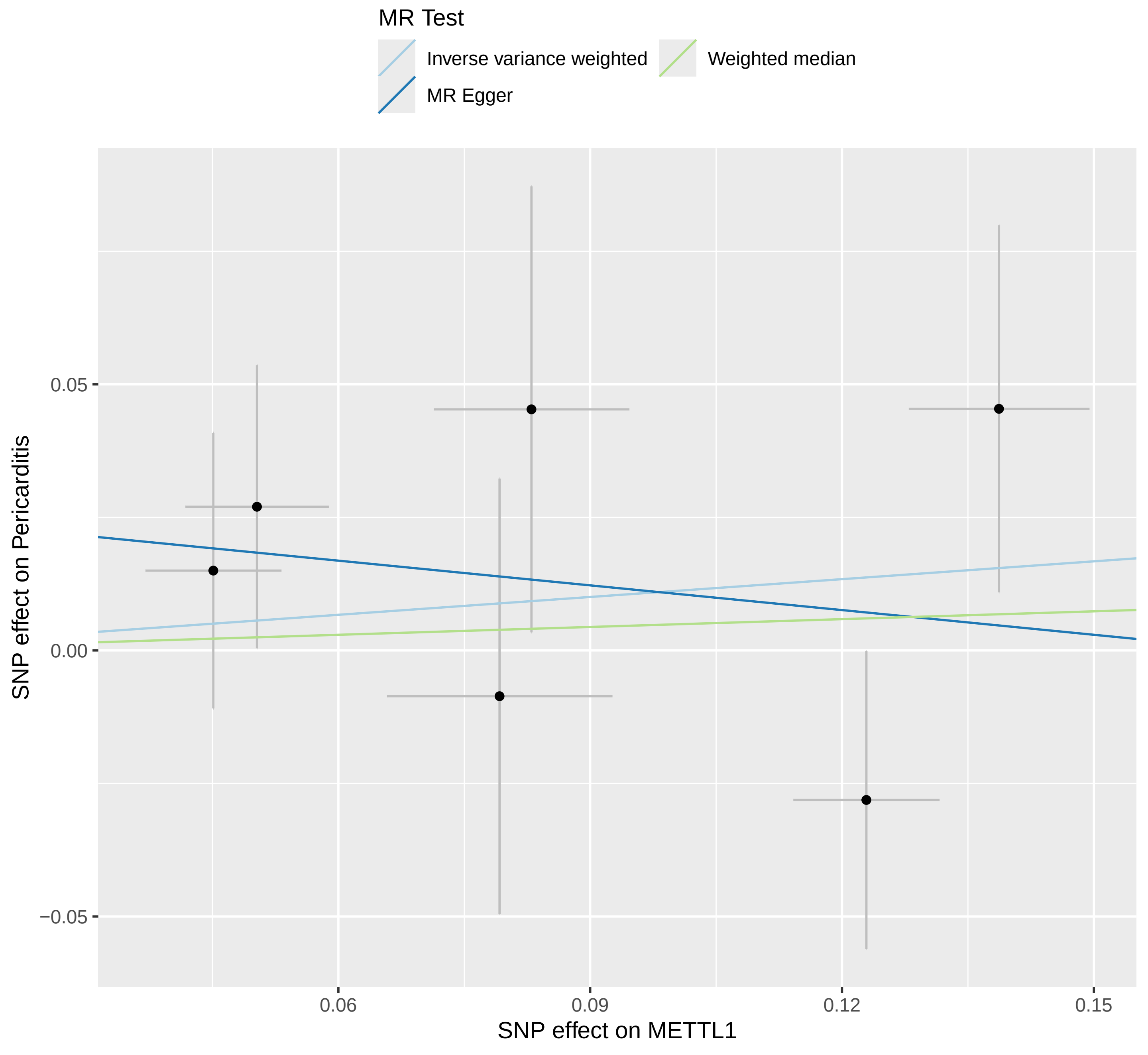
MR Test
Inverse variance weighted
MR Egger
Weighted median
SNP effect on Pericarditis
0.05
0.00
−0.05
0.06
0.09
0.12
0.15
SNP effect on METTL1

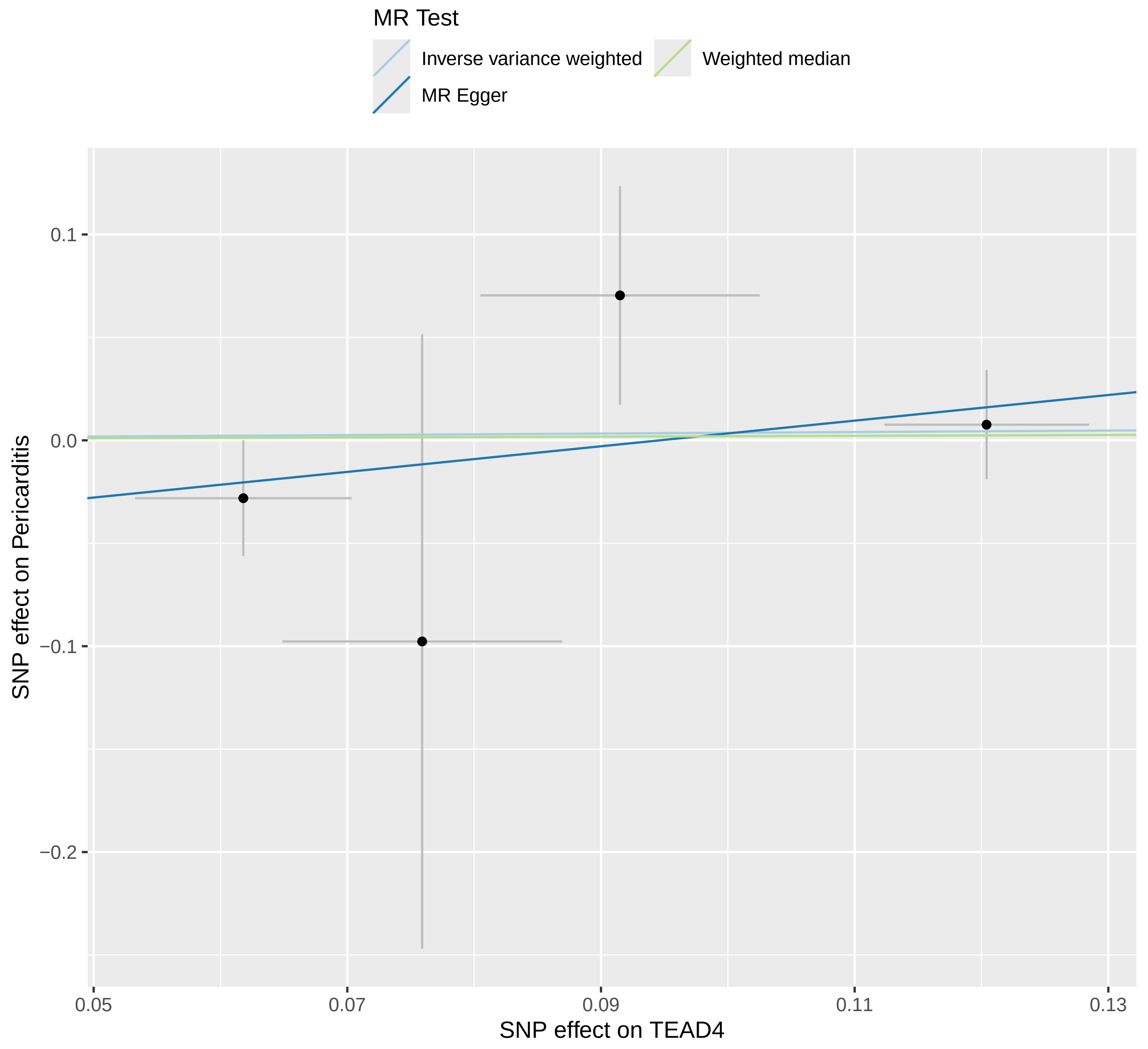

MR Test
Inverse variance weighted
Weighted median
MR Egger
0.1
0.0
−0.1
−0.2
SNP effect on Pericarditis
0.05
0.07
0.09
0.11
0.13
SNP effect on TEAD4

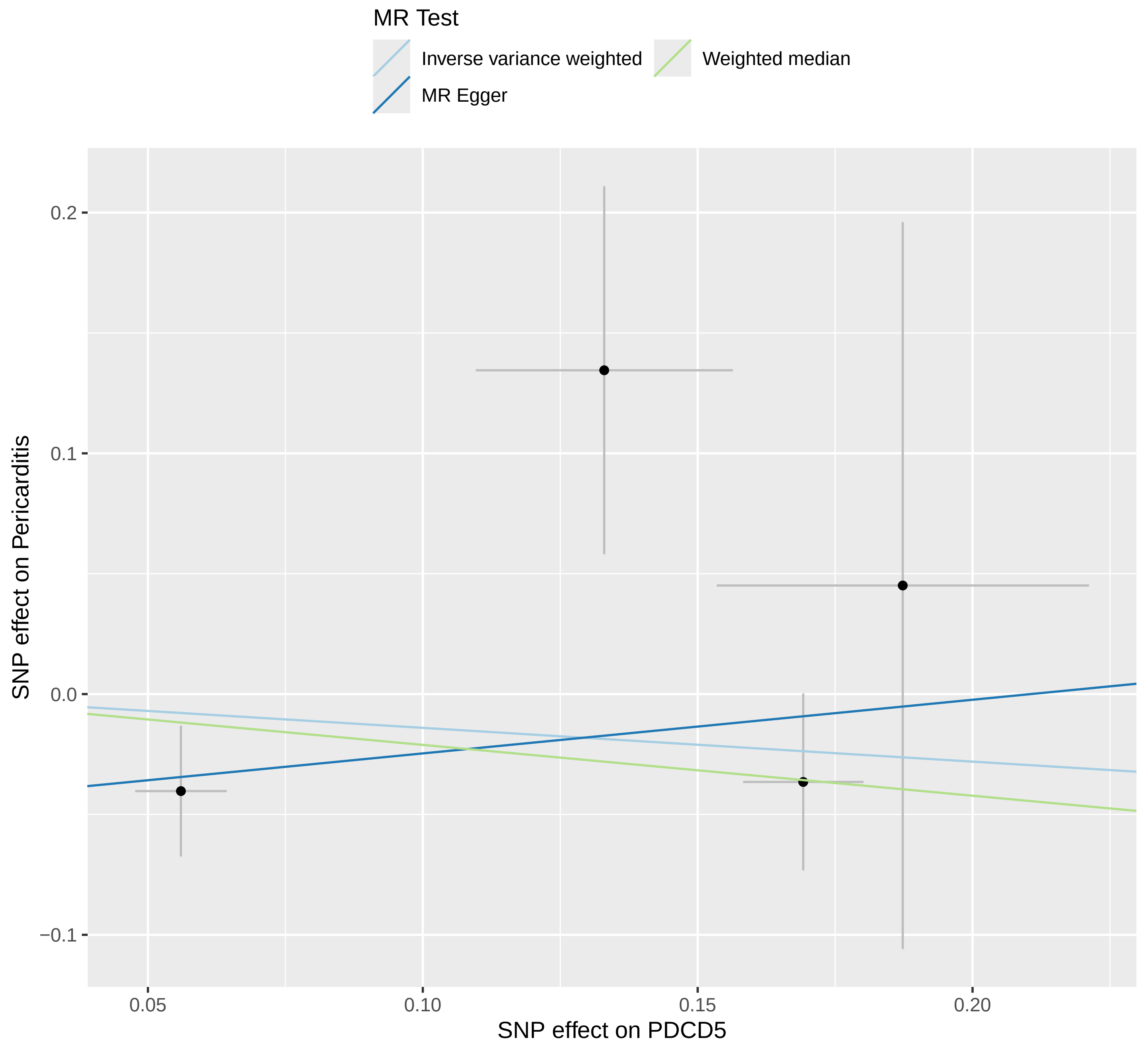

MR Test
Inverse variance weighted
Weighted median
MR Egger
0.2
0.1
0.0
−0.1
SNP effect on Pericarditis
0.05
0.10
0.15
0.20
SNP effect on PDCD5

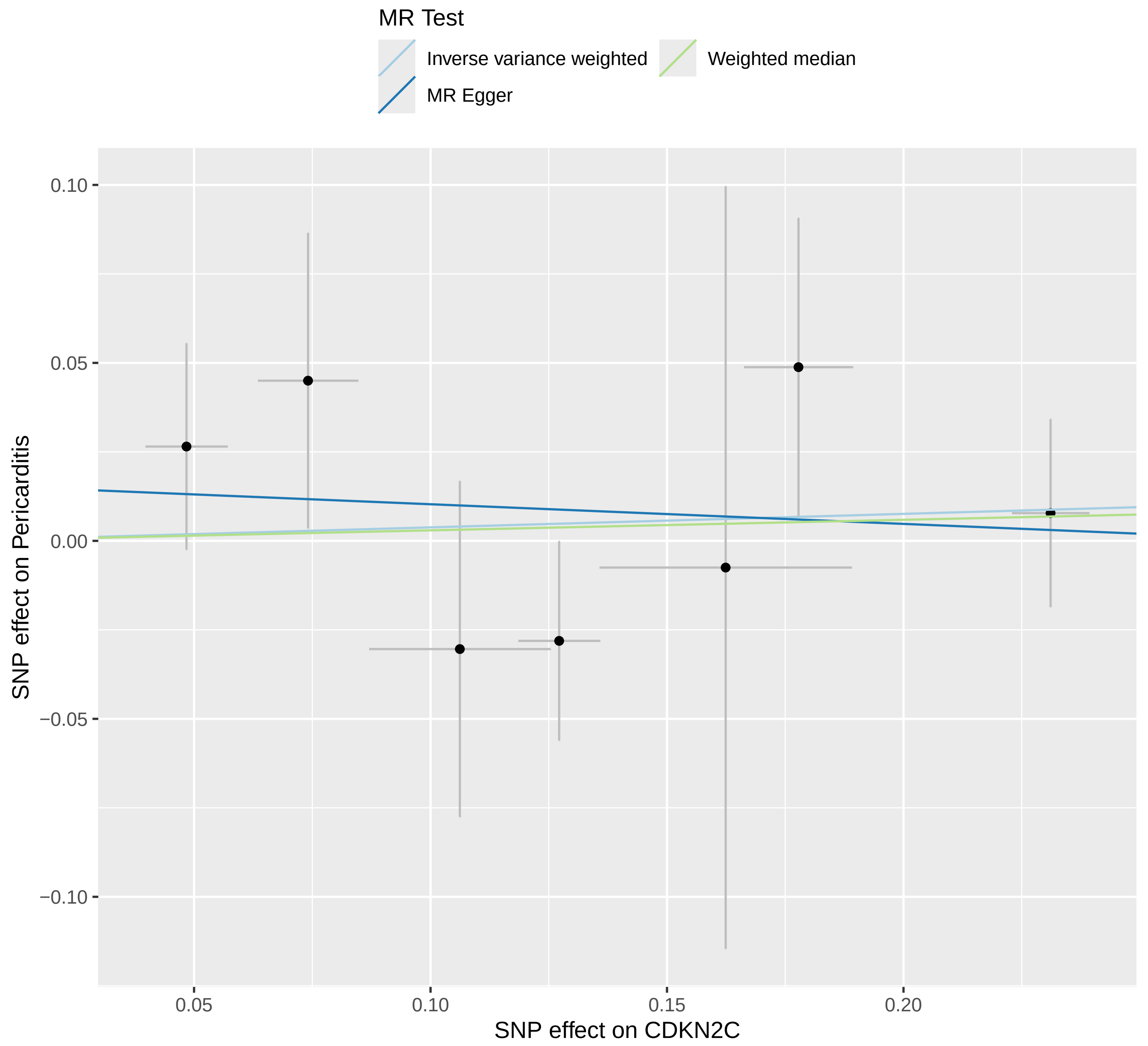
MR Test
Inverse variance weighted
MR Egger
Weighted median
0.10
0.05
0.00
−0.05
−0.10
SNP effect on Pericarditis
0.05
0.10
0.15
0.20
SNP effect on CDKN2C

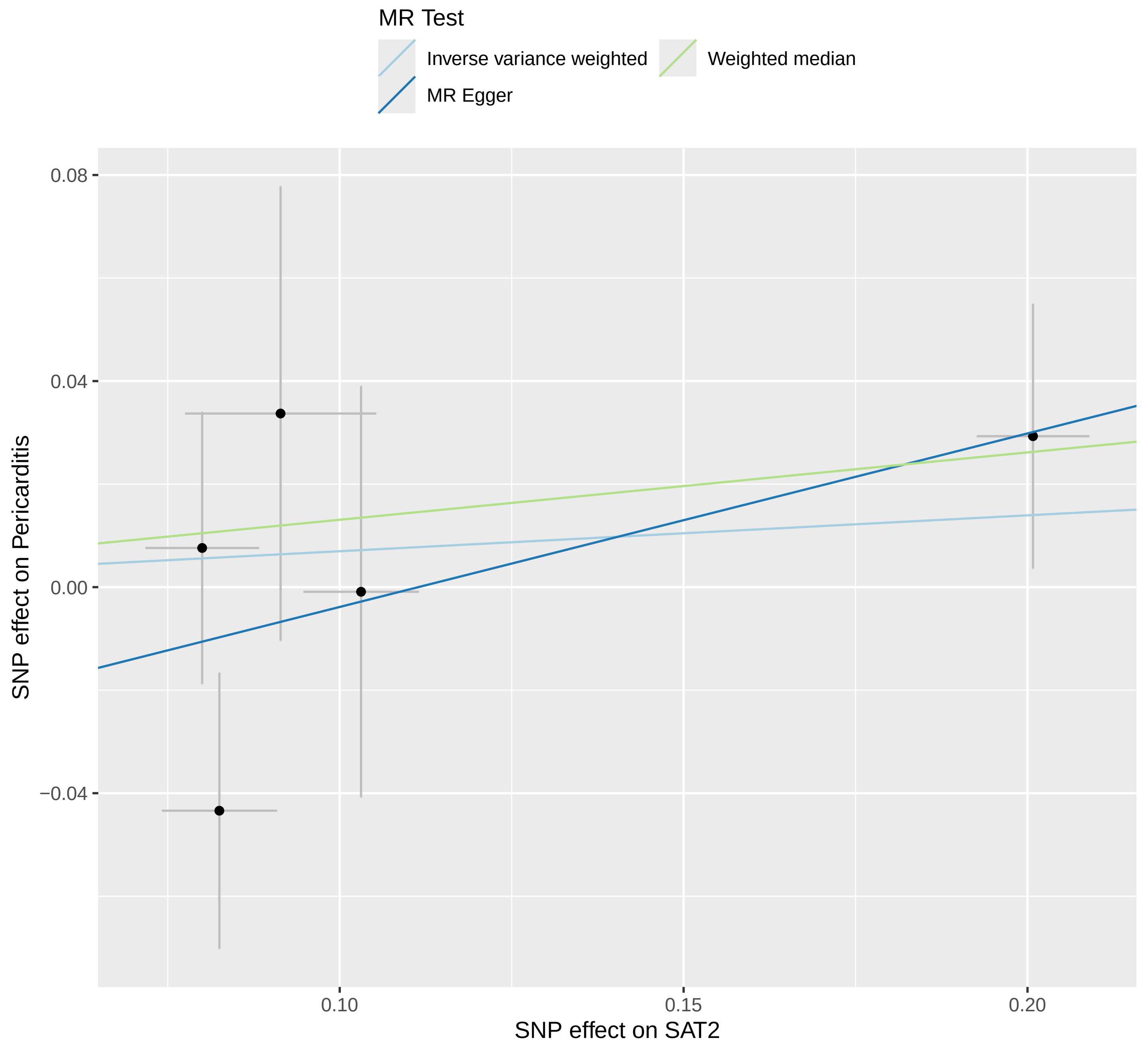
MR Test
Inverse variance weighted
Weighted median
MR Egger
SNP effect on Pericarditis
0.08
0.04
0.00
−0.04
0.10
0.15
0.20
SNP effect on SAT2

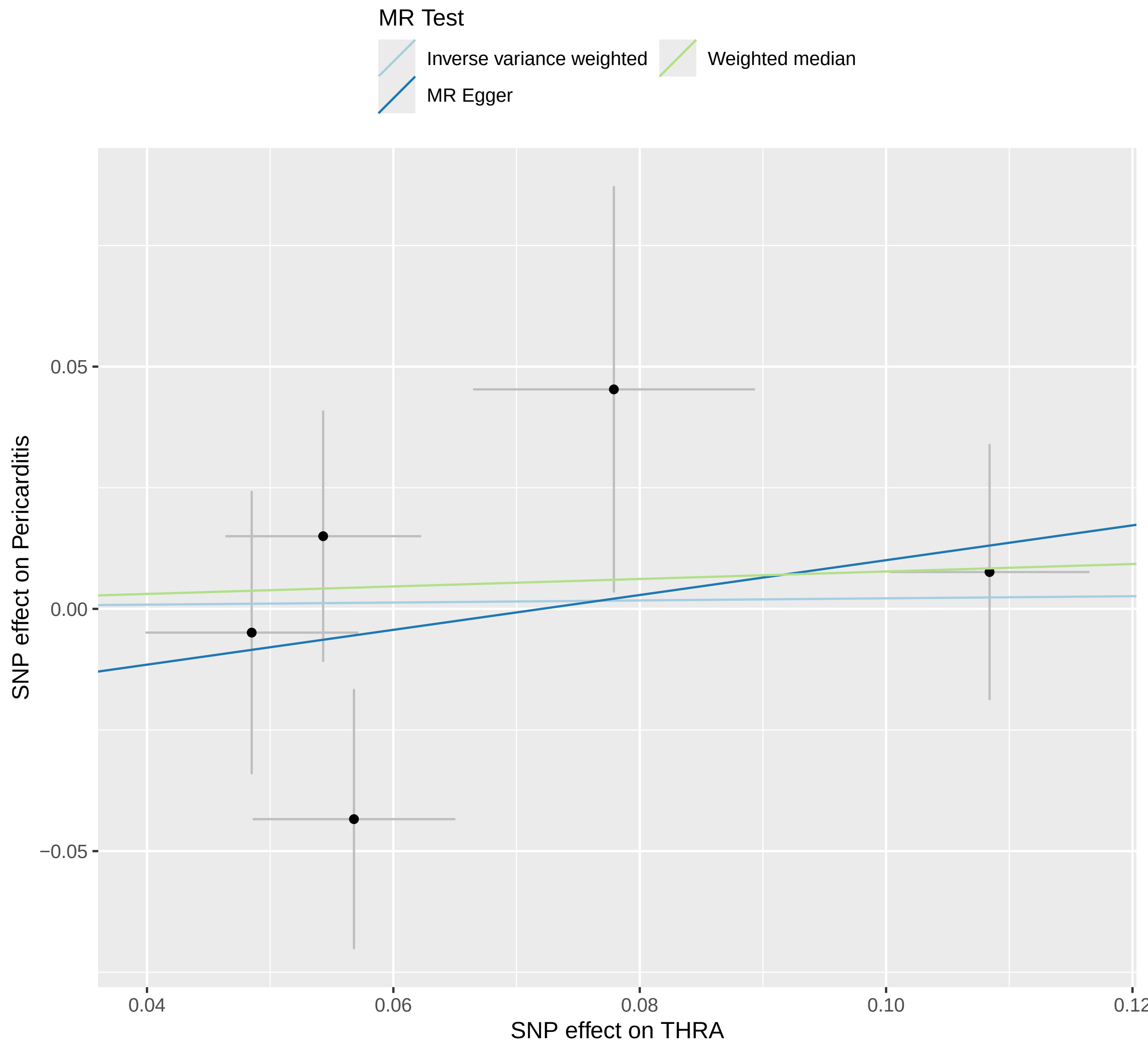

MR Test
Inverse variance weighted
Weighted median
MR Egger
SNP effect on Pericarditis
0.05
0.00
−0.05
0.04
0.06
0.08
0.10
0.12
SNP effect on THRA

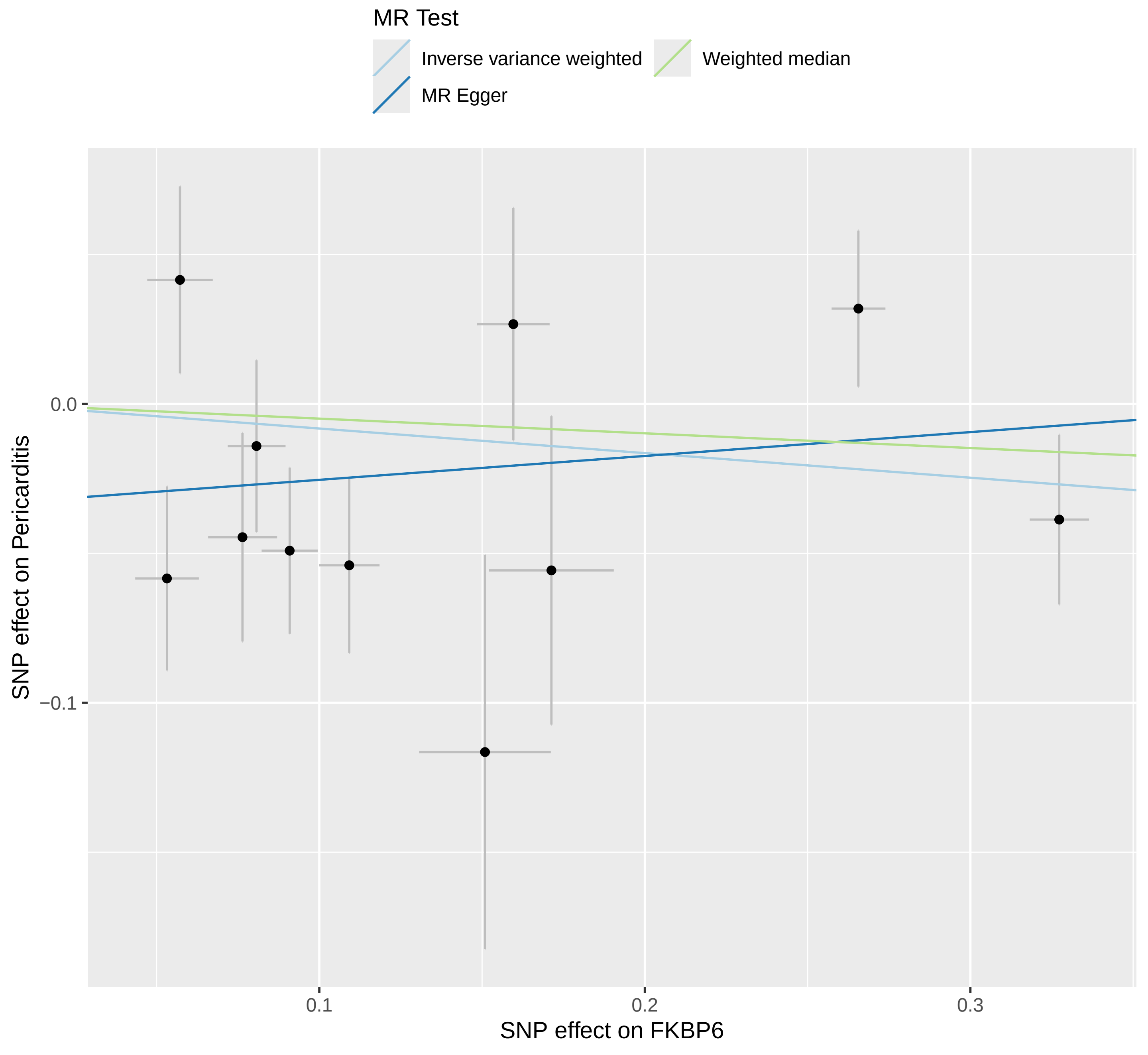

MR Test
Inverse variance weighted
Weighted median
MR Egger
0.0
−0.1
SNP effect on Pericarditis
0.1
0.2
0.3
SNP effect on FKBP6

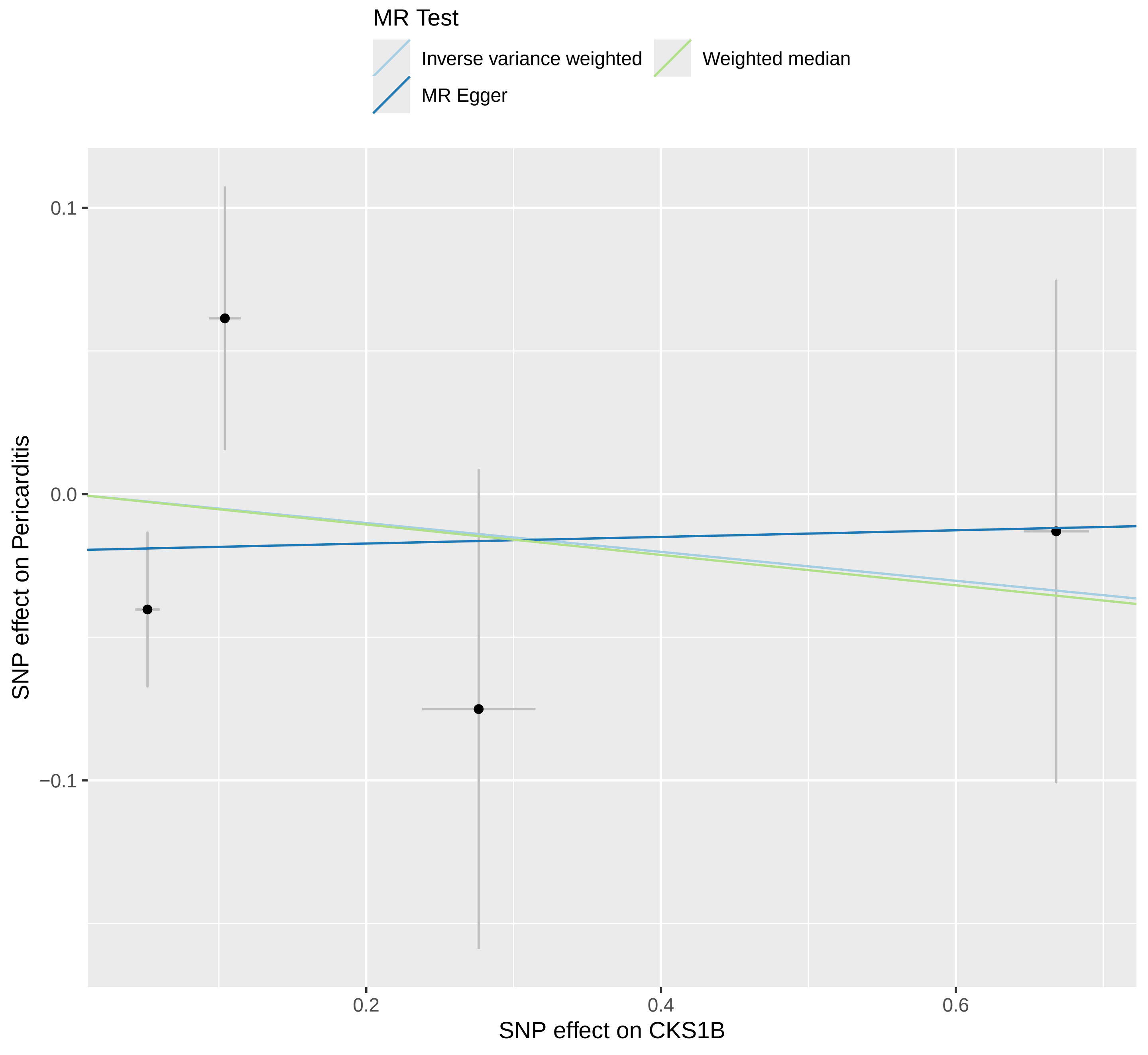
MR Test
Inverse variance weighted
Weighted median
MR Egger
0.1
0.0
−0.1
0.2
0.4
0.6
SNP effect on Pericarditis
SNP effect on CKS1B

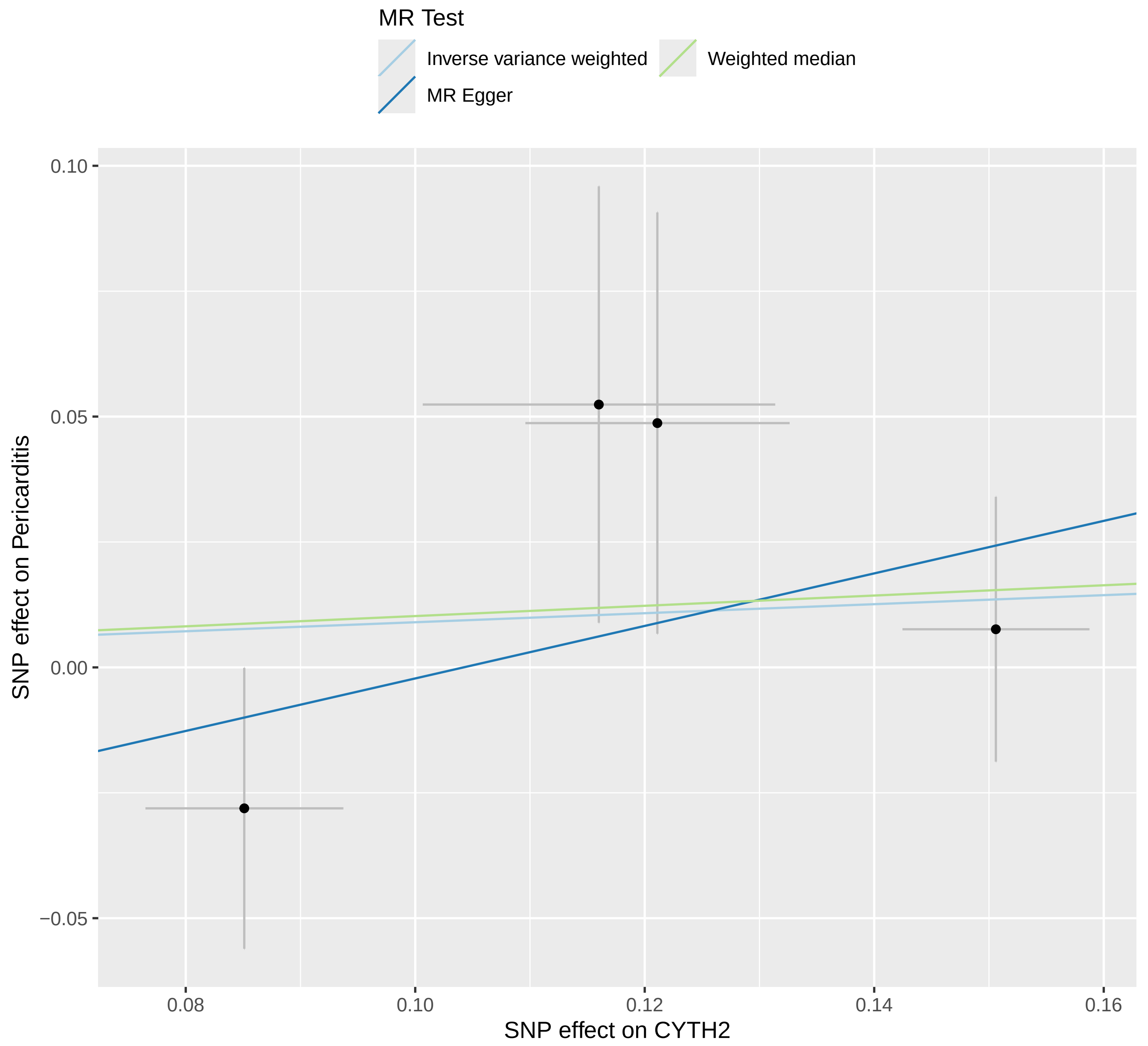
MR Test
Inverse variance weighted
MR Egger
Weighted median
0.10
0.05
0.00
−0.05
SNP effect on Pericarditis
0.08
0.10
0.12
0.14
0.16
SNP effect on CYTH2

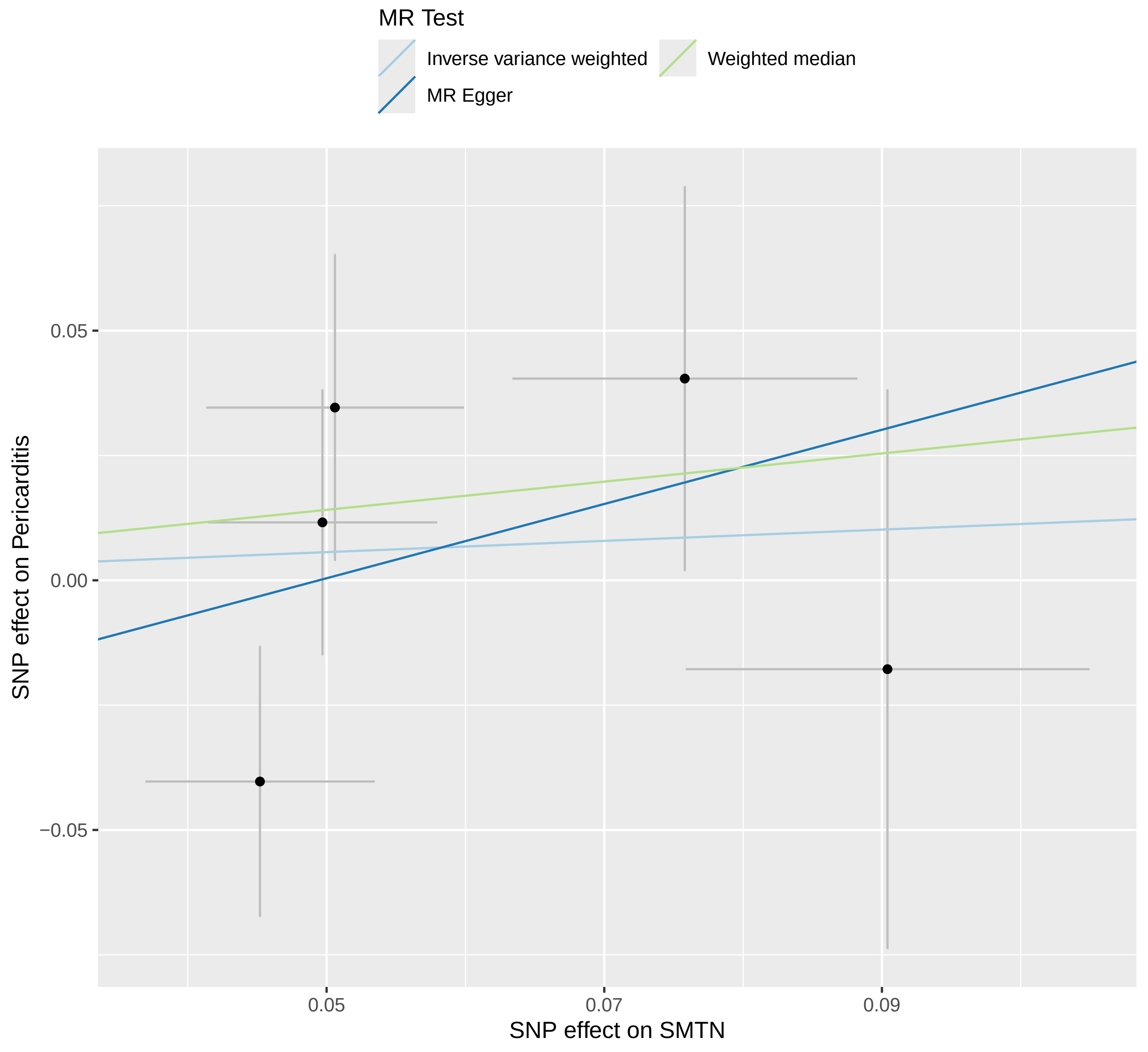
MR Test
Inverse variance weighted
MR Egger
Weighted median
0.05
0.00
−0.05
SNP effect on Pericarditis
0.05
0.07
0.09
SNP effect on SMTN

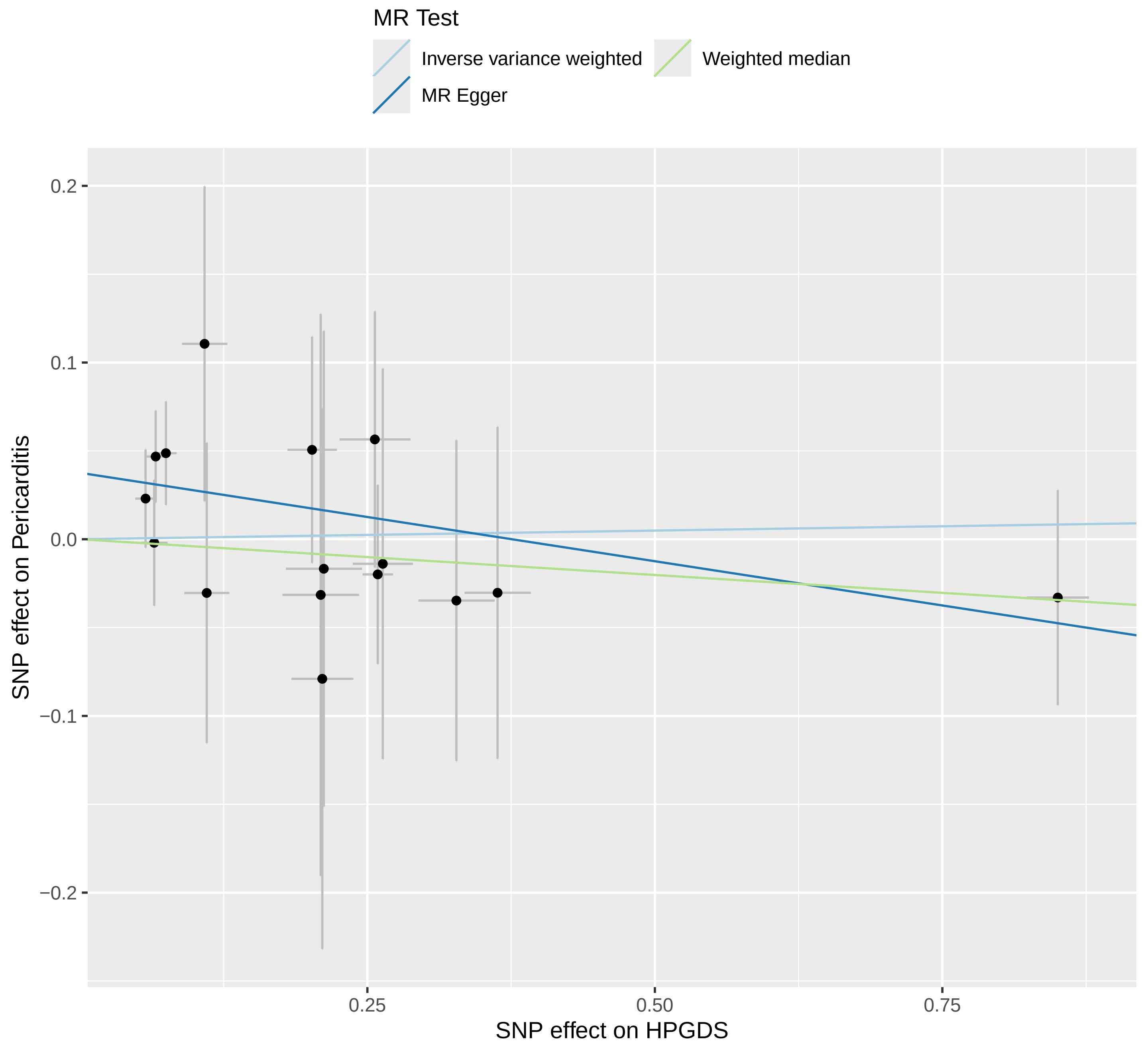

MR Test
Inverse variance weighted
Weighted median
MR Egger
0.2
0.1
0.0
−0.1
−0.2
SNP effect on Pericarditis
0.25
0.50
0.75
SNP effect on HPGDS

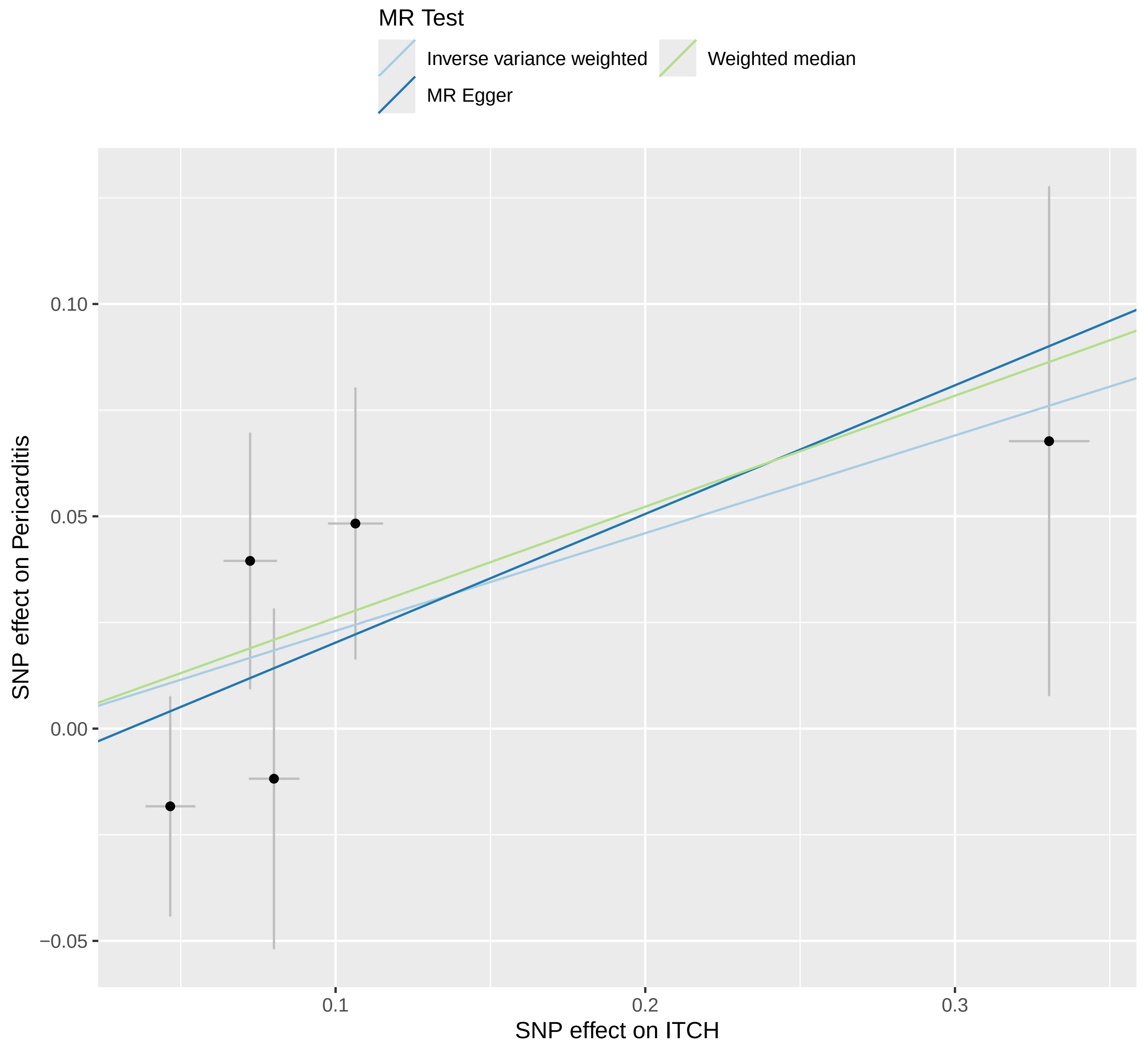
MR Test
Inverse variance weighted
MR Egger
Weighted median
0.10
0.05
0.00
−0.05
SNP effect on Pericarditis
0.1
0.2
0.3
SNP effect on ITCH

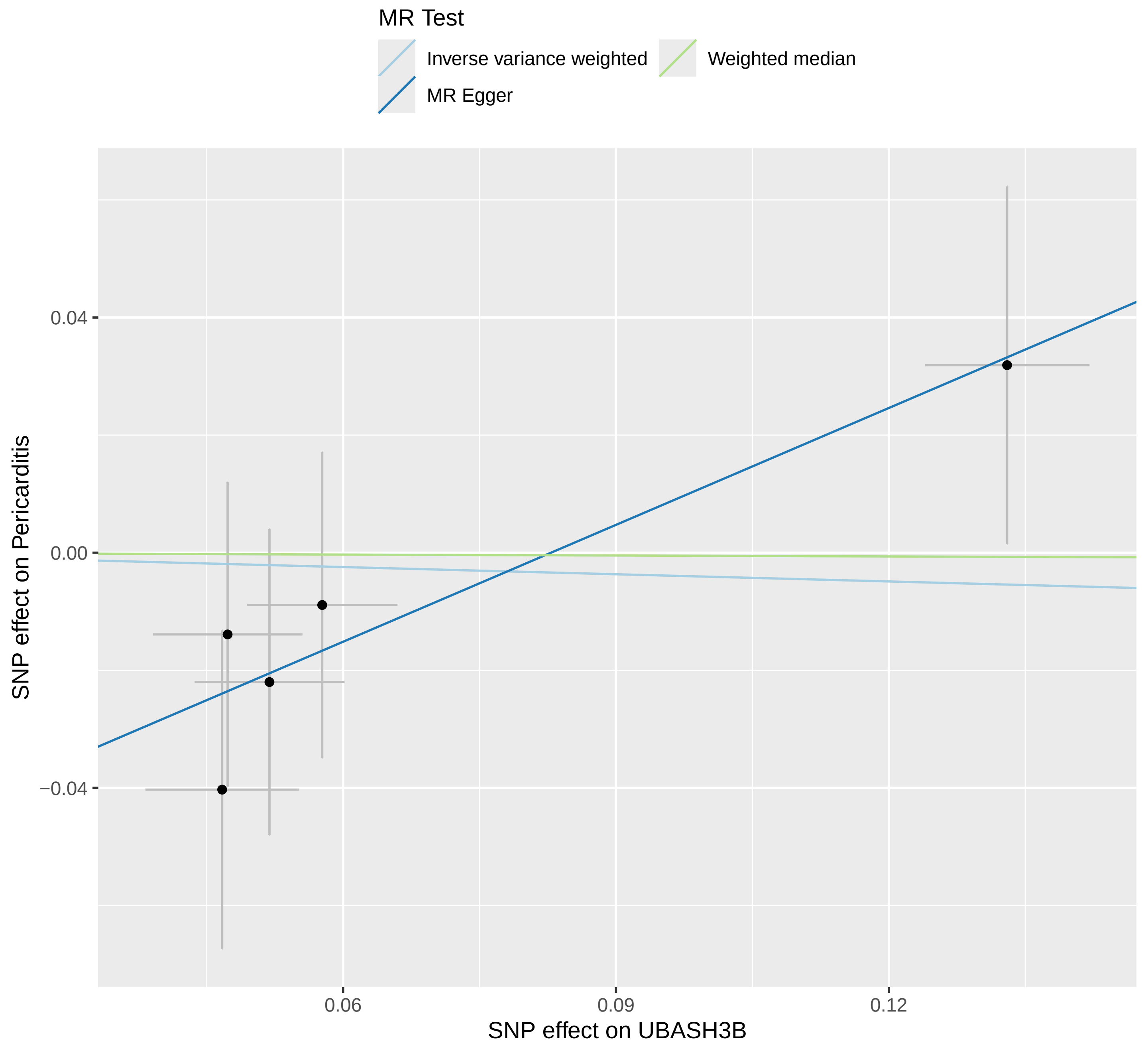

MR Test
Inverse variance weighted
Weighted median
MR Egger
0.04
0.00
−0.04
0.06
0.09
0.12
SNP effect on Pericarditis
SNP effect on UBASH3B

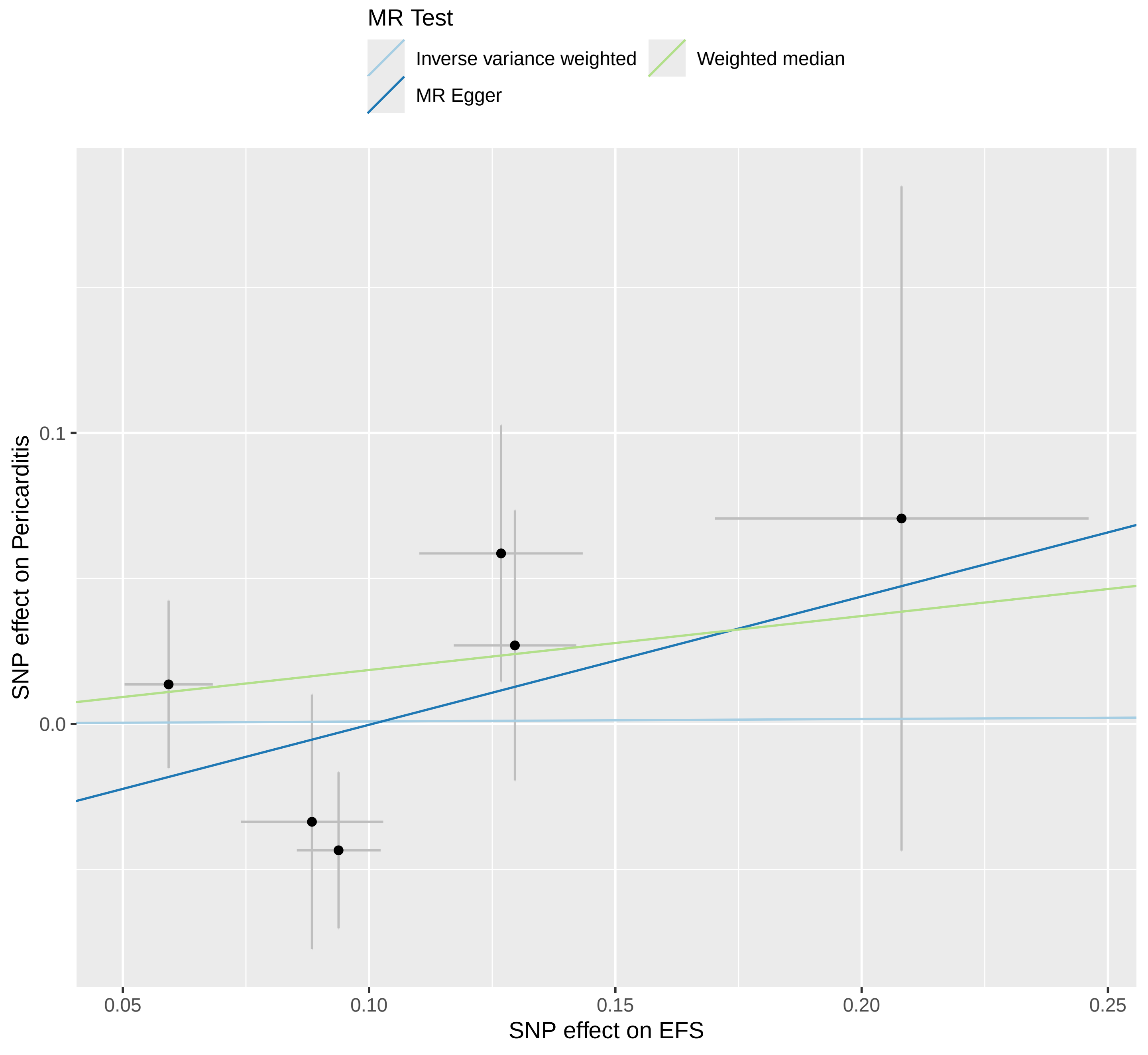
MR Test
Inverse variance weighted
Weighted median
MR Egger
SNP effect on Pericarditis
0.1
0.0
0.05
0.10
0.15
0.20
0.25
SNP effect on EFS

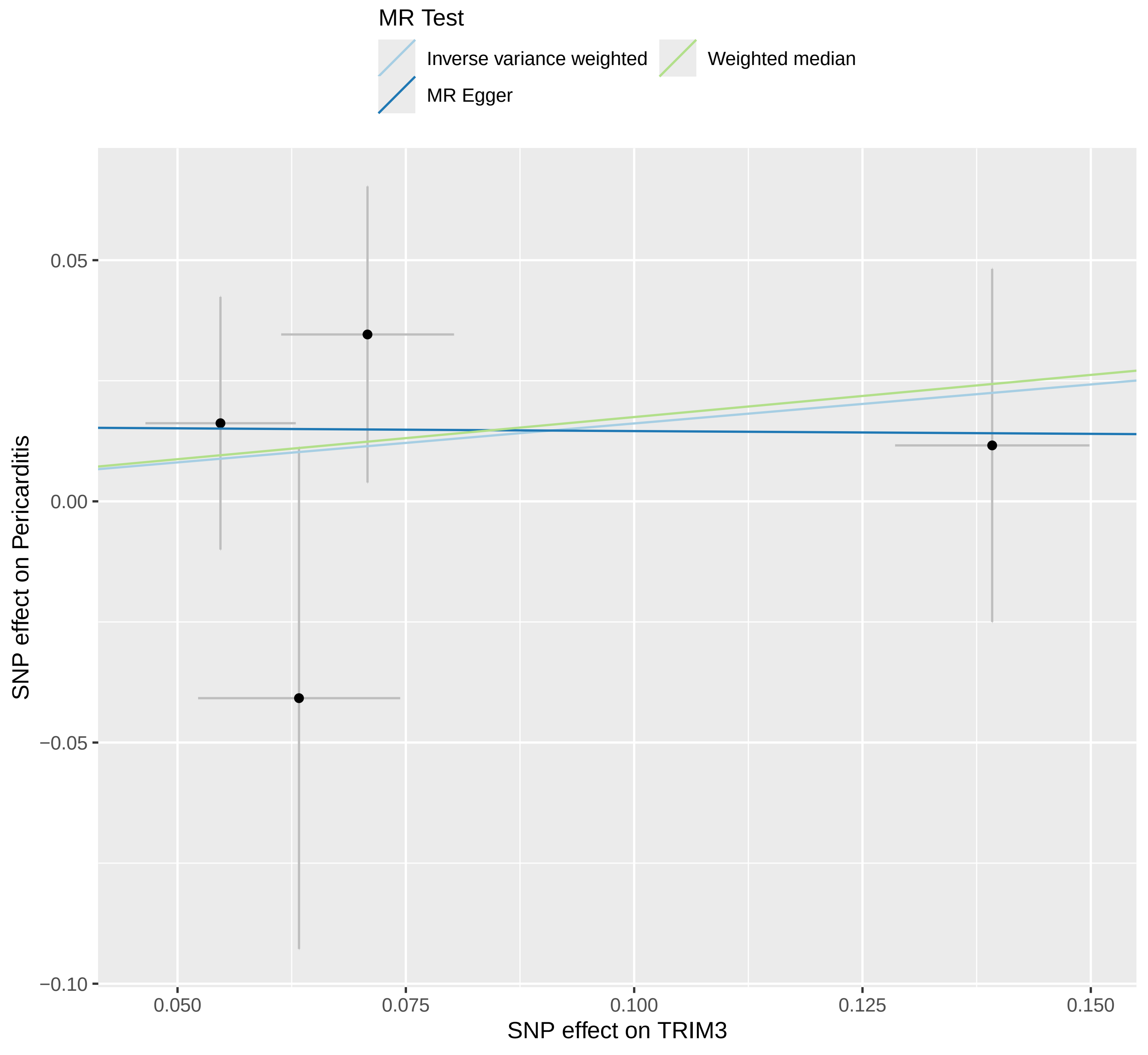
MR Test
Inverse variance weighted
Weighted median
MR Egger
SNP effect on Pericarditis
0.05
0.00
−0.05
−0.10
0.050
0.075
0.100
0.125
0.150
SNP effect on TRIM3

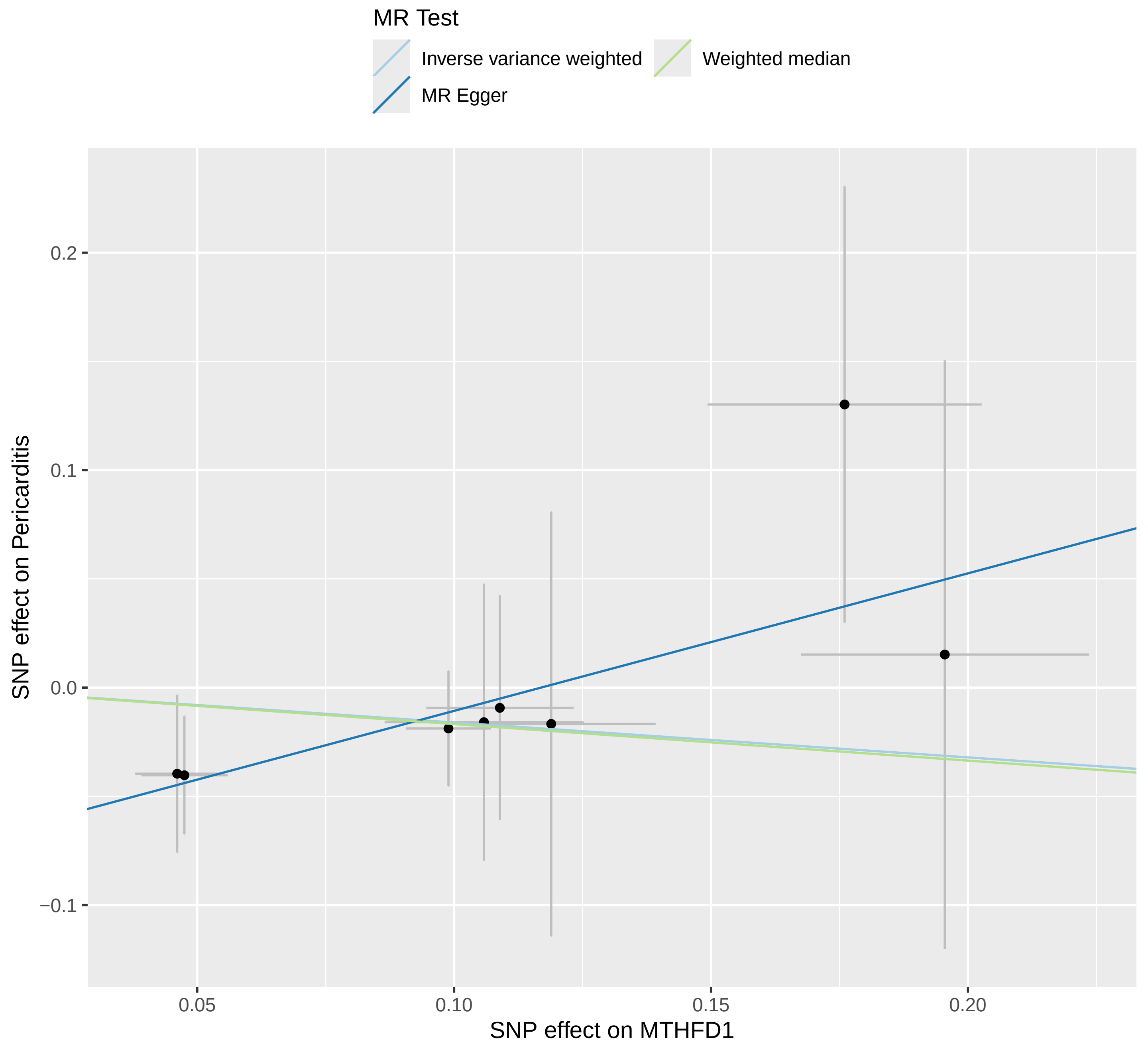
MR Test
Inverse variance weighted
Weighted median
MR Egger
0.2
0.1
0.0
−0.1
0.05
0.10
0.15
0.20
SNP effect on Pericarditis
SNP effect on MTHFD1

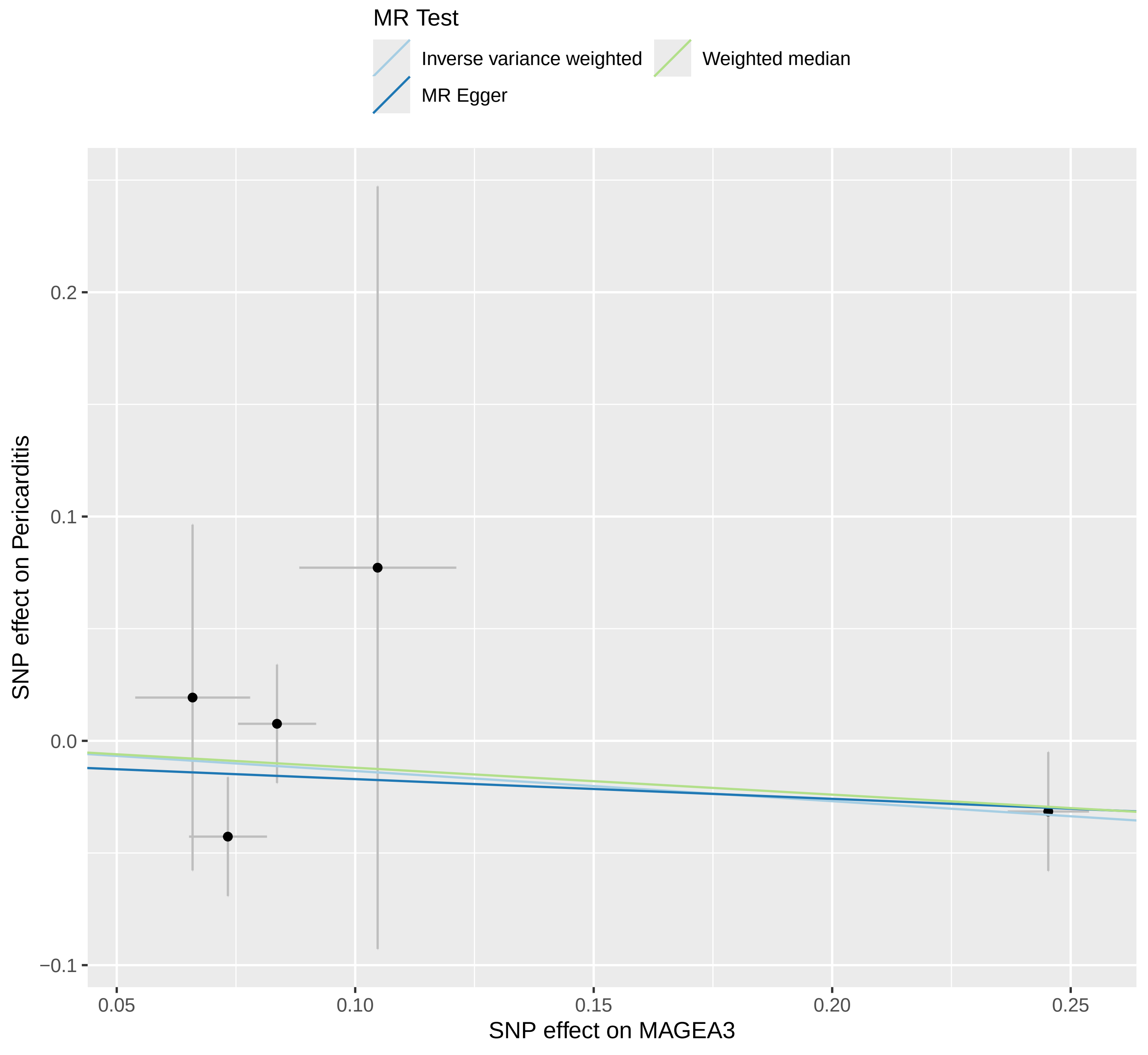
MR Test
Inverse variance weighted
Weighted median
MR Egger
0.2
0.1
0.0
−0.1
SNP effect on Pericarditis
0.05
0.10
0.15
0.20
0.25
SNP effect on MAGEA3

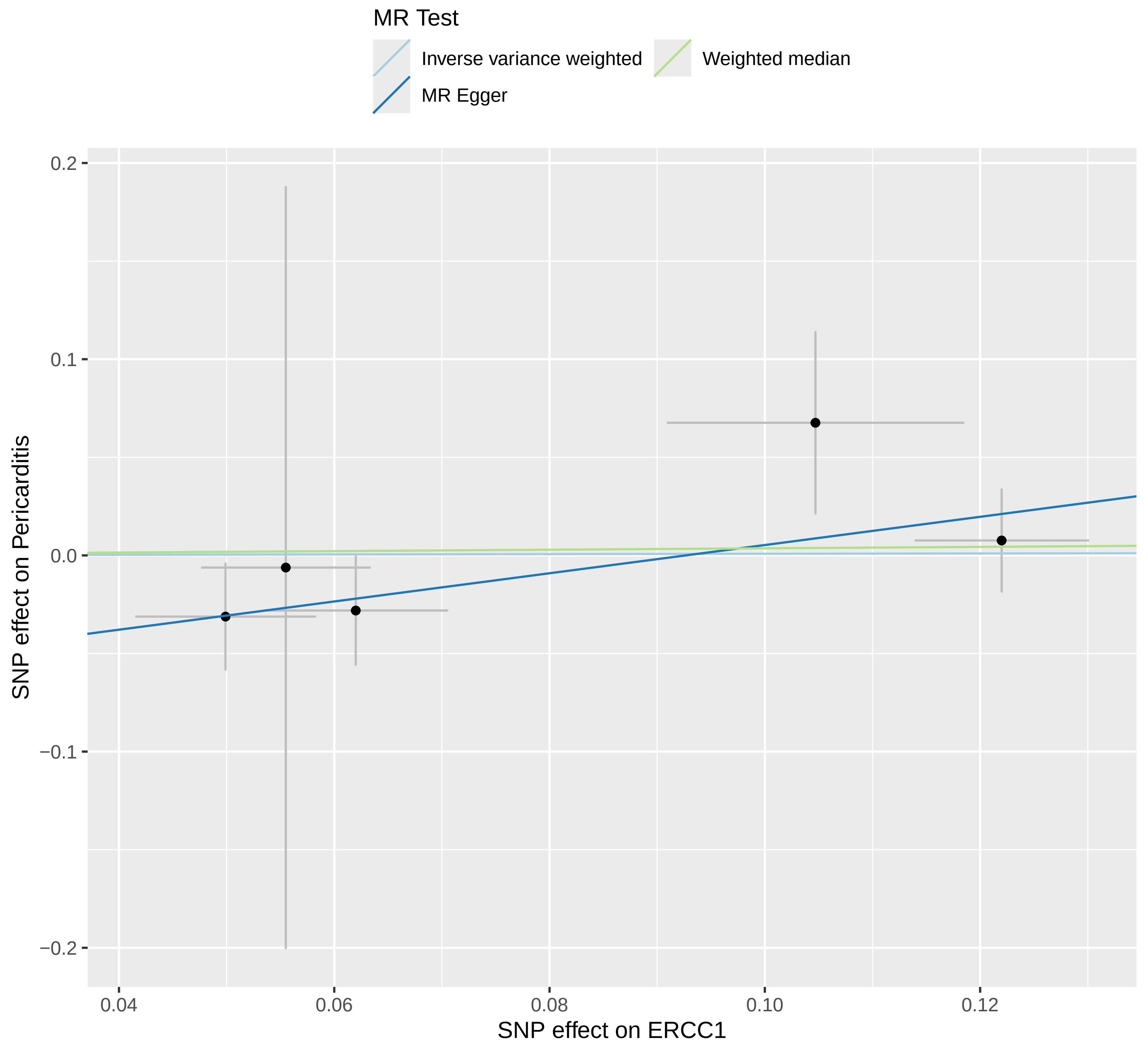

MR Test
Inverse variance weighted
Weighted median
MR Egger
SNP effect on Pericarditis
0.2
0.1
0.0
−0.1
−0.2
0.04
0.06
0.08
0.10
0.12
SNP effect on ERCC1

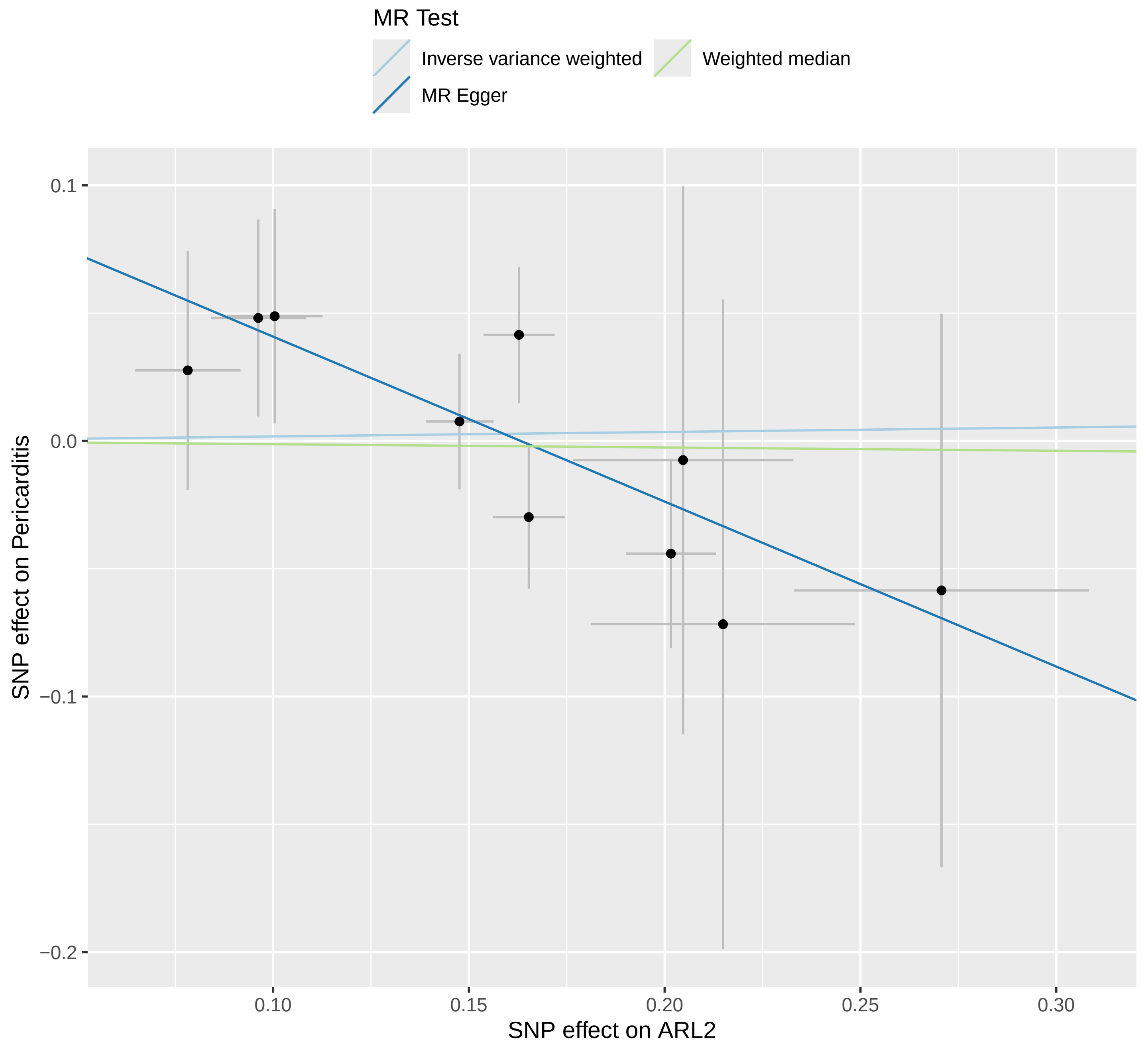
MR Test
Inverse variance weighted
MR Egger
Weighted median
SNP effect on Pericarditis
0.1
0.0
−0.1
−0.2
0.10
0.15
0.20
0.25
0.30
SNP effect on ARL2

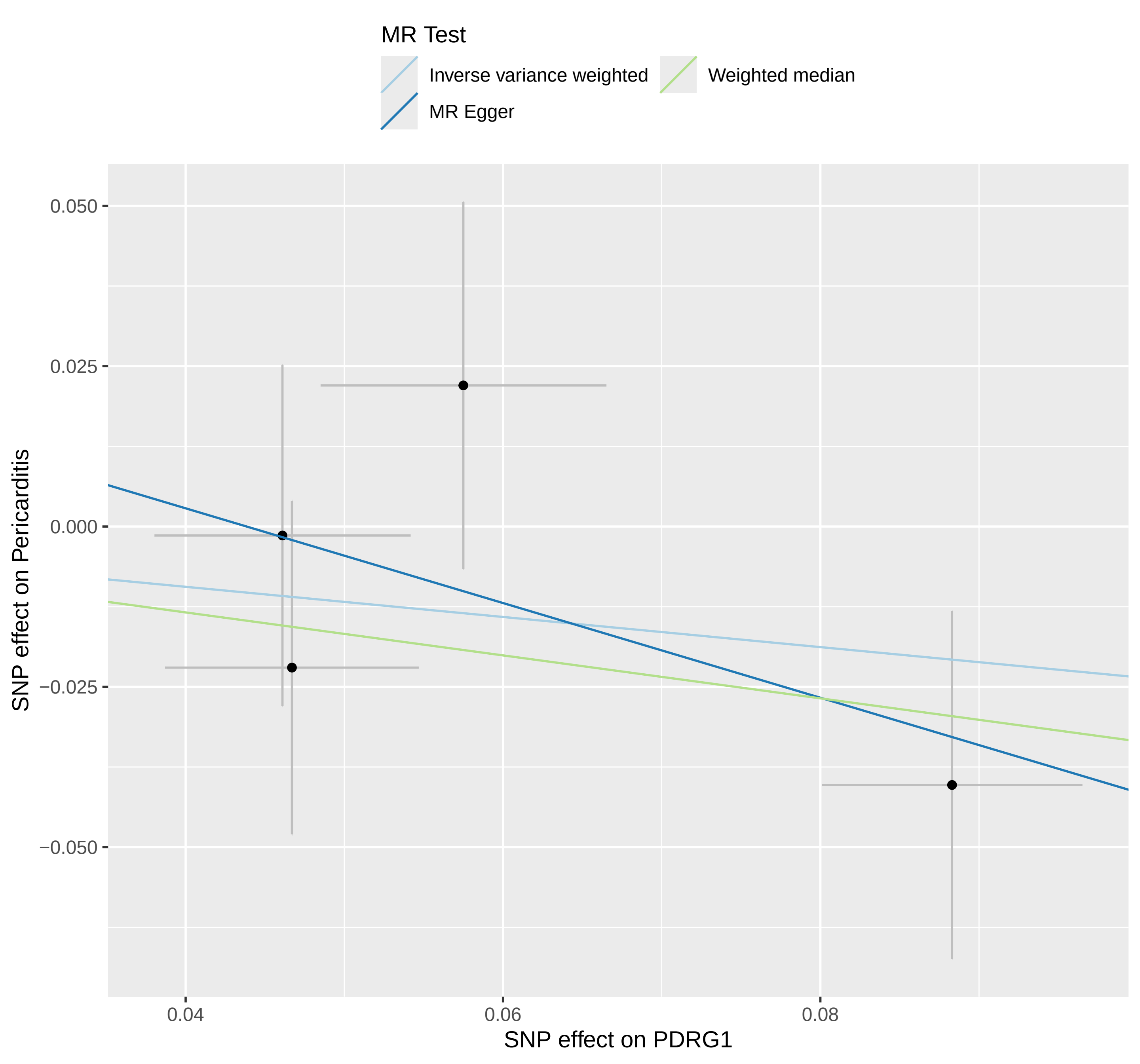
MR Test
Inverse variance weighted
MR Egger
Weighted median
0.050
0.025
0.000
−0.025
−0.050
SNP effect on Pericarditis
0.04
0.06
0.08
SNP effect on PDRG1

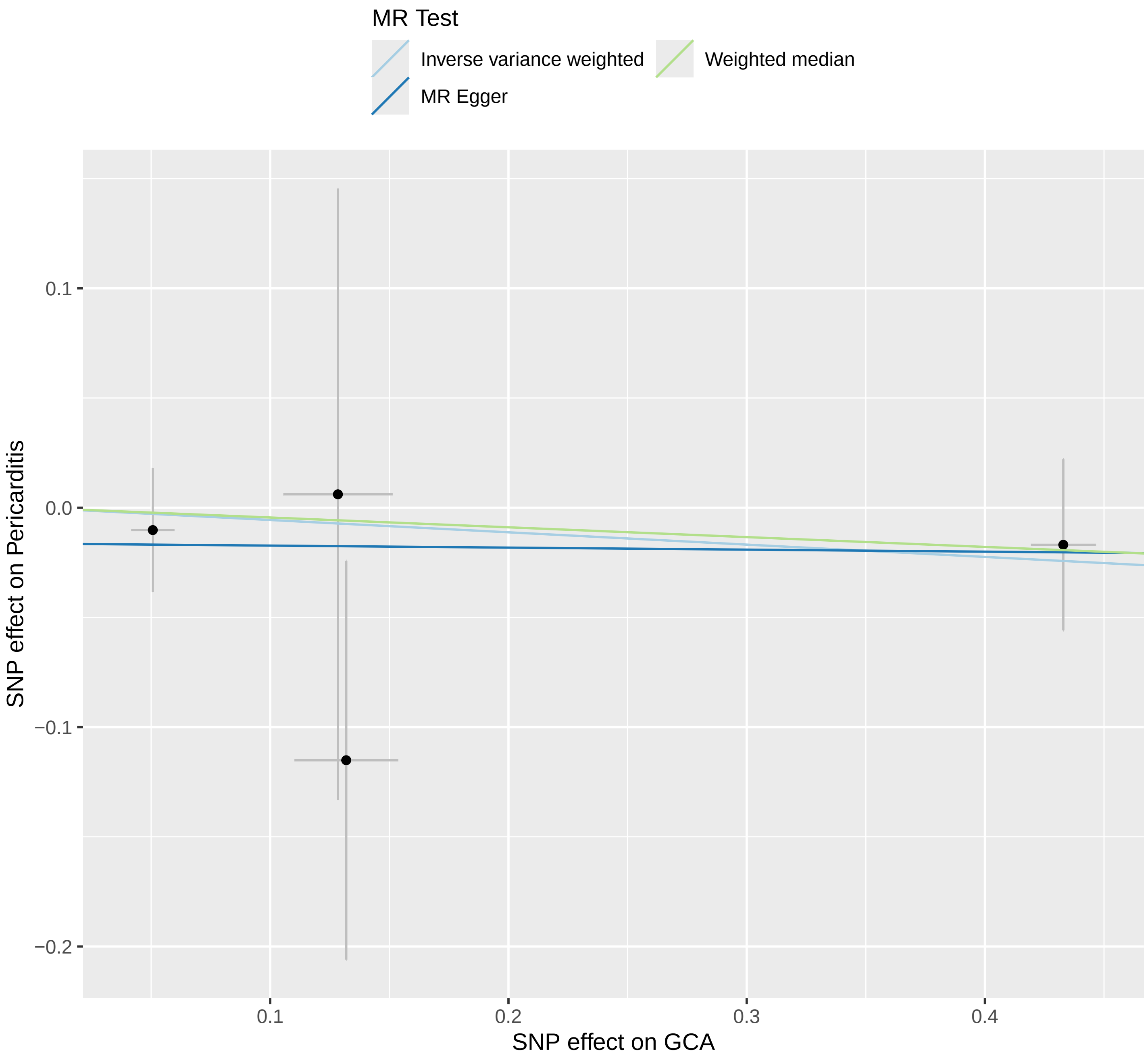

MR Test
Inverse variance weighted
Weighted median
MR Egger
0.1
0.0
−0.1
−0.2
SNP effect on Pericarditis
0.1
0.2
0.3
0.4
SNP effect on GCA

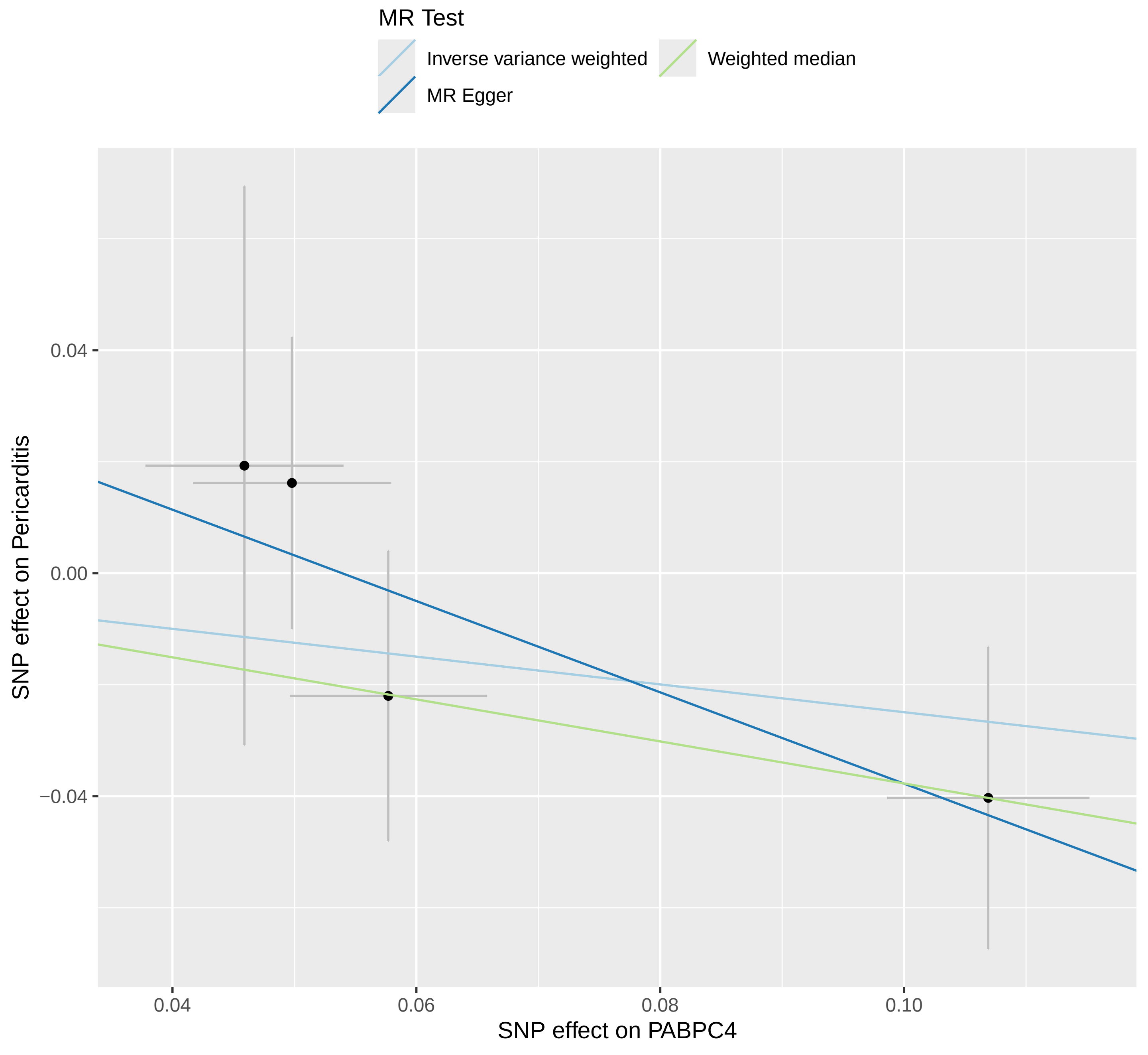

MR Test
Inverse variance weighted
Weighted median
MR Egger
0.04
0.00
−0.04
SNP effect on Pericarditis
0.04
0.06
0.08
0.10
SNP effect on PABPC4

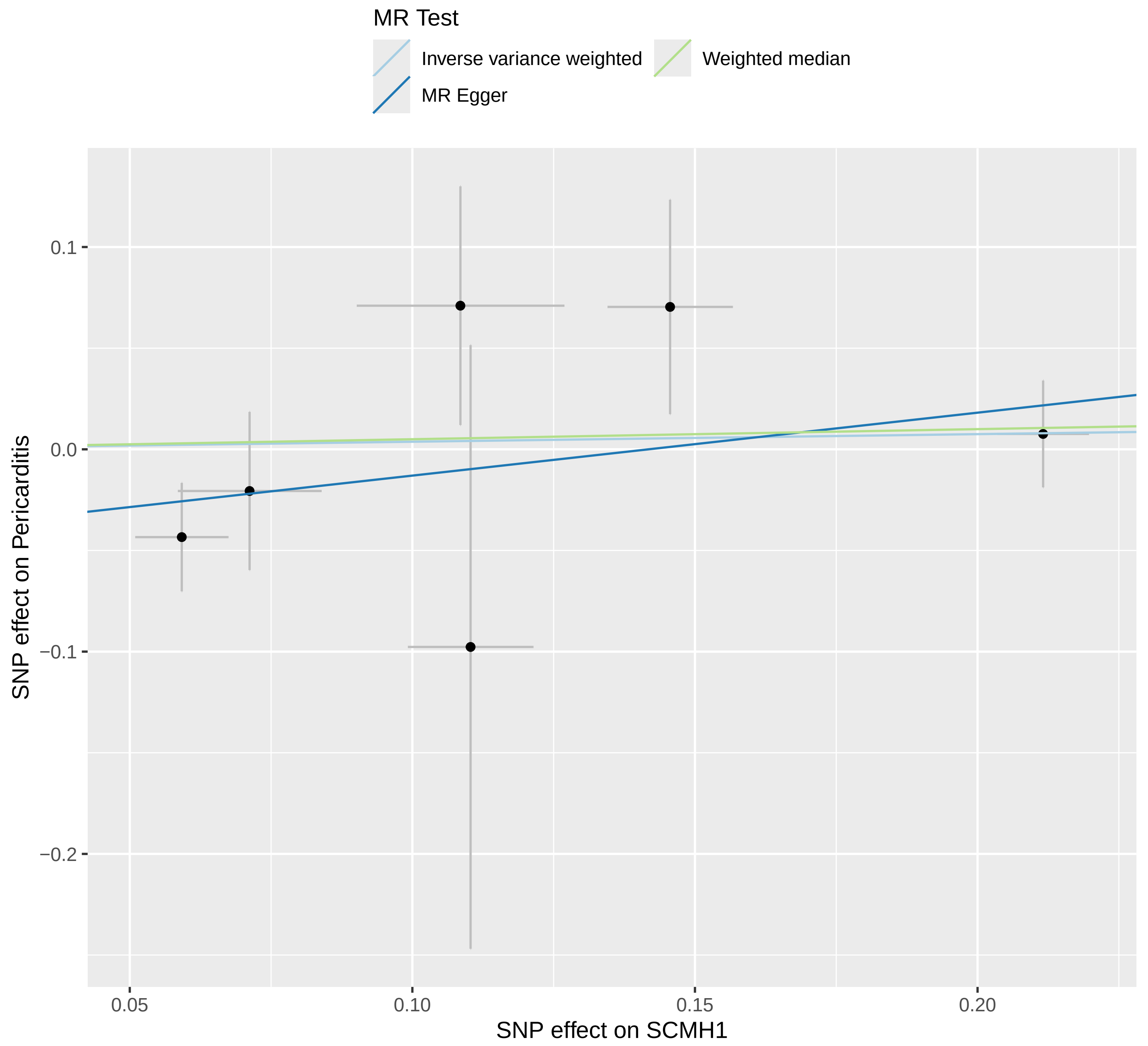
MR Test
Inverse variance weighted
MR Egger
Weighted median
0.1
0.0
−0.1
−0.2
SNP effect on Pericarditis
0.05
0.10
0.15
0.20
SNP effect on SCMH1

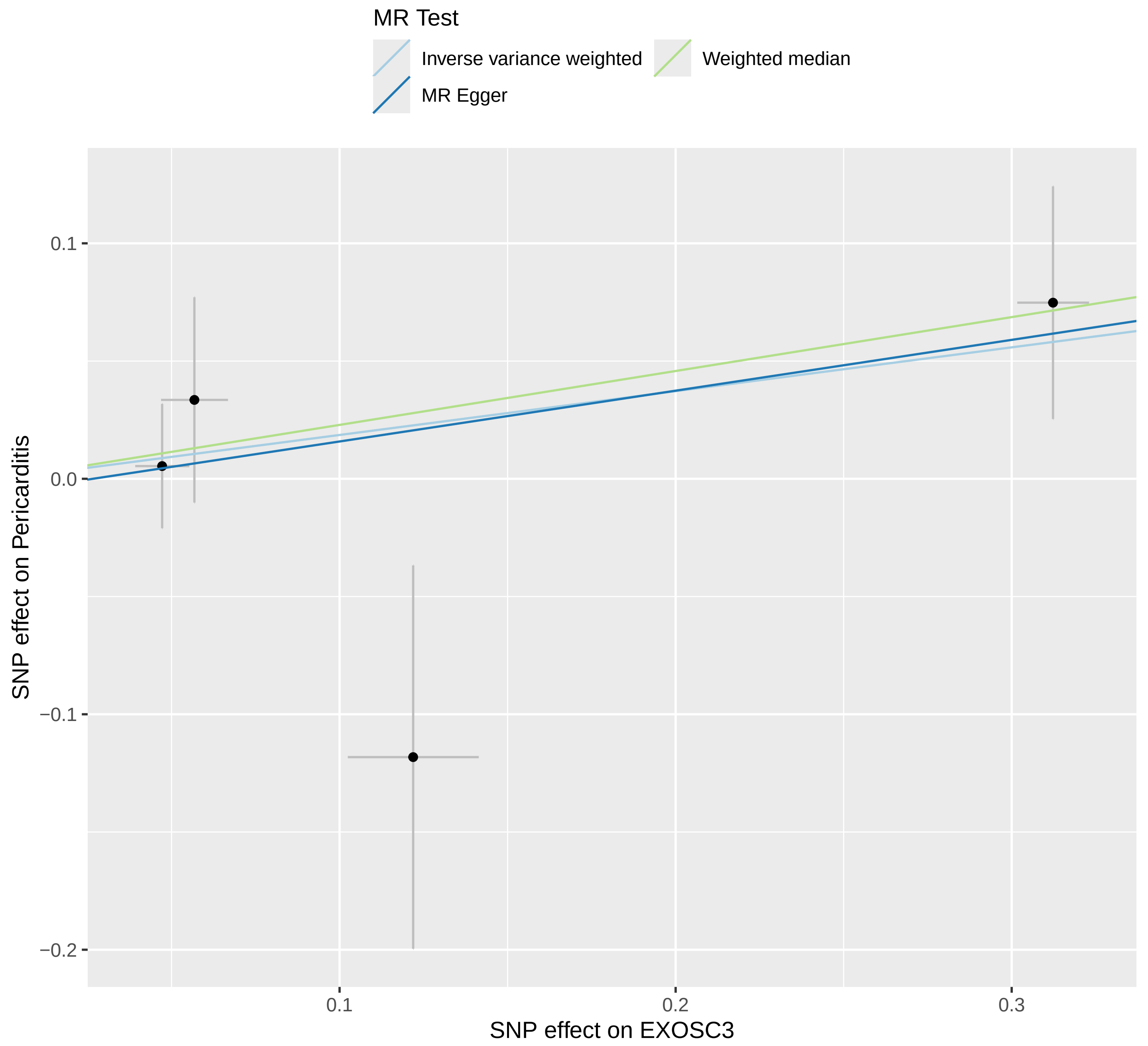
MR Test
Inverse variance weighted
Weighted median
MR Egger
0.1
0.0
−0.1
−0.2
SNP effect on Pericarditis
0.1
0.2
0.3
SNP effect on EXOSC3

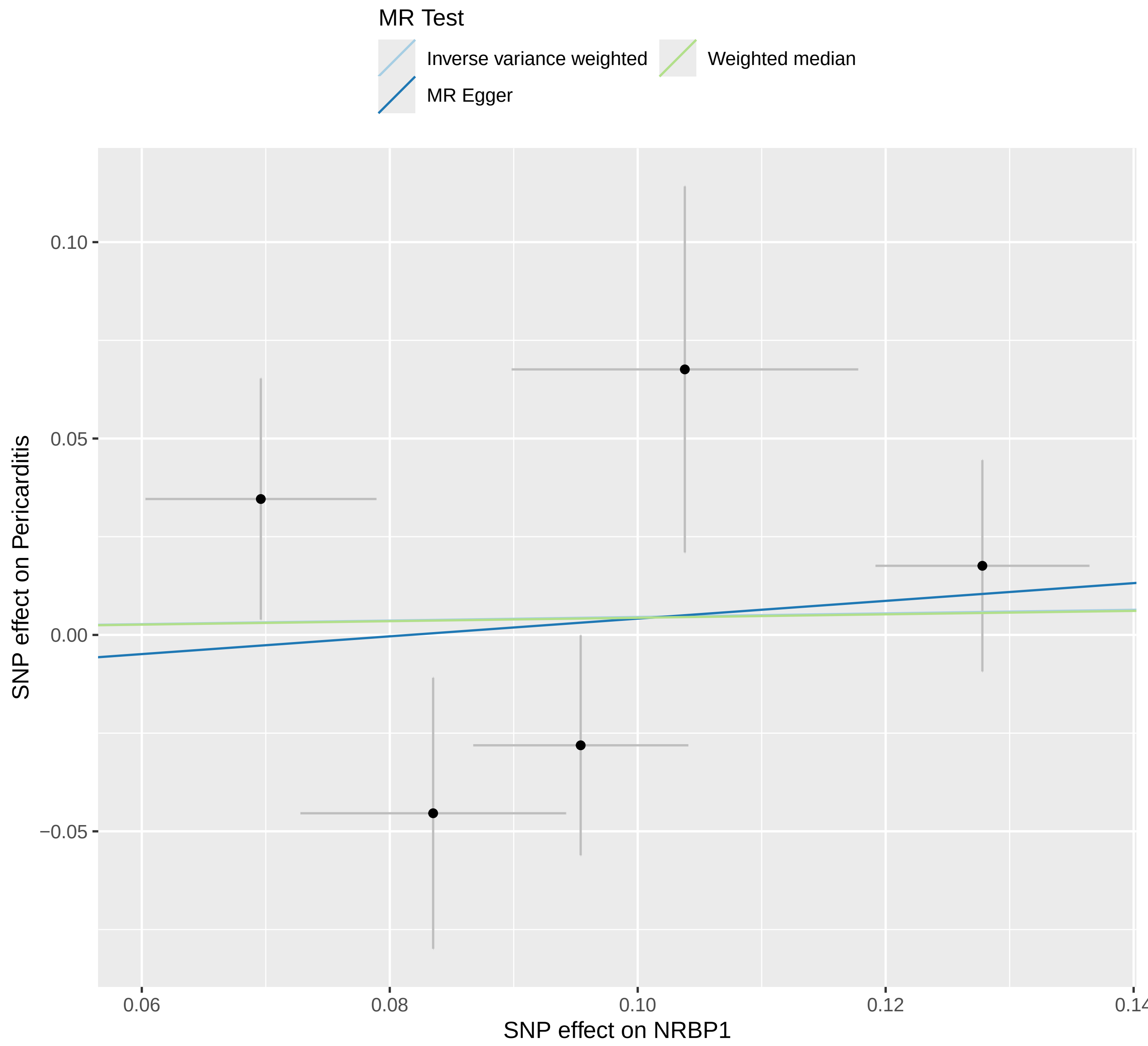
MR Test
Inverse variance weighted
Weighted median
MR Egger
SNP effect on Pericarditis
0.10
0.05
0.00
−0.05
0.06
0.08
0.10
0.12
0.14
SNP effect on NRBP1

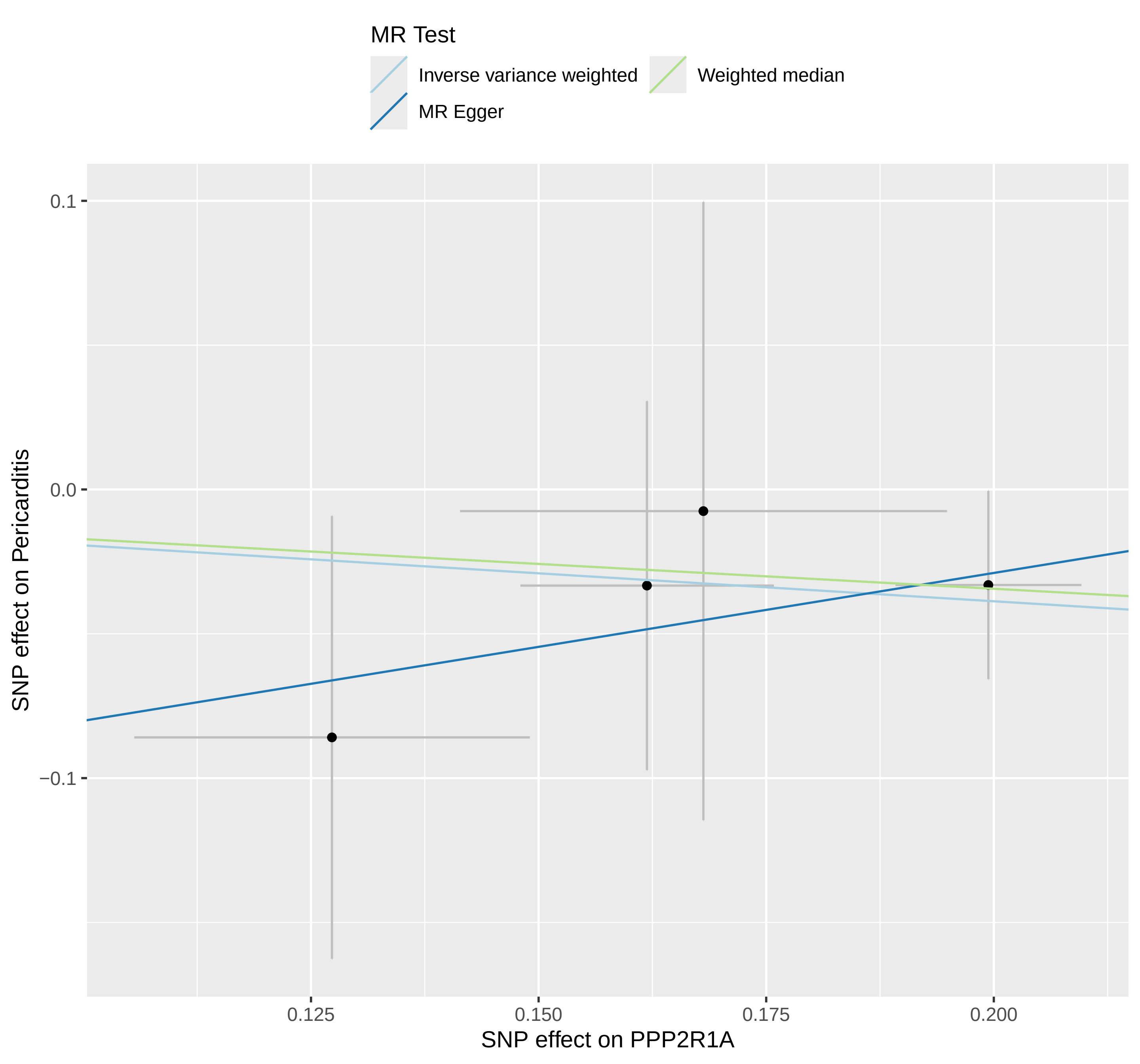

MR Test
Inverse variance weighted
Weighted median
MR Egger
0.1
0.0
−0.1
SNP effect on Pericarditis
0.125
0.150
0.175
0.200
SNP effect on PPP2R1A

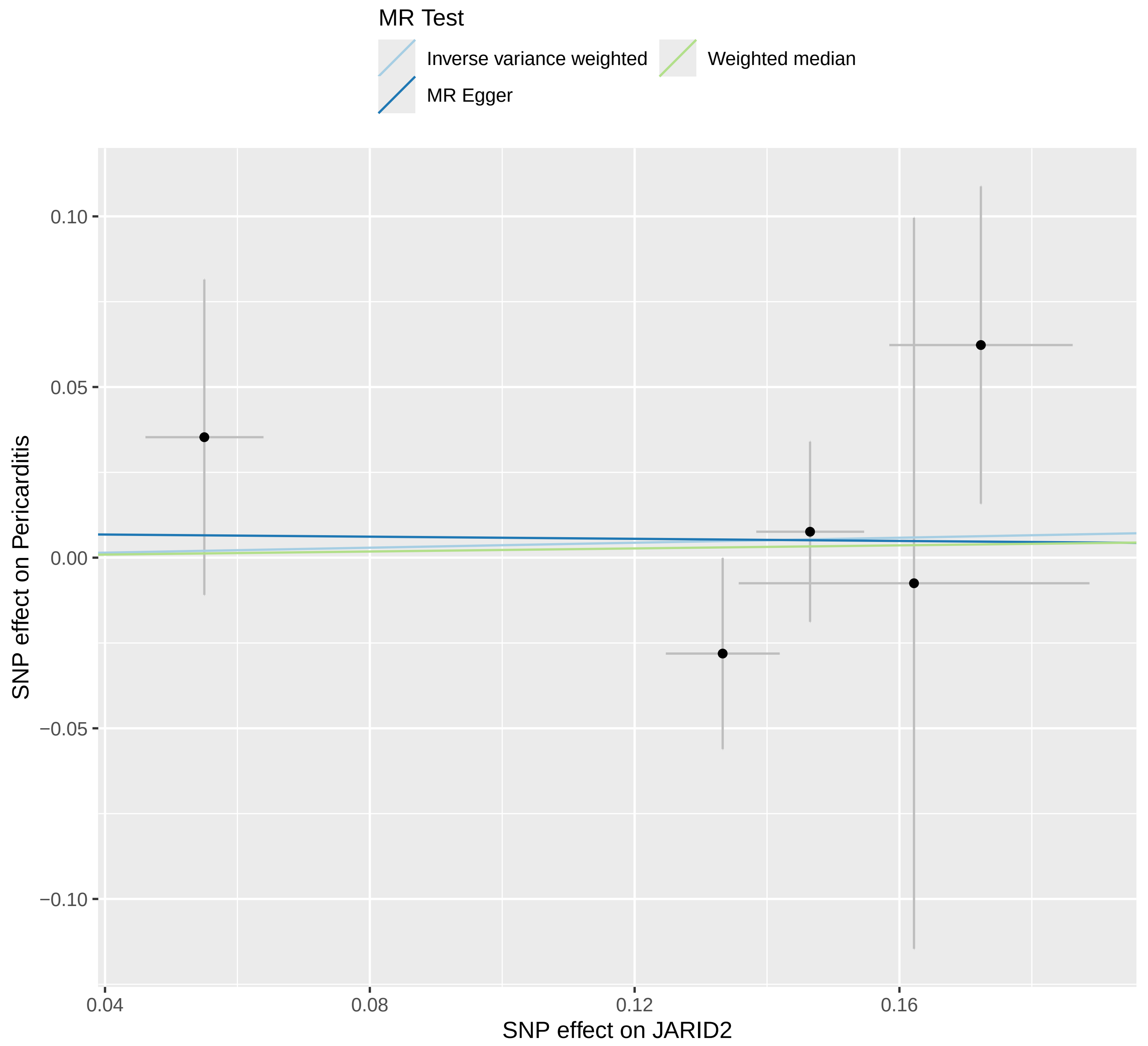
MR Test
Inverse variance weighted
MR Egger
Weighted median
0.10
0.05
0.00
−0.05
−0.10
SNP effect on Pericarditis
0.04
0.08
0.12
0.16
SNP effect on JARID2

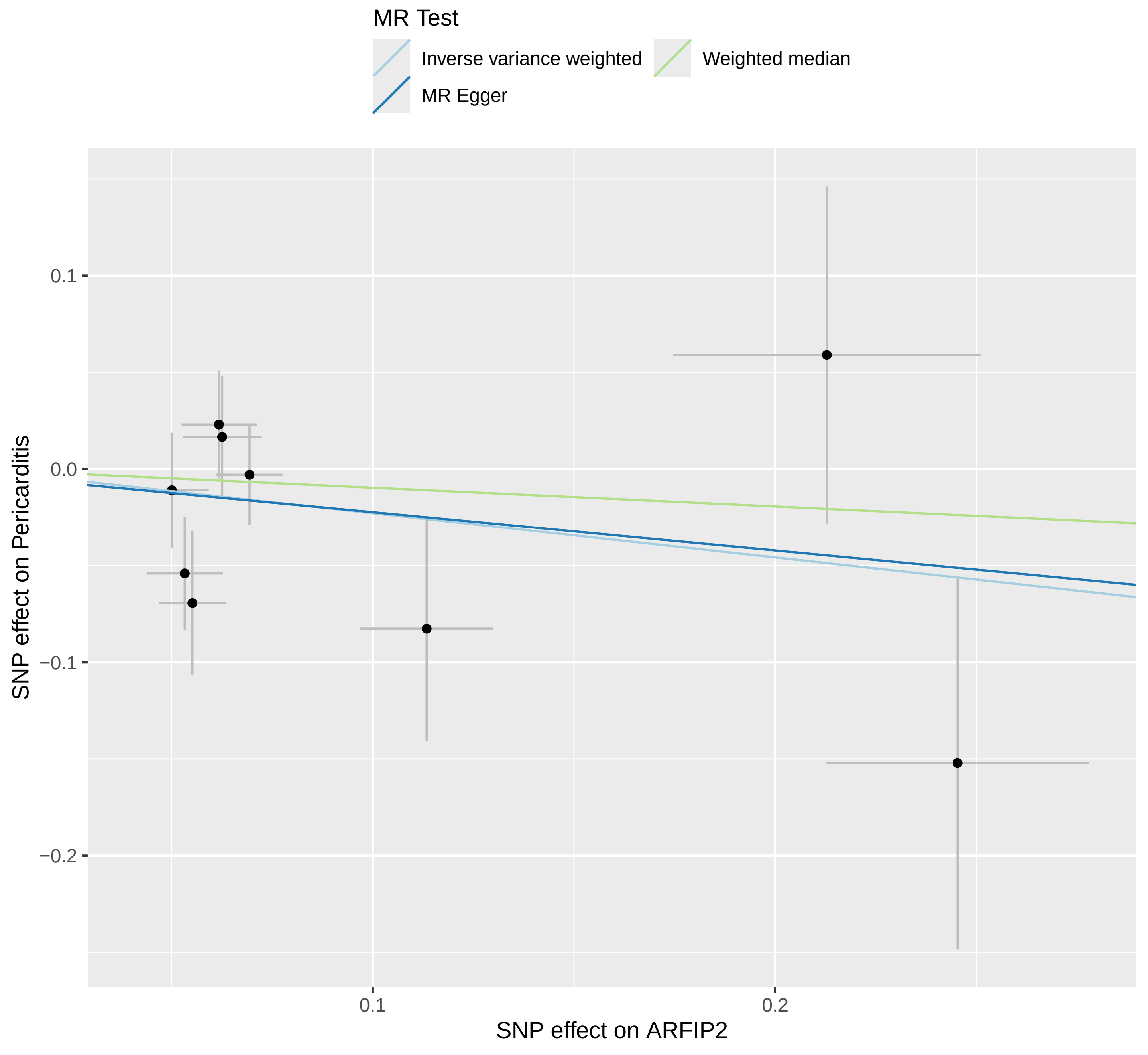

MR Test
Inverse variance weighted
Weighted median
MR Egger
0.1
0.0
−0.1
−0.2
SNP effect on Pericarditis
0.1
0.2
SNP effect on ARFIP2

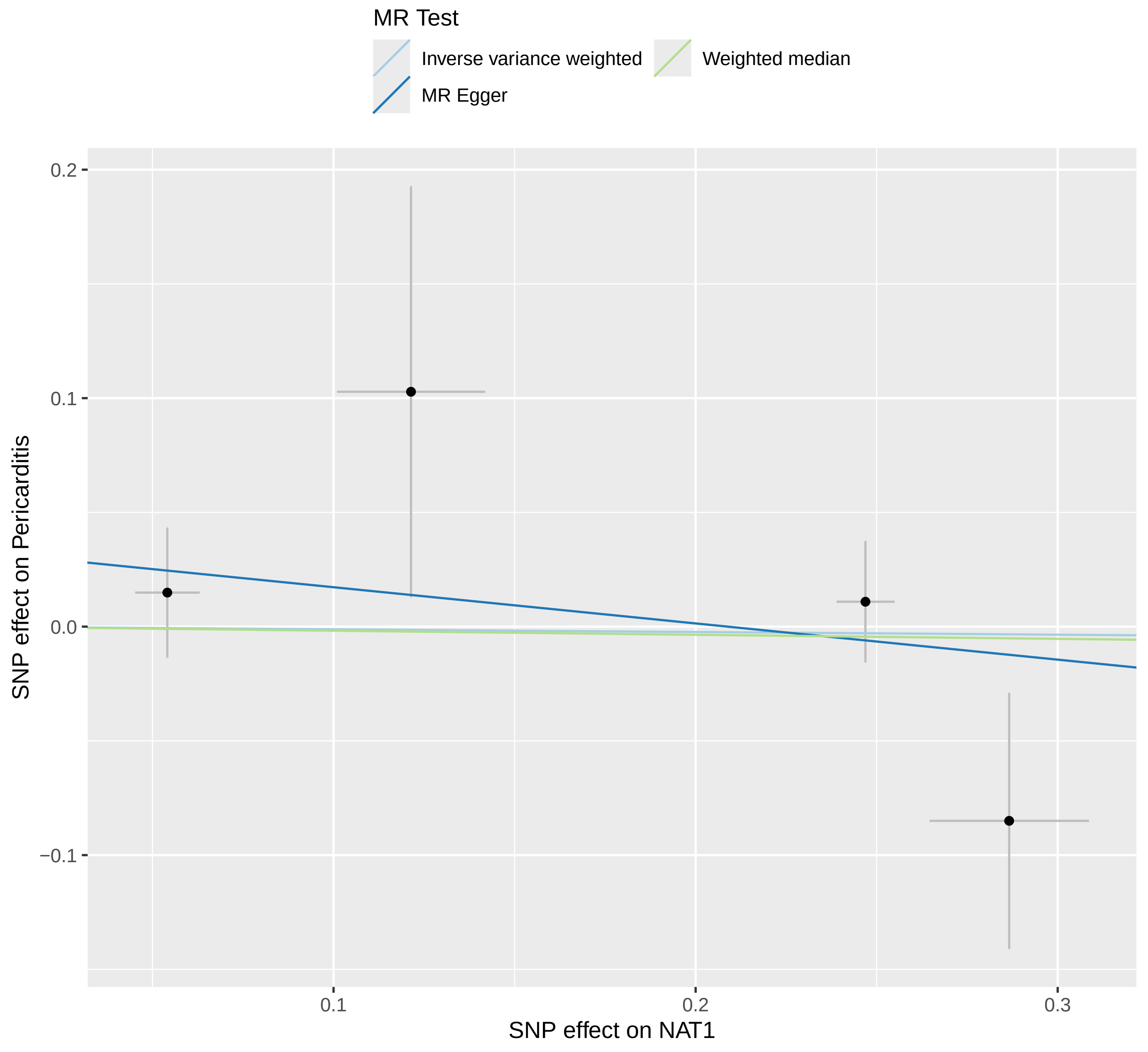
MR Test
Inverse variance weighted
Weighted median
MR Egger
SNP effect on Pericarditis
0.2
0.1
0.0
−0.1
0.1
0.2
0.3
SNP effect on NAT1

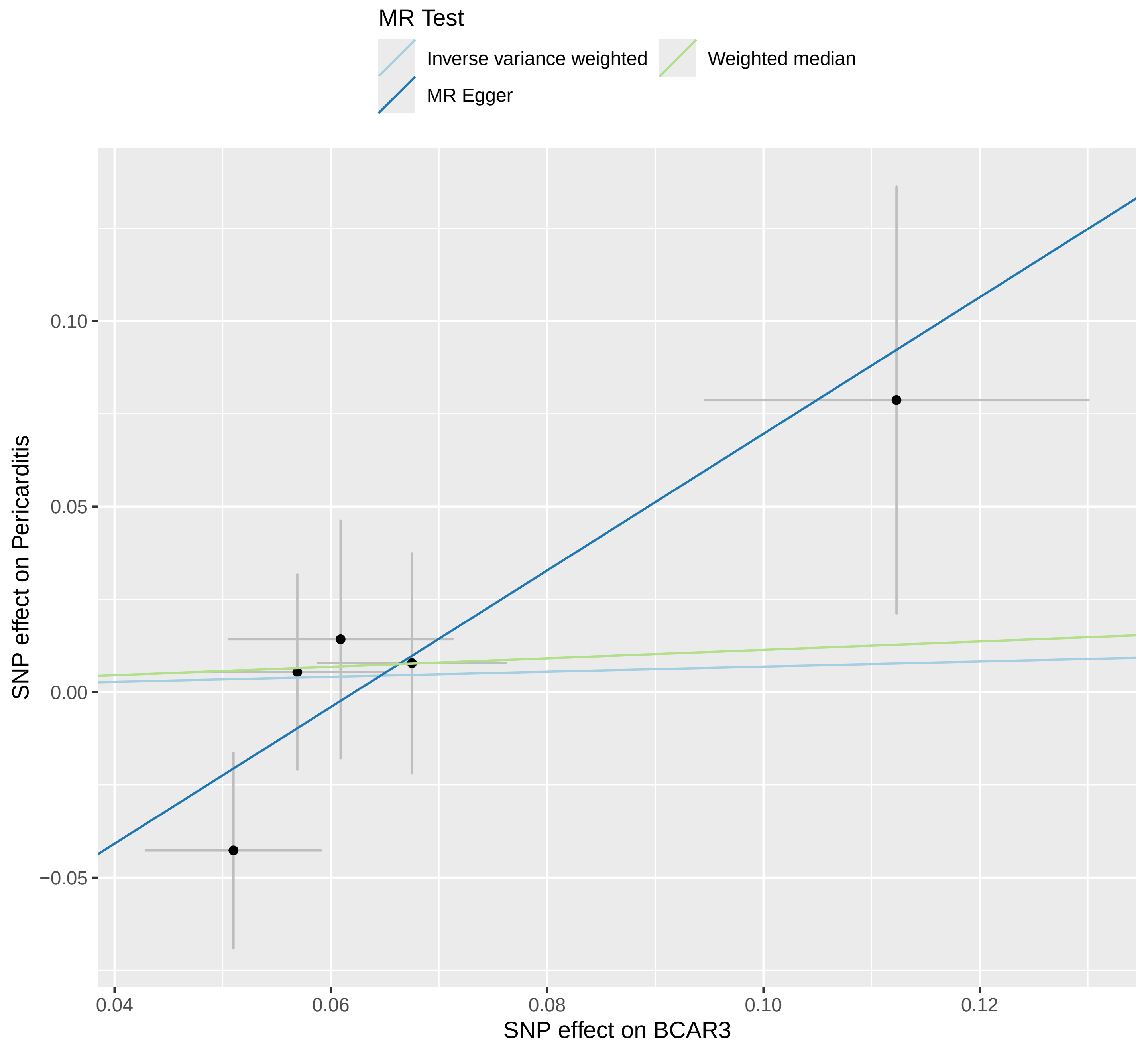
MR Test
Inverse variance weighted
MR Egger
Weighted median
0.10
0.05
0.00
−0.05
SNP effect on Pericarditis
0.04
0.06
0.08
0.10
0.12
SNP effect on BCAR3

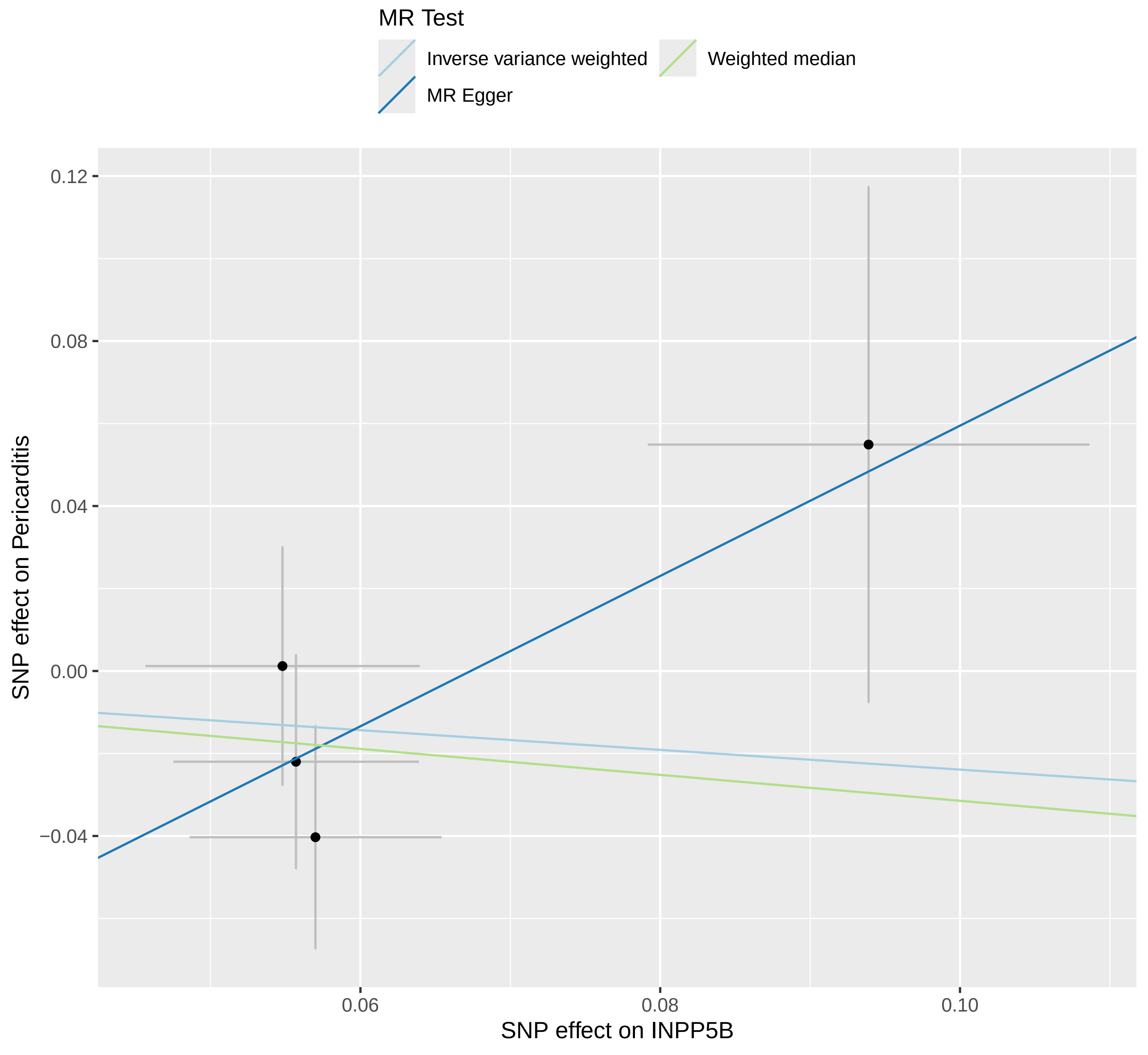
MR Test
Inverse variance weighted
MR Egger
Weighted median
0.12
0.08
0.04
0.00
−0.04
0.06
0.08
0.10
SNP effect on Pericarditis
SNP effect on INPP5B

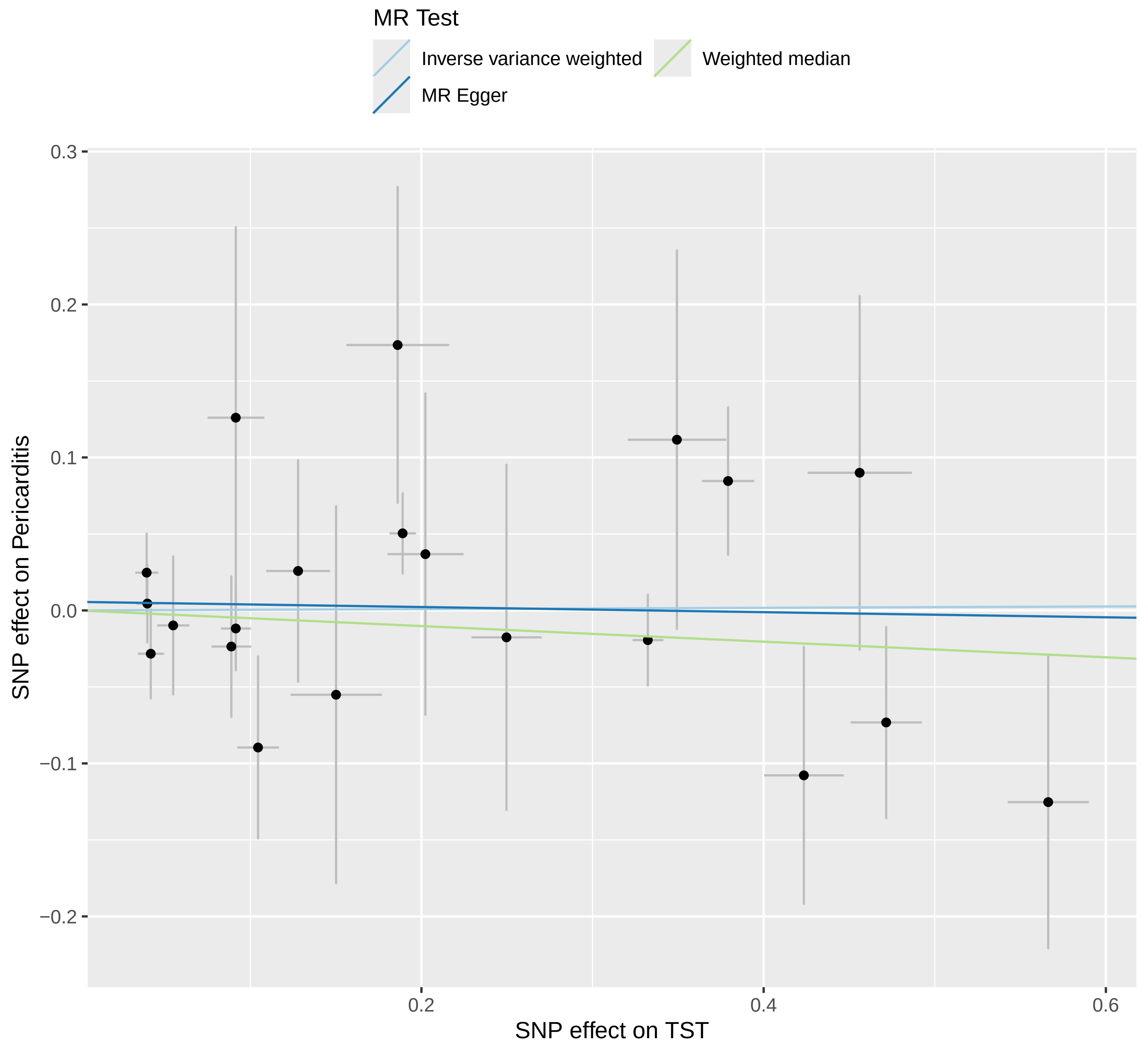

MR Test
Inverse variance weighted
Weighted median
MR Egger
0.3
0.2
0.1
0.0
−0.1
−0.2
SNP effect on Pericarditis
0.2
0.4
0.6
SNP effect on TST

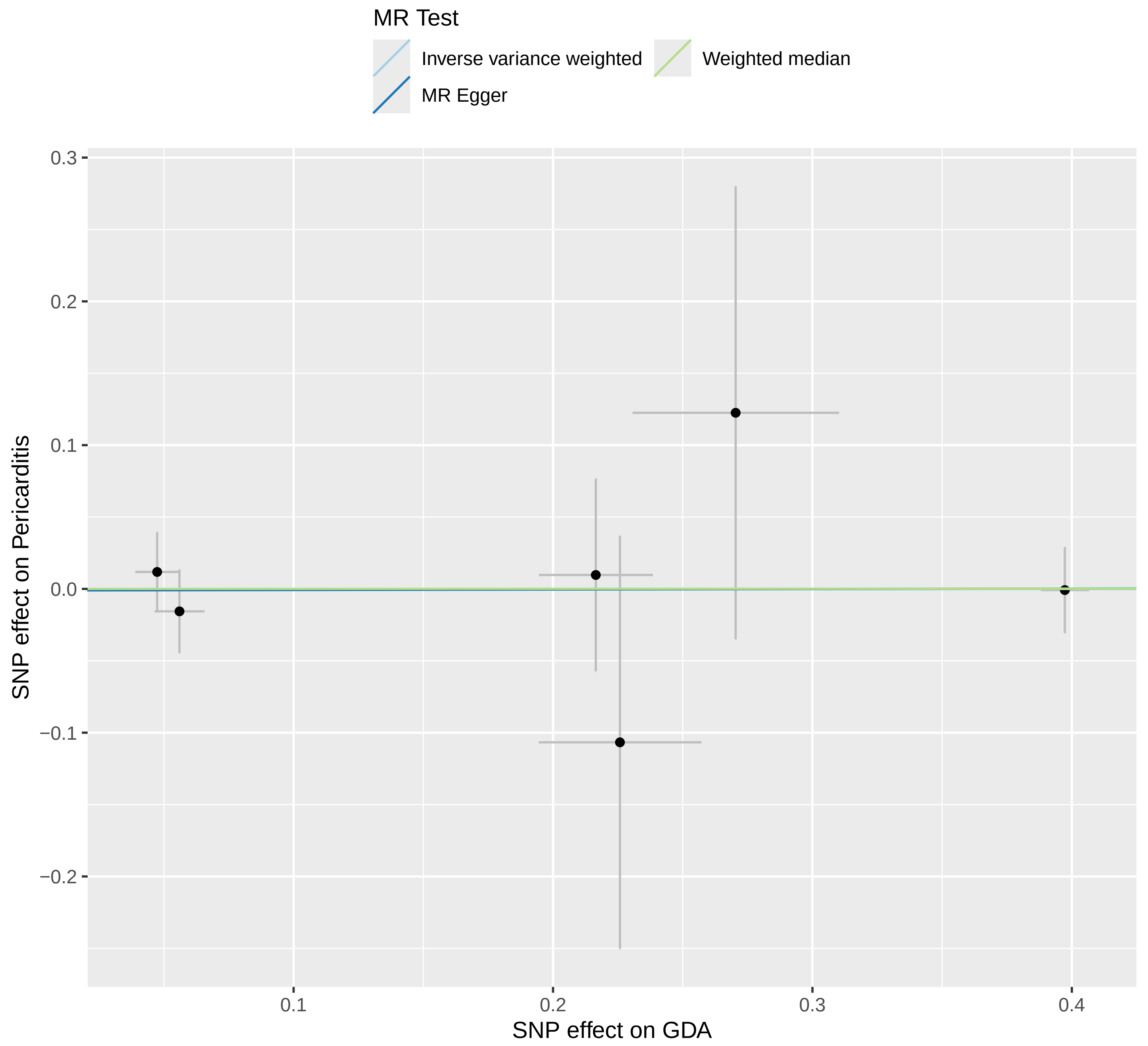
MR Test
Inverse variance weighted
Weighted median
MR Egger
0.3
0.2
0.1
0.0
−0.1
−0.2
0.1
0.2
0.3
0.4
SNP effect on Pericarditis
SNP effect on GDA

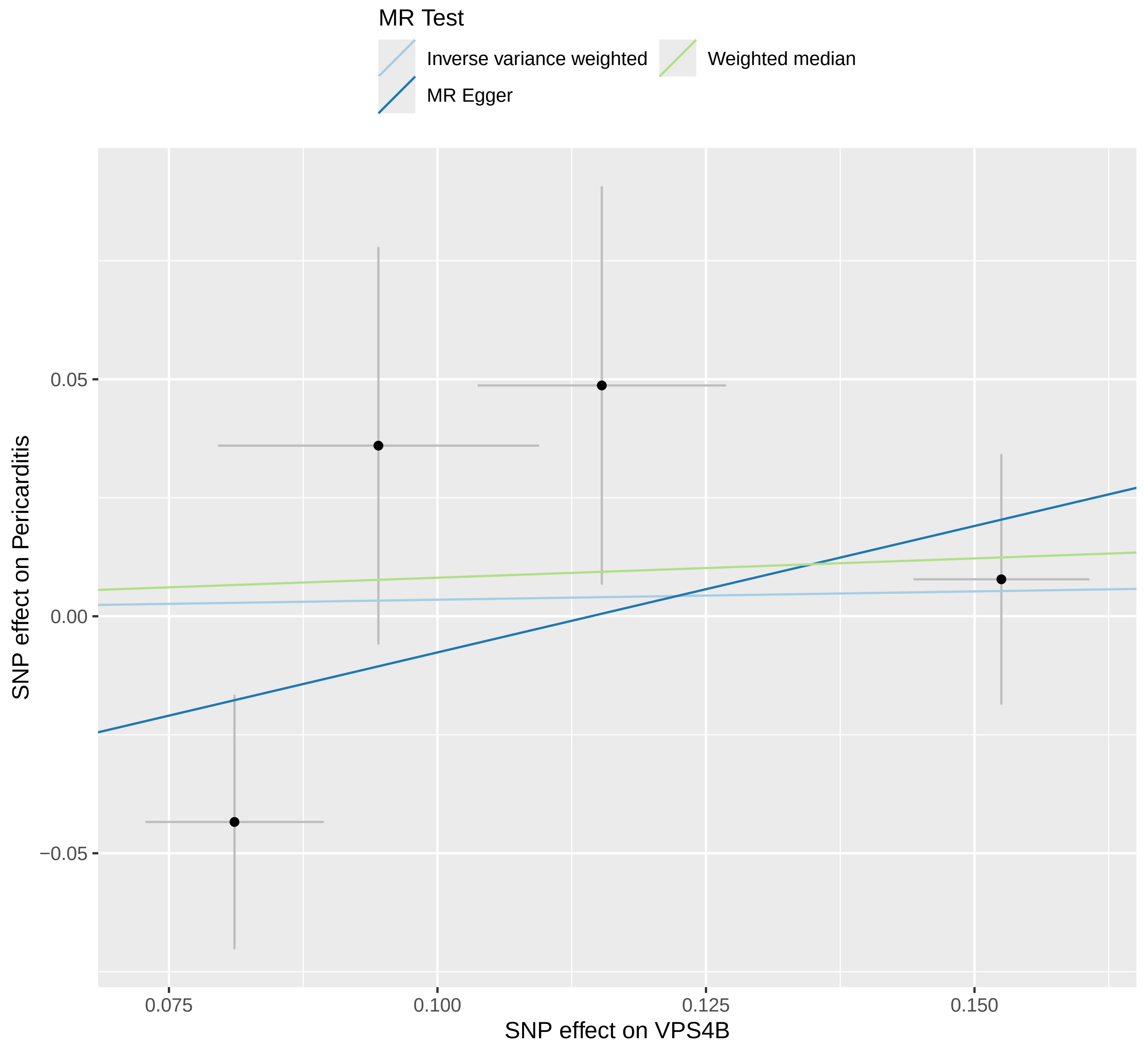
MR Test
Inverse variance weighted
Weighted median
MR Egger
0.05
0.00
−0.05
SNP effect on Pericarditis
0.075
0.100
0.125
0.150
SNP effect on VPS4B

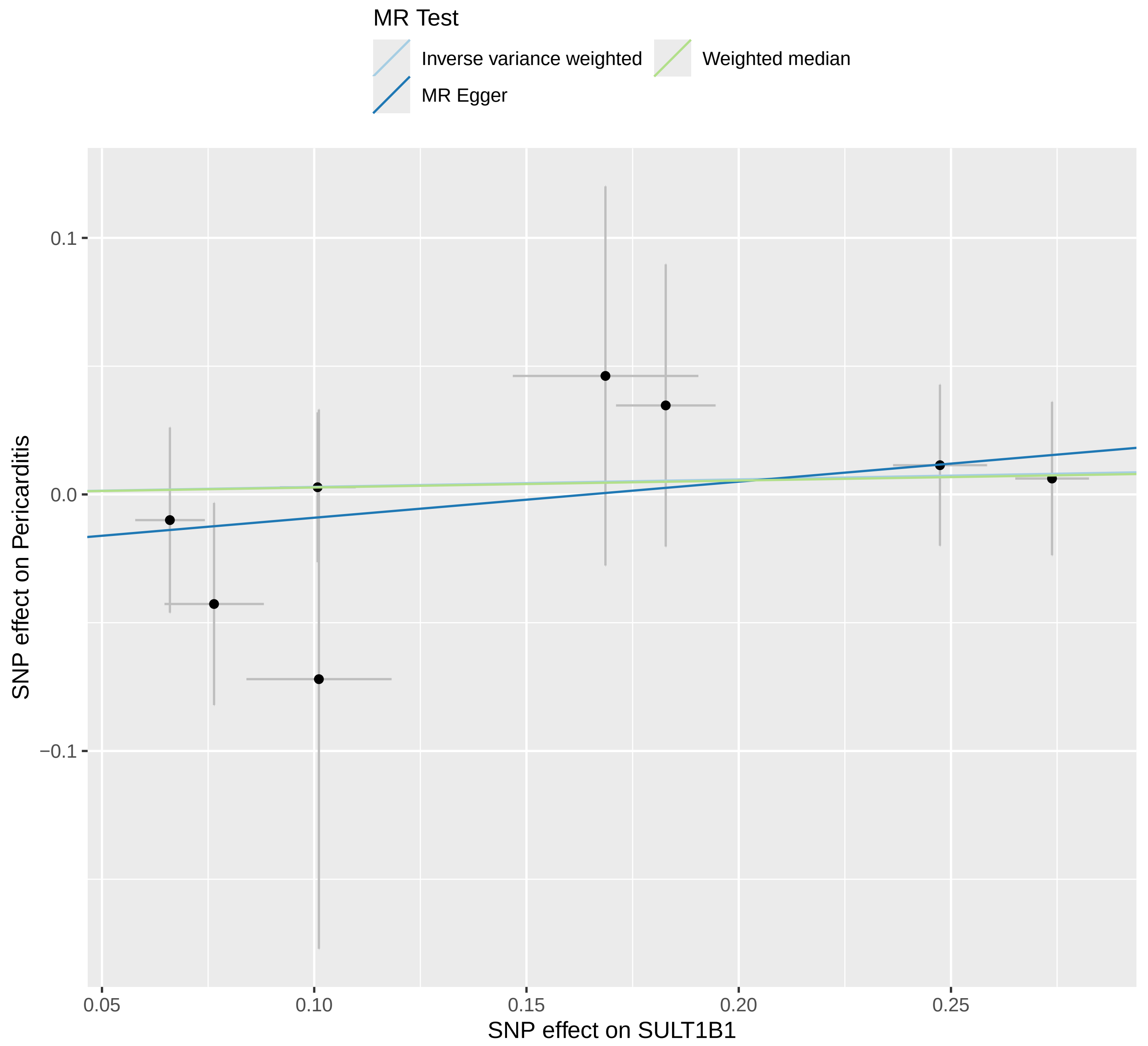

MR Test
Inverse variance weighted
Weighted median
MR Egger
0.1
0.0
−0.1
SNP effect on Pericarditis
0.05
0.10
0.15
0.20
0.25
SNP effect on SULT1B1

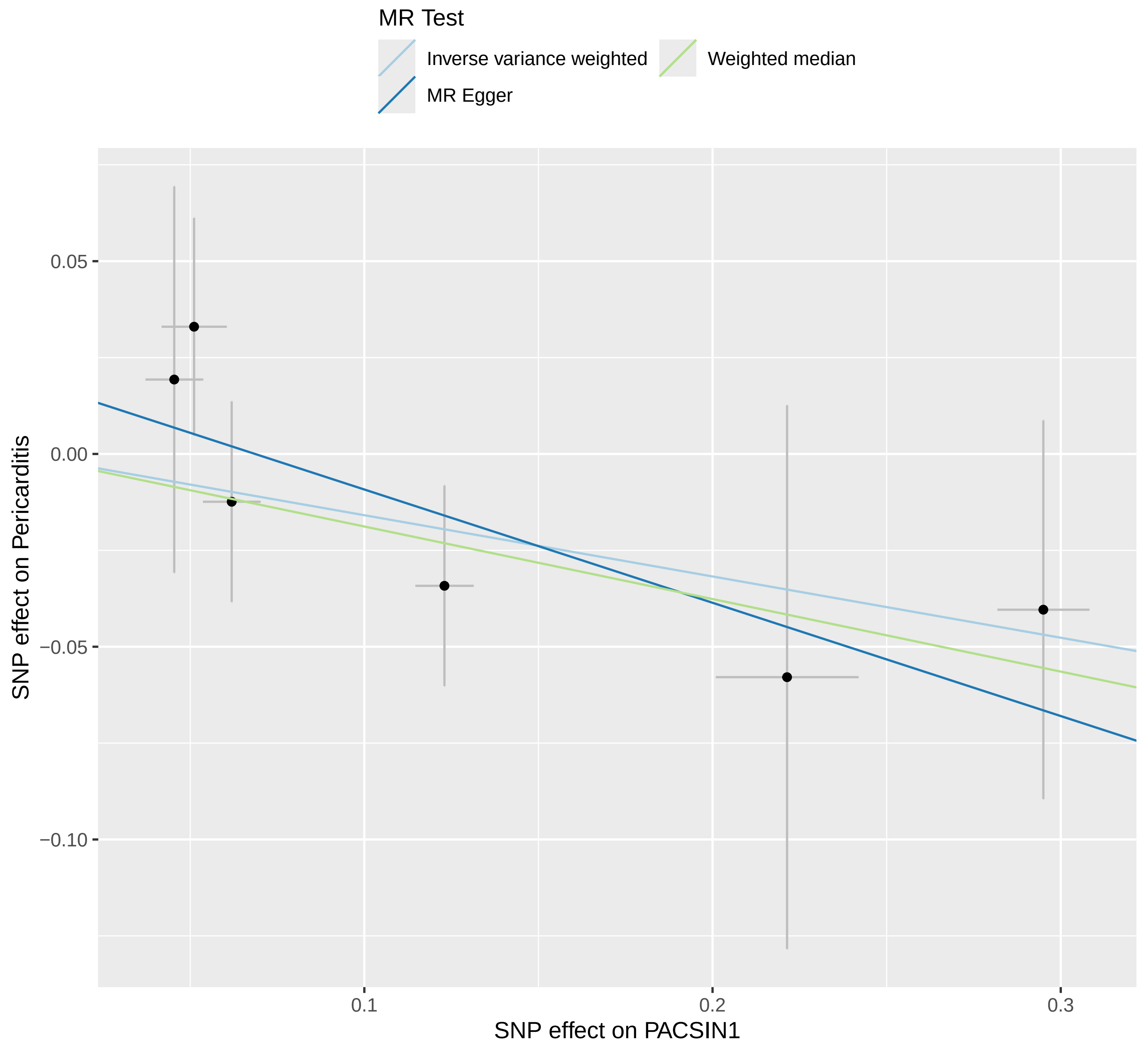

MR Test
Inverse variance weighted
MR Egger
Weighted median
0.05
0.00
−0.05
−0.10
0.1
0.2
0.3
SNP effect on Pericarditis
SNP effect on PACSIN1

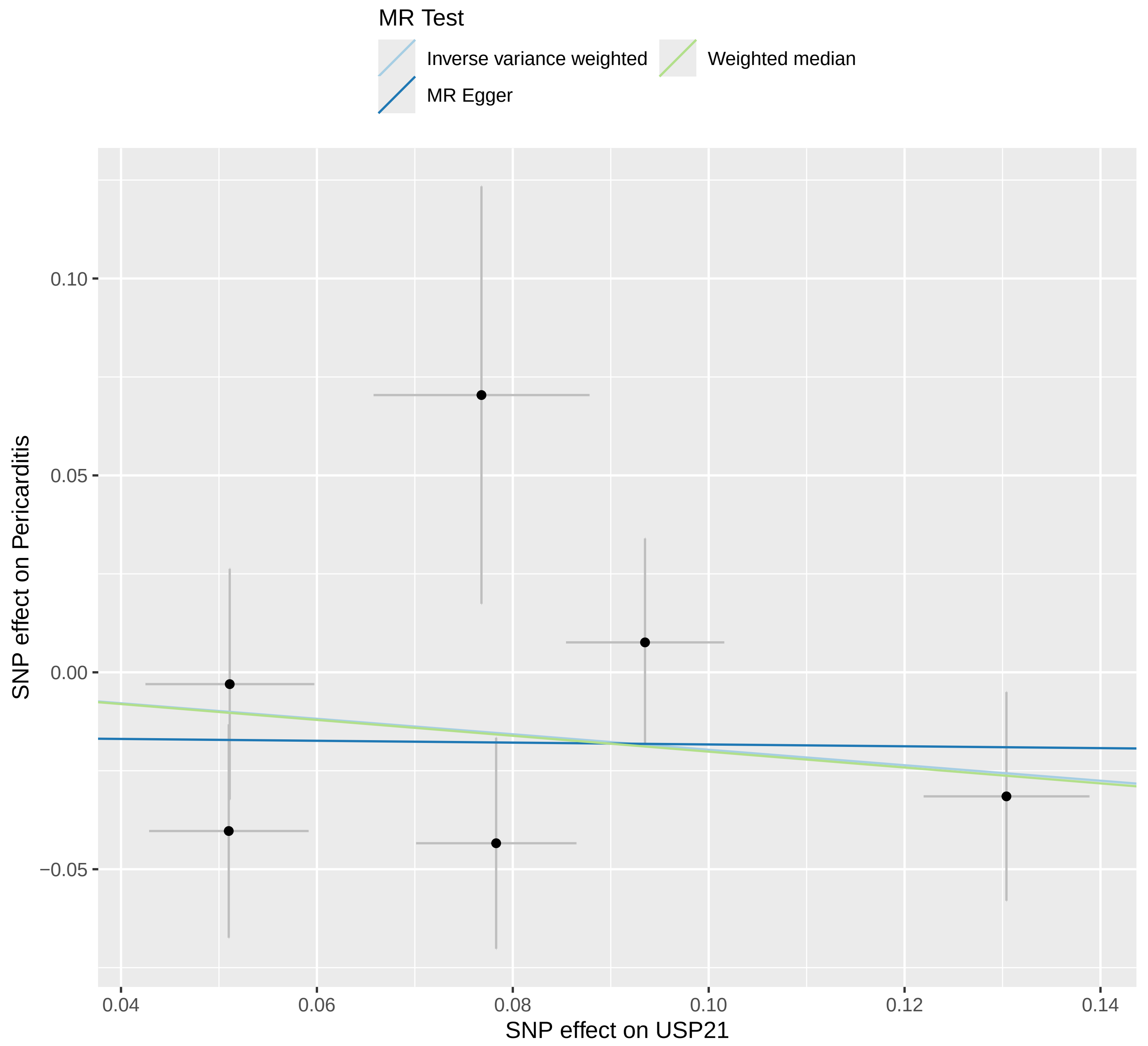
MR Test
Inverse variance weighted
Weighted median
MR Egger
0.10
0.05
0.00
−0.05
SNP effect on Pericarditis
0.04
0.06
0.08
0.10
0.12
0.14
SNP effect on USP21

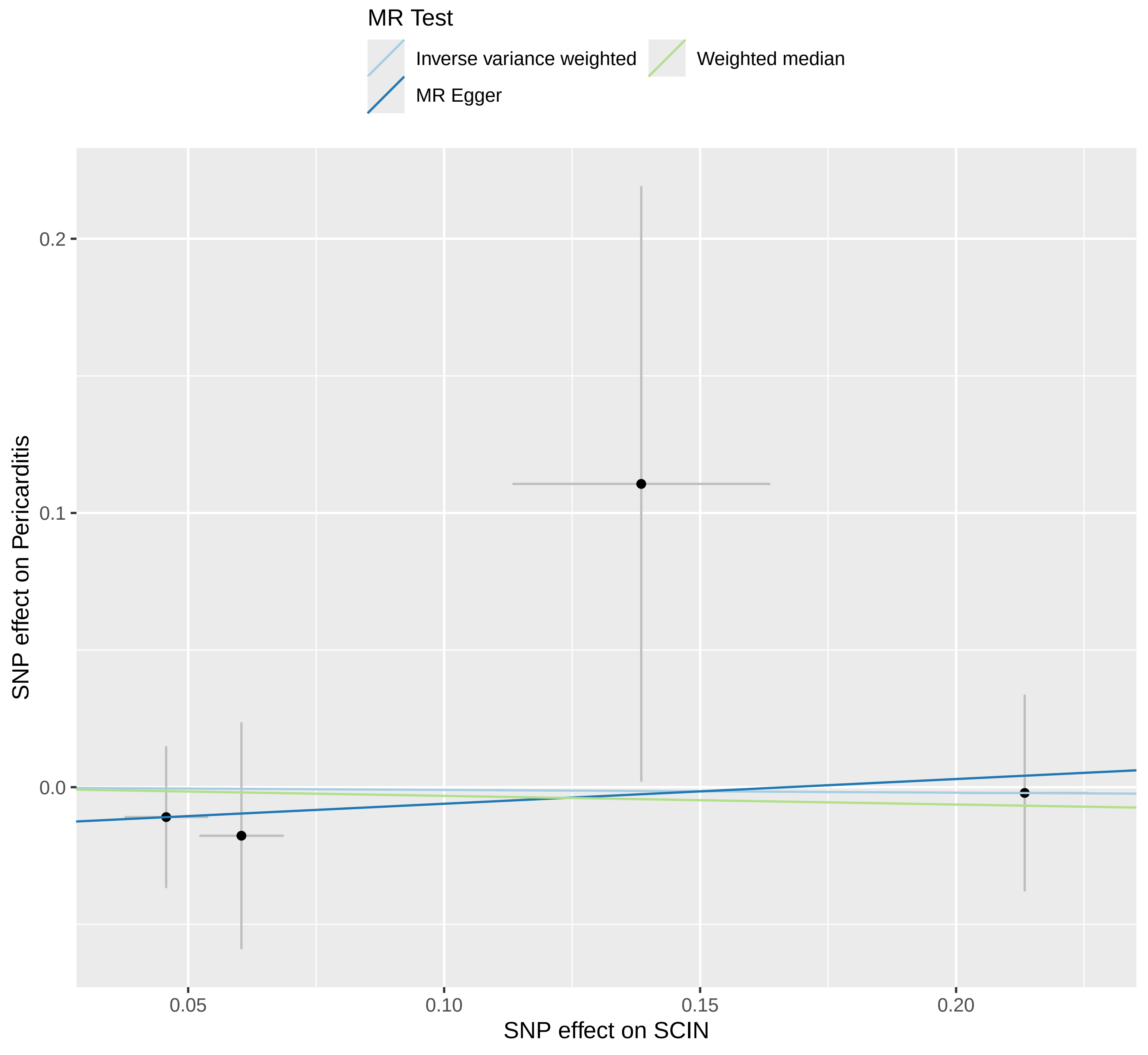

MR Test
Inverse variance weighted
Weighted median
MR Egger
SNP effect on Pericarditis
0.2
0.1
0.0
0.05
0.10
0.15
0.20
SNP effect on SCIN

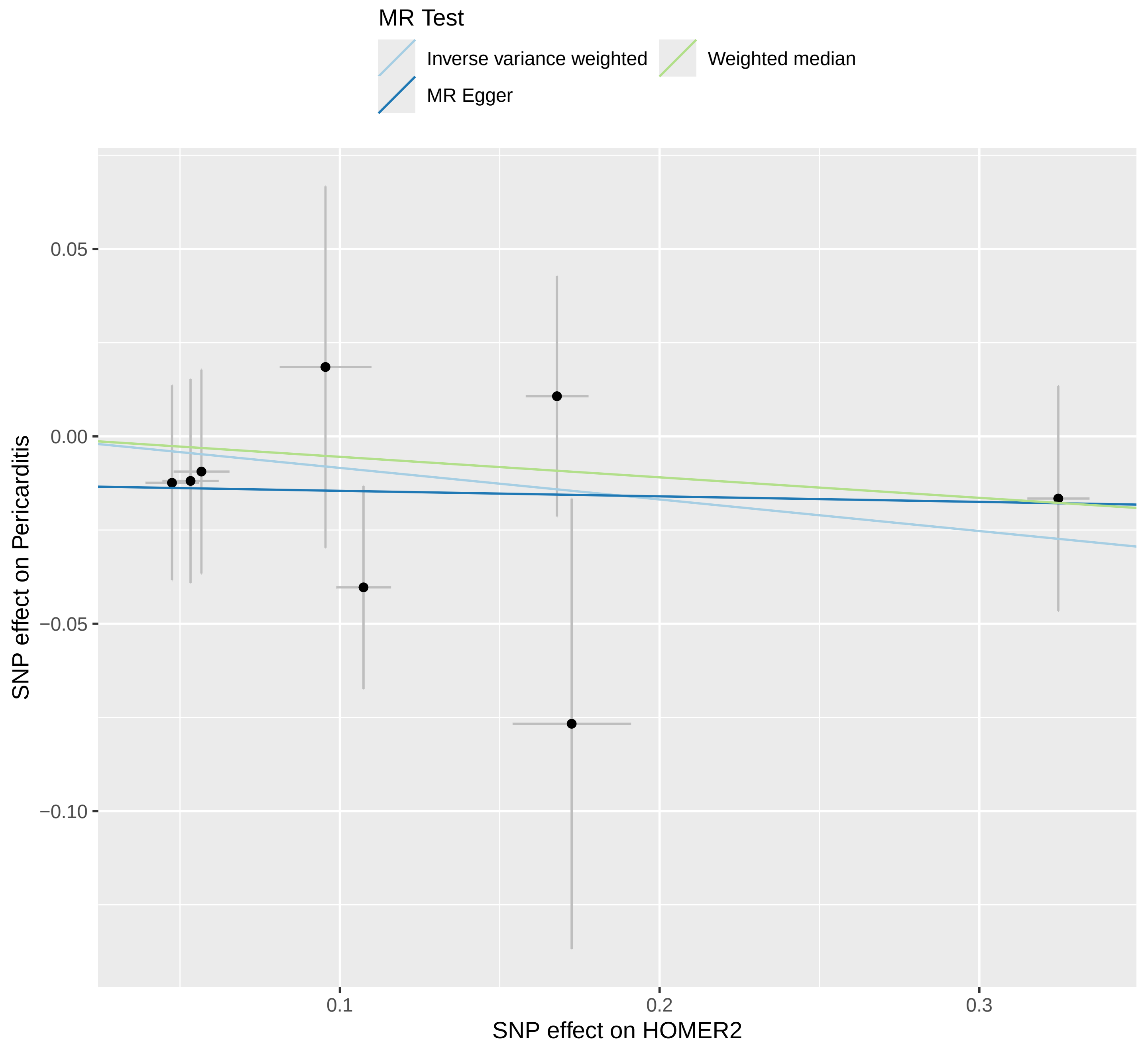

MR Test
Inverse variance weighted
MR Egger
Weighted median
0.05
0.00
−0.05
−0.10
SNP effect on Pericarditis
0.1
0.2
0.3
SNP effect on HOMER2

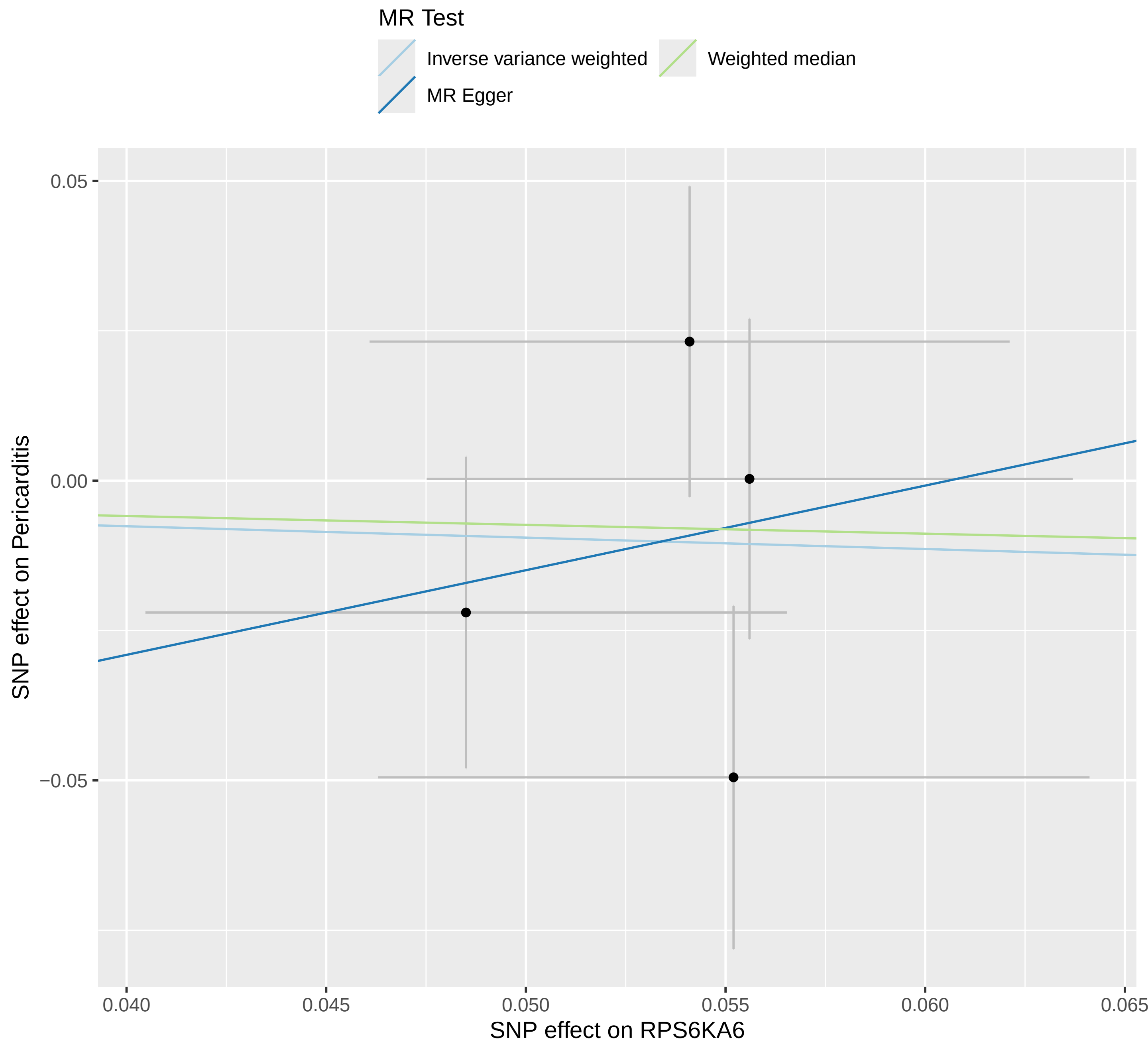
MR Test
Inverse variance weighted
MR Egger
Weighted median
0.05
0.00
−0.05
SNP effect on Pericarditis
0.040
0.045
0.050
0.055
0.060
0.065
SNP effect on RPS6KA6

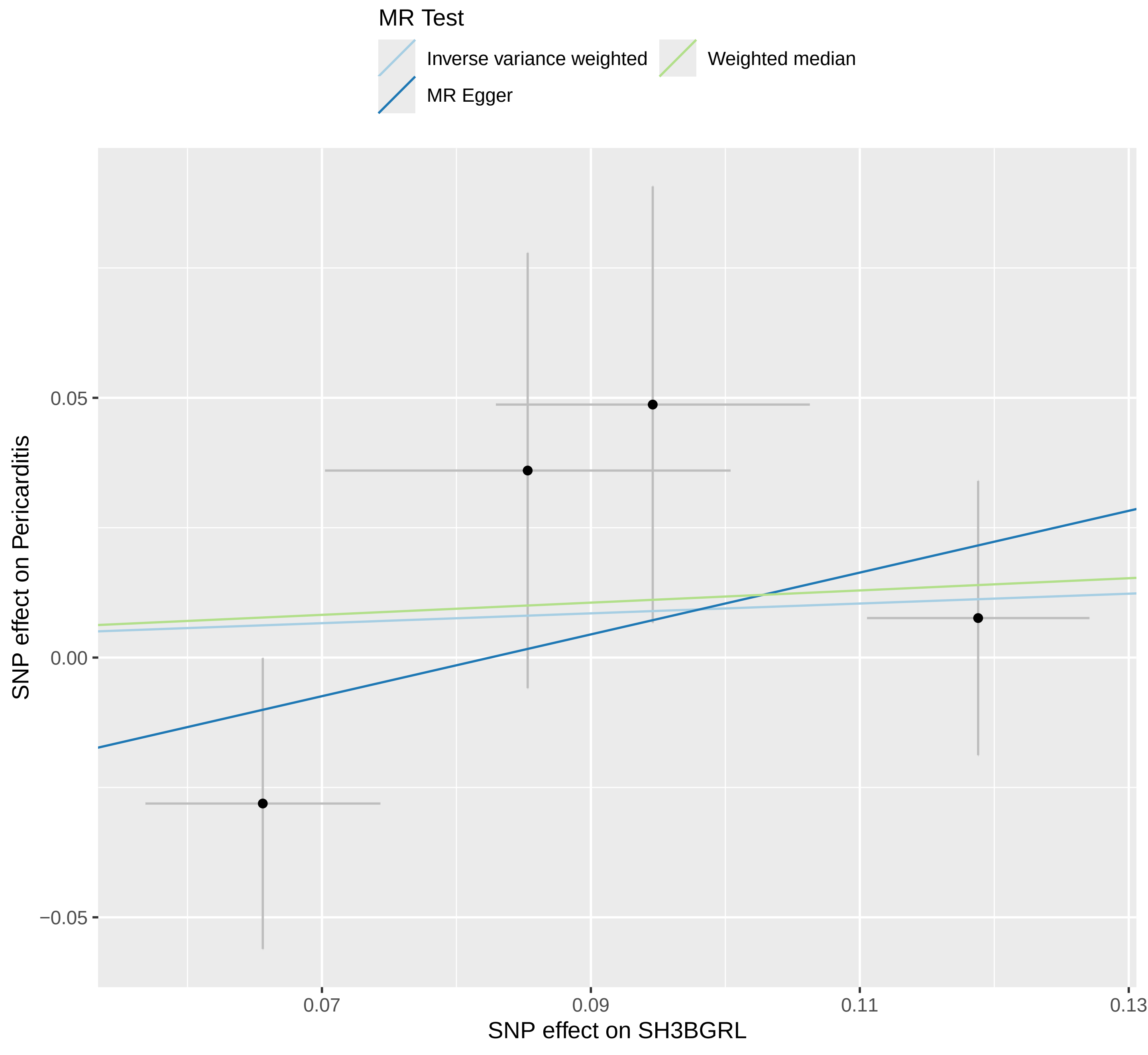
MR Test
Inverse variance weighted
Weighted median
MR Egger
SNP effect on Pericarditis
0.05
0.00
−0.05
0.07
0.09
0.11
0.13
SNP effect on SH3BGRL

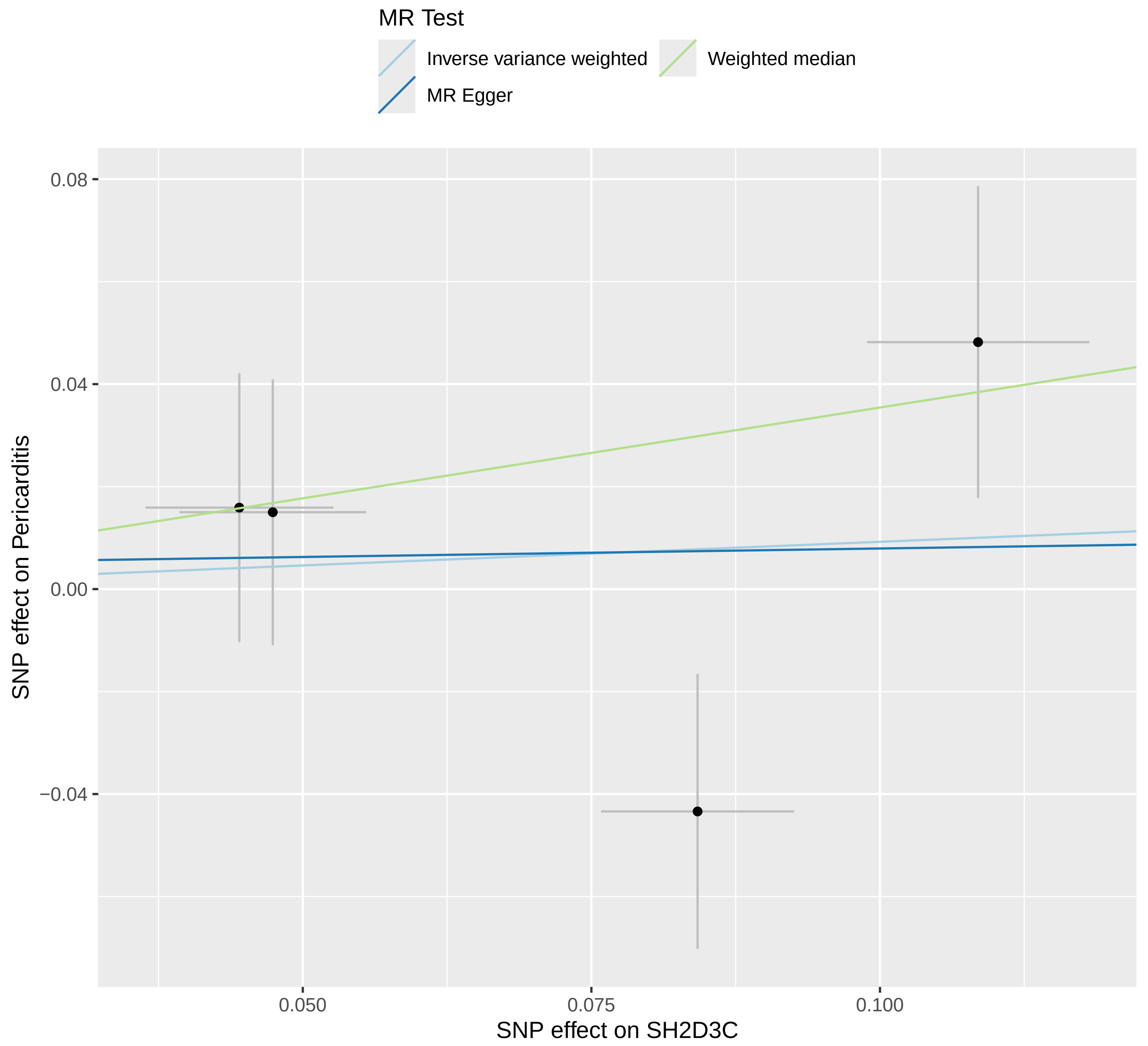

MR Test
Inverse variance weighted
MR Egger
Weighted median
0.08
0.04
0.00
−0.04
SNP effect on Pericarditis
0.050
0.075
0.100
SNP effect on SH2D3C

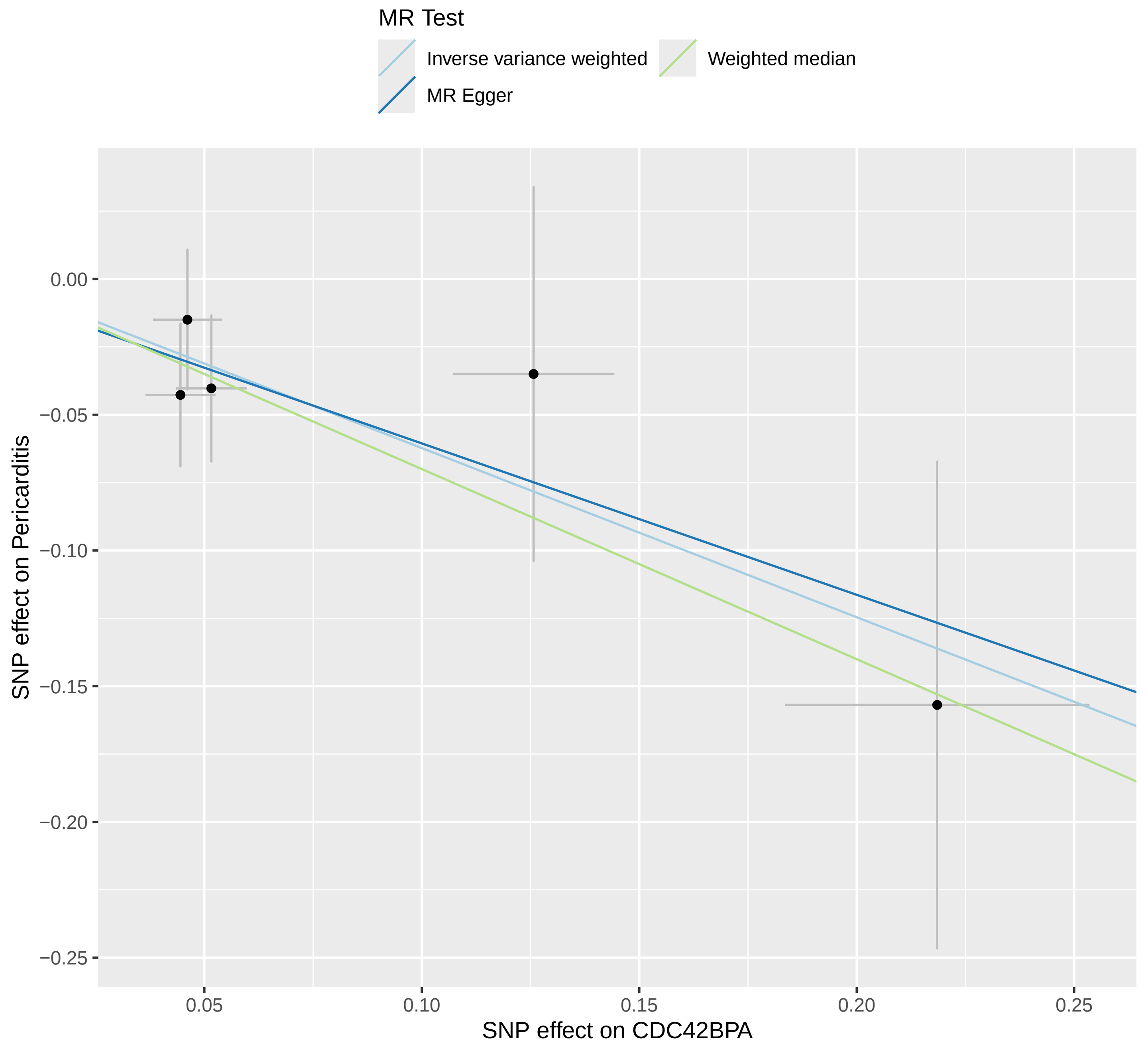
MR Test
Inverse variance weighted
MR Egger
Weighted median
SNP effect on Pericarditis
0.00
−0.05
−0.10
−0.15
−0.20
−0.25
0.05
0.10
0.15
0.20
0.25
SNP effect on CDC42BPA

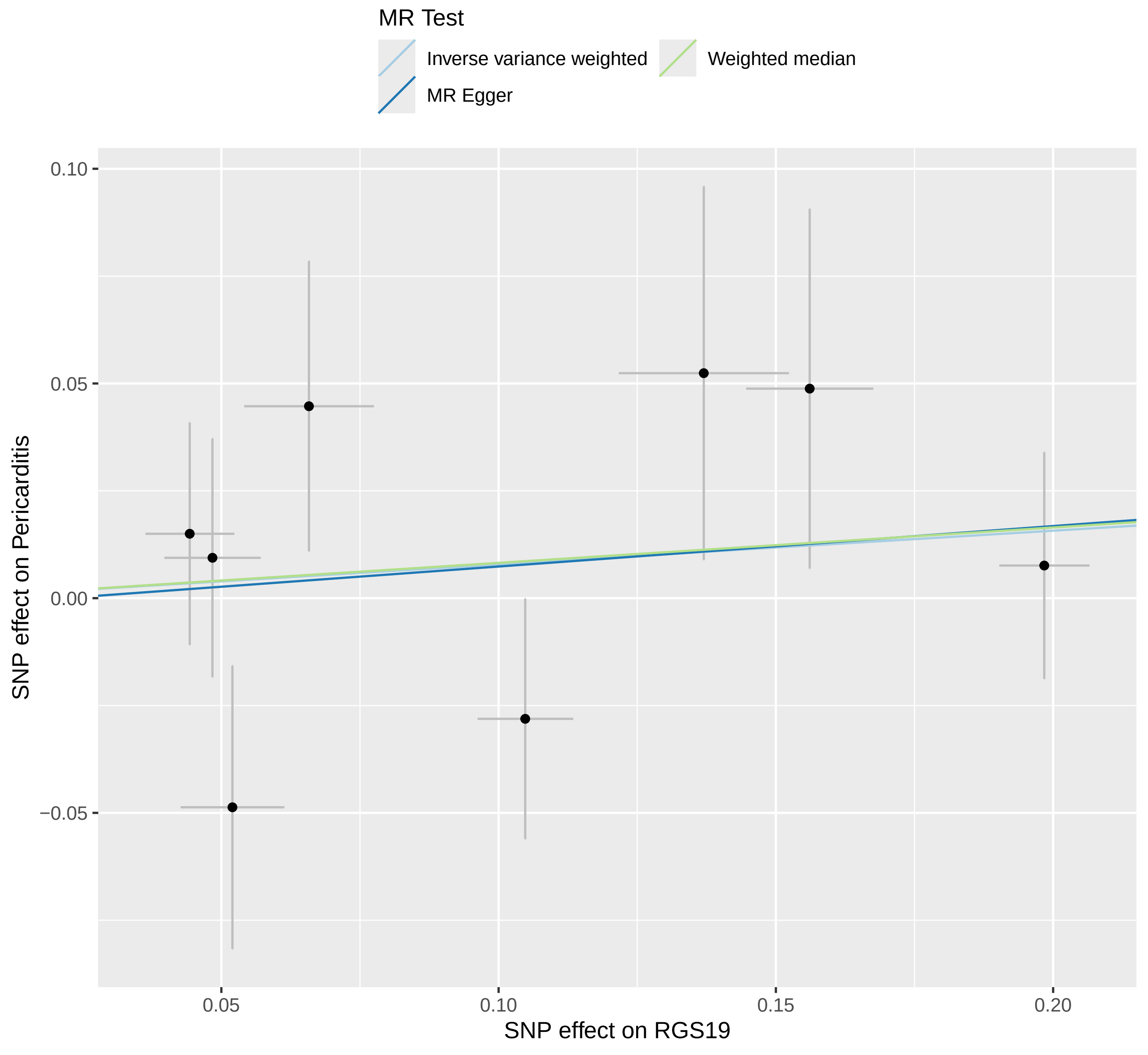
MR Test
Inverse variance weighted
Weighted median
MR Egger
0.10
0.05
0.00
−0.05
SNP effect on Pericarditis
0.05
0.10
0.15
0.20
SNP effect on RGS19

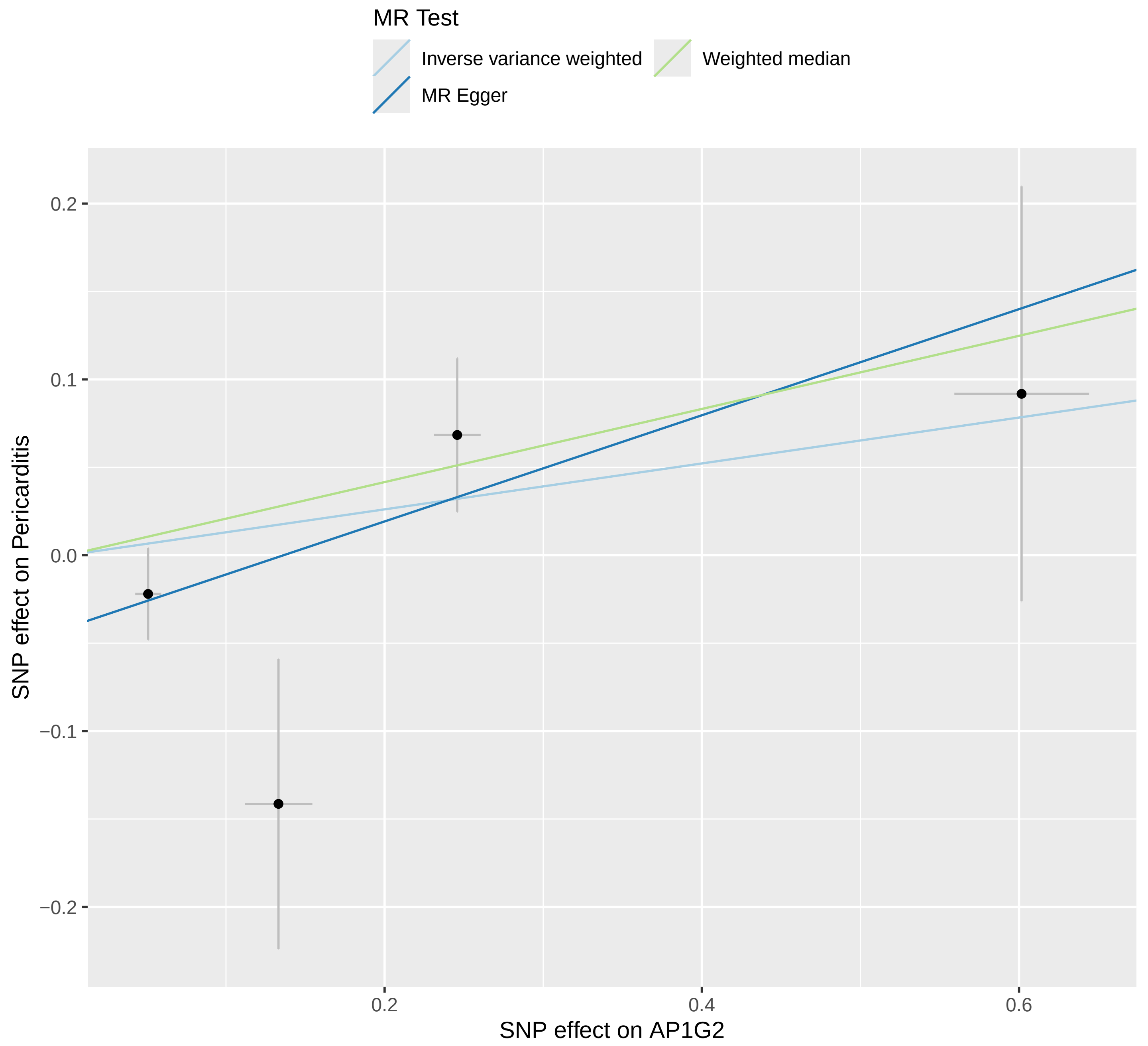

MR Test
Inverse variance weighted
Weighted median
MR Egger
0.2
0.1
0.0
−0.1
−0.2
SNP effect on Pericarditis
0.2
0.4
0.6
SNP effect on AP1G2

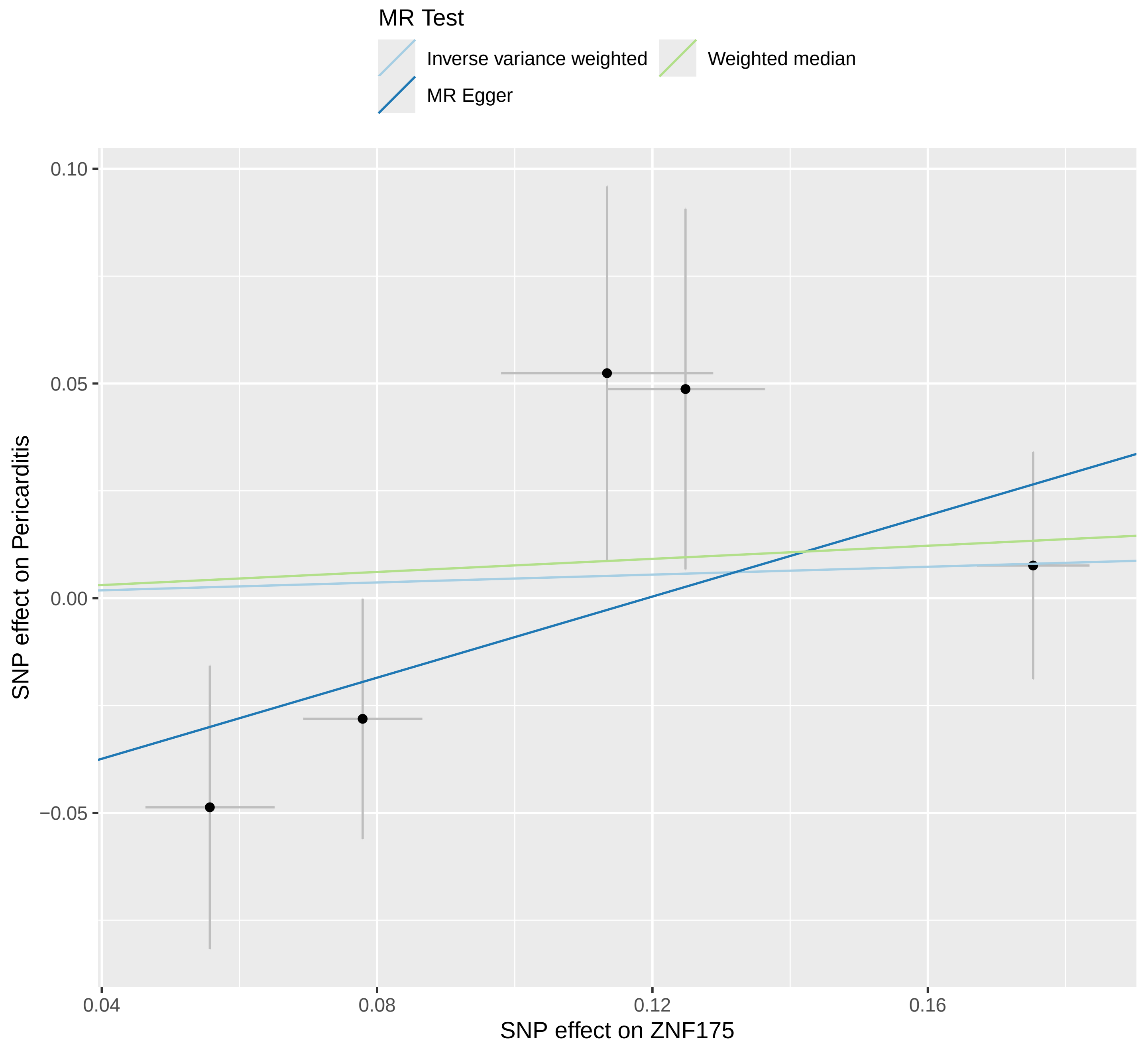

MR Test
Inverse variance weighted
Weighted median
MR Egger
0.10
0.05
0.00
−0.05
SNP effect on Pericarditis
0.04
0.08
0.12
0.16
SNP effect on ZNF175

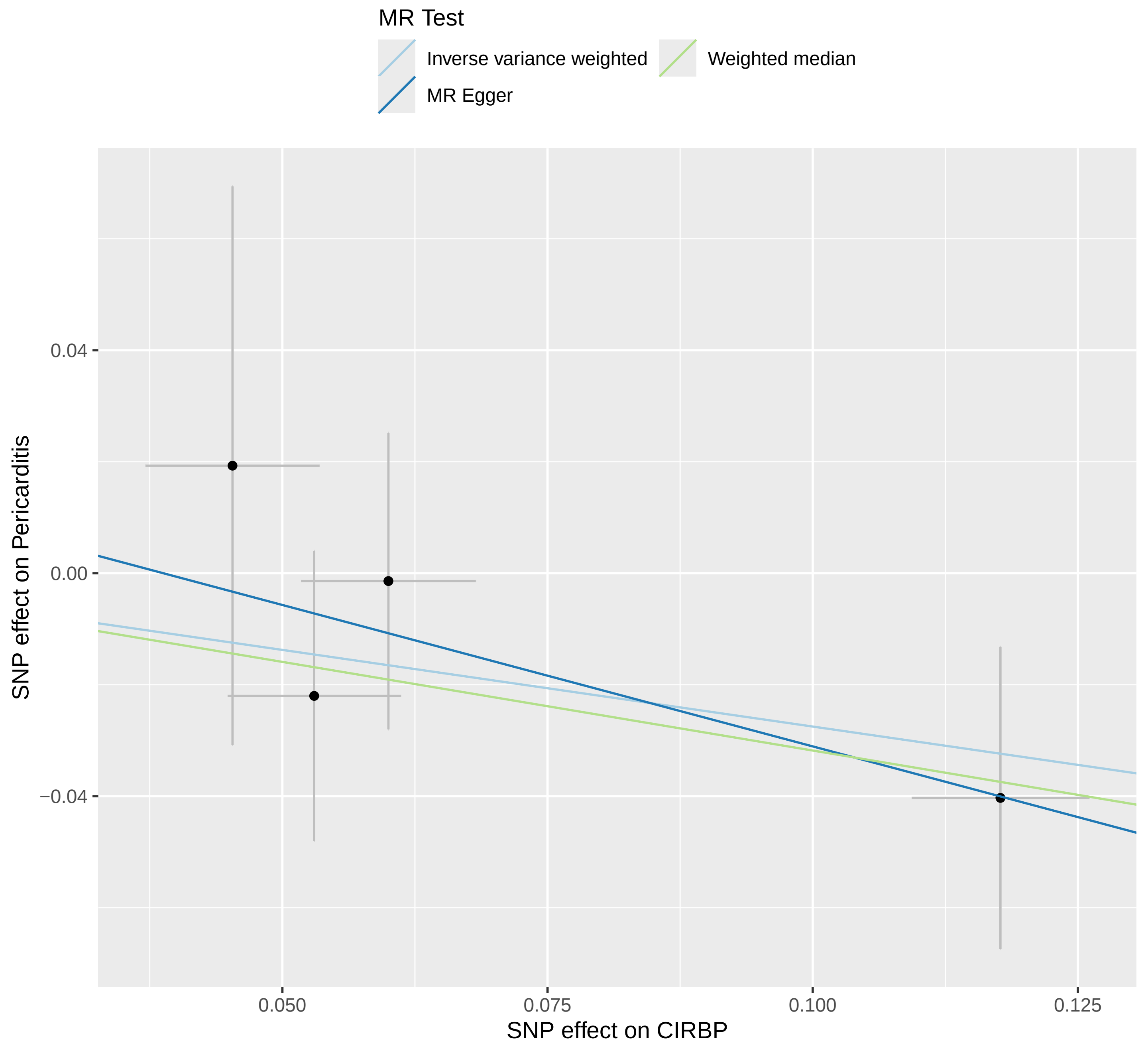

MR Test
Inverse variance weighted
MR Egger
Weighted median
0.04
0.00
−0.04
0.050
0.075
0.100
0.125
SNP effect on Pericarditis
SNP effect on CIRBP

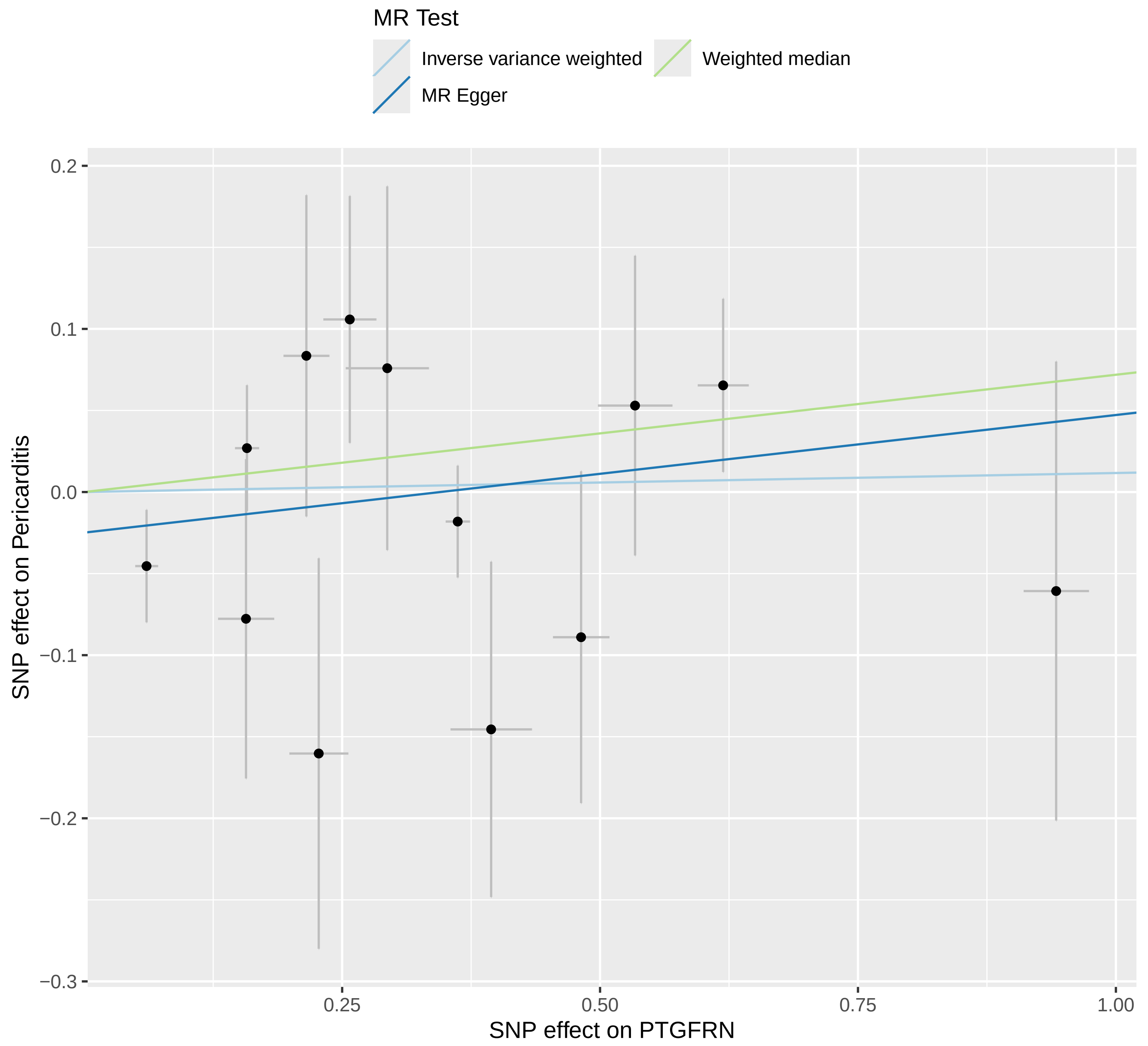
MR Test
Inverse variance weighted
Weighted median
MR Egger
0.2
0.1
0.0
−0.1
−0.2
−0.3
SNP effect on Pericarditis
0.25
0.50
0.75
1.00
SNP effect on PTGFRN

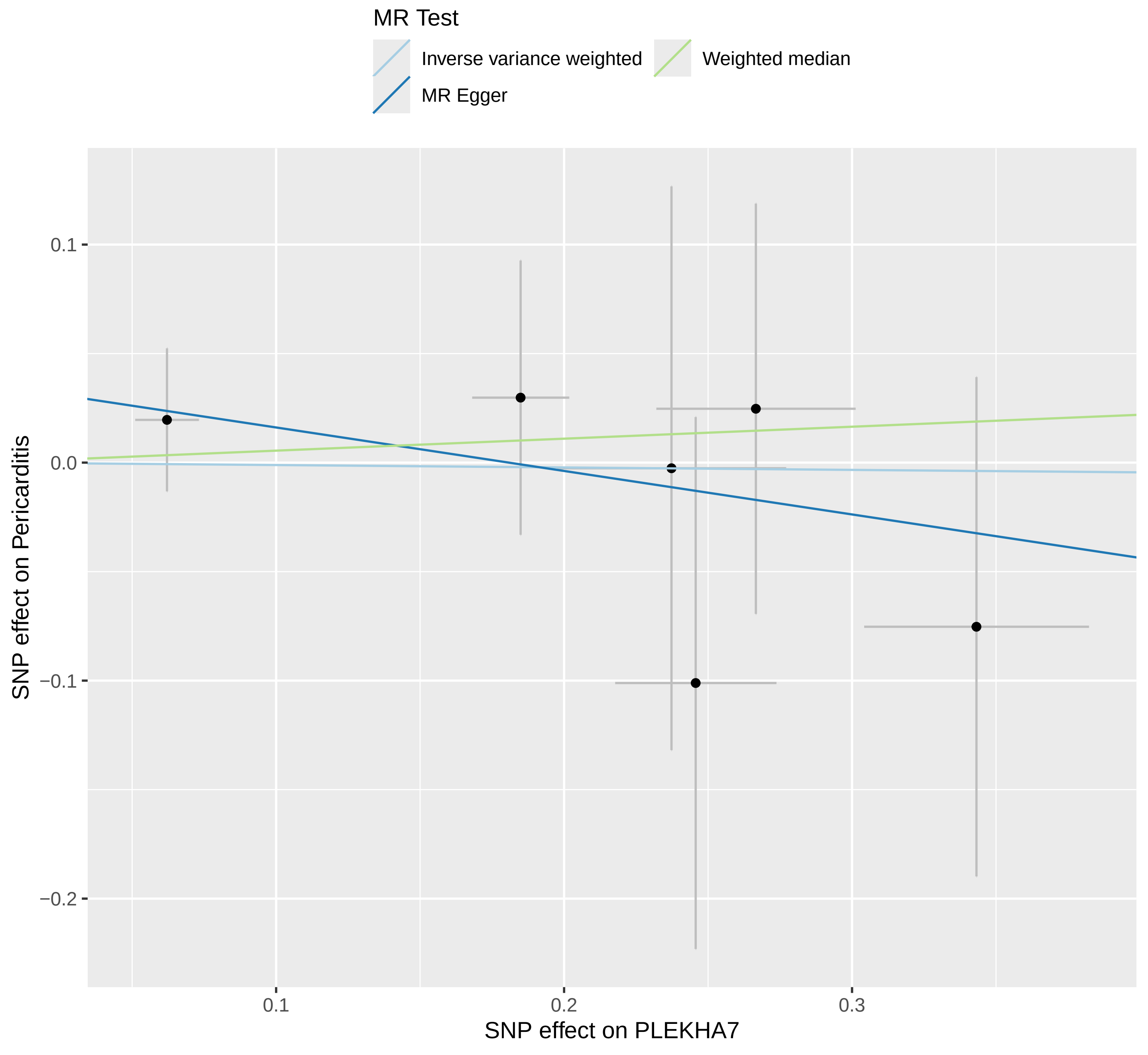

MR Test
Inverse variance weighted
Weighted median
MR Egger
0.1
0.0
−0.1
−0.2
SNP effect on Pericarditis
0.1
0.2
0.3
SNP effect on PLEKHA7

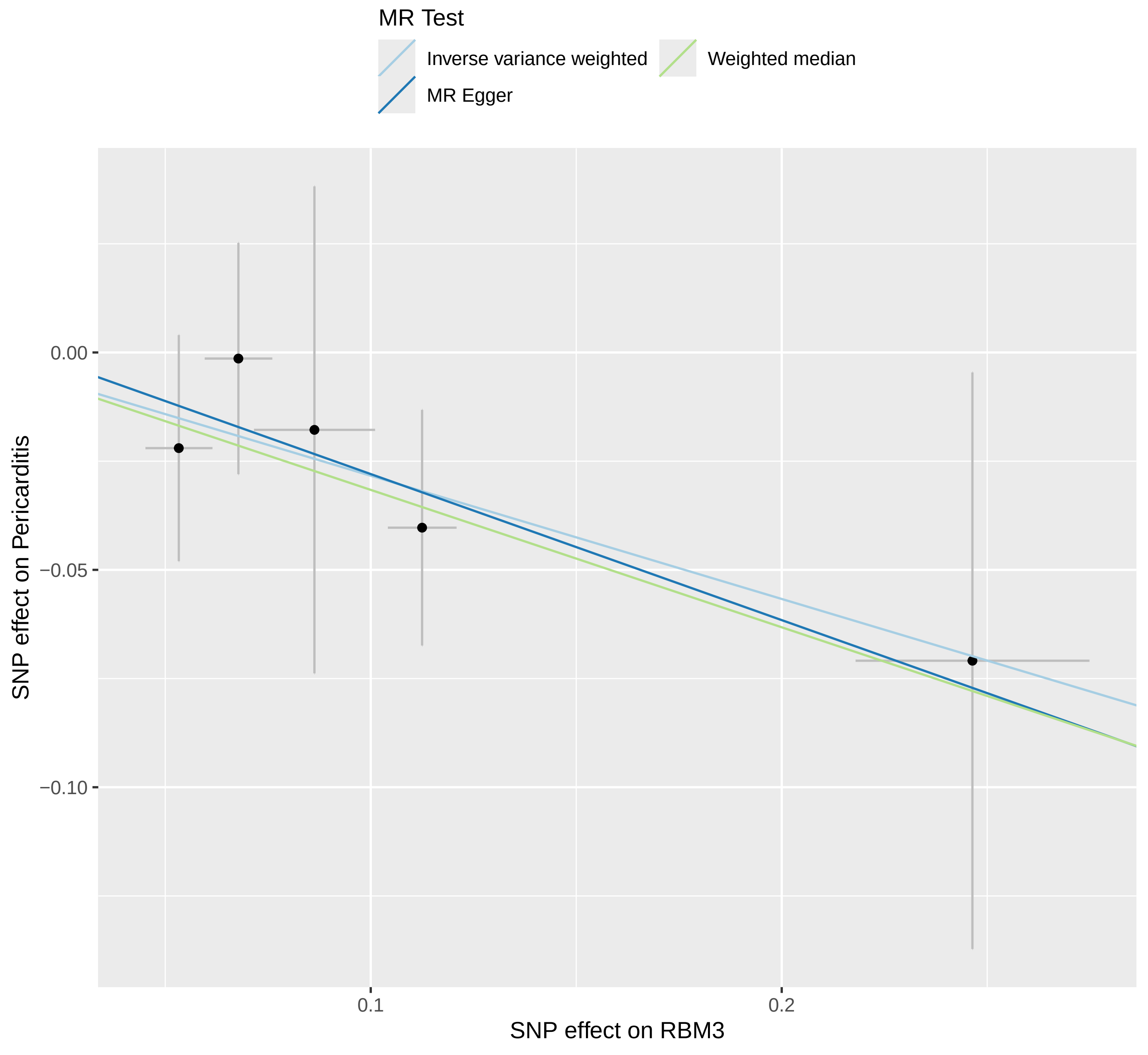

MR Test
Inverse variance weighted
MR Egger
Weighted median
0.00
−0.05
−0.10
0.1
0.2
SNP effect on Pericarditis
SNP effect on RBM3

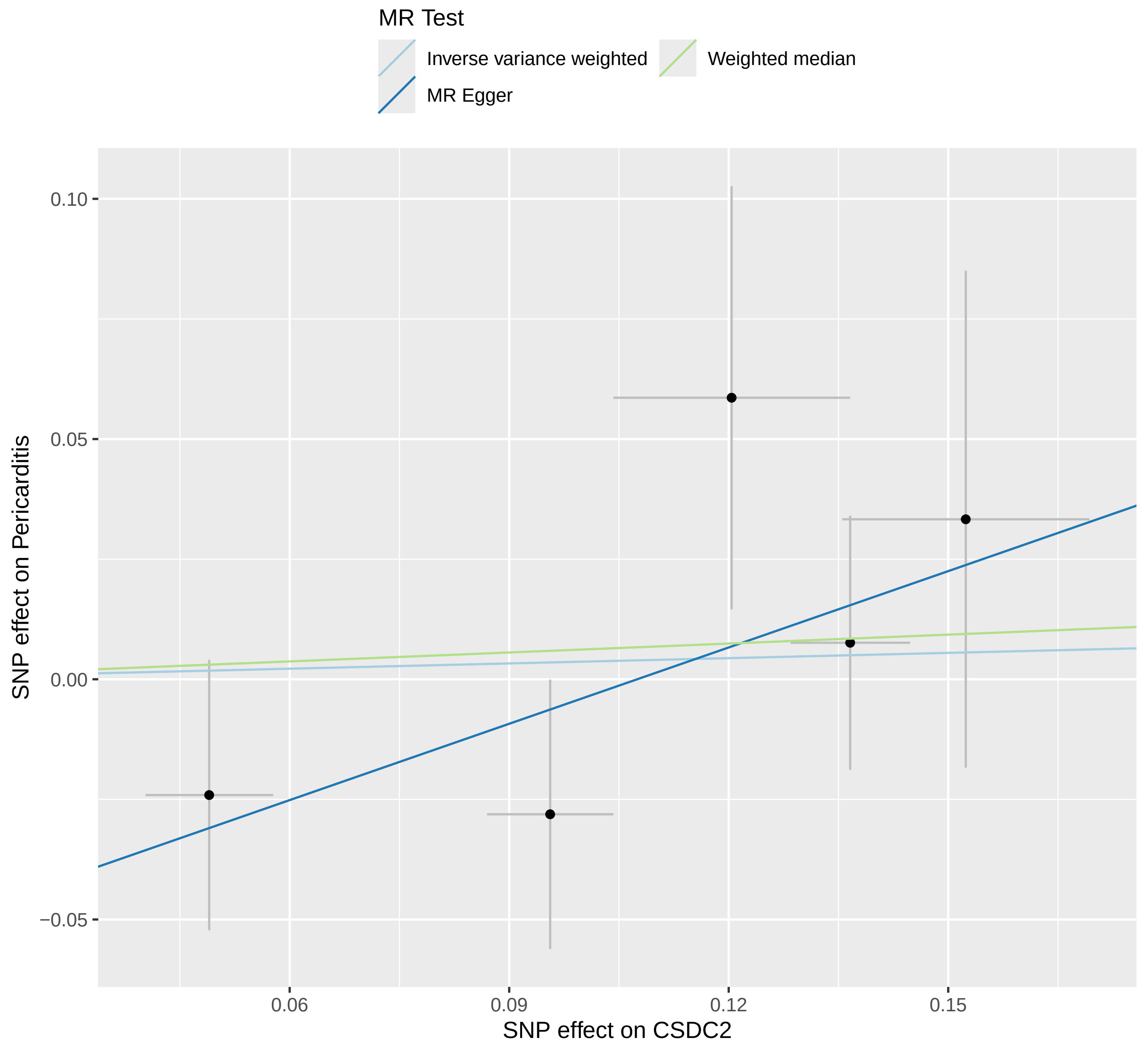
MR Test
Inverse variance weighted
MR Egger
Weighted median
0.10
0.05
0.00
−0.05
SNP effect on Pericarditis
0.06
0.09
0.12
0.15
SNP effect on CSDC2

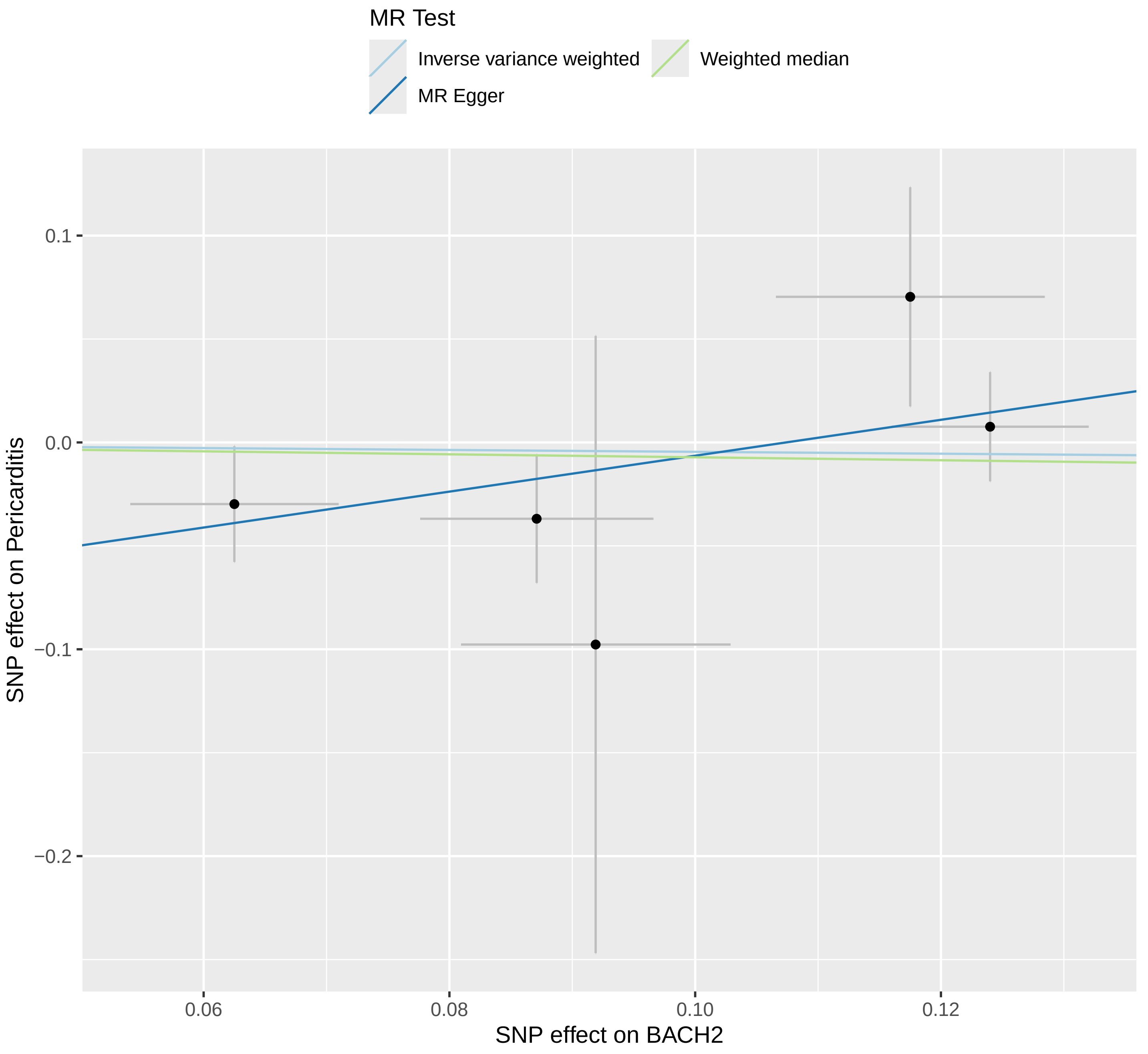
MR Test
Inverse variance weighted
MR Egger
Weighted median
0.1
0.0
−0.1
−0.2
SNP effect on Pericarditis
0.06
0.08
0.10
0.12
SNP effect on BACH2

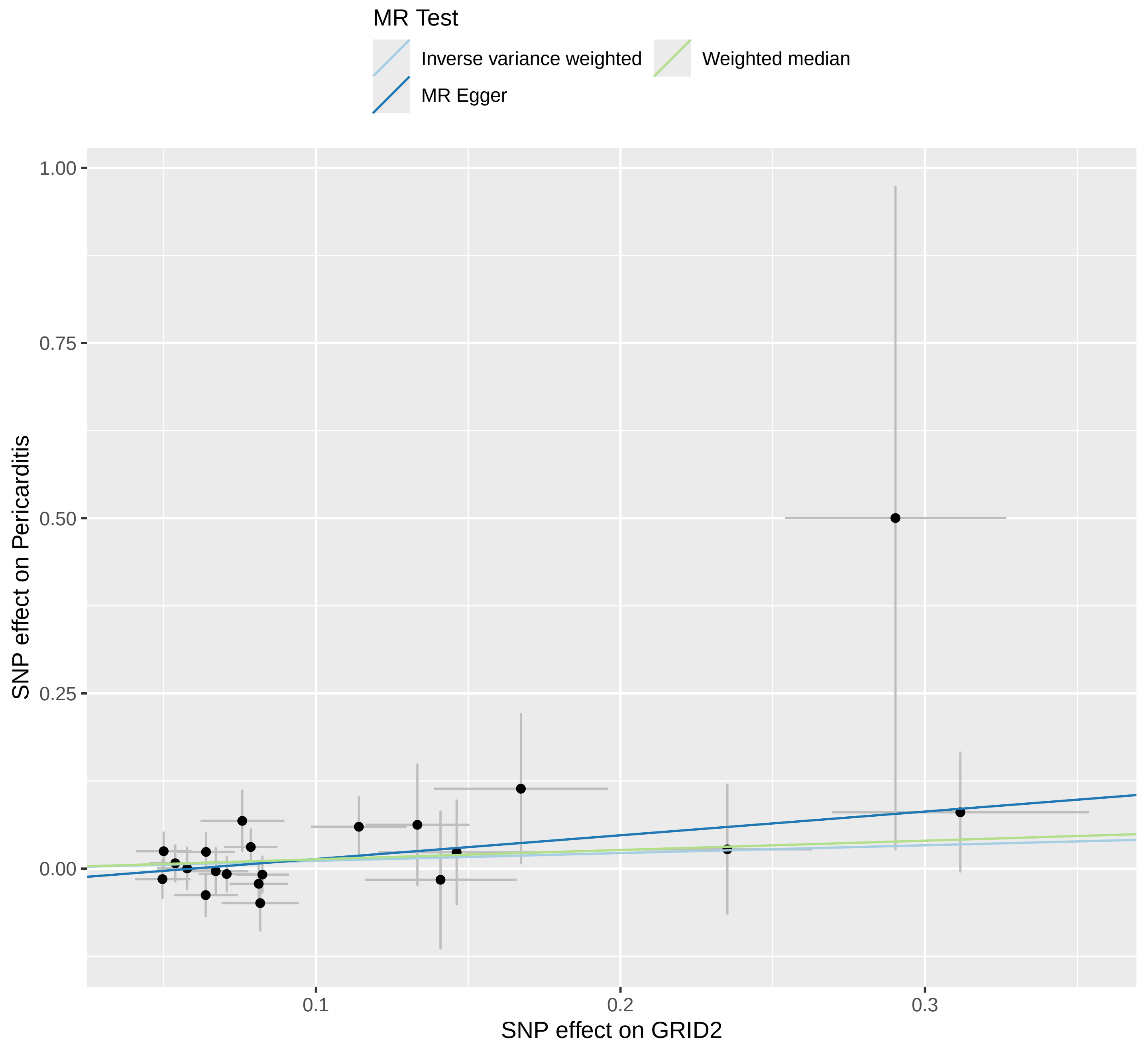
MR Test
Inverse variance weighted
Weighted median
MR Egger
1.00
0.75
0.50
0.25
0.00
0.1
0.2
0.3
SNP effect on Pericarditis
SNP effect on GRID2

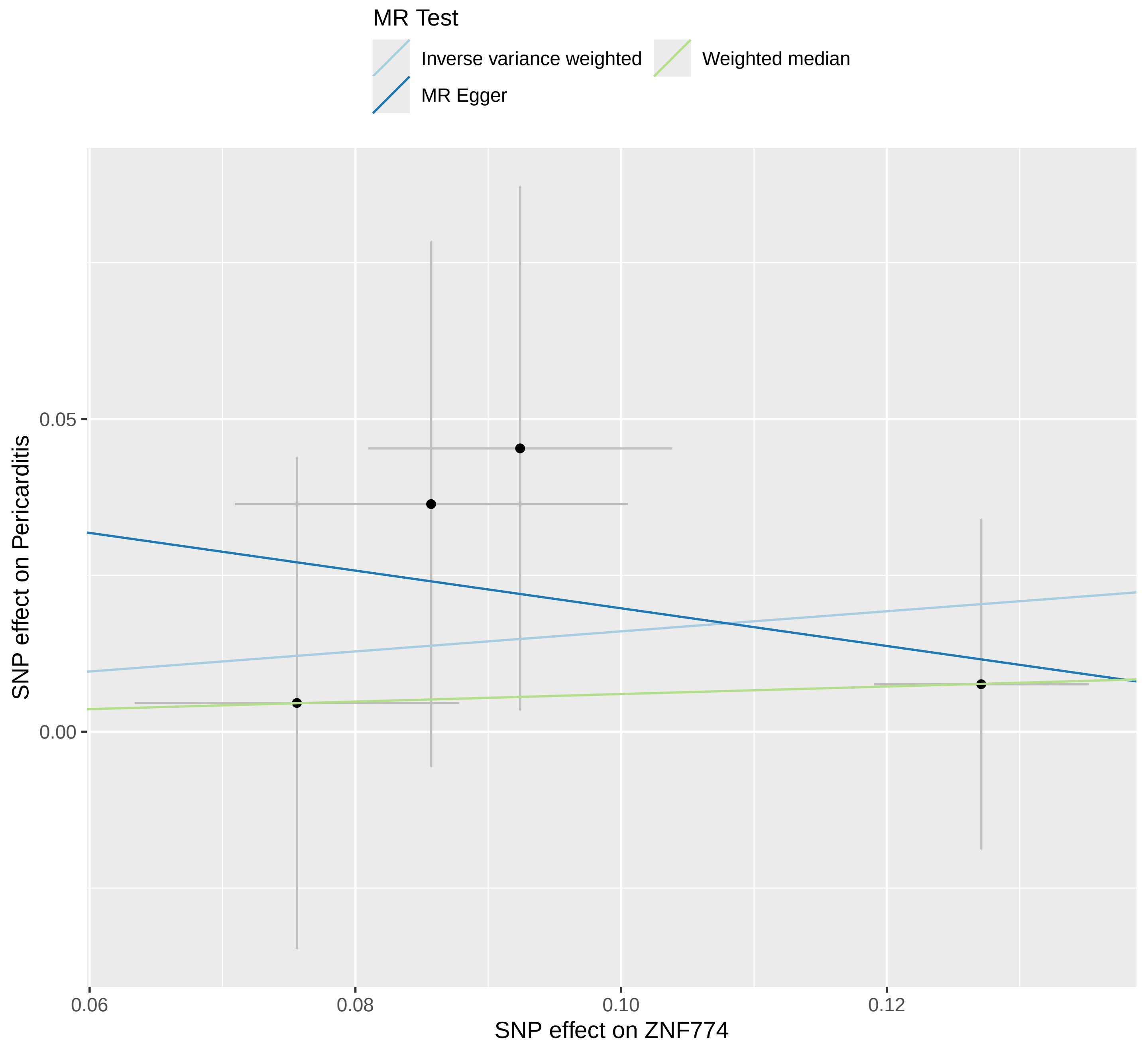
MR Test
Inverse variance weighted
Weighted median
MR Egger
0.05
0.00
SNP effect on Pericarditis
0.06
0.08
0.10
0.12
SNP effect on ZNF774

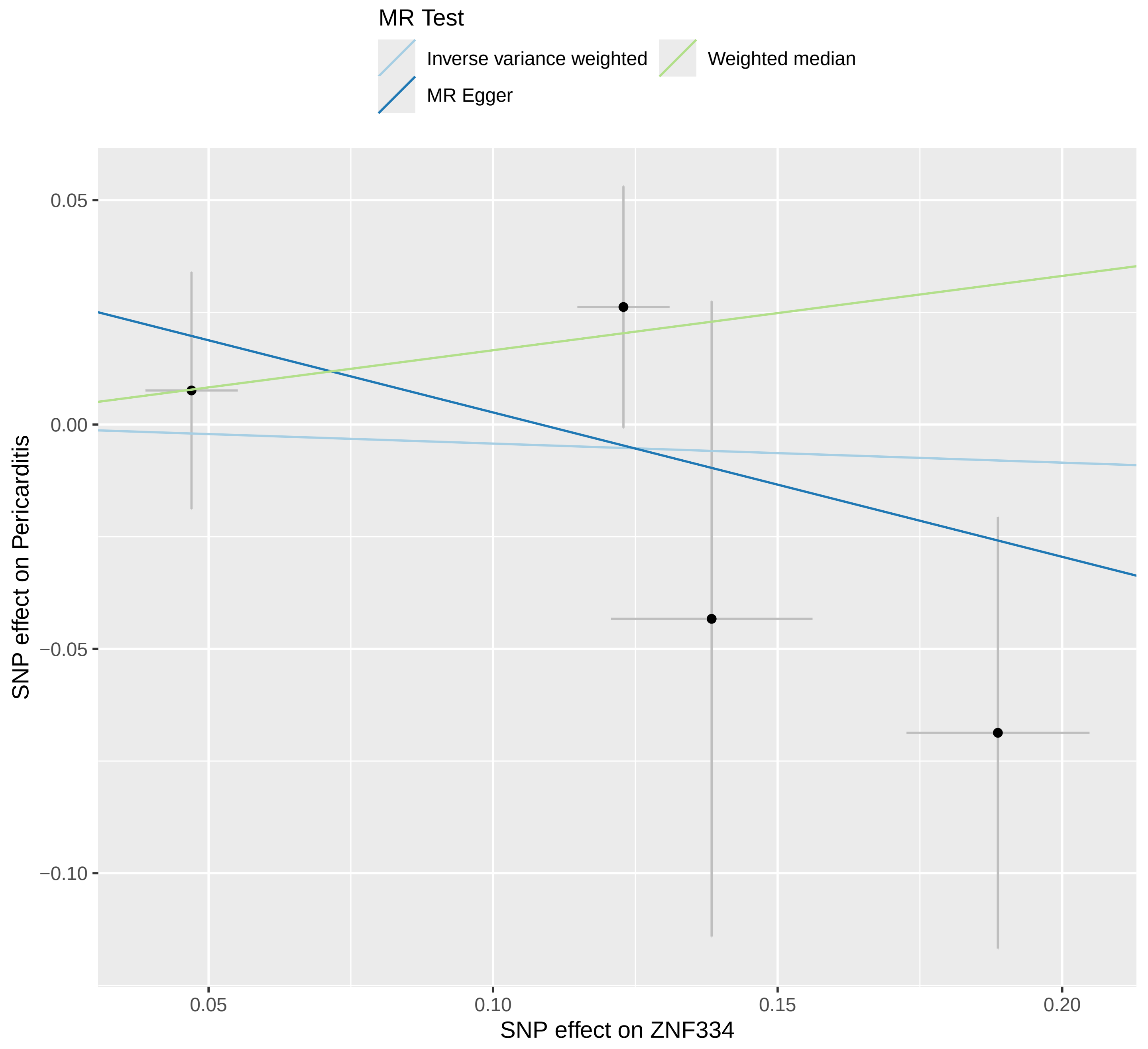
MR Test
Inverse variance weighted
Weighted median
MR Egger
0.05
0.00
−0.05
−0.10
SNP effect on Pericarditis
0.05
0.10
0.15
0.20
SNP effect on ZNF334

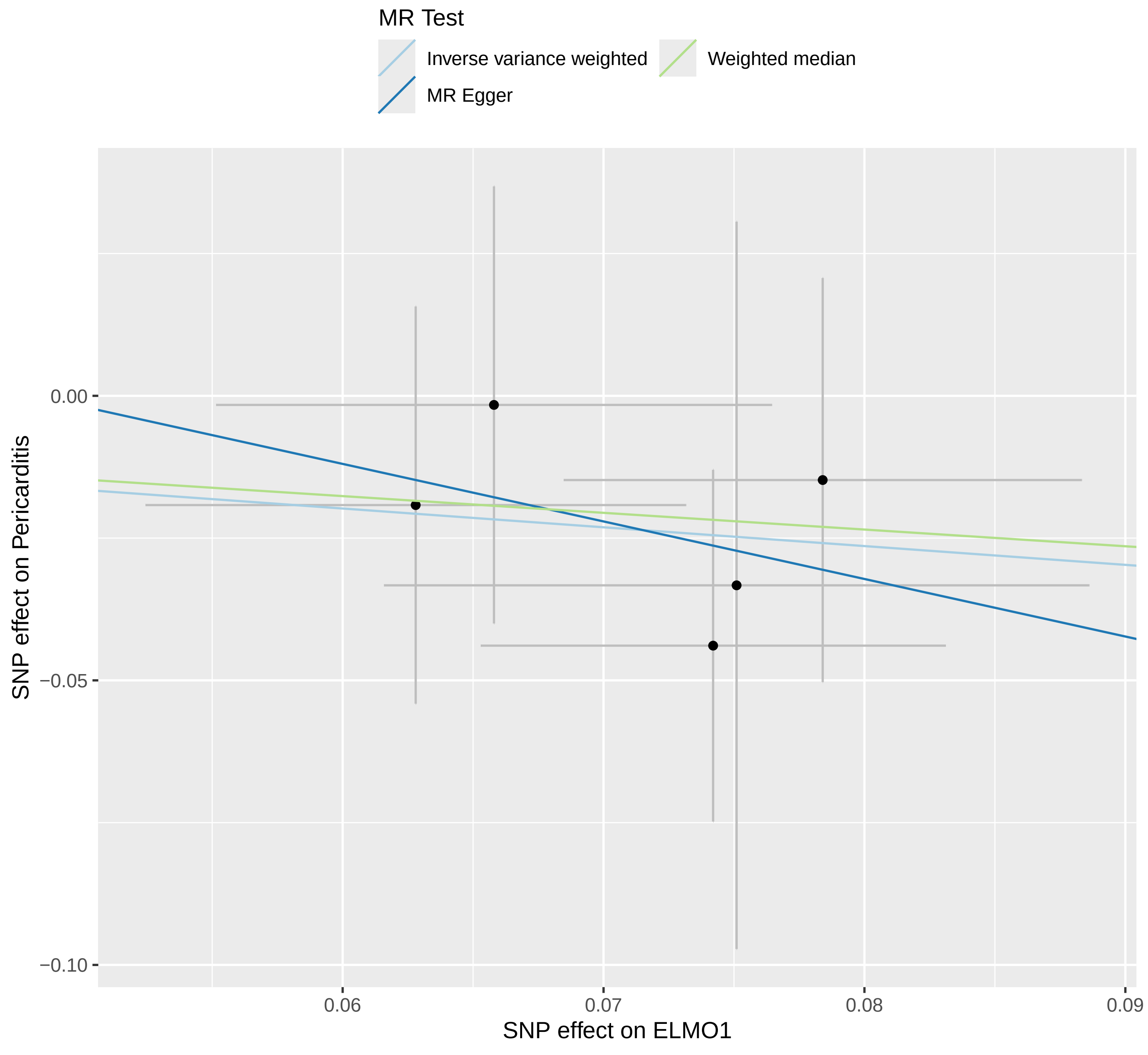
MR Test
Inverse variance weighted
MR Egger
Weighted median
0.00
−0.05
−0.10
0.06
0.07
0.08
0.09
SNP effect on Pericarditis
SNP effect on ELMO1

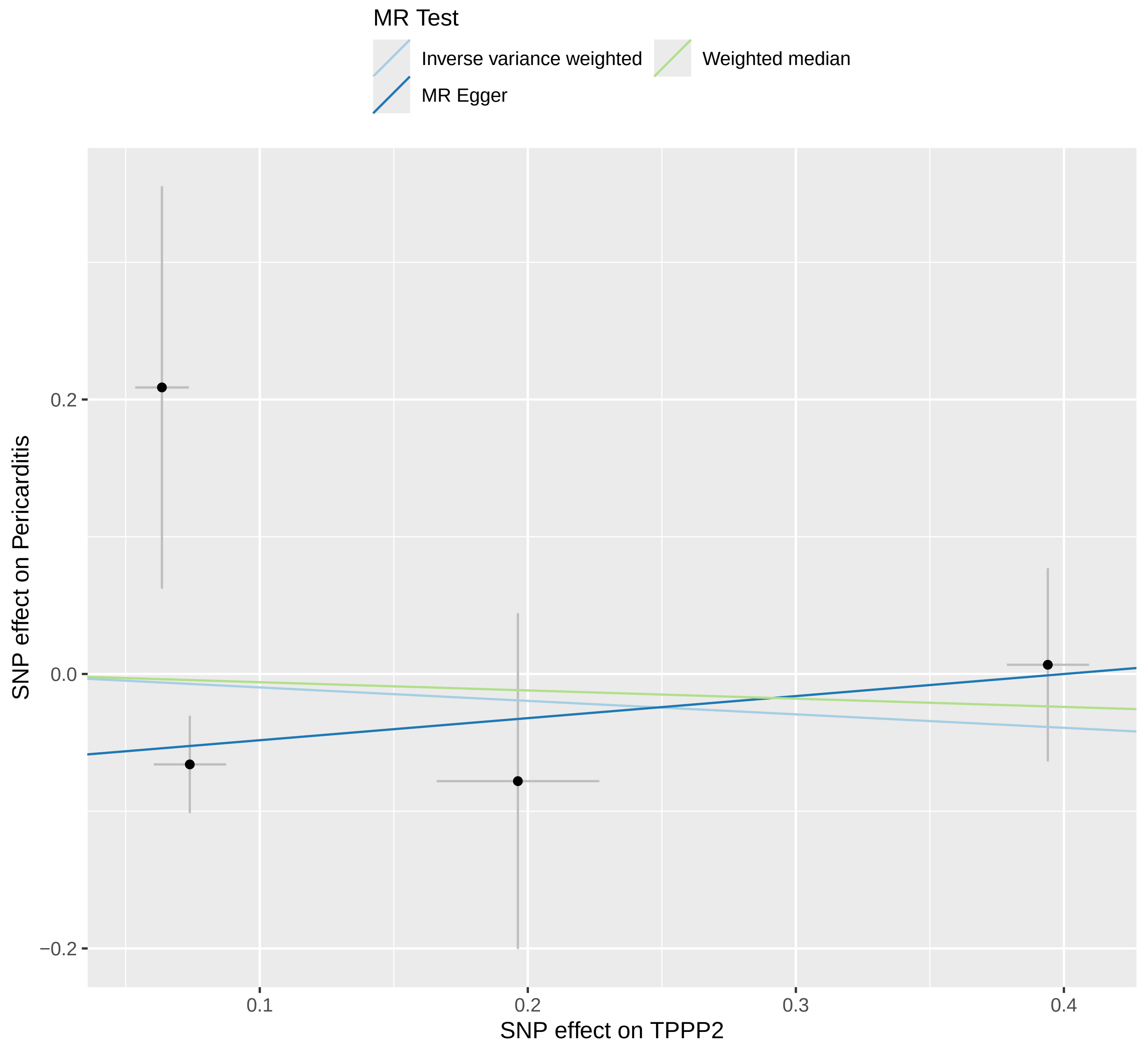

MR Test
Inverse variance weighted
Weighted median
MR Egger
0.2
0.0
−0.2
0.1
0.2
0.3
0.4
SNP effect on TPPP2
SNP effect on Pericarditis

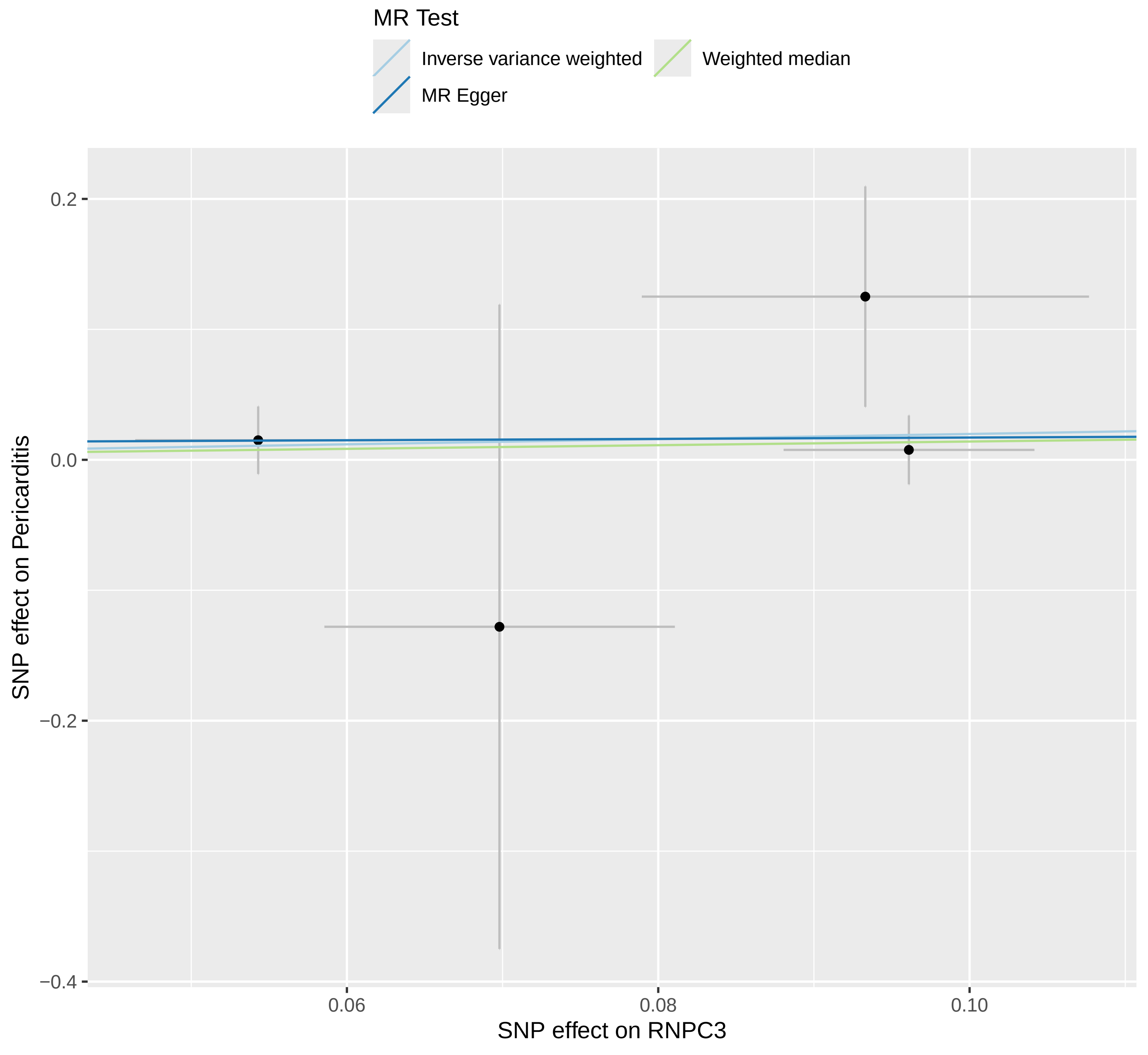

MR Test
Inverse variance weighted
Weighted median
MR Egger
0.2
0.0
−0.2
−0.4
0.06
0.08
0.10
SNP effect on Pericarditis
SNP effect on RNPC3

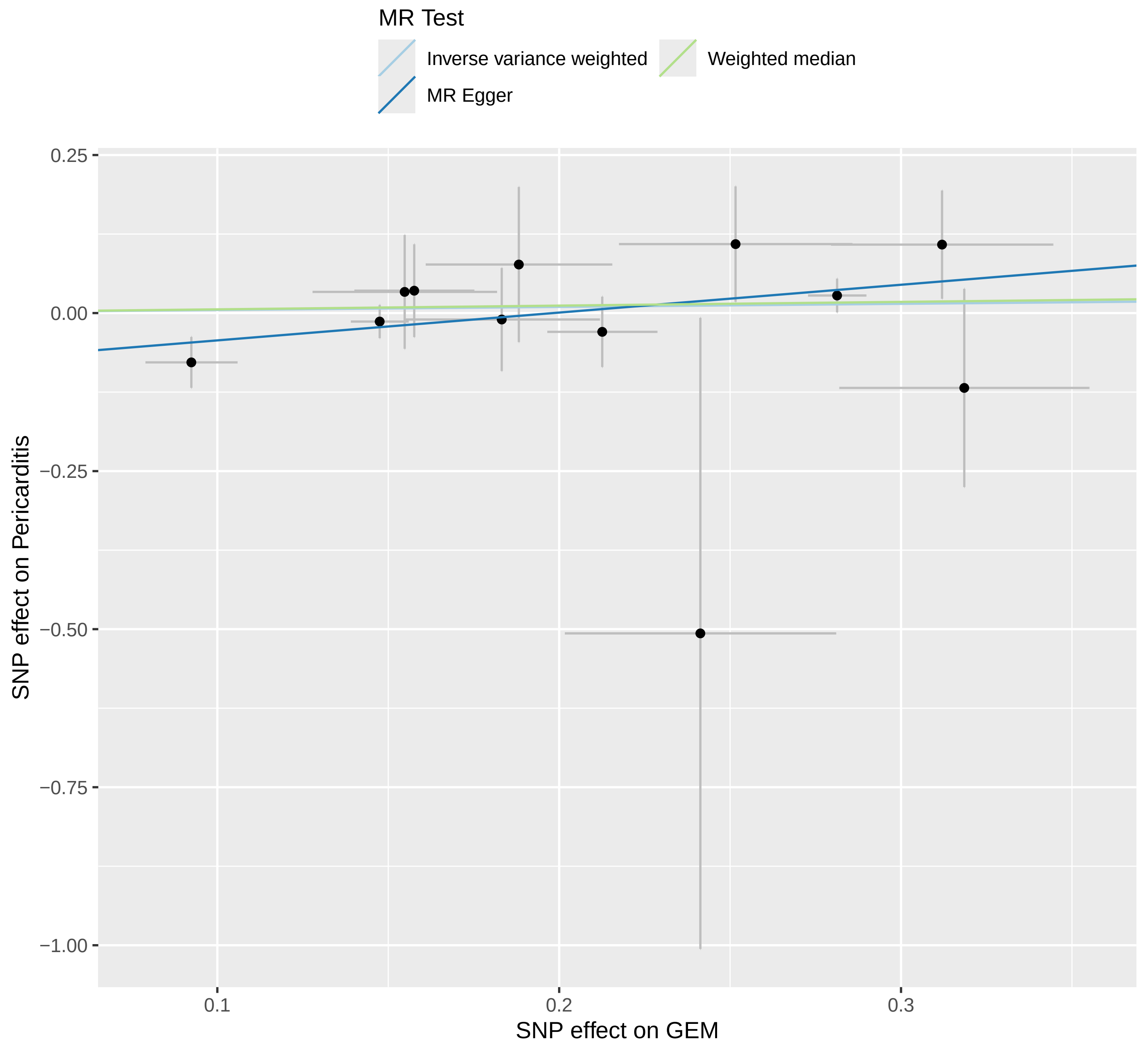
MR Test
Inverse variance weighted
MR Egger
Weighted median
0.25
0.00
−0.25
−0.50
−0.75
−1.00
SNP effect on Pericarditis
0.1
0.2
0.3
SNP effect on GEM

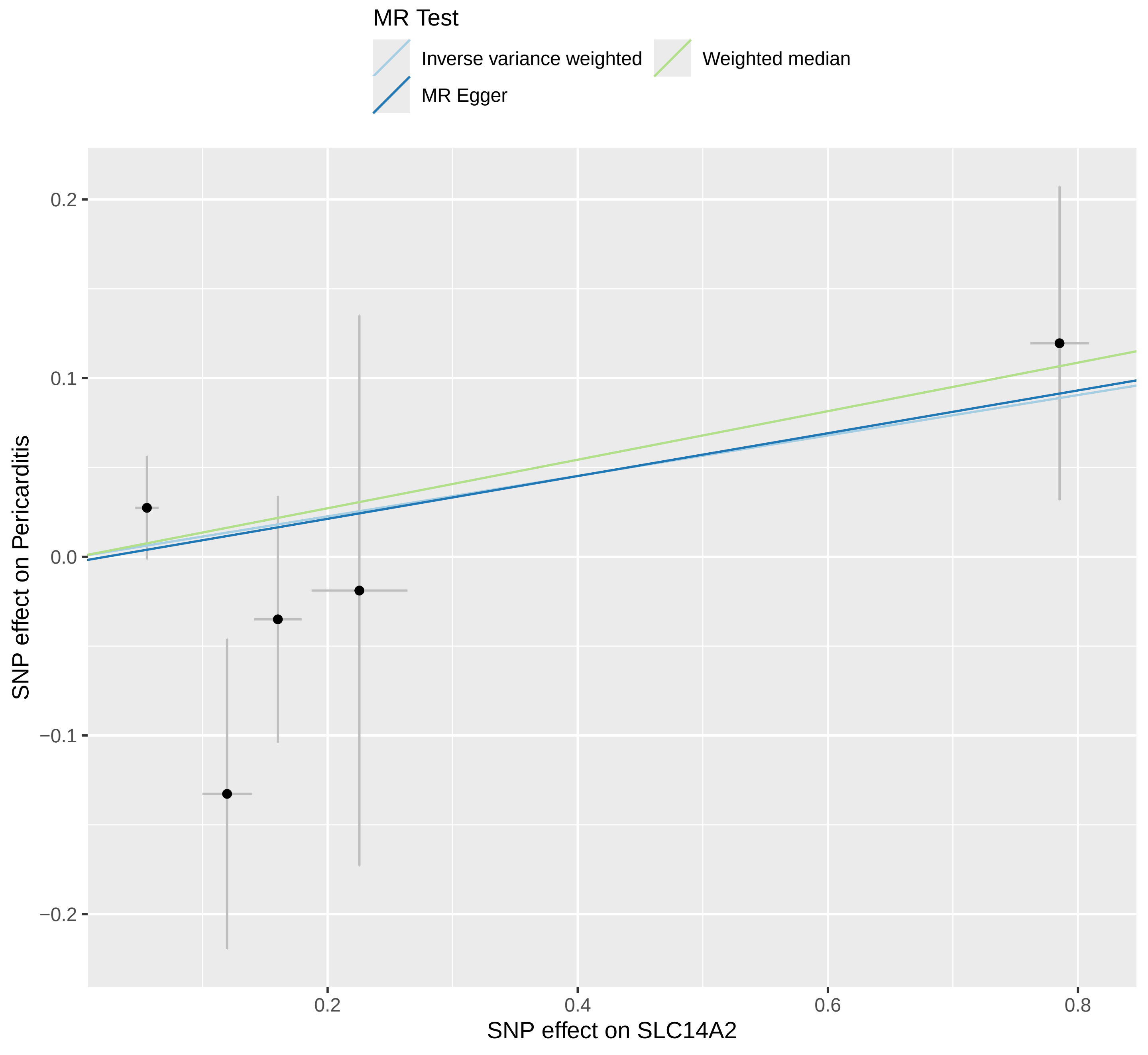

MR Test
Inverse variance weighted
Weighted median
MR Egger
0.2
0.1
0.0
−0.1
−0.2
SNP effect on Pericarditis
0.2
0.4
0.6
0.8
SNP effect on SLC14A2

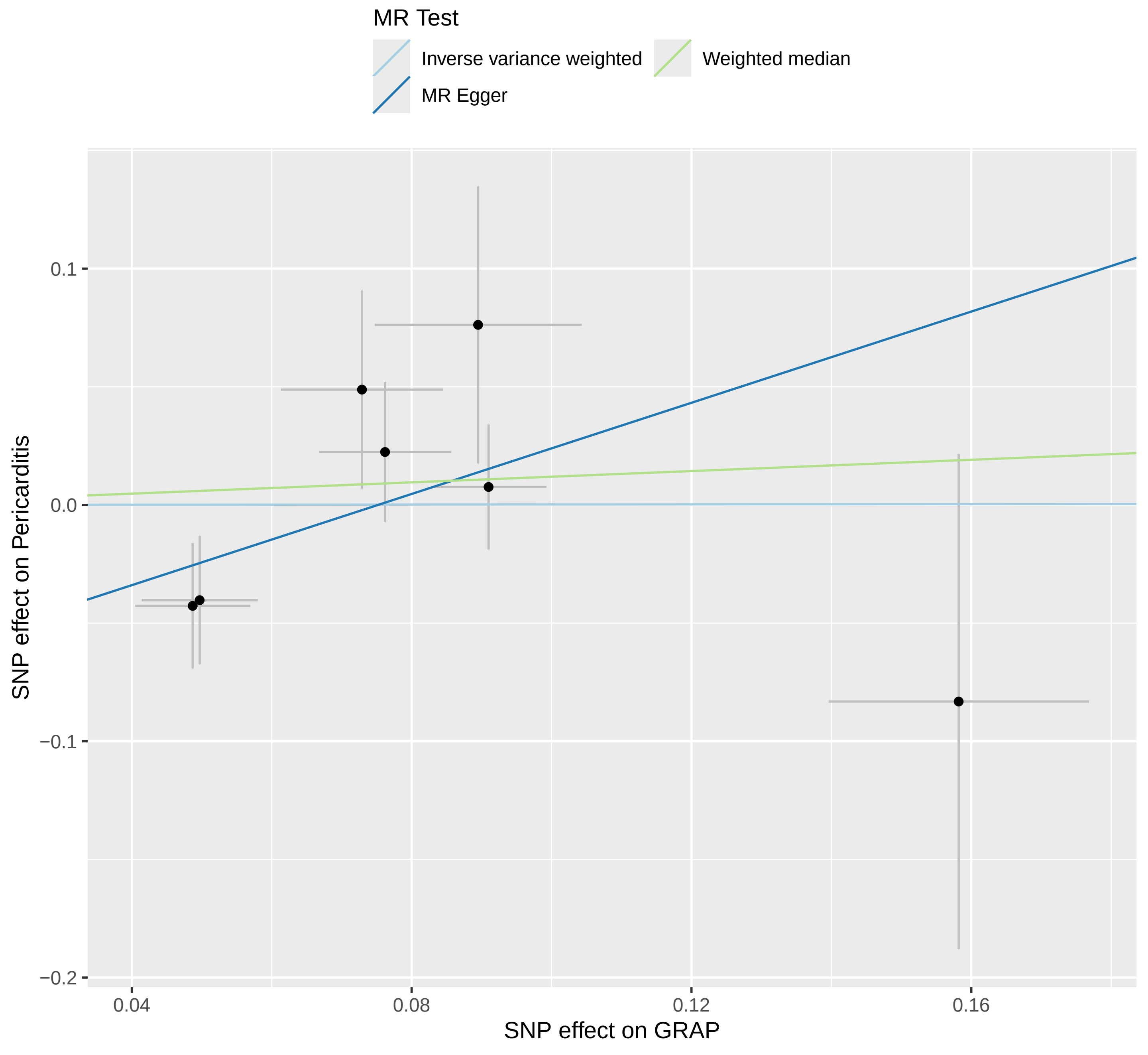
MR Test
Inverse variance weighted
Weighted median
MR Egger
0.1
0.0
−0.1
−0.2
0.04
0.08
0.12
0.16
SNP effect on Pericarditis
SNP effect on GRAP

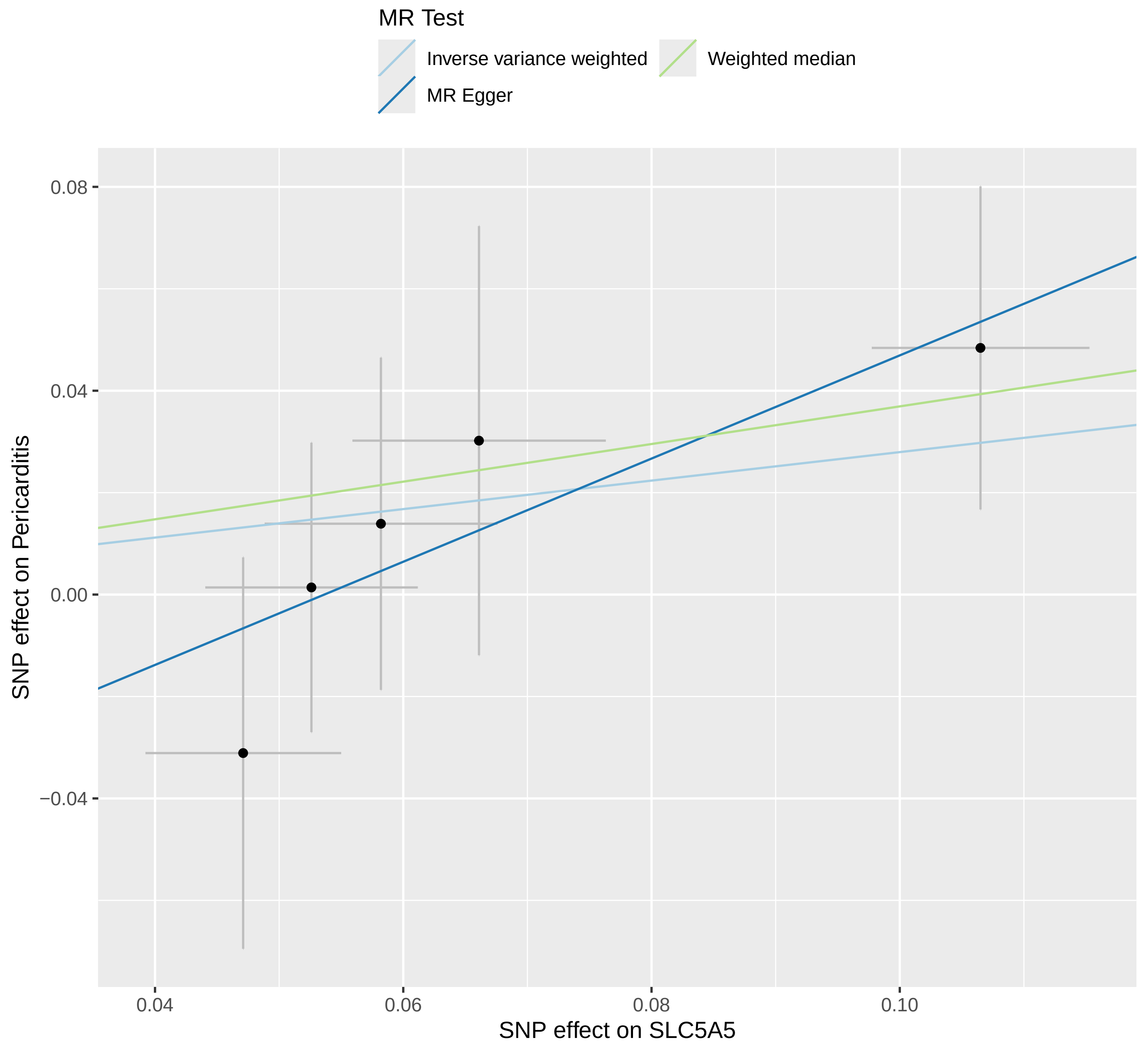
MR Test
Inverse variance weighted
Weighted median
MR Egger
0.08
0.04
0.00
−0.04
0.04
0.06
0.08
0.10
SNP effect on Pericarditis
SNP effect on SLC5A5

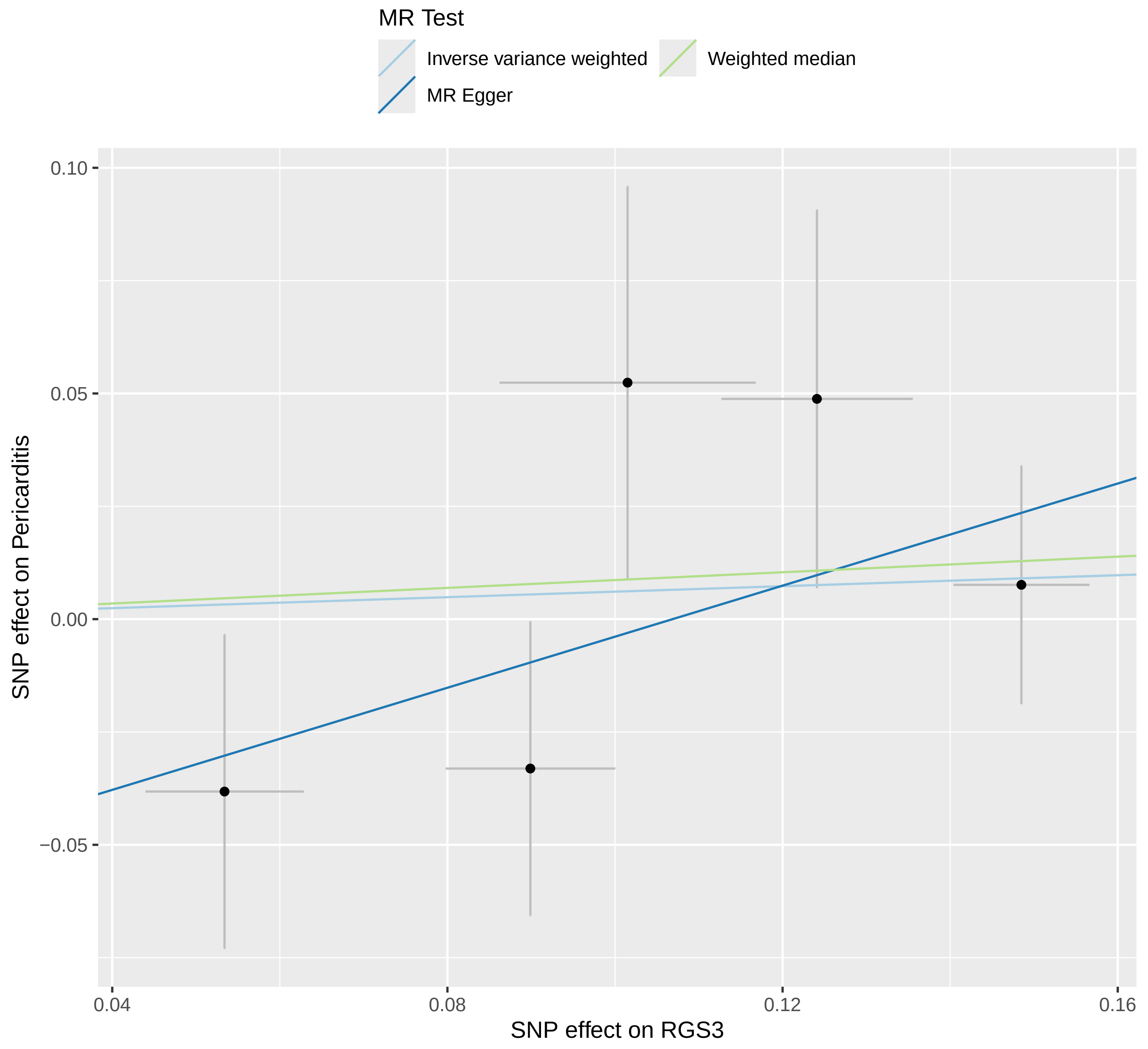
MR Test
Inverse variance weighted
MR Egger
Weighted median
0.10
0.05
0.00
−0.05
SNP effect on Pericarditis
0.04
0.08
0.12
0.16
SNP effect on RGS3

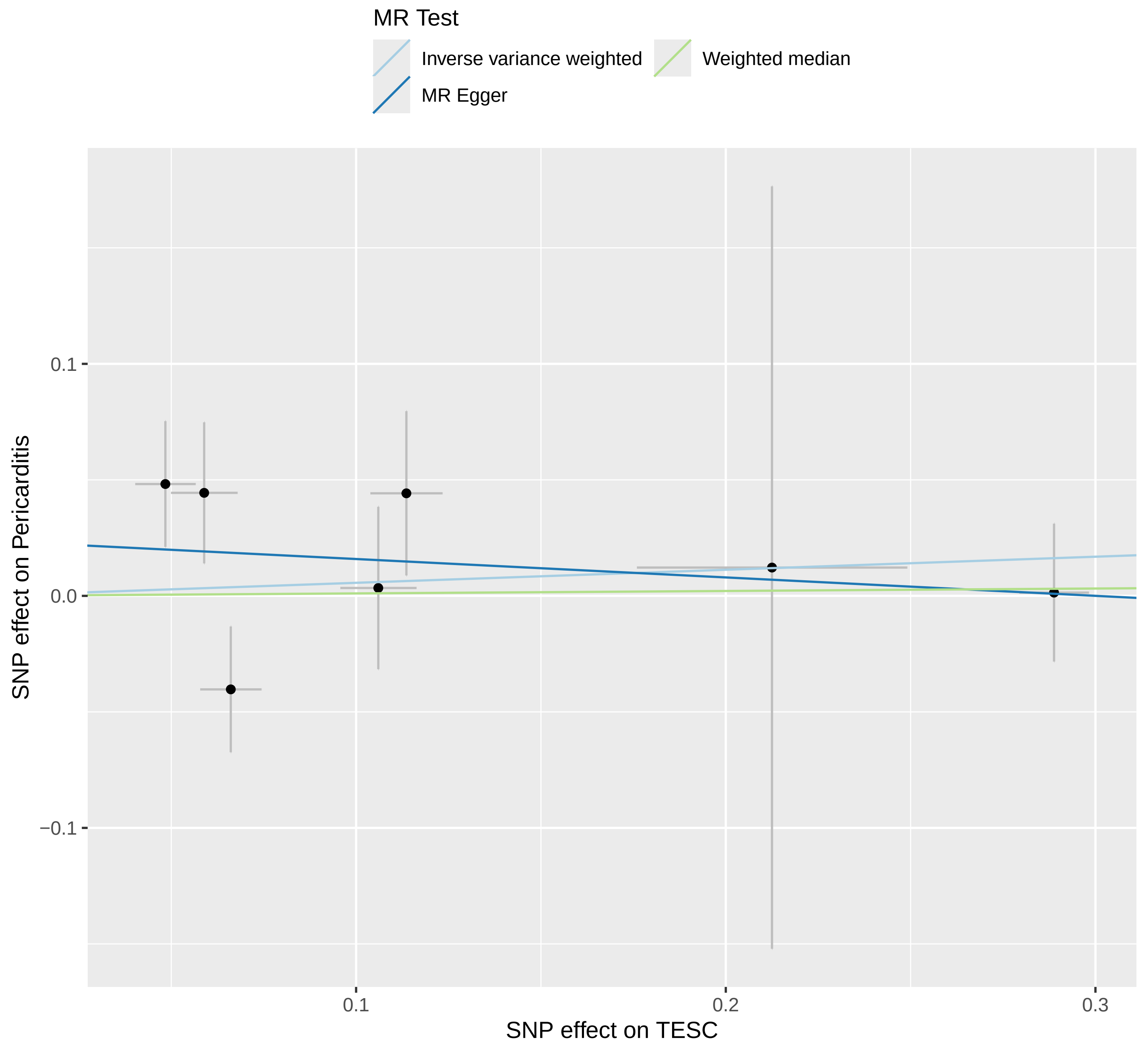
MR Test
Inverse variance weighted
Weighted median
MR Egger
SNP effect on Pericarditis
0.1
0.0
−0.1
0.1
0.2
0.3
SNP effect on TESC

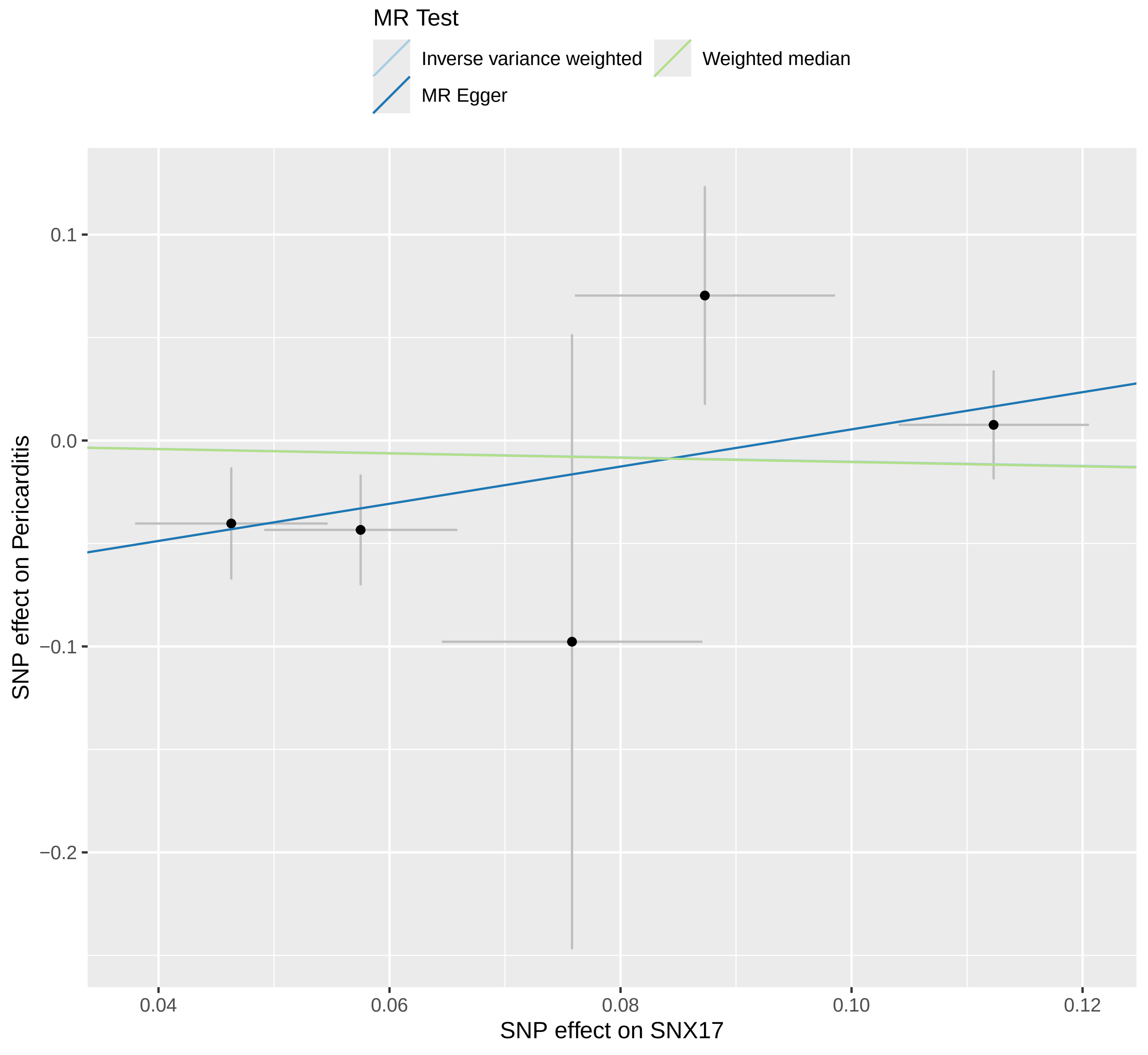
MR Test
Inverse variance weighted
Weighted median
MR Egger
SNP effect on Pericarditis
0.1
0.0
−0.1
−0.2
0.04
0.06
0.08
0.10
0.12
SNP effect on SNX17

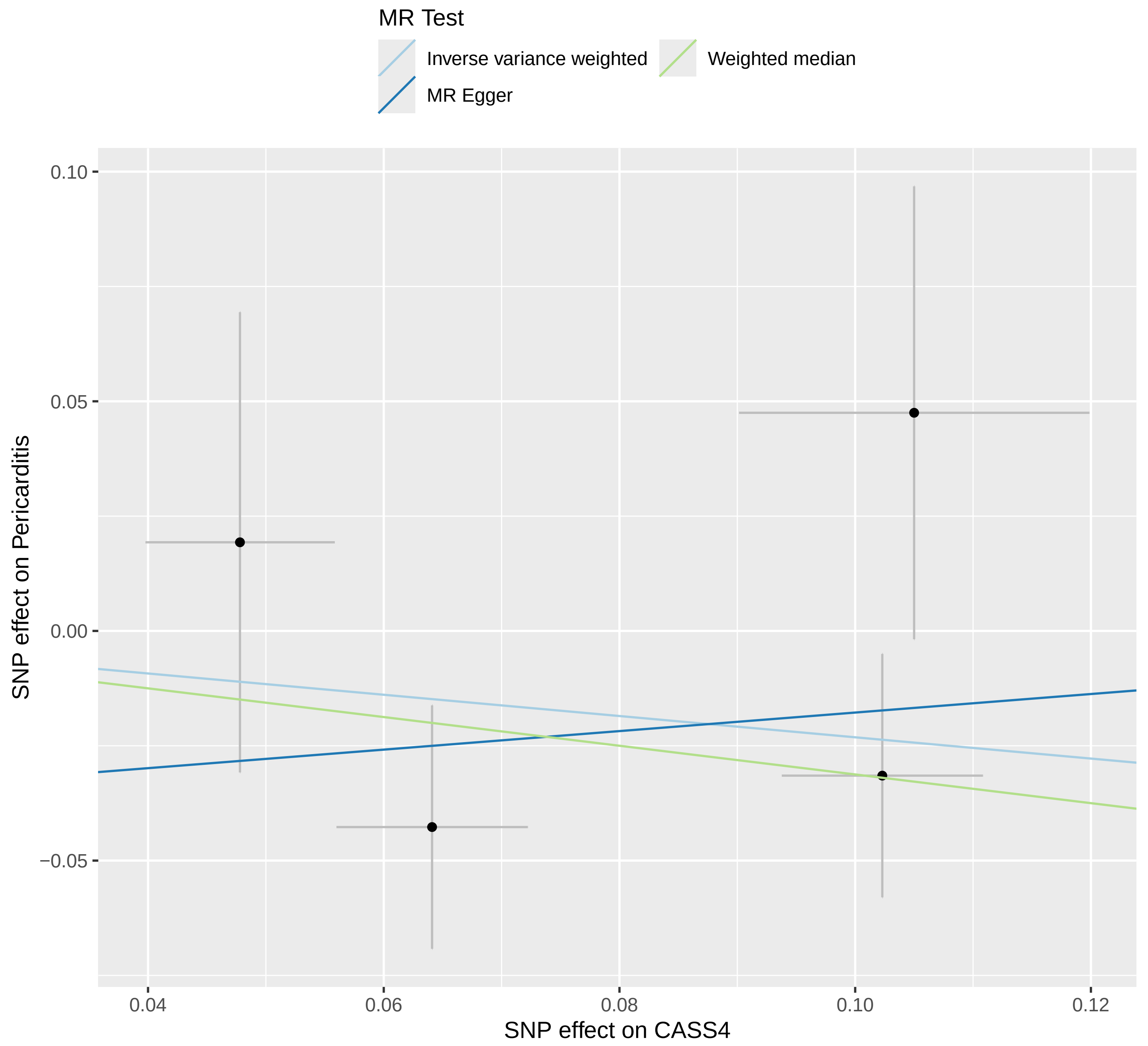

MR Test
Inverse variance weighted
MR Egger
Weighted median
0.10
0.05
0.00
−0.05
SNP effect on Pericarditis
0.04
0.06
0.08
0.10
0.12
SNP effect on CASS4

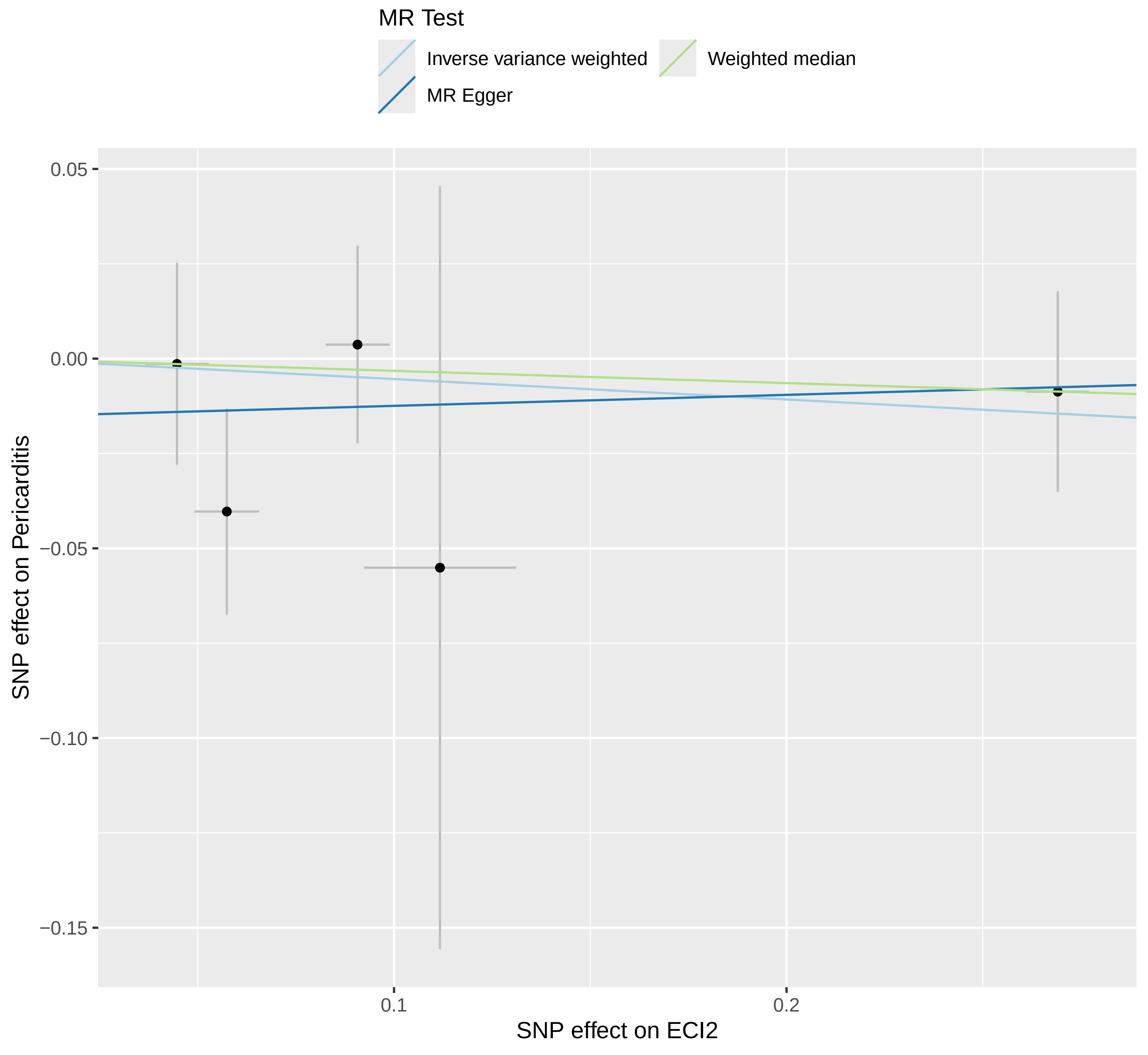

MR Test
Inverse variance weighted
Weighted median
MR Egger
0.05
0.00
−0.05
−0.10
−0.15
SNP effect on Pericarditis
0.1
0.2
SNP effect on ECI2

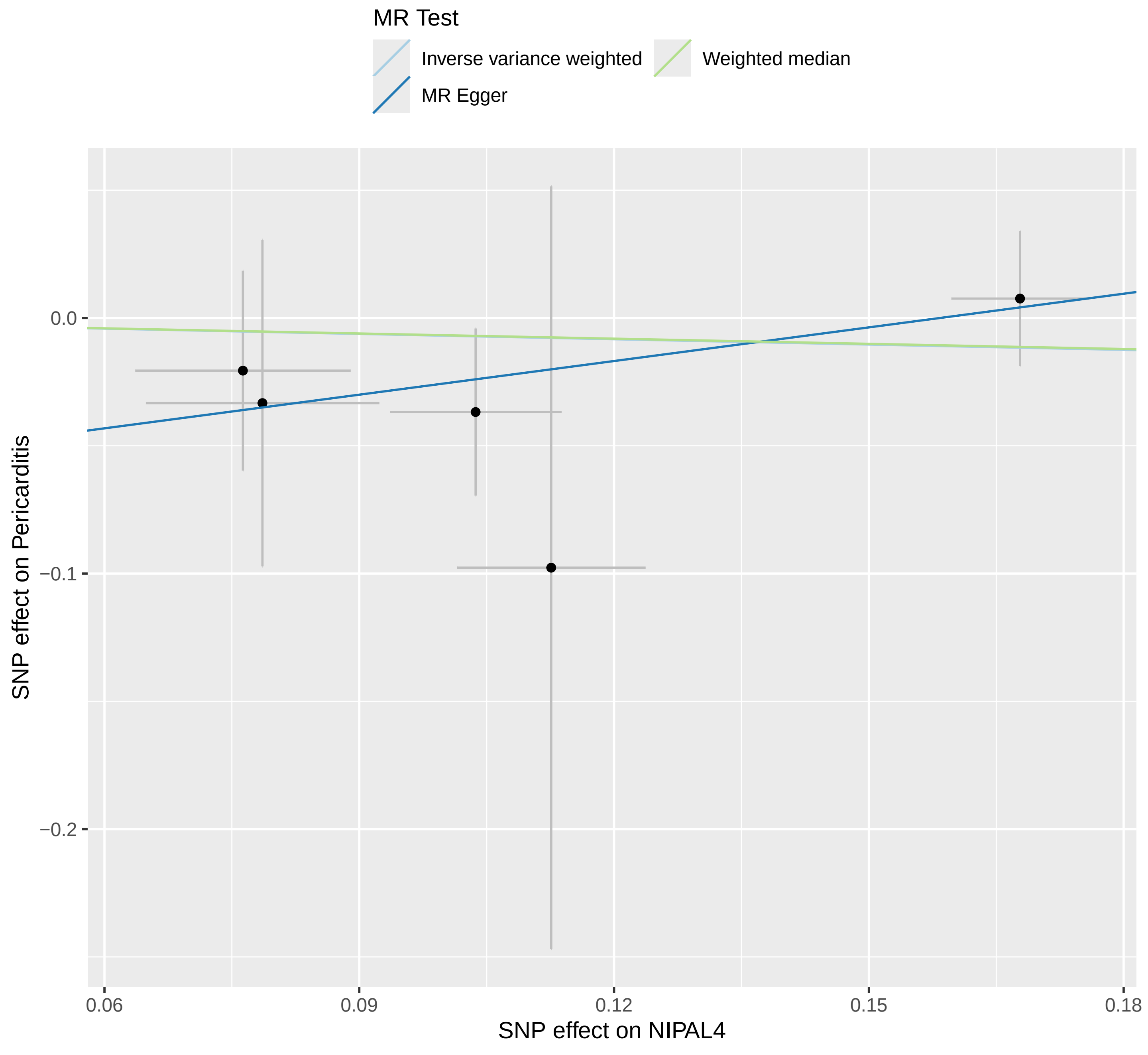
MR Test
Inverse variance weighted
Weighted median
MR Egger
SNP effect on Pericarditis
0.0
−0.1
−0.2
0.06
0.09
0.12
0.15
0.18
SNP effect on NIPAL4

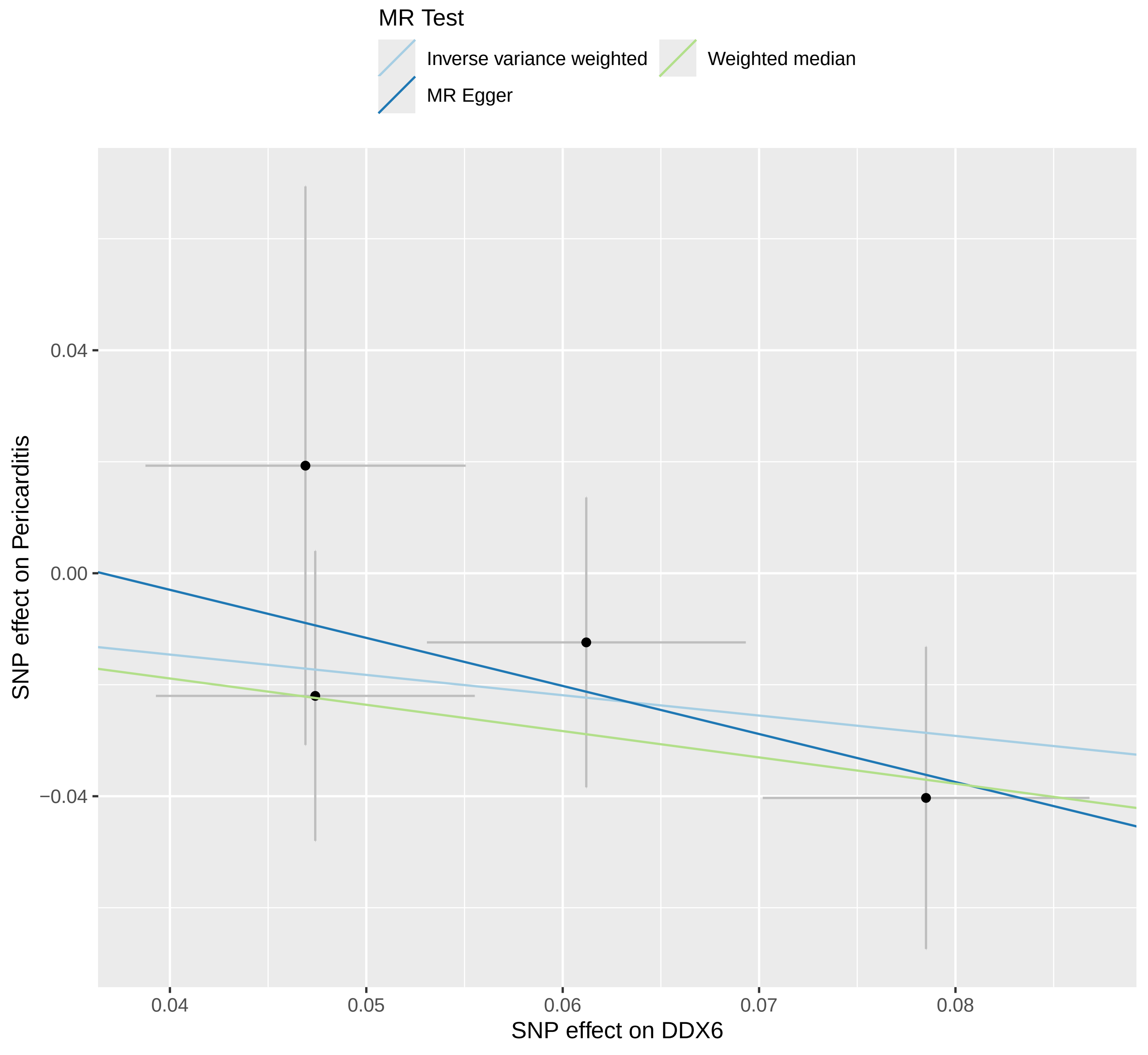

MR Test
Inverse variance weighted
MR Egger
Weighted median
SNP effect on Pericarditis
0.04
0.00
−0.04
0.04
0.05
0.06
0.07
0.08
SNP effect on DDX6

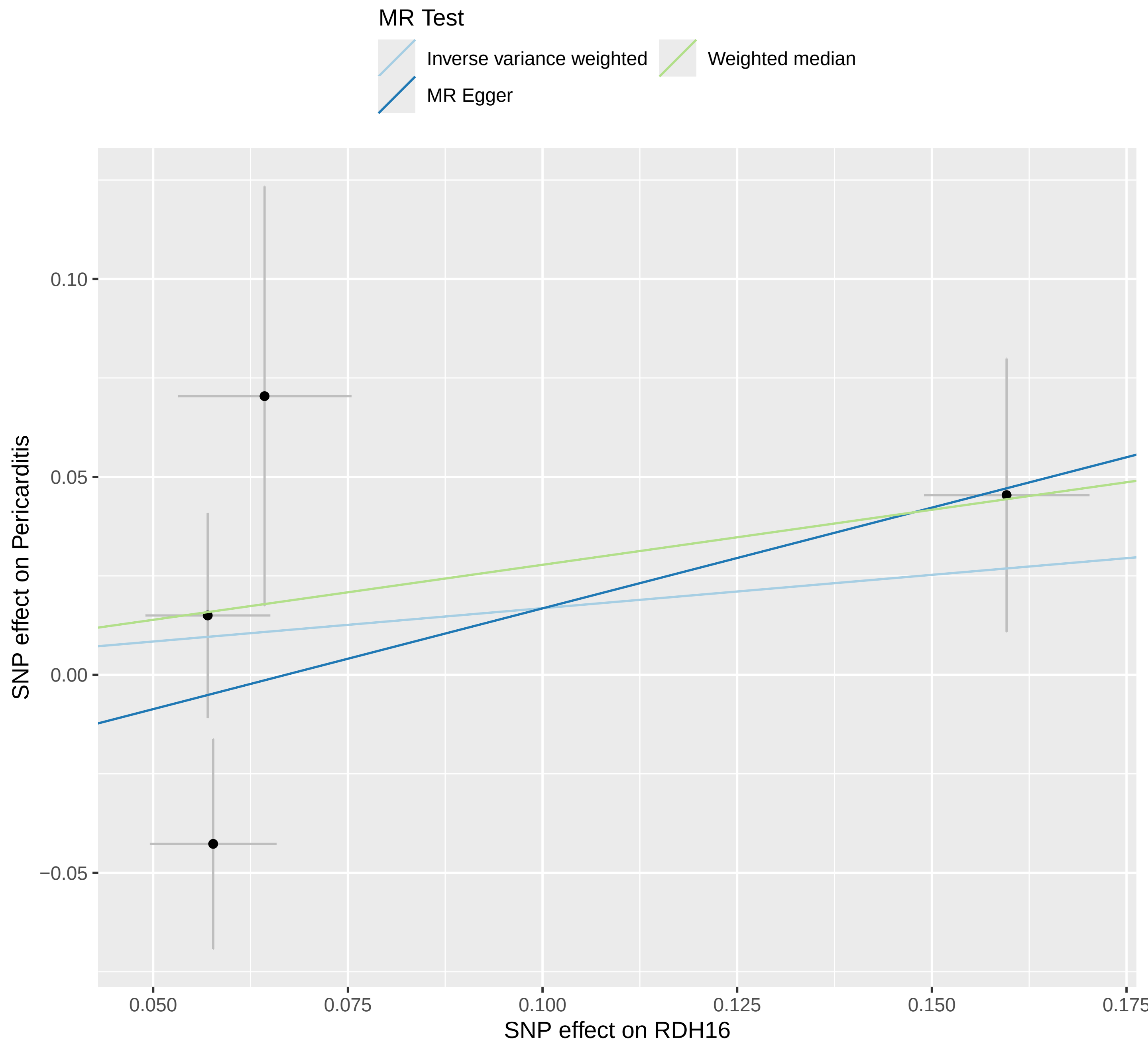
MR Test
Inverse variance weighted
Weighted median
MR Egger
0.10
0.05
0.00
−0.05
SNP effect on Pericarditis
0.050
0.075
0.100
0.125
0.150
0.175
SNP effect on RDH16

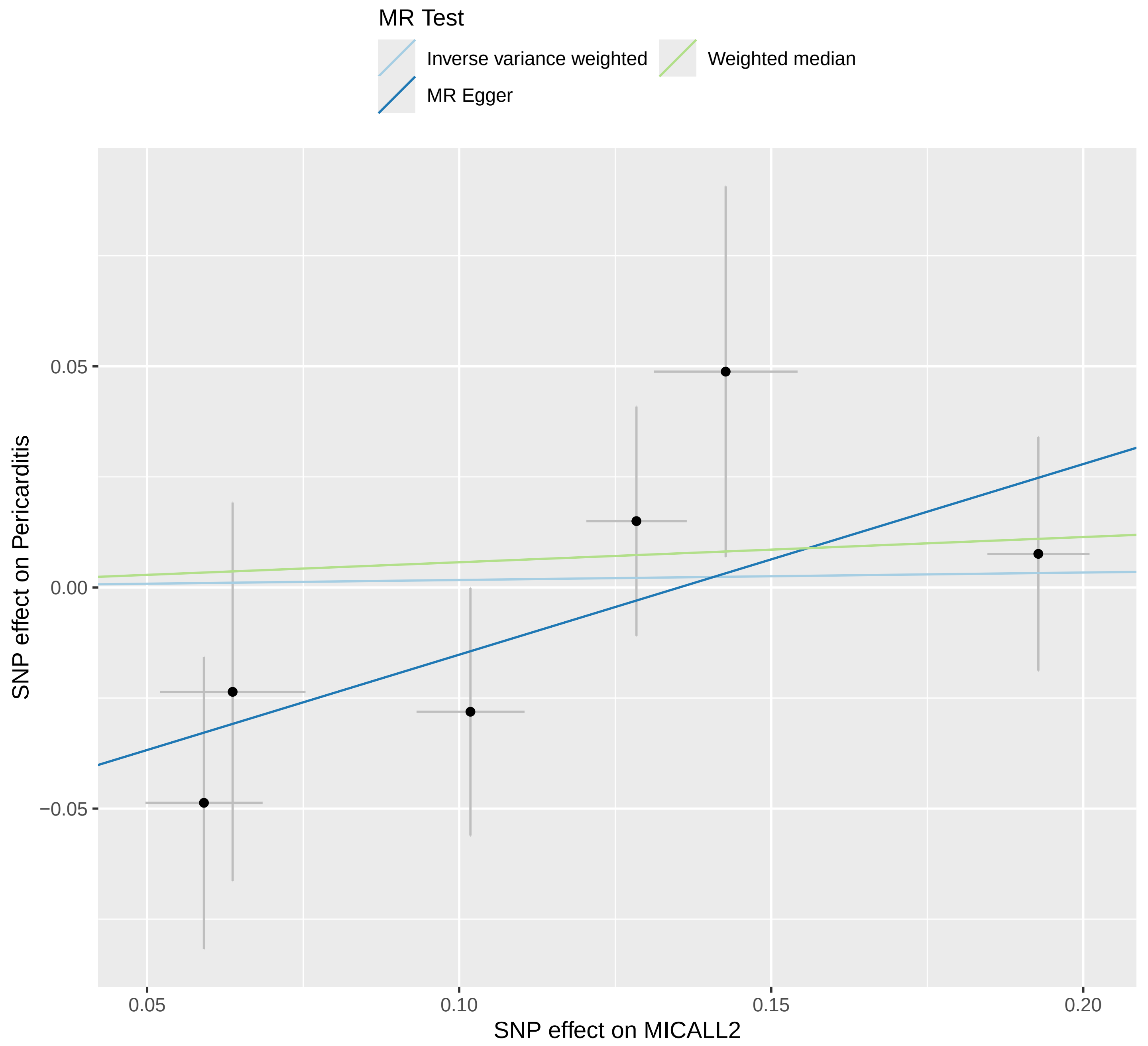
MR Test
Inverse variance weighted
MR Egger
Weighted median
SNP effect on Pericarditis
0.05
0.00
−0.05
SNP effect on MICALL2
0.05
0.10
0.15
0.20

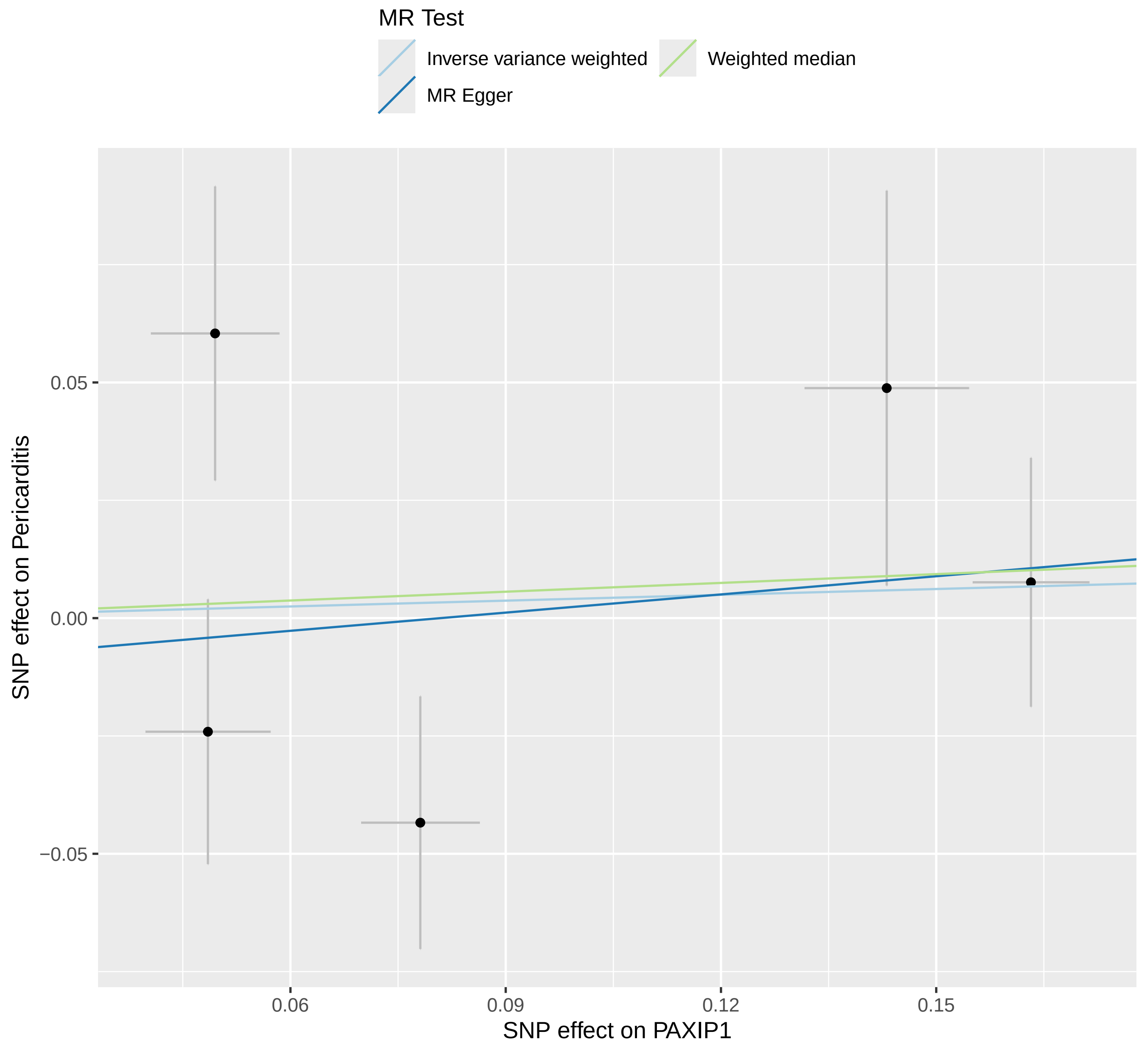
MR Test
Inverse variance weighted
MR Egger
Weighted median
0.05
0.00
−0.05
0.06
0.09
0.12
0.15
SNP effect on PAXIP1
SNP effect on Pericarditis

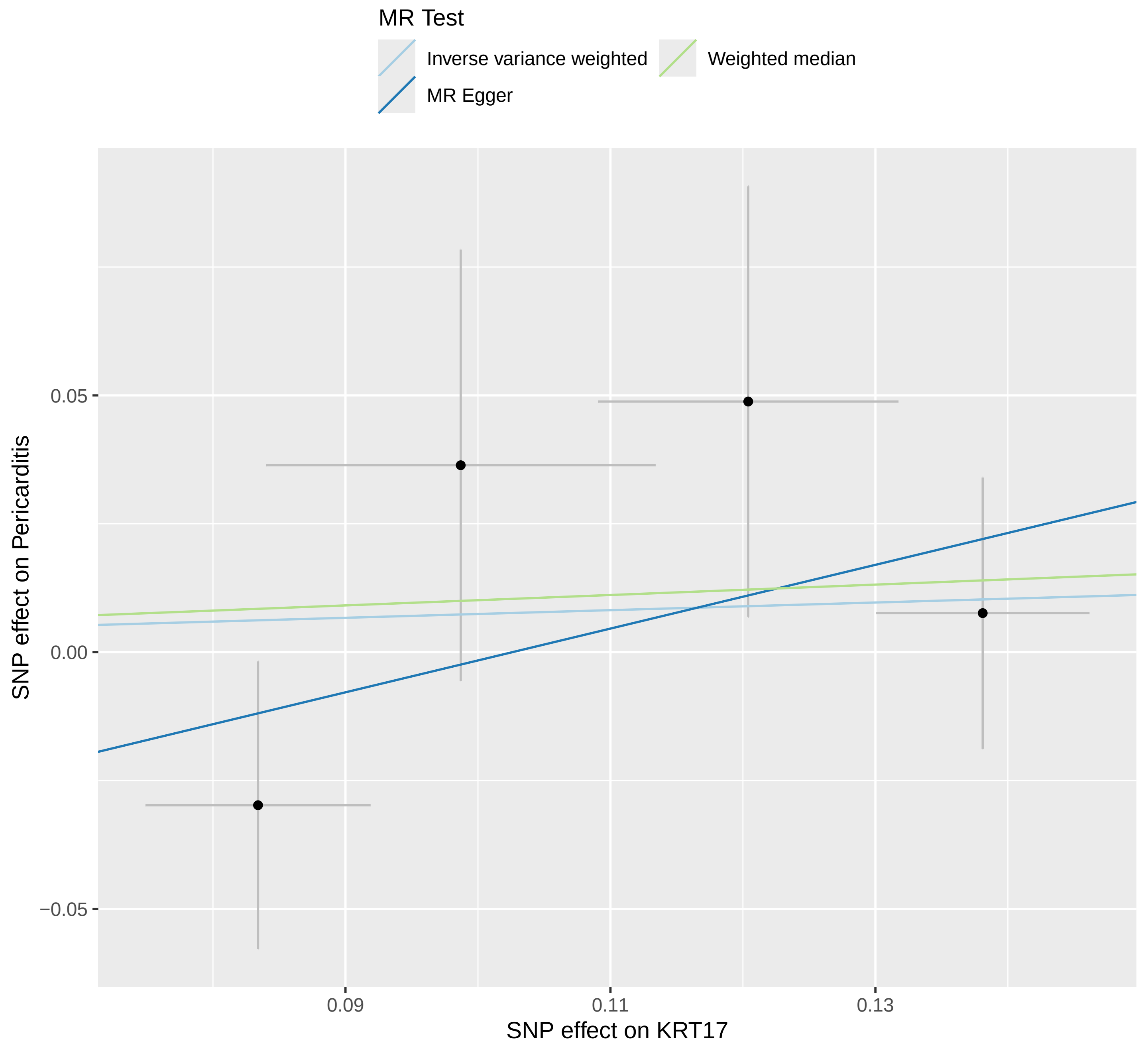
MR Test
Inverse variance weighted
MR Egger
Weighted median
SNP effect on Pericarditis
0.05
0.00
−0.05
0.09
0.11
0.13
SNP effect on KRT17

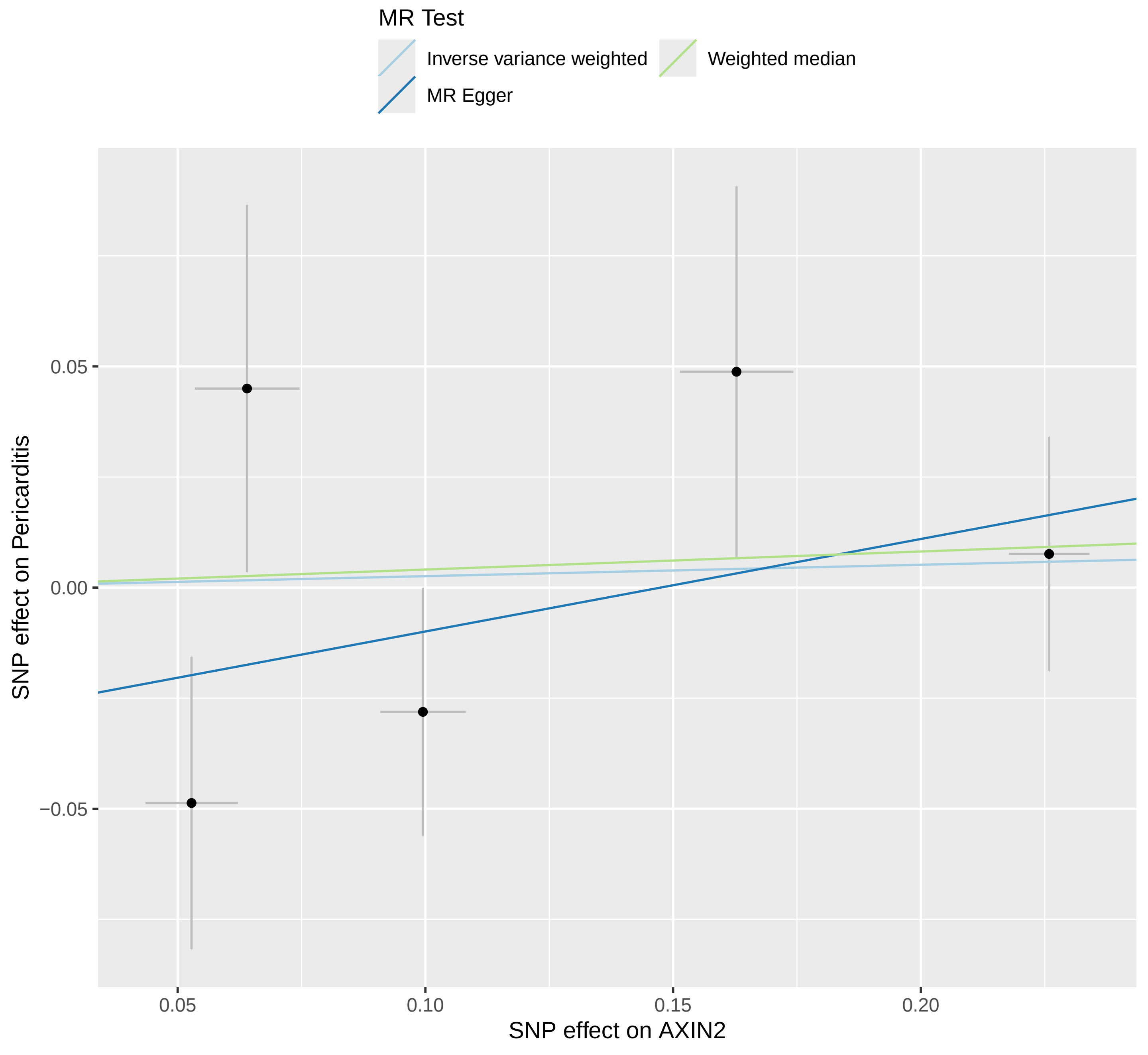

MR Test
Inverse variance weighted
Weighted median
MR Egger
SNP effect on Pericarditis
0.05
0.00
−0.05
0.05
0.10
0.15
0.20
SNP effect on AXIN2

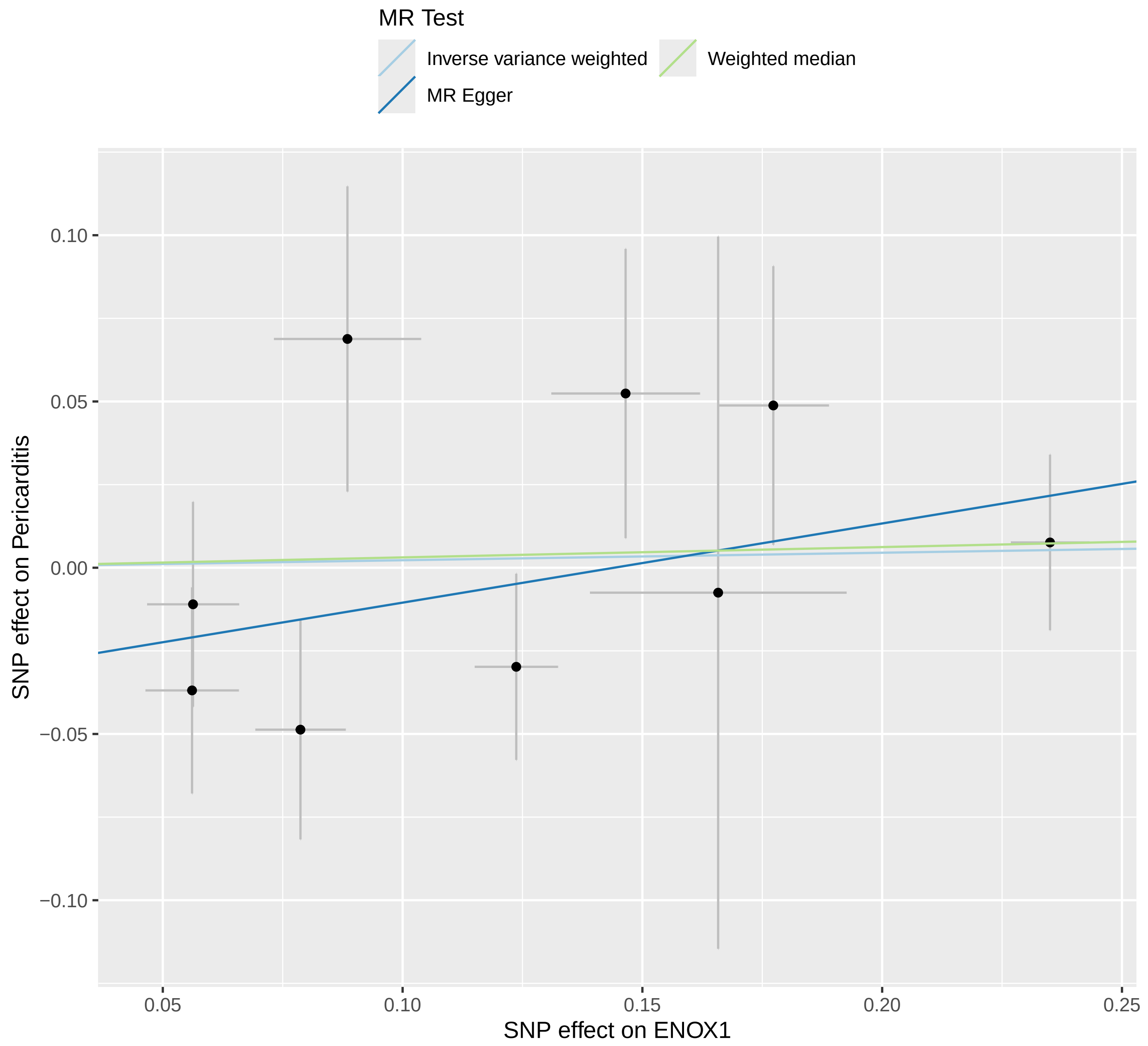
MR Test
Inverse variance weighted
MR Egger
Weighted median
SNP effect on Pericarditis
0.10
0.05
0.00
−0.05
−0.10
0.05
0.10
0.15
0.20
0.25
SNP effect on ENOX1

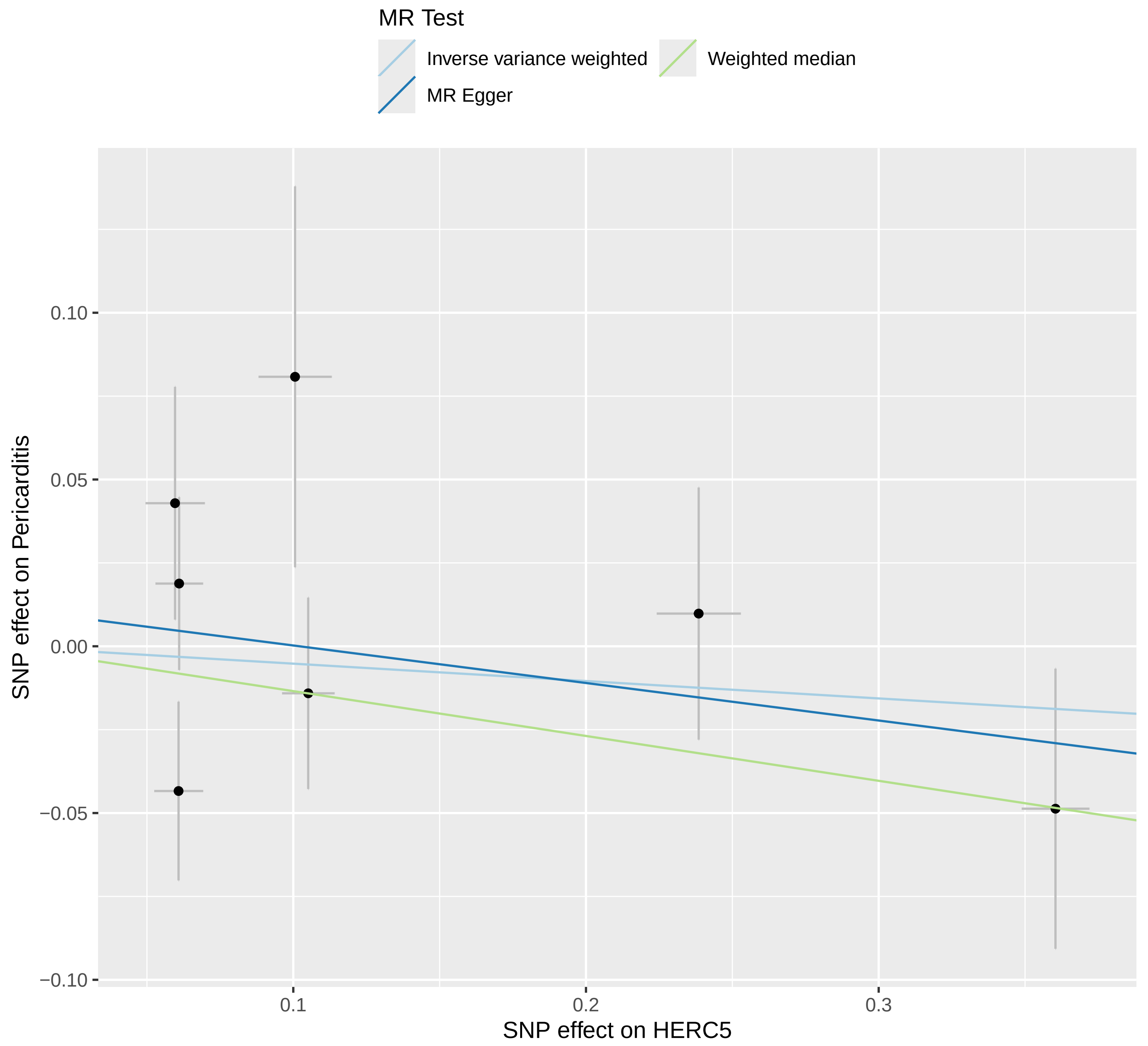

MR Test
Inverse variance weighted
MR Egger
Weighted median
0.10
0.05
0.00
−0.05
−0.10
0.1
0.2
0.3
SNP effect on Pericarditis
SNP effect on HERC5

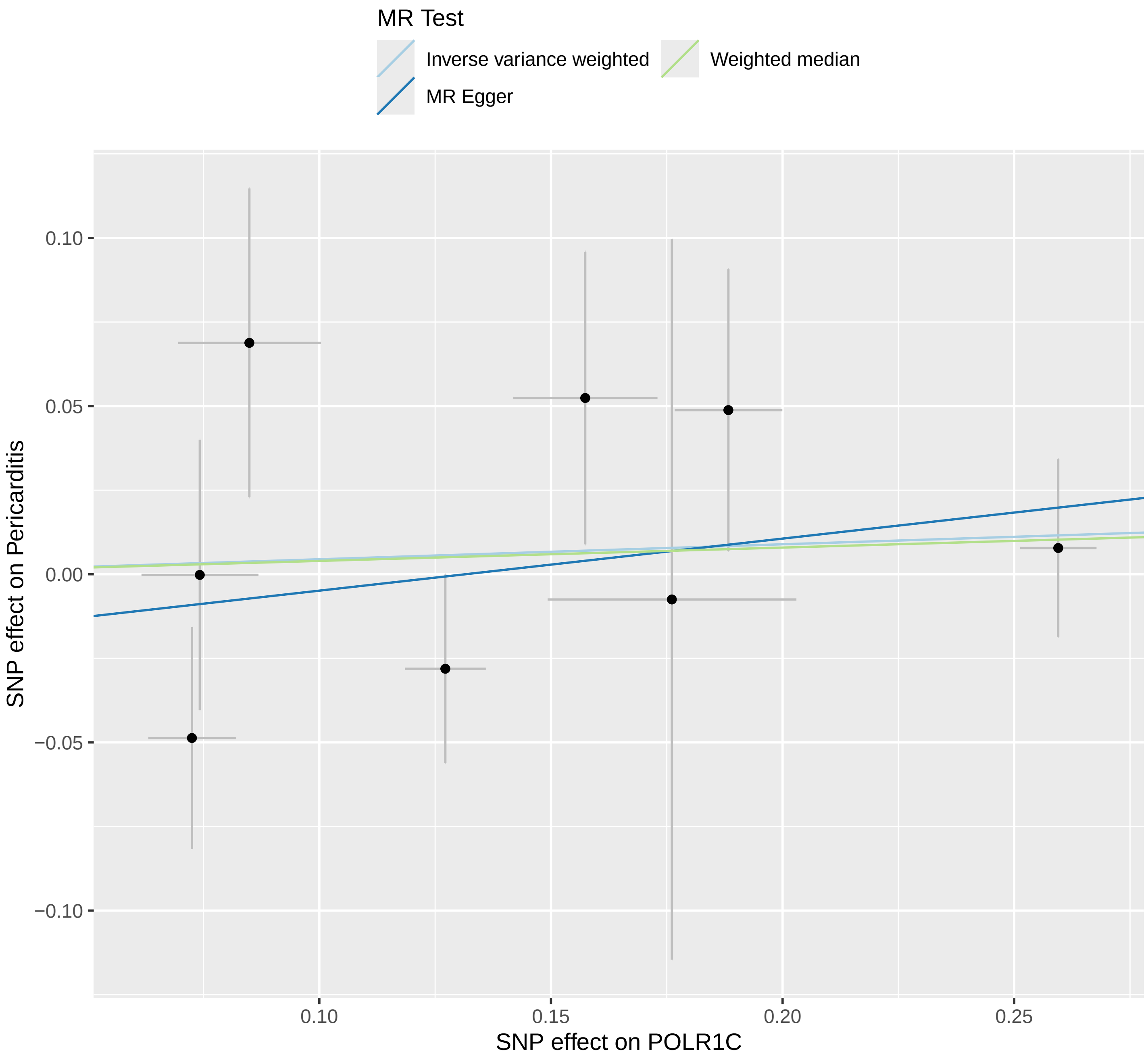

MR Test
Inverse variance weighted
Weighted median
MR Egger
0.10
0.05
0.00
−0.05
−0.10
SNP effect on Pericarditis
0.10
0.15
0.20
0.25
SNP effect on POLR1C

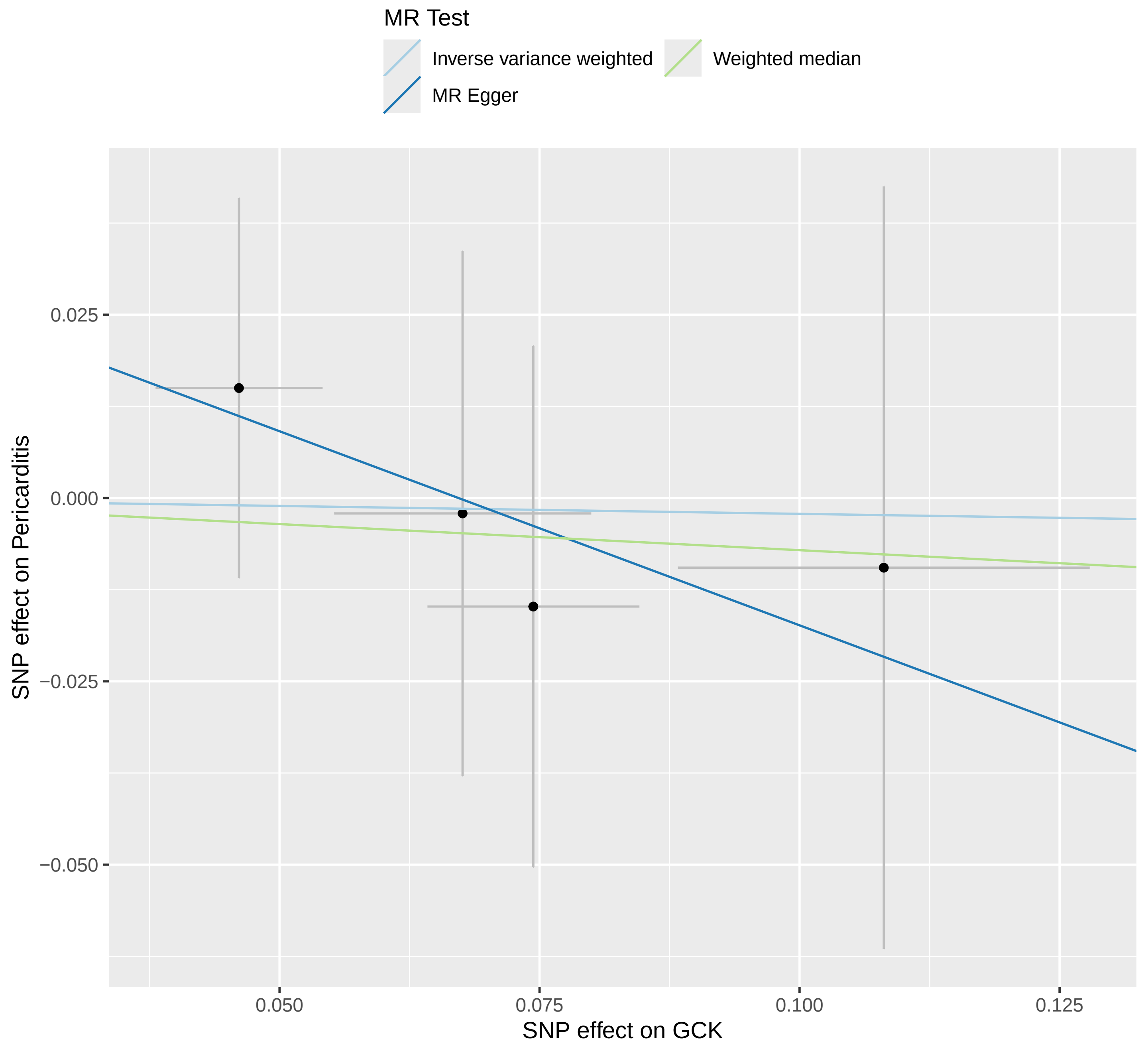

MR Test
Inverse variance weighted
Weighted median
MR Egger
0.025
0.000
−0.025
−0.050
SNP effect on Pericarditis
0.050
0.075
0.100
0.125
SNP effect on GCK

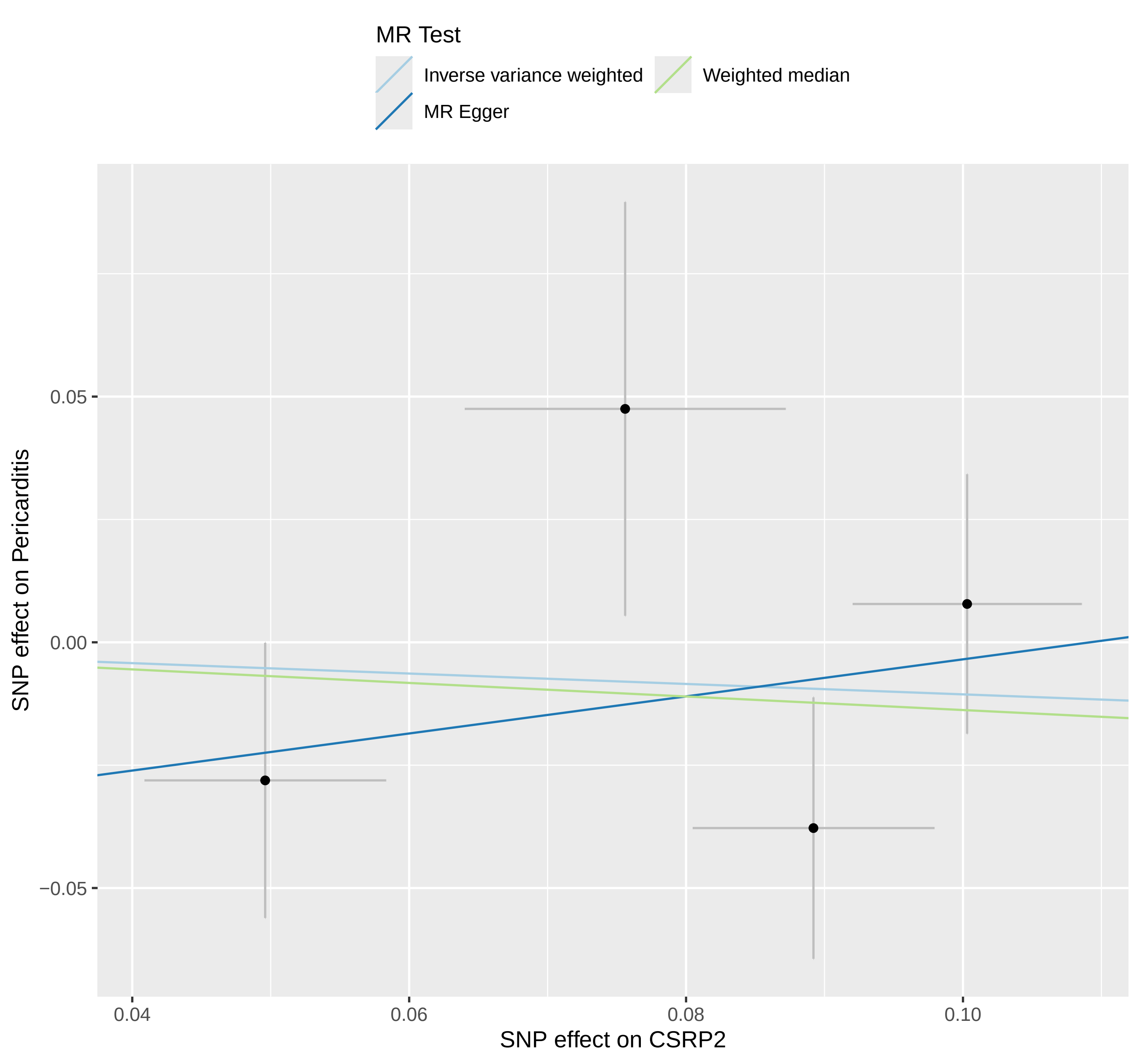
MR Test
Inverse variance weighted
MR Egger
Weighted median
SNP effect on Pericarditis
0.05
0.00
−0.05
0.04
0.06
0.08
0.10
SNP effect on CSRP2

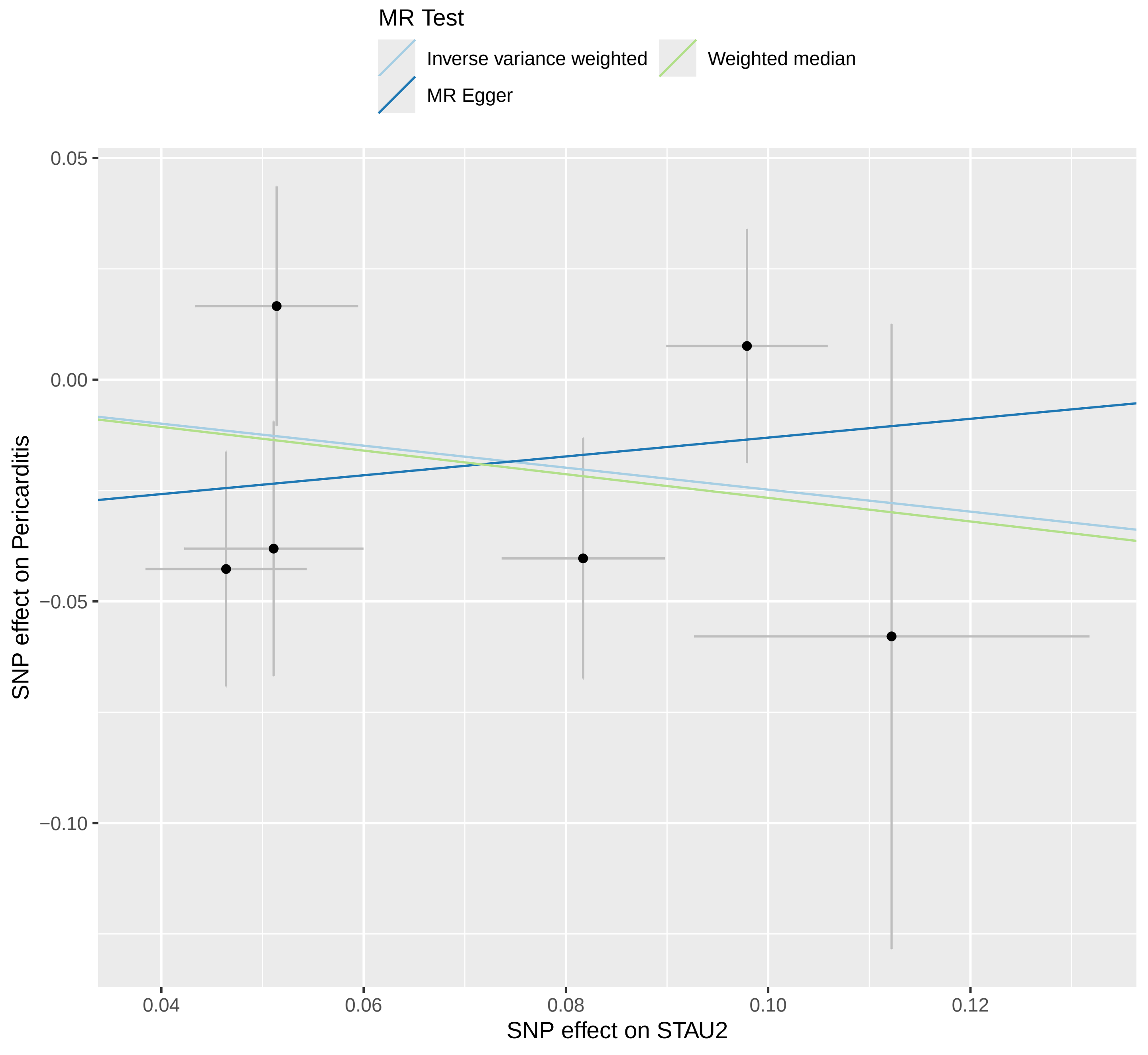
MR Test
Inverse variance weighted
MR Egger
Weighted median
0.05
0.00
−0.05
−0.10
0.04
0.06
0.08
0.10
0.12
SNP effect on STAU2
SNP effect on Pericarditis

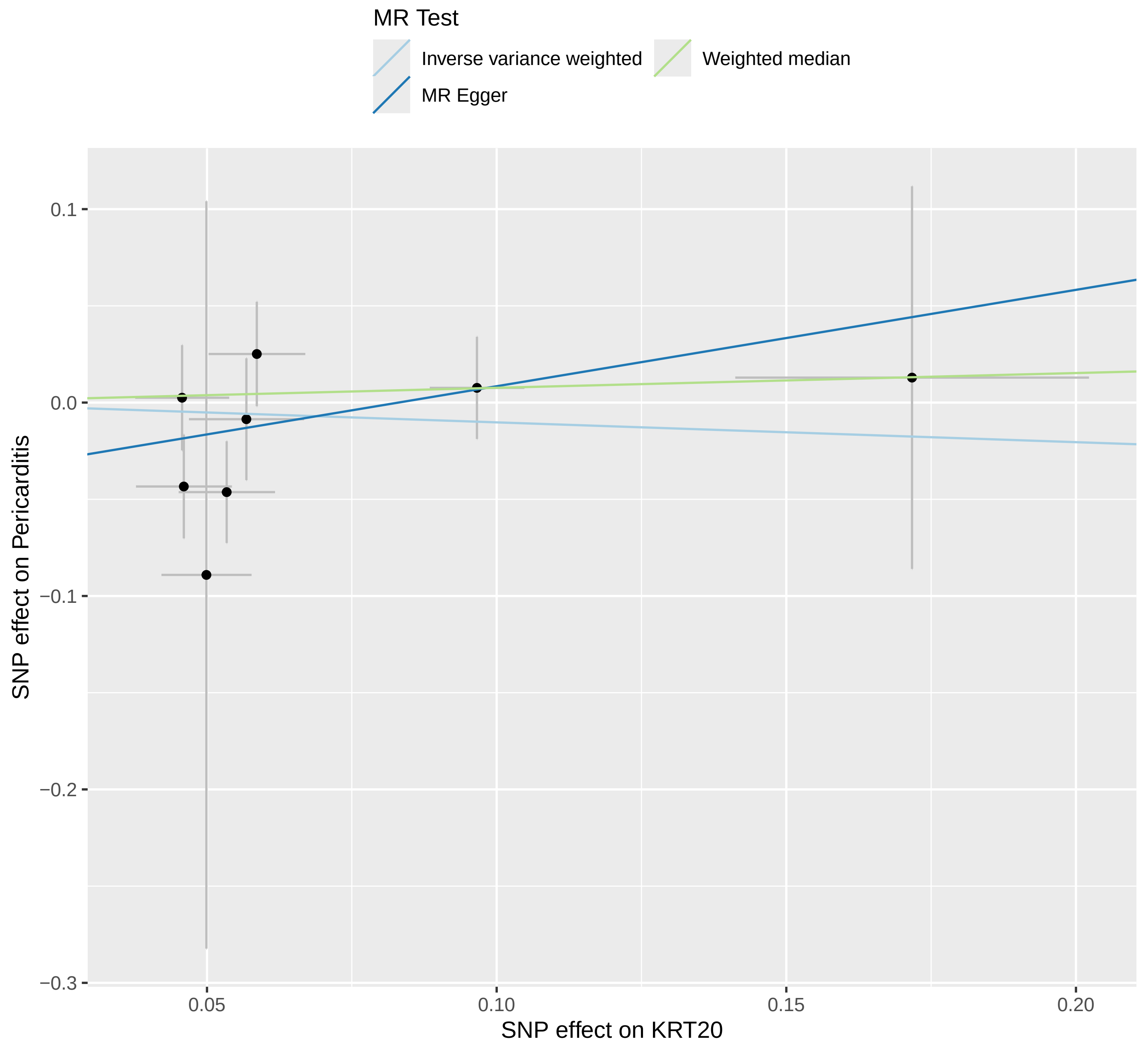
MR Test
Inverse variance weighted
MR Egger
Weighted median
0.1
0.0
−0.1
−0.2
−0.3
SNP effect on Pericarditis
0.05
0.10
0.15
0.20
SNP effect on KRT20

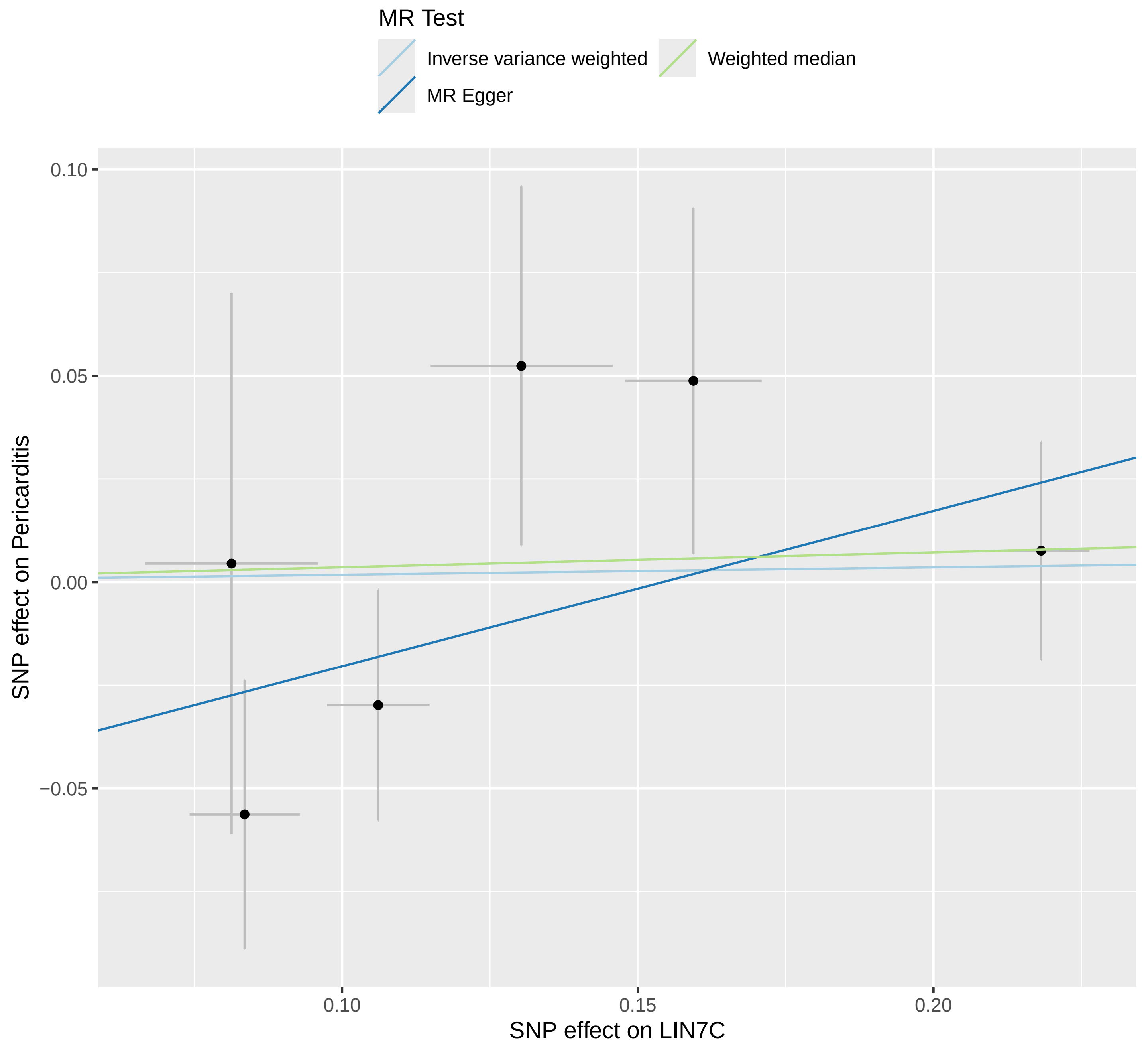
MR Test
Inverse variance weighted
MR Egger
Weighted median
0.10
0.05
0.00
−0.05
SNP effect on Pericarditis
0.10
0.15
0.20
SNP effect on LIN7C

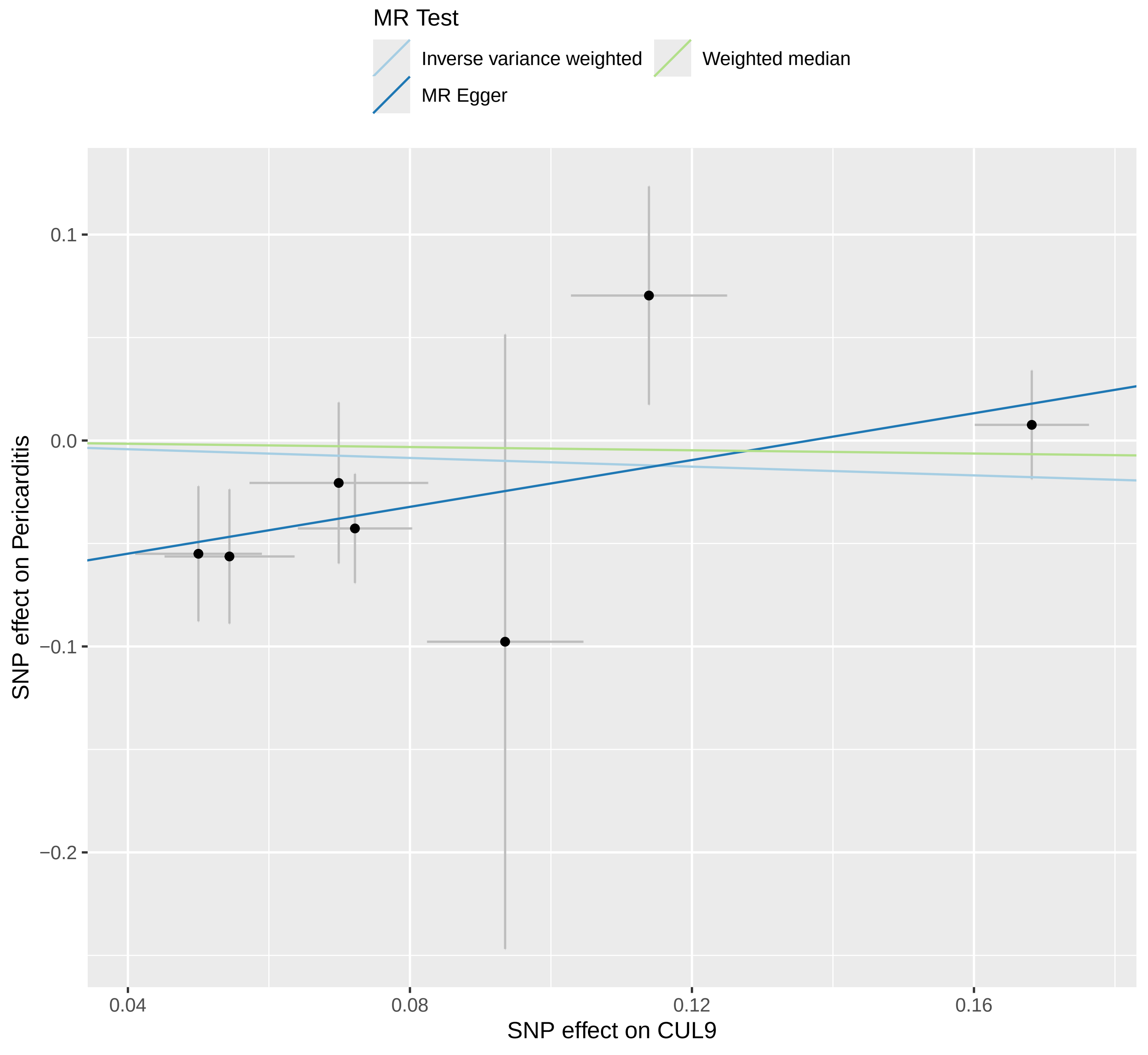

MR Test
Inverse variance weighted
Weighted median
MR Egger
0.1
0.0
−0.1
−0.2
SNP effect on Pericarditis
0.04
0.08
0.12
0.16
SNP effect on CUL9

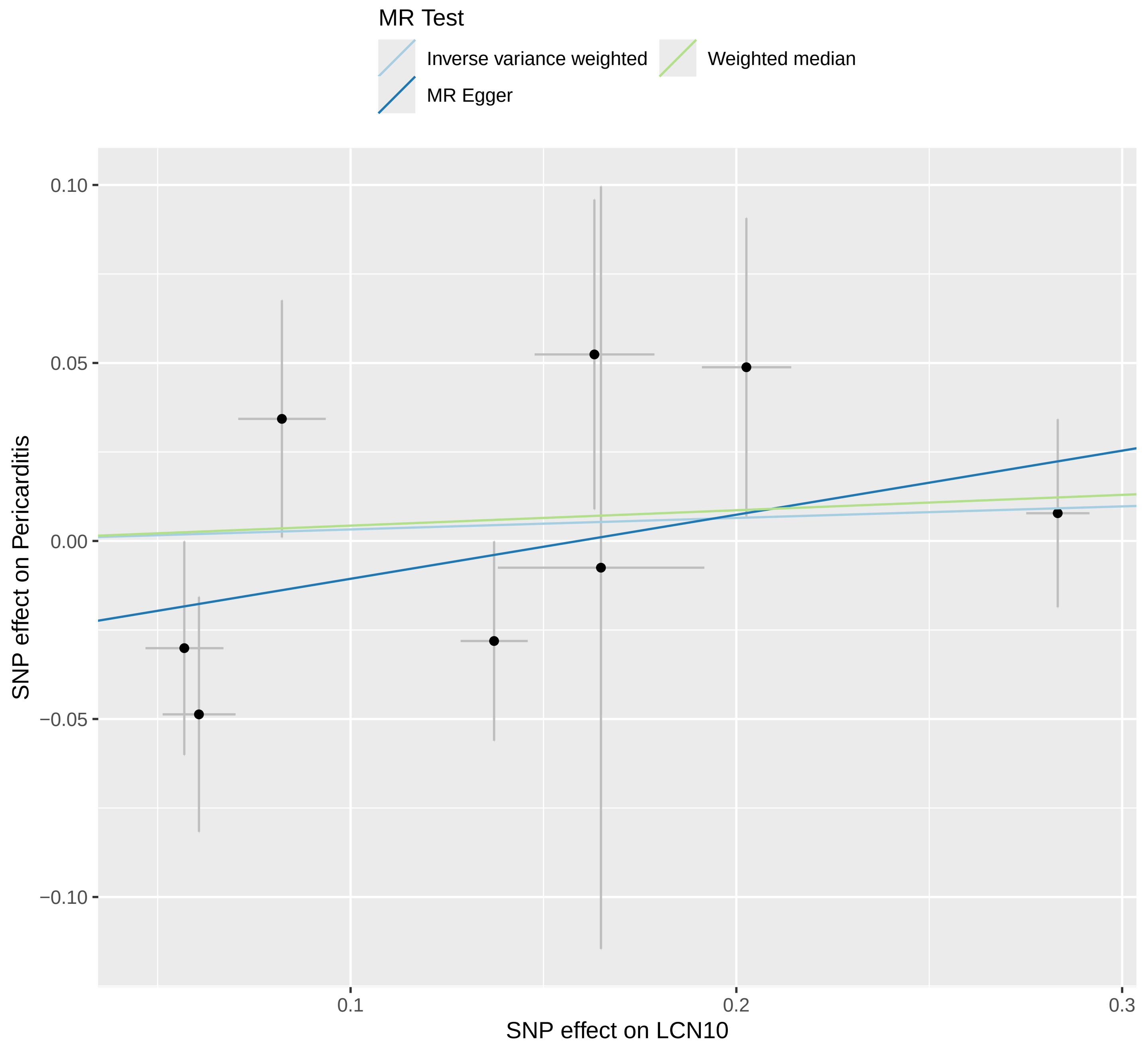

MR Test
Inverse variance weighted
Weighted median
MR Egger
0.10
0.05
0.00
−0.05
−0.10
SNP effect on Pericarditis
0.1
0.2
0.3
SNP effect on LCN10

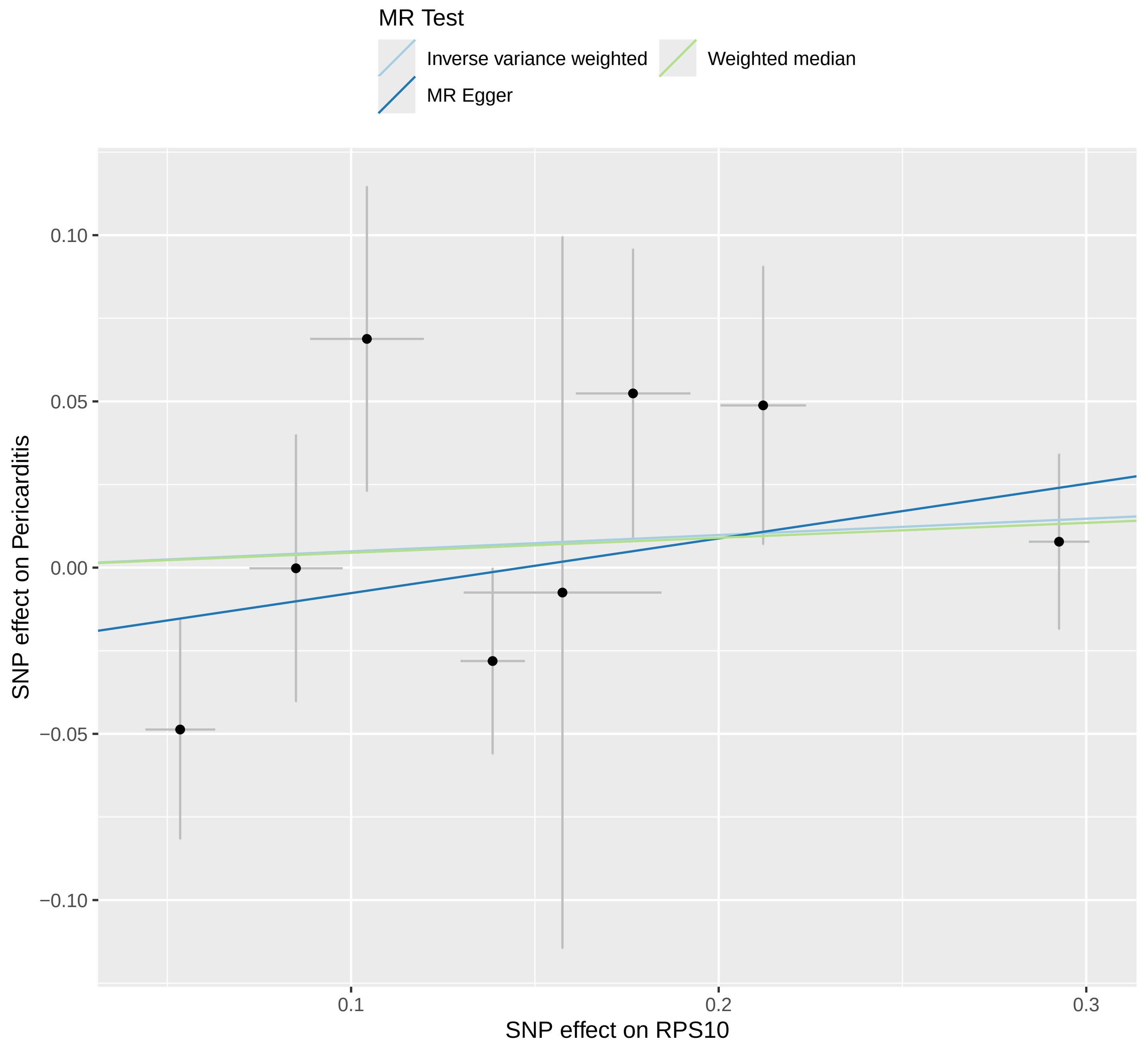
MR Test
Inverse variance weighted
Weighted median
MR Egger
0.10
0.05
0.00
−0.05
−0.10
SNP effect on Pericarditis
0.1
0.2
0.3
SNP effect on RPS10

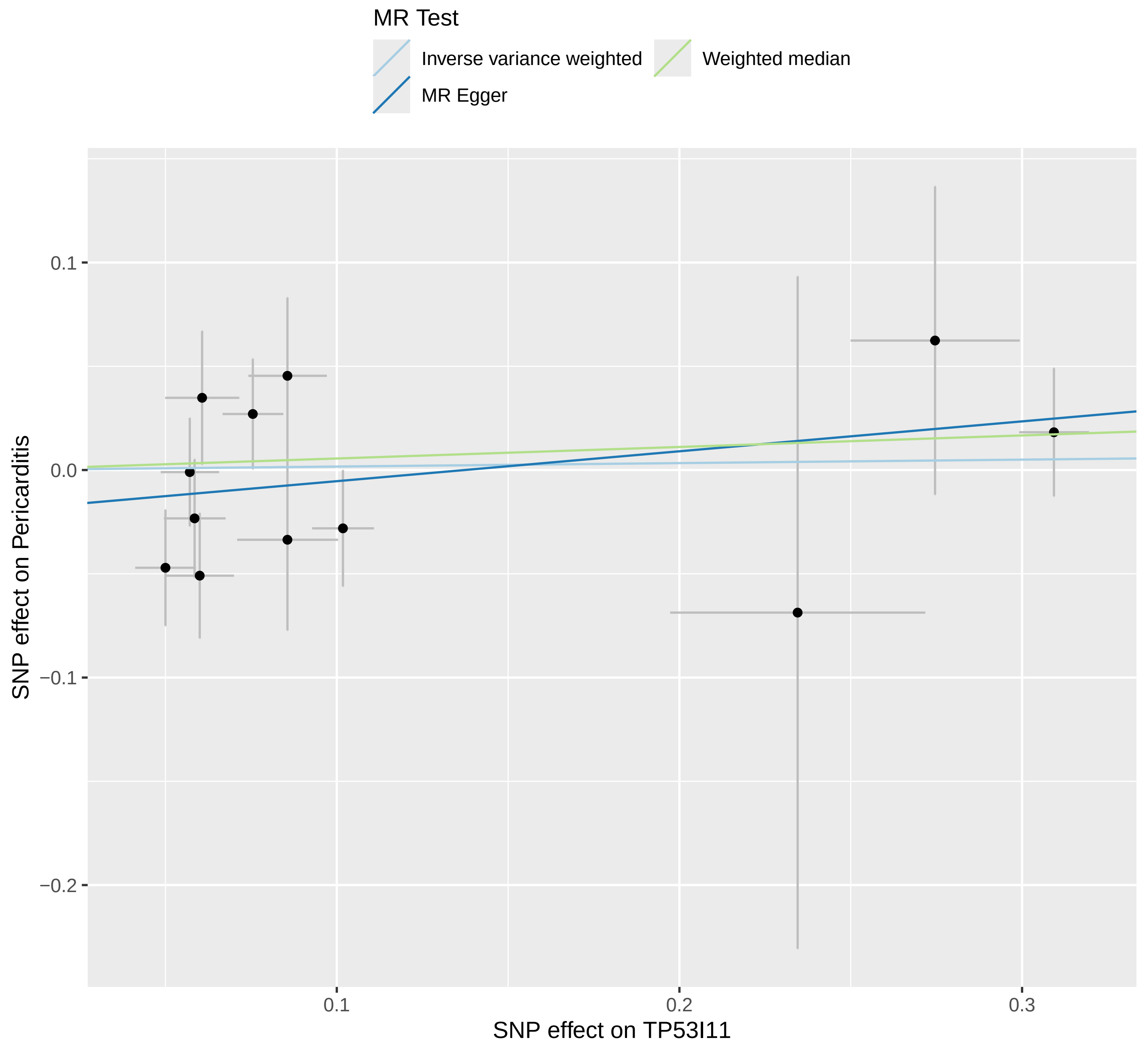

MR Test
Inverse variance weighted
Weighted median
MR Egger
0.1
0.0
−0.1
−0.2
SNP effect on Pericarditis
0.1
0.2
0.3
SNP effect on TP53I11

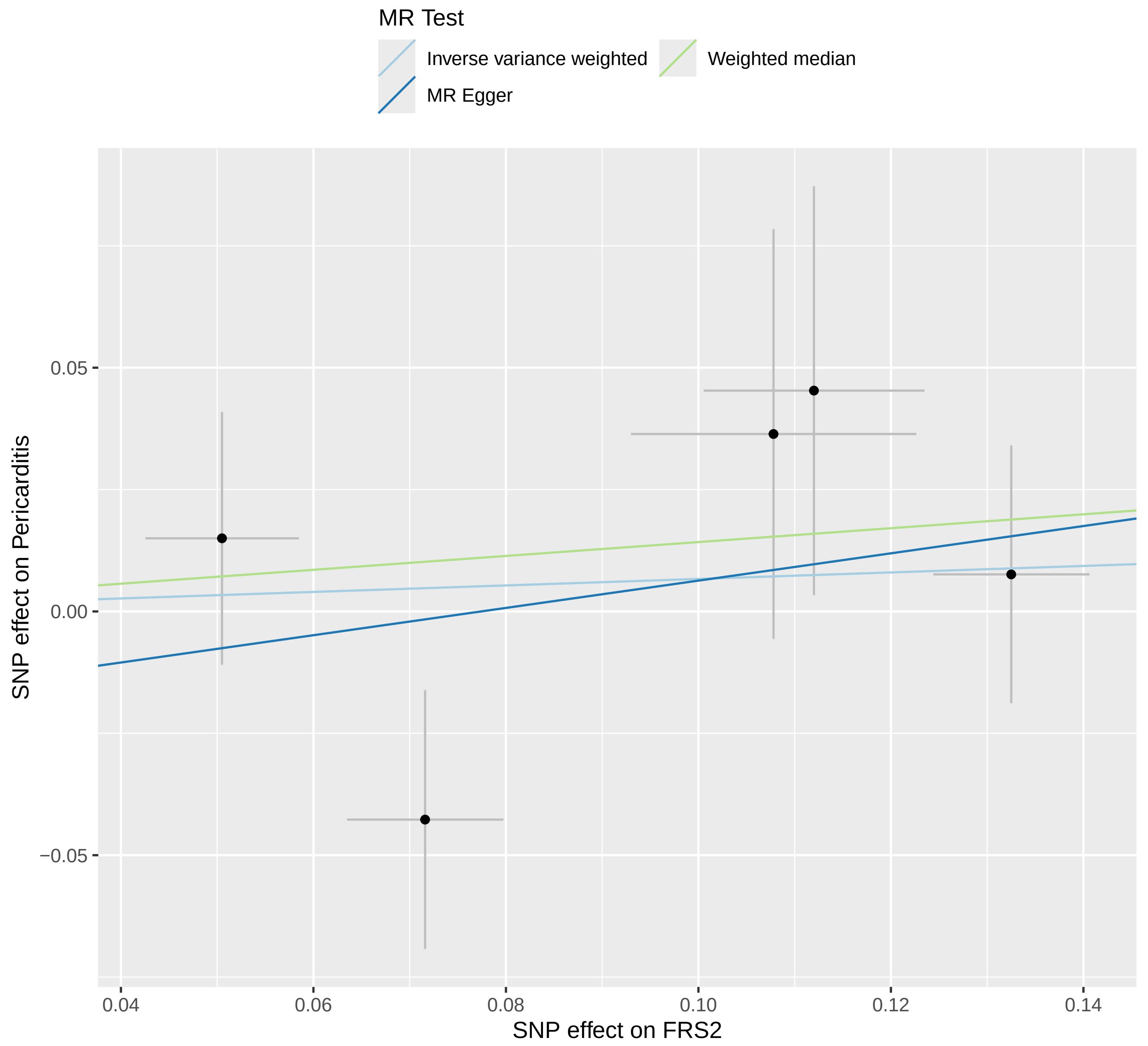

MR Test
Inverse variance weighted
MR Egger
Weighted median
0.05
0.00
−0.05
SNP effect on Pericarditis
0.04
0.06
0.08
0.10
0.12
0.14
SNP effect on FRS2

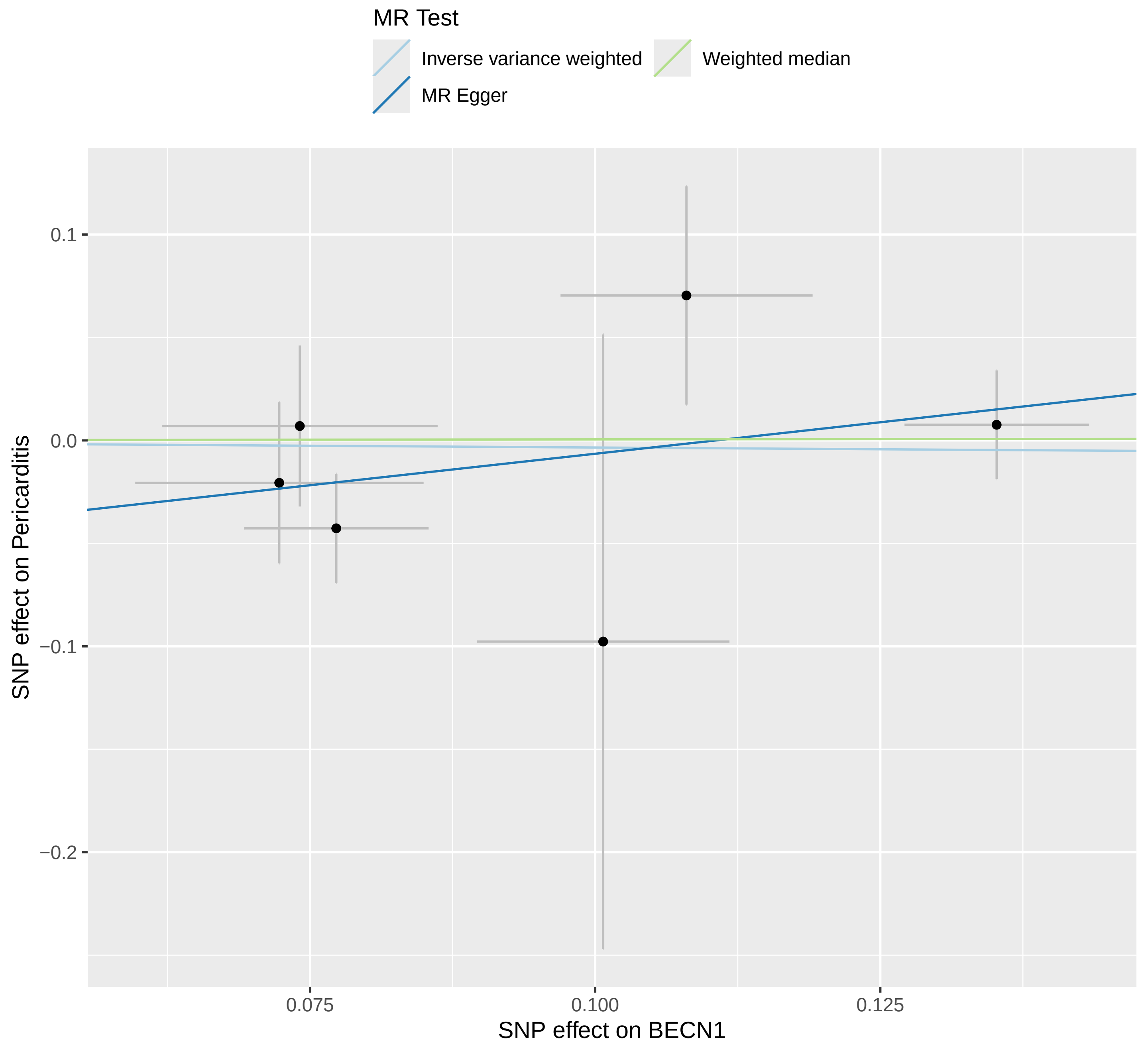

MR Test
Inverse variance weighted
Weighted median
MR Egger
0.1
0.0
−0.1
−0.2
0.075
0.100
0.125
SNP effect on BECN1
SNP effect on Pericarditis

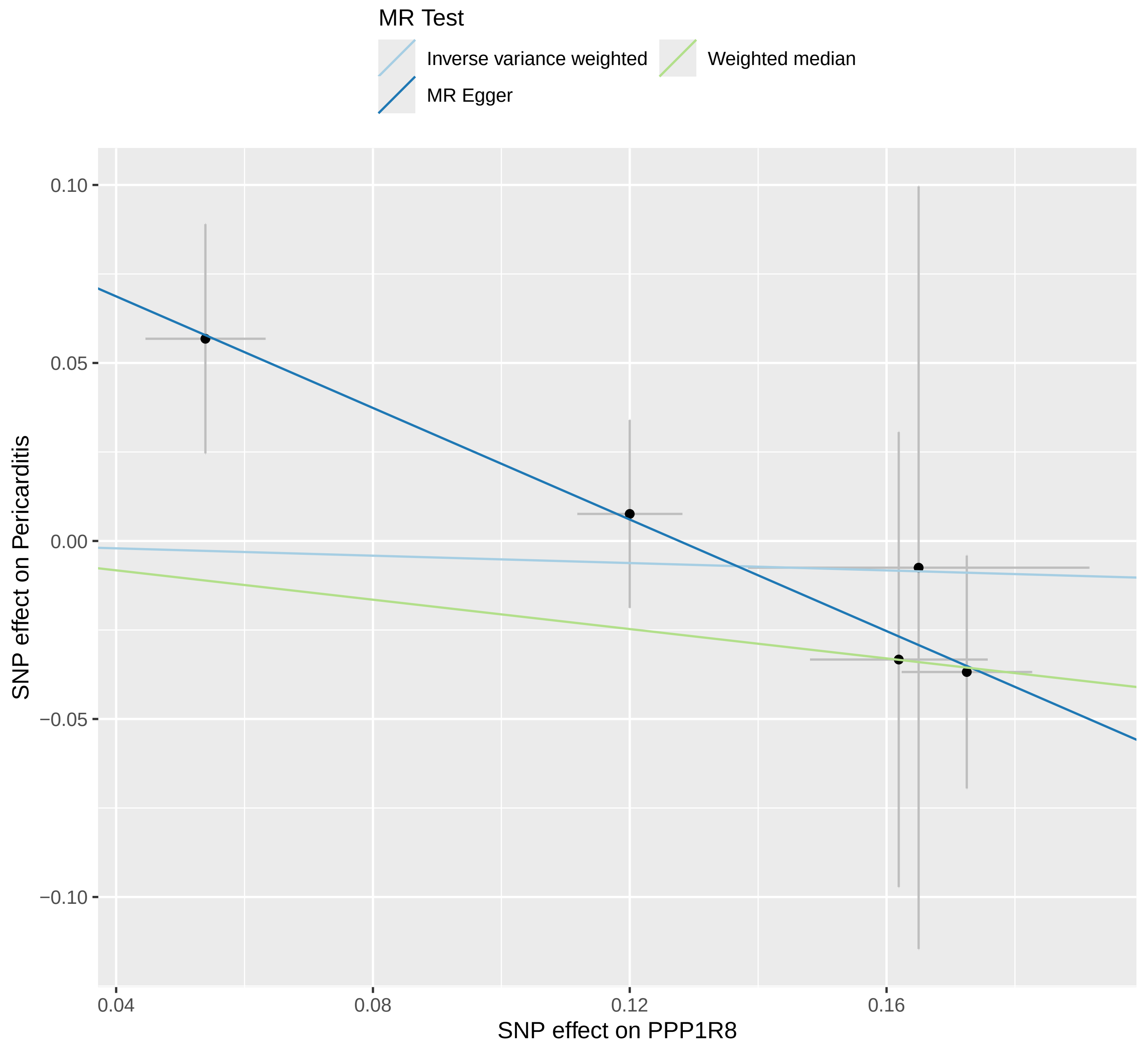
MR Test
Inverse variance weighted
Weighted median
MR Egger
0.10
0.05
0.00
−0.05
−0.10
SNP effect on Pericarditis
0.04
0.08
0.12
0.16
SNP effect on PPP1R8

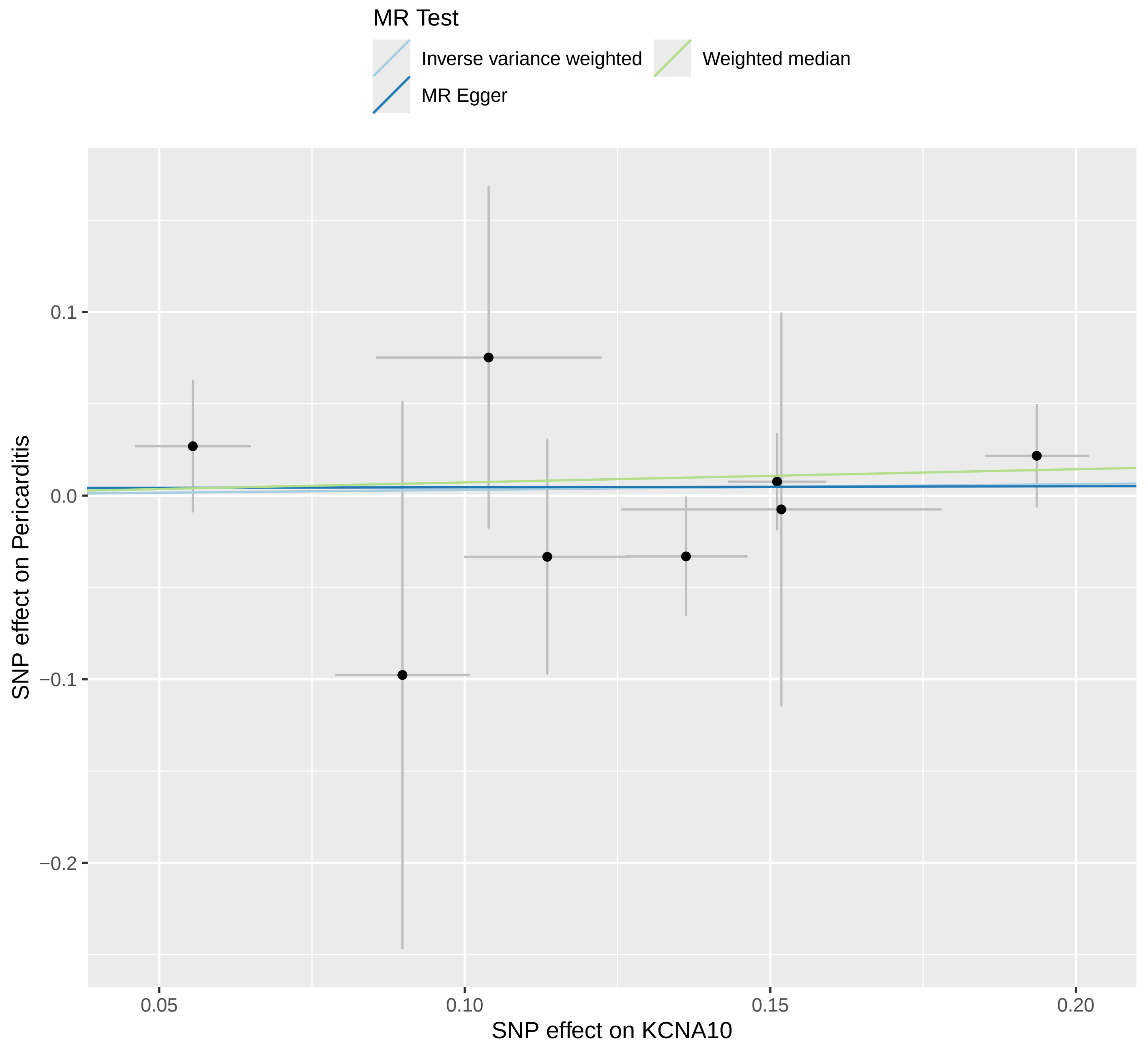
MR Test
Inverse variance weighted
Weighted median
MR Egger
SNP effect on Pericarditis
0.1
0.0
−0.1
−0.2
0.05
0.10
0.15
0.20
SNP effect on KCNA10

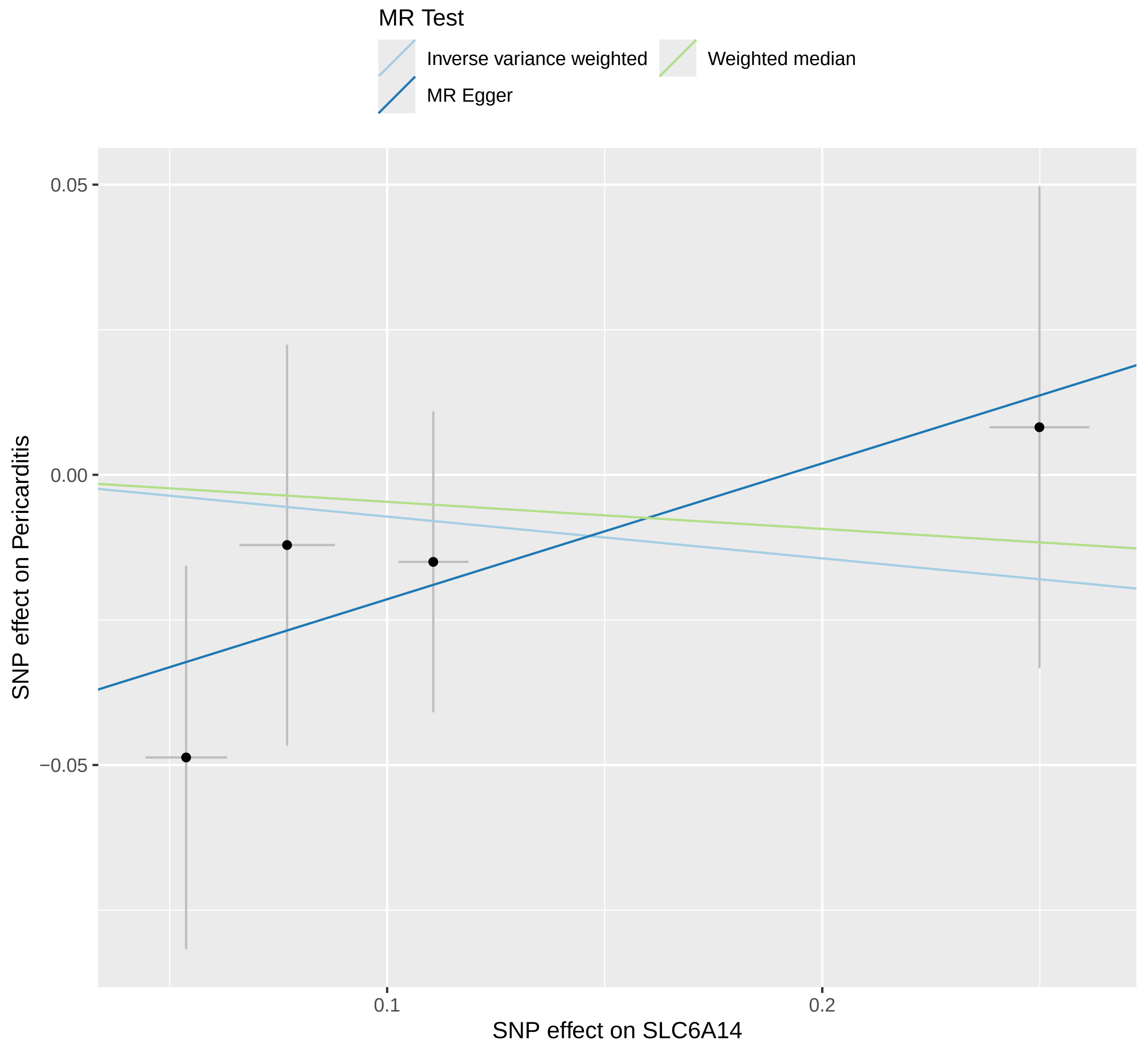
MR Test
Inverse variance weighted
Weighted median
MR Egger
0.05
0.00
−0.05
0.1
0.2
SNP effect on Pericarditis
SNP effect on SLC6A14

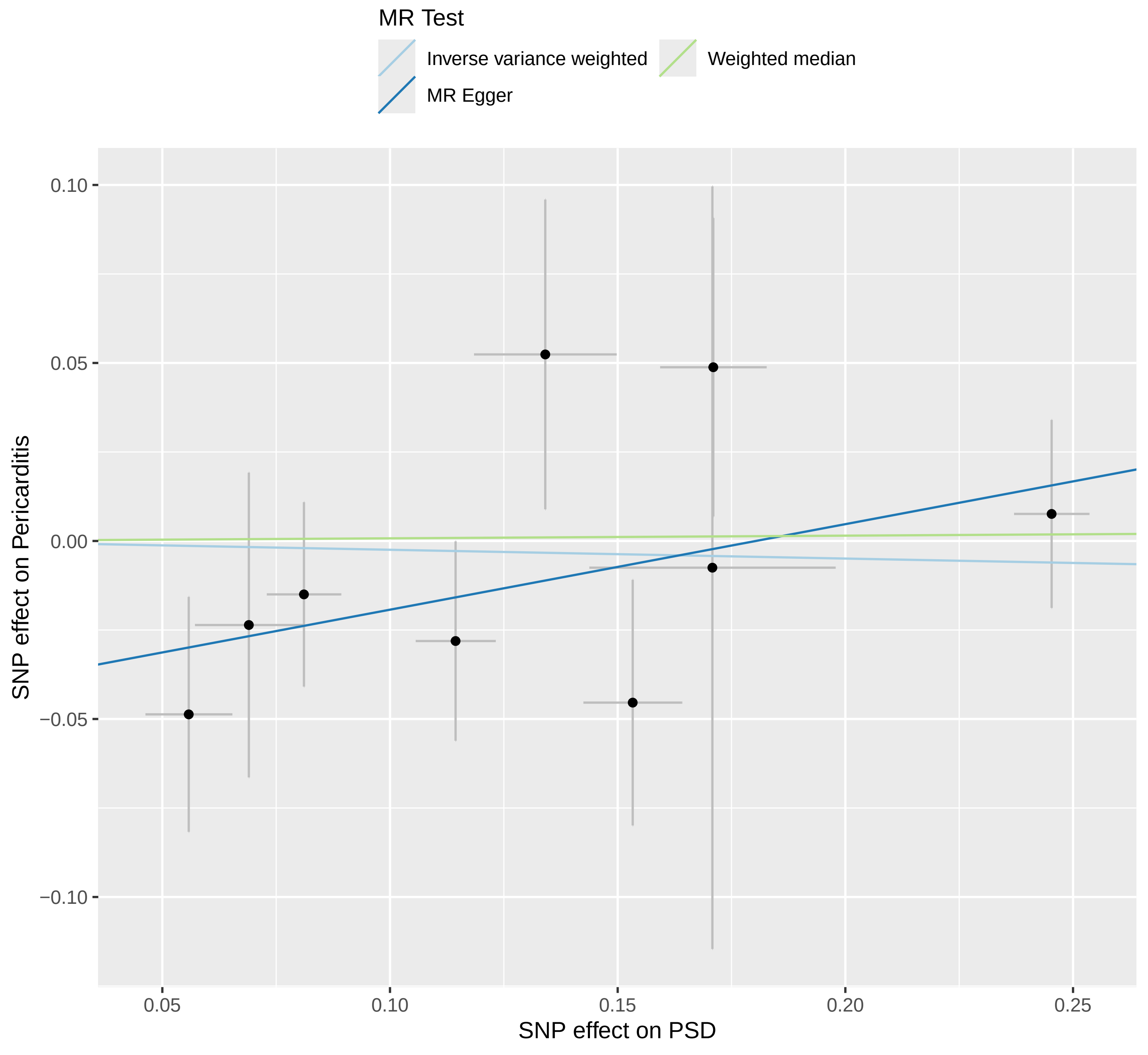

MR Test
Inverse variance weighted
Weighted median
MR Egger
0.10
0.05
0.00
−0.05
−0.10
SNP effect on Pericarditis
0.05
0.10
0.15
0.20
0.25
SNP effect on PSD

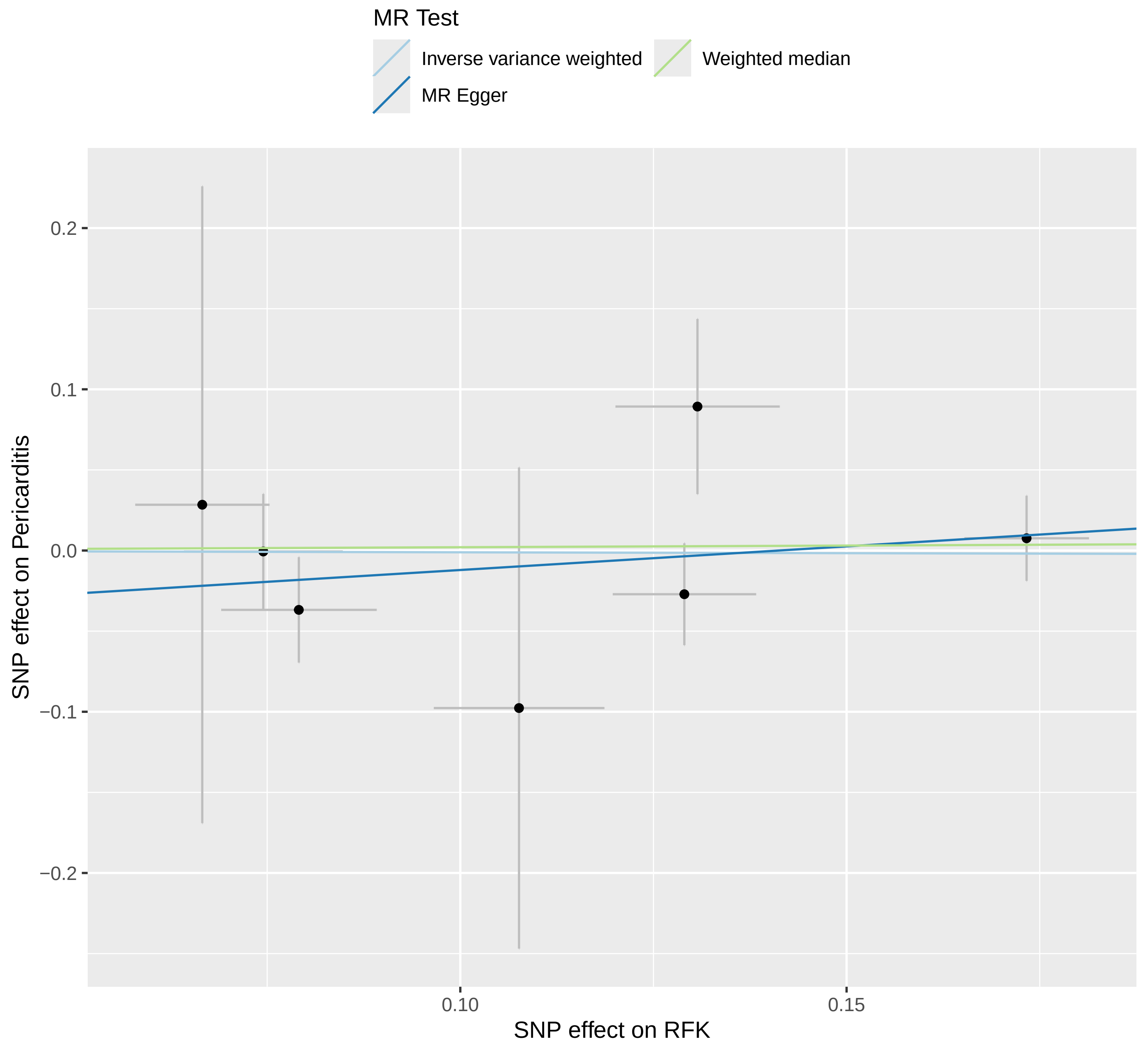

MR Test
Inverse variance weighted
Weighted median
MR Egger
SNP effect on Pericarditis
0.2
0.1
0.0
−0.1
−0.2
0.10
0.15
SNP effect on RFK

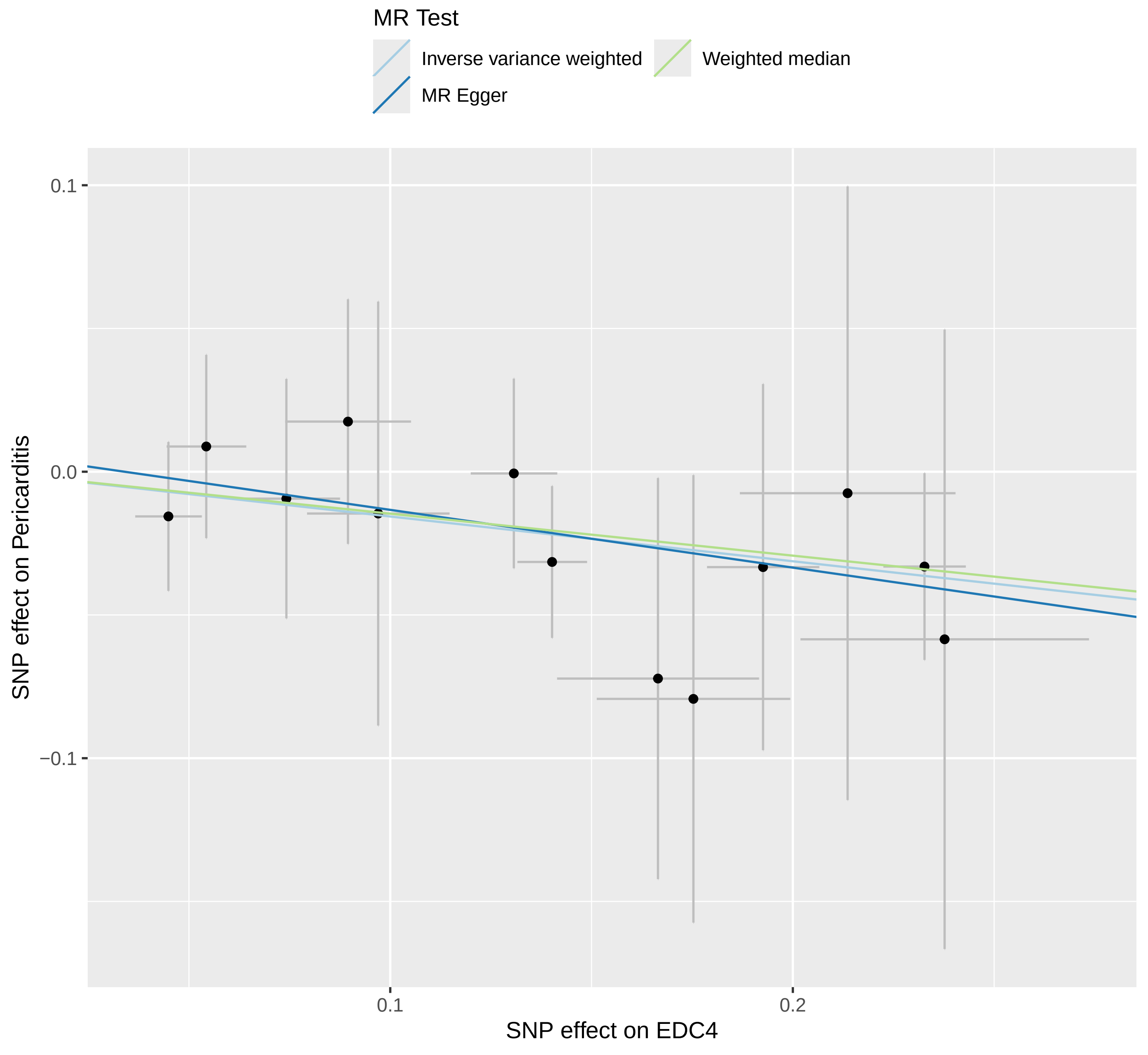
MR Test
Inverse variance weighted
Weighted median
MR Egger
0.1
0.0
−0.1
0.1
0.2
SNP effect on Pericarditis
SNP effect on EDC4

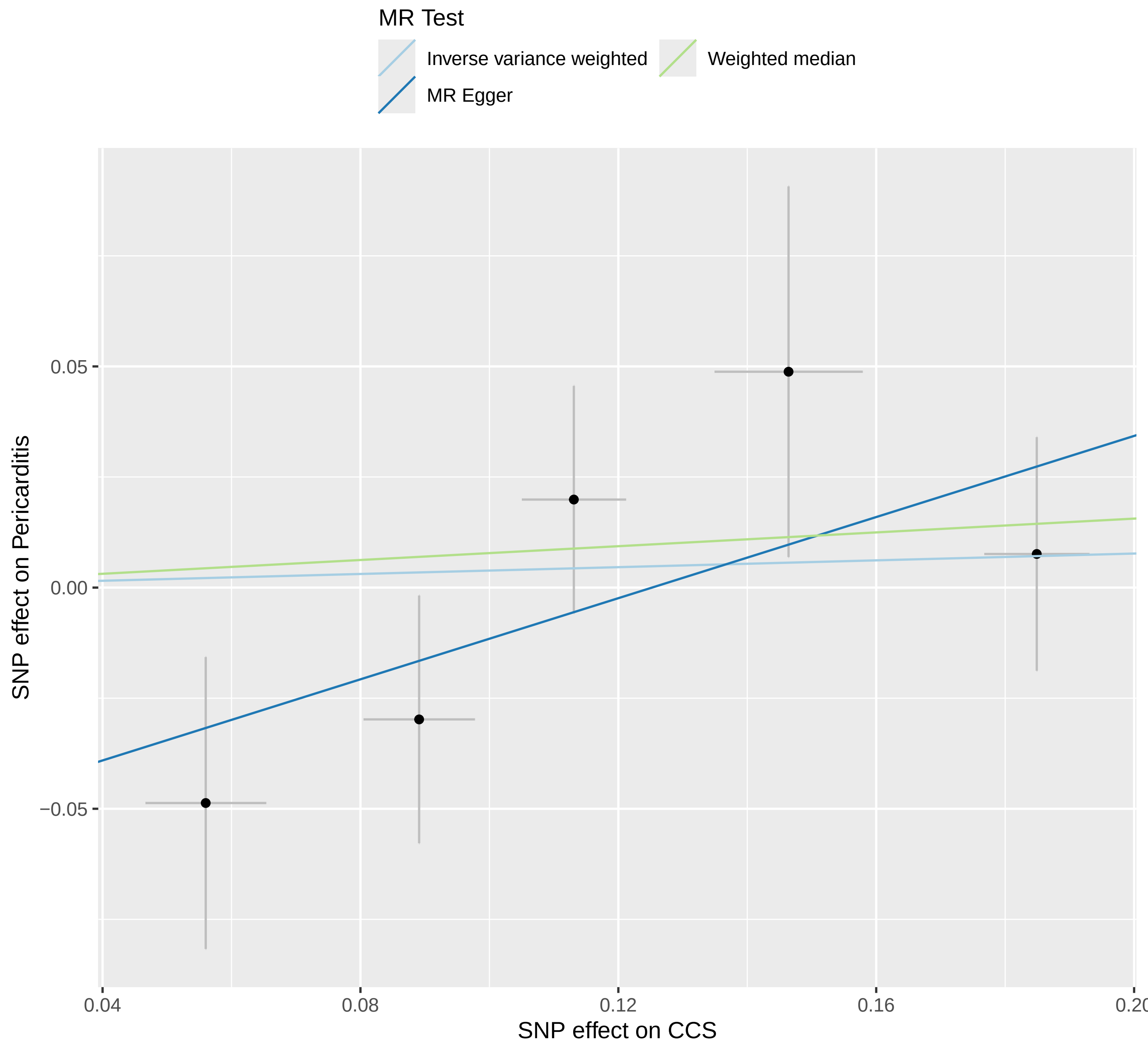

MR Test
Inverse variance weighted
Weighted median
MR Egger
SNP effect on Pericarditis
0.05
0.00
−0.05
0.04
0.08
0.12
0.16
0.20
SNP effect on CCS

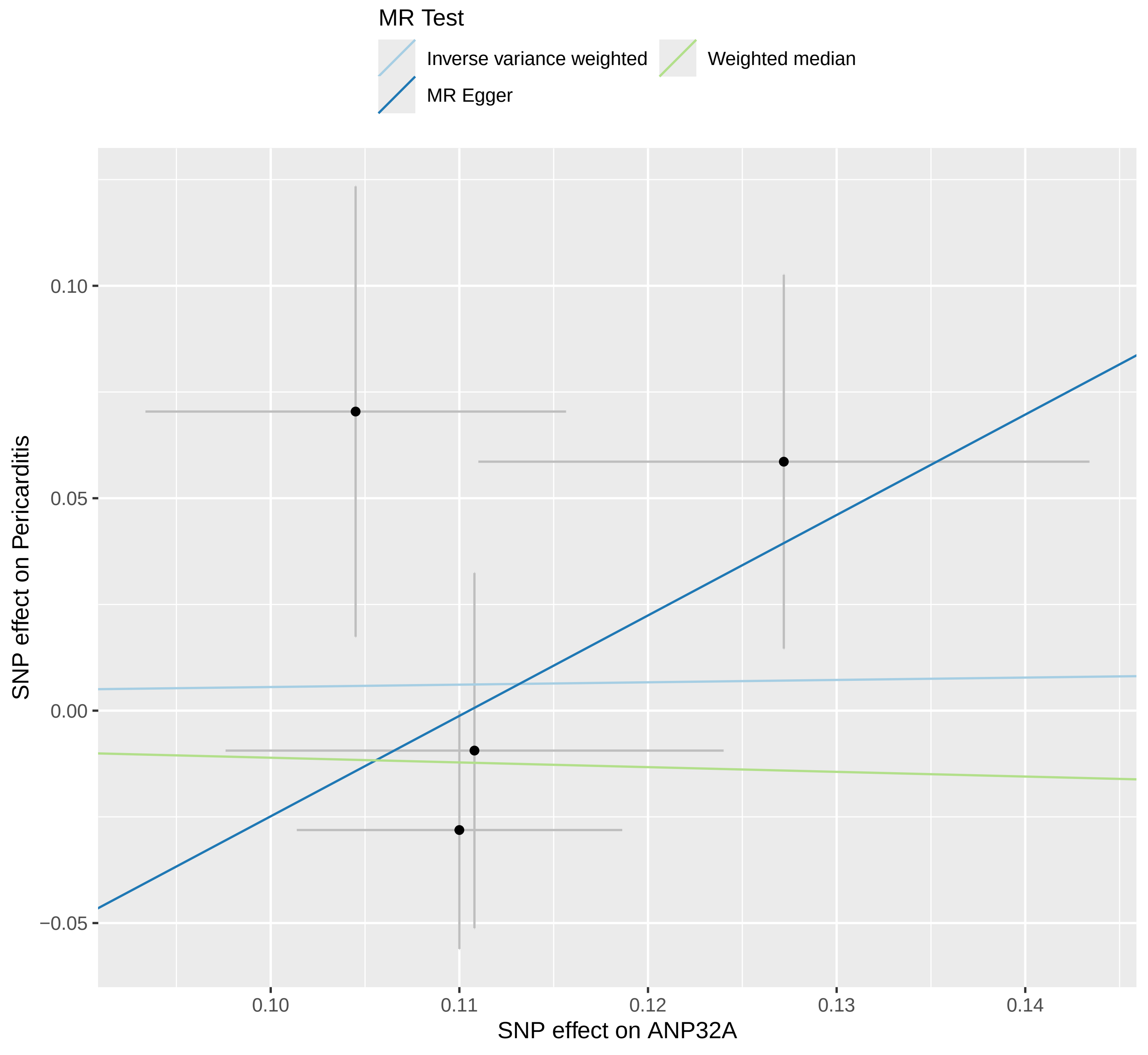
MR Test
Inverse variance weighted
Weighted median
MR Egger
SNP effect on Pericarditis
0.10
0.05
0.00
−0.05
0.10
0.11
0.12
0.13
0.14
SNP effect on ANP32A

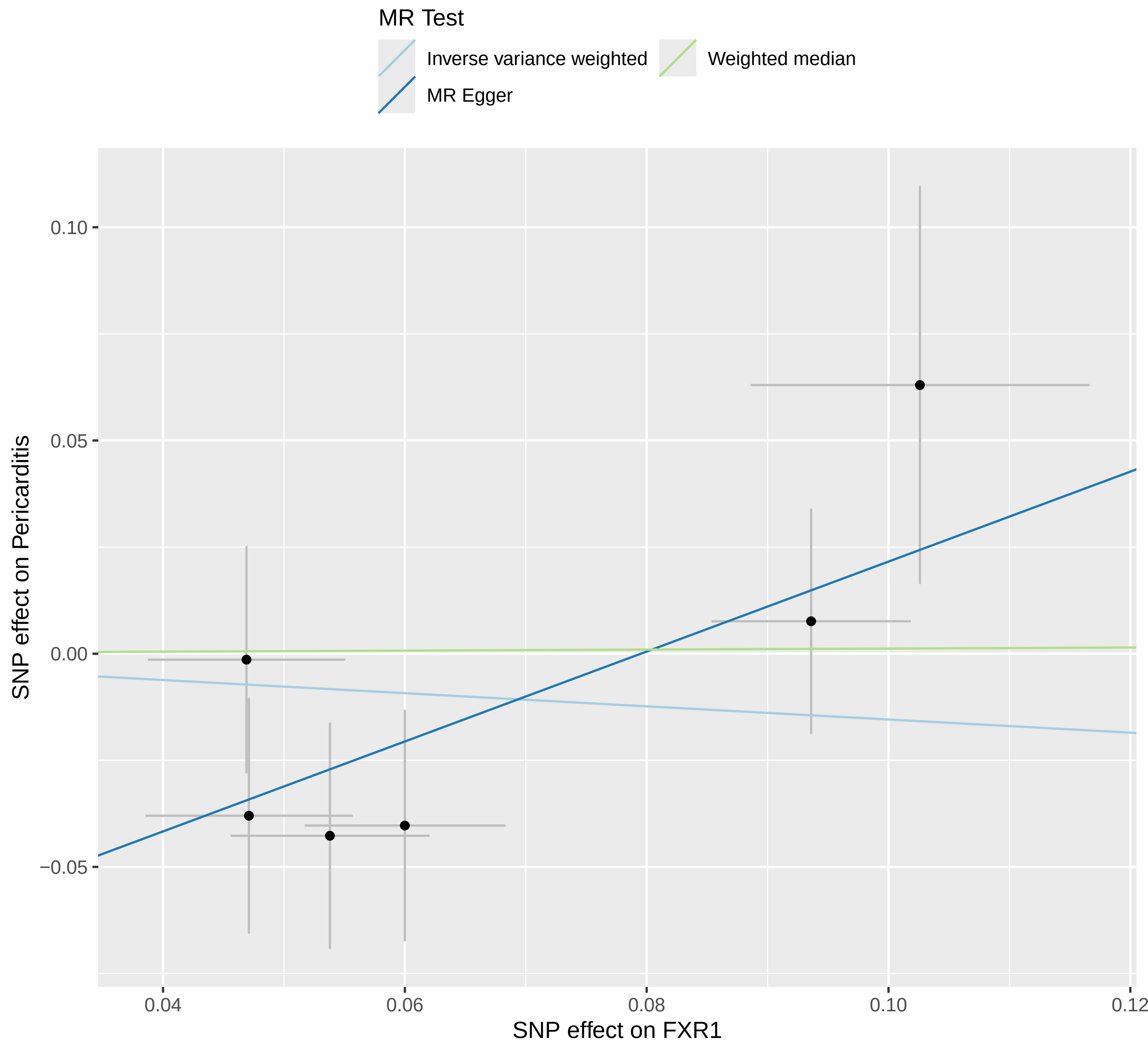
MR Test
Inverse variance weighted
MR Egger
Weighted median
SNP effect on Pericarditis
0.10
0.05
0.00
−0.05
0.04
0.06
0.08
0.10
0.12
SNP effect on FXR1

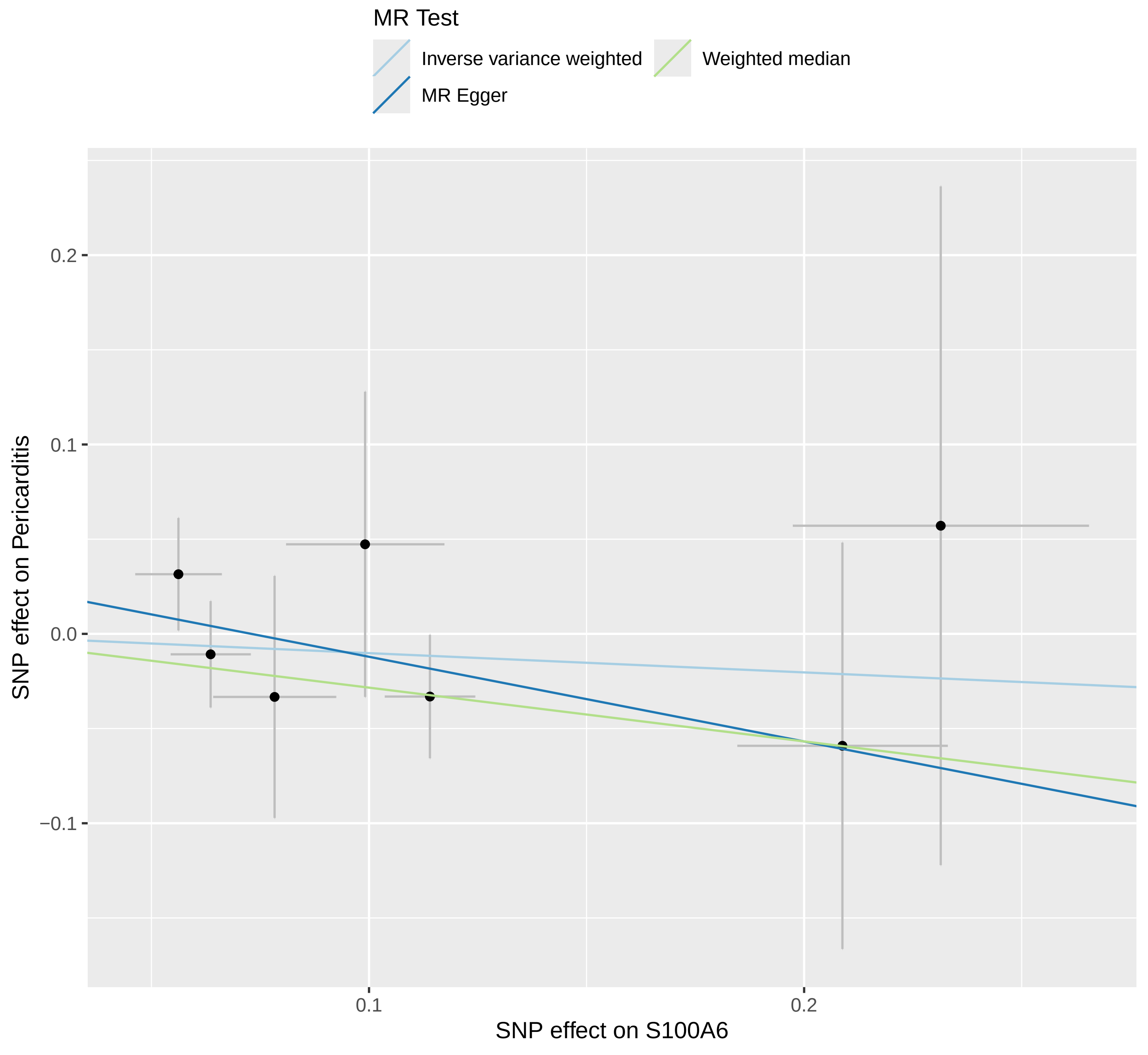

MR Test
Inverse variance weighted
Weighted median
MR Egger
0.2
0.1
0.0
−0.1
0.1
0.2
SNP effect on Pericarditis
SNP effect on S100A6

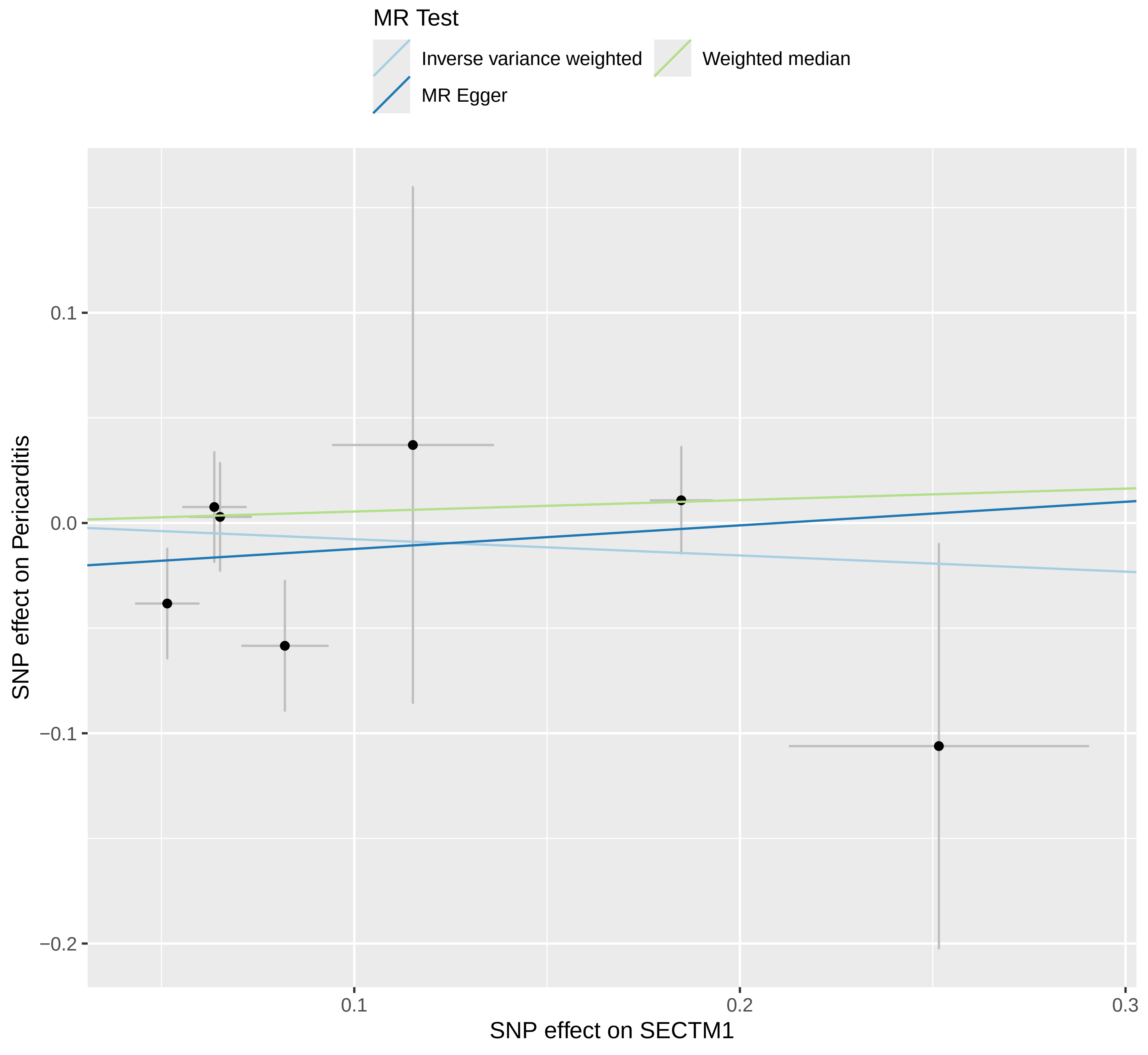

MR Test
Inverse variance weighted
Weighted median
MR Egger
SNP effect on Pericarditis
0.1
0.0
−0.1
−0.2
0.1
0.2
0.3
SNP effect on SECTM1

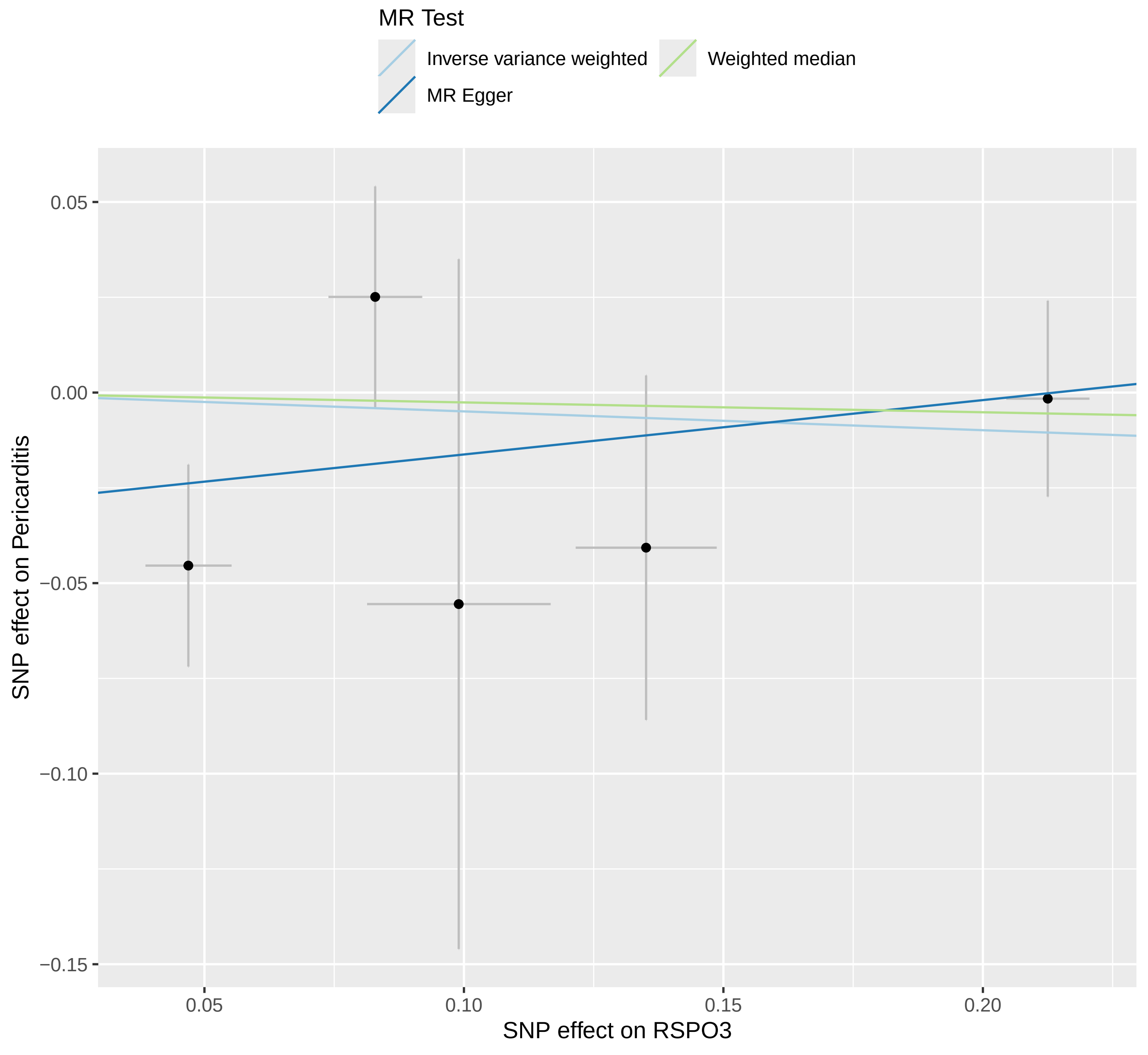
MR Test
Inverse variance weighted
Weighted median
MR Egger
0.05
0.00
−0.05
−0.10
−0.15
SNP effect on Pericarditis
0.05
0.10
0.15
0.20
SNP effect on RSPO3

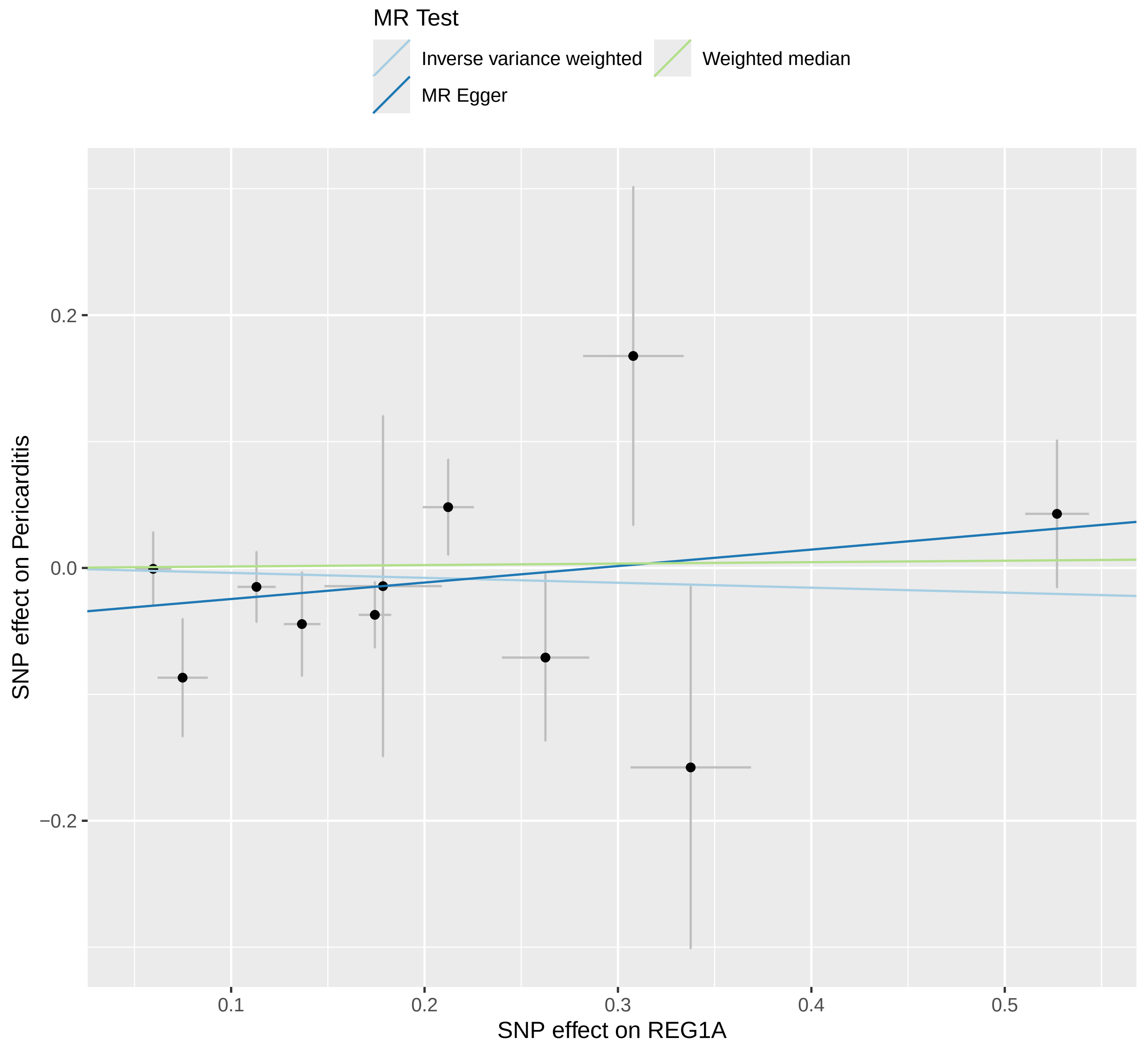
MR Test
Inverse variance weighted
Weighted median
MR Egger
0.2
0.0
−0.2
SNP effect on Pericarditis
0.1
0.2
0.3
0.4
0.5
SNP effect on REG1A

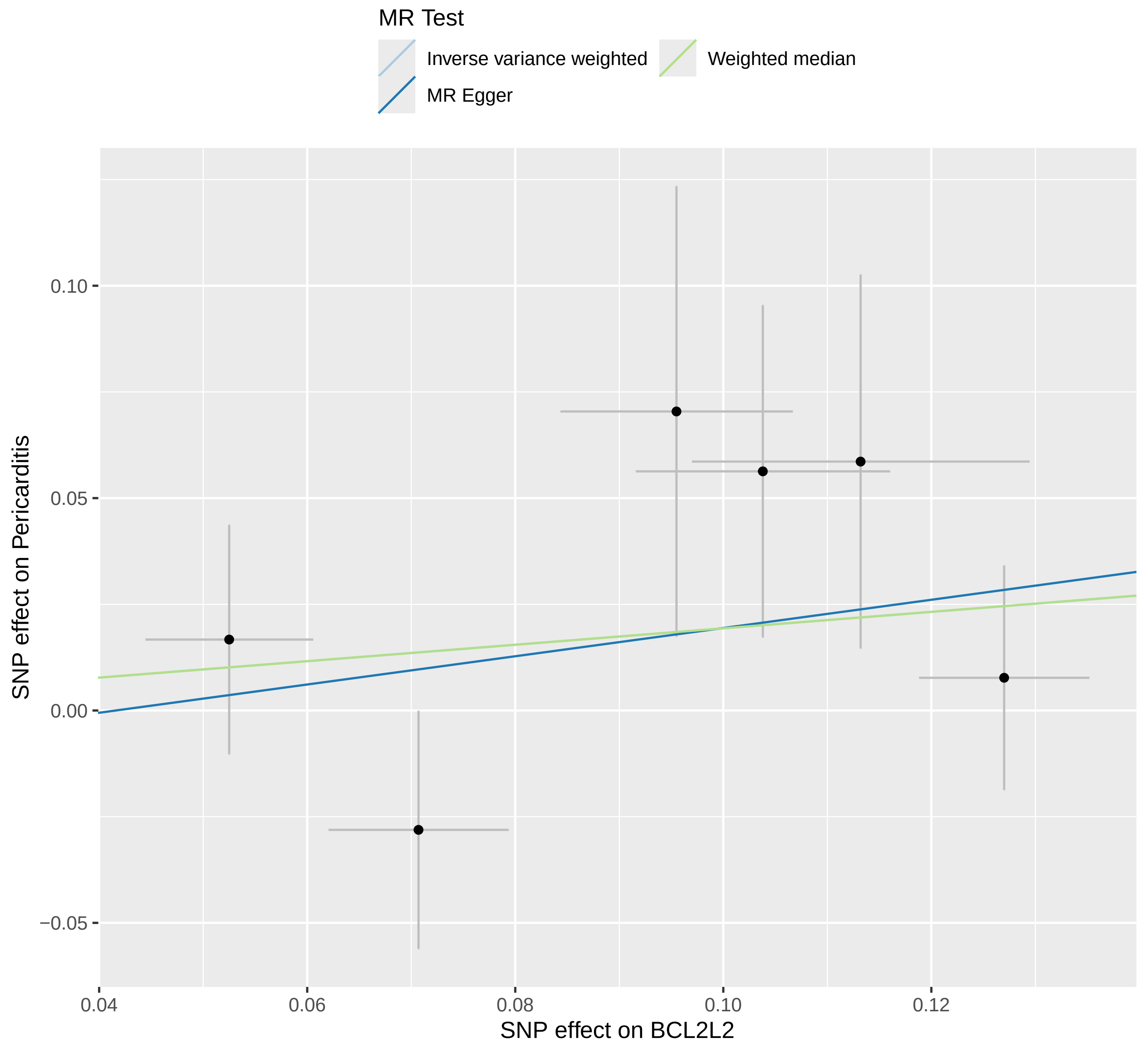
MR Test
Inverse variance weighted
Weighted median
MR Egger
SNP effect on Pericarditis
0.10
0.05
0.00
−0.05
0.04
0.06
0.08
0.10
0.12
SNP effect on BCL2L2

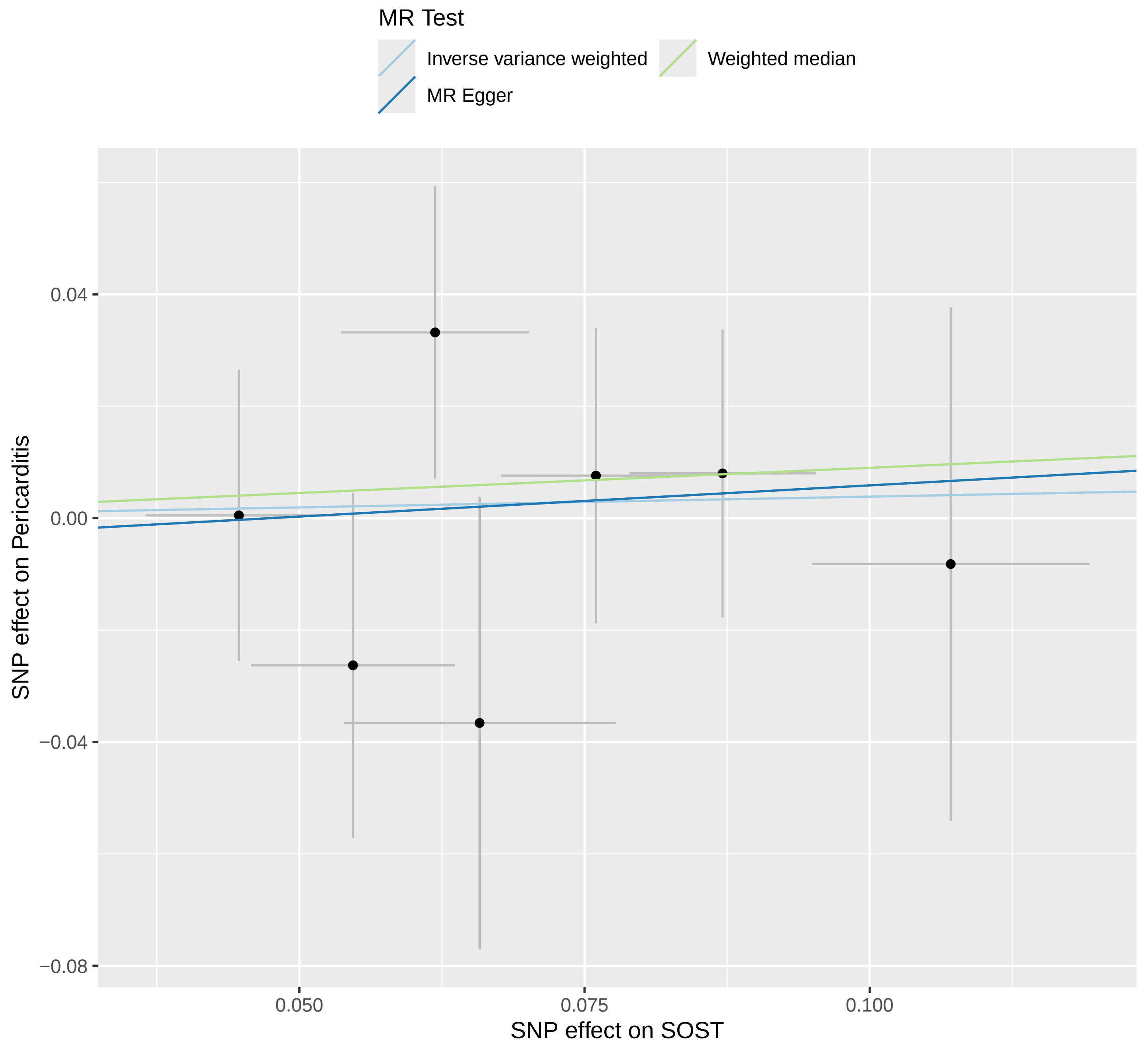
MR Test
Inverse variance weighted
Weighted median
MR Egger
SNP effect on Pericarditis
0.04
0.00
−0.04
−0.08
0.050
0.075
0.100
SNP effect on SOST

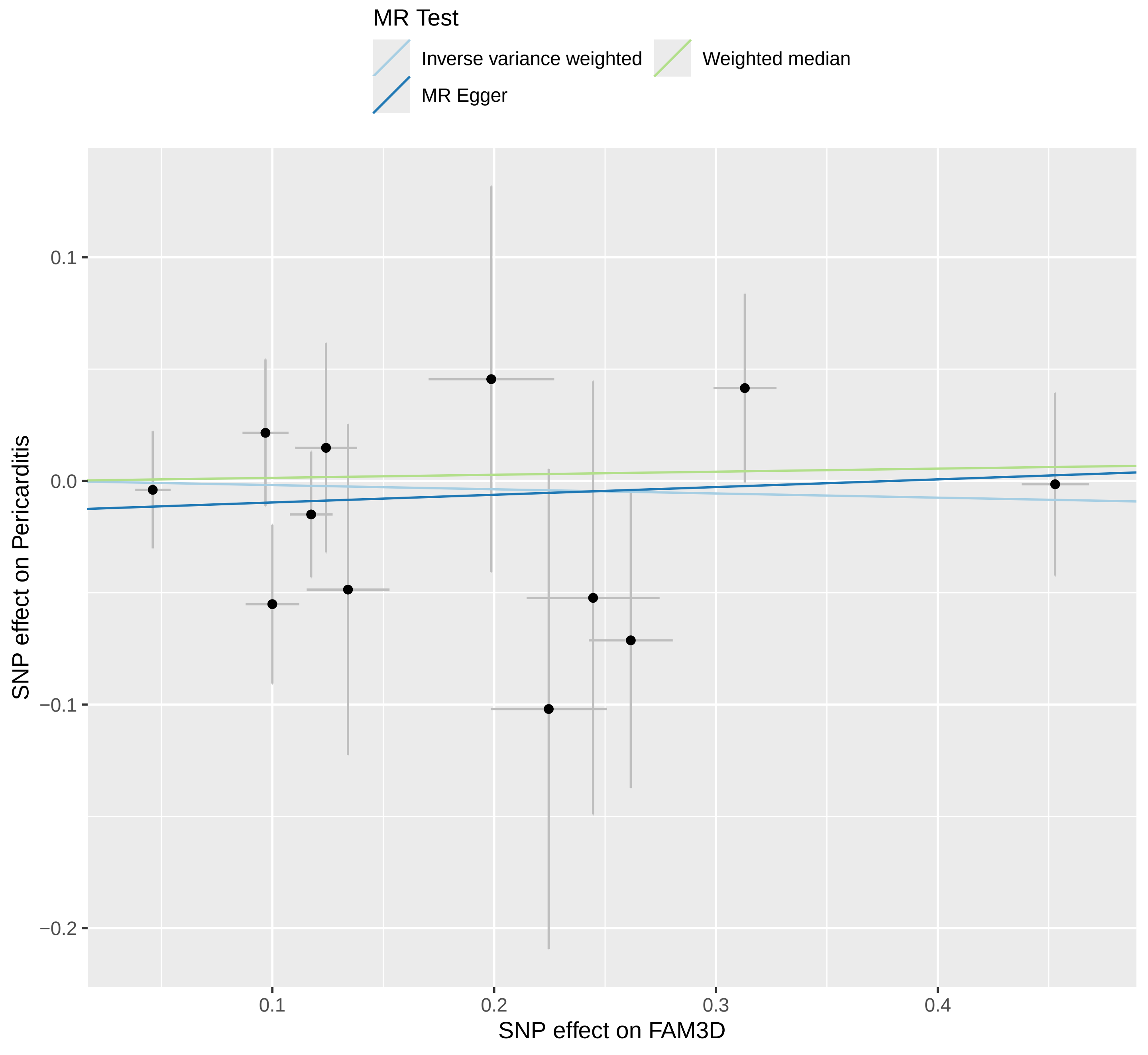
MR Test
Inverse variance weighted
Weighted median
MR Egger
0.1
0.0
−0.1
−0.2
SNP effect on Pericarditis
0.1
0.2
0.3
0.4
SNP effect on FAM3D

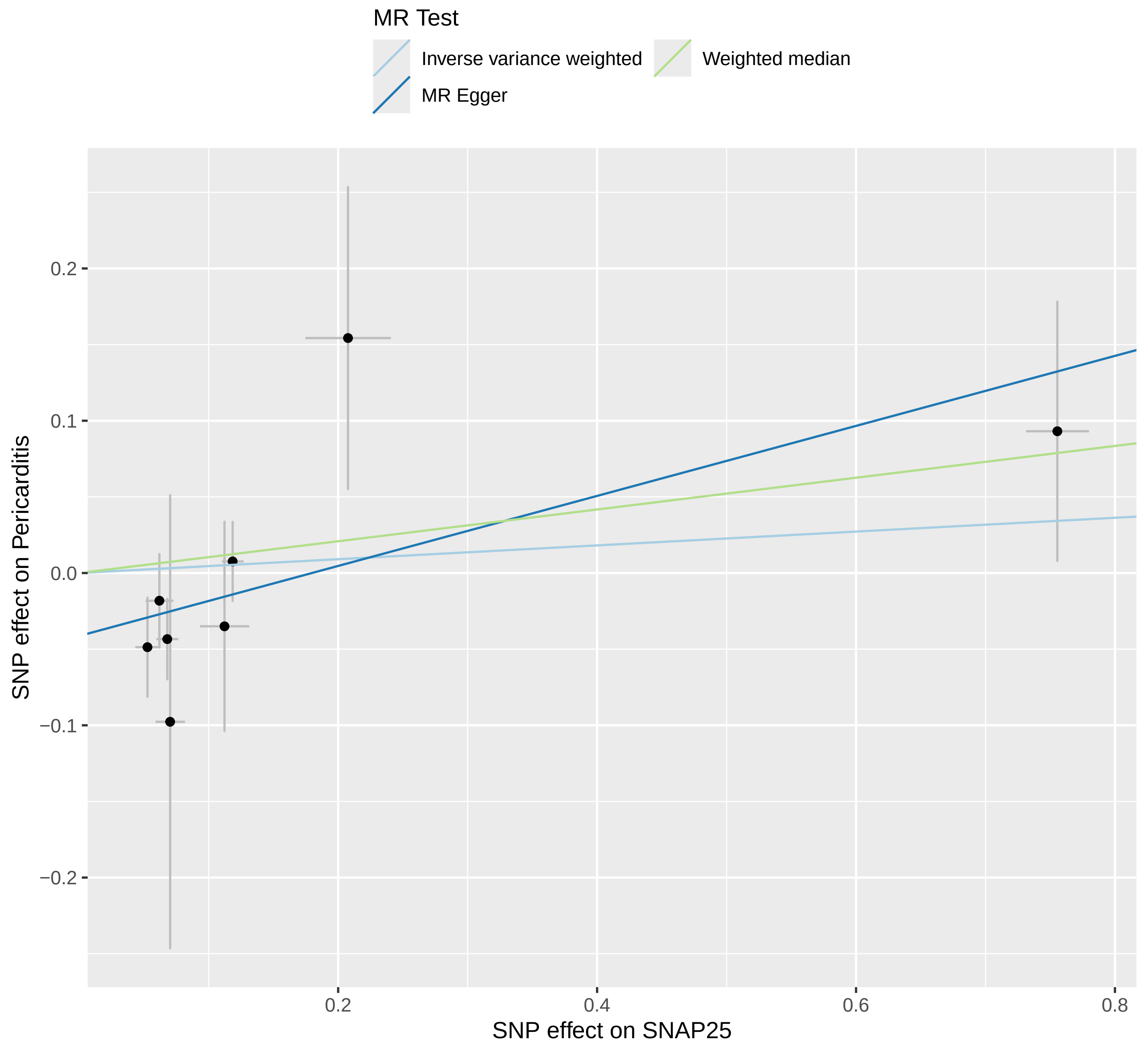
MR Test
Inverse variance weighted
Weighted median
MR Egger
0.2
0.1
0.0
−0.1
−0.2
SNP effect on Pericarditis
0.2
0.4
0.6
0.8
SNP effect on SNAP25

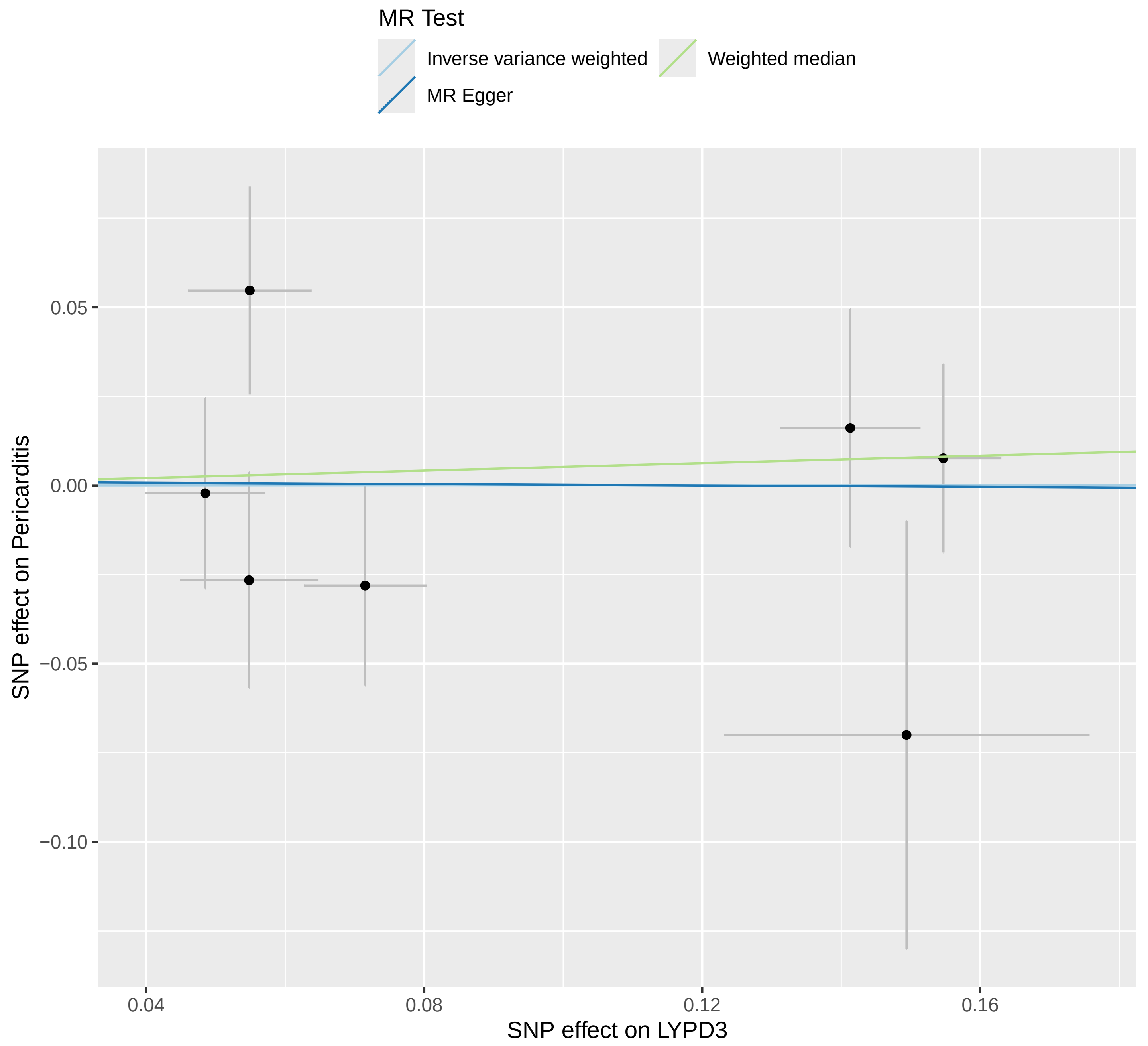
MR Test
Inverse variance weighted
Weighted median
MR Egger
0.05
0.00
−0.05
−0.10
SNP effect on Pericarditis
0.04
0.08
0.12
0.16
SNP effect on LYPD3

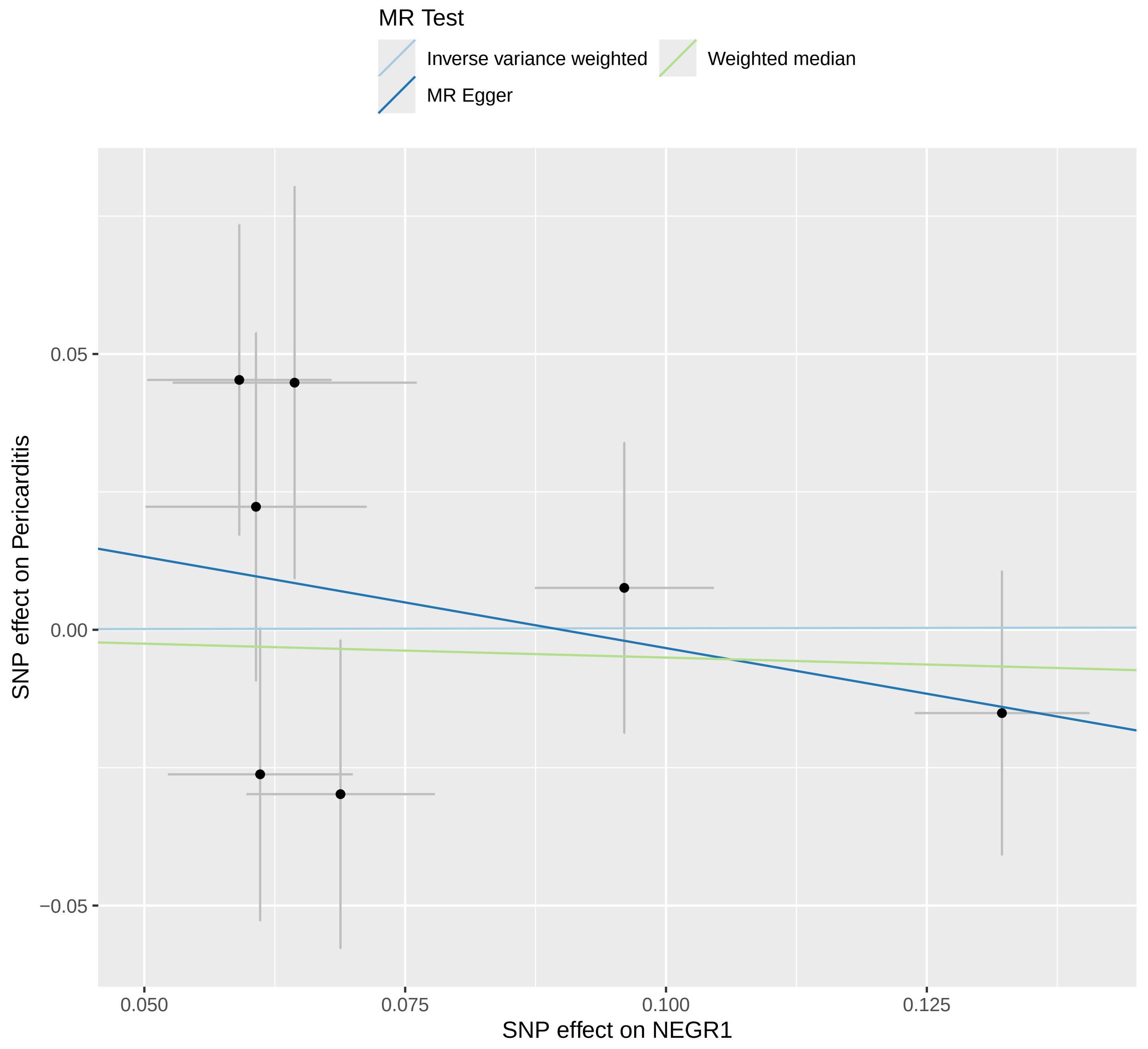
MR Test
Inverse variance weighted
MR Egger
Weighted median
SNP effect on Pericarditis
0.05
0.00
−0.05
0.050
0.075
0.100
0.125
SNP effect on NEGR1

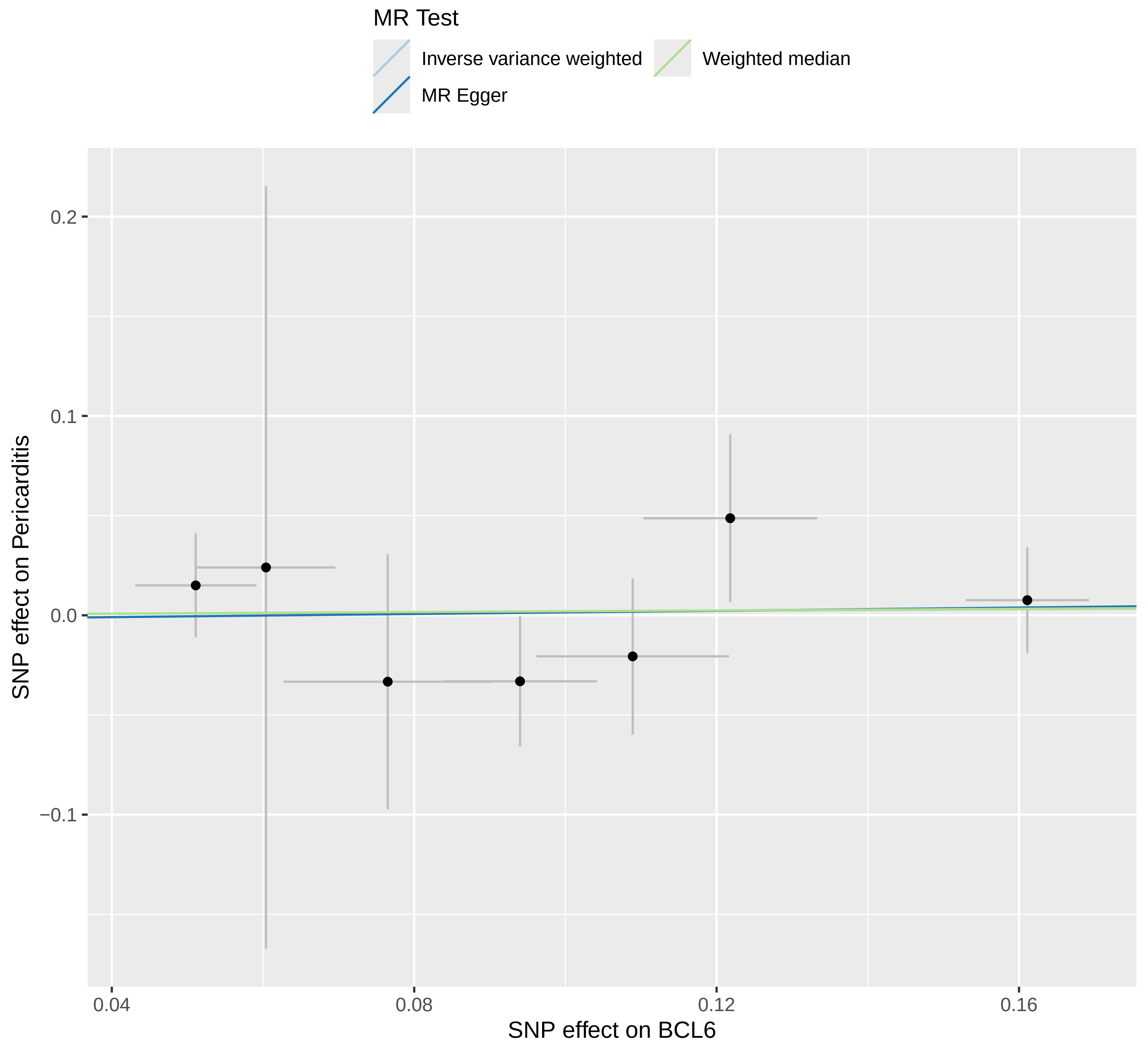

MR Test
Inverse variance weighted
Weighted median
MR Egger
0.2
0.1
0.0
−0.1
0.04
0.08
0.12
0.16
SNP effect on Pericarditis
SNP effect on BCL6

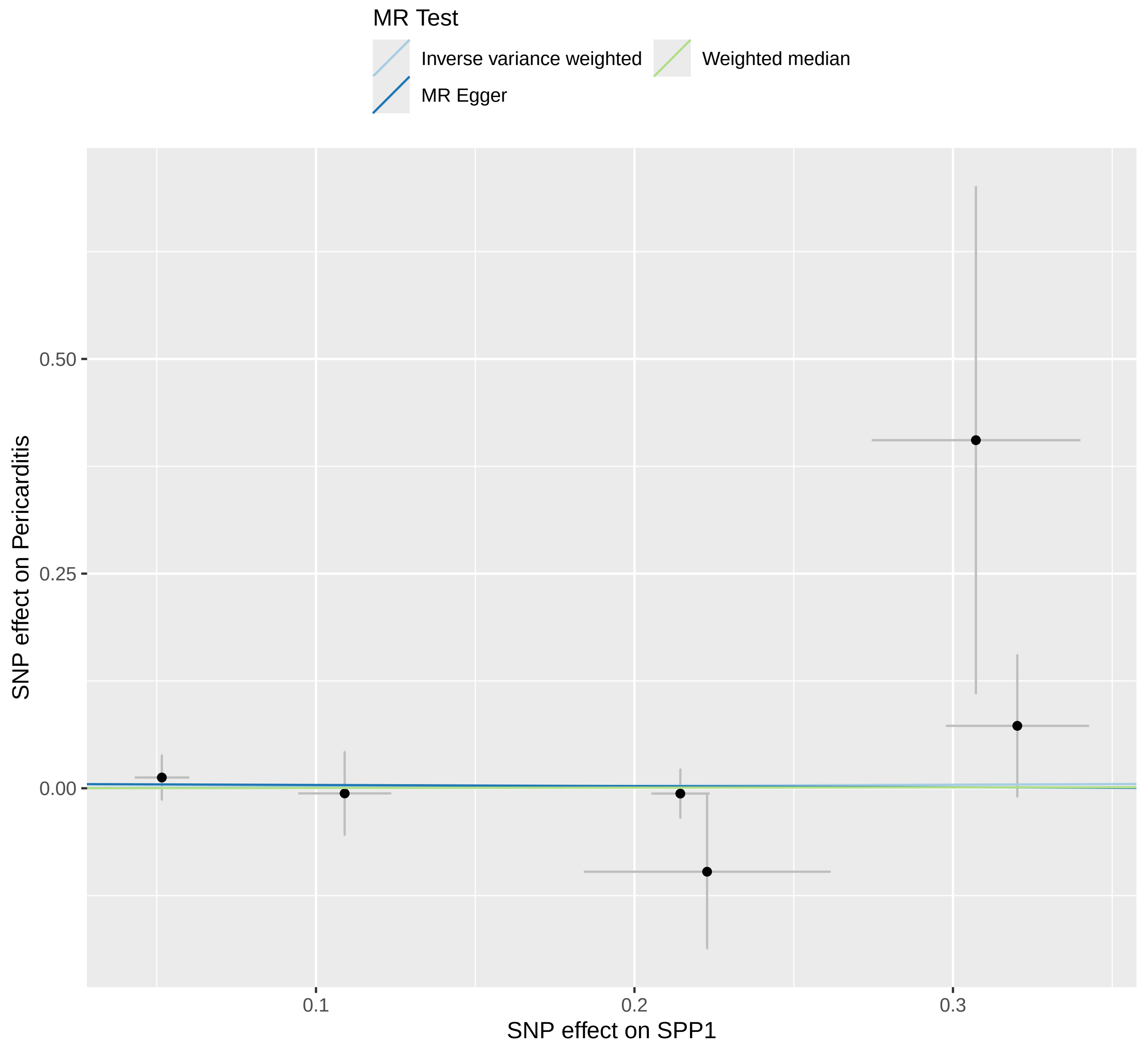
MR Test
Inverse variance weighted
Weighted median
MR Egger
0.50
0.25
0.00
0.1
0.2
0.3
SNP effect on Pericarditis
SNP effect on SPP1

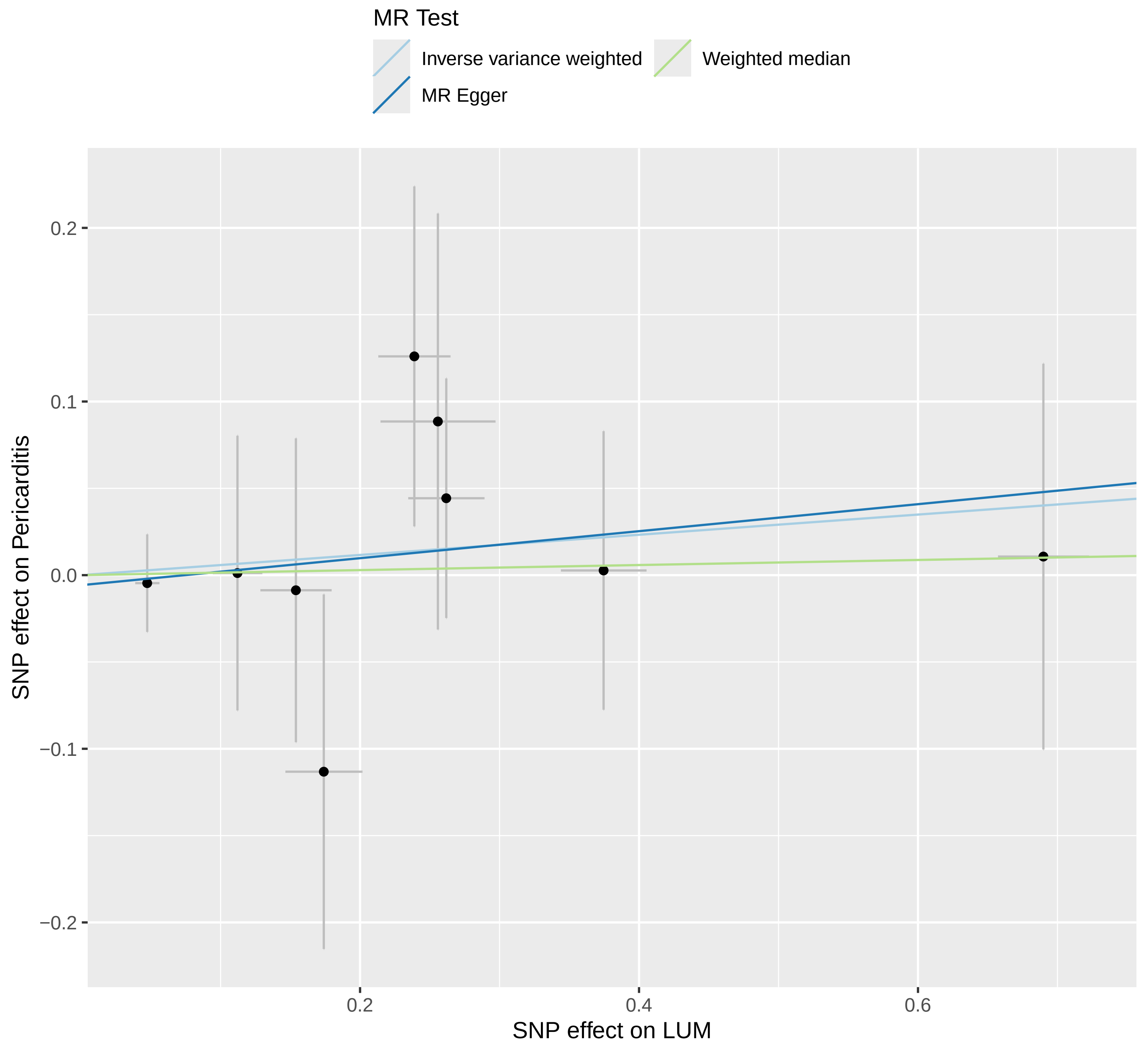

MR Test
Inverse variance weighted
Weighted median
MR Egger
SNP effect on Pericarditis
0.2
0.1
0.0
−0.1
−0.2
0.2
0.4
0.6
SNP effect on LUM

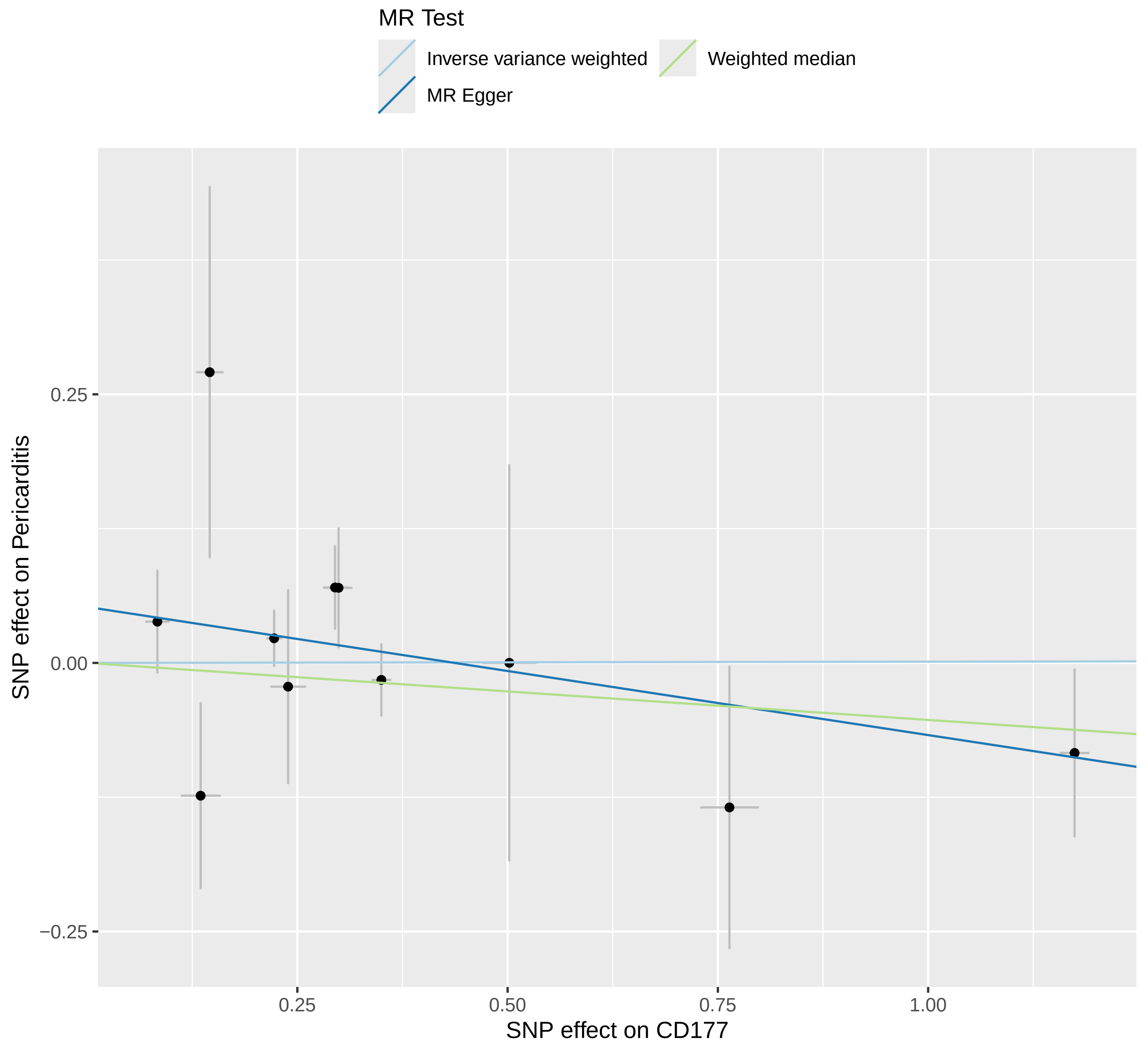
MR Test
Inverse variance weighted
Weighted median
MR Egger
SNP effect on Pericarditis
0.25
0.00
−0.25
0.25
0.50
0.75
1.00
SNP effect on CD177

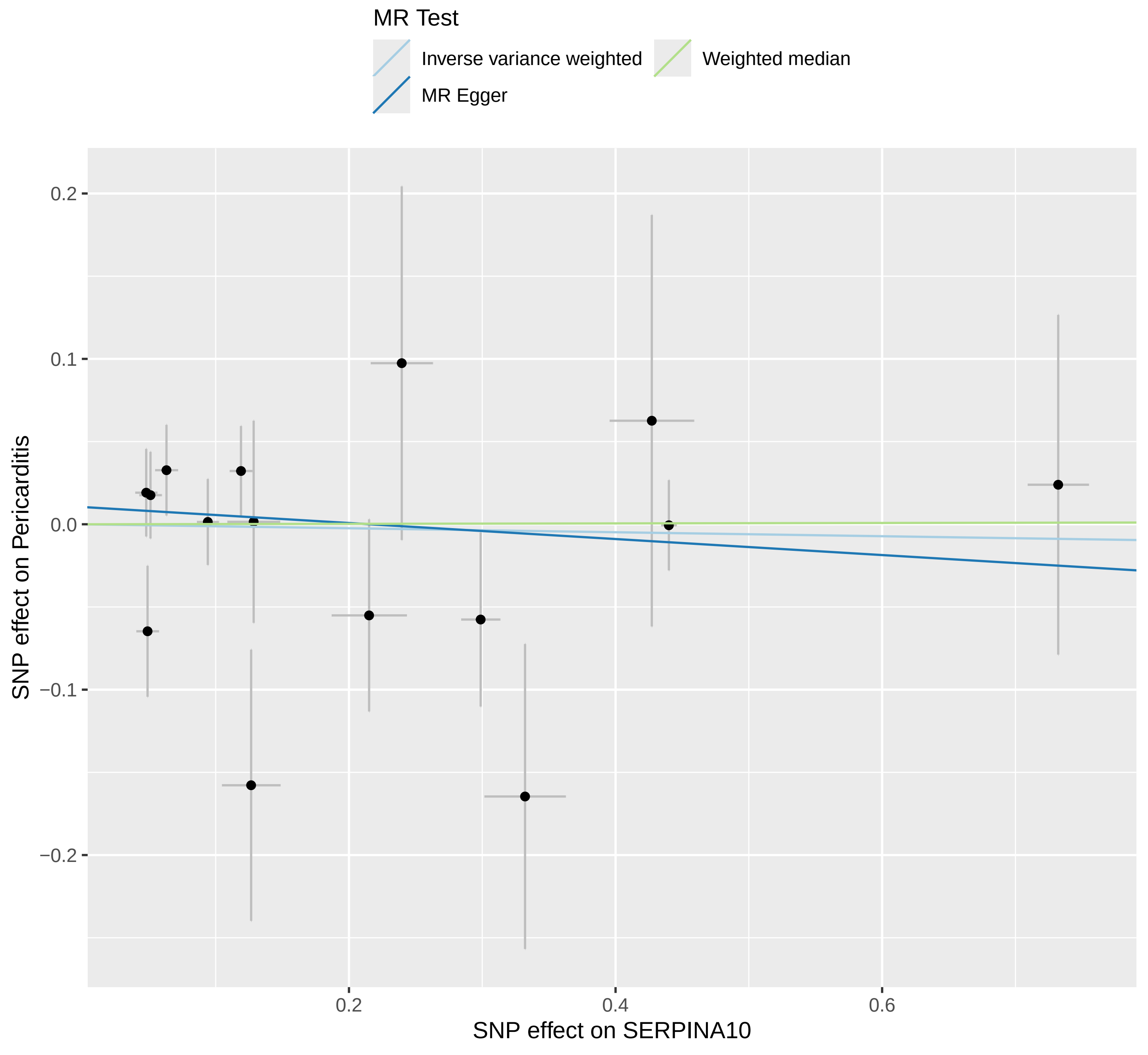

MR Test
Inverse variance weighted
Weighted median
MR Egger
SNP effect on Pericarditis
0.2
0.1
0.0
−0.1
−0.2
0.2
0.4
0.6
SNP effect on SERPINA10

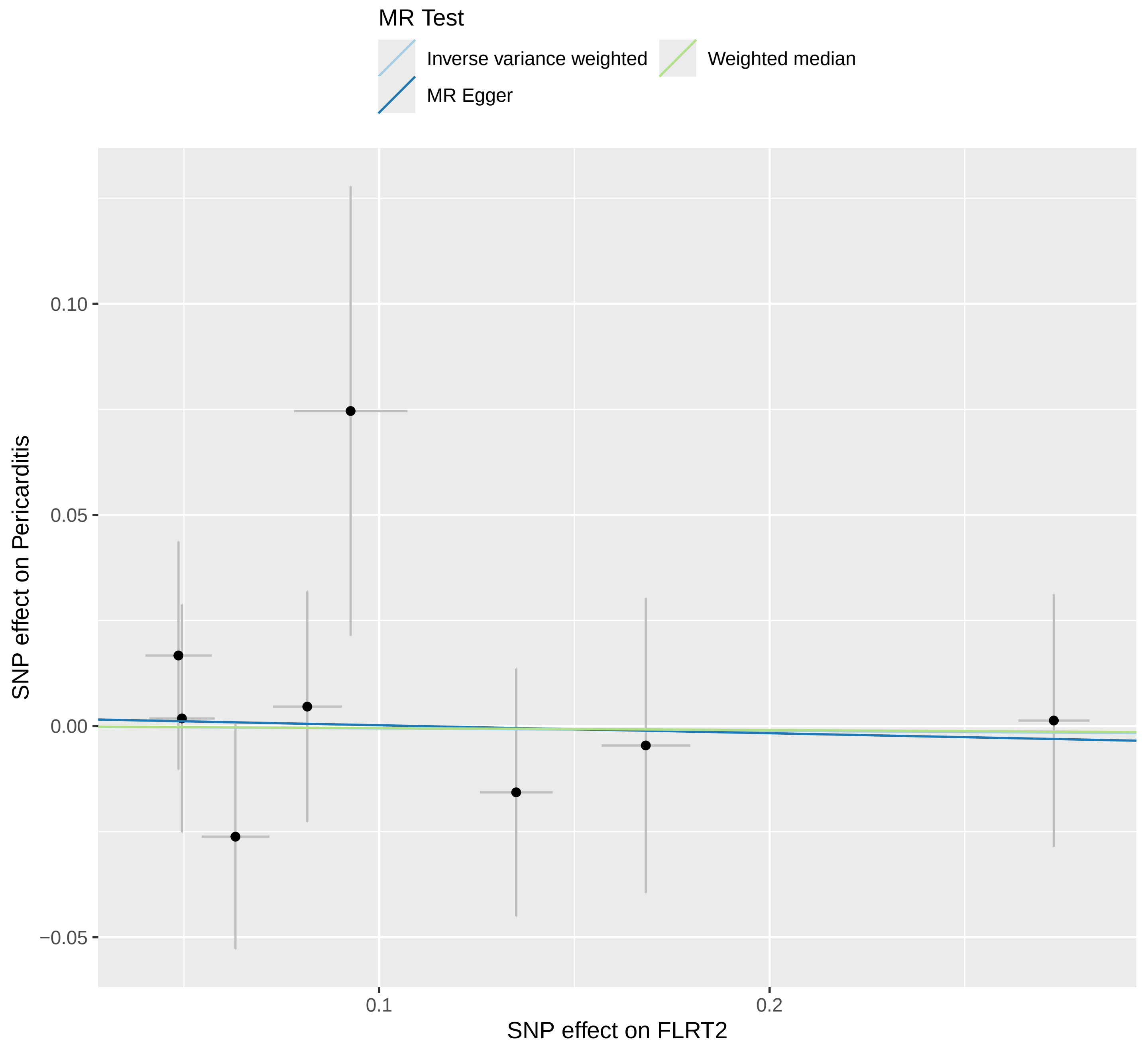

MR Test
Inverse variance weighted
Weighted median
MR Egger
0.10
0.05
0.00
−0.05
0.1
0.2
SNP effect on Pericarditis
SNP effect on FLRT2

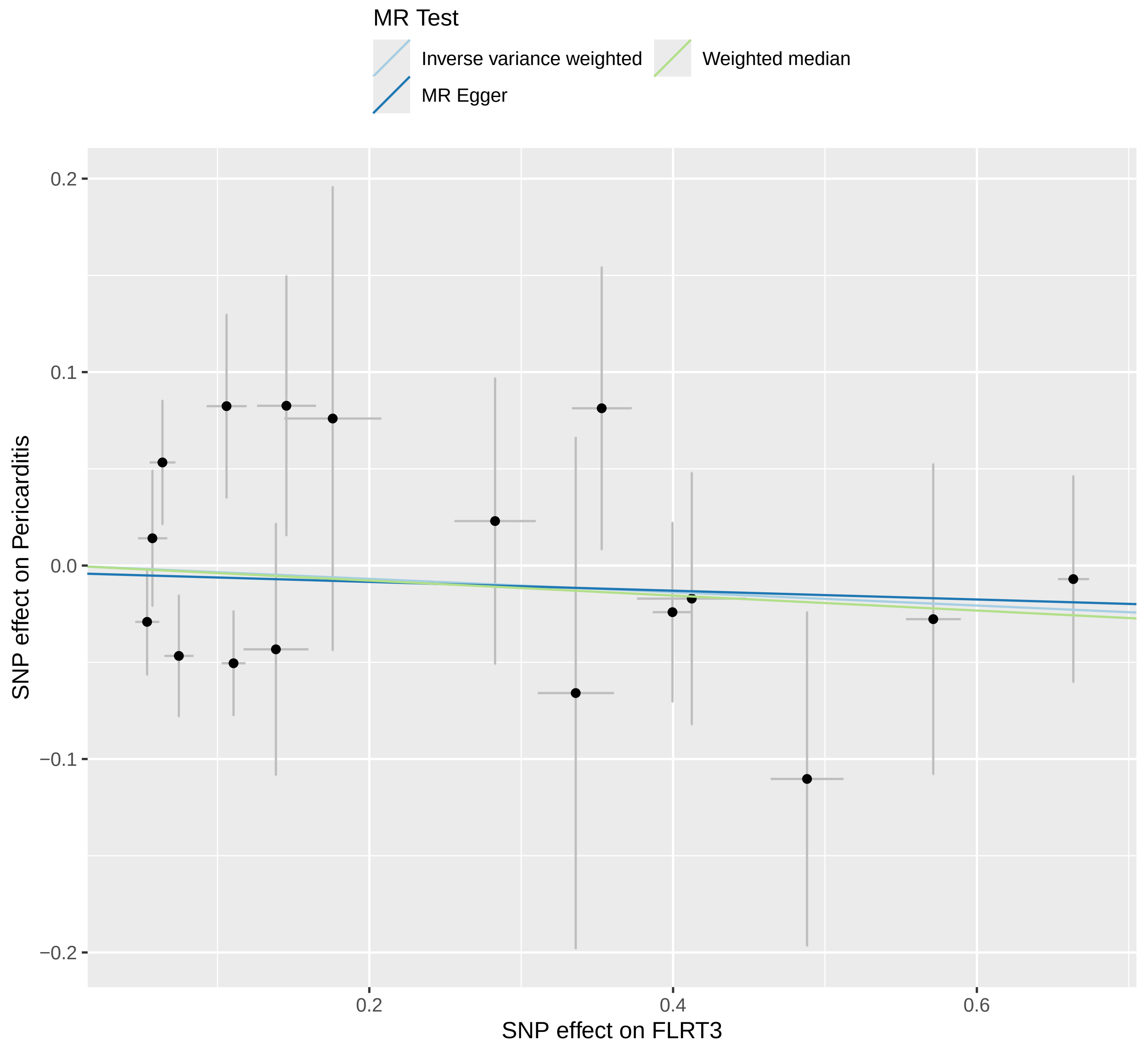
MR Test
Inverse variance weighted
Weighted median
MR Egger
0.2
0.1
0.0
−0.1
−0.2
0.2
0.4
0.6
SNP effect on Pericarditis
SNP effect on FLRT3

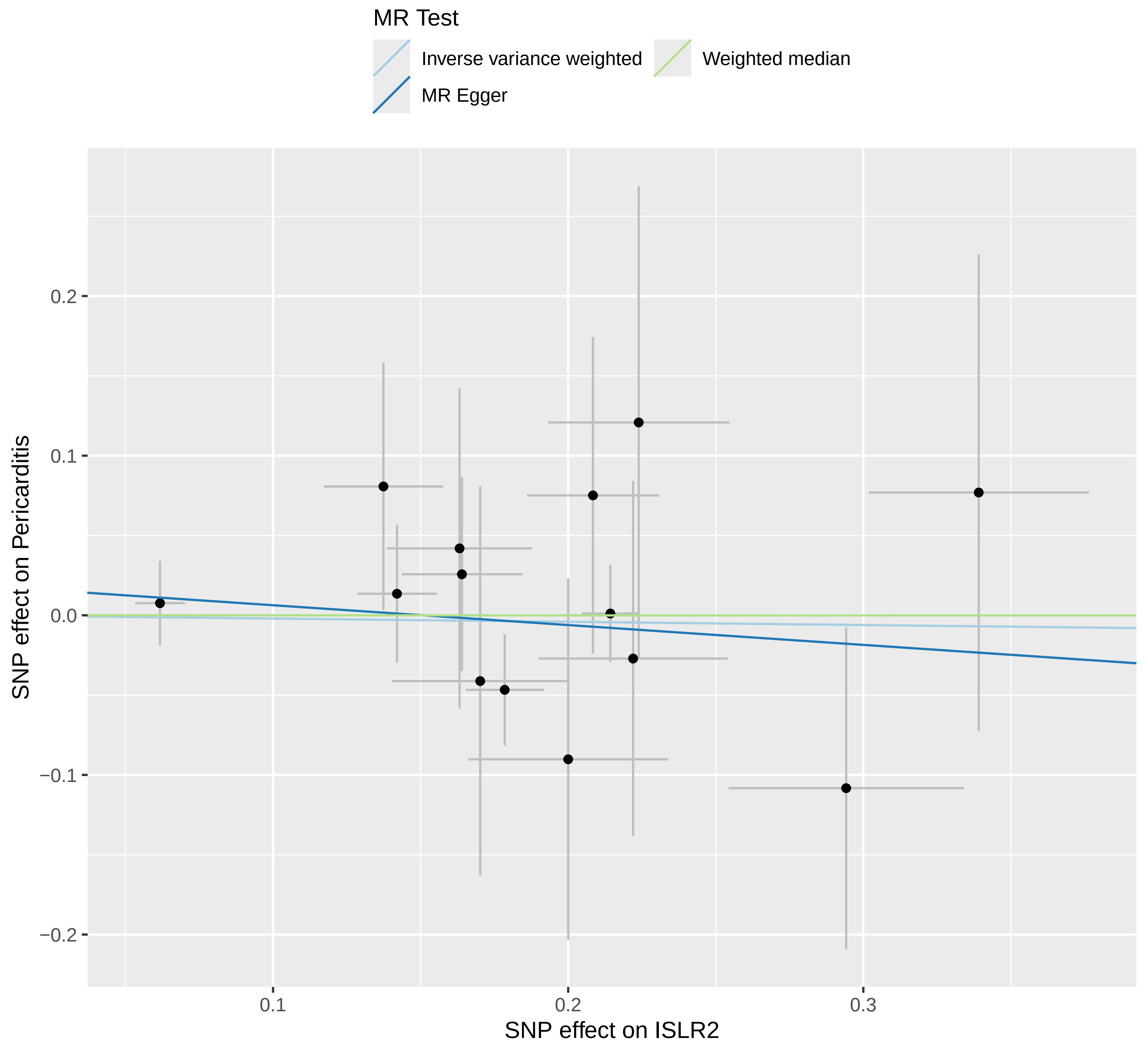

MR Test
Inverse variance weighted
Weighted median
MR Egger
0.2
0.1
0.0
−0.1
−0.2
SNP effect on Pericarditis
0.1
0.2
0.3
SNP effect on ISLR2

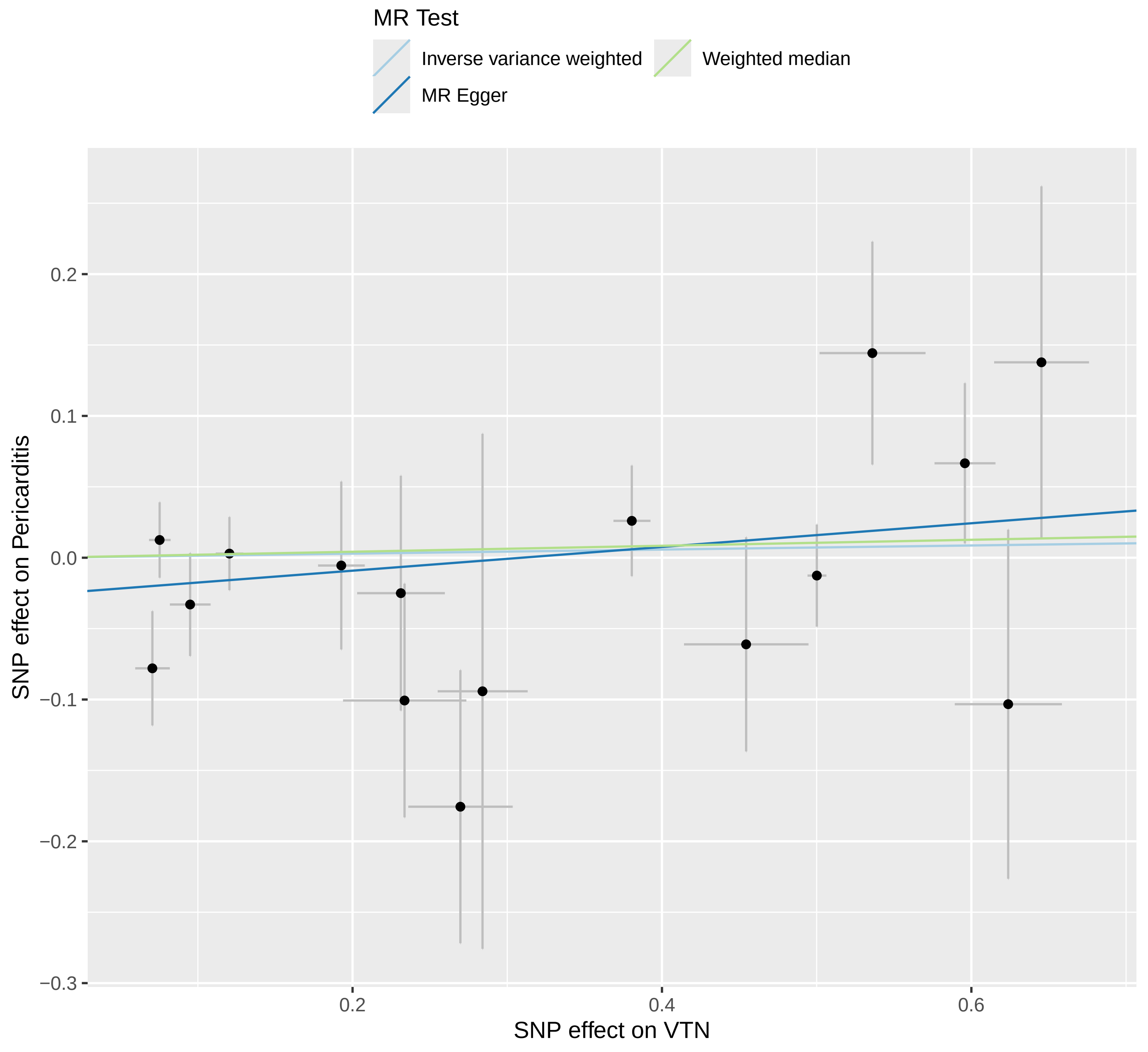
MR Test
Inverse variance weighted
Weighted median
MR Egger
SNP effect on Pericarditis
0.2
0.1
0.0
−0.1
−0.2
−0.3
0.2
0.4
0.6
SNP effect on VTN

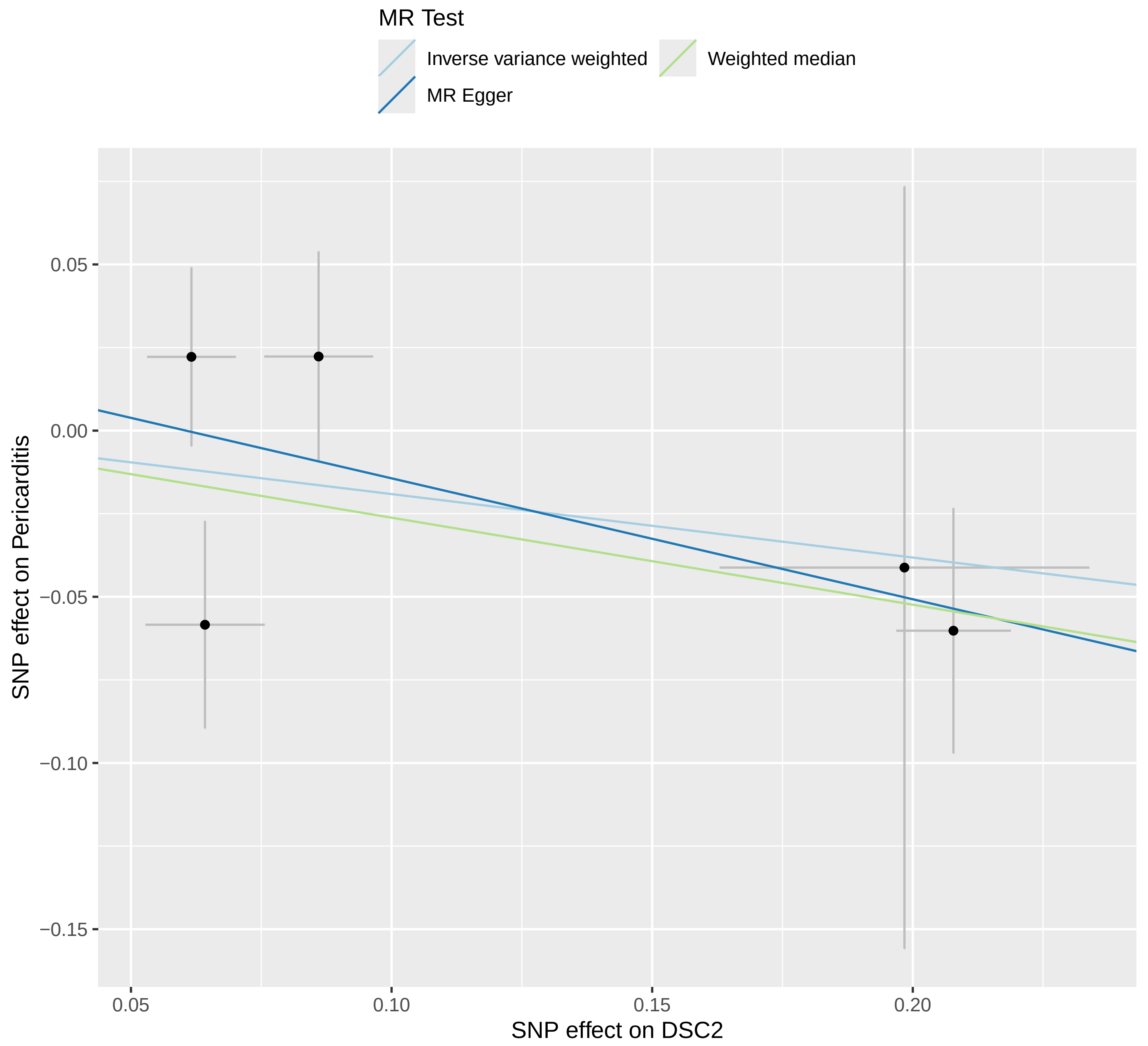
MR Test
Inverse variance weighted
MR Egger
Weighted median
SNP effect on Pericarditis
0.05
0.00
−0.05
−0.10
−0.15
0.05
0.10
0.15
0.20
SNP effect on DSC2

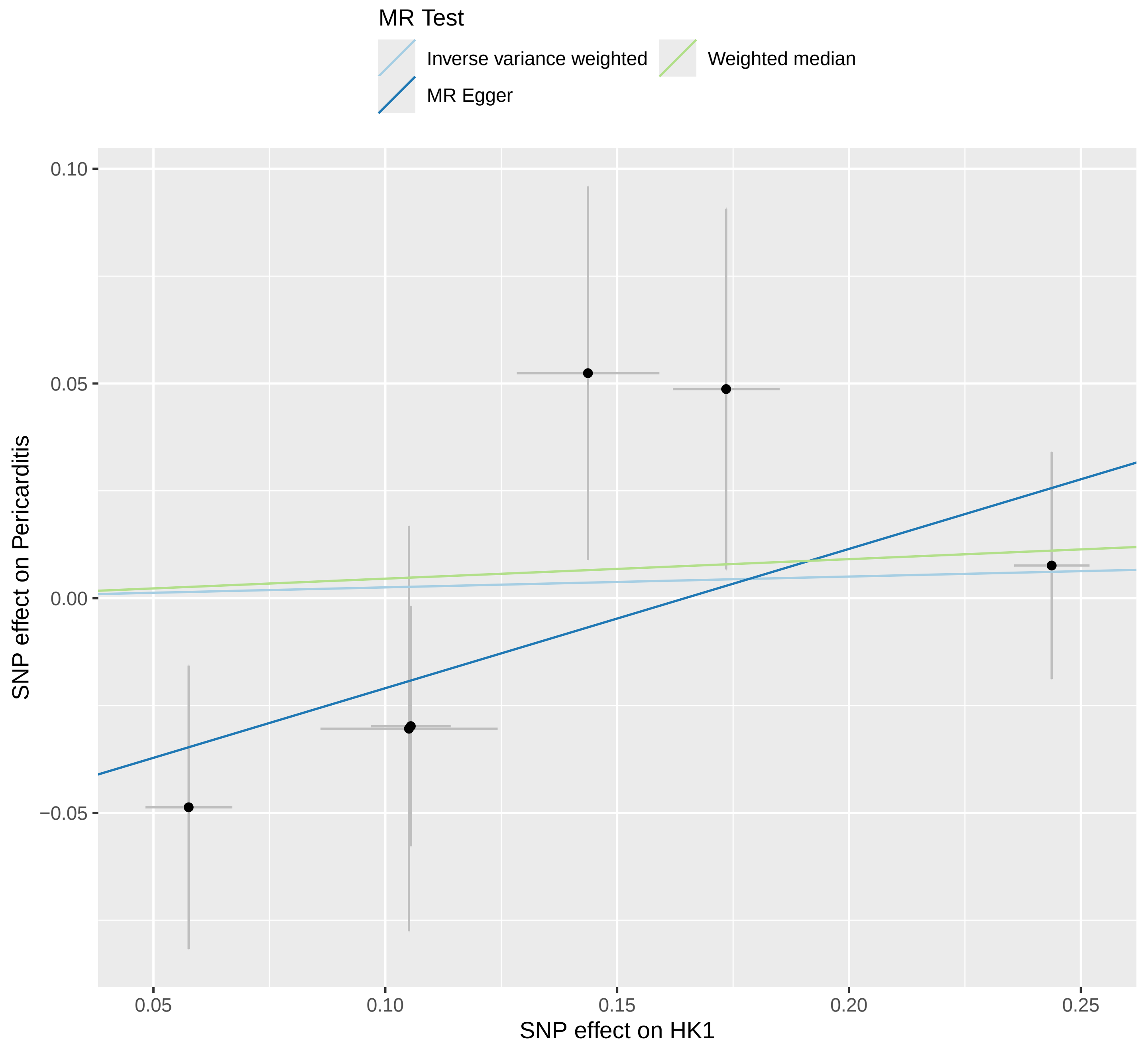
MR Test
Inverse variance weighted
Weighted median
MR Egger
0.10
0.05
0.00
−0.05
SNP effect on Pericarditis
0.05
0.10
0.15
0.20
0.25
SNP effect on HK1

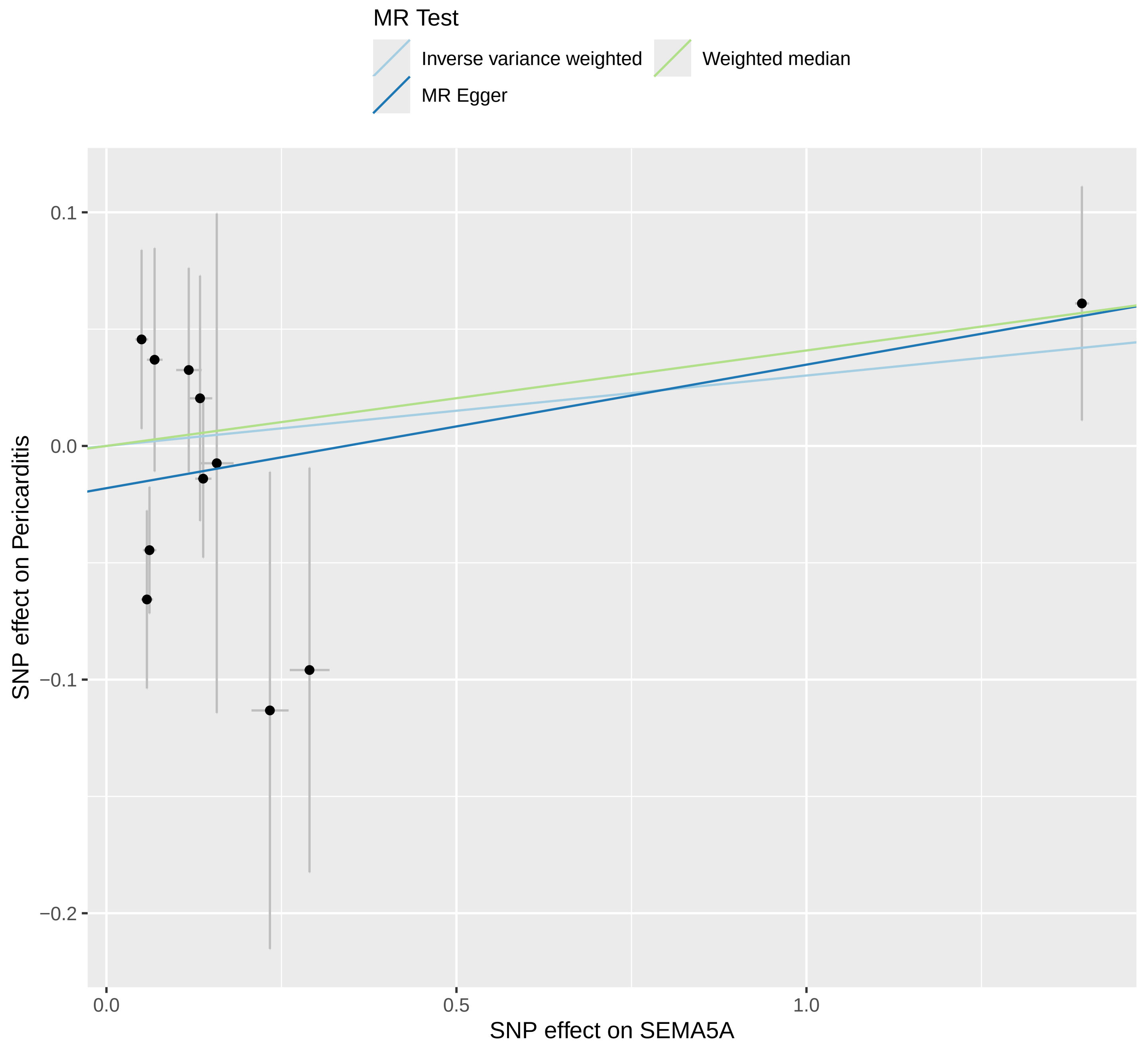

MR Test
Inverse variance weighted
Weighted median
MR Egger
0.1
0.0
−0.1
−0.2
0.0
0.5
1.0
SNP effect on Pericarditis
SNP effect on SEMA5A

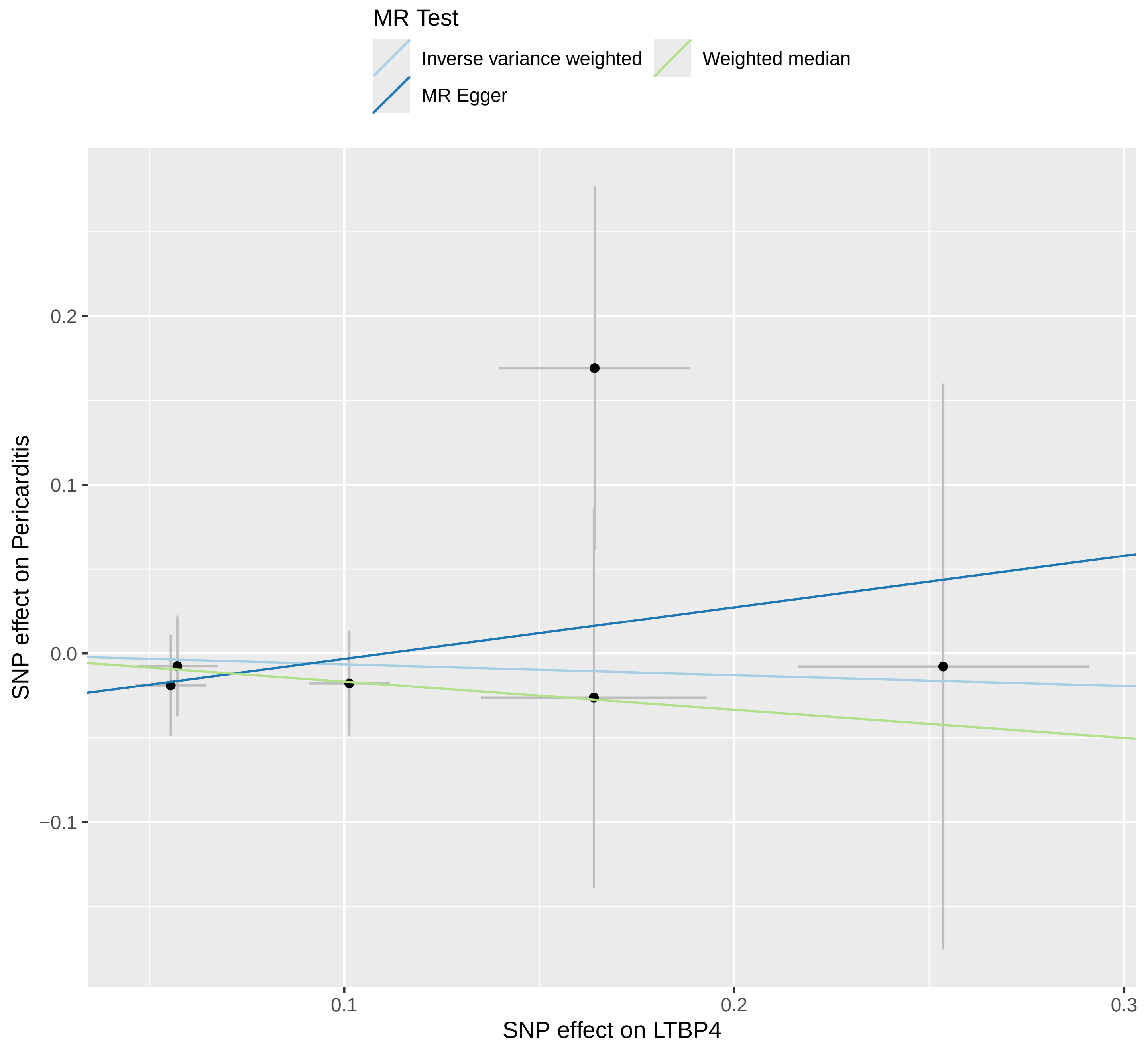

MR Test
Inverse variance weighted
Weighted median
MR Egger
SNP effect on Pericarditis
0.2
0.1
0.0
−0.1
0.1
0.2
0.3
SNP effect on LTBP4

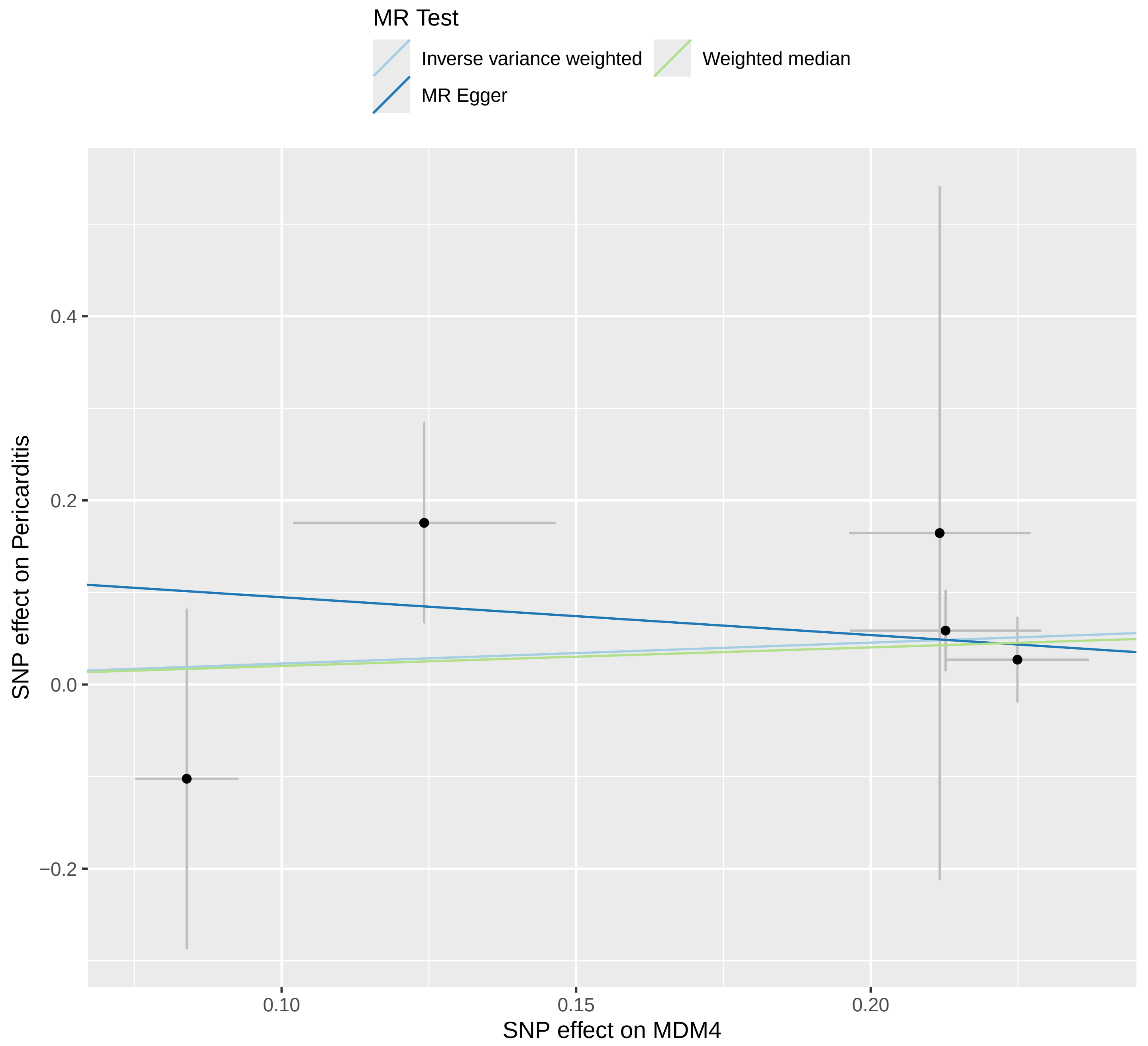

MR Test
Inverse variance weighted
MR Egger
Weighted median
SNP effect on Pericarditis
0.4
0.2
0.0
−0.2
0.10
0.15
0.20
SNP effect on MDM4

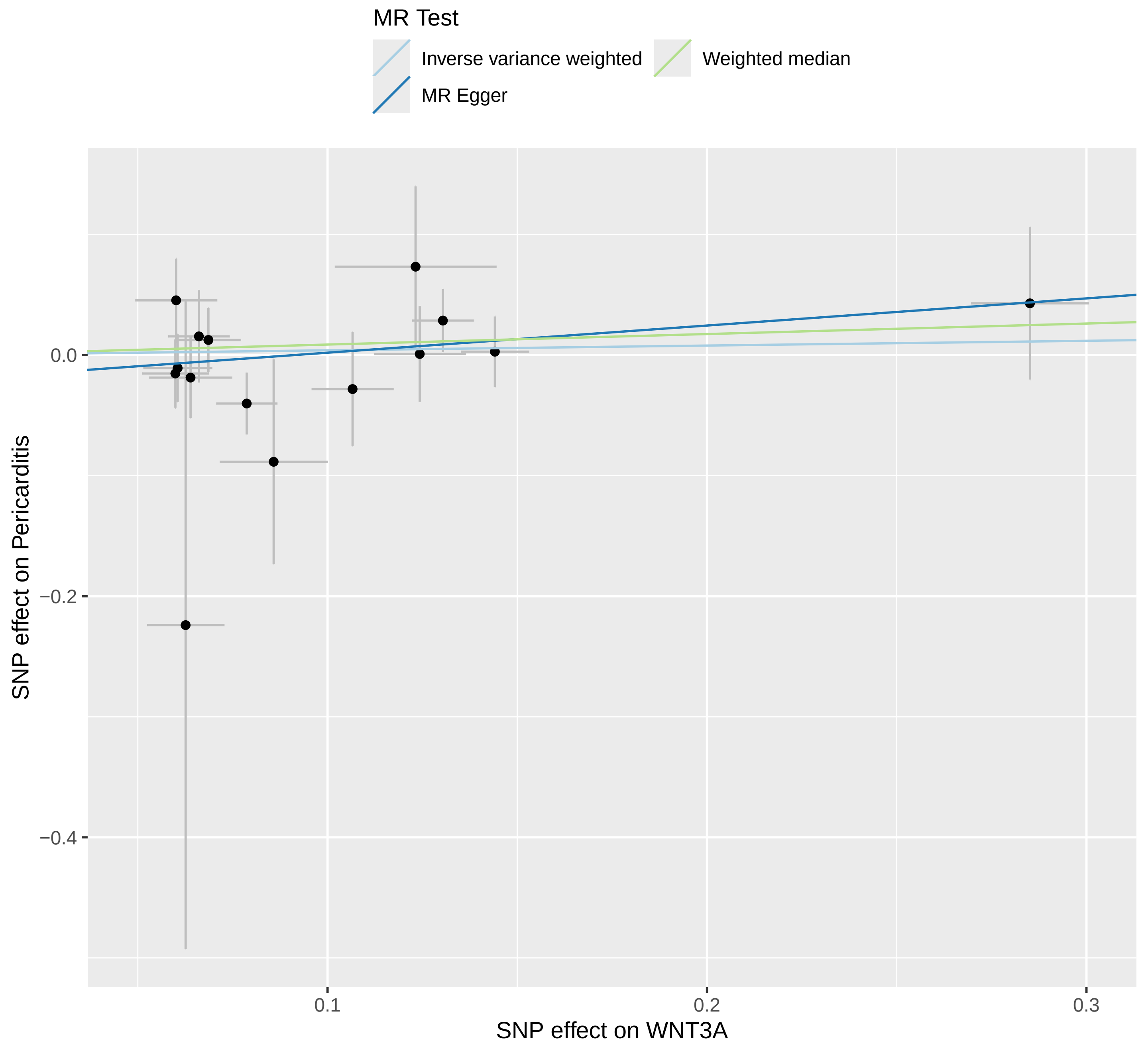

MR Test
Inverse variance weighted
Weighted median
MR Egger
0.0
−0.2
−0.4
SNP effect on Pericarditis
0.1
0.2
0.3
SNP effect on WNT3A

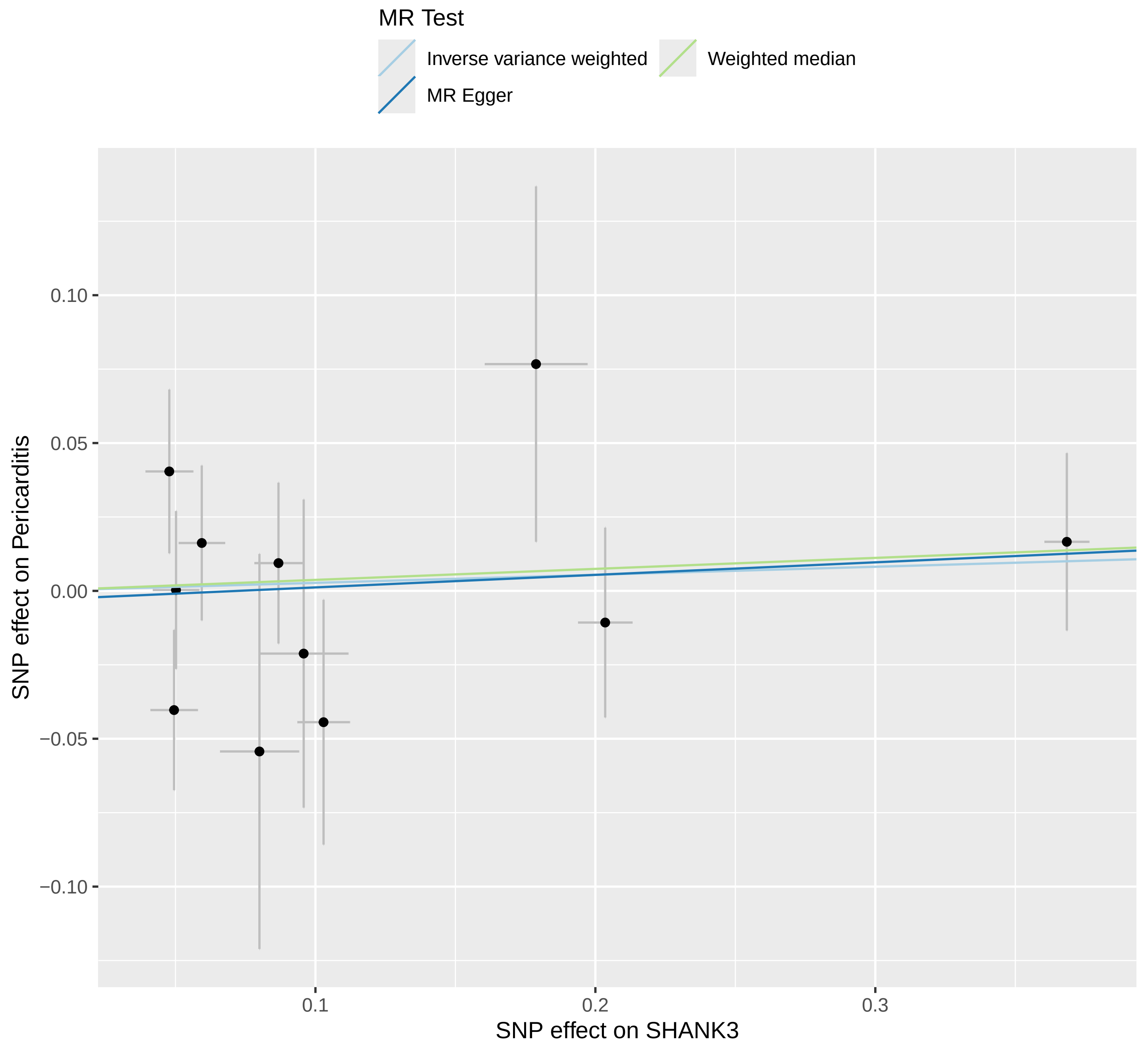

MR Test
Inverse variance weighted
MR Egger
Weighted median
SNP effect on Pericarditis
0.10
0.05
0.00
−0.05
−0.10
0.1
0.2
0.3
SNP effect on SHANK3

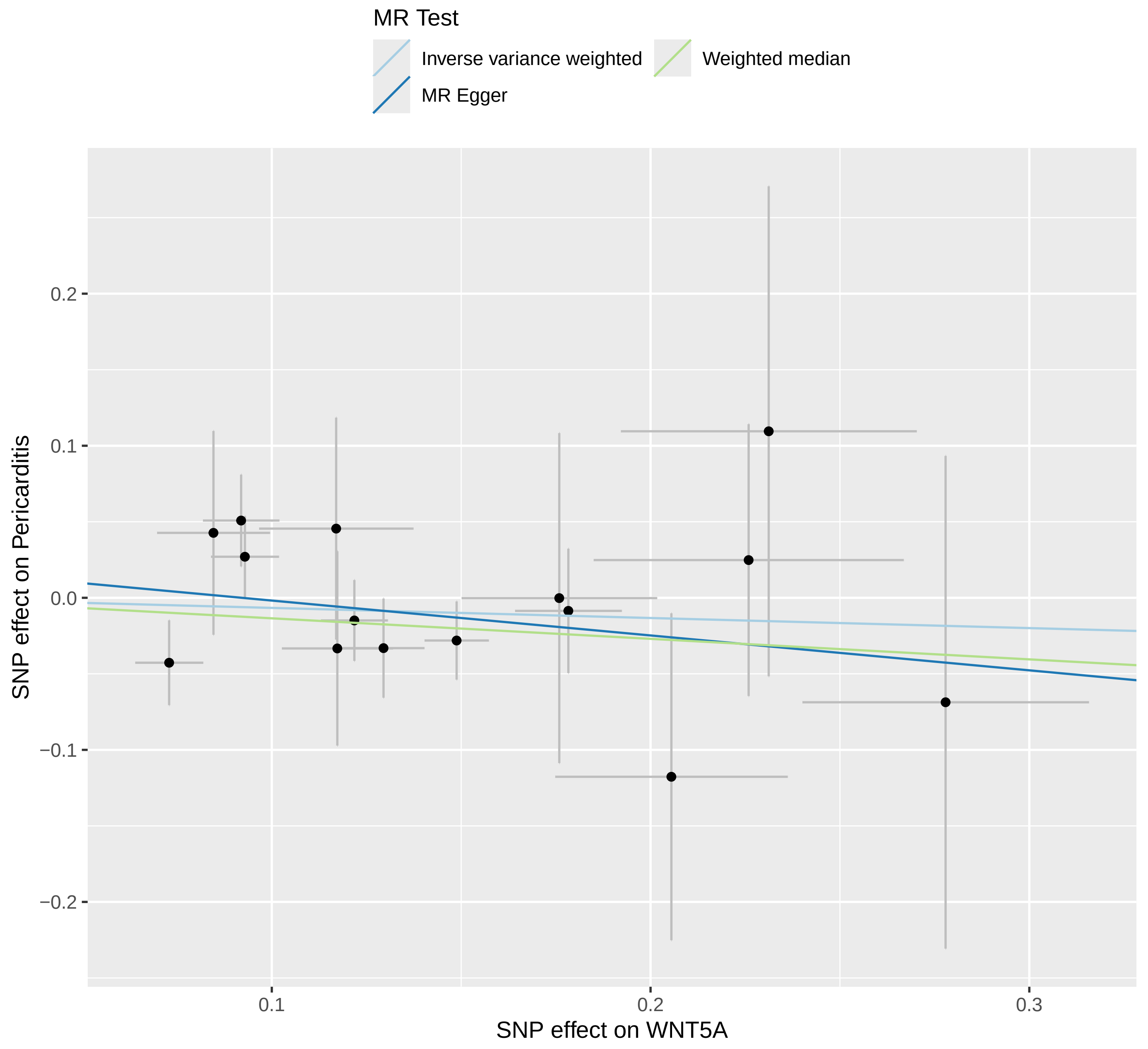
MR Test
Inverse variance weighted
Weighted median
MR Egger
0.2
0.1
0.0
−0.1
−0.2
SNP effect on Pericarditis
0.1
0.2
0.3
SNP effect on WNT5A

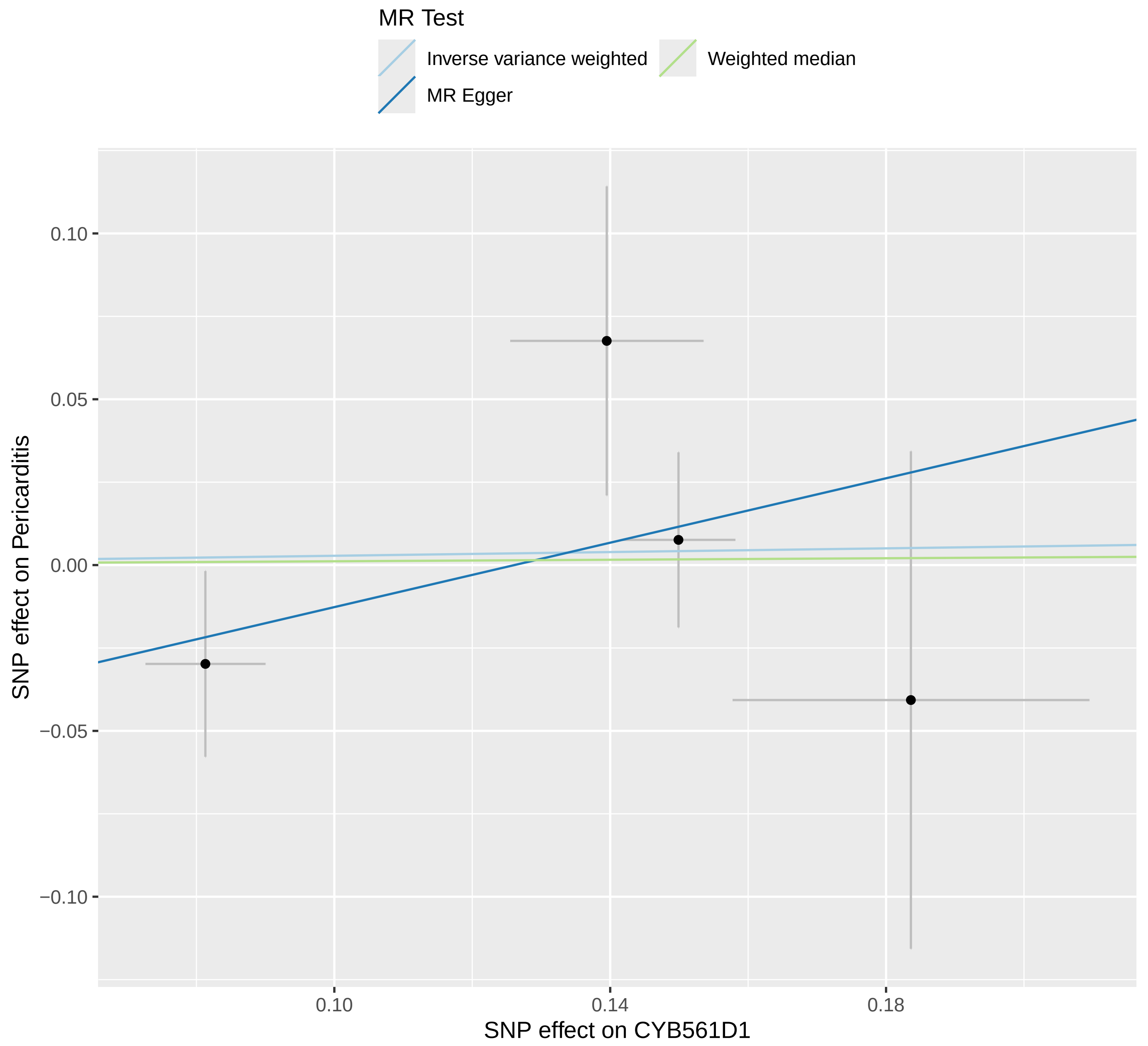
MR Test
Inverse variance weighted
Weighted median
MR Egger
0.10
0.05
0.00
−0.05
−0.10
SNP effect on Pericarditis
0.10
0.14
0.18
SNP effect on CYB561D1

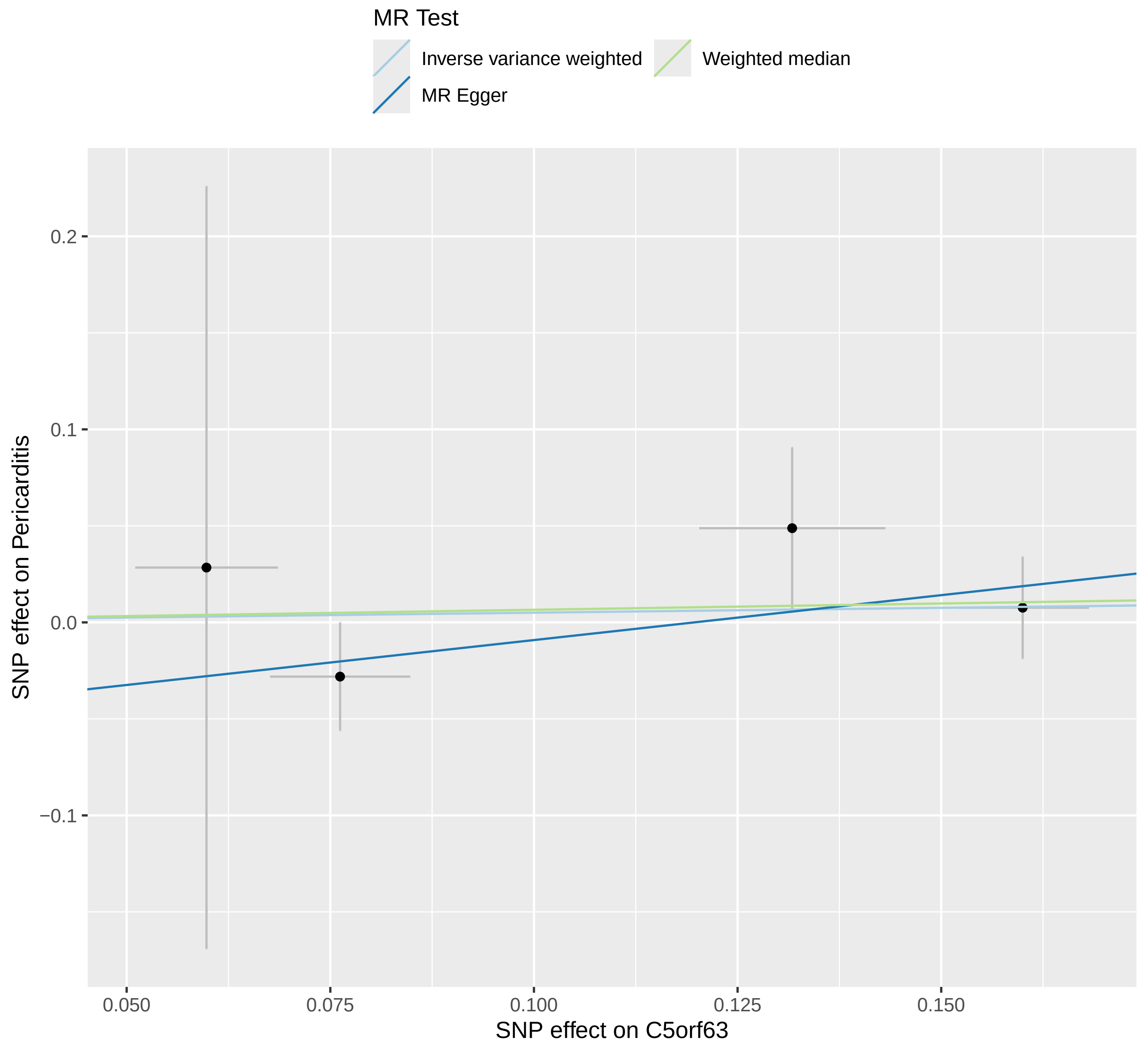
MR Test
Inverse variance weighted
Weighted median
MR Egger
0.2
0.1
0.0
−0.1
SNP effect on Pericarditis
0.050
0.075
0.100
0.125
0.150
SNP effect on C5orf63

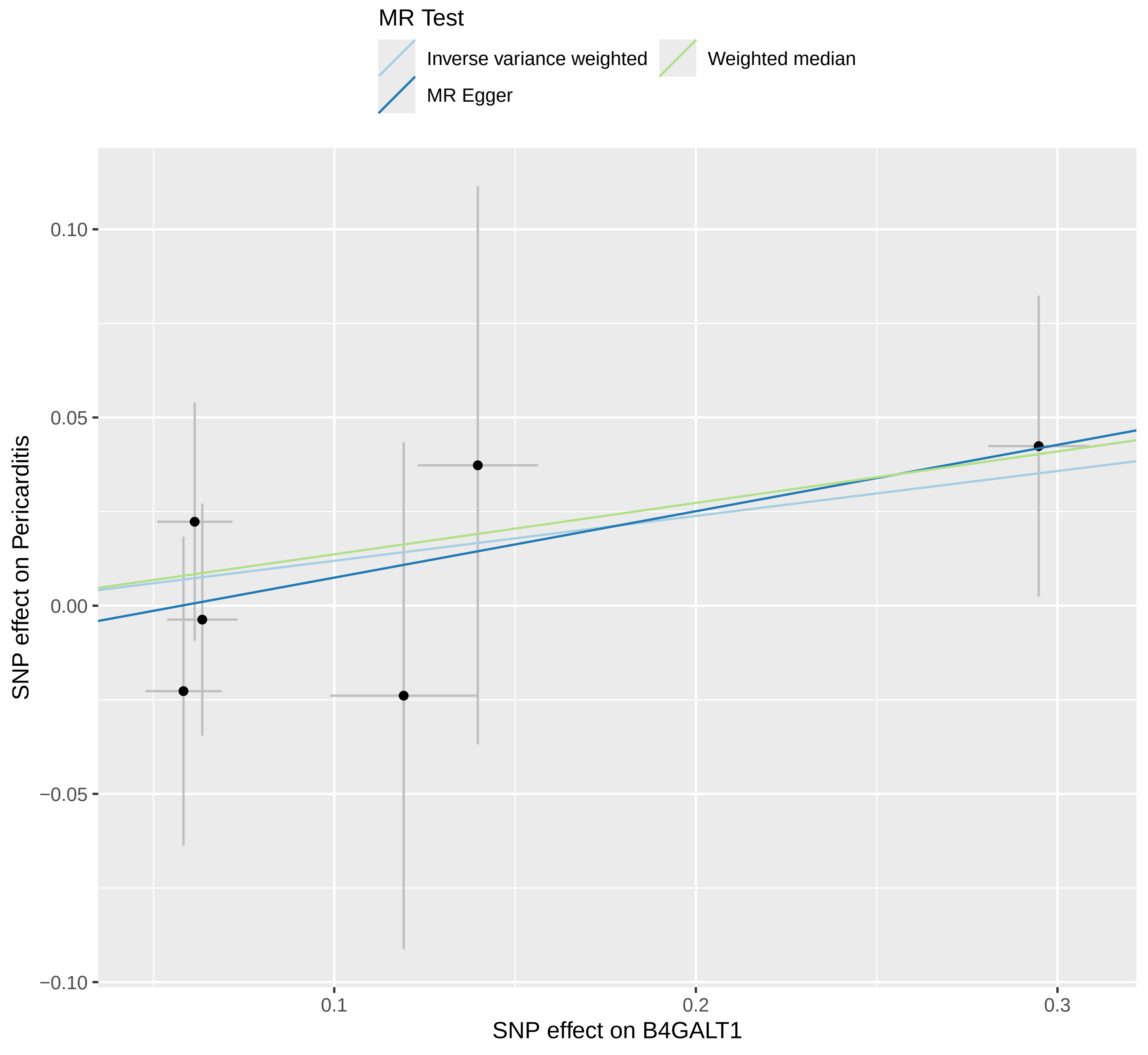

MR Test
Inverse variance weighted
MR Egger
Weighted median
0.10
0.05
0.00
−0.05
−0.10
SNP effect on Pericarditis
0.1
0.2
0.3
SNP effect on B4GALT1

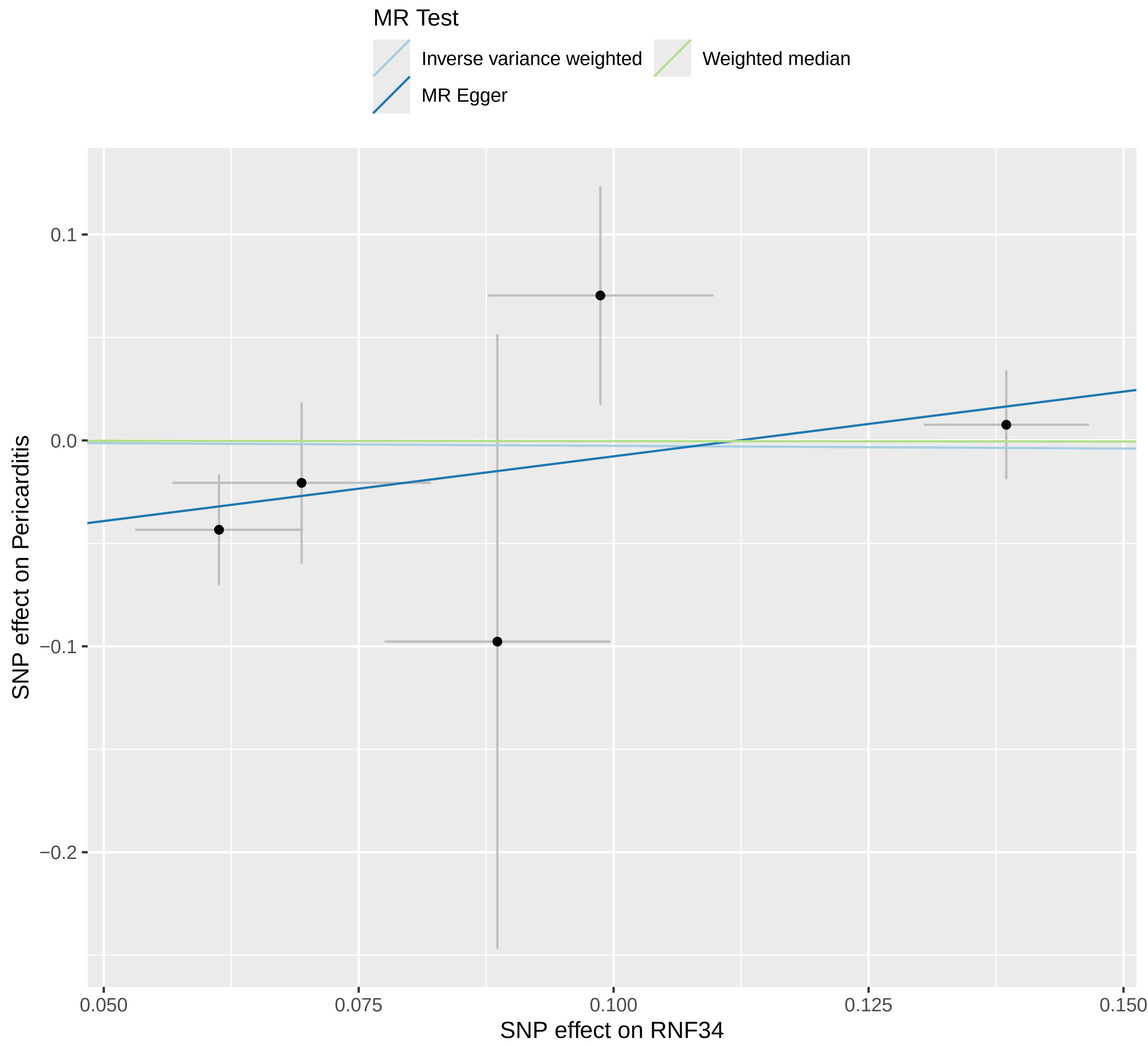

MR Test
Inverse variance weighted
Weighted median
MR Egger
0.1
0.0
−0.1
−0.2
SNP effect on Pericarditis
0.050
0.075
0.100
0.125
0.150
SNP effect on RNF34

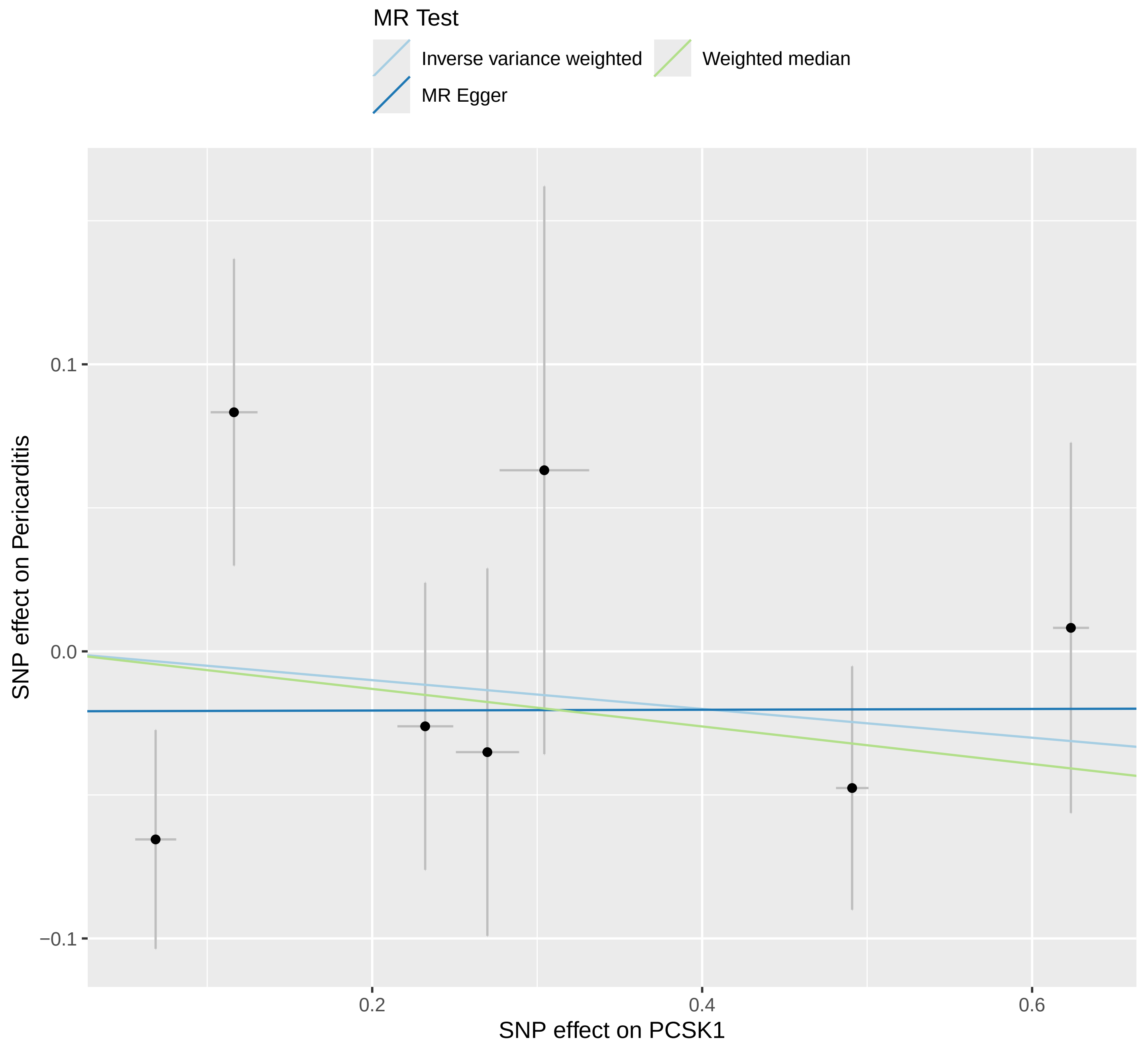
MR Test
Inverse variance weighted
MR Egger
Weighted median
0.1
0.0
−0.1
SNP effect on Pericarditis
0.2
0.4
0.6
SNP effect on PCSK1

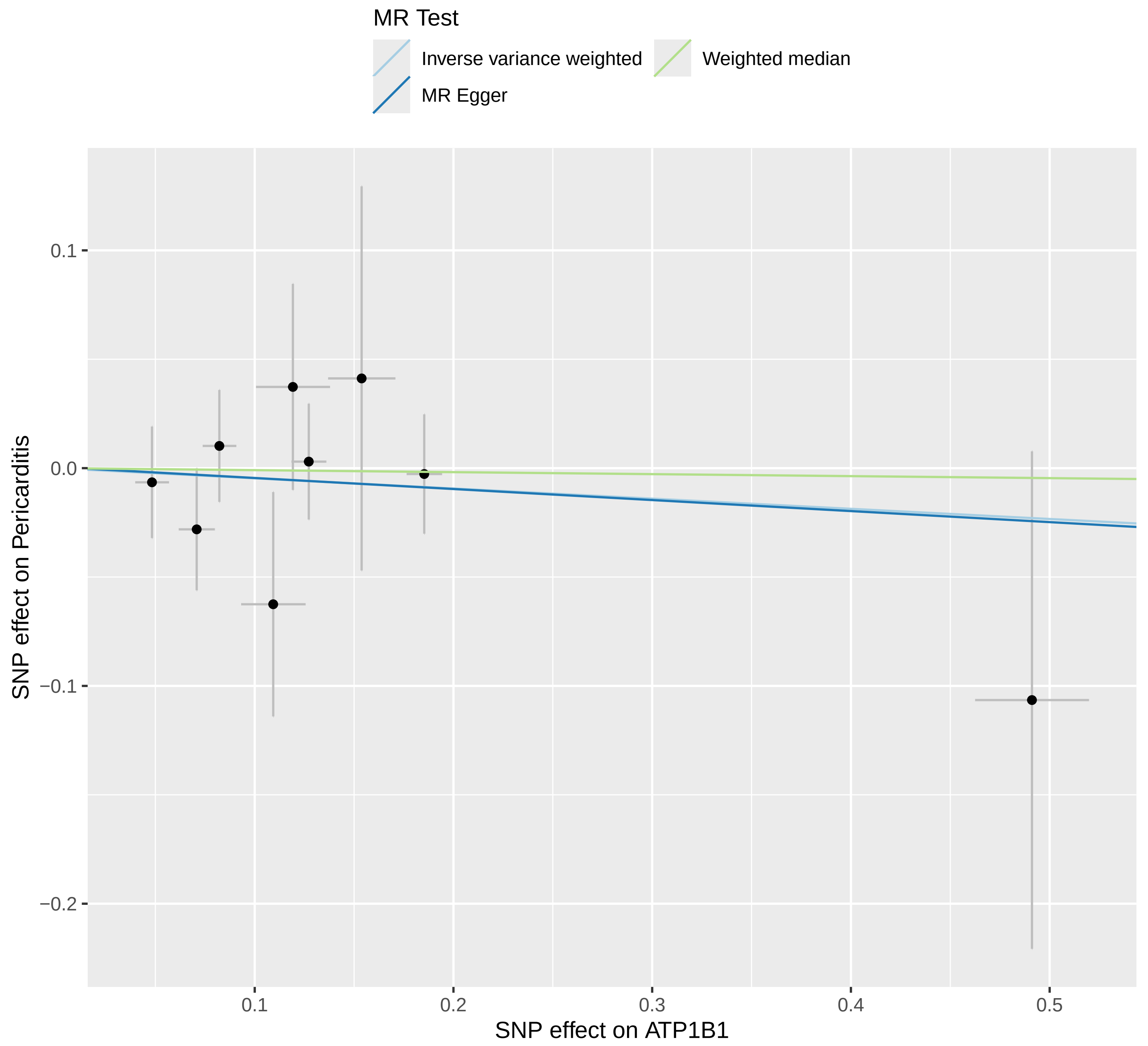
MR Test
Inverse variance weighted
Weighted median
MR Egger
0.1
0.0
−0.1
−0.2
SNP effect on Pericarditis
0.1
0.2
0.3
0.4
0.5
SNP effect on ATP1B1

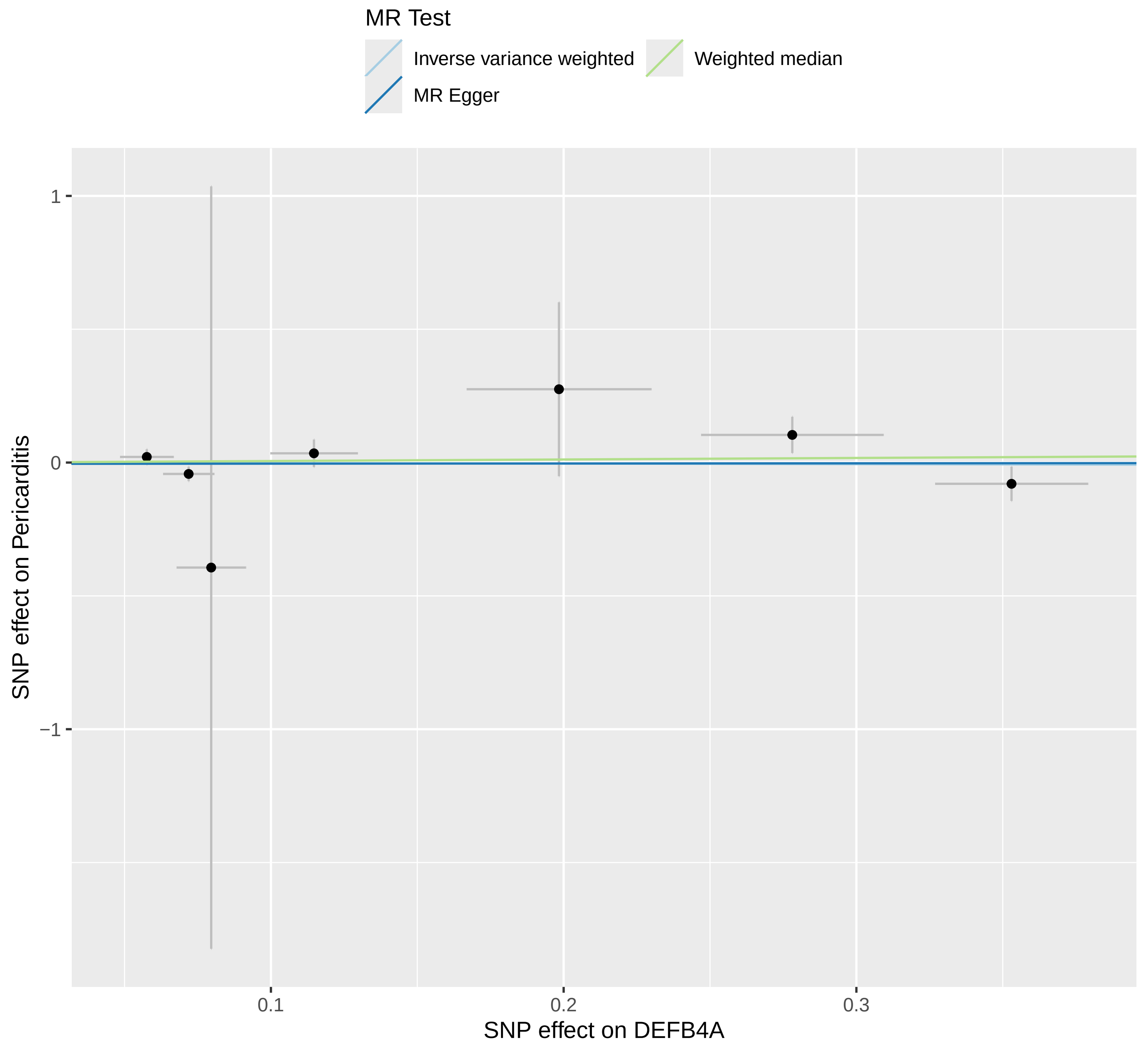
MR Test
Inverse variance weighted
MR Egger
Weighted median
1
0
−1
SNP effect on Pericarditis
0.1
0.2
0.3
SNP effect on DEFB4A

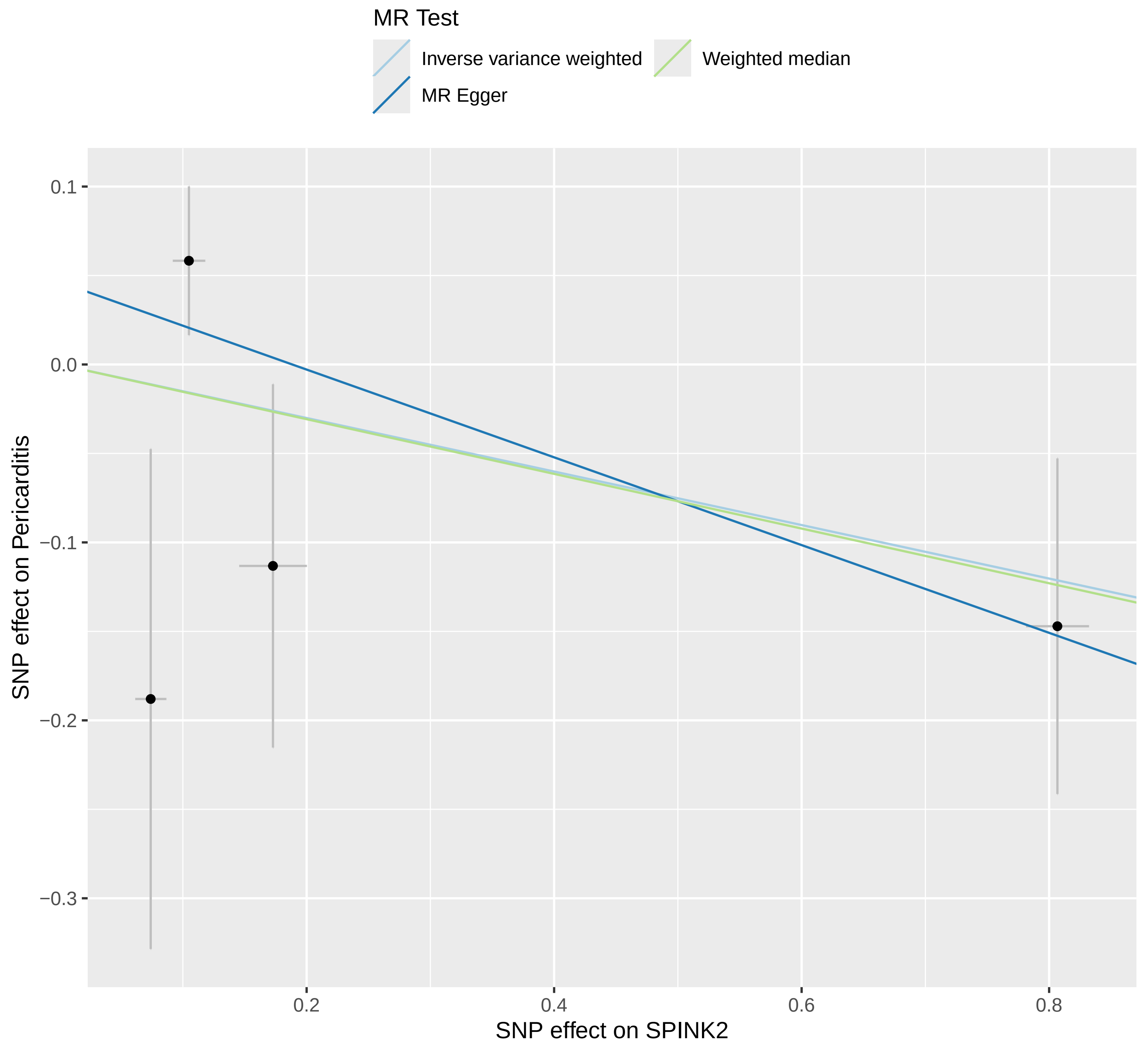
MR Test
Inverse variance weighted
Weighted median
MR Egger
0.1
0.0
−0.1
−0.2
−0.3
SNP effect on Pericarditis
0.2
0.4
0.6
0.8
SNP effect on SPINK2

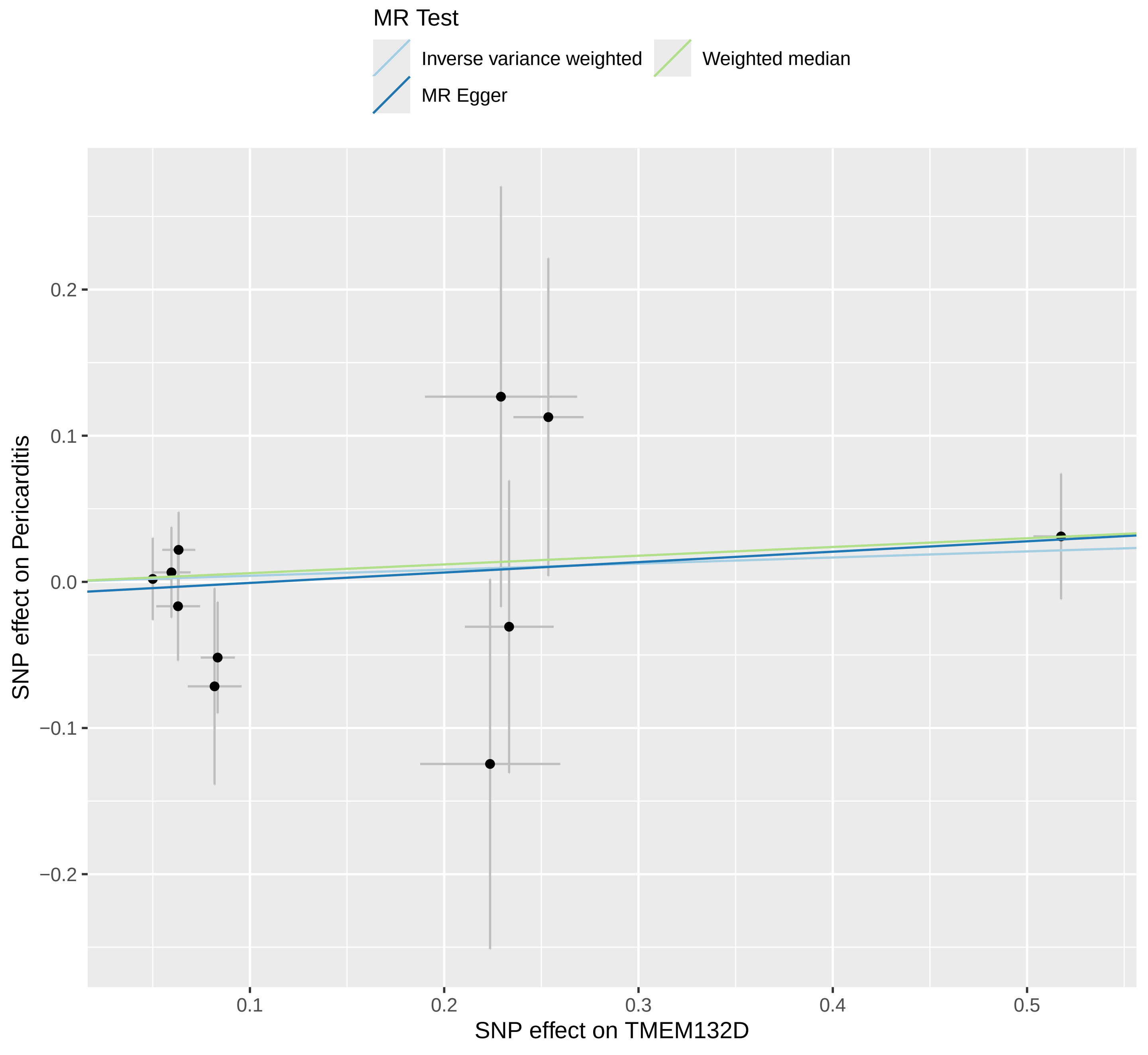
MR Test
Inverse variance weighted
MR Egger
Weighted median
SNP effect on Pericarditis
SNP effect on TMEM132D
0.2
0.1
0.0
−0.1
−0.2
0.1
0.2
0.3
0.4
0.5

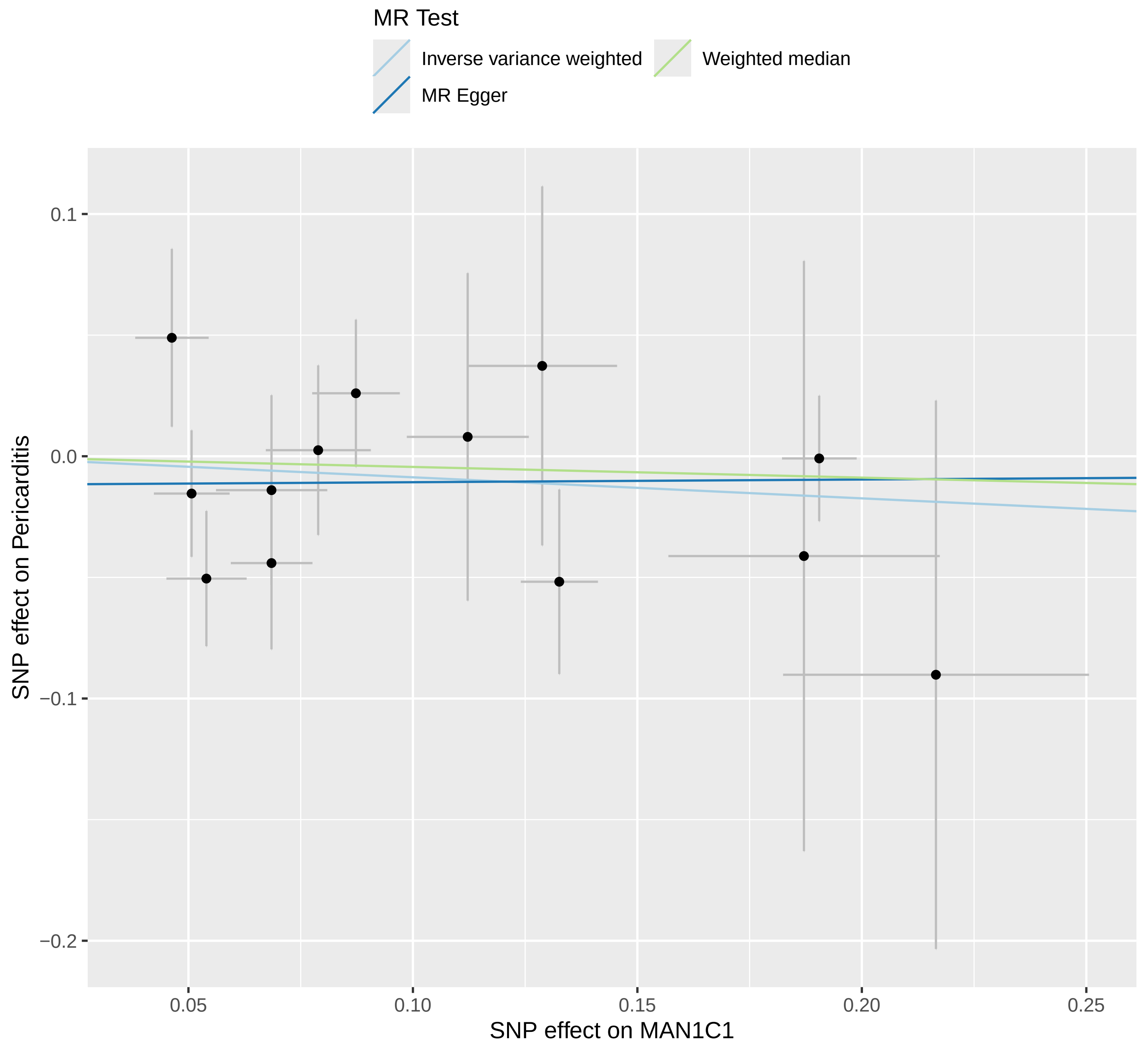
MR Test
Inverse variance weighted
Weighted median
MR Egger
0.1
0.0
−0.1
−0.2
0.05
0.10
0.15
0.20
0.25
SNP effect on Pericarditis
SNP effect on MAN1C1

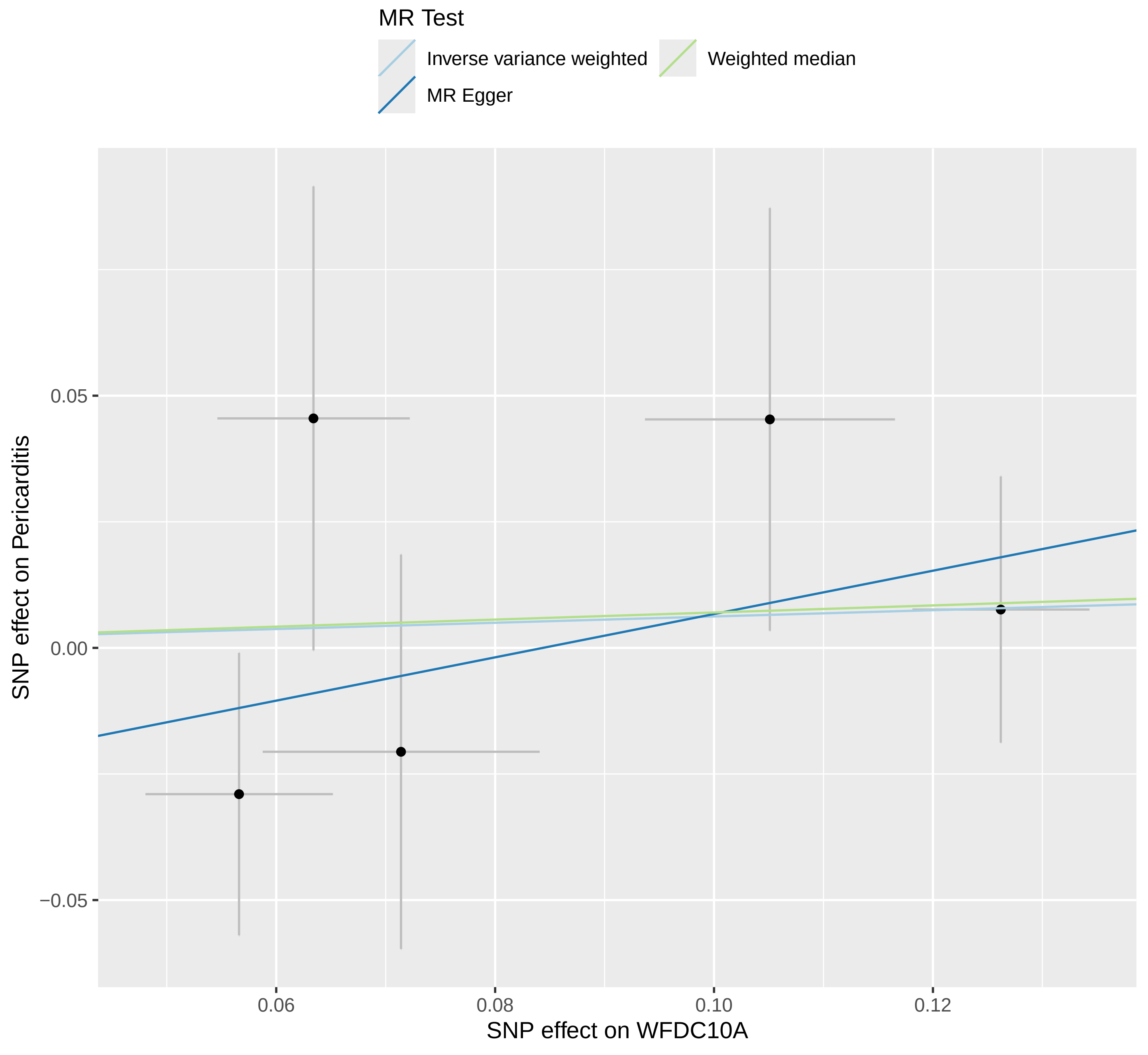
MR Test
Inverse variance weighted
MR Egger
Weighted median
0.05
0.00
−0.05
SNP effect on Pericarditis
0.06
0.08
0.10
0.12
SNP effect on WFDC10A

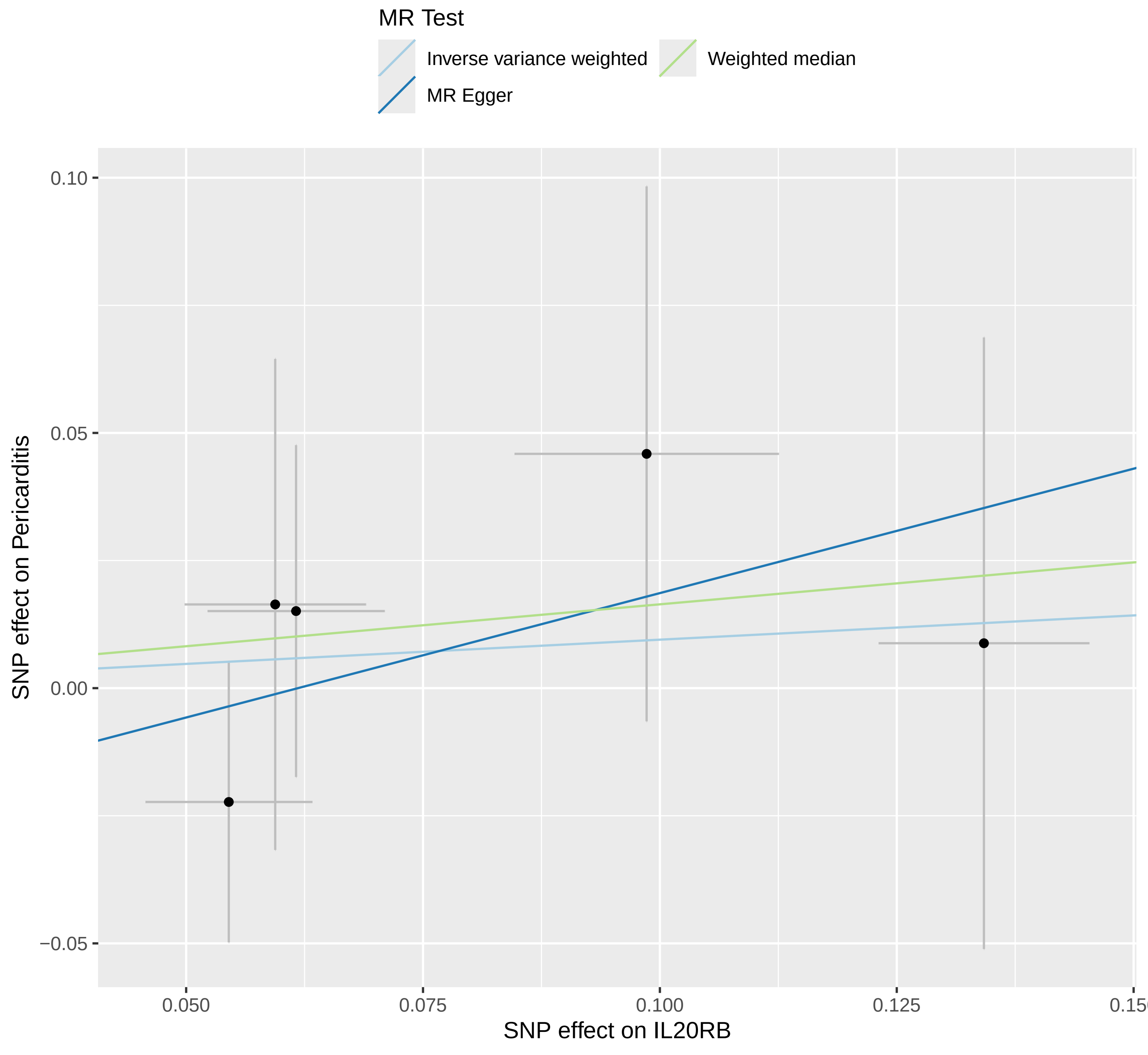

MR Test
Inverse variance weighted
Weighted median
MR Egger
0.10
0.05
0.00
−0.05
SNP effect on Pericarditis
0.050
0.075
0.100
0.125
SNP effect on IL20RB

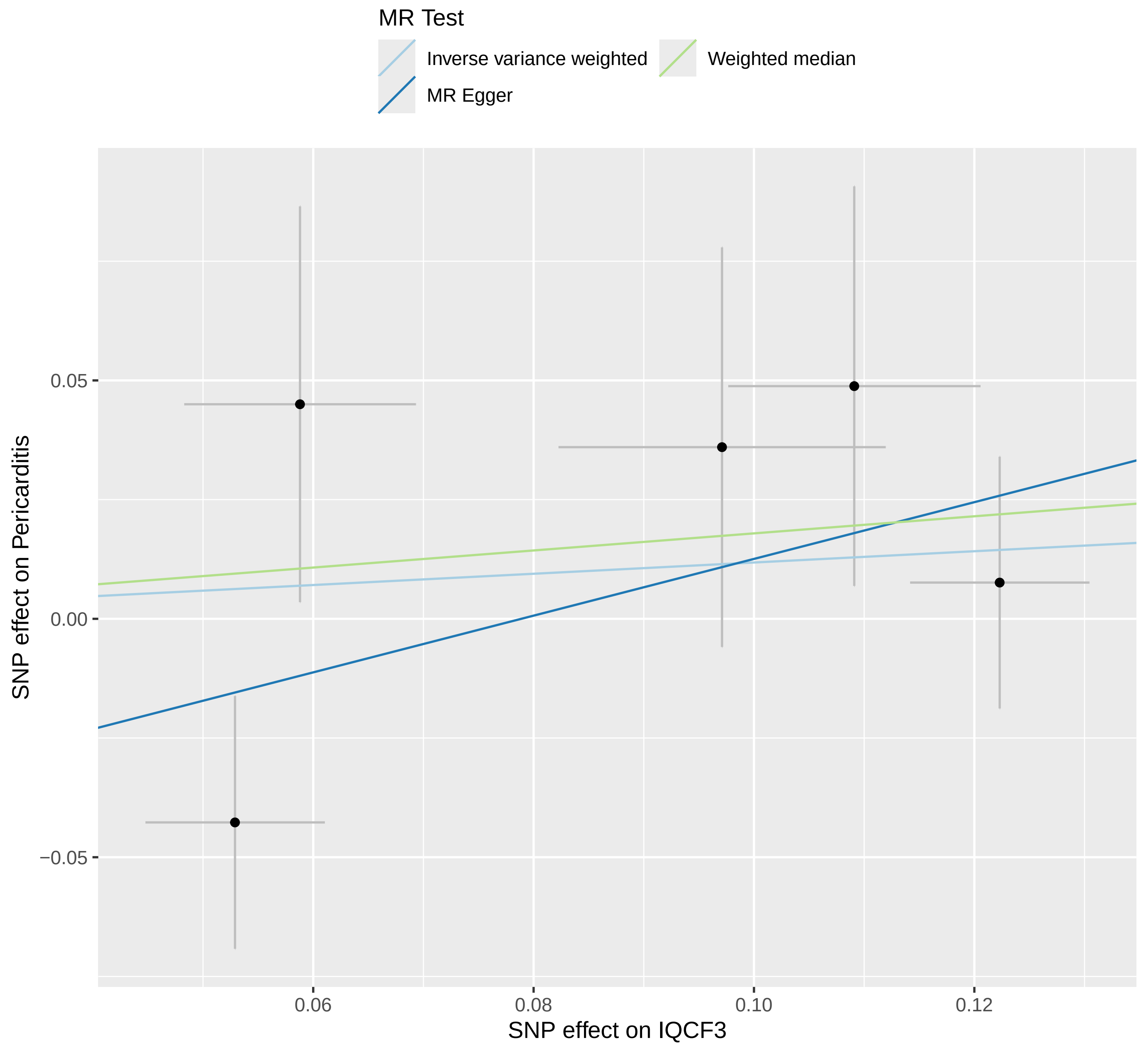

MR Test
Inverse variance weighted
MR Egger
Weighted median
0.05
0.00
−0.05
0.06
0.08
0.10
0.12
SNP effect on Pericarditis
SNP effect on IQCF3

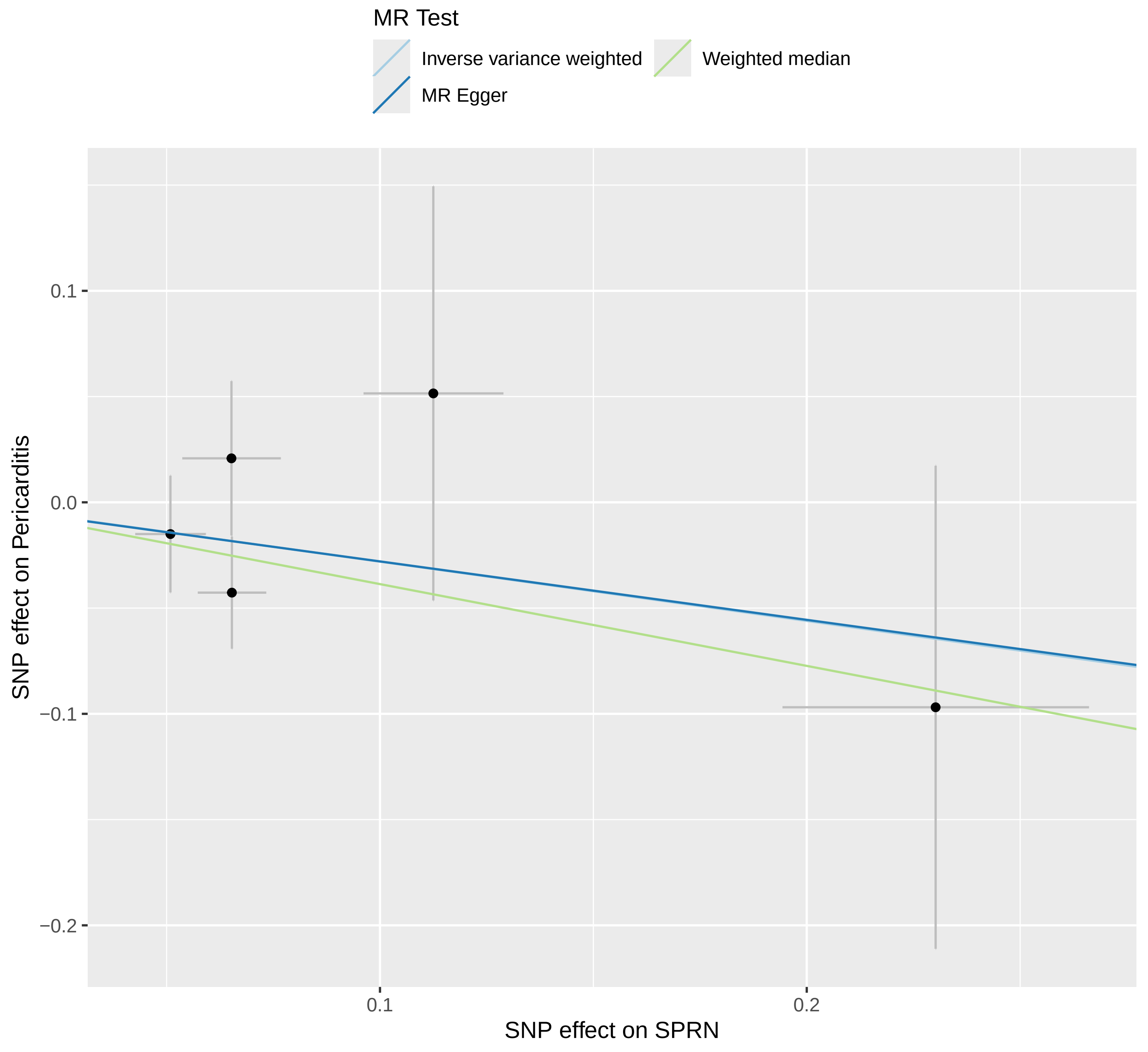
MR Test
Inverse variance weighted
Weighted median
MR Egger
0.1
0.0
−0.1
−0.2
SNP effect on Pericarditis
0.1
0.2
SNP effect on SPRN

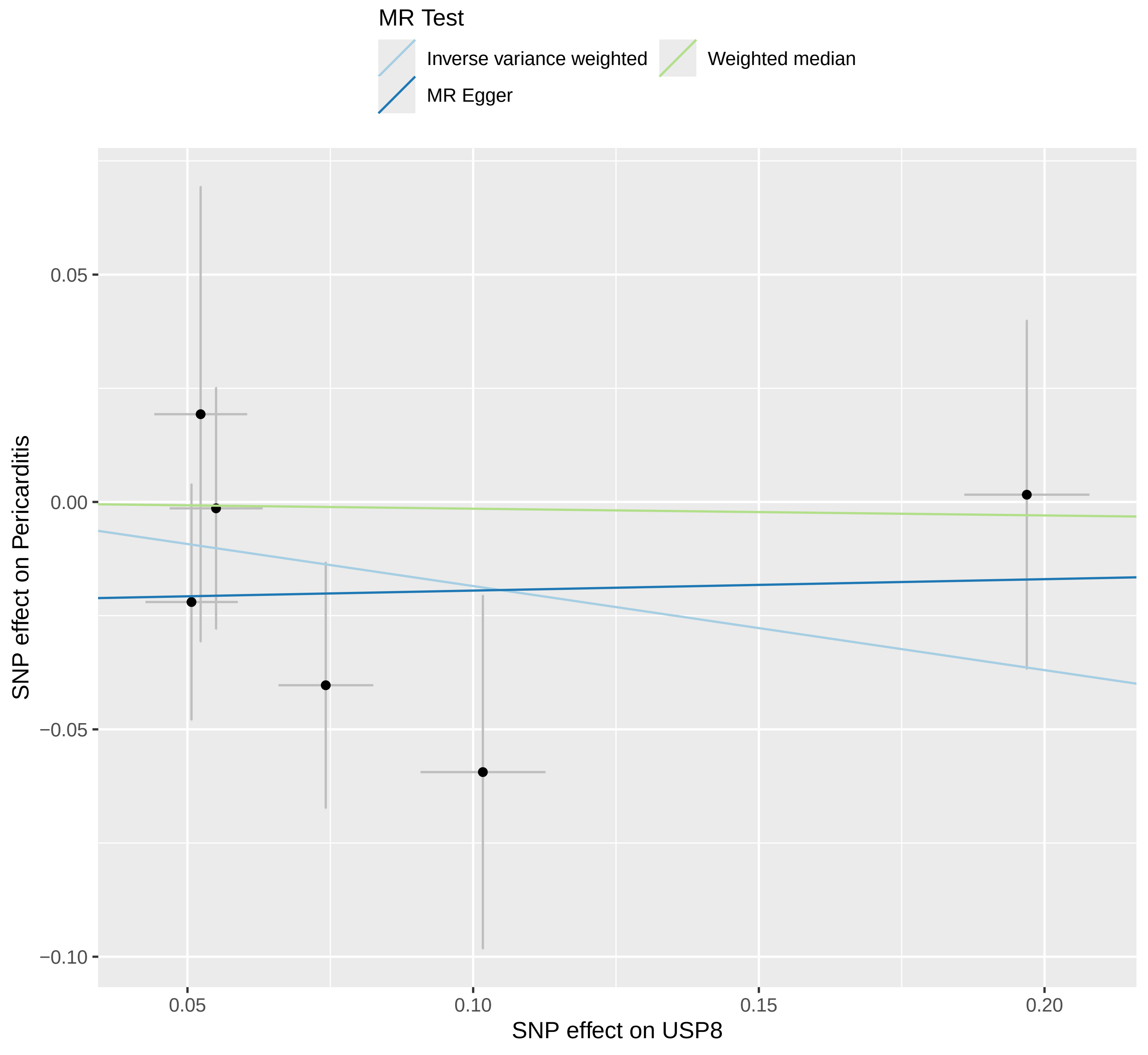
MR Test
Inverse variance weighted
MR Egger
Weighted median
0.05
0.00
−0.05
−0.10
0.05
0.10
0.15
0.20
SNP effect on USP8
SNP effect on Pericarditis

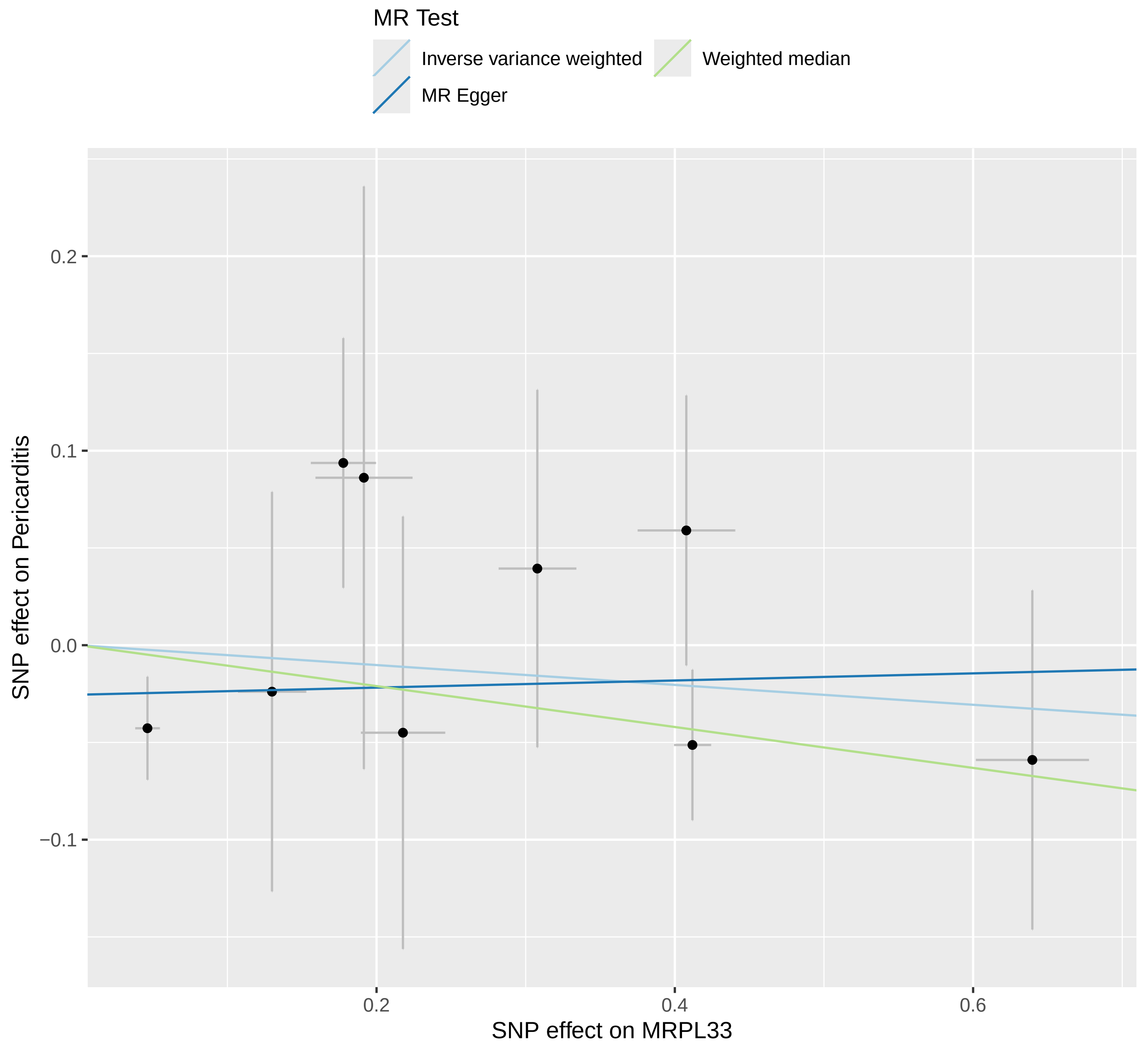
MR Test
Inverse variance weighted
Weighted median
MR Egger
0.2
0.1
0.0
−0.1
SNP effect on Pericarditis
0.2
0.4
0.6
SNP effect on MRPL33

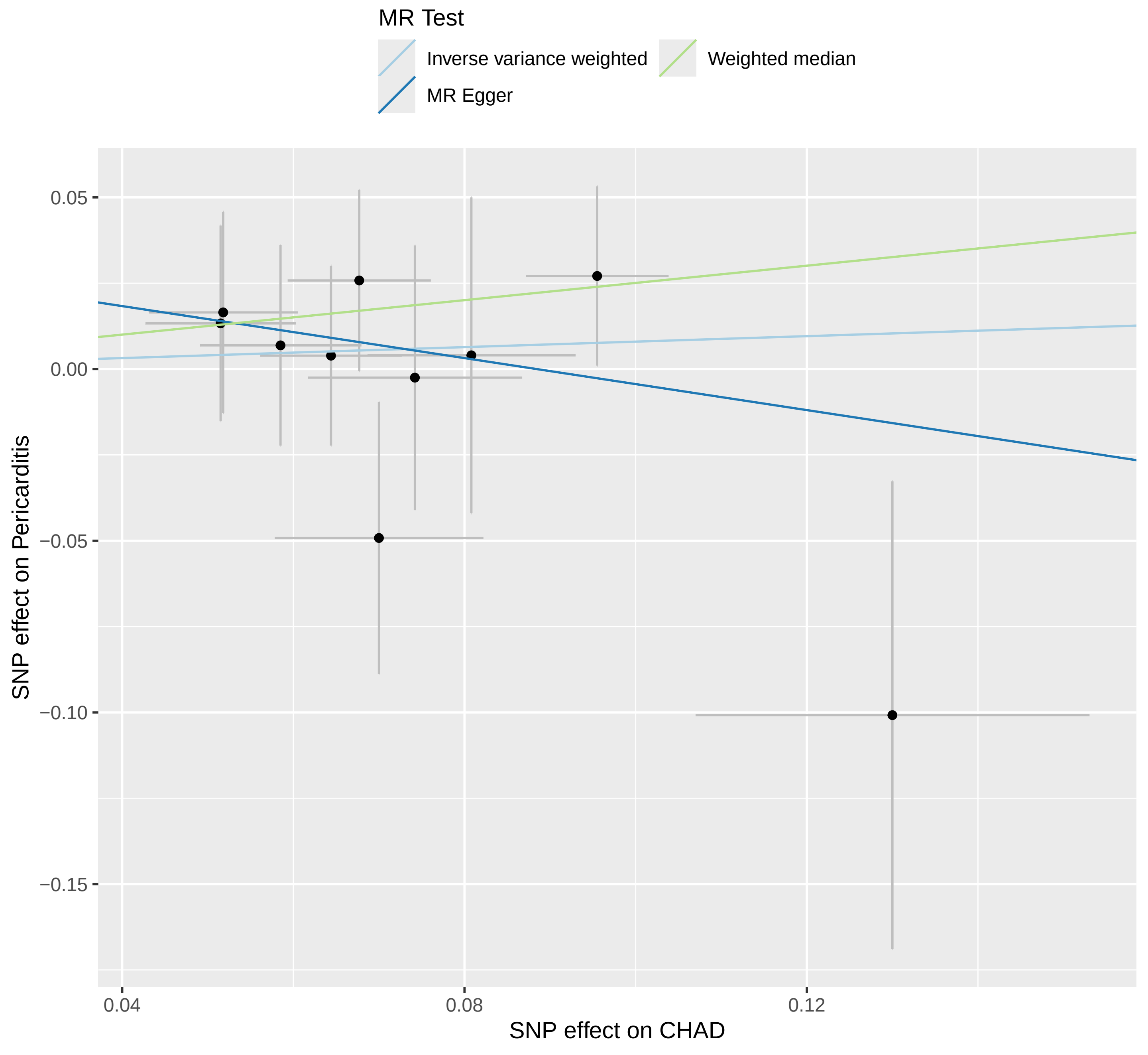

MR Test
Inverse variance weighted
Weighted median
MR Egger
0.05
0.00
−0.05
−0.10
−0.15
SNP effect on Pericarditis
0.04
0.08
0.12
SNP effect on CHAD

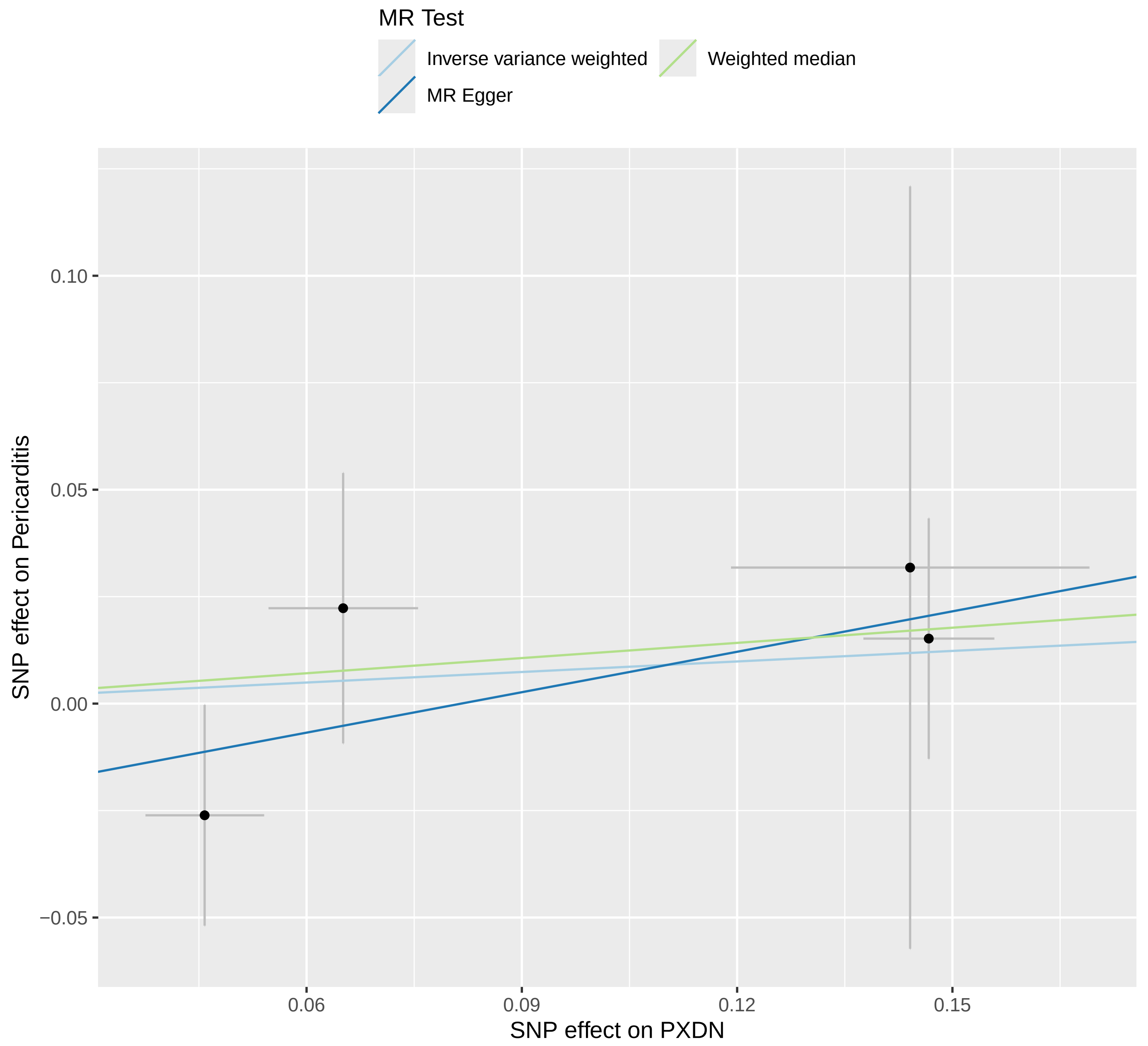

MR Test
Inverse variance weighted
MR Egger
Weighted median
0.10
0.05
0.00
−0.05
SNP effect on Pericarditis
0.06
0.09
0.12
0.15
SNP effect on PXDN

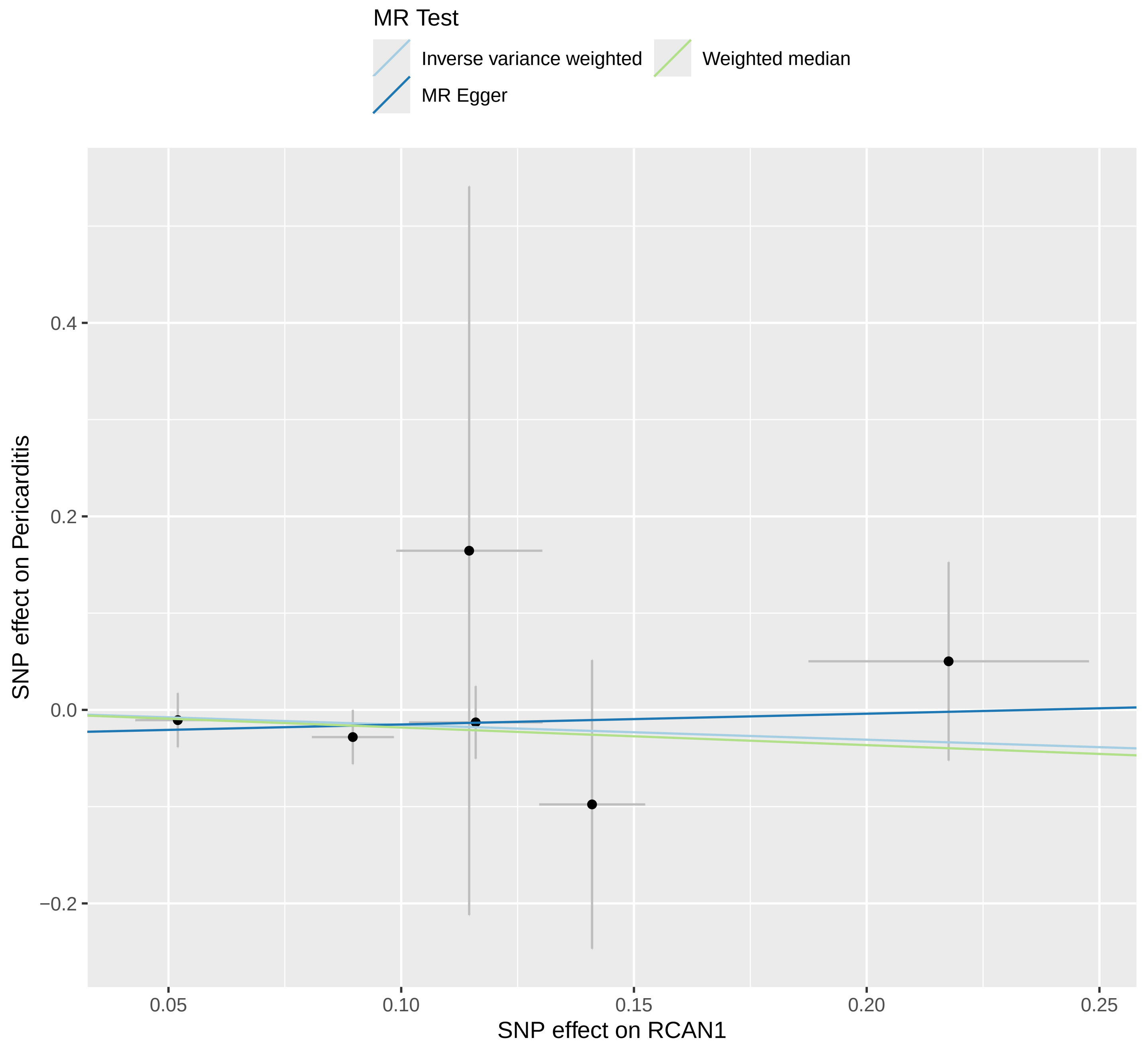
MR Test
Inverse variance weighted
MR Egger
Weighted median
SNP effect on Pericarditis
0.4
0.2
0.0
−0.2
0.05
0.10
0.15
0.20
0.25
SNP effect on RCAN1

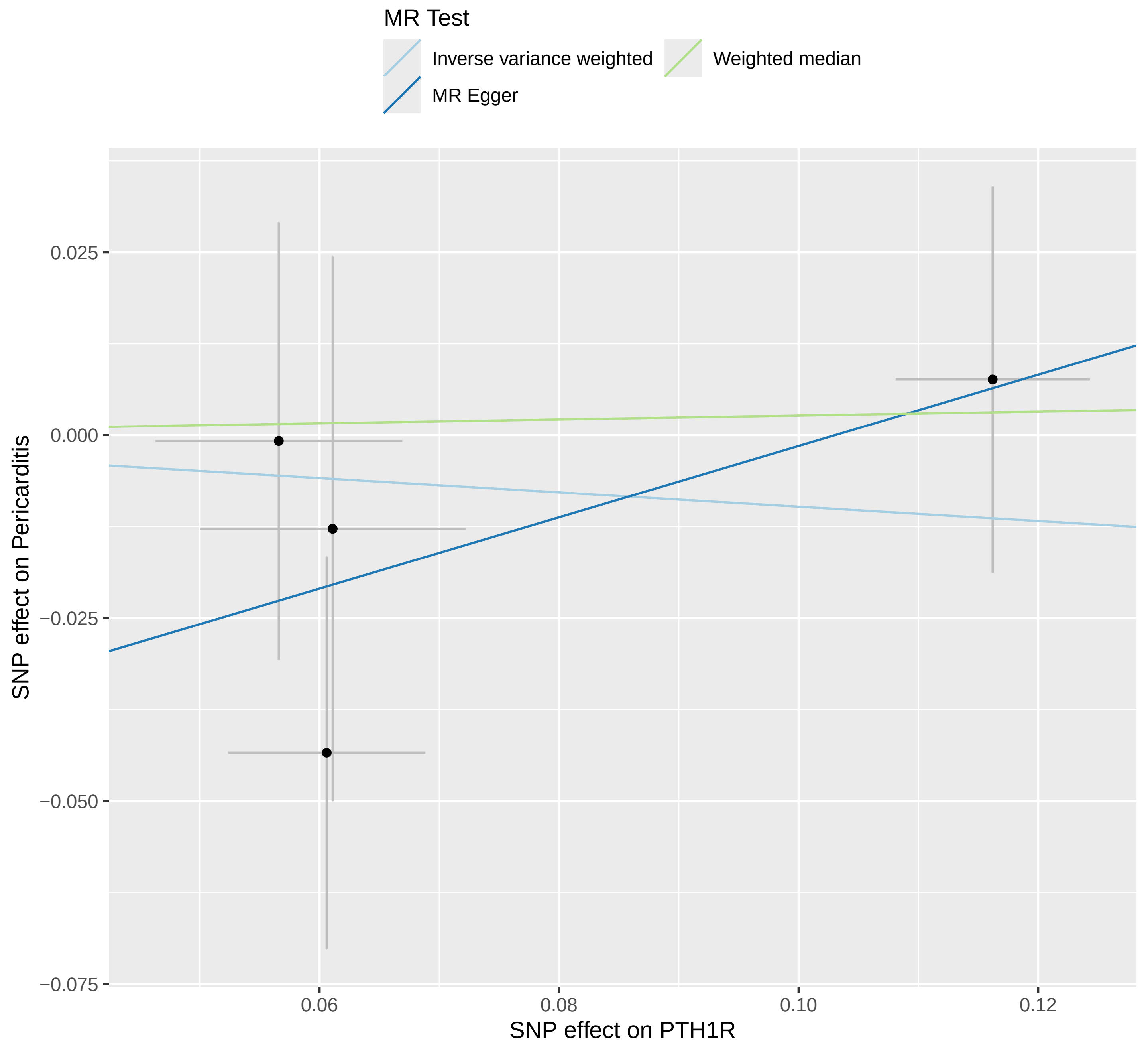

MR Test
Inverse variance weighted
Weighted median
MR Egger
0.025
0.000
−0.025
−0.050
−0.075
SNP effect on Pericarditis
0.06
0.08
0.10
0.12
SNP effect on PTH1R

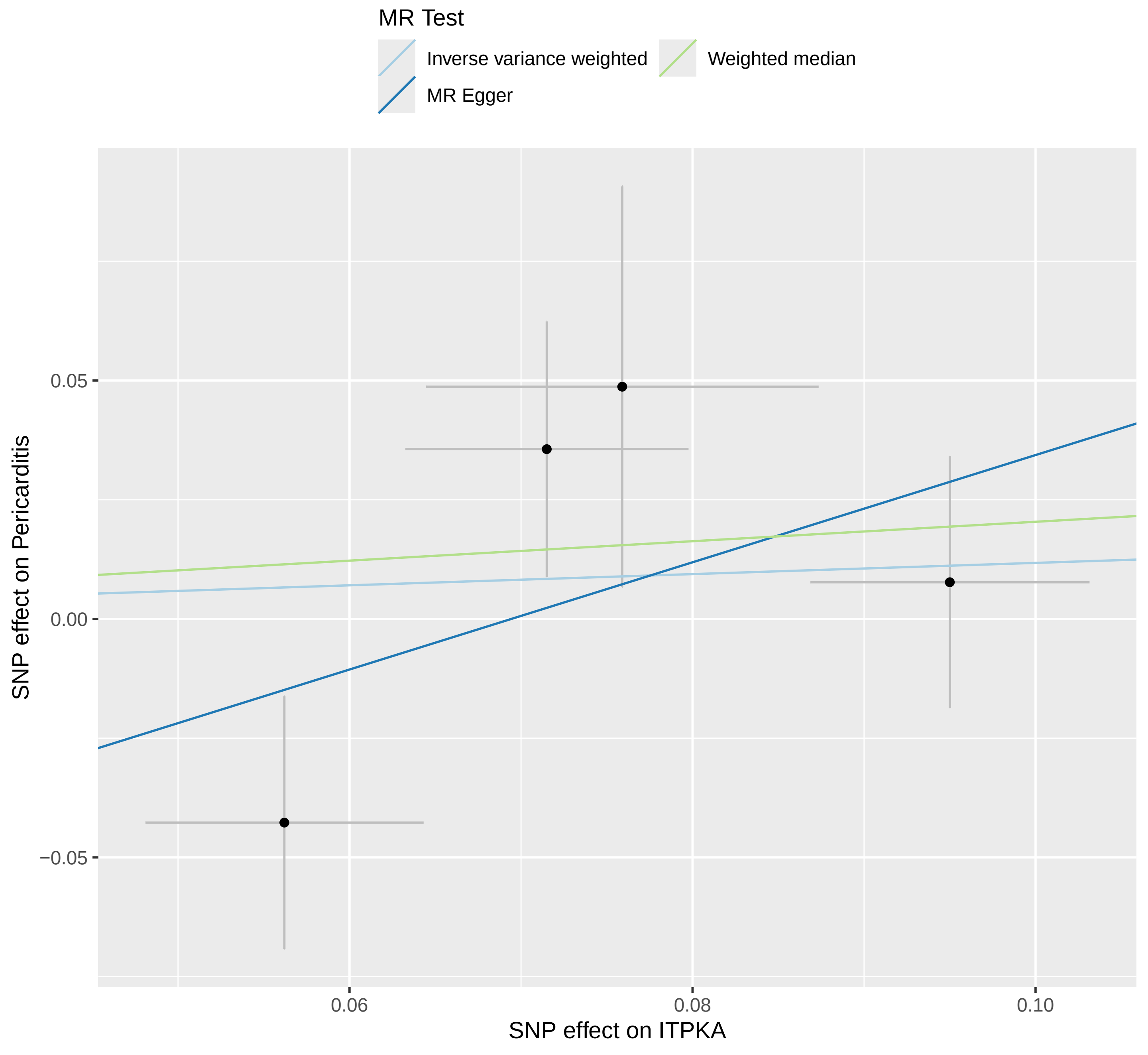
MR Test
Inverse variance weighted
MR Egger
Weighted median
SNP effect on Pericarditis
0.05
0.00
−0.05
0.06
0.08
0.10
SNP effect on ITPKA

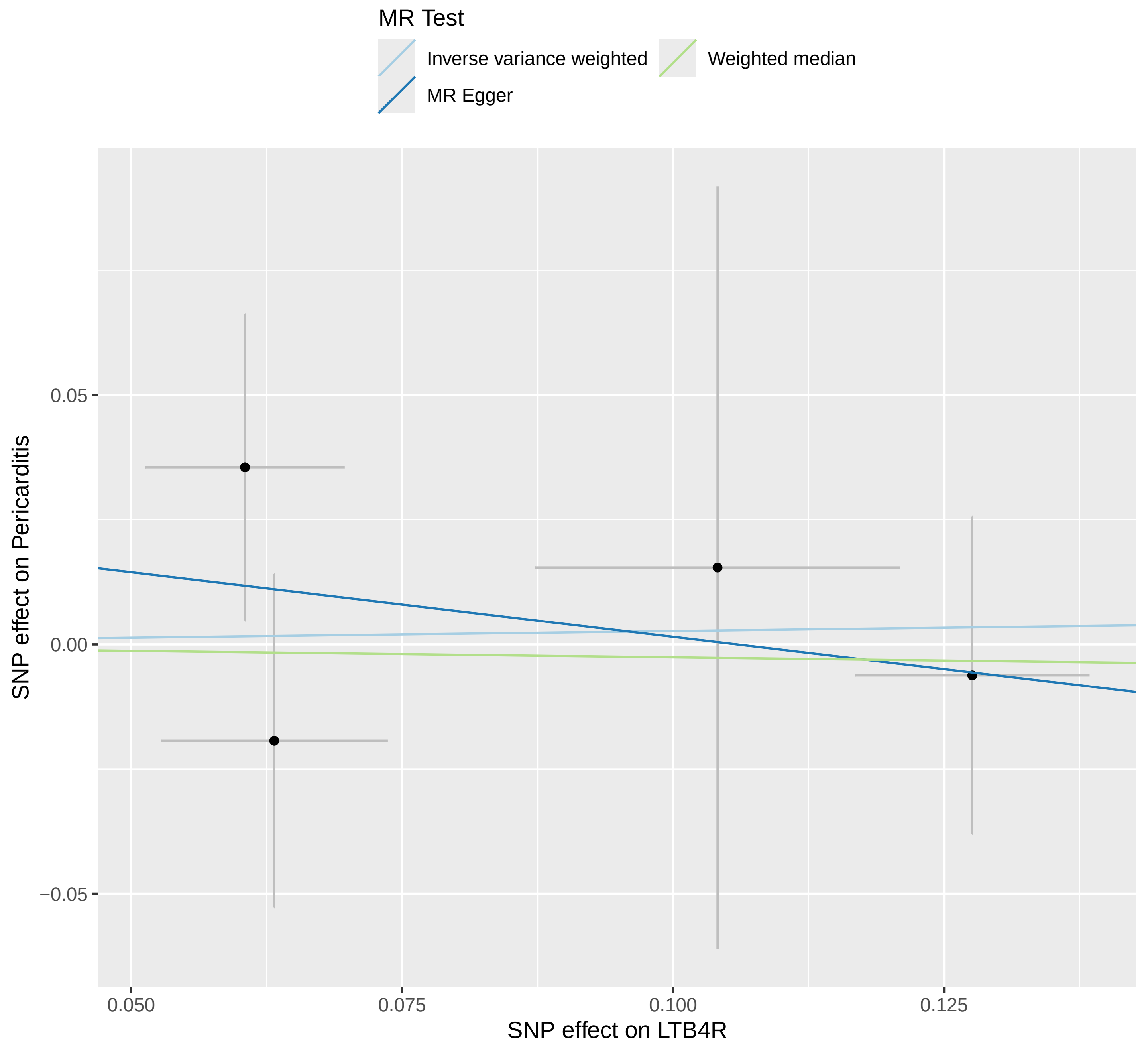

MR Test
Inverse variance weighted
Weighted median
MR Egger
0.05
0.00
−0.05
SNP effect on Pericarditis
0.050
0.075
0.100
0.125
SNP effect on LTB4R

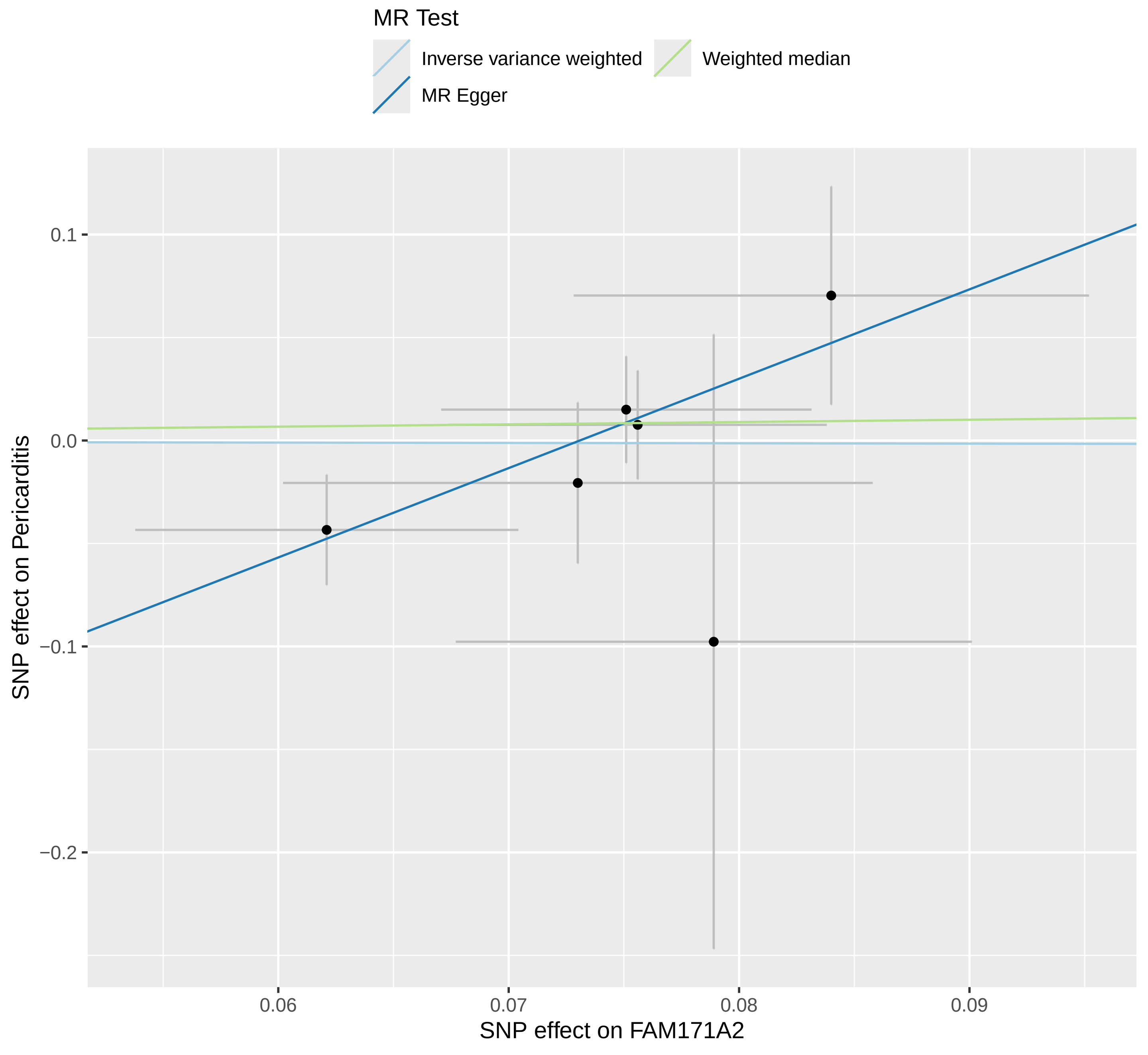
MR Test
Inverse variance weighted
Weighted median
MR Egger
0.1
0.0
−0.1
−0.2
SNP effect on Pericarditis
0.06
0.07
0.08
0.09
SNP effect on FAM171A2

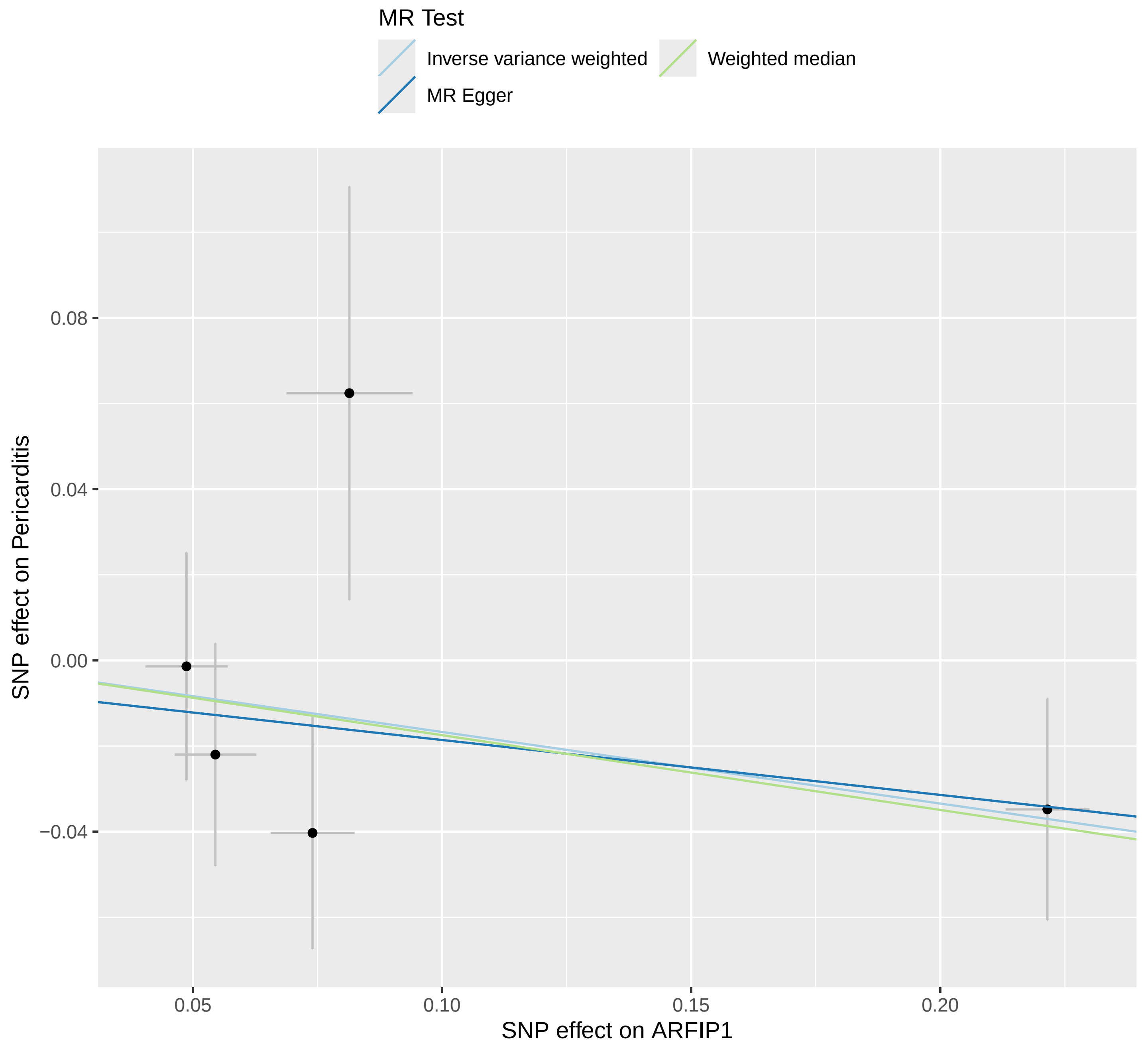

MR Test
Inverse variance weighted
Weighted median
MR Egger
0.08
0.04
0.00
−0.04
0.05
0.10
0.15
0.20
SNP effect on ARFIP1
SNP effect on Pericarditis

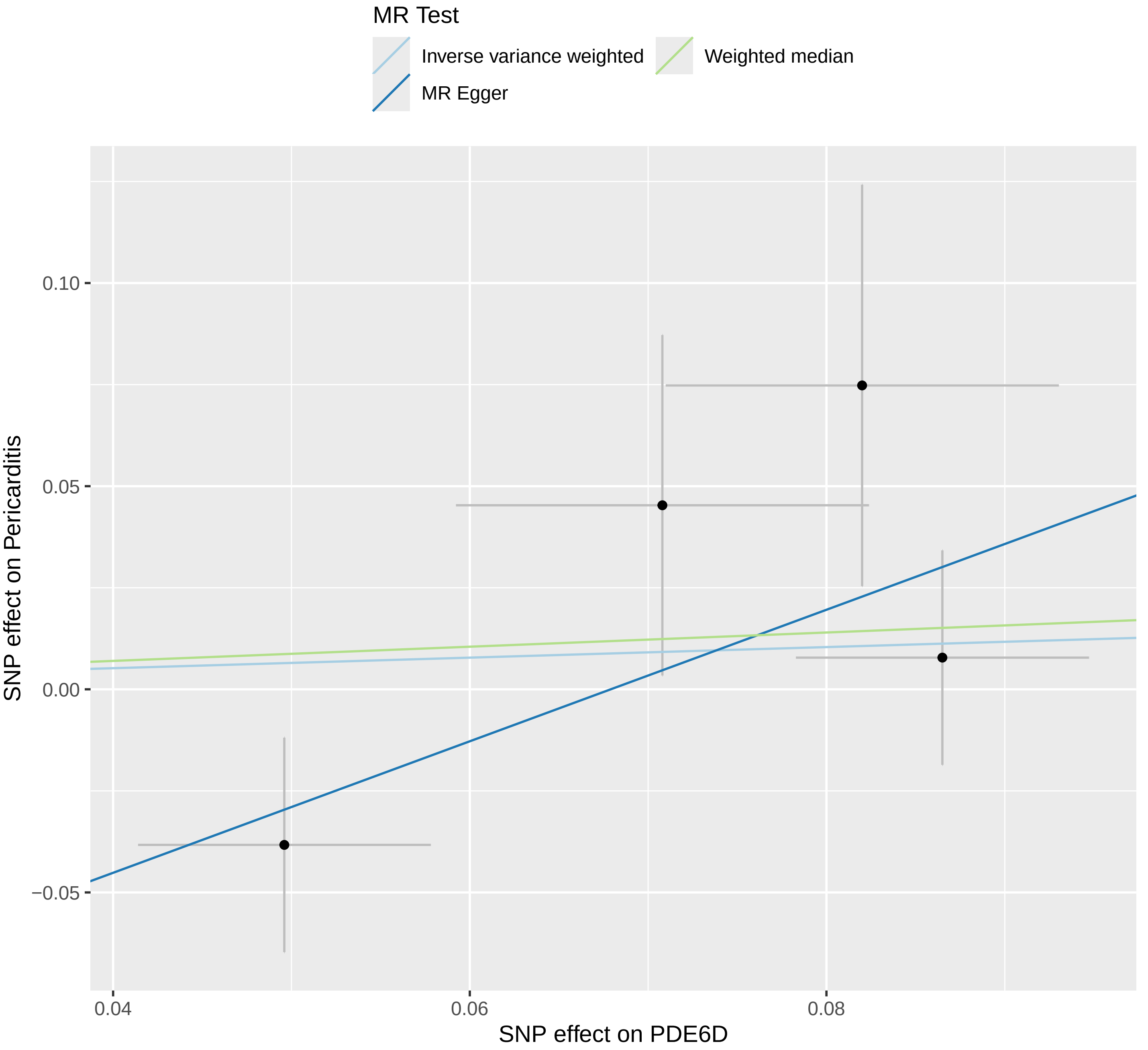

MR Test
Inverse variance weighted
Weighted median
MR Egger
0.10
0.05
0.00
−0.05
SNP effect on Pericarditis
0.04
0.06
0.08
SNP effect on PDE6D

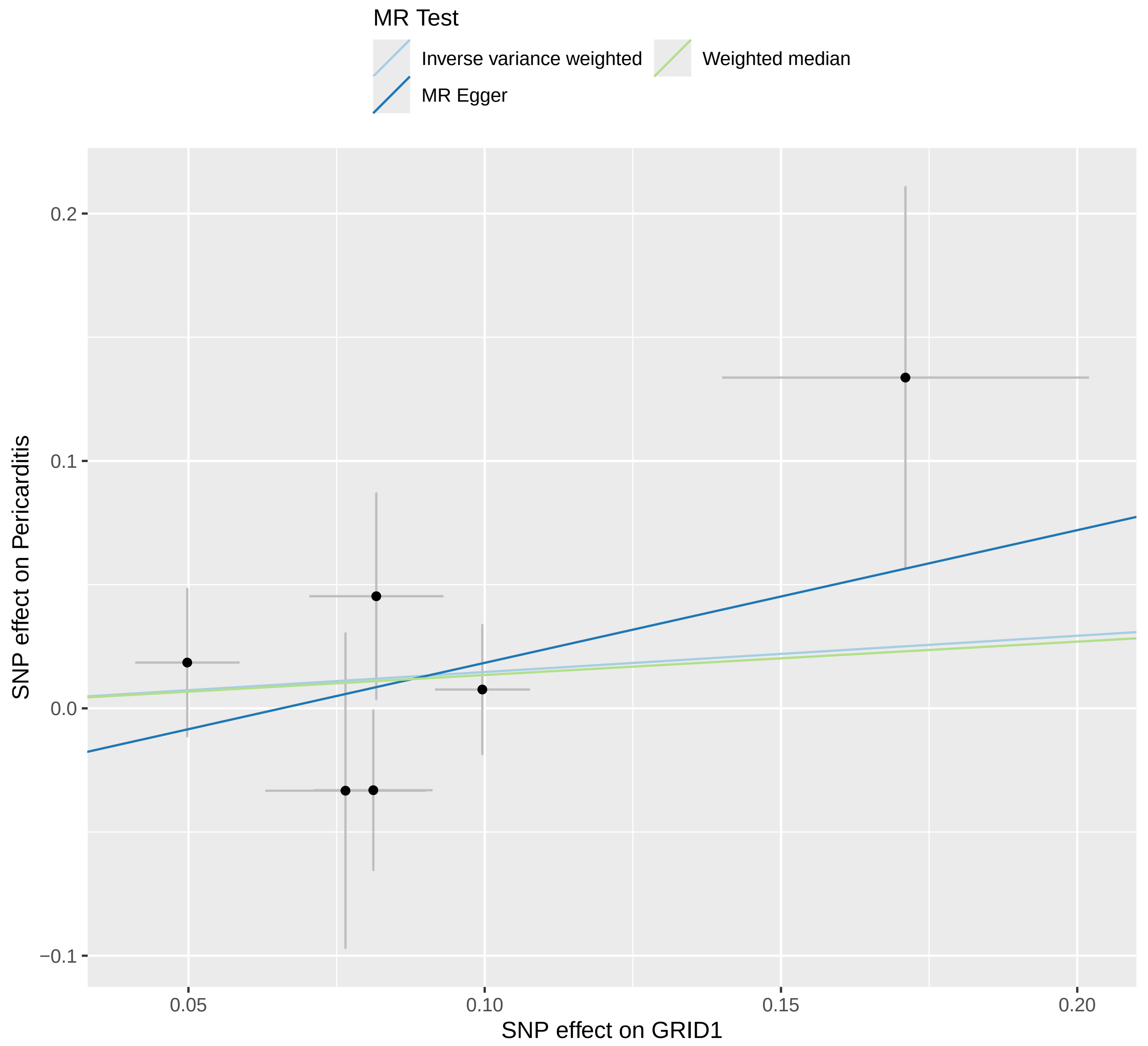

MR Test
Inverse variance weighted
Weighted median
MR Egger
SNP effect on Pericarditis
0.2
0.1
0.0
−0.1
0.05
0.10
0.15
0.20
SNP effect on GRID1

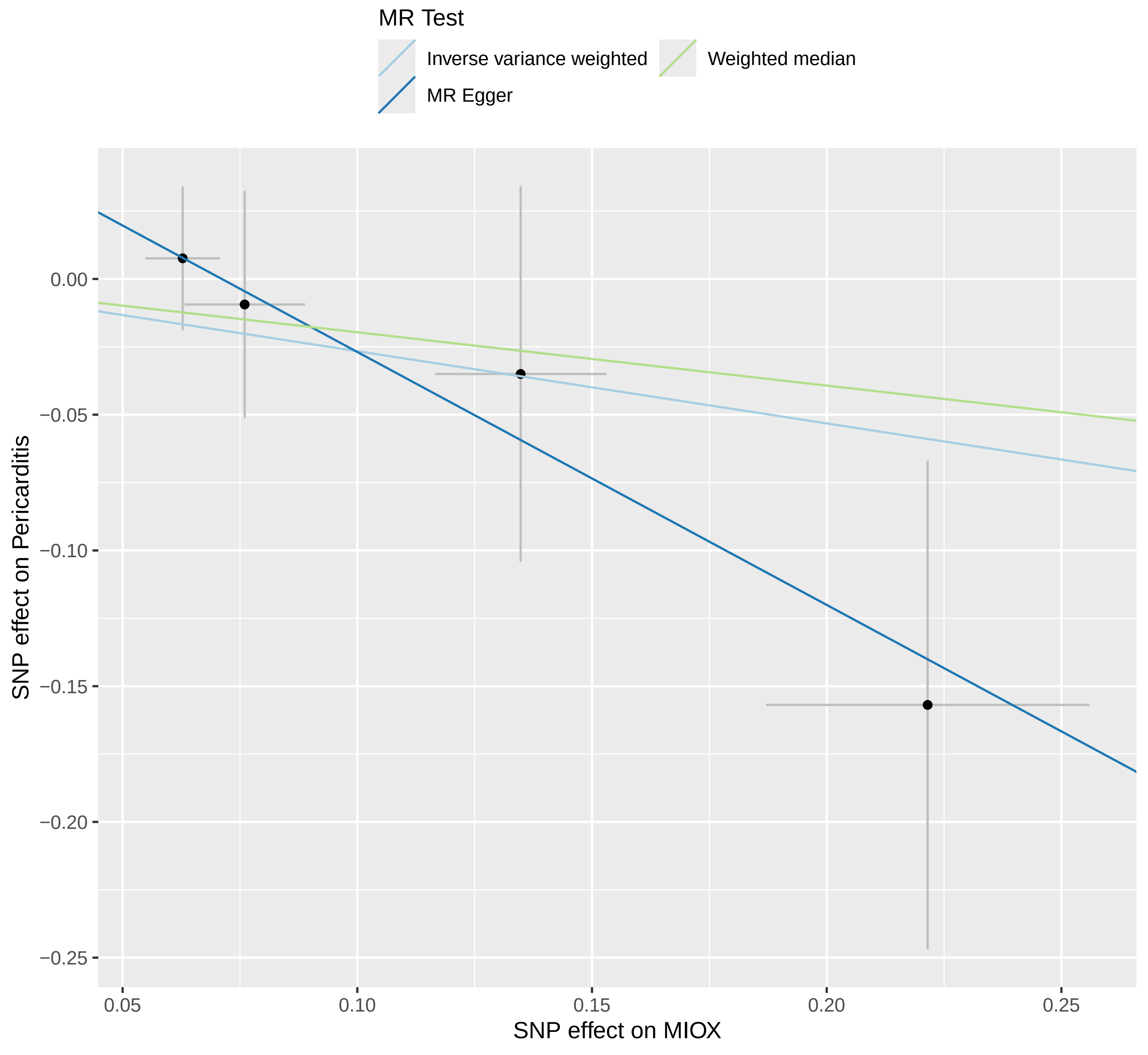
MR Test
Inverse variance weighted
Weighted median
MR Egger
0.00
−0.05
−0.10
−0.15
−0.20
−0.25
SNP effect on Pericarditis
0.05
0.10
0.15
0.20
0.25
SNP effect on MIOX

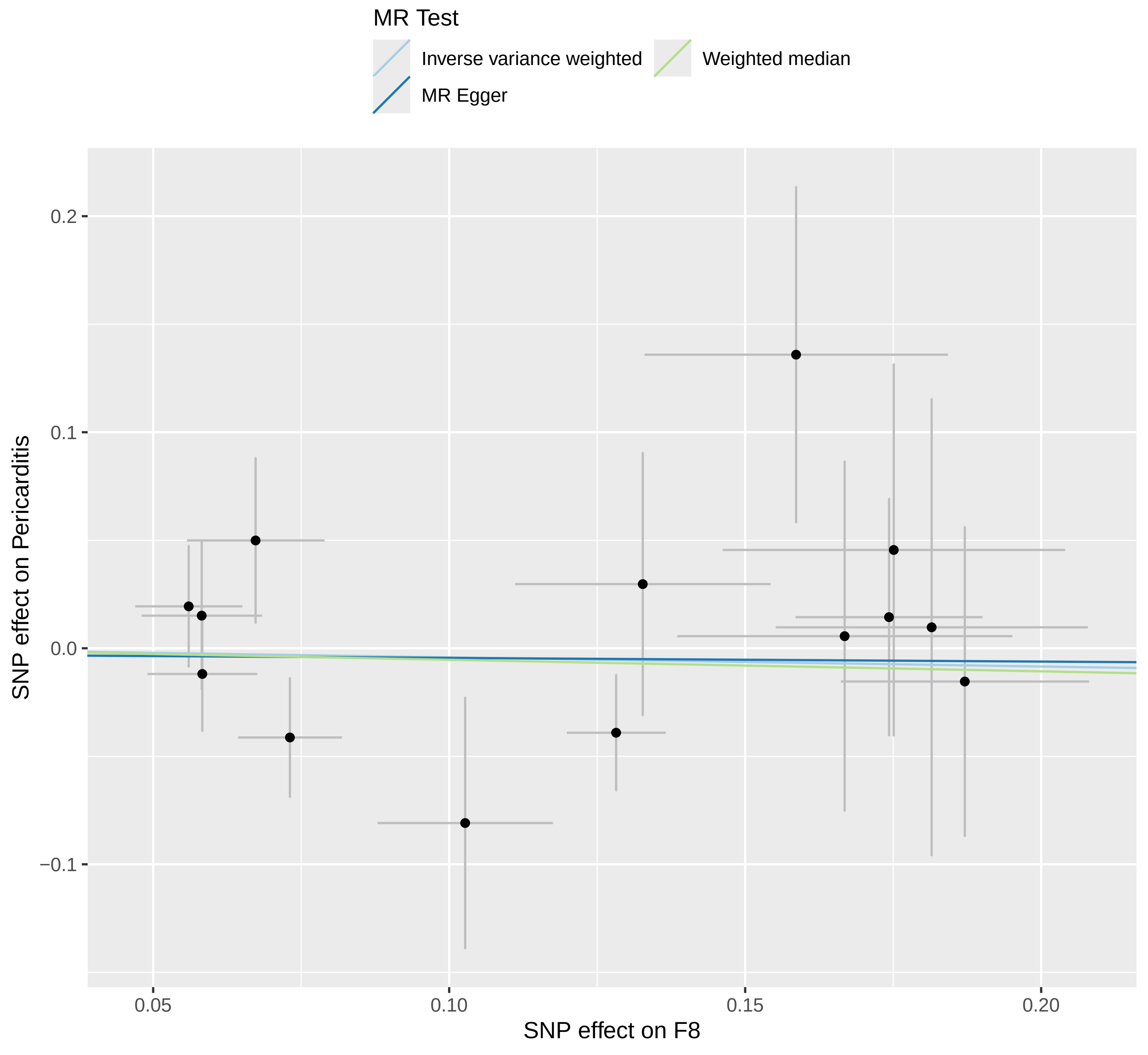
MR Test
Inverse variance weighted
Weighted median
MR Egger
0.2
0.1
0.0
−0.1
SNP effect on Pericarditis
0.05
0.10
0.15
0.20
SNP effect on F8

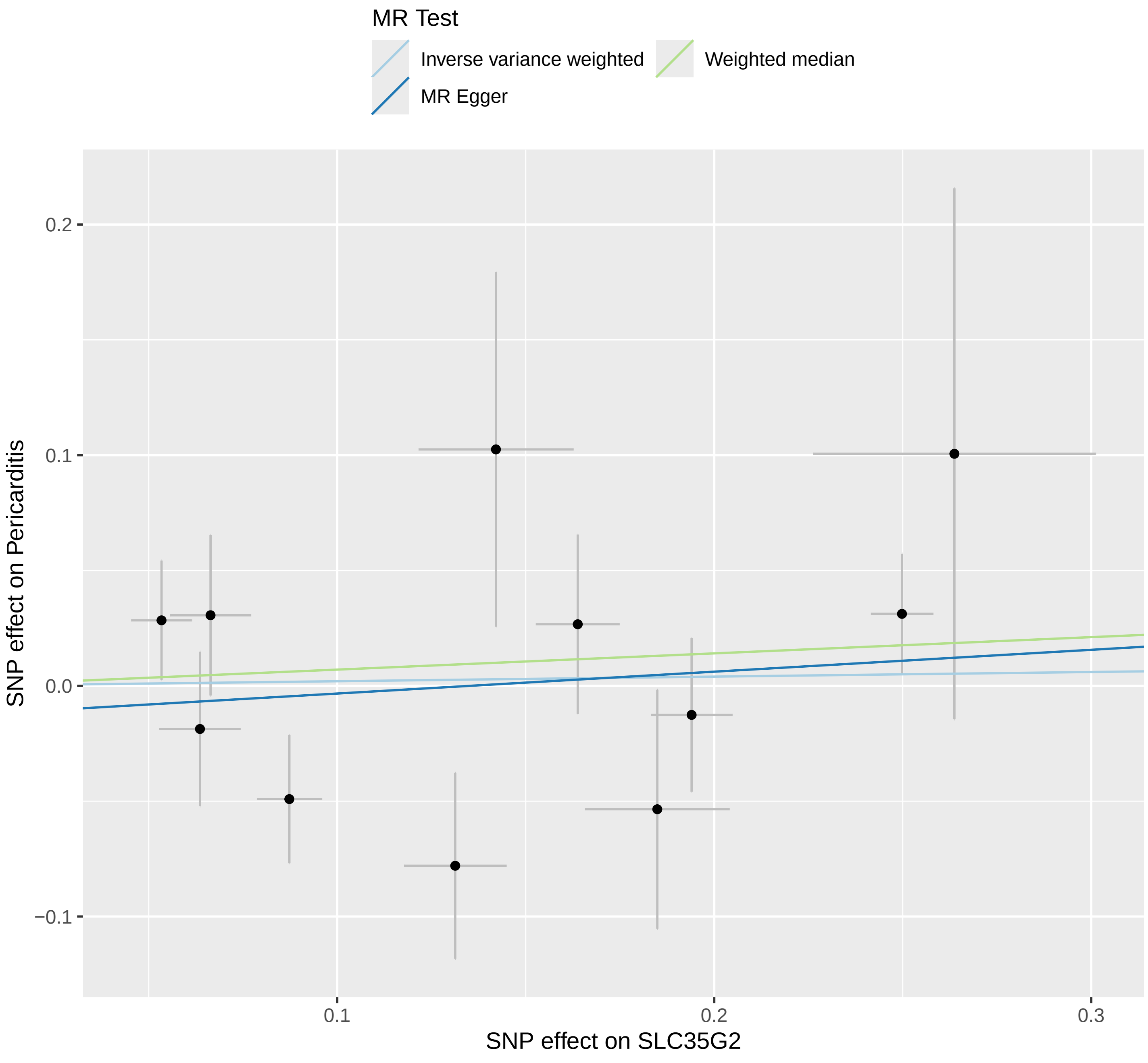

MR Test
Inverse variance weighted
Weighted median
MR Egger
0.2
0.1
0.0
−0.1
0.1
0.2
0.3
SNP effect on Pericarditis
SNP effect on SLC35G2

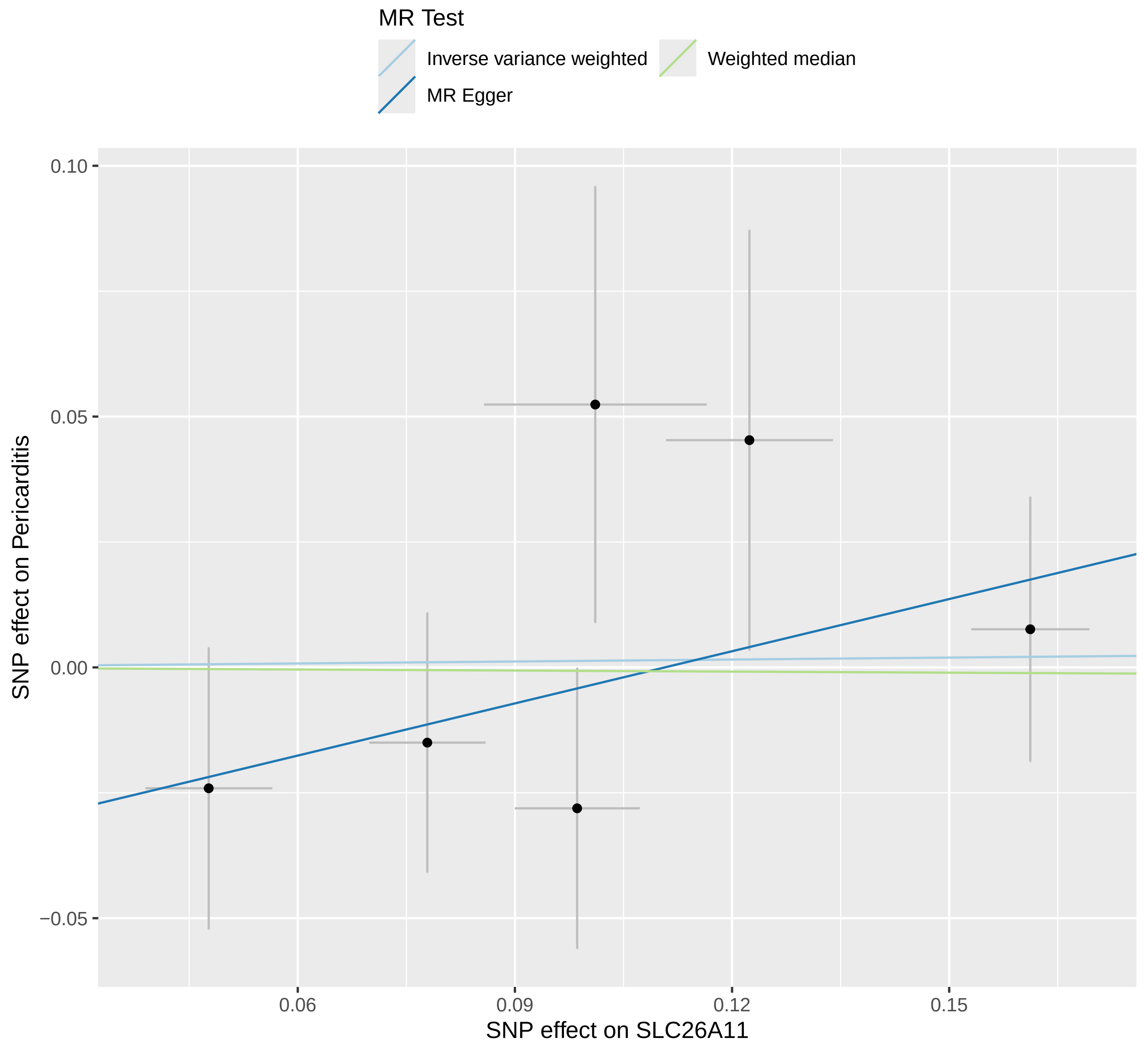
MR Test
Inverse variance weighted
MR Egger
Weighted median
0.10
0.05
0.00
−0.05
SNP effect on Pericarditis
0.06
0.09
0.12
0.15
SNP effect on SLC26A11

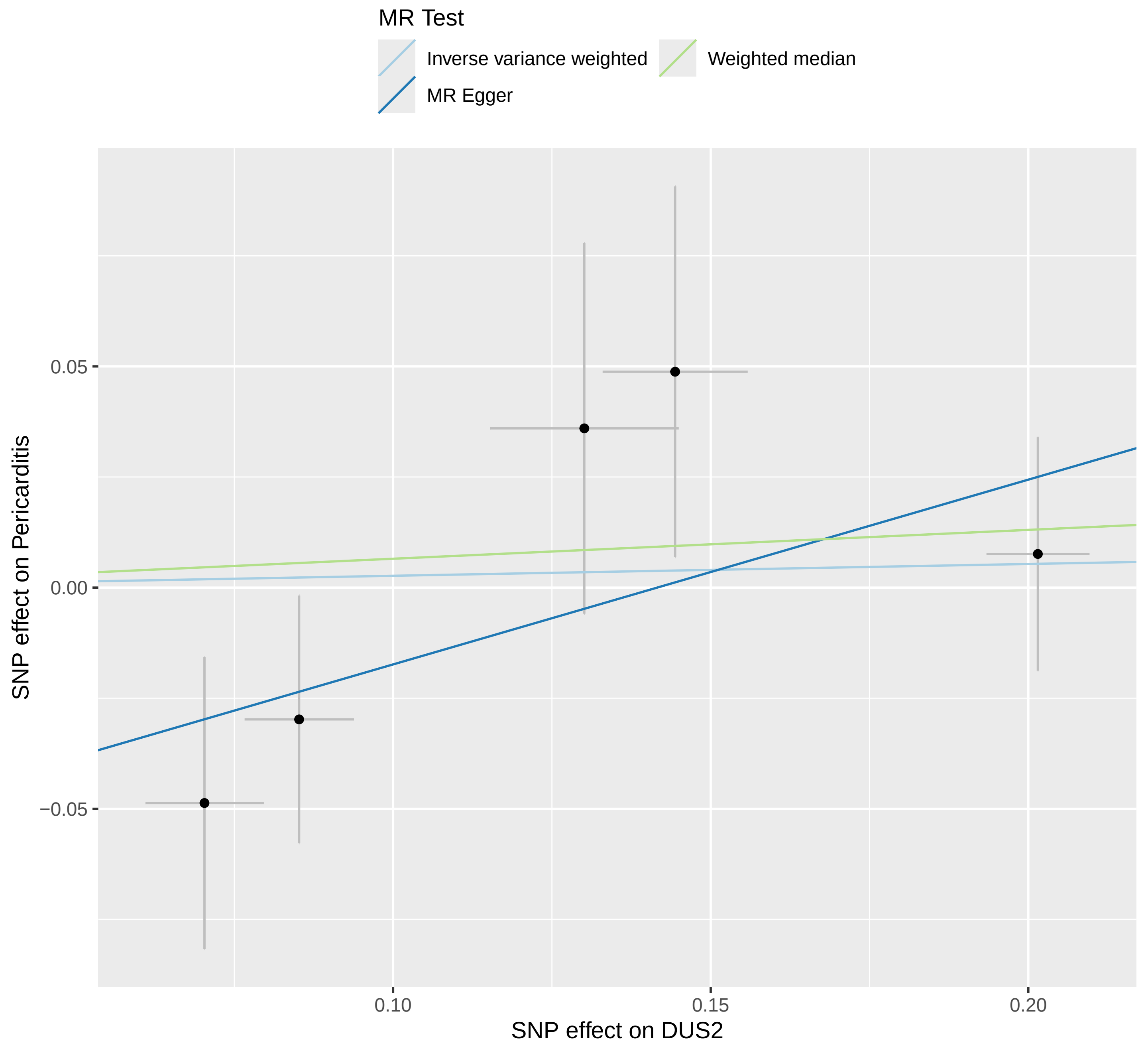
MR Test
Inverse variance weighted
MR Egger
Weighted median
SNP effect on Pericarditis
0.05
0.00
−0.05
0.10
0.15
0.20
SNP effect on DUS2

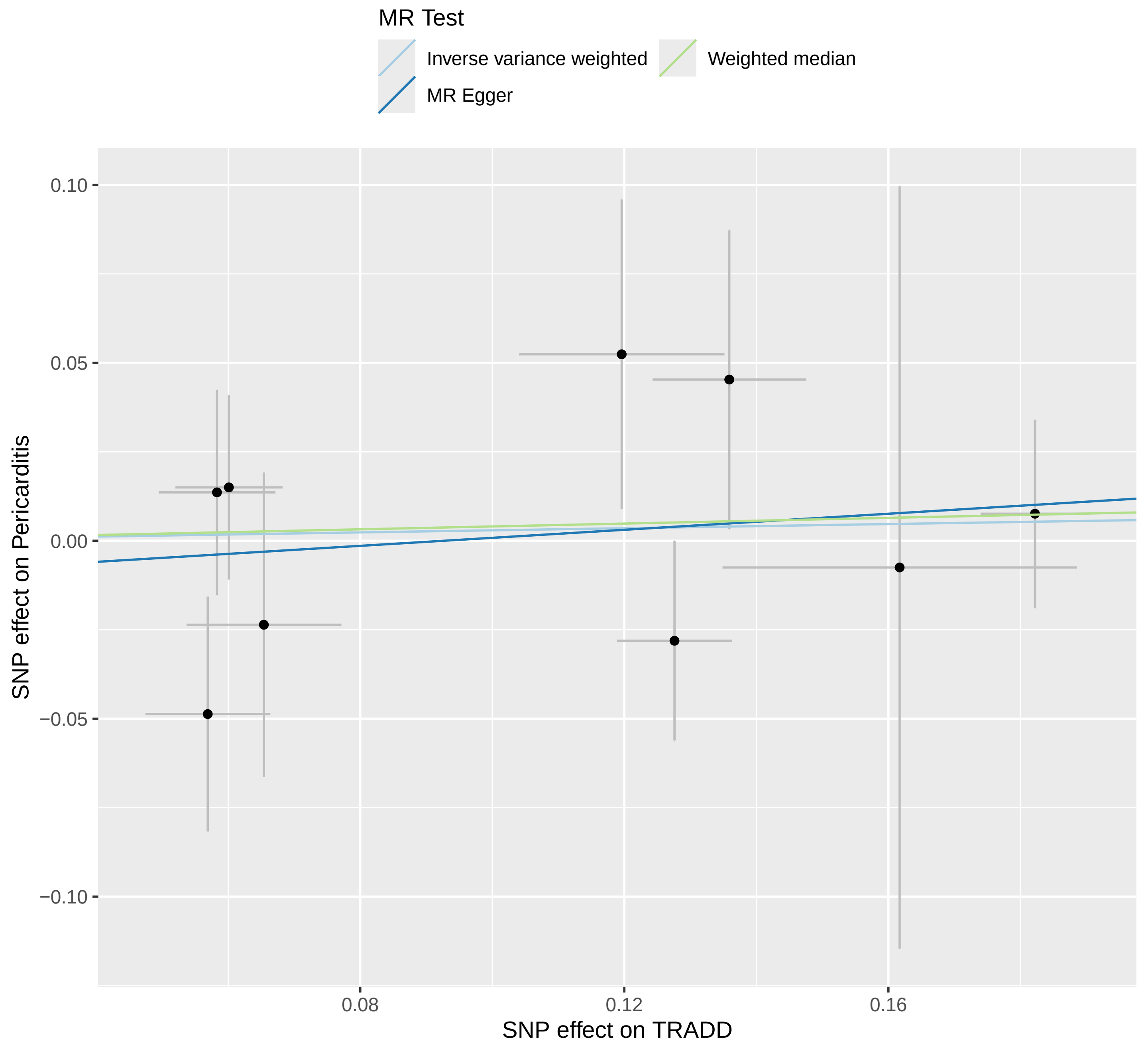
MR Test
Inverse variance weighted
MR Egger
Weighted median
SNP effect on Pericarditis
0.10
0.05
0.00
−0.05
−0.10
0.08
0.12
0.16
SNP effect on TRADD

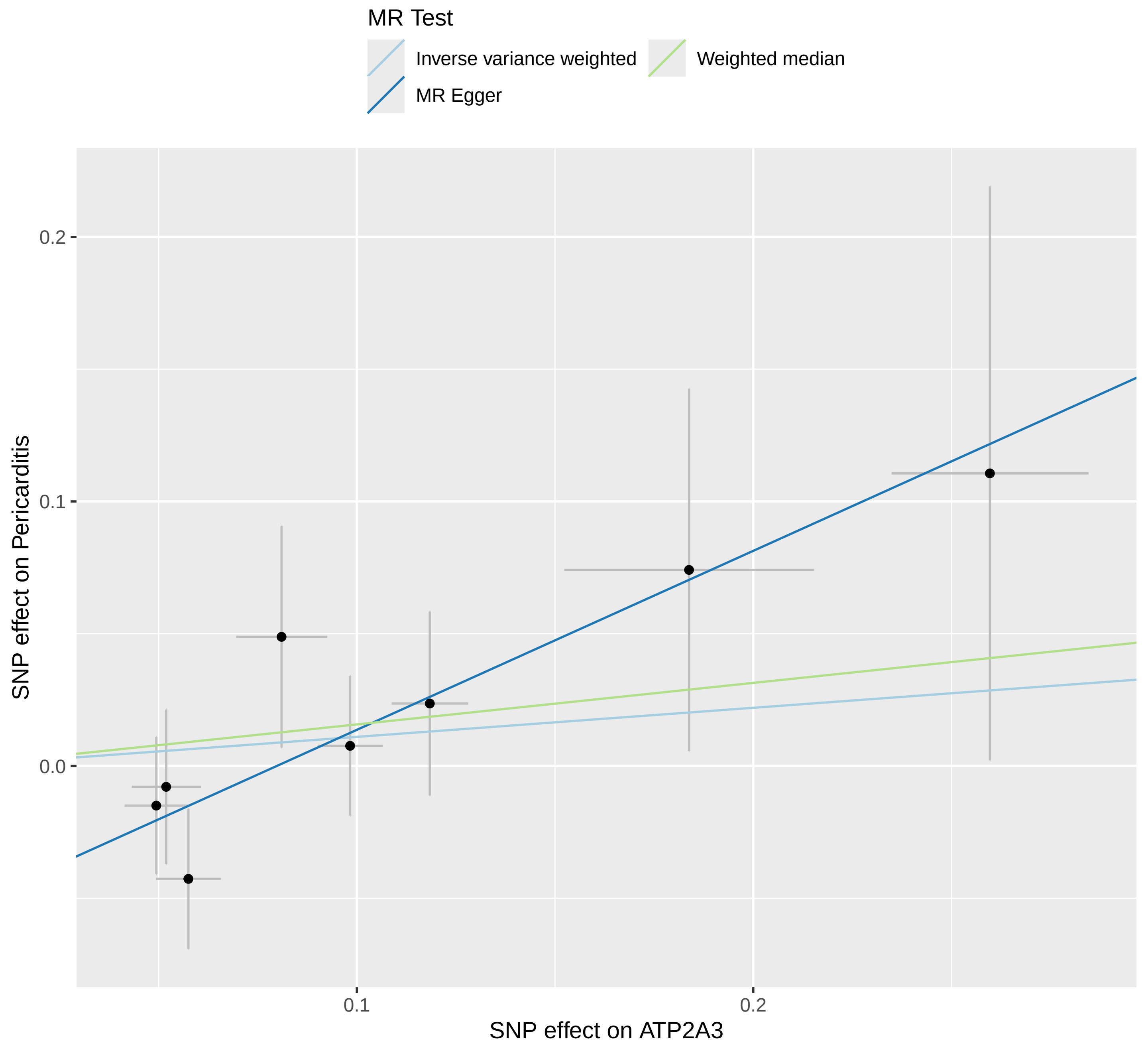
MR Test
Inverse variance weighted
MR Egger
Weighted median
SNP effect on Pericarditis
0.2
0.1
0.0
0.1
0.2
SNP effect on ATP2A3

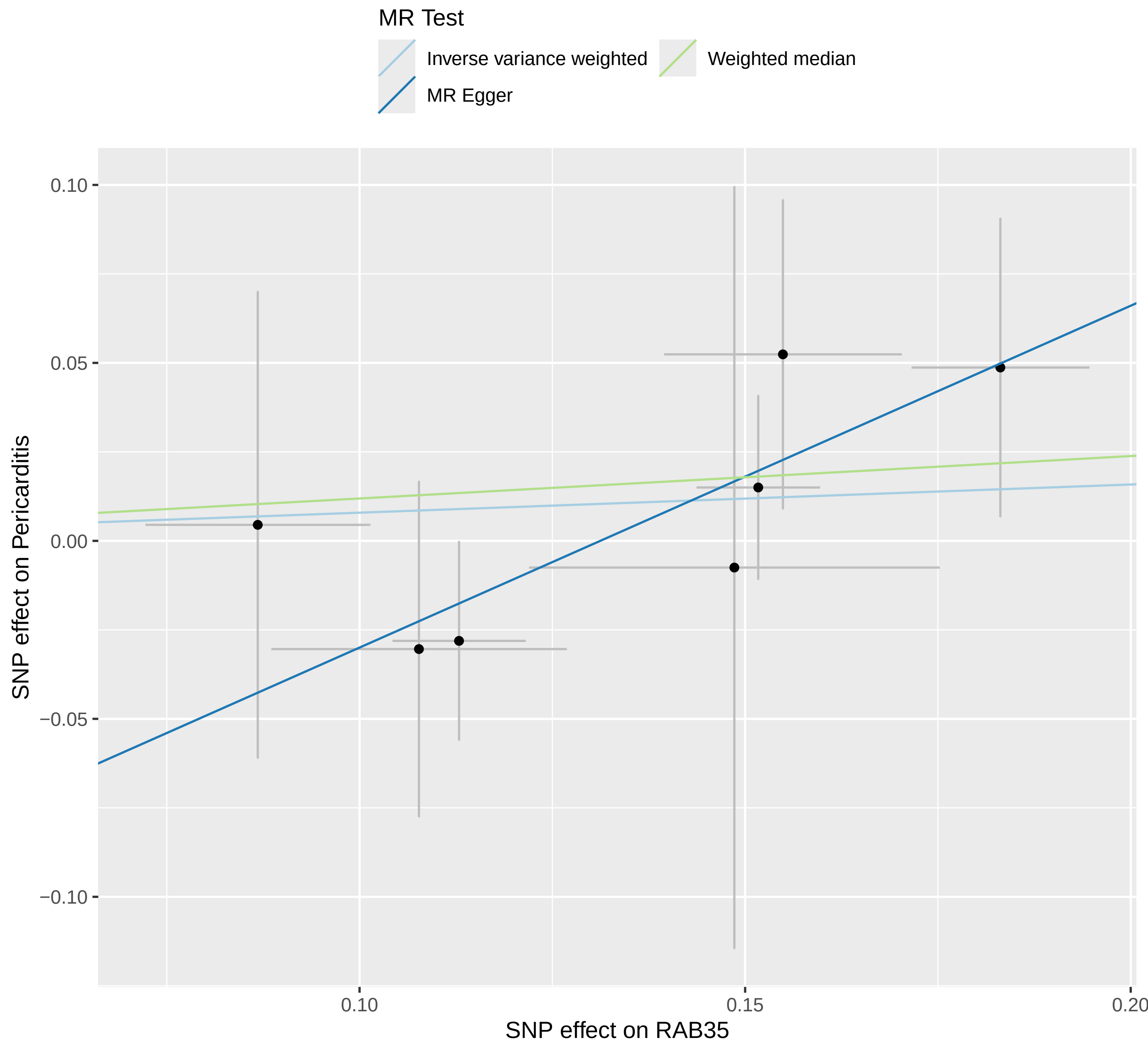
MR Test
Inverse variance weighted
Weighted median
MR Egger
0.10
0.05
0.00
−0.05
−0.10
SNP effect on Pericarditis
0.10
0.15
0.20
SNP effect on RAB35

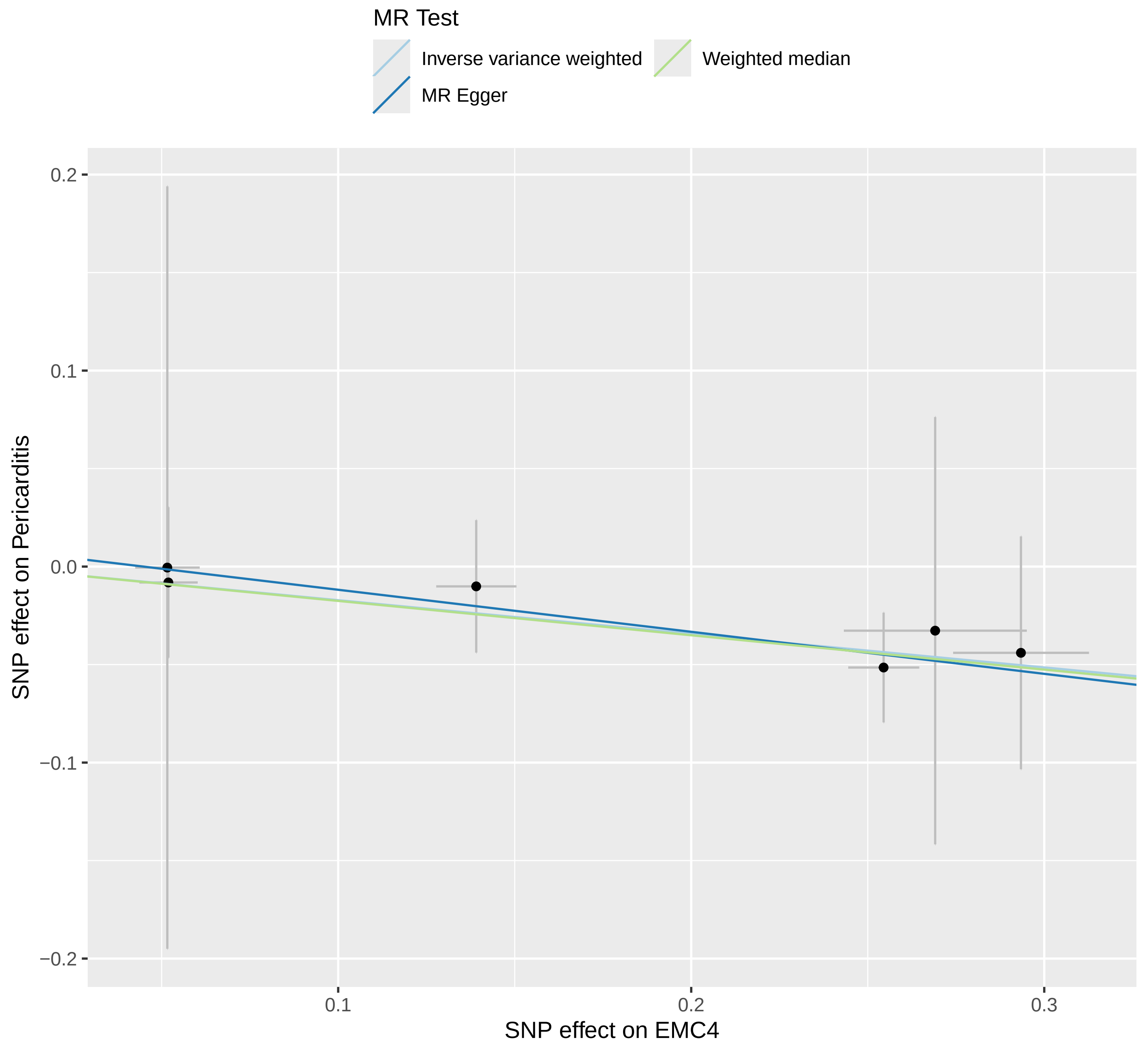

MR Test
Inverse variance weighted
Weighted median
MR Egger
0.2
0.1
0.0
−0.1
−0.2
SNP effect on Pericarditis
0.1
0.2
0.3
SNP effect on EMC4

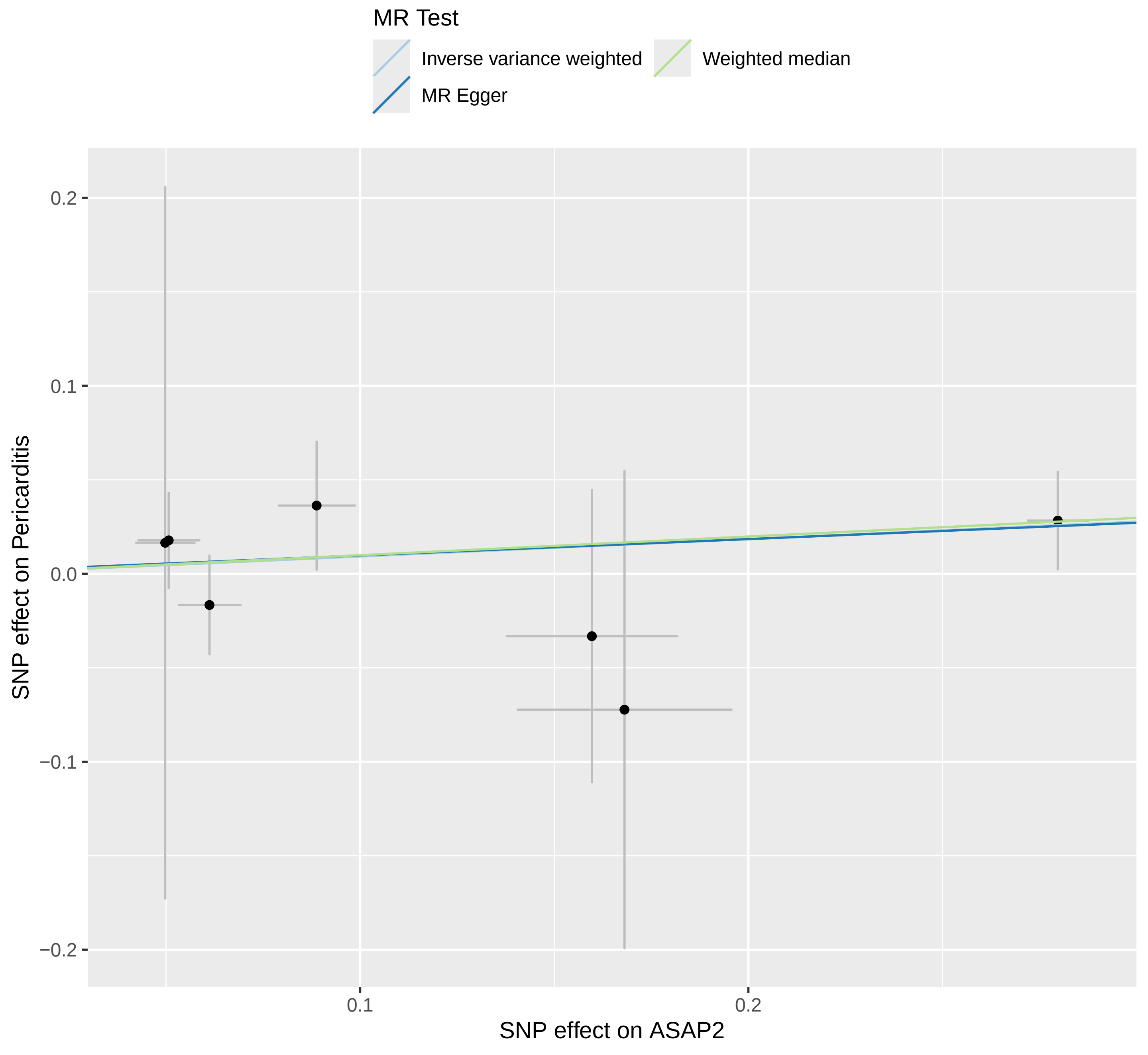

MR Test
Inverse variance weighted
Weighted median
MR Egger
0.2
0.1
0.0
−0.1
−0.2
0.1
0.2
SNP effect on Pericarditis
SNP effect on ASAP2

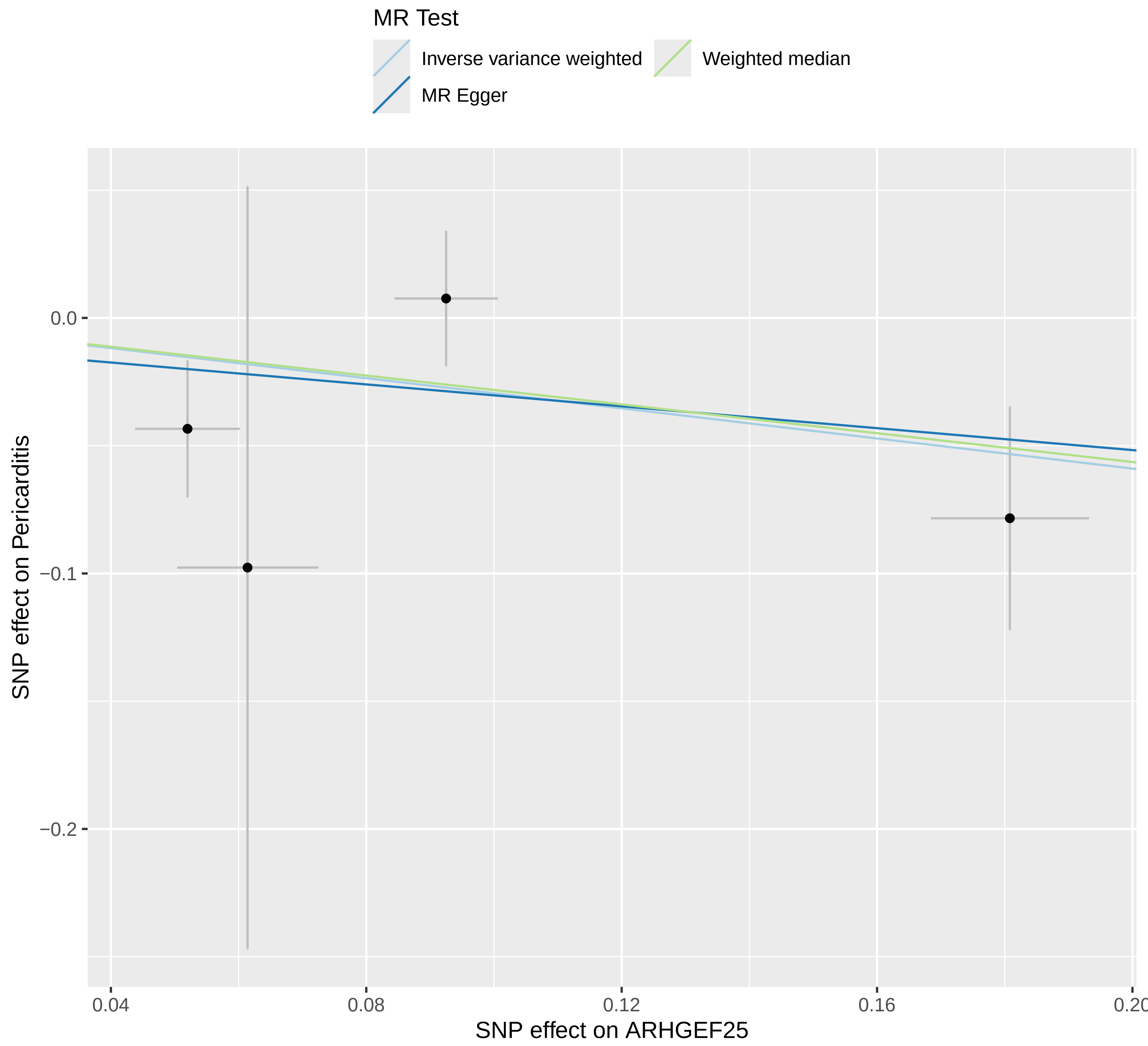
MR Test
Inverse variance weighted
Weighted median
MR Egger
0.0
−0.1
−0.2
0.04
0.08
0.12
0.16
0.20
SNP effect on ARHGEF25
SNP effect on Pericarditis

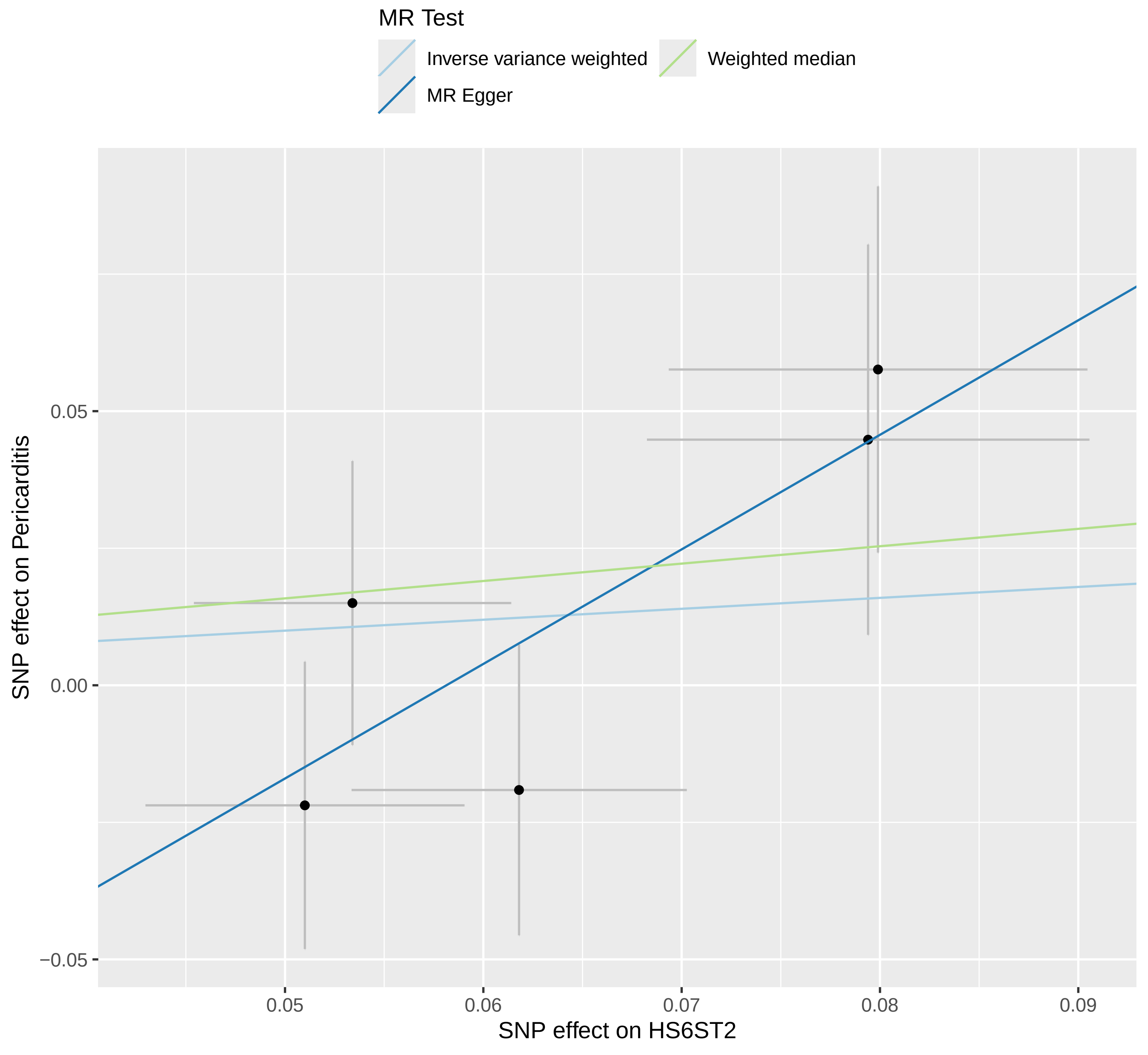
MR Test
Inverse variance weighted
MR Egger
Weighted median
SNP effect on Pericarditis
0.05
0.00
−0.05
0.05
0.06
0.07
0.08
0.09
SNP effect on HS6ST2

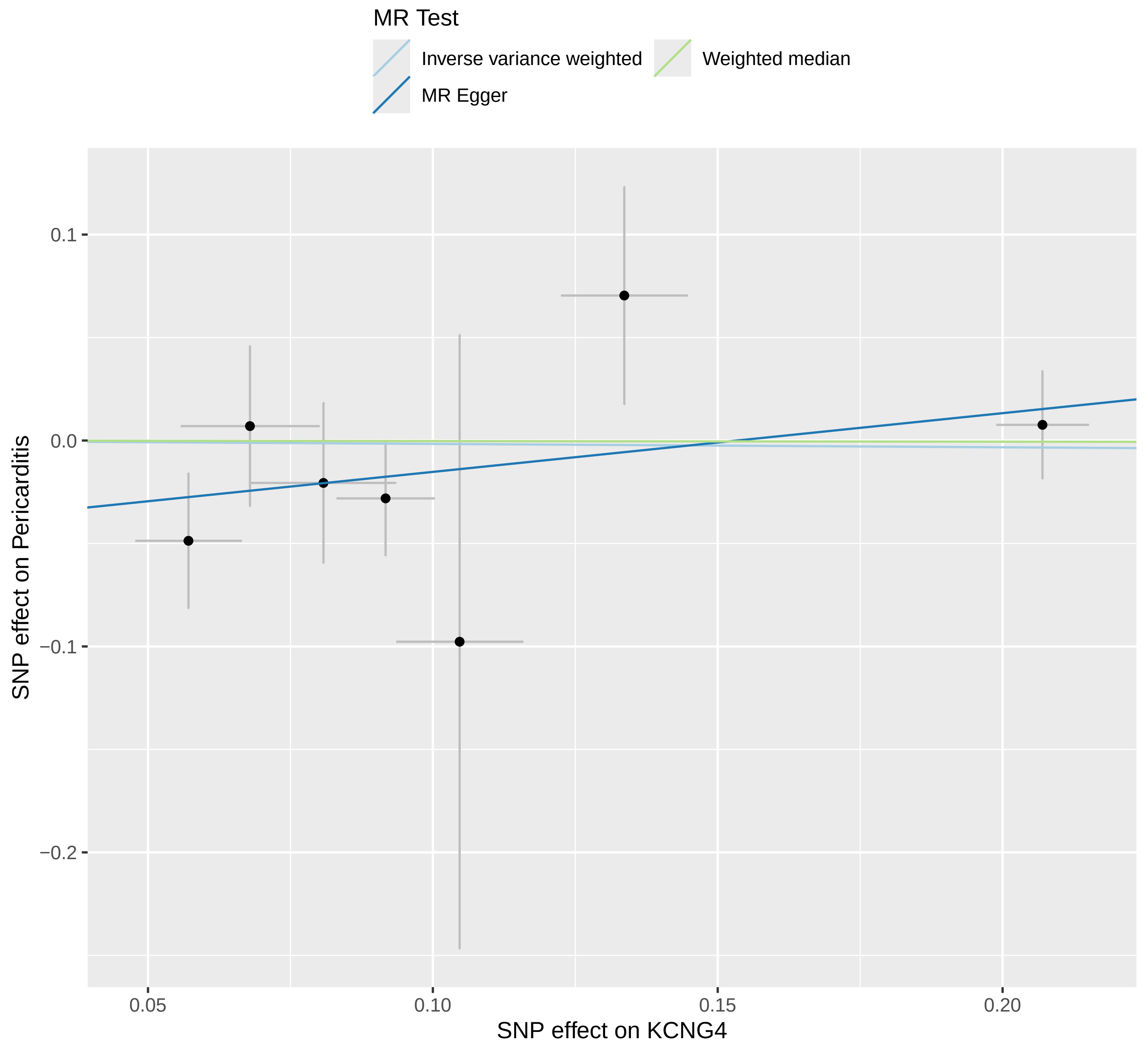

MR Test
Inverse variance weighted
Weighted median
MR Egger
0.1
0.0
−0.1
−0.2
SNP effect on Pericarditis
0.05
0.10
0.15
0.20
SNP effect on KCNG4

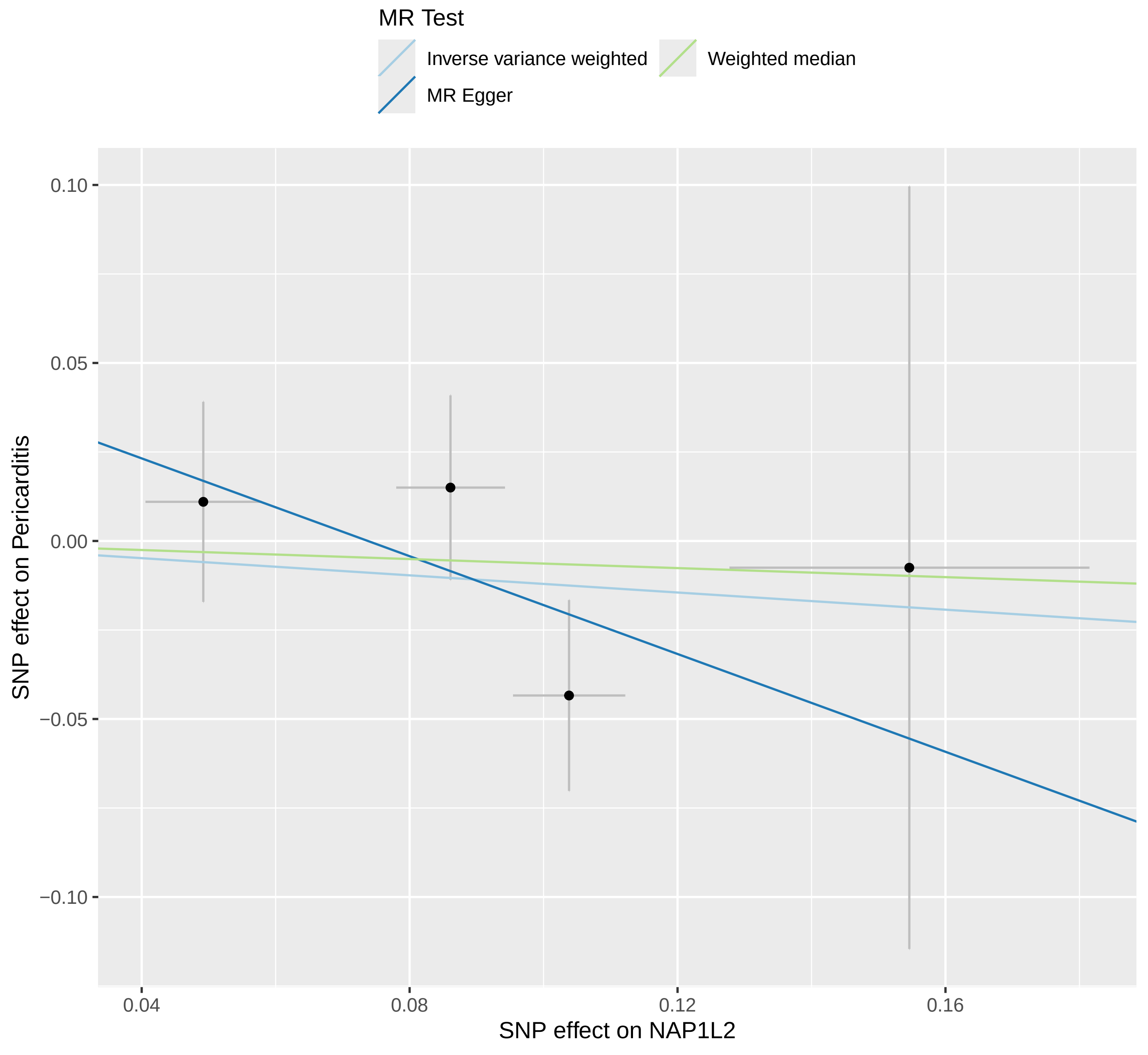
MR Test
Inverse variance weighted
Weighted median
MR Egger
0.10
0.05
0.00
−0.05
−0.10
SNP effect on Pericarditis
0.04
0.08
0.12
0.16
SNP effect on NAP1L2

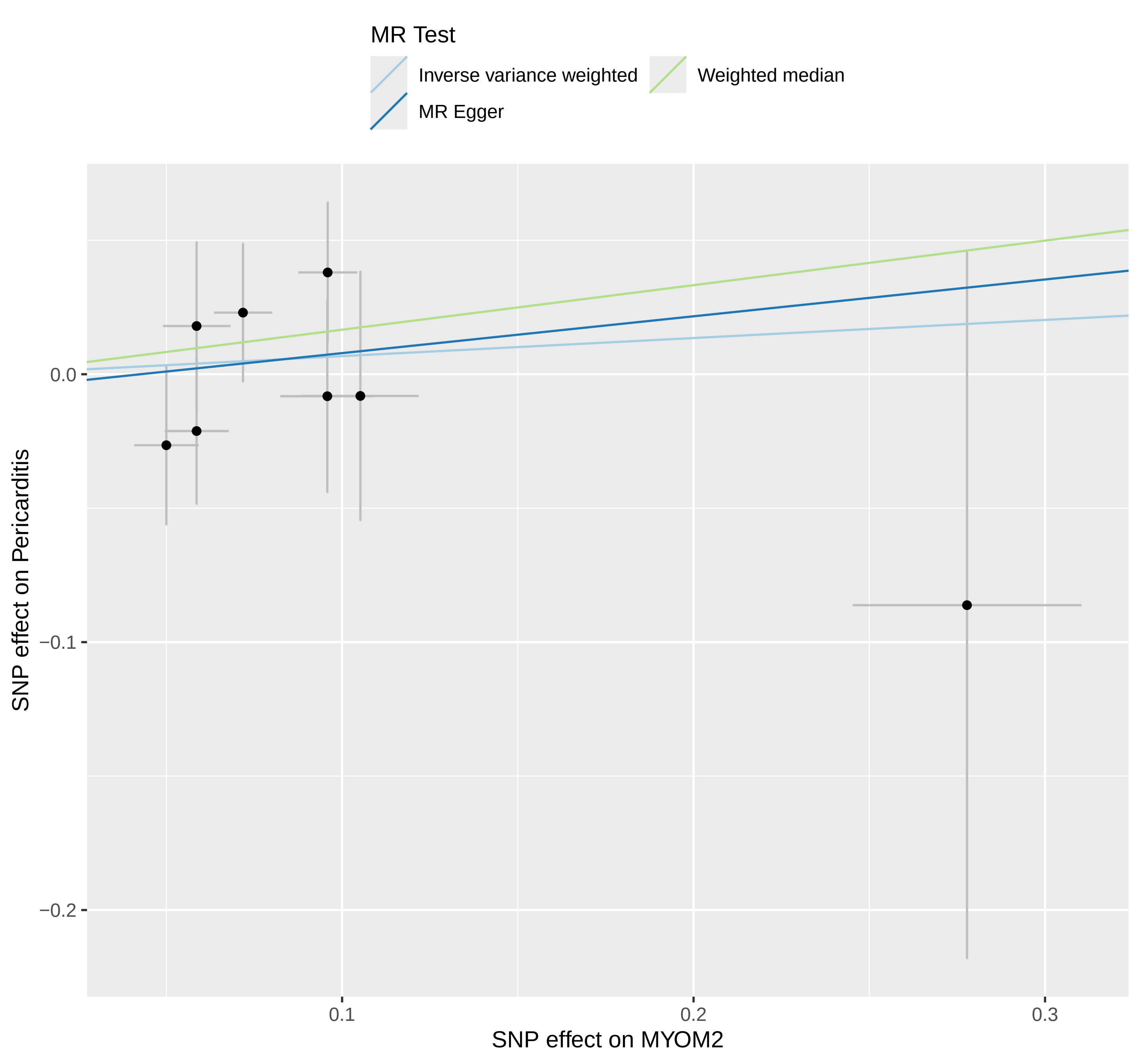

MR Test
Inverse variance weighted
Weighted median
MR Egger
0.0
−0.1
−0.2
0.1
0.2
0.3
SNP effect on Pericarditis
SNP effect on MYOM2

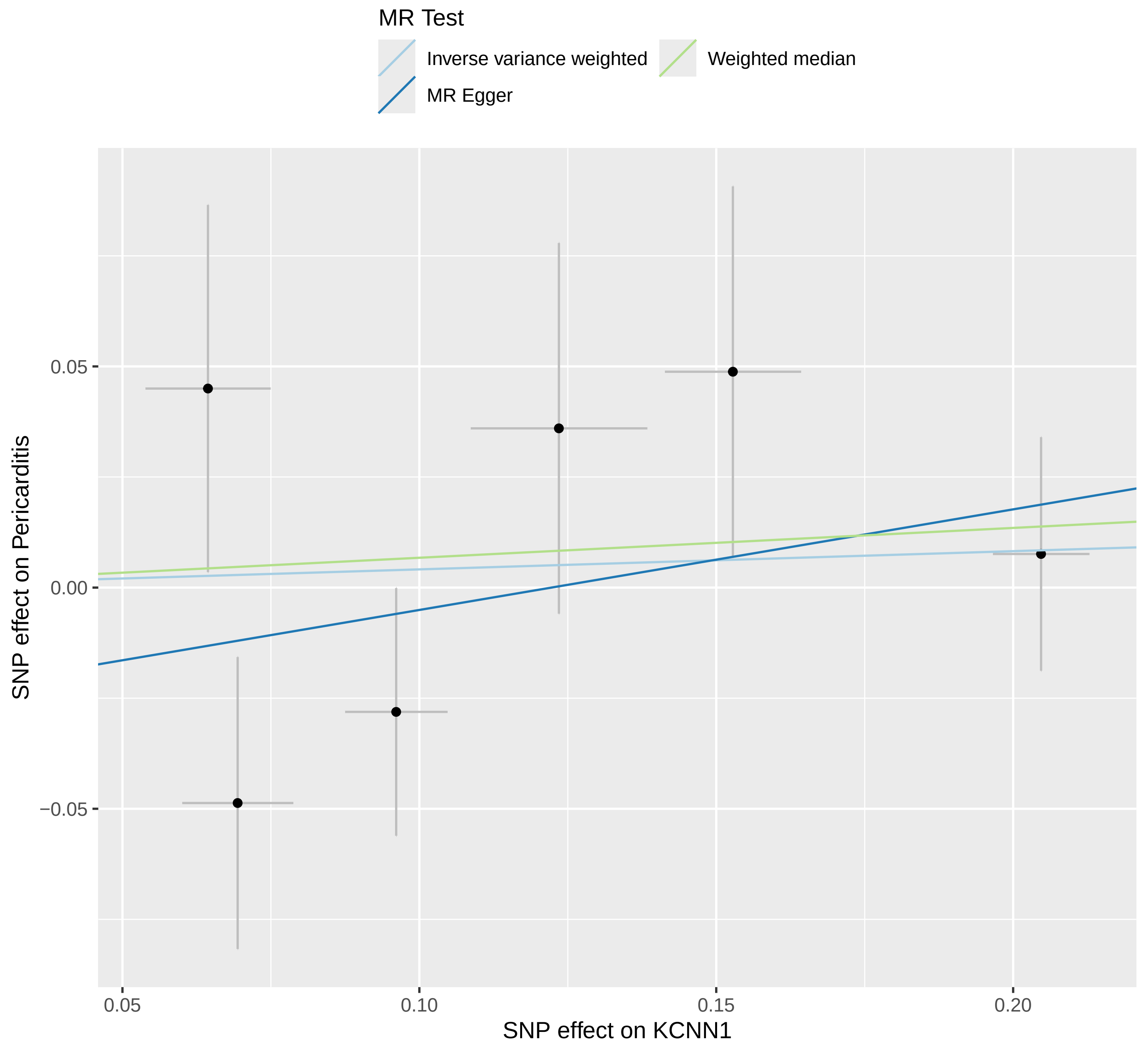
MR Test
Inverse variance weighted
Weighted median
MR Egger
0.05
0.00
−0.05
SNP effect on Pericarditis
0.05
0.10
0.15
0.20
SNP effect on KCNN1

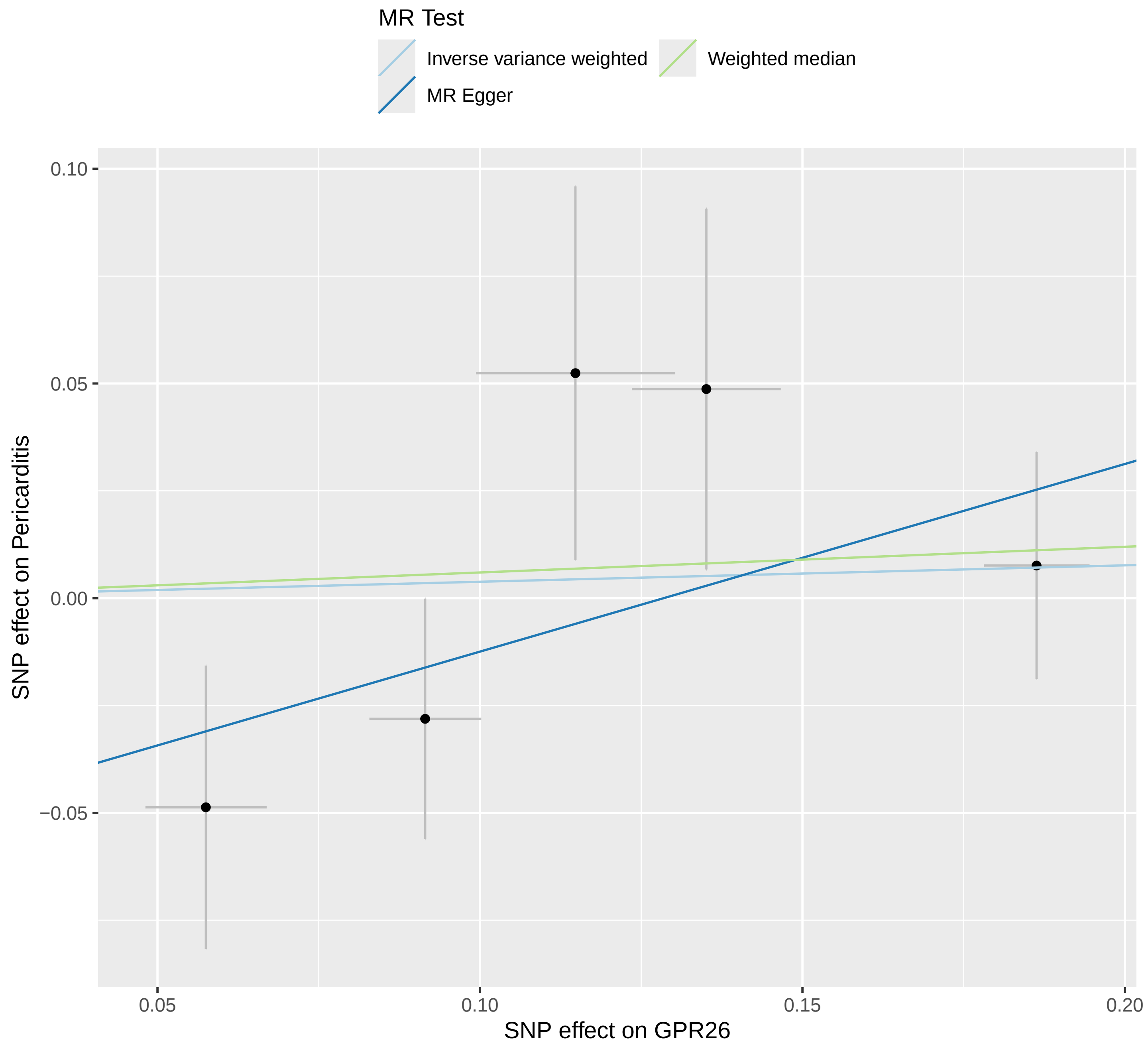

MR Test
Inverse variance weighted
MR Egger
Weighted median
0.10
0.05
0.00
−0.05
SNP effect on Pericarditis
0.05
0.10
0.15
0.20
SNP effect on GPR26

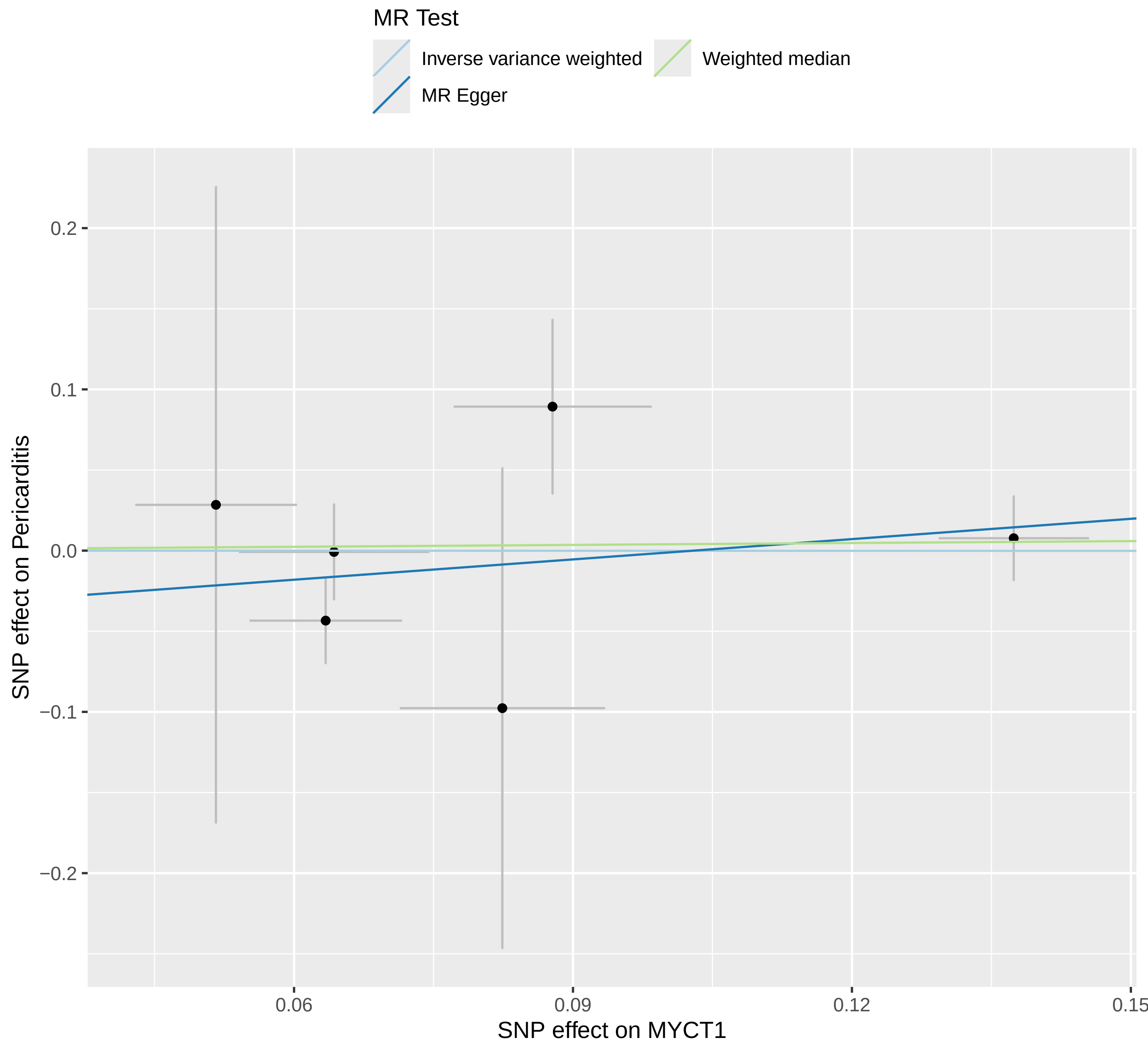
MR Test
Inverse variance weighted
MR Egger
Weighted median
SNP effect on Pericarditis
0.2
0.1
0.0
−0.1
−0.2
0.06
0.09
0.12
0.15
SNP effect on MYCT1

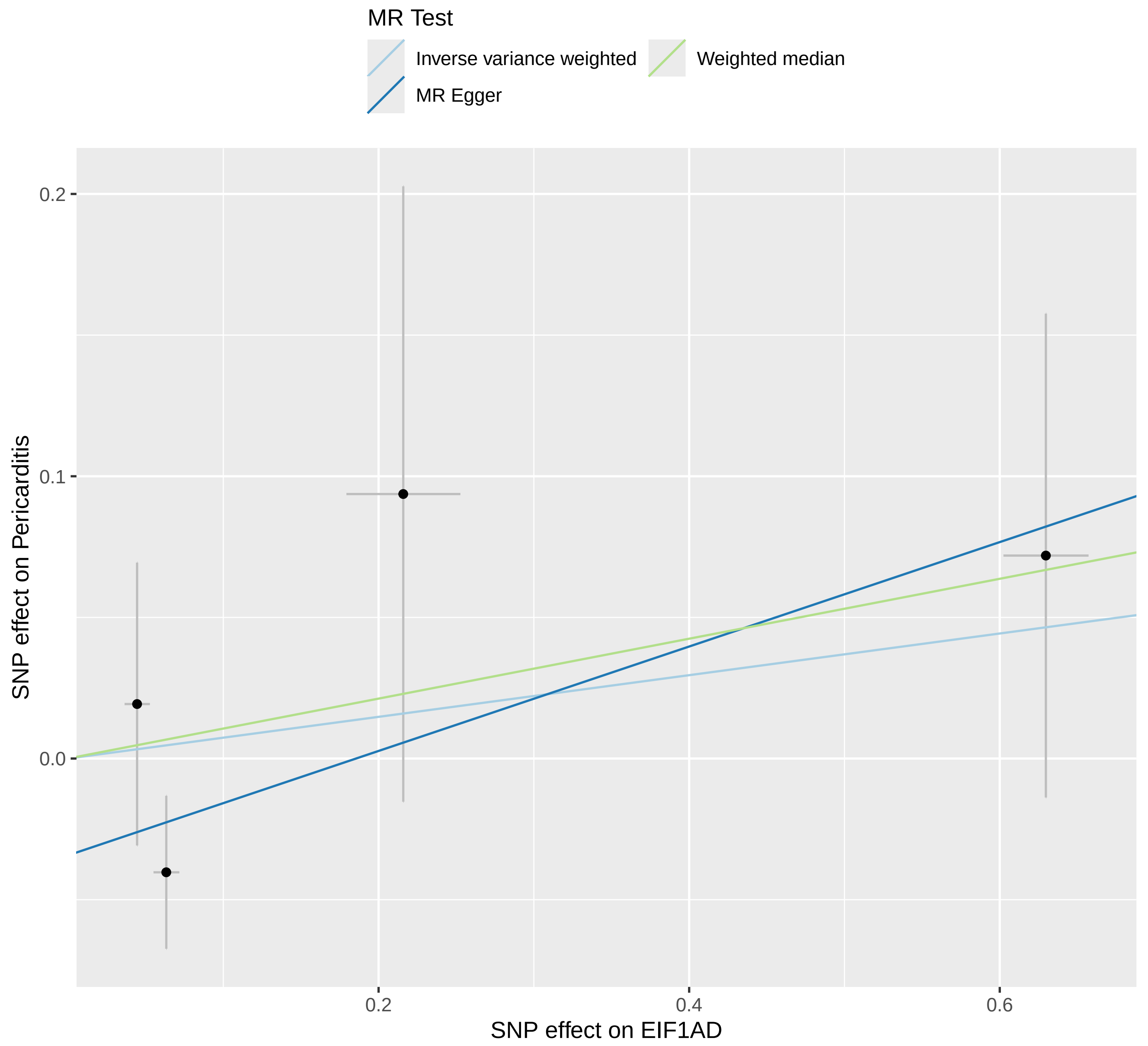
MR Test
Inverse variance weighted
Weighted median
MR Egger
0.2
0.1
0.0
SNP effect on Pericarditis
0.2
0.4
0.6
SNP effect on EIF1AD

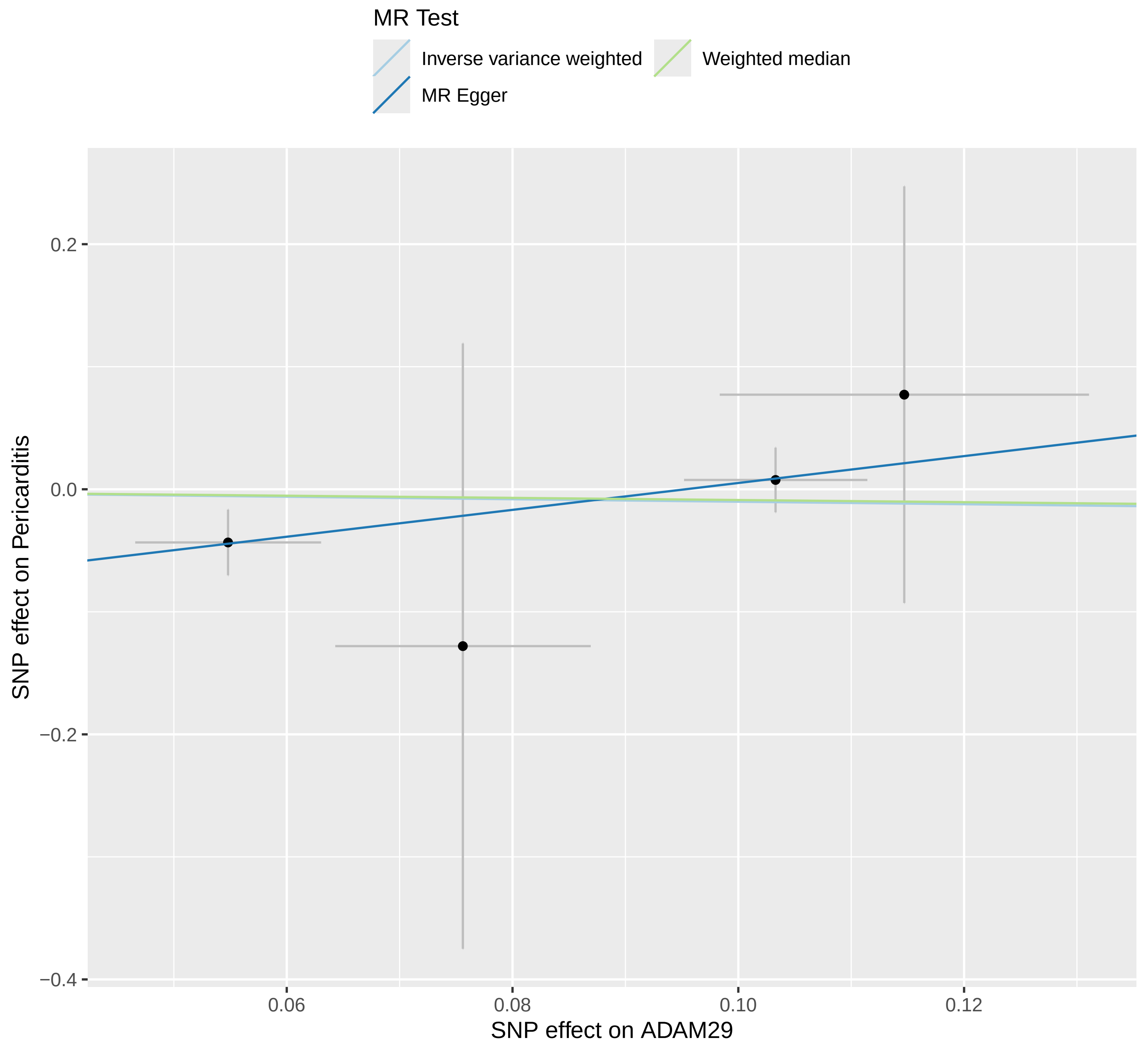
MR Test
Inverse variance weighted
Weighted median
MR Egger
SNP effect on Pericarditis
0.2
0.0
−0.2
−0.4
0.06
0.08
0.10
0.12
SNP effect on ADAM29

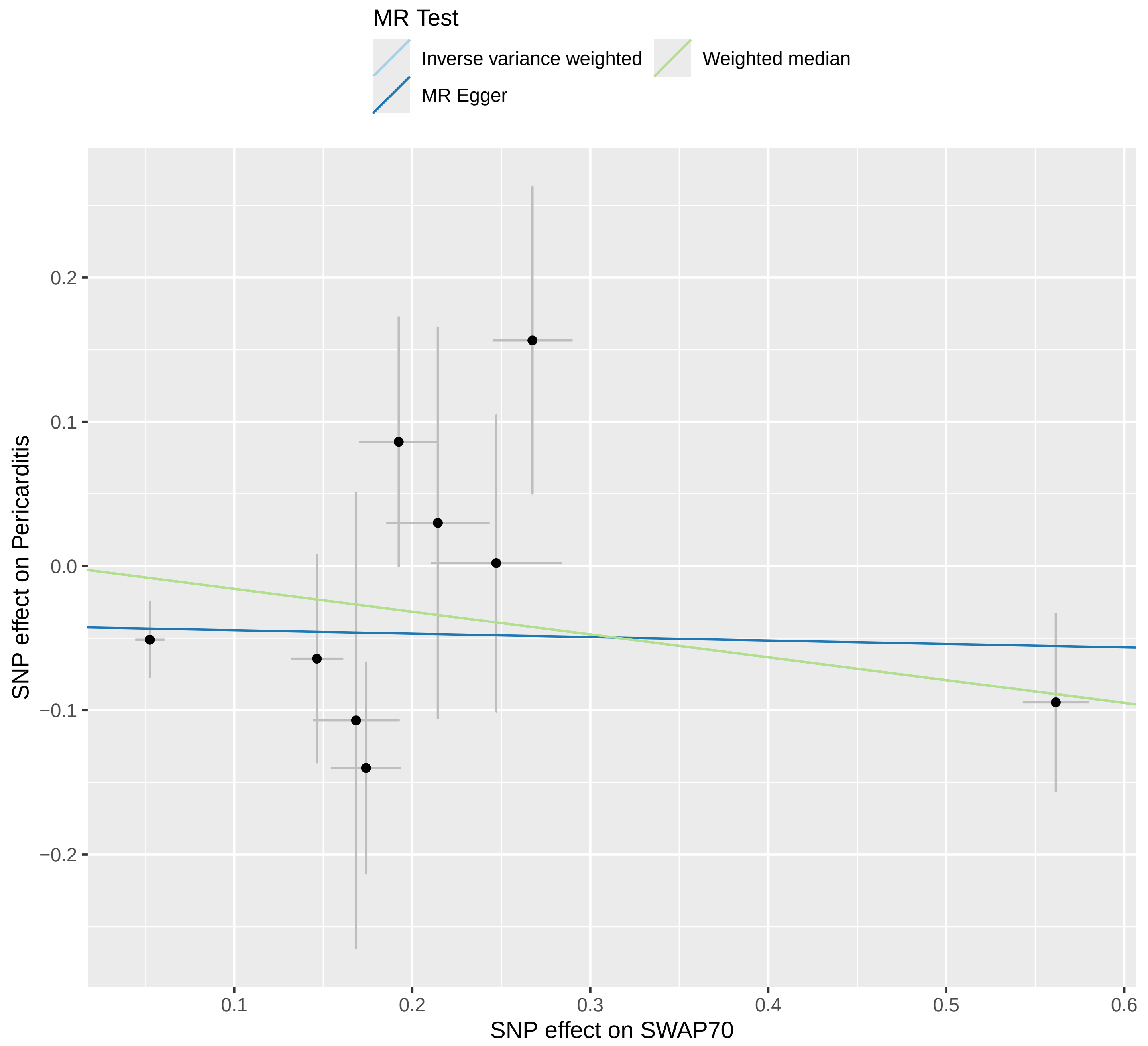
MR Test
Inverse variance weighted
Weighted median
MR Egger
SNP effect on Pericarditis
0.2
0.1
0.0
−0.1
−0.2
0.1
0.2
0.3
0.4
0.5
0.6
SNP effect on SWAP70

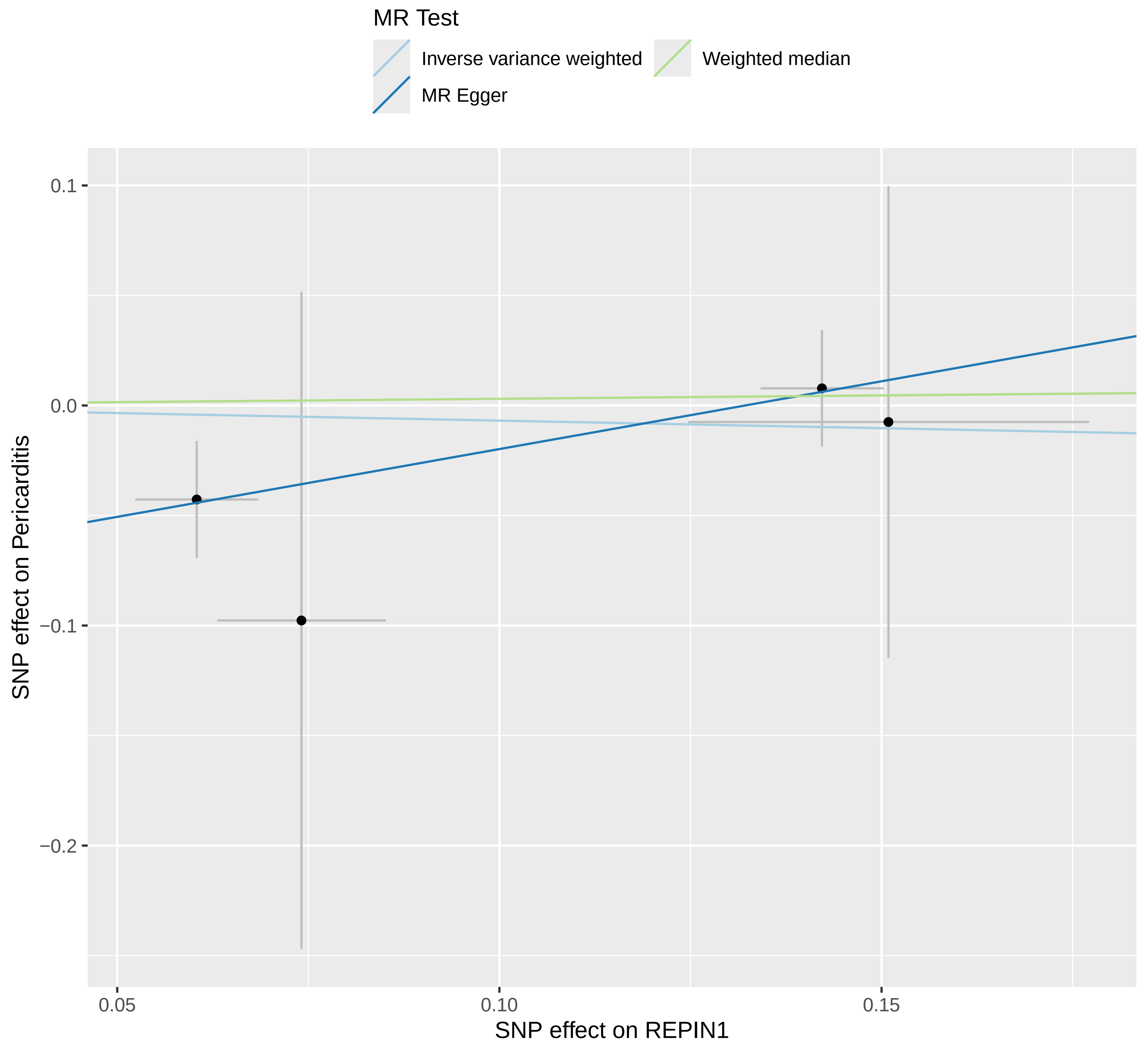

MR Test
Inverse variance weighted
Weighted median
MR Egger
0.1
0.0
−0.1
−0.2
SNP effect on Pericarditis
0.05
0.10
0.15
SNP effect on REPIN1

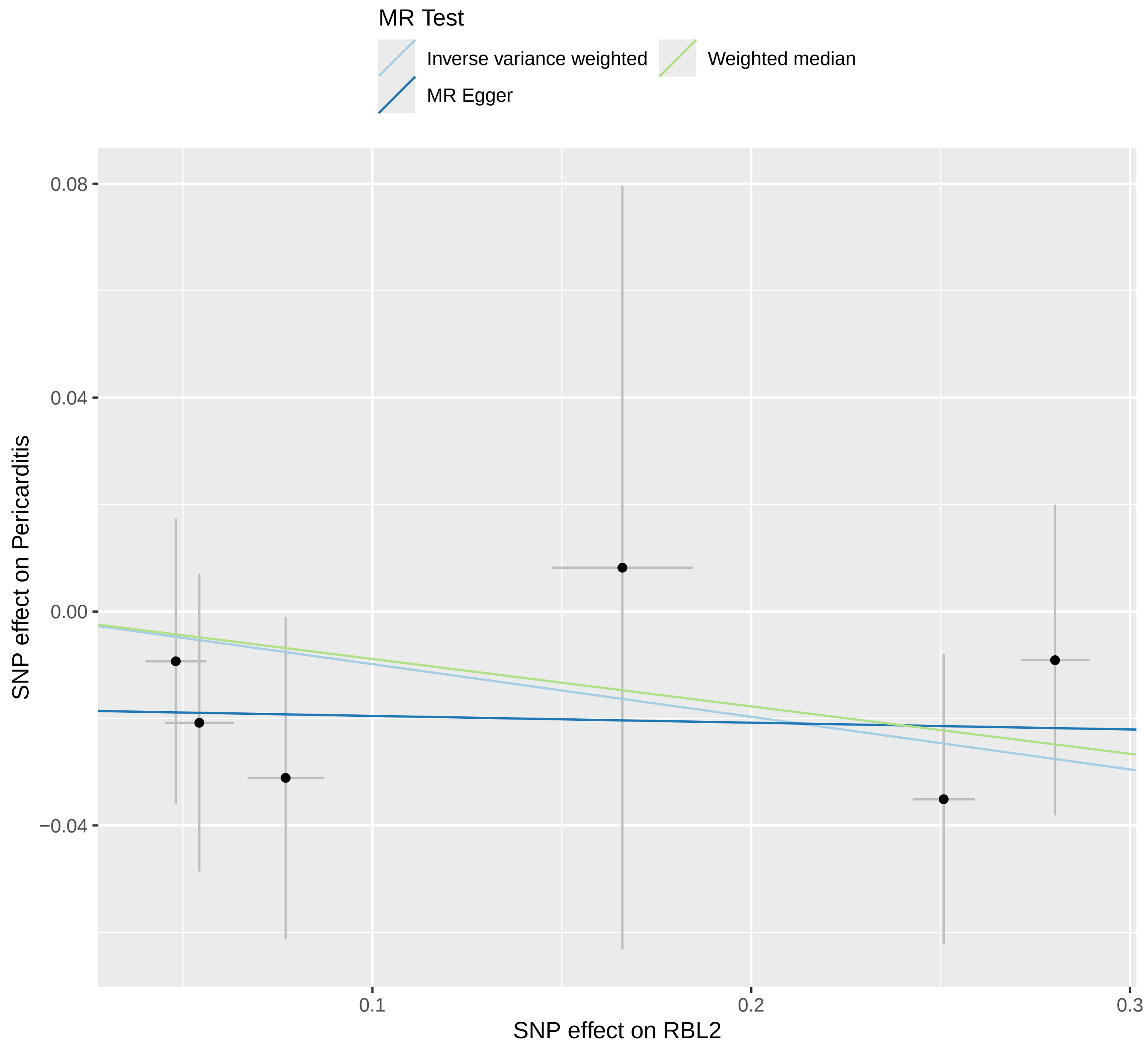
MR Test
Inverse variance weighted
MR Egger
Weighted median
0.08
0.04
0.00
−0.04
SNP effect on Pericarditis
0.1
0.2
0.3
SNP effect on RBL2

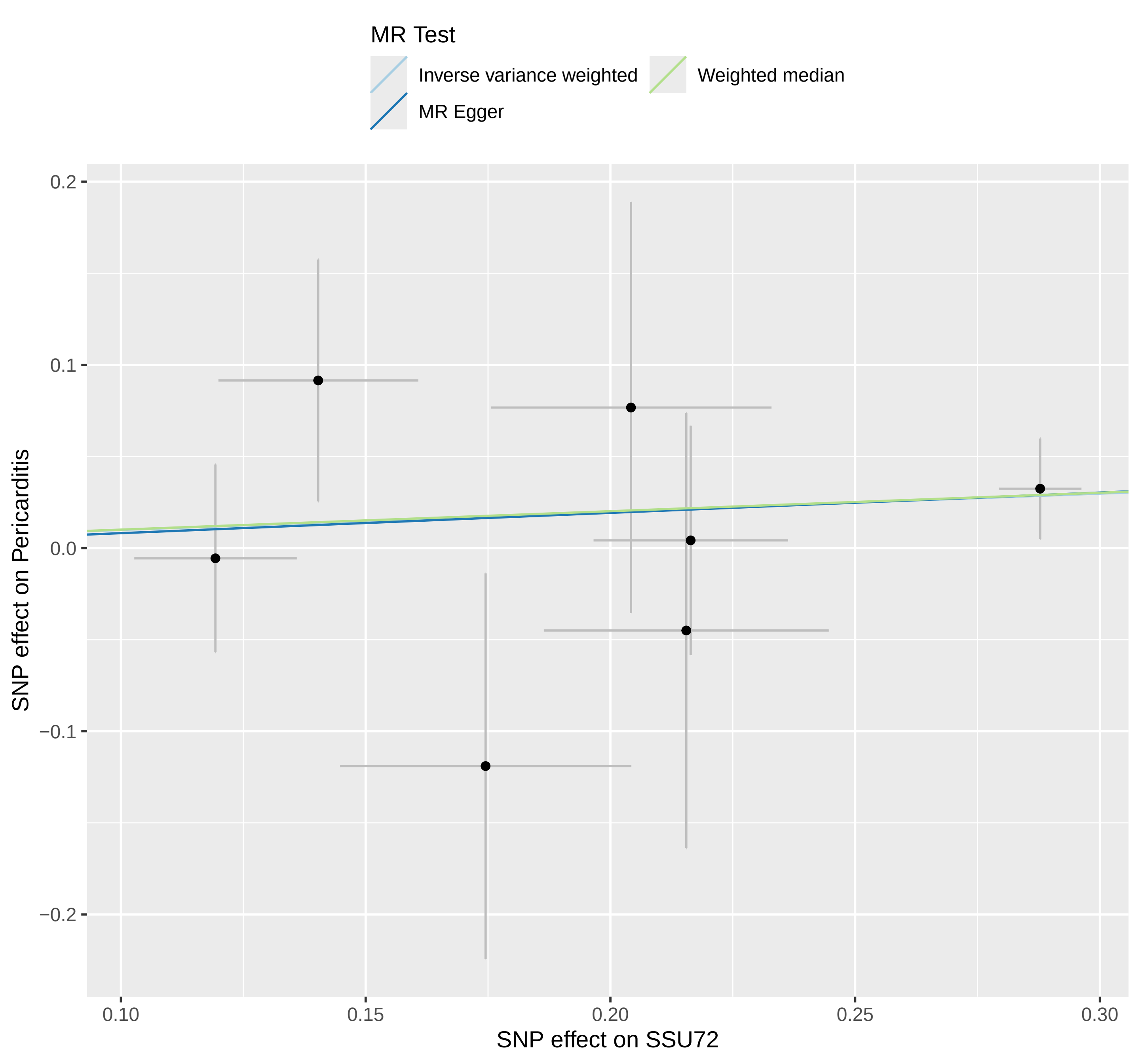
MR Test
Inverse variance weighted
Weighted median
MR Egger
0.2
0.1
0.0
−0.1
−0.2
SNP effect on Pericarditis
0.10
0.15
0.20
0.25
0.30
SNP effect on SSU72

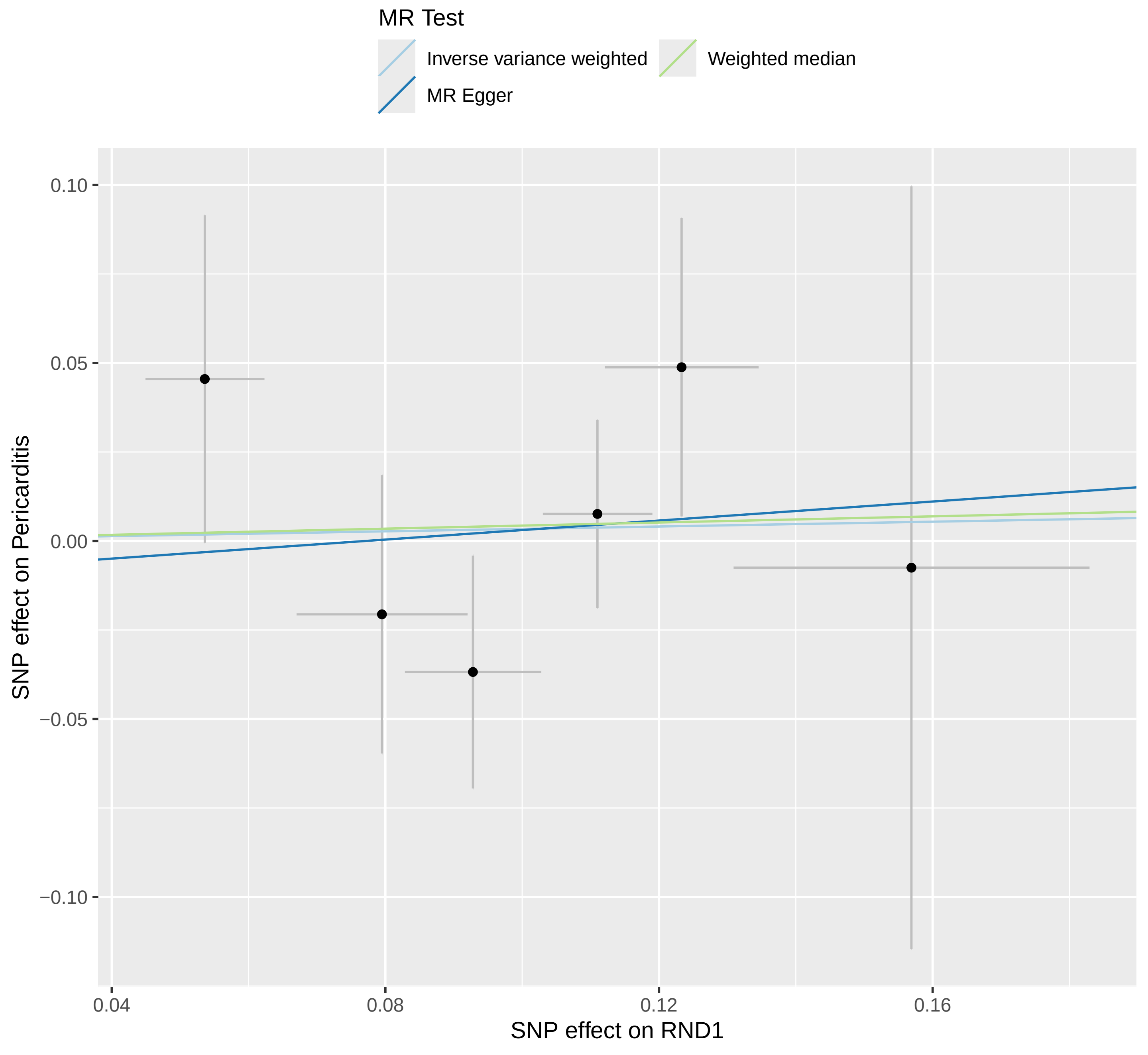
MR Test
Inverse variance weighted
MR Egger
Weighted median
SNP effect on Pericarditis
0.10
0.05
0.00
−0.05
−0.10
0.04
0.08
0.12
0.16
SNP effect on RND1

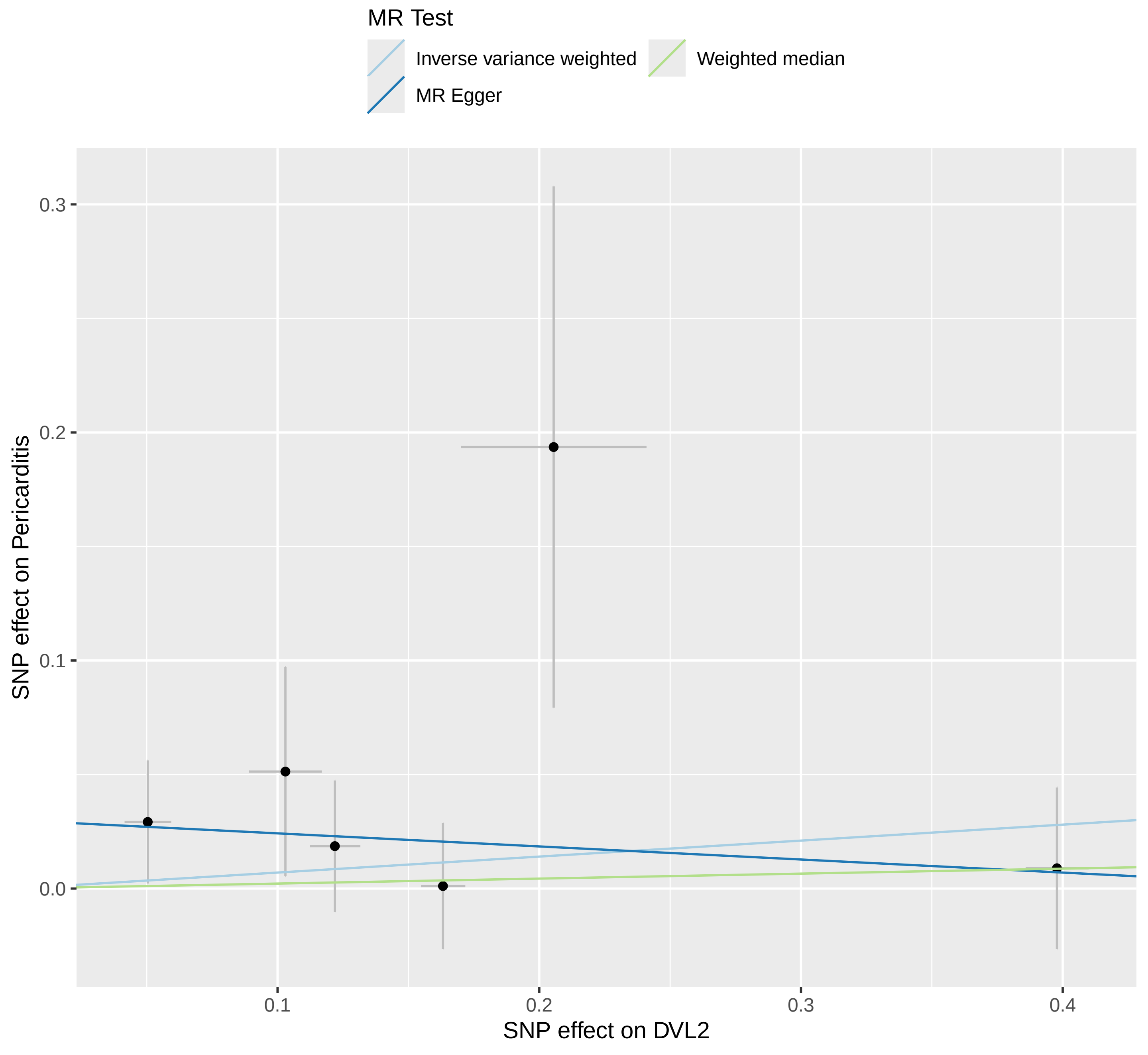
MR Test
Inverse variance weighted
MR Egger
Weighted median
SNP effect on Pericarditis
0.3
0.2
0.1
0.0
0.1
0.2
0.3
0.4
SNP effect on DVL2

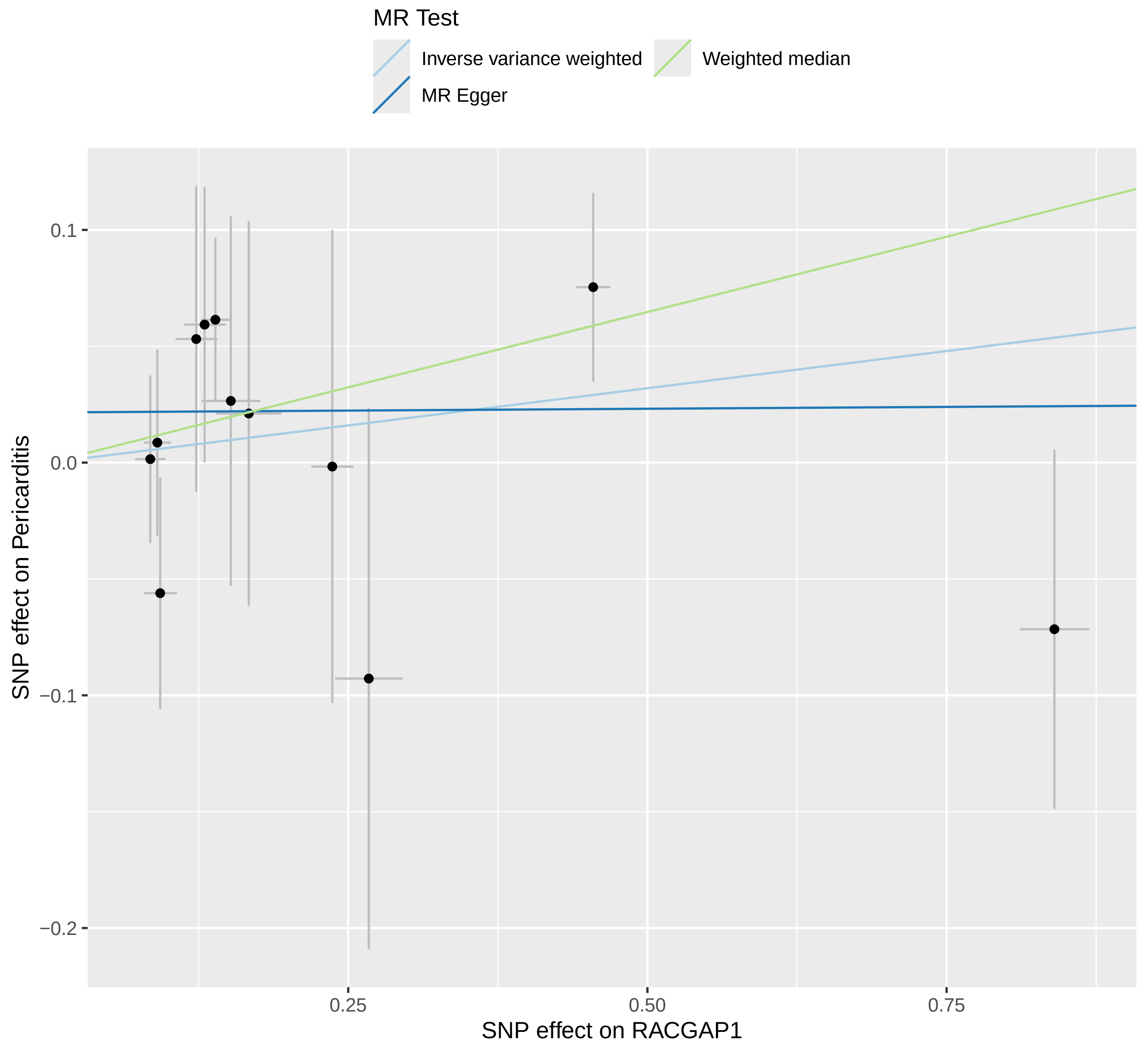
MR Test
Inverse variance weighted
Weighted median
MR Egger
0.1
0.0
−0.1
−0.2
SNP effect on Pericarditis
0.25
0.50
0.75
SNP effect on RACGAP1

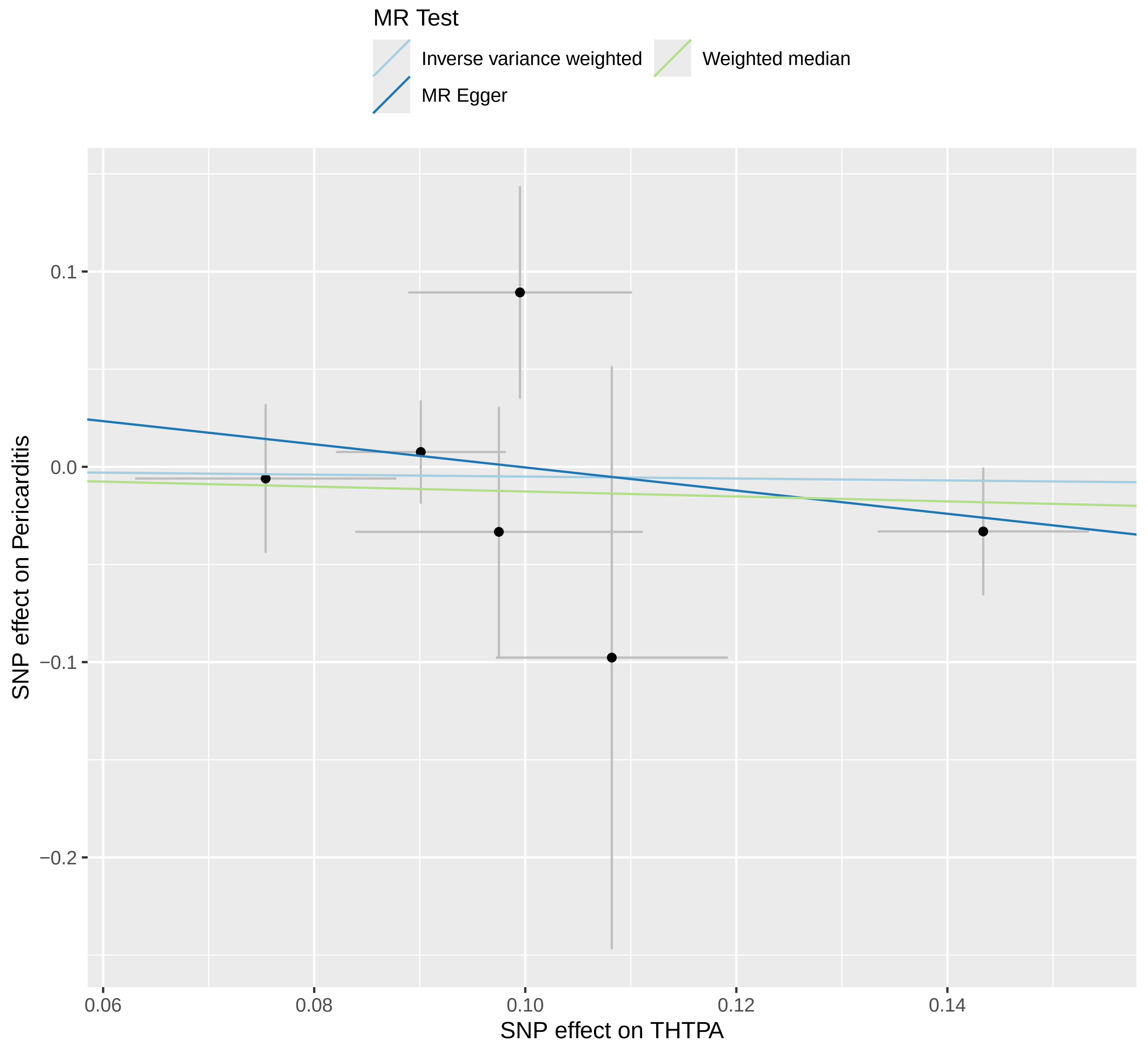

MR Test
Inverse variance weighted
Weighted median
MR Egger
0.1
0.0
−0.1
−0.2
SNP effect on Pericarditis
0.06
0.08
0.10
0.12
0.14
SNP effect on THTPA

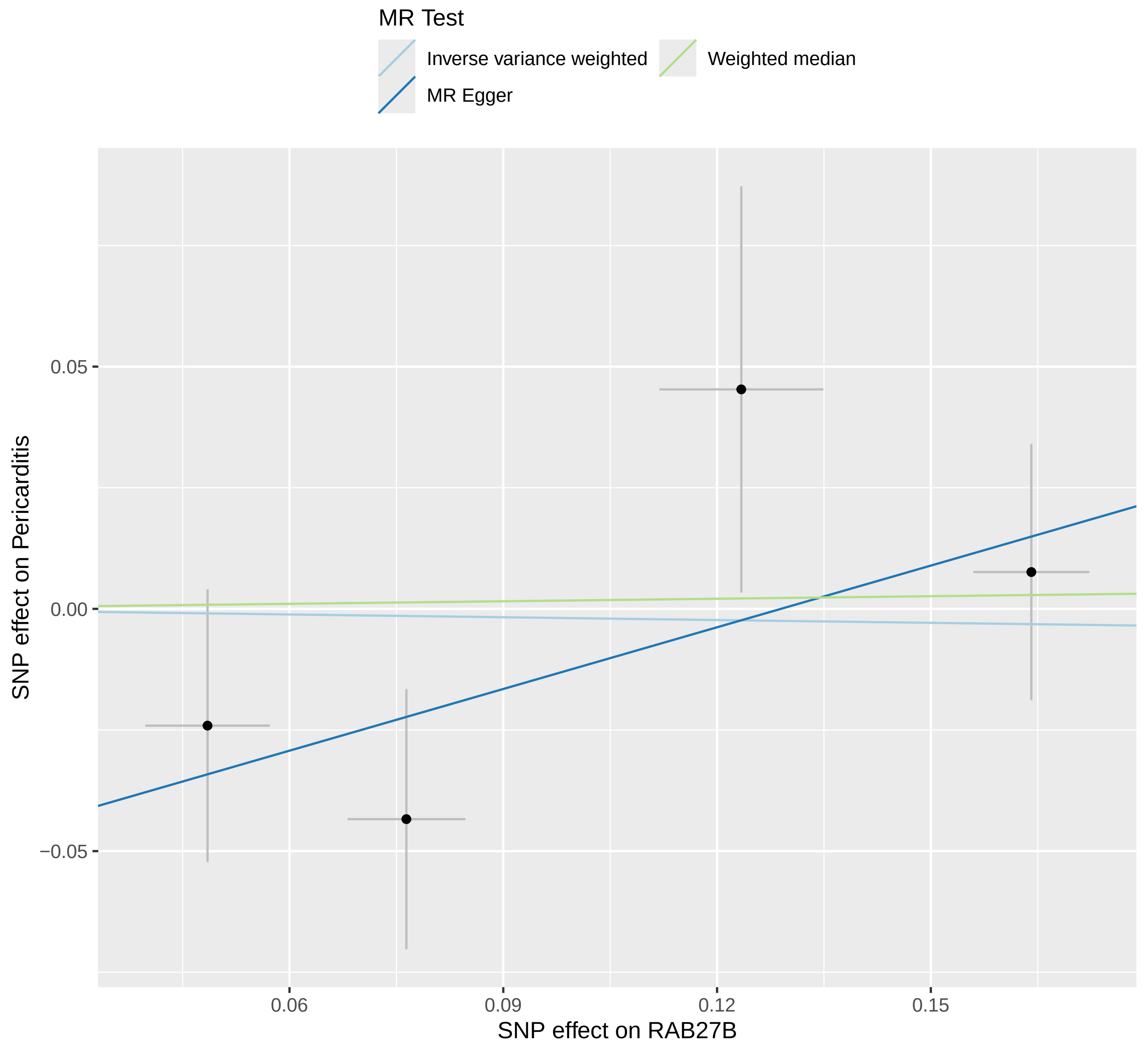
MR Test
Inverse variance weighted
MR Egger
Weighted median
0.05
0.00
−0.05
SNP effect on Pericarditis
0.06
0.09
0.12
0.15
SNP effect on RAB27B

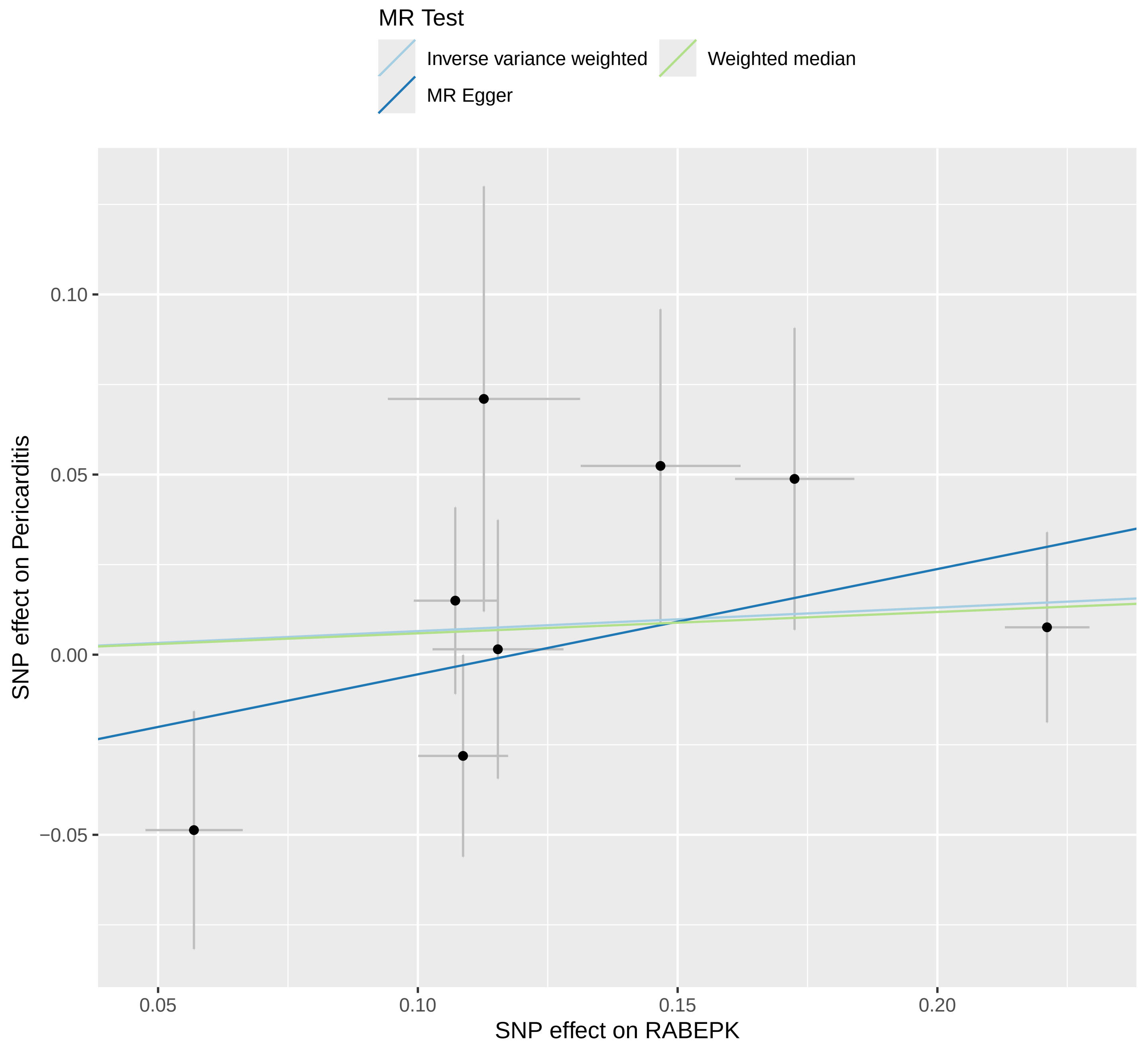

MR Test
Inverse variance weighted
MR Egger
Weighted median
0.10
0.05
0.00
−0.05
SNP effect on Pericarditis
0.05
0.10
0.15
0.20
SNP effect on RABEPK

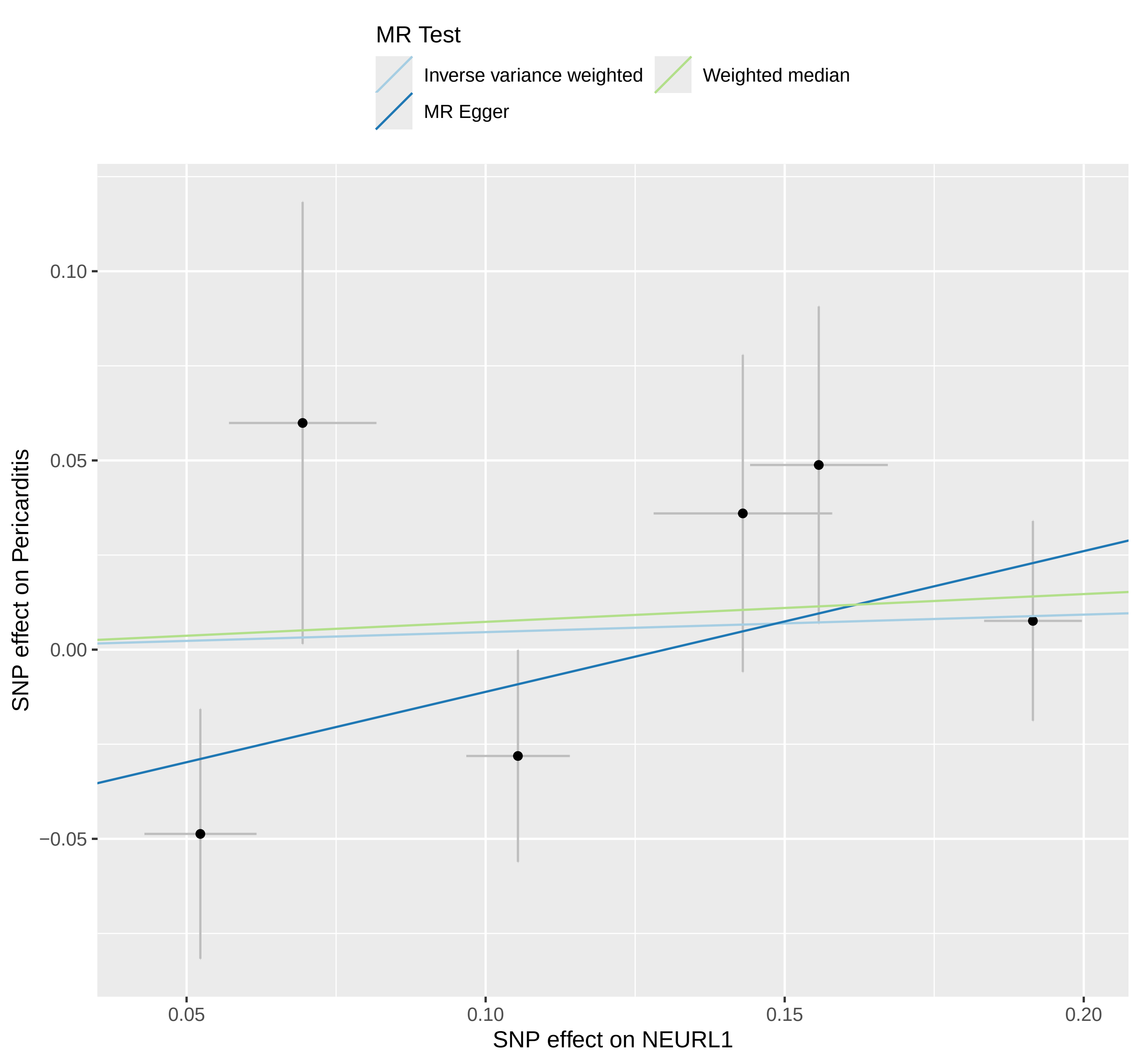
MR Test
Inverse variance weighted
Weighted median
MR Egger
0.10
0.05
0.00
−0.05
SNP effect on Pericarditis
0.05
0.10
0.15
0.20
SNP effect on NEURL1

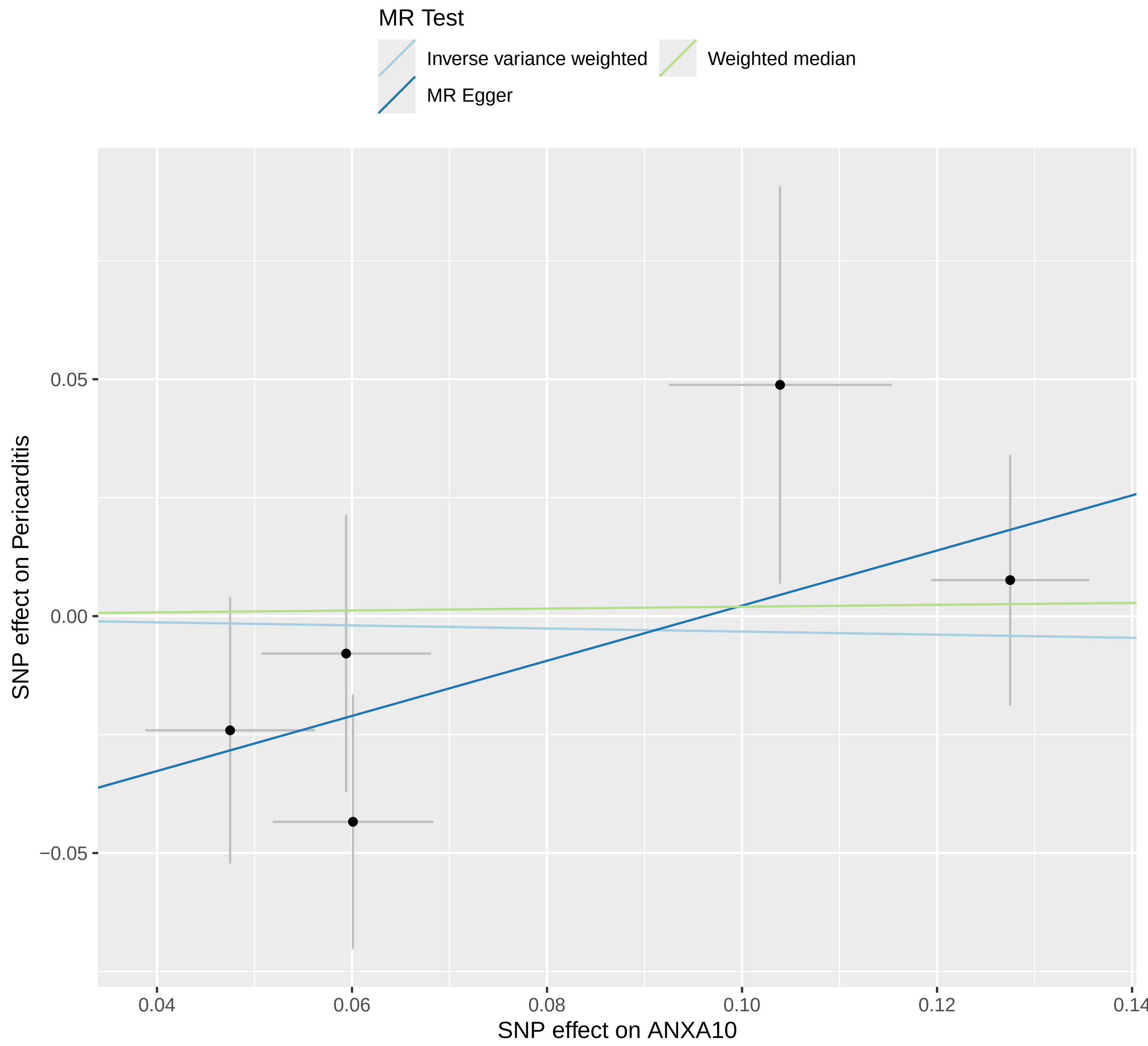
MR Test
Inverse variance weighted
MR Egger
Weighted median
SNP effect on Pericarditis
0.05
0.00
−0.05
0.04
0.06
0.08
0.10
0.12
0.14
SNP effect on ANXA10

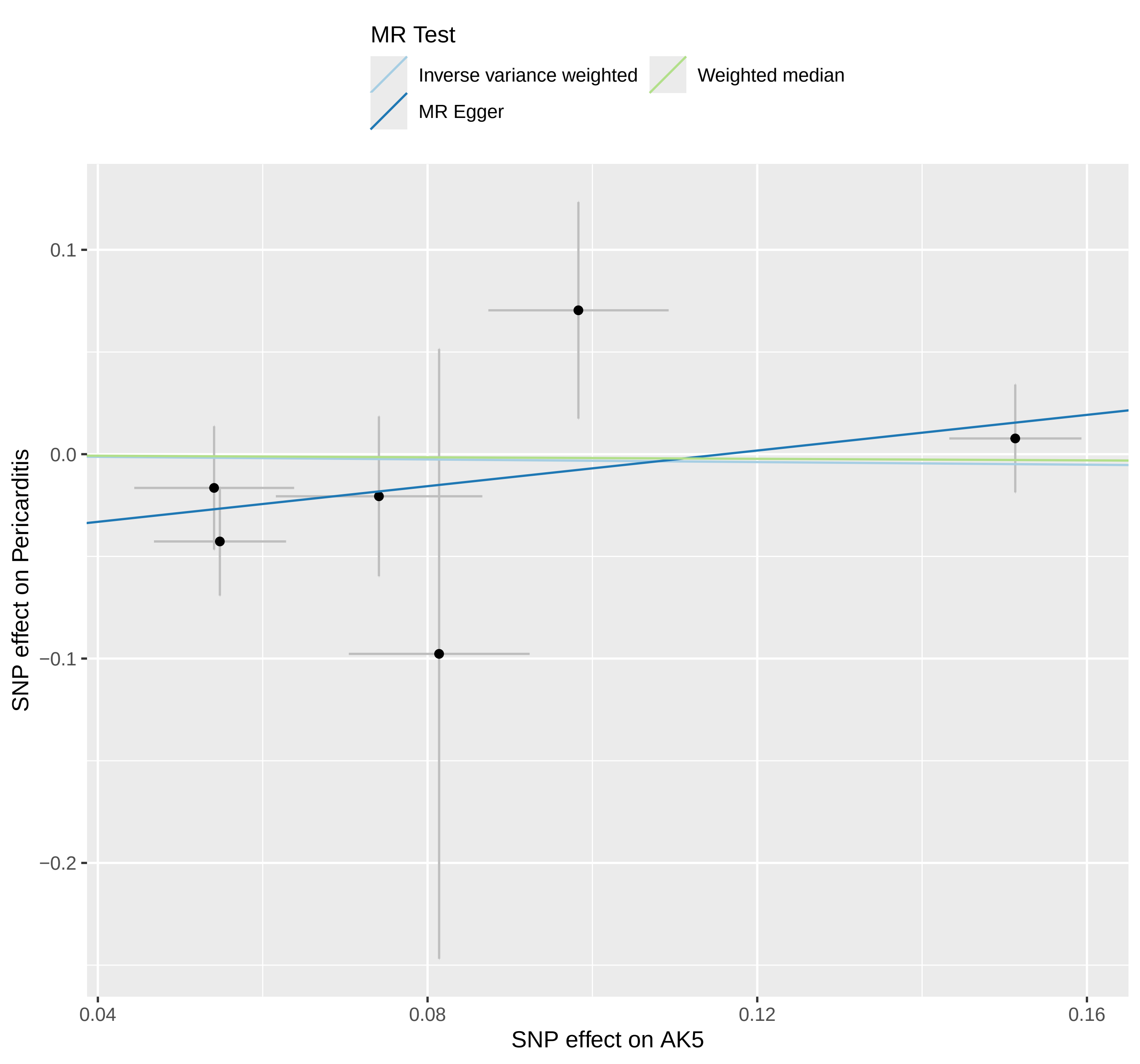

MR Test
Inverse variance weighted
Weighted median
MR Egger
0.1
0.0
−0.1
−0.2
SNP effect on Pericarditis
0.04
0.08
0.12
0.16
SNP effect on AK5

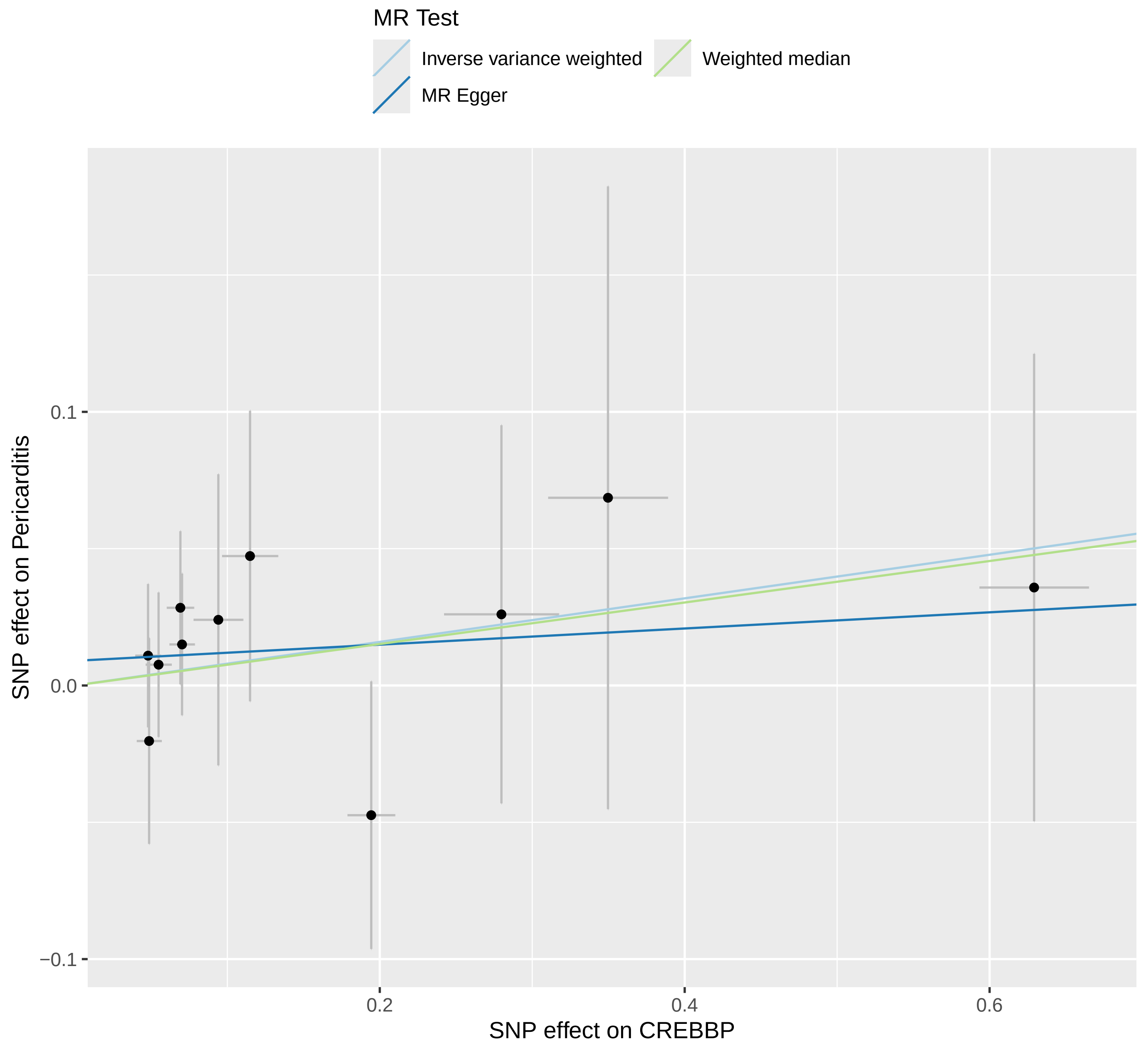
MR Test
Inverse variance weighted
Weighted median
MR Egger
SNP effect on Pericarditis
0.1
0.0
−0.1
0.2
0.4
0.6
SNP effect on CREBBP

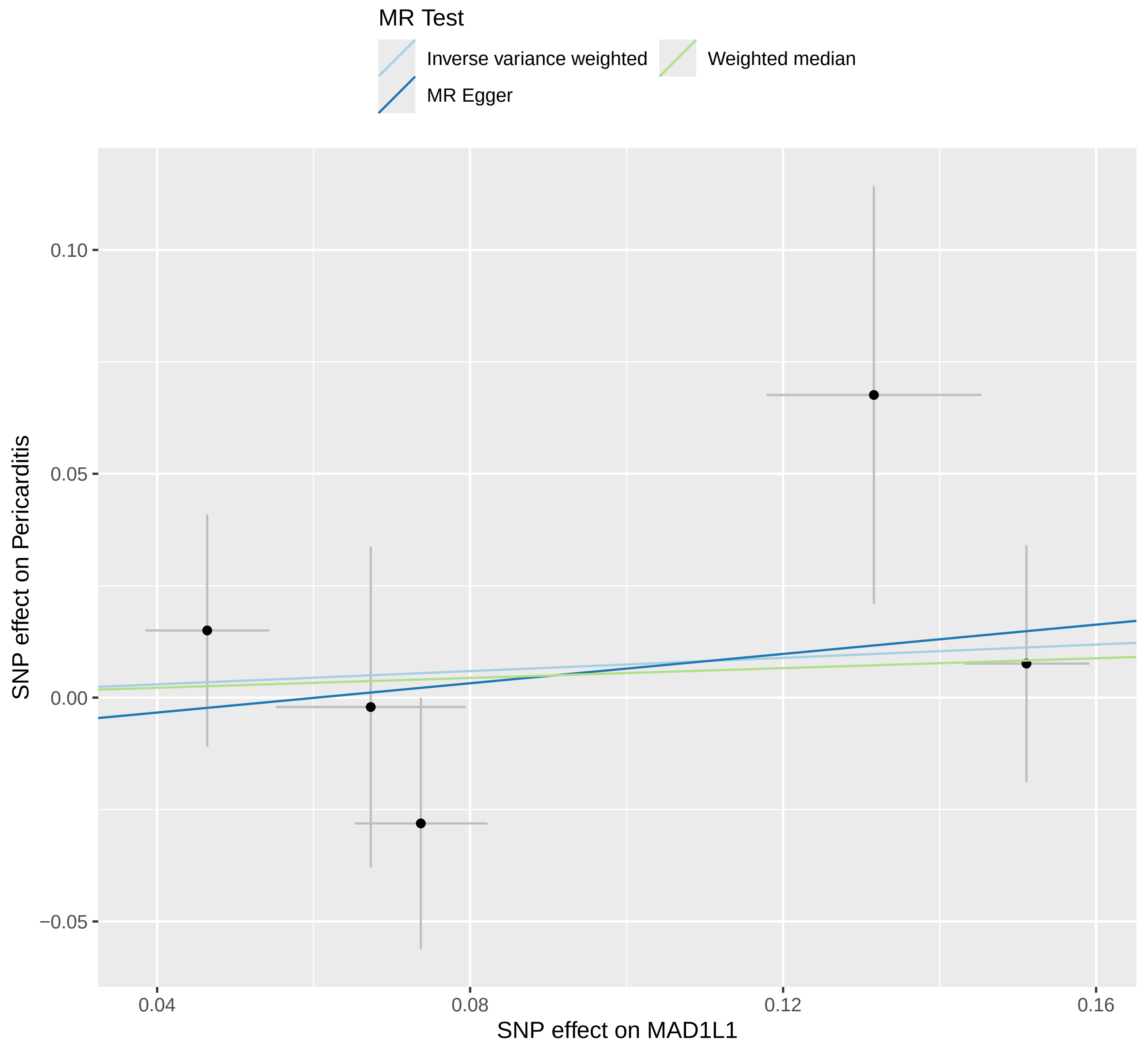
MR Test
Inverse variance weighted
Weighted median
MR Egger
0.10
0.05
0.00
−0.05
SNP effect on Pericarditis
0.04
0.08
0.12
0.16
SNP effect on MAD1L1

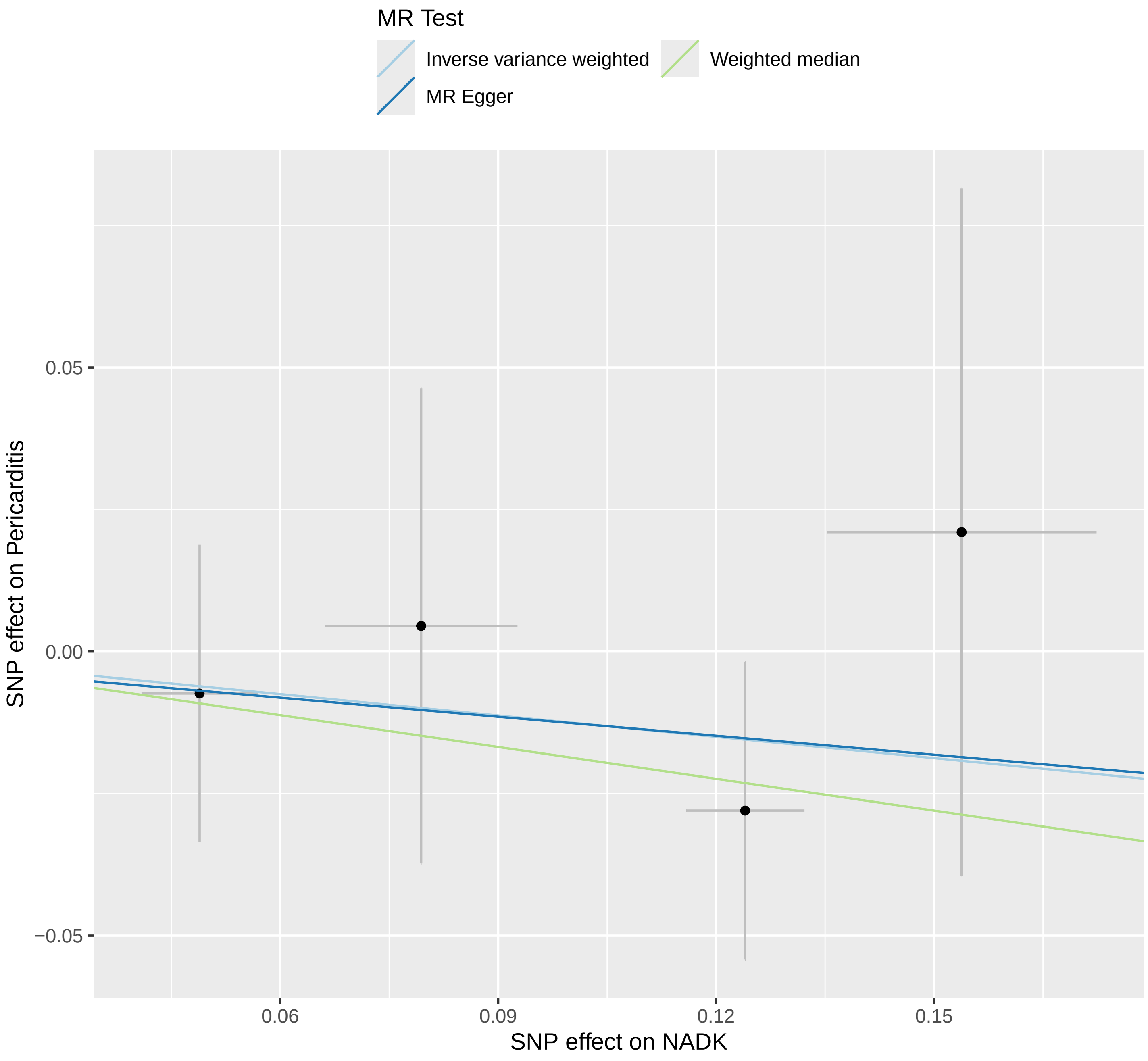
MR Test
Inverse variance weighted
MR Egger
Weighted median
SNP effect on Pericarditis
0.05
0.00
−0.05
0.06
0.09
0.12
0.15
SNP effect on NADK

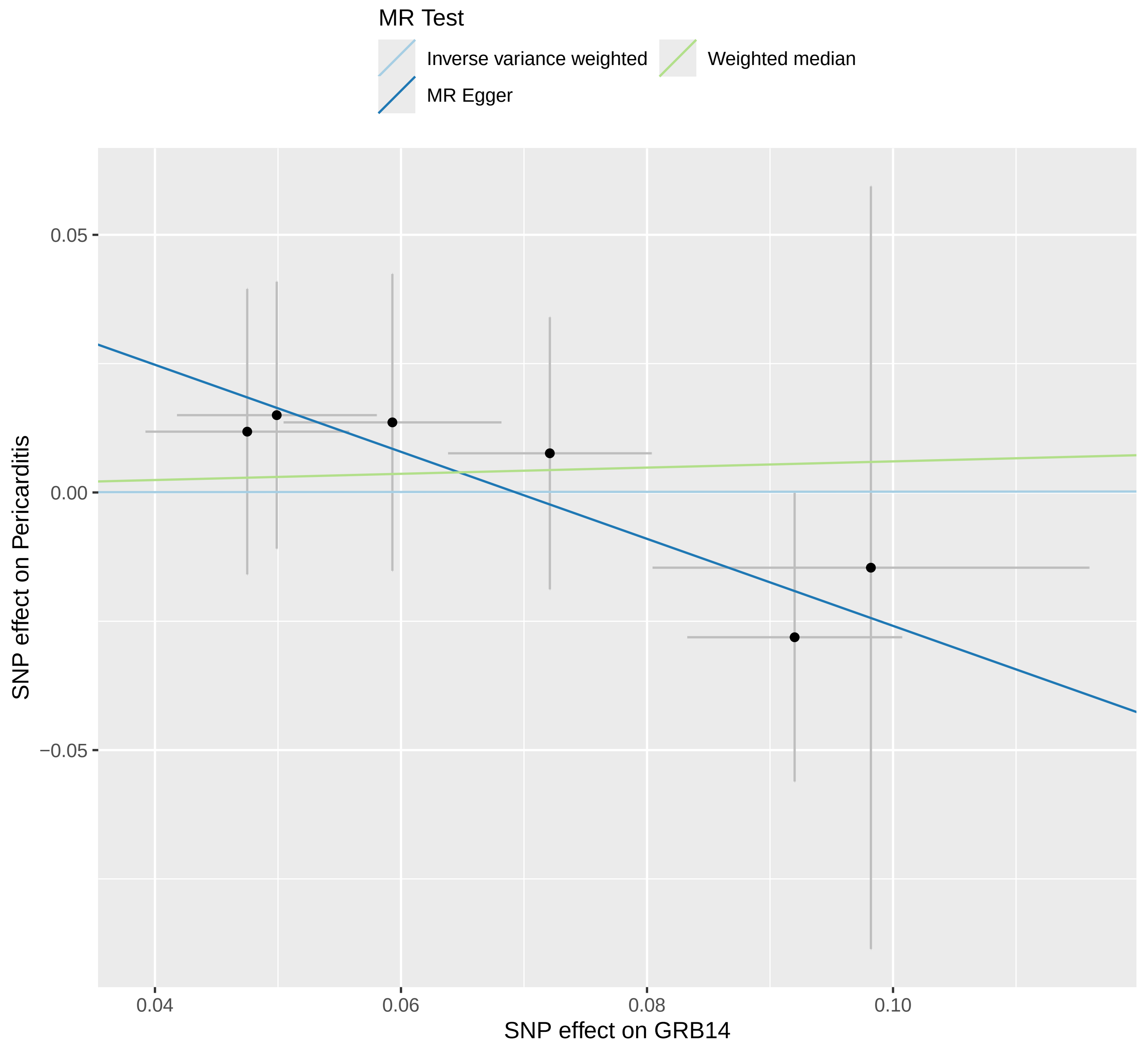
MR Test
Inverse variance weighted
Weighted median
MR Egger
0.05
0.00
−0.05
SNP effect on Pericarditis
0.04
0.06
0.08
0.10
SNP effect on GRB14

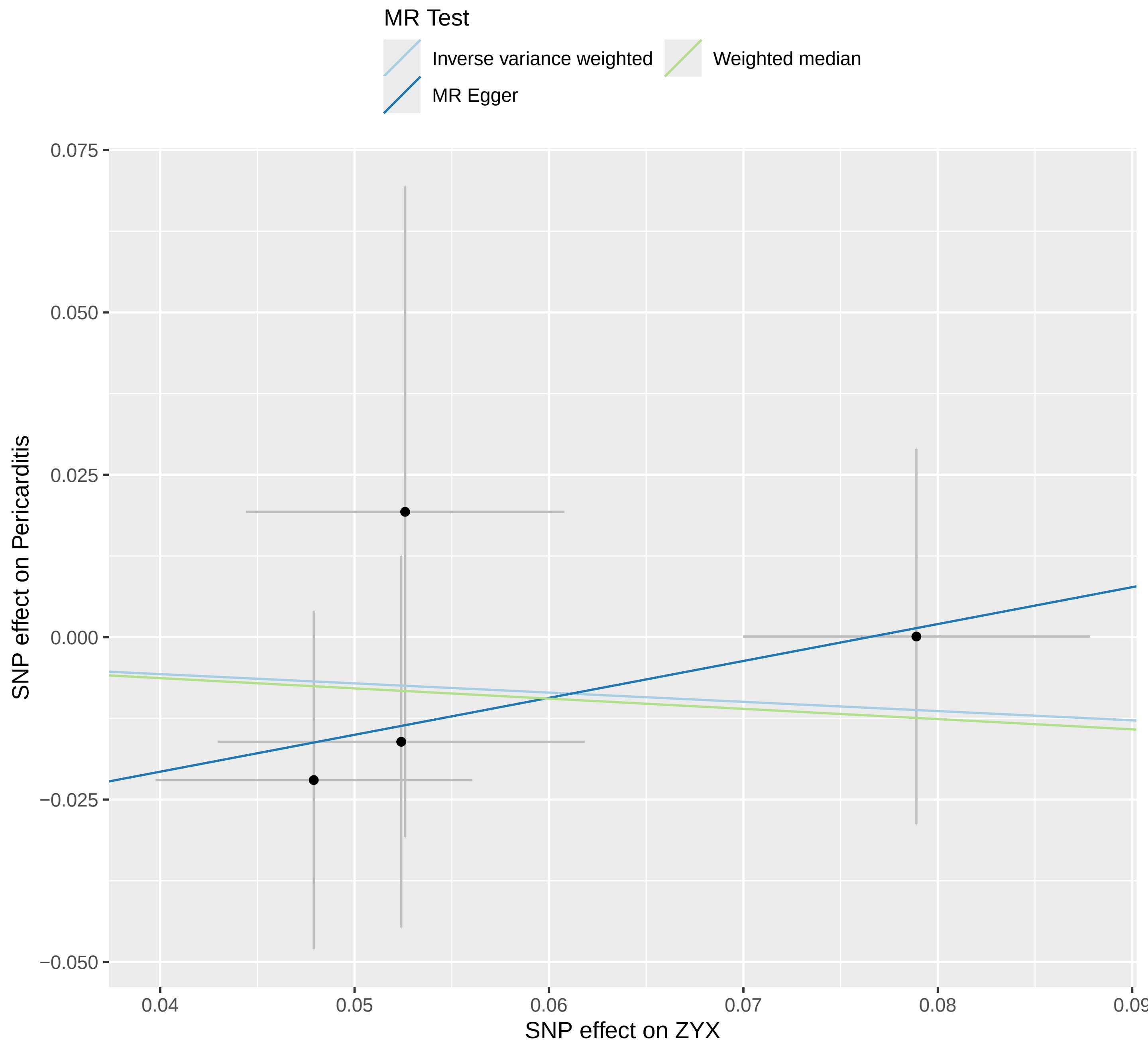

MR Test
Inverse variance weighted
MR Egger
Weighted median
0.075
0.050
0.025
0.000
−0.025
−0.050
SNP effect on Pericarditis
0.04
0.05
0.06
0.07
0.08
0.09
SNP effect on ZYX

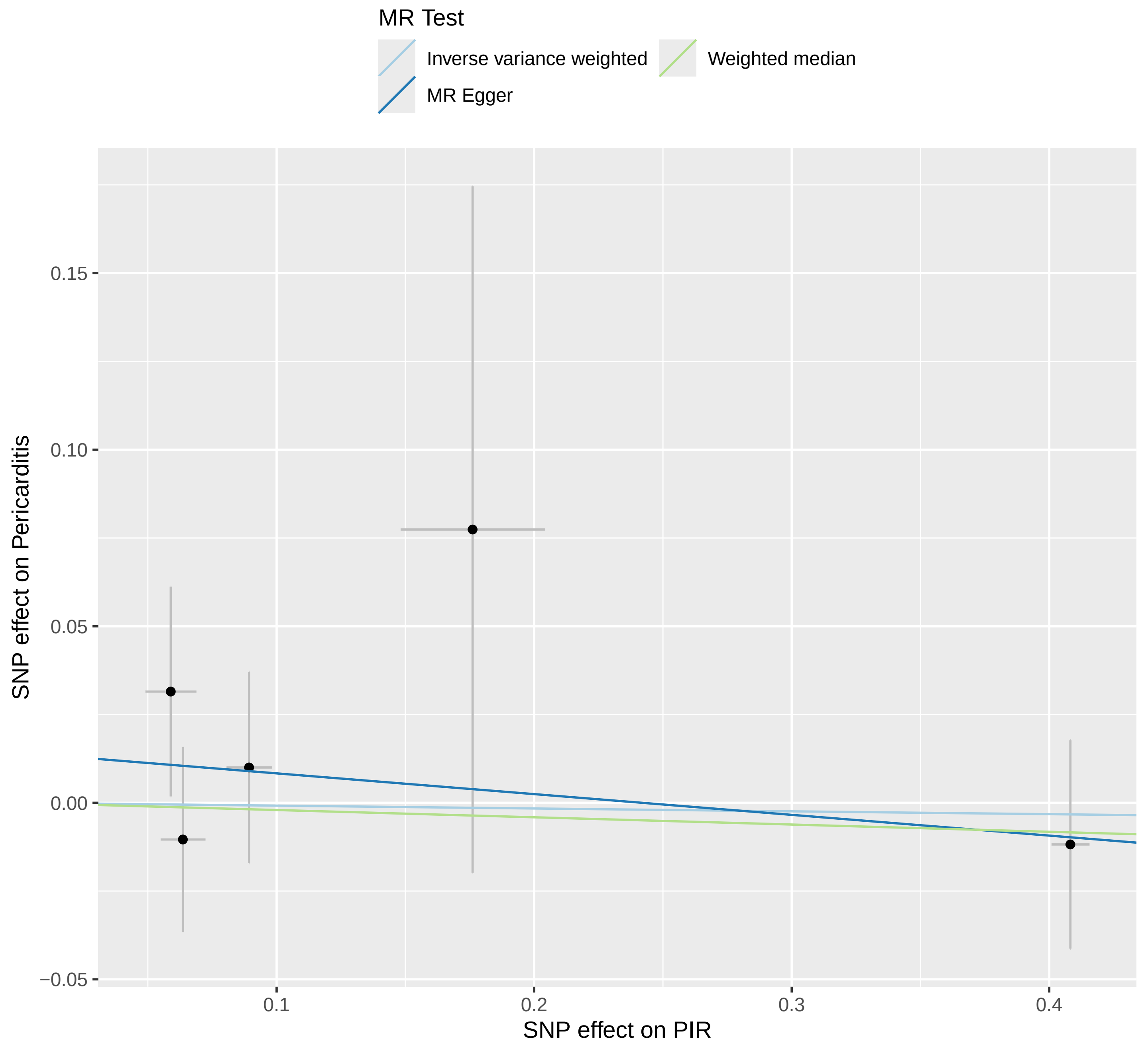
MR Test
Inverse variance weighted
Weighted median
MR Egger
0.15
0.10
0.05
0.00
−0.05
0.1
0.2
0.3
0.4
SNP effect on Pericarditis
SNP effect on PIR

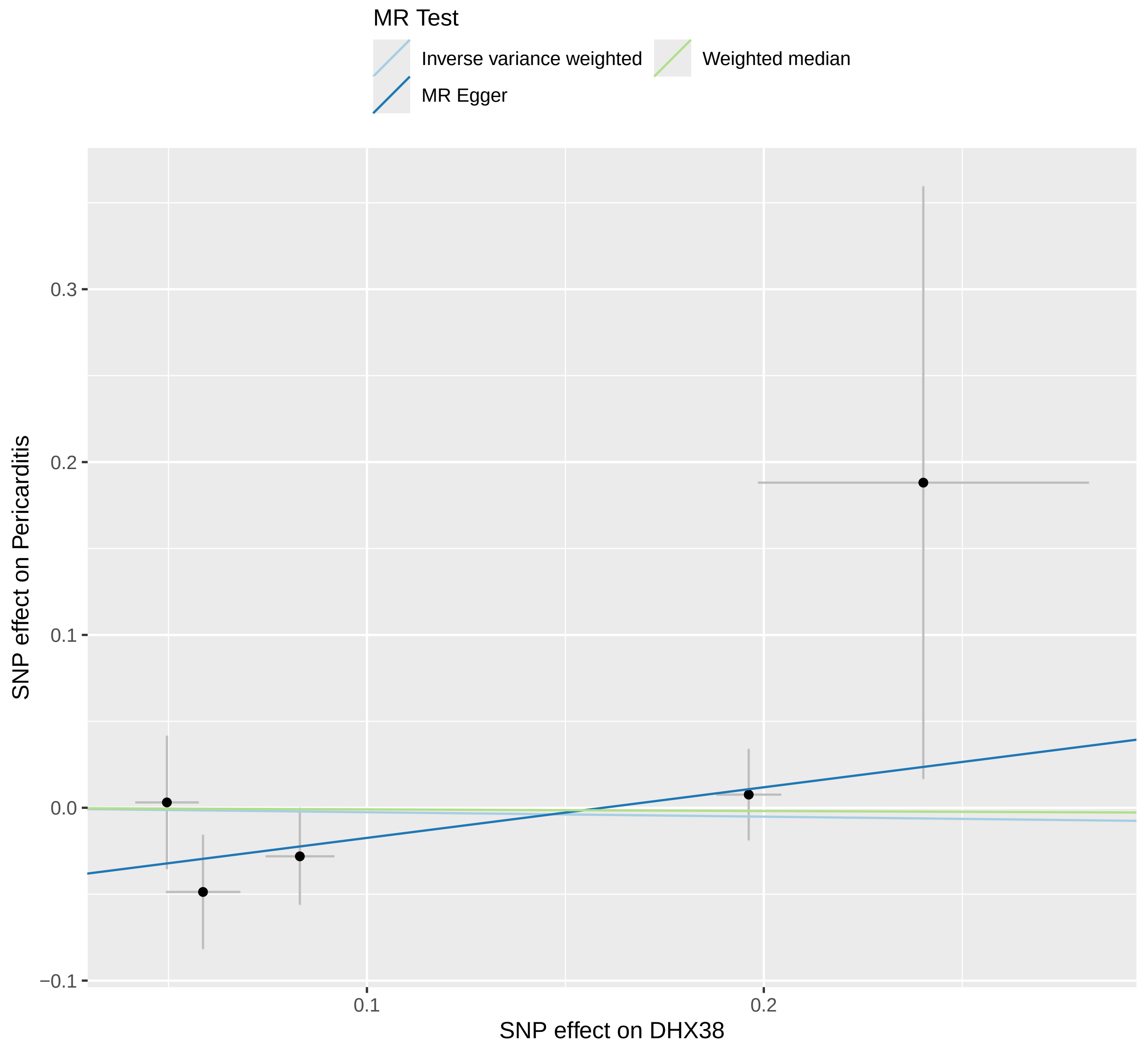
MR Test
Inverse variance weighted
Weighted median
MR Egger
0.3
0.2
0.1
0.0
−0.1
SNP effect on Pericarditis
0.1
0.2
SNP effect on DHX38

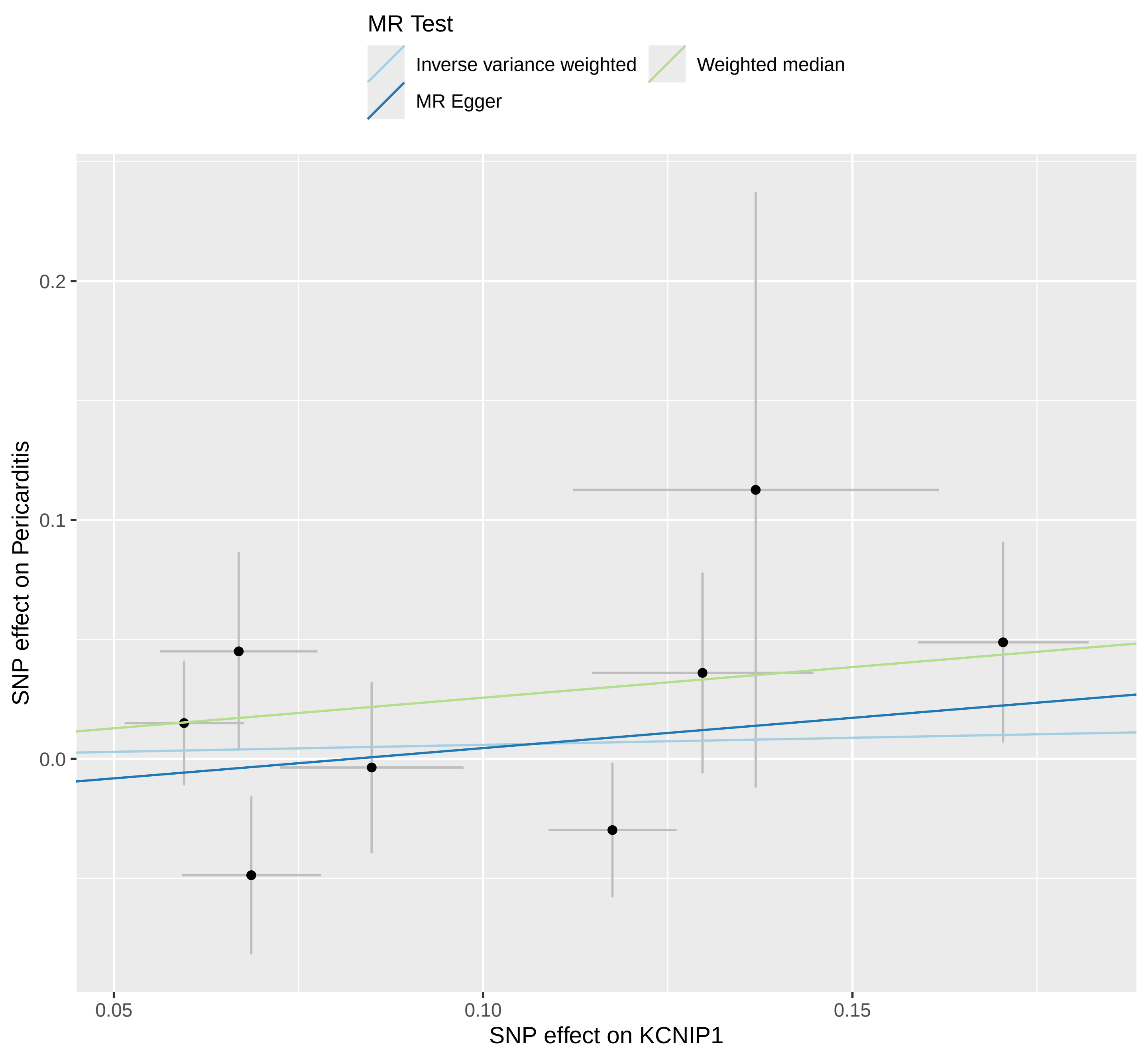
MR Test
Inverse variance weighted
Weighted median
MR Egger
0.2
0.1
0.0
SNP effect on Pericarditis
0.05
0.10
0.15
SNP effect on KCNIP1

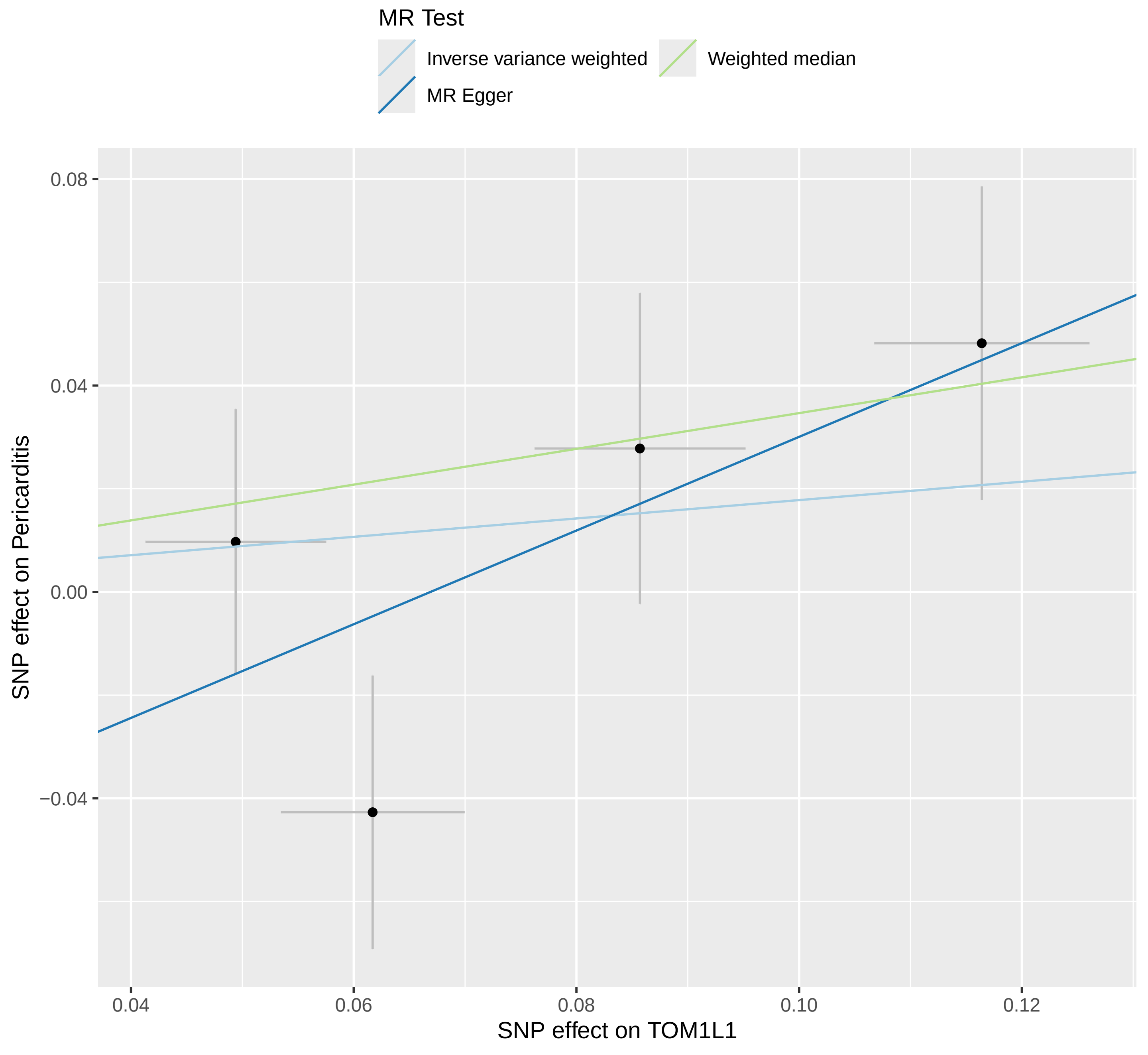
MR Test
Inverse variance weighted
Weighted median
MR Egger
0.08
0.04
0.00
−0.04
SNP effect on Pericarditis
0.04
0.06
0.08
0.10
0.12
SNP effect on TOM1L1

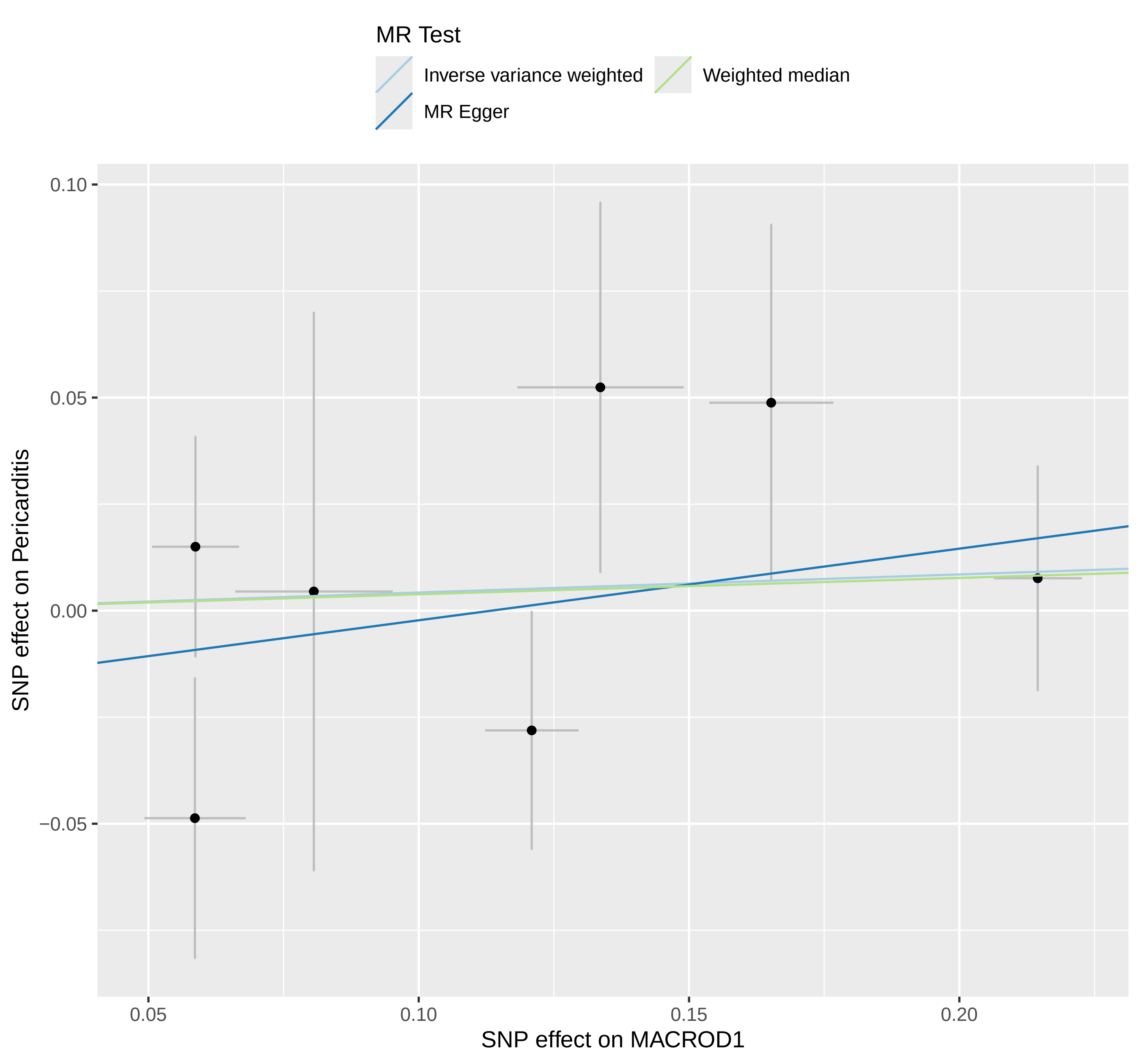
MR Test
Inverse variance weighted
MR Egger
Weighted median
0.10
0.05
0.00
−0.05
SNP effect on Pericarditis
0.05
0.10
0.15
0.20
SNP effect on MACROD1

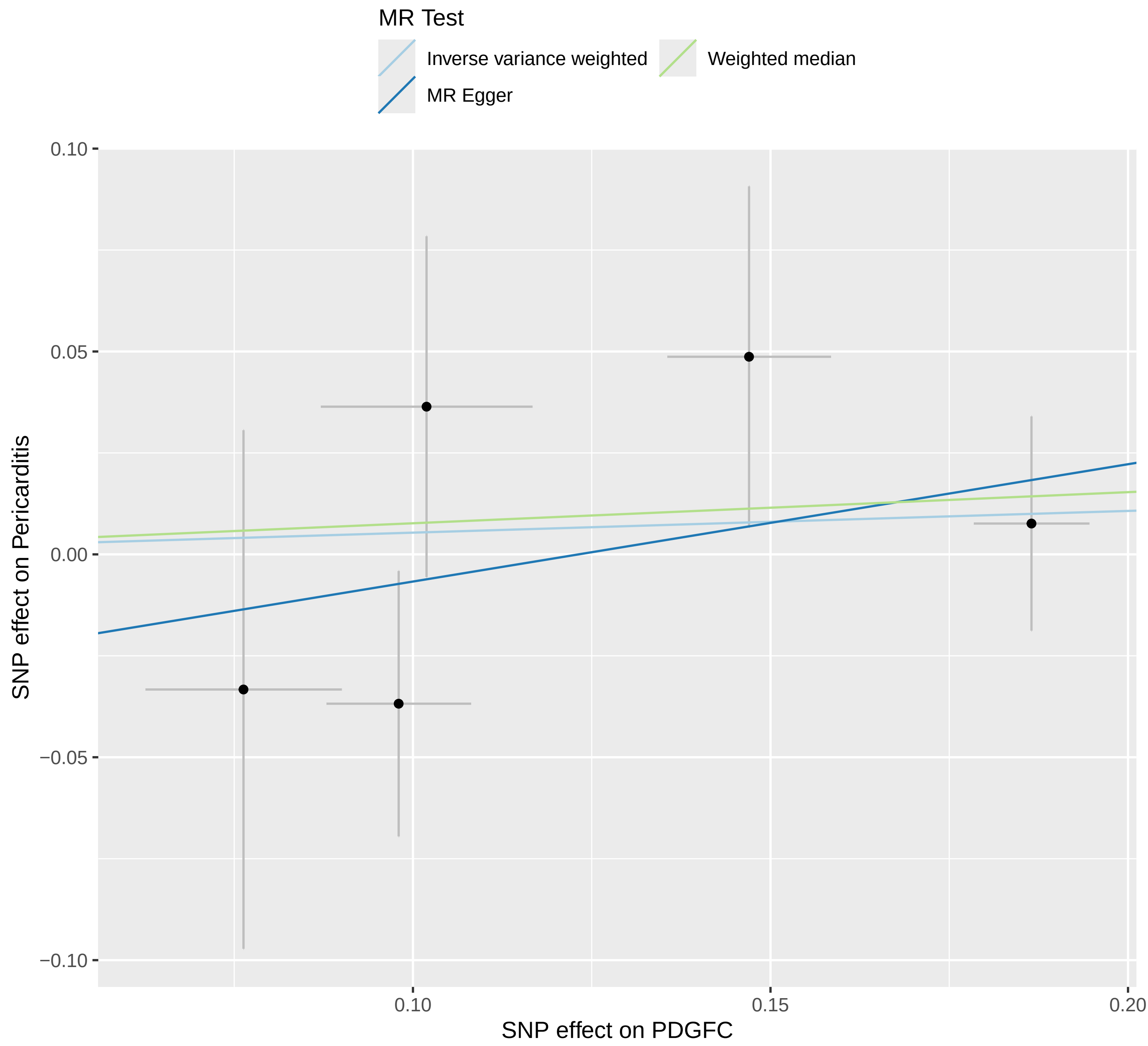
MR Test
Inverse variance weighted
Weighted median
MR Egger
0.10
0.05
0.00
−0.05
−0.10
SNP effect on Pericarditis
0.10
0.15
0.20
SNP effect on PDGFC

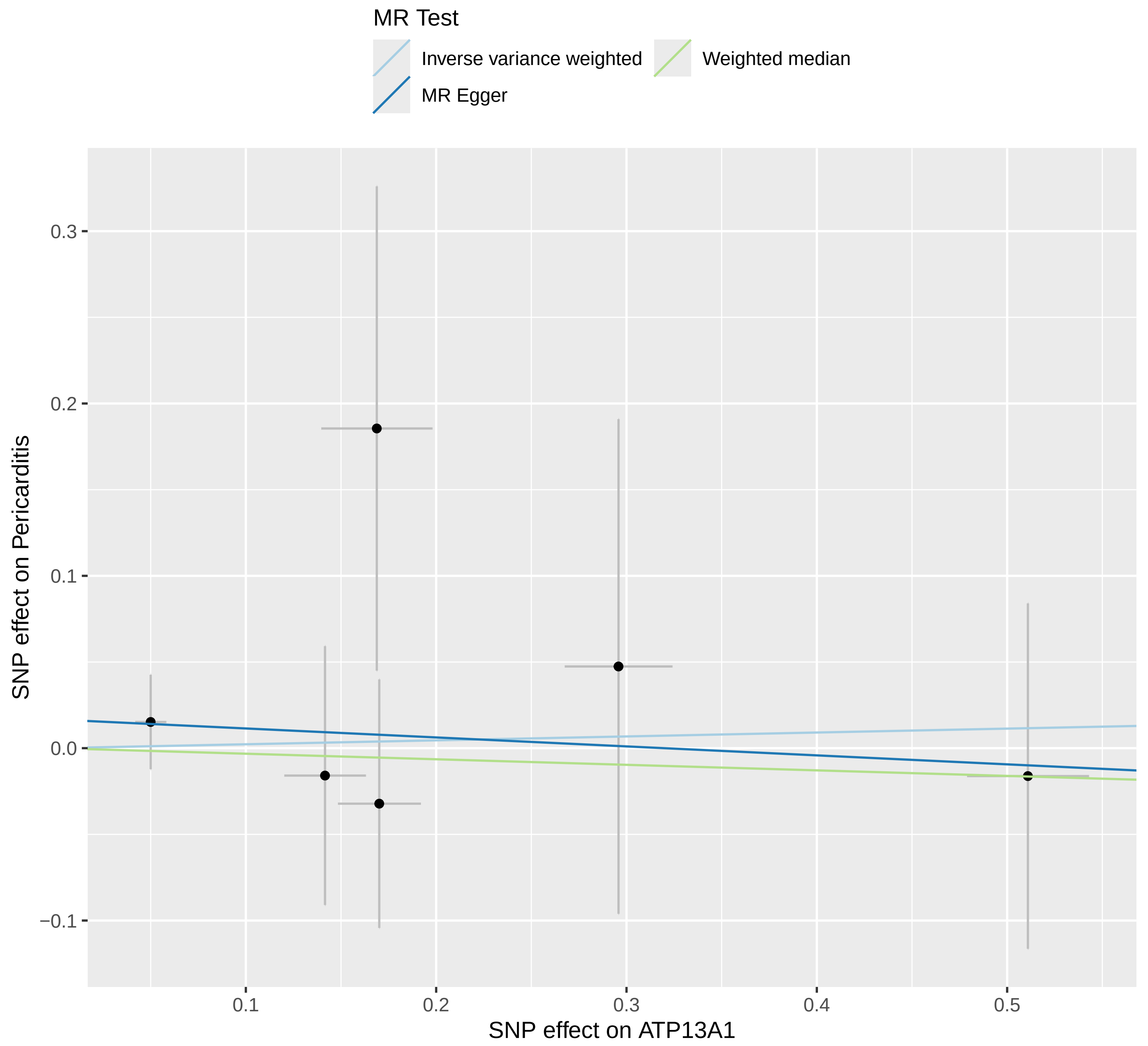

MR Test
Inverse variance weighted
Weighted median
MR Egger
SNP effect on Pericarditis
0.3
0.2
0.1
0.0
−0.1
0.1
0.2
0.3
0.4
0.5
SNP effect on ATP13A1

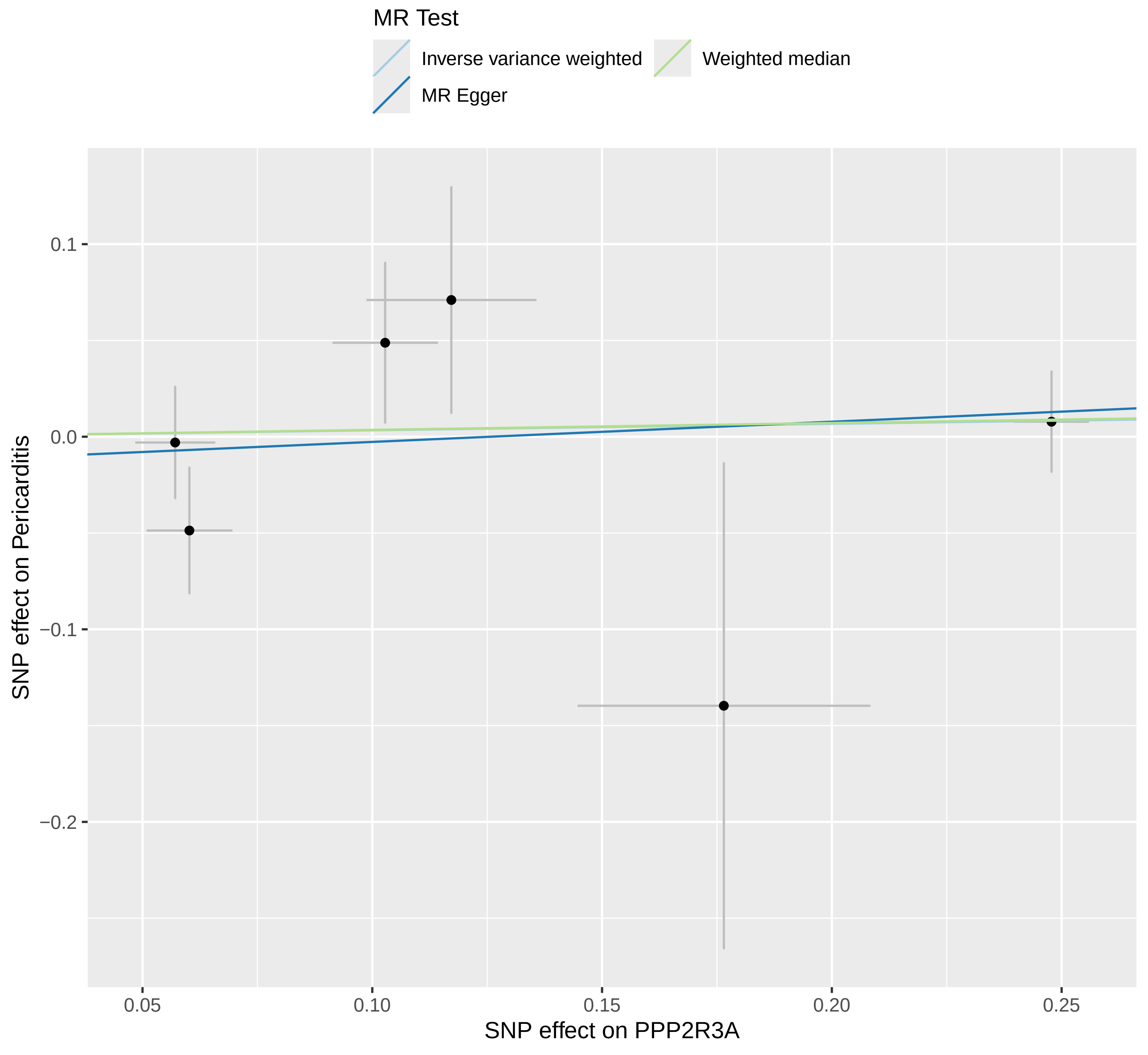
MR Test
Inverse variance weighted
MR Egger
Weighted median
0.1
0.0
−0.1
−0.2
SNP effect on Pericarditis
0.05
0.10
0.15
0.20
0.25
SNP effect on PPP2R3A

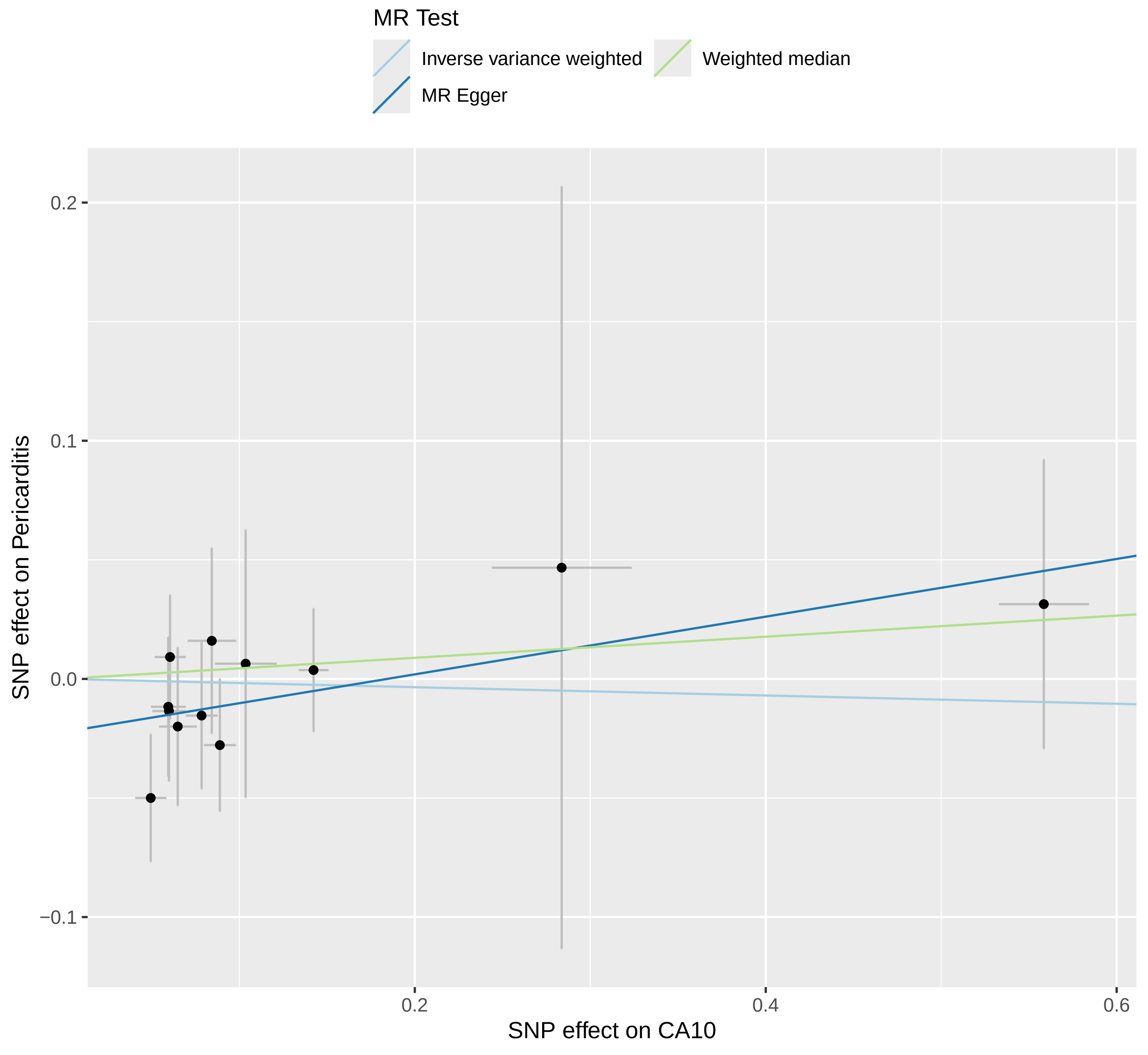

MR Test
Inverse variance weighted
Weighted median
MR Egger
SNP effect on Pericarditis
0.2
0.1
0.0
−0.1
0.2
0.4
0.6
SNP effect on CA10

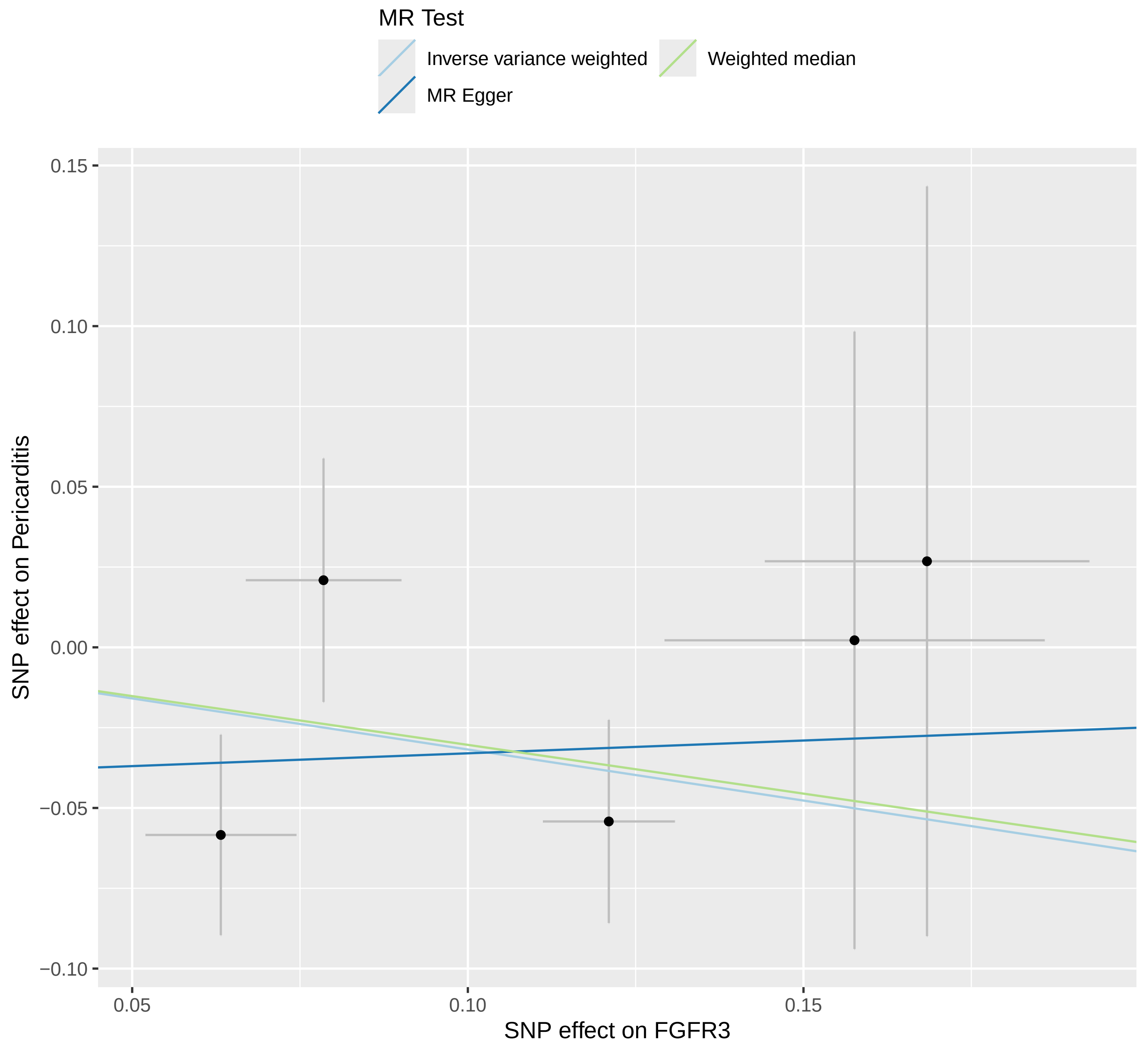

MR Test
Inverse variance weighted
MR Egger
Weighted median
0.15
0.10
0.05
0.00
−0.05
−0.10
SNP effect on Pericarditis
0.05
0.10
0.15
SNP effect on FGFR3

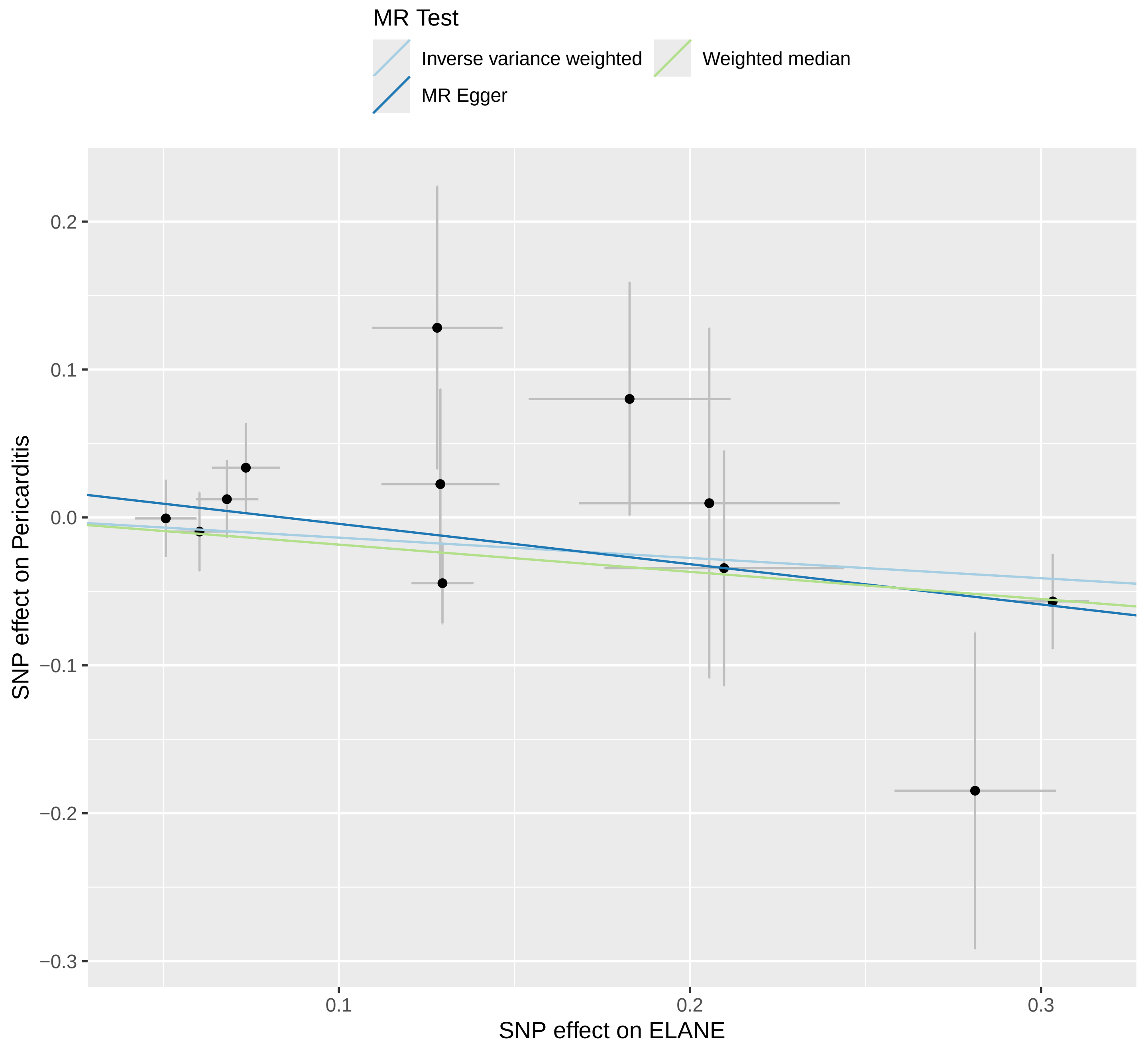
MR Test
Inverse variance weighted
MR Egger
Weighted median
SNP effect on Pericarditis
0.2
0.1
0.0
−0.1
−0.2
−0.3
0.1
0.2
0.3
SNP effect on ELANE

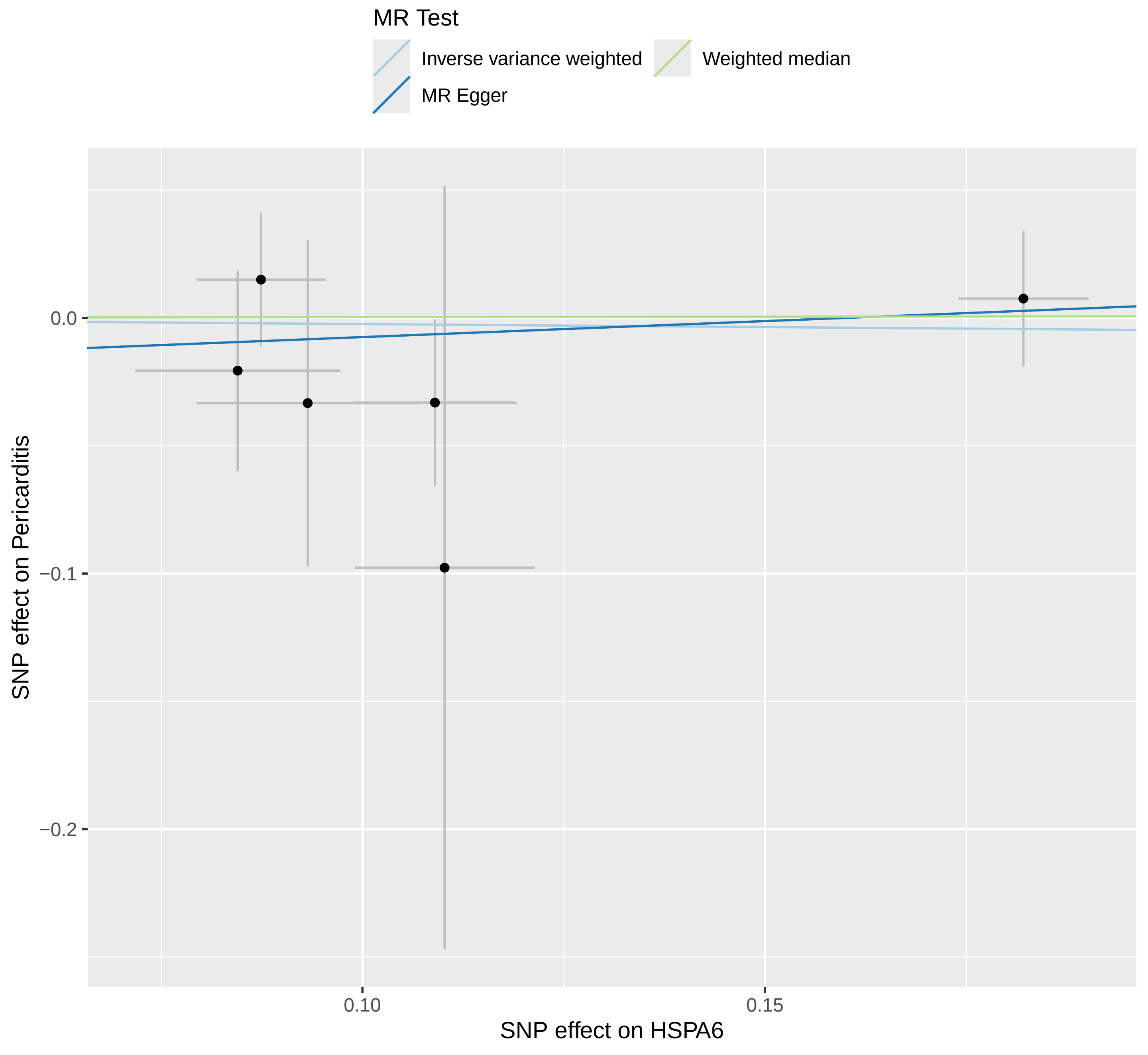

MR Test
Inverse variance weighted
Weighted median
MR Egger
SNP effect on Pericarditis
0.0
−0.1
−0.2
0.10
0.15
SNP effect on HSPA6

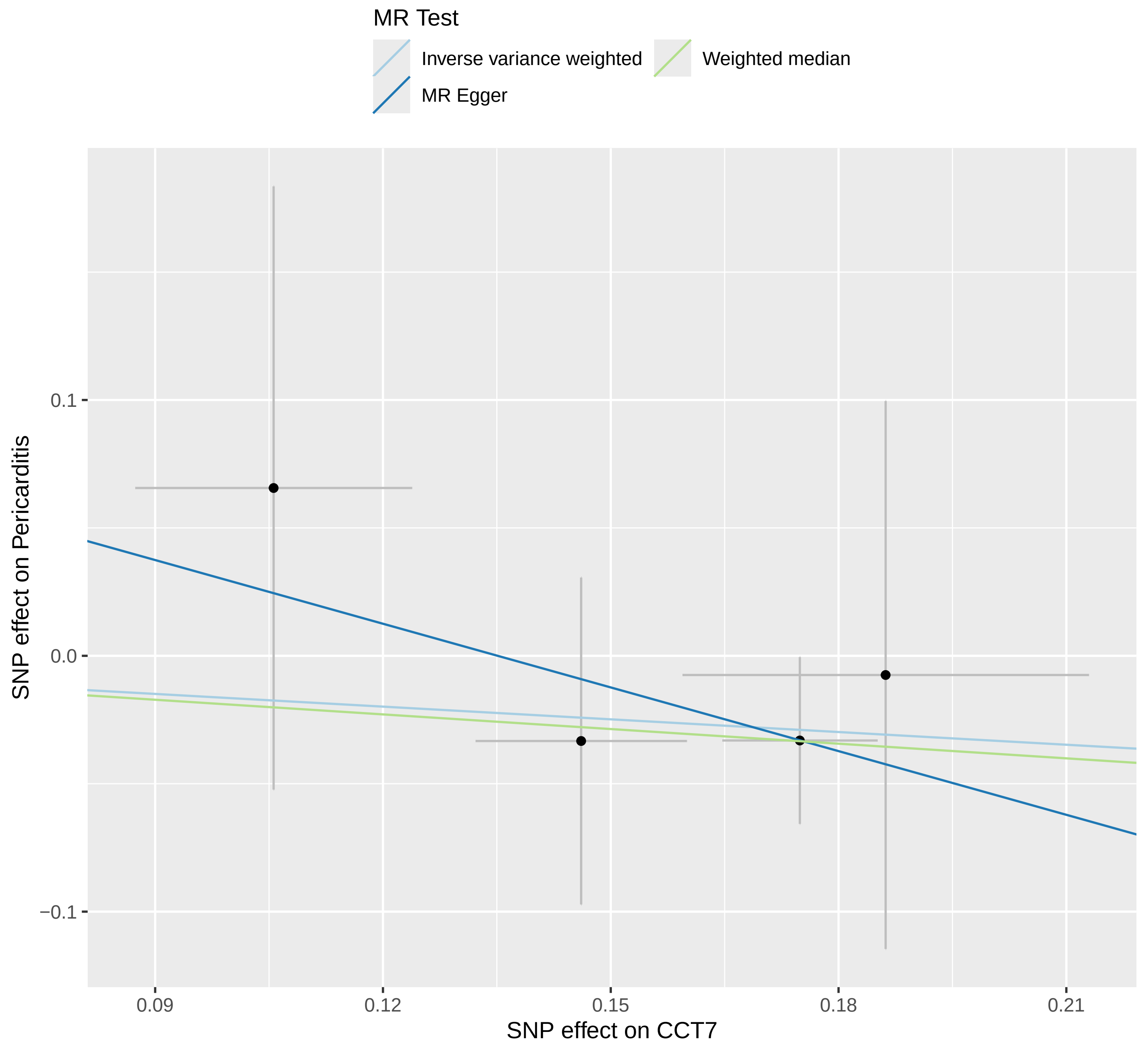

MR Test
Inverse variance weighted
Weighted median
MR Egger
0.1
0.0
−0.1
SNP effect on Pericarditis
0.09
0.12
0.15
0.18
0.21
SNP effect on CCT7

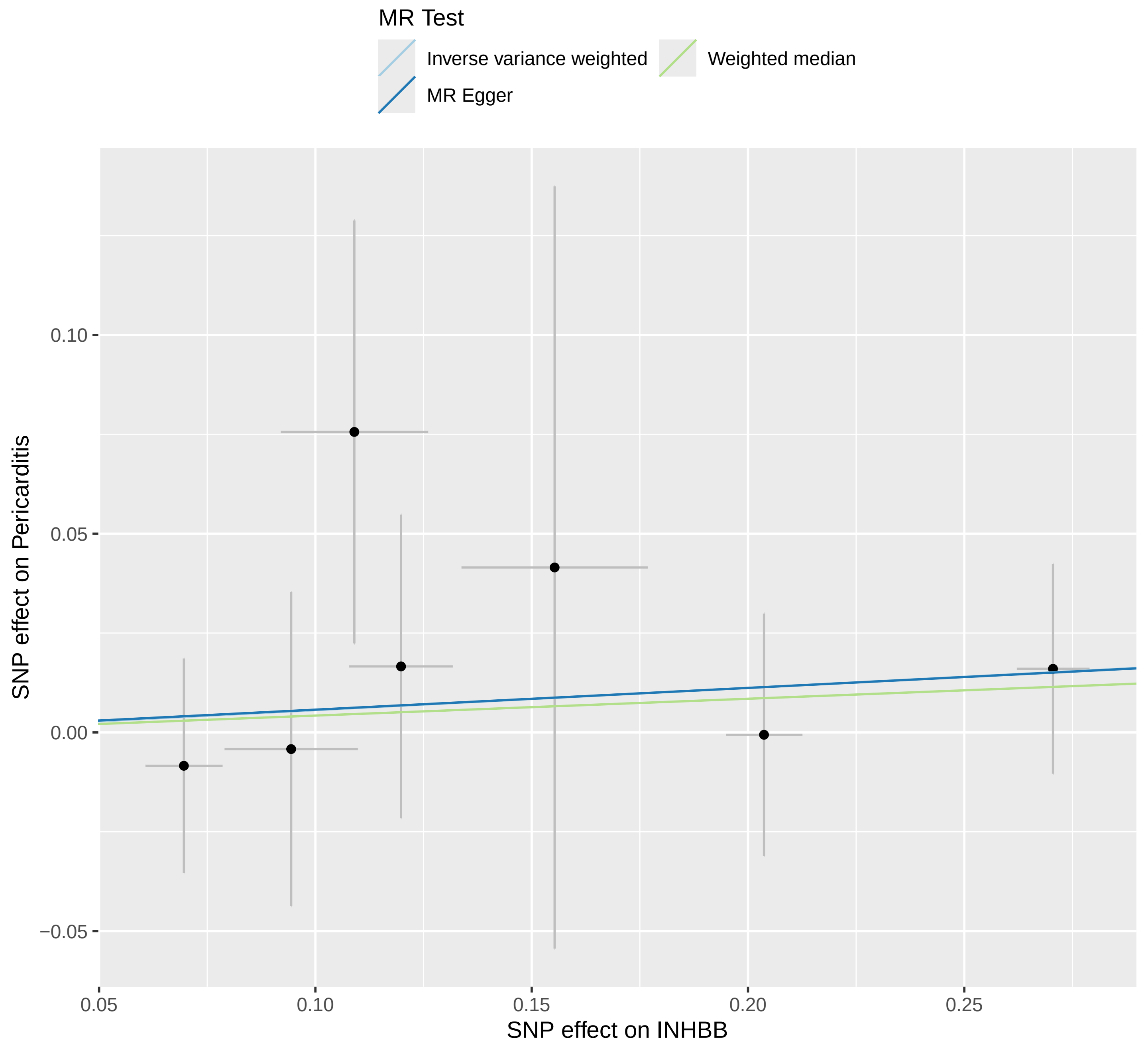
MR Test
Inverse variance weighted
Weighted median
MR Egger
0.10
0.05
0.00
−0.05
SNP effect on Pericarditis
0.05
0.10
0.15
0.20
0.25
SNP effect on INHBB

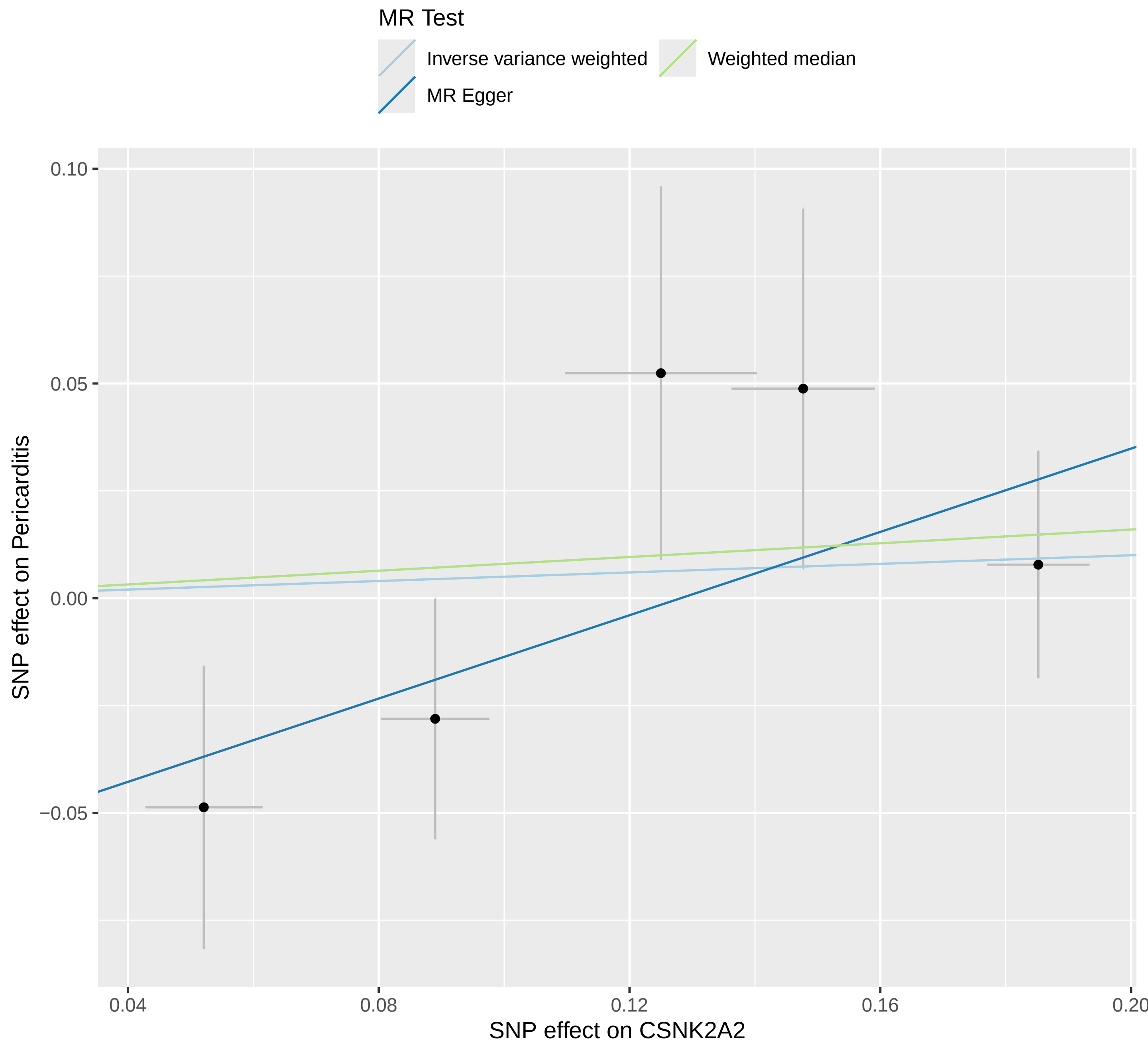

MR Test
Inverse variance weighted
Weighted median
MR Egger
0.10
0.05
0.00
−0.05
0.04
0.08
0.12
0.16
0.20
SNP effect on Pericarditis
SNP effect on CSNK2A2

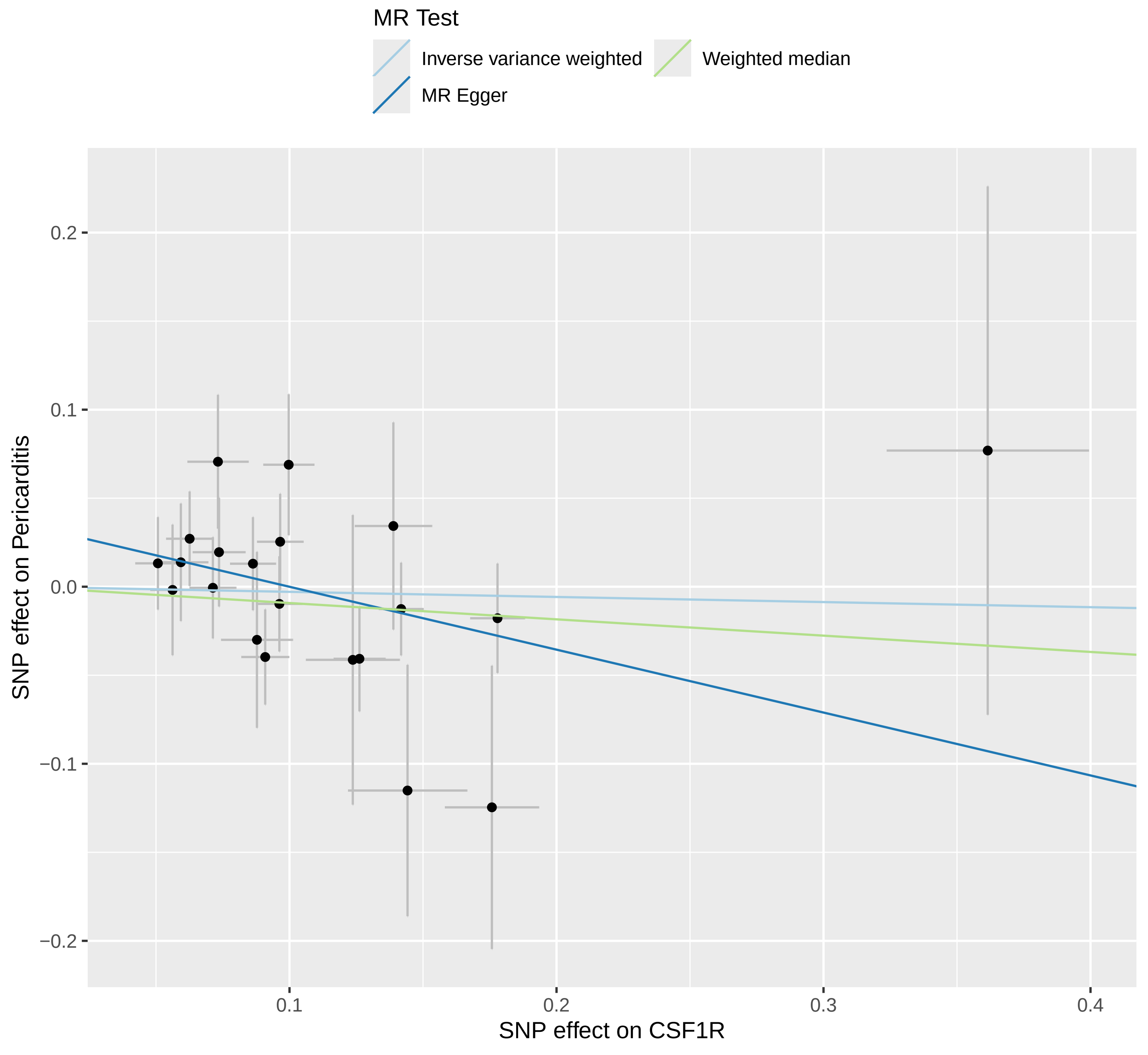
MR Test
Inverse variance weighted
MR Egger
Weighted median
SNP effect on Pericarditis
SNP effect on CSF1R
0.2
0.1
0.0
−0.1
−0.2
0.1
0.2
0.3
0.4

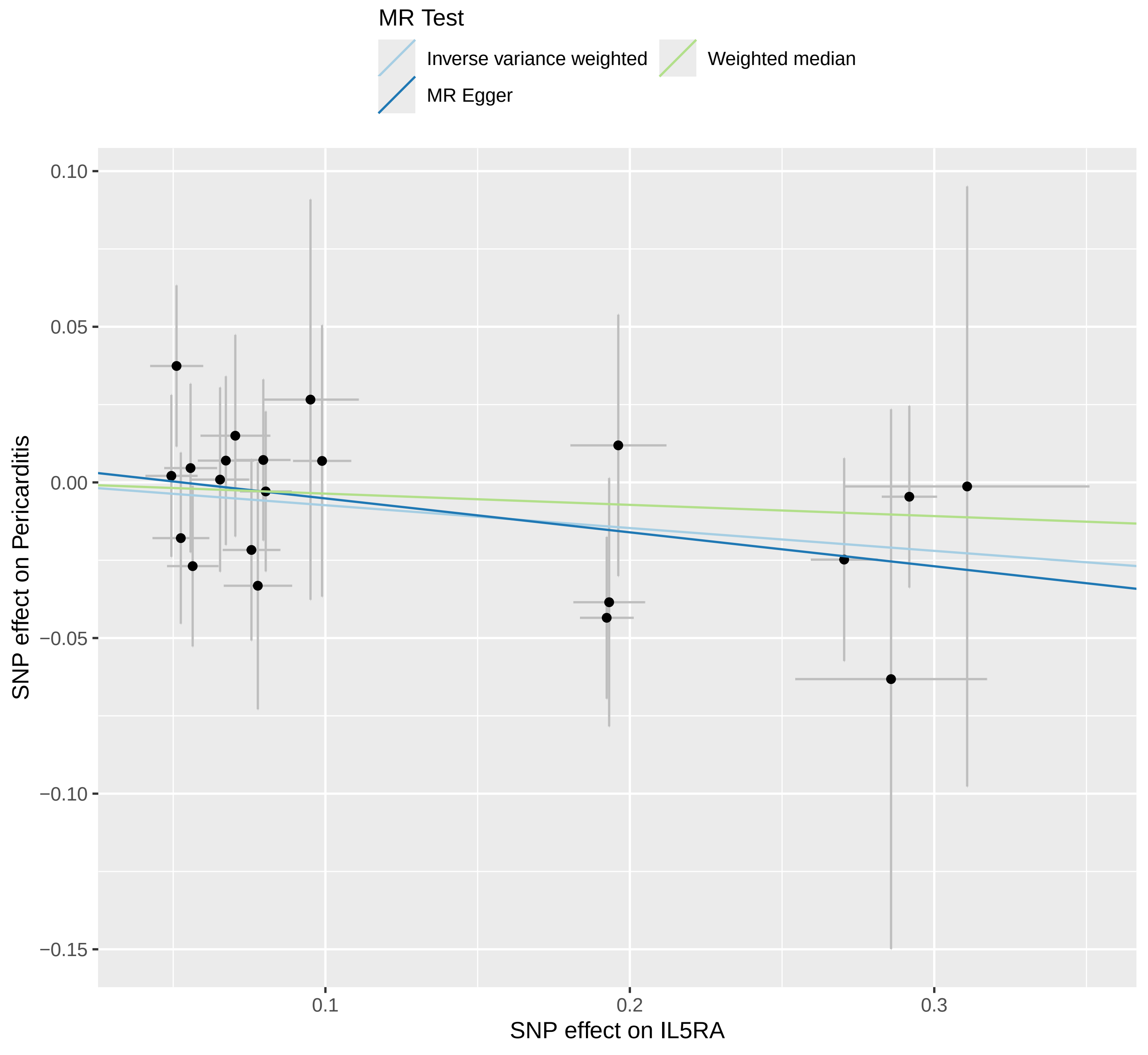
MR Test
Inverse variance weighted
MR Egger
Weighted median
0.10
0.05
0.00
−0.05
−0.10
−0.15
SNP effect on Pericarditis
0.1
0.2
0.3
SNP effect on IL5RA

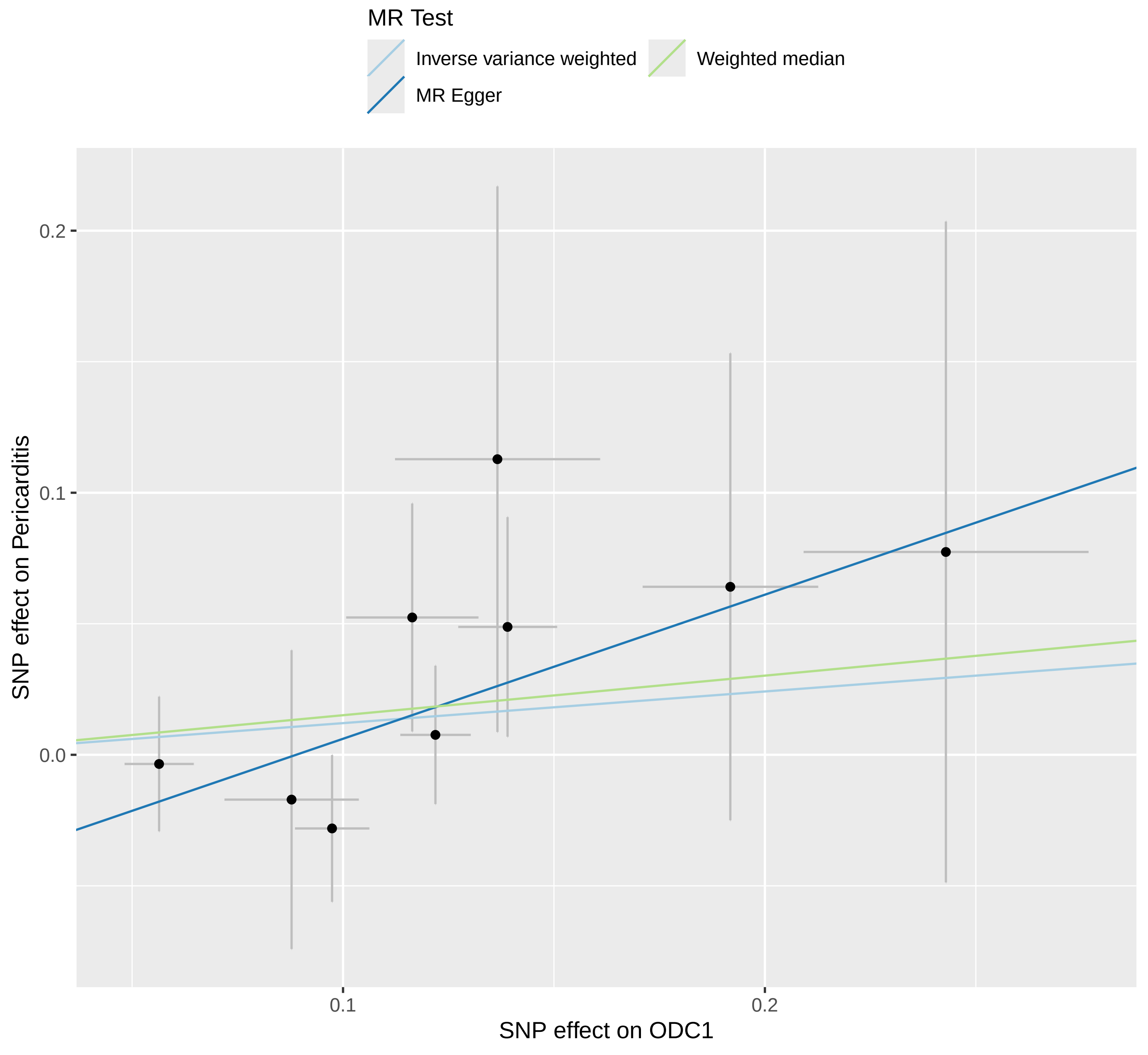
MR Test
Inverse variance weighted
Weighted median
MR Egger
0.2
0.1
0.0
SNP effect on Pericarditis
0.1
0.2
SNP effect on ODC1

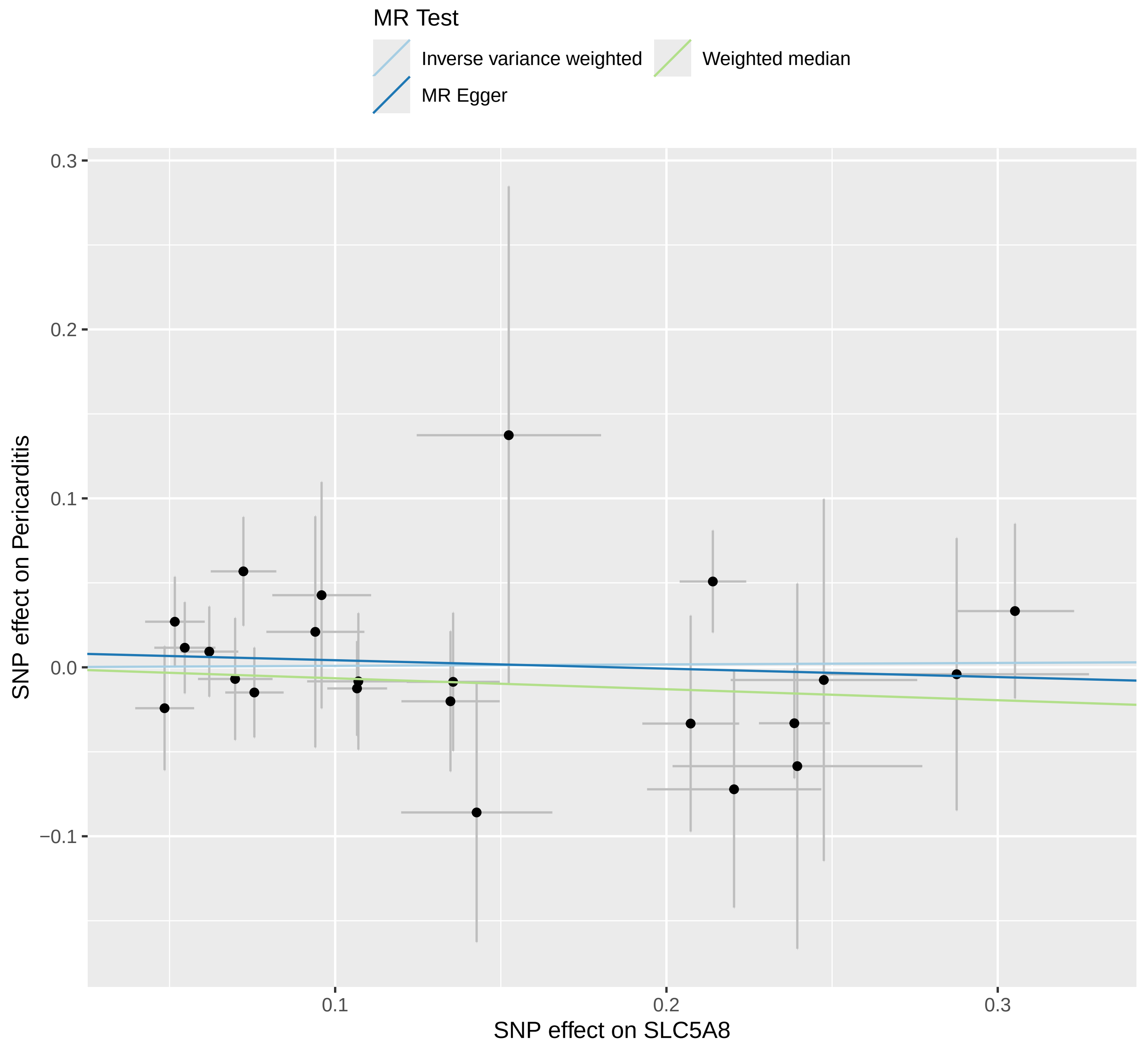

MR Test
Inverse variance weighted
Weighted median
MR Egger
0.3
0.2
0.1
0.0
−0.1
SNP effect on Pericarditis
0.1
0.2
0.3
SNP effect on SLC5A8

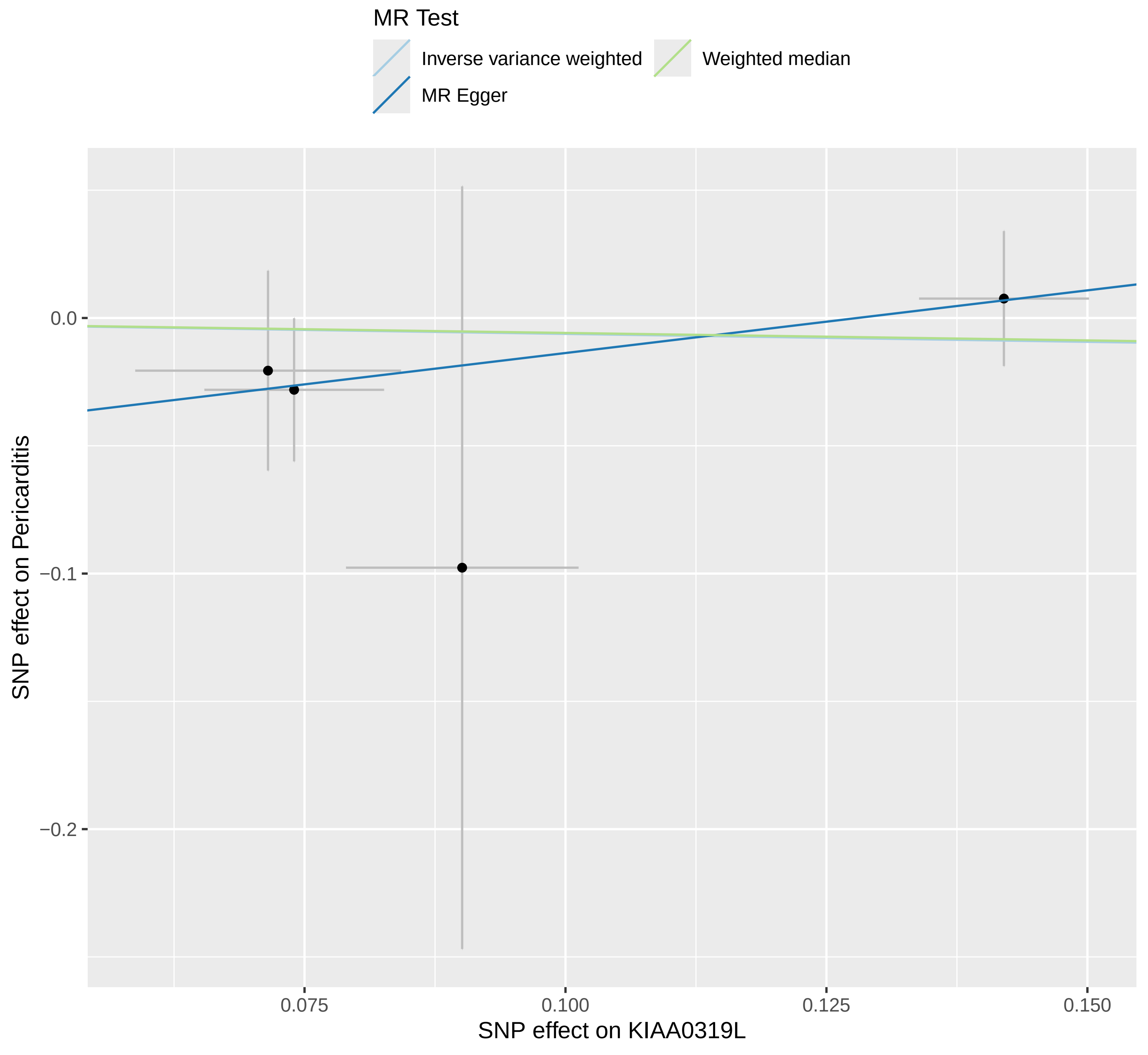

MR Test
Inverse variance weighted
MR Egger
Weighted median
0.0
−0.1
−0.2
0.075
0.100
0.125
0.150
SNP effect on Pericarditis
SNP effect on KIAA0319L

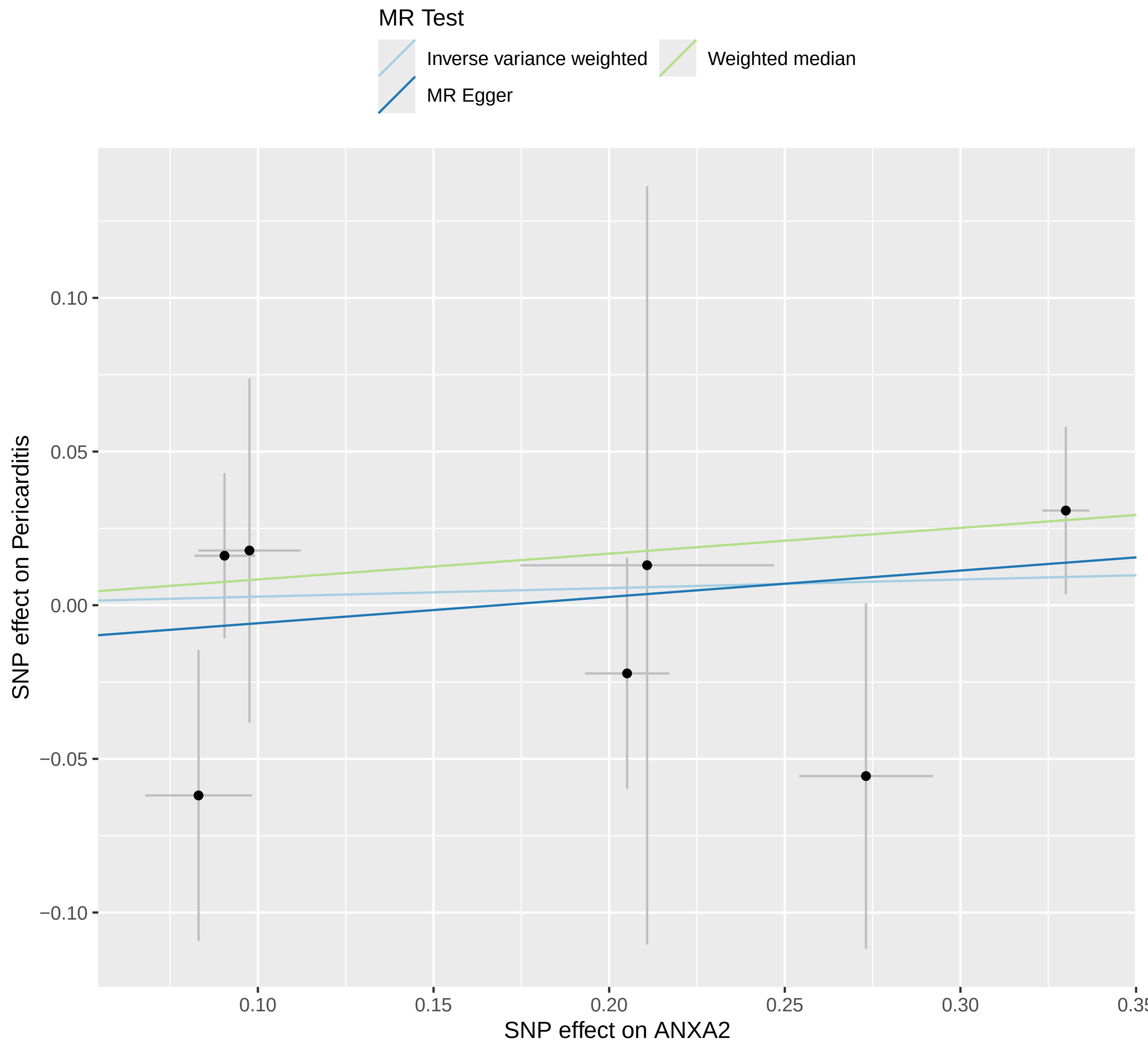

MR Test
Inverse variance weighted
Weighted median
MR Egger
0.10
0.05
0.00
−0.05
−0.10
0.10
0.15
0.20
0.25
0.30
SNP effect on Pericarditis
SNP effect on ANXA2

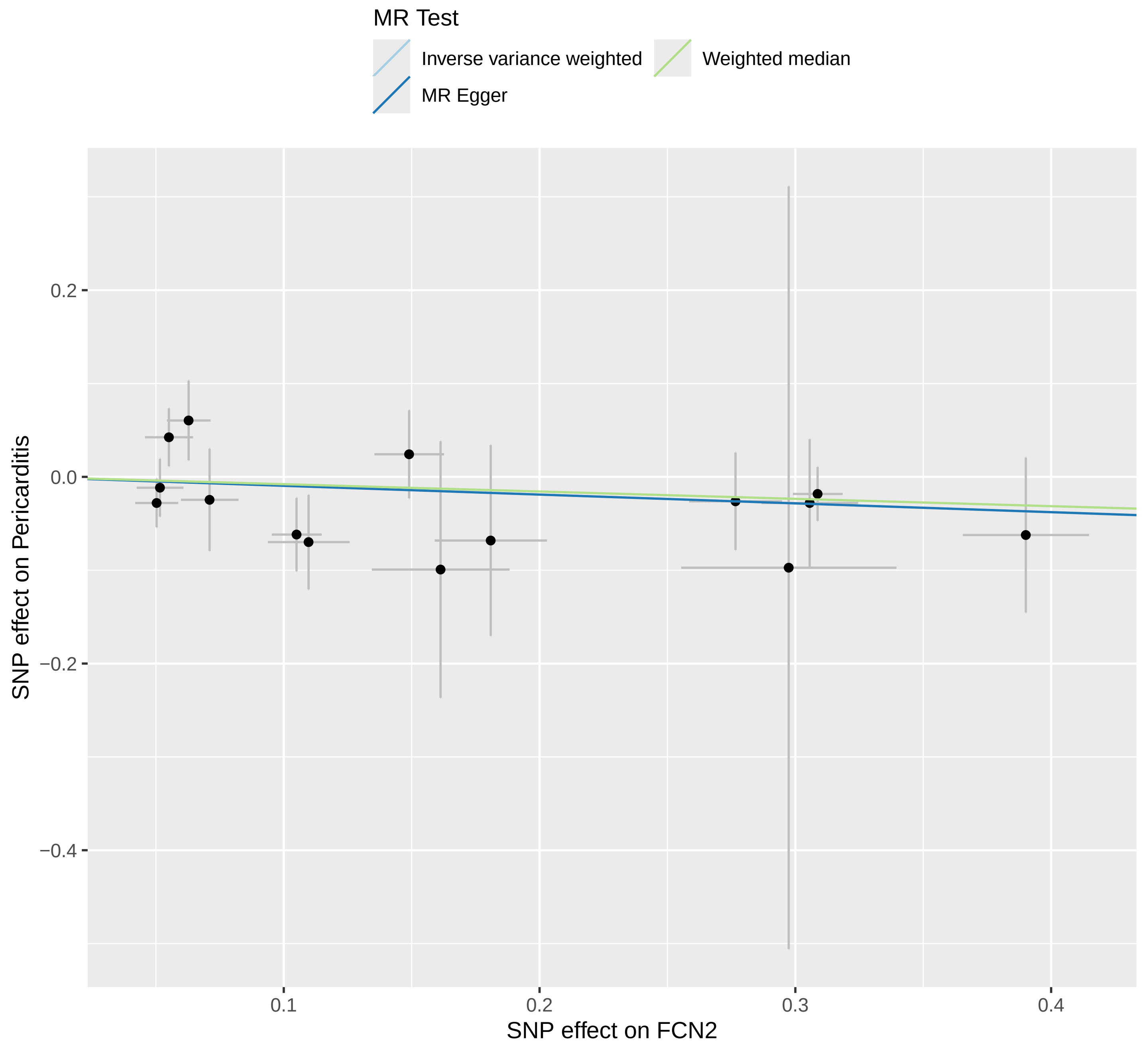
MR Test
Inverse variance weighted
Weighted median
MR Egger
SNP effect on Pericarditis
0.2
0.0
−0.2
−0.4
SNP effect on FCN2
0.1
0.2
0.3
0.4

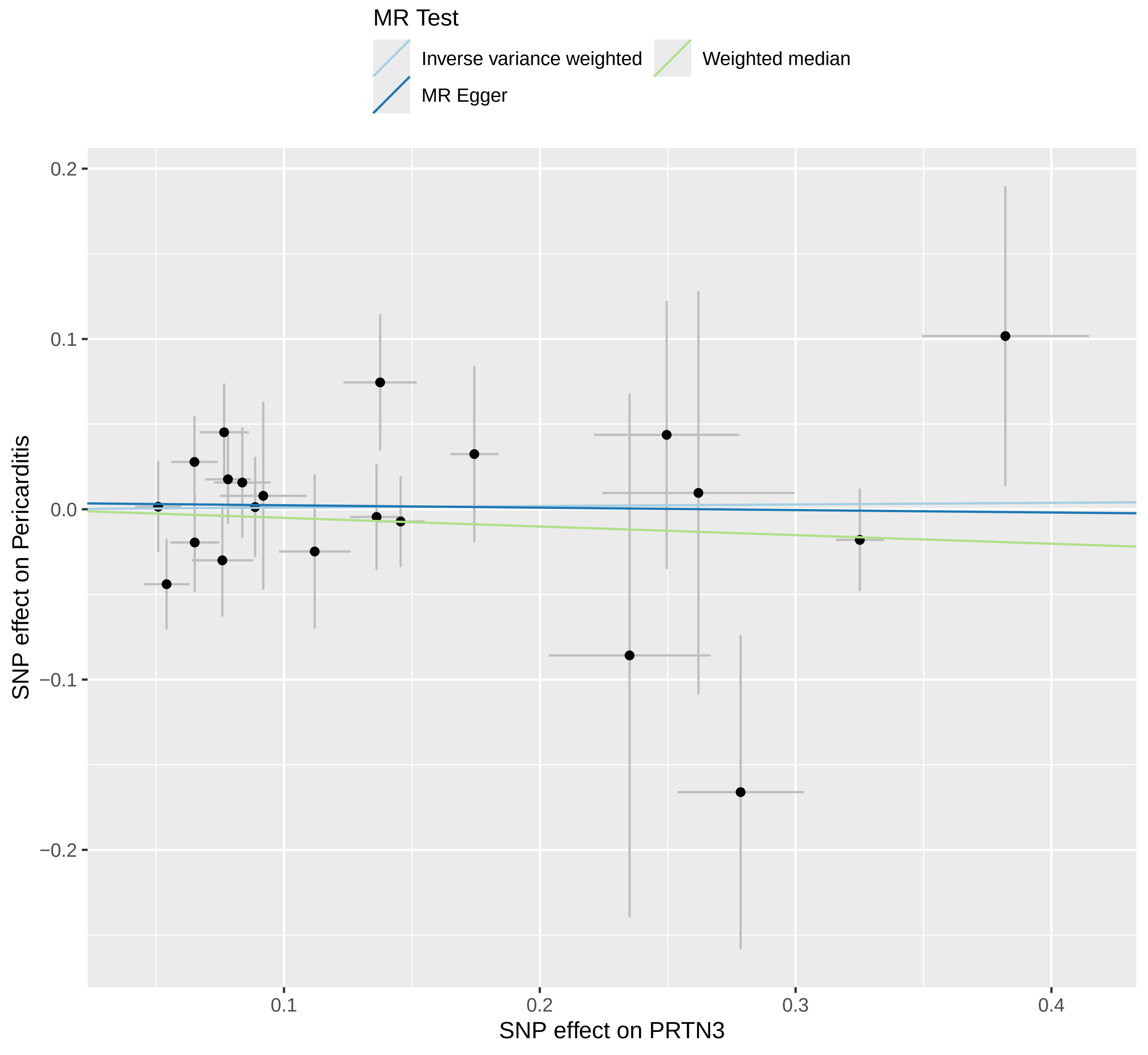
MR Test
Inverse variance weighted
Weighted median
MR Egger
0.2
0.1
0.0
−0.1
−0.2
0.1
0.2
0.3
0.4
SNP effect on Pericarditis
SNP effect on PRTN3

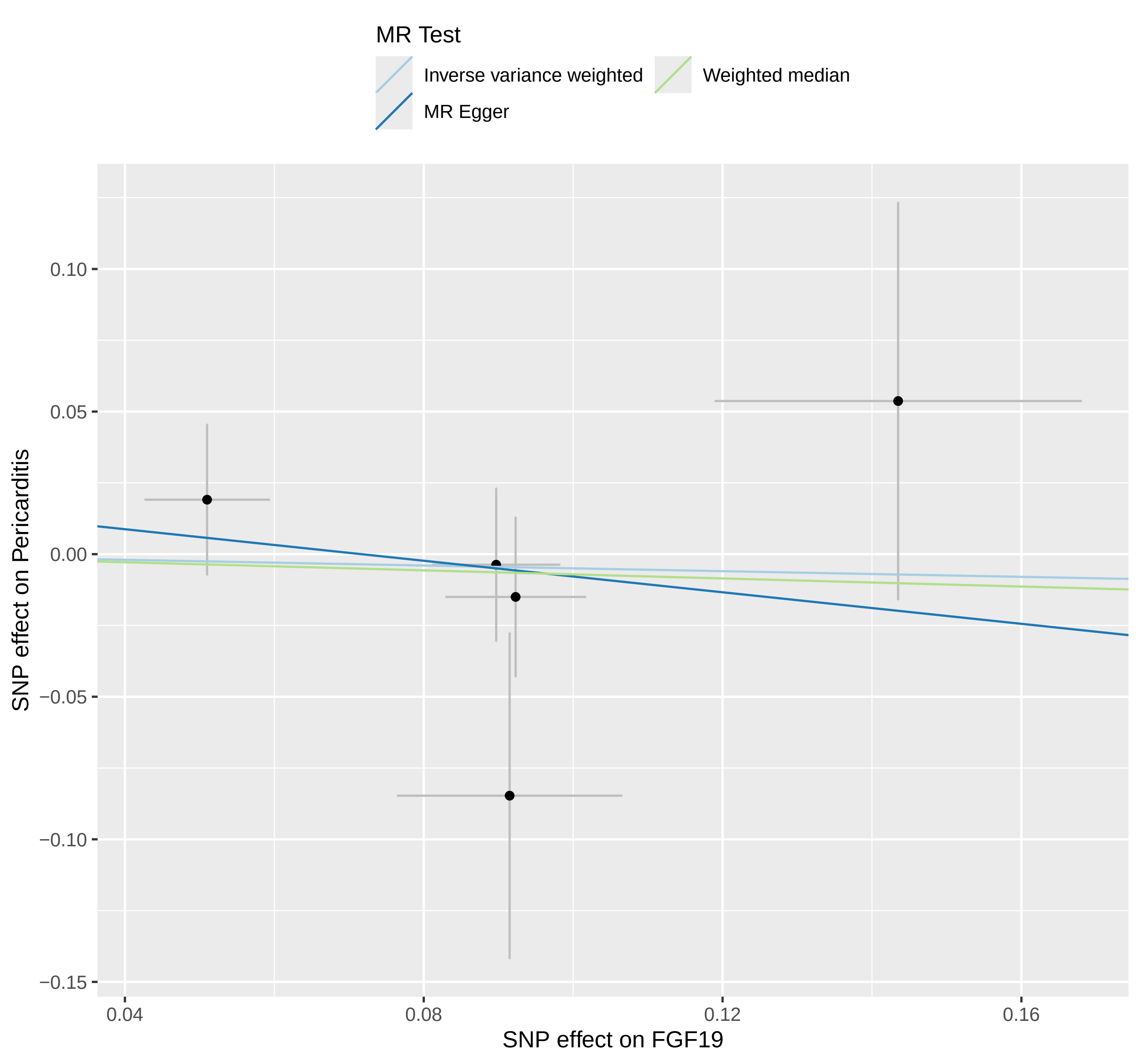
MR Test
Inverse variance weighted
Weighted median
MR Egger
0.10
0.05
0.00
−0.05
−0.10
−0.15
SNP effect on Pericarditis
0.04
0.08
0.12
0.16
SNP effect on FGF19

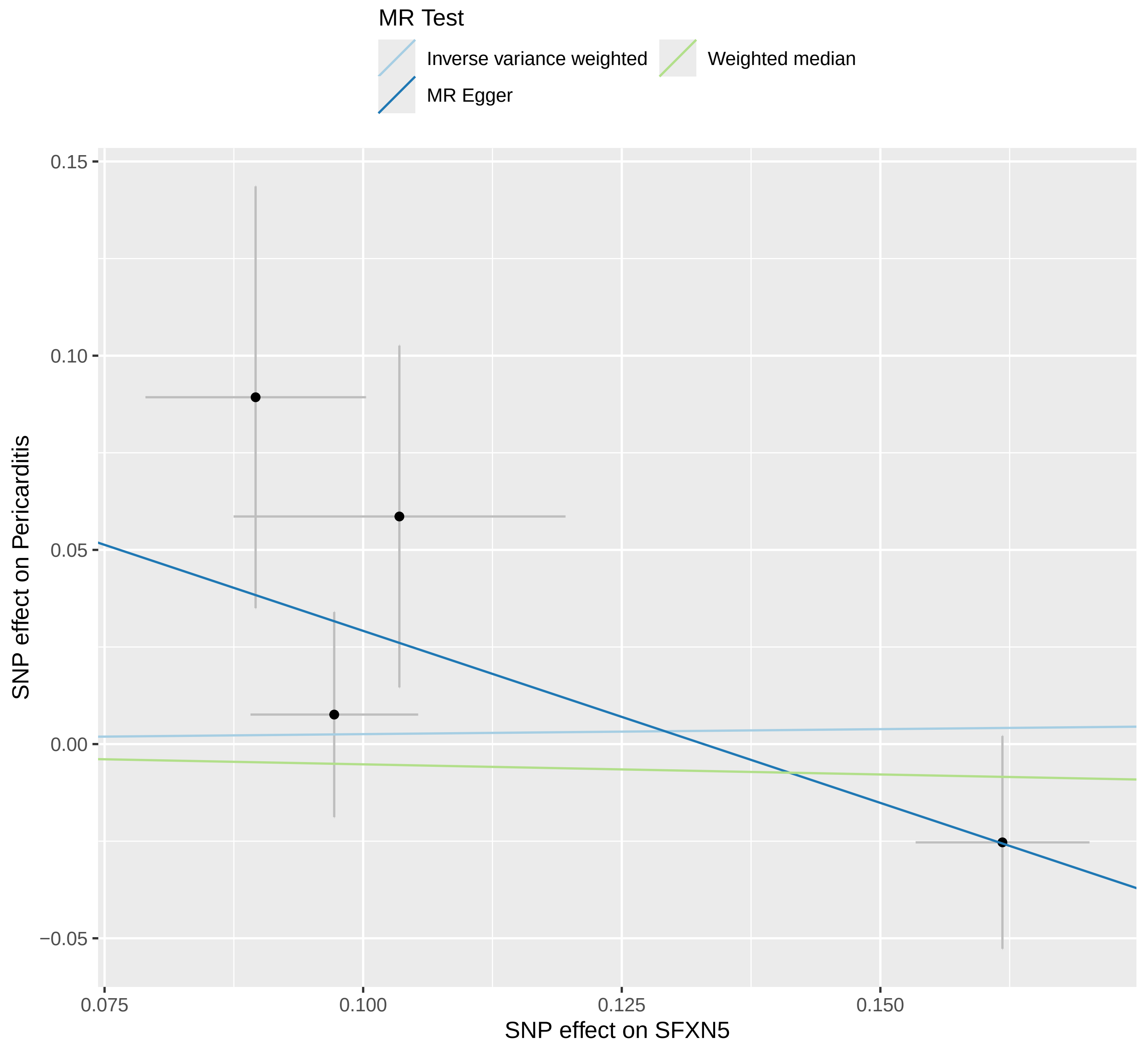
MR Test
Inverse variance weighted
MR Egger
Weighted median
0.15
0.10
0.05
0.00
−0.05
SNP effect on Pericarditis
0.075
0.100
0.125
0.150
SNP effect on SFXN5

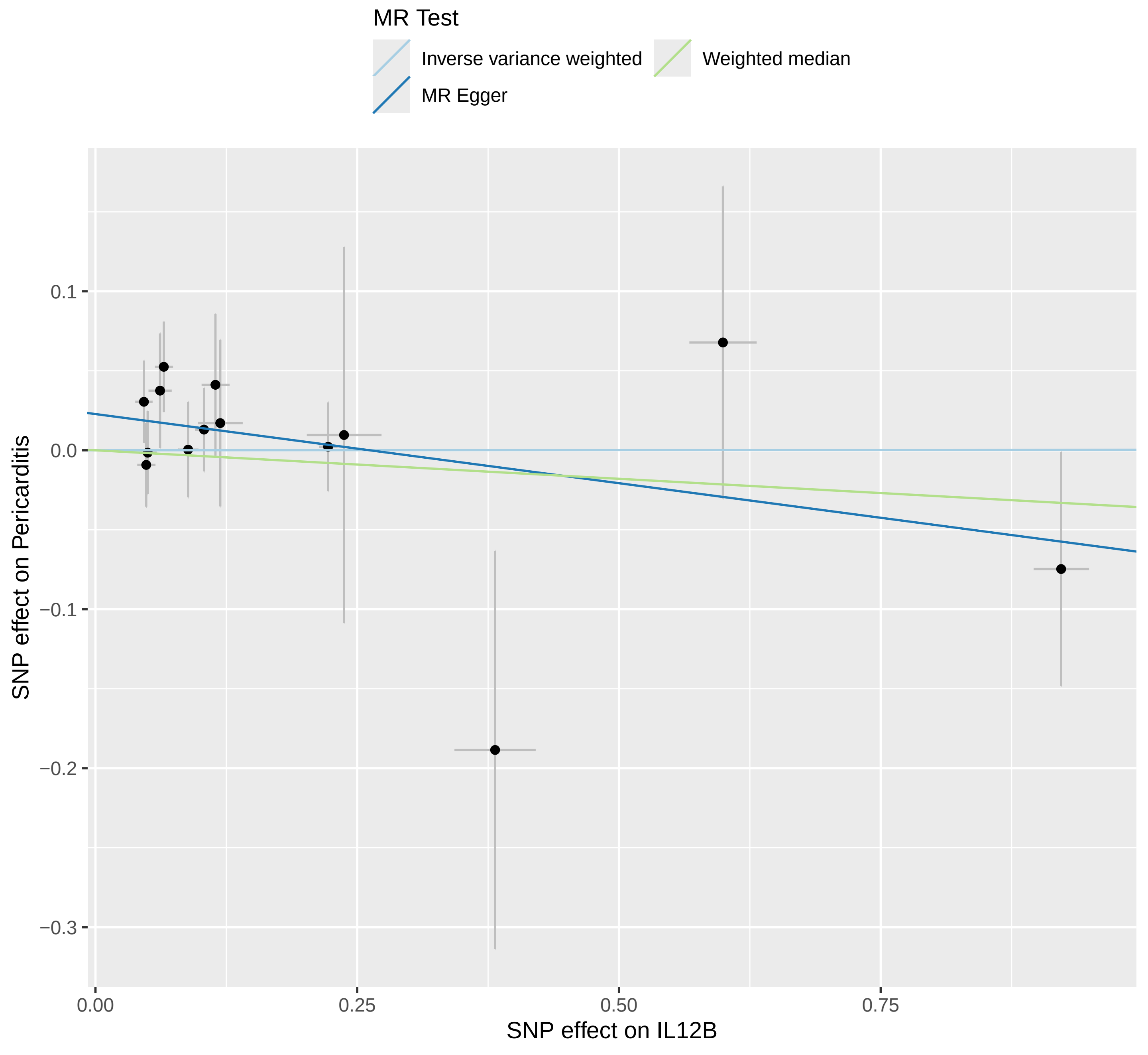

MR Test
Inverse variance weighted
Weighted median
MR Egger
SNP effect on Pericarditis
SNP effect on IL12B
0.1
0.0
−0.1
−0.2
−0.3
0.00
0.25
0.50
0.75

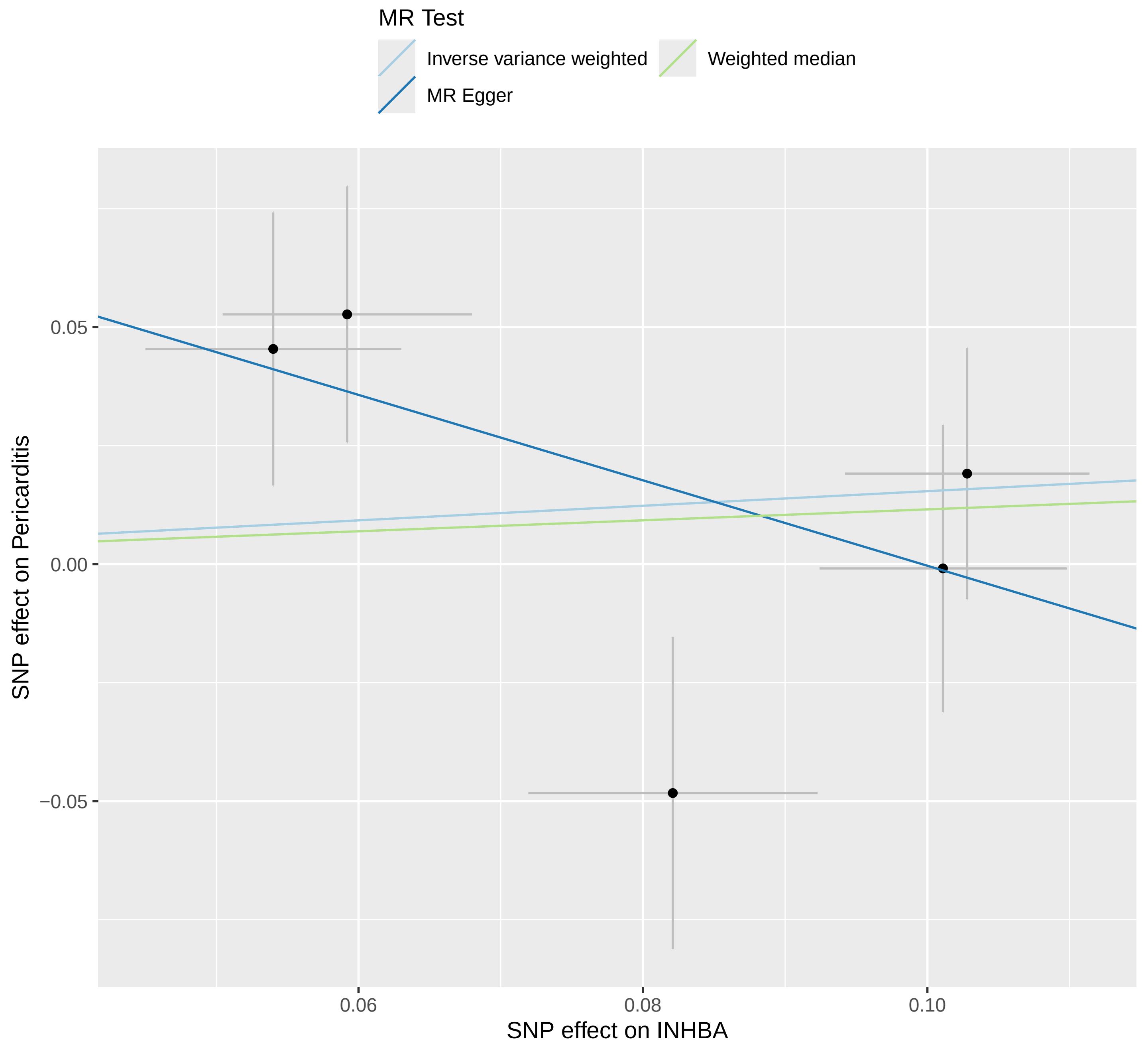

MR Test
Inverse variance weighted
Weighted median
MR Egger
0.05
0.00
−0.05
0.06
0.08
0.10
SNP effect on Pericarditis
SNP effect on INHBA

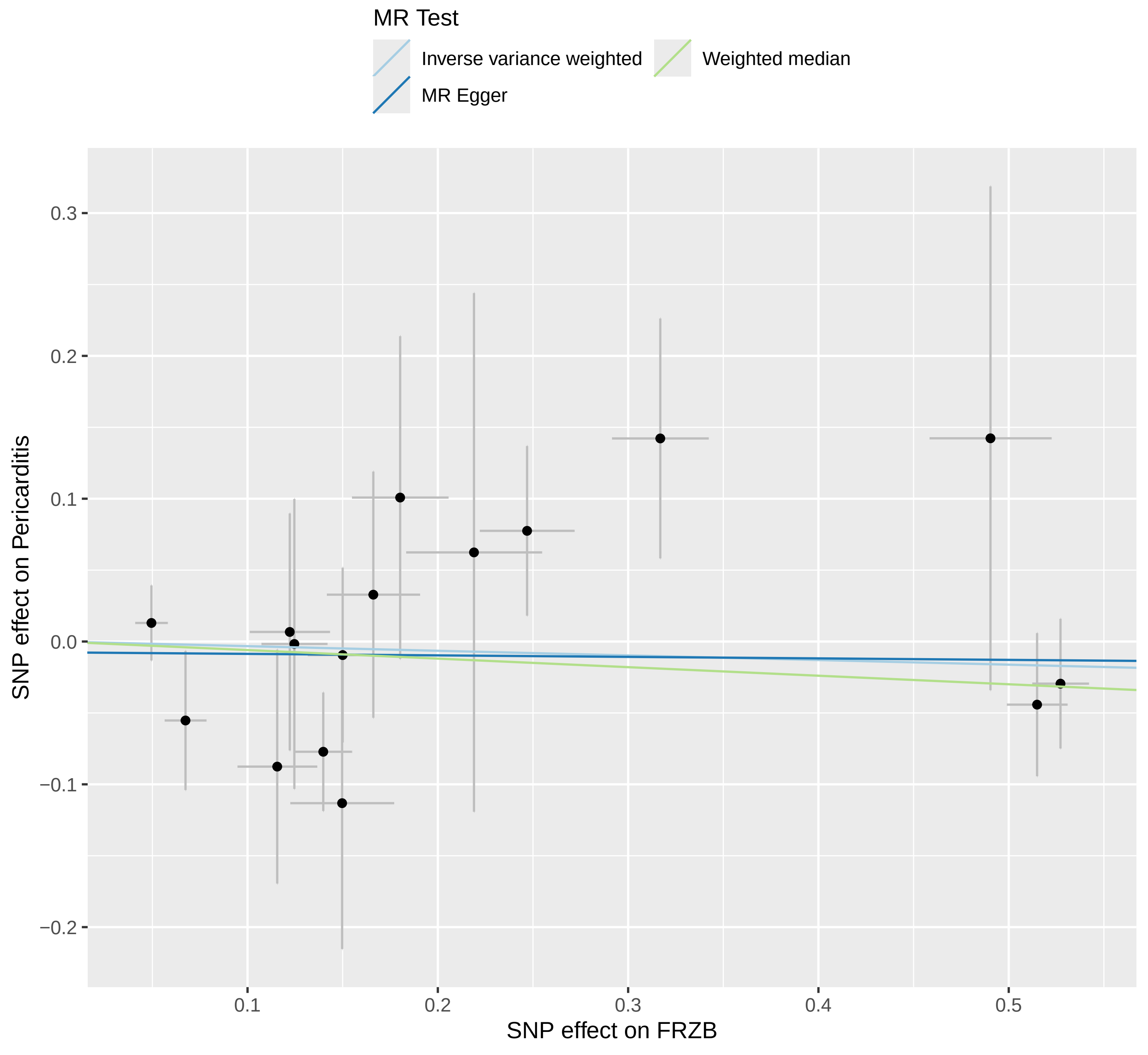
MR Test
Inverse variance weighted
Weighted median
MR Egger
0.3
0.2
0.1
0.0
−0.1
−0.2
SNP effect on Pericarditis
0.1
0.2
0.3
0.4
0.5
SNP effect on FRZB

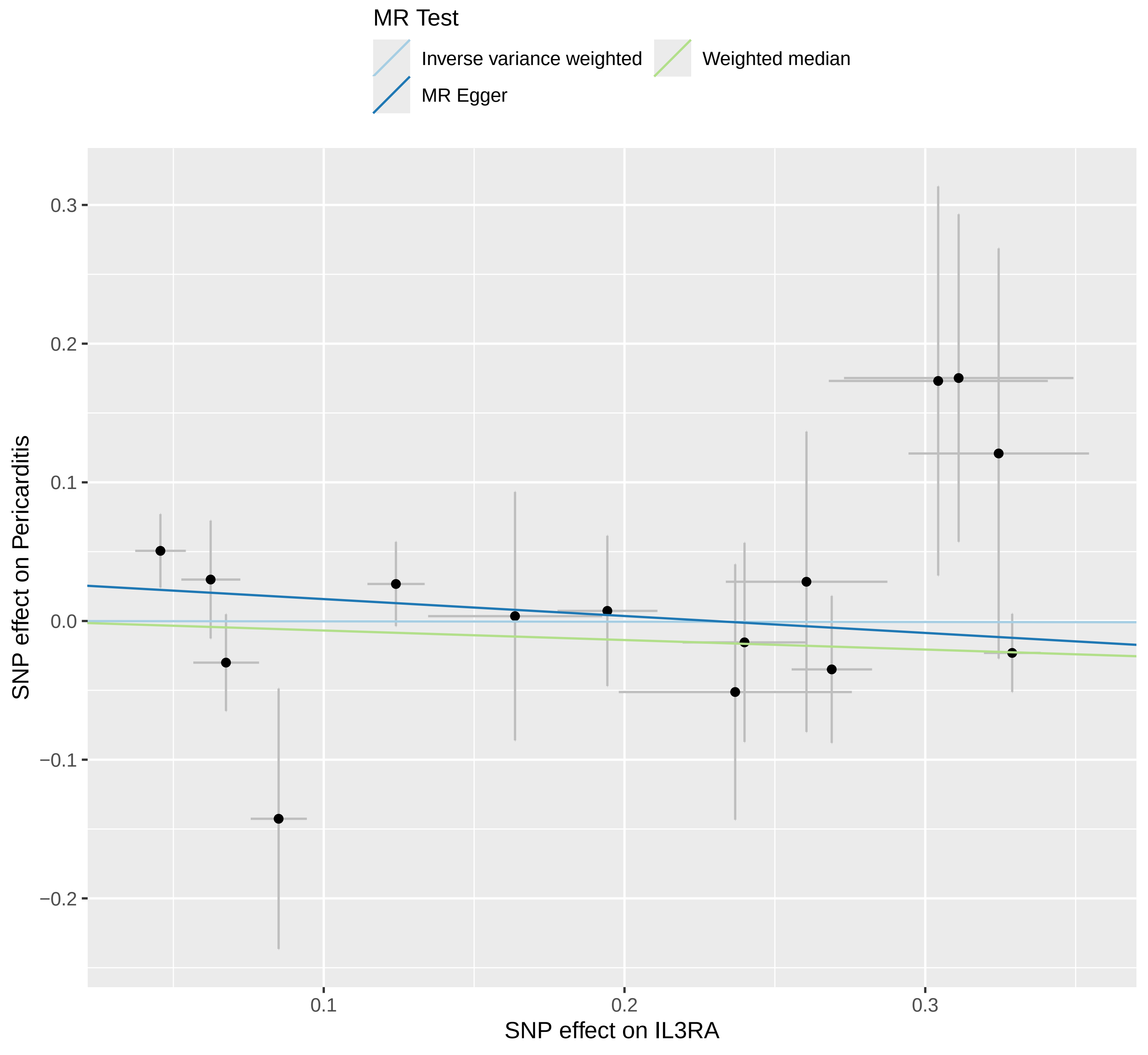
MR Test
Inverse variance weighted
Weighted median
MR Egger
SNP effect on Pericarditis
SNP effect on IL3RA
0.3
0.2
0.1
0.0
−0.1
−0.2
0.1
0.2
0.3

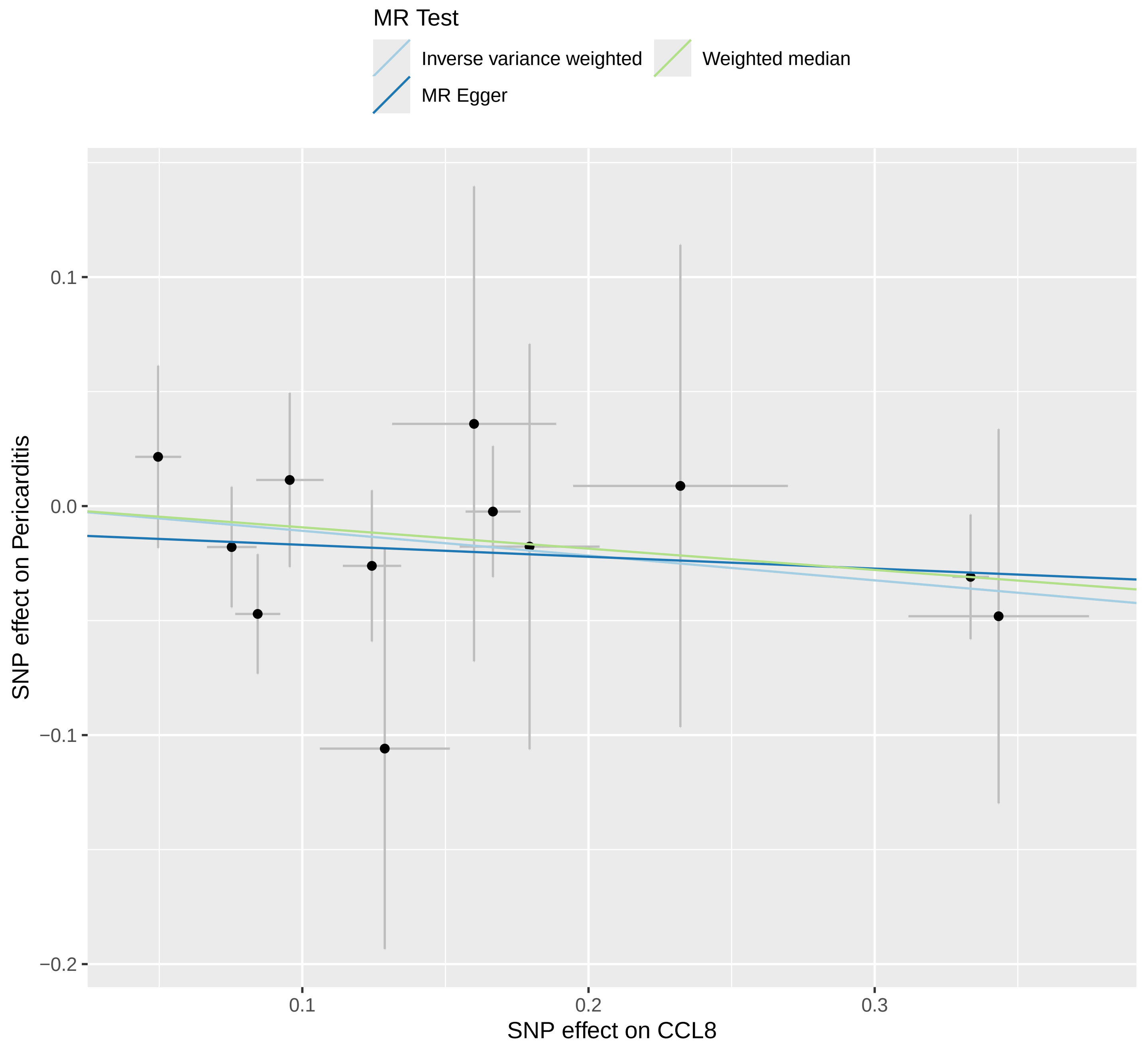
MR Test
Inverse variance weighted
Weighted median
MR Egger
SNP effect on Pericarditis
0.1
0.0
−0.1
−0.2
0.1
0.2
0.3
SNP effect on CCL8

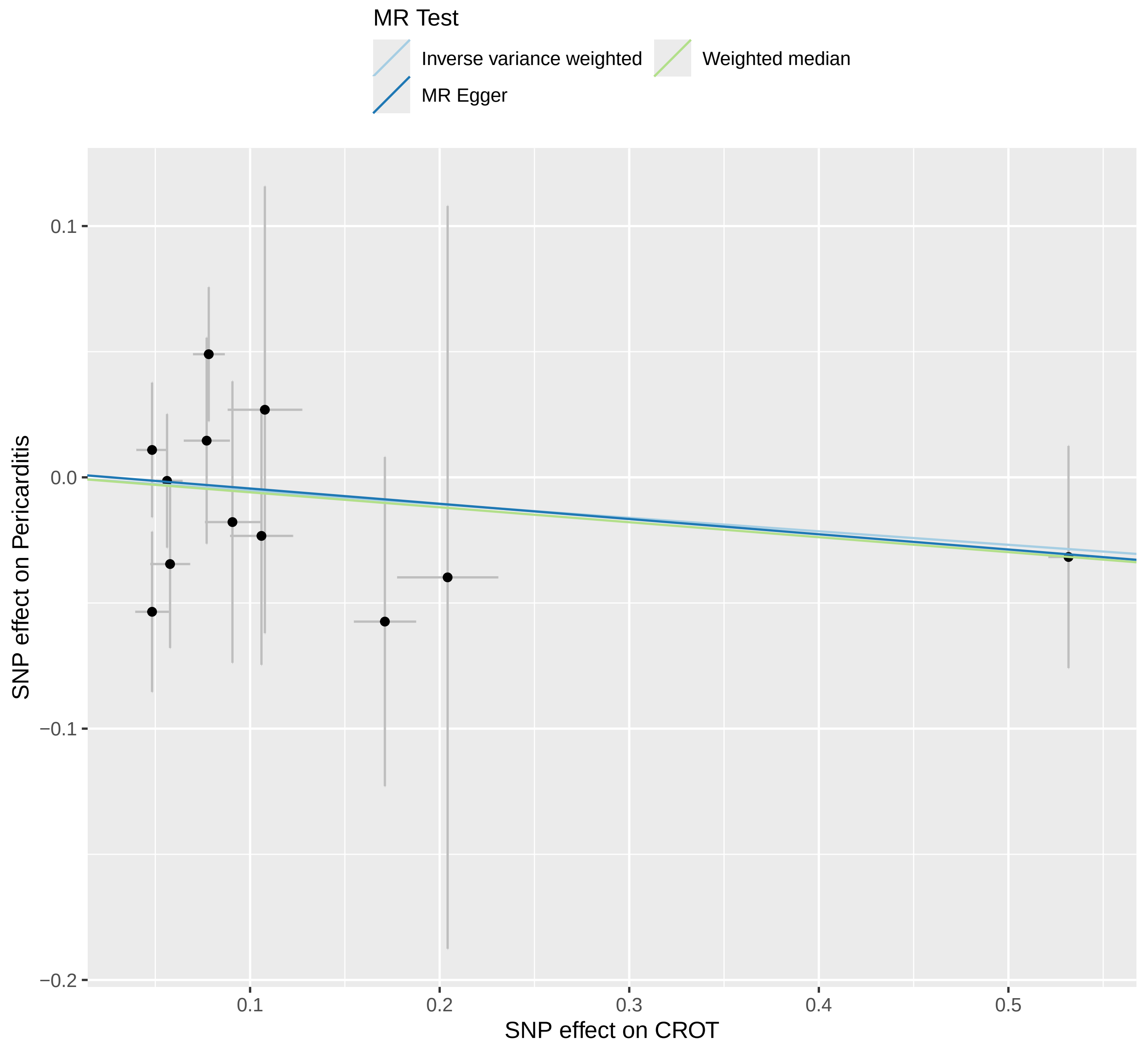
MR Test
Inverse variance weighted
Weighted median
MR Egger
SNP effect on Pericarditis
0.1
0.0
−0.1
−0.2
0.1
0.2
0.3
0.4
0.5
SNP effect on CROT

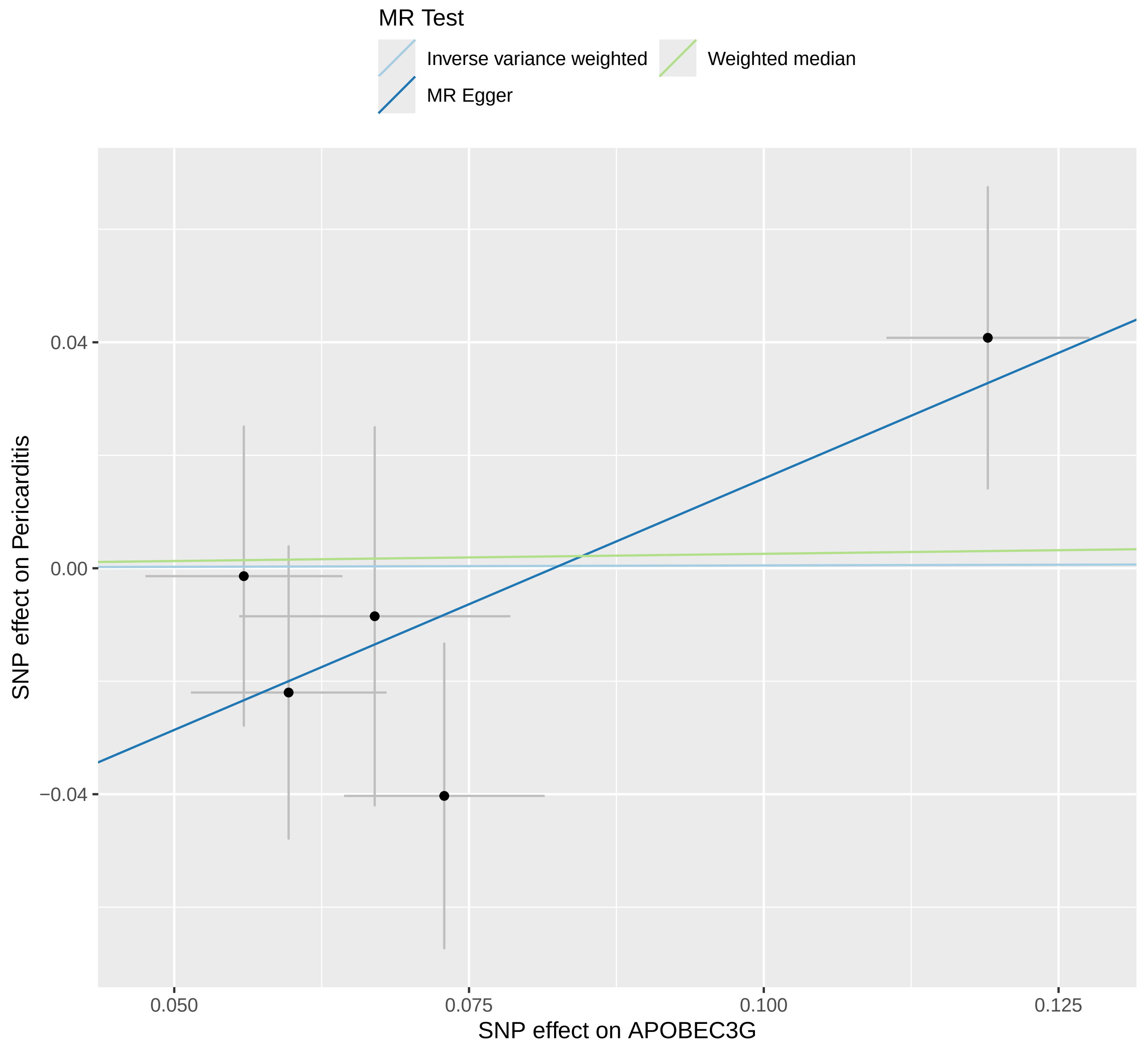
MR Test
Inverse variance weighted
Weighted median
MR Egger
0.04
0.00
−0.04
SNP effect on Pericarditis
0.050
0.075
0.100
0.125
SNP effect on APOBEC3G

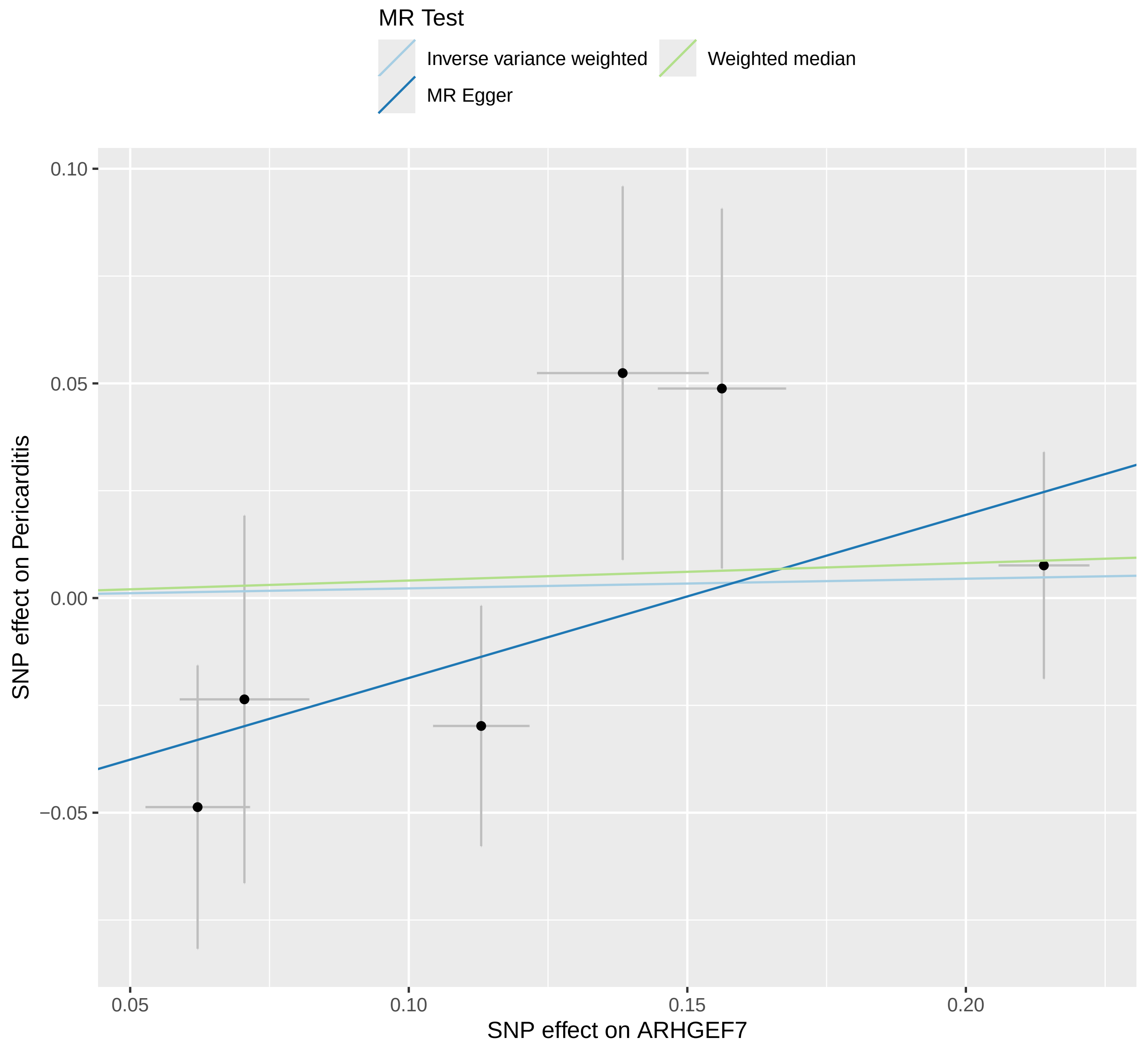
MR Test
Inverse variance weighted
Weighted median
MR Egger
0.10
0.05
0.00
−0.05
SNP effect on Pericarditis
0.05
0.10
0.15
0.20
SNP effect on ARHGEF7

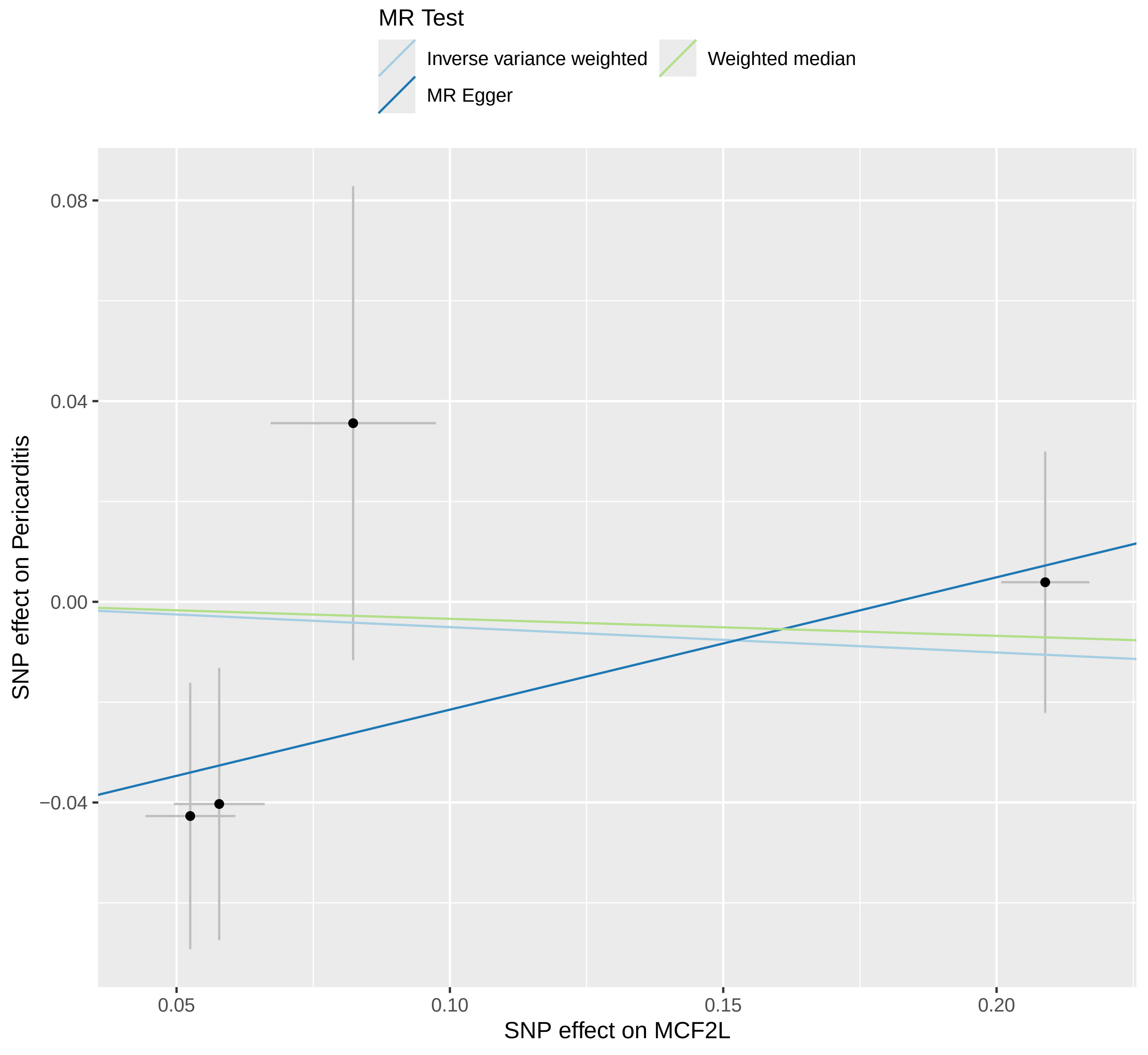
MR Test
Inverse variance weighted
MR Egger
Weighted median
0.08
0.04
0.00
−0.04
SNP effect on Pericarditis
0.05
0.10
0.15
0.20
SNP effect on MCF2L

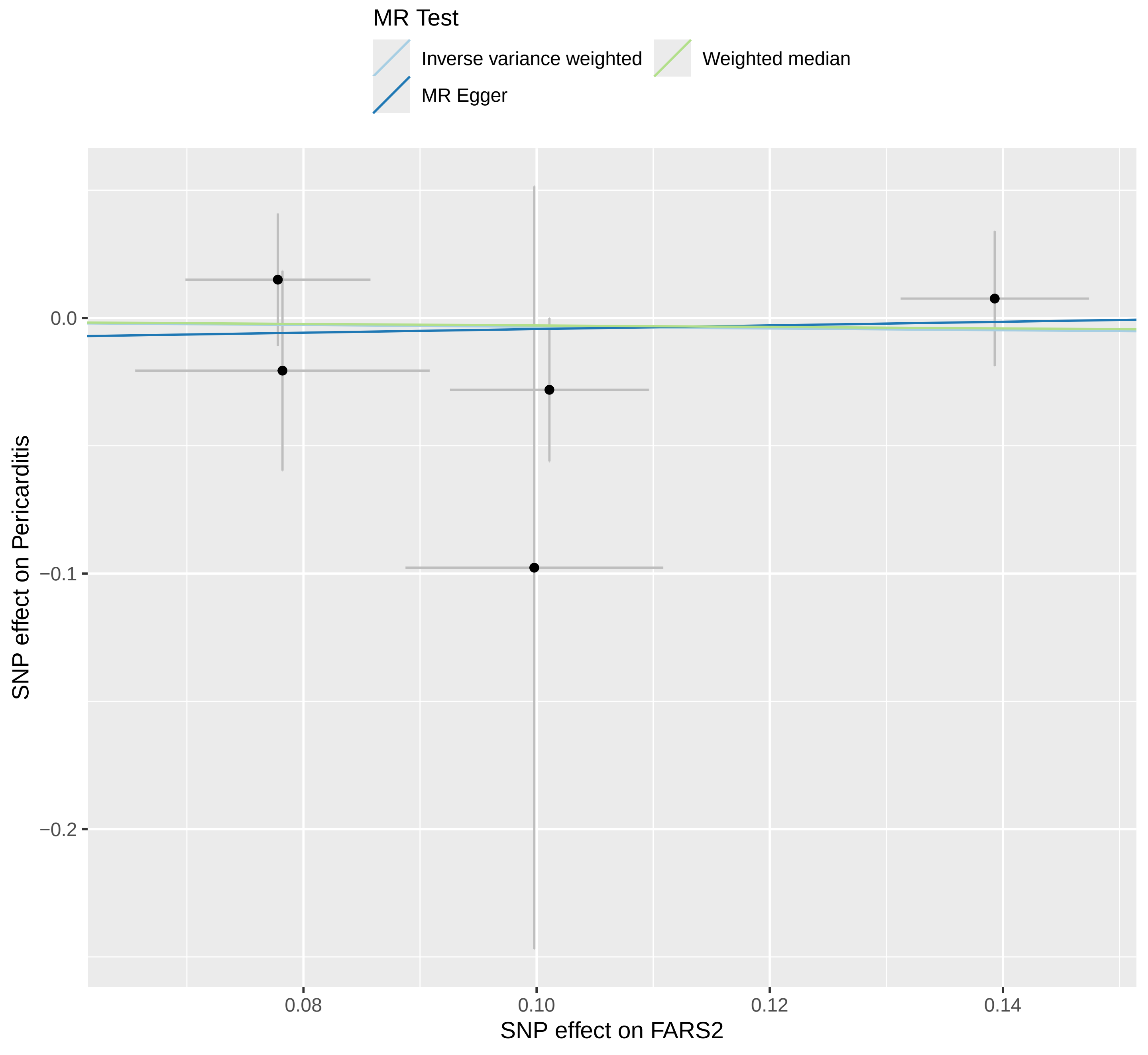
MR Test
Inverse variance weighted
Weighted median
MR Egger
SNP effect on Pericarditis
0.0
−0.1
−0.2
0.08
0.10
0.12
0.14
SNP effect on FARS2

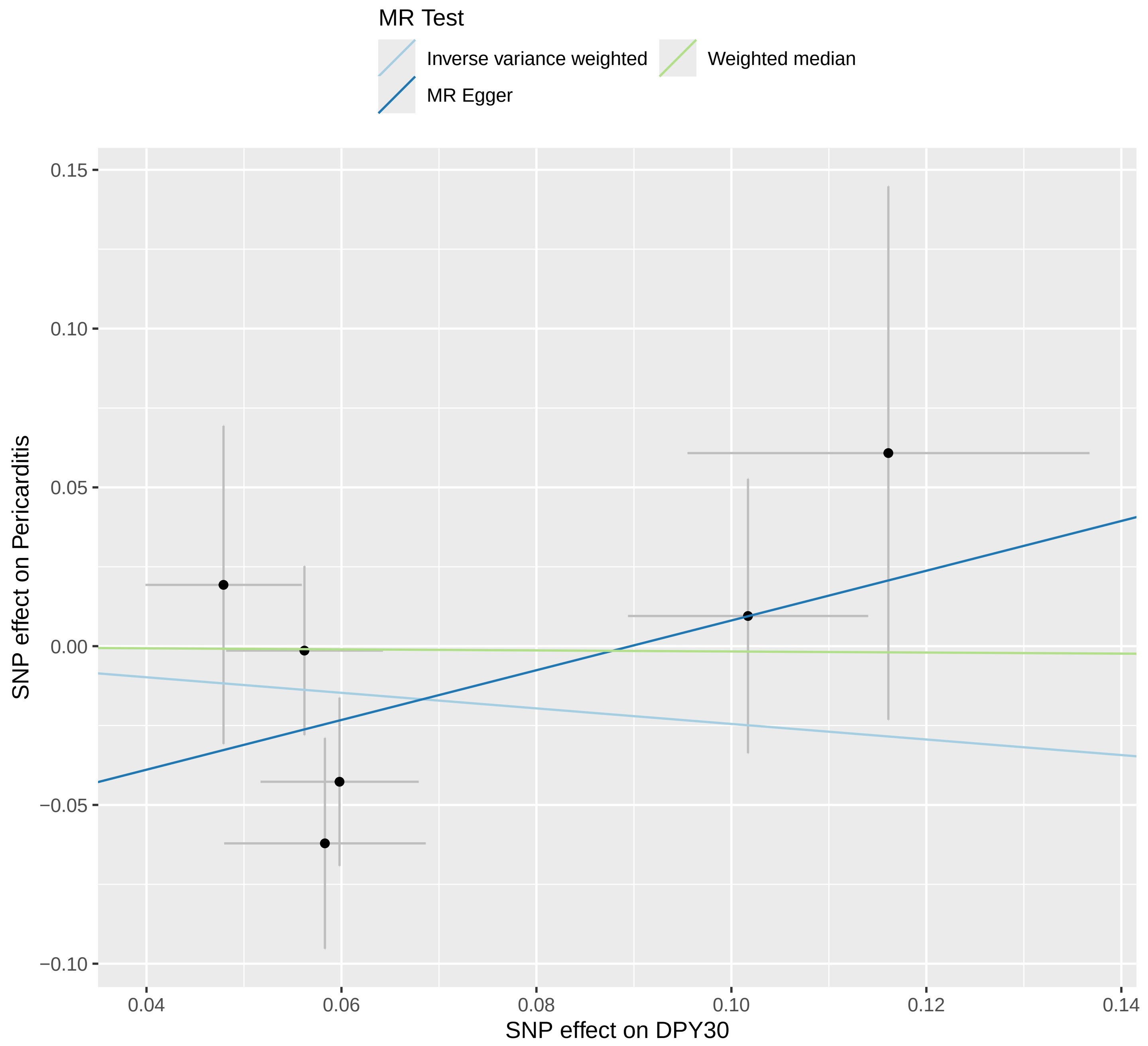

MR Test
Inverse variance weighted
Weighted median
MR Egger
0.15
0.10
0.05
0.00
−0.05
−0.10
SNP effect on Pericarditis
0.04
0.06
0.08
0.10
0.12
0.14
SNP effect on DPY30

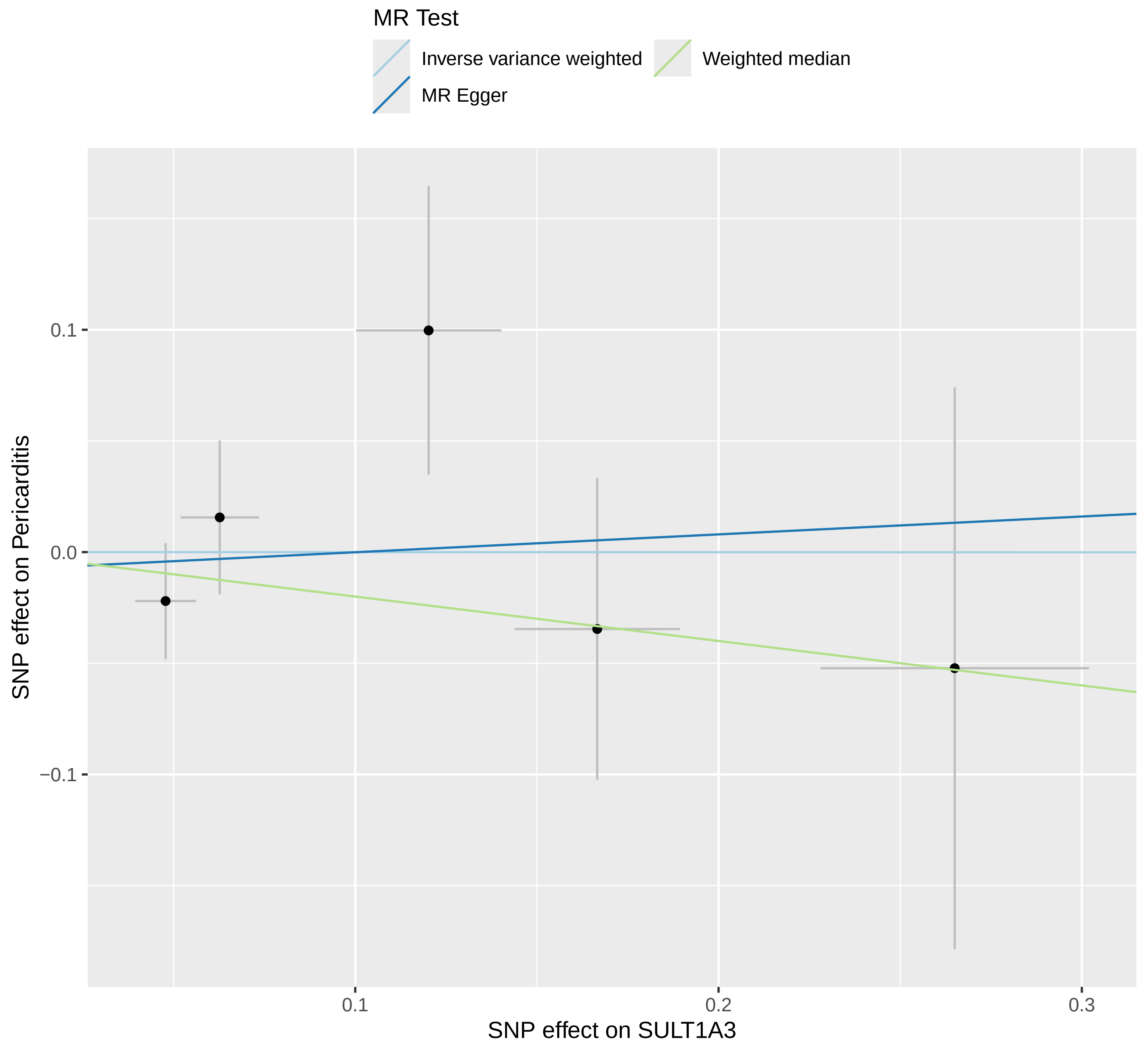
MR Test
Inverse variance weighted
Weighted median
MR Egger
0.1
0.0
−0.1
SNP effect on Pericarditis
0.1
0.2
0.3
SNP effect on SULT1A3

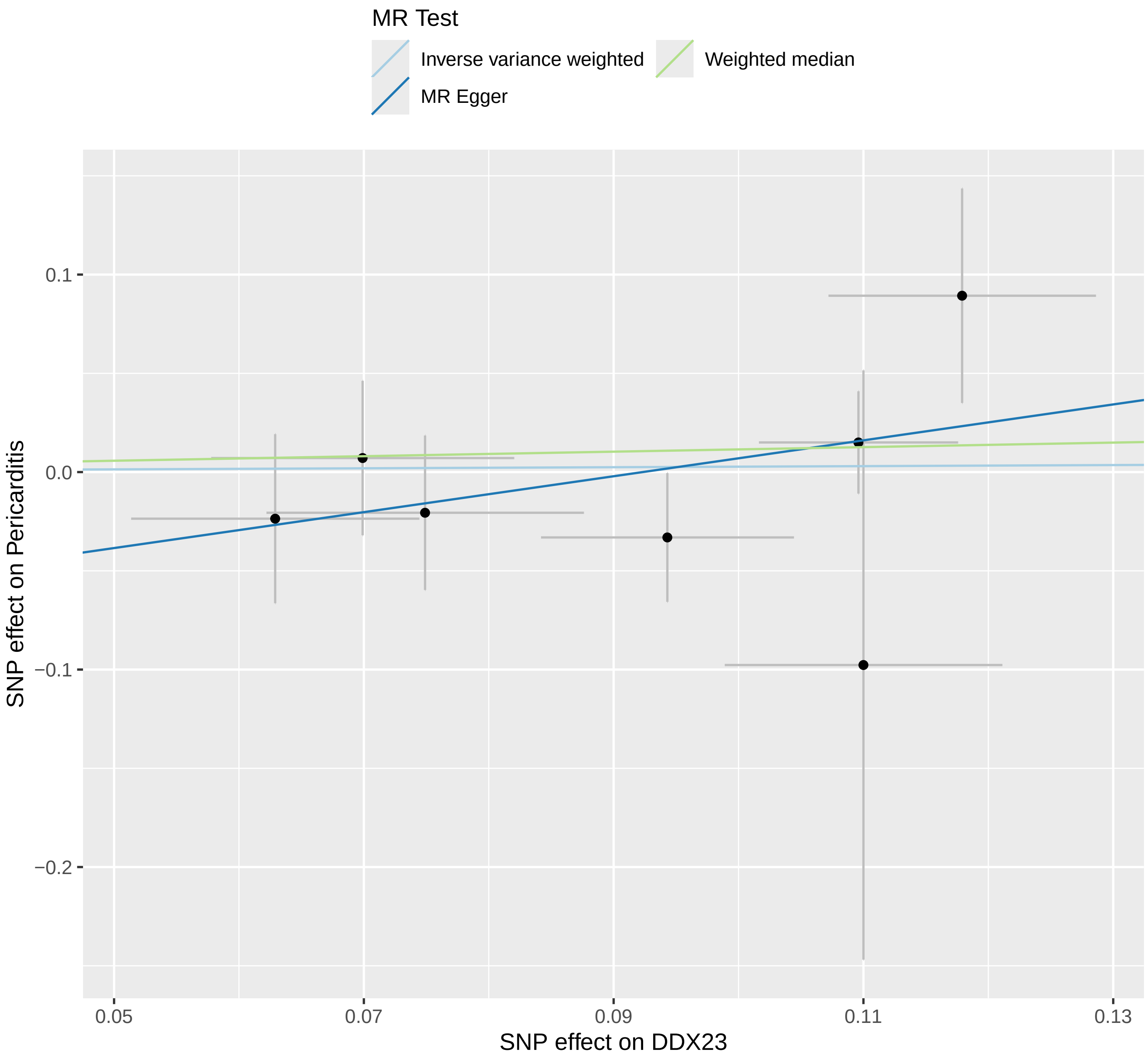

MR Test
Inverse variance weighted
Weighted median
MR Egger
SNP effect on Pericarditis
0.1
0.0
−0.1
−0.2
0.05
0.07
0.09
0.11
0.13
SNP effect on DDX23

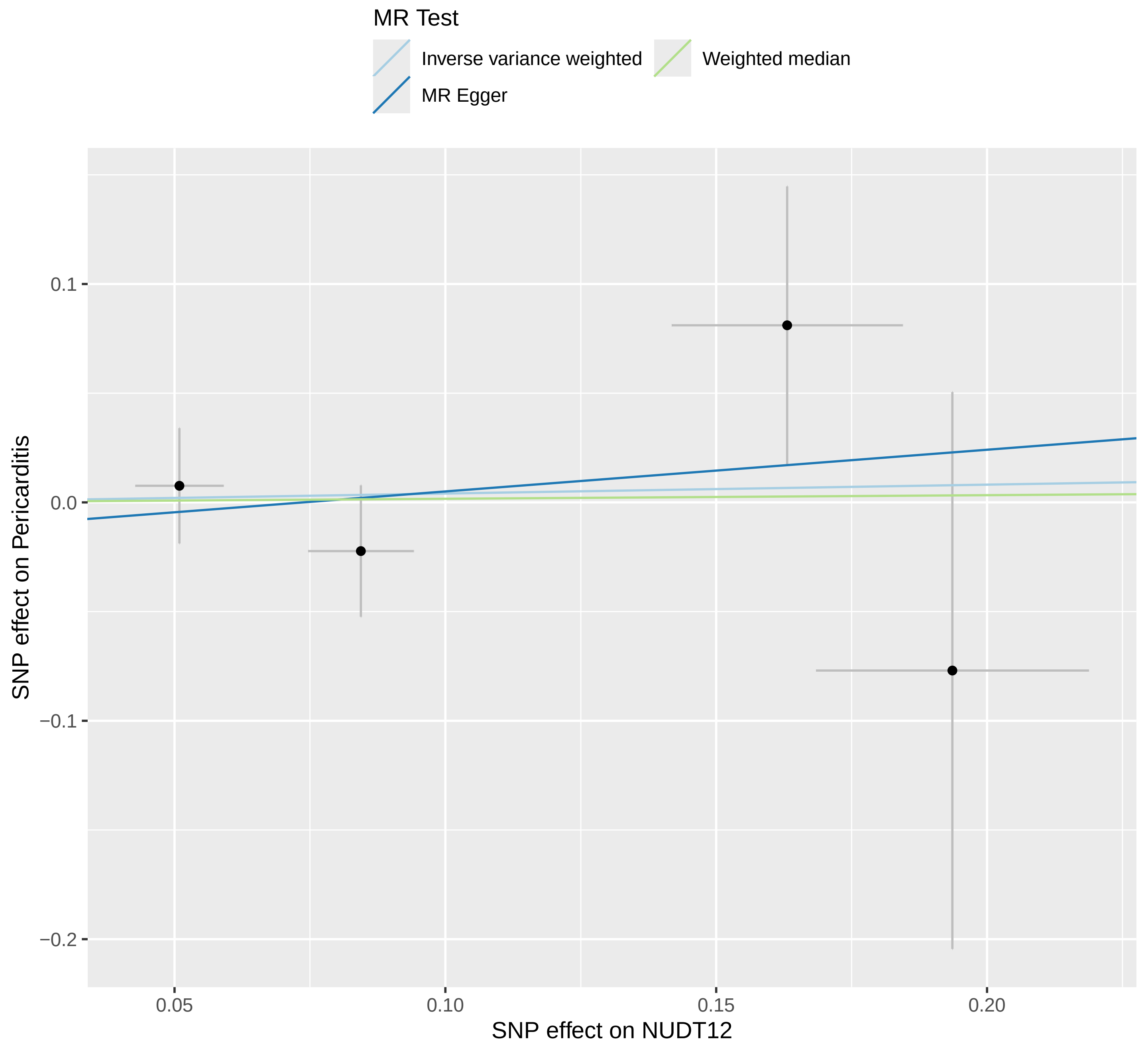

MR Test
Inverse variance weighted
Weighted median
MR Egger
0.1
0.0
−0.1
−0.2
SNP effect on Pericarditis
0.05
0.10
0.15
0.20
SNP effect on NUDT12

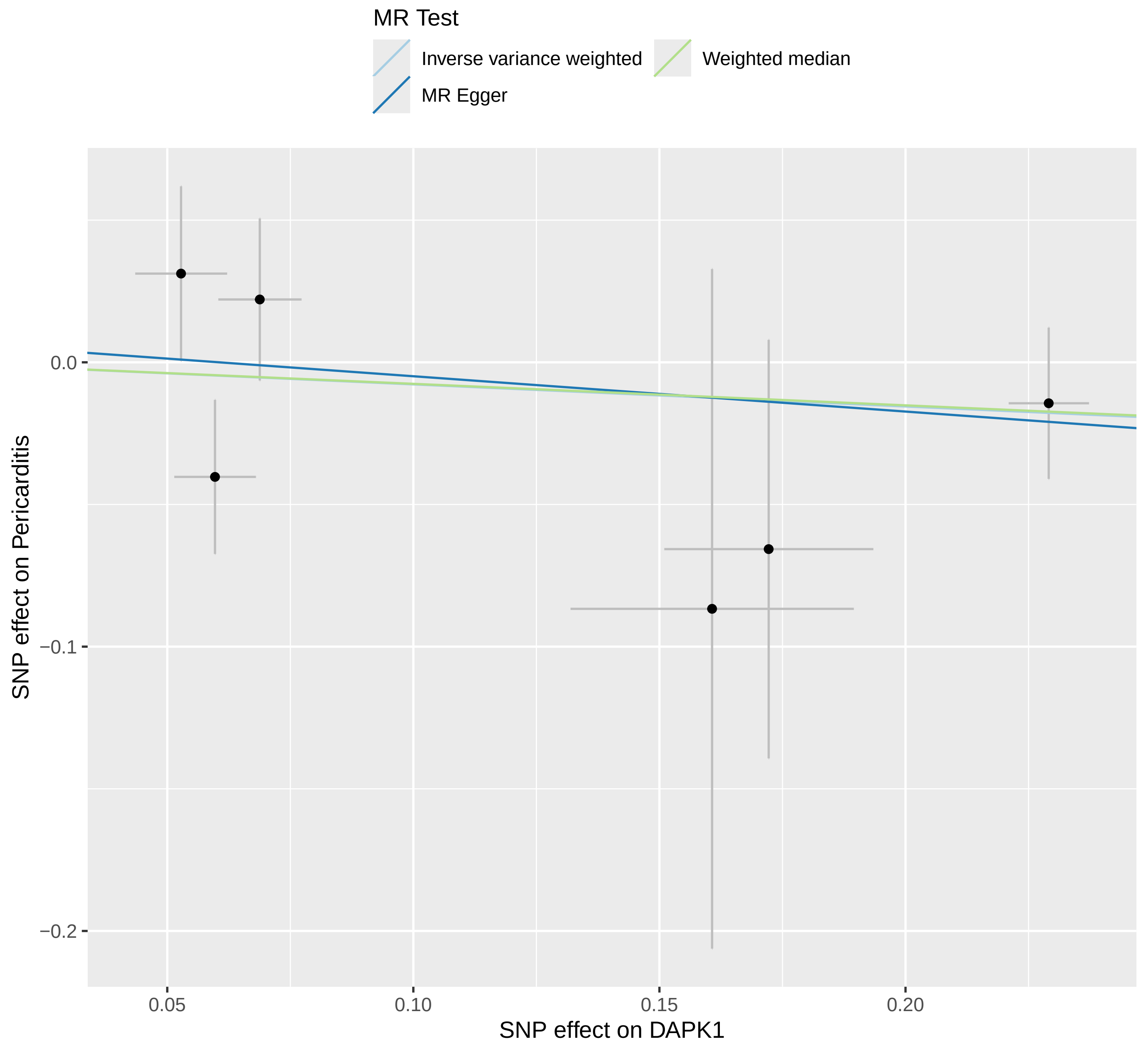
MR Test
Inverse variance weighted
Weighted median
MR Egger
SNP effect on Pericarditis
0.0
−0.1
−0.2
0.05
0.10
0.15
0.20
SNP effect on DAPK1

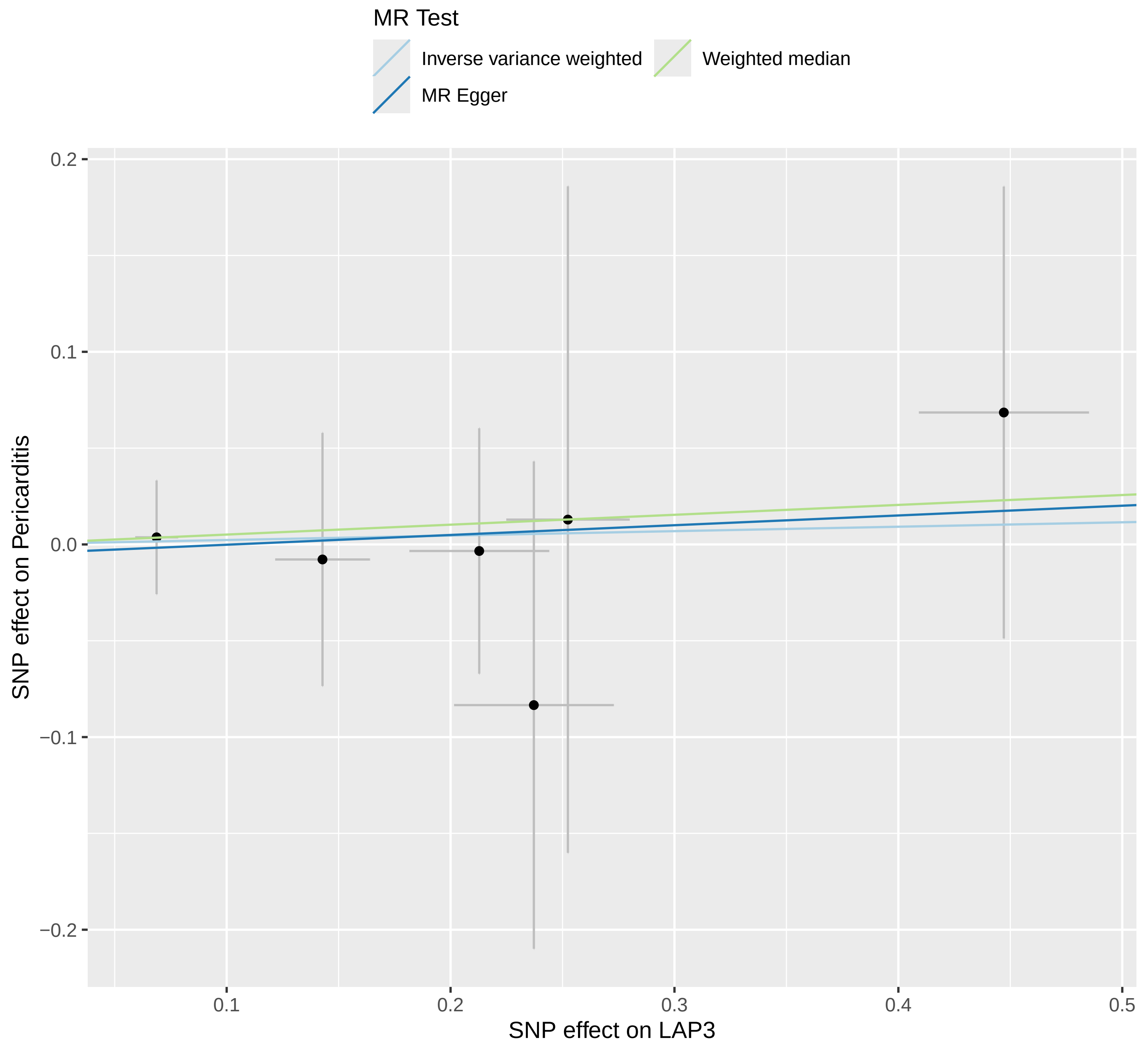
MR Test
Inverse variance weighted
Weighted median
MR Egger
SNP effect on Pericarditis
0.2
0.1
0.0
−0.1
−0.2
0.1
0.2
0.3
0.4
0.5
SNP effect on LAP3

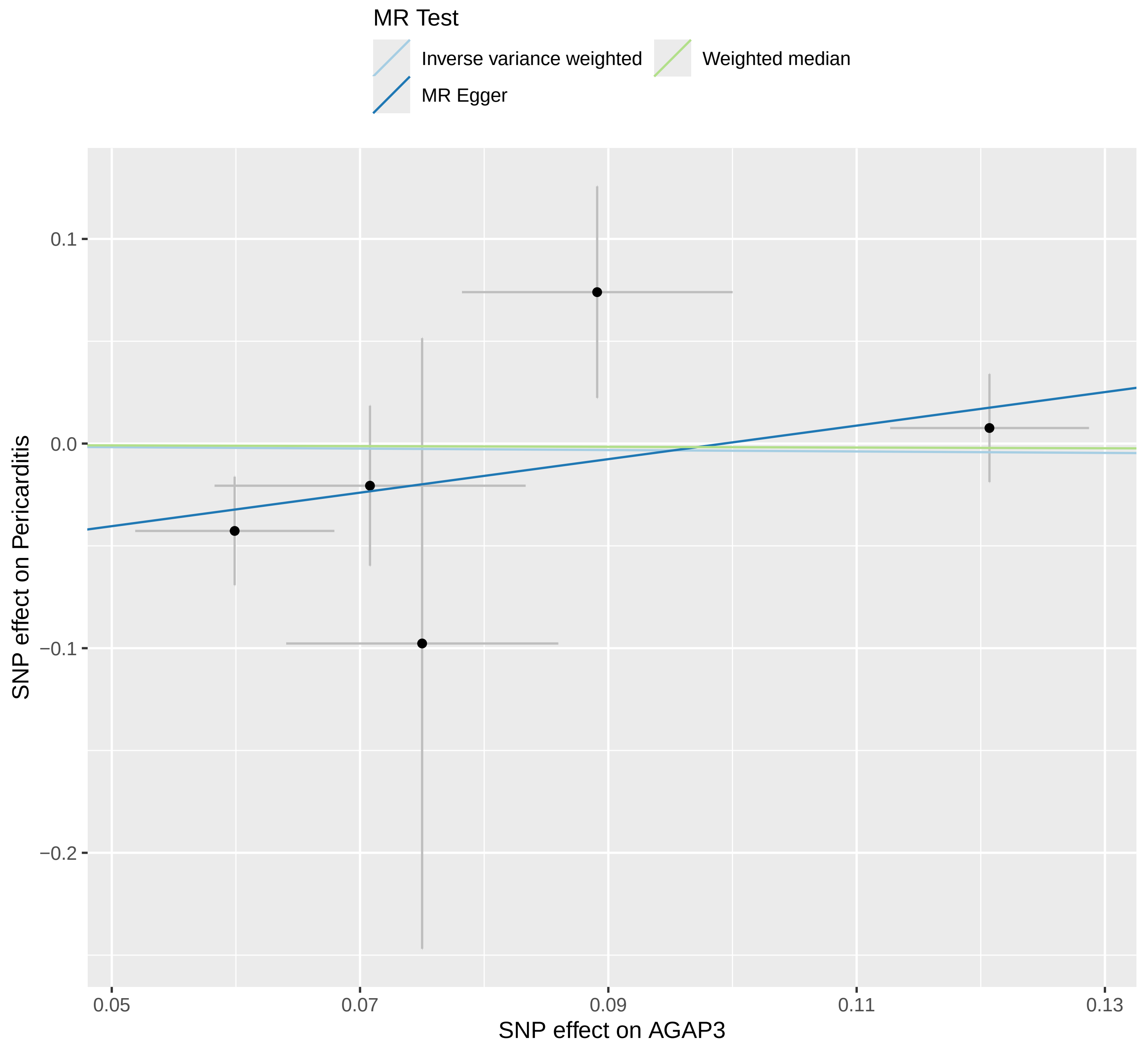
MR Test
Inverse variance weighted
Weighted median
MR Egger
0.1
0.0
−0.1
−0.2
0.05
0.07
0.09
0.11
0.13
SNP effect on Pericarditis
SNP effect on AGAP3

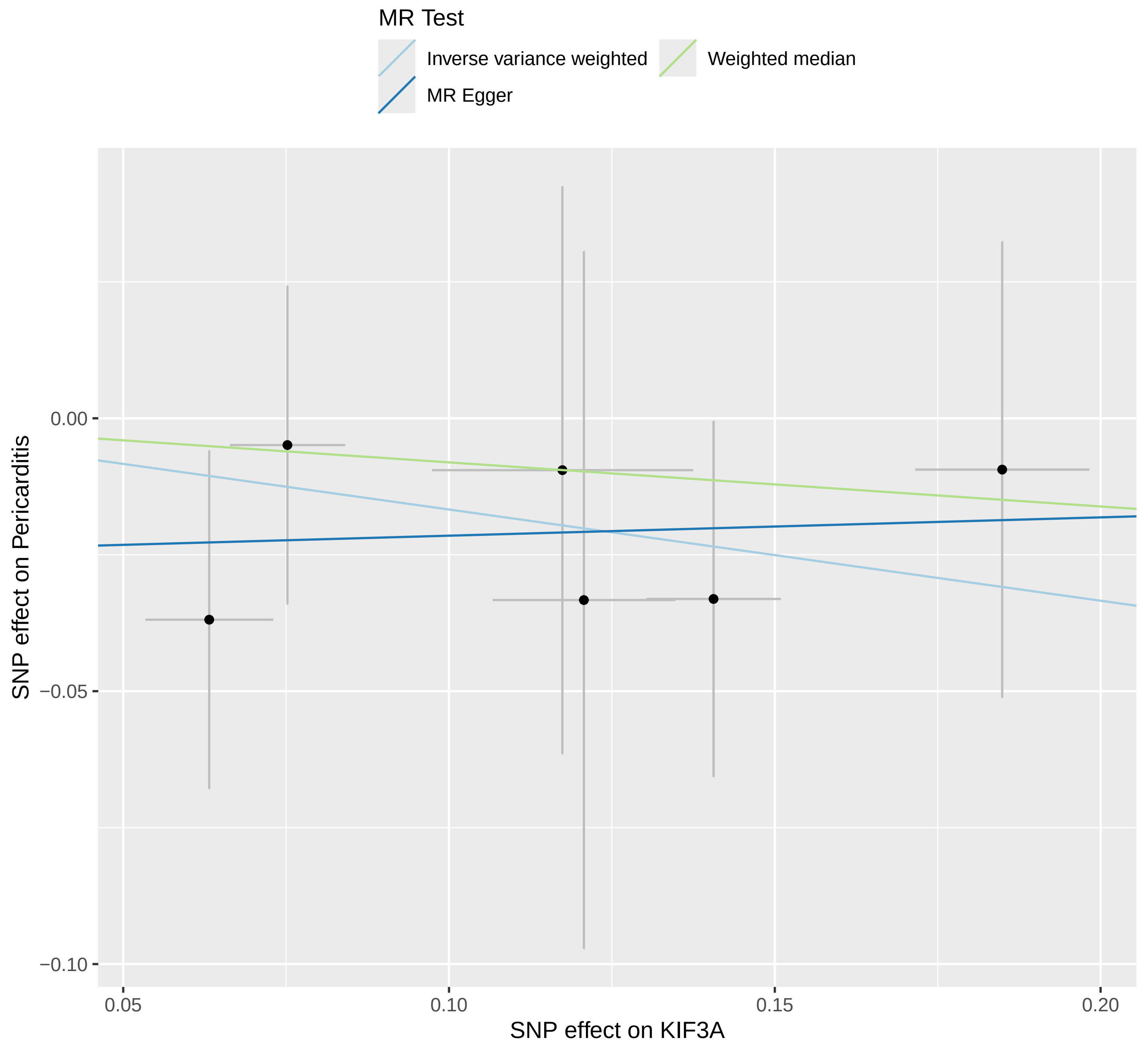
MR Test
Inverse variance weighted
Weighted median
MR Egger
SNP effect on Pericarditis
0.00
−0.05
−0.10
0.05
0.10
0.15
0.20
SNP effect on KIF3A

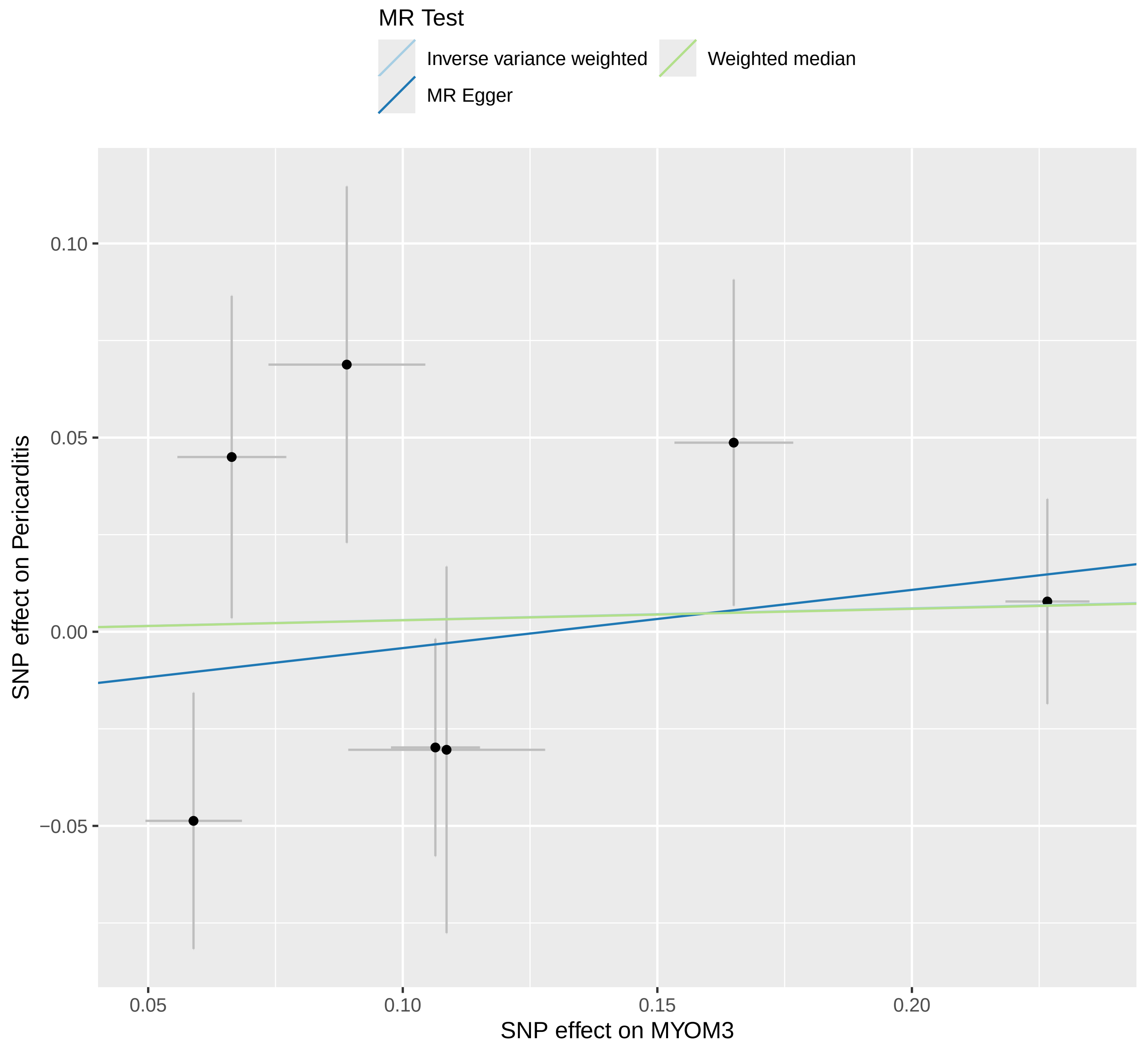
MR Test
Inverse variance weighted
Weighted median
MR Egger
SNP effect on Pericarditis
0.10
0.05
0.00
−0.05
0.05
0.10
0.15
0.20
SNP effect on MYOM3

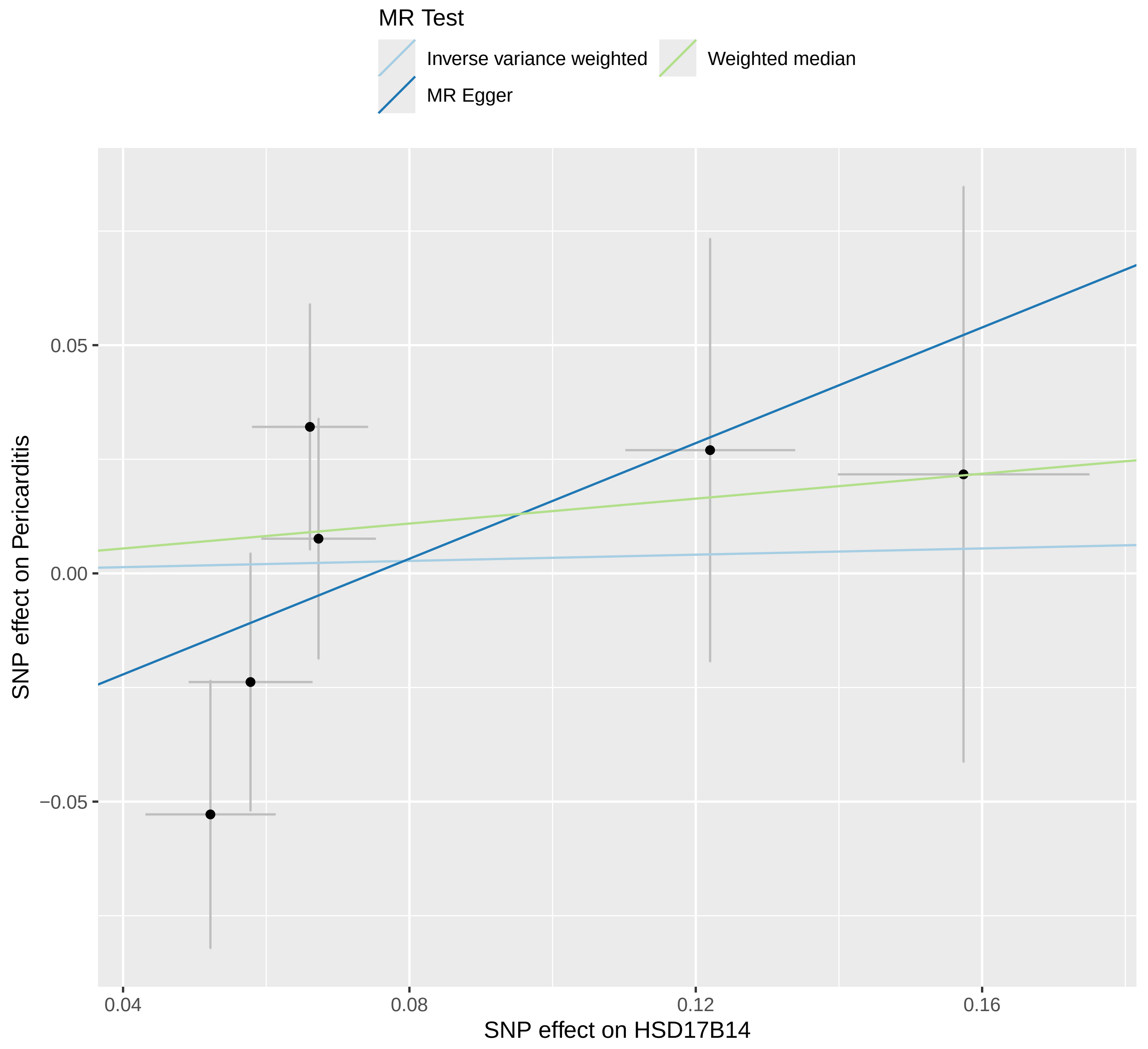
MR Test
Inverse variance weighted
Weighted median
MR Egger
0.05
0.00
−0.05
SNP effect on Pericarditis
0.04
0.08
0.12
0.16
SNP effect on HSD17B14

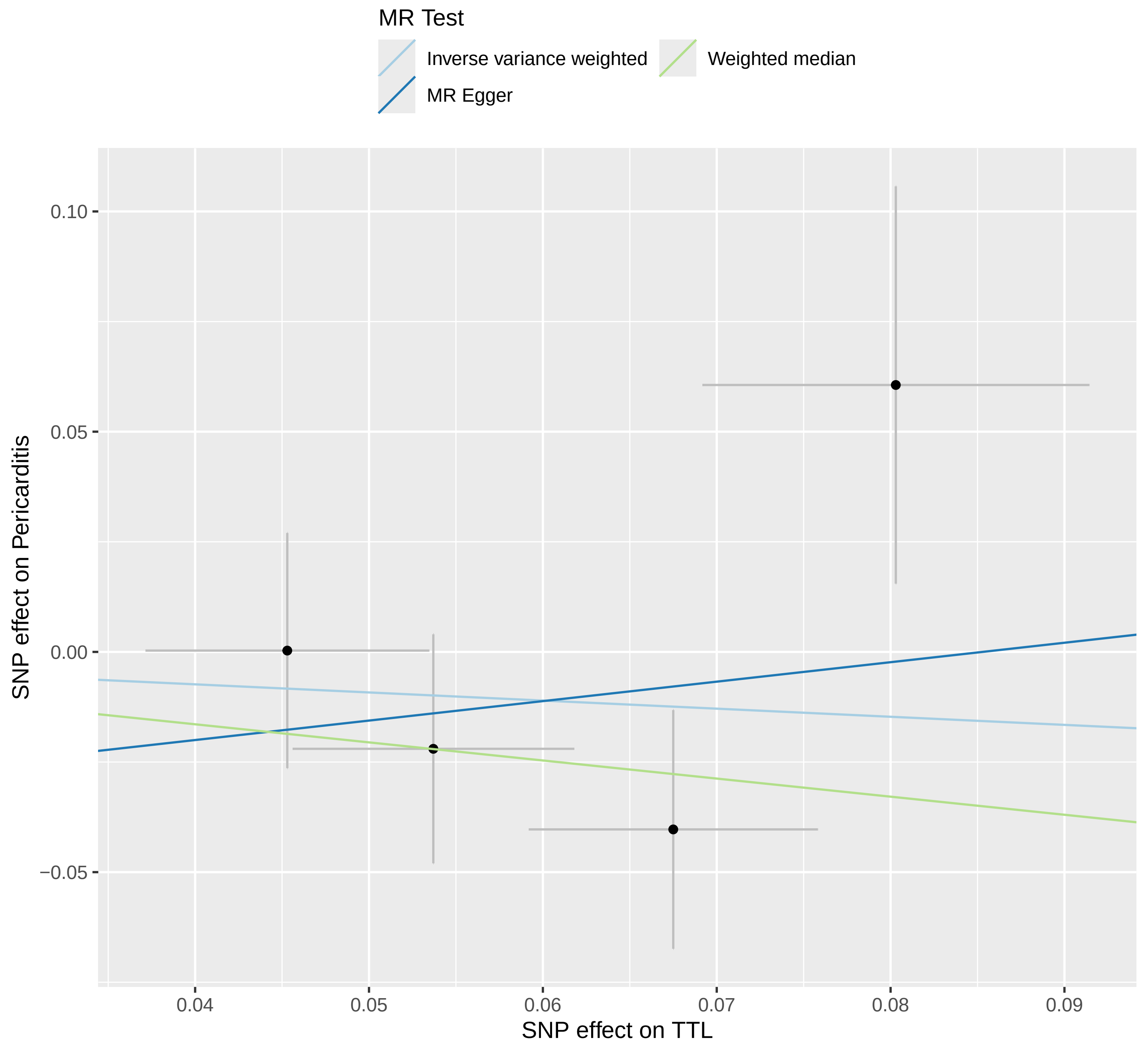
MR Test
Inverse variance weighted
MR Egger
Weighted median
0.10
0.05
0.00
−0.05
SNP effect on Pericarditis
0.04
0.05
0.06
0.07
0.08
0.09
SNP effect on TTL

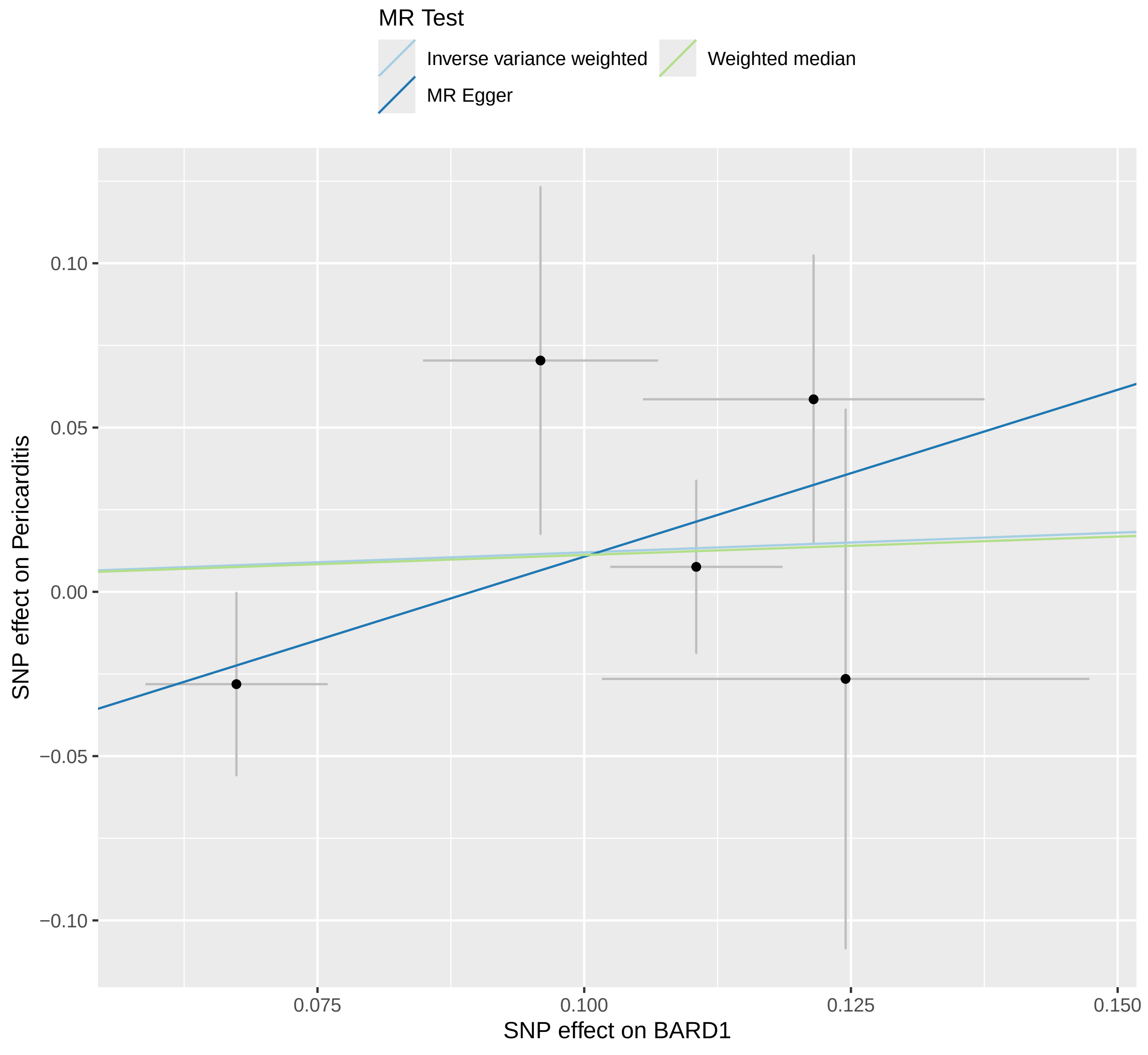

MR Test
Inverse variance weighted
Weighted median
MR Egger
SNP effect on Pericarditis
0.10
0.05
0.00
−0.05
−0.10
0.075
0.100
0.125
0.150
SNP effect on BARD1

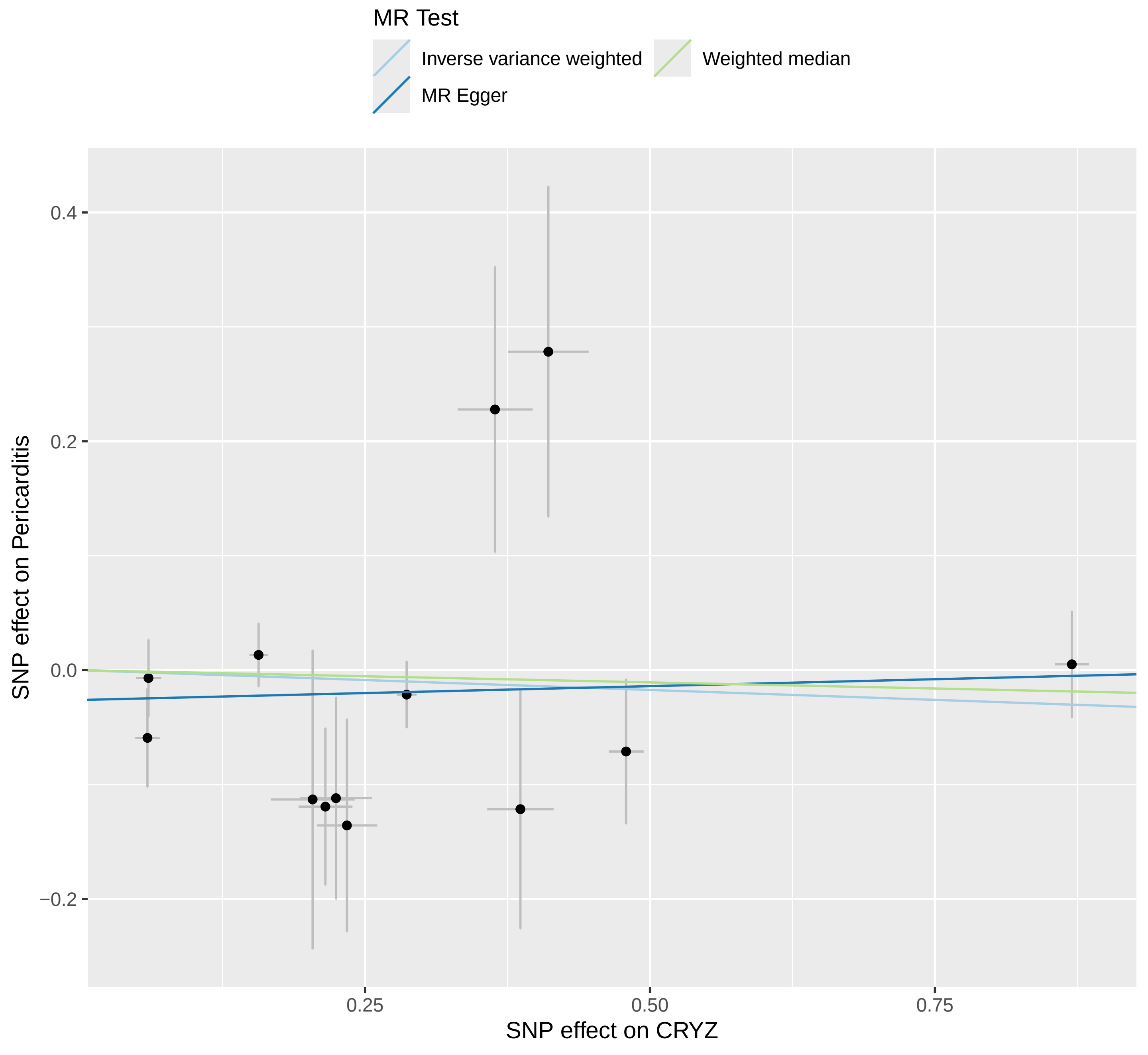
MR Test
Inverse variance weighted
Weighted median
MR Egger
SNP effect on Pericarditis
0.4
0.2
0.0
−0.2
0.25
0.50
0.75
SNP effect on CRYZ

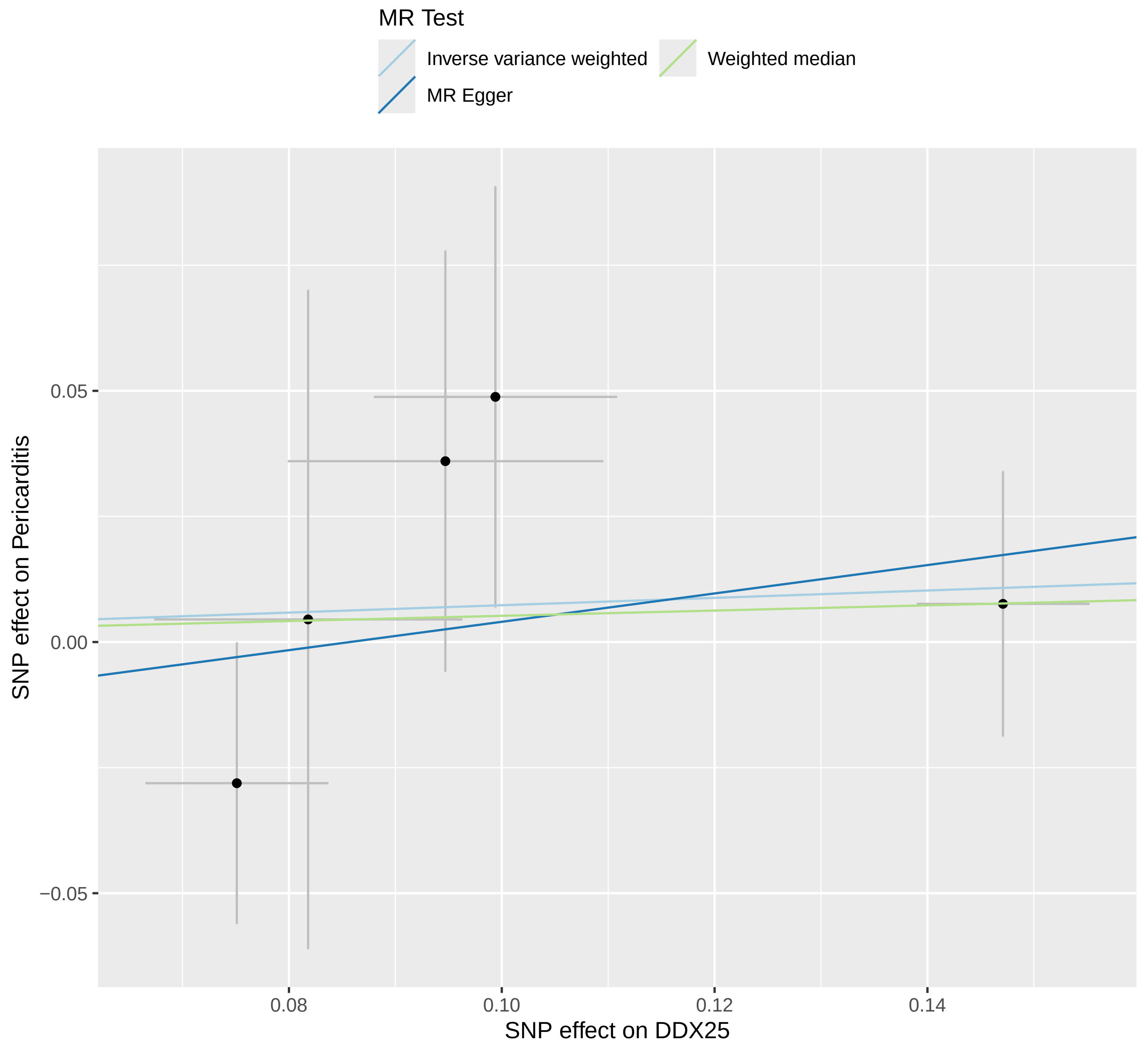
MR Test
Inverse variance weighted
MR Egger
Weighted median
0.05
0.00
−0.05
SNP effect on Pericarditis
0.08
0.10
0.12
0.14
SNP effect on DDX25

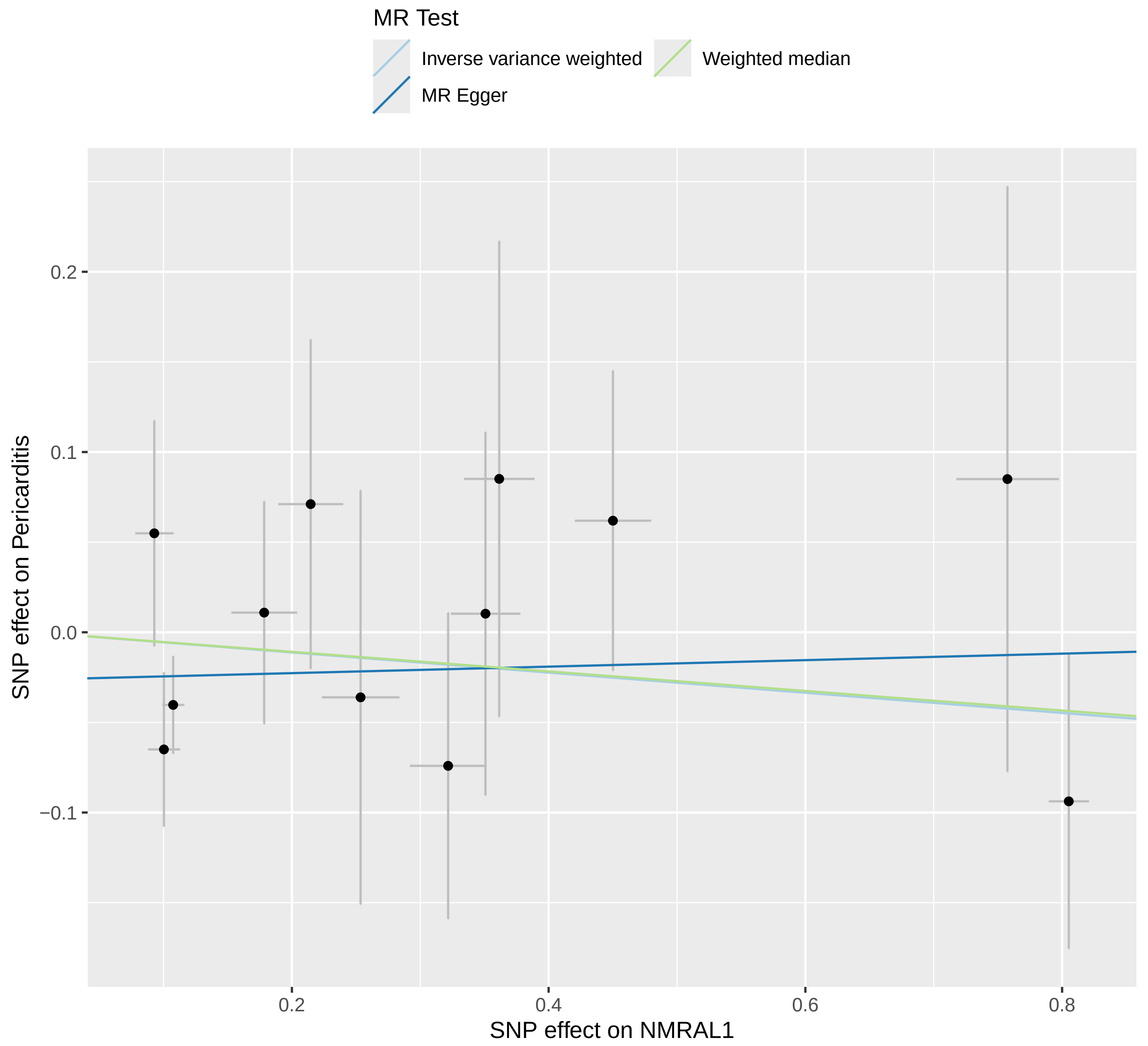
MR Test
Inverse variance weighted
Weighted median
MR Egger
SNP effect on Pericarditis
0.2
0.1
0.0
−0.1
0.2
0.4
0.6
0.8
SNP effect on NMRAL1

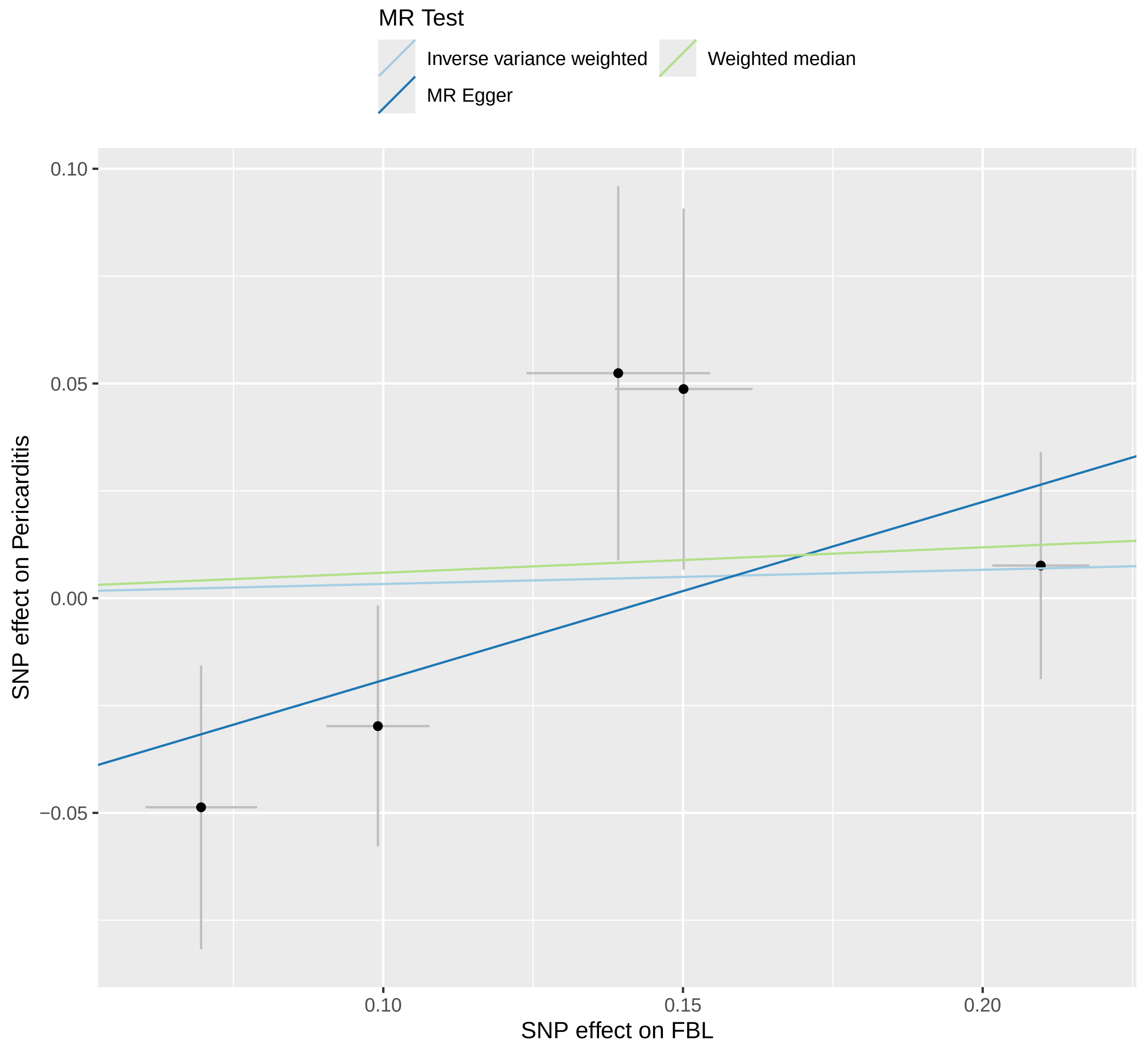
MR Test
Inverse variance weighted
MR Egger
Weighted median
0.10
0.05
0.00
−0.05
SNP effect on Pericarditis
0.10
0.15
0.20
SNP effect on FBL

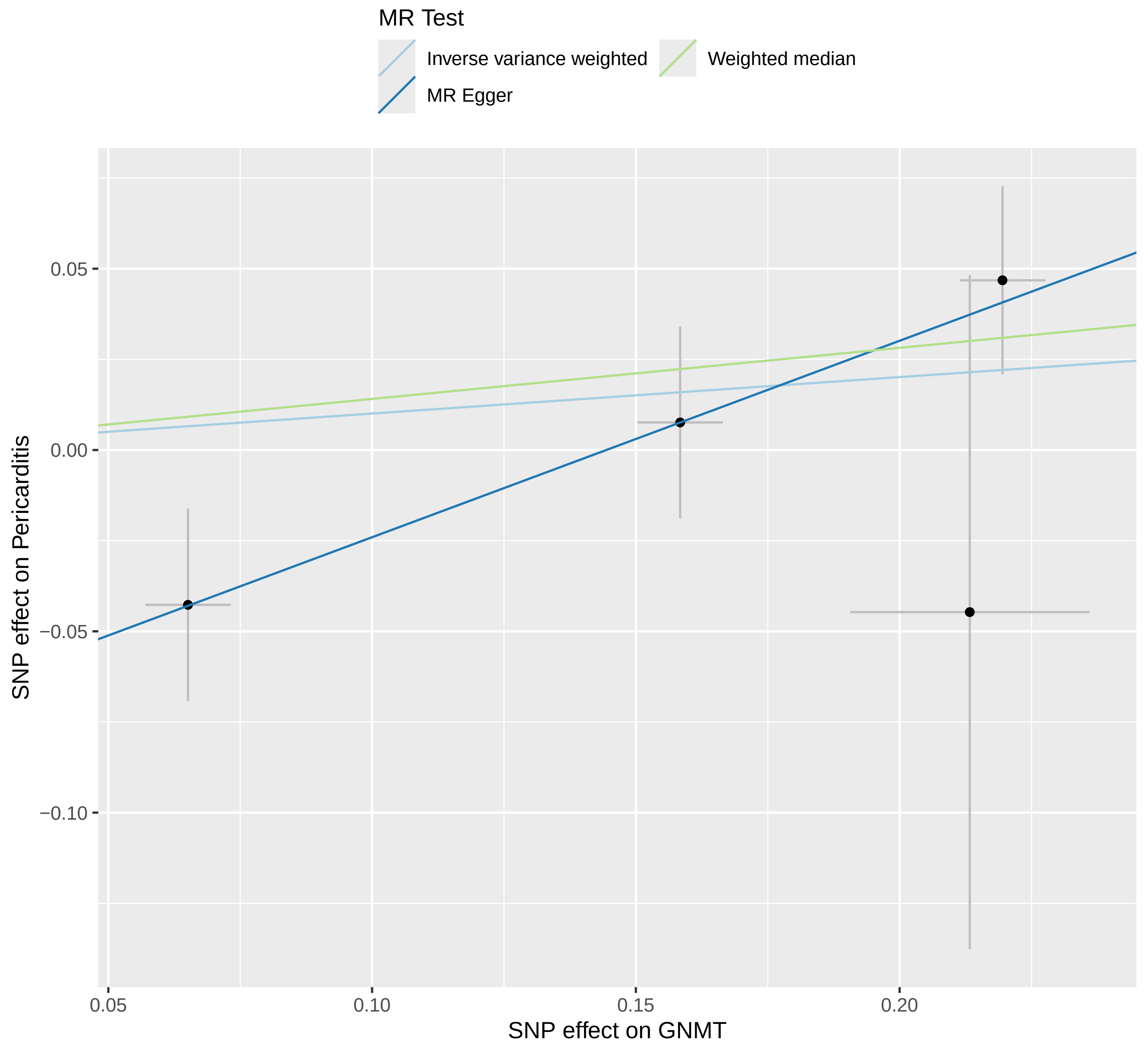

MR Test
Inverse variance weighted
Weighted median
MR Egger
SNP effect on Pericarditis
0.05
0.00
−0.05
−0.10
0.05
0.10
0.15
0.20
SNP effect on GNMT

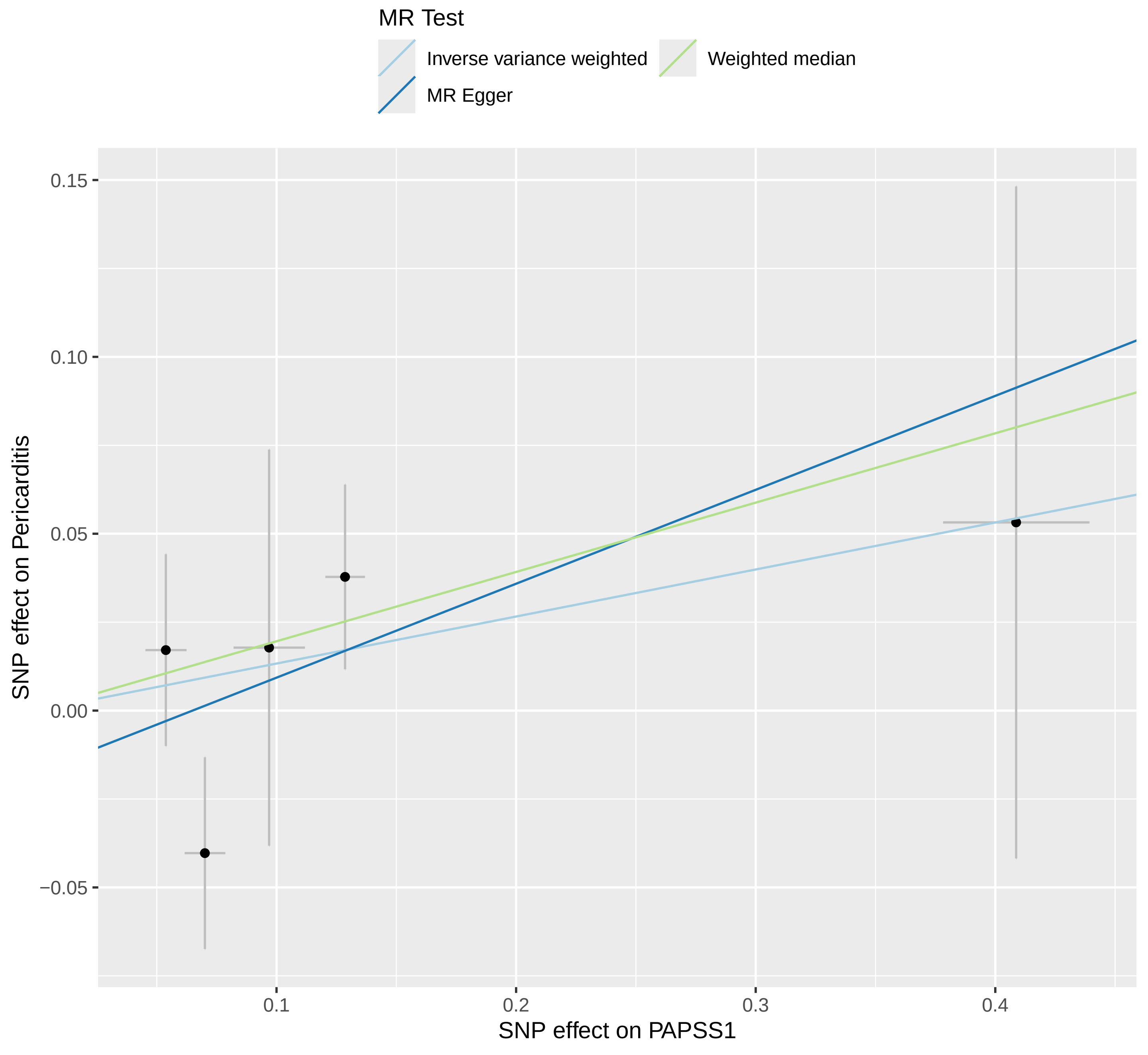
MR Test
Inverse variance weighted
MR Egger
Weighted median
0.15
0.10
0.05
0.00
−0.05
0.1
0.2
0.3
0.4
SNP effect on Pericarditis
SNP effect on PAPSS1

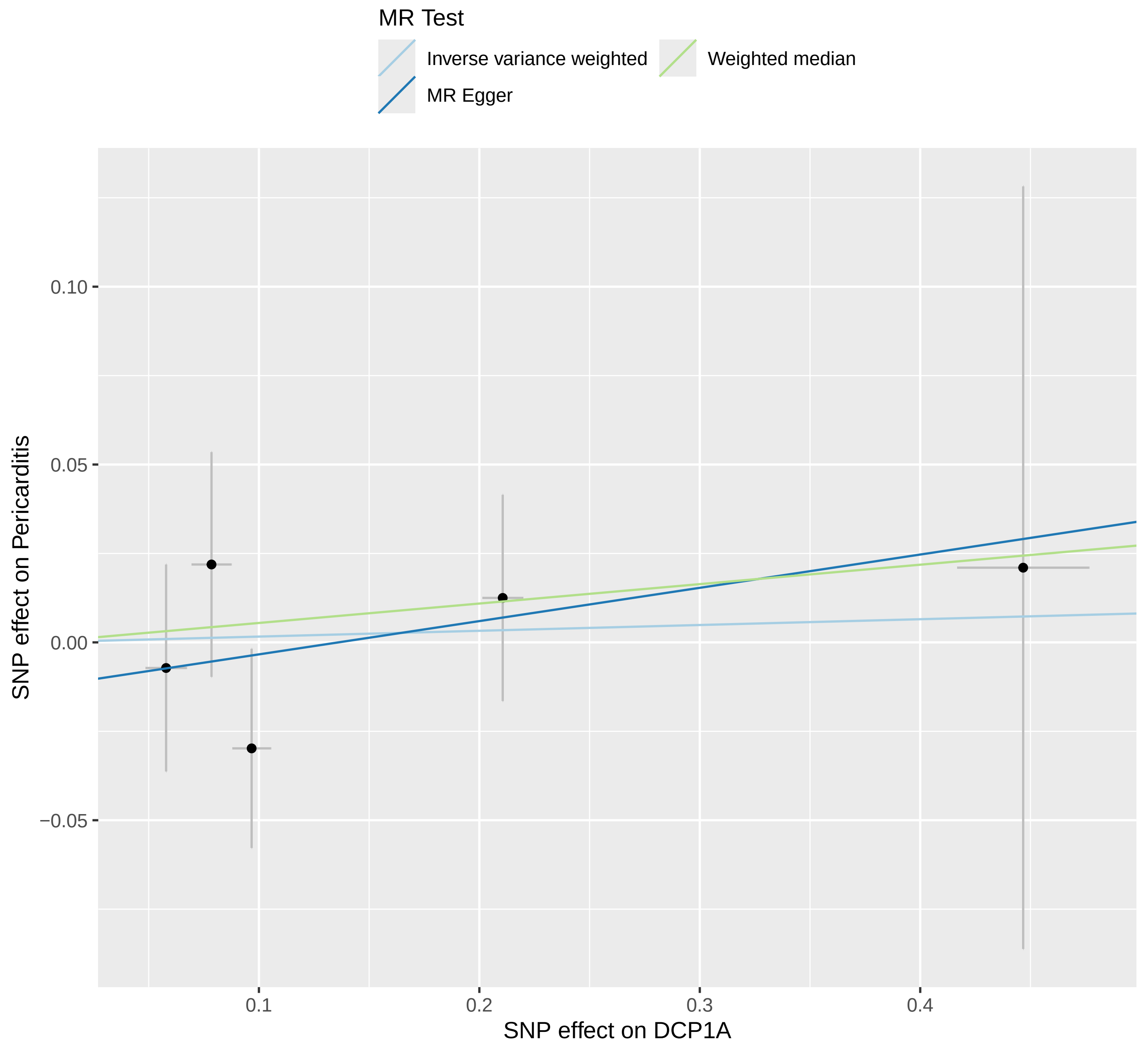

MR Test
Inverse variance weighted
Weighted median
MR Egger
0.10
0.05
0.00
−0.05
SNP effect on Pericarditis
0.1
0.2
0.3
0.4
SNP effect on DCP1A

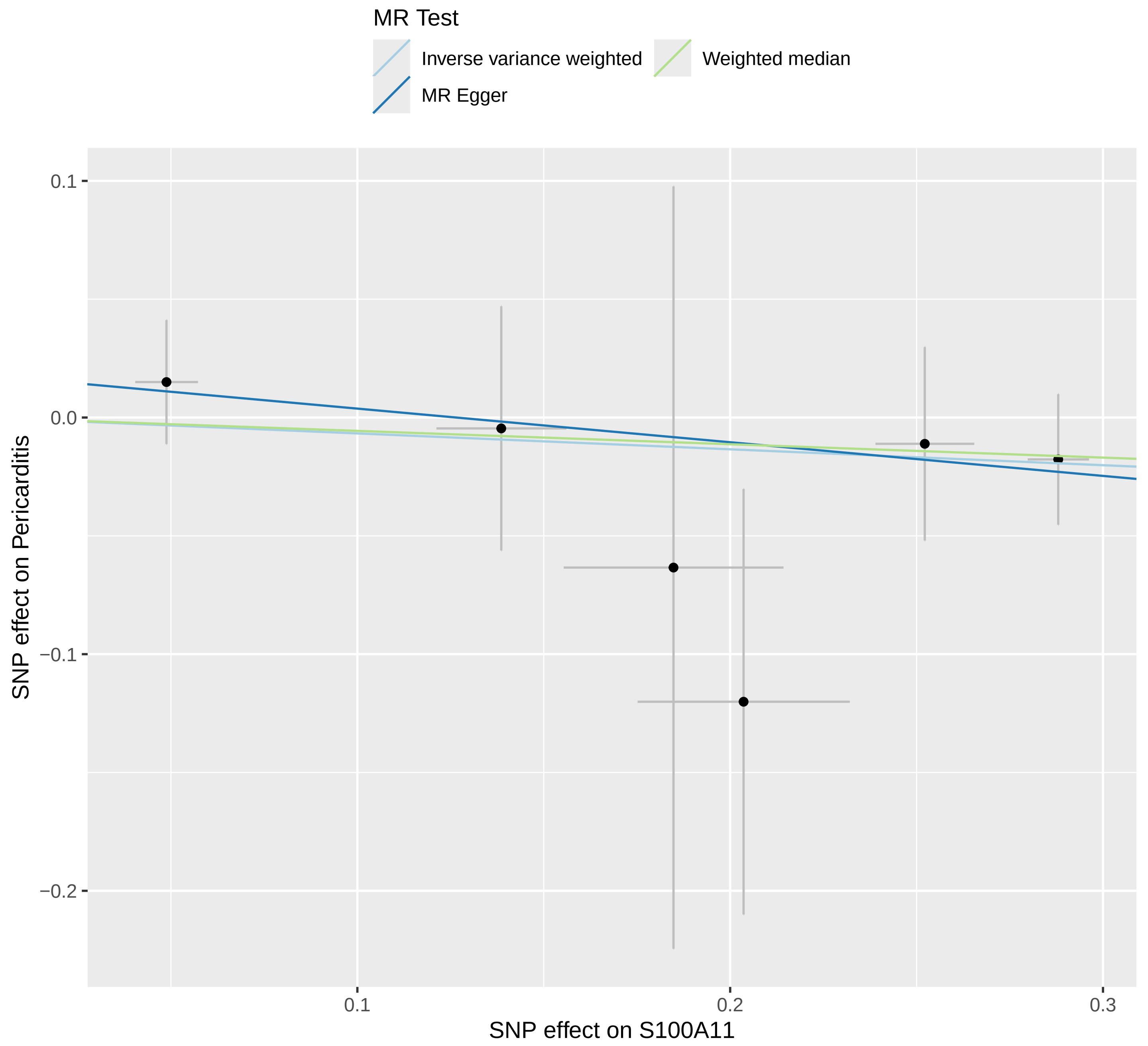
MR Test
Inverse variance weighted
Weighted median
MR Egger
SNP effect on Pericarditis
0.1
0.0
−0.1
−0.2
SNP effect on S100A11
0.1
0.2
0.3

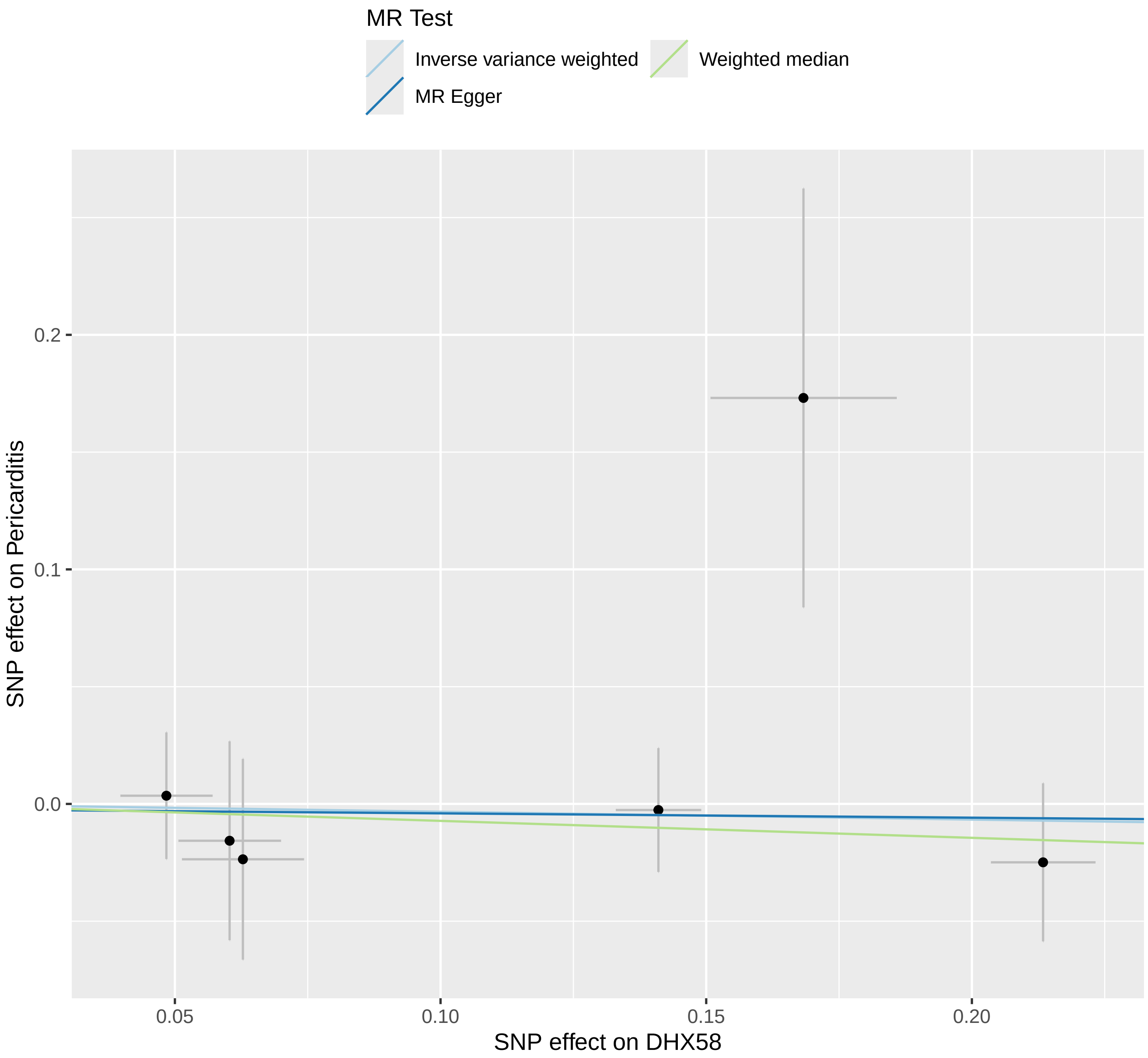
MR Test
Inverse variance weighted
Weighted median
MR Egger
SNP effect on Pericarditis
0.2
0.1
0.0
0.05
0.10
0.15
0.20
SNP effect on DHX58

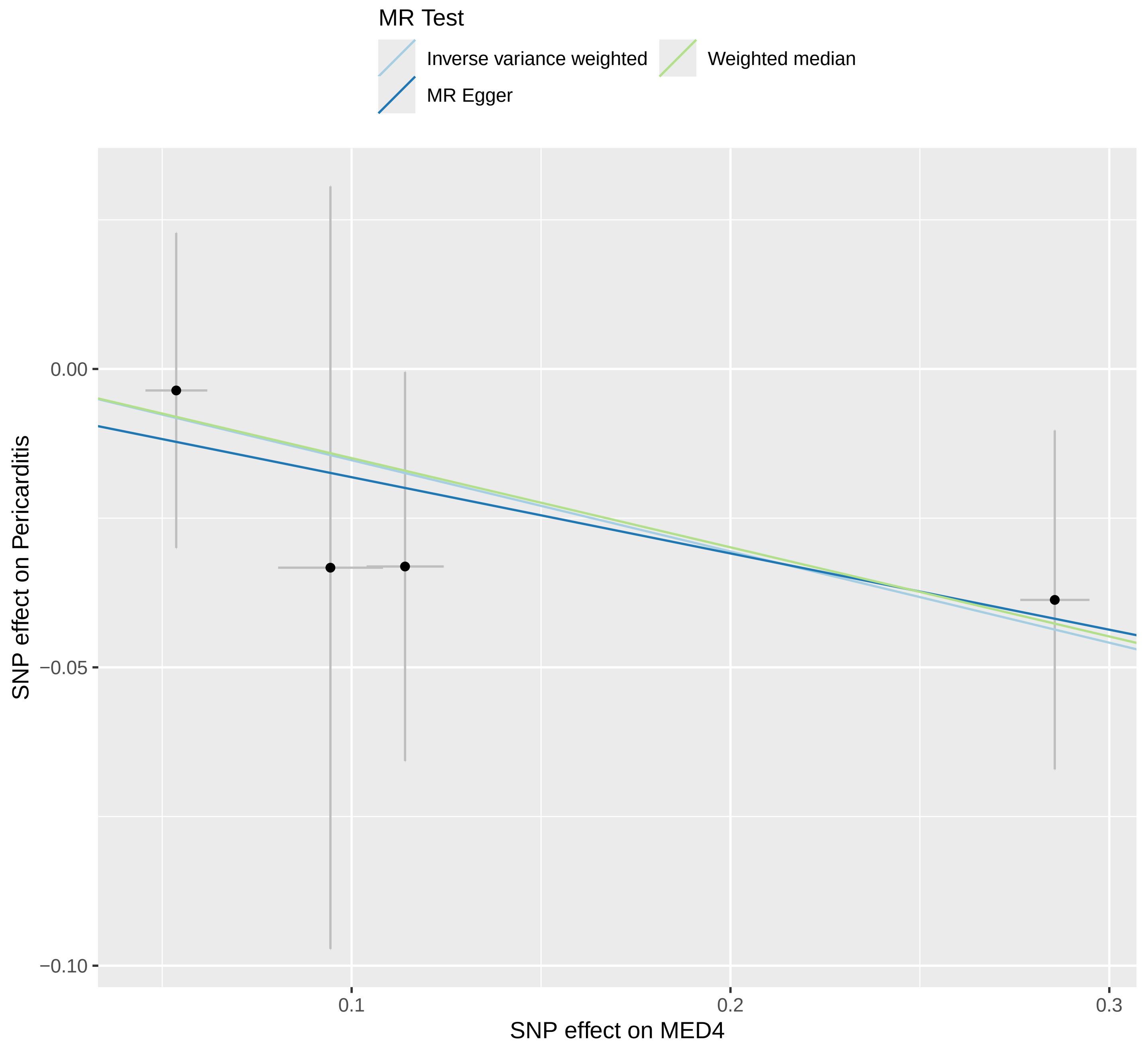
MR Test
Inverse variance weighted
MR Egger
Weighted median
0.00
−0.05
−0.10
SNP effect on Pericarditis
0.1
0.2
0.3
SNP effect on MED4

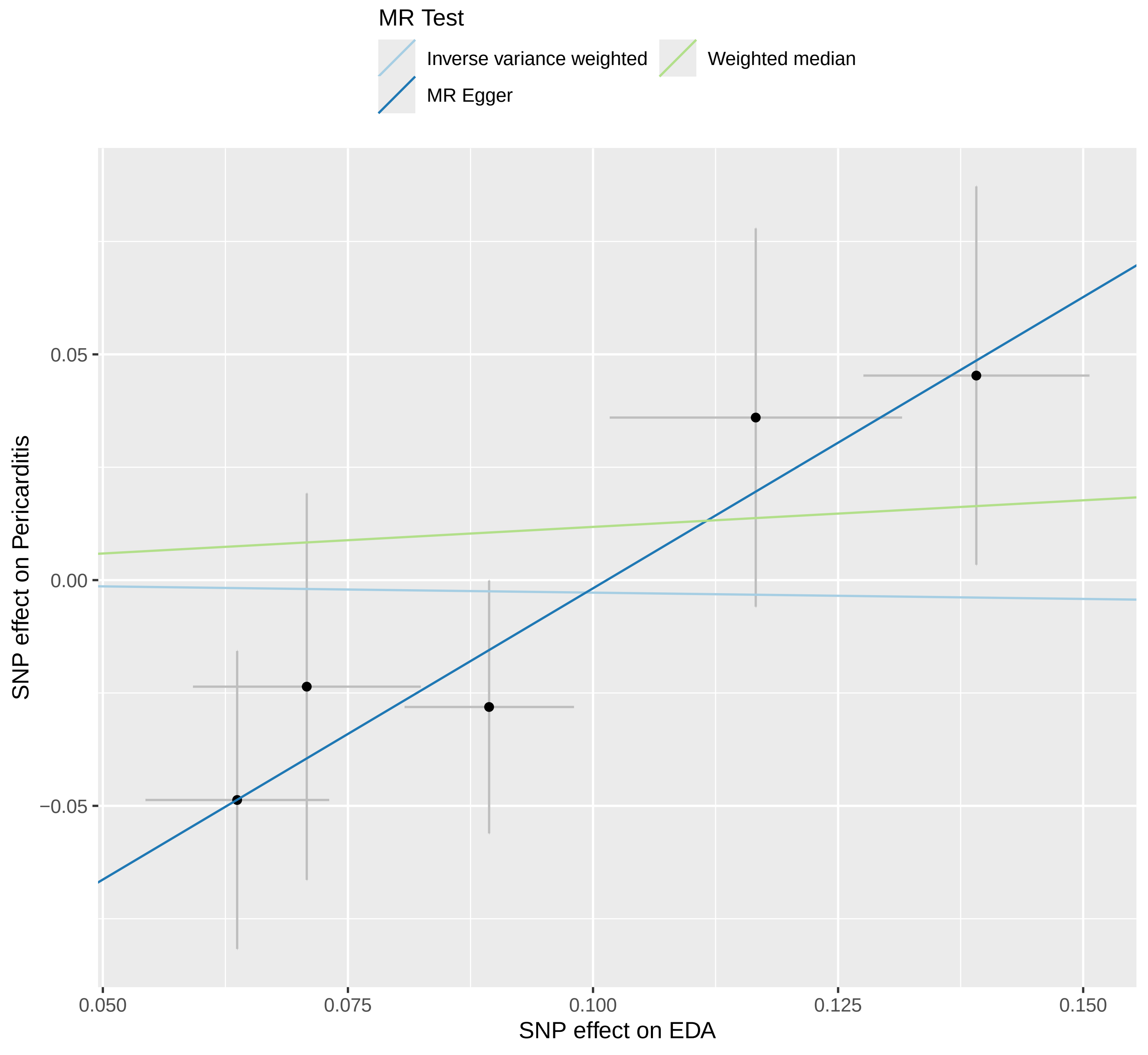

MR Test
Inverse variance weighted
MR Egger
Weighted median
0.05
0.00
−0.05
SNP effect on Pericarditis
0.050
0.075
0.100
0.125
0.150
SNP effect on EDA

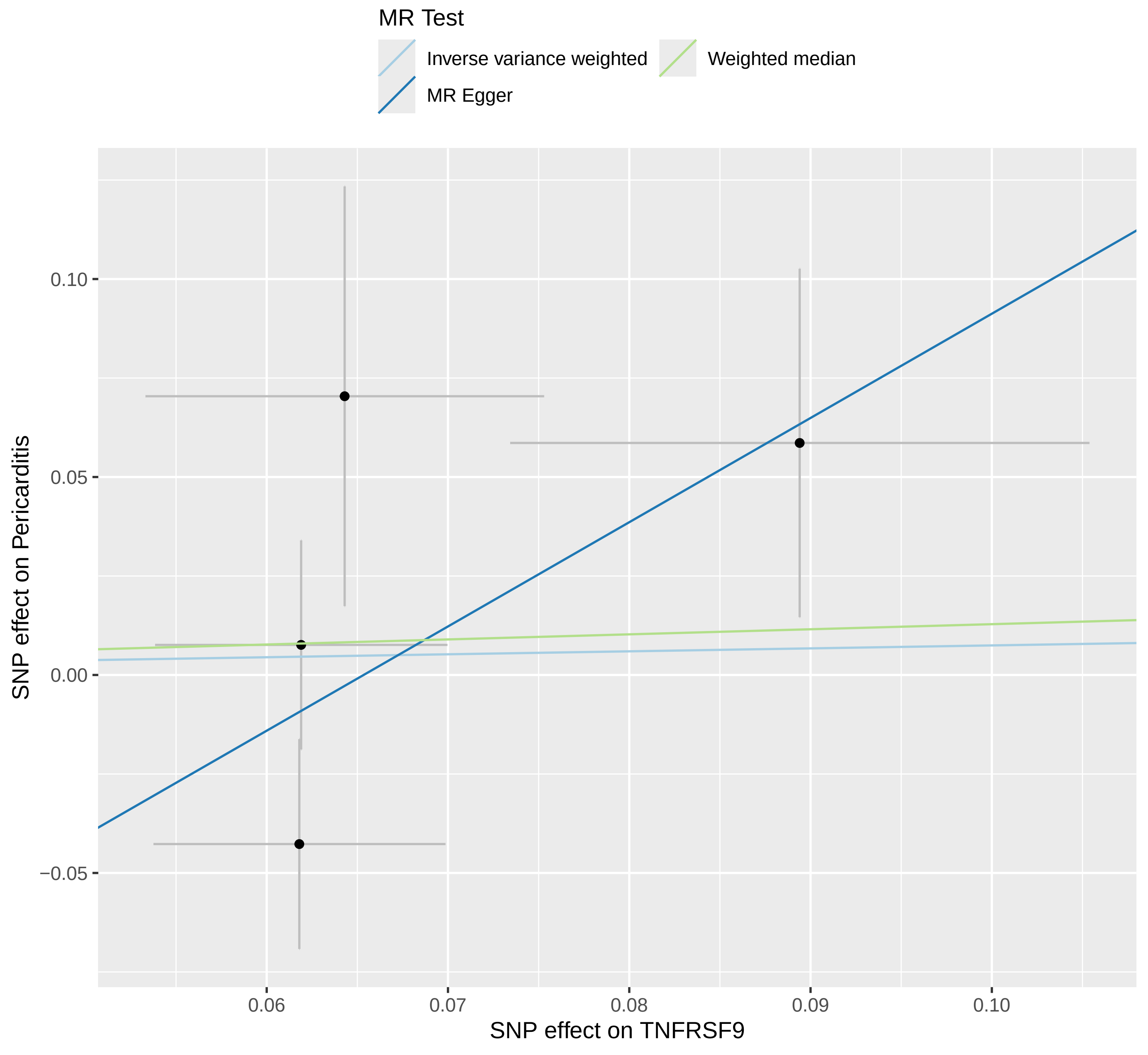

MR Test
Inverse variance weighted
MR Egger
Weighted median
SNP effect on Pericarditis
0.10
0.05
0.00
−0.05
0.06
0.07
0.08
0.09
0.10
SNP effect on TNFRSF9

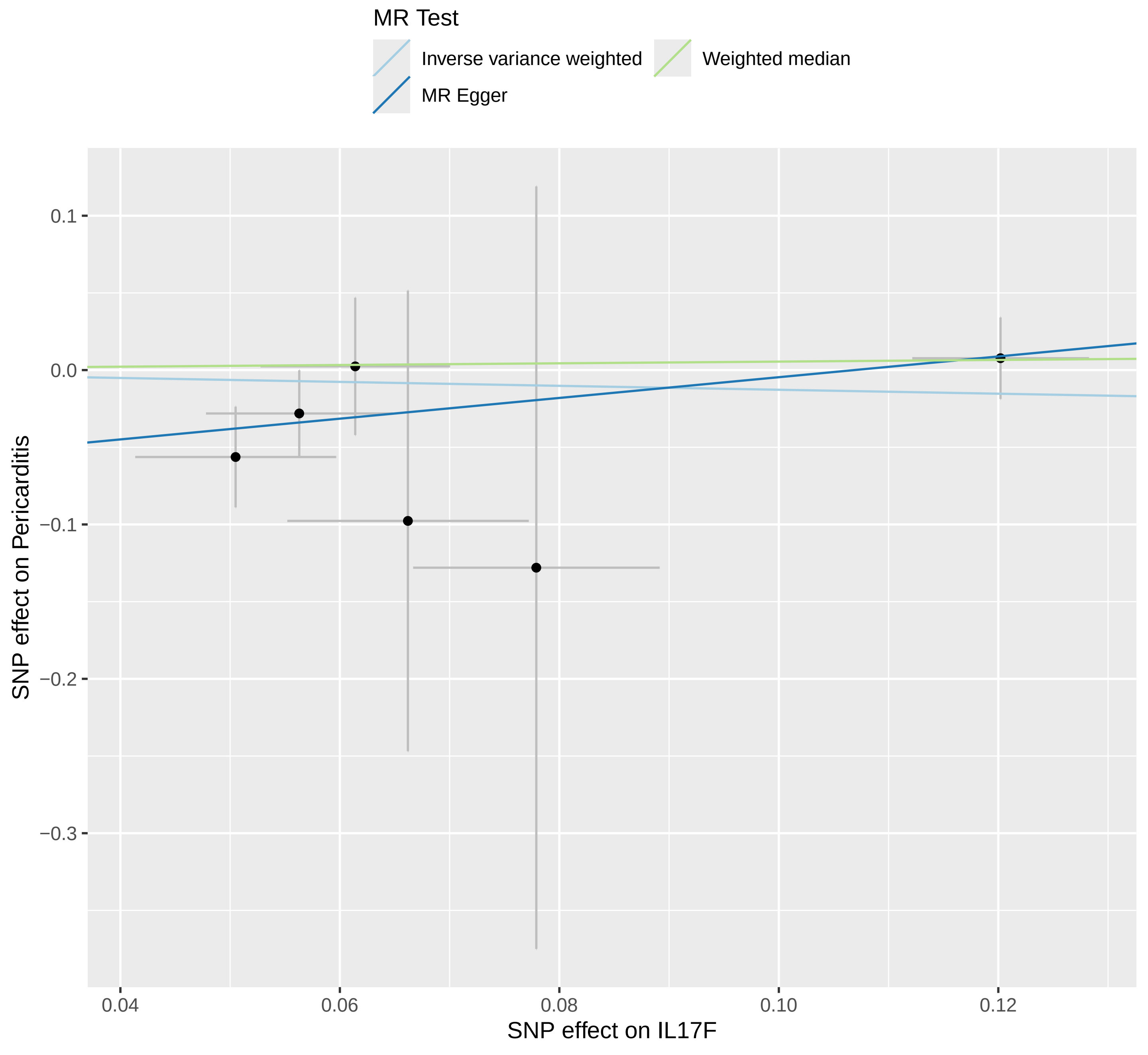
MR Test
Inverse variance weighted
Weighted median
MR Egger
0.1
0.0
−0.1
−0.2
−0.3
SNP effect on Pericarditis
0.04
0.06
0.08
0.10
0.12
SNP effect on IL17F

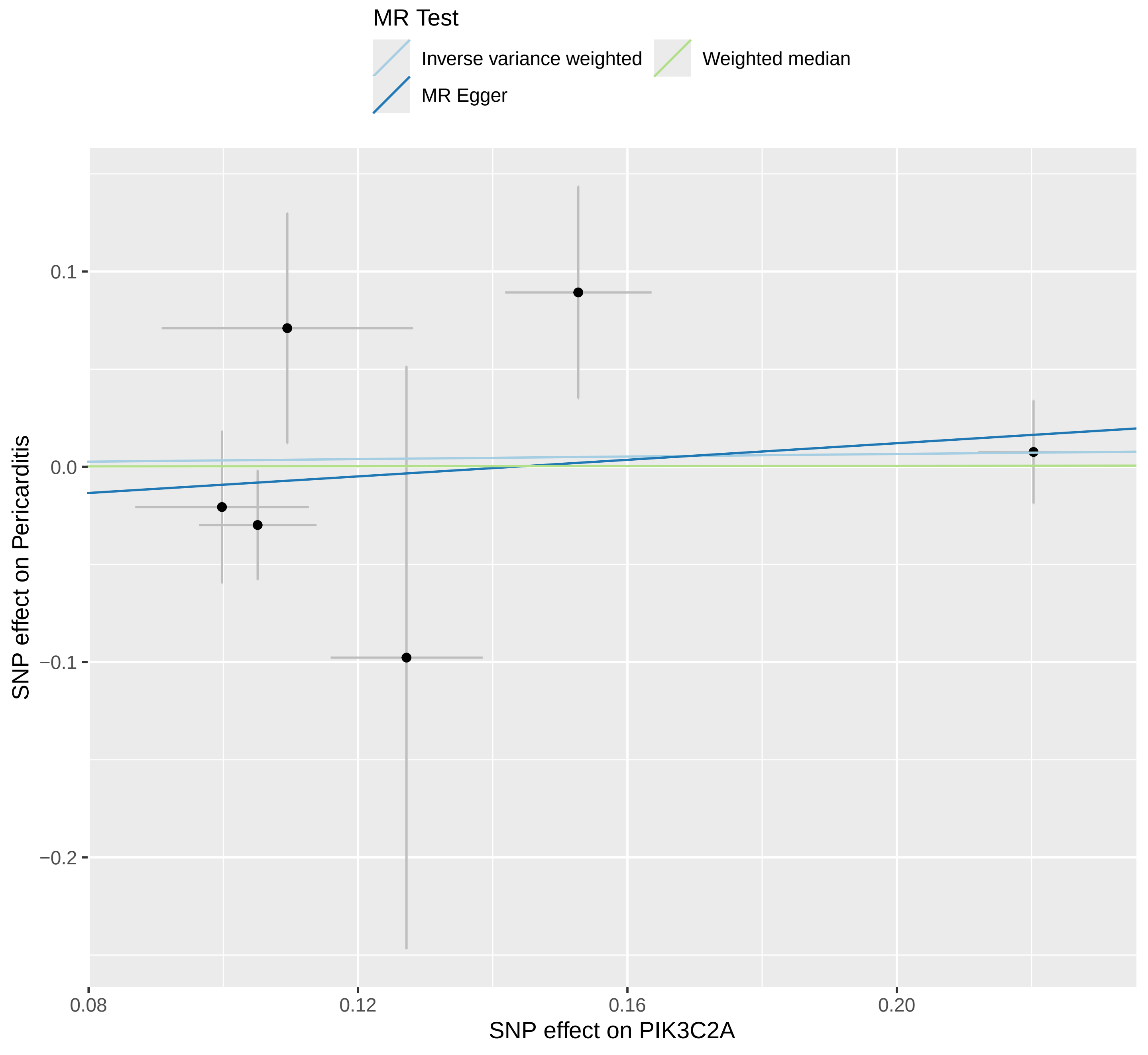
MR Test
Inverse variance weighted
Weighted median
MR Egger
0.1
0.0
−0.1
−0.2
SNP effect on Pericarditis
0.08
0.12
0.16
0.20
SNP effect on PIK3C2A

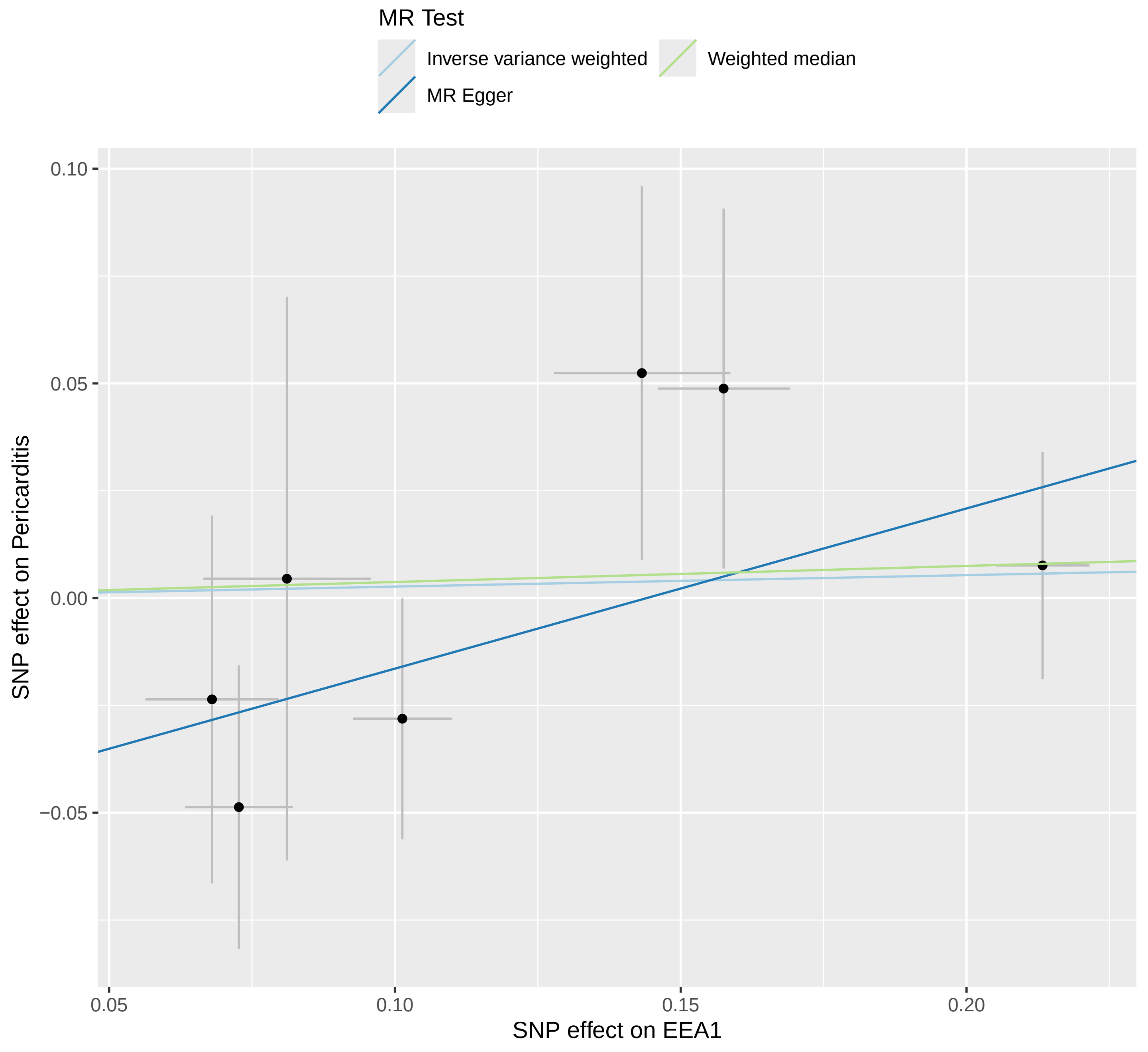
MR Test
Inverse variance weighted
Weighted median
MR Egger
0.10
0.05
0.00
−0.05
SNP effect on Pericarditis
0.05
0.10
0.15
0.20
SNP effect on EEA1

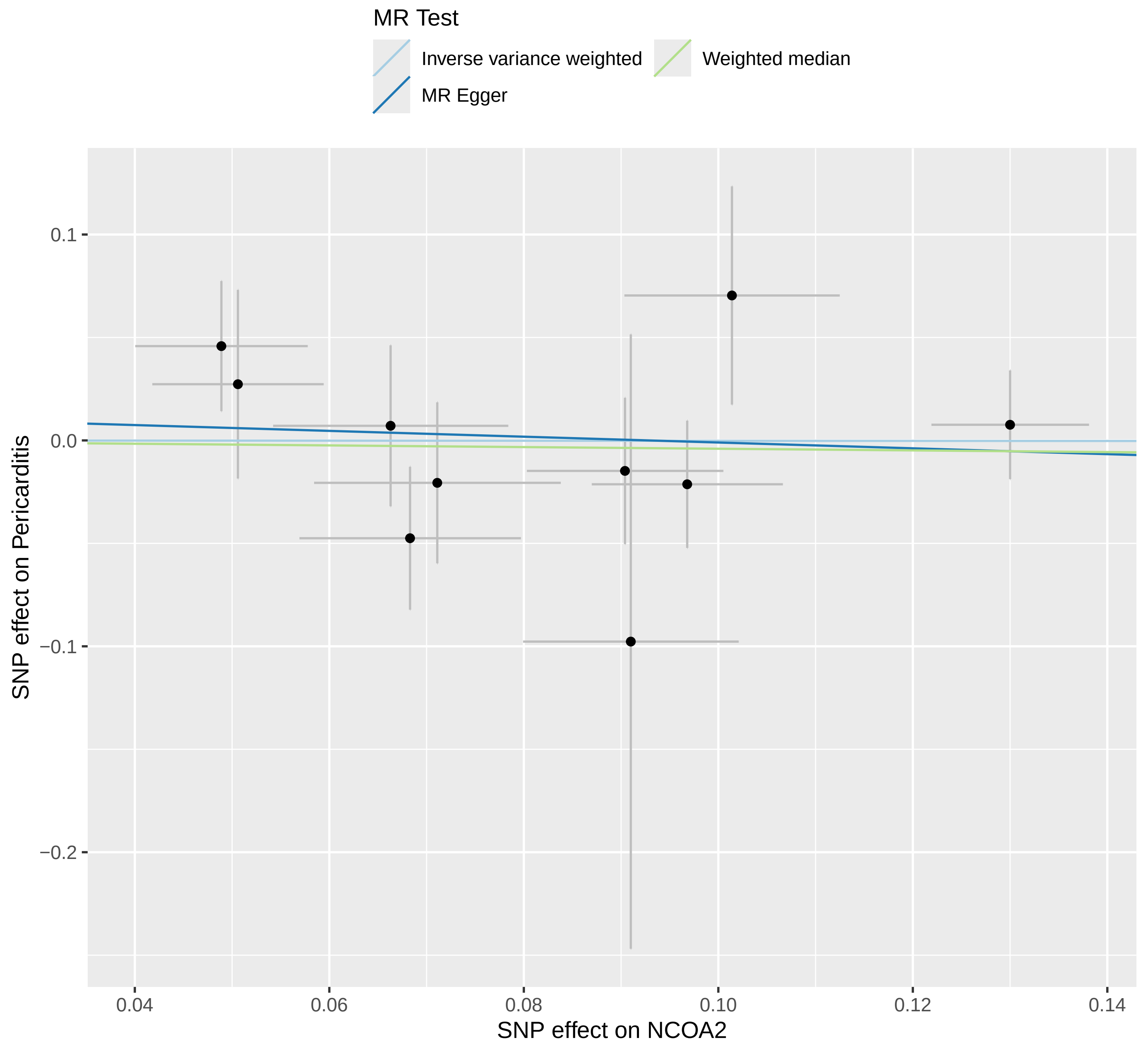
MR Test
Inverse variance weighted
Weighted median
MR Egger
0.1
0.0
−0.1
−0.2
SNP effect on Pericarditis
0.04
0.06
0.08
0.10
0.12
0.14
SNP effect on NCOA2

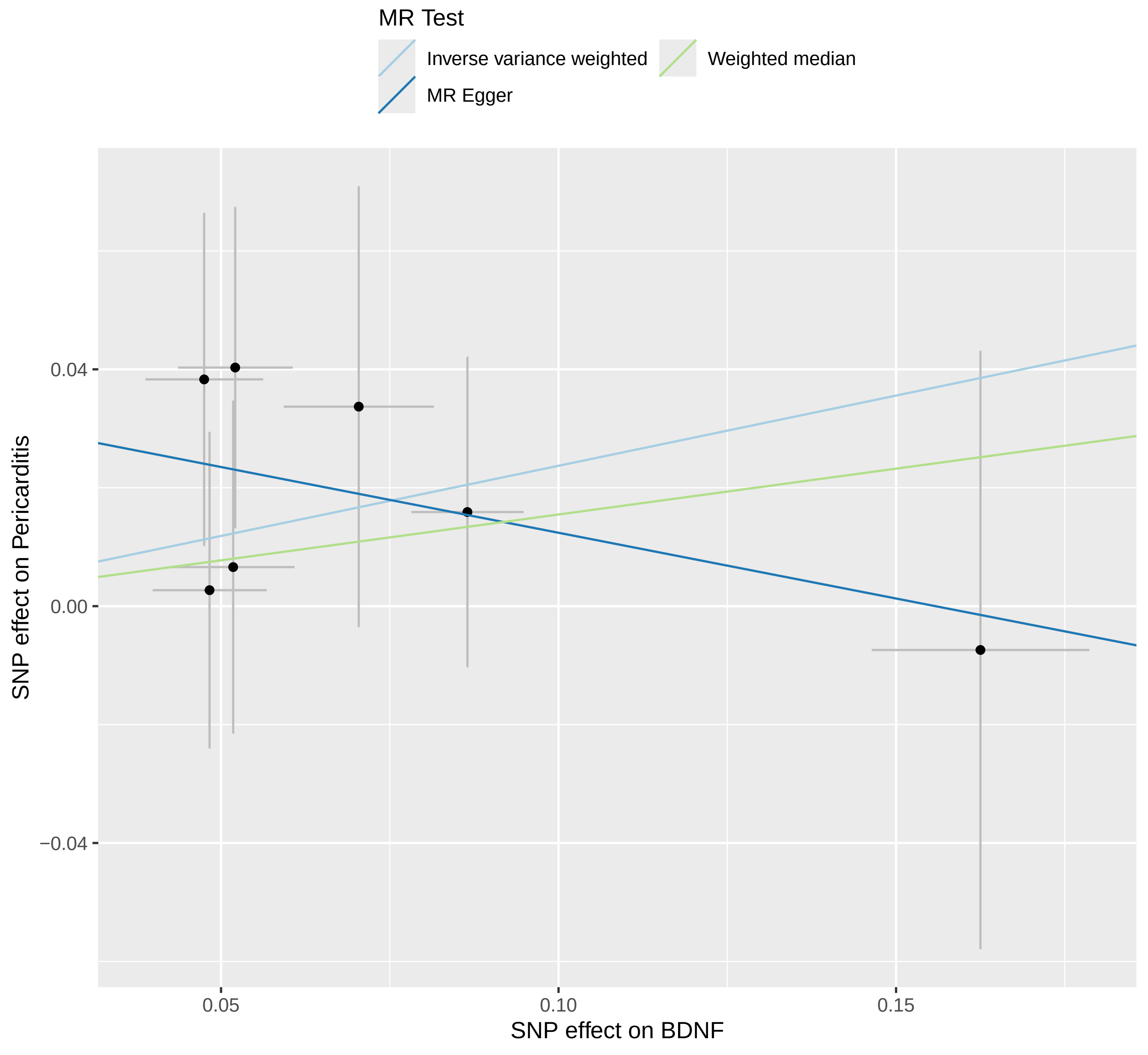

MR Test
Inverse variance weighted
MR Egger
Weighted median
0.04
0.00
−0.04
SNP effect on Pericarditis
0.05
0.10
0.15
SNP effect on BDNF

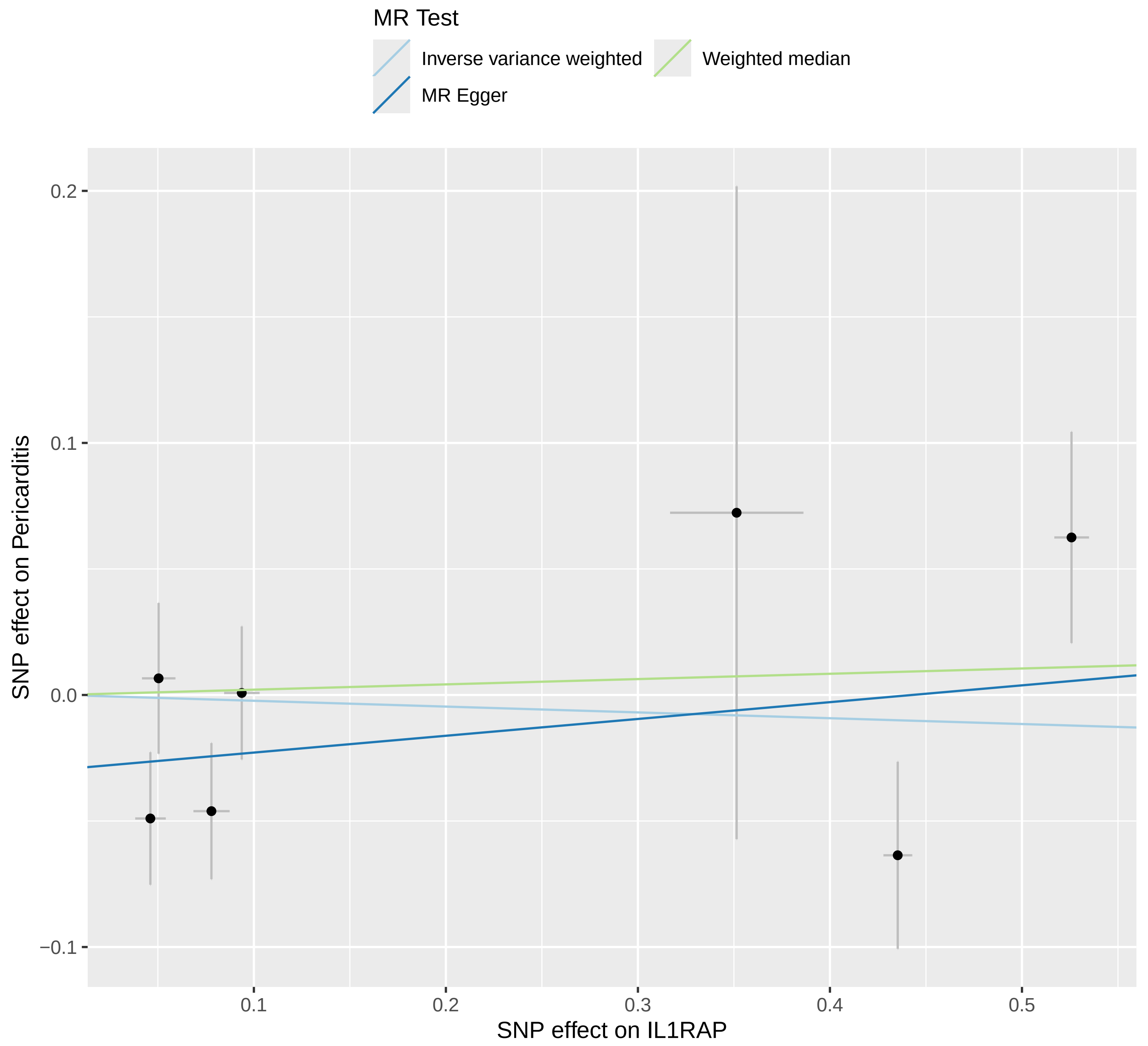

MR Test
Inverse variance weighted
MR Egger
Weighted median
0.2
0.1
0.0
−0.1
SNP effect on Pericarditis
0.1
0.2
0.3
0.4
0.5
SNP effect on IL1RAP

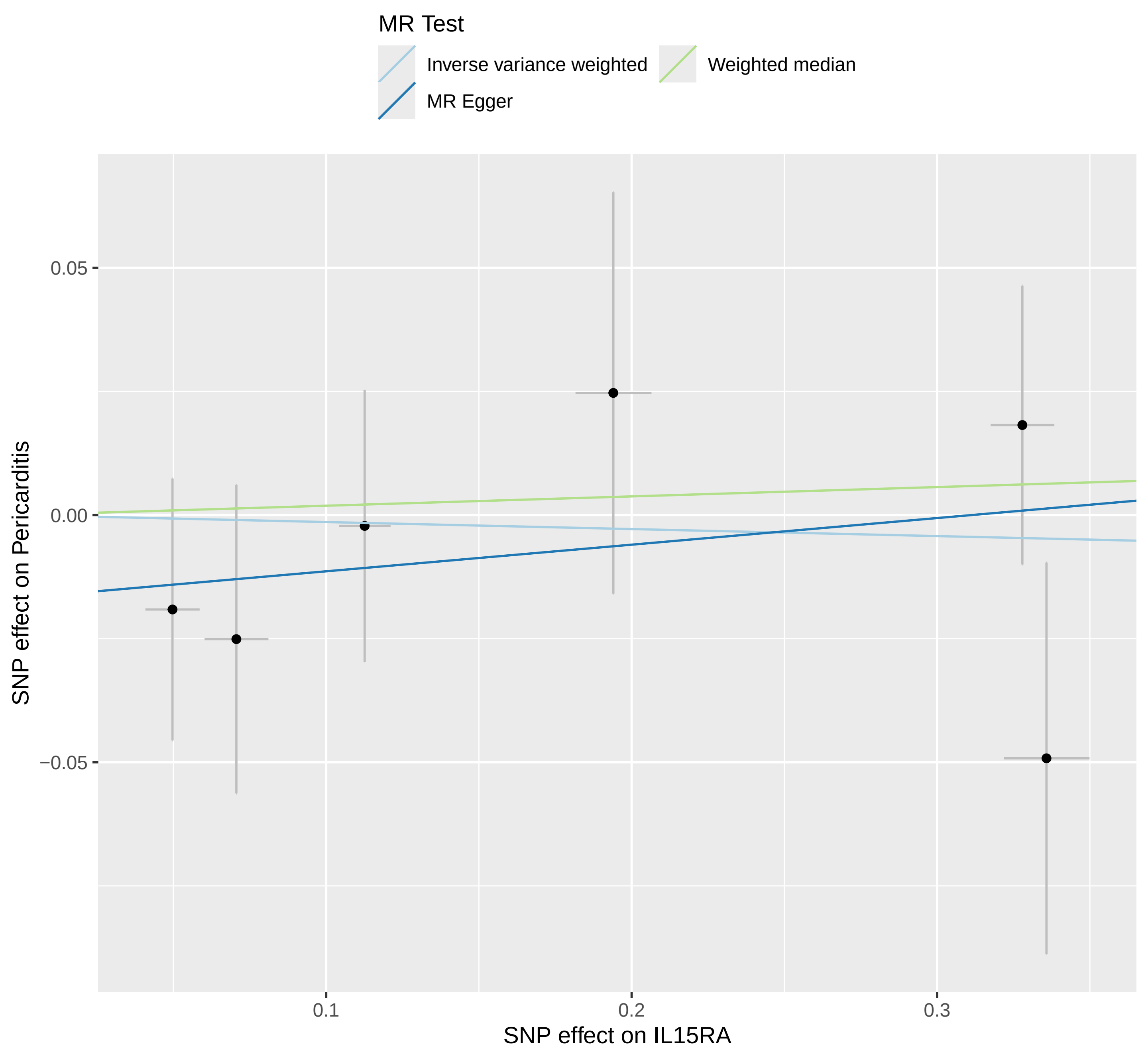
MR Test
Inverse variance weighted
MR Egger
Weighted median
0.05
0.00
−0.05
0.1
0.2
0.3
SNP effect on Pericarditis
SNP effect on IL15RA

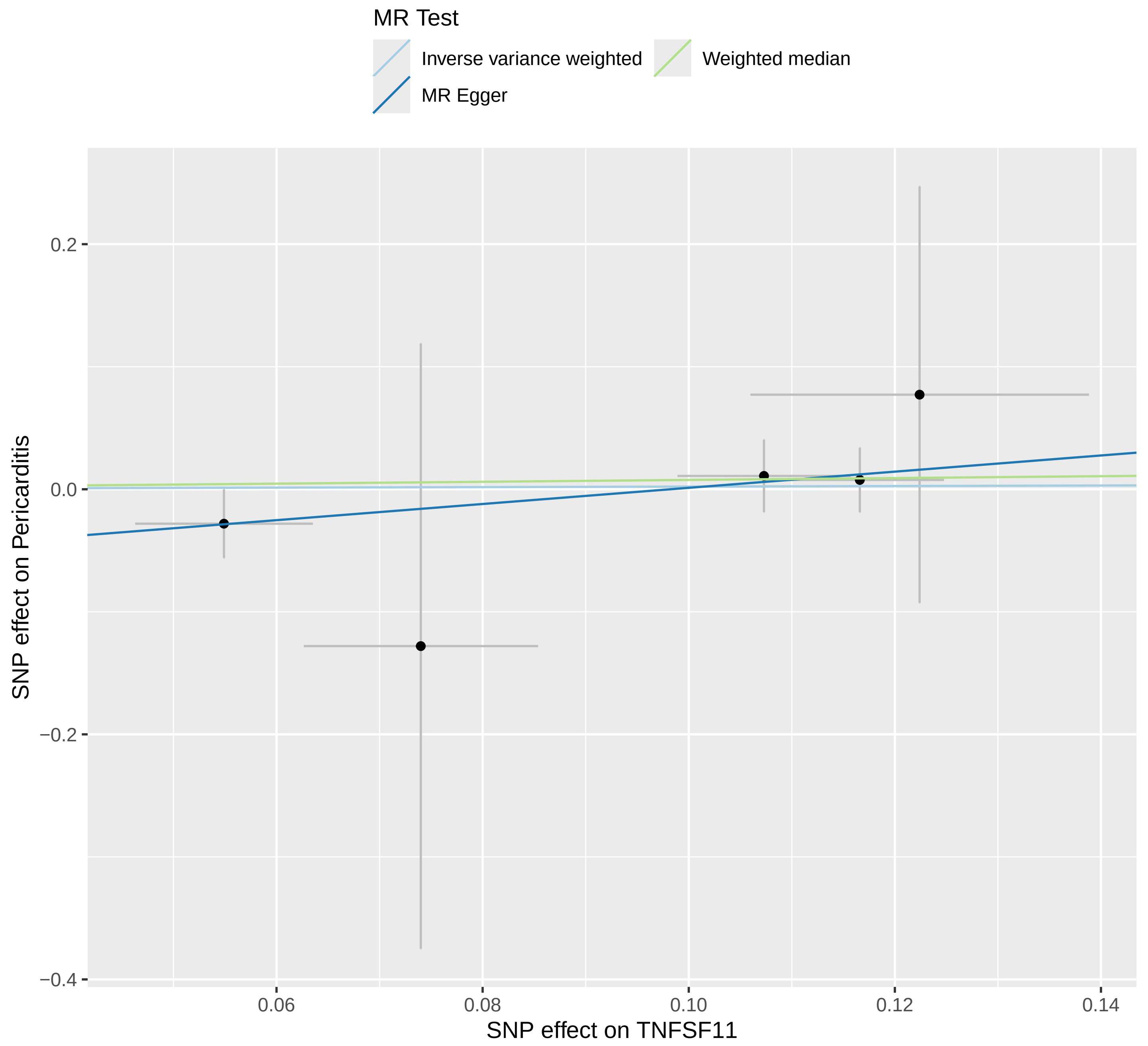

MR Test
Inverse variance weighted
Weighted median
MR Egger
0.2
0.0
−0.2
−0.4
SNP effect on Pericarditis
0.06
0.08
0.10
0.12
0.14
SNP effect on TNFSF11

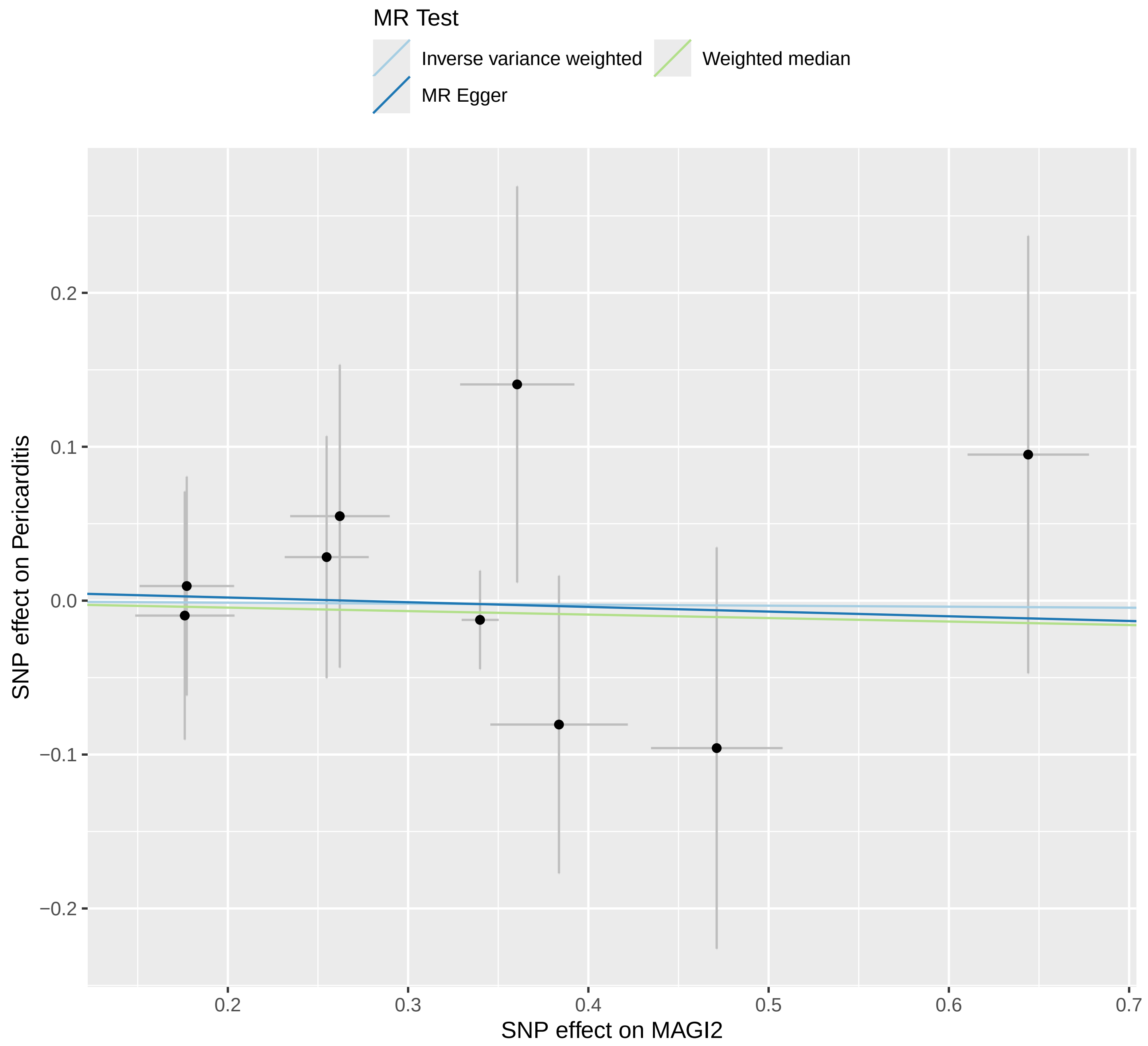
MR Test
Inverse variance weighted
MR Egger
Weighted median
SNP effect on Pericarditis
0.2
0.1
0.0
−0.1
−0.2
0.2
0.3
0.4
0.5
0.6
0.7
SNP effect on MAGI2

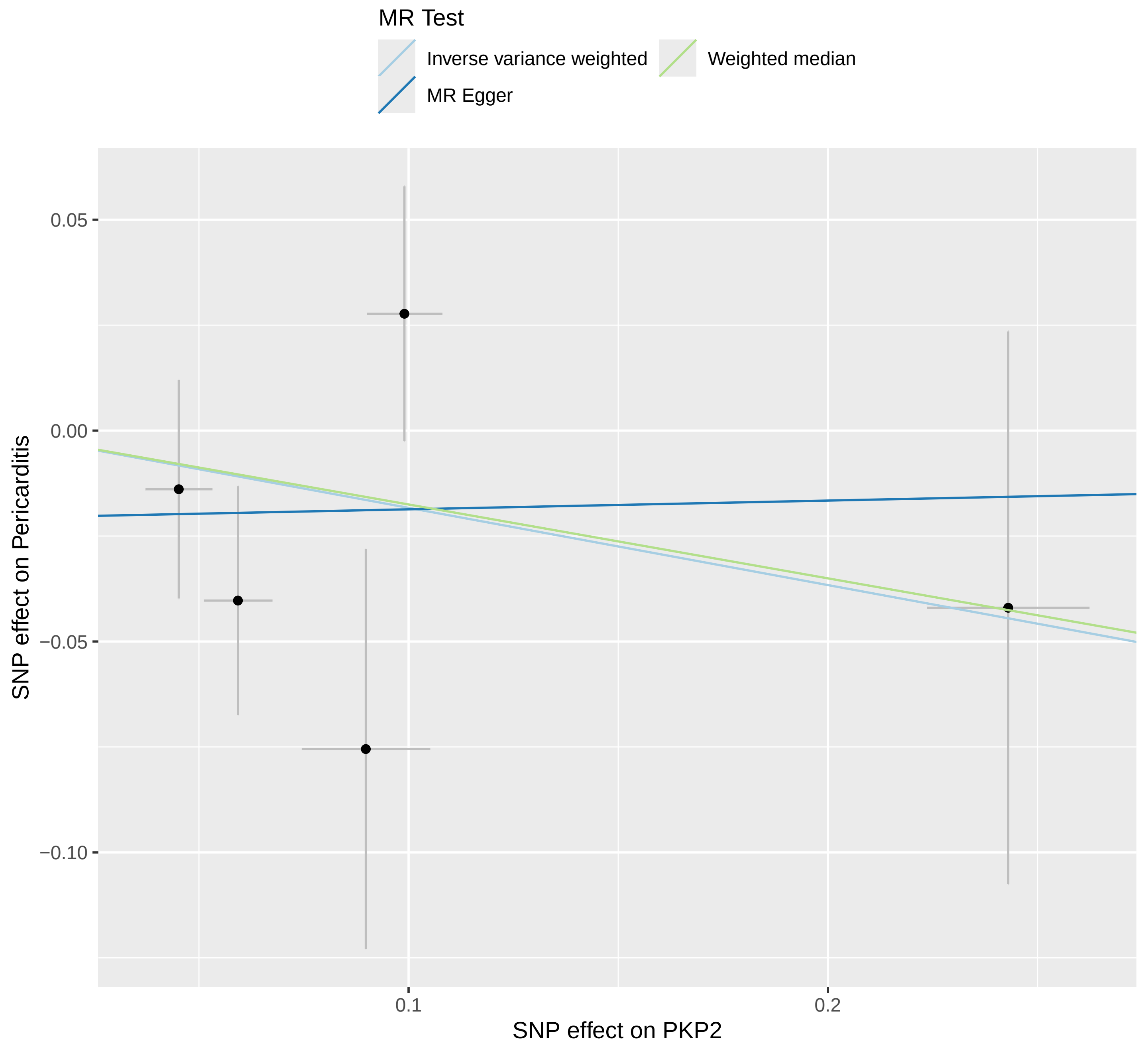

MR Test
Inverse variance weighted
Weighted median
MR Egger
0.05
0.00
−0.05
−0.10
SNP effect on Pericarditis
0.1
0.2
SNP effect on PKP2

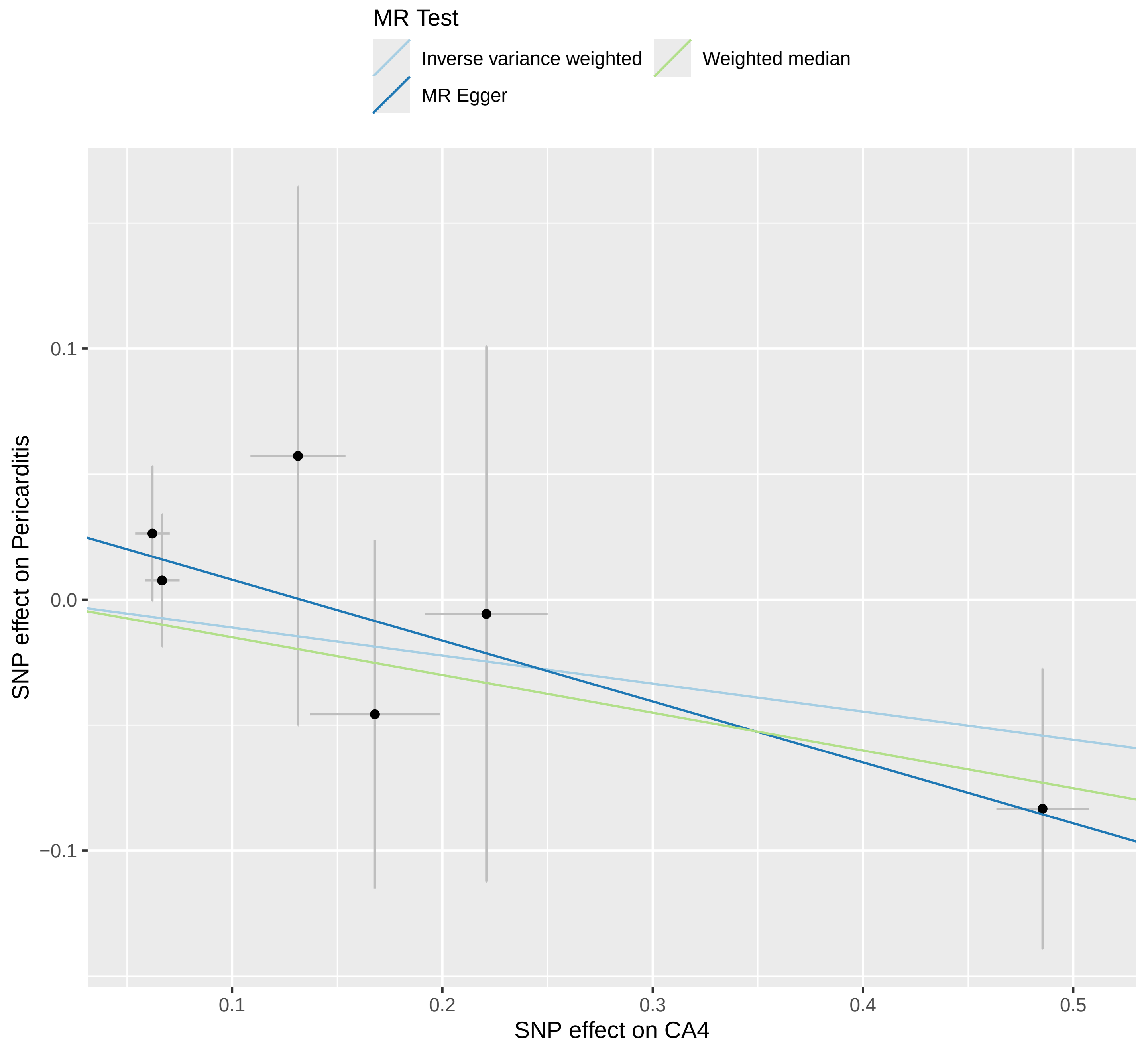
MR Test
Inverse variance weighted
Weighted median
MR Egger
0.1
0.0
−0.1
SNP effect on Pericarditis
0.1
0.2
0.3
0.4
0.5
SNP effect on CA4

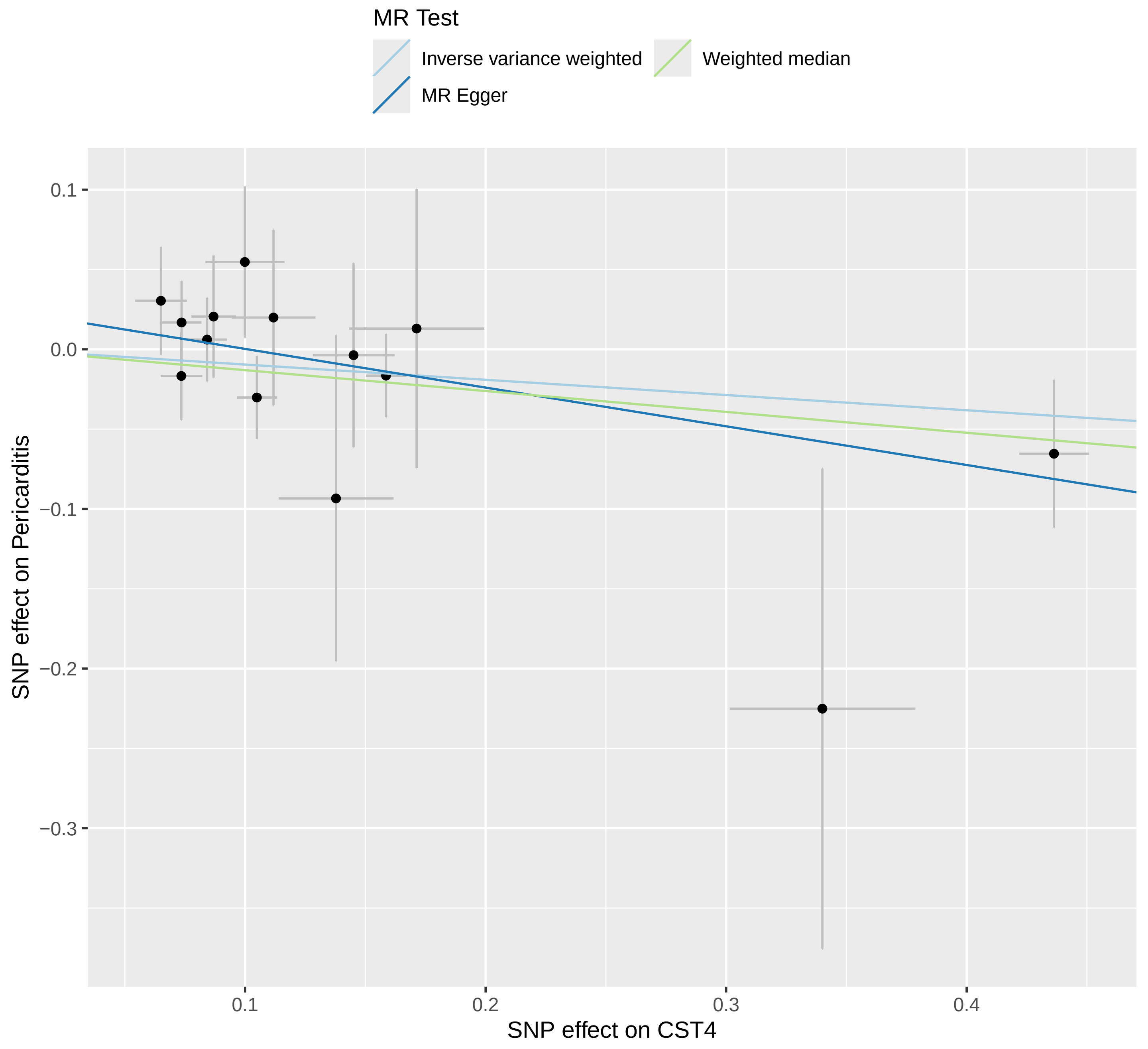

MR Test
Inverse variance weighted
Weighted median
MR Egger
0.1
0.0
−0.1
−0.2
−0.3
SNP effect on Pericarditis
0.1
0.2
0.3
0.4
SNP effect on CST4

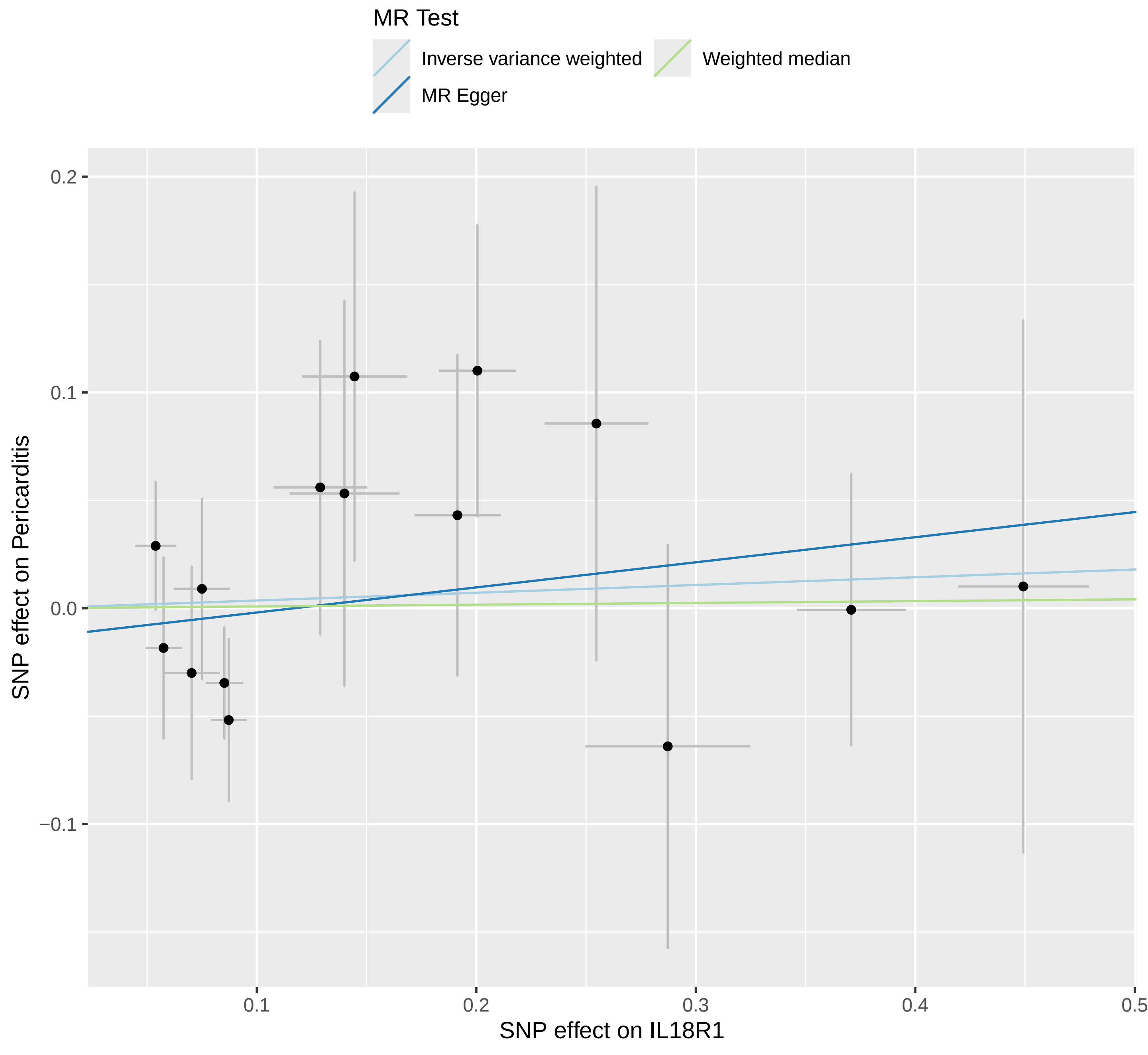

MR Test
Inverse variance weighted
Weighted median
MR Egger
0.2
0.1
0.0
−0.1
0.1
0.2
0.3
0.4
0.5
SNP effect on Pericarditis
SNP effect on IL18R1

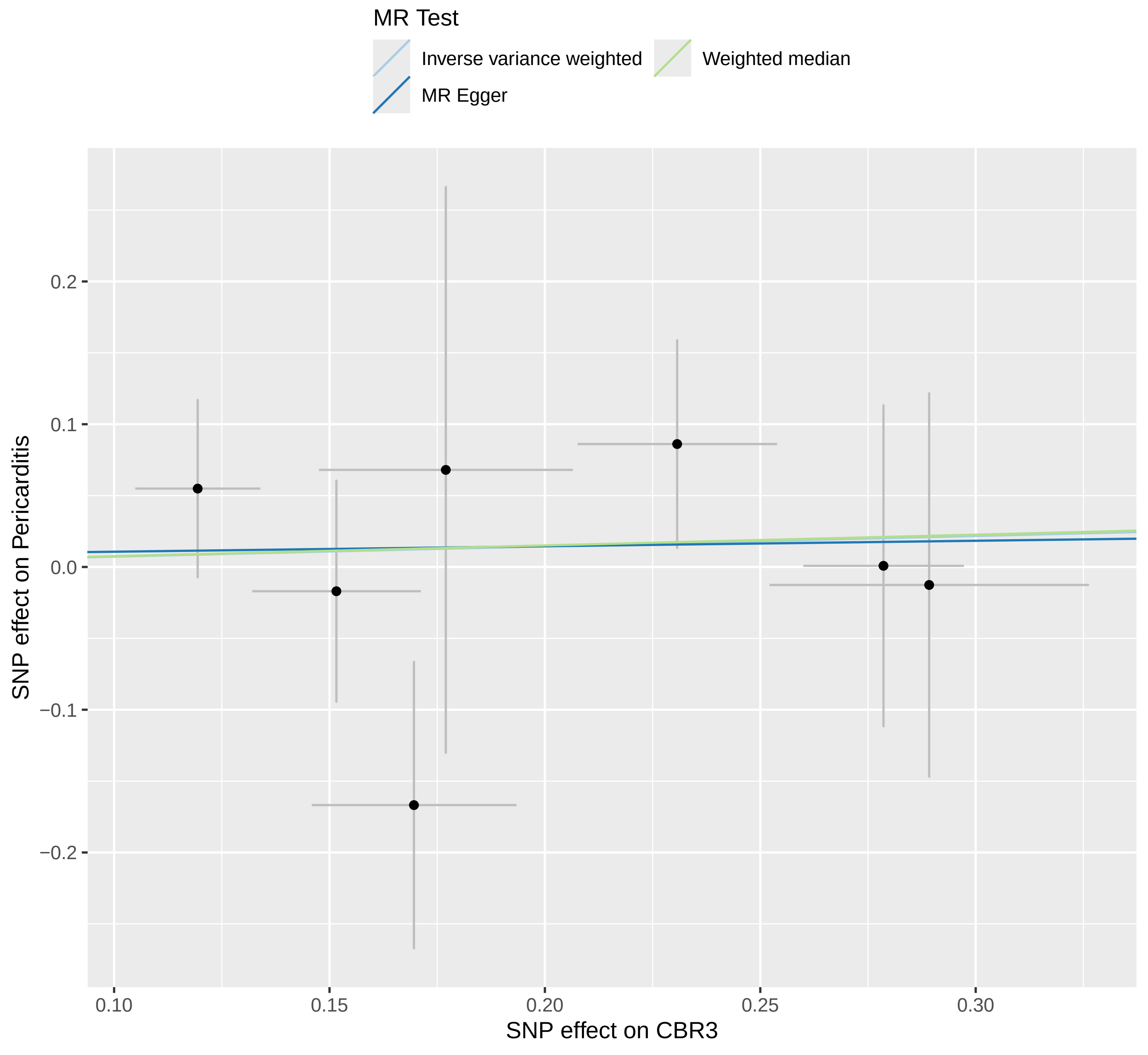
MR Test
Inverse variance weighted
Weighted median
MR Egger
SNP effect on Pericarditis
SNP effect on CBR3
0.2
0.1
0.0
−0.1
−0.2
0.10
0.15
0.20
0.25
0.30

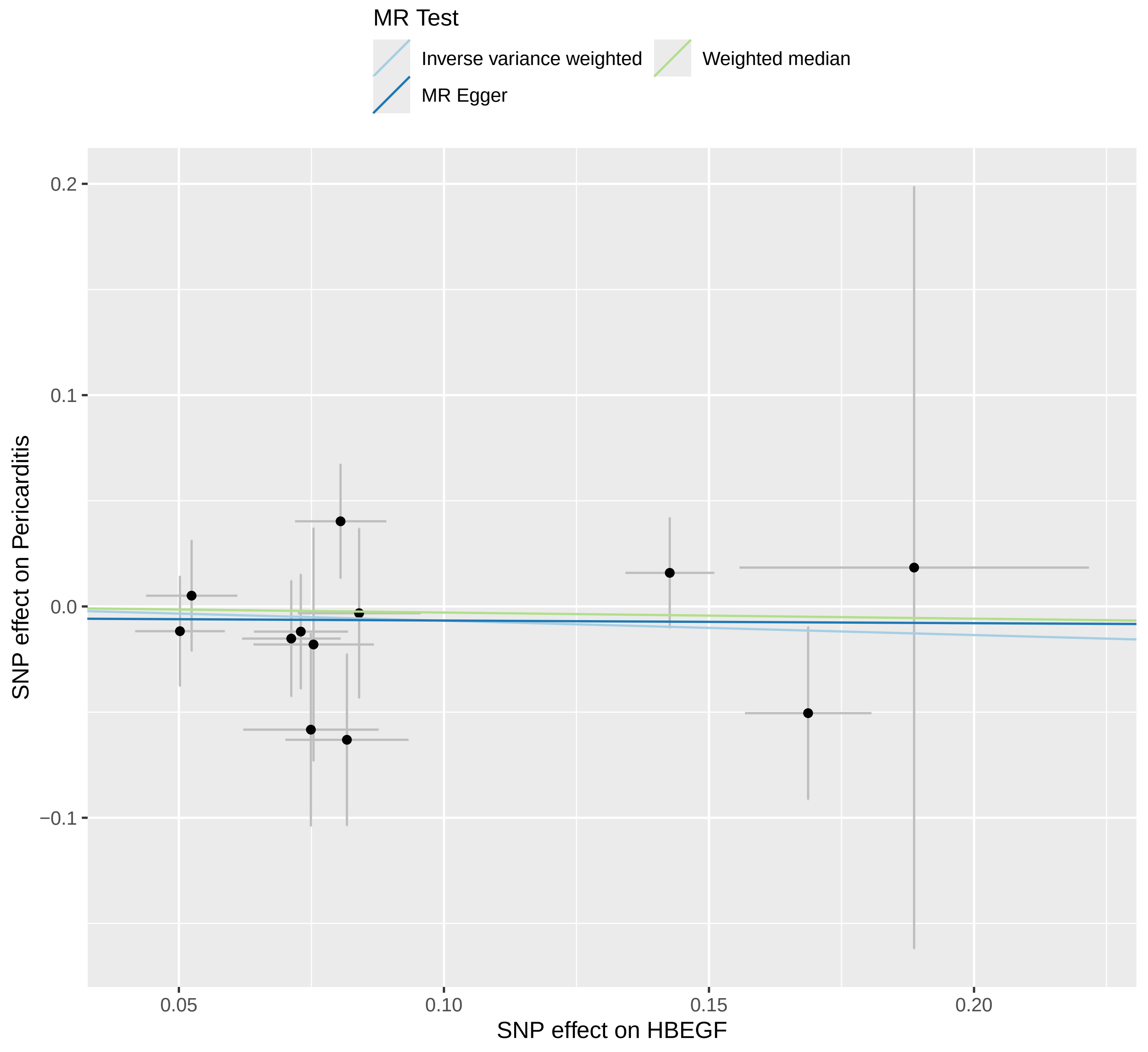
MR Test
Inverse variance weighted
Weighted median
MR Egger
0.2
0.1
0.0
−0.1
SNP effect on Pericarditis
0.05
0.10
0.15
0.20
SNP effect on HBEGF

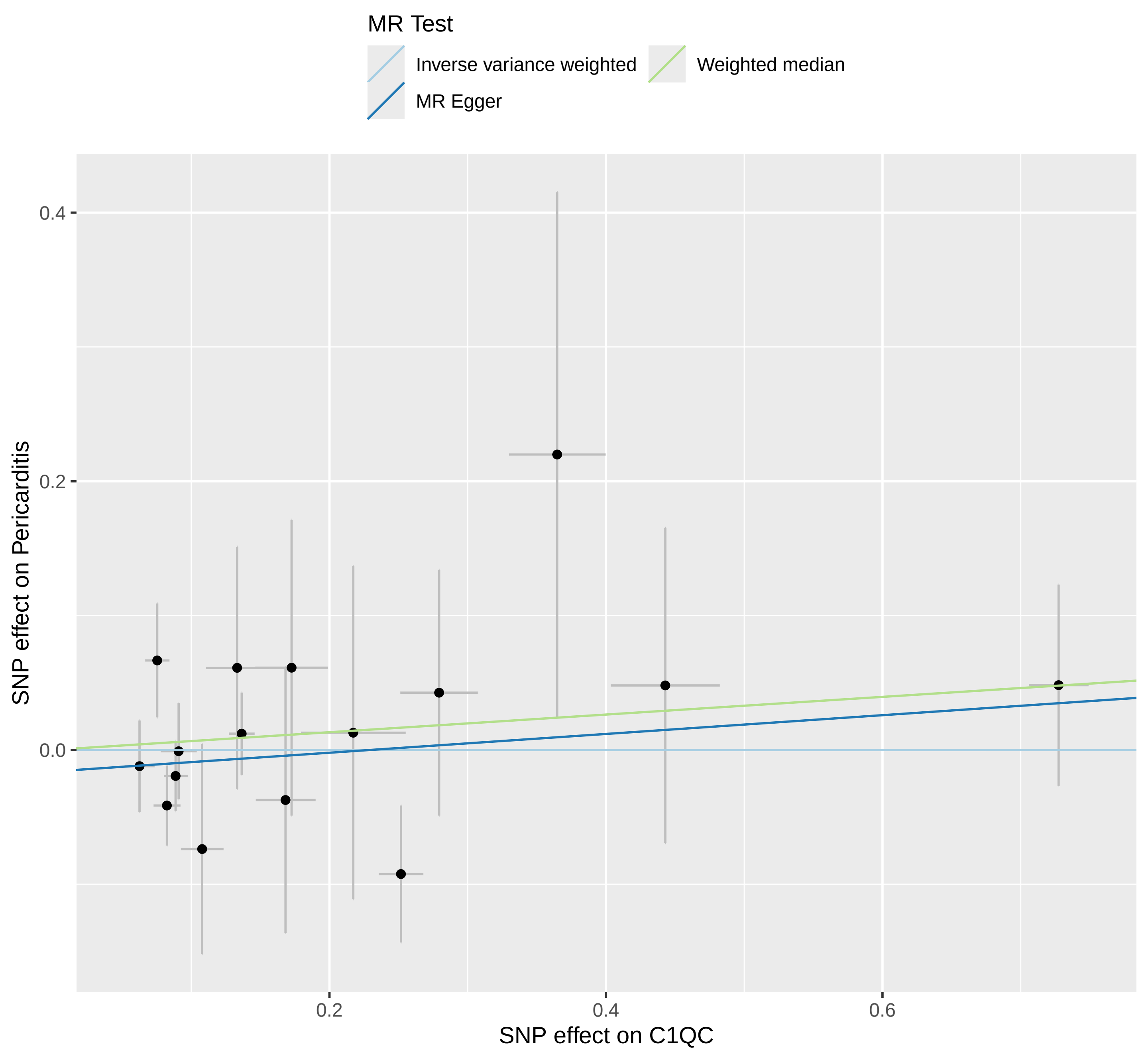
MR Test
Inverse variance weighted
MR Egger
Weighted median
0.4
0.2
0.0
0.2
0.4
0.6
SNP effect on Pericarditis
SNP effect on C1QC

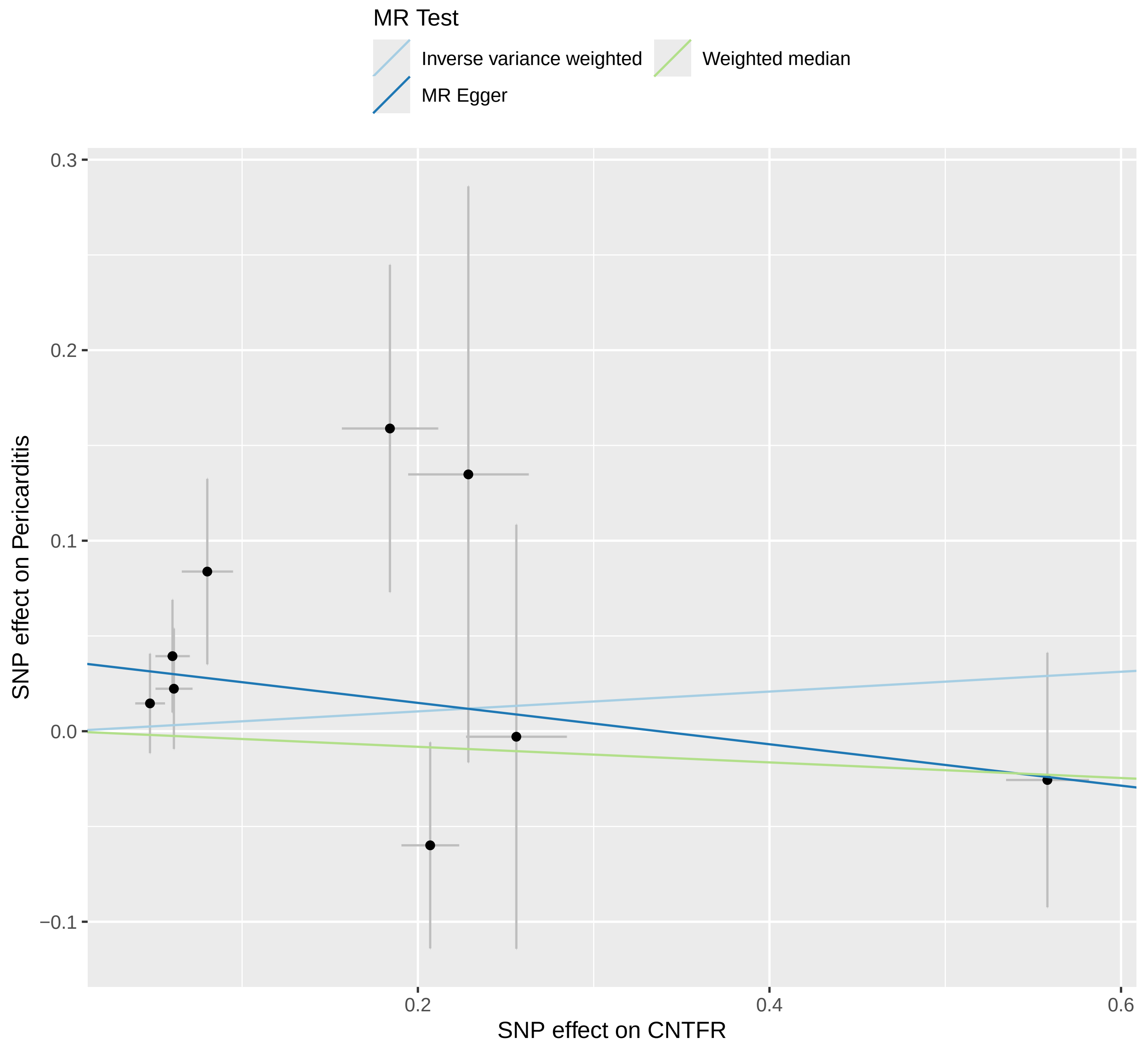
MR Test
Inverse variance weighted
Weighted median
MR Egger
0.3
0.2
0.1
0.0
−0.1
SNP effect on Pericarditis
0.2
0.4
0.6
SNP effect on CNTFR

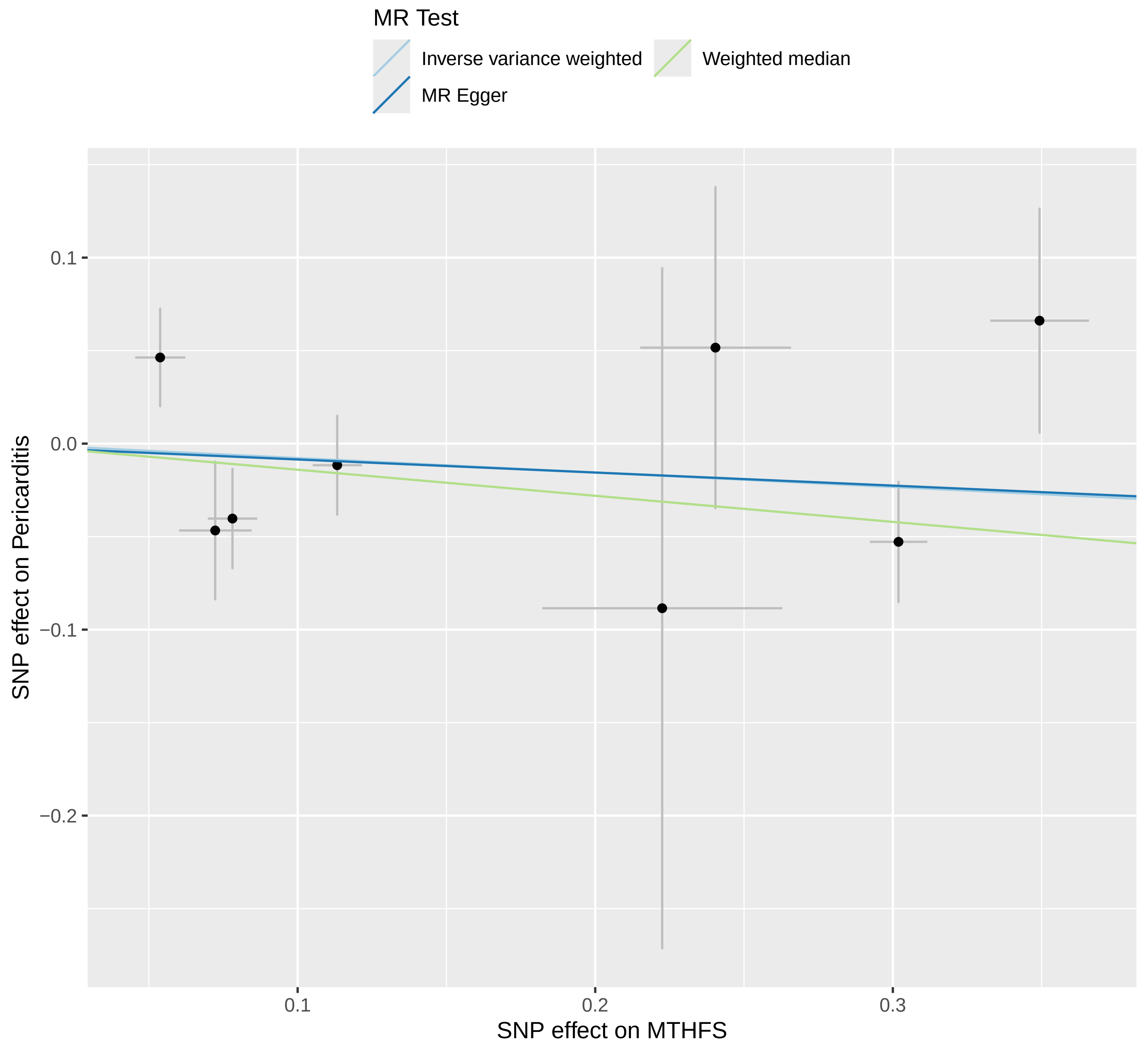

MR Test
Inverse variance weighted
Weighted median
MR Egger
0.1
0.0
−0.1
−0.2
SNP effect on Pericarditis
0.1
0.2
0.3
SNP effect on MTHFS

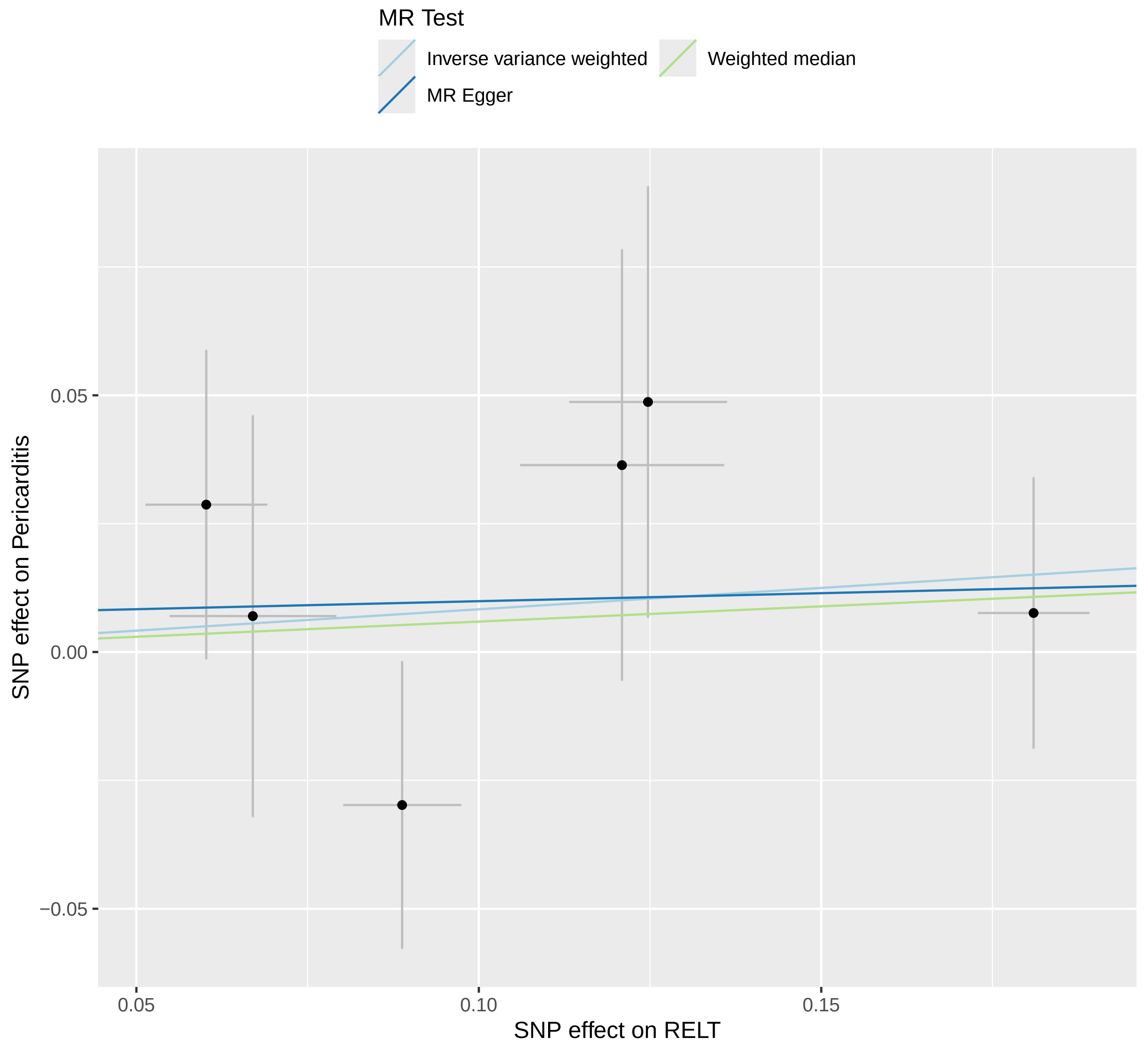
MR Test
Inverse variance weighted
MR Egger
Weighted median
SNP effect on Pericarditis
0.05
0.00
−0.05
0.05
0.10
0.15
SNP effect on RELT

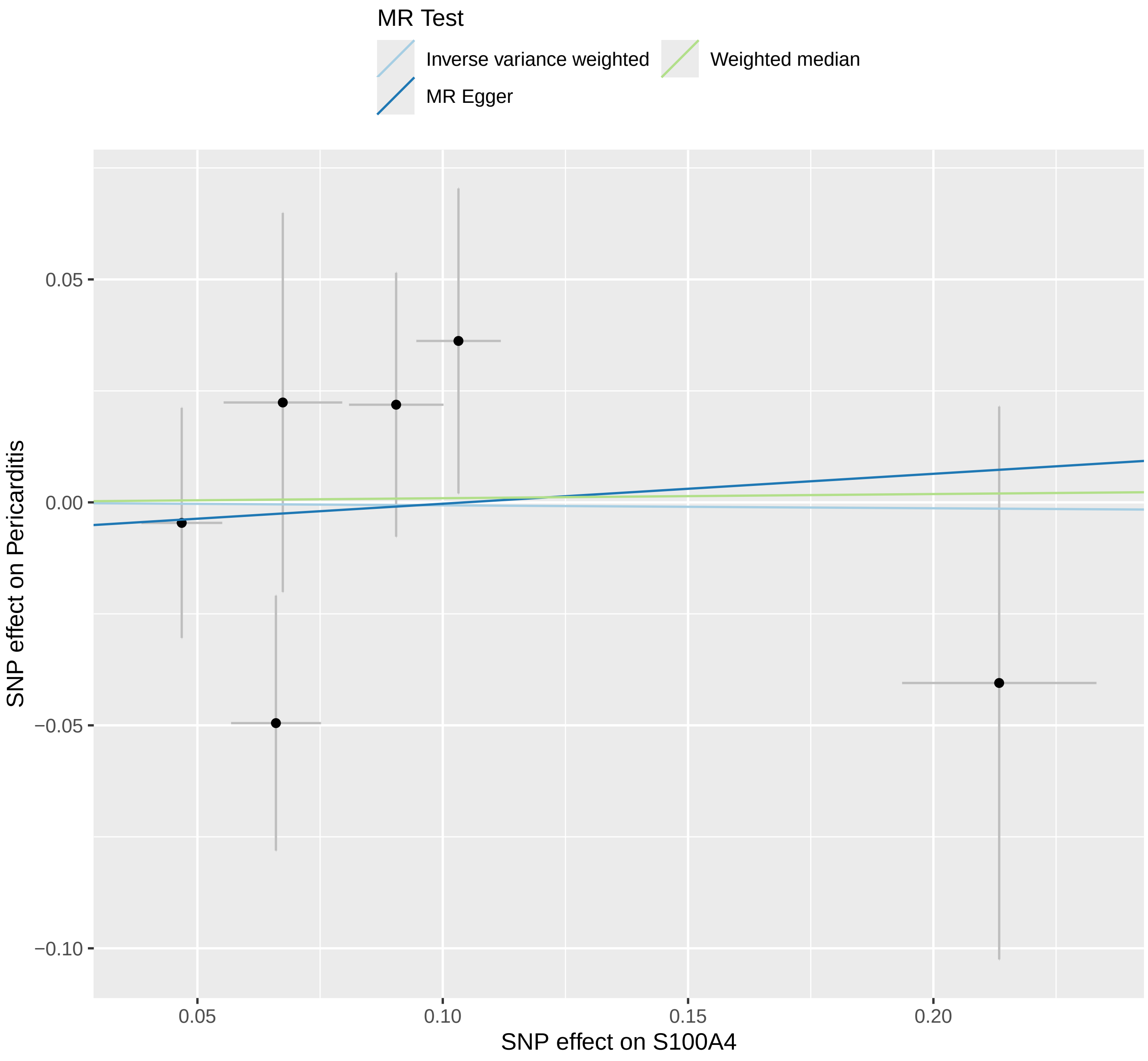

MR Test
Inverse variance weighted
Weighted median
MR Egger
0.05
0.00
−0.05
−0.10
SNP effect on Pericarditis
0.05
0.10
0.15
0.20
SNP effect on S100A4

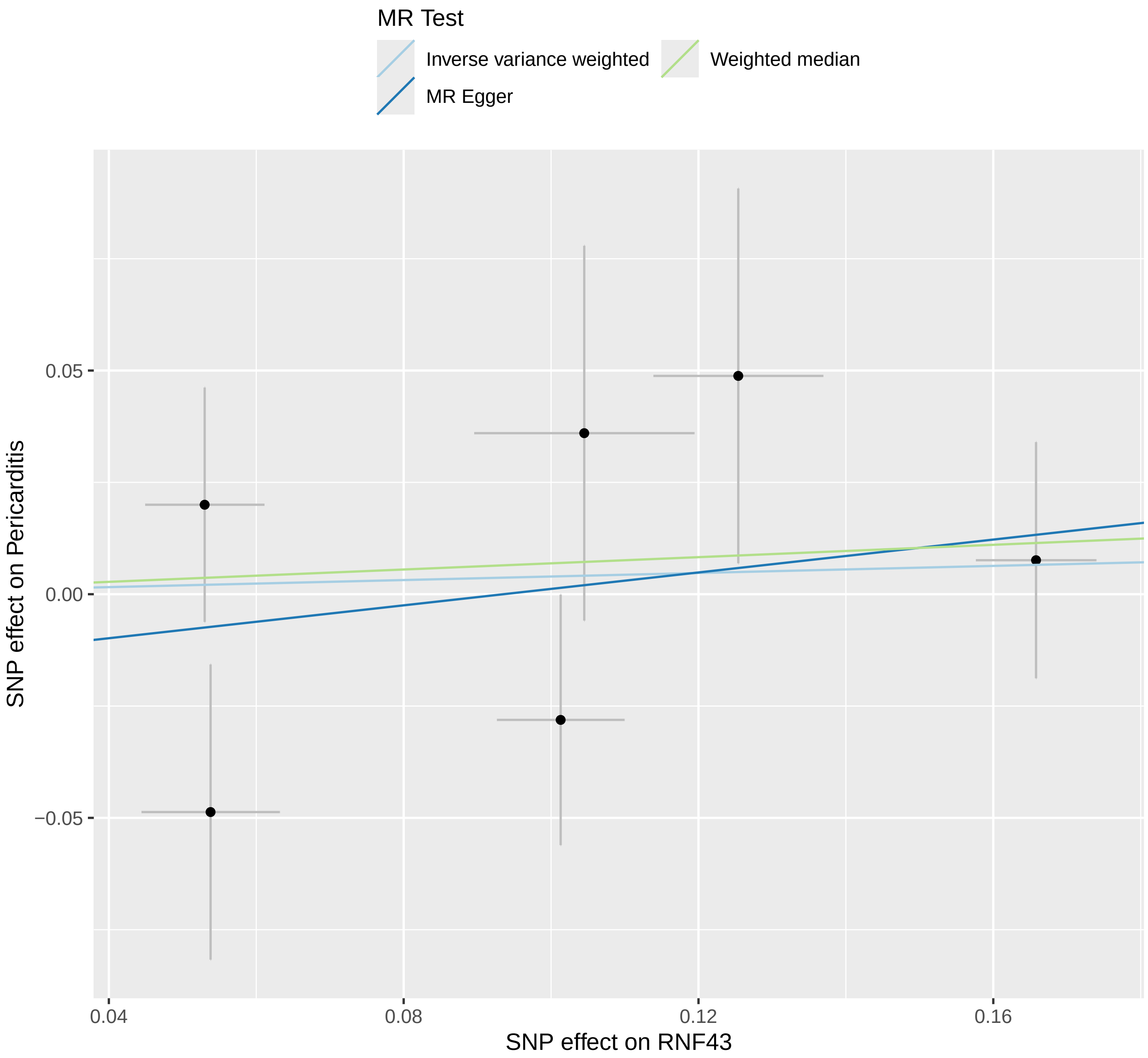

MR Test
Inverse variance weighted
MR Egger
Weighted median
0.05
0.00
−0.05
0.04
0.08
0.12
0.16
SNP effect on RNF43
SNP effect on Pericarditis

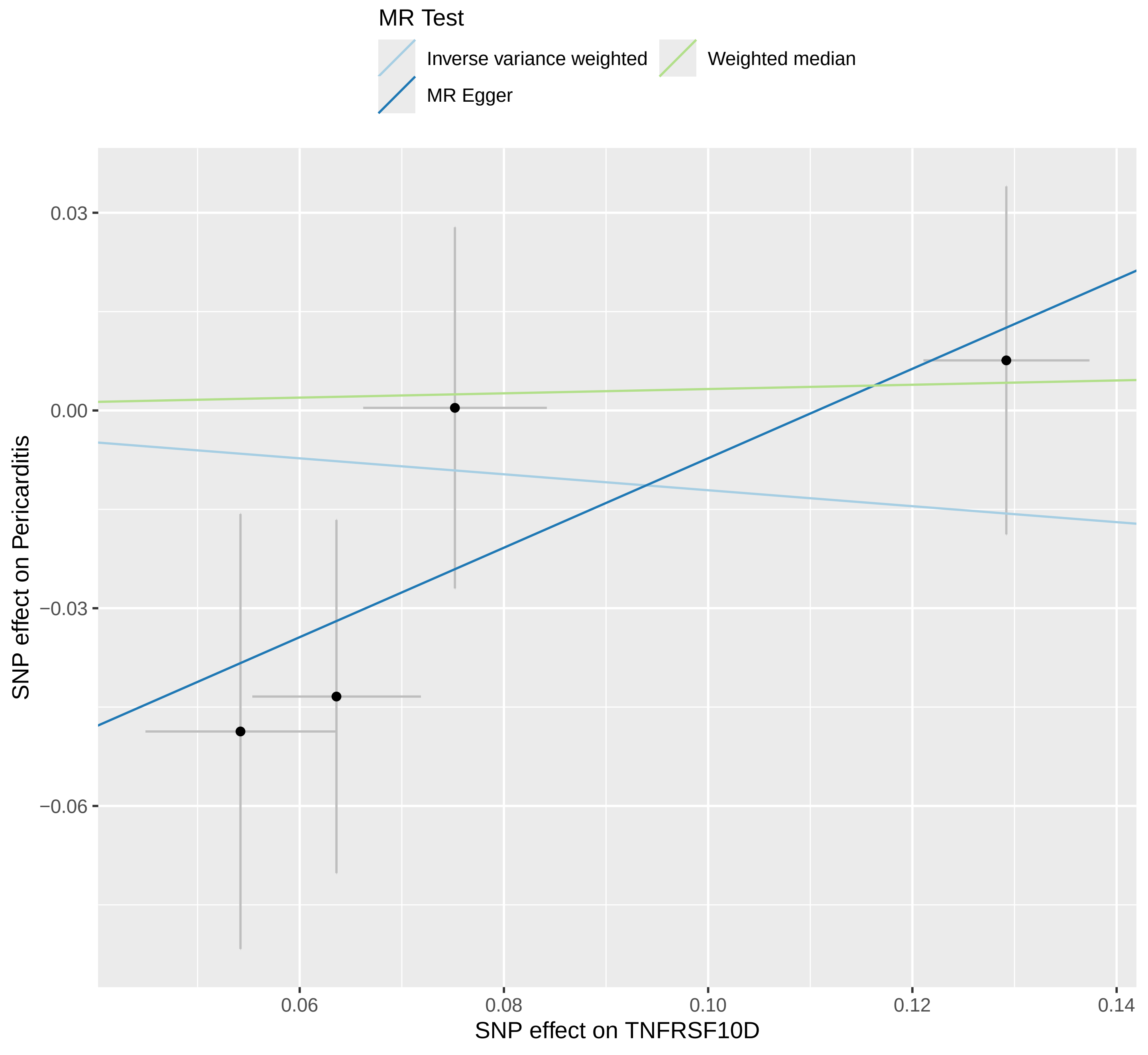
MR Test
Inverse variance weighted
Weighted median
MR Egger
0.03
0.00
−0.03
−0.06
0.06
0.08
0.10
0.12
0.14
SNP effect on Pericarditis
SNP effect on TNFRSF10D

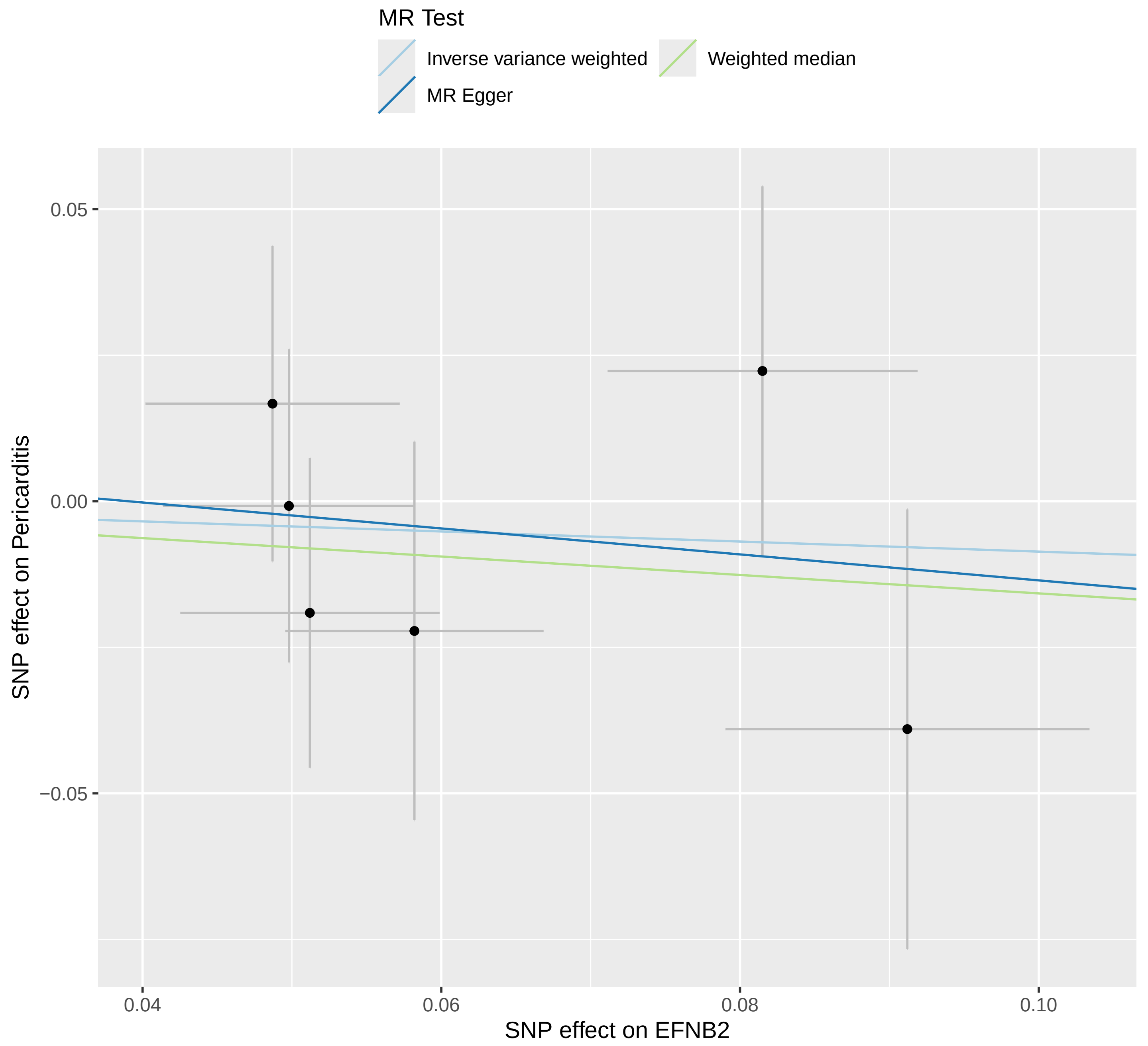
MR Test
Inverse variance weighted
Weighted median
MR Egger
0.05
0.00
−0.05
0.04
0.06
0.08
0.10
SNP effect on Pericarditis
SNP effect on EFNB2

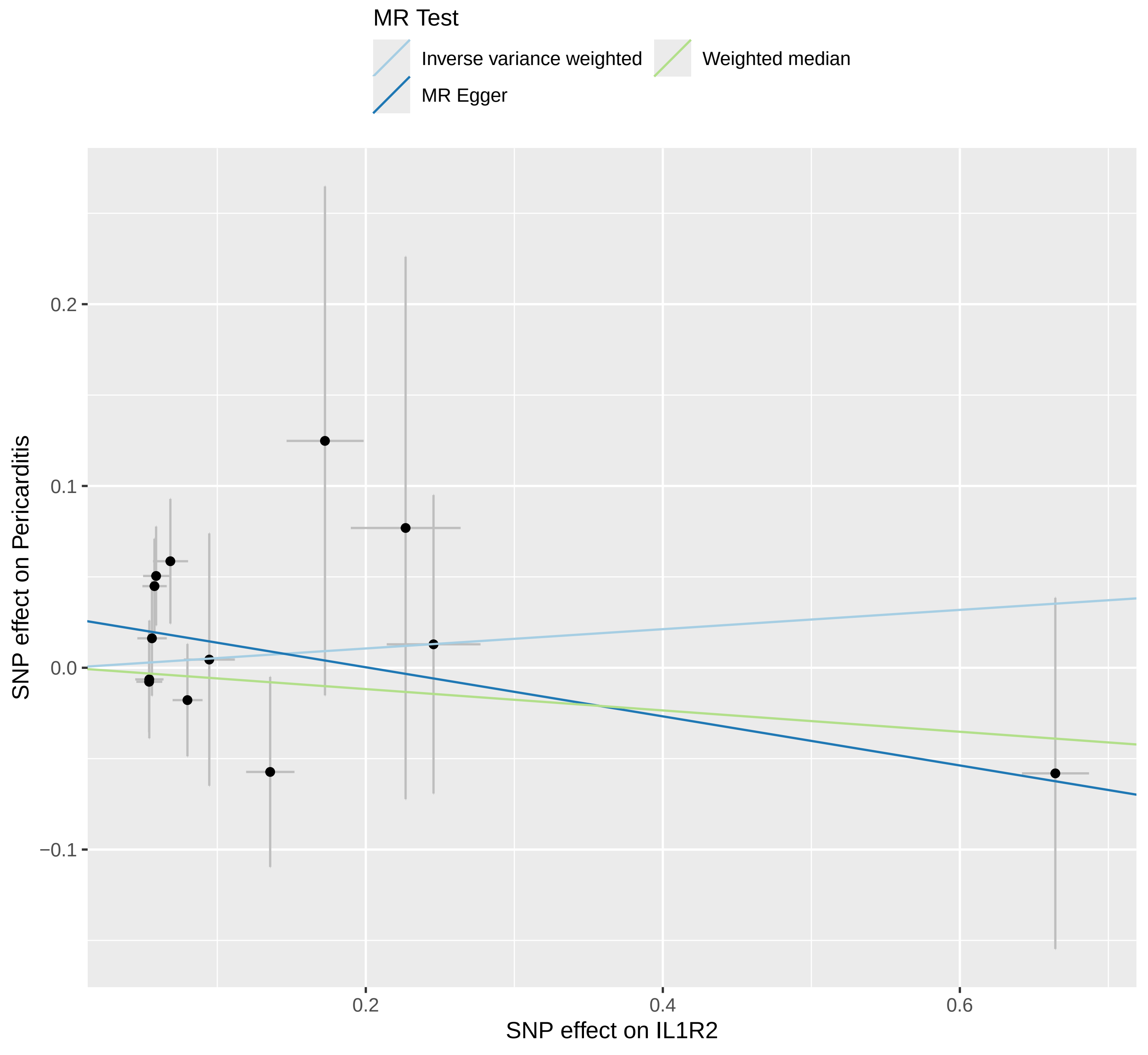
MR Test
Inverse variance weighted
Weighted median
MR Egger
SNP effect on Pericarditis
0.2
0.1
0.0
−0.1
0.2
0.4
0.6
SNP effect on IL1R2

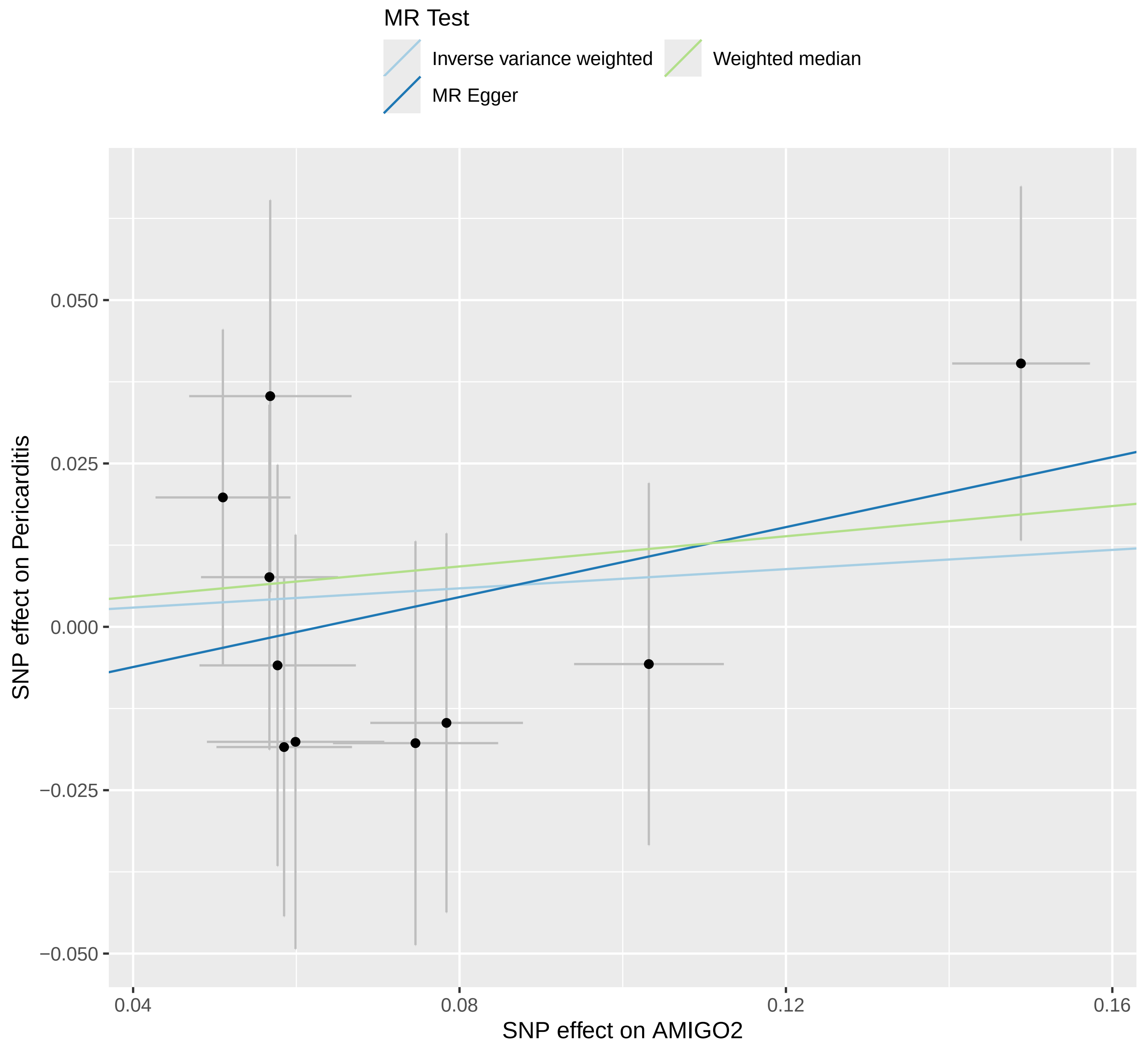
MR Test
Inverse variance weighted
Weighted median
MR Egger
0.050
0.025
0.000
−0.025
−0.050
SNP effect on Pericarditis
0.04
0.08
0.12
0.16
SNP effect on AMIGO2

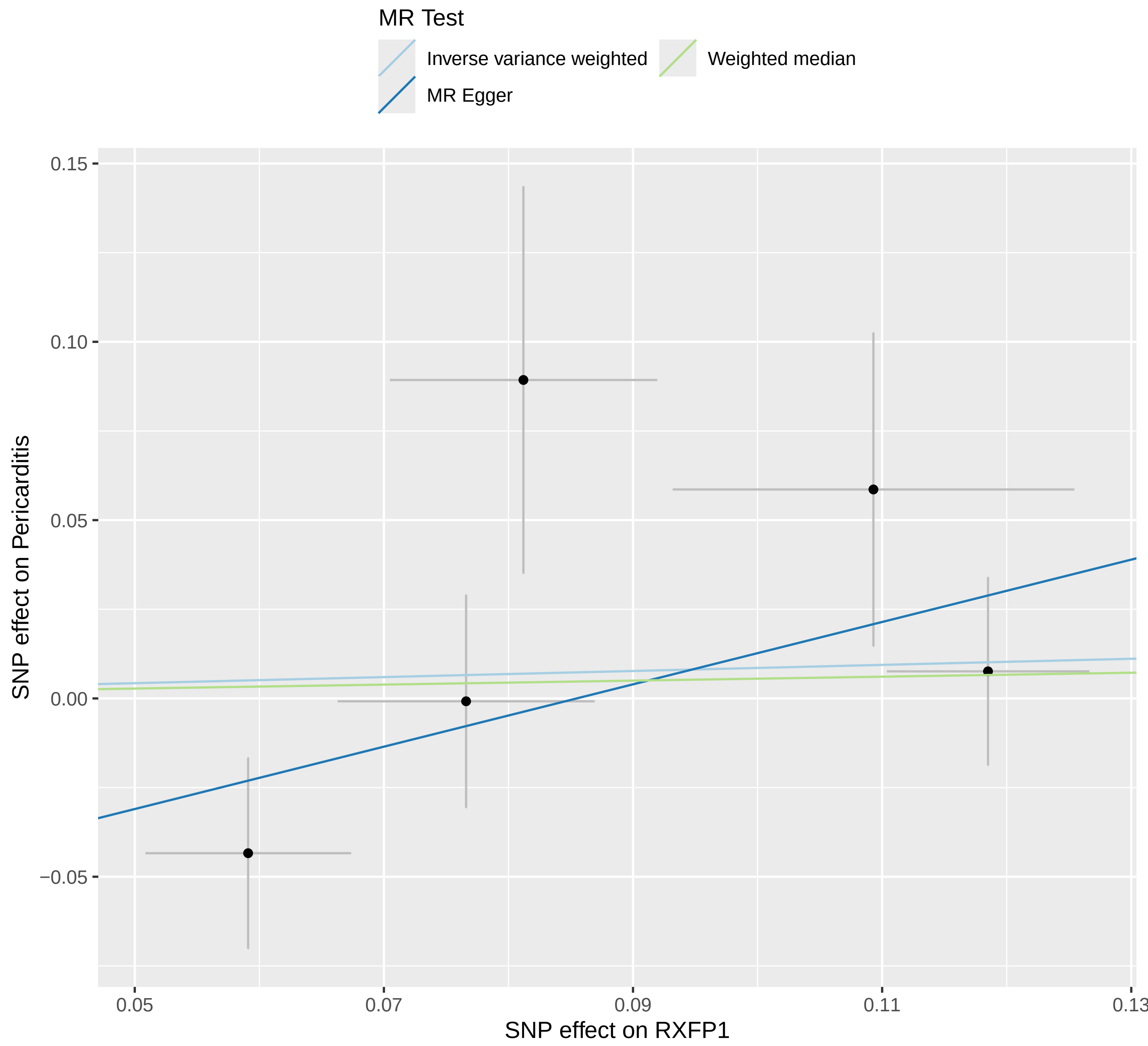
MR Test
Inverse variance weighted
MR Egger
Weighted median
0.15
0.10
0.05
0.00
−0.05
SNP effect on Pericarditis
0.05
0.07
0.09
0.11
0.13
SNP effect on RXFP1

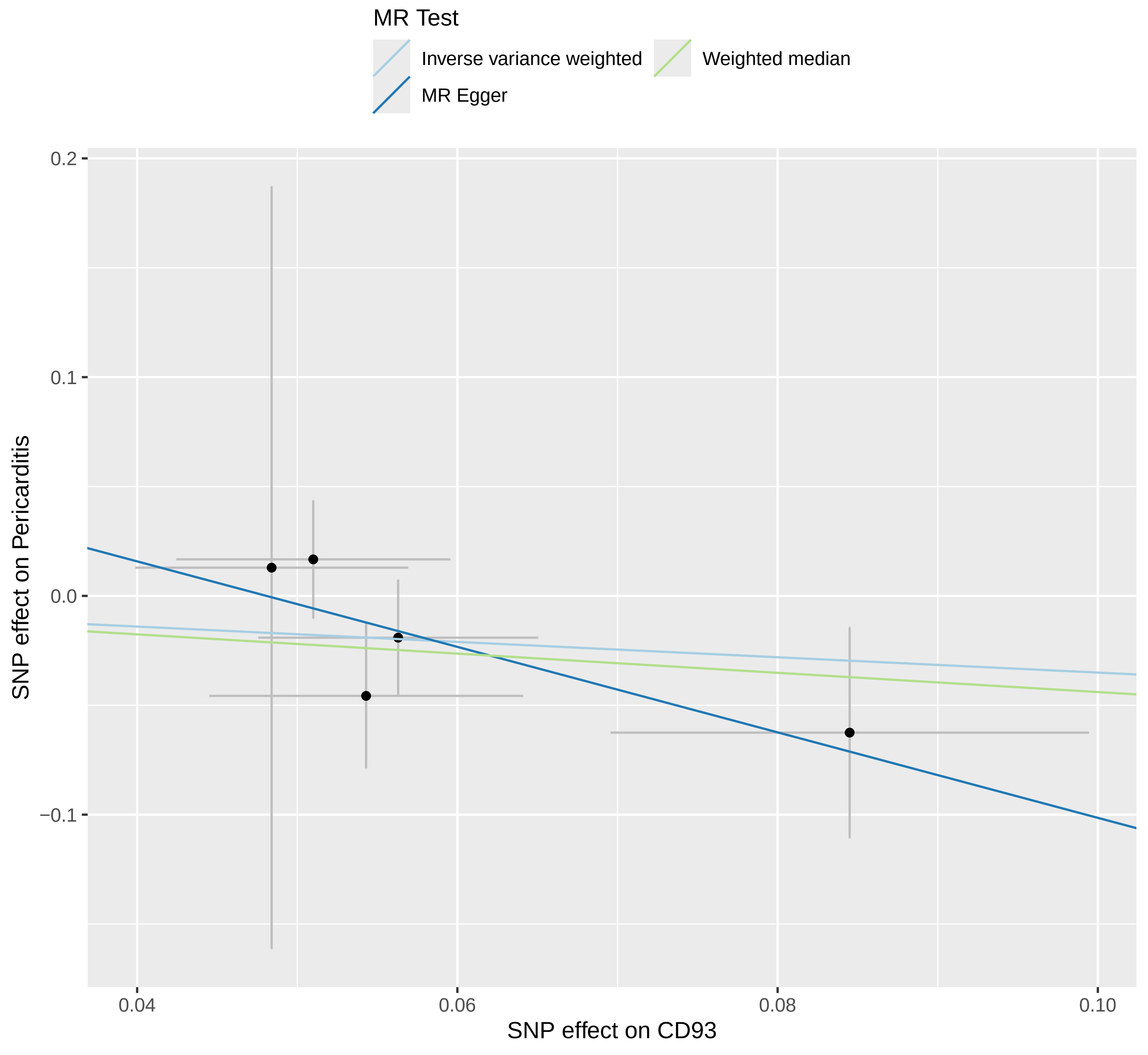

MR Test
Inverse variance weighted
Weighted median
MR Egger
0.2
0.1
0.0
−0.1
SNP effect on Pericarditis
0.04
0.06
0.08
0.10
SNP effect on CD93

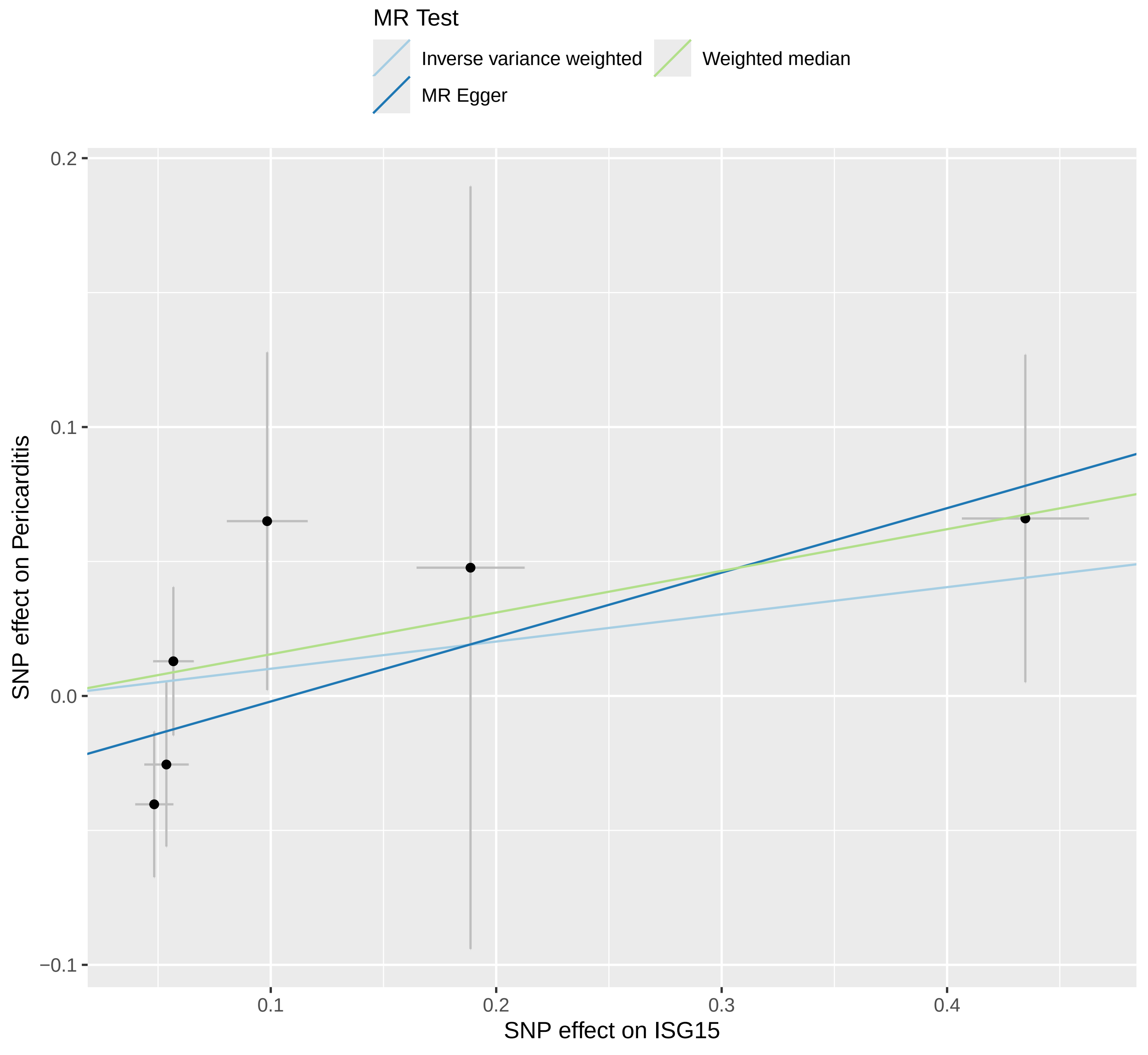

MR Test
Inverse variance weighted
Weighted median
MR Egger
0.2
0.1
0.0
−0.1
0.1
0.2
0.3
0.4
SNP effect on Pericarditis
SNP effect on ISG15

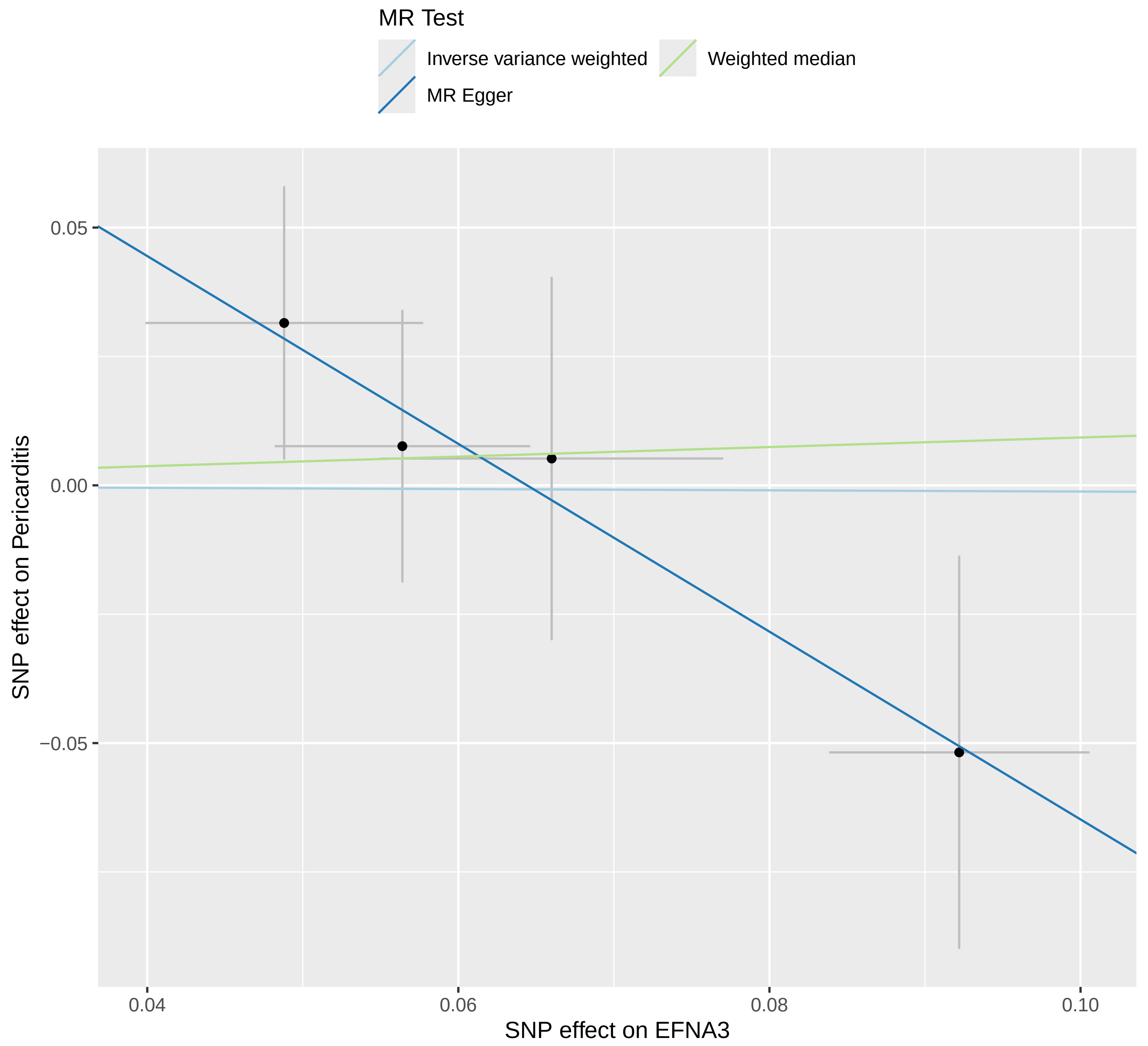
MR Test
Inverse variance weighted
Weighted median
MR Egger
0.05
0.00
−0.05
0.04
0.06
0.08
0.10
SNP effect on Pericarditis
SNP effect on EFNA3

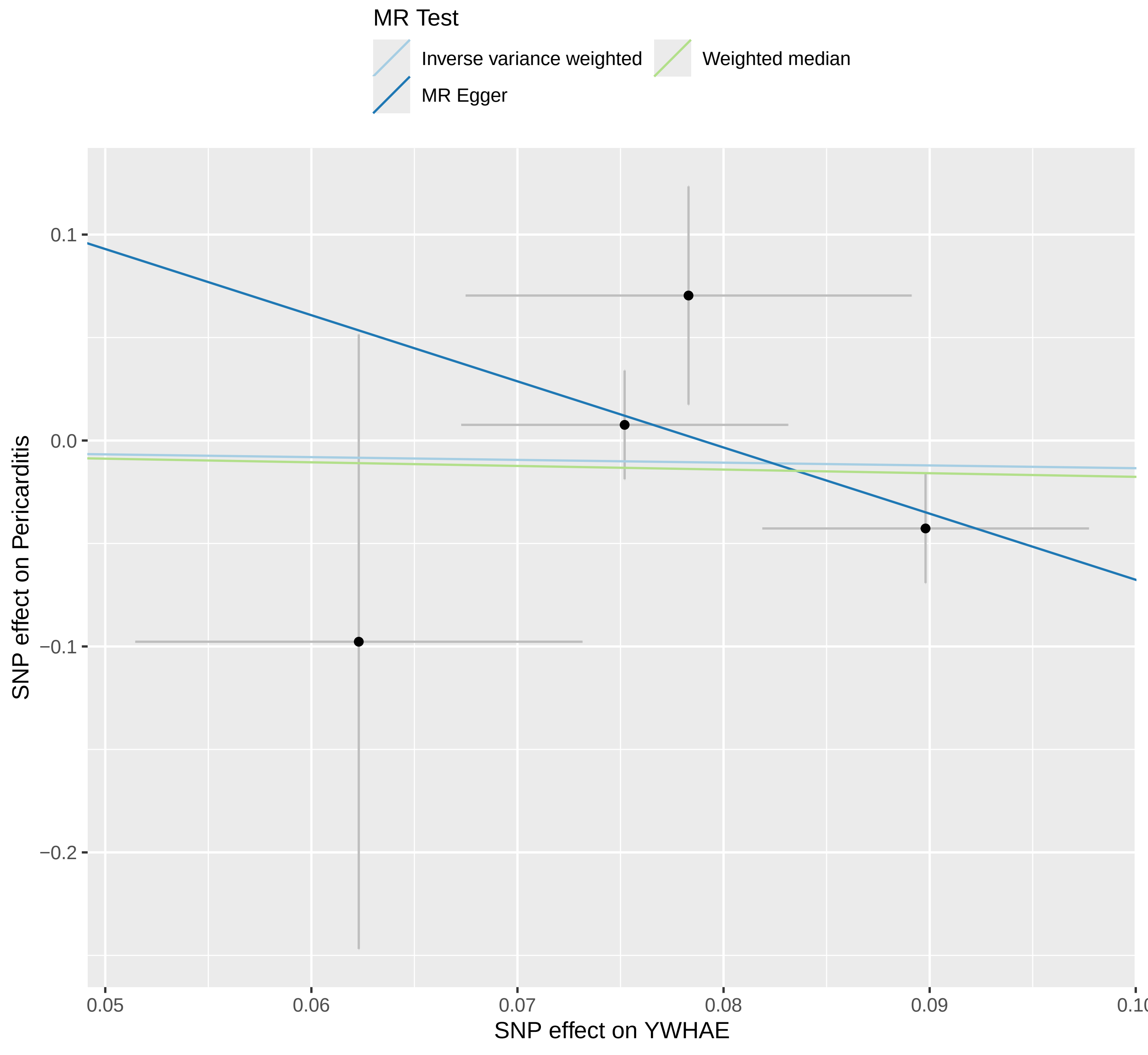

MR Test
Inverse variance weighted
Weighted median
MR Egger
0.1
0.0
−0.1
−0.2
SNP effect on Pericarditis
0.05
0.06
0.07
0.08
0.09
0.10
SNP effect on YWHAE

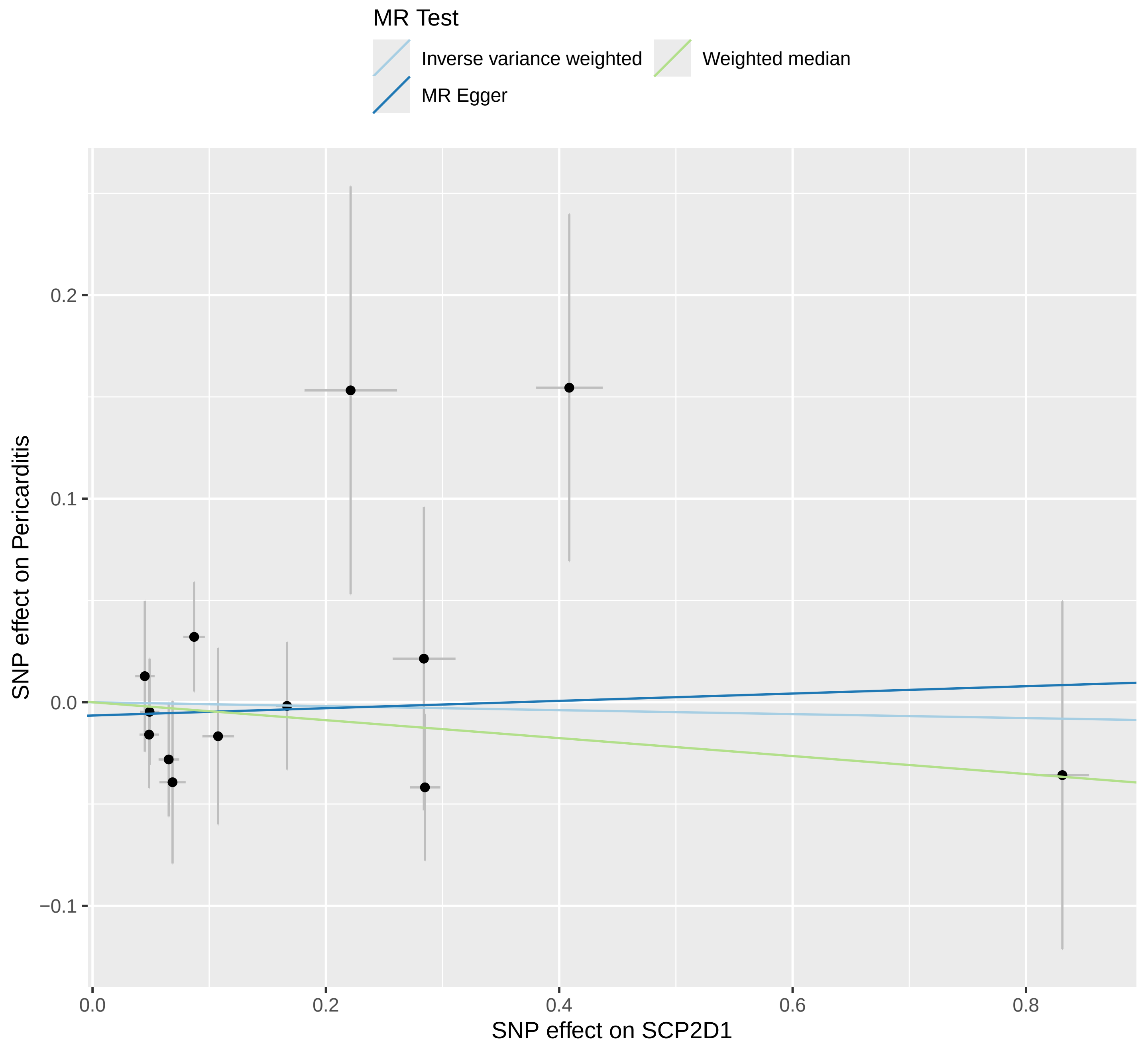
MR Test
Inverse variance weighted
Weighted median
MR Egger
0.2
0.1
0.0
−0.1
SNP effect on Pericarditis
0.0
0.2
0.4
0.6
0.8
SNP effect on SCP2D1

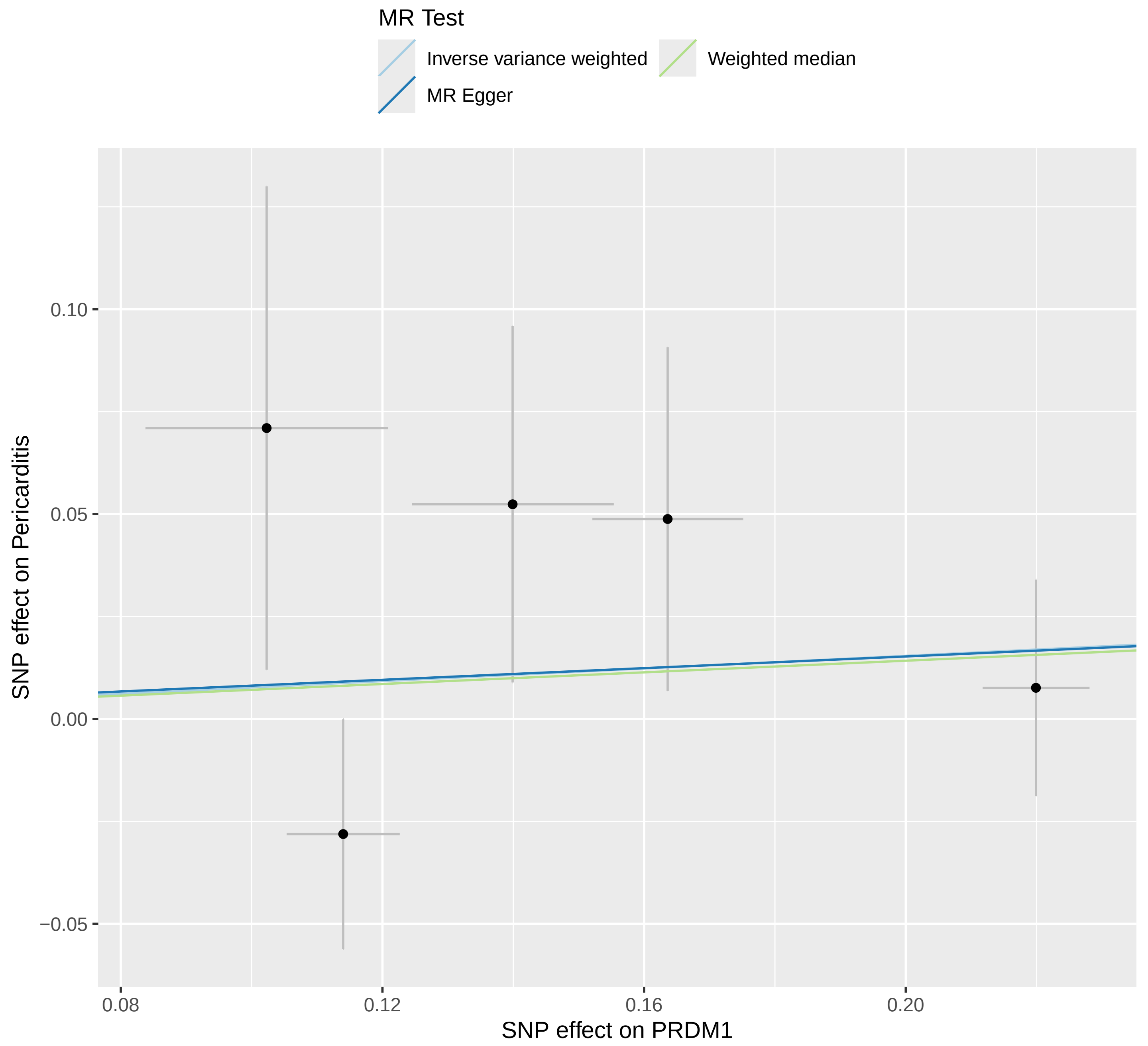
MR Test
Inverse variance weighted
MR Egger
Weighted median
SNP effect on Pericarditis
0.10
0.05
0.00
−0.05
0.08
0.12
0.16
0.20
SNP effect on PRDM1

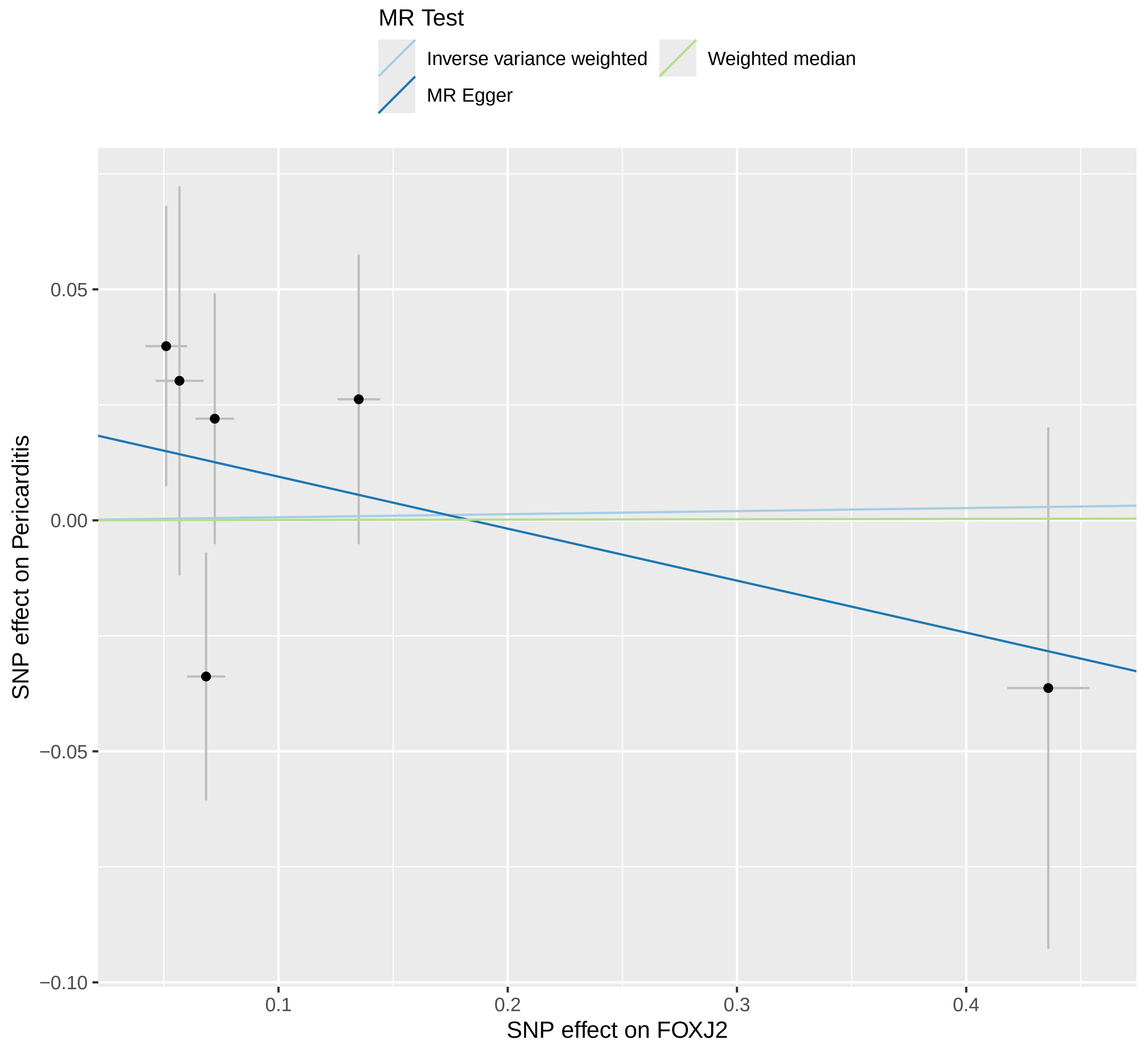
MR Test
Inverse variance weighted
Weighted median
MR Egger
0.05
0.00
−0.05
−0.10
0.1
0.2
0.3
0.4
SNP effect on Pericarditis
SNP effect on FOXJ2

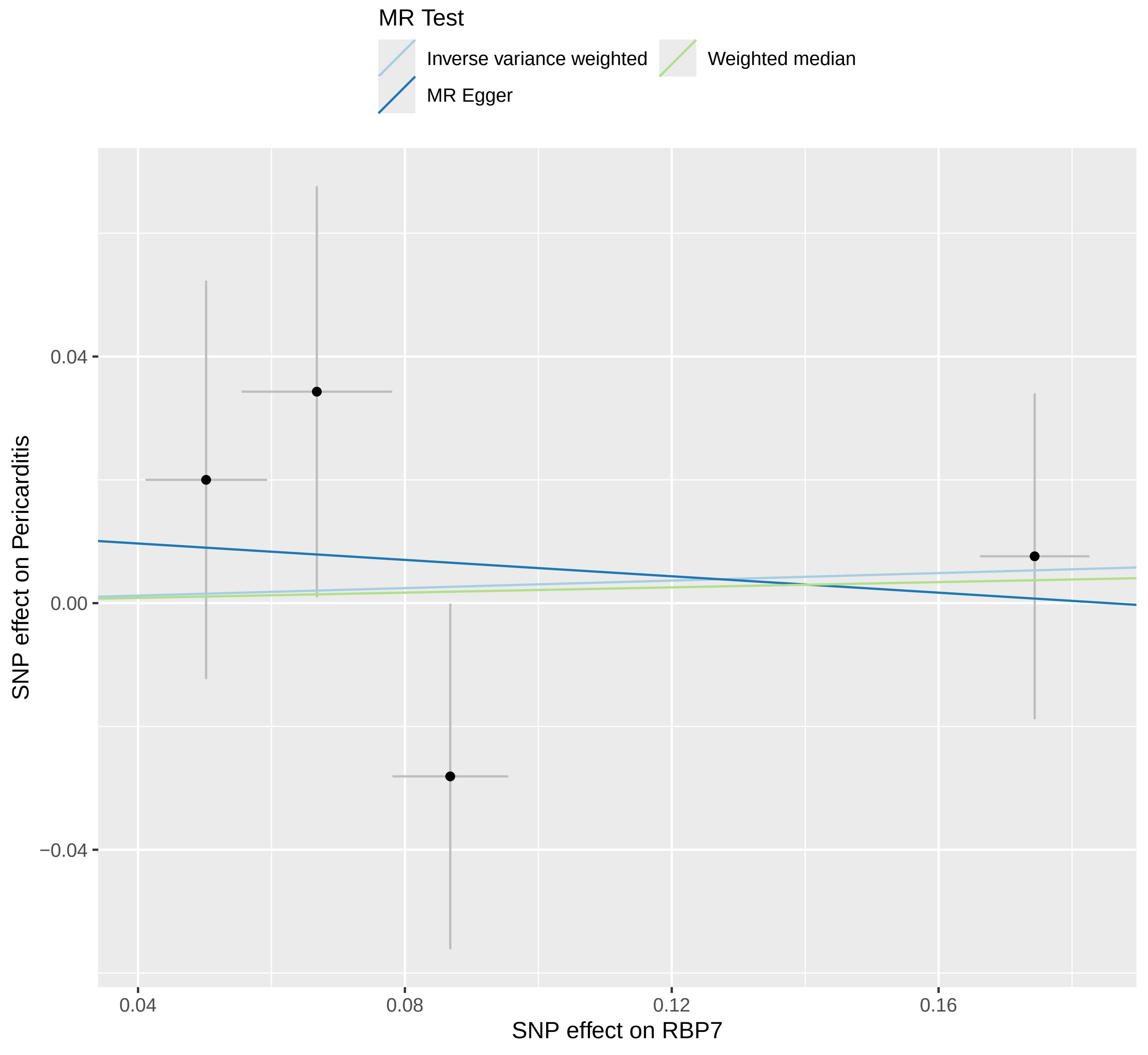
MR Test
Inverse variance weighted
MR Egger
Weighted median
SNP effect on Pericarditis
0.04
0.00
−0.04
0.04
0.08
0.12
0.16
SNP effect on RBP7

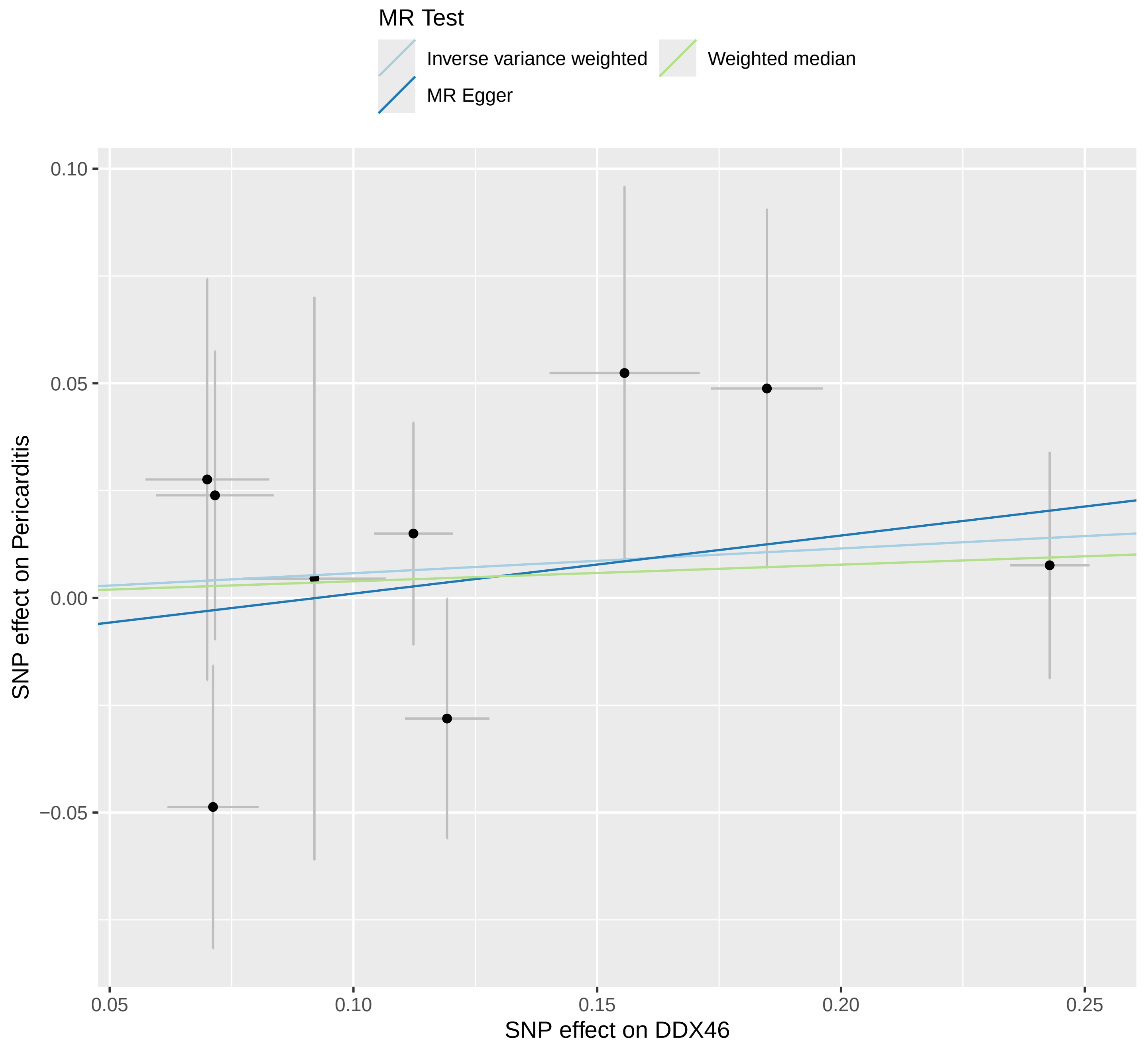
MR Test
Inverse variance weighted
MR Egger
Weighted median
0.10
0.05
0.00
−0.05
SNP effect on Pericarditis
0.05
0.10
0.15
0.20
0.25
SNP effect on DDX46

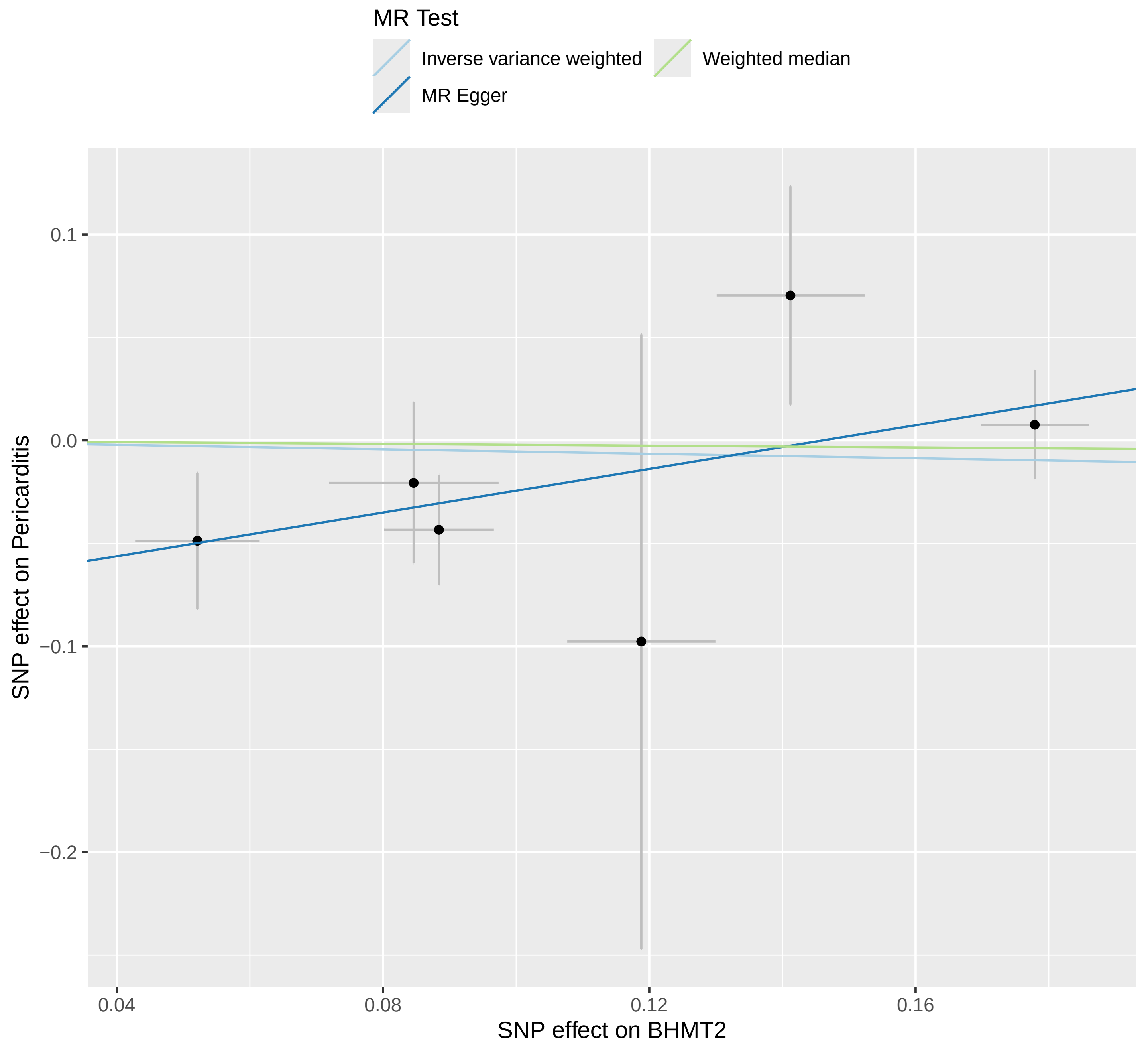
MR Test
Inverse variance weighted
Weighted median
MR Egger
0.1
0.0
−0.1
−0.2
0.04
0.08
0.12
0.16
SNP effect on BHMT2
SNP effect on Pericarditis

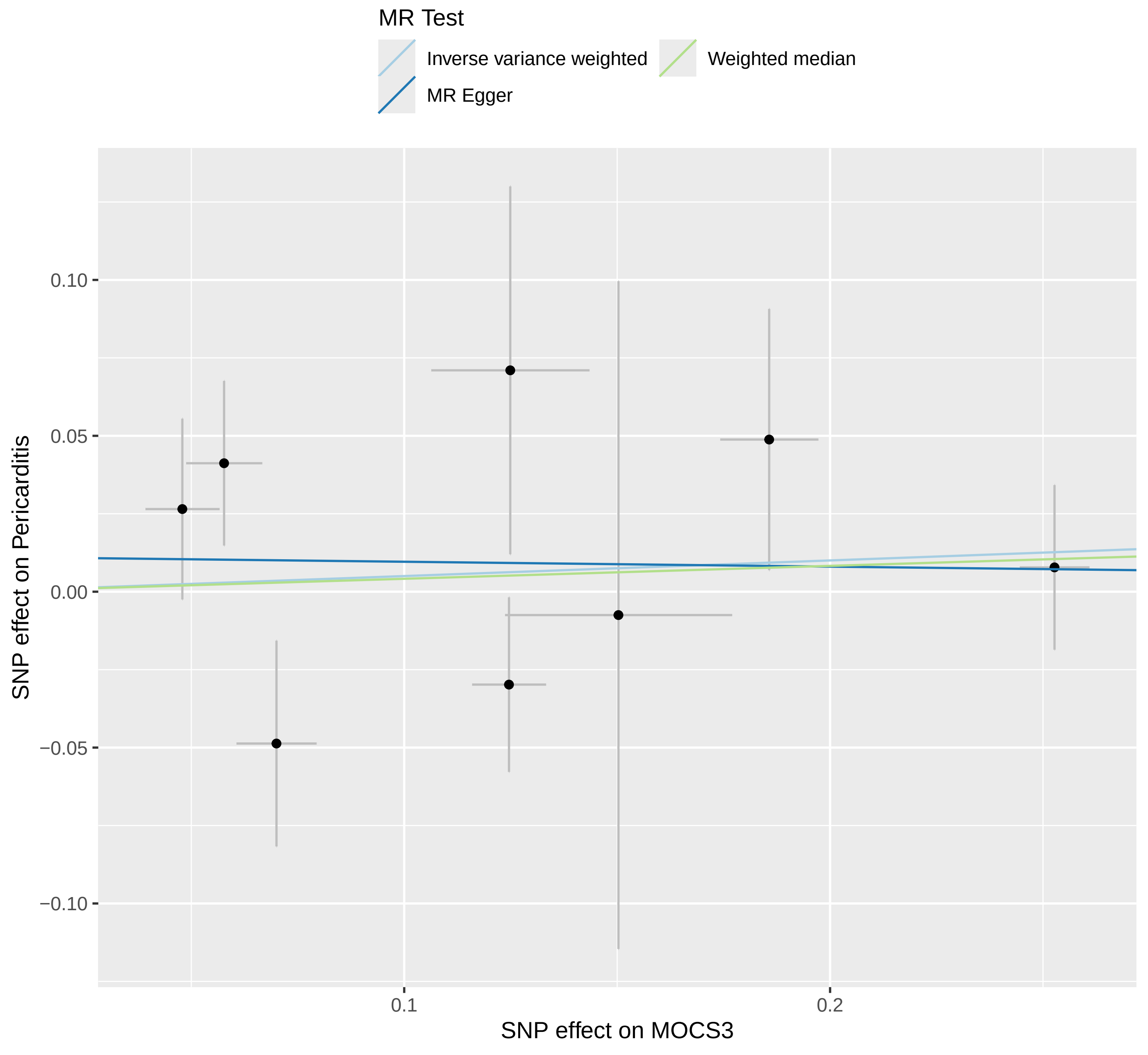
MR Test
Inverse variance weighted
MR Egger
Weighted median
0.10
0.05
0.00
−0.05
−0.10
SNP effect on Pericarditis
0.1
0.2
SNP effect on MOCS3

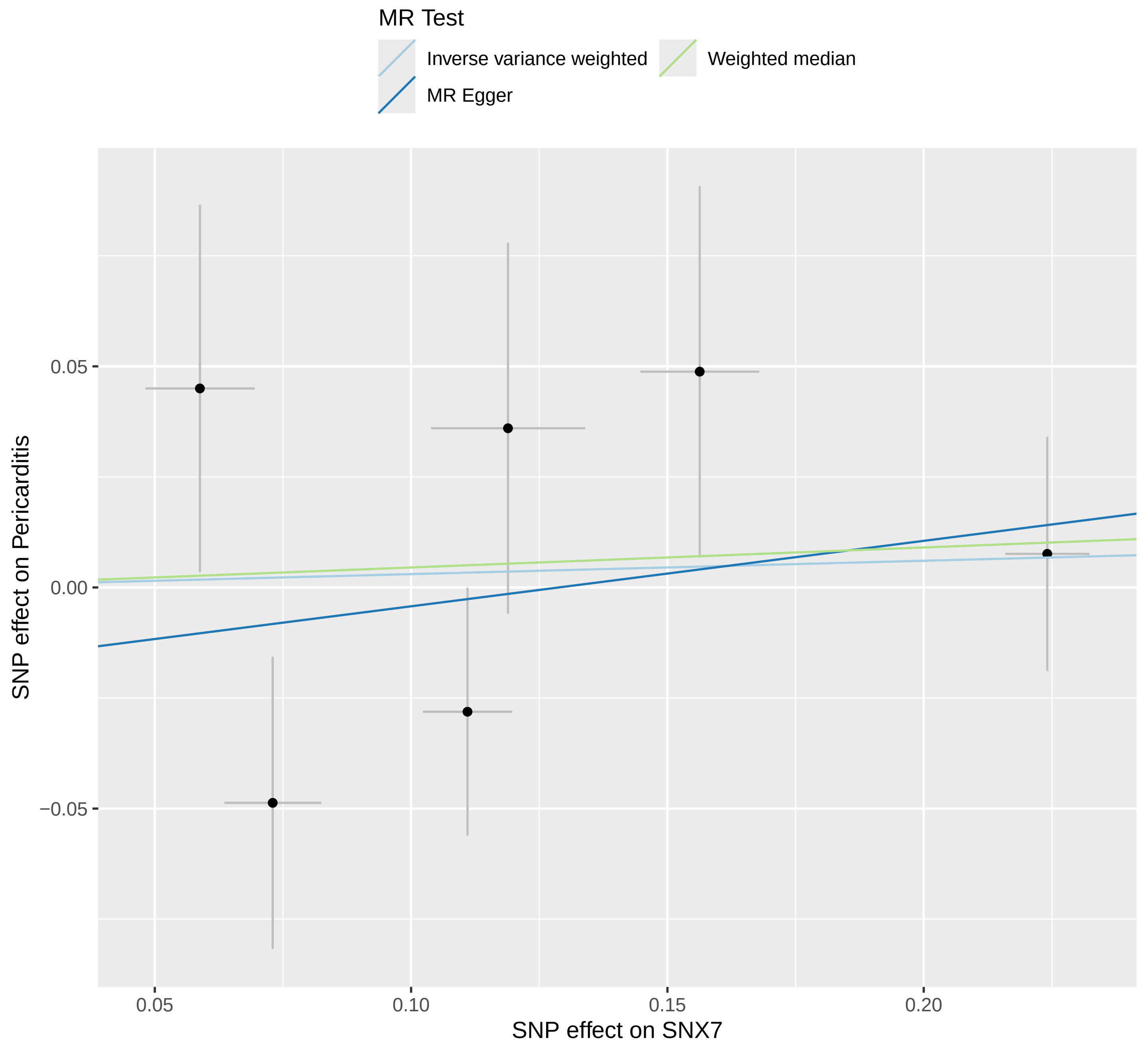
MR Test
Inverse variance weighted
Weighted median
MR Egger
0.05
0.00
−0.05
SNP effect on Pericarditis
0.05
0.10
0.15
0.20
SNP effect on SNX7

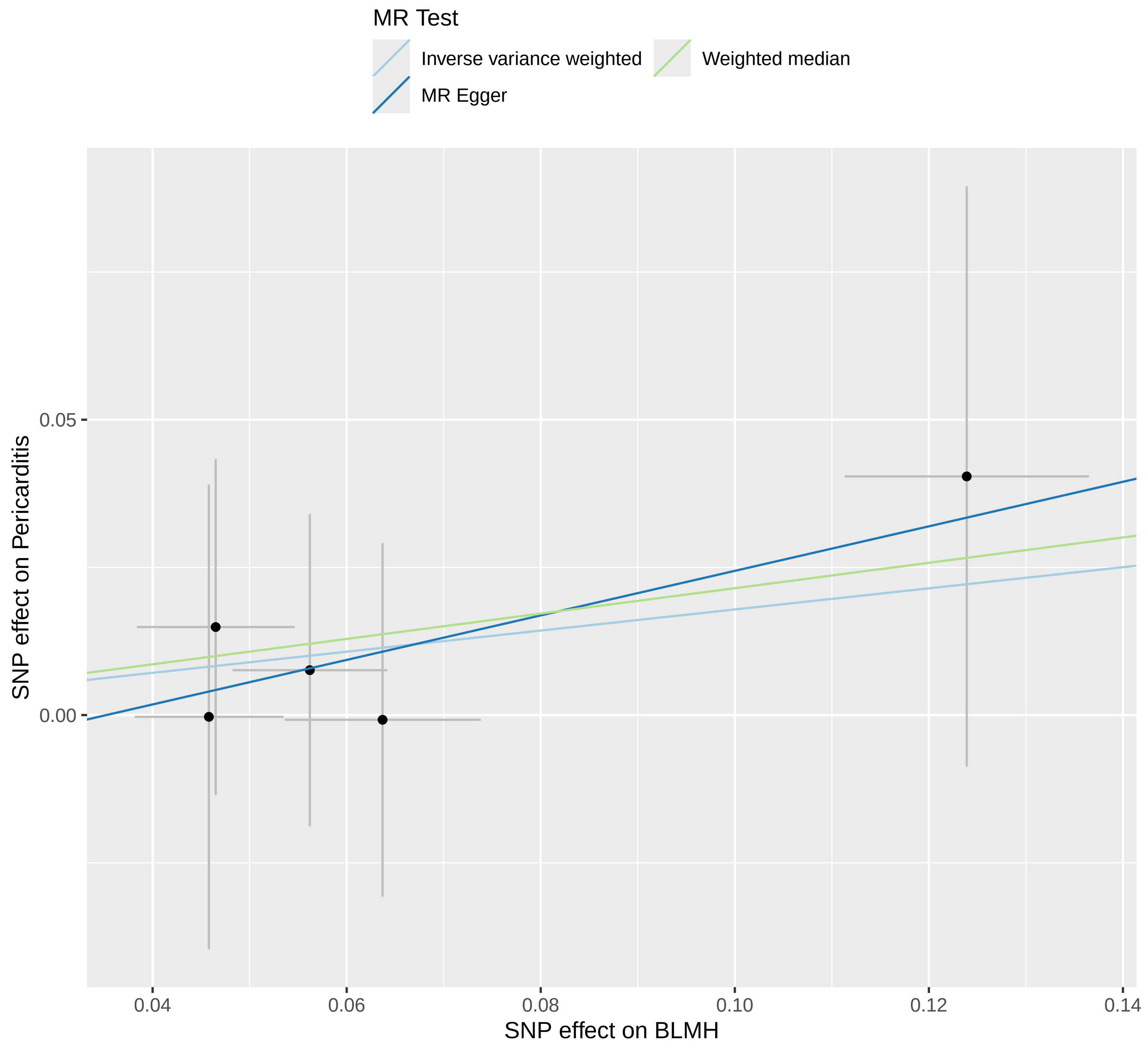
MR Test
Inverse variance weighted
MR Egger
Weighted median
0.05
0.00
SNP effect on Pericarditis
0.04
0.06
0.08
0.10
0.12
0.14
SNP effect on BLMH

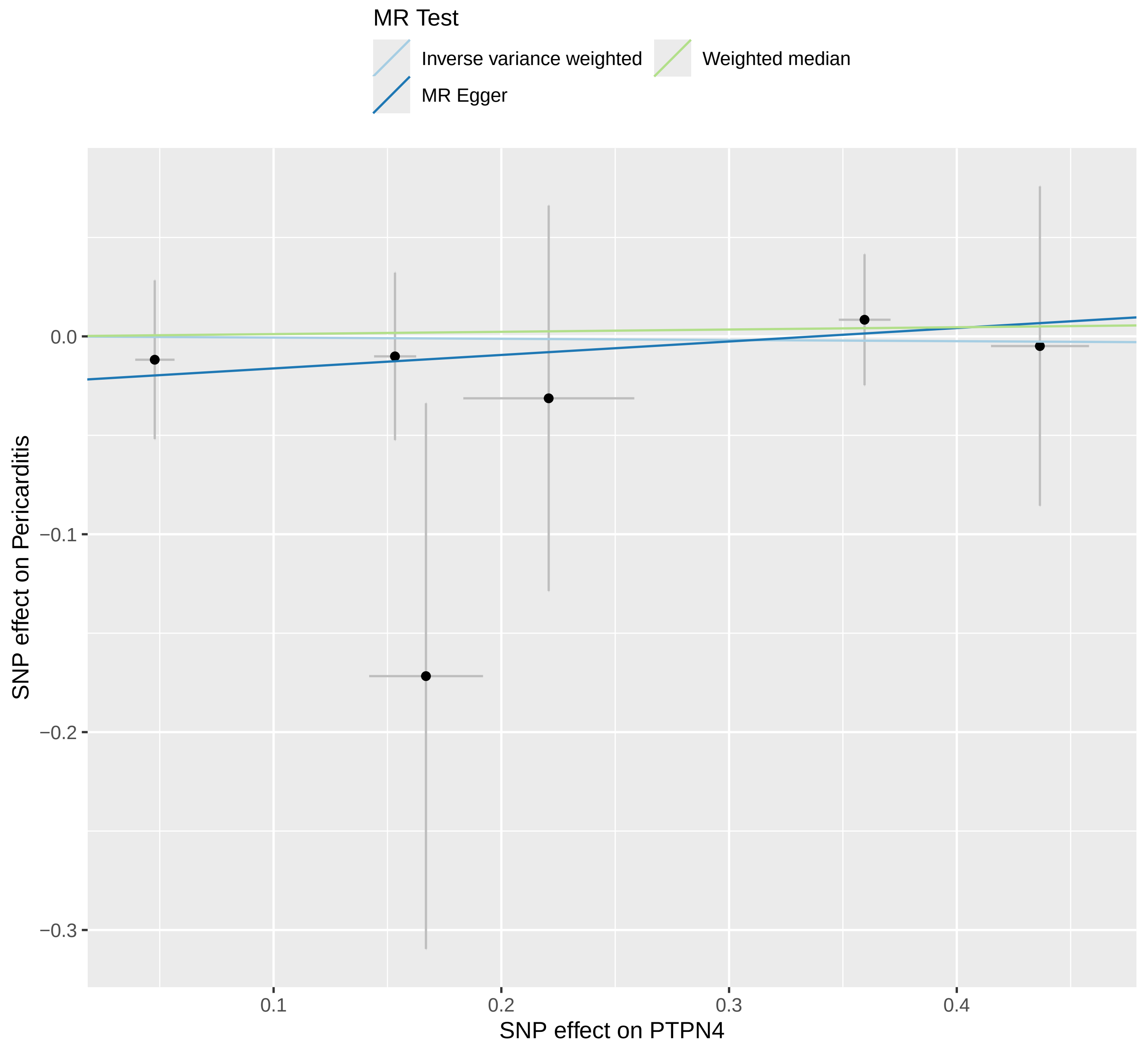
MR Test
Inverse variance weighted
Weighted median
MR Egger
SNP effect on Pericarditis
0.0
−0.1
−0.2
−0.3
0.1
0.2
0.3
0.4
SNP effect on PTPN4

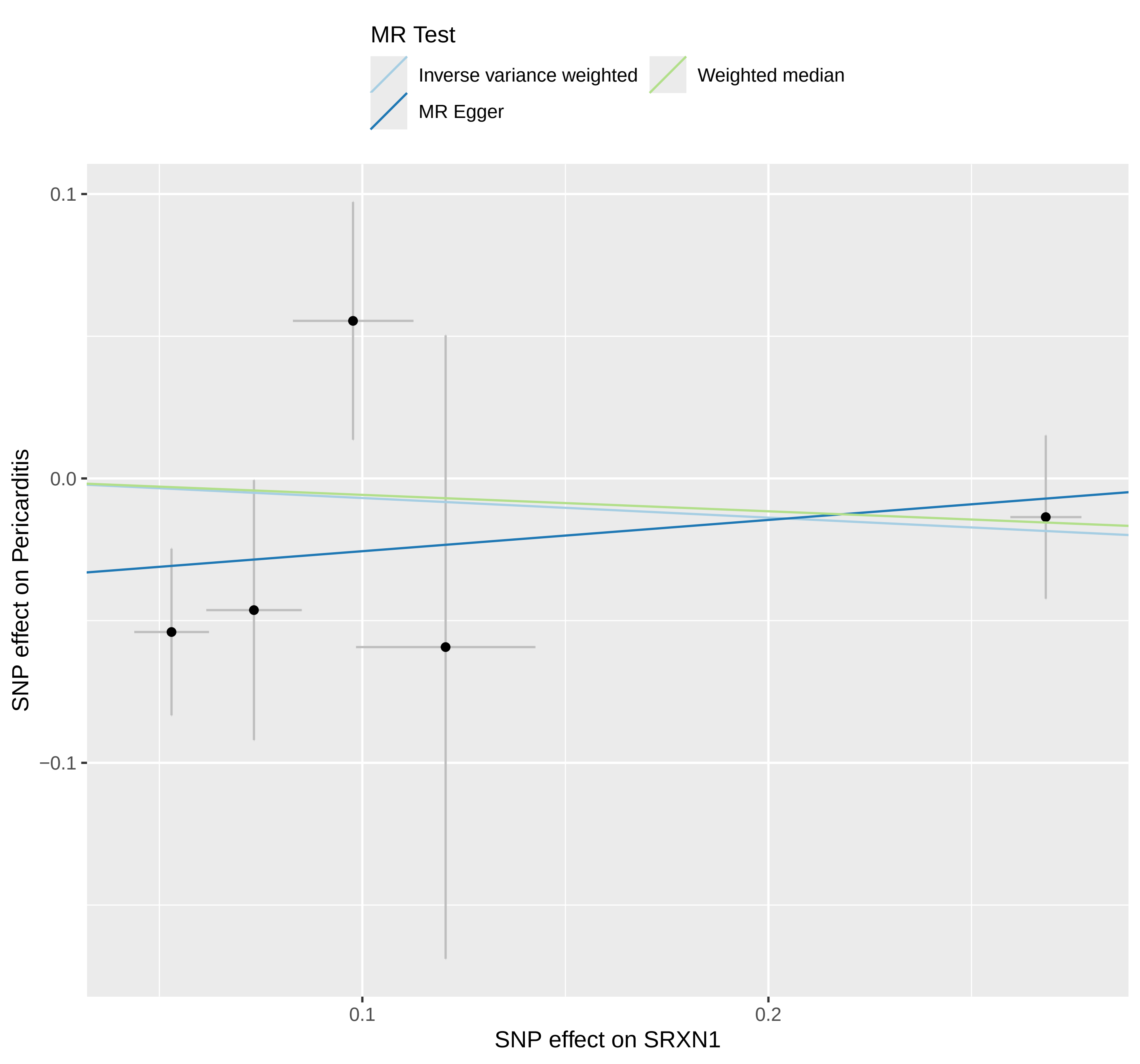

MR Test
Inverse variance weighted
Weighted median
MR Egger
0.1
0.0
−0.1
0.1
0.2
SNP effect on Pericarditis
SNP effect on SRXN1

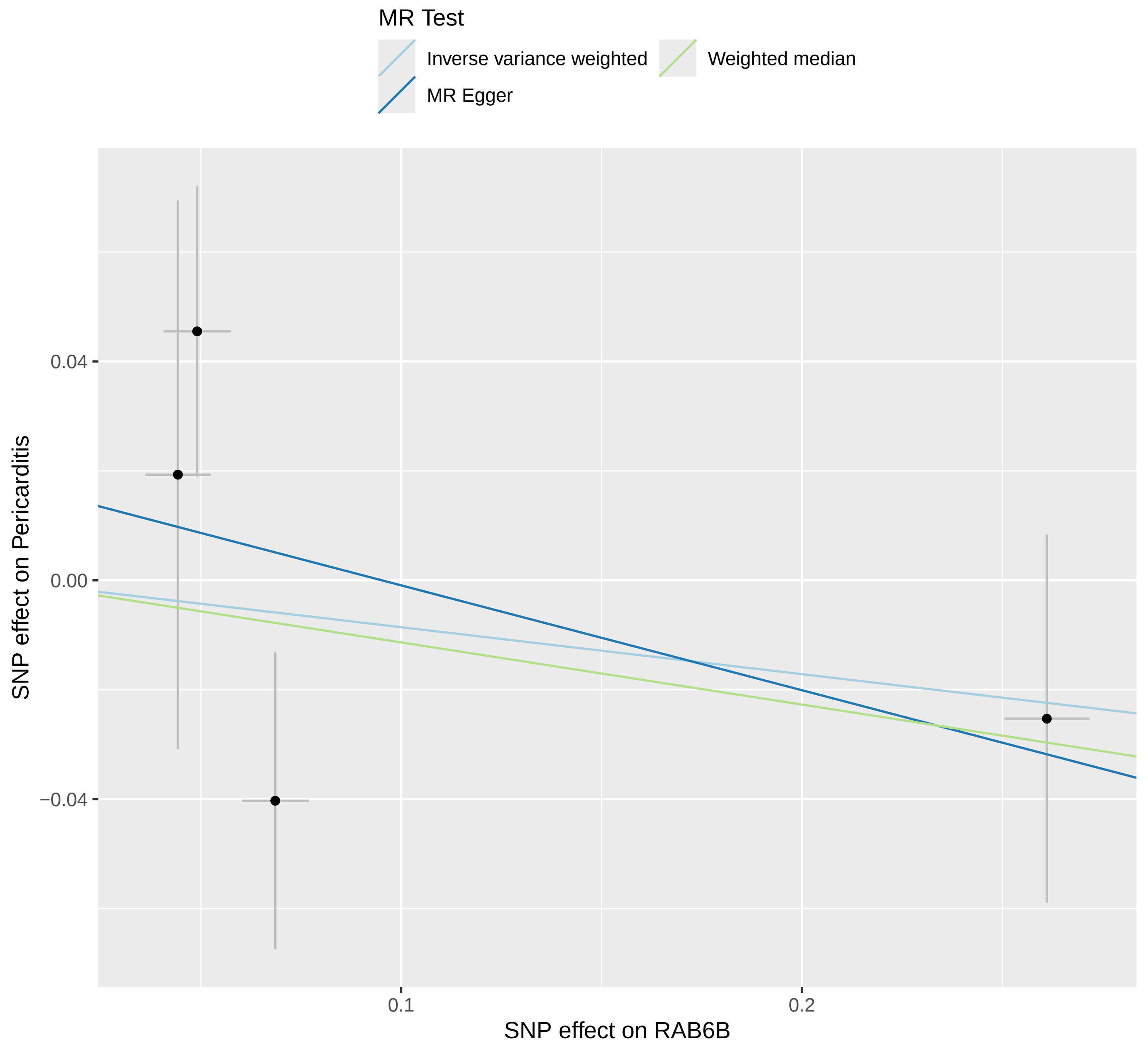
MR Test
Inverse variance weighted
Weighted median
MR Egger
0.04
0.00
−0.04
0.1
0.2
SNP effect on Pericarditis
SNP effect on RAB6B

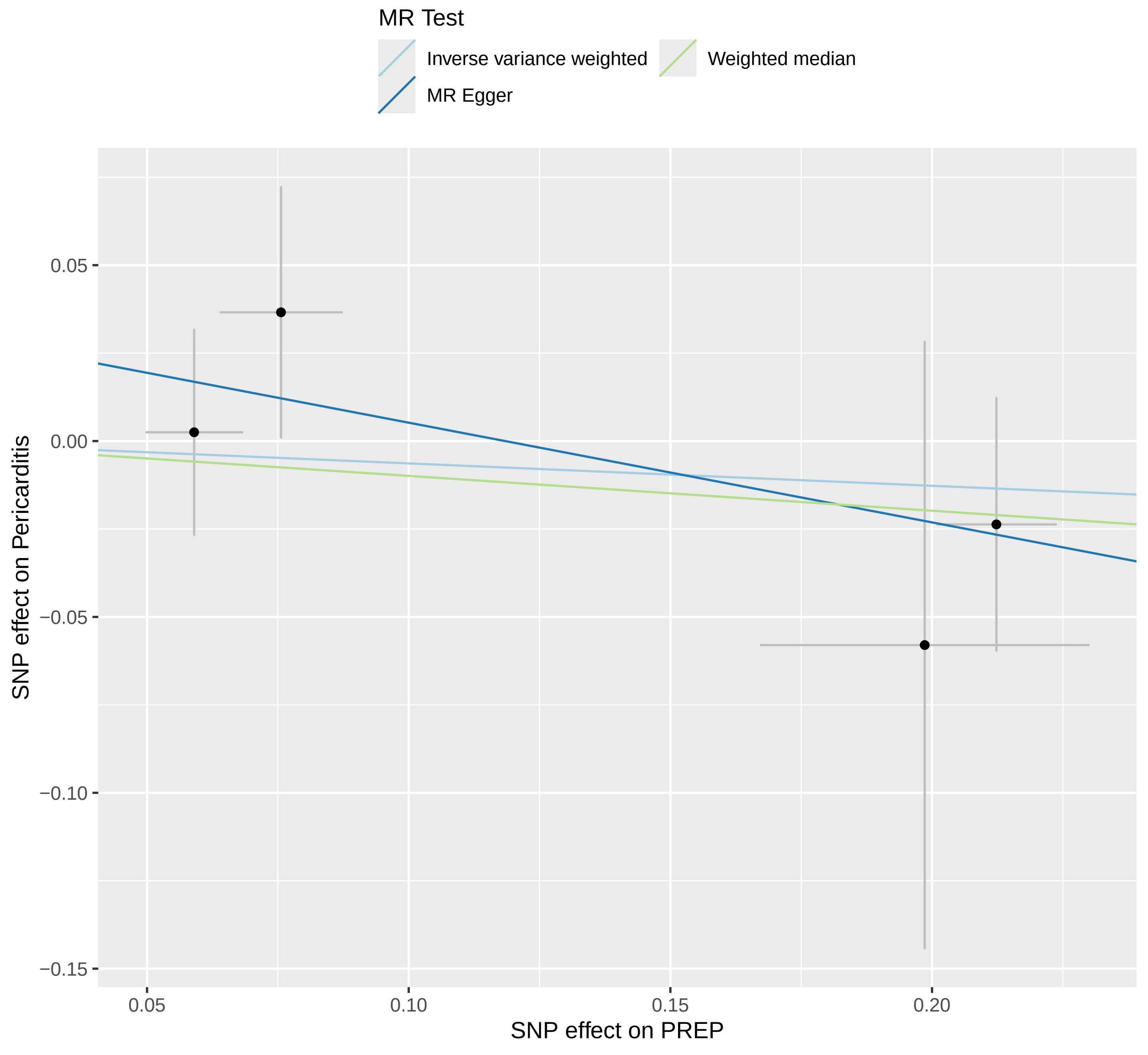

MR Test
Inverse variance weighted
Weighted median
MR Egger
0.05
0.00
−0.05
−0.10
−0.15
SNP effect on Pericarditis
0.05
0.10
0.15
0.20
SNP effect on PREP

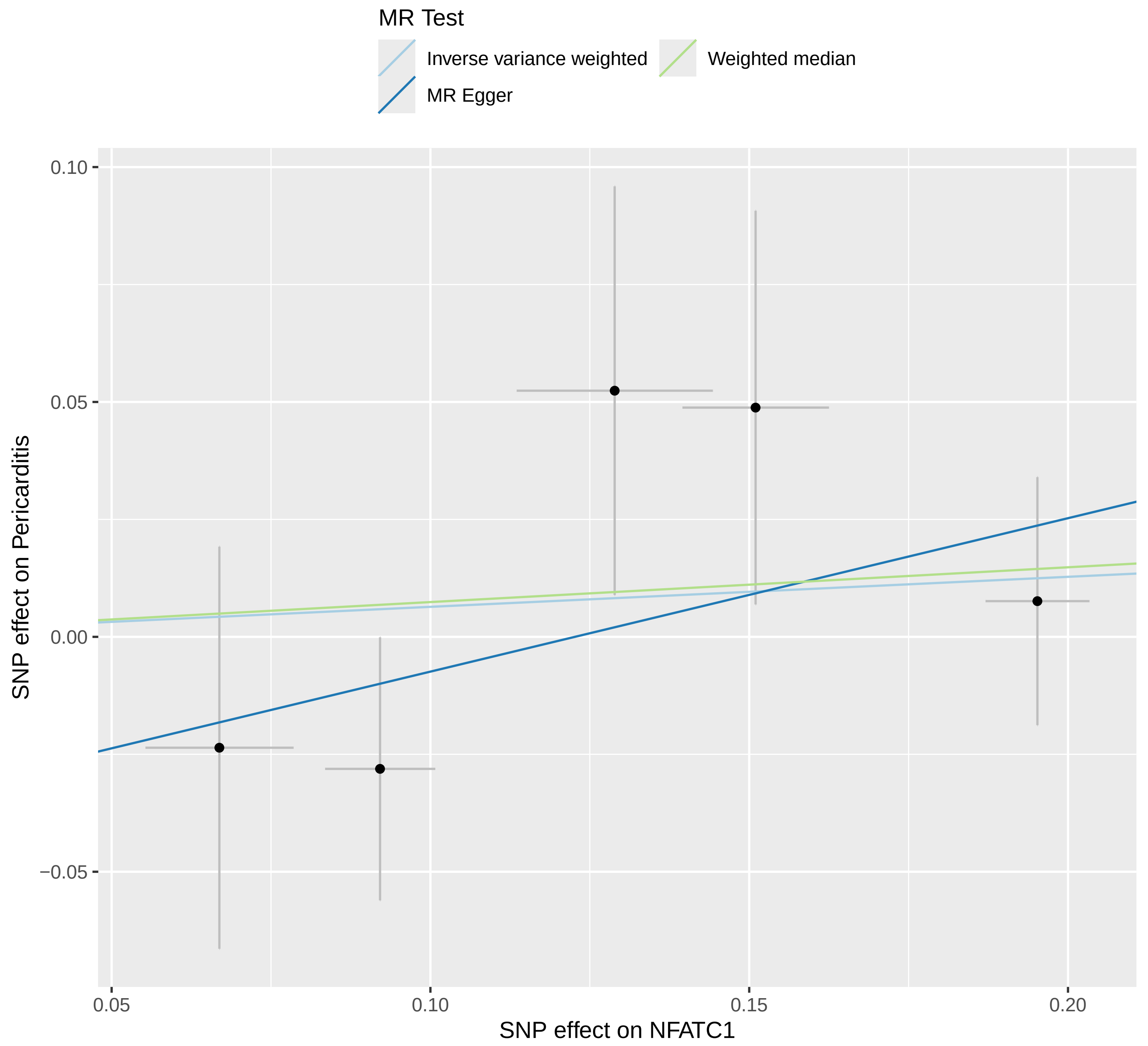

MR Test
Inverse variance weighted
MR Egger
Weighted median
0.10
0.05
0.00
−0.05
SNP effect on Pericarditis
0.05
0.10
0.15
0.20
SNP effect on NFATC1

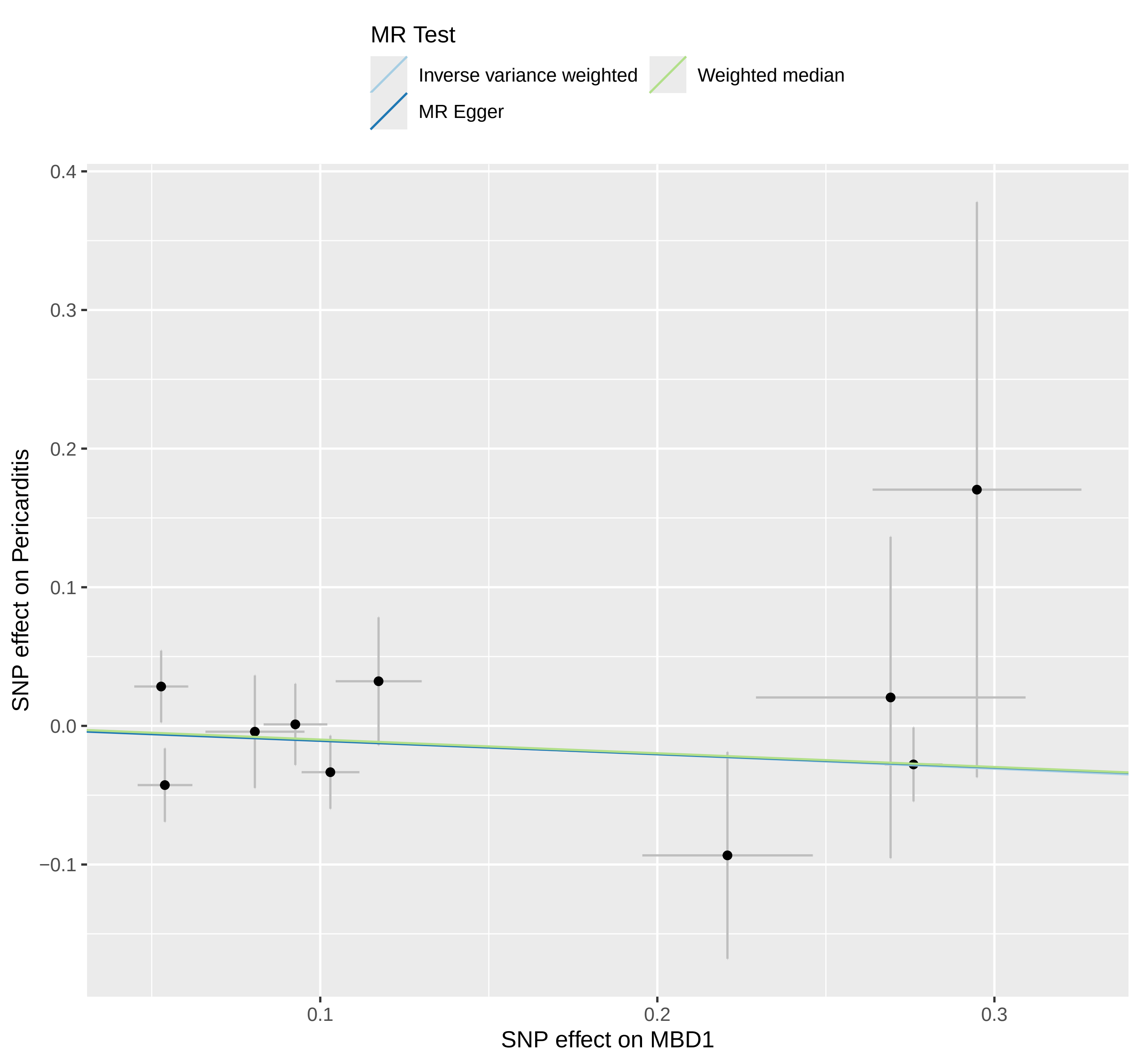
MR Test
Inverse variance weighted
Weighted median
MR Egger
0.4
0.3
0.2
0.1
0.0
−0.1
SNP effect on Pericarditis
0.1
0.2
0.3
SNP effect on MBD1

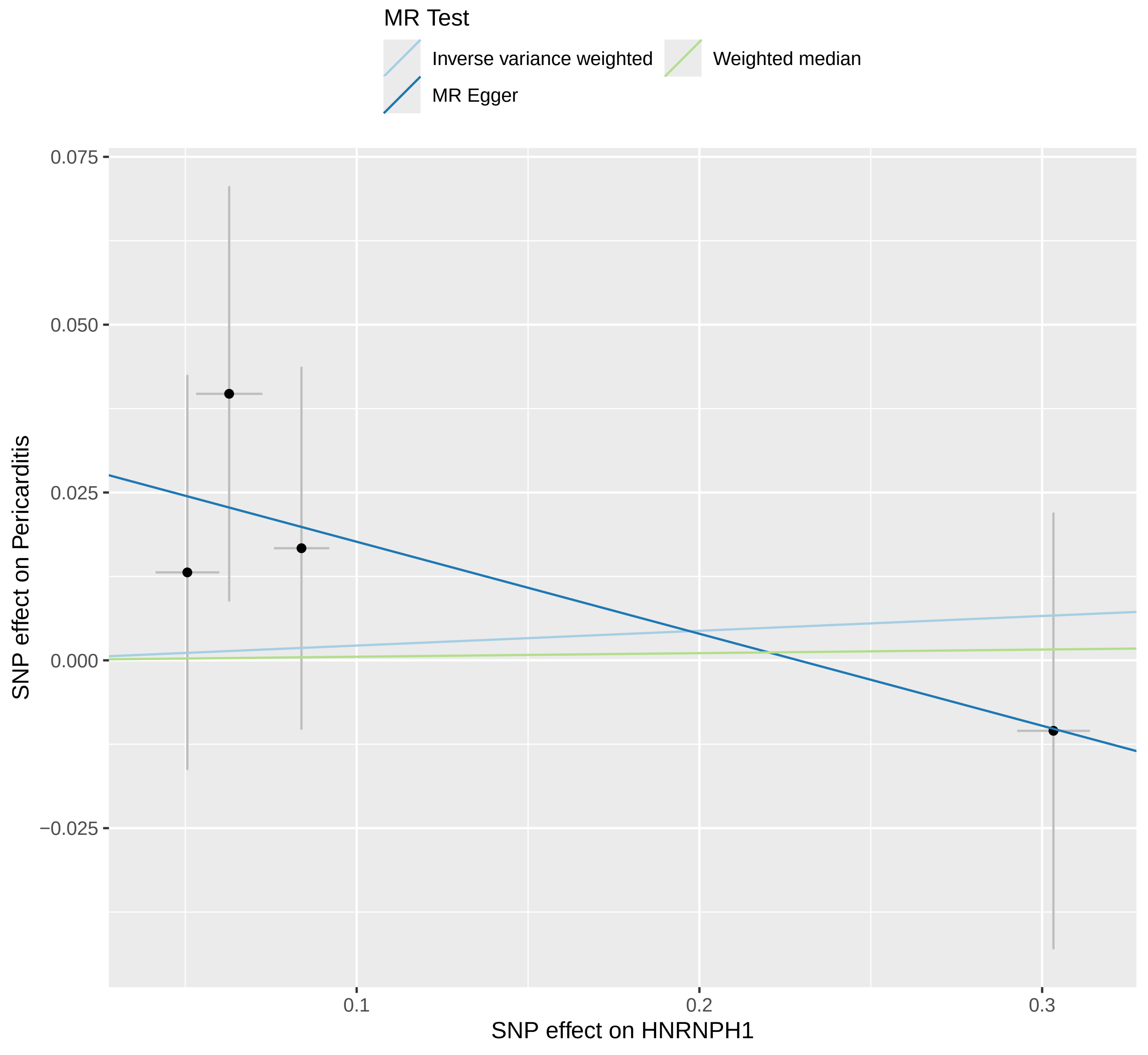
MR Test
Inverse variance weighted
Weighted median
MR Egger
0.075
0.050
0.025
0.000
−0.025
0.1
0.2
0.3
SNP effect on Pericarditis
SNP effect on HNRNPH1

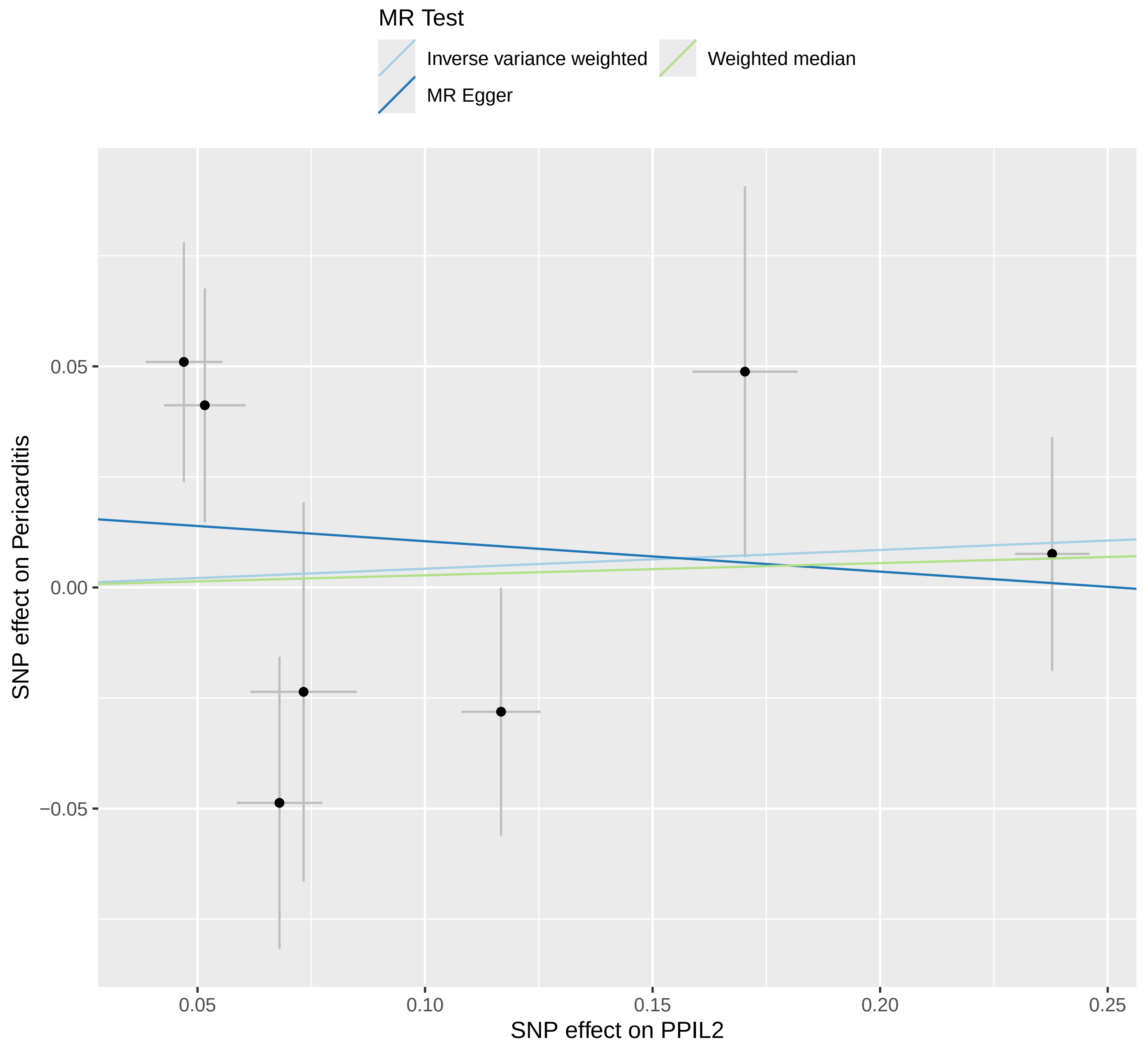

MR Test
Inverse variance weighted
Weighted median
MR Egger
0.05
0.00
−0.05
SNP effect on Pericarditis
0.05
0.10
0.15
0.20
0.25
SNP effect on PPIL2

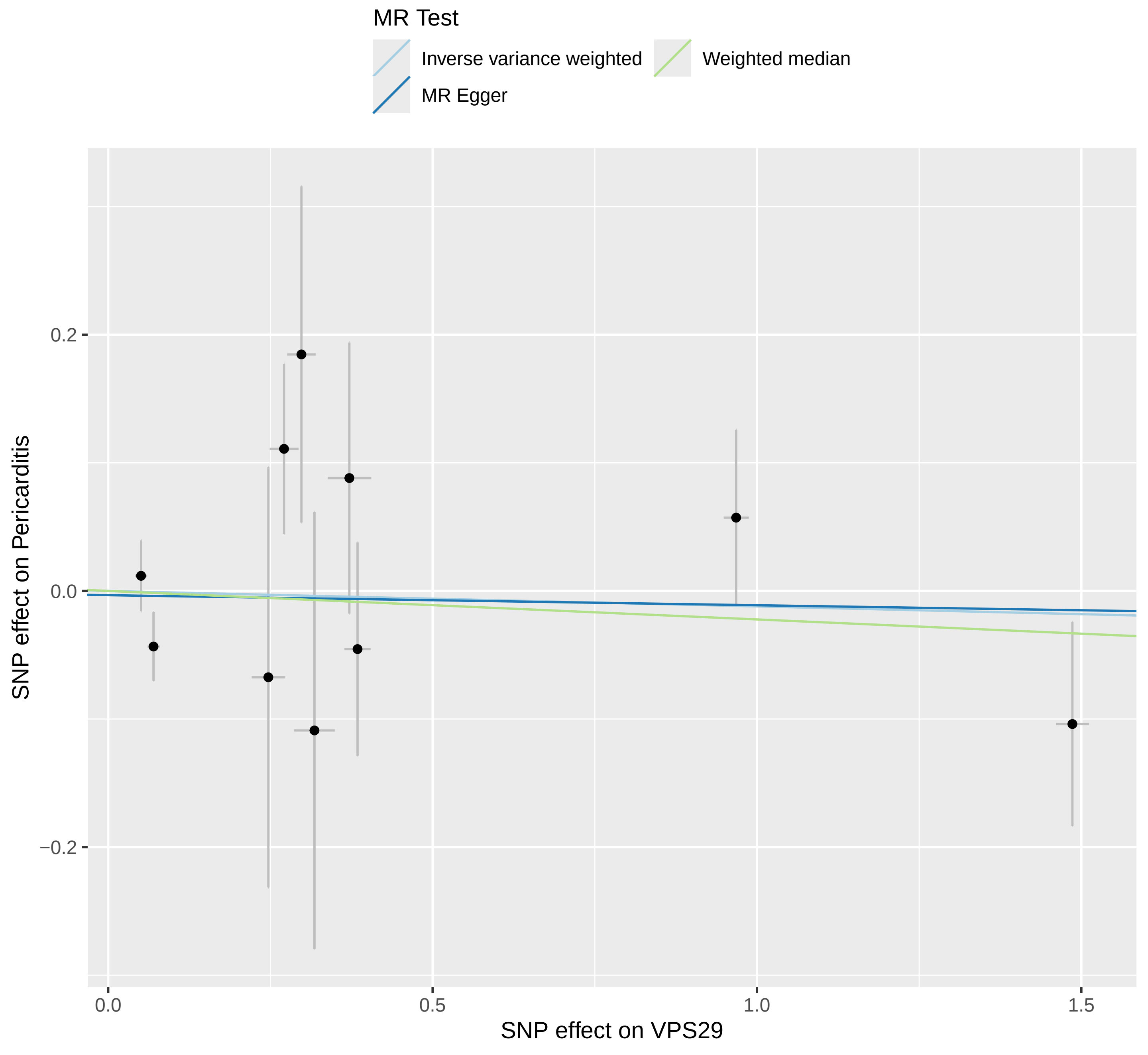

MR Test
Inverse variance weighted
Weighted median
MR Egger
0.2
0.0
−0.2
0.0
0.5
1.0
1.5
SNP effect on Pericarditis
SNP effect on VPS29

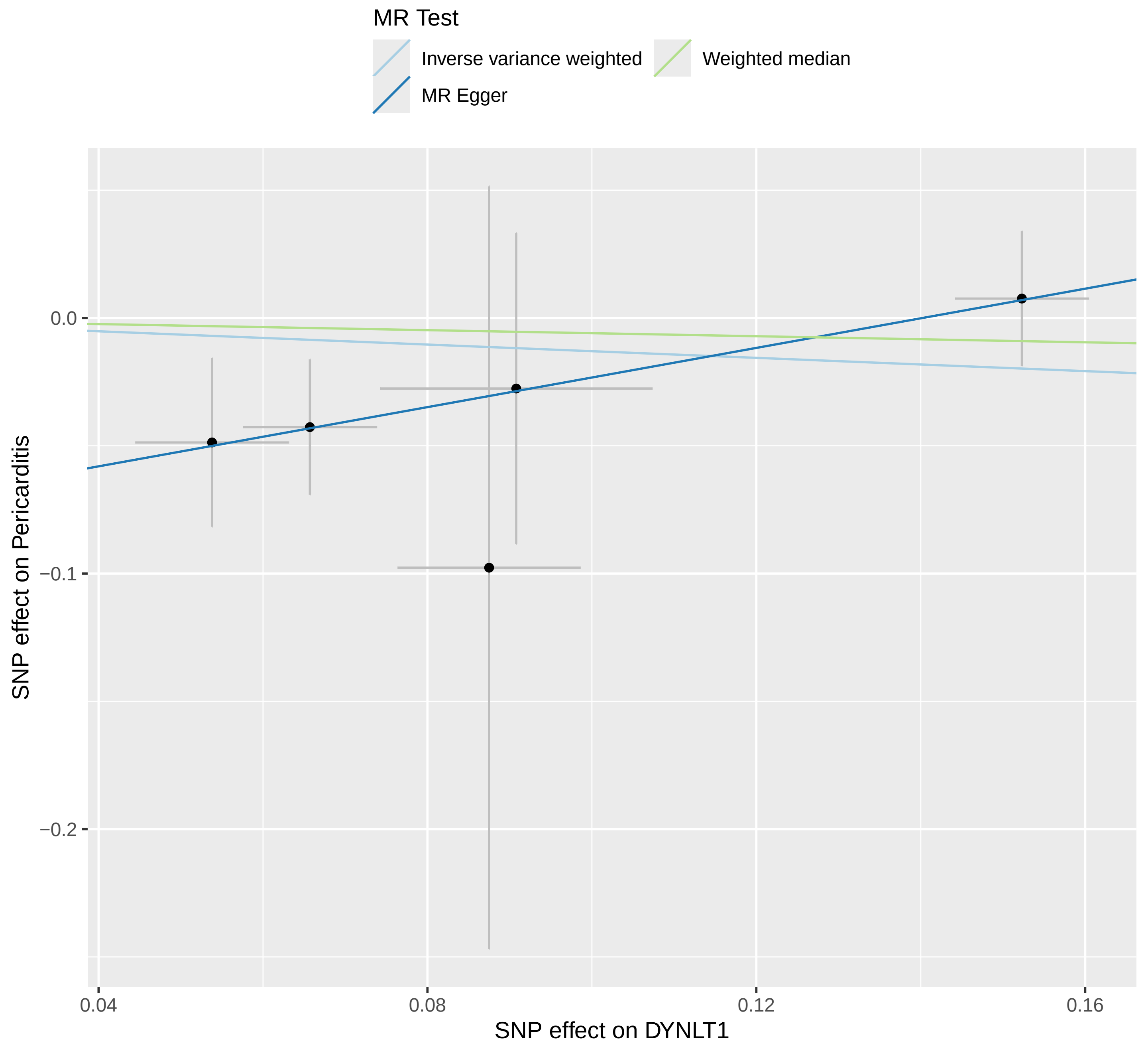
MR Test
Inverse variance weighted
Weighted median
MR Egger
0.0
−0.1
−0.2
0.04
0.08
0.12
0.16
SNP effect on Pericarditis
SNP effect on DYNLT1

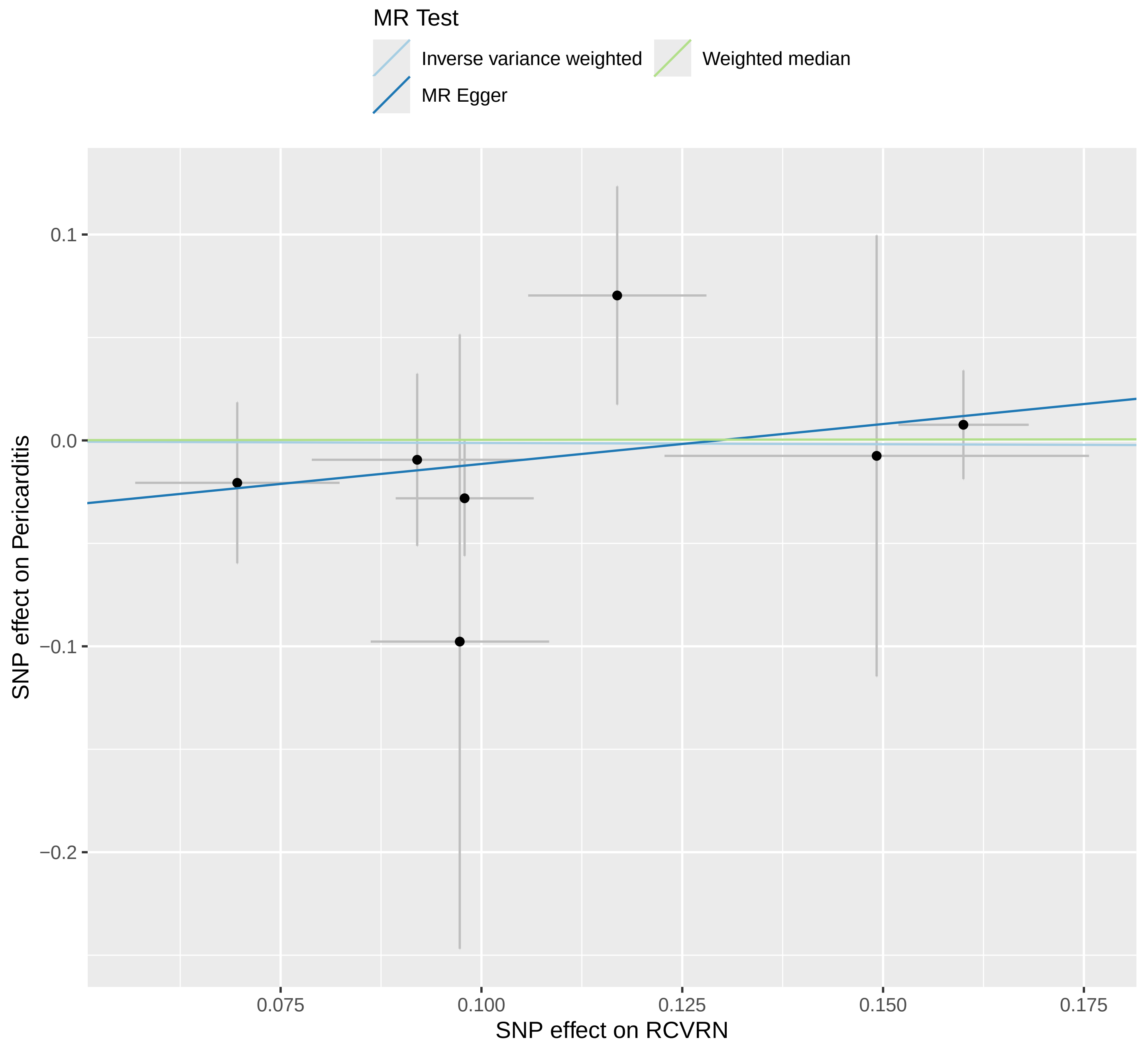
MR Test
Inverse variance weighted
Weighted median
MR Egger
0.1
0.0
−0.1
−0.2
0.075
0.100
0.125
0.150
0.175
SNP effect on RCVRN
SNP effect on Pericarditis

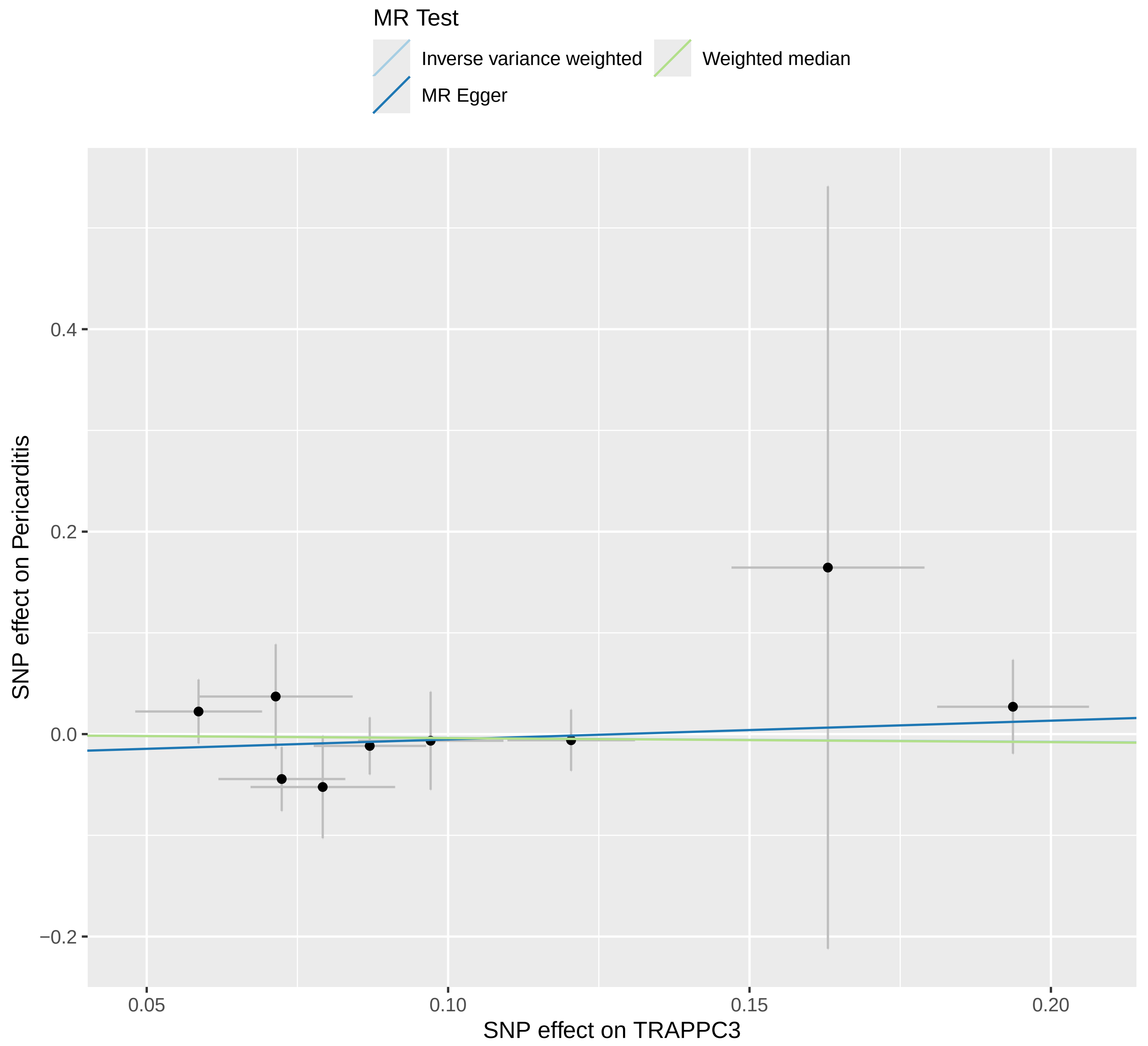

MR Test
Inverse variance weighted
Weighted median
MR Egger
SNP effect on Pericarditis
0.4
0.2
0.0
−0.2
0.05
0.10
0.15
0.20
SNP effect on TRAPPC3

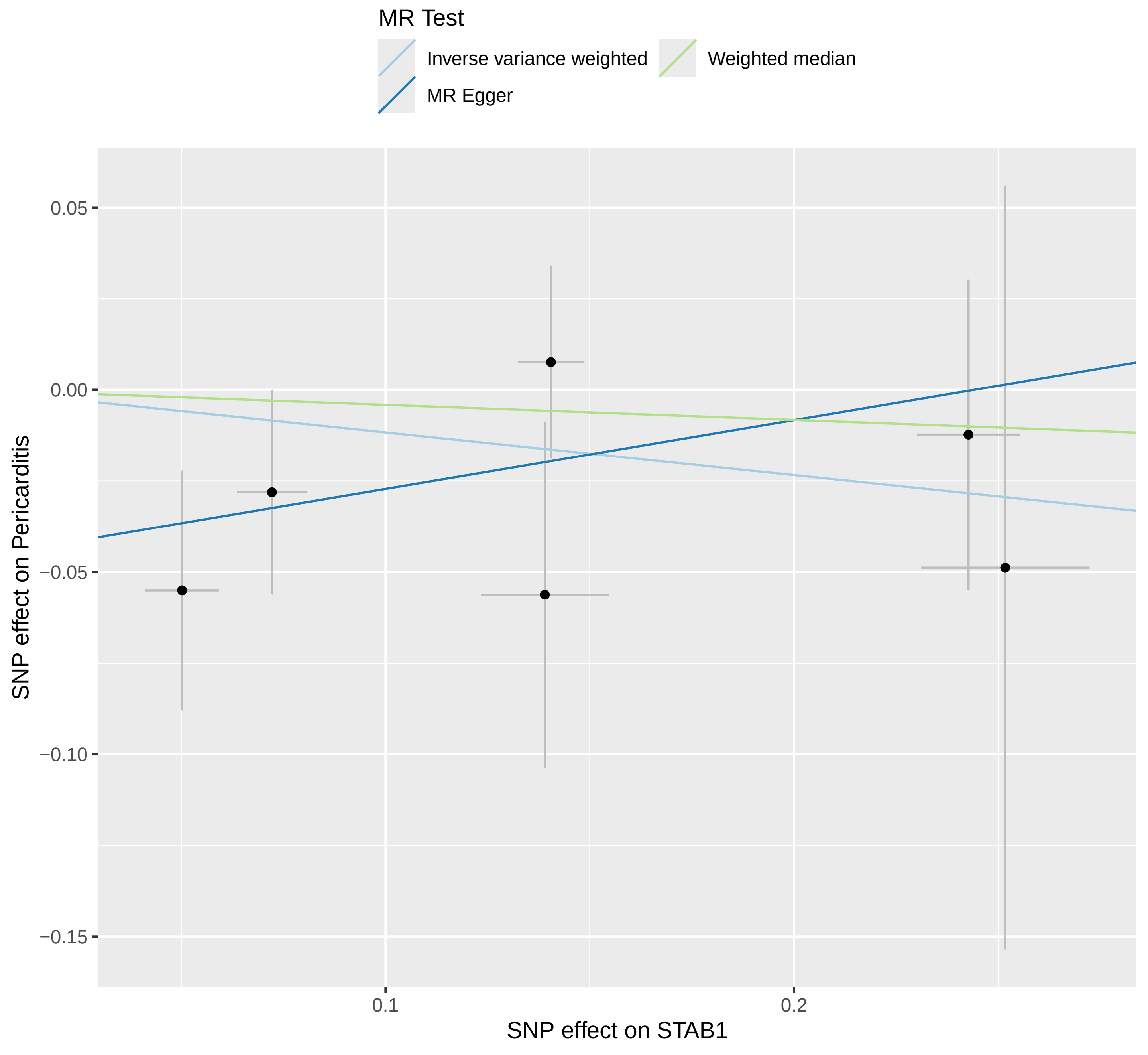
MR Test
Inverse variance weighted
Weighted median
MR Egger
0.05
0.00
−0.05
−0.10
−0.15
0.1
0.2
SNP effect on Pericarditis
SNP effect on STAB1

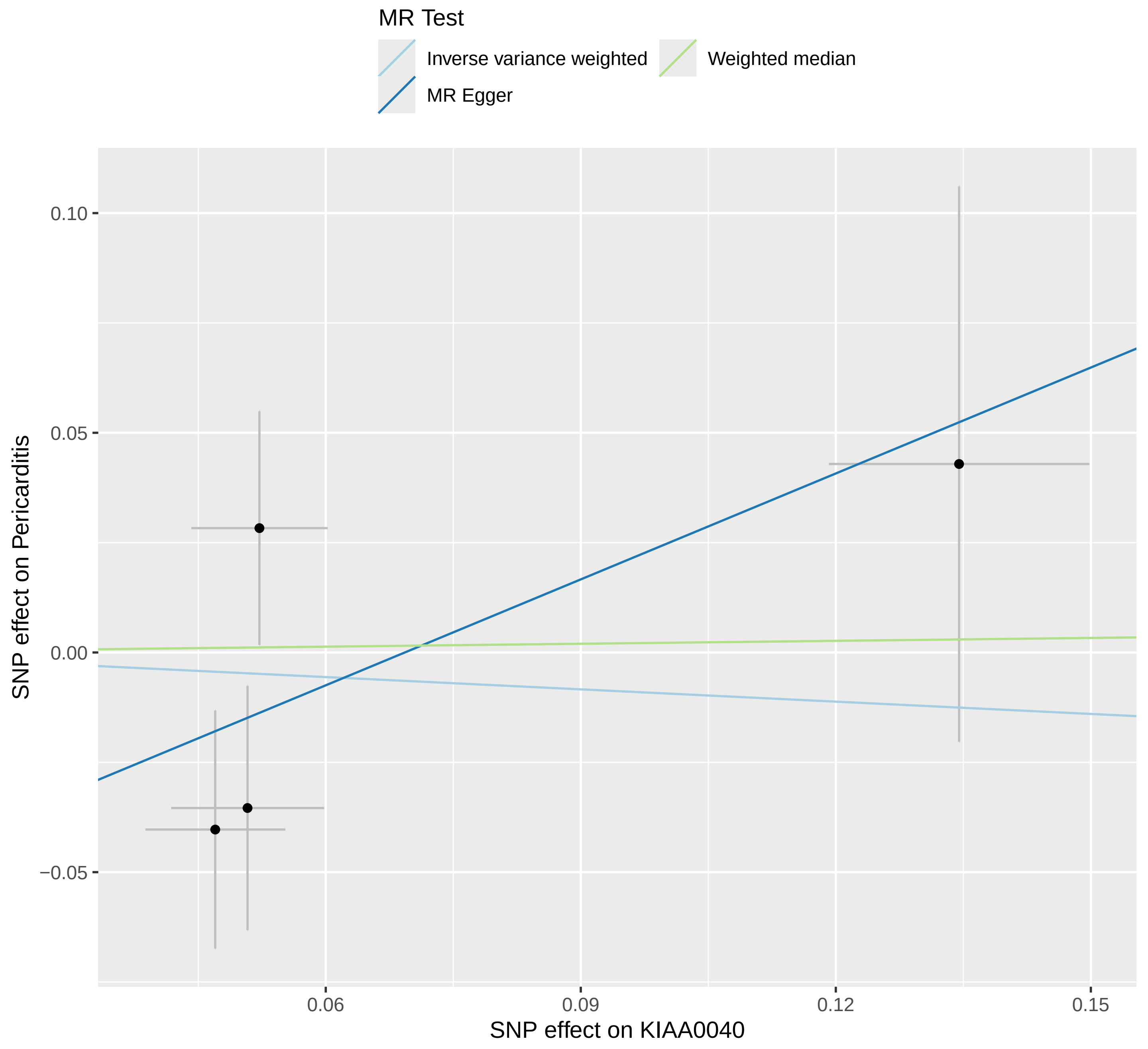

MR Test
Inverse variance weighted
Weighted median
MR Egger
0.10
0.05
0.00
−0.05
0.06
0.09
0.12
0.15
SNP effect on Pericarditis
SNP effect on KIAA0040

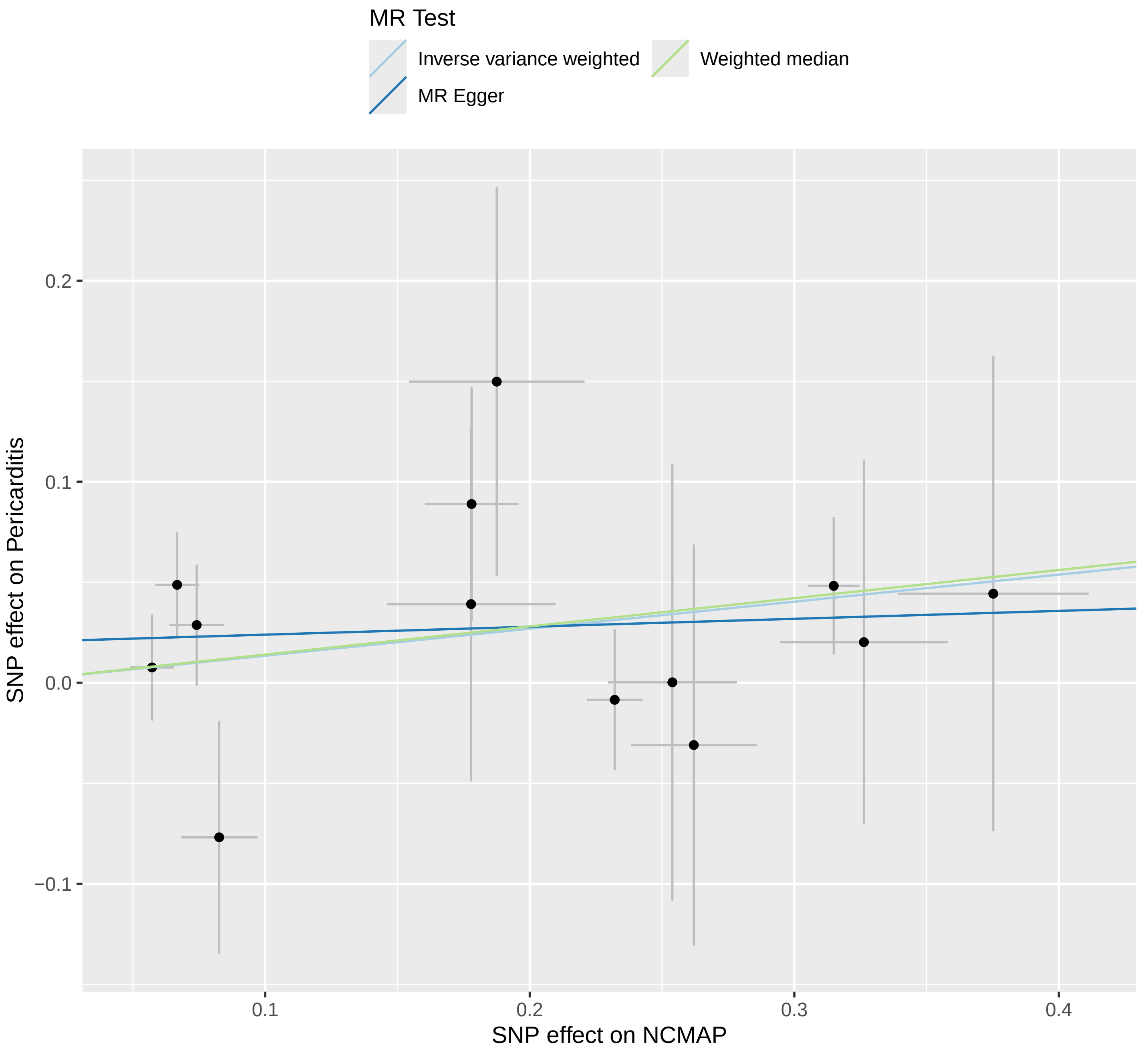
MR Test
Inverse variance weighted
Weighted median
MR Egger
SNP effect on Pericarditis
0.2
0.1
0.0
−0.1
0.1
0.2
0.3
0.4
SNP effect on NCMAP

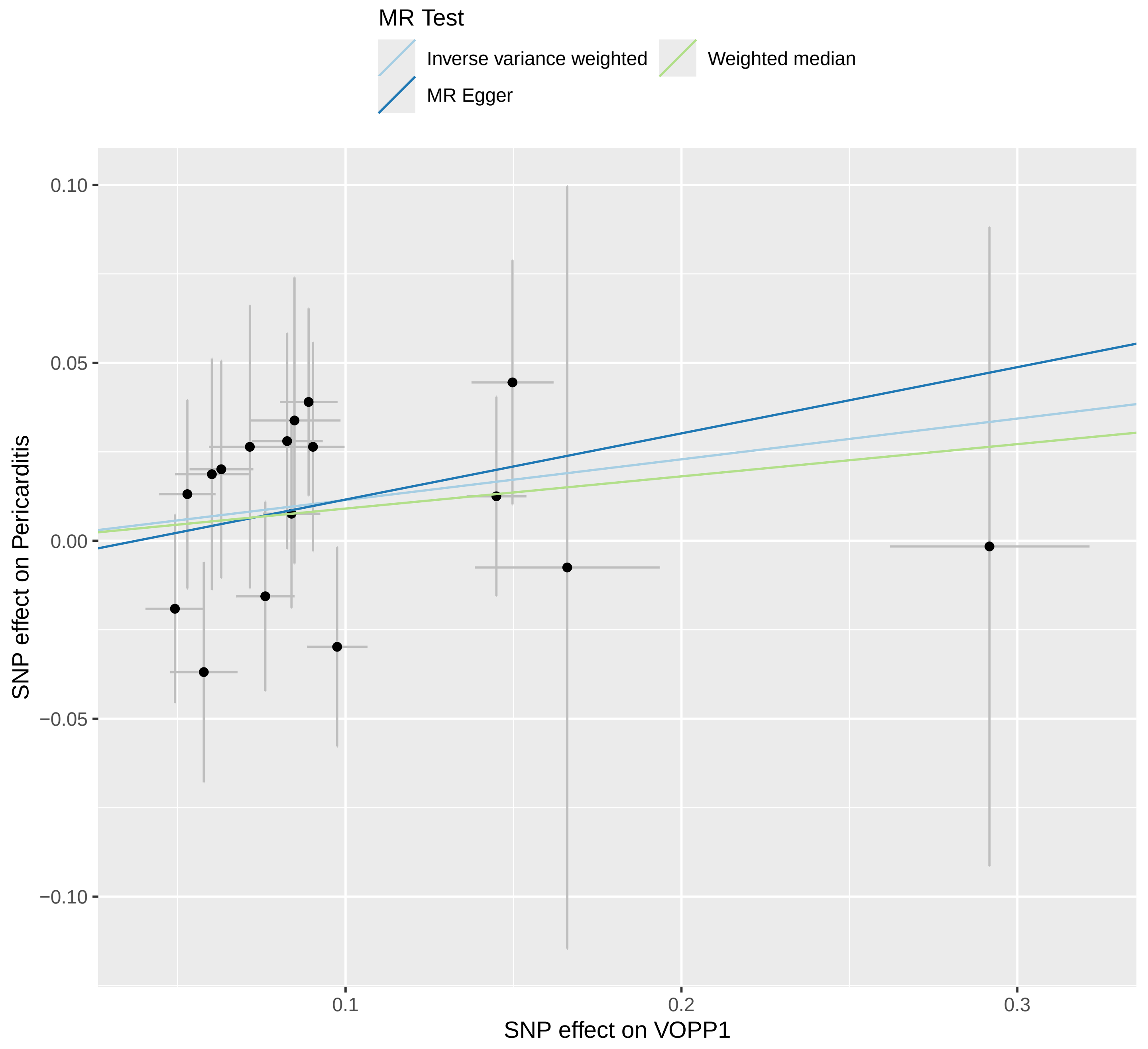
MR Test
Inverse variance weighted
MR Egger
Weighted median
0.10
0.05
0.00
−0.05
−0.10
SNP effect on Pericarditis
0.1
0.2
0.3
SNP effect on VOPP1

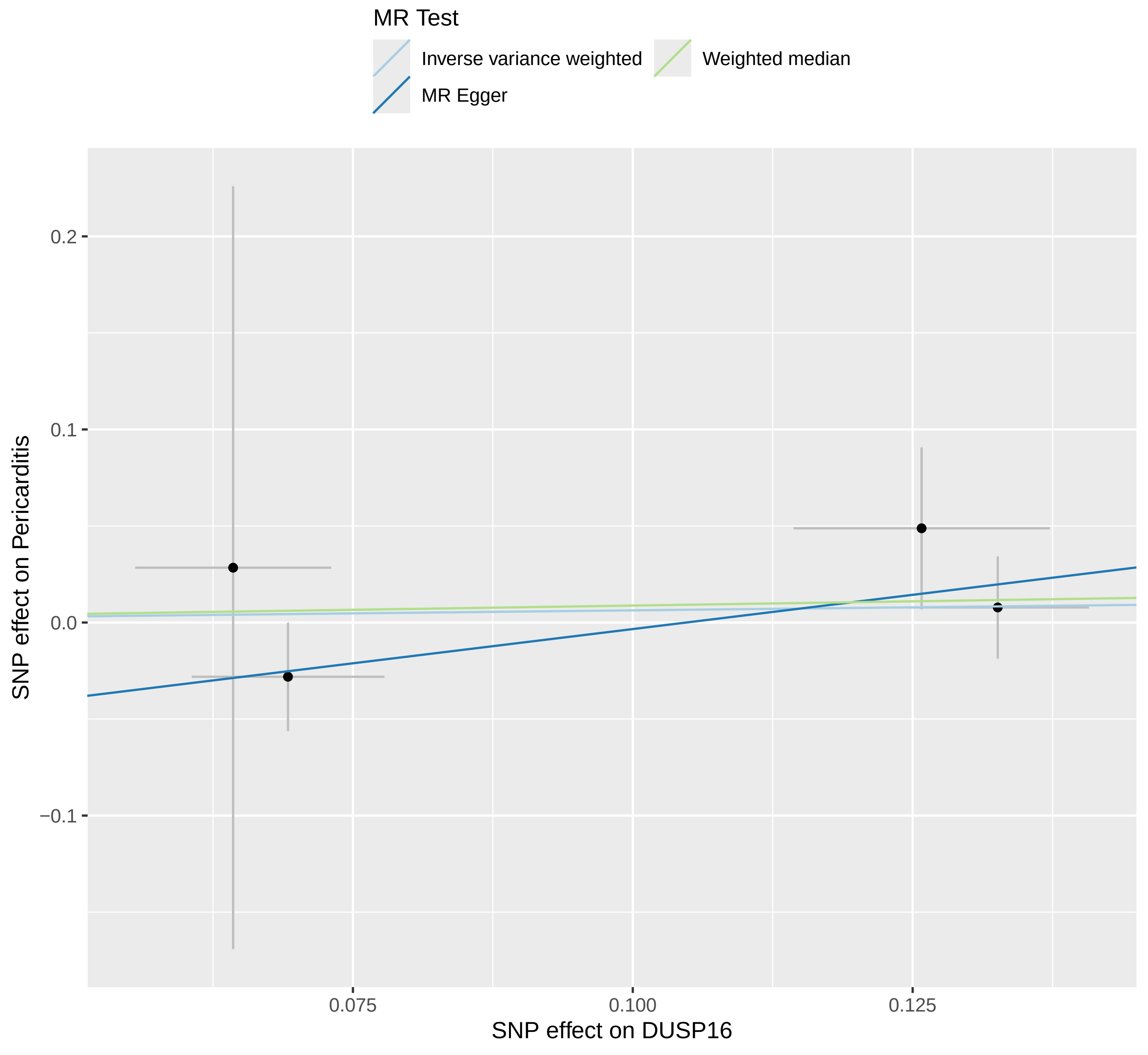
MR Test
Inverse variance weighted
Weighted median
MR Egger
0.2
0.1
0.0
−0.1
SNP effect on Pericarditis
0.075
0.100
0.125
SNP effect on DUSP16

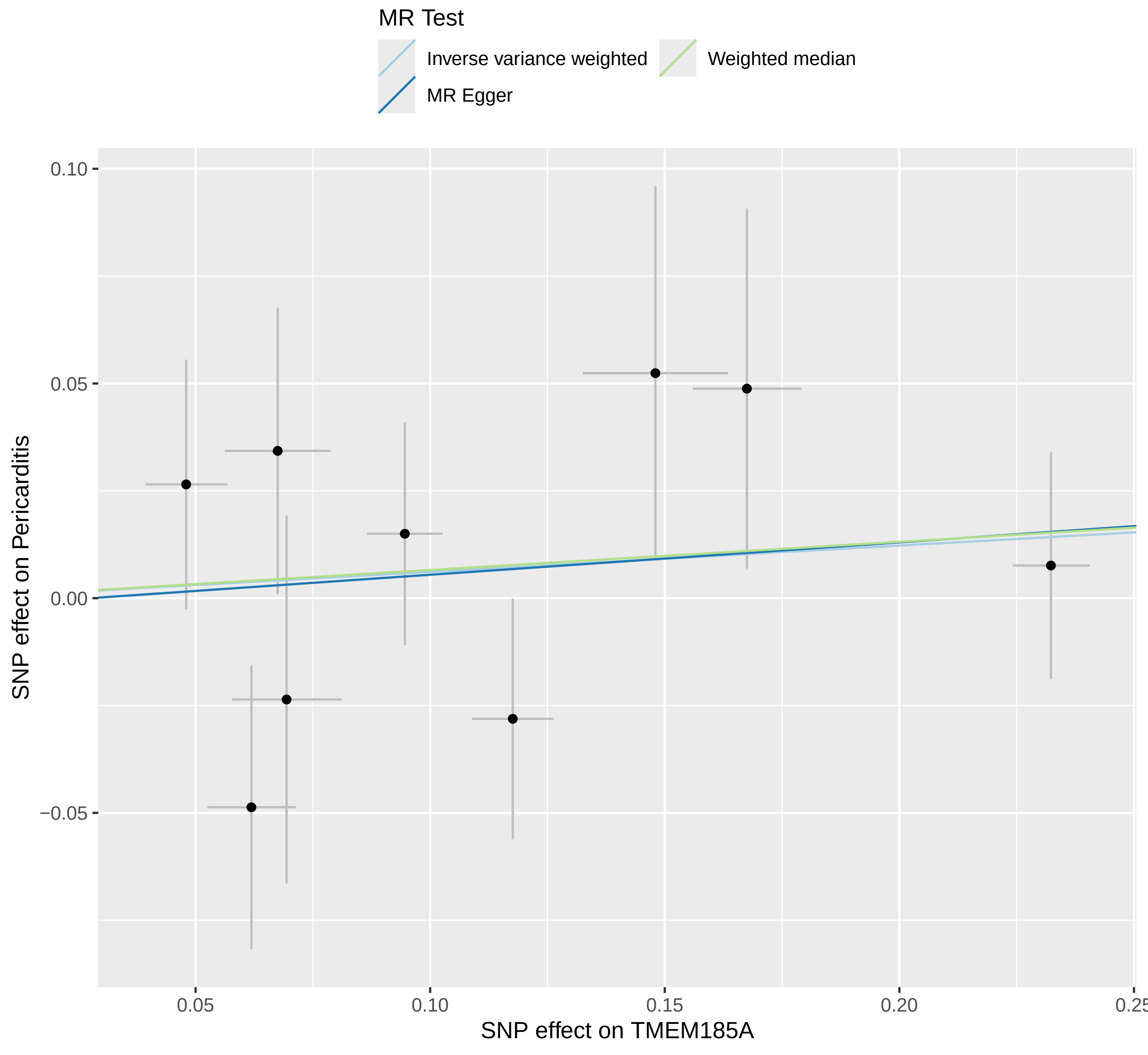
MR Test
Inverse variance weighted
Weighted median
MR Egger
0.10
0.05
0.00
−0.05
0.05
0.10
0.15
0.20
0.25
SNP effect on Pericarditis
SNP effect on TMEM185A

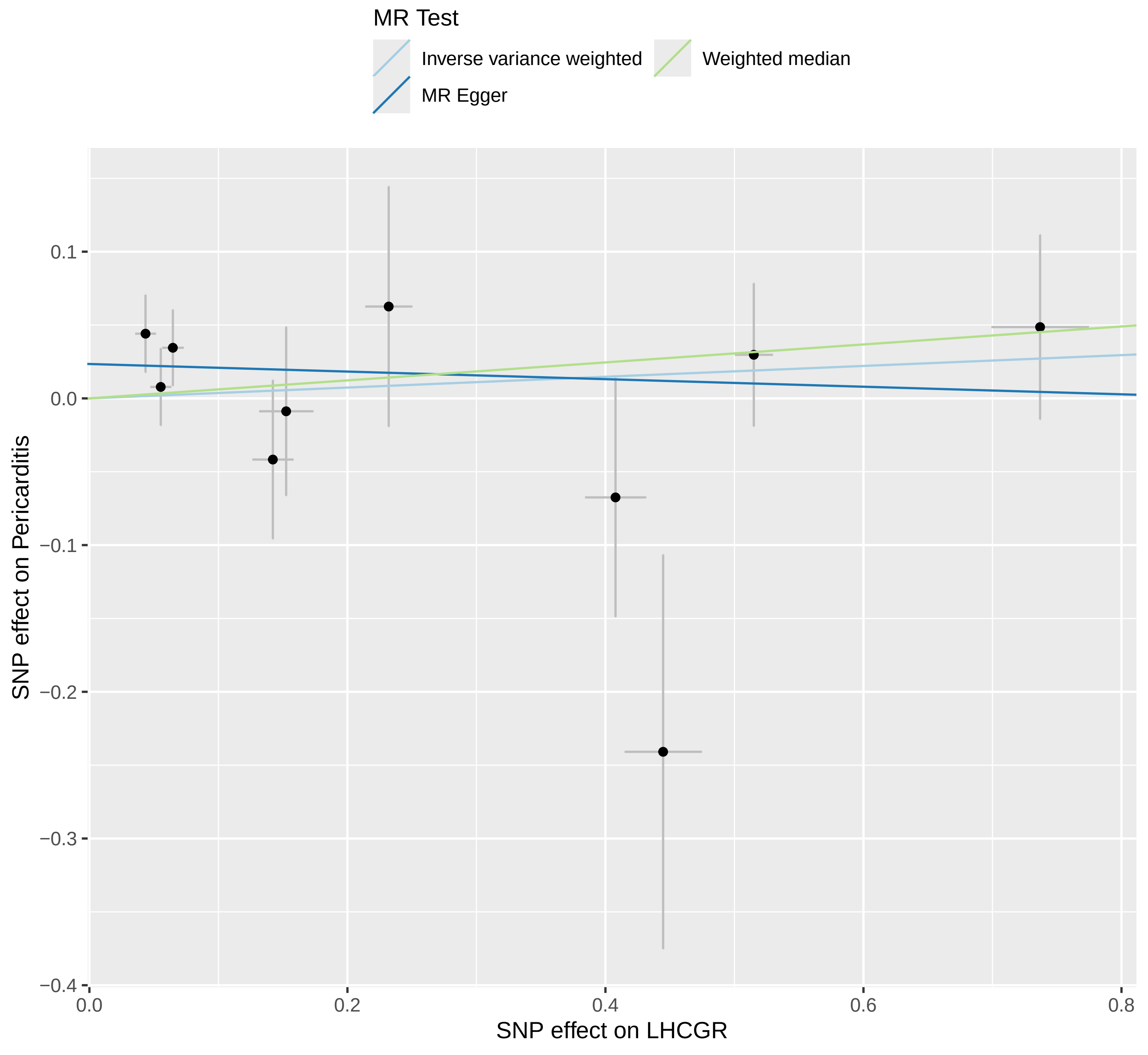
MR Test
Inverse variance weighted
Weighted median
MR Egger
SNP effect on Pericarditis
SNP effect on LHCGR
0.1
0.0
−0.1
−0.2
−0.3
−0.4
0.0
0.2
0.4
0.6
0.8

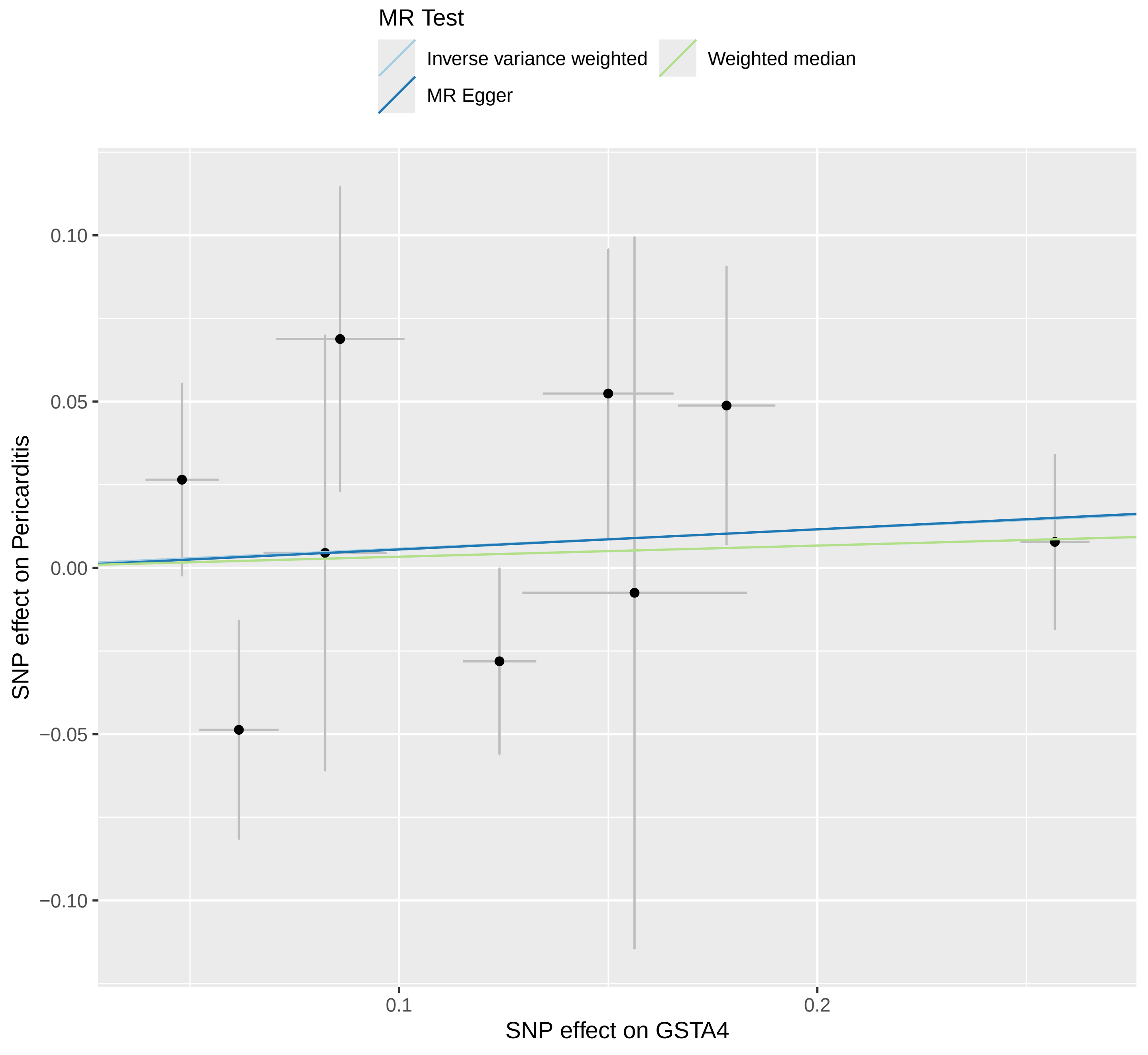
MR Test
Inverse variance weighted
Weighted median
MR Egger
0.10
0.05
0.00
−0.05
−0.10
SNP effect on Pericarditis
0.1
0.2
SNP effect on GSTA4

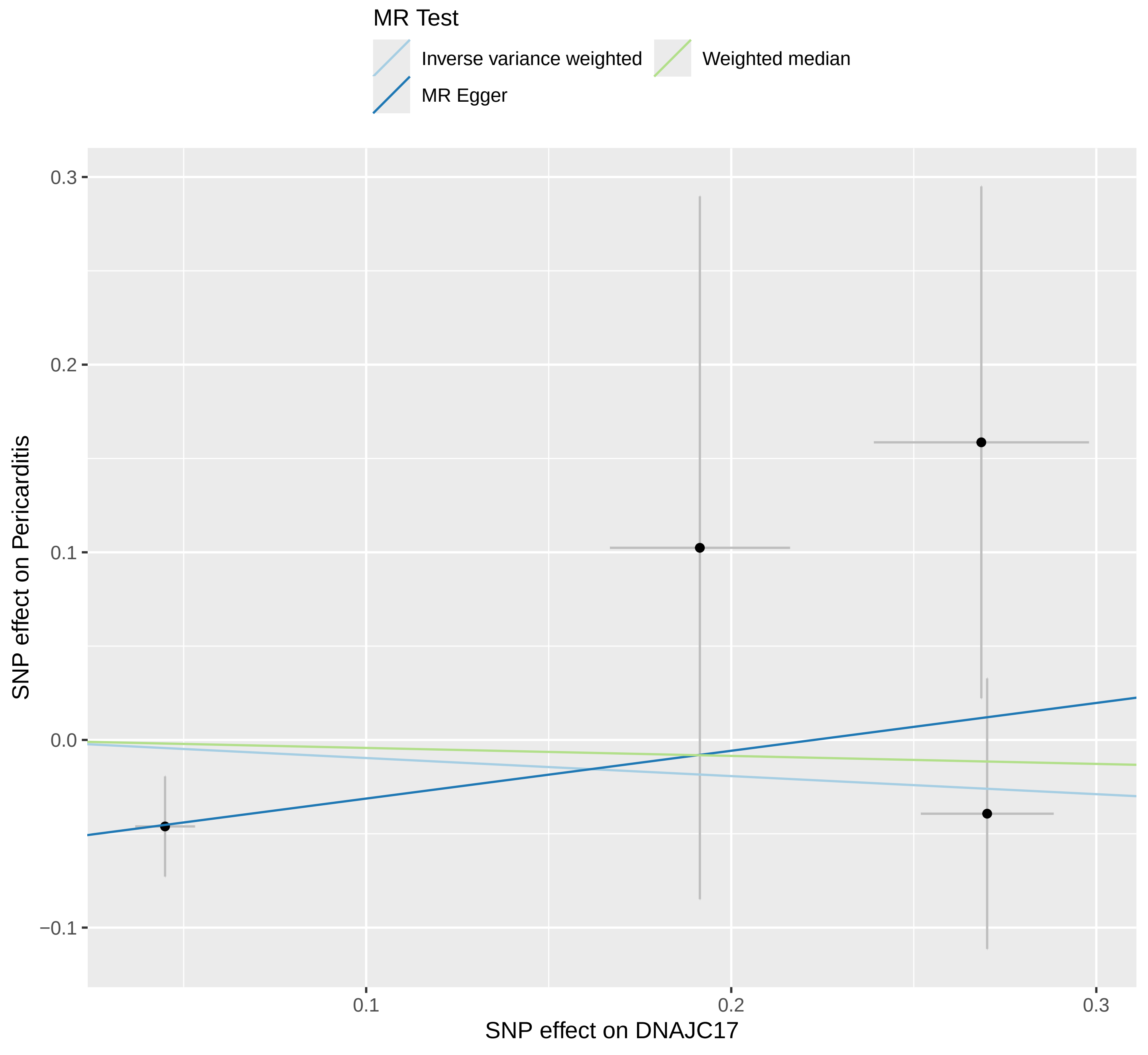
MR Test
Inverse variance weighted
Weighted median
MR Egger
0.3
0.2
0.1
0.0
−0.1
0.1
0.2
0.3
SNP effect on Pericarditis
SNP effect on DNAJC17

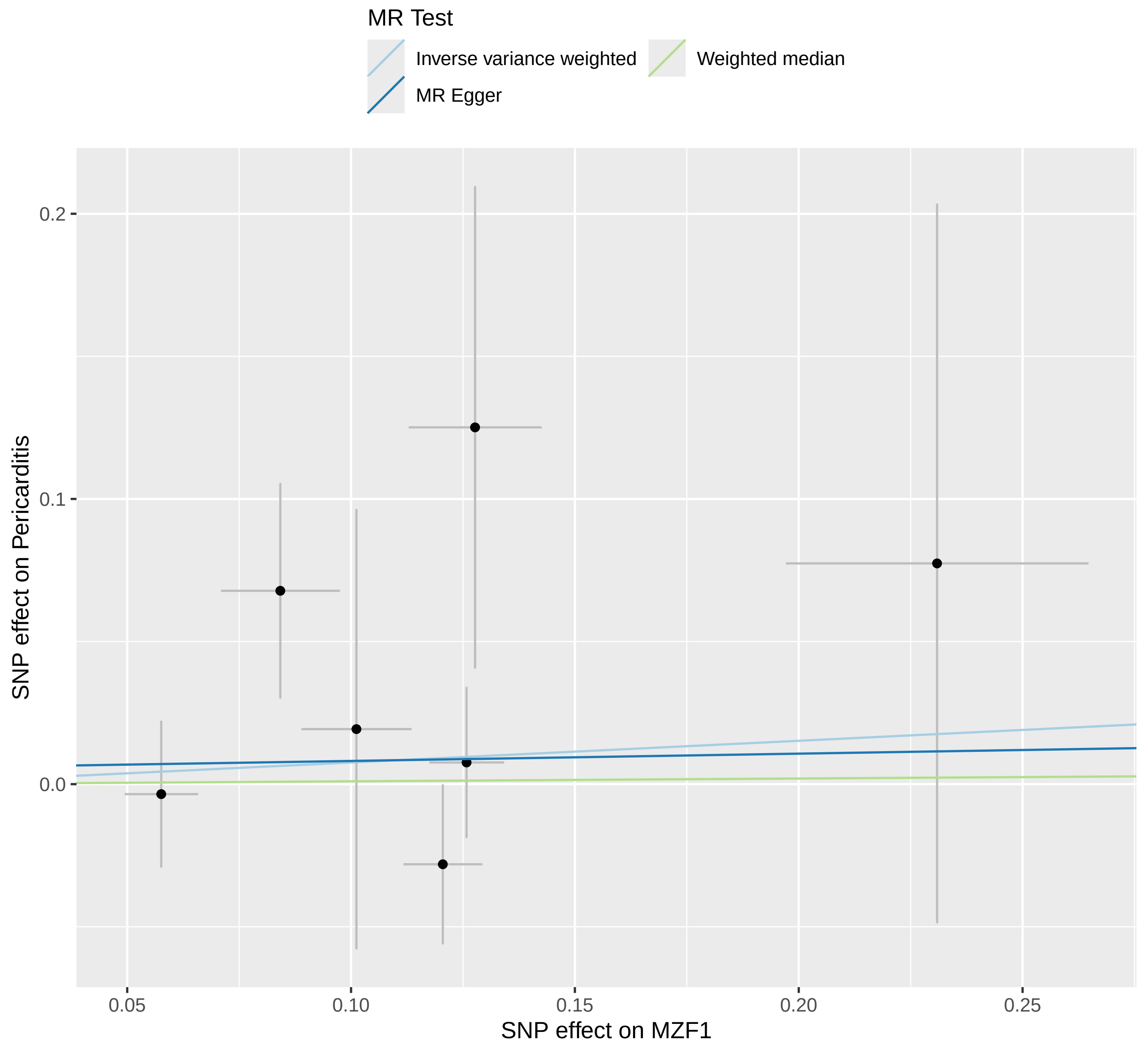
MR Test
Inverse variance weighted
Weighted median
MR Egger
0.2
0.1
0.0
SNP effect on Pericarditis
0.05
0.10
0.15
0.20
0.25
SNP effect on MZF1

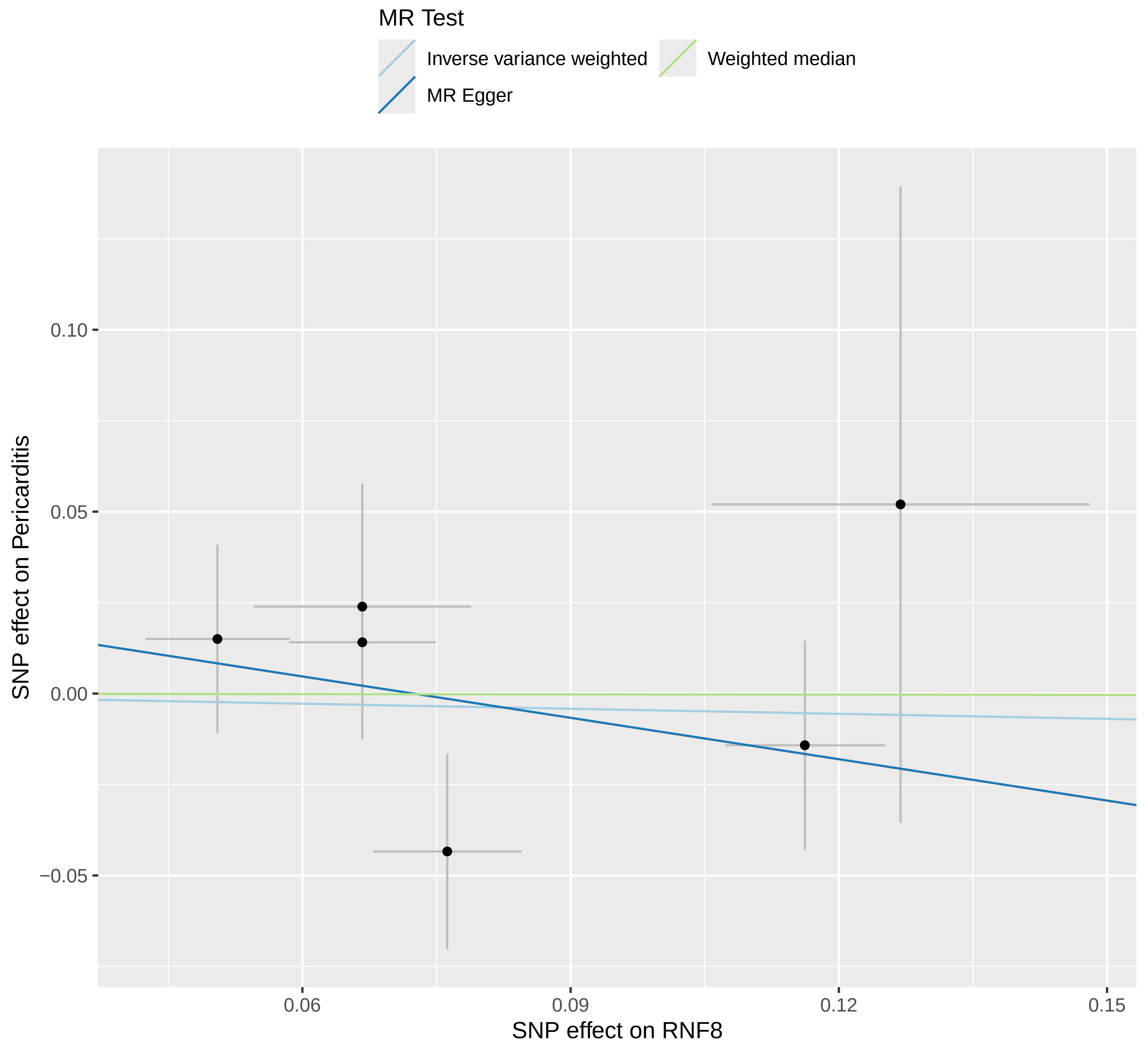

MR Test
Inverse variance weighted
MR Egger
Weighted median
SNP effect on Pericarditis
0.10
0.05
0.00
−0.05
0.06
0.09
0.12
0.15
SNP effect on RNF8

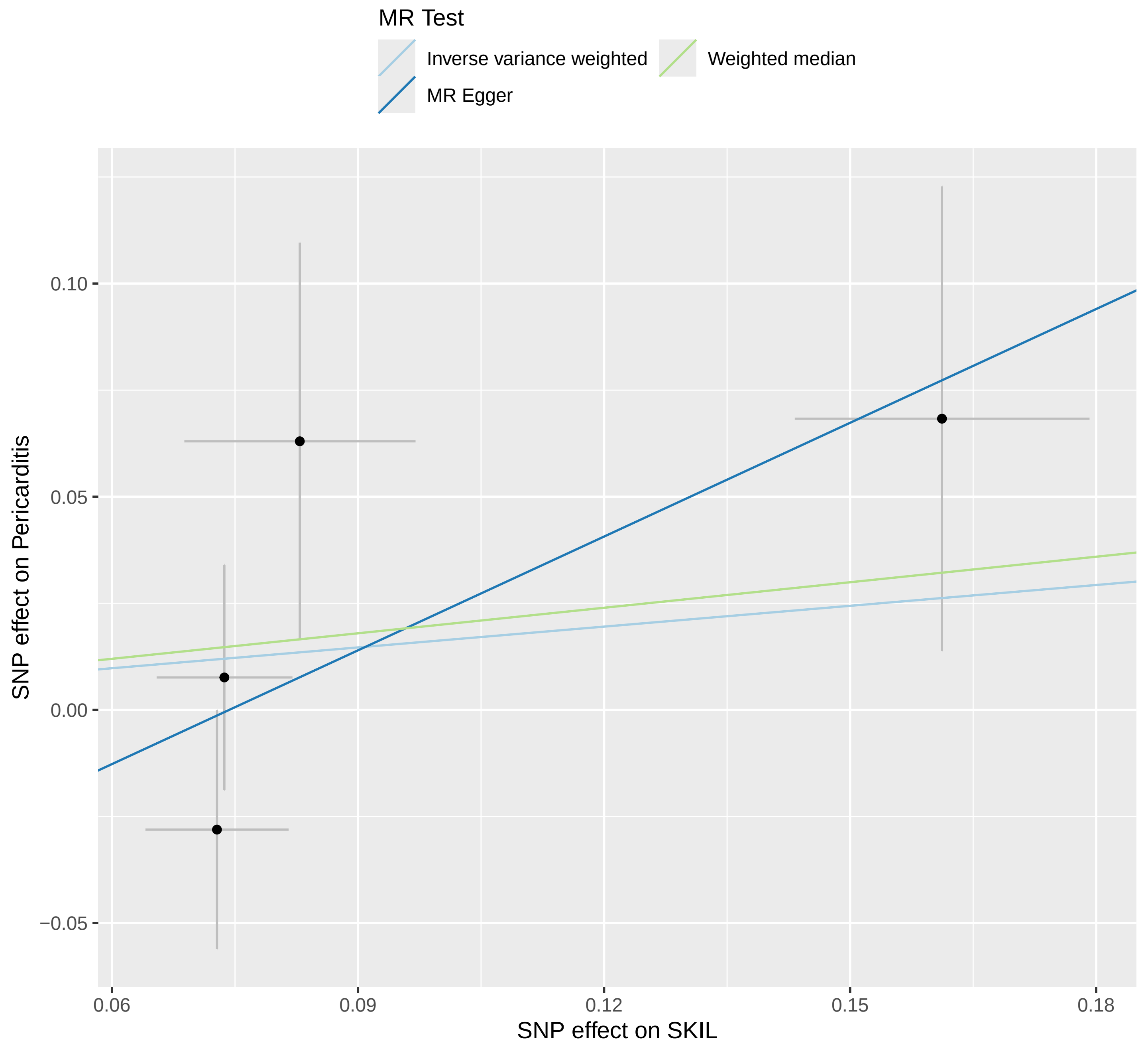

MR Test
Inverse variance weighted
Weighted median
MR Egger
0.10
0.05
0.00
−0.05
SNP effect on Pericarditis
0.06
0.09
0.12
0.15
0.18
SNP effect on SKIL

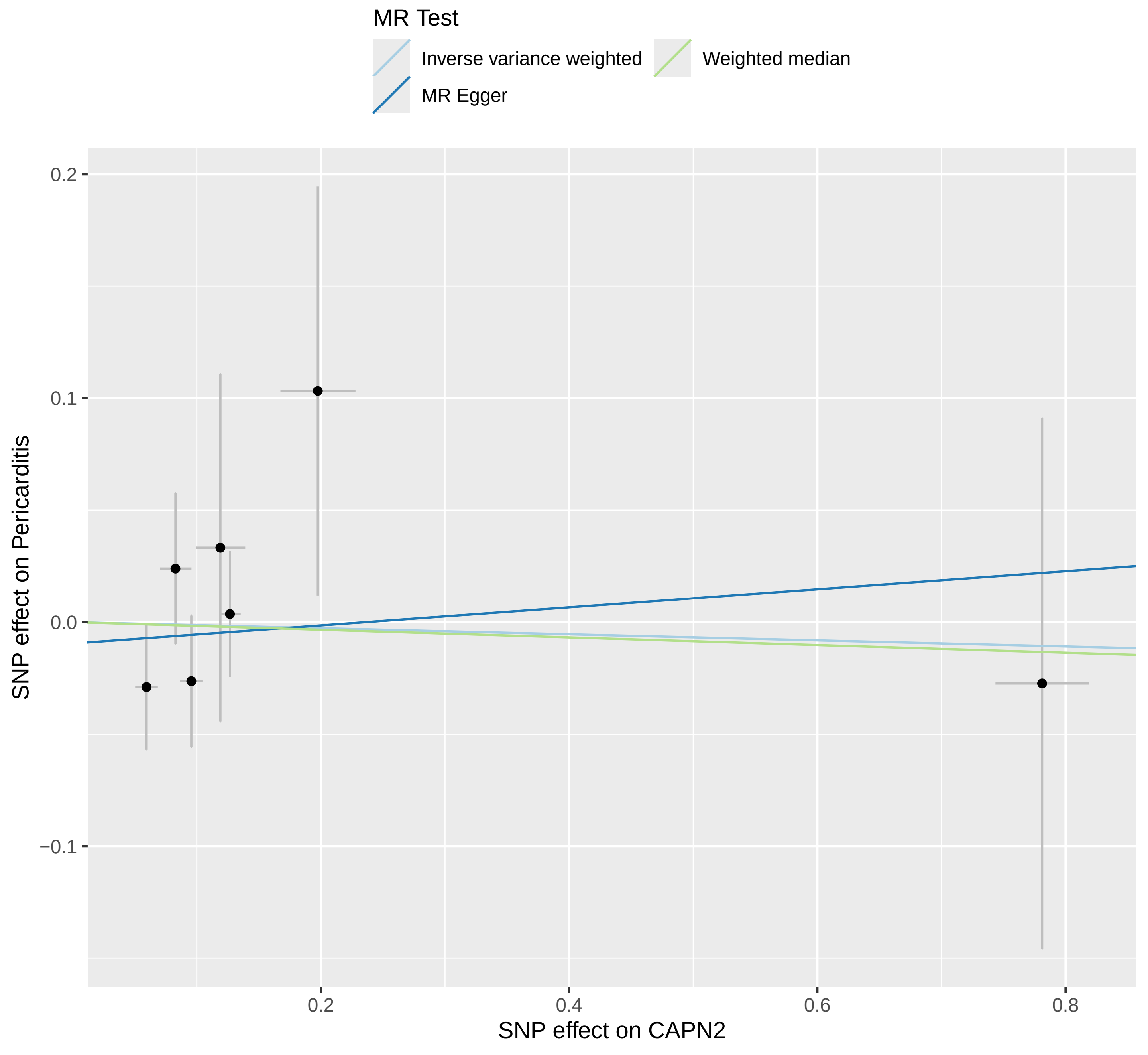
MR Test
Inverse variance weighted
Weighted median
MR Egger
0.2
0.1
0.0
−0.1
0.2
0.4
0.6
0.8
SNP effect on Pericarditis
SNP effect on CAPN2

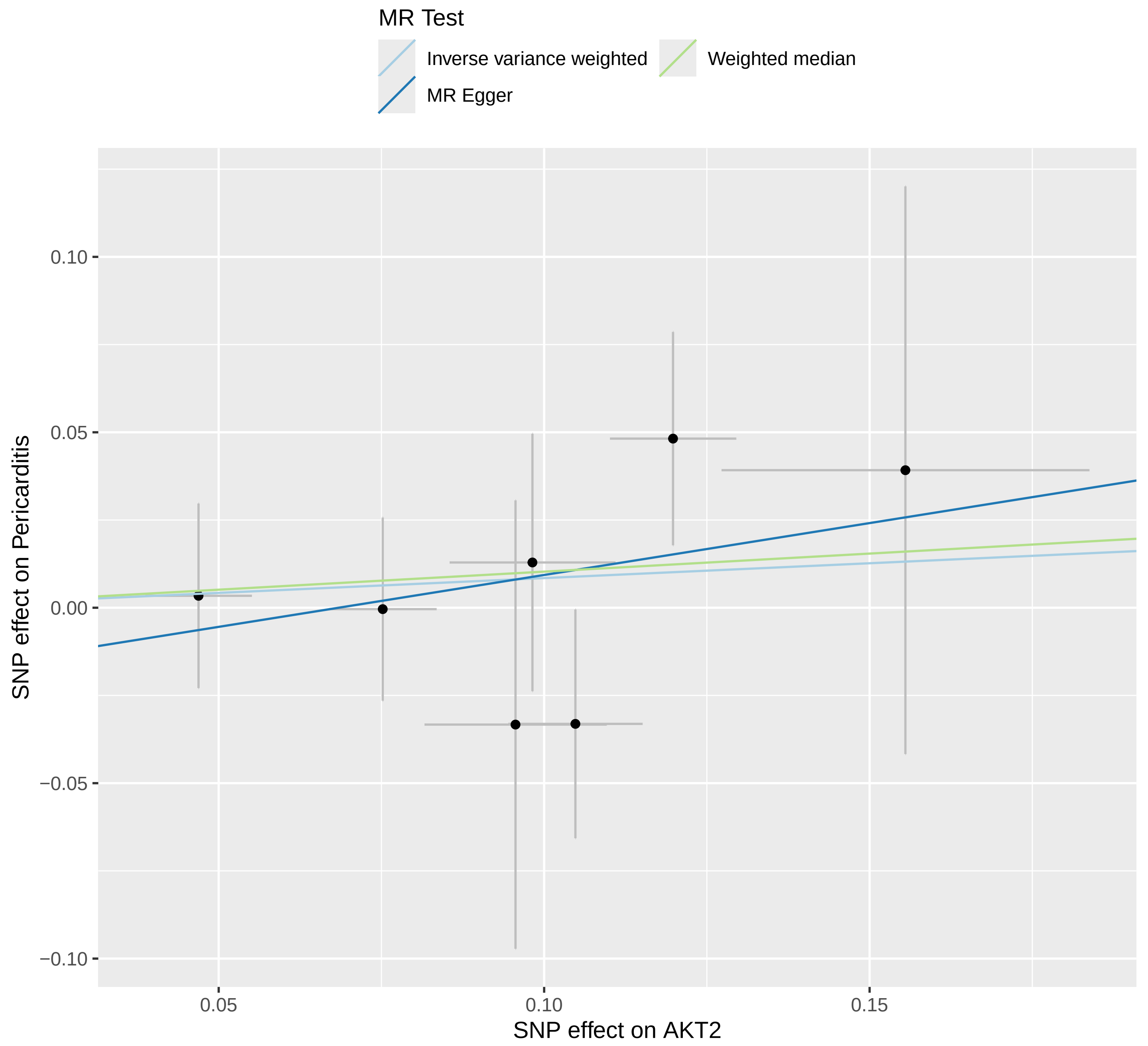
MR Test
Inverse variance weighted
MR Egger
Weighted median
0.10
0.05
0.00
−0.05
−0.10
SNP effect on Pericarditis
0.05
0.10
0.15
SNP effect on AKT2

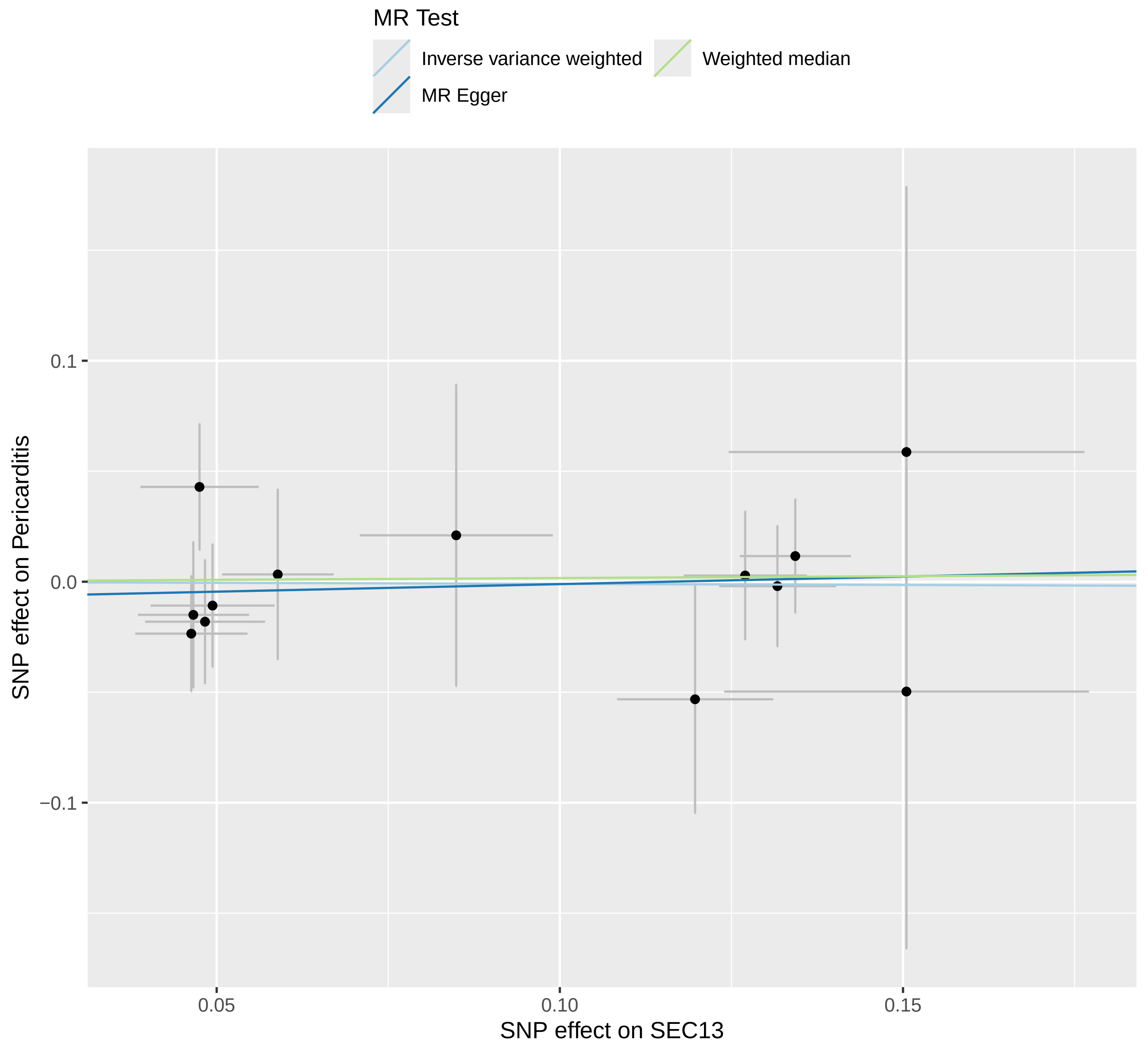
MR Test
Inverse variance weighted
Weighted median
MR Egger
SNP effect on Pericarditis
0.1
0.0
−0.1
0.05
0.10
0.15
SNP effect on SEC13

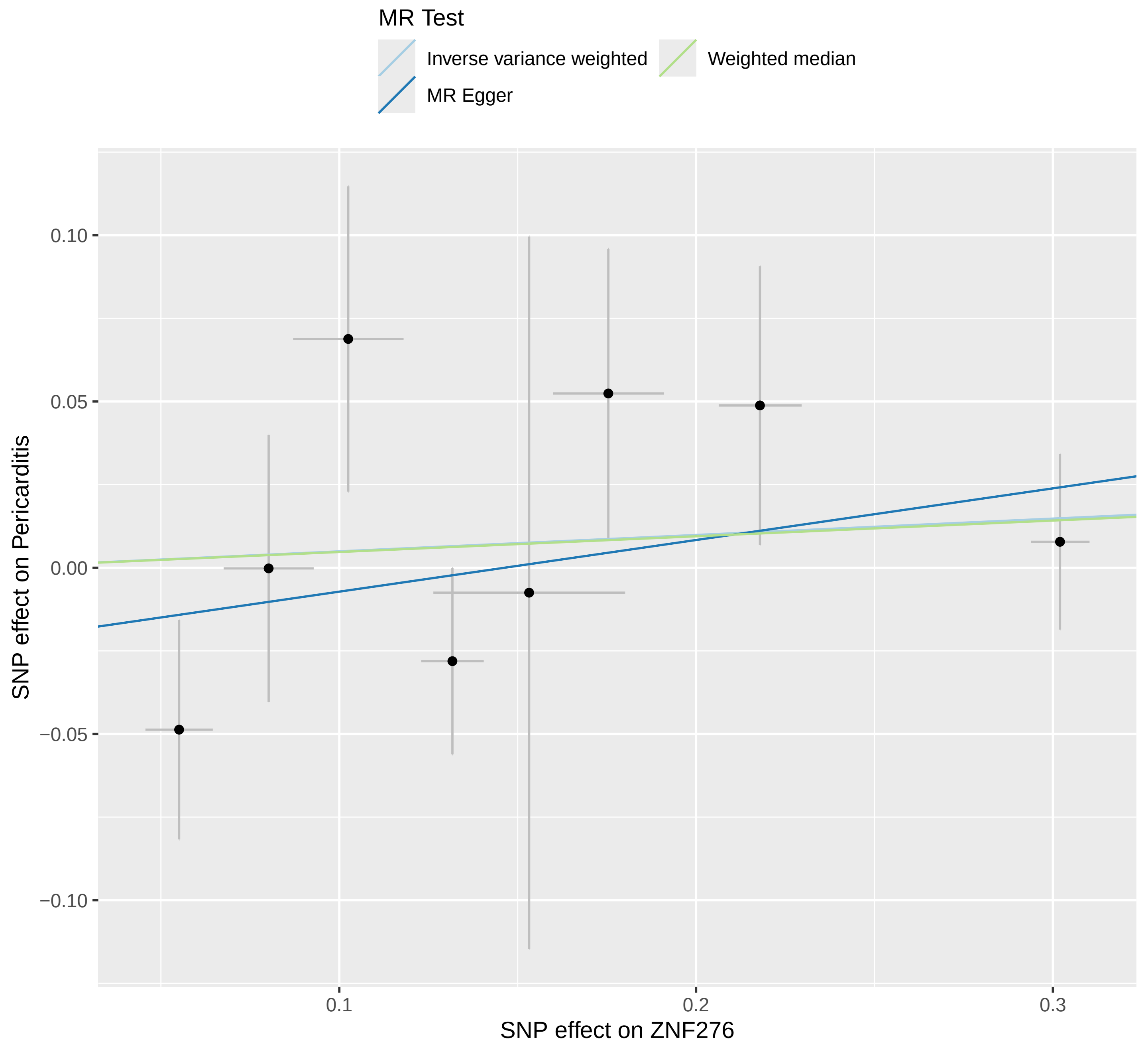
MR Test
Inverse variance weighted
MR Egger
Weighted median
0.10
0.05
0.00
−0.05
−0.10
SNP effect on Pericarditis
0.1
0.2
0.3
SNP effect on ZNF276

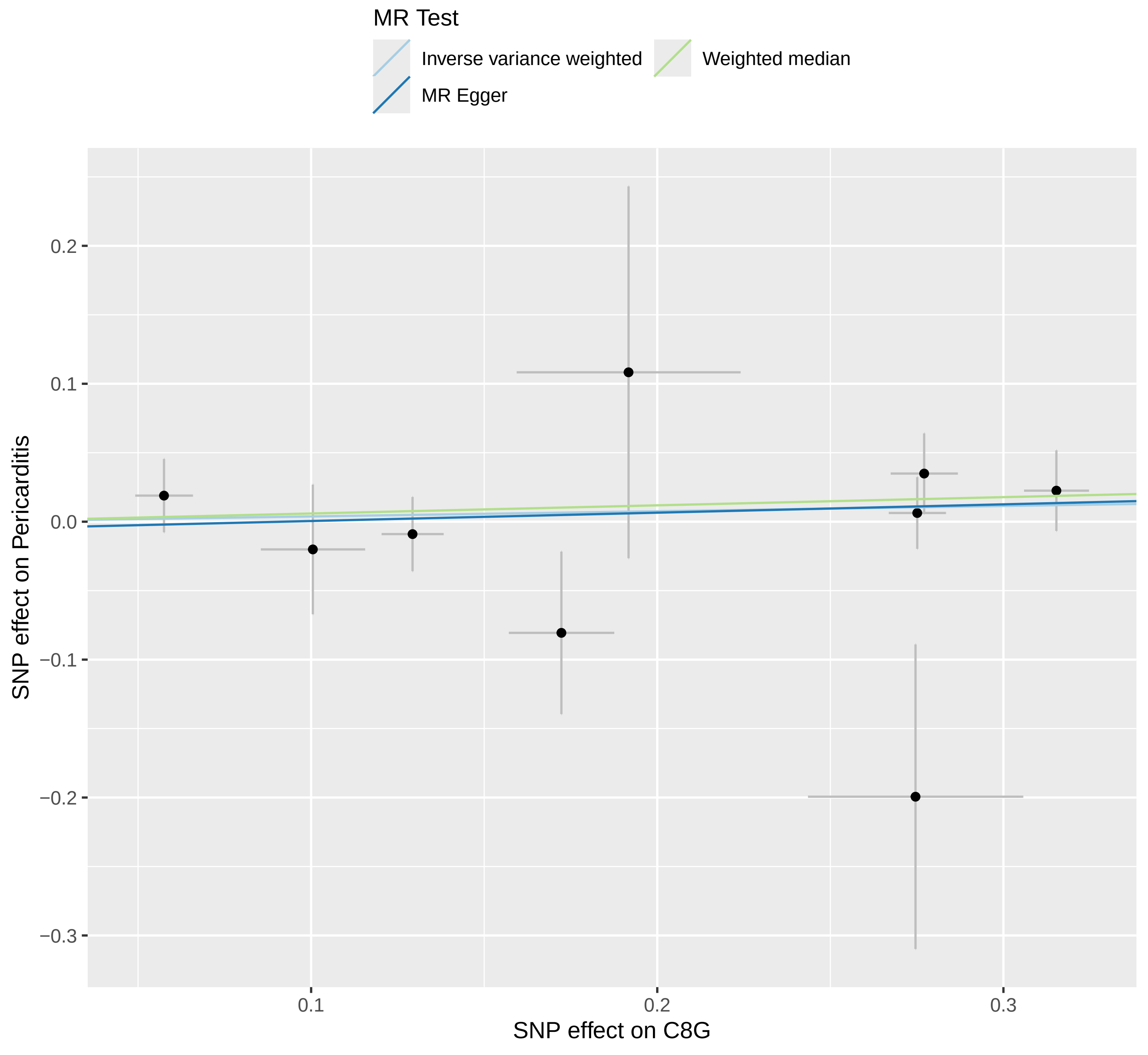

MR Test
Inverse variance weighted
Weighted median
MR Egger
0.2
0.1
0.0
−0.1
−0.2
−0.3
SNP effect on Pericarditis
0.1
0.2
0.3
SNP effect on C8G

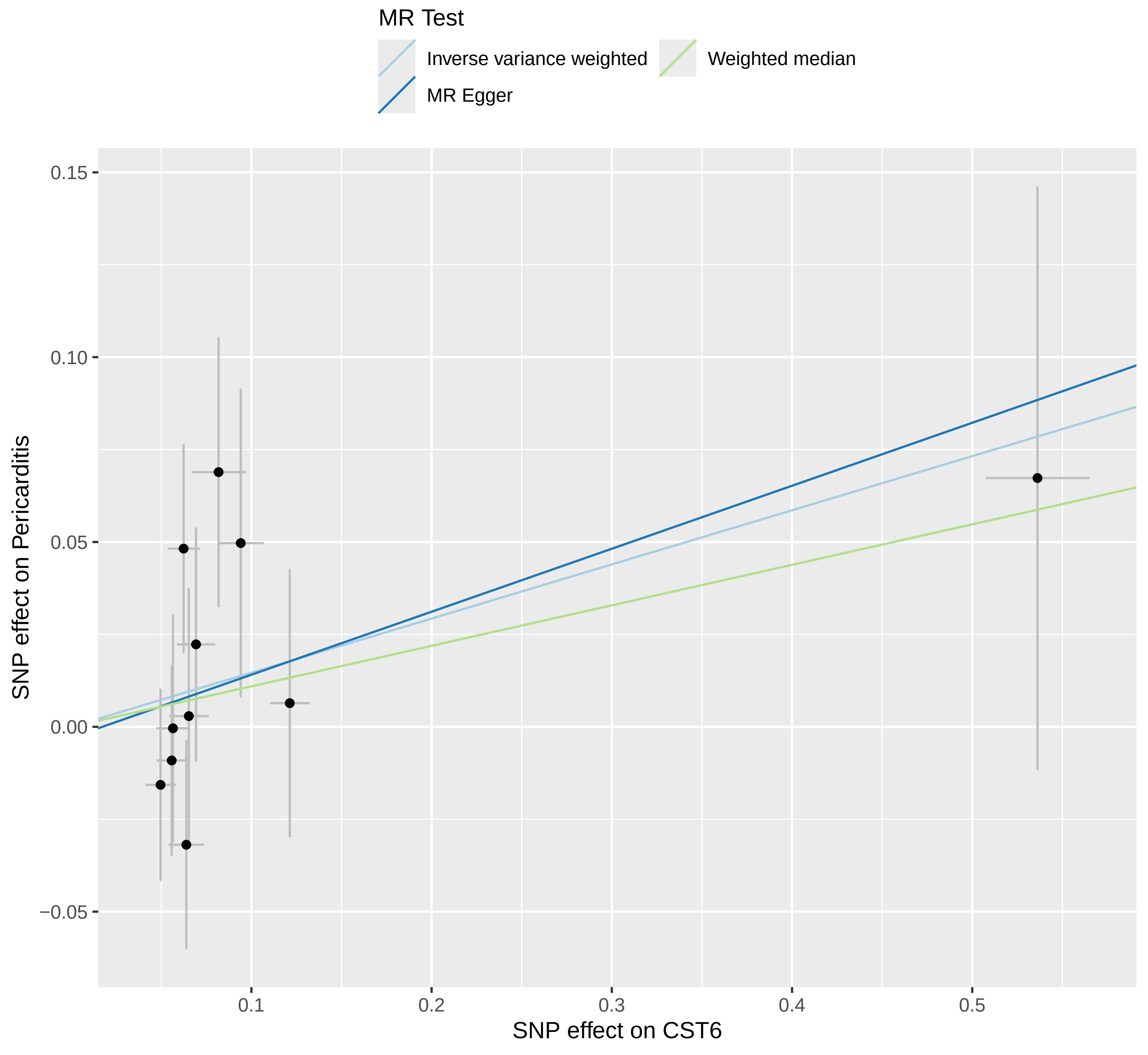

MR Test
Inverse variance weighted
MR Egger
Weighted median
SNP effect on Pericarditis
0.15
0.10
0.05
0.00
−0.05
0.1
0.2
0.3
0.4
0.5
SNP effect on CST6

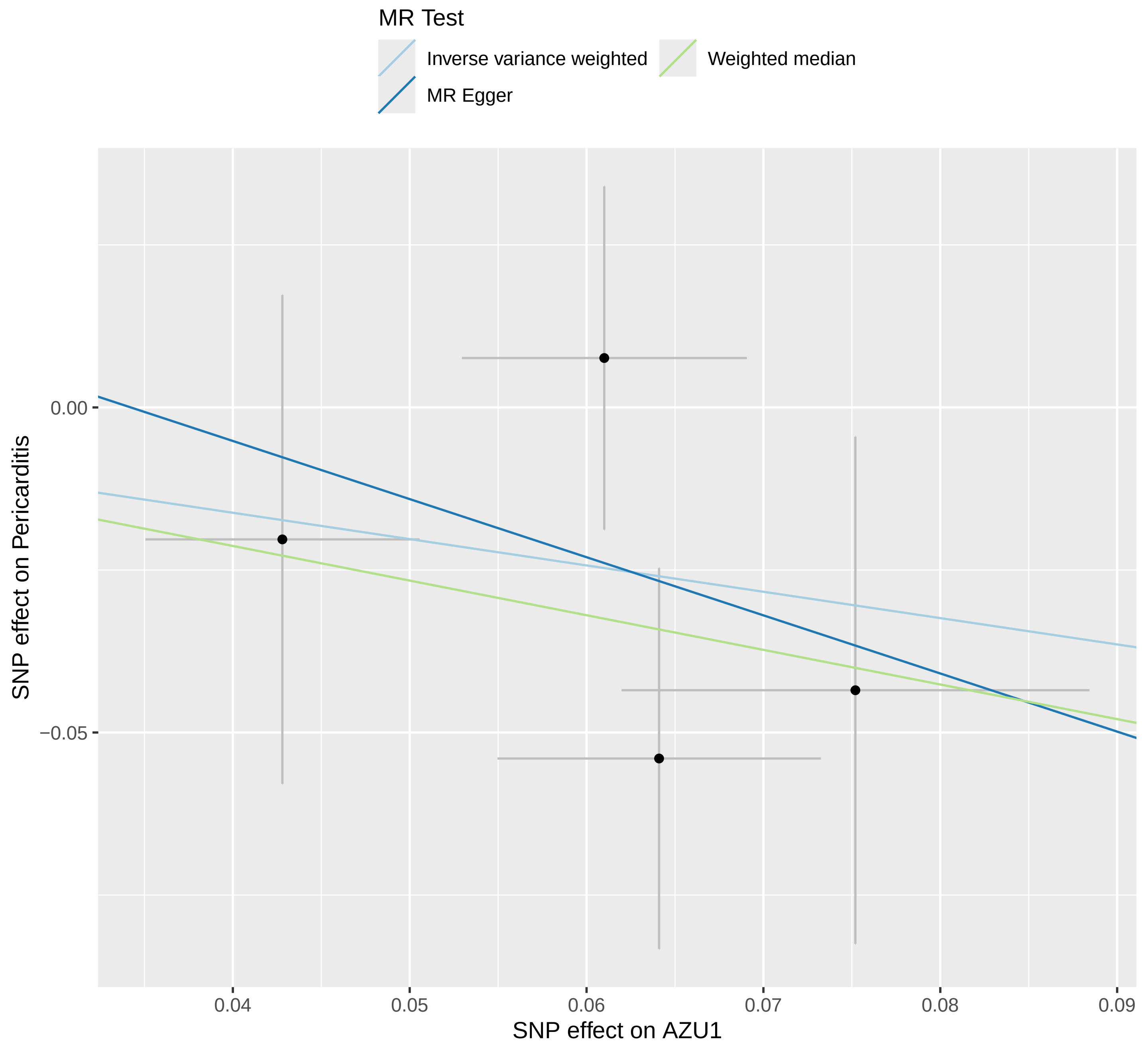
MR Test
Inverse variance weighted
Weighted median
MR Egger
0.00
−0.05
SNP effect on Pericarditis
0.04
0.05
0.06
0.07
0.08
0.09
SNP effect on AZU1

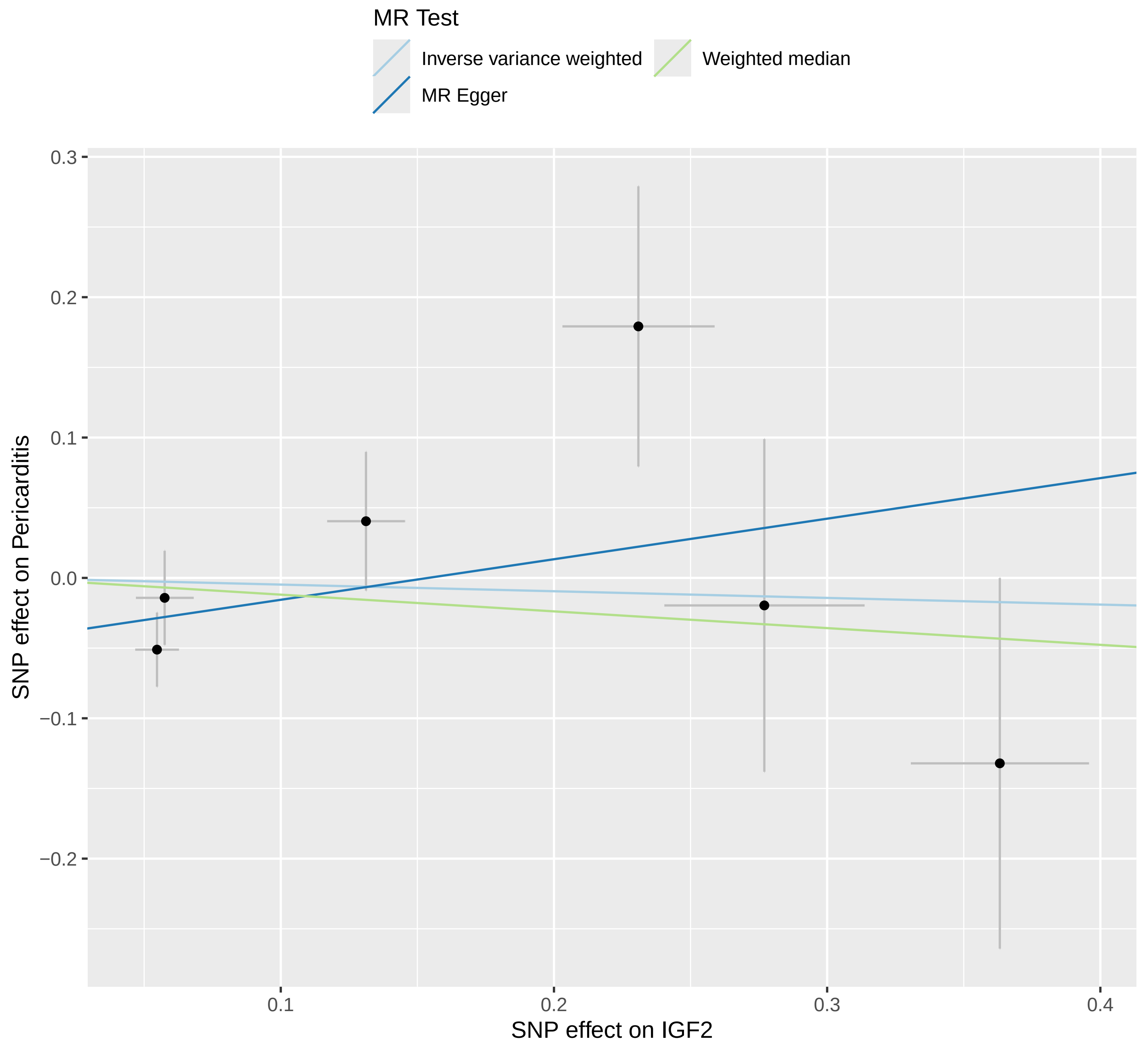
MR Test
Inverse variance weighted
Weighted median
MR Egger
SNP effect on Pericarditis
0.3
0.2
0.1
0.0
−0.1
−0.2
0.1
0.2
0.3
0.4
SNP effect on IGF2

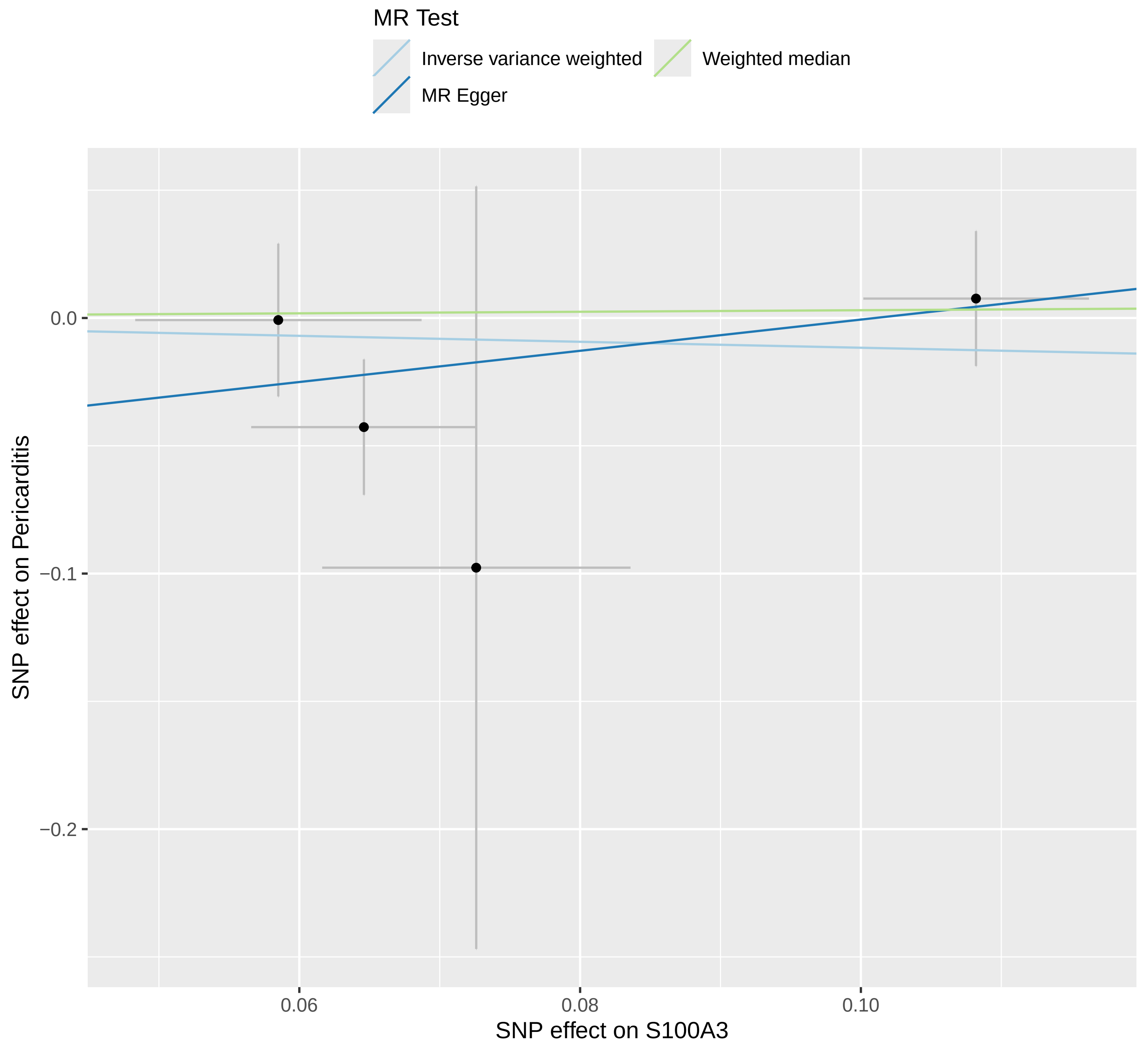
MR Test
Inverse variance weighted
MR Egger
Weighted median
SNP effect on Pericarditis
0.0
−0.1
−0.2
0.06
0.08
0.10
SNP effect on S100A3

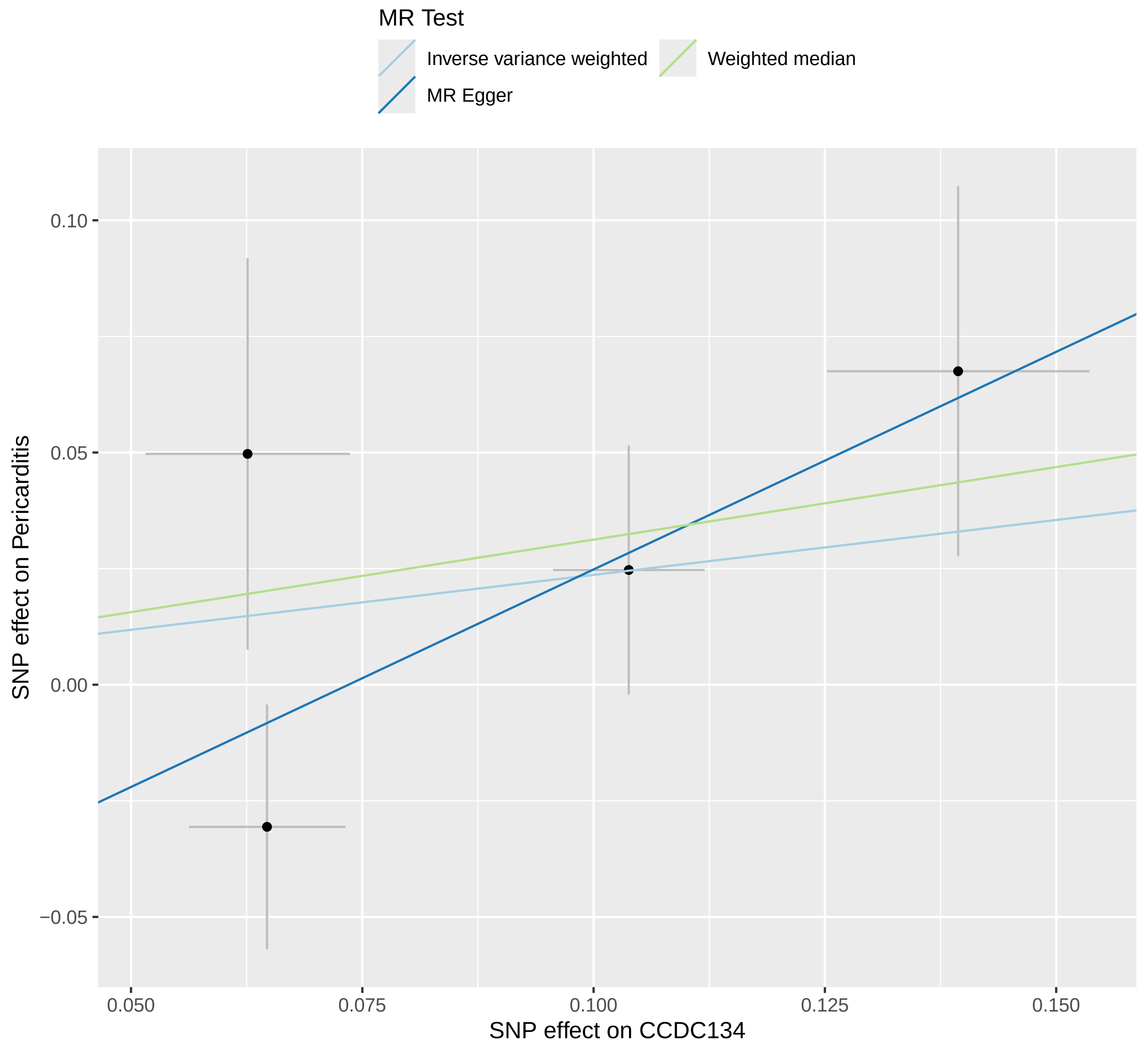
MR Test
Inverse variance weighted
MR Egger
Weighted median
0.10
0.05
0.00
−0.05
0.050
0.075
0.100
0.125
0.150
SNP effect on Pericarditis
SNP effect on CCDC134

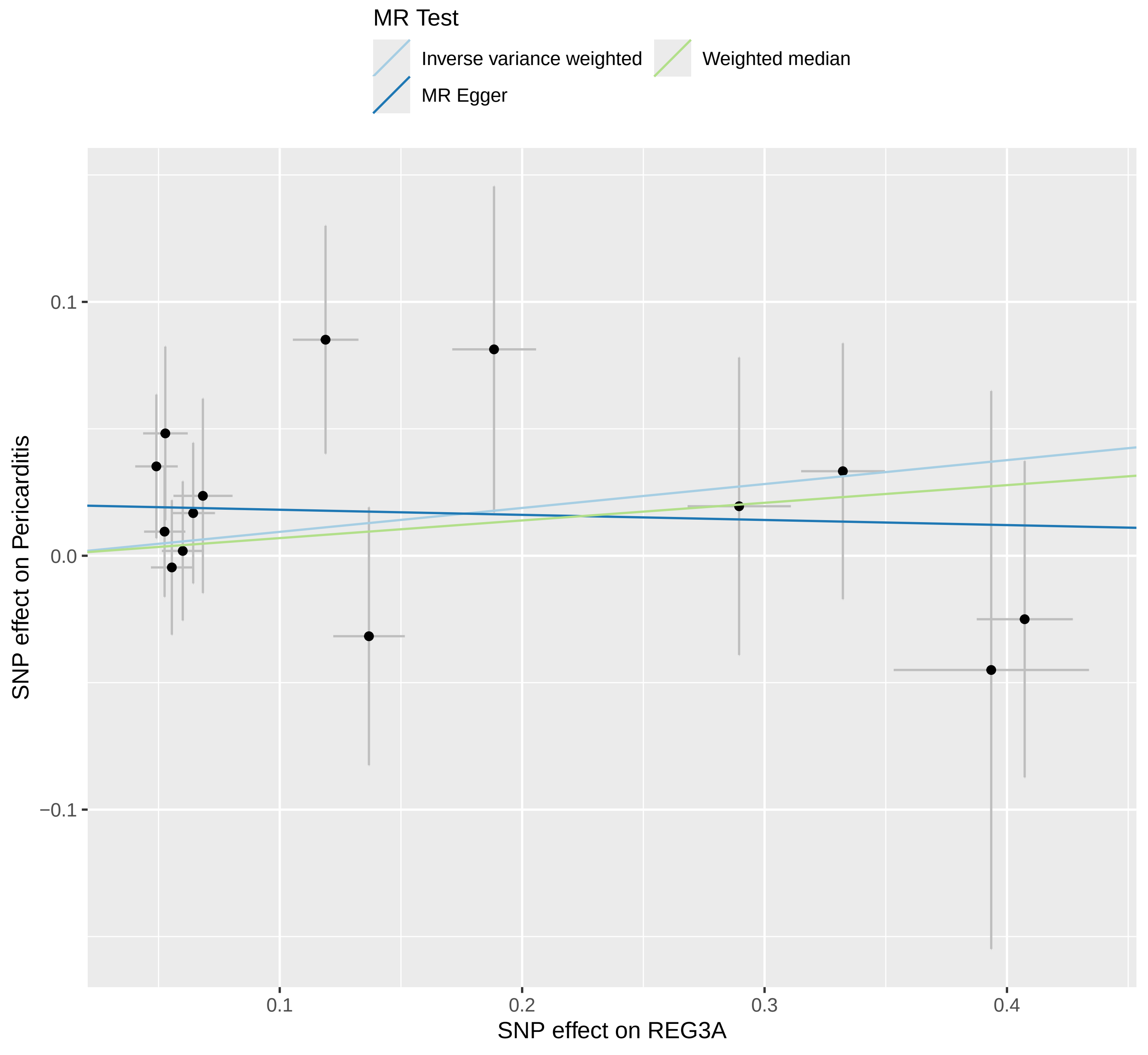
MR Test
Inverse variance weighted
Weighted median
MR Egger
SNP effect on Pericarditis
0.1
0.0
−0.1
0.1
0.2
0.3
0.4
SNP effect on REG3A

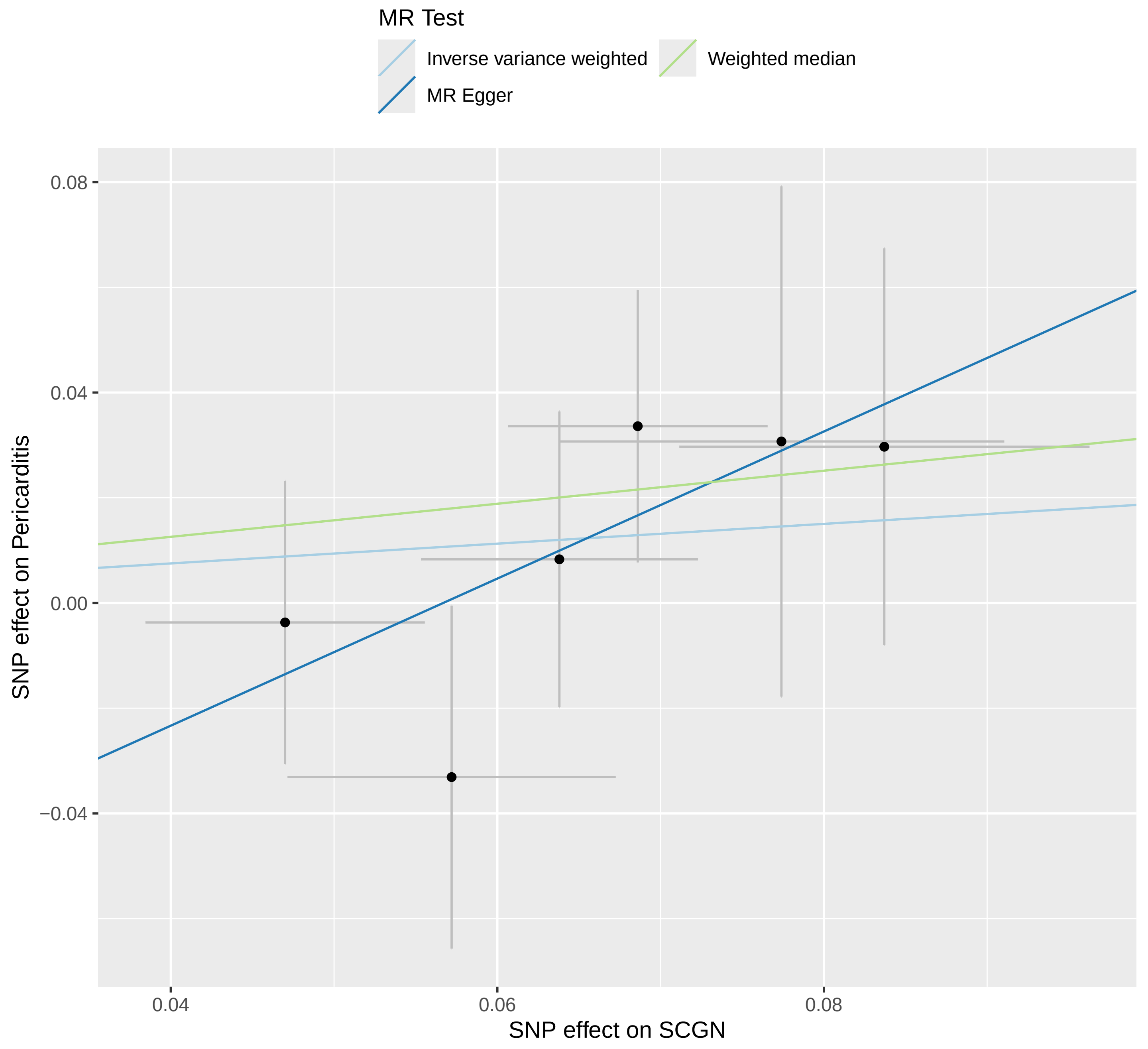
MR Test
Inverse variance weighted
Weighted median
MR Egger
0.08
0.04
0.00
−0.04
SNP effect on Pericarditis
0.04
0.06
0.08
SNP effect on SCGN

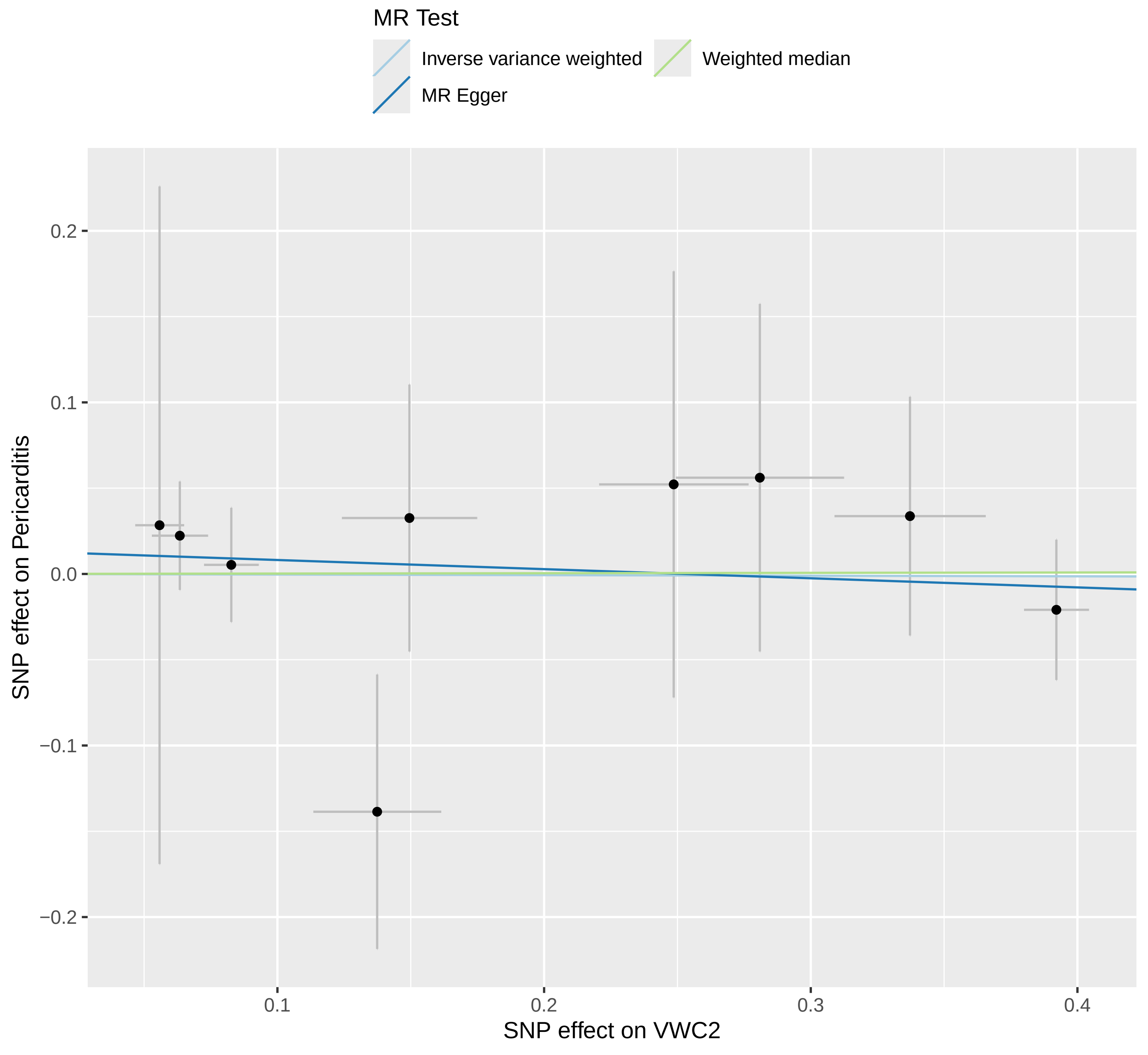
MR Test
Inverse variance weighted
Weighted median
MR Egger
SNP effect on Pericarditis
0.2
0.1
0.0
−0.1
−0.2
0.1
0.2
0.3
0.4
SNP effect on VWC2

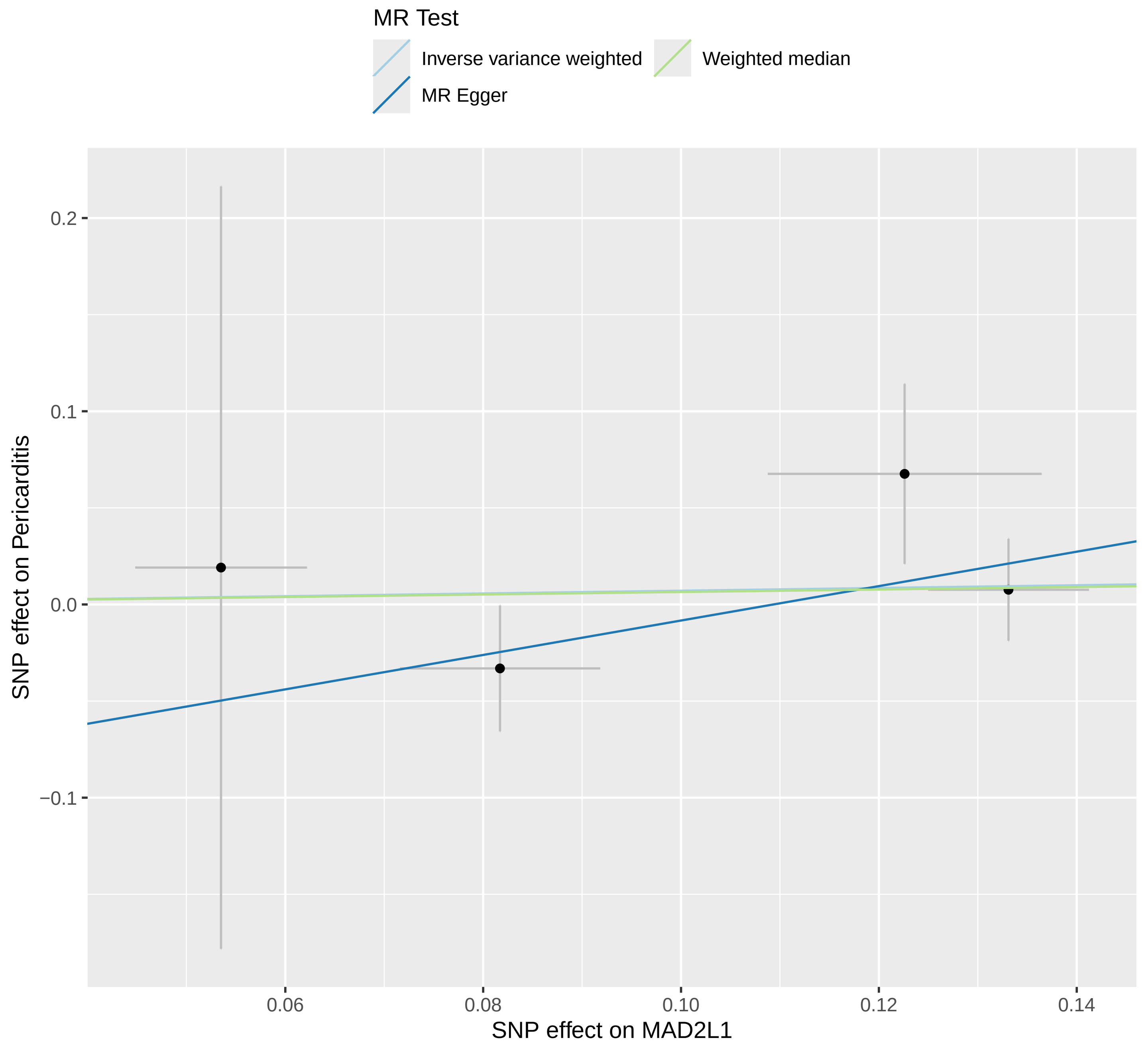
MR Test
Inverse variance weighted
Weighted median
MR Egger
SNP effect on Pericarditis
0.2
0.1
0.0
−0.1
0.06
0.08
0.10
0.12
0.14
SNP effect on MAD2L1

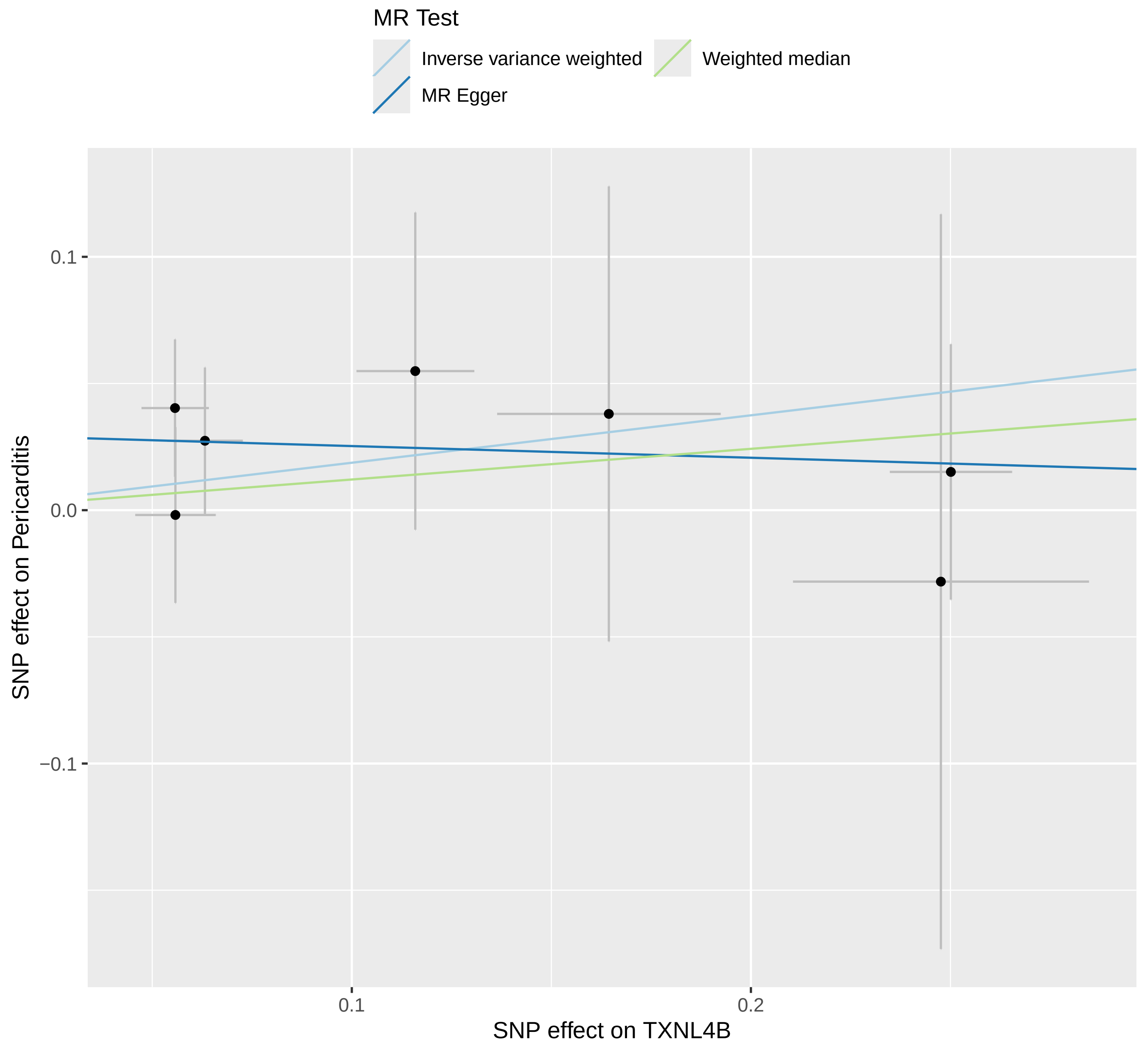

MR Test
Inverse variance weighted
Weighted median
MR Egger
0.1
0.0
−0.1
0.1
0.2
SNP effect on Pericarditis
SNP effect on TXNL4B

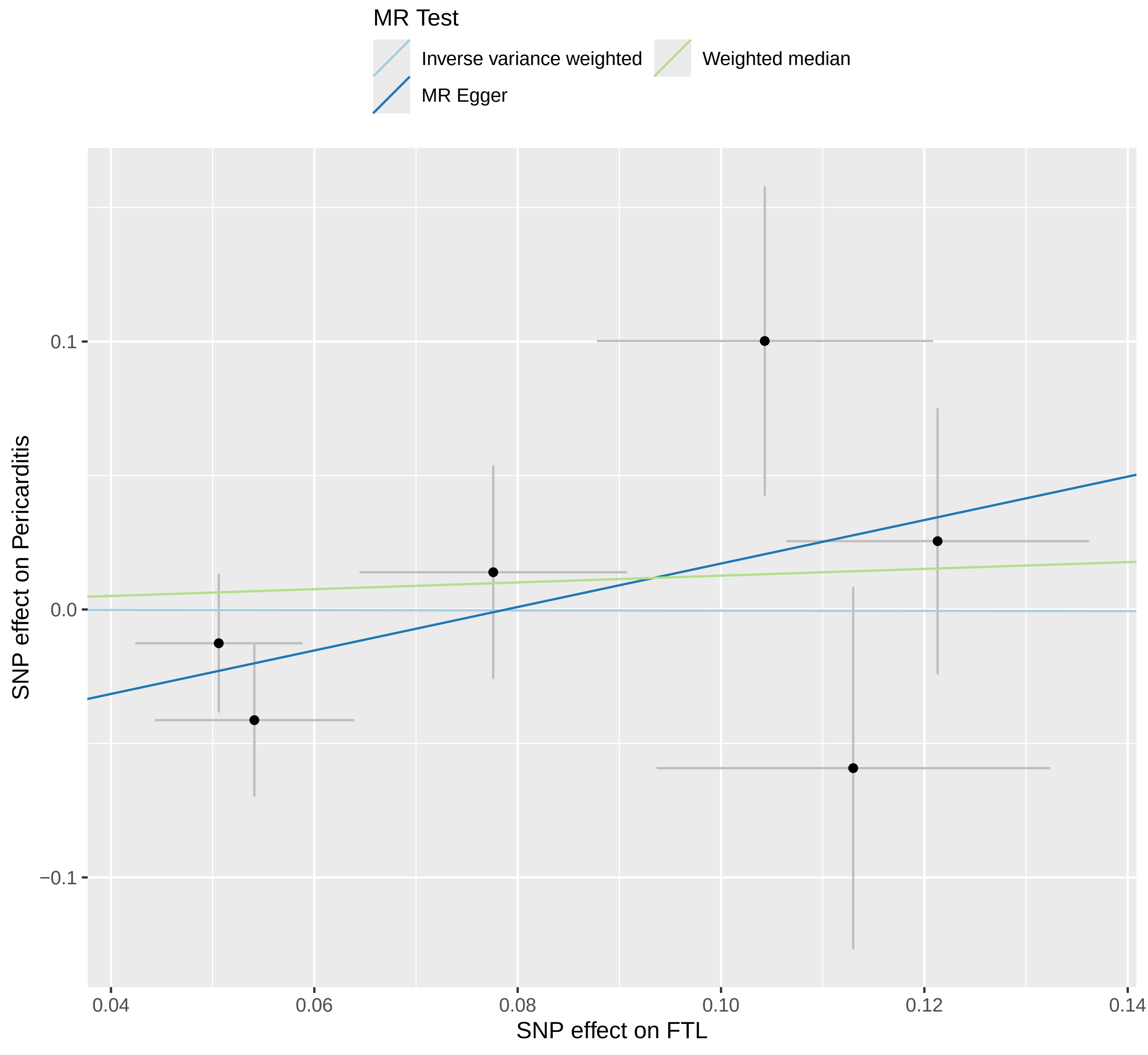

MR Test
Inverse variance weighted
Weighted median
MR Egger
SNP effect on Pericarditis
0.1
0.0
−0.1
0.04
0.06
0.08
0.10
0.12
0.14
SNP effect on FTL

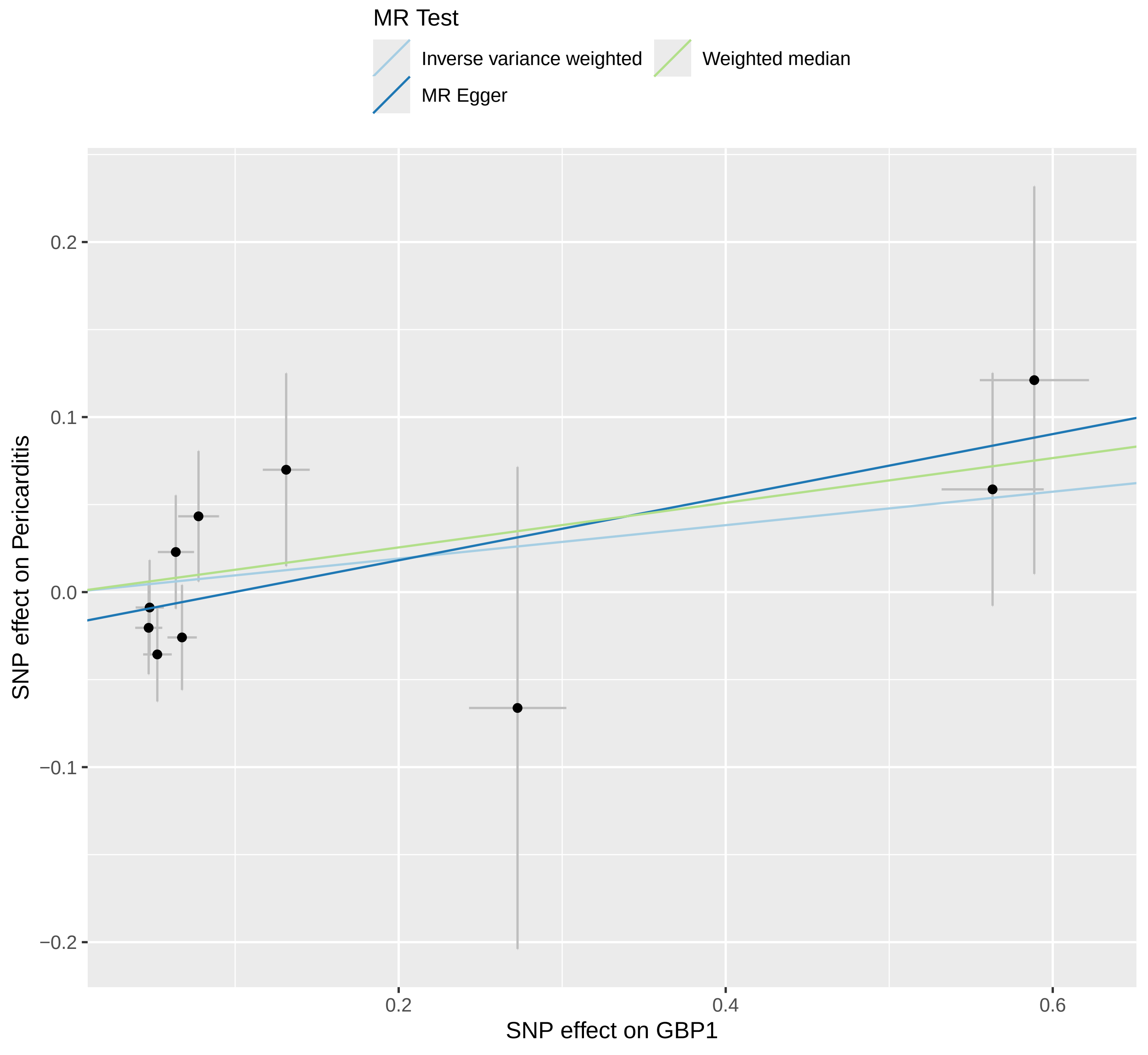

MR Test
Inverse variance weighted
MR Egger
Weighted median
0.2
0.1
0.0
−0.1
−0.2
SNP effect on Pericarditis
0.2
0.4
0.6
SNP effect on GBP1

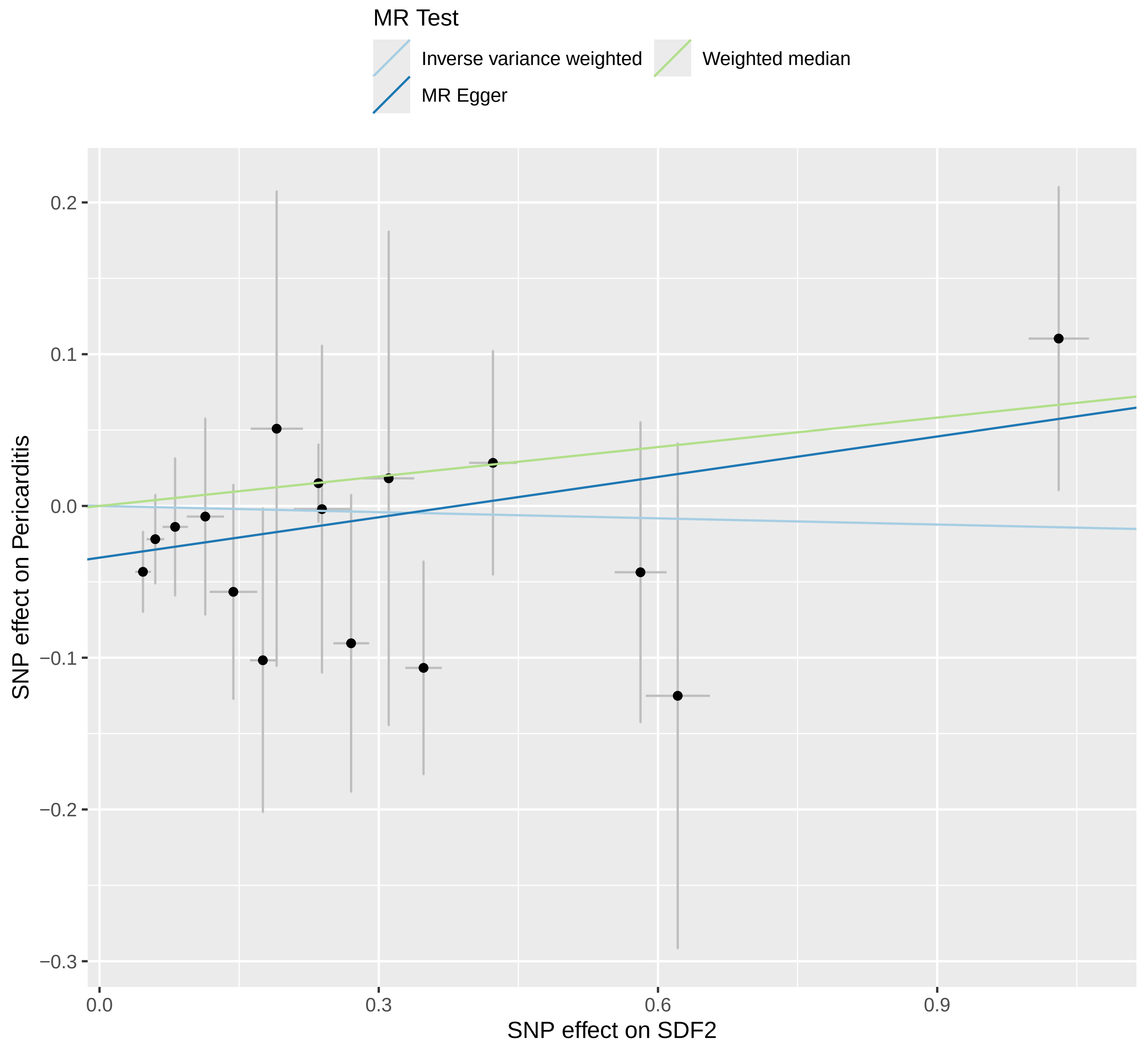

MR Test
Inverse variance weighted
Weighted median
MR Egger
SNP effect on Pericarditis
0.2
0.1
0.0
−0.1
−0.2
−0.3
0.0
0.3
0.6
0.9
SNP effect on SDF2

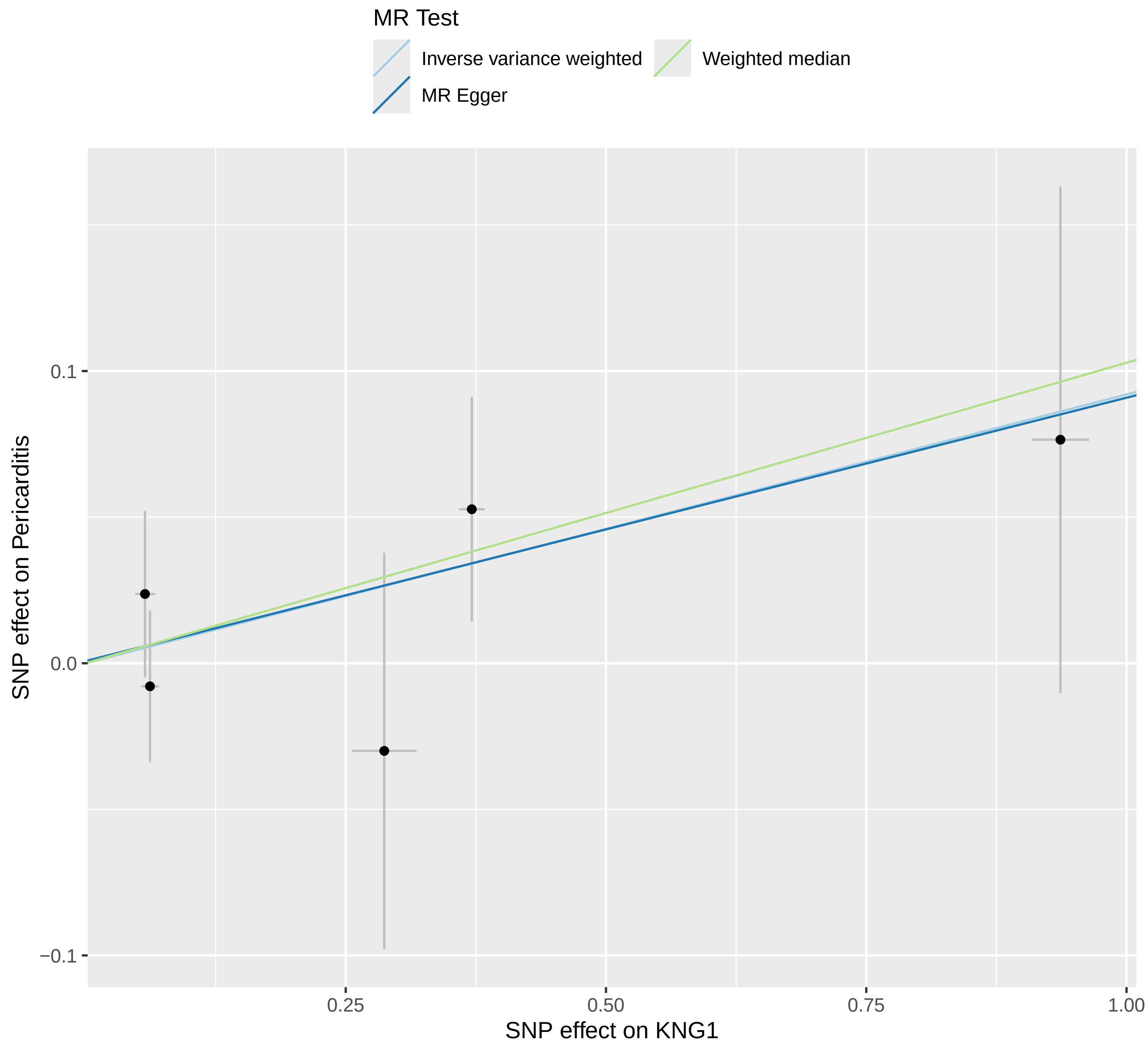
MR Test
Inverse variance weighted
Weighted median
MR Egger
0.1
0.0
−0.1
0.25
0.50
0.75
1.00
SNP effect on Pericarditis
SNP effect on KNG1

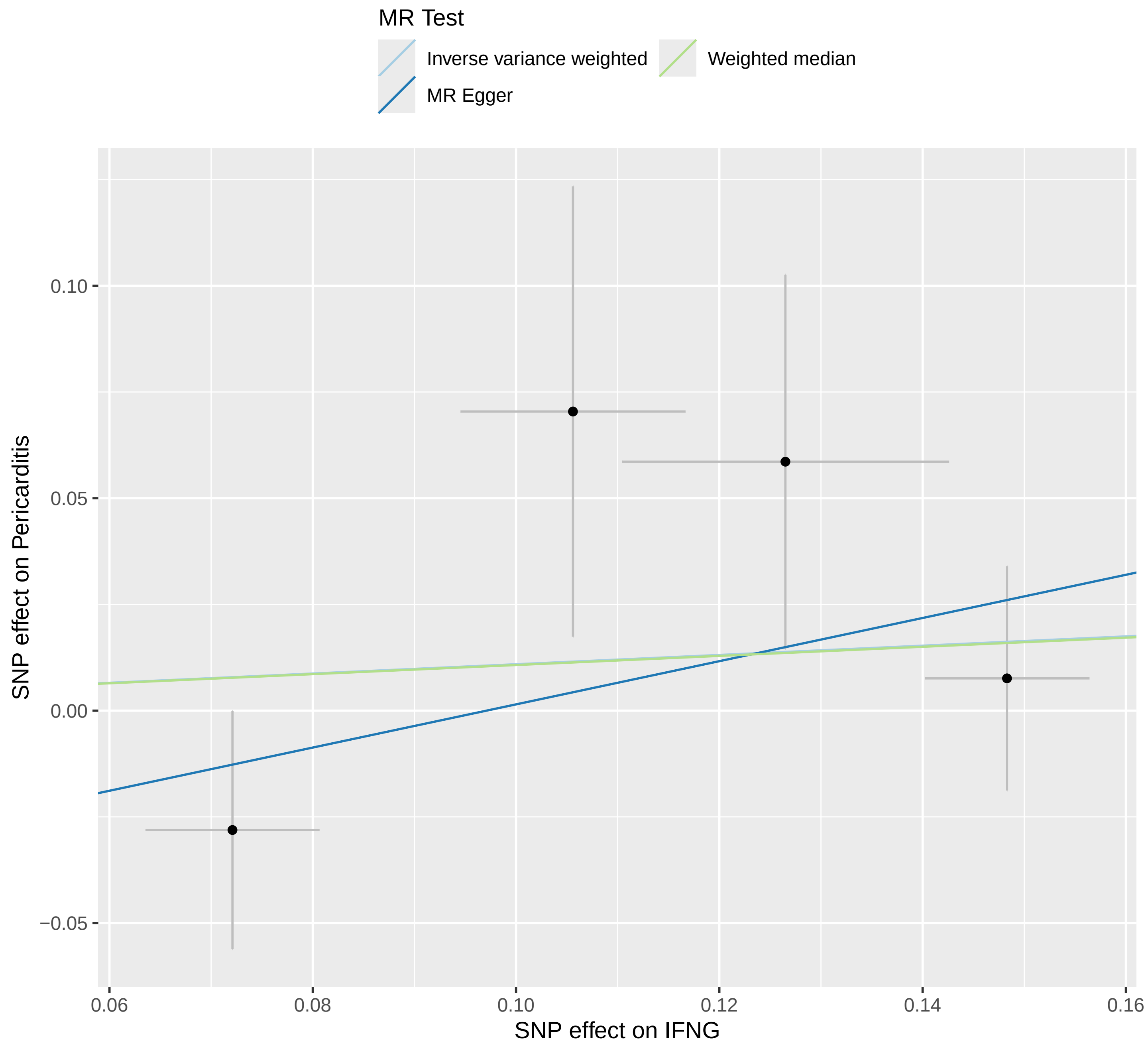

MR Test
Inverse variance weighted
MR Egger
Weighted median
SNP effect on Pericarditis
0.10
0.05
0.00
−0.05
0.06
0.08
0.10
0.12
0.14
0.16
SNP effect on IFNG

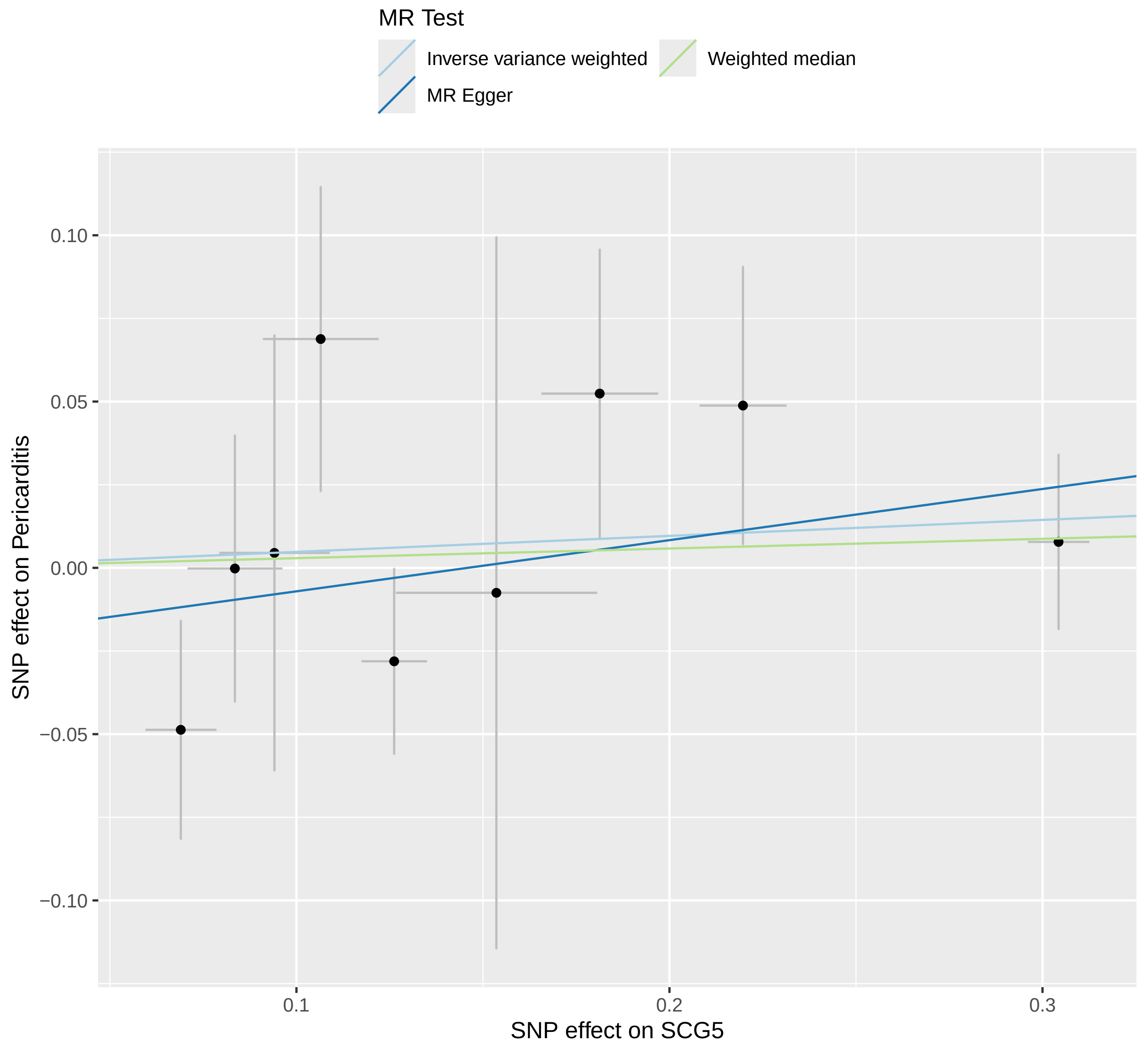
MR Test
Inverse variance weighted
MR Egger
Weighted median
0.10
0.05
0.00
−0.05
−0.10
SNP effect on Pericarditis
0.1
0.2
0.3
SNP effect on SCG5

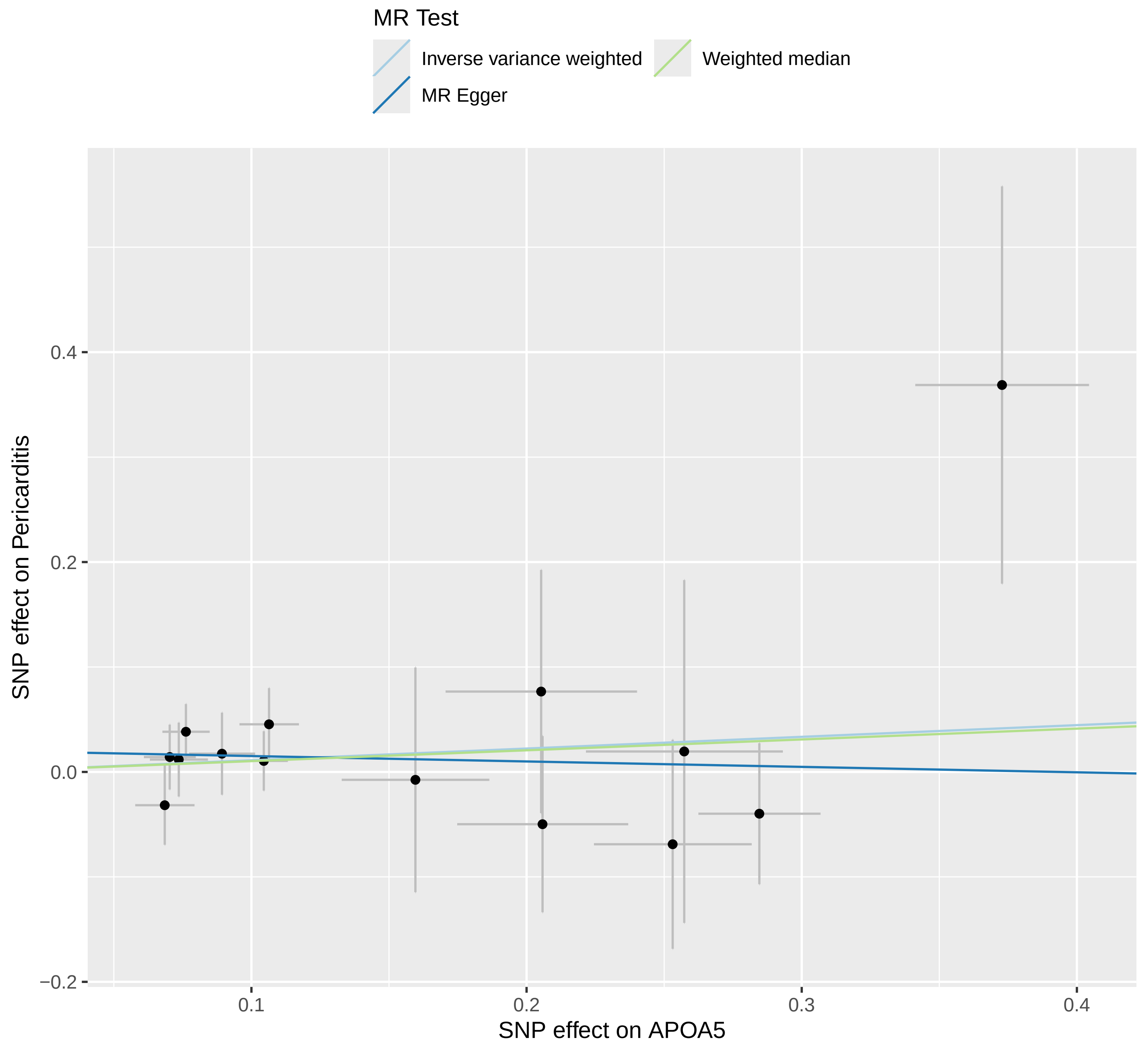

MR Test
Inverse variance weighted
Weighted median
MR Egger
SNP effect on Pericarditis
0.4
0.2
0.0
−0.2
0.1
0.2
0.3
0.4
SNP effect on APOA5

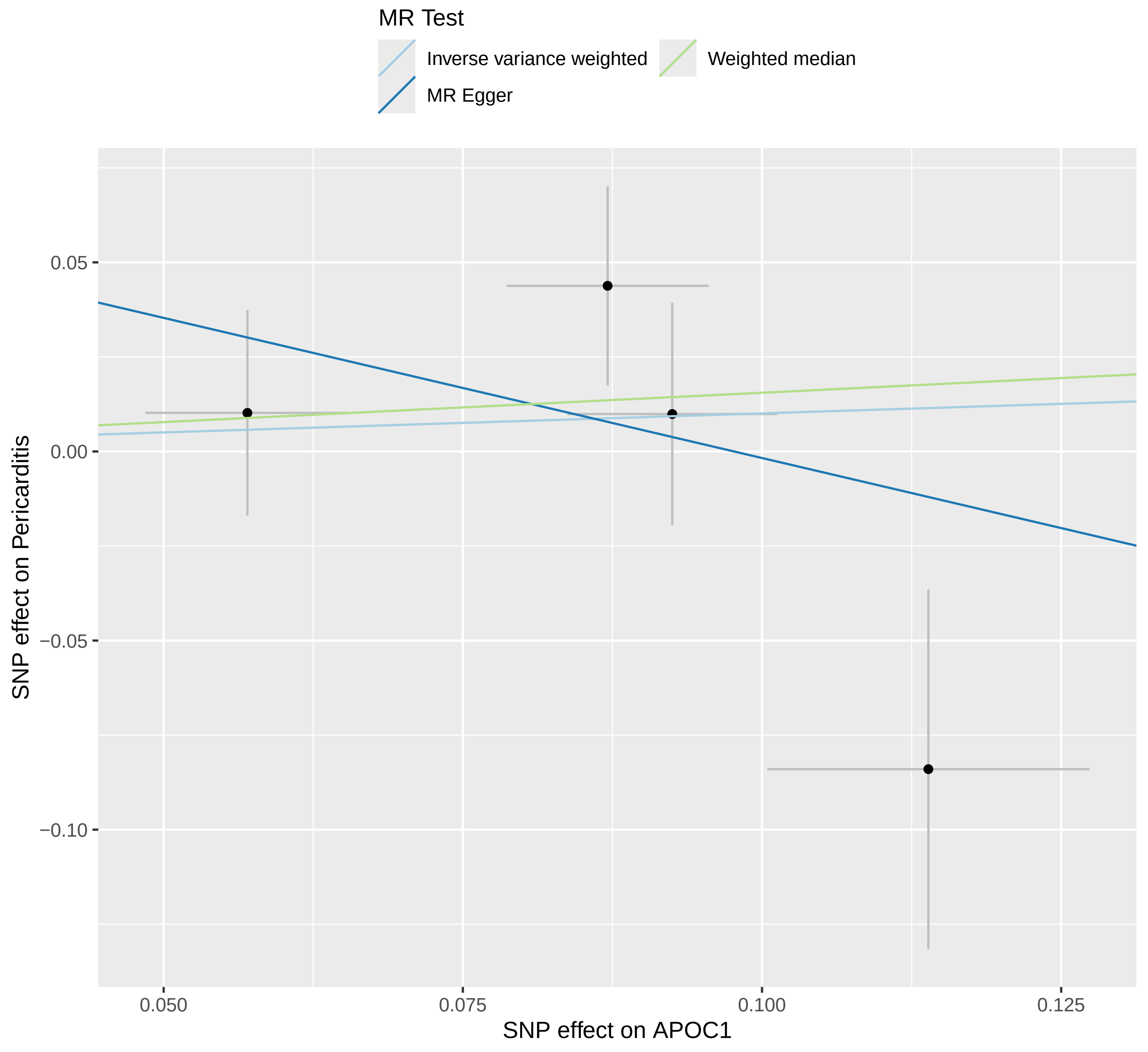
MR Test
Inverse variance weighted
Weighted median
MR Egger
SNP effect on Pericarditis
0.05
0.00
−0.05
−0.10
0.050
0.075
0.100
0.125
SNP effect on APOC1

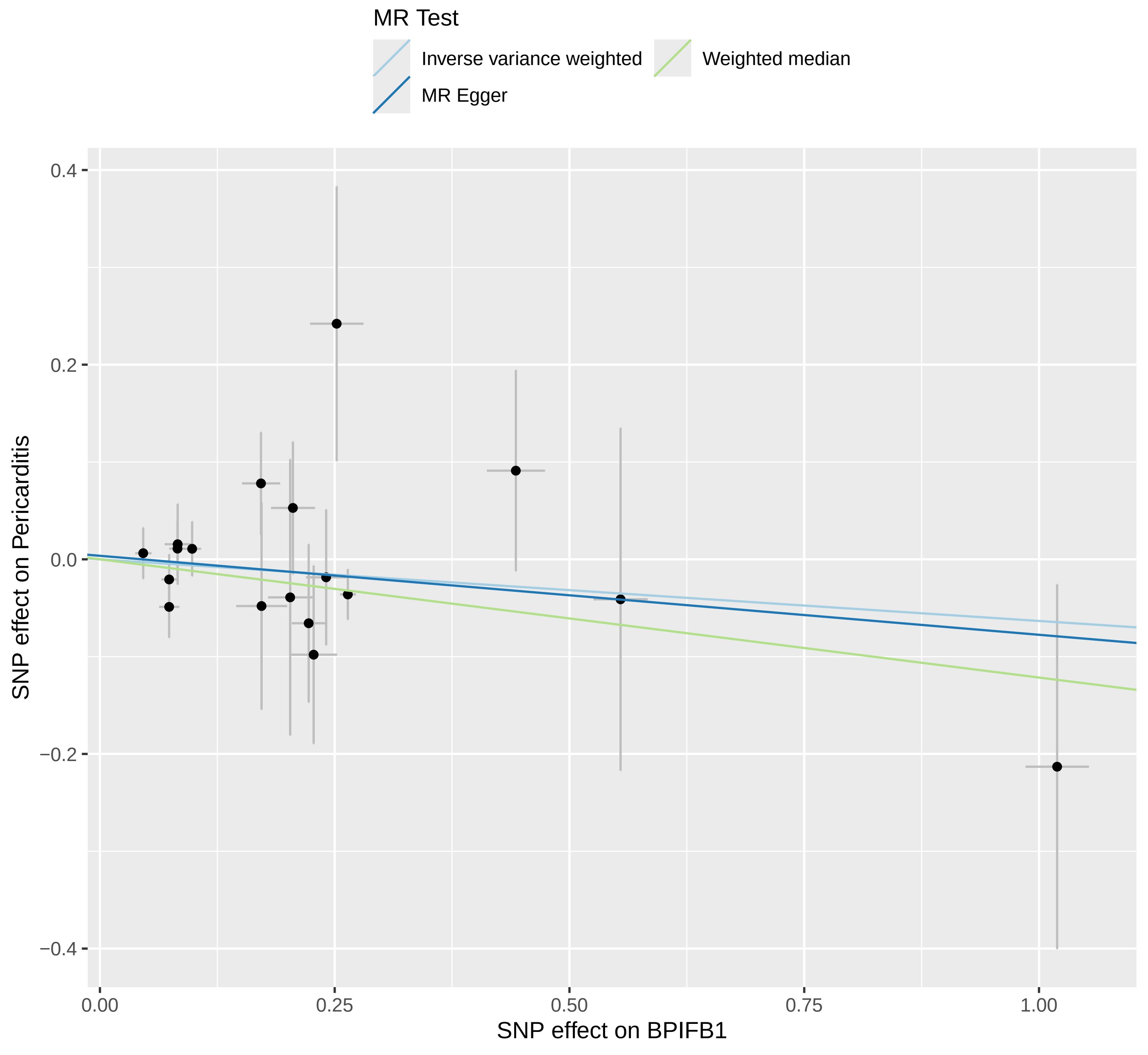

MR Test
Inverse variance weighted
MR Egger
Weighted median
0.4
0.2
0.0
−0.2
−0.4
SNP effect on Pericarditis
0.00
0.25
0.50
0.75
1.00
SNP effect on BPIFB1

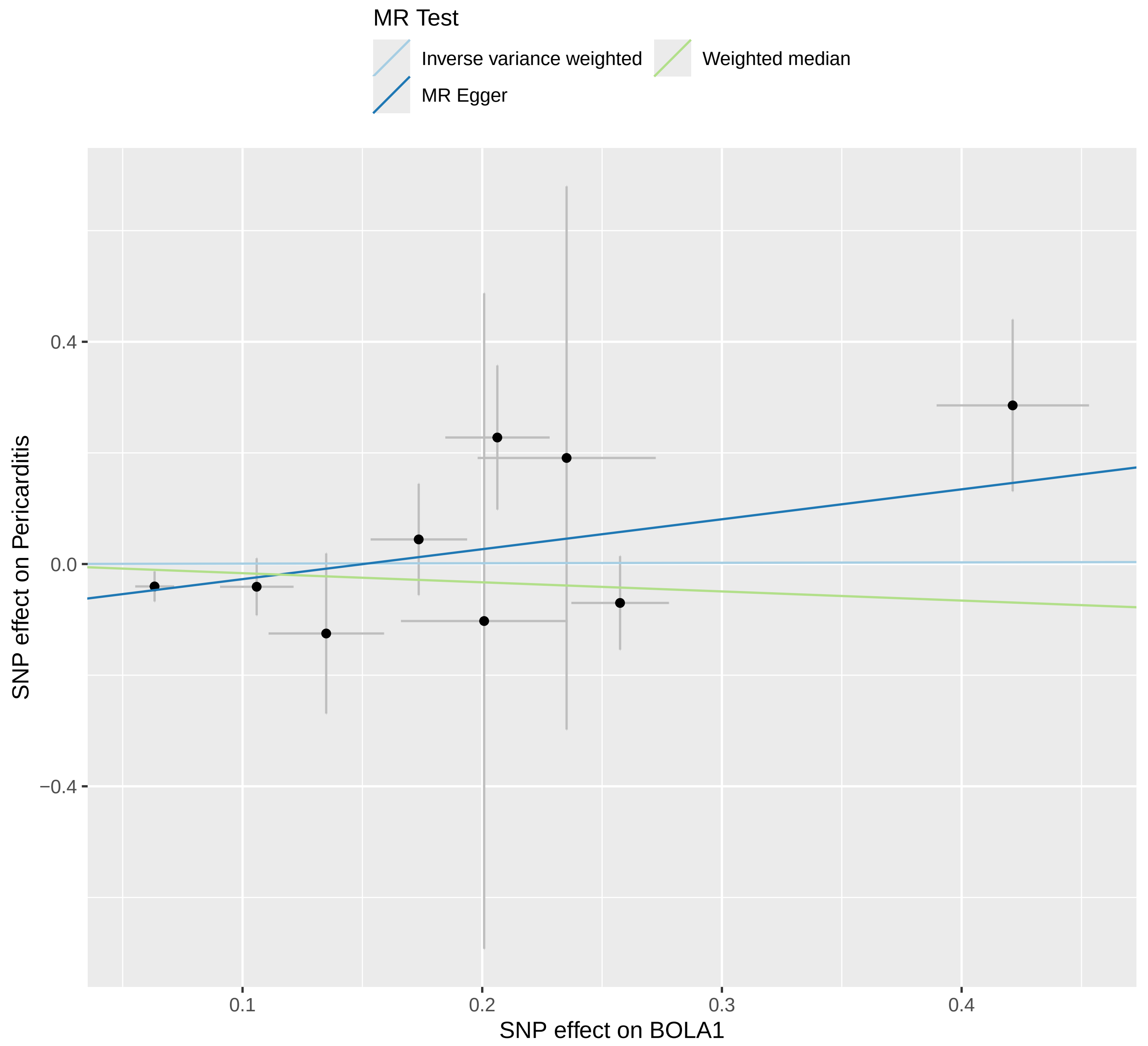
MR Test
Inverse variance weighted
MR Egger
Weighted median
0.4
0.0
−0.4
SNP effect on Pericarditis
0.1
0.2
0.3
0.4
SNP effect on BOLA1

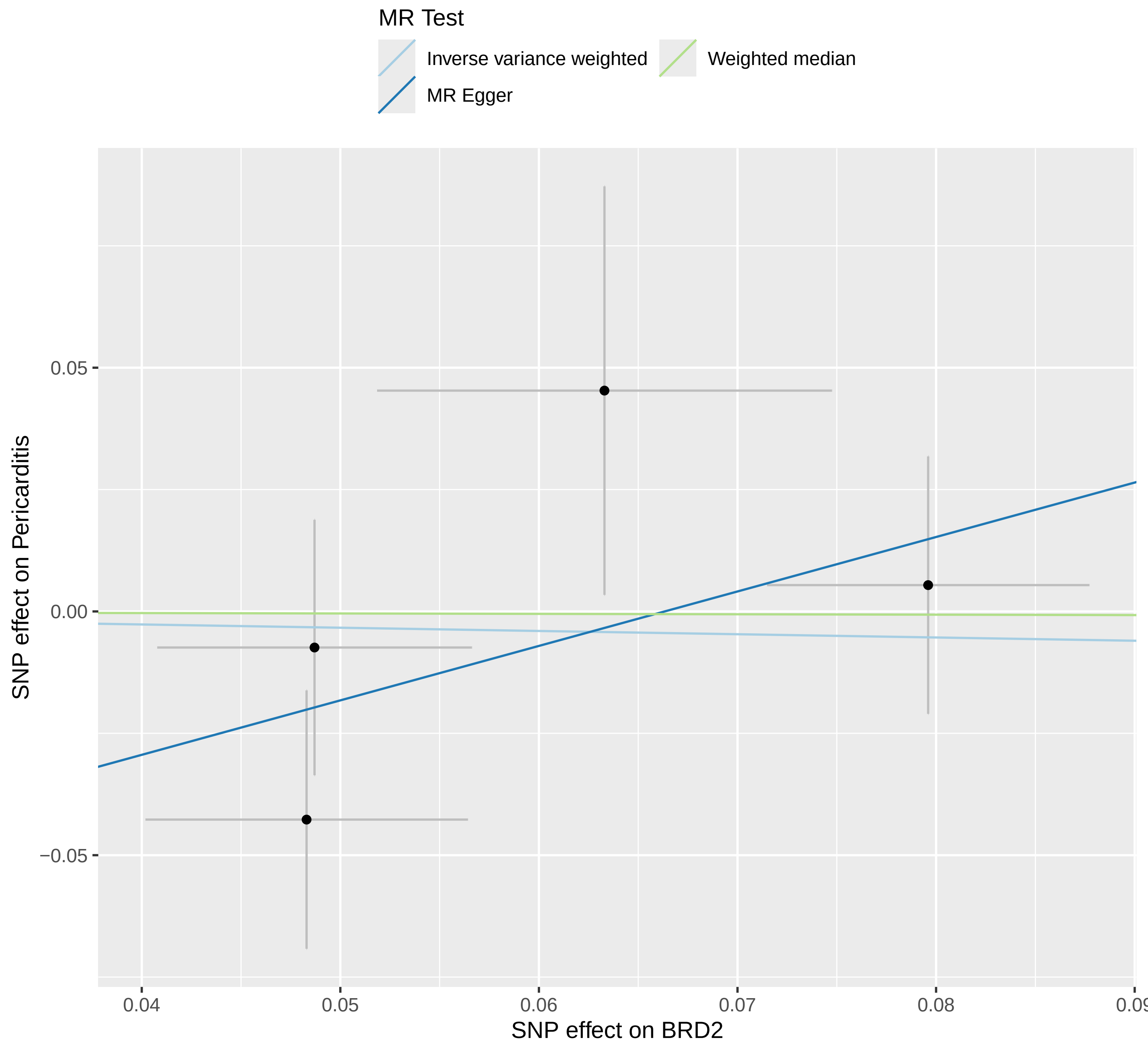
MR Test
Inverse variance weighted
Weighted median
MR Egger
0.05
0.00
−0.05
SNP effect on Pericarditis
0.04
0.05
0.06
0.07
0.08
0.09
SNP effect on BRD2

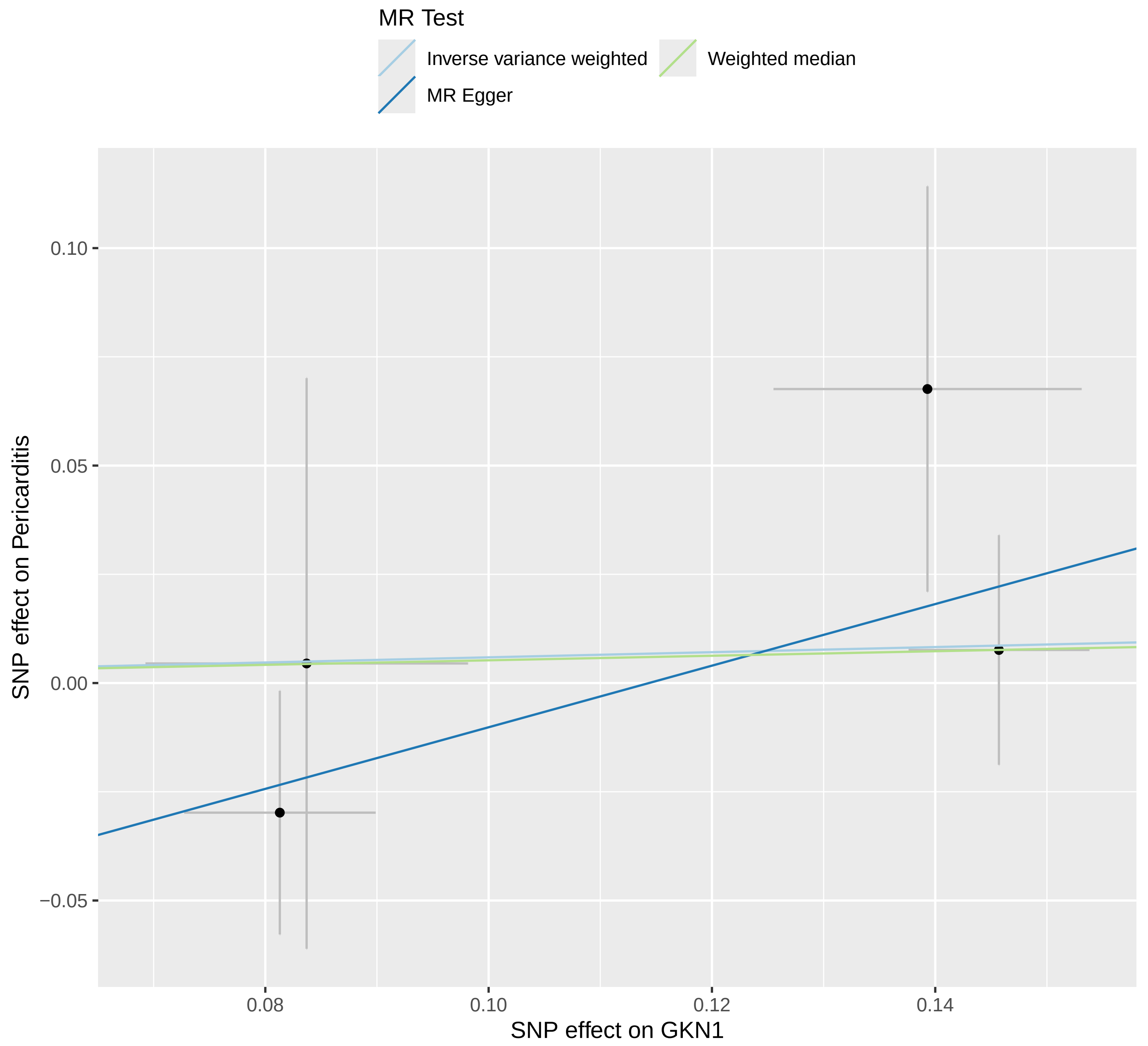

MR Test
Inverse variance weighted
MR Egger
Weighted median
0.10
0.05
0.00
−0.05
SNP effect on Pericarditis
0.08
0.10
0.12
0.14
SNP effect on GKN1

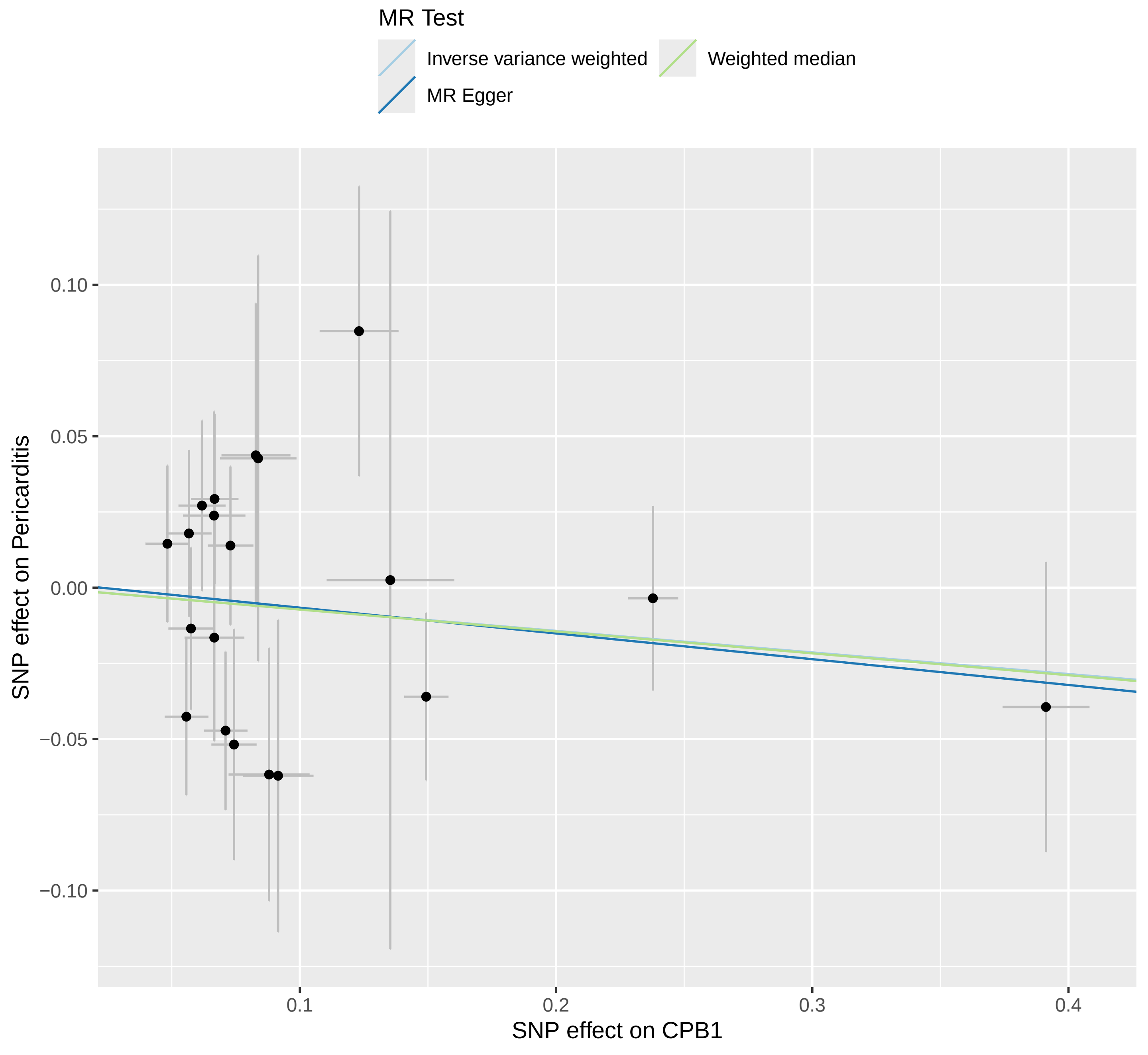

MR Test
Inverse variance weighted
Weighted median
MR Egger
SNP effect on Pericarditis
0.10
0.05
0.00
−0.05
−0.10
0.1
0.2
0.3
0.4
SNP effect on CPB1

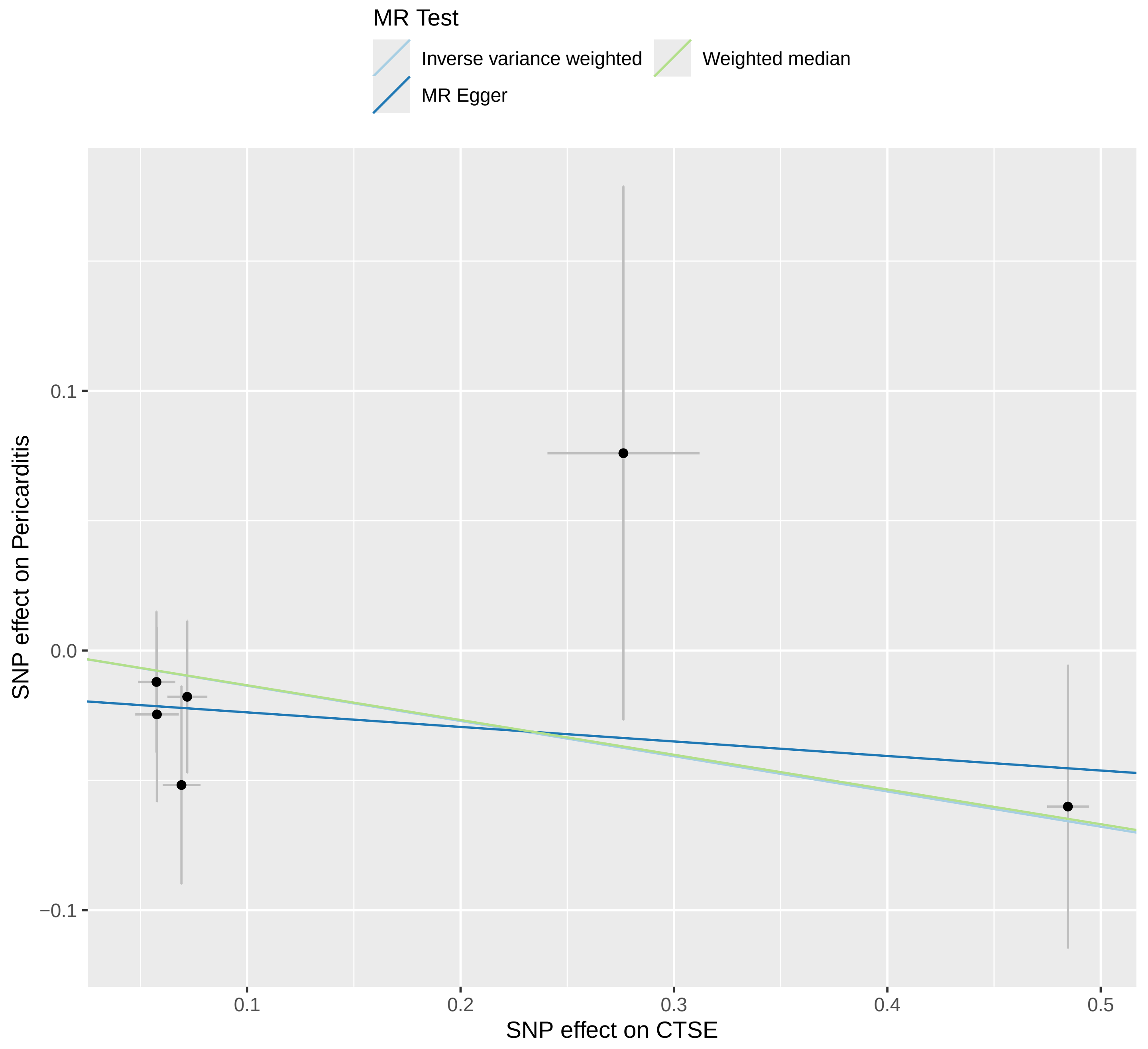
MR Test
Inverse variance weighted
MR Egger
Weighted median
SNP effect on Pericarditis
0.1
0.0
−0.1
0.1
0.2
0.3
0.4
0.5
SNP effect on CTSE

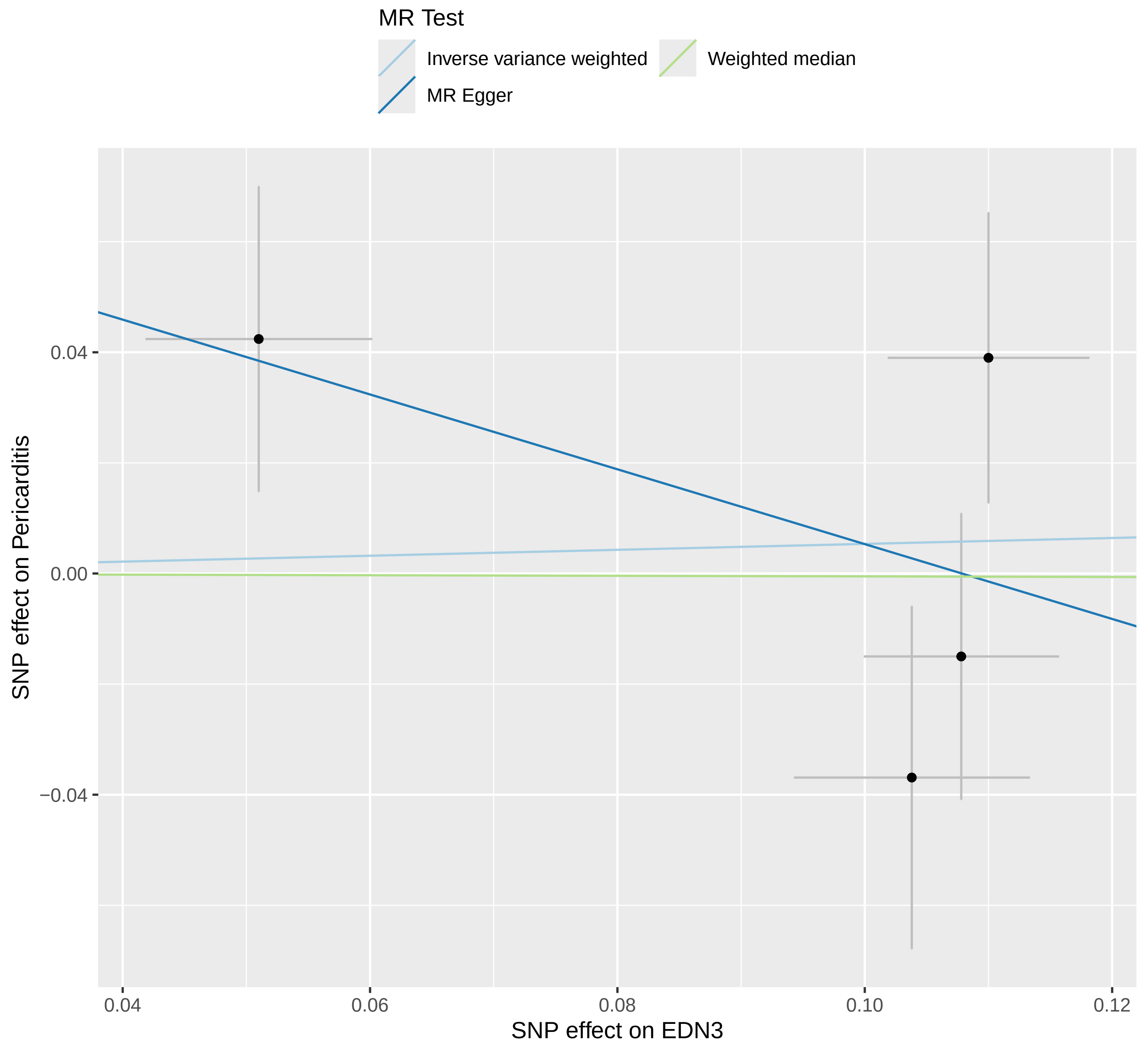
MR Test
Inverse variance weighted
Weighted median
MR Egger
0.04
0.00
−0.04
0.04
0.06
0.08
0.10
0.12
SNP effect on Pericarditis
SNP effect on EDN3

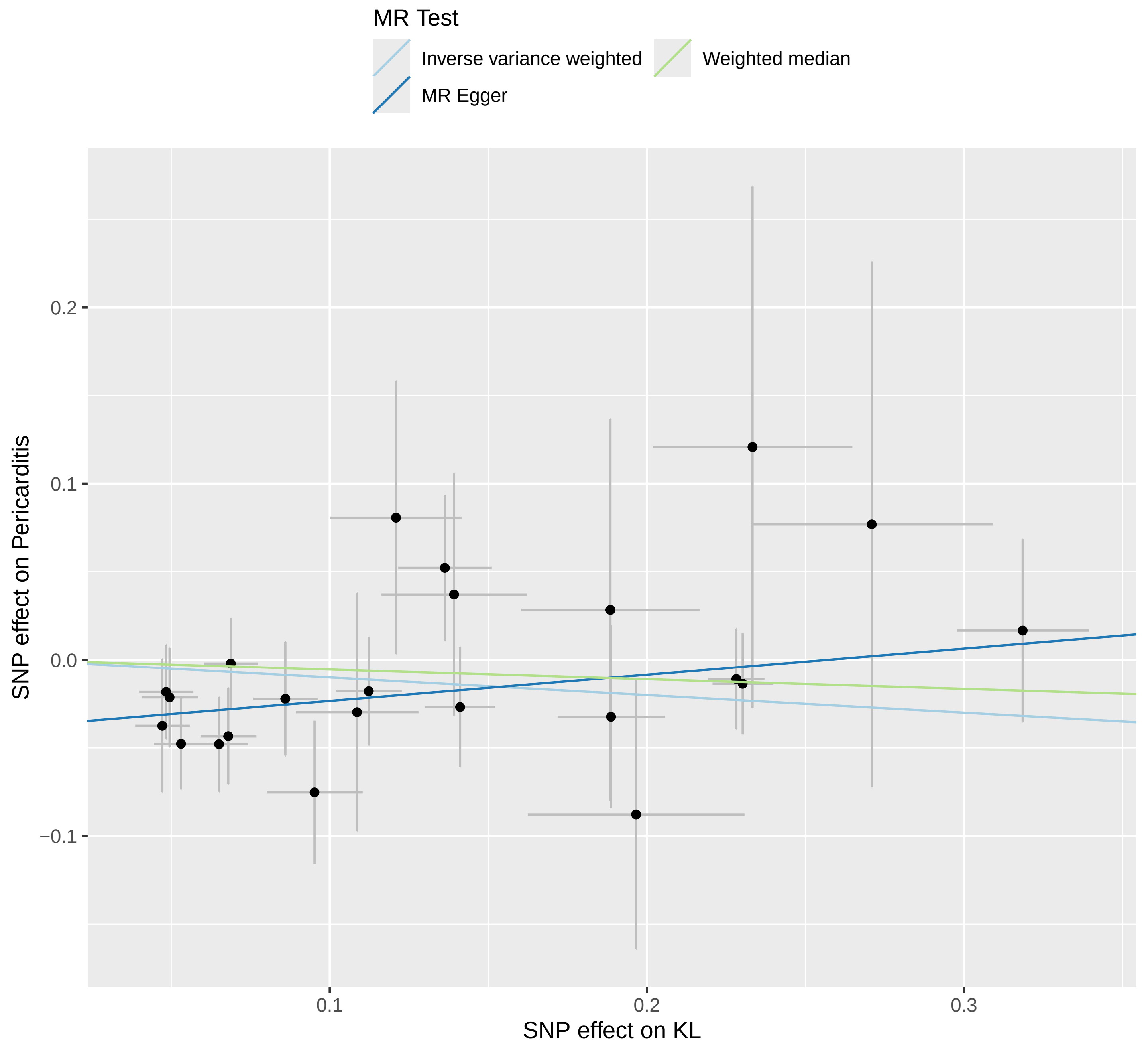
MR Test
Inverse variance weighted
Weighted median
MR Egger
SNP effect on Pericarditis
0.2
0.1
0.0
−0.1
0.1
0.2
0.3
SNP effect on KL

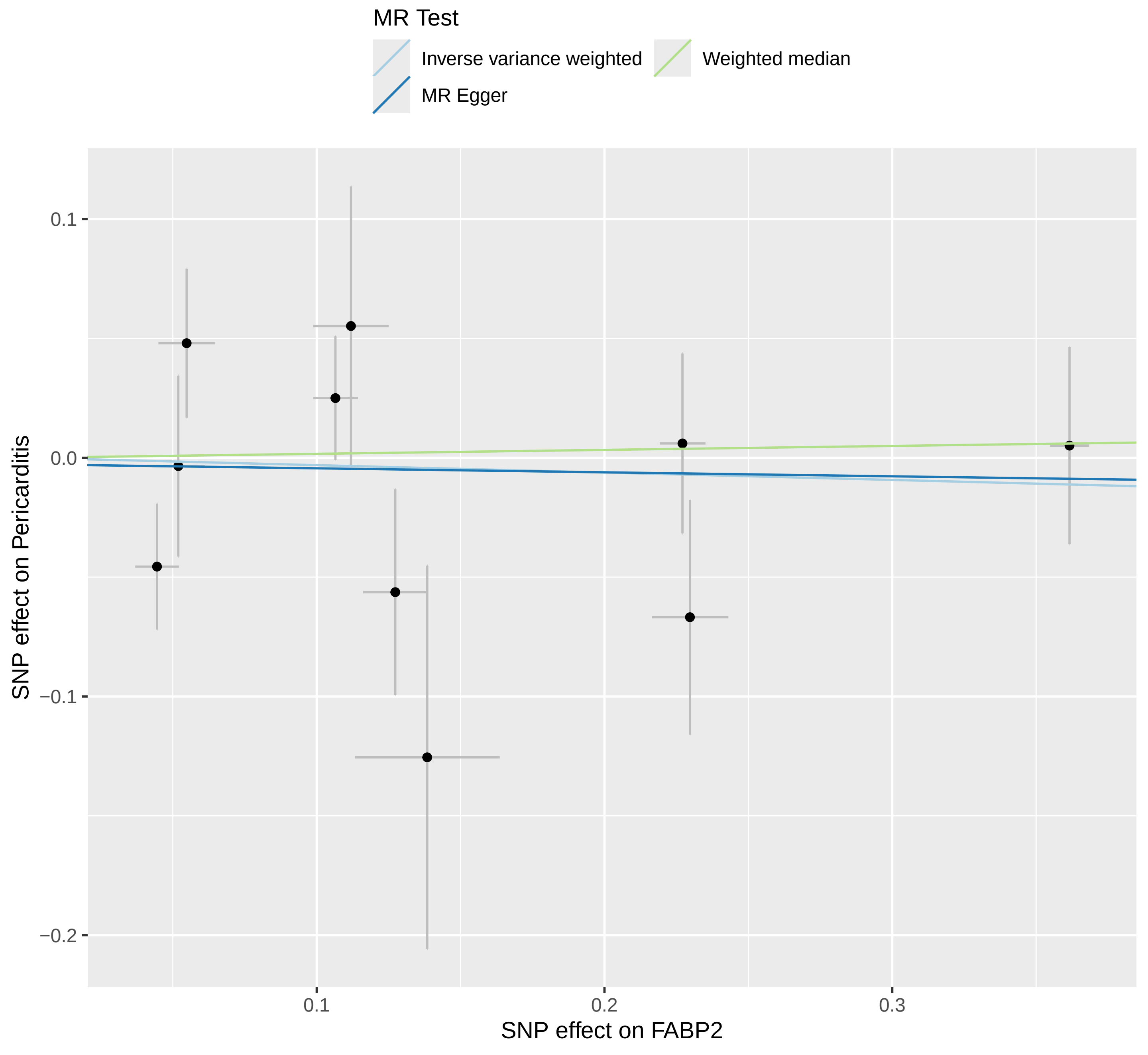

MR Test
Inverse variance weighted
MR Egger
Weighted median
SNP effect on Pericarditis
0.1
0.0
−0.1
−0.2
SNP effect on FABP2
0.1
0.2
0.3

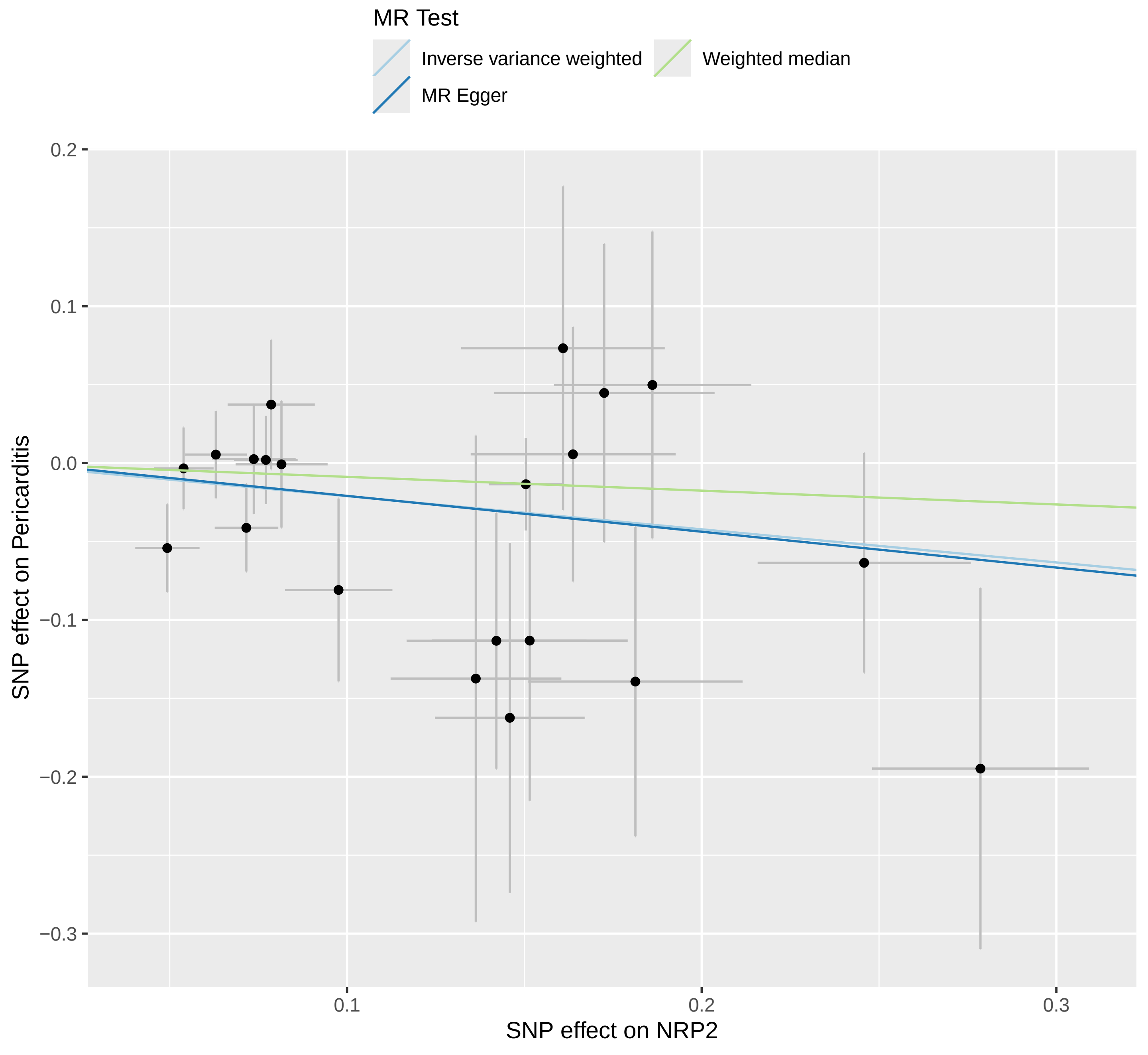
MR Test
Inverse variance weighted
Weighted median
MR Egger
0.2
0.1
0.0
−0.1
−0.2
−0.3
SNP effect on Pericarditis
0.1
0.2
0.3
SNP effect on NRP2

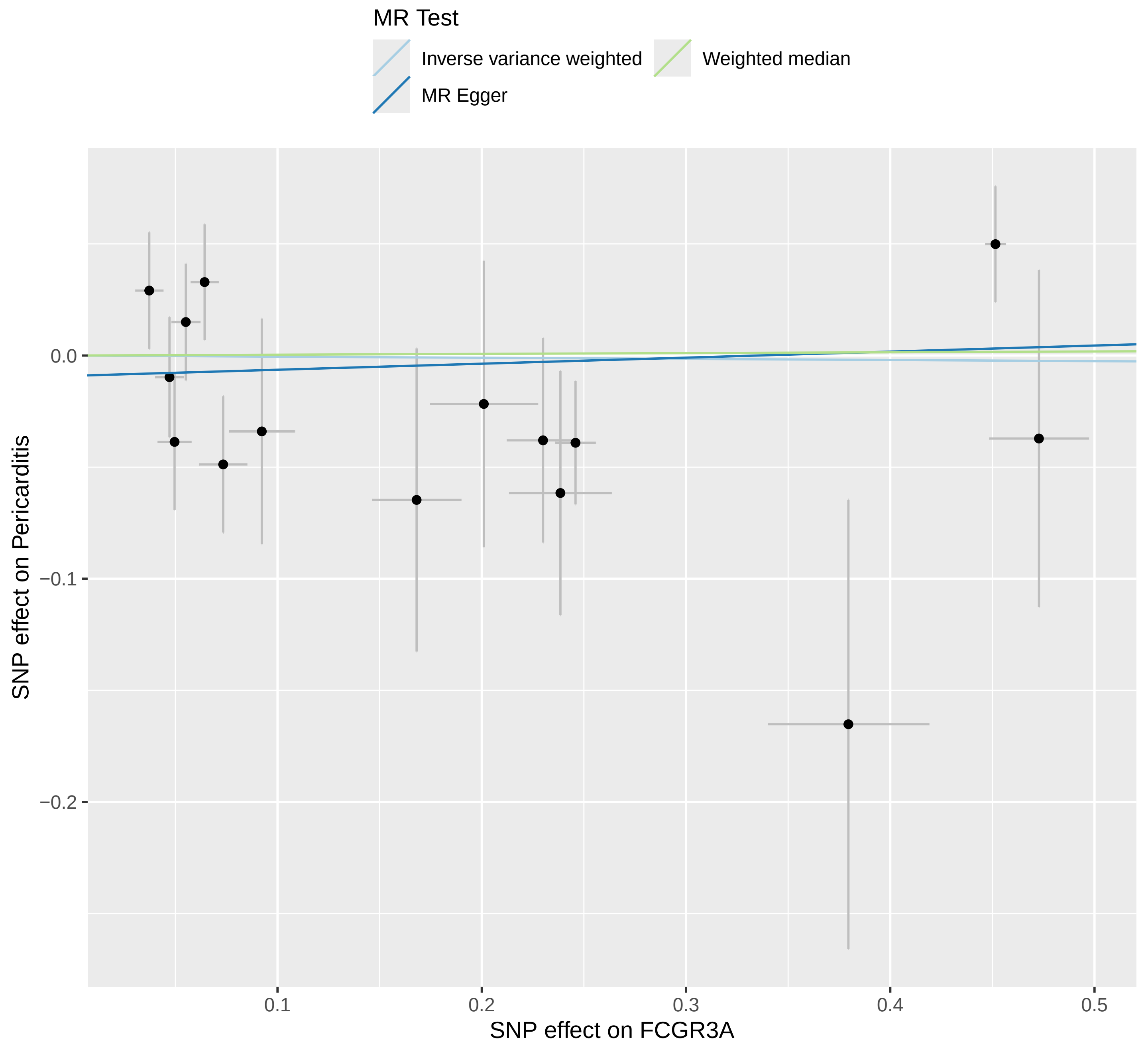

MR Test
Inverse variance weighted
Weighted median
MR Egger
0.0
−0.1
−0.2
0.1
0.2
0.3
0.4
0.5
SNP effect on Pericarditis
SNP effect on FCGR3A

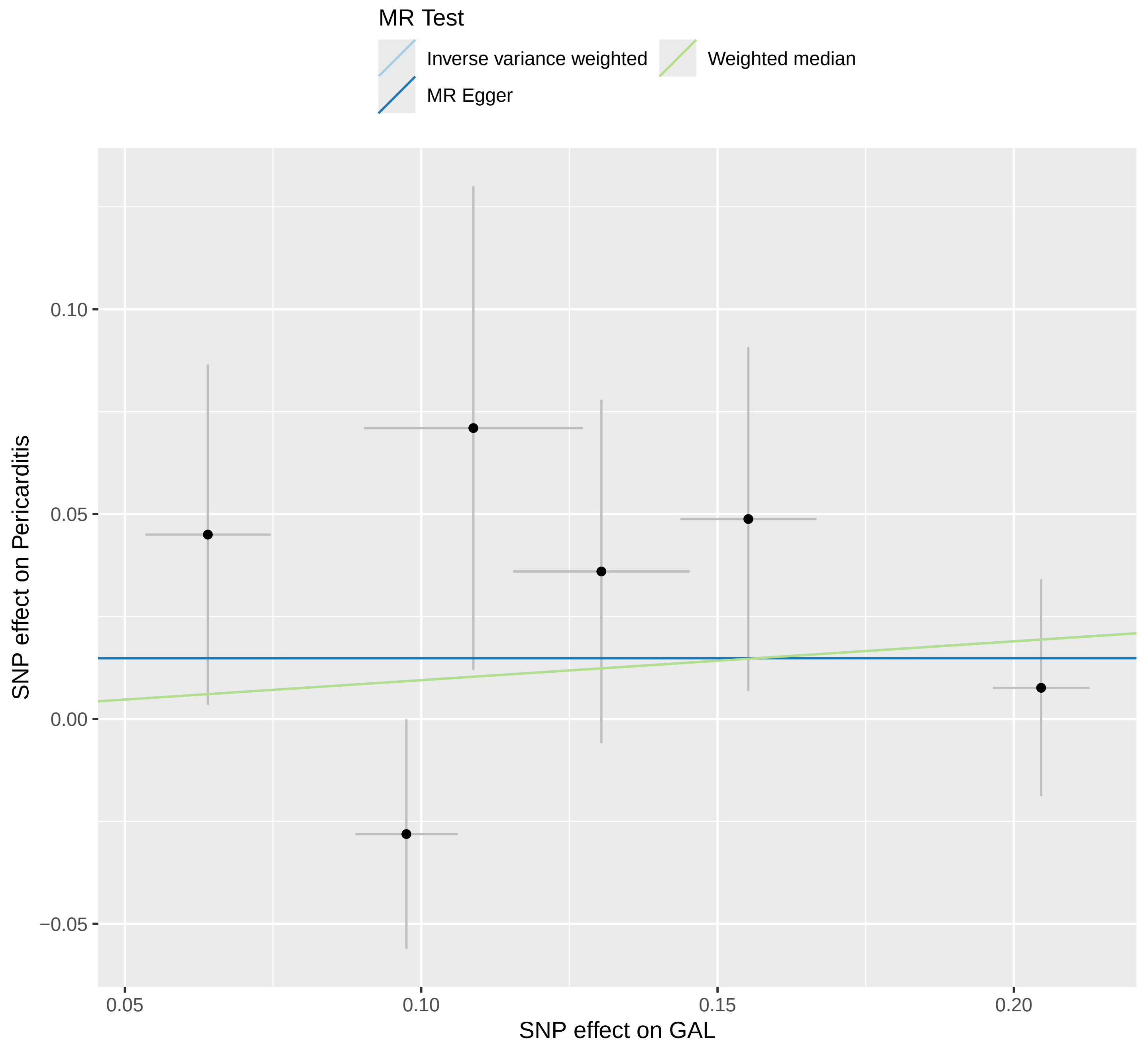

MR Test
Inverse variance weighted
Weighted median
MR Egger
0.10
0.05
0.00
−0.05
0.05
0.10
0.15
0.20
SNP effect on Pericarditis
SNP effect on GAL

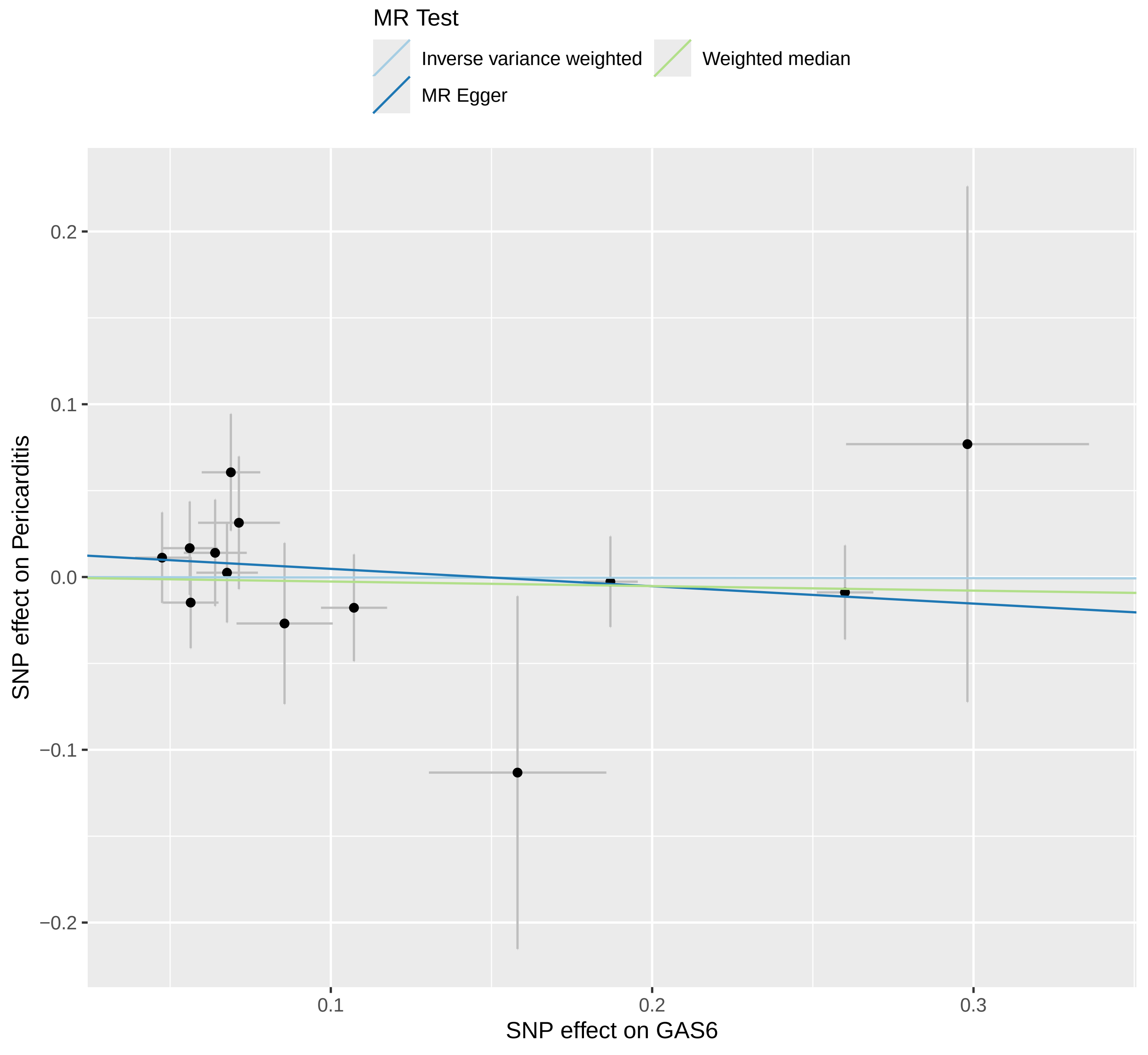
MR Test
Inverse variance weighted
Weighted median
MR Egger
SNP effect on Pericarditis
0.2
0.1
0.0
−0.1
−0.2
0.1
0.2
0.3
SNP effect on GAS6

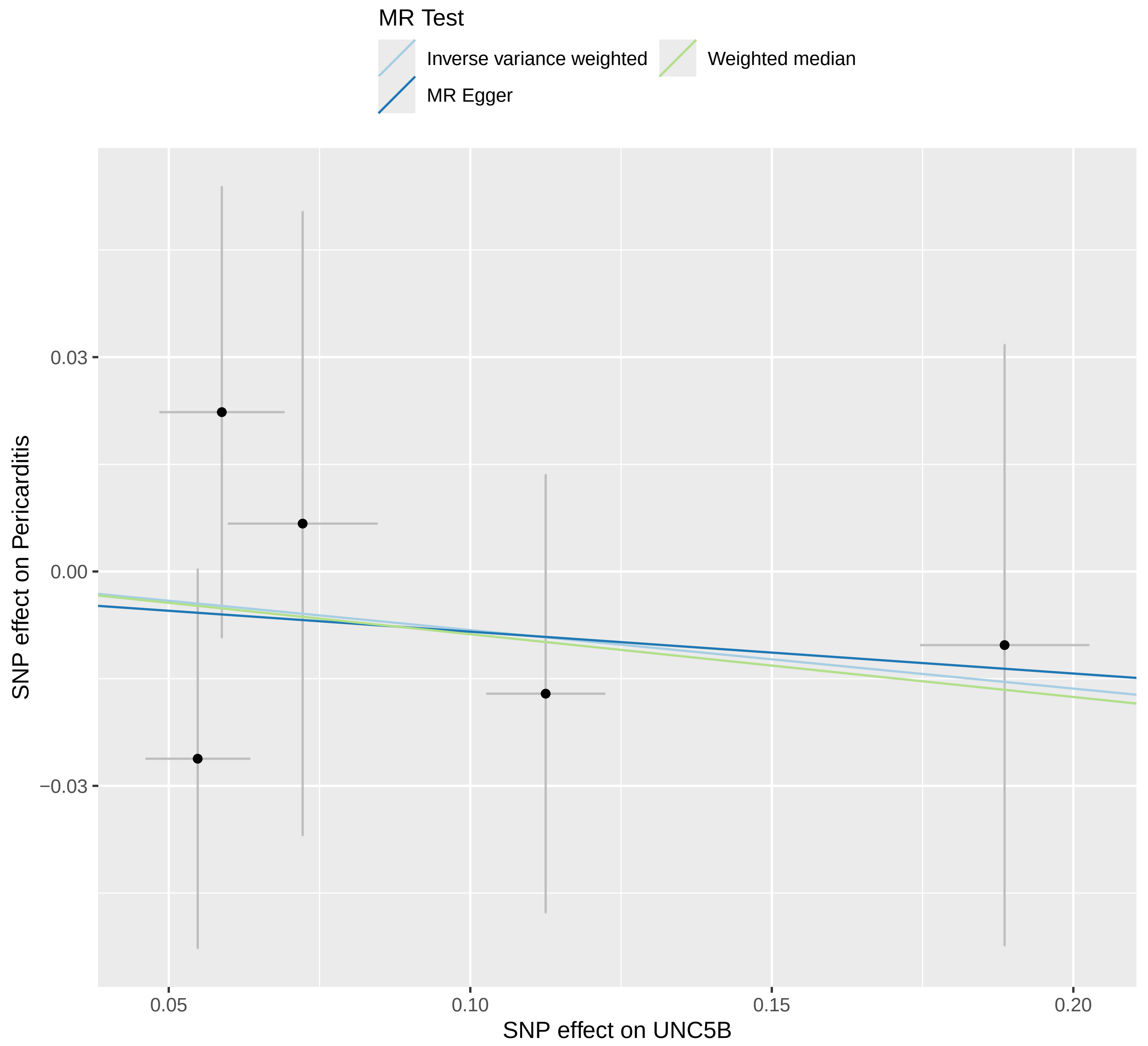
MR Test
Inverse variance weighted
Weighted median
MR Egger
0.03
0.00
−0.03
0.05
0.10
0.15
0.20
SNP effect on Pericarditis
SNP effect on UNC5B

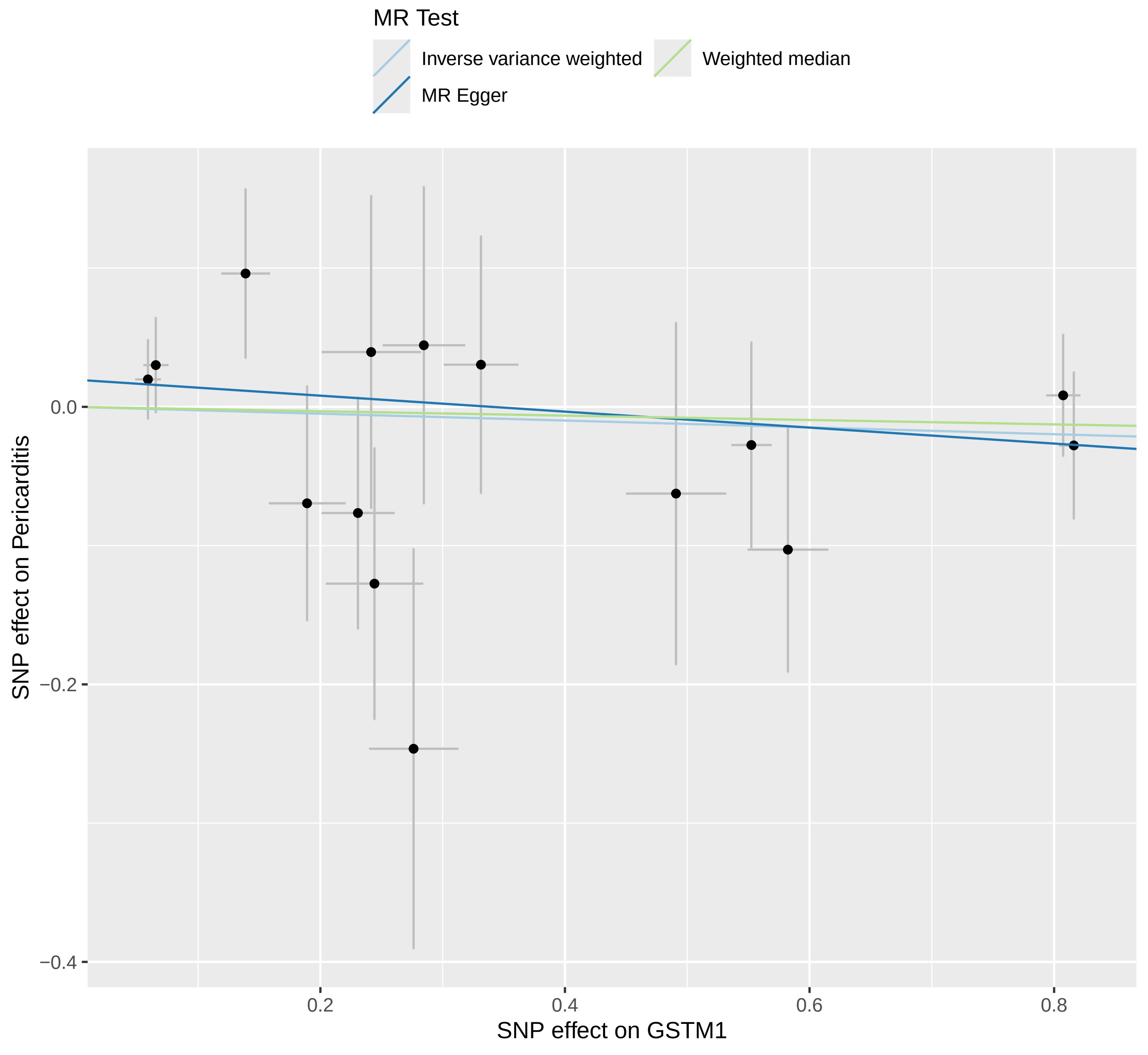

MR Test
Inverse variance weighted
Weighted median
MR Egger
SNP effect on Pericarditis
0.0
−0.2
−0.4
0.2
0.4
0.6
0.8
SNP effect on GSTM1

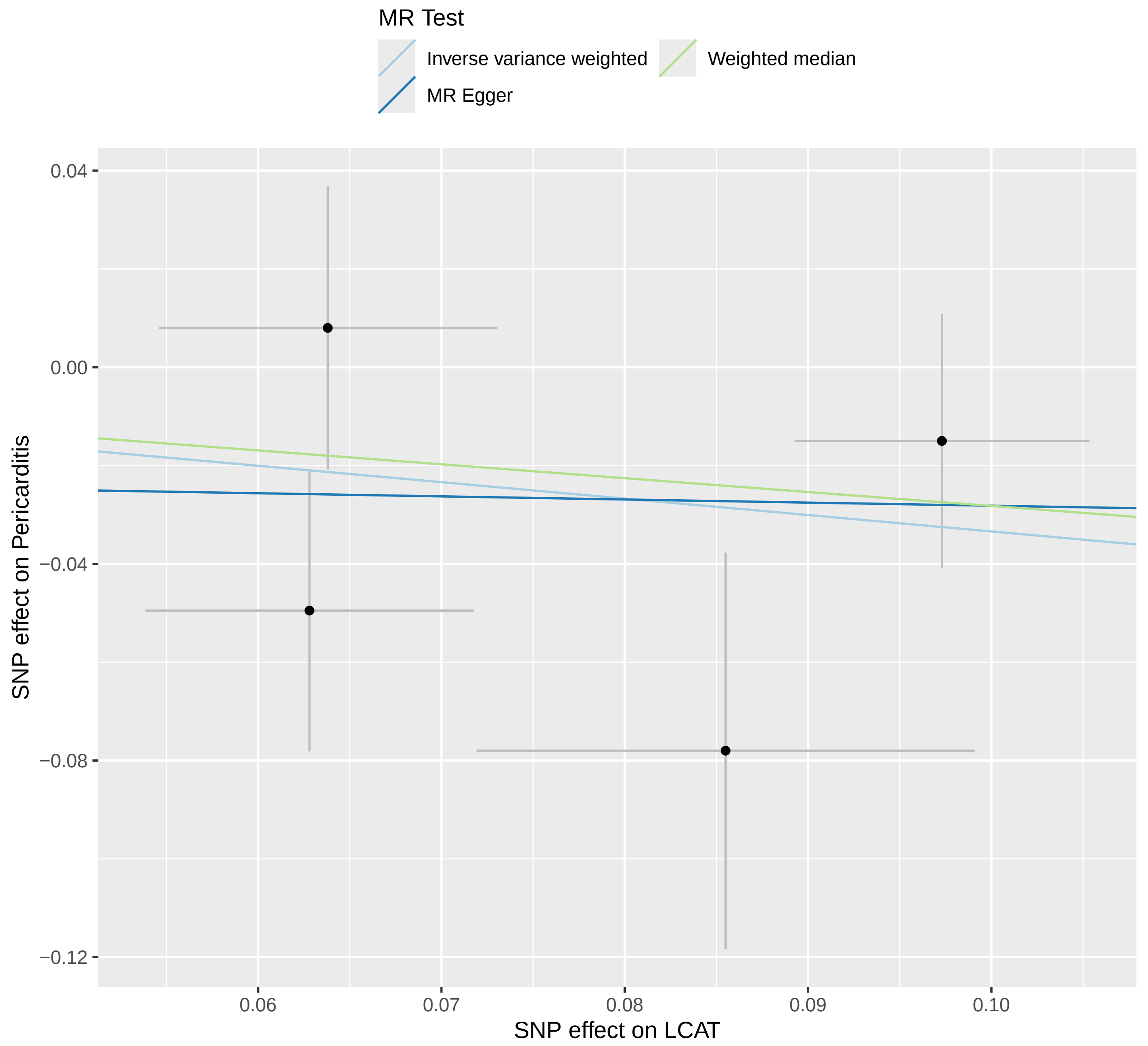
MR Test
Inverse variance weighted
Weighted median
MR Egger
0.04
0.00
−0.04
−0.08
−0.12
SNP effect on Pericarditis
0.06
0.07
0.08
0.09
0.10
SNP effect on LCAT

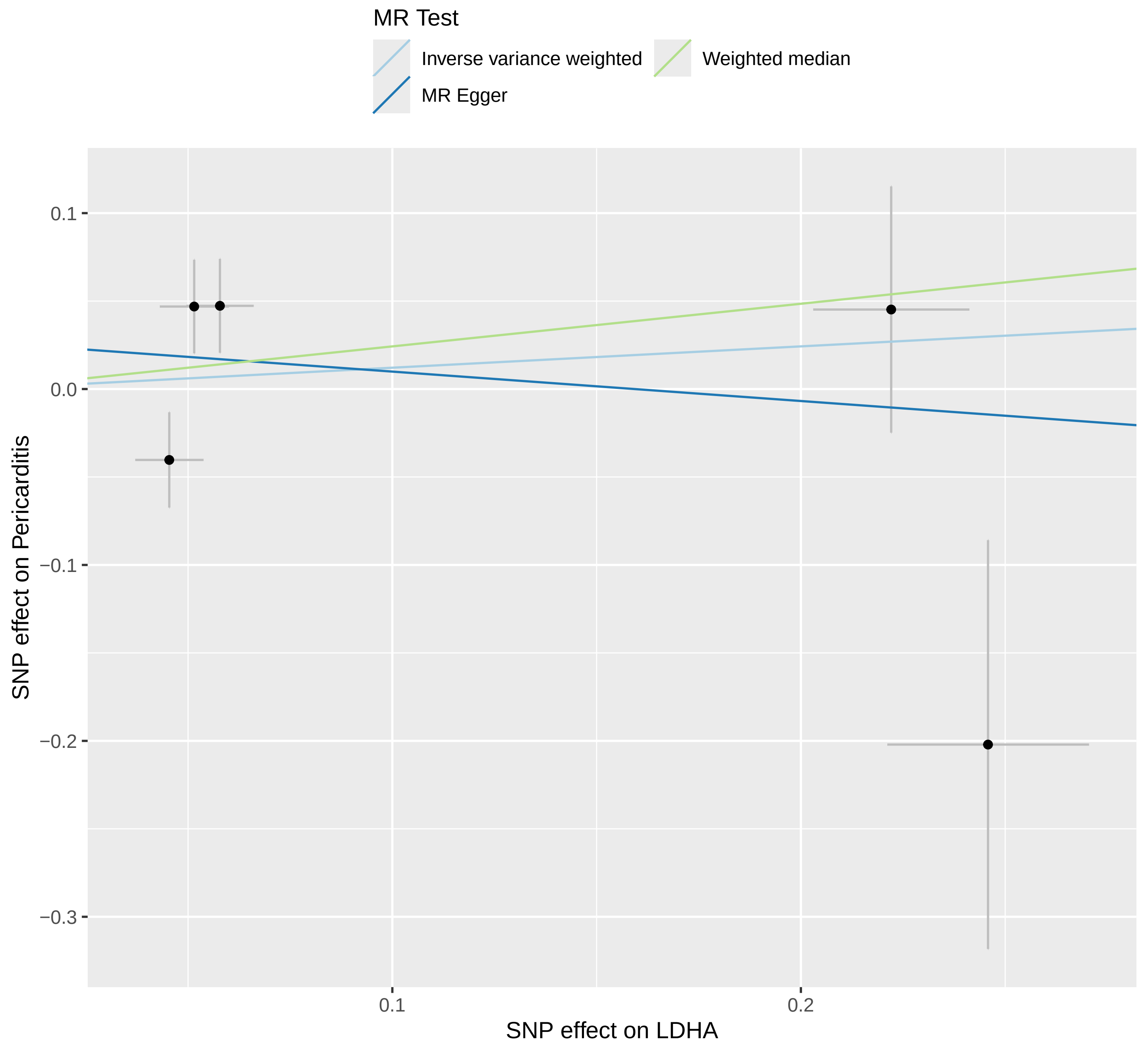
MR Test
Inverse variance weighted
Weighted median
MR Egger
0.1
0.0
−0.1
−0.2
−0.3
SNP effect on Pericarditis
0.1
0.2
SNP effect on LDHA

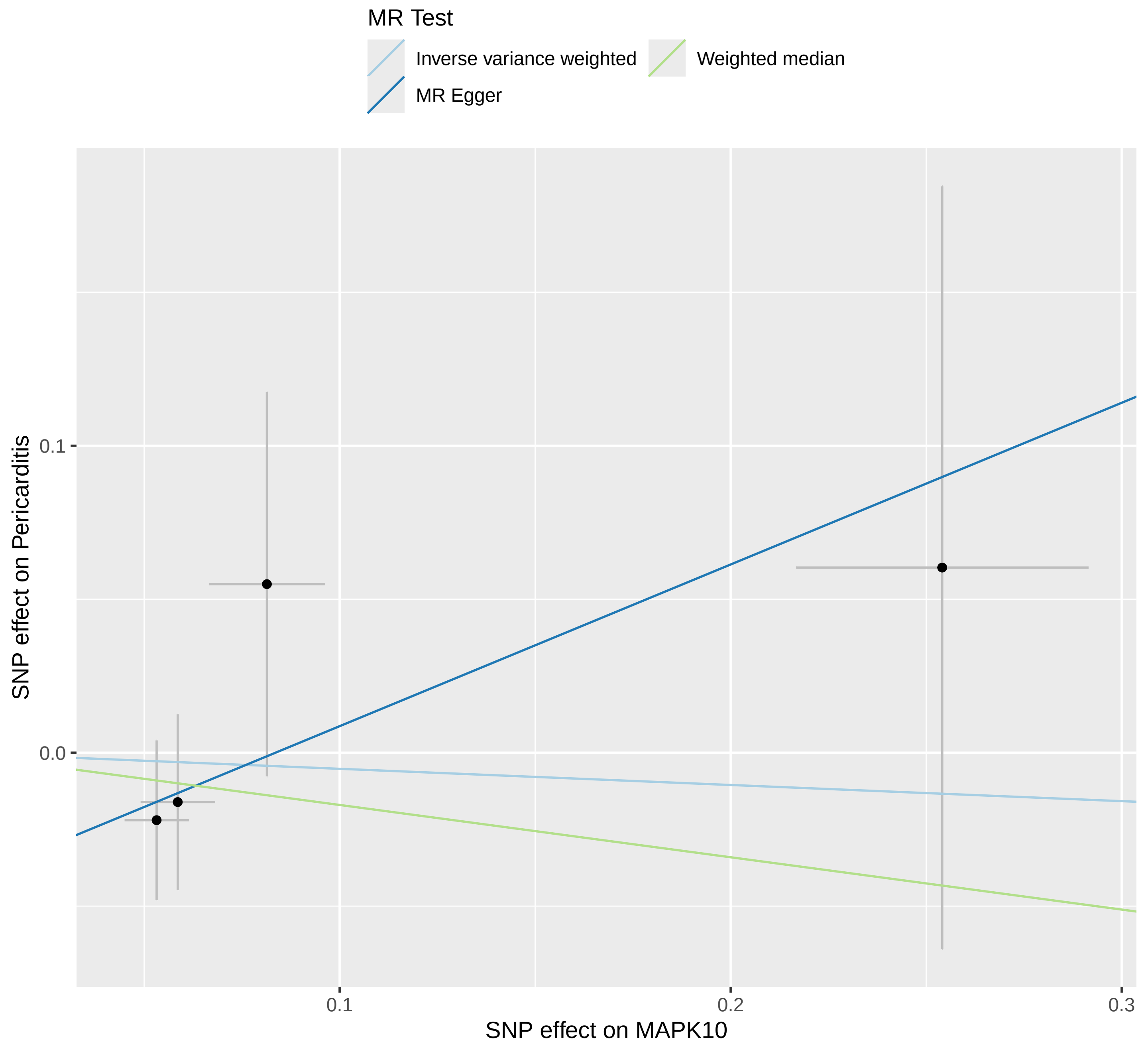
MR Test
Inverse variance weighted
MR Egger
Weighted median
SNP effect on Pericarditis
0.1
0.0
0.1
0.2
0.3
SNP effect on MAPK10

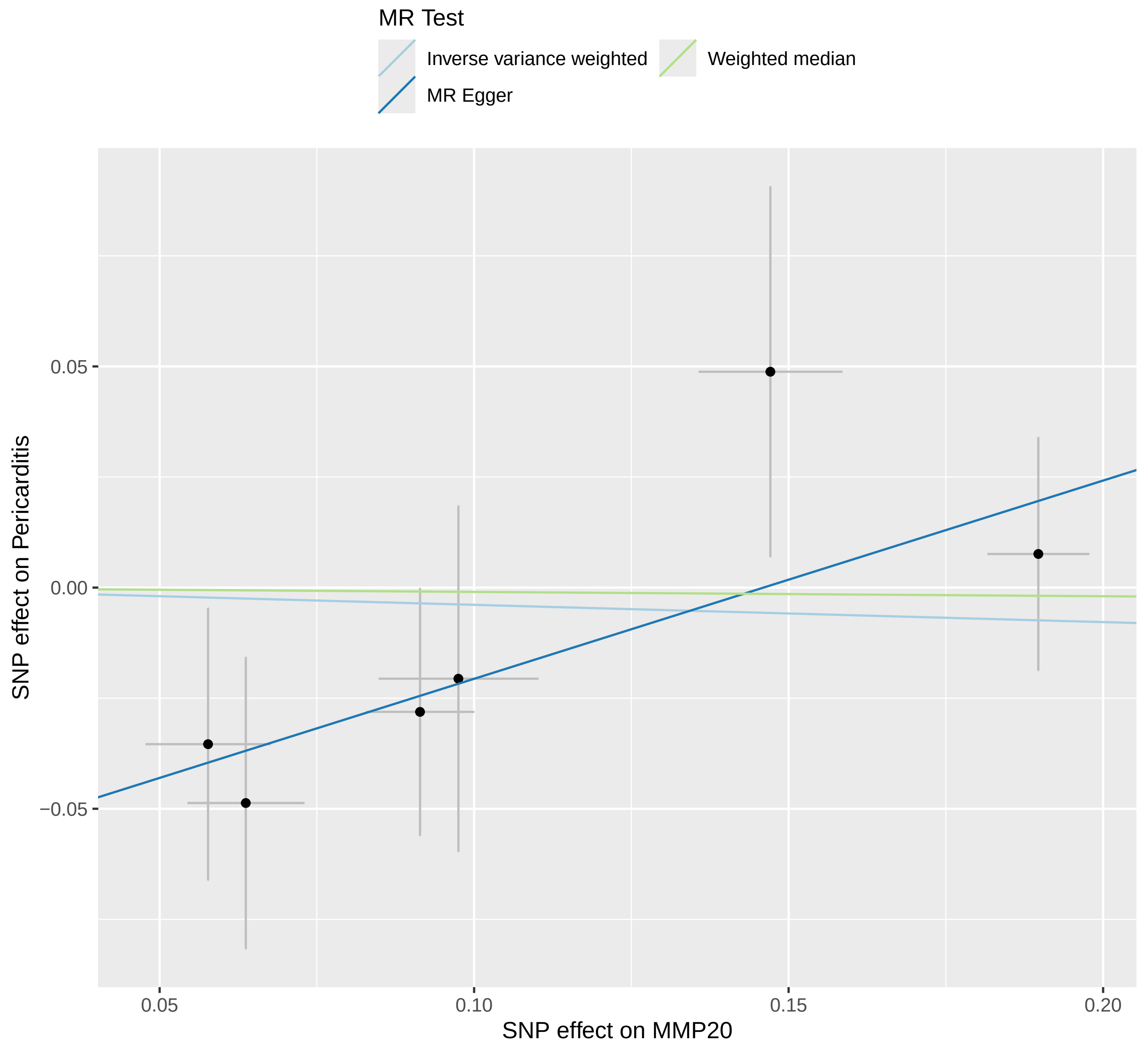
MR Test
Inverse variance weighted
Weighted median
MR Egger
0.05
0.00
−0.05
SNP effect on Pericarditis
0.05
0.10
0.15
0.20
SNP effect on MMP20

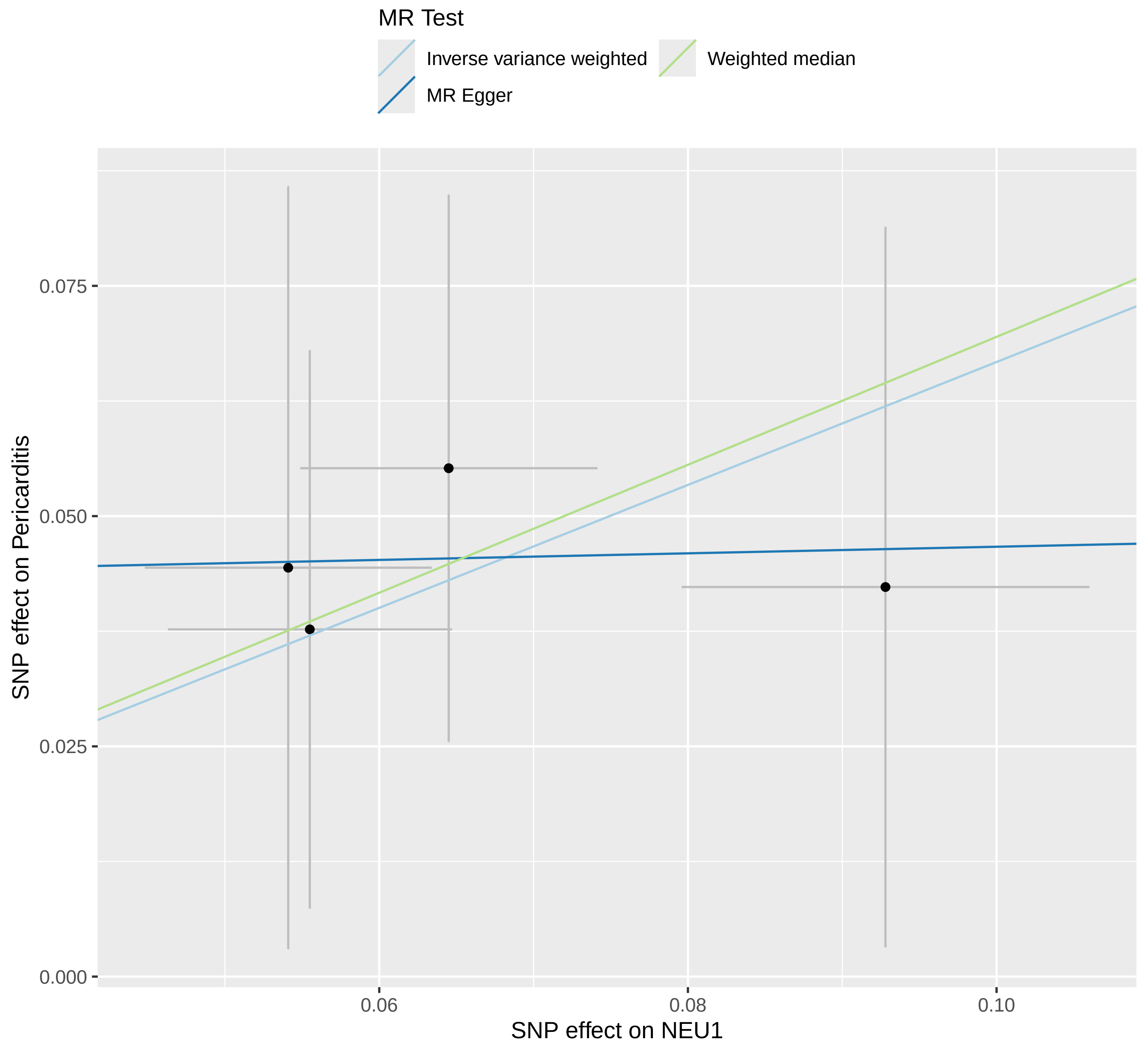
MR Test
Inverse variance weighted
Weighted median
MR Egger
0.075
0.050
0.025
0.000
0.06
0.08
0.10
SNP effect on Pericarditis
SNP effect on NEU1

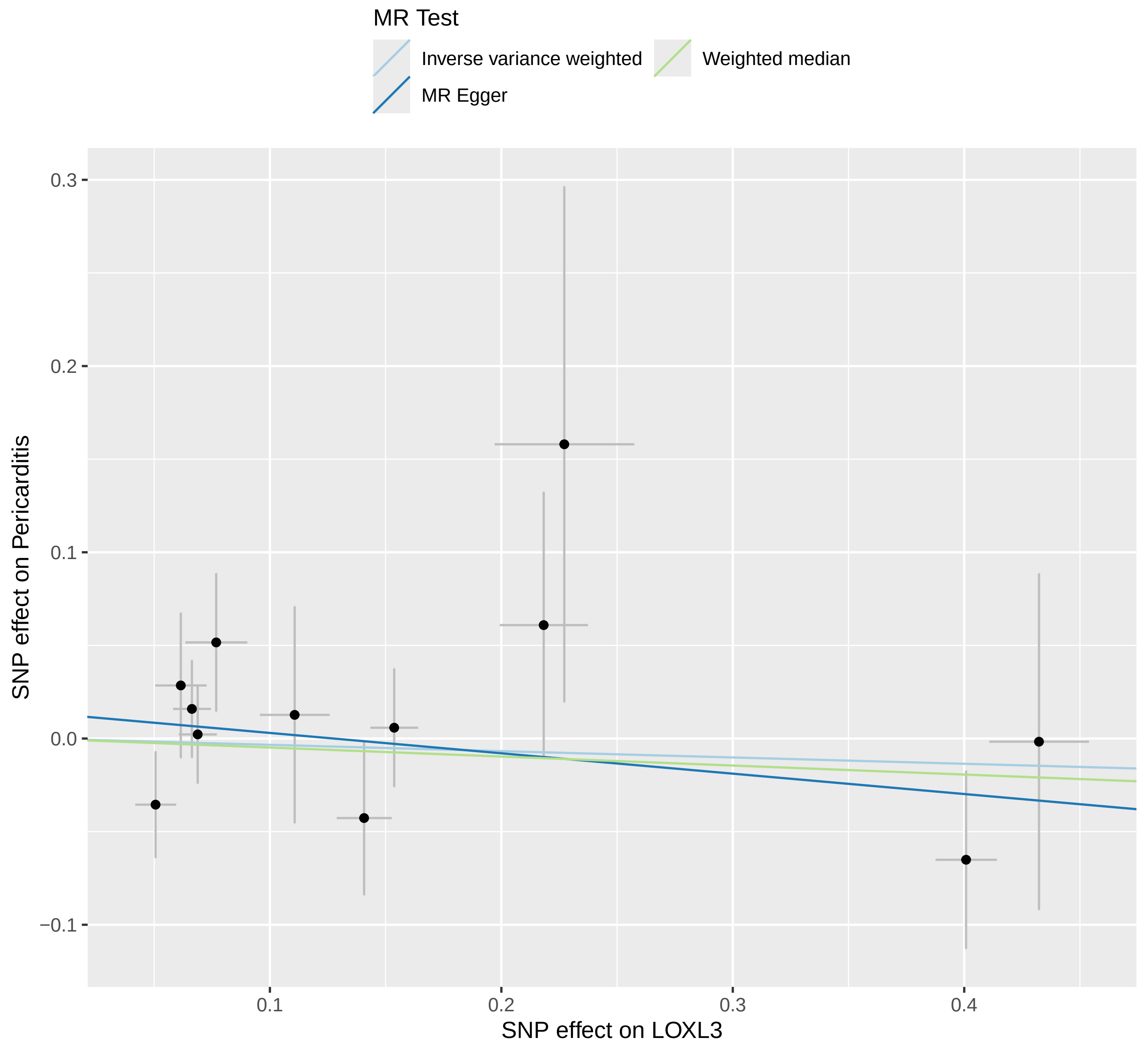
MR Test
Inverse variance weighted
MR Egger
Weighted median
0.3
0.2
0.1
0.0
−0.1
SNP effect on Pericarditis
0.1
0.2
0.3
0.4
SNP effect on LOXL3

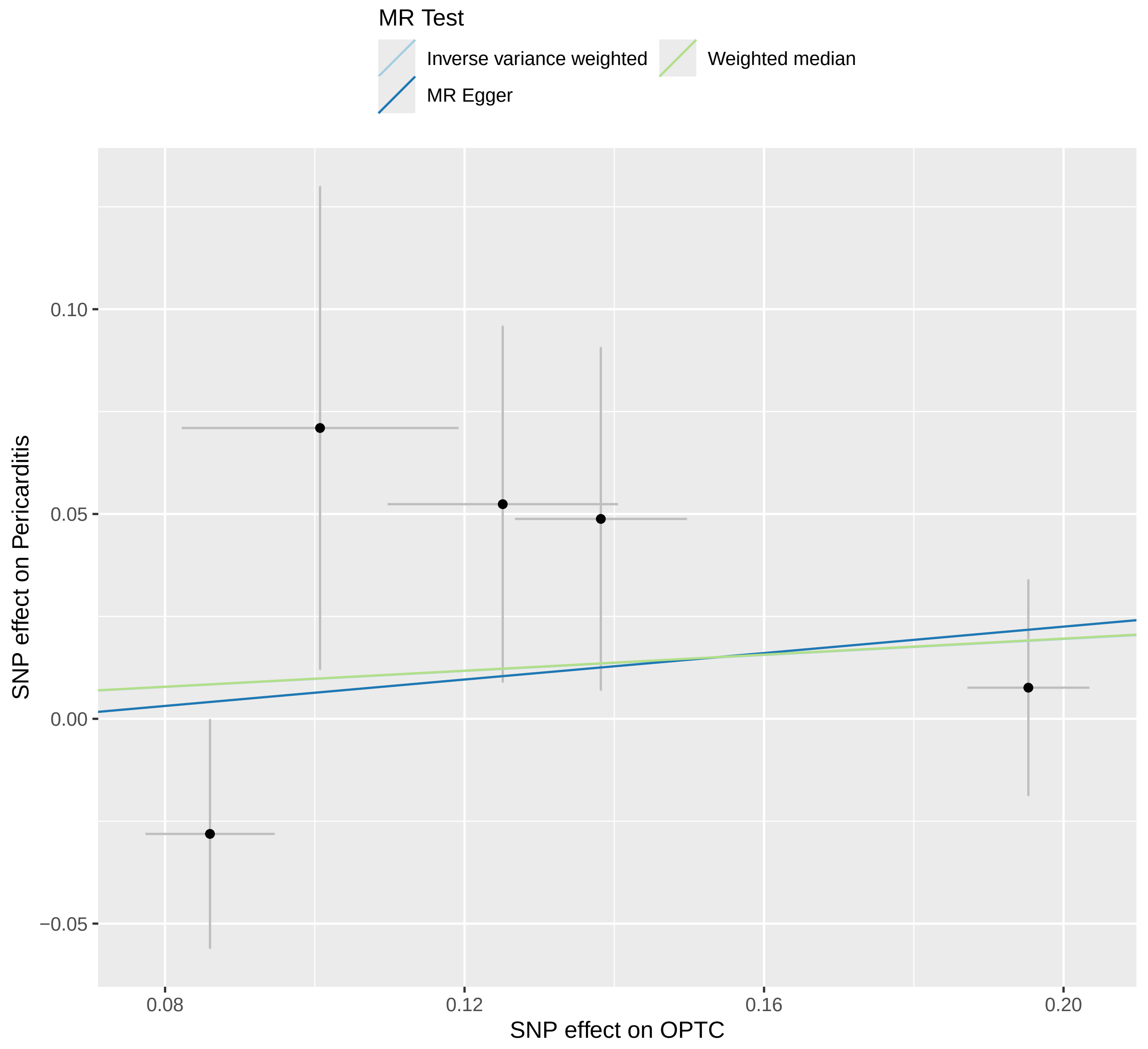

MR Test
Inverse variance weighted
MR Egger
Weighted median
0.10
0.05
0.00
−0.05
SNP effect on Pericarditis
0.08
0.12
0.16
0.20
SNP effect on OPTC

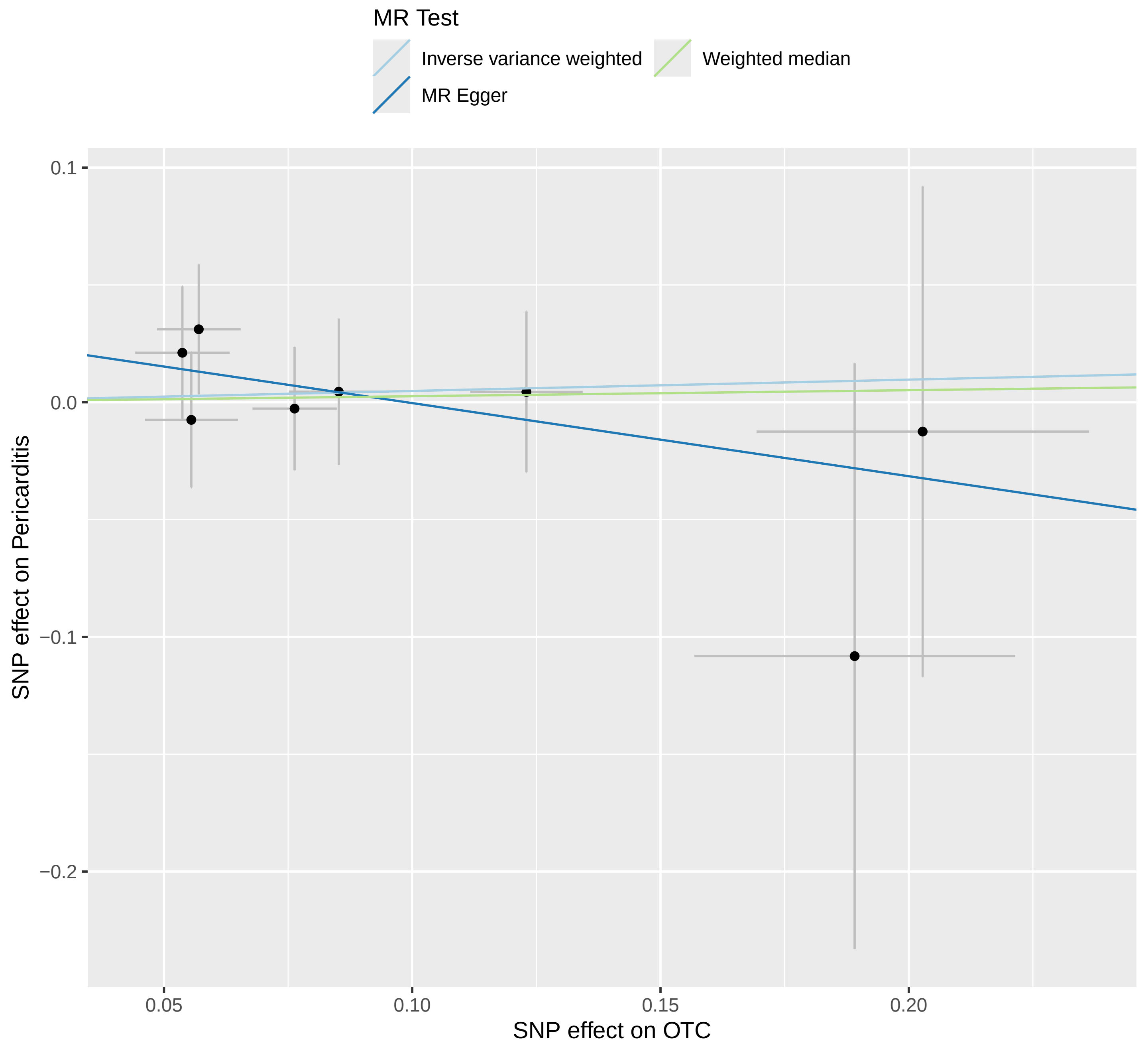
MR Test
Inverse variance weighted
Weighted median
MR Egger
0.1
0.0
−0.1
−0.2
SNP effect on Pericarditis
0.05
0.10
0.15
0.20
SNP effect on OTC

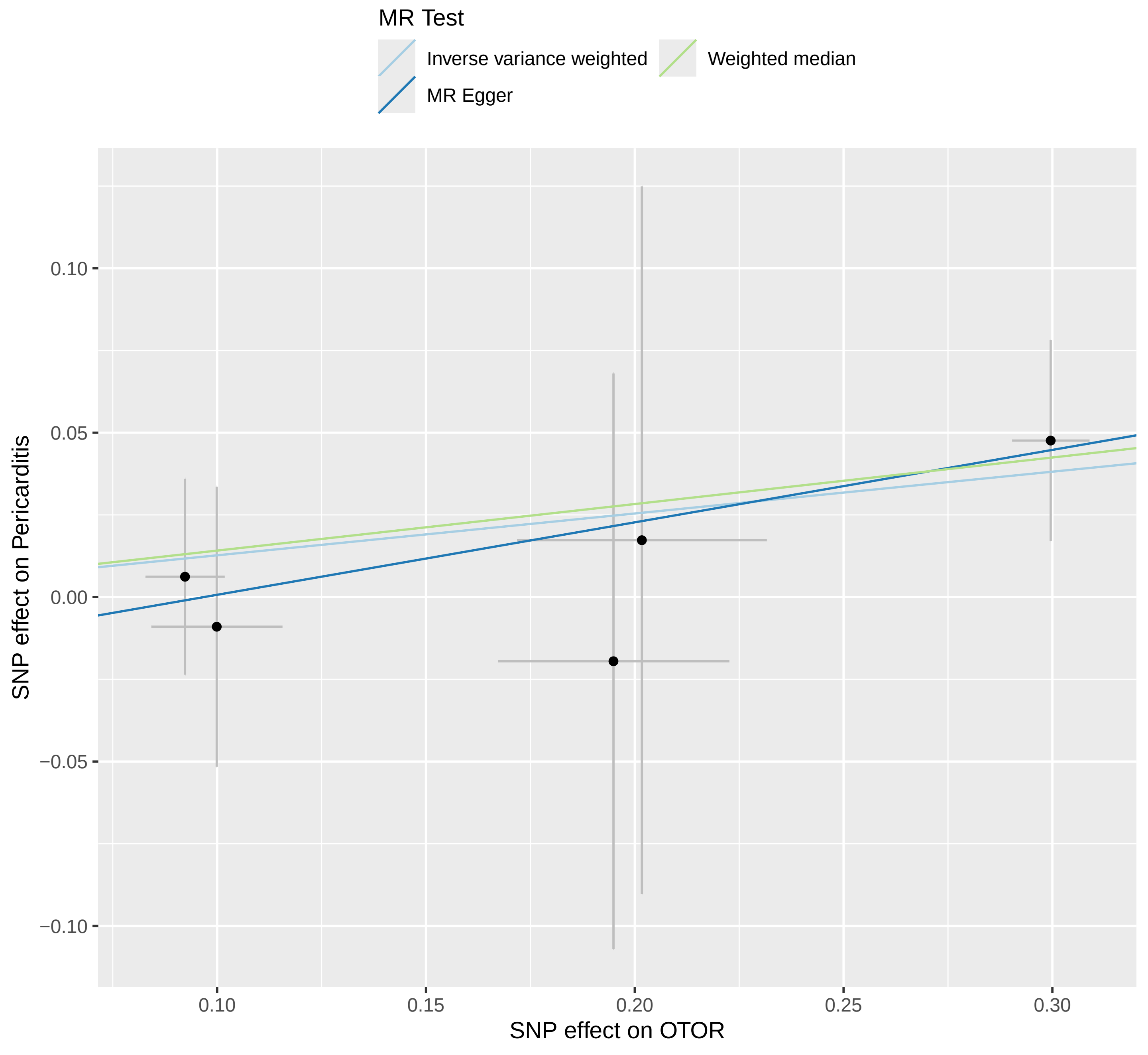
MR Test
Inverse variance weighted
Weighted median
MR Egger
0.10
0.05
0.00
−0.05
−0.10
SNP effect on Pericarditis
0.10
0.15
0.20
0.25
0.30
SNP effect on OTOR

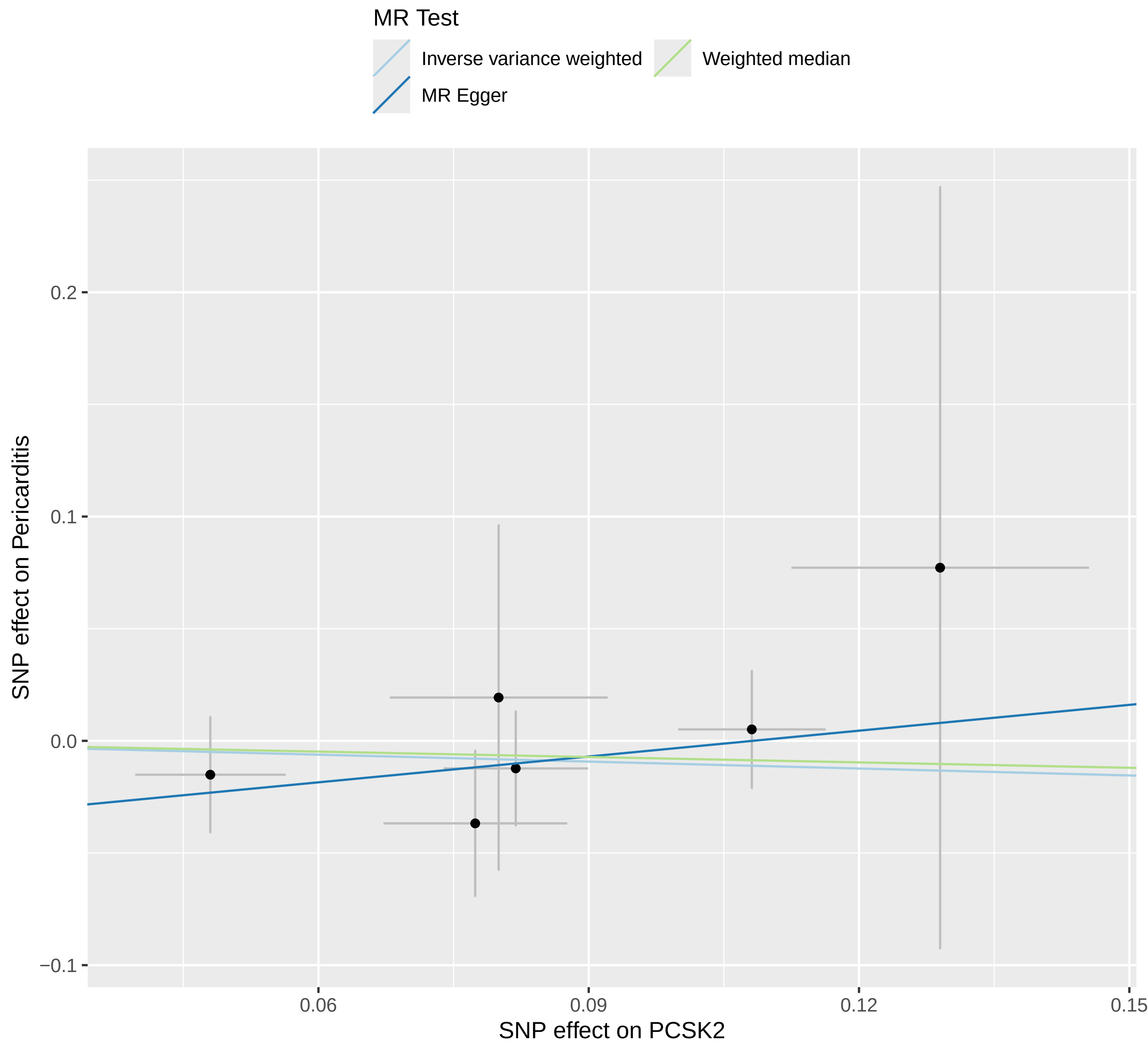

MR Test
Inverse variance weighted
Weighted median
MR Egger
SNP effect on Pericarditis
0.2
0.1
0.0
−0.1
0.06
0.09
0.12
0.15
SNP effect on PCSK2

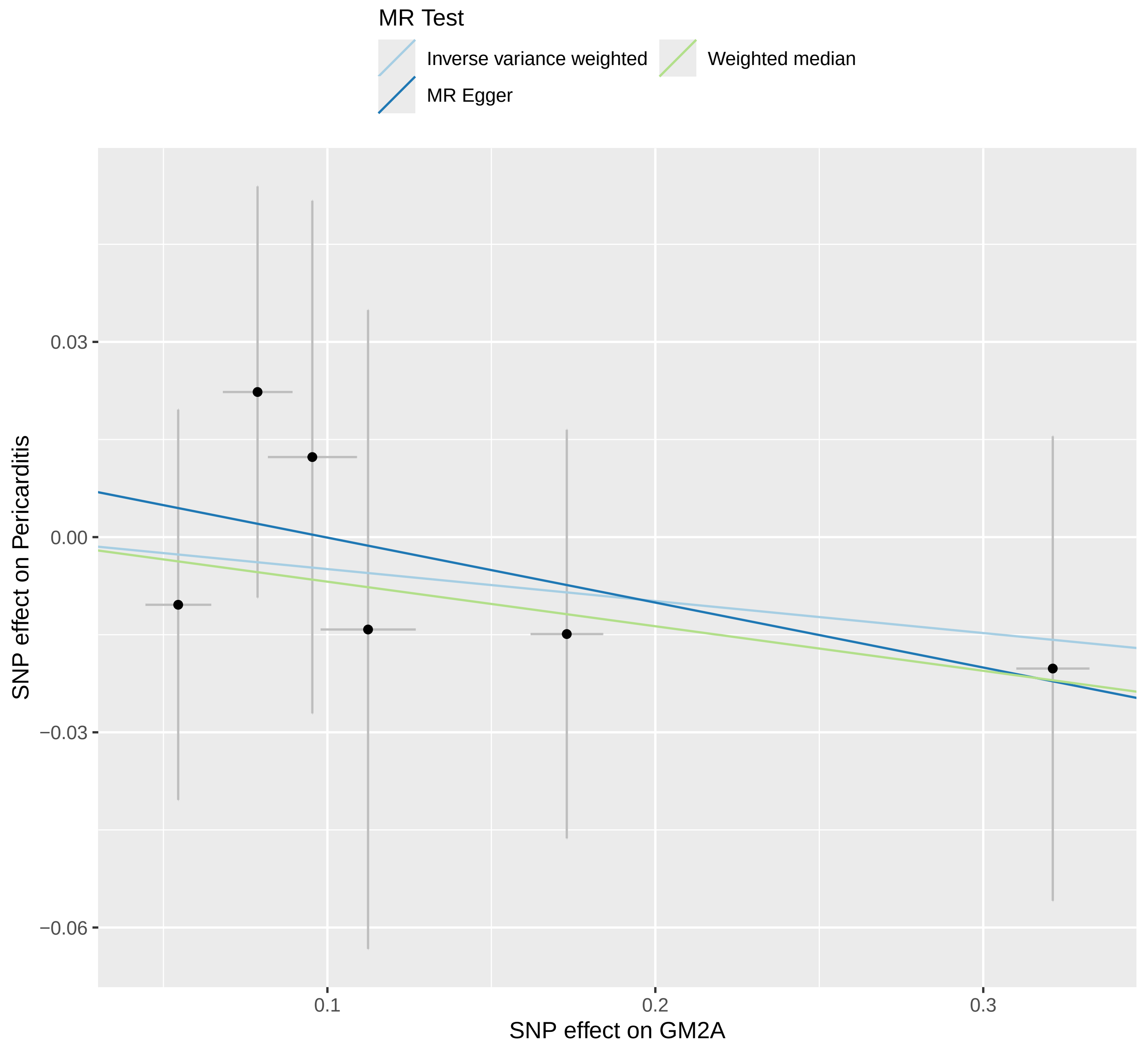
MR Test
Inverse variance weighted
Weighted median
MR Egger
SNP effect on Pericarditis
0.03
0.00
−0.03
−0.06
0.1
0.2
0.3
SNP effect on GM2A

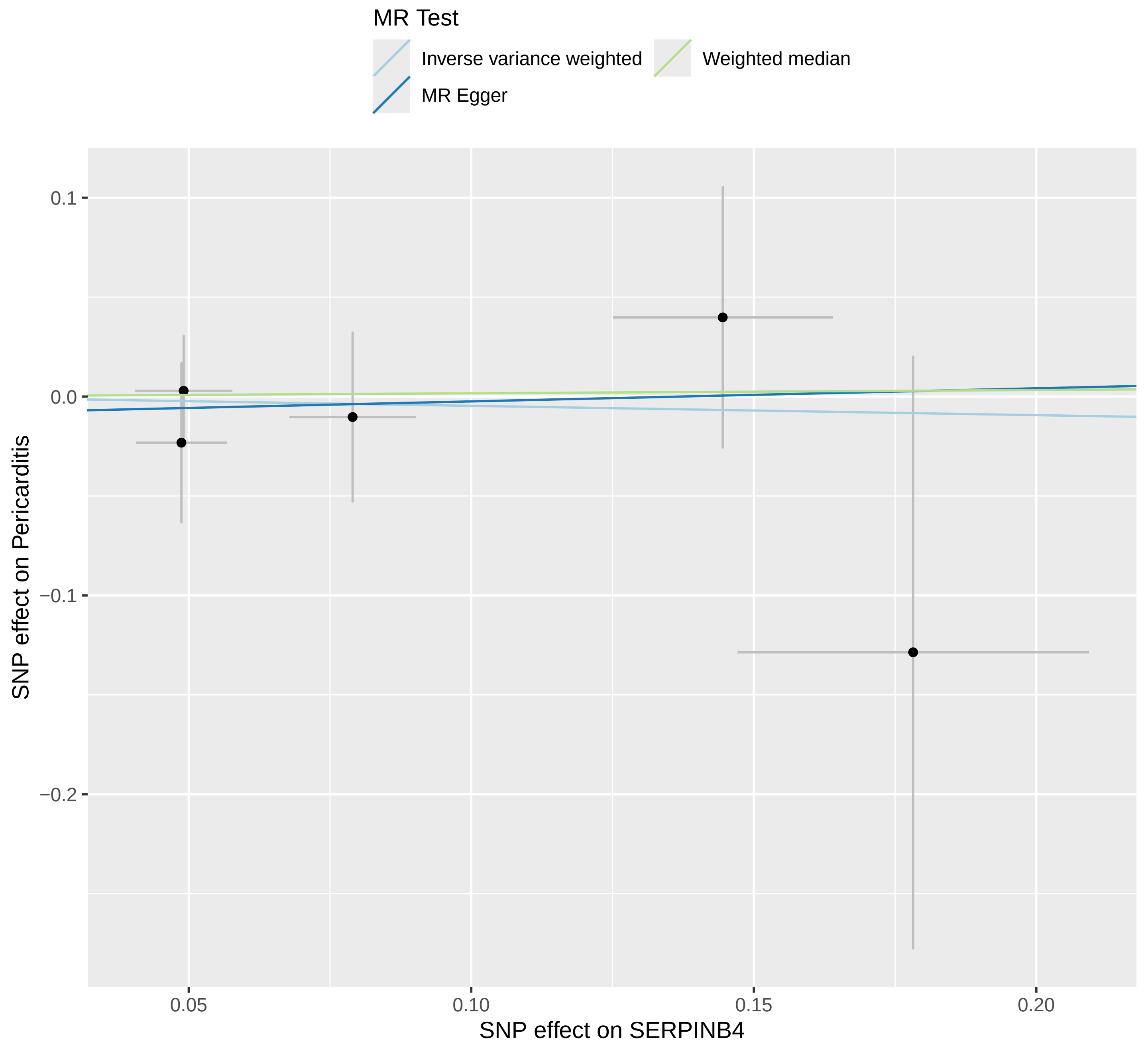

MR Test
Inverse variance weighted
MR Egger
Weighted median
0.1
0.0
−0.1
−0.2
SNP effect on Pericarditis
0.05
0.10
0.15
0.20
SNP effect on SERPINB4

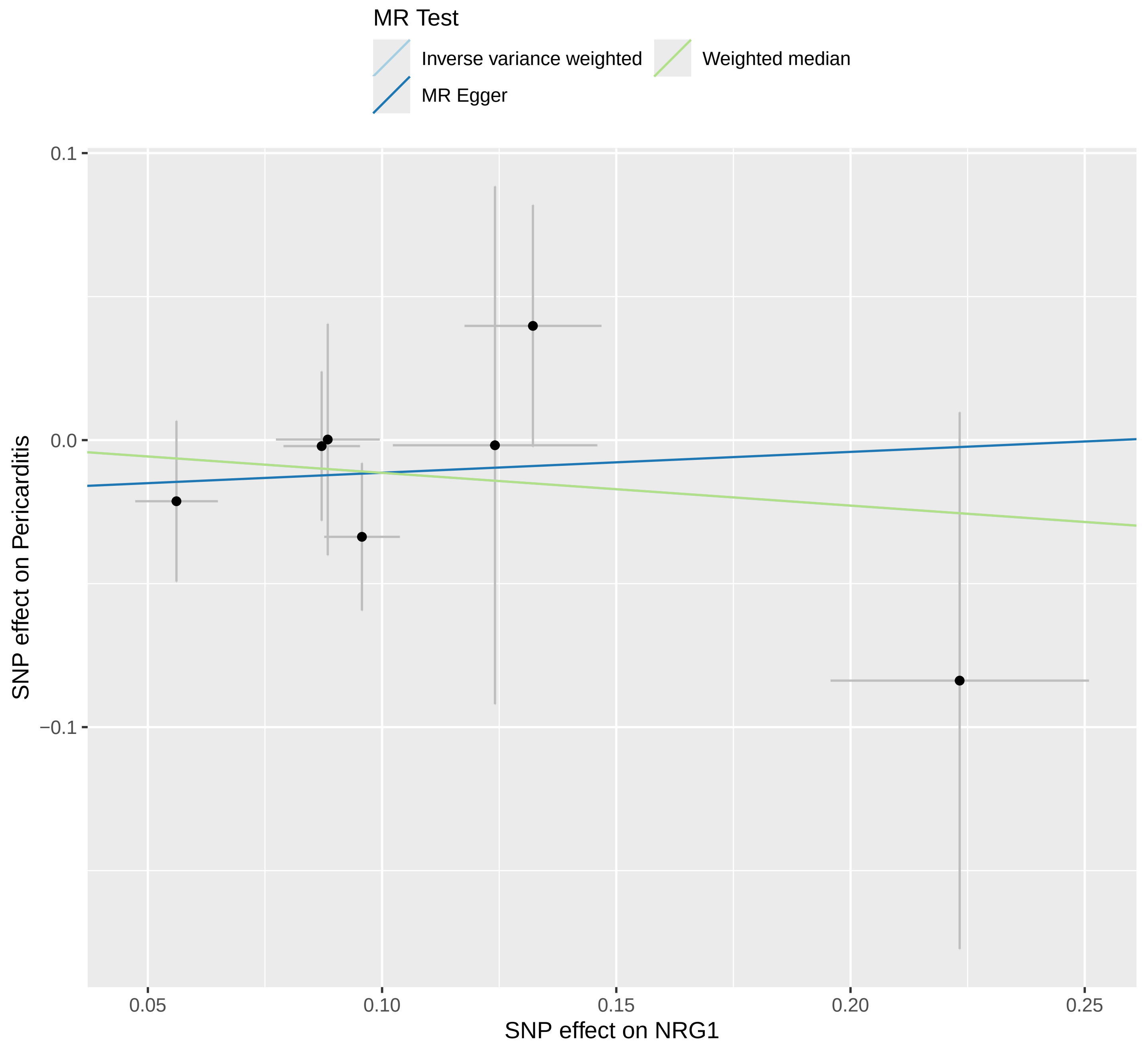
MR Test
Inverse variance weighted
Weighted median
MR Egger
SNP effect on Pericarditis
0.1
0.0
−0.1
0.05
0.10
0.15
0.20
0.25
SNP effect on NRG1

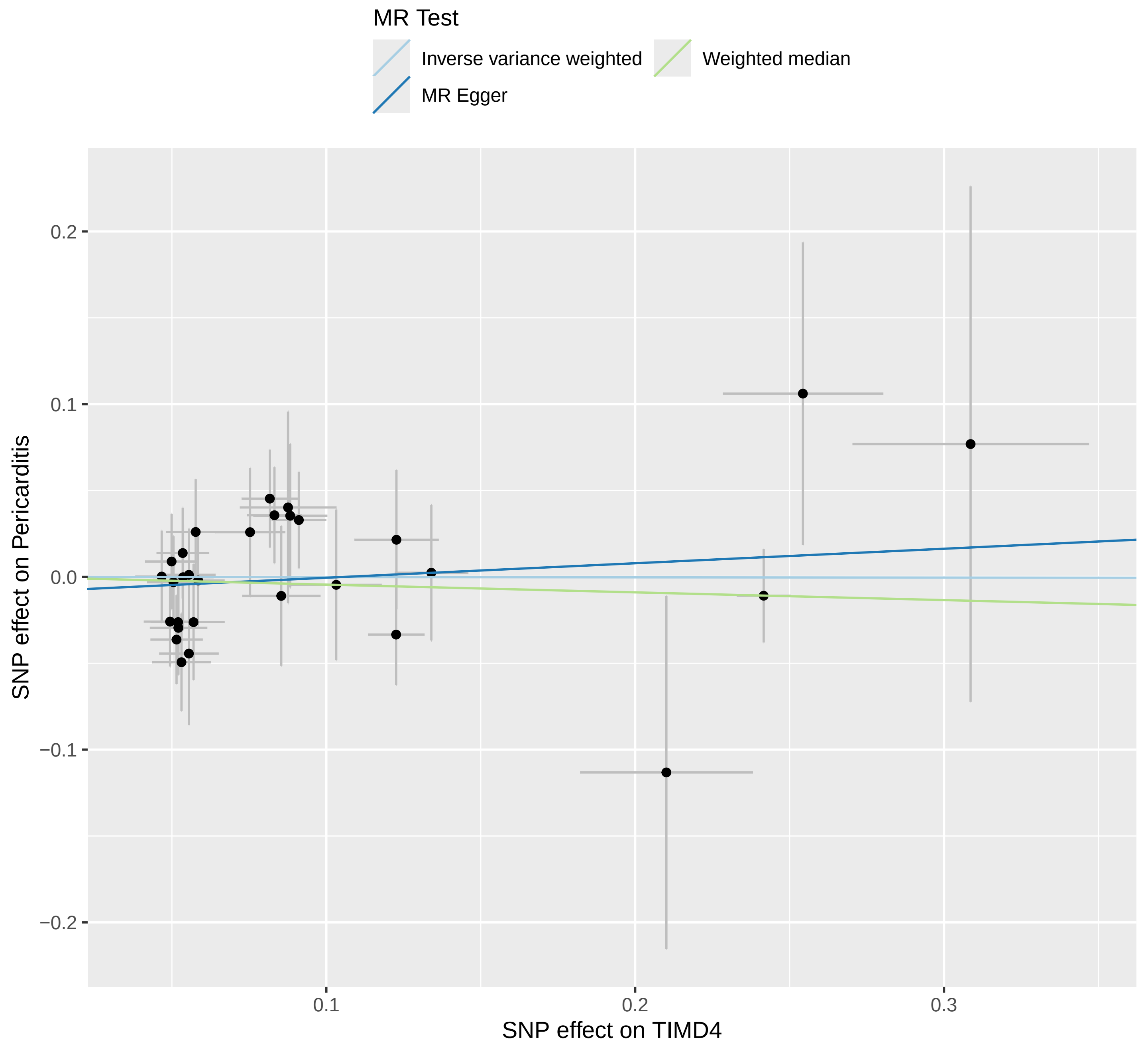
MR Test
Inverse variance weighted
Weighted median
MR Egger
0.2
0.1
0.0
−0.1
−0.2
SNP effect on Pericarditis
0.1
0.2
0.3
SNP effect on TIMD4

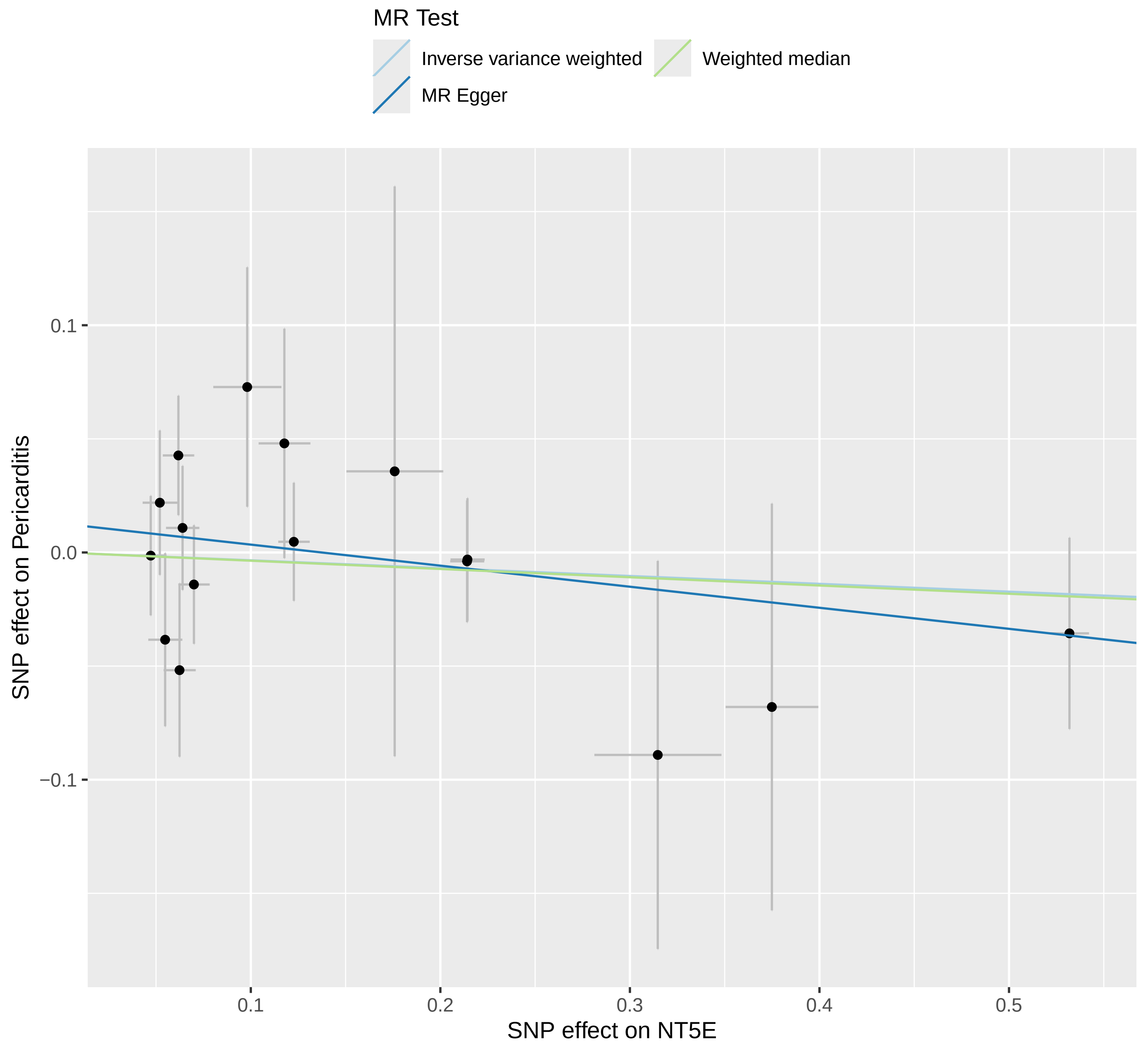
MR Test
Inverse variance weighted
Weighted median
MR Egger
0.1
0.0
−0.1
SNP effect on Pericarditis
0.1
0.2
0.3
0.4
0.5
SNP effect on NT5E

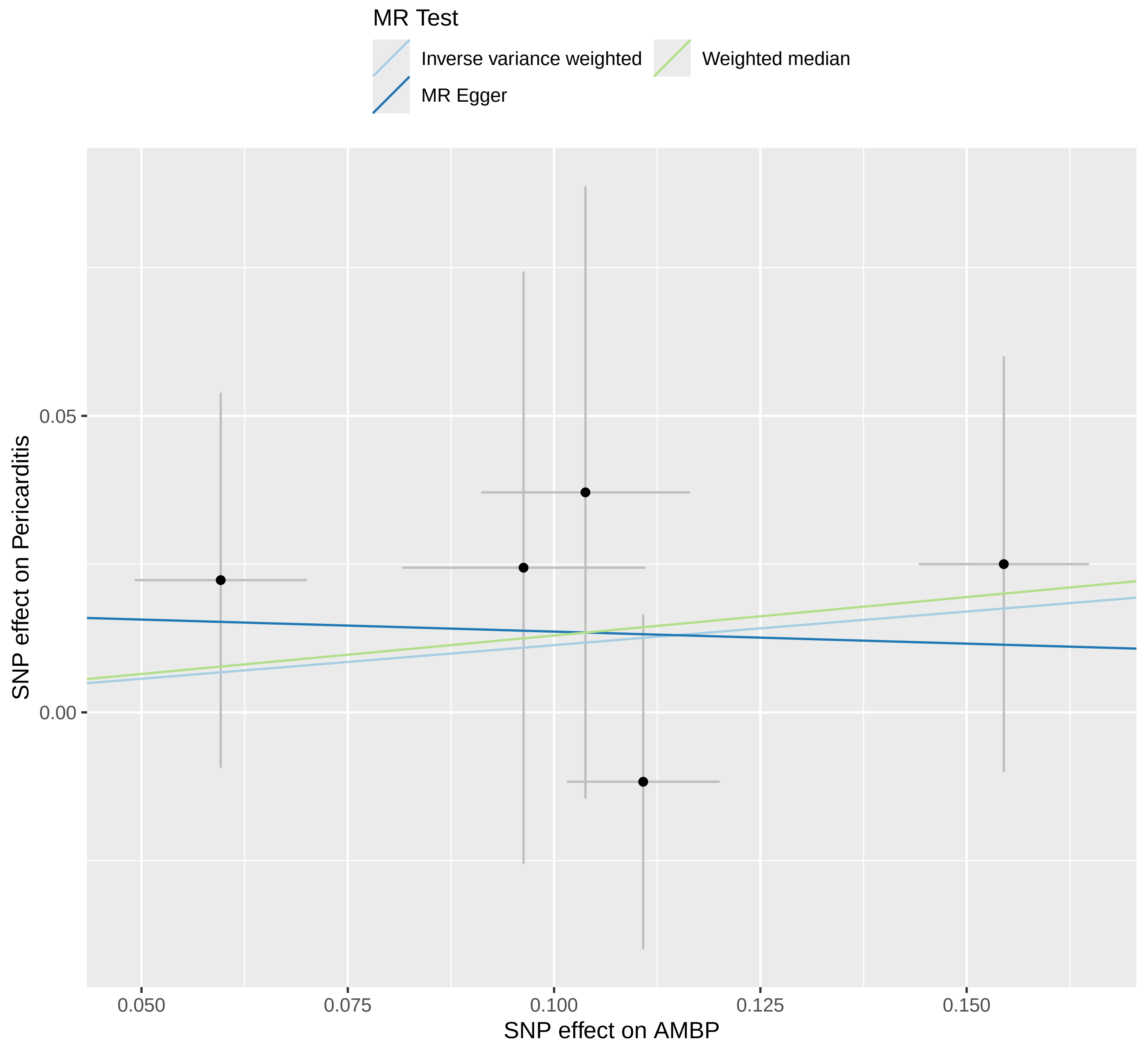
MR Test
Inverse variance weighted
MR Egger
Weighted median
SNP effect on Pericarditis
0.05
0.00
0.050
0.075
0.100
0.125
0.150
SNP effect on AMBP

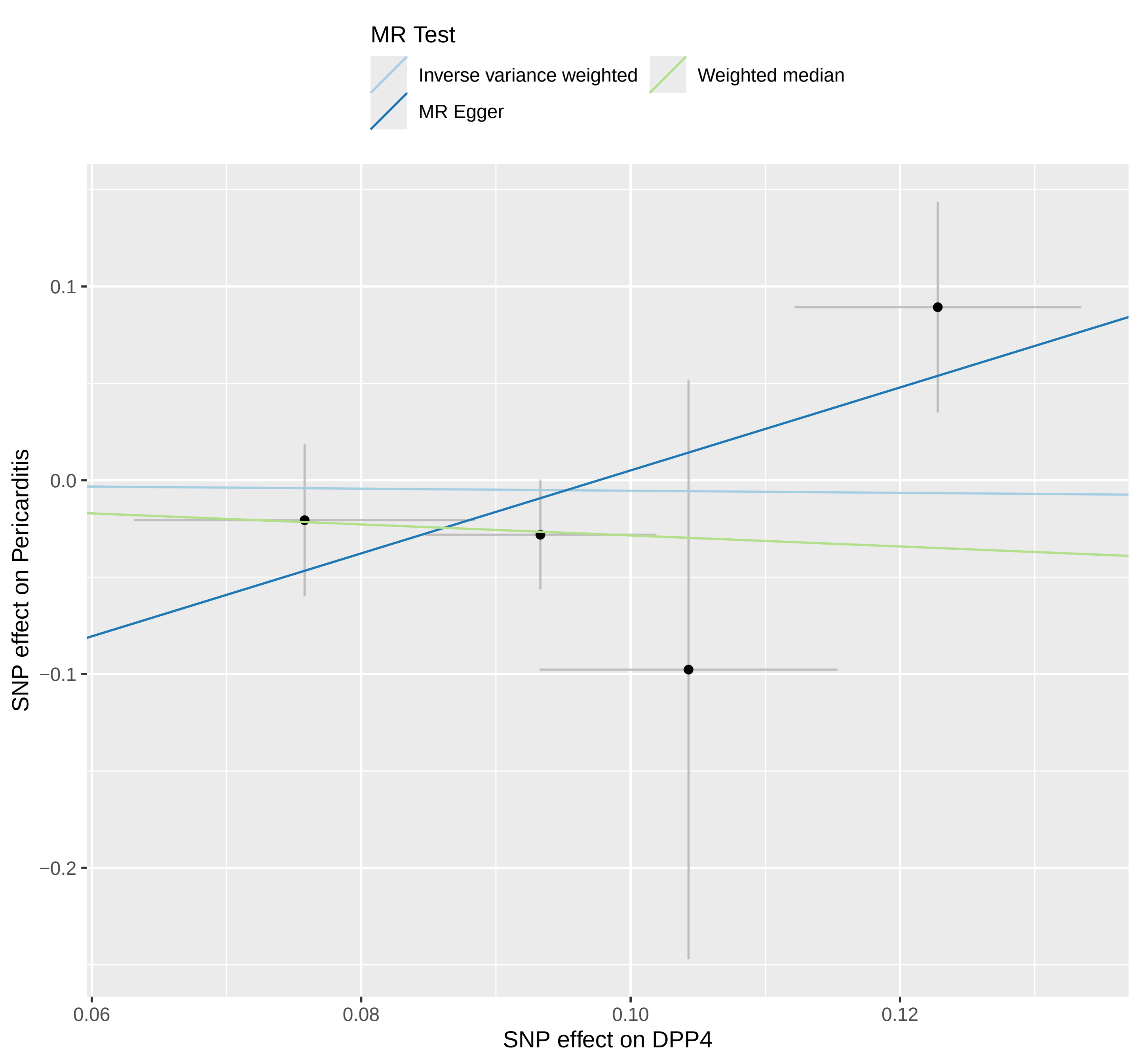

MR Test
Inverse variance weighted
Weighted median
MR Egger
0.1
0.0
−0.1
−0.2
SNP effect on Pericarditis
0.06
0.08
0.10
0.12
SNP effect on DPP4

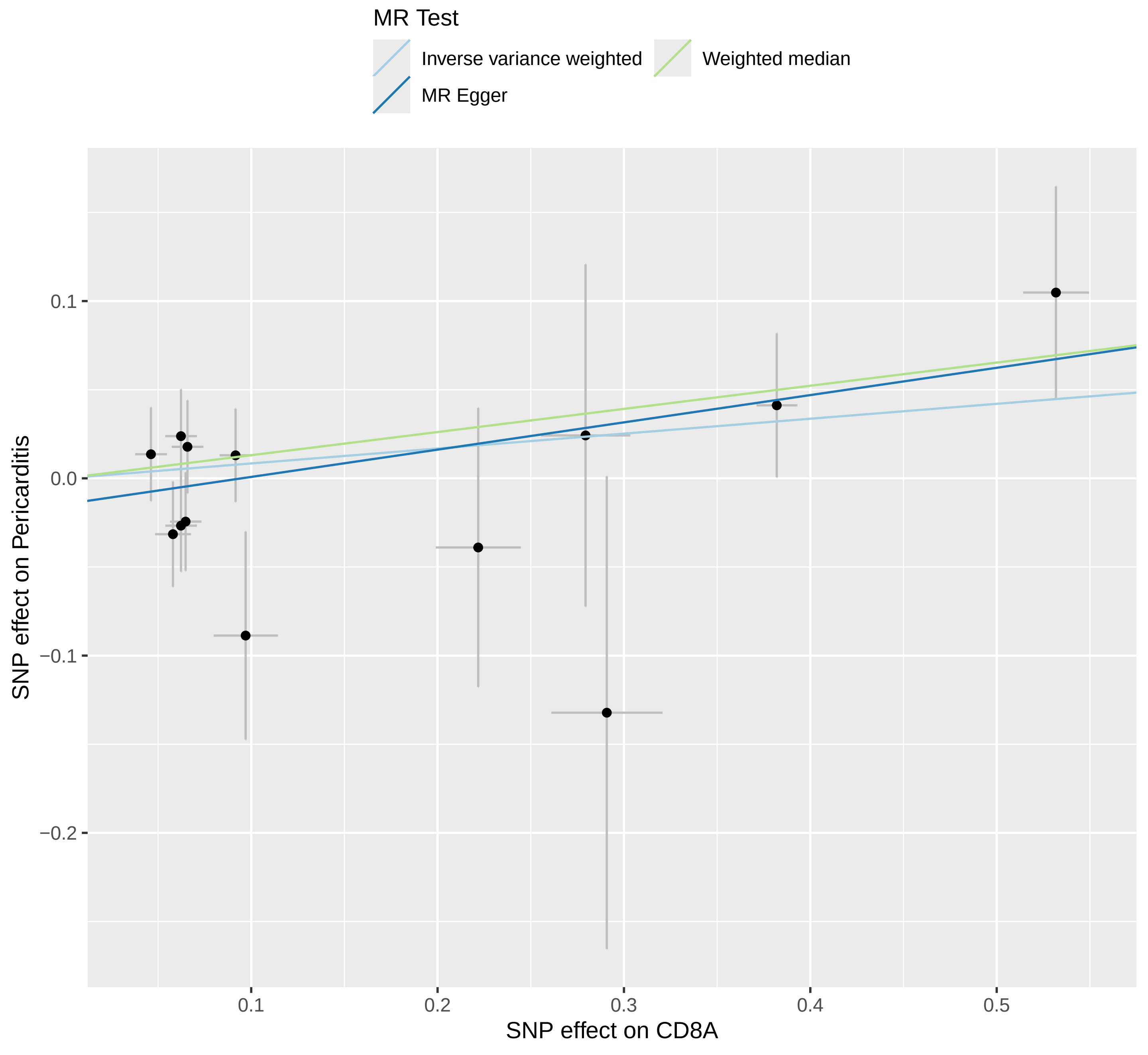
MR Test
Inverse variance weighted
Weighted median
MR Egger
0.1
0.0
−0.1
−0.2
0.1
0.2
0.3
0.4
0.5
SNP effect on Pericarditis
SNP effect on CD8A

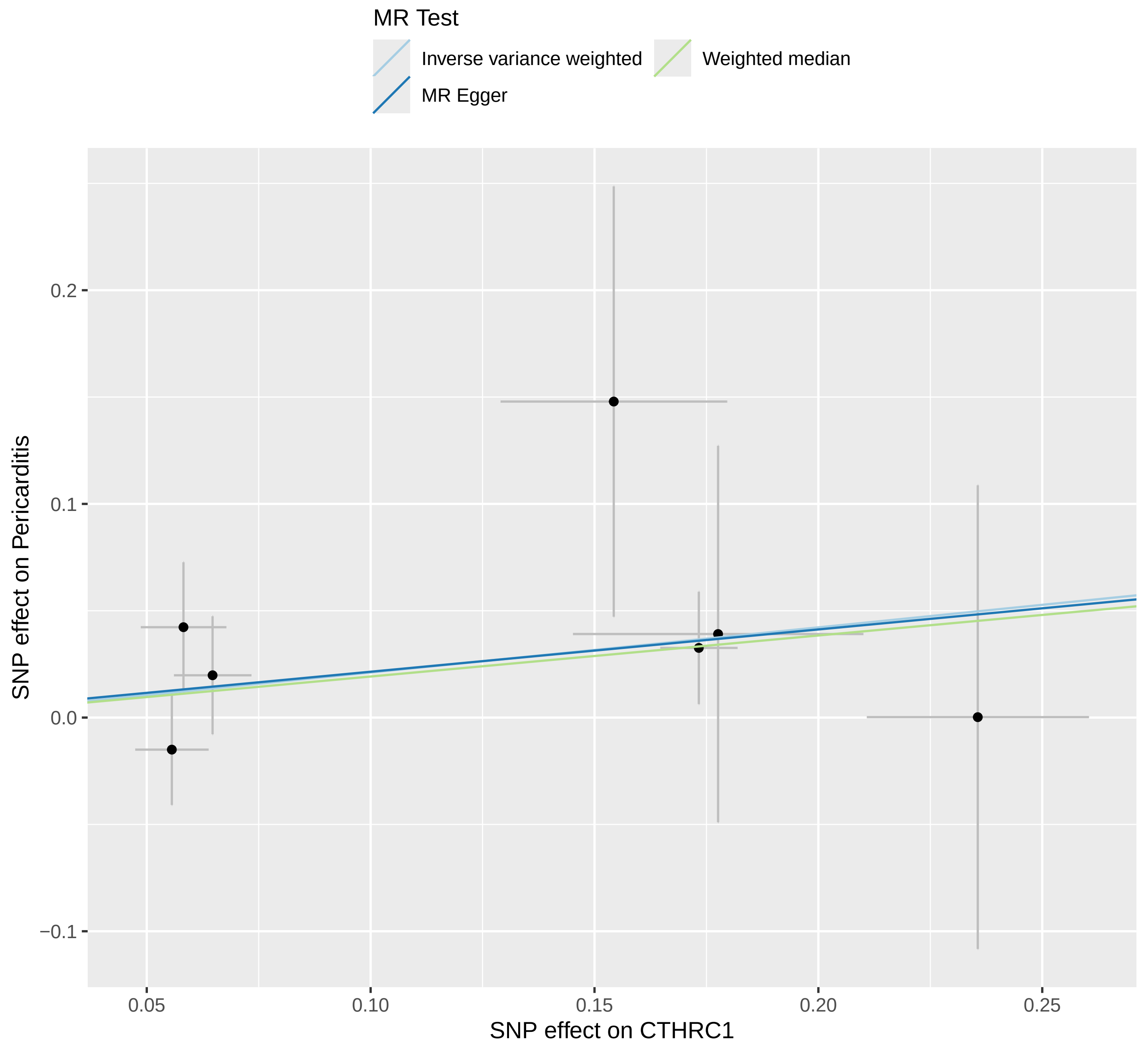
MR Test
Inverse variance weighted
Weighted median
MR Egger
SNP effect on Pericarditis
0.2
0.1
0.0
−0.1
0.05
0.10
0.15
0.20
0.25
SNP effect on CTHRC1

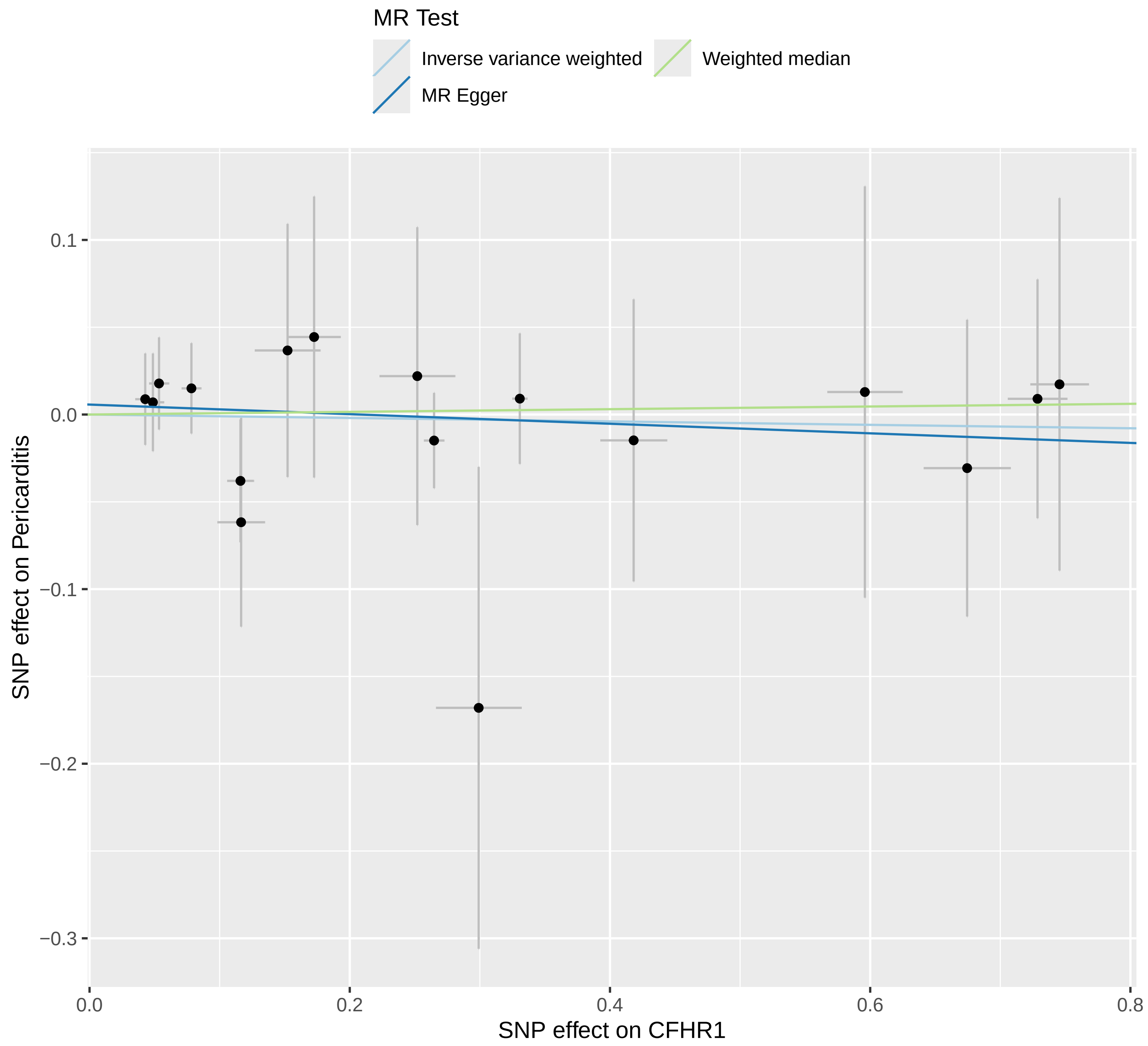
MR Test
Inverse variance weighted
Weighted median
MR Egger
0.1
0.0
−0.1
−0.2
−0.3
SNP effect on Pericarditis
0.0
0.2
0.4
0.6
0.8
SNP effect on CFHR1

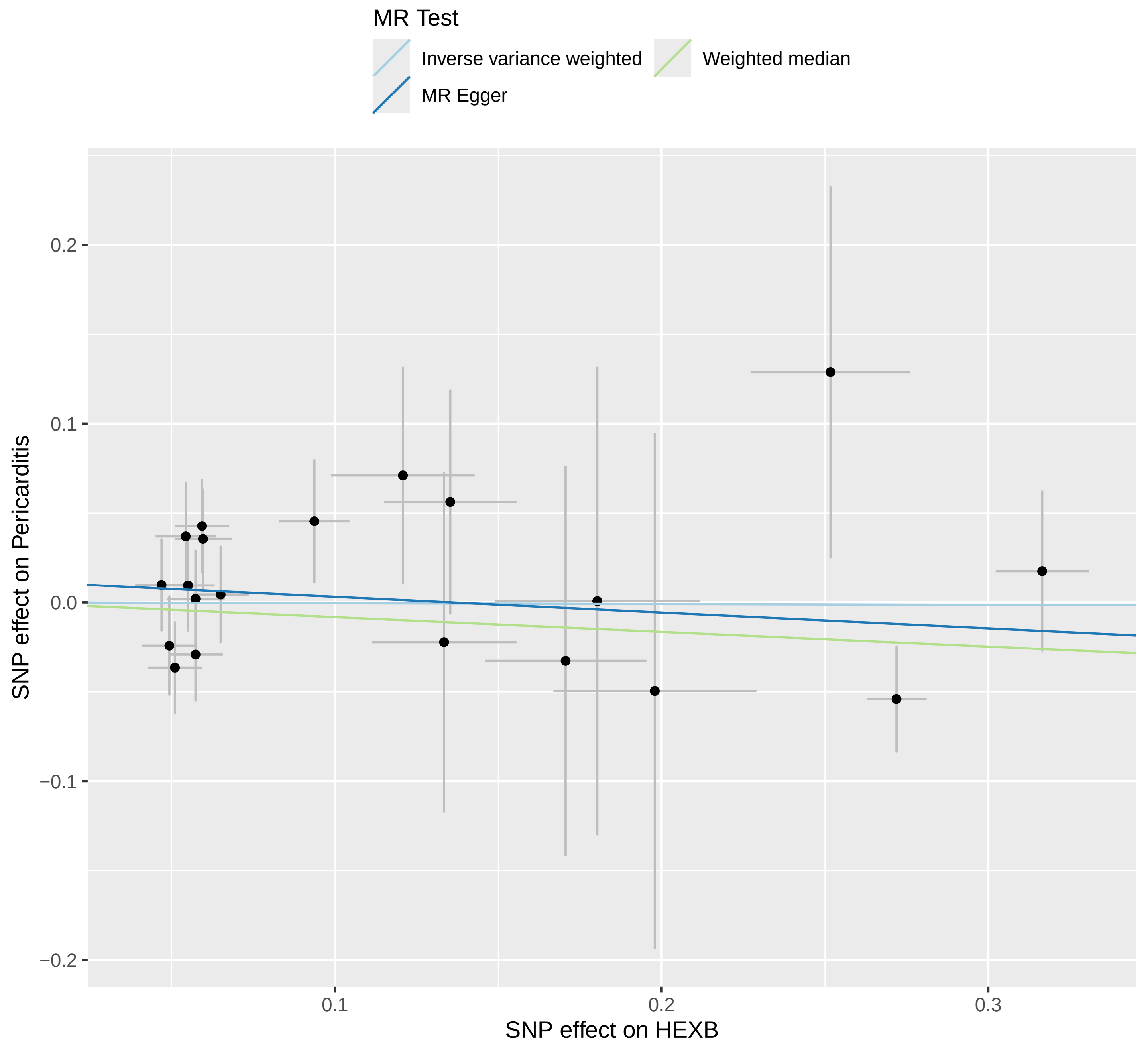

MR Test
Inverse variance weighted
Weighted median
MR Egger
SNP effect on Pericarditis
0.2
0.1
0.0
−0.1
−0.2
0.1
0.2
0.3
SNP effect on HEXB

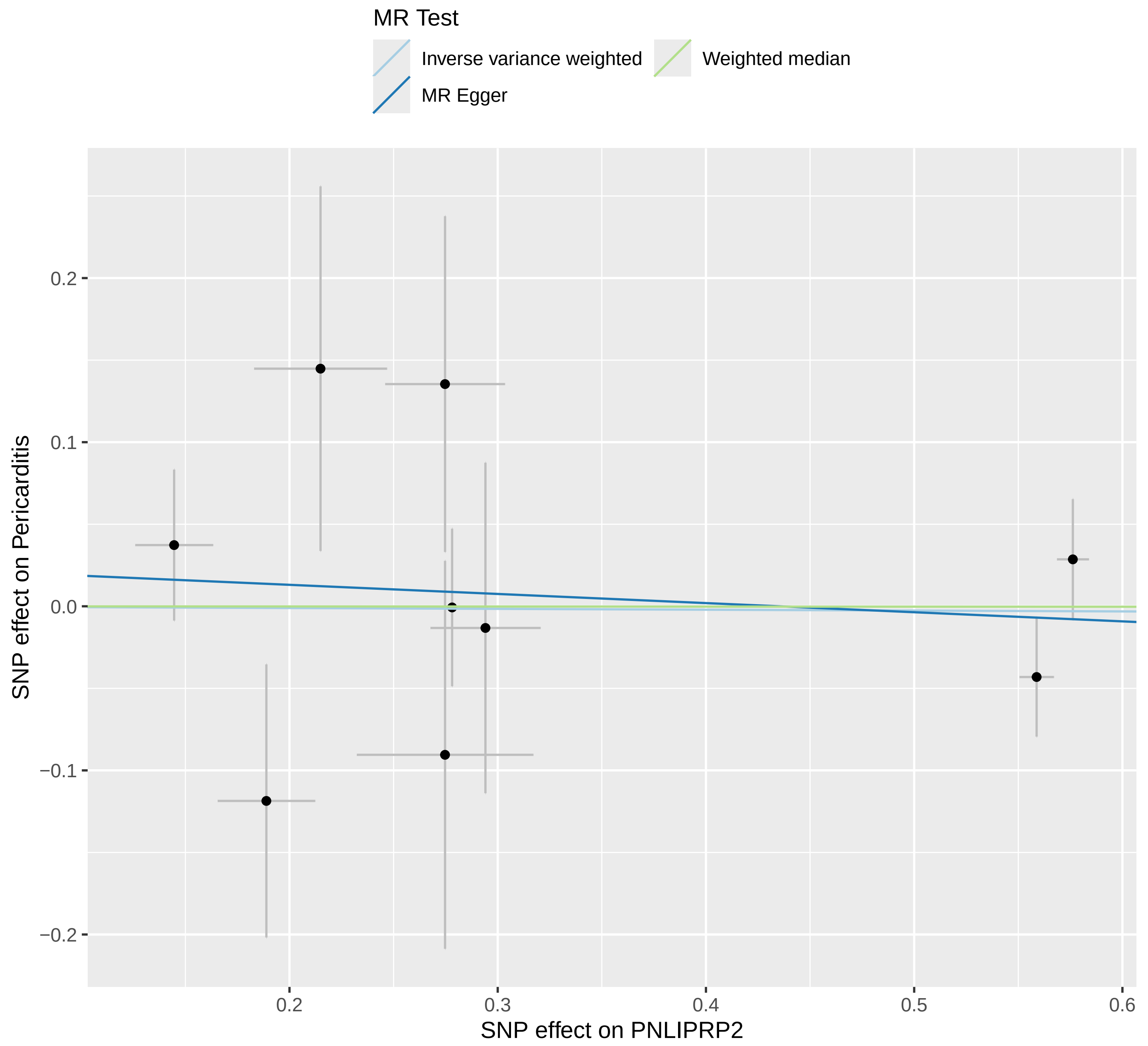
MR Test
Inverse variance weighted
Weighted median
MR Egger
SNP effect on Pericarditis
0.2
0.1
0.0
−0.1
−0.2
0.2
0.3
0.4
0.5
0.6
SNP effect on PNLIPRP2

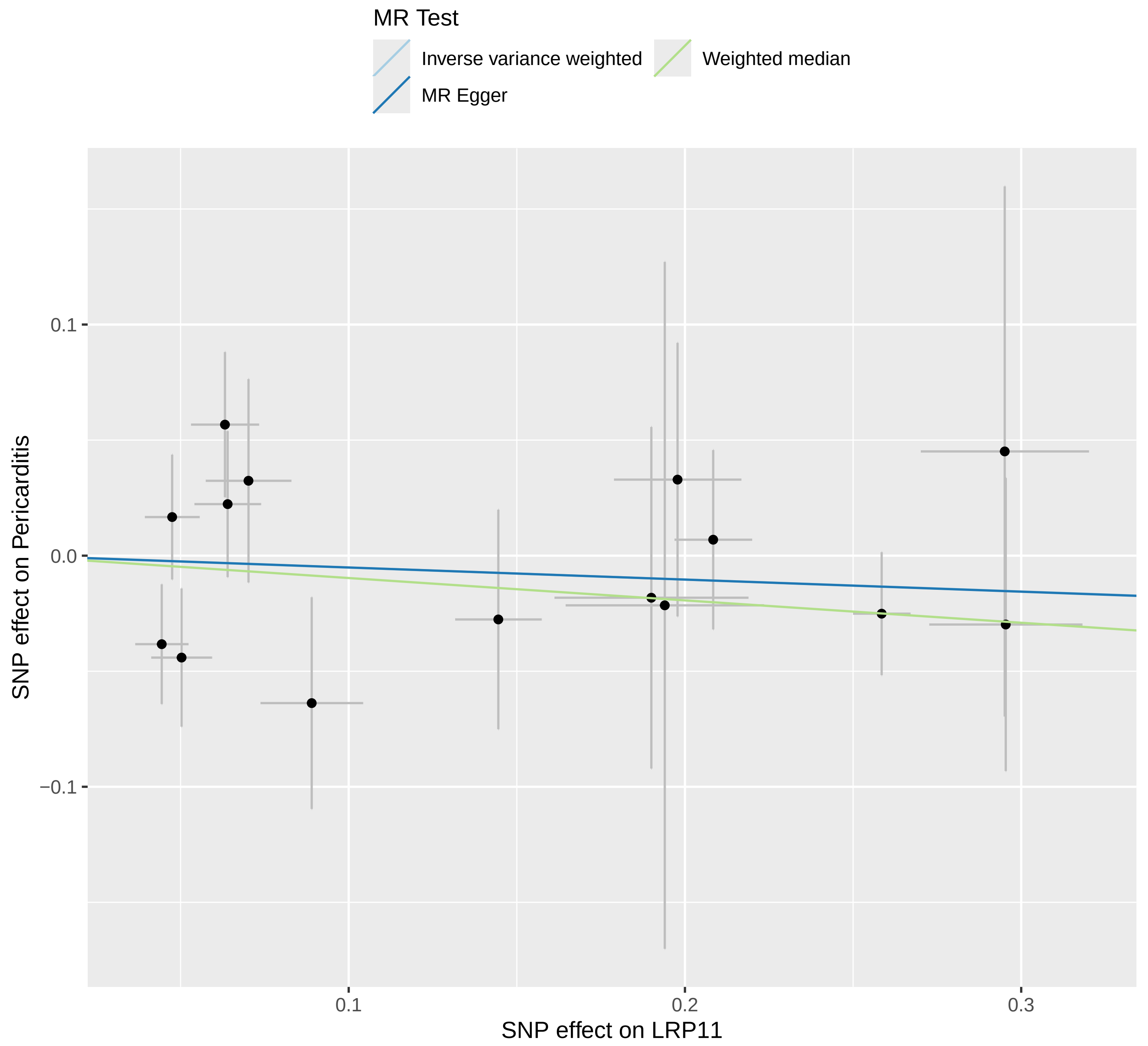
MR Test
Inverse variance weighted
Weighted median
MR Egger
SNP effect on Pericarditis
0.1
0.0
−0.1
0.1
0.2
0.3
SNP effect on LRP11

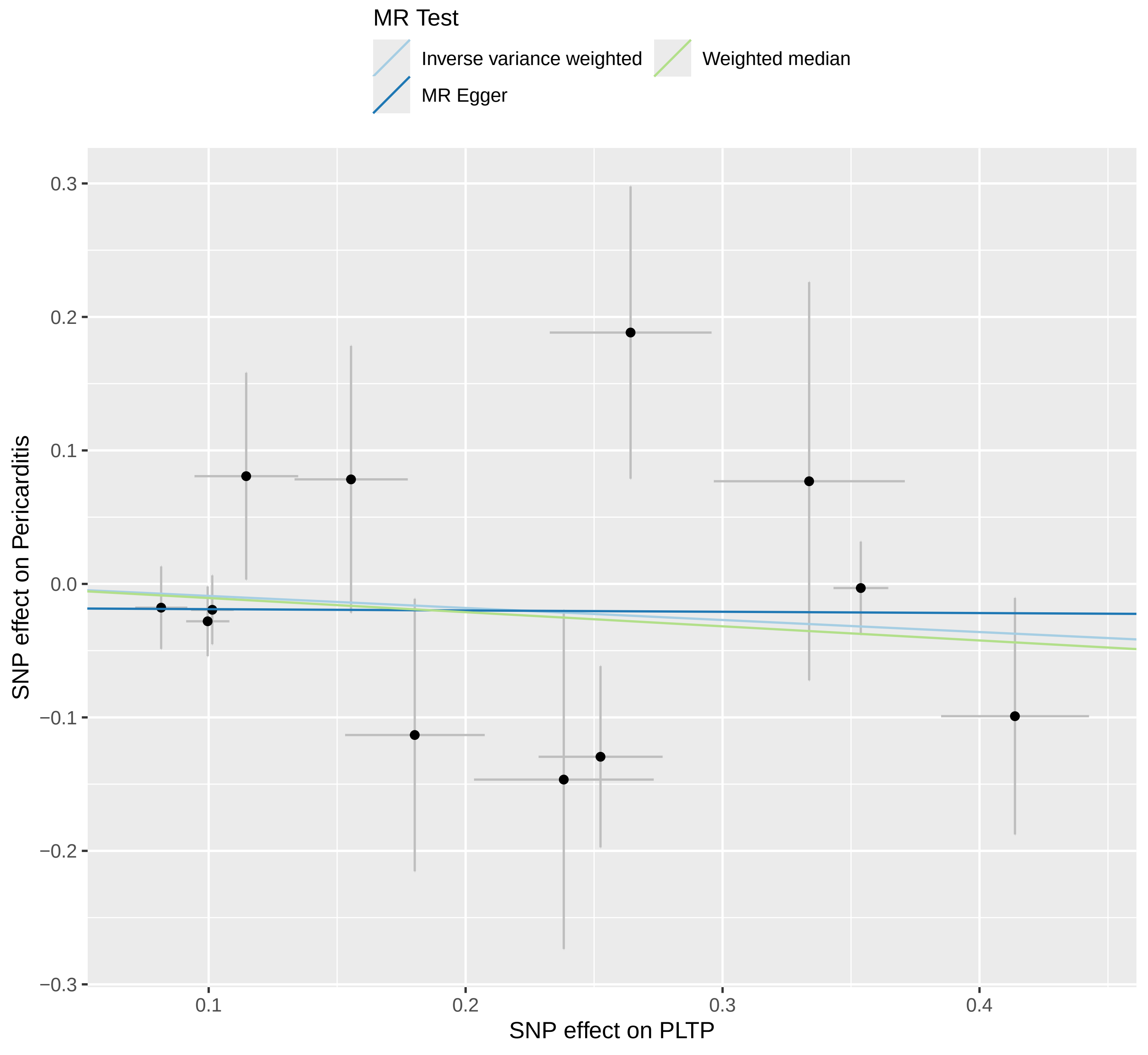
MR Test
Inverse variance weighted
MR Egger
Weighted median
SNP effect on Pericarditis
0.3
0.2
0.1
0.0
−0.1
−0.2
−0.3
0.1
0.2
0.3
0.4
SNP effect on PLTP

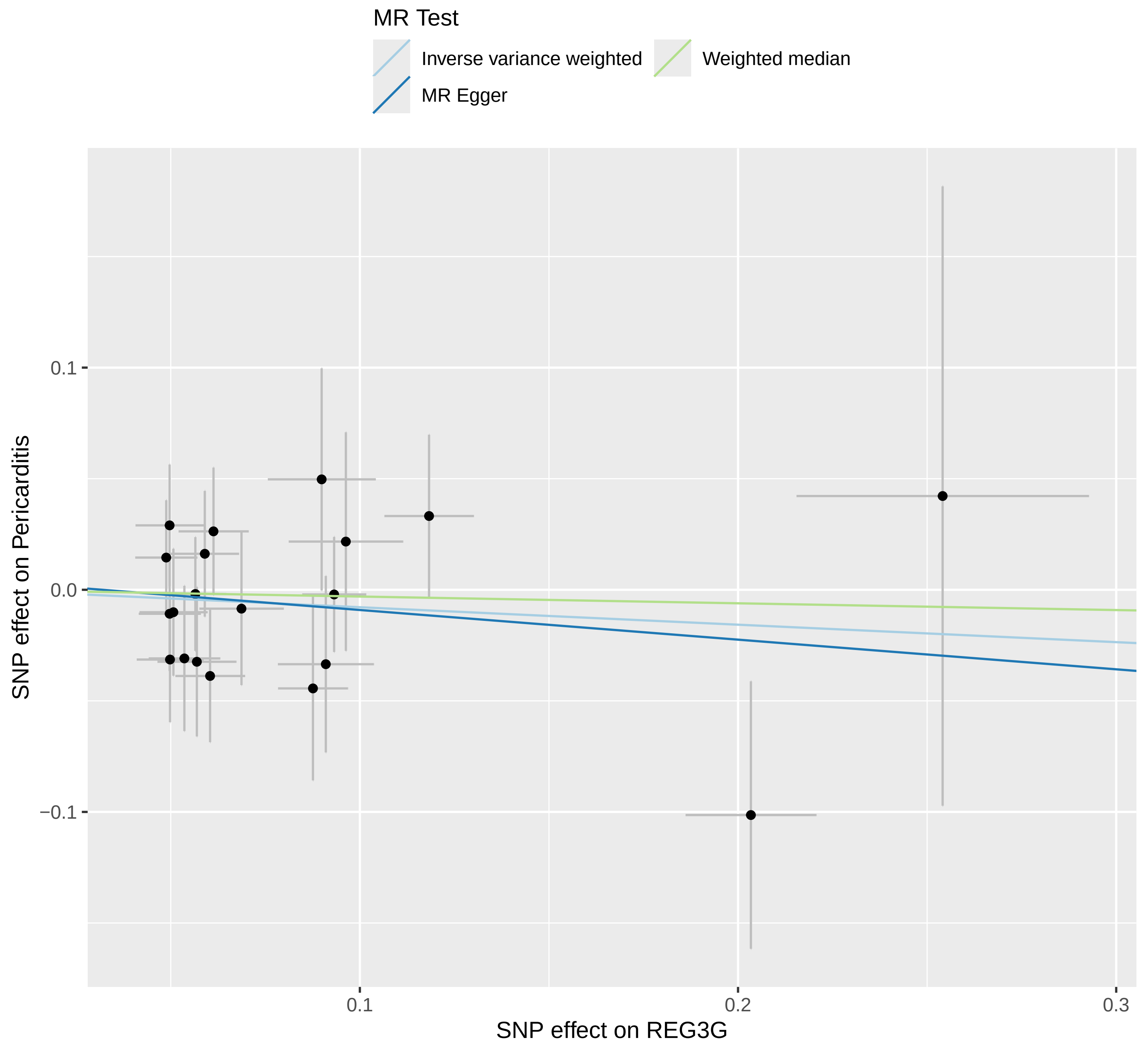

MR Test
Inverse variance weighted
Weighted median
MR Egger
0.1
0.0
−0.1
0.1
0.2
0.3
SNP effect on Pericarditis
SNP effect on REG3G

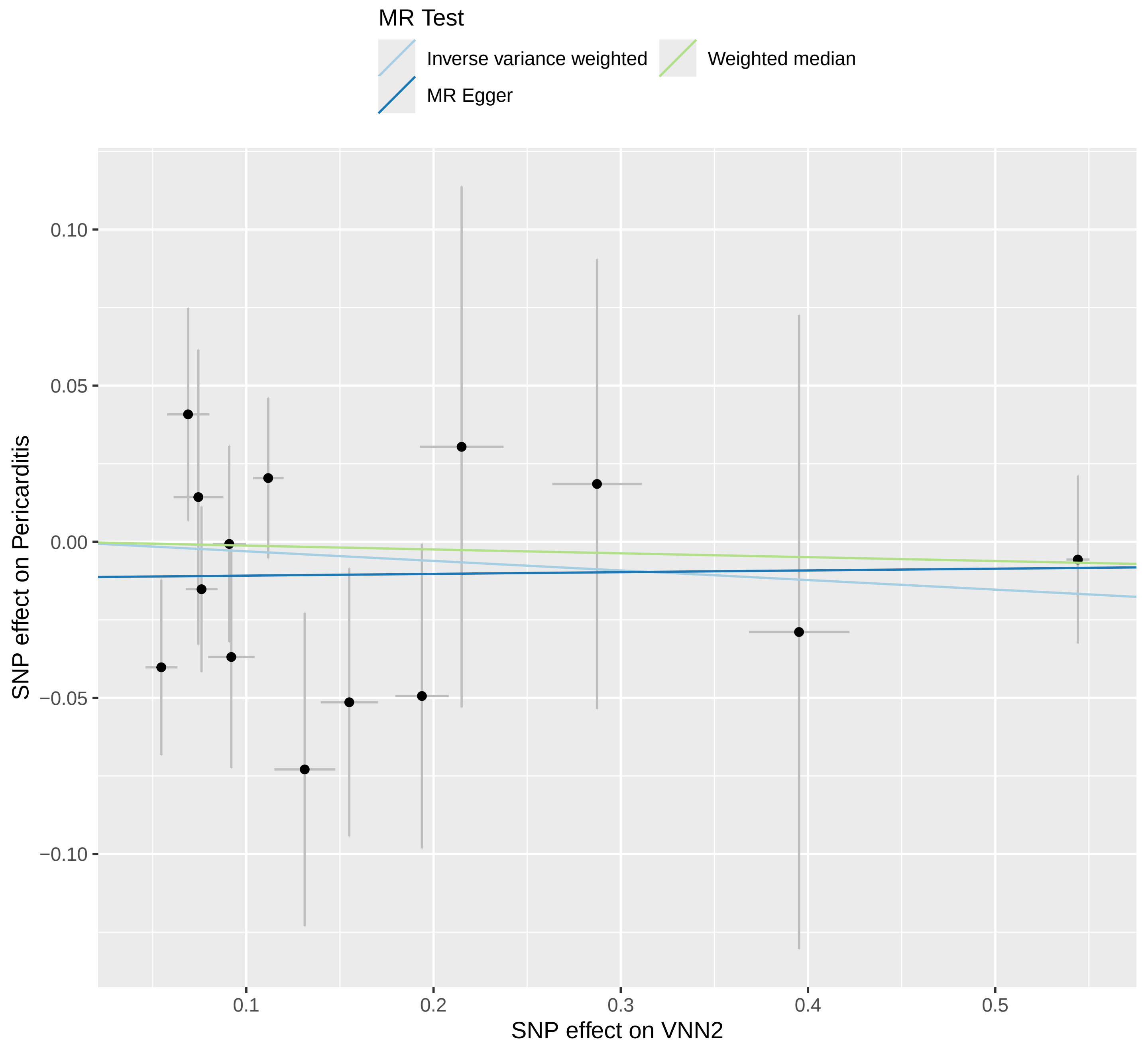

MR Test
Inverse variance weighted
MR Egger
Weighted median
0.10
0.05
0.00
−0.05
−0.10
SNP effect on Pericarditis
0.1
0.2
0.3
0.4
0.5
SNP effect on VNN2

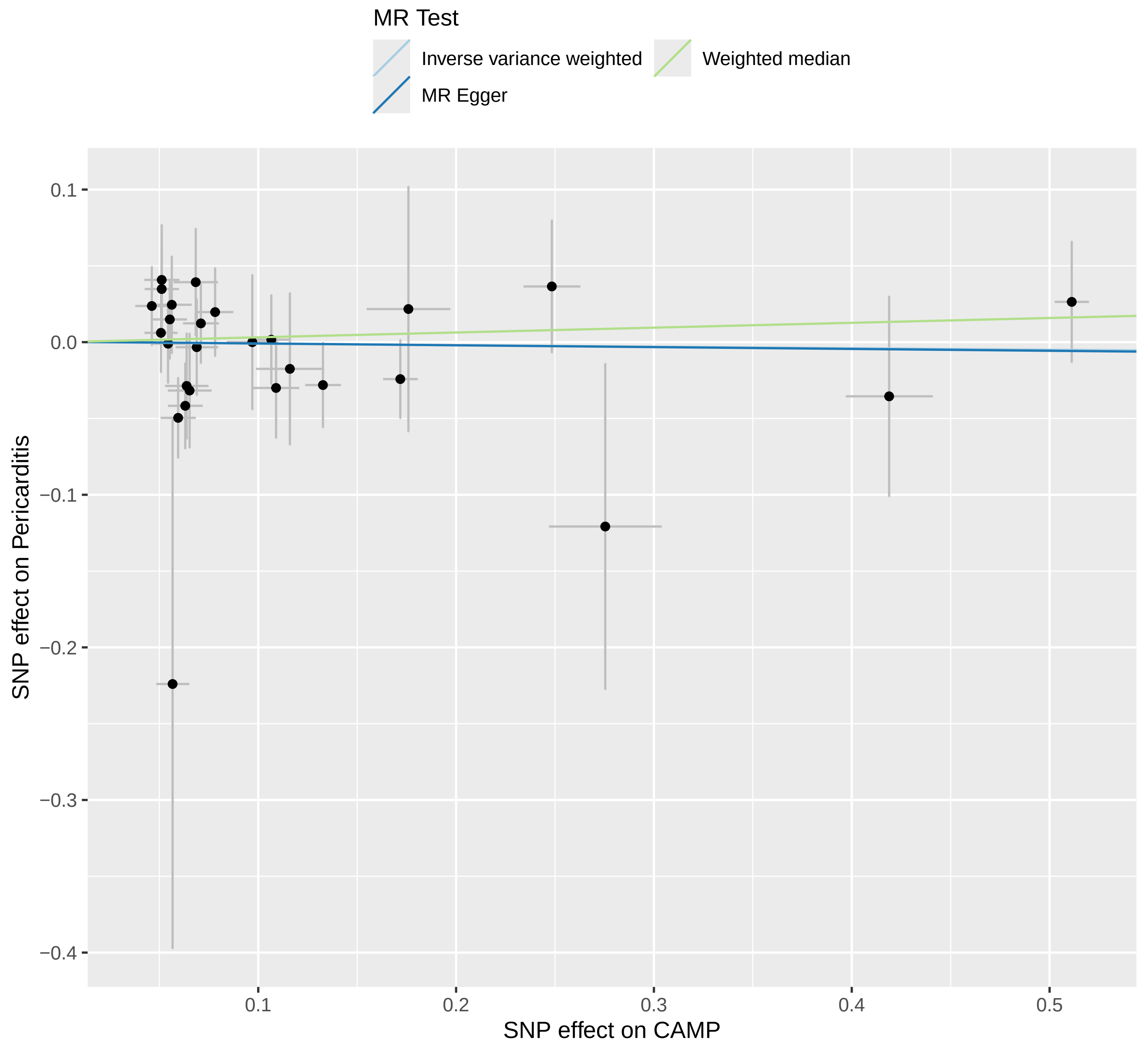
MR Test
Inverse variance weighted
Weighted median
MR Egger
SNP effect on Pericarditis
SNP effect on CAMP
0.1
0.0
−0.1
−0.2
−0.3
−0.4
0.1
0.2
0.3
0.4
0.5

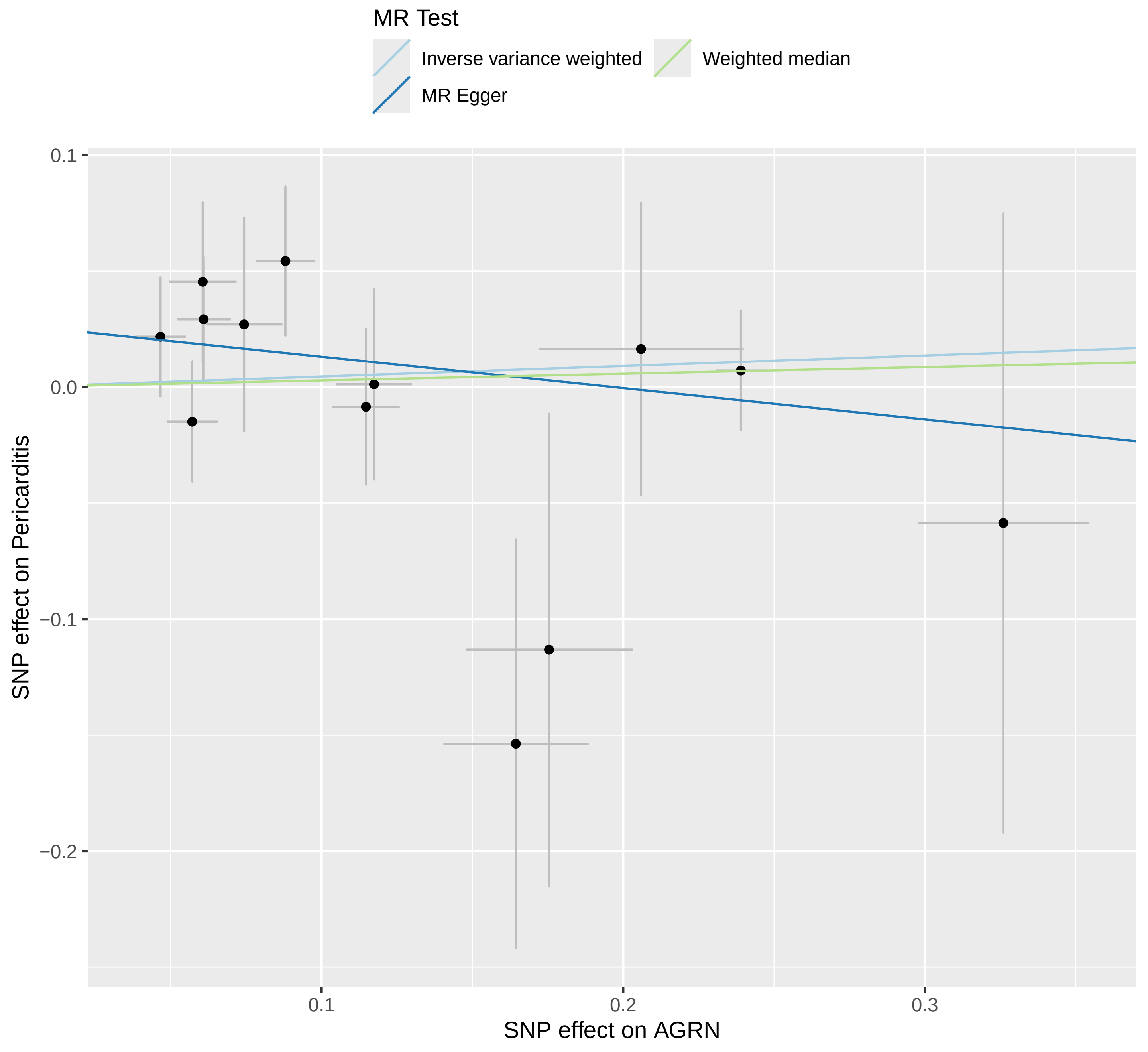
MR Test
Inverse variance weighted
Weighted median
MR Egger
0.1
0.0
−0.1
−0.2
SNP effect on Pericarditis
0.1
0.2
0.3
SNP effect on AGRN

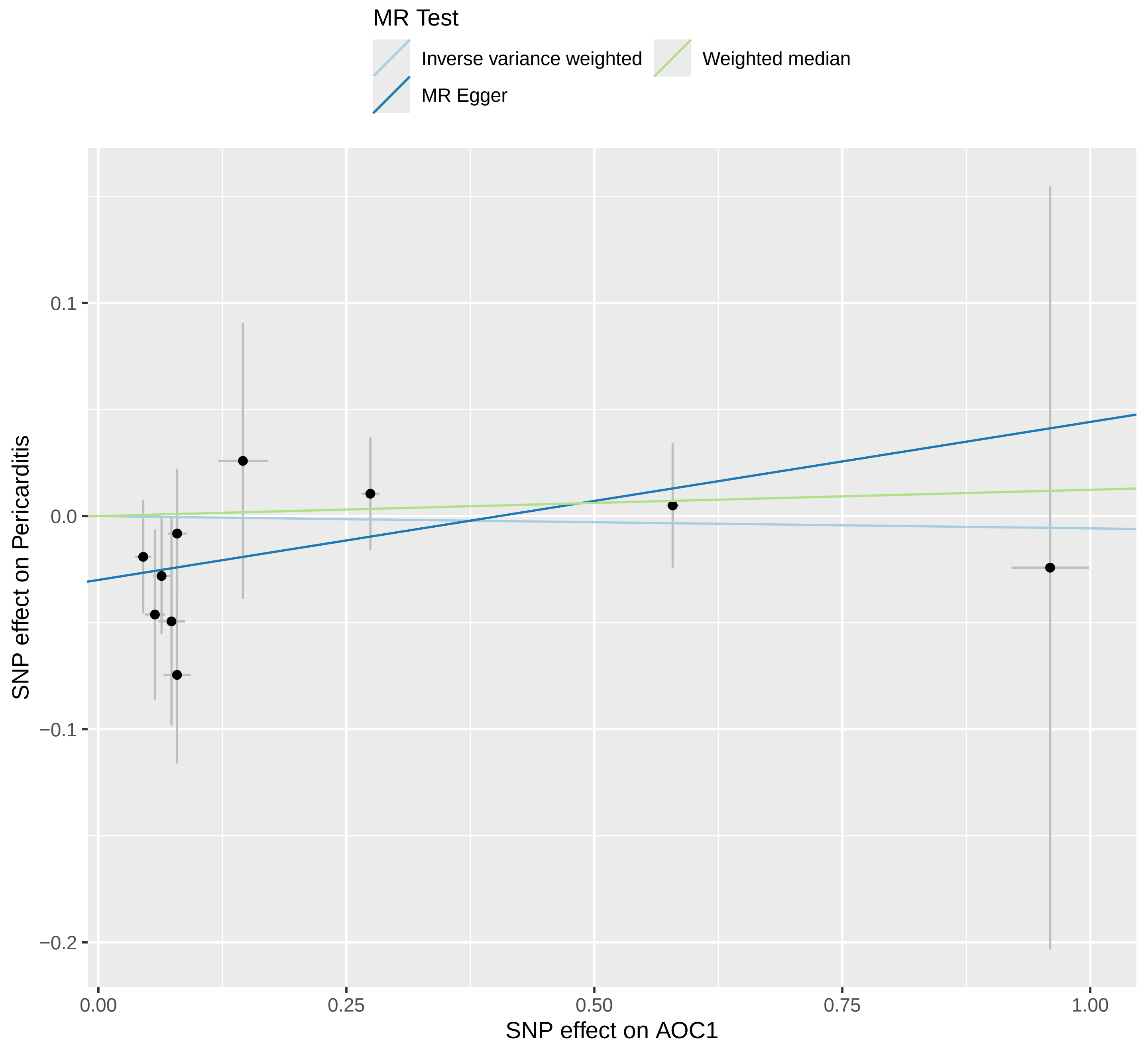

MR Test
Inverse variance weighted
Weighted median
MR Egger
SNP effect on Pericarditis
0.1
0.0
−0.1
−0.2
0.00
0.25
0.50
0.75
1.00
SNP effect on AOC1

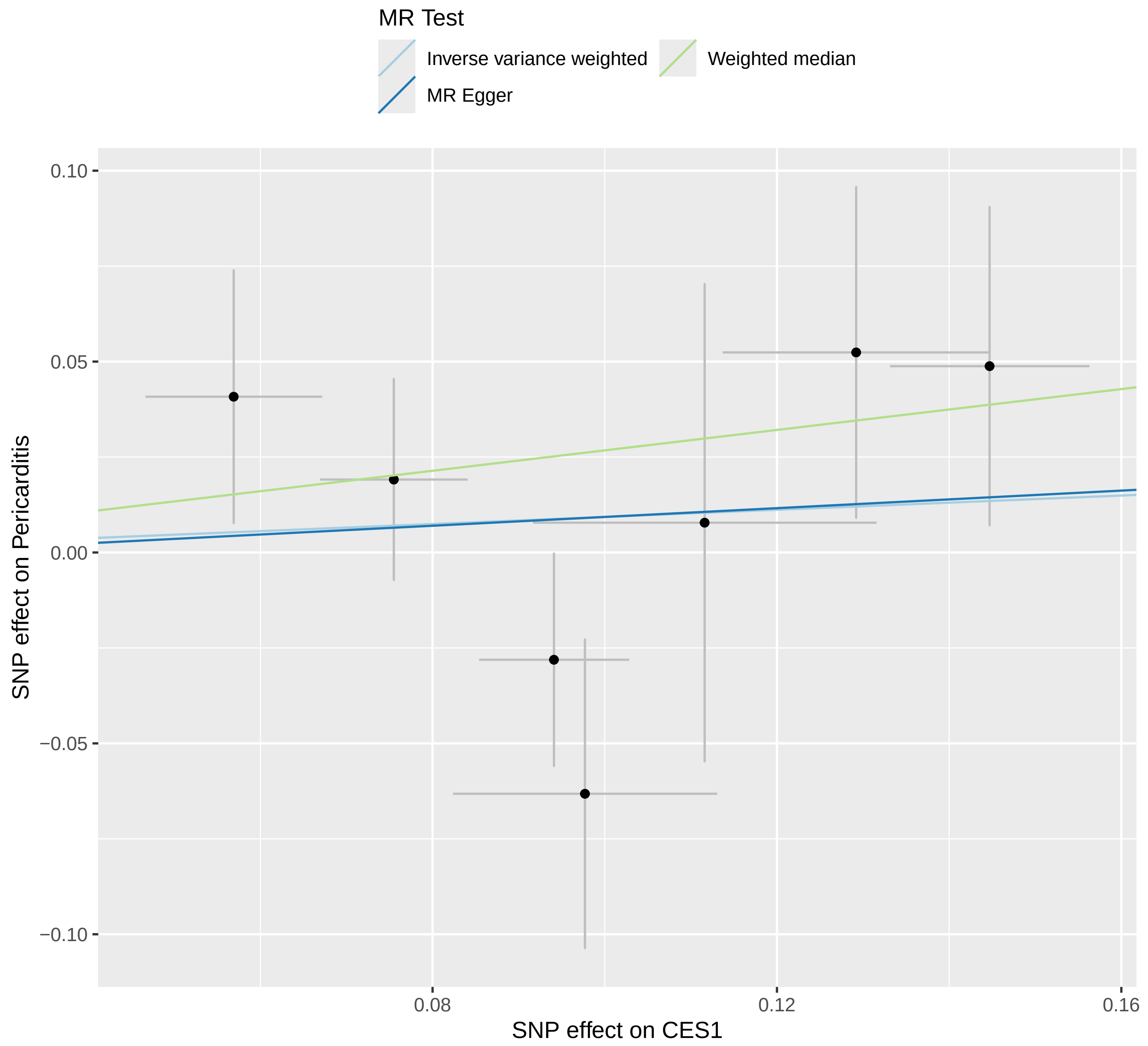

MR Test
Inverse variance weighted
MR Egger
Weighted median
0.10
0.05
0.00
−0.05
−0.10
SNP effect on Pericarditis
0.08
0.12
0.16
SNP effect on CES1

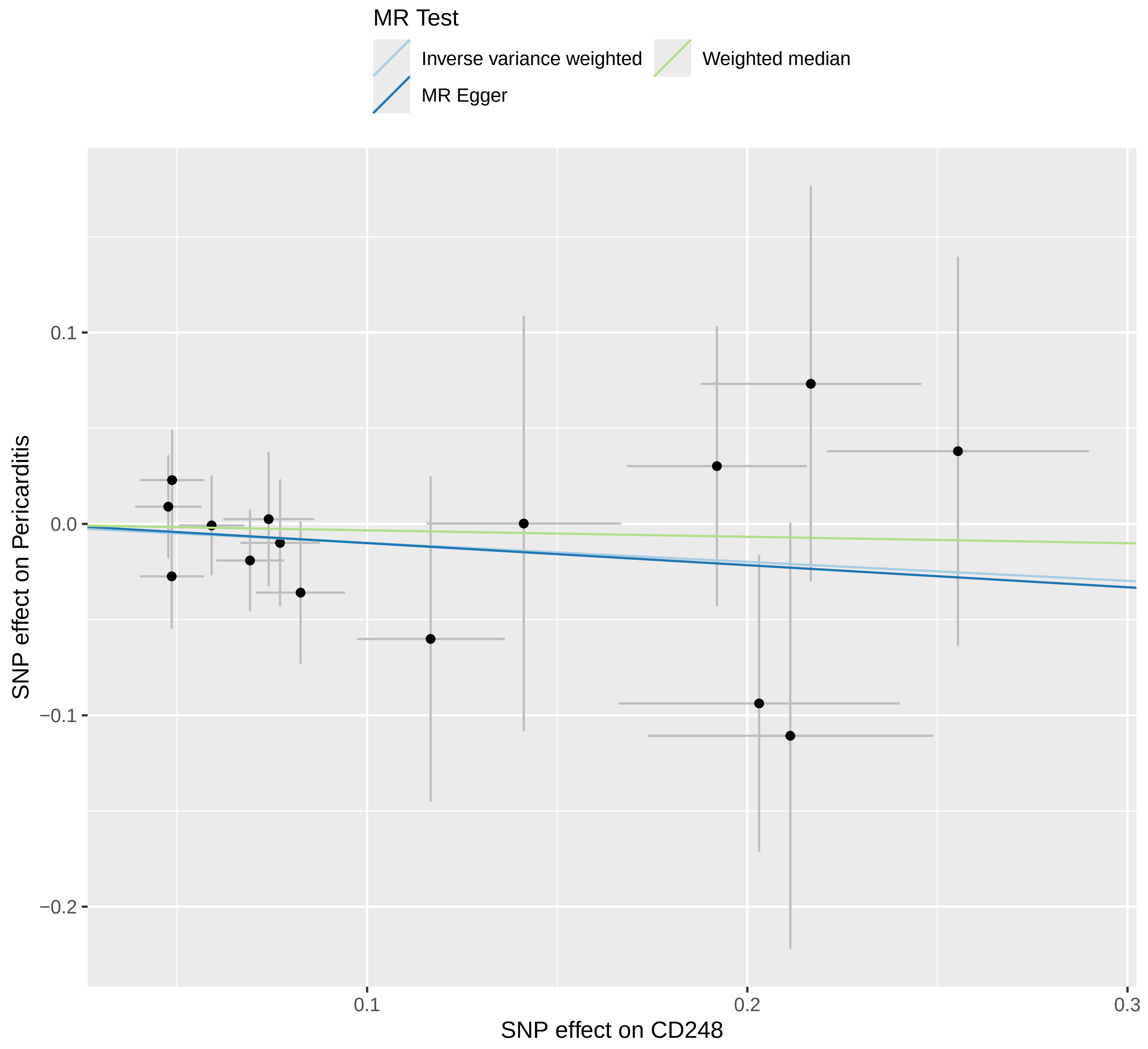
MR Test
Inverse variance weighted
Weighted median
MR Egger
SNP effect on Pericarditis
0.1
0.0
−0.1
−0.2
0.1
0.2
0.3
SNP effect on CD248

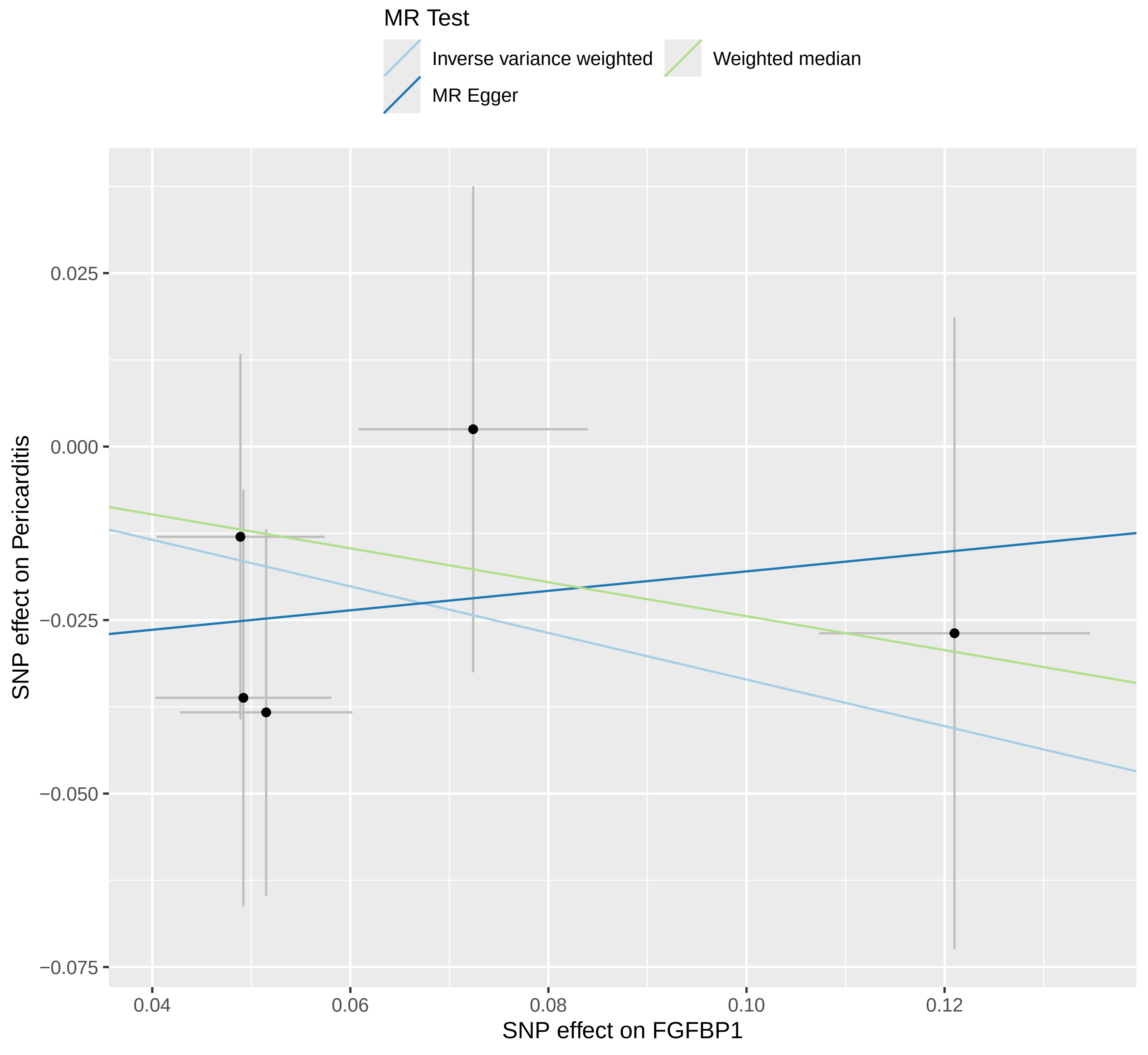
MR Test
Inverse variance weighted
Weighted median
MR Egger
0.025
0.000
−0.025
−0.050
−0.075
0.04
0.06
0.08
0.10
0.12
SNP effect on FGFBP1
SNP effect on Pericarditis

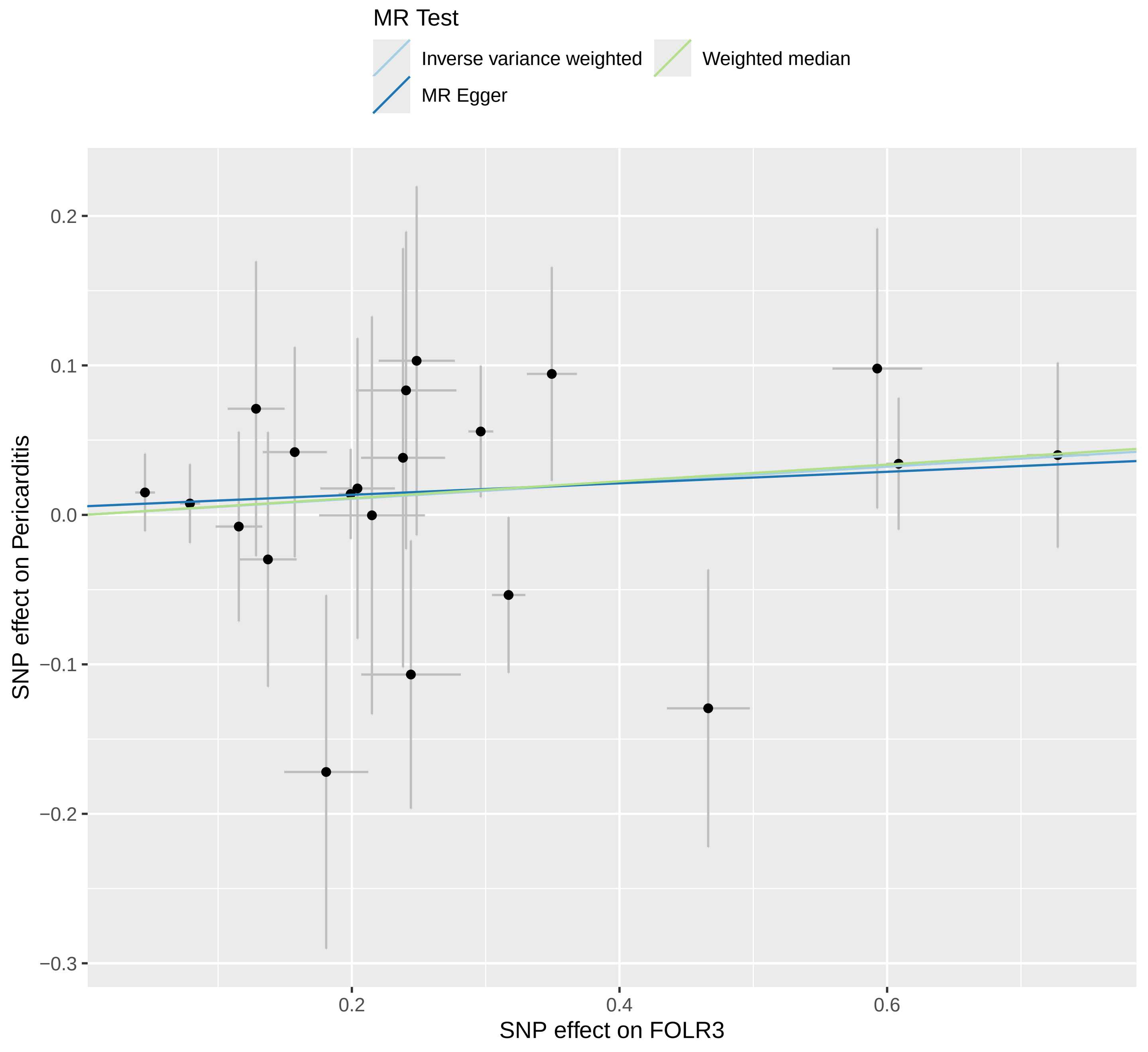

MR Test
Inverse variance weighted
Weighted median
MR Egger
0.2
0.1
0.0
−0.1
−0.2
−0.3
SNP effect on Pericarditis
0.2
0.4
0.6
SNP effect on FOLR3

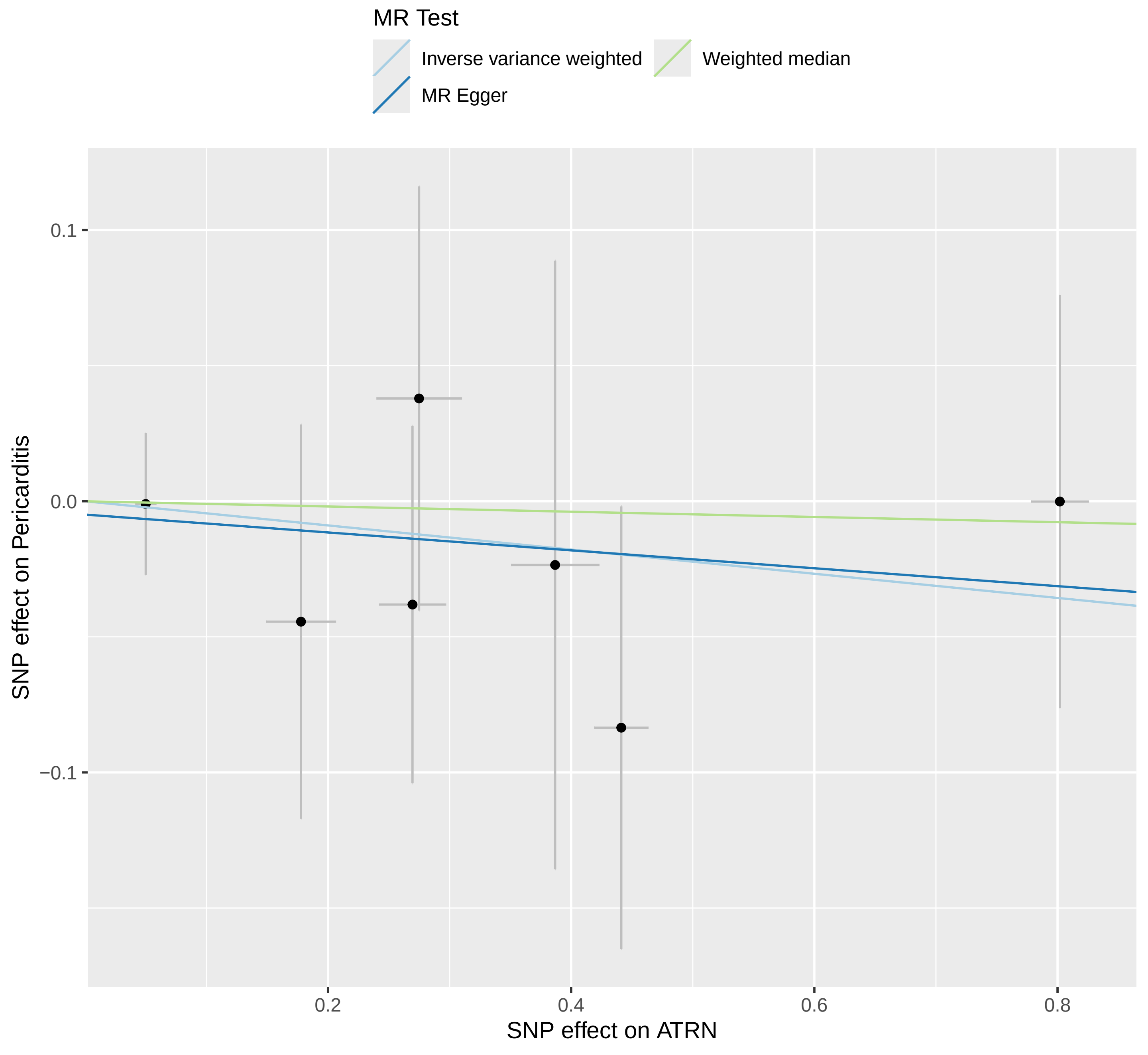
MR Test
Inverse variance weighted
Weighted median
MR Egger
0.1
0.0
−0.1
SNP effect on Pericarditis
0.2
0.4
0.6
0.8
SNP effect on ATRN

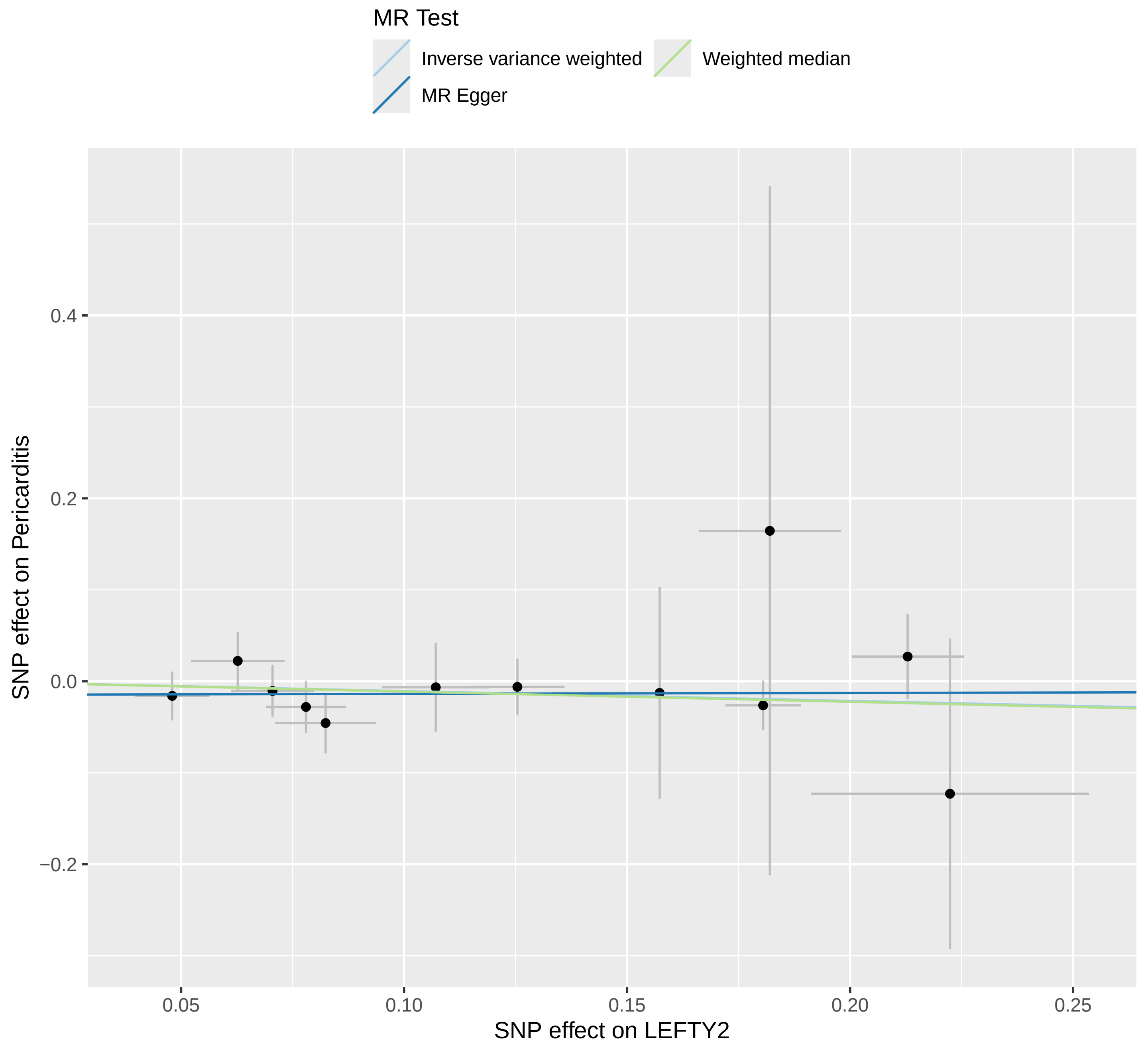

MR Test
Inverse variance weighted
Weighted median
MR Egger
0.4
0.2
0.0
−0.2
SNP effect on Pericarditis
0.05
0.10
0.15
0.20
0.25
SNP effect on LEFTY2

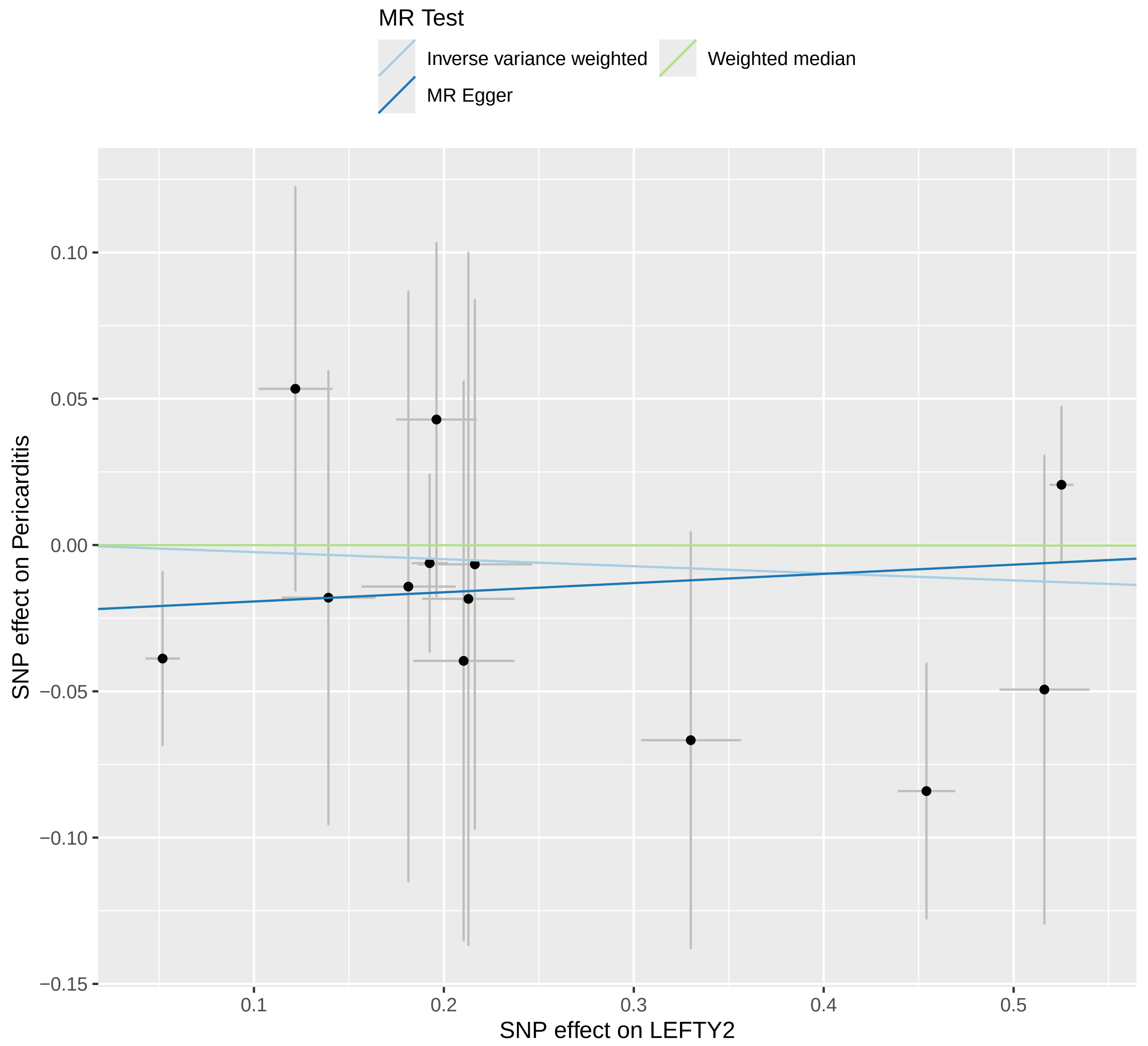
MR Test
Inverse variance weighted
Weighted median
MR Egger
SNP effect on Pericarditis
SNP effect on LEFTY2
0.10
0.05
0.00
−0.05
−0.10
−0.15
0.1
0.2
0.3
0.4
0.5

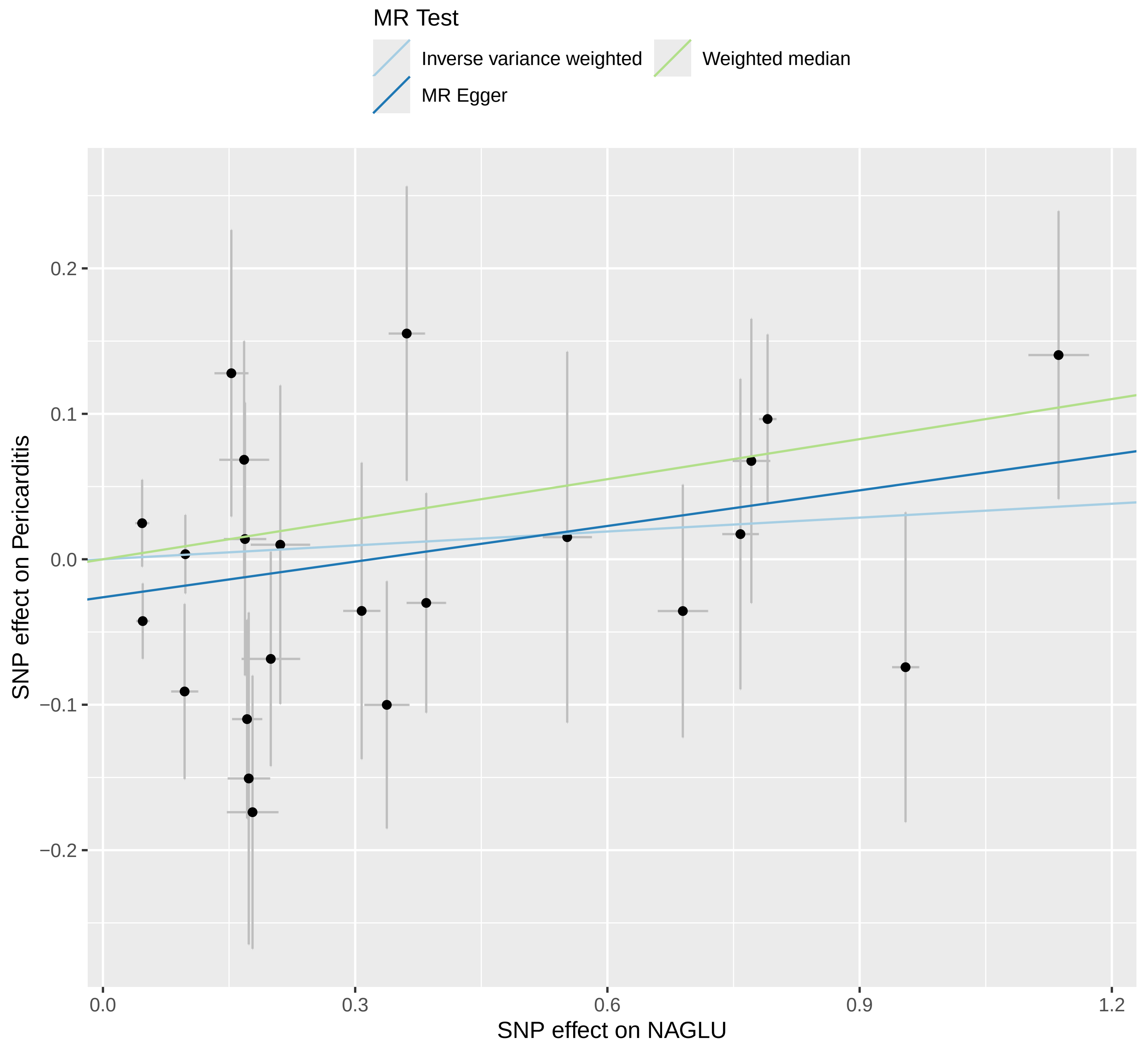

MR Test
Inverse variance weighted
Weighted median
MR Egger
SNP effect on Pericarditis
0.2
0.1
0.0
−0.1
−0.2
0.0
0.3
0.6
0.9
1.2
SNP effect on NAGLU

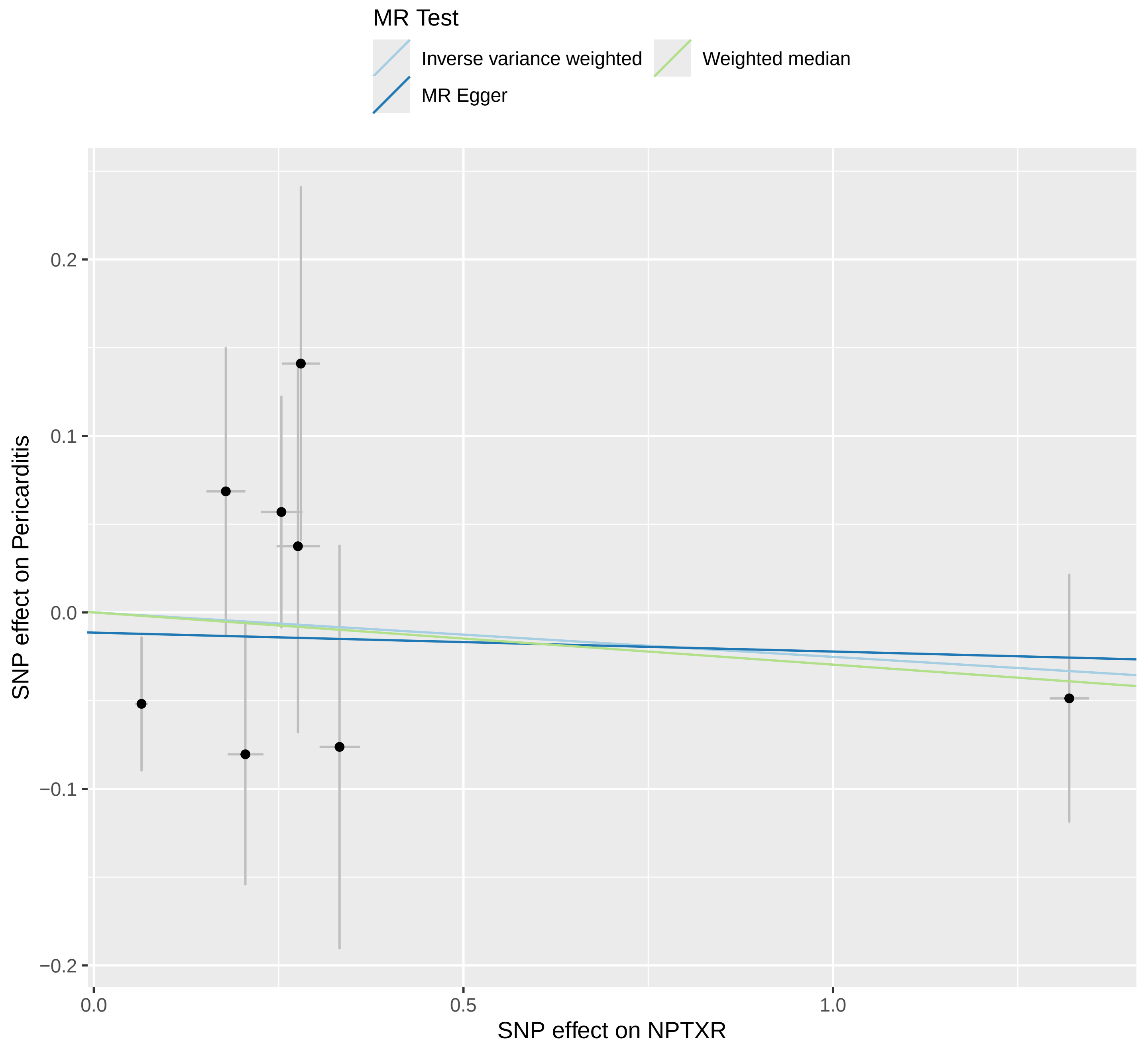

MR Test
Inverse variance weighted
Weighted median
MR Egger
0.2
0.1
0.0
−0.1
−0.2
0.0
0.5
1.0
SNP effect on Pericarditis
SNP effect on NPTXR

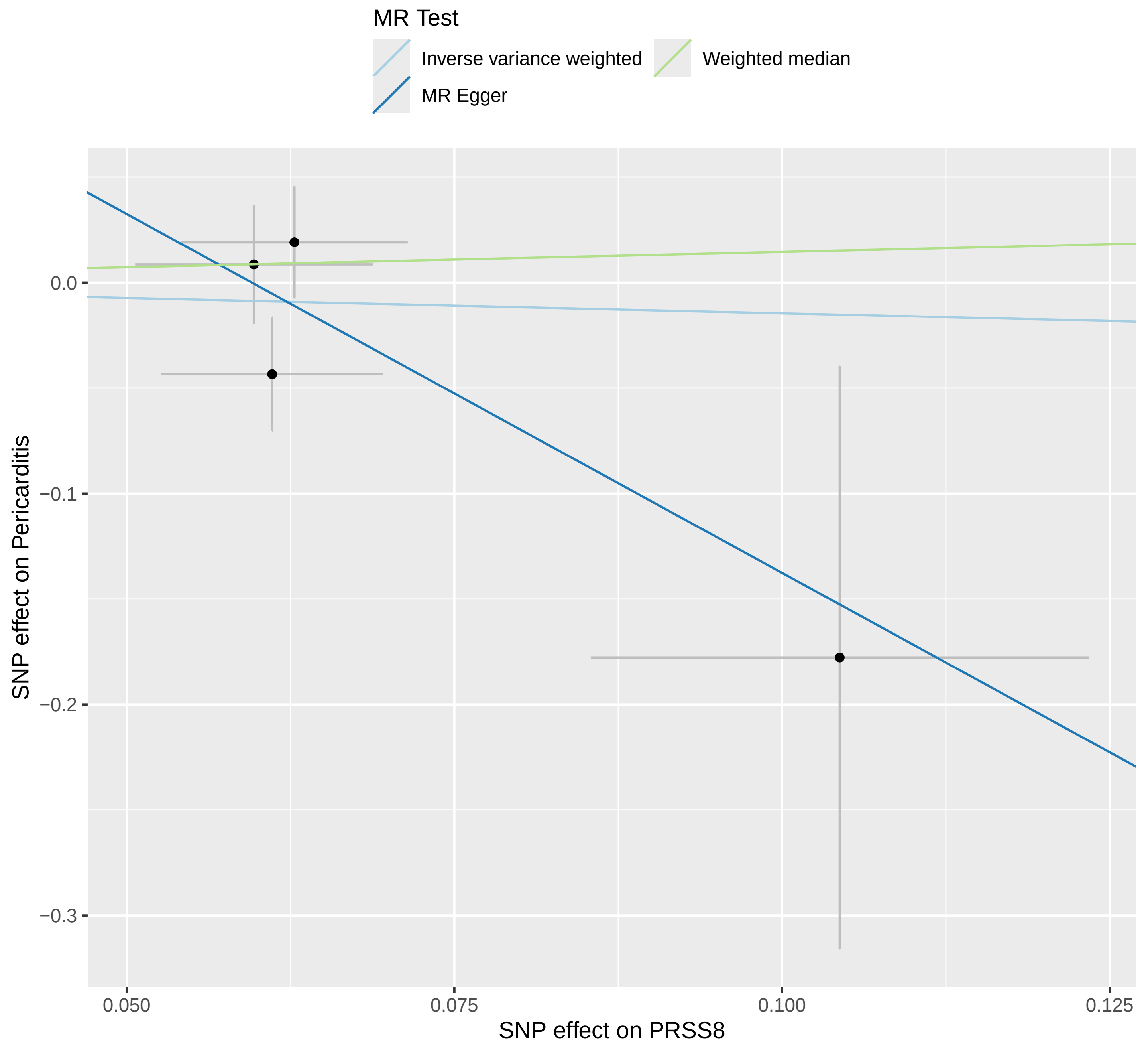

MR Test
Inverse variance weighted
Weighted median
MR Egger
0.0
−0.1
−0.2
−0.3
SNP effect on Pericarditis
0.050
0.075
0.100
0.125
SNP effect on PRSS8

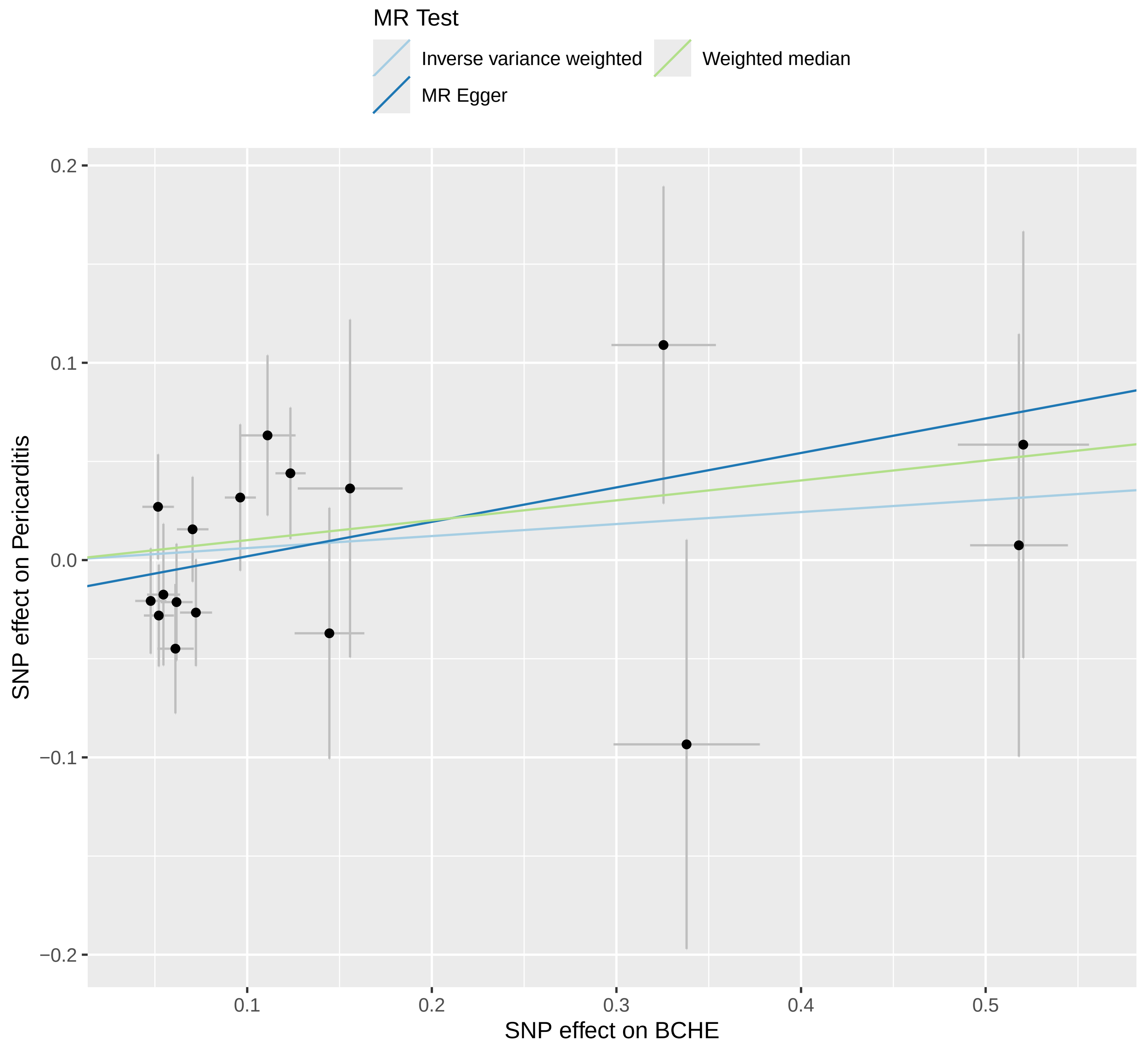
MR Test
Inverse variance weighted
Weighted median
MR Egger
0.2
0.1
0.0
−0.1
−0.2
SNP effect on Pericarditis
0.1
0.2
0.3
0.4
0.5
SNP effect on BCHE

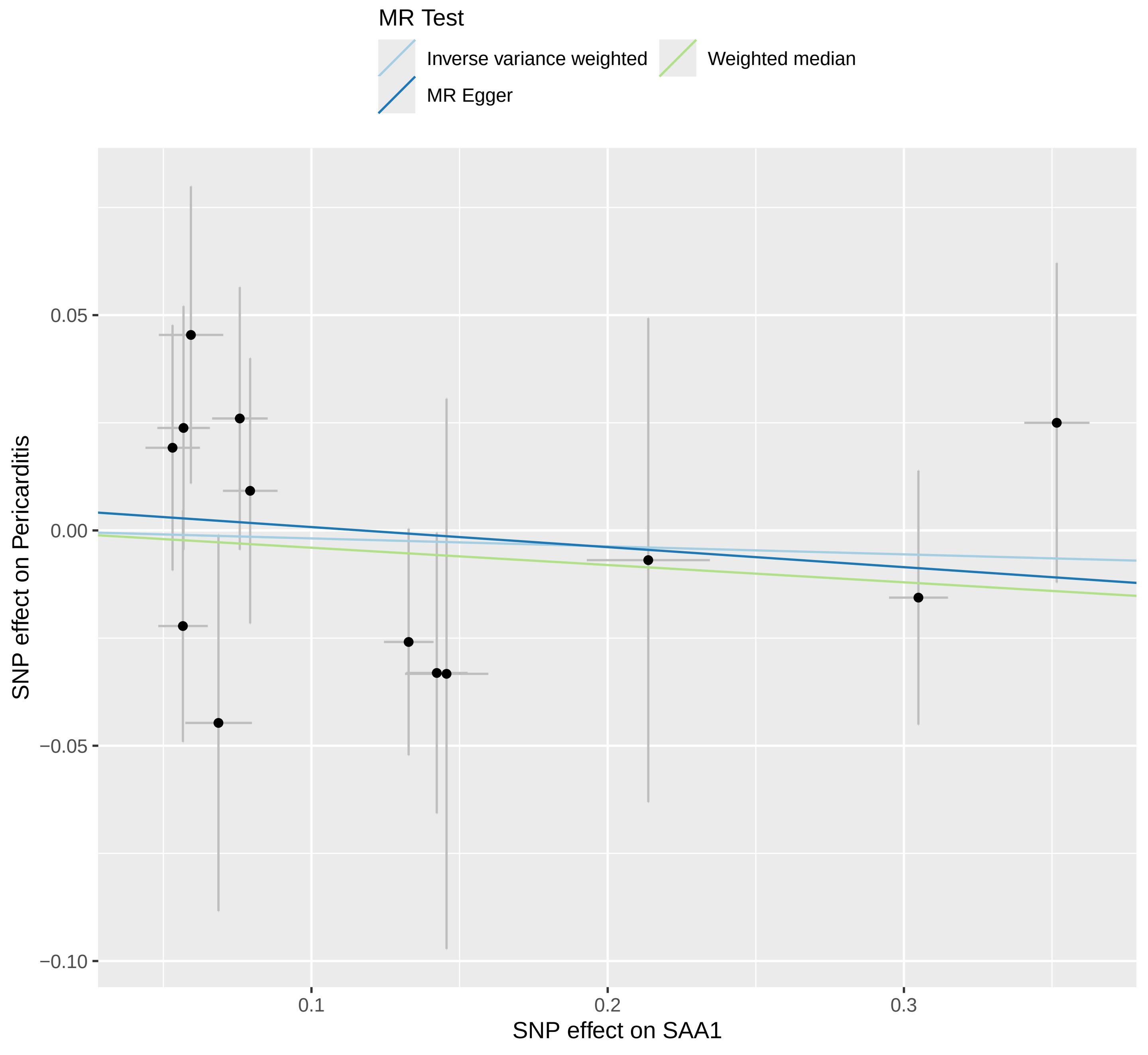
MR Test
Inverse variance weighted
Weighted median
MR Egger
SNP effect on Pericarditis
0.05
0.00
−0.05
−0.10
0.1
0.2
0.3
SNP effect on SAA1

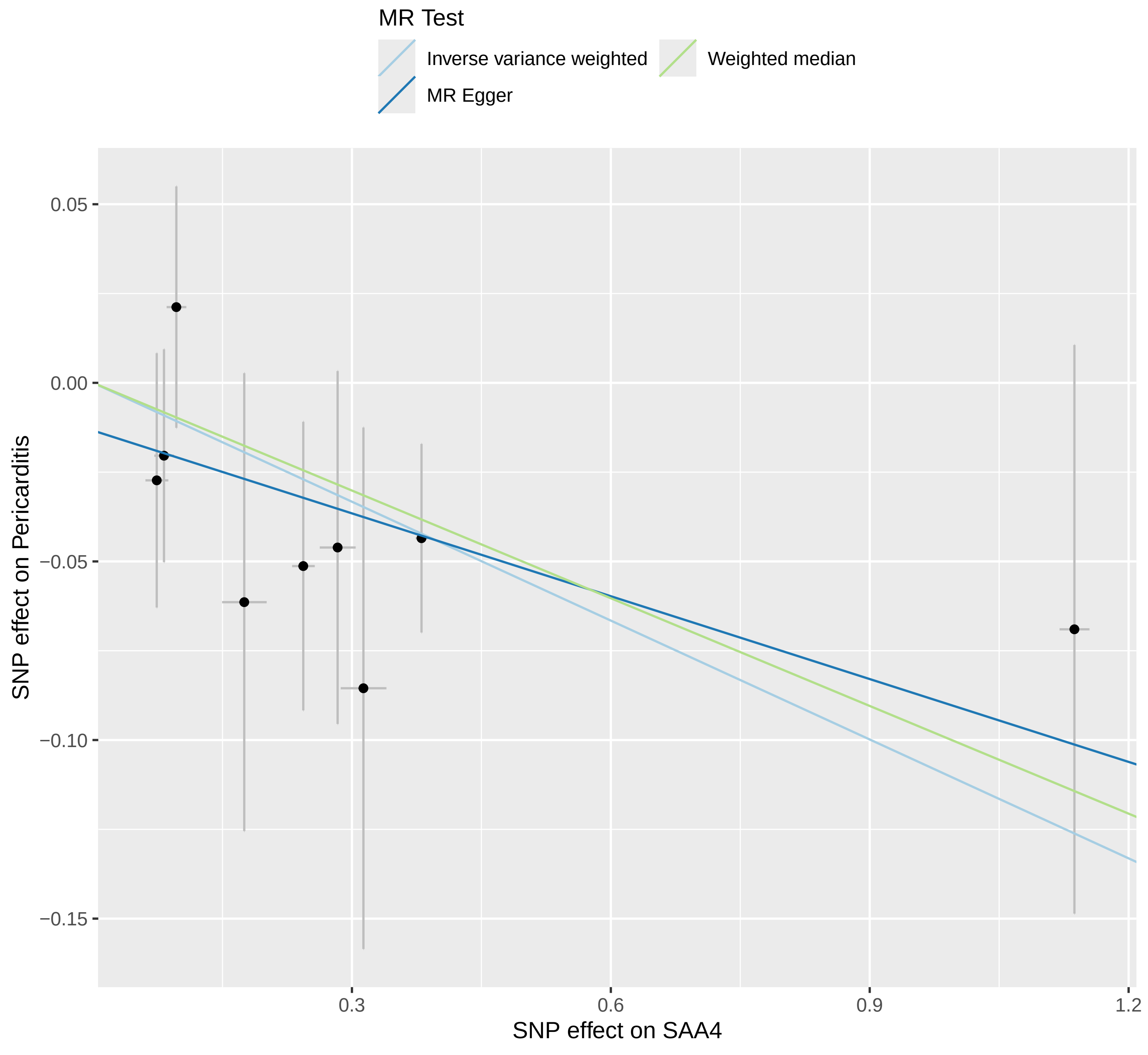
MR Test
Inverse variance weighted
Weighted median
MR Egger
0.05
0.00
−0.05
−0.10
−0.15
0.3
0.6
0.9
1.2
SNP effect on SAA4
SNP effect on Pericarditis

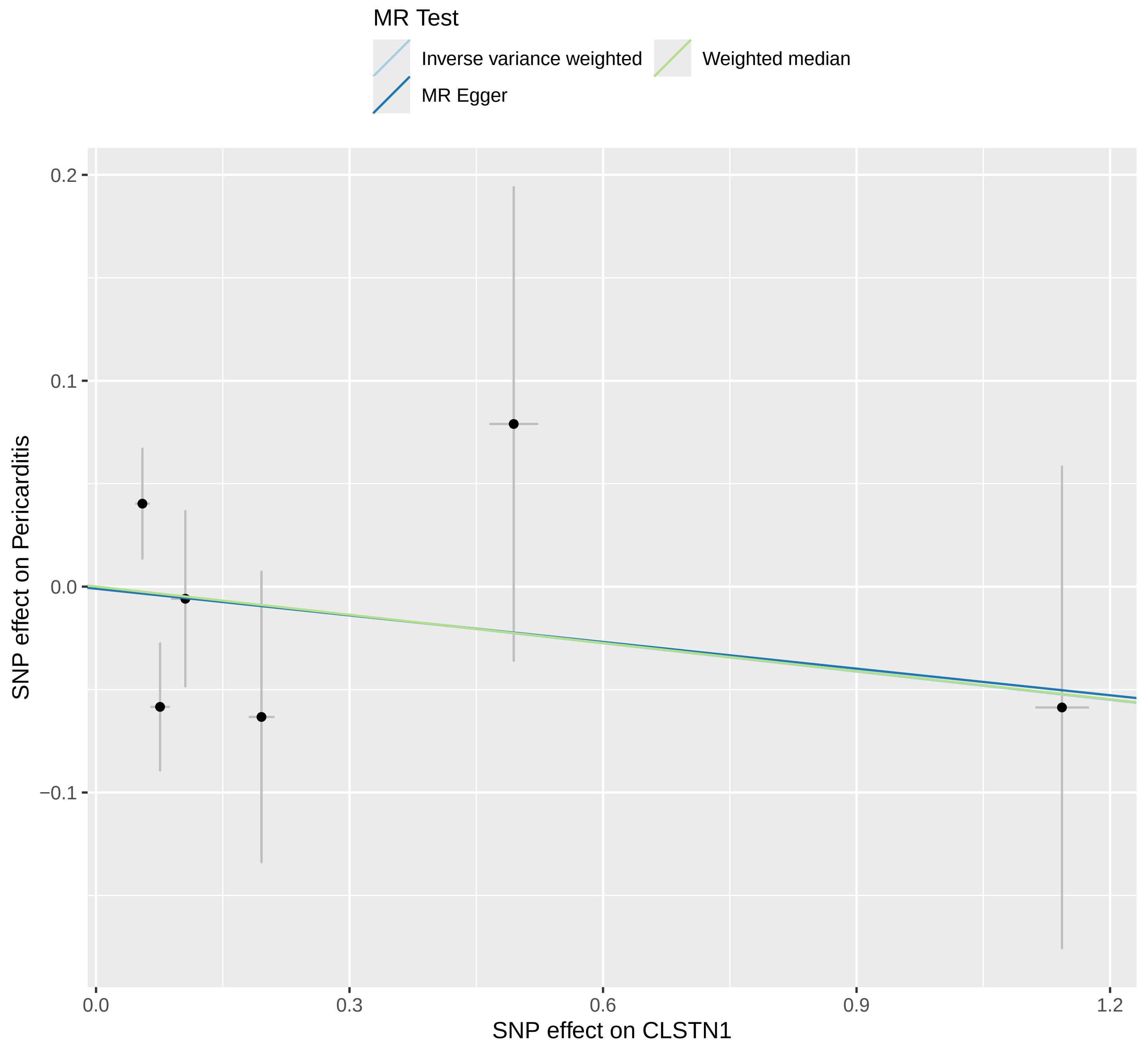

MR Test
Inverse variance weighted
Weighted median
MR Egger
0.2
0.1
0.0
−0.1
SNP effect on Pericarditis
0.0
0.3
0.6
0.9
1.2
SNP effect on CLSTN1

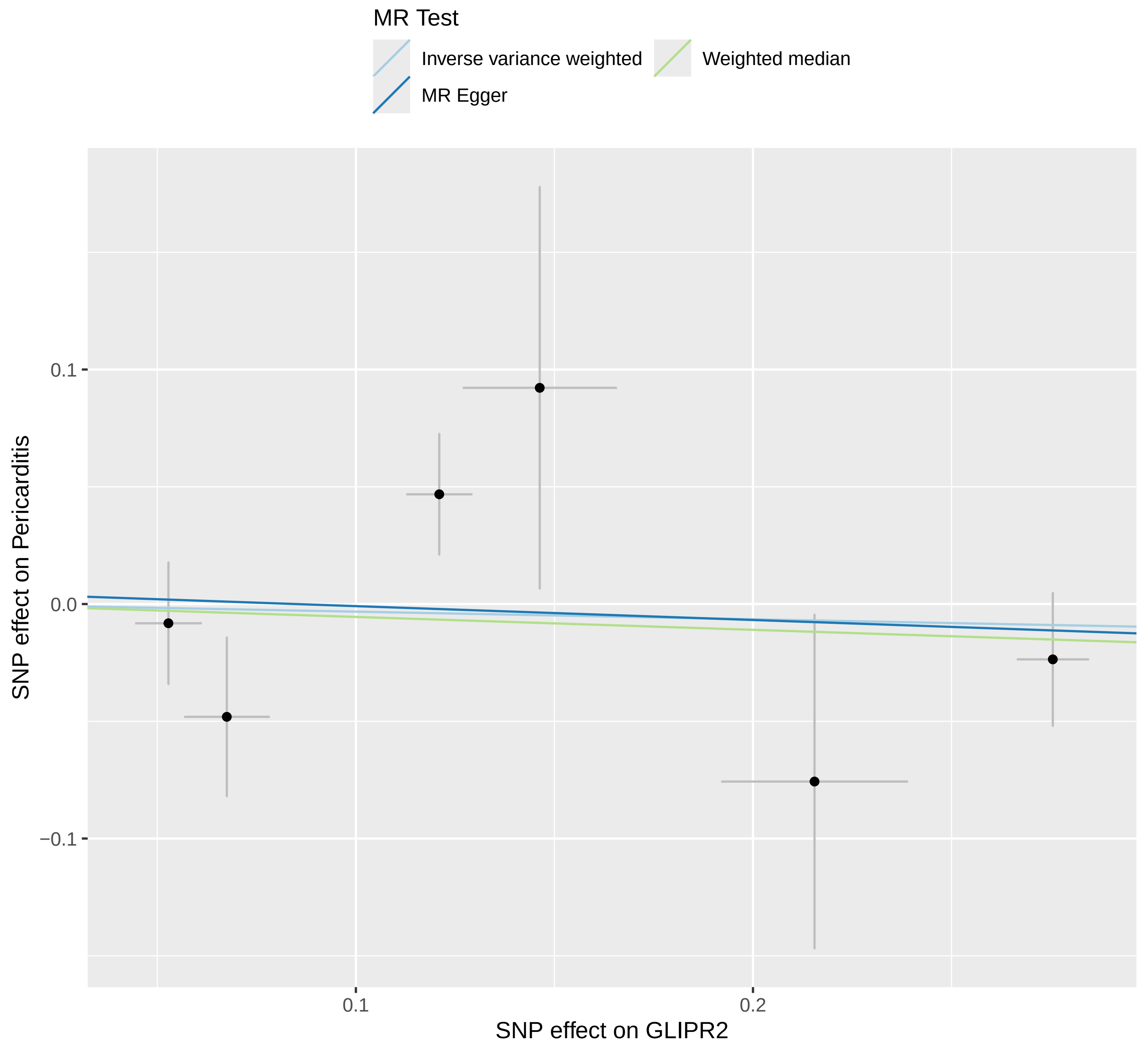

MR Test
Inverse variance weighted
MR Egger
Weighted median
SNP effect on Pericarditis
0.1
0.0
−0.1
SNP effect on GLIPR2
0.1
0.2

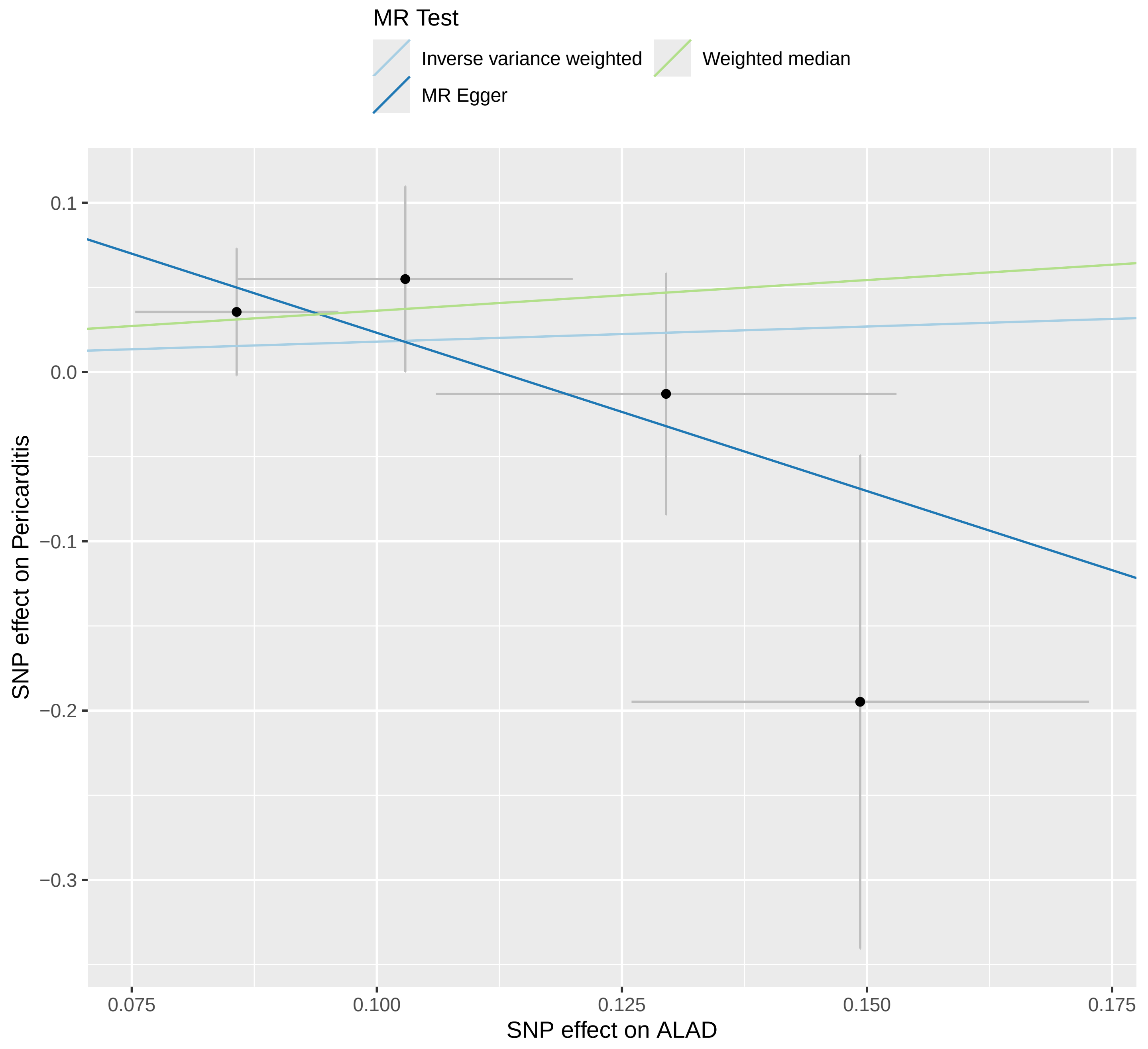
MR Test
Inverse variance weighted
Weighted median
MR Egger
SNP effect on Pericarditis
0.1
0.0
−0.1
−0.2
−0.3
0.075
0.100
0.125
0.150
0.175
SNP effect on ALAD

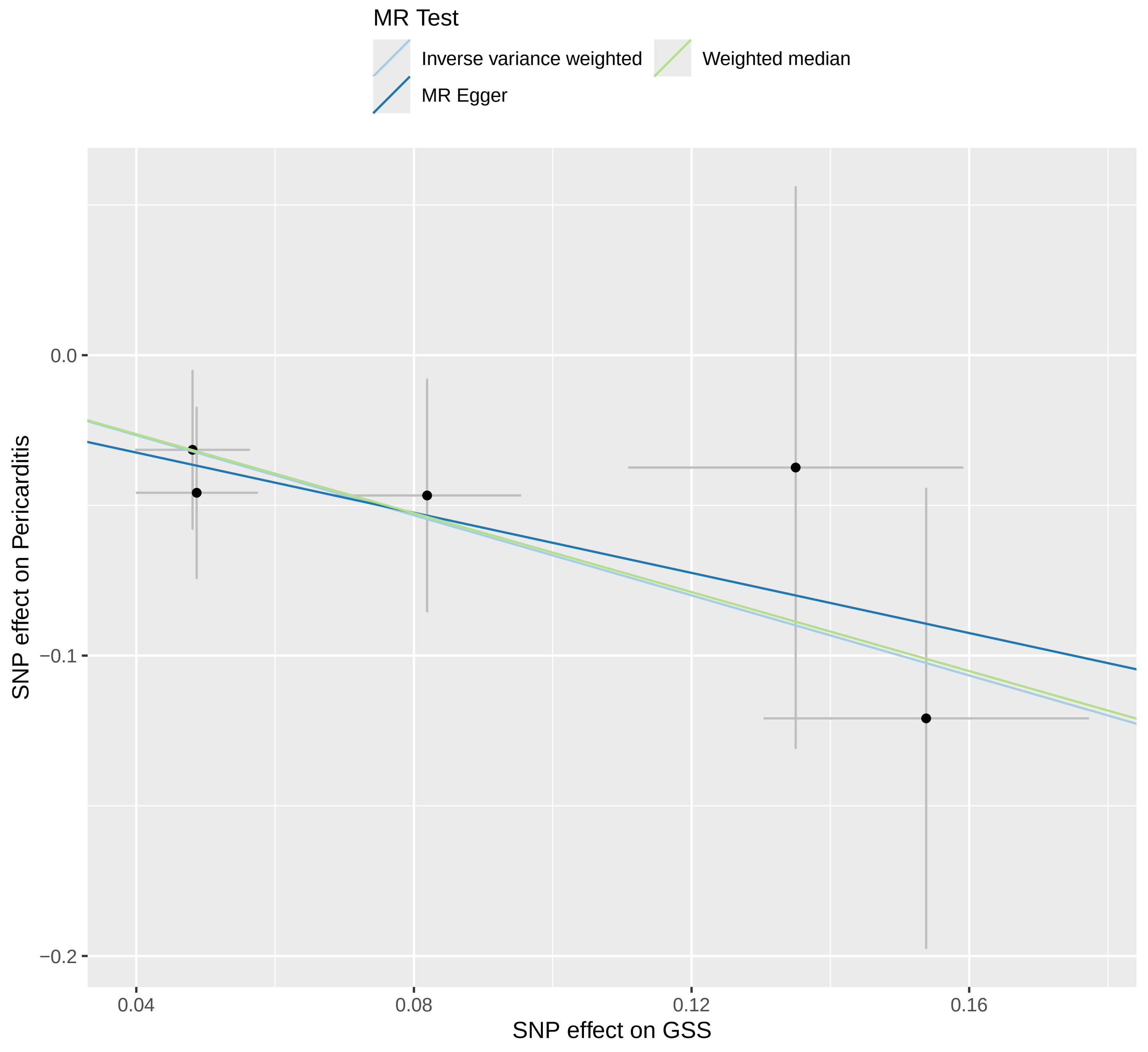

MR Test
Inverse variance weighted
Weighted median
MR Egger
0.0
−0.1
−0.2
0.04
0.08
0.12
0.16
SNP effect on Pericarditis
SNP effect on GSS

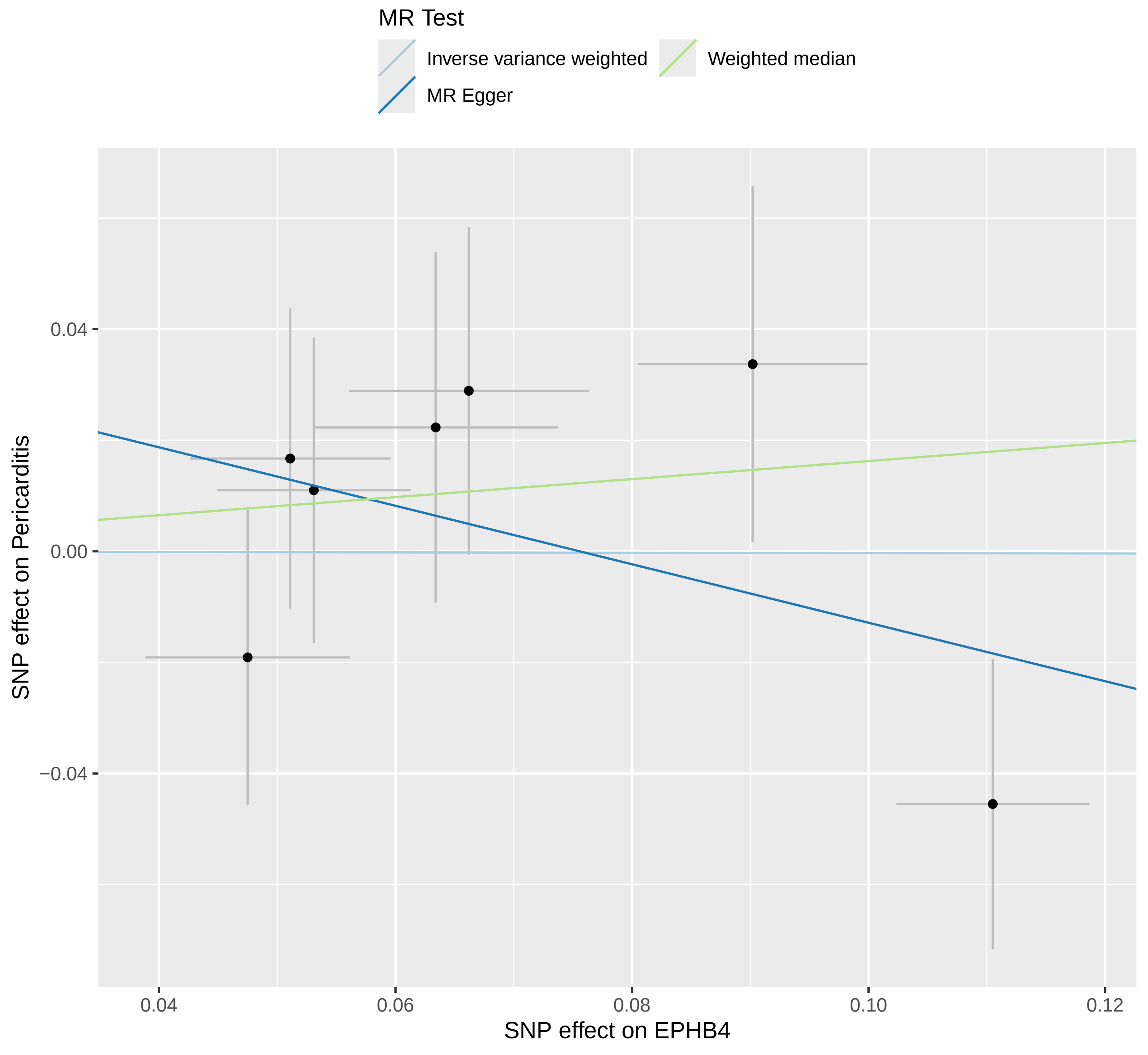
MR Test
Inverse variance weighted
Weighted median
MR Egger
0.04
0.00
−0.04
SNP effect on Pericarditis
0.04
0.06
0.08
0.10
0.12
SNP effect on EPHB4

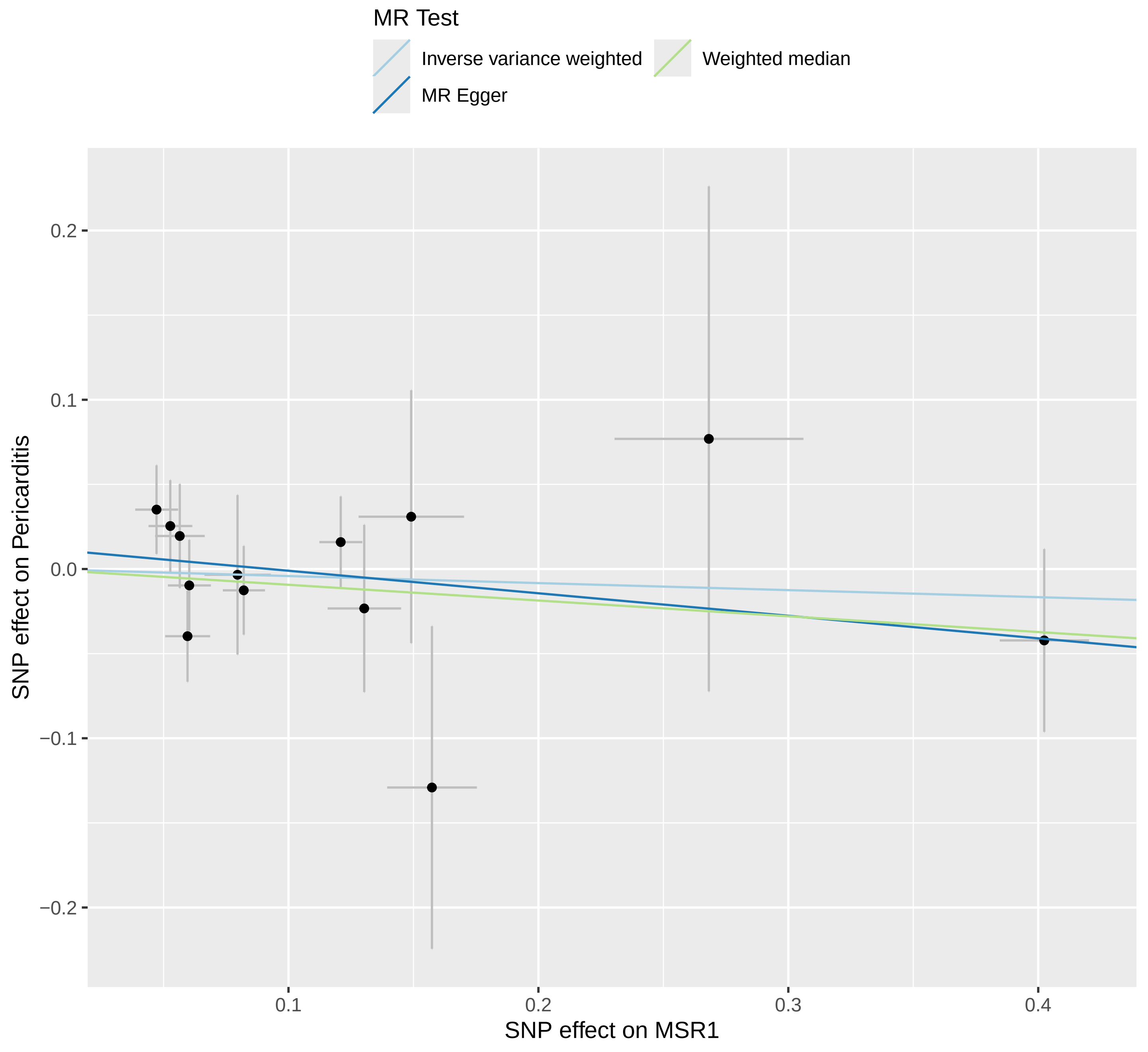
MR Test
Inverse variance weighted
Weighted median
MR Egger
SNP effect on Pericarditis
0.2
0.1
0.0
−0.1
−0.2
0.1
0.2
0.3
0.4
SNP effect on MSR1

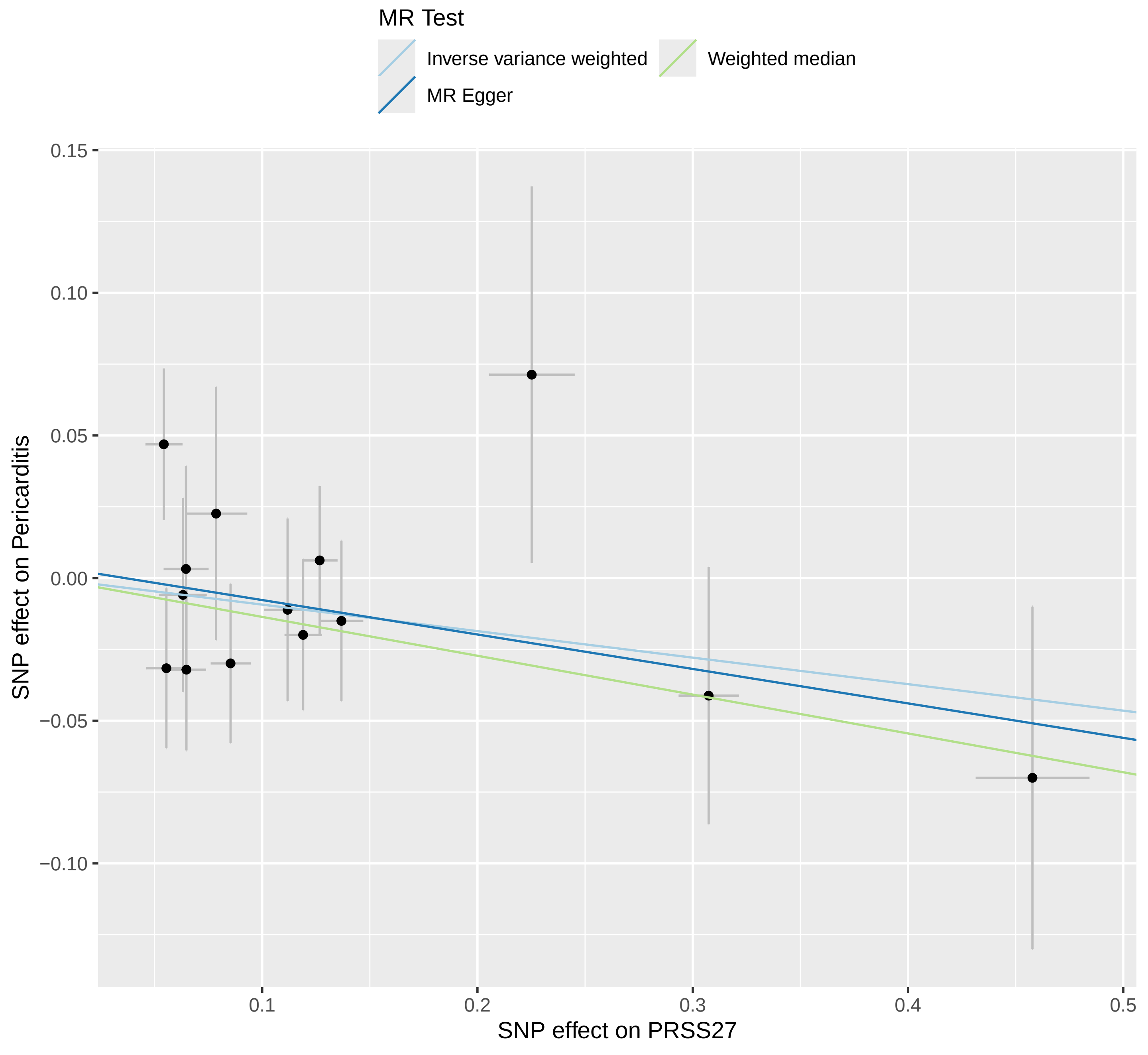

MR Test
Inverse variance weighted
Weighted median
MR Egger
0.15
0.10
0.05
0.00
−0.05
−0.10
SNP effect on Pericarditis
0.1
0.2
0.3
0.4
0.5
SNP effect on PRSS27

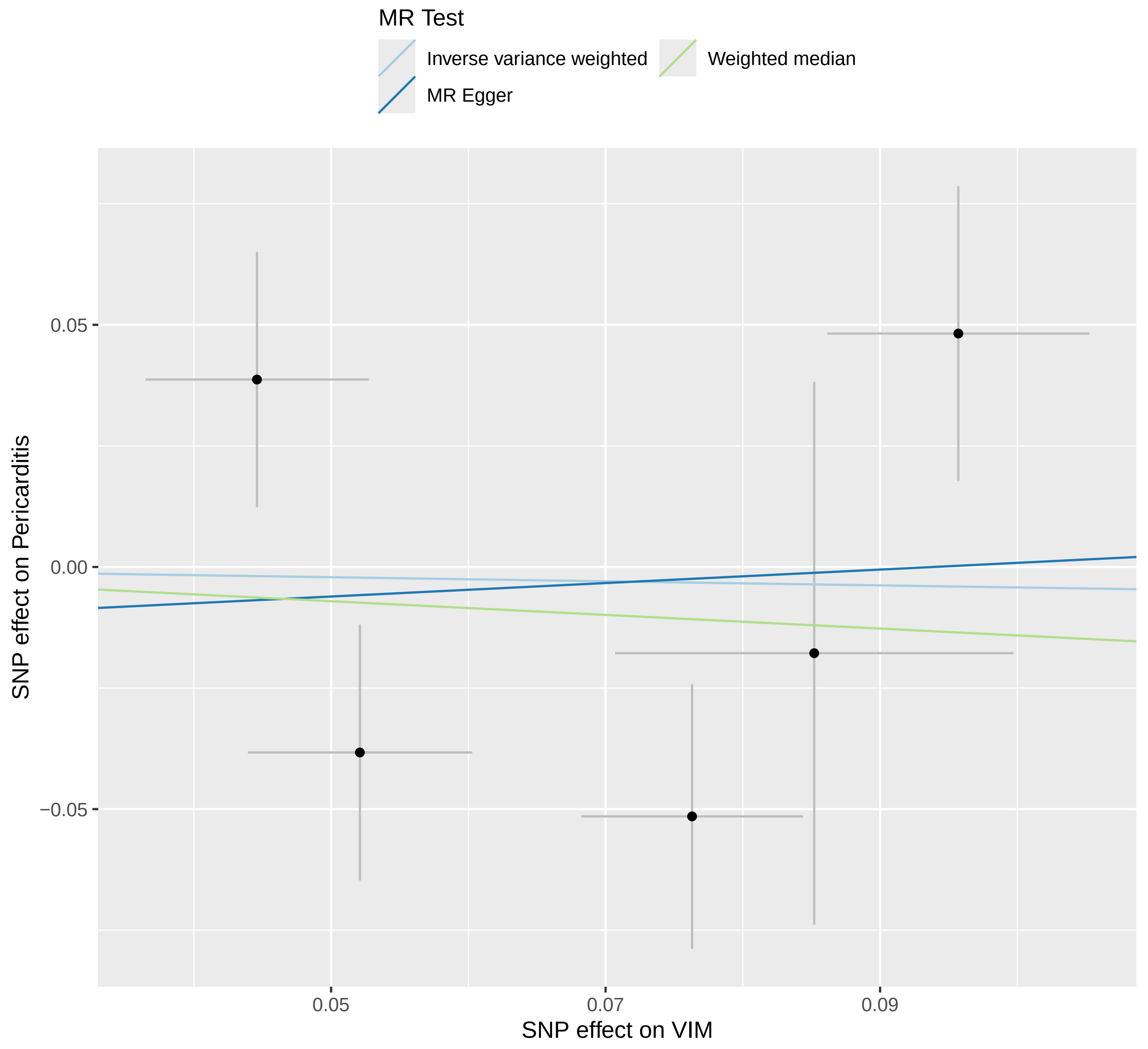

MR Test
Inverse variance weighted
MR Egger
Weighted median
0.05
0.00
−0.05
SNP effect on Pericarditis
0.05
0.07
0.09
SNP effect on VIM

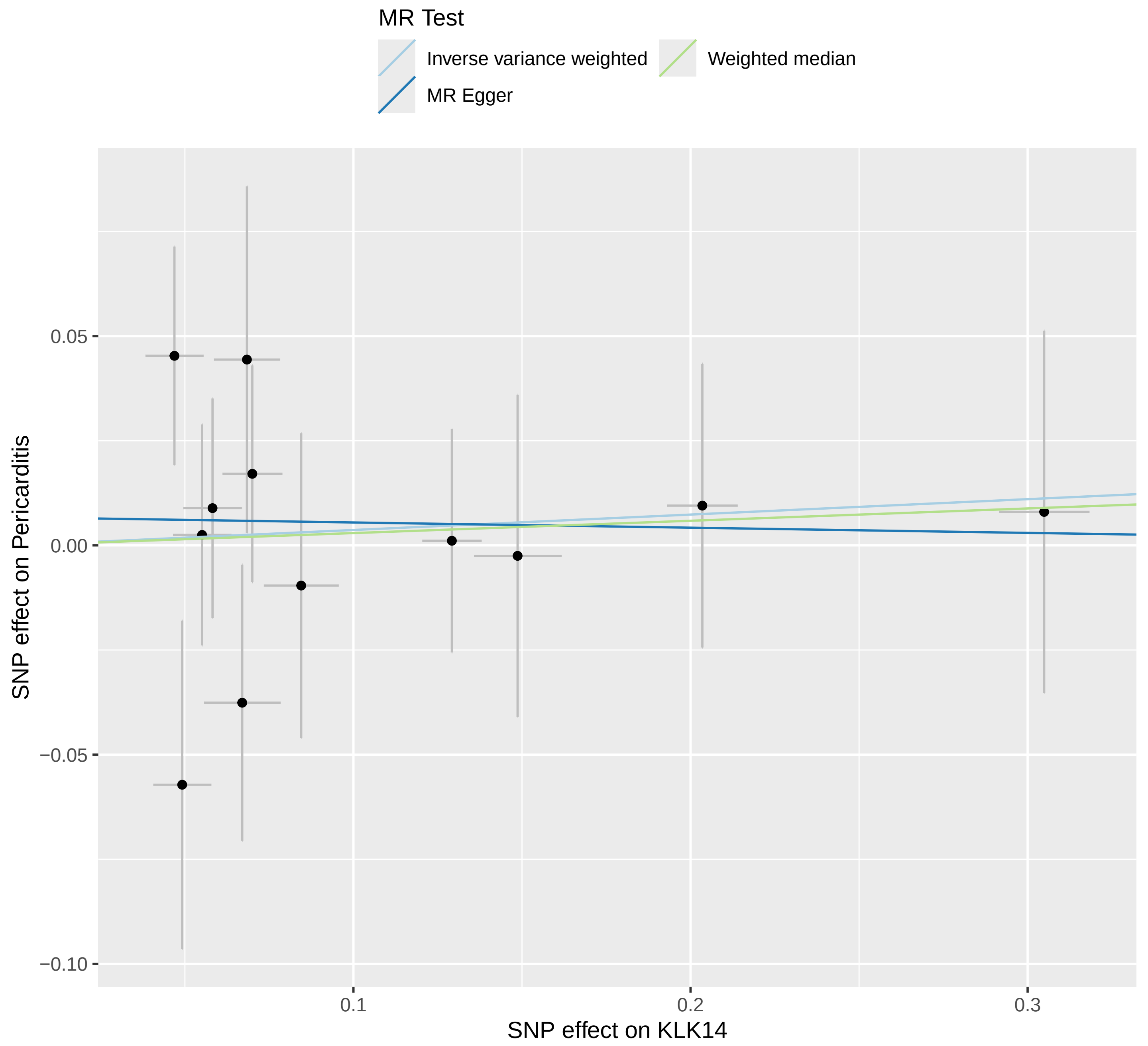

MR Test
Inverse variance weighted
Weighted median
MR Egger
SNP effect on Pericarditis
0.05
0.00
−0.05
−0.10
0.1
0.2
0.3
SNP effect on KLK14

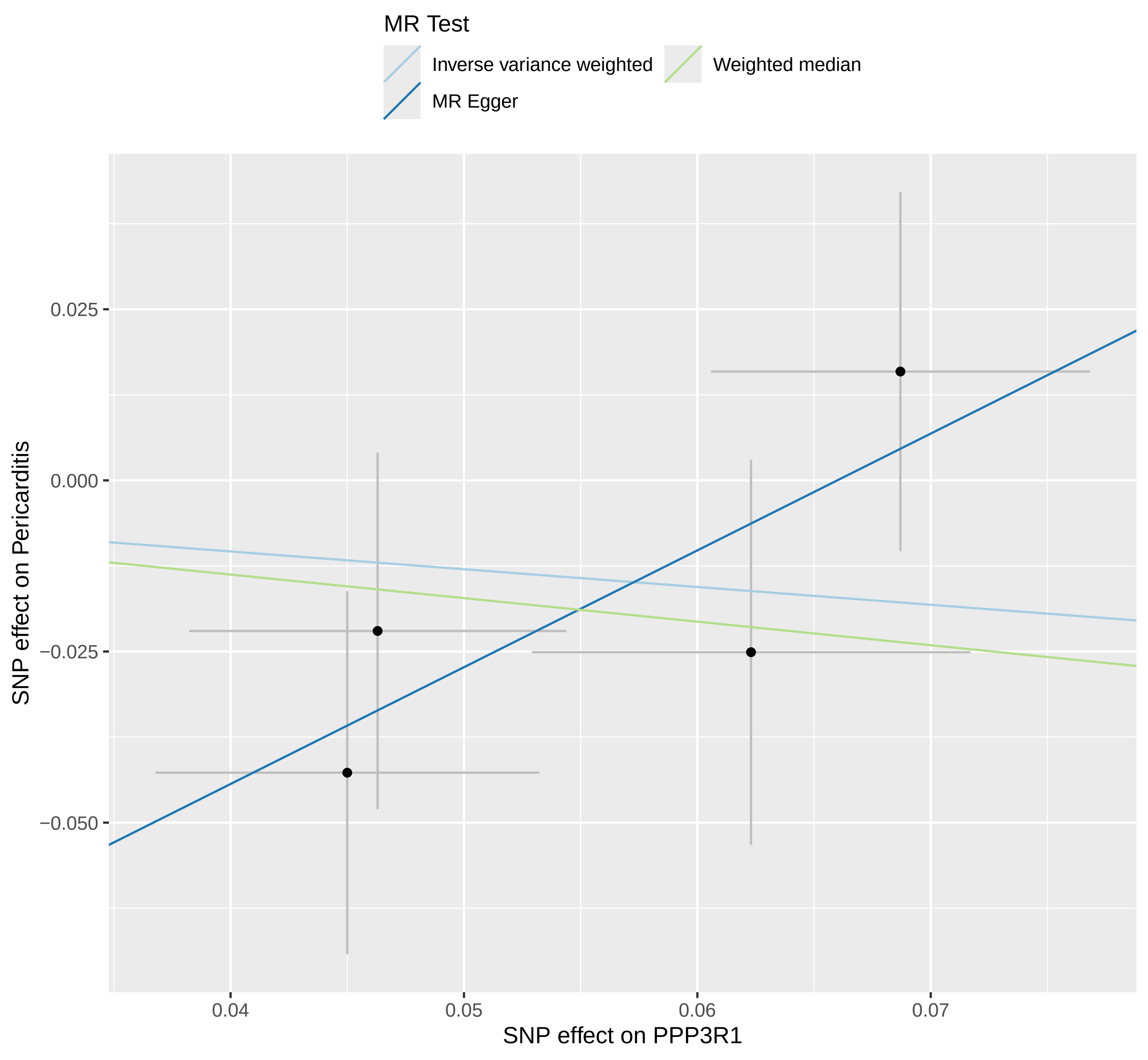

MR Test
Inverse variance weighted
Weighted median
MR Egger
SNP effect on Pericarditis
0.025
0.000
−0.025
−0.050
0.04
0.05
0.06
0.07
SNP effect on PPP3R1

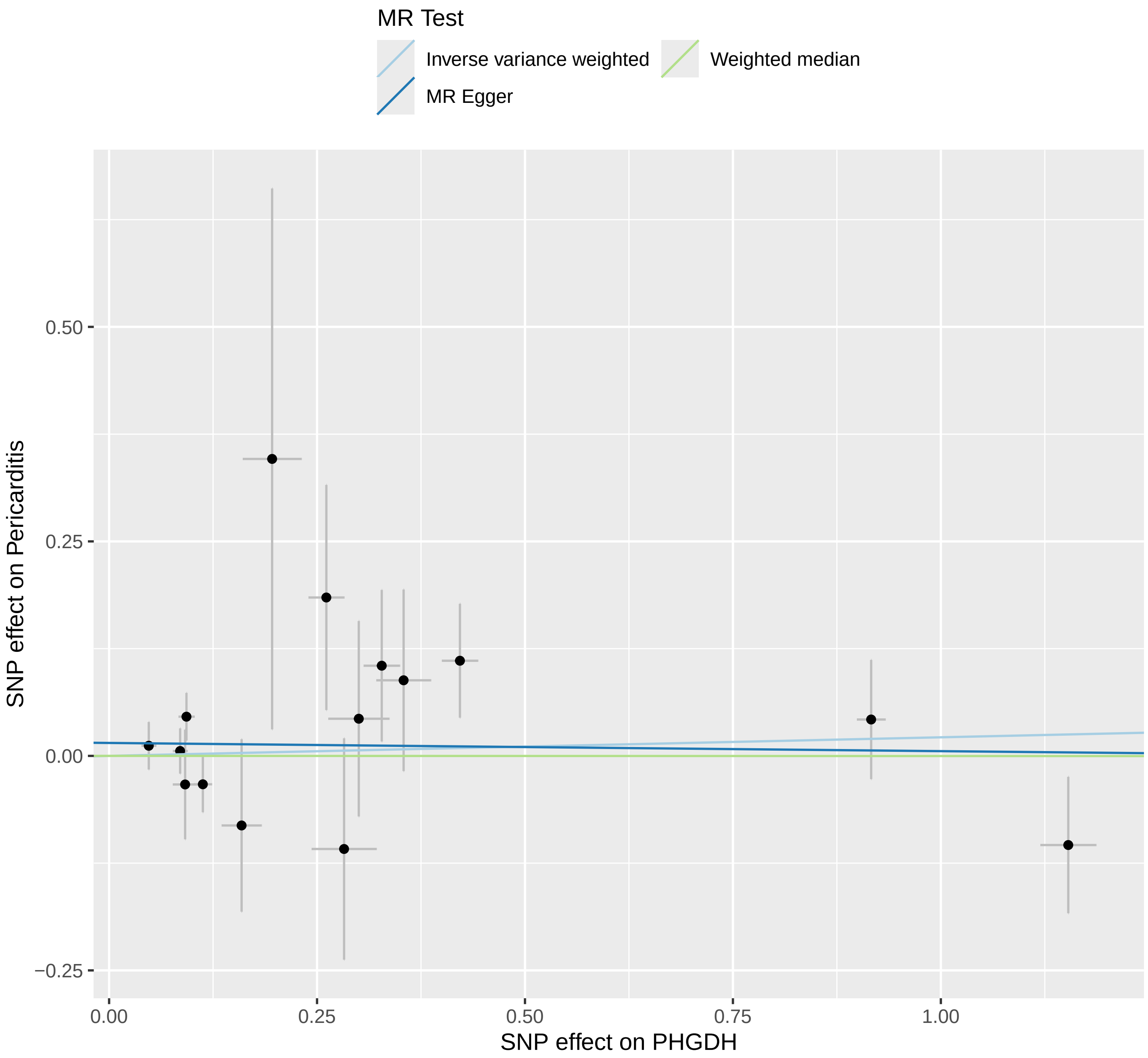
MR Test
Inverse variance weighted
Weighted median
MR Egger
SNP effect on Pericarditis
0.50
0.25
0.00
−0.25
0.00
0.25
0.50
0.75
1.00
SNP effect on PHGDH

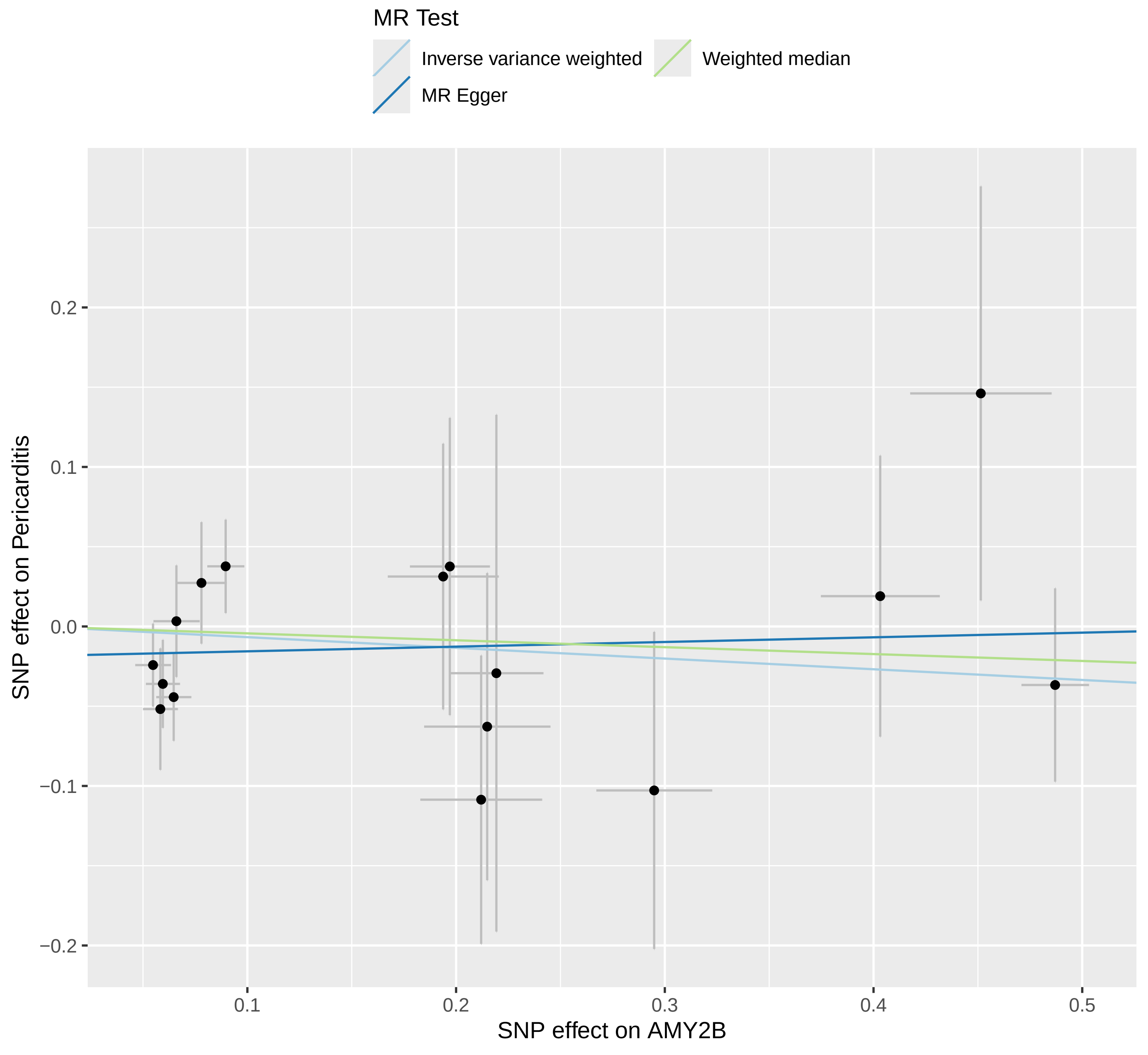
MR Test
Inverse variance weighted
Weighted median
MR Egger
SNP effect on Pericarditis
0.2
0.1
0.0
−0.1
−0.2
0.1
0.2
0.3
0.4
0.5
SNP effect on AMY2B

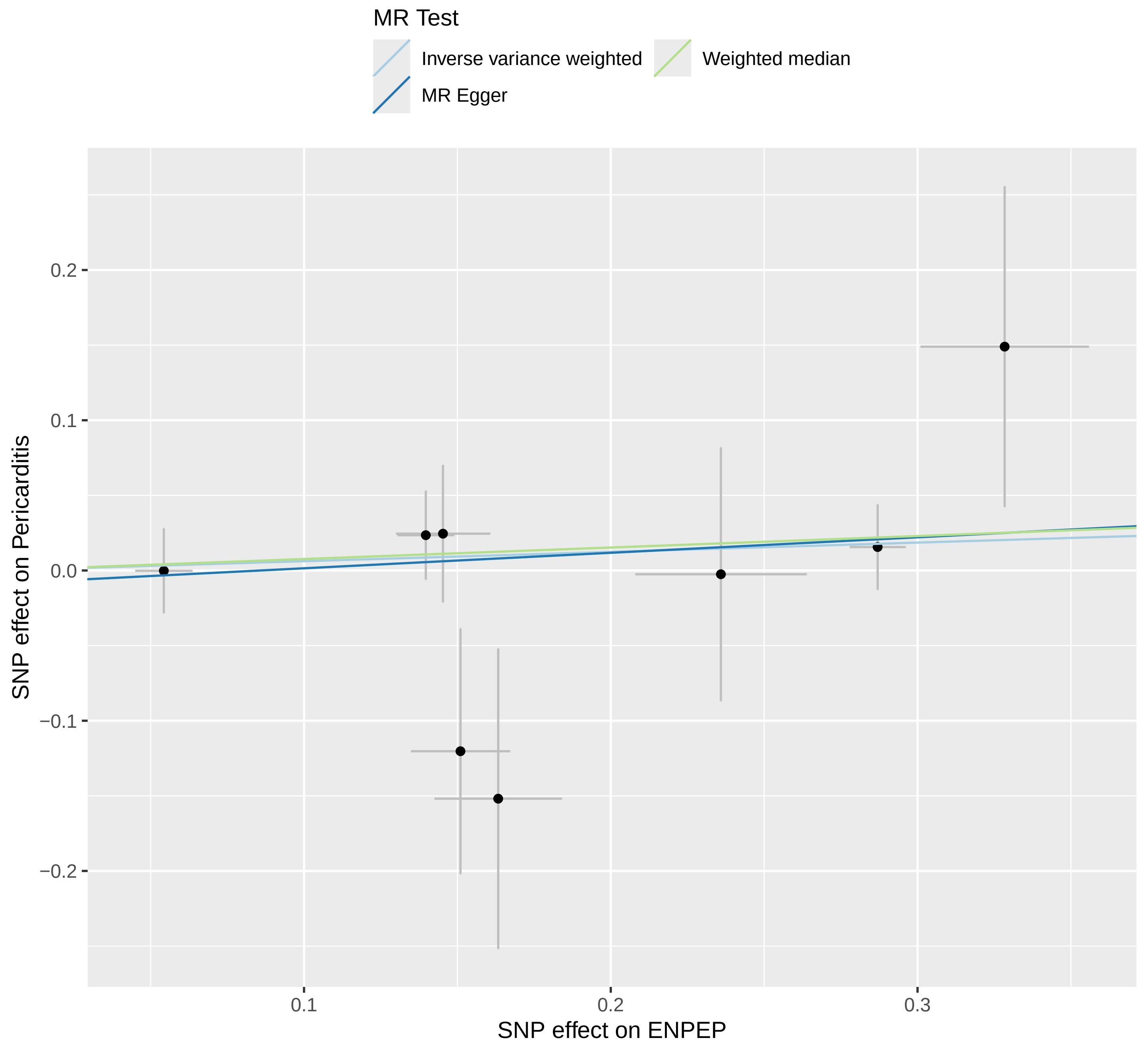
MR Test
Inverse variance weighted
Weighted median
MR Egger
SNP effect on Pericarditis
0.2
0.1
0.0
−0.1
−0.2
0.1
0.2
0.3
SNP effect on ENPEP

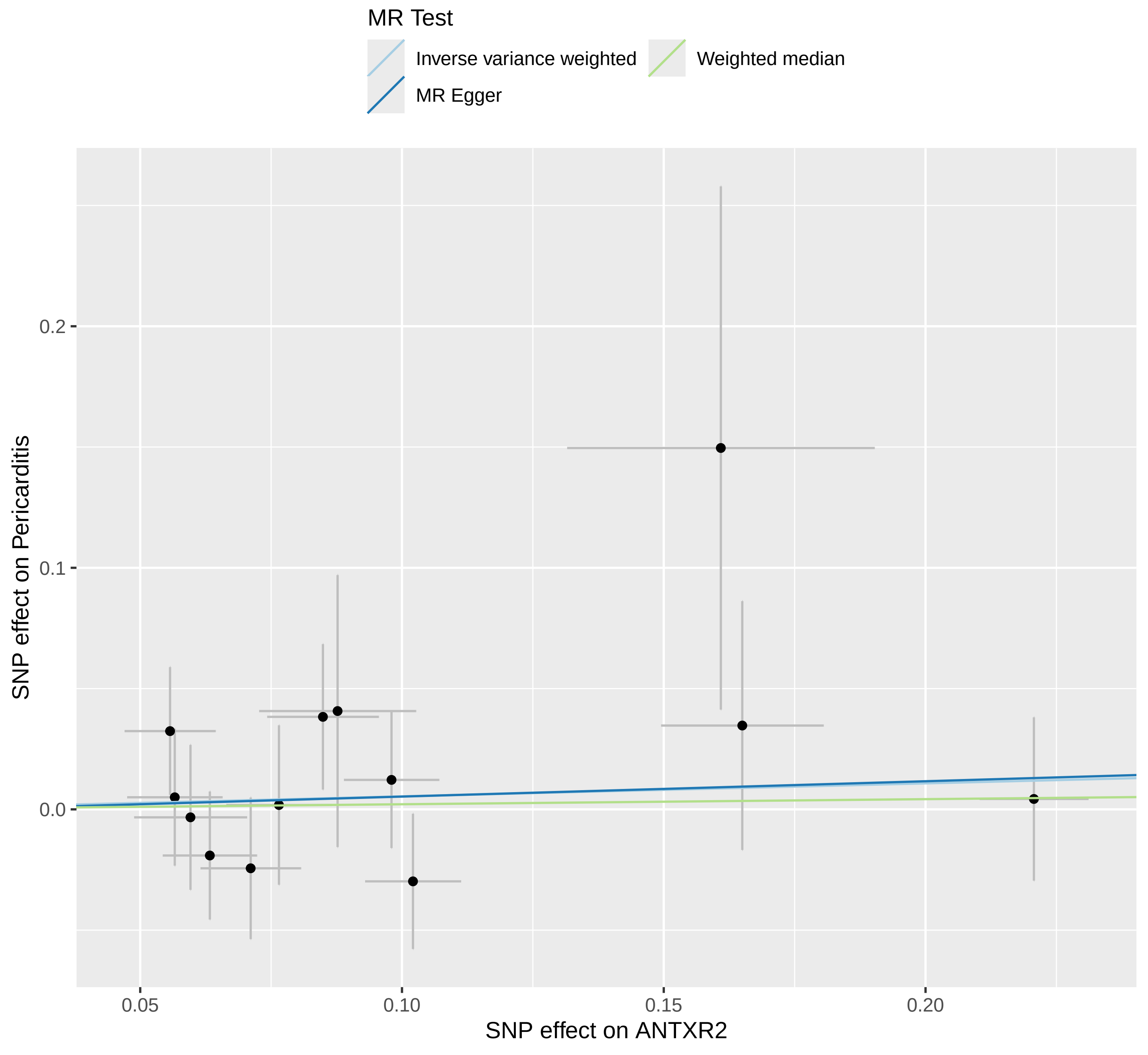
MR Test
Inverse variance weighted
Weighted median
MR Egger
SNP effect on Pericarditis
0.2
0.1
0.0
0.05
0.10
0.15
0.20
SNP effect on ANTXR2

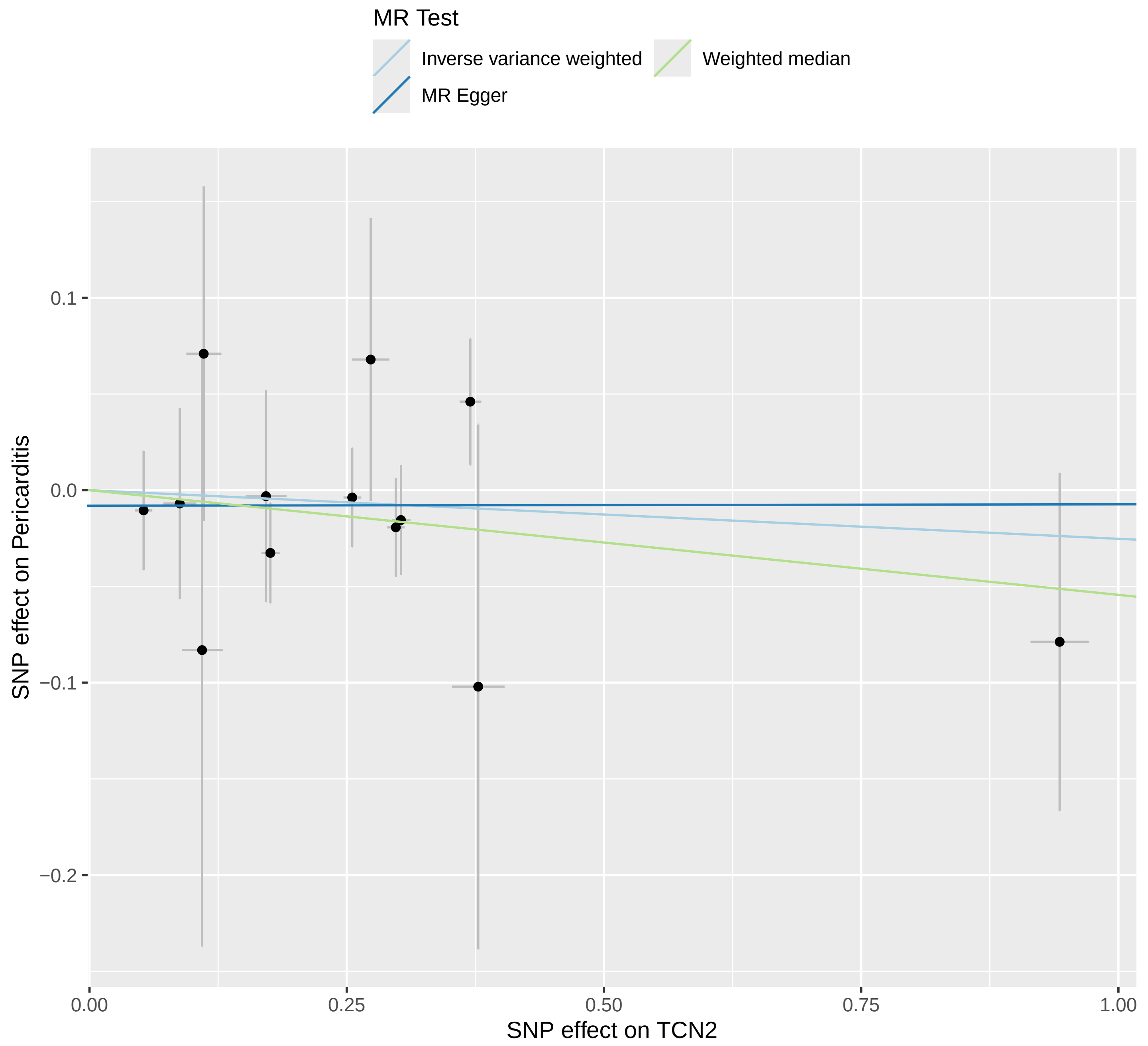
MR Test
Inverse variance weighted
Weighted median
MR Egger
SNP effect on Pericarditis
0.1
0.0
−0.1
−0.2
0.00
0.25
0.50
0.75
1.00
SNP effect on TCN2

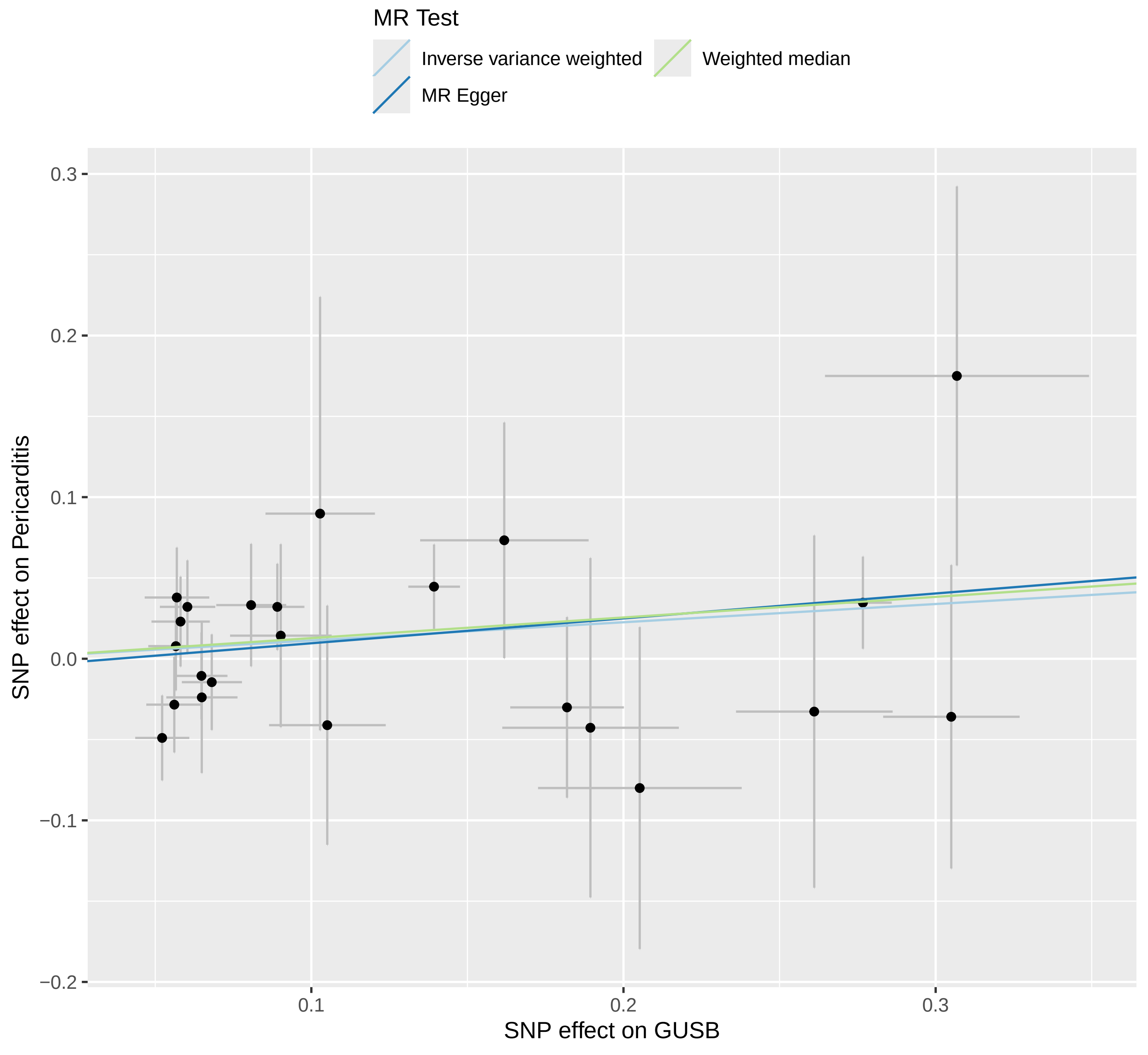
MR Test
Inverse variance weighted
Weighted median
MR Egger
0.3
0.2
0.1
0.0
−0.1
−0.2
SNP effect on Pericarditis
0.1
0.2
0.3
SNP effect on GUSB

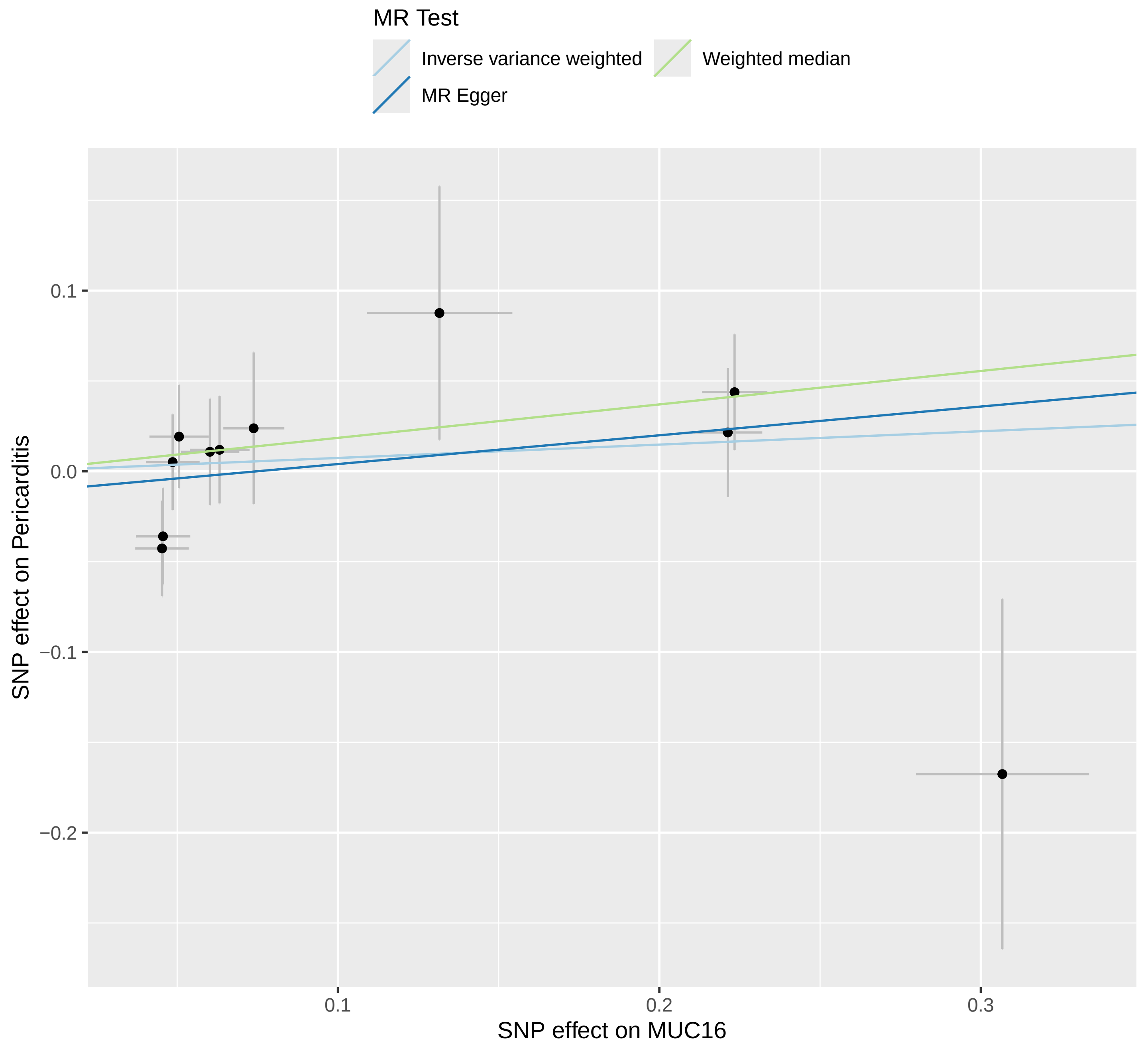
MR Test
Inverse variance weighted
Weighted median
MR Egger
0.1
0.0
−0.1
−0.2
0.1
0.2
0.3
SNP effect on Pericarditis
SNP effect on MUC16

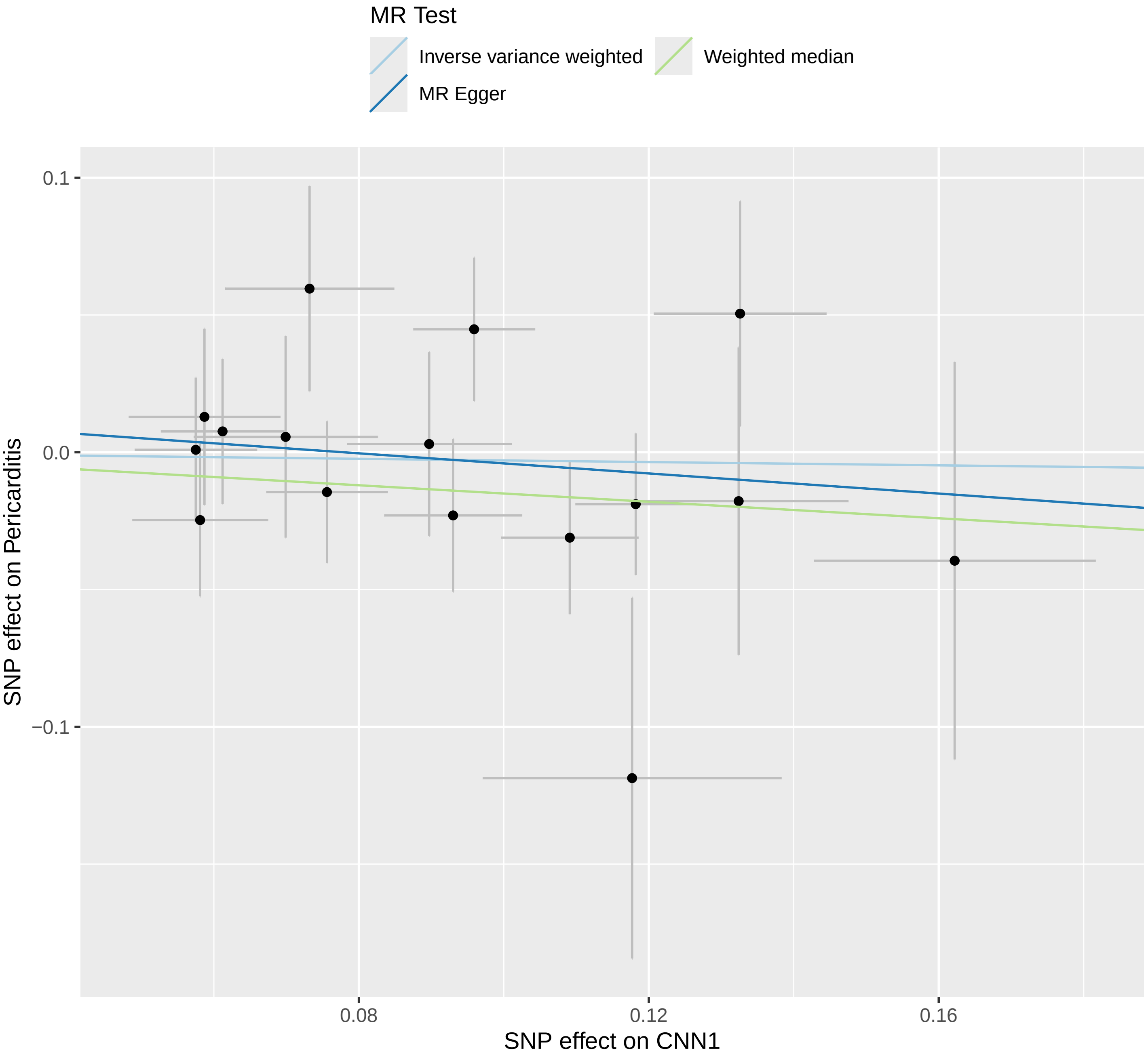
MR Test
Inverse variance weighted
Weighted median
MR Egger
0.1
0.0
−0.1
0.08
0.12
0.16
SNP effect on Pericarditis
SNP effect on CNN1

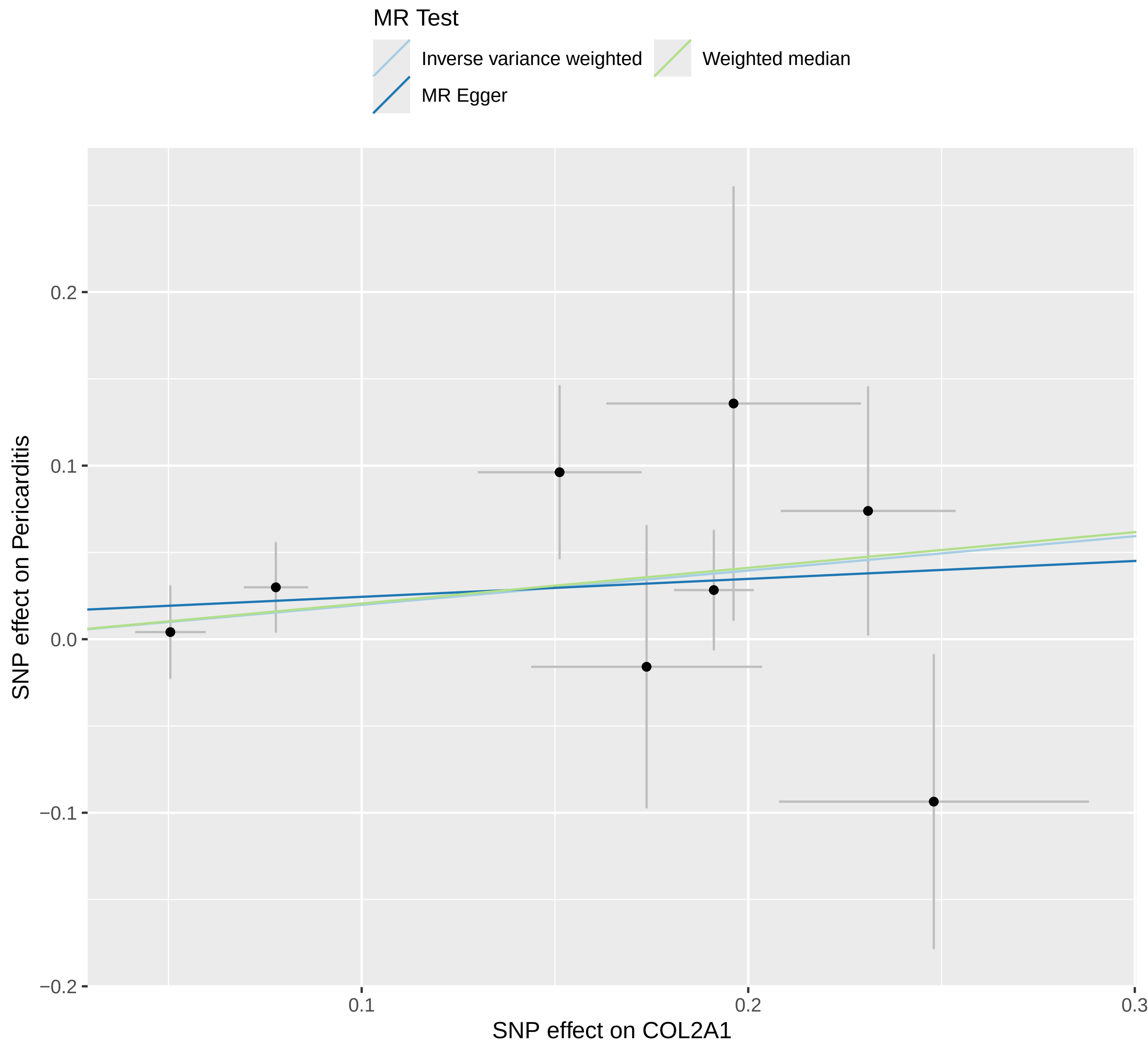
MR Test
Inverse variance weighted
Weighted median
MR Egger
SNP effect on Pericarditis
0.2
0.1
0.0
−0.1
−0.2
0.1
0.2
0.3
SNP effect on COL2A1

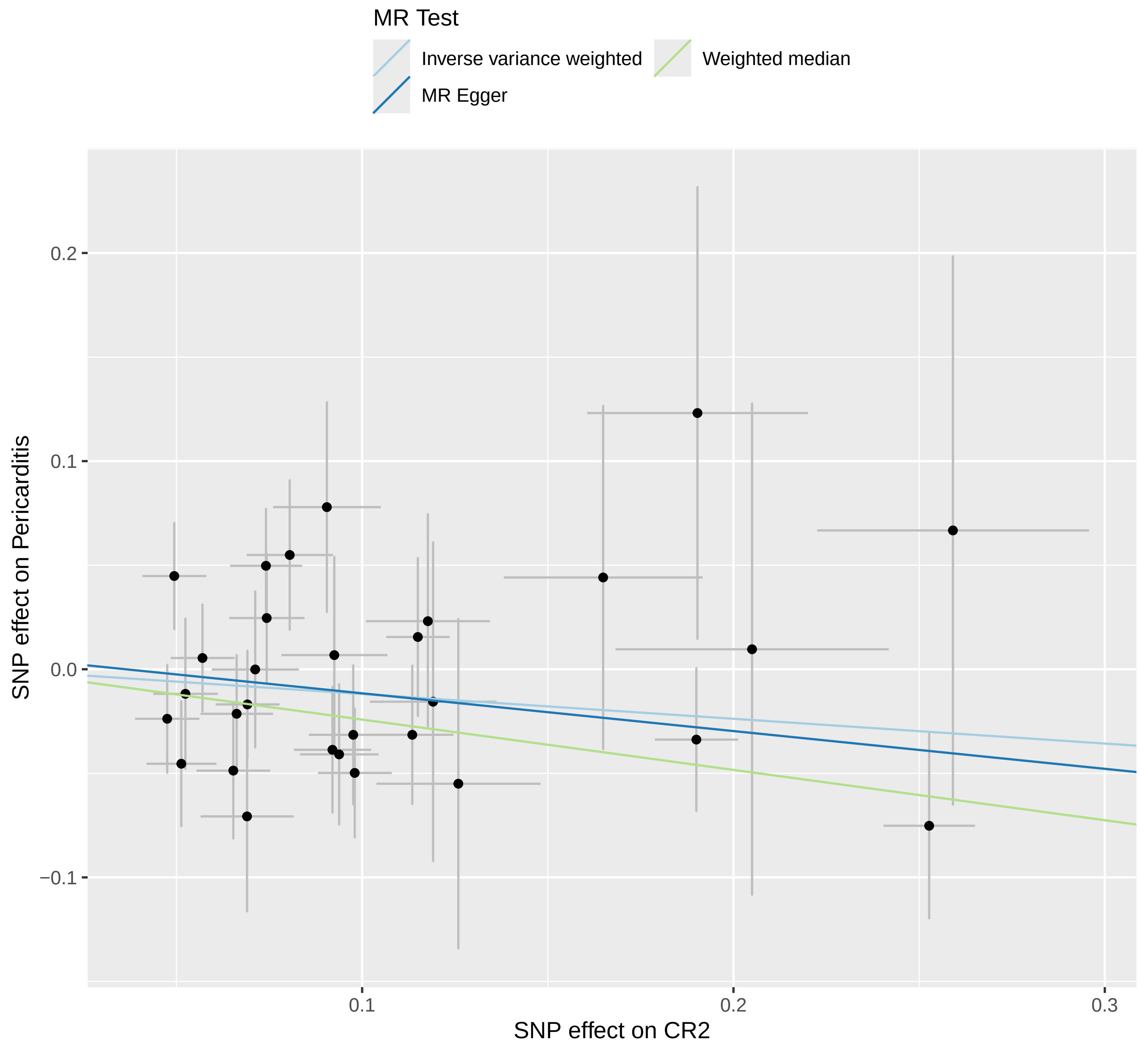

MR Test
Inverse variance weighted
MR Egger
Weighted median
SNP effect on Pericarditis
0.2
0.1
0.0
−0.1
0.1
0.2
0.3
SNP effect on CR2

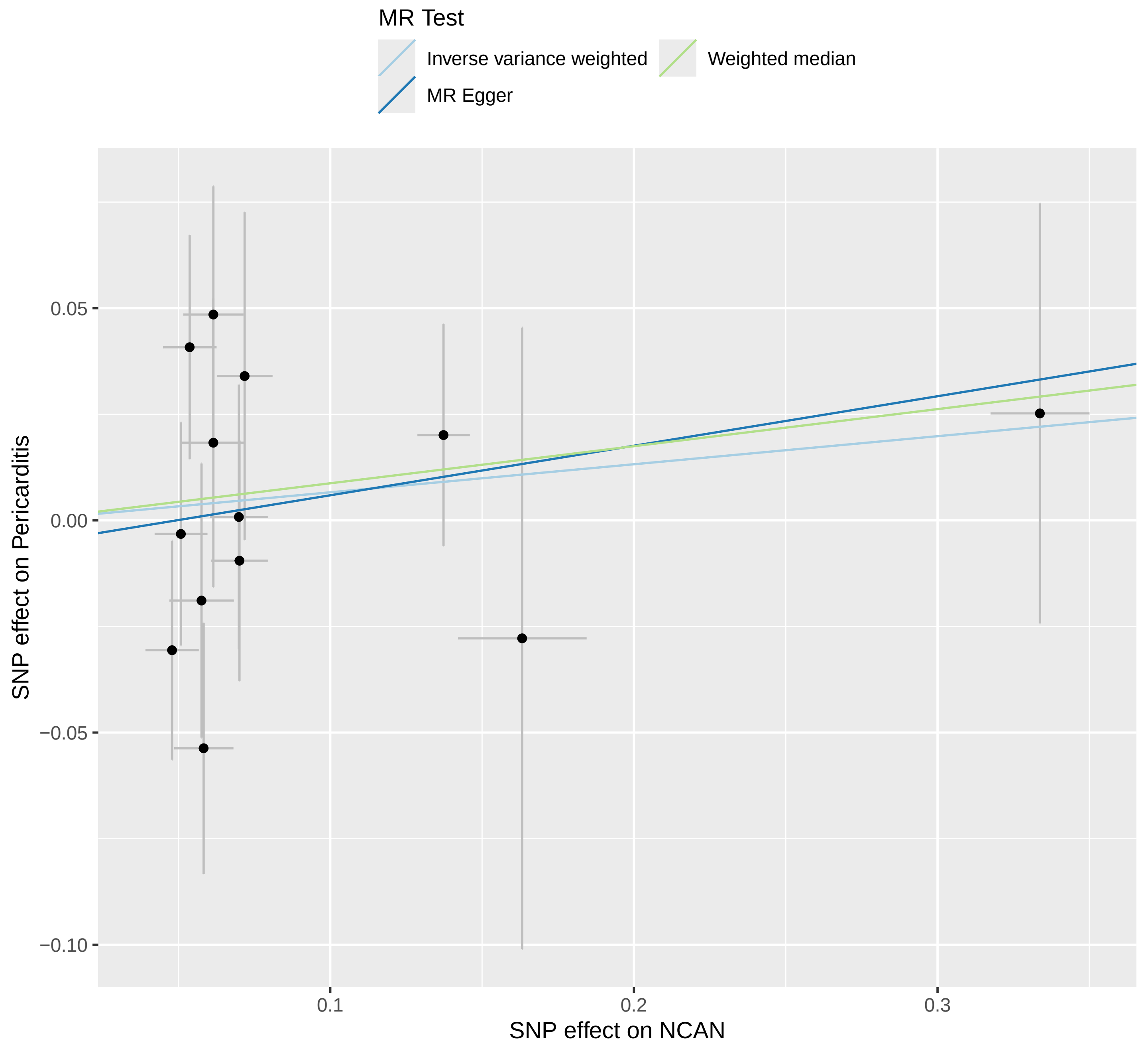
MR Test
Inverse variance weighted
MR Egger
Weighted median
SNP effect on Pericarditis
0.05
0.00
−0.05
−0.10
0.1
0.2
0.3
SNP effect on NCAN

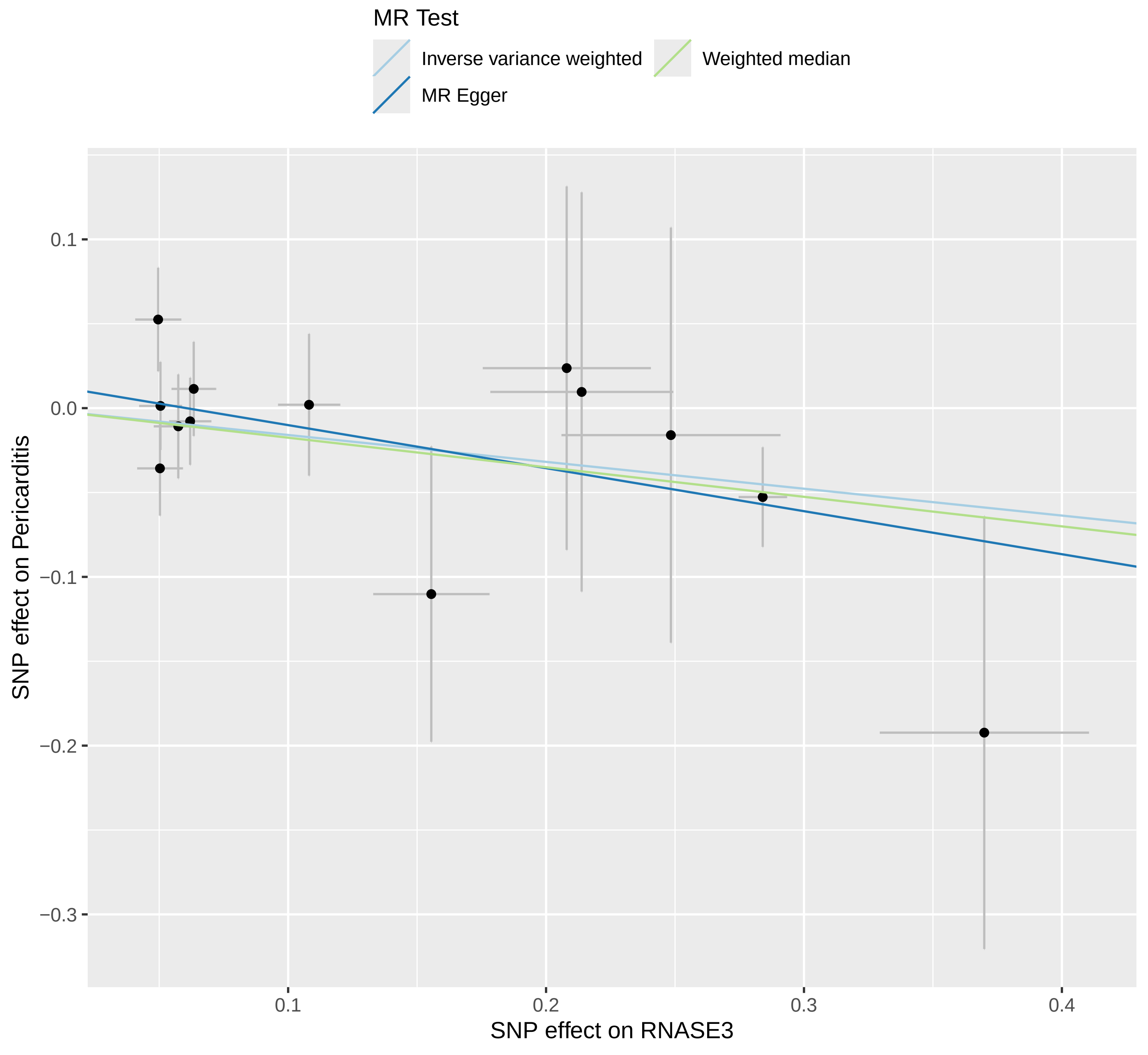
MR Test
Inverse variance weighted
Weighted median
MR Egger
0.1
0.0
−0.1
−0.2
−0.3
SNP effect on Pericarditis
0.1
0.2
0.3
0.4
SNP effect on RNASE3

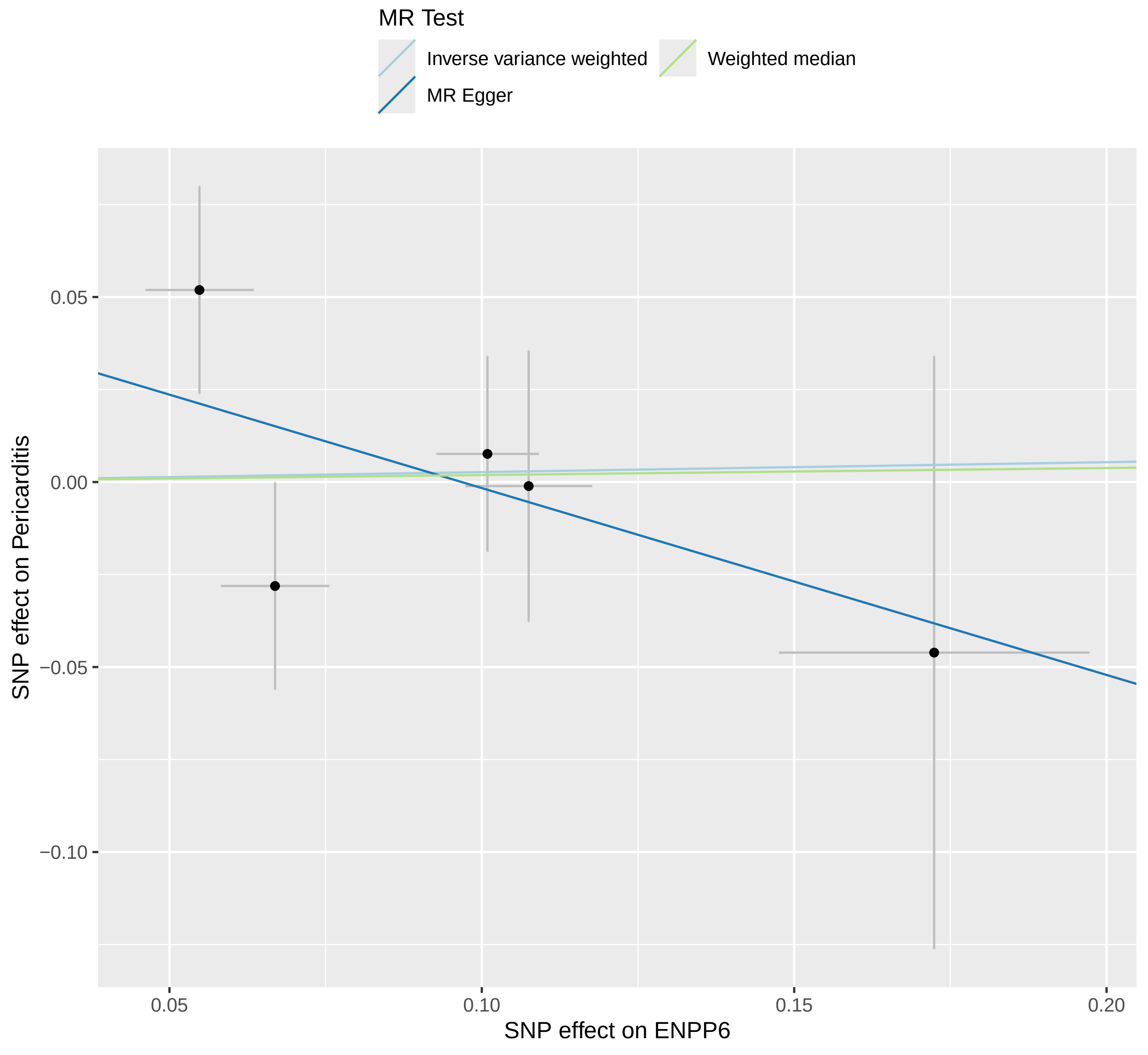
MR Test
Inverse variance weighted
Weighted median
MR Egger
0.05
0.00
−0.05
−0.10
SNP effect on Pericarditis
0.05
0.10
0.15
0.20
SNP effect on ENPP6

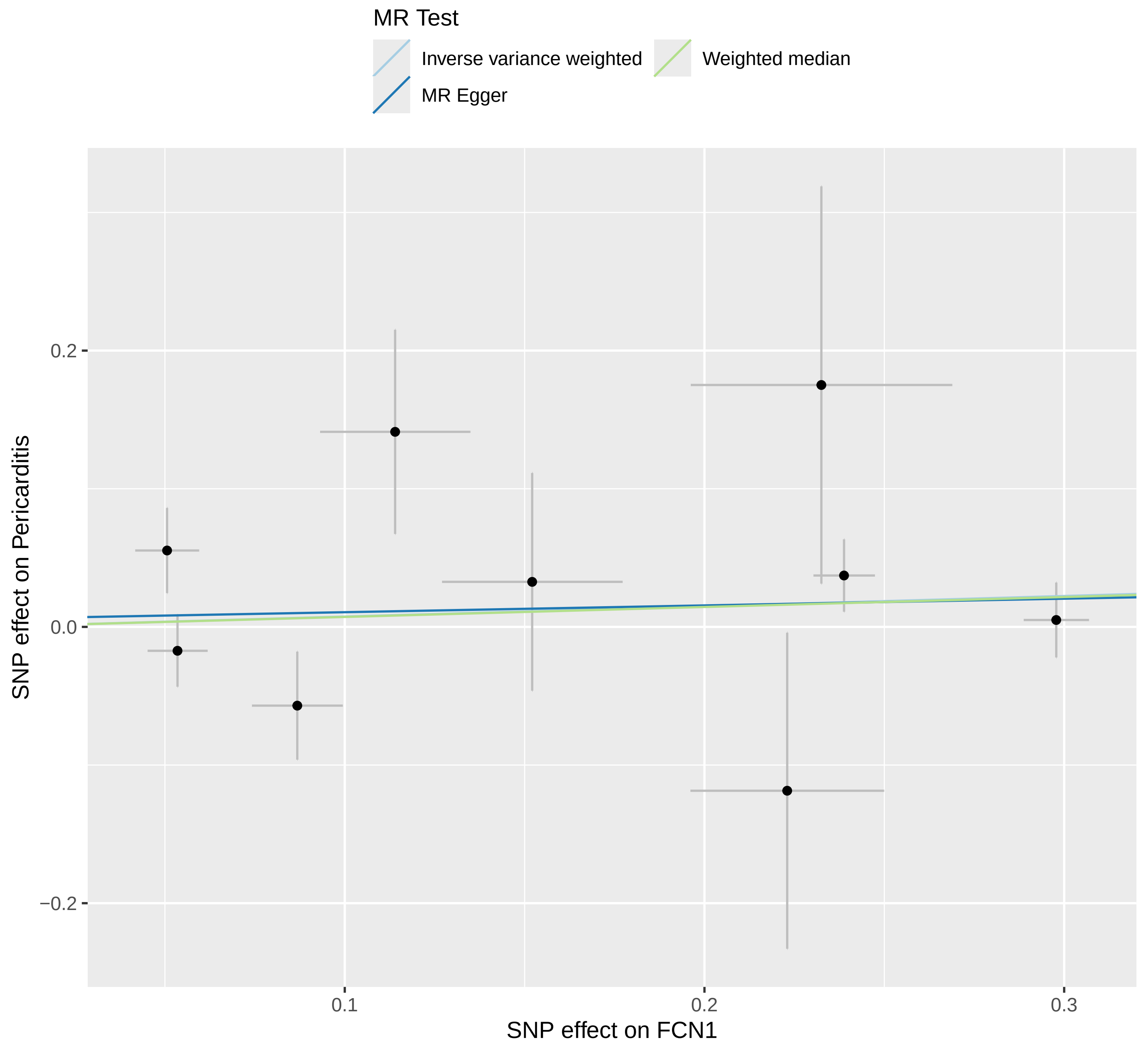

MR Test
Inverse variance weighted
Weighted median
MR Egger
0.2
0.0
−0.2
SNP effect on Pericarditis
0.1
0.2
0.3
SNP effect on FCN1

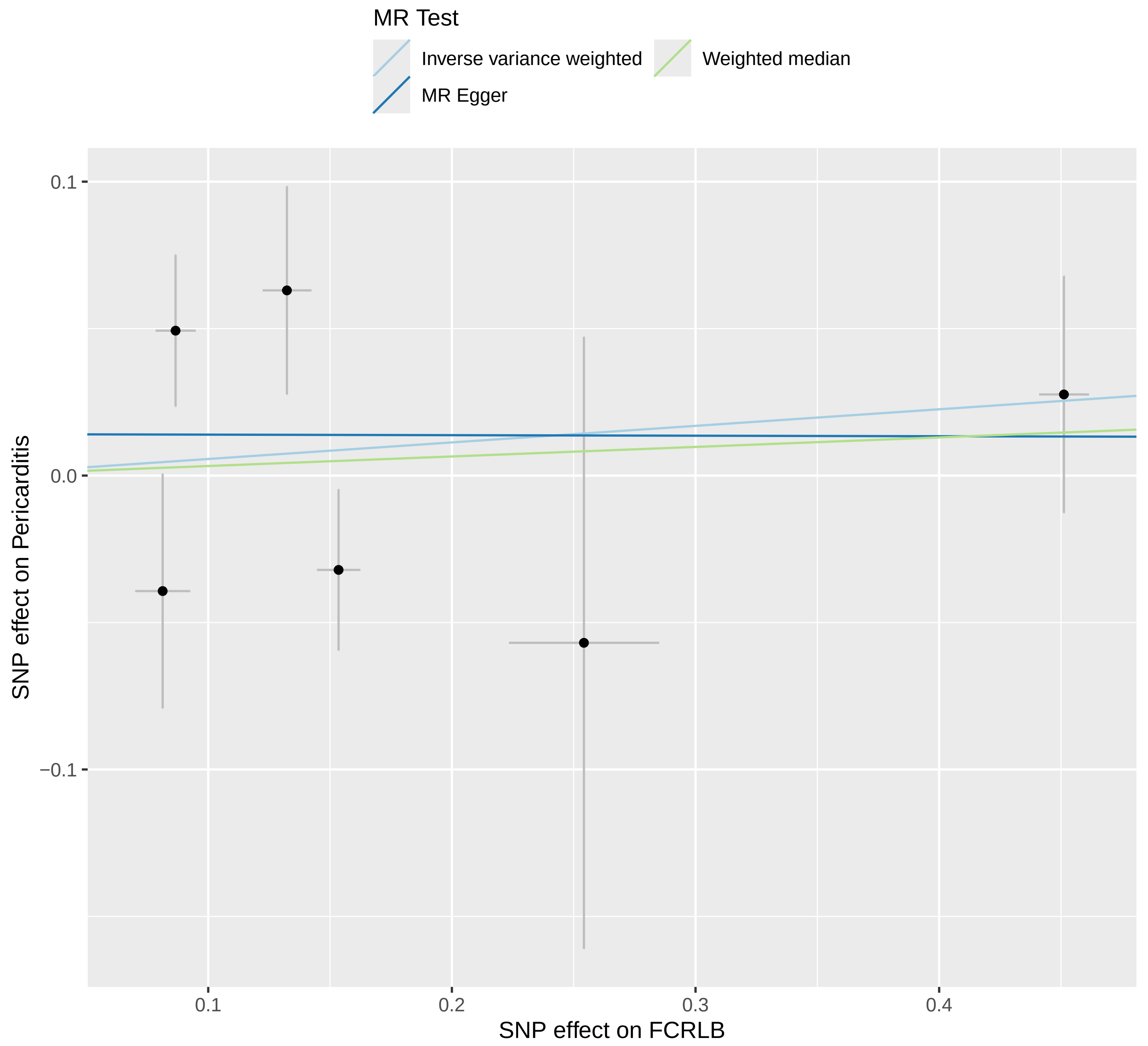

MR Test
Inverse variance weighted
Weighted median
MR Egger
0.1
0.0
−0.1
SNP effect on Pericarditis
0.1
0.2
0.3
0.4
SNP effect on FCRLB

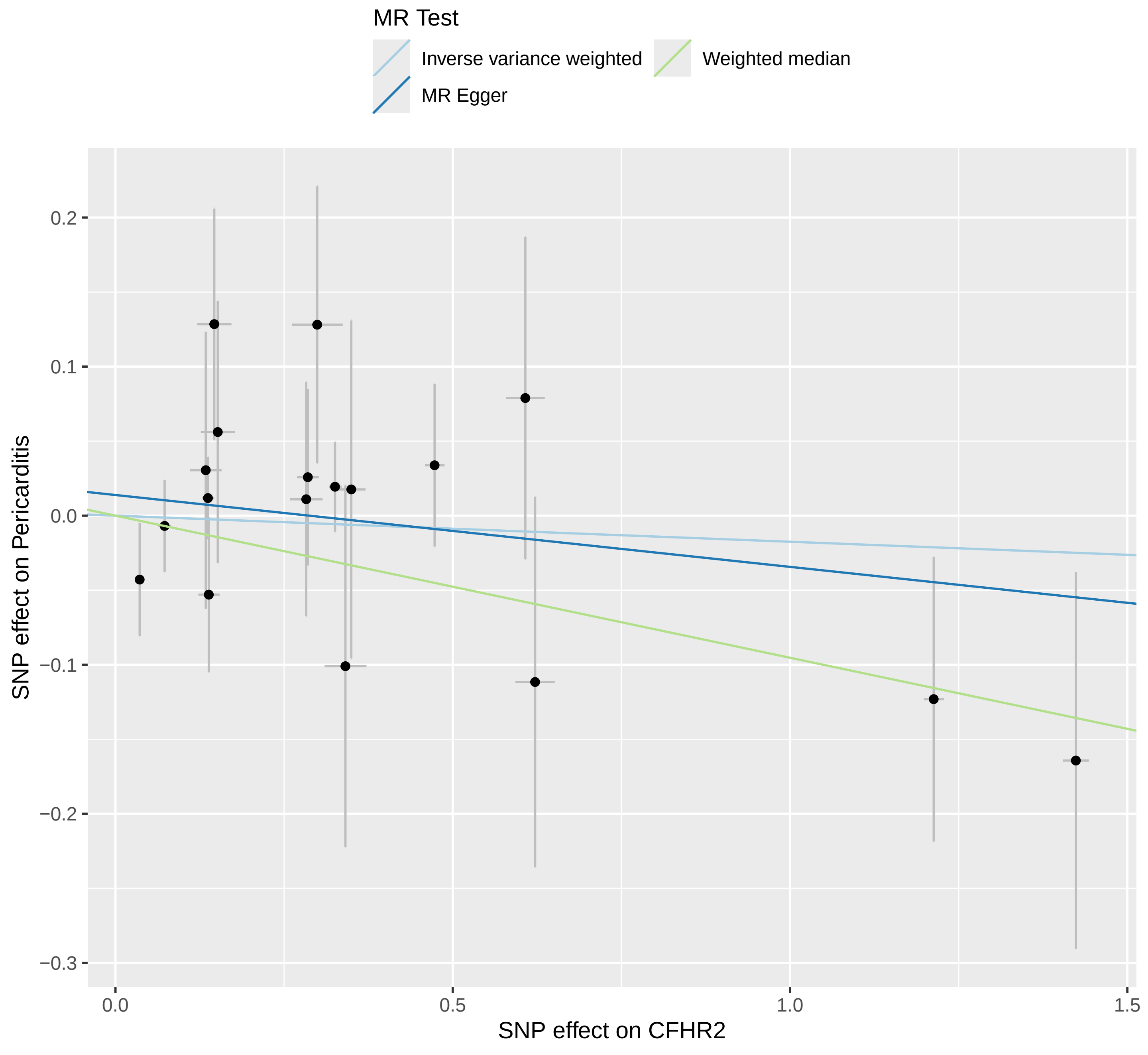

MR Test
Inverse variance weighted
Weighted median
MR Egger
SNP effect on Pericarditis
SNP effect on CFHR2
0.2
0.1
0.0
−0.1
−0.2
−0.3
0.0
0.5
1.0
1.5

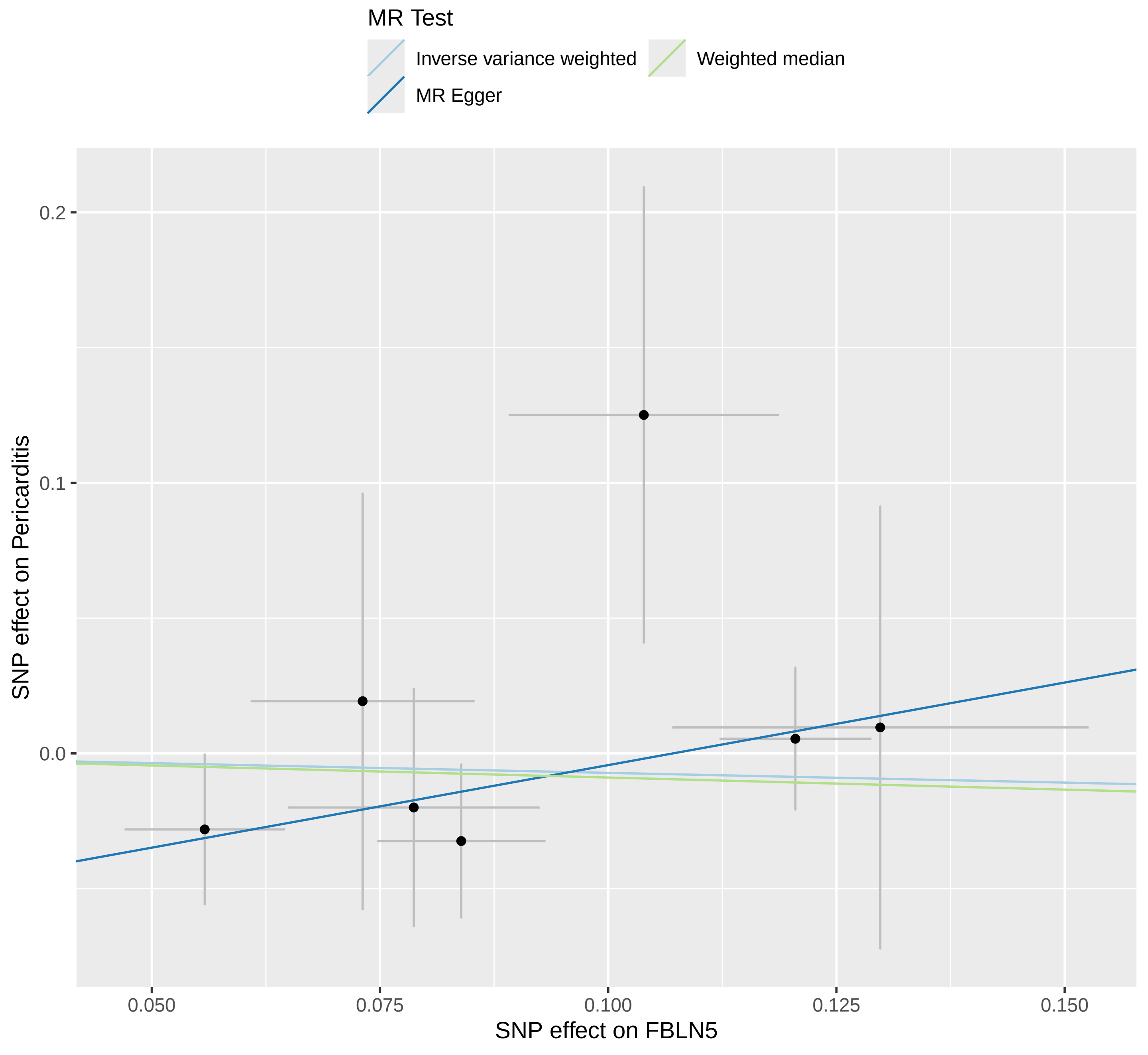

MR Test
Inverse variance weighted
Weighted median
MR Egger
0.2
0.1
0.0
SNP effect on Pericarditis
0.050
0.075
0.100
0.125
0.150
SNP effect on FBLN5

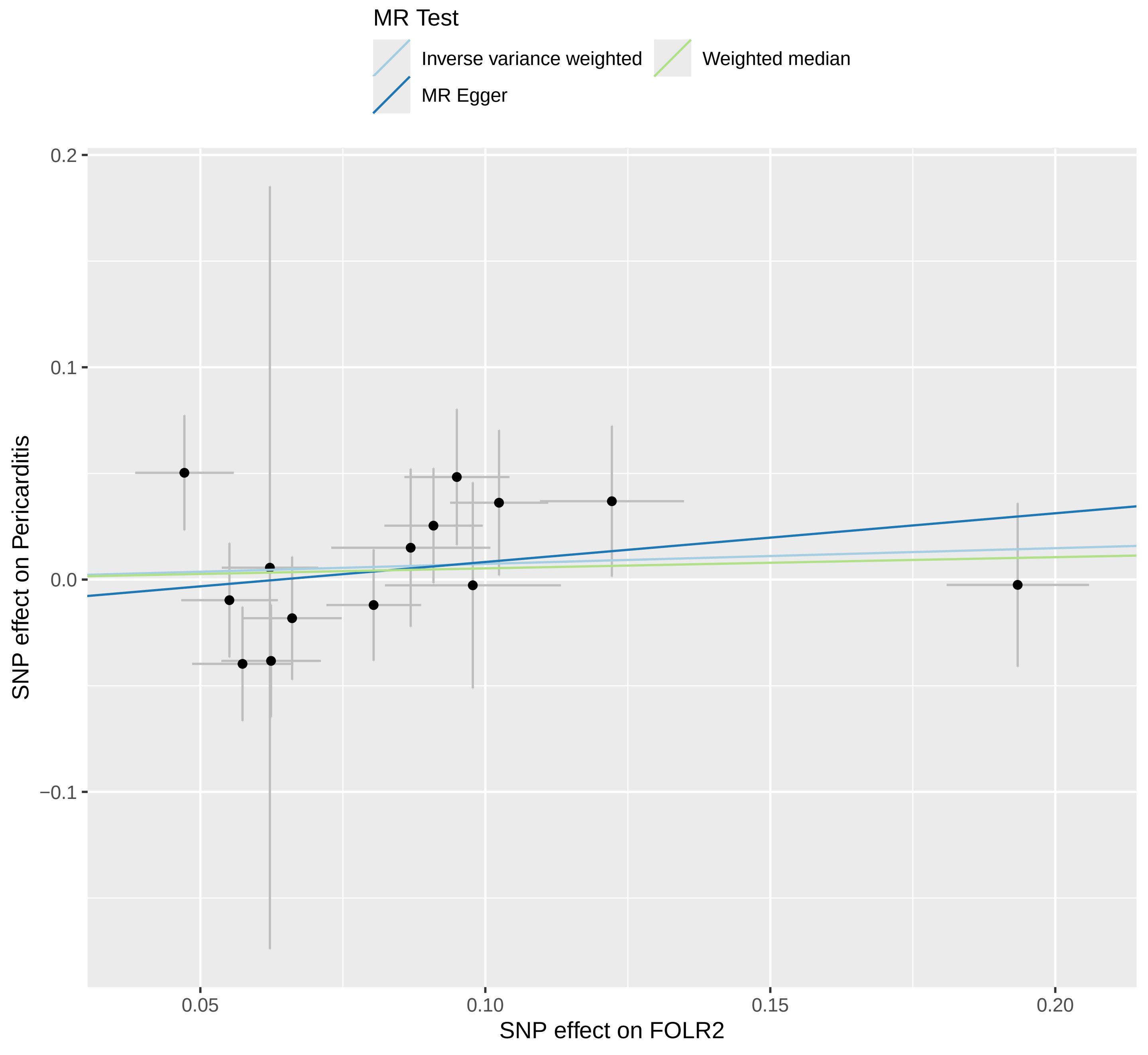
MR Test
Inverse variance weighted
MR Egger
Weighted median
0.2
0.1
0.0
−0.1
SNP effect on Pericarditis
0.05
0.10
0.15
0.20
SNP effect on FOLR2

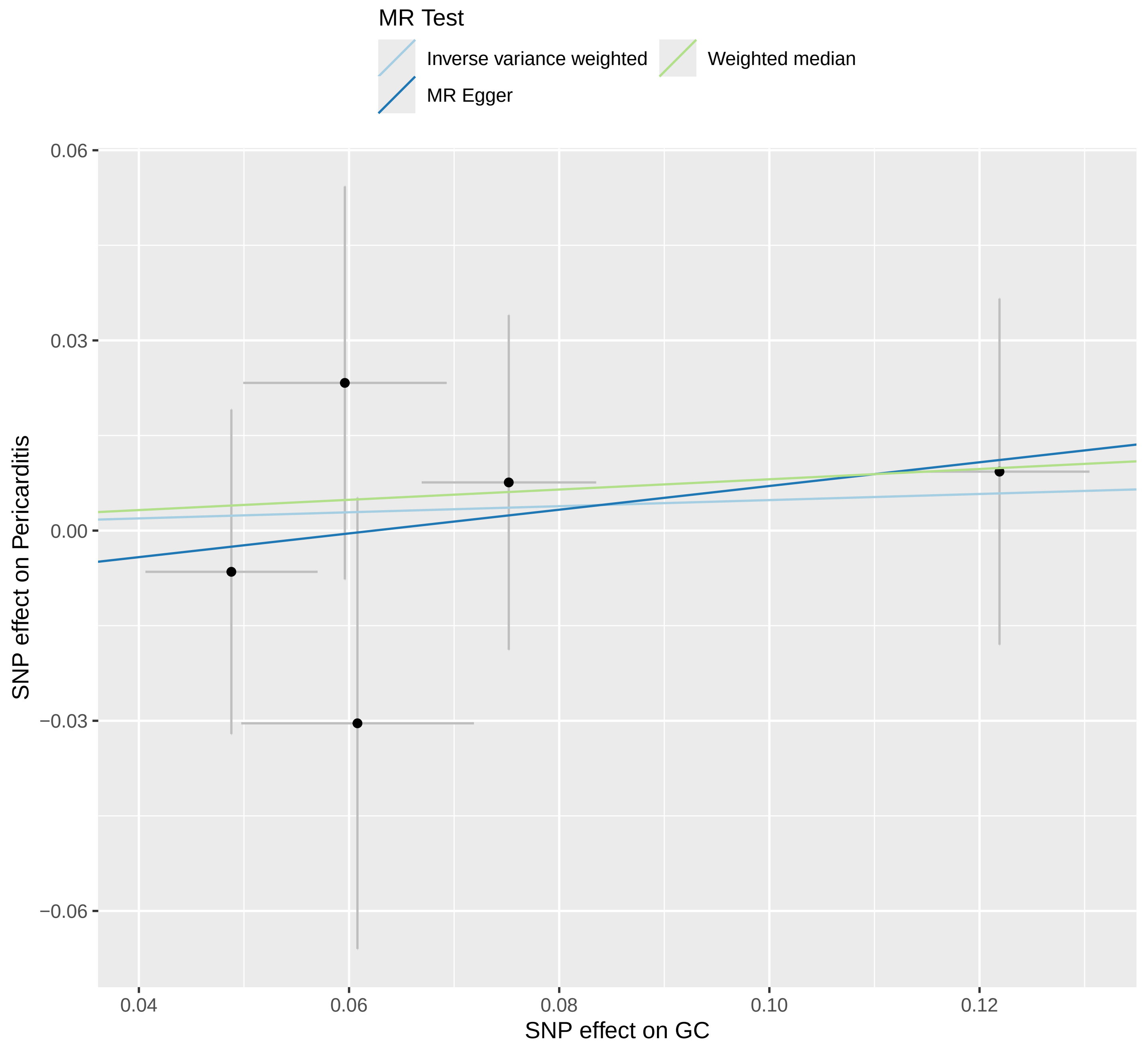
MR Test
Inverse variance weighted
Weighted median
MR Egger
0.06
0.03
0.00
−0.03
−0.06
SNP effect on Pericarditis
0.04
0.06
0.08
0.10
0.12
SNP effect on GC

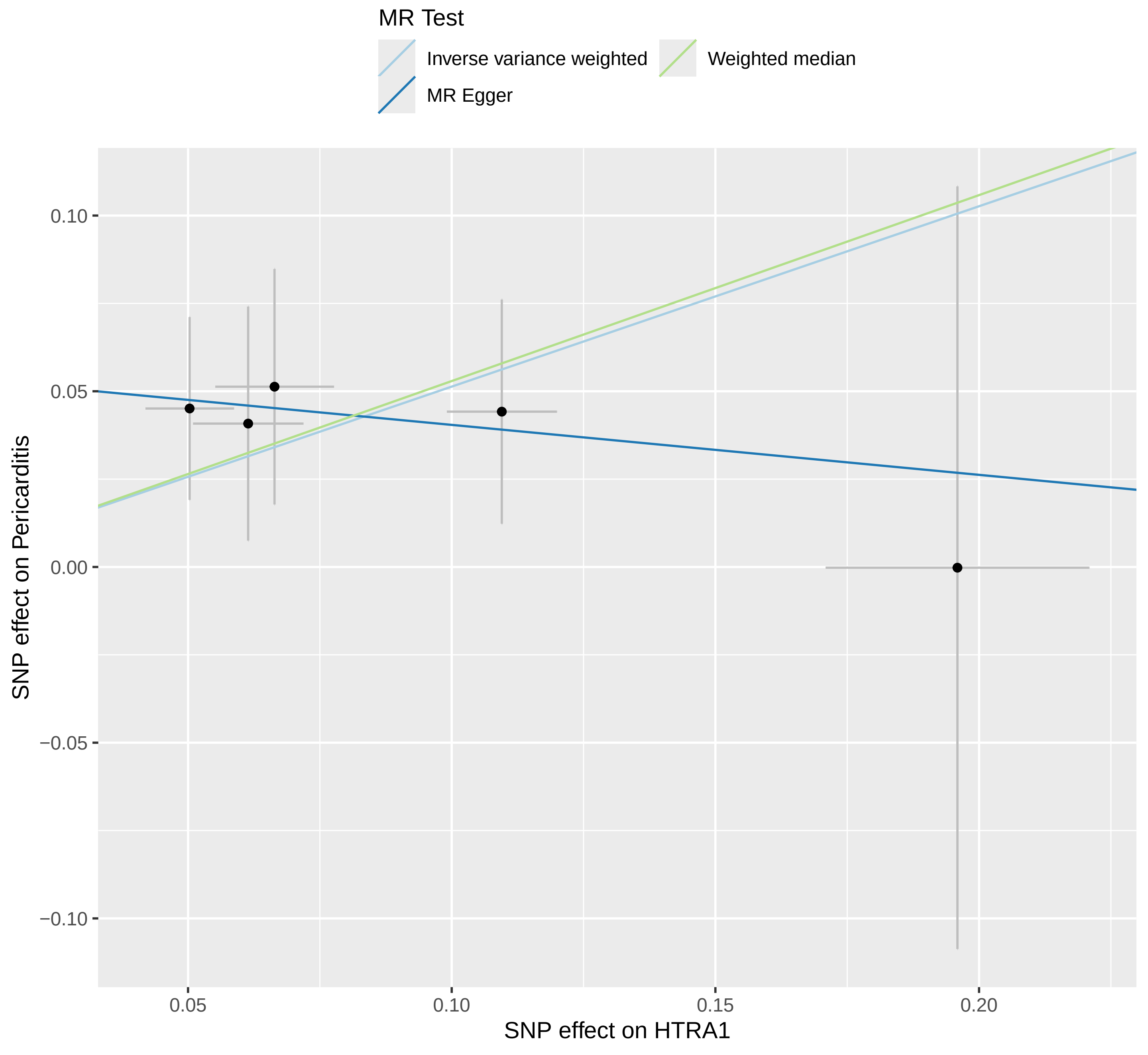
MR Test
Inverse variance weighted
MR Egger
Weighted median
SNP effect on Pericarditis
0.10
0.05
0.00
−0.05
−0.10
0.05
0.10
0.15
0.20
SNP effect on HTRA1

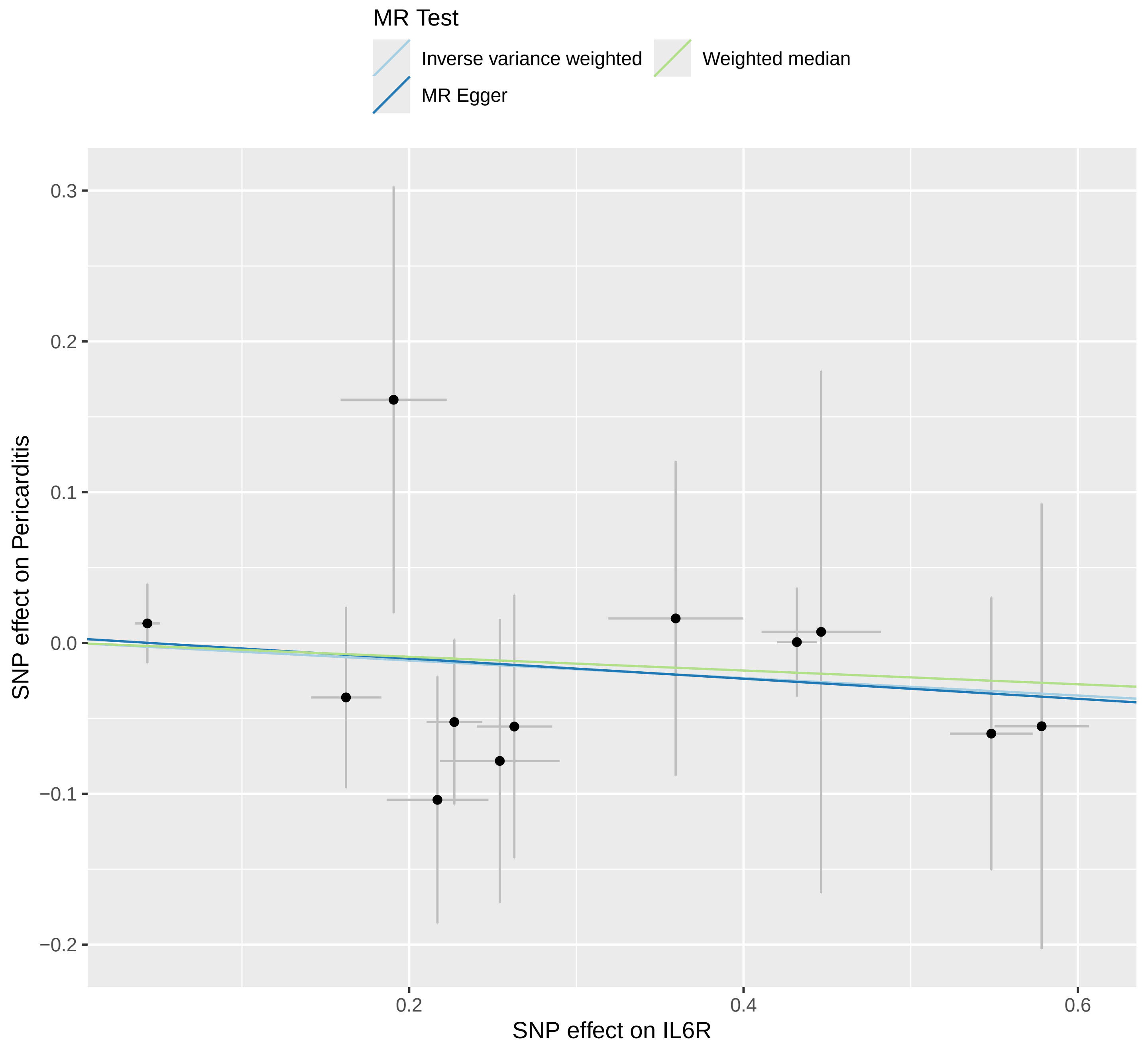
MR Test
Inverse variance weighted
Weighted median
MR Egger
SNP effect on Pericarditis
0.3
0.2
0.1
0.0
−0.1
−0.2
0.2
0.4
0.6
SNP effect on IL6R

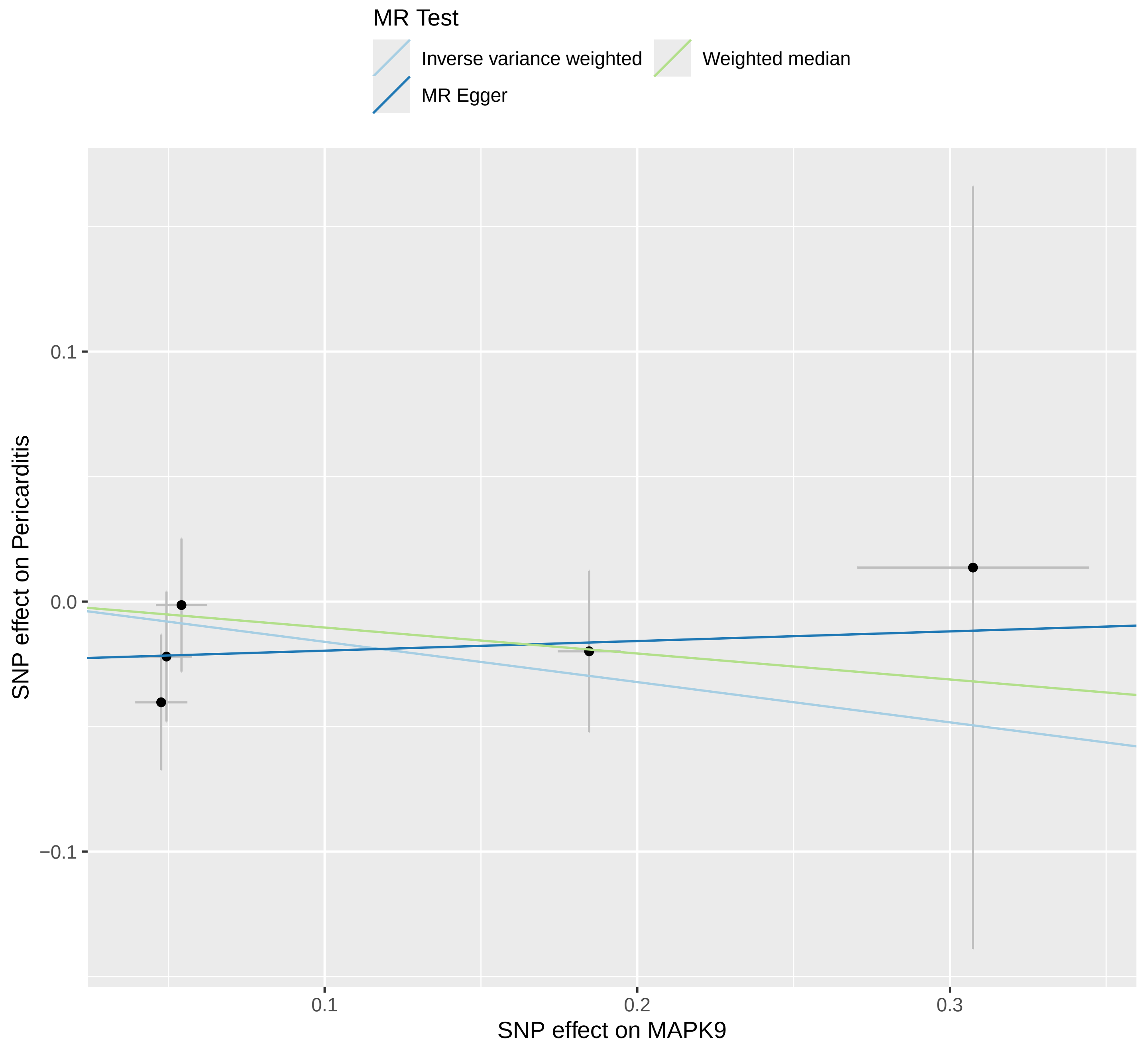
MR Test
Inverse variance weighted
Weighted median
MR Egger
0.1
0.0
−0.1
SNP effect on Pericarditis
0.1
0.2
0.3
SNP effect on MAPK9

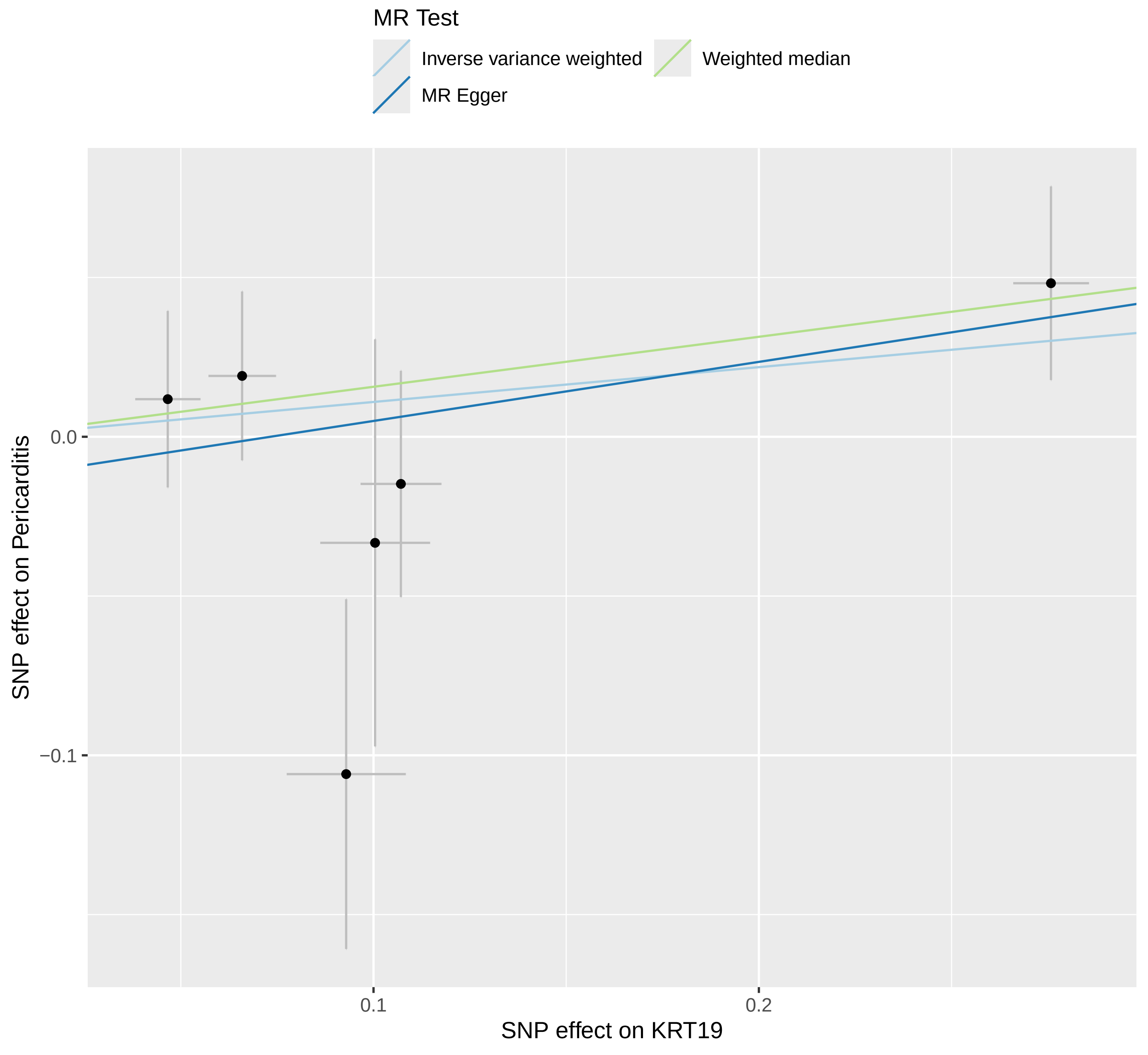
MR Test
Inverse variance weighted
Weighted median
MR Egger
SNP effect on Pericarditis
0.0
−0.1
0.1
0.2
SNP effect on KRT19

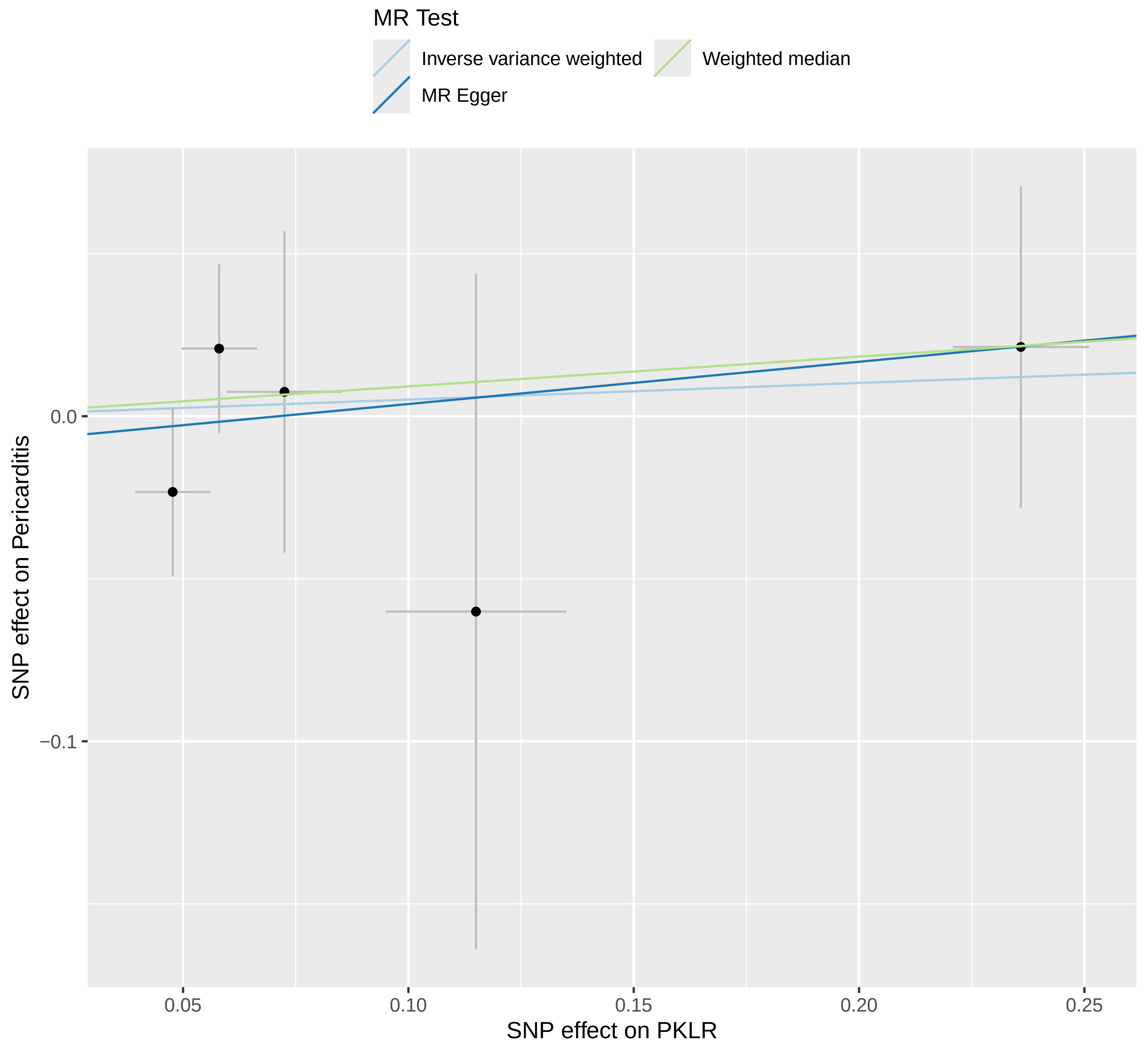
MR Test
Inverse variance weighted
Weighted median
MR Egger
0.0
−0.1
SNP effect on Pericarditis
0.05
0.10
0.15
0.20
0.25
SNP effect on PKLR

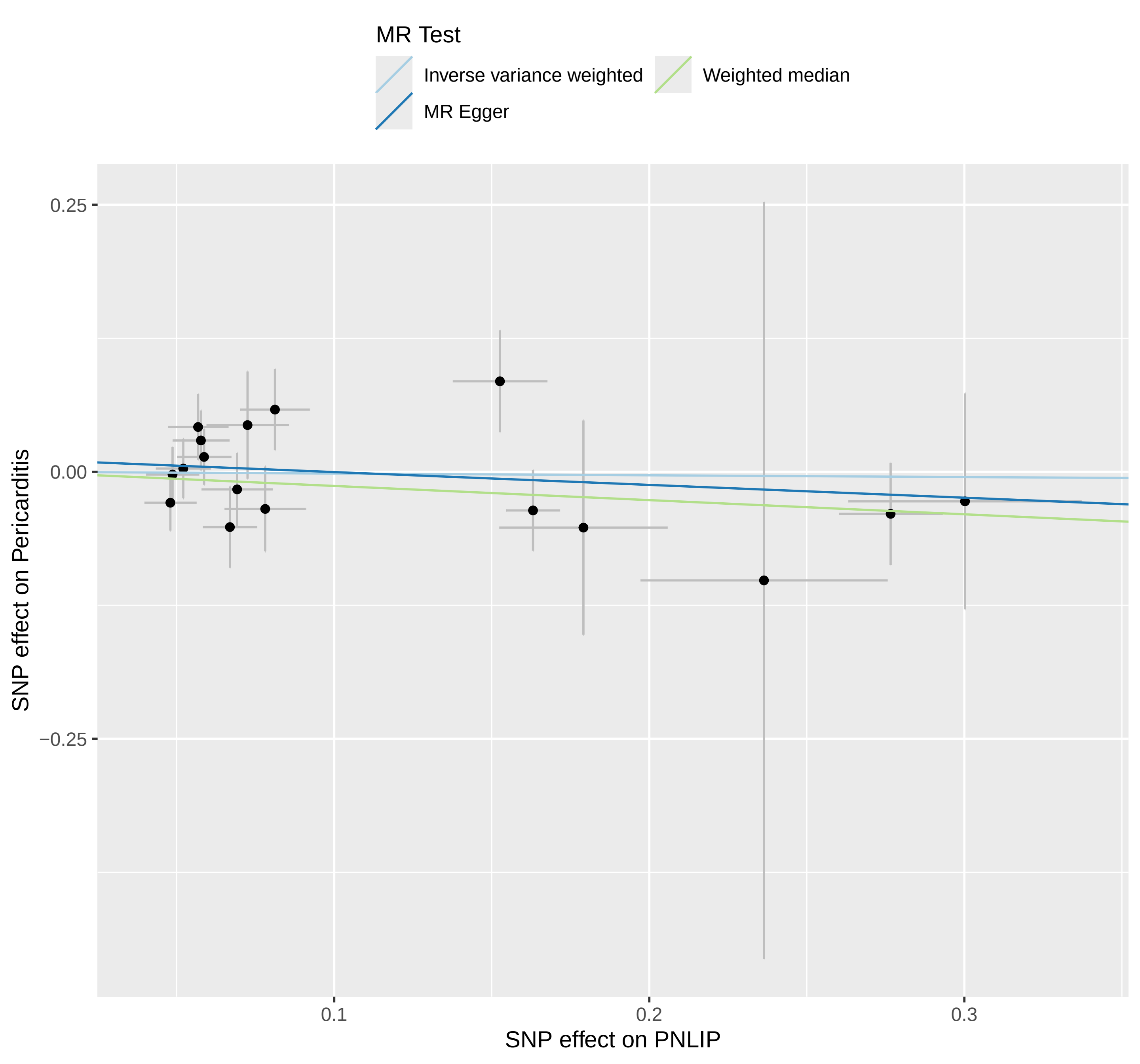
MR Test
Inverse variance weighted
MR Egger
Weighted median
0.25
0.00
−0.25
SNP effect on Pericarditis
0.1
0.2
0.3
SNP effect on PNLIP

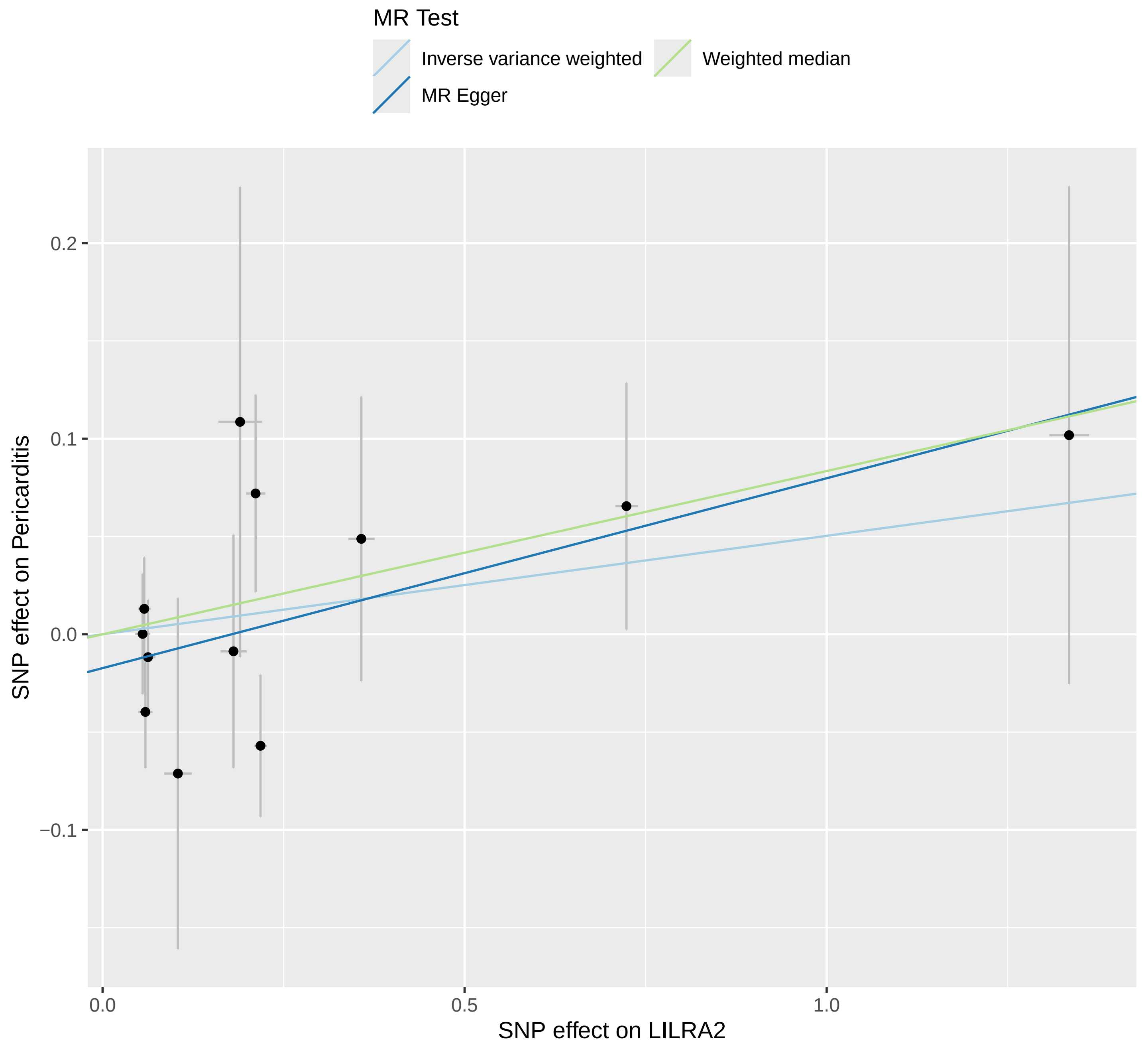
MR Test
Inverse variance weighted
Weighted median
MR Egger
0.2
0.1
0.0
−0.1
SNP effect on Pericarditis
0.0
0.5
1.0
SNP effect on LILRA2

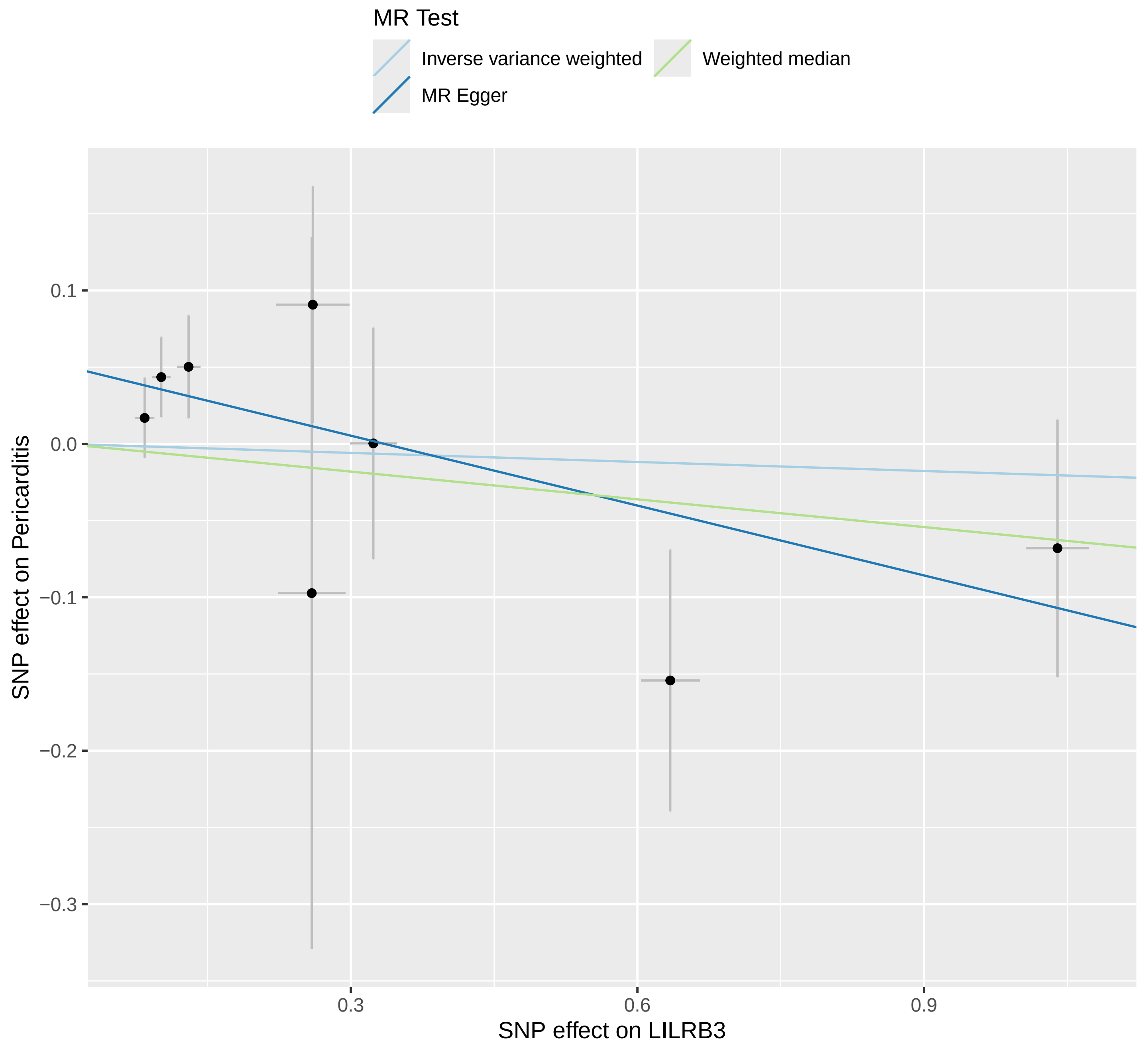
MR Test
Inverse variance weighted
Weighted median
MR Egger
SNP effect on Pericarditis
0.1
0.0
−0.1
−0.2
−0.3
0.3
0.6
0.9
SNP effect on LILRB3

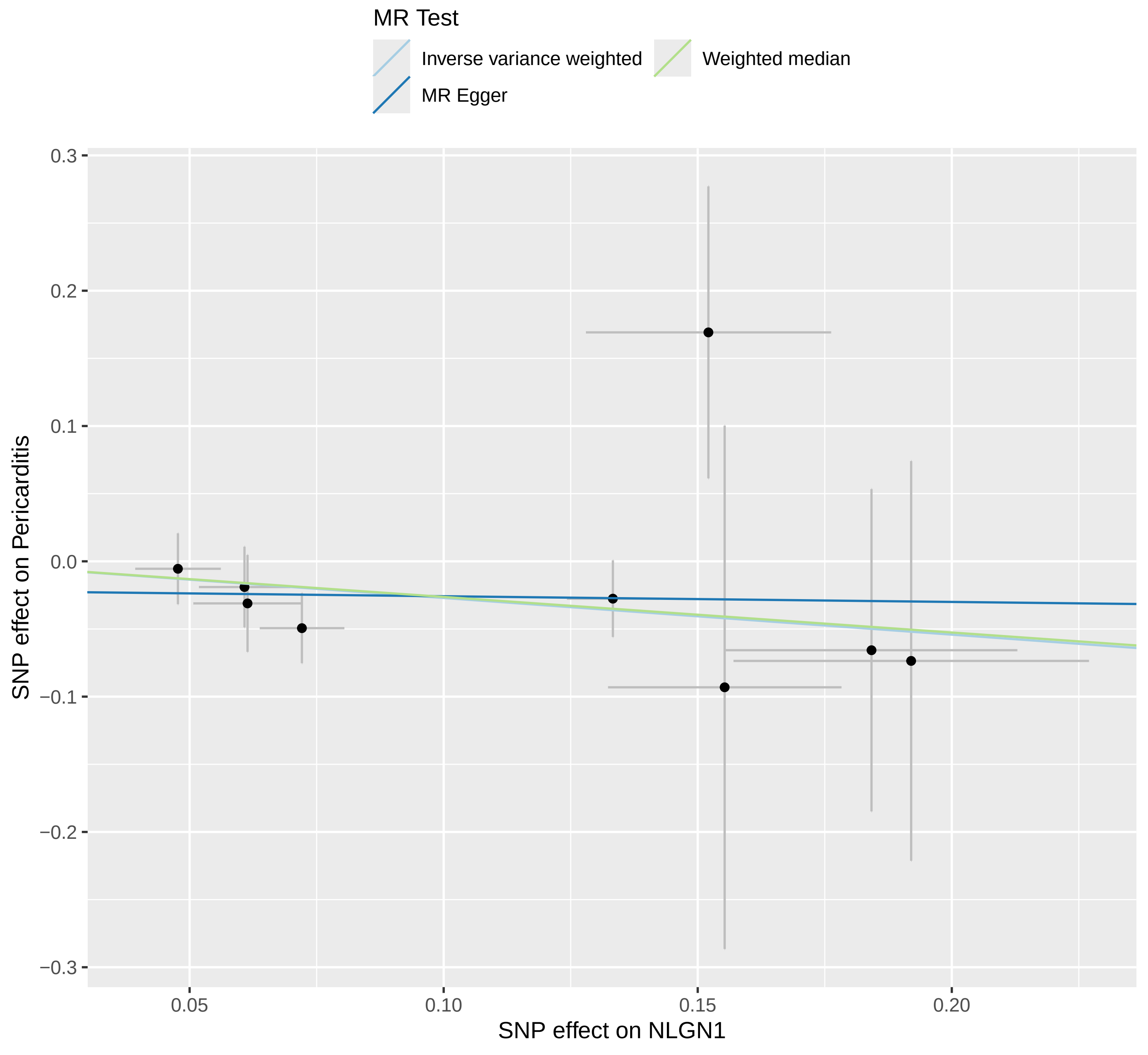

MR Test
Inverse variance weighted
Weighted median
MR Egger
0.3
0.2
0.1
0.0
−0.1
−0.2
−0.3
SNP effect on Pericarditis
0.05
0.10
0.15
0.20
SNP effect on NLGN1

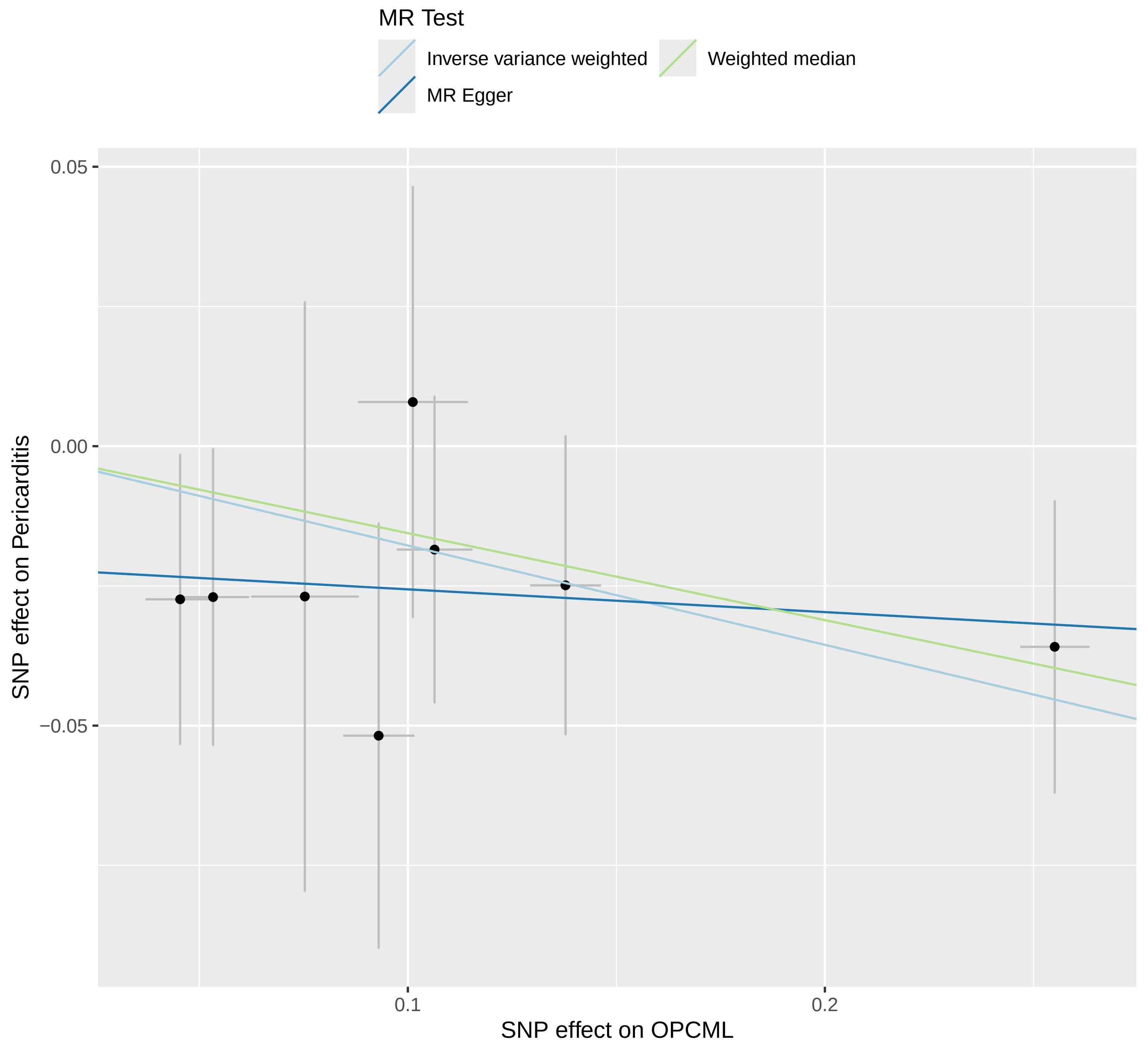
MR Test
Inverse variance weighted
Weighted median
MR Egger
0.05
0.00
−0.05
0.1
0.2
SNP effect on Pericarditis
SNP effect on OPCML

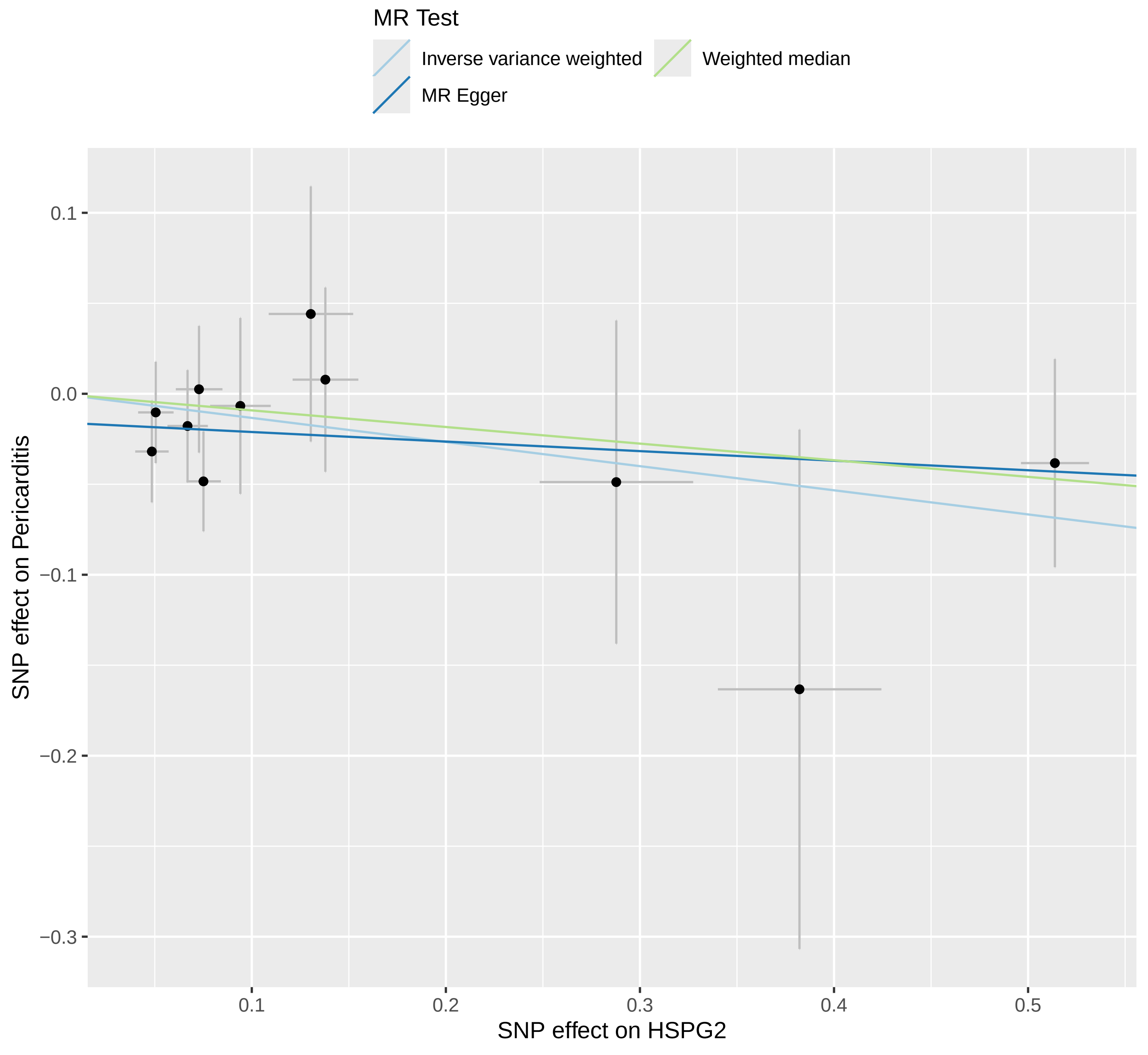
MR Test
Inverse variance weighted
MR Egger
Weighted median
0.1
0.0
−0.1
−0.2
−0.3
SNP effect on Pericarditis
0.1
0.2
0.3
0.4
0.5
SNP effect on HSPG2

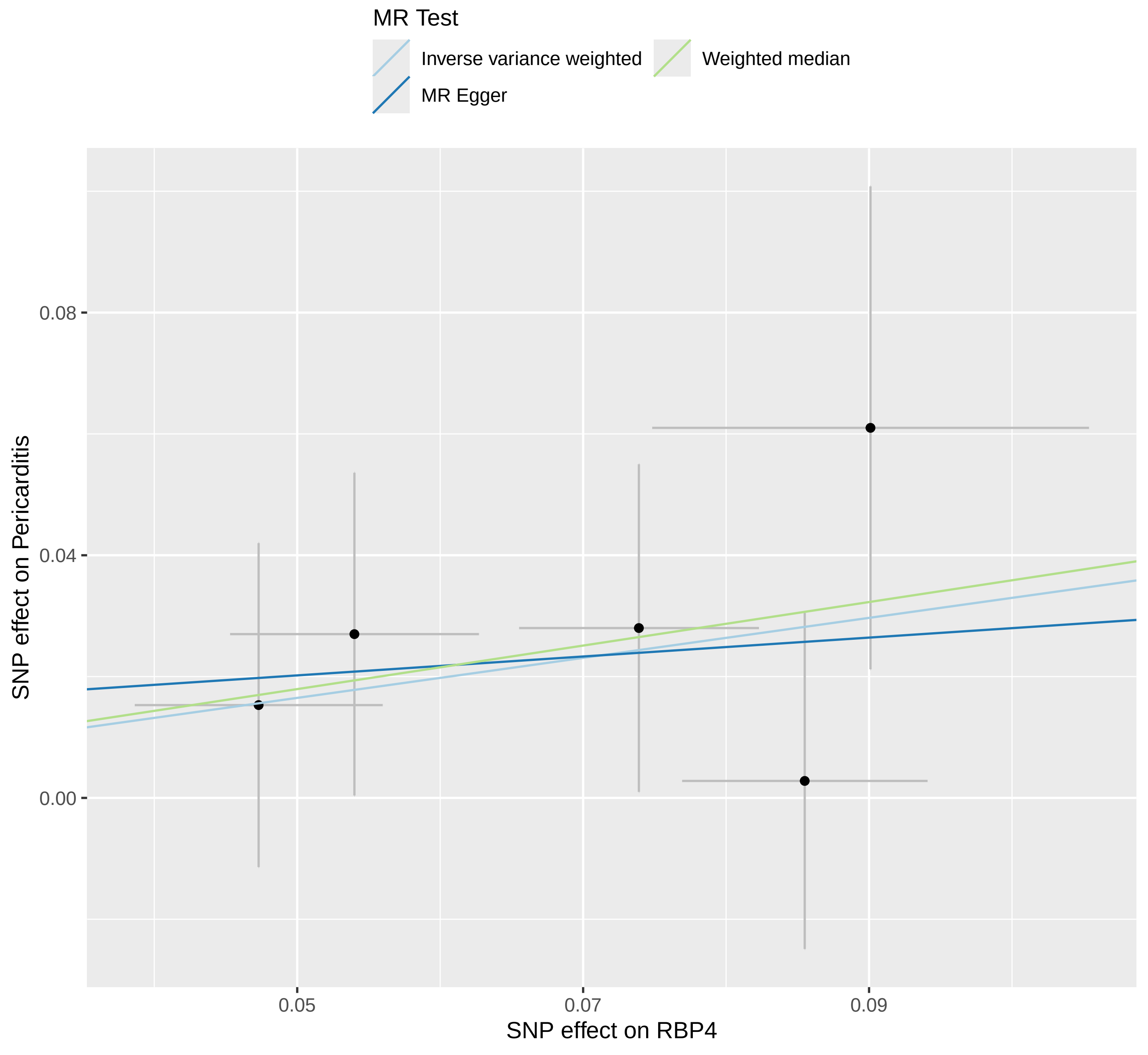

MR Test
Inverse variance weighted
Weighted median
MR Egger
0.08
0.04
0.00
0.05
0.07
0.09
SNP effect on Pericarditis
SNP effect on RBP4

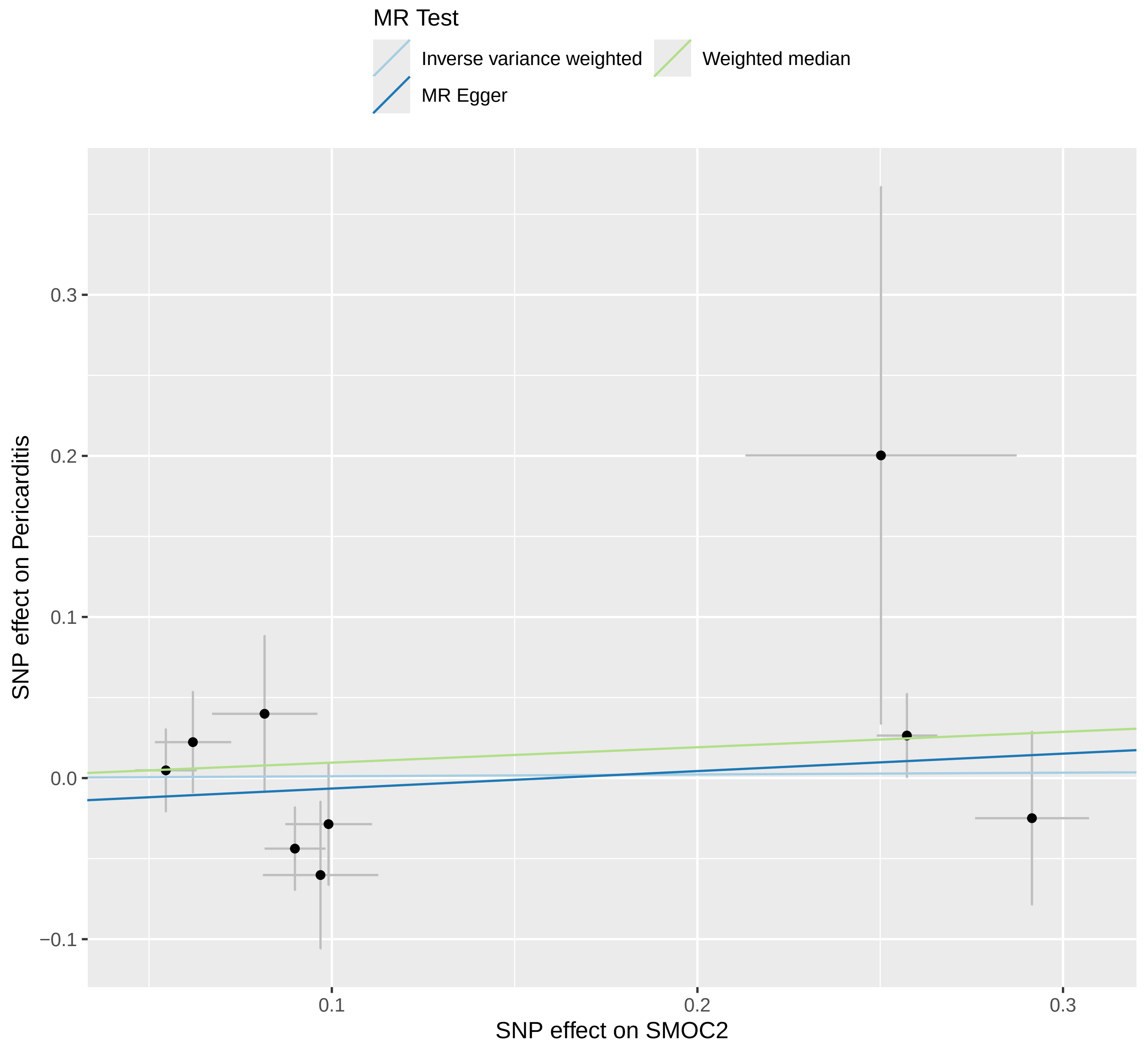

MR Test
Inverse variance weighted
Weighted median
MR Egger
SNP effect on Pericarditis
0.3
0.2
0.1
0.0
−0.1
0.1
0.2
0.3
SNP effect on SMOC2

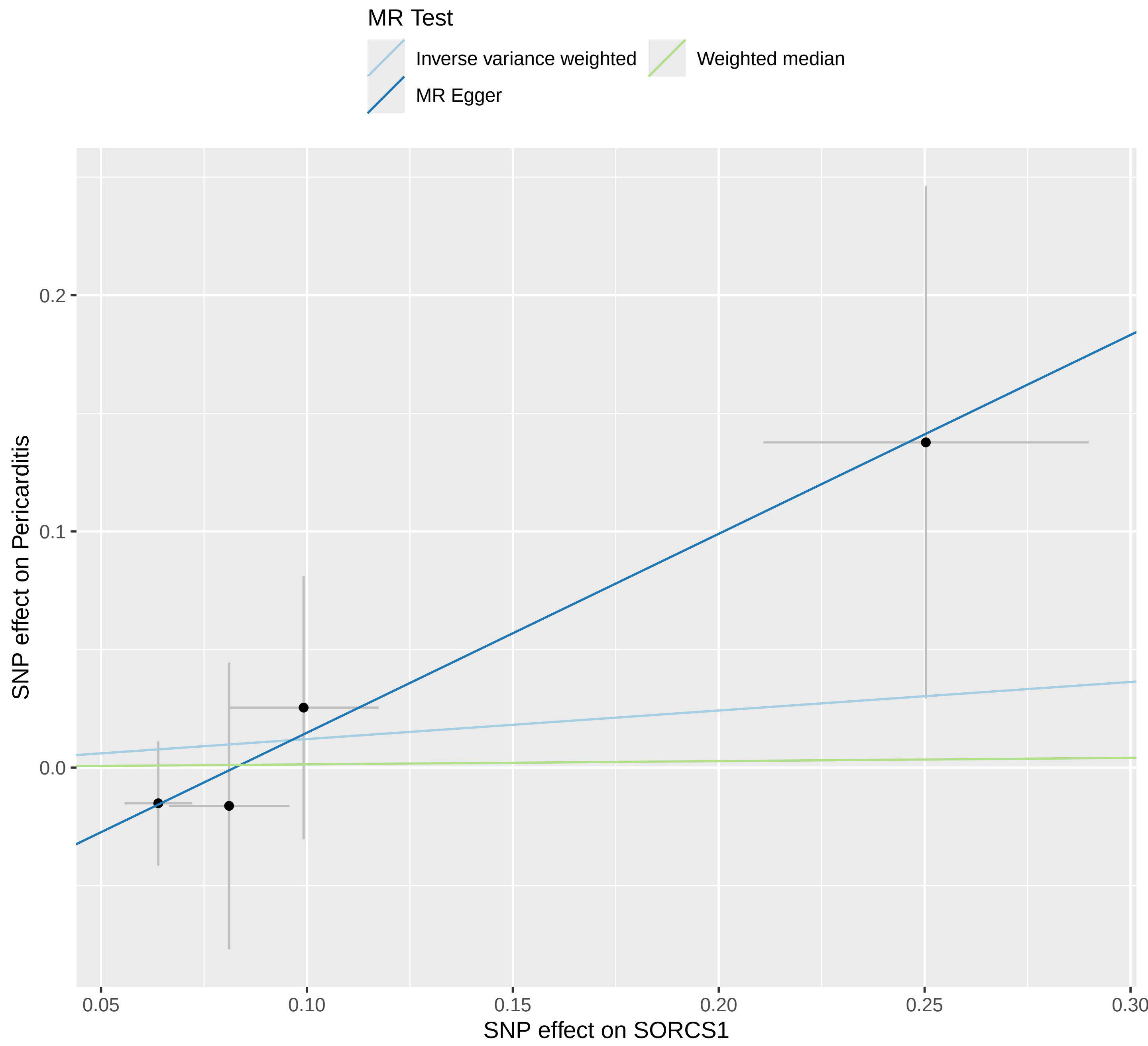

MR Test
Inverse variance weighted
MR Egger
Weighted median
0.2
0.1
0.0
0.05
0.10
0.15
0.20
0.25
0.30
SNP effect on SORCS1
SNP effect on Pericarditis

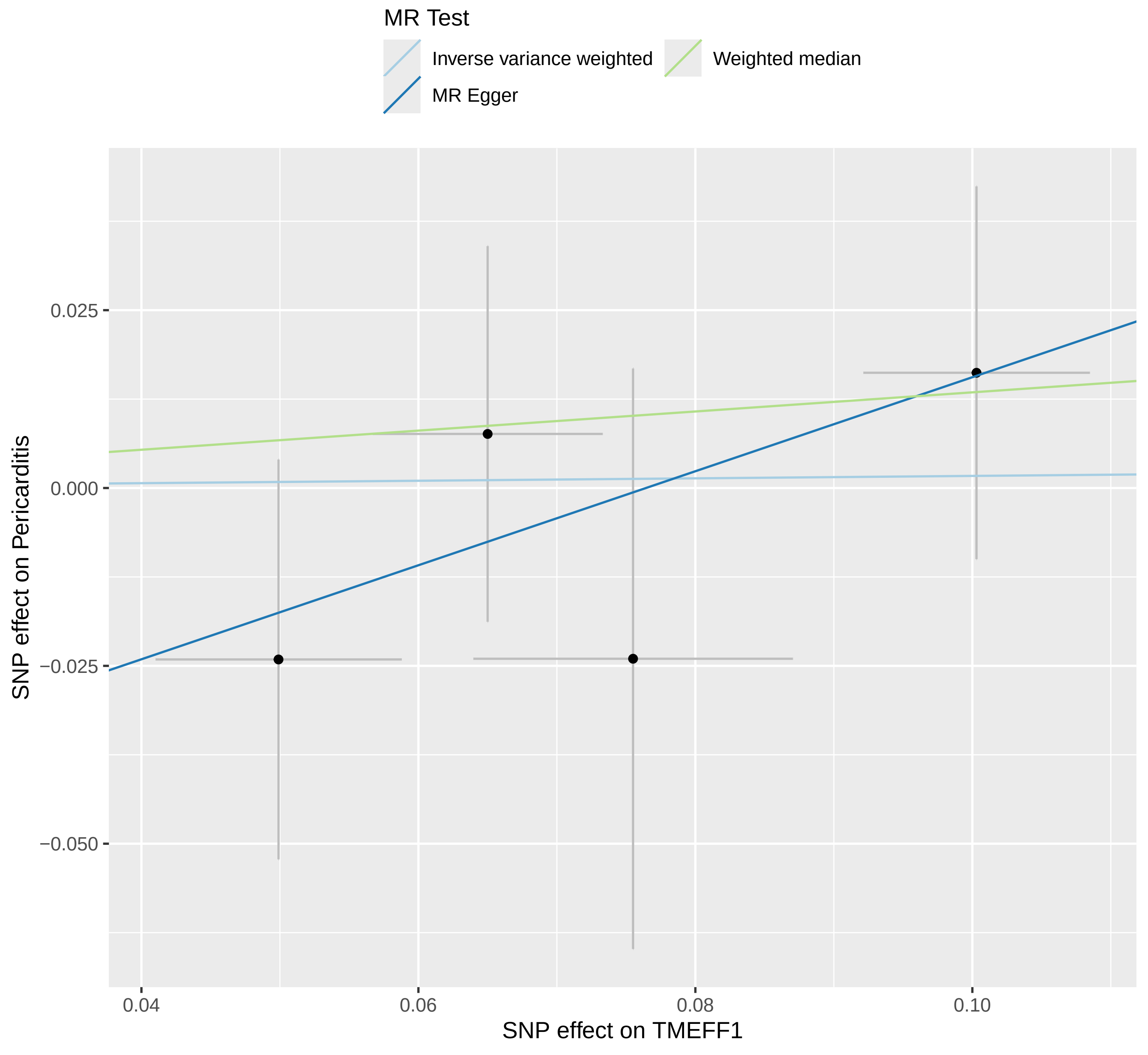
MR Test
Inverse variance weighted
MR Egger
Weighted median
0.025
0.000
−0.025
−0.050
SNP effect on Pericarditis
0.04
0.06
0.08
0.10
SNP effect on TMEFF1

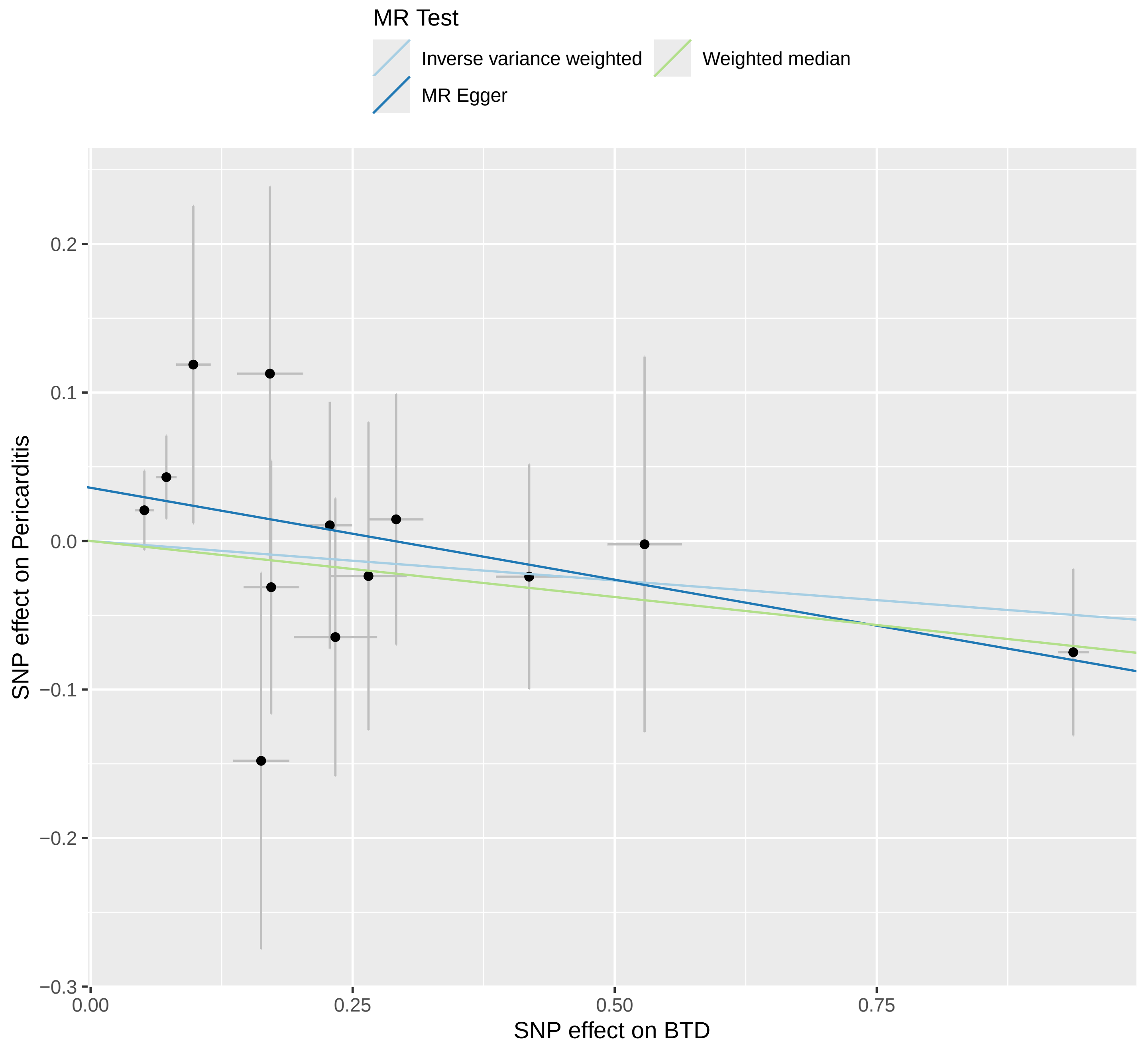
MR Test
Inverse variance weighted
Weighted median
MR Egger
SNP effect on Pericarditis
SNP effect on BTD
0.2
0.1
0.0
−0.1
−0.2
−0.3
0.00
0.25
0.50
0.75

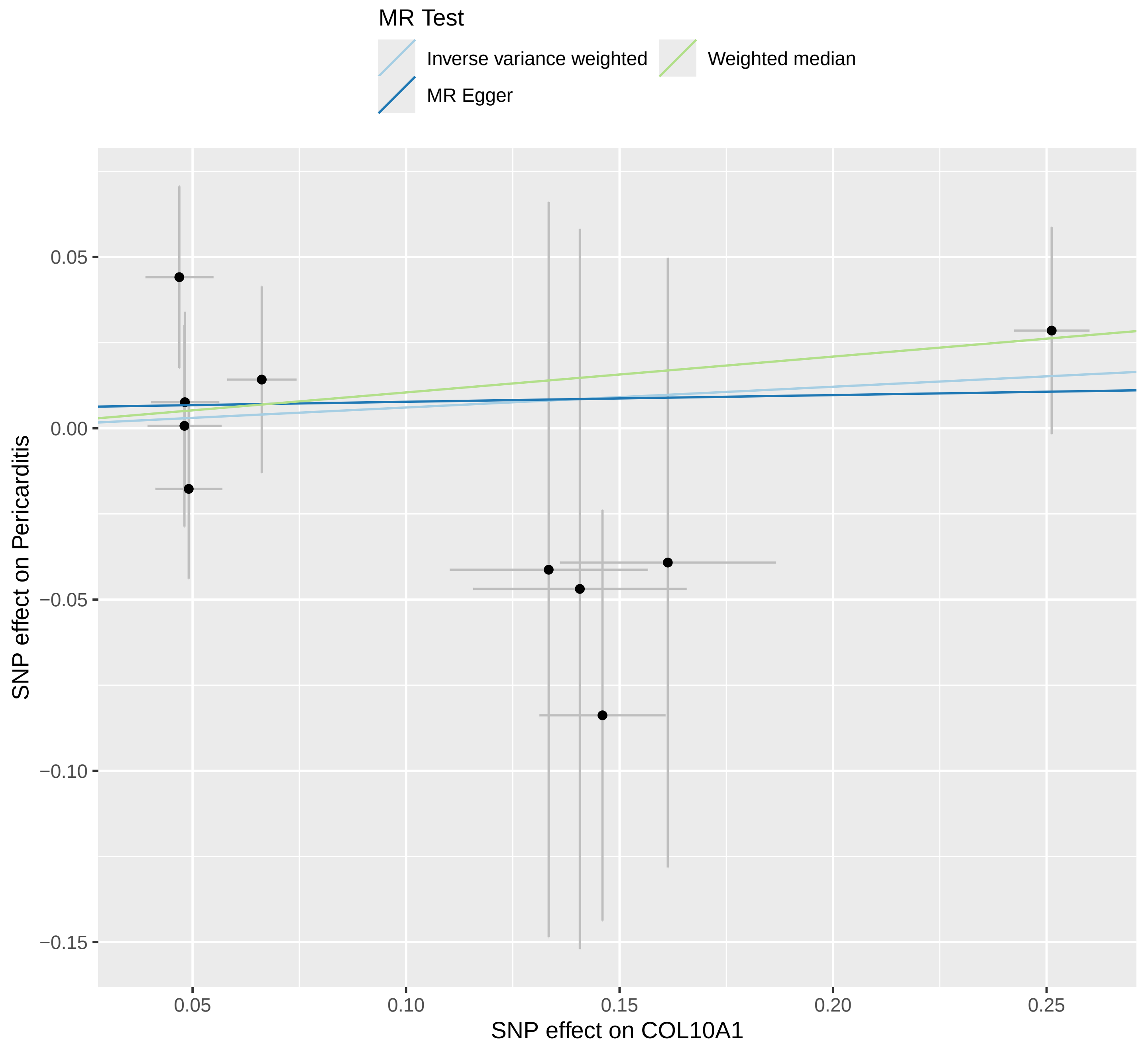
MR Test
Inverse variance weighted
MR Egger
Weighted median
SNP effect on Pericarditis
SNP effect on COL10A1
0.05
0.00
−0.05
−0.10
−0.15
0.05
0.10
0.15
0.20
0.25

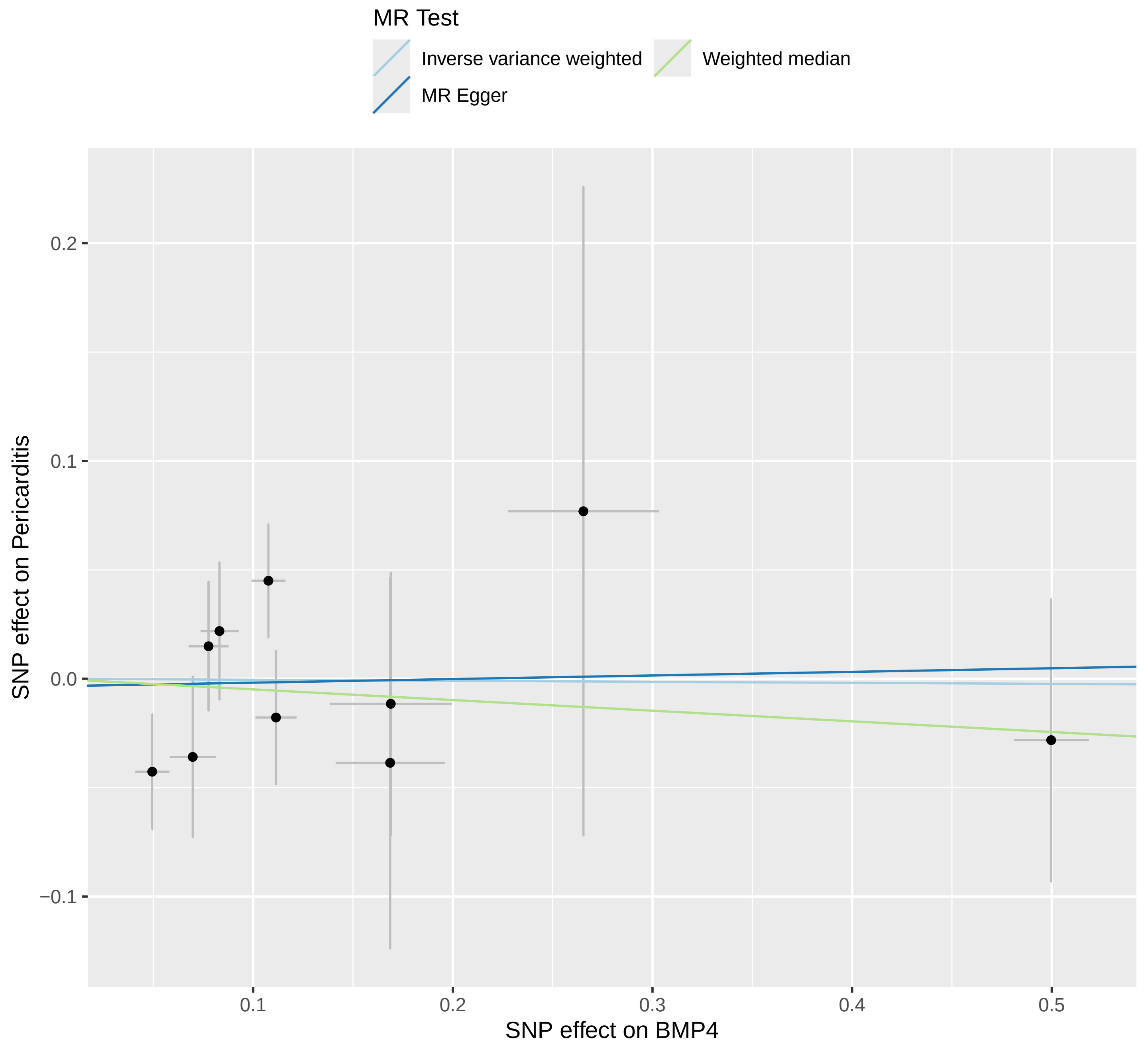

MR Test
Inverse variance weighted
Weighted median
MR Egger
0.2
0.1
0.0
−0.1
0.1
0.2
0.3
0.4
0.5
SNP effect on Pericarditis
SNP effect on BMP4

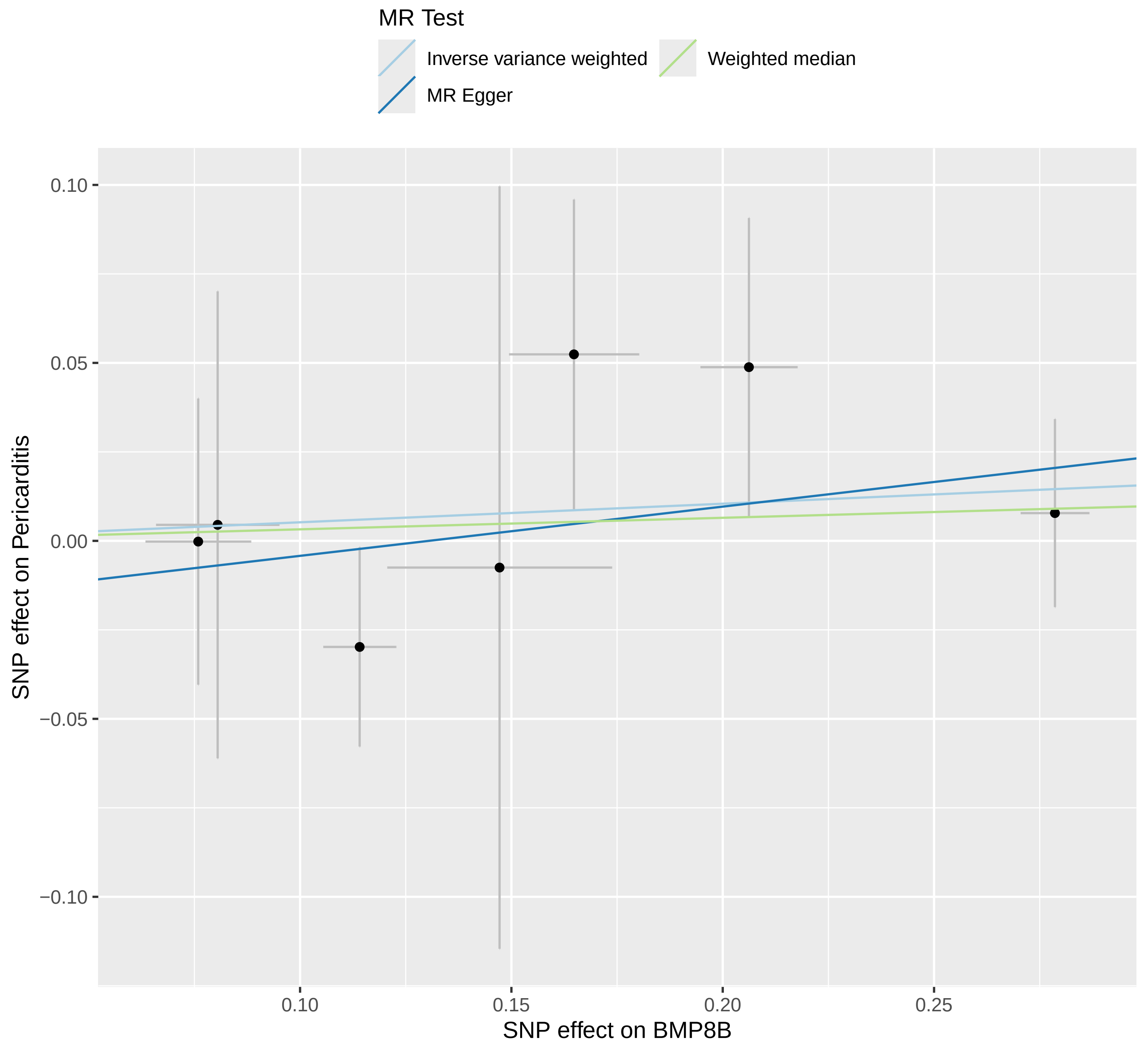

MR Test
Inverse variance weighted
MR Egger
Weighted median
0.10
0.05
0.00
−0.05
−0.10
SNP effect on Pericarditis
0.10
0.15
0.20
0.25
SNP effect on BMP8B

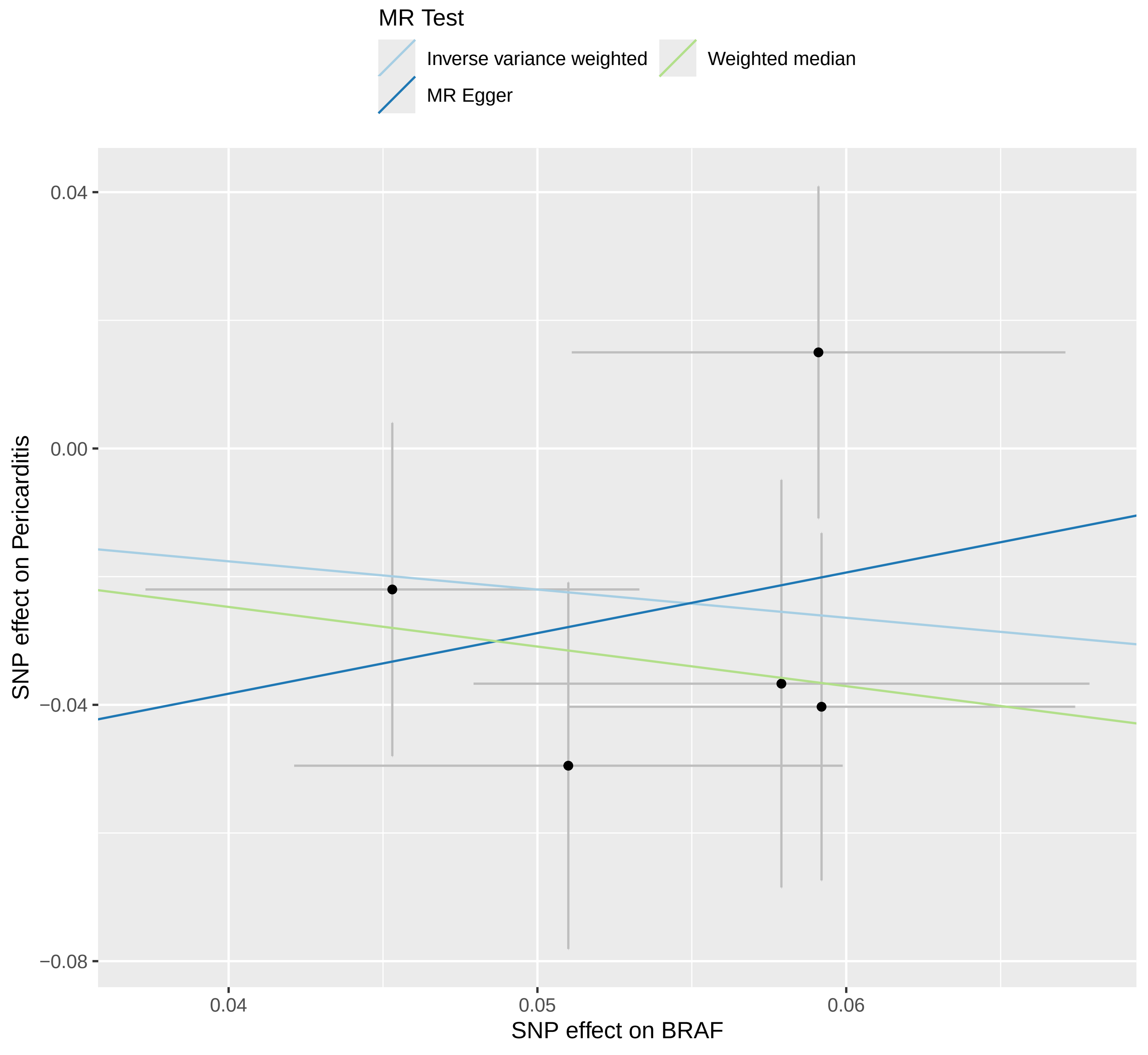
MR Test
Inverse variance weighted
Weighted median
MR Egger
0.04
0.00
−0.04
−0.08
SNP effect on Pericarditis
0.04
0.05
0.06
SNP effect on BRAF

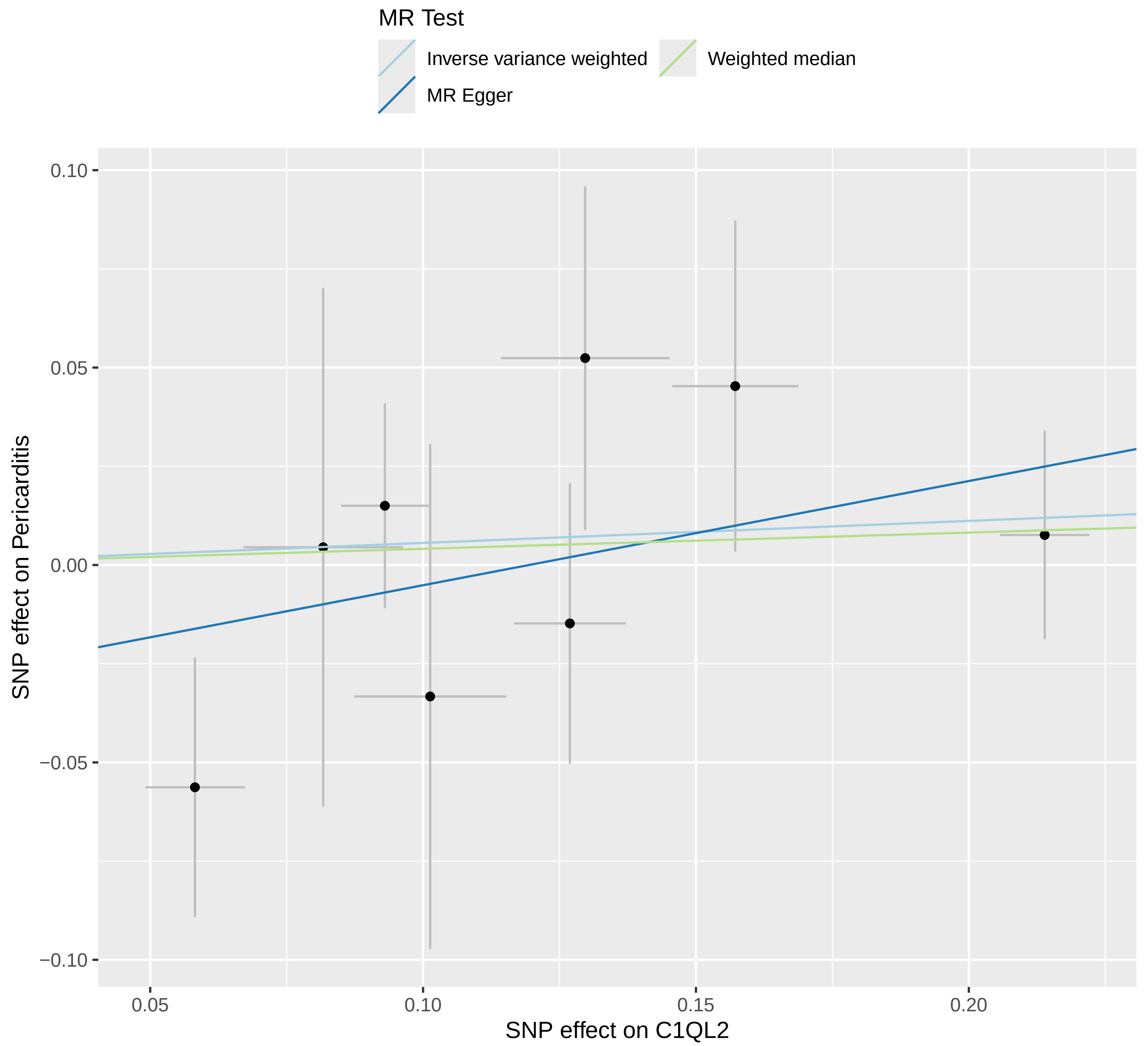

MR Test
Inverse variance weighted
MR Egger
Weighted median
0.10
0.05
0.00
−0.05
−0.10
SNP effect on Pericarditis
0.05
0.10
0.15
0.20
SNP effect on C1QL2

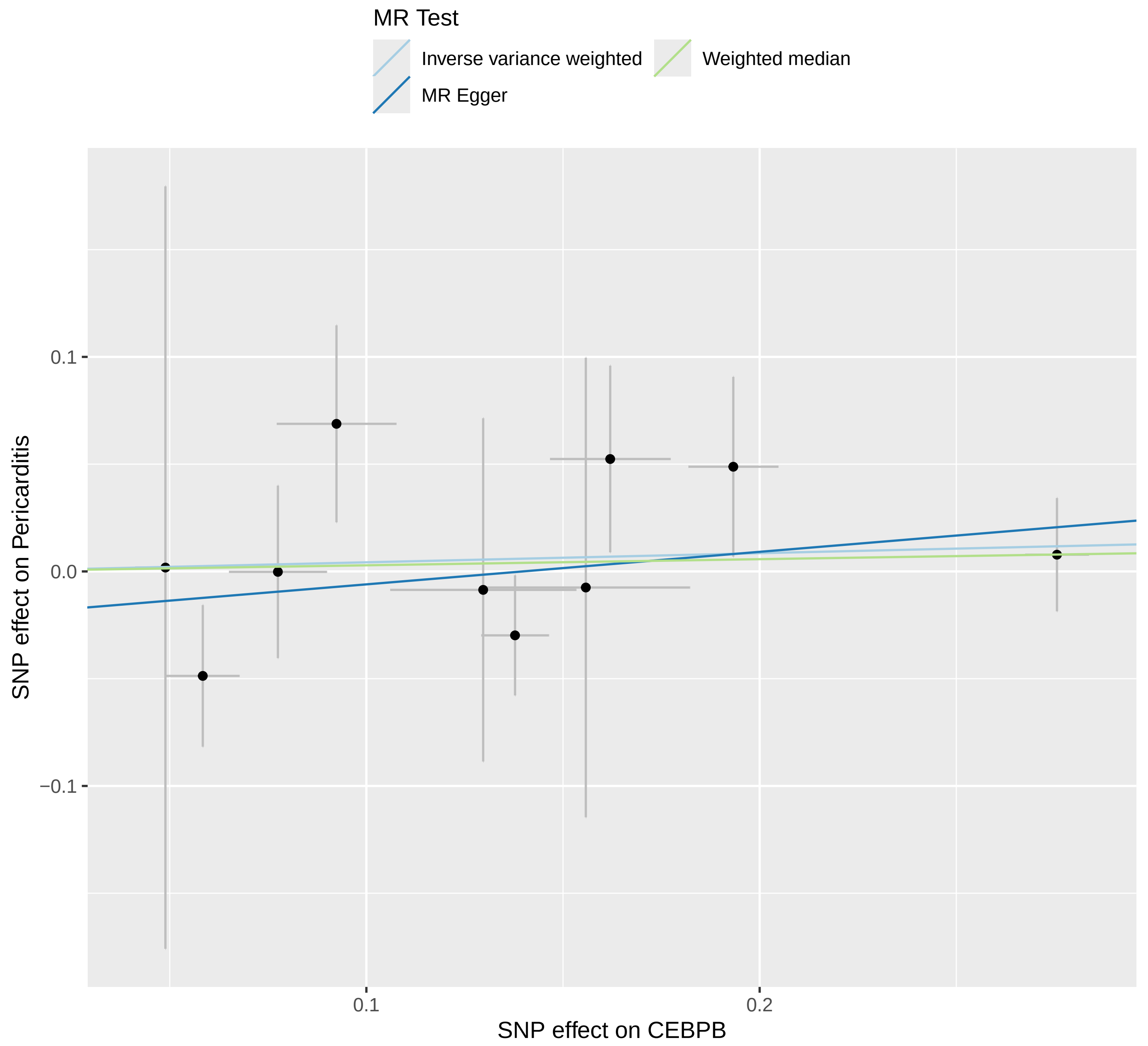
MR Test
Inverse variance weighted
Weighted median
MR Egger
0.1
0.0
−0.1
SNP effect on Pericarditis
0.1
0.2
SNP effect on CEBPB

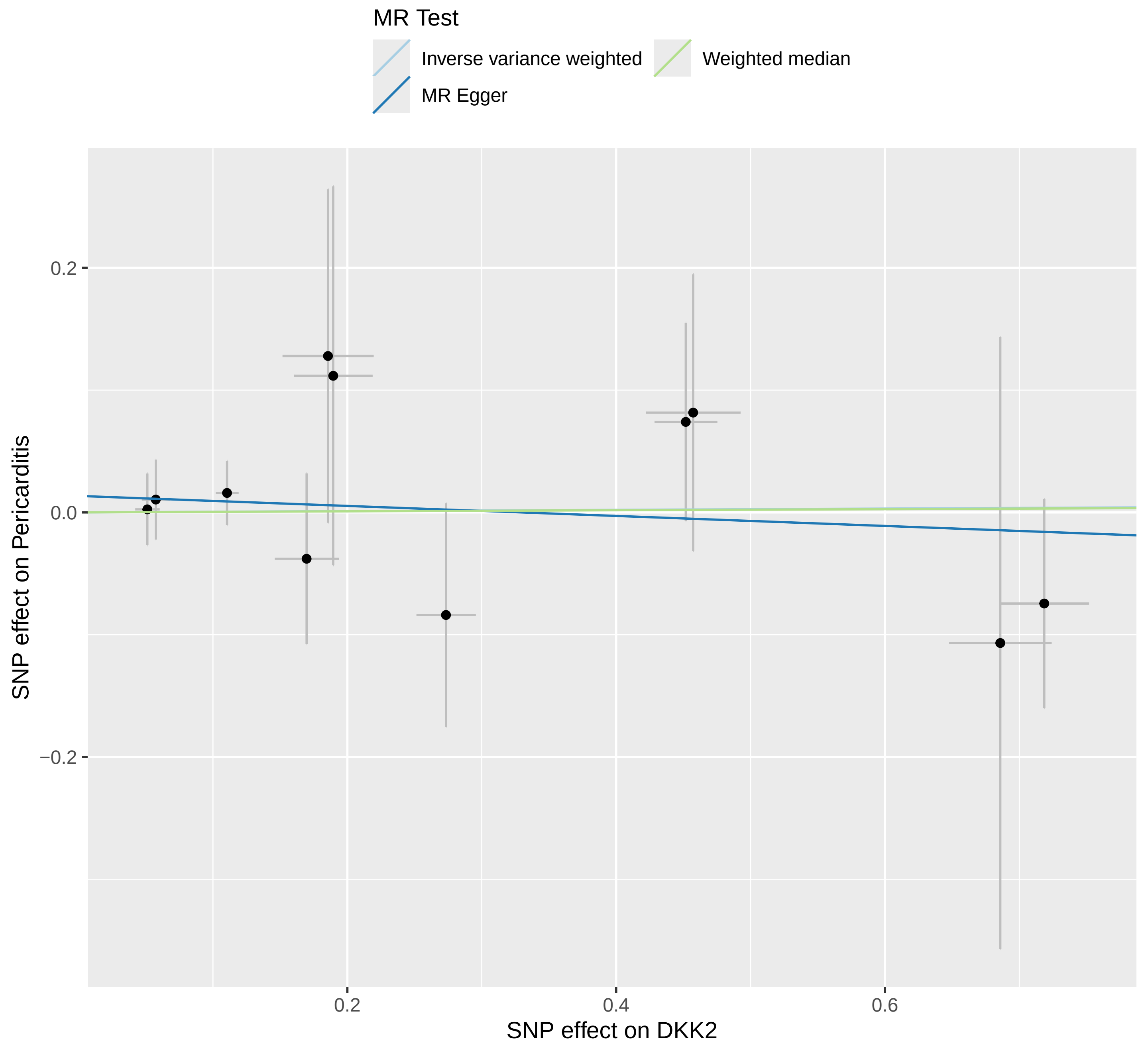
MR Test
Inverse variance weighted
Weighted median
MR Egger
0.2
0.0
−0.2
0.2
0.4
0.6
SNP effect on Pericarditis
SNP effect on DKK2

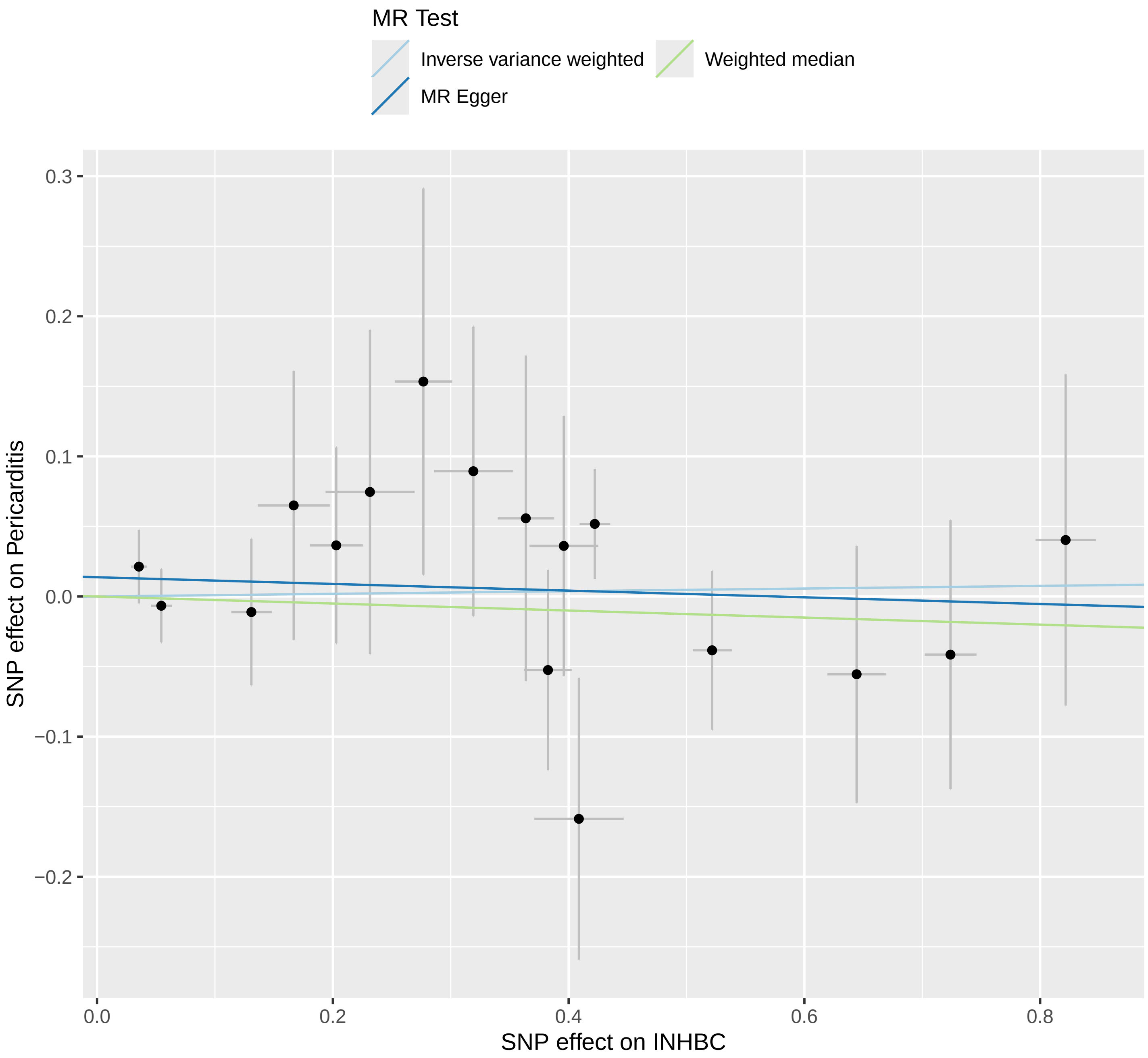

MR Test
Inverse variance weighted
Weighted median
MR Egger
0.3
0.2
0.1
0.0
−0.1
−0.2
SNP effect on Pericarditis
0.0
0.2
0.4
0.6
0.8
SNP effect on INHBC

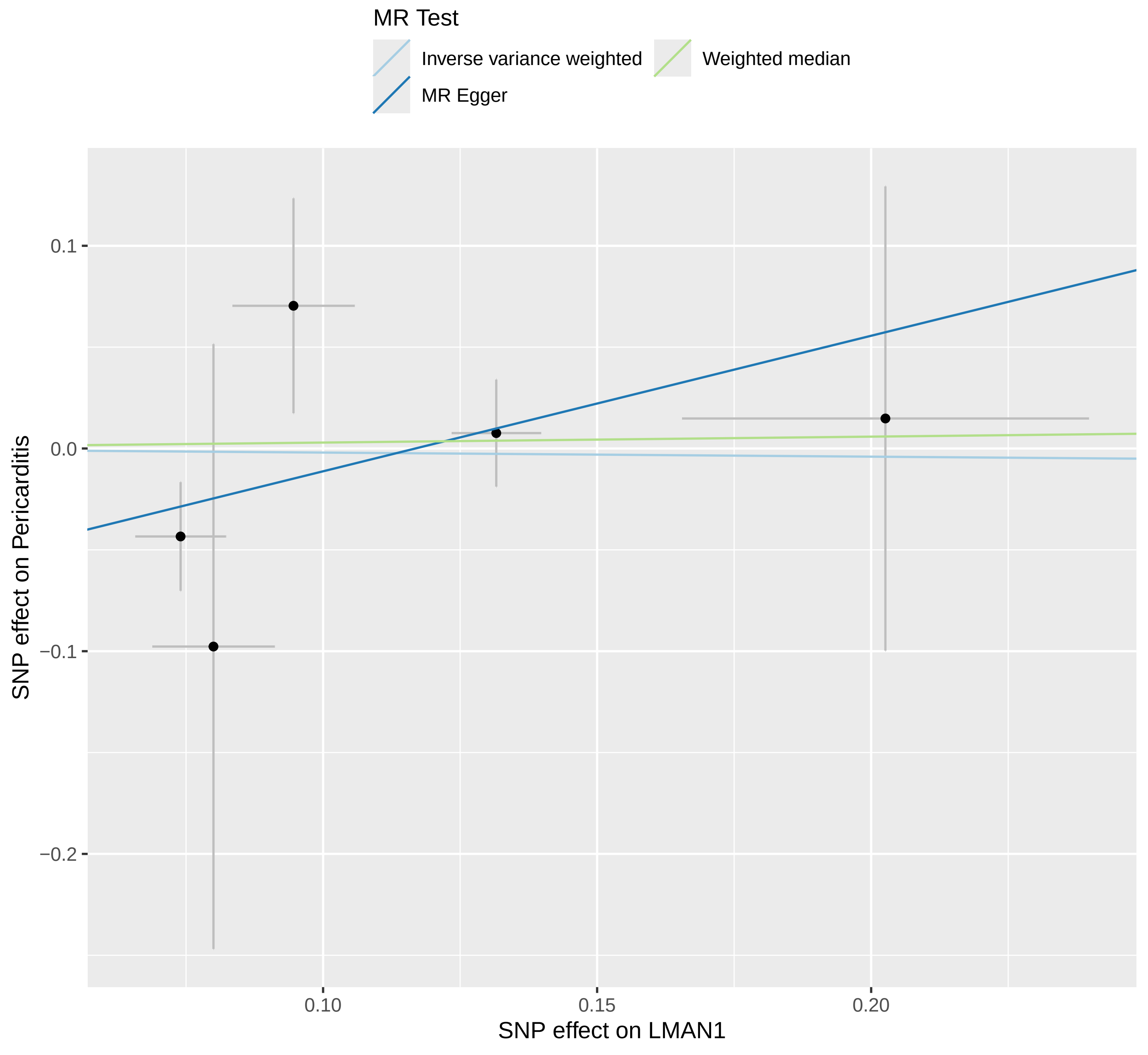
MR Test
Inverse variance weighted
Weighted median
MR Egger
SNP effect on Pericarditis
0.1
0.0
−0.1
−0.2
0.10
0.15
0.20
SNP effect on LMAN1

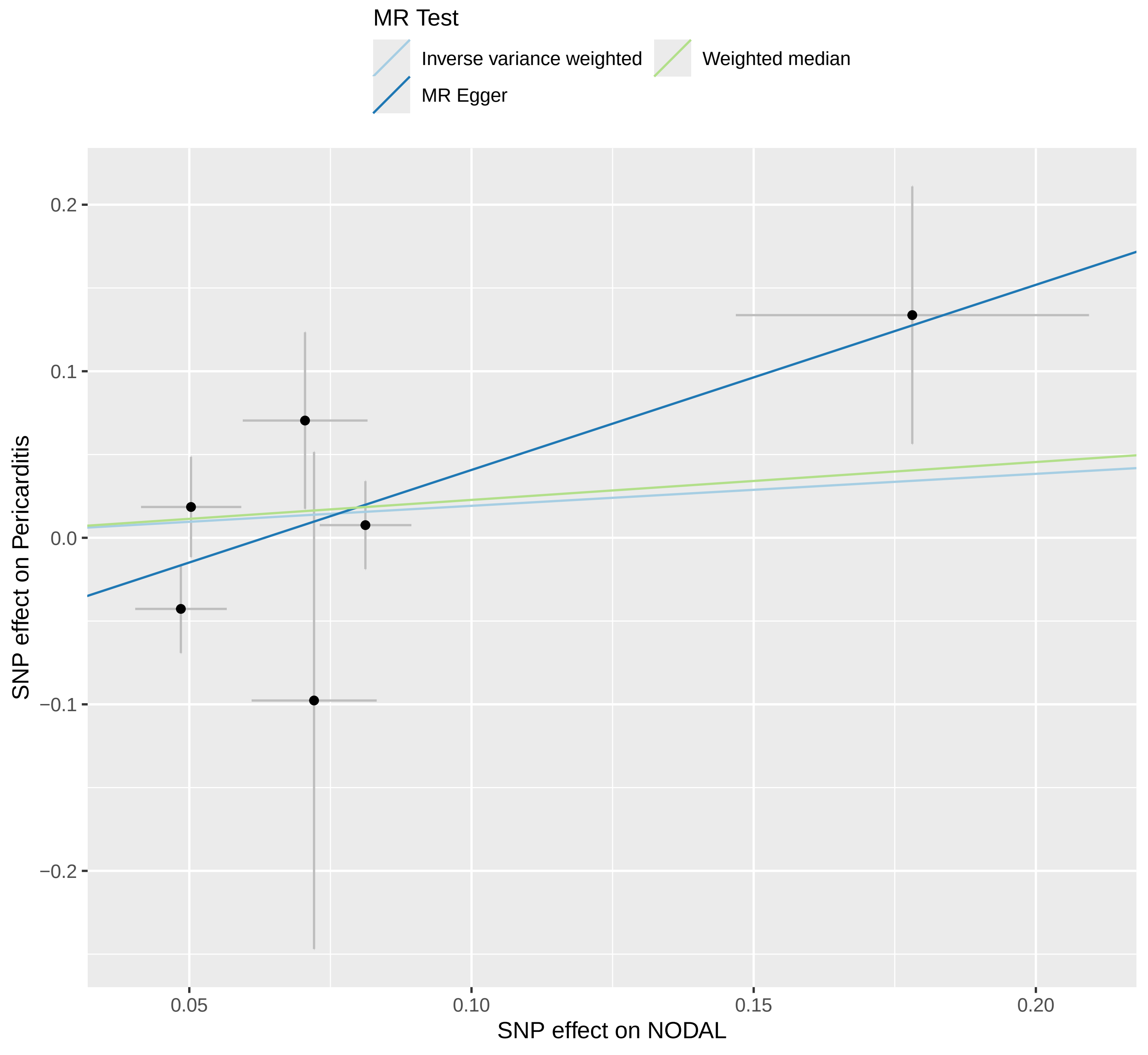
MR Test
Inverse variance weighted
Weighted median
MR Egger
SNP effect on Pericarditis
0.2
0.1
0.0
−0.1
−0.2
0.05
0.10
0.15
0.20
SNP effect on NODAL

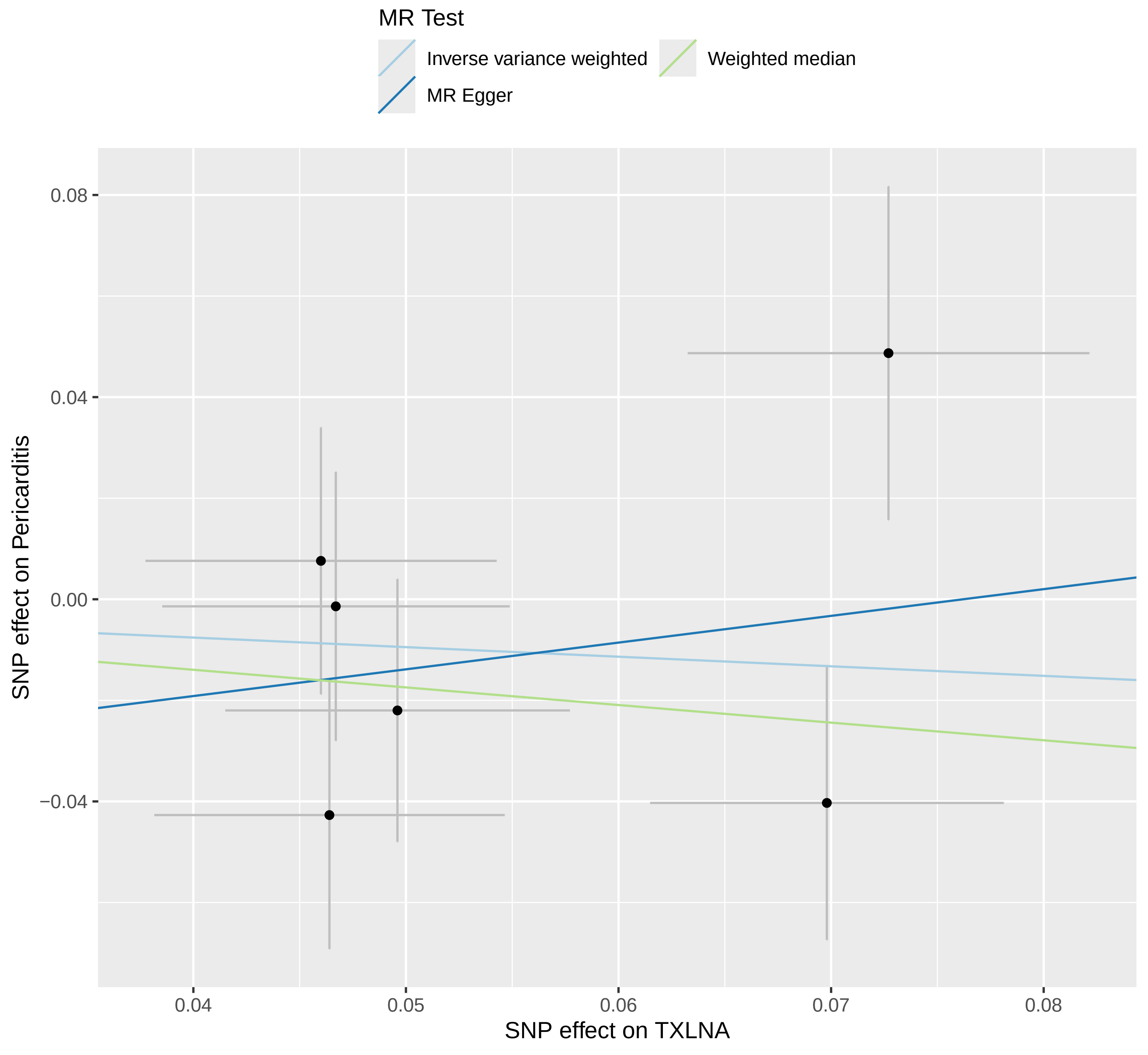
MR Test
Inverse variance weighted
MR Egger
Weighted median
0.08
0.04
0.00
−0.04
0.04
0.05
0.06
0.07
0.08
SNP effect on Pericarditis
SNP effect on TXLNA

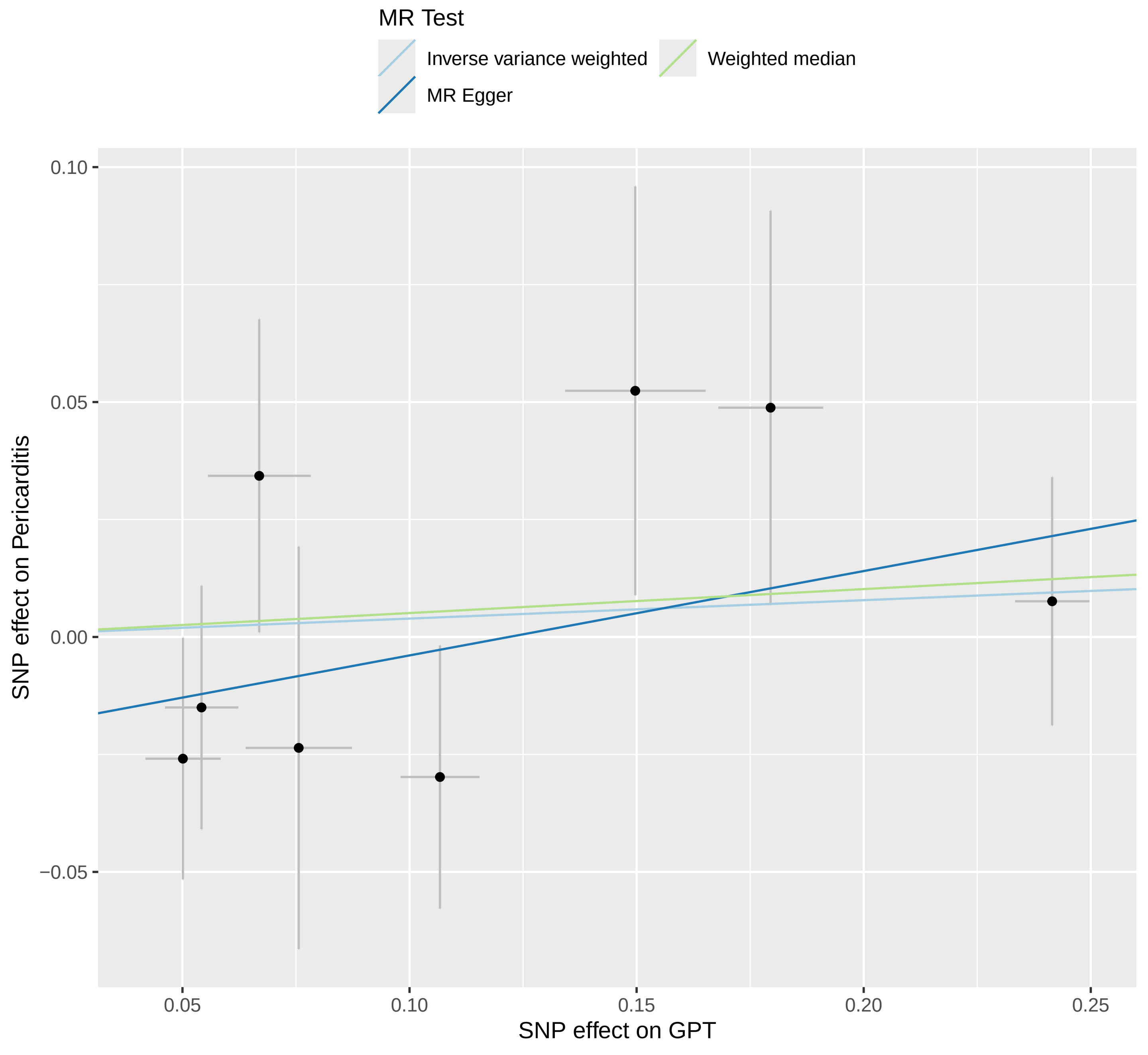

MR Test
Inverse variance weighted
Weighted median
MR Egger
0.10
0.05
0.00
−0.05
SNP effect on Pericarditis
0.05
0.10
0.15
0.20
0.25
SNP effect on GPT

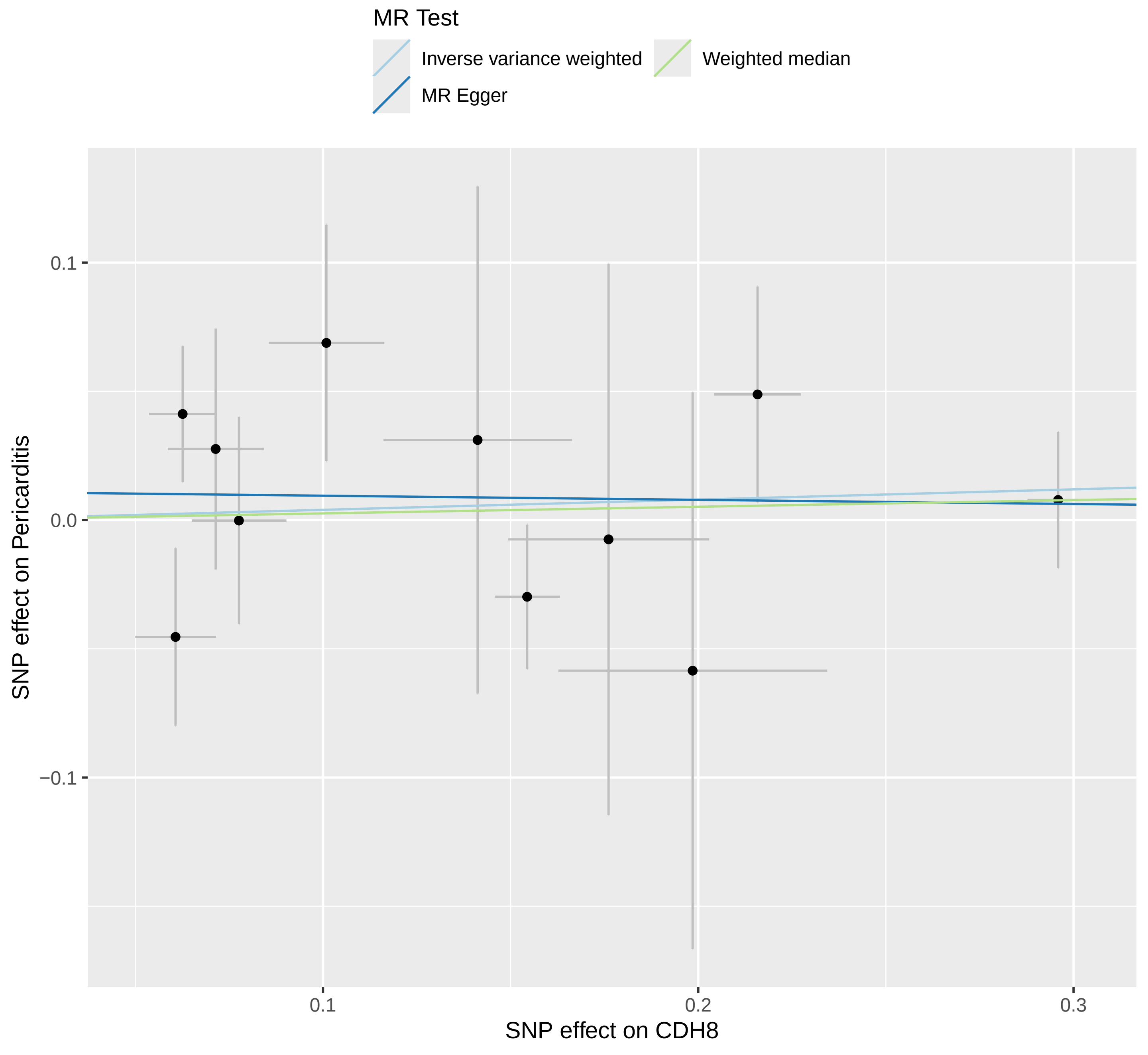
MR Test
Inverse variance weighted
Weighted median
MR Egger
0.1
0.0
−0.1
SNP effect on Pericarditis
0.1
0.2
0.3
SNP effect on CDH8

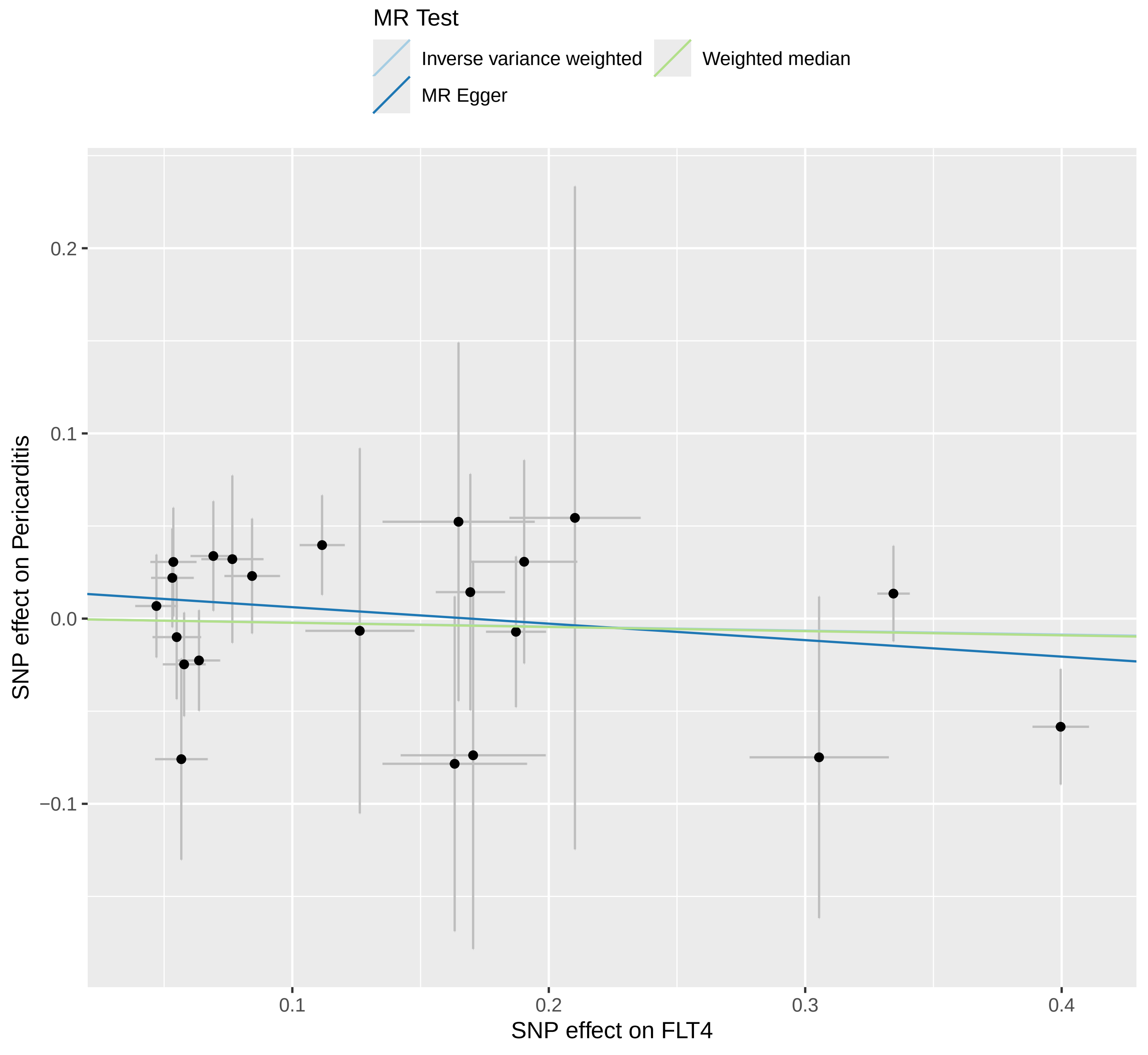

MR Test
Inverse variance weighted
MR Egger
Weighted median
0.2
0.1
0.0
−0.1
SNP effect on Pericarditis
0.1
0.2
0.3
0.4
SNP effect on FLT4

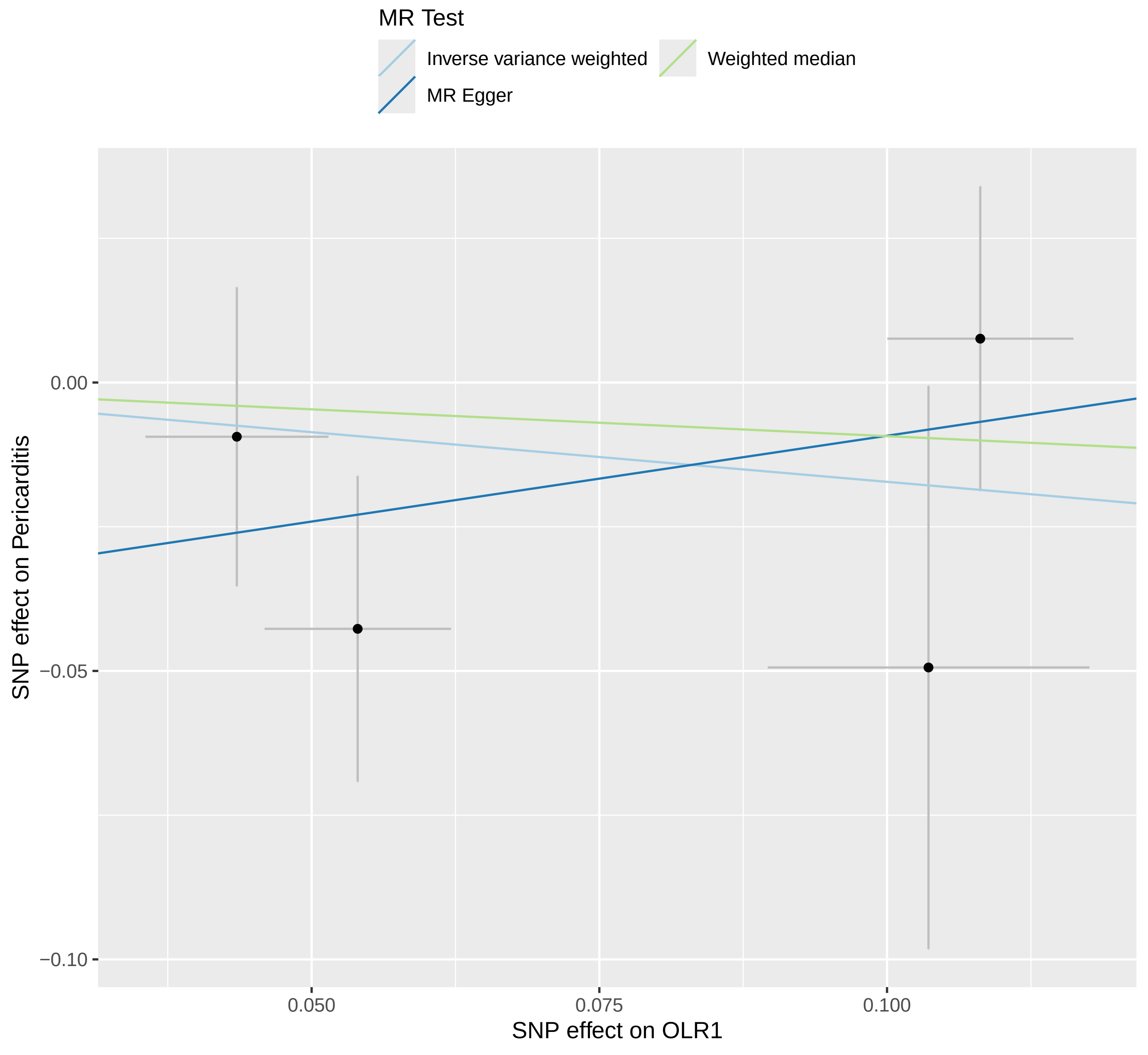
MR Test
Inverse variance weighted
MR Egger
Weighted median
0.00
−0.05
−0.10
SNP effect on Pericarditis
0.050
0.075
0.100
SNP effect on OLR1

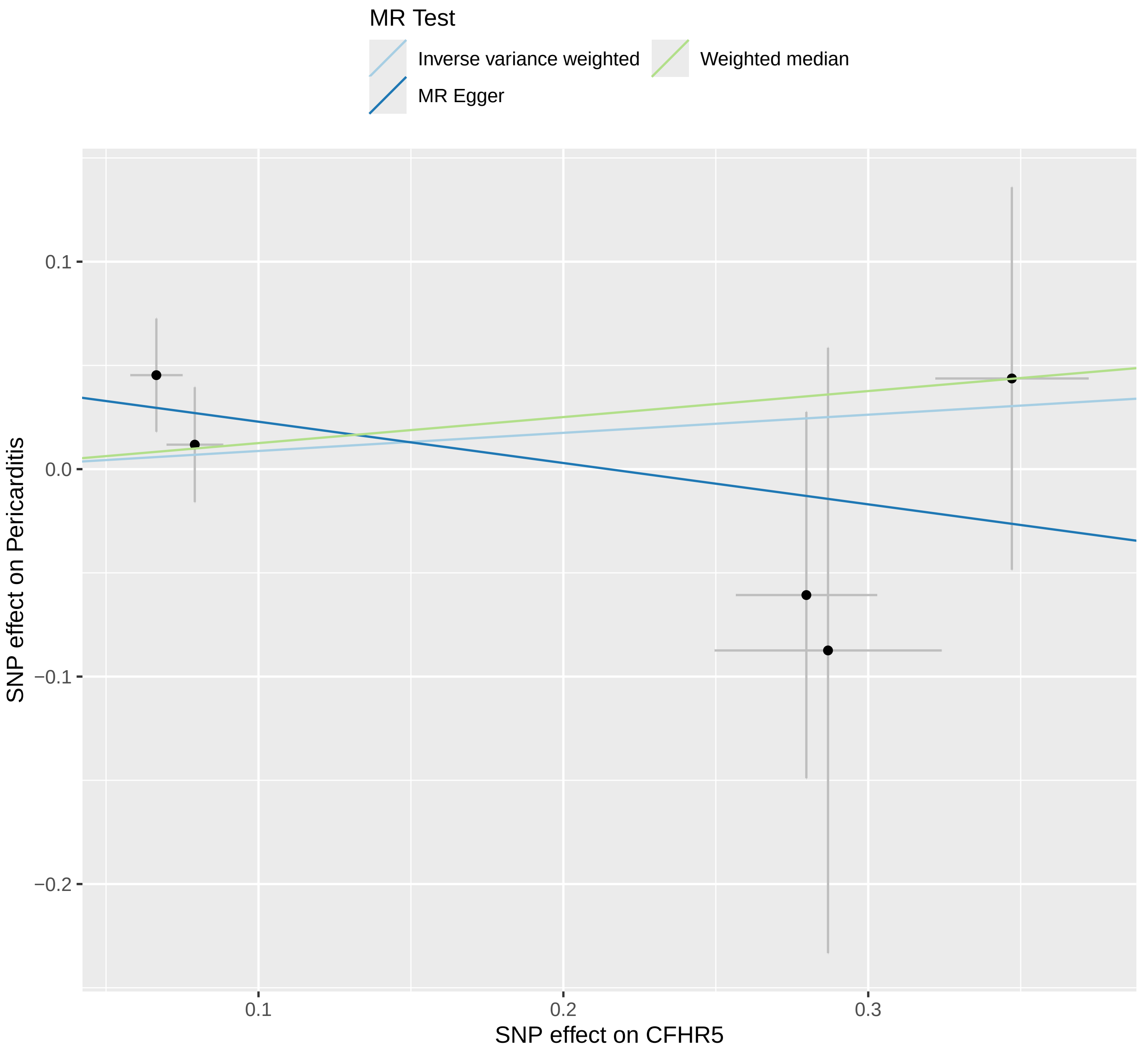
MR Test
Inverse variance weighted
Weighted median
MR Egger
0.1
0.0
−0.1
−0.2
SNP effect on Pericarditis
0.1
0.2
0.3
SNP effect on CFHR5

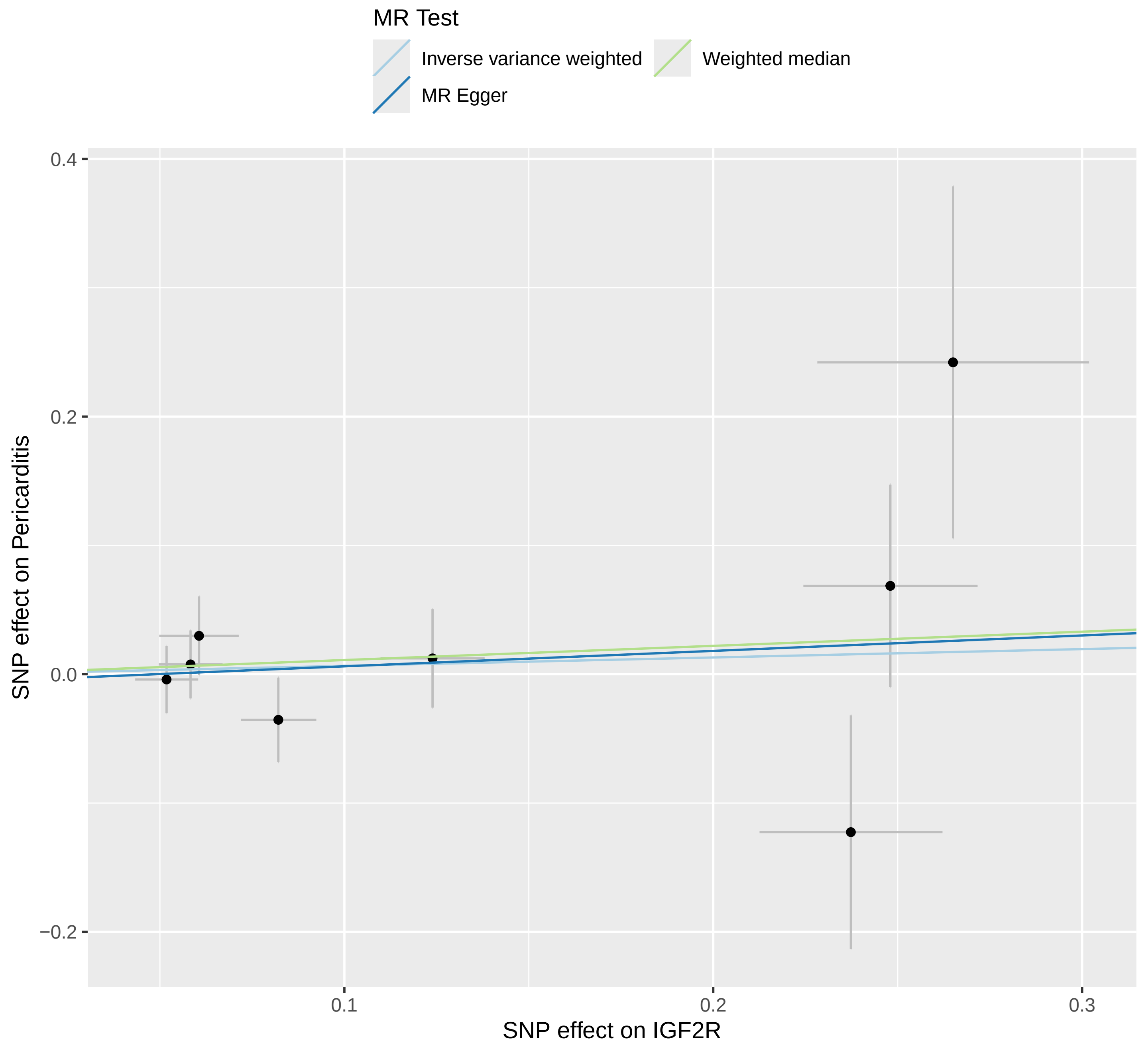

MR Test
Inverse variance weighted
MR Egger
Weighted median
0.4
0.2
0.0
−0.2
0.1
0.2
0.3
SNP effect on Pericarditis
SNP effect on IGF2R

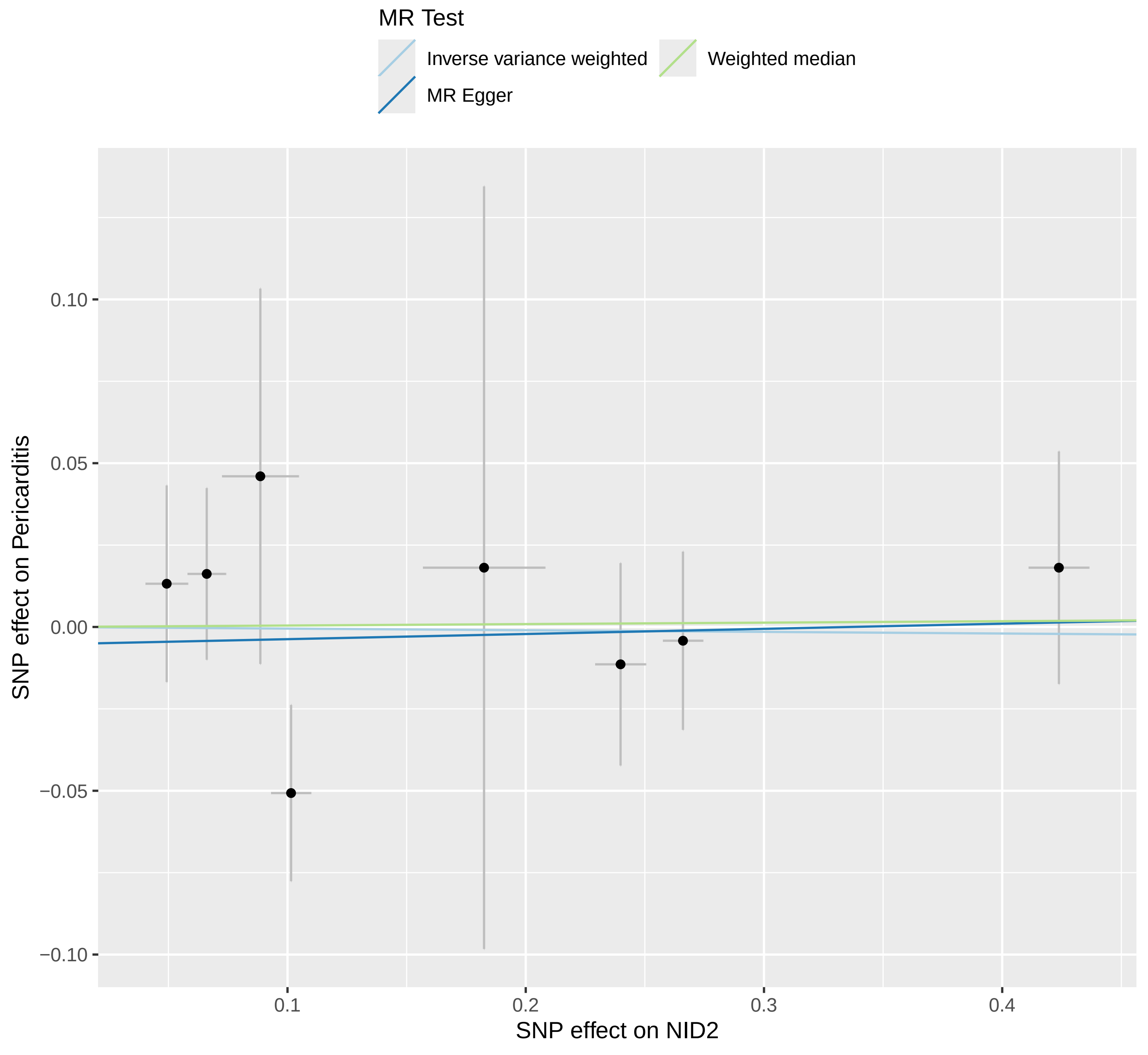
MR Test
Inverse variance weighted
MR Egger
Weighted median
SNP effect on Pericarditis
0.10
0.05
0.00
−0.05
−0.10
0.1
0.2
0.3
0.4
SNP effect on NID2

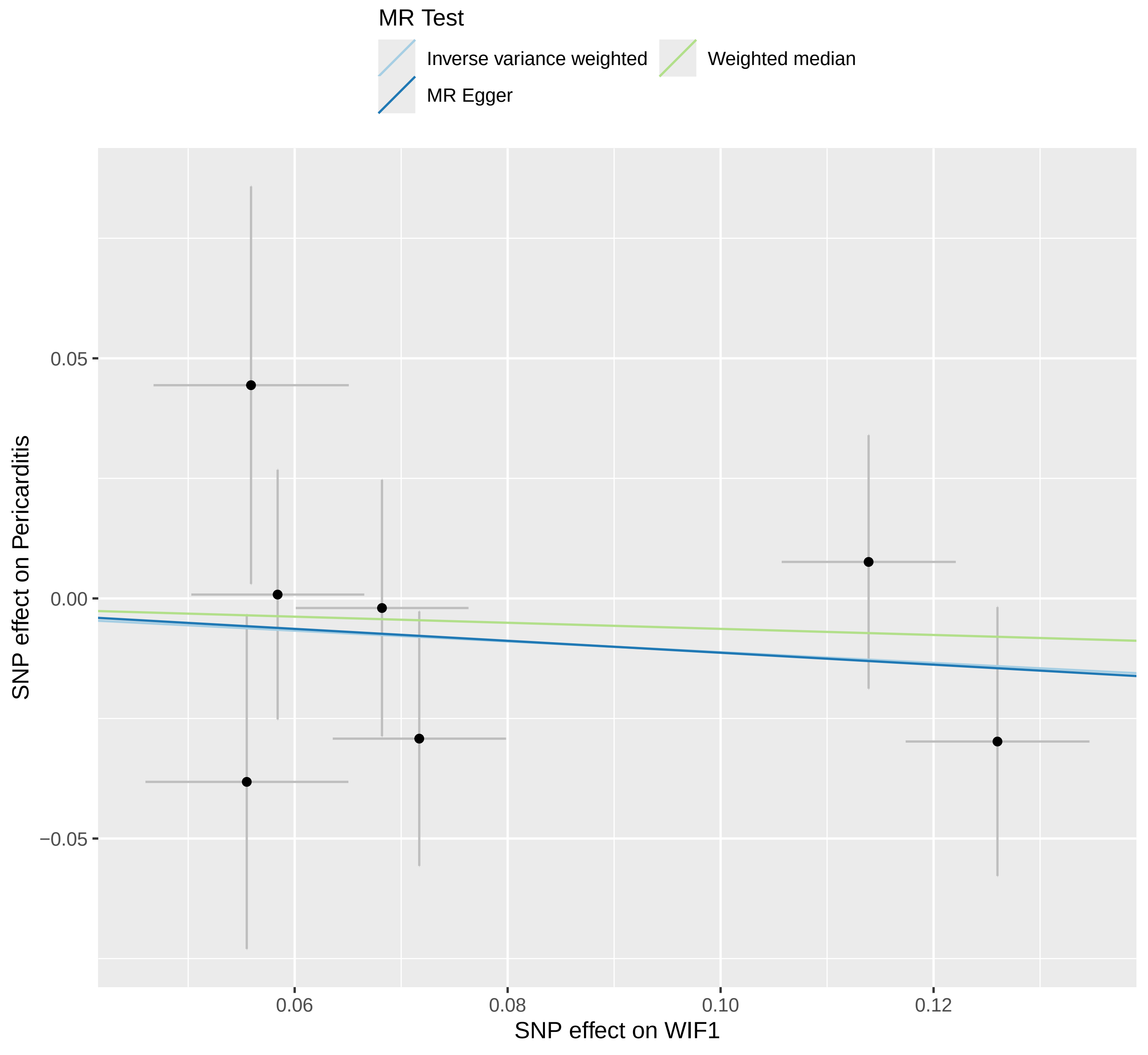
MR Test
Inverse variance weighted
MR Egger
Weighted median
0.05
0.00
−0.05
SNP effect on Pericarditis
0.06
0.08
0.10
0.12
SNP effect on WIF1

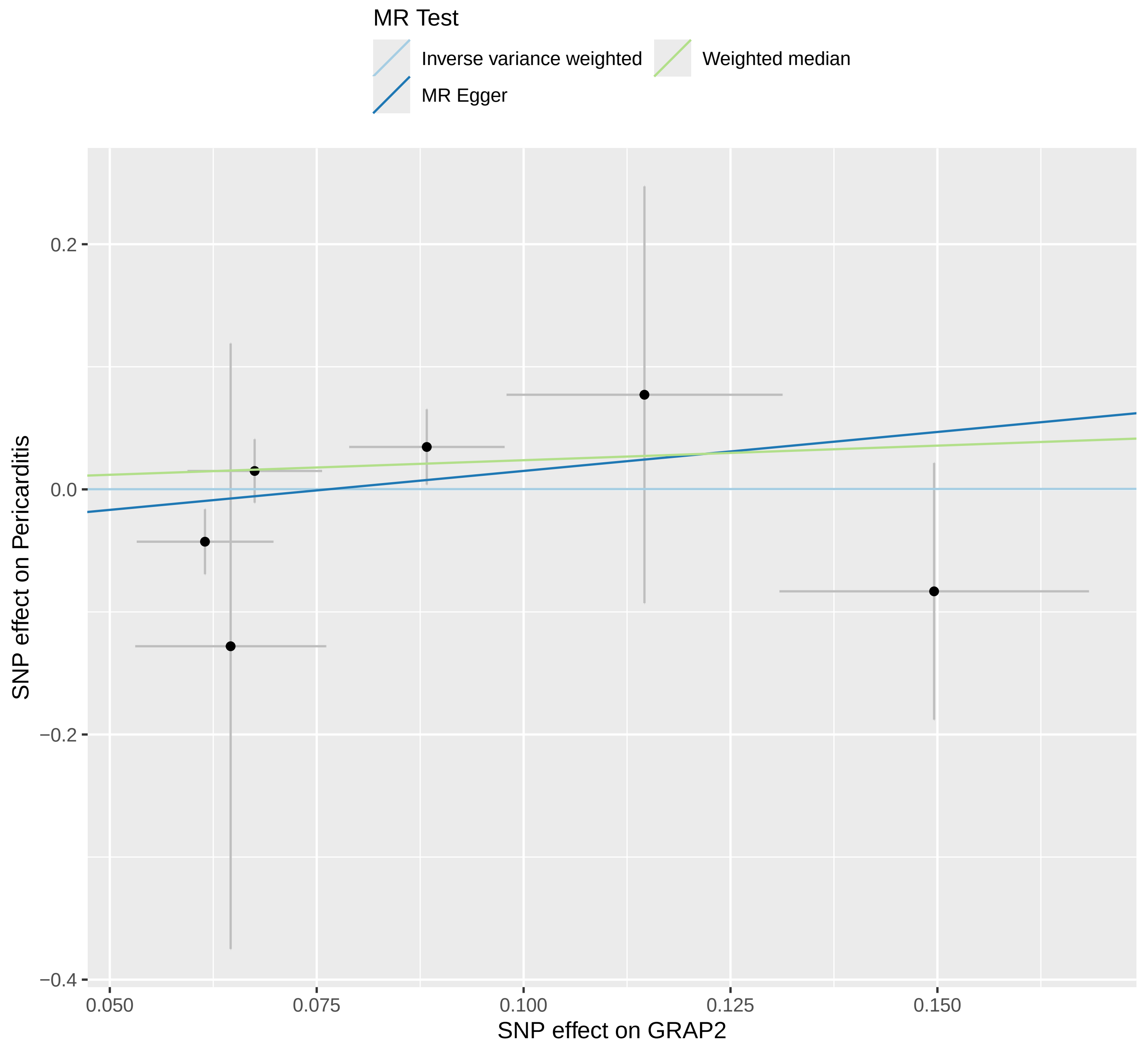
MR Test
Inverse variance weighted
Weighted median
MR Egger
SNP effect on Pericarditis
0.2
0.0
−0.2
−0.4
0.050
0.075
0.100
0.125
0.150
SNP effect on GRAP2

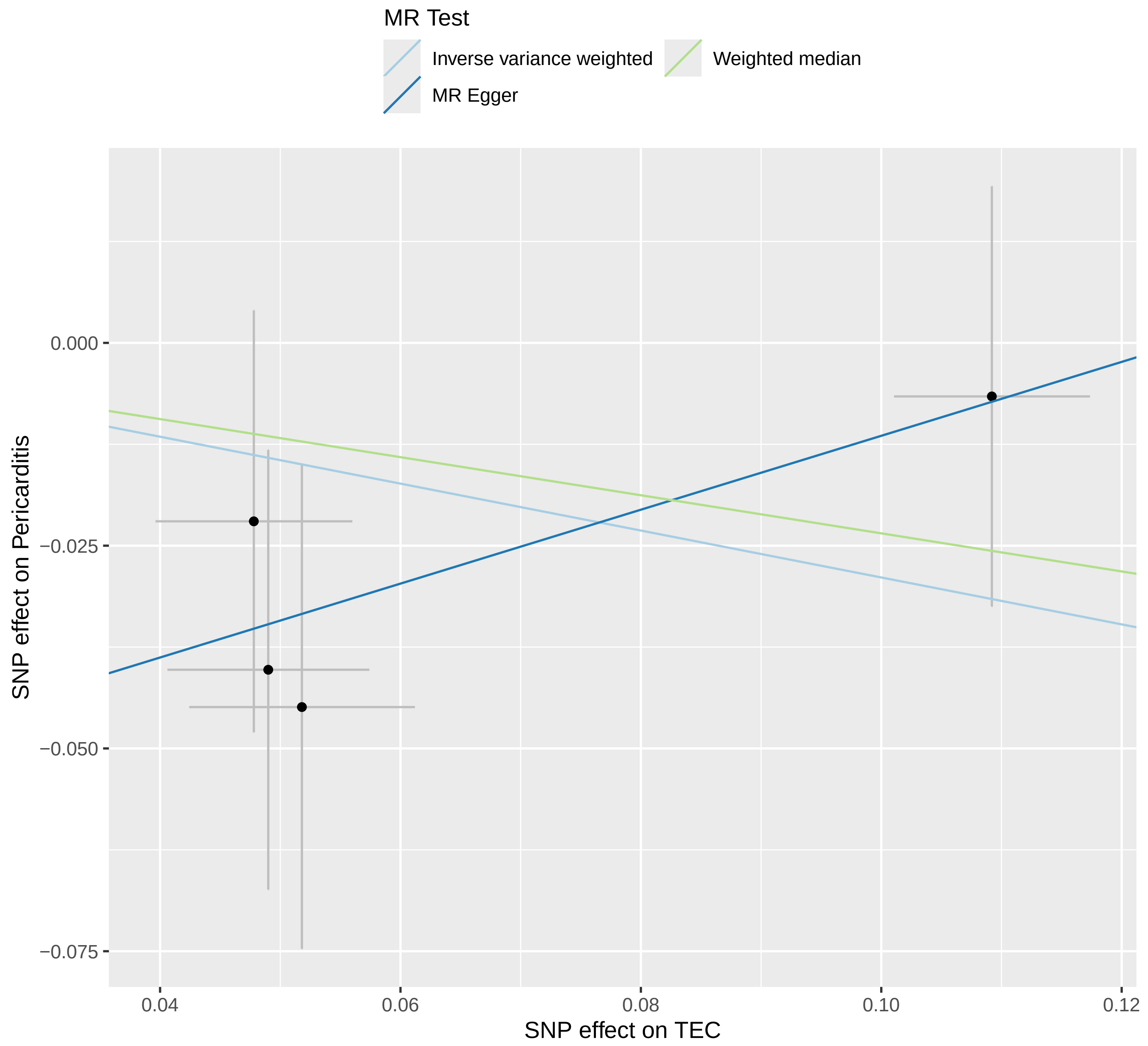

MR Test
Inverse variance weighted
Weighted median
MR Egger
0.000
−0.025
−0.050
−0.075
SNP effect on Pericarditis
0.04
0.06
0.08
0.10
0.12
SNP effect on TEC

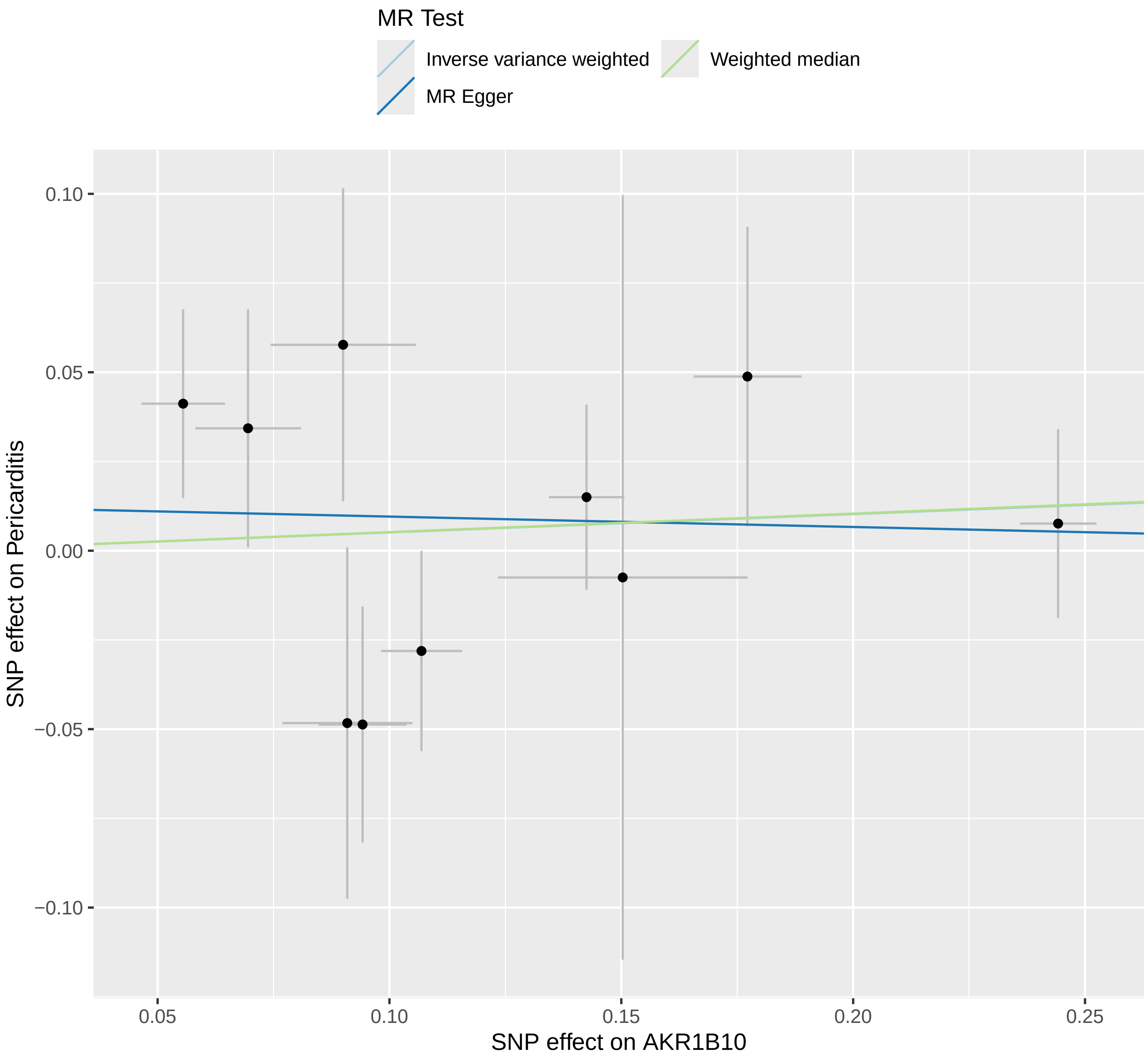
MR Test
Inverse variance weighted
MR Egger
Weighted median
0.10
0.05
0.00
−0.05
−0.10
SNP effect on Pericarditis
0.05
0.10
0.15
0.20
0.25
SNP effect on AKR1B10

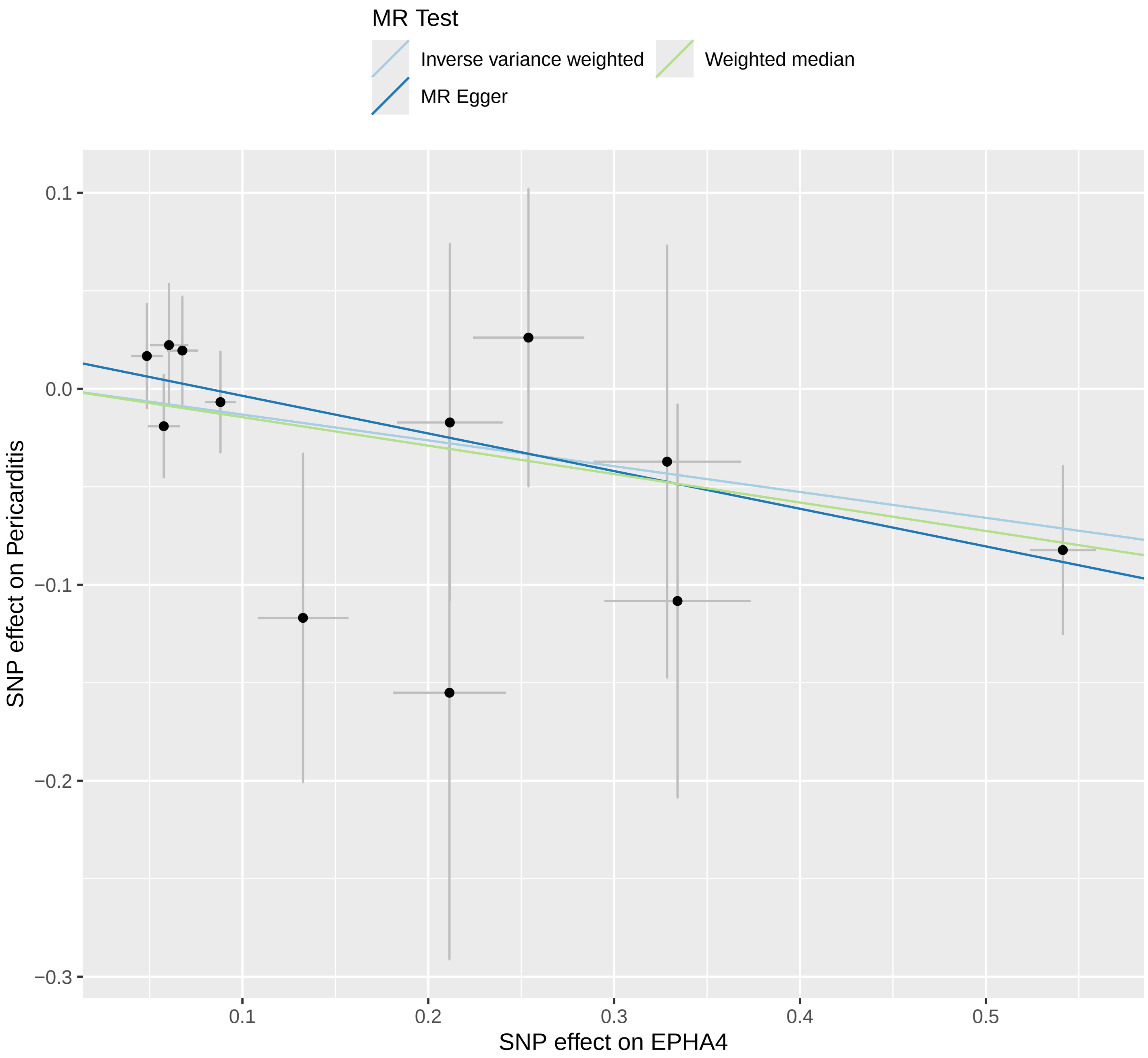
MR Test
Inverse variance weighted
Weighted median
MR Egger
0.1
0.0
−0.1
−0.2
−0.3
SNP effect on Pericarditis
0.1
0.2
0.3
0.4
0.5
SNP effect on EPHA4

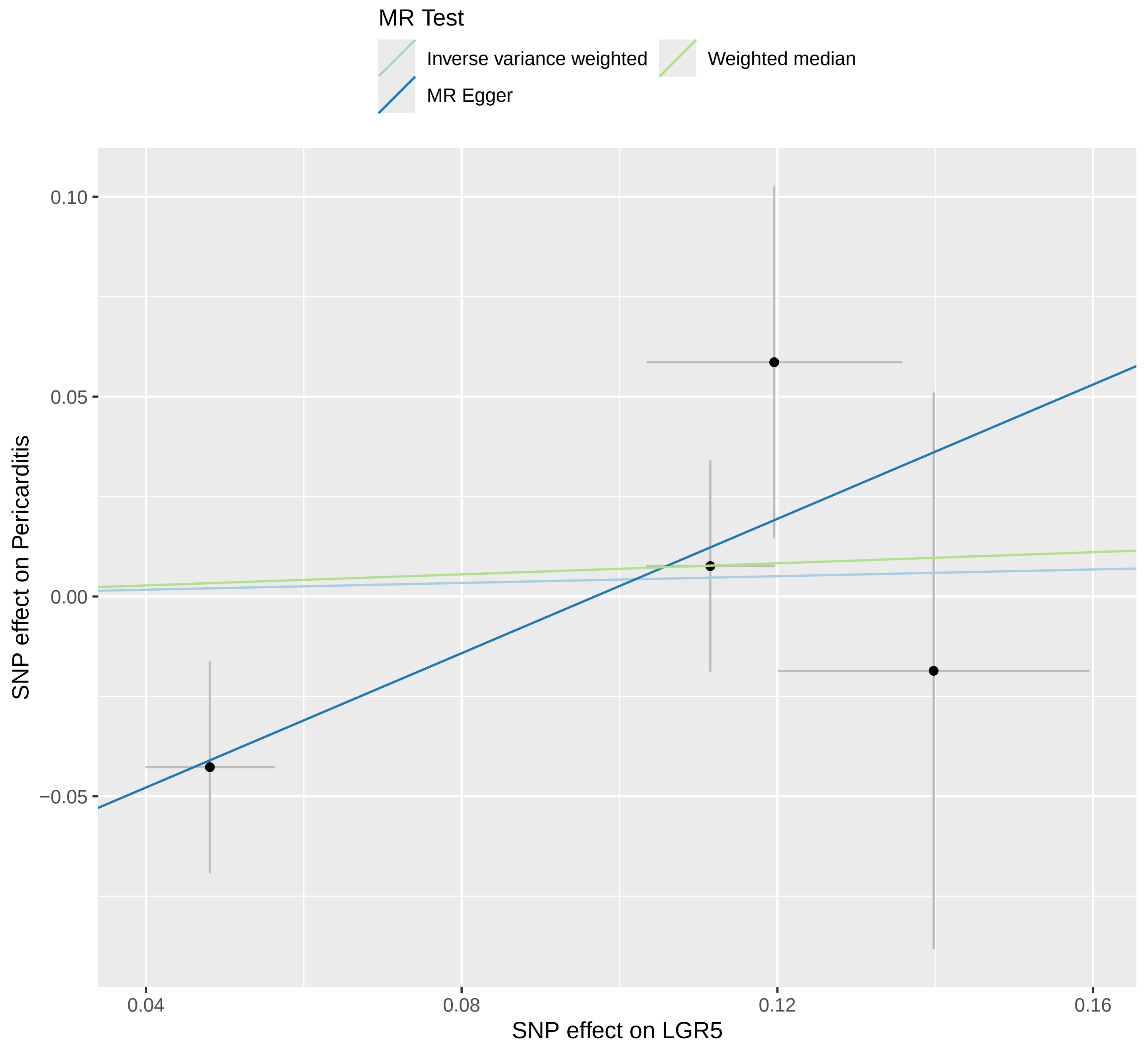
MR Test
Inverse variance weighted
Weighted median
MR Egger
SNP effect on Pericarditis
0.10
0.05
0.00
−0.05
0.04
0.08
0.12
0.16
SNP effect on LGR5

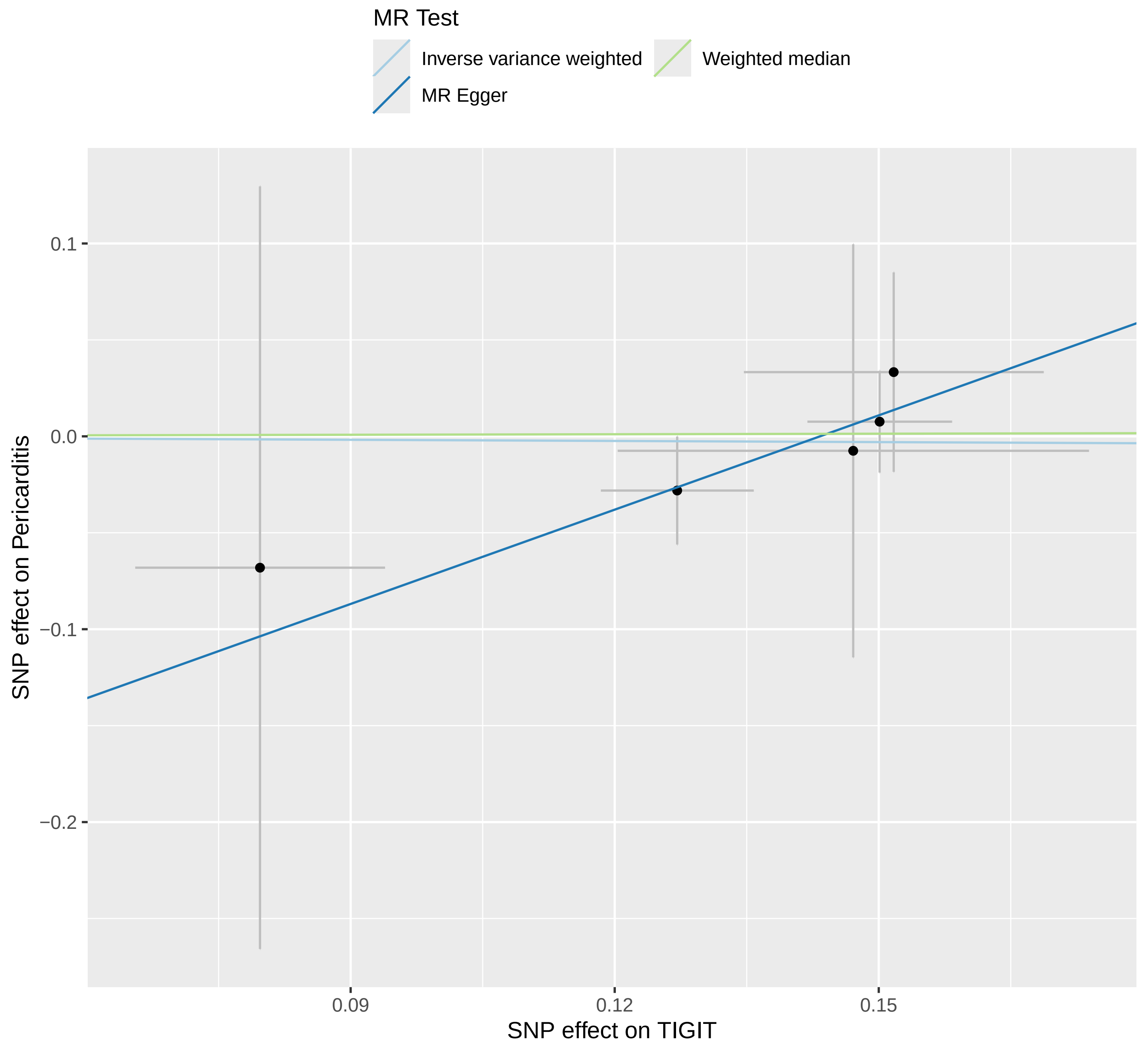
MR Test
Inverse variance weighted
MR Egger
Weighted median
0.1
0.0
−0.1
−0.2
SNP effect on Pericarditis
0.09
0.12
0.15
SNP effect on TIGIT

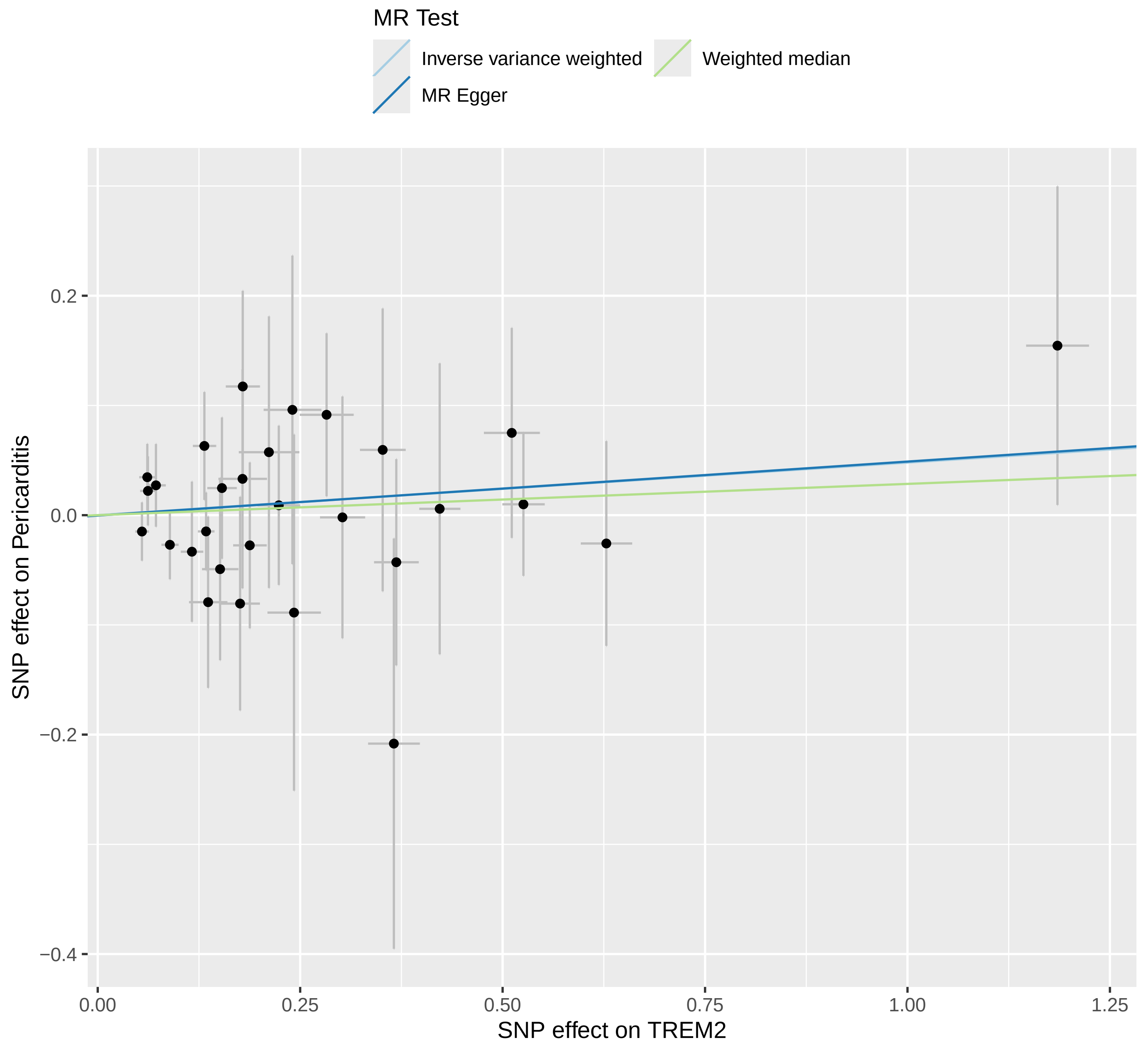
MR Test
Inverse variance weighted
Weighted median
MR Egger
0.2
0.0
−0.2
−0.4
SNP effect on Pericarditis
0.00
0.25
0.50
0.75
1.00
1.25
SNP effect on TREM2

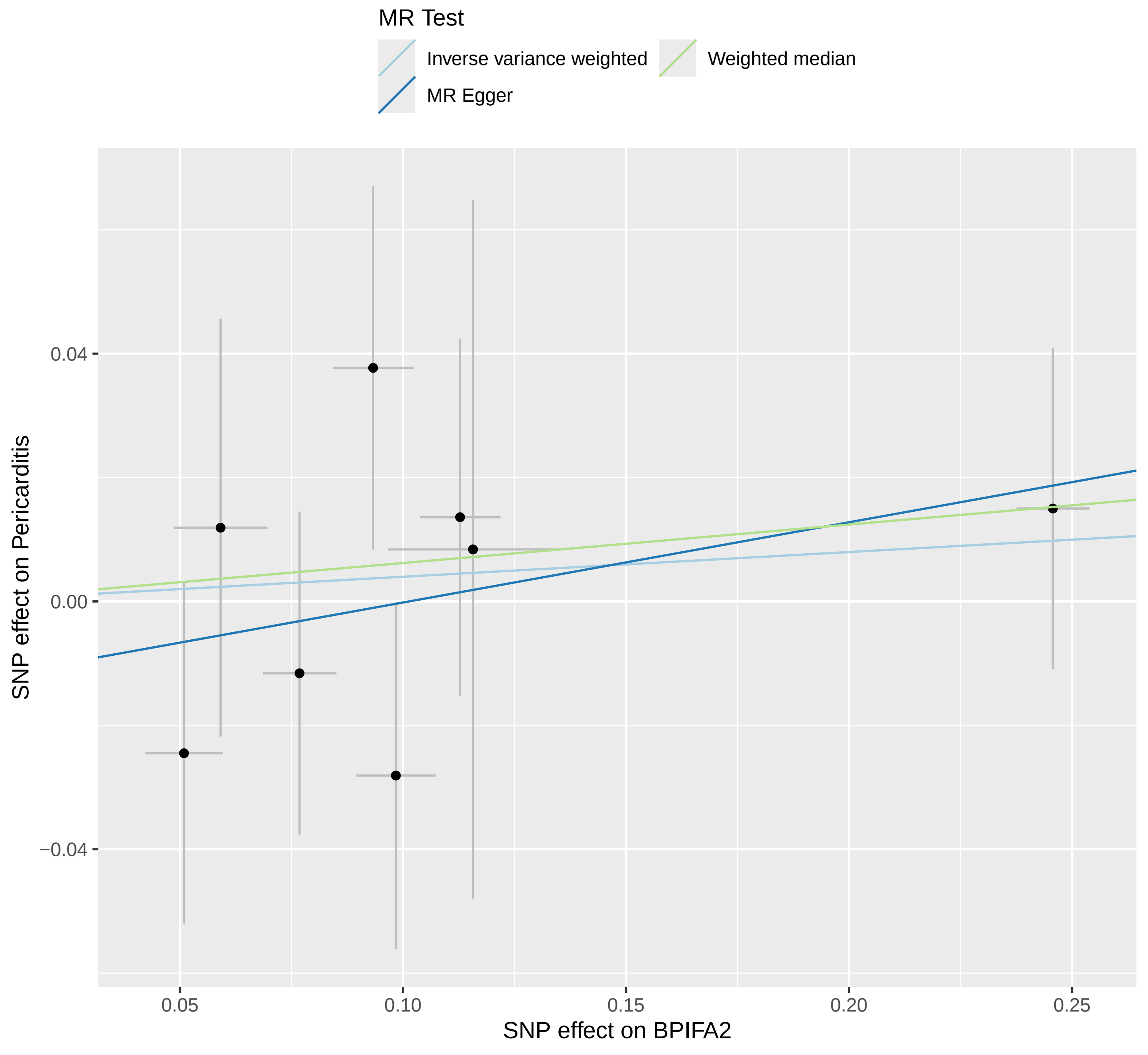

MR Test
Inverse variance weighted
Weighted median
MR Egger
SNP effect on Pericarditis
0.04
0.00
−0.04
0.05
0.10
0.15
0.20
0.25
SNP effect on BPIFA2

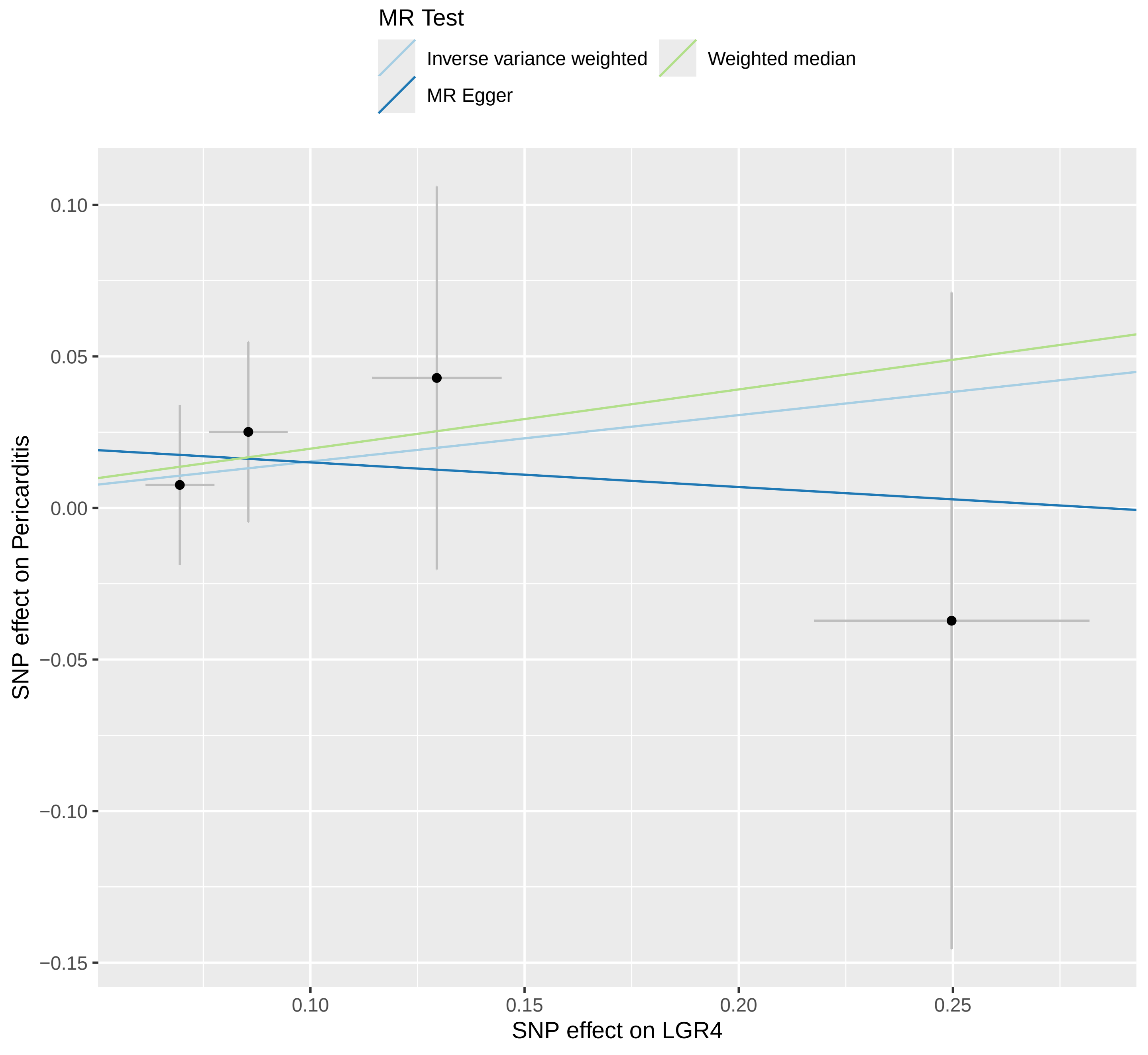

MR Test
Inverse variance weighted
Weighted median
MR Egger
SNP effect on Pericarditis
0.10
0.05
0.00
−0.05
−0.10
−0.15
0.10
0.15
0.20
0.25
SNP effect on LGR4

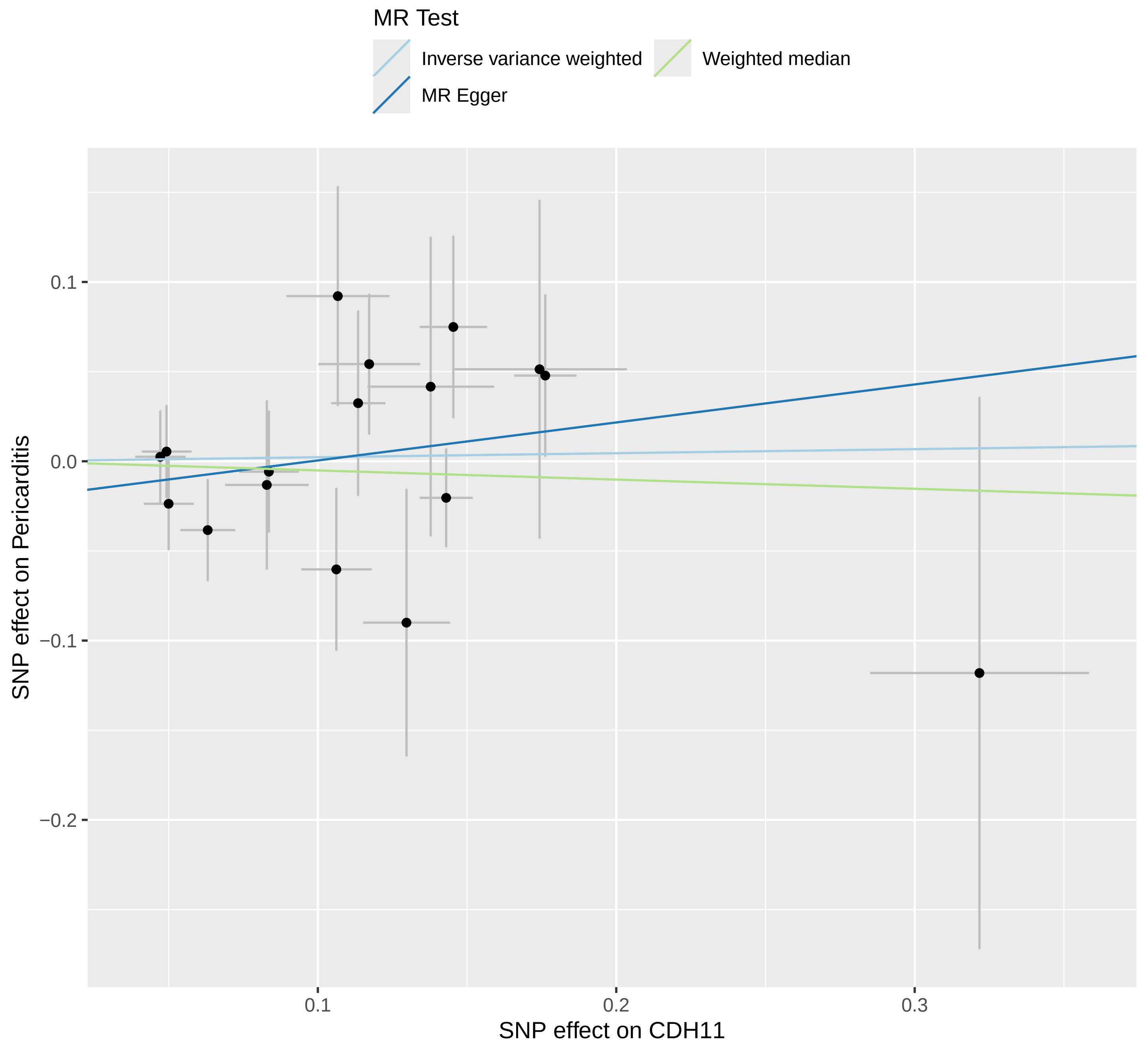
MR Test
Inverse variance weighted
Weighted median
MR Egger
0.1
0.0
−0.1
−0.2
SNP effect on Pericarditis
0.1
0.2
0.3
SNP effect on CDH11

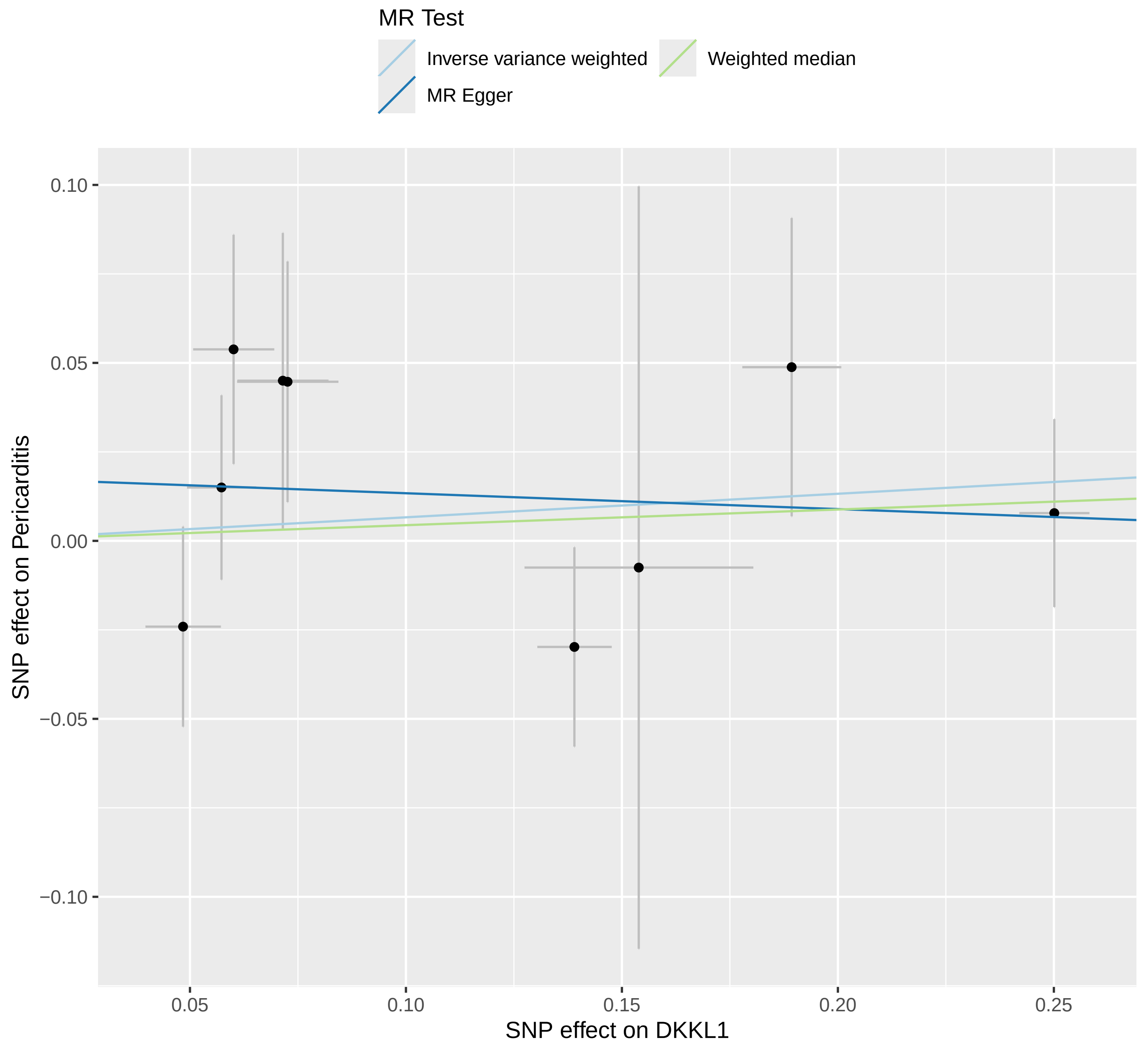
MR Test
Inverse variance weighted
MR Egger
Weighted median
0.10
0.05
0.00
−0.05
−0.10
SNP effect on Pericarditis
0.05
0.10
0.15
0.20
0.25
SNP effect on DKKL1

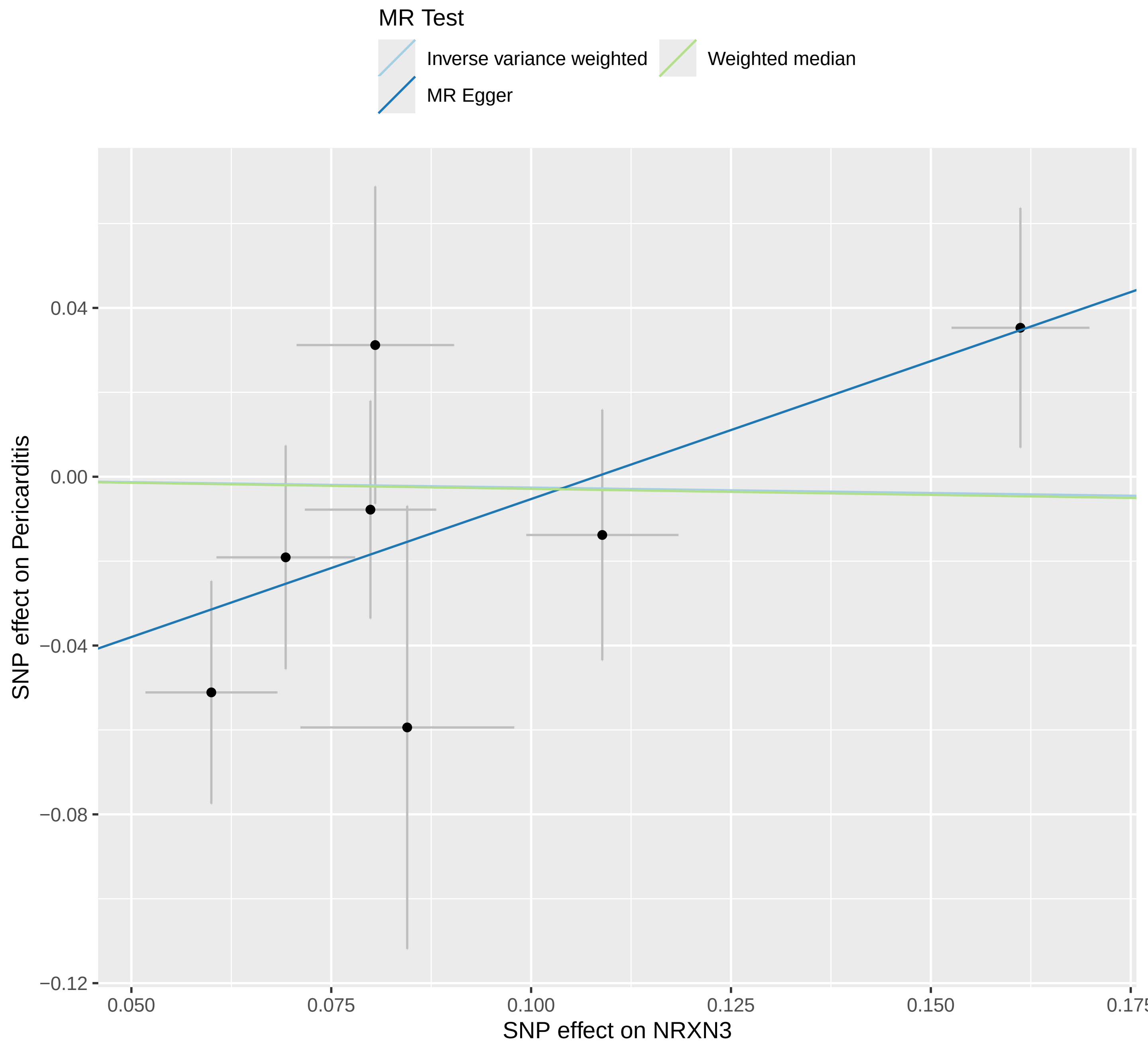

MR Test
Inverse variance weighted
Weighted median
MR Egger
SNP effect on Pericarditis
0.04
0.00
−0.04
−0.08
−0.12
0.050
0.075
0.100
0.125
0.150
0.175
SNP effect on NRXN3

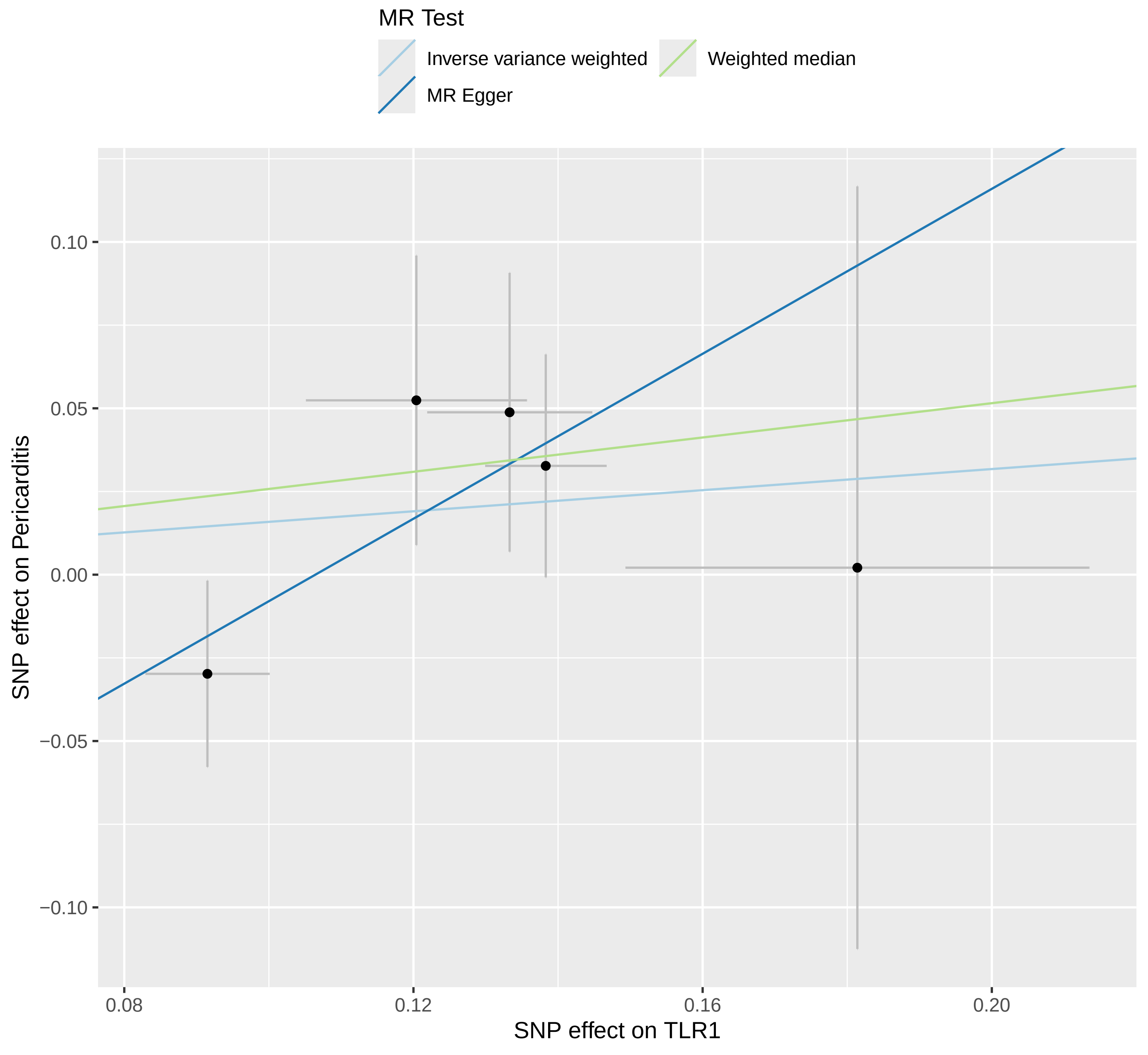
MR Test
Inverse variance weighted
Weighted median
MR Egger
0.10
0.05
0.00
−0.05
−0.10
SNP effect on Pericarditis
0.08
0.12
0.16
0.20
SNP effect on TLR1

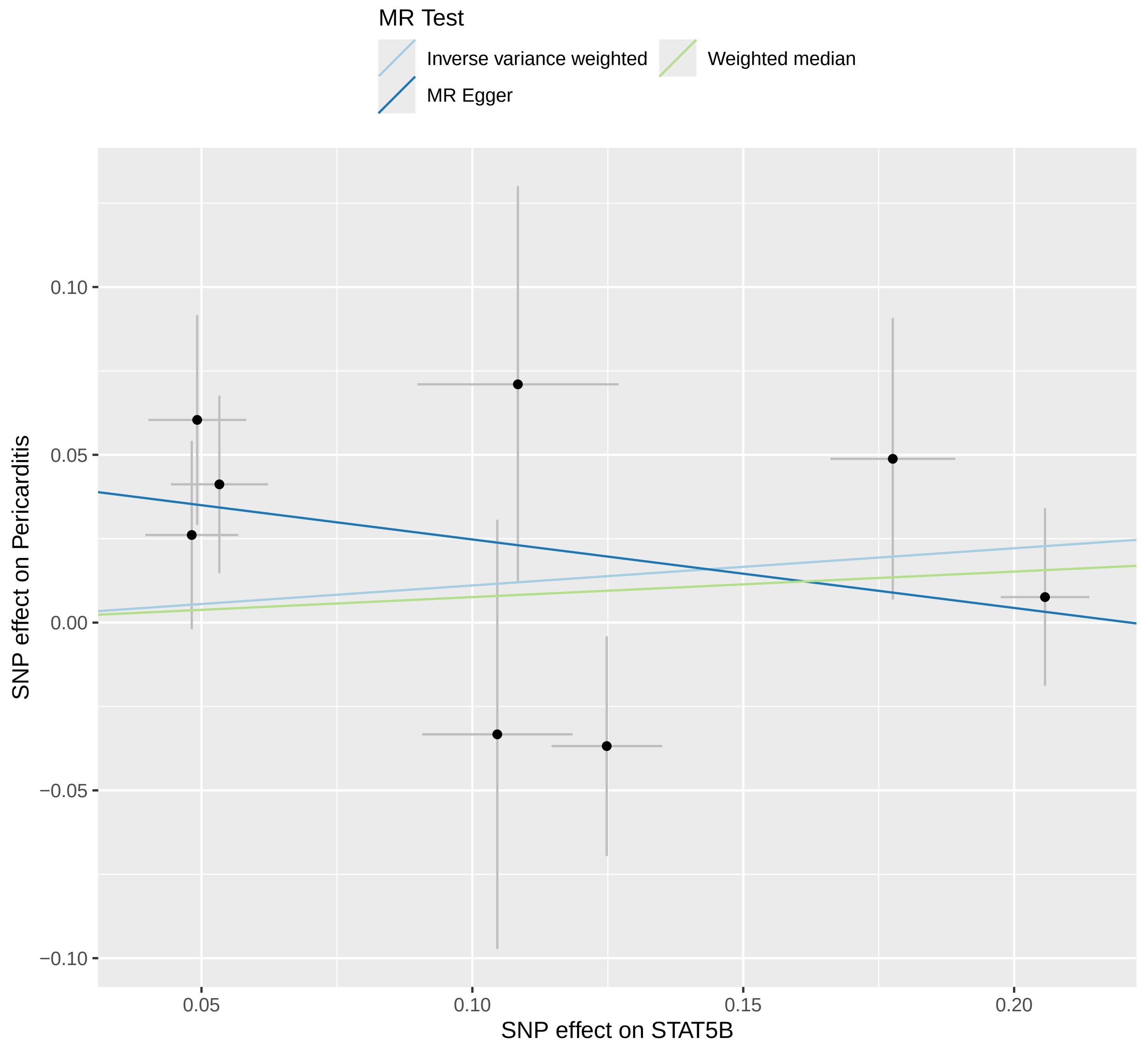
MR Test
Inverse variance weighted
Weighted median
MR Egger
0.10
0.05
0.00
−0.05
−0.10
SNP effect on Pericarditis
0.05
0.10
0.15
0.20
SNP effect on STAT5B

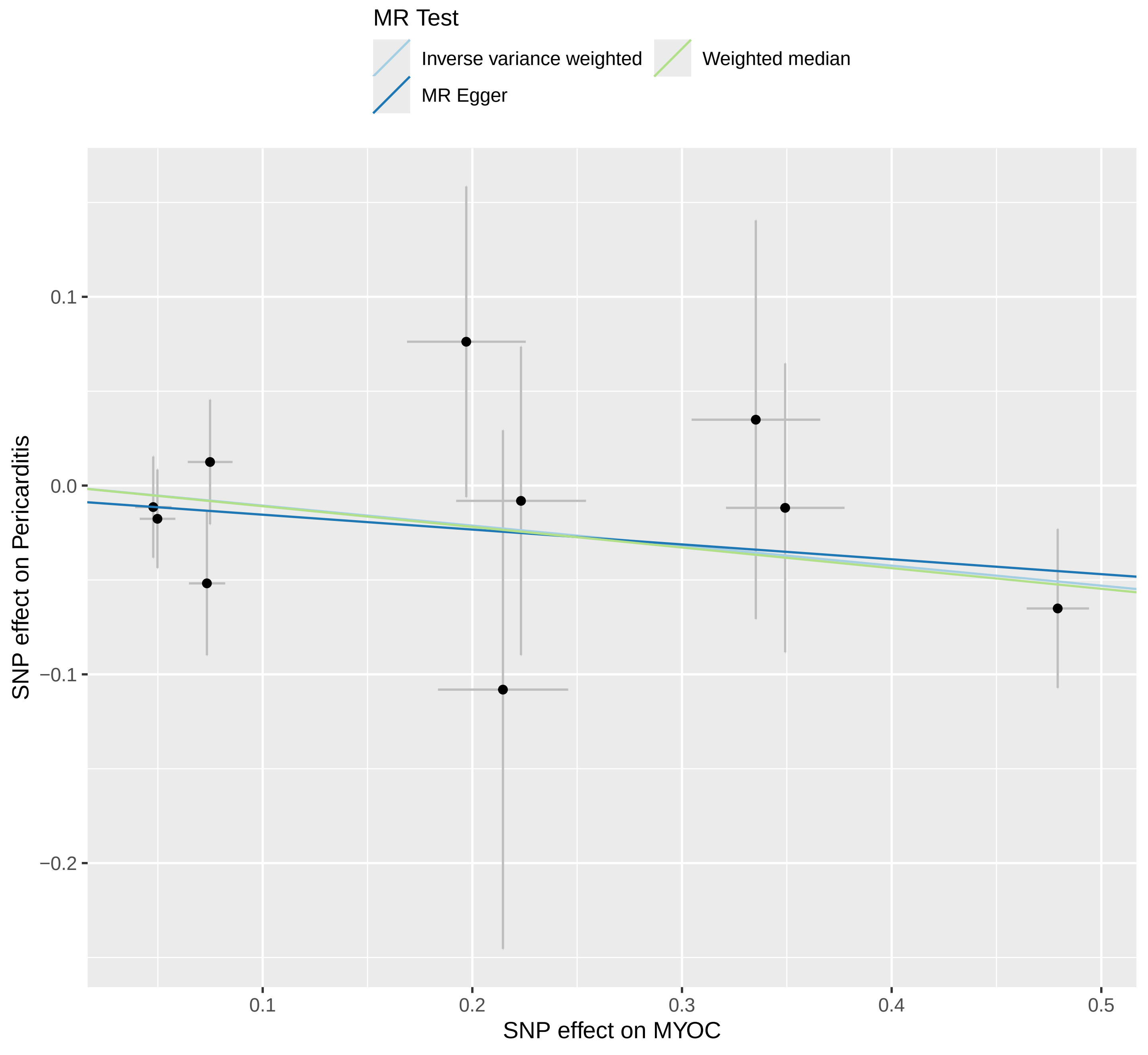
MR Test
Inverse variance weighted
Weighted median
MR Egger
0.1
0.0
−0.1
−0.2
SNP effect on Pericarditis
0.1
0.2
0.3
0.4
0.5
SNP effect on MYOC

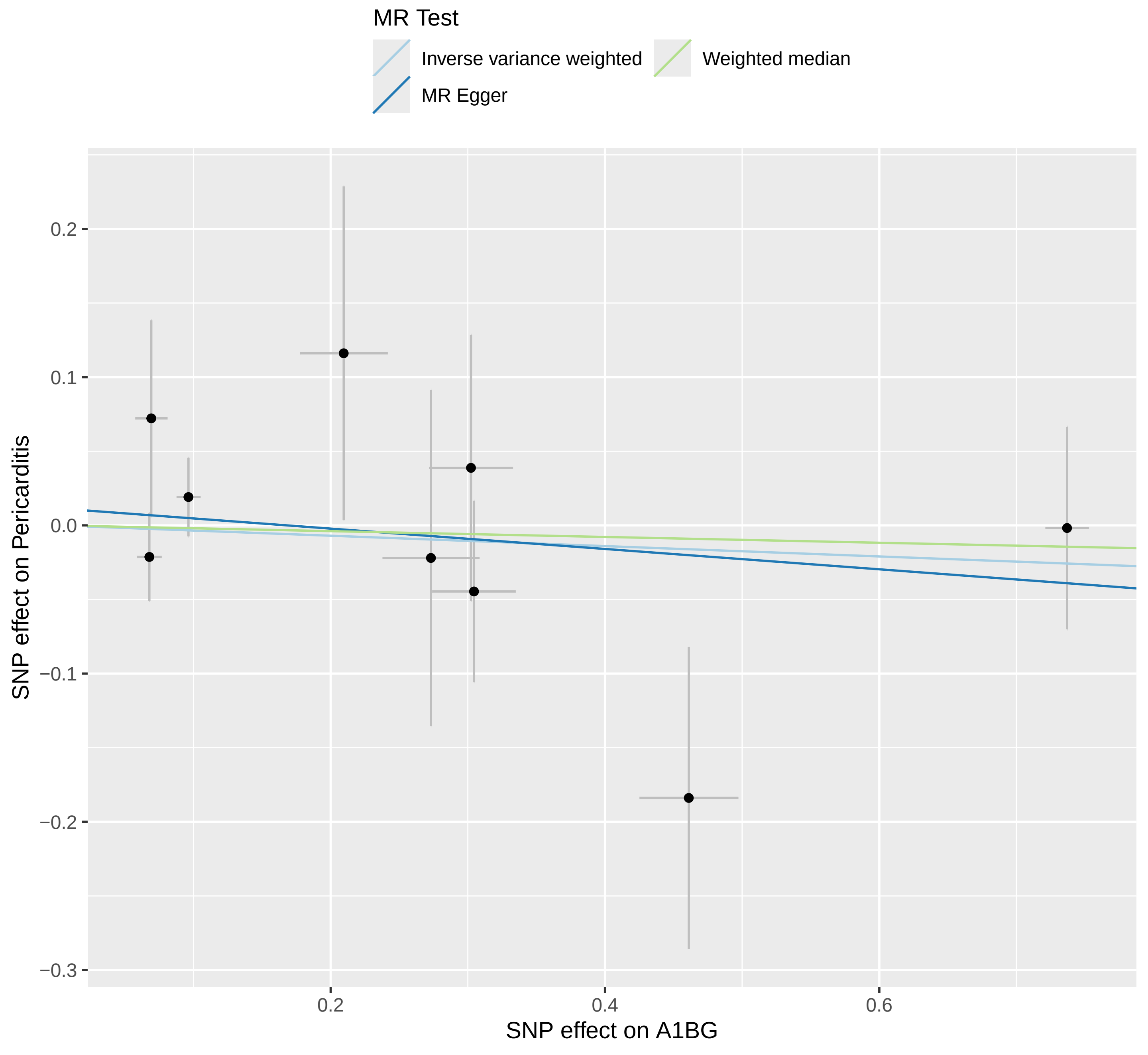

MR Test
Inverse variance weighted
MR Egger
Weighted median
SNP effect on Pericarditis
0.2
0.1
0.0
−0.1
−0.2
−0.3
0.2
0.4
0.6
SNP effect on A1BG

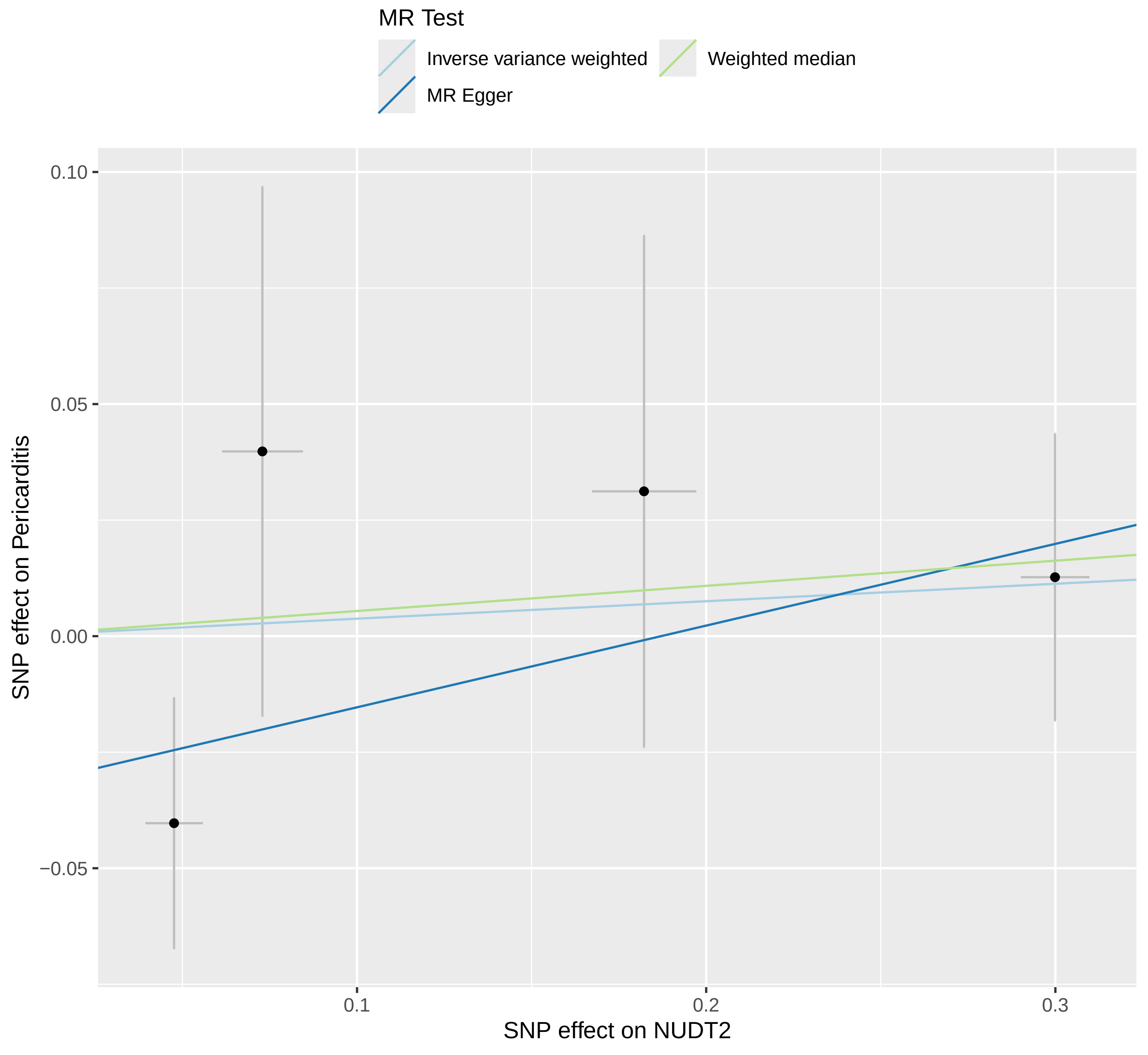

MR Test
Inverse variance weighted
Weighted median
MR Egger
SNP effect on Pericarditis
0.10
0.05
0.00
−0.05
0.1
0.2
0.3
SNP effect on NUDT2

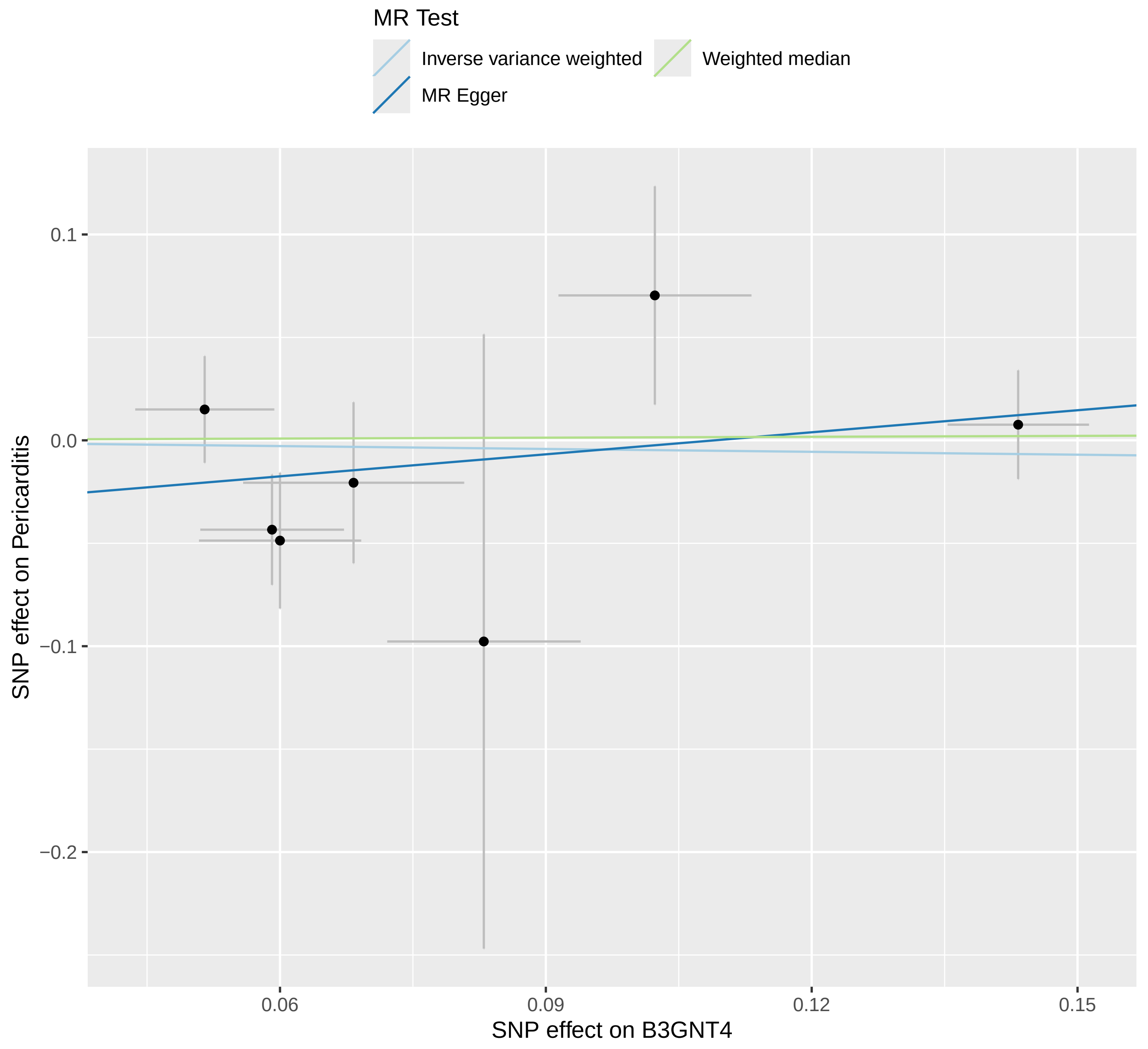
MR Test
Inverse variance weighted
Weighted median
MR Egger
0.1
0.0
−0.1
−0.2
SNP effect on Pericarditis
0.06
0.09
0.12
0.15
SNP effect on B3GNT4

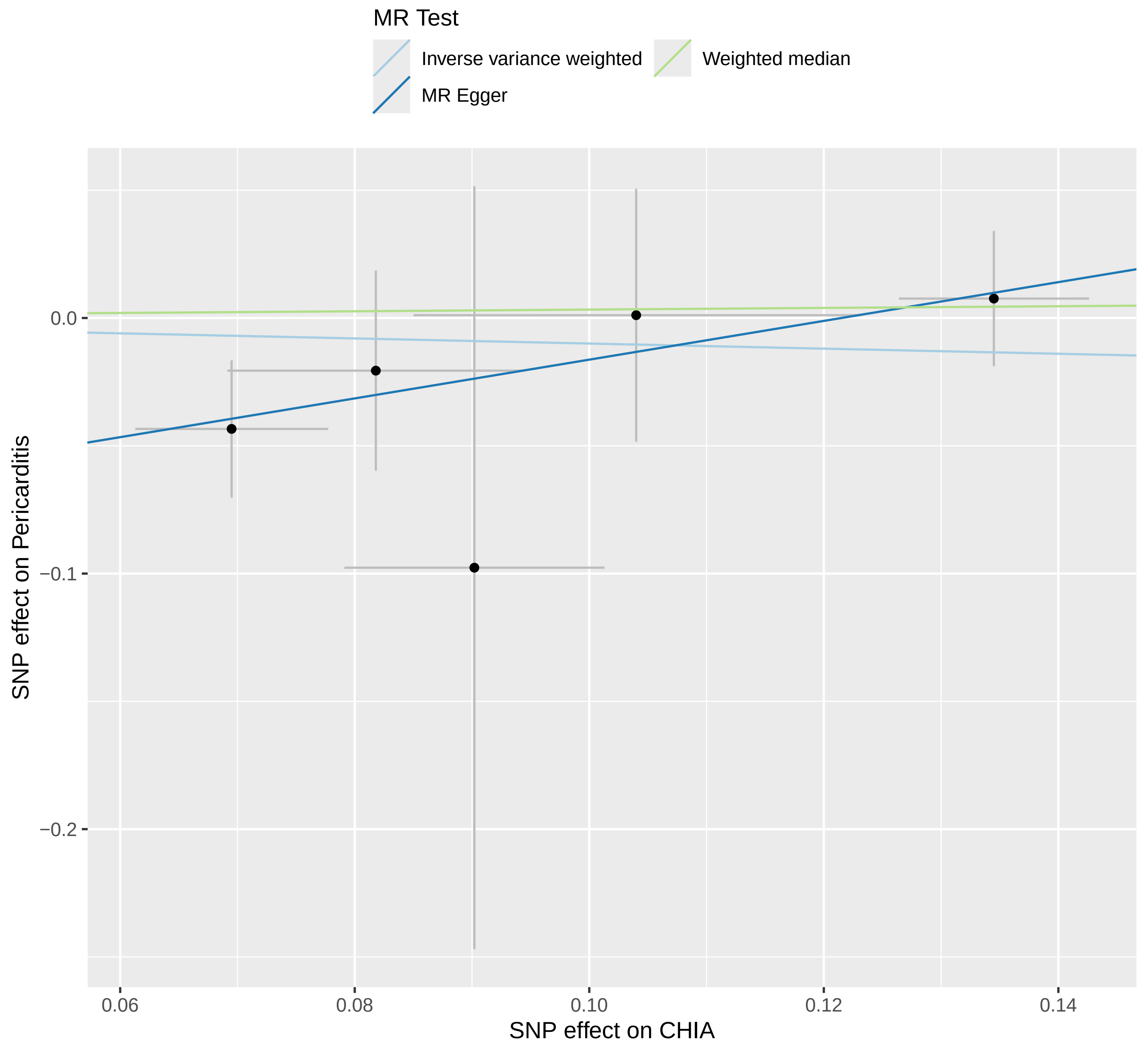

MR Test
Inverse variance weighted
Weighted median
MR Egger
SNP effect on Pericarditis
0.0
−0.1
−0.2
0.06
0.08
0.10
0.12
0.14
SNP effect on CHIA

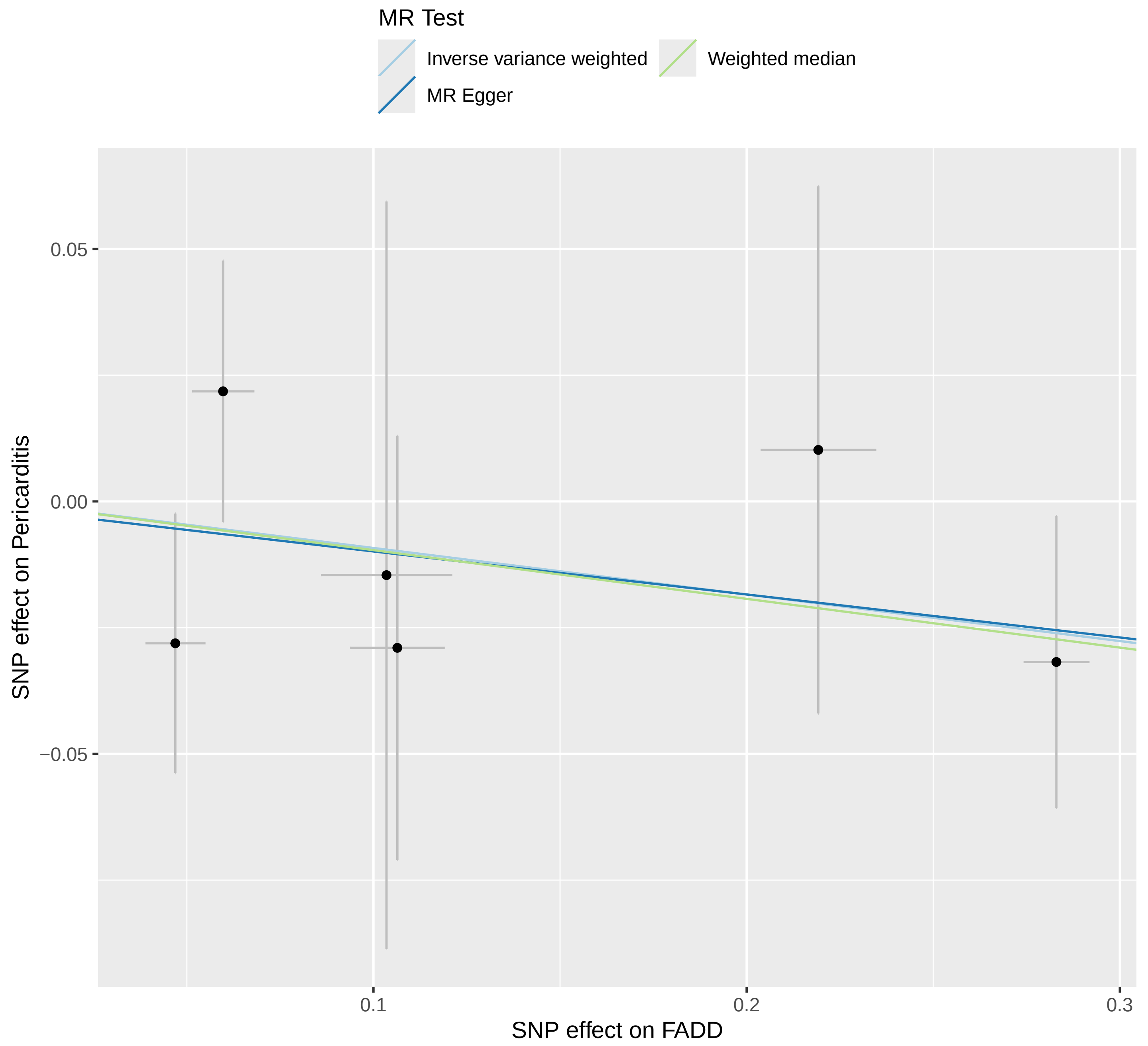

MR Test
Inverse variance weighted
MR Egger
Weighted median
0.05
0.00
−0.05
SNP effect on Pericarditis
0.1
0.2
0.3
SNP effect on FADD

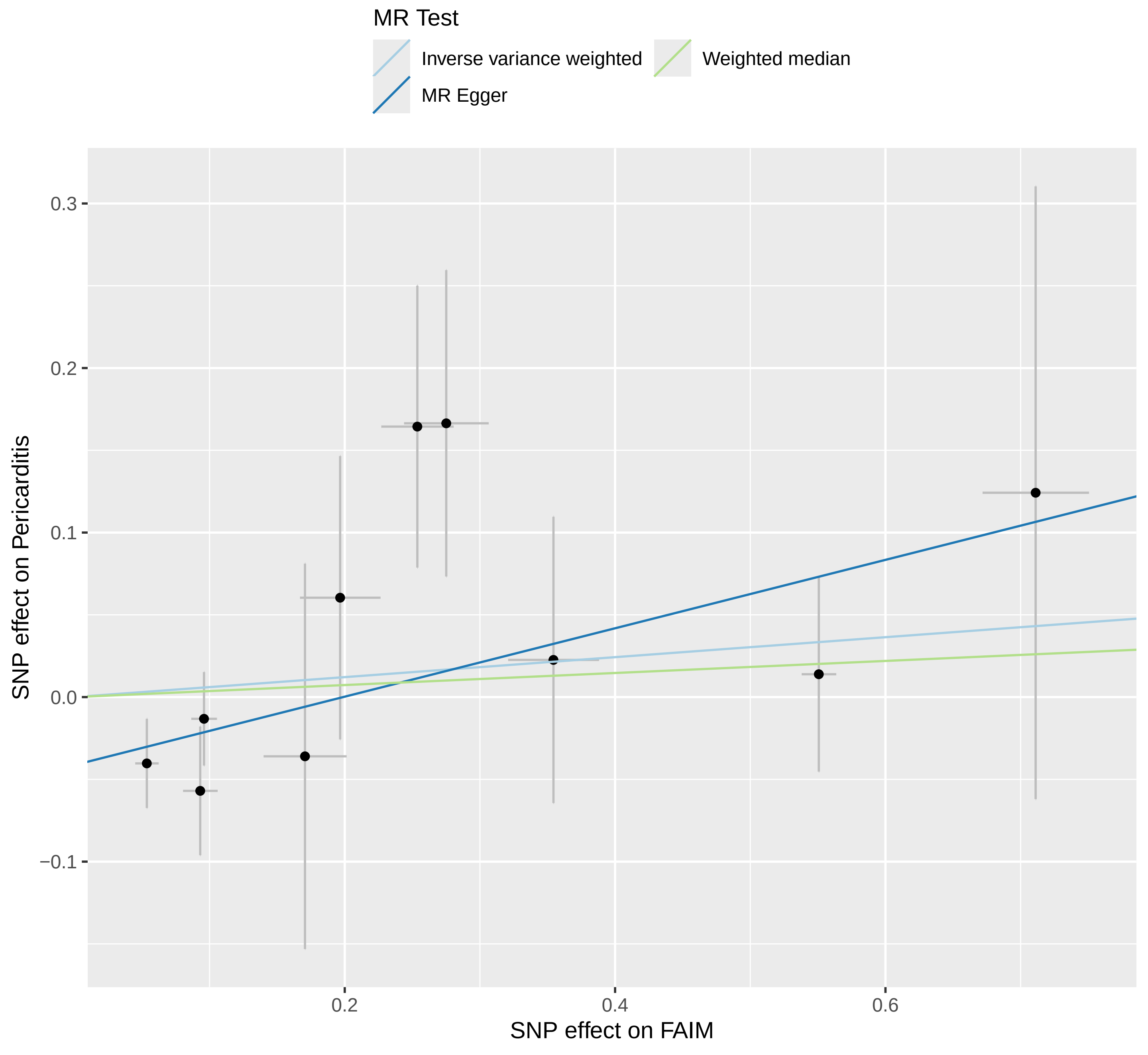
MR Test
Inverse variance weighted
Weighted median
MR Egger
0.3
0.2
0.1
0.0
−0.1
0.2
0.4
0.6
SNP effect on Pericarditis
SNP effect on FAIM

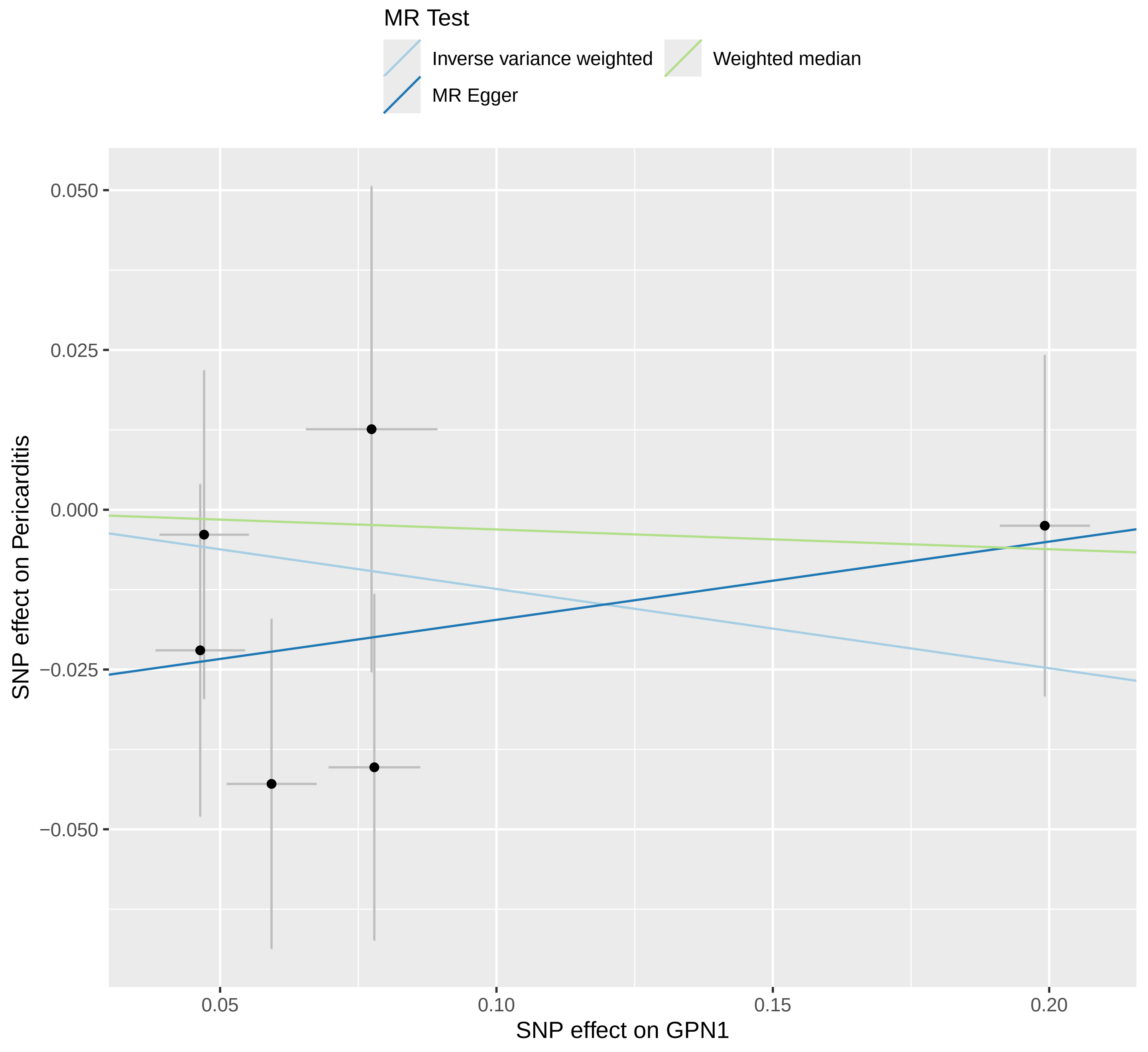

MR Test
Inverse variance weighted
MR Egger
Weighted median
0.050
0.025
0.000
−0.025
−0.050
SNP effect on Pericarditis
0.05
0.10
0.15
0.20
SNP effect on GPN1

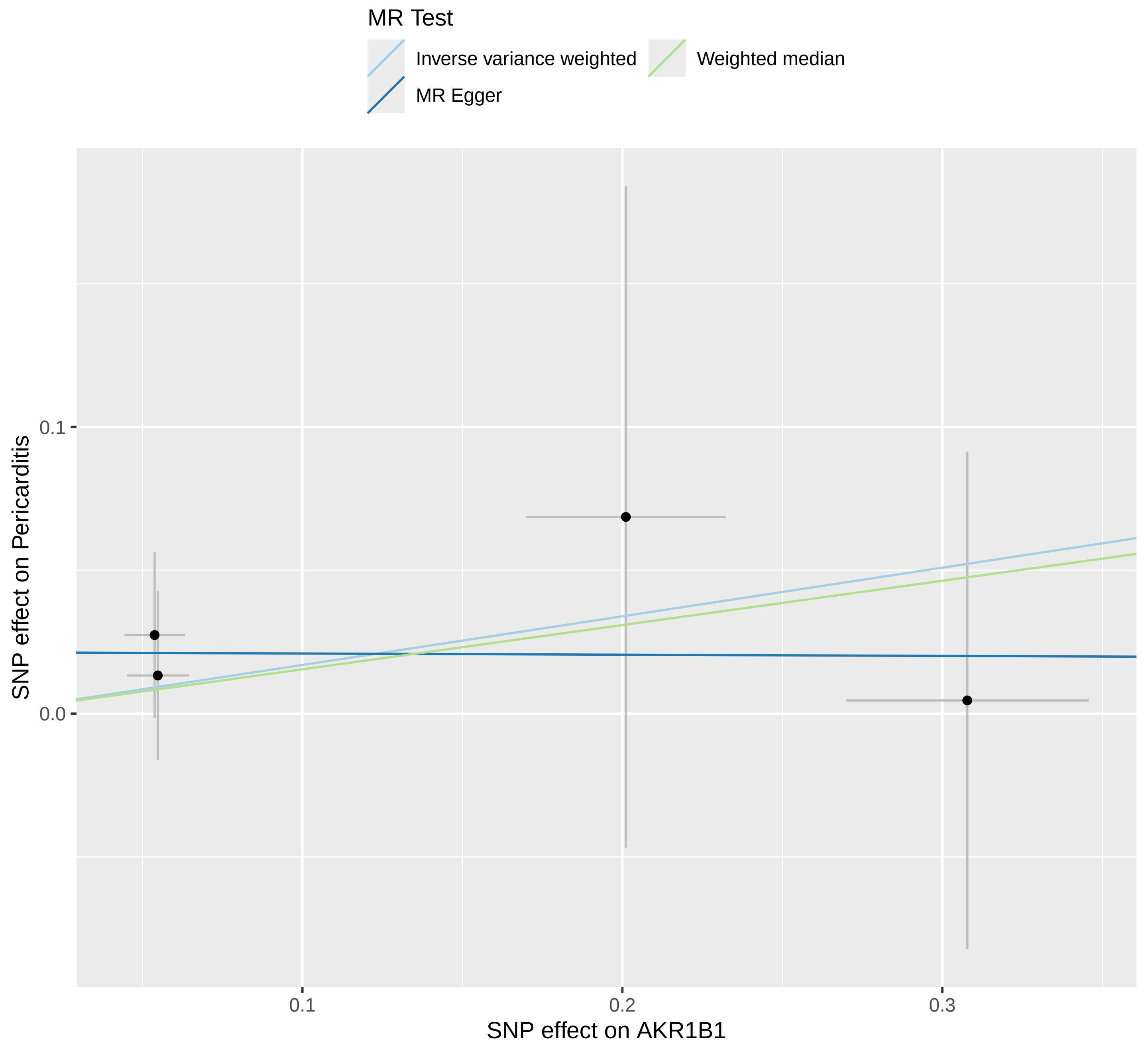

MR Test
Inverse variance weighted
Weighted median
MR Egger
SNP effect on Pericarditis
0.1
0.0
0.1
0.2
0.3
SNP effect on AKR1B1

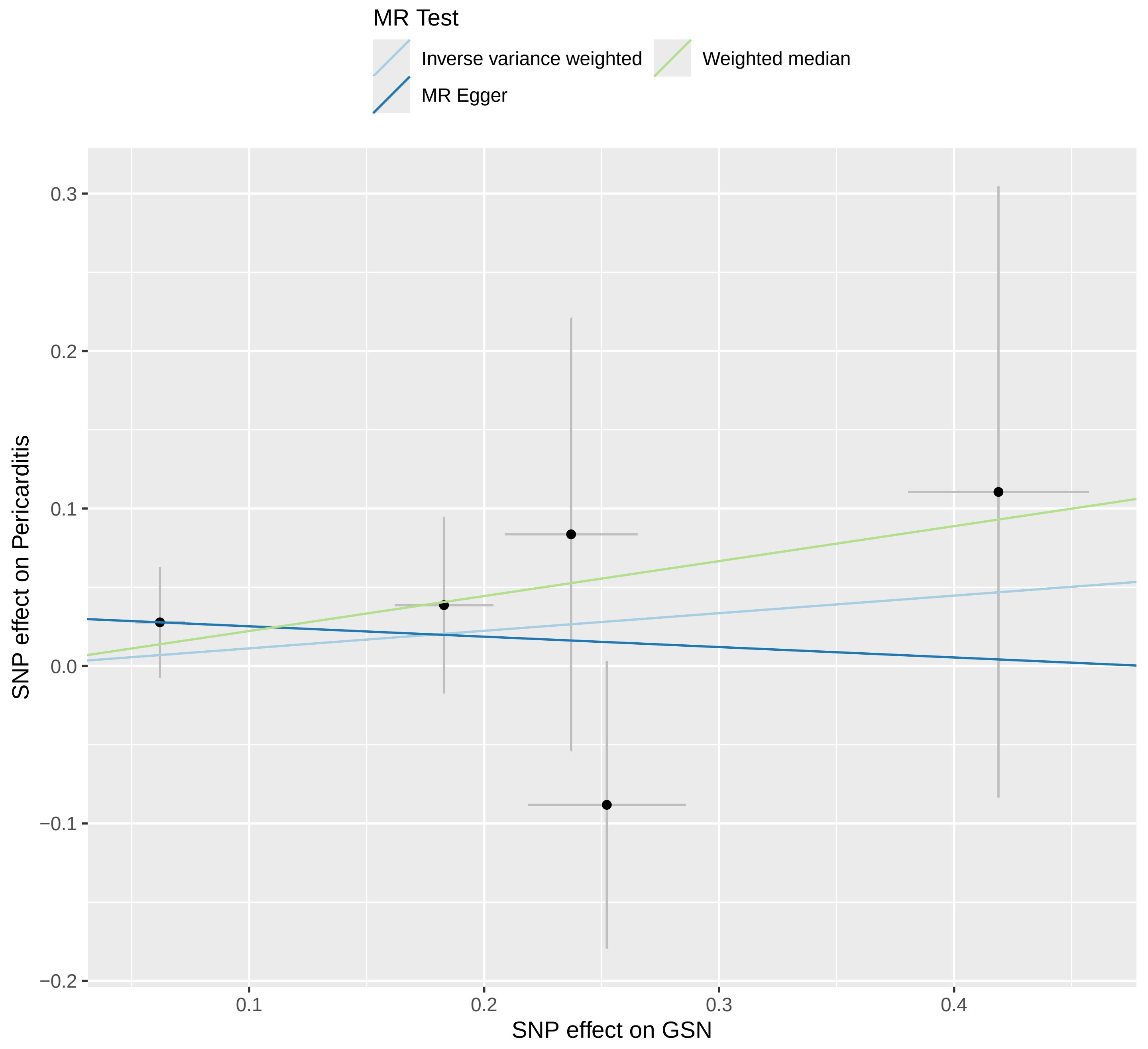
MR Test
Inverse variance weighted
Weighted median
MR Egger
0.3
0.2
0.1
0.0
−0.1
−0.2
SNP effect on Pericarditis
0.1
0.2
0.3
0.4
SNP effect on GSN

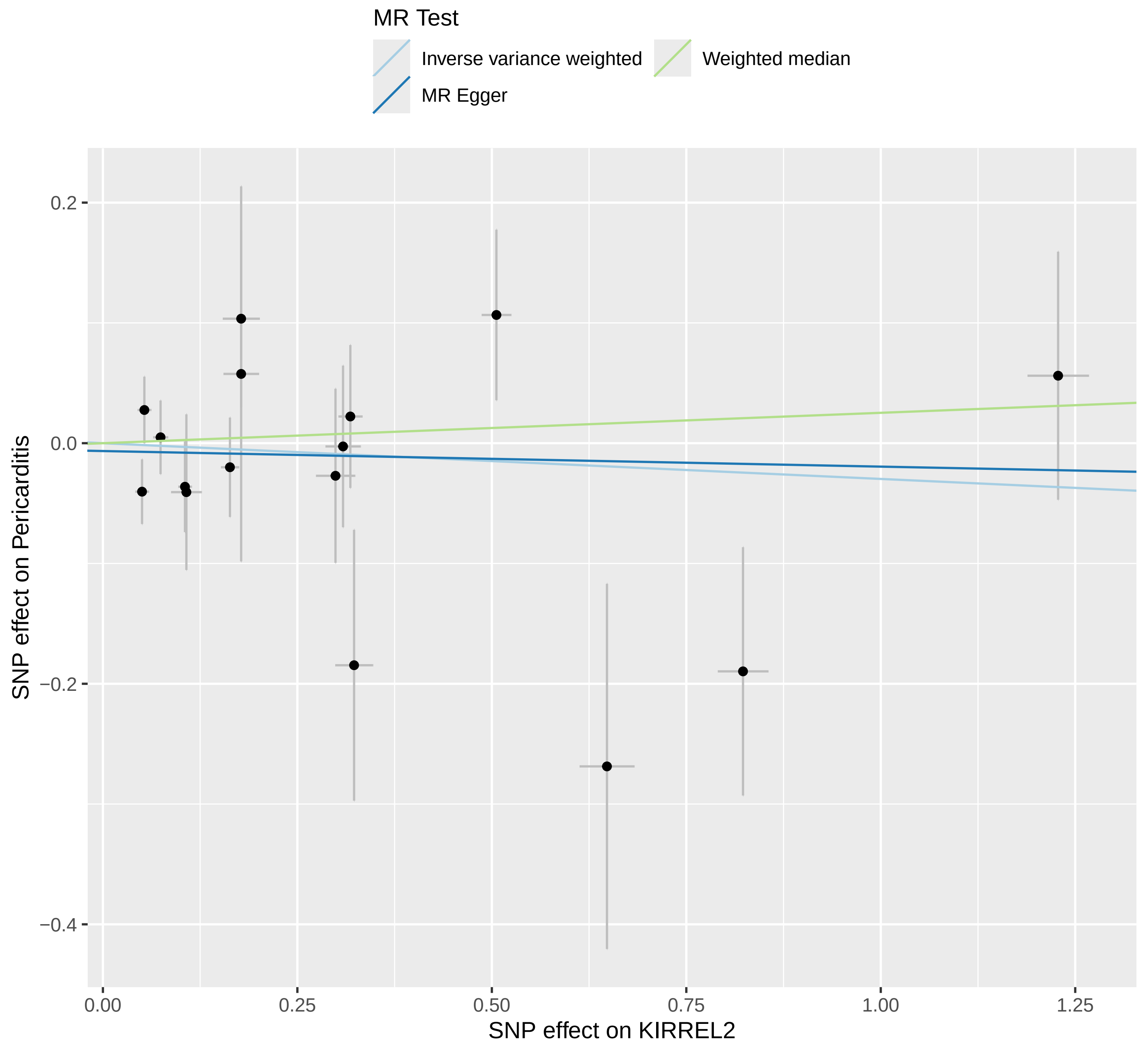

MR Test
Inverse variance weighted
Weighted median
MR Egger
0.2
0.0
−0.2
−0.4
SNP effect on Pericarditis
0.00
0.25
0.50
0.75
1.00
1.25
SNP effect on KIRREL2

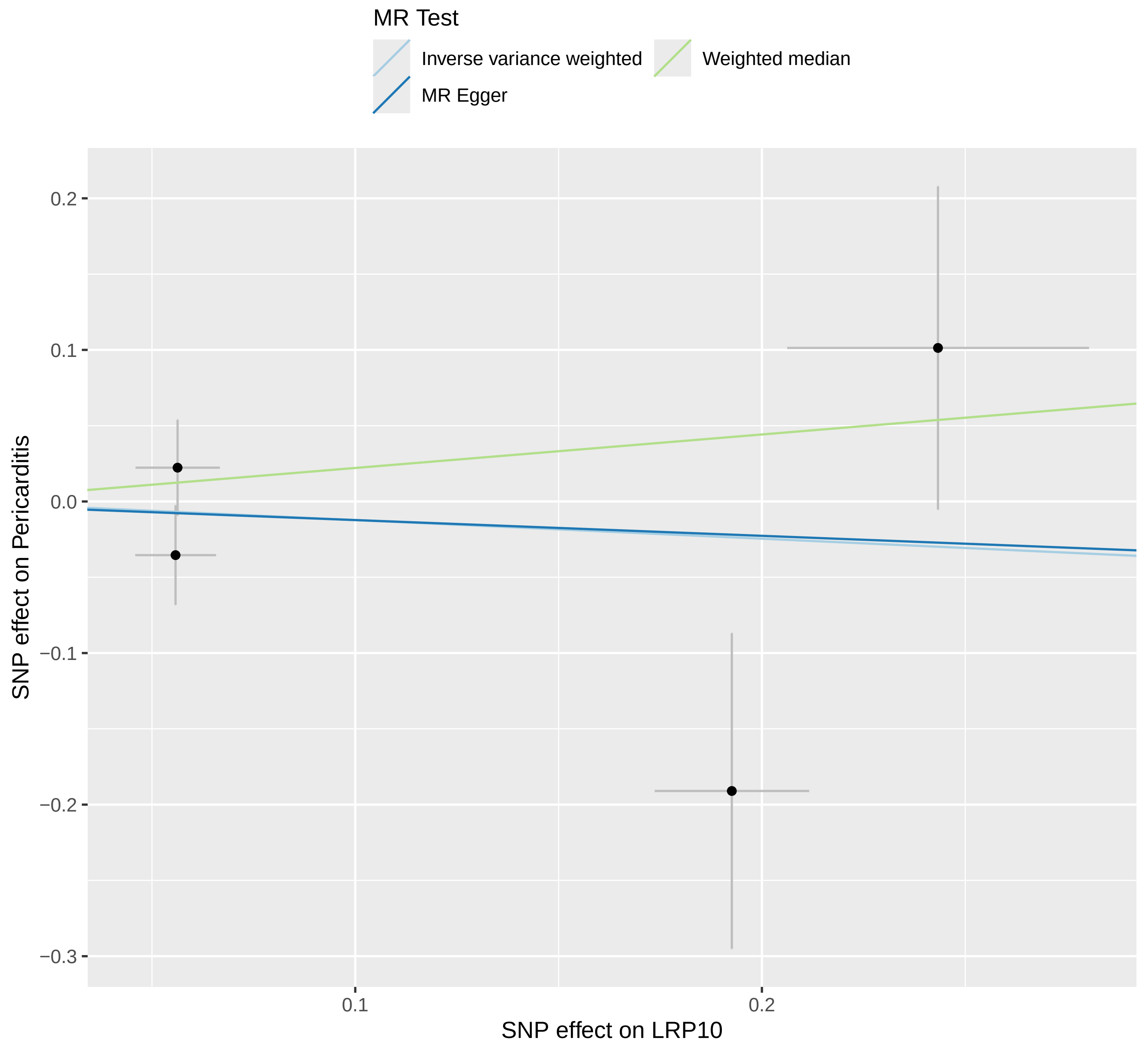

MR Test
Inverse variance weighted
Weighted median
MR Egger
0.2
0.1
0.0
−0.1
−0.2
−0.3
0.1
0.2
SNP effect on LRP10
SNP effect on Pericarditis

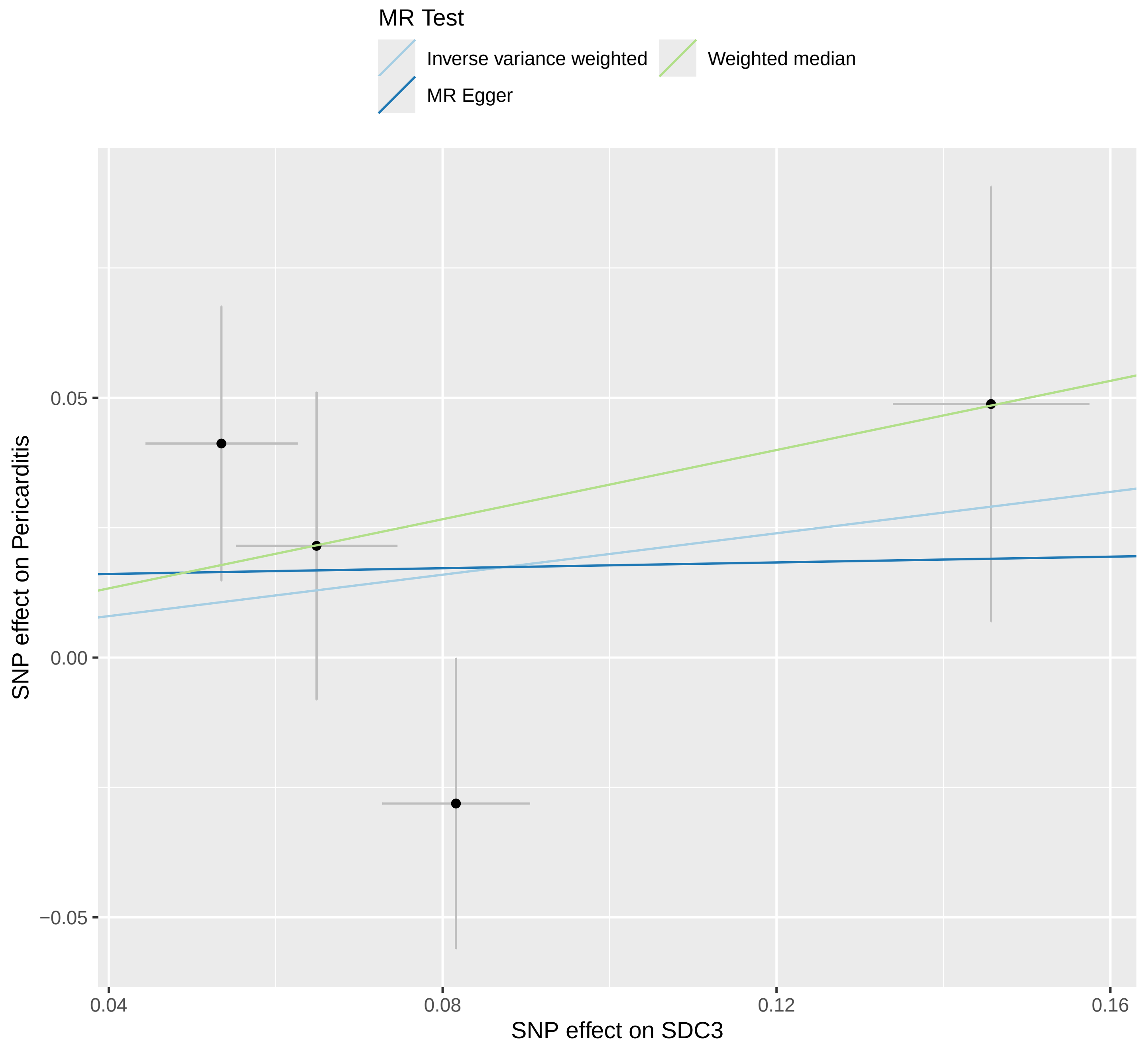

MR Test
Inverse variance weighted
MR Egger
Weighted median
SNP effect on Pericarditis
0.05
0.00
−0.05
0.04
0.08
0.12
0.16
SNP effect on SDC3

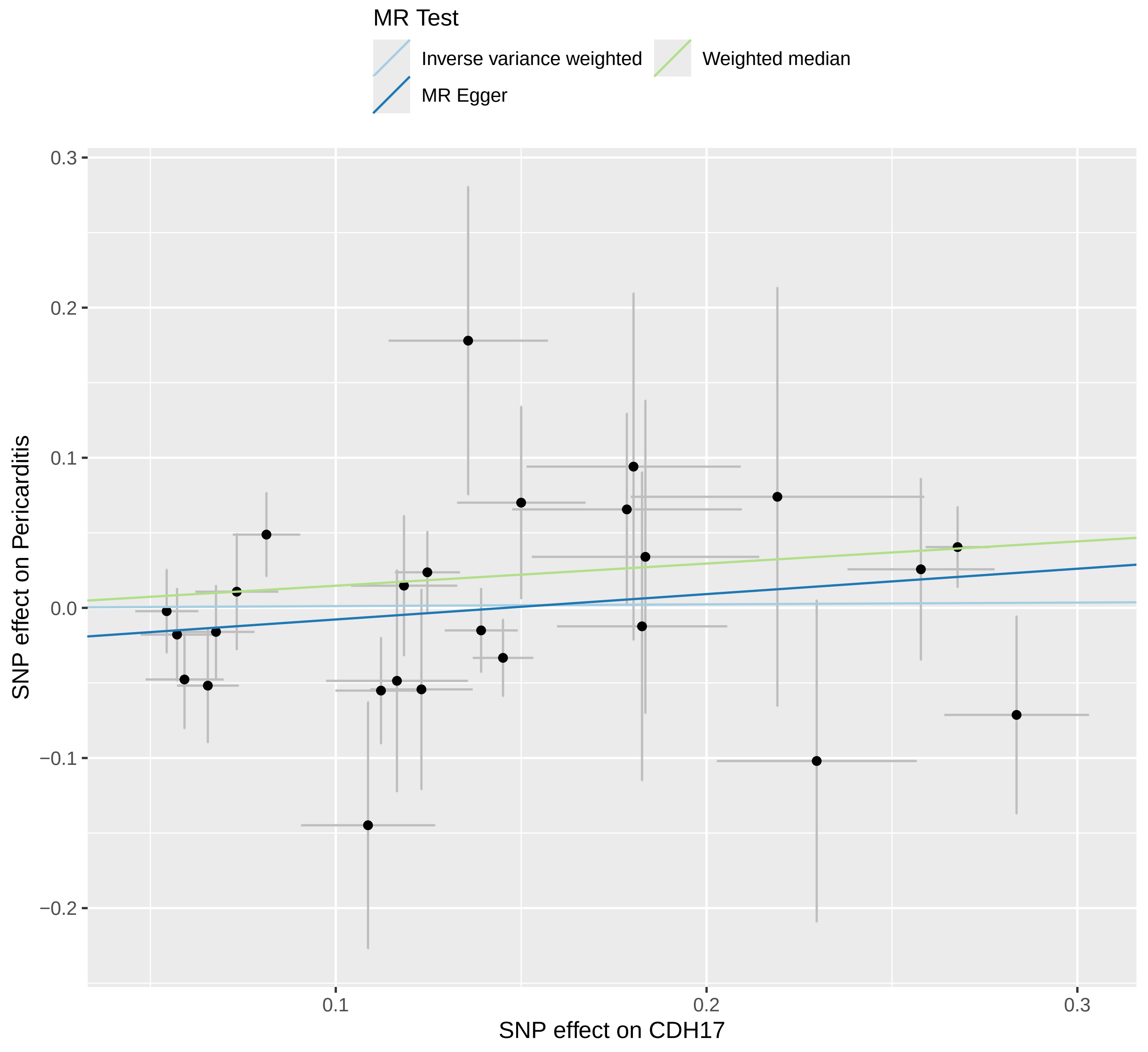

MR Test
Inverse variance weighted
Weighted median
MR Egger
0.3
0.2
0.1
0.0
−0.1
−0.2
SNP effect on Pericarditis
0.1
0.2
0.3
SNP effect on CDH17

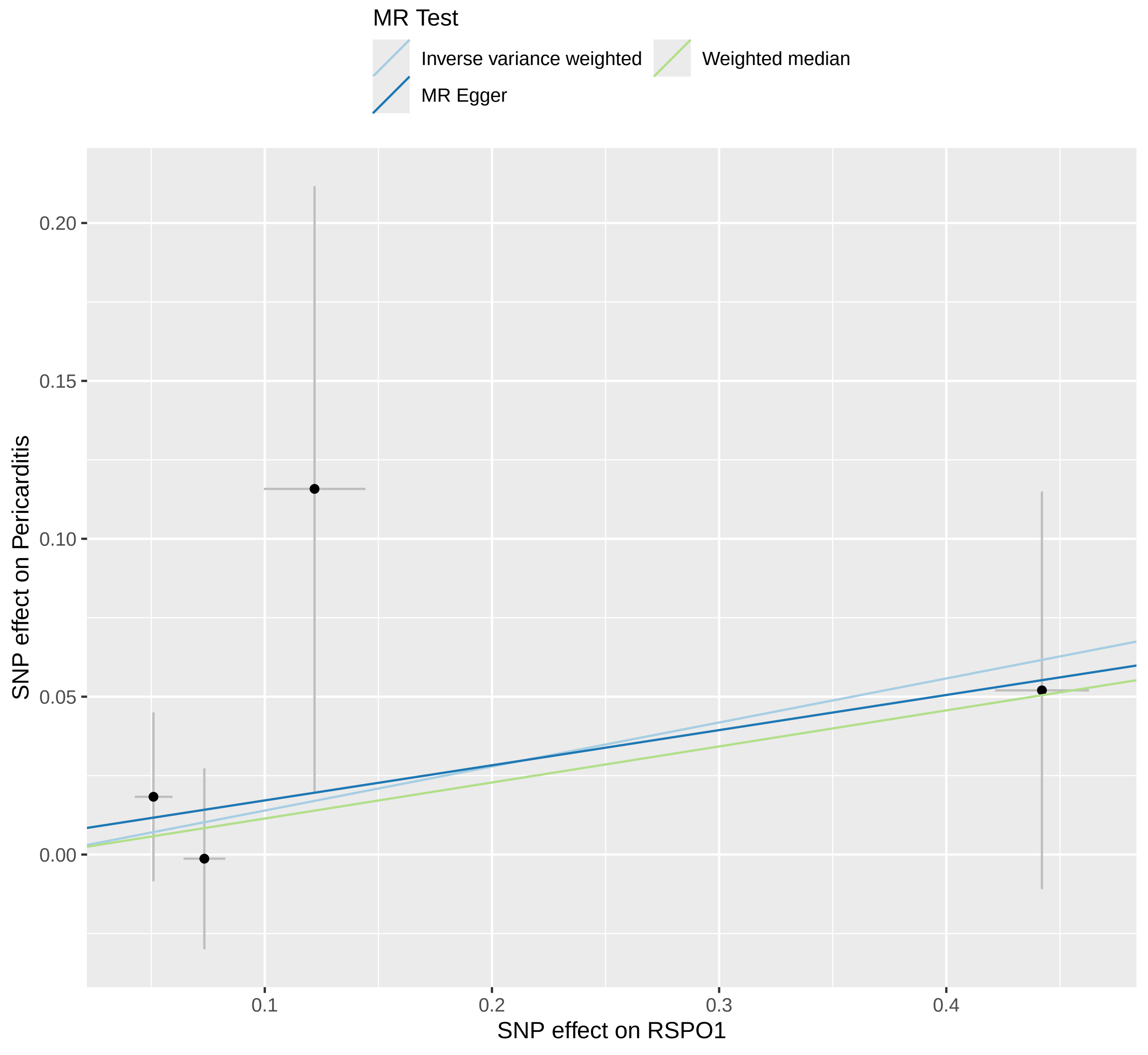
MR Test
Inverse variance weighted
Weighted median
MR Egger
0.20
0.15
0.10
0.05
0.00
SNP effect on Pericarditis
0.1
0.2
0.3
0.4
SNP effect on RSPO1

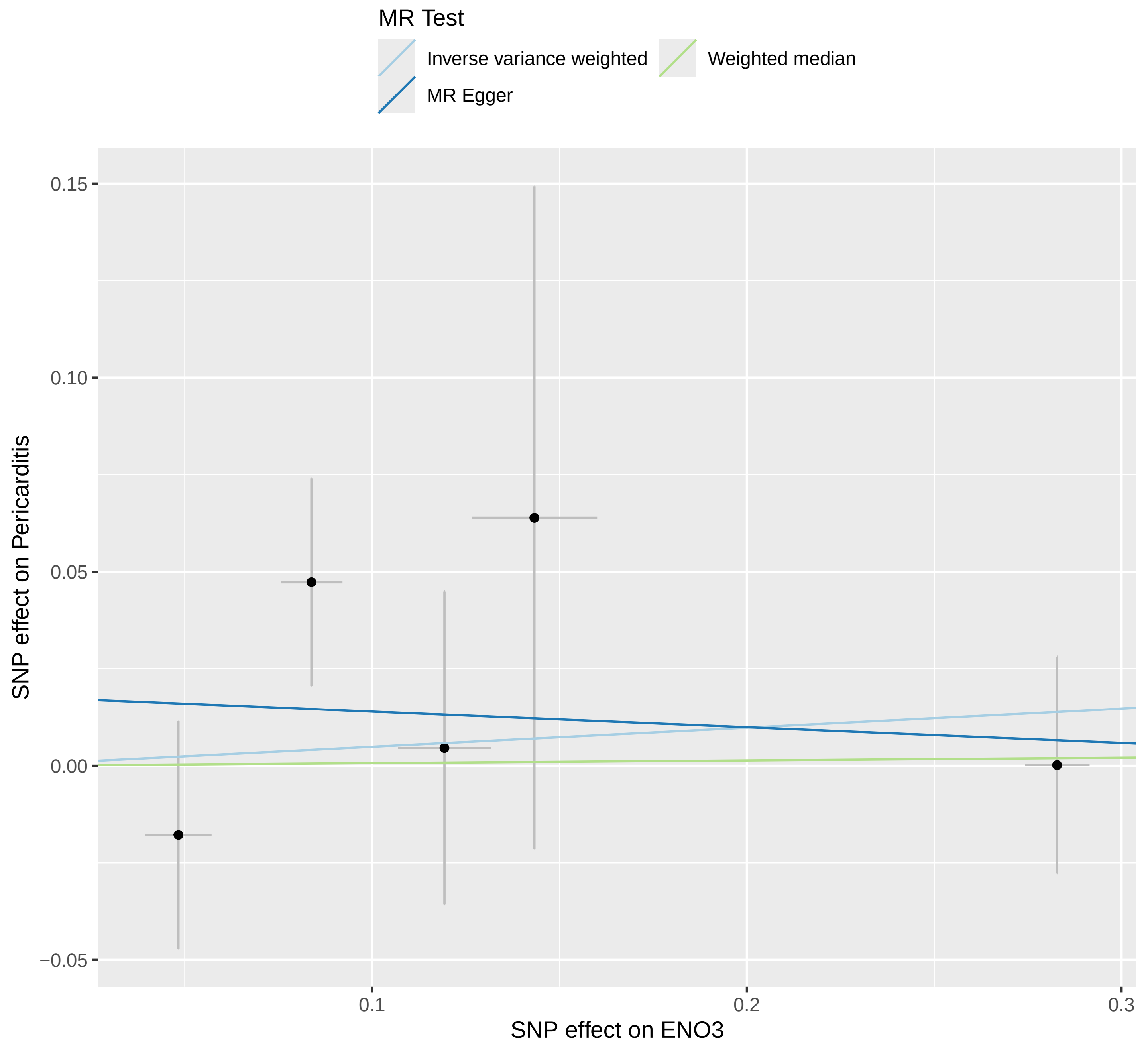

MR Test
Inverse variance weighted
MR Egger
Weighted median
0.15
0.10
0.05
0.00
−0.05
0.1
0.2
0.3
SNP effect on Pericarditis
SNP effect on ENO3

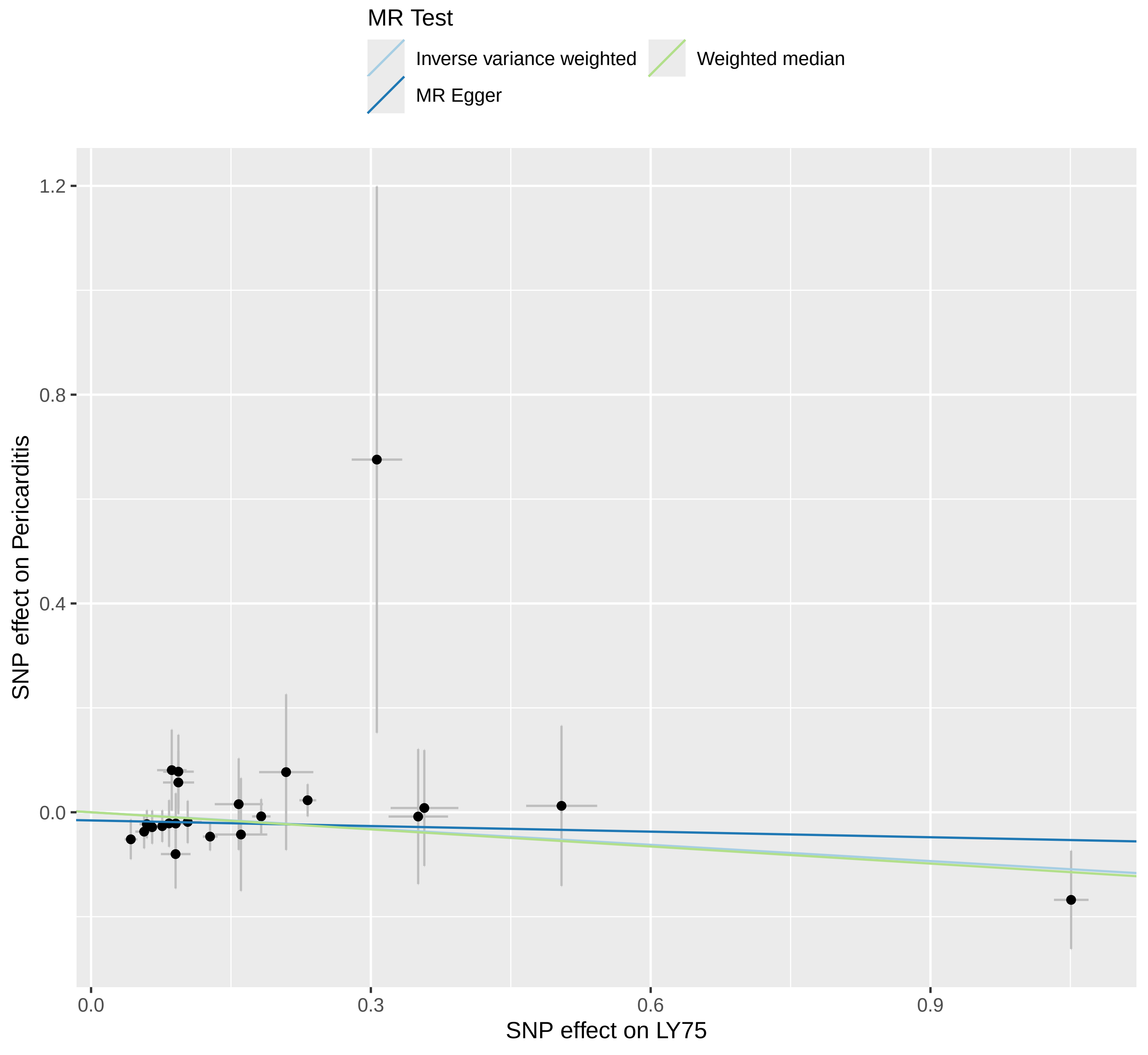
MR Test
Inverse variance weighted
Weighted median
MR Egger
1.2
0.8
0.4
0.0
0.0
0.3
0.6
0.9
SNP effect on Pericarditis
SNP effect on LY75

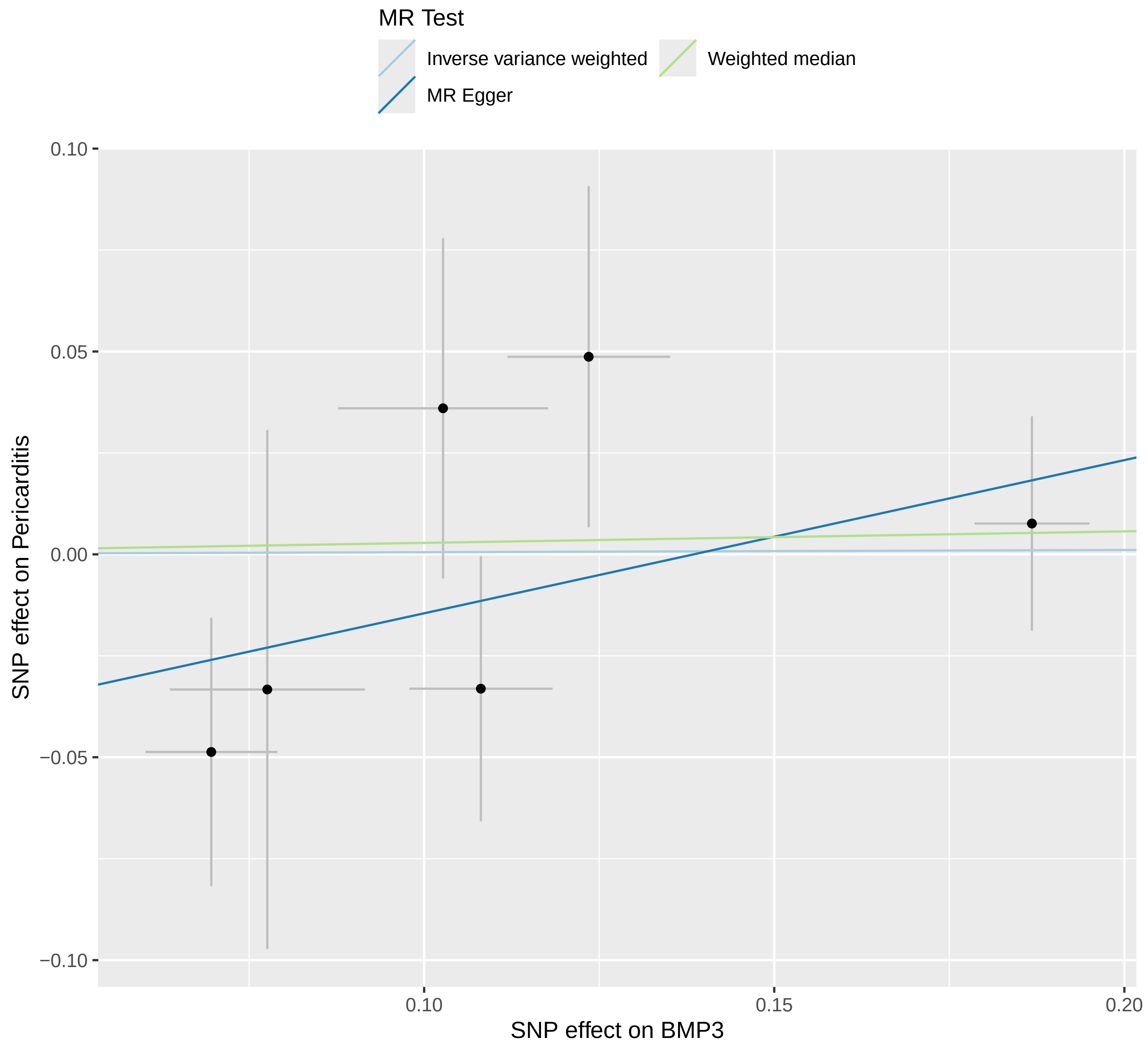
MR Test
Inverse variance weighted
Weighted median
MR Egger
0.10
0.05
0.00
−0.05
−0.10
SNP effect on Pericarditis
0.10
0.15
0.20
SNP effect on BMP3

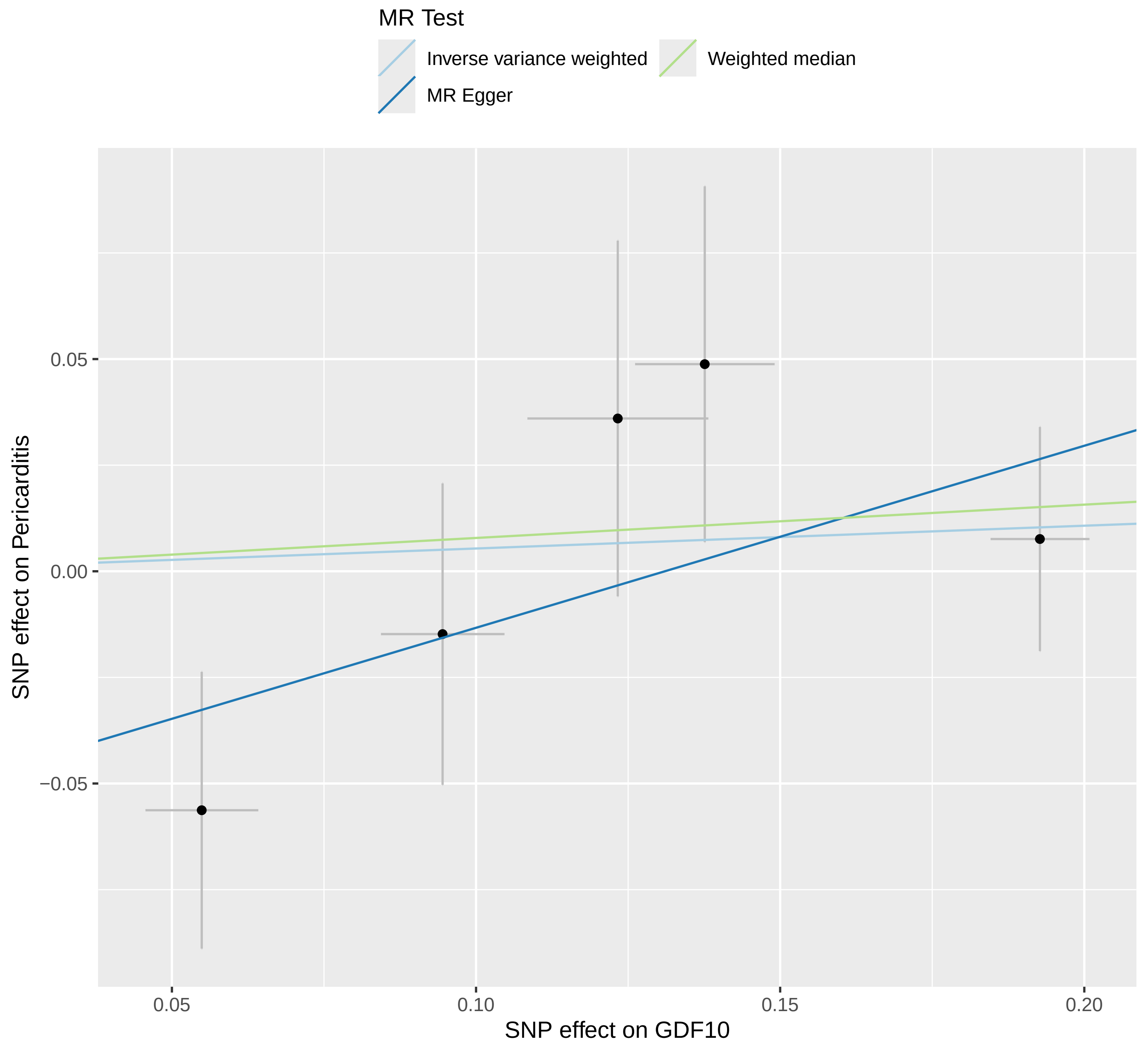

MR Test
Inverse variance weighted
Weighted median
MR Egger
SNP effect on Pericarditis
0.05
0.00
−0.05
0.05
0.10
0.15
0.20
SNP effect on GDF10

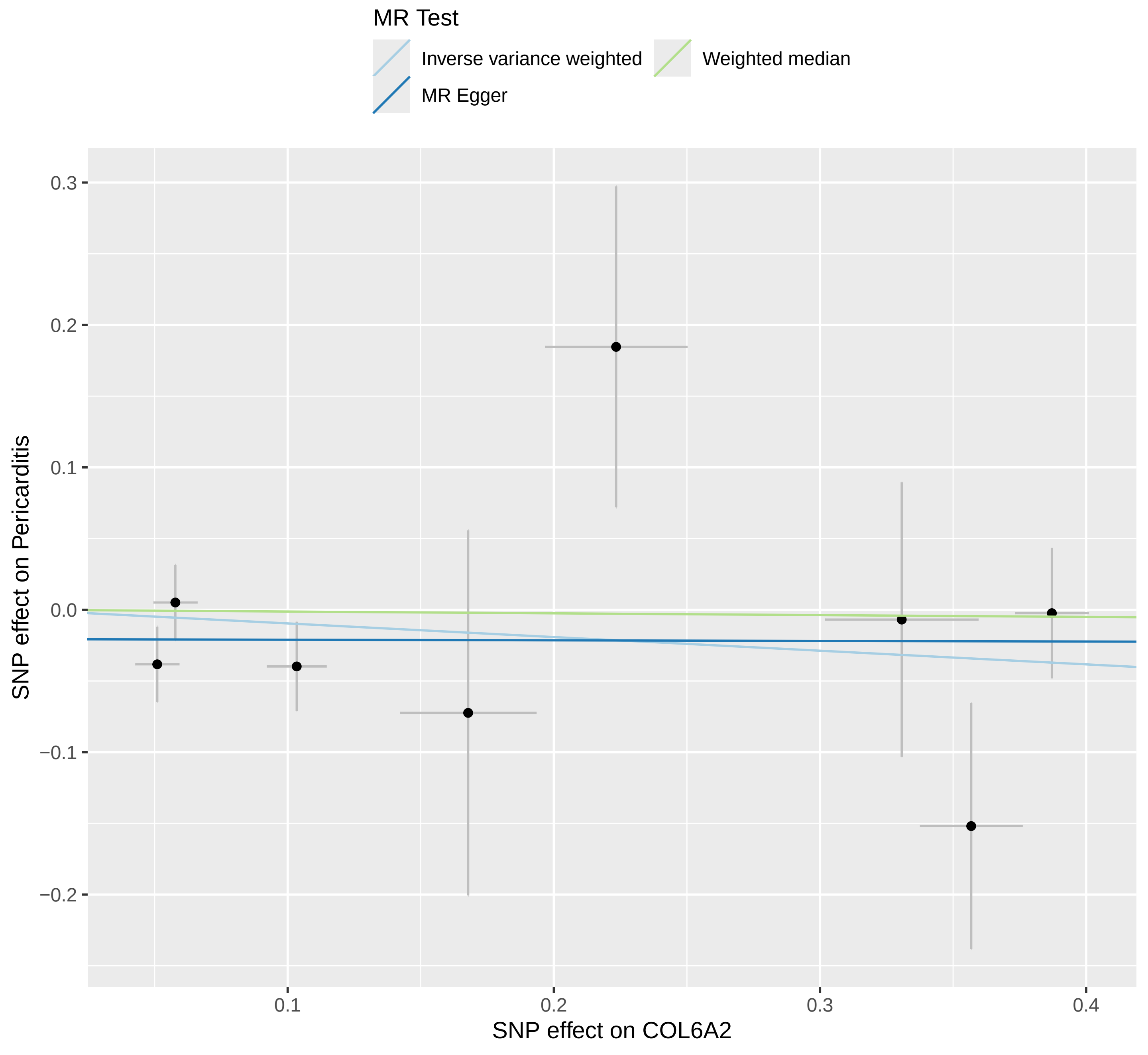

MR Test
Inverse variance weighted
Weighted median
MR Egger
0.3
0.2
0.1
0.0
−0.1
−0.2
0.1
0.2
0.3
0.4
SNP effect on Pericarditis
SNP effect on COL6A2

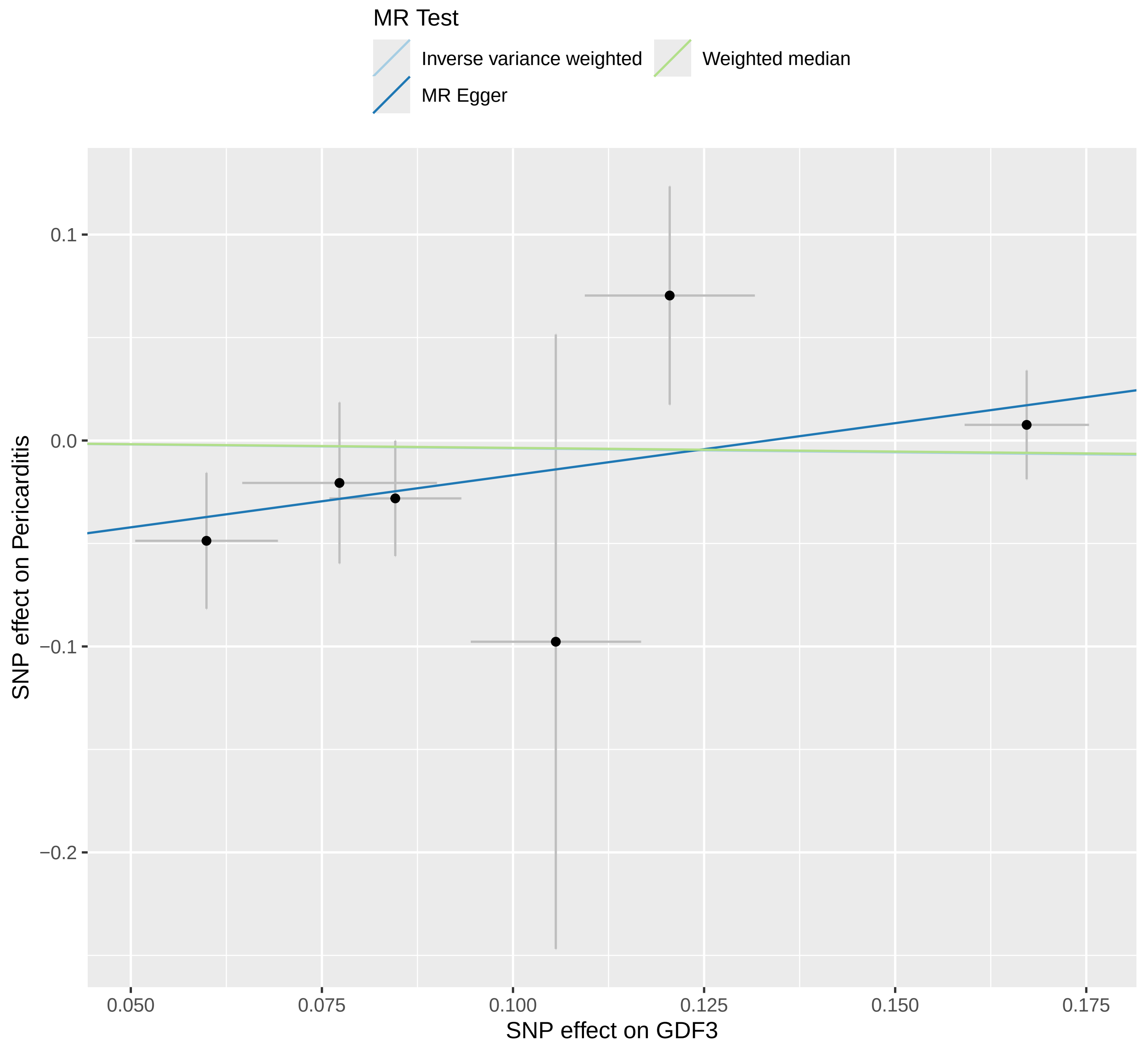
MR Test
Inverse variance weighted
MR Egger
Weighted median
SNP effect on Pericarditis
0.1
0.0
−0.1
−0.2
0.050
0.075
0.100
0.125
0.150
0.175
SNP effect on GDF3

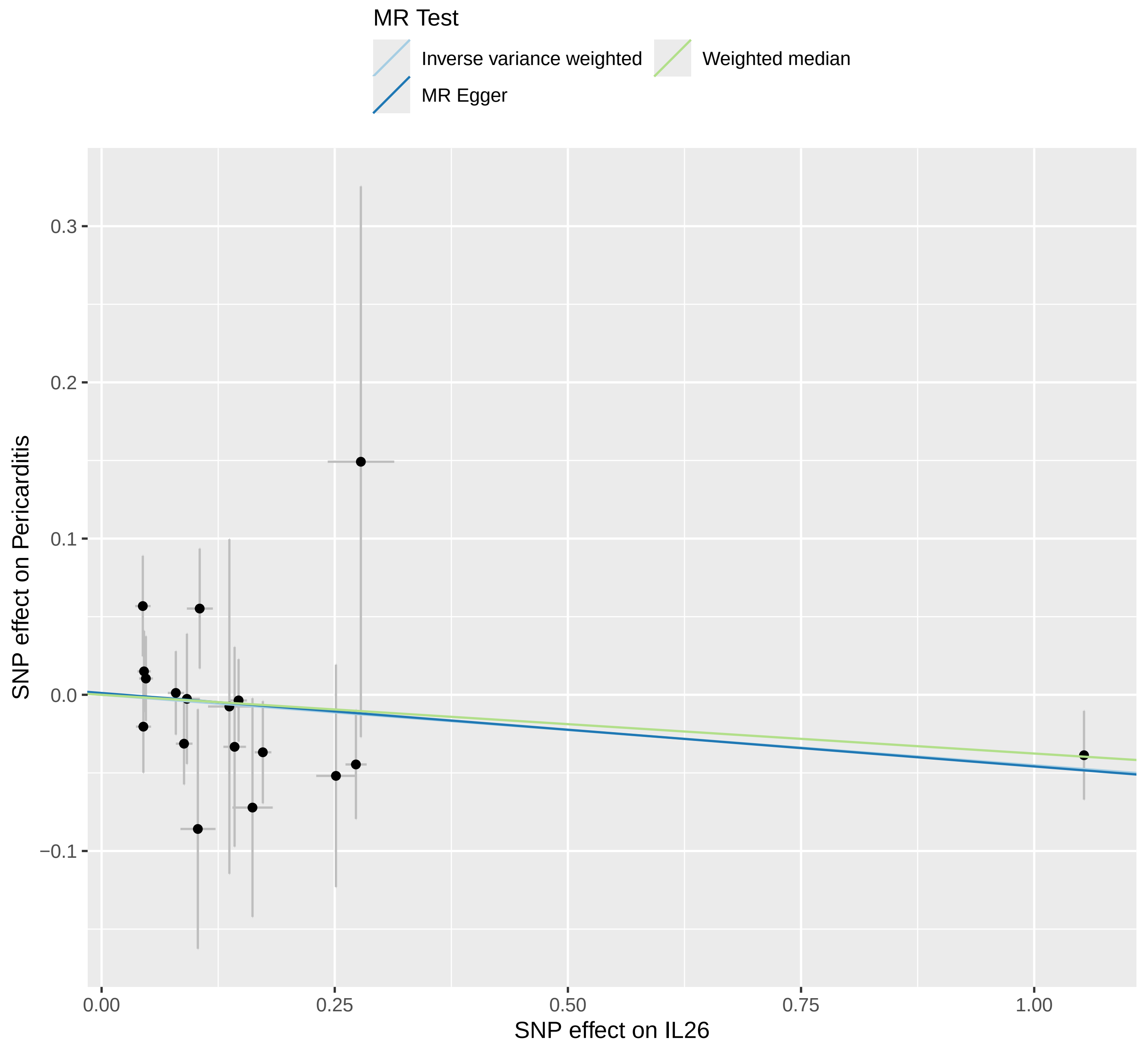

MR Test
Inverse variance weighted
Weighted median
MR Egger
0.3
0.2
0.1
0.0
−0.1
SNP effect on Pericarditis
0.00
0.25
0.50
0.75
1.00
SNP effect on IL26

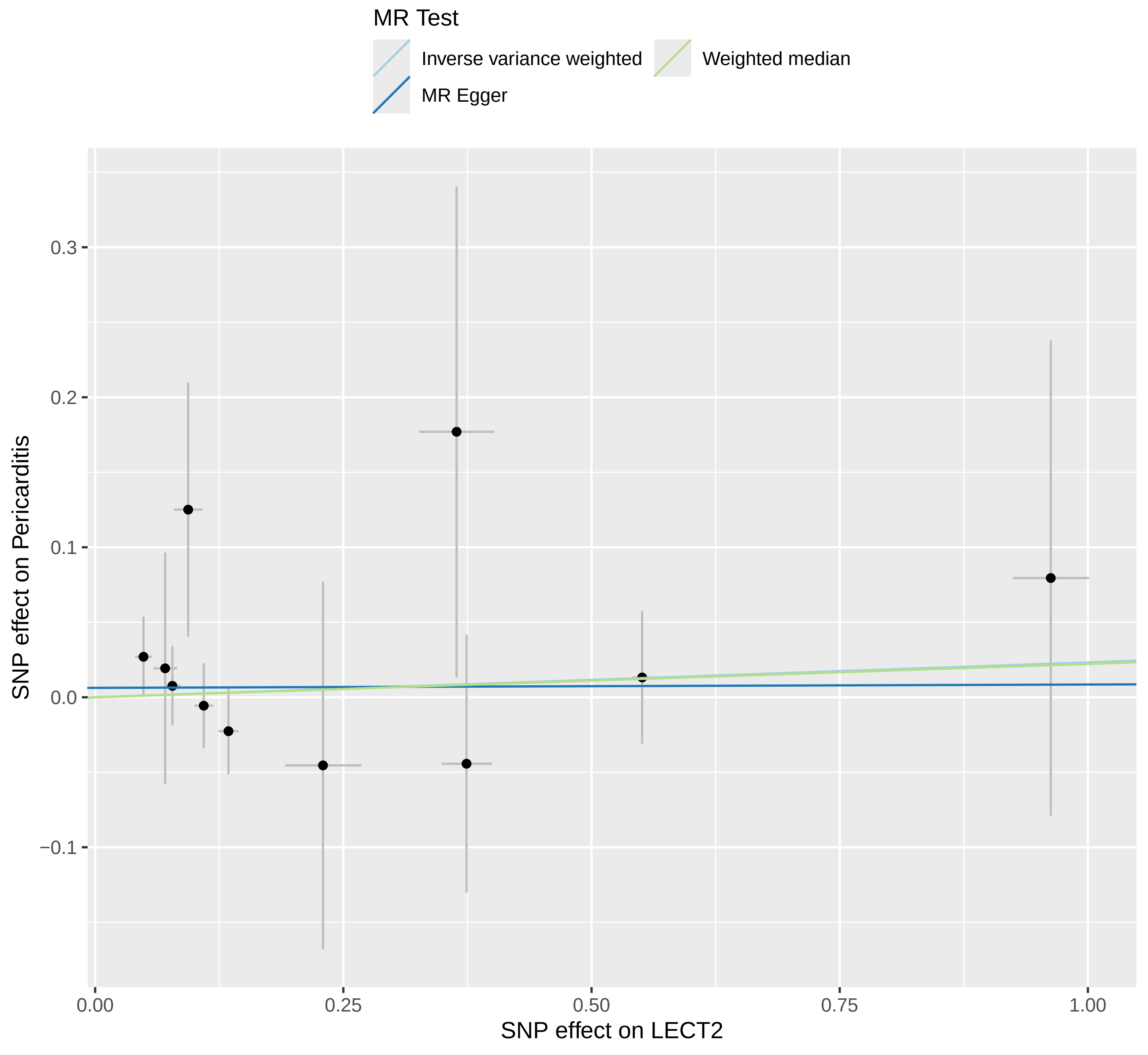
MR Test
Inverse variance weighted
Weighted median
MR Egger
SNP effect on Pericarditis
0.3
0.2
0.1
0.0
−0.1
0.00
0.25
0.50
0.75
1.00
SNP effect on LECT2

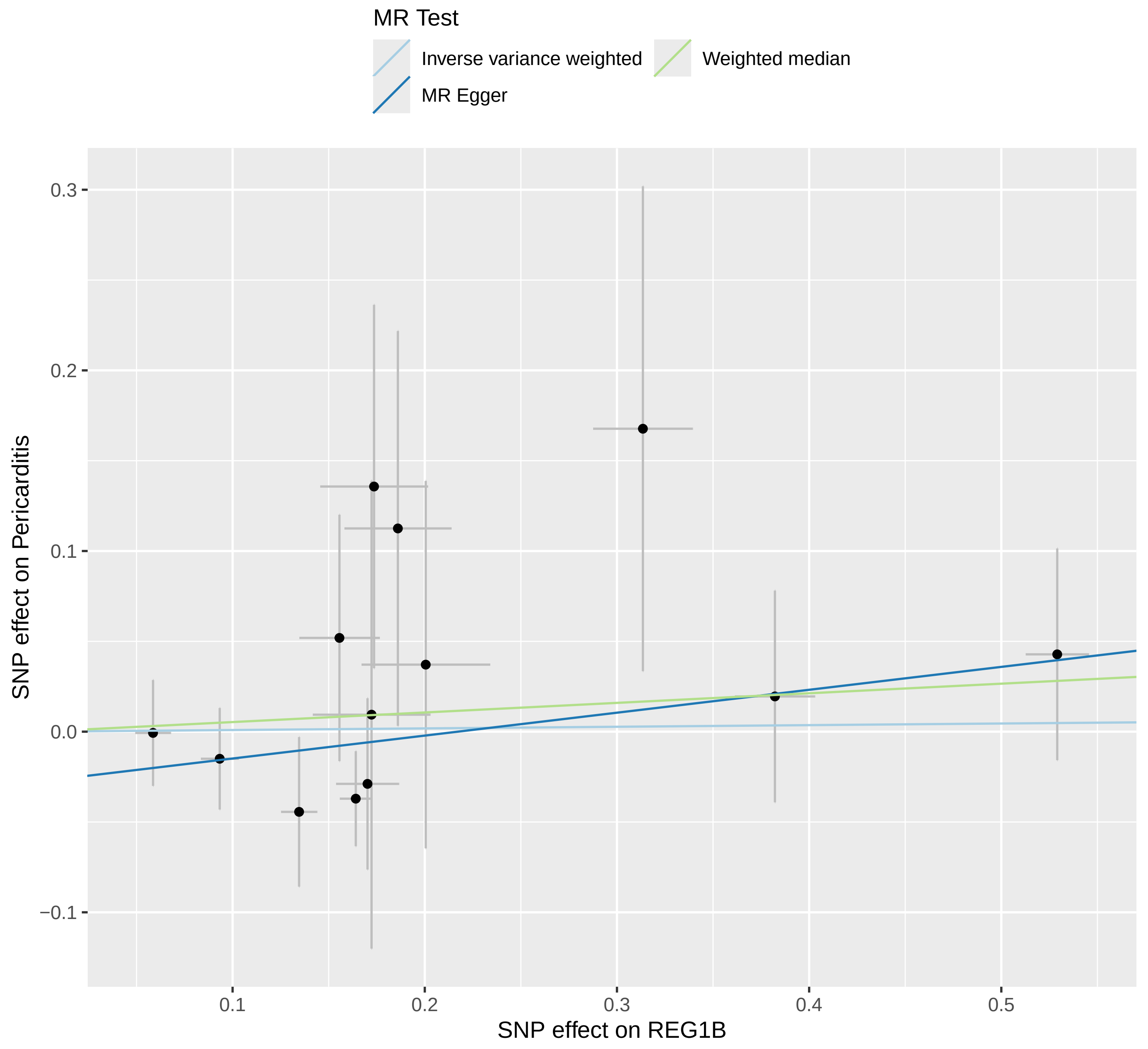

MR Test
Inverse variance weighted
Weighted median
MR Egger
SNP effect on Pericarditis
SNP effect on REG1B
0.3
0.2
0.1
0.0
−0.1
0.1
0.2
0.3
0.4
0.5

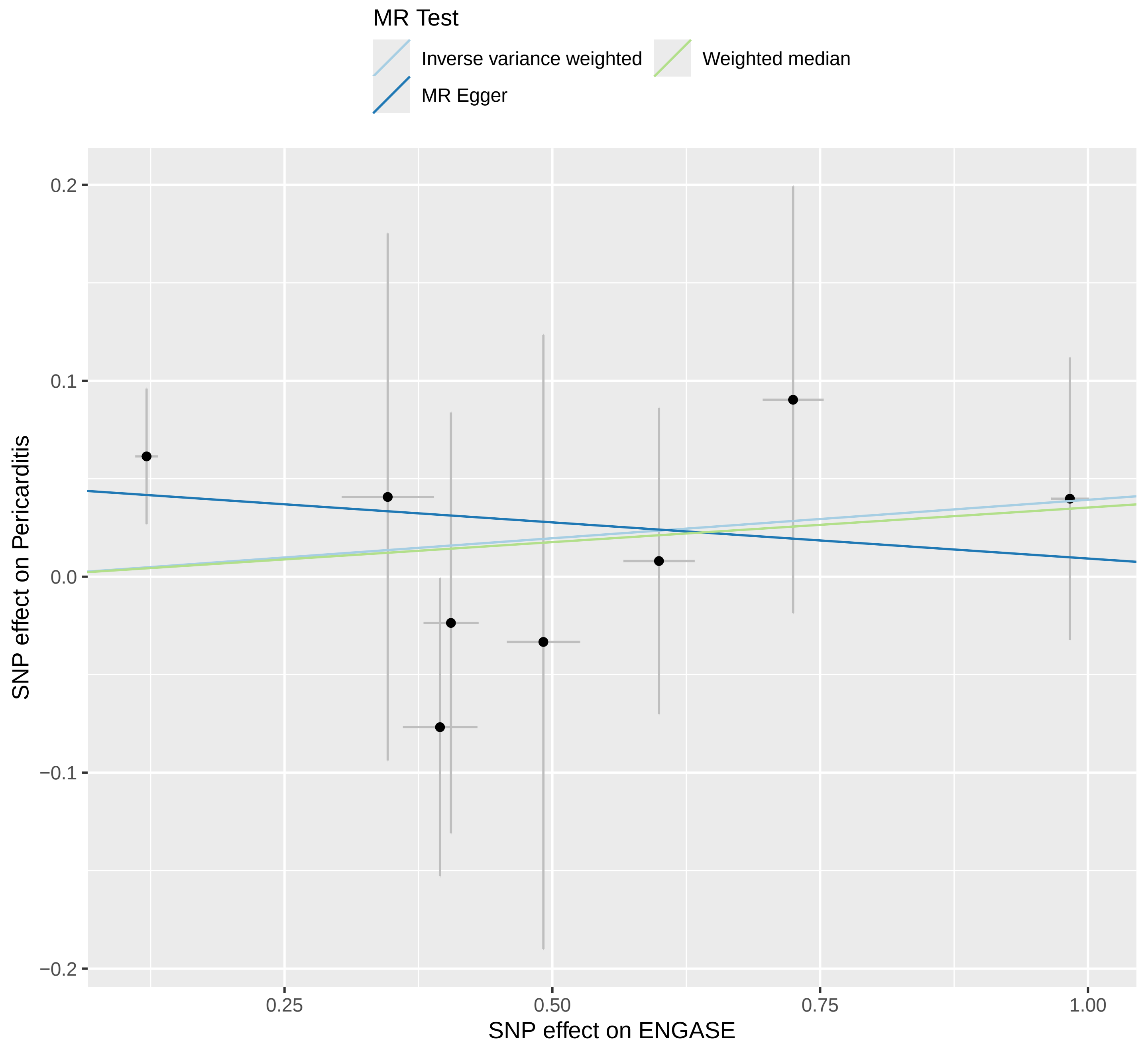
MR Test
Inverse variance weighted
Weighted median
MR Egger
SNP effect on Pericarditis
0.2
0.1
0.0
−0.1
−0.2
0.25
0.50
0.75
1.00
SNP effect on ENGASE

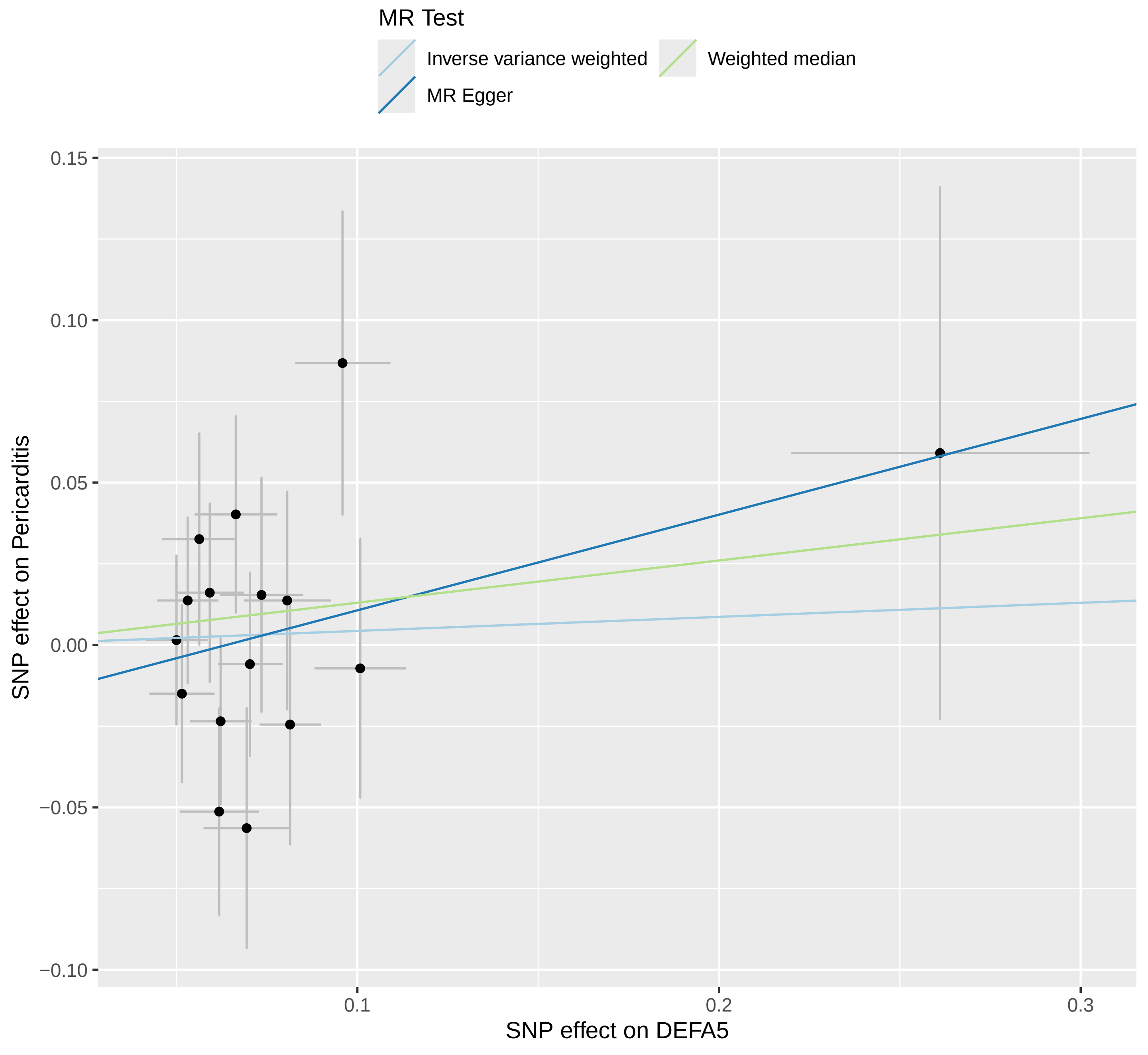
MR Test
Inverse variance weighted
Weighted median
MR Egger
SNP effect on Pericarditis
0.15
0.10
0.05
0.00
−0.05
−0.10
0.1
0.2
0.3
SNP effect on DEFA5

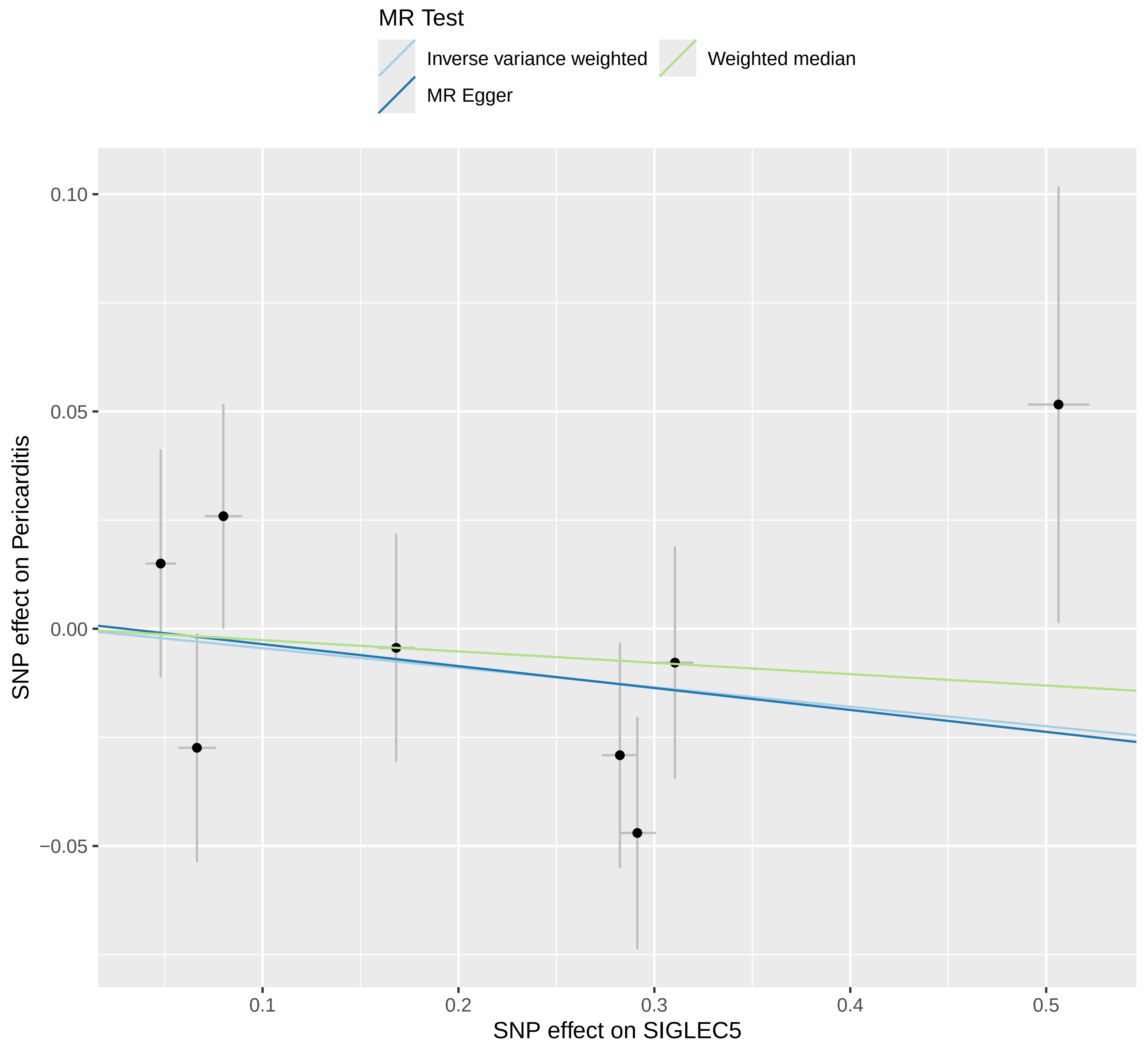
MR Test
Inverse variance weighted
MR Egger
Weighted median
0.10
0.05
0.00
−0.05
SNP effect on Pericarditis
0.1
0.2
0.3
0.4
0.5
SNP effect on SIGLEC5

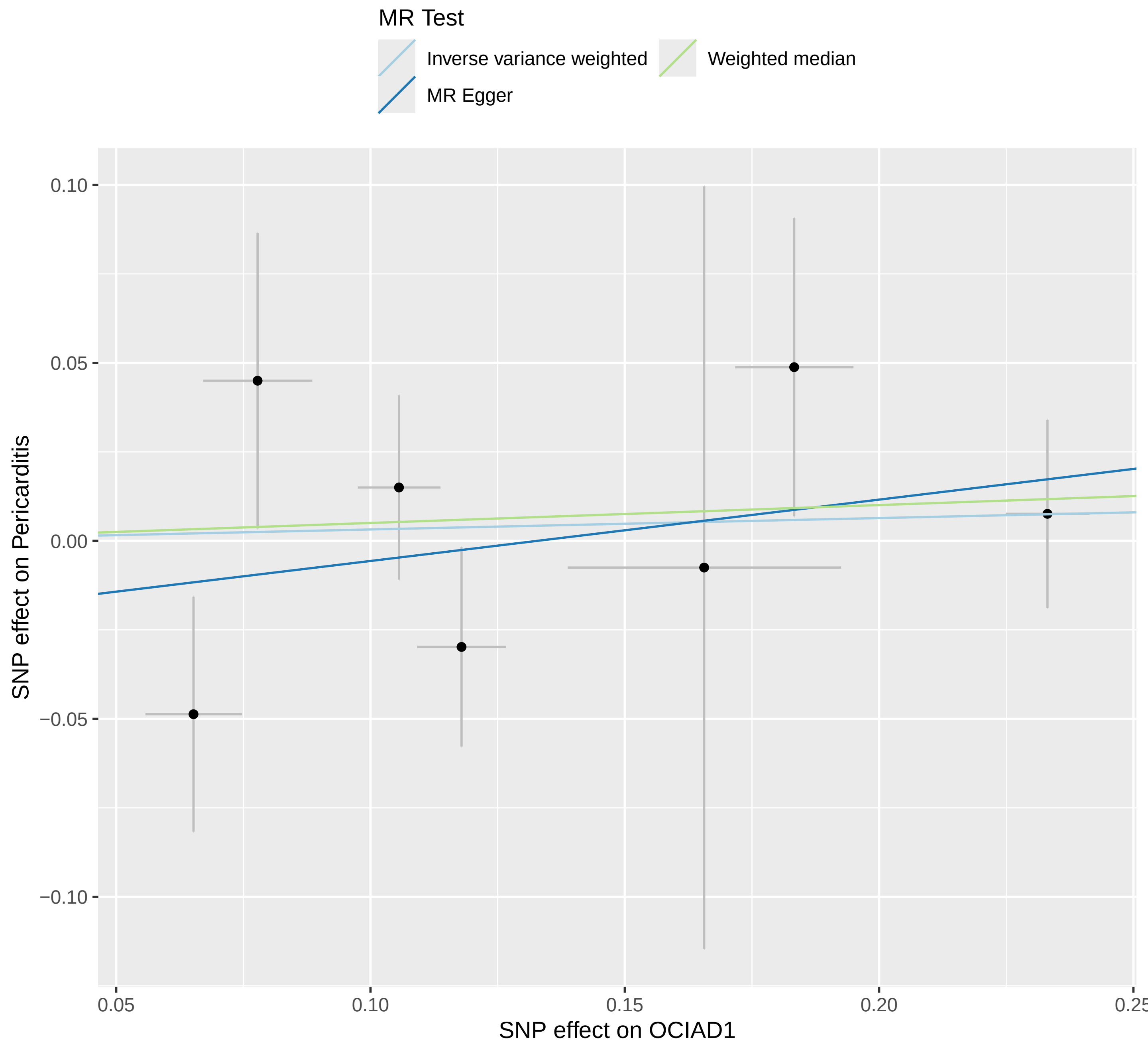
MR Test
Inverse variance weighted
MR Egger
Weighted median
0.10
0.05
0.00
−0.05
−0.10
SNP effect on Pericarditis
0.05
0.10
0.15
0.20
0.25
SNP effect on OCIAD1

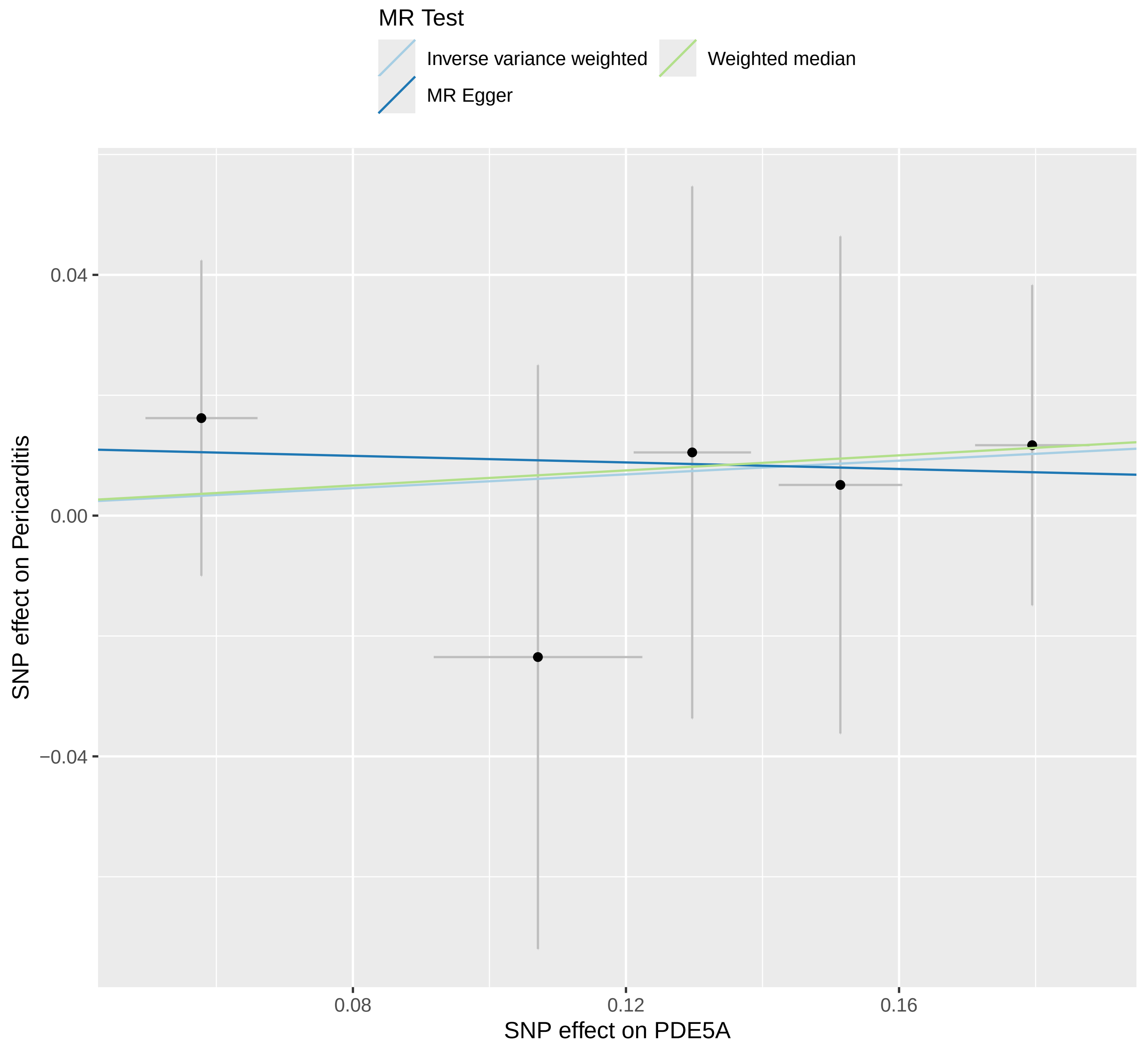

MR Test
Inverse variance weighted
MR Egger
Weighted median
0.04
0.00
−0.04
0.08
0.12
0.16
SNP effect on Pericarditis
SNP effect on PDE5A

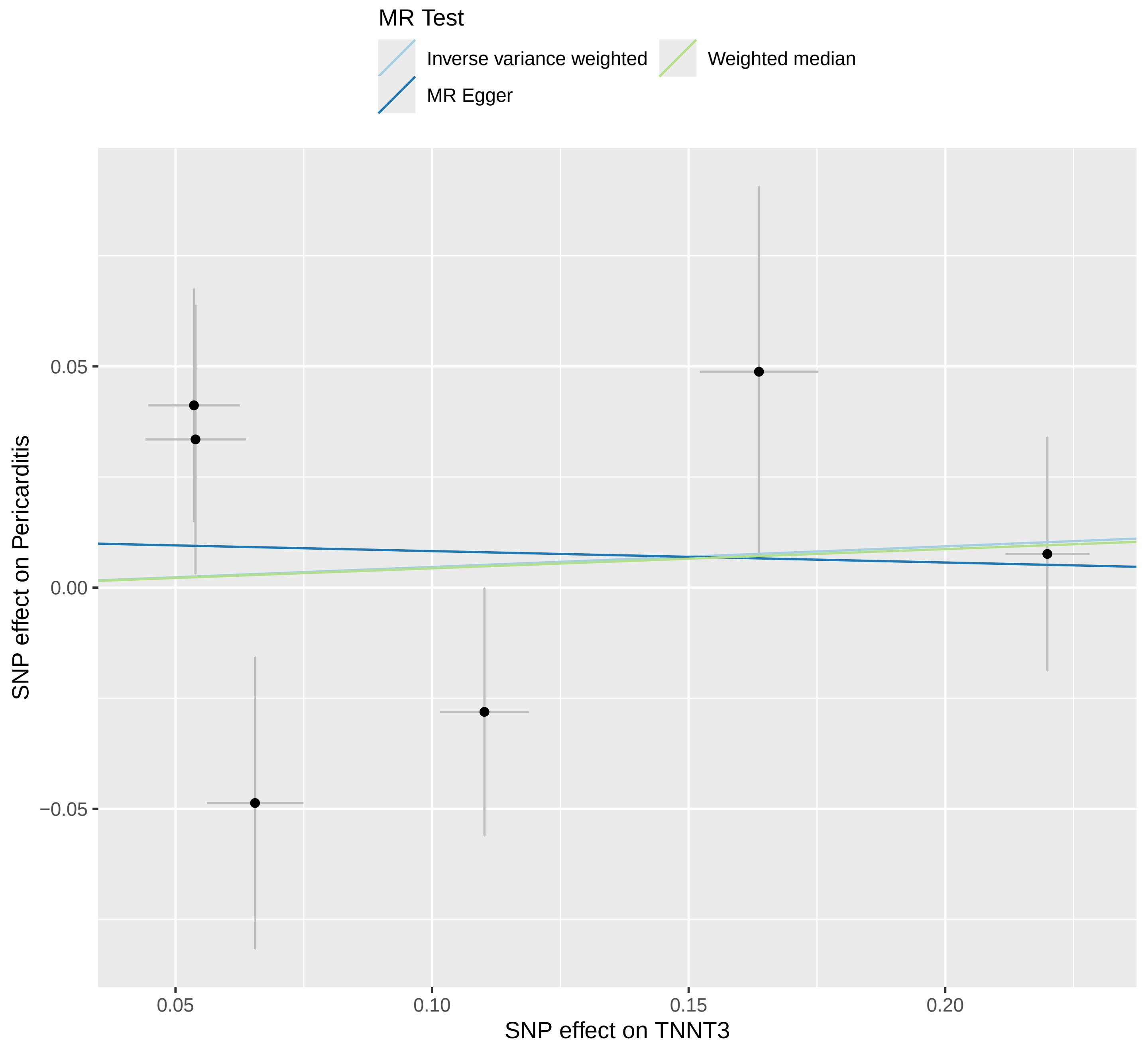
MR Test
Inverse variance weighted
MR Egger
Weighted median
SNP effect on Pericarditis
0.05
0.00
−0.05
0.05
0.10
0.15
0.20
SNP effect on TNNT3

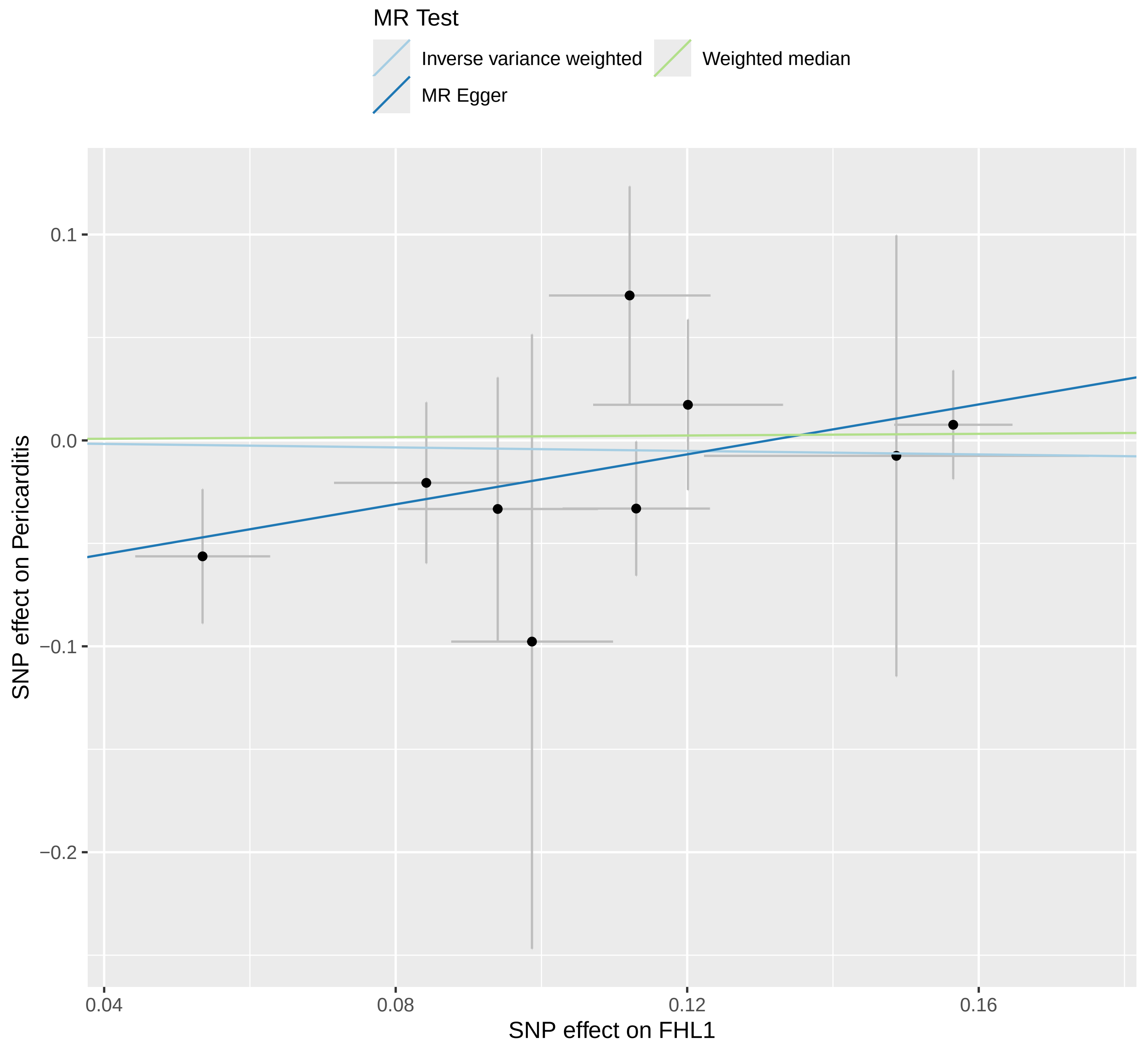
MR Test
Inverse variance weighted
Weighted median
MR Egger
0.1
0.0
−0.1
−0.2
SNP effect on Pericarditis
0.04
0.08
0.12
0.16
SNP effect on FHL1

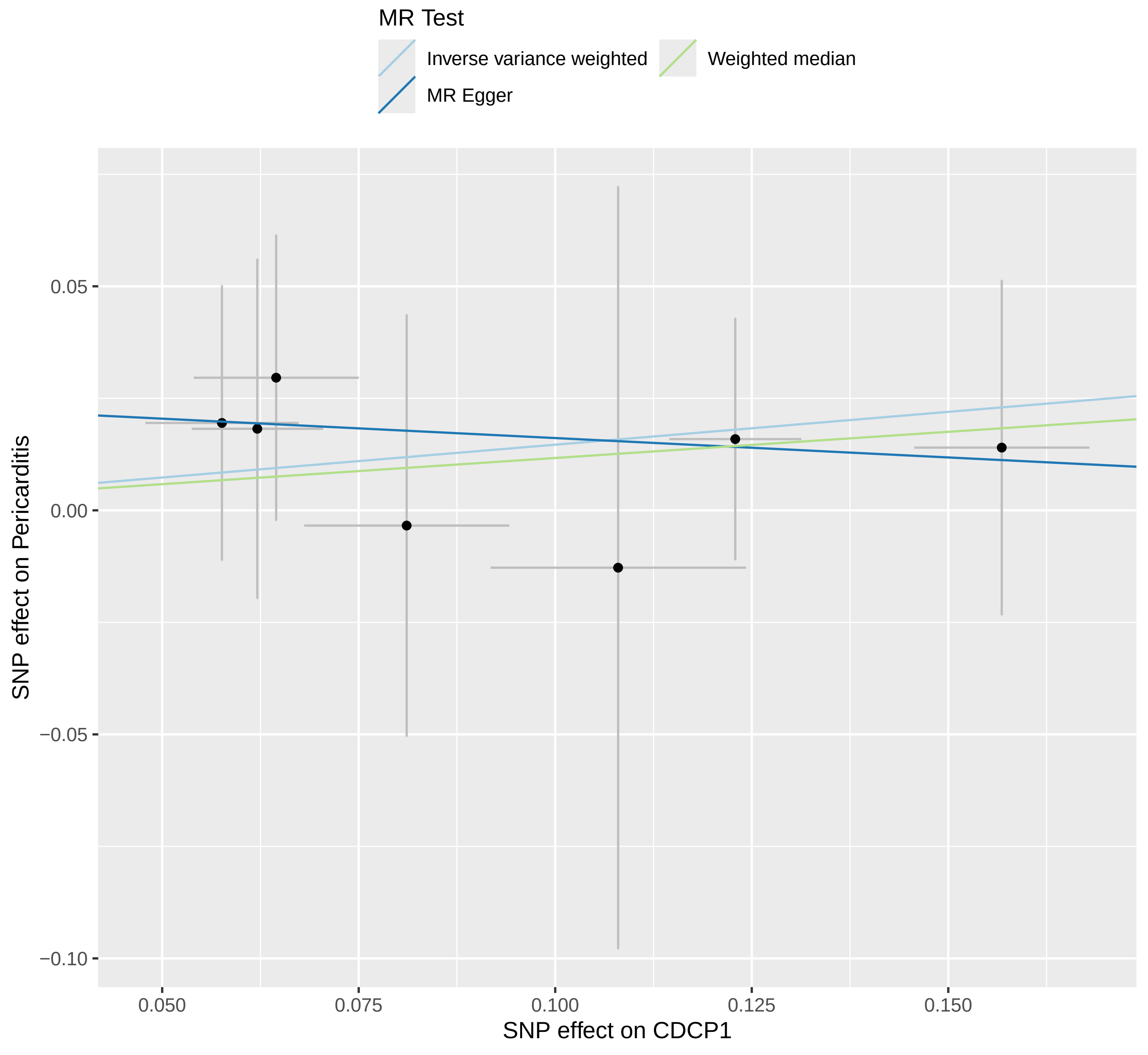
MR Test
Inverse variance weighted
Weighted median
MR Egger
0.05
0.00
−0.05
−0.10
SNP effect on Pericarditis
0.050
0.075
0.100
0.125
0.150
SNP effect on CDCP1

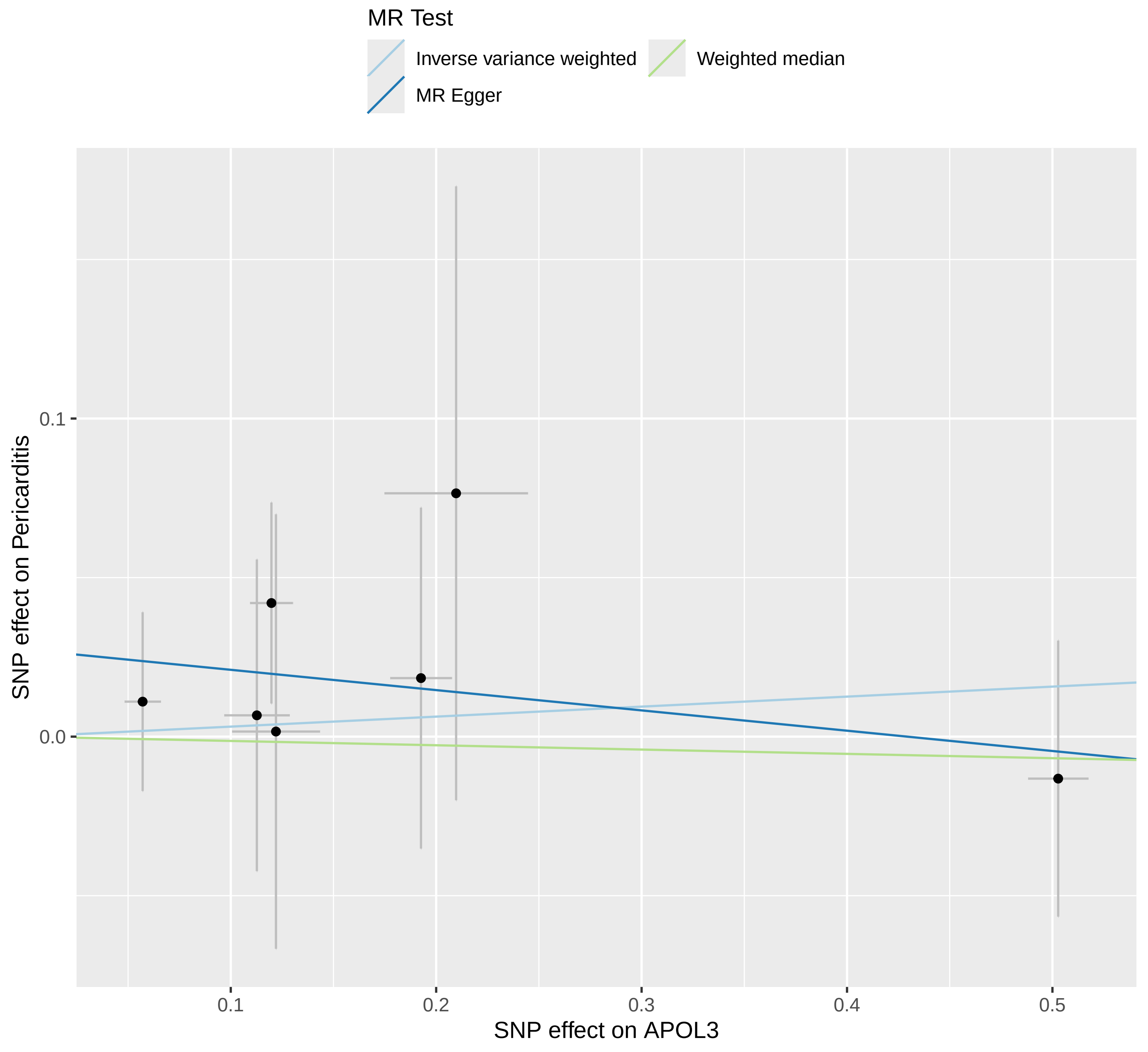
MR Test
Inverse variance weighted
MR Egger
Weighted median
SNP effect on Pericarditis
0.1
0.0
0.1
0.2
0.3
0.4
0.5
SNP effect on APOL3

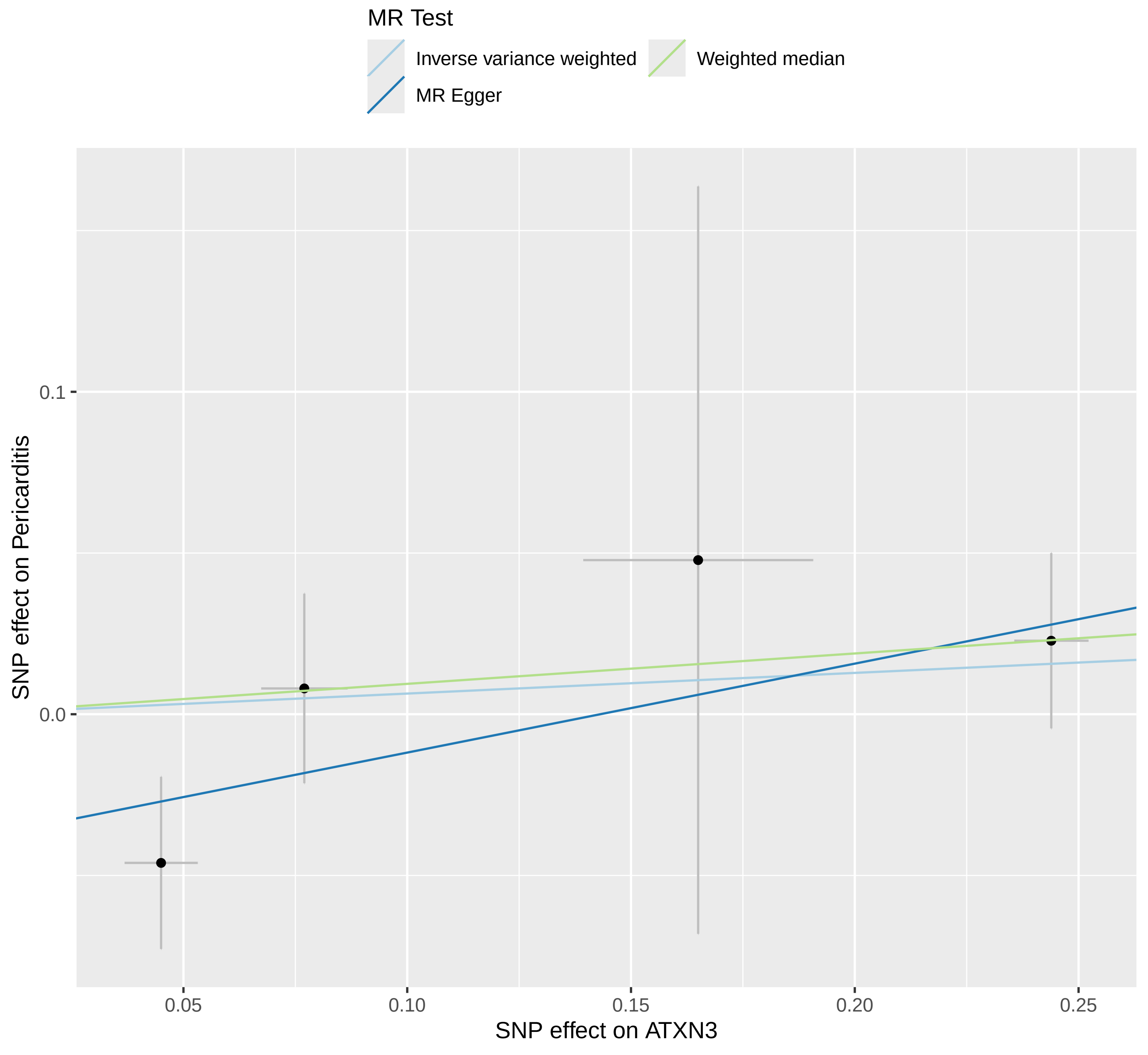
MR Test
Inverse variance weighted
Weighted median
MR Egger
0.1
0.0
SNP effect on Pericarditis
0.05
0.10
0.15
0.20
0.25
SNP effect on ATXN3

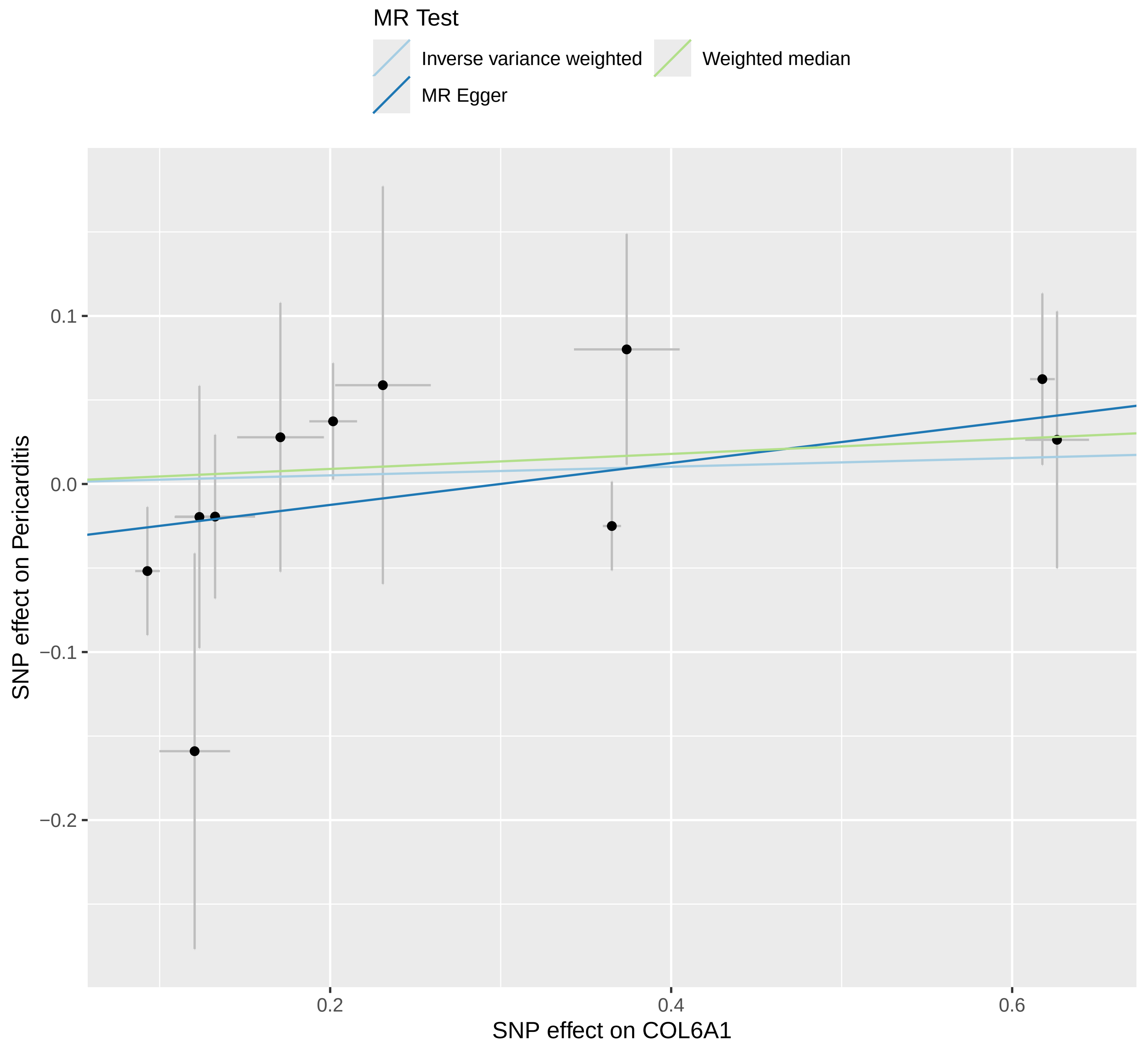
MR Test
Inverse variance weighted
MR Egger
Weighted median
0.1
0.0
−0.1
−0.2
SNP effect on Pericarditis
0.2
0.4
0.6
SNP effect on COL6A1

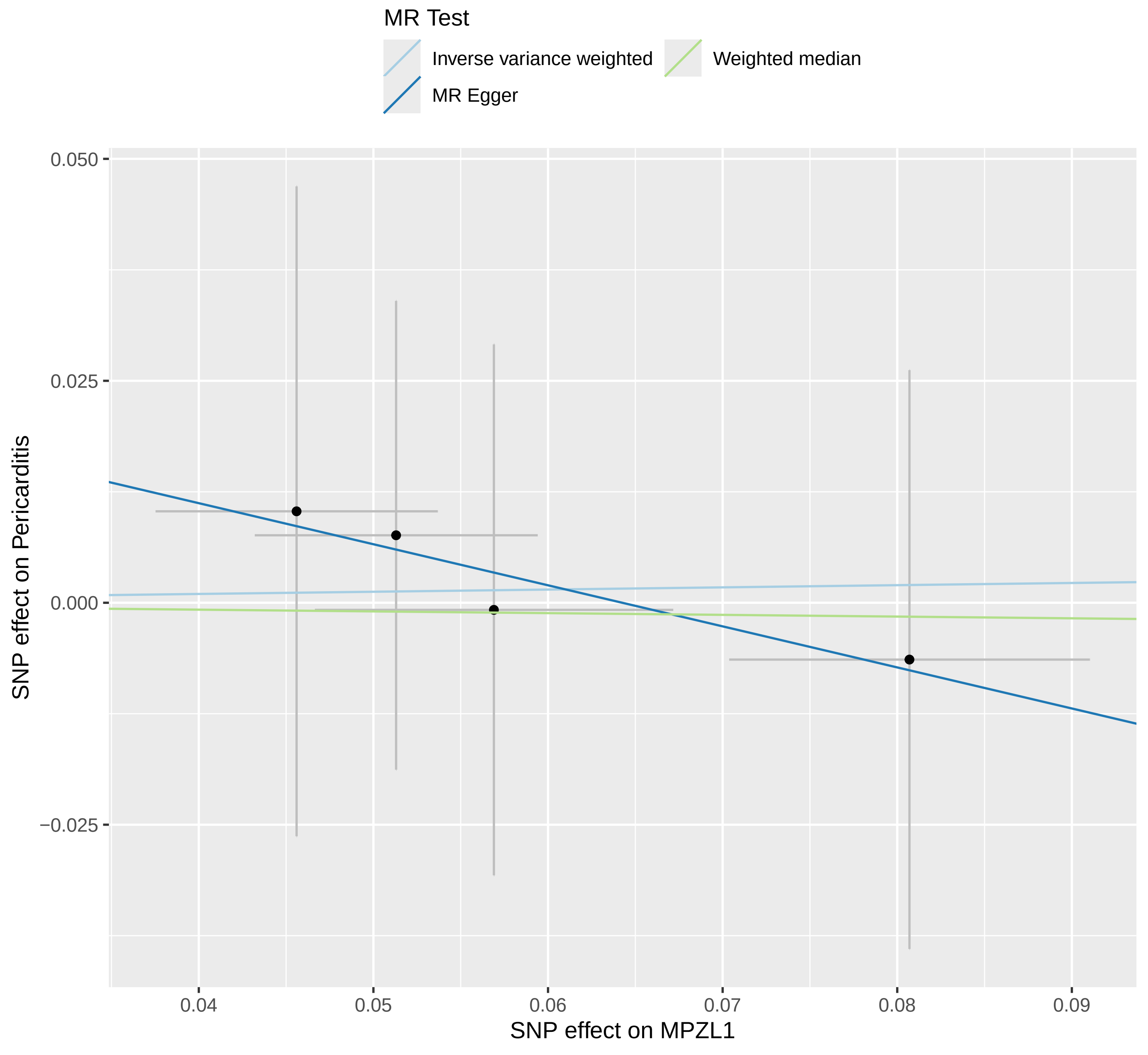
MR Test
Inverse variance weighted
Weighted median
MR Egger
0.050
0.025
0.000
−0.025
SNP effect on Pericarditis
0.04
0.05
0.06
0.07
0.08
0.09
SNP effect on MPZL1

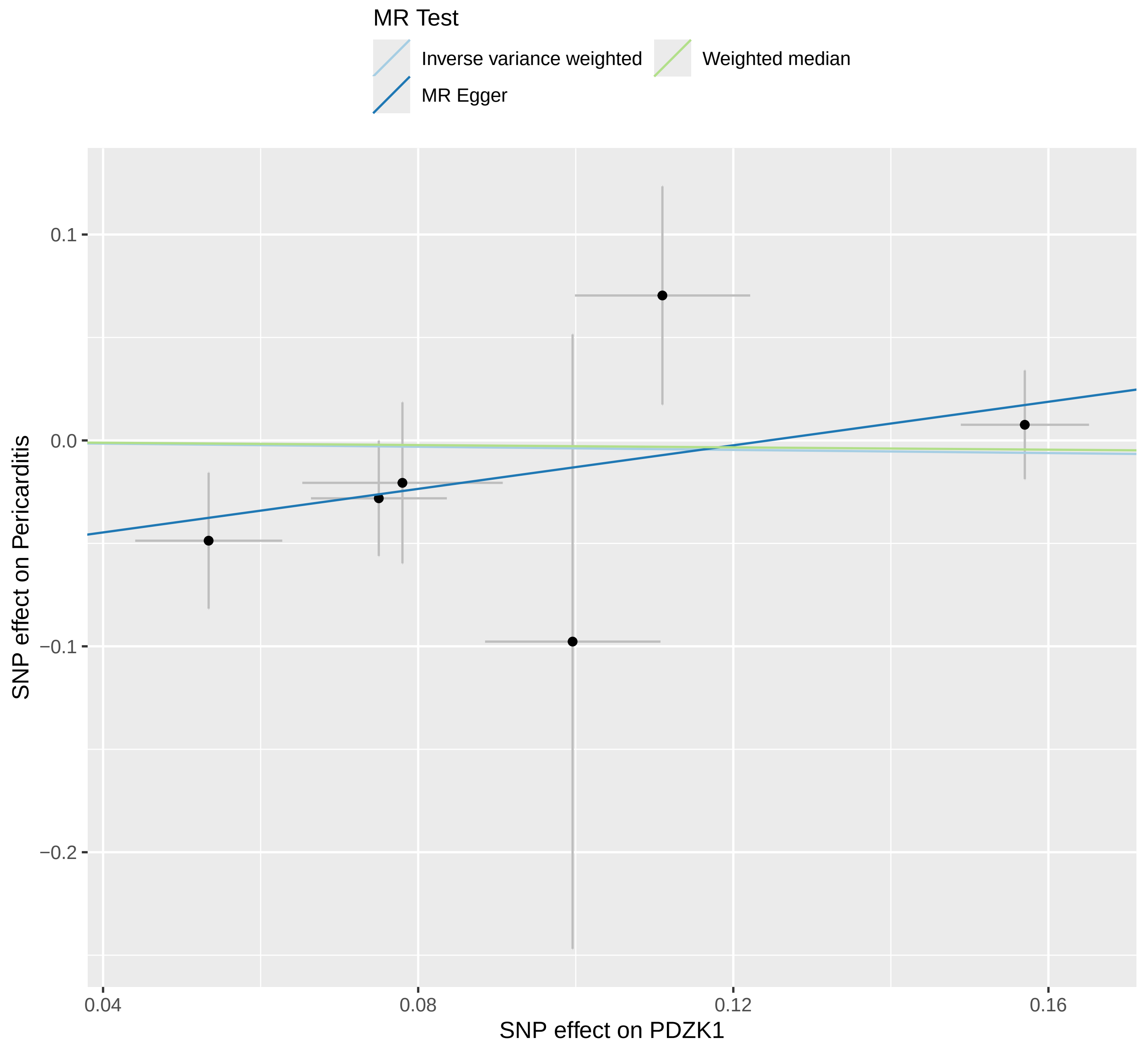
MR Test
Inverse variance weighted
Weighted median
MR Egger
0.1
0.0
−0.1
−0.2
SNP effect on Pericarditis
0.04
0.08
0.12
0.16
SNP effect on PDZK1

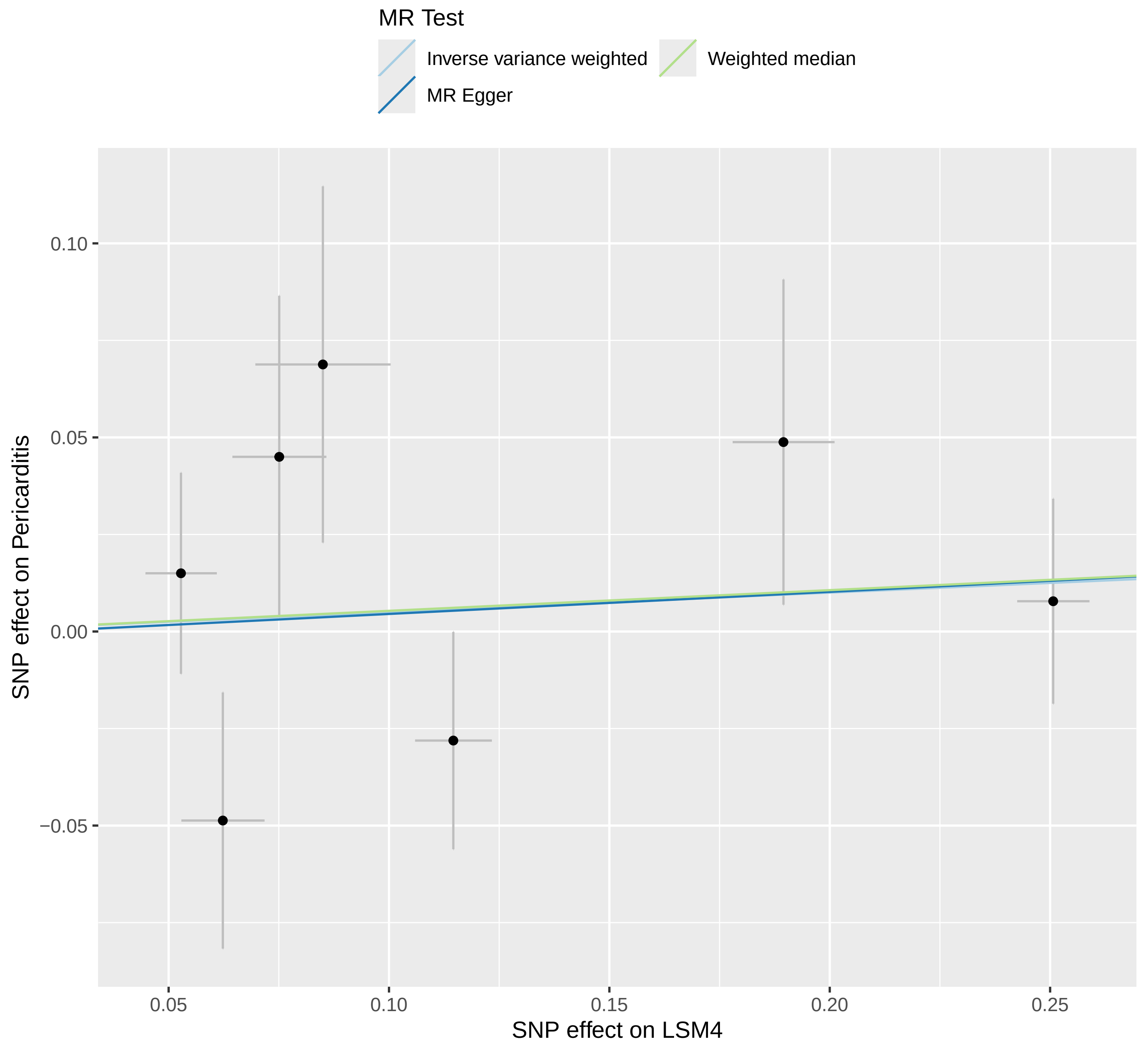
MR Test
Inverse variance weighted
Weighted median
MR Egger
SNP effect on Pericarditis
0.10
0.05
0.00
−0.05
0.05
0.10
0.15
0.20
0.25
SNP effect on LSM4

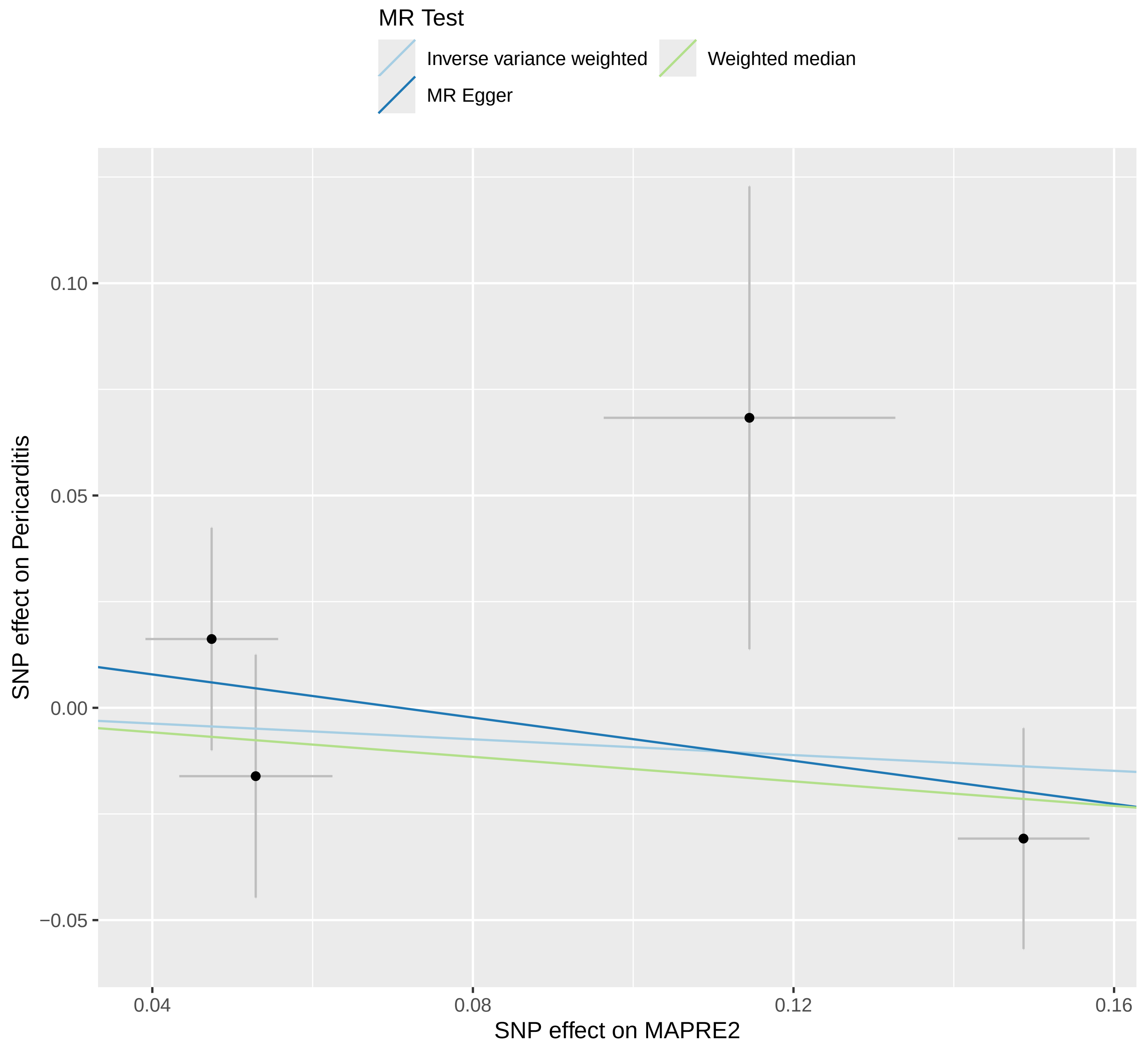

MR Test
Inverse variance weighted
MR Egger
Weighted median
0.10
0.05
0.00
−0.05
SNP effect on Pericarditis
0.04
0.08
0.12
0.16
SNP effect on MAPRE2

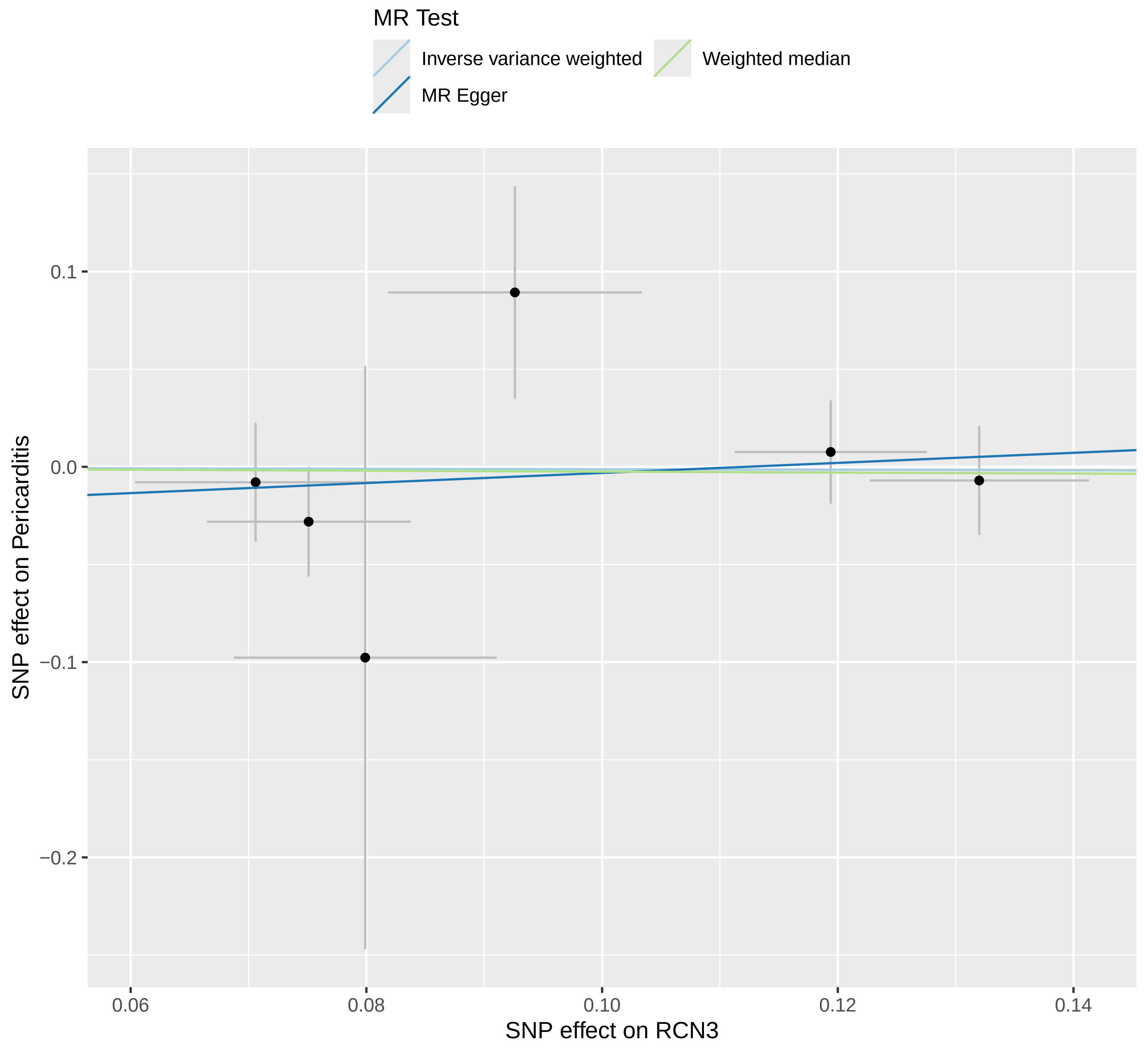
MR Test
Inverse variance weighted
Weighted median
MR Egger
0.1
0.0
−0.1
−0.2
0.06
0.08
0.10
0.12
0.14
SNP effect on Pericarditis
SNP effect on RCN3

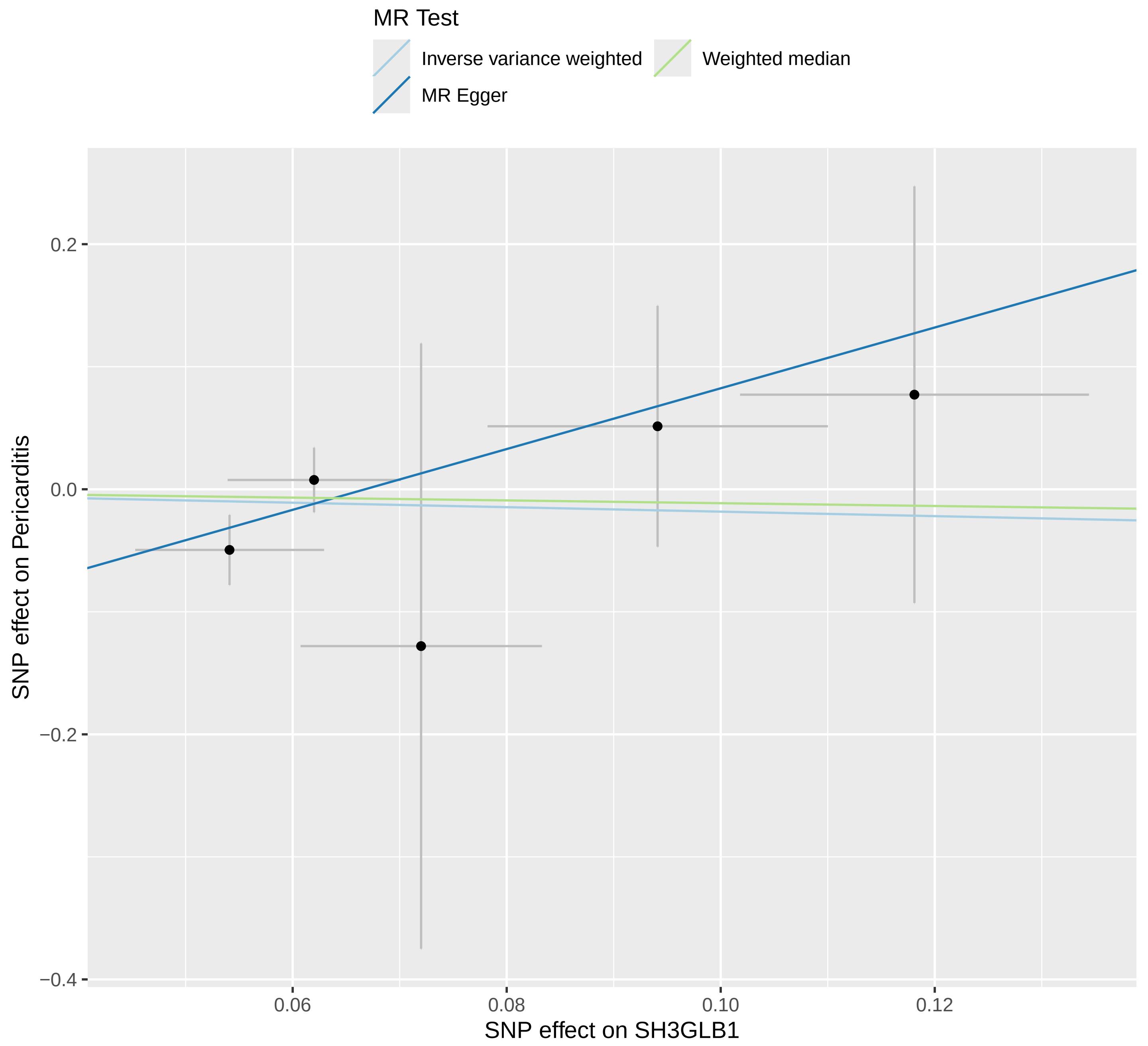
MR Test
Inverse variance weighted
Weighted median
MR Egger
SNP effect on Pericarditis
0.2
0.0
−0.2
−0.4
0.06
0.08
0.10
0.12
SNP effect on SH3GLB1

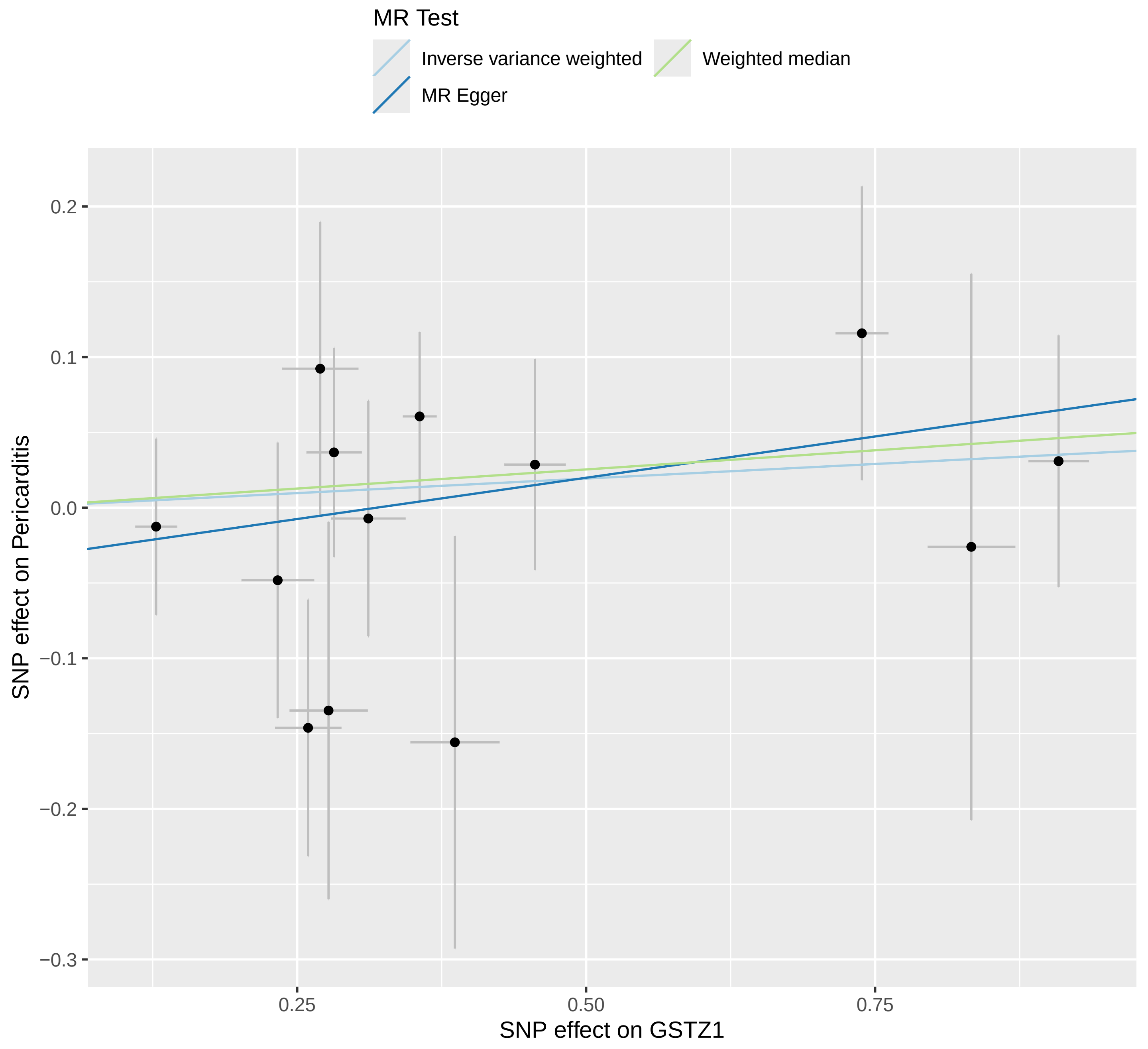
MR Test
Inverse variance weighted
MR Egger
Weighted median
0.2
0.1
0.0
−0.1
−0.2
−0.3
0.25
0.50
0.75
SNP effect on Pericarditis
SNP effect on GSTZ1

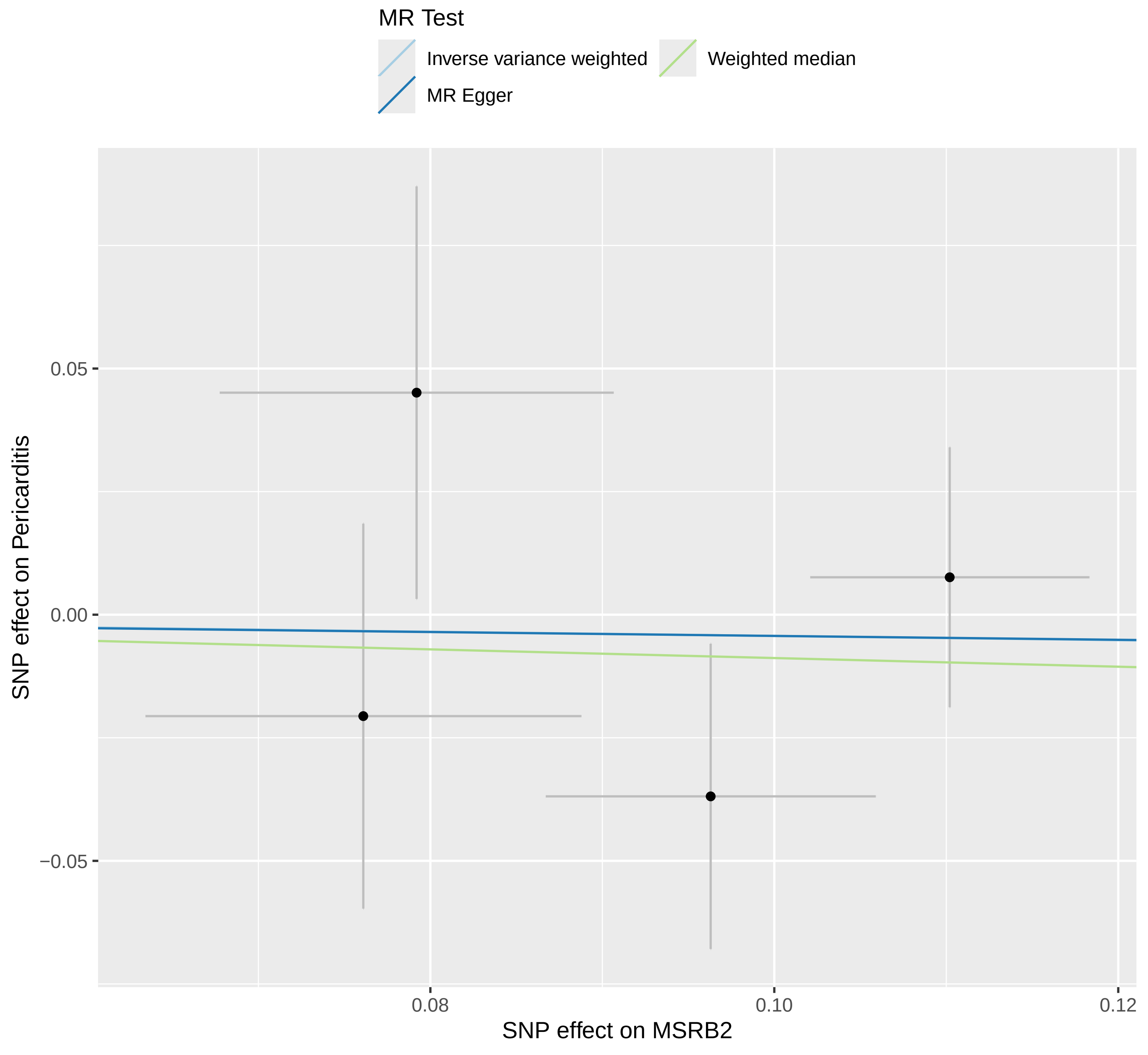
MR Test
Inverse variance weighted
Weighted median
MR Egger
0.05
0.00
−0.05
SNP effect on Pericarditis
0.08
0.10
0.12
SNP effect on MSRB2

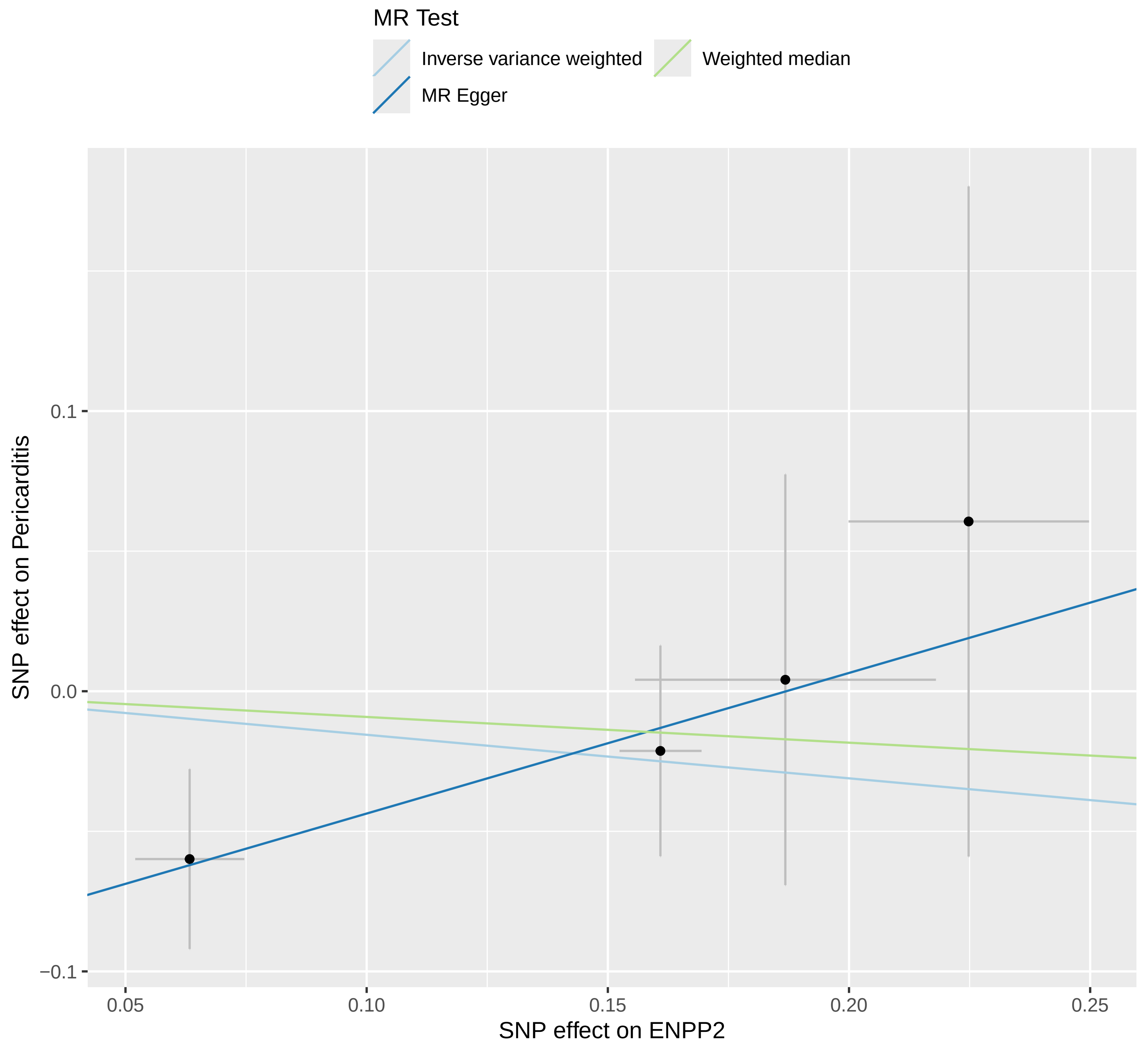

MR Test
Inverse variance weighted
Weighted median
MR Egger
0.1
0.0
−0.1
SNP effect on Pericarditis
0.05
0.10
0.15
0.20
0.25
SNP effect on ENPP2

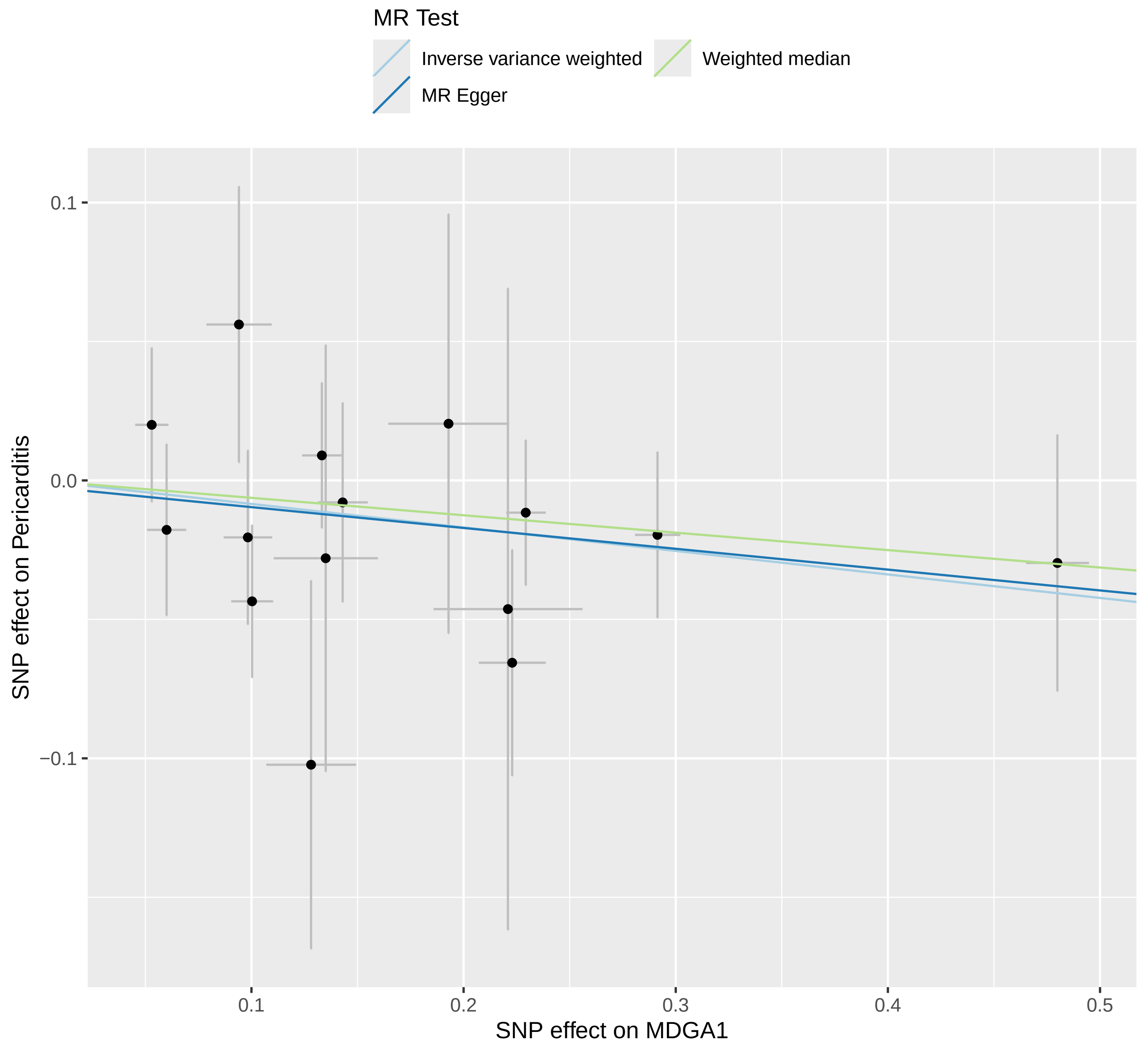
MR Test
Inverse variance weighted
Weighted median
MR Egger
0.1
0.0
−0.1
SNP effect on Pericarditis
0.1
0.2
0.3
0.4
0.5
SNP effect on MDGA1

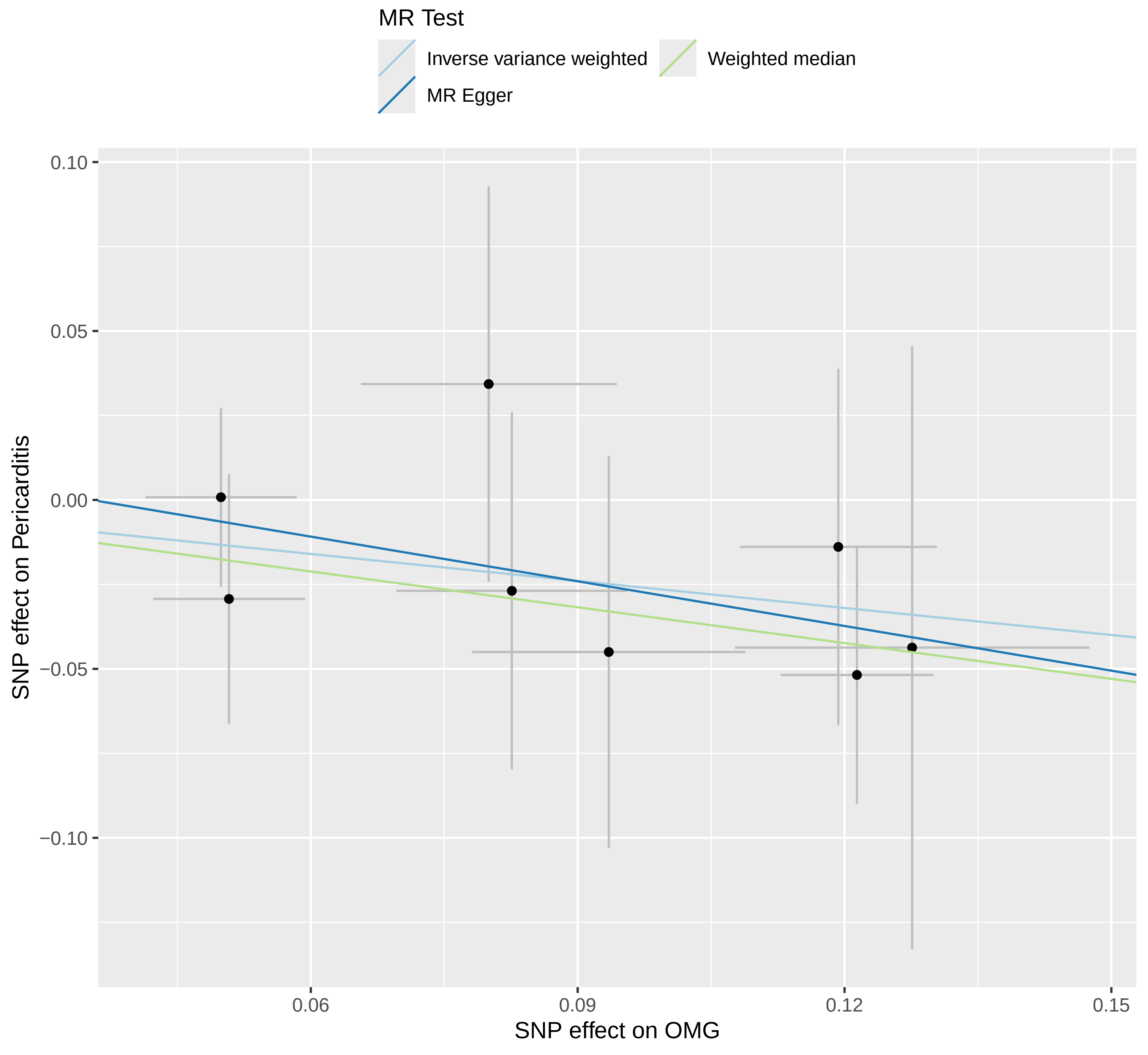
MR Test
Inverse variance weighted
MR Egger
Weighted median
0.10
0.05
0.00
−0.05
−0.10
SNP effect on Pericarditis
0.06
0.09
0.12
0.15
SNP effect on OMG

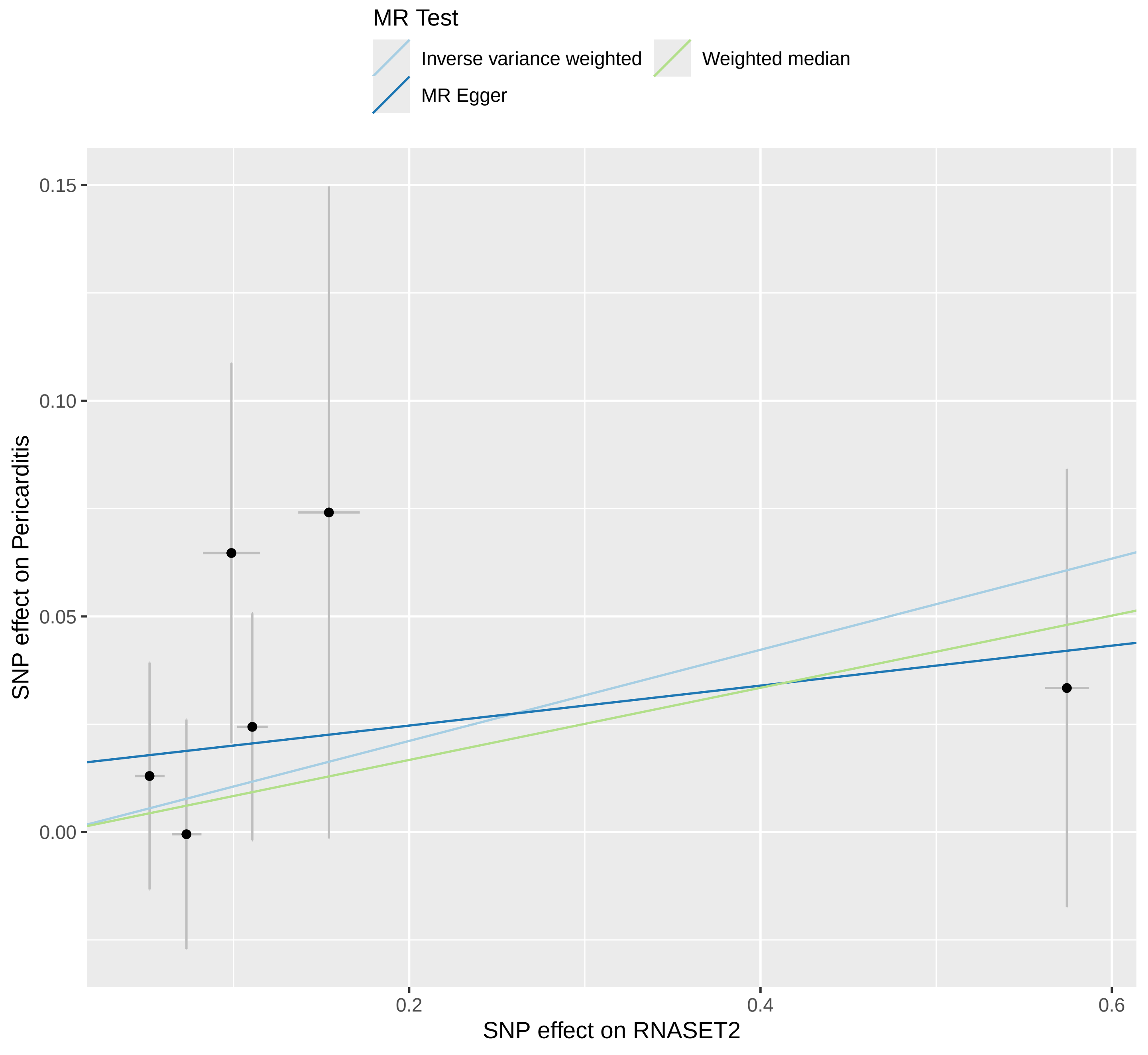
MR Test
Inverse variance weighted
Weighted median
MR Egger
0.15
0.10
0.05
0.00
SNP effect on Pericarditis
0.2
0.4
0.6
SNP effect on RNASET2

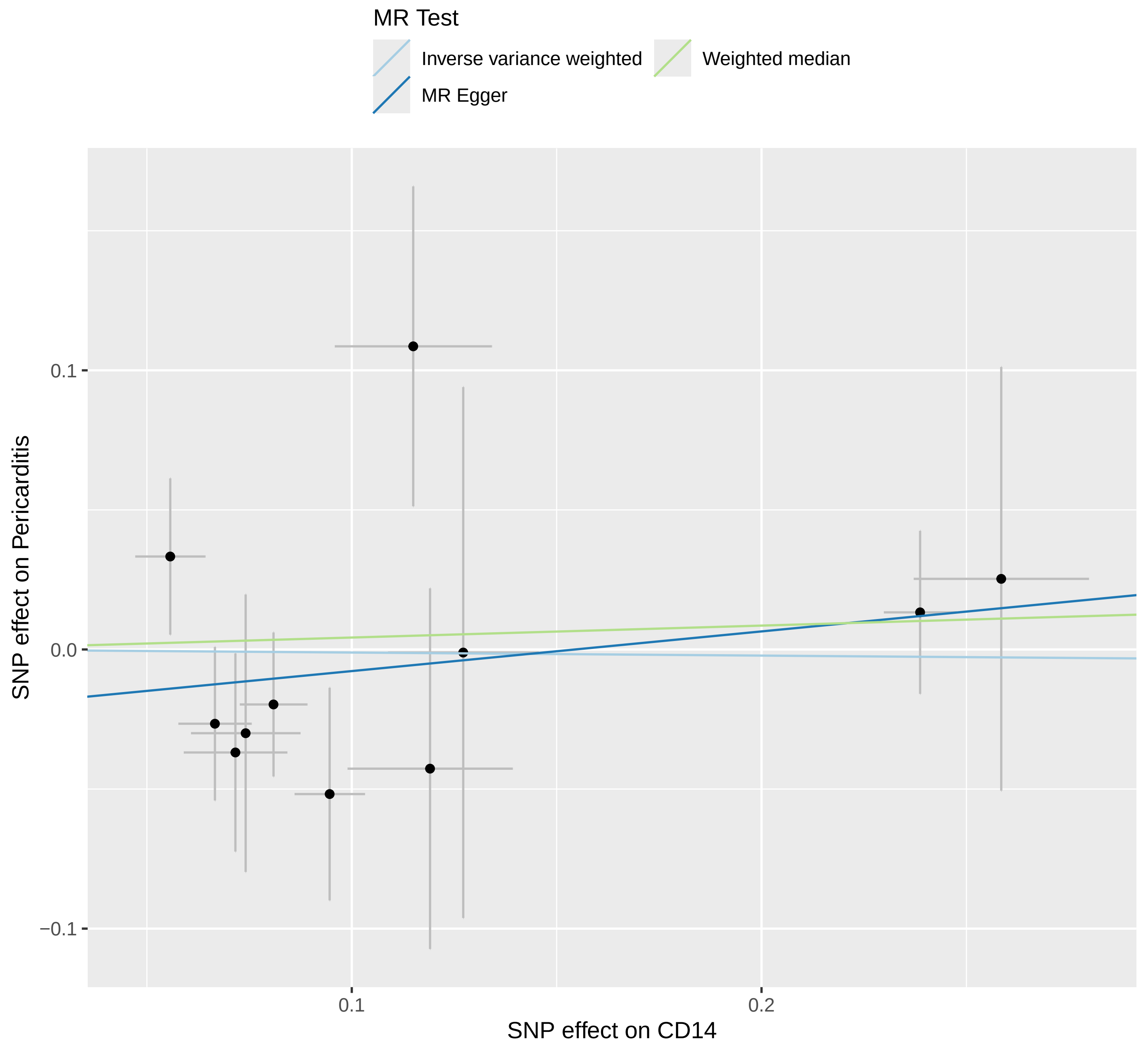

MR Test
Inverse variance weighted
Weighted median
MR Egger
SNP effect on Pericarditis
0.1
0.0
−0.1
0.1
0.2
SNP effect on CD14

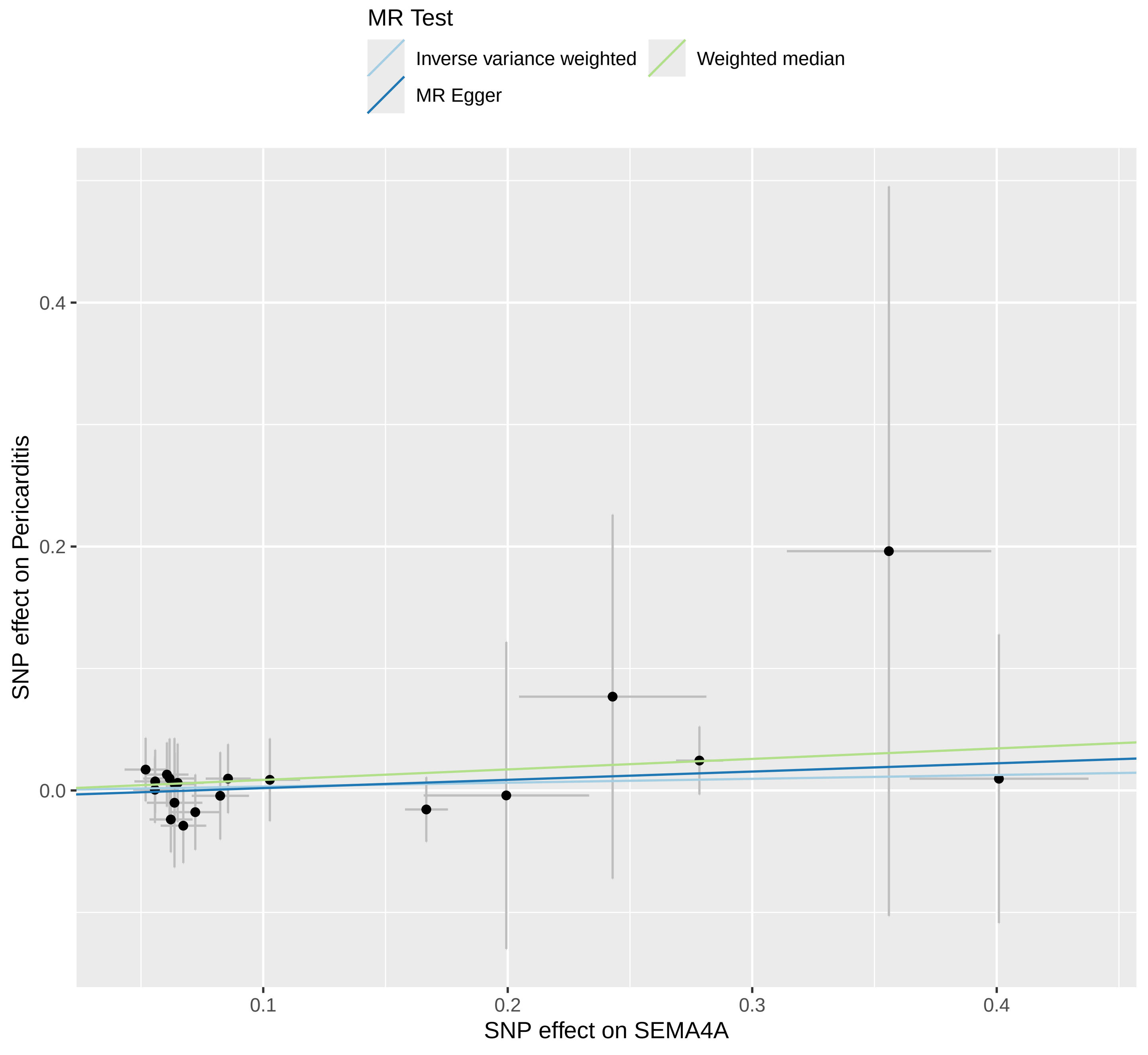
MR Test
Inverse variance weighted
Weighted median
MR Egger
SNP effect on Pericarditis
0.4
0.2
0.0
0.1
0.2
0.3
0.4
SNP effect on SEMA4A

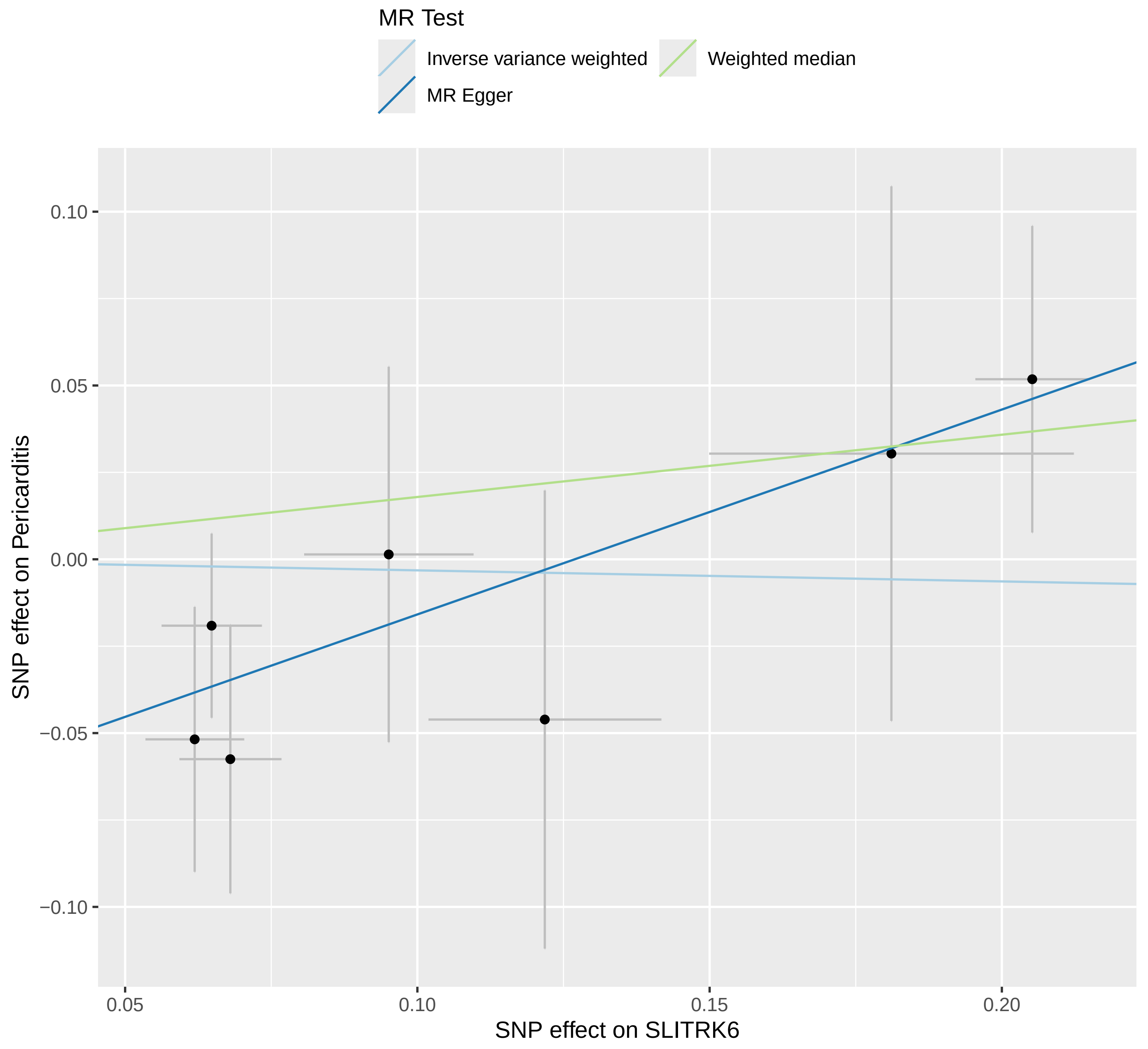

MR Test
Inverse variance weighted
MR Egger
Weighted median
0.10
0.05
0.00
−0.05
−0.10
SNP effect on Pericarditis
0.05
0.10
0.15
0.20
SNP effect on SLITRK6

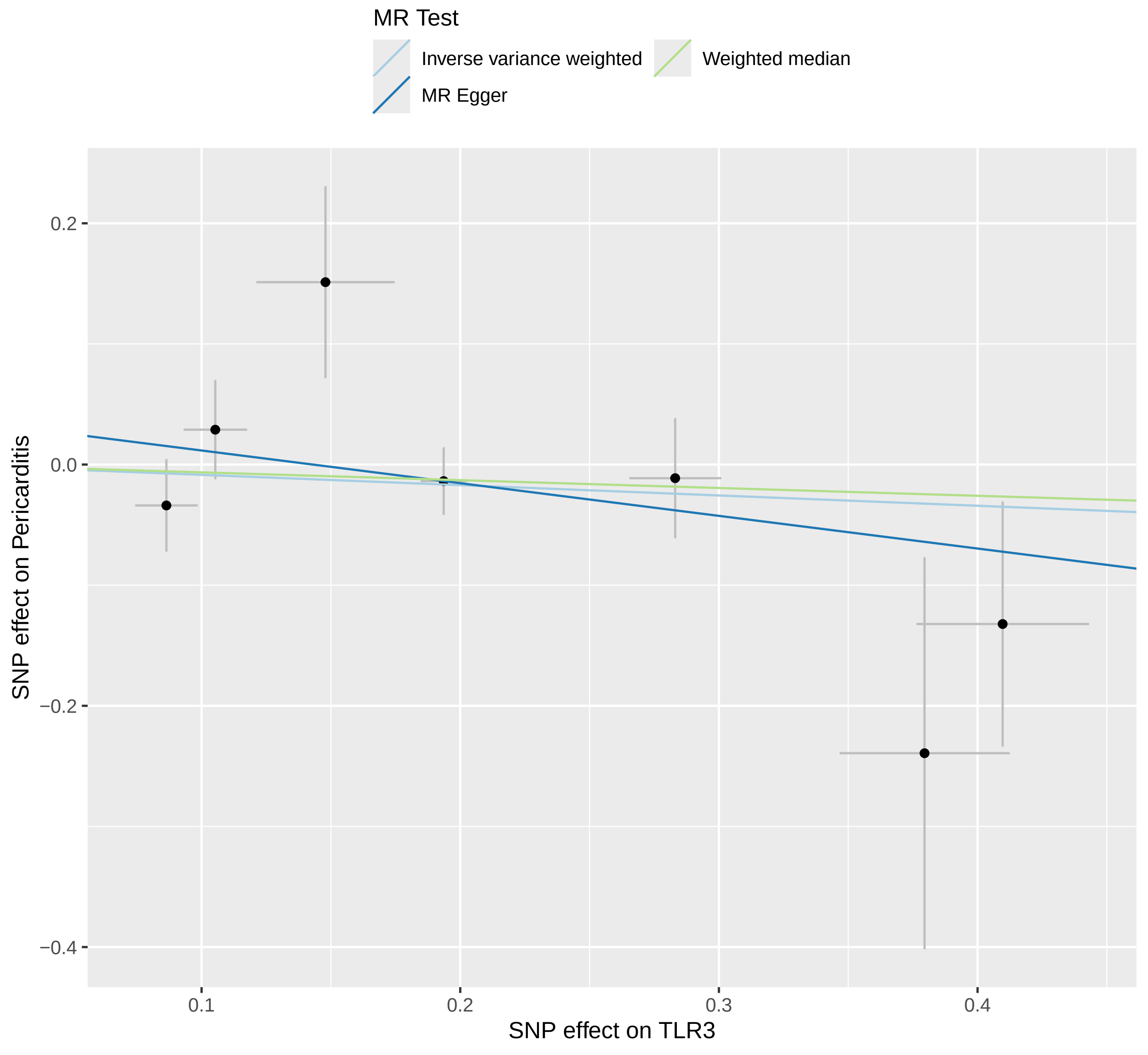

MR Test
Inverse variance weighted
Weighted median
MR Egger
0.2
0.0
−0.2
−0.4
SNP effect on Pericarditis
0.1
0.2
0.3
0.4
SNP effect on TLR3

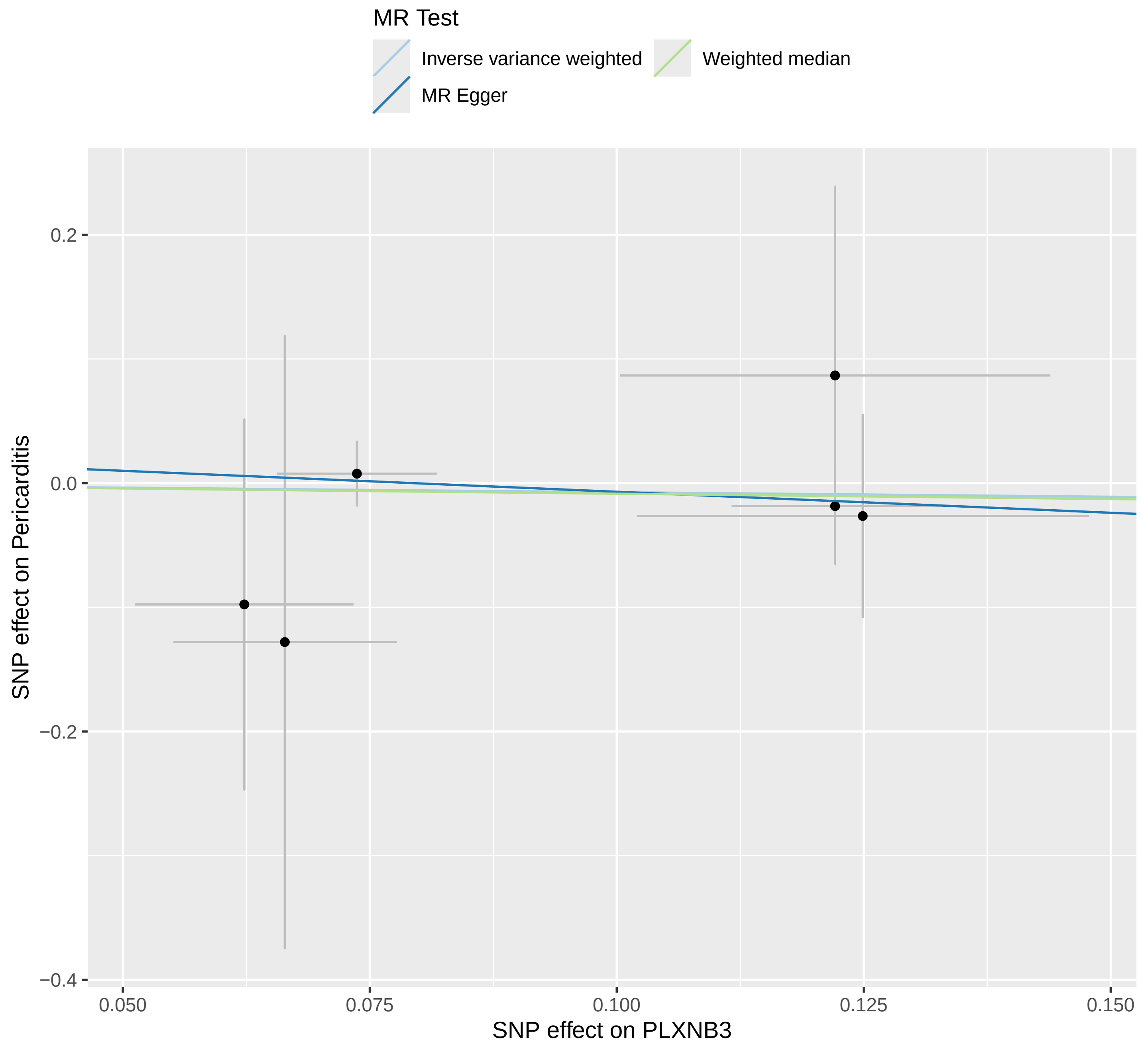

MR Test
Inverse variance weighted
Weighted median
MR Egger
0.2
0.0
−0.2
−0.4
SNP effect on Pericarditis
0.050
0.075
0.100
0.125
0.150
SNP effect on PLXNB3

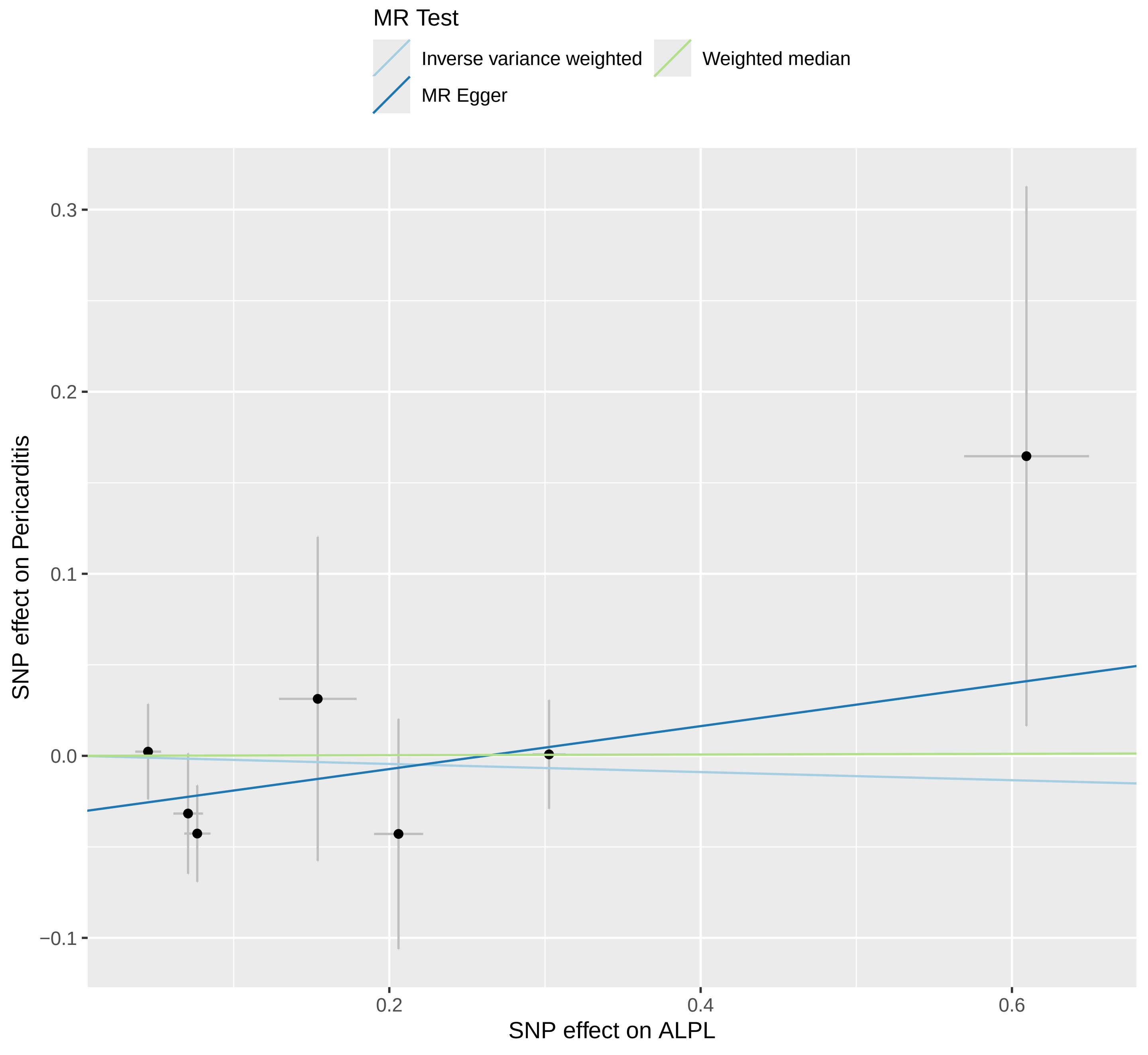

MR Test
Inverse variance weighted
Weighted median
MR Egger
0.3
0.2
0.1
0.0
−0.1
SNP effect on Pericarditis
0.2
0.4
0.6
SNP effect on ALPL

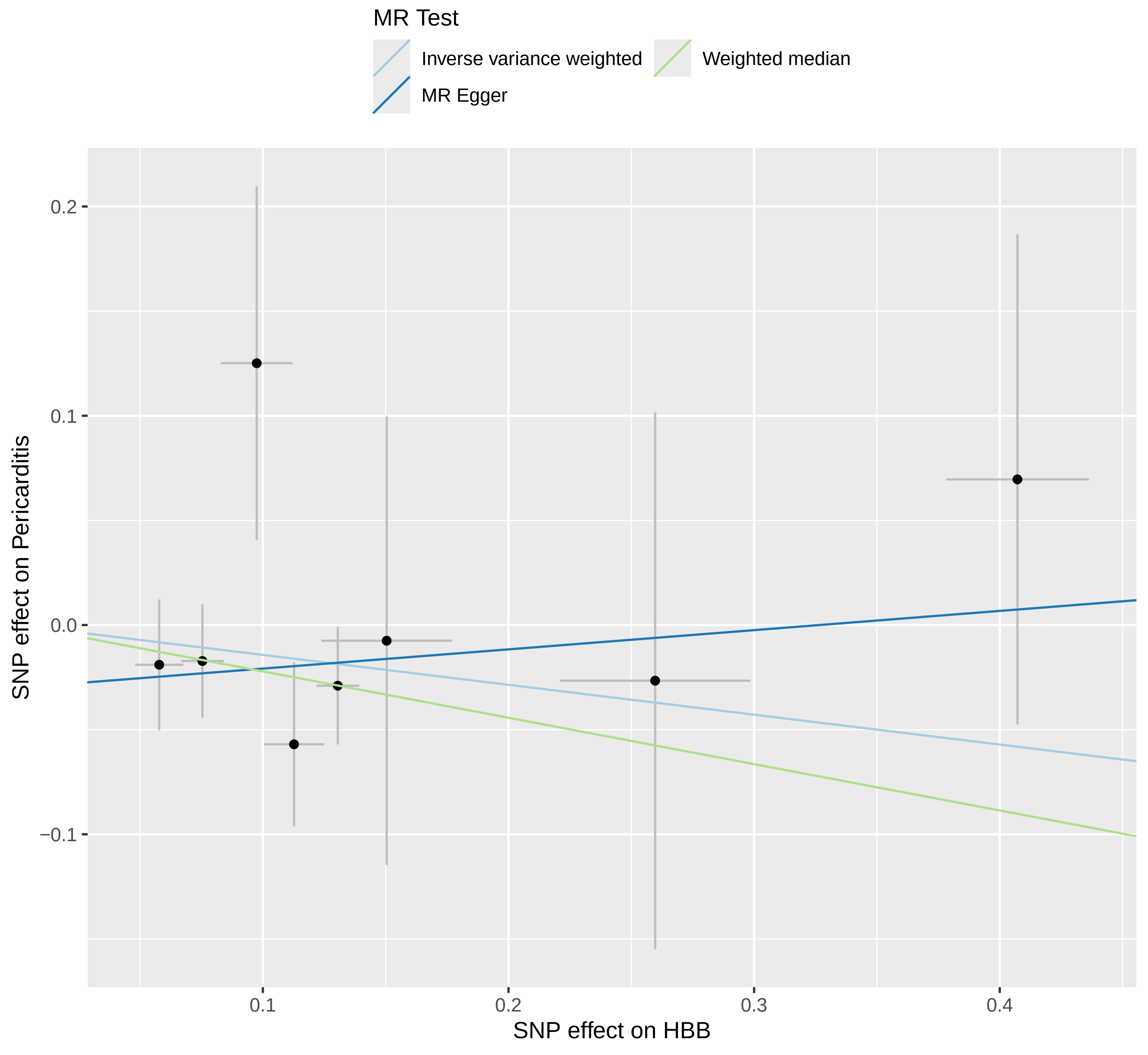

MR Test
Inverse variance weighted
Weighted median
MR Egger
0.2
0.1
0.0
−0.1
SNP effect on Pericarditis
0.1
0.2
0.3
0.4
SNP effect on HBB

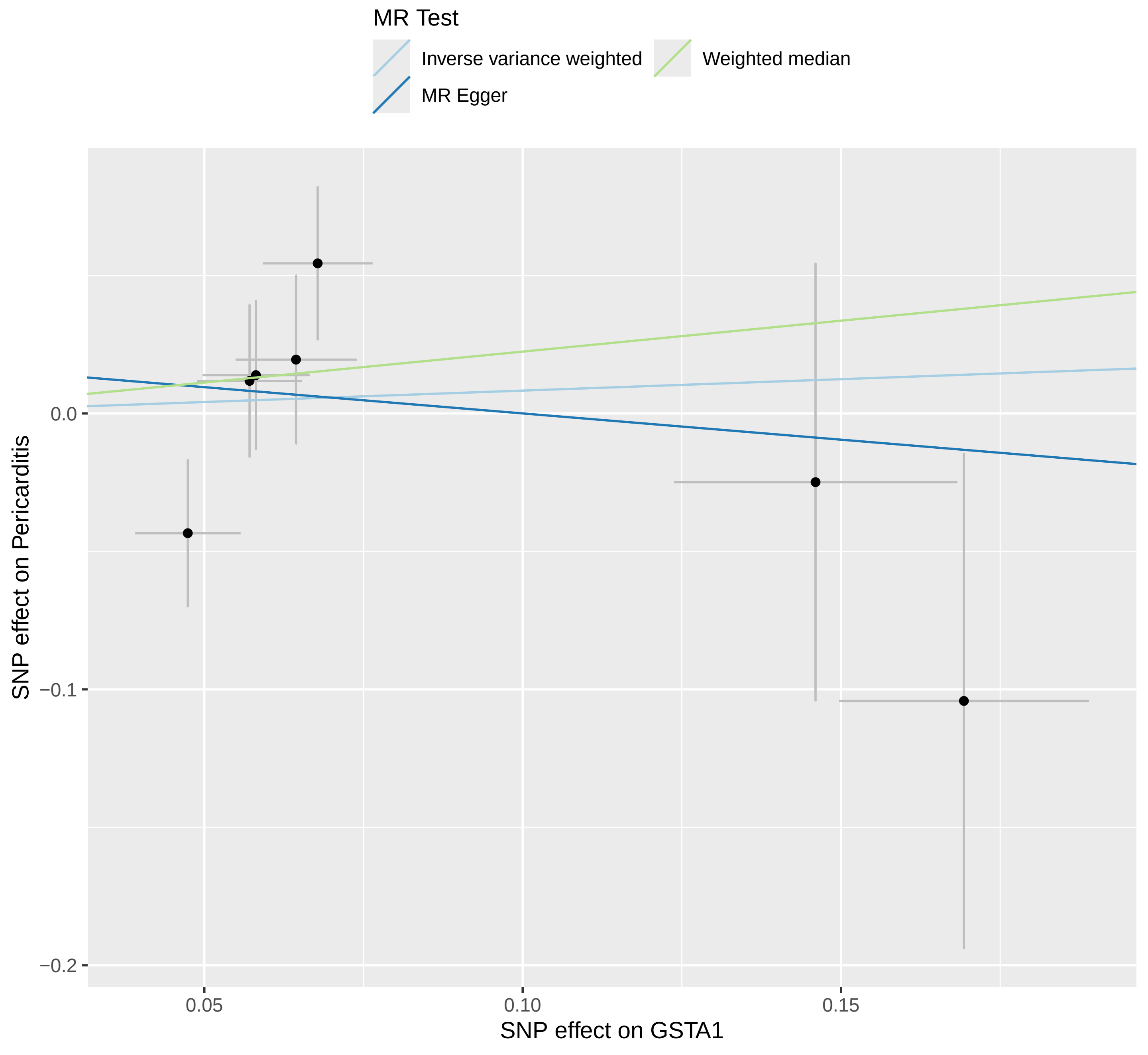
MR Test
Inverse variance weighted
Weighted median
MR Egger
SNP effect on Pericarditis
0.0
−0.1
−0.2
0.05
0.10
0.15
SNP effect on GSTA1

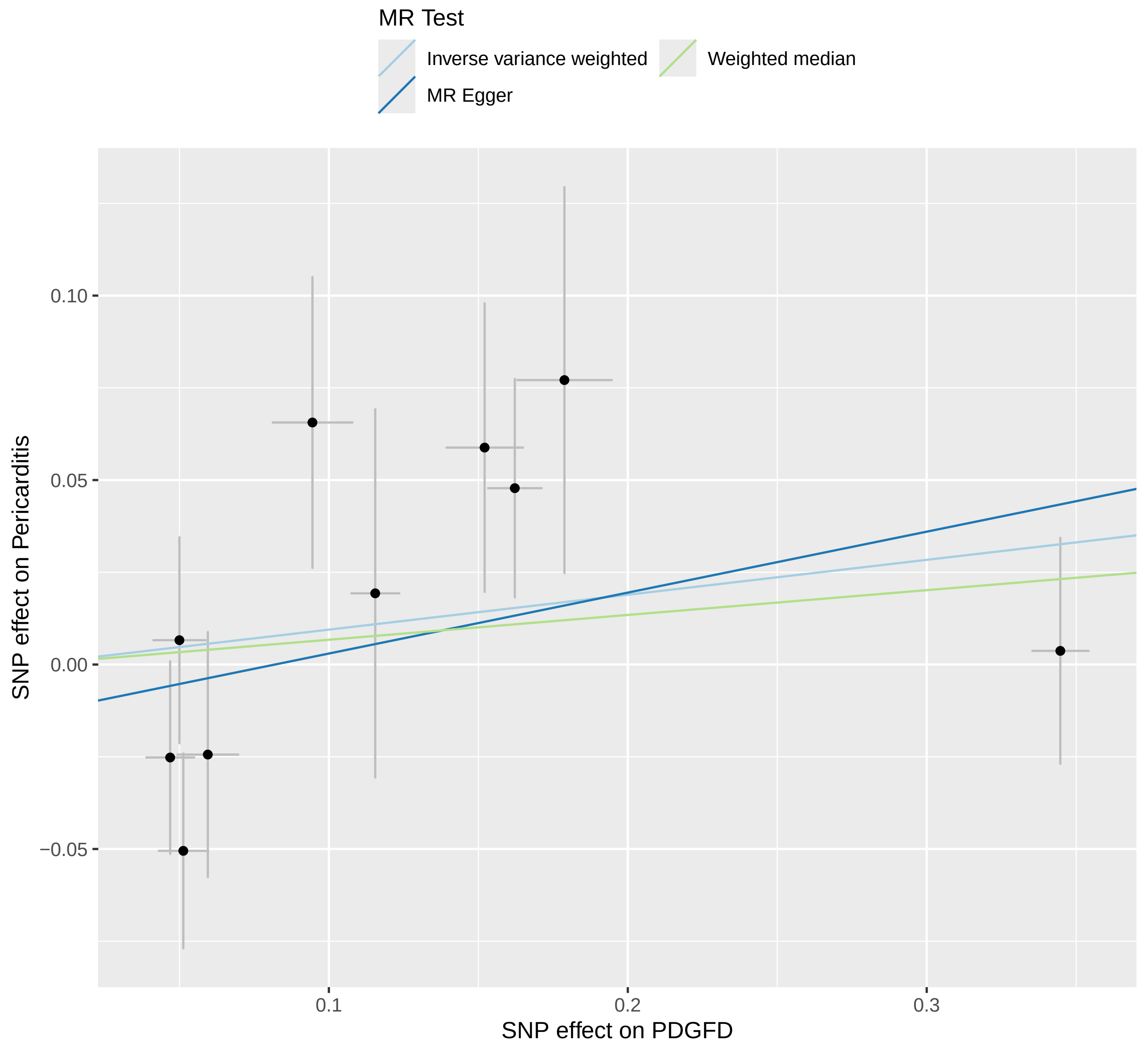
MR Test
Inverse variance weighted
MR Egger
Weighted median
SNP effect on Pericarditis
0.10
0.05
0.00
−0.05
0.1
0.2
0.3
SNP effect on PDGFD

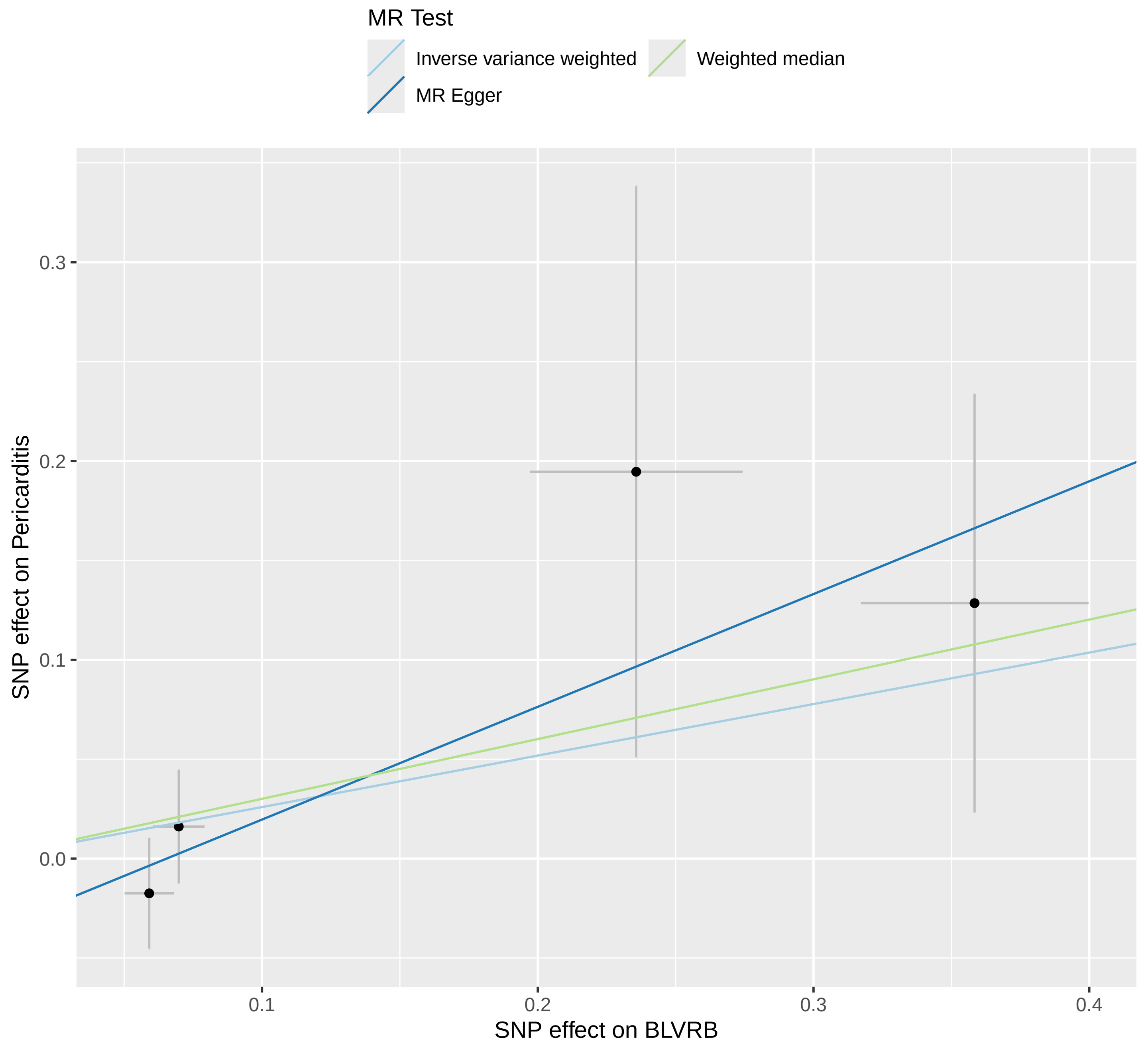

MR Test
Inverse variance weighted
Weighted median
MR Egger
SNP effect on Pericarditis
0.0
0.1
0.2
0.3
SNP effect on BLVRB
0.1
0.2
0.3
0.4

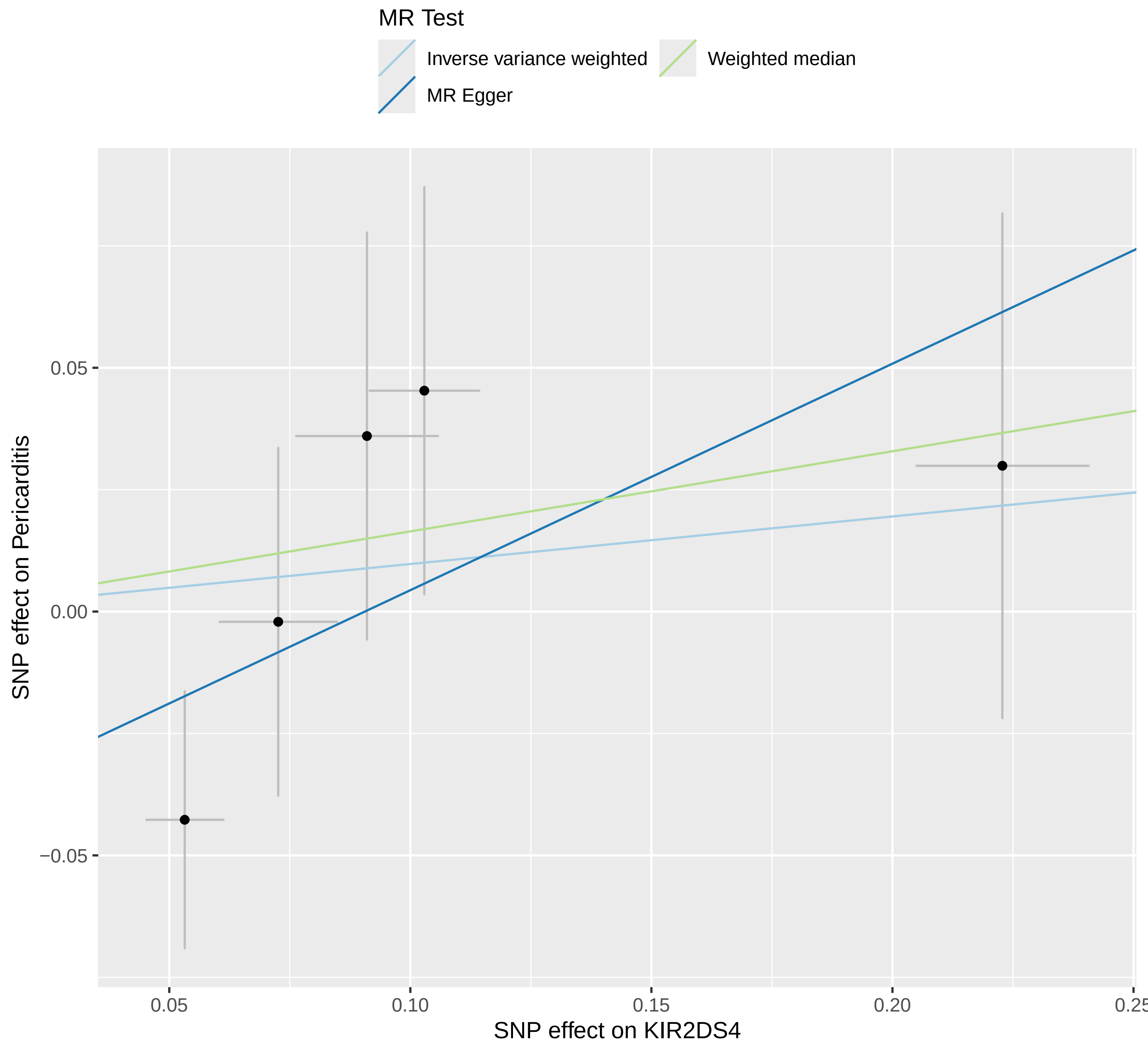
MR Test
Inverse variance weighted
MR Egger
Weighted median
SNP effect on Pericarditis
0.05
0.00
−0.05
SNP effect on KIR2DS4
0.05
0.10
0.15
0.20
0.25

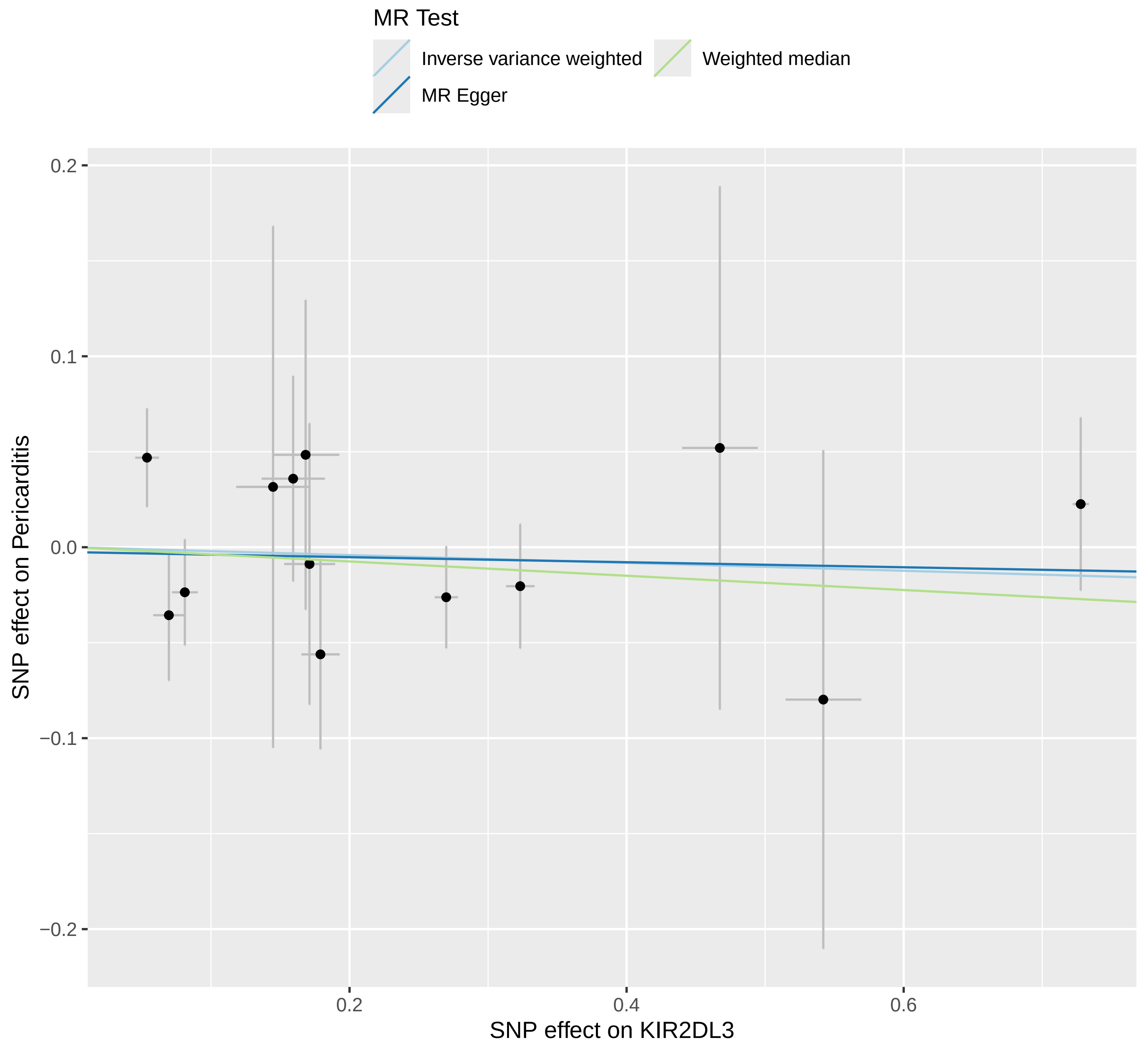
MR Test
Inverse variance weighted
Weighted median
MR Egger
0.2
0.1
0.0
−0.1
−0.2
SNP effect on Pericarditis
0.2
0.4
0.6
SNP effect on KIR2DL3

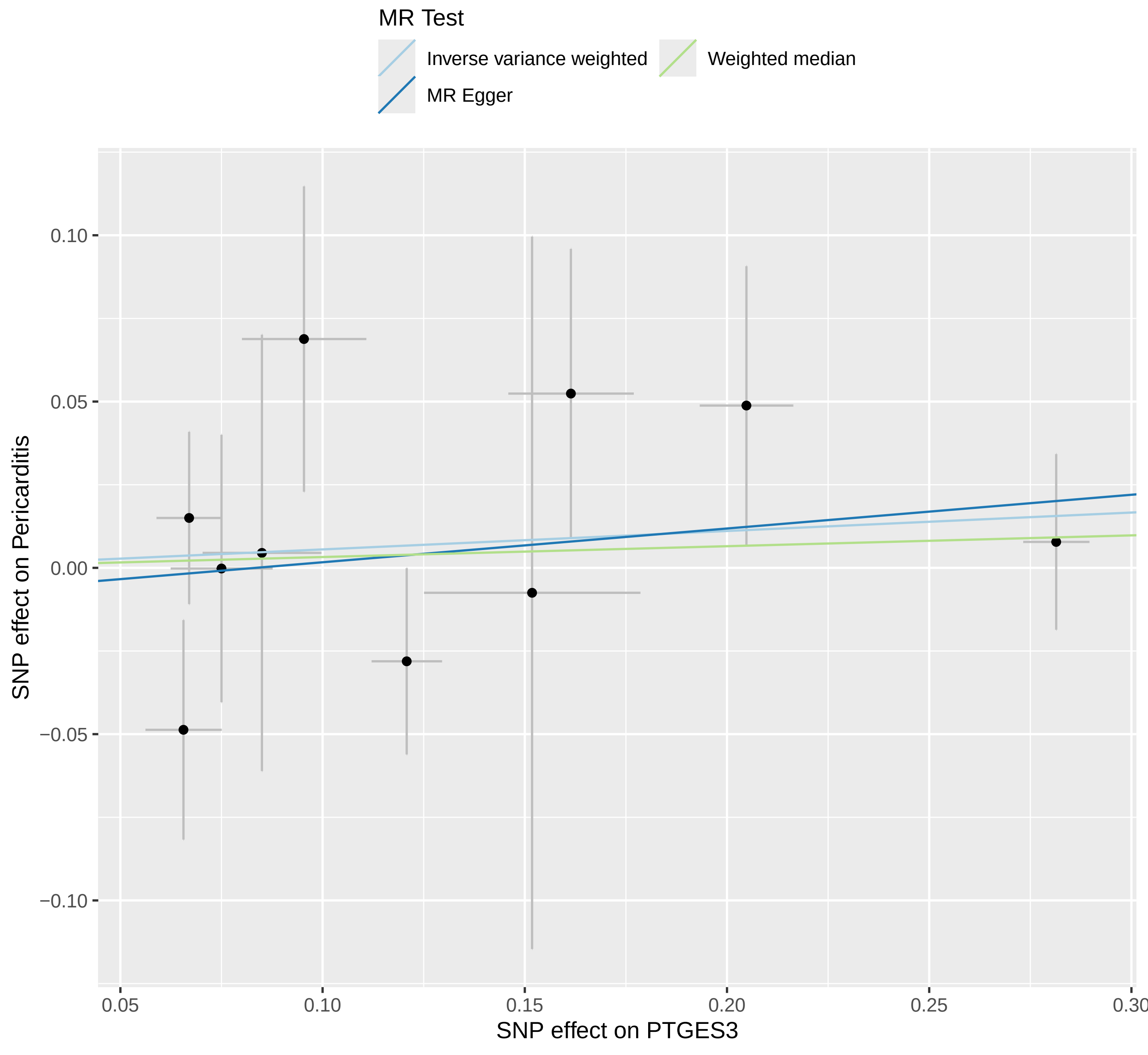
MR Test
Inverse variance weighted
MR Egger
Weighted median
SNP effect on Pericarditis
0.10
0.05
0.00
−0.05
−0.10
0.05
0.10
0.15
0.20
0.25
0.30
SNP effect on PTGES3

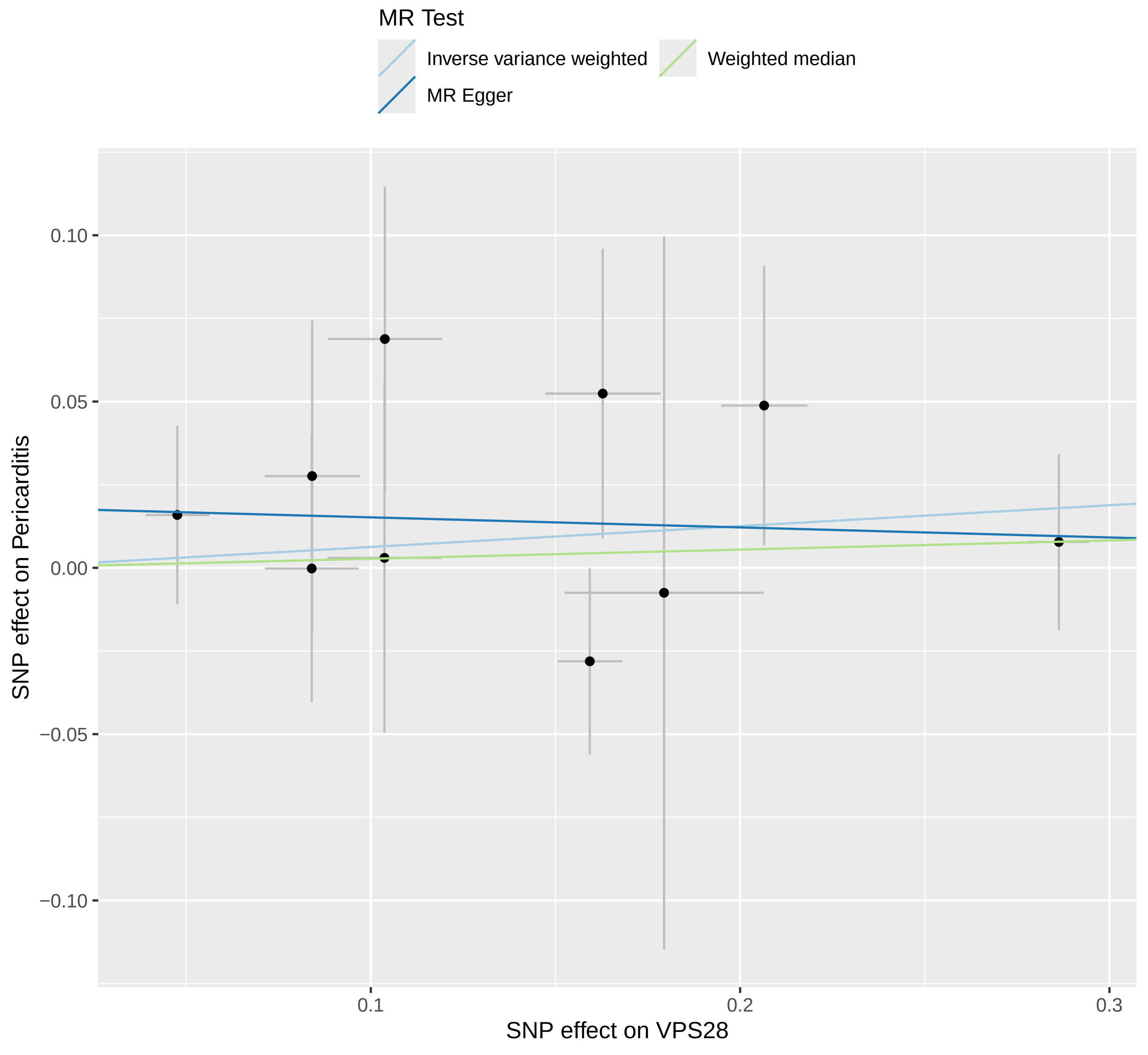

MR Test
Inverse variance weighted
MR Egger
Weighted median
0.10
0.05
0.00
−0.05
−0.10
SNP effect on Pericarditis
0.1
0.2
0.3
SNP effect on VPS28

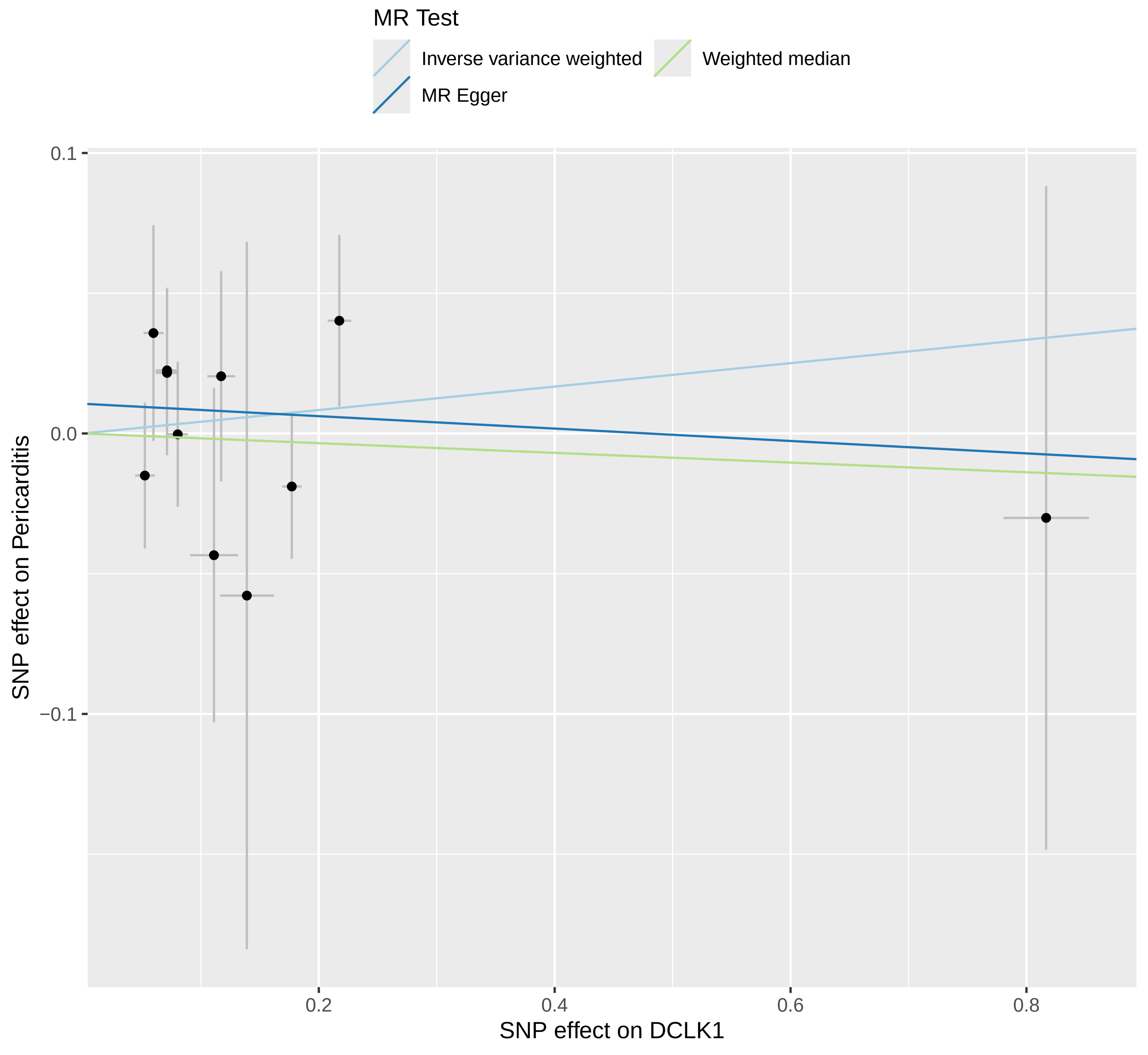

MR Test
Inverse variance weighted
Weighted median
MR Egger
0.1
0.0
−0.1
SNP effect on Pericarditis
0.2
0.4
0.6
0.8
SNP effect on DCLK1

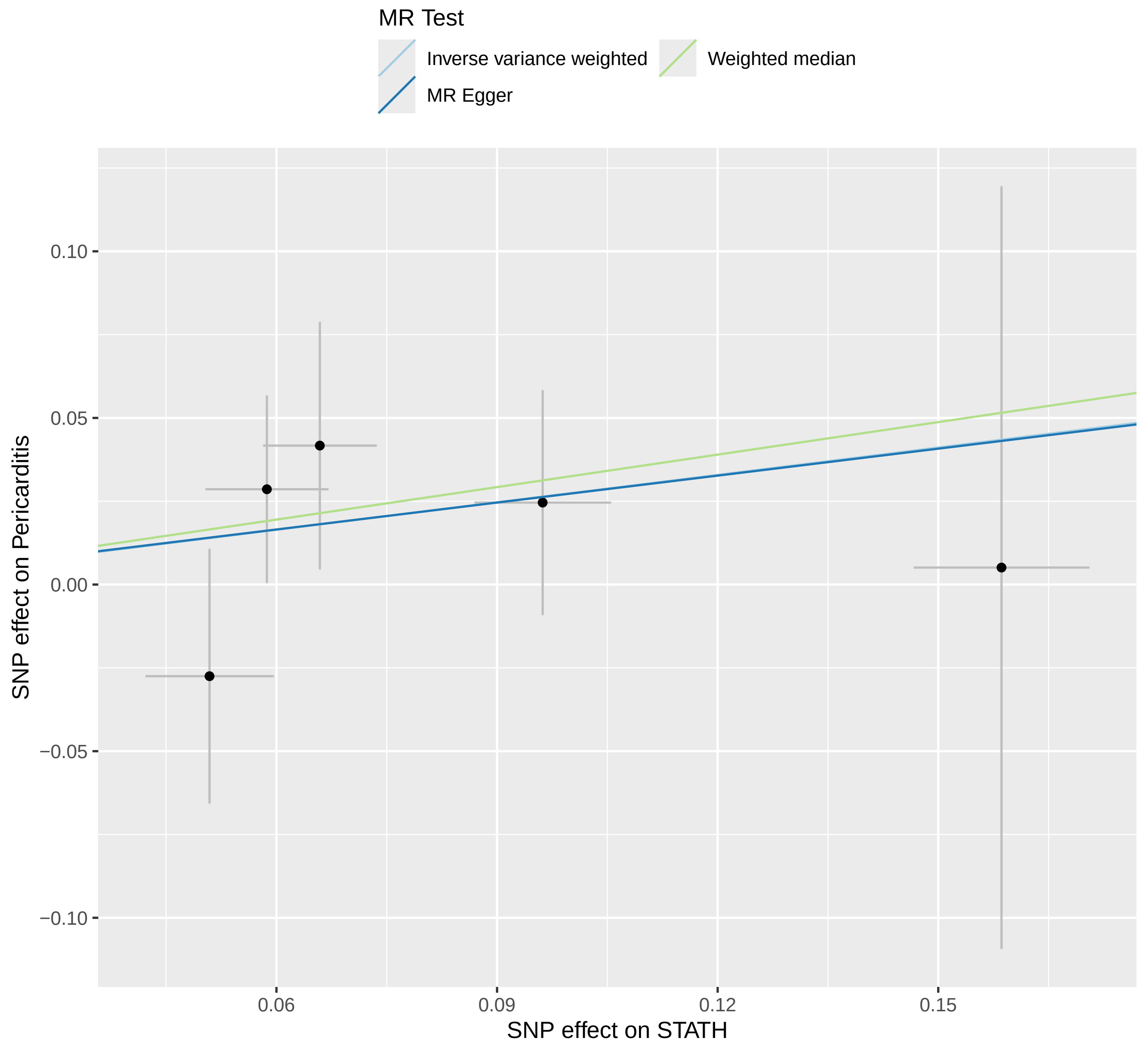
MR Test
Inverse variance weighted
MR Egger
Weighted median
0.10
0.05
0.00
−0.05
−0.10
SNP effect on Pericarditis
0.06
0.09
0.12
0.15
SNP effect on STATH

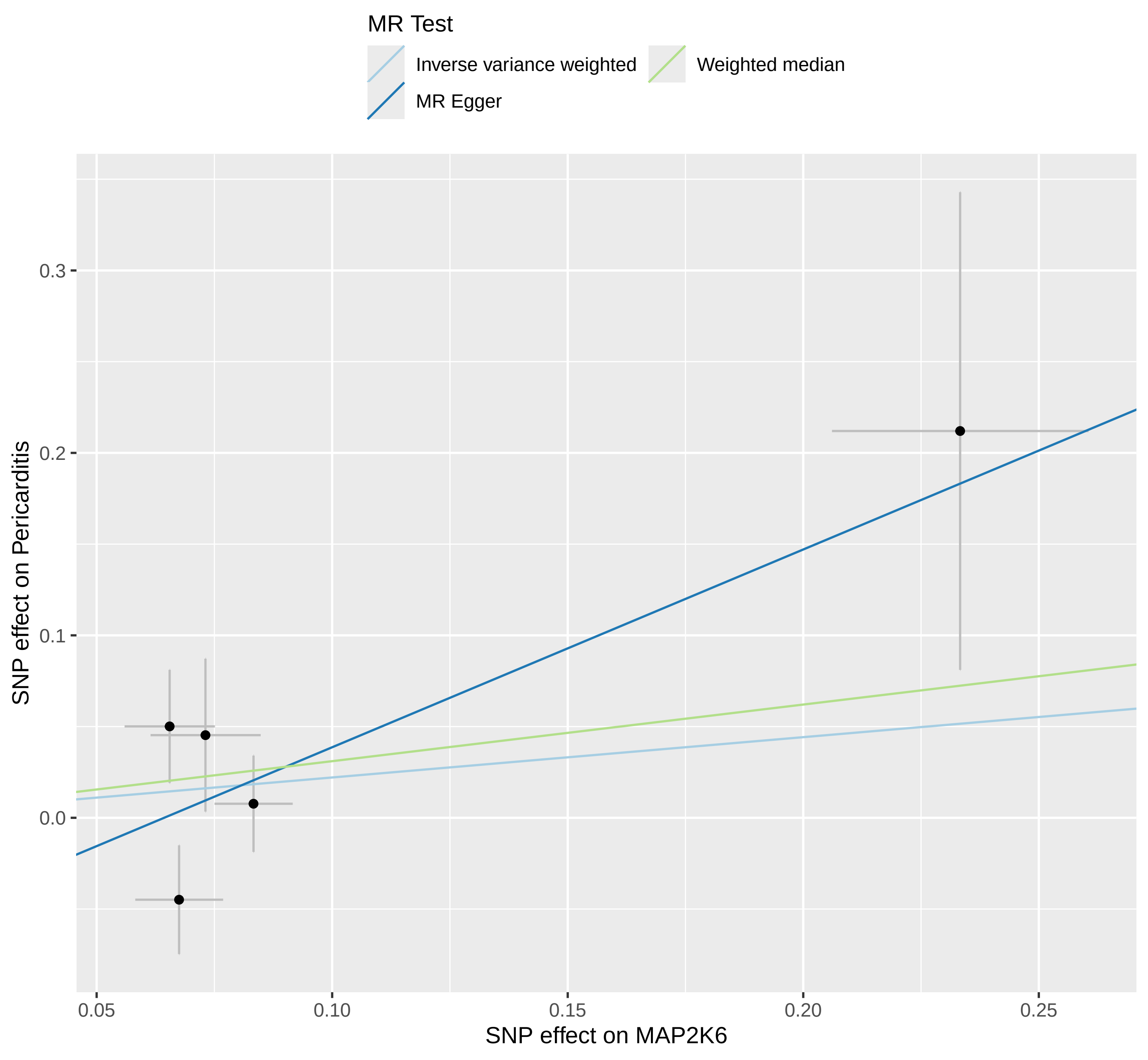

MR Test
Inverse variance weighted
MR Egger
Weighted median
SNP effect on Pericarditis
0.3
0.2
0.1
0.0
0.05
0.10
0.15
0.20
0.25
SNP effect on MAP2K6

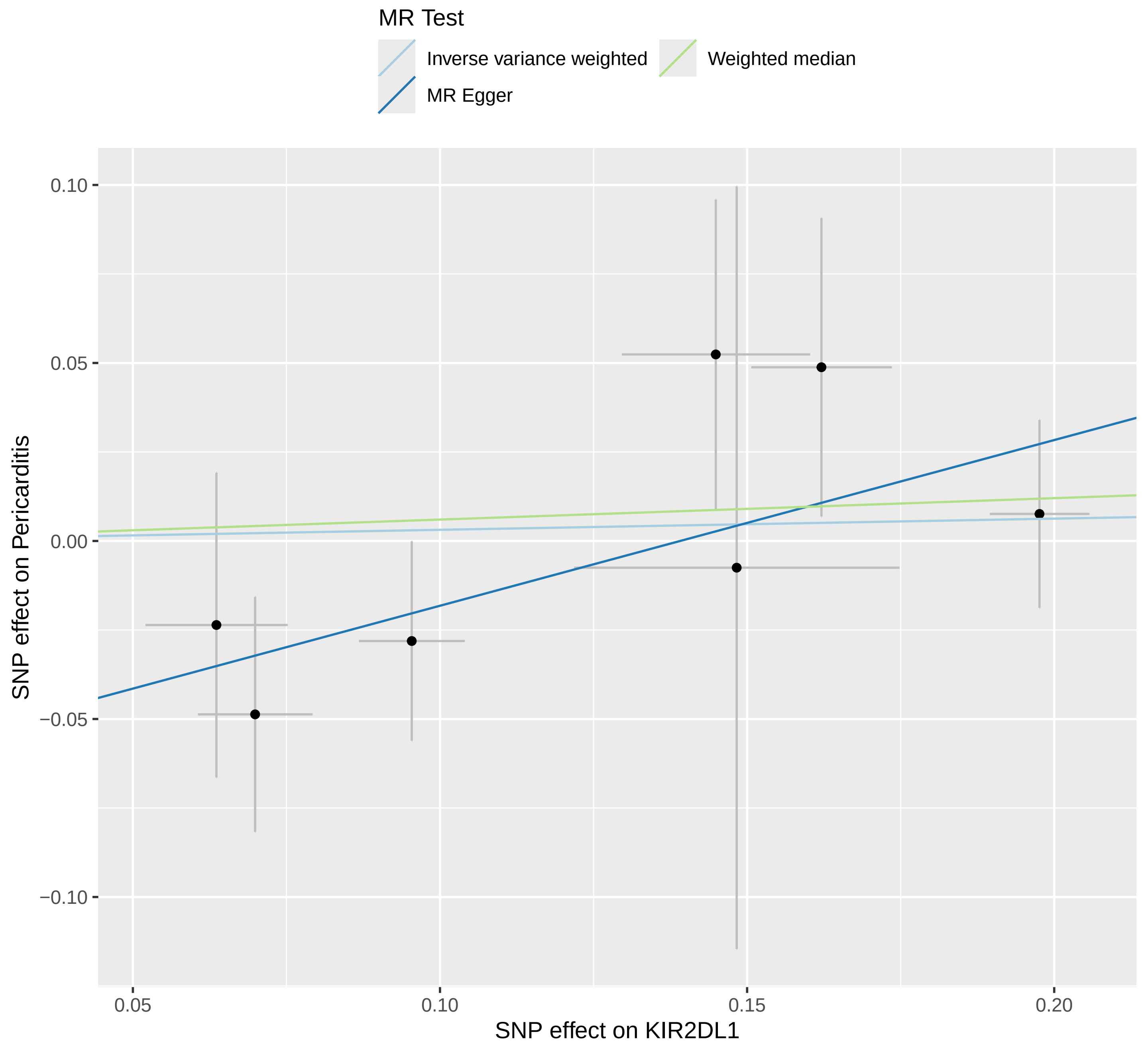

MR Test
Inverse variance weighted
Weighted median
MR Egger
0.10
0.05
0.00
−0.05
−0.10
SNP effect on Pericarditis
0.05
0.10
0.15
0.20
SNP effect on KIR2DL1

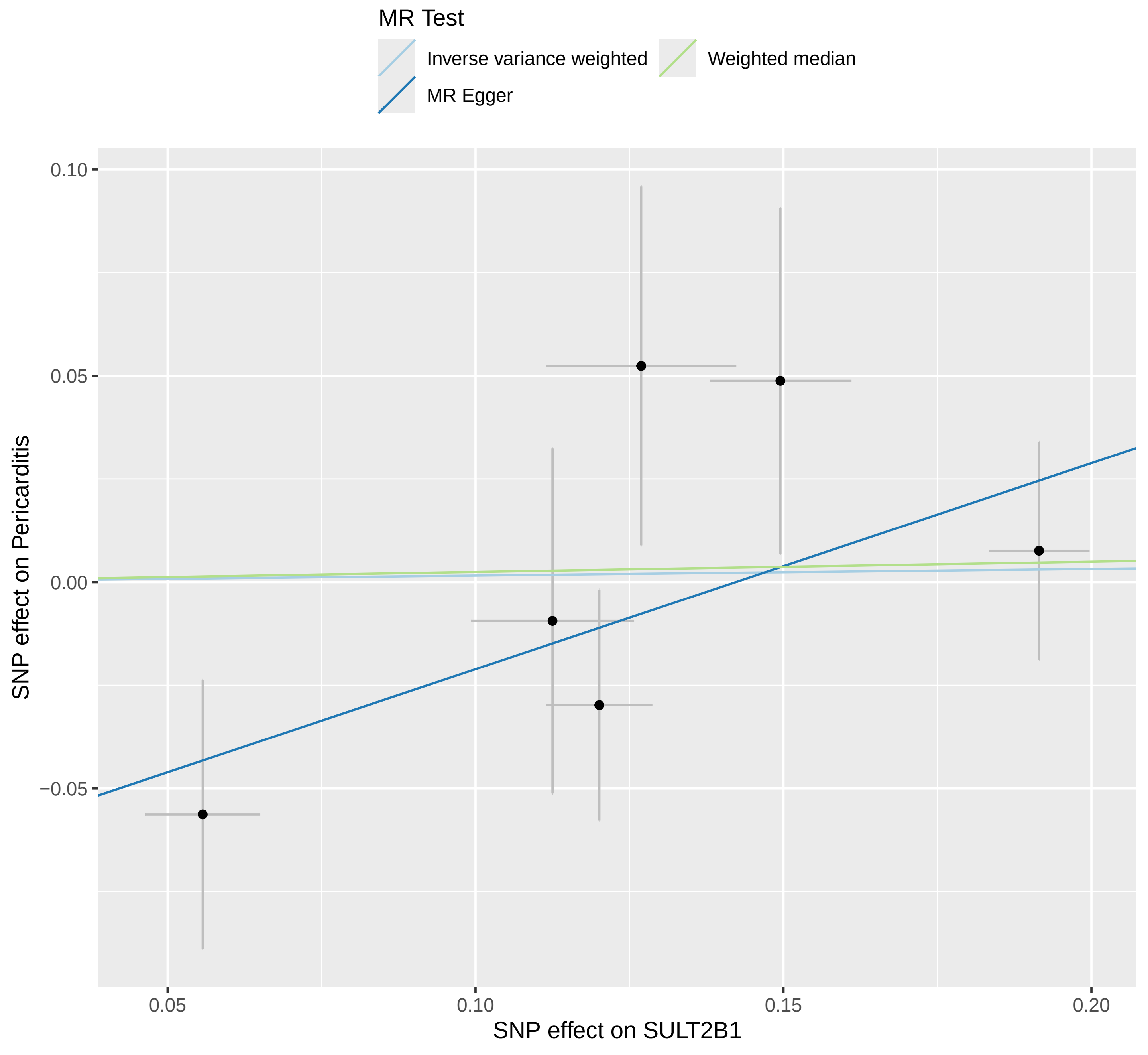

MR Test
Inverse variance weighted
MR Egger
Weighted median
0.10
0.05
0.00
−0.05
SNP effect on Pericarditis
0.05
0.10
0.15
0.20
SNP effect on SULT2B1

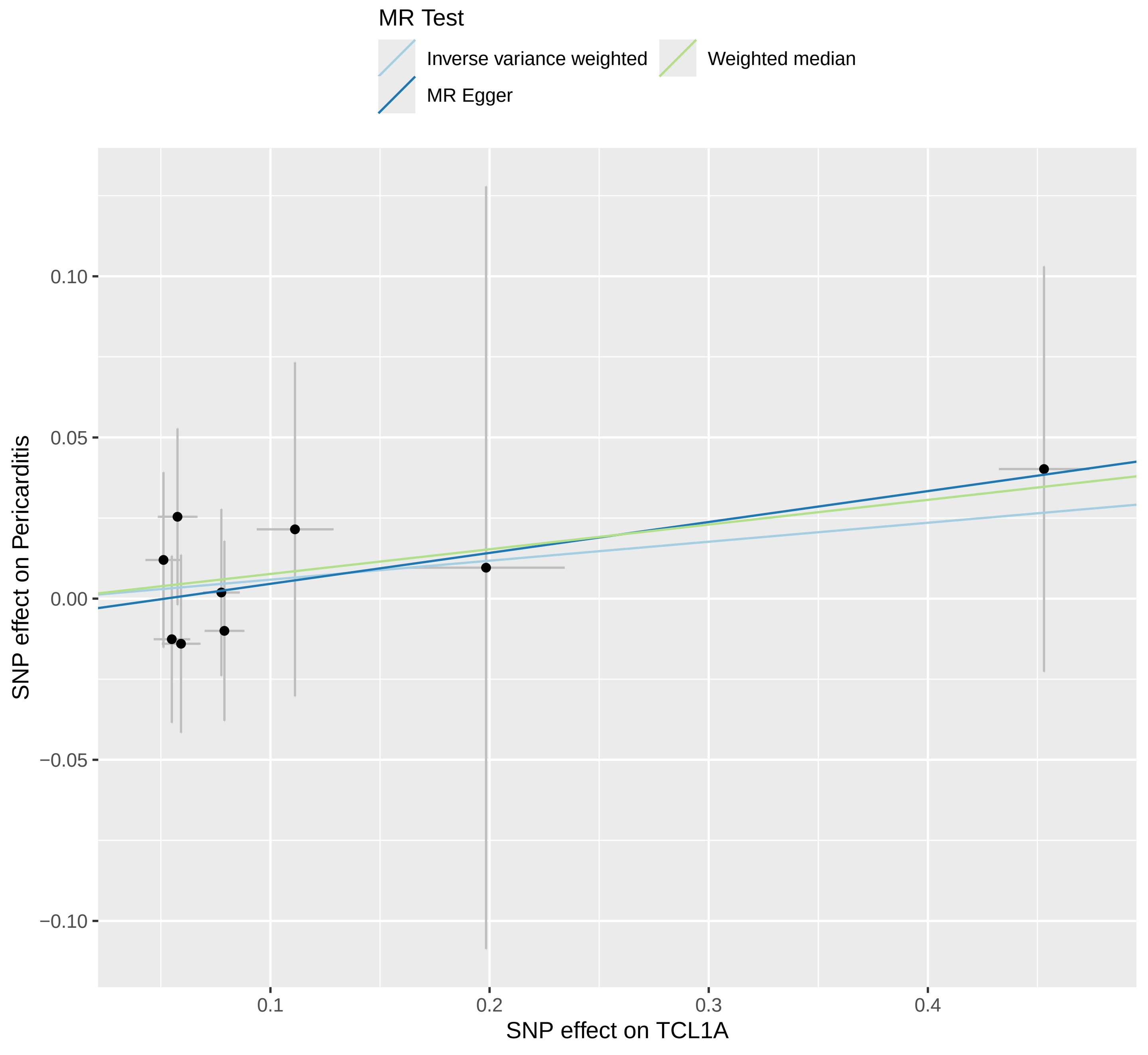
MR Test
Inverse variance weighted
Weighted median
MR Egger
0.10
0.05
0.00
−0.05
−0.10
SNP effect on Pericarditis
0.1
0.2
0.3
0.4
SNP effect on TCL1A

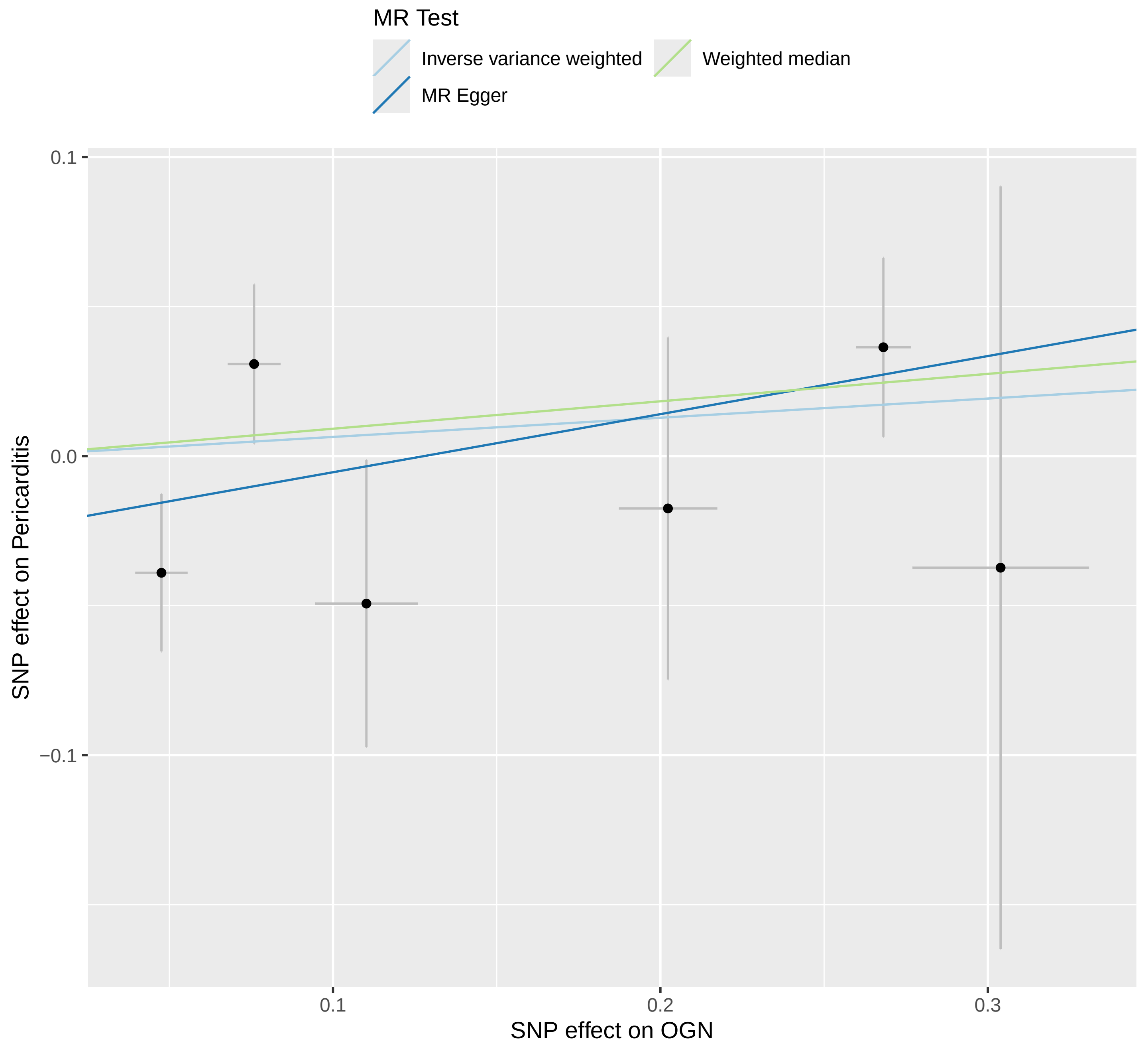
MR Test
Inverse variance weighted
Weighted median
MR Egger
0.1
0.0
−0.1
SNP effect on Pericarditis
0.1
0.2
0.3
SNP effect on OGN

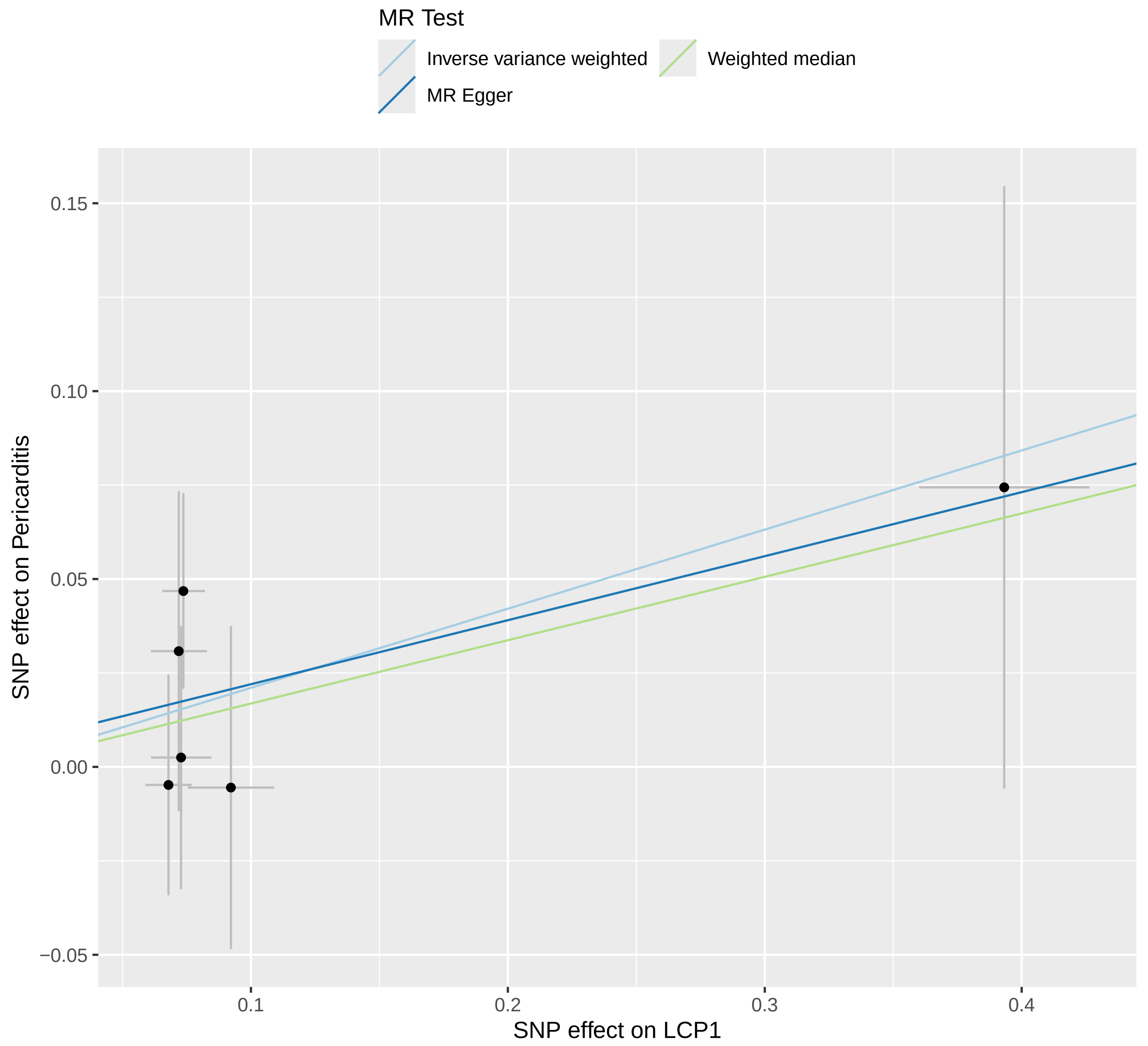
MR Test
Inverse variance weighted
MR Egger
Weighted median
0.15
0.10
0.05
0.00
−0.05
SNP effect on Pericarditis
0.1
0.2
0.3
0.4
SNP effect on LCP1

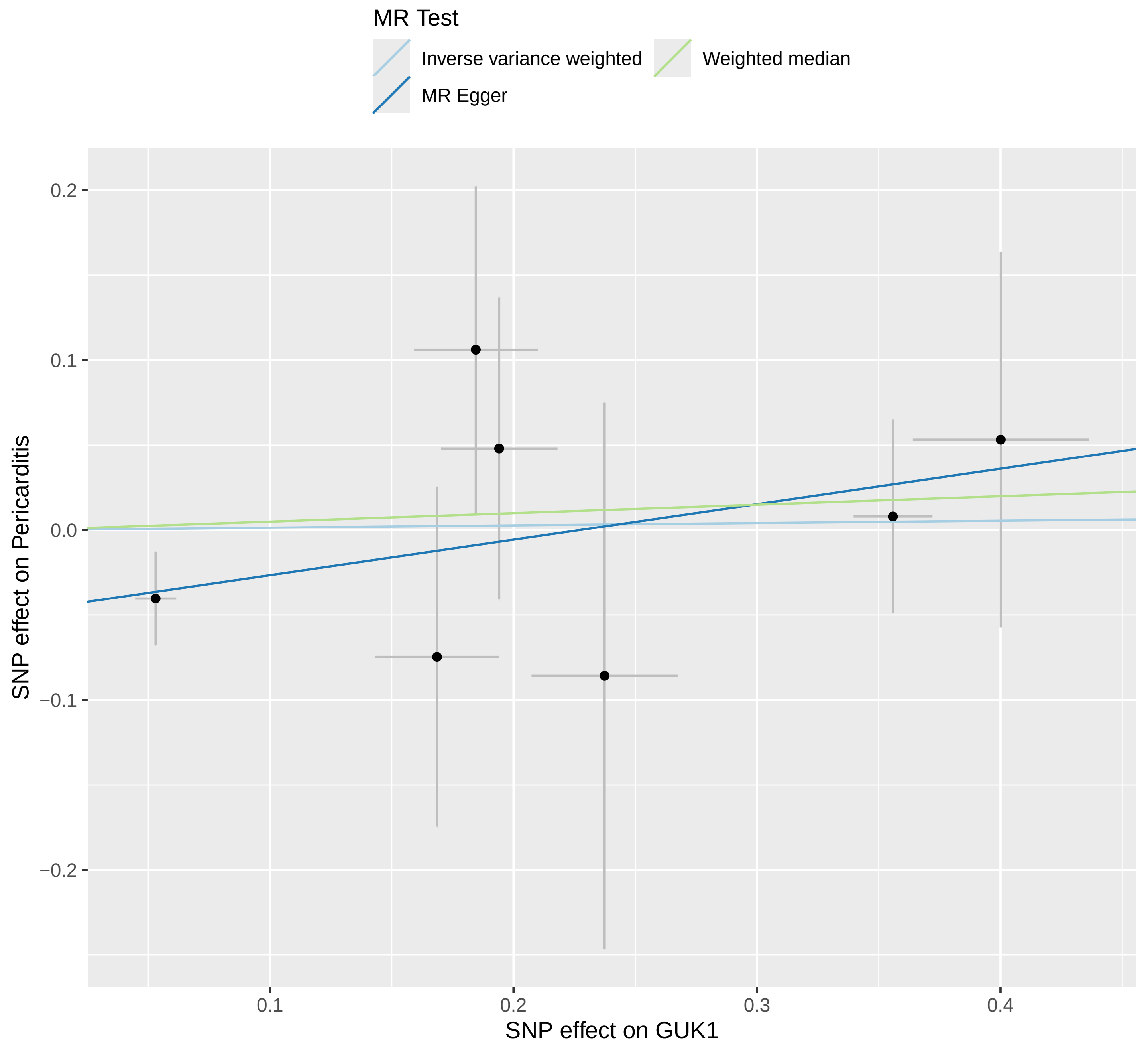
MR Test
Inverse variance weighted
Weighted median
MR Egger
0.2
0.1
0.0
−0.1
−0.2
SNP effect on Pericarditis
0.1
0.2
0.3
0.4
SNP effect on GUK1

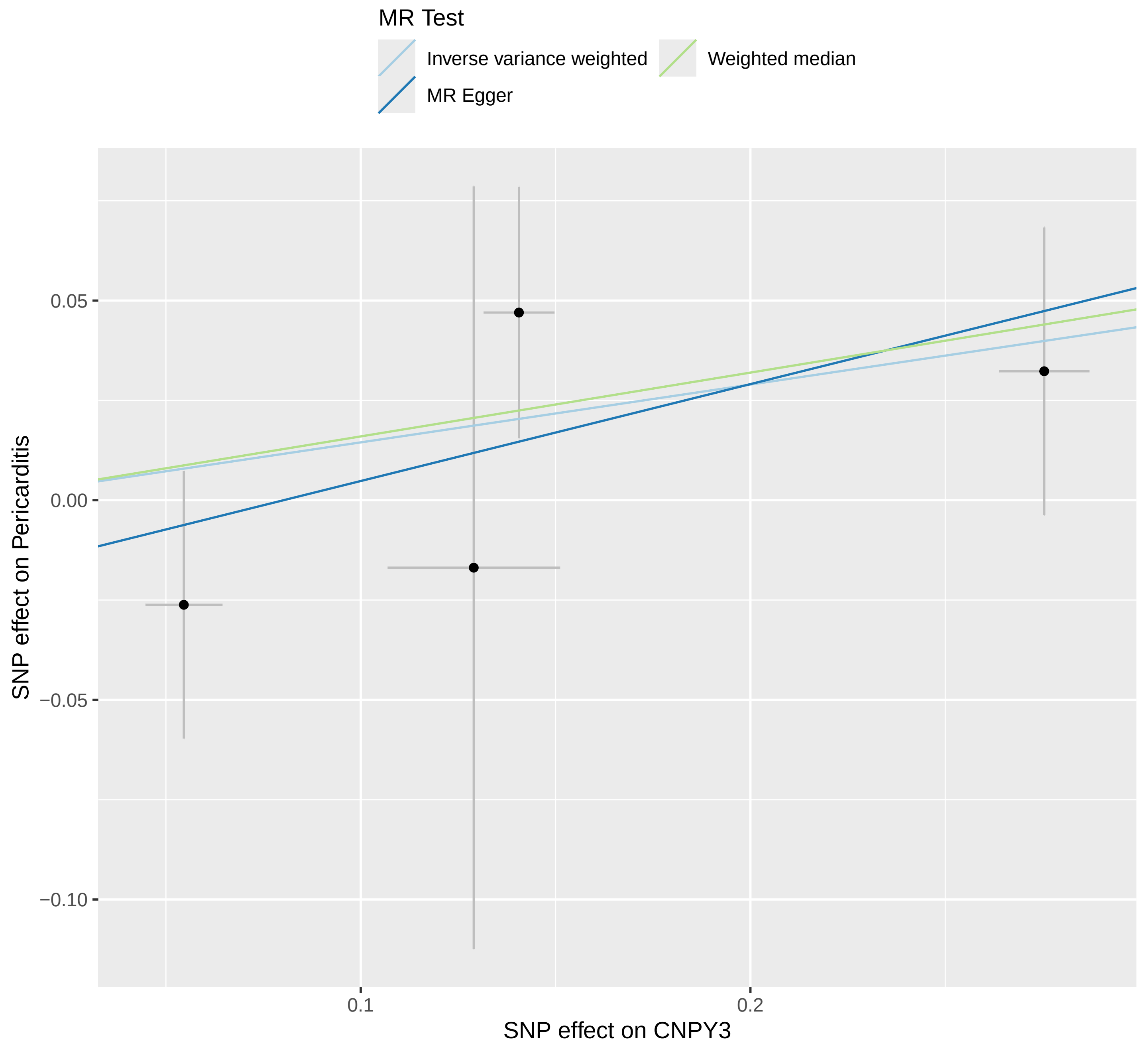
MR Test
Inverse variance weighted
Weighted median
MR Egger
0.05
0.00
−0.05
−0.10
0.1
0.2
SNP effect on Pericarditis
SNP effect on CNPY3

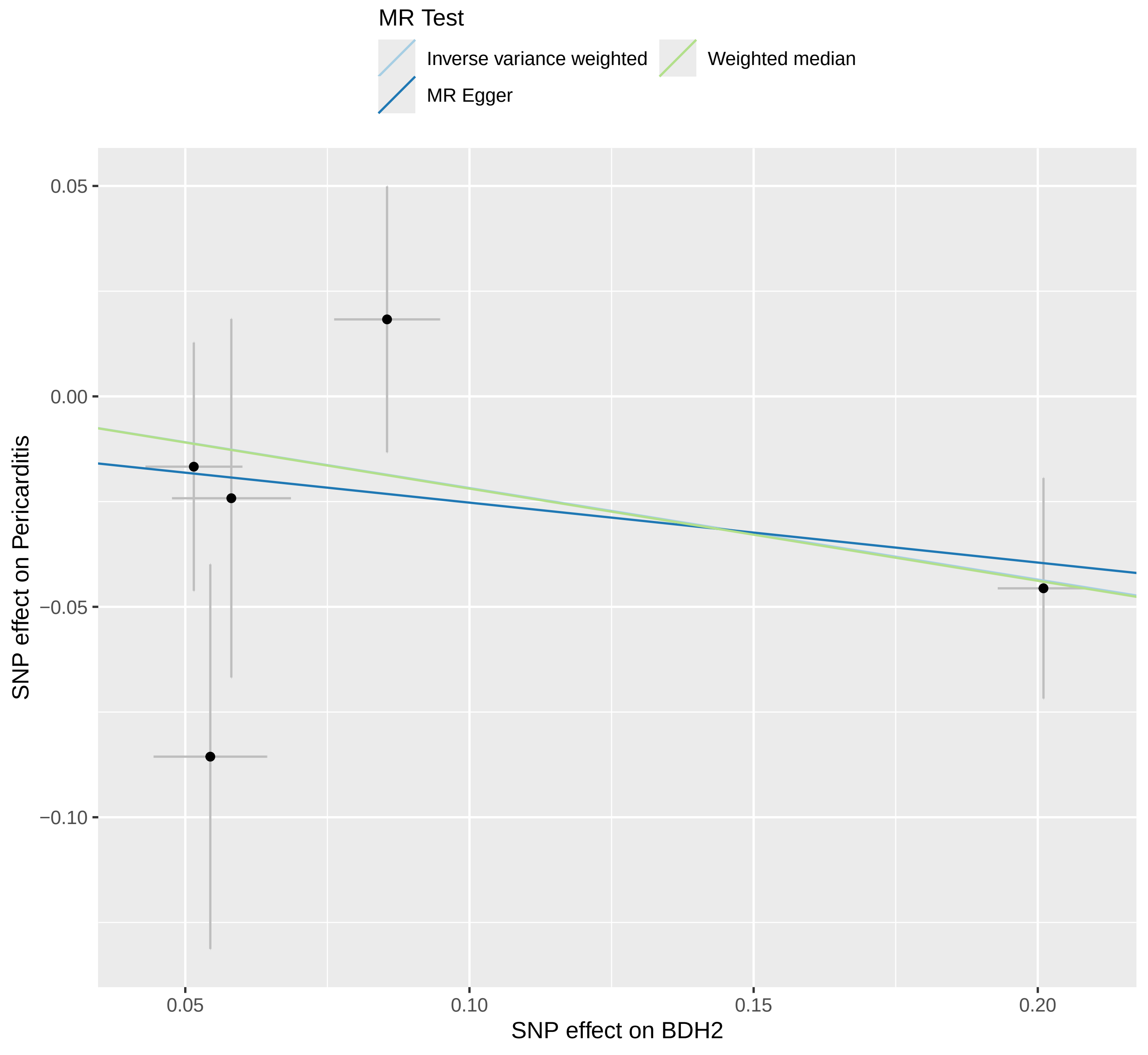

MR Test
Inverse variance weighted
Weighted median
MR Egger
0.05
0.00
−0.05
−0.10
SNP effect on Pericarditis
0.05
0.10
0.15
0.20
SNP effect on BDH2

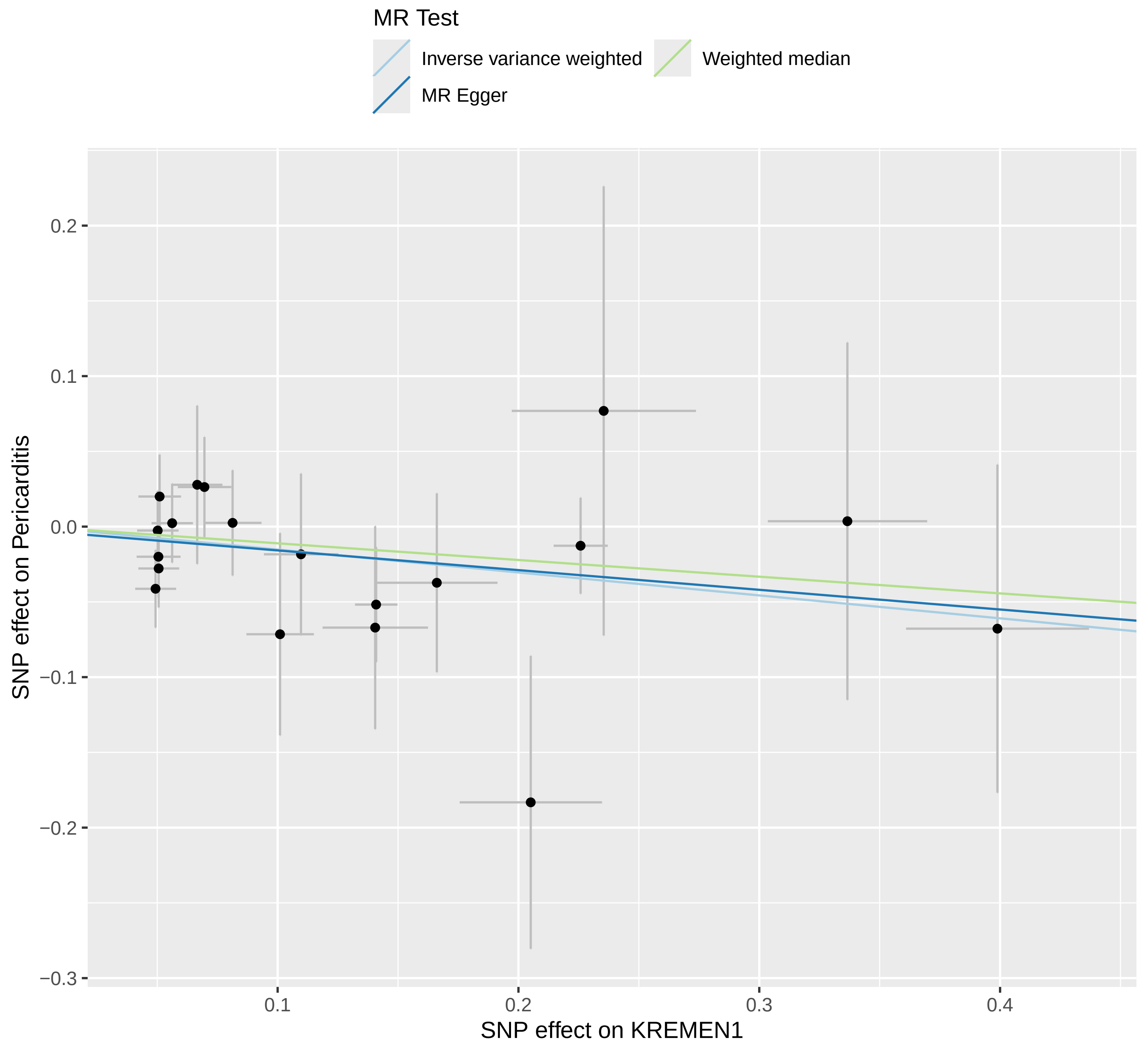

MR Test
Inverse variance weighted
Weighted median
MR Egger
0.2
0.1
0.0
−0.1
−0.2
−0.3
SNP effect on Pericarditis
0.1
0.2
0.3
0.4
SNP effect on KREMEN1

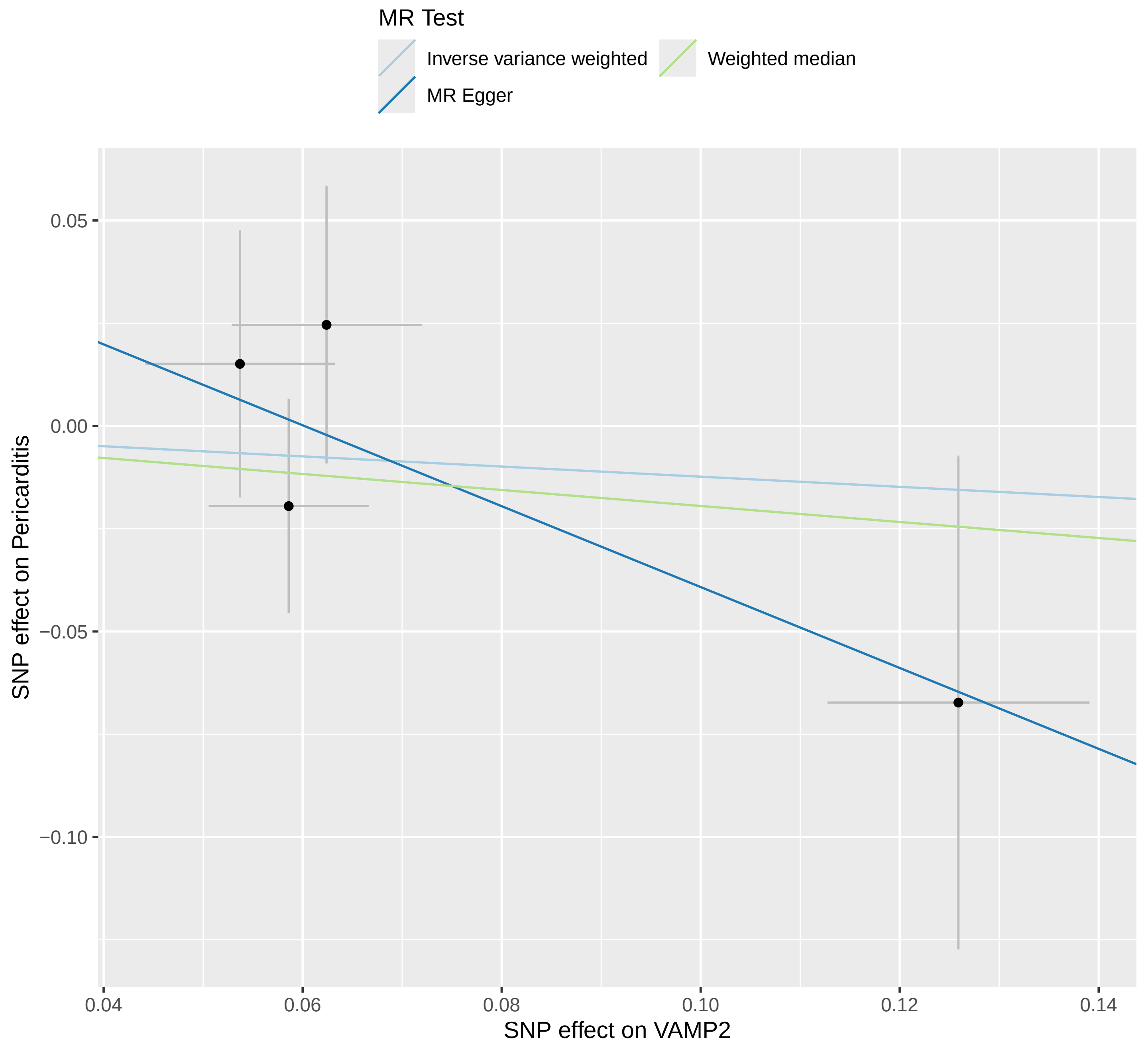

MR Test
Inverse variance weighted
Weighted median
MR Egger
0.05
0.00
−0.05
−0.10
SNP effect on Pericarditis
0.04
0.06
0.08
0.10
0.12
0.14
SNP effect on VAMP2

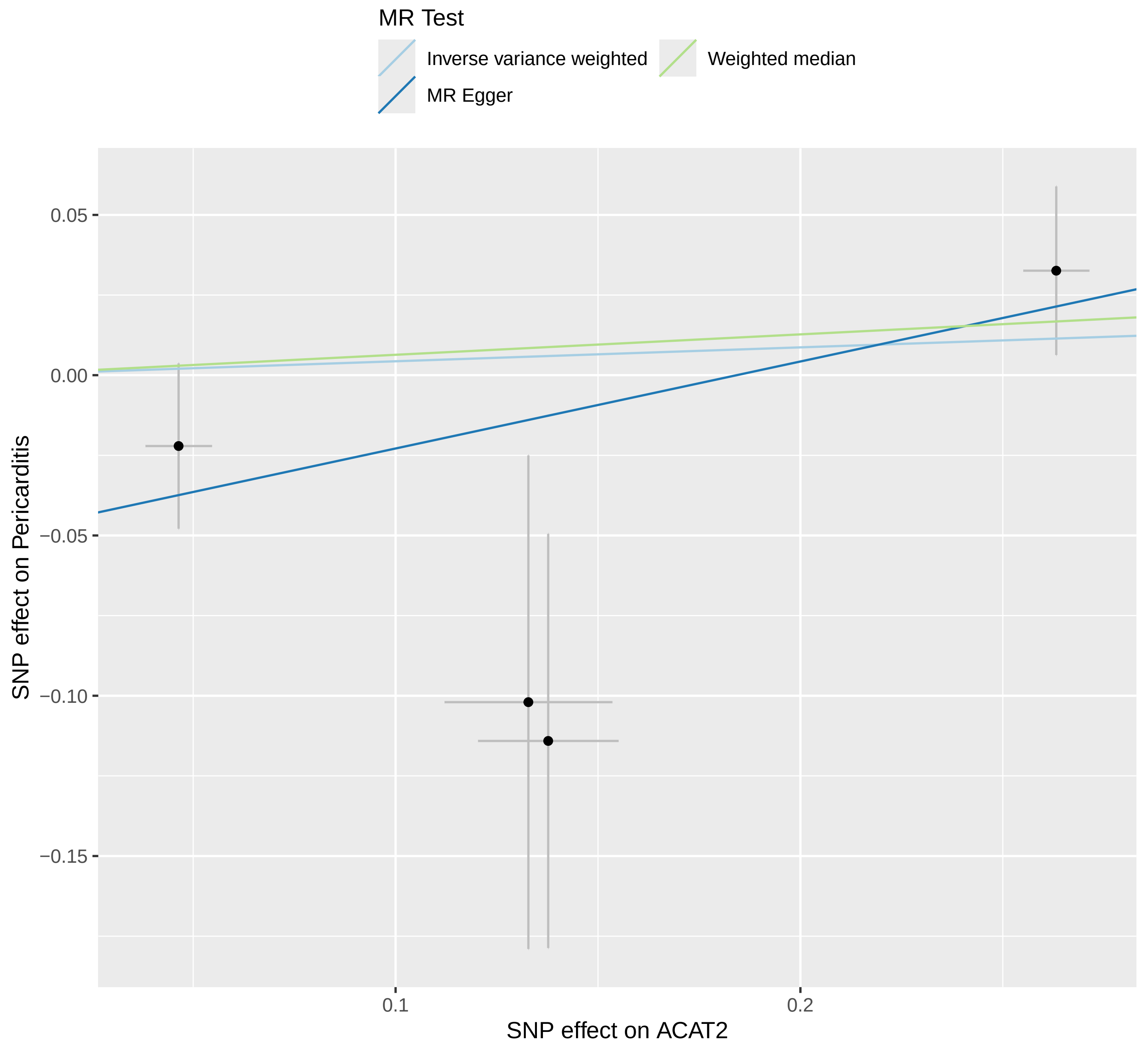
MR Test
Inverse variance weighted
MR Egger
Weighted median
SNP effect on Pericarditis
0.05
0.00
−0.05
−0.10
−0.15
0.1
0.2
SNP effect on ACAT2

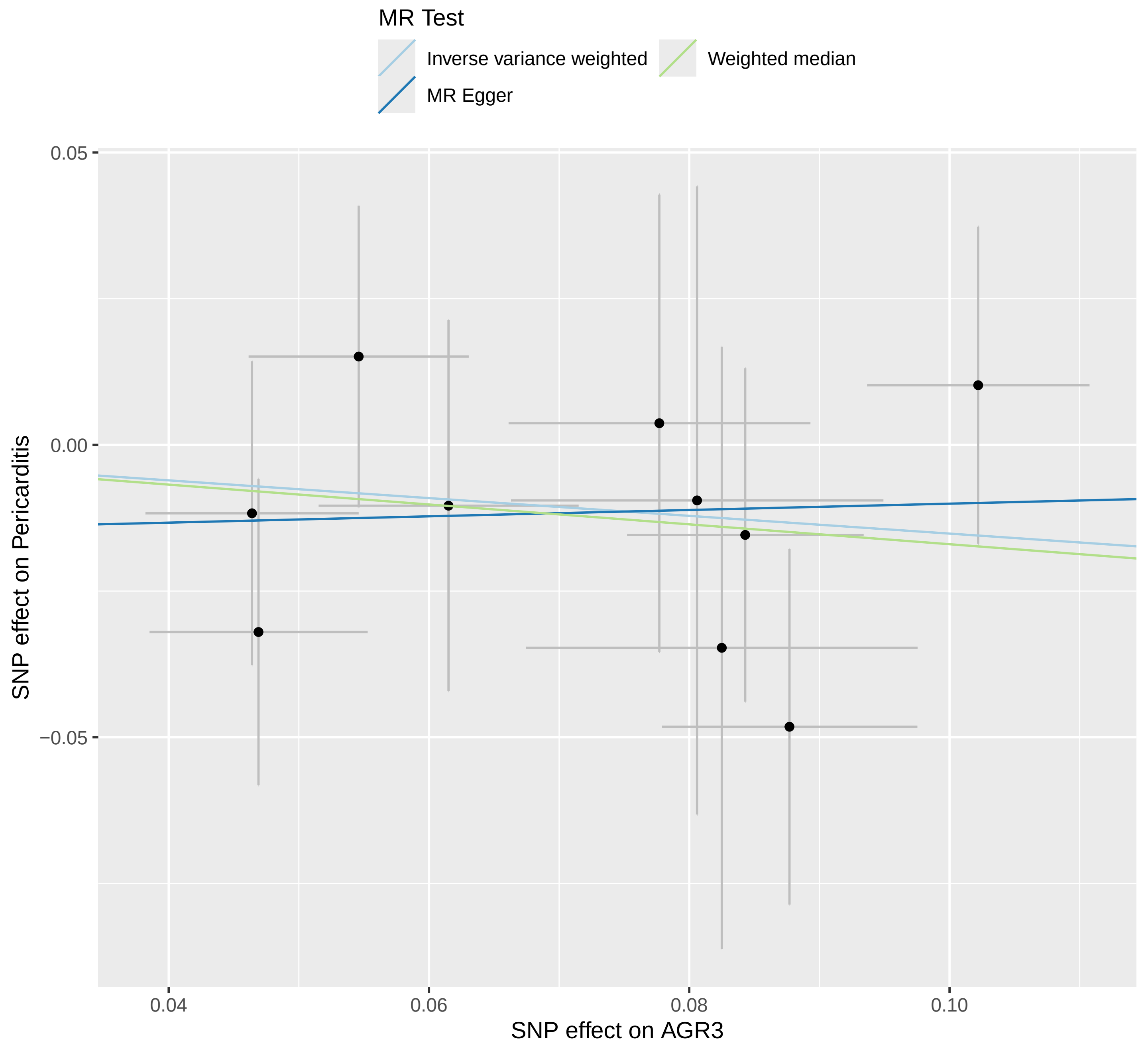

MR Test
Inverse variance weighted
MR Egger
Weighted median
0.05
0.00
−0.05
SNP effect on Pericarditis
0.04
0.06
0.08
0.10
SNP effect on AGR3

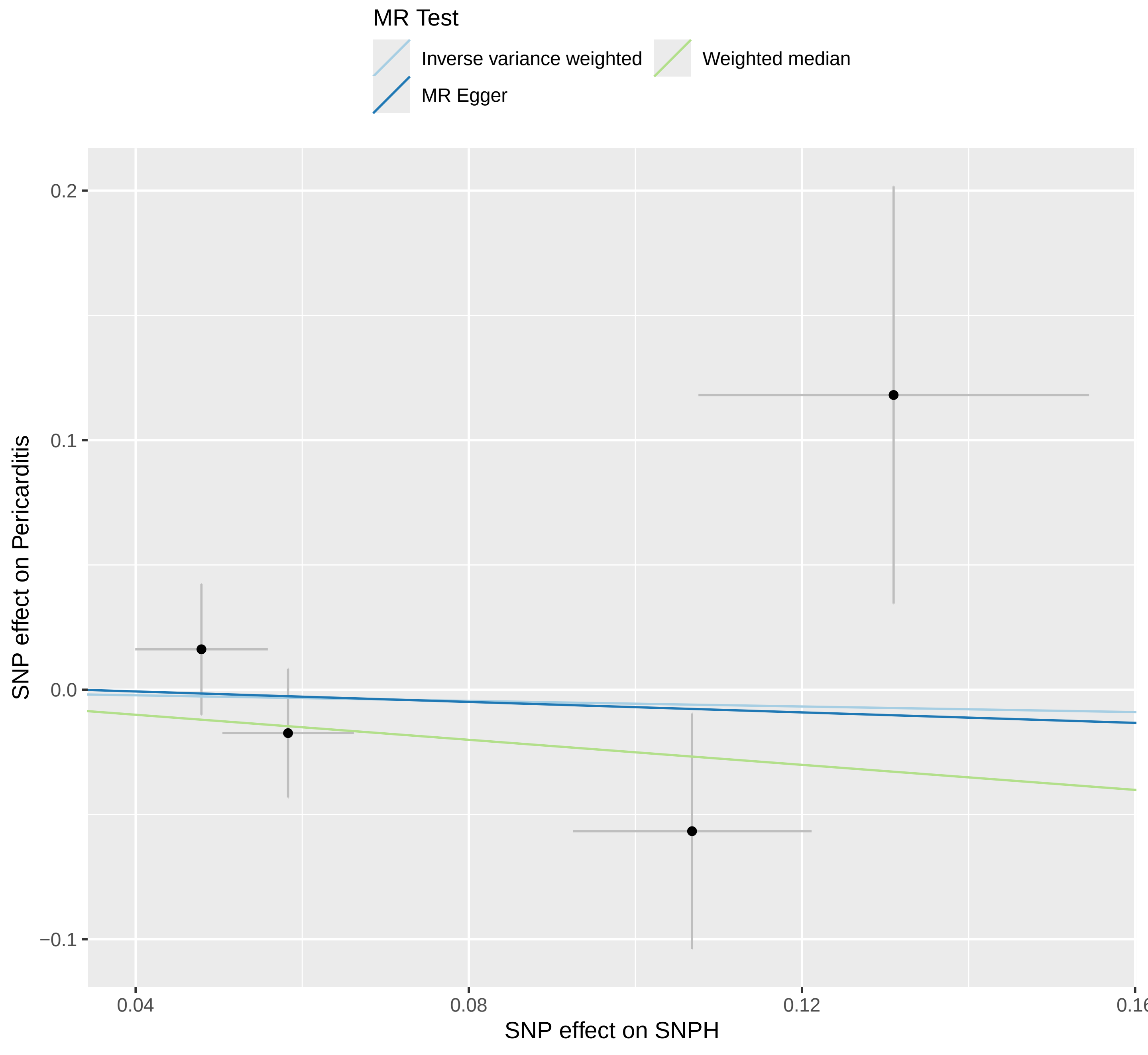
MR Test
Inverse variance weighted
Weighted median
MR Egger
0.2
0.1
0.0
−0.1
0.04
0.08
0.12
0.16
SNP effect on Pericarditis
SNP effect on SNPH

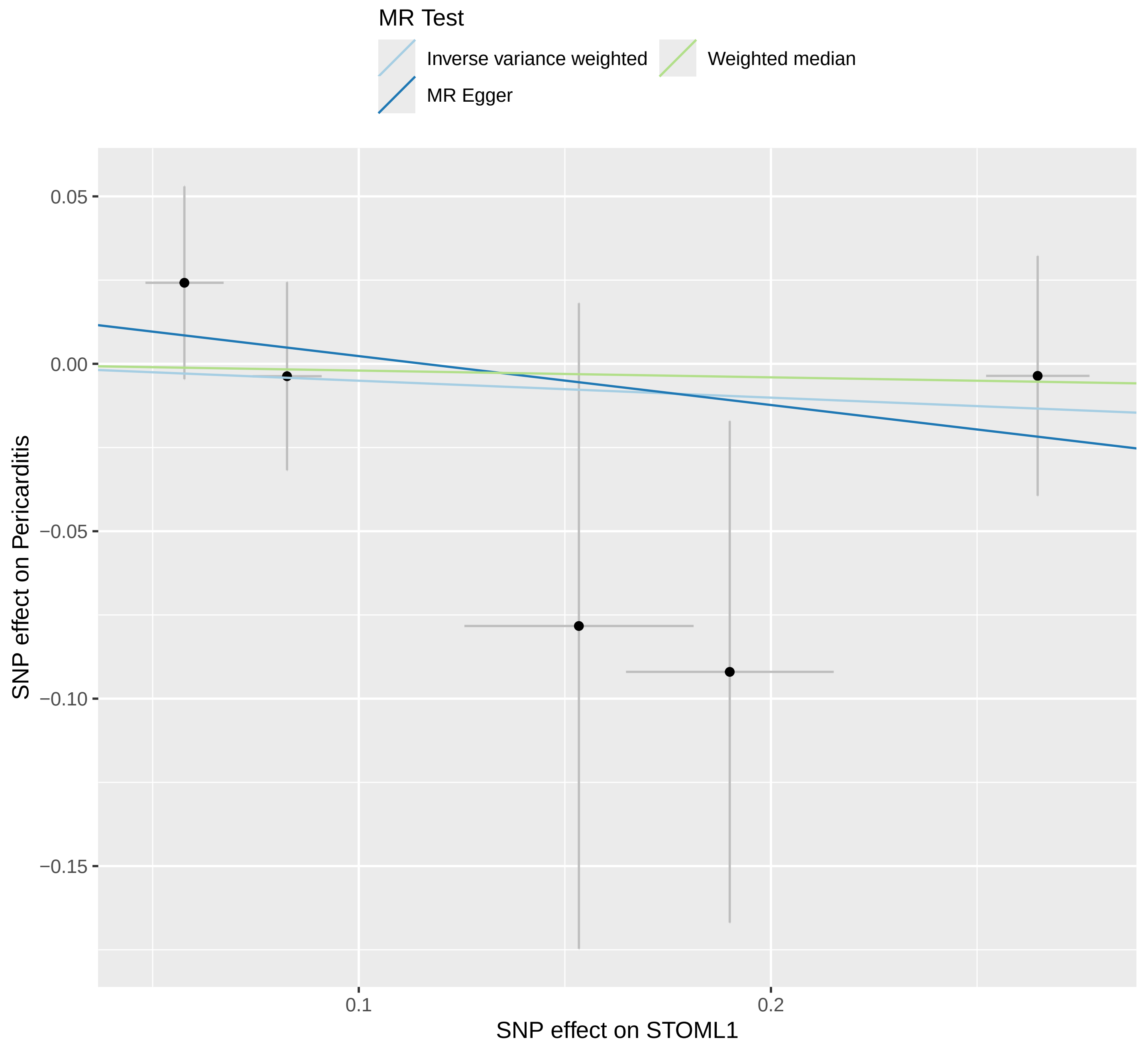
MR Test
Inverse variance weighted
Weighted median
MR Egger
0.05
0.00
−0.05
−0.10
−0.15
SNP effect on Pericarditis
0.1
0.2
SNP effect on STOML1

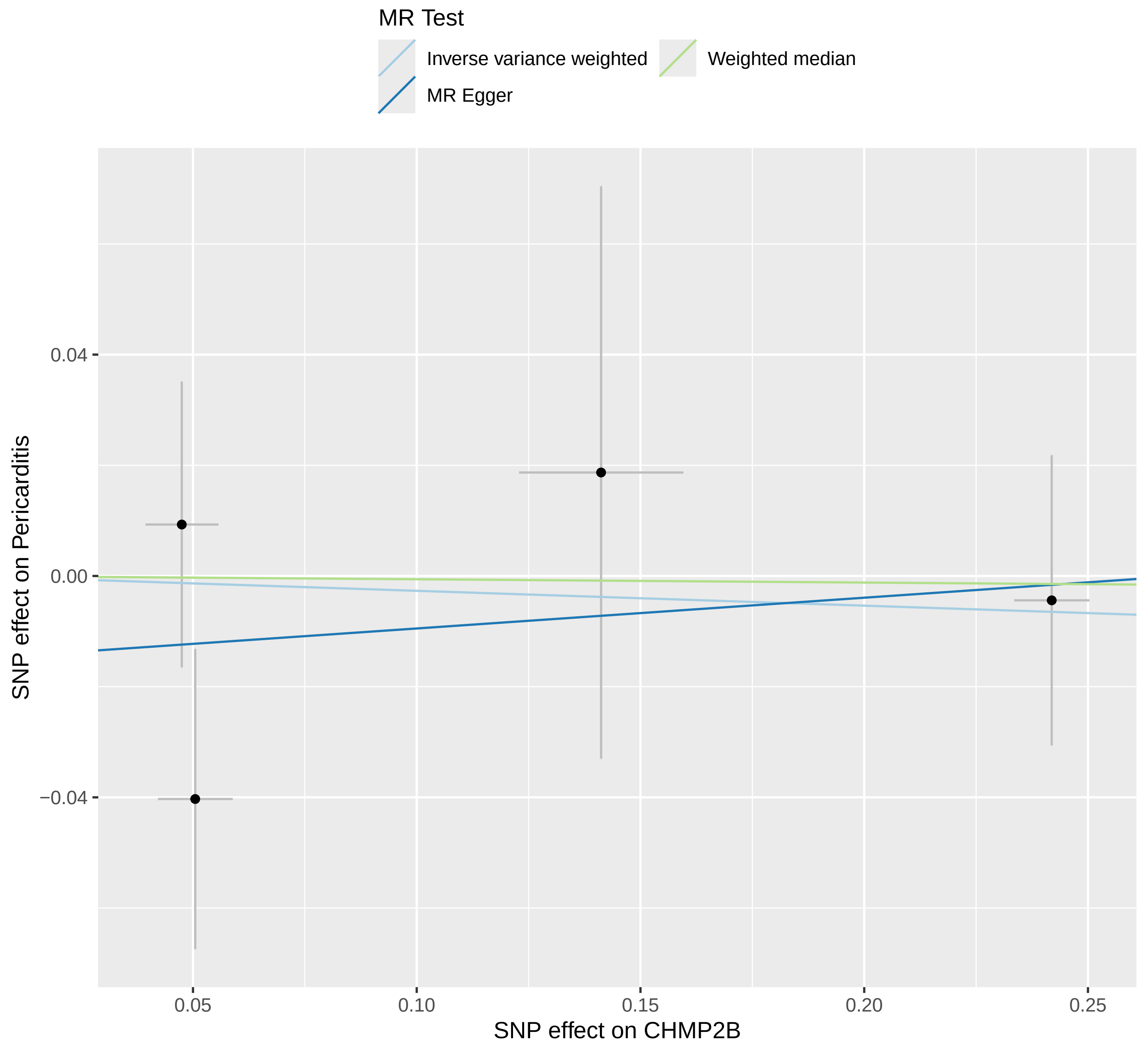

MR Test
Inverse variance weighted
Weighted median
MR Egger
0.04
0.00
−0.04
0.05
0.10
0.15
0.20
0.25
SNP effect on Pericarditis
SNP effect on CHMP2B

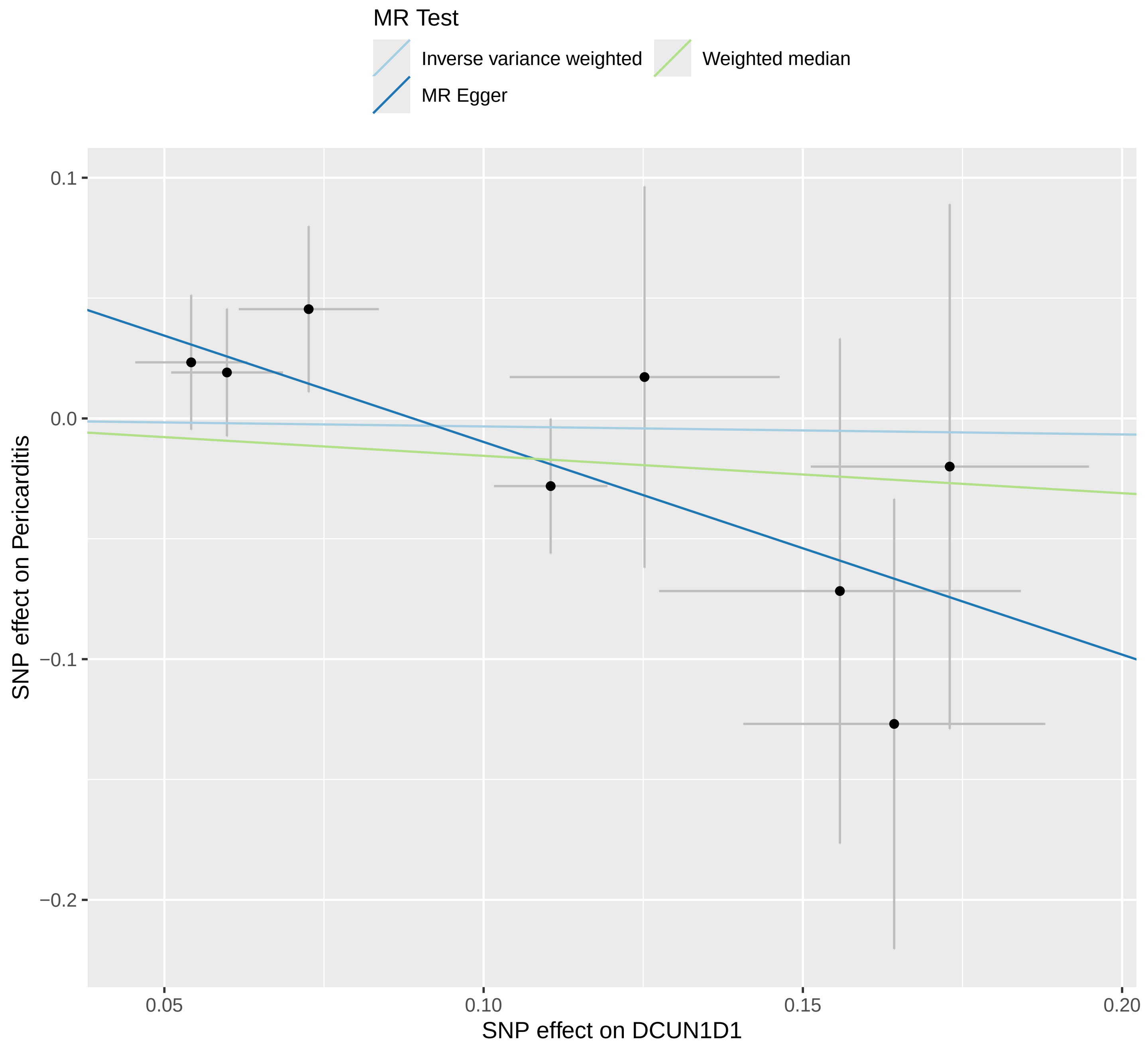
MR Test
Inverse variance weighted
Weighted median
MR Egger
SNP effect on Pericarditis
0.1
0.0
−0.1
−0.2
0.05
0.10
0.15
0.20
SNP effect on DCUN1D1

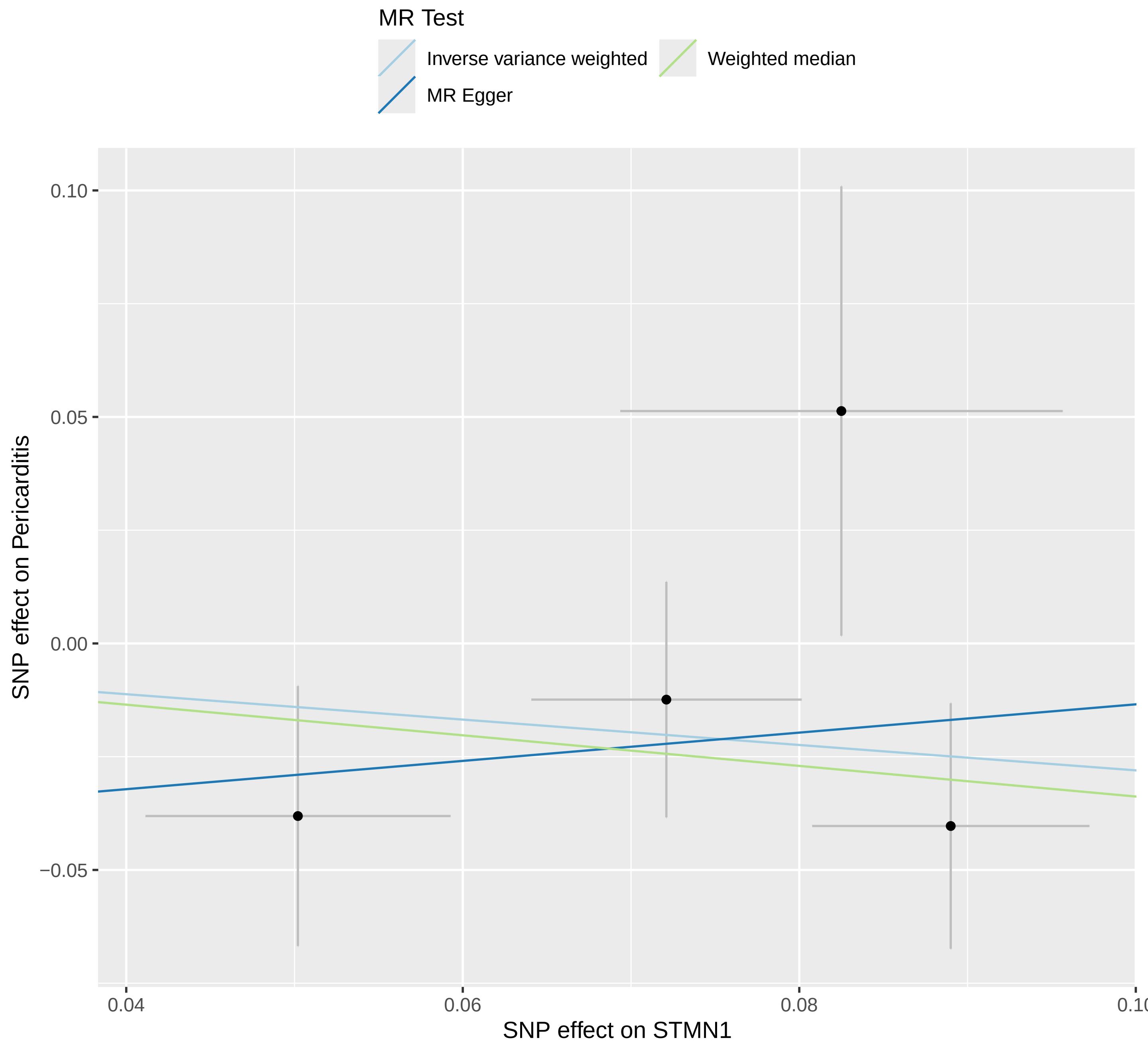
MR Test
Inverse variance weighted
Weighted median
MR Egger
SNP effect on Pericarditis
0.10
0.05
0.00
−0.05
0.04
0.06
0.08
0.10
SNP effect on STMN1

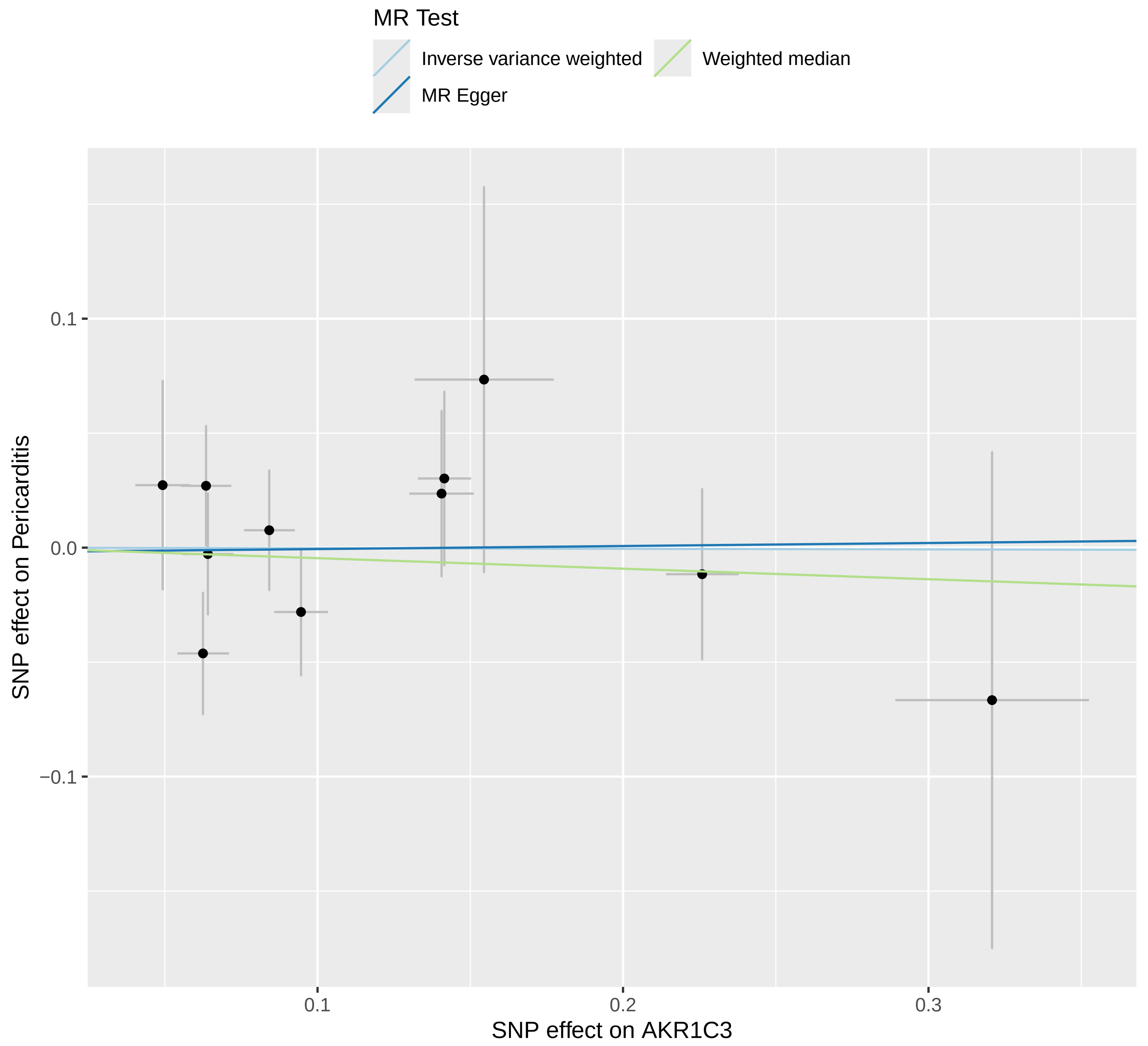
MR Test
Inverse variance weighted
Weighted median
MR Egger
0.1
0.0
−0.1
SNP effect on Pericarditis
0.1
0.2
0.3
SNP effect on AKR1C3

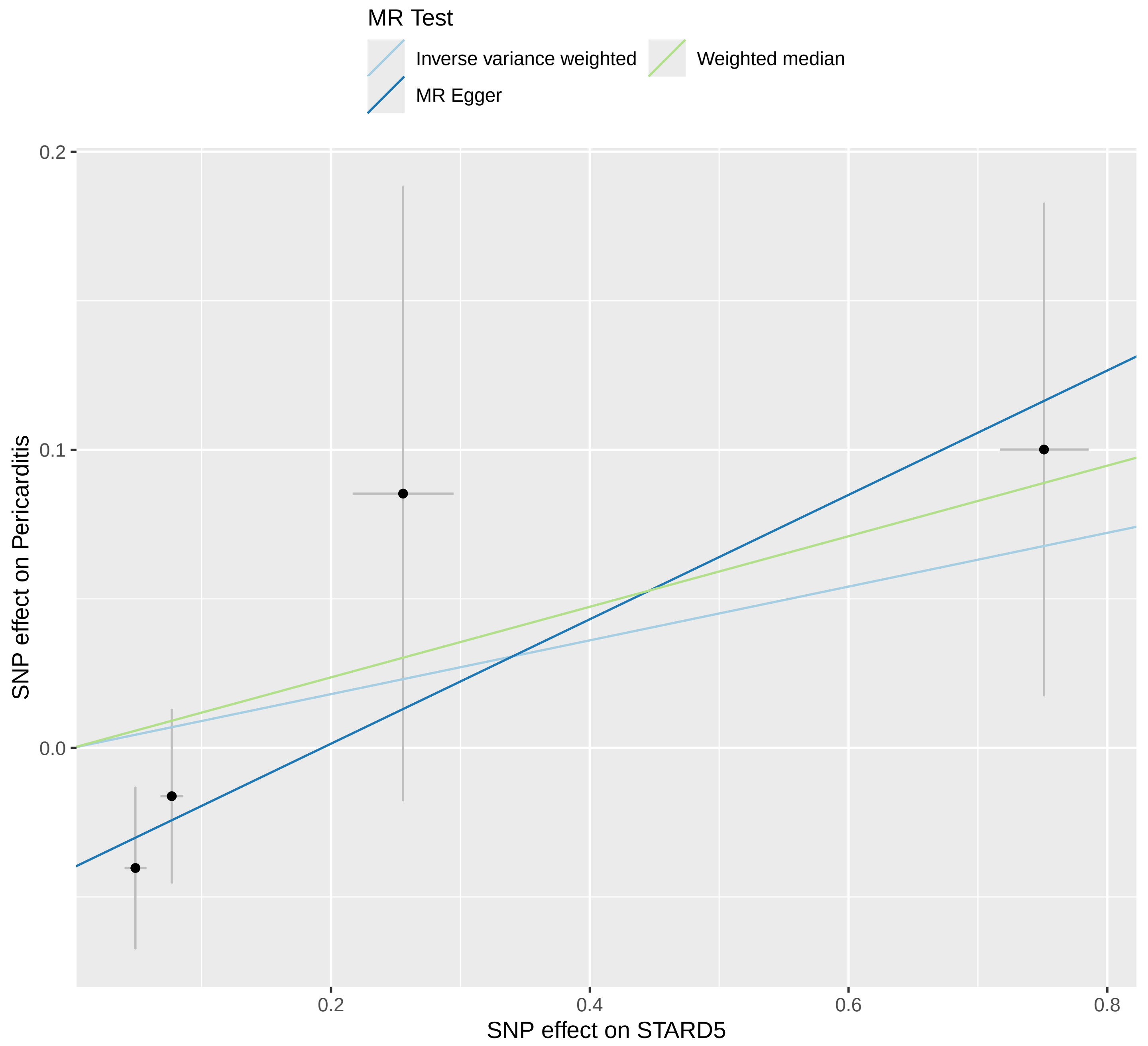
MR Test
Inverse variance weighted
MR Egger
Weighted median
0.2
0.1
0.0
SNP effect on Pericarditis
0.2
0.4
0.6
0.8
SNP effect on STARD5

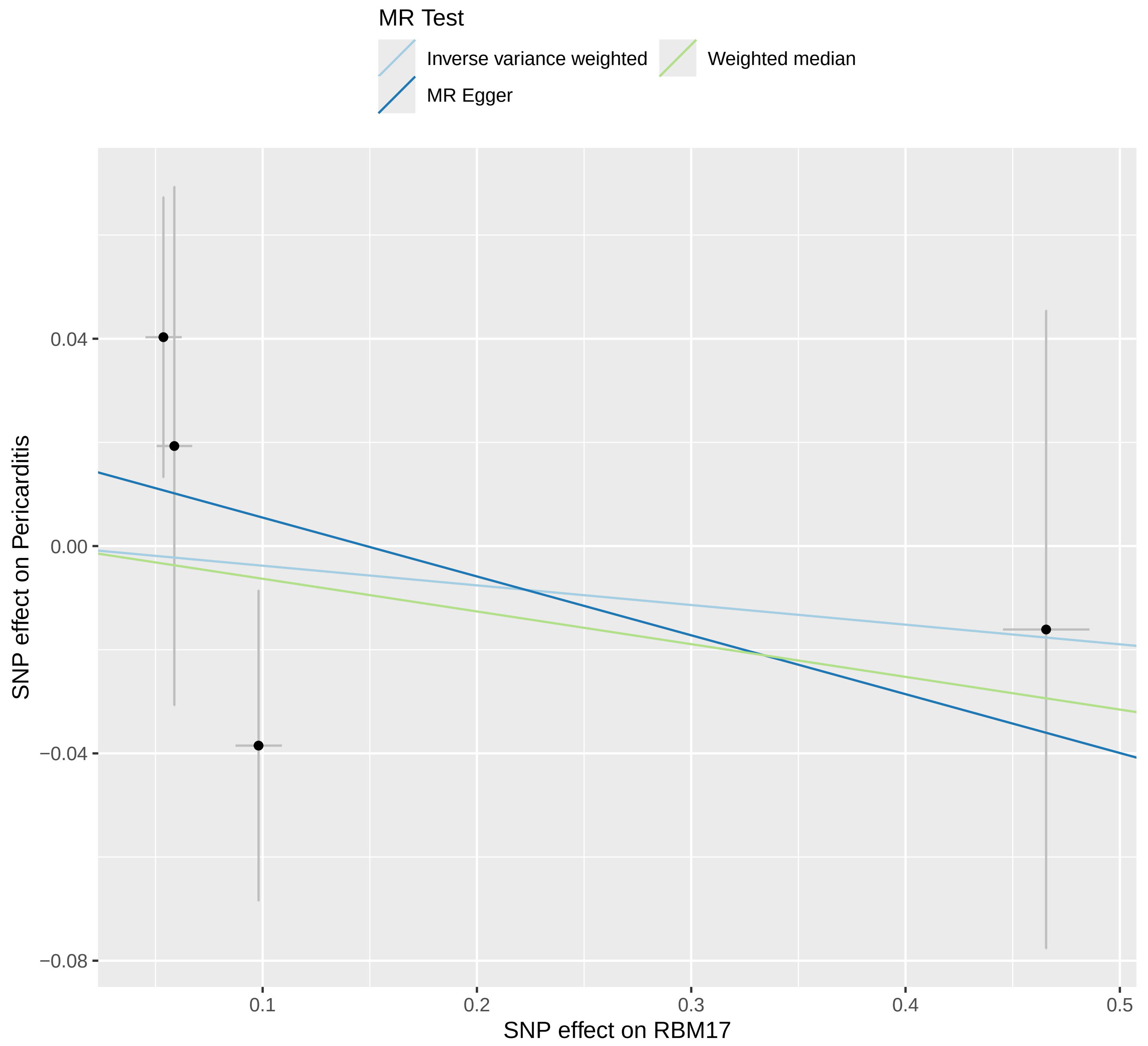

MR Test
Inverse variance weighted
Weighted median
MR Egger
SNP effect on Pericarditis
0.04
0.00
−0.04
−0.08
0.1
0.2
0.3
0.4
0.5
SNP effect on RBM17

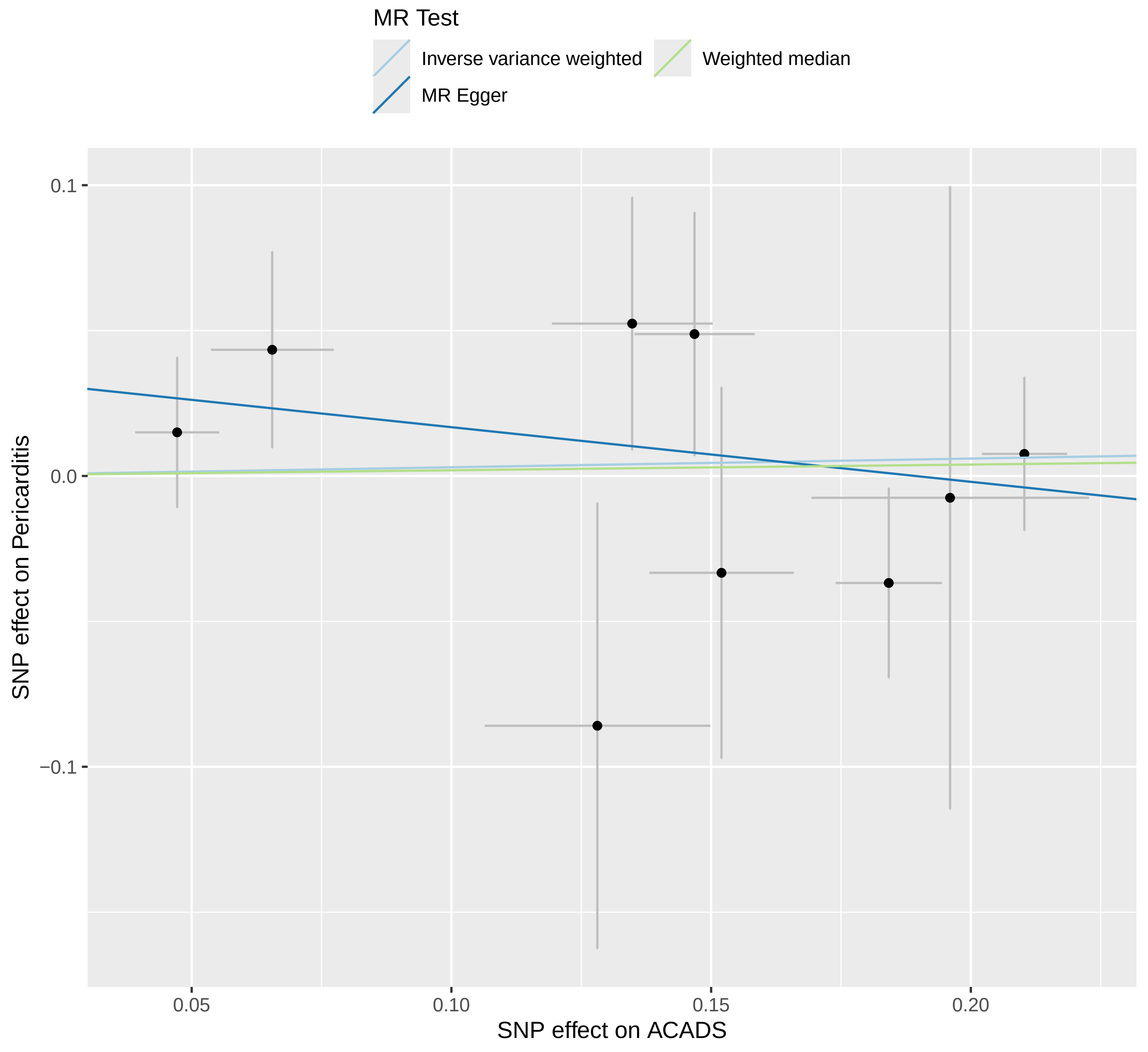
MR Test
Inverse variance weighted
Weighted median
MR Egger
0.1
0.0
−0.1
SNP effect on Pericarditis
0.05
0.10
0.15
0.20
SNP effect on ACADS

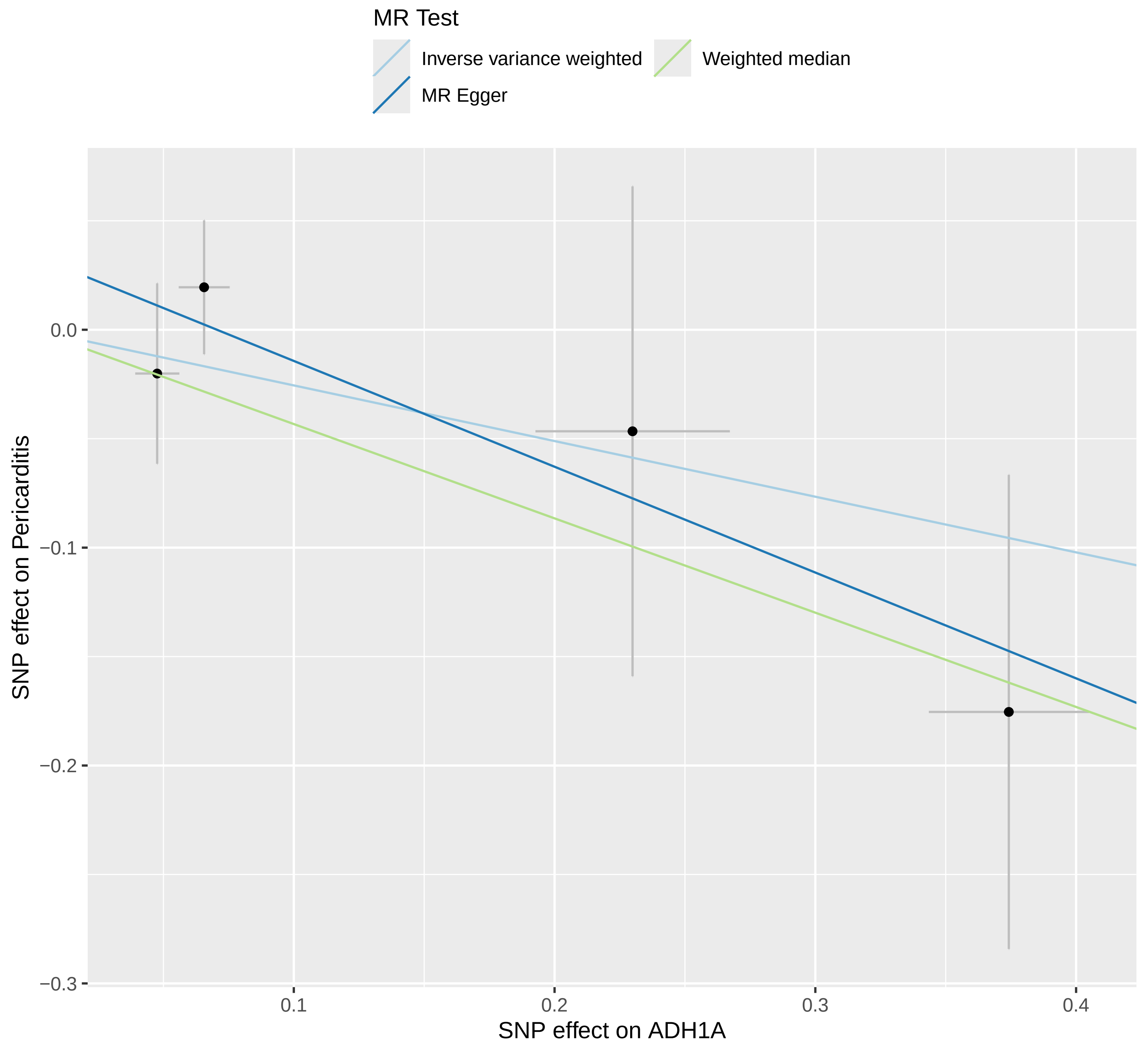
MR Test
Inverse variance weighted
Weighted median
MR Egger
SNP effect on Pericarditis
0.0
−0.1
−0.2
−0.3
0.1
0.2
0.3
0.4
SNP effect on ADH1A

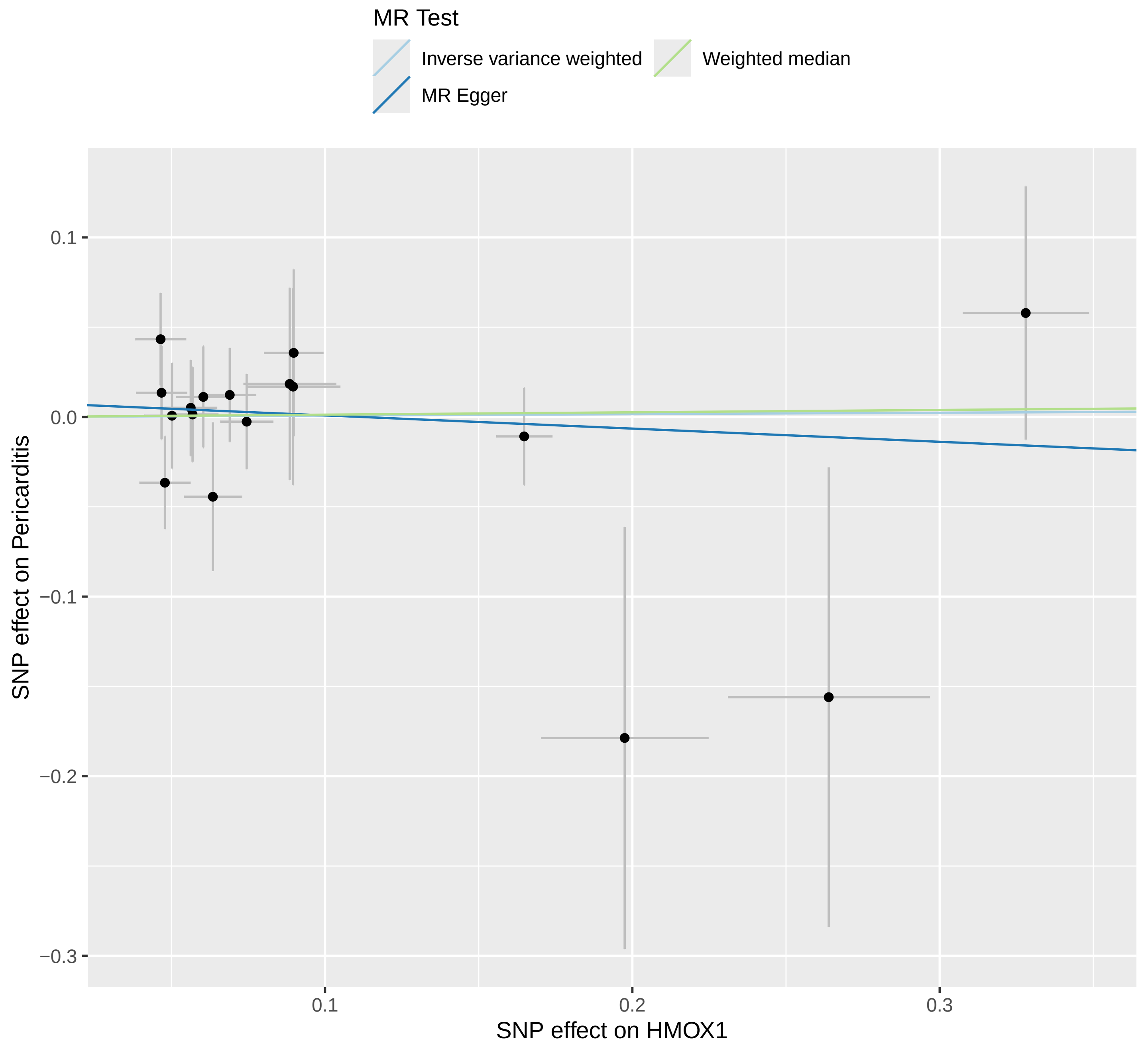

MR Test
Inverse variance weighted
Weighted median
MR Egger
0.1
0.0
−0.1
−0.2
−0.3
SNP effect on Pericarditis
0.1
0.2
0.3
SNP effect on HMOX1

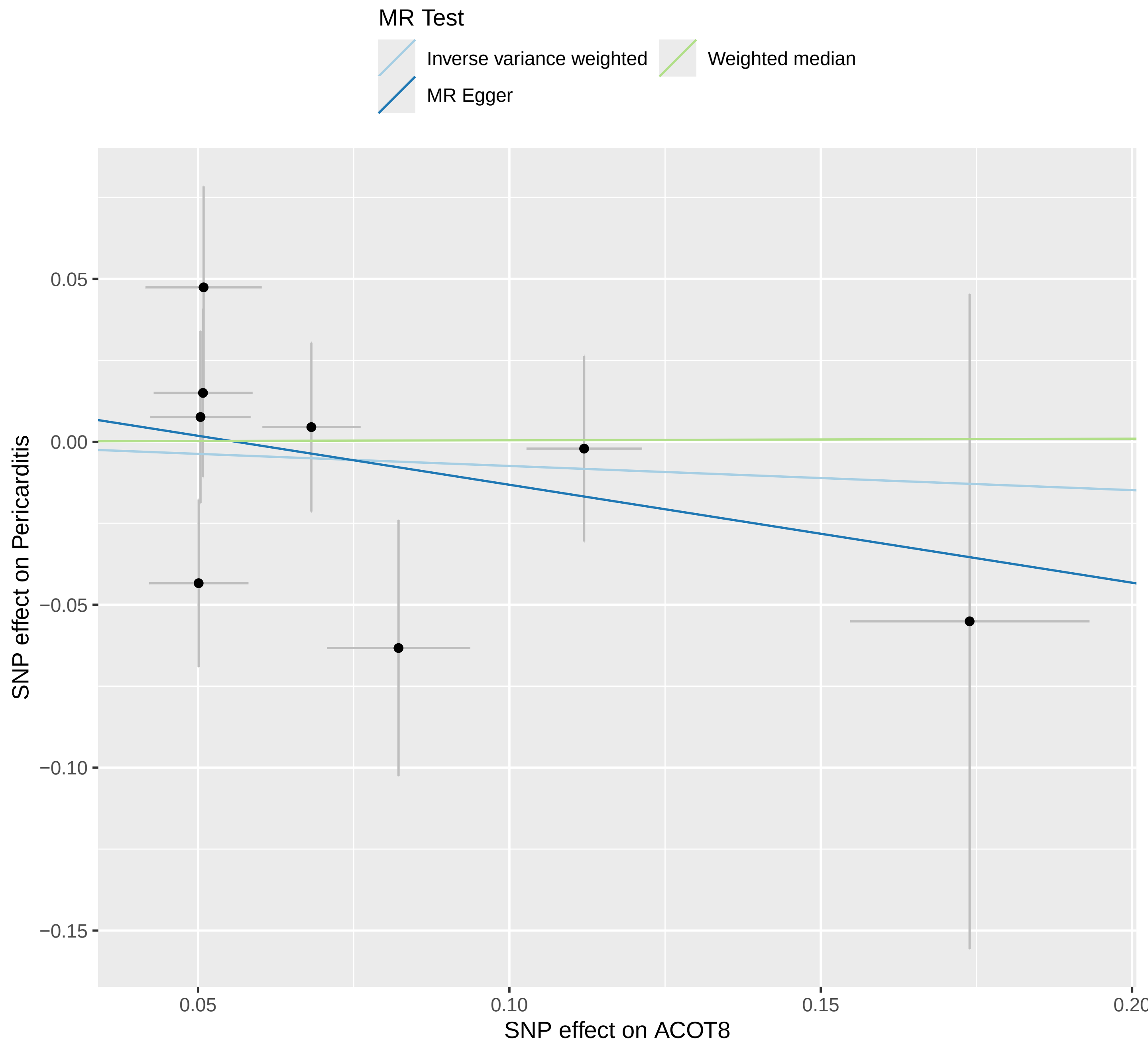

MR Test
Inverse variance weighted
Weighted median
MR Egger
0.05
0.00
−0.05
−0.10
−0.15
SNP effect on Pericarditis
0.05
0.10
0.15
0.20
SNP effect on ACOT8

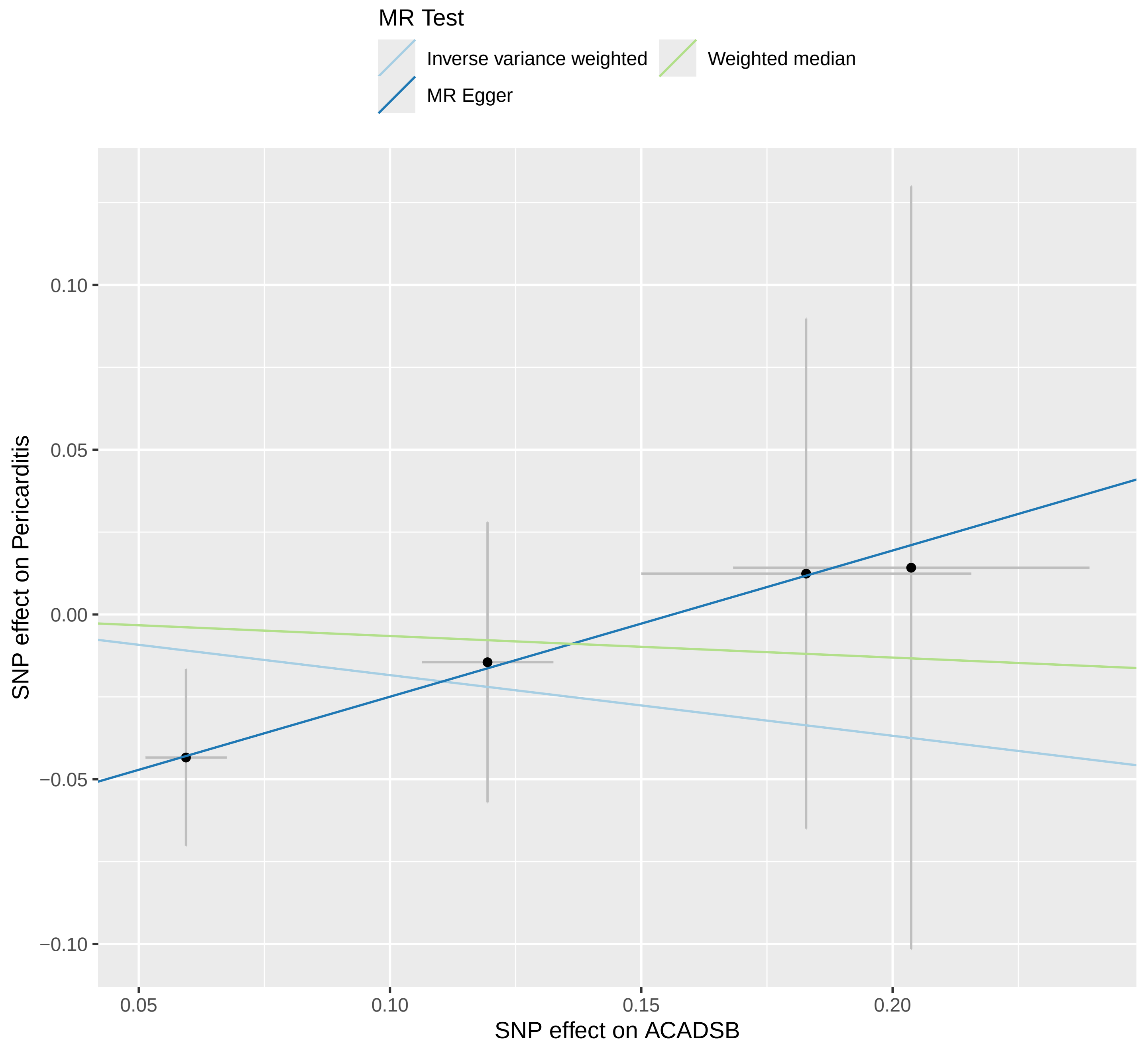
MR Test
Inverse variance weighted
MR Egger
Weighted median
SNP effect on Pericarditis
SNP effect on ACADSB
0.10
0.05
0.00
−0.05
−0.10
0.05
0.10
0.15
0.20

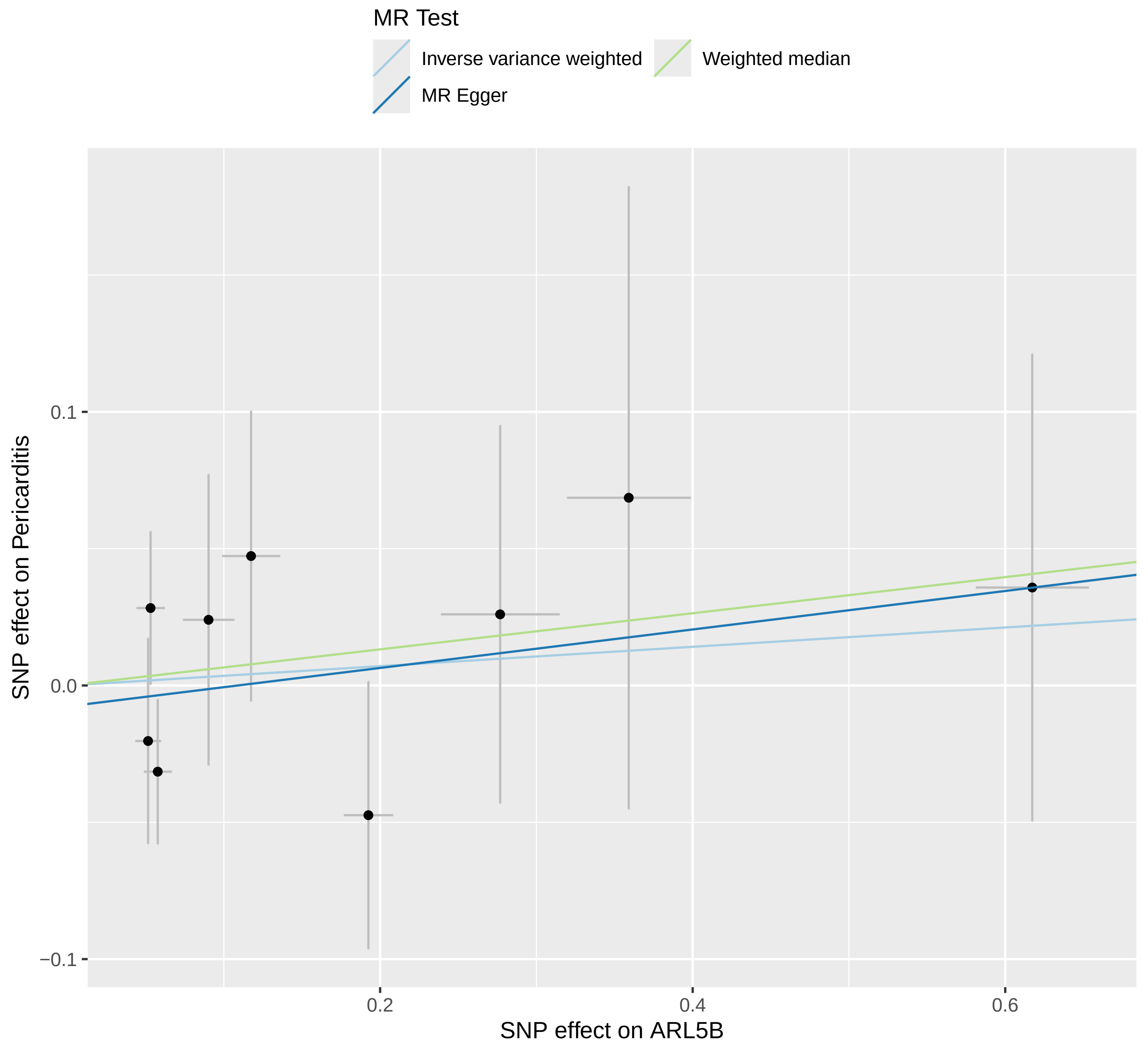
MR Test
Inverse variance weighted
Weighted median
MR Egger
SNP effect on Pericarditis
0.1
0.0
−0.1
0.2
0.4
0.6
SNP effect on ARL5B

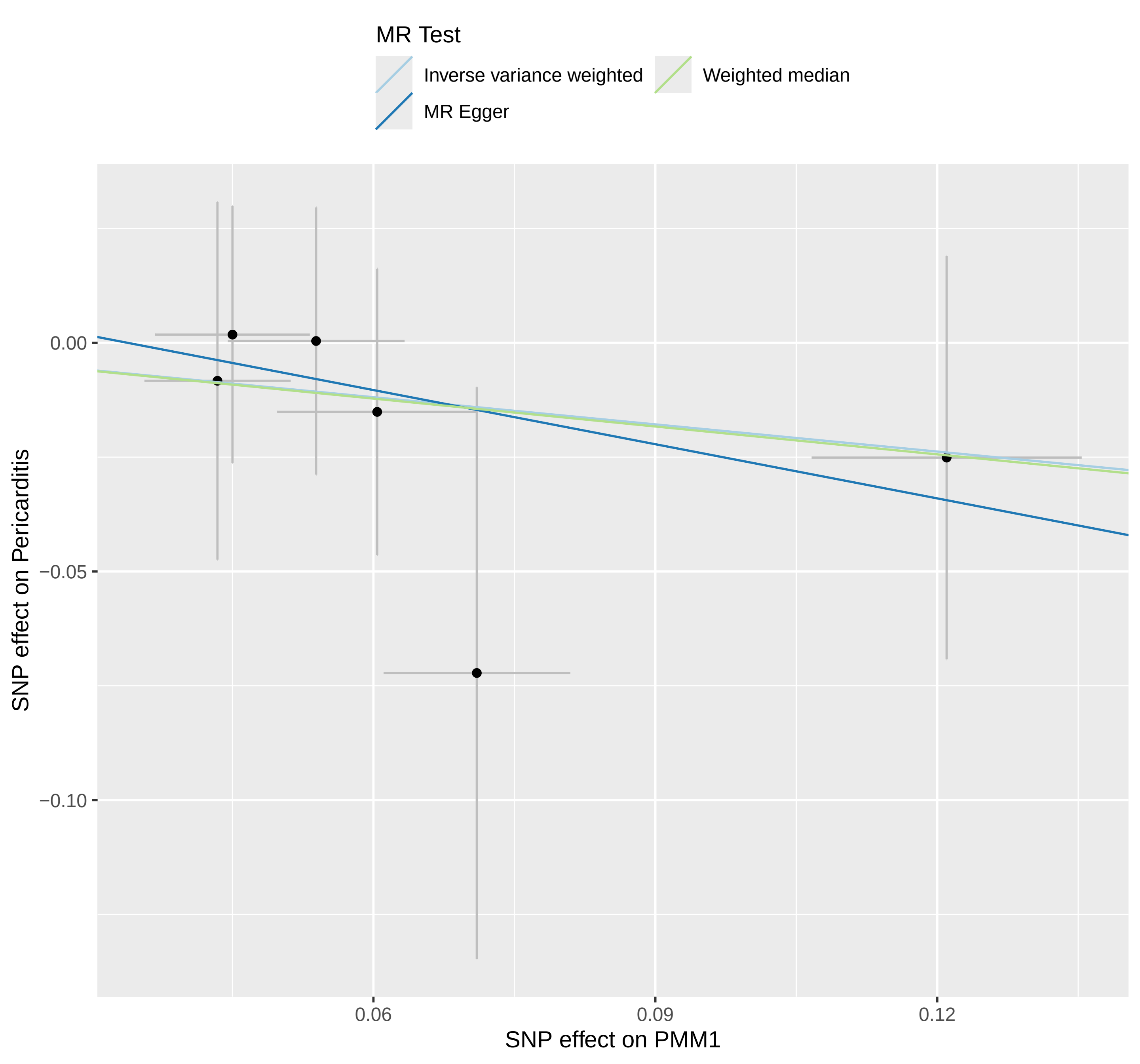

MR Test
Inverse variance weighted
Weighted median
MR Egger
0.00
−0.05
−0.10
SNP effect on Pericarditis
0.06
0.09
0.12
SNP effect on PMM1

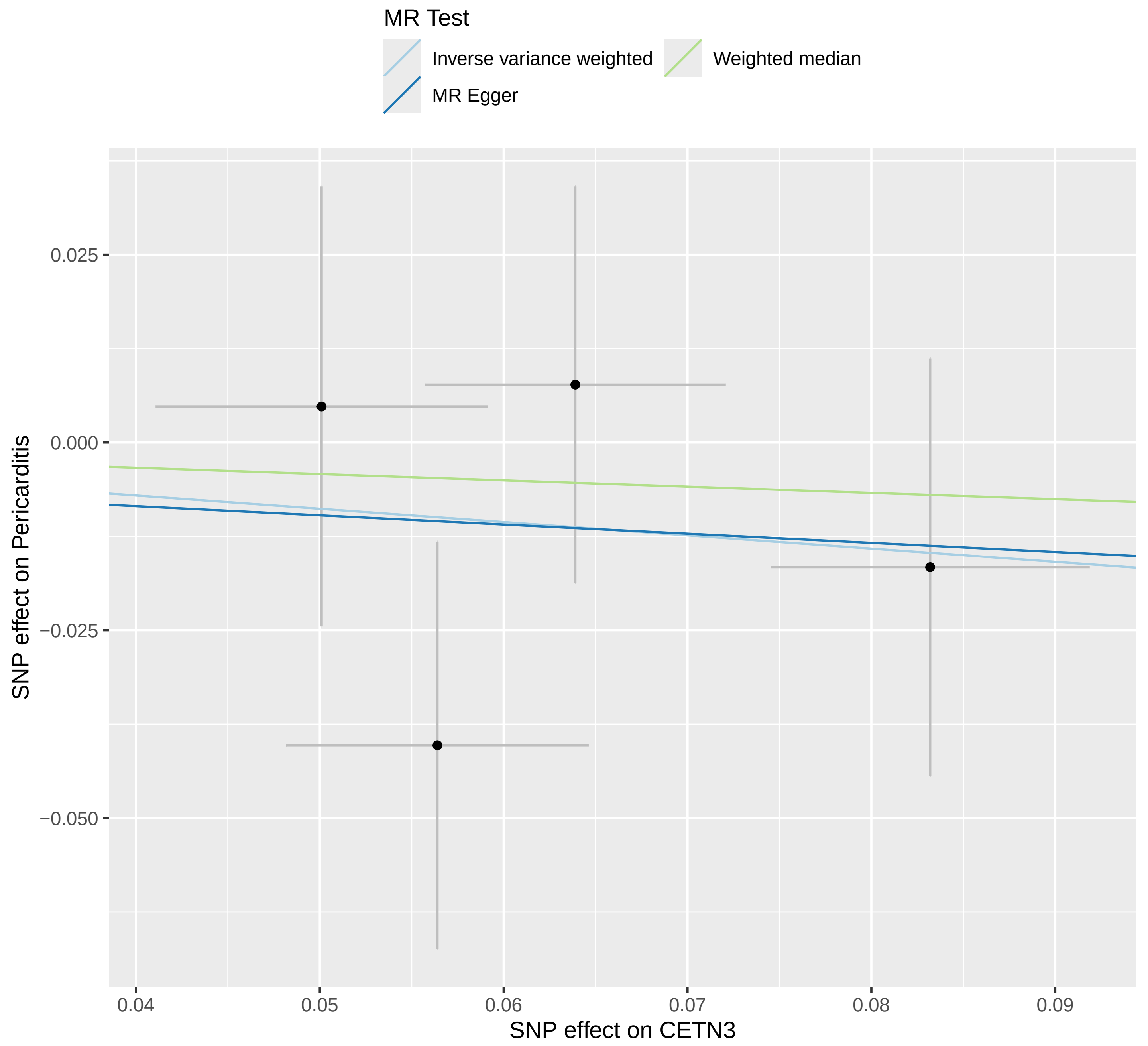
MR Test
Inverse variance weighted
Weighted median
MR Egger
0.025
0.000
−0.025
−0.050
SNP effect on Pericarditis
0.04
0.05
0.06
0.07
0.08
0.09
SNP effect on CETN3

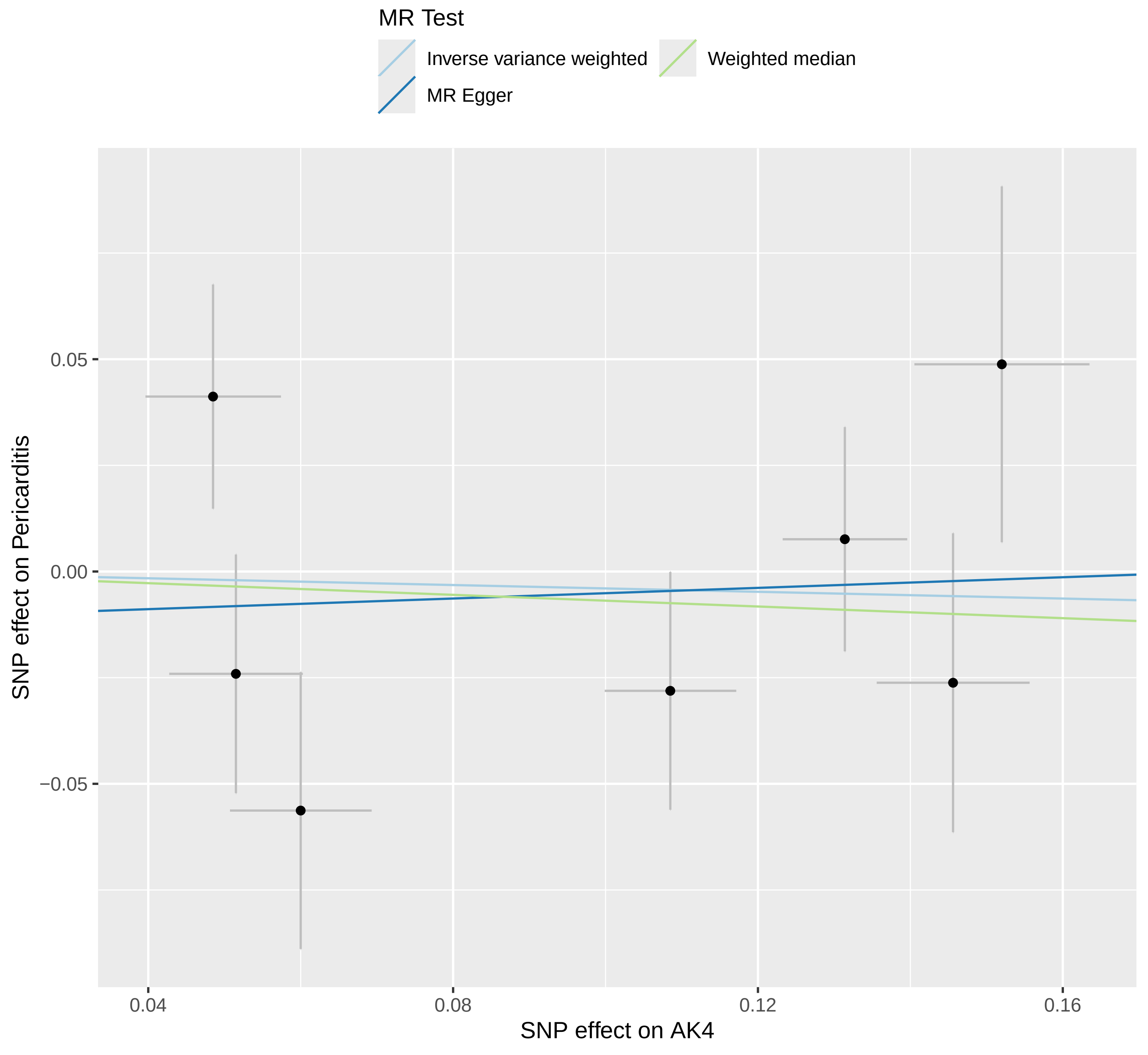
MR Test
Inverse variance weighted
Weighted median
MR Egger
0.05
0.00
−0.05
SNP effect on Pericarditis
0.04
0.08
0.12
0.16
SNP effect on AK4

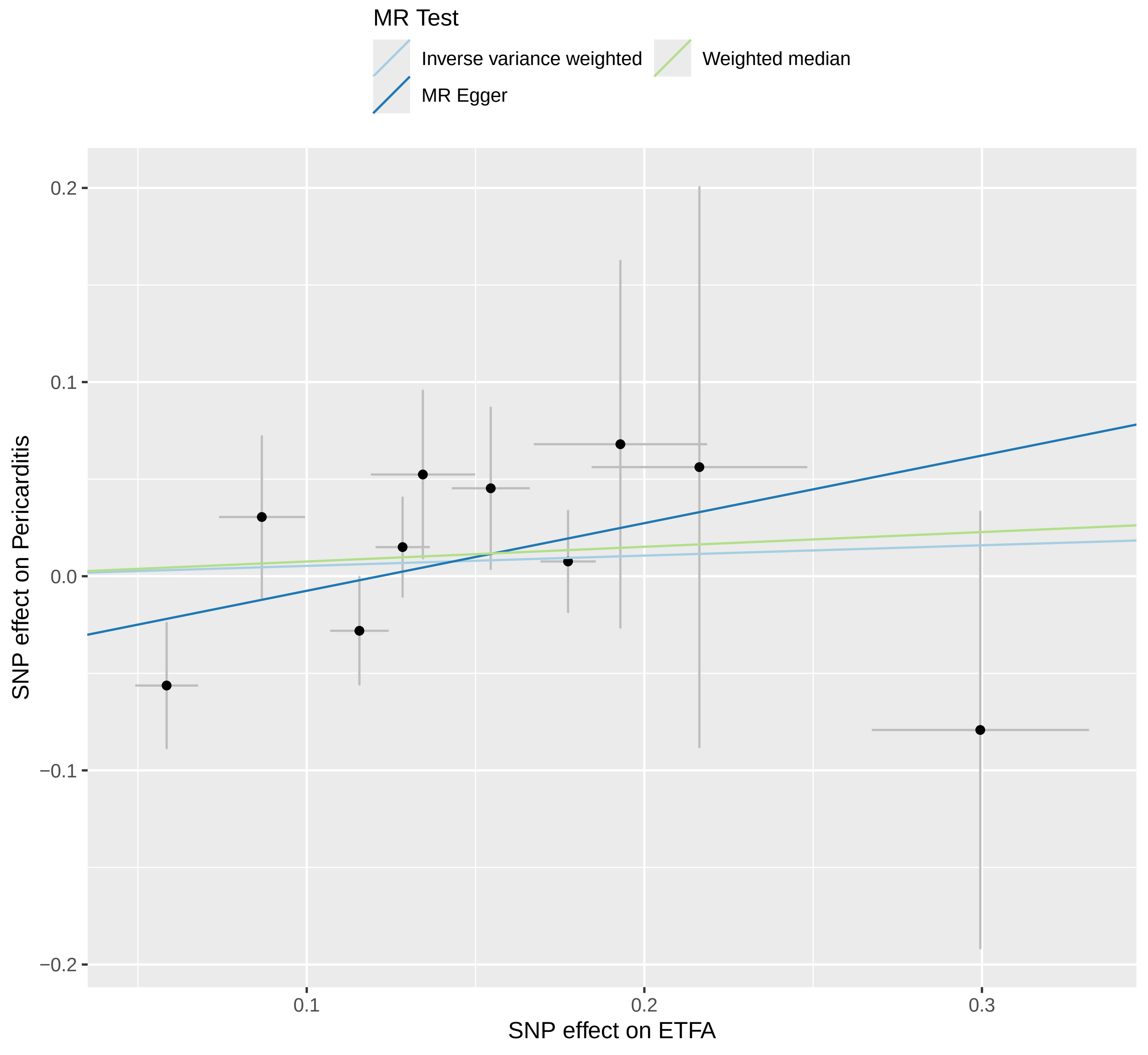
MR Test
Inverse variance weighted
Weighted median
MR Egger
0.2
0.1
0.0
−0.1
−0.2
SNP effect on Pericarditis
0.1
0.2
0.3
SNP effect on ETFA

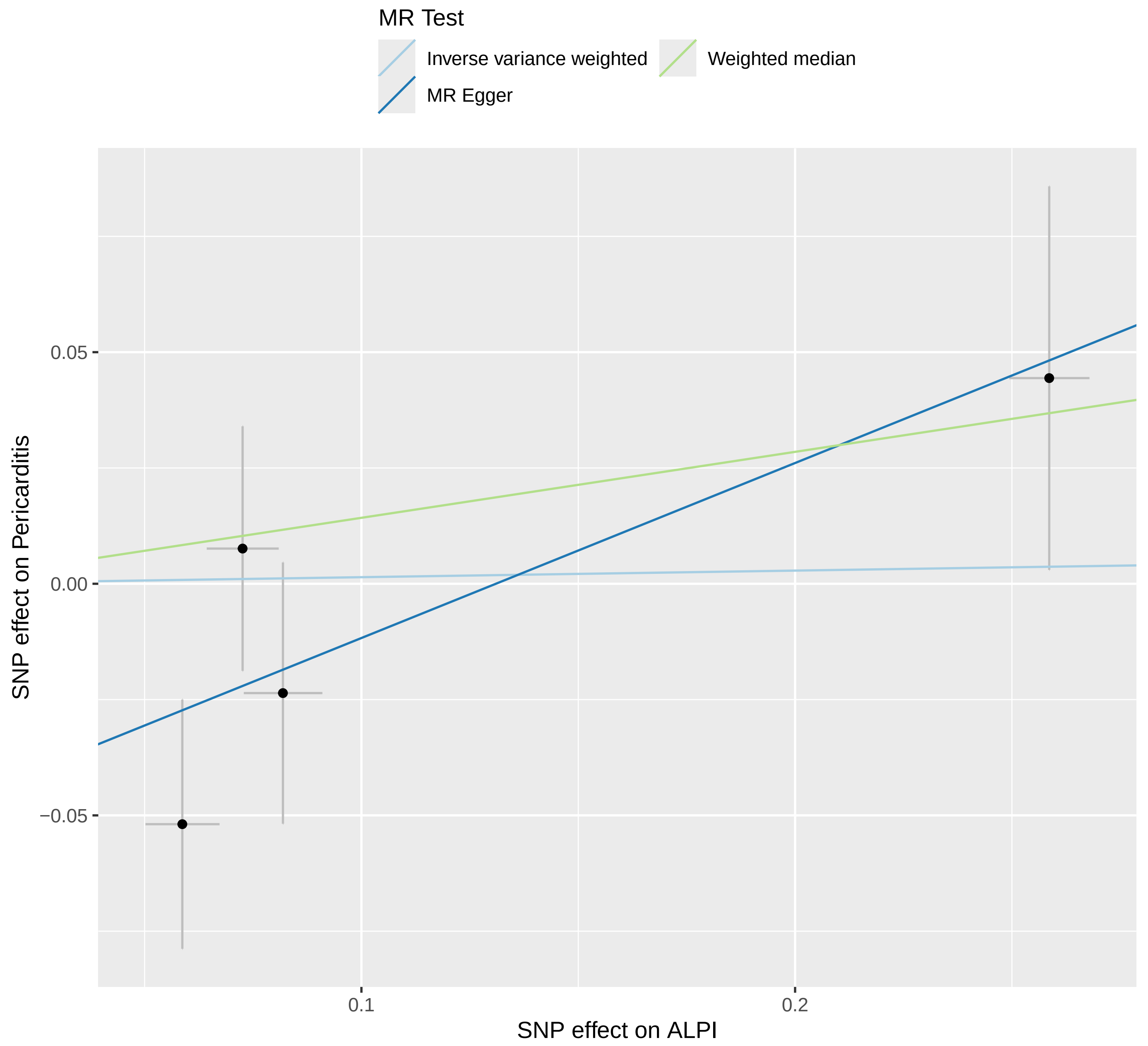
MR Test
Inverse variance weighted
Weighted median
MR Egger
0.05
0.00
−0.05
0.1
0.2
SNP effect on ALPI
SNP effect on Pericarditis

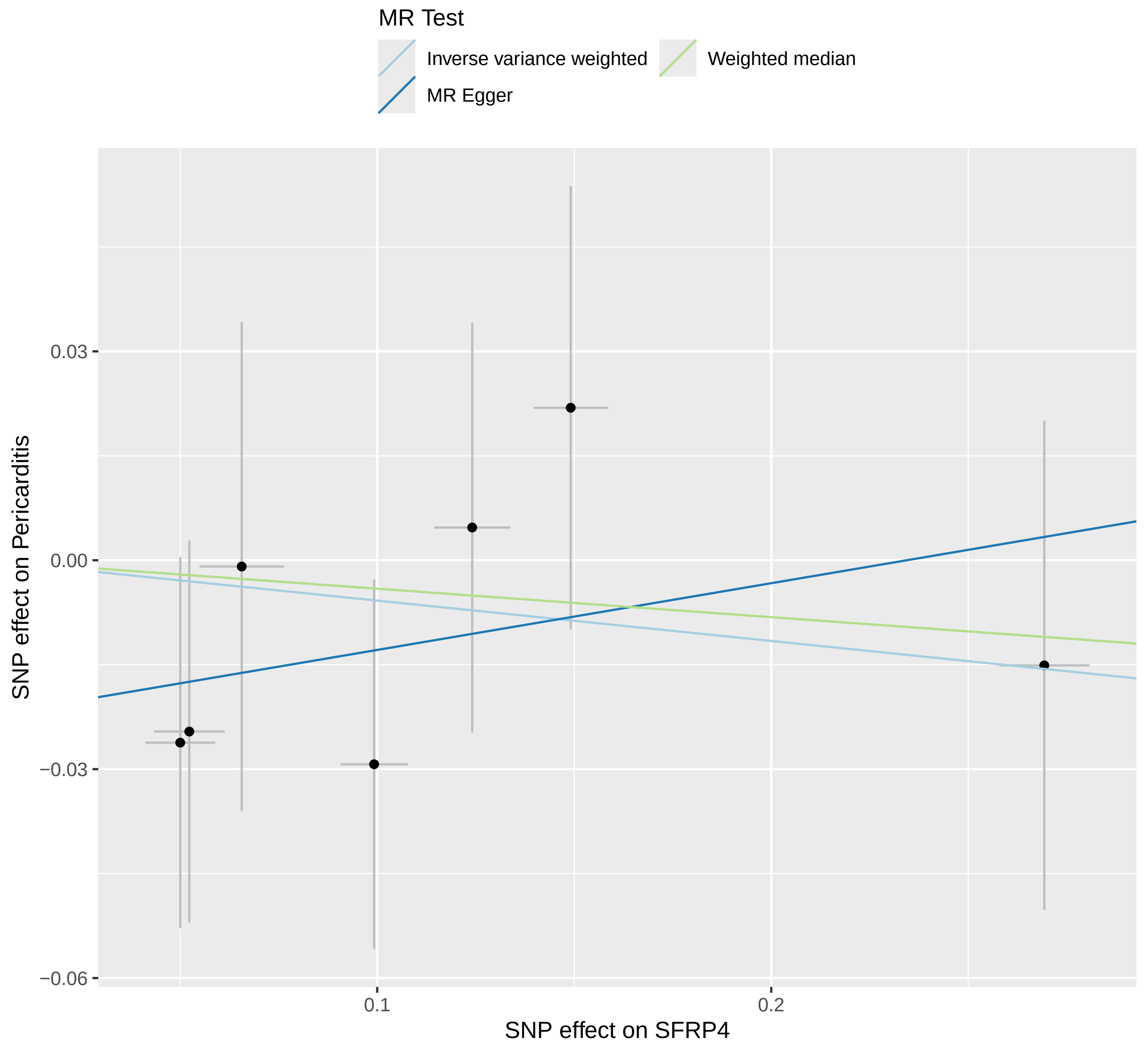

MR Test
Inverse variance weighted
Weighted median
MR Egger
0.03
0.00
−0.03
−0.06
0.1
0.2
SNP effect on SFRP4
SNP effect on Pericarditis

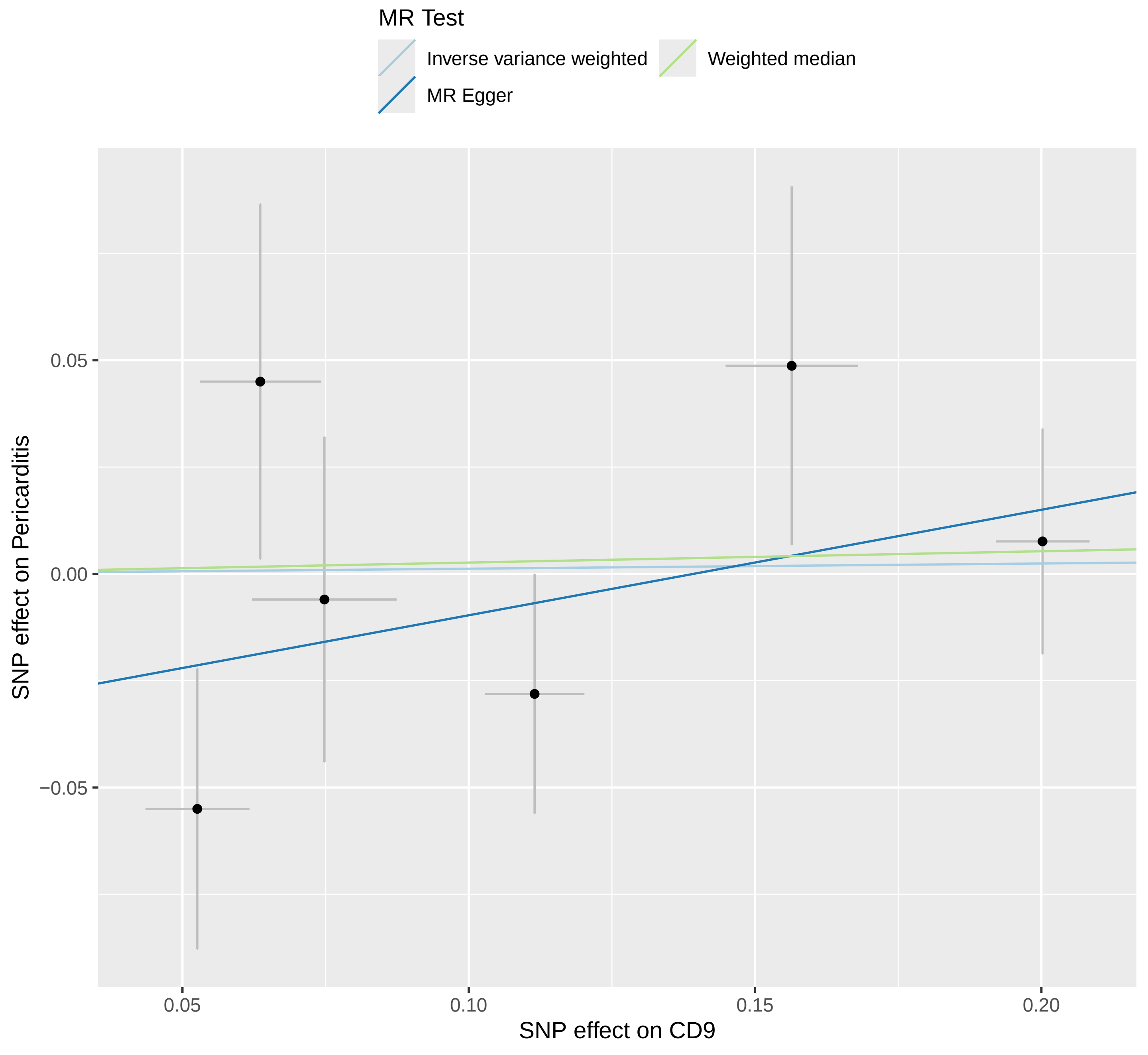

MR Test
Inverse variance weighted
Weighted median
MR Egger
0.05
0.00
−0.05
SNP effect on Pericarditis
0.05
0.10
0.15
0.20
SNP effect on CD9

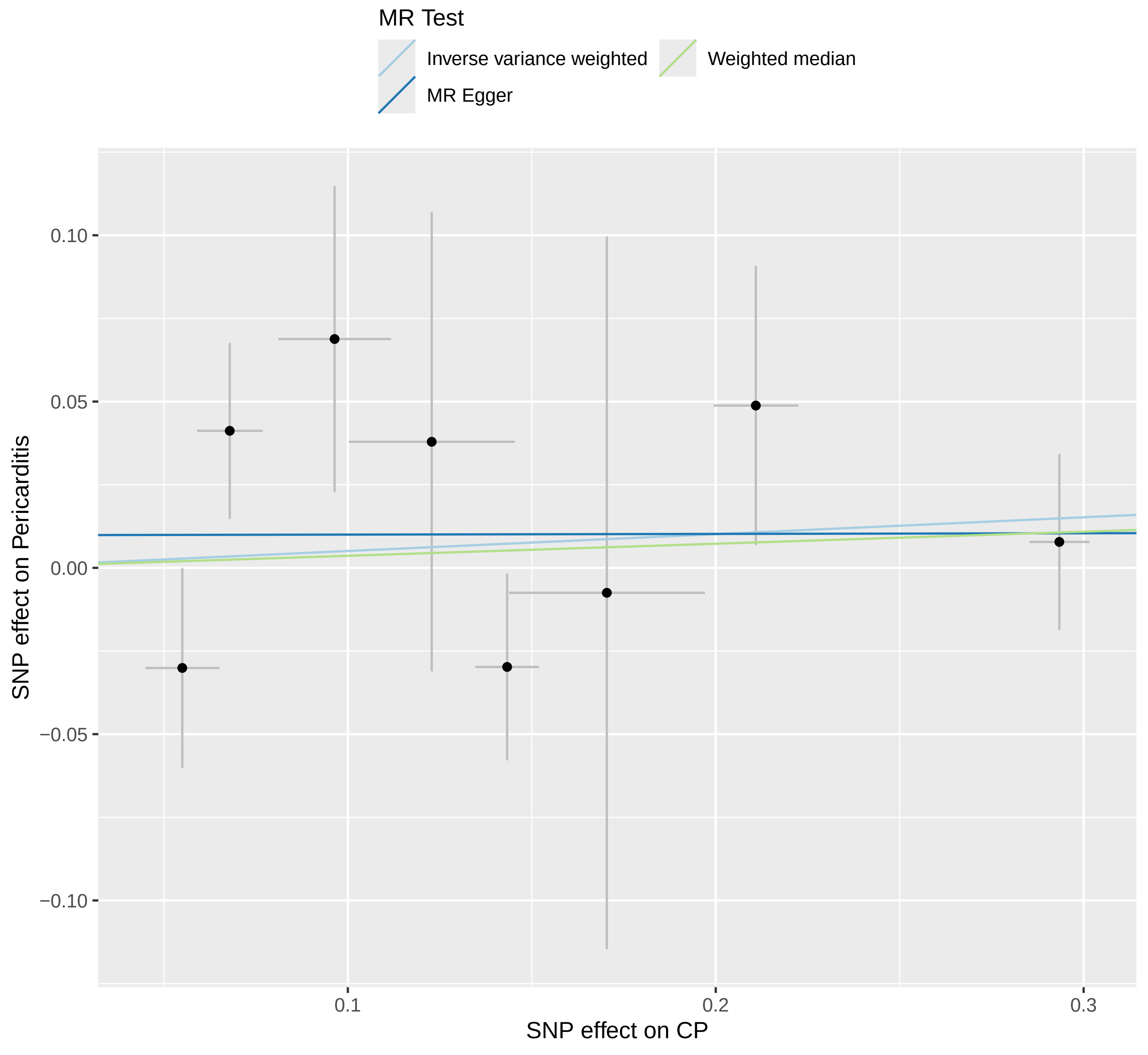
MR Test
Inverse variance weighted
Weighted median
MR Egger
0.10
0.05
0.00
−0.05
−0.10
SNP effect on Pericarditis
0.1
0.2
0.3
SNP effect on CP

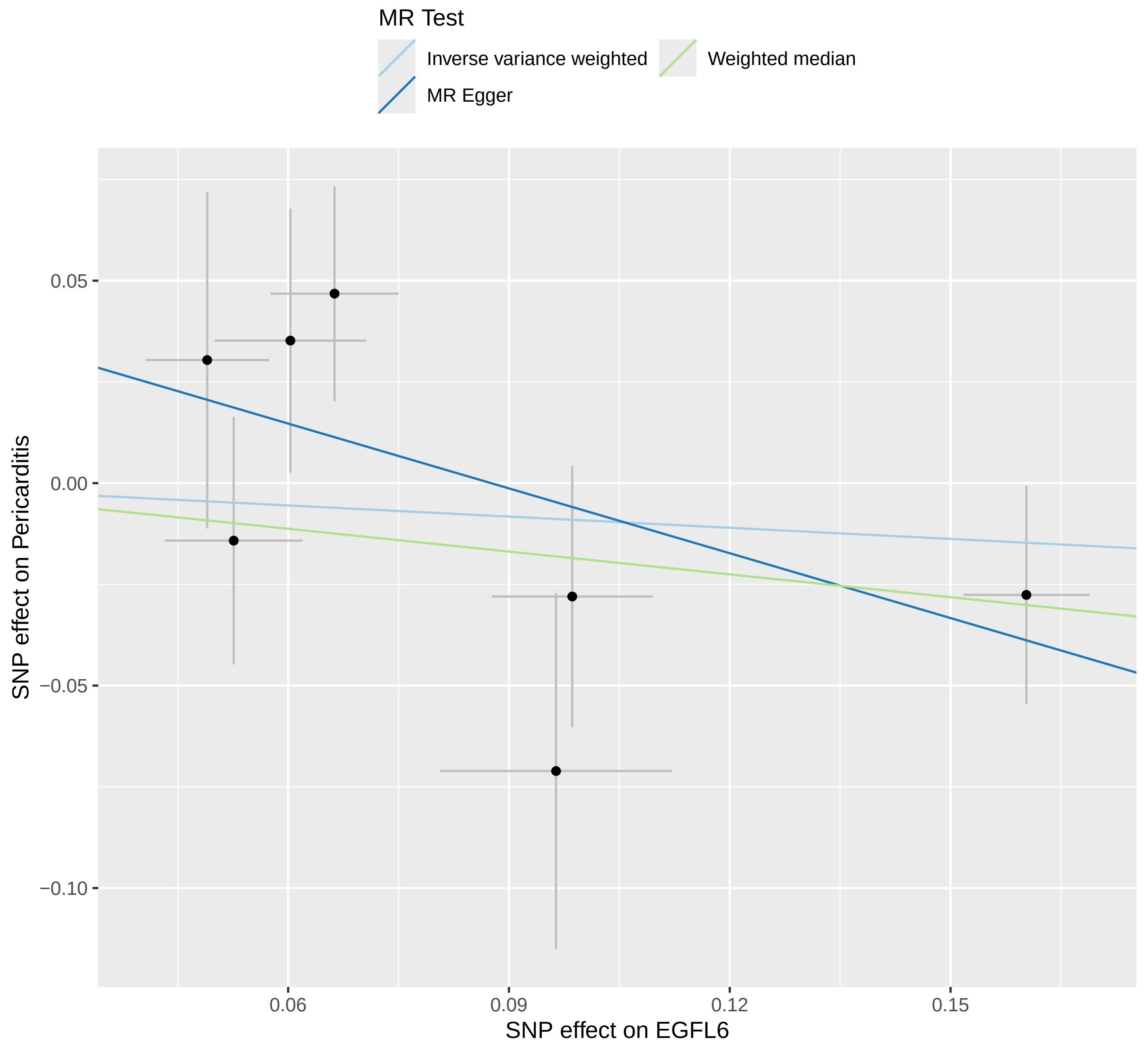

MR Test
Inverse variance weighted
Weighted median
MR Egger
0.05
0.00
−0.05
−0.10
SNP effect on Pericarditis
0.06
0.09
0.12
0.15
SNP effect on EGFL6

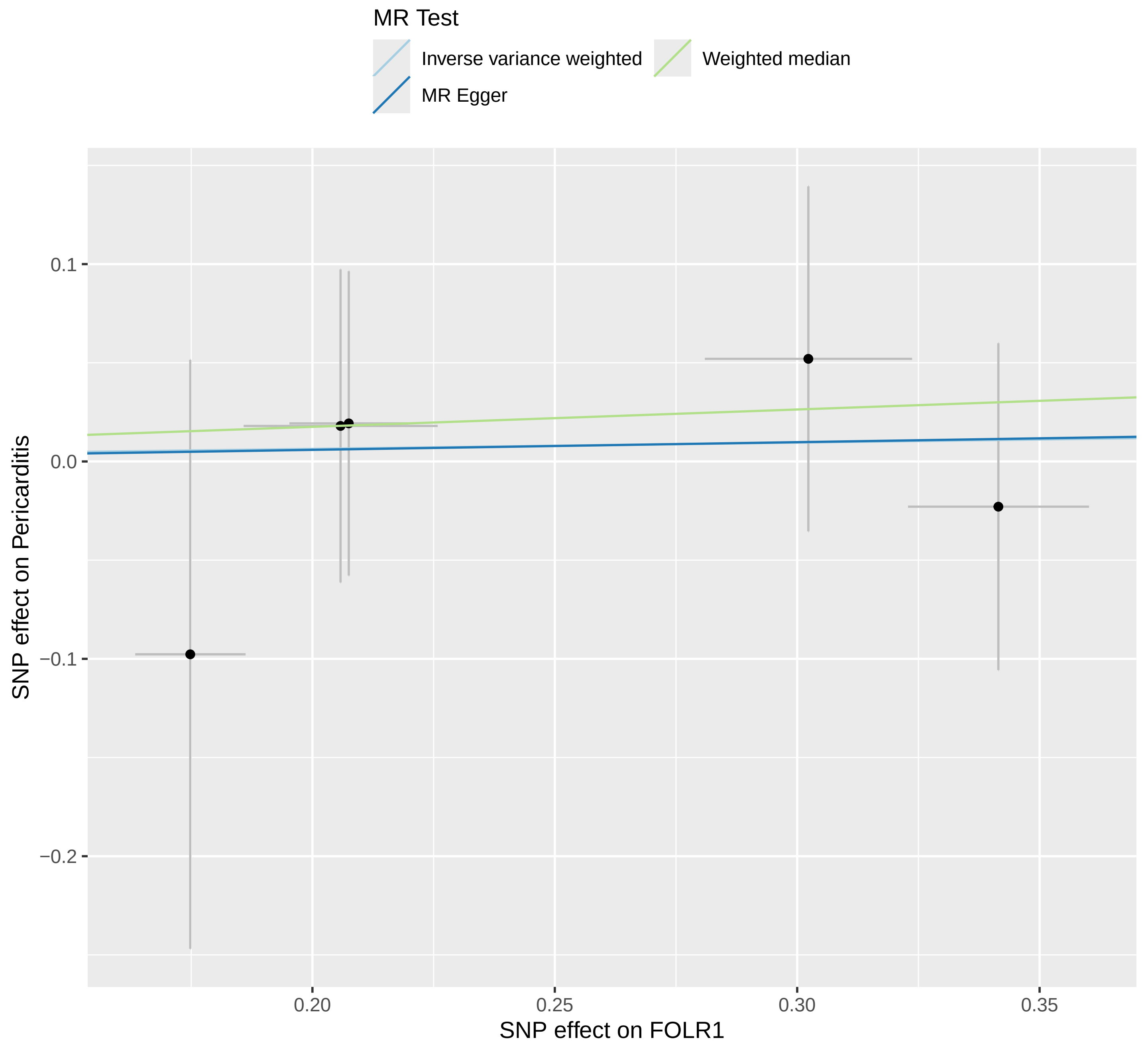

MR Test
Inverse variance weighted
MR Egger
Weighted median
0.1
0.0
−0.1
−0.2
SNP effect on Pericarditis
0.20
0.25
0.30
0.35
SNP effect on FOLR1

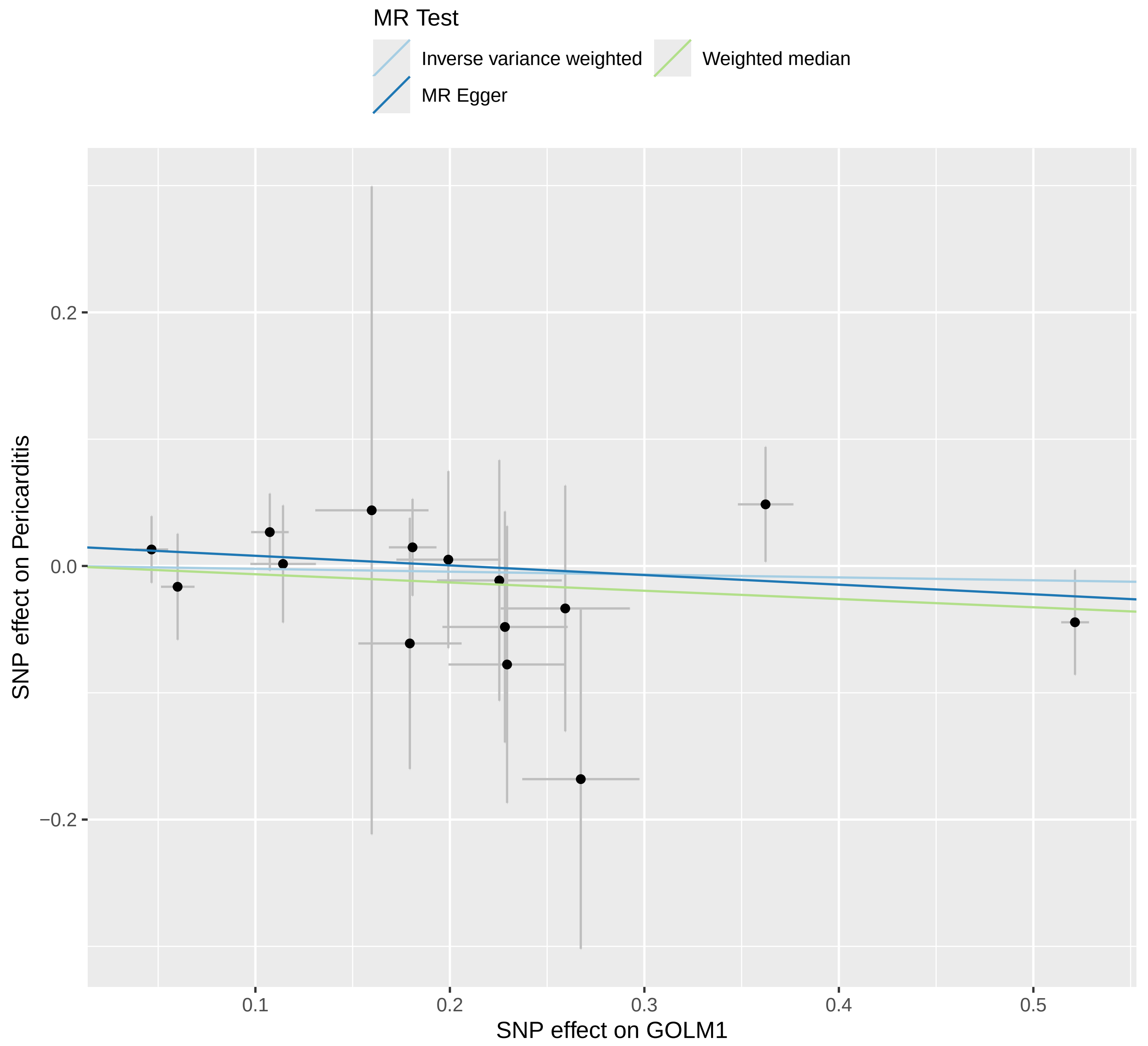
MR Test
Inverse variance weighted
Weighted median
MR Egger
SNP effect on Pericarditis
0.2
0.0
−0.2
0.1
0.2
0.3
0.4
0.5
SNP effect on GOLM1

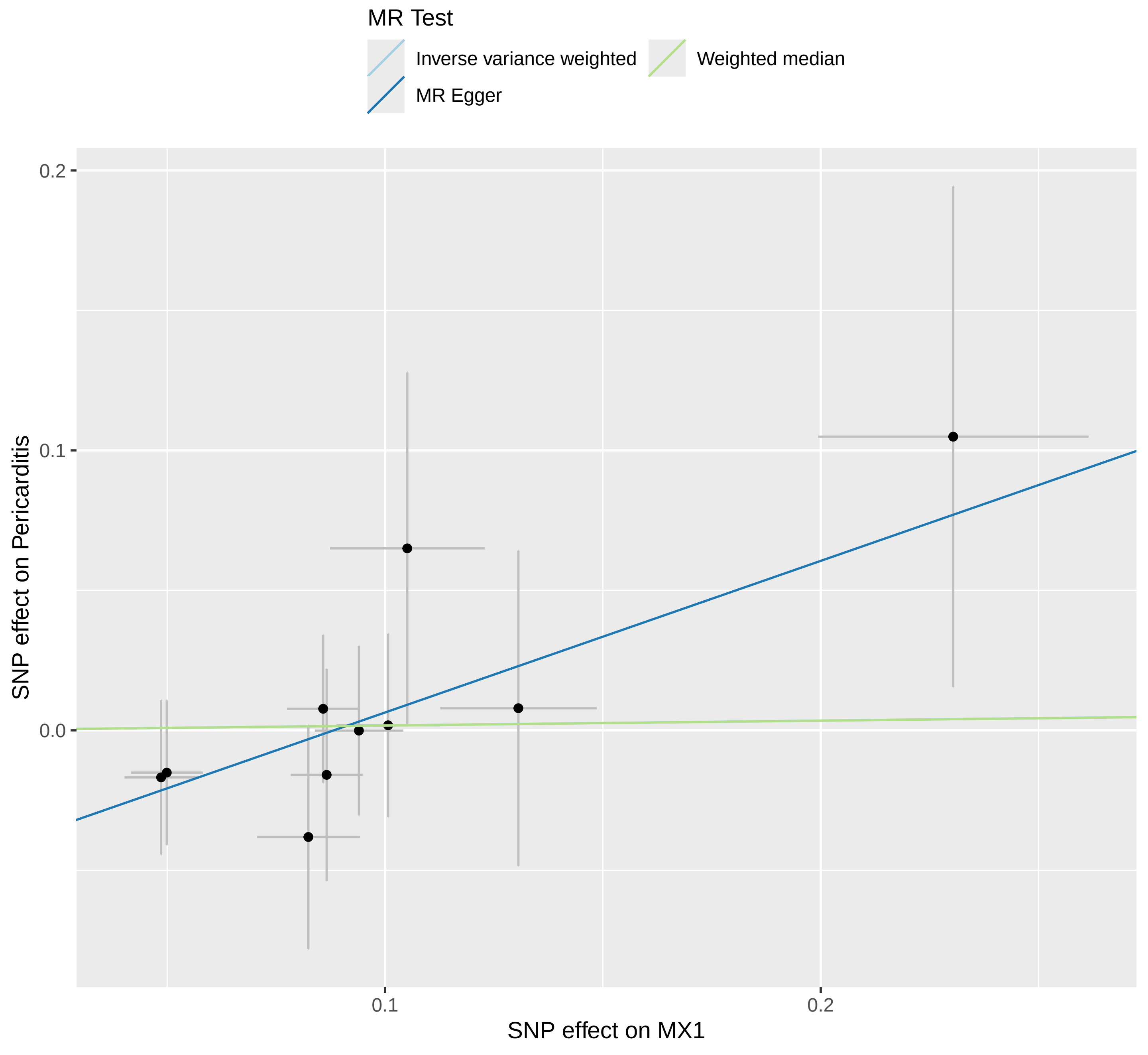
MR Test
Inverse variance weighted
MR Egger
Weighted median
0.2
0.1
0.0
0.1
0.2
SNP effect on Pericarditis
SNP effect on MX1

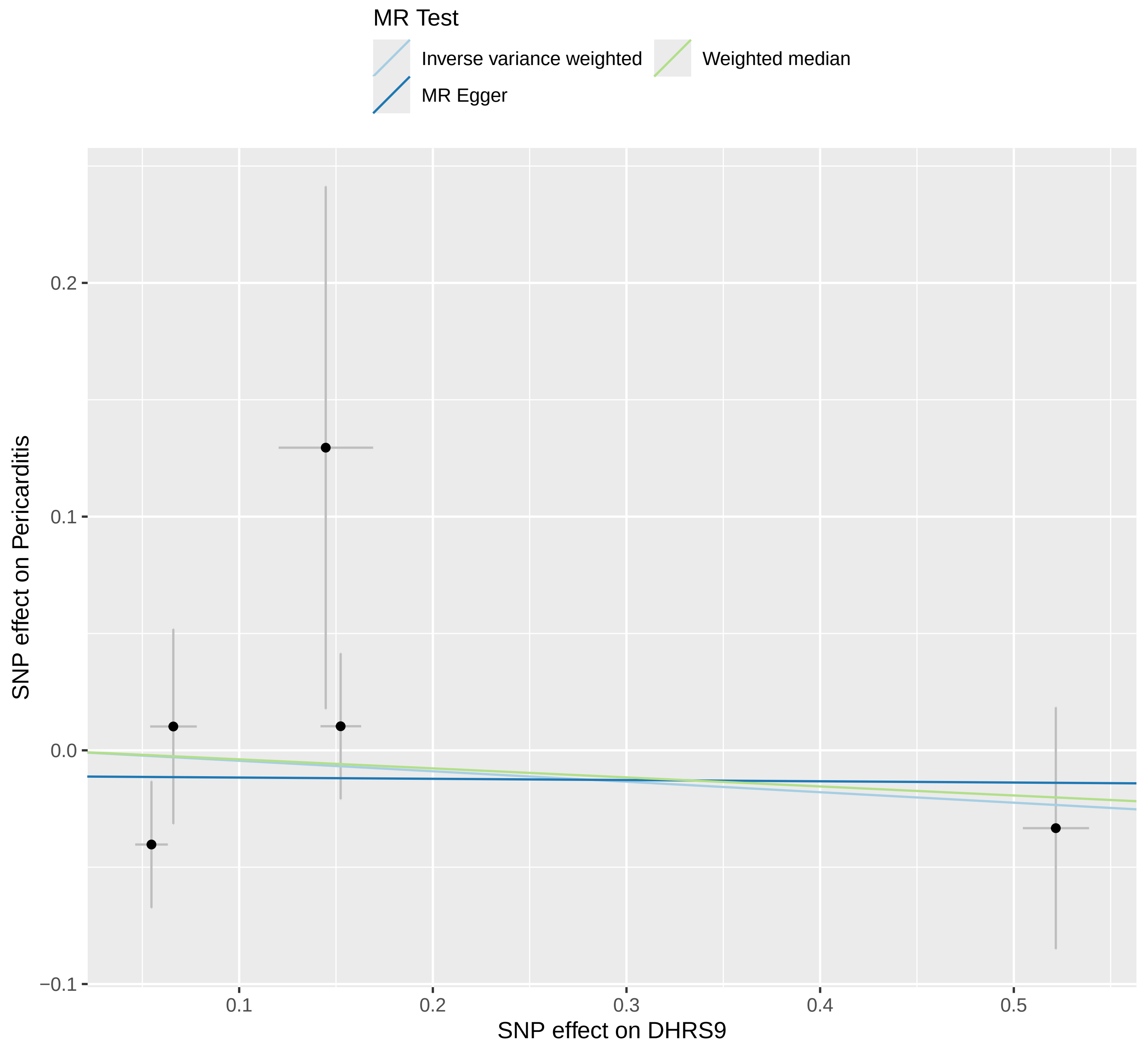
MR Test
Inverse variance weighted
Weighted median
MR Egger
SNP effect on Pericarditis
0.2
0.1
0.0
−0.1
0.1
0.2
0.3
0.4
0.5
SNP effect on DHRS9

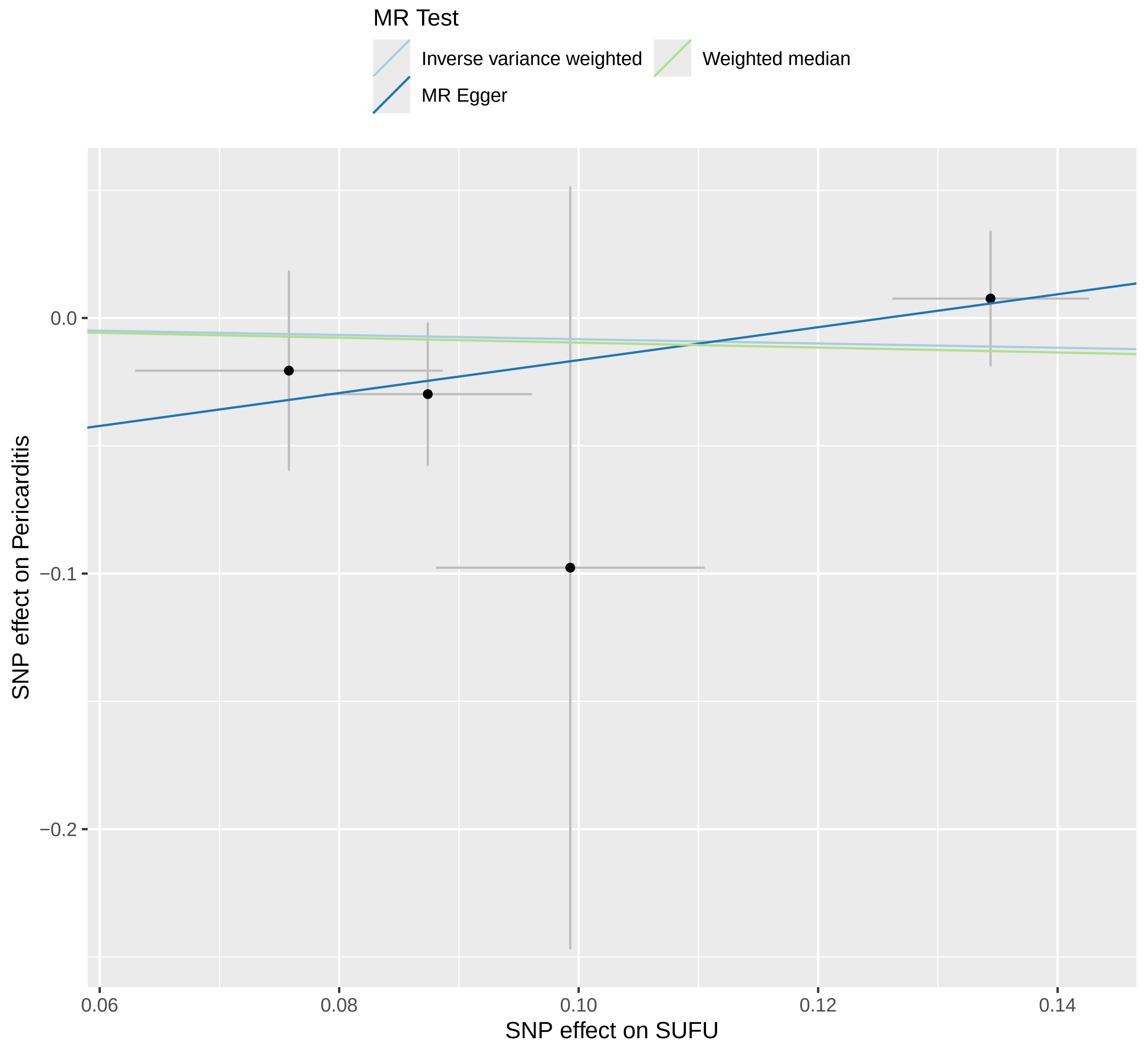
MR Test
Inverse variance weighted
Weighted median
MR Egger
SNP effect on Pericarditis
0.0
−0.1
−0.2
0.06
0.08
0.10
0.12
0.14
SNP effect on SUFU

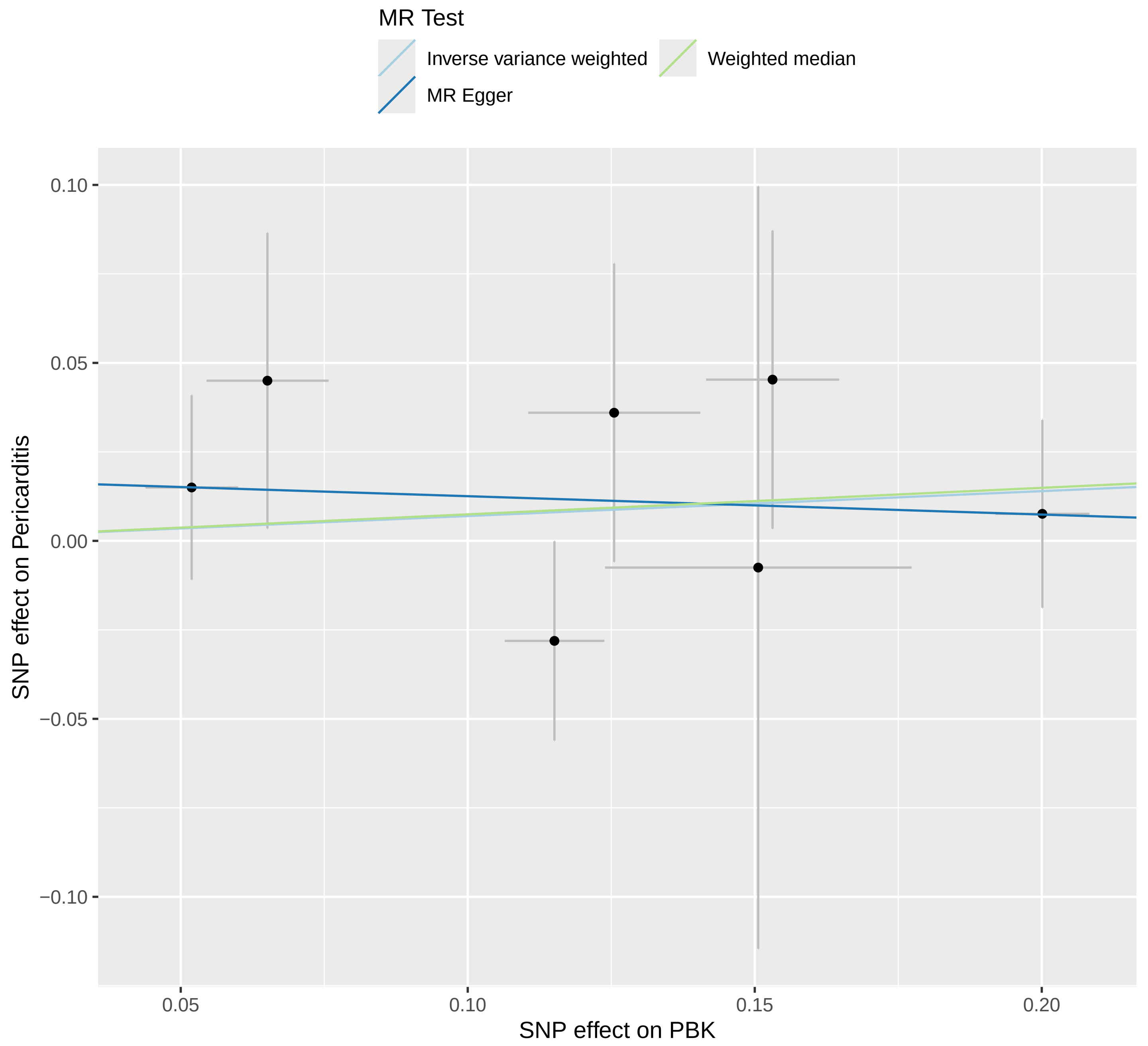
MR Test
Inverse variance weighted
MR Egger
Weighted median
0.10
0.05
0.00
−0.05
−0.10
SNP effect on Pericarditis
0.05
0.10
0.15
0.20
SNP effect on PBK

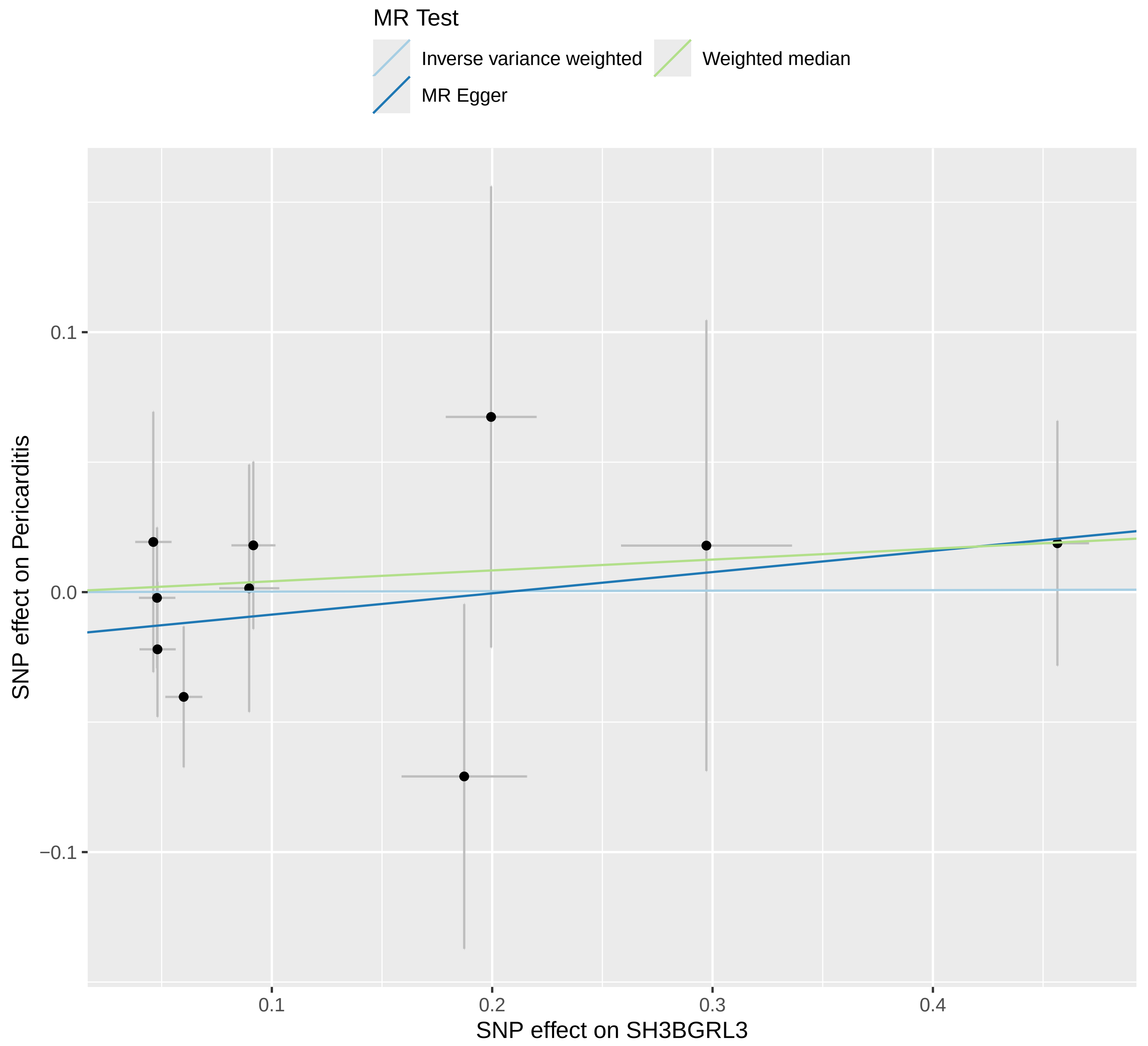

MR Test
Inverse variance weighted
Weighted median
MR Egger
SNP effect on Pericarditis
0.1
0.0
−0.1
0.1
0.2
0.3
0.4
SNP effect on SH3BGRL3

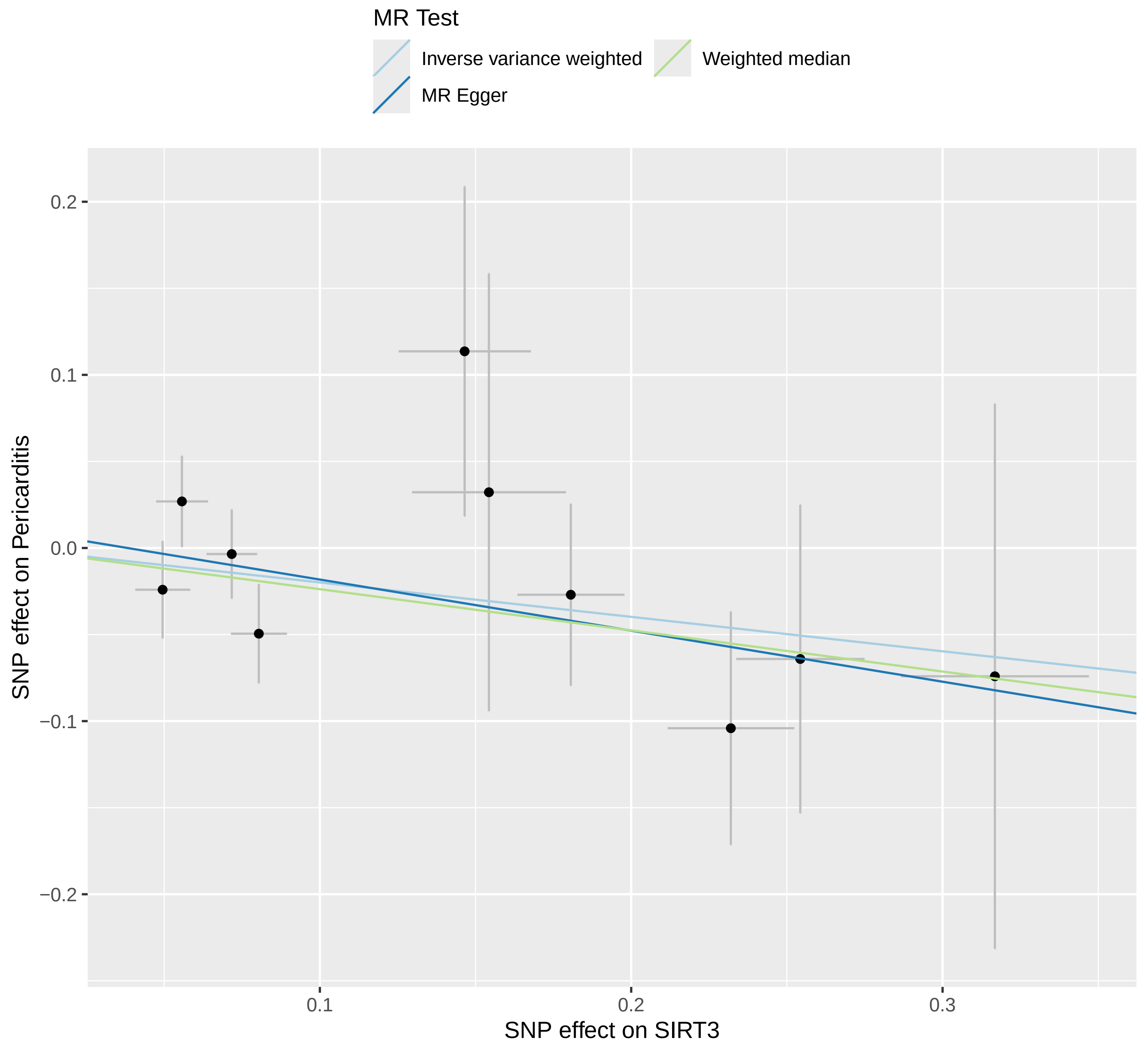

MR Test
Inverse variance weighted
MR Egger
Weighted median
SNP effect on Pericarditis
0.2
0.1
0.0
−0.1
−0.2
0.1
0.2
0.3
SNP effect on SIRT3

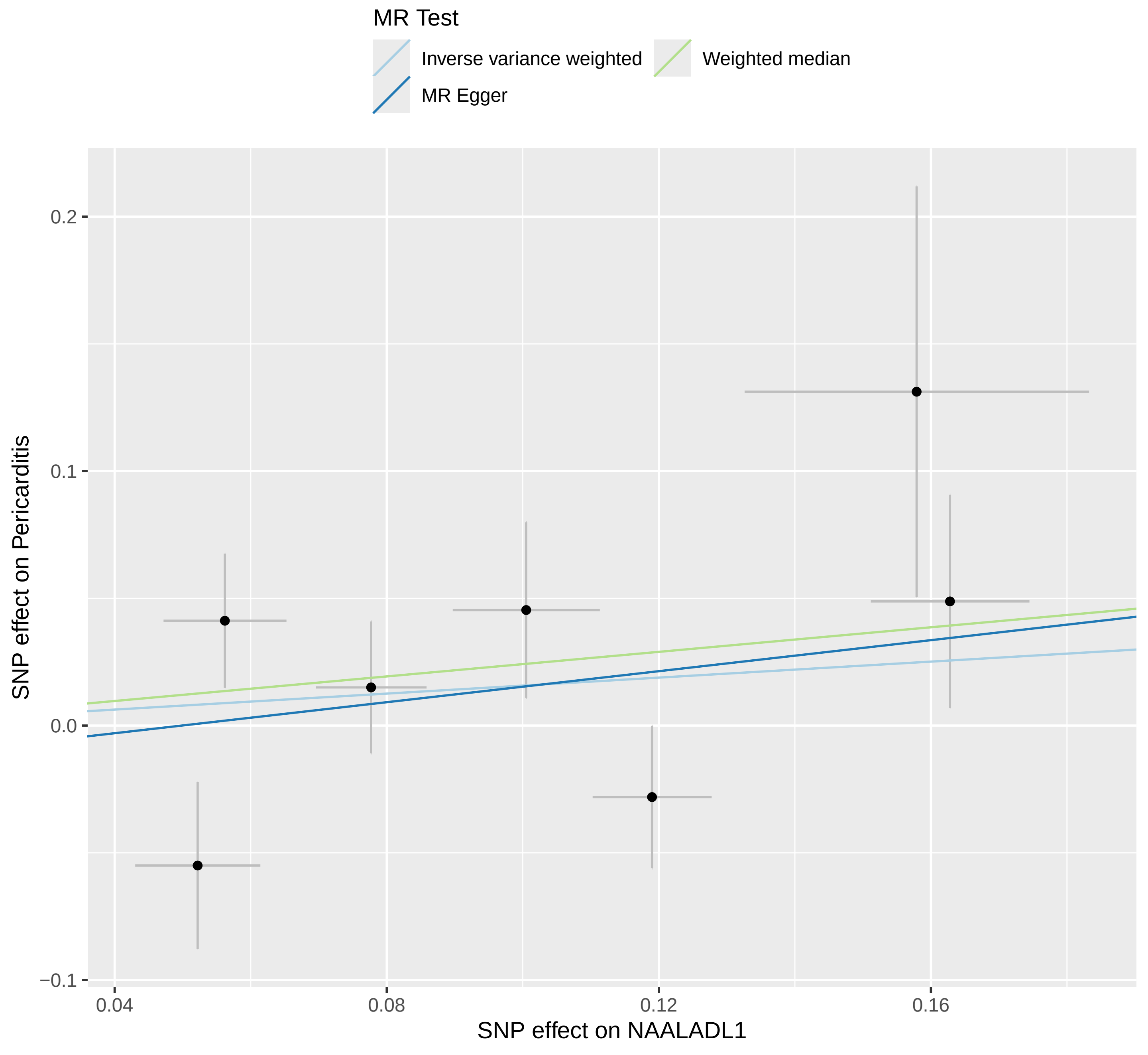
MR Test
Inverse variance weighted
Weighted median
MR Egger
0.2
0.1
0.0
−0.1
0.04
0.08
0.12
0.16
SNP effect on Pericarditis
SNP effect on NAALADL1

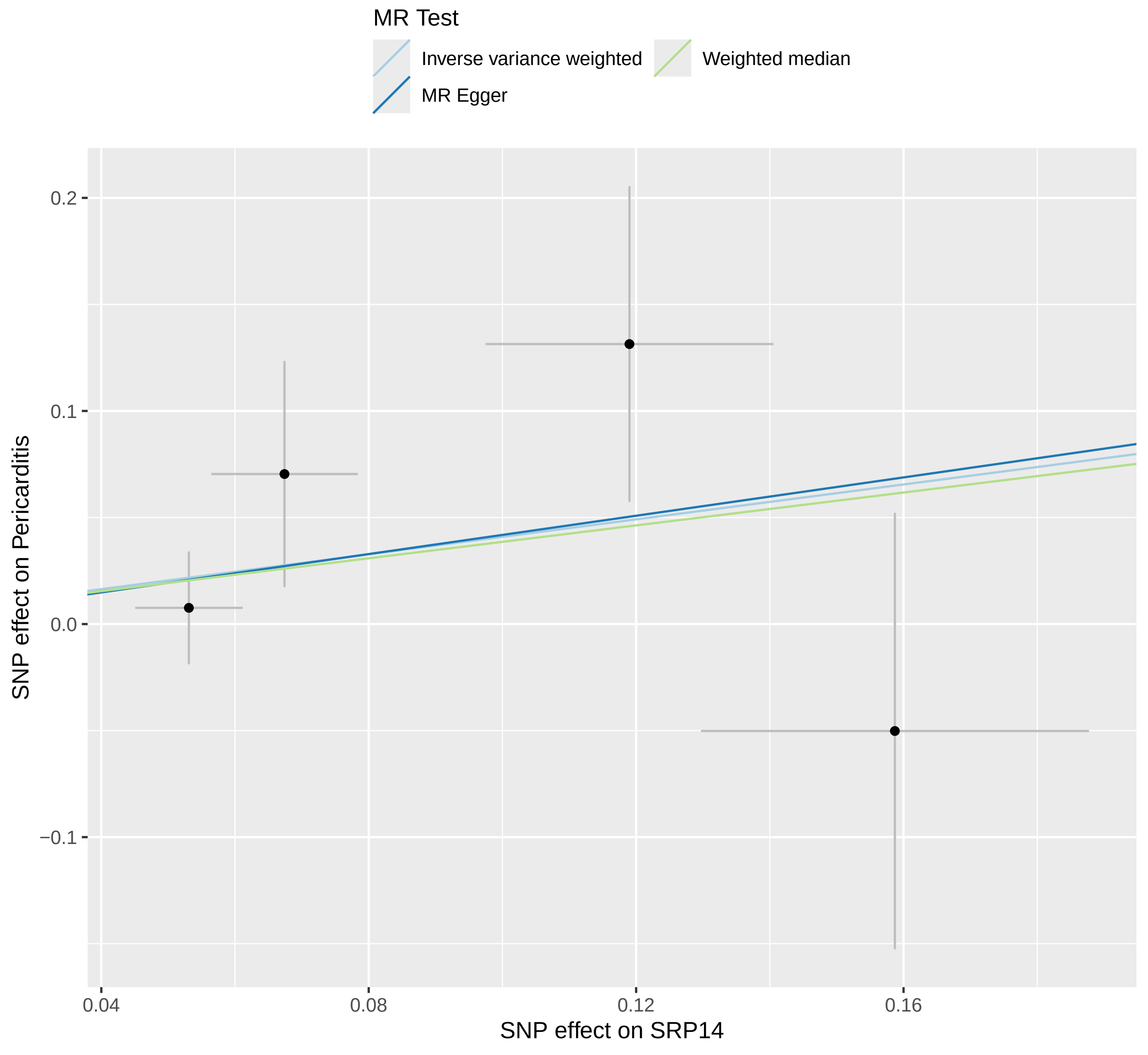
MR Test
Inverse variance weighted
Weighted median
MR Egger
0.2
0.1
0.0
−0.1
SNP effect on Pericarditis
0.04
0.08
0.12
0.16
SNP effect on SRP14

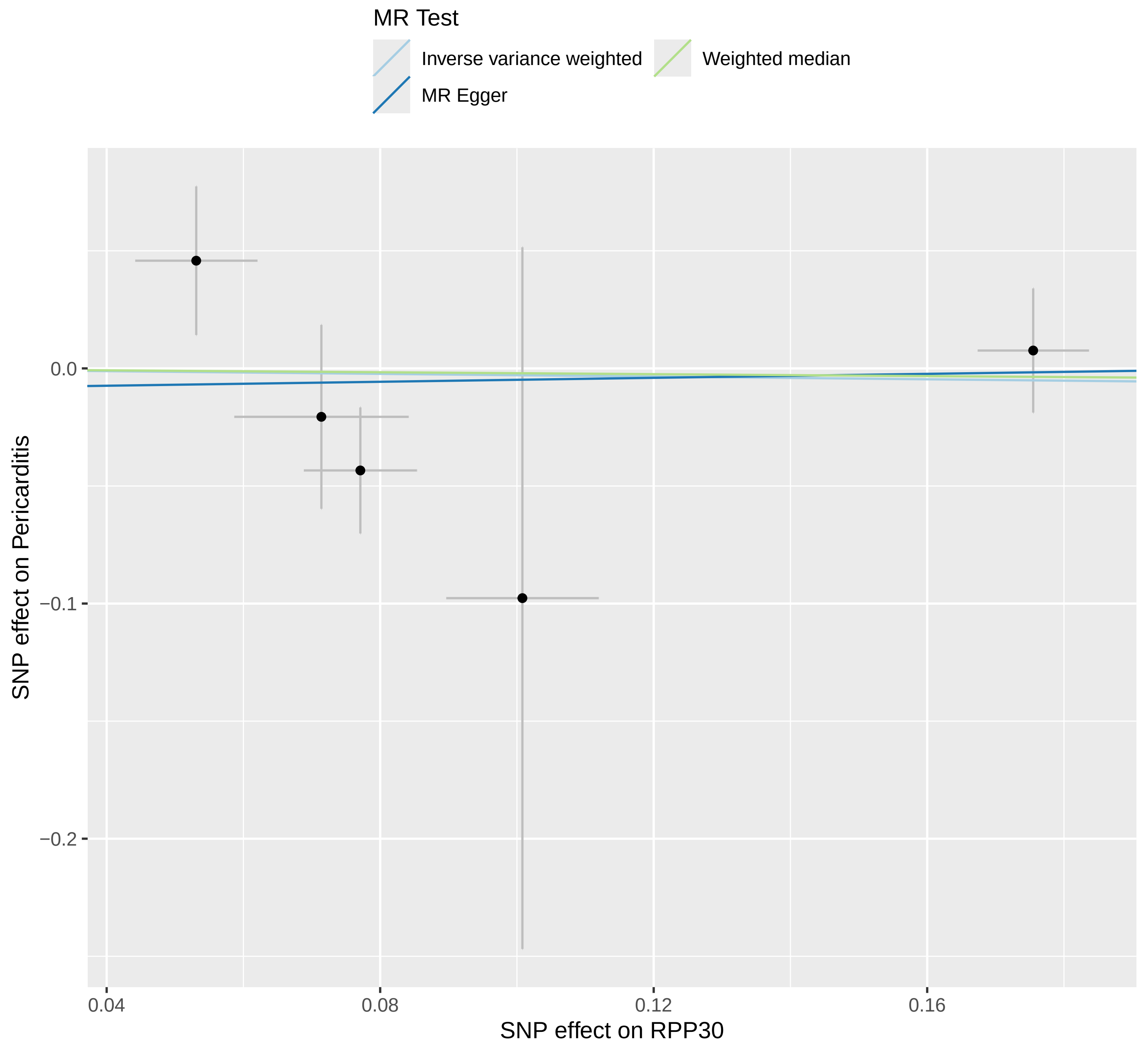
MR Test
Inverse variance weighted
Weighted median
MR Egger
0.0
−0.1
−0.2
SNP effect on Pericarditis
0.04
0.08
0.12
0.16
SNP effect on RPP30

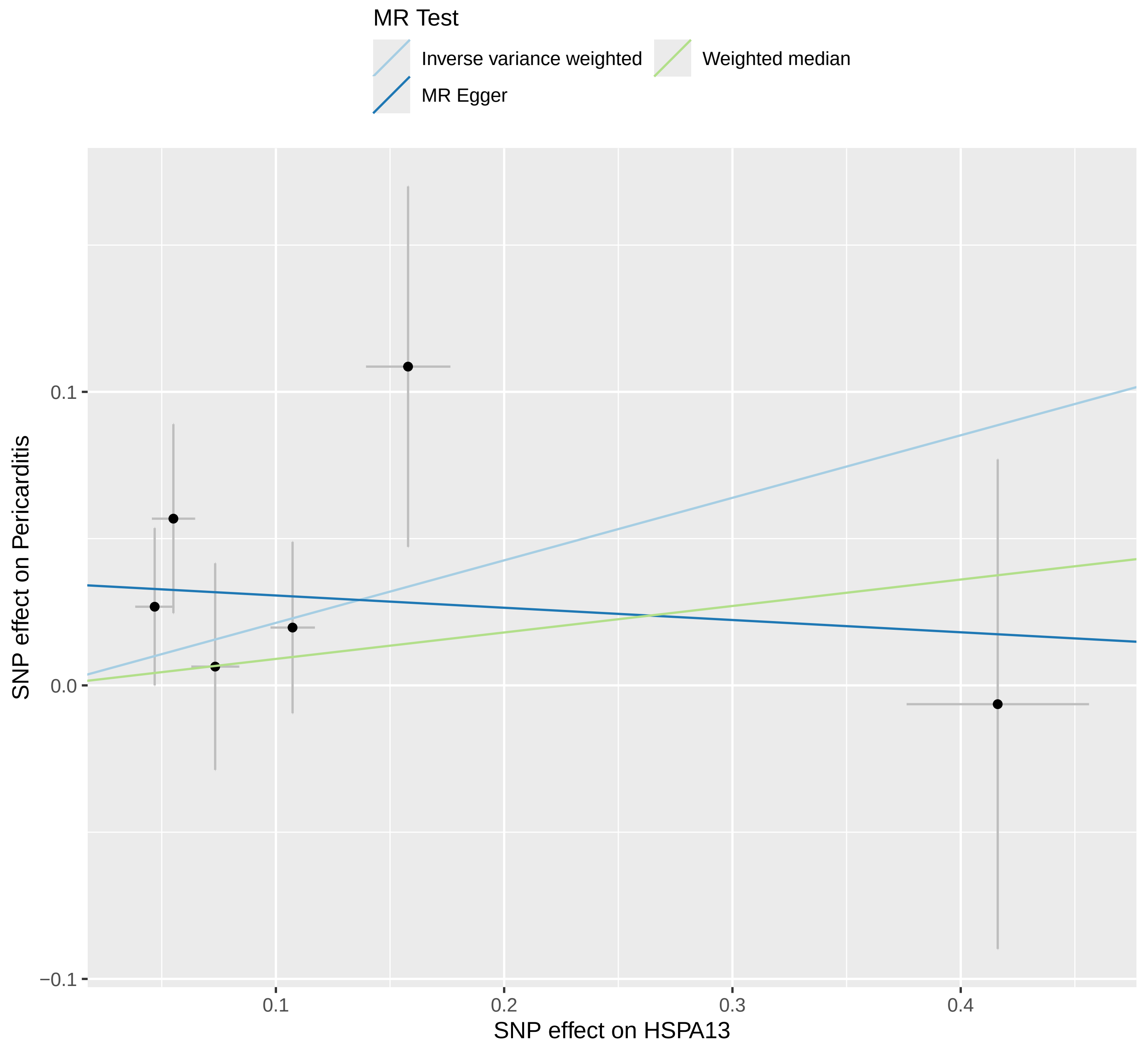
MR Test
Inverse variance weighted
Weighted median
MR Egger
SNP effect on Pericarditis
0.1
0.0
−0.1
0.1
0.2
0.3
0.4
SNP effect on HSPA13

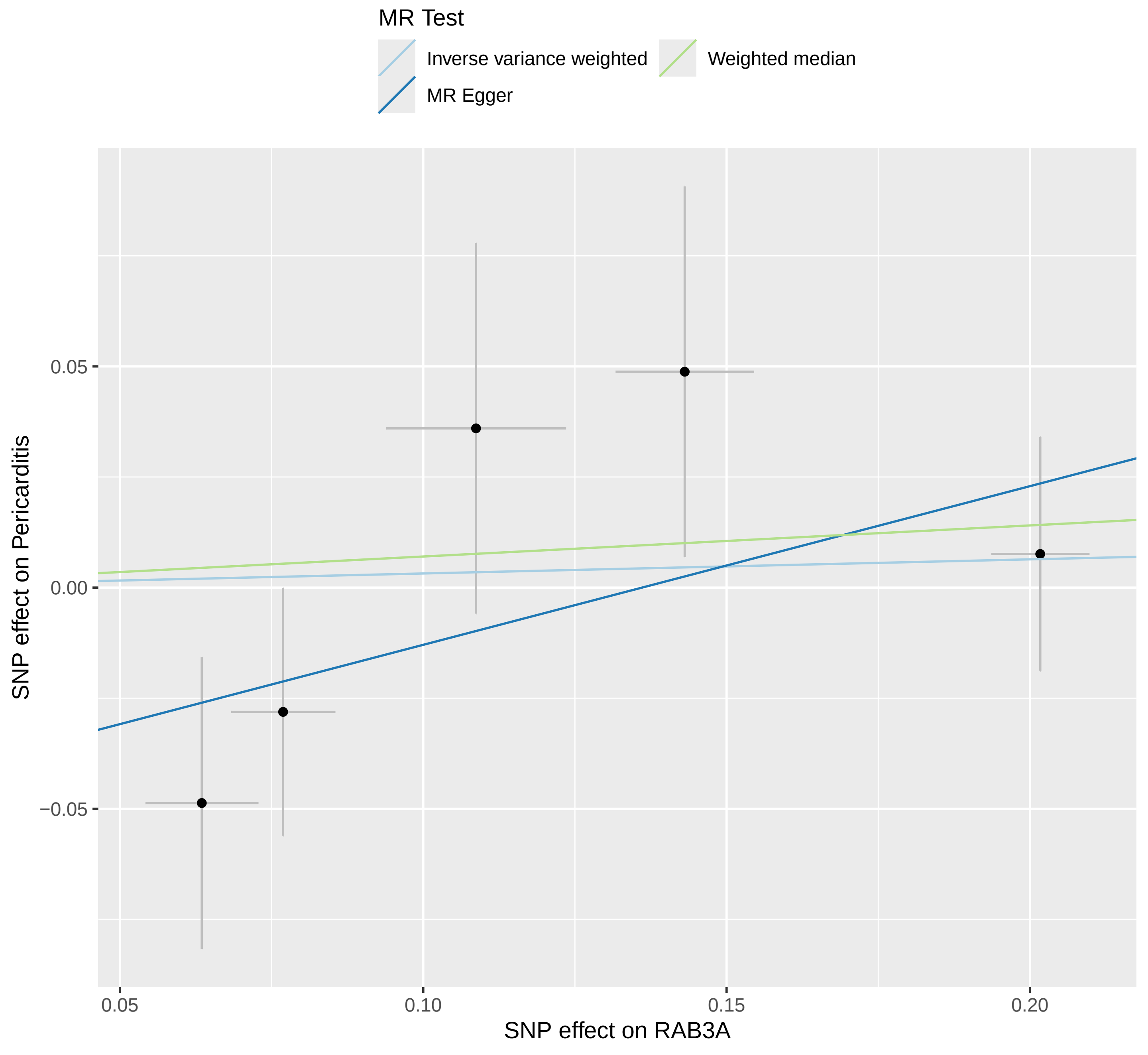
MR Test
Inverse variance weighted
MR Egger
Weighted median
SNP effect on Pericarditis
0.05
0.00
−0.05
0.05
0.10
0.15
0.20
SNP effect on RAB3A

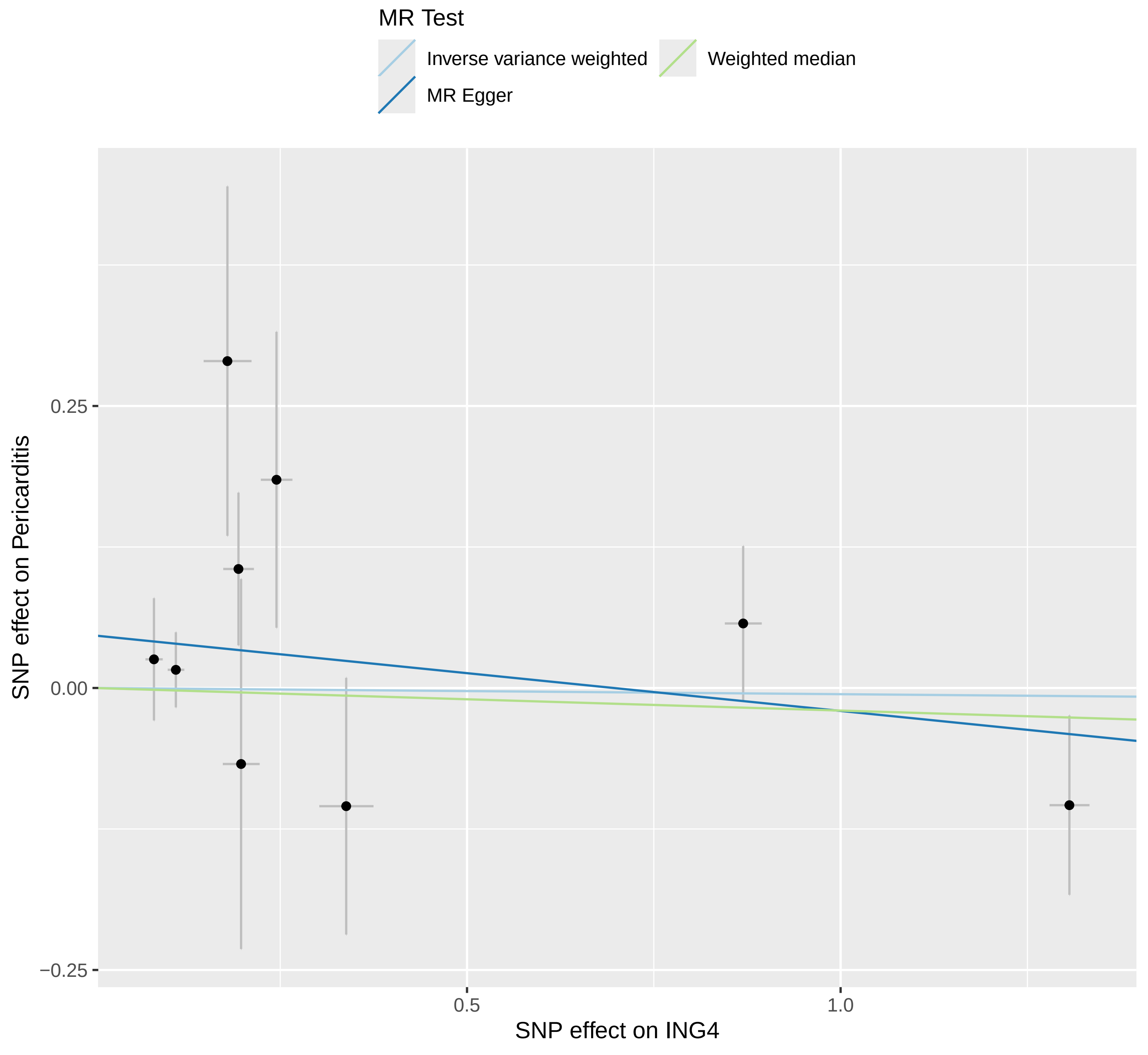
MR Test
Inverse variance weighted
MR Egger
Weighted median
0.25
0.00
−0.25
0.5
1.0
SNP effect on Pericarditis
SNP effect on ING4

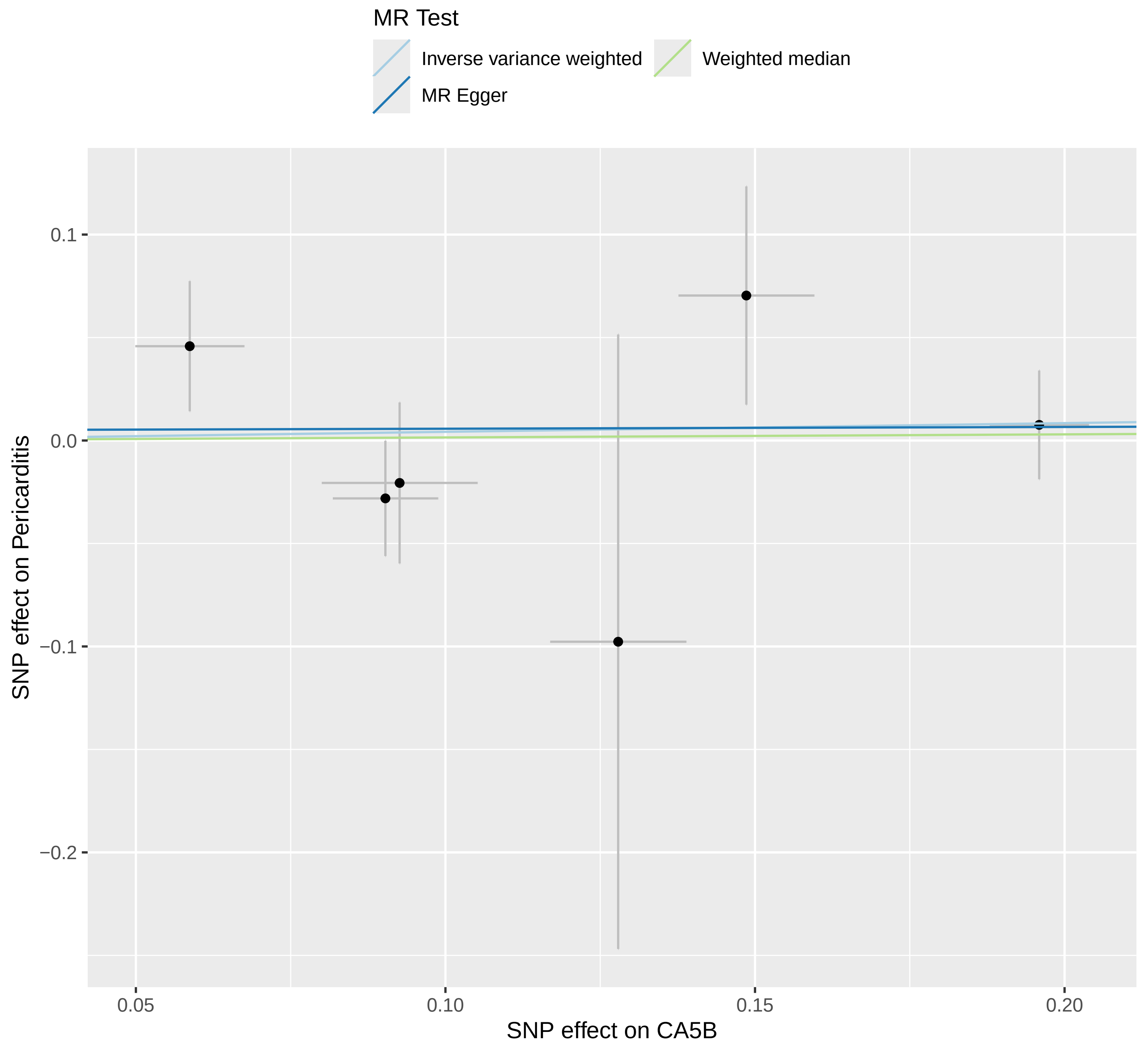

MR Test
Inverse variance weighted
Weighted median
MR Egger
0.1
0.0
−0.1
−0.2
SNP effect on Pericarditis
0.05
0.10
0.15
0.20
SNP effect on CA5B

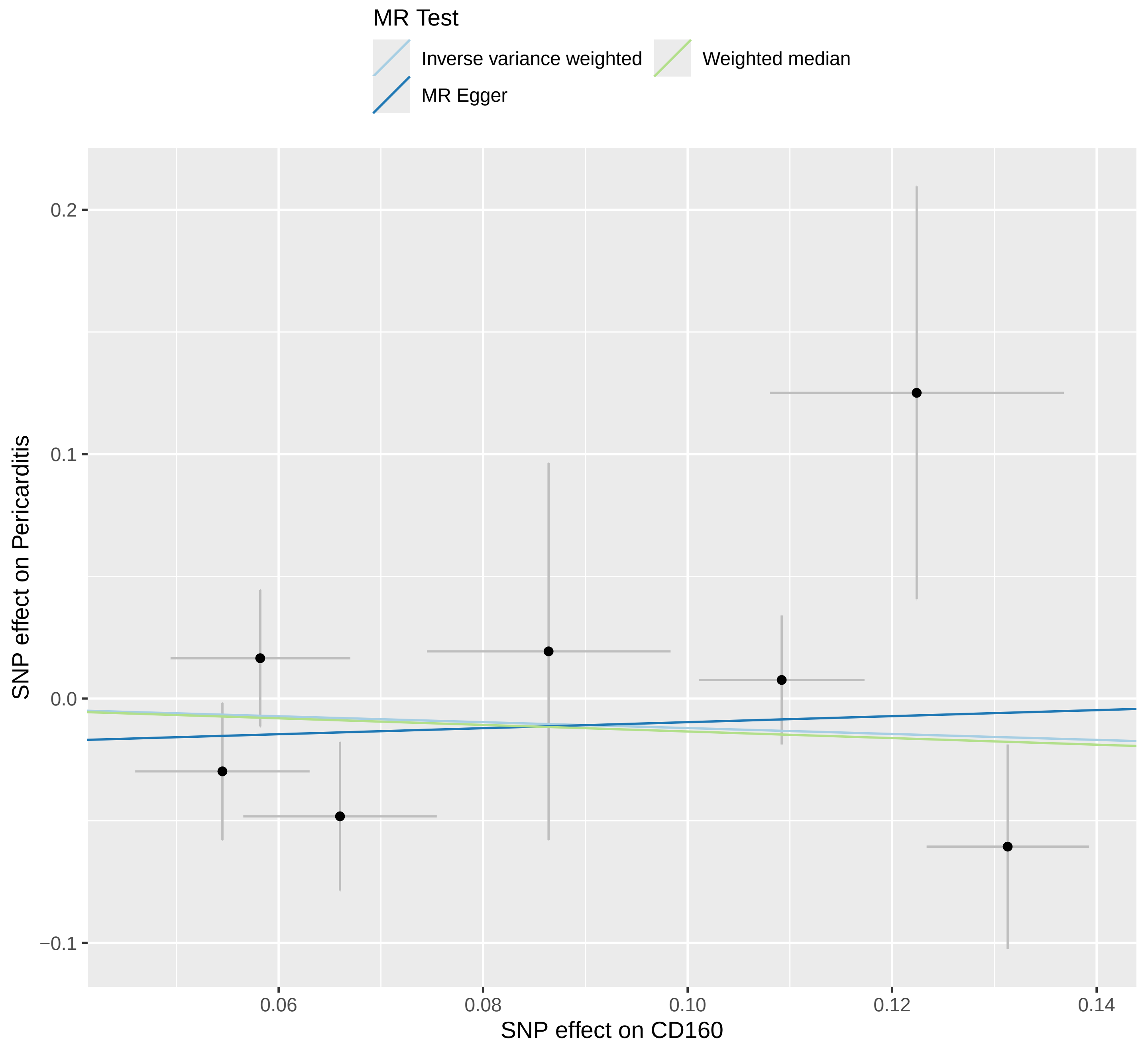

MR Test
Inverse variance weighted
Weighted median
MR Egger
SNP effect on Pericarditis
0.2
0.1
0.0
−0.1
0.06
0.08
0.10
0.12
0.14
SNP effect on CD160

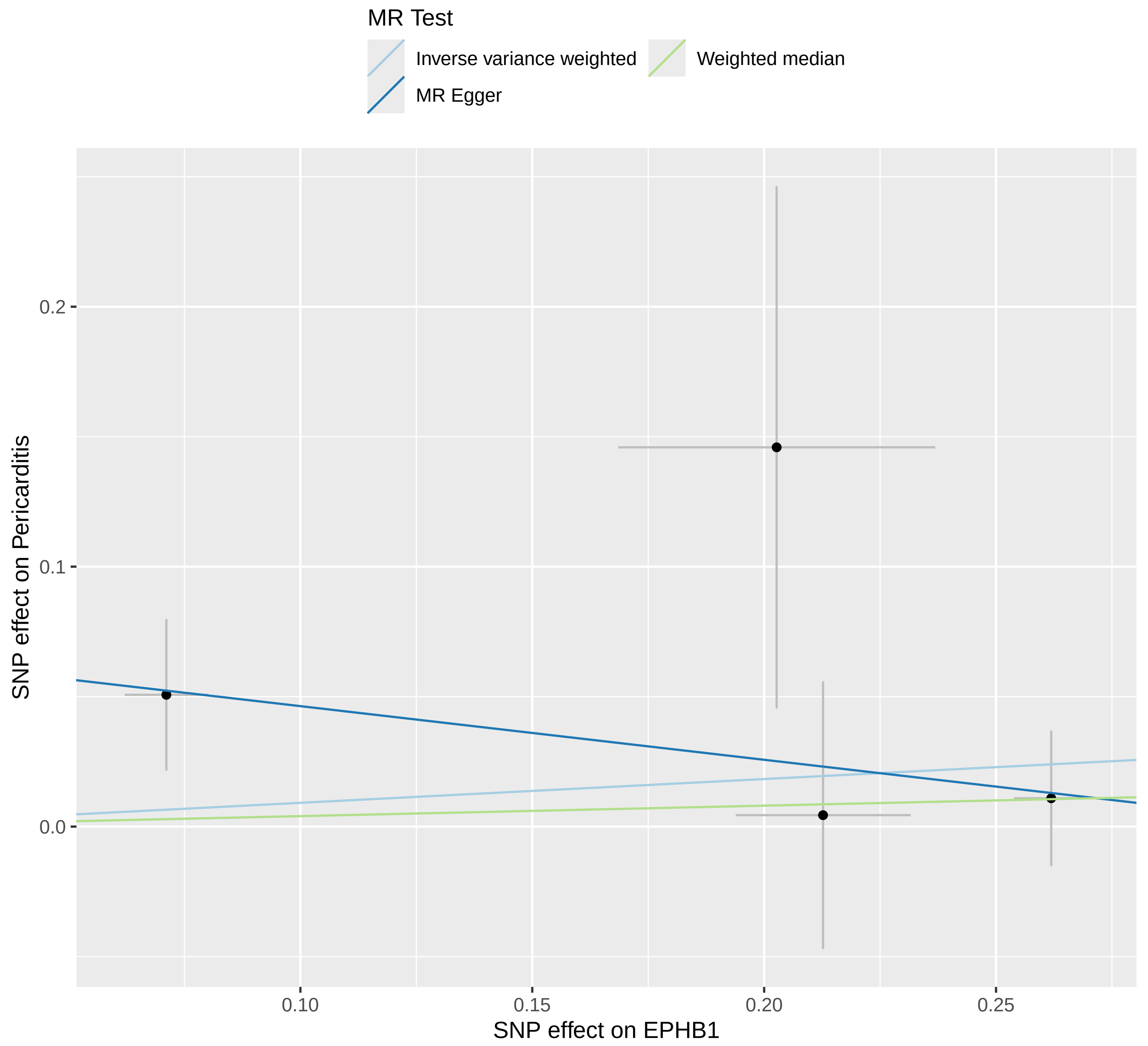

MR Test
Inverse variance weighted
Weighted median
MR Egger
0.2
0.1
0.0
0.10
0.15
0.20
0.25
SNP effect on Pericarditis
SNP effect on EPHB1

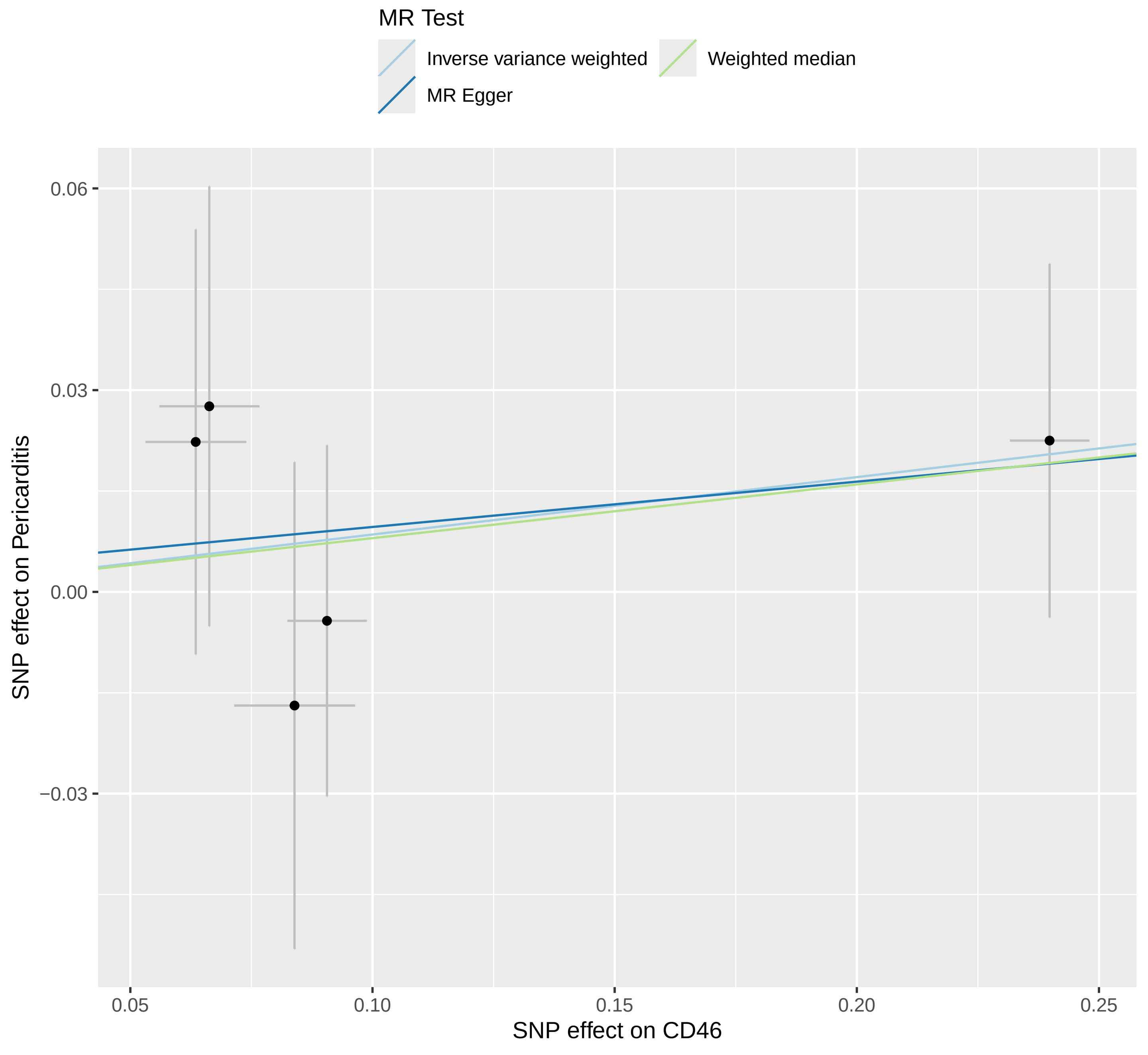

MR Test
Inverse variance weighted
Weighted median
MR Egger
0.06
0.03
0.00
−0.03
SNP effect on Pericarditis
0.05
0.10
0.15
0.20
0.25
SNP effect on CD46

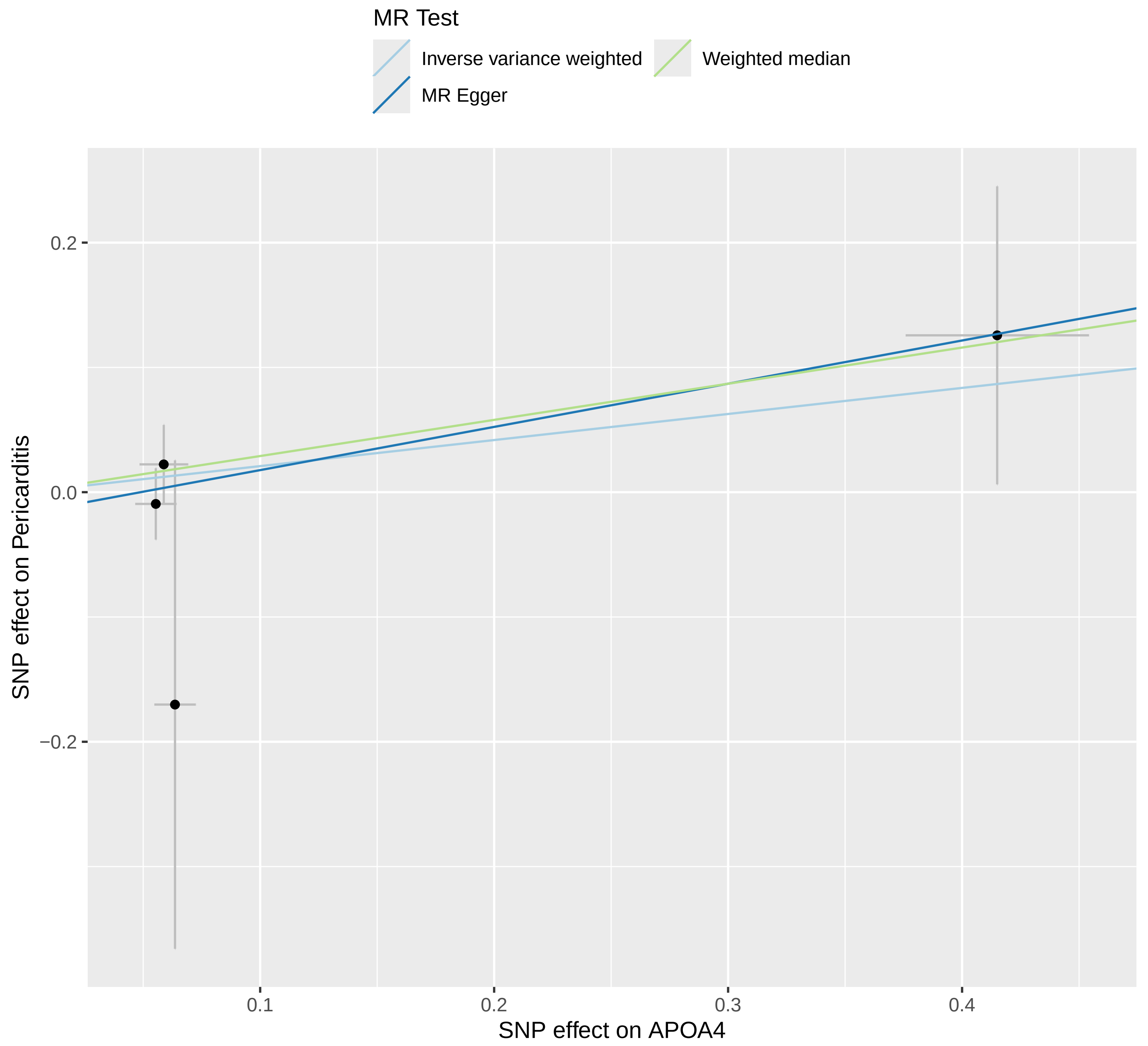
MR Test
Inverse variance weighted
Weighted median
MR Egger
0.2
0.0
−0.2
0.1
0.2
0.3
0.4
SNP effect on Pericarditis
SNP effect on APOA4

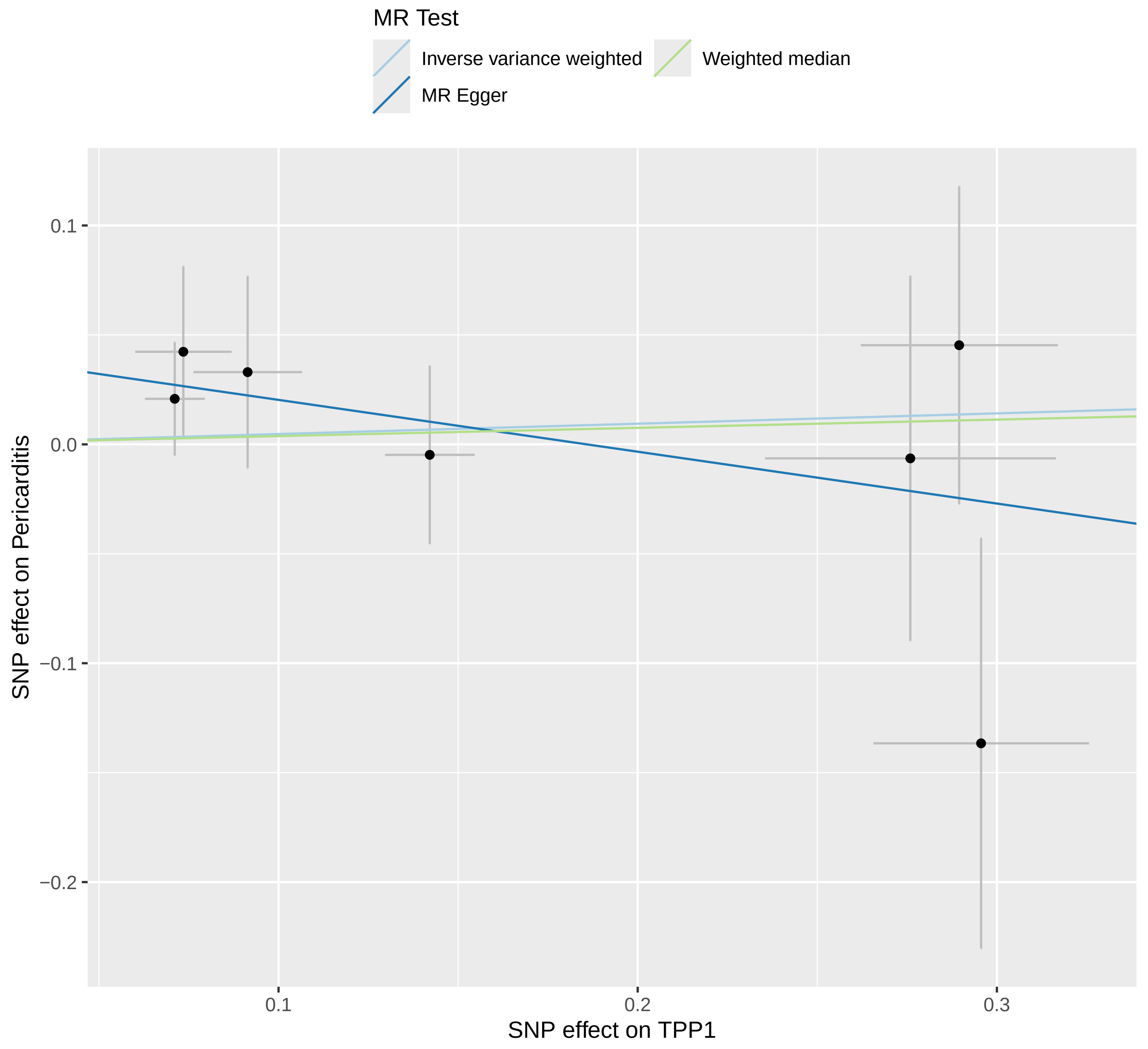
MR Test
Inverse variance weighted
Weighted median
MR Egger
0.1
0.0
−0.1
−0.2
SNP effect on Pericarditis
0.1
0.2
0.3
SNP effect on TPP1

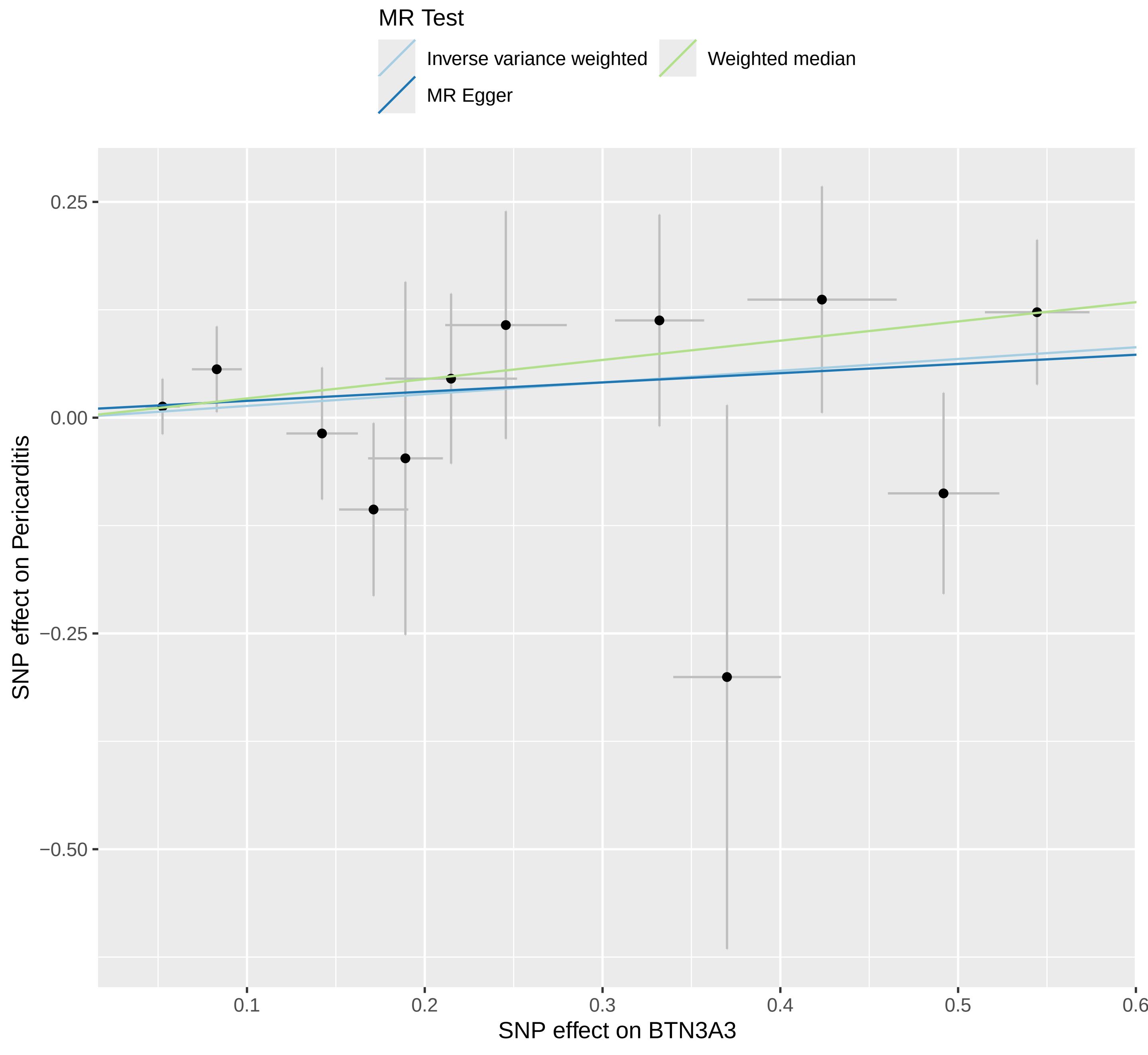
MR Test
Inverse variance weighted
Weighted median
MR Egger
SNP effect on Pericarditis
0.25
0.00
−0.25
−0.50
0.1
0.2
0.3
0.4
0.5
0.6
SNP effect on BTN3A3

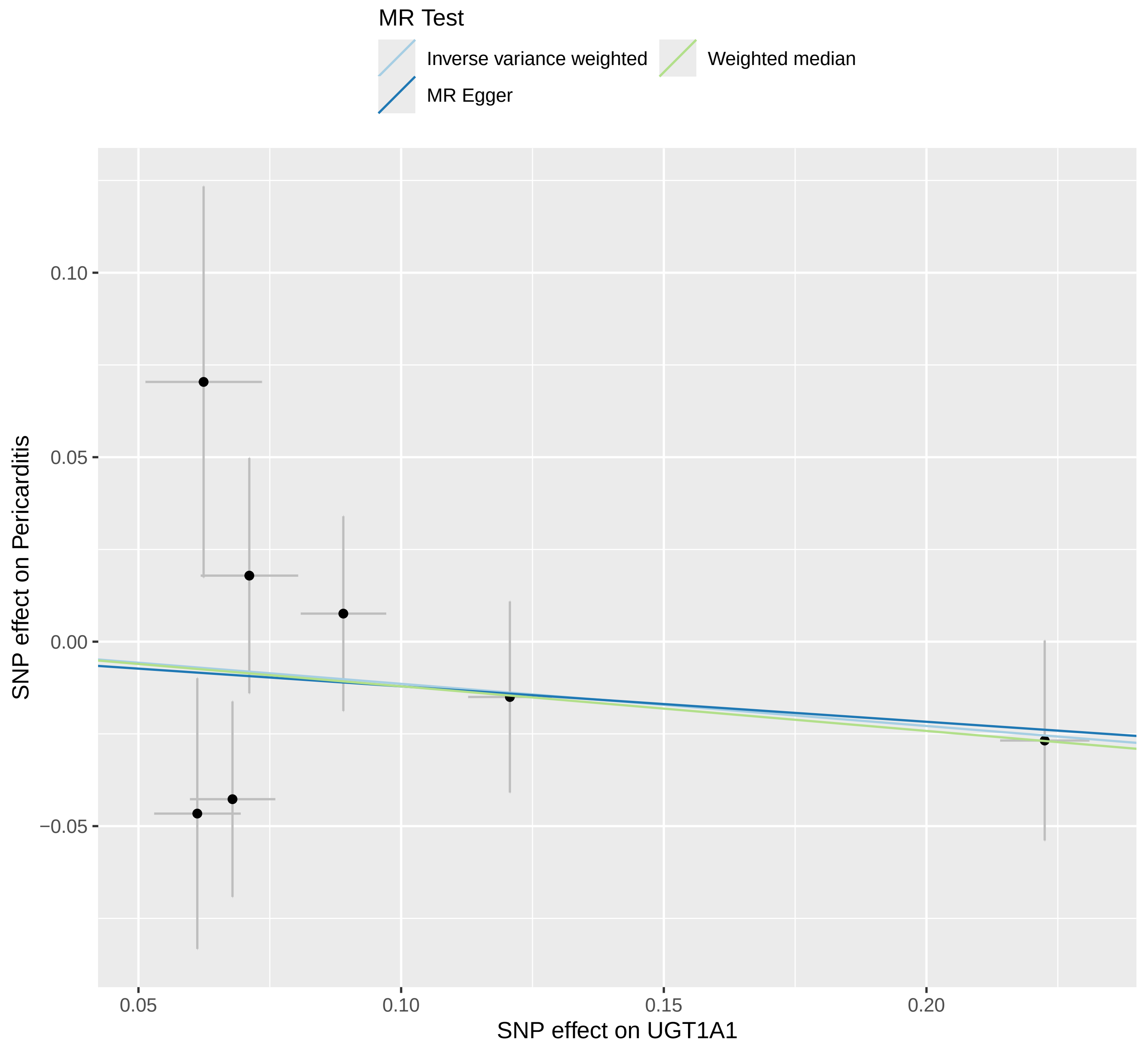
MR Test
Inverse variance weighted
Weighted median
MR Egger
0.10
0.05
0.00
−0.05
SNP effect on Pericarditis
0.05
0.10
0.15
0.20
SNP effect on UGT1A1

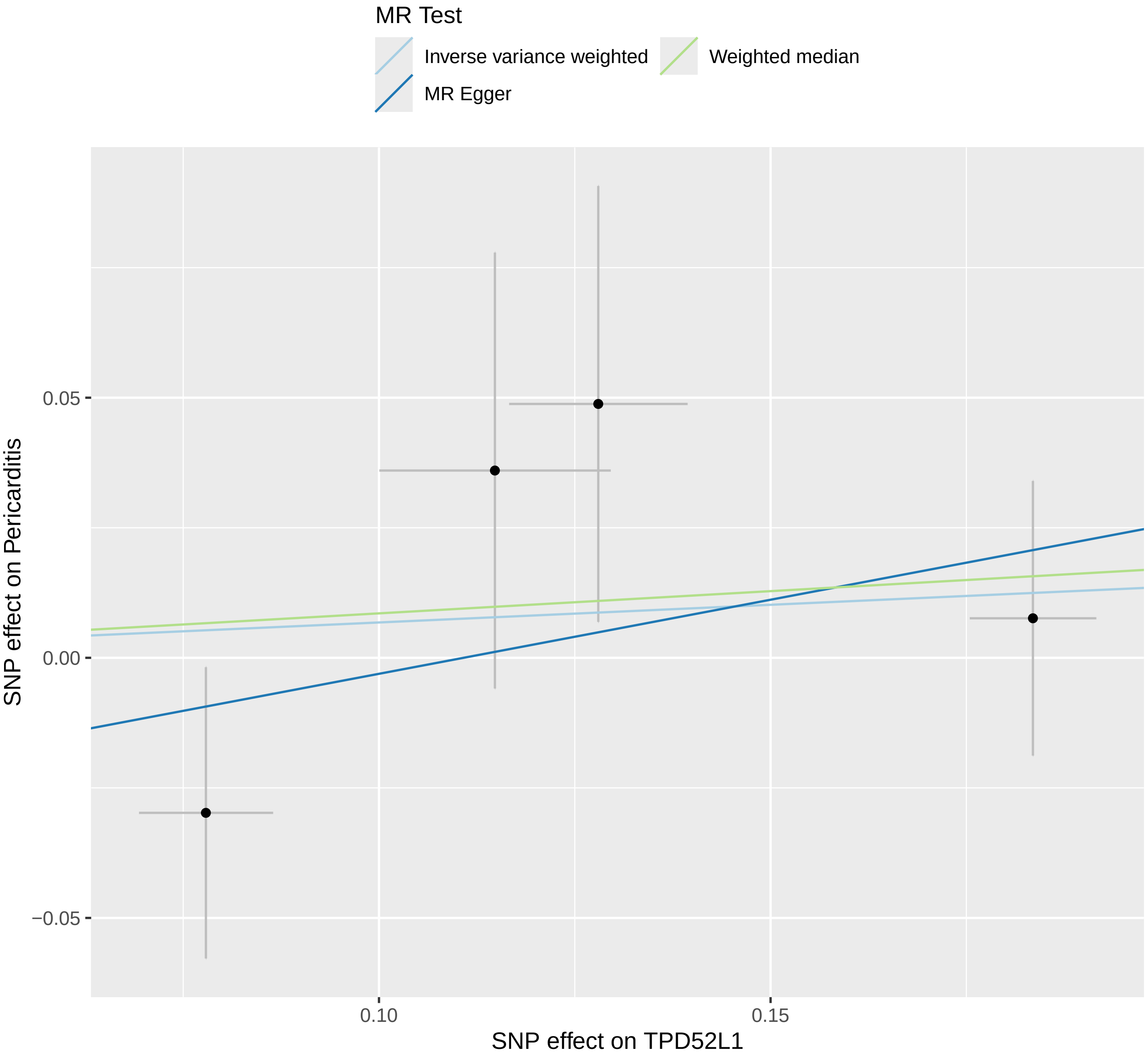

MR Test
Inverse variance weighted
MR Egger
Weighted median
0.05
0.00
−0.05
SNP effect on Pericarditis
0.10
0.15
SNP effect on TPD52L1

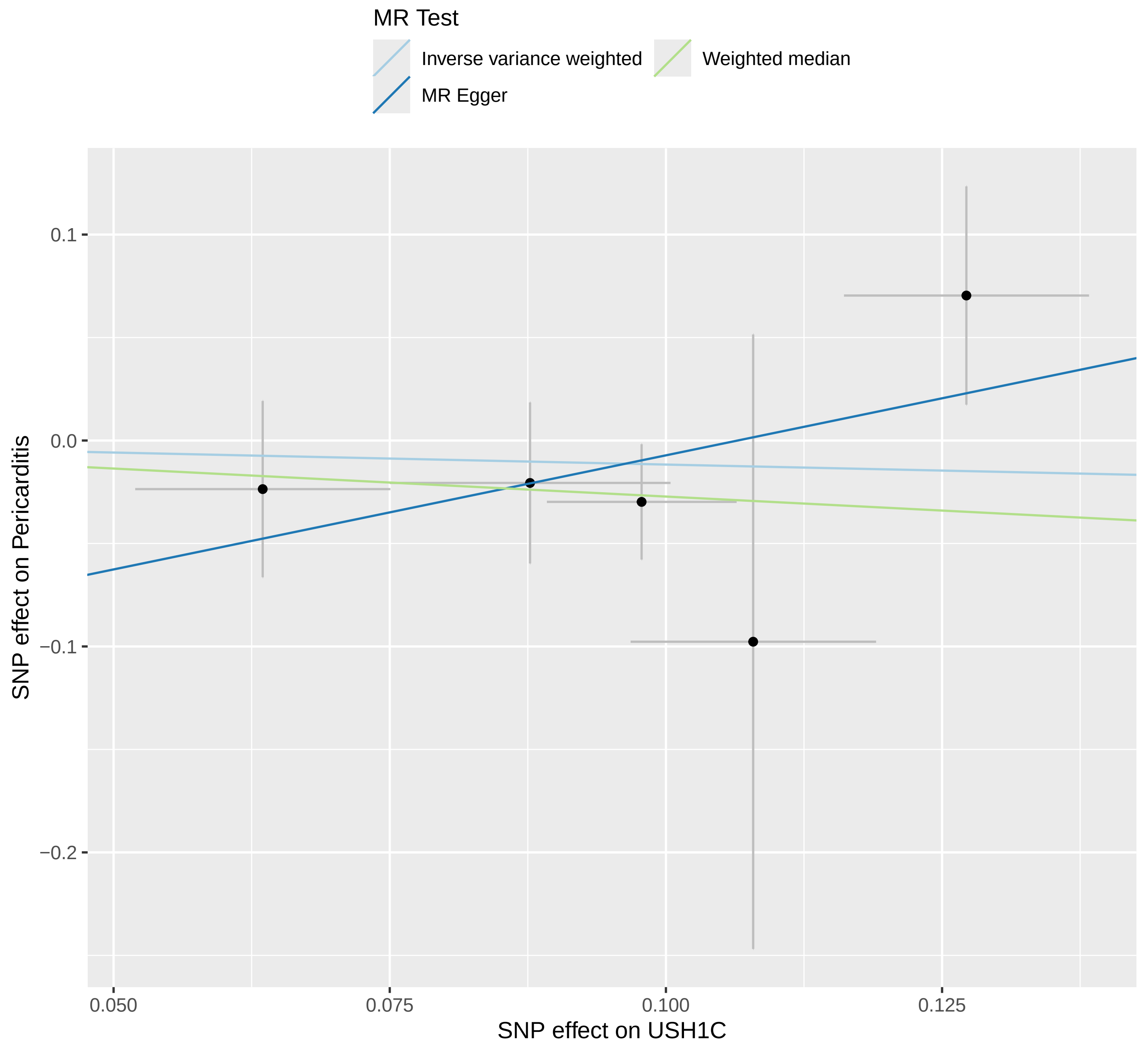
MR Test
Inverse variance weighted
Weighted median
MR Egger
0.1
0.0
−0.1
−0.2
SNP effect on Pericarditis
0.050
0.075
0.100
0.125
SNP effect on USH1C

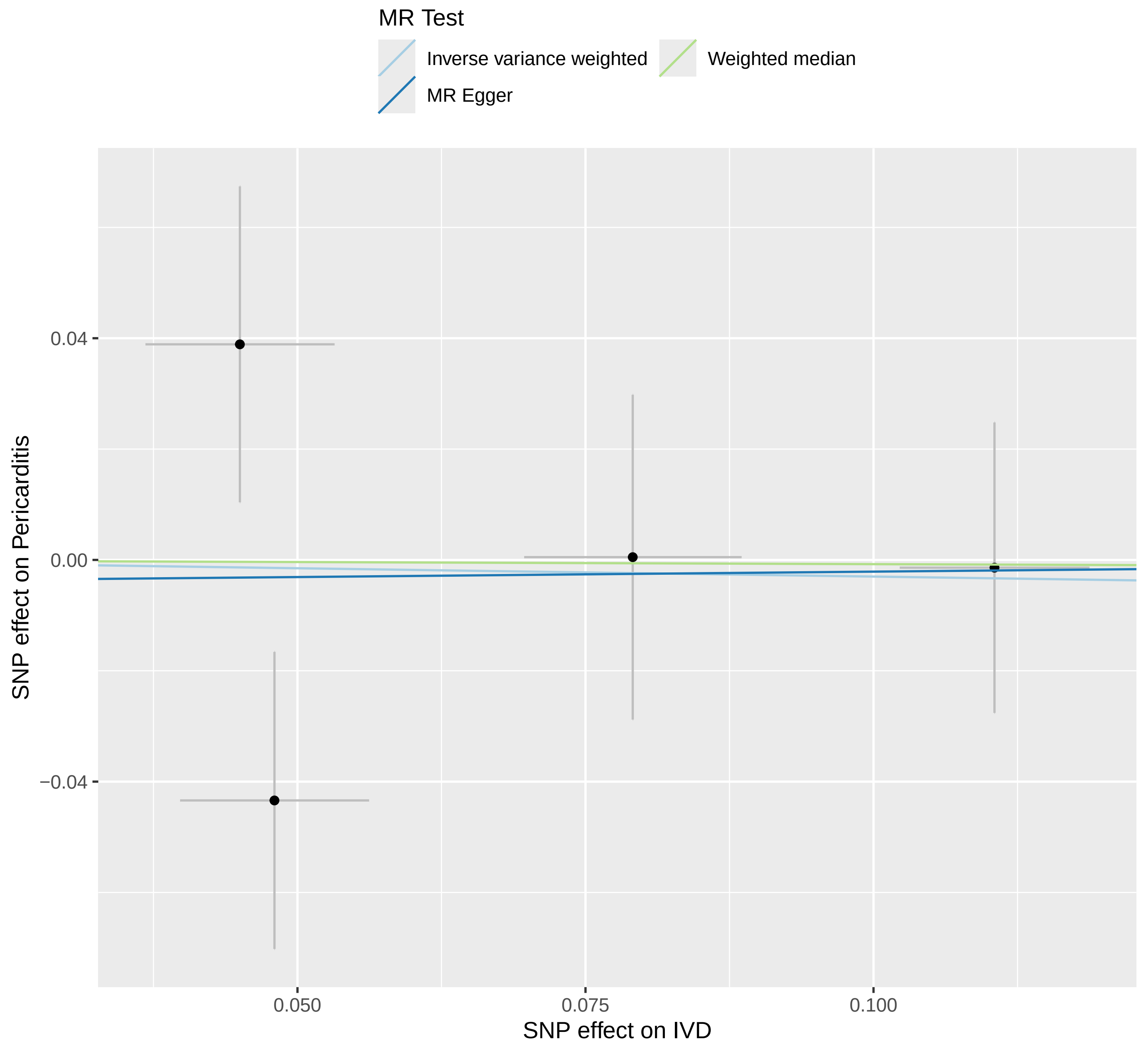

MR Test
Inverse variance weighted
MR Egger
Weighted median
SNP effect on Pericarditis
0.04
0.00
−0.04
0.050
0.075
0.100
SNP effect on IVD

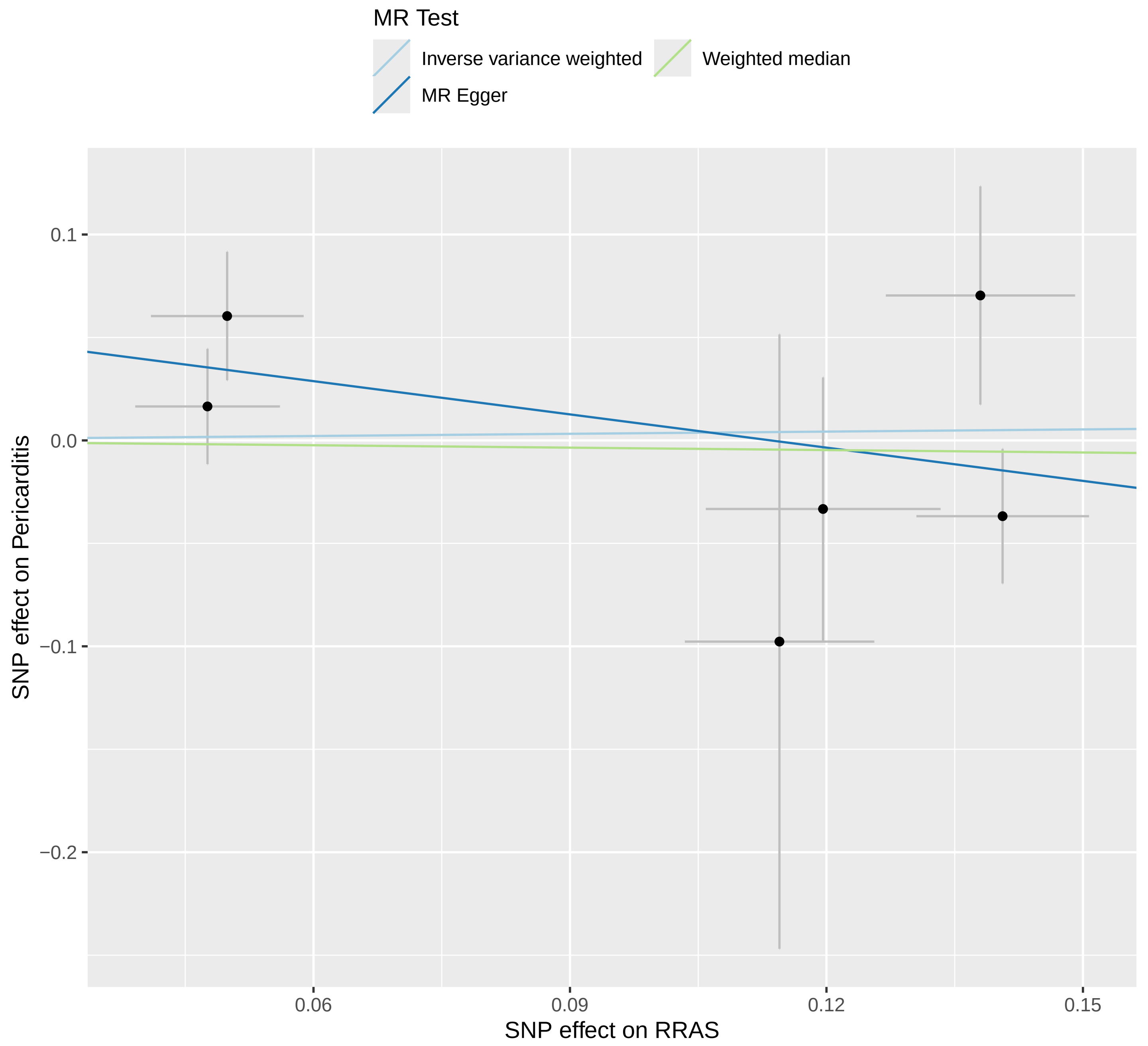
MR Test
Inverse variance weighted
Weighted median
MR Egger
0.1
0.0
−0.1
−0.2
SNP effect on Pericarditis
0.06
0.09
0.12
0.15
SNP effect on RRAS

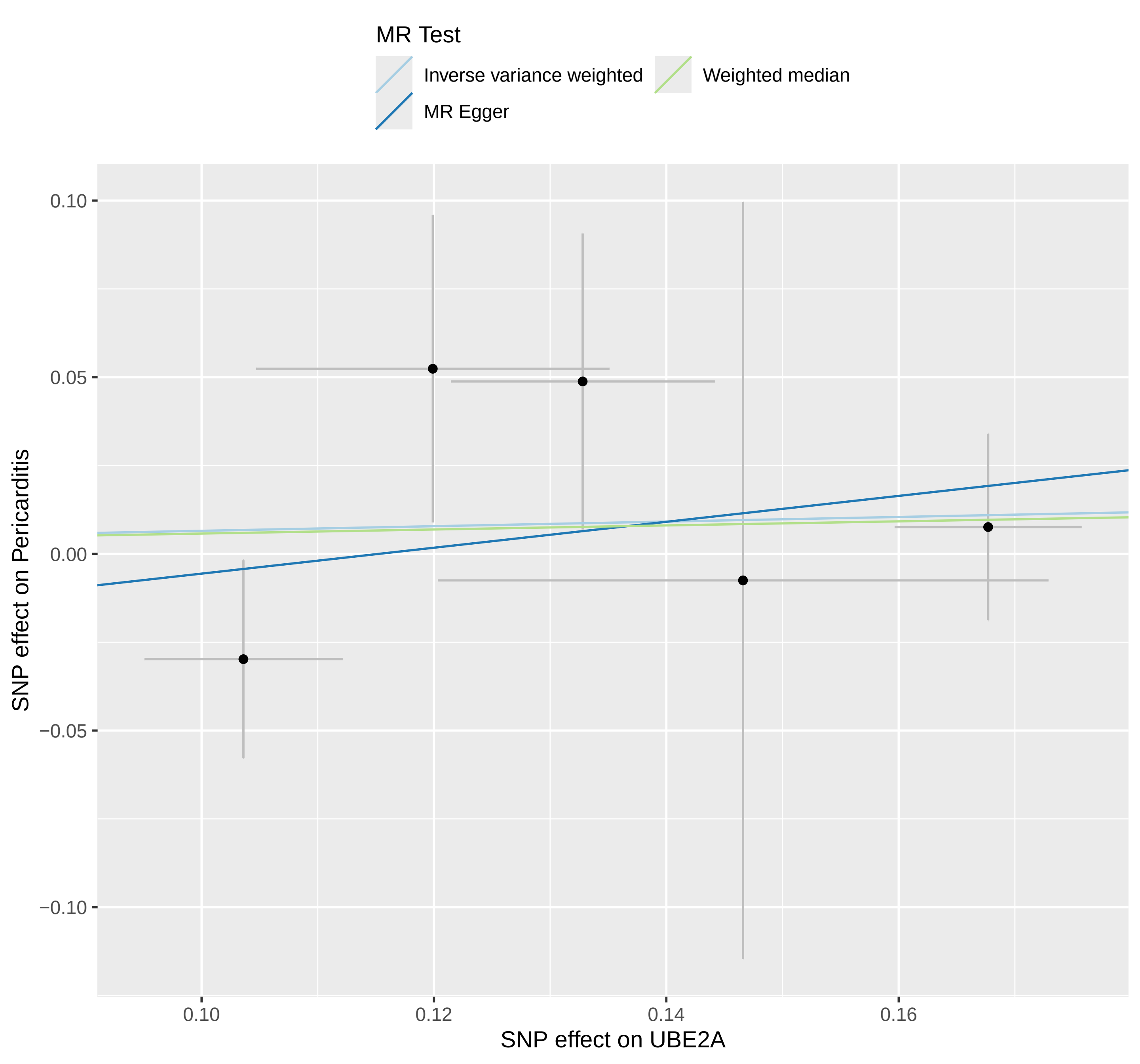

MR Test
Inverse variance weighted
MR Egger
Weighted median
SNP effect on Pericarditis
0.10
0.05
0.00
−0.05
−0.10
0.10
0.12
0.14
0.16
SNP effect on UBE2A

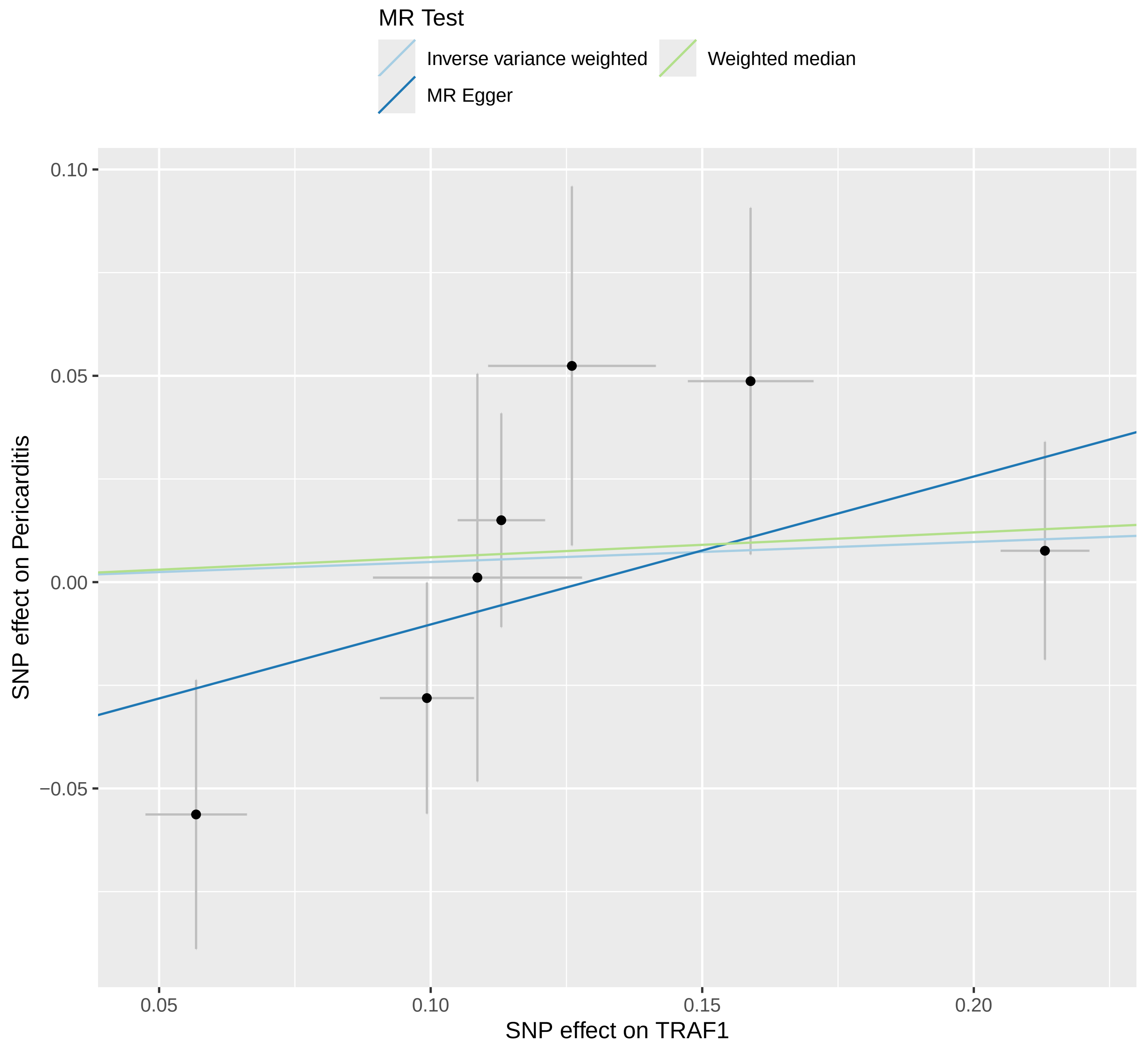

MR Test
Inverse variance weighted
Weighted median
MR Egger
0.10
0.05
0.00
−0.05
SNP effect on Pericarditis
0.05
0.10
0.15
0.20
SNP effect on TRAF1

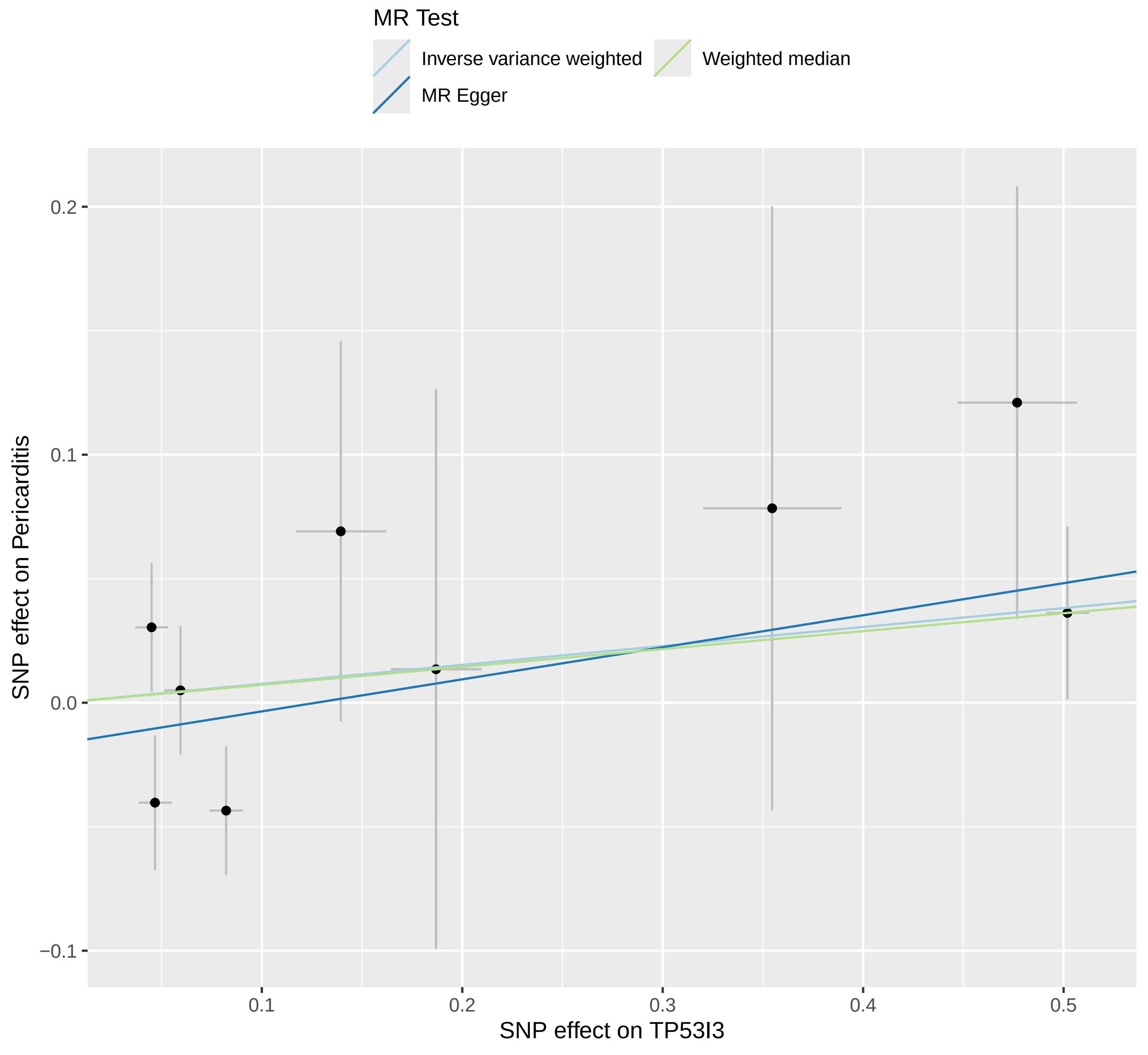

MR Test
Inverse variance weighted
Weighted median
MR Egger
SNP effect on Pericarditis
0.2
0.1
0.0
−0.1
0.1
0.2
0.3
0.4
0.5
SNP effect on TP53I3

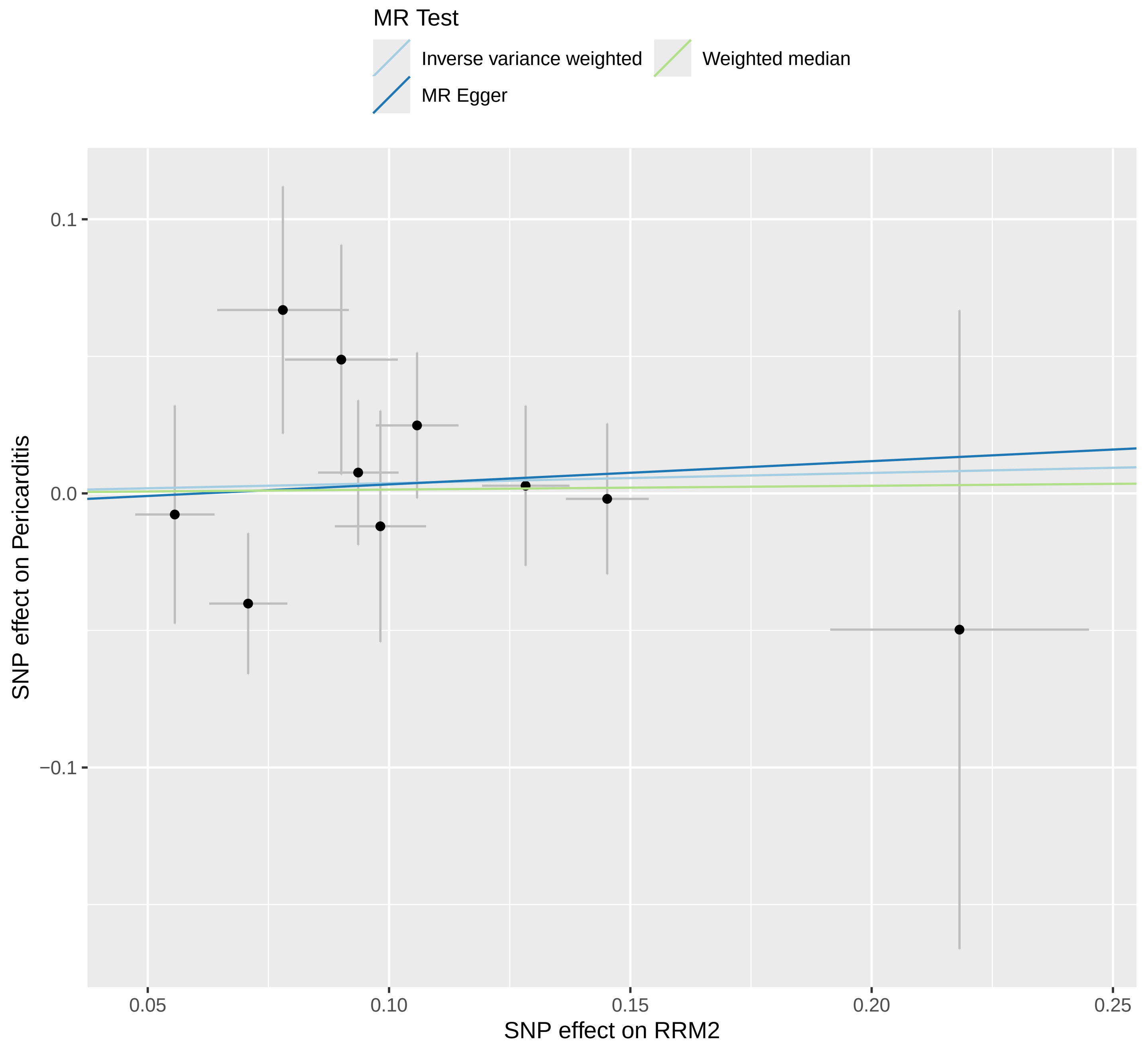
MR Test
Inverse variance weighted
Weighted median
MR Egger
0.1
0.0
−0.1
SNP effect on Pericarditis
0.05
0.10
0.15
0.20
0.25
SNP effect on RRM2

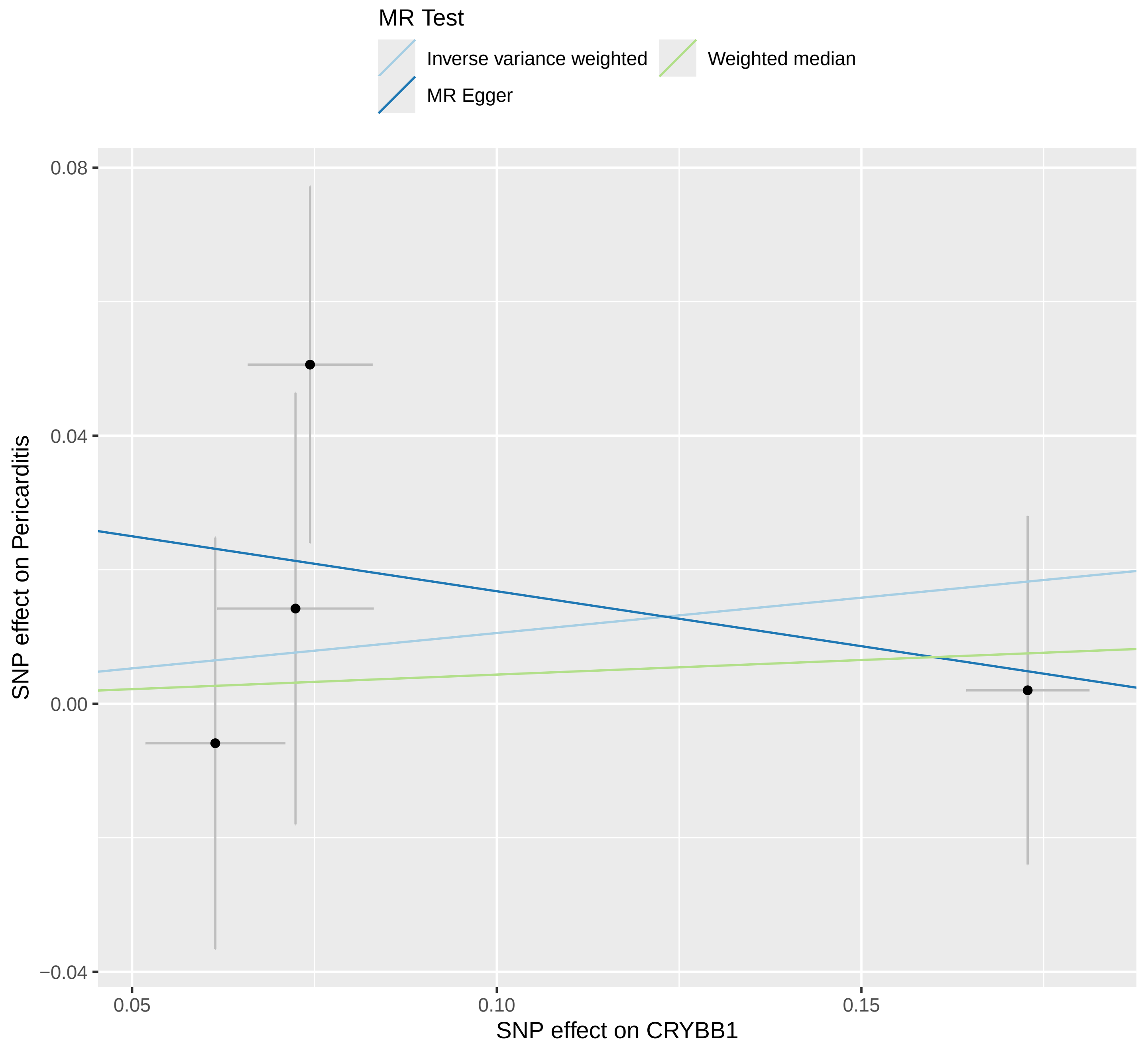

MR Test
Inverse variance weighted
MR Egger
Weighted median
0.08
0.04
0.00
−0.04
SNP effect on Pericarditis
0.05
0.10
0.15
SNP effect on CRYBB1

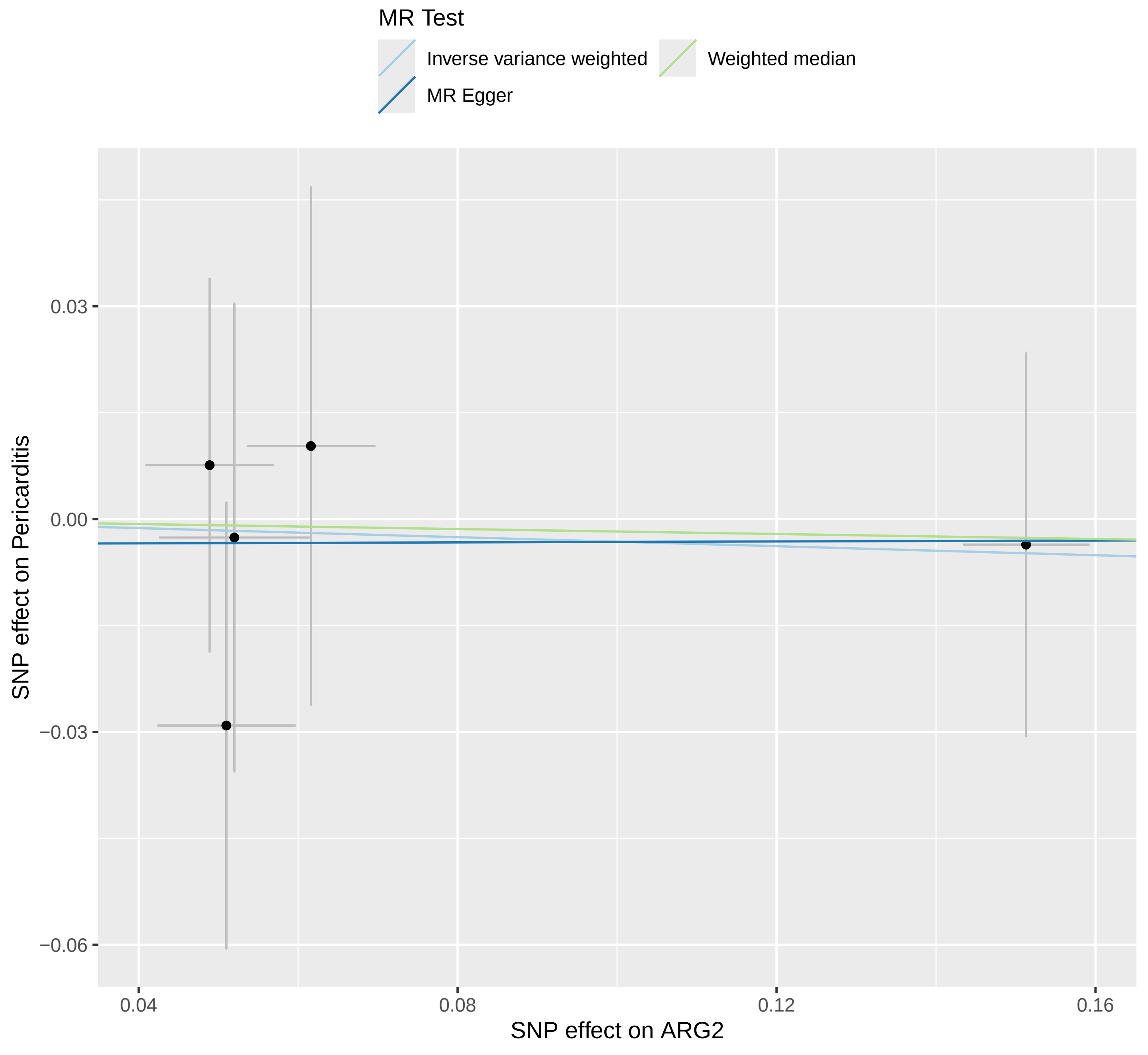
MR Test
Inverse variance weighted
Weighted median
MR Egger
SNP effect on Pericarditis
0.03
0.00
−0.03
−0.06
0.04
0.08
0.12
0.16
SNP effect on ARG2

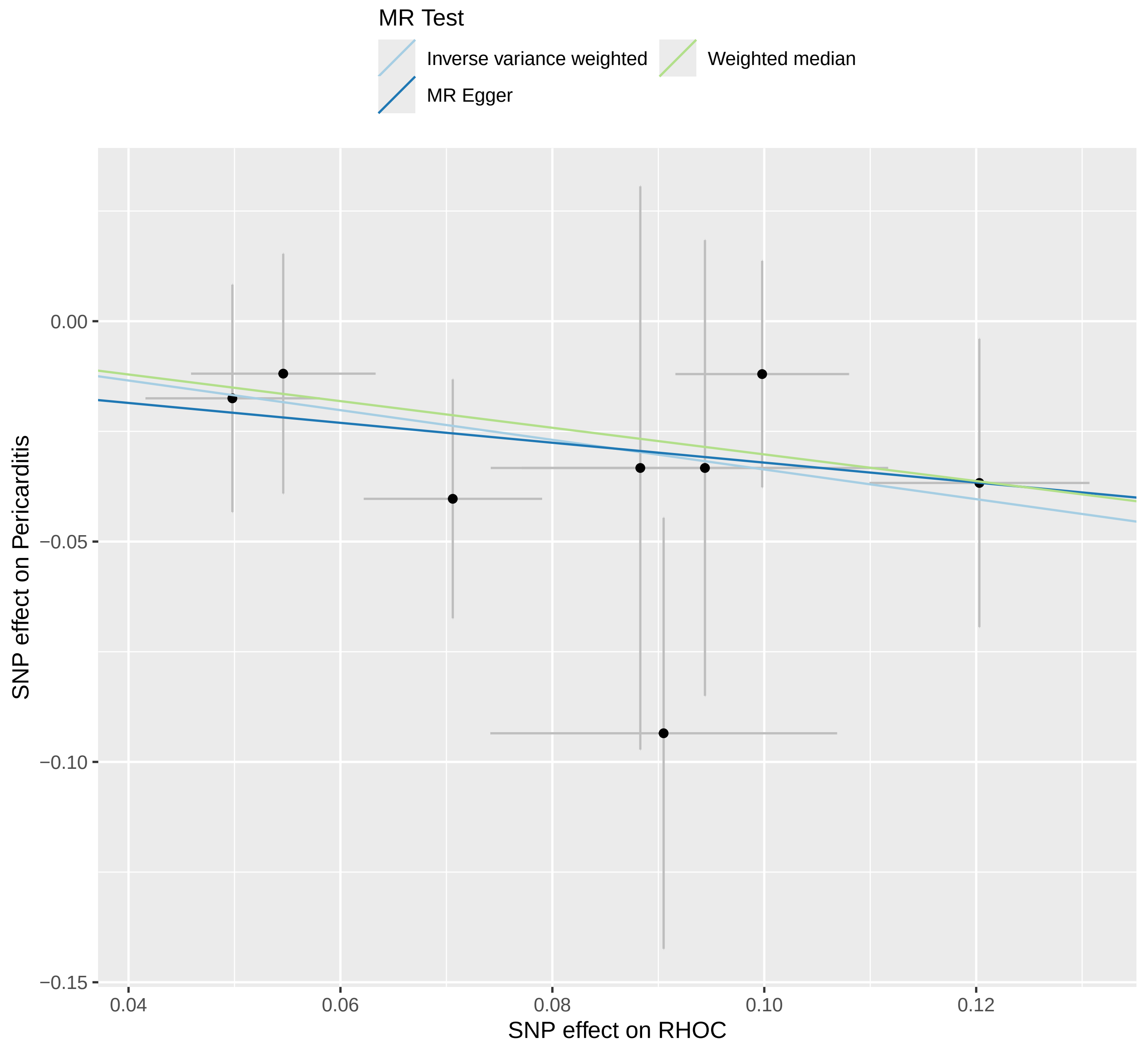

MR Test
Inverse variance weighted
Weighted median
MR Egger
0.00
−0.05
−0.10
−0.15
0.04
0.06
0.08
0.10
0.12
SNP effect on Pericarditis
SNP effect on RHOC

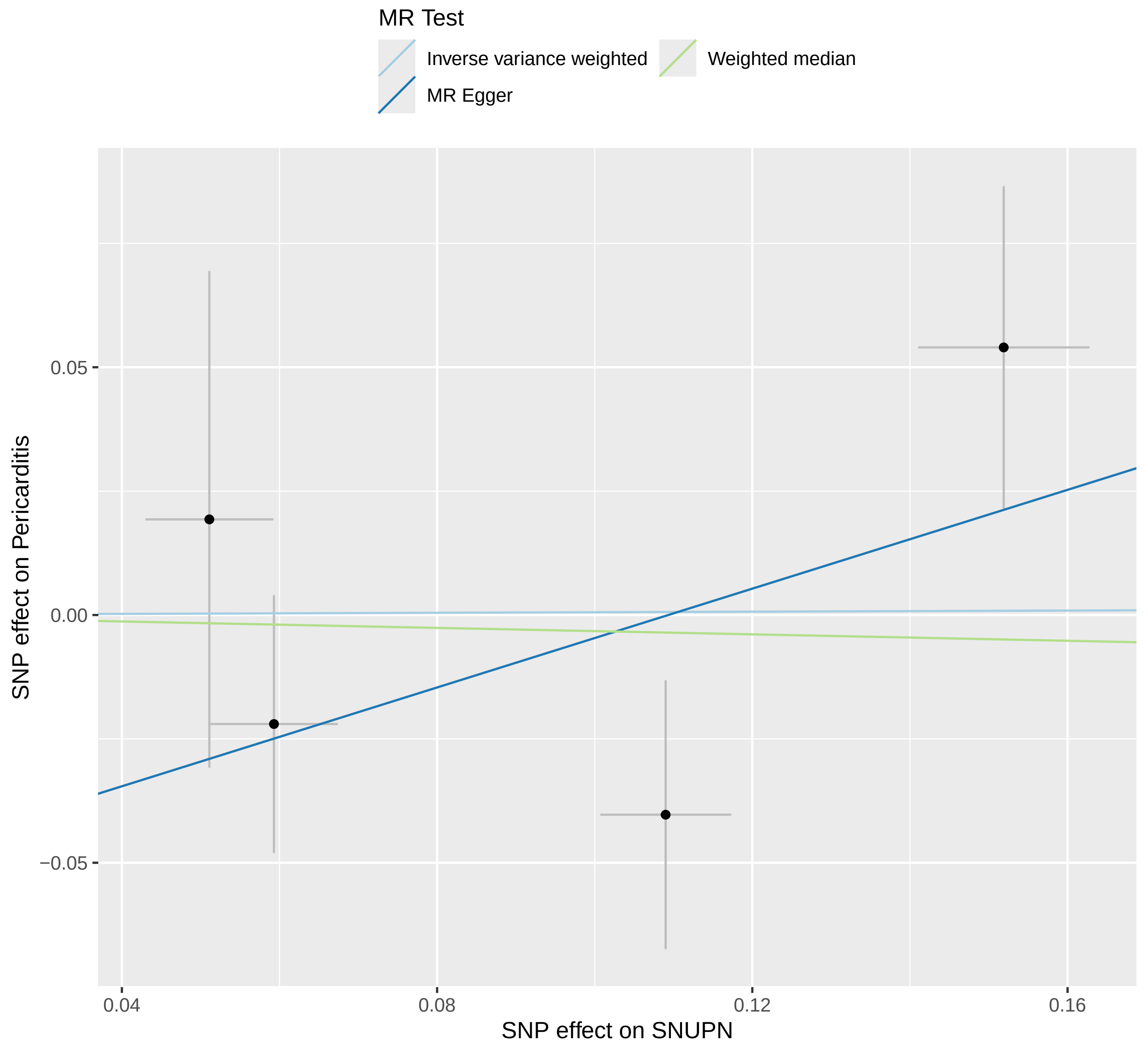

MR Test
Inverse variance weighted
Weighted median
MR Egger
0.05
0.00
−0.05
0.04
0.08
0.12
0.16
SNP effect on Pericarditis
SNP effect on SNUPN

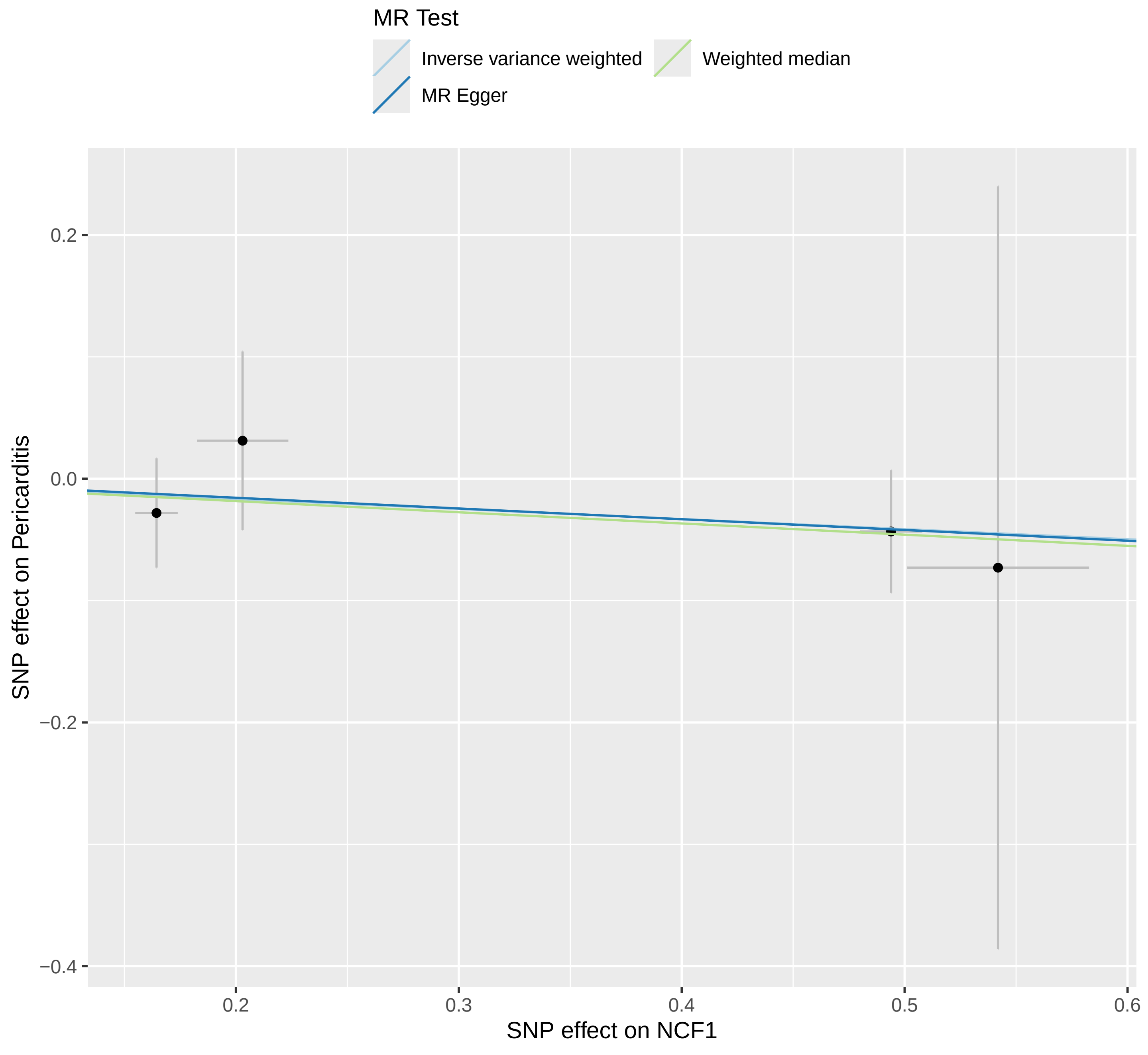
MR Test
Inverse variance weighted
Weighted median
MR Egger
SNP effect on Pericarditis
0.2
0.0
−0.2
−0.4
0.2
0.3
0.4
0.5
0.6
SNP effect on NCF1

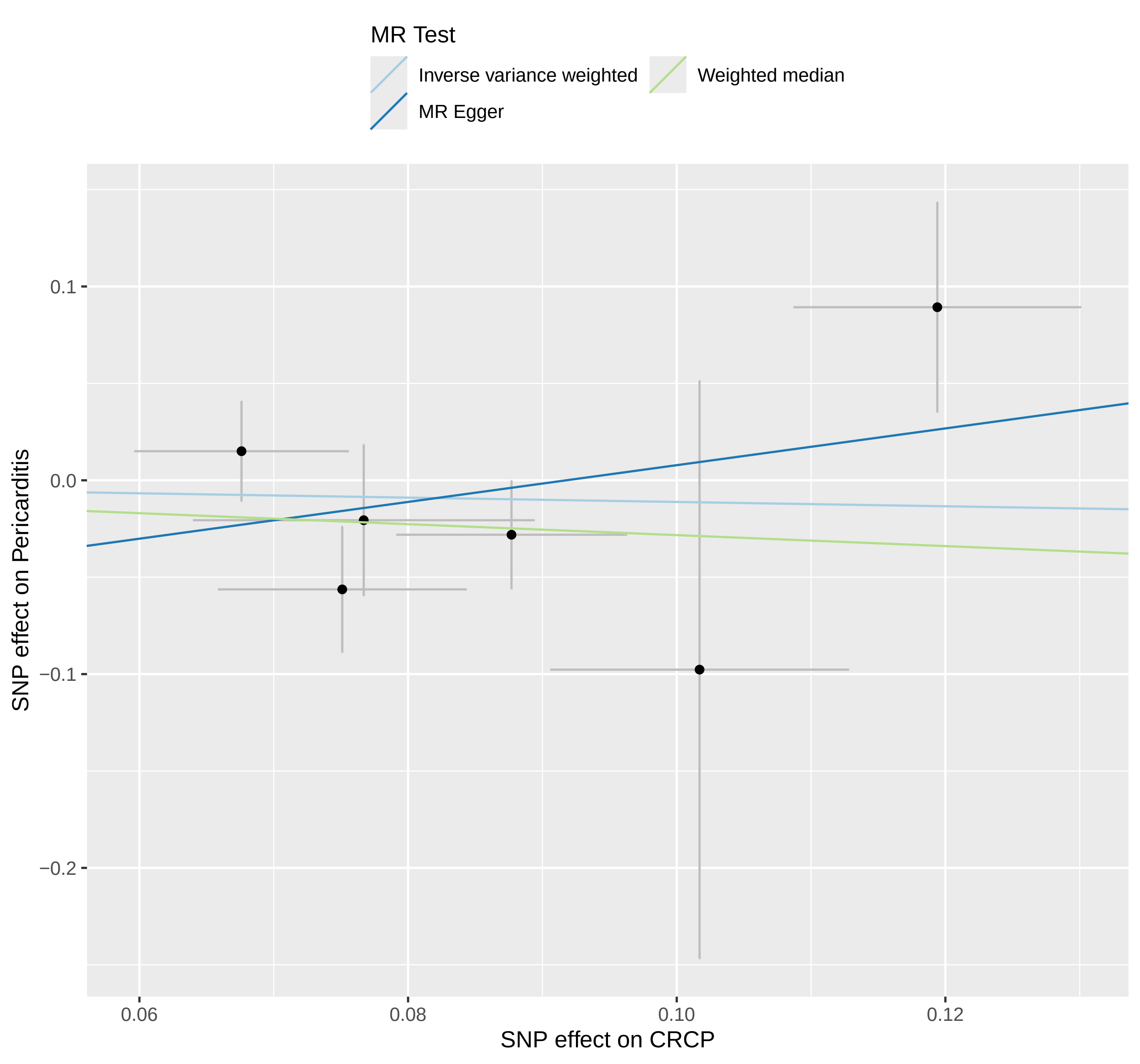
MR Test
Inverse variance weighted
Weighted median
MR Egger
0.1
0.0
−0.1
−0.2
SNP effect on Pericarditis
0.06
0.08
0.10
0.12
SNP effect on CRCP

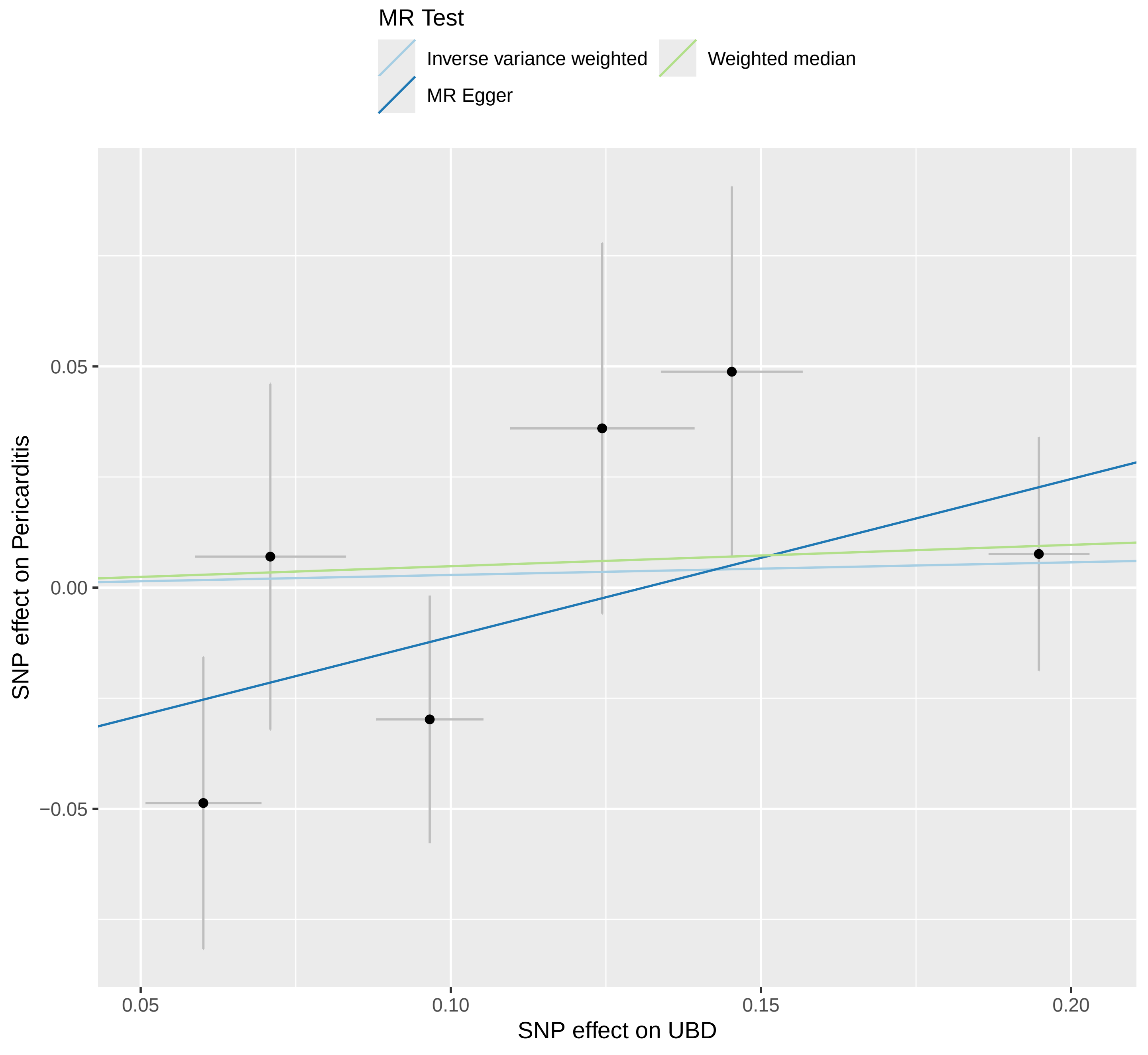
MR Test
Inverse variance weighted
MR Egger
Weighted median
0.05
0.00
−0.05
0.05
0.10
0.15
0.20
SNP effect on UBD
SNP effect on Pericarditis

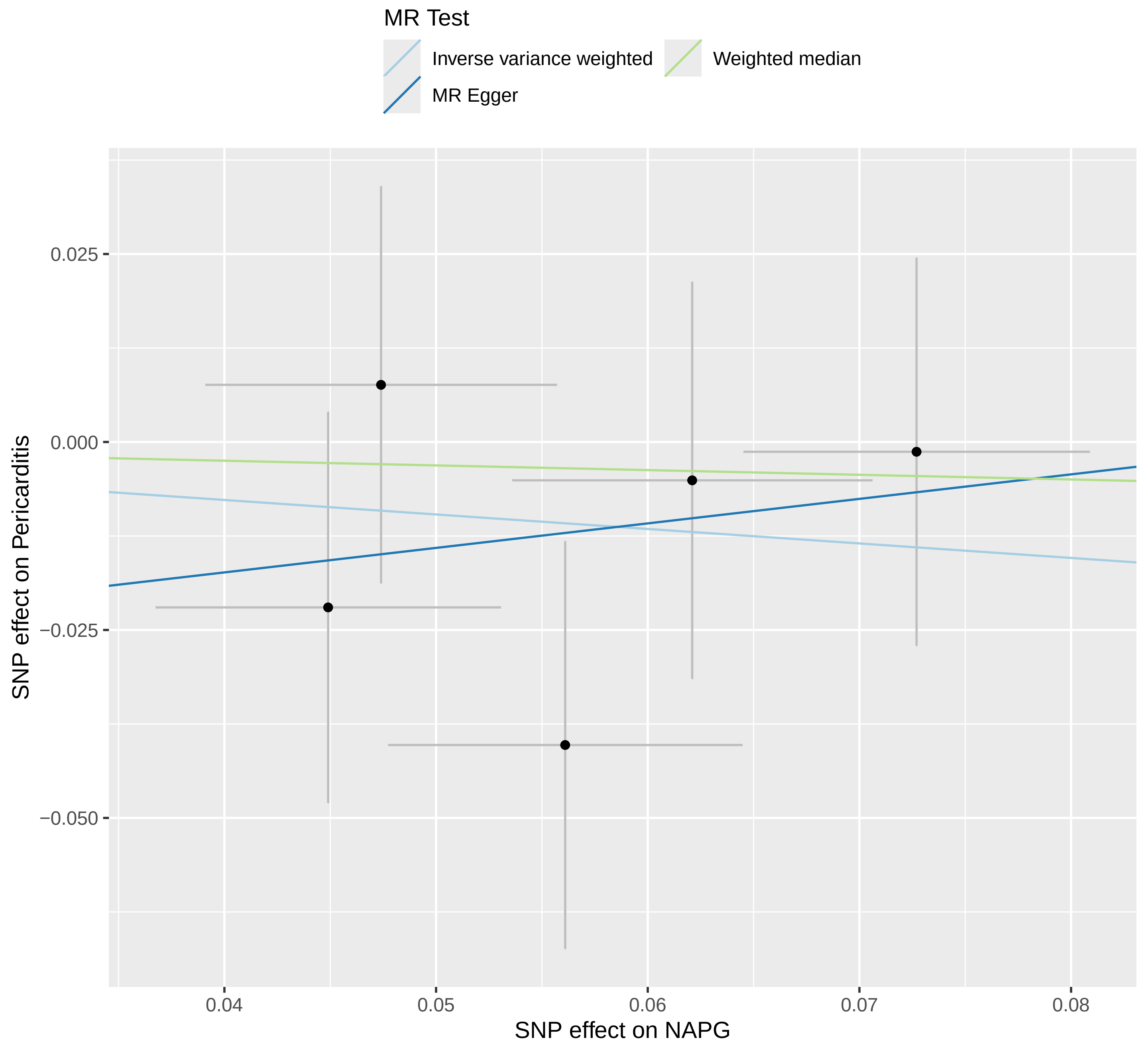

MR Test
Inverse variance weighted
MR Egger
Weighted median
0.025
0.000
−0.025
−0.050
SNP effect on Pericarditis
0.04
0.05
0.06
0.07
0.08
SNP effect on NAPG

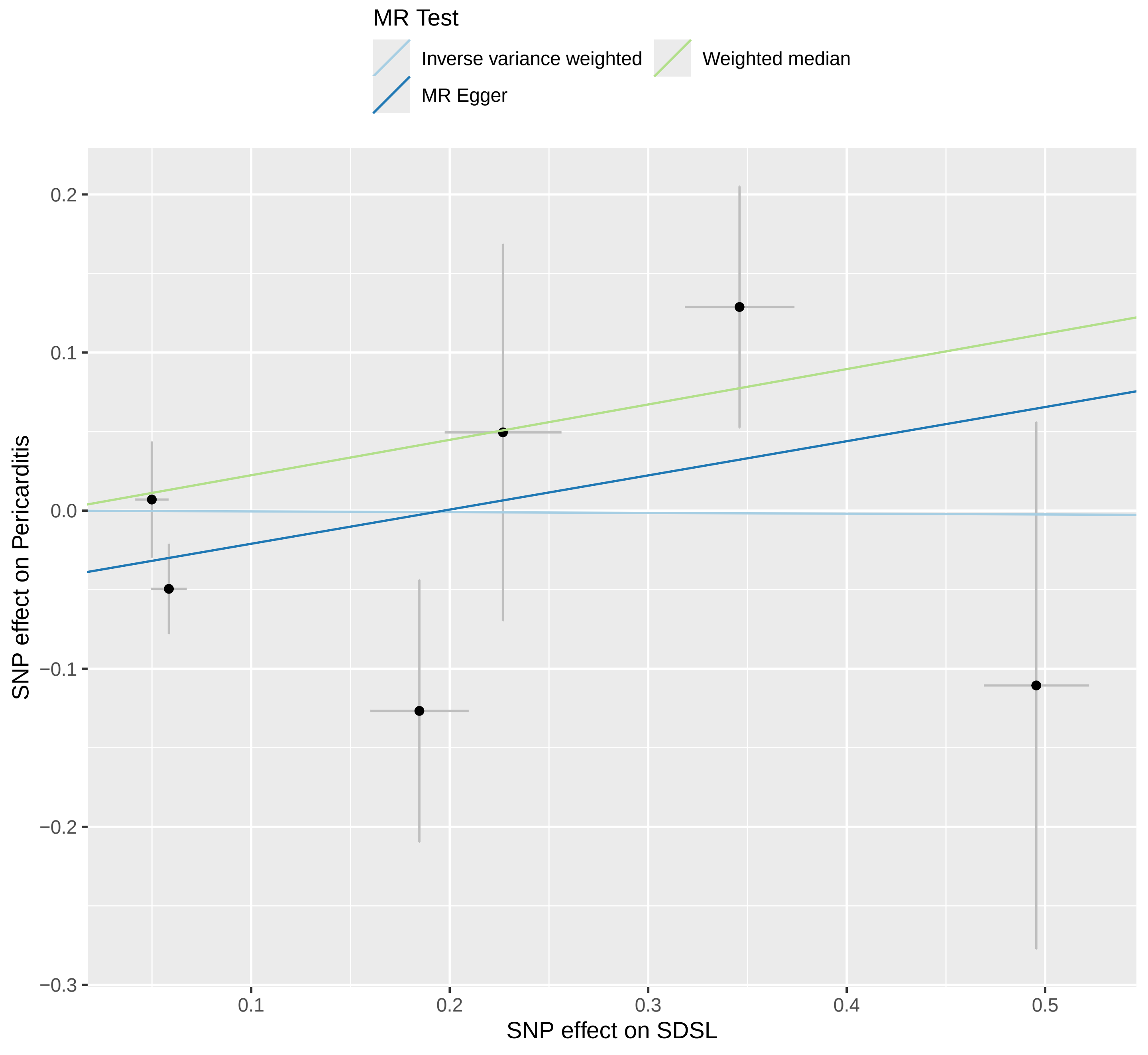
MR Test
Inverse variance weighted
Weighted median
MR Egger
0.2
0.1
0.0
−0.1
−0.2
−0.3
0.1
0.2
0.3
0.4
0.5
SNP effect on Pericarditis
SNP effect on SDSL

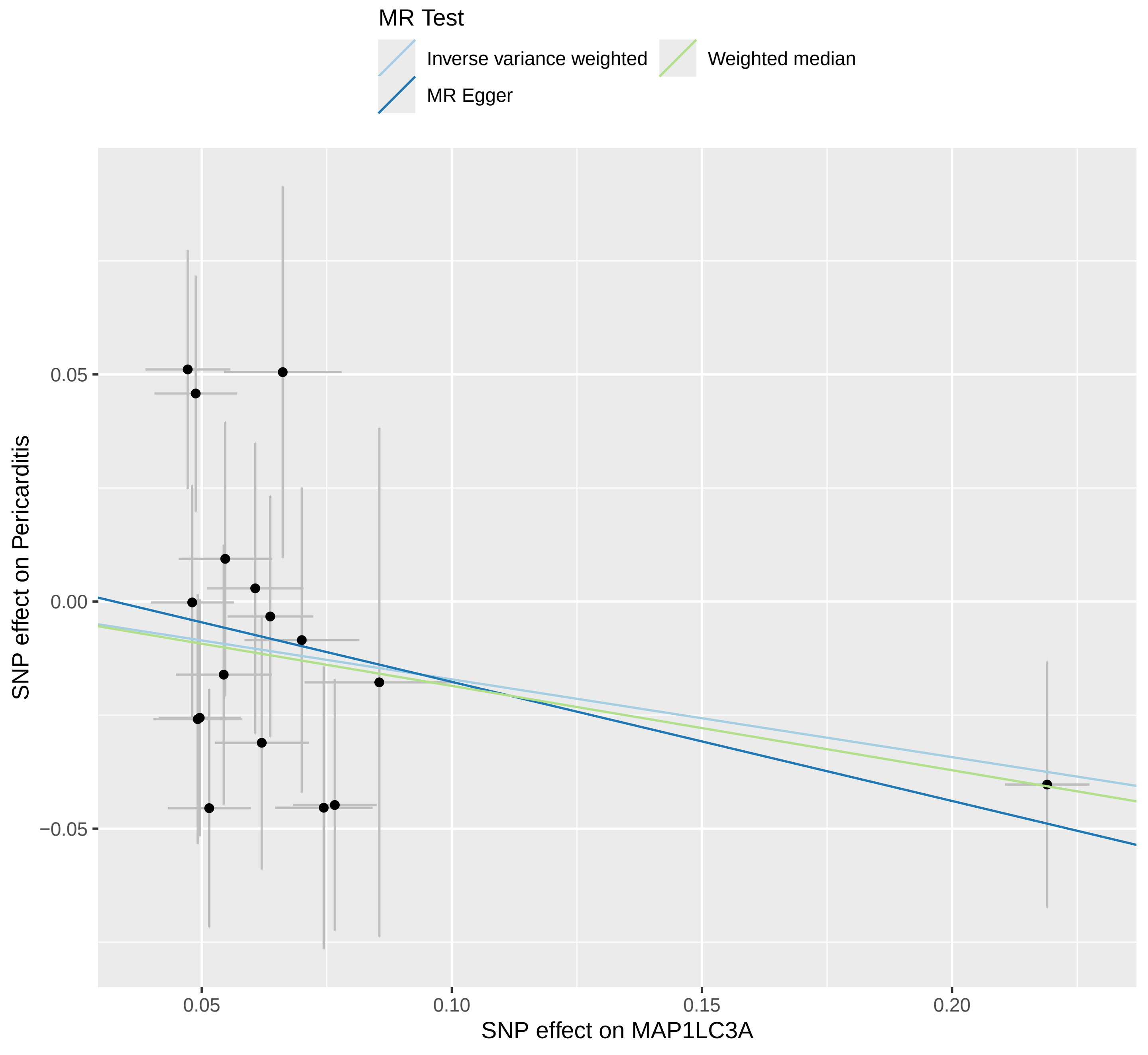
MR Test
Inverse variance weighted
Weighted median
MR Egger
SNP effect on Pericarditis
0.05
0.00
−0.05
0.05
0.10
0.15
0.20
SNP effect on MAP1LC3A

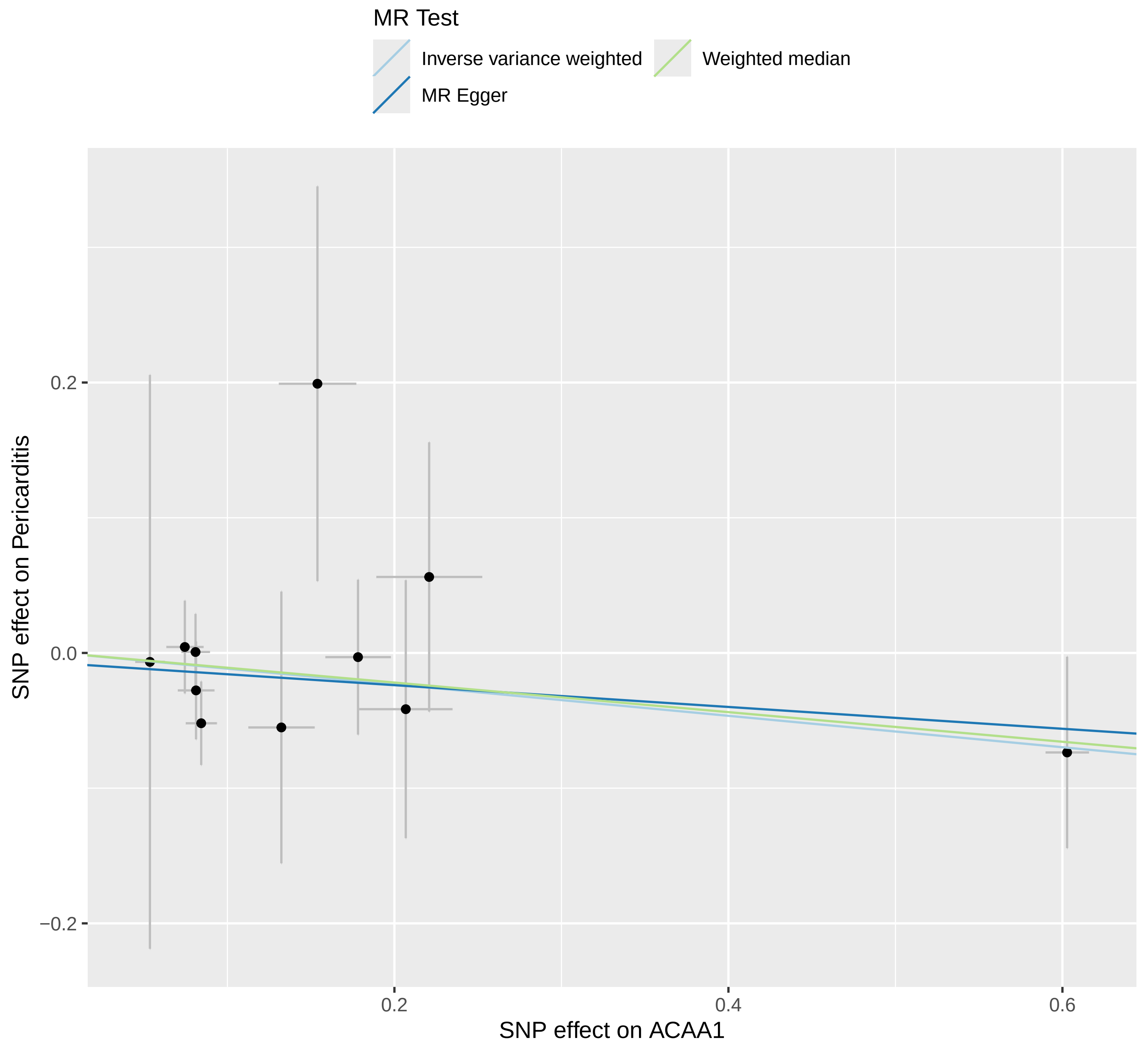

MR Test
Inverse variance weighted
Weighted median
MR Egger
SNP effect on Pericarditis
0.2
0.0
−0.2
SNP effect on ACAA1
0.2
0.4
0.6

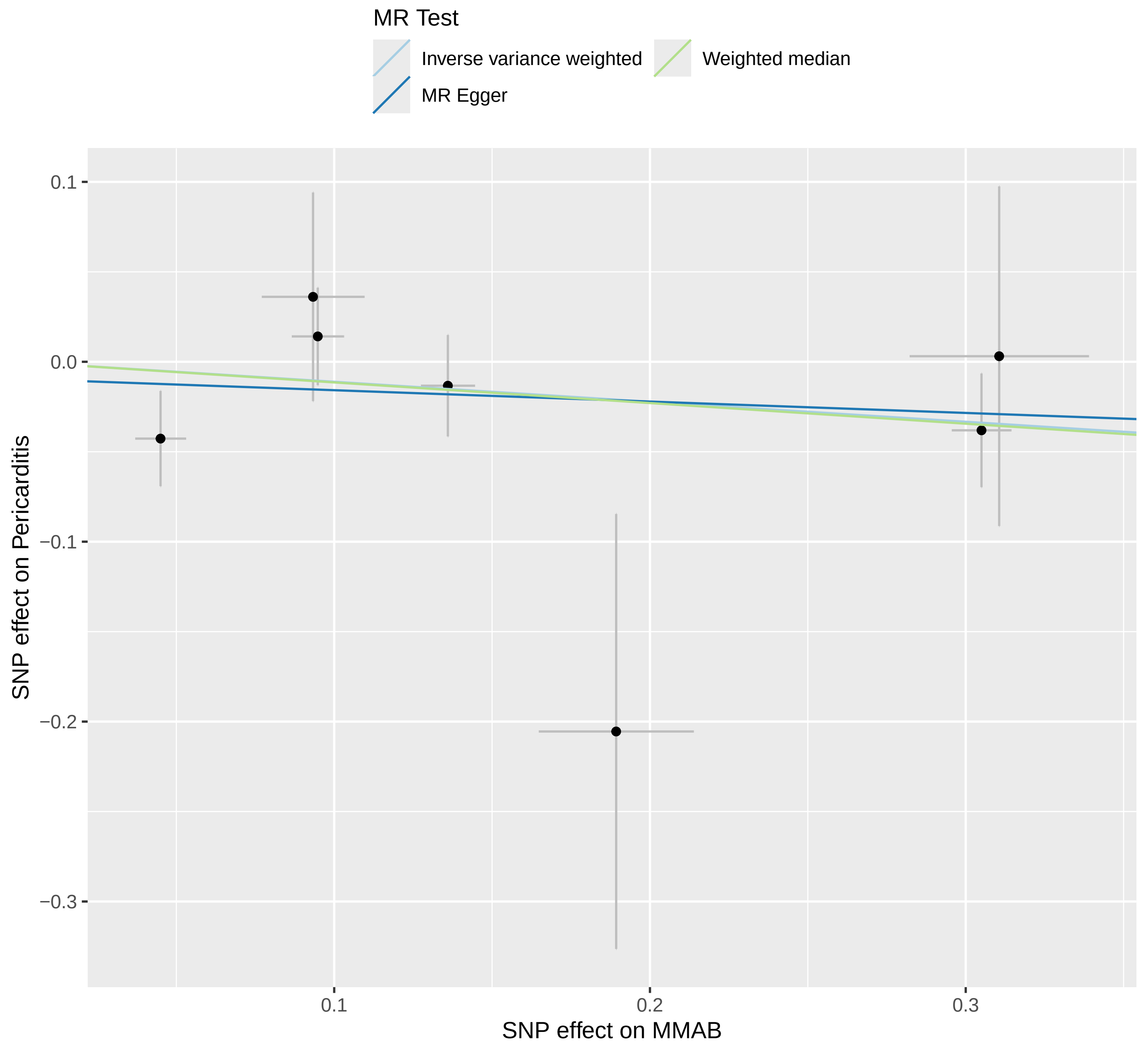

MR Test
Inverse variance weighted
Weighted median
MR Egger
0.1
0.0
−0.1
−0.2
−0.3
SNP effect on Pericarditis
0.1
0.2
0.3
SNP effect on MMAB

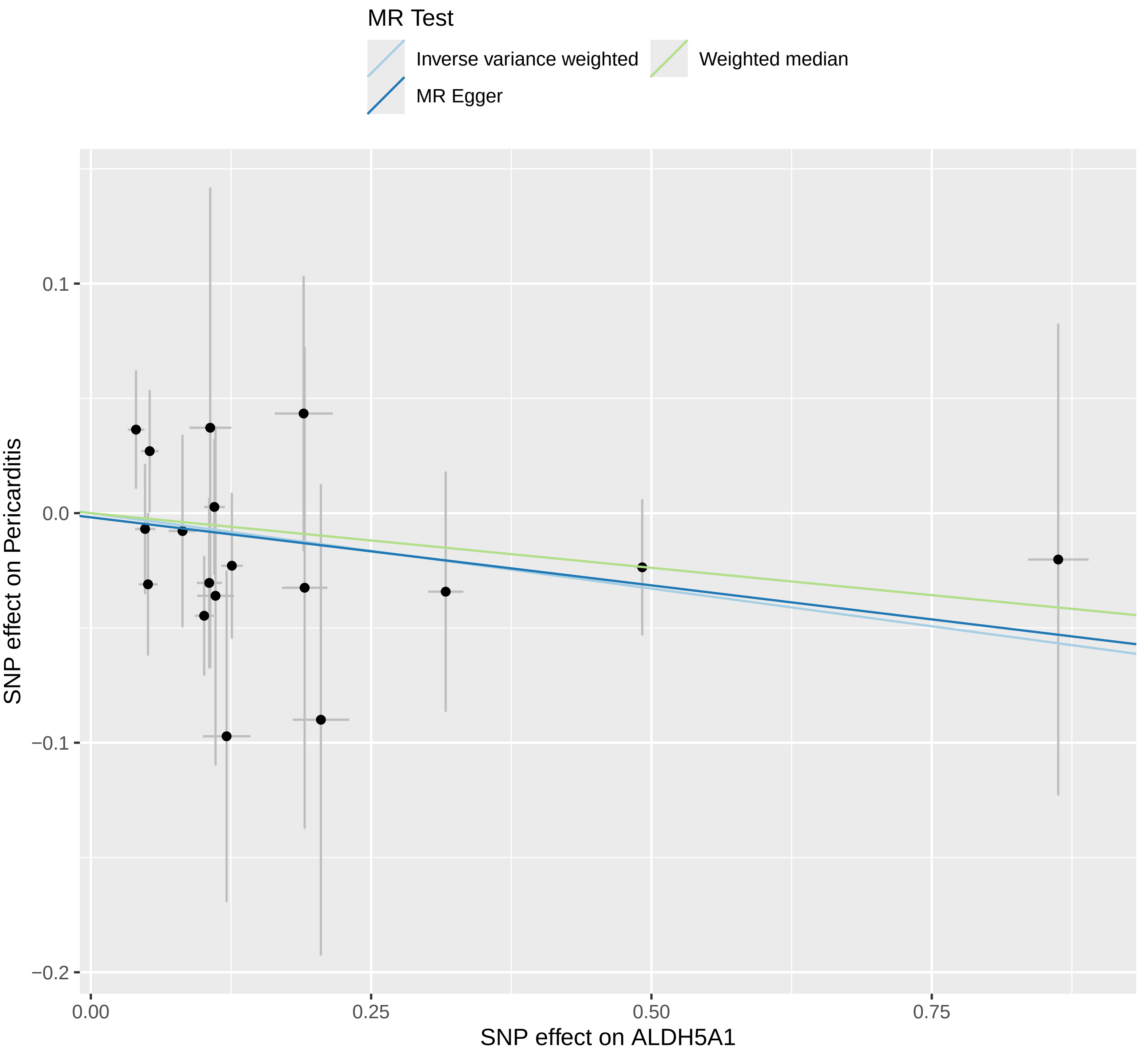
MR Test
Inverse variance weighted
Weighted median
MR Egger
SNP effect on Pericarditis
0.1
0.0
−0.1
−0.2
0.00
0.25
0.50
0.75
SNP effect on ALDH5A1

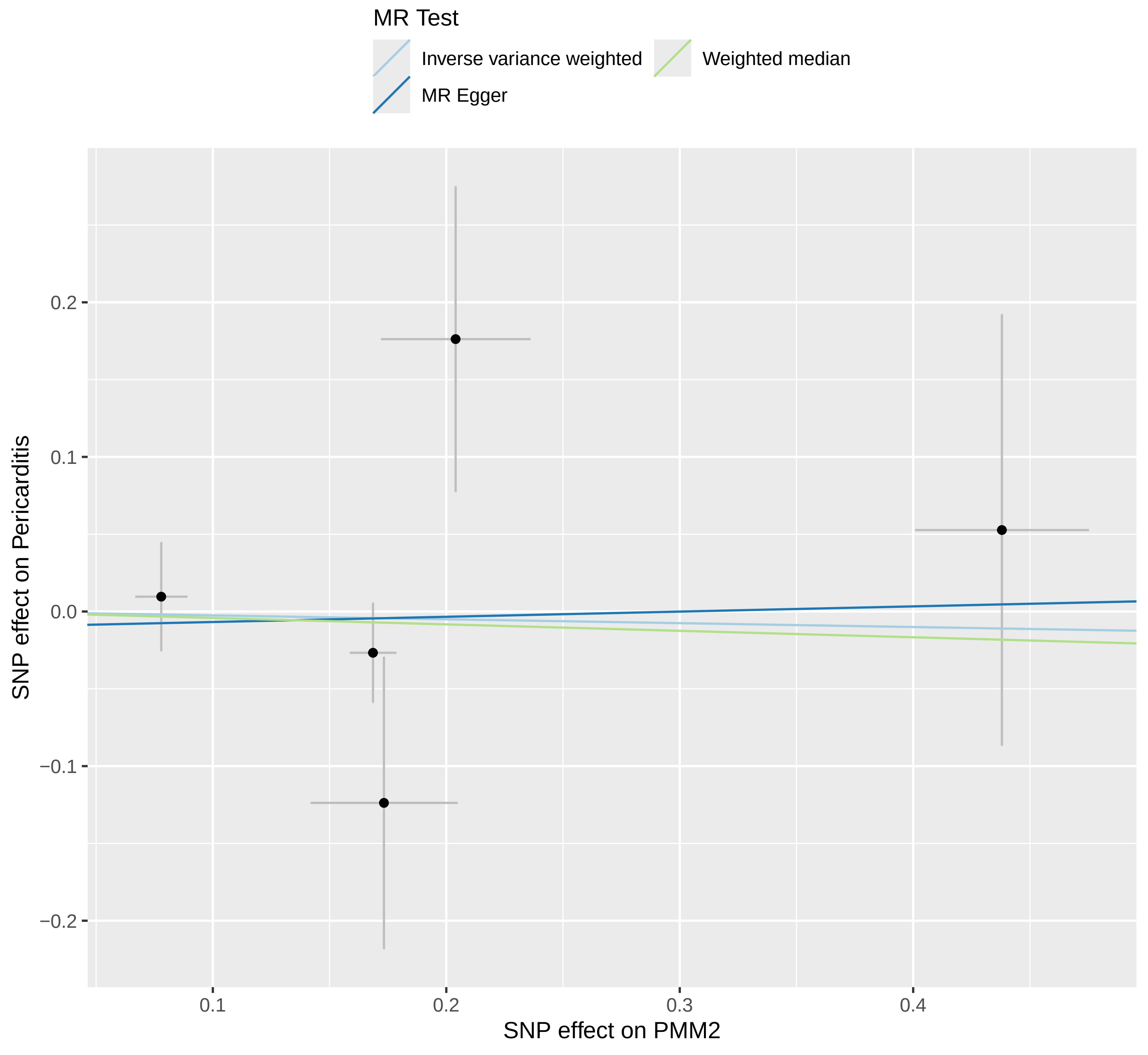

MR Test
Inverse variance weighted
Weighted median
MR Egger
0.2
0.1
0.0
−0.1
−0.2
SNP effect on Pericarditis
0.1
0.2
0.3
0.4
SNP effect on PMM2

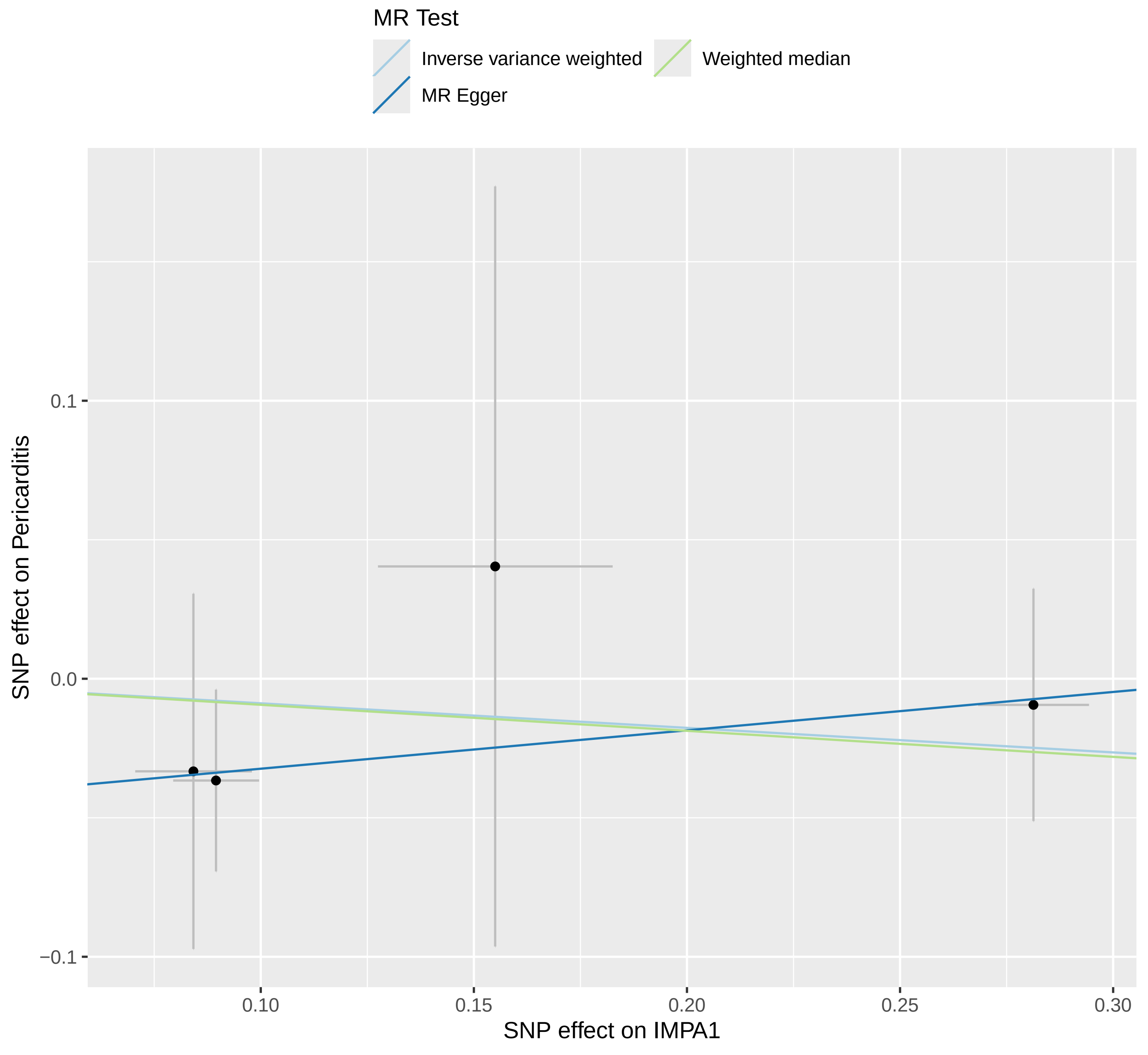
MR Test
Inverse variance weighted
MR Egger
Weighted median
SNP effect on Pericarditis
0.1
0.0
−0.1
0.10
0.15
0.20
0.25
0.30
SNP effect on IMPA1

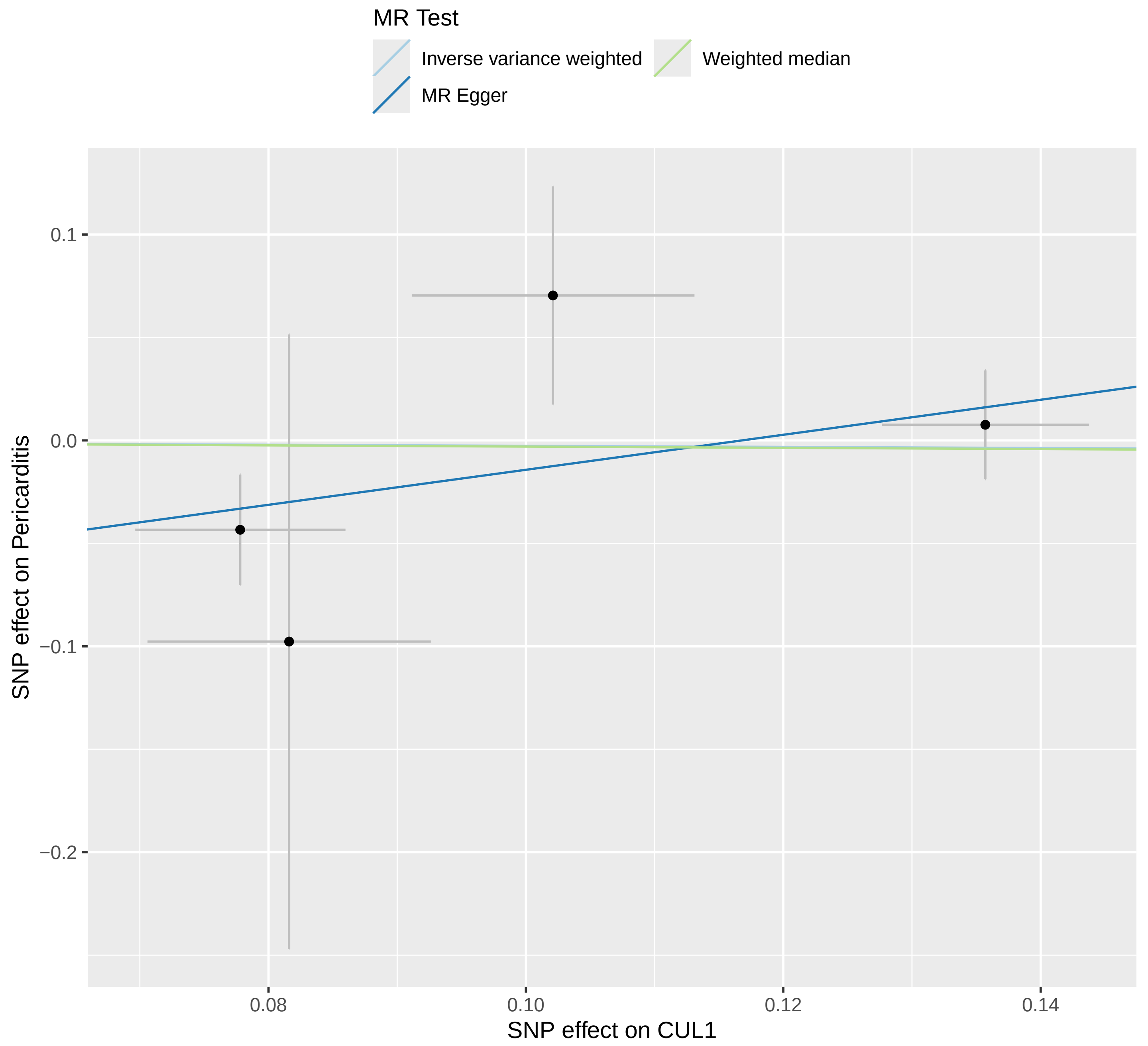

MR Test
Inverse variance weighted
MR Egger
Weighted median
0.1
0.0
−0.1
−0.2
SNP effect on Pericarditis
0.08
0.10
0.12
0.14
SNP effect on CUL1

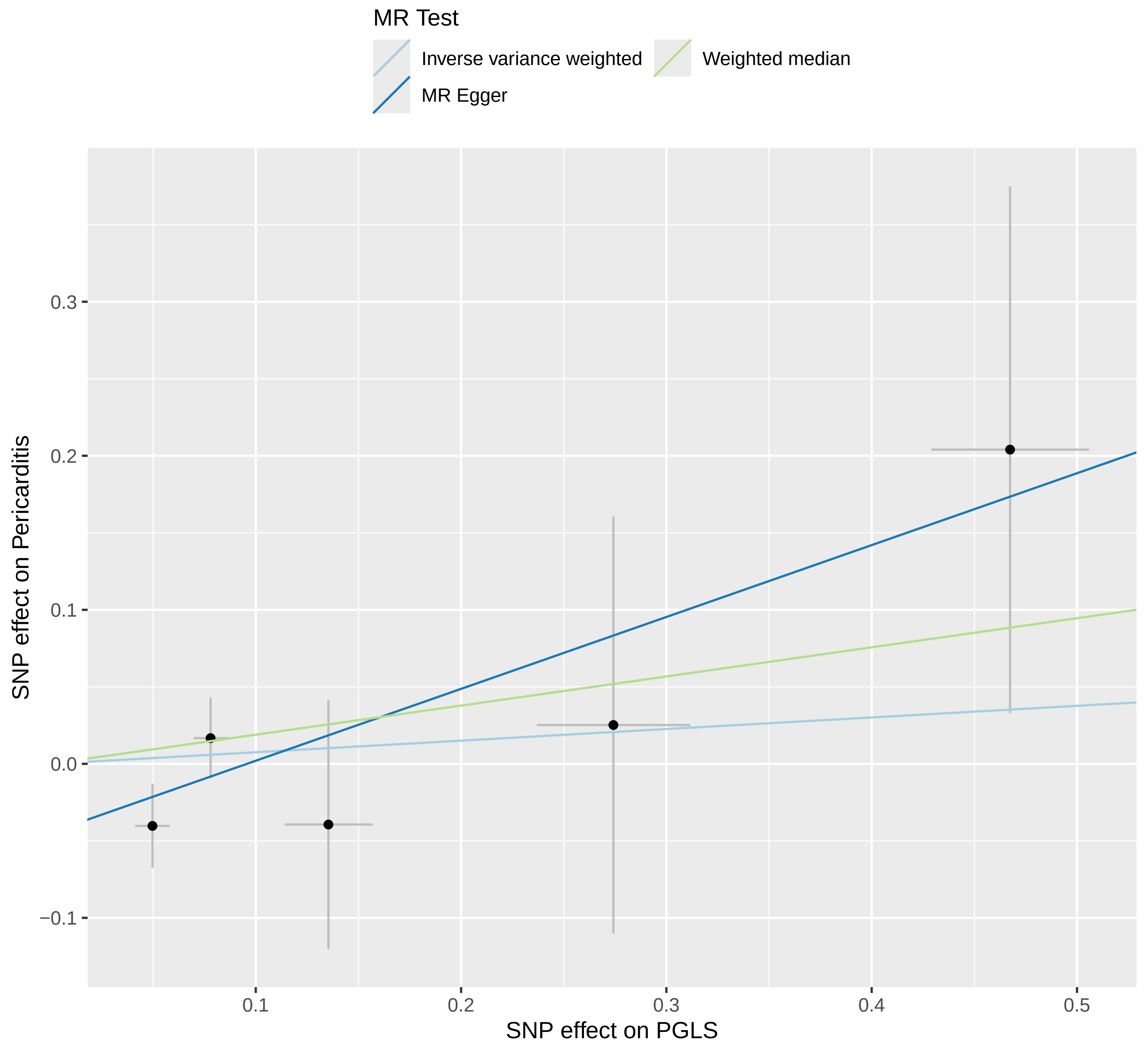

MR Test
Inverse variance weighted
Weighted median
MR Egger
SNP effect on Pericarditis
SNP effect on PGLS
0.3
0.2
0.1
0.0
−0.1
0.1
0.2
0.3
0.4
0.5

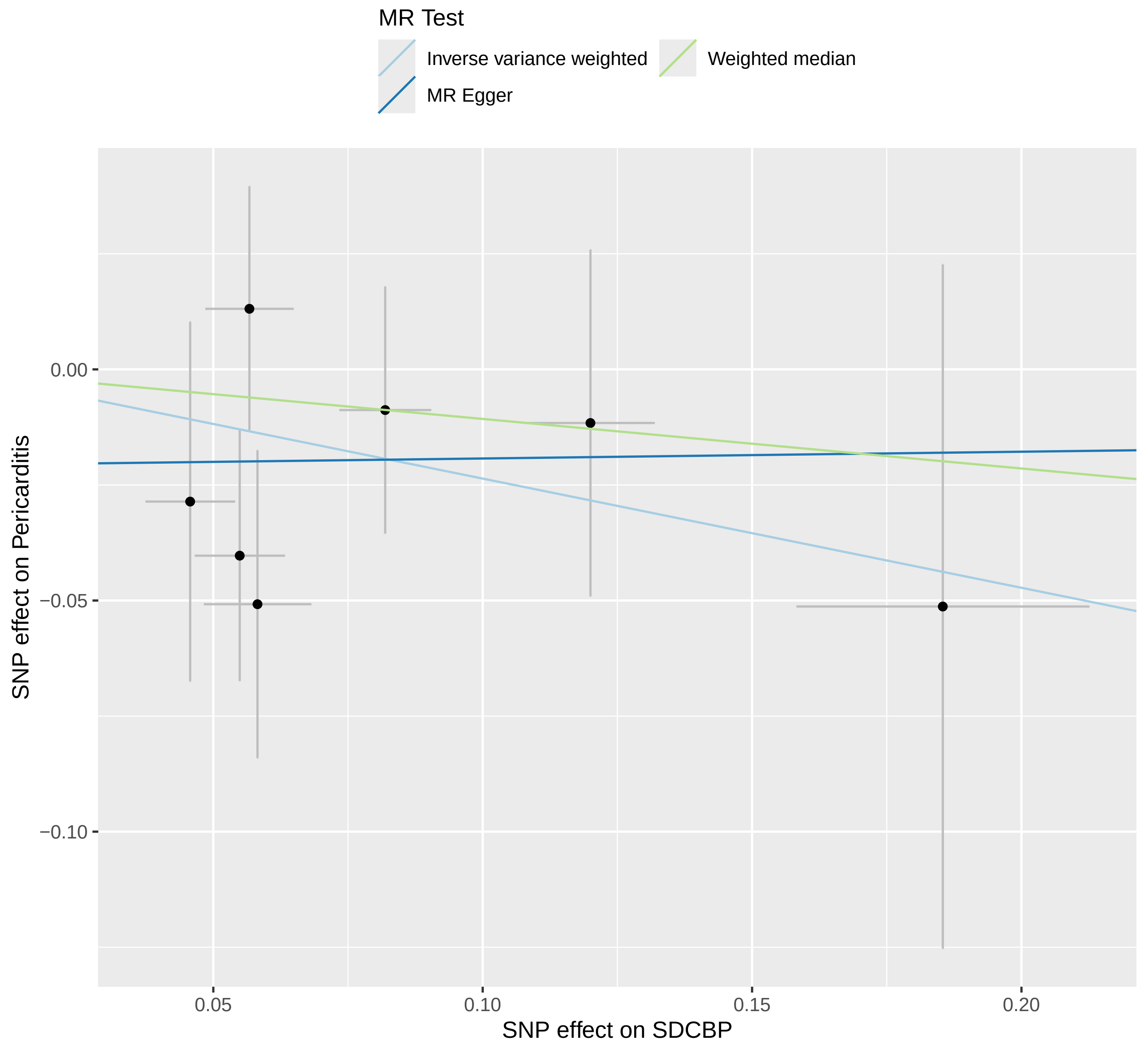
MR Test
Inverse variance weighted
Weighted median
MR Egger
0.00
−0.05
−0.10
SNP effect on Pericarditis
0.05
0.10
0.15
0.20
SNP effect on SDCBP

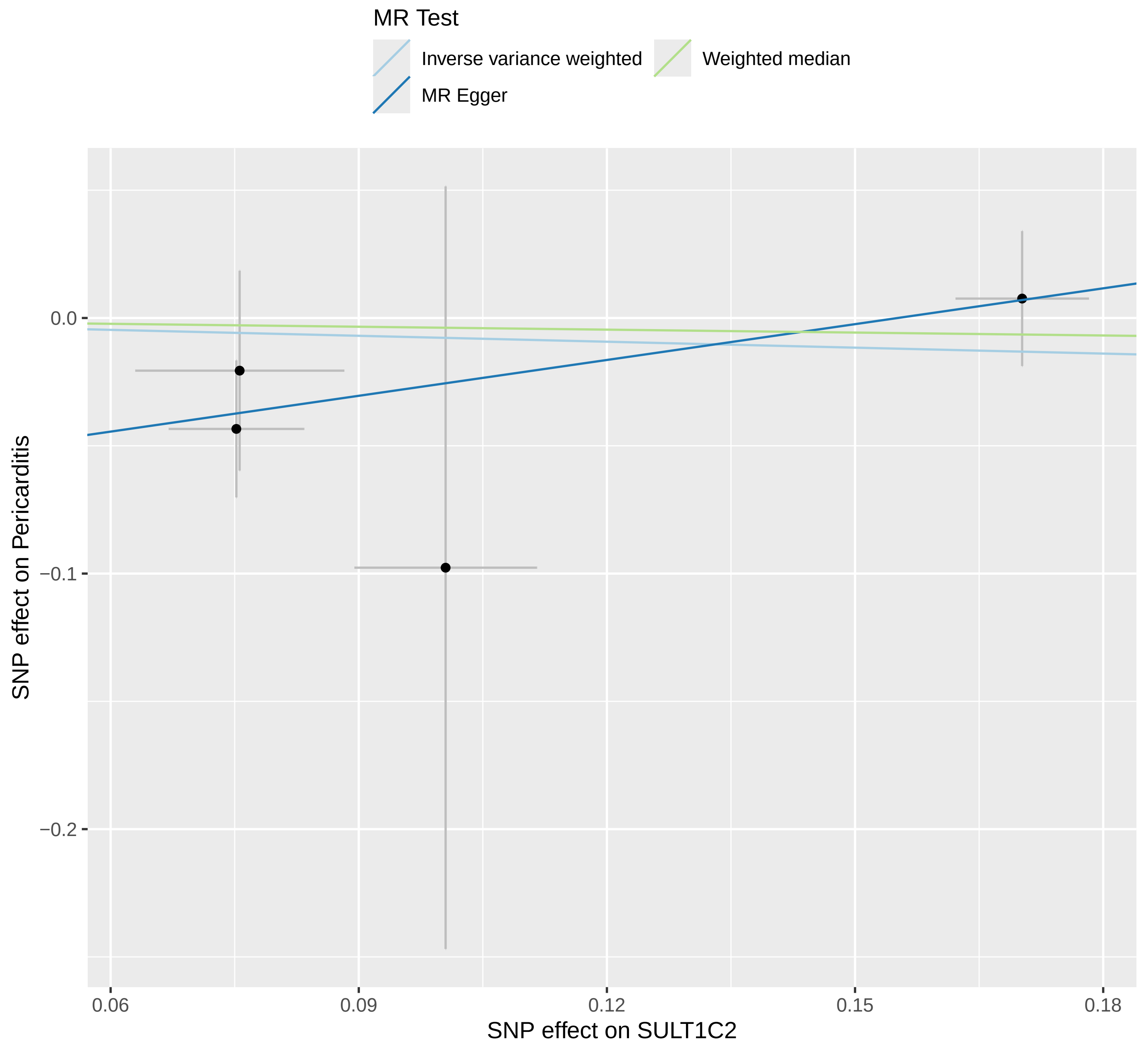

MR Test
Inverse variance weighted
Weighted median
MR Egger
0.0
−0.1
−0.2
SNP effect on Pericarditis
0.06
0.09
0.12
0.15
0.18
SNP effect on SULT1C2

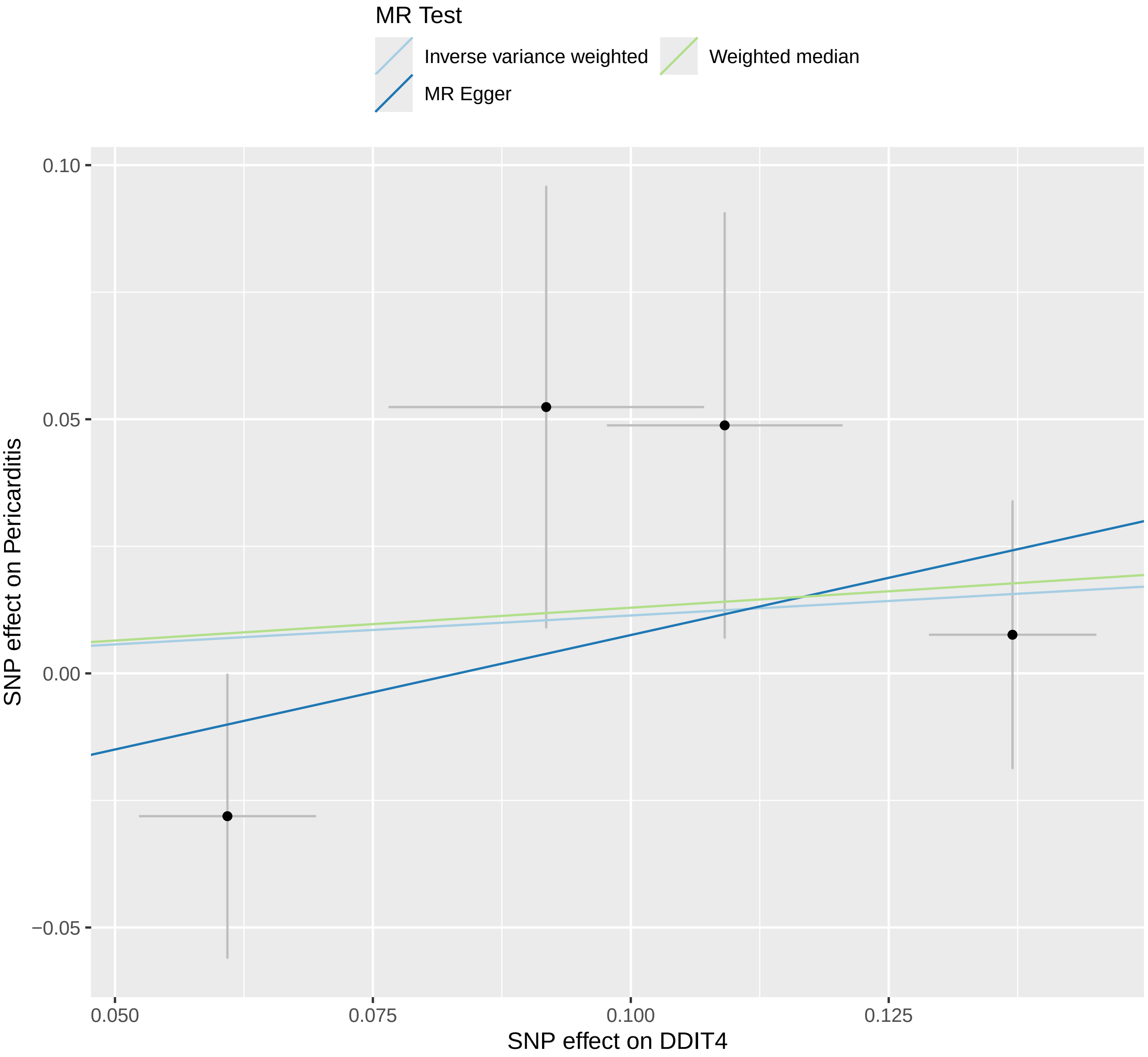
MR Test
Inverse variance weighted
Weighted median
MR Egger
0.10
0.05
0.00
−0.05
SNP effect on Pericarditis
0.050
0.075
0.100
0.125
SNP effect on DDIT4

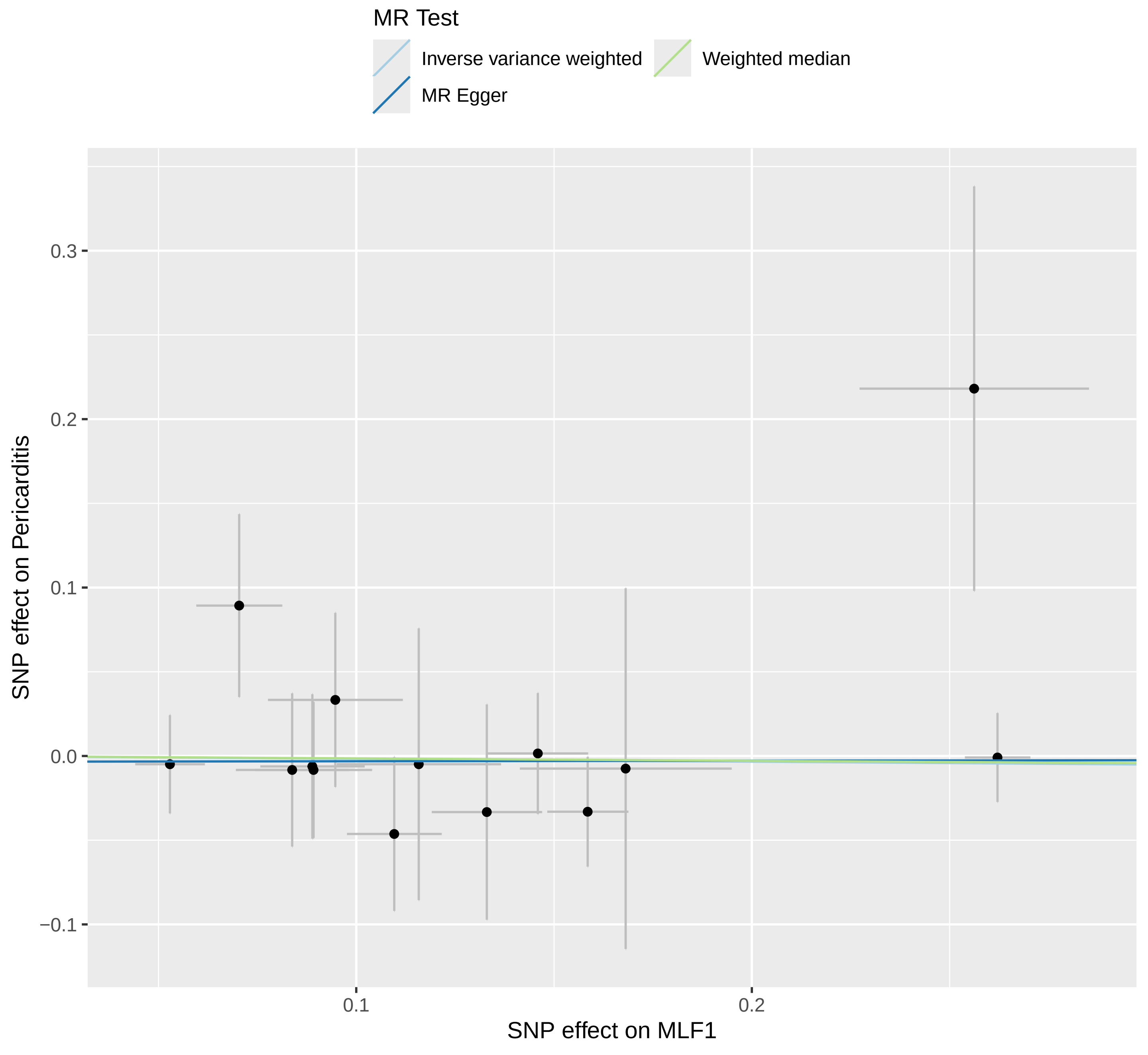

MR Test
Inverse variance weighted
Weighted median
MR Egger
0.3
0.2
0.1
0.0
−0.1
0.1
0.2
SNP effect on Pericarditis
SNP effect on MLF1

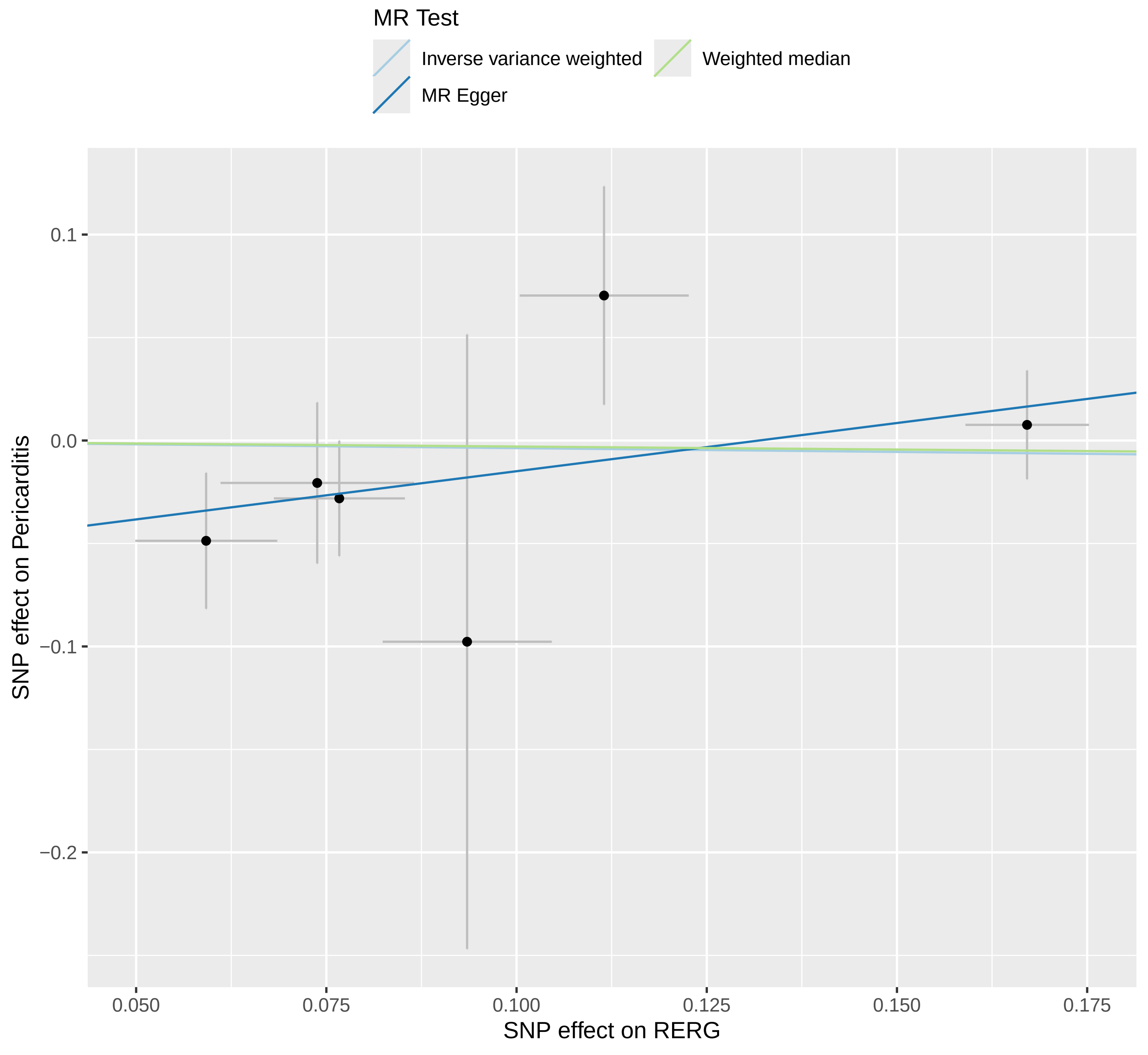
MR Test
Inverse variance weighted
MR Egger
Weighted median
0.1
0.0
−0.1
−0.2
SNP effect on Pericarditis
0.050
0.075
0.100
0.125
0.150
0.175
SNP effect on RERG

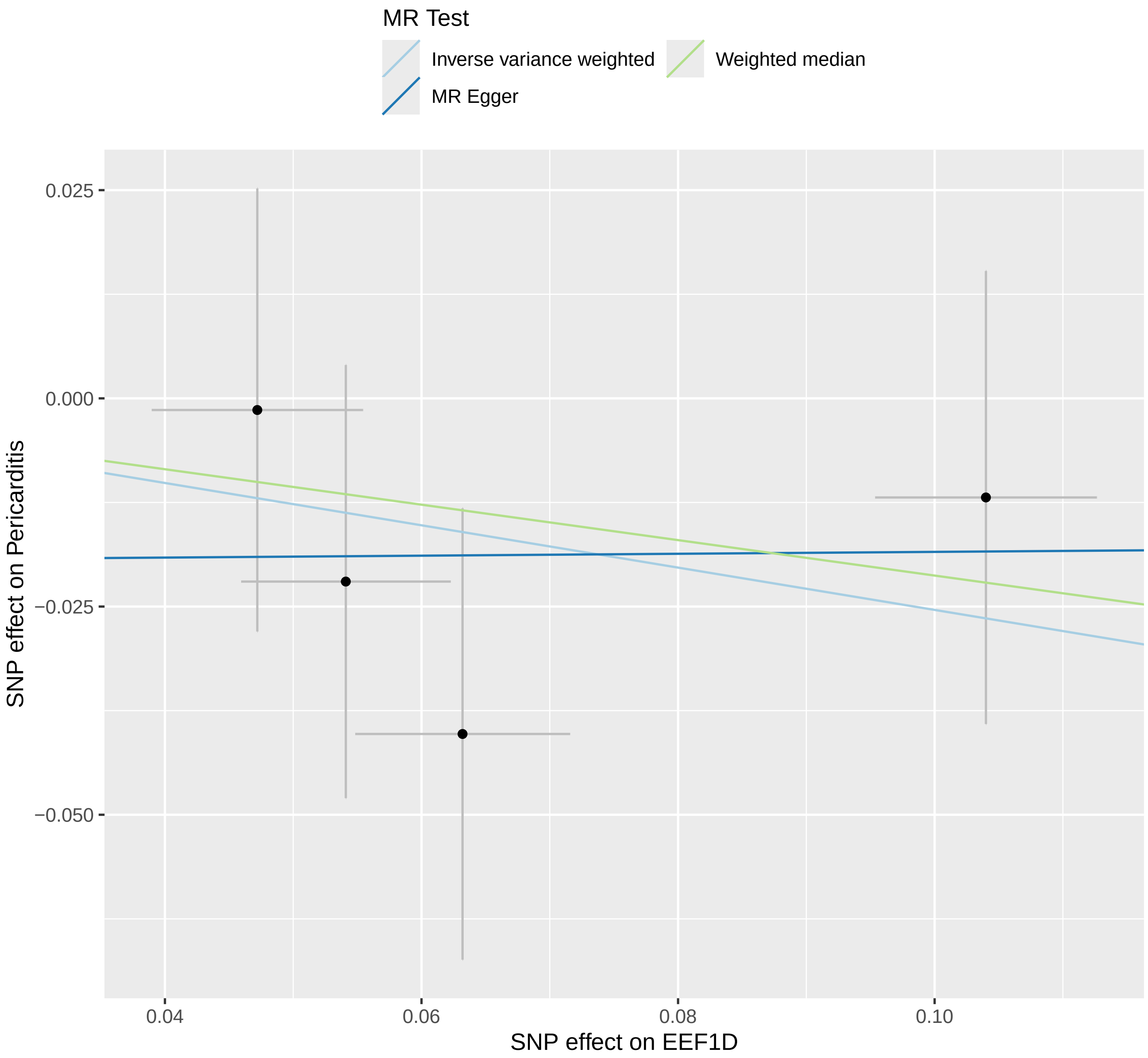
MR Test
Inverse variance weighted
MR Egger
Weighted median
0.025
0.000
−0.025
−0.050
SNP effect on Pericarditis
0.04
0.06
0.08
0.10
SNP effect on EEF1D

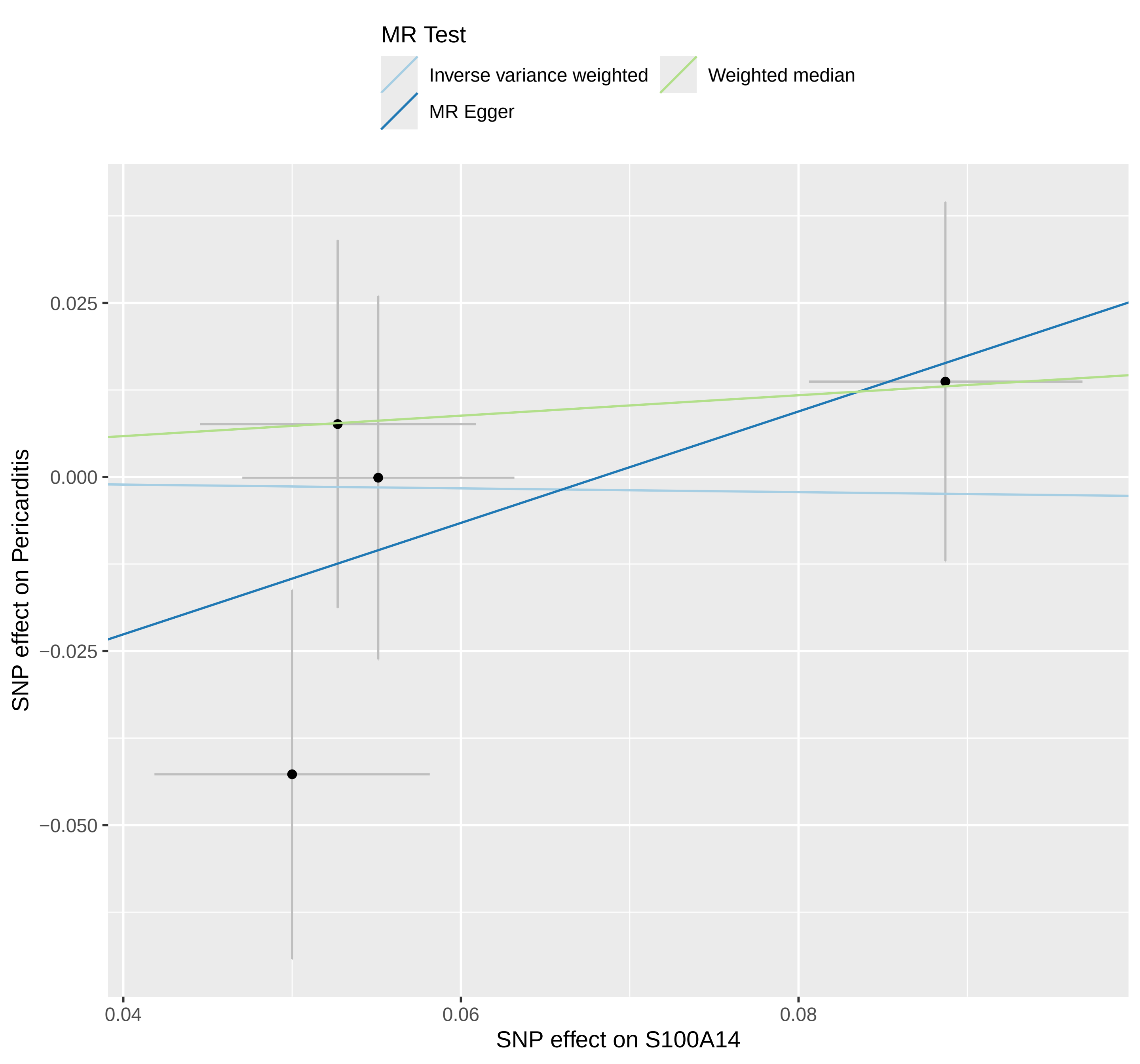

MR Test
Inverse variance weighted
Weighted median
MR Egger
0.025
0.000
−0.025
−0.050
0.04
0.06
0.08
SNP effect on S100A14
SNP effect on Pericarditis

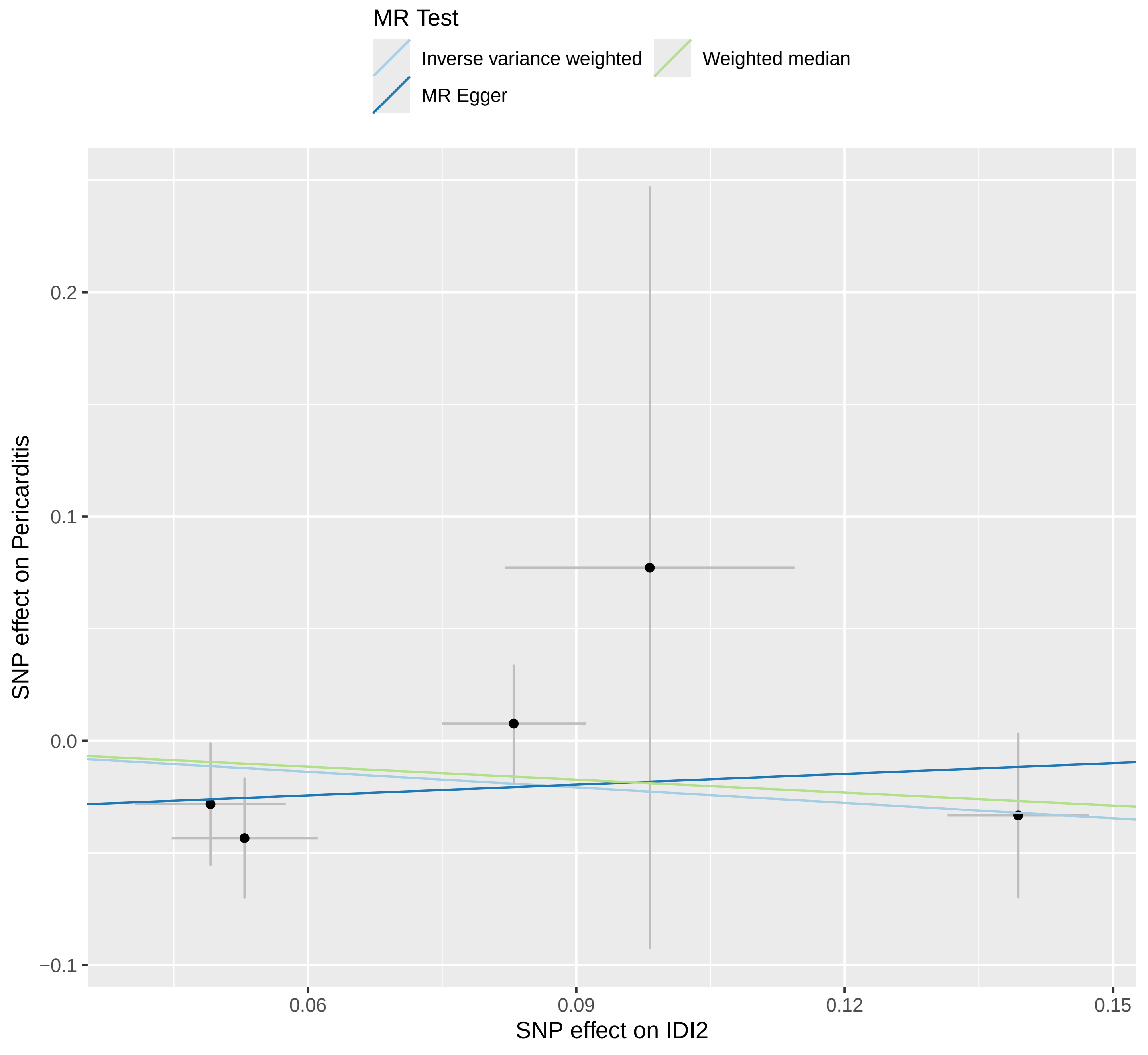
MR Test
Inverse variance weighted
MR Egger
Weighted median
SNP effect on Pericarditis
0.2
0.1
0.0
−0.1
0.06
0.09
0.12
0.15
SNP effect on IDI2

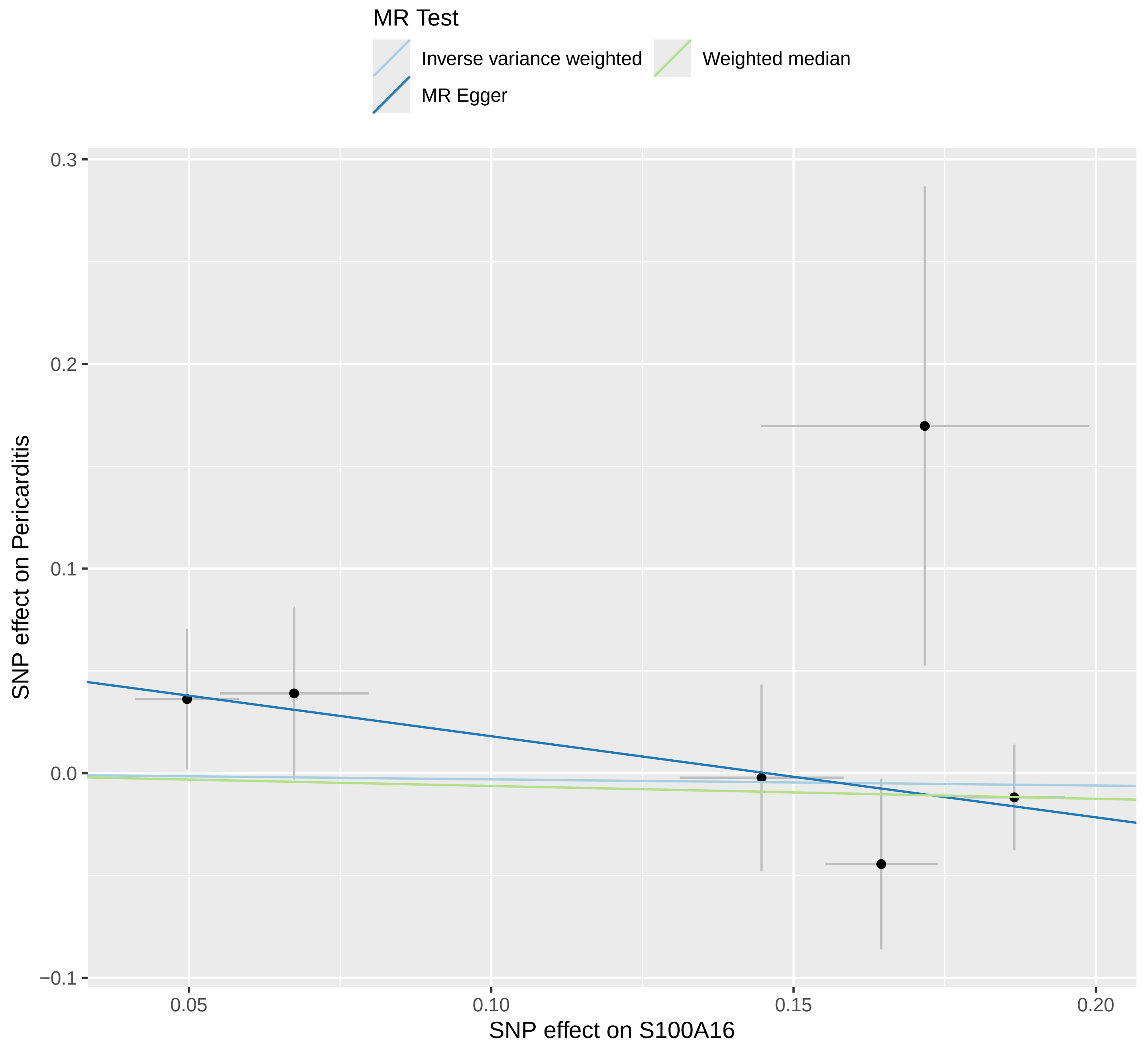
MR Test
Inverse variance weighted
Weighted median
MR Egger
0.3
0.2
0.1
0.0
−0.1
SNP effect on Pericarditis
0.05
0.10
0.15
0.20
SNP effect on S100A16

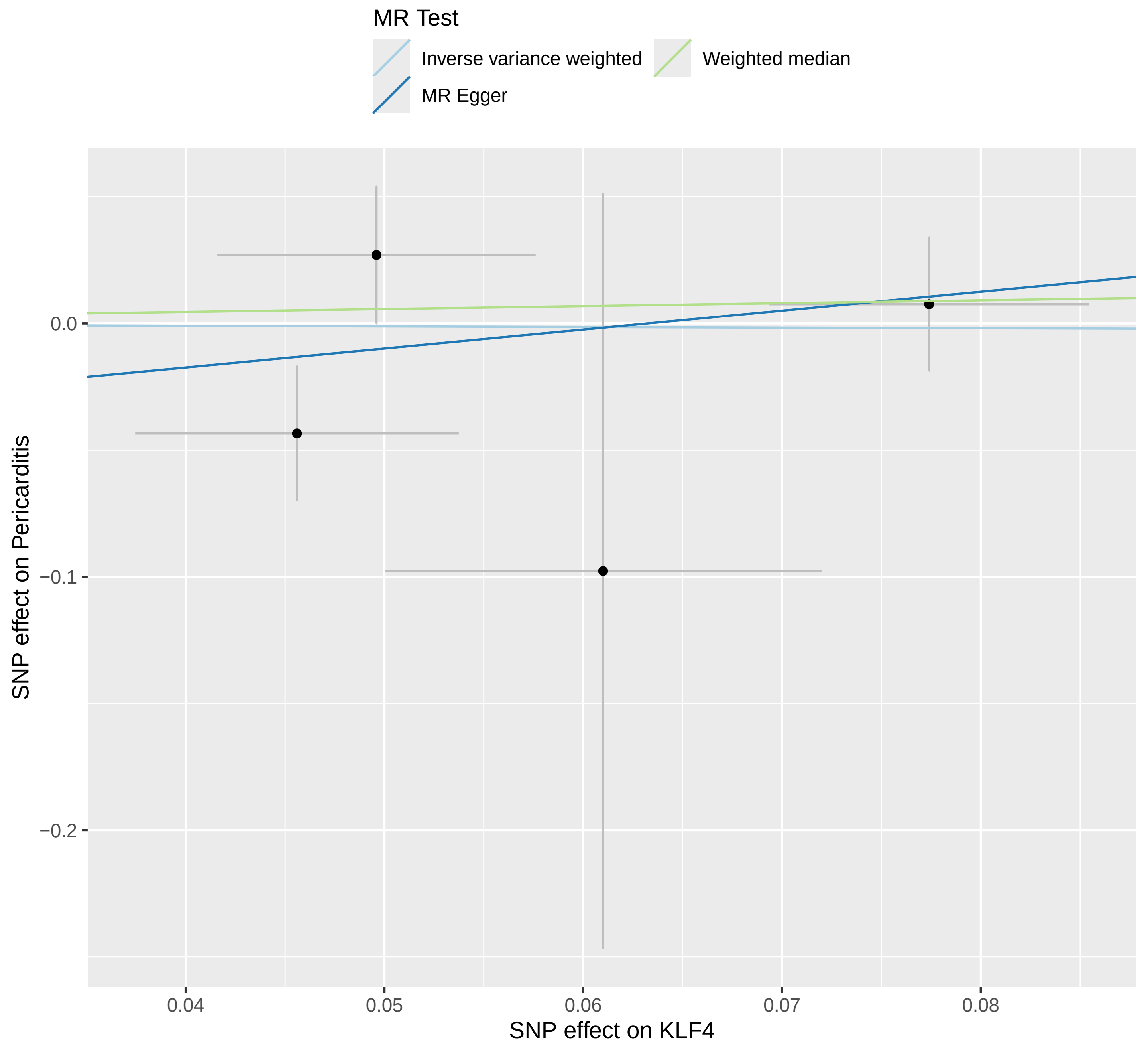

MR Test
Inverse variance weighted
Weighted median
MR Egger
0.0
−0.1
−0.2
0.04
0.05
0.06
0.07
0.08
SNP effect on KLF4
SNP effect on Pericarditis

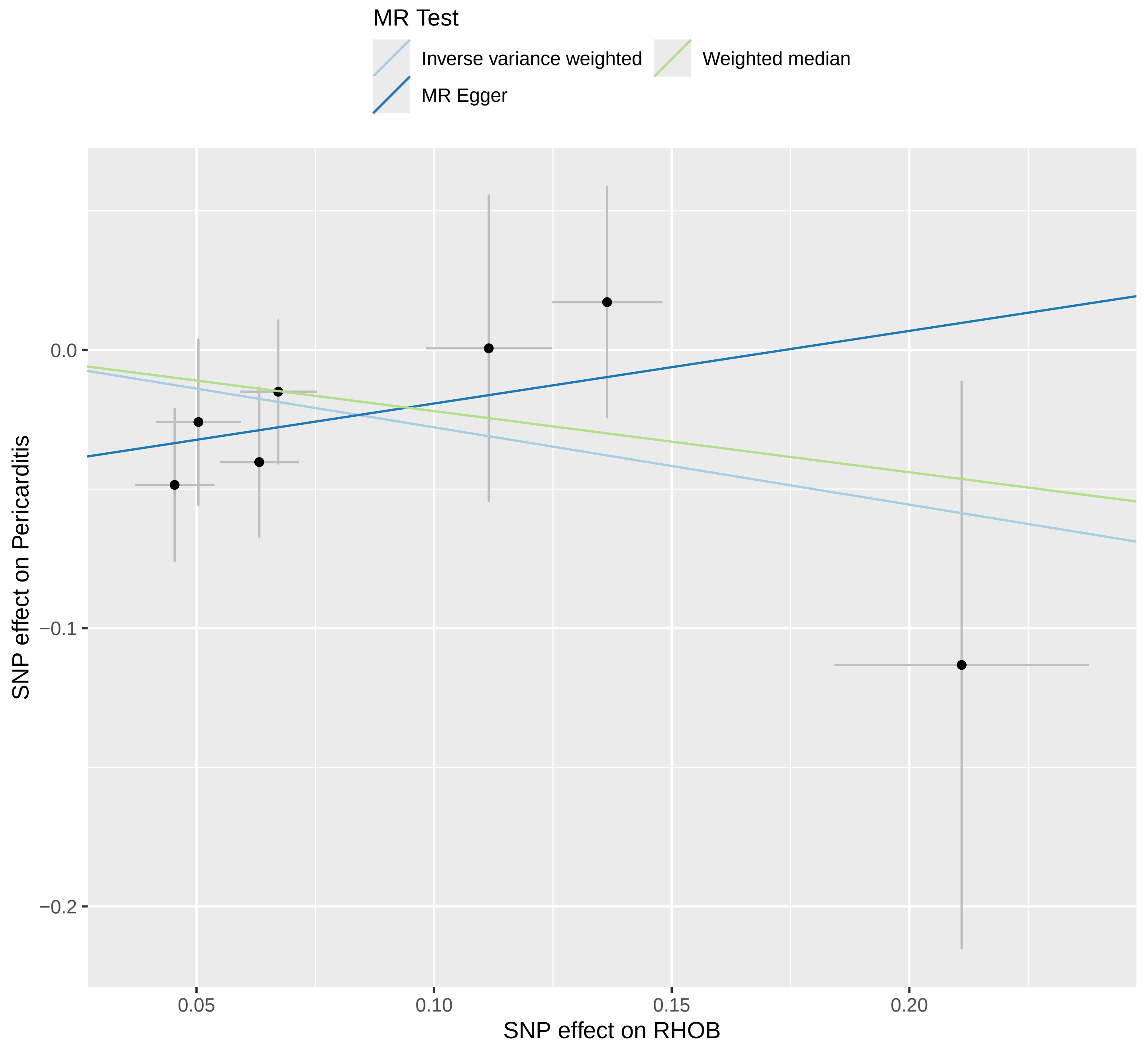
MR Test
Inverse variance weighted
MR Egger
Weighted median
0.0
−0.1
−0.2
SNP effect on Pericarditis
0.05
0.10
0.15
0.20
SNP effect on RHOB

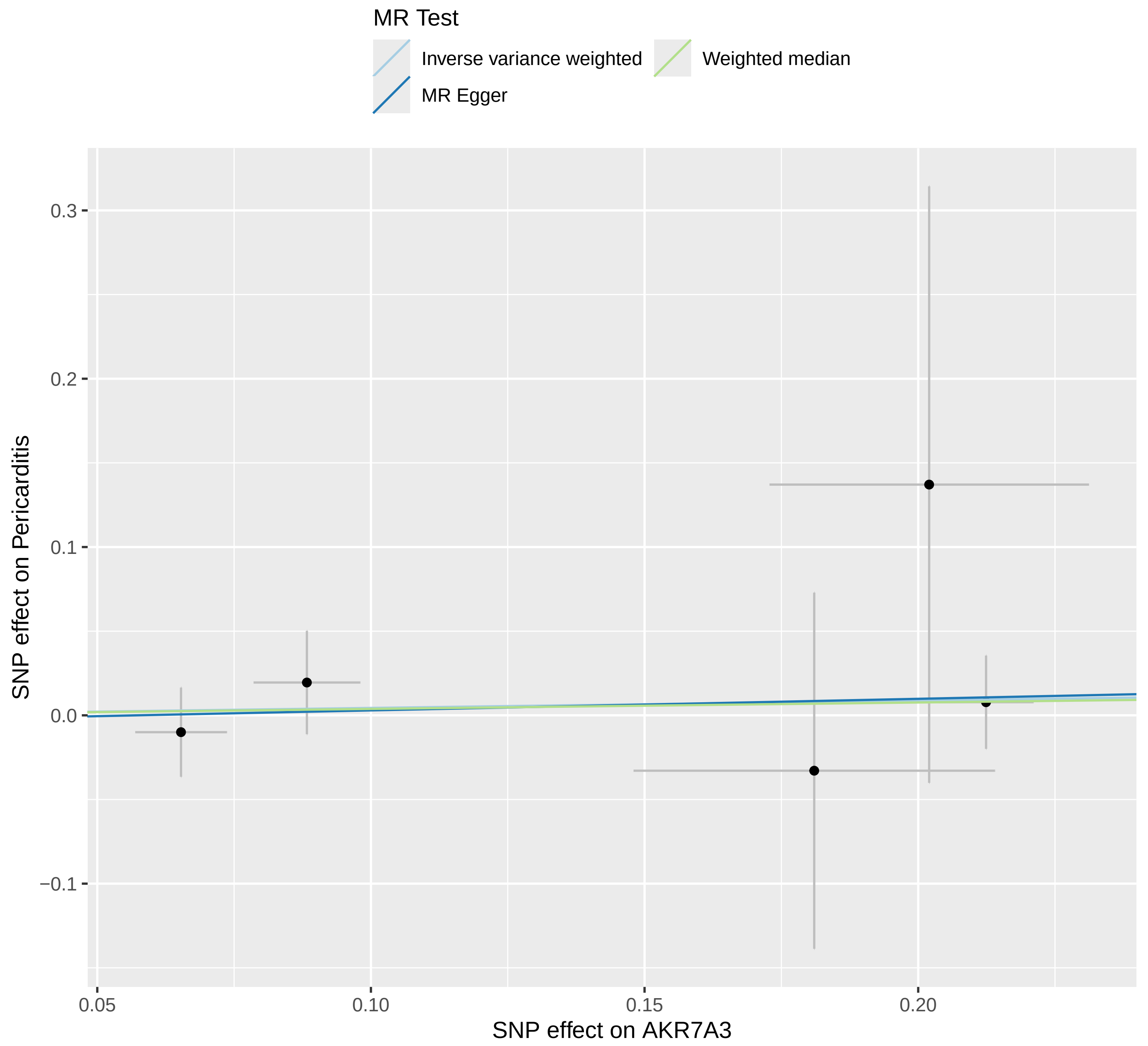
MR Test
Inverse variance weighted
Weighted median
MR Egger
SNP effect on Pericarditis
0.3
0.2
0.1
0.0
−0.1
0.05
0.10
0.15
0.20
SNP effect on AKR7A3

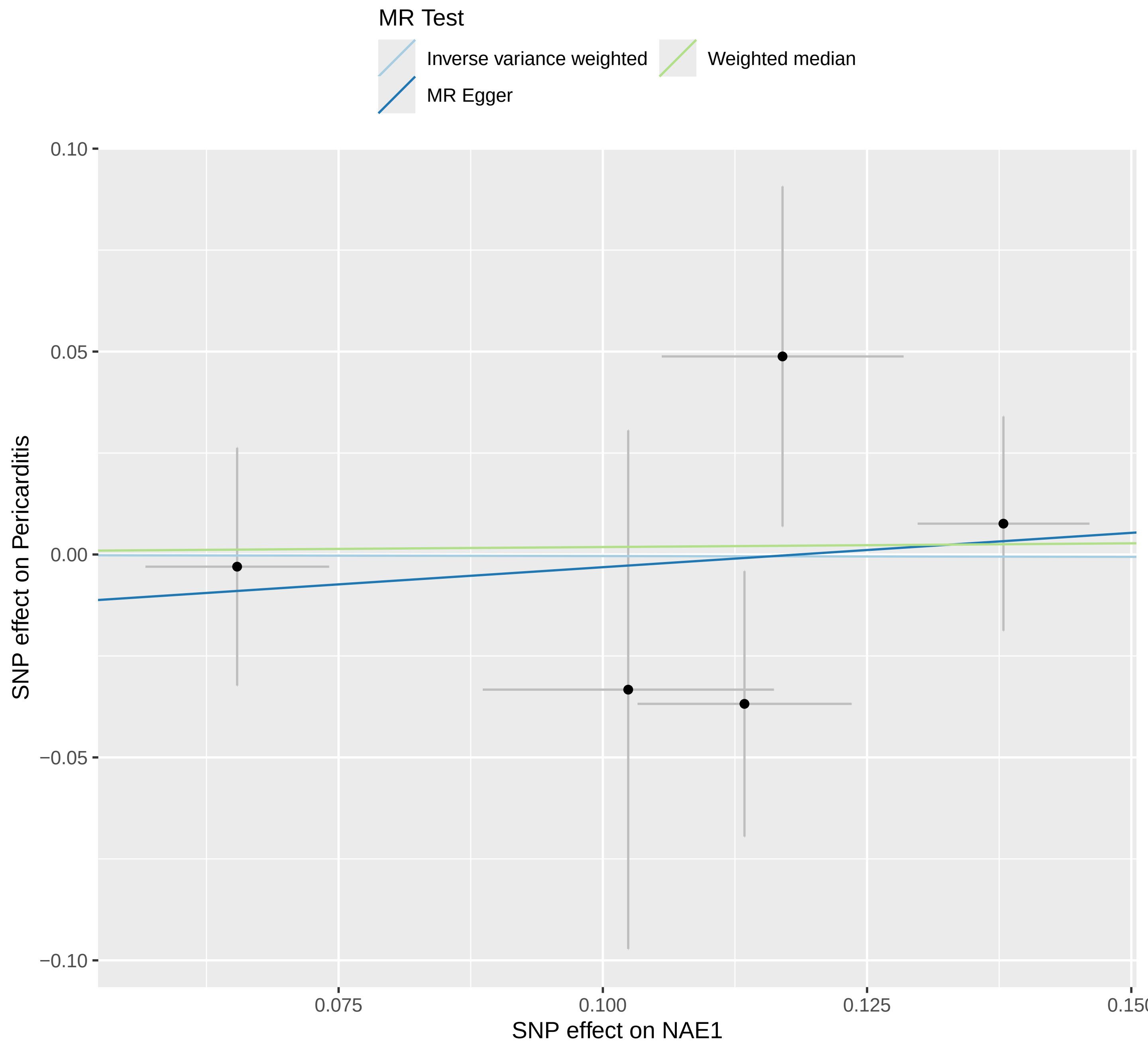

MR Test
Inverse variance weighted
Weighted median
MR Egger
0.10
0.05
0.00
−0.05
−0.10
SNP effect on Pericarditis
0.075
0.100
0.125
0.150
SNP effect on NAE1

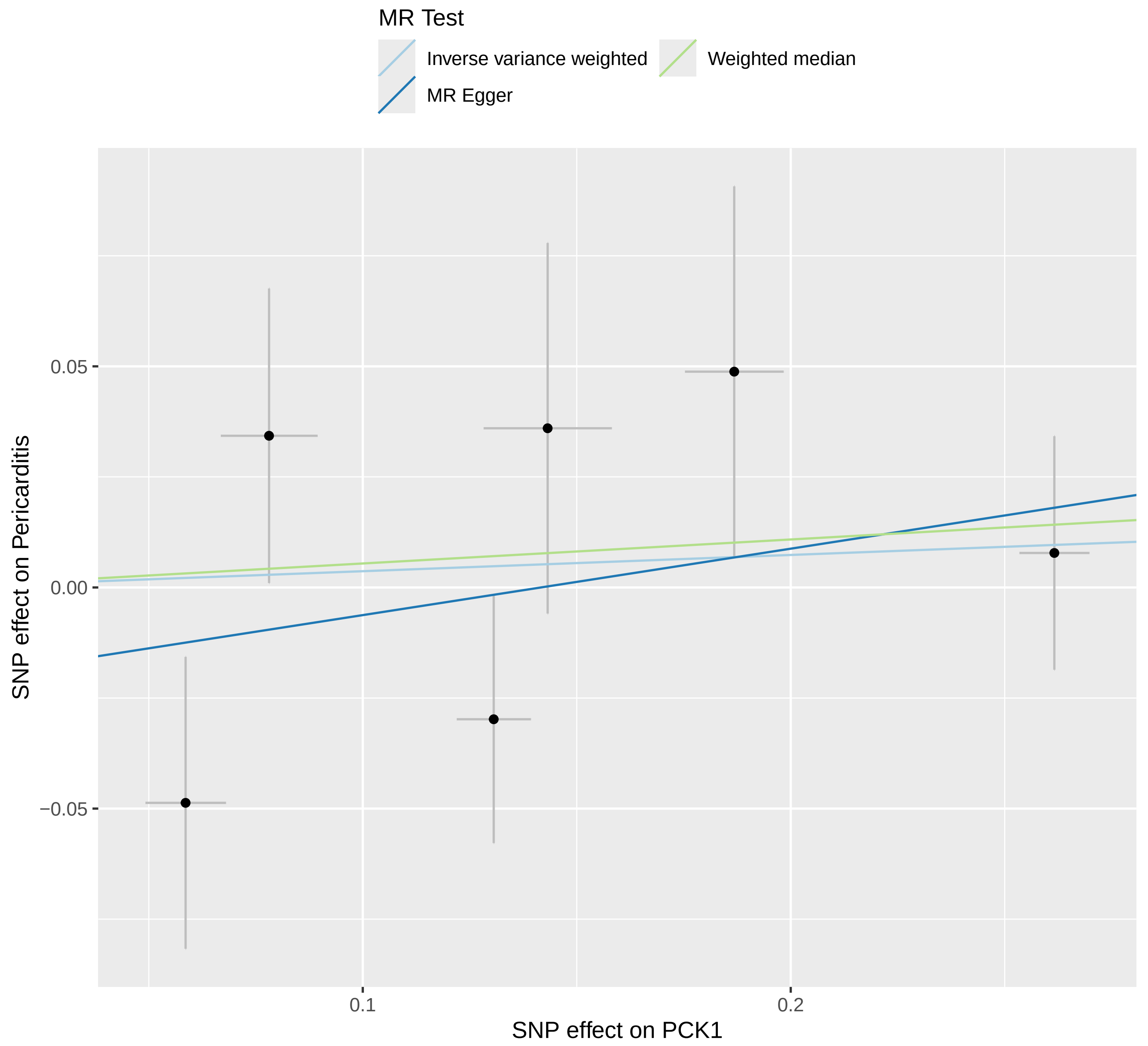
MR Test
Inverse variance weighted
Weighted median
MR Egger
0.05
0.00
−0.05
SNP effect on Pericarditis
0.1
0.2
SNP effect on PCK1

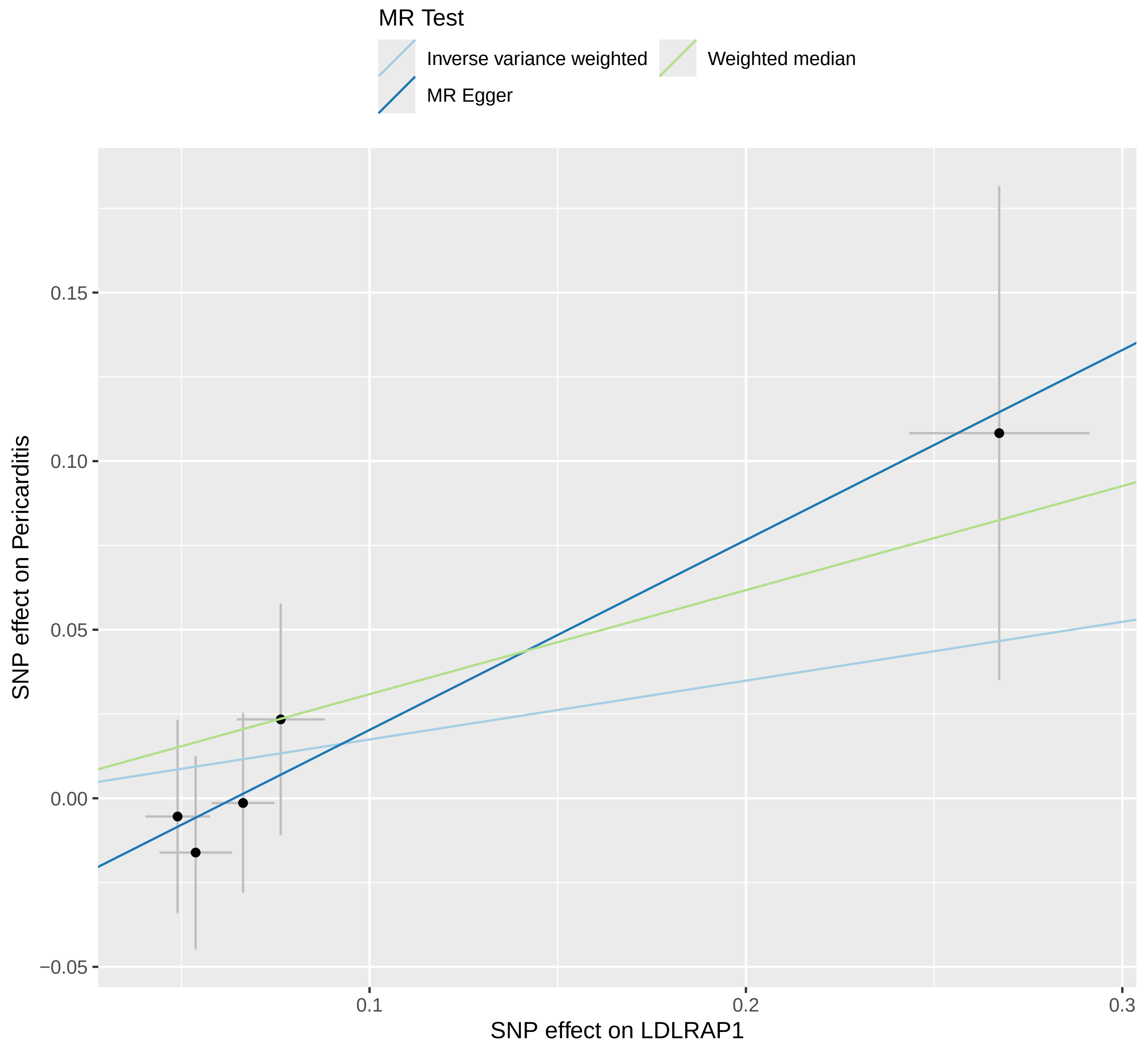
MR Test
Inverse variance weighted
Weighted median
MR Egger
0.15
0.10
0.05
0.00
−0.05
0.1
0.2
0.3
SNP effect on Pericarditis
SNP effect on LDLRAP1

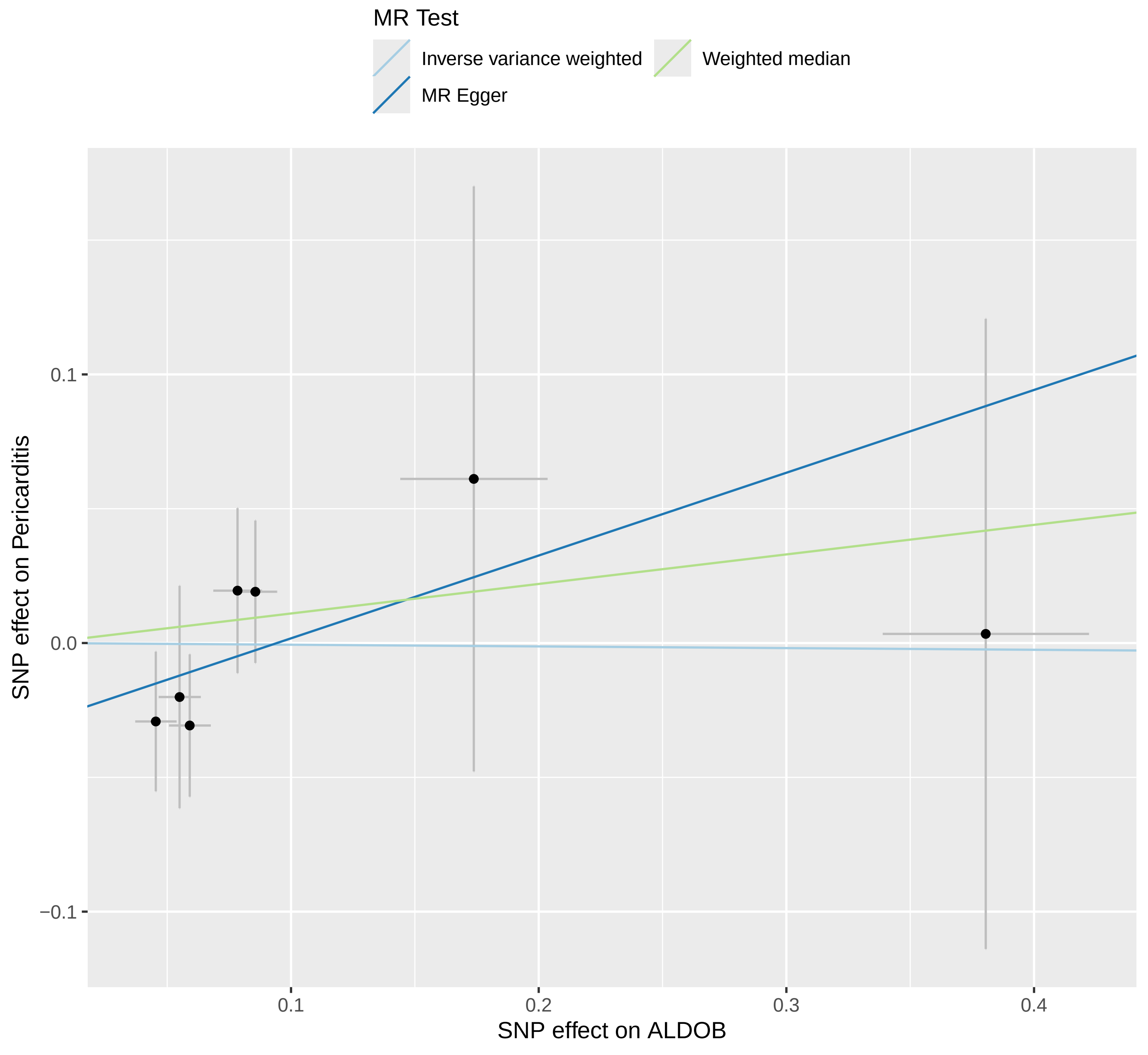
MR Test
Inverse variance weighted
Weighted median
MR Egger
0.1
0.0
−0.1
SNP effect on Pericarditis
0.1
0.2
0.3
0.4
SNP effect on ALDOB

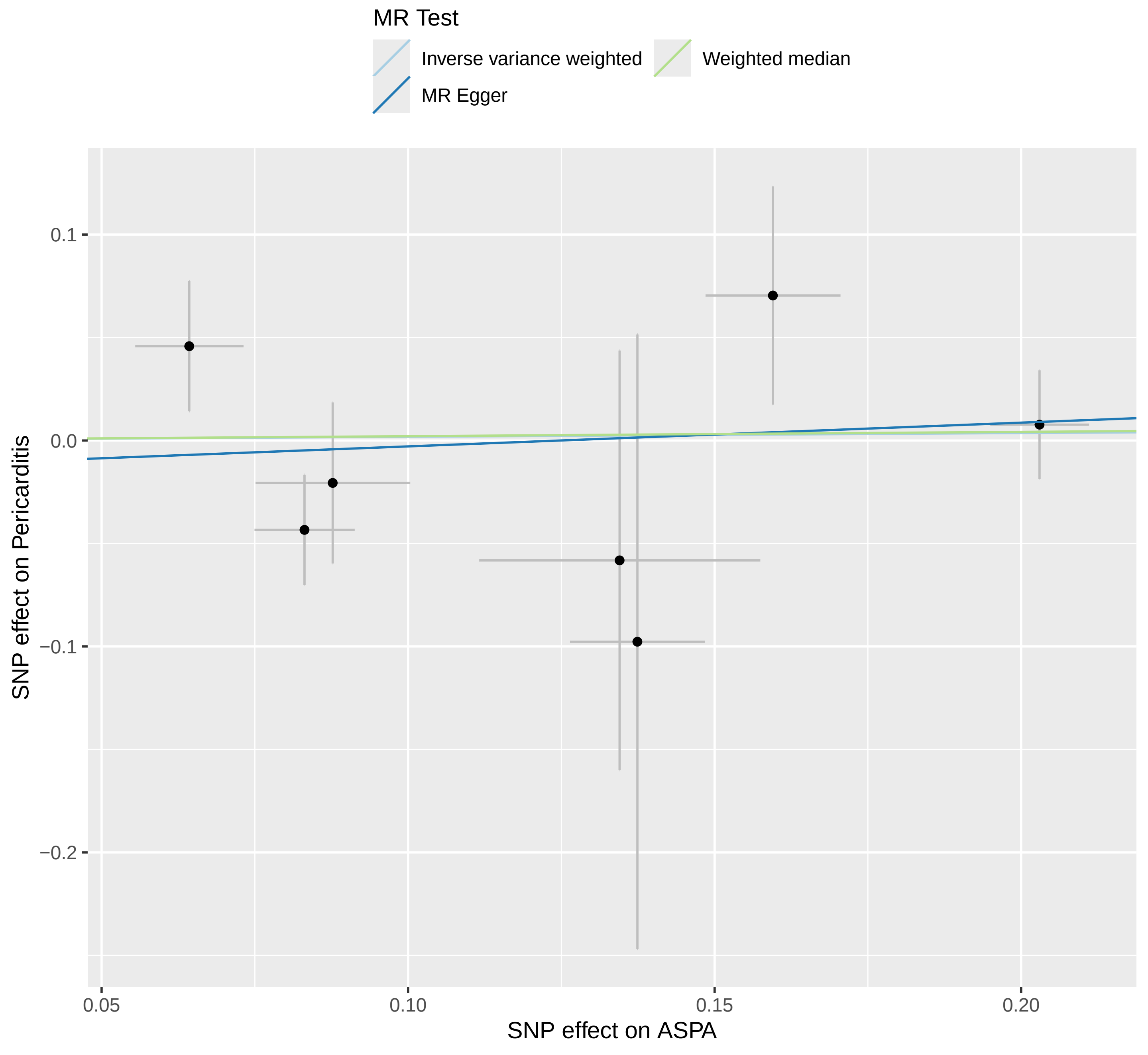
MR Test
Inverse variance weighted
Weighted median
MR Egger
SNP effect on Pericarditis
0.1
0.0
−0.1
−0.2
0.05
0.10
0.15
0.20
SNP effect on ASPA

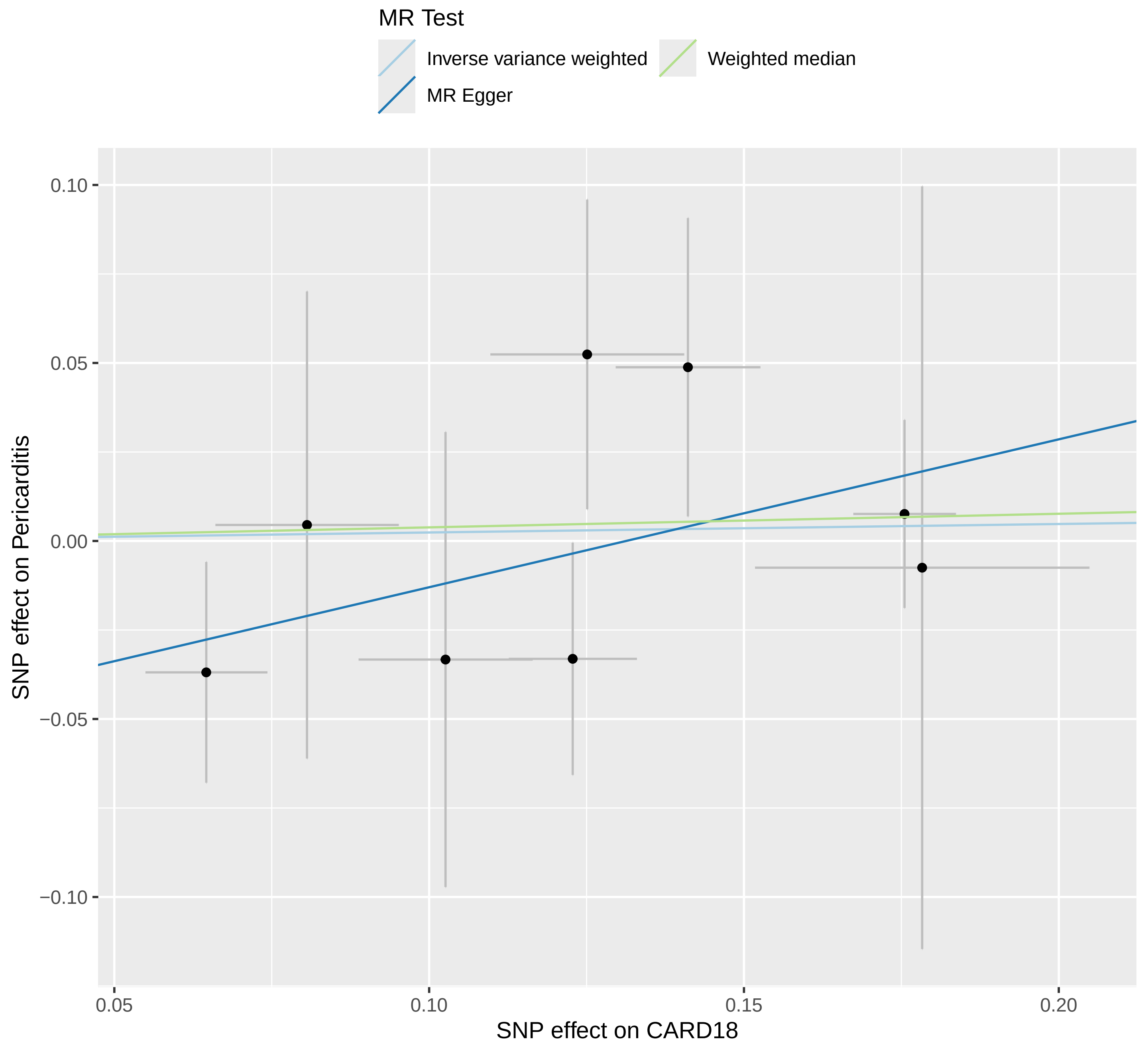
MR Test
Inverse variance weighted
MR Egger
Weighted median
SNP effect on Pericarditis
0.10
0.05
0.00
−0.05
−0.10
0.05
0.10
0.15
0.20
SNP effect on CARD18

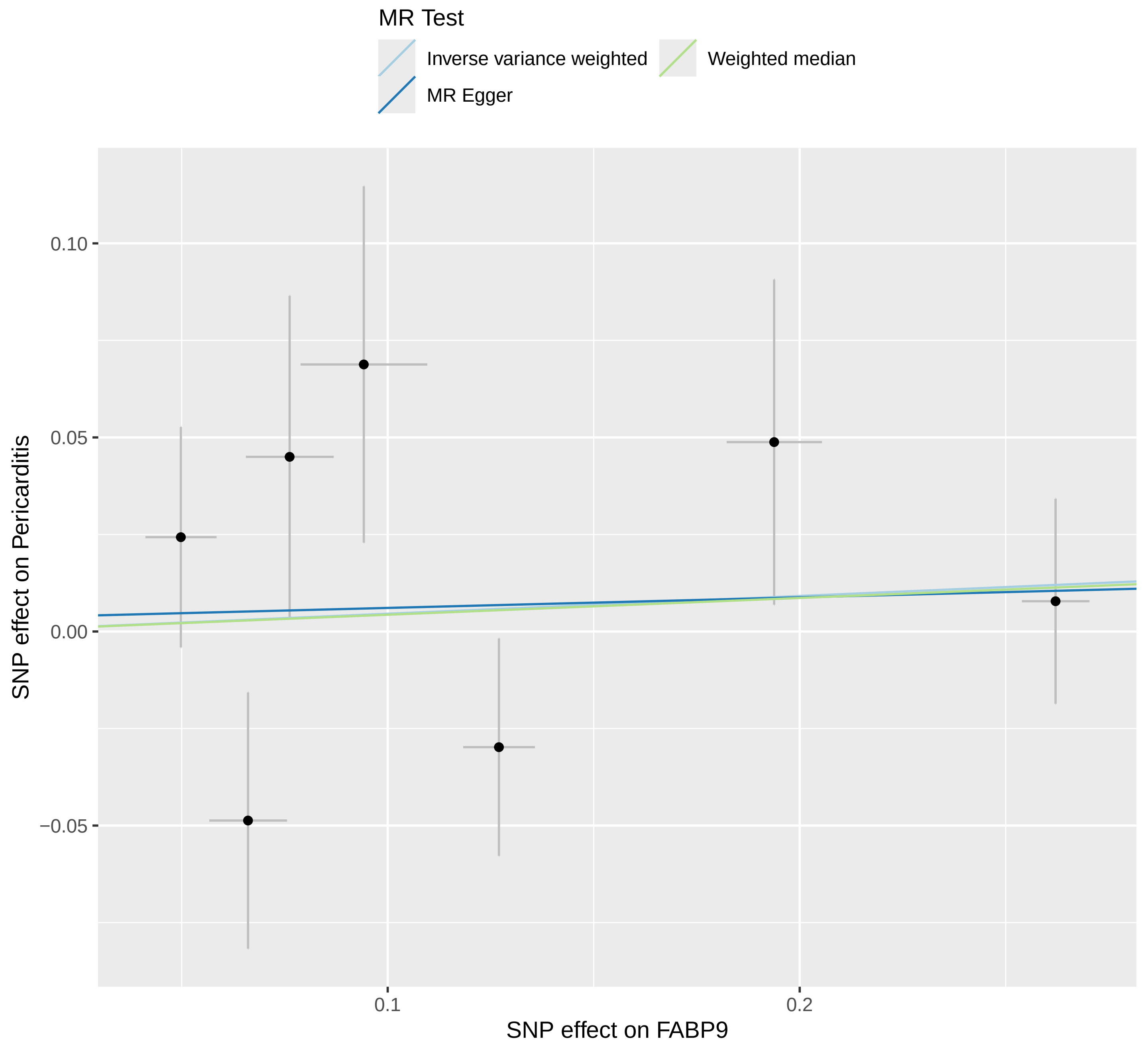

MR Test
Inverse variance weighted
MR Egger
Weighted median
0.10
0.05
0.00
−0.05
SNP effect on Pericarditis
0.1
0.2
SNP effect on FABP9

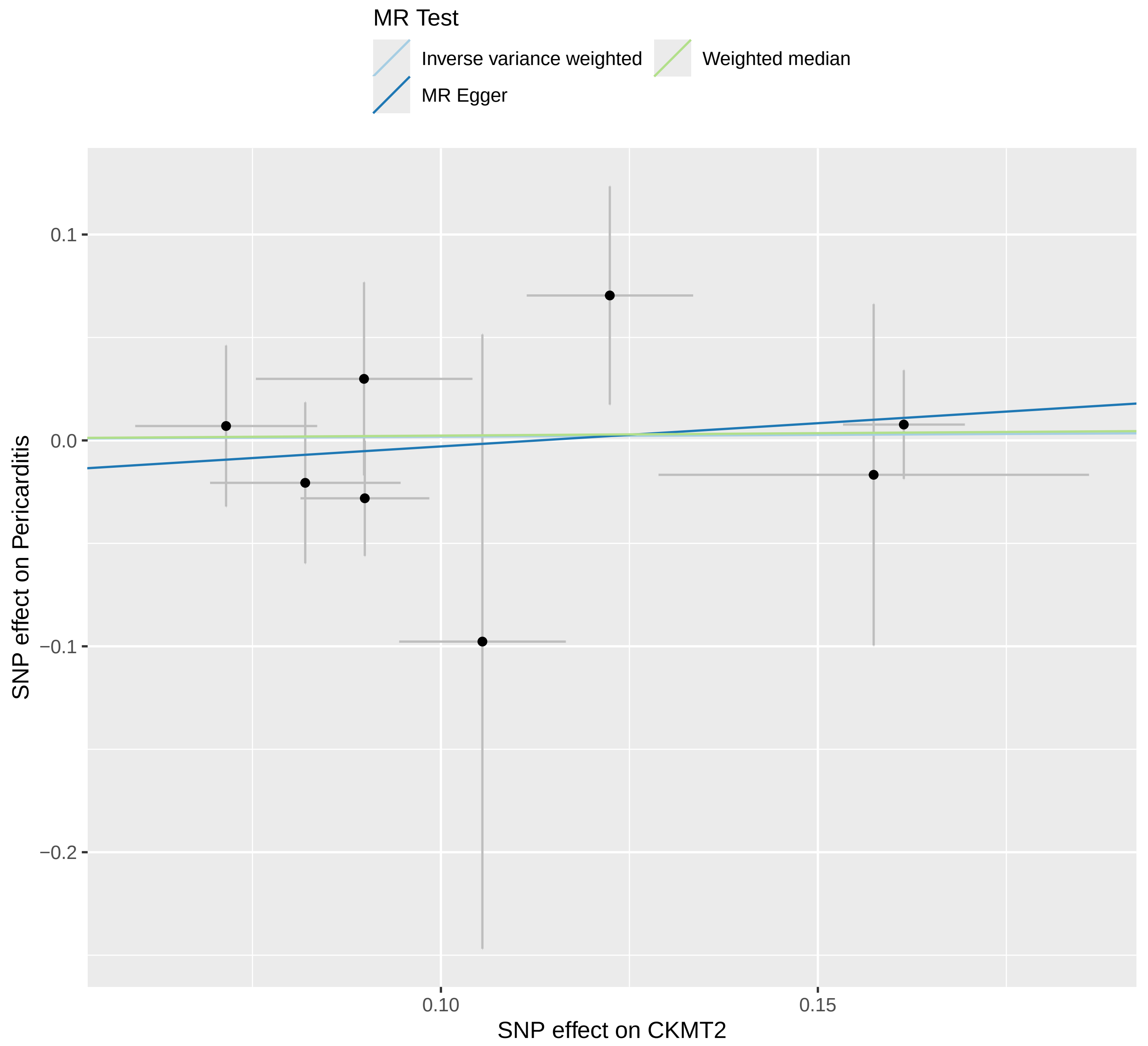
MR Test
Inverse variance weighted
Weighted median
MR Egger
SNP effect on Pericarditis
0.1
0.0
−0.1
−0.2
0.10
0.15
SNP effect on CKMT2

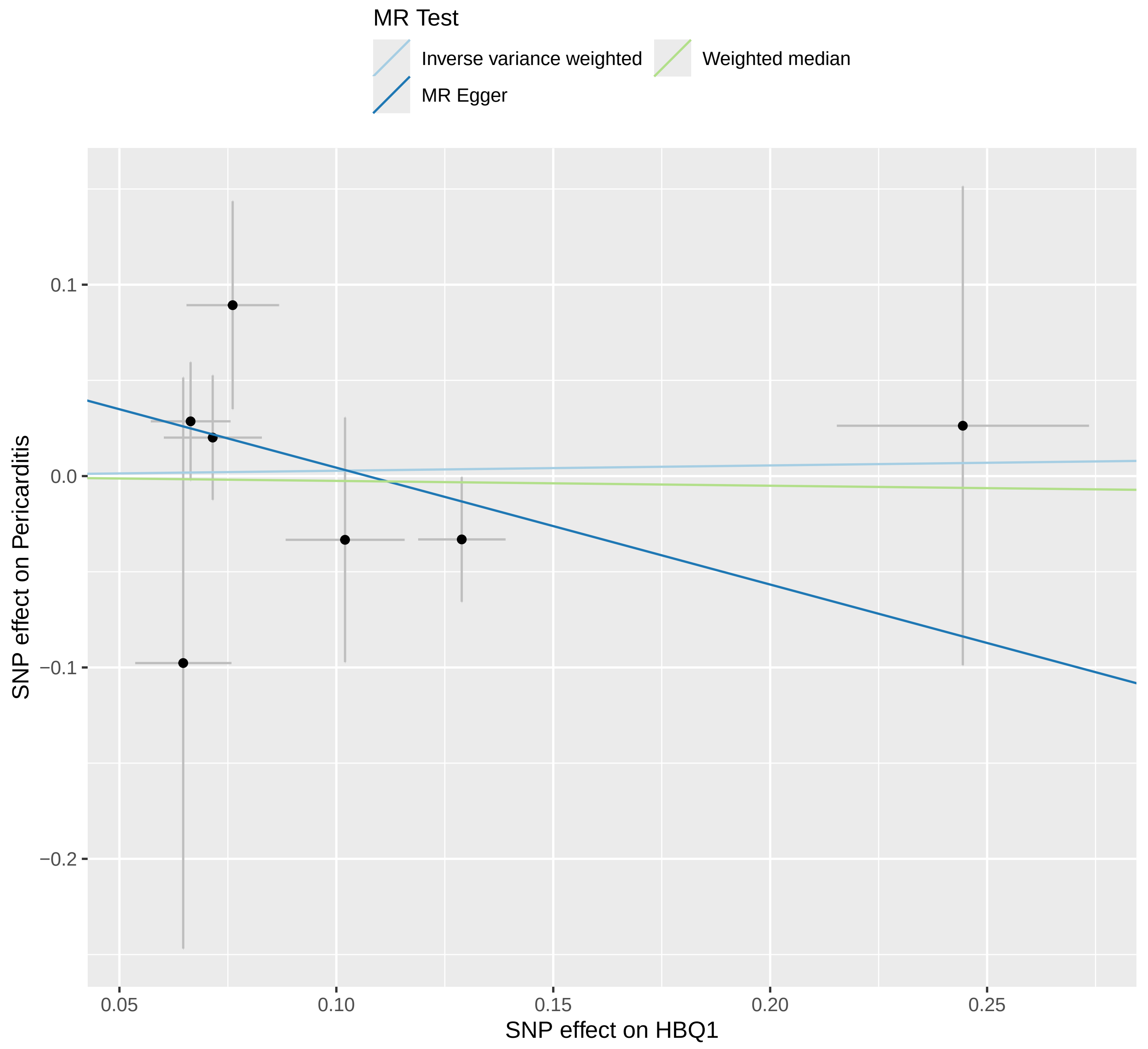

MR Test
Inverse variance weighted
Weighted median
MR Egger
0.1
0.0
−0.1
−0.2
SNP effect on Pericarditis
0.05
0.10
0.15
0.20
0.25
SNP effect on HBQ1

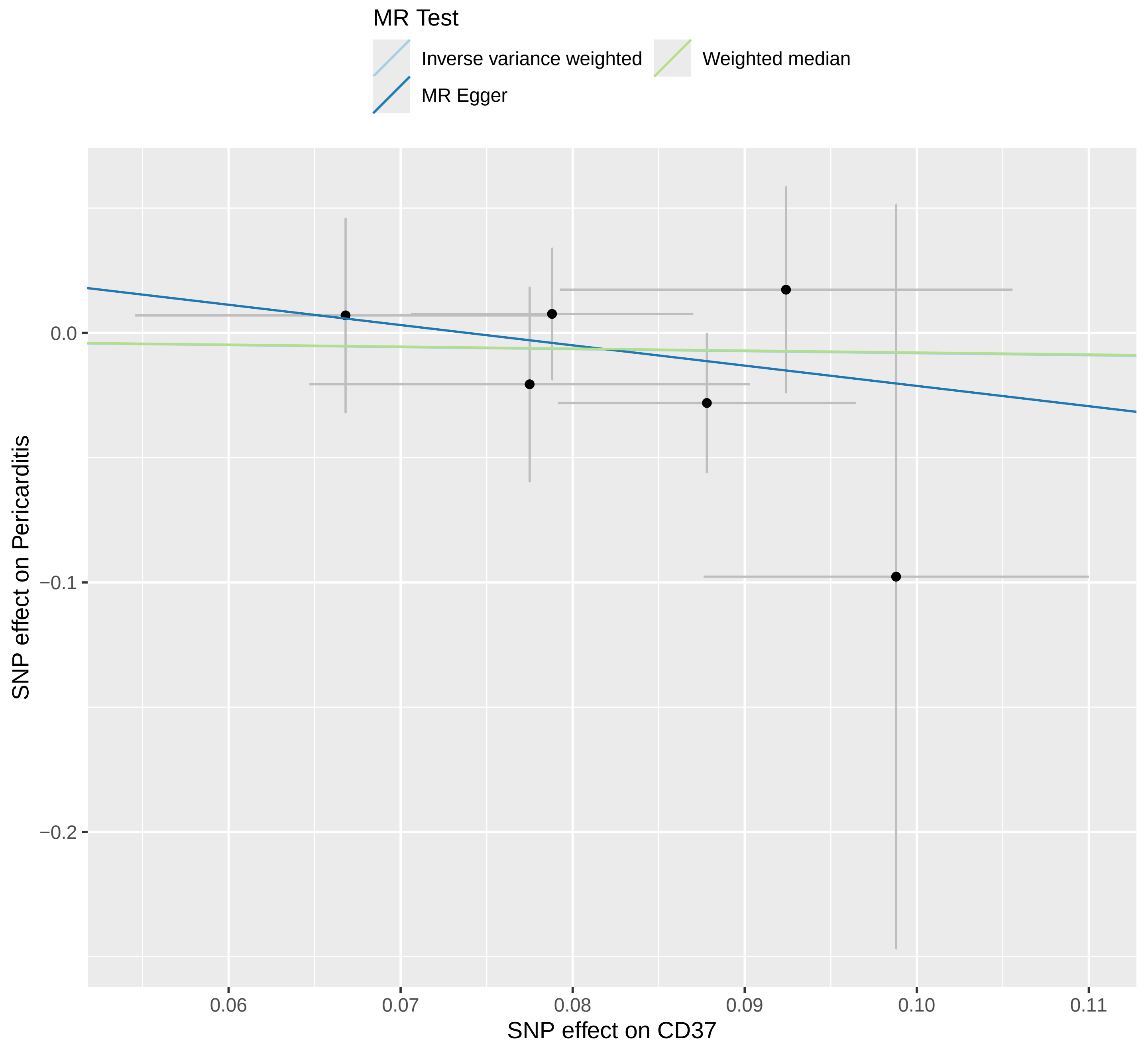
MR Test
Inverse variance weighted
Weighted median
MR Egger
0.0
−0.1
−0.2
0.06
0.07
0.08
0.09
0.10
0.11
SNP effect on CD37
SNP effect on Pericarditis

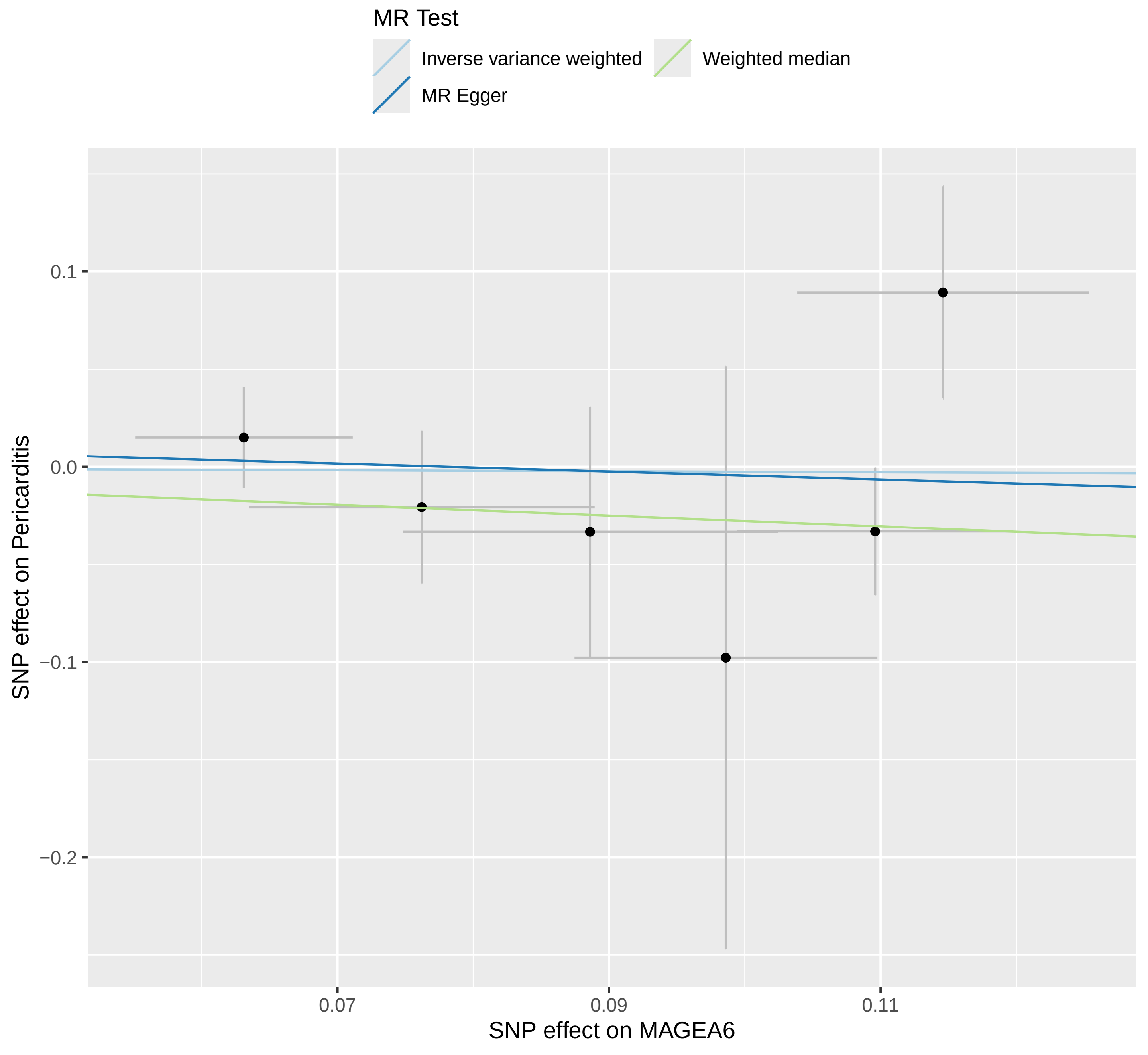

MR Test
Inverse variance weighted
MR Egger
Weighted median
0.1
0.0
−0.1
−0.2
SNP effect on Pericarditis
0.07
0.09
0.11
SNP effect on MAGEA6

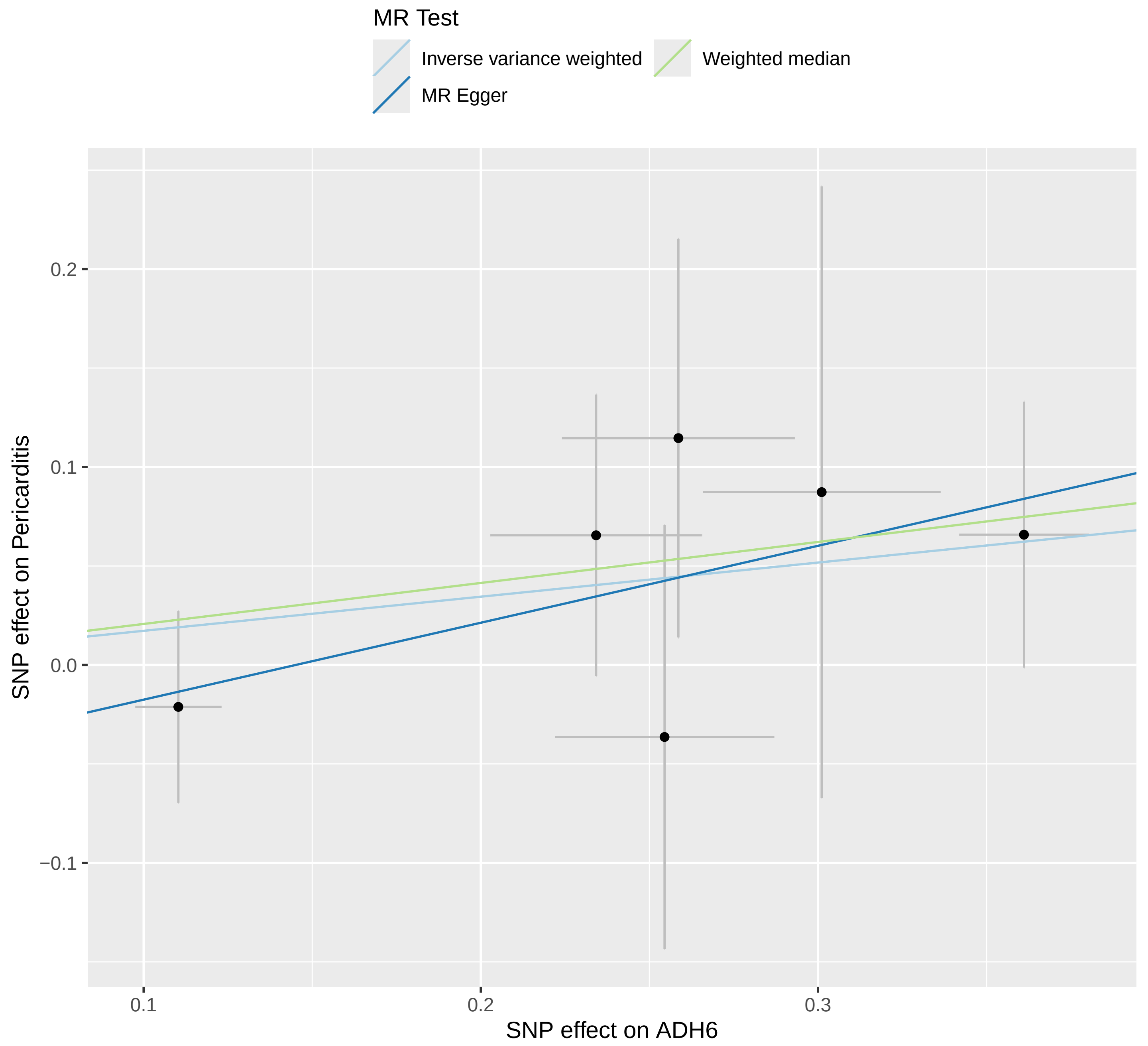
MR Test
Inverse variance weighted
Weighted median
MR Egger
0.2
0.1
0.0
−0.1
SNP effect on Pericarditis
0.1
0.2
0.3
SNP effect on ADH6

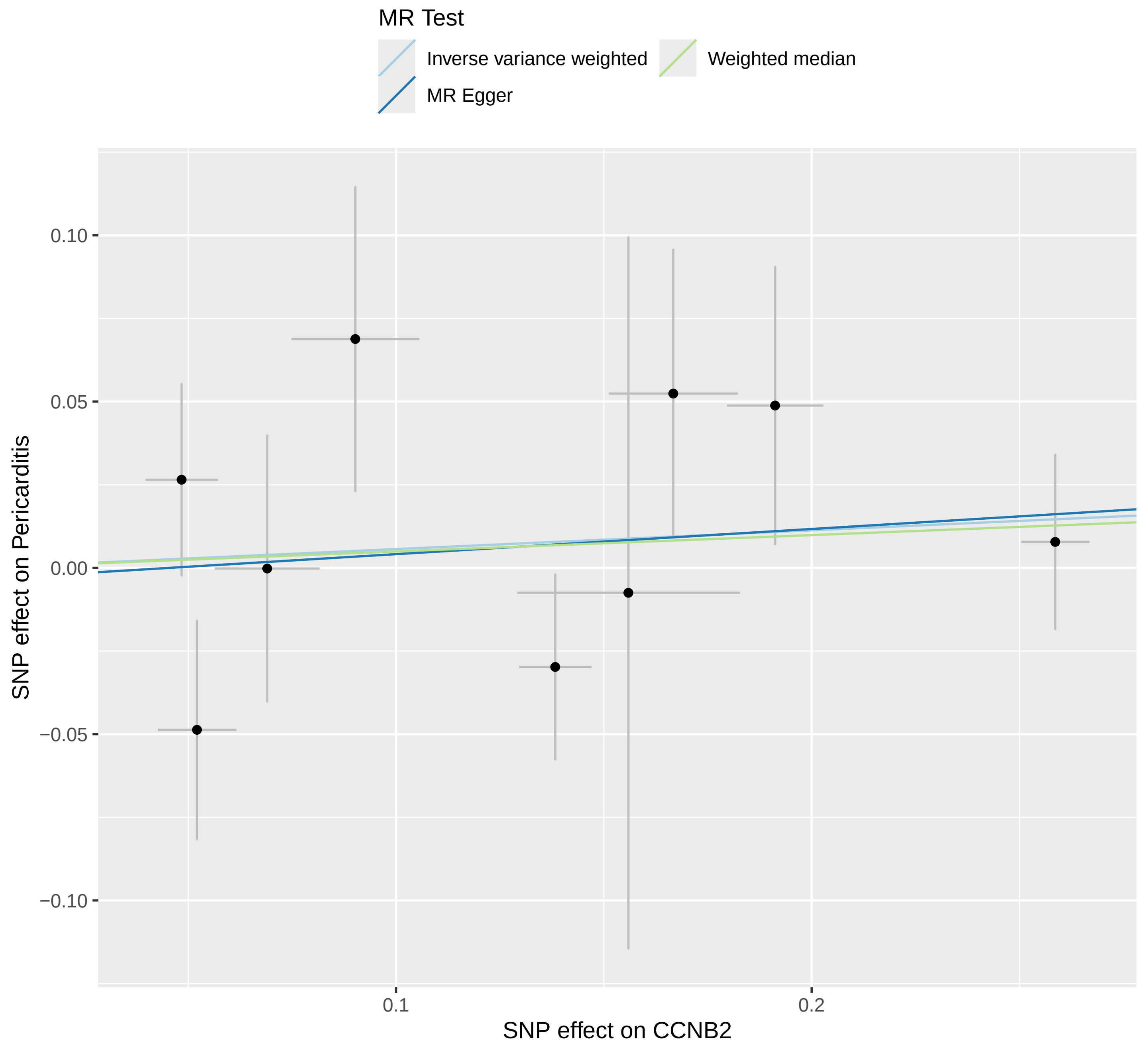
MR Test
Inverse variance weighted
MR Egger
Weighted median
0.10
0.05
0.00
−0.05
−0.10
SNP effect on Pericarditis
0.1
0.2
SNP effect on CCNB2

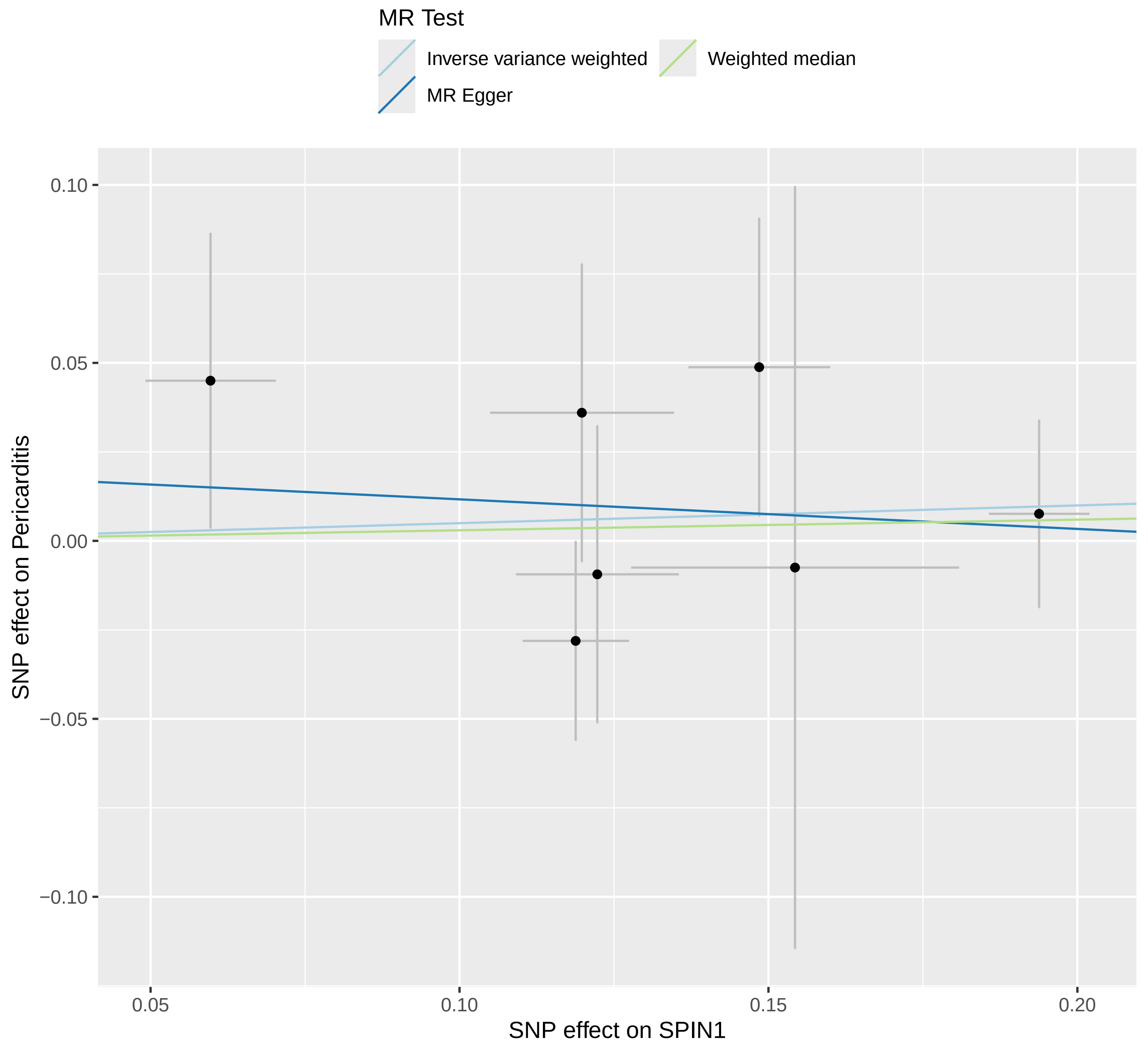
MR Test
Inverse variance weighted
MR Egger
Weighted median
0.10
0.05
0.00
−0.05
−0.10
SNP effect on Pericarditis
0.05
0.10
0.15
0.20
SNP effect on SPIN1

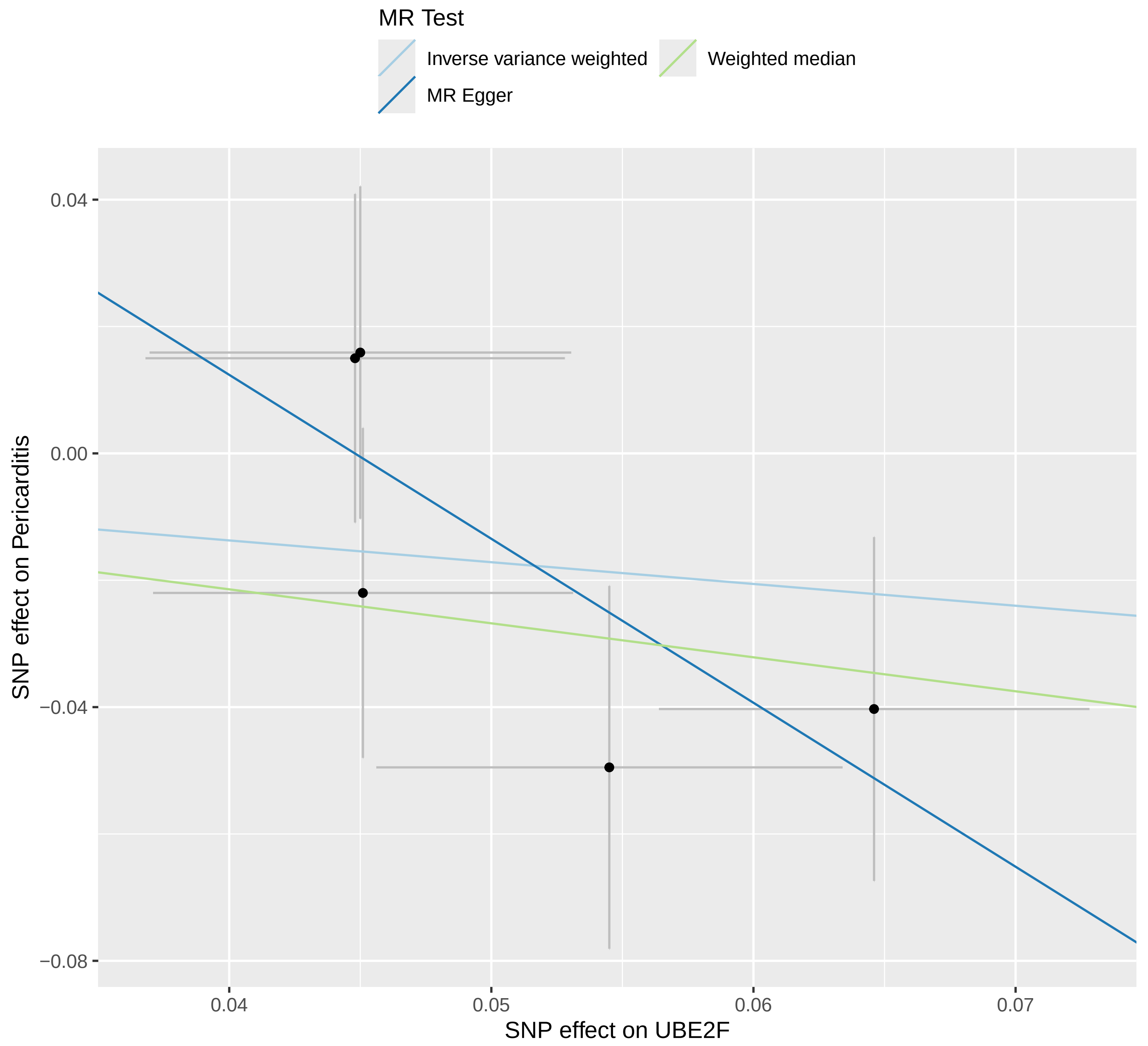

MR Test
Inverse variance weighted
Weighted median
MR Egger
SNP effect on Pericarditis
0.04
0.00
−0.04
−0.08
0.04
0.05
0.06
0.07
SNP effect on UBE2F

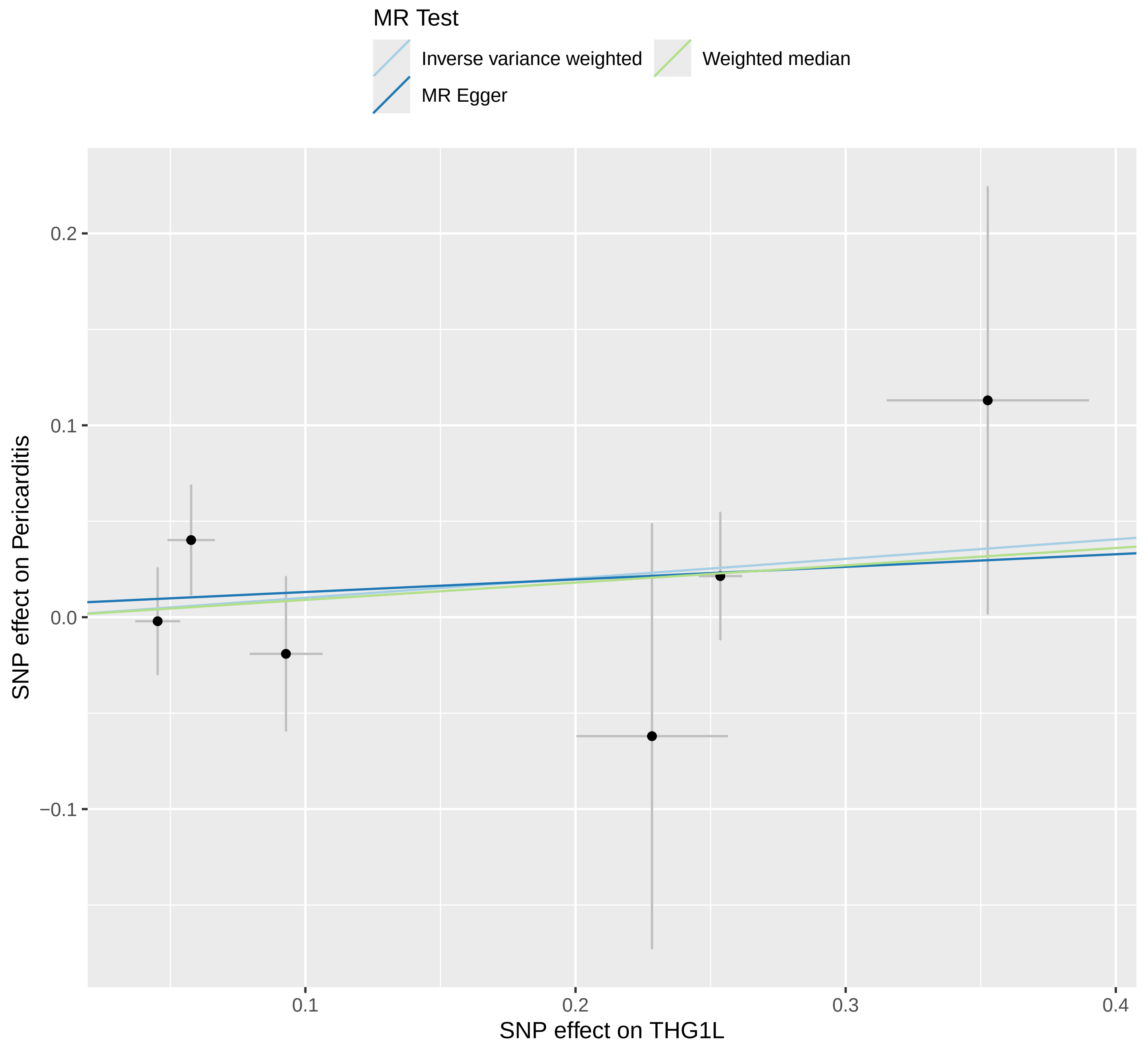
MR Test
Inverse variance weighted
Weighted median
MR Egger
0.2
0.1
0.0
−0.1
SNP effect on Pericarditis
0.1
0.2
0.3
0.4
SNP effect on THG1L

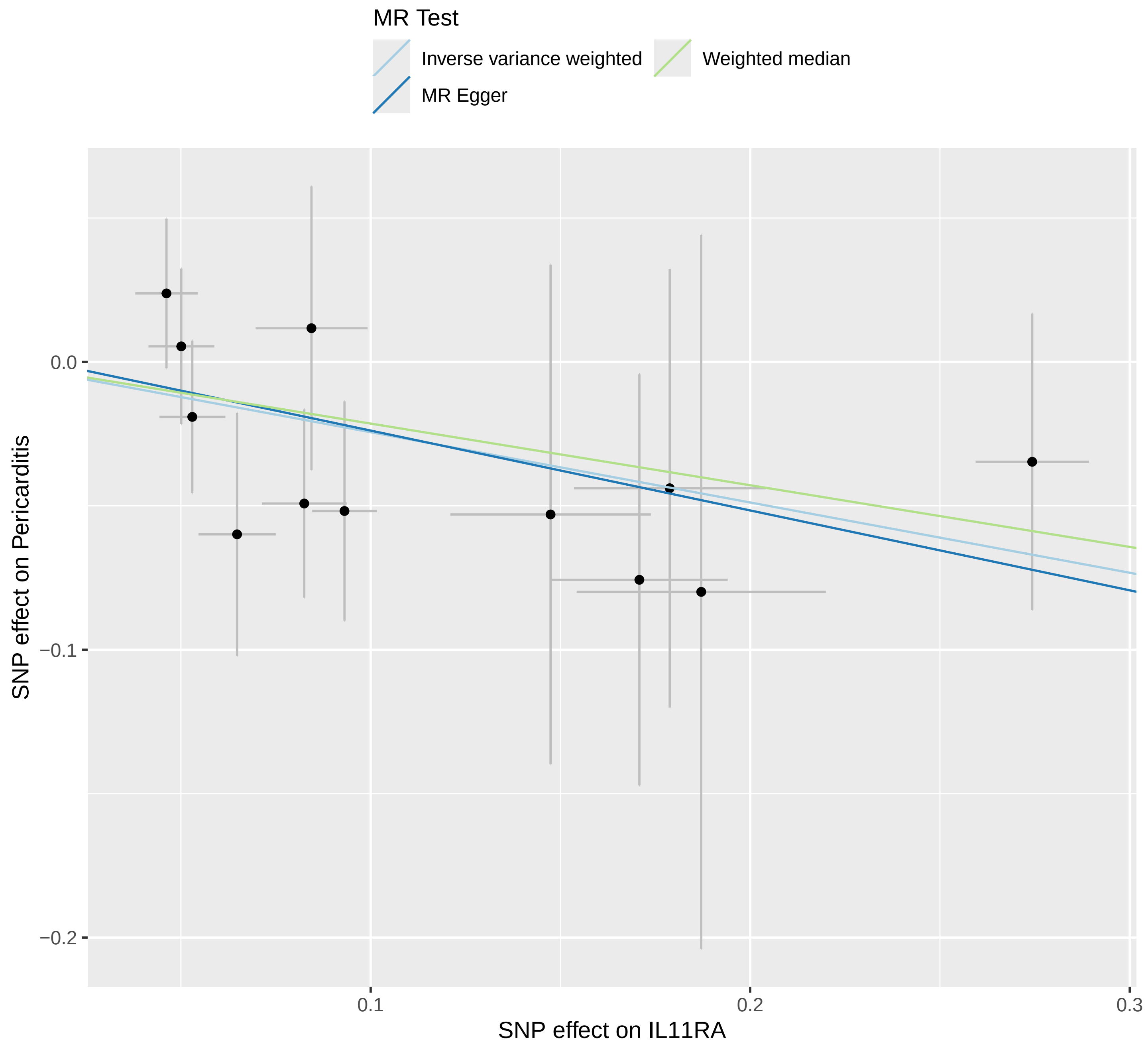
MR Test
Inverse variance weighted
Weighted median
MR Egger
SNP effect on Pericarditis
0.0
−0.1
−0.2
0.1
0.2
0.3
SNP effect on IL11RA

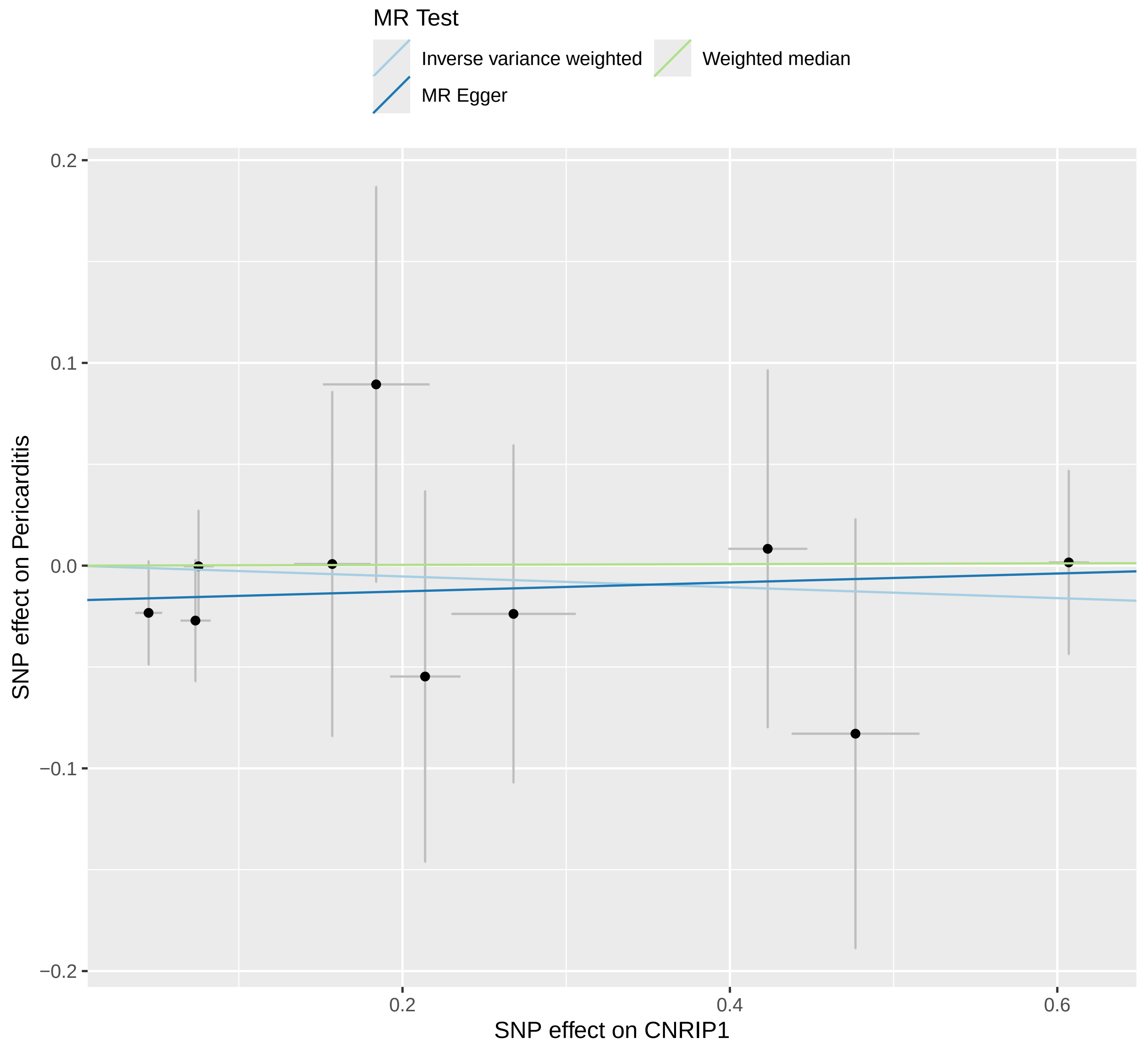
MR Test
Inverse variance weighted
Weighted median
MR Egger
0.2
0.1
0.0
−0.1
−0.2
SNP effect on Pericarditis
0.2
0.4
0.6
SNP effect on CNRIP1

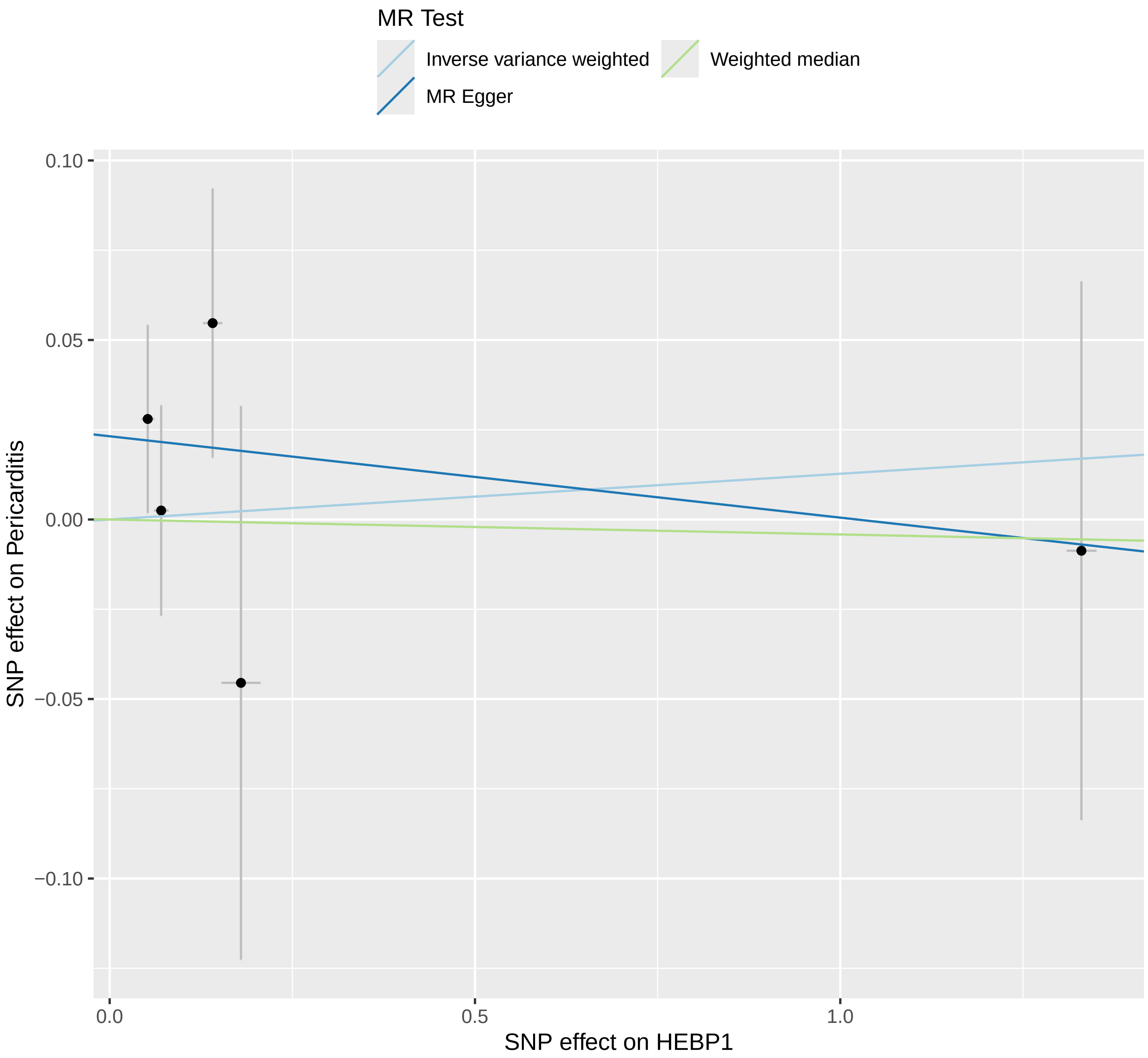
MR Test
Inverse variance weighted
MR Egger
Weighted median
0.10
0.05
0.00
−0.05
−0.10
SNP effect on Pericarditis
0.0
0.5
1.0
SNP effect on HEBP1

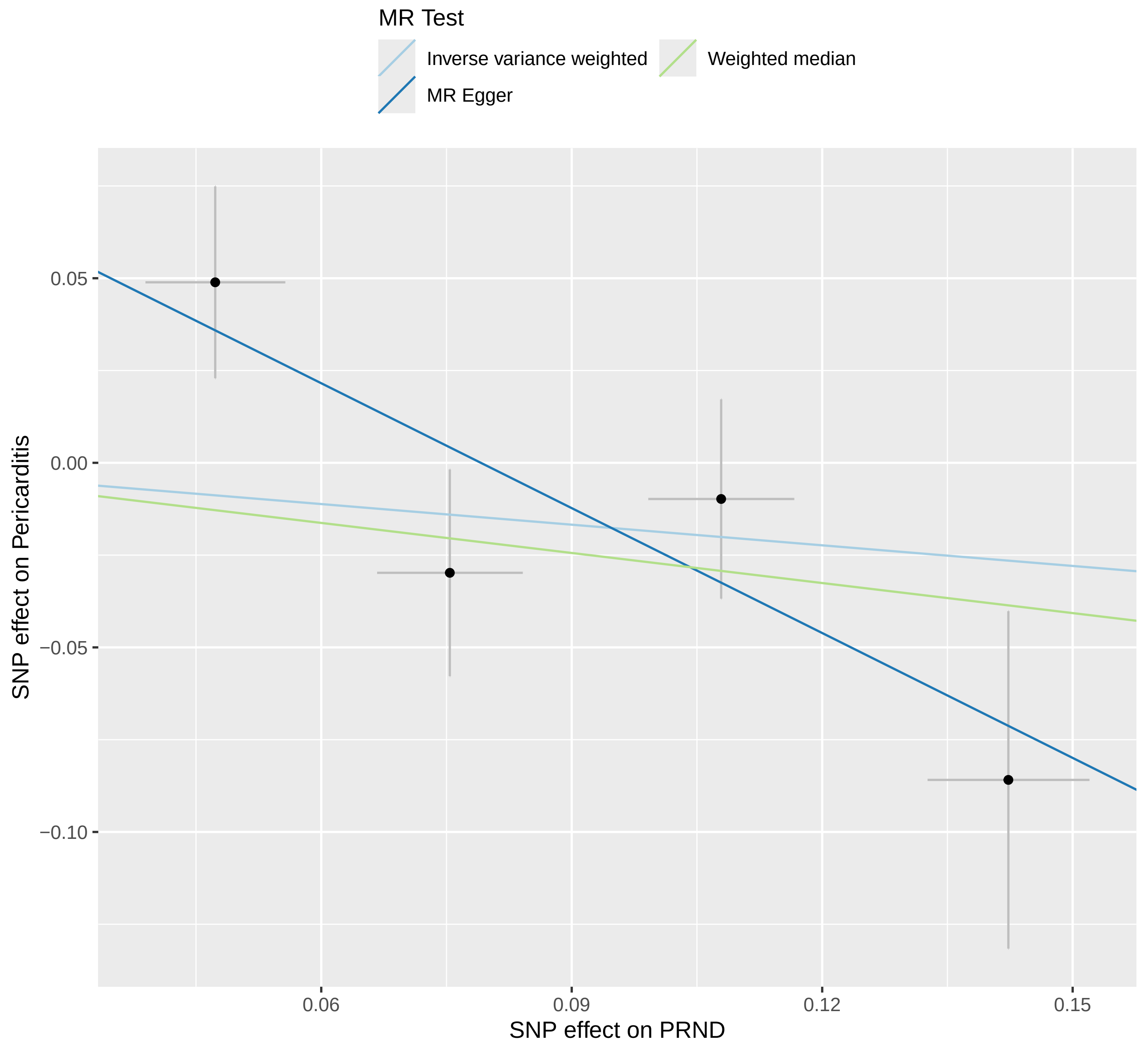

MR Test
Inverse variance weighted
Weighted median
MR Egger
0.05
0.00
−0.05
−0.10
SNP effect on Pericarditis
0.06
0.09
0.12
0.15
SNP effect on PRND

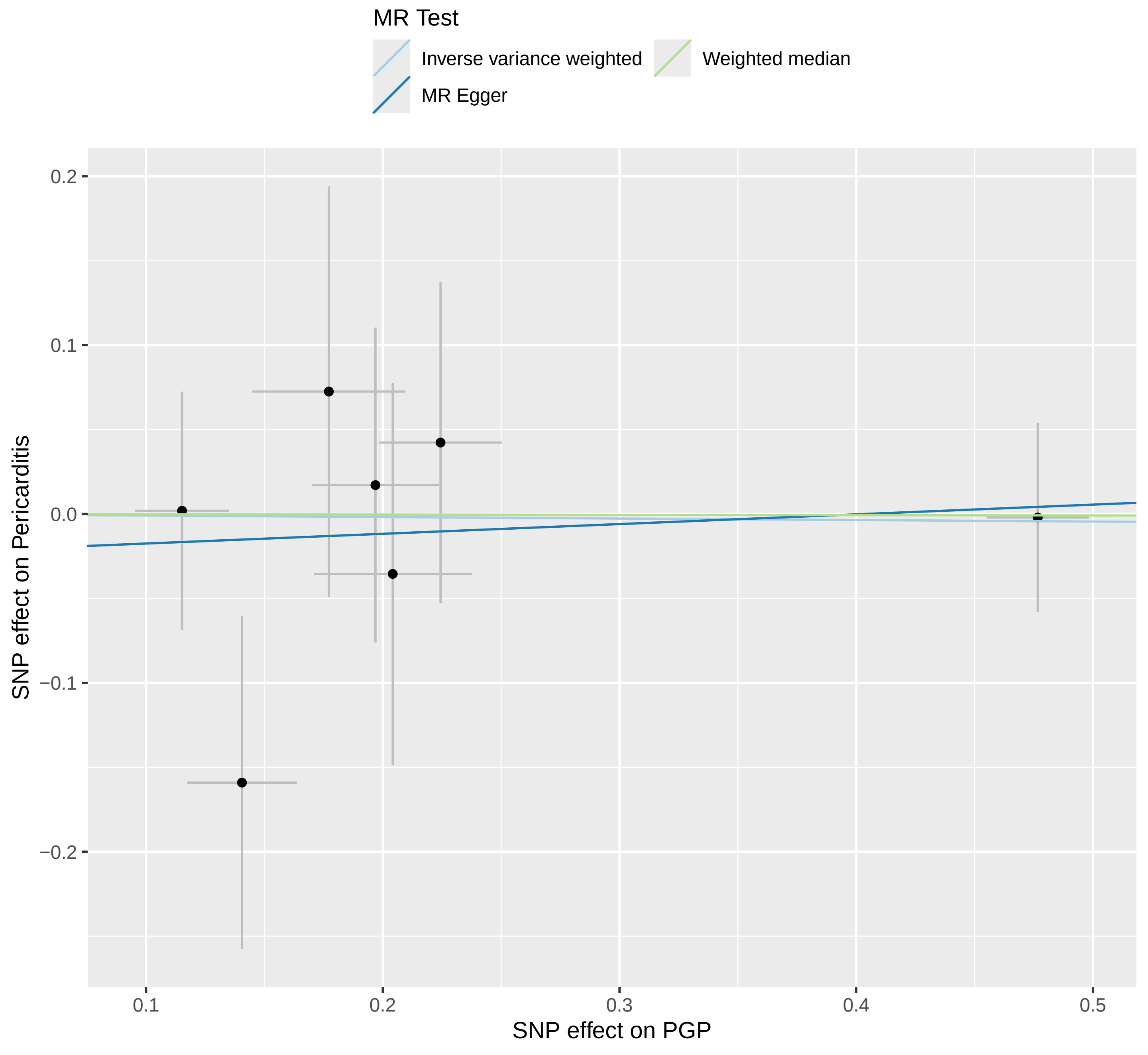
MR Test
Inverse variance weighted
Weighted median
MR Egger
SNP effect on Pericarditis
0.2
0.1
0.0
−0.1
−0.2
0.1
0.2
0.3
0.4
0.5
SNP effect on PGP

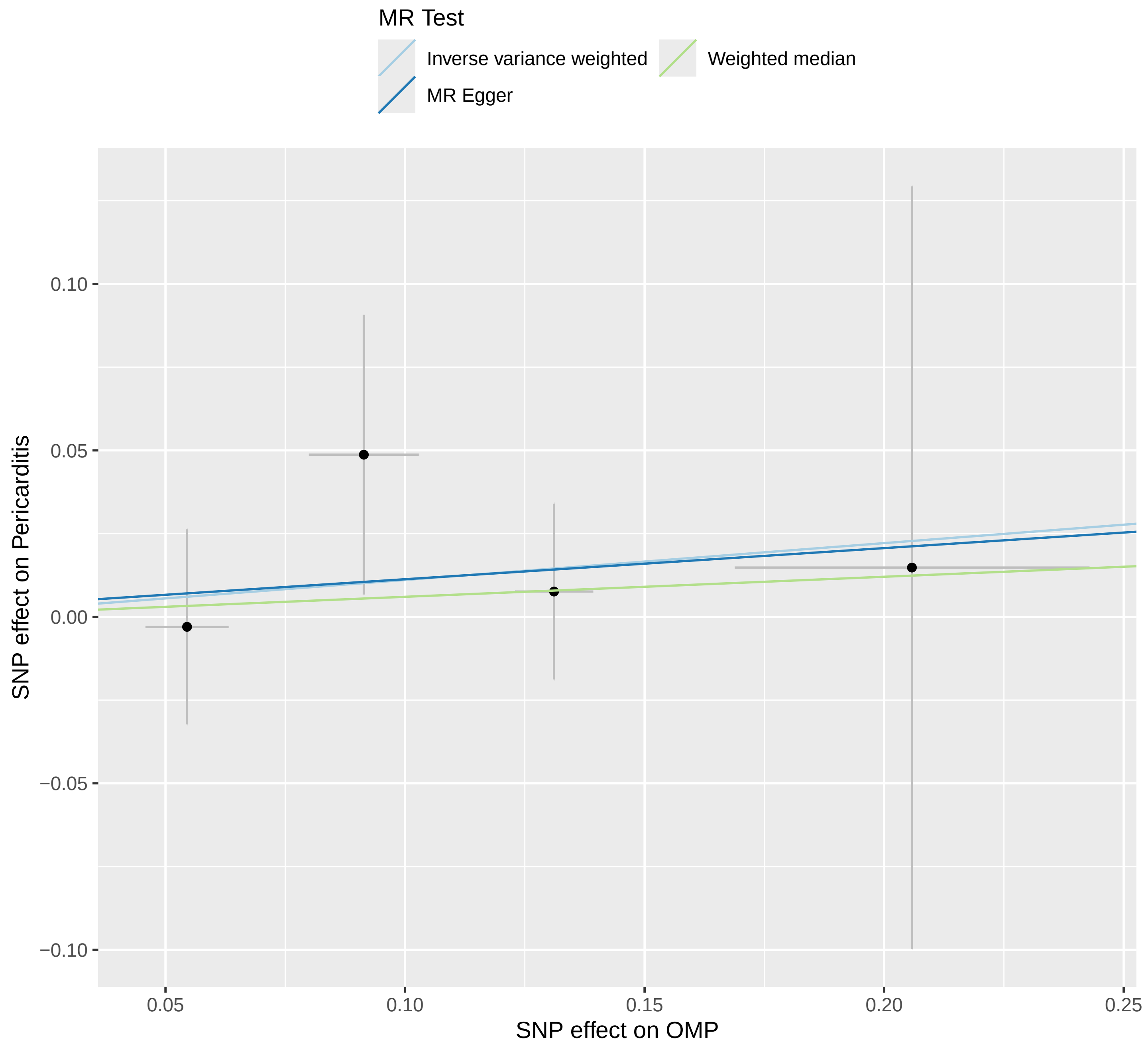
MR Test
Inverse variance weighted
MR Egger
Weighted median
0.10
0.05
0.00
−0.05
−0.10
SNP effect on Pericarditis
0.05
0.10
0.15
0.20
0.25
SNP effect on OMP

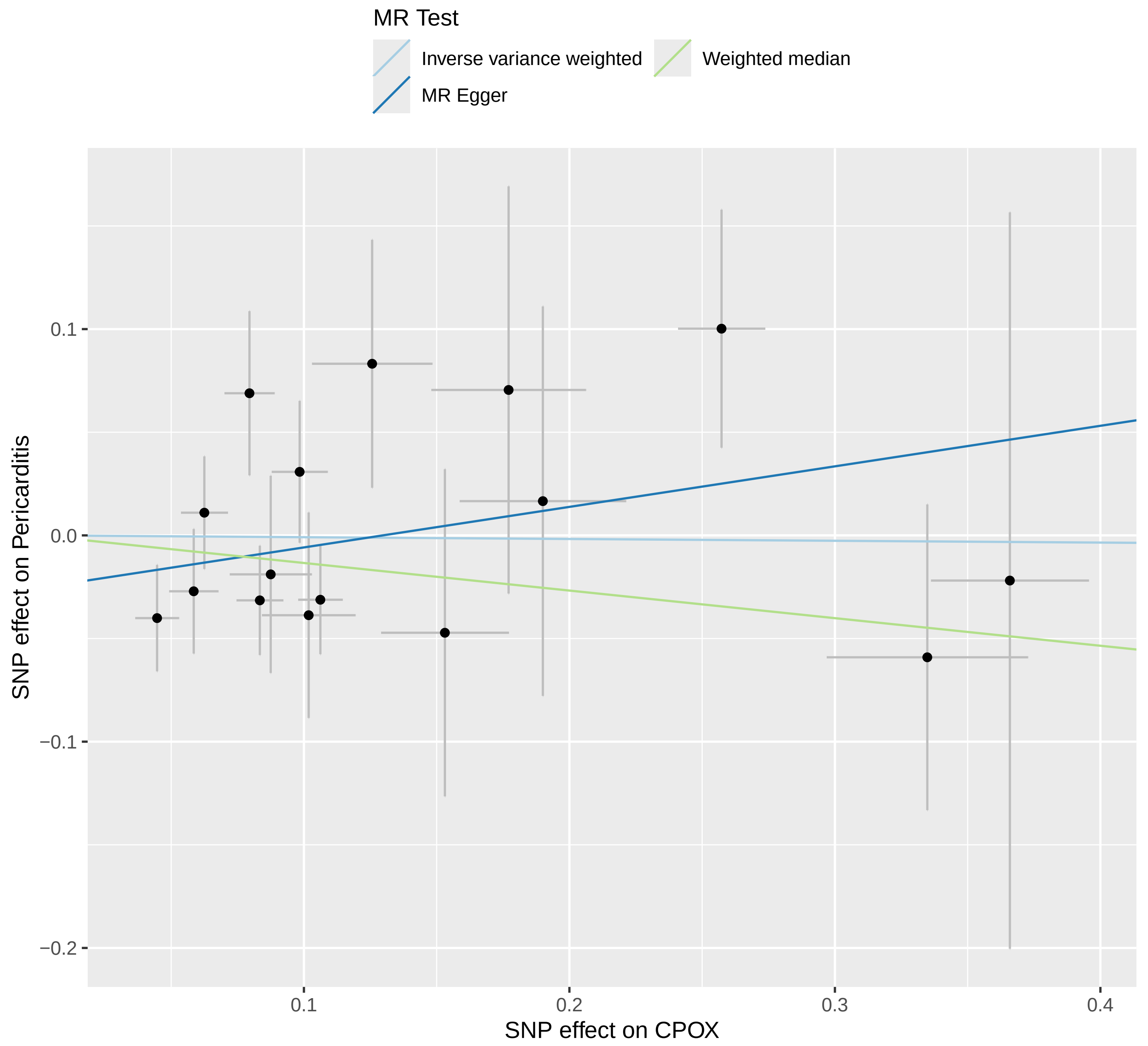
MR Test
Inverse variance weighted
MR Egger
Weighted median
0.1
0.0
−0.1
−0.2
0.1
0.2
0.3
0.4
SNP effect on Pericarditis
SNP effect on CPOX

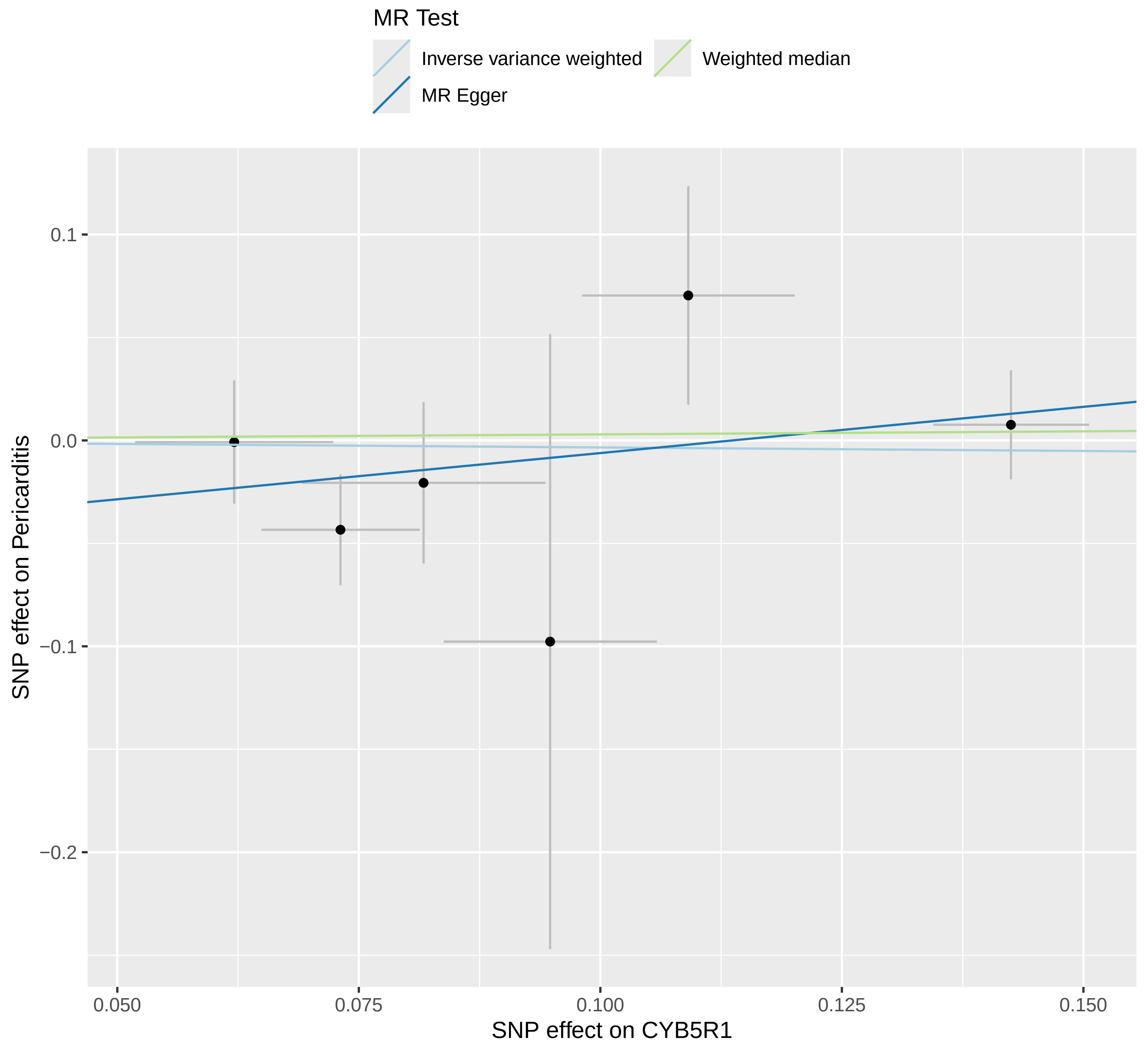
MR Test
Inverse variance weighted
Weighted median
MR Egger
SNP effect on Pericarditis
0.1
0.0
−0.1
−0.2
0.050
0.075
0.100
0.125
0.150
SNP effect on CYB5R1

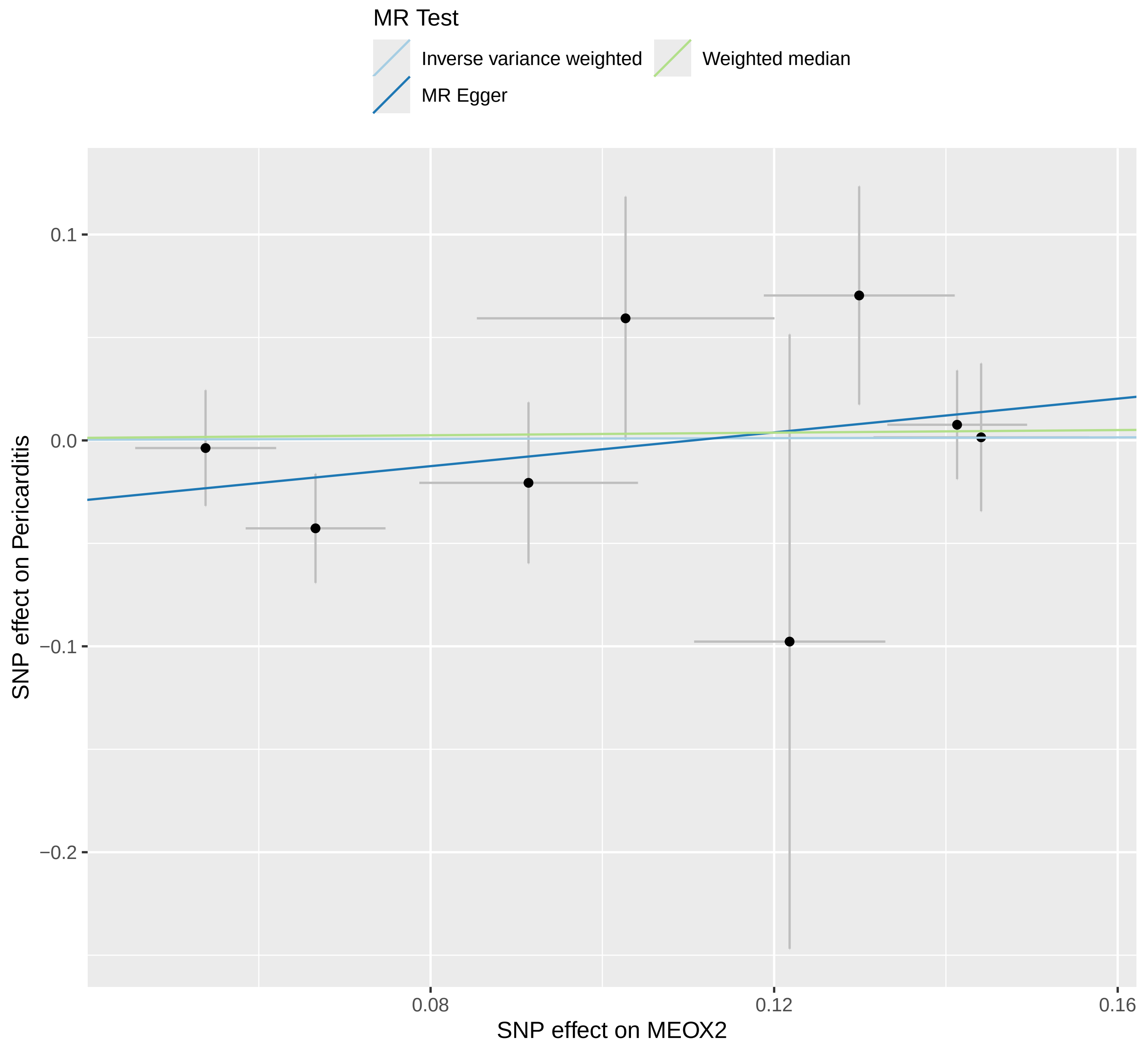
MR Test
Inverse variance weighted
Weighted median
MR Egger
SNP effect on Pericarditis
0.1
0.0
−0.1
−0.2
0.08
0.12
0.16
SNP effect on MEOX2

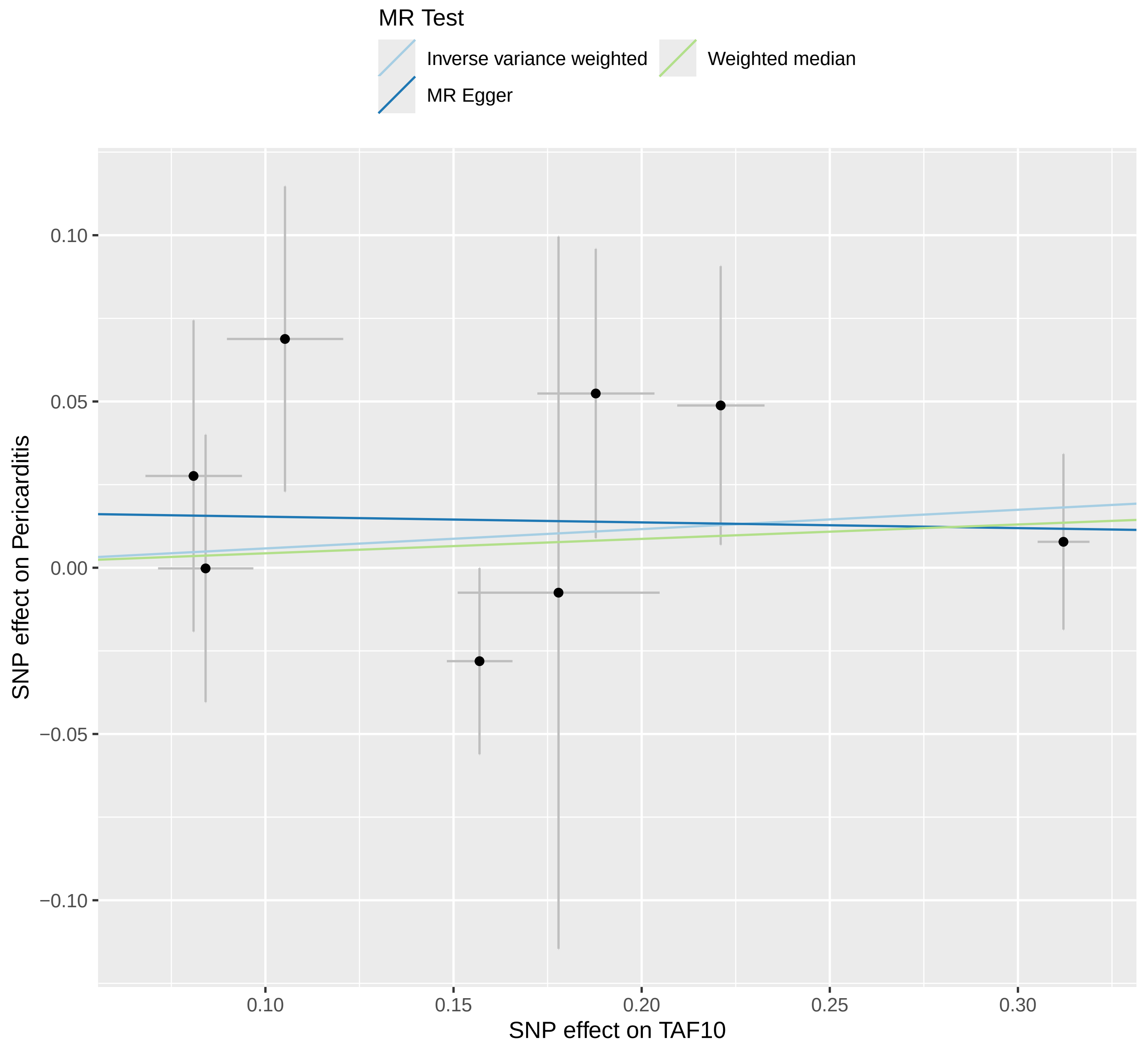
MR Test
Inverse variance weighted
MR Egger
Weighted median
0.10
0.05
0.00
−0.05
−0.10
SNP effect on Pericarditis
0.10
0.15
0.20
0.25
0.30
SNP effect on TAF10

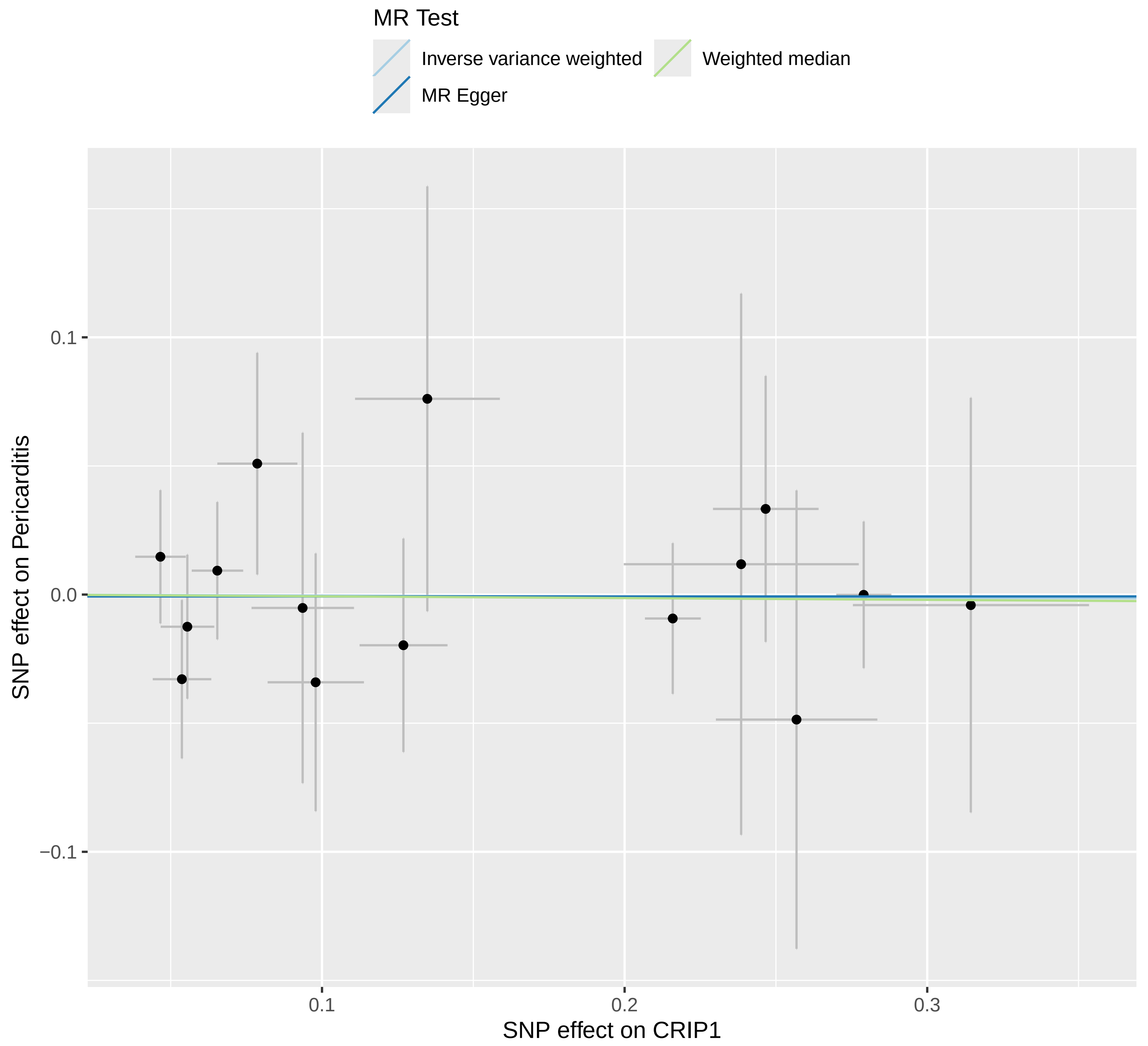
MR Test
Inverse variance weighted
Weighted median
MR Egger
0.1
0.0
−0.1
SNP effect on Pericarditis
0.1
0.2
0.3
SNP effect on CRIP1

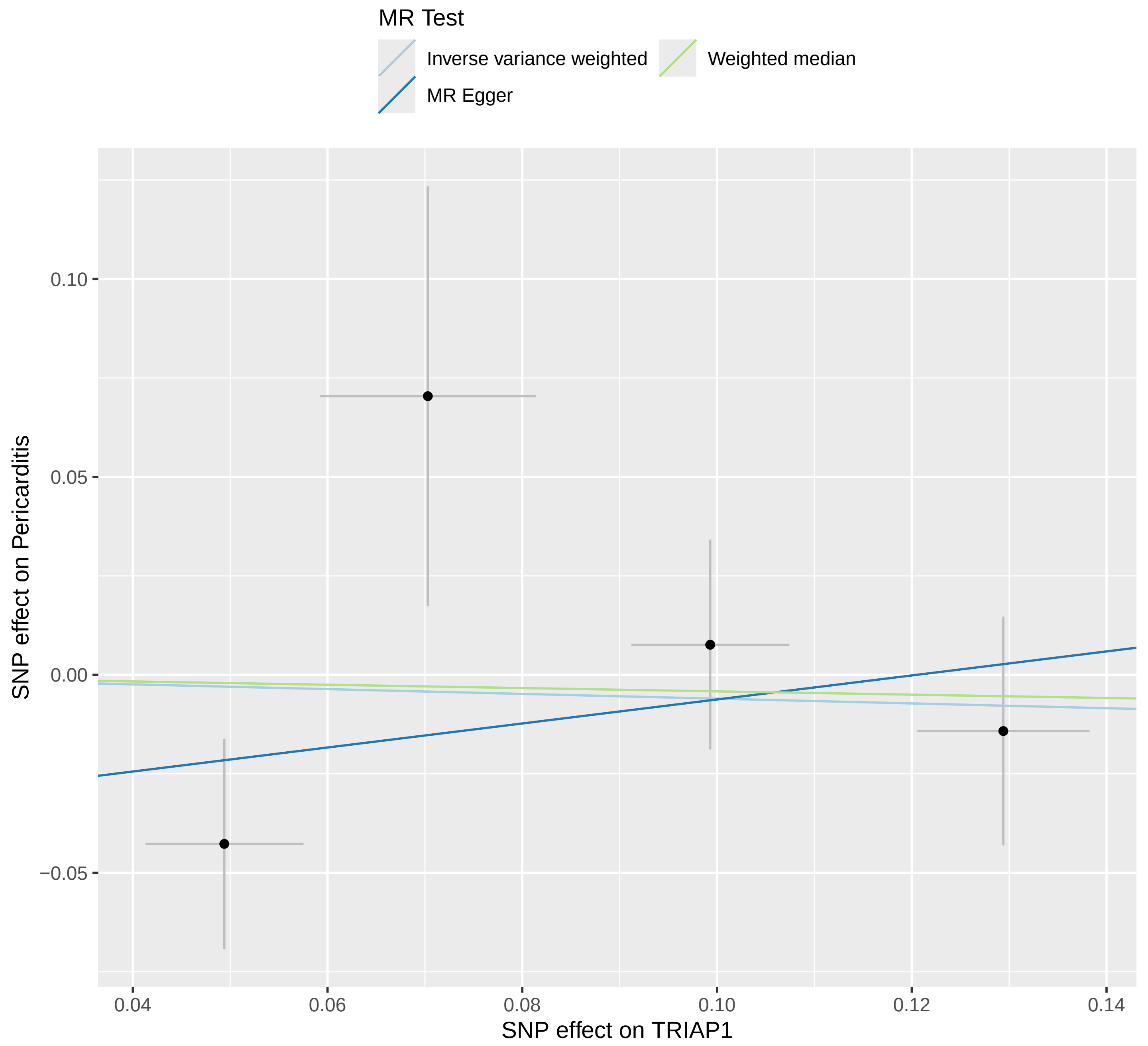
MR Test
Inverse variance weighted
Weighted median
MR Egger
SNP effect on Pericarditis
0.10
0.05
0.00
−0.05
0.04
0.06
0.08
0.10
0.12
0.14
SNP effect on TRIAP1

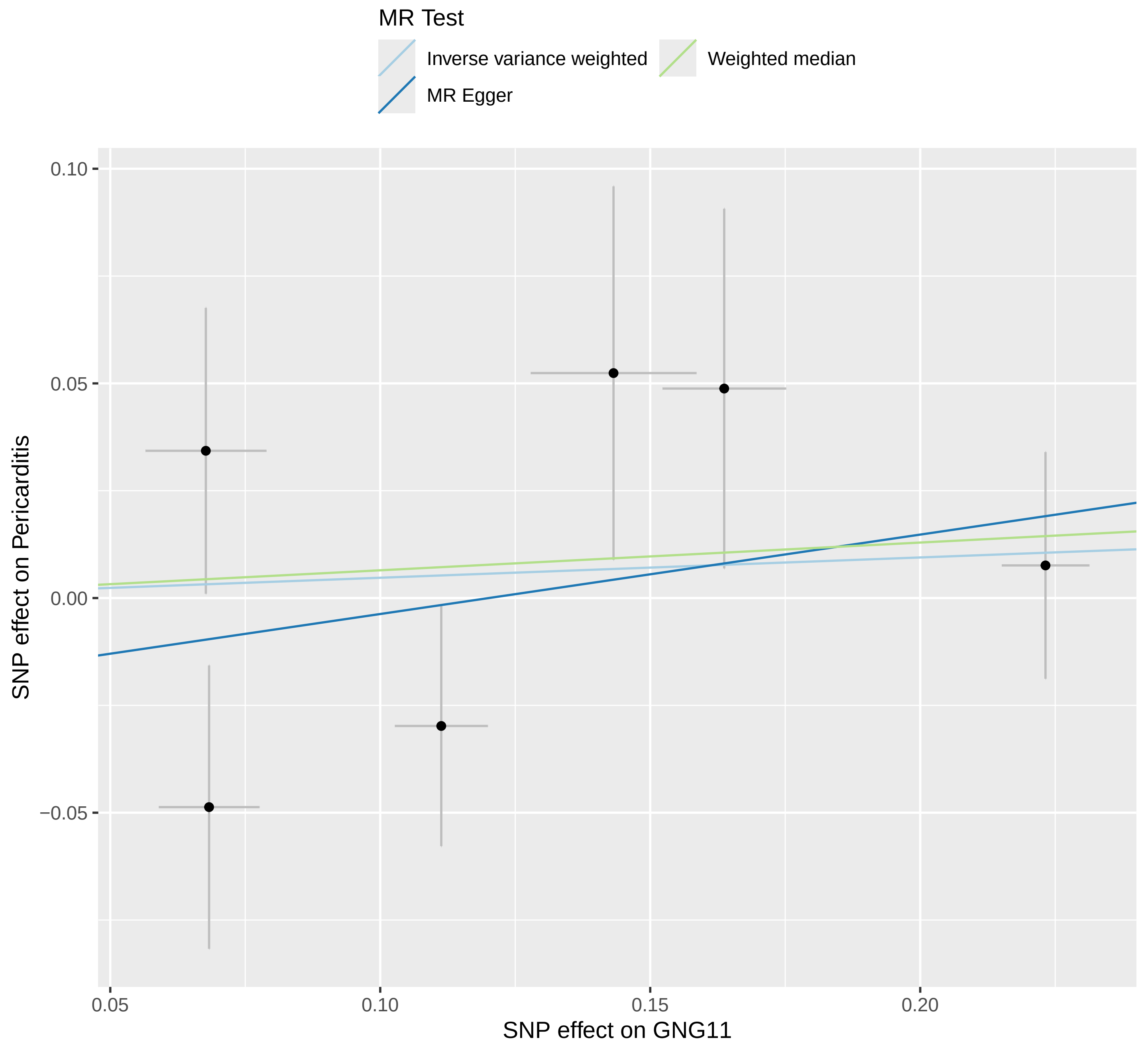

MR Test
Inverse variance weighted
MR Egger
Weighted median
SNP effect on Pericarditis
0.10
0.05
0.00
−0.05
0.05
0.10
0.15
0.20
SNP effect on GNG11

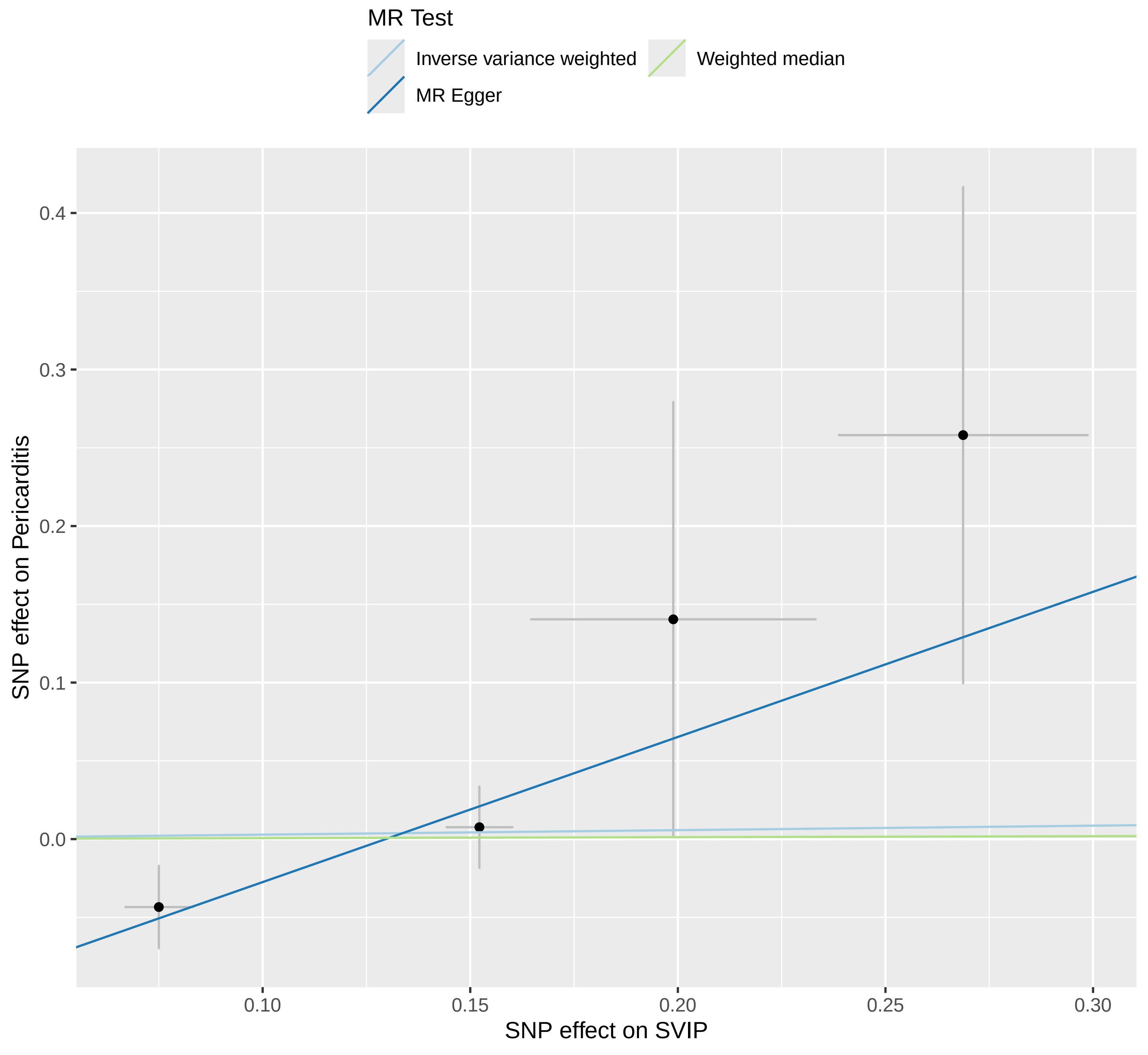
MR Test
Inverse variance weighted
MR Egger
Weighted median
SNP effect on Pericarditis
0.4
0.3
0.2
0.1
0.0
0.10
0.15
0.20
0.25
0.30
SNP effect on SVIP

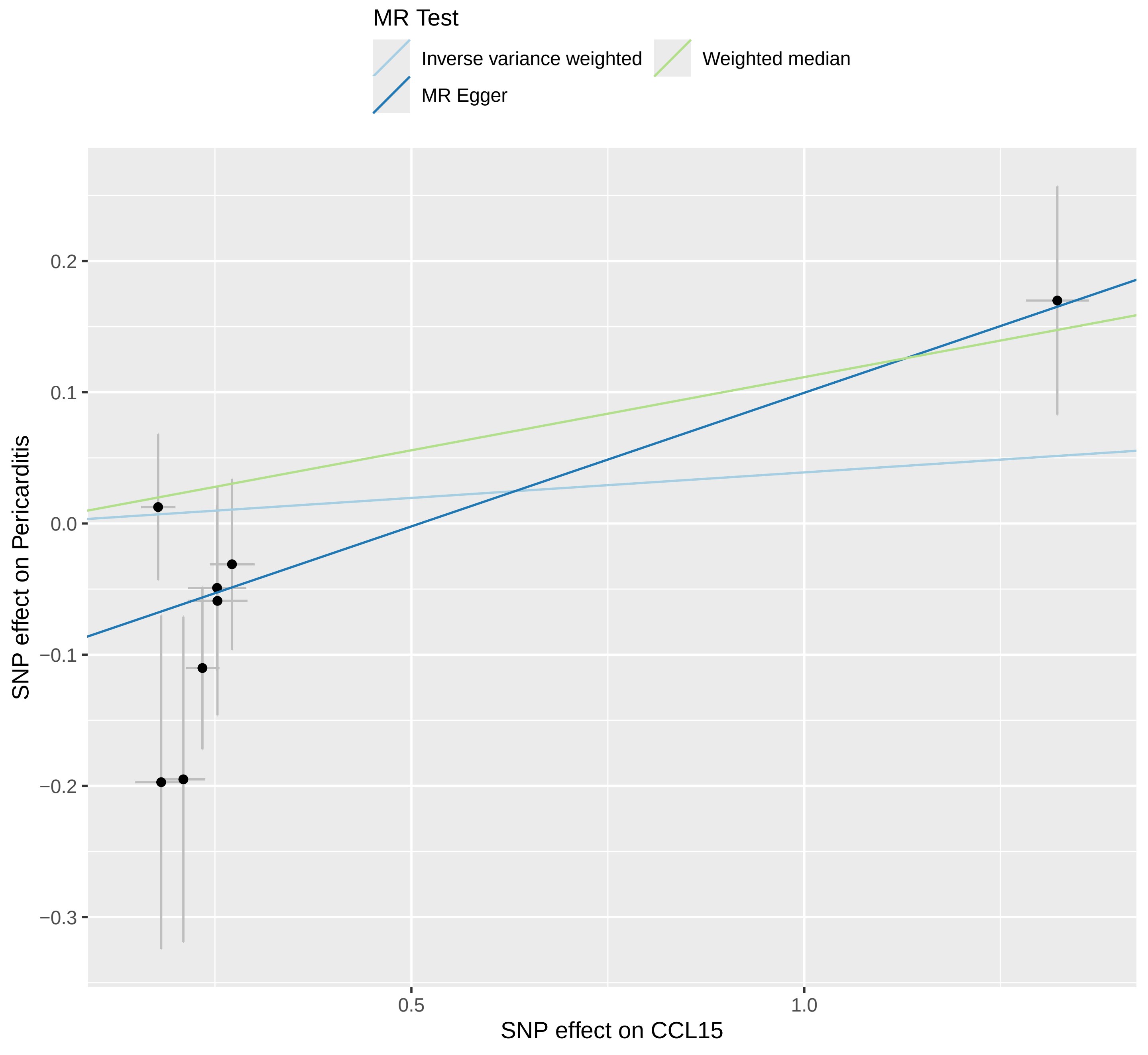

MR Test
Inverse variance weighted
Weighted median
MR Egger
0.2
0.1
0.0
−0.1
−0.2
−0.3
SNP effect on Pericarditis
0.5
1.0
SNP effect on CCL15

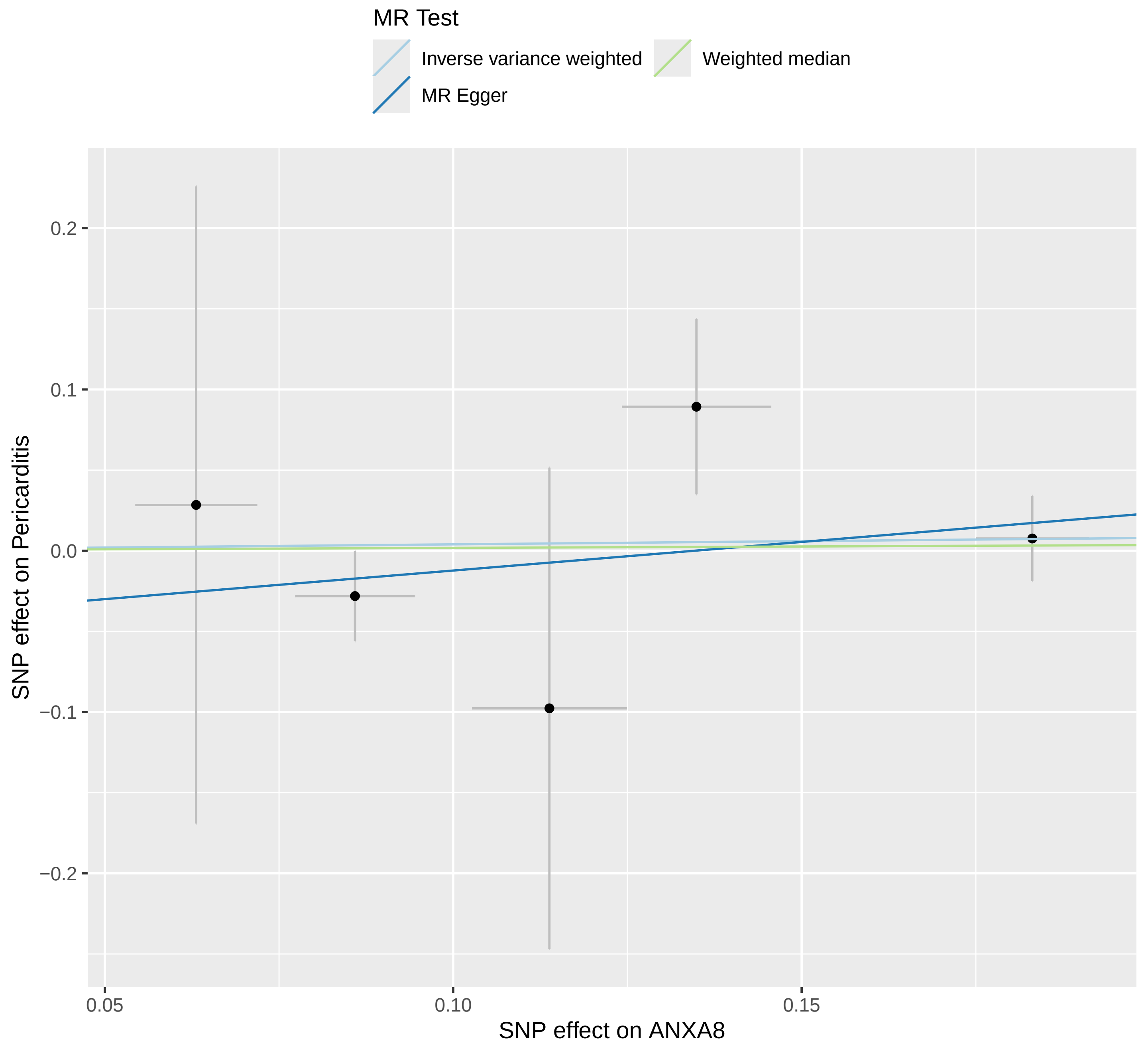
MR Test
Inverse variance weighted
Weighted median
MR Egger
0.2
0.1
0.0
−0.1
−0.2
SNP effect on Pericarditis
0.05
0.10
0.15
SNP effect on ANXA8

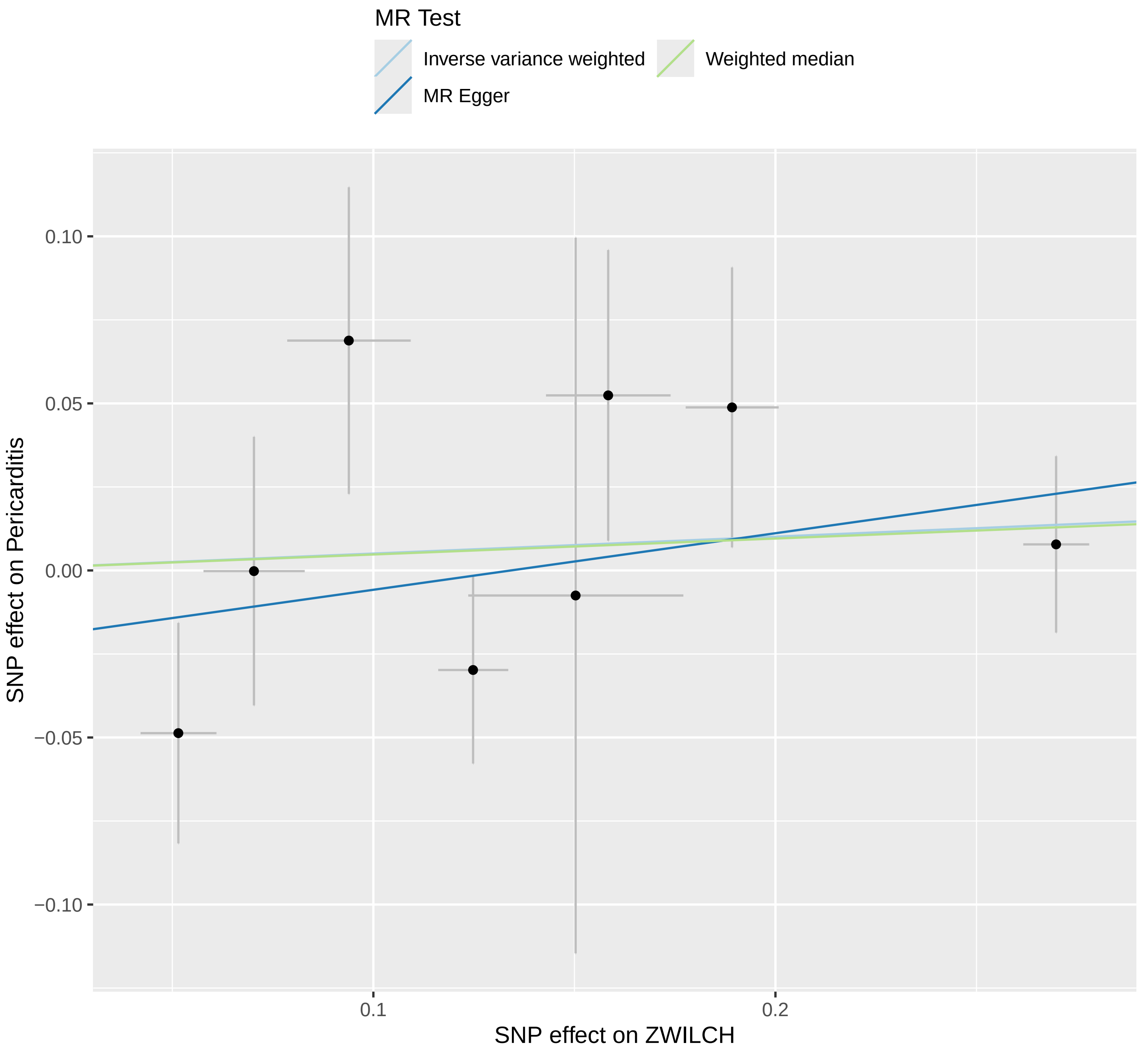
MR Test
Inverse variance weighted
Weighted median
MR Egger
0.10
0.05
0.00
−0.05
−0.10
SNP effect on Pericarditis
0.1
0.2
SNP effect on ZWILCH

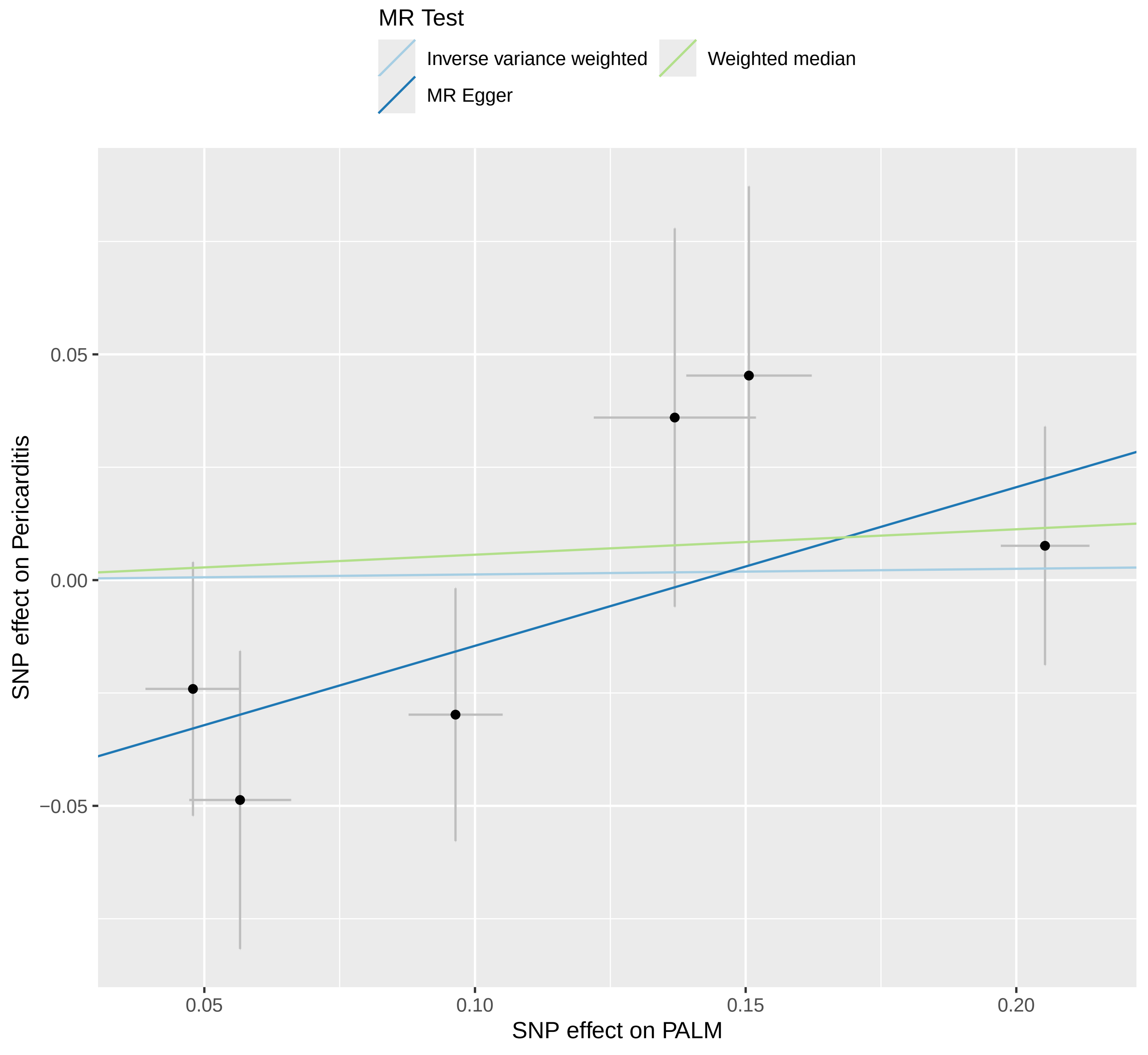
MR Test
Inverse variance weighted
Weighted median
MR Egger
SNP effect on Pericarditis
0.05
0.00
−0.05
0.05
0.10
0.15
0.20
SNP effect on PALM

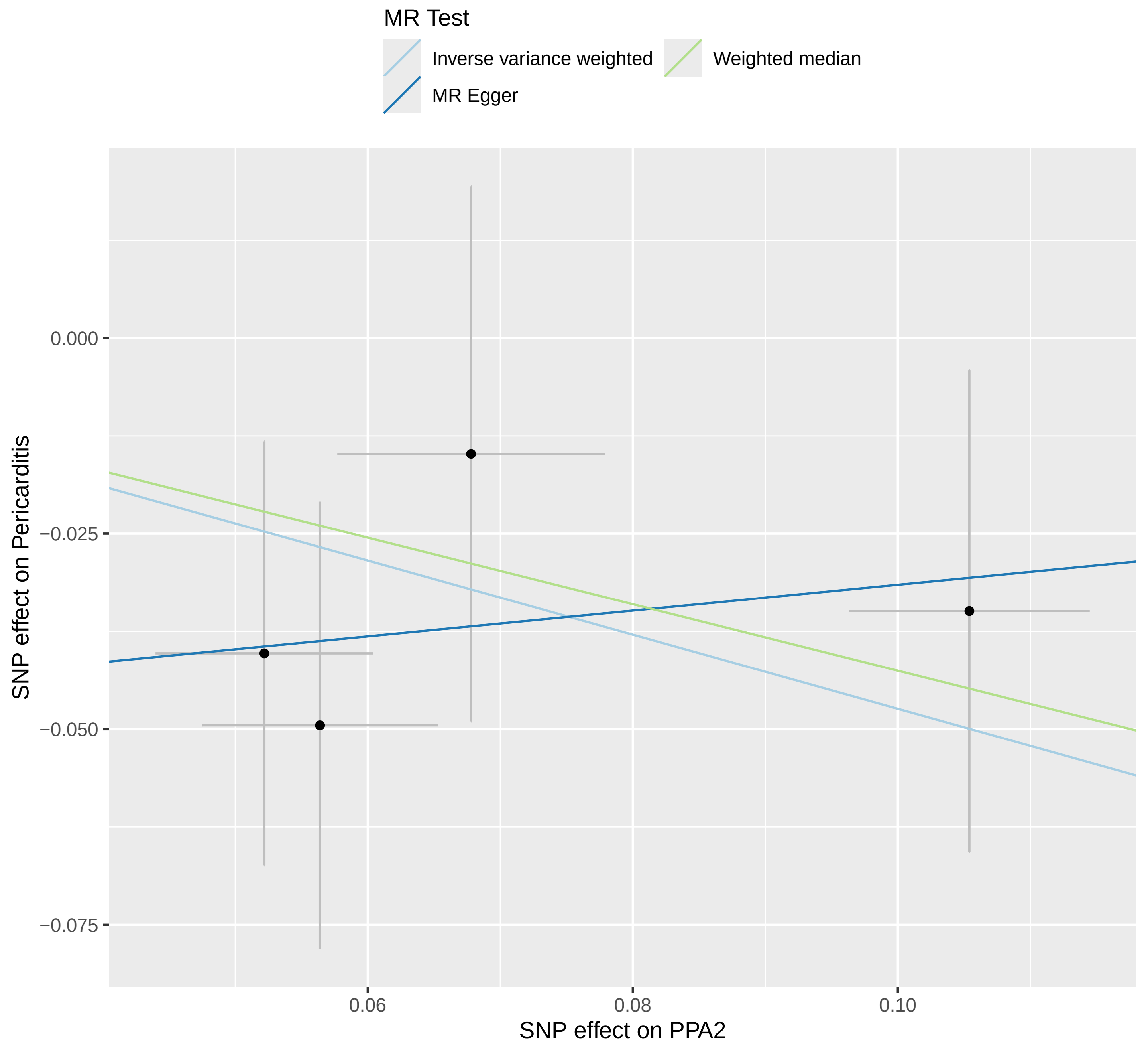

MR Test
Inverse variance weighted
MR Egger
Weighted median
0.000
−0.025
−0.050
−0.075
SNP effect on Pericarditis
0.06
0.08
0.10
SNP effect on PPA2

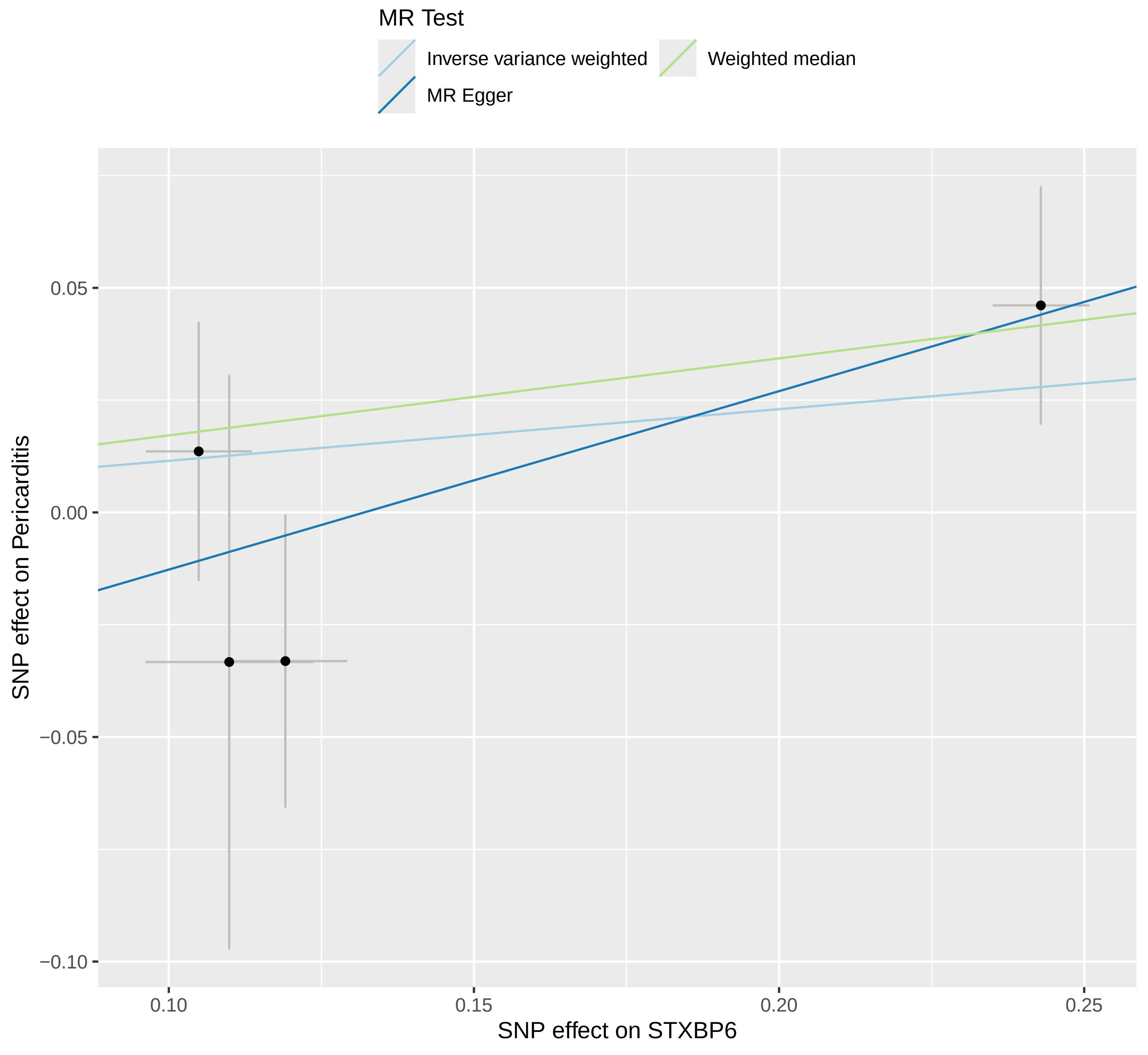
MR Test
Inverse variance weighted
Weighted median
MR Egger
SNP effect on Pericarditis
0.05
0.00
−0.05
−0.10
0.10
0.15
0.20
0.25
SNP effect on STXBP6

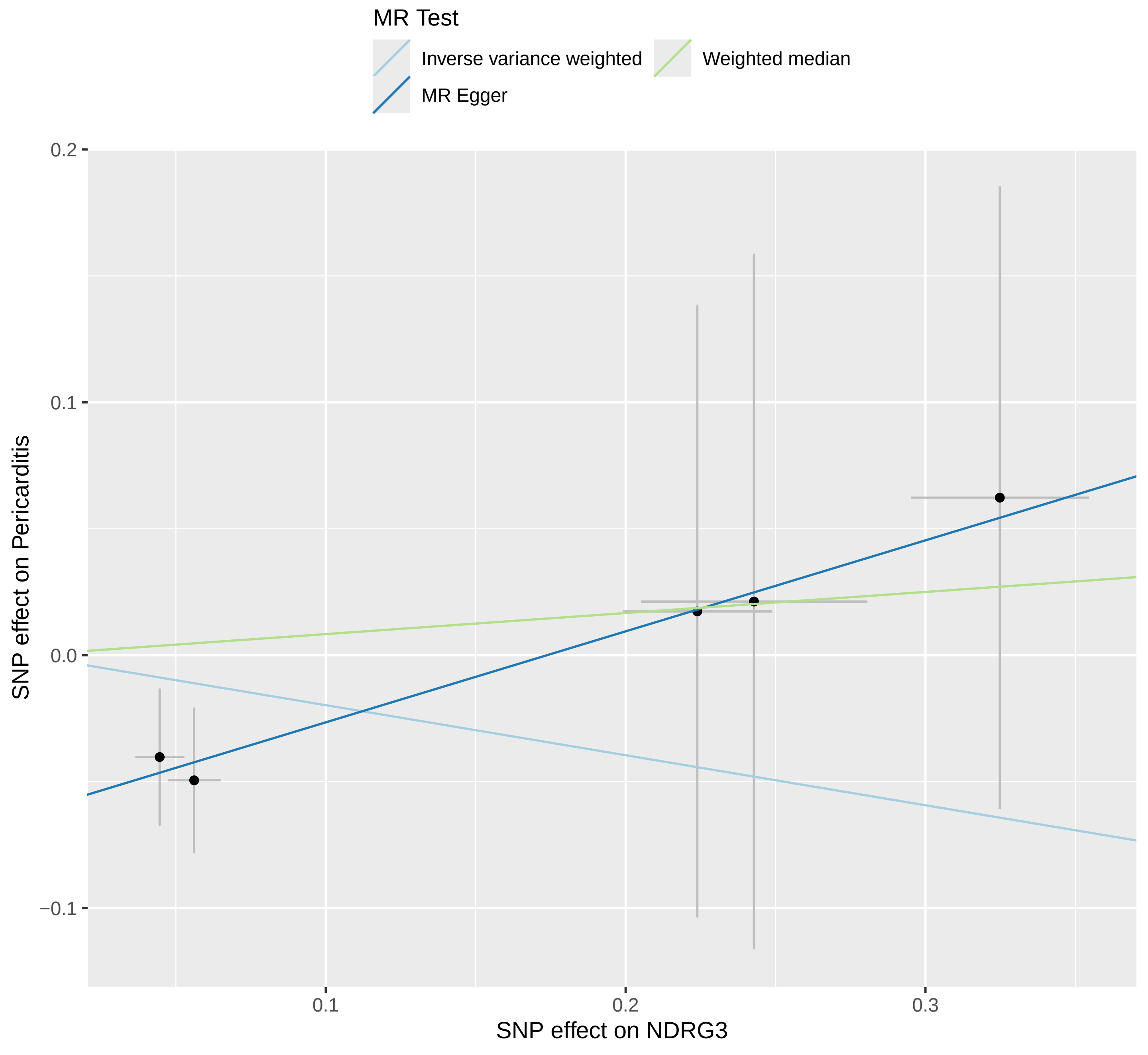

MR Test
Inverse variance weighted
Weighted median
MR Egger
0.2
0.1
0.0
−0.1
SNP effect on Pericarditis
0.1
0.2
0.3
SNP effect on NDRG3

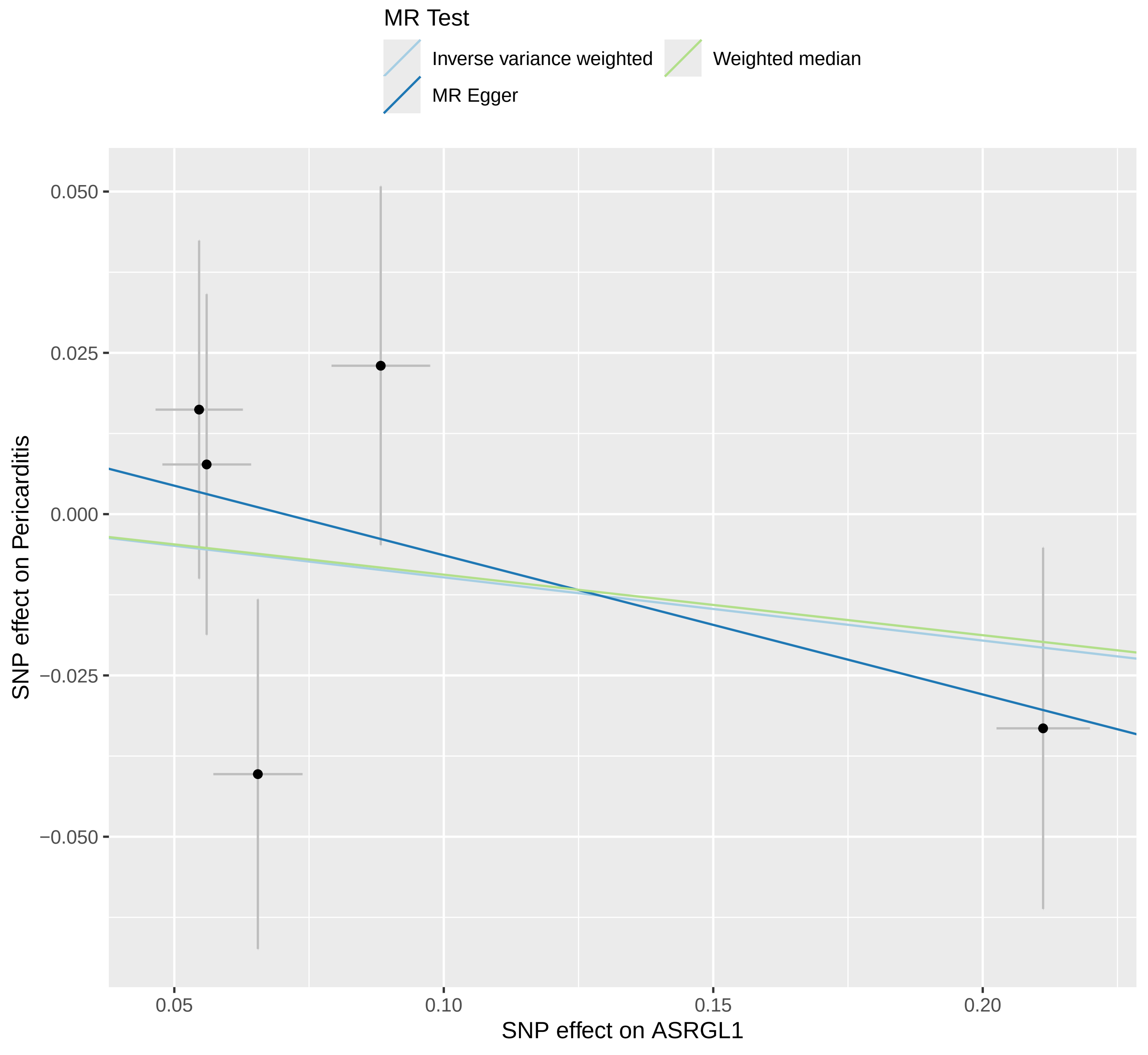

MR Test
Inverse variance weighted
Weighted median
MR Egger
0.050
0.025
0.000
−0.025
−0.050
SNP effect on Pericarditis
0.05
0.10
0.15
0.20
SNP effect on ASRGL1

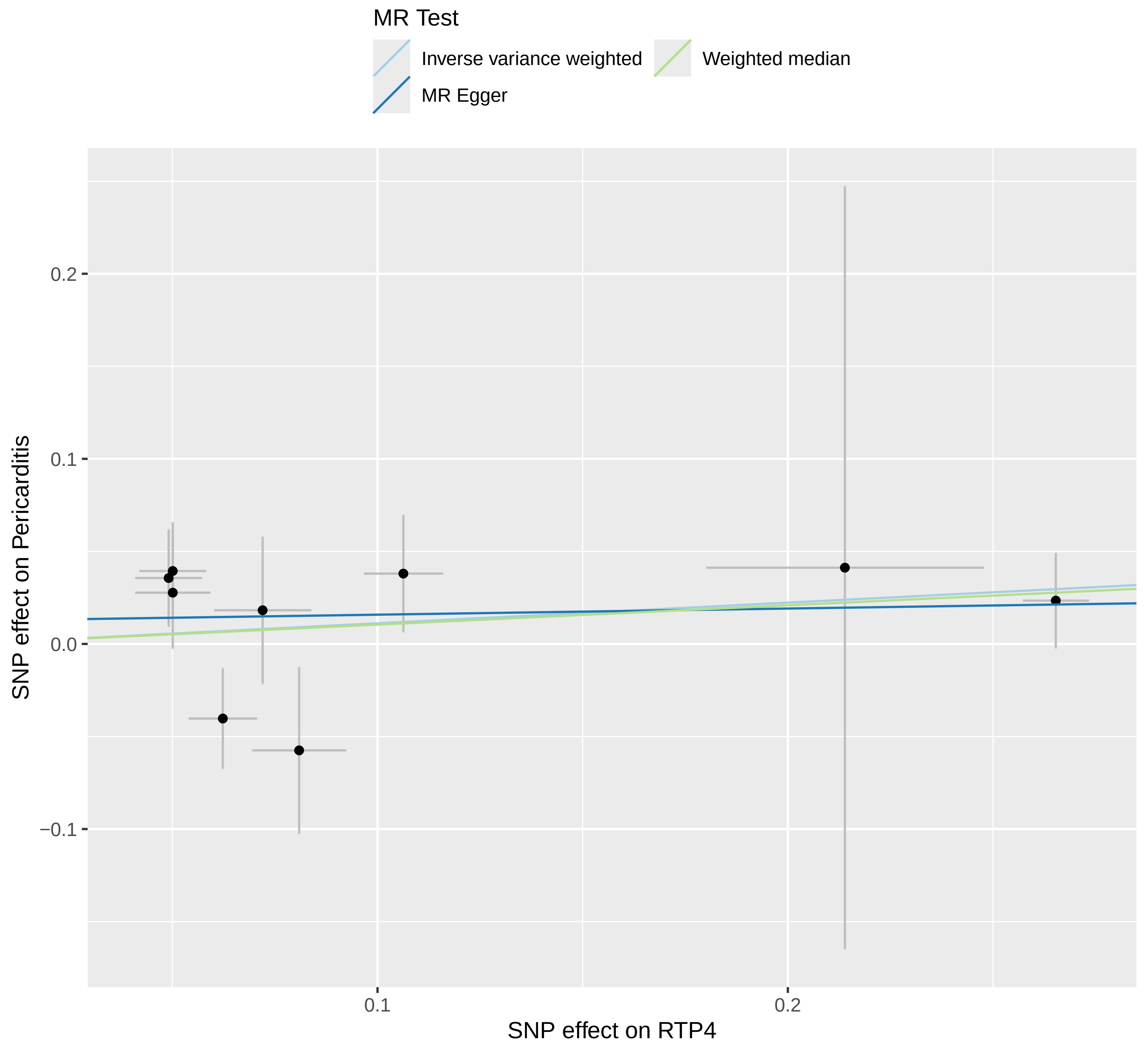
MR Test
Inverse variance weighted
Weighted median
MR Egger
SNP effect on Pericarditis
0.2
0.1
0.0
−0.1
0.1
0.2
SNP effect on RTP4

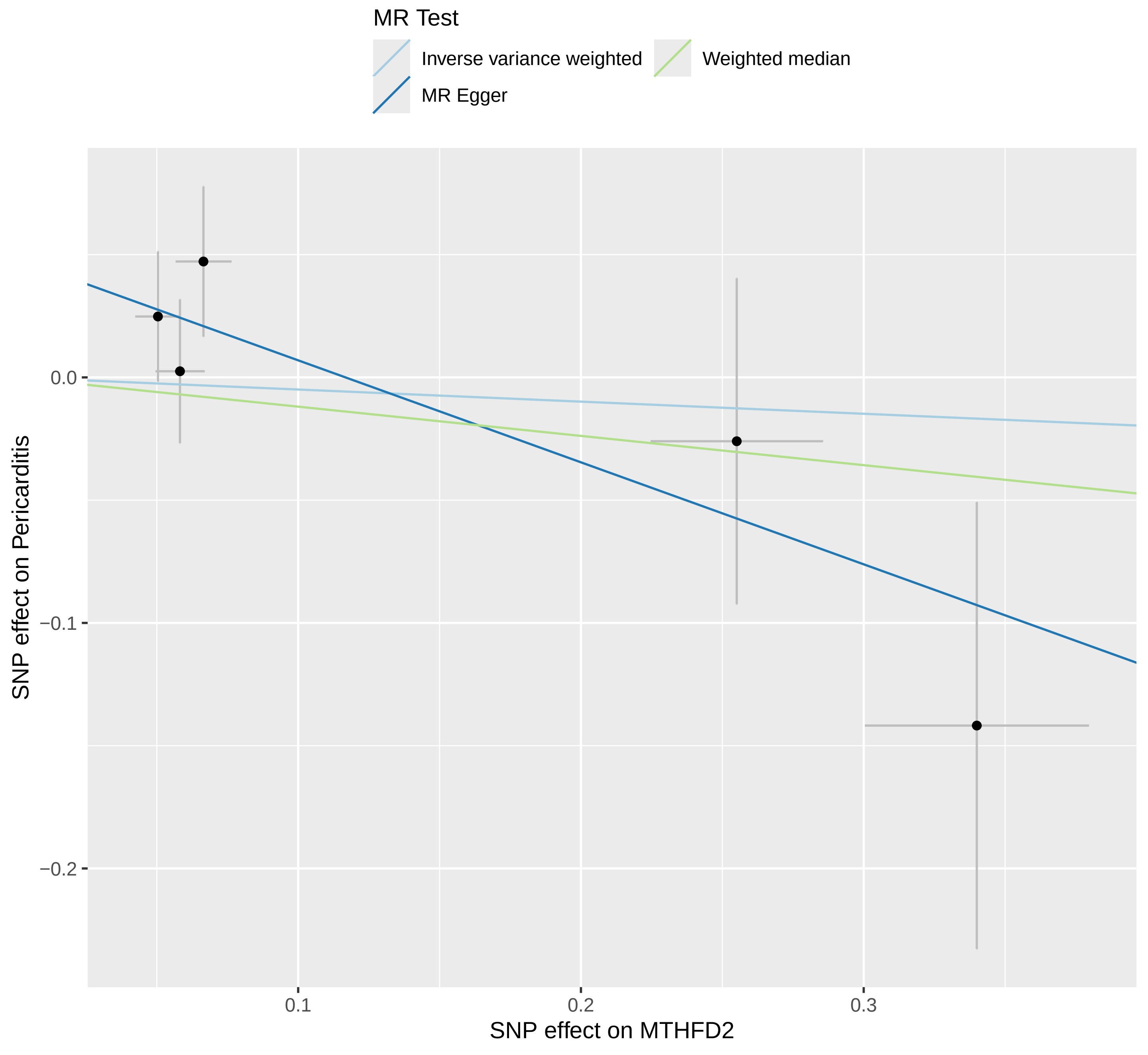
MR Test
Inverse variance weighted
Weighted median
MR Egger
0.0
−0.1
−0.2
0.1
0.2
0.3
SNP effect on Pericarditis
SNP effect on MTHFD2

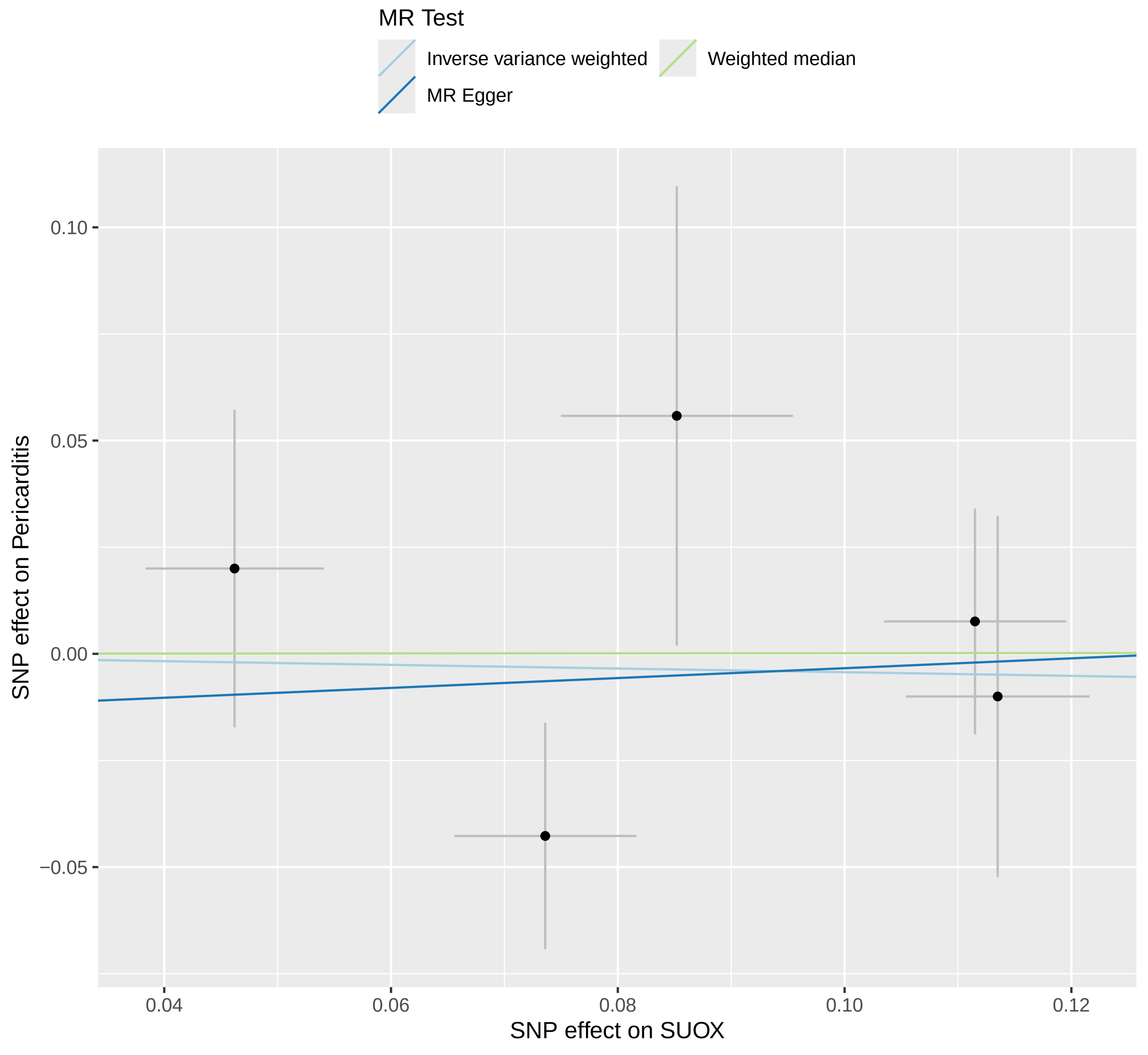
MR Test
Inverse variance weighted
Weighted median
MR Egger
0.10
0.05
0.00
−0.05
SNP effect on Pericarditis
0.04
0.06
0.08
0.10
0.12
SNP effect on SUOX

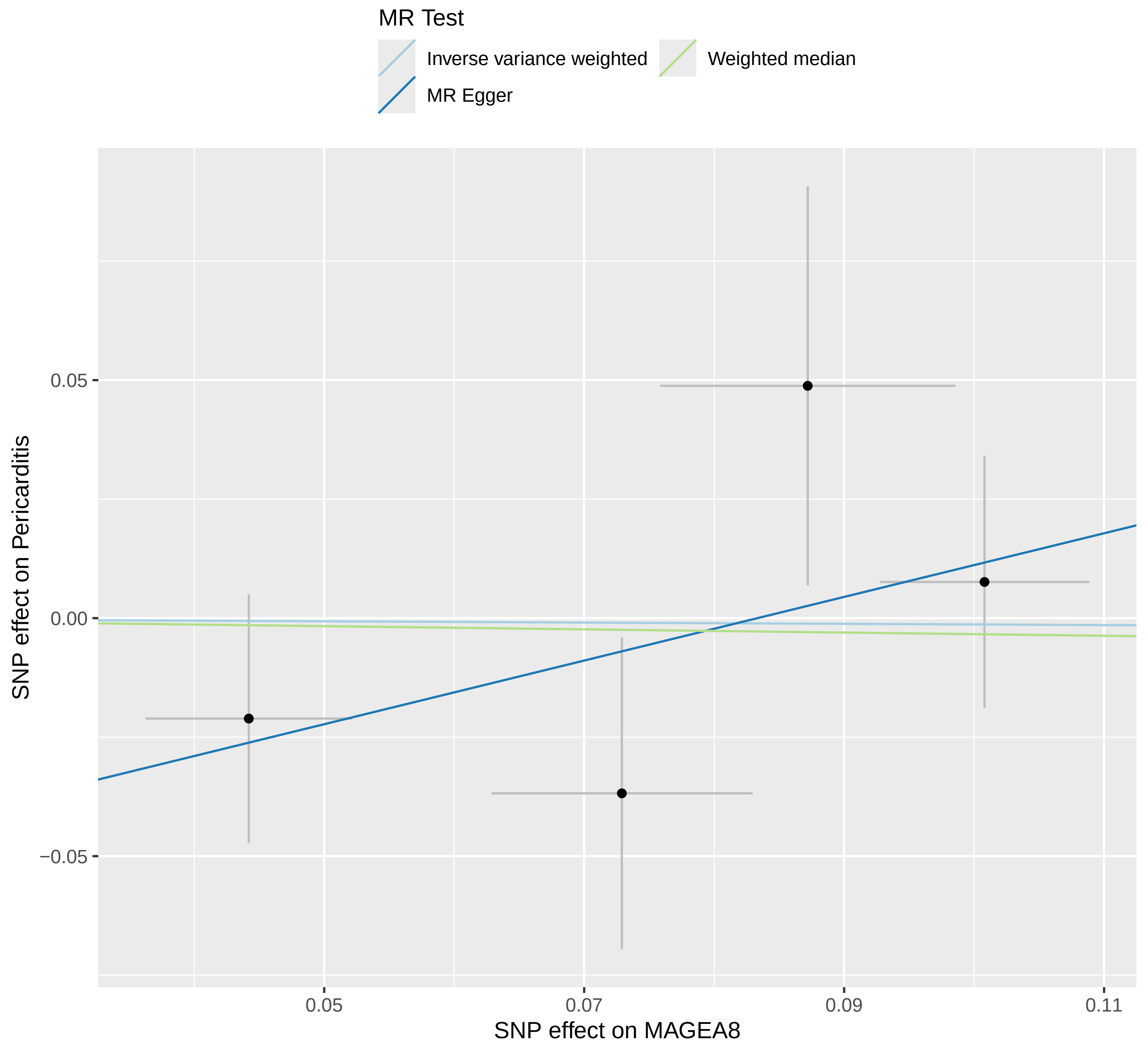
MR Test
Inverse variance weighted
Weighted median
MR Egger
0.05
0.00
−0.05
SNP effect on Pericarditis
0.05
0.07
0.09
0.11
SNP effect on MAGEA8

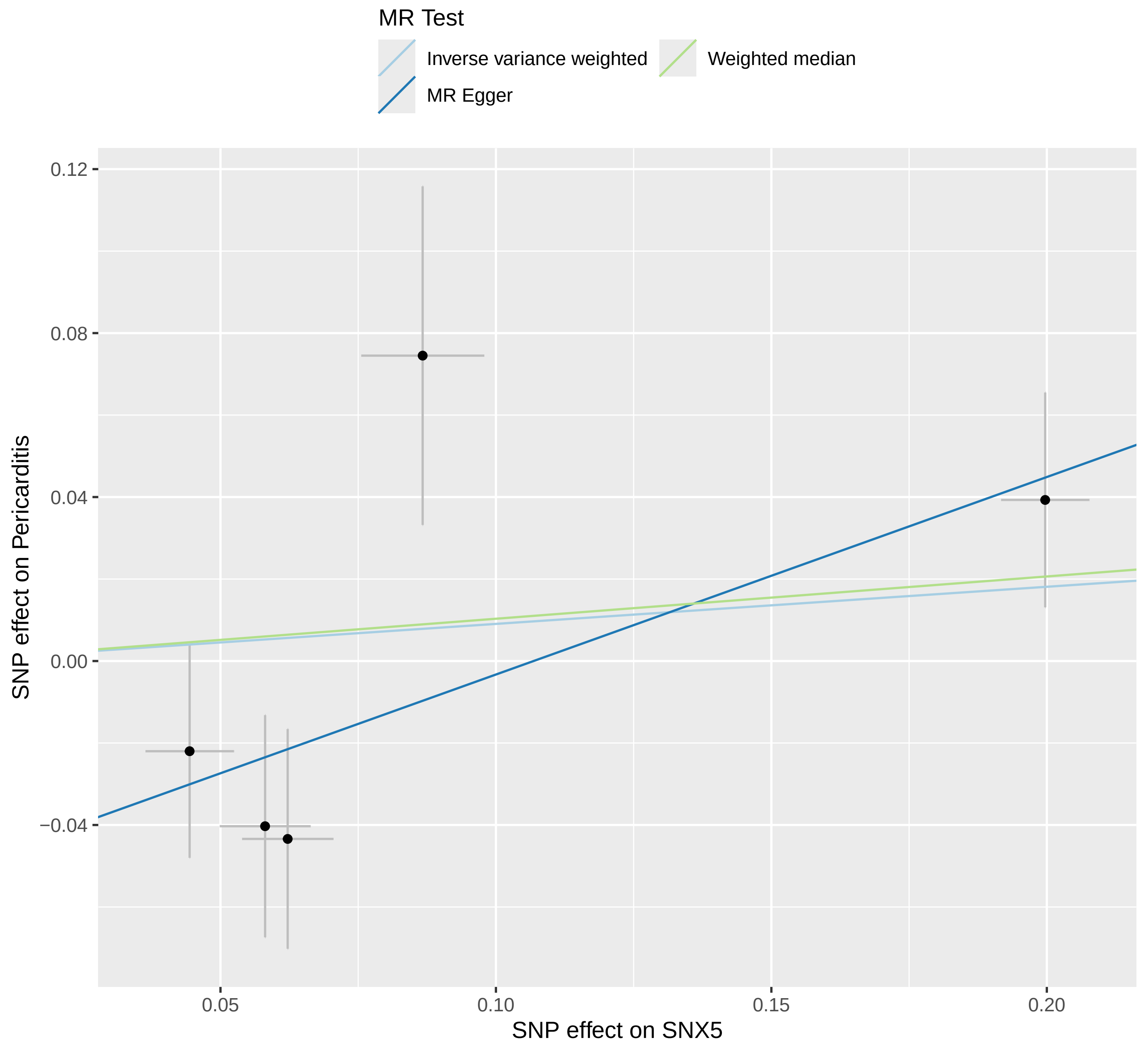

MR Test
Inverse variance weighted
Weighted median
MR Egger
0.12
0.08
0.04
0.00
−0.04
SNP effect on Pericarditis
0.05
0.10
0.15
0.20
SNP effect on SNX5

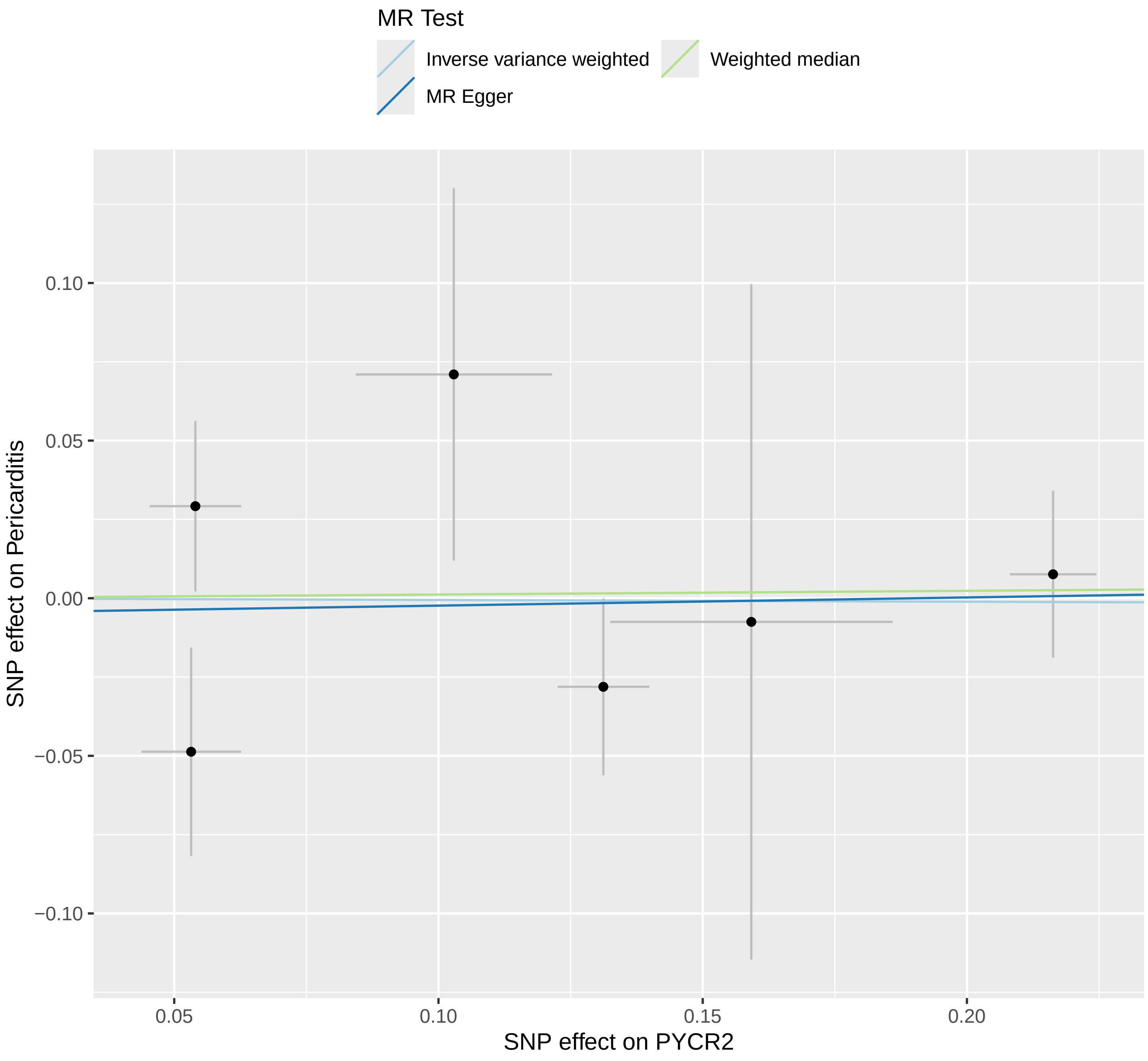
MR Test
Inverse variance weighted
Weighted median
MR Egger
0.10
0.05
0.00
−0.05
−0.10
SNP effect on Pericarditis
0.05
0.10
0.15
0.20
SNP effect on PYCR2

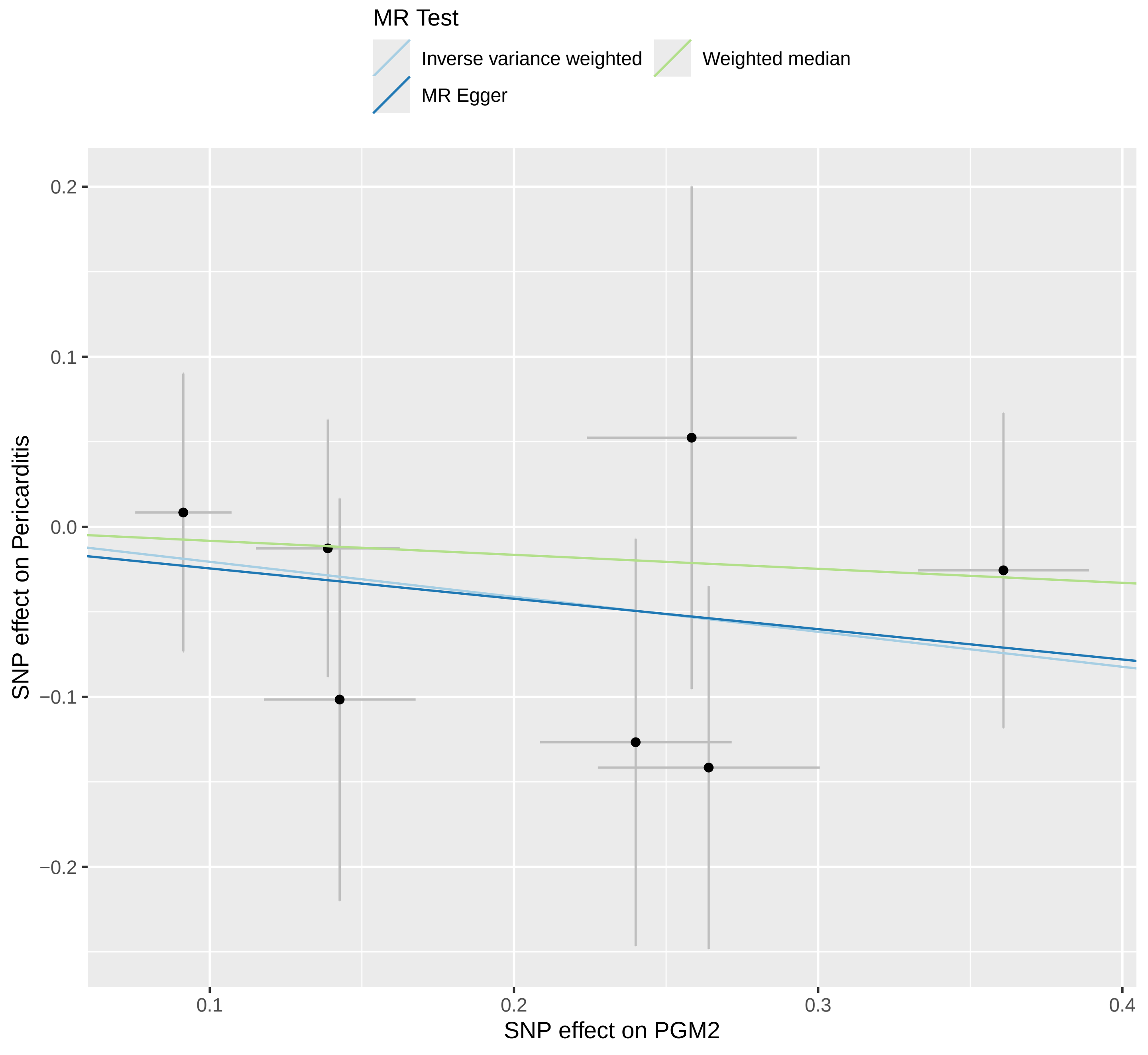
MR Test
Inverse variance weighted
MR Egger
Weighted median
0.2
0.1
0.0
−0.1
−0.2
SNP effect on Pericarditis
0.1
0.2
0.3
0.4
SNP effect on PGM2

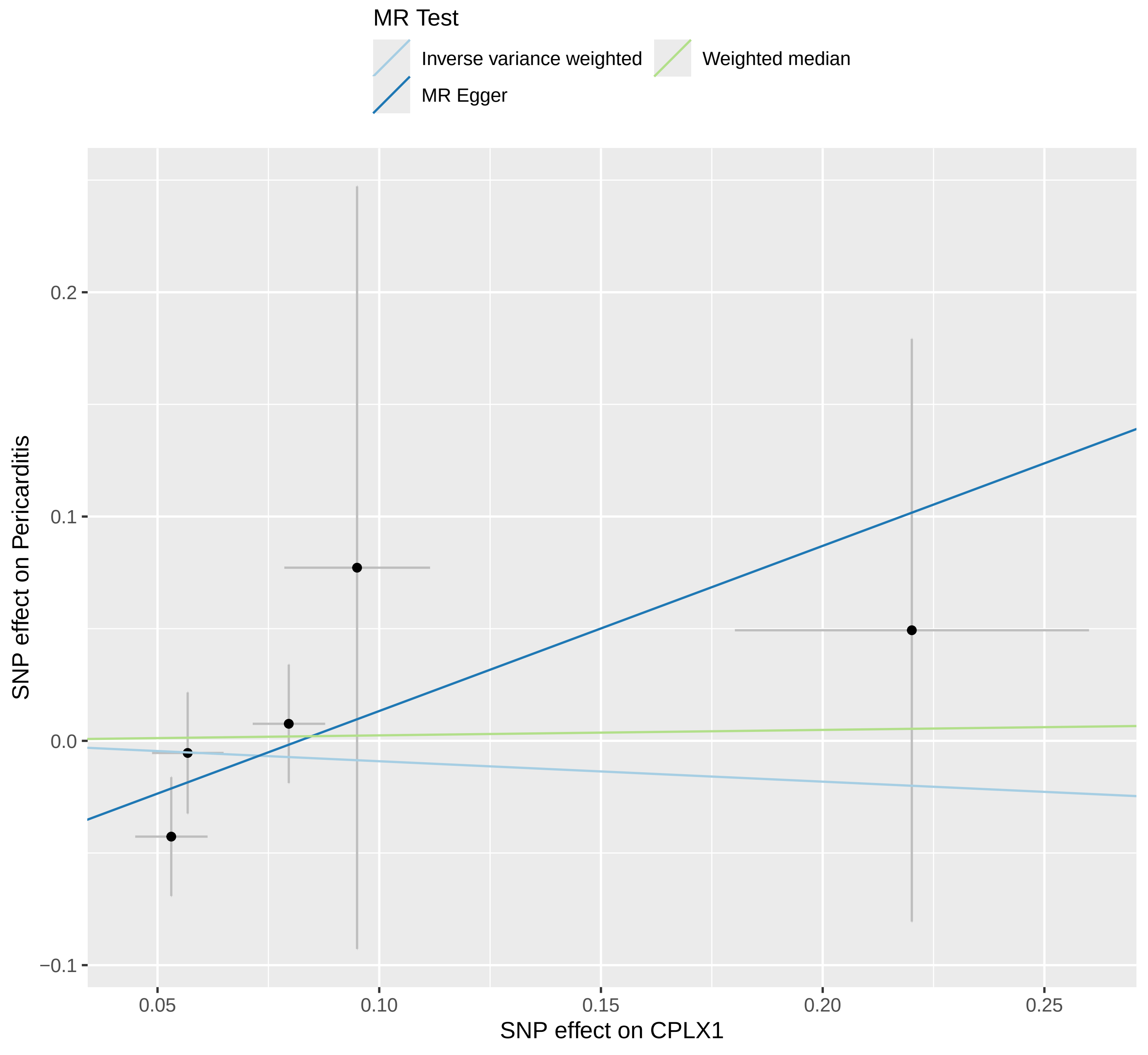
MR Test
Inverse variance weighted
Weighted median
MR Egger
0.2
0.1
0.0
−0.1
SNP effect on Pericarditis
0.05
0.10
0.15
0.20
0.25
SNP effect on CPLX1

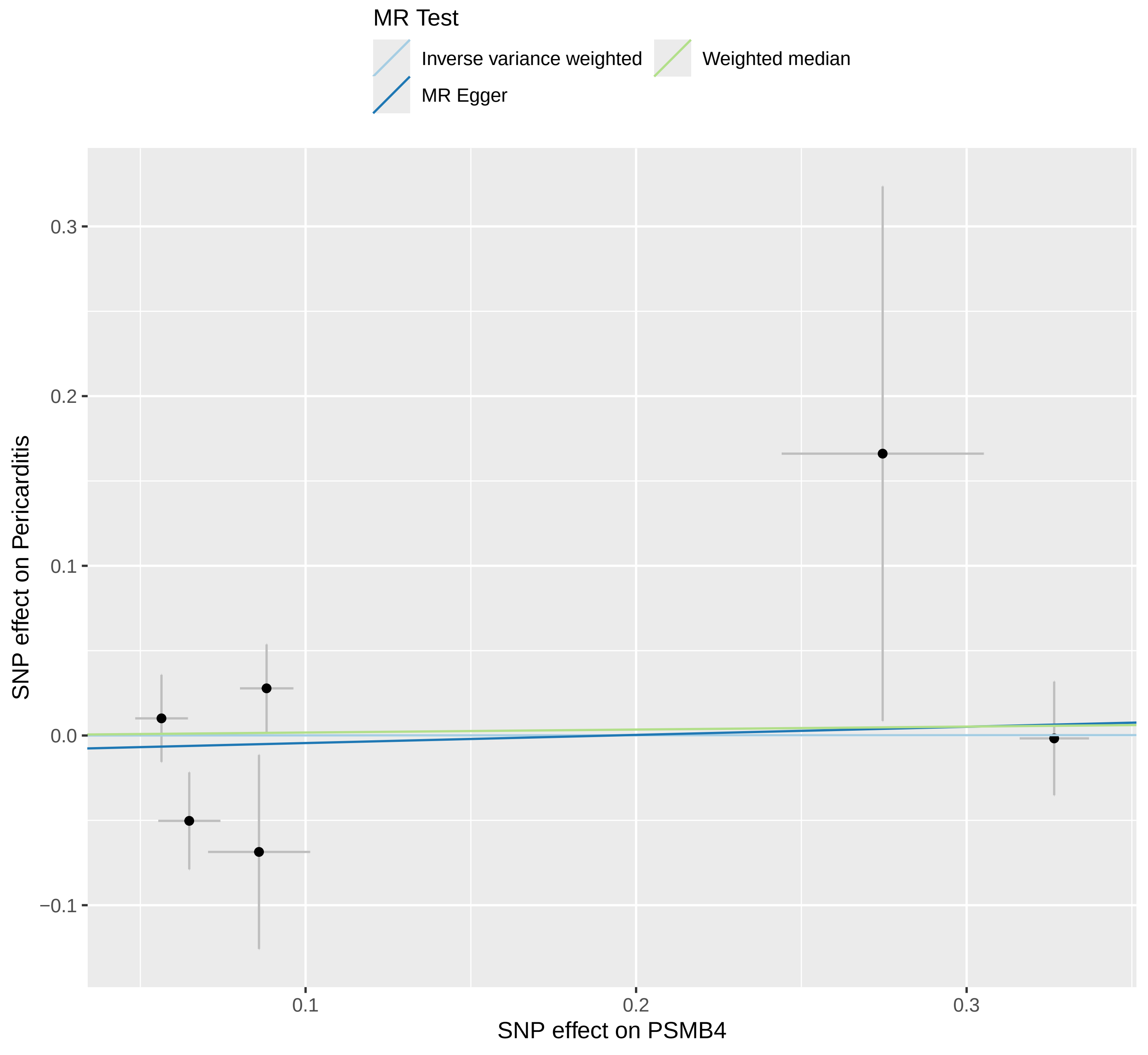
MR Test
Inverse variance weighted
Weighted median
MR Egger
0.3
0.2
0.1
0.0
−0.1
SNP effect on Pericarditis
0.1
0.2
0.3
SNP effect on PSMB4

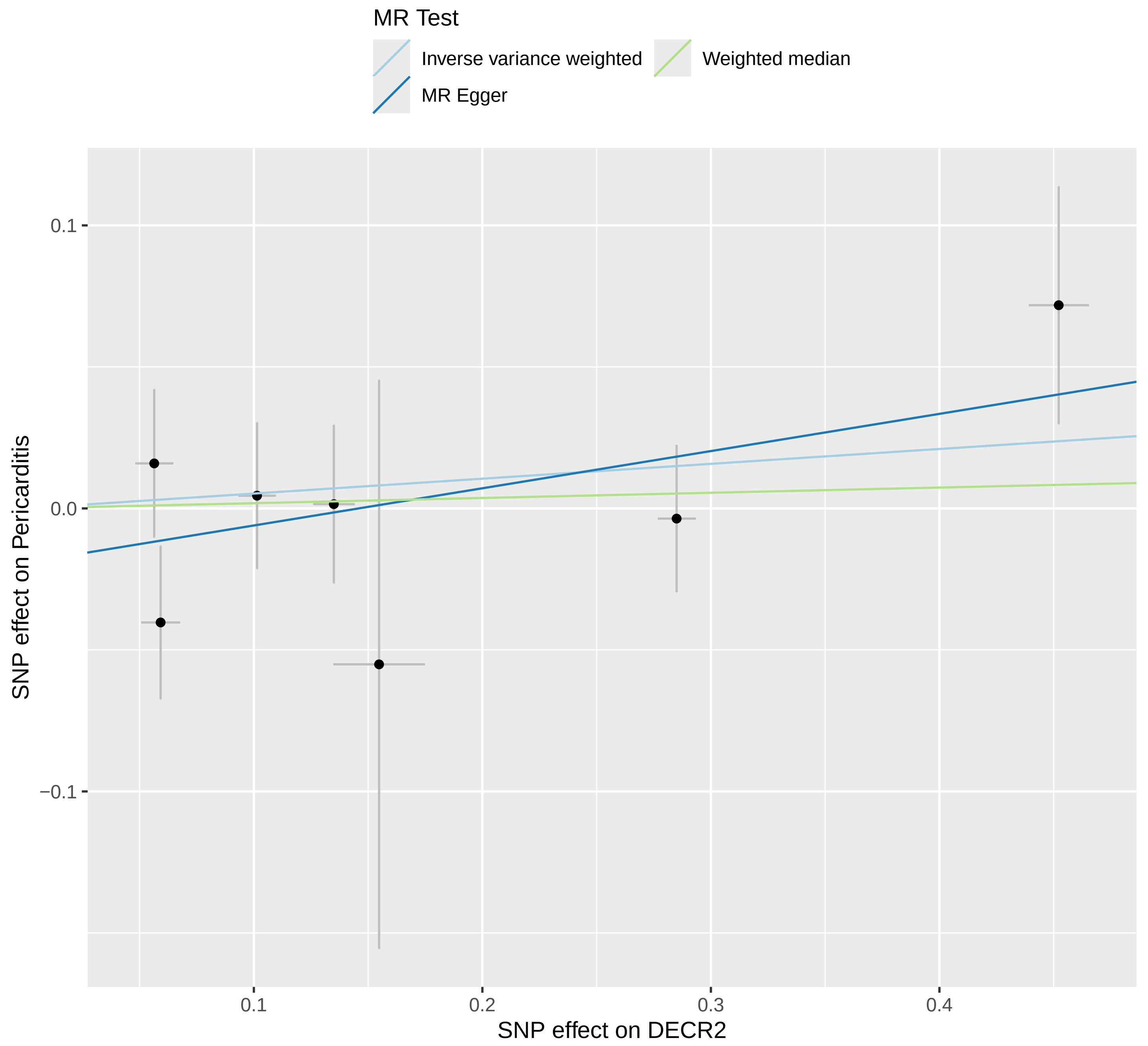

MR Test
Inverse variance weighted
Weighted median
MR Egger
0.1
0.0
−0.1
SNP effect on Pericarditis
0.1
0.2
0.3
0.4
SNP effect on DECR2

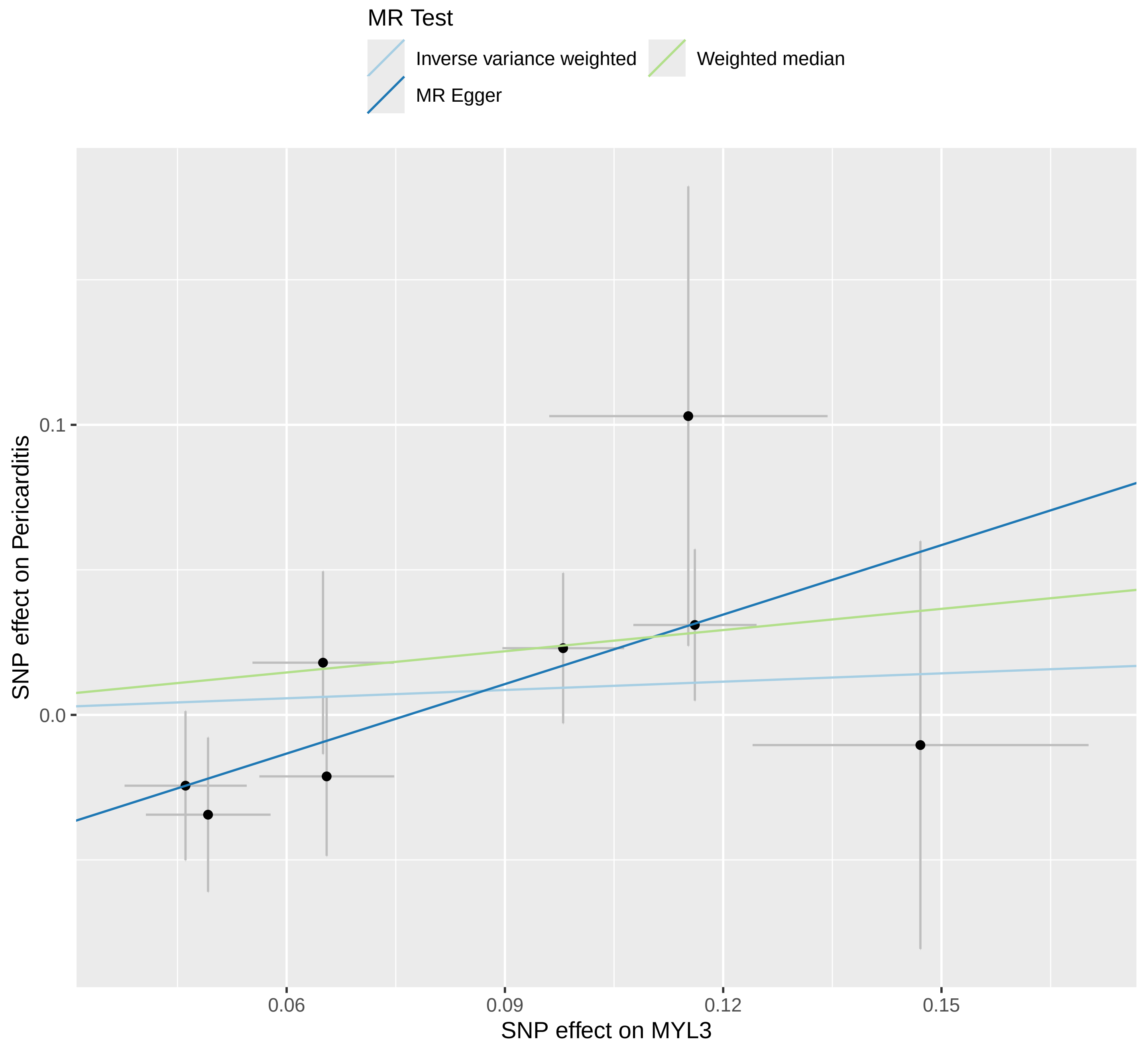
MR Test
Inverse variance weighted
Weighted median
MR Egger
0.1
0.0
SNP effect on Pericarditis
0.06
0.09
0.12
0.15
SNP effect on MYL3

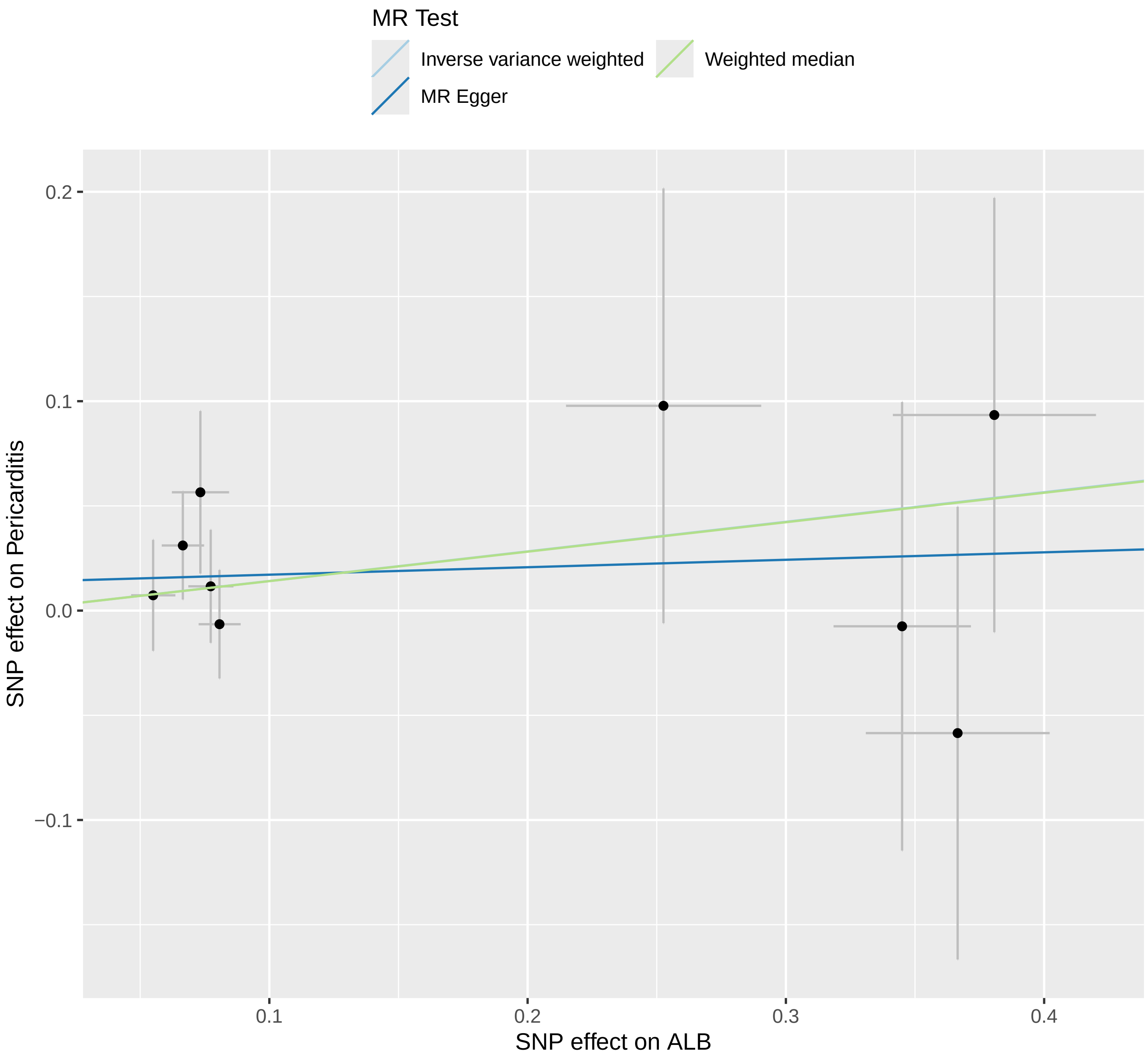

MR Test
Inverse variance weighted
Weighted median
MR Egger
0.2
0.1
0.0
−0.1
0.1
0.2
0.3
0.4
SNP effect on Pericarditis
SNP effect on ALB

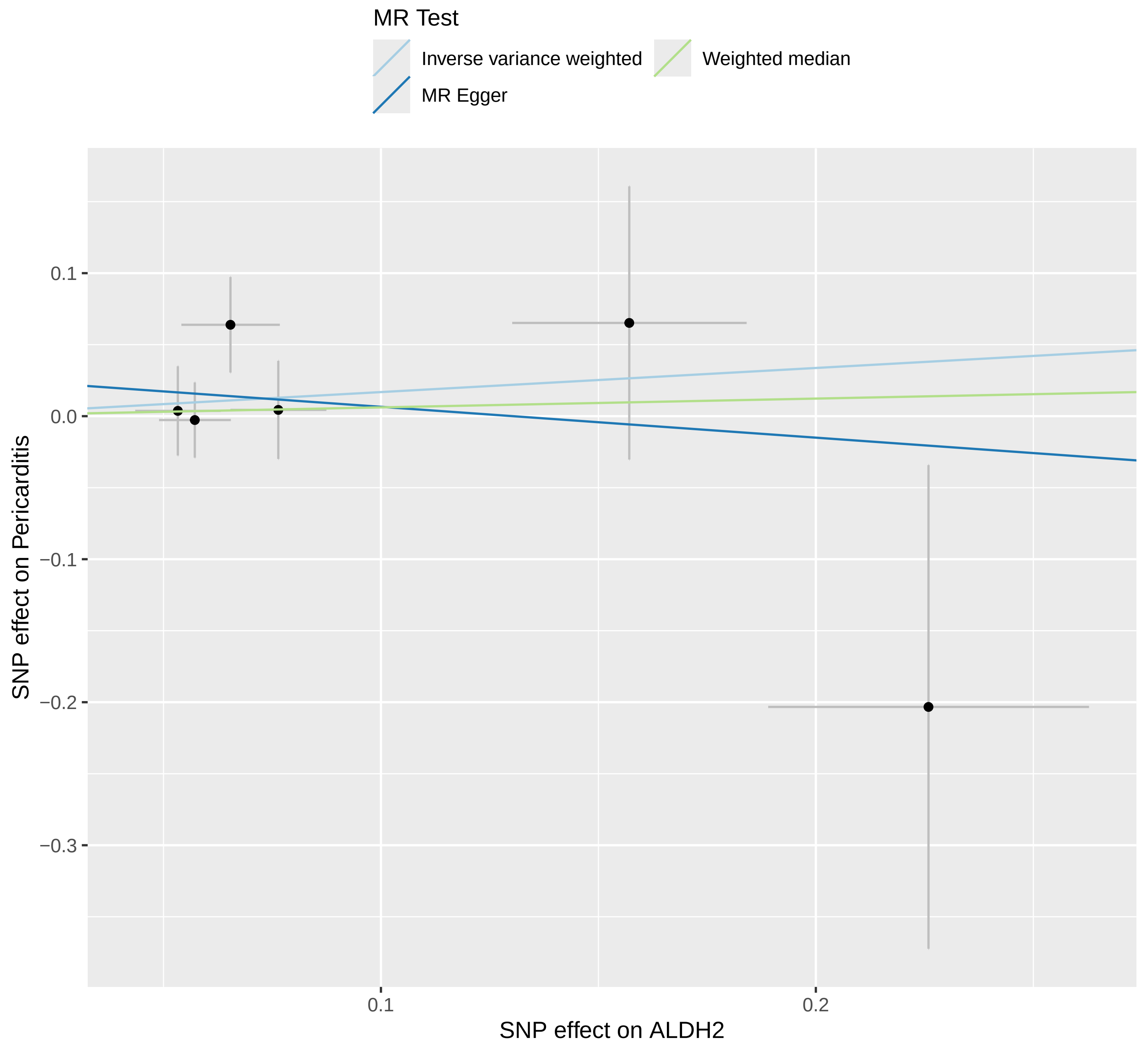
MR Test
Inverse variance weighted
Weighted median
MR Egger
0.1
0.0
−0.1
−0.2
−0.3
SNP effect on Pericarditis
0.1
0.2
SNP effect on ALDH2

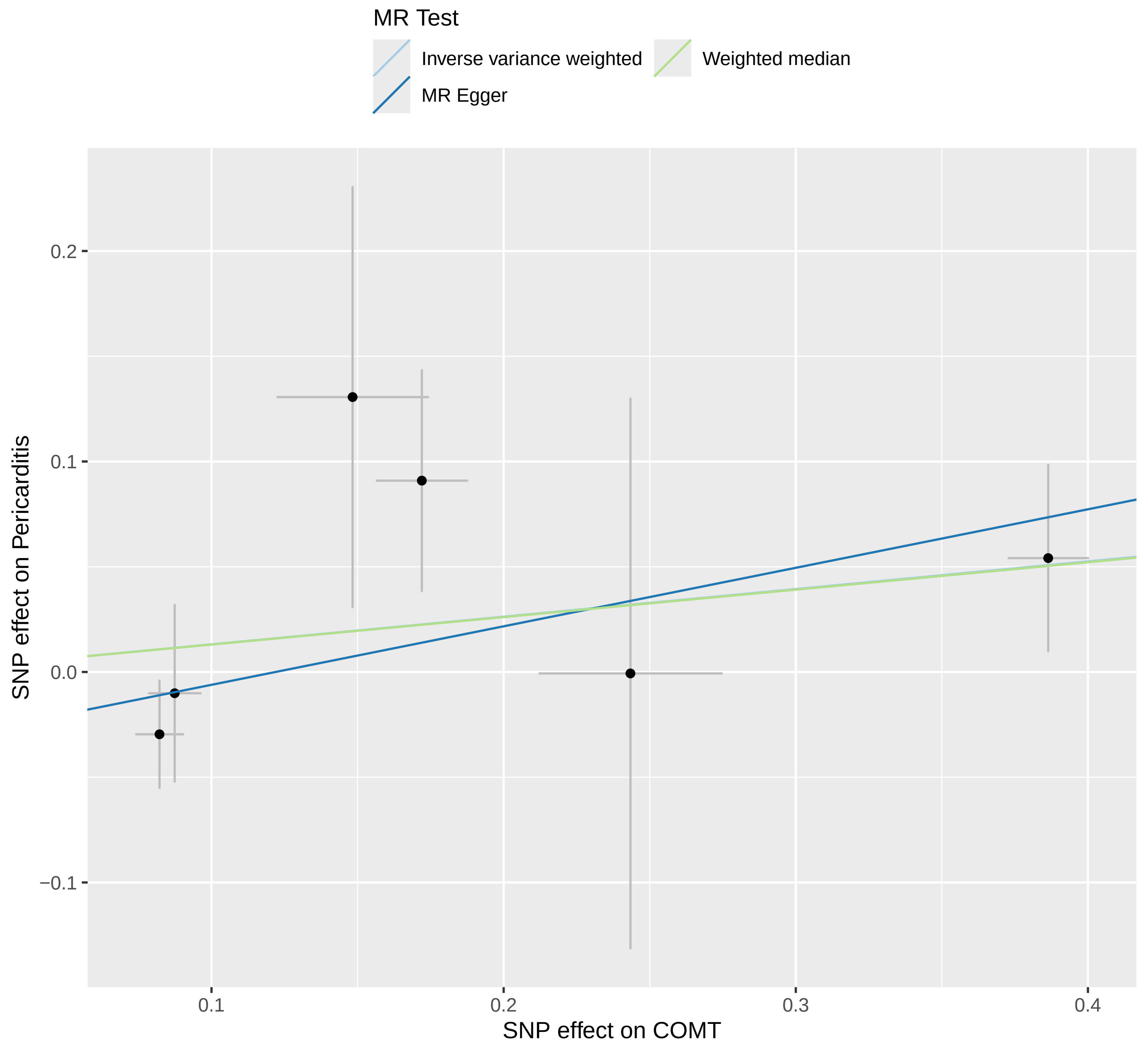

MR Test
Inverse variance weighted
Weighted median
MR Egger
SNP effect on Pericarditis
0.2
0.1
0.0
−0.1
0.1
0.2
0.3
0.4
SNP effect on COMT

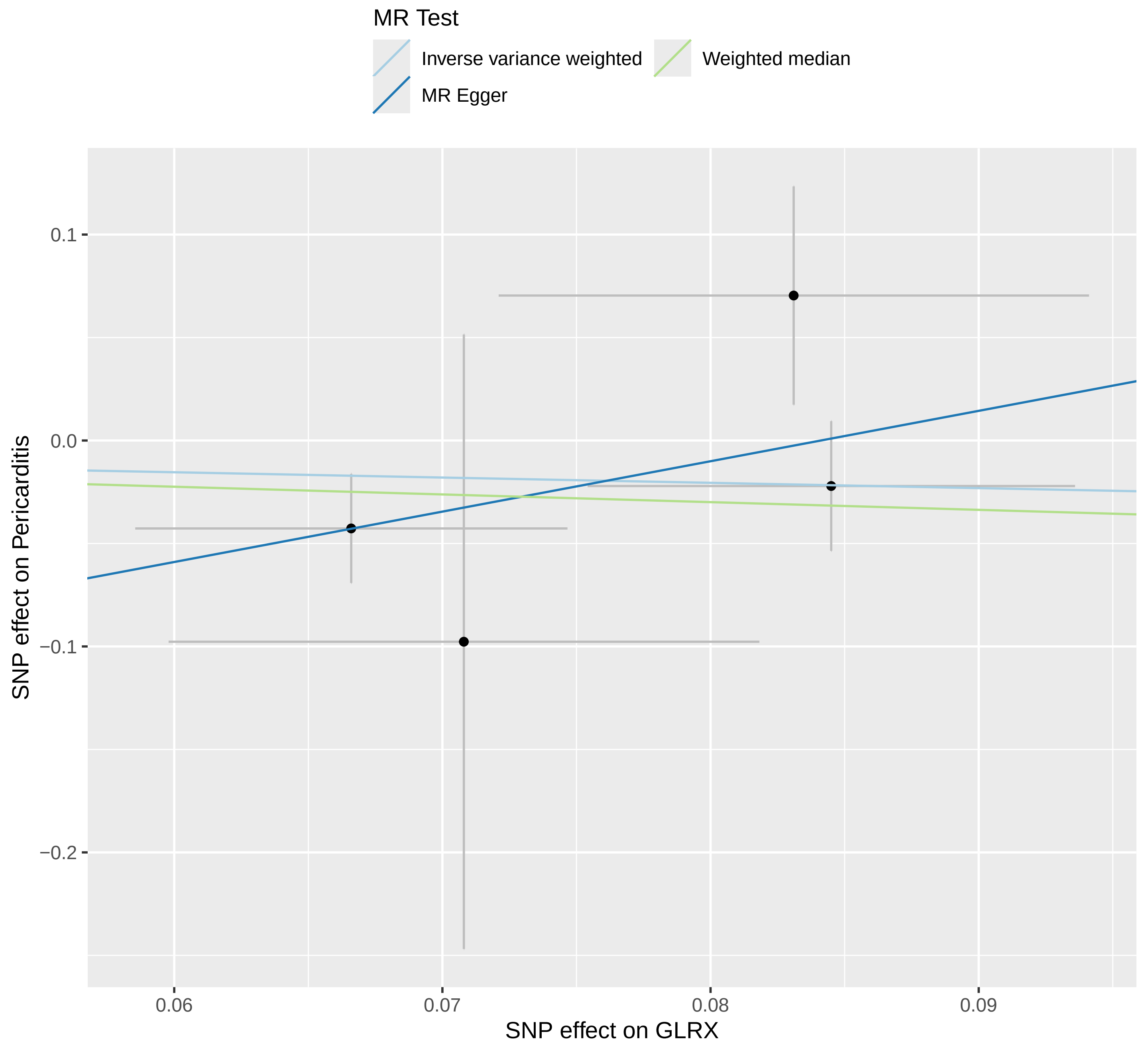
MR Test
Inverse variance weighted
Weighted median
MR Egger
0.1
0.0
−0.1
−0.2
SNP effect on Pericarditis
0.06
0.07
0.08
0.09
SNP effect on GLRX

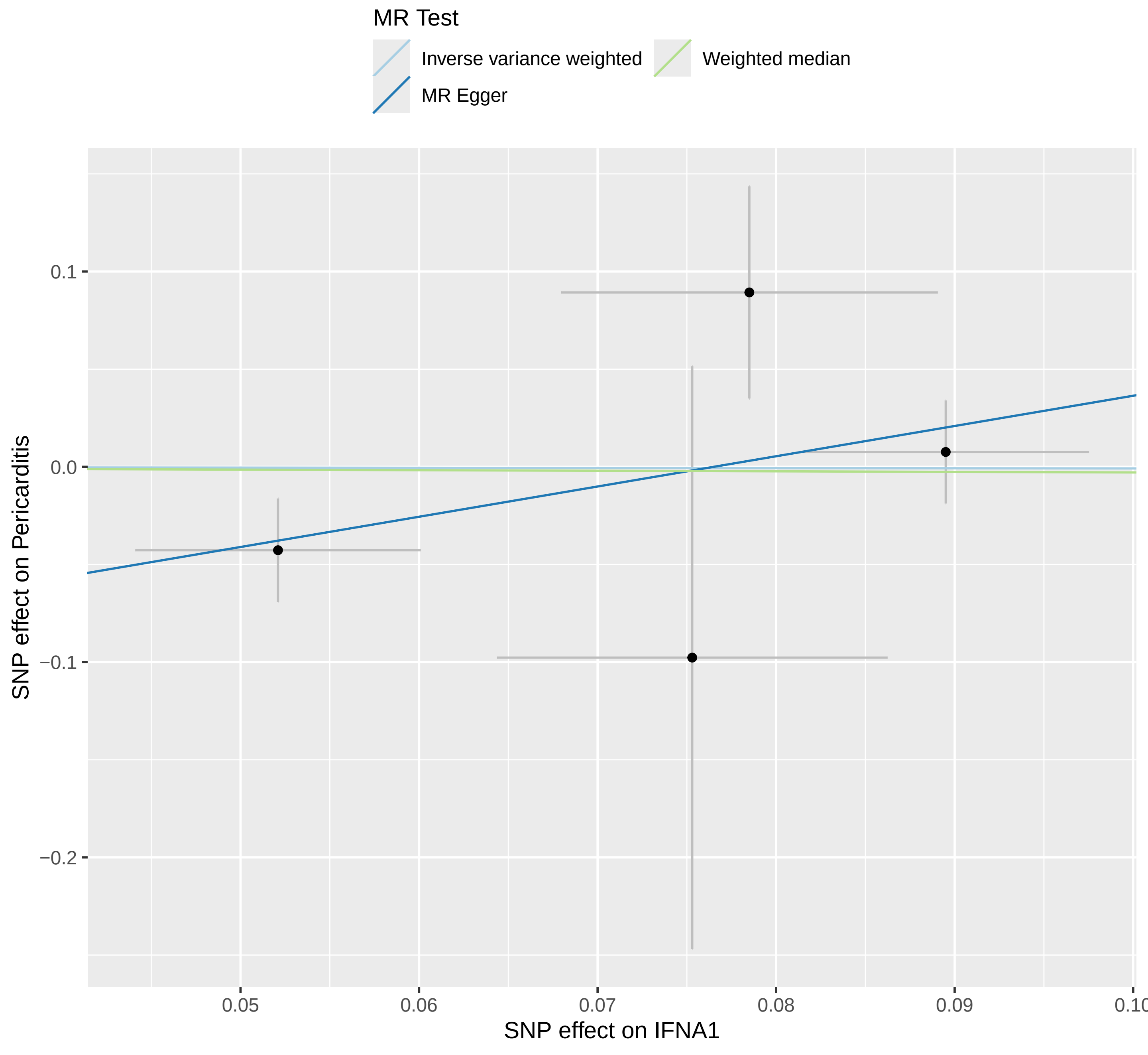

MR Test
Inverse variance weighted
Weighted median
MR Egger
0.1
0.0
−0.1
−0.2
0.05
0.06
0.07
0.08
0.09
0.10
SNP effect on Pericarditis
SNP effect on IFNA1

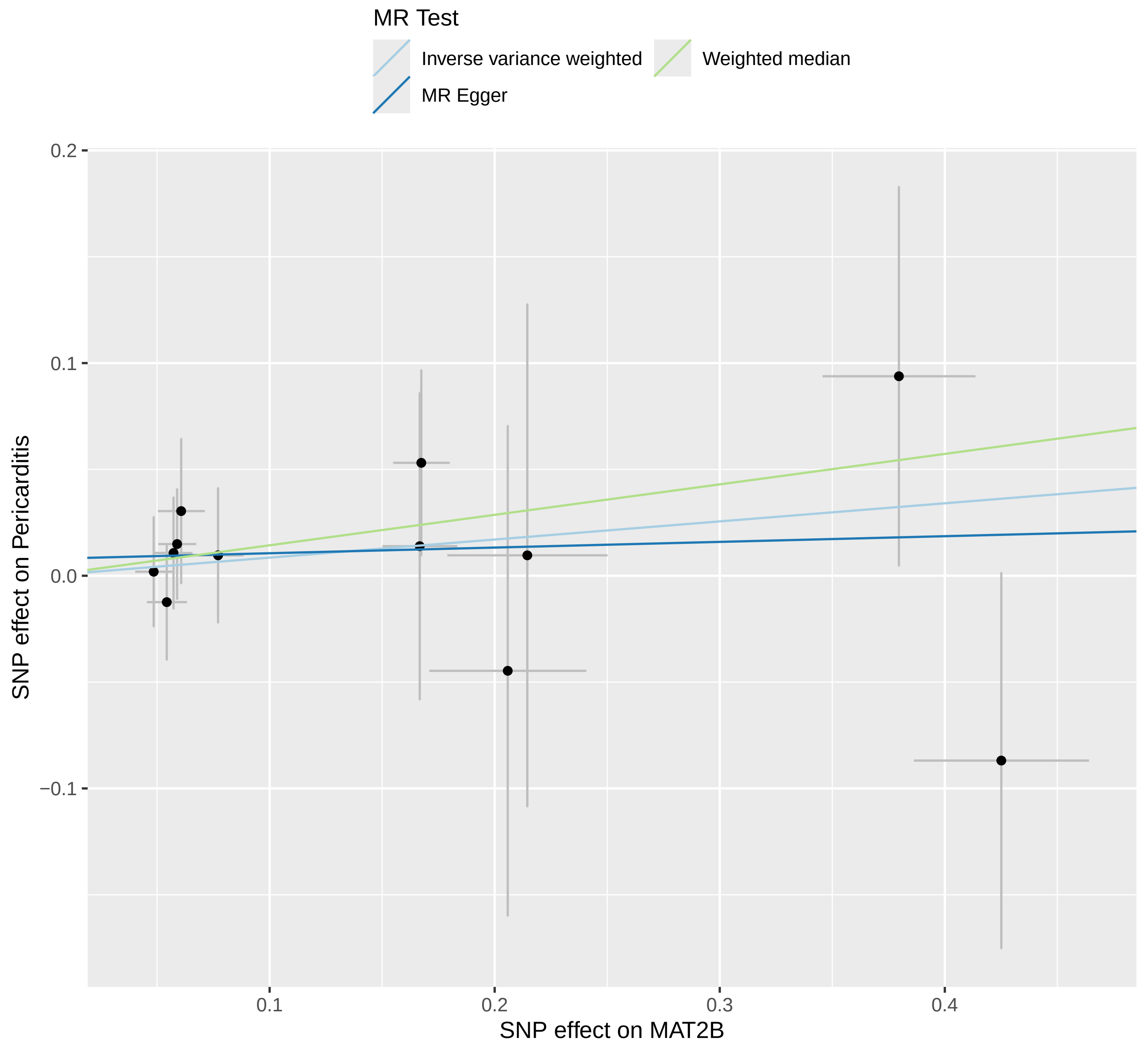

MR Test
Inverse variance weighted
Weighted median
MR Egger
0.2
0.1
0.0
−0.1
0.1
0.2
0.3
0.4
SNP effect on MAT2B
SNP effect on Pericarditis

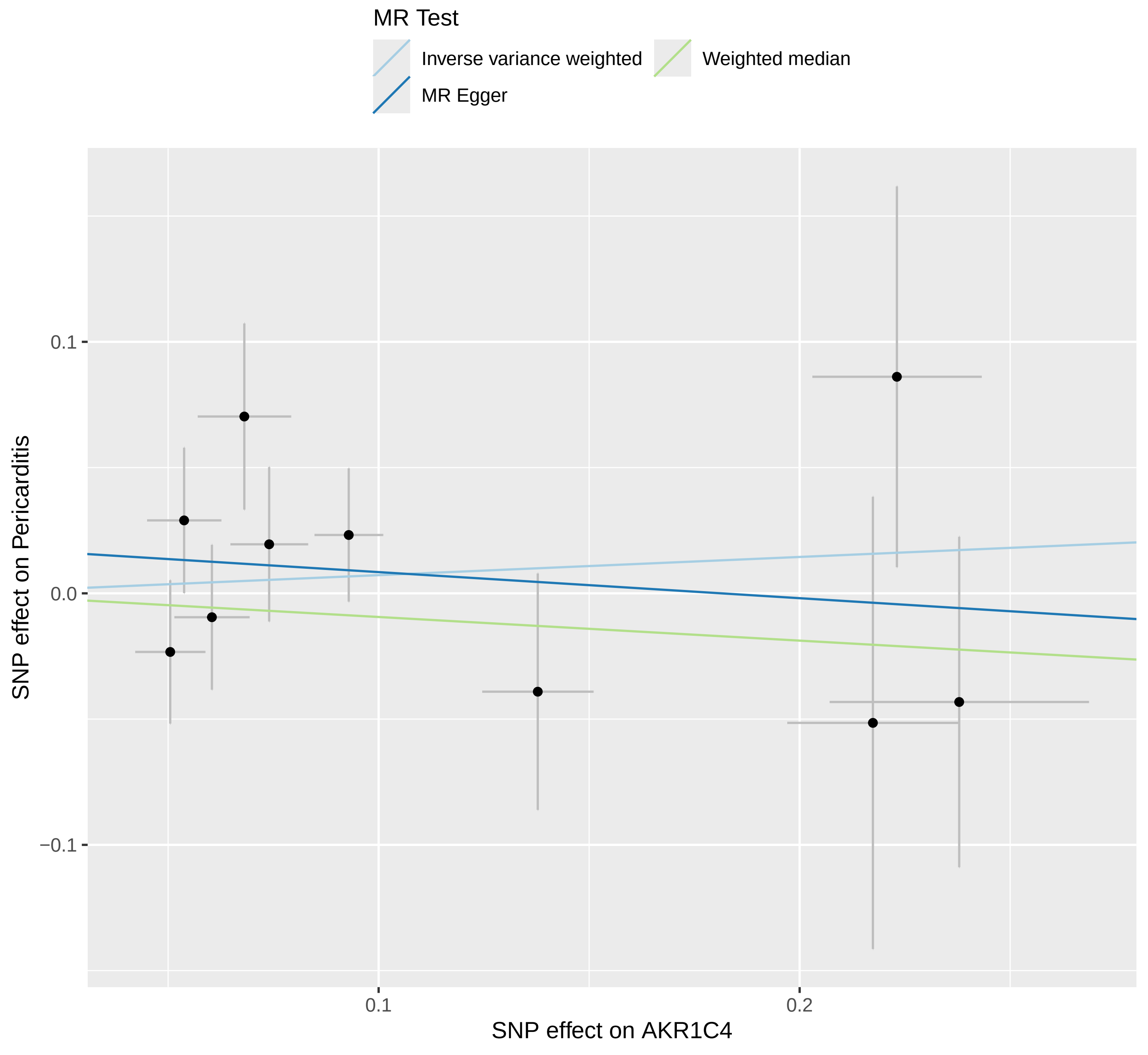

MR Test
Inverse variance weighted
Weighted median
MR Egger
0.1
0.0
−0.1
SNP effect on Pericarditis
0.1
0.2
SNP effect on AKR1C4

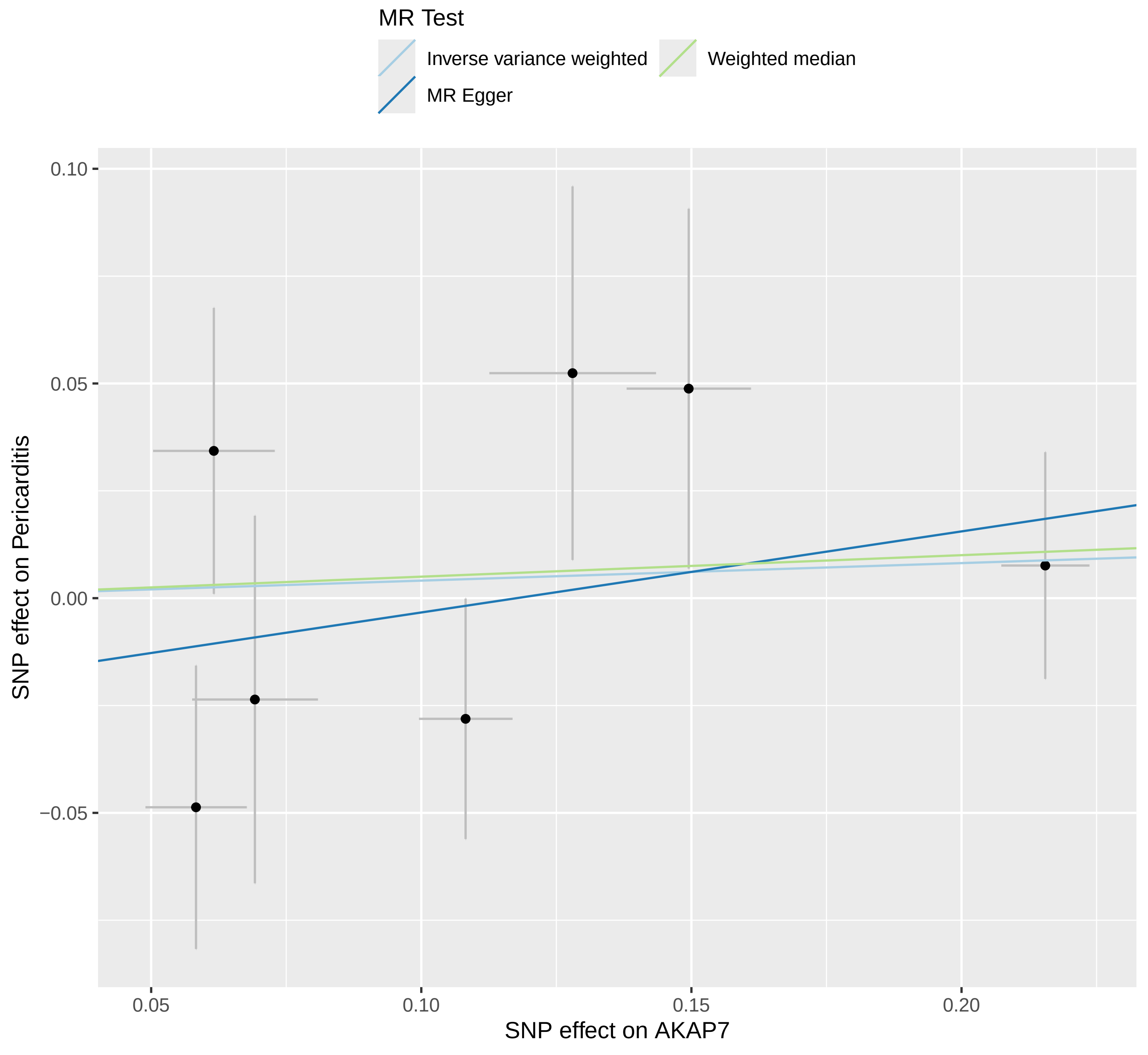
MR Test
Inverse variance weighted
MR Egger
Weighted median
0.10
0.05
0.00
−0.05
SNP effect on Pericarditis
0.05
0.10
0.15
0.20
SNP effect on AKAP7

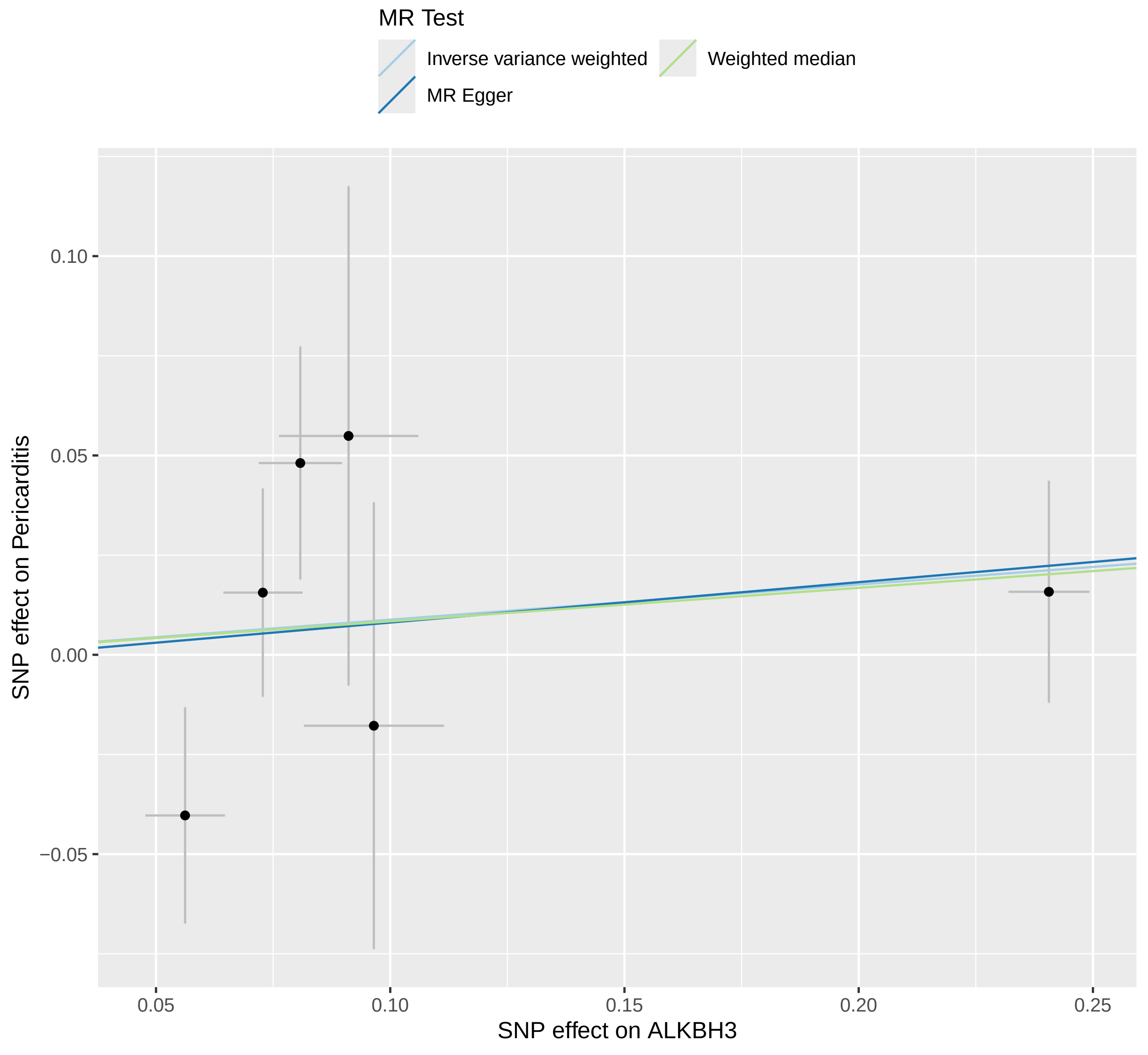
MR Test
Inverse variance weighted
Weighted median
MR Egger
0.10
0.05
0.00
−0.05
SNP effect on Pericarditis
0.05
0.10
0.15
0.20
0.25
SNP effect on ALKBH3

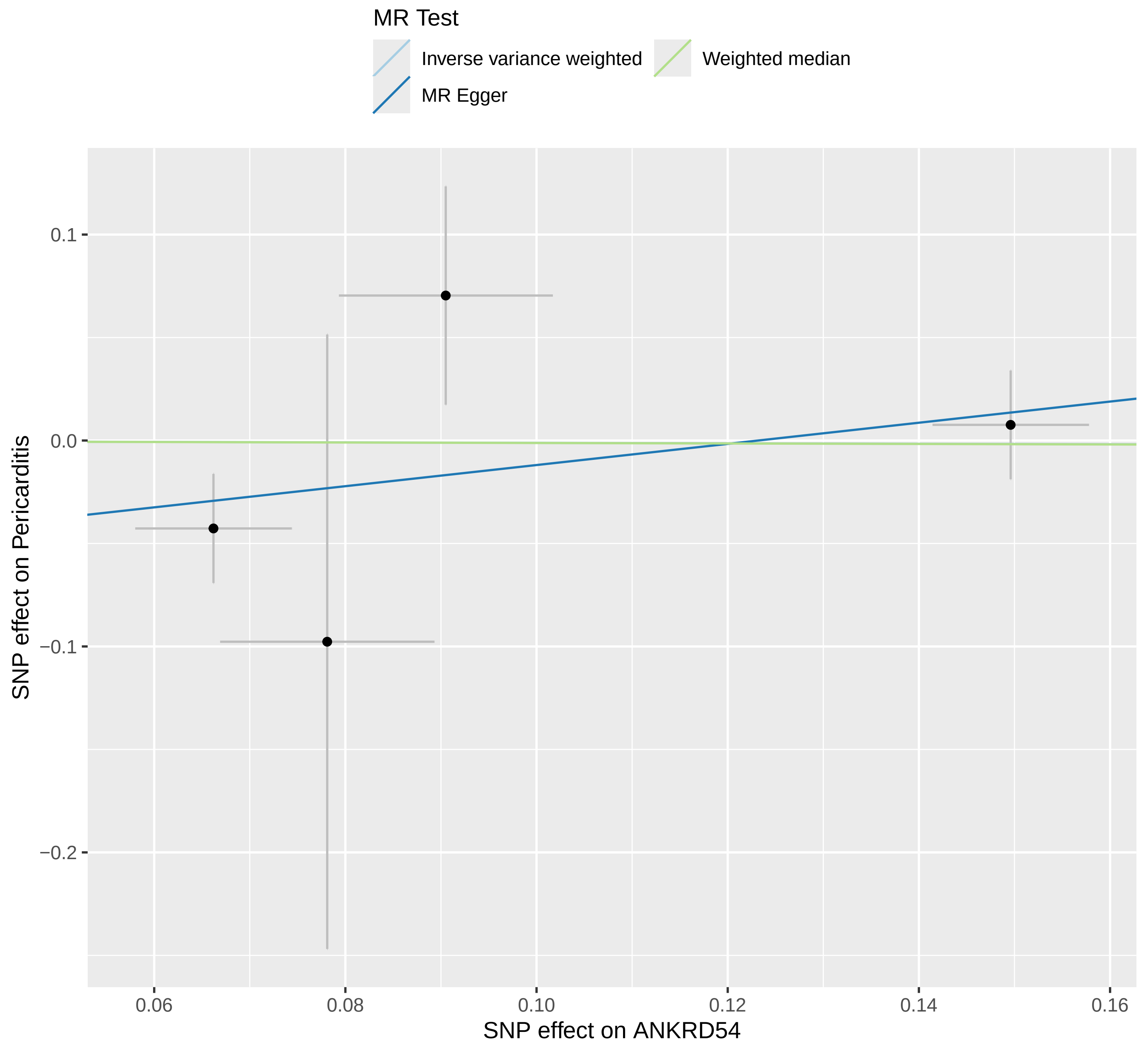
MR Test
Inverse variance weighted
Weighted median
MR Egger
0.1
0.0
−0.1
−0.2
SNP effect on Pericarditis
0.06
0.08
0.10
0.12
0.14
0.16
SNP effect on ANKRD54

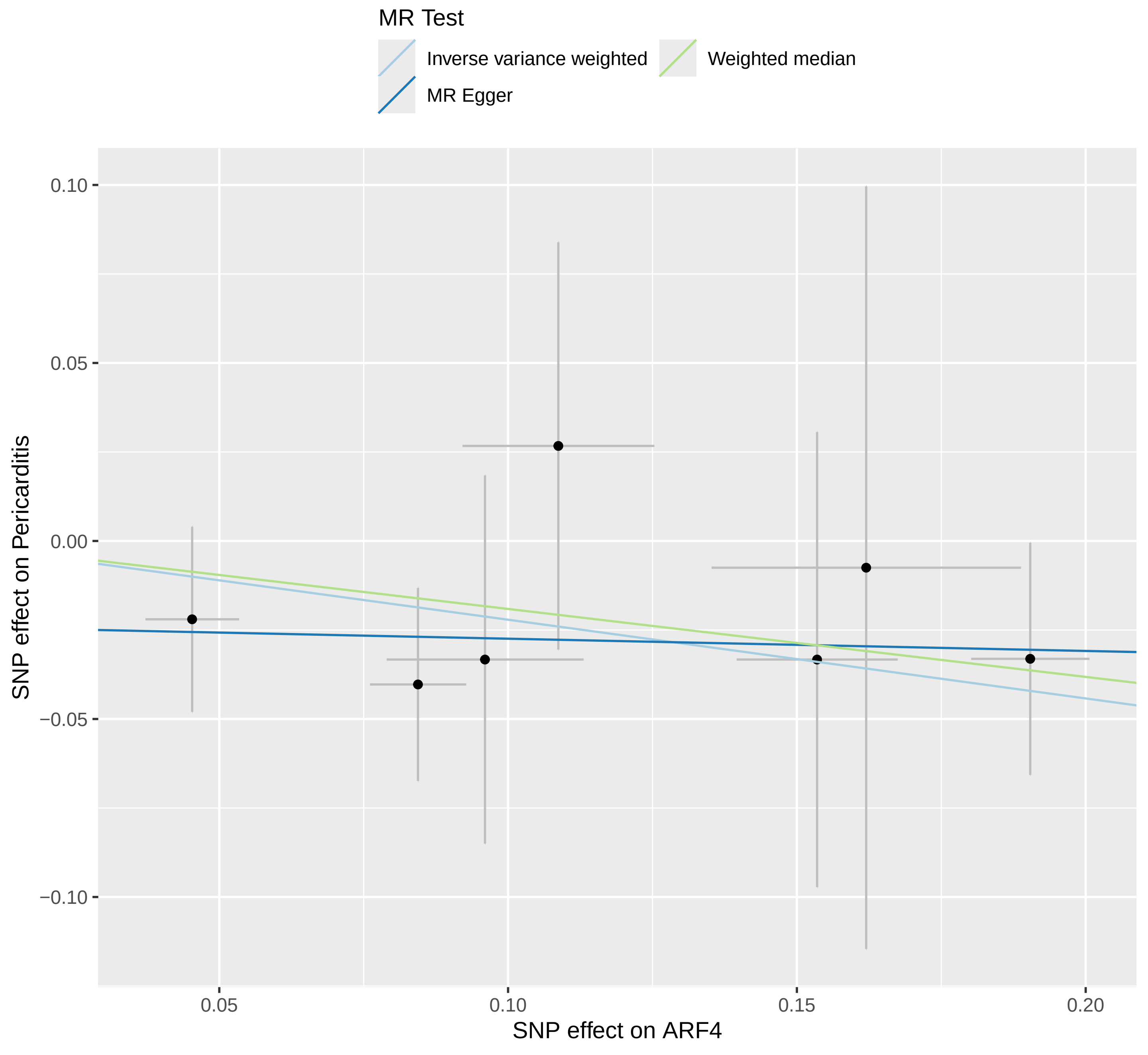
MR Test
Inverse variance weighted
MR Egger
Weighted median
0.10
0.05
0.00
−0.05
−0.10
0.05
0.10
0.15
0.20
SNP effect on Pericarditis
SNP effect on ARF4

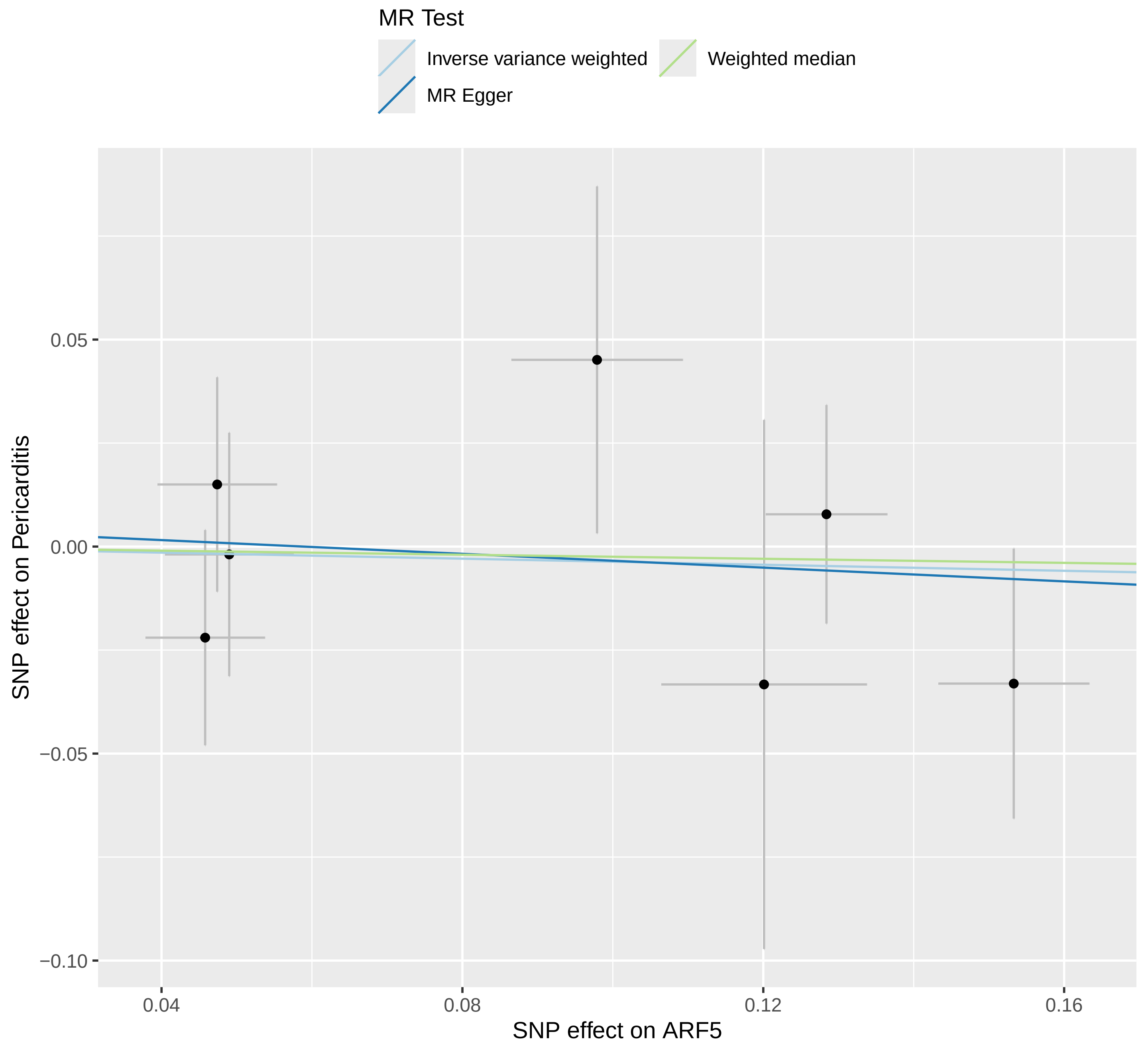

MR Test
Inverse variance weighted
MR Egger
Weighted median
SNP effect on Pericarditis
0.05
0.00
−0.05
−0.10
0.04
0.08
0.12
0.16
SNP effect on ARF5

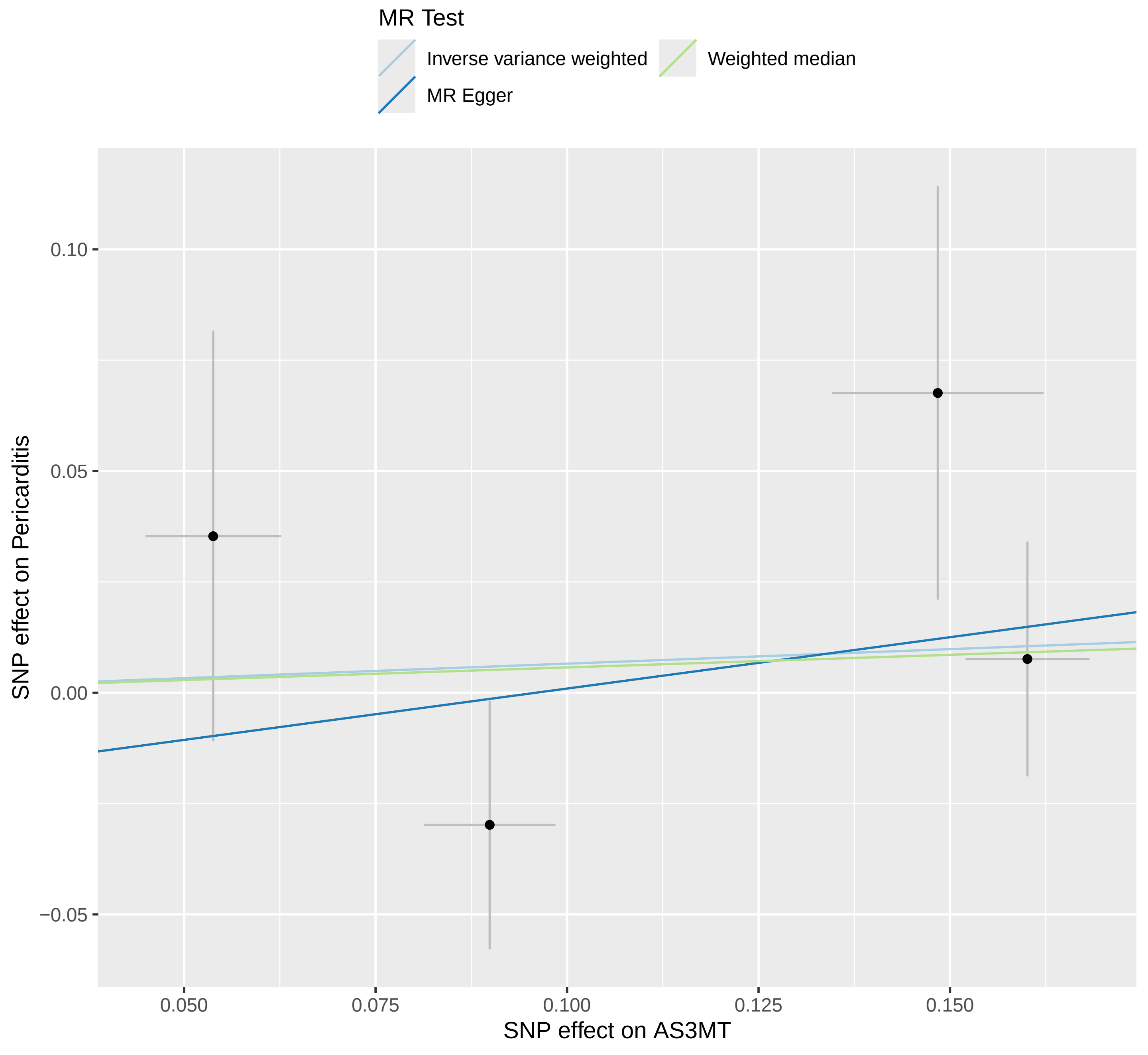

MR Test
Inverse variance weighted
Weighted median
MR Egger
0.10
0.05
0.00
−0.05
SNP effect on Pericarditis
0.050
0.075
0.100
0.125
0.150
SNP effect on AS3MT

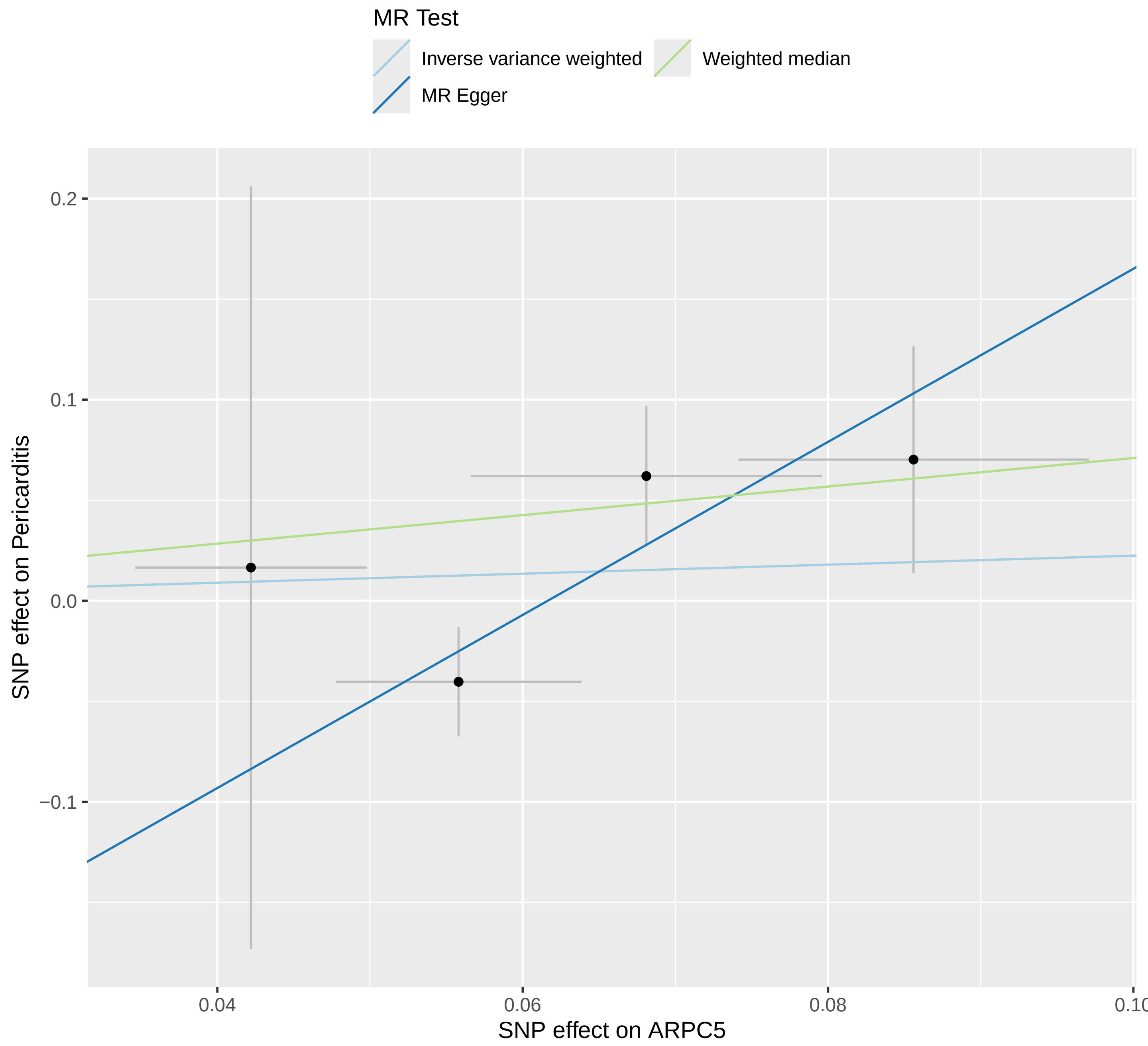
MR Test
Inverse variance weighted
Weighted median
MR Egger
0.2
0.1
0.0
−0.1
SNP effect on Pericarditis
0.04
0.06
0.08
0.10
SNP effect on ARPC5

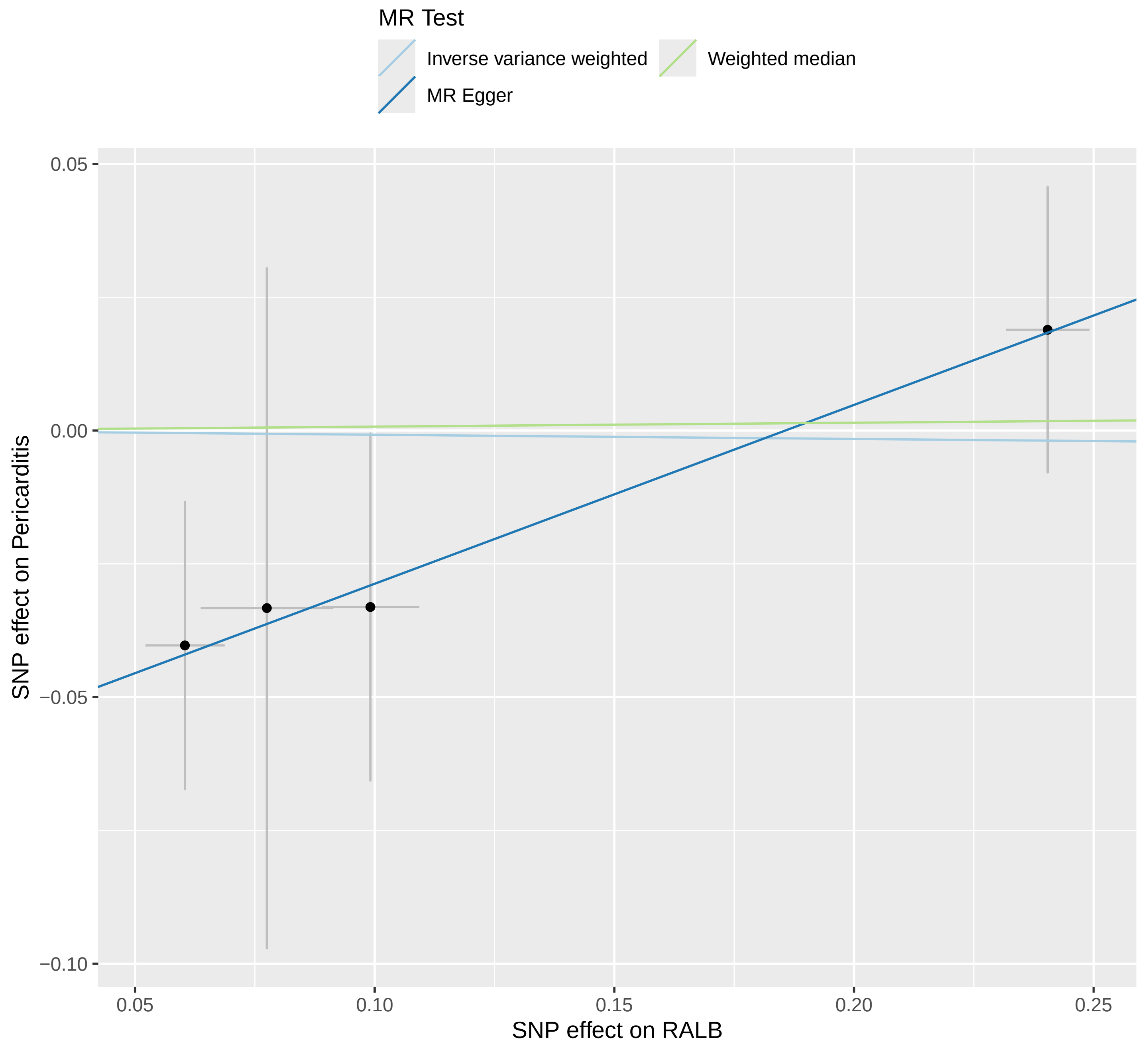
MR Test
Inverse variance weighted
MR Egger
Weighted median
0.05
0.00
−0.05
−0.10
SNP effect on Pericarditis
0.05
0.10
0.15
0.20
0.25
SNP effect on RALB

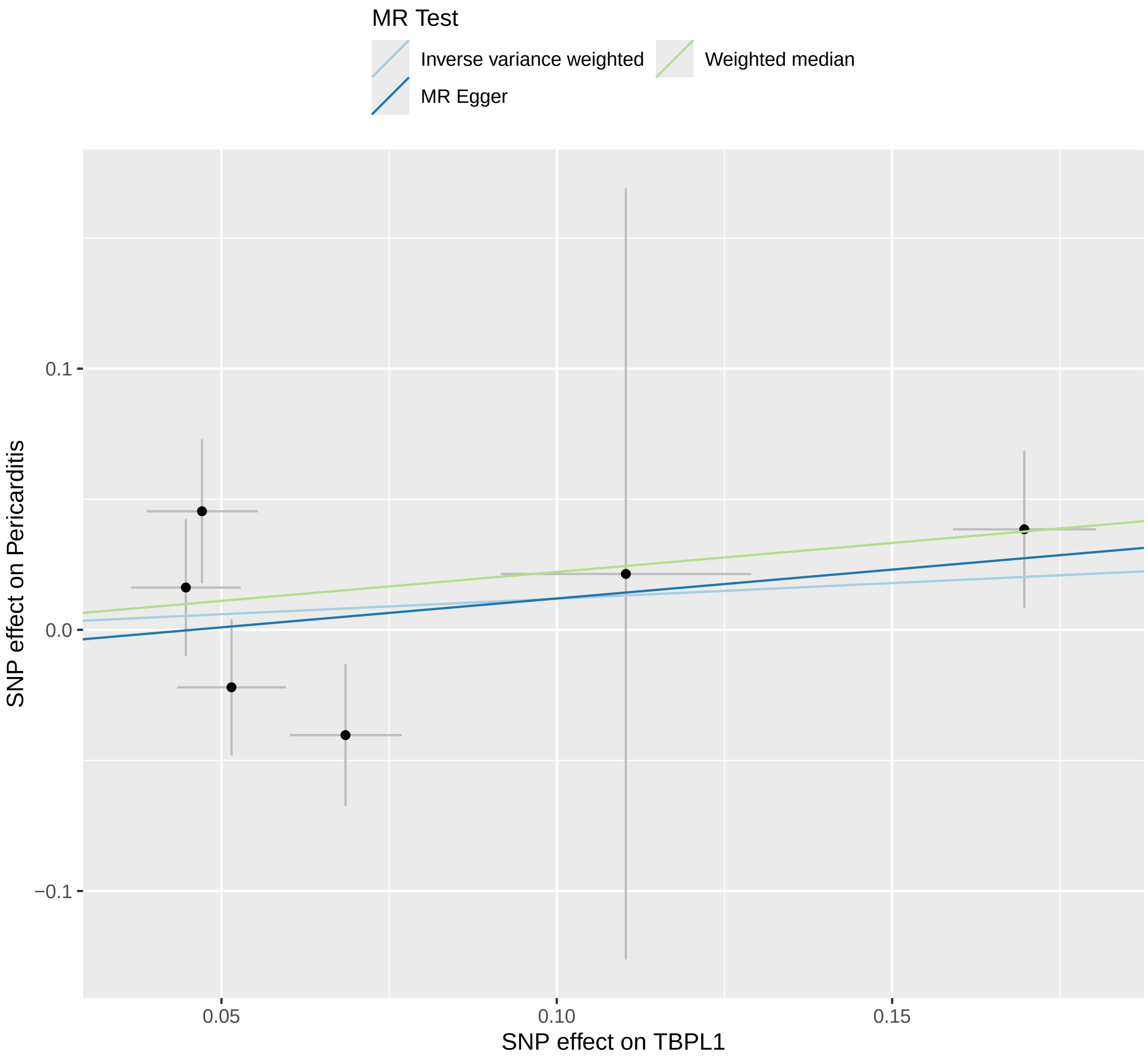
MR Test
Inverse variance weighted
Weighted median
MR Egger
0.1
0.0
−0.1
0.05
0.10
0.15
SNP effect on Pericarditis
SNP effect on TBPL1

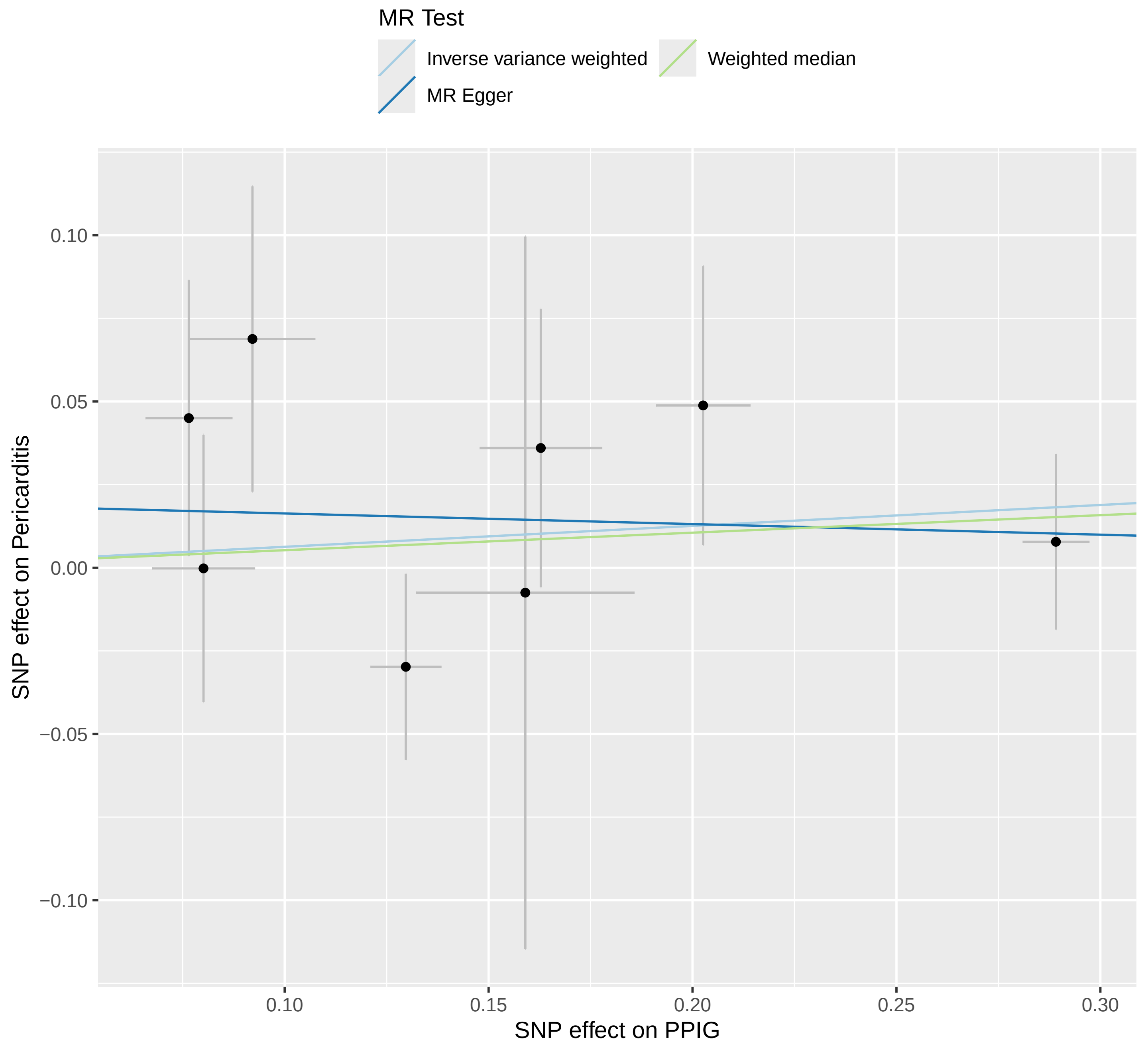

MR Test
Inverse variance weighted
MR Egger
Weighted median
0.10
0.05
0.00
−0.05
−0.10
SNP effect on Pericarditis
0.10
0.15
0.20
0.25
0.30
SNP effect on PPIG
