# Supplementary material for: Causal Association Between Plasma Proteins and Pericarditis: A Mendelian Randomization Study With Therapeutic Target Identification
Source: Mediators Inflamm. 2026 Feb 9;2026:4659271. doi: 10.1155/mi/4659271 (PMC12887433; doi:10.1155/mi/4659271)
Supplement: Supplementary file 6 — Supporting Information 6 Figure S2.Leave‐one‐out results of MR analysis between plasmas proteins and Pericarditis [file MI-2026-4659271-s005.pdf]

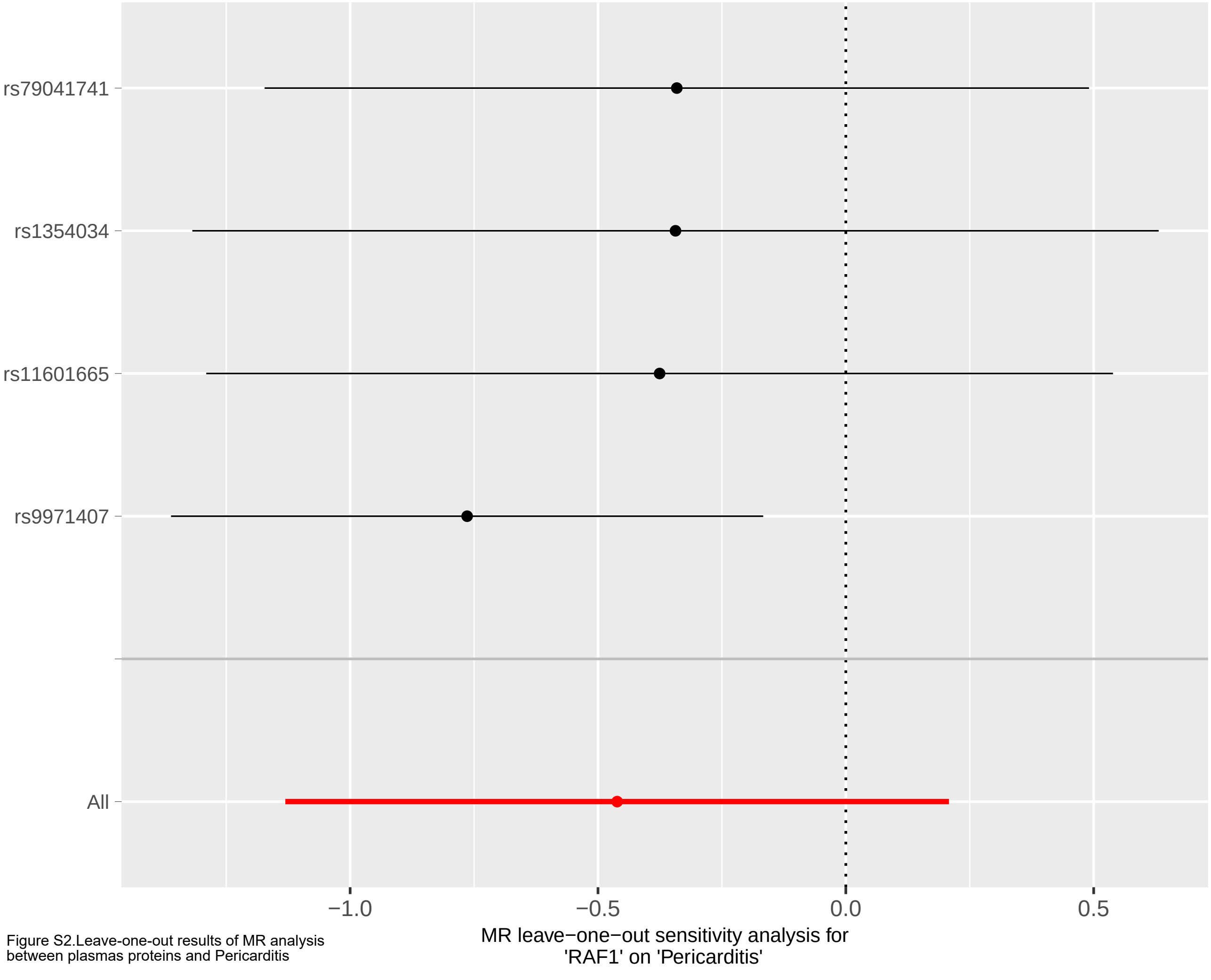

Figure S2.Leave-one-out results of MR analysis
between plasmas proteins and Pericarditis

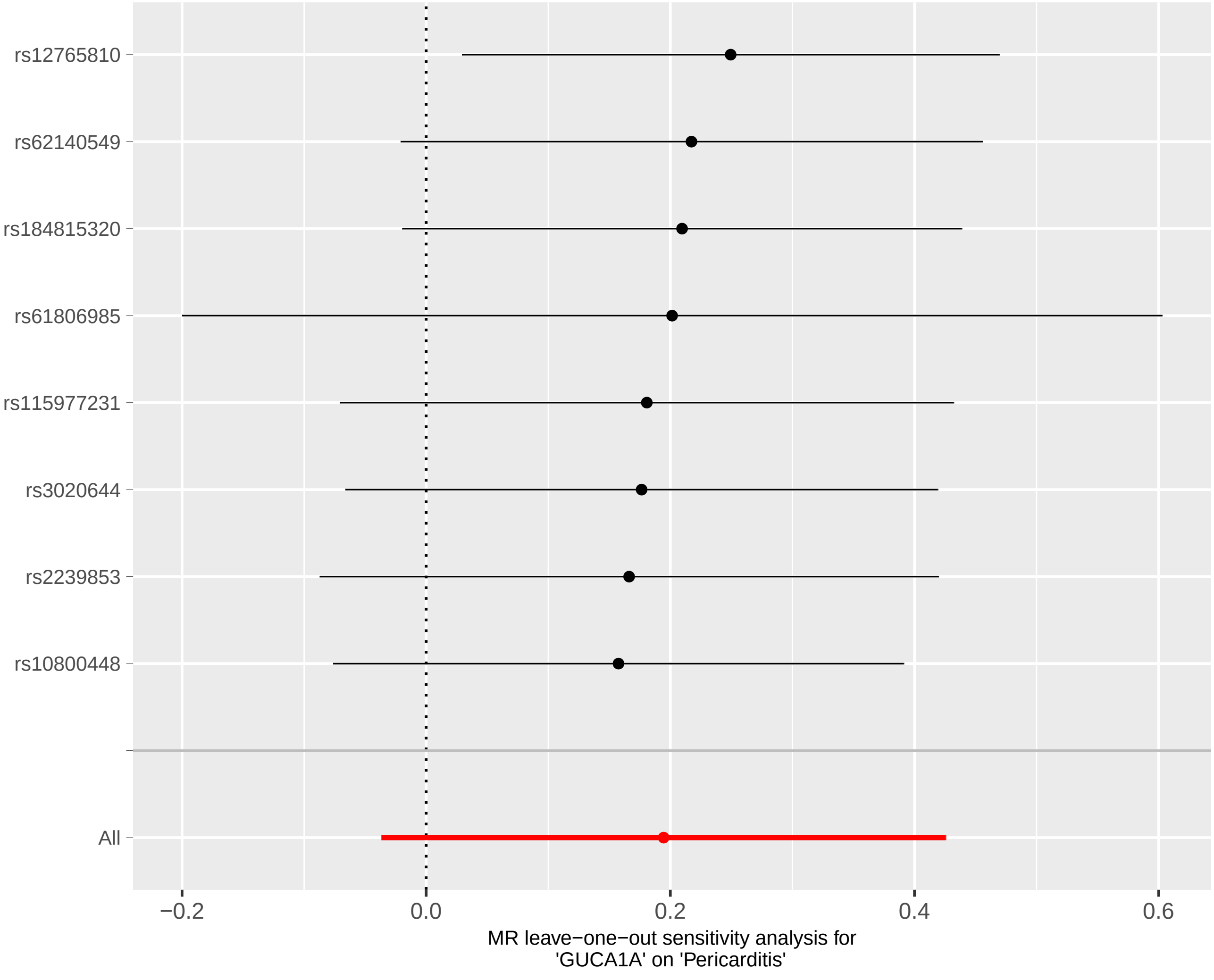
rs12765810
rs62140549
rs184815320
rs61806985
rs115977231
rs3020644
rs2239853
rs10800448
All
−0.2
0.0
0.2
0.4
0.6
MR leave−one−out sensitivity analysis for
'GUCA1A' on 'Pericarditis'

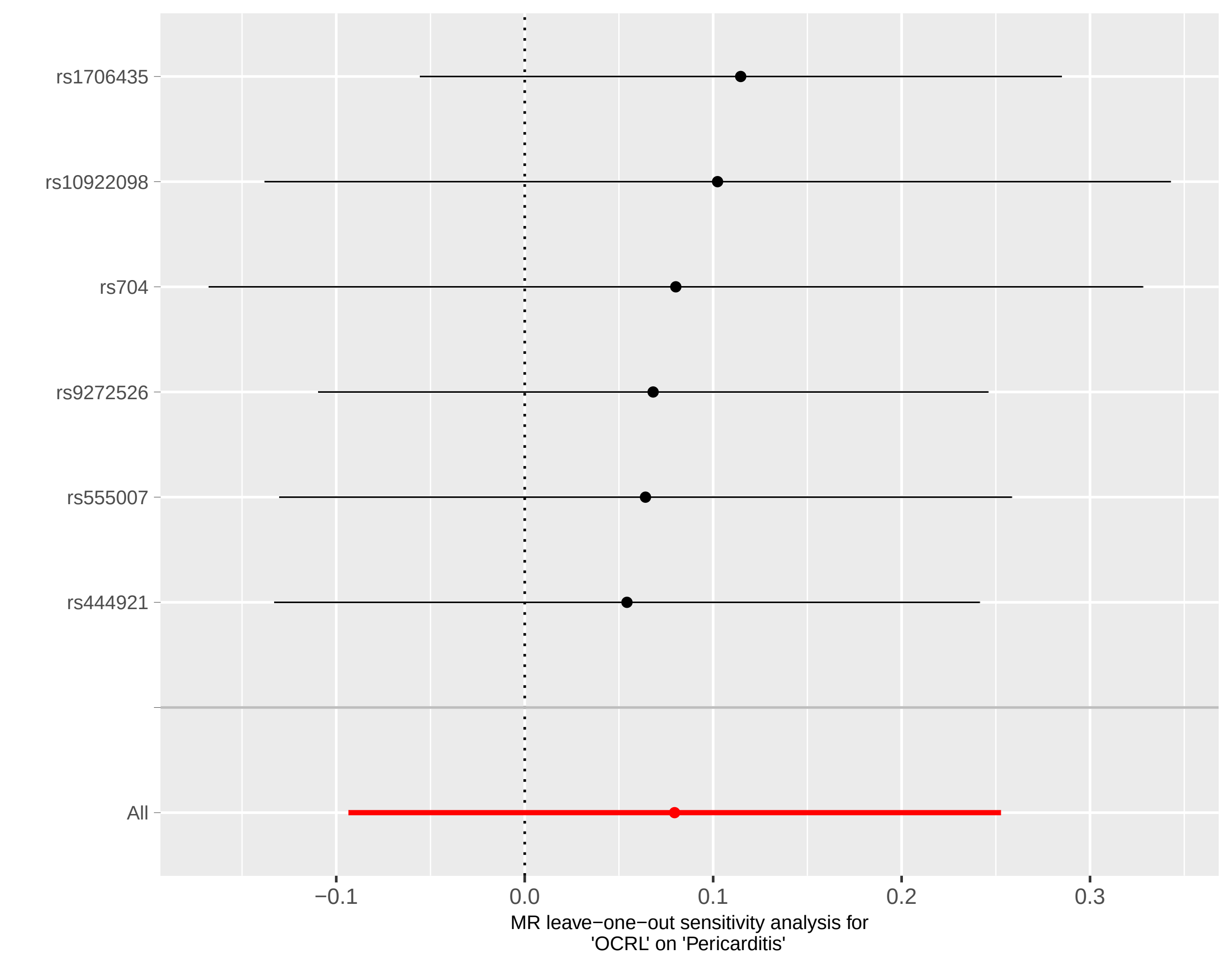

rs1706435
rs10922098
rs704
rs9272526
rs555007
rs444921
All
−0.1
0.0
0.1
0.2
0.3
MR leave−one−out sensitivity analysis for
'OCRL' on 'Pericarditis'

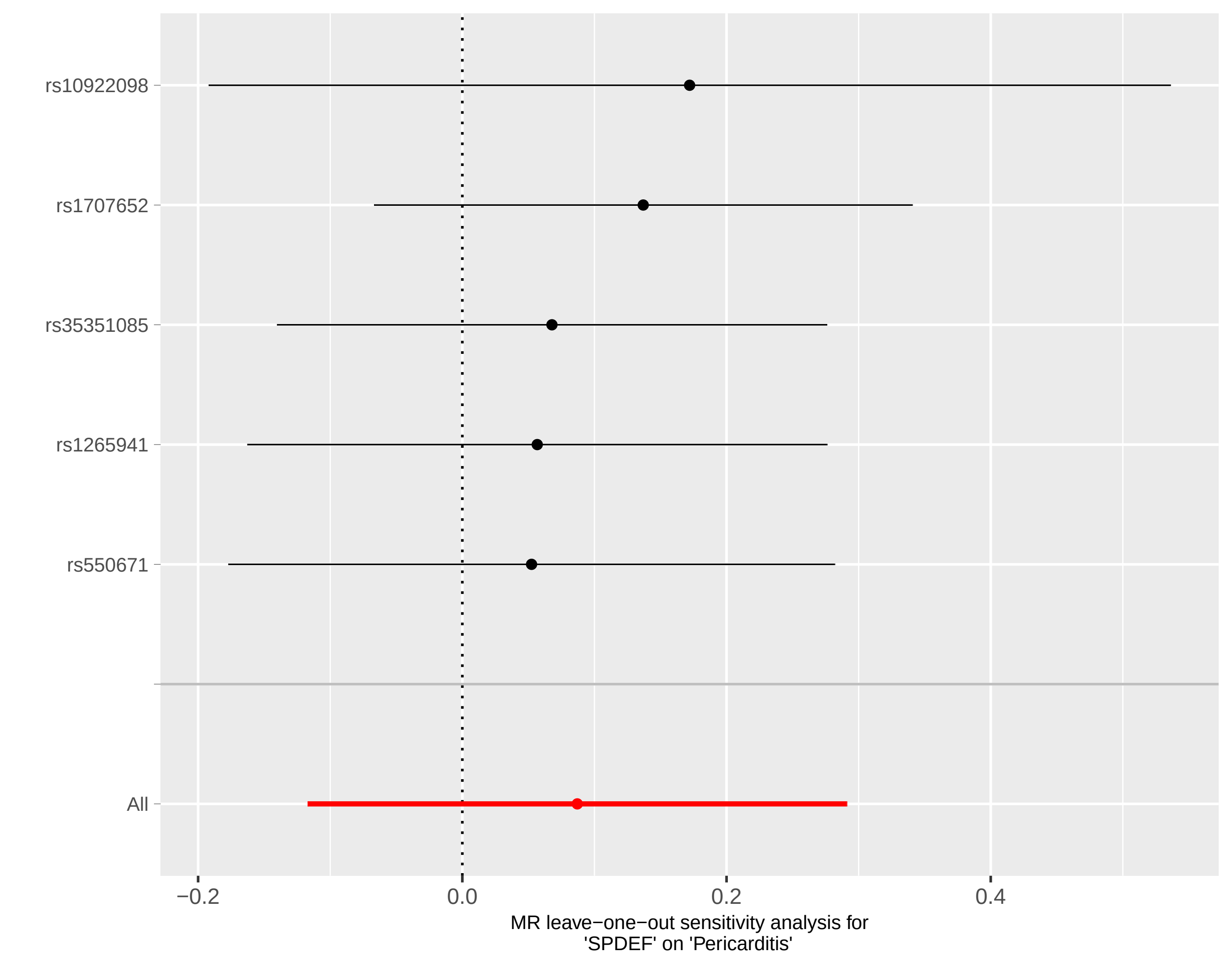
rs10922098
rs1707652
rs35351085
rs1265941
rs550671
All
−0.2
0.0
0.2
0.4
MR leave−one−out sensitivity analysis for
'SPDEF' on 'Pericarditis'

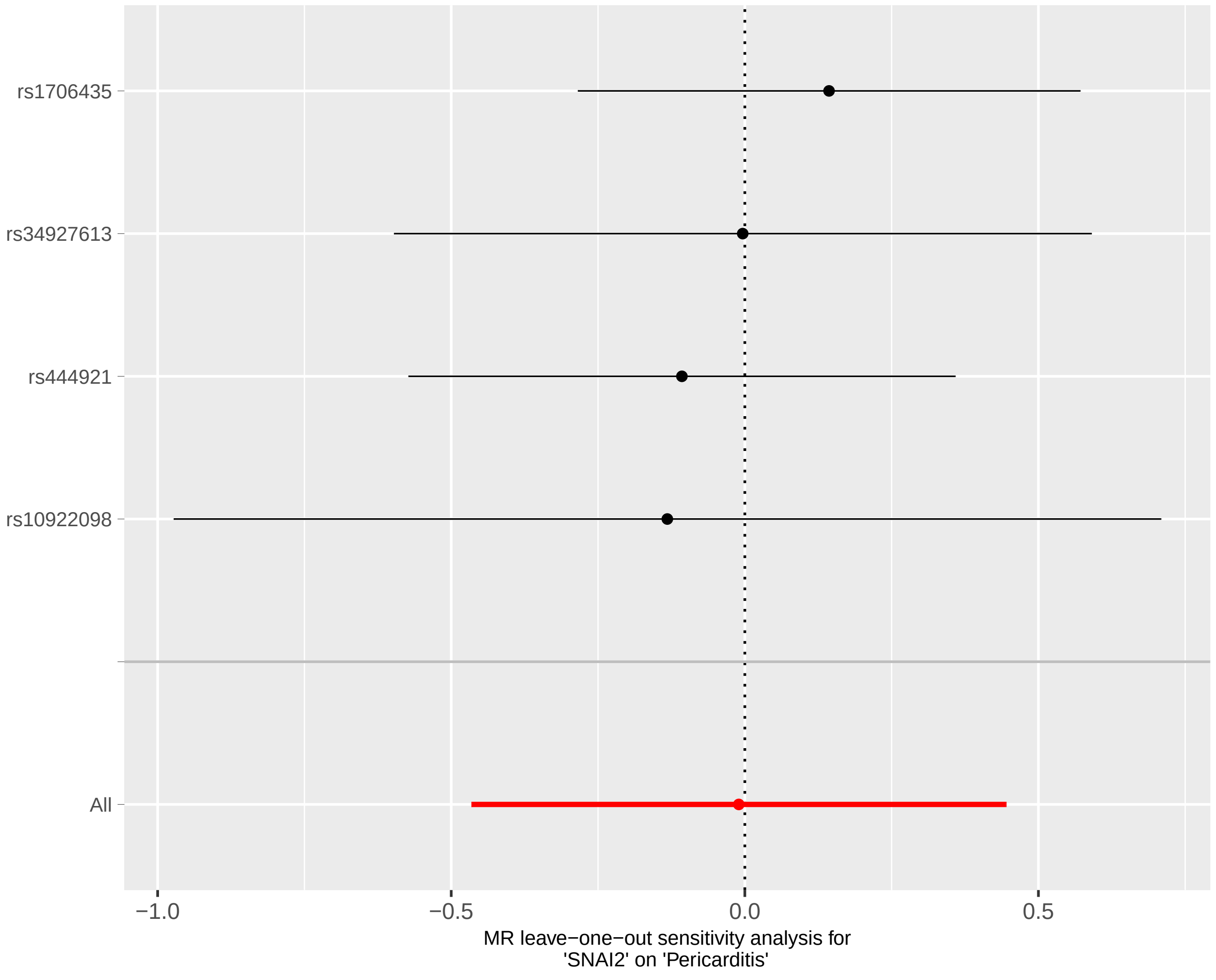
rs1706435
rs34927613
rs444921
rs10922098
All
−1.0
−0.5
0.0
0.5
MR leave−one−out sensitivity analysis for
'SNAI2' on 'Pericarditis'

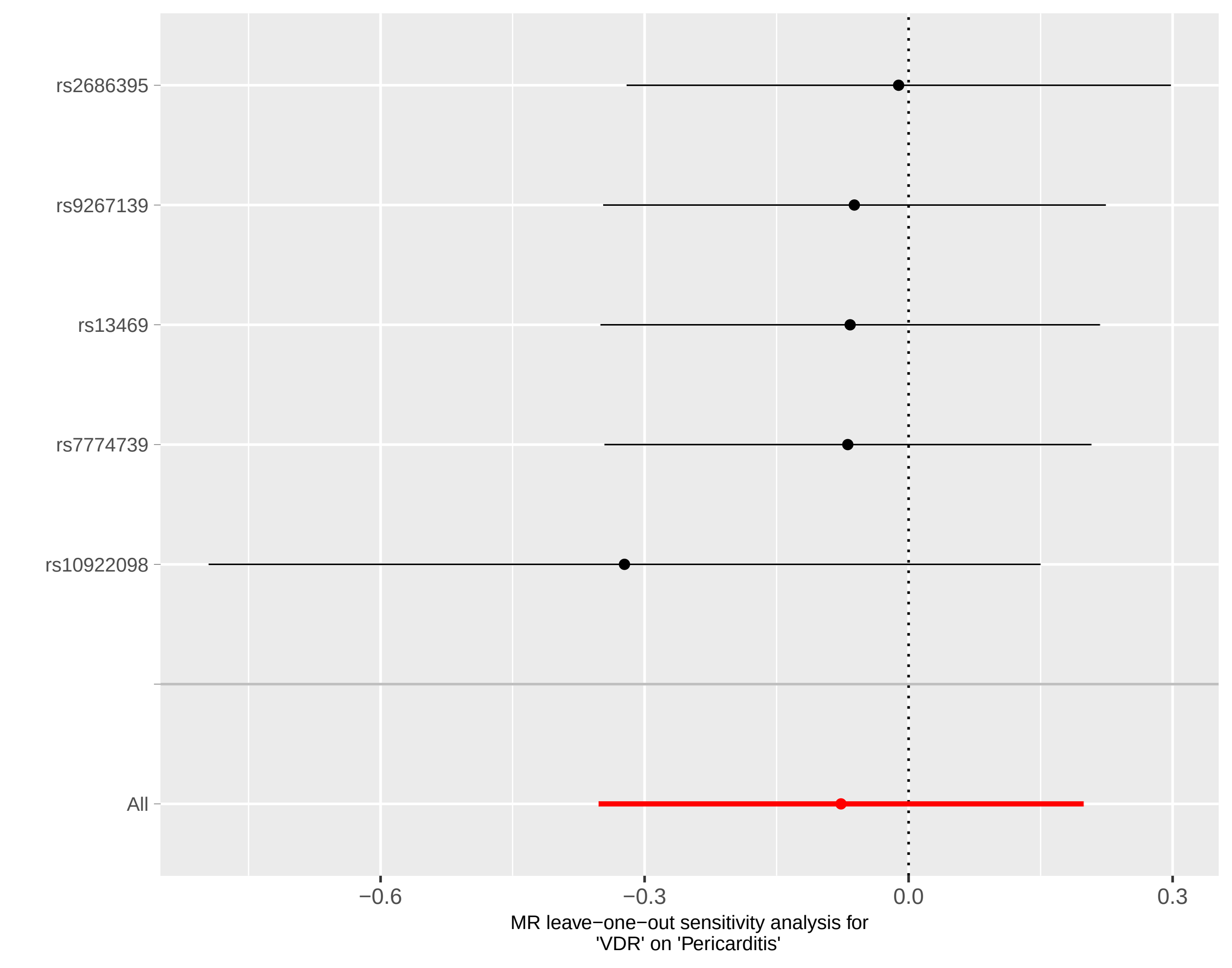

rs2686395
rs9267139
rs13469
rs7774739
rs10922098
All
−0.6
−0.3
0.0
0.3
MR leave−one−out sensitivity analysis for
'VDR' on 'Pericarditis'

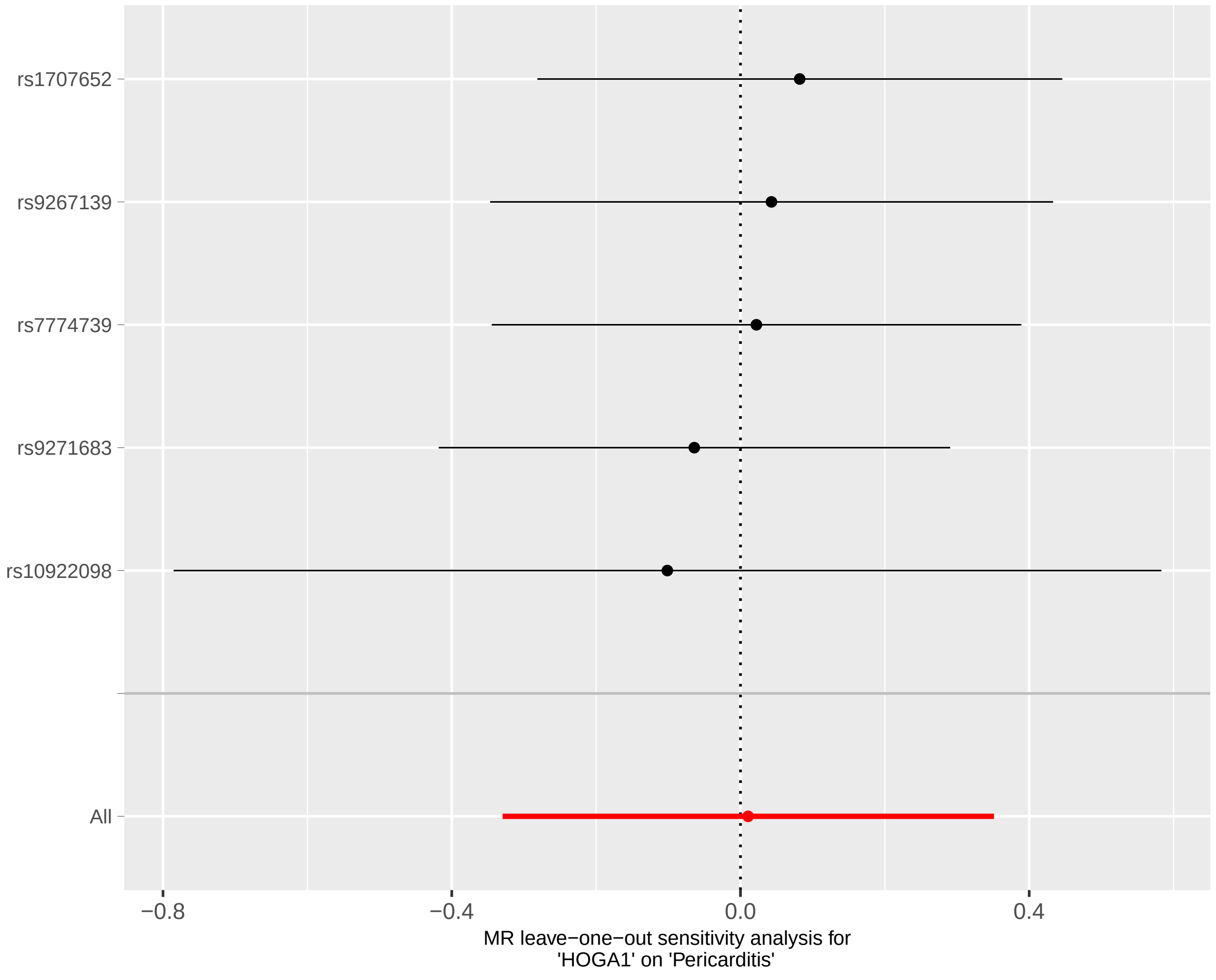
rs1707652
rs9267139
rs7774739
rs9271683
rs10922098
All
−0.8
−0.4
0.0
0.4
MR leave−one−out sensitivity analysis for
'HOGA1' on 'Pericarditis'

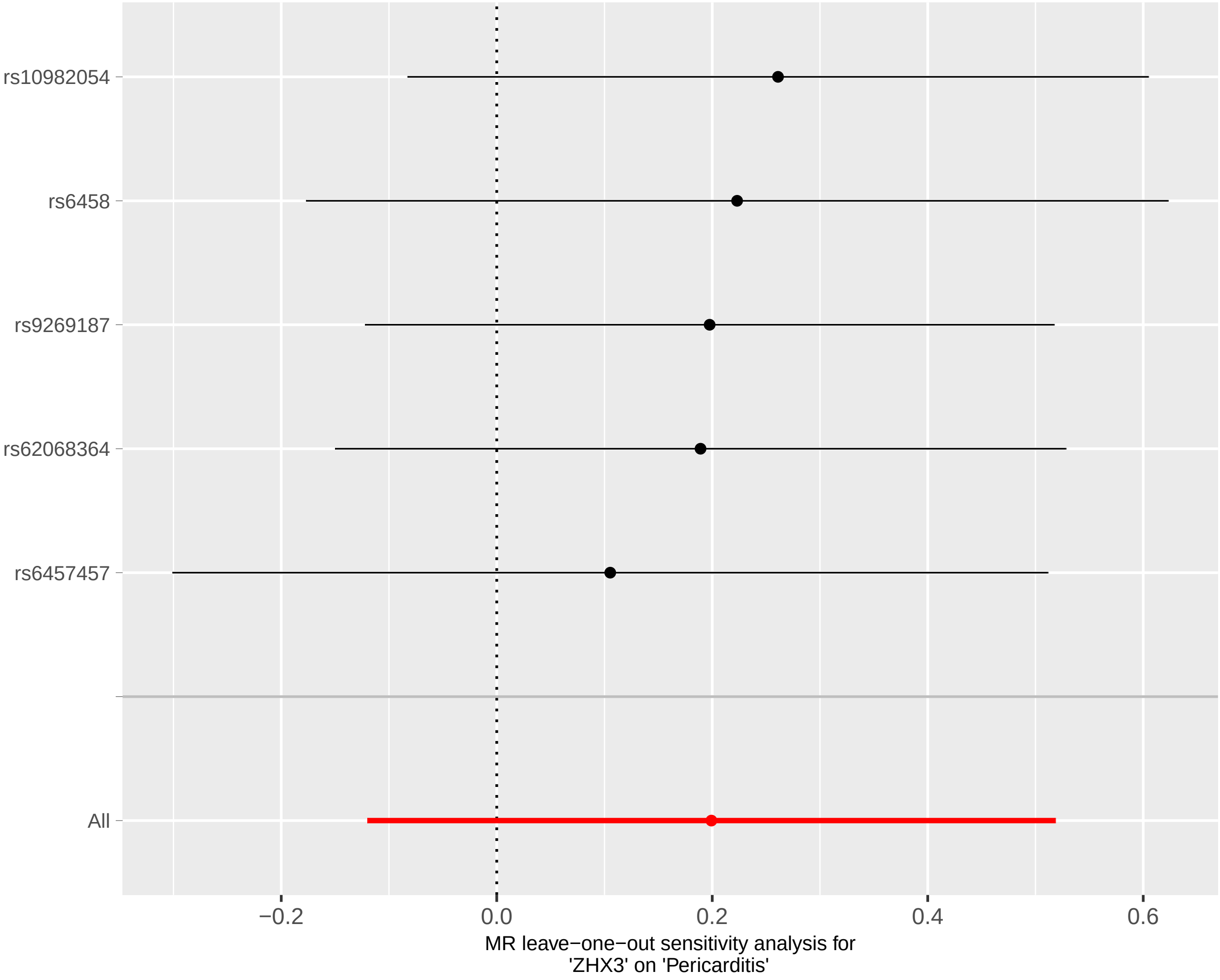

rs10982054
rs6458
rs9269187
rs62068364
rs6457457
All
−0.2
0.0
0.2
0.4
0.6
MR leave−one−out sensitivity analysis for
'ZHX3' on 'Pericarditis'

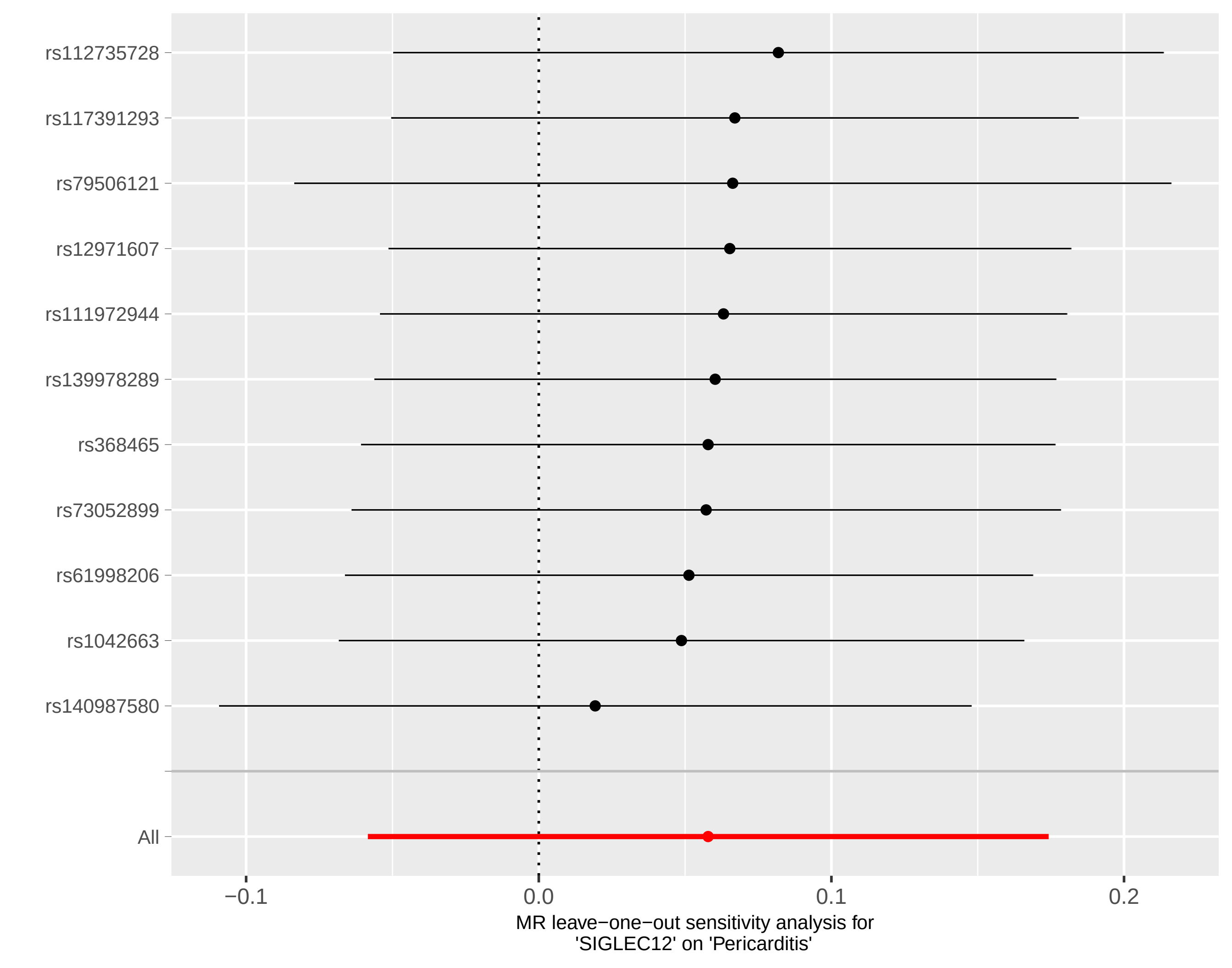
rs112735728
rs117391293
rs79506121
rs12971607
rs111972944
rs139978289
rs368465
rs73052899
rs61998206
rs1042663
rs140987580
All
−0.1
0.0
0.1
0.2
MR leave−one−out sensitivity analysis for
'SIGLEC12' on 'Pericarditis'

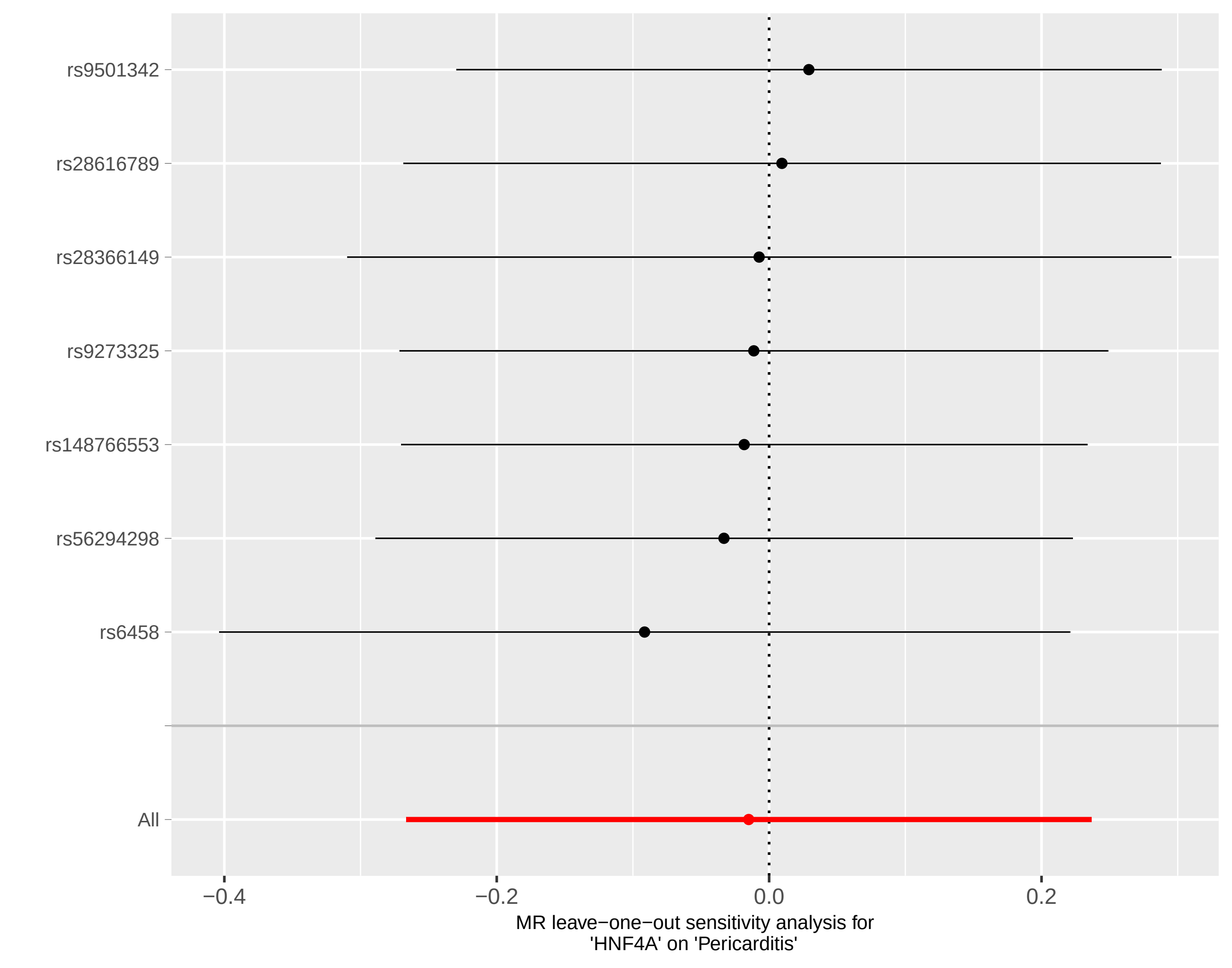
rs9501342
rs28616789
rs28366149
rs9273325
rs148766553
rs56294298
rs6458
All
−0.4
−0.2
0.0
0.2
MR leave−one−out sensitivity analysis for
'HNF4A' on 'Pericarditis'

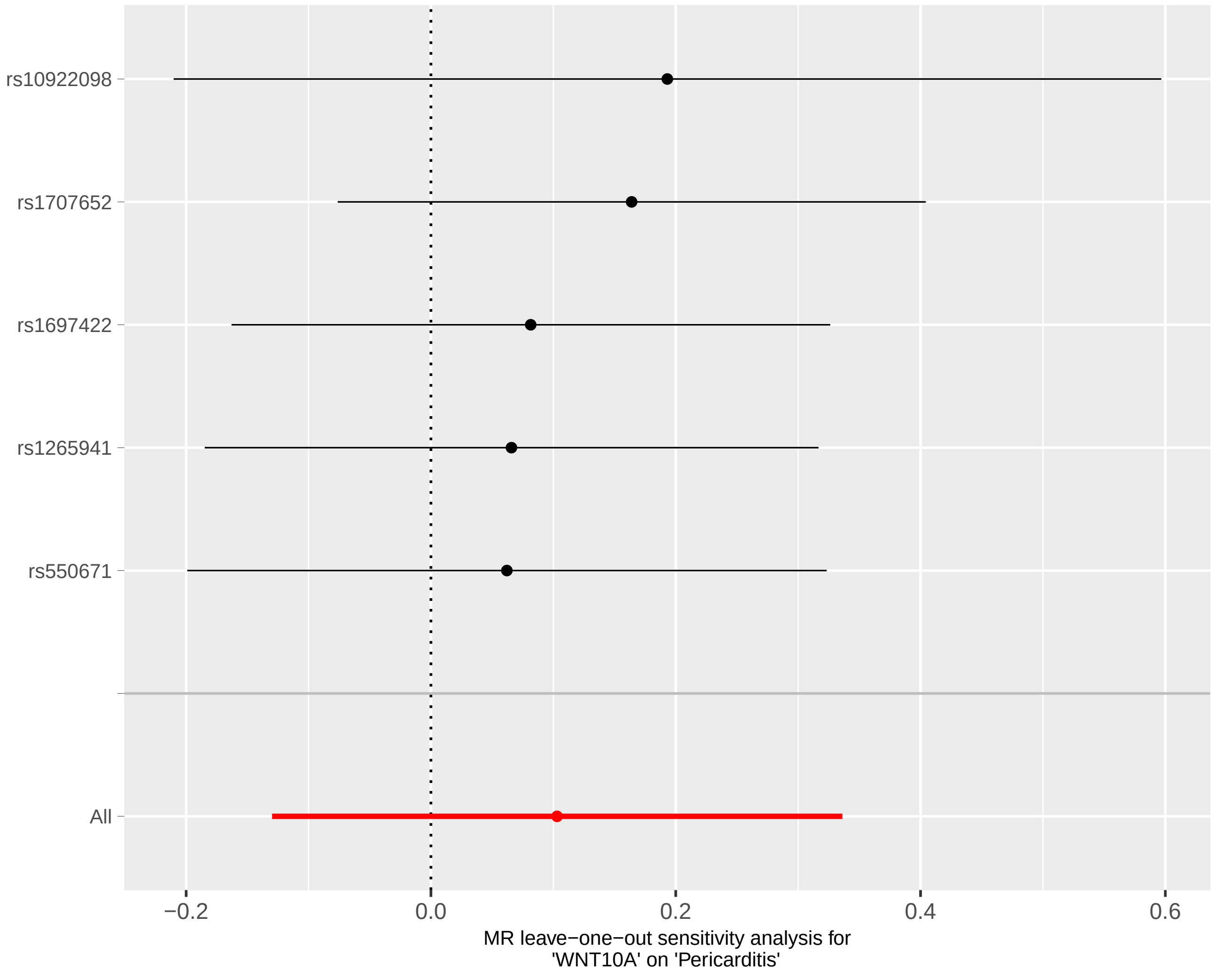

rs10922098
rs1707652
rs1697422
rs1265941
rs550671
All
−0.2
0.0
0.2
0.4
0.6
MR leave−one−out sensitivity analysis for
'WNT10A' on 'Pericarditis'

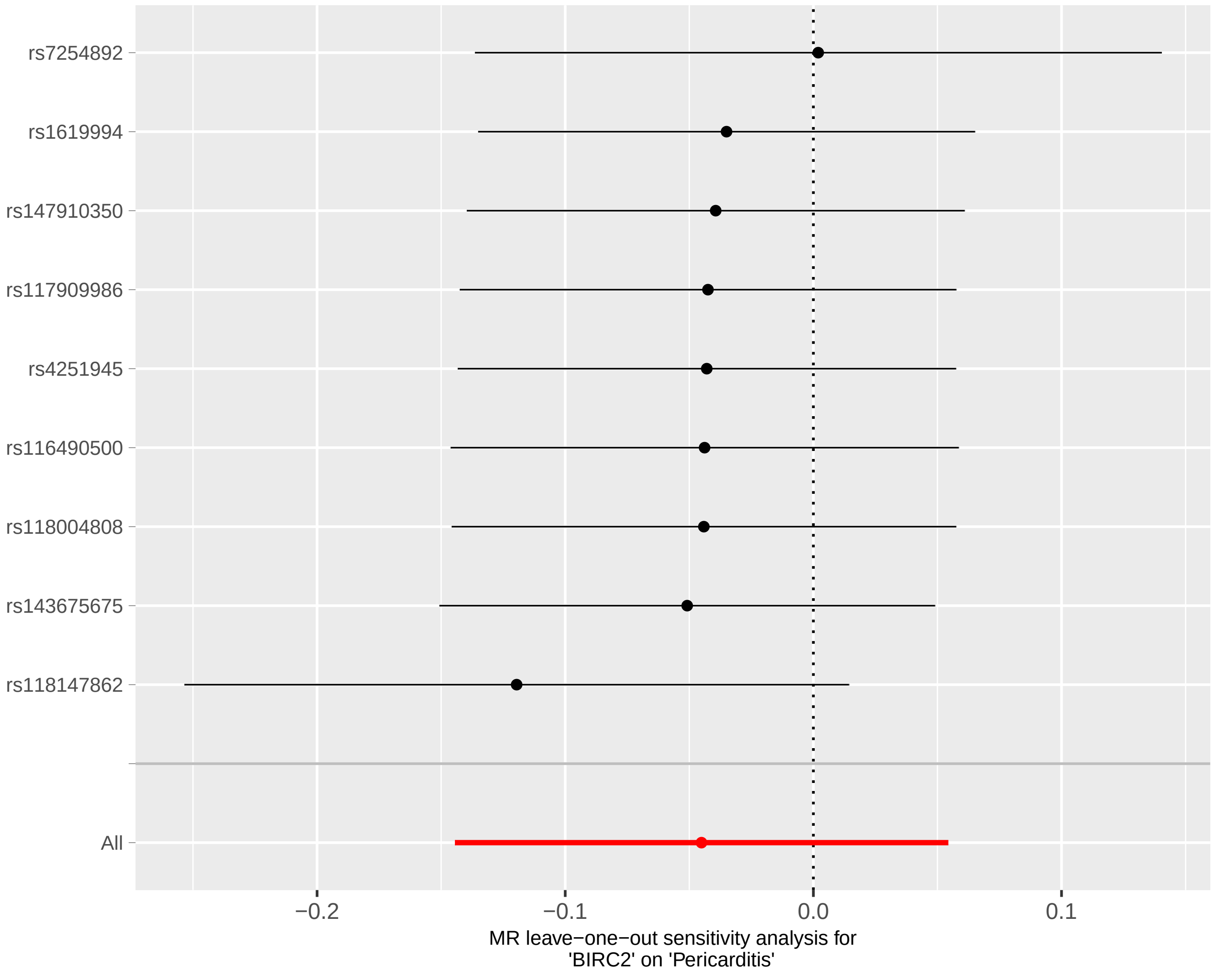
rs7254892
rs1619994
rs147910350
rs117909986
rs4251945
rs116490500
rs118004808
rs143675675
rs118147862
All
−0.2
−0.1
0.0
0.1
MR leave−one−out sensitivity analysis for
'BIRC2' on 'Pericarditis'

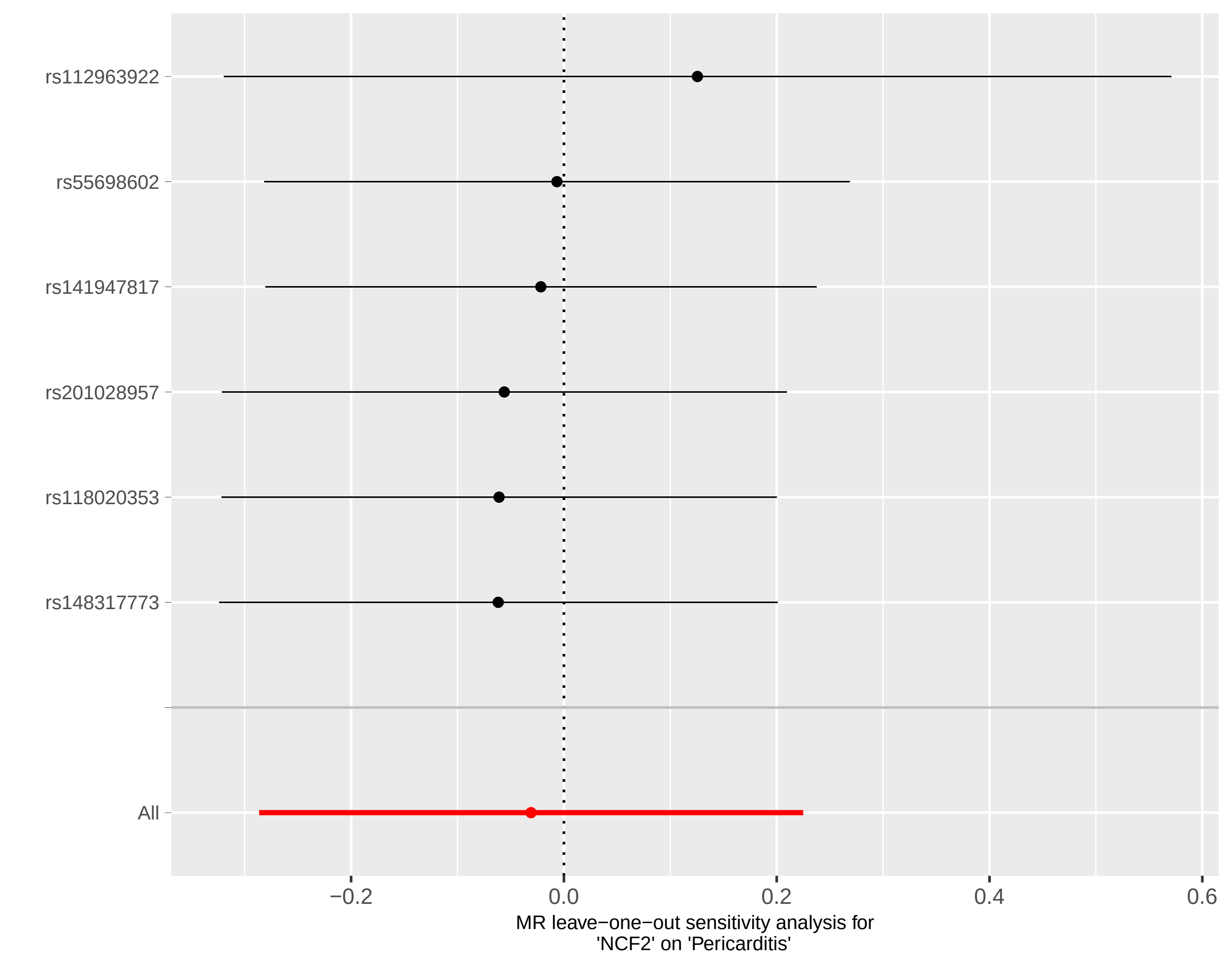
rs112963922
rs55698602
rs141947817
rs201028957
rs118020353
rs148317773
All
−0.2
0.0
0.2
0.4
0.6
MR leave−one−out sensitivity analysis for
'NCF2' on 'Pericarditis'

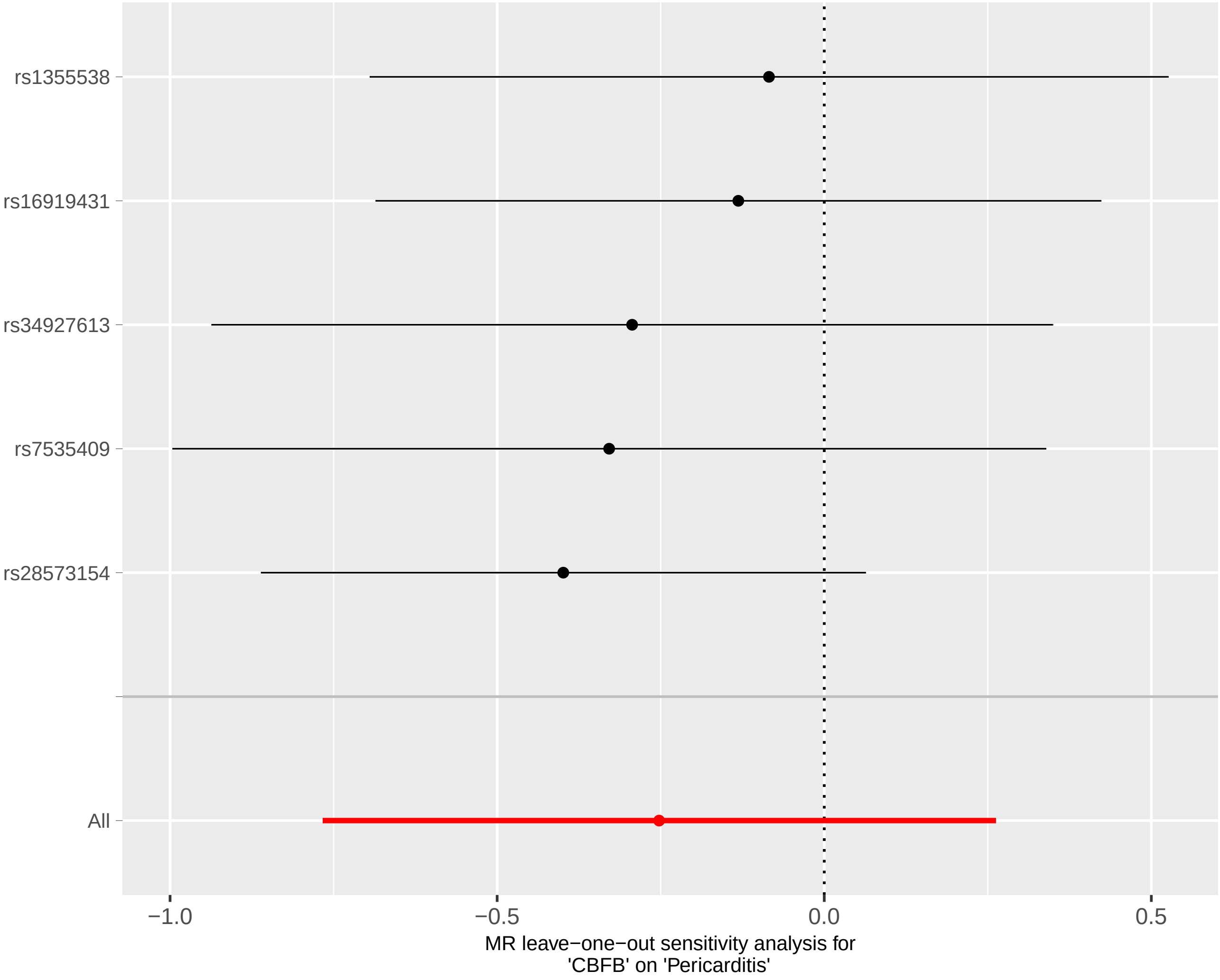
rs1355538
rs16919431
rs34927613
rs7535409
rs28573154
All
−1.0
−0.5
0.0
0.5
MR leave−one−out sensitivity analysis for
'CBFB' on 'Pericarditis'

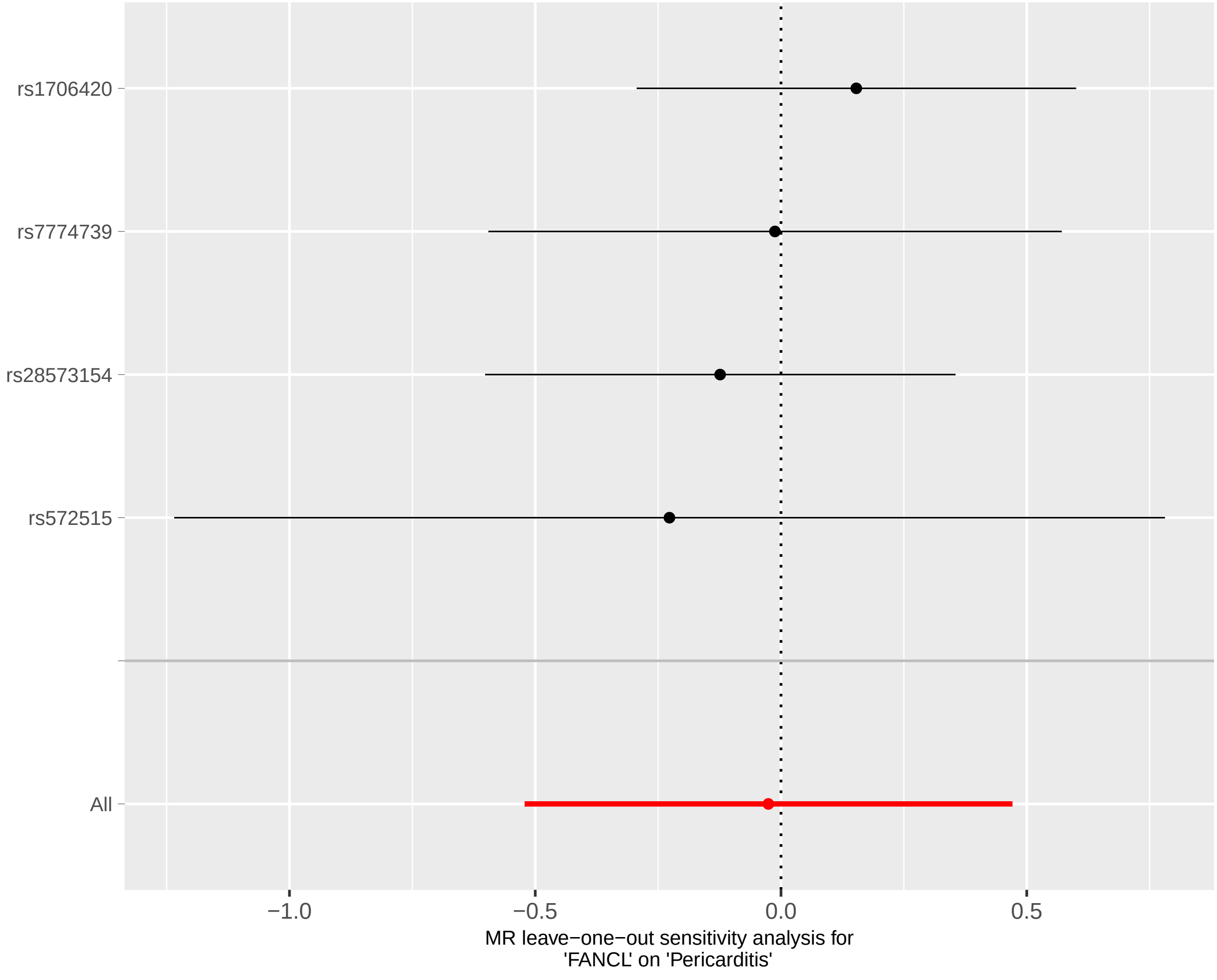

rs1706420
rs7774739
rs28573154
rs572515
All
−1.0
−0.5
0.0
0.5
MR leave−one−out sensitivity analysis for
'FANCL' on 'Pericarditis'

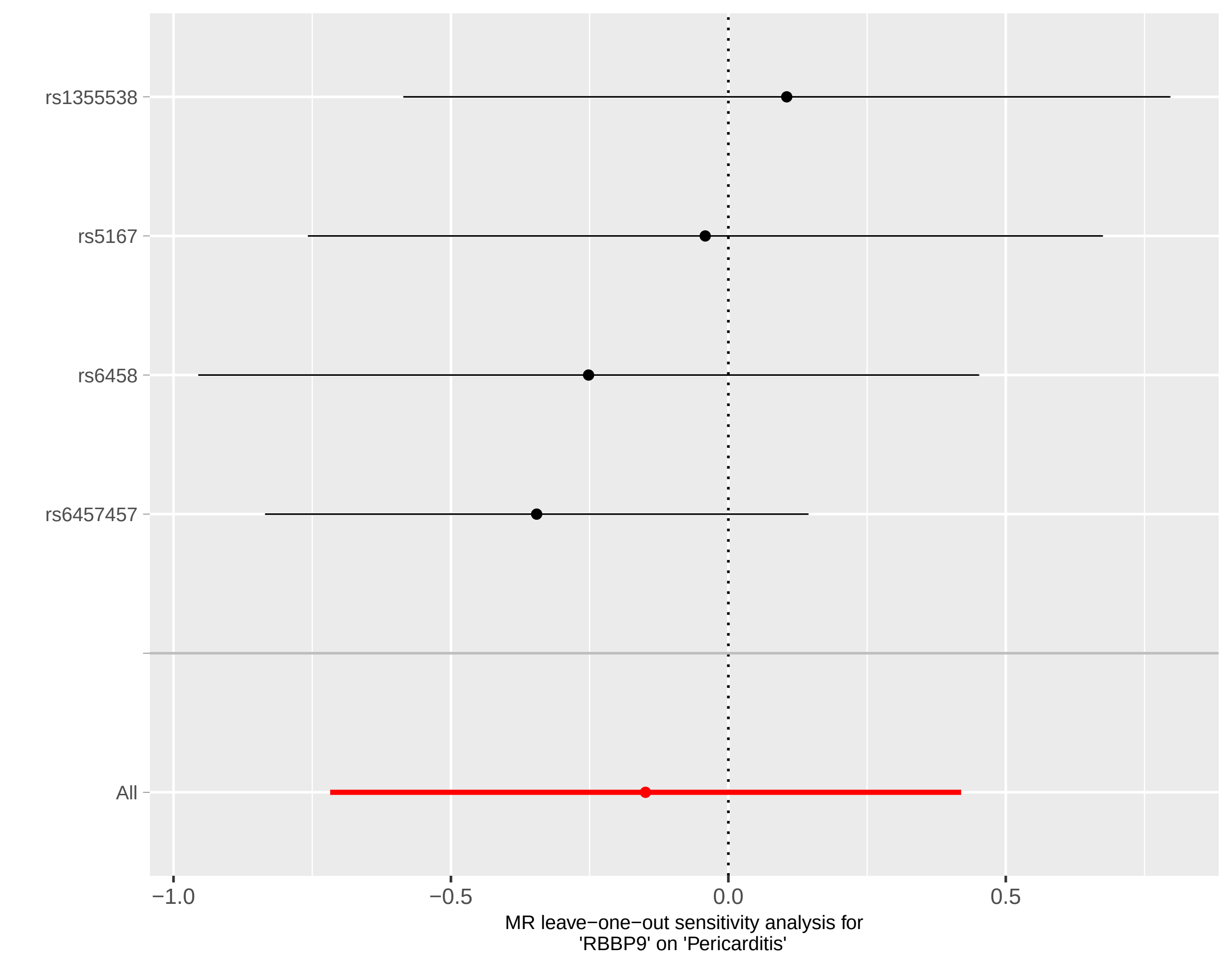

rs1355538
rs5167
rs6458
rs6457457
All
−1.0
−0.5
0.0
0.5
MR leave−one−out sensitivity analysis for
'RBBP9' on 'Pericarditis'

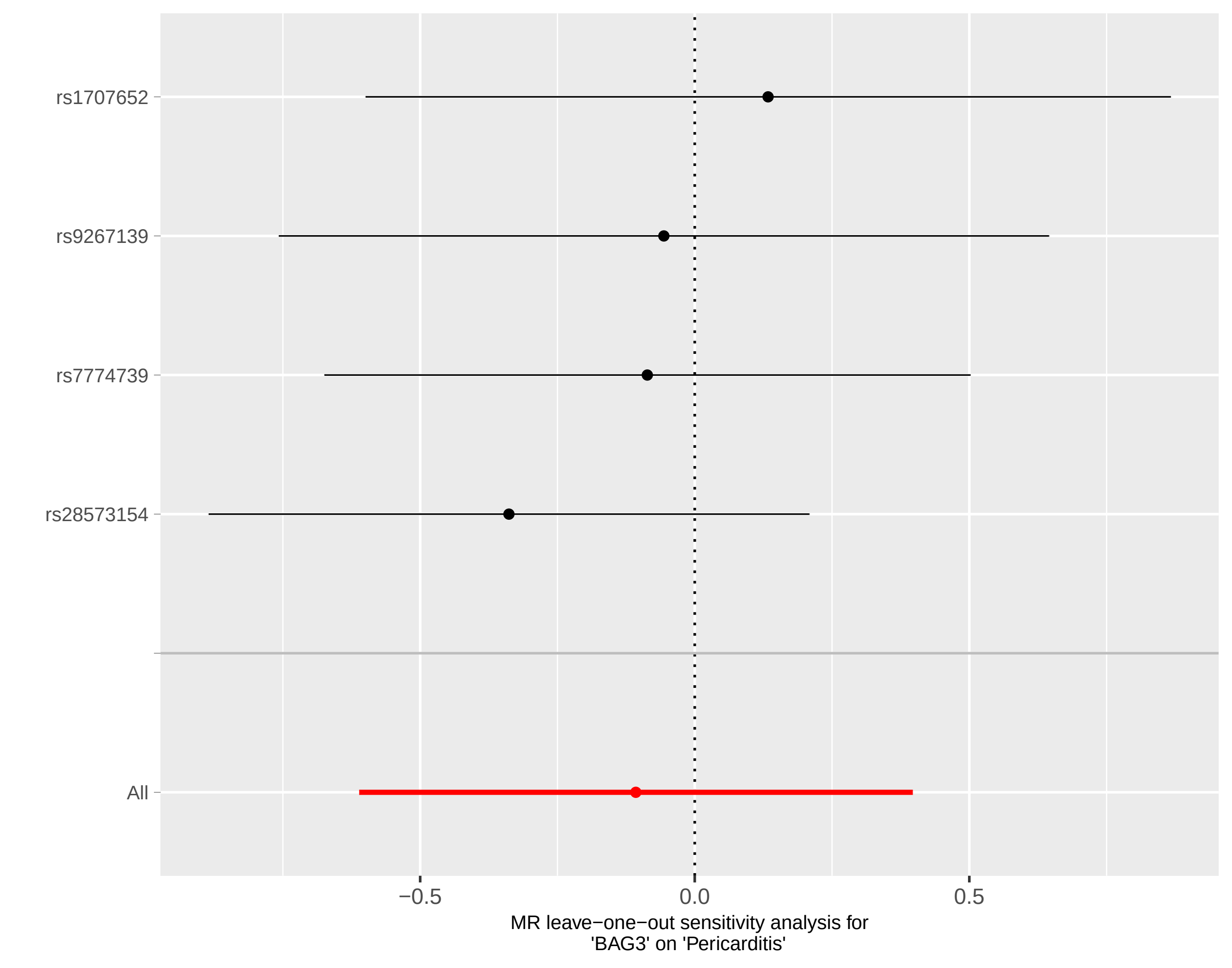
rs1707652
rs9267139
rs7774739
rs28573154
All
−0.5
0.0
0.5
MR leave−one−out sensitivity analysis for
'BAG3' on 'Pericarditis'

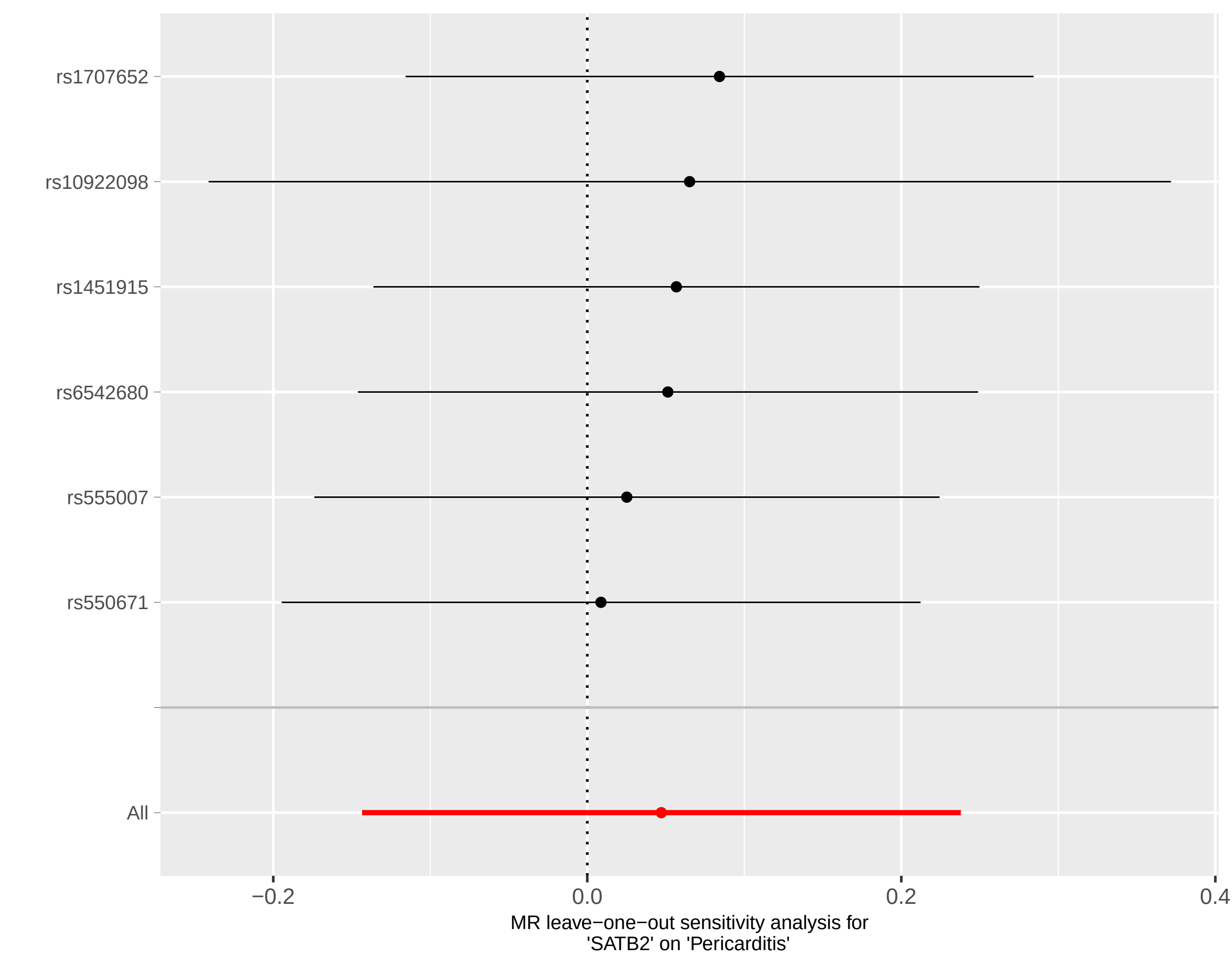
rs1707652
rs10922098
rs1451915
rs6542680
rs555007
rs550671
All
−0.2
0.0
0.2
0.4
MR leave−one−out sensitivity analysis for
'SATB2' on 'Pericarditis'

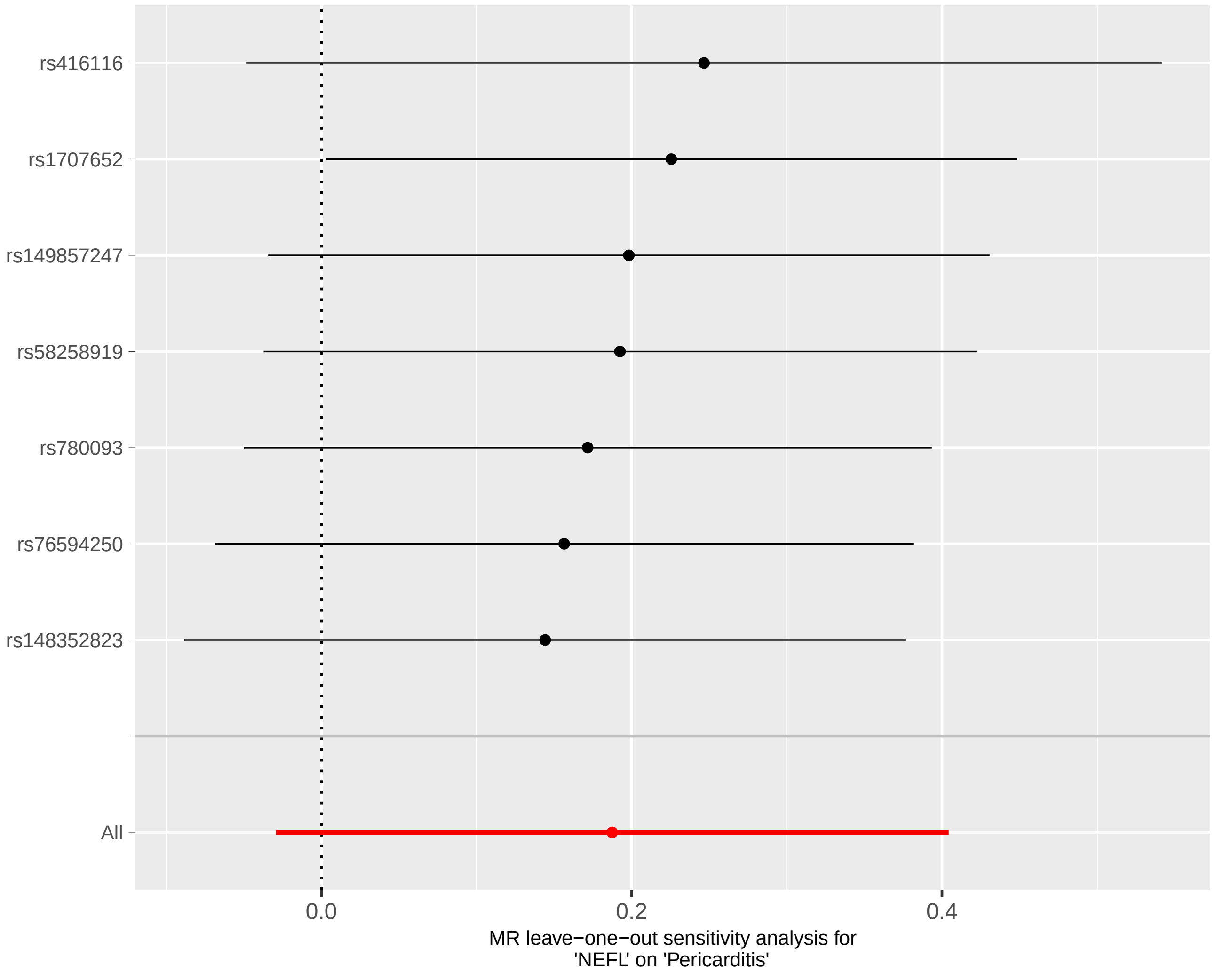
rs416116
rs1707652
rs149857247
rs58258919
rs780093
rs76594250
rs148352823
All
0.0
0.2
0.4
MR leave−one−out sensitivity analysis for
'NEFL' on 'Pericarditis'

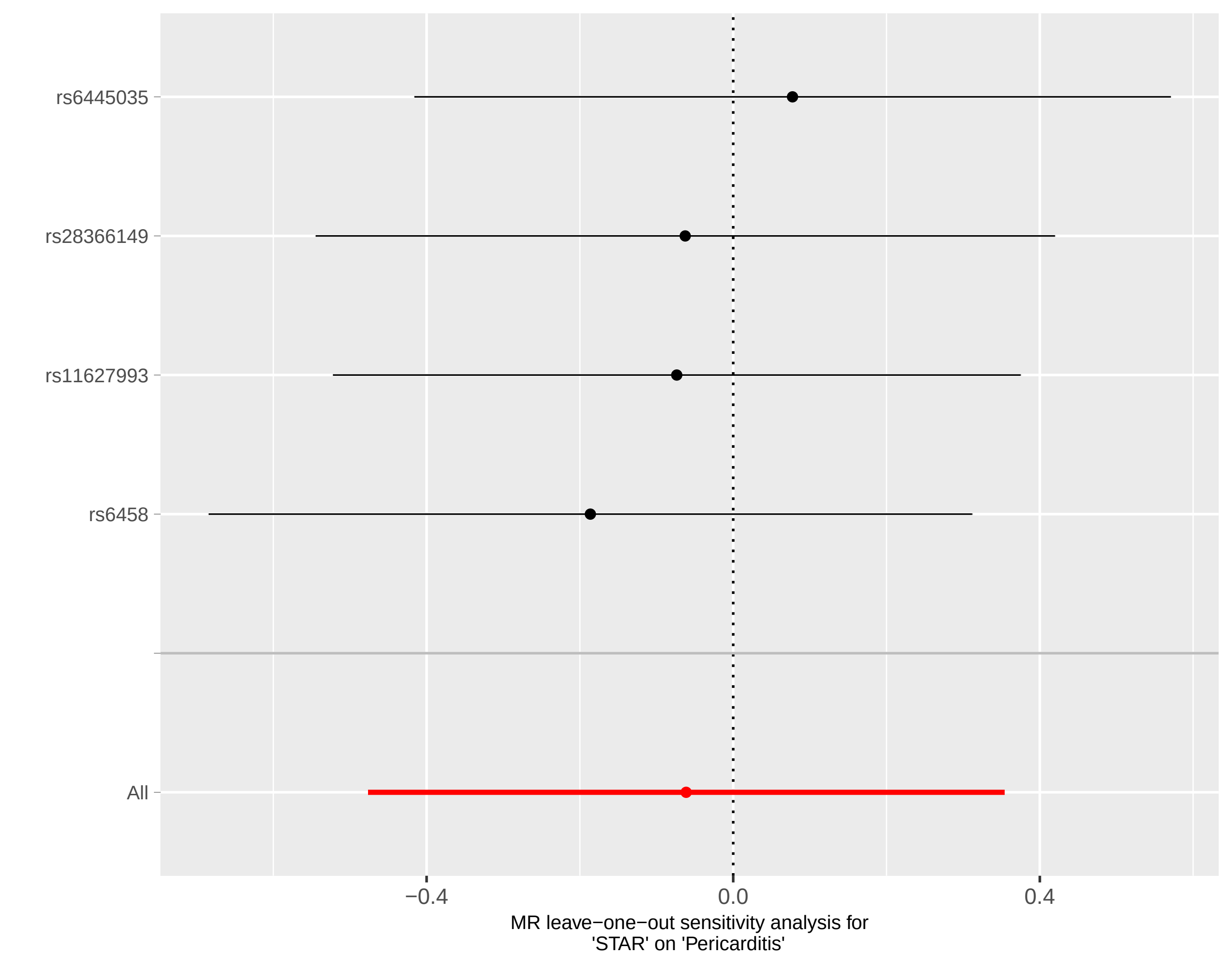

rs6445035
rs28366149
rs11627993
rs6458
All
−0.4
0.0
0.4
MR leave−one−out sensitivity analysis for
'STAR' on 'Pericarditis'

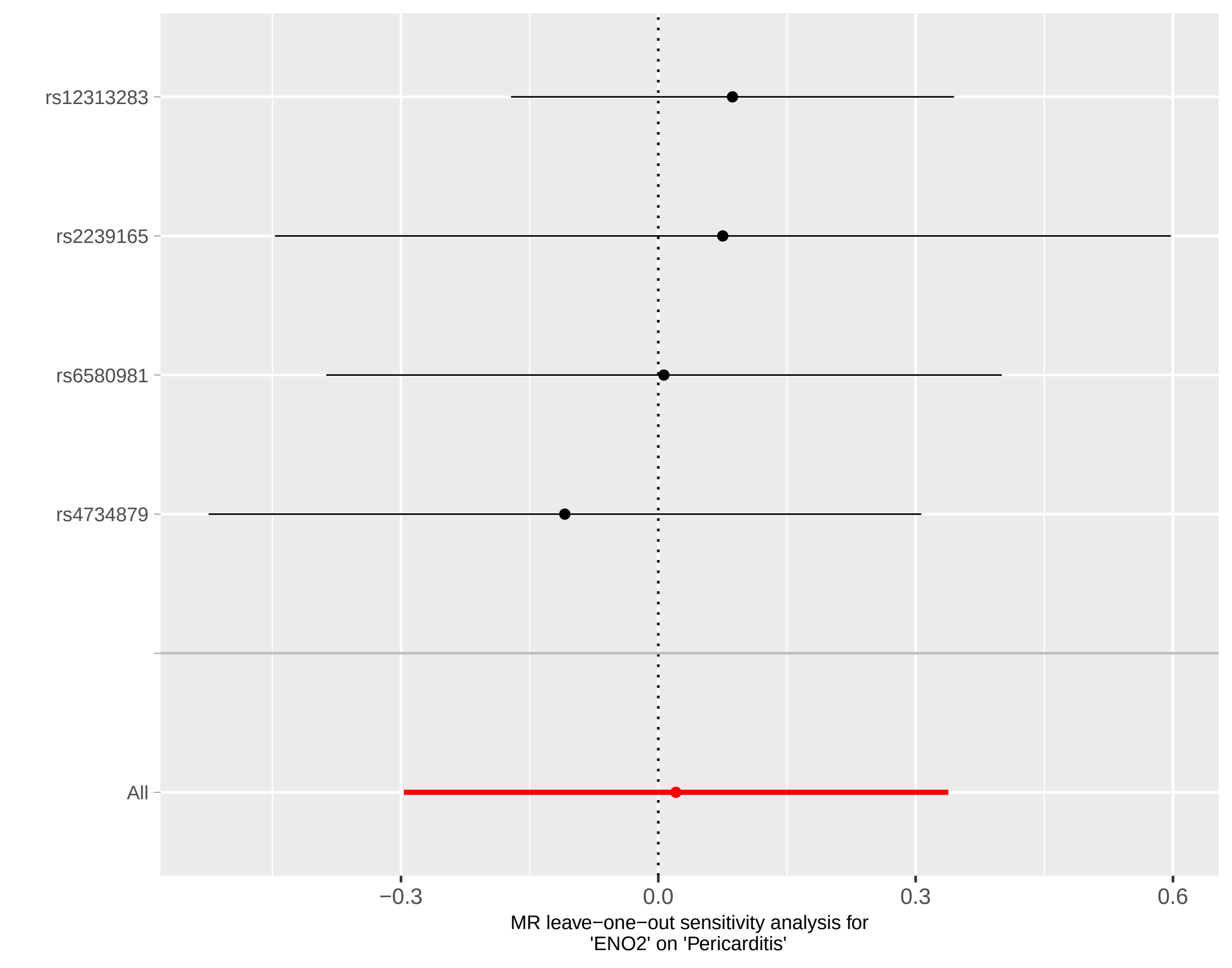
rs12313283
rs2239165
rs6580981
rs4734879
All
−0.3
0.0
0.3
0.6
MR leave−one−out sensitivity analysis for
'ENO2' on 'Pericarditis'

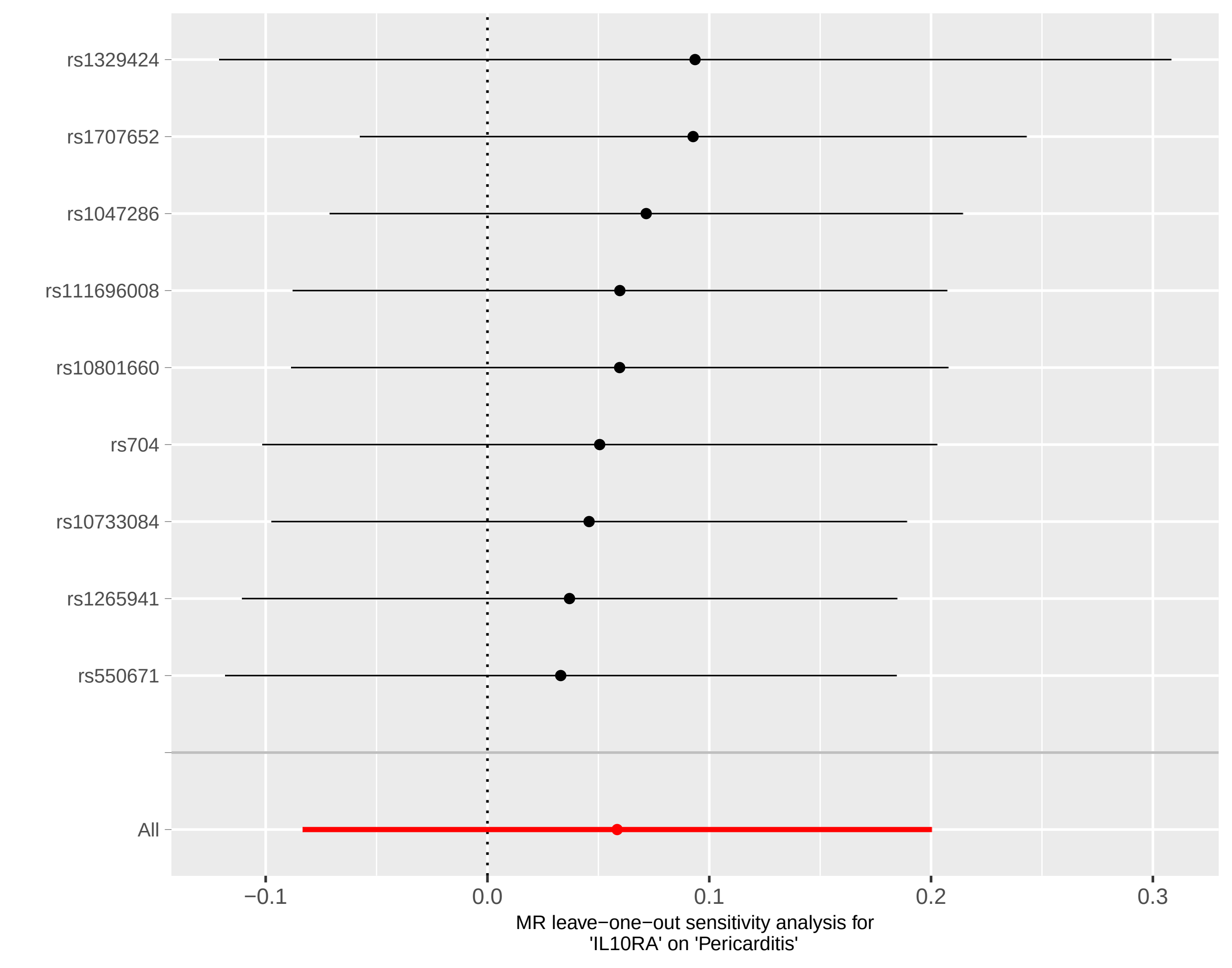
rs1329424
rs1707652
rs1047286
rs111696008
rs10801660
rs704
rs10733084
rs1265941
rs550671
All
−0.1
0.0
0.1
0.2
0.3
MR leave−one−out sensitivity analysis for
'IL10RA' on 'Pericarditis'

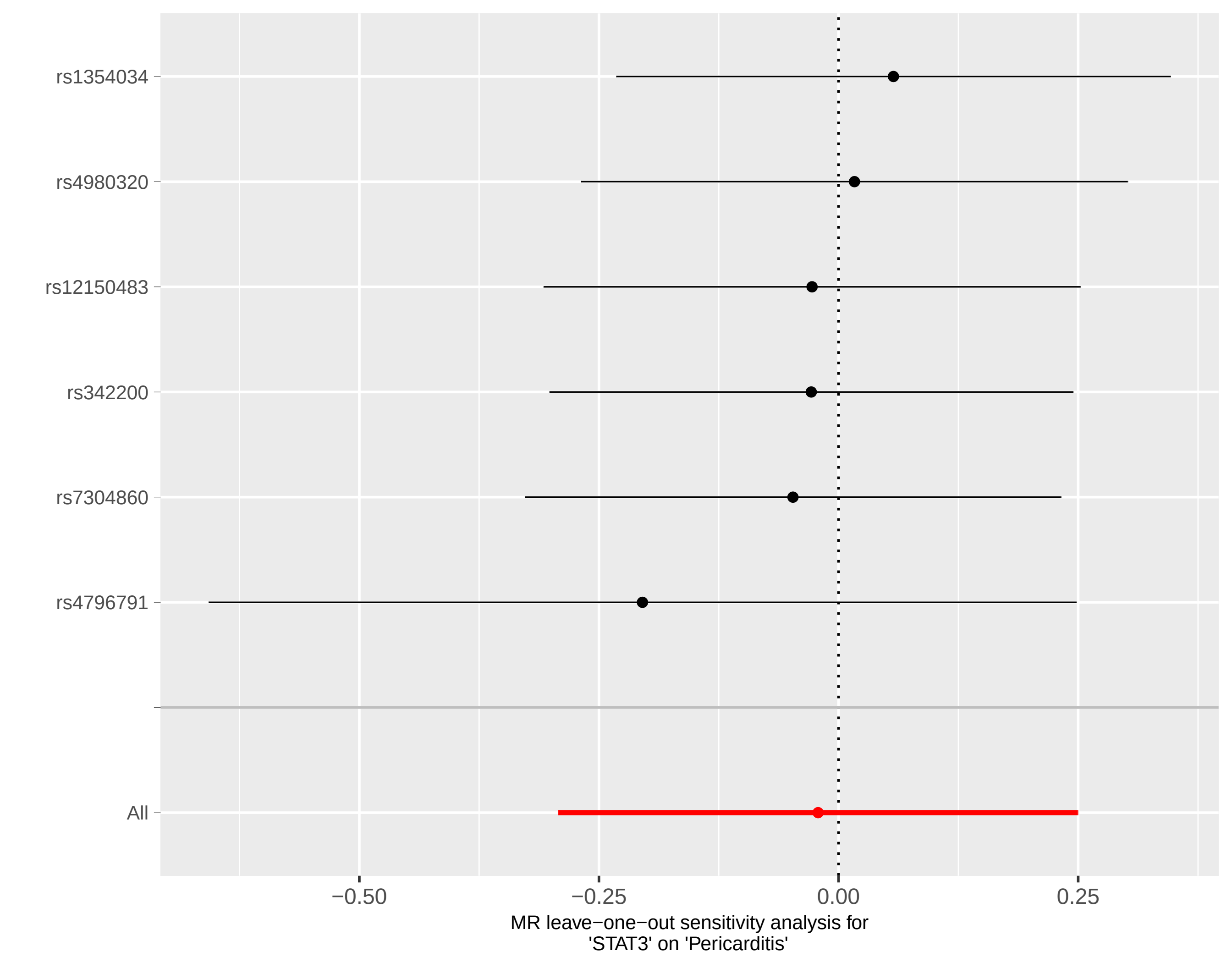
rs1354034
rs4980320
rs12150483
rs342200
rs7304860
rs4796791
All
−0.50
−0.25
0.00
0.25
MR leave−one−out sensitivity analysis for
'STAT3' on 'Pericarditis'

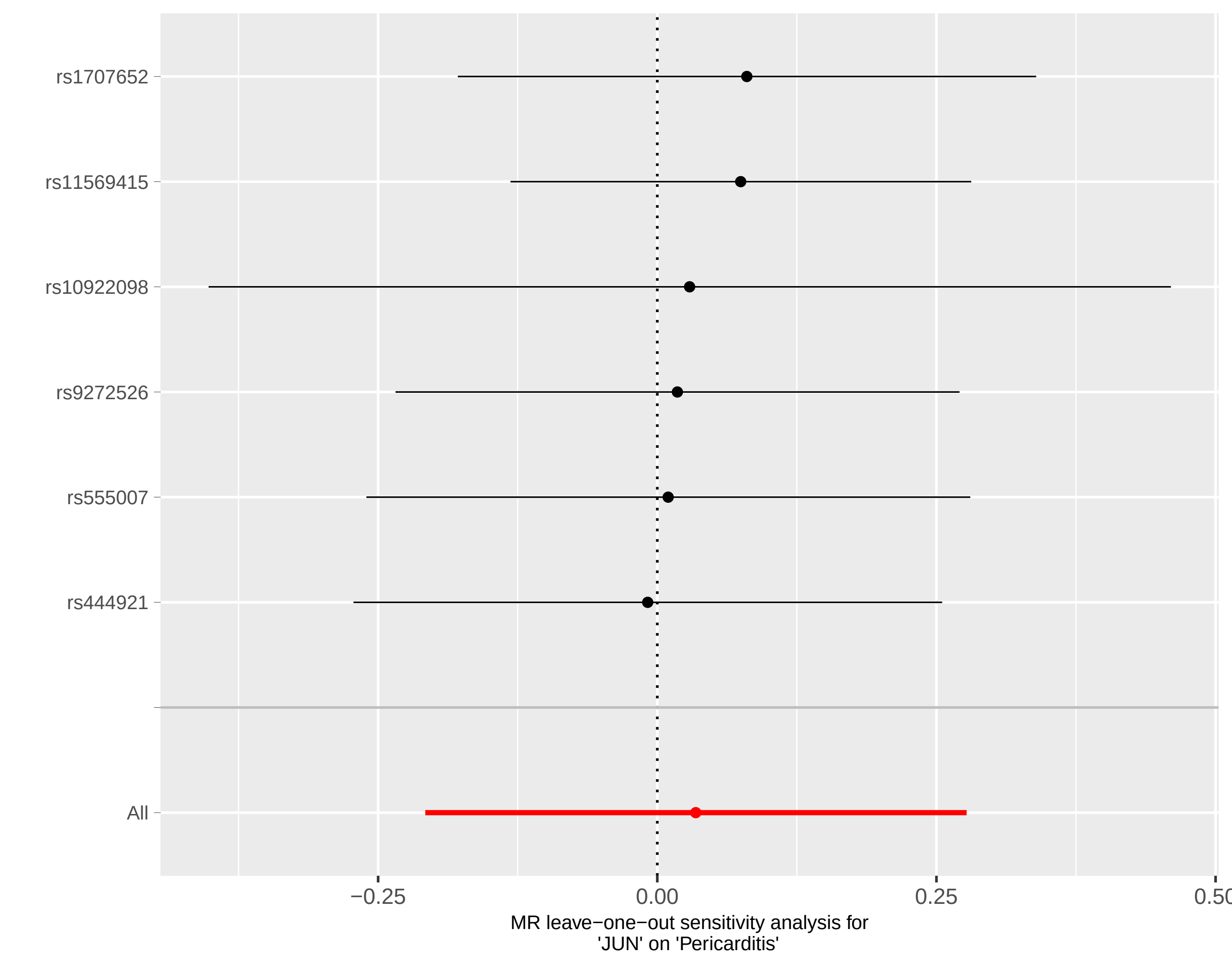
rs1707652
rs11569415
rs10922098
rs9272526
rs555007
rs444921
All
−0.25
0.00
0.25
0.50
MR leave−one−out sensitivity analysis for
'JUN' on 'Pericarditis'

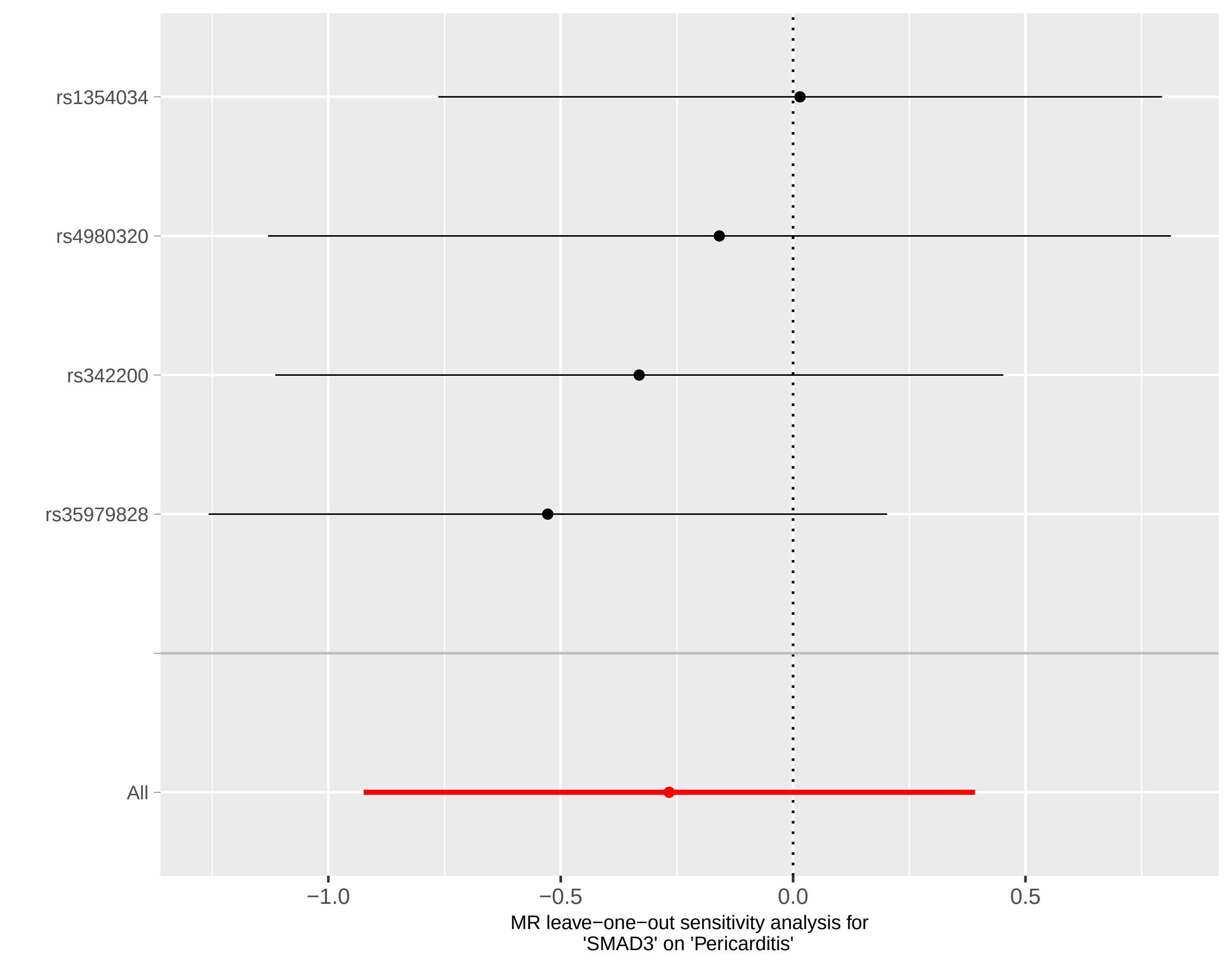
rs1354034
rs4980320
rs342200
rs35979828
All
−1.0
−0.5
0.0
0.5
MR leave−one−out sensitivity analysis for
'SMAD3' on 'Pericarditis'

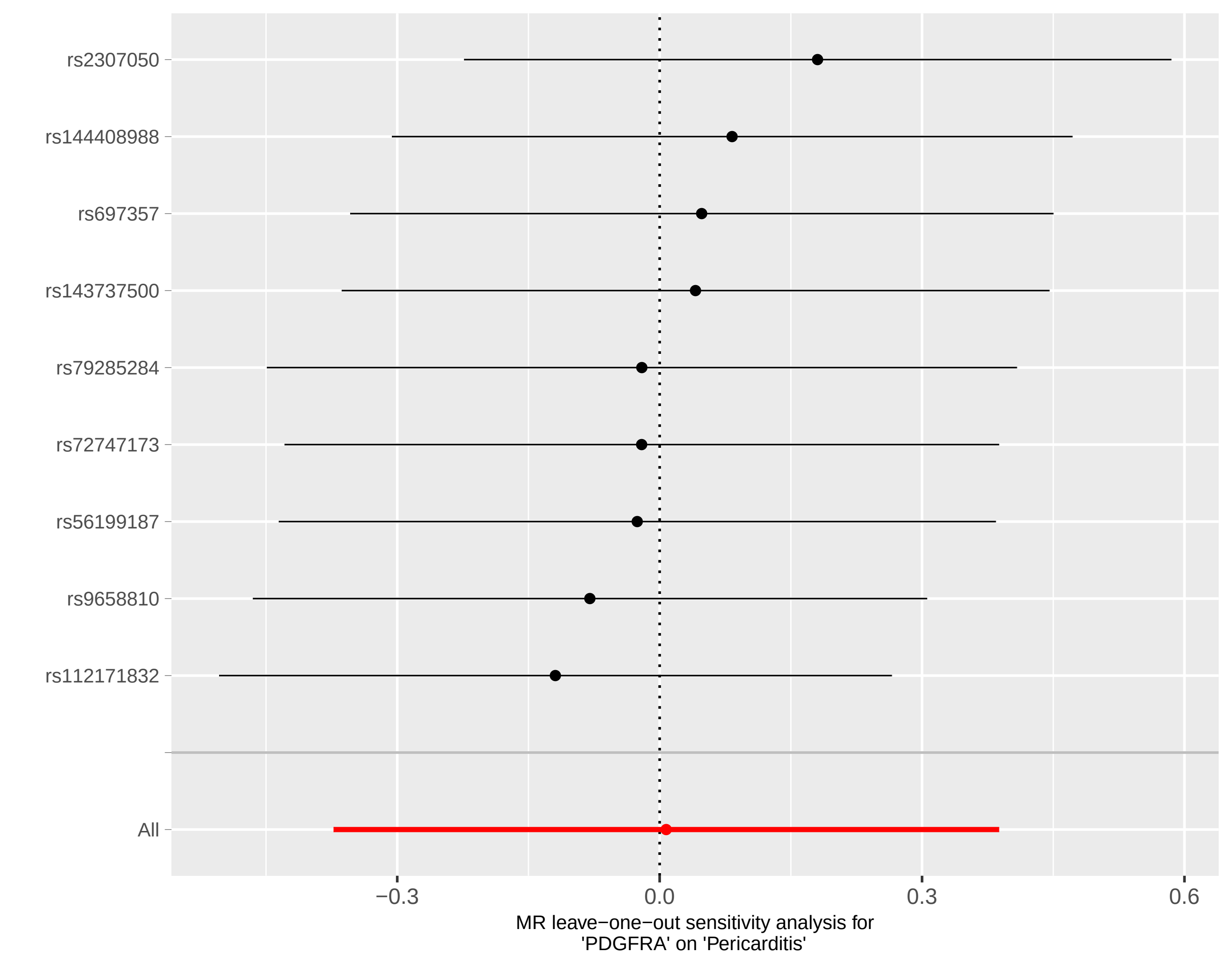

rs2307050
rs144408988
rs697357
rs143737500
rs79285284
rs72747173
rs56199187
rs9658810
rs112171832
All
−0.3
0.0
0.3
0.6
MR leave−one−out sensitivity analysis for
'PDGFRA' on 'Pericarditis'

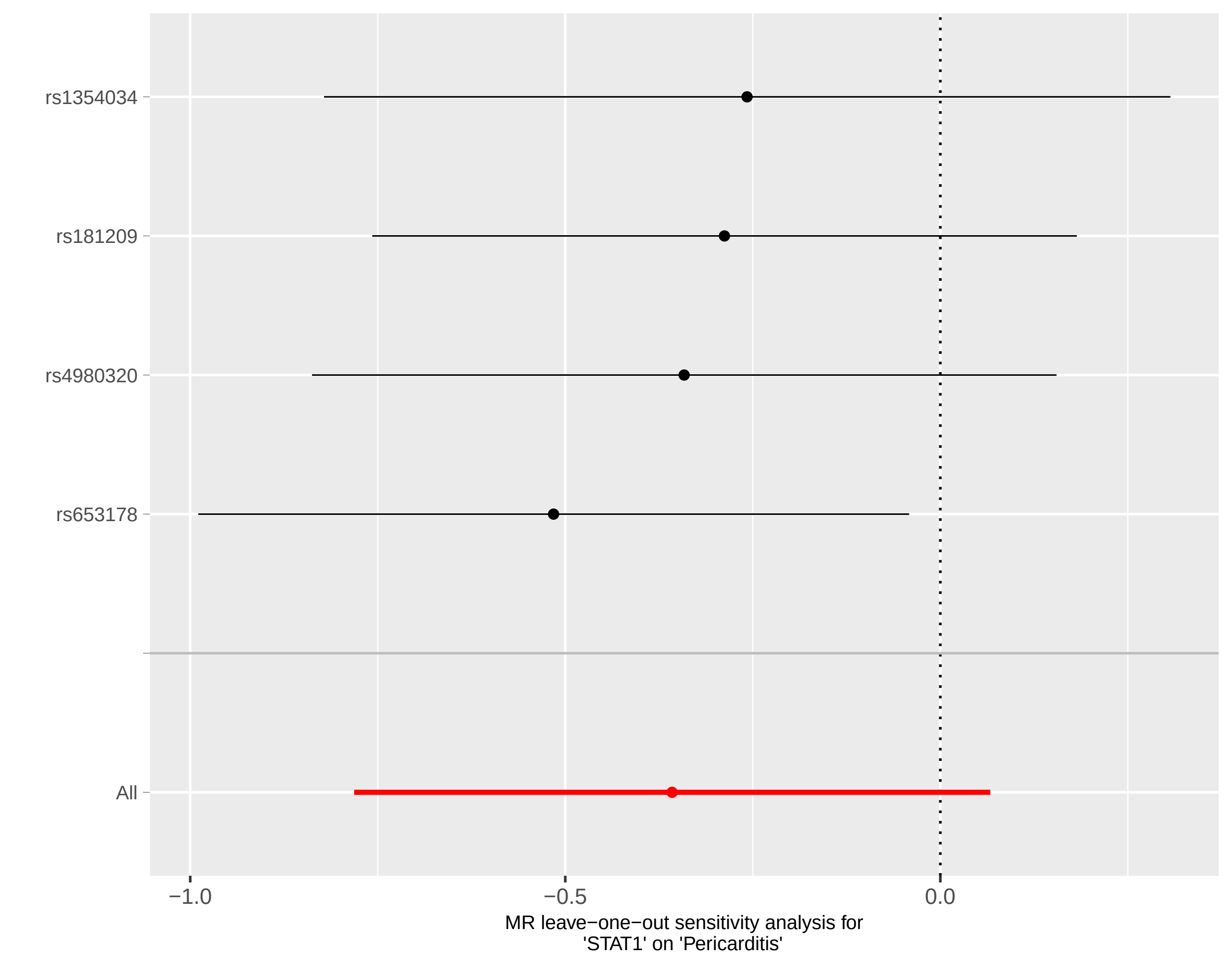
rs1354034
rs181209
rs4980320
rs653178
All
−1.0
−0.5
0.0
MR leave−one−out sensitivity analysis for
'STAT1' on 'Pericarditis'

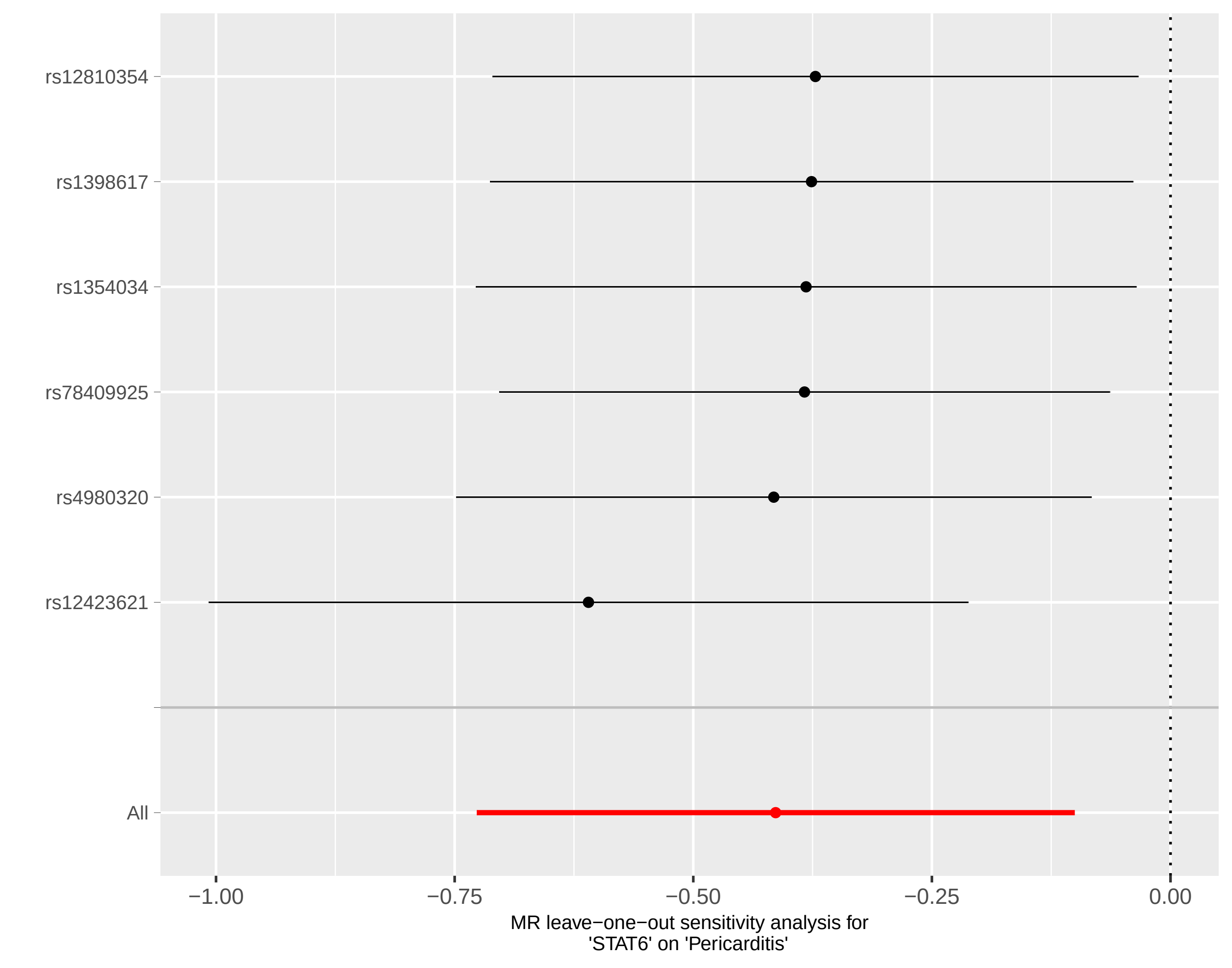
rs12810354
rs1398617
rs1354034
rs78409925
rs4980320
rs12423621
All
−1.00
−0.75
−0.50
−0.25
0.00
MR leave−one−out sensitivity analysis for
'STAT6' on 'Pericarditis'

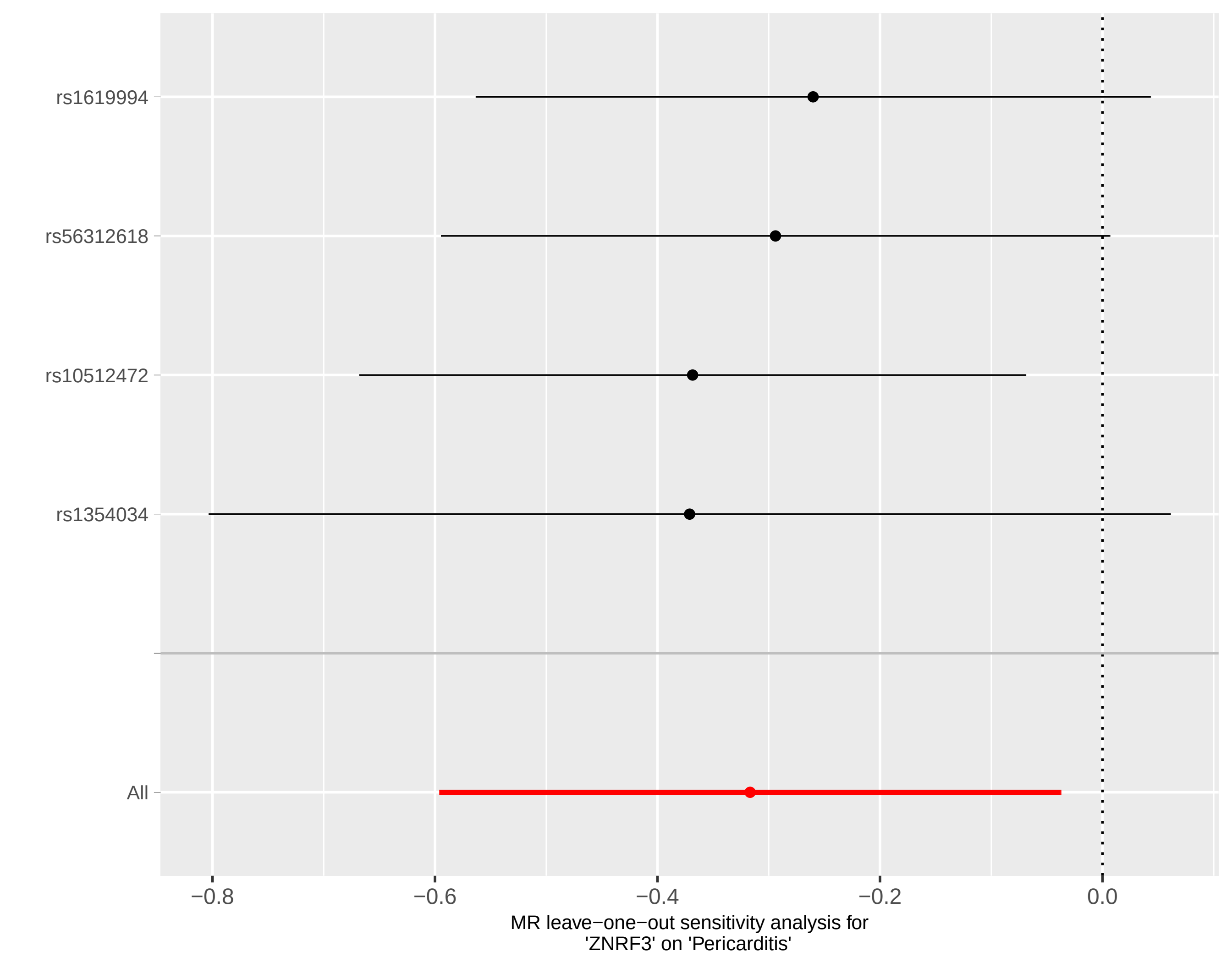
rs1619994
rs56312618
rs10512472
rs1354034
All
−0.8
−0.6
−0.4
−0.2
0.0
MR leave−one−out sensitivity analysis for
'ZNRF3' on 'Pericarditis'

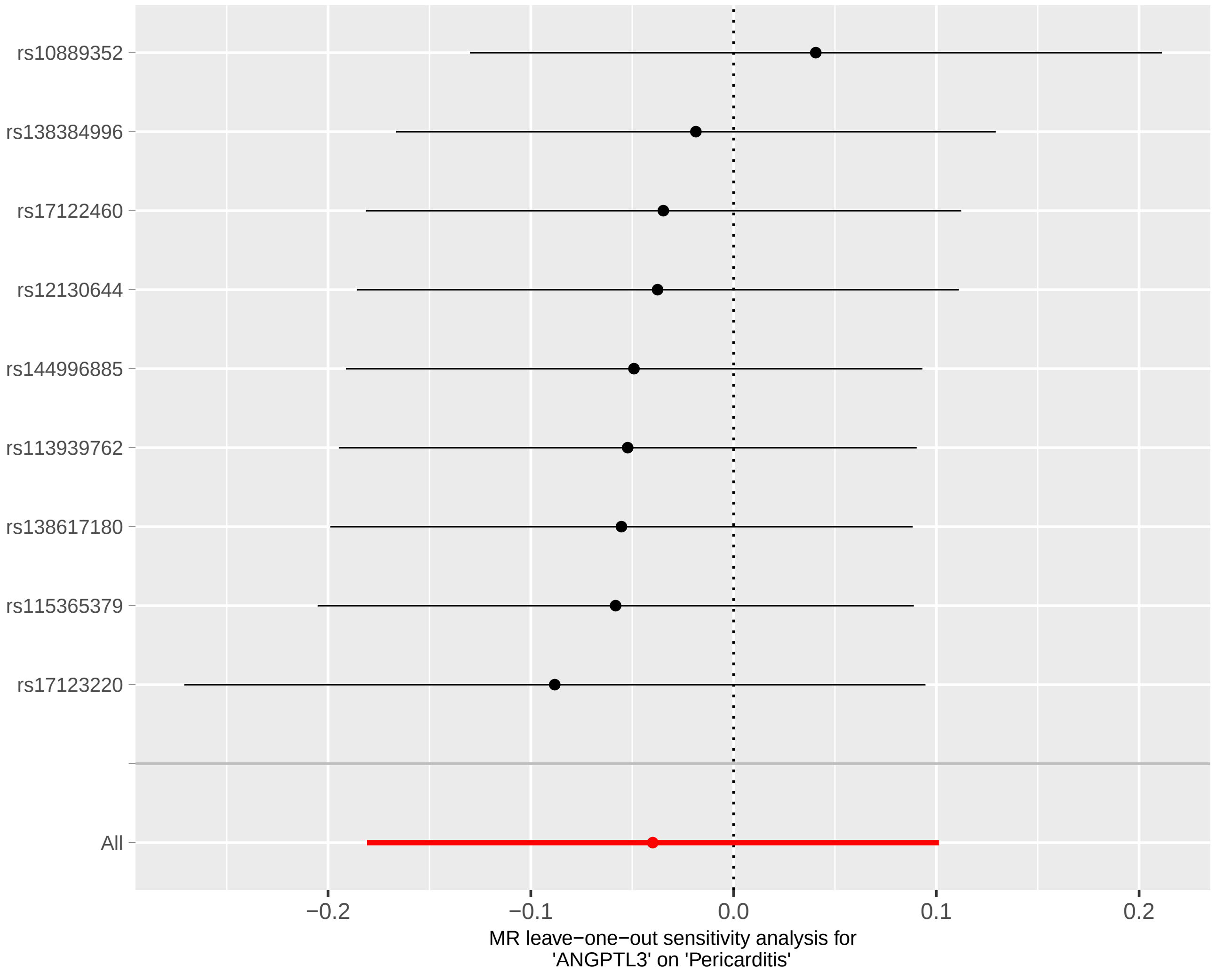

rs10889352
rs138384996
rs17122460
rs12130644
rs144996885
rs113939762
rs138617180
rs115365379
rs17123220
All
−0.2
−0.1
0.0
0.1
0.2
MR leave−one−out sensitivity analysis for
'ANGPTL3' on 'Pericarditis'

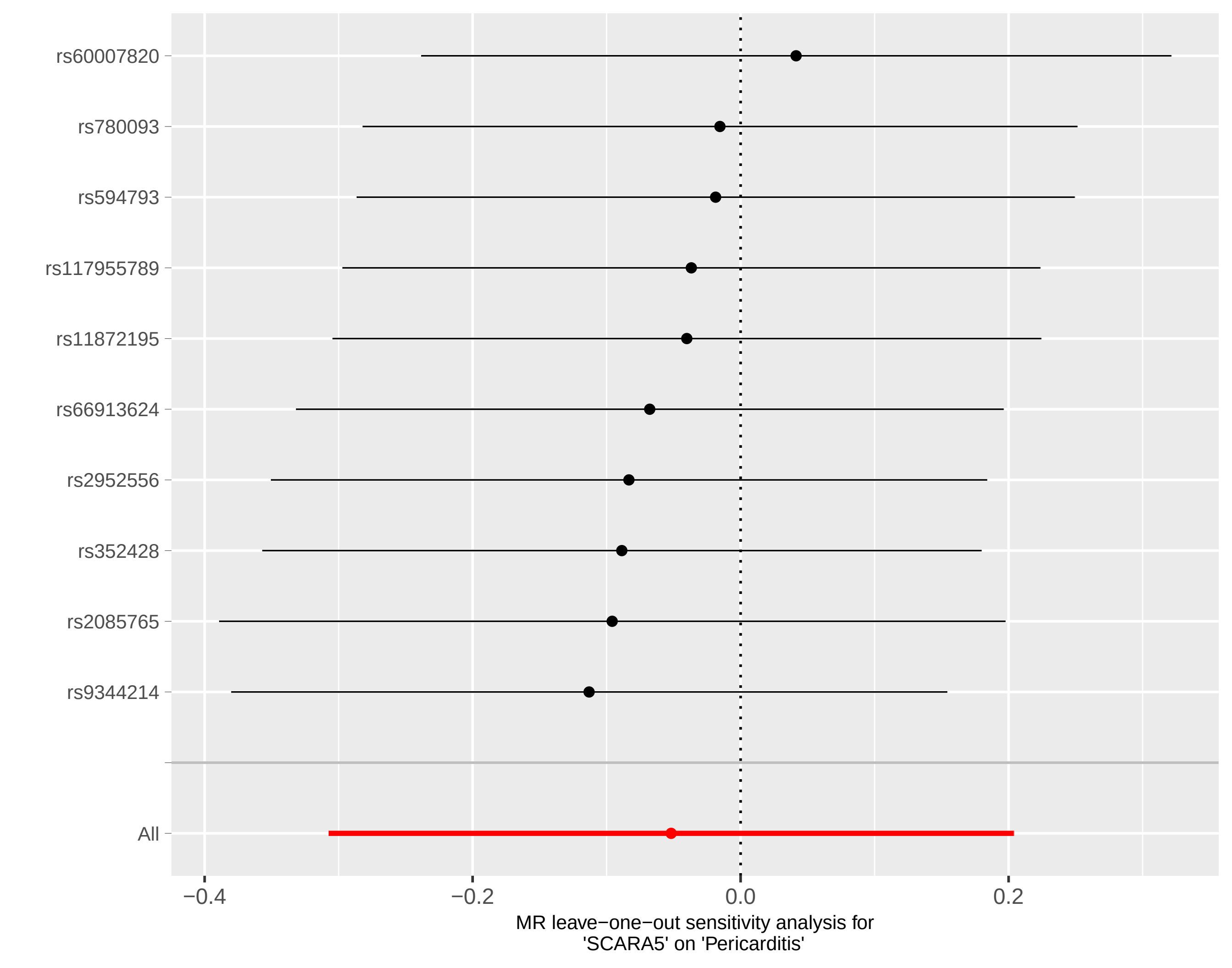
rs60007820
rs780093
rs594793
rs117955789
rs11872195
rs66913624
rs2952556
rs352428
rs2085765
rs9344214
All
−0.4
−0.2
0.0
0.2
MR leave−one−out sensitivity analysis for
'SCARA5' on 'Pericarditis'

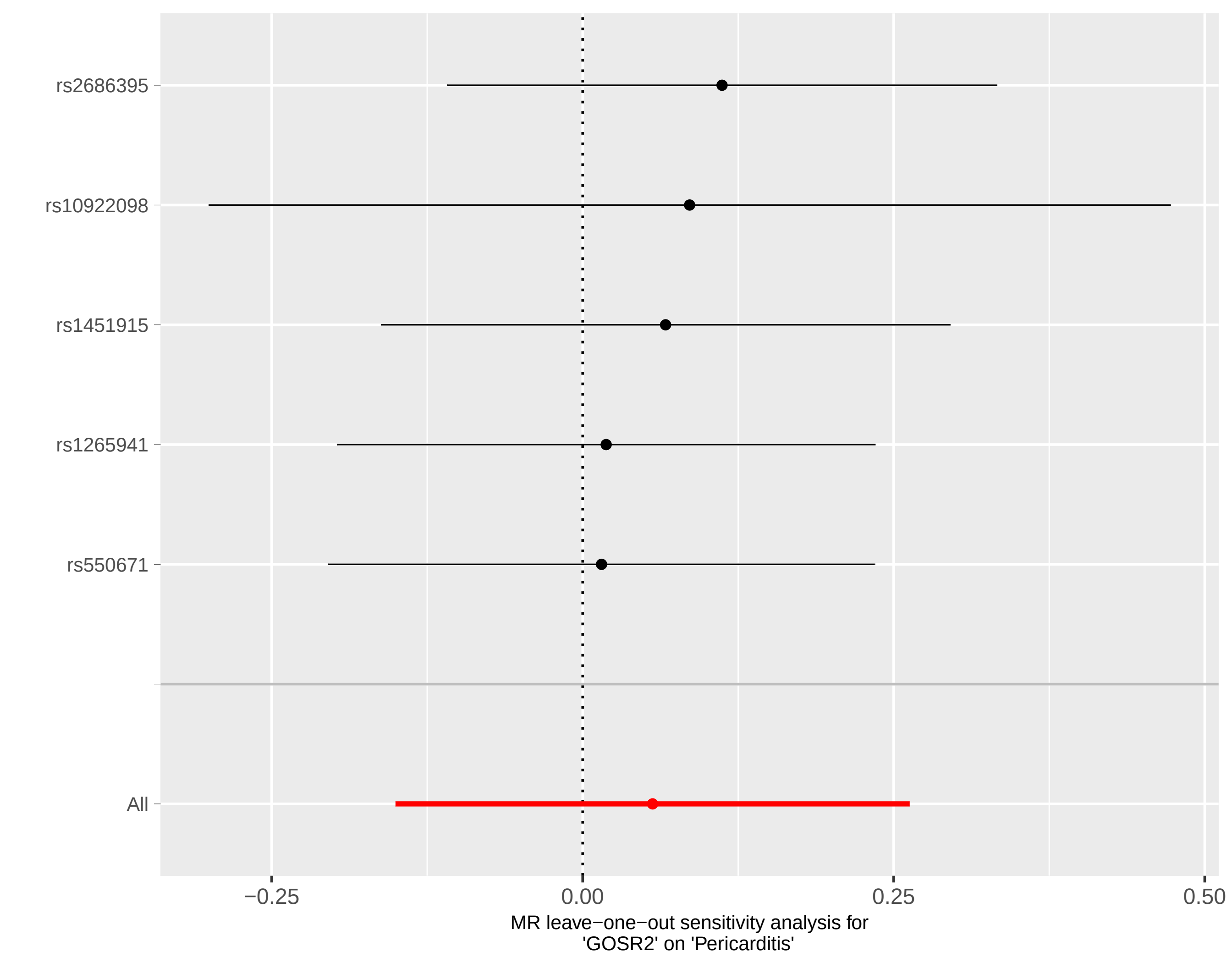

rs2686395
rs10922098
rs1451915
rs1265941
rs550671
All
−0.25
0.00
0.25
0.50
MR leave−one−out sensitivity analysis for
'GOSR2' on 'Pericarditis'

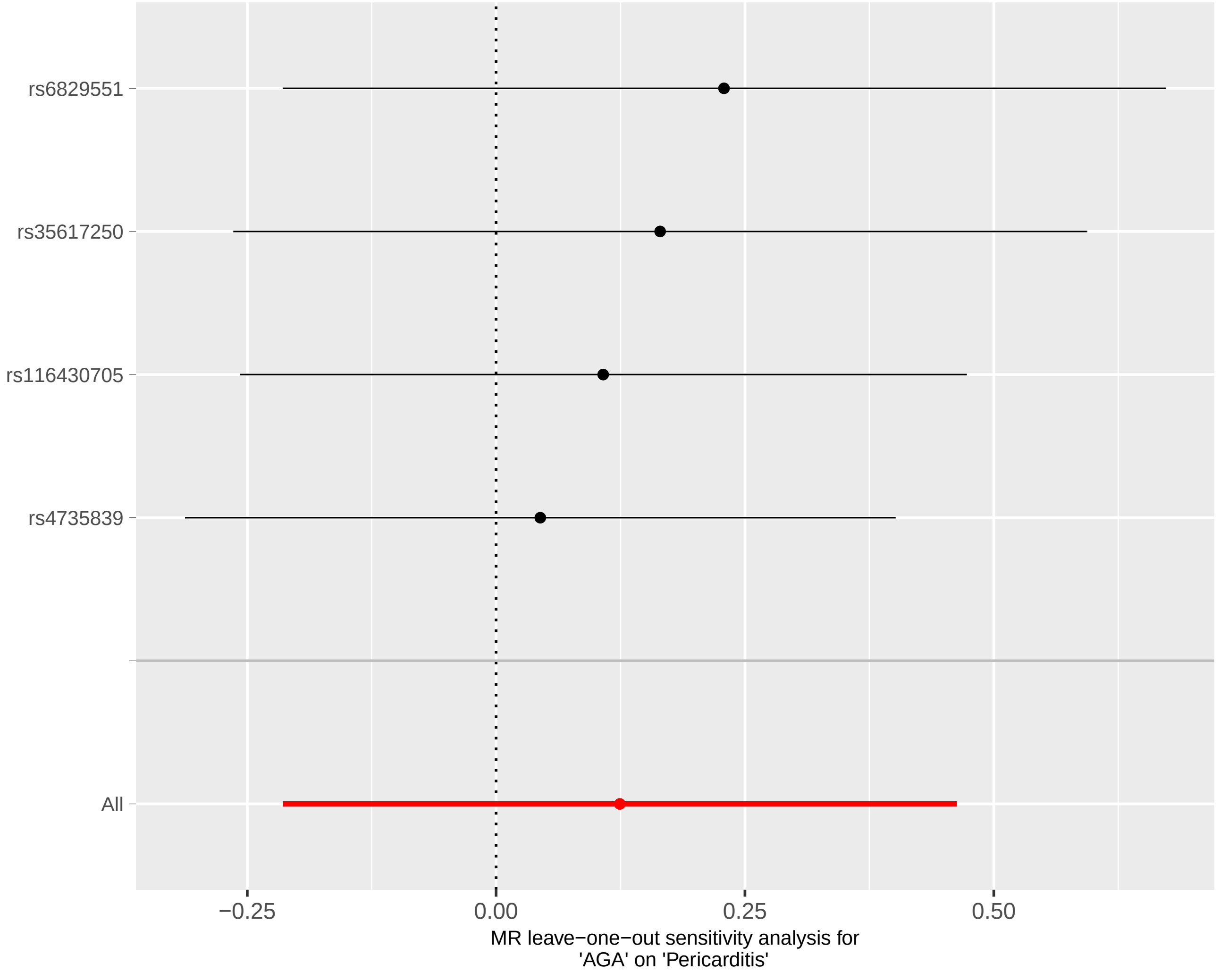
rs6829551
rs35617250
rs116430705
rs4735839
All
−0.25
0.00
0.25
0.50
MR leave−one−out sensitivity analysis for
'AGA' on 'Pericarditis'

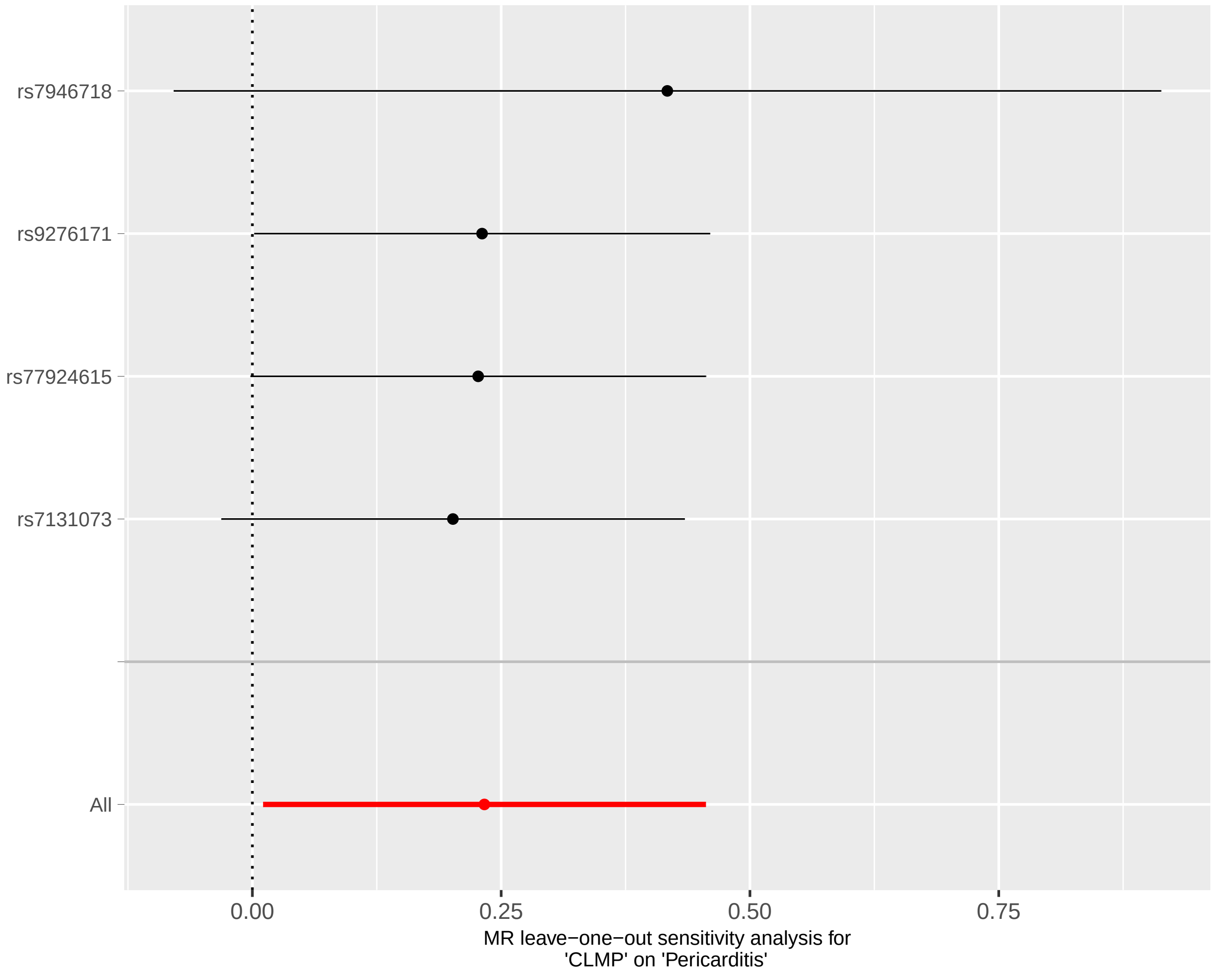
rs7946718
rs9276171
rs77924615
rs7131073
All
0.00
0.25
0.50
0.75
MR leave−one−out sensitivity analysis for
'CLMP' on 'Pericarditis'

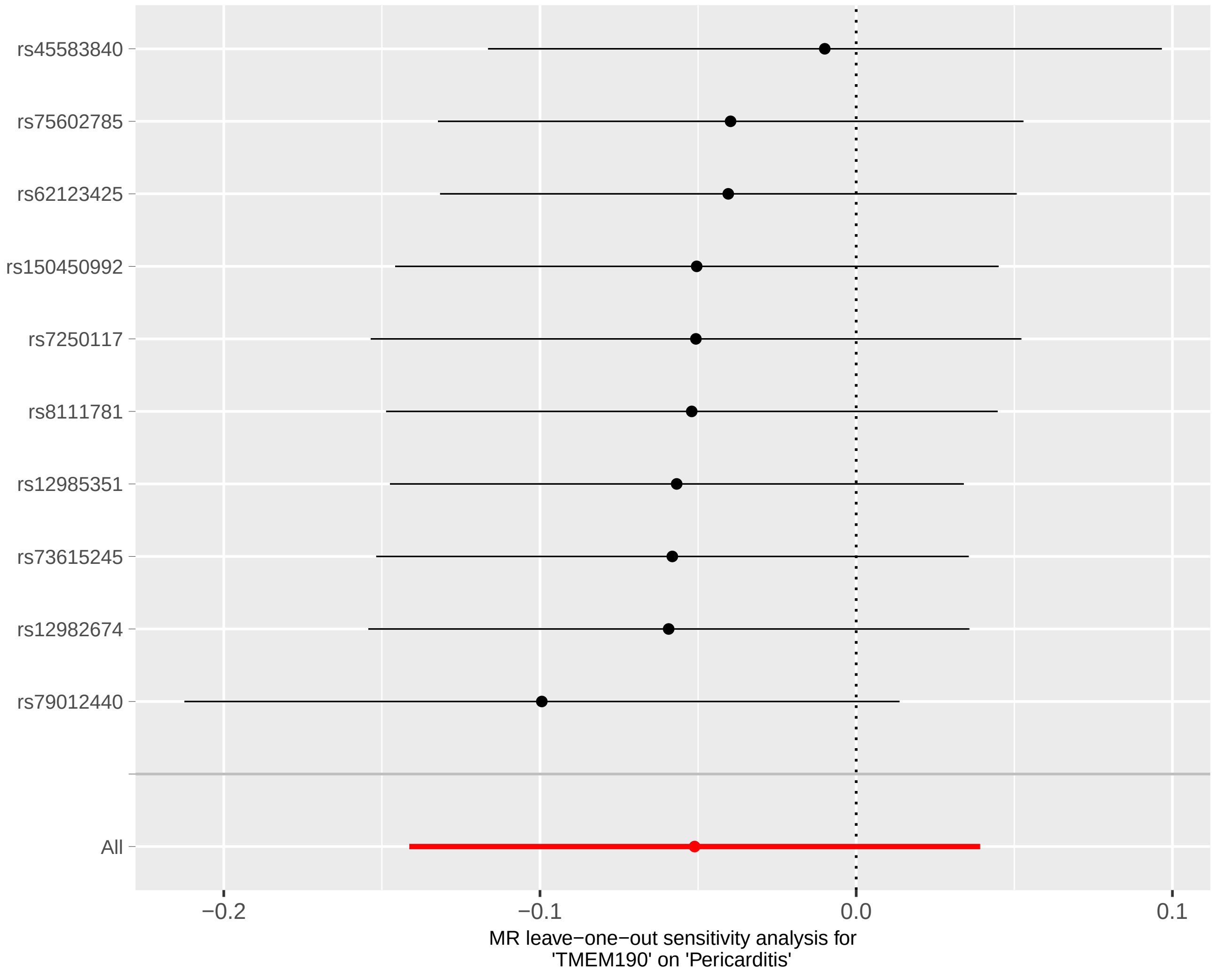
rs45583840
rs75602785
rs62123425
rs150450992
rs7250117
rs8111781
rs12985351
rs73615245
rs12982674
rs79012440
All
−0.2
−0.1
0.0
0.1
MR leave−one−out sensitivity analysis for
'TMEM190' on 'Pericarditis'

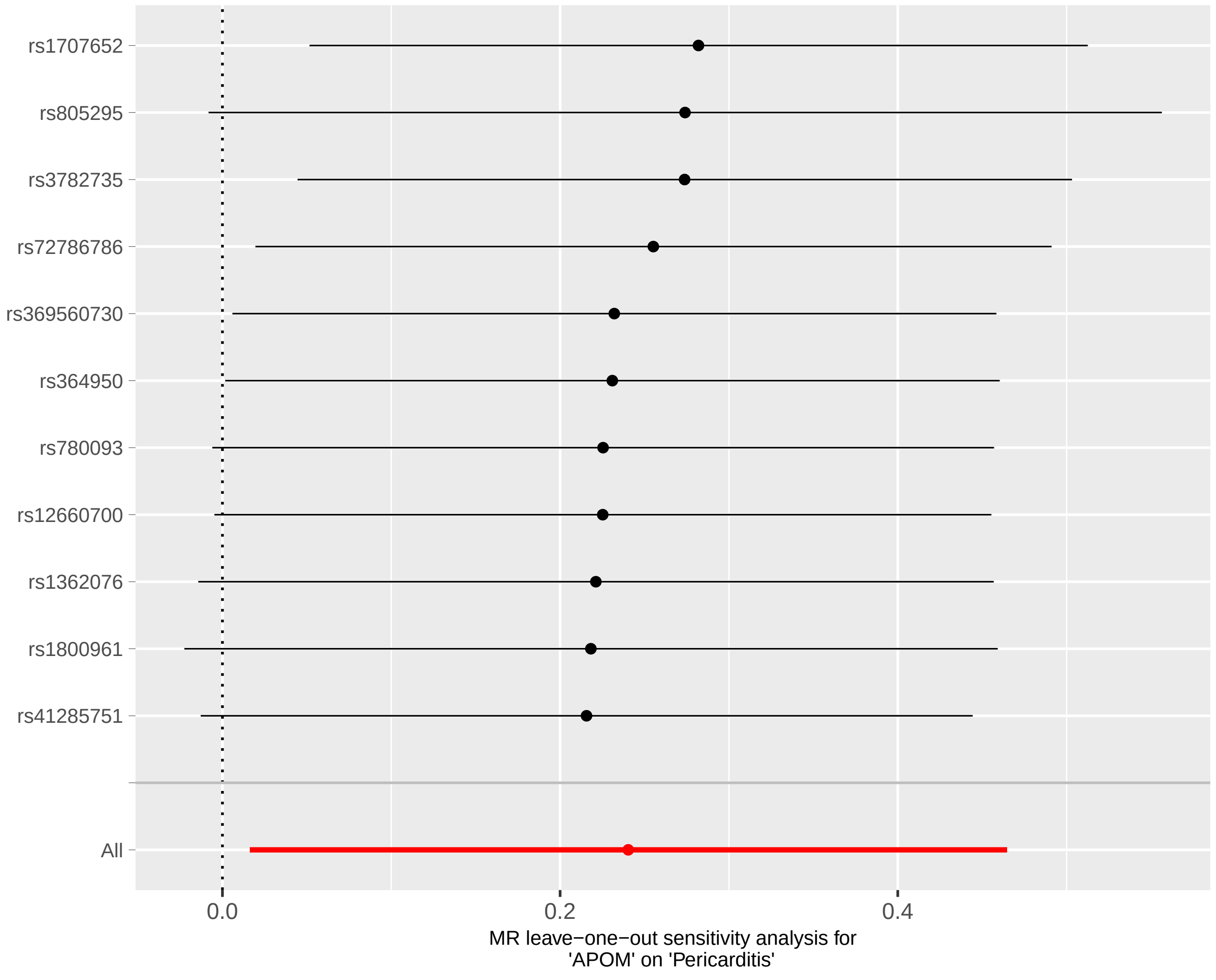

rs1707652
rs805295
rs3782735
rs72786786
rs369560730
rs364950
rs780093
rs12660700
rs1362076
rs1800961
rs41285751
All
0.0
0.2
0.4
MR leave−one−out sensitivity analysis for
'APOM' on 'Pericarditis'

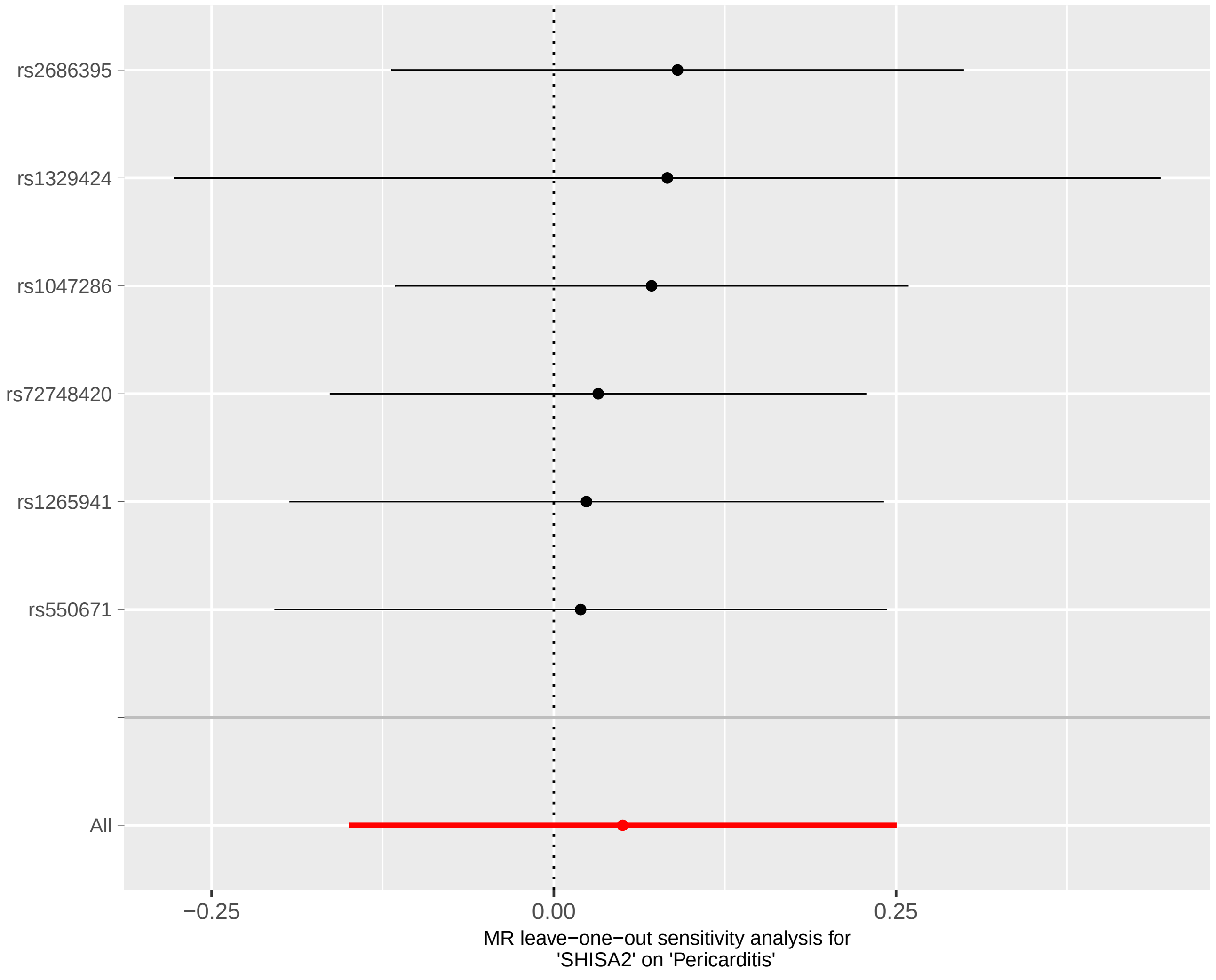

rs2686395
rs1329424
rs1047286
rs72748420
rs1265941
rs550671
All
−0.25
0.00
0.25
MR leave−one−out sensitivity analysis for
'SHISA2' on 'Pericarditis'

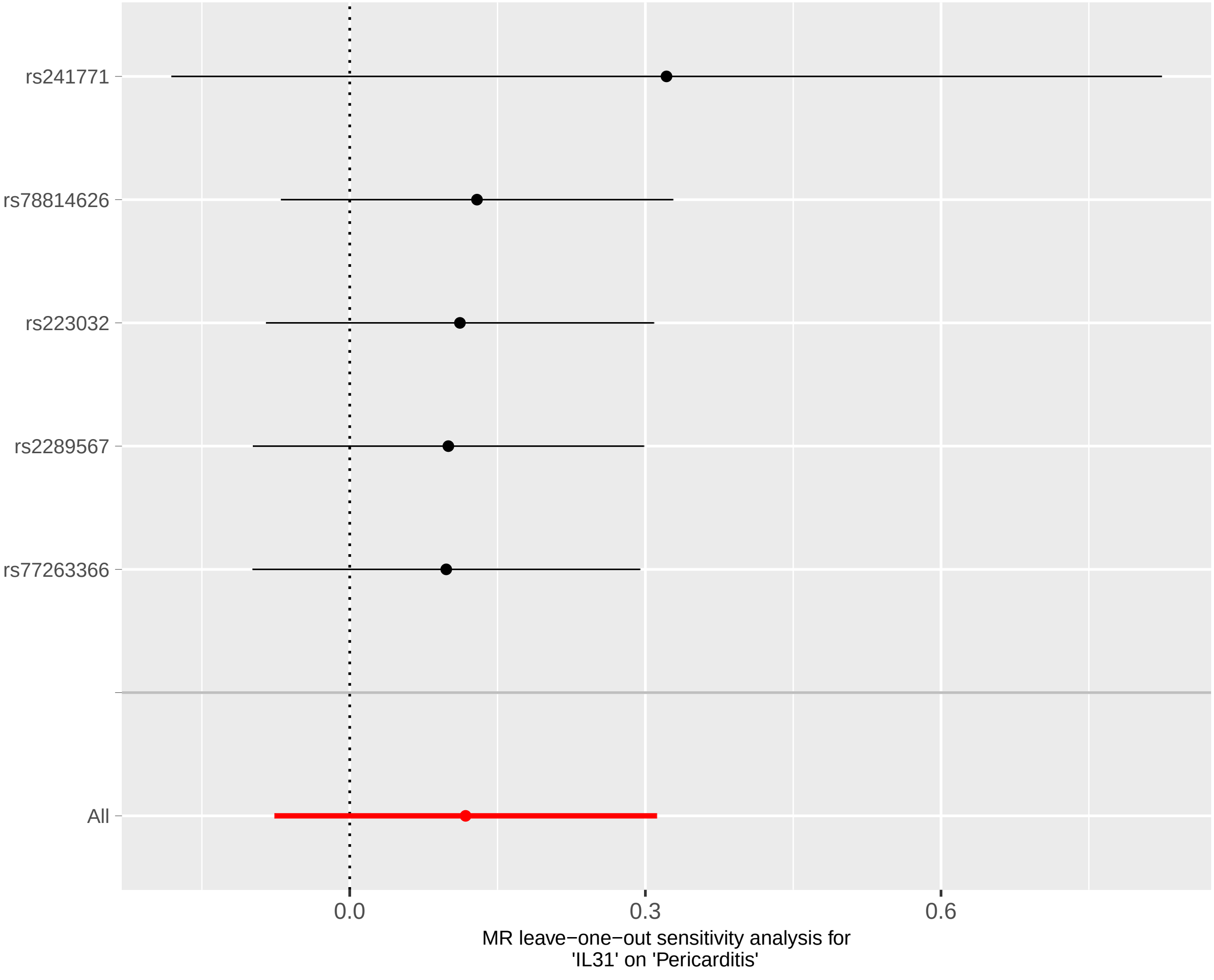
rs241771
rs78814626
rs223032
rs2289567
rs77263366
All
0.0
0.3
0.6
MR leave−one−out sensitivity analysis for
'IL31' on 'Pericarditis'

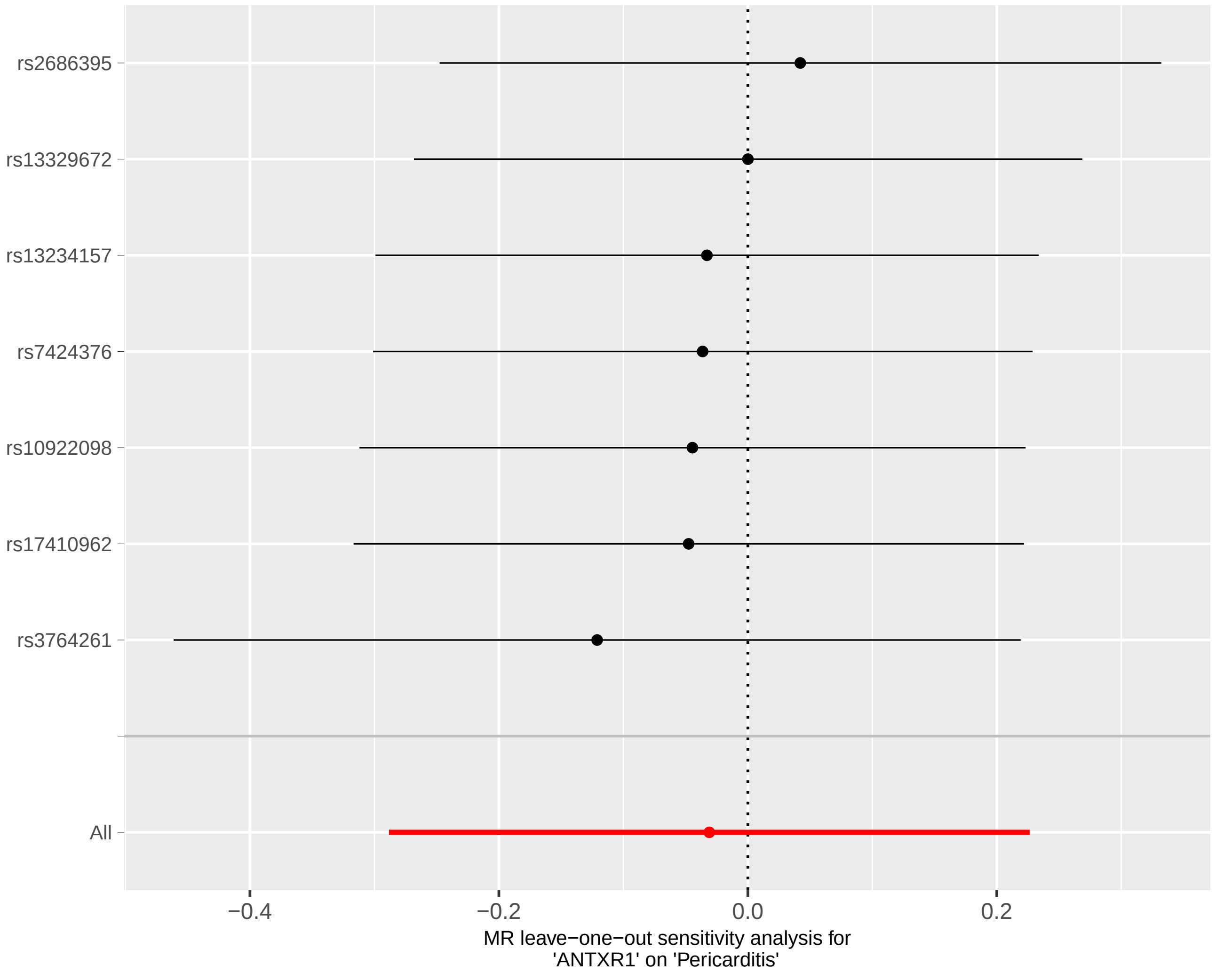

rs2686395
rs13329672
rs13234157
rs7424376
rs10922098
rs17410962
rs3764261
All
−0.4
−0.2
0.0
0.2
MR leave−one−out sensitivity analysis for
'ANTXR1' on 'Pericarditis'

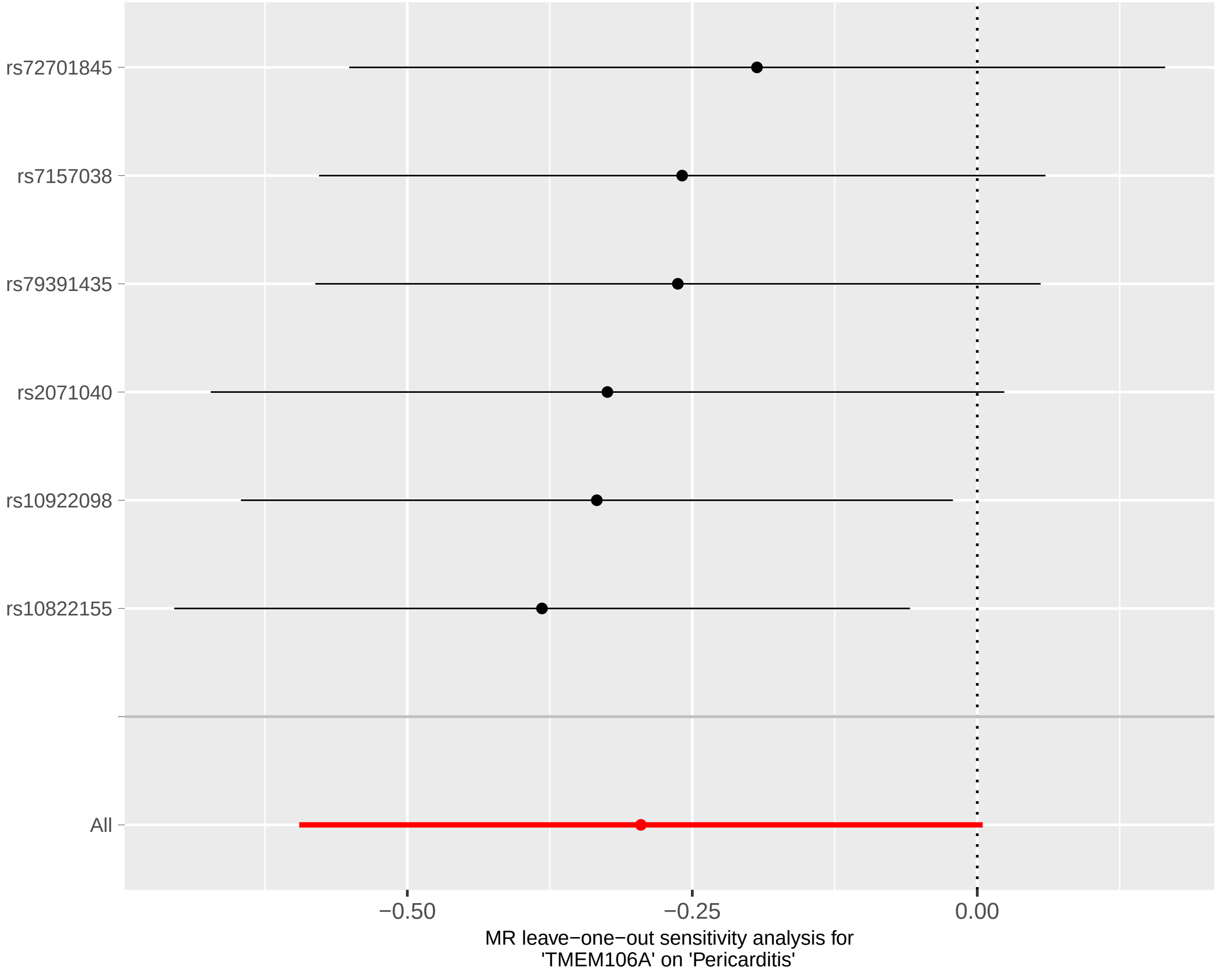
rs72701845
rs7157038
rs79391435
rs2071040
rs10922098
rs10822155
All
−0.50
−0.25
0.00
MR leave−one−out sensitivity analysis for
'TMEM106A' on 'Pericarditis'

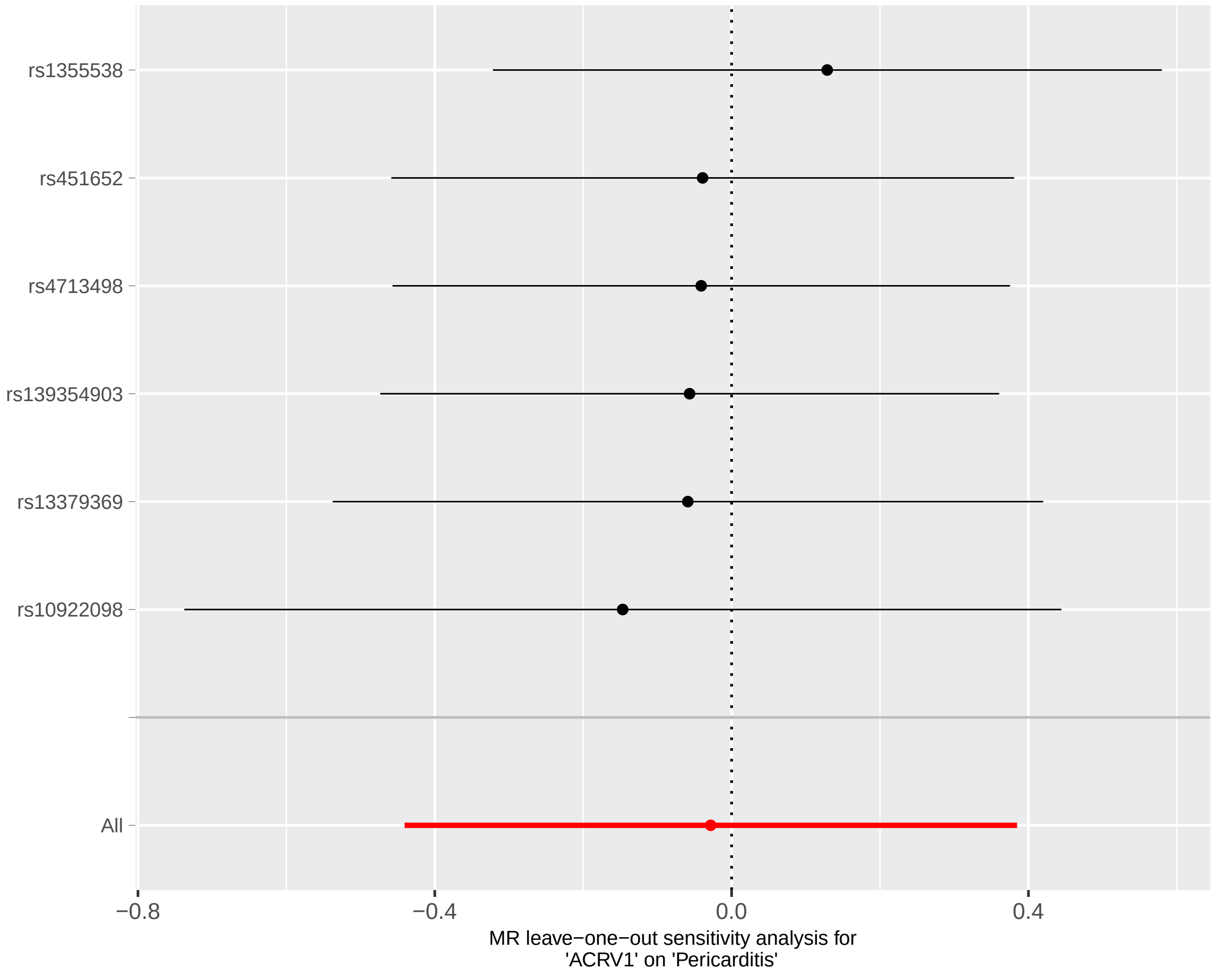
rs1355538
rs451652
rs4713498
rs139354903
rs13379369
rs10922098
All
−0.8
−0.4
0.0
0.4
MR leave−one−out sensitivity analysis for
'ACRV1' on 'Pericarditis'

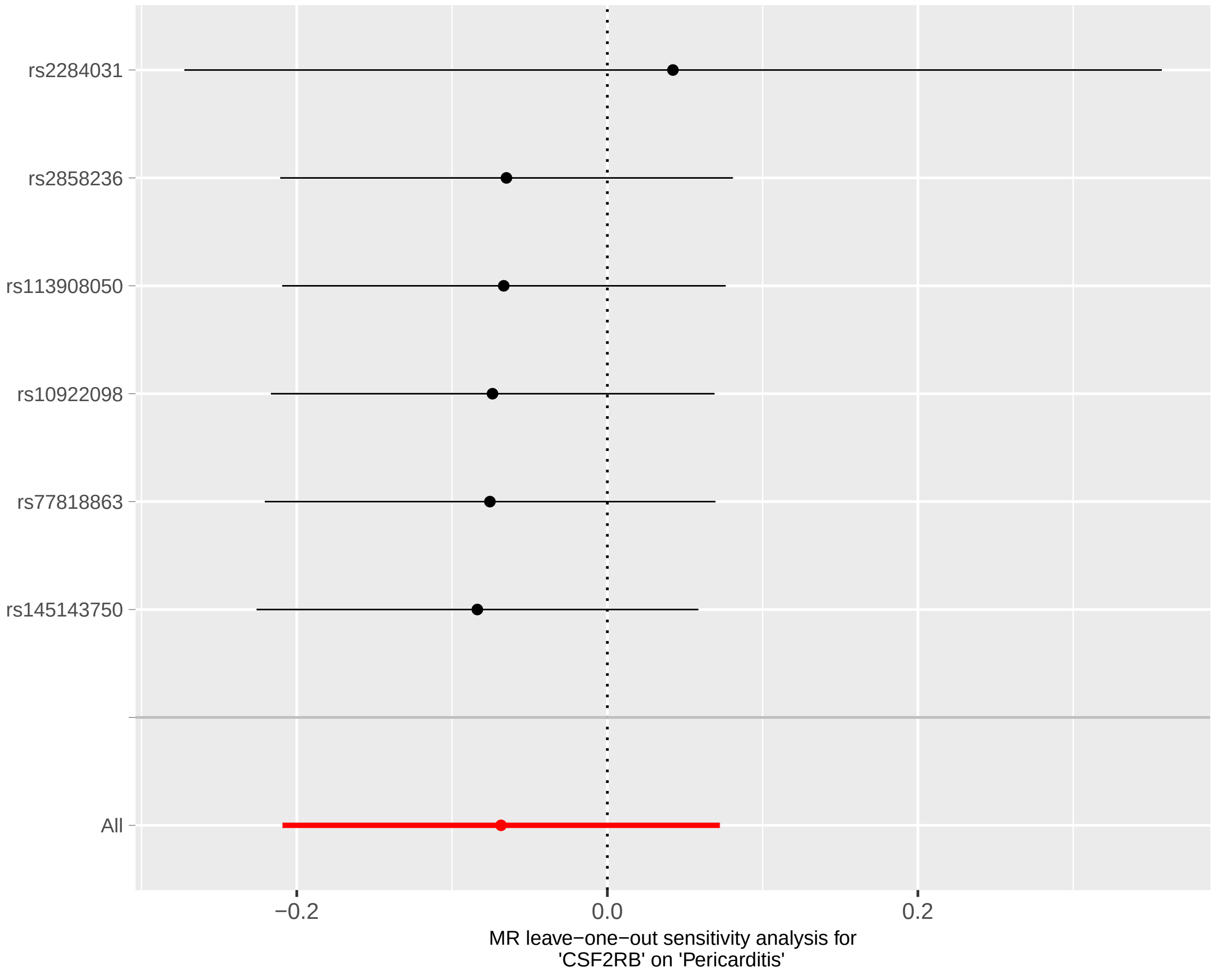
rs2284031
rs2858236
rs113908050
rs10922098
rs77818863
rs145143750
All
−0.2
0.0
0.2
MR leave−one−out sensitivity analysis for
'CSF2RB' on 'Pericarditis'

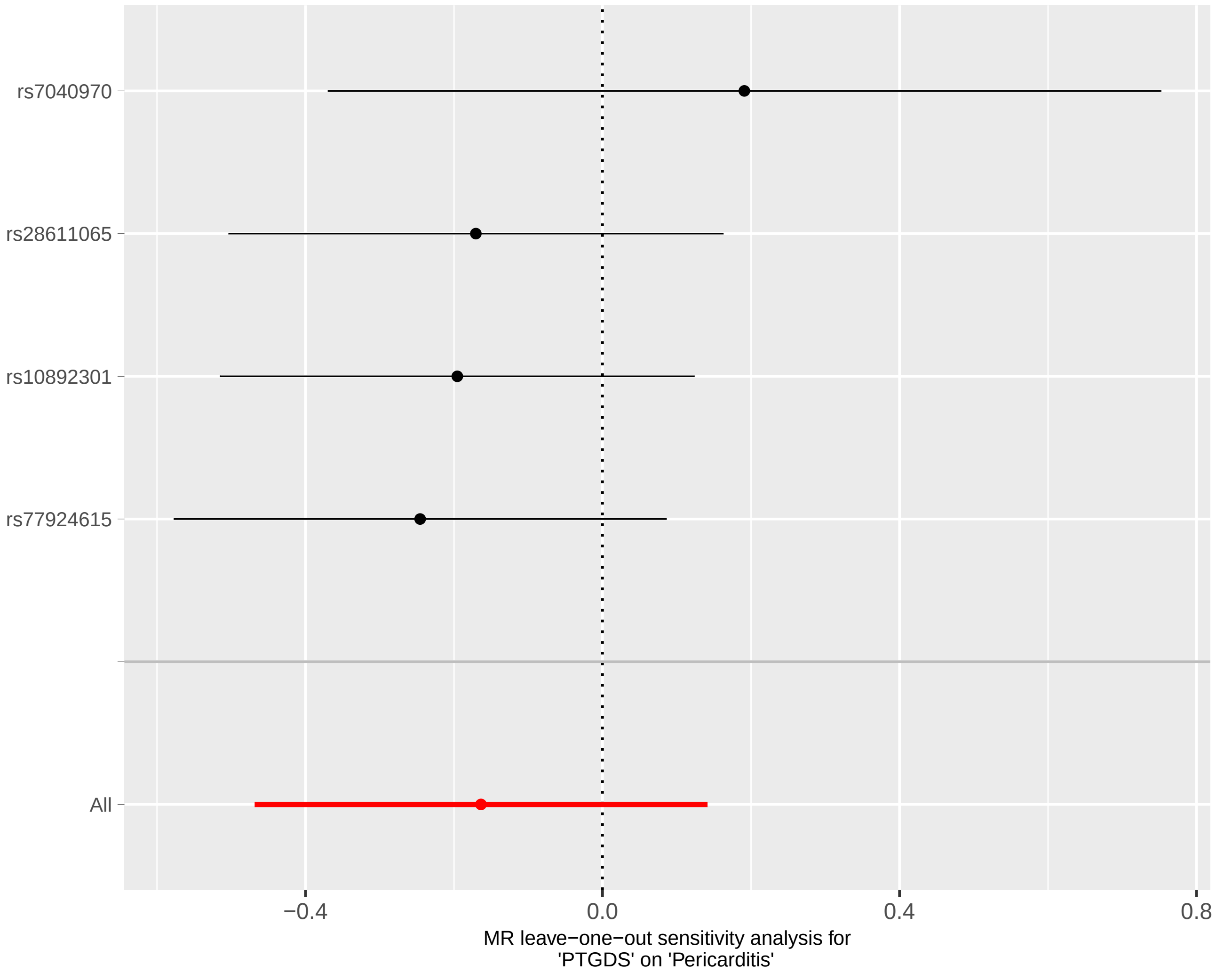
rs7040970
rs28611065
rs10892301
rs77924615
All
−0.4
0.0
0.4
0.8
MR leave−one−out sensitivity analysis for
'PTGDS' on 'Pericarditis'

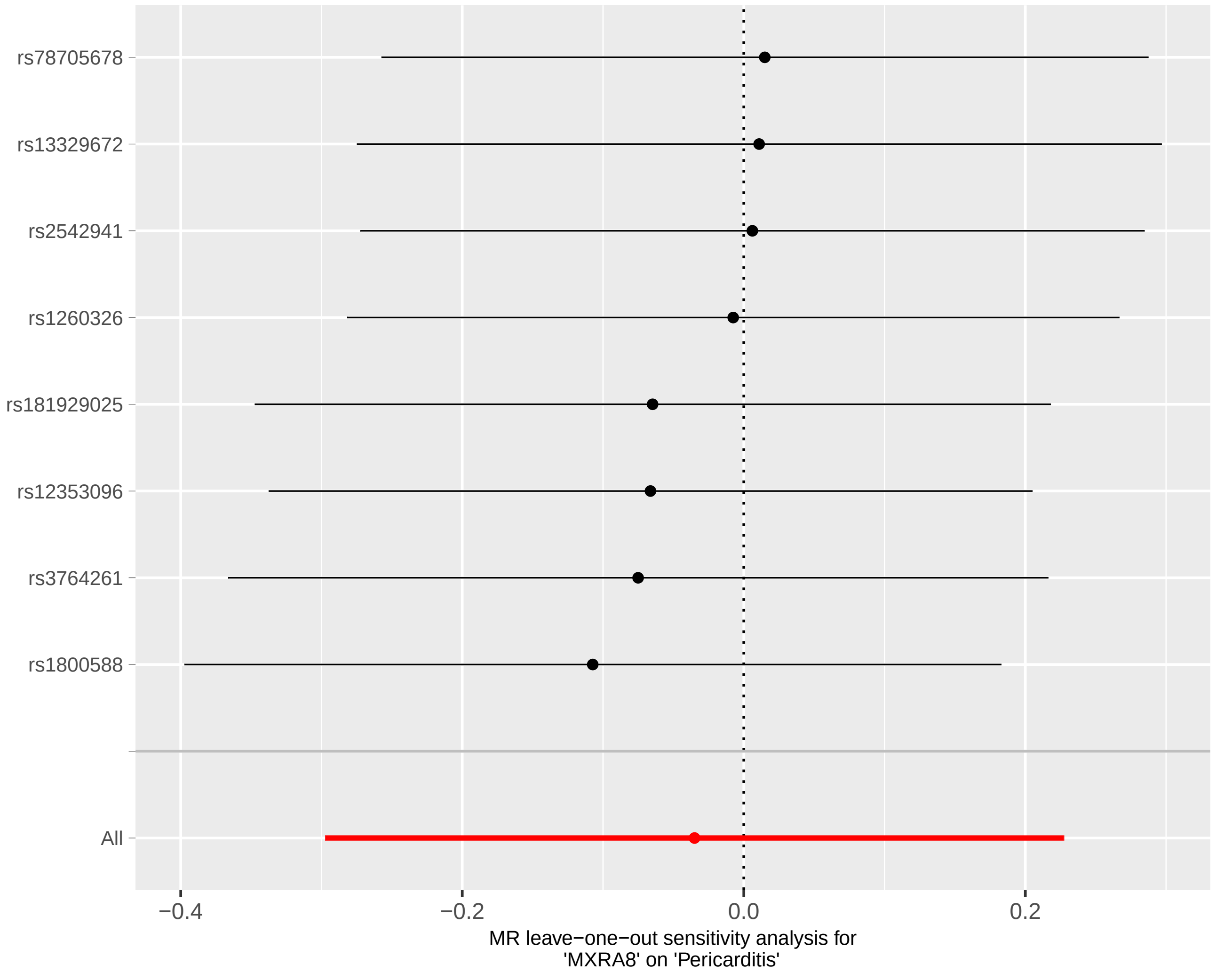
rs78705678
rs13329672
rs2542941
rs1260326
rs181929025
rs12353096
rs3764261
rs1800588
All
−0.4
−0.2
0.0
0.2
MR leave−one−out sensitivity analysis for
'MXRA8' on 'Pericarditis'

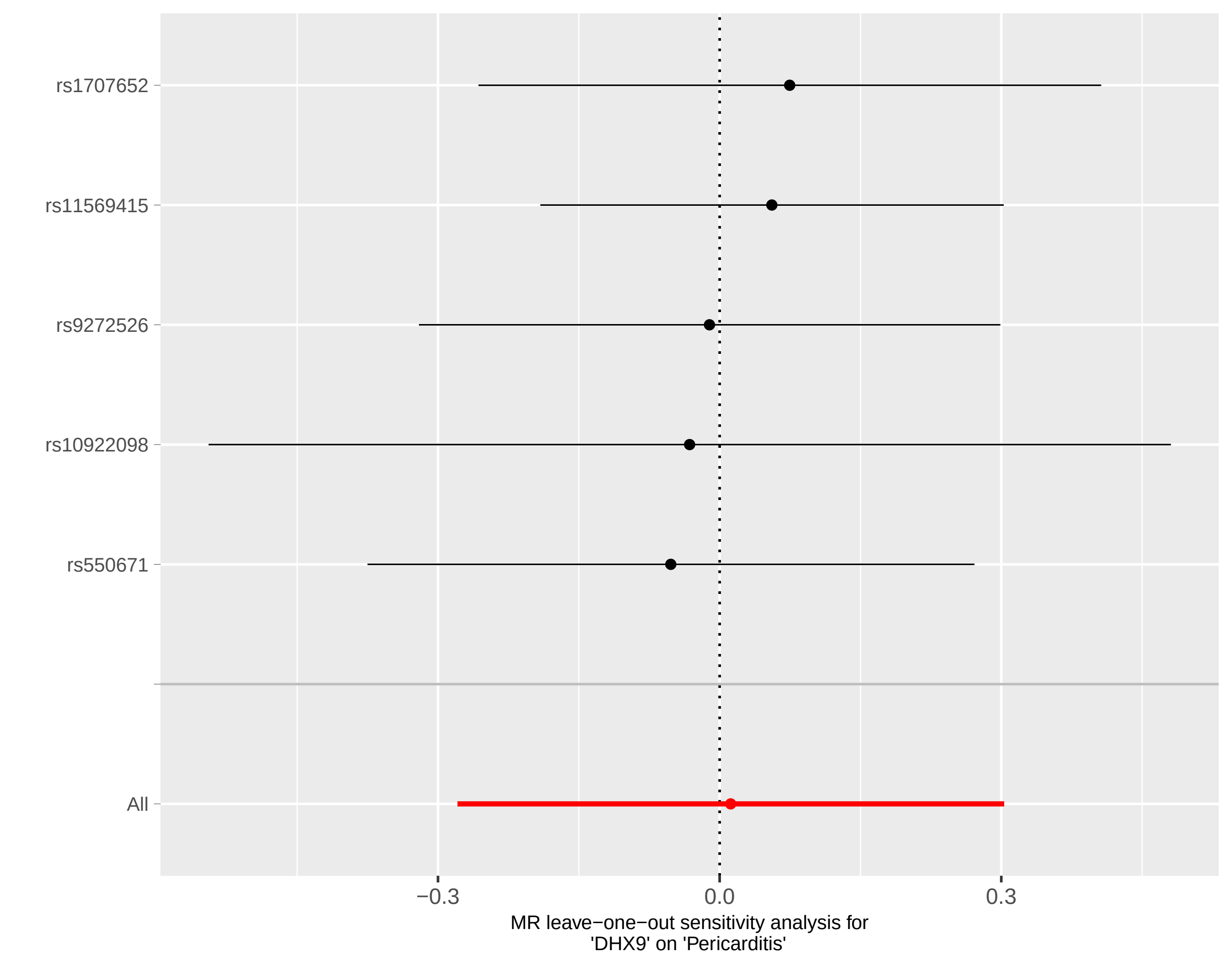
rs1707652
rs11569415
rs9272526
rs10922098
rs550671
All
−0.3
0.0
0.3
MR leave−one−out sensitivity analysis for
'DHX9' on 'Pericarditis'

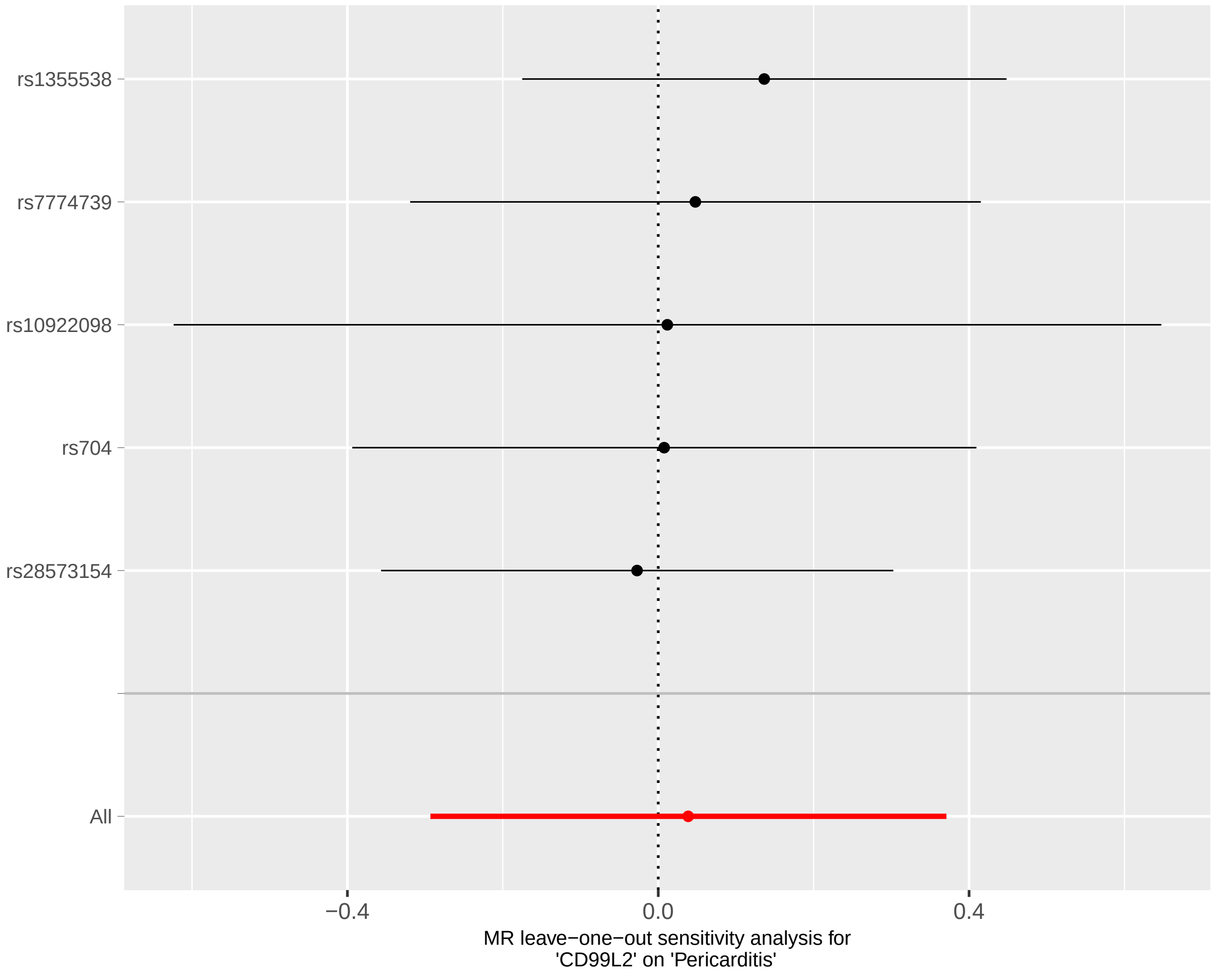
rs1355538
rs7774739
rs10922098
rs704
rs28573154
All
−0.4
0.0
0.4
MR leave−one−out sensitivity analysis for
'CD99L2' on 'Pericarditis'

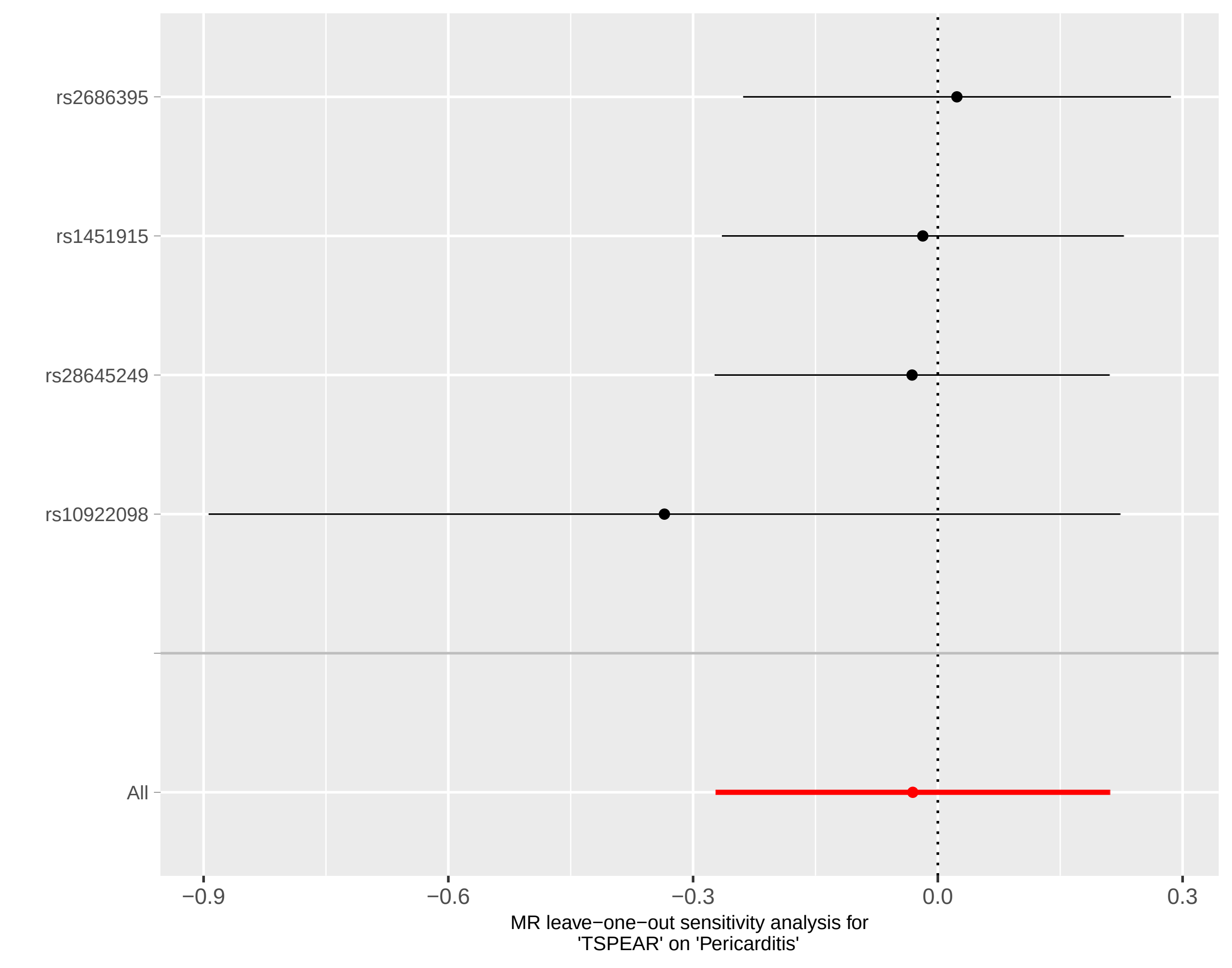
rs2686395
rs1451915
rs28645249
rs10922098
All
−0.9
−0.6
−0.3
0.0
0.3
MR leave−one−out sensitivity analysis for
'TSPEAR' on 'Pericarditis'

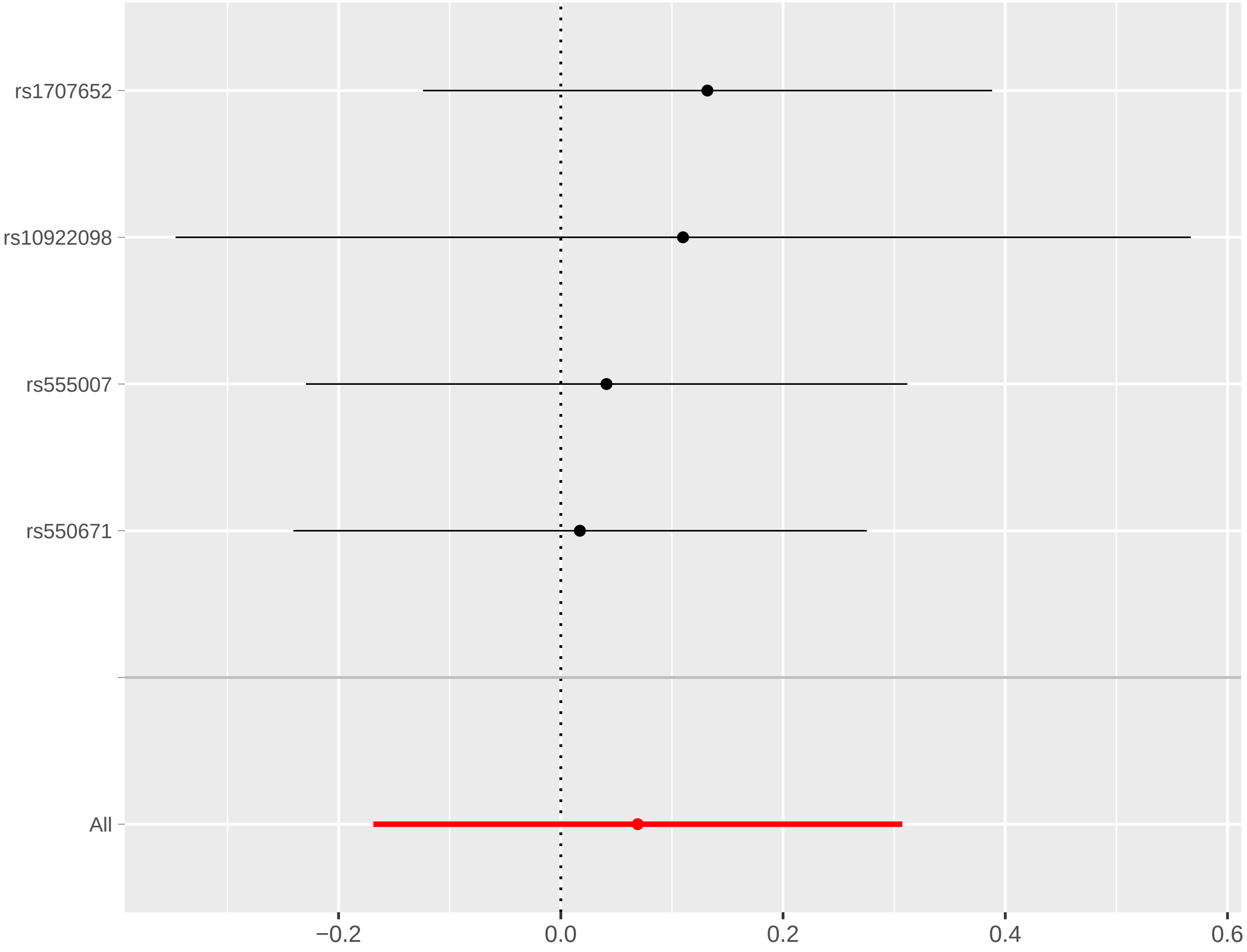

MR leave−one−out sensitivity analysis for
'BMPR1B' on 'Pericarditis'

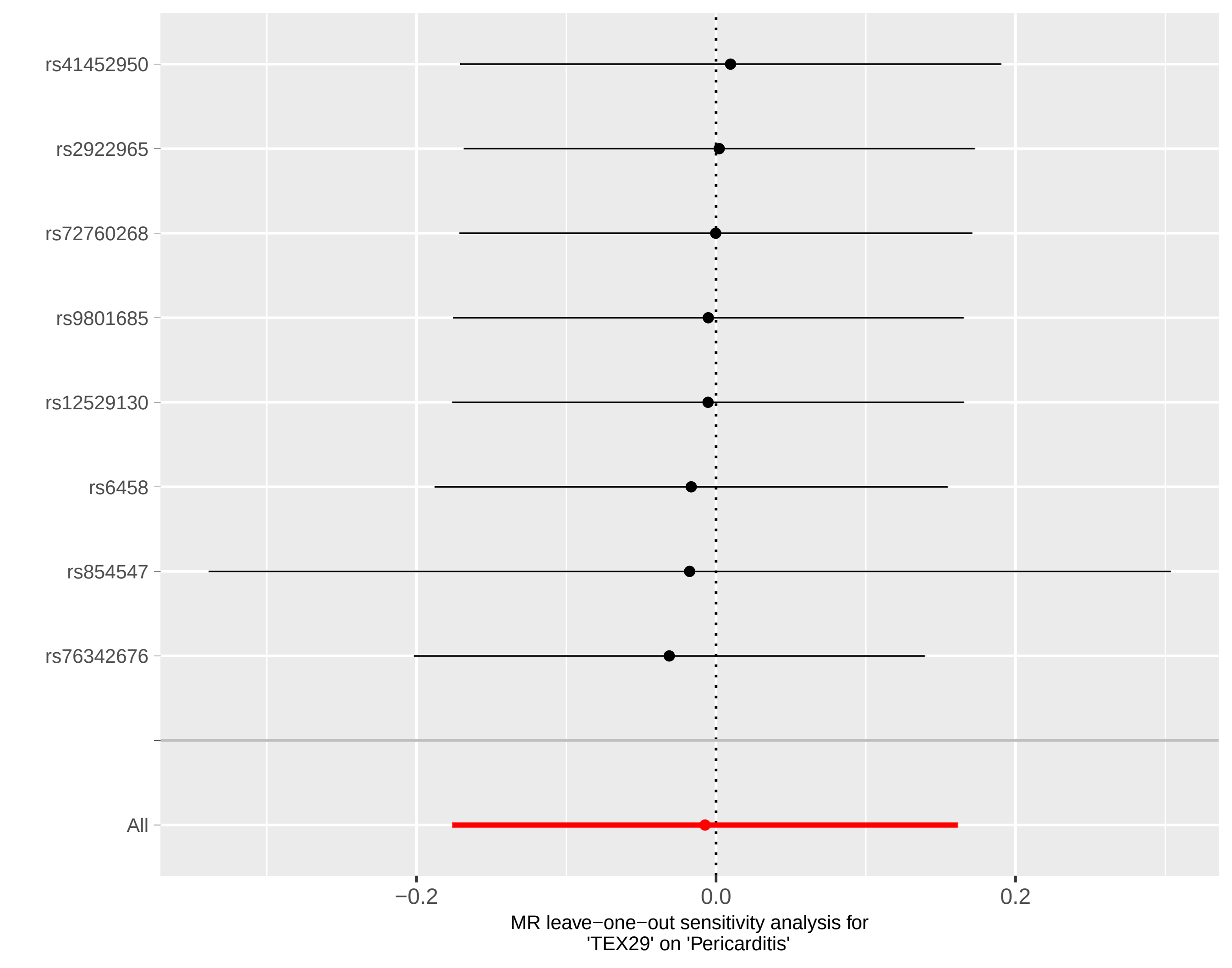
rs41452950
rs2922965
rs72760268
rs9801685
rs12529130
rs6458
rs854547
rs76342676
All
−0.2
0.0
0.2
MR leave−one−out sensitivity analysis for
'TEX29' on 'Pericarditis'

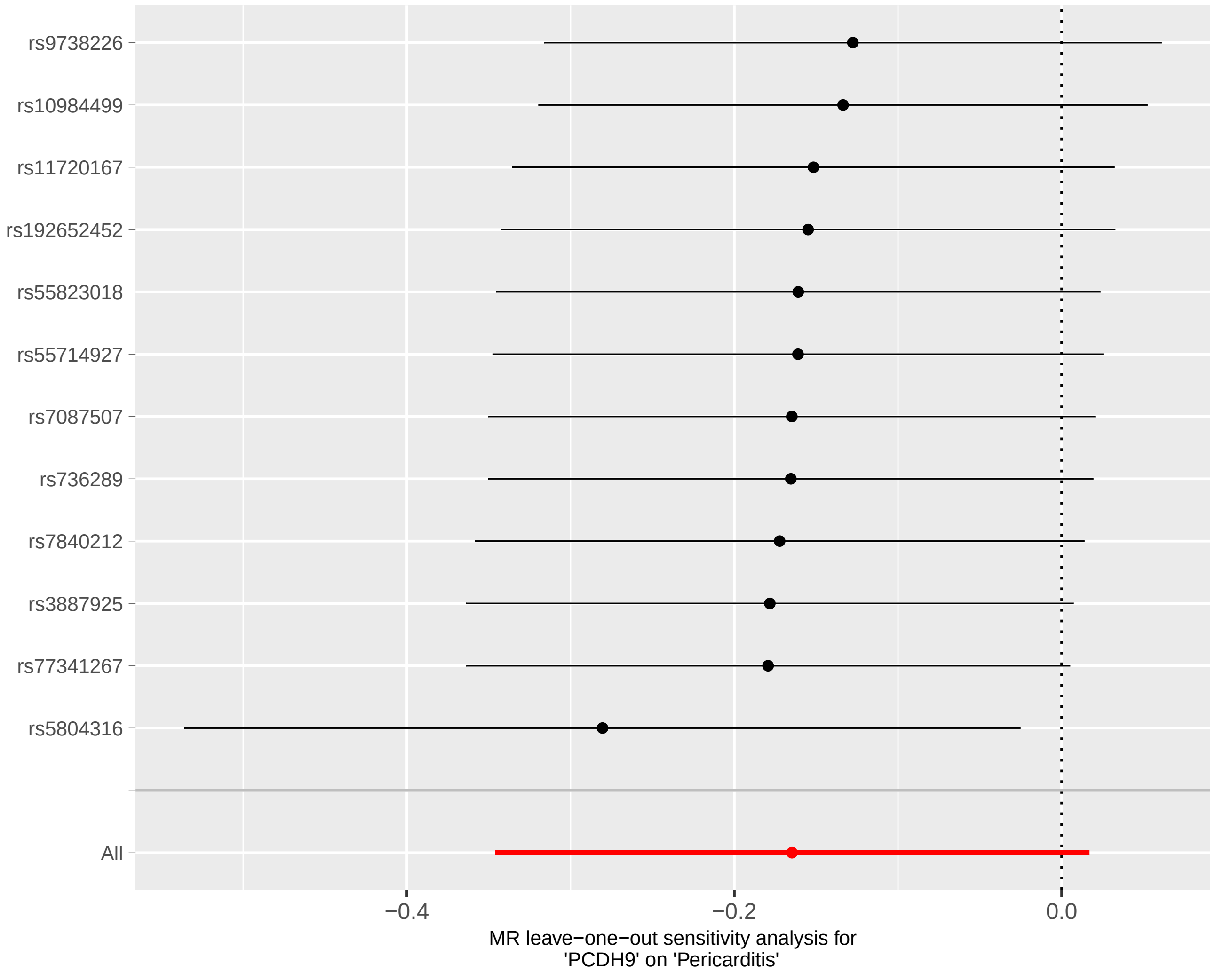
rs9738226
rs10984499
rs11720167
rs192652452
rs55823018
rs55714927
rs7087507
rs736289
rs7840212
rs3887925
rs77341267
rs5804316
All
−0.4
−0.2
0.0
MR leave−one−out sensitivity analysis for
'PCDH9' on 'Pericarditis'

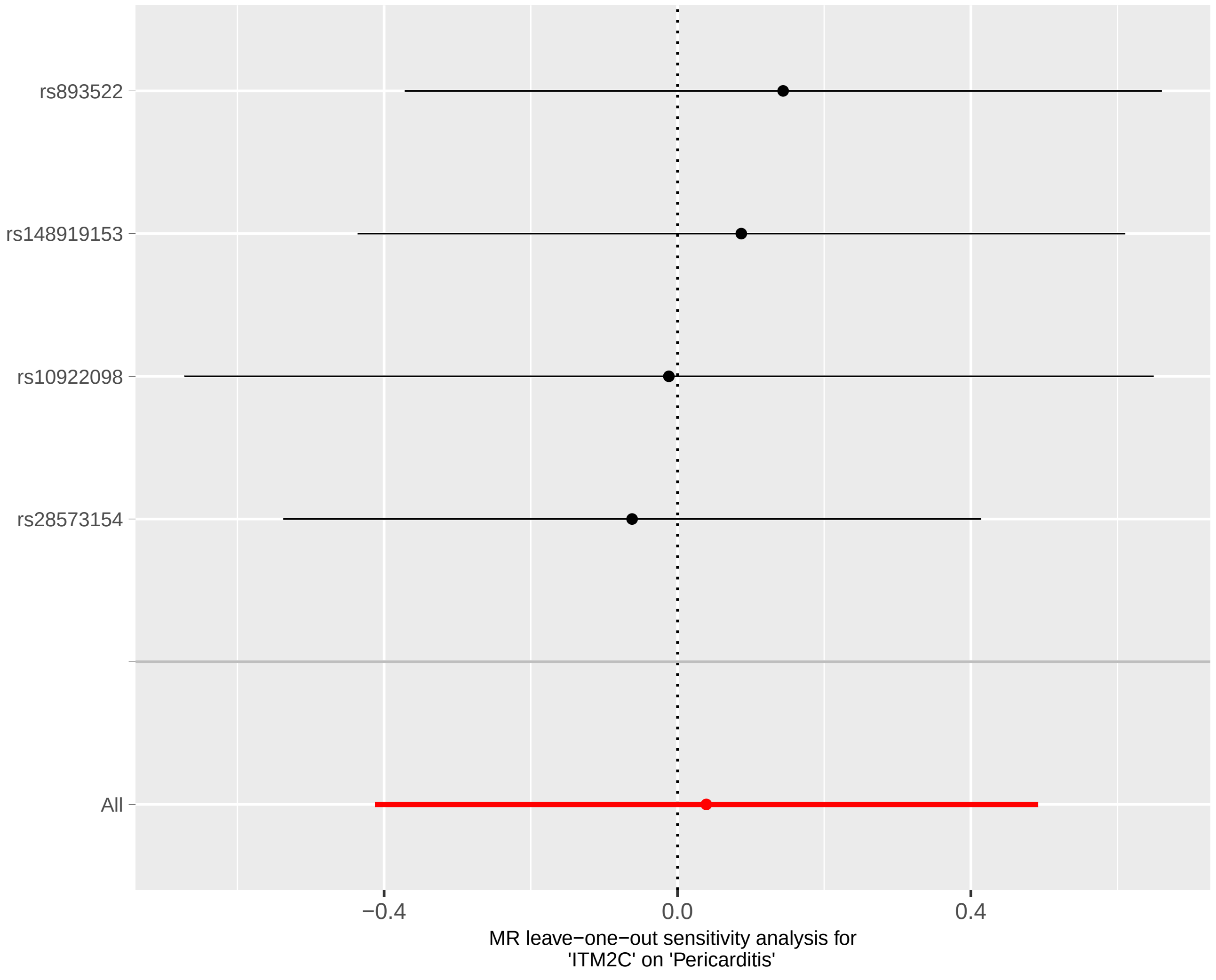
rs893522
rs148919153
rs10922098
rs28573154
All
−0.4
0.0
0.4
MR leave−one−out sensitivity analysis for
'ITM2C' on 'Pericarditis'

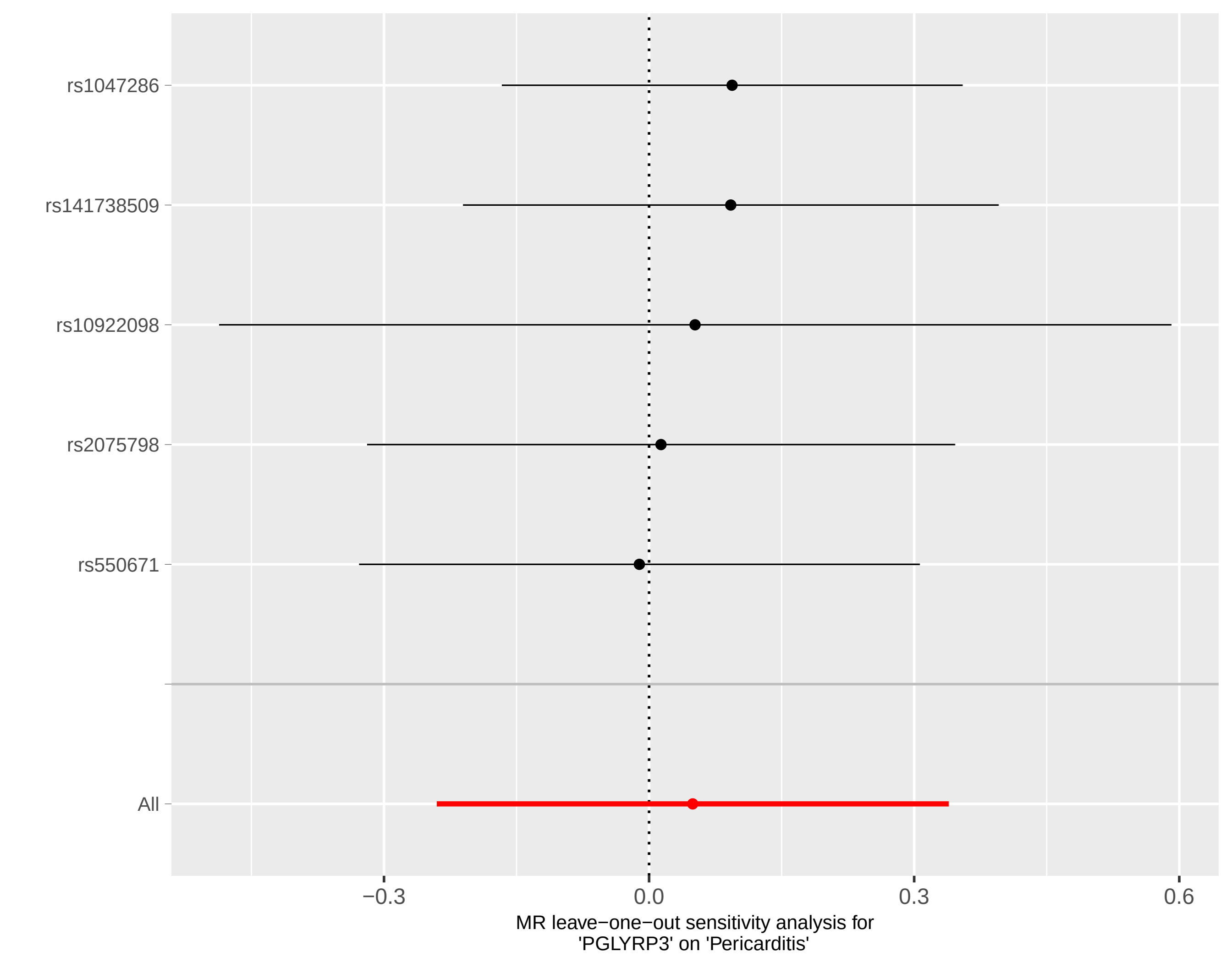
rs1047286
rs141738509
rs10922098
rs2075798
rs550671
All
−0.3
0.0
0.3
0.6
MR leave−one−out sensitivity analysis for
'PGLYRP3' on 'Pericarditis'

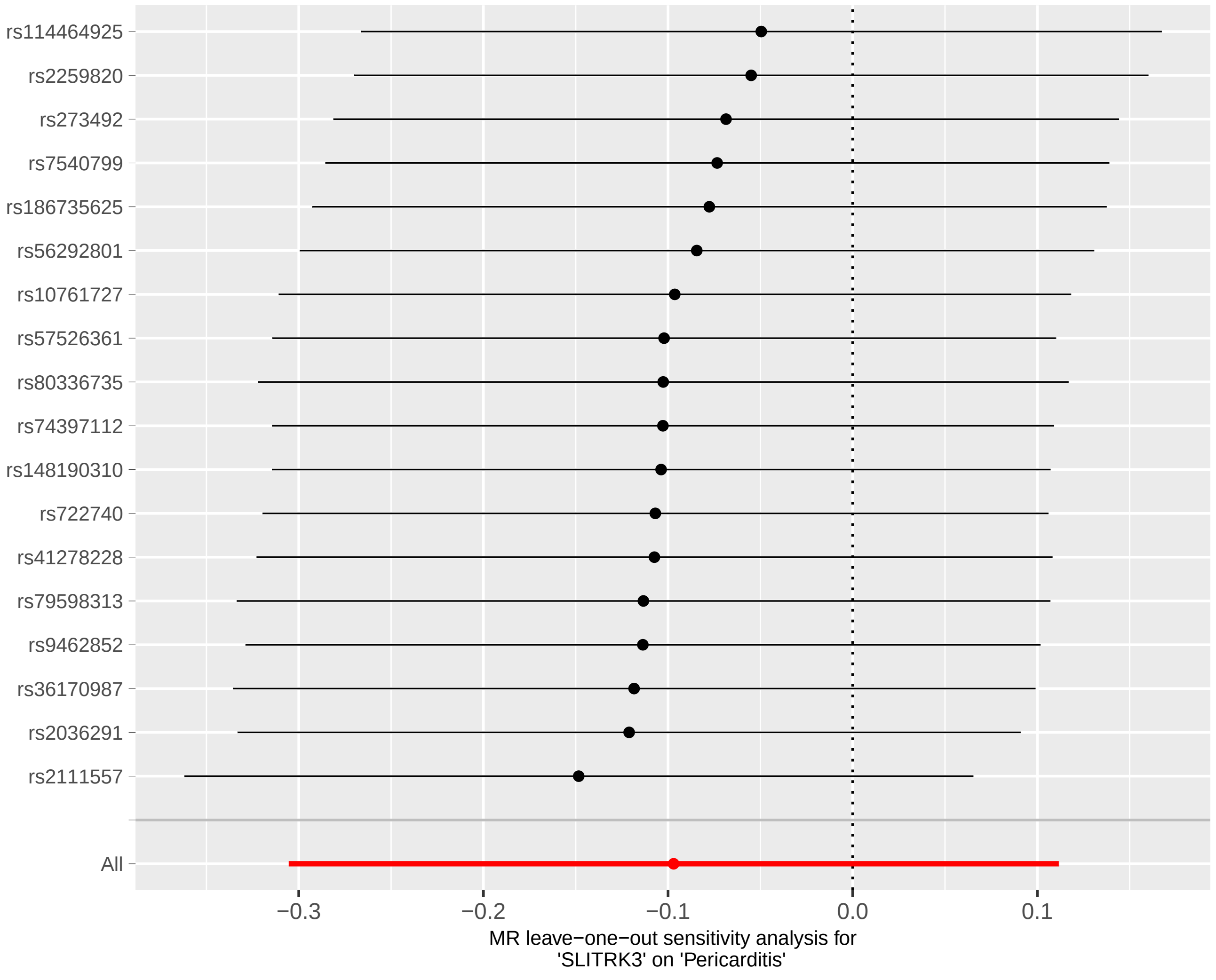
rs114464925
rs2259820
rs273492
rs7540799
rs186735625
rs56292801
rs10761727
rs57526361
rs80336735
rs74397112
rs148190310
rs722740
rs41278228
rs79598313
rs9462852
rs36170987
rs2036291
rs2111557
All
−0.3
−0.2
−0.1
0.0
0.1
MR leave−one−out sensitivity analysis for
'SLITRK3' on 'Pericarditis'

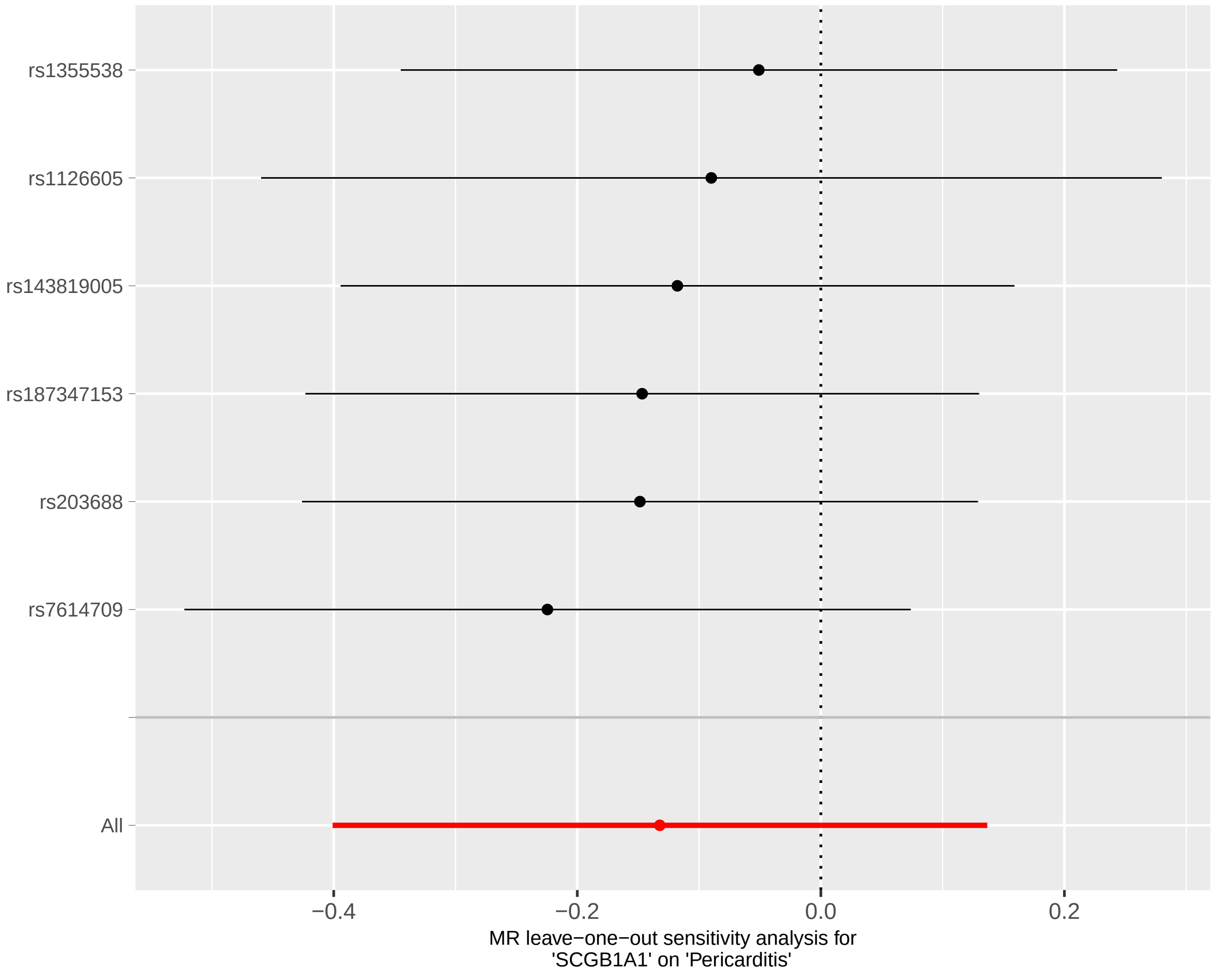
rs1355538
rs1126605
rs143819005
rs187347153
rs203688
rs7614709
All
−0.4
−0.2
0.0
0.2
MR leave−one−out sensitivity analysis for
'SCGB1A1' on 'Pericarditis'

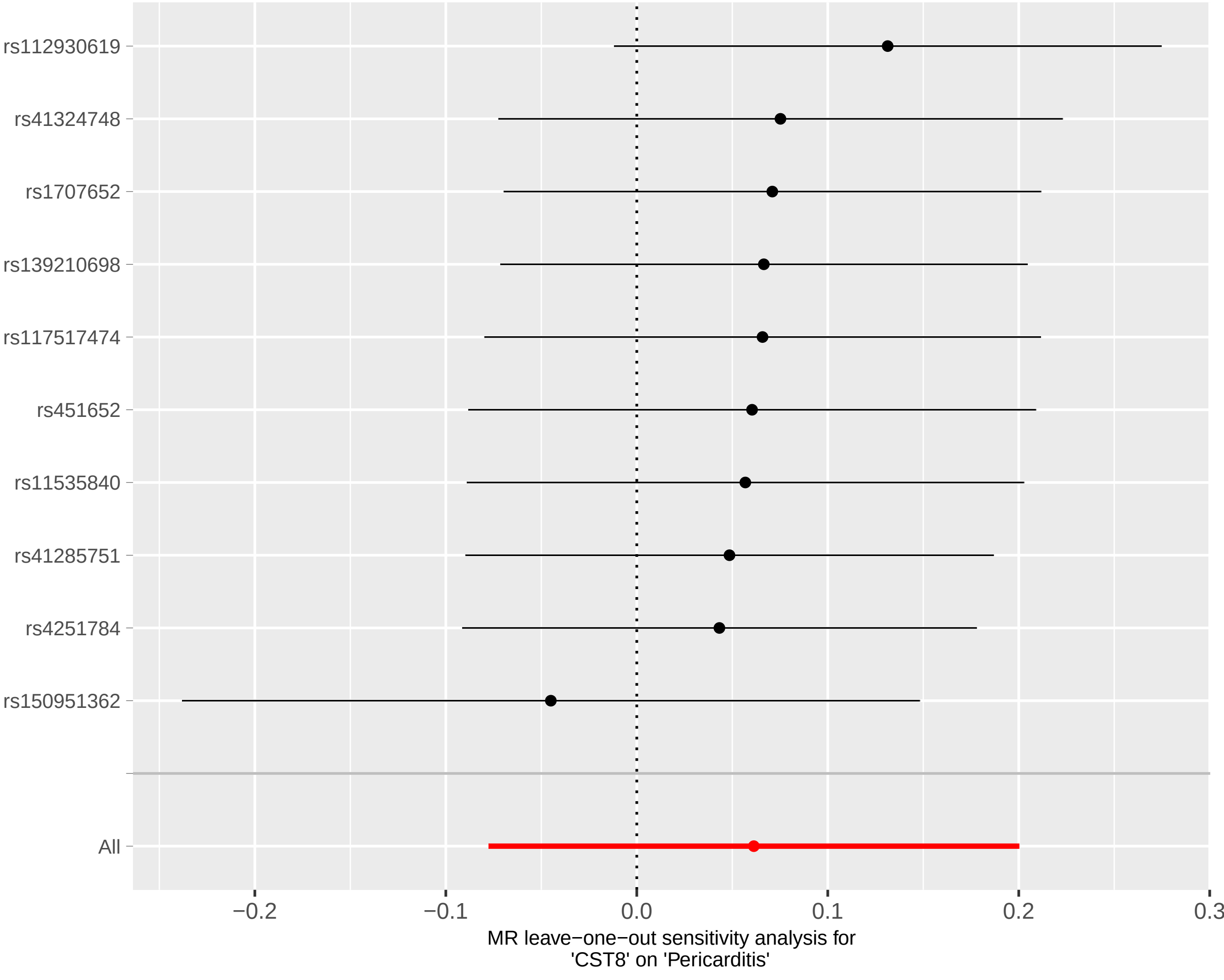
rs112930619
rs41324748
rs1707652
rs139210698
rs117517474
rs451652
rs11535840
rs41285751
rs4251784
rs150951362
All
−0.2
−0.1
0.0
0.1
0.2
0.3
MR leave−one−out sensitivity analysis for
'CST8' on 'Pericarditis'

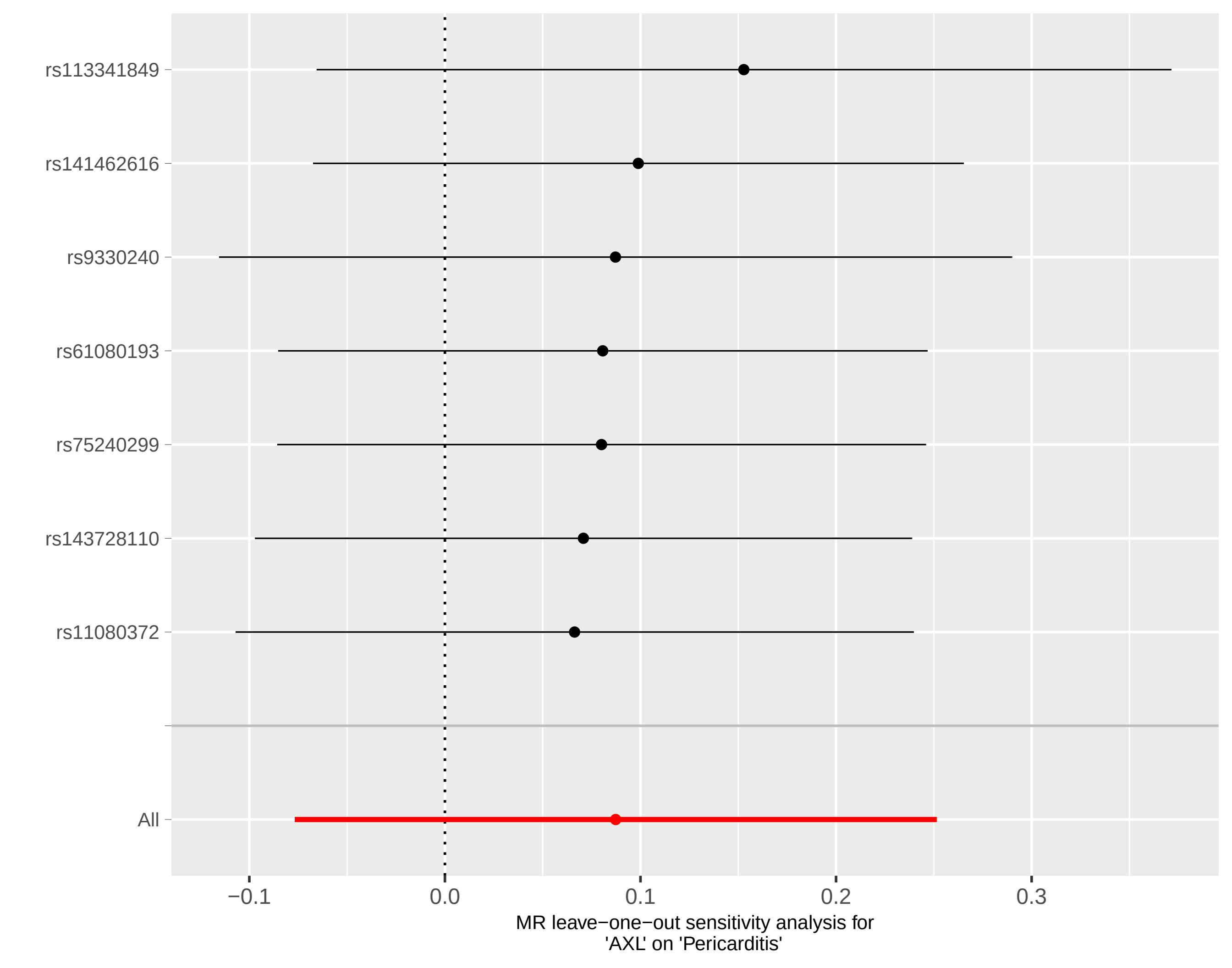

rs113341849
rs141462616
rs9330240
rs61080193
rs75240299
rs143728110
rs11080372
All
−0.1
0.0
0.1
0.2
0.3
MR leave−one−out sensitivity analysis for
'AXL' on 'Pericarditis'

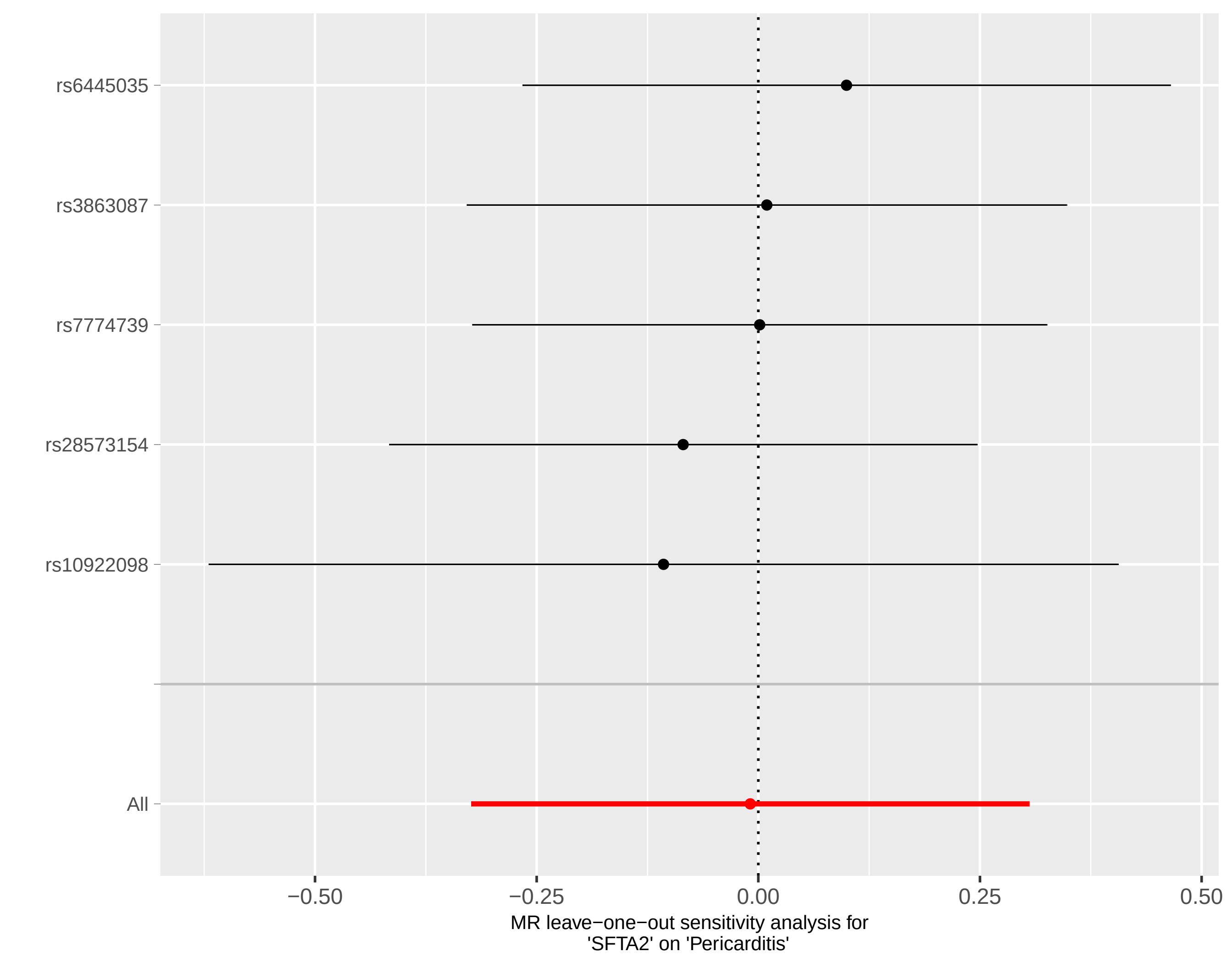
rs6445035
rs3863087
rs7774739
rs28573154
rs10922098
All
−0.50
−0.25
0.00
0.25
0.50
MR leave−one−out sensitivity analysis for
'SFTA2' on 'Pericarditis'

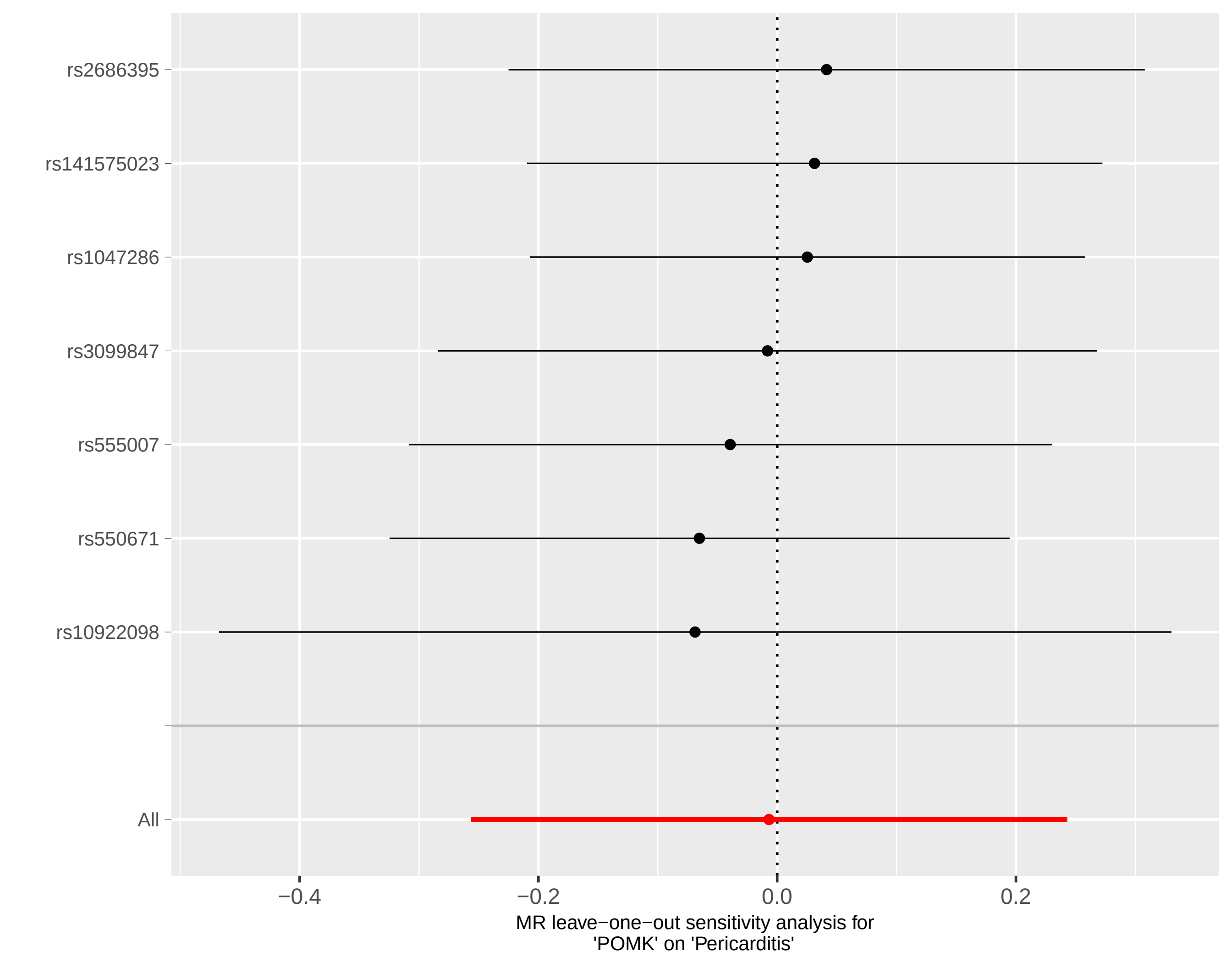

rs2686395
rs141575023
rs1047286
rs3099847
rs555007
rs550671
rs10922098
All
−0.4
−0.2
0.0
0.2
MR leave−one−out sensitivity analysis for
'POMK' on 'Pericarditis'

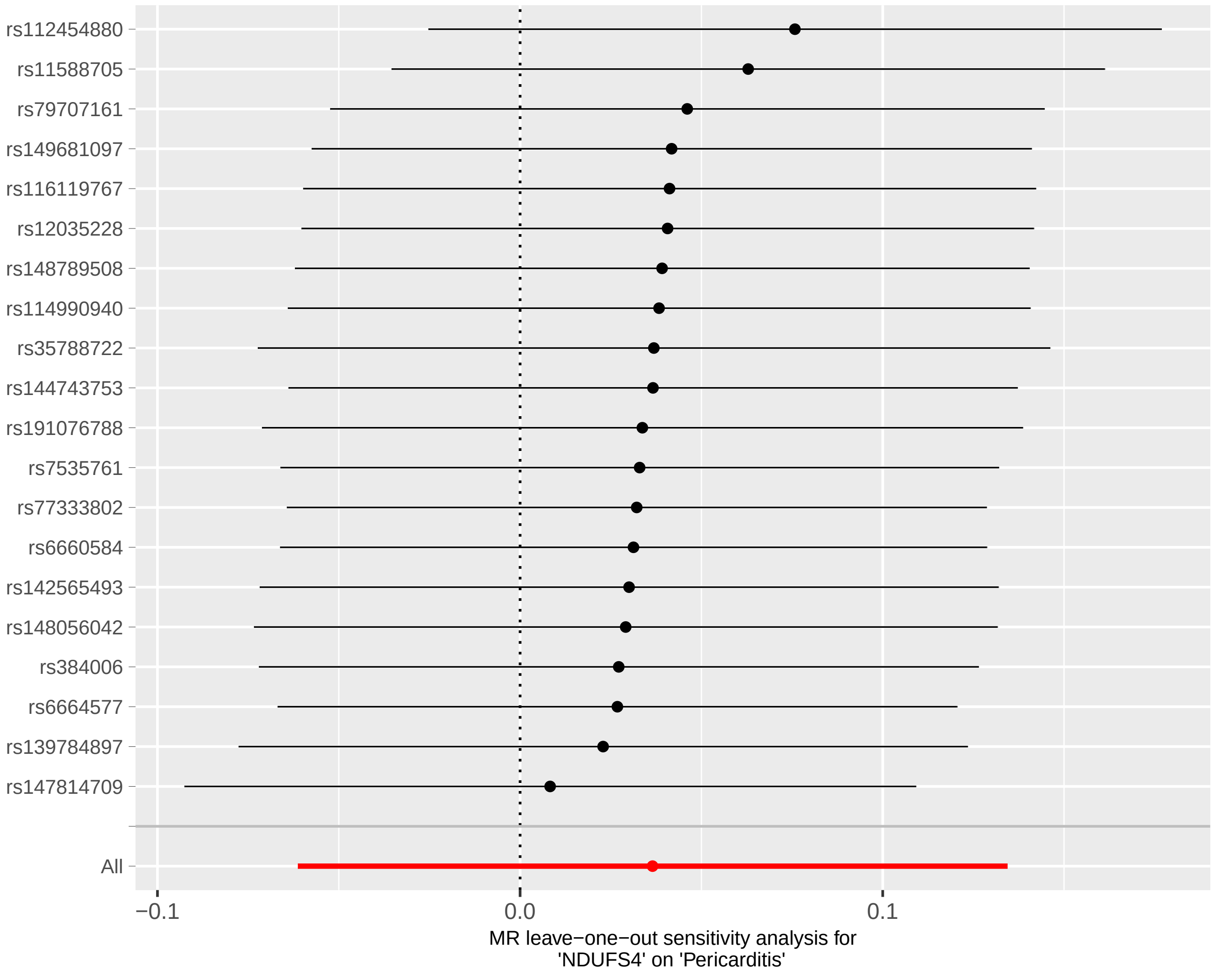

rs112454880
rs11588705
rs79707161
rs149681097
rs116119767
rs12035228
rs148789508
rs114990940
rs35788722
rs144743753
rs191076788
rs7535761
rs77333802
rs6660584
rs142565493
rs148056042
rs384006
rs6664577
rs139784897
rs147814709
All
−0.1
0.0
0.1
MR leave−one−out sensitivity analysis for
'NDUFS4' on 'Pericarditis'

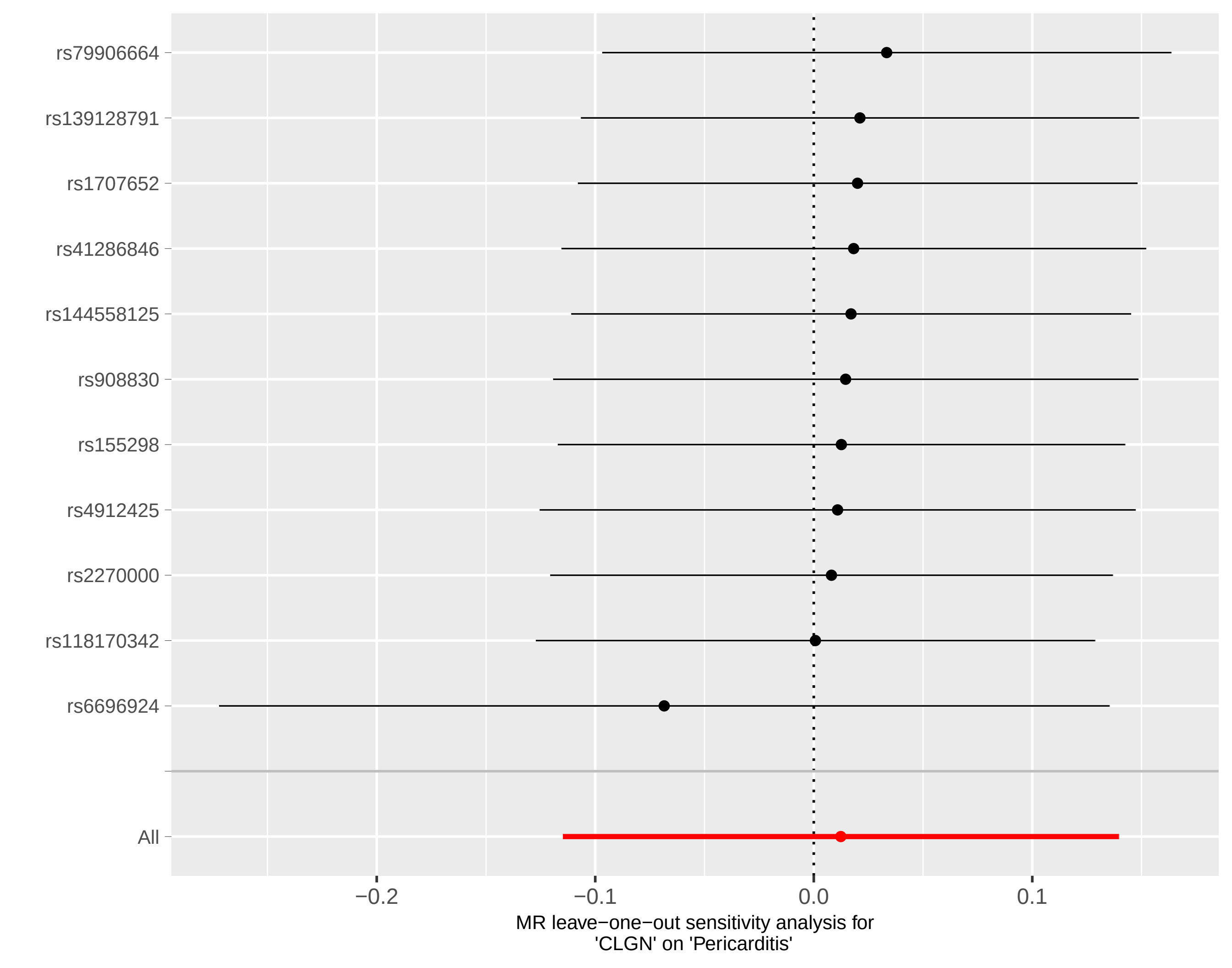

rs79906664
rs139128791
rs1707652
rs41286846
rs144558125
rs908830
rs155298
rs4912425
rs2270000
rs118170342
rs6696924
All
−0.2
−0.1
0.0
0.1
MR leave−one−out sensitivity analysis for
'CLGN' on 'Pericarditis'

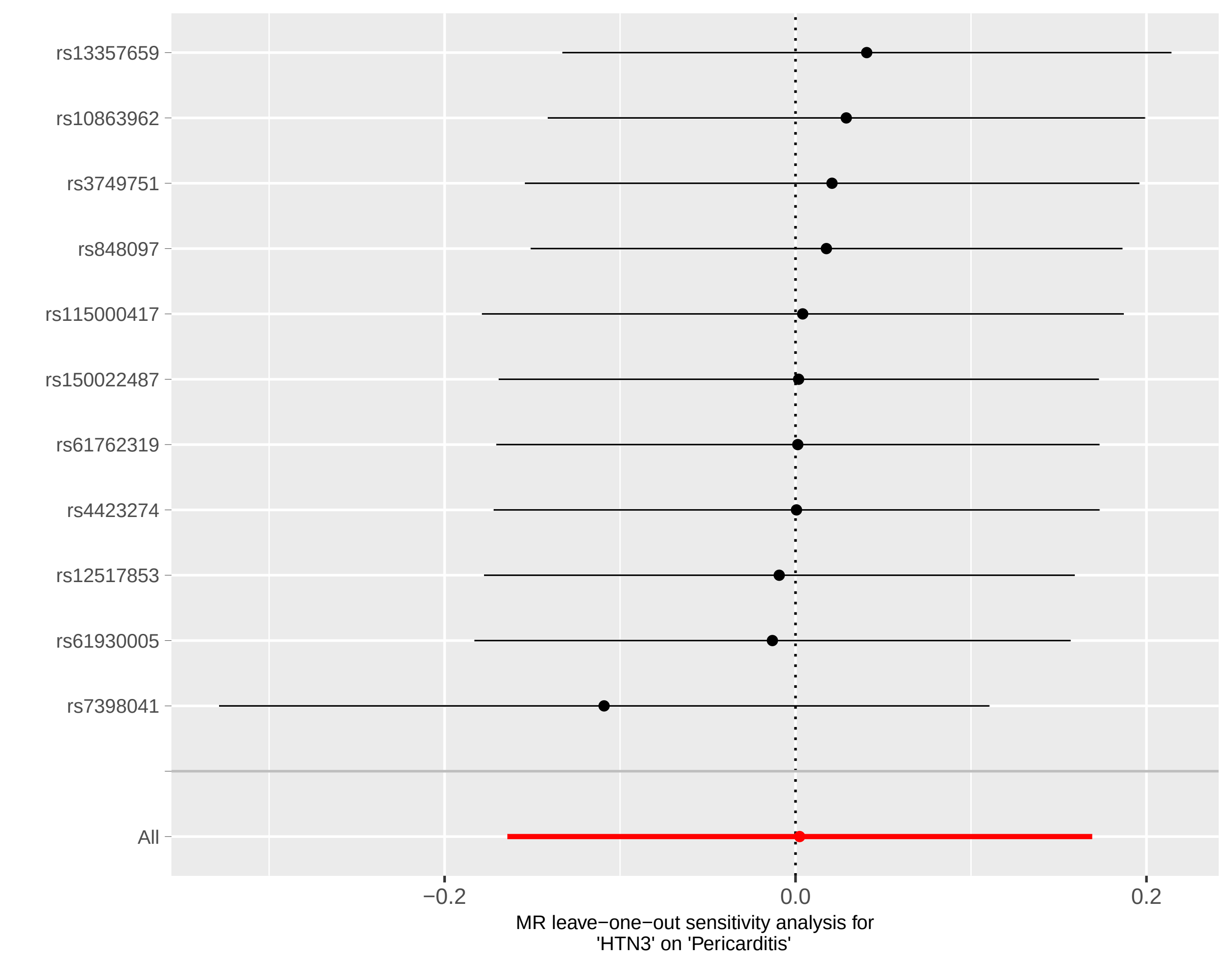

rs13357659
rs10863962
rs3749751
rs848097
rs115000417
rs150022487
rs61762319
rs4423274
rs12517853
rs61930005
rs7398041
All
−0.2
0.0
0.2
MR leave−one−out sensitivity analysis for
'HTN3' on 'Pericarditis'

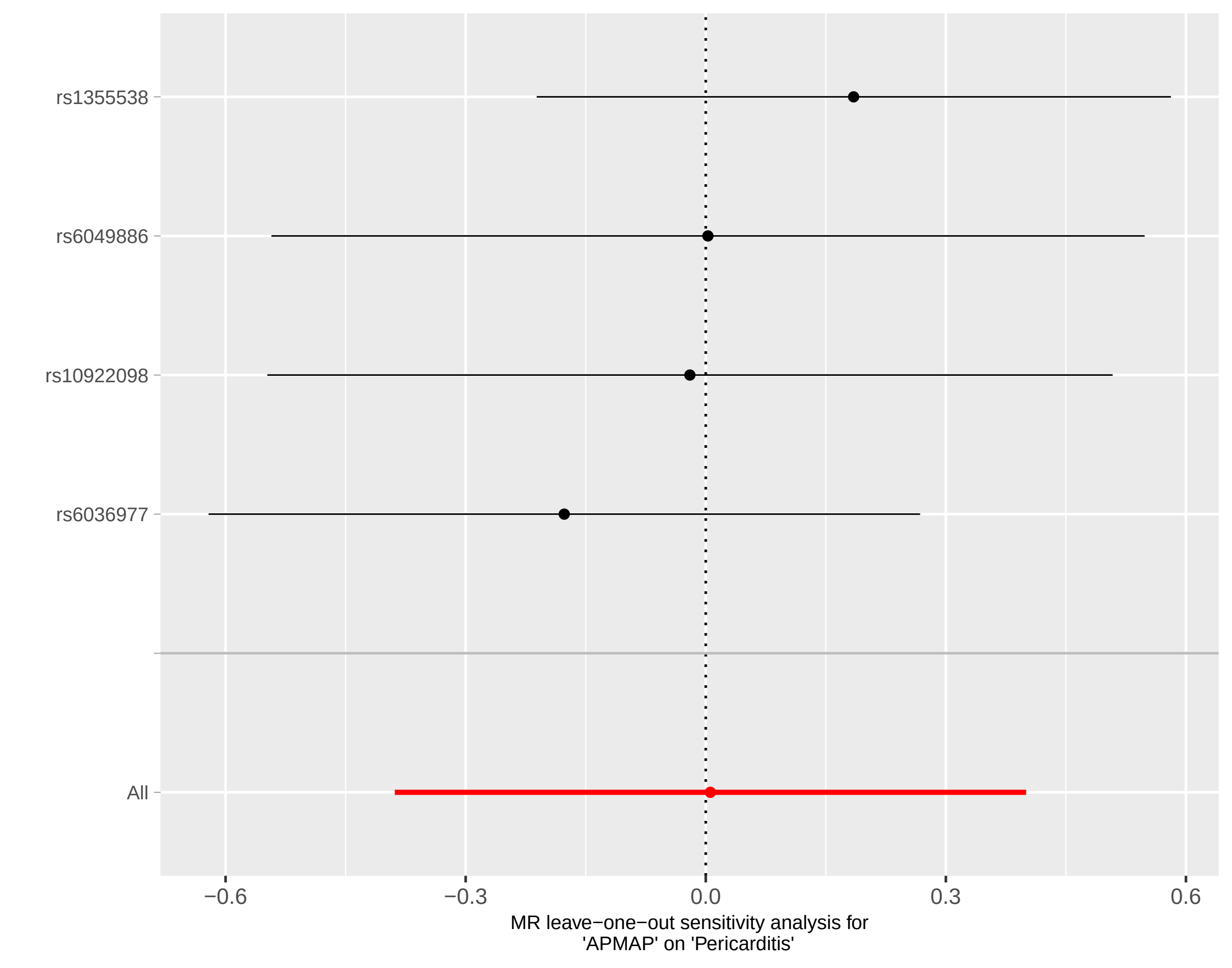
rs1355538
rs6049886
rs10922098
rs6036977
All
−0.6
−0.3
0.0
0.3
0.6
MR leave−one−out sensitivity analysis for
'APMAP' on 'Pericarditis'

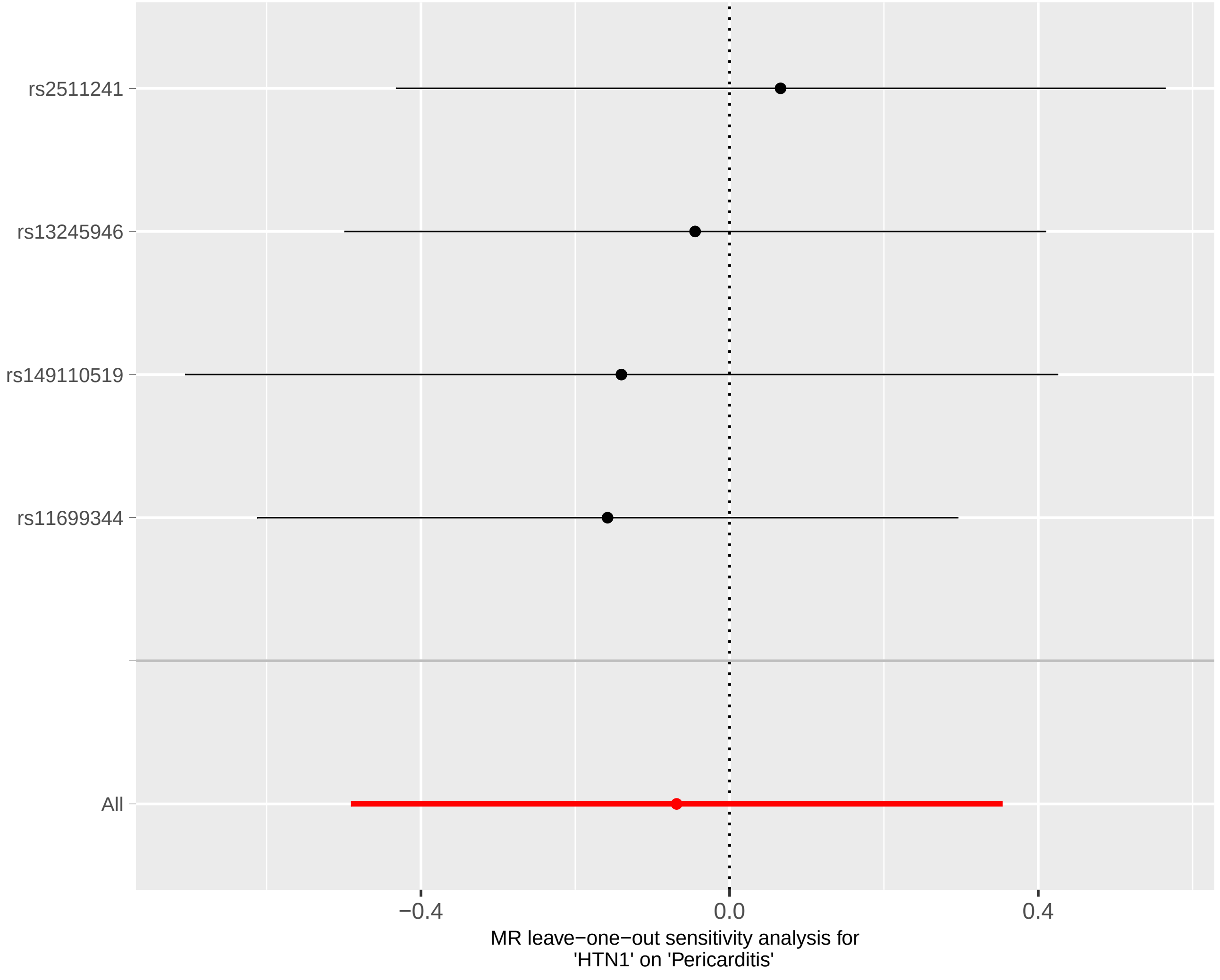
rs2511241
rs13245946
rs149110519
rs11699344
All
−0.4
0.0
0.4
MR leave−one−out sensitivity analysis for
'HTN1' on 'Pericarditis'

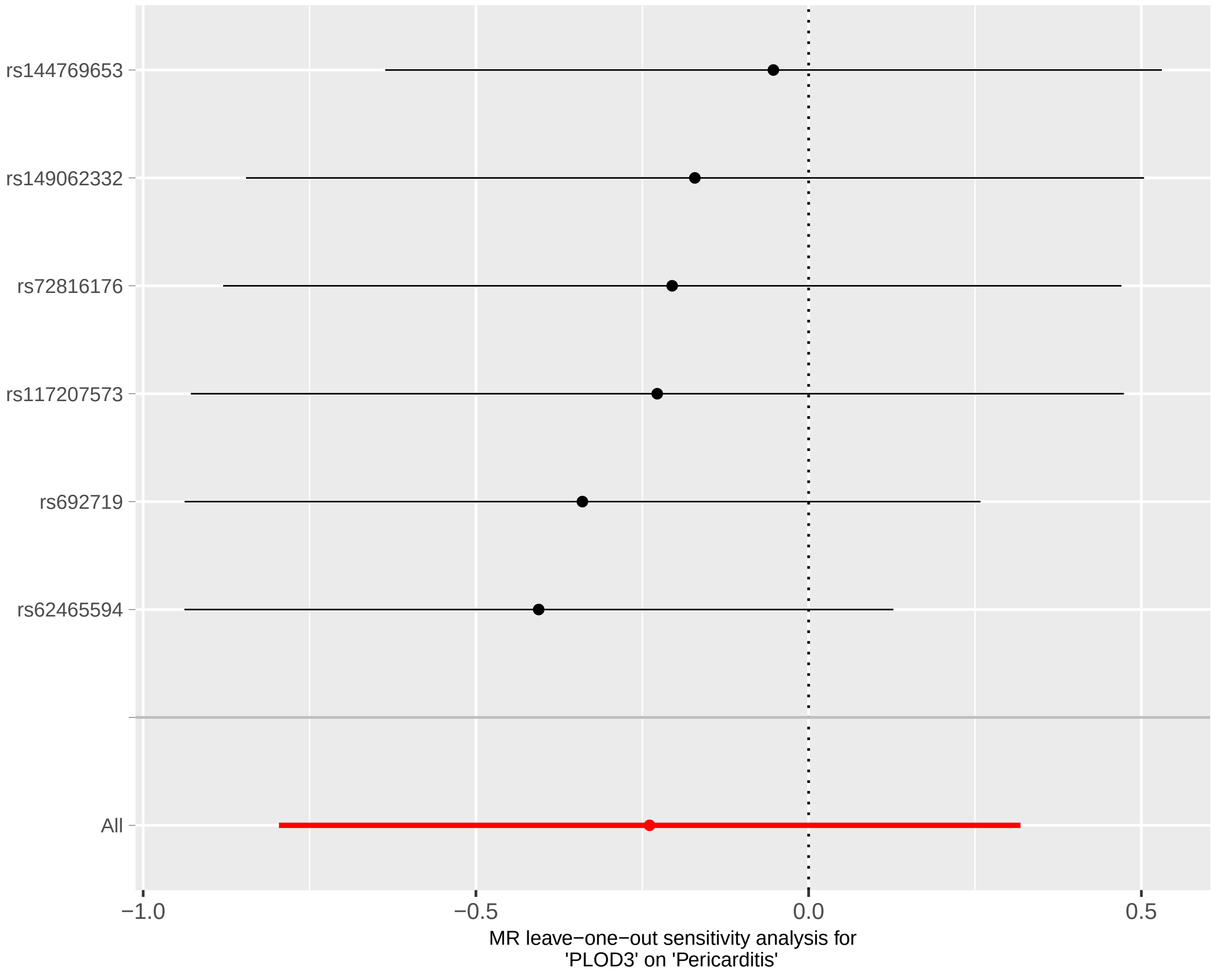

rs144769653
rs149062332
rs72816176
rs117207573
rs692719
rs62465594
All
−1.0
−0.5
0.0
0.5
MR leave−one−out sensitivity analysis for
'PLOD3' on 'Pericarditis'

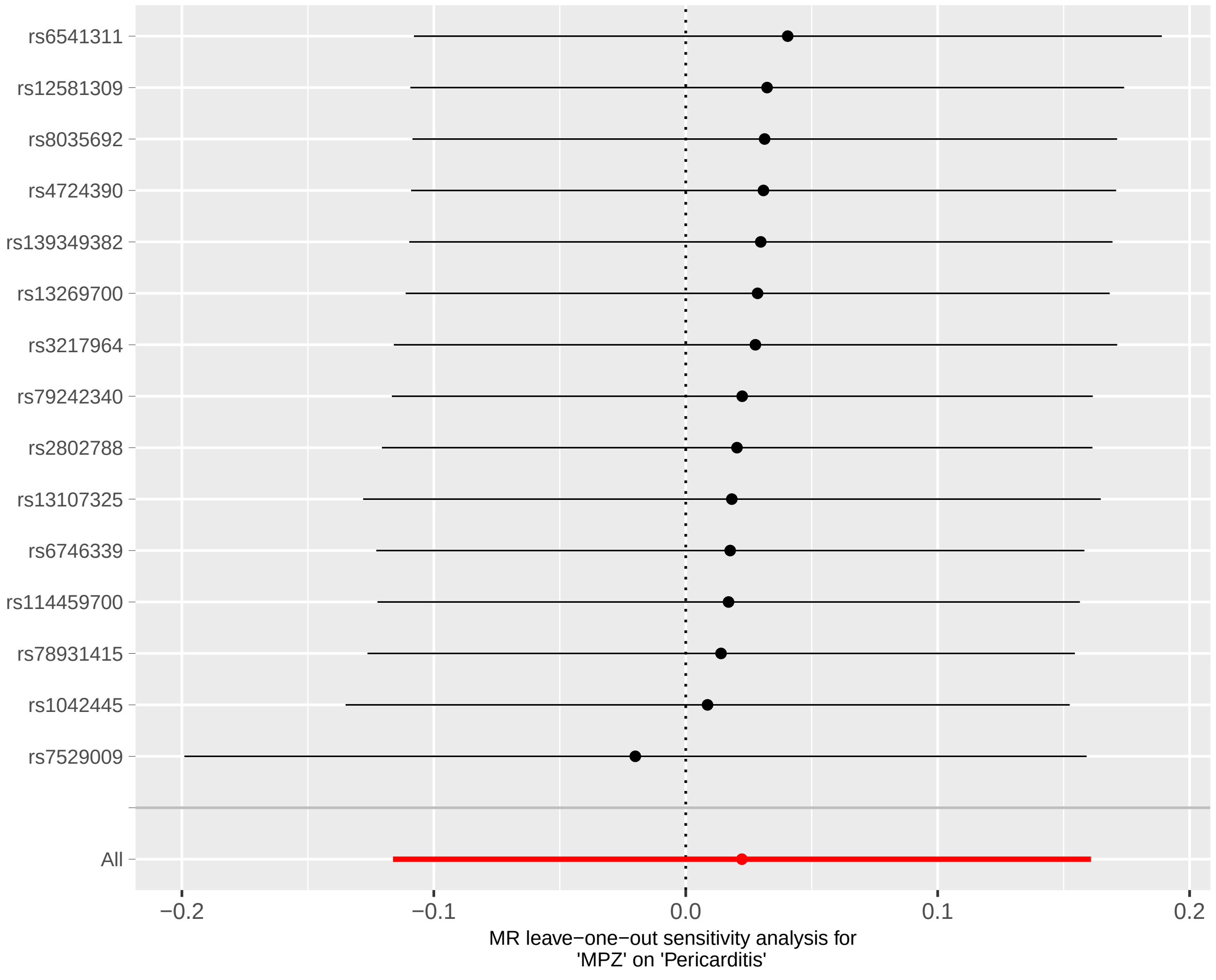

rs6541311
rs12581309
rs8035692
rs4724390
rs139349382
rs13269700
rs3217964
rs79242340
rs2802788
rs13107325
rs6746339
rs114459700
rs78931415
rs1042445
rs7529009
All
−0.2
−0.1
0.0
0.1
0.2
MR leave−one−out sensitivity analysis for
'MPZ' on 'Pericarditis'

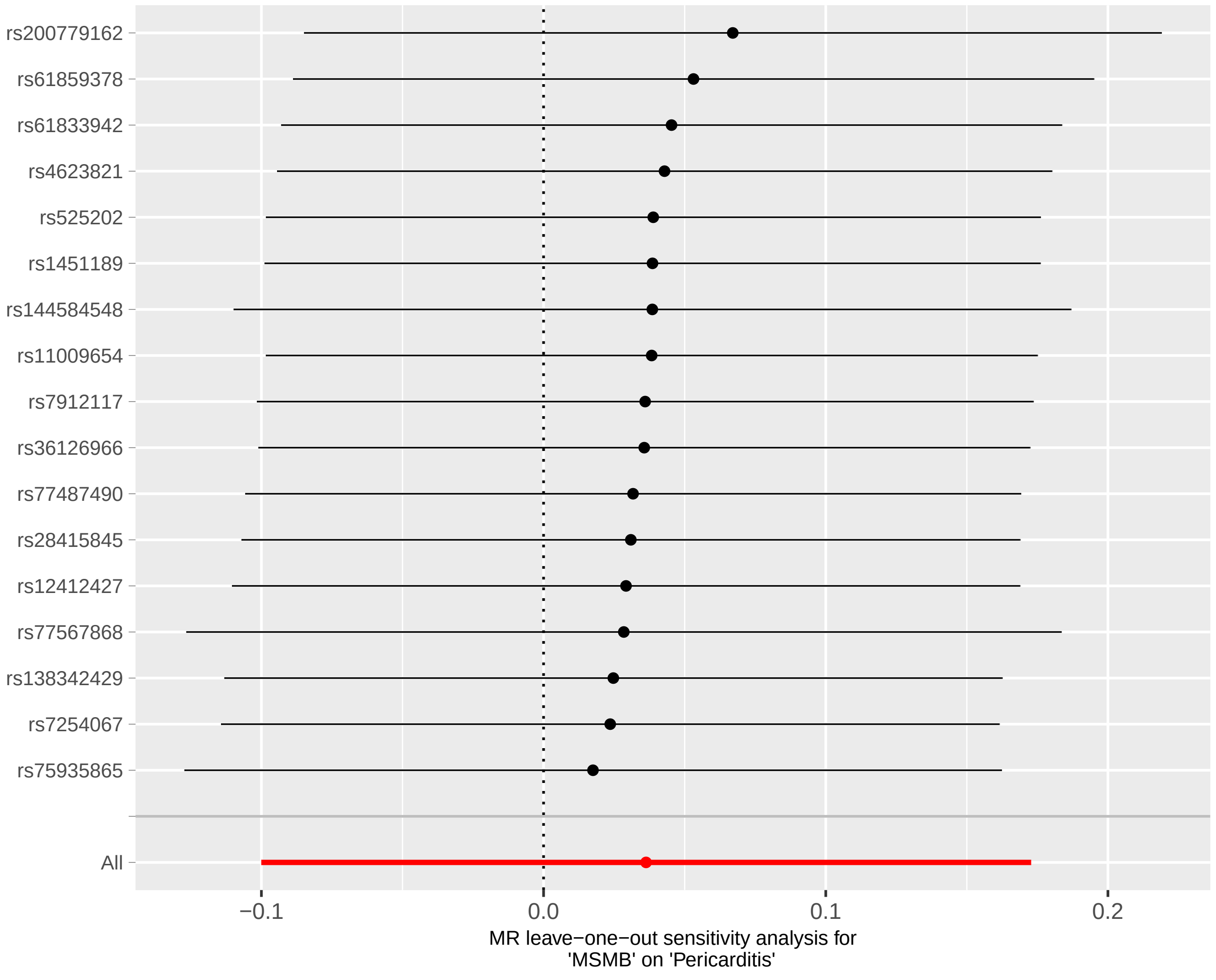

rs200779162
rs61859378
rs61833942
rs4623821
rs525202
rs1451189
rs144584548
rs11009654
rs7912117
rs36126966
rs77487490
rs28415845
rs12412427
rs77567868
rs138342429
rs7254067
rs75935865
All
−0.1
0.0
0.1
0.2
MR leave−one−out sensitivity analysis for
'MSMB' on 'Pericarditis'

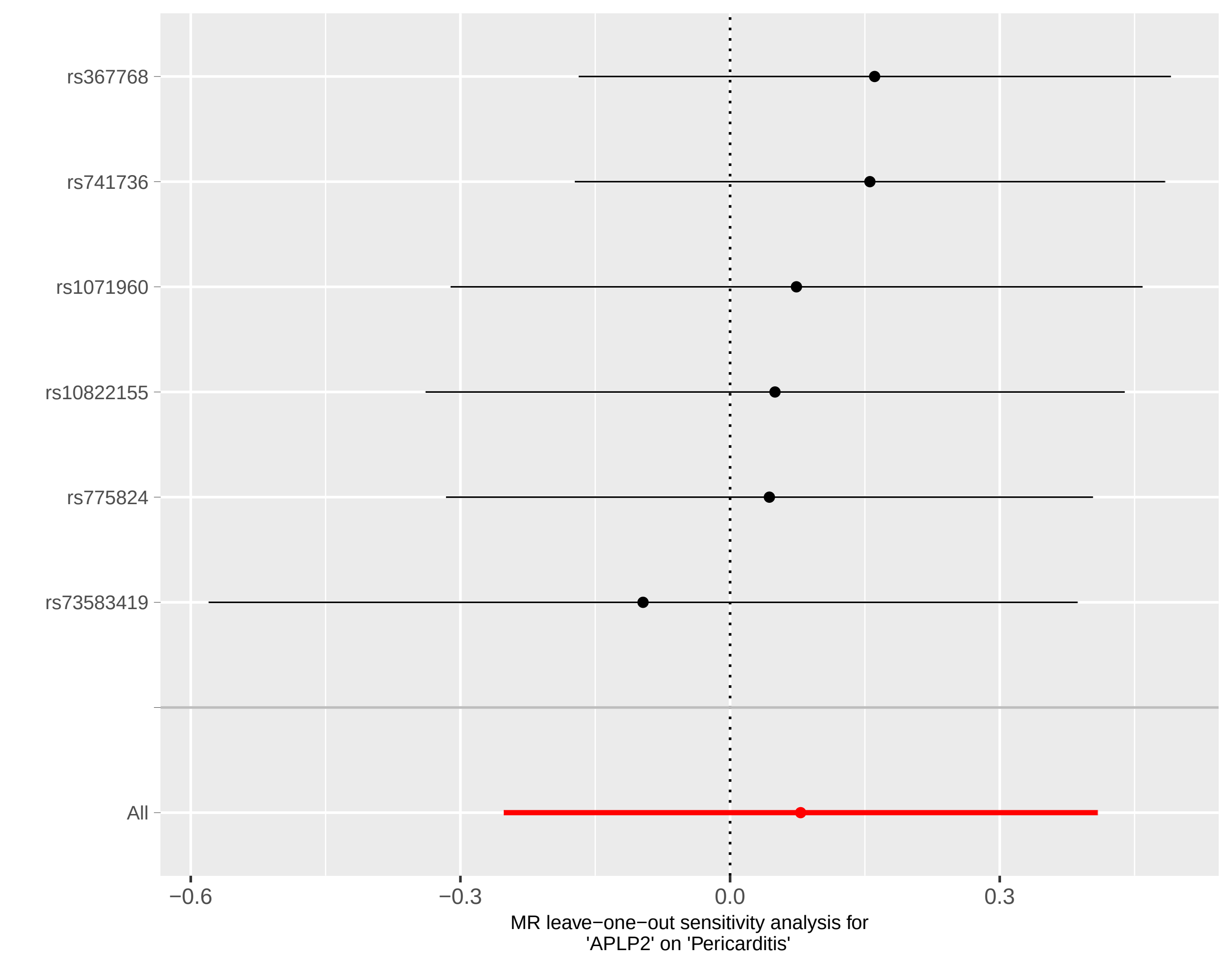

rs367768
rs741736
rs1071960
rs10822155
rs775824
rs73583419
All
−0.6
−0.3
0.0
0.3
MR leave−one−out sensitivity analysis for
'APLP2' on 'Pericarditis'

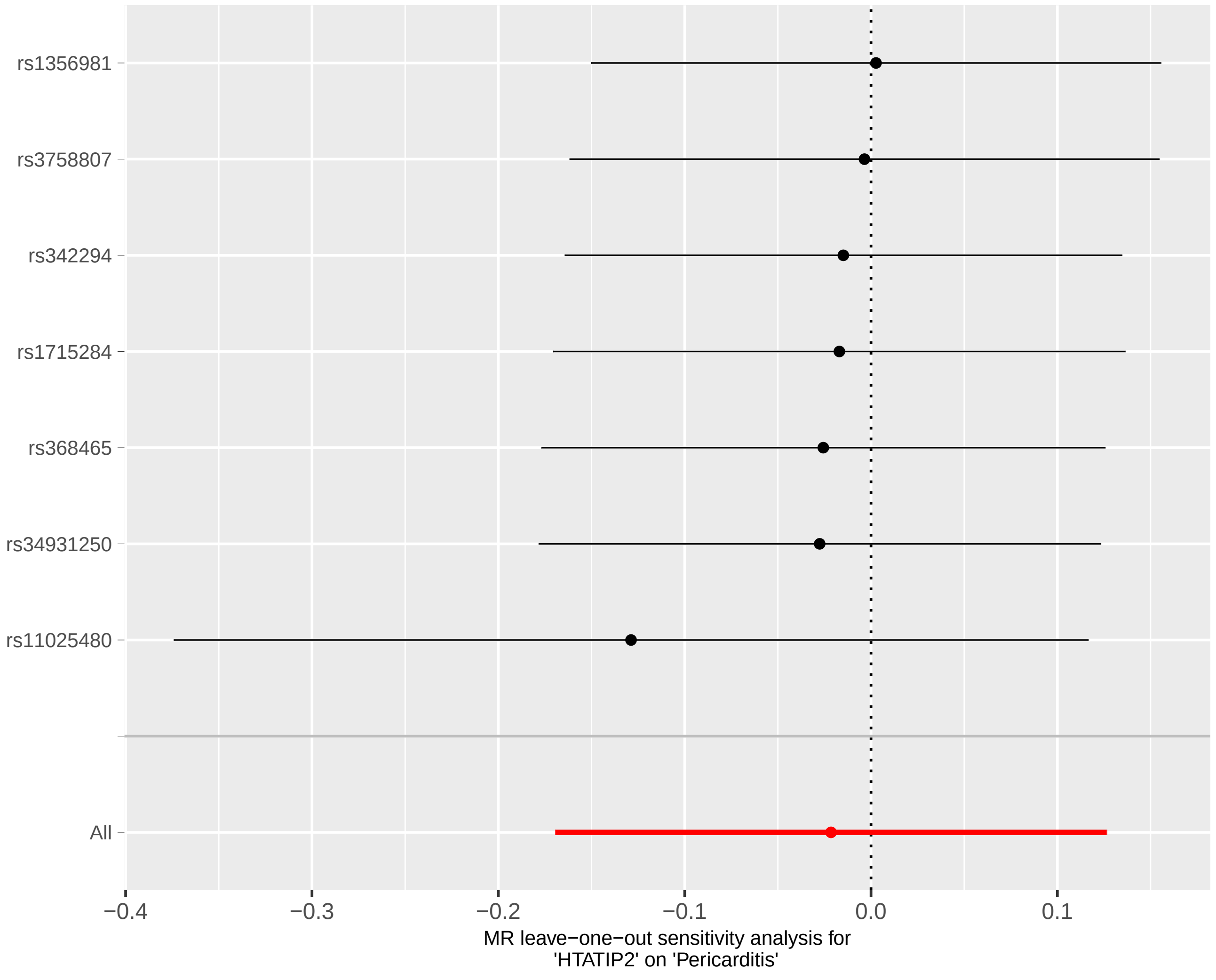

rs1356981
rs3758807
rs342294
rs1715284
rs368465
rs34931250
rs11025480
All
−0.4
−0.3
−0.2
−0.1
0.0
0.1
MR leave−one−out sensitivity analysis for
'HTATIP2' on 'Pericarditis'

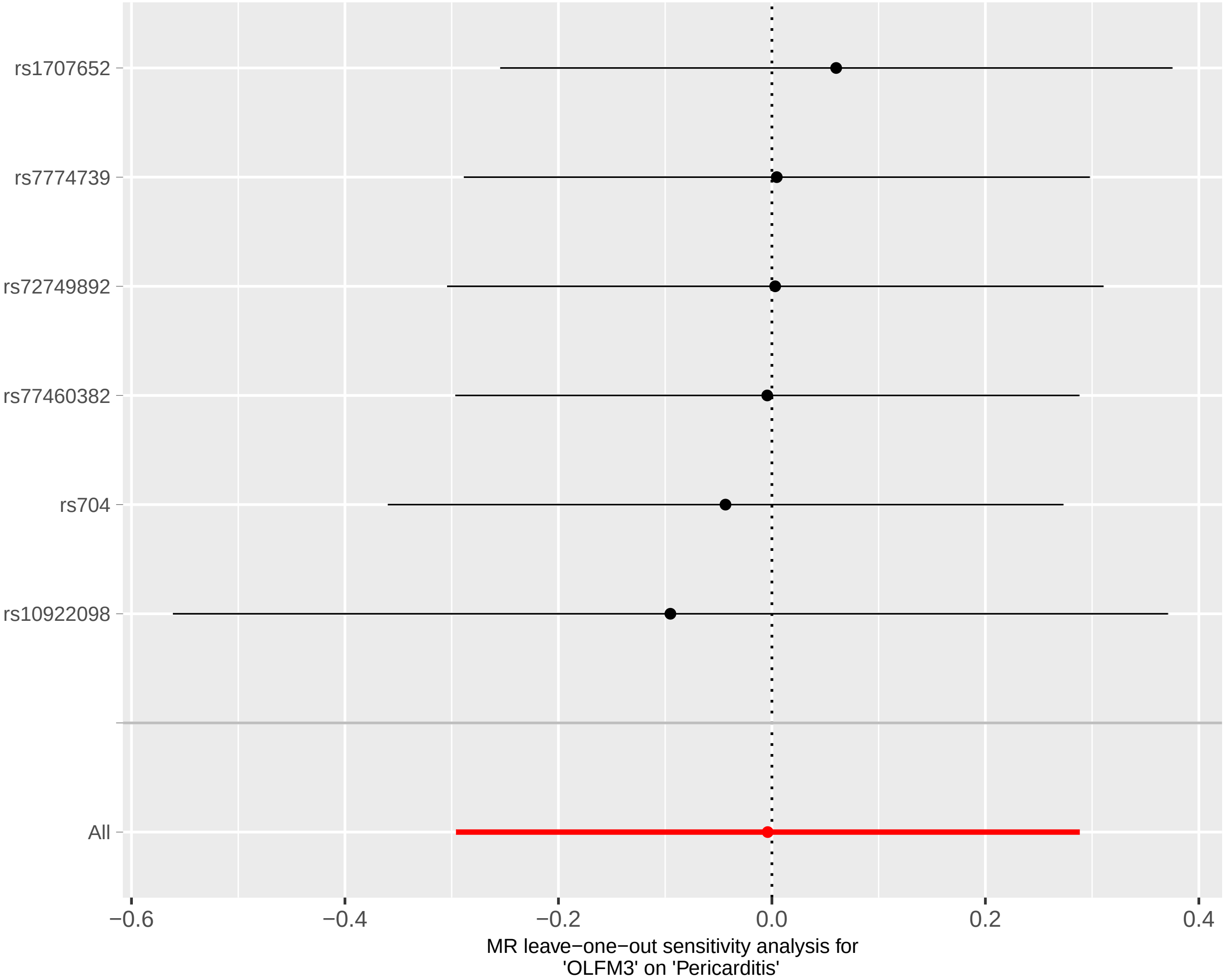
rs1707652
rs7774739
rs72749892
rs77460382
rs704
rs10922098
All
−0.6
−0.4
−0.2
0.0
0.2
0.4
MR leave−one−out sensitivity analysis for
'OLFM3' on 'Pericarditis'

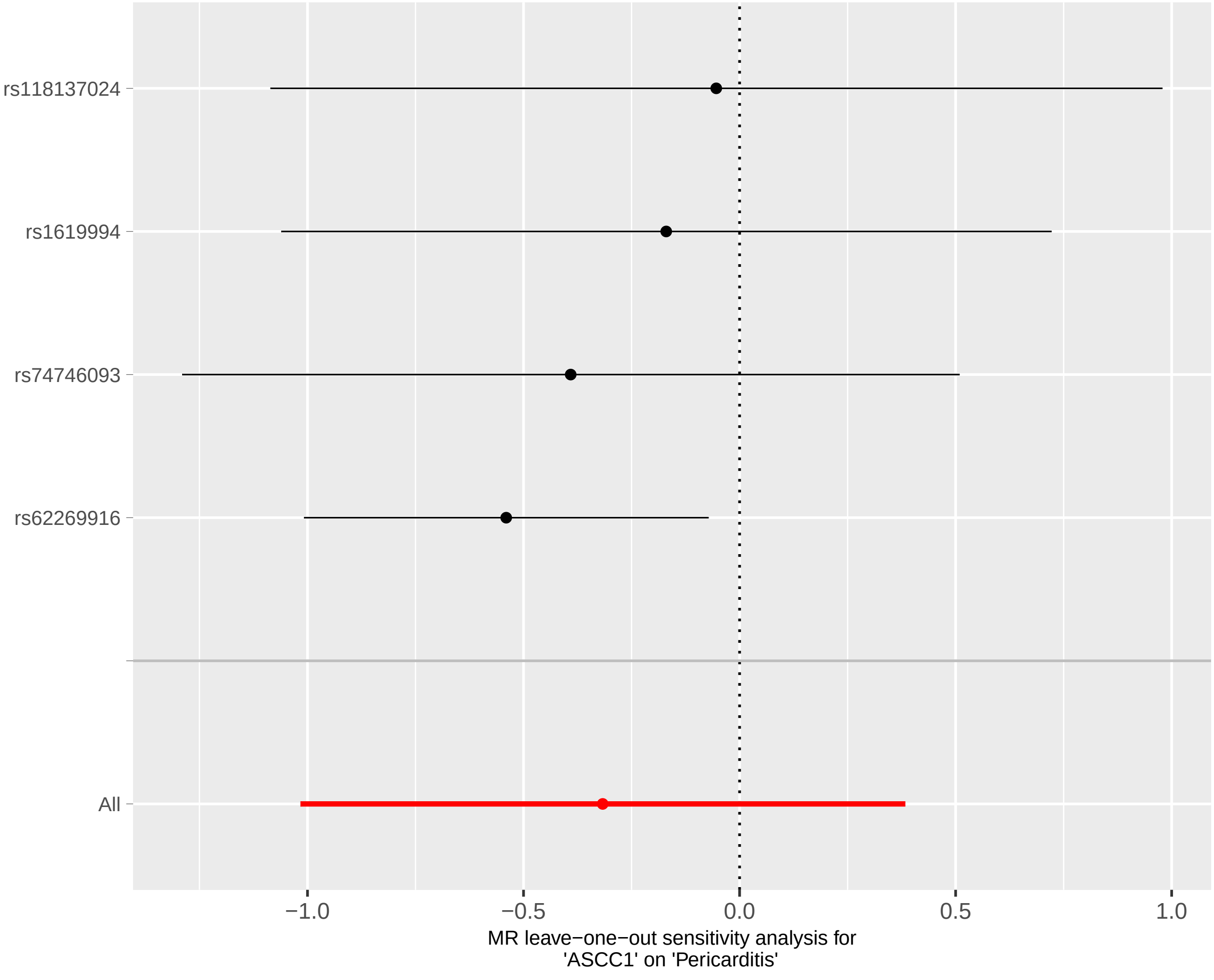

rs118137024
rs1619994
rs74746093
rs62269916
All
−1.0
−0.5
0.0
0.5
1.0
MR leave−one−out sensitivity analysis for
'ASCC1' on 'Pericarditis'

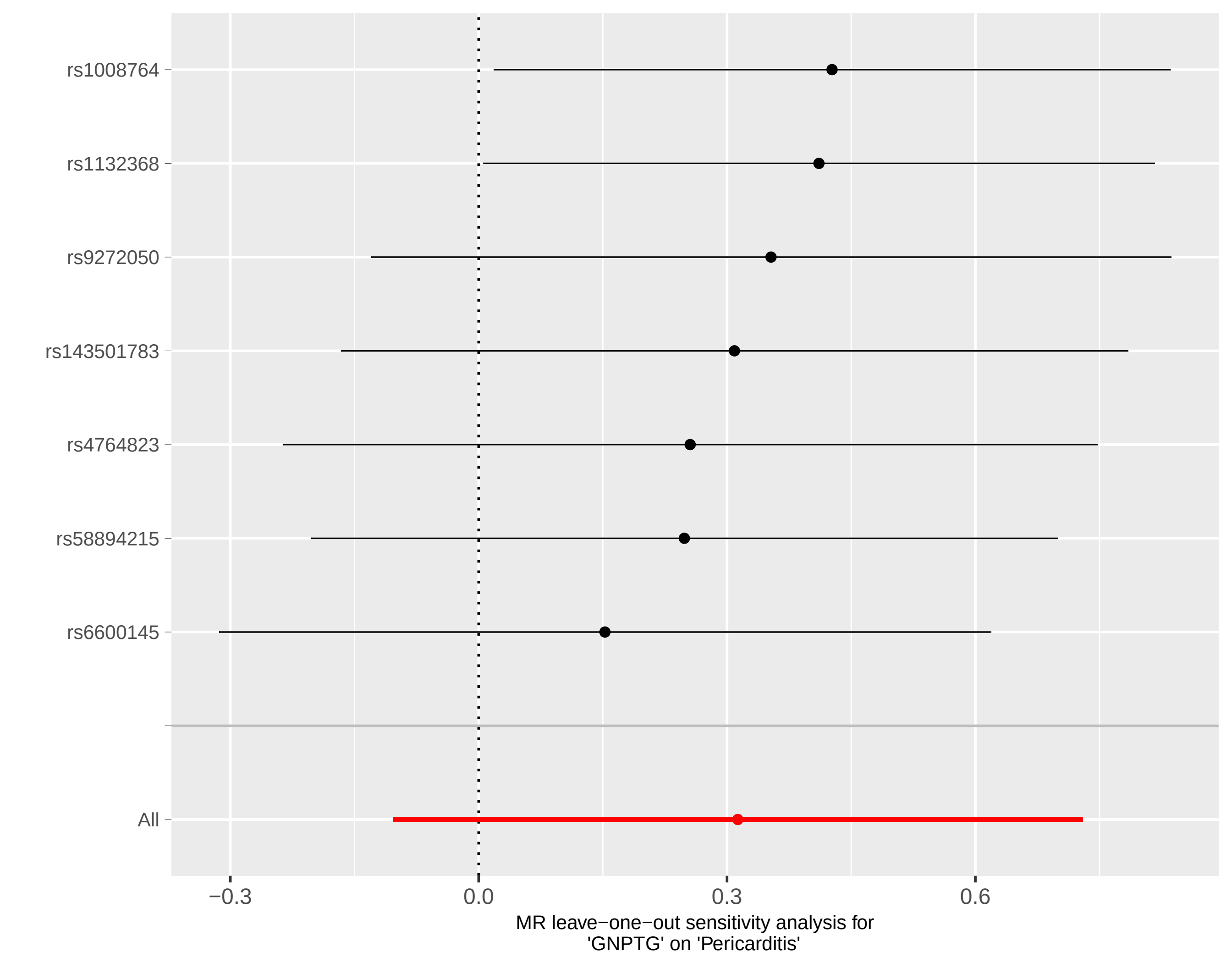
rs1008764
rs1132368
rs9272050
rs143501783
rs4764823
rs58894215
rs6600145
All
−0.3
0.0
0.3
0.6
MR leave−one−out sensitivity analysis for
'GNPTG' on 'Pericarditis'

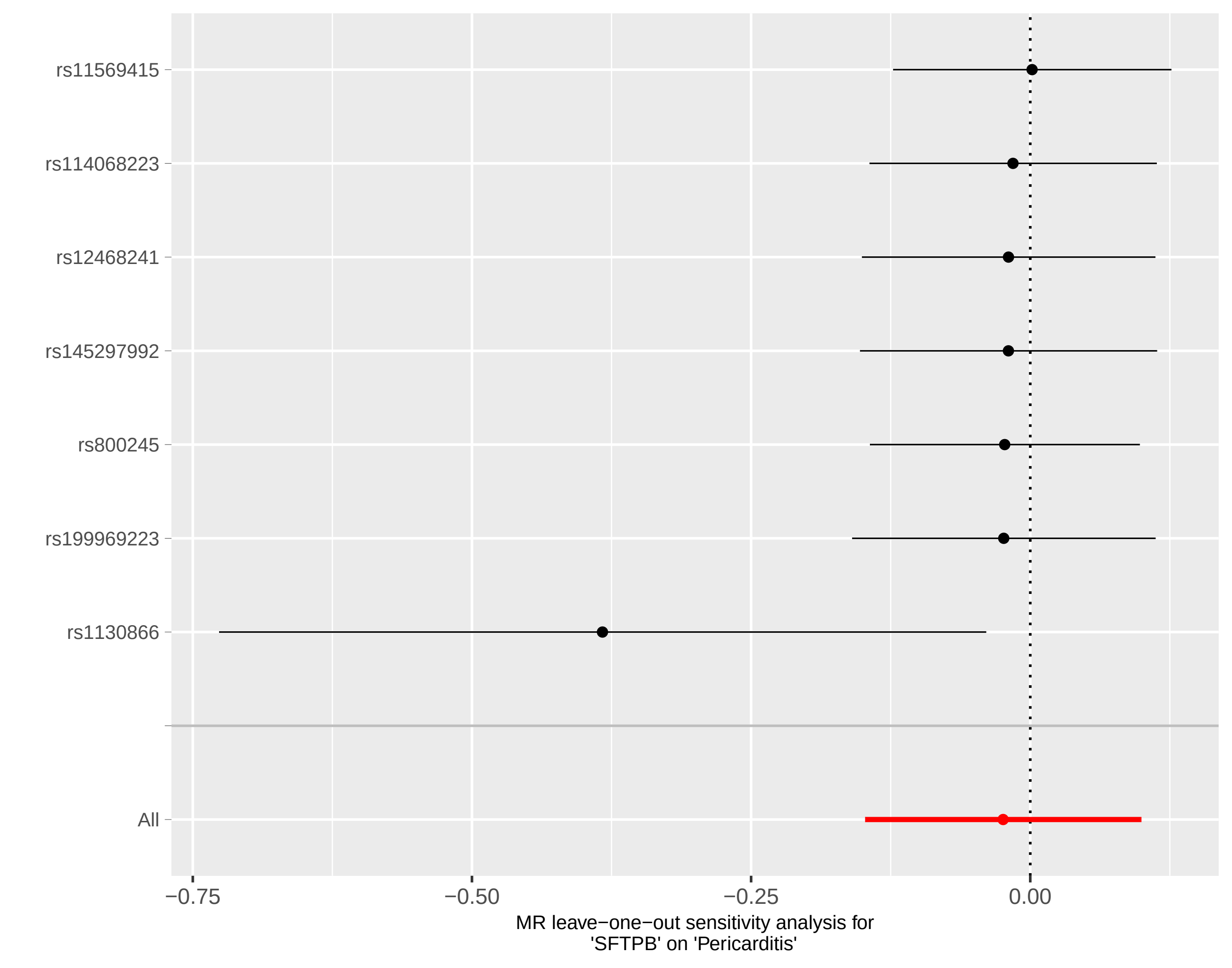
rs11569415
rs114068223
rs12468241
rs145297992
rs800245
rs199969223
rs1130866
All
−0.75
−0.50
−0.25
0.00
MR leave−one−out sensitivity analysis for
'SFTPB' on 'Pericarditis'

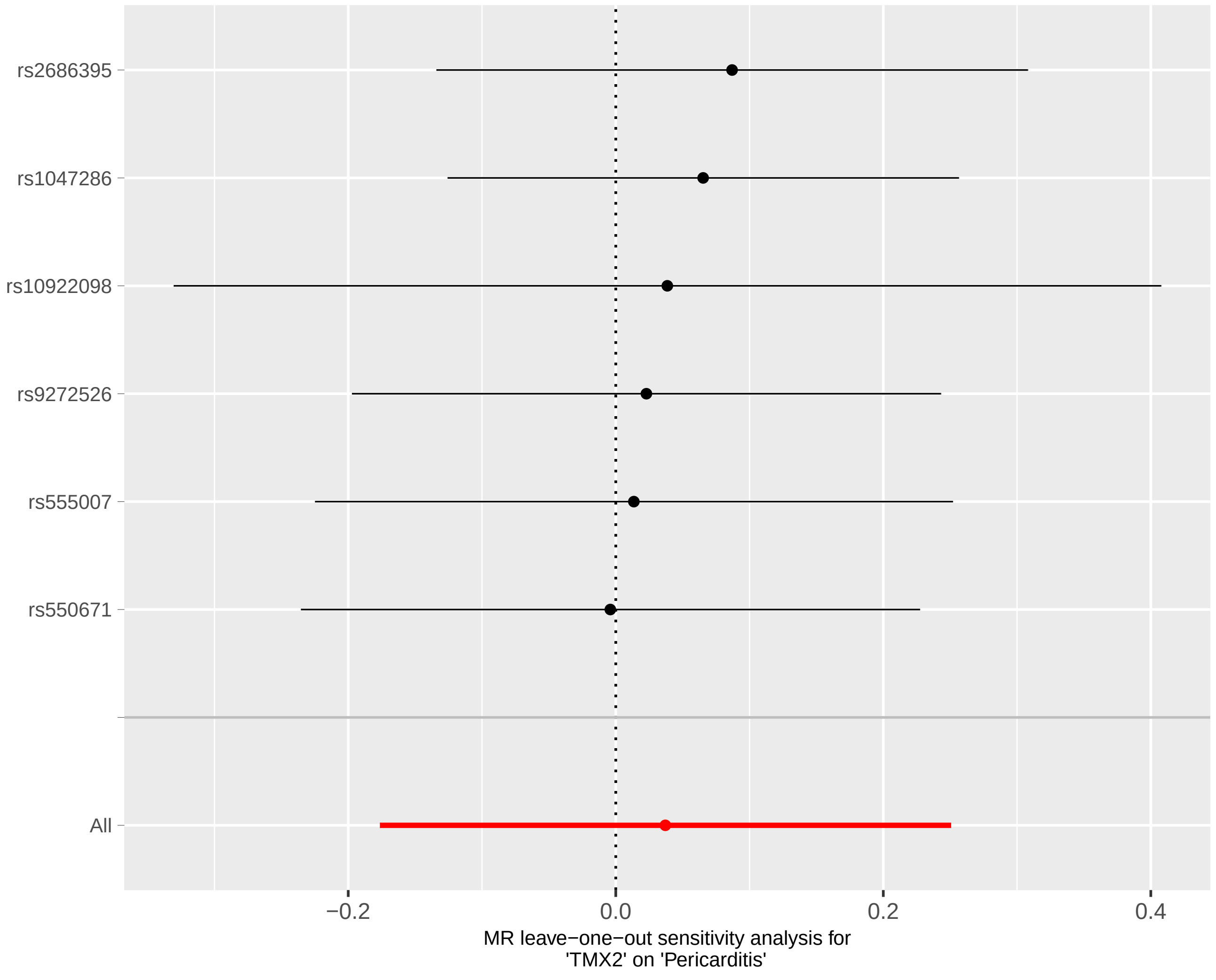

rs2686395
rs1047286
rs10922098
rs9272526
rs555007
rs550671
All
−0.2
0.0
0.2
0.4
MR leave−one−out sensitivity analysis for
'TMX2' on 'Pericarditis'

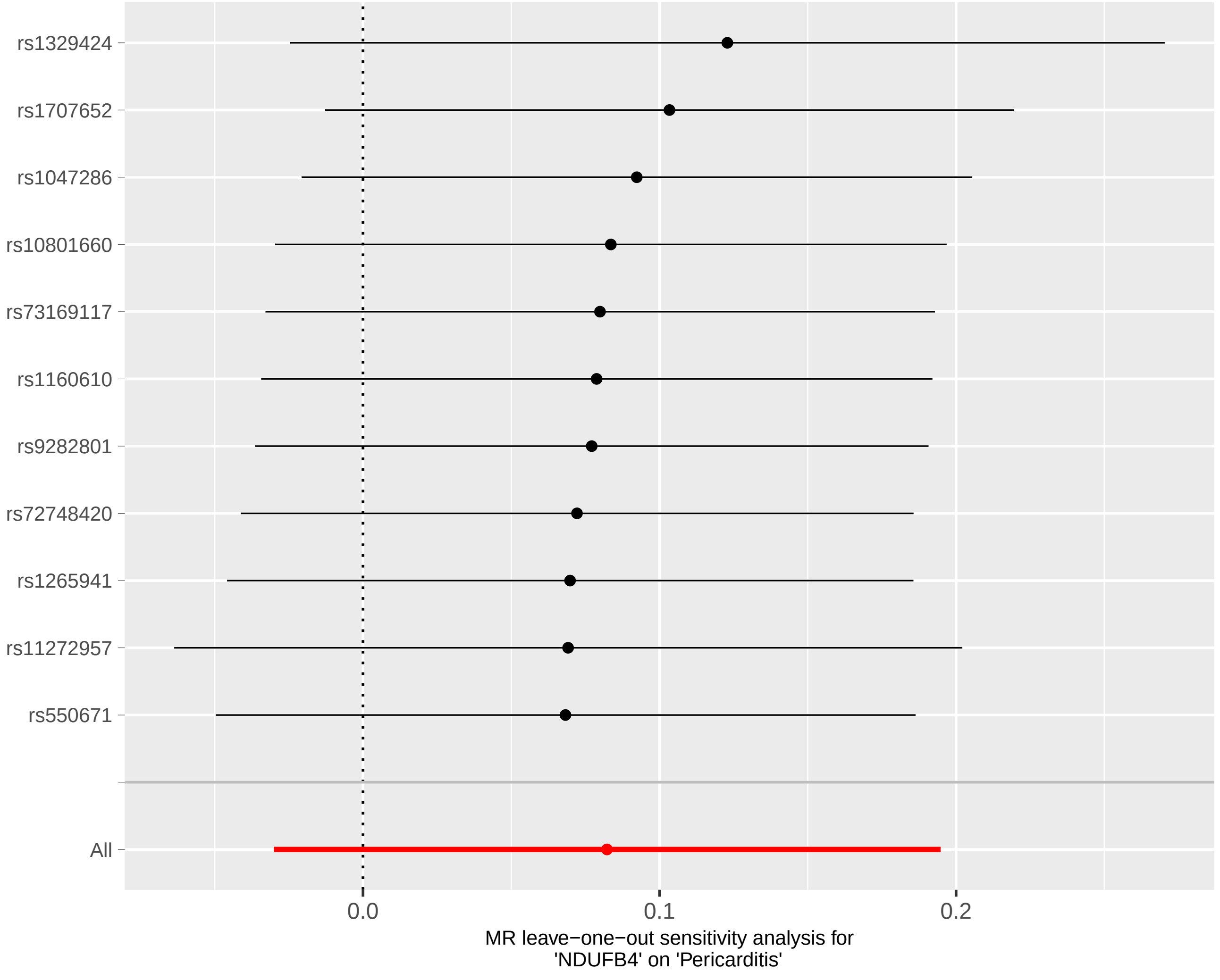
rs1329424
rs1707652
rs1047286
rs10801660
rs73169117
rs1160610
rs9282801
rs72748420
rs1265941
rs11272957
rs550671
All
0.0
0.1
0.2
MR leave−one−out sensitivity analysis for
'NDUFB4' on 'Pericarditis'

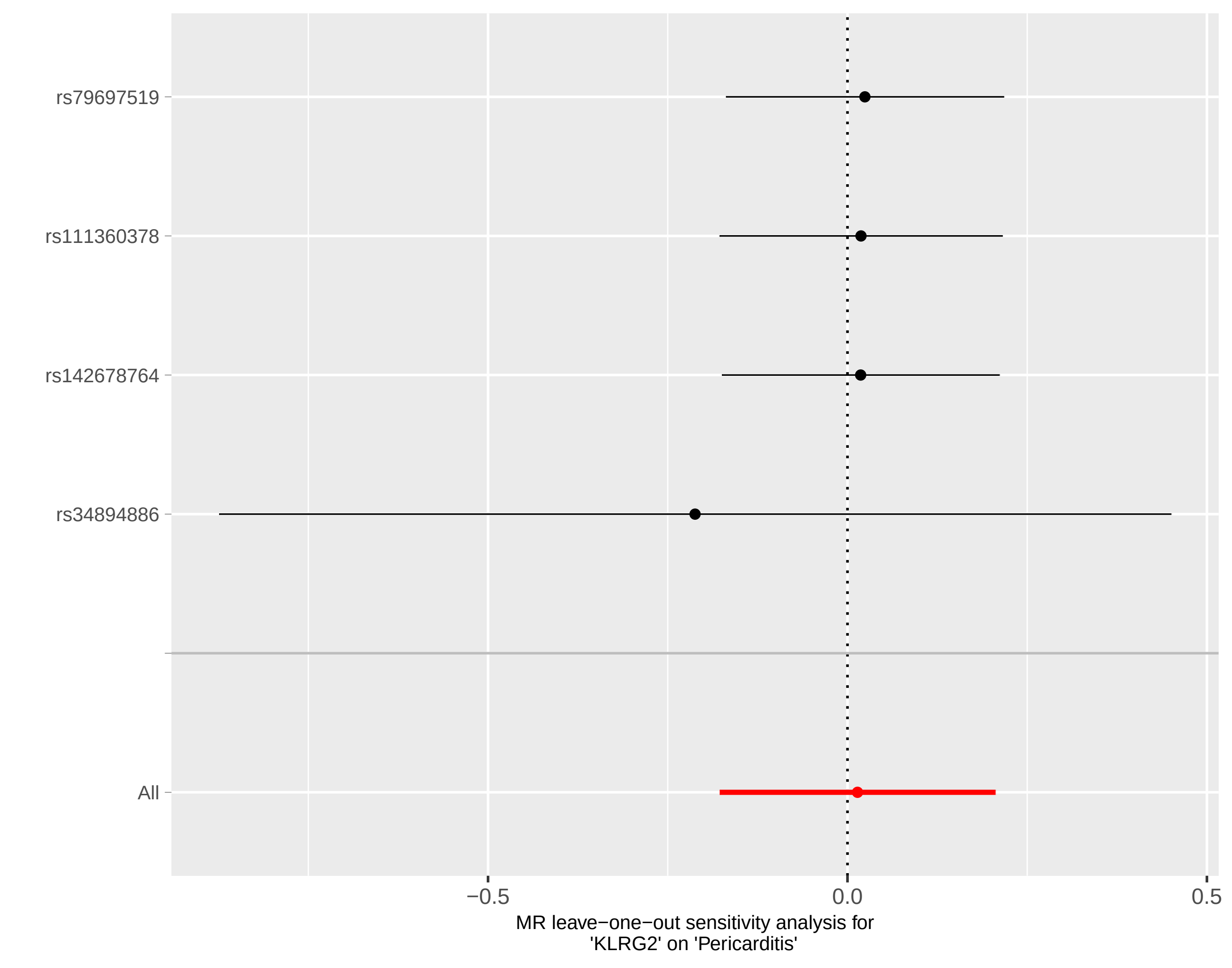
rs79697519
rs111360378
rs142678764
rs34894886
All
−0.5
0.0
0.5
MR leave−one−out sensitivity analysis for
'KLRG2' on 'Pericarditis'

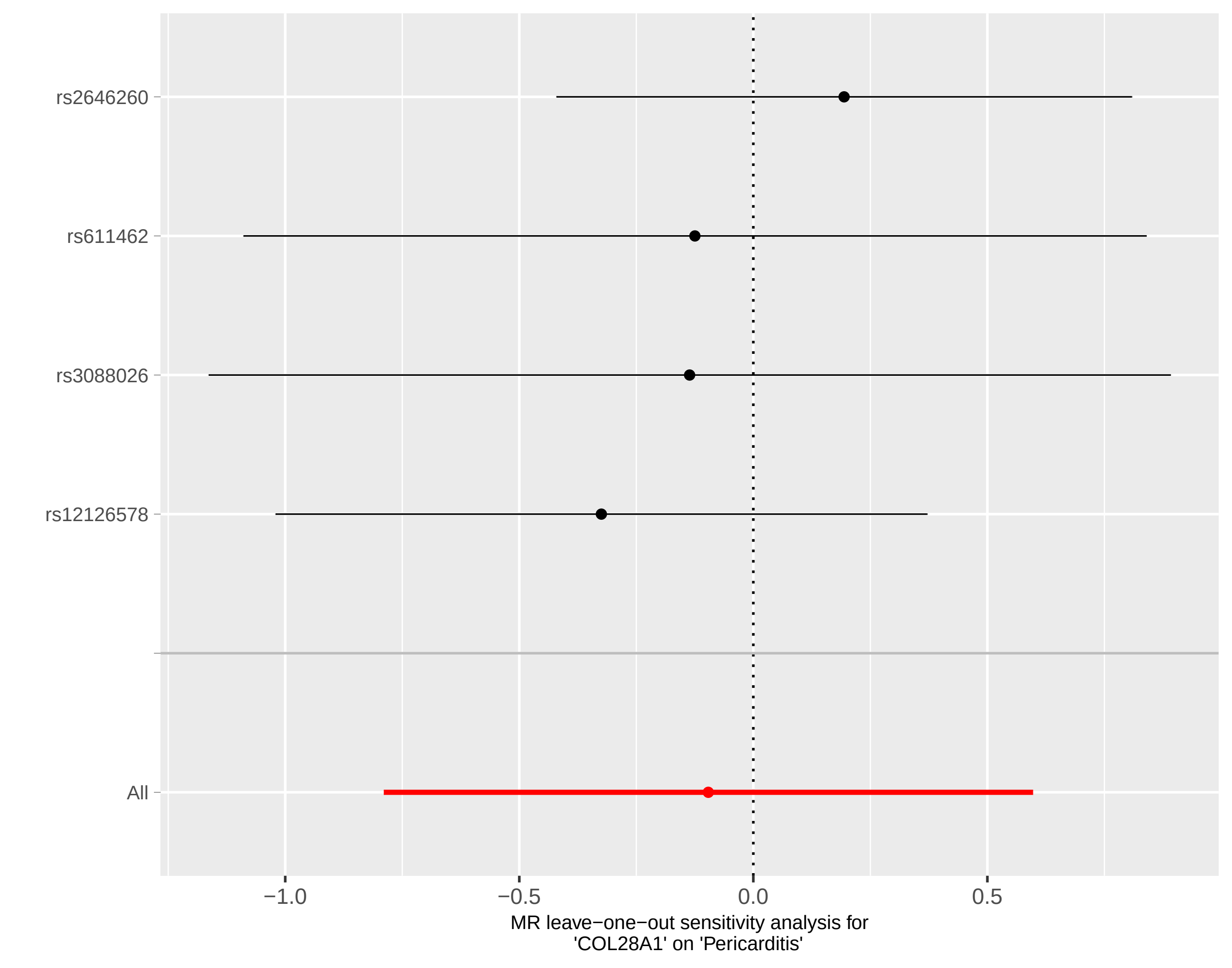
rs2646260
rs611462
rs3088026
rs12126578
All
−1.0
−0.5
0.0
0.5
MR leave−one−out sensitivity analysis for
'COL28A1' on 'Pericarditis'

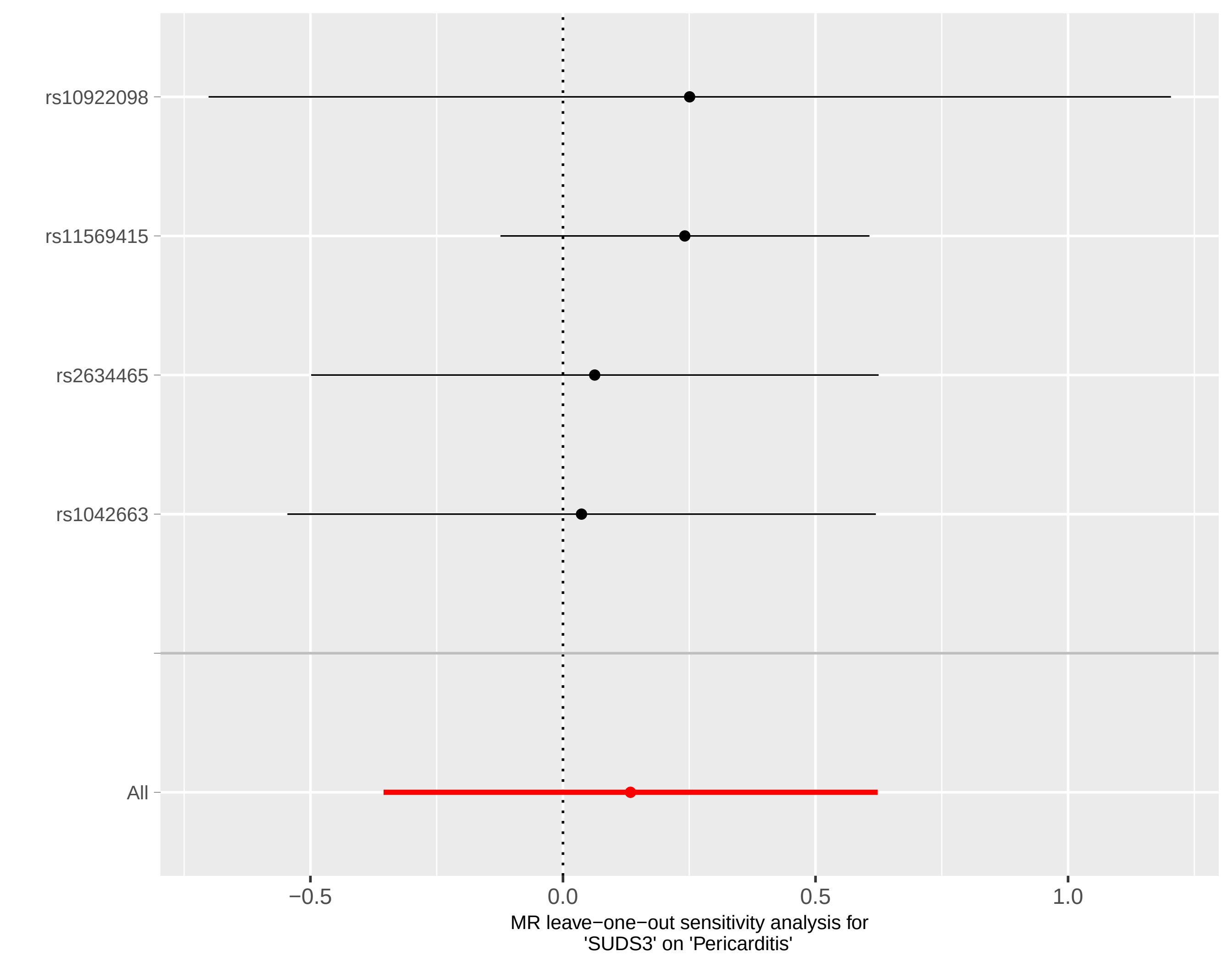

rs10922098
rs11569415
rs2634465
rs1042663
All
−0.5
0.0
0.5
1.0
MR leave−one−out sensitivity analysis for
'SUDS3' on 'Pericarditis'

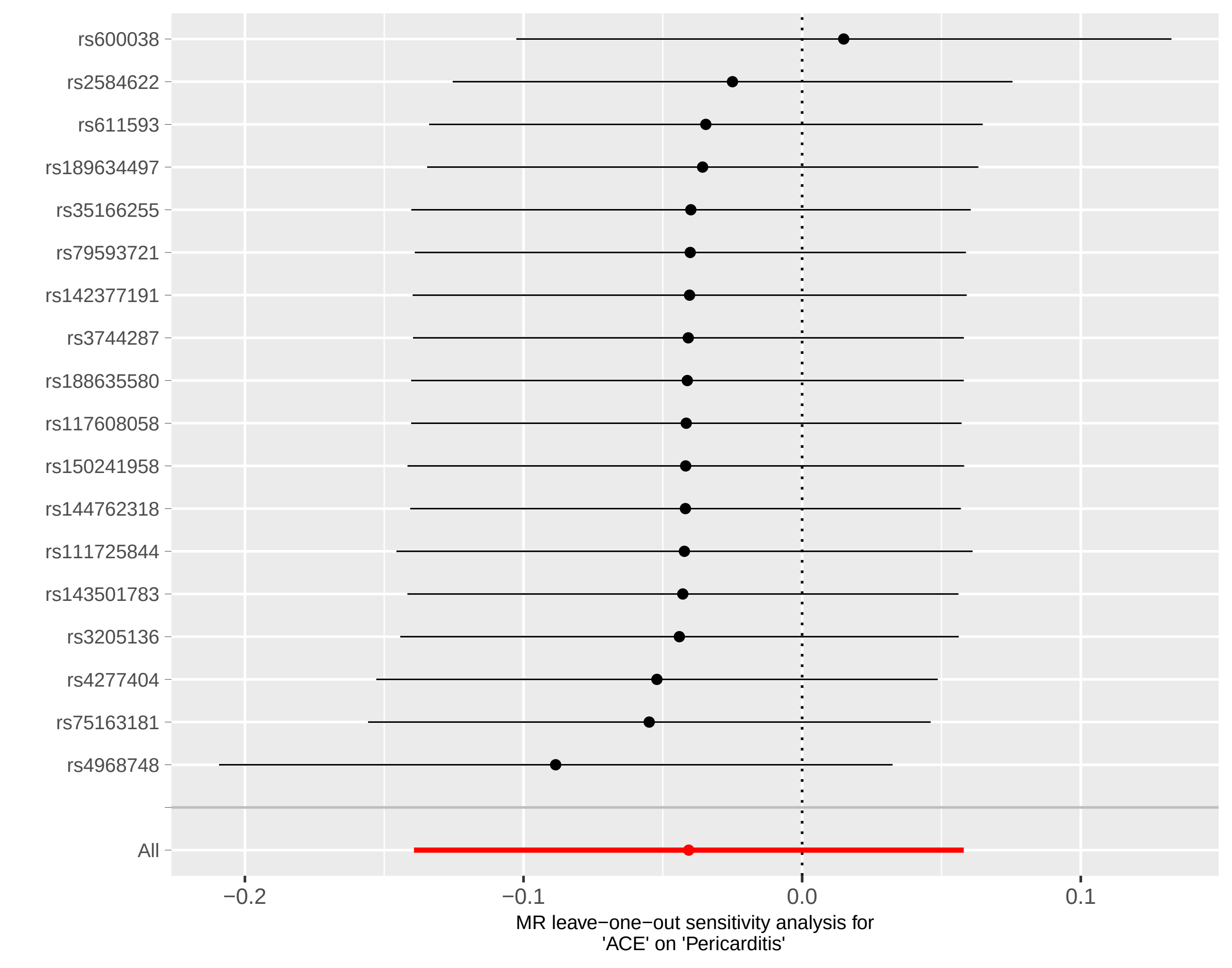
rs600038
rs2584622
rs611593
rs189634497
rs35166255
rs79593721
rs142377191
rs3744287
rs188635580
rs117608058
rs150241958
rs144762318
rs111725844
rs143501783
rs3205136
rs4277404
rs75163181
rs4968748
All
−0.2
−0.1
0.0
0.1
MR leave−one−out sensitivity analysis for
'ACE' on 'Pericarditis'

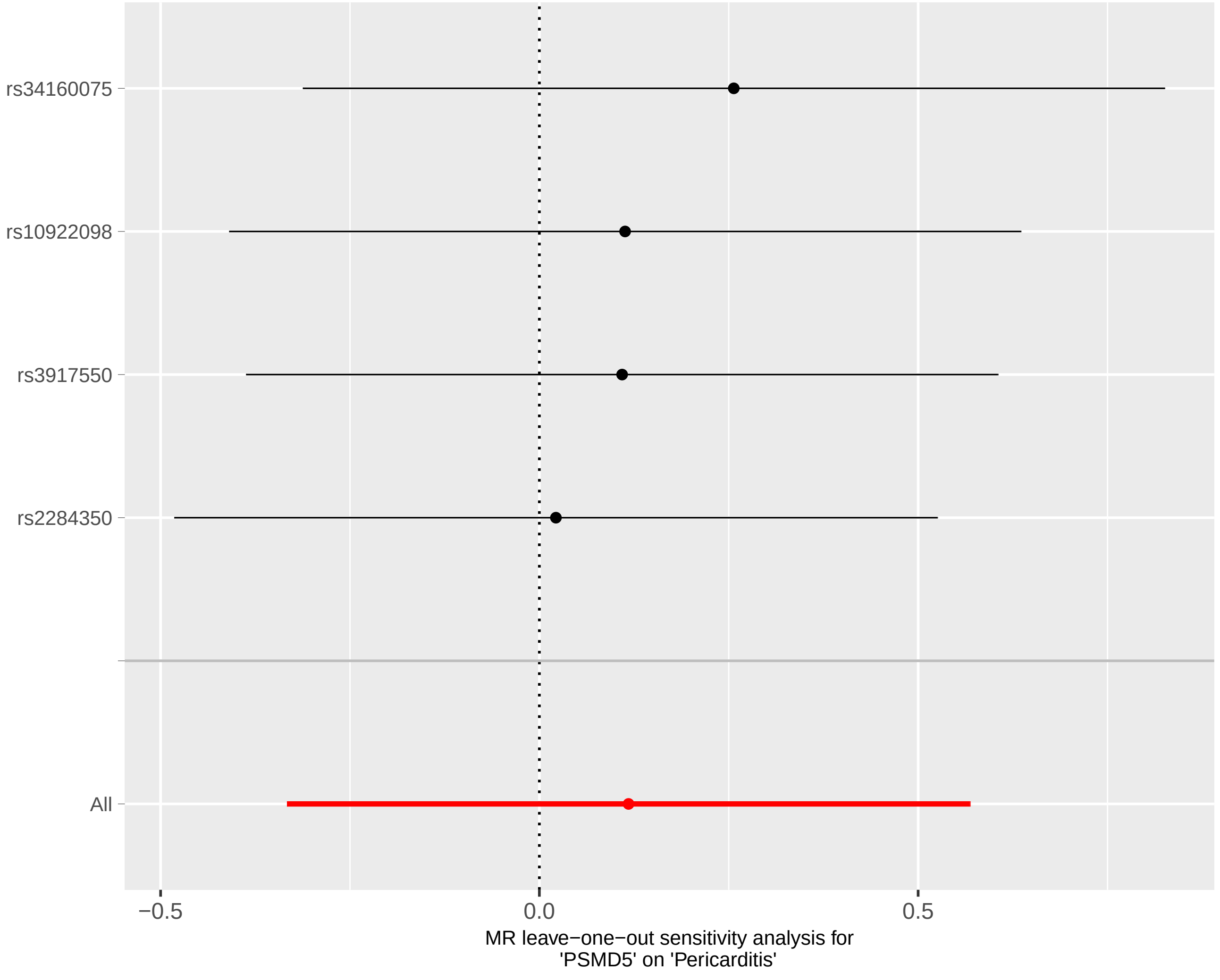

rs34160075
rs10922098
rs3917550
rs2284350
All
−0.5
0.0
0.5
MR leave−one−out sensitivity analysis for
'PSMD5' on 'Pericarditis'

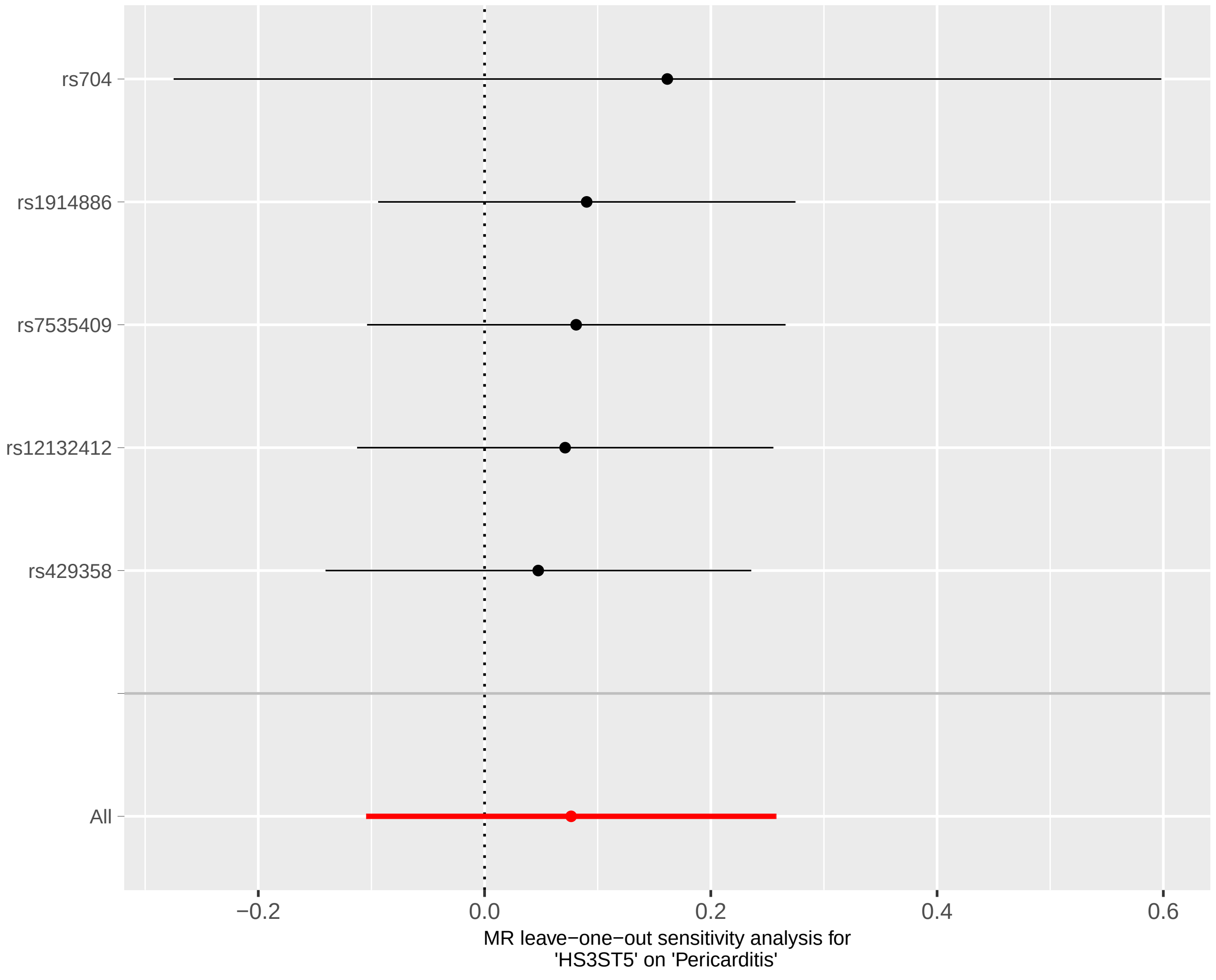

rs704
rs1914886
rs7535409
rs12132412
rs429358
All
−0.2
0.0
0.2
0.4
0.6
MR leave−one−out sensitivity analysis for
'HS3ST5' on 'Pericarditis'

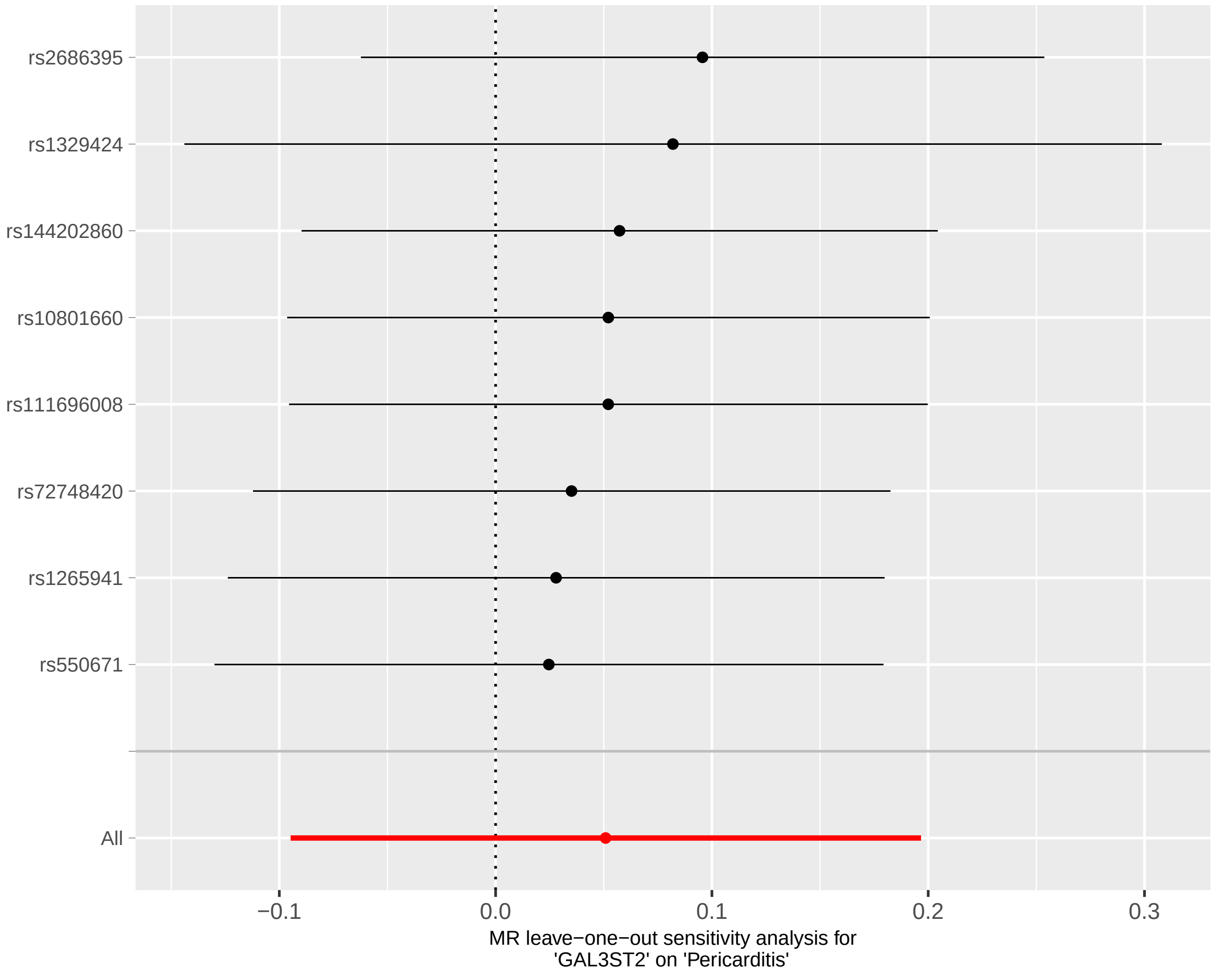
rs2686395
rs1329424
rs144202860
rs10801660
rs111696008
rs72748420
rs1265941
rs550671
All
−0.1
0.0
0.1
0.2
0.3
MR leave−one−out sensitivity analysis for
'GAL3ST2' on 'Pericarditis'

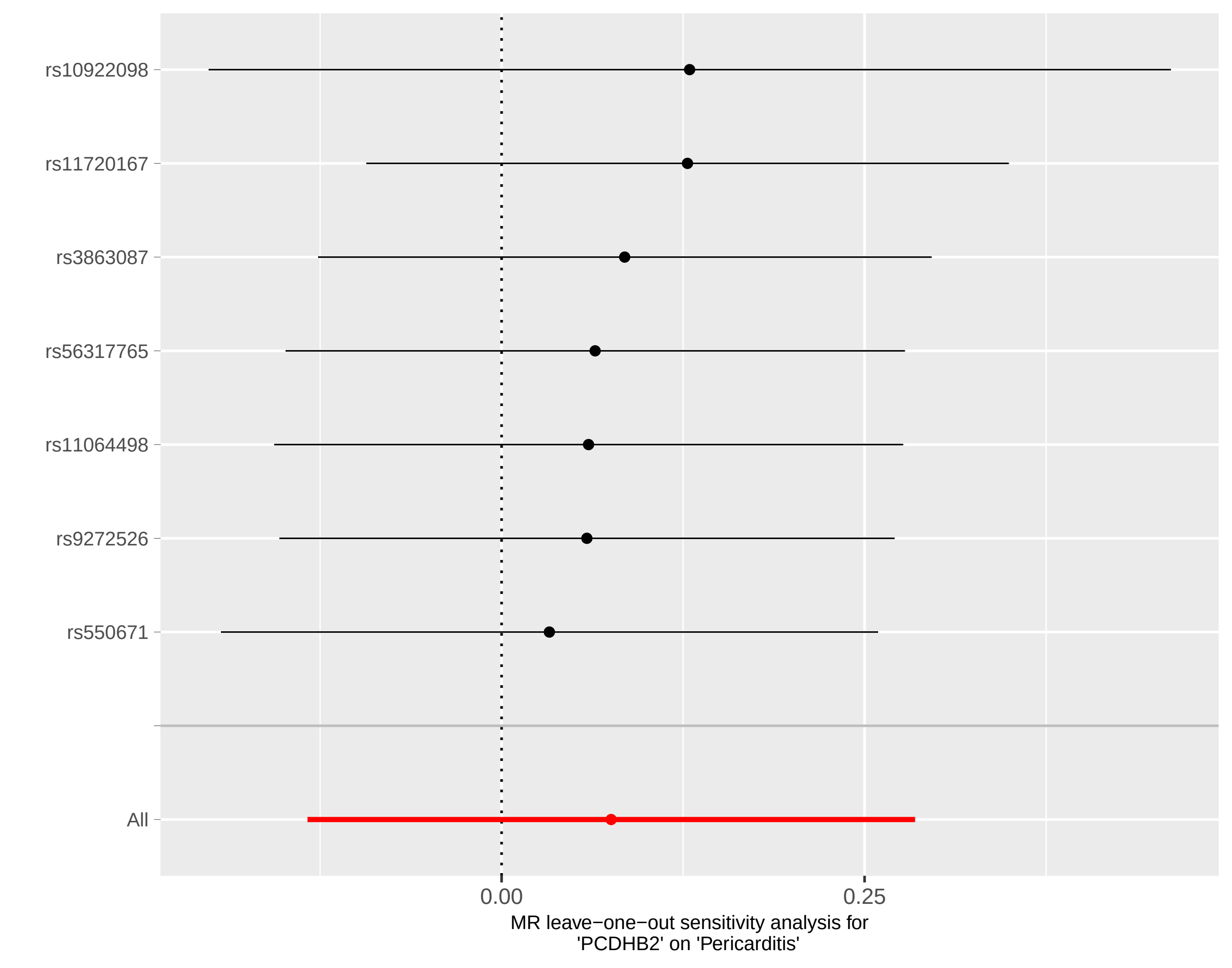

rs10922098
rs11720167
rs3863087
rs56317765
rs11064498
rs9272526
rs550671
All
0.00
0.25
MR leave−one−out sensitivity analysis for
'PCDHB2' on 'Pericarditis'

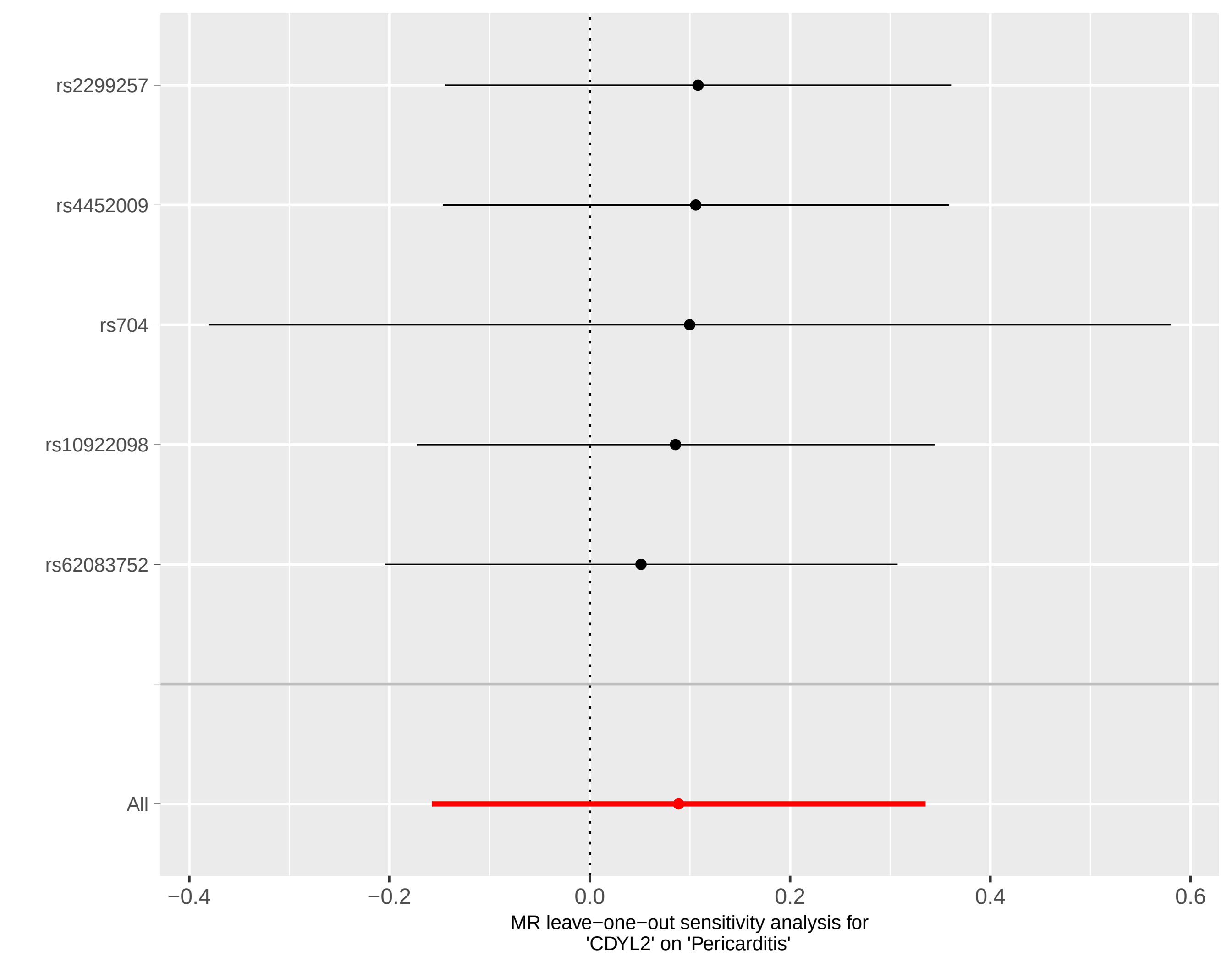
rs2299257
rs4452009
rs704
rs10922098
rs62083752
All
−0.4
−0.2
0.0
0.2
0.4
0.6
MR leave−one−out sensitivity analysis for
'CDYL2' on 'Pericarditis'

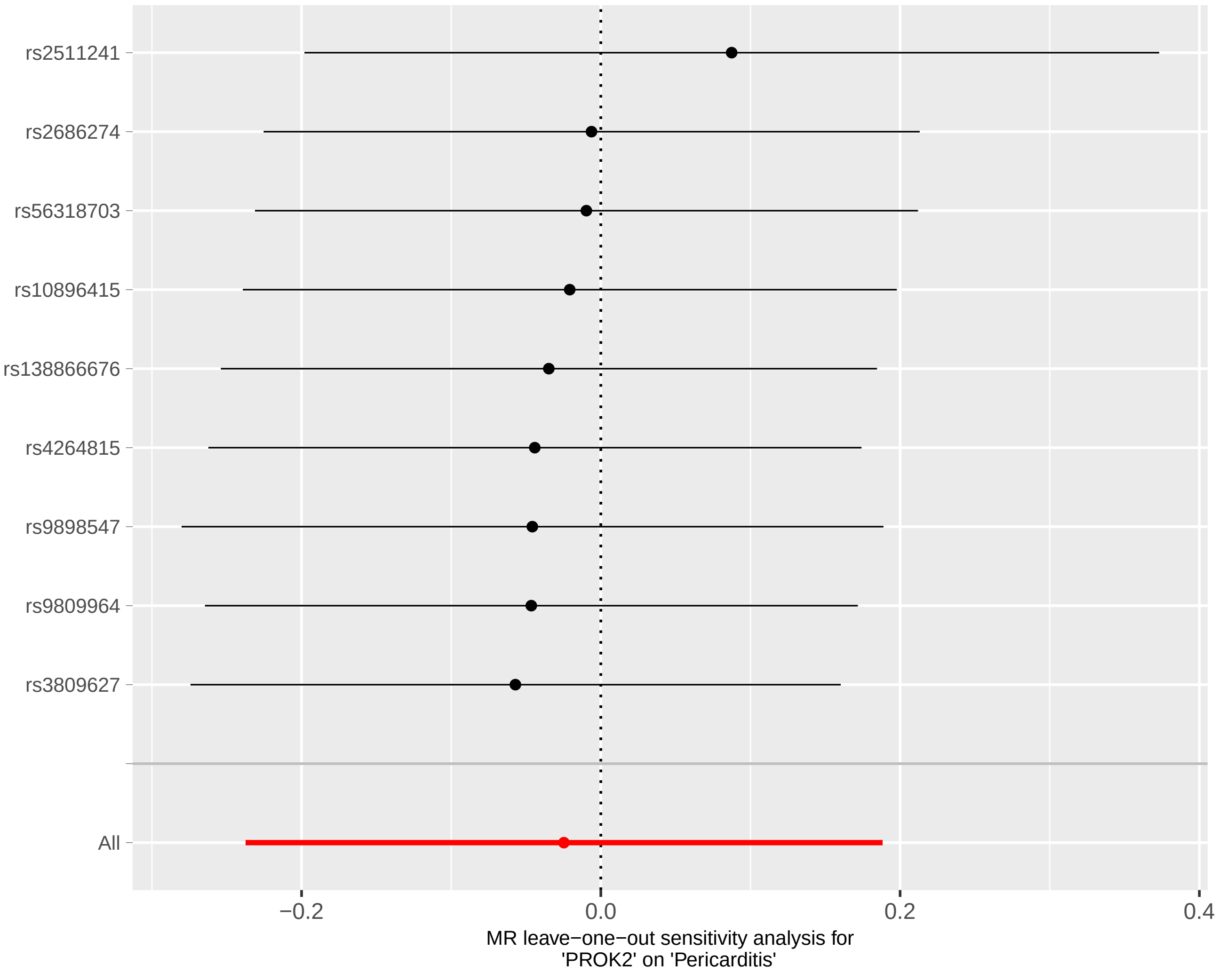
rs2511241
rs2686274
rs56318703
rs10896415
rs138866676
rs4264815
rs9898547
rs9809964
rs3809627
All
−0.2
0.0
0.2
0.4
MR leave−one−out sensitivity analysis for
'PROK2' on 'Pericarditis'

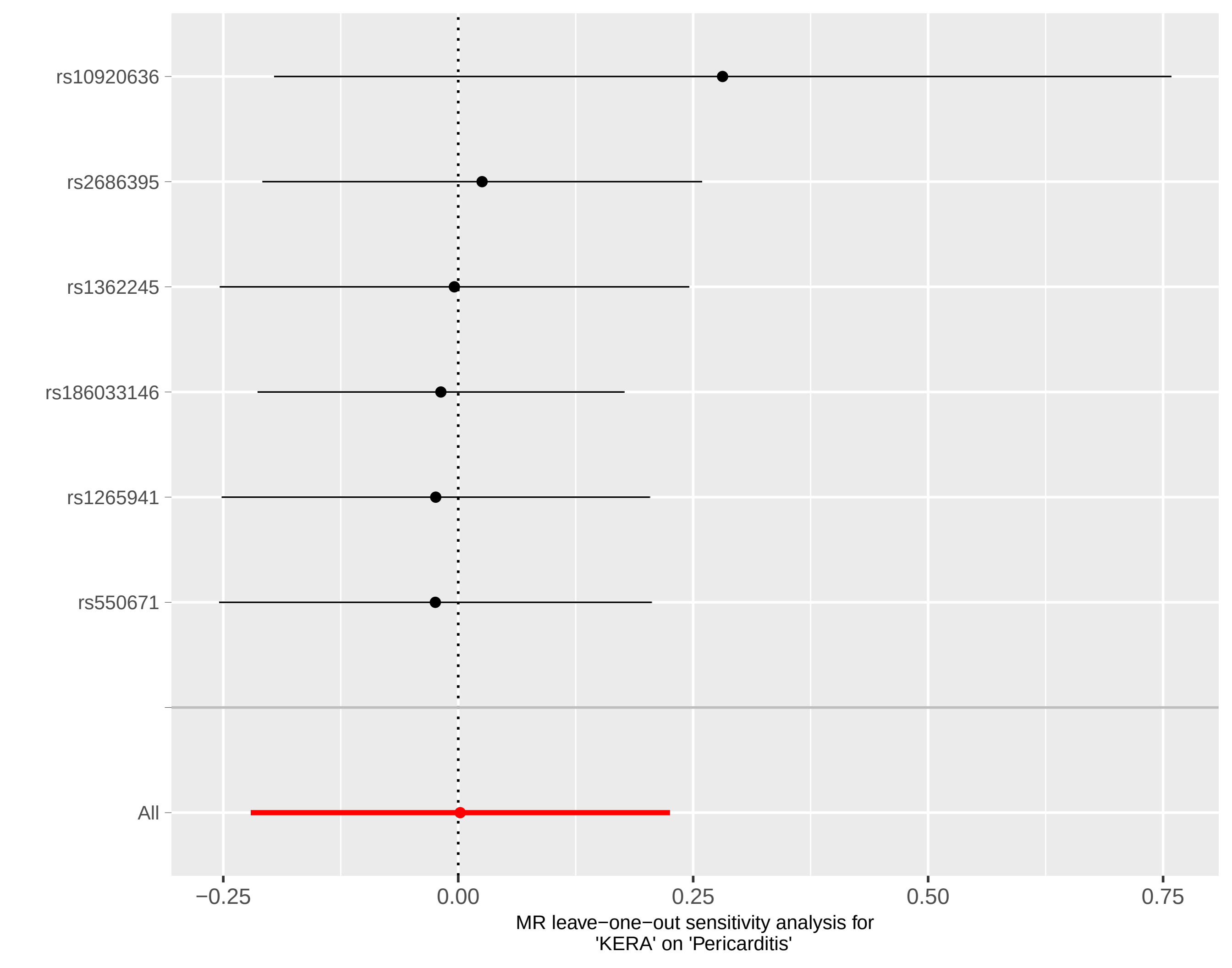
rs10920636
rs2686395
rs1362245
rs186033146
rs1265941
rs550671
All
−0.25
0.00
0.25
0.50
0.75
MR leave−one−out sensitivity analysis for
'KERA' on 'Pericarditis'

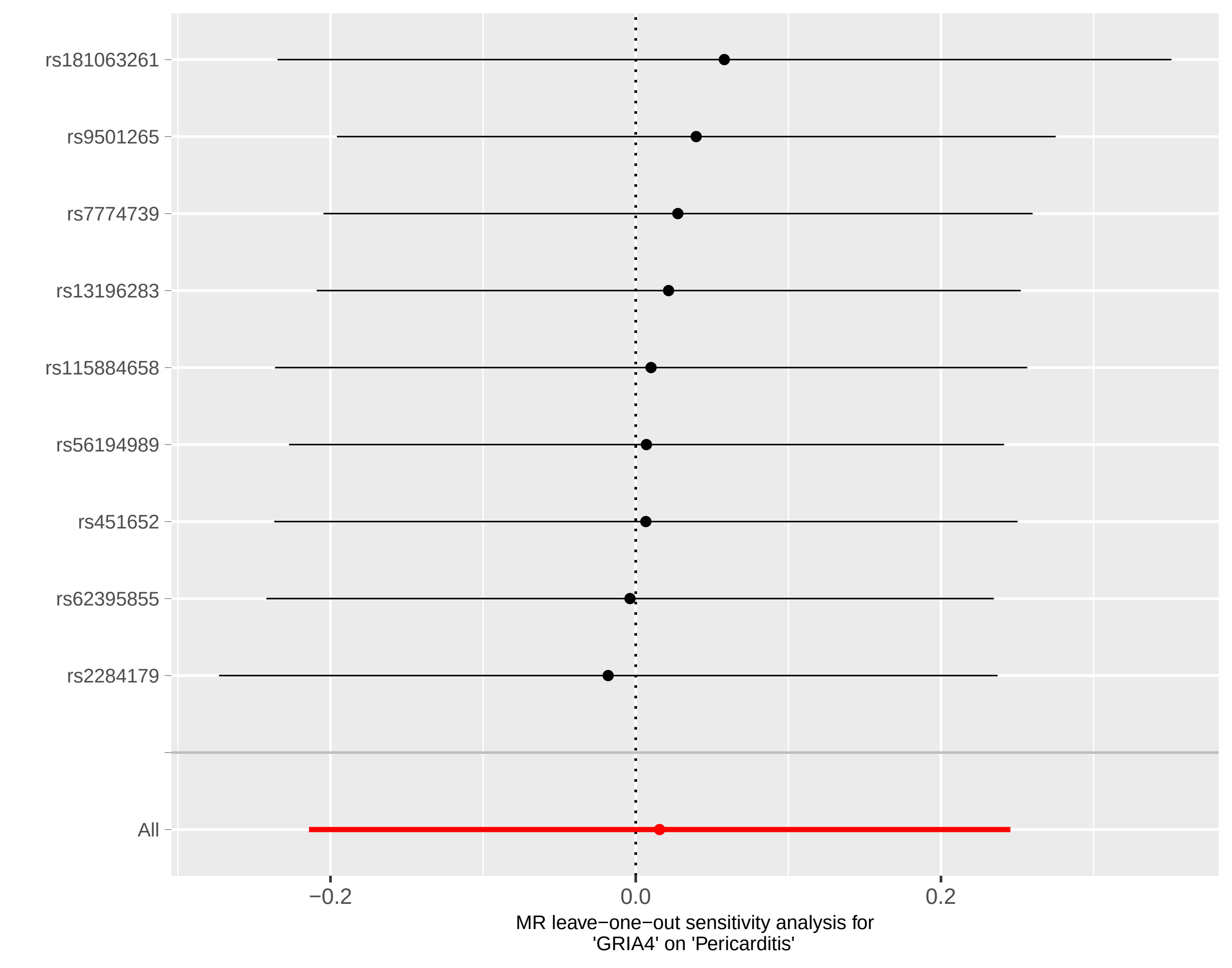
rs181063261
rs9501265
rs7774739
rs13196283
rs115884658
rs56194989
rs451652
rs62395855
rs2284179
All
−0.2
0.0
0.2
MR leave−one−out sensitivity analysis for
'GRIA4' on 'Pericarditis'

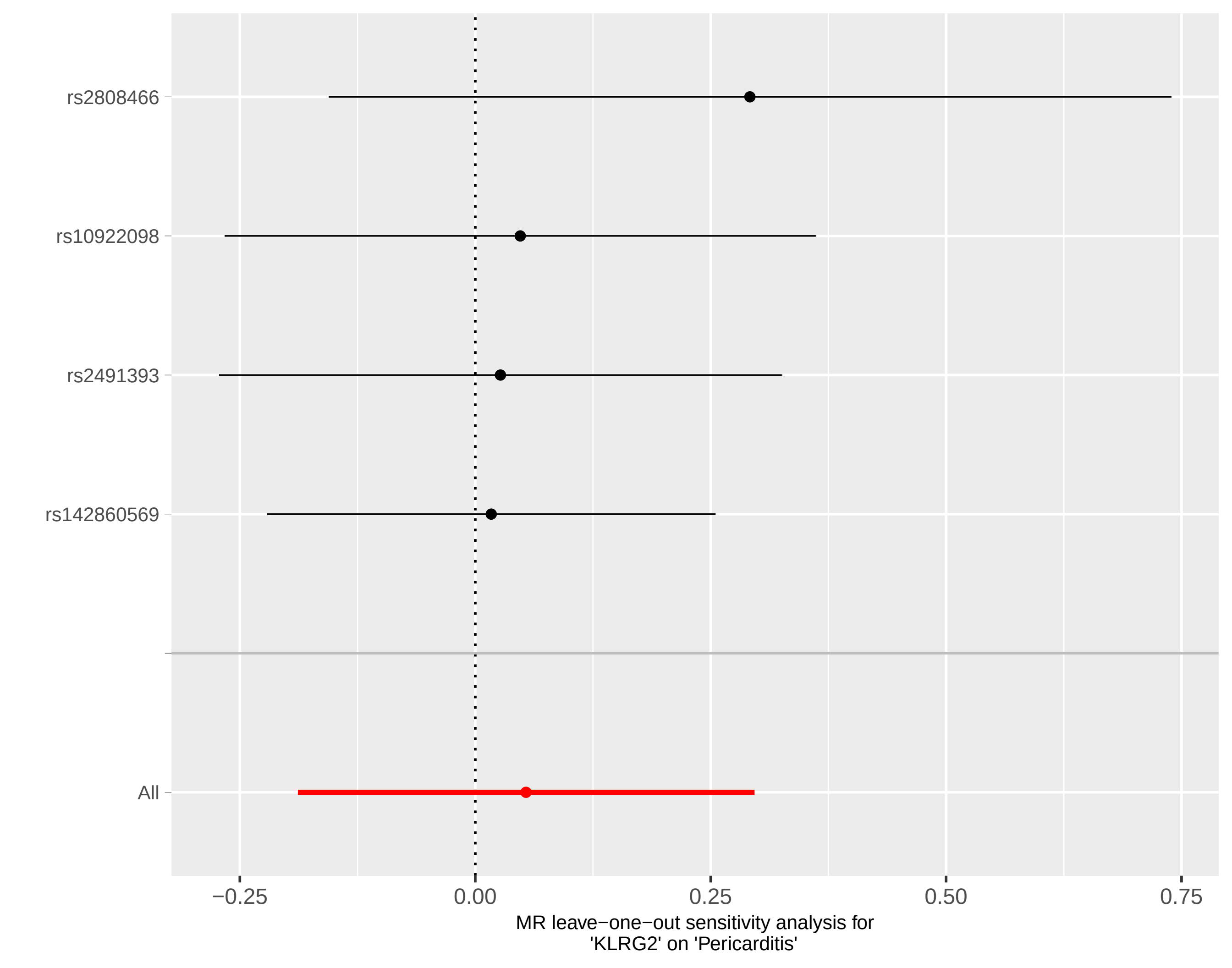
rs2808466
rs10922098
rs2491393
rs142860569
All
−0.25
0.00
0.25
0.50
0.75
MR leave−one−out sensitivity analysis for
'KLRG2' on 'Pericarditis'

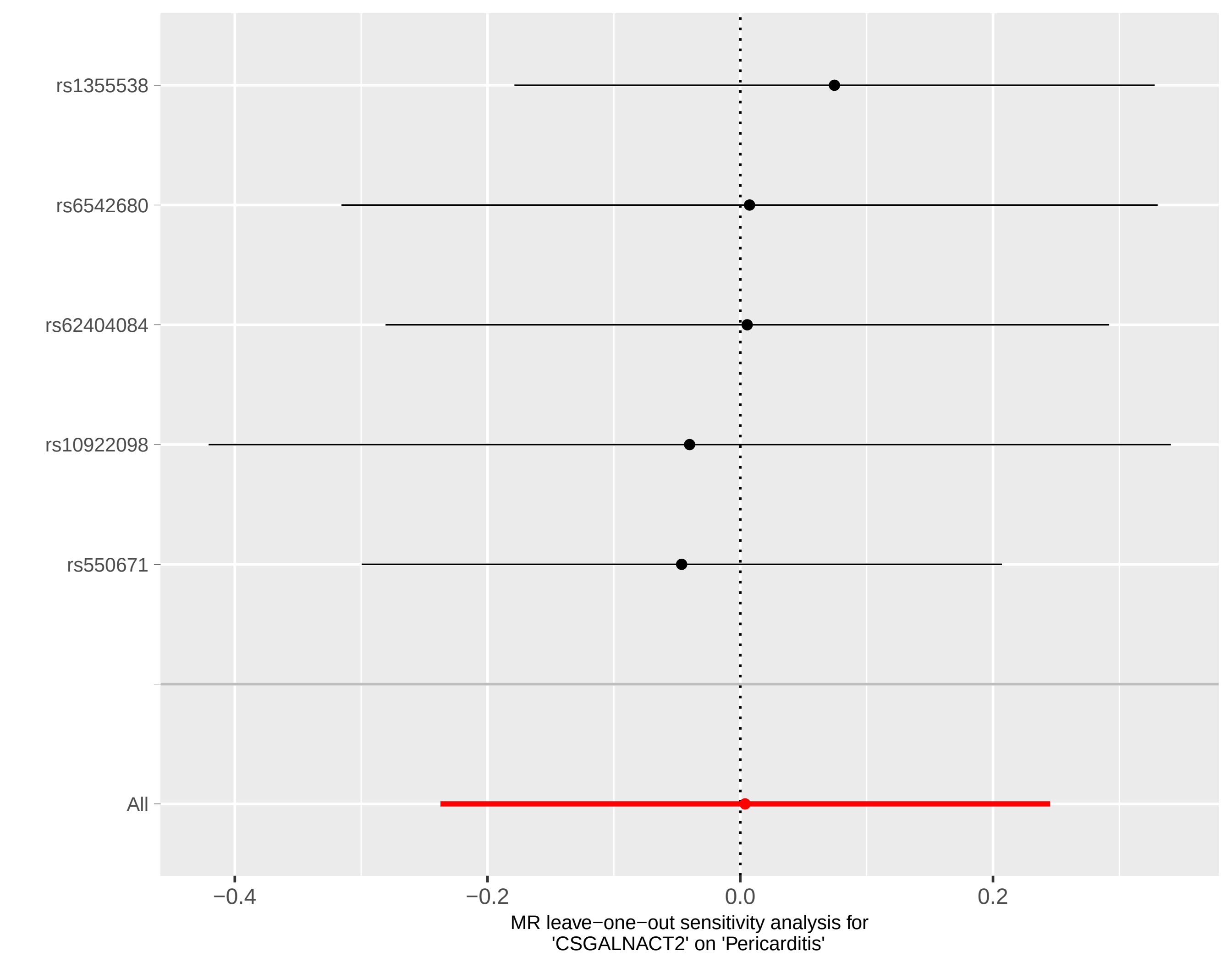

rs1355538
rs6542680
rs62404084
rs10922098
rs550671
All
−0.4
−0.2
0.0
0.2
MR leave−one−out sensitivity analysis for
'CSGALNACT2' on 'Pericarditis'

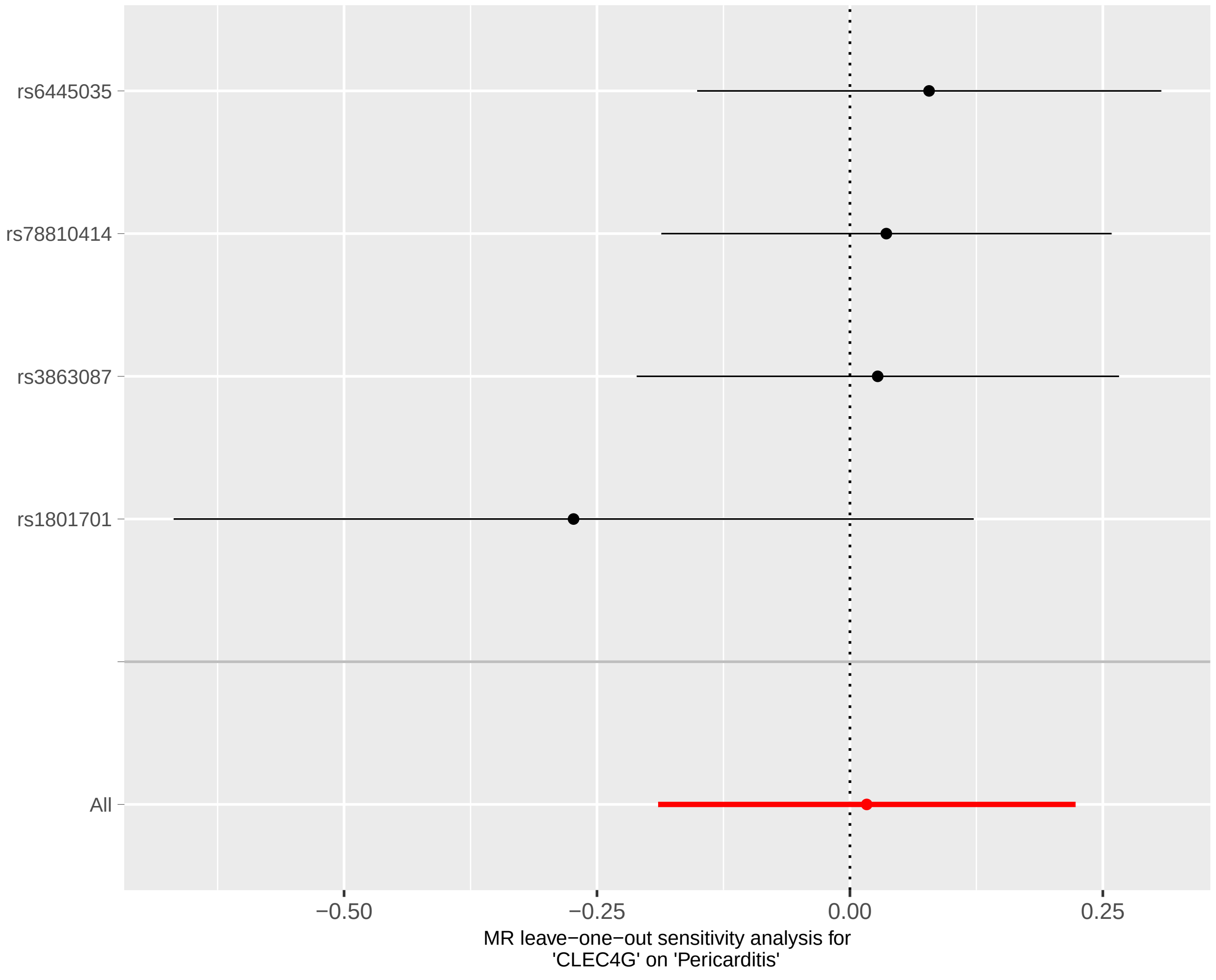

rs6445035
rs78810414
rs3863087
rs1801701
All
−0.50
−0.25
0.00
0.25
MR leave−one−out sensitivity analysis for
'CLEC4G' on 'Pericarditis'

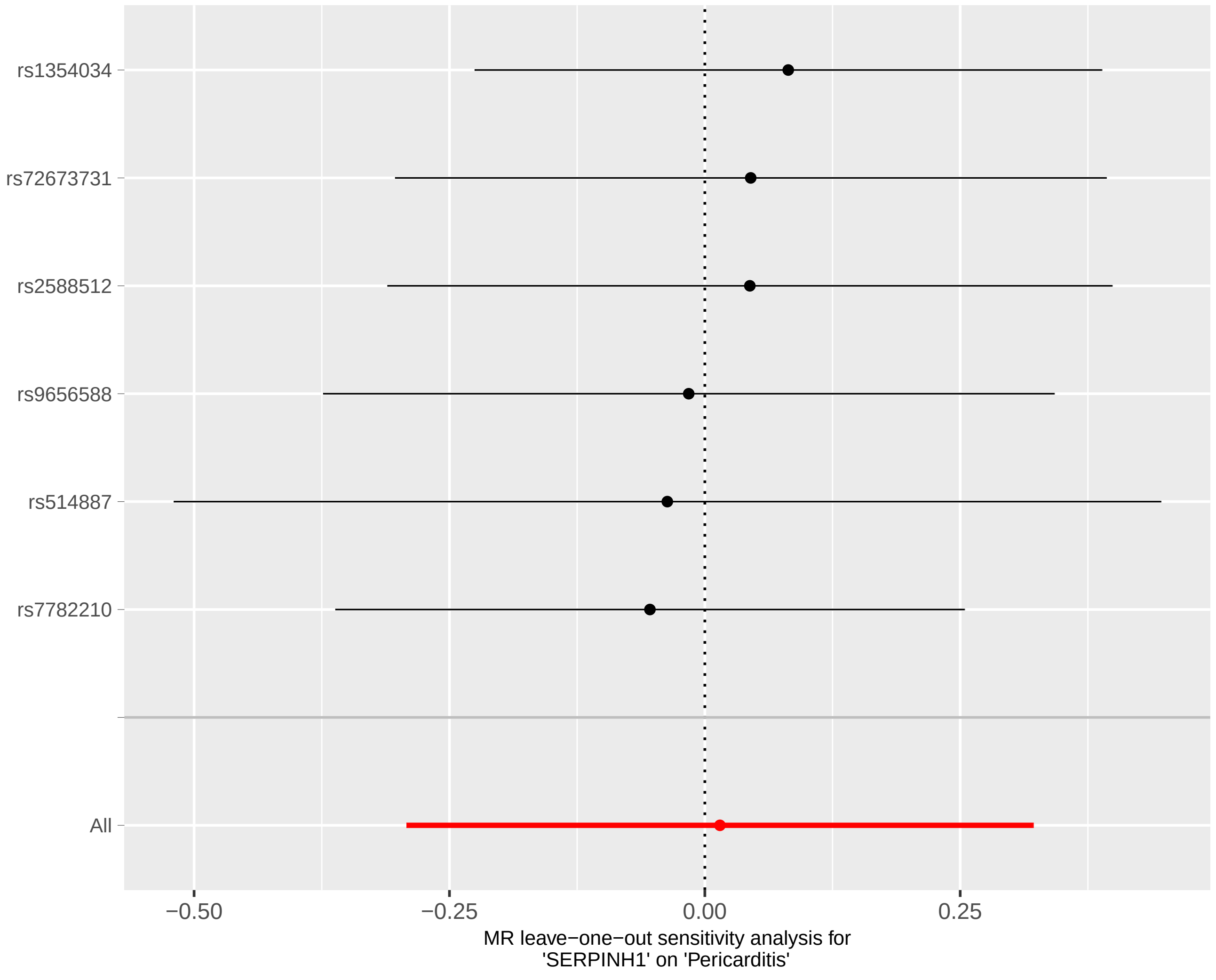
rs1354034
rs72673731
rs2588512
rs9656588
rs514887
rs7782210
All
−0.50
−0.25
0.00
0.25
MR leave−one−out sensitivity analysis for
'SERPINH1' on 'Pericarditis'

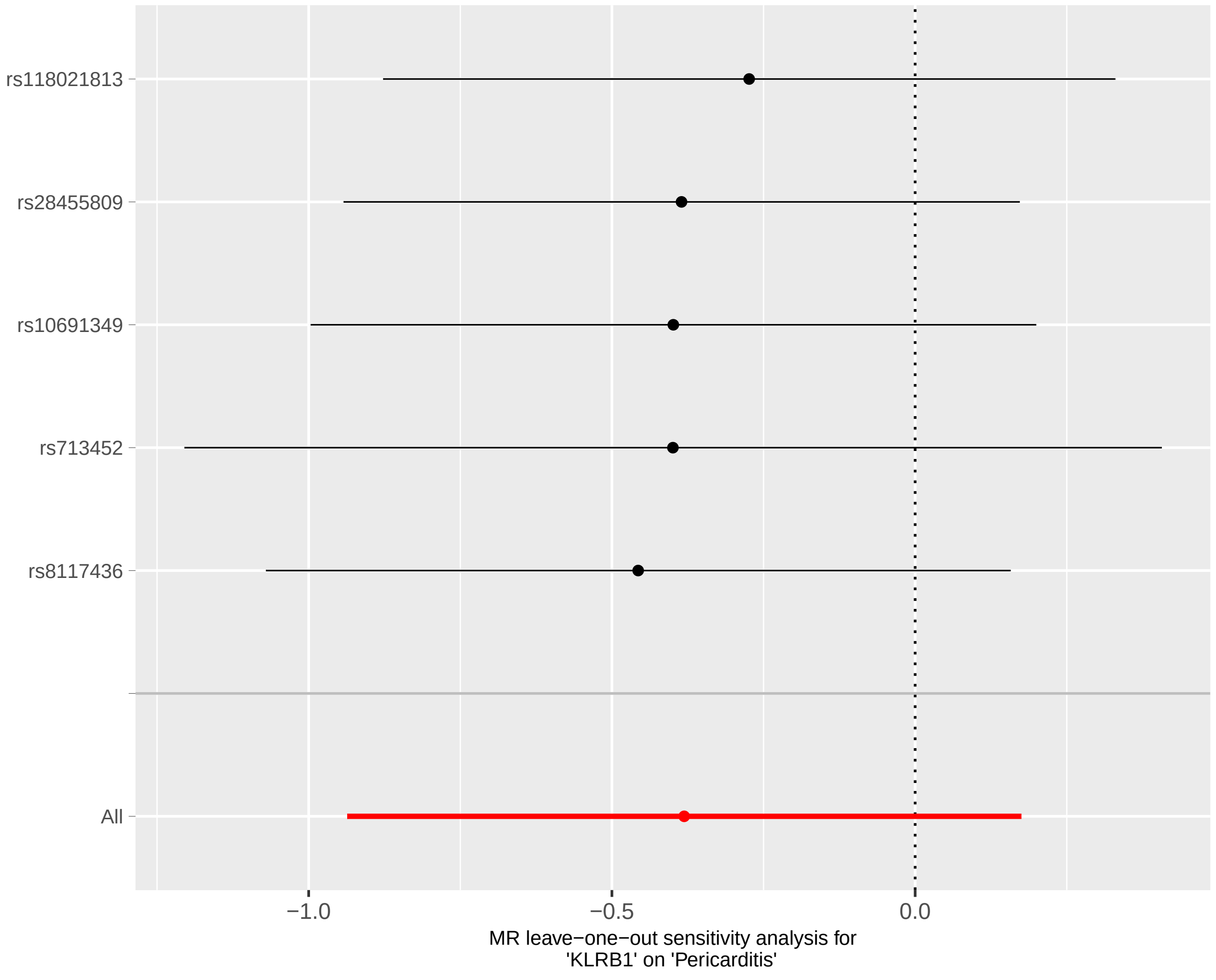
rs118021813
rs28455809
rs10691349
rs713452
rs8117436
All
−1.0
−0.5
0.0
MR leave−one−out sensitivity analysis for
'KLRB1' on 'Pericarditis'

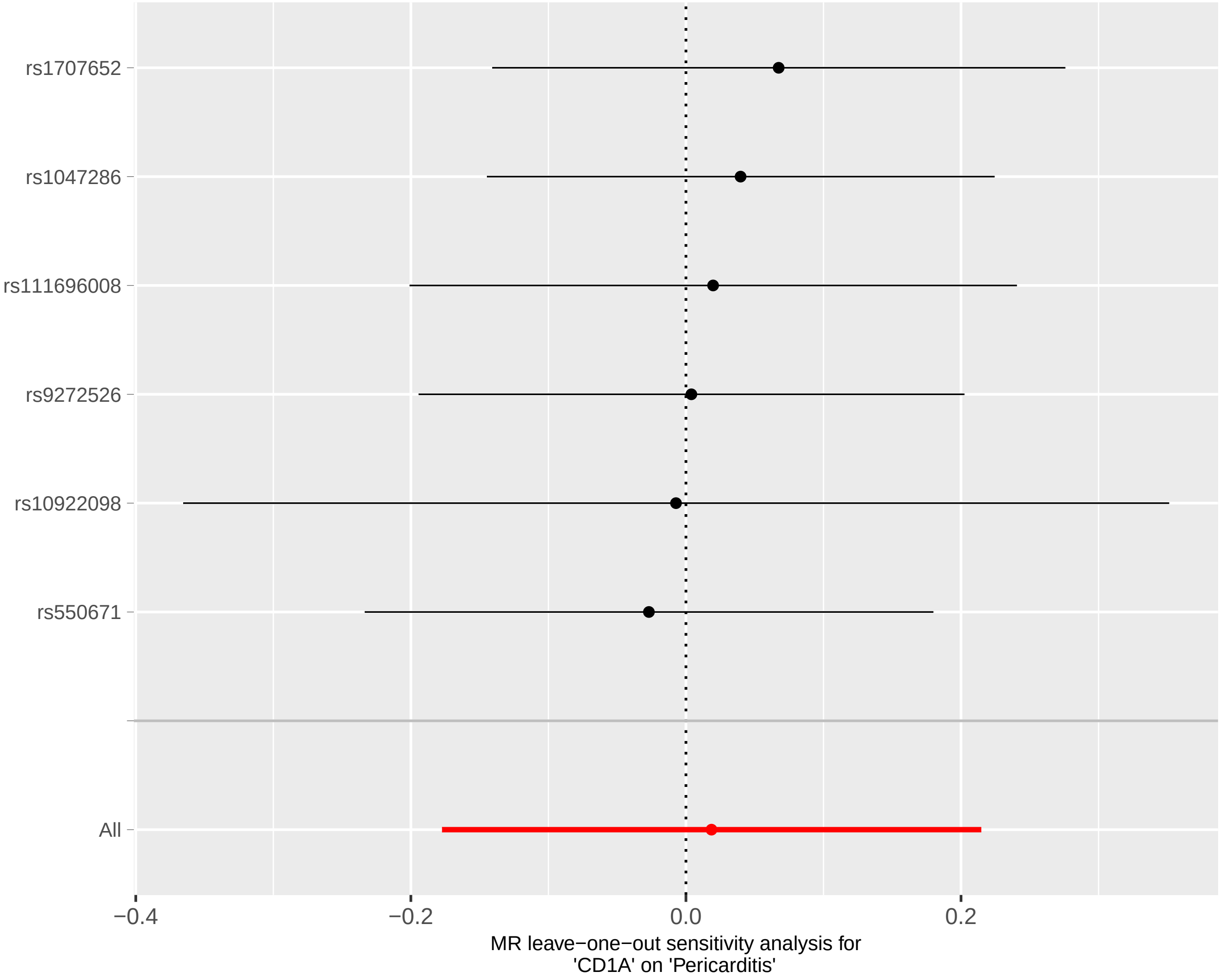

rs1707652
rs1047286
rs111696008
rs9272526
rs10922098
rs550671
All
−0.4
−0.2
0.0
0.2
MR leave−one−out sensitivity analysis for
'CD1A' on 'Pericarditis'

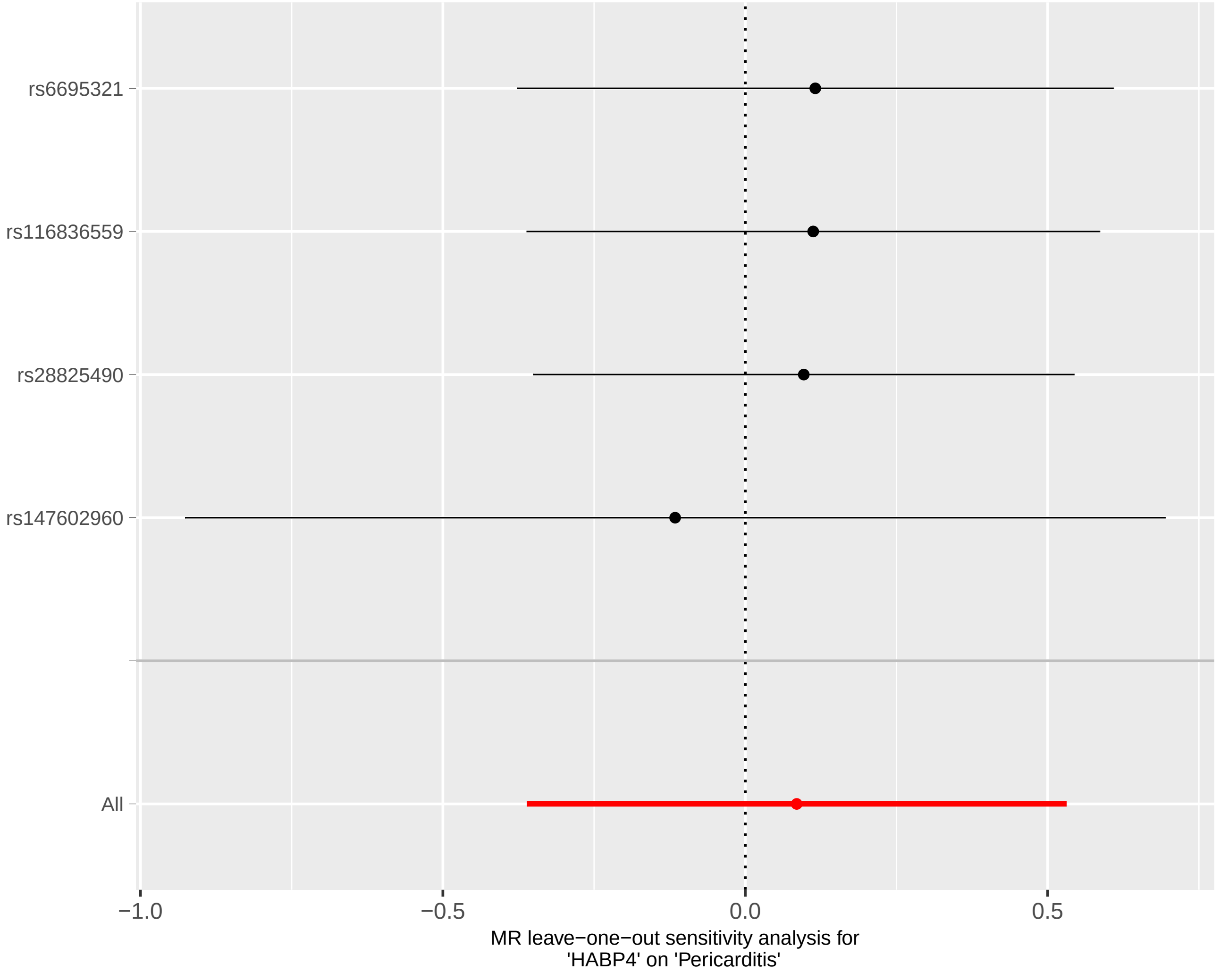

rs6695321
rs116836559
rs28825490
rs147602960
All
−1.0
−0.5
0.0
0.5
MR leave−one−out sensitivity analysis for
'HABP4' on 'Pericarditis'

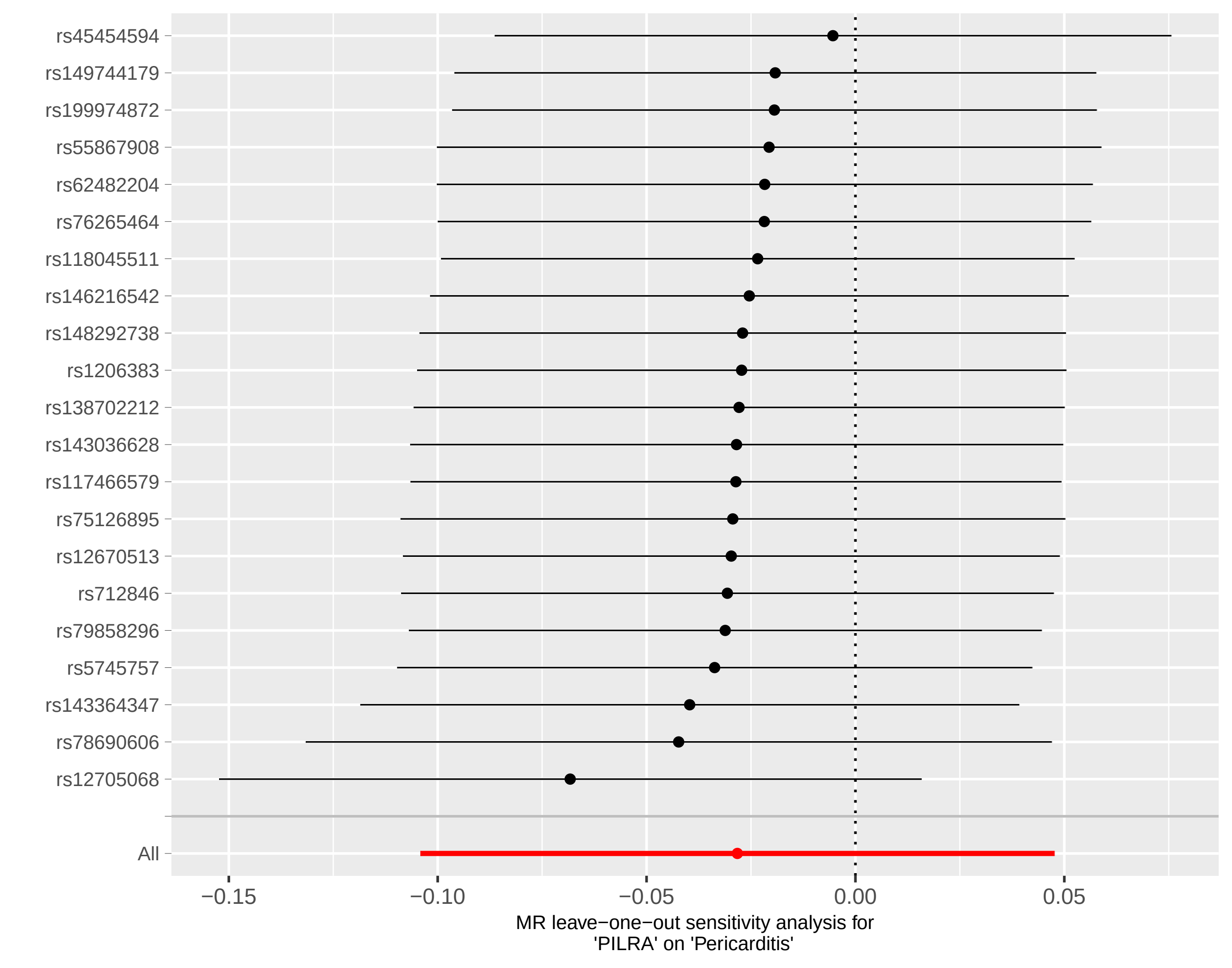
rs45454594
rs149744179
rs199974872
rs55867908
rs62482204
rs76265464
rs118045511
rs146216542
rs148292738
rs1206383
rs138702212
rs143036628
rs117466579
rs75126895
rs12670513
rs712846
rs79858296
rs5745757
rs143364347
rs78690606
rs12705068
All
−0.15
−0.10
−0.05
0.00
0.05
MR leave−one−out sensitivity analysis for
'PILRA' on 'Pericarditis'

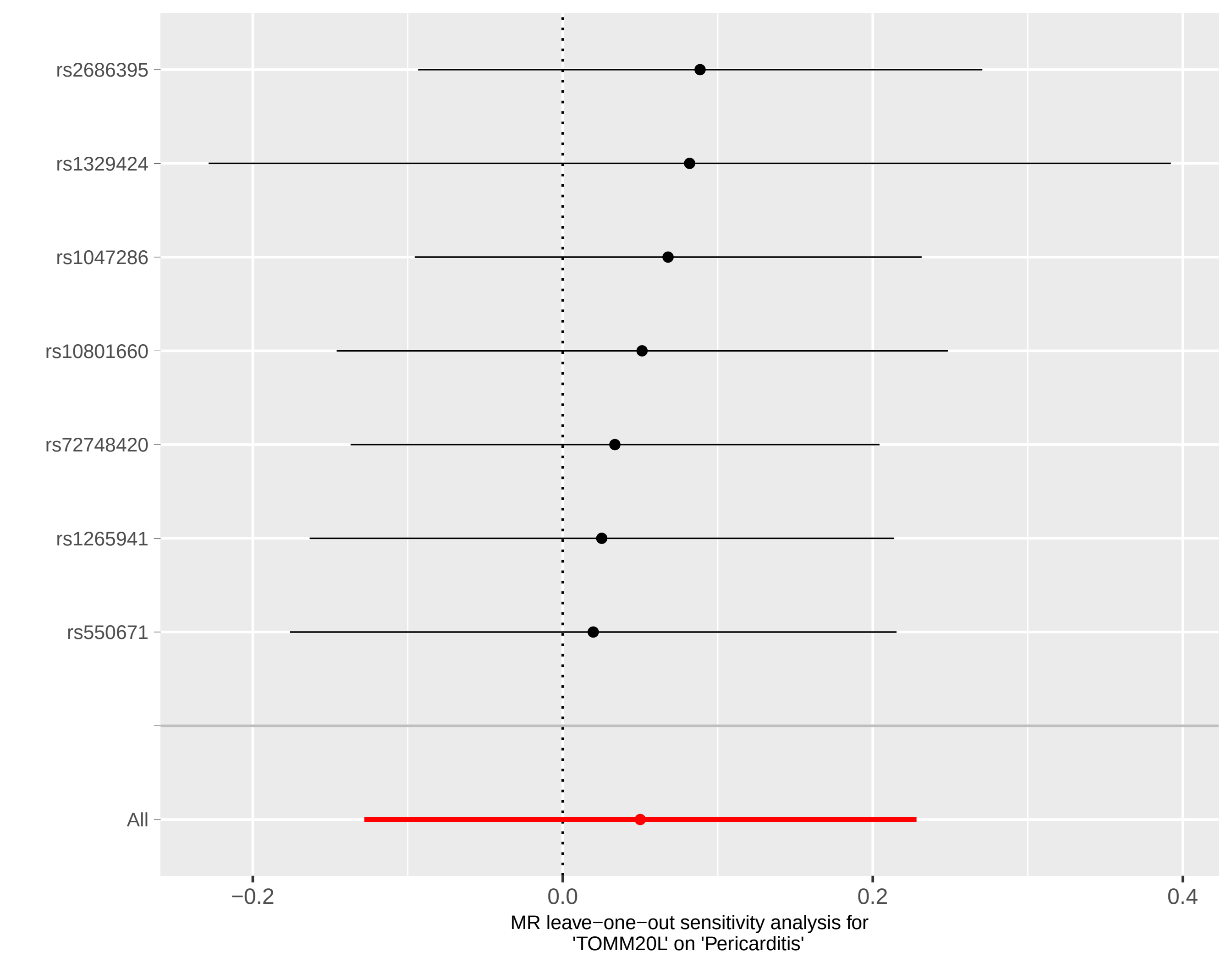
rs2686395
rs1329424
rs1047286
rs10801660
rs72748420
rs1265941
rs550671
All
−0.2
0.0
0.2
0.4
MR leave−one−out sensitivity analysis for
'TOMM20L' on 'Pericarditis'

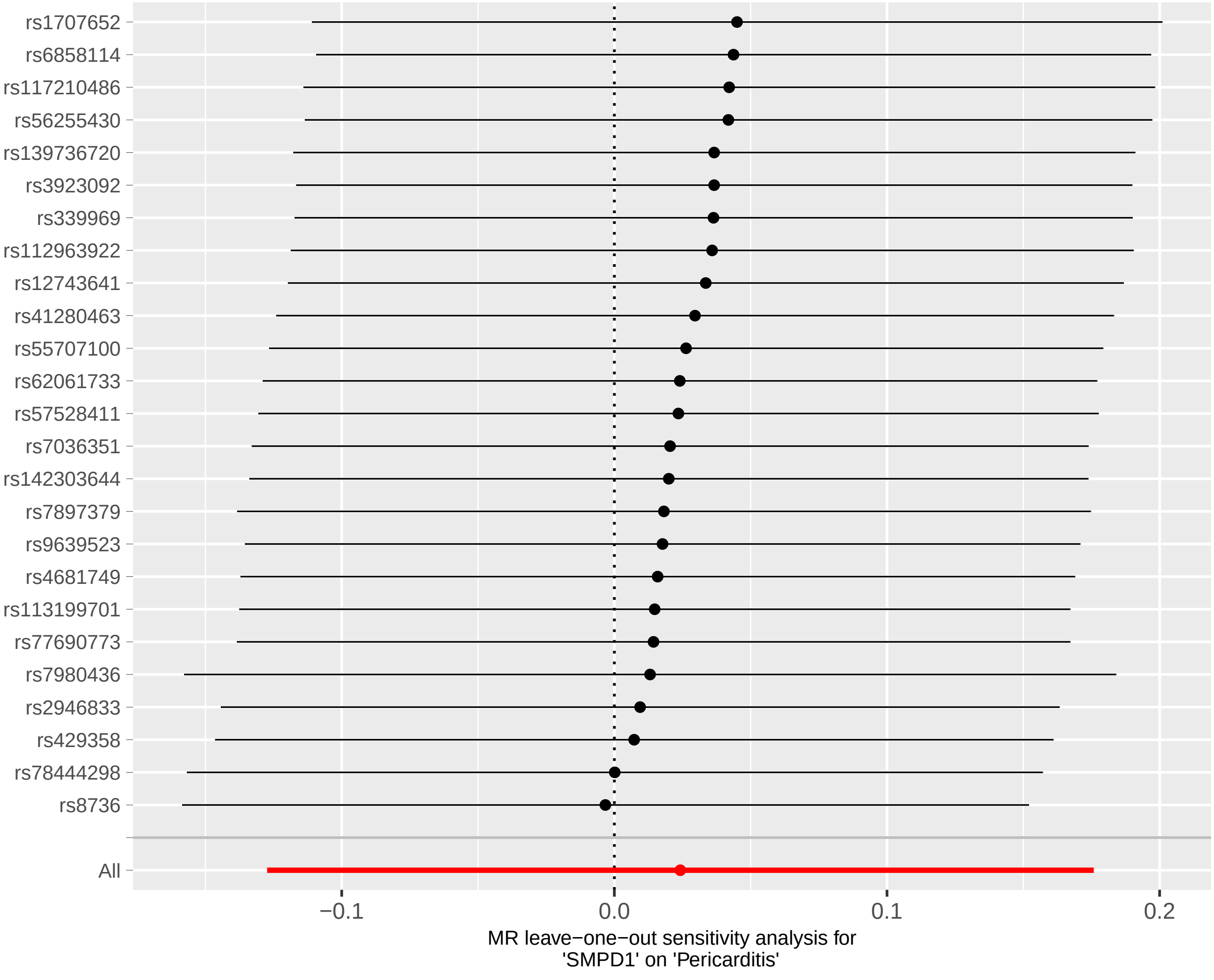
rs1707652
rs6858114
rs117210486
rs56255430
rs139736720
rs3923092
rs339969
rs112963922
rs12743641
rs41280463
rs55707100
rs62061733
rs57528411
rs7036351
rs142303644
rs7897379
rs9639523
rs4681749
rs113199701
rs77690773
rs7980436
rs2946833
rs429358
rs78444298
rs8736
All
−0.1
0.0
0.1
0.2
MR leave−one−out sensitivity analysis for
'SMPD1' on 'Pericarditis'

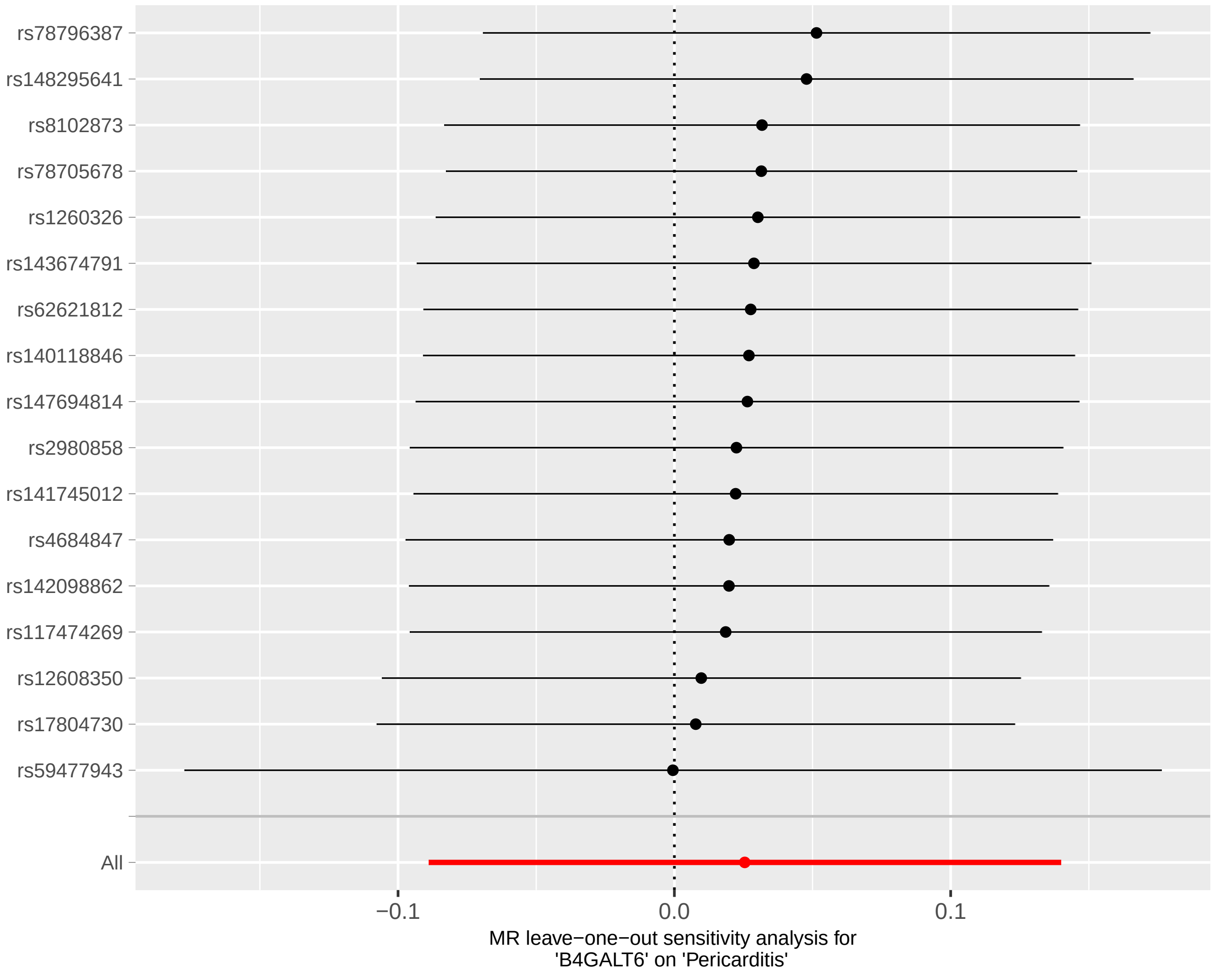

rs78796387
rs148295641
rs8102873
rs78705678
rs1260326
rs143674791
rs62621812
rs140118846
rs147694814
rs2980858
rs141745012
rs4684847
rs142098862
rs117474269
rs12608350
rs17804730
rs59477943
All
−0.1
0.0
0.1
MR leave−one−out sensitivity analysis for
'B4GALT6' on 'Pericarditis'

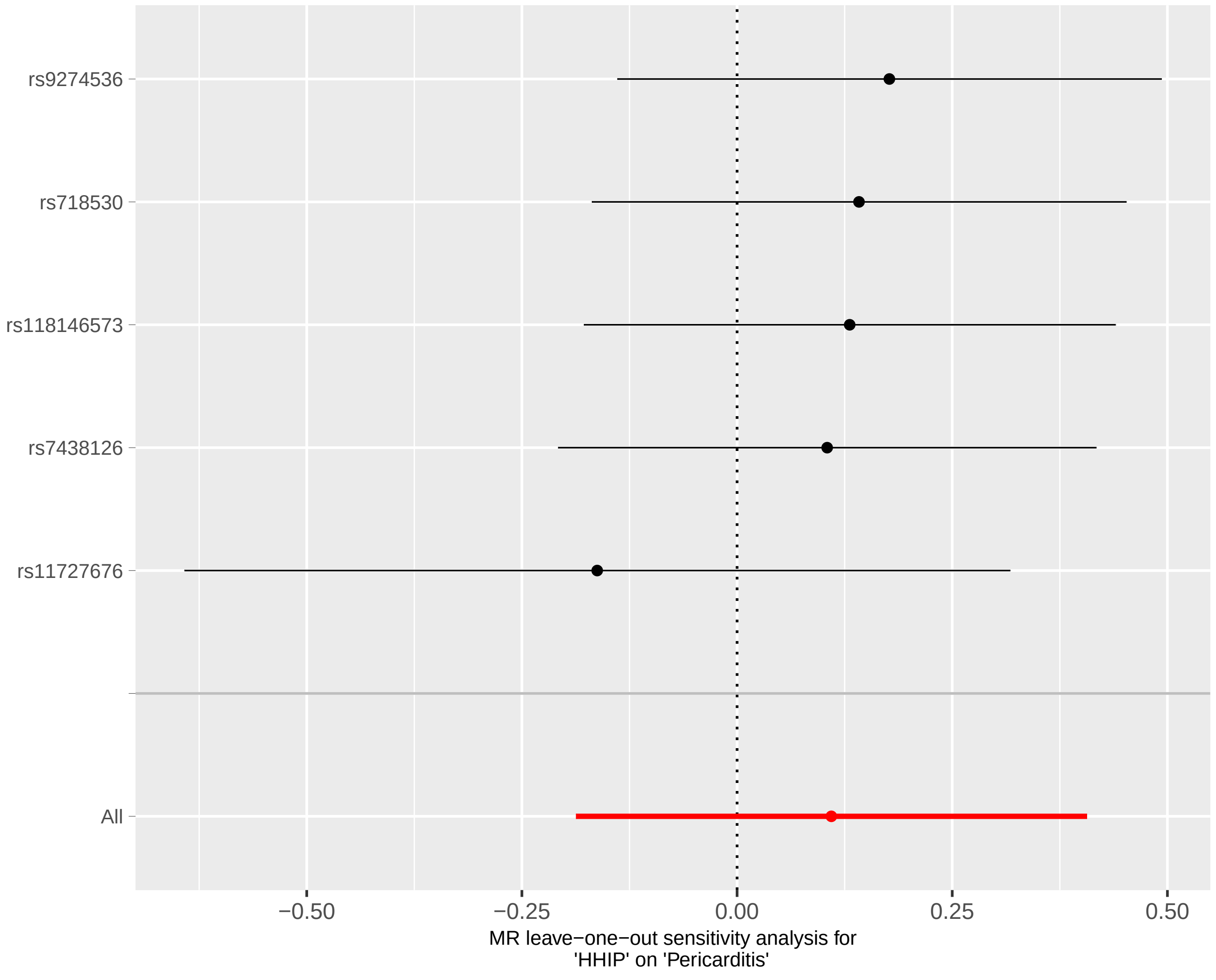
rs9274536
rs718530
rs118146573
rs7438126
rs11727676
All
−0.50
−0.25
0.00
0.25
0.50
MR leave−one−out sensitivity analysis for
'HHIP' on 'Pericarditis'

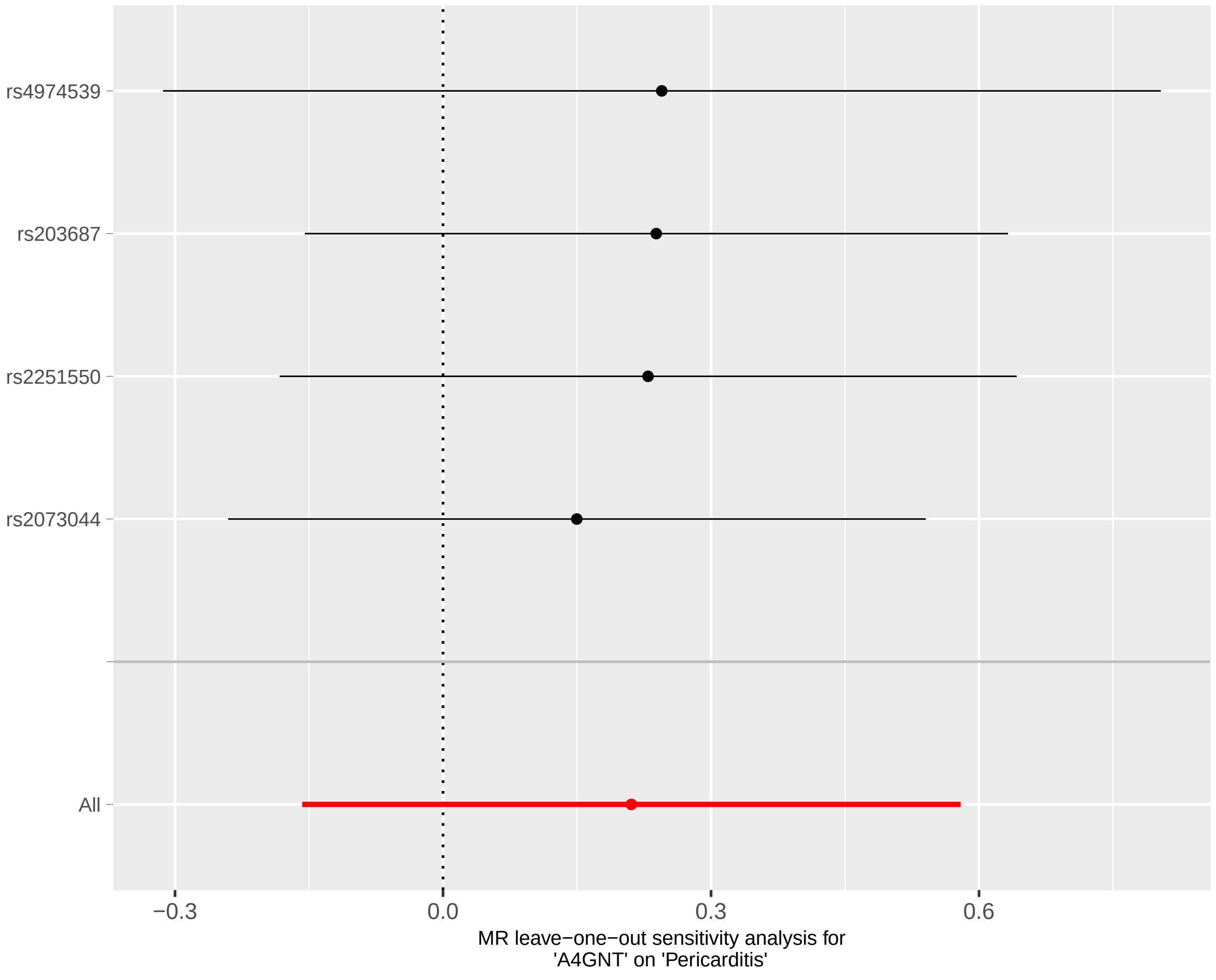

rs4974539
rs203687
rs2251550
rs2073044
All
−0.3
0.0
0.3
0.6
MR leave−one−out sensitivity analysis for
'A4GNT' on 'Pericarditis'

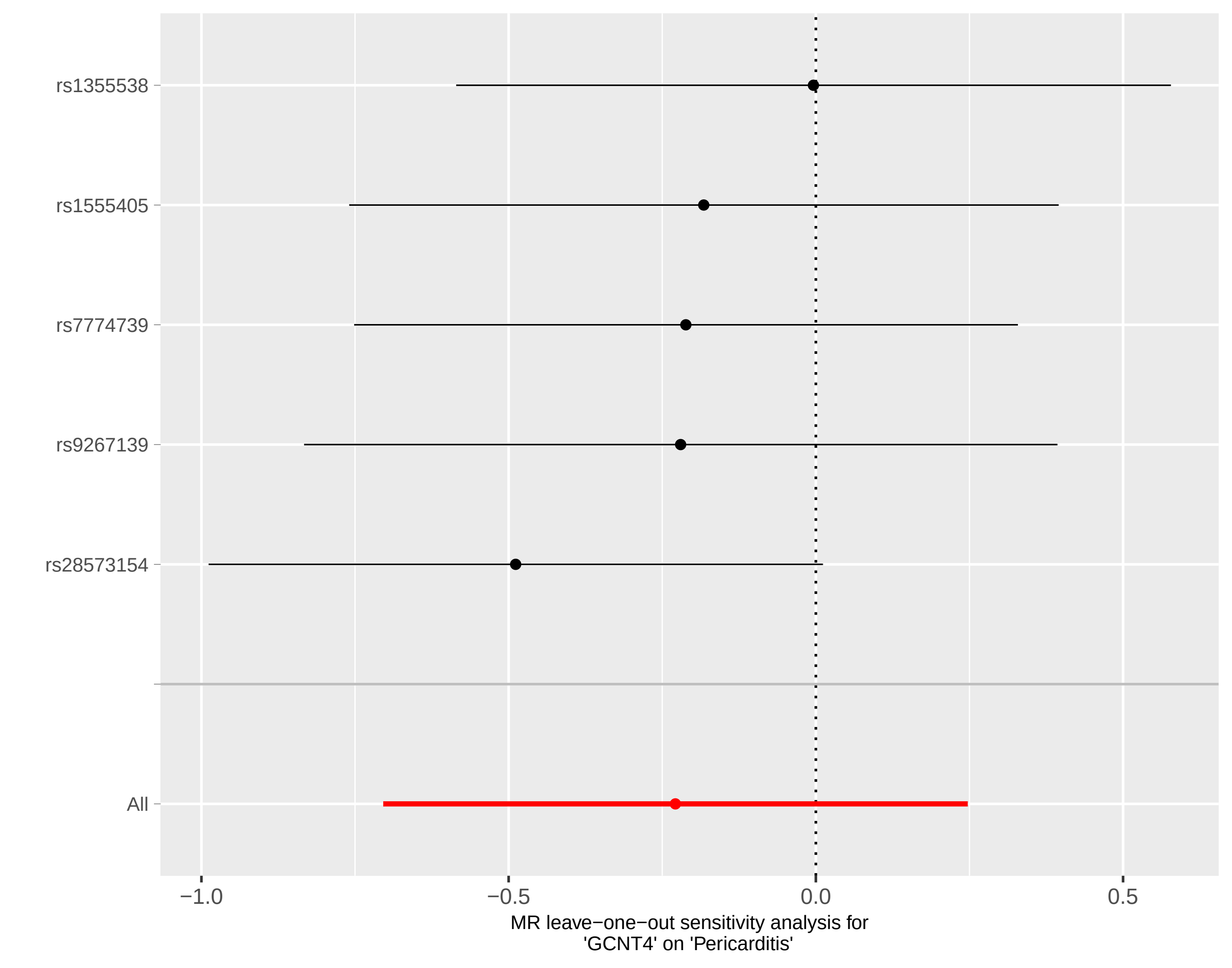

rs1355538
rs1555405
rs7774739
rs9267139
rs28573154
All
−1.0
−0.5
0.0
0.5
MR leave−one−out sensitivity analysis for
'GCNT4' on 'Pericarditis'

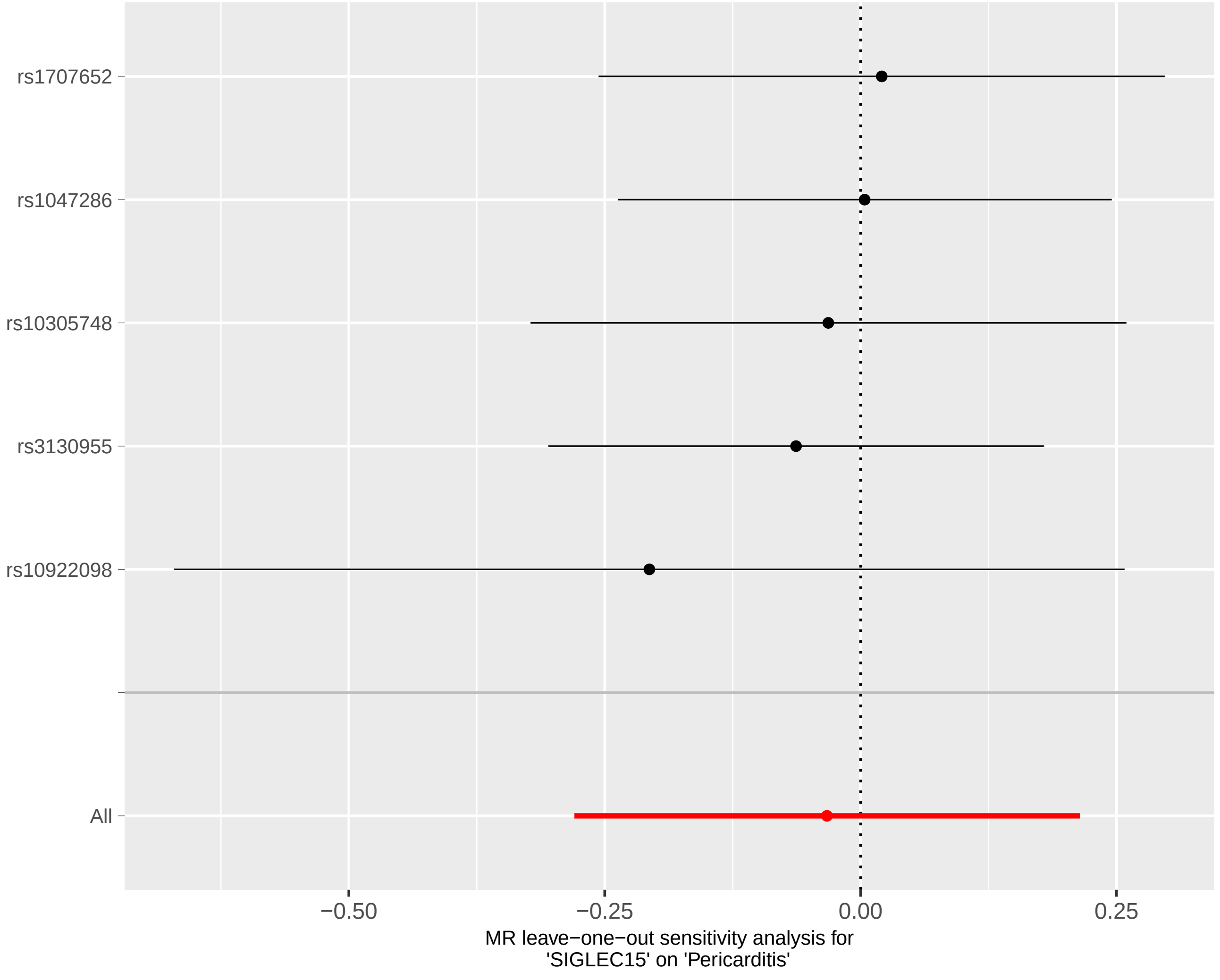

rs1707652
rs1047286
rs10305748
rs3130955
rs10922098
All
−0.50
−0.25
0.00
0.25
MR leave−one−out sensitivity analysis for
'SIGLEC15' on 'Pericarditis'

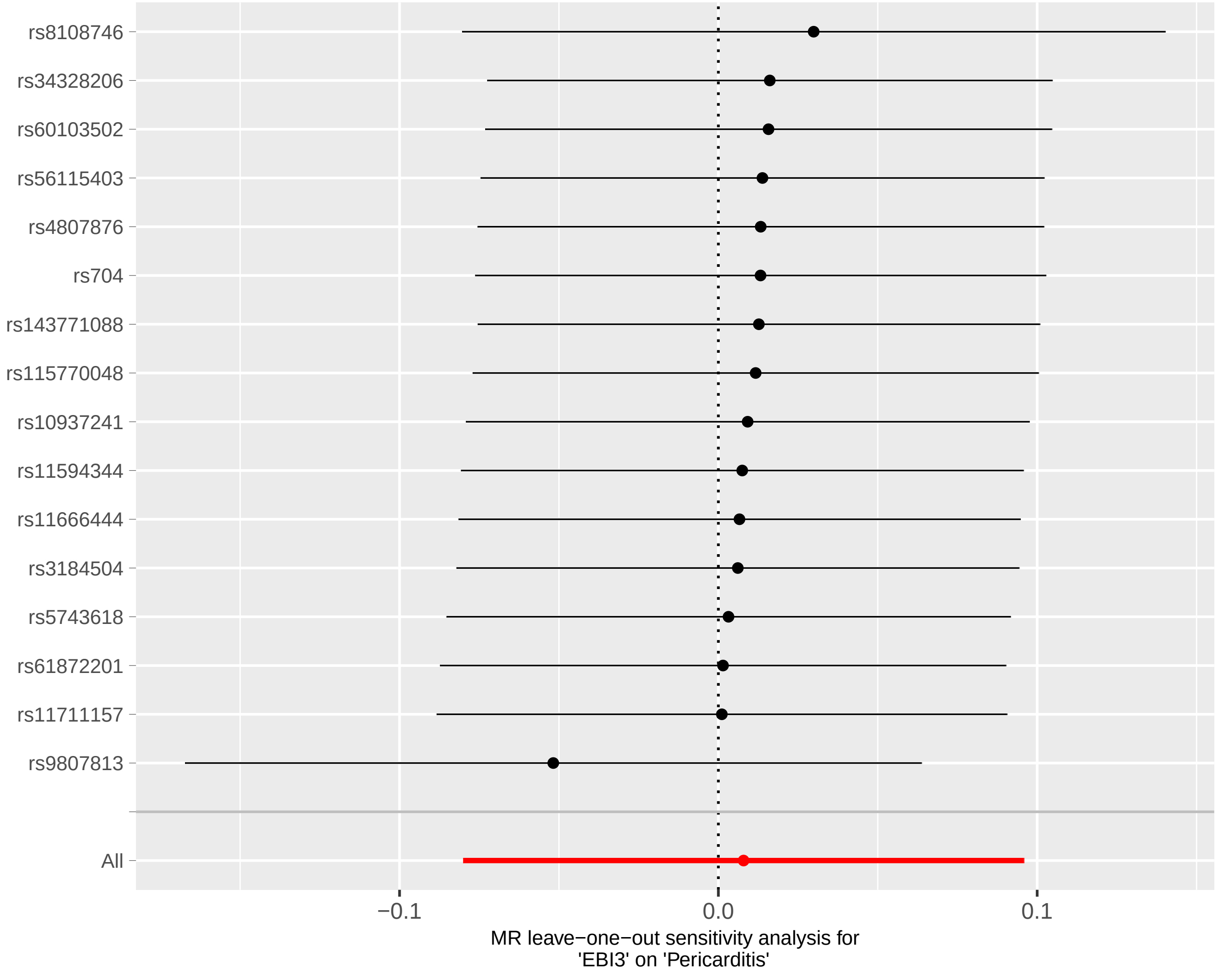

rs8108746
rs34328206
rs60103502
rs56115403
rs4807876
rs704
rs143771088
rs115770048
rs10937241
rs11594344
rs11666444
rs3184504
rs5743618
rs61872201
rs11711157
rs9807813
All
−0.1
0.0
0.1
MR leave−one−out sensitivity analysis for
'EBI3' on 'Pericarditis'

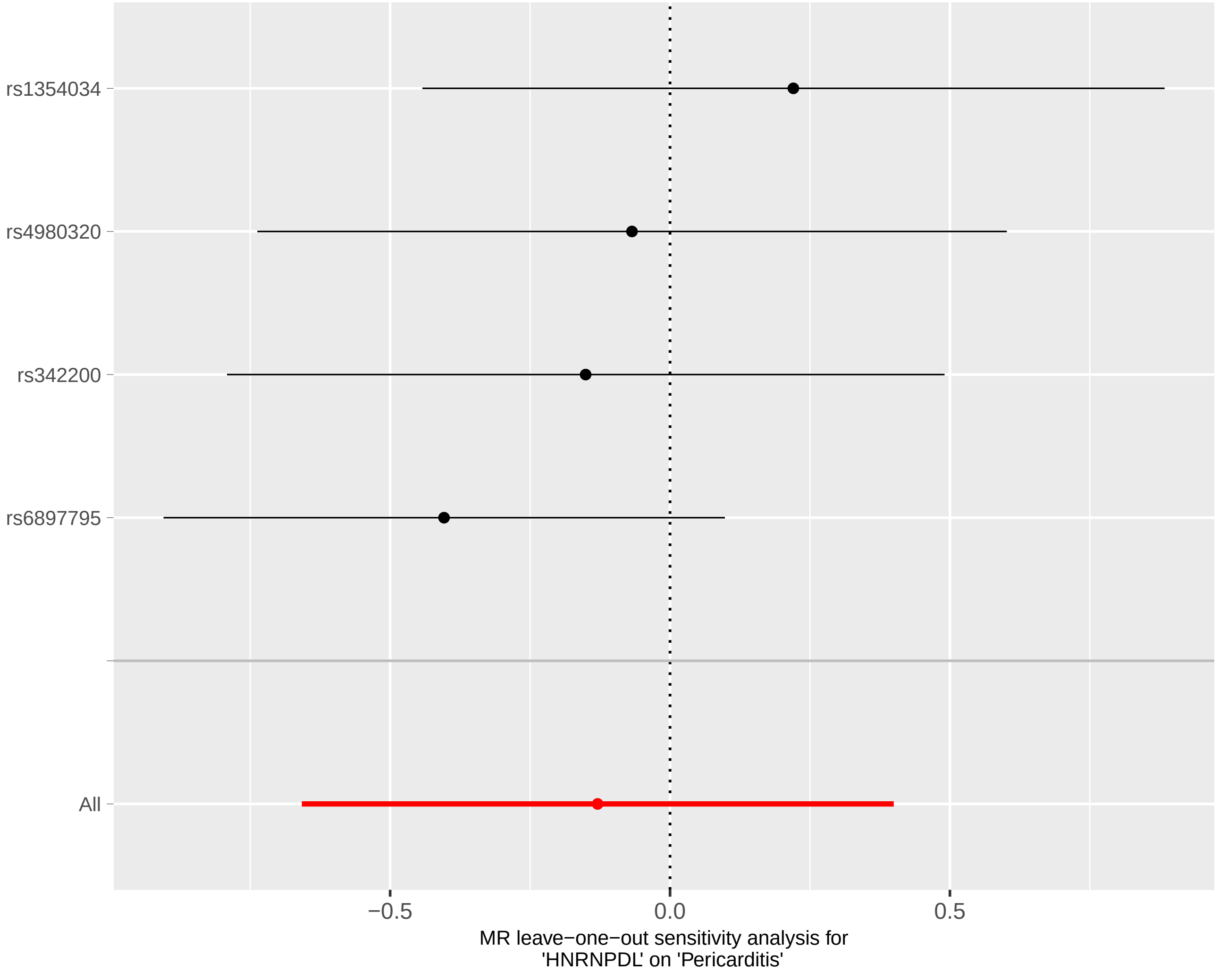
rs1354034
rs4980320
rs342200
rs6897795
All
−0.5
0.0
0.5
MR leave−one−out sensitivity analysis for
'HNRNPDL' on 'Pericarditis'

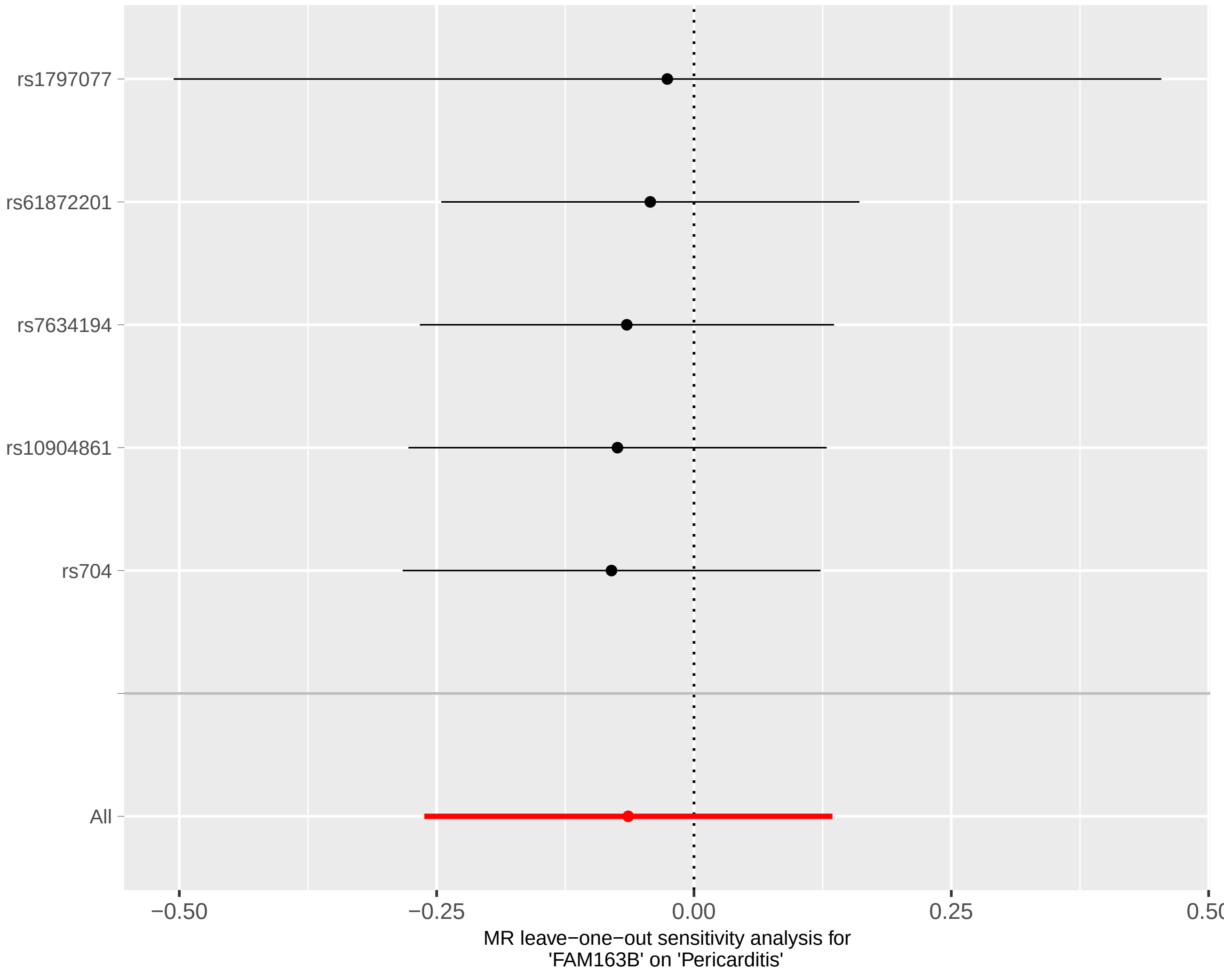

rs1797077
rs61872201
rs7634194
rs10904861
rs704
All
−0.50
−0.25
0.00
0.25
0.50
MR leave−one−out sensitivity analysis for
'FAM163B' on 'Pericarditis'

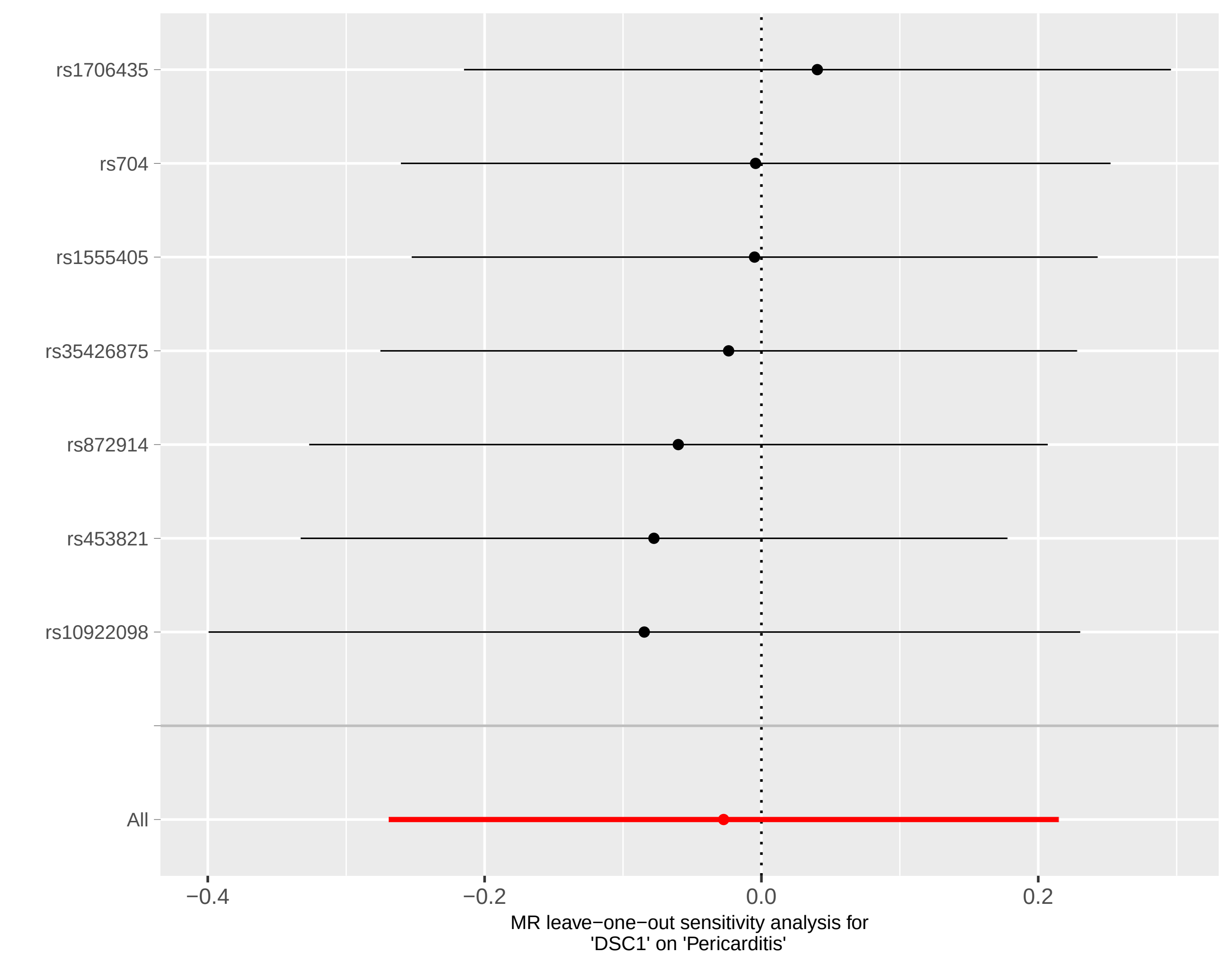
rs1706435
rs704
rs1555405
rs35426875
rs872914
rs453821
rs10922098
All
−0.4
−0.2
0.0
0.2
MR leave−one−out sensitivity analysis for
'DSC1' on 'Pericarditis'

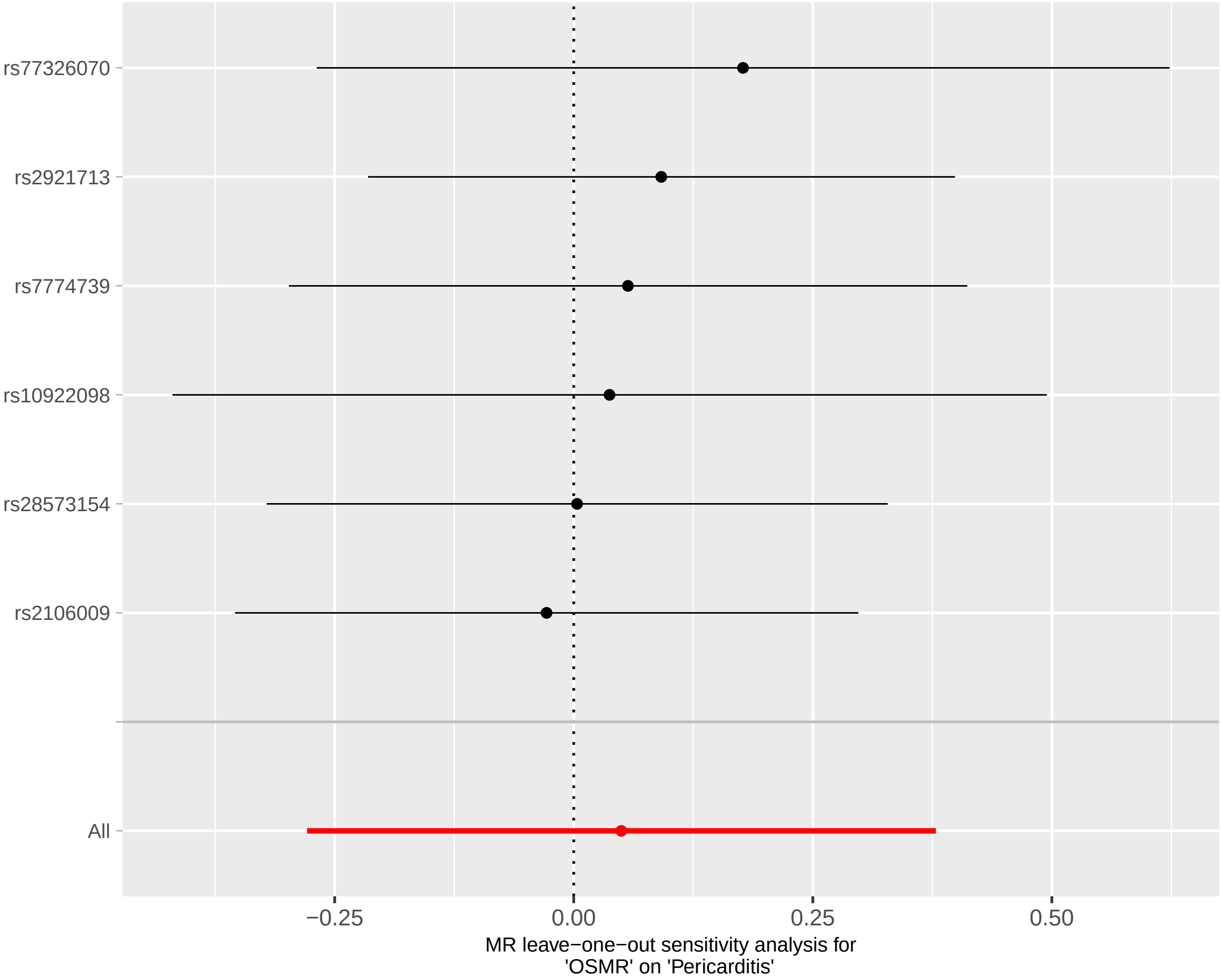

rs77326070
rs2921713
rs7774739
rs10922098
rs28573154
rs2106009
All
−0.25
0.00
0.25
0.50
MR leave−one−out sensitivity analysis for
'OSMR' on 'Pericarditis'

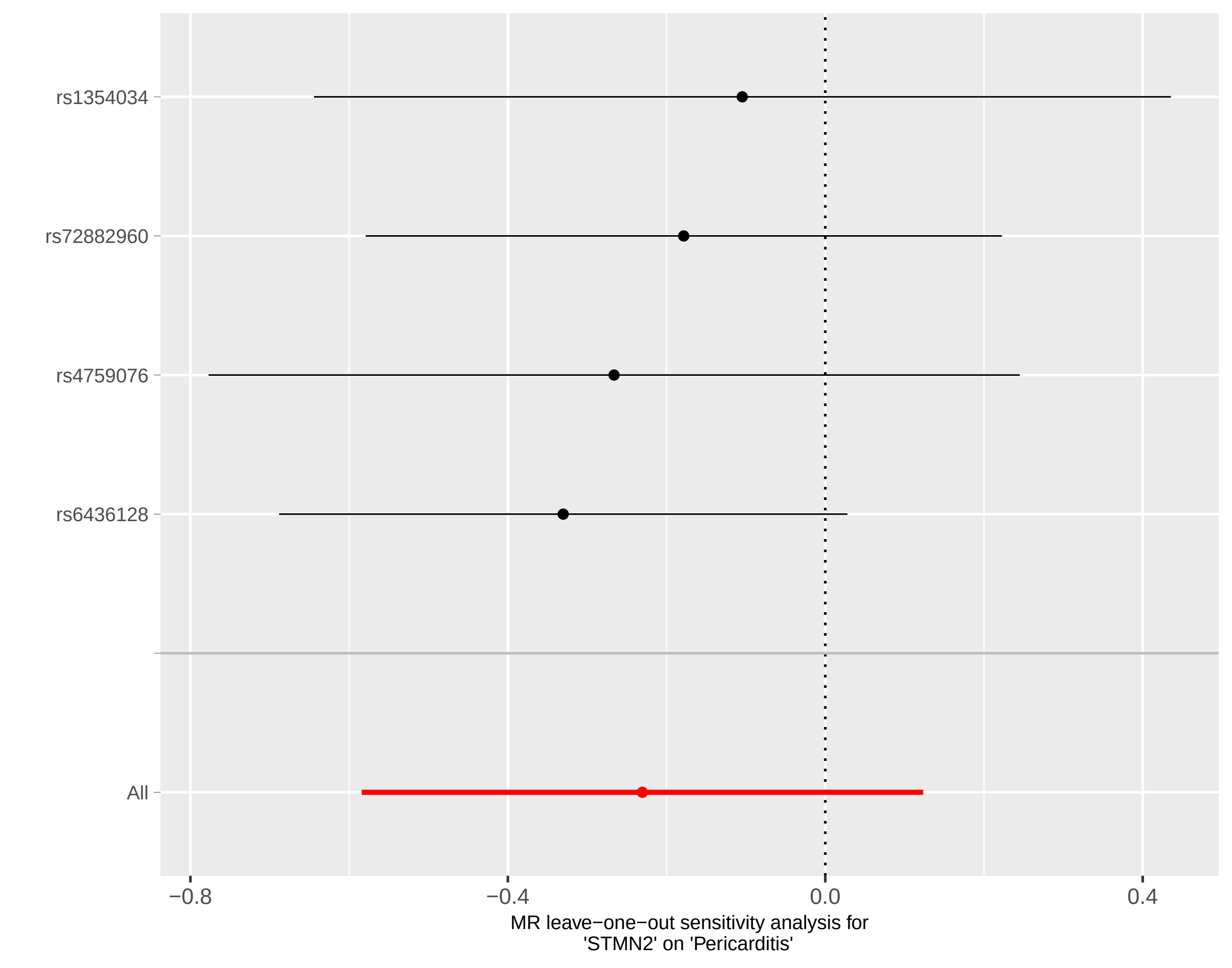

rs1354034
rs72882960
rs4759076
rs6436128
All
−0.8
−0.4
0.0
0.4
MR leave−one−out sensitivity analysis for
'STMN2' on 'Pericarditis'

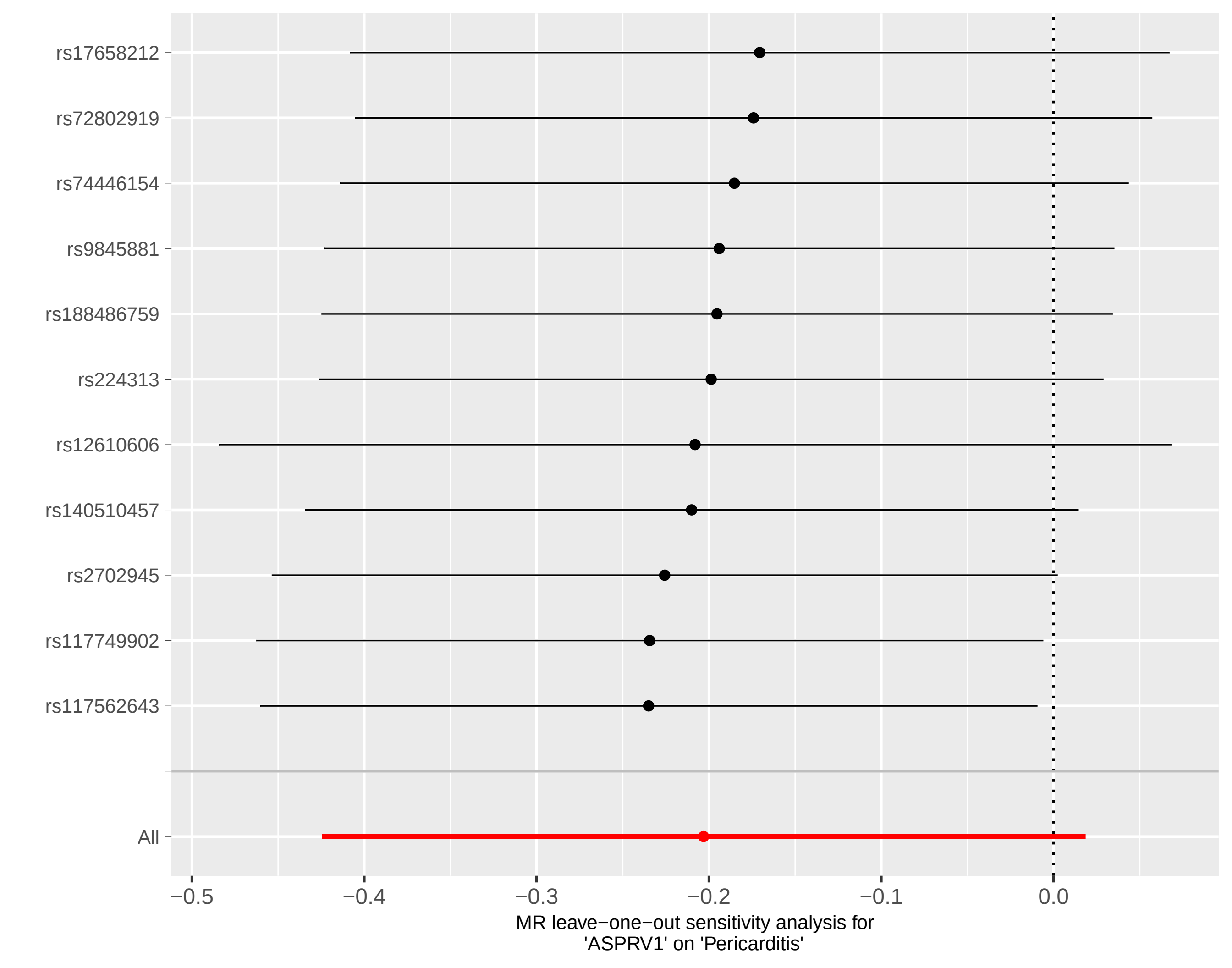
rs17658212
rs72802919
rs74446154
rs9845881
rs188486759
rs224313
rs12610606
rs140510457
rs2702945
rs117749902
rs117562643
All
−0.5
−0.4
−0.3
−0.2
−0.1
0.0
MR leave−one−out sensitivity analysis for
'ASPRV1' on 'Pericarditis'

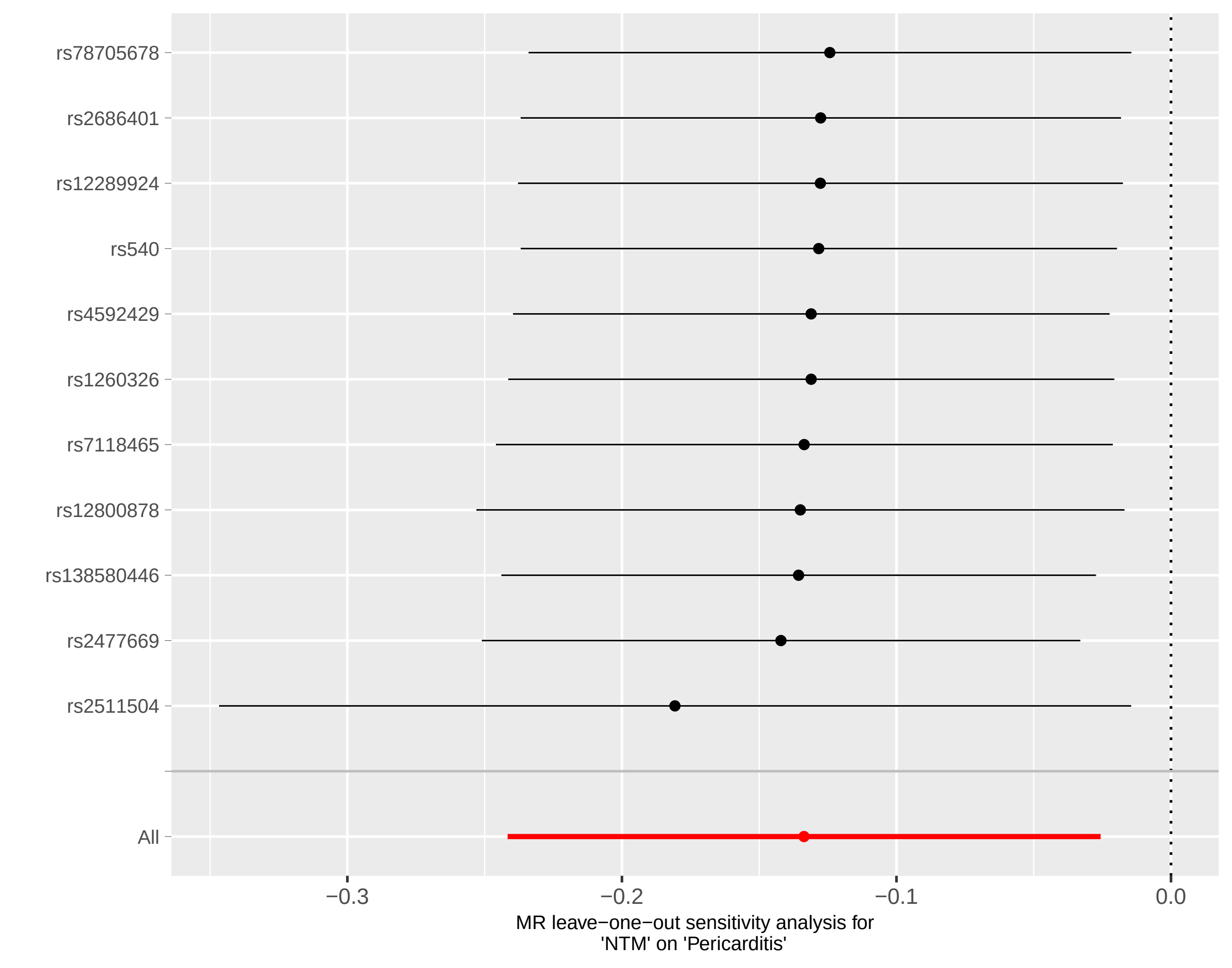
rs78705678
rs2686401
rs12289924
rs540
rs4592429
rs1260326
rs7118465
rs12800878
rs138580446
rs2477669
rs2511504
All
−0.3
−0.2
−0.1
0.0
MR leave−one−out sensitivity analysis for
'NTM' on 'Pericarditis'

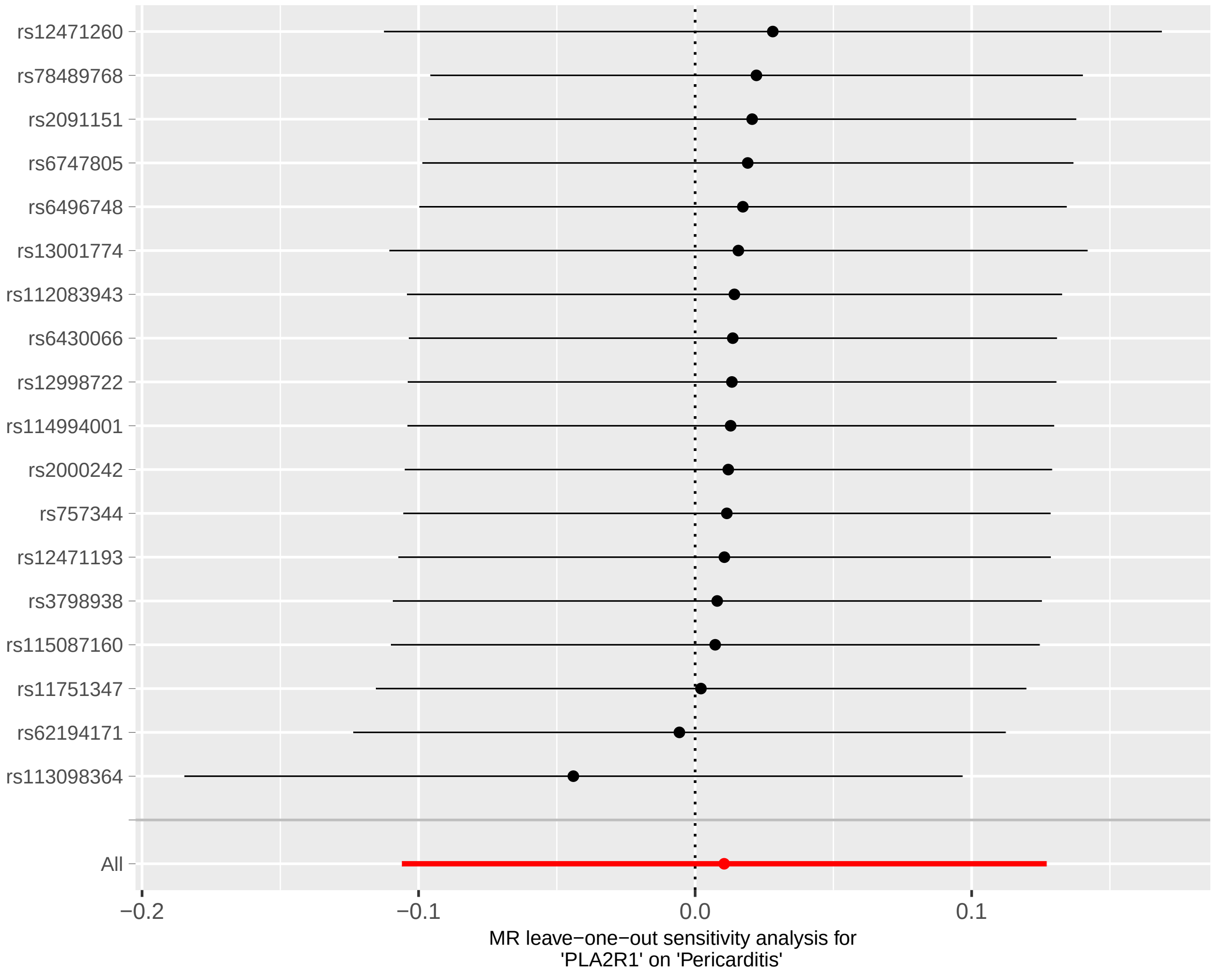

rs12471260
rs78489768
rs2091151
rs6747805
rs6496748
rs13001774
rs112083943
rs6430066
rs12998722
rs114994001
rs2000242
rs757344
rs12471193
rs3798938
rs115087160
rs11751347
rs62194171
rs113098364
All
−0.2
−0.1
0.0
0.1
MR leave−one−out sensitivity analysis for
'PLA2R1' on 'Pericarditis'

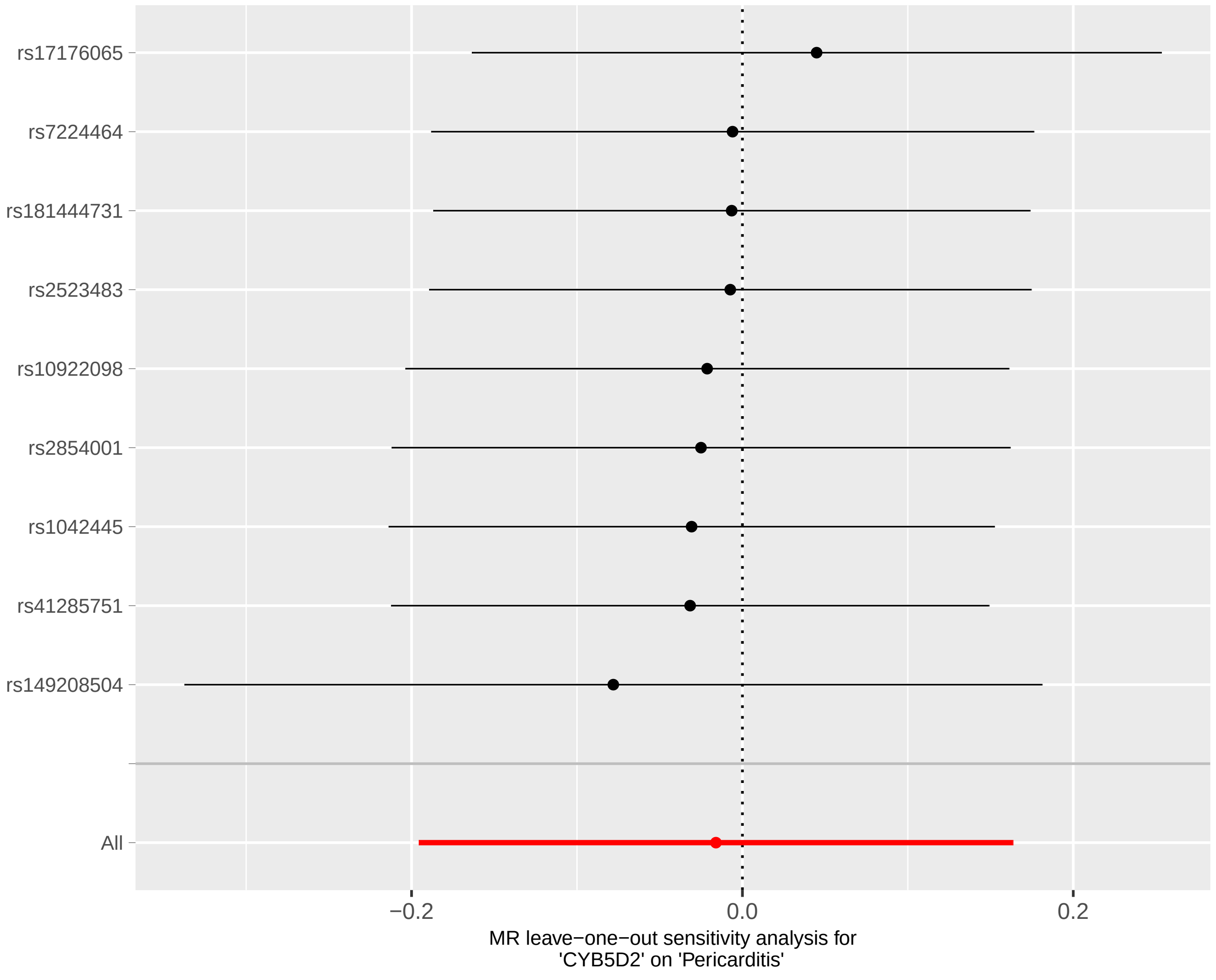

rs17176065
rs7224464
rs181444731
rs2523483
rs10922098
rs2854001
rs1042445
rs41285751
rs149208504
All
−0.2
0.0
0.2
MR leave−one−out sensitivity analysis for
'CYB5D2' on 'Pericarditis'

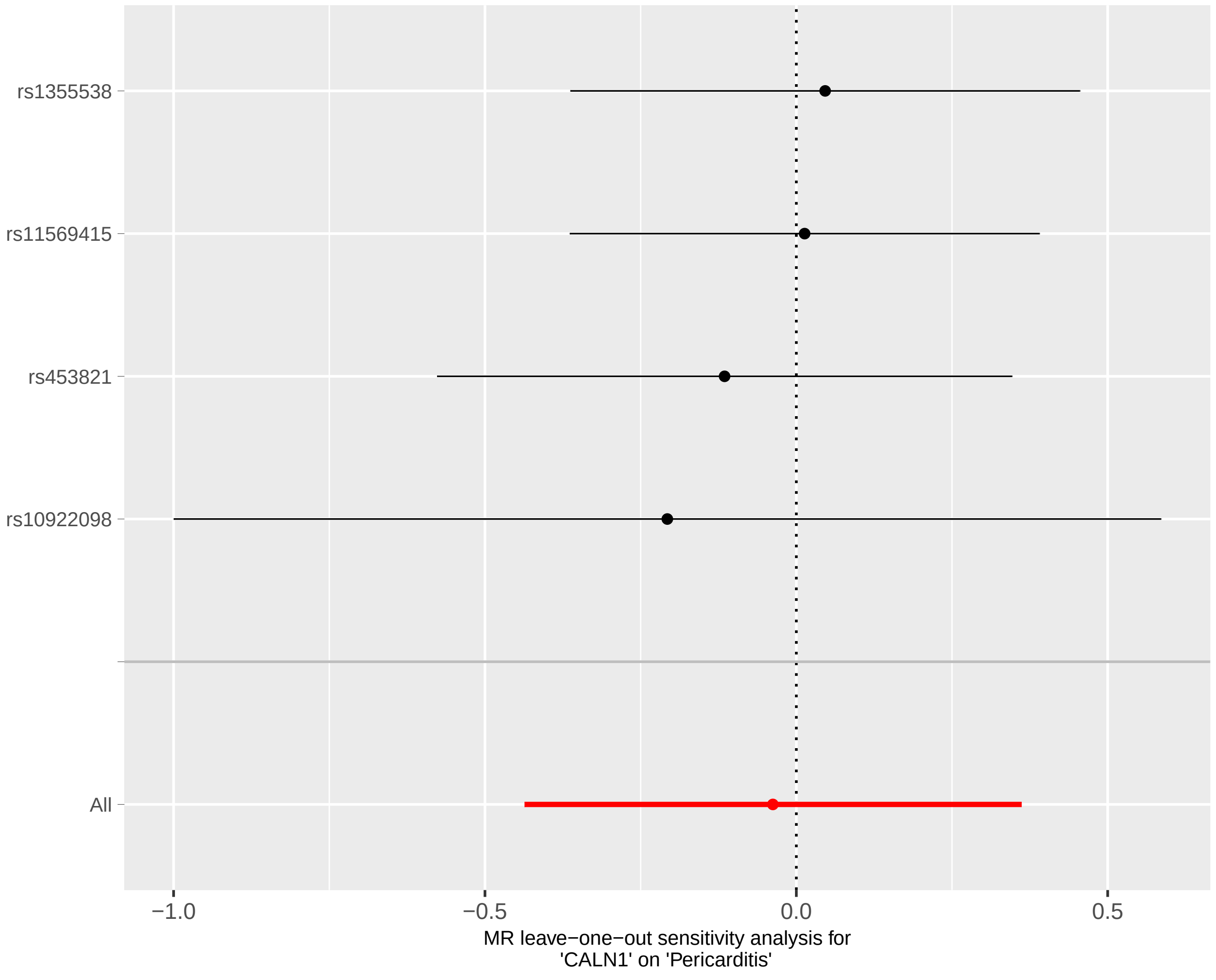

rs1355538
rs11569415
rs453821
rs10922098
All
−1.0
−0.5
0.0
0.5
MR leave−one−out sensitivity analysis for
'CALN1' on 'Pericarditis'

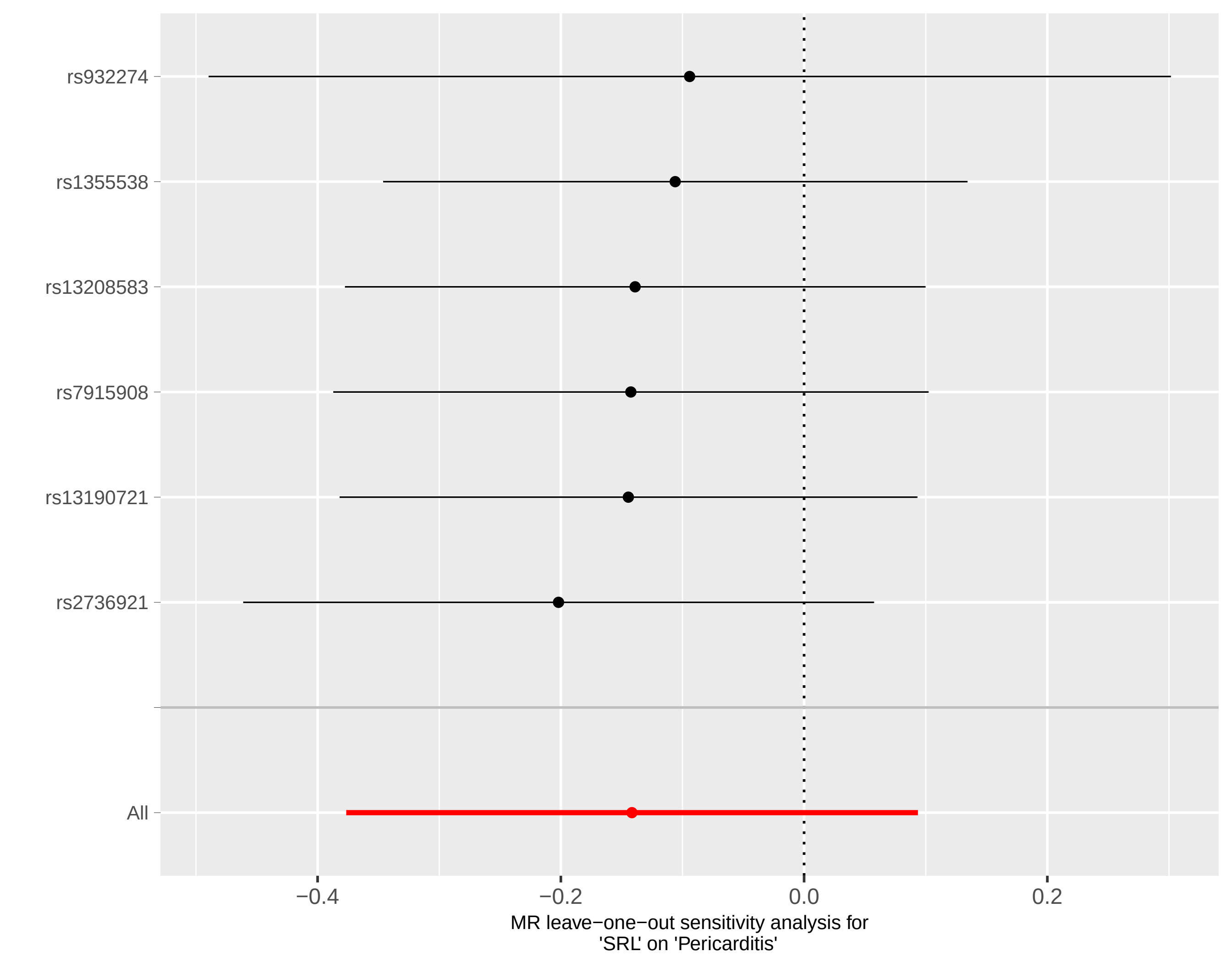

rs932274
rs1355538
rs13208583
rs7915908
rs13190721
rs2736921
All
−0.4
−0.2
0.0
0.2
MR leave−one−out sensitivity analysis for
'SRL' on 'Pericarditis'

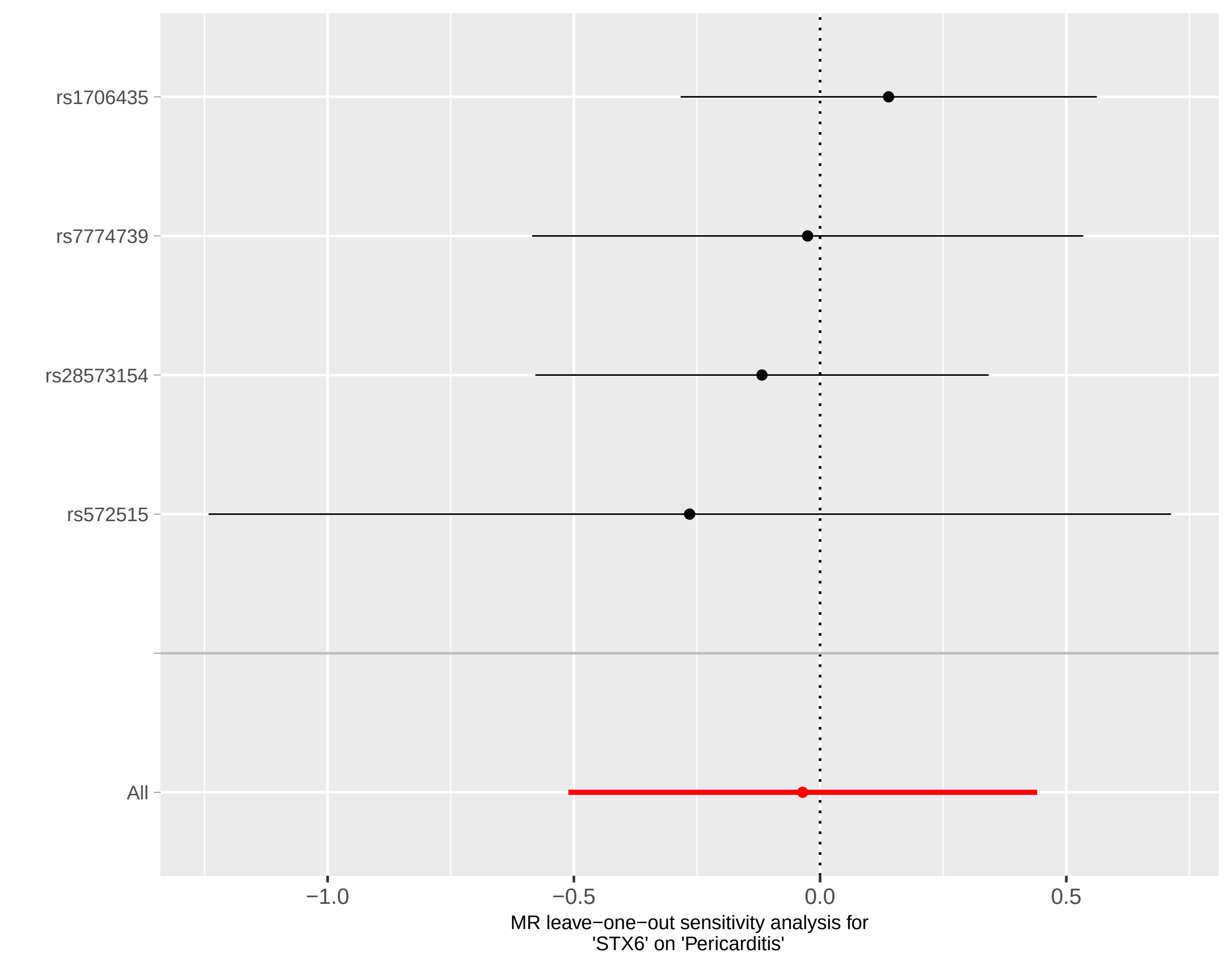

rs1706435
rs7774739
rs28573154
rs572515
All
−1.0
−0.5
0.0
0.5
MR leave−one−out sensitivity analysis for
'STX6' on 'Pericarditis'

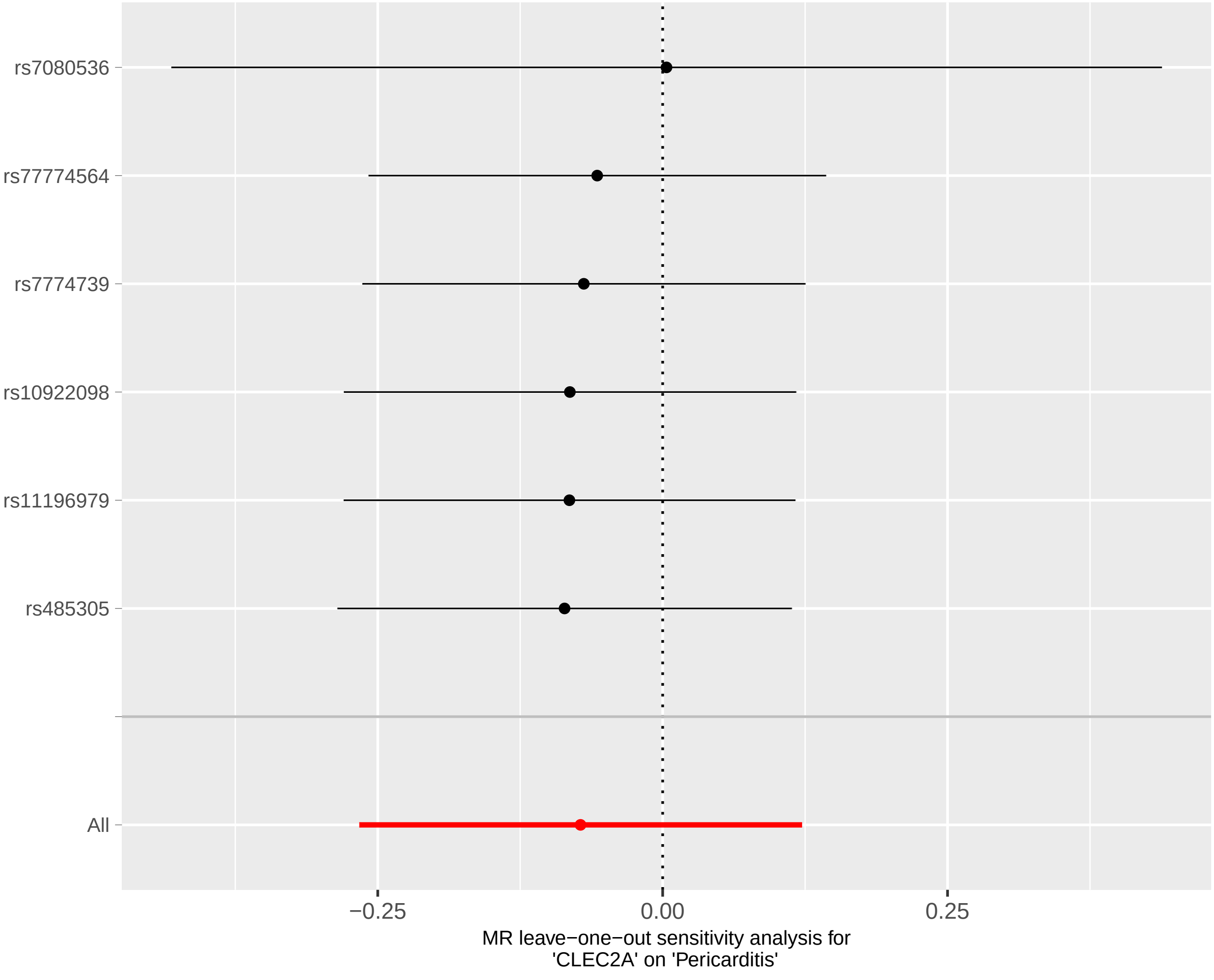

rs7080536
rs77774564
rs7774739
rs10922098
rs11196979
rs485305
All
−0.25
0.00
0.25
MR leave−one−out sensitivity analysis for
'CLEC2A' on 'Pericarditis'

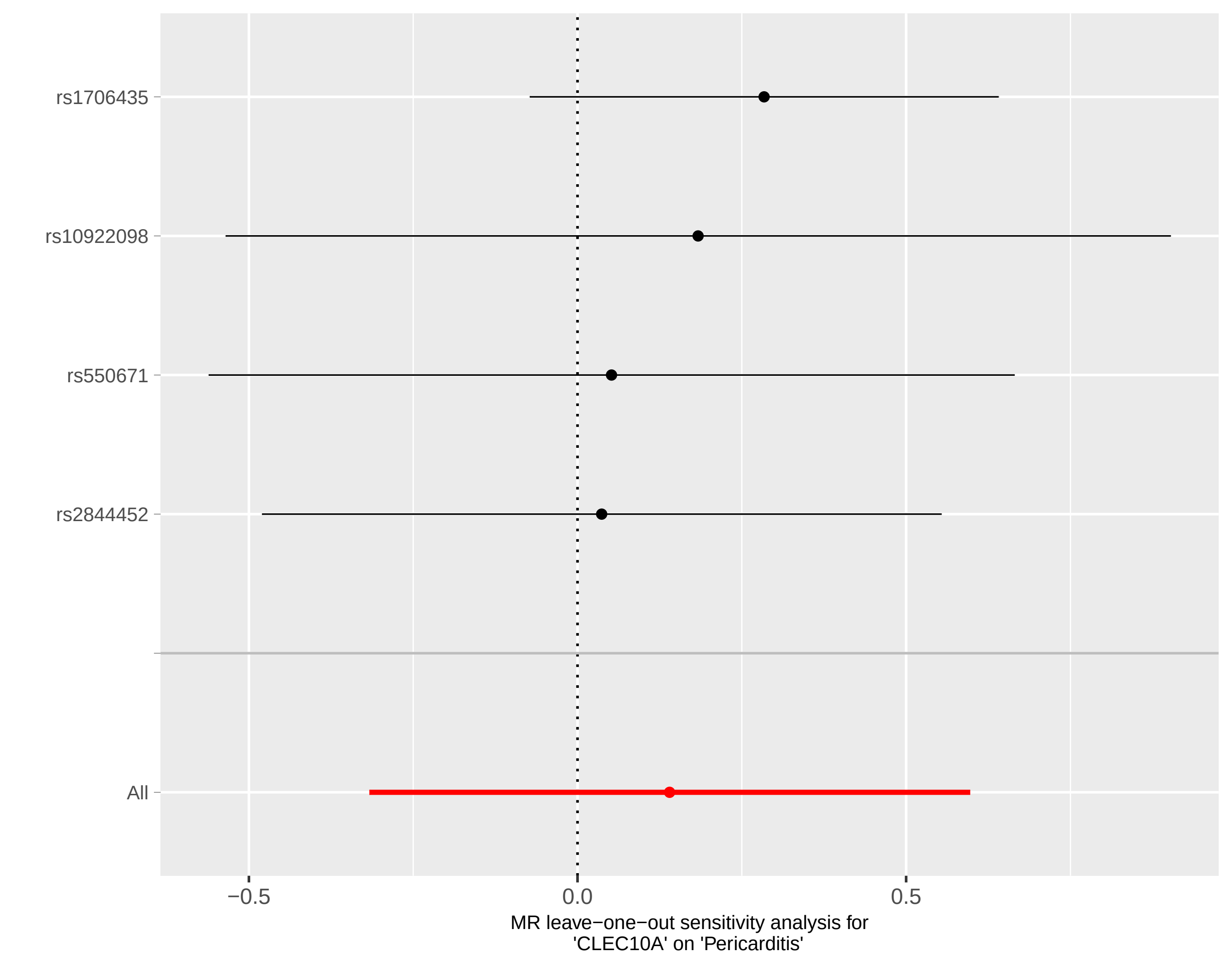

rs1706435
rs10922098
rs550671
rs2844452
All
−0.5
0.0
0.5
MR leave−one−out sensitivity analysis for
'CLEC10A' on 'Pericarditis'

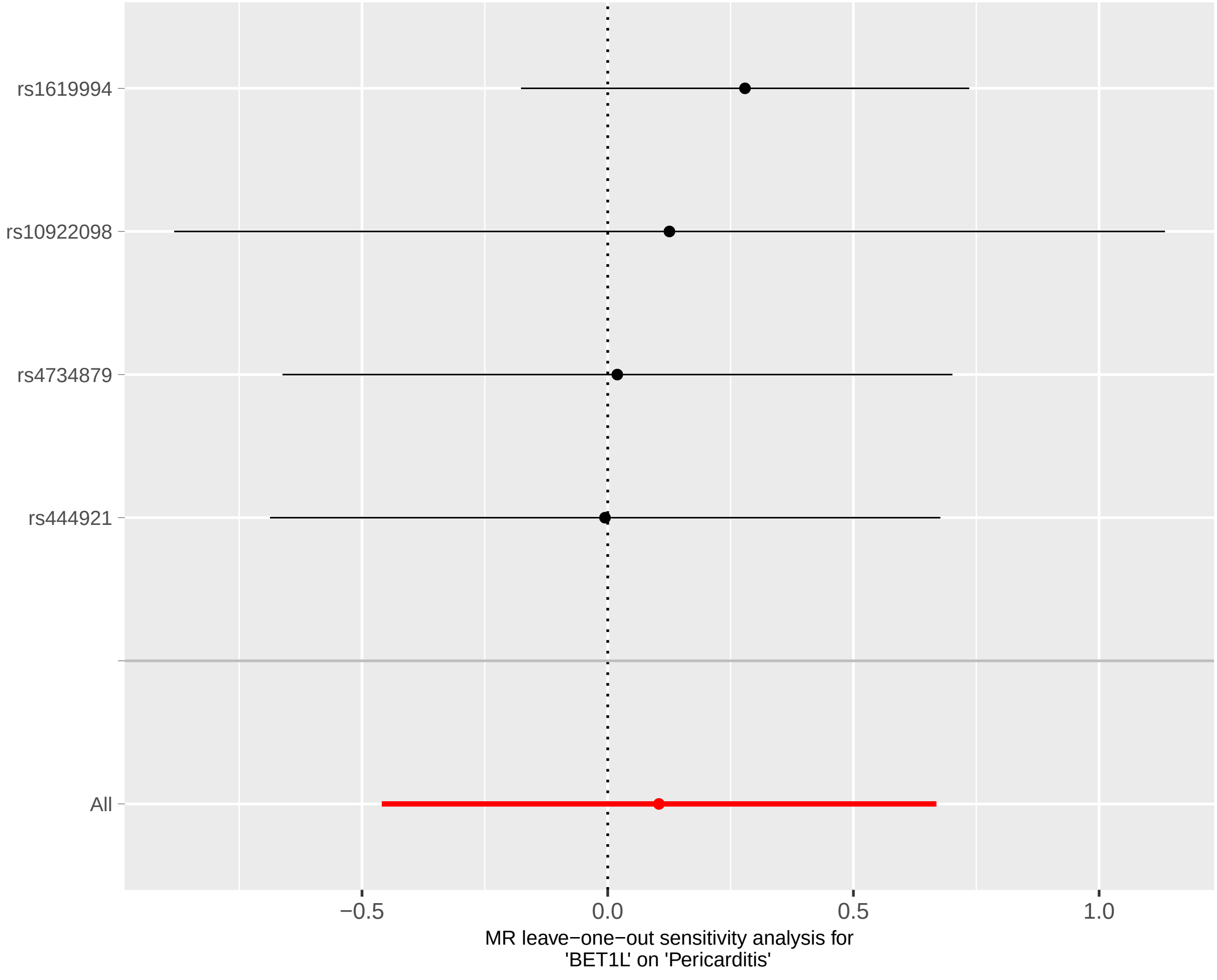

rs1619994
rs10922098
rs4734879
rs444921
All
−0.5
0.0
0.5
1.0
MR leave−one−out sensitivity analysis for
'BET1L' on 'Pericarditis'

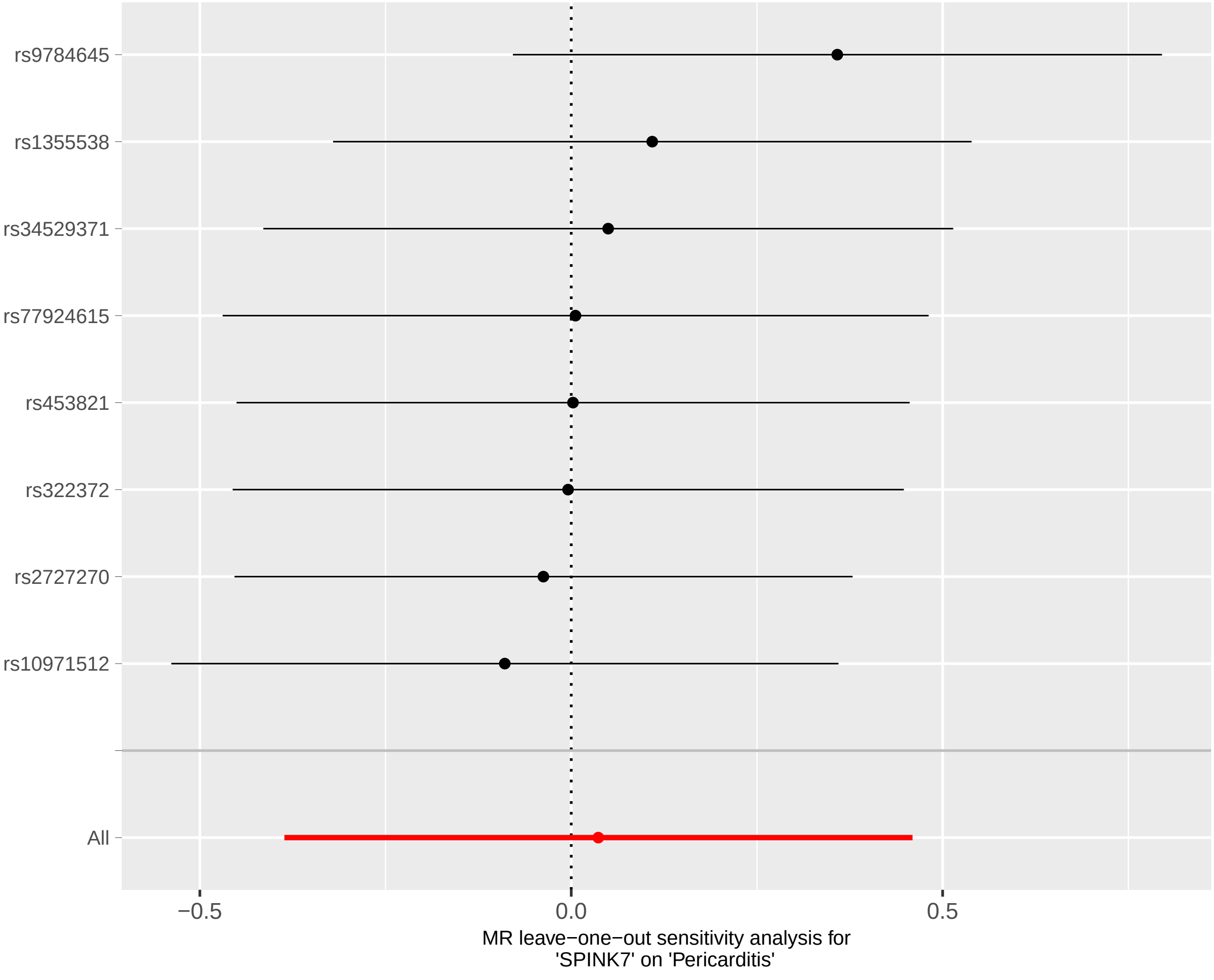
rs9784645
rs1355538
rs34529371
rs77924615
rs453821
rs322372
rs2727270
rs10971512
All
−0.5
0.0
0.5
MR leave−one−out sensitivity analysis for
'SPINK7' on 'Pericarditis'

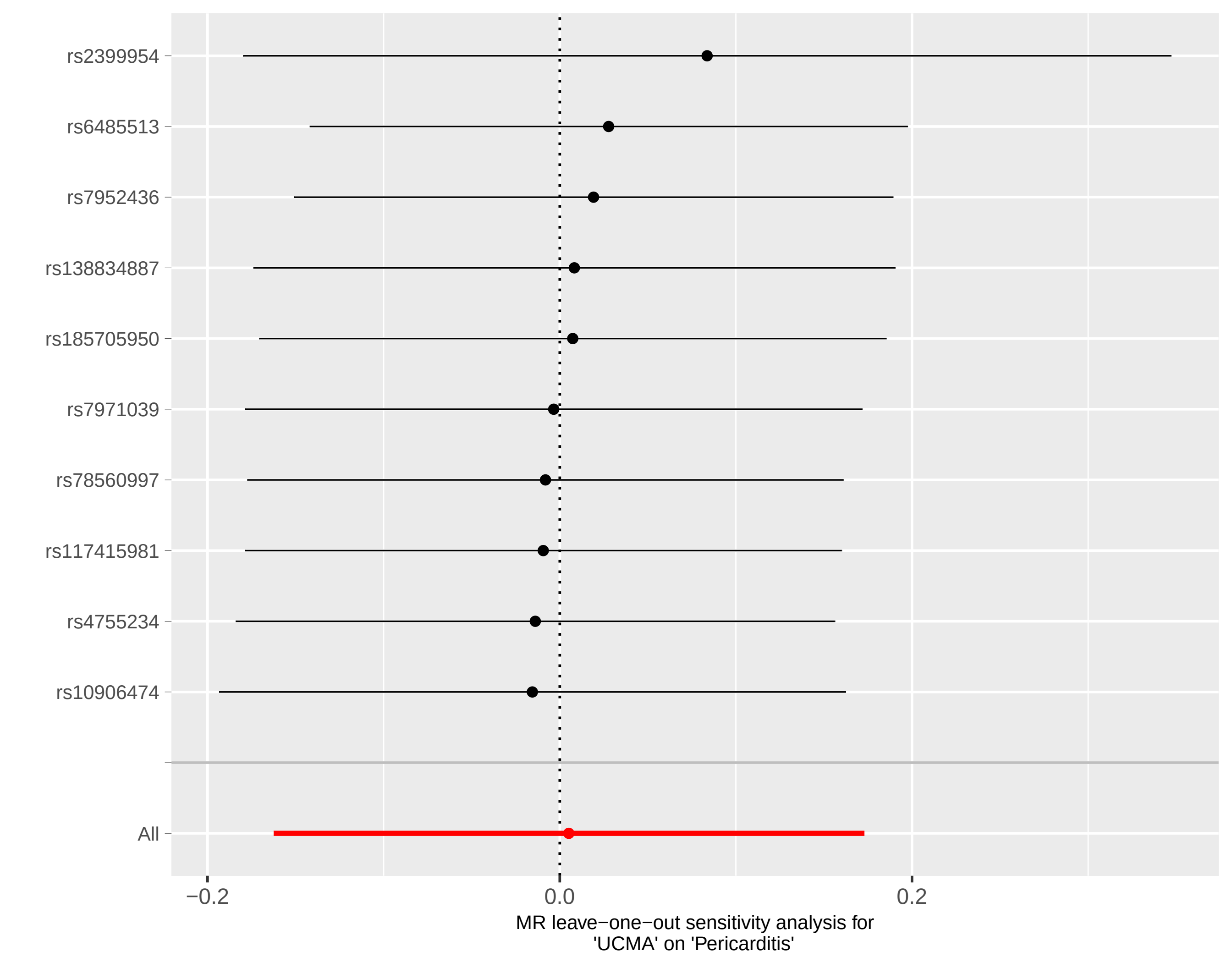

rs2399954
rs6485513
rs7952436
rs138834887
rs185705950
rs7971039
rs78560997
rs117415981
rs4755234
rs10906474
All
−0.2
0.0
0.2
MR leave−one−out sensitivity analysis for
'UCMA' on 'Pericarditis'

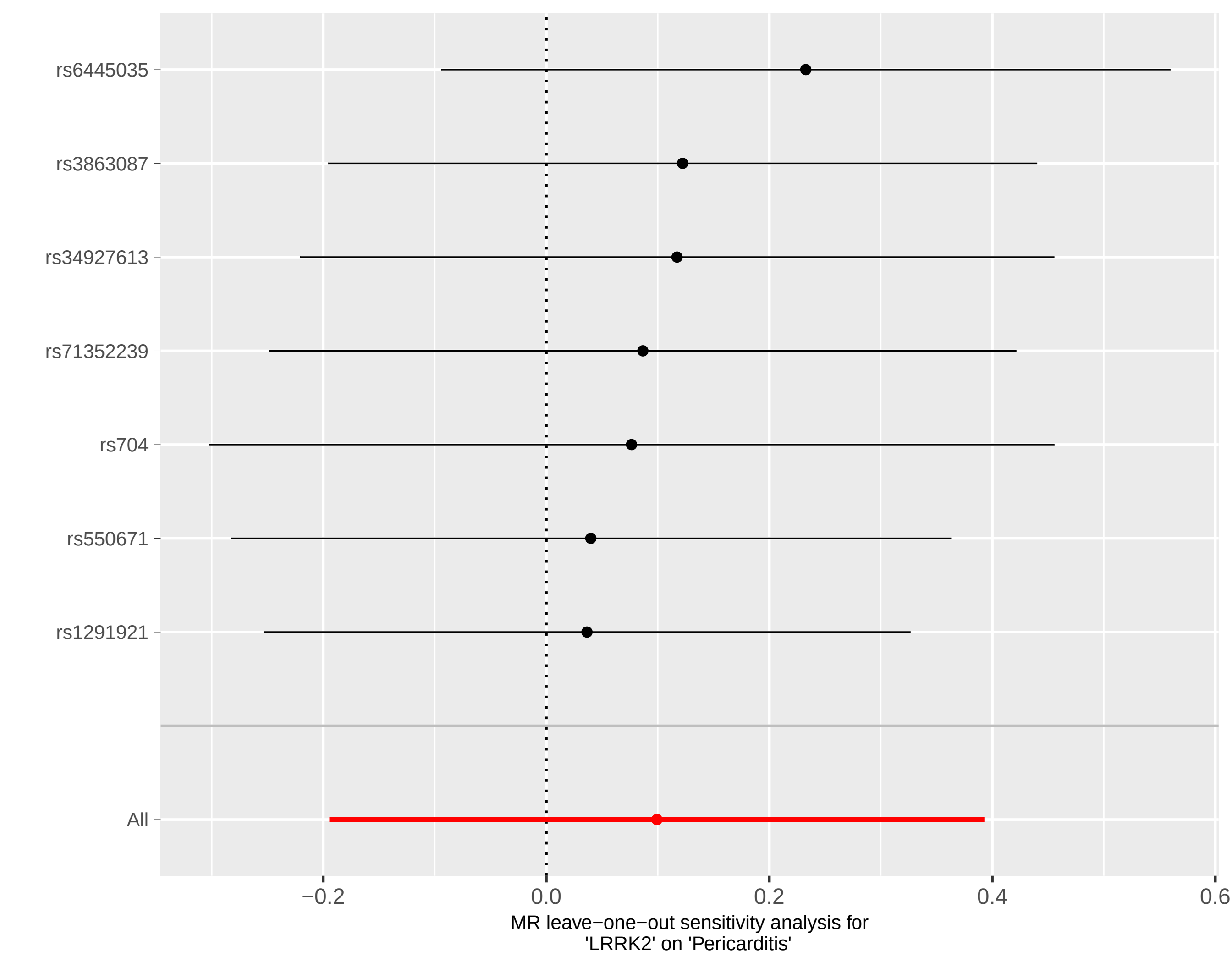

rs6445035
rs3863087
rs34927613
rs71352239
rs704
rs550671
rs1291921
All
−0.2
0.0
0.2
0.4
0.6
MR leave−one−out sensitivity analysis for
'LRRK2' on 'Pericarditis'

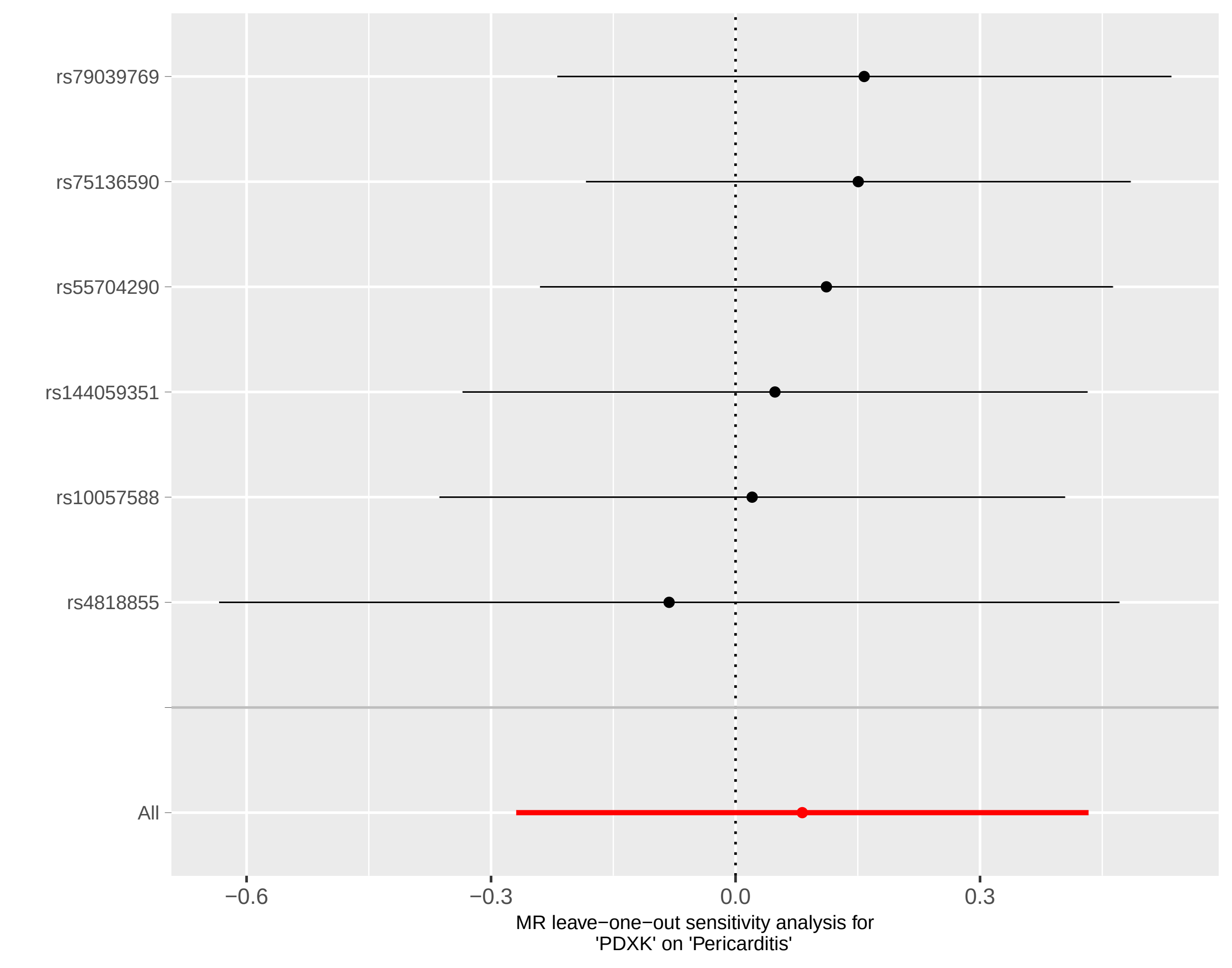
rs79039769
rs75136590
rs55704290
rs144059351
rs10057588
rs4818855
All
−0.6
−0.3
0.0
0.3
MR leave−one−out sensitivity analysis for
'PDXK' on 'Pericarditis'

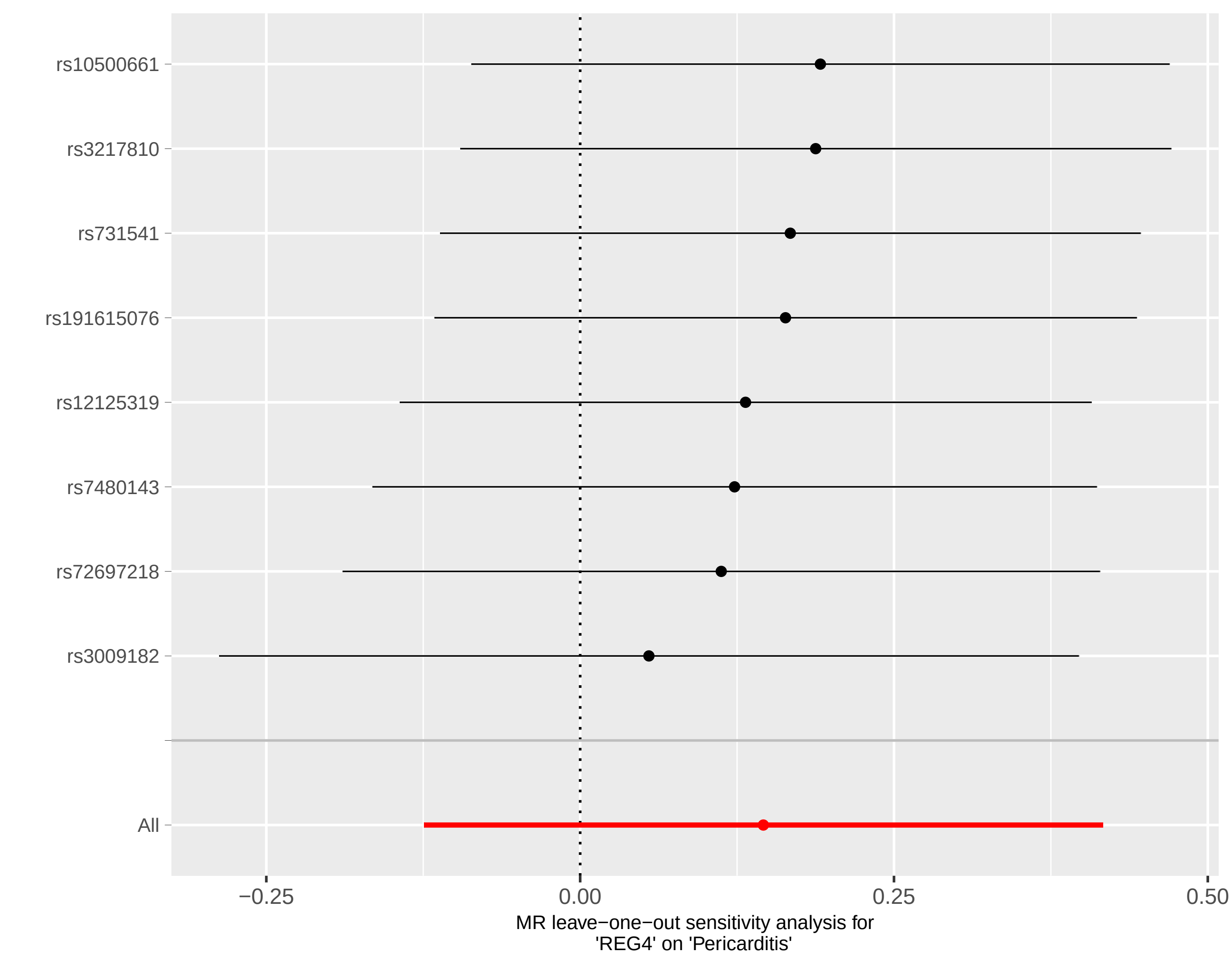

rs10500661
rs3217810
rs731541
rs191615076
rs12125319
rs7480143
rs72697218
rs3009182
All
−0.25
0.00
0.25
0.50
MR leave−one−out sensitivity analysis for
'REG4' on 'Pericarditis'

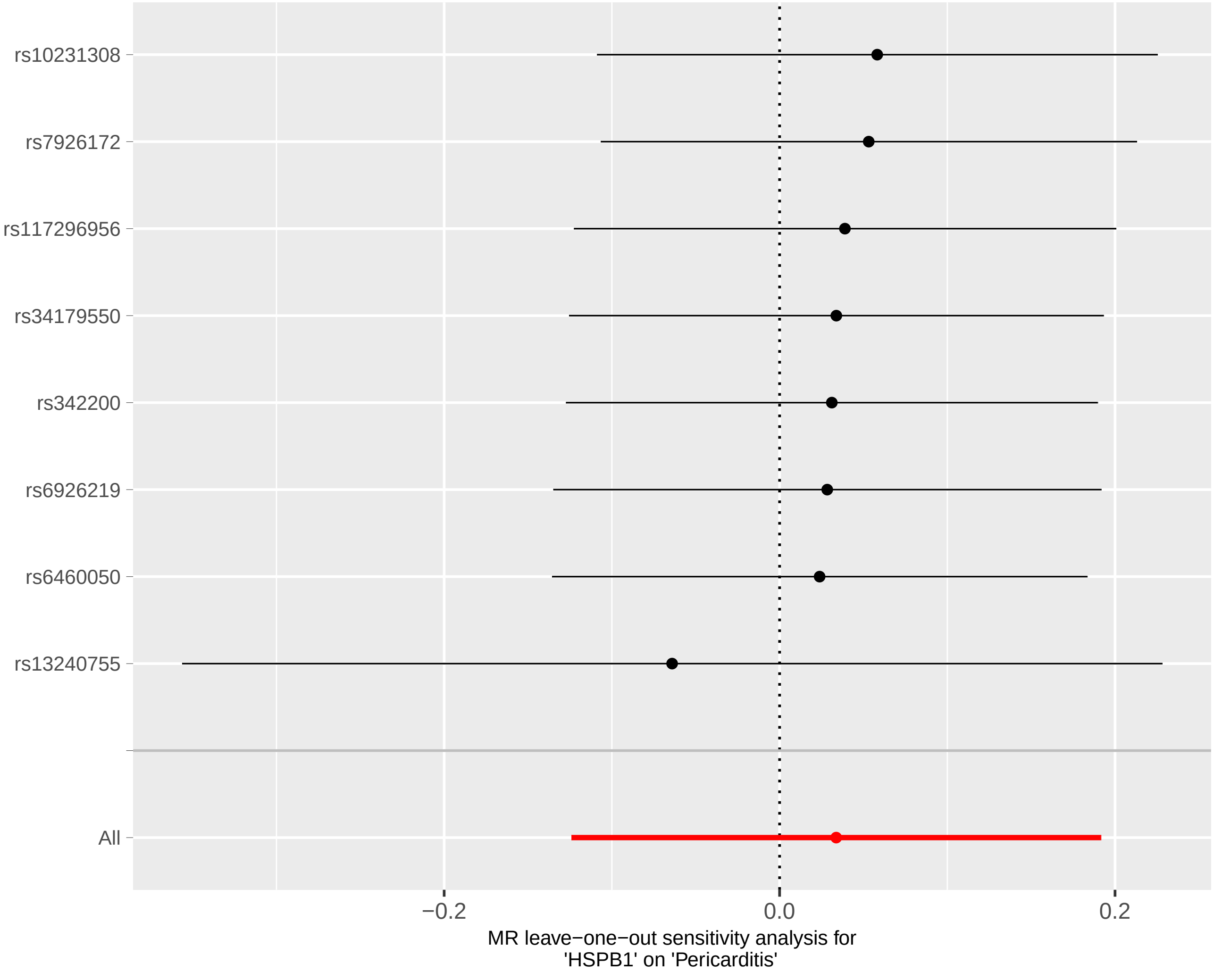
rs10231308
rs7926172
rs117296956
rs34179550
rs342200
rs6926219
rs6460050
rs13240755
All
−0.2
0.0
0.2
MR leave−one−out sensitivity analysis for
'HSPB1' on 'Pericarditis'

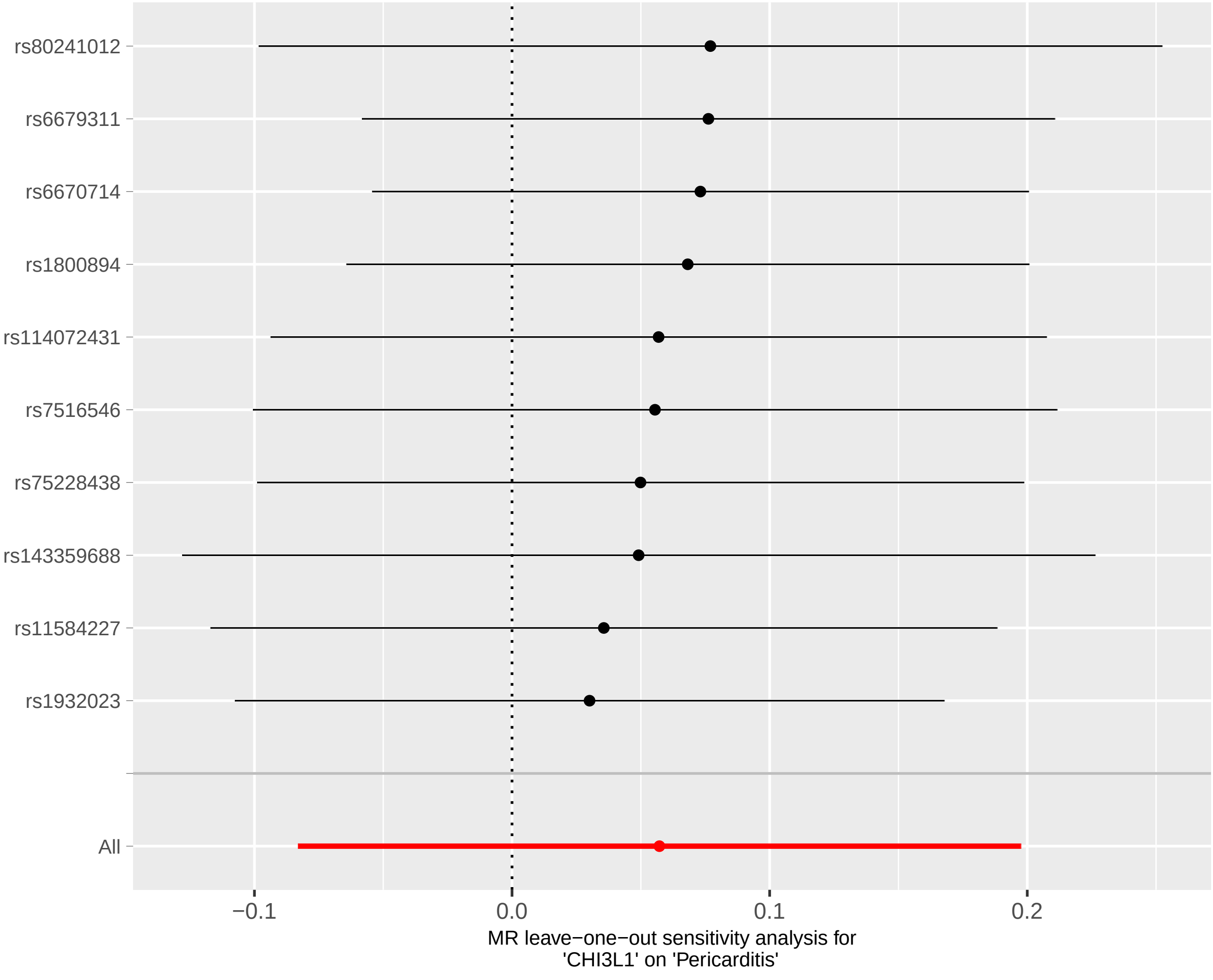
rs80241012
rs6679311
rs6670714
rs1800894
rs114072431
rs7516546
rs75228438
rs143359688
rs11584227
rs1932023
All
−0.1
0.0
0.1
0.2
MR leave−one−out sensitivity analysis for
'CHI3L1' on 'Pericarditis'

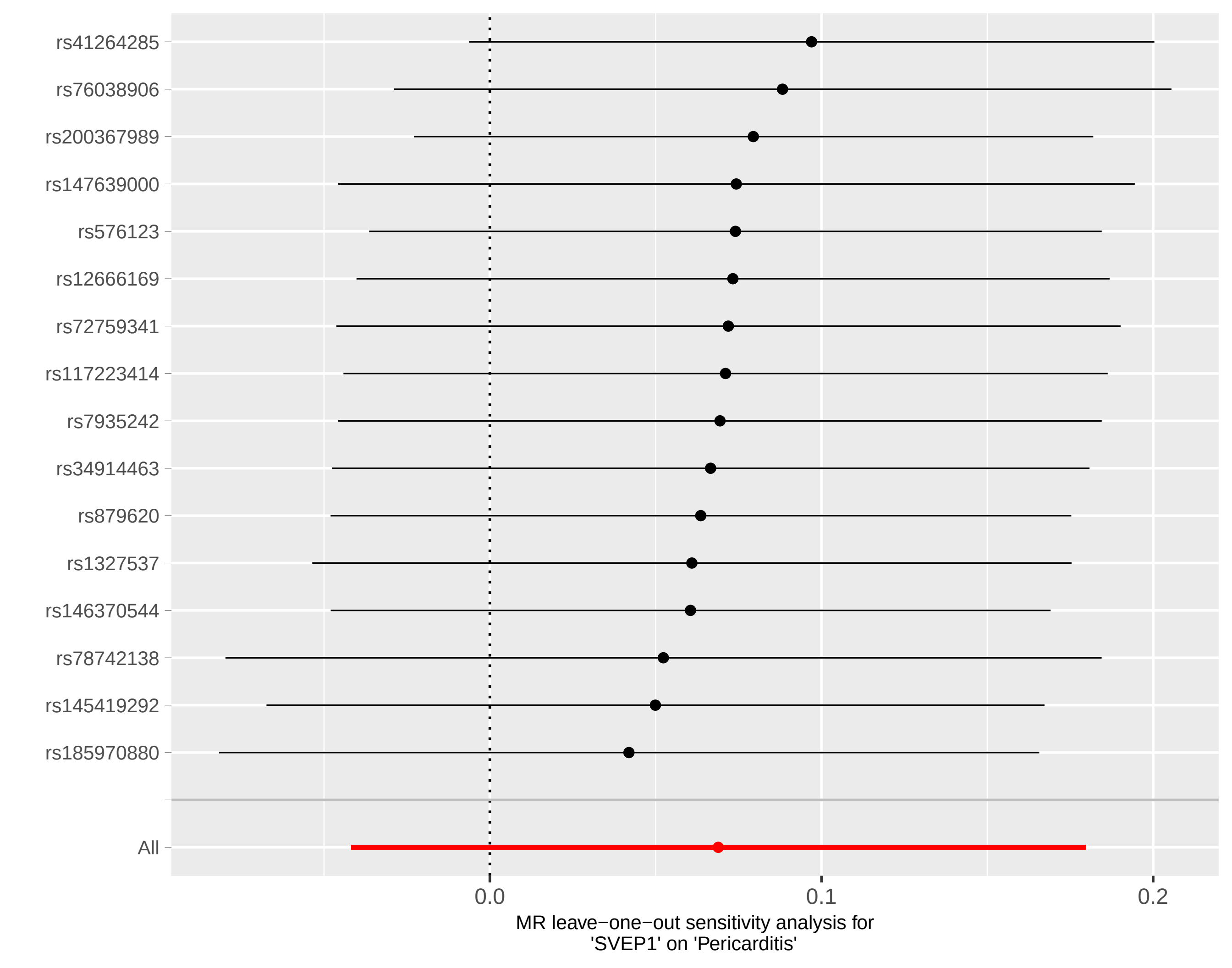
rs41264285
rs76038906
rs200367989
rs147639000
rs576123
rs12666169
rs72759341
rs117223414
rs7935242
rs34914463
rs879620
rs1327537
rs146370544
rs78742138
rs145419292
rs185970880
All
0.0
0.1
0.2
MR leave−one−out sensitivity analysis for
'SVEP1' on 'Pericarditis'

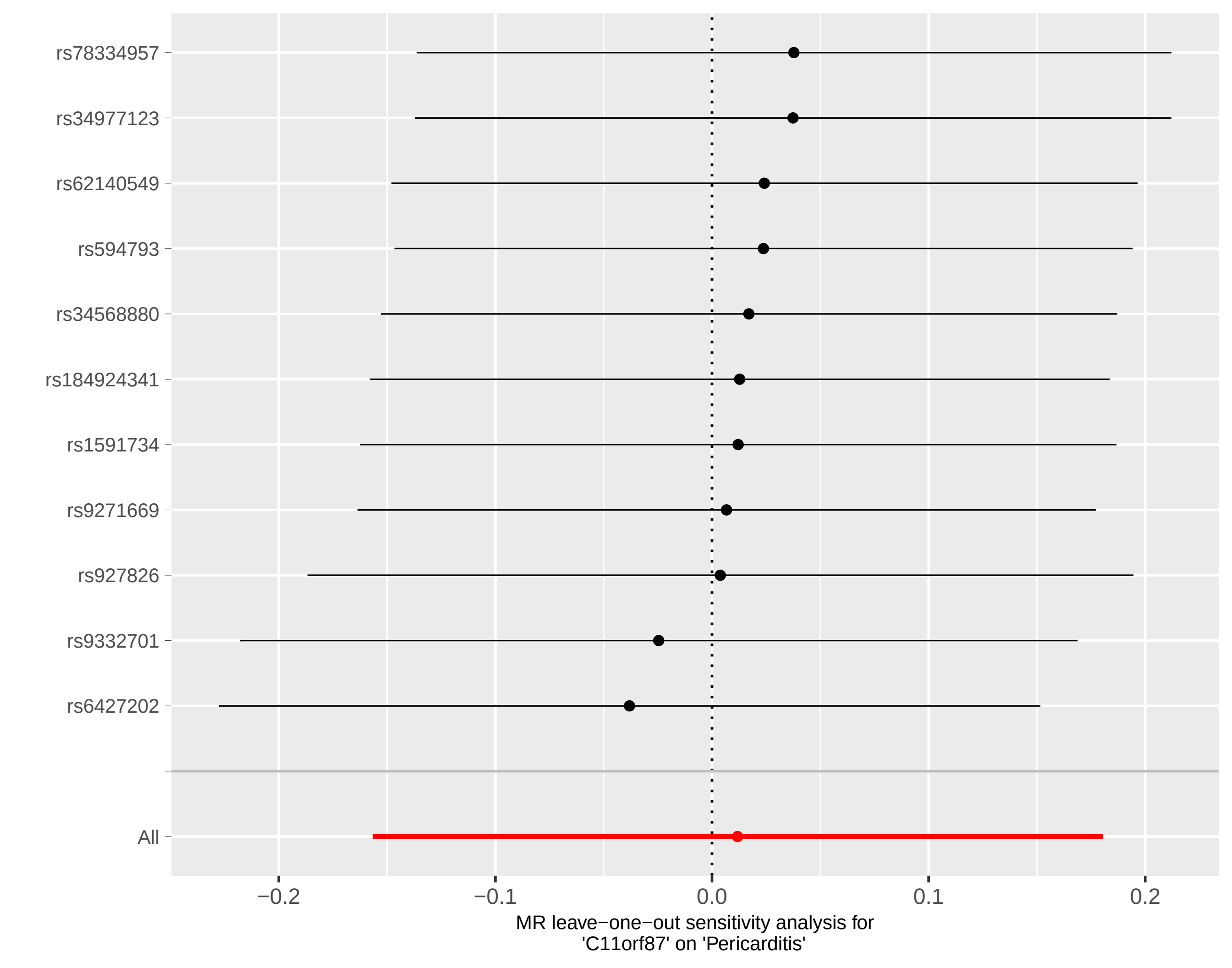

rs78334957
rs34977123
rs62140549
rs594793
rs34568880
rs184924341
rs1591734
rs9271669
rs927826
rs9332701
rs6427202
All
−0.2
−0.1
0.0
0.1
0.2
MR leave−one−out sensitivity analysis for
'C11orf87' on 'Pericarditis'

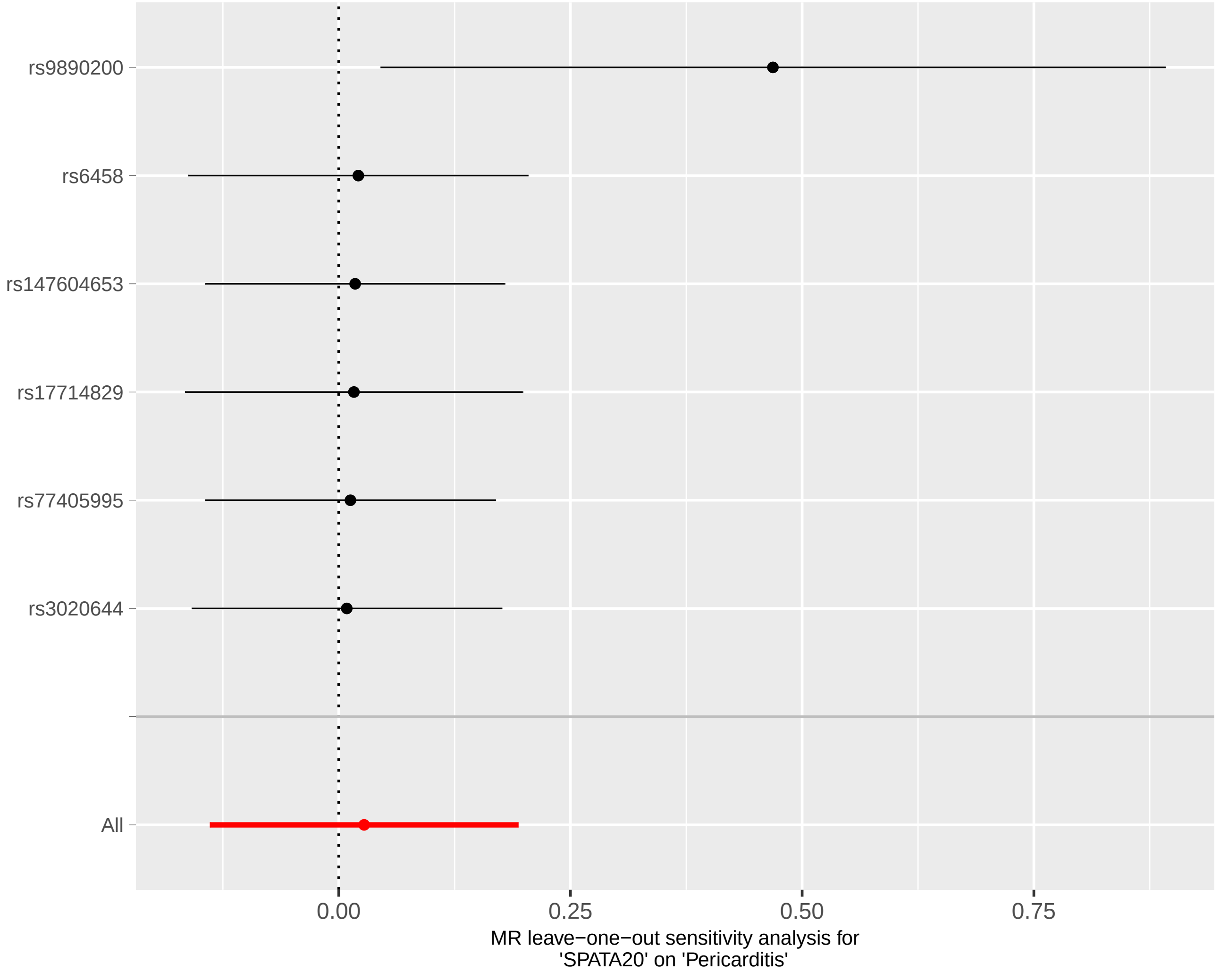
rs9890200
rs6458
rs147604653
rs17714829
rs77405995
rs3020644
All
0.00
0.25
0.50
0.75
MR leave−one−out sensitivity analysis for
'SPATA20' on 'Pericarditis'

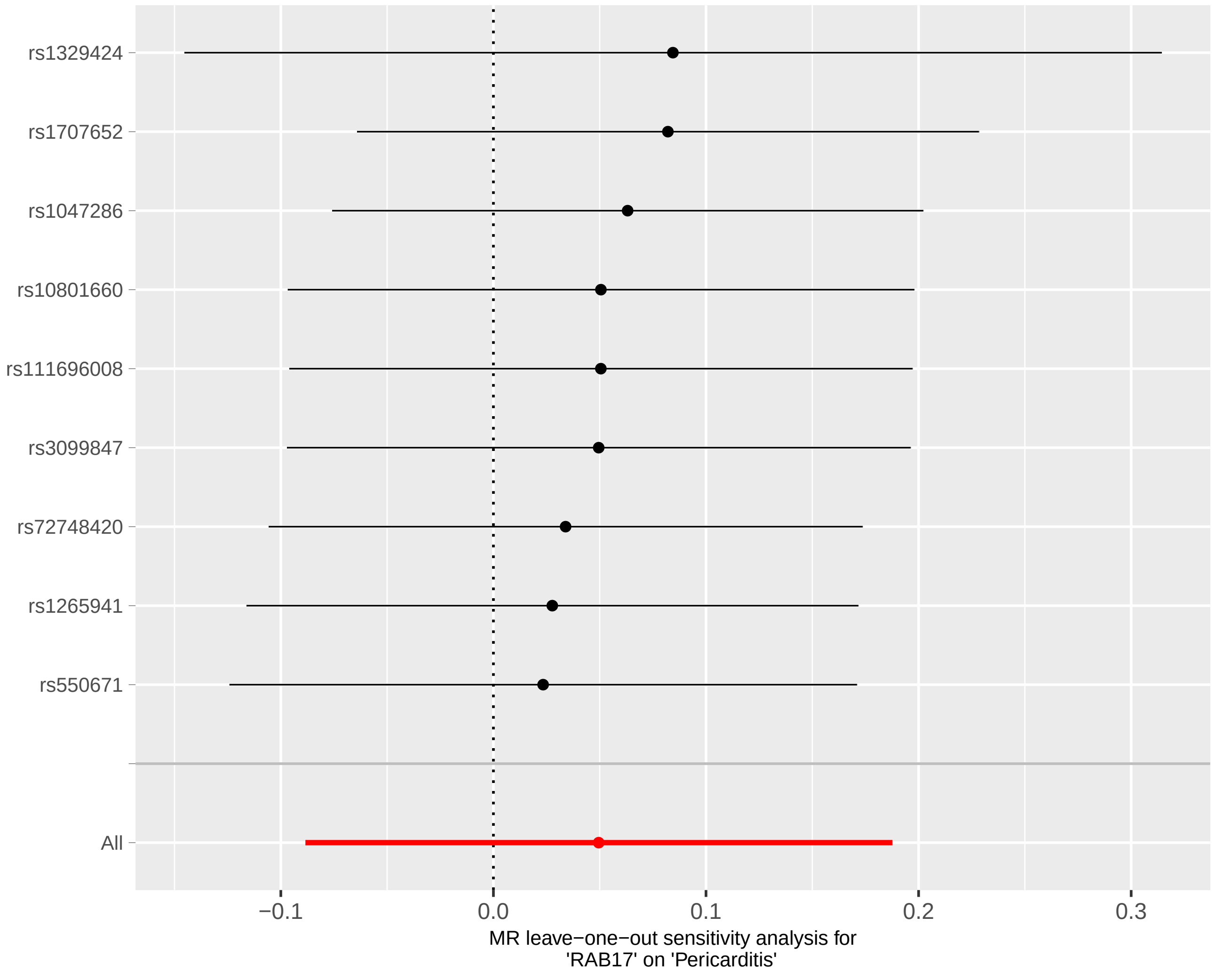
rs1329424
rs1707652
rs1047286
rs10801660
rs111696008
rs3099847
rs72748420
rs1265941
rs550671
All
−0.1
0.0
0.1
0.2
0.3
MR leave−one−out sensitivity analysis for
'RAB17' on 'Pericarditis'

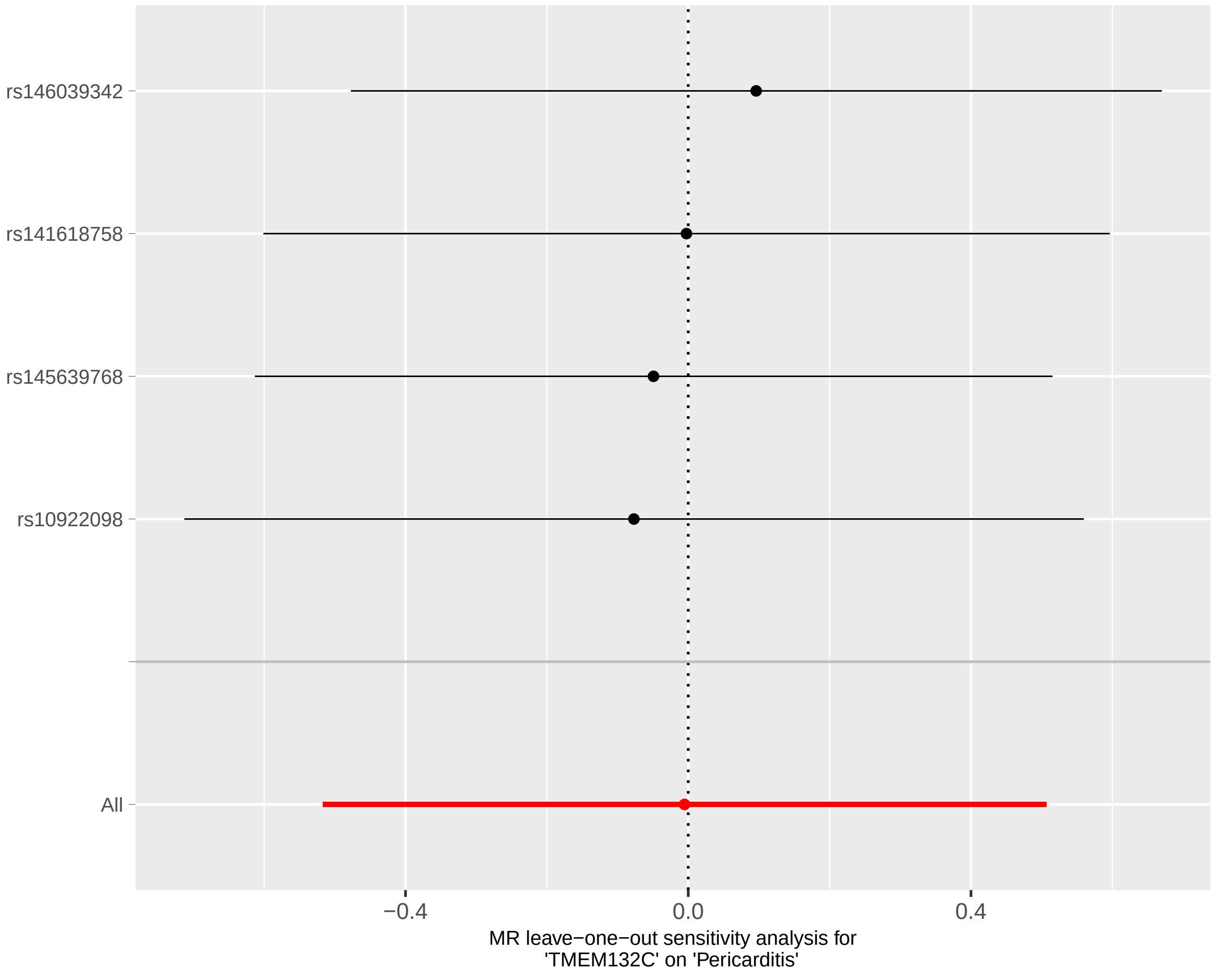
rs146039342
rs141618758
rs145639768
rs10922098
All
−0.4
0.0
0.4
MR leave−one−out sensitivity analysis for
'TMEM132C' on 'Pericarditis'

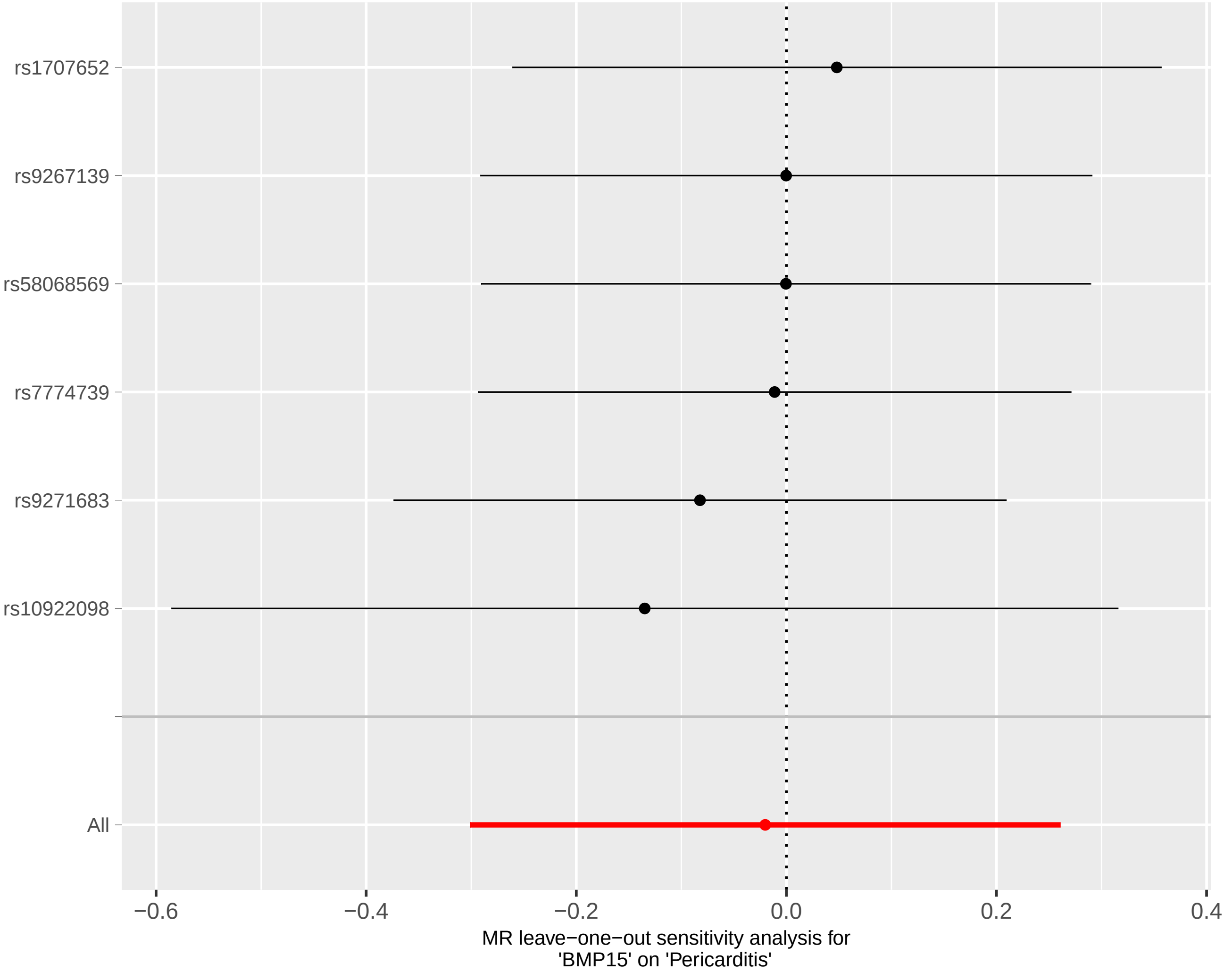
rs1707652
rs9267139
rs58068569
rs7774739
rs9271683
rs10922098
All
−0.6
−0.4
−0.2
0.0
0.2
0.4
MR leave−one−out sensitivity analysis for
'BMP15' on 'Pericarditis'

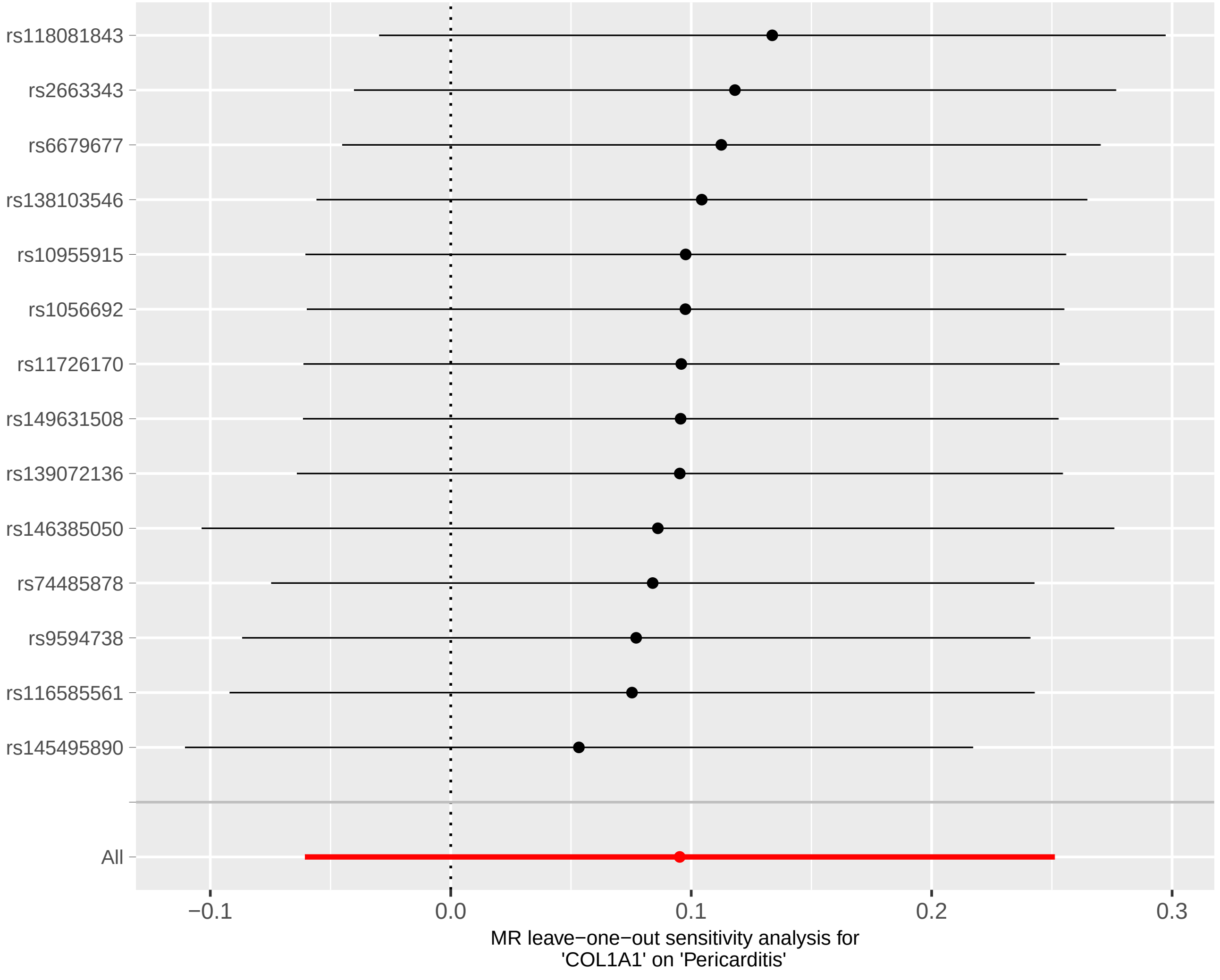
rs118081843
rs2663343
rs6679677
rs138103546
rs10955915
rs1056692
rs11726170
rs149631508
rs139072136
rs146385050
rs74485878
rs9594738
rs116585561
rs145495890
All
−0.1
0.0
0.1
0.2
0.3
MR leave−one−out sensitivity analysis for
'COL1A1' on 'Pericarditis'

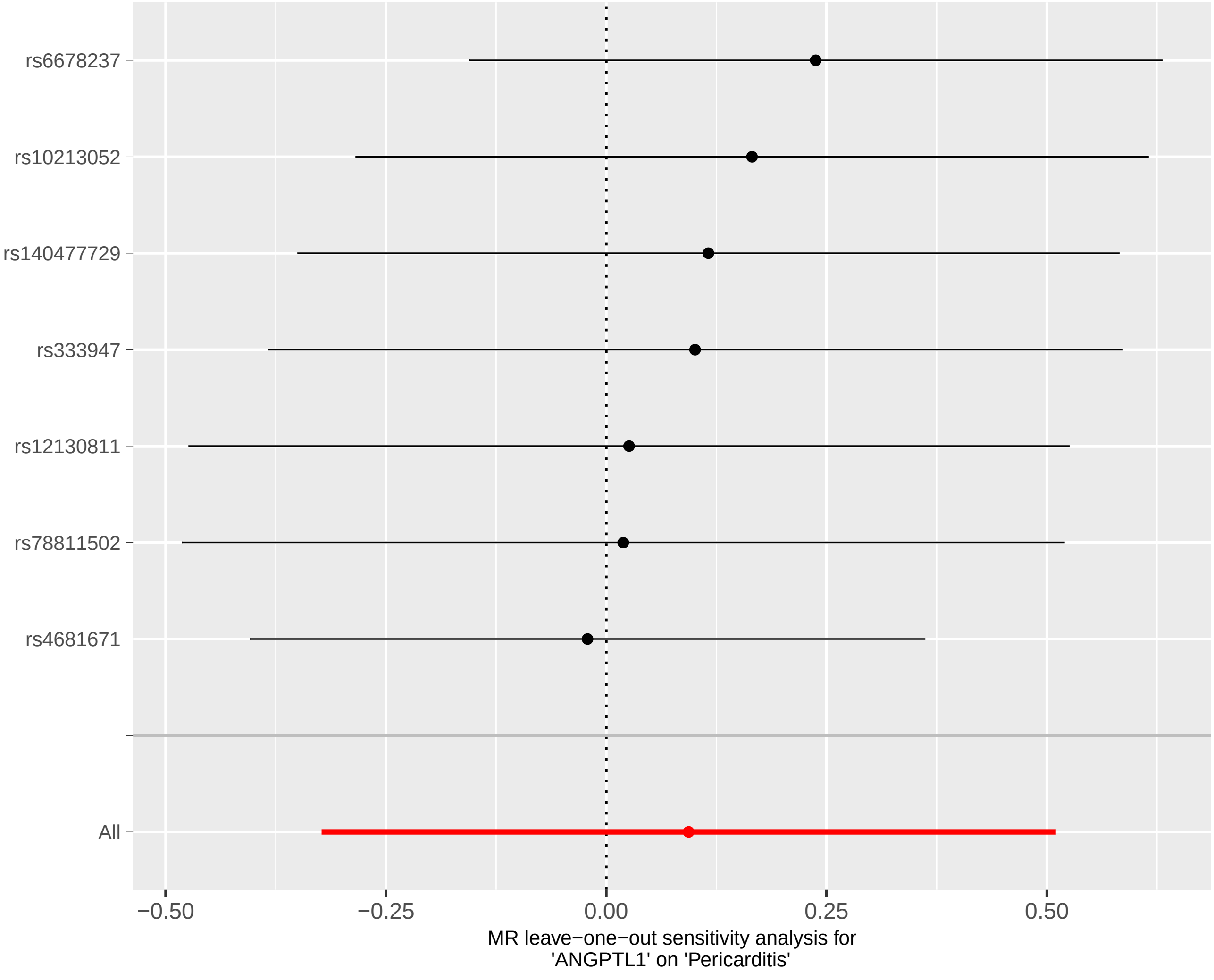

rs6678237
rs10213052
rs140477729
rs333947
rs12130811
rs78811502
rs4681671
All
−0.50
−0.25
0.00
0.25
0.50
MR leave−one−out sensitivity analysis for
'ANGPTL1' on 'Pericarditis'

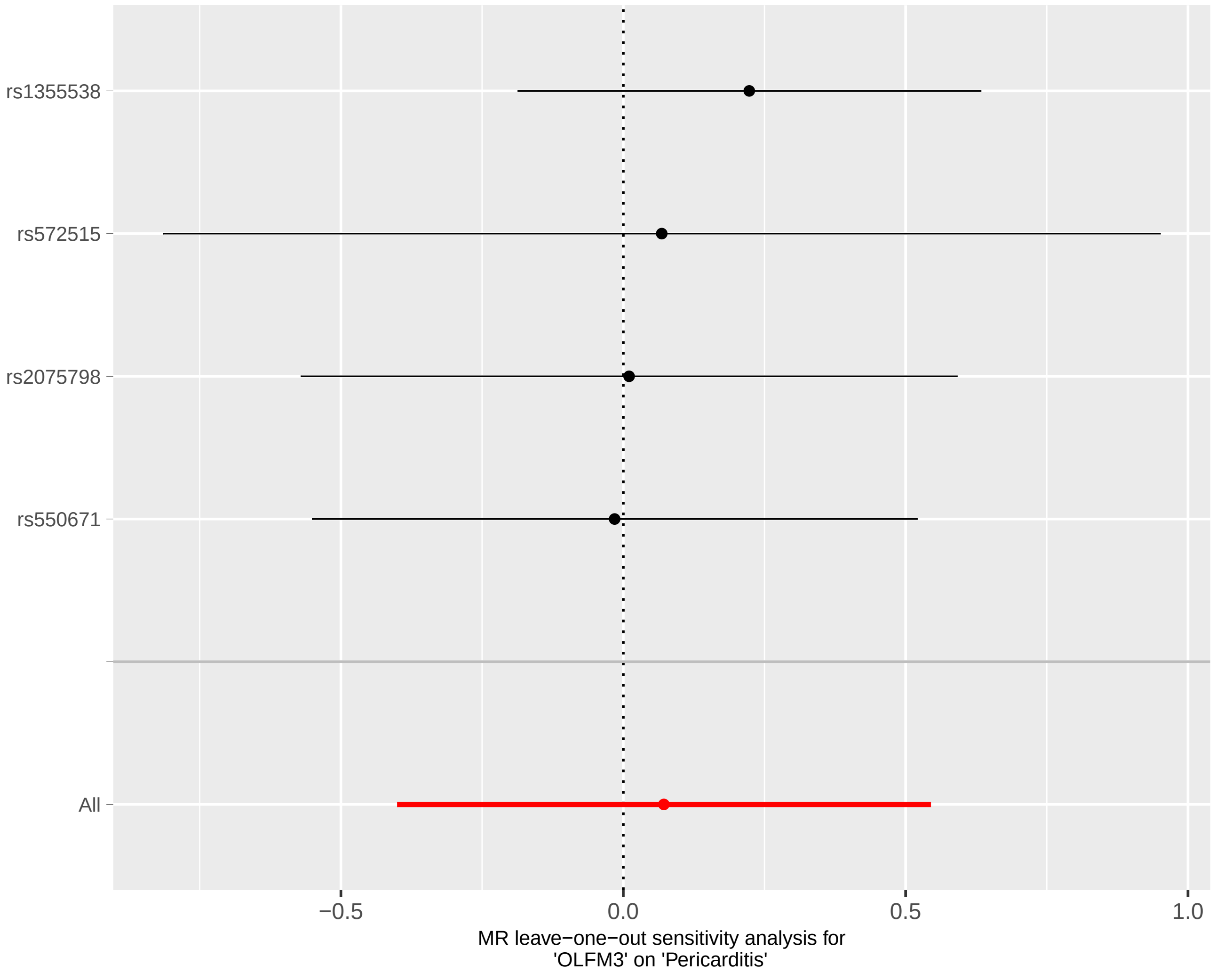
rs1355538
rs572515
rs2075798
rs550671
All
−0.5
0.0
0.5
1.0
MR leave−one−out sensitivity analysis for
'OLFM3' on 'Pericarditis'

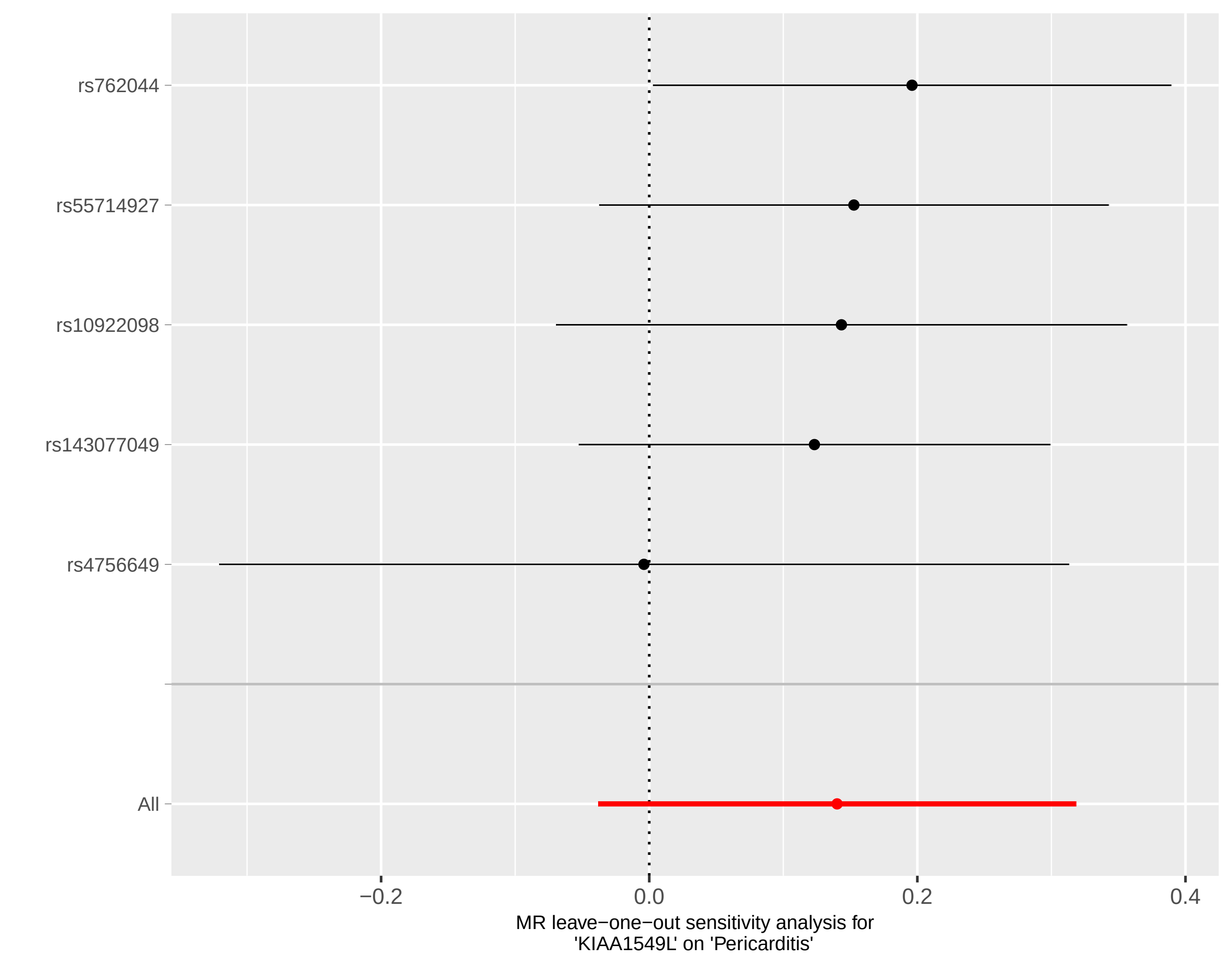

rs762044
rs55714927
rs10922098
rs143077049
rs4756649
All
−0.2
0.0
0.2
0.4
MR leave−one−out sensitivity analysis for
'KIAA1549L' on 'Pericarditis'

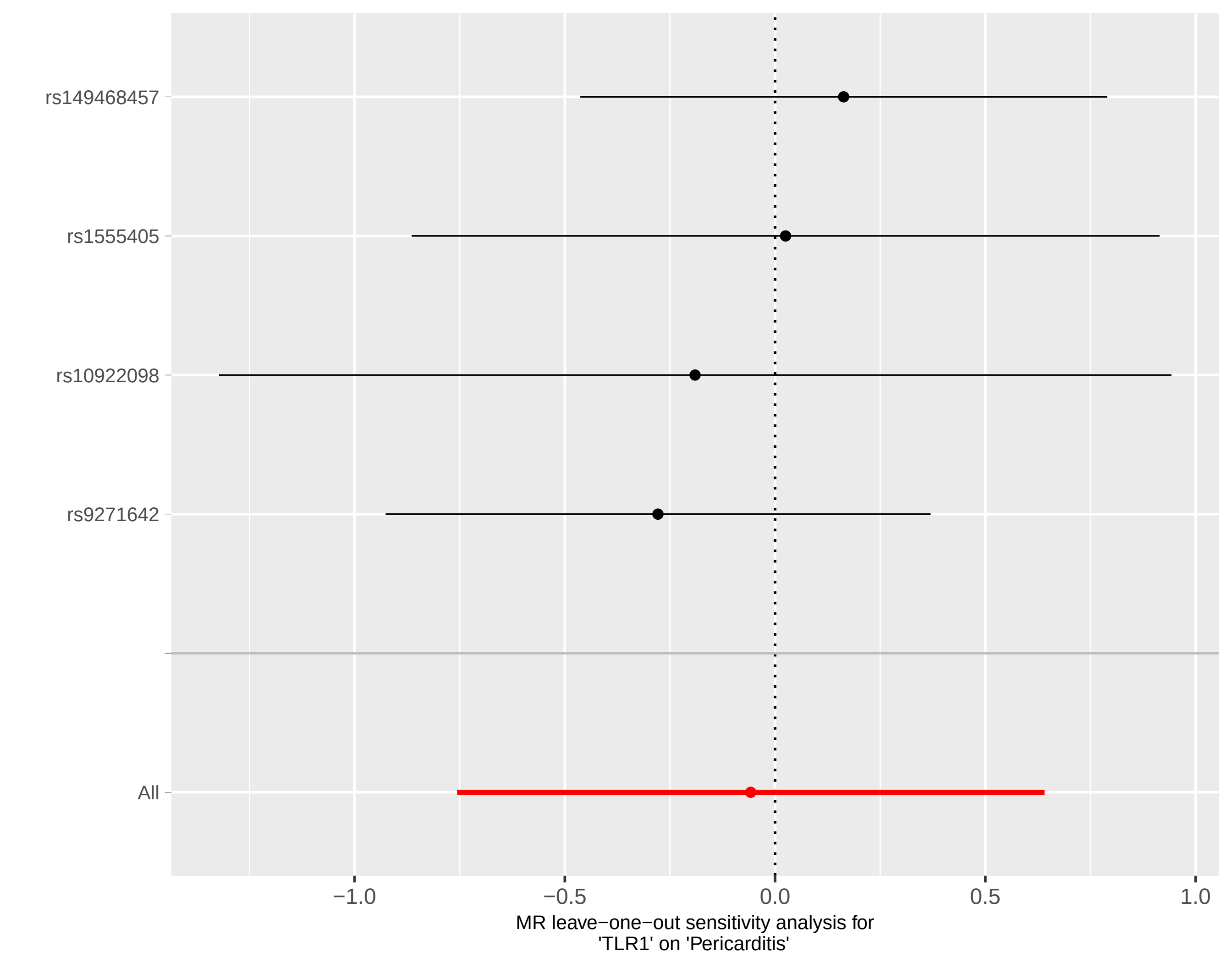
rs149468457
rs1555405
rs10922098
rs9271642
All
−1.0
−0.5
0.0
0.5
1.0
MR leave−one−out sensitivity analysis for
'TLR1' on 'Pericarditis'

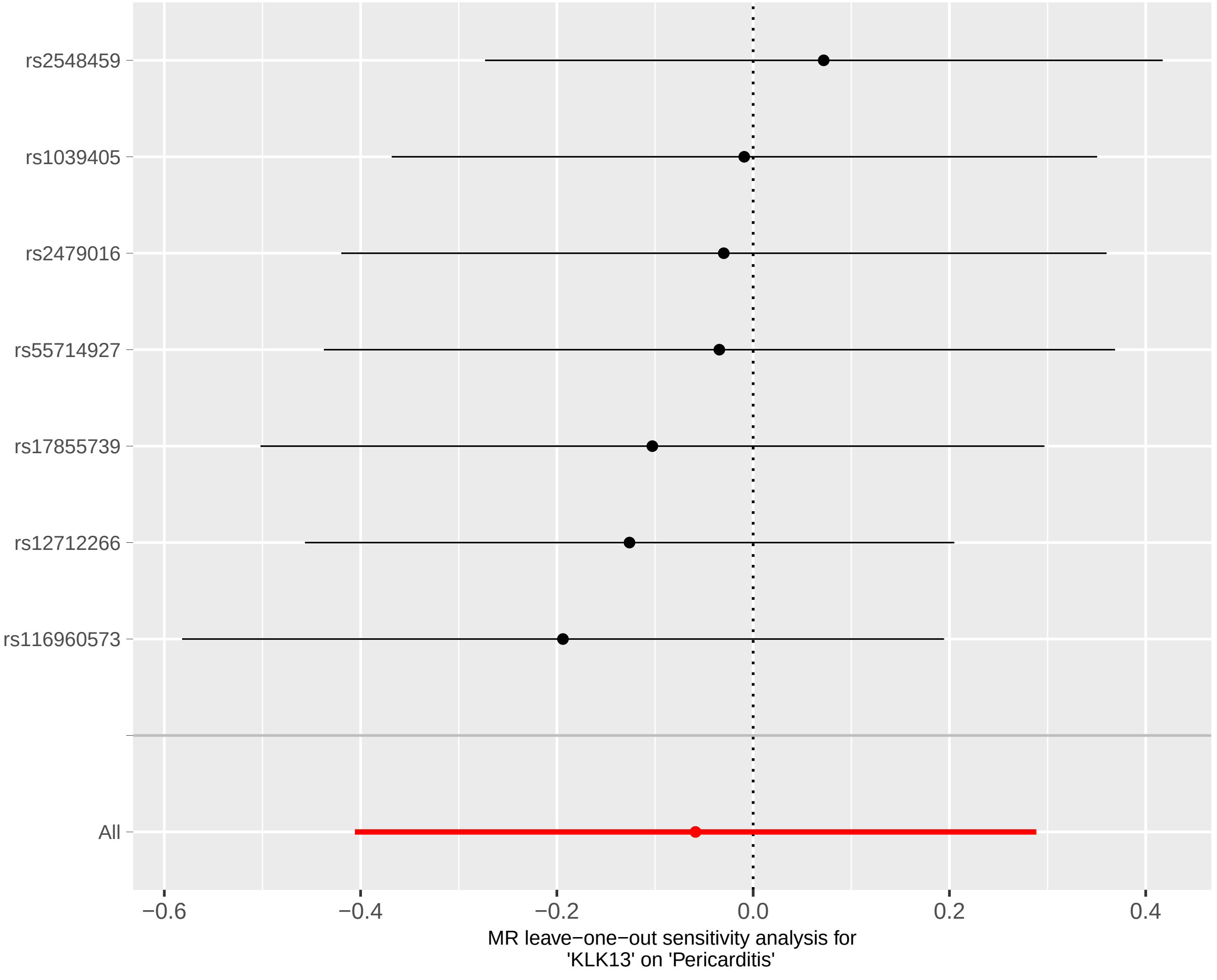

rs2548459
rs1039405
rs2479016
rs55714927
rs17855739
rs12712266
rs116960573
All
−0.6
−0.4
−0.2
0.0
0.2
0.4
MR leave−one−out sensitivity analysis for
'KLK13' on 'Pericarditis'

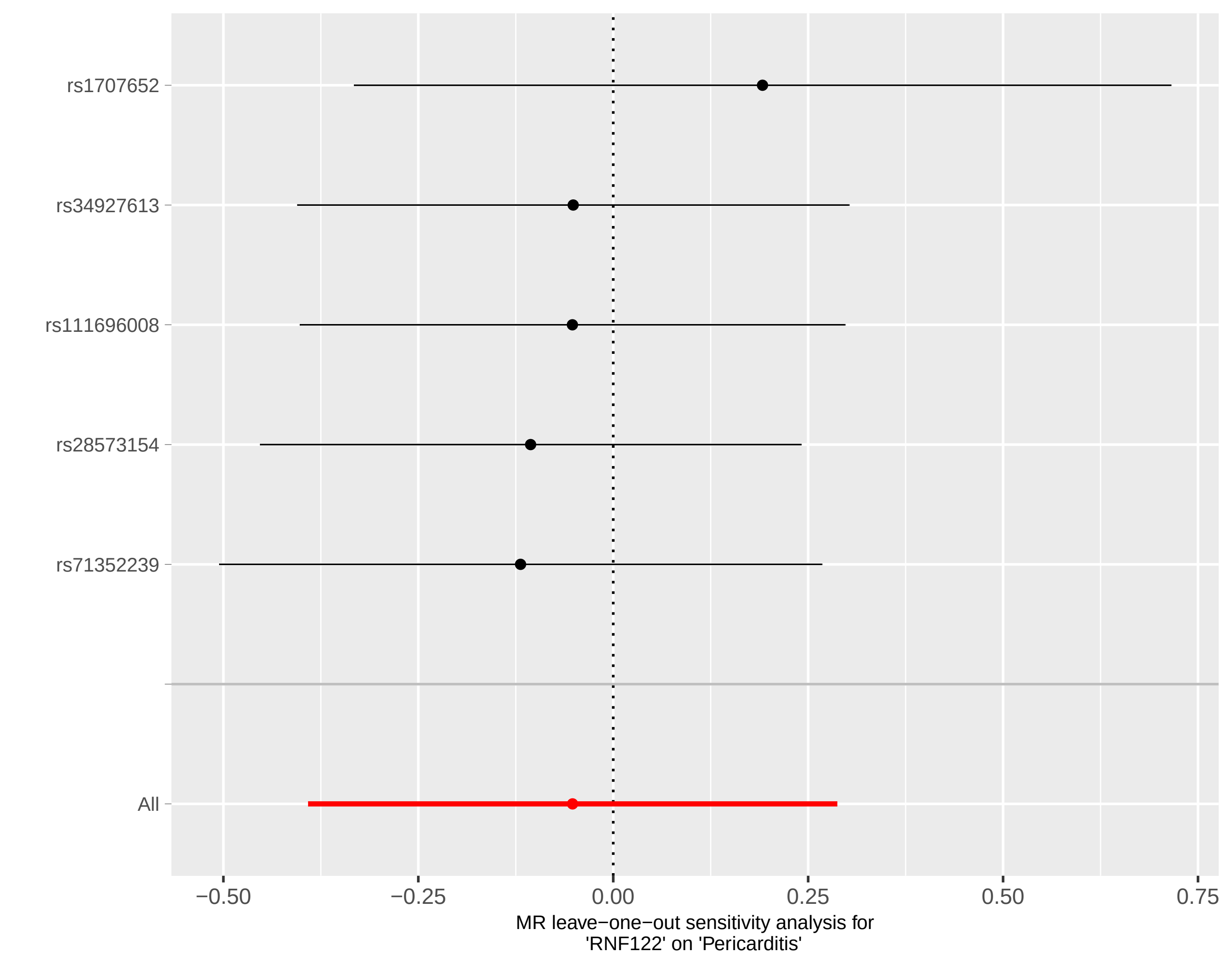
rs1707652
rs34927613
rs111696008
rs28573154
rs71352239
All
−0.50
−0.25
0.00
0.25
0.50
0.75
MR leave−one−out sensitivity analysis for
'RNF122' on 'Pericarditis'

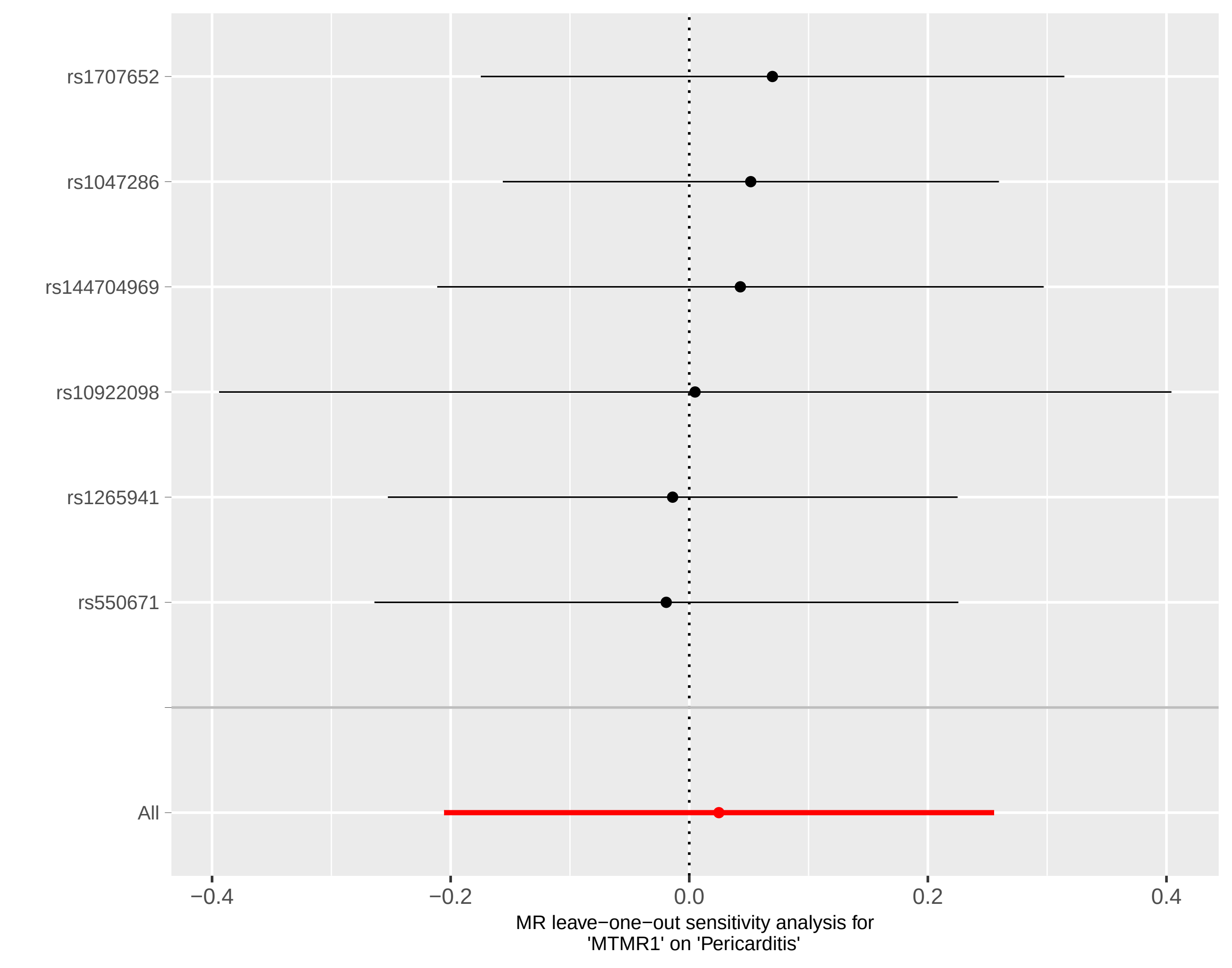

rs1707652
rs1047286
rs144704969
rs10922098
rs1265941
rs550671
All
−0.4
−0.2
0.0
0.2
0.4
MR leave−one−out sensitivity analysis for
'MTMR1' on 'Pericarditis'

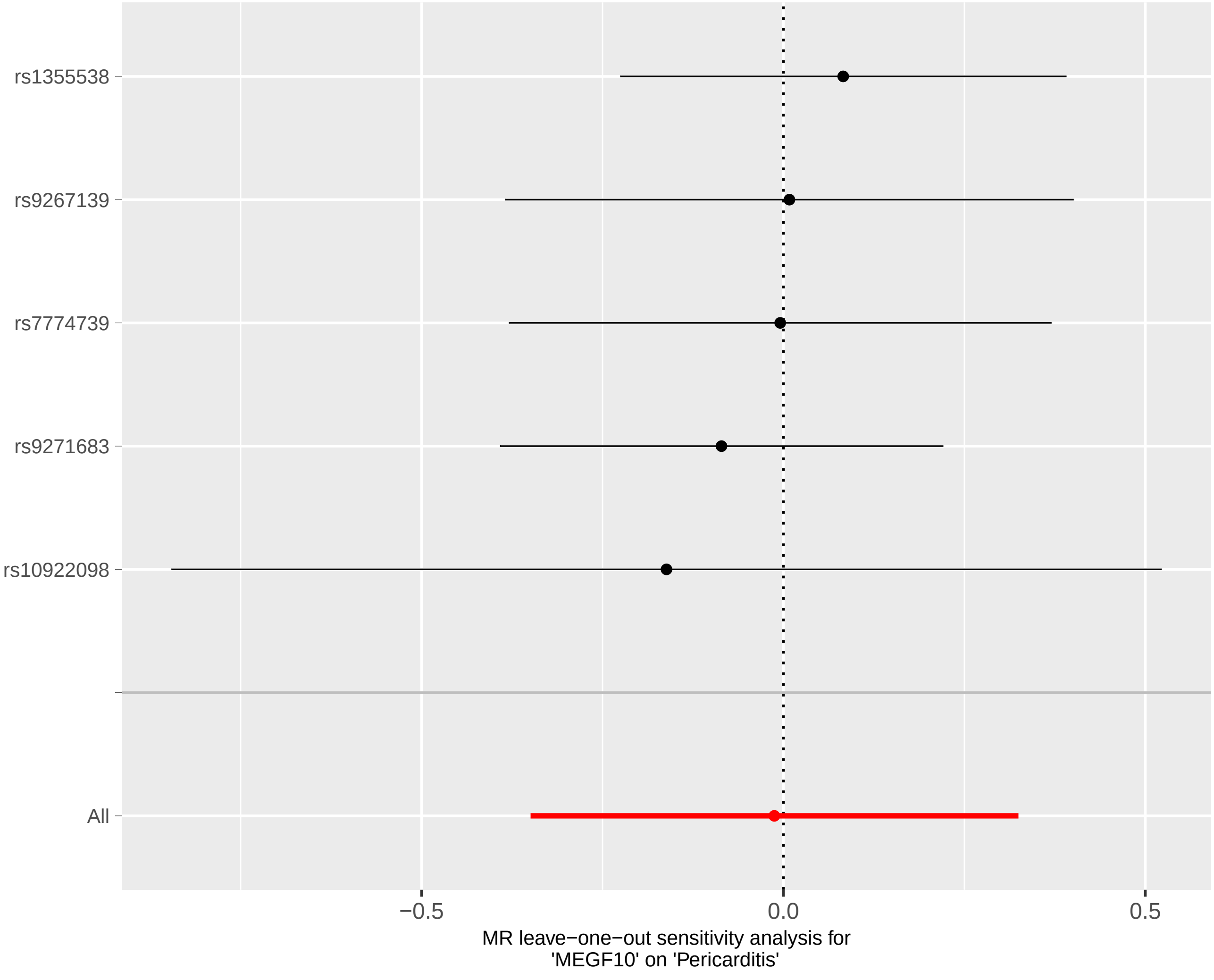
rs1355538
rs9267139
rs7774739
rs9271683
rs10922098
All
−0.5
0.0
0.5
MR leave−one−out sensitivity analysis for
'MEGF10' on 'Pericarditis'

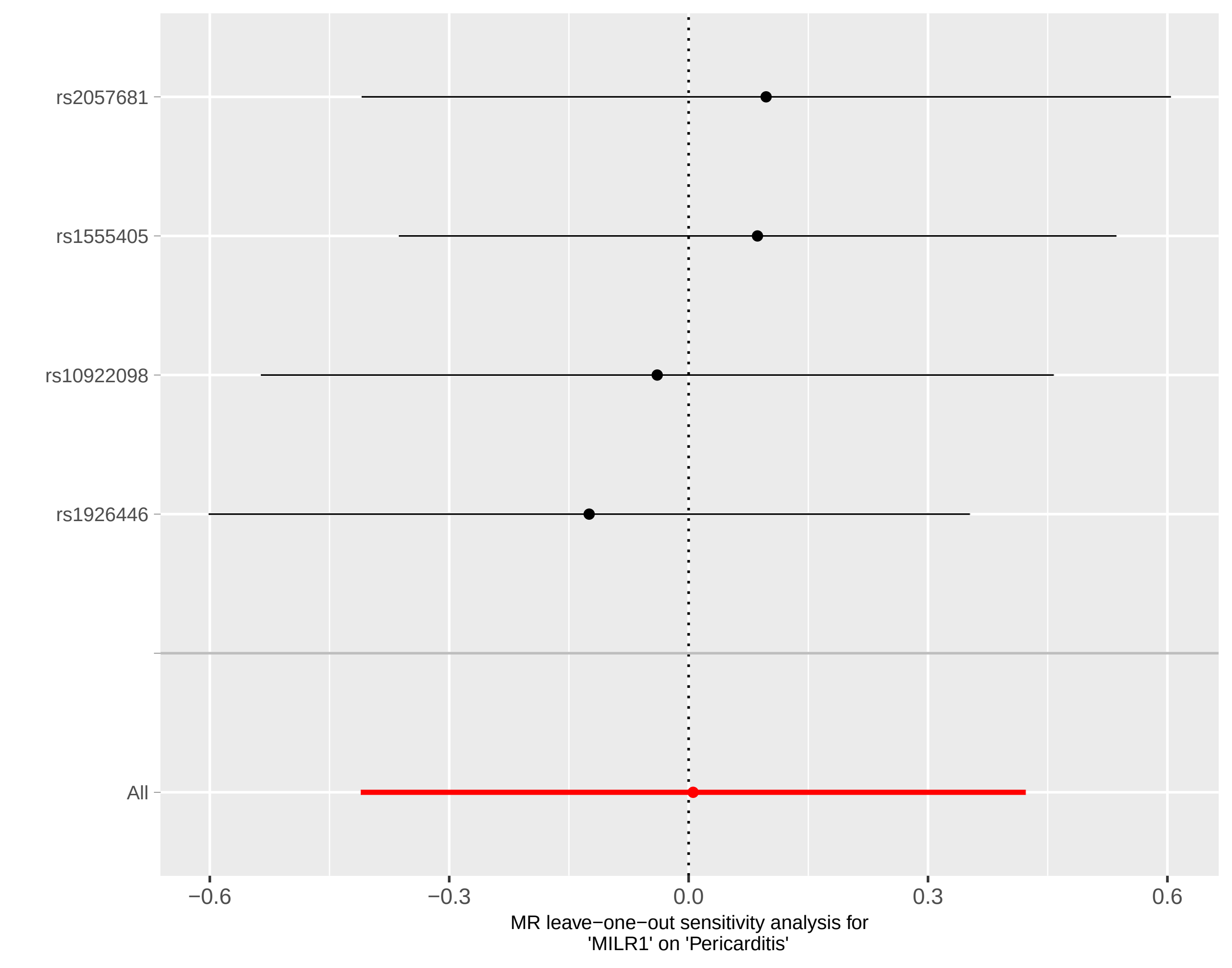

rs2057681
rs1555405
rs10922098
rs1926446
All
−0.6
−0.3
0.0
0.3
0.6
MR leave−one−out sensitivity analysis for
'MILR1' on 'Pericarditis'

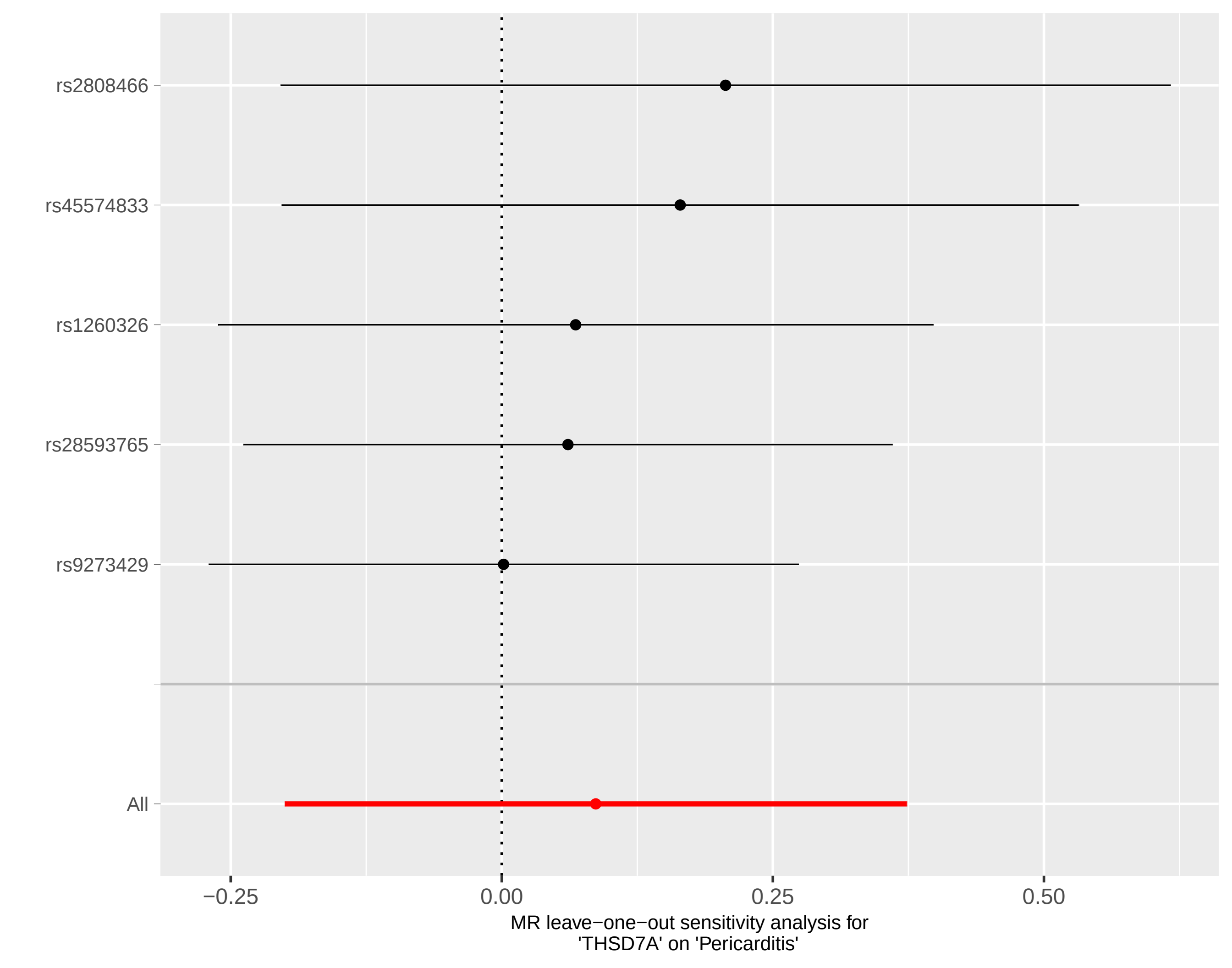

rs2808466
rs45574833
rs1260326
rs28593765
rs9273429
All
−0.25
0.00
0.25
0.50
MR leave−one−out sensitivity analysis for
'THSD7A' on 'Pericarditis'

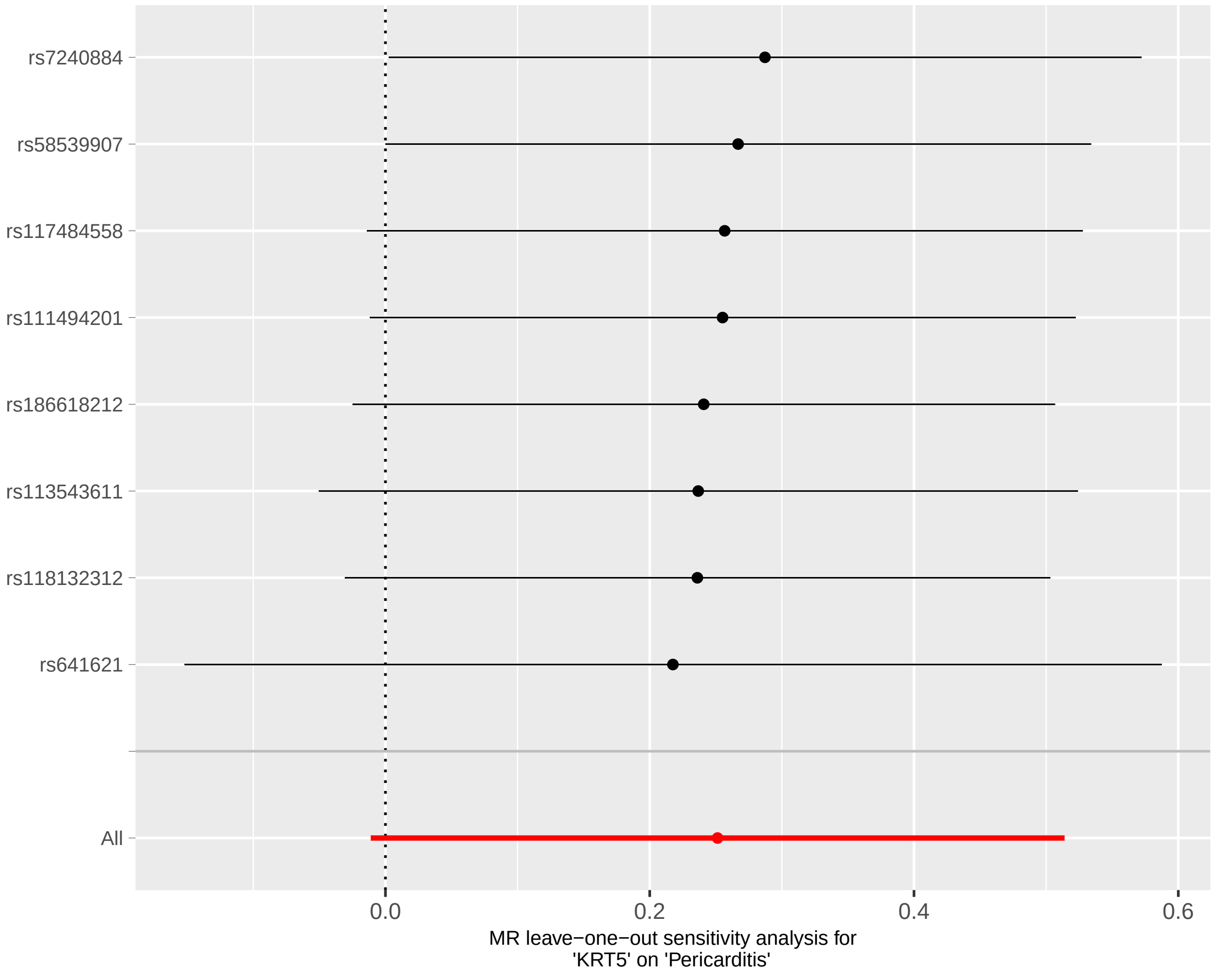
rs7240884
rs58539907
rs117484558
rs111494201
rs186618212
rs113543611
rs118132312
rs641621
All
0.0
0.2
0.4
0.6
MR leave−one−out sensitivity analysis for
'KRT5' on 'Pericarditis'

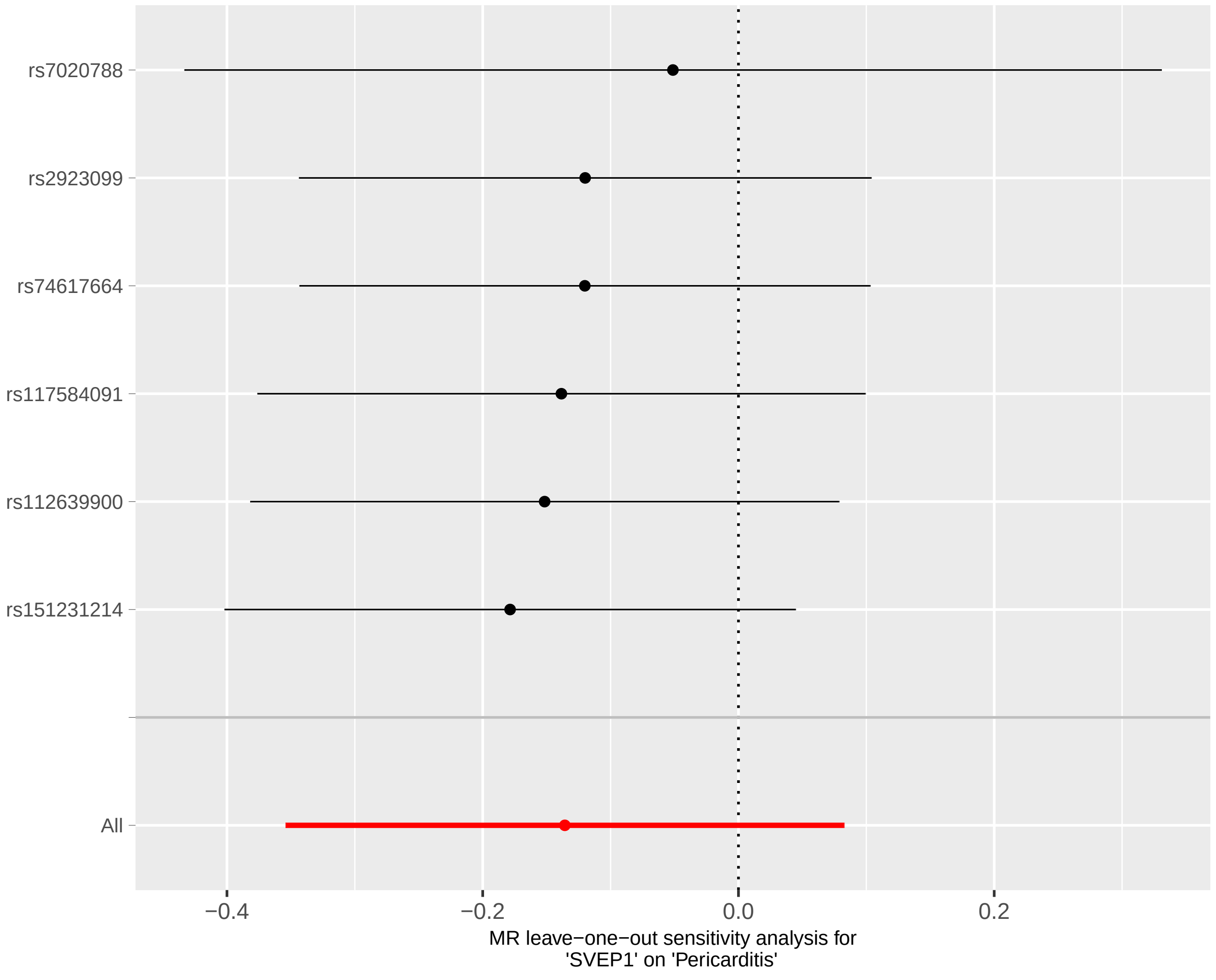

rs7020788
rs2923099
rs74617664
rs117584091
rs112639900
rs151231214
All
−0.4
−0.2
0.0
0.2
MR leave−one−out sensitivity analysis for
'SVEP1' on 'Pericarditis'

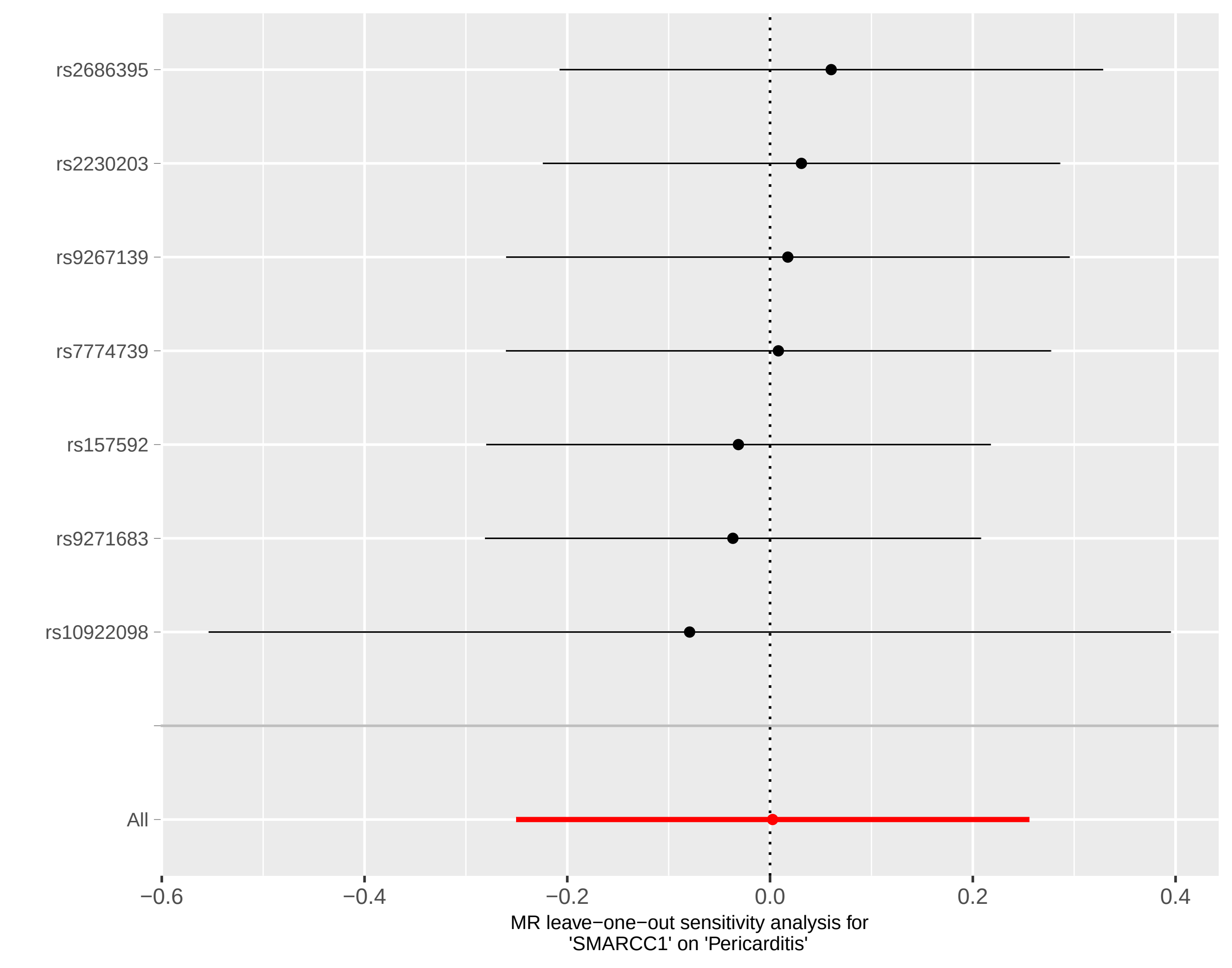
rs2686395
rs2230203
rs9267139
rs7774739
rs157592
rs9271683
rs10922098
All
−0.6
−0.4
−0.2
0.0
0.2
0.4
MR leave−one−out sensitivity analysis for
'SMARCC1' on 'Pericarditis'

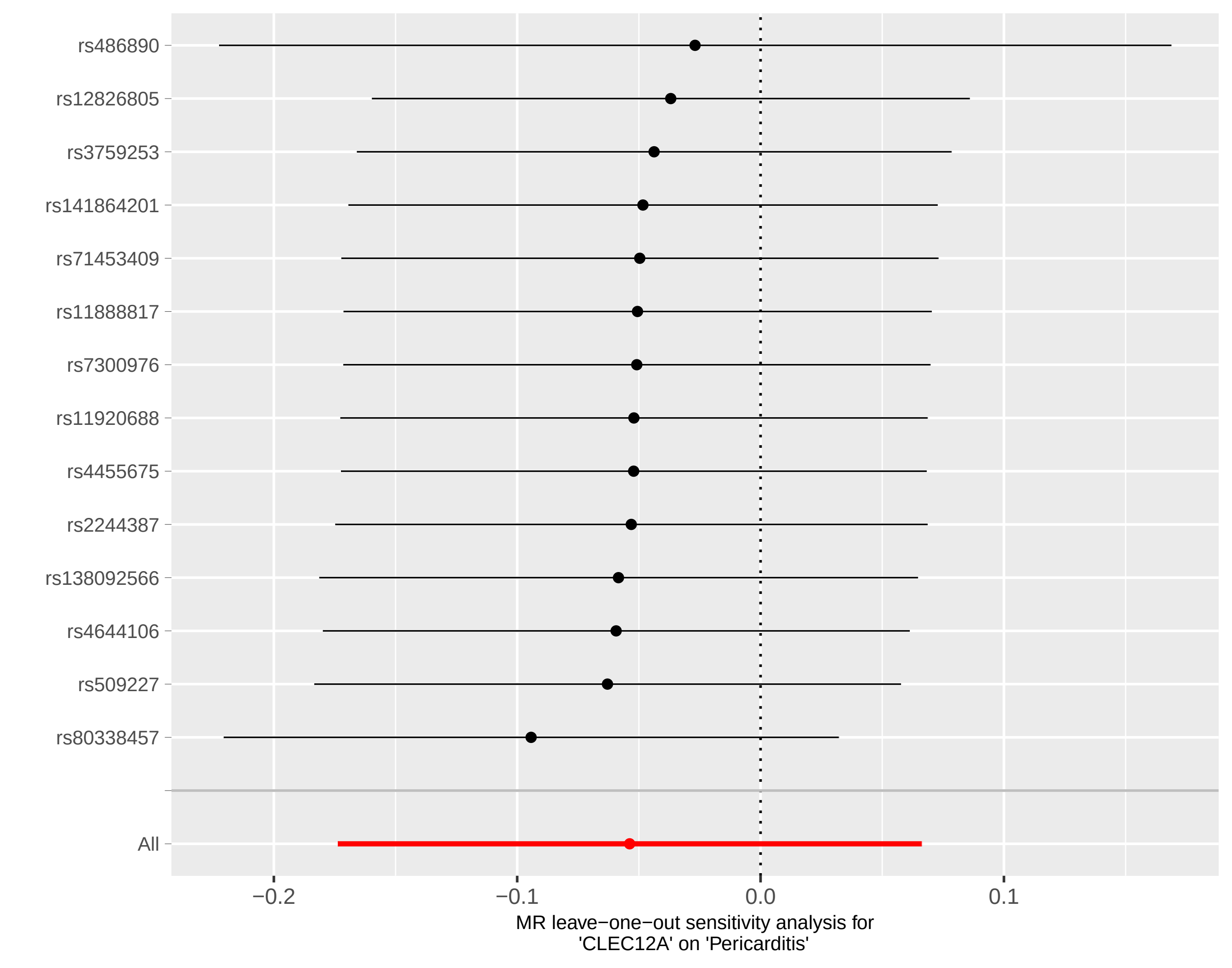
rs486890
rs12826805
rs3759253
rs141864201
rs71453409
rs11888817
rs7300976
rs11920688
rs4455675
rs2244387
rs138092566
rs4644106
rs509227
rs80338457
All
−0.2
−0.1
0.0
0.1
MR leave−one−out sensitivity analysis for
'CLEC12A' on 'Pericarditis'

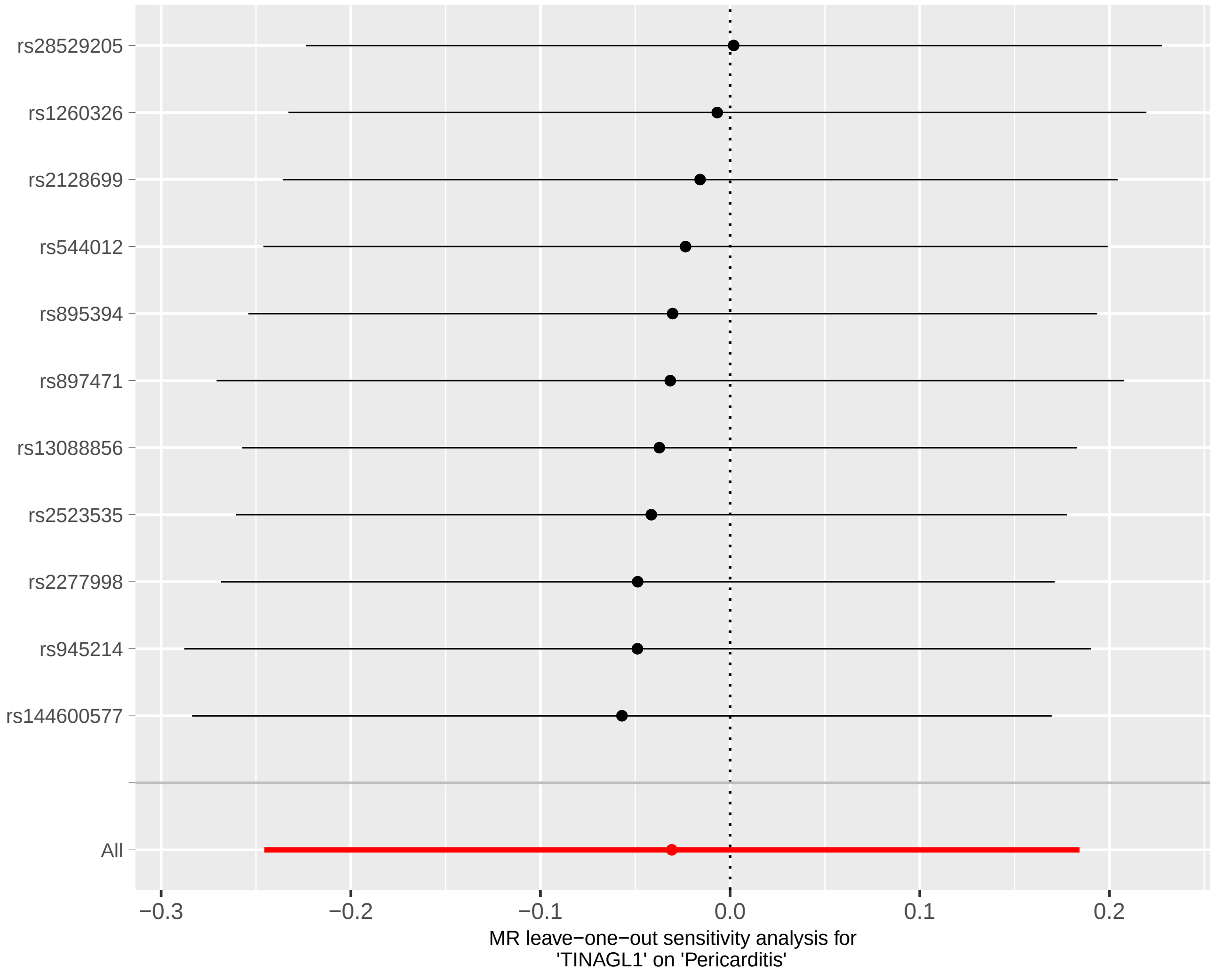

rs28529205
rs1260326
rs2128699
rs544012
rs895394
rs897471
rs13088856
rs2523535
rs2277998
rs945214
rs144600577
All
−0.3
−0.2
−0.1
0.0
0.1
0.2
MR leave−one−out sensitivity analysis for
'TINAGL1' on 'Pericarditis'

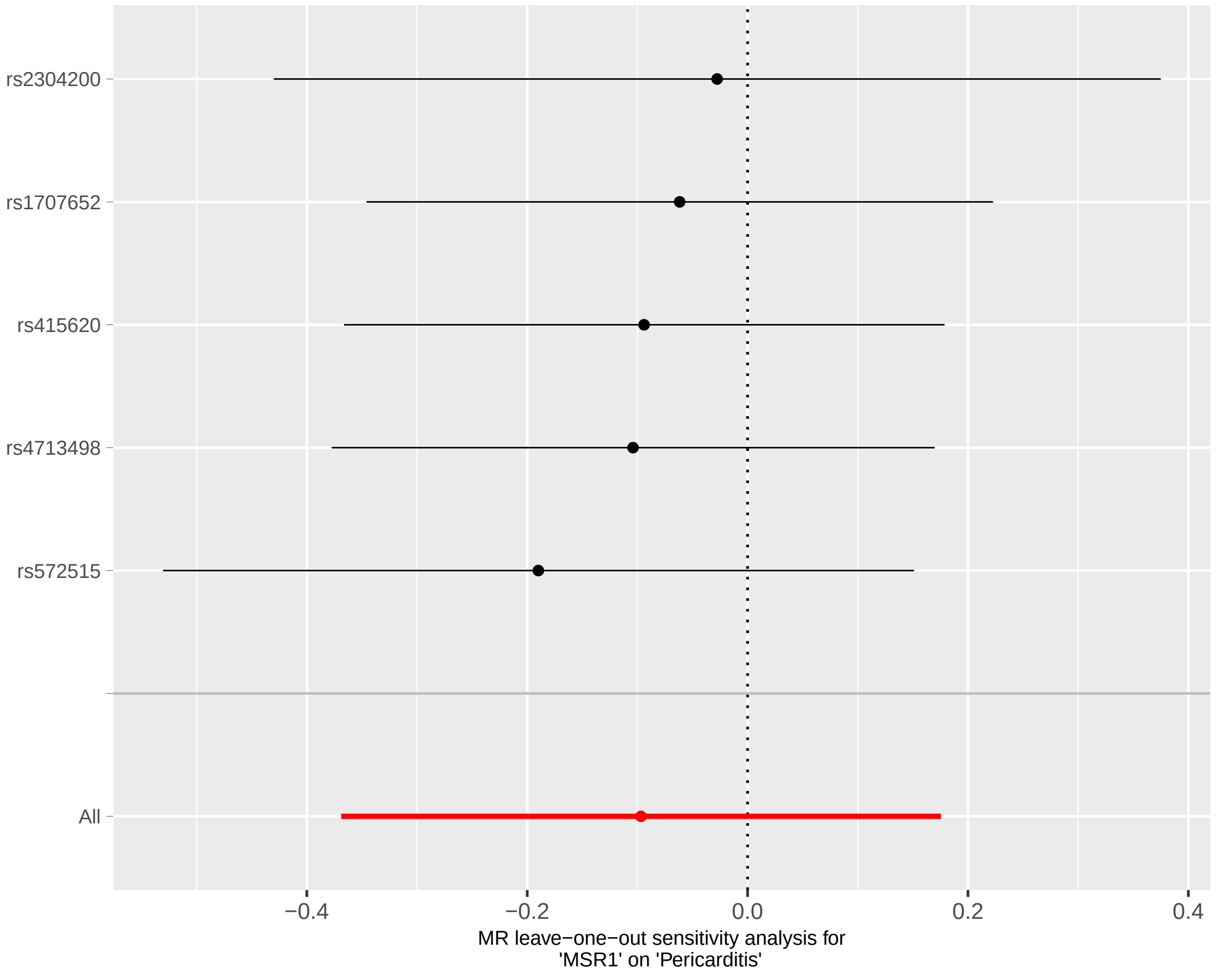

rs2304200
rs1707652
rs415620
rs4713498
rs572515
All
−0.4
−0.2
0.0
0.2
0.4
MR leave−one−out sensitivity analysis for
'MSR1' on 'Pericarditis'

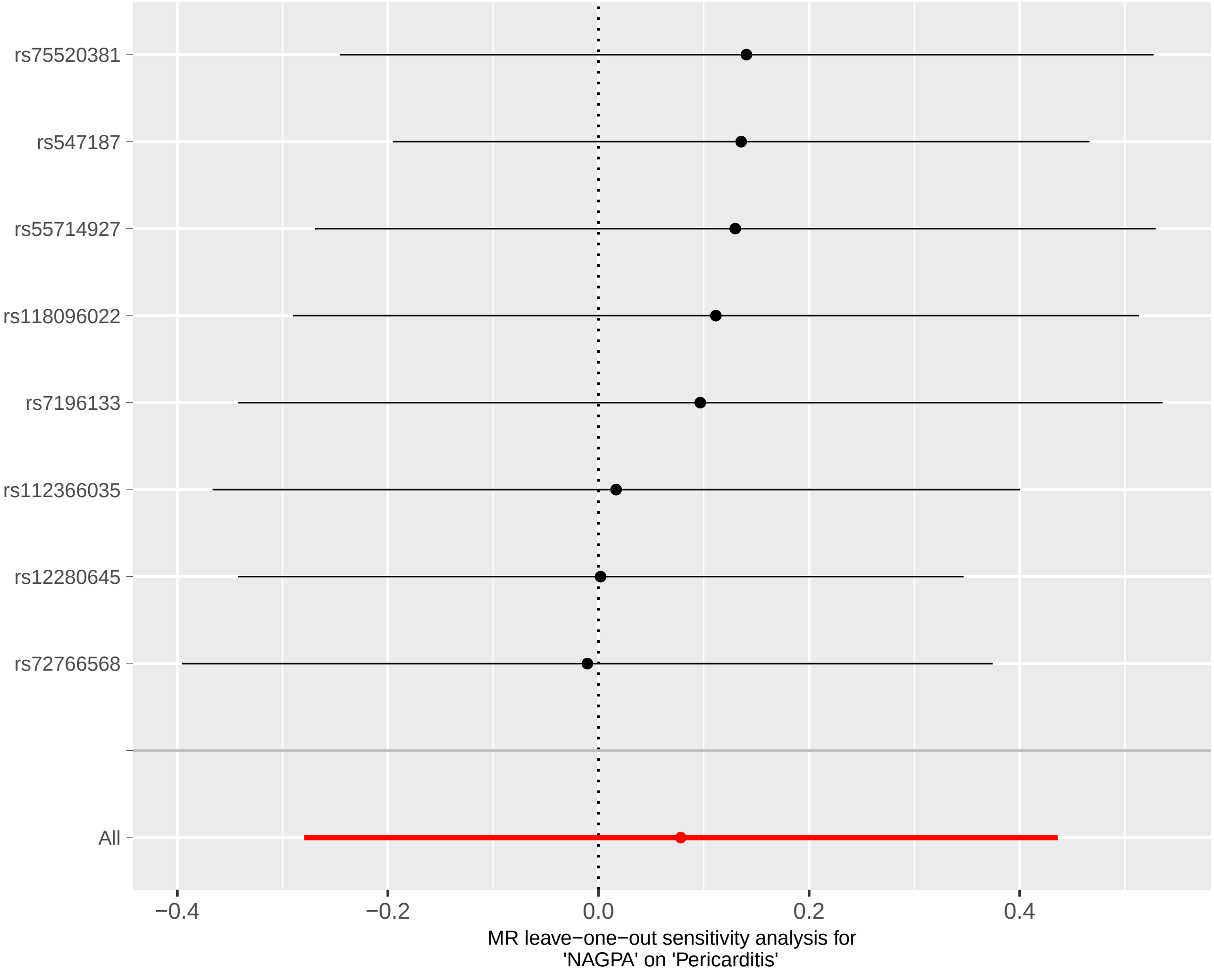
rs75520381
rs547187
rs55714927
rs118096022
rs7196133
rs112366035
rs12280645
rs72766568
All
−0.4
−0.2
0.0
0.2
0.4
MR leave−one−out sensitivity analysis for
'NAGPA' on 'Pericarditis'

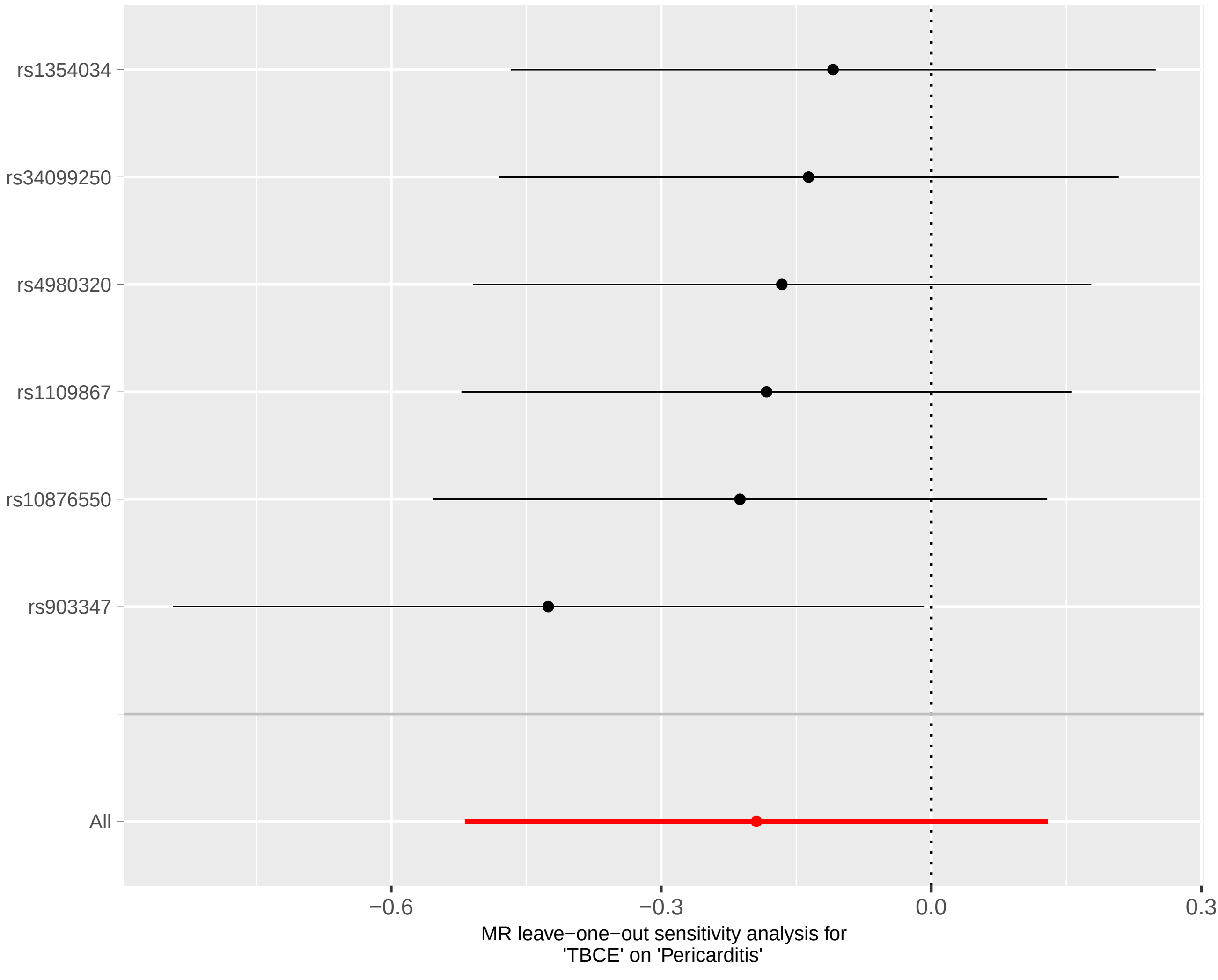
rs1354034
rs34099250
rs4980320
rs1109867
rs10876550
rs903347
All
−0.6
−0.3
0.0
0.3
MR leave−one−out sensitivity analysis for
'TBCE' on 'Pericarditis'

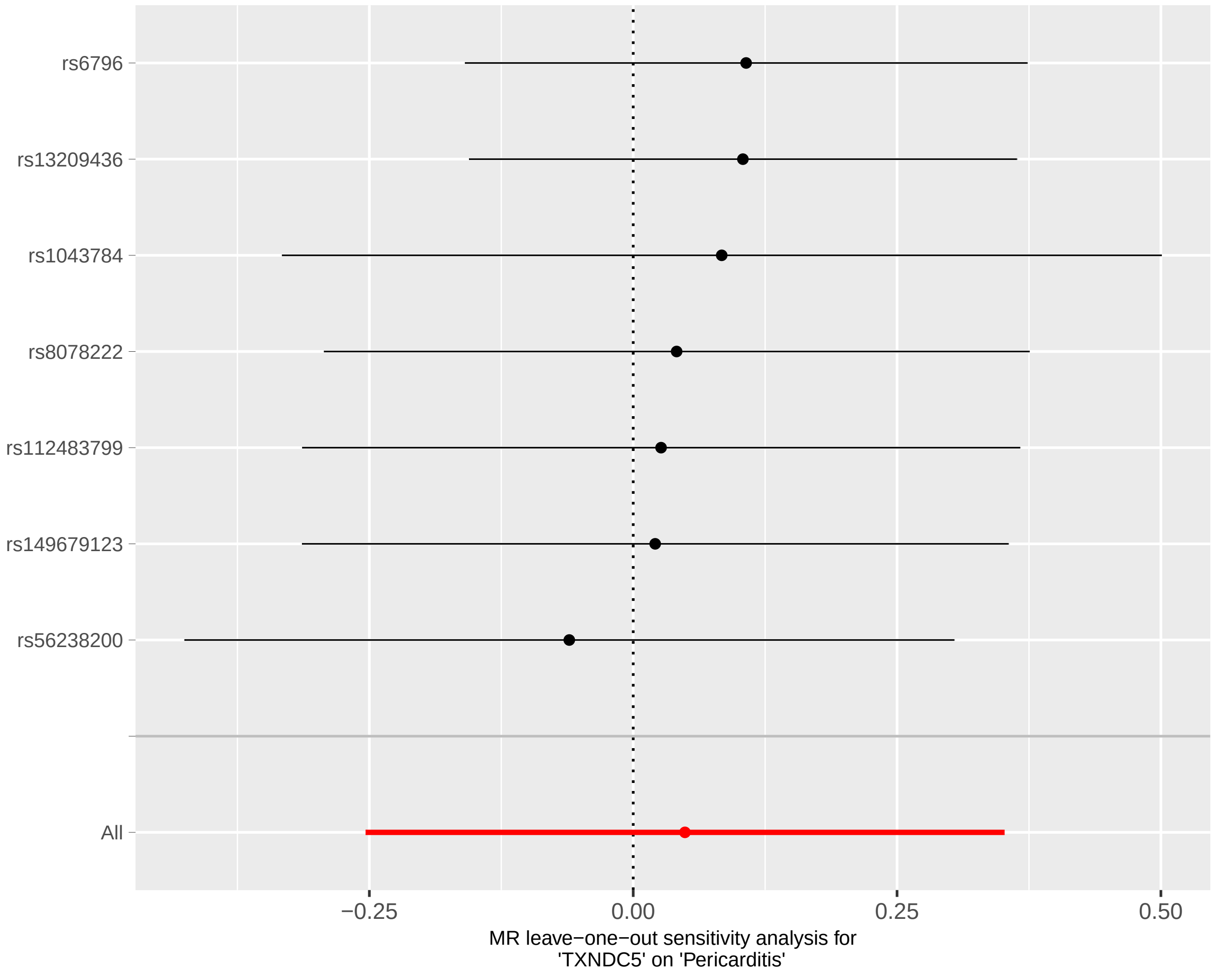
rs6796
rs13209436
rs1043784
rs8078222
rs112483799
rs149679123
rs56238200
All
−0.25
0.00
0.25
0.50
MR leave−one−out sensitivity analysis for
'TXNDC5' on 'Pericarditis'

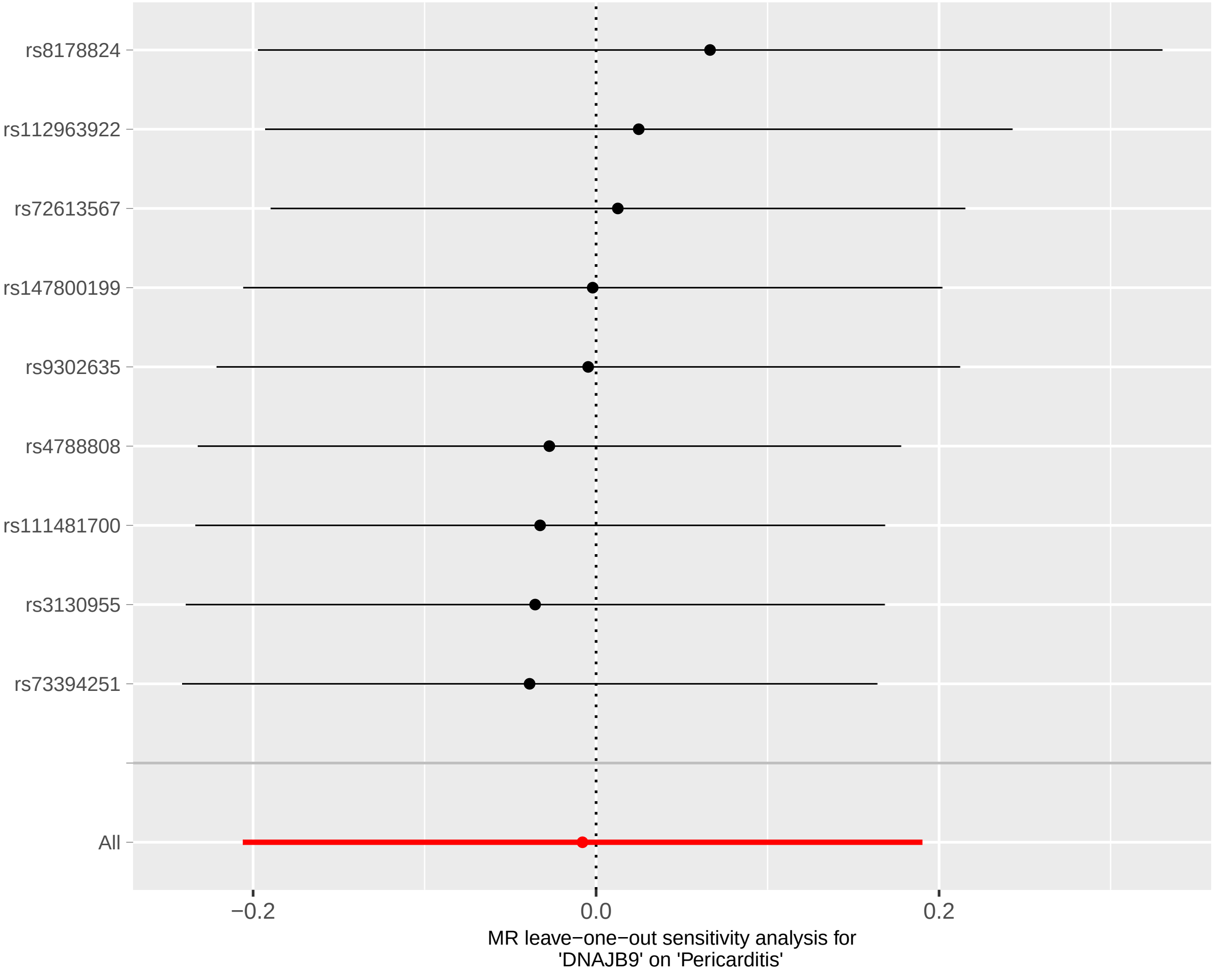
rs8178824
rs112963922
rs72613567
rs147800199
rs9302635
rs4788808
rs111481700
rs3130955
rs73394251
All
−0.2
0.0
0.2
MR leave−one−out sensitivity analysis for
'DNAJB9' on 'Pericarditis'

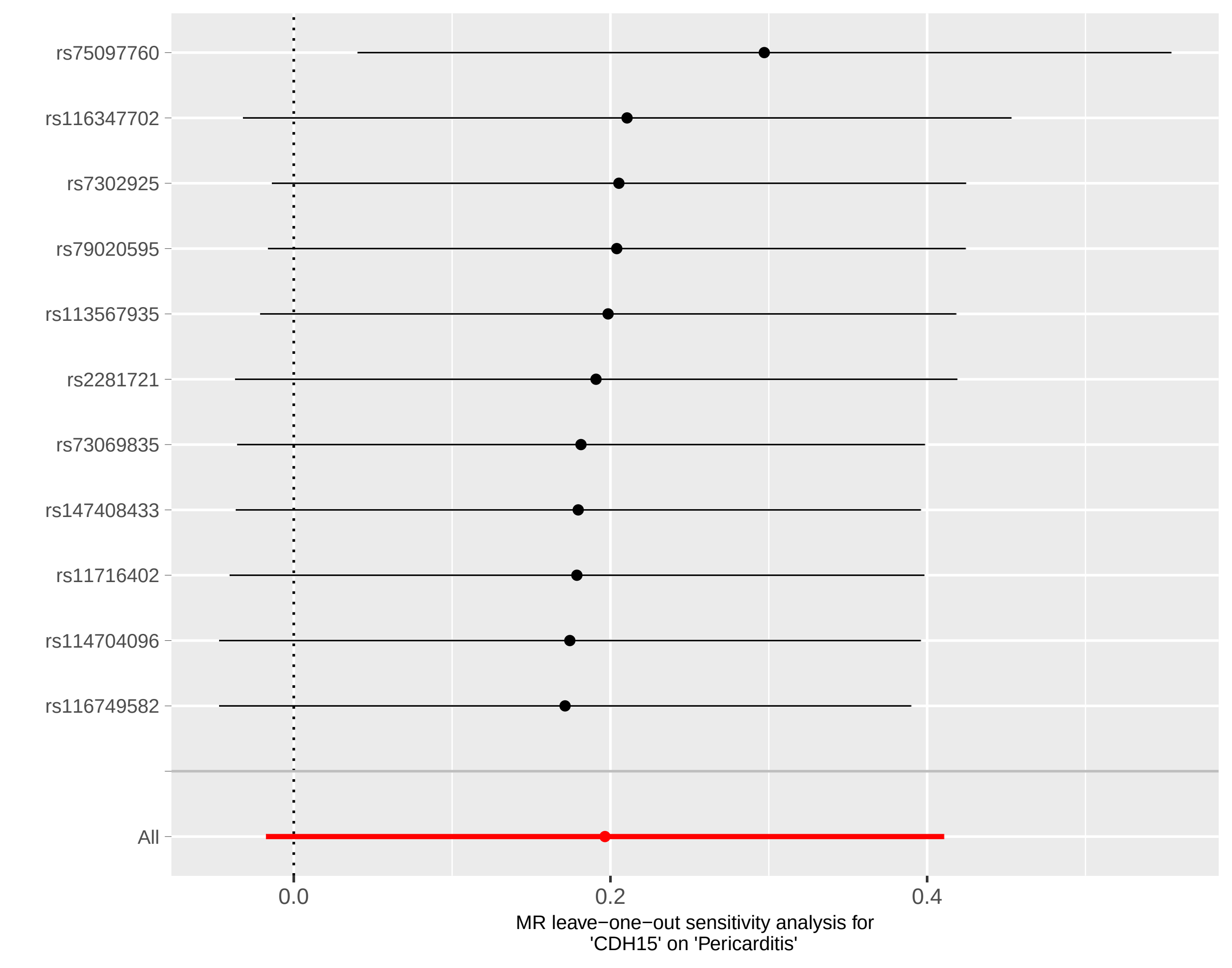
rs75097760
rs116347702
rs7302925
rs79020595
rs113567935
rs2281721
rs73069835
rs147408433
rs11716402
rs114704096
rs116749582
All
0.0
0.2
0.4
MR leave−one−out sensitivity analysis for
'CDH15' on 'Pericarditis'

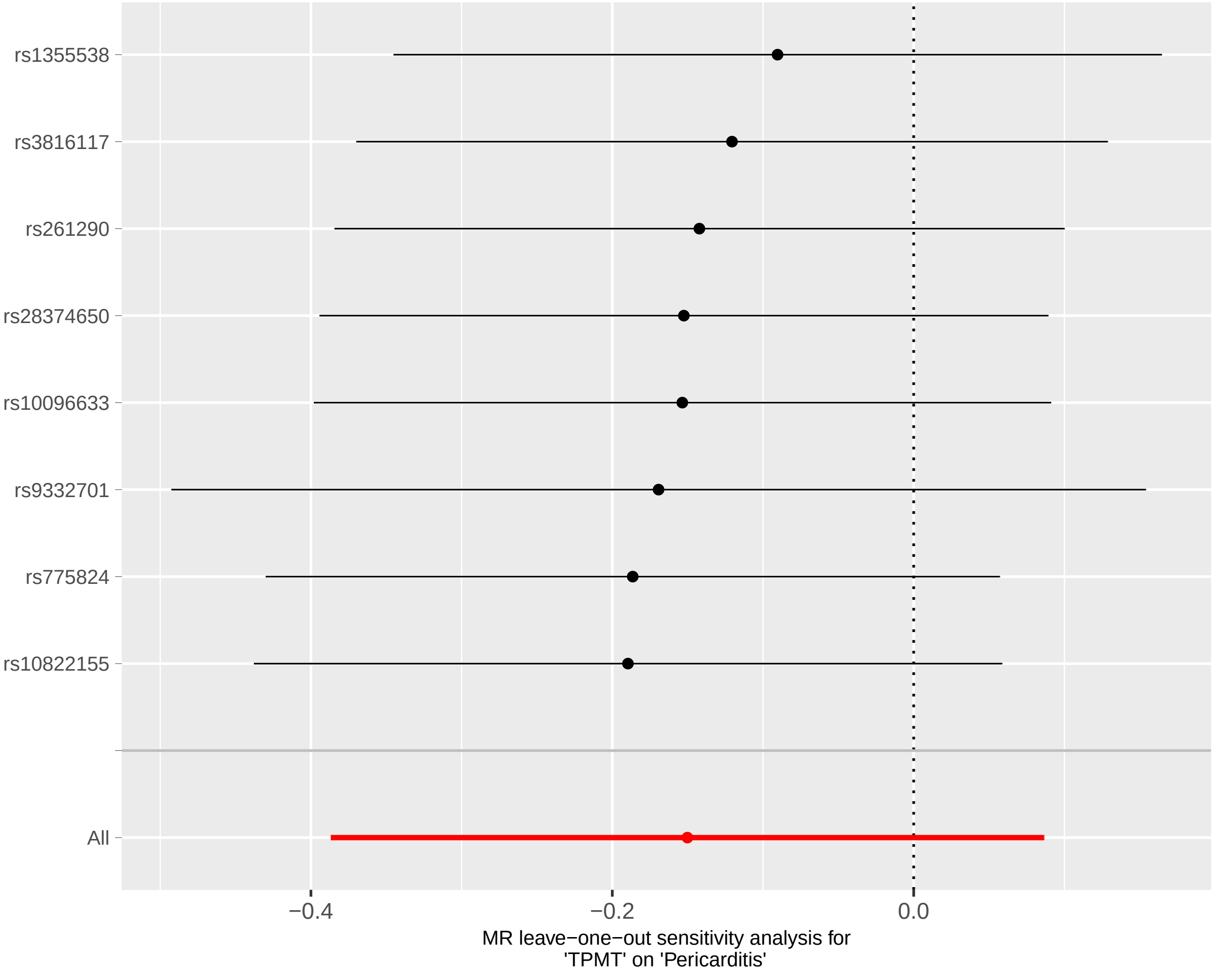
rs1355538
rs3816117
rs261290
rs28374650
rs10096633
rs9332701
rs775824
rs10822155
All
−0.4
−0.2
0.0
MR leave−one−out sensitivity analysis for
'TPMT' on 'Pericarditis'

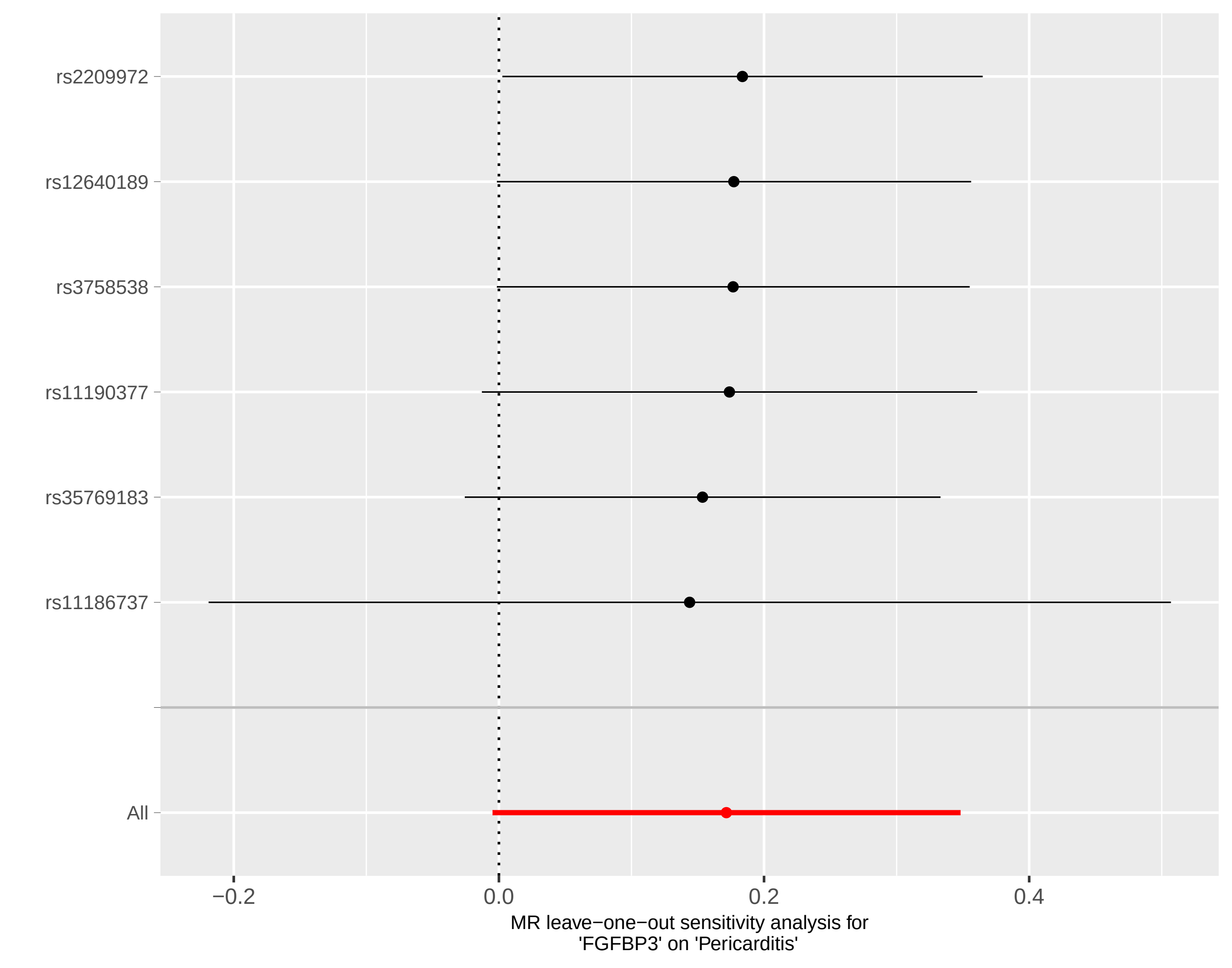

rs2209972
rs12640189
rs3758538
rs11190377
rs35769183
rs11186737
All
−0.2
0.0
0.2
0.4
MR leave−one−out sensitivity analysis for
'FGFBP3' on 'Pericarditis'

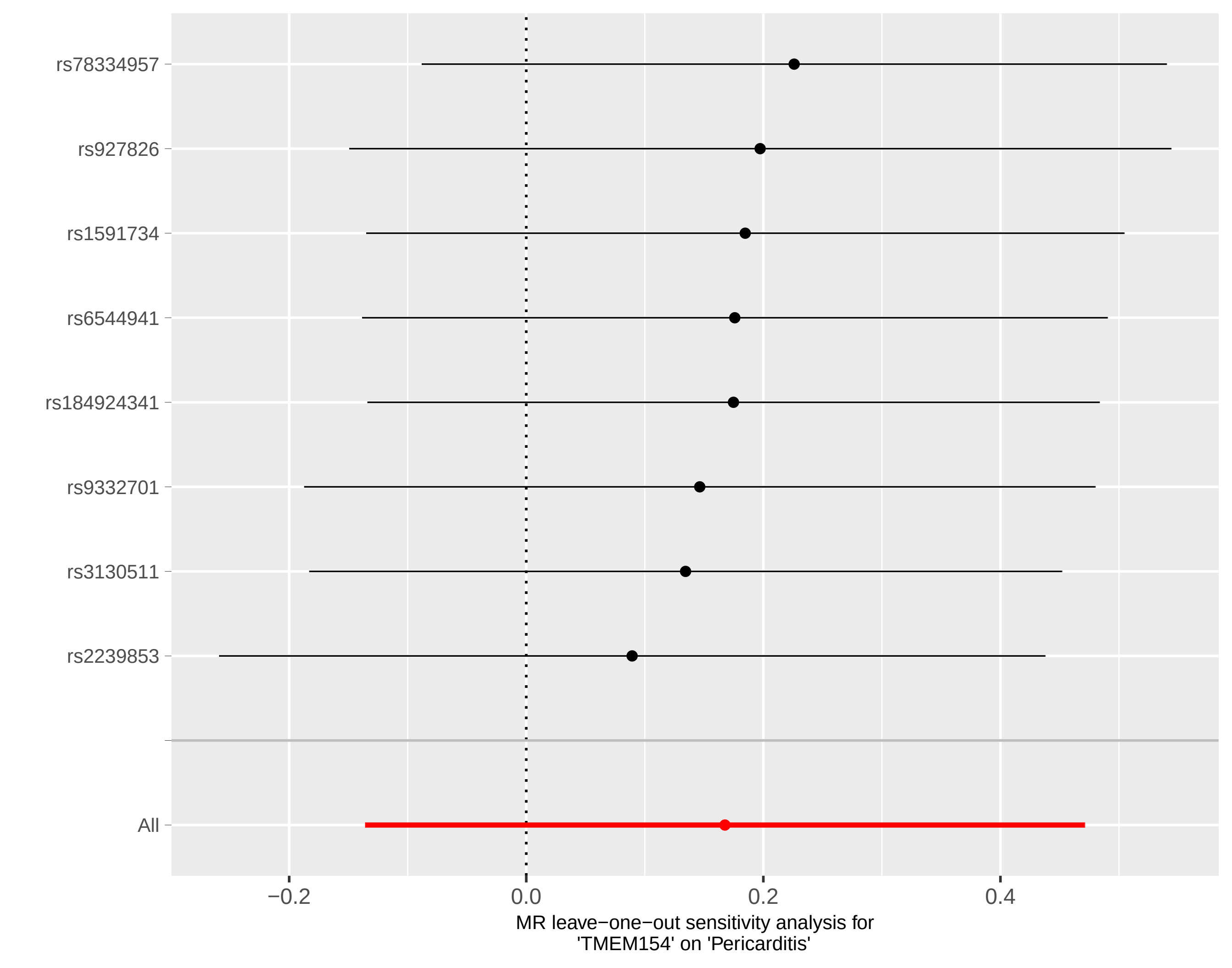
rs78334957
rs927826
rs1591734
rs6544941
rs184924341
rs9332701
rs3130511
rs2239853
All
−0.2
0.0
0.2
0.4
MR leave−one−out sensitivity analysis for
'TMEM154' on 'Pericarditis'

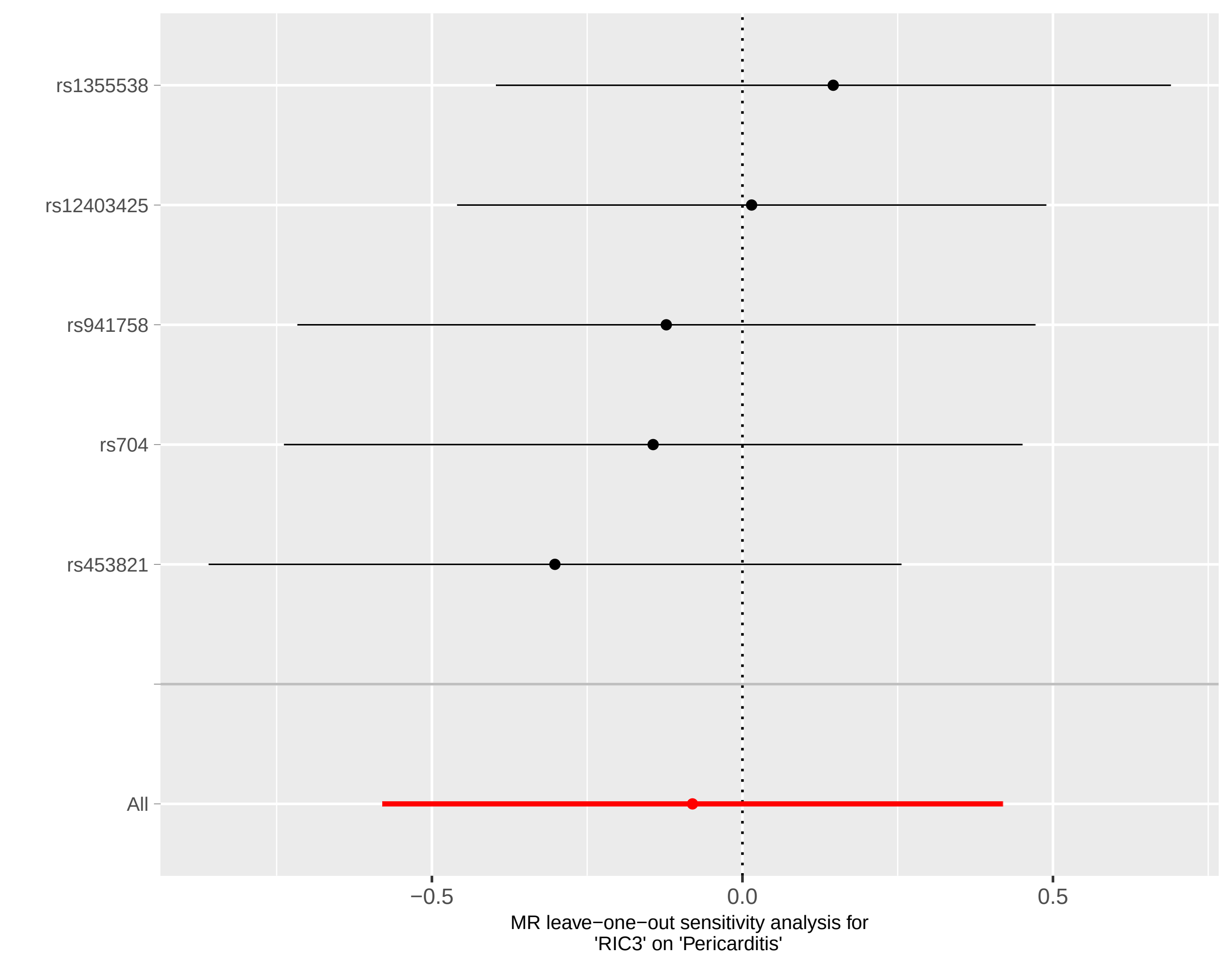
rs1355538
rs12403425
rs941758
rs704
rs453821
All
−0.5
0.0
0.5
MR leave−one−out sensitivity analysis for
'RIC3' on 'Pericarditis'

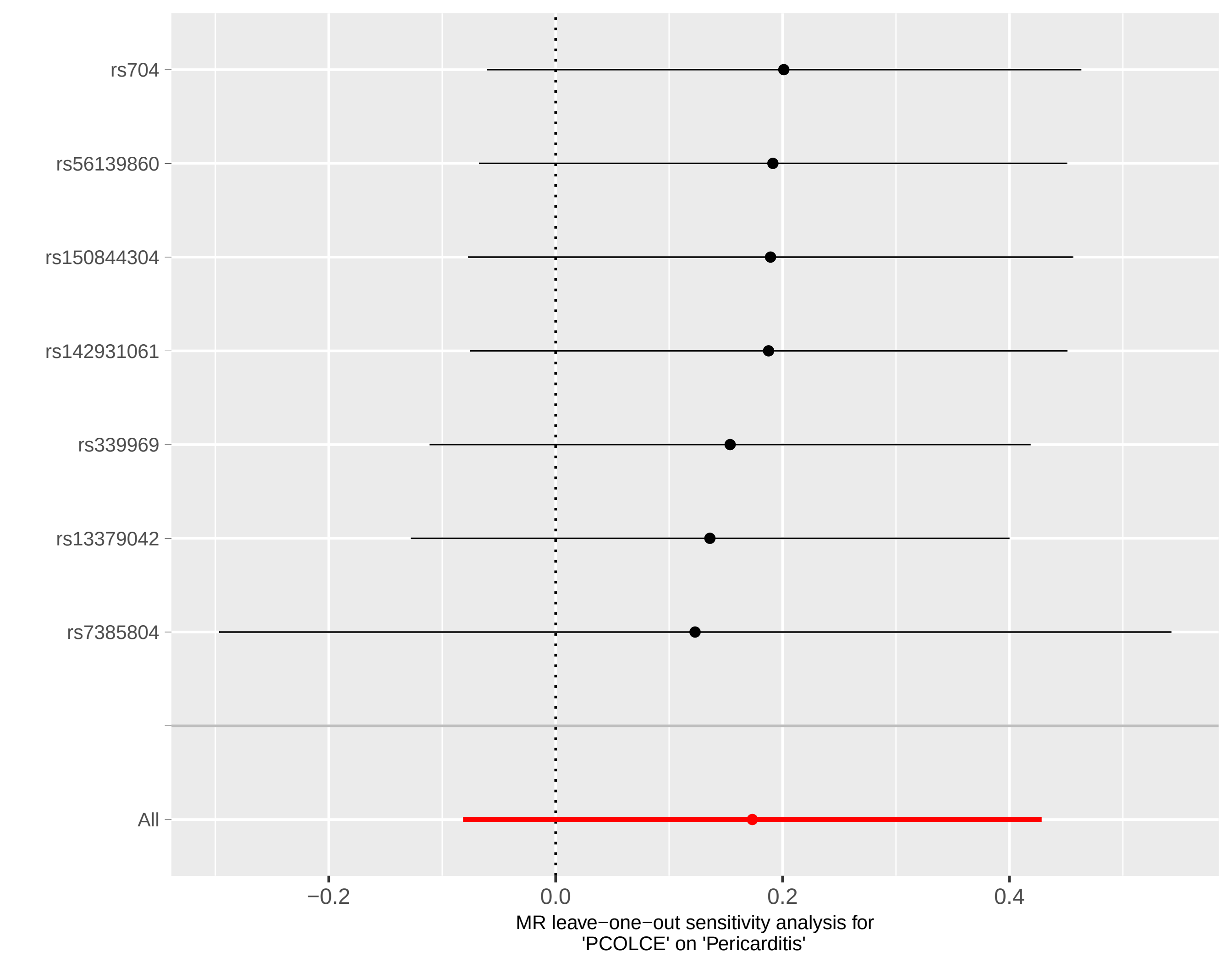

rs704
rs56139860
rs150844304
rs142931061
rs339969
rs13379042
rs7385804
All
−0.2
0.0
0.2
0.4
MR leave−one−out sensitivity analysis for
'PCOLCE' on 'Pericarditis'

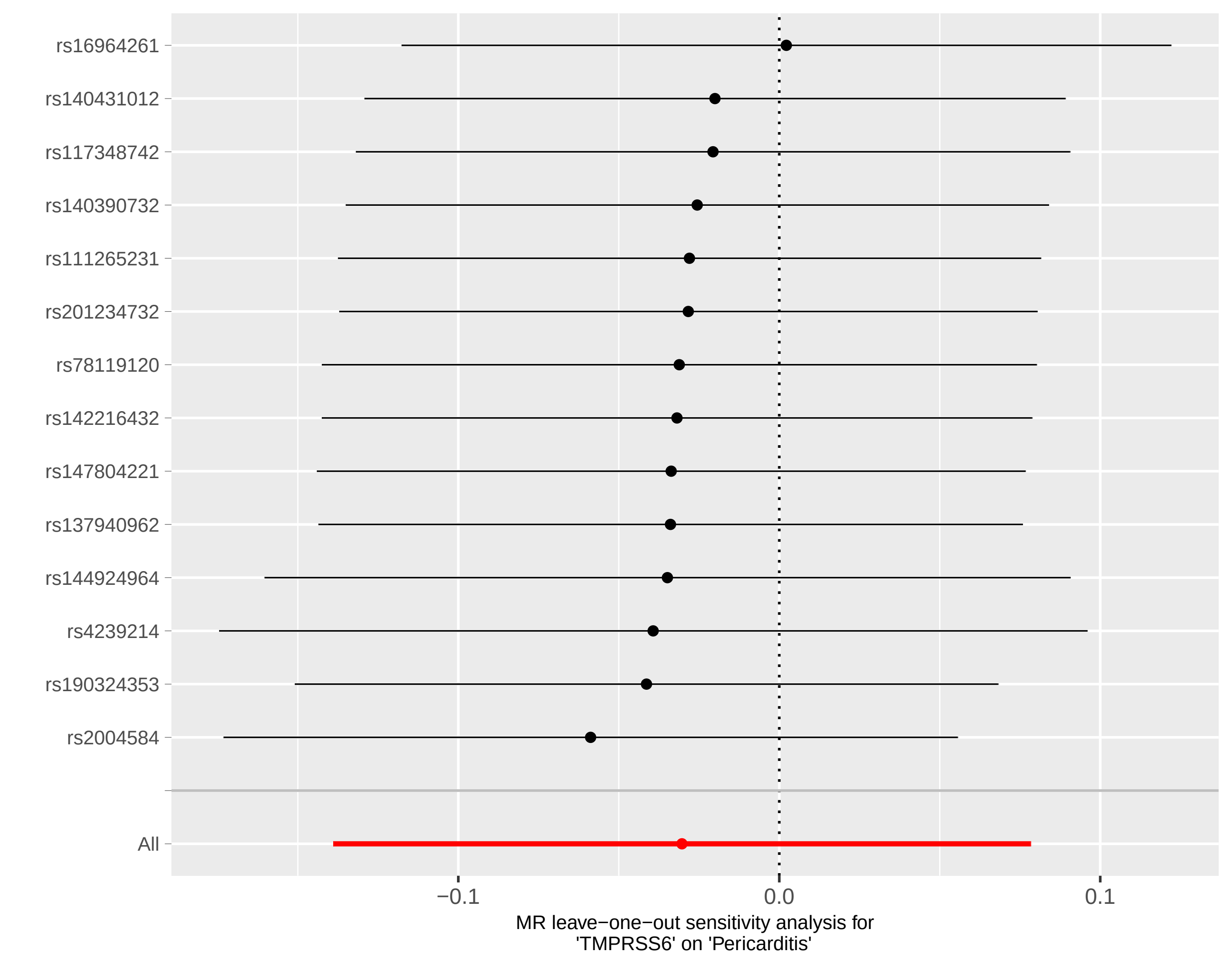
rs16964261
rs140431012
rs117348742
rs140390732
rs111265231
rs201234732
rs78119120
rs142216432
rs147804221
rs137940962
rs144924964
rs4239214
rs190324353
rs2004584
All
−0.1
0.0
0.1
MR leave−one−out sensitivity analysis for
'TMPRSS6' on 'Pericarditis'

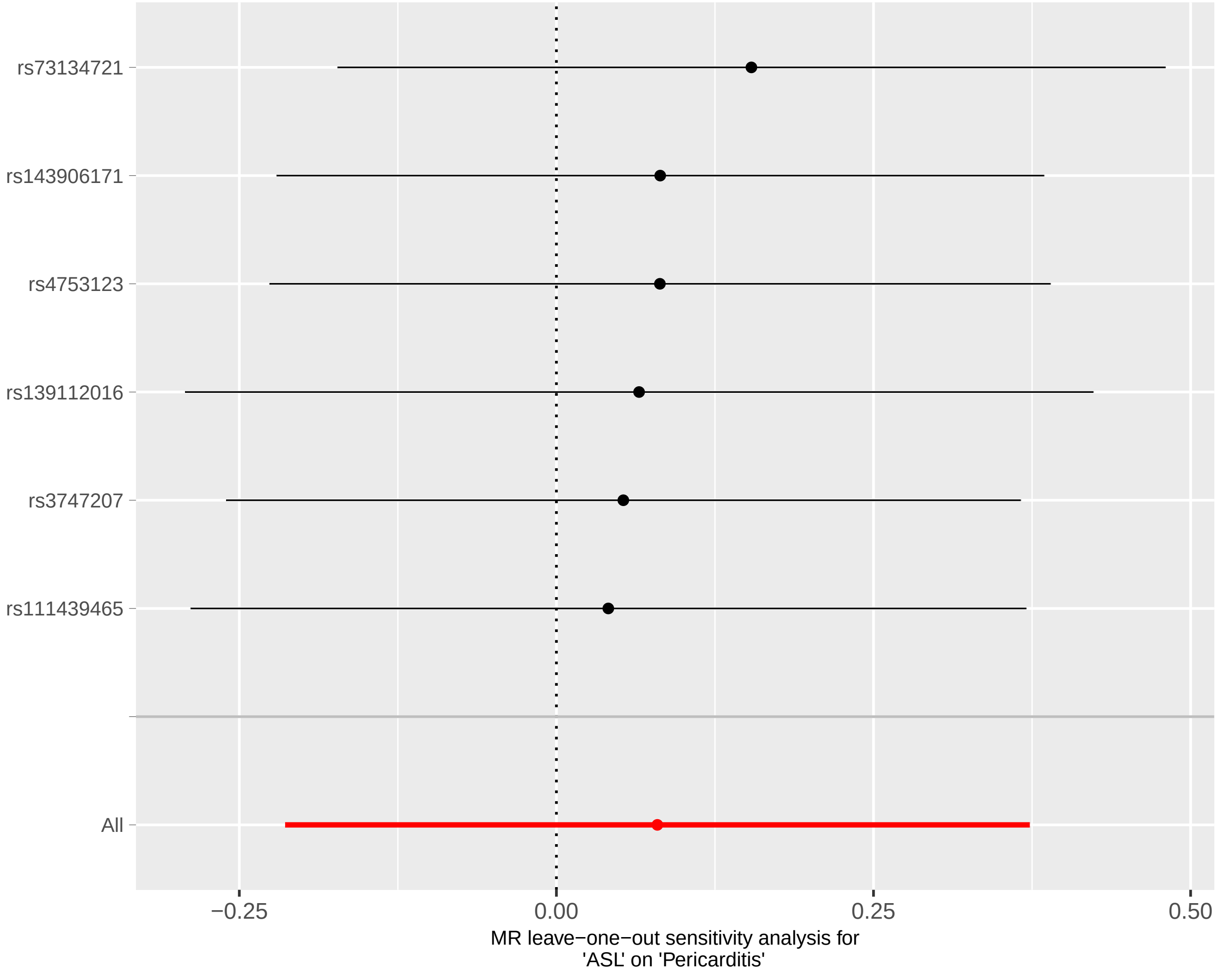

rs73134721
rs143906171
rs4753123
rs139112016
rs3747207
rs111439465
All
−0.25
0.00
0.25
0.50
MR leave−one−out sensitivity analysis for
'ASL' on 'Pericarditis'

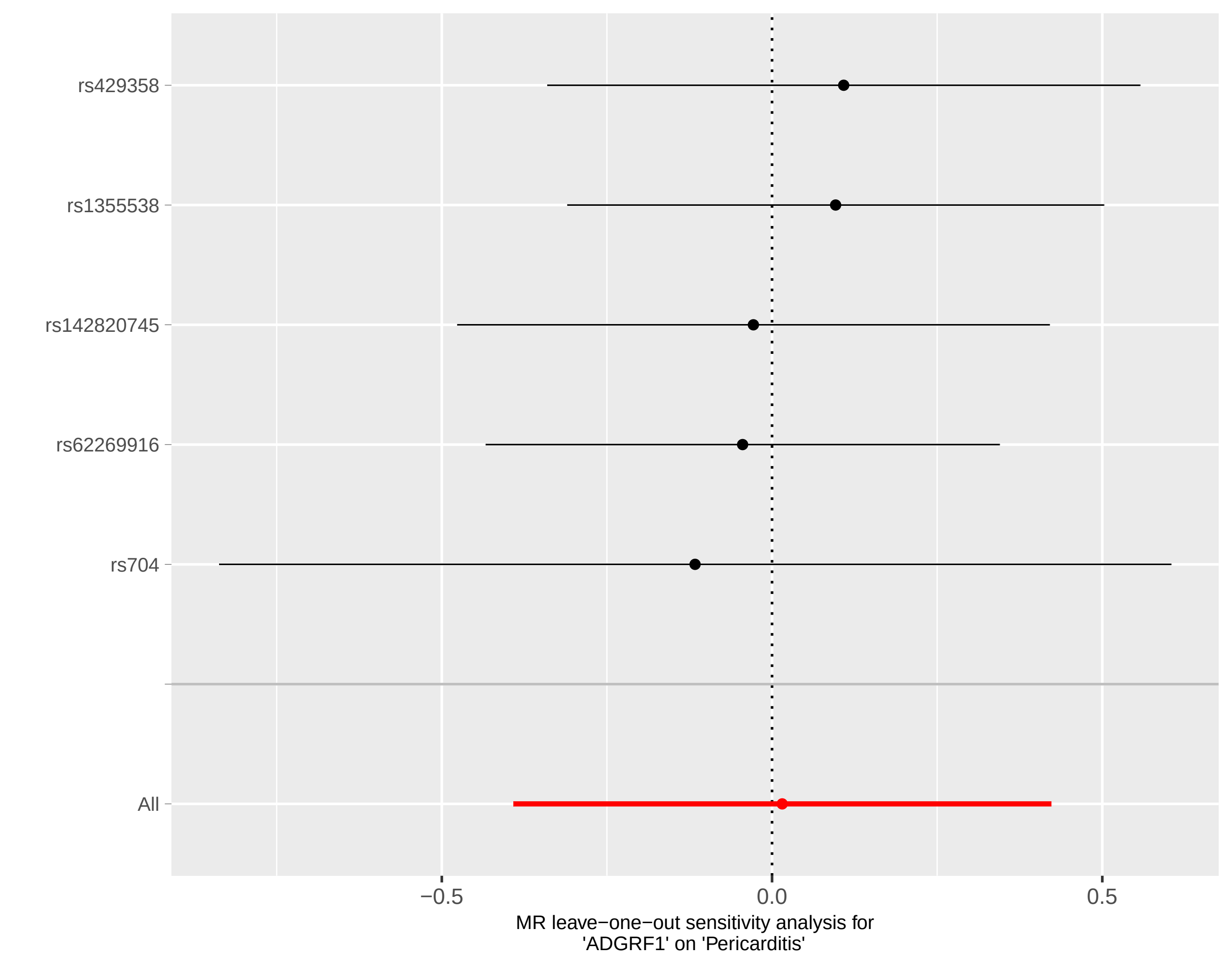
rs429358
rs1355538
rs142820745
rs62269916
rs704
All
−0.5
0.0
0.5
MR leave−one−out sensitivity analysis for
'ADGRF1' on 'Pericarditis'

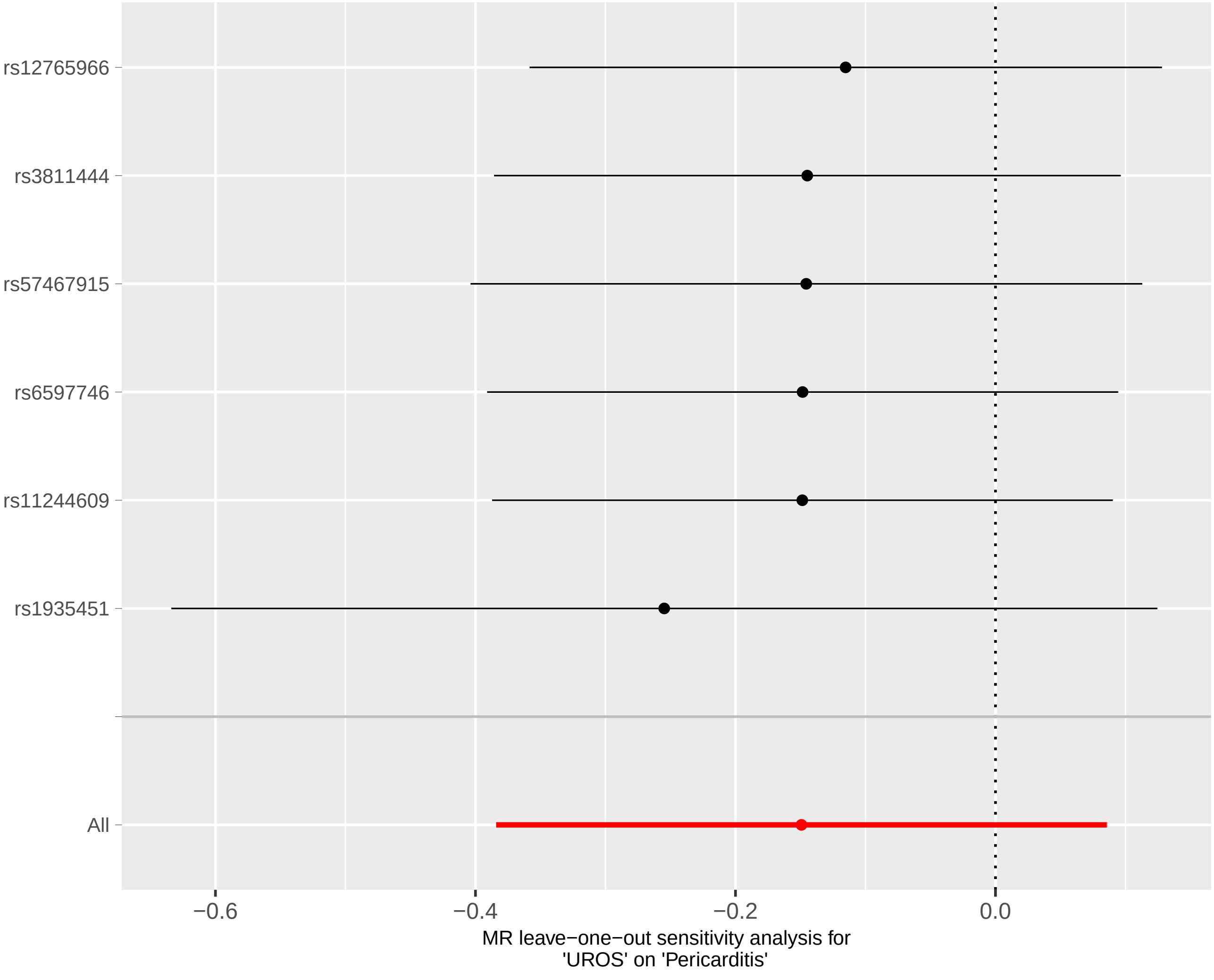
rs12765966
rs3811444
rs57467915
rs6597746
rs11244609
rs1935451
All
−0.6
−0.4
−0.2
0.0
MR leave−one−out sensitivity analysis for
'UROS' on 'Pericarditis'

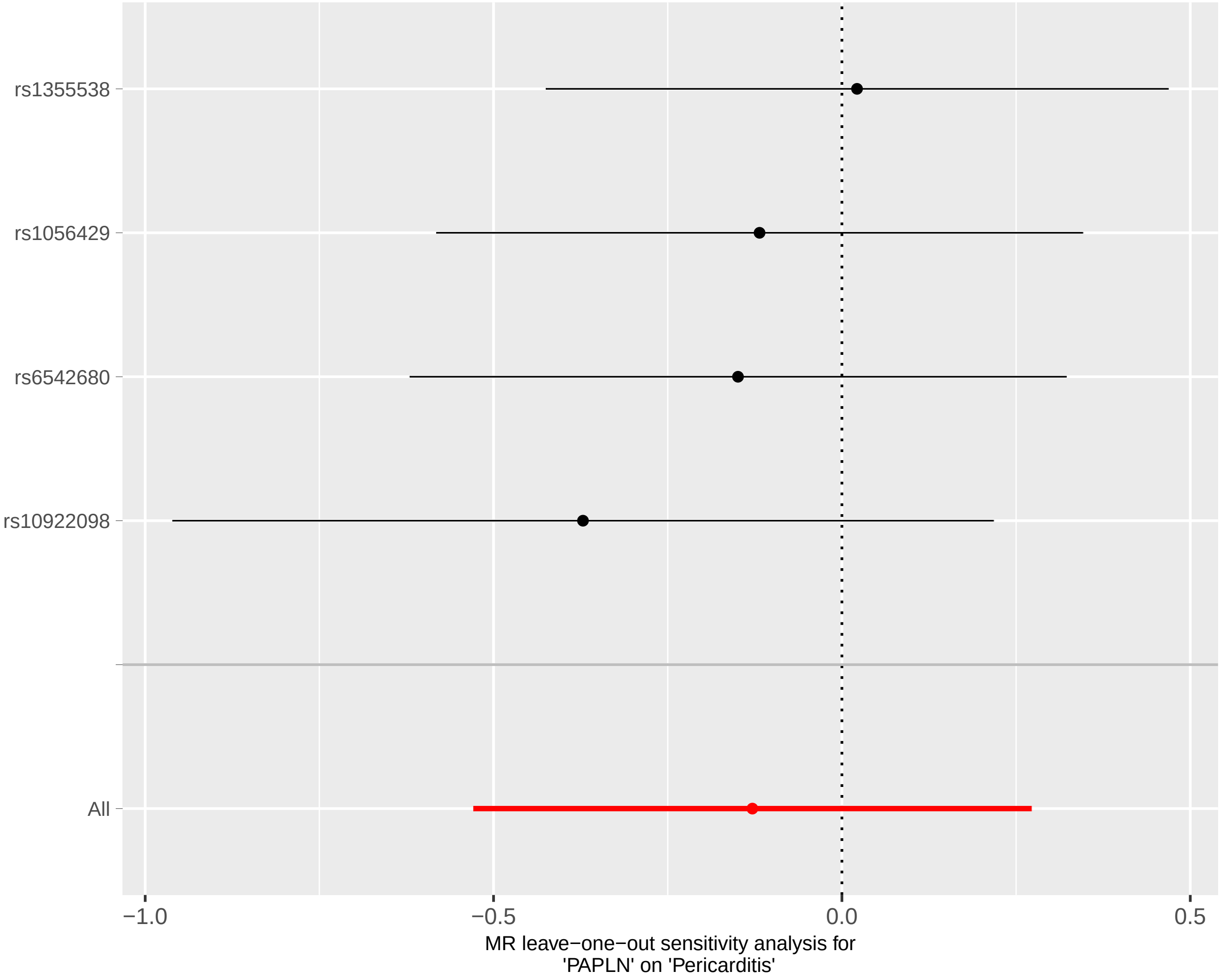

rs1355538
rs1056429
rs6542680
rs10922098
All
−1.0
−0.5
0.0
0.5
MR leave−one−out sensitivity analysis for
'PAPLN' on 'Pericarditis'

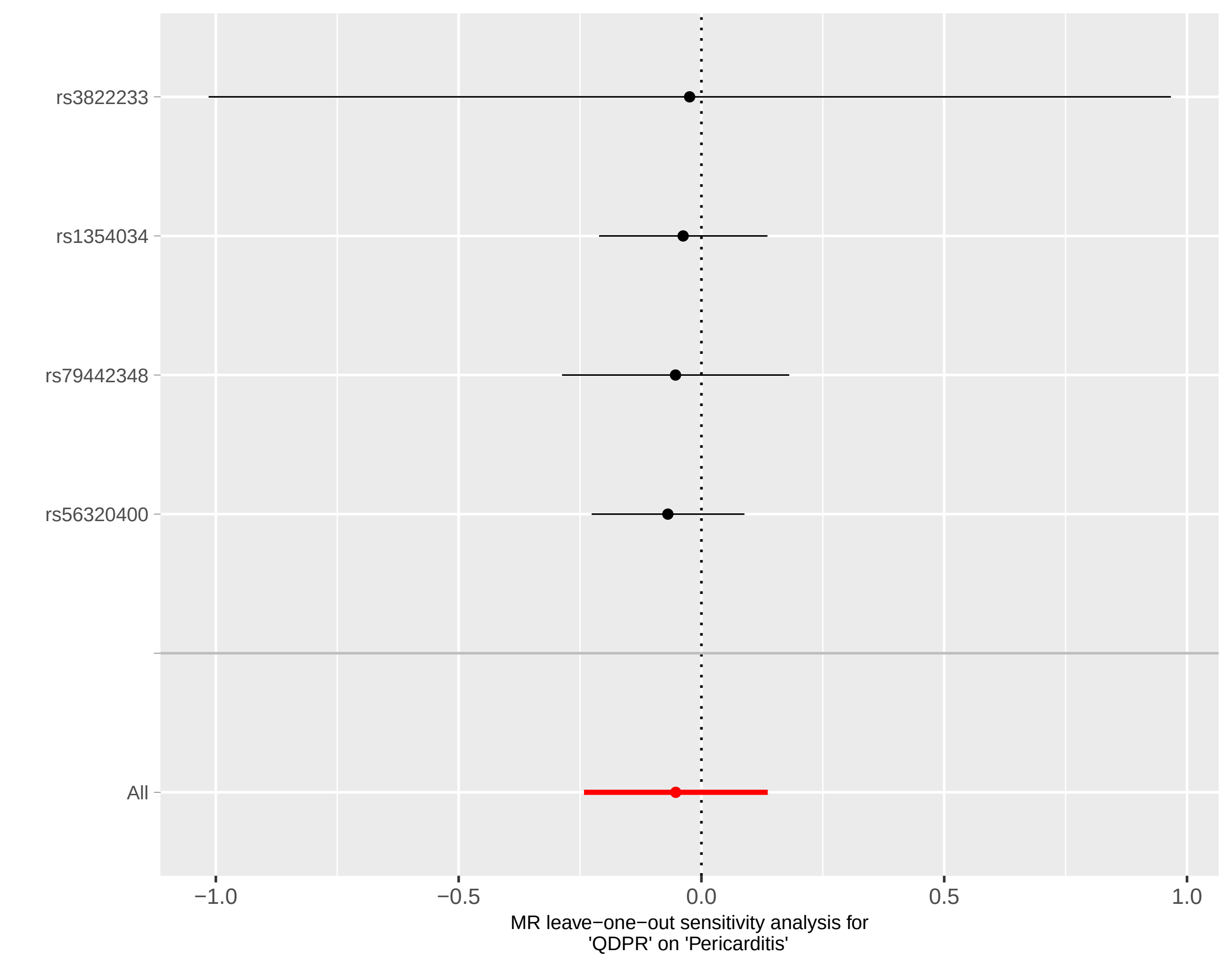
rs3822233
rs1354034
rs79442348
rs56320400
All
−1.0
−0.5
0.0
0.5
1.0
MR leave−one−out sensitivity analysis for
'QDPR' on 'Pericarditis'

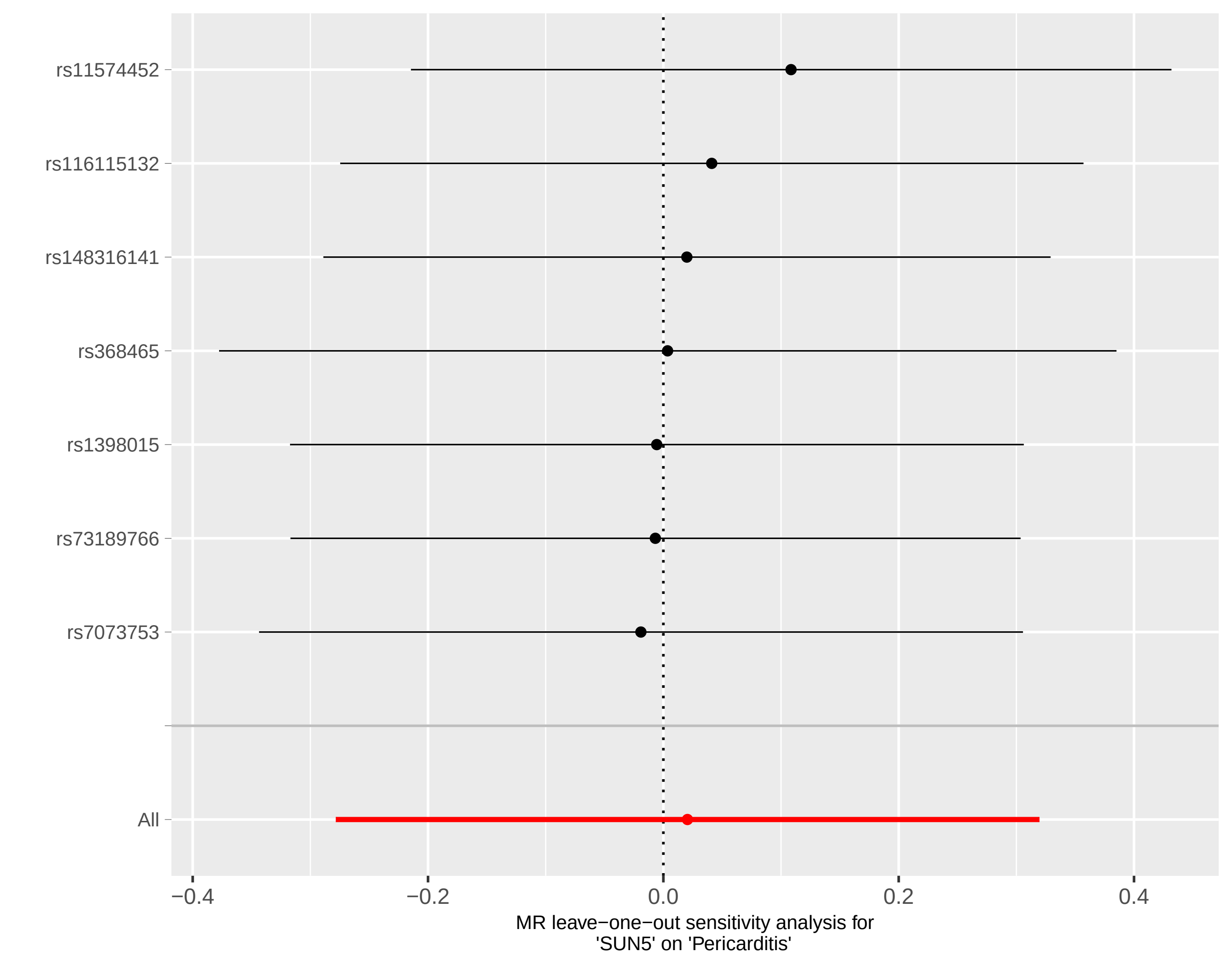
rs11574452
rs116115132
rs148316141
rs368465
rs1398015
rs73189766
rs7073753
All
−0.4
−0.2
0.0
0.2
0.4
MR leave−one−out sensitivity analysis for
'SUN5' on 'Pericarditis'

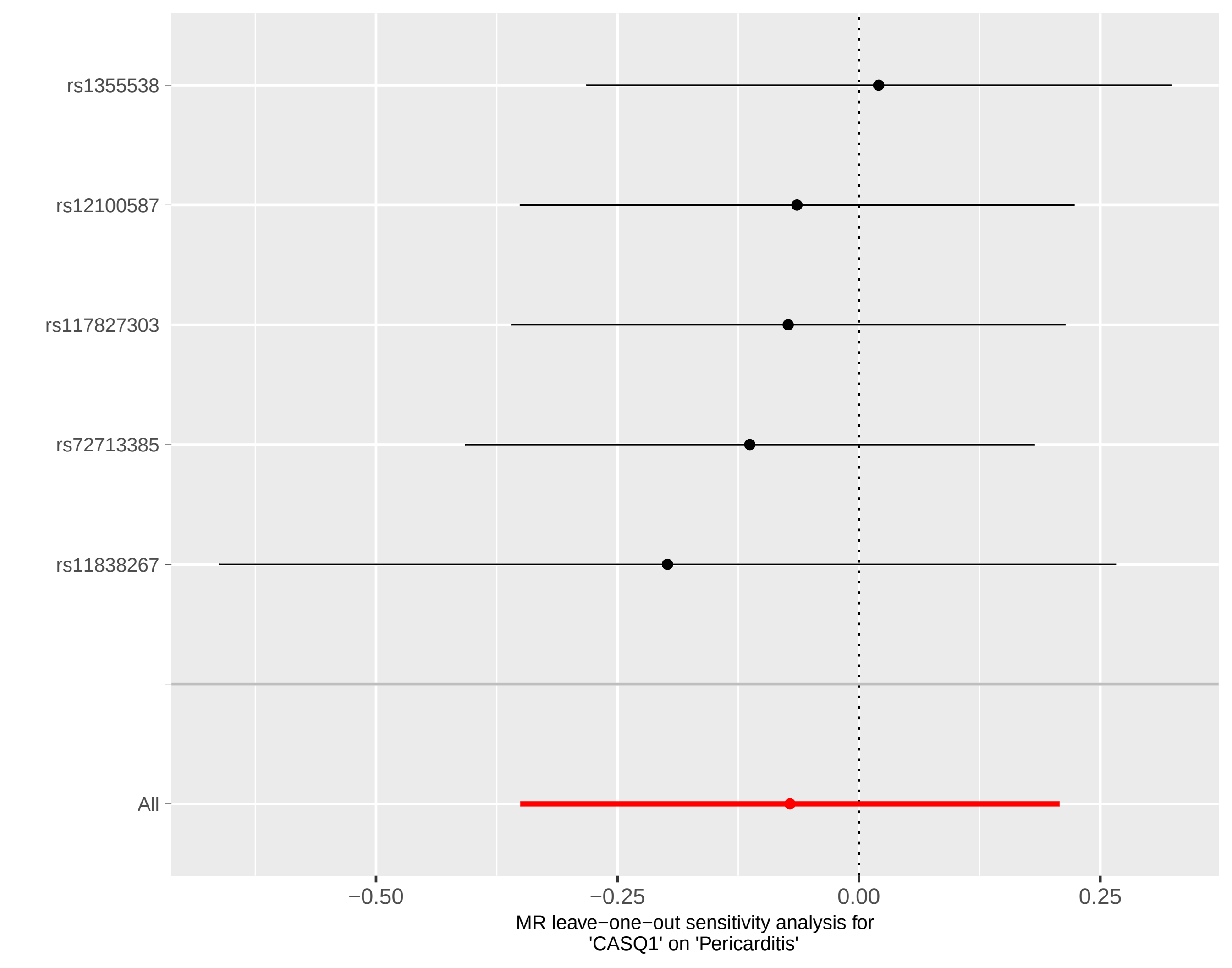

rs1355538
rs12100587
rs117827303
rs72713385
rs11838267
All
−0.50
−0.25
0.00
0.25
MR leave−one−out sensitivity analysis for
'CASQ1' on 'Pericarditis'

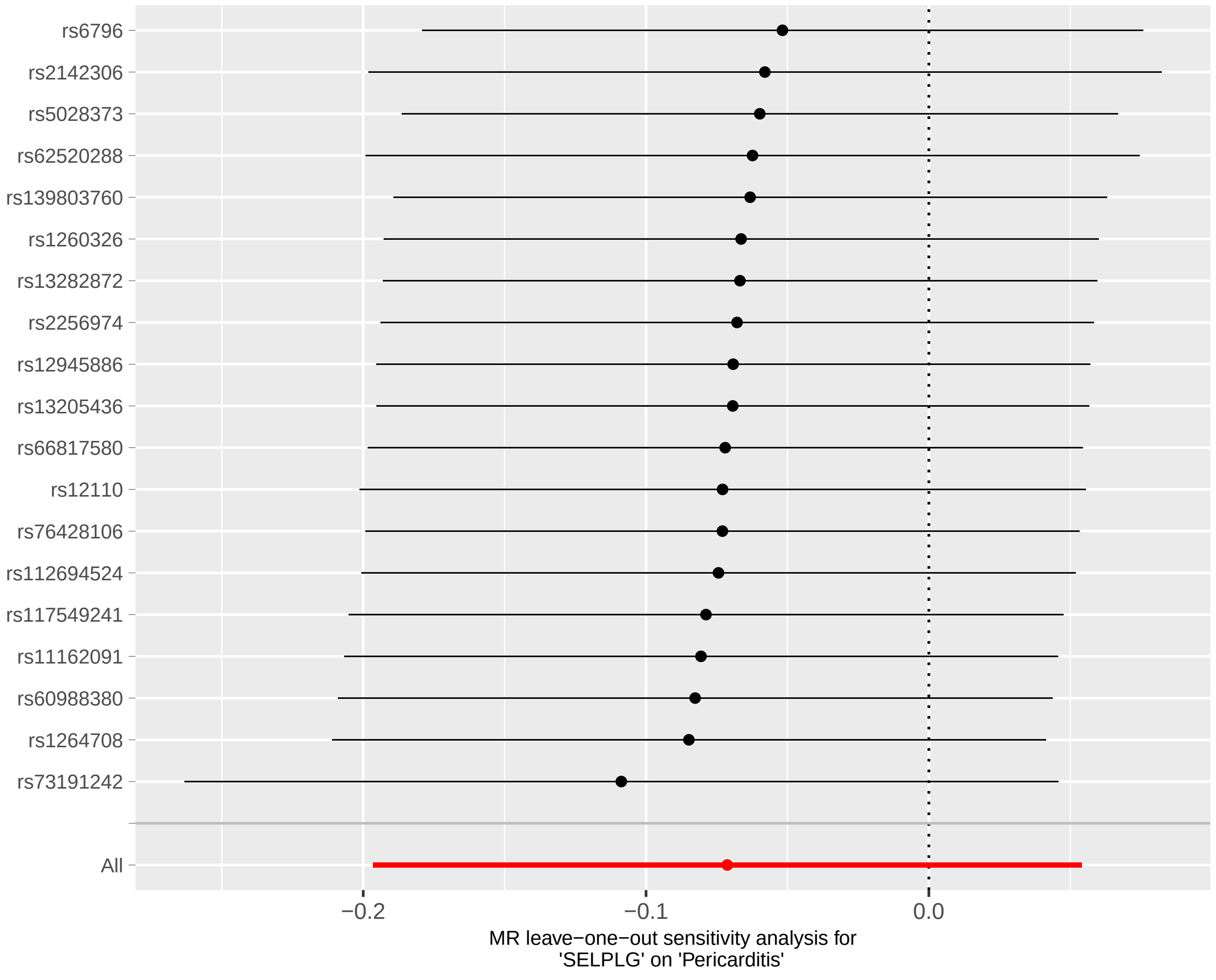
rs6796
rs2142306
rs5028373
rs62520288
rs139803760
rs1260326
rs13282872
rs2256974
rs12945886
rs13205436
rs66817580
rs12110
rs76428106
rs112694524
rs117549241
rs11162091
rs60988380
rs1264708
rs73191242
All
−0.2
−0.1
0.0
MR leave−one−out sensitivity analysis for
'SELPLG' on 'Pericarditis'

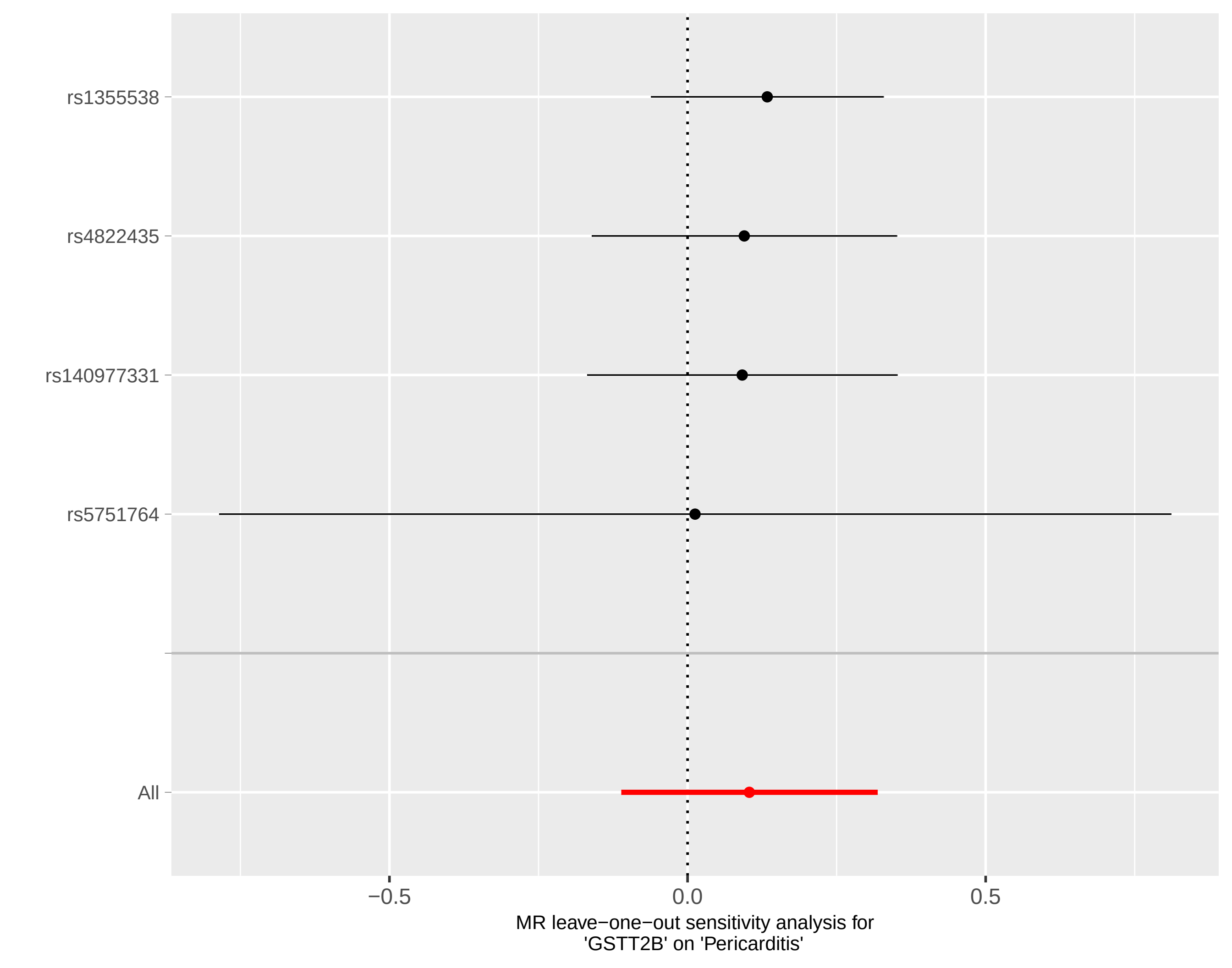
rs1355538
rs4822435
rs140977331
rs5751764
All
−0.5
0.0
0.5
MR leave−one−out sensitivity analysis for
'GSTT2B' on 'Pericarditis'

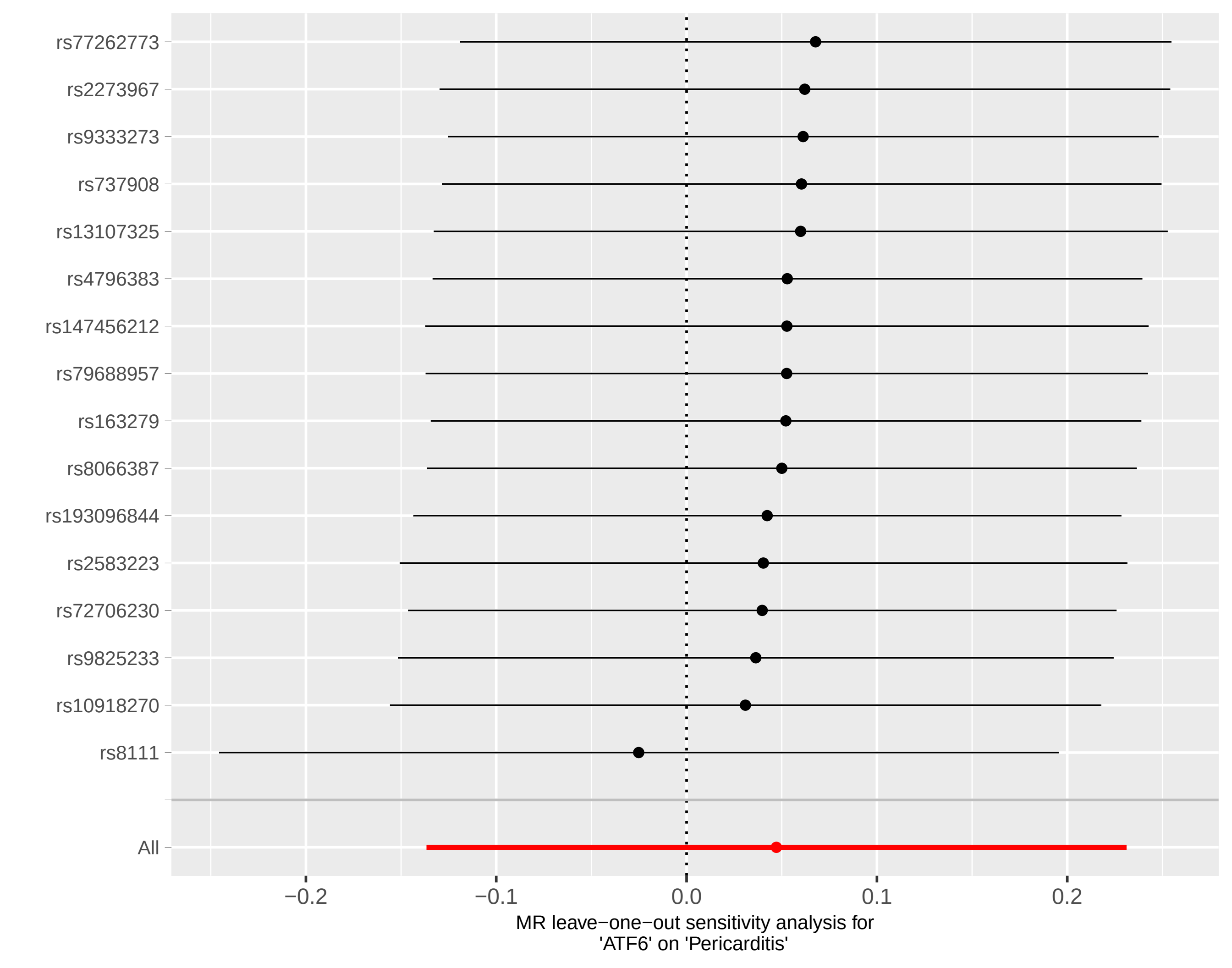
rs77262773
rs2273967
rs9333273
rs737908
rs13107325
rs4796383
rs147456212
rs79688957
rs163279
rs8066387
rs193096844
rs2583223
rs72706230
rs9825233
rs10918270
rs8111
All
−0.2
−0.1
0.0
0.1
0.2
MR leave−one−out sensitivity analysis for
'ATF6' on 'Pericarditis'

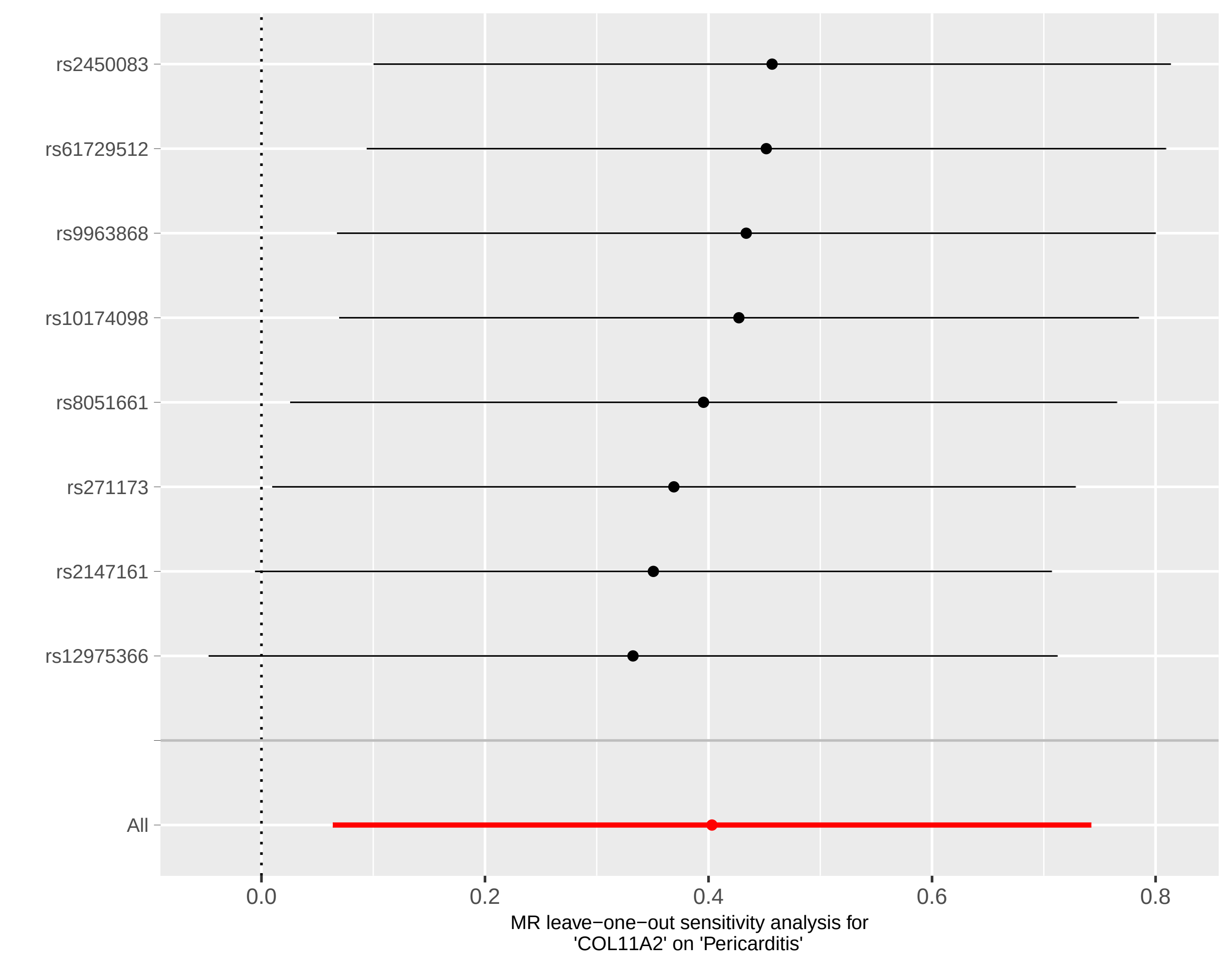

rs2450083
rs61729512
rs9963868
rs10174098
rs8051661
rs271173
rs2147161
rs12975366
All
0.0
0.2
0.4
0.6
0.8
MR leave−one−out sensitivity analysis for
'COL11A2' on 'Pericarditis'

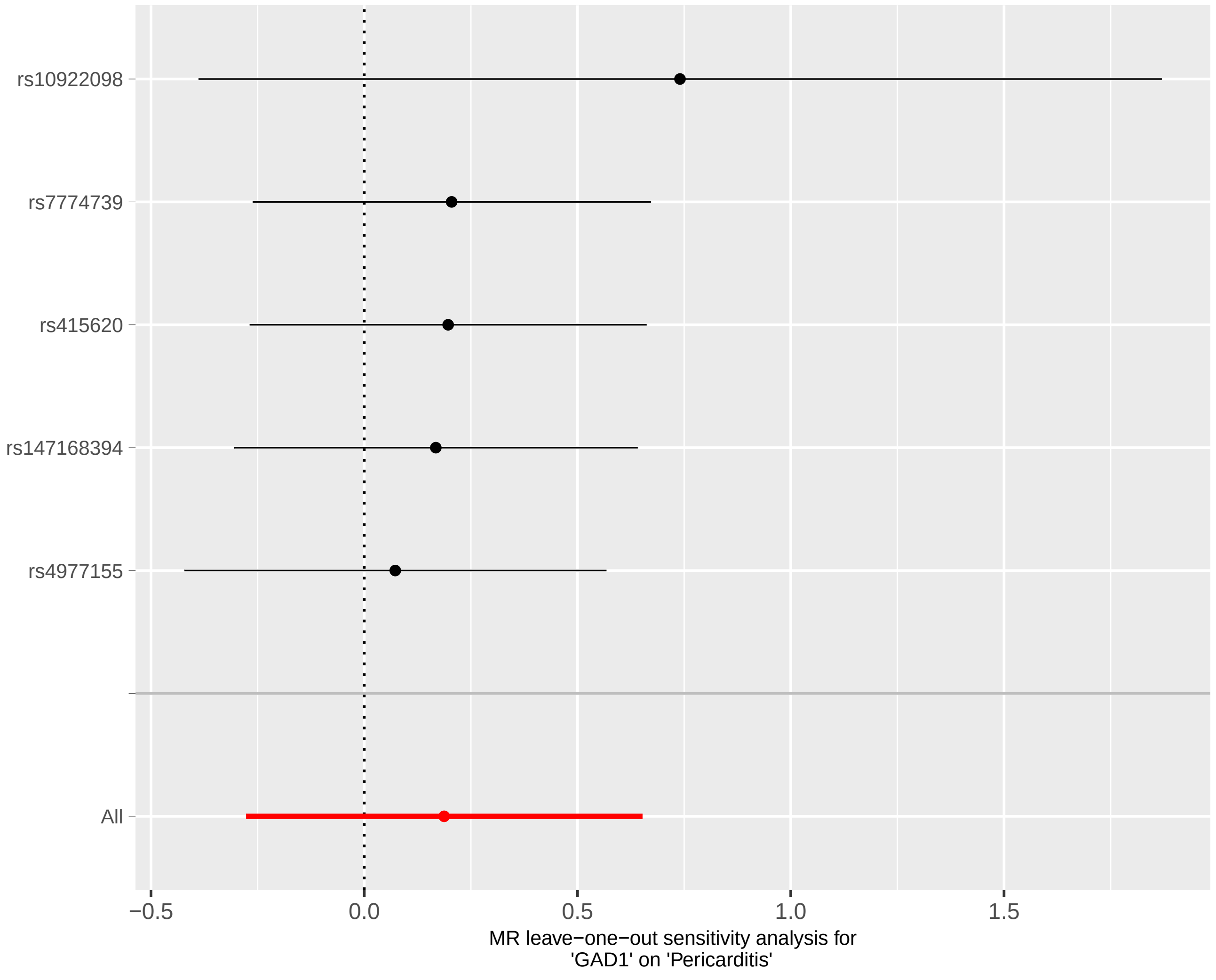

rs10922098
rs7774739
rs415620
rs147168394
rs4977155
All
−0.5
0.0
0.5
1.0
1.5
MR leave−one−out sensitivity analysis for
'GAD1' on 'Pericarditis'

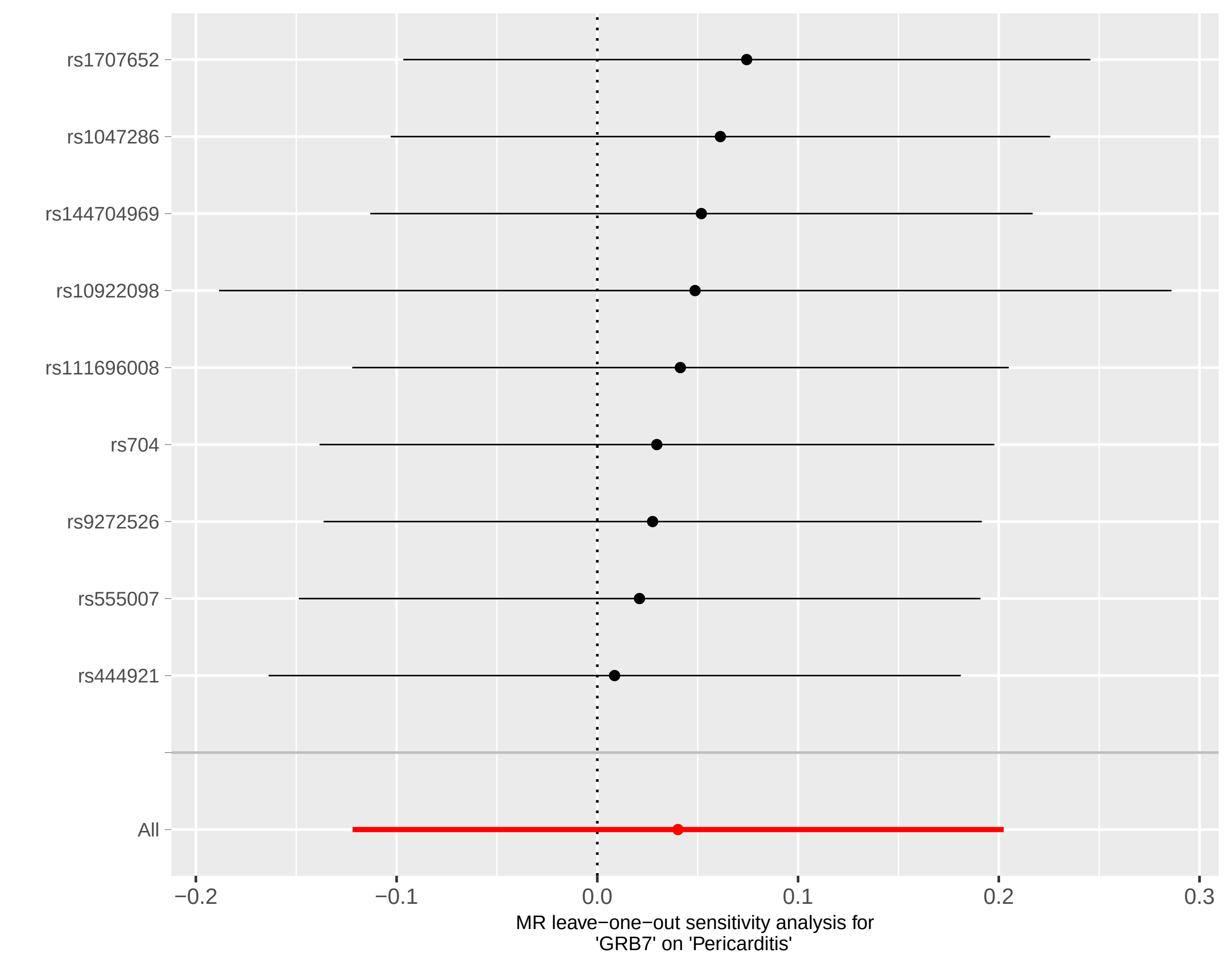
rs1707652
rs1047286
rs144704969
rs10922098
rs111696008
rs704
rs9272526
rs555007
rs444921
All
−0.2
−0.1
0.0
0.1
0.2
0.3
MR leave−one−out sensitivity analysis for
'GRB7' on 'Pericarditis'

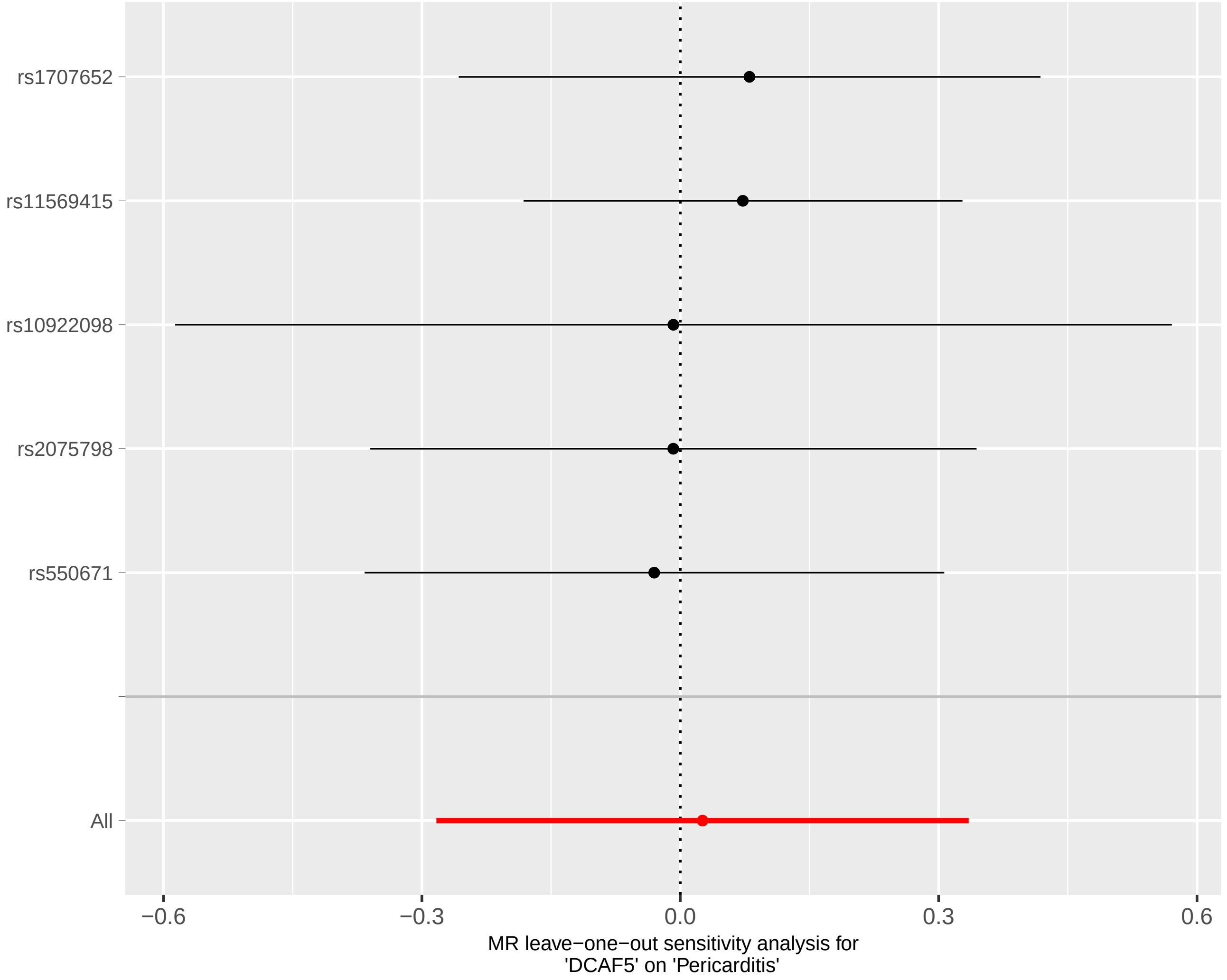
rs1707652
rs11569415
rs10922098
rs2075798
rs550671
All
−0.6
−0.3
0.0
0.3
0.6
MR leave−one−out sensitivity analysis for
'DCAF5' on 'Pericarditis'

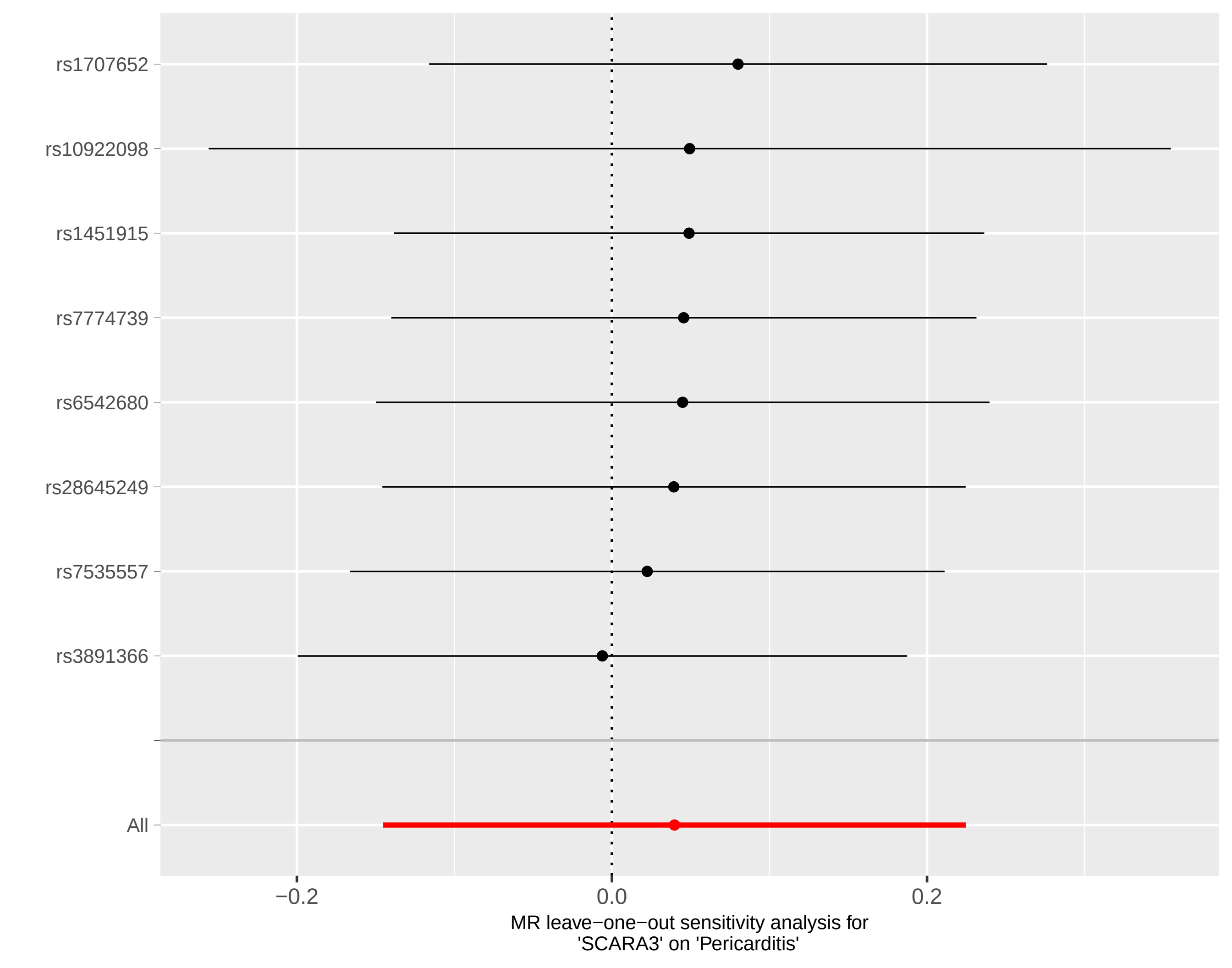

rs1707652
rs10922098
rs1451915
rs7774739
rs6542680
rs28645249
rs7535557
rs3891366
All
−0.2
0.0
0.2
MR leave−one−out sensitivity analysis for
'SCARA3' on 'Pericarditis'

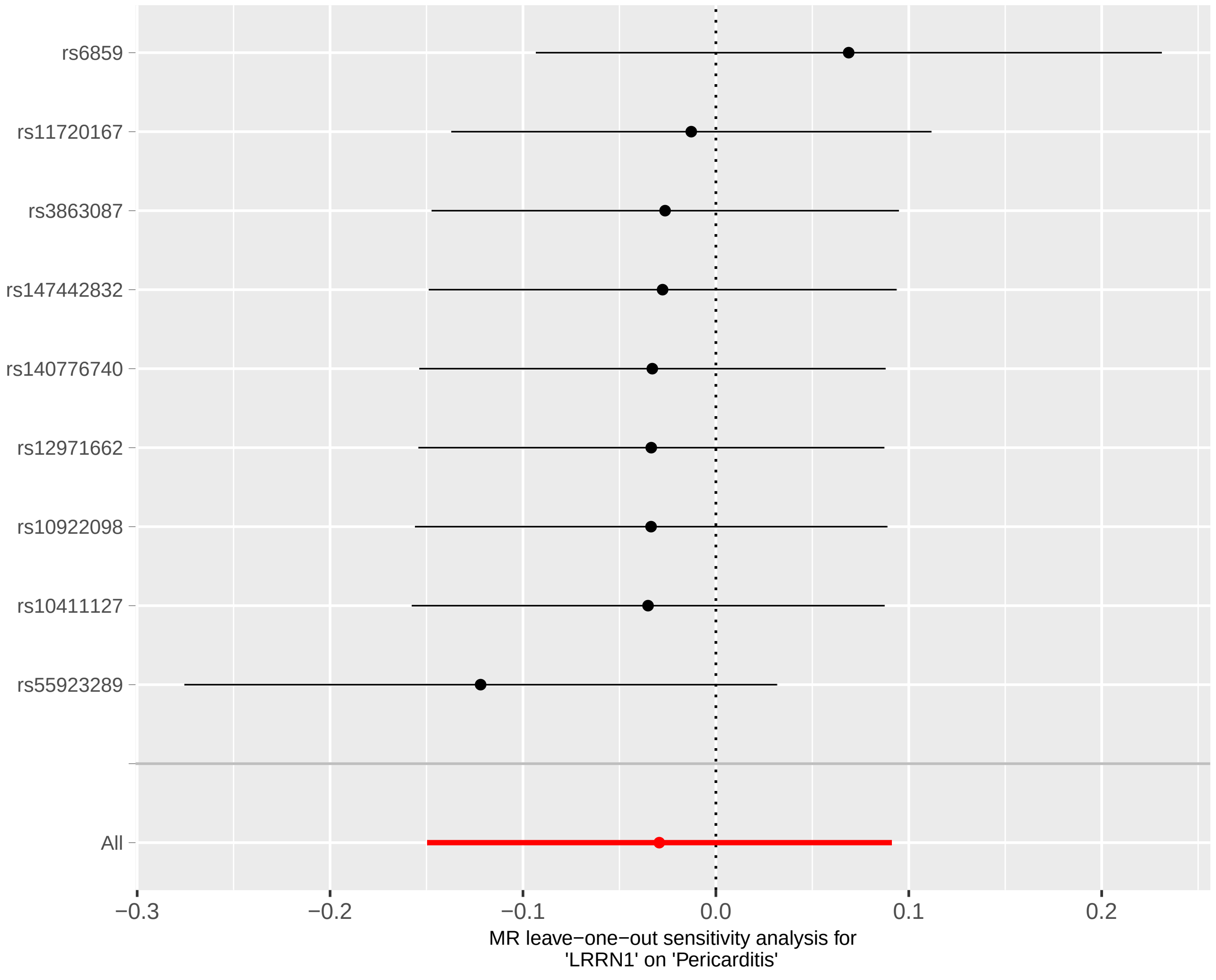

rs6859
rs11720167
rs3863087
rs147442832
rs140776740
rs12971662
rs10922098
rs10411127
rs55923289
All
−0.3
−0.2
−0.1
0.0
0.1
0.2
MR leave−one−out sensitivity analysis for
'LRRN1' on 'Pericarditis'

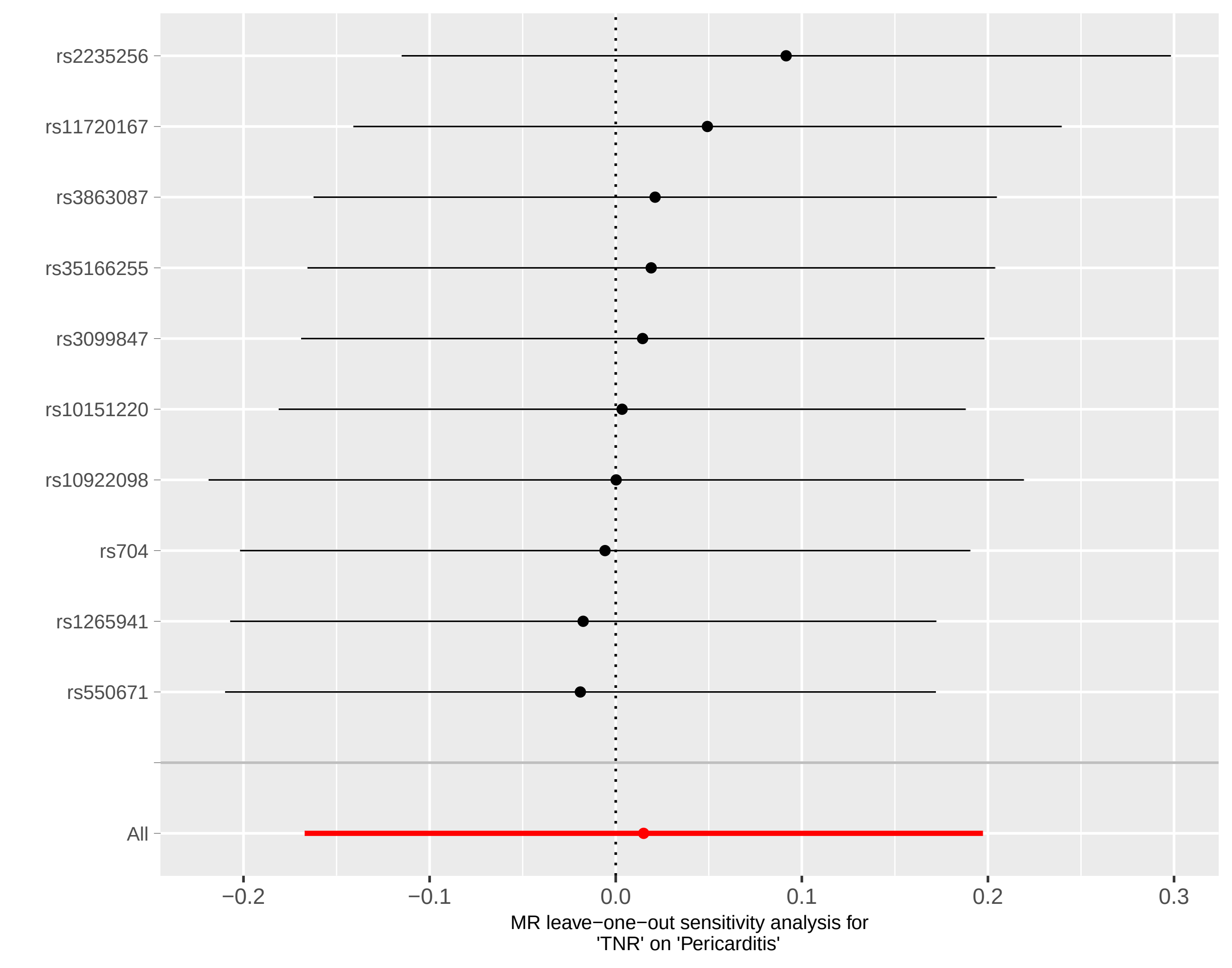
rs2235256
rs11720167
rs3863087
rs35166255
rs3099847
rs10151220
rs10922098
rs704
rs1265941
rs550671
All
−0.2
−0.1
0.0
0.1
0.2
0.3
MR leave−one−out sensitivity analysis for
'TNR' on 'Pericarditis'

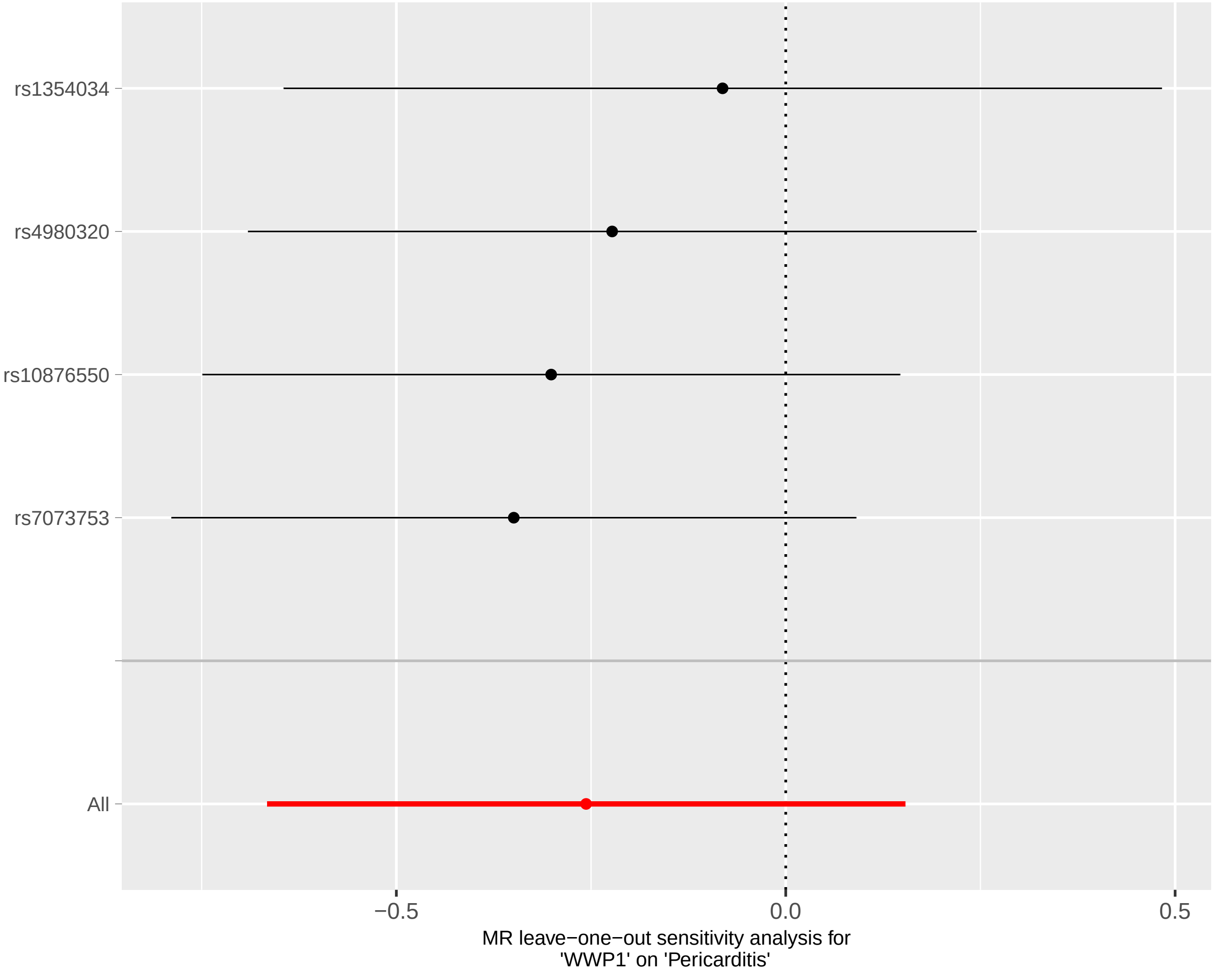
rs1354034
rs4980320
rs10876550
rs7073753
All
−0.5
0.0
0.5
MR leave−one−out sensitivity analysis for
'WWP1' on 'Pericarditis'

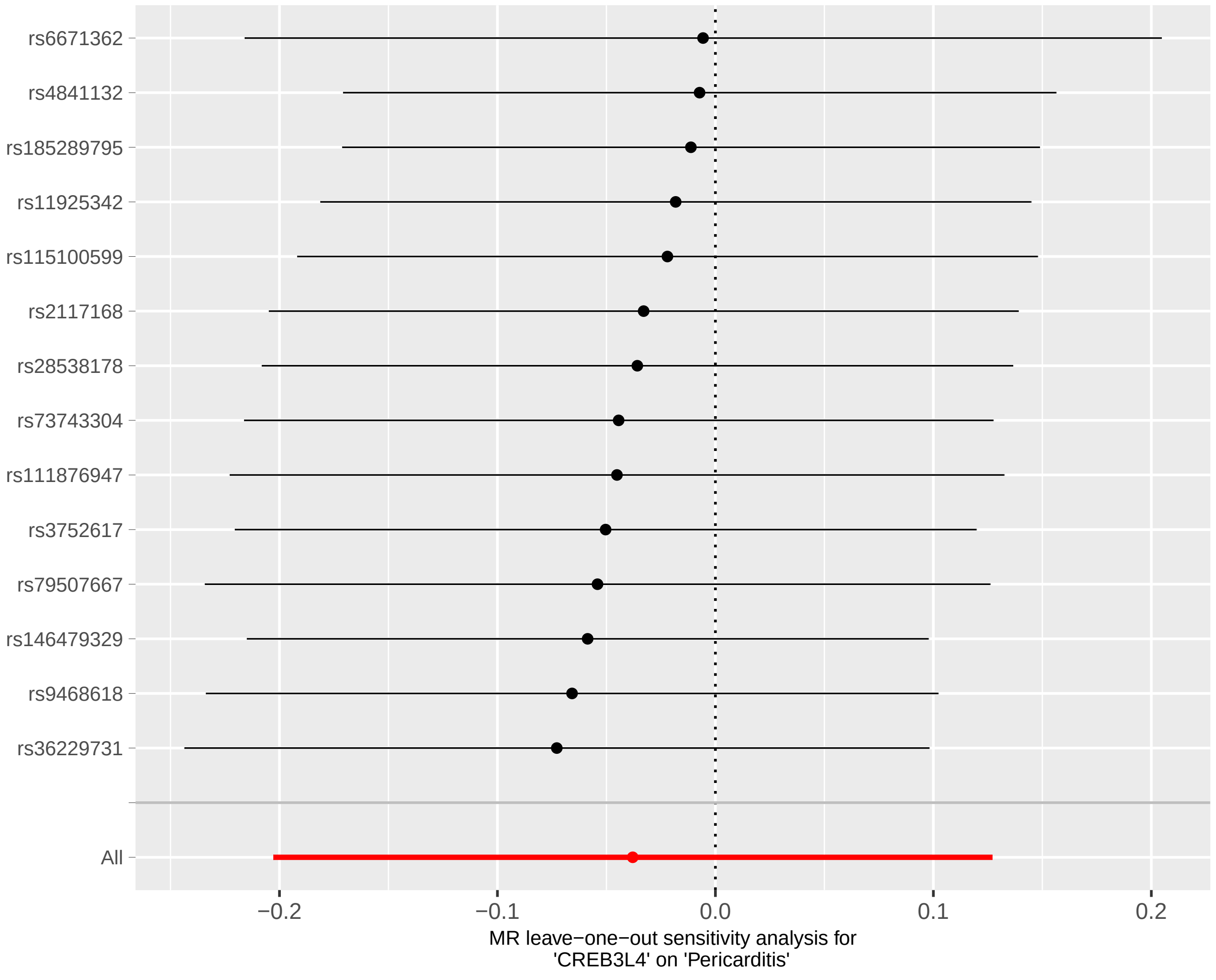
rs6671362
rs4841132
rs185289795
rs11925342
rs115100599
rs2117168
rs28538178
rs73743304
rs111876947
rs3752617
rs79507667
rs146479329
rs9468618
rs36229731
All
−0.2
−0.1
0.0
0.1
0.2
MR leave−one−out sensitivity analysis for
'CREB3L4' on 'Pericarditis'

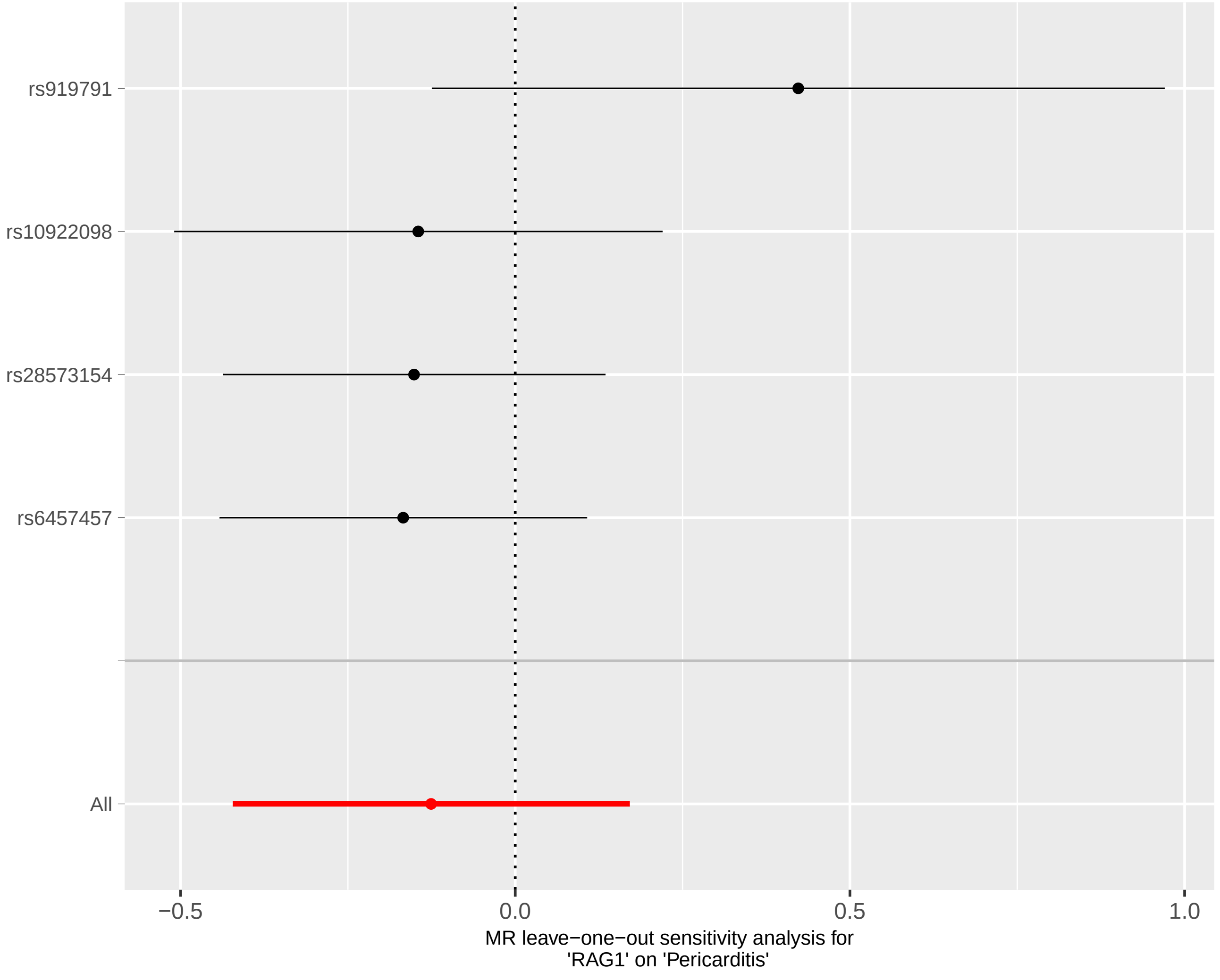

rs919791
rs10922098
rs28573154
rs6457457
All
−0.5
0.0
0.5
1.0
MR leave−one−out sensitivity analysis for
'RAG1' on 'Pericarditis'

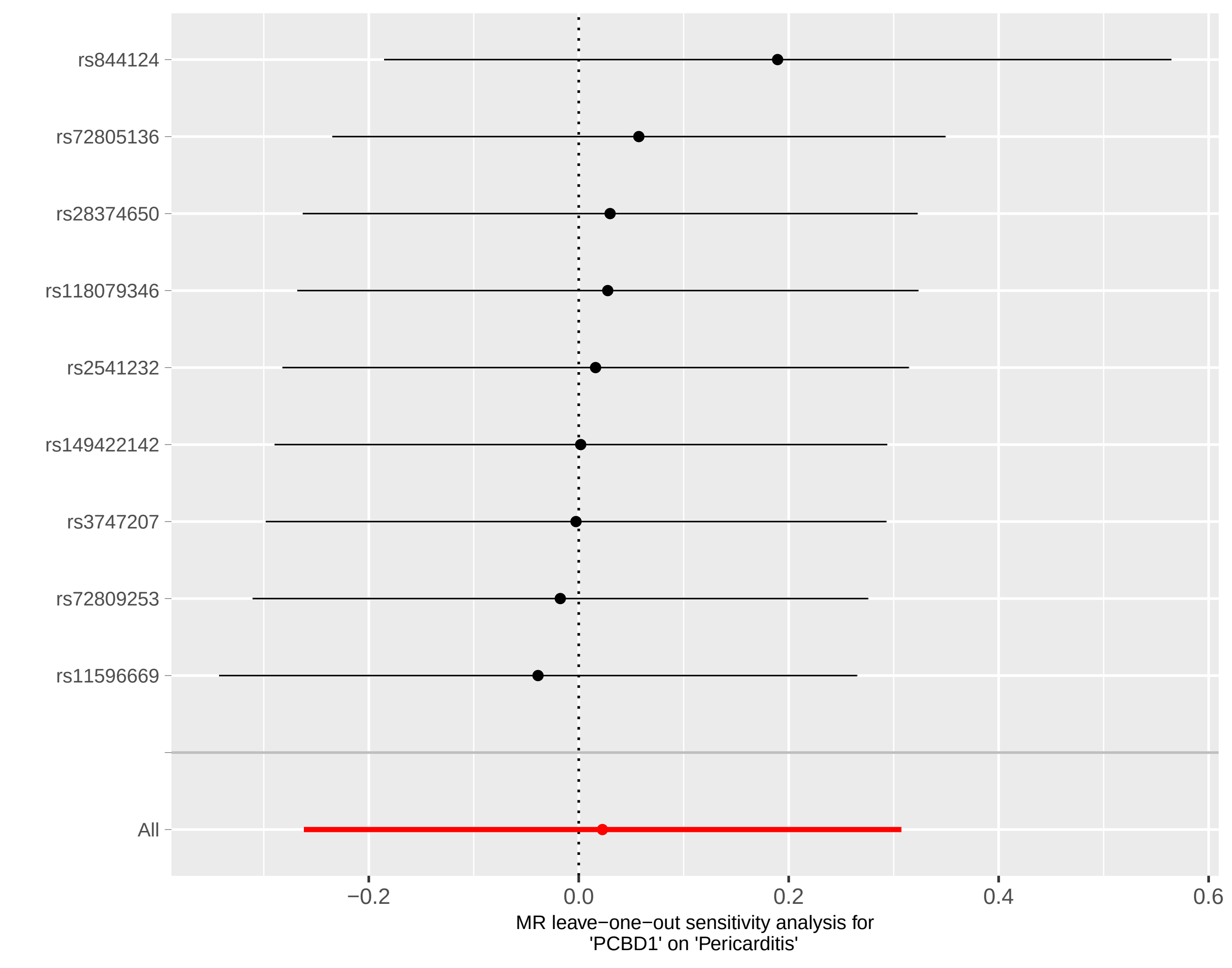

rs844124
rs72805136
rs28374650
rs118079346
rs2541232
rs149422142
rs3747207
rs72809253
rs11596669
All
−0.2
0.0
0.2
0.4
0.6
MR leave−one−out sensitivity analysis for
'PCBD1' on 'Pericarditis'

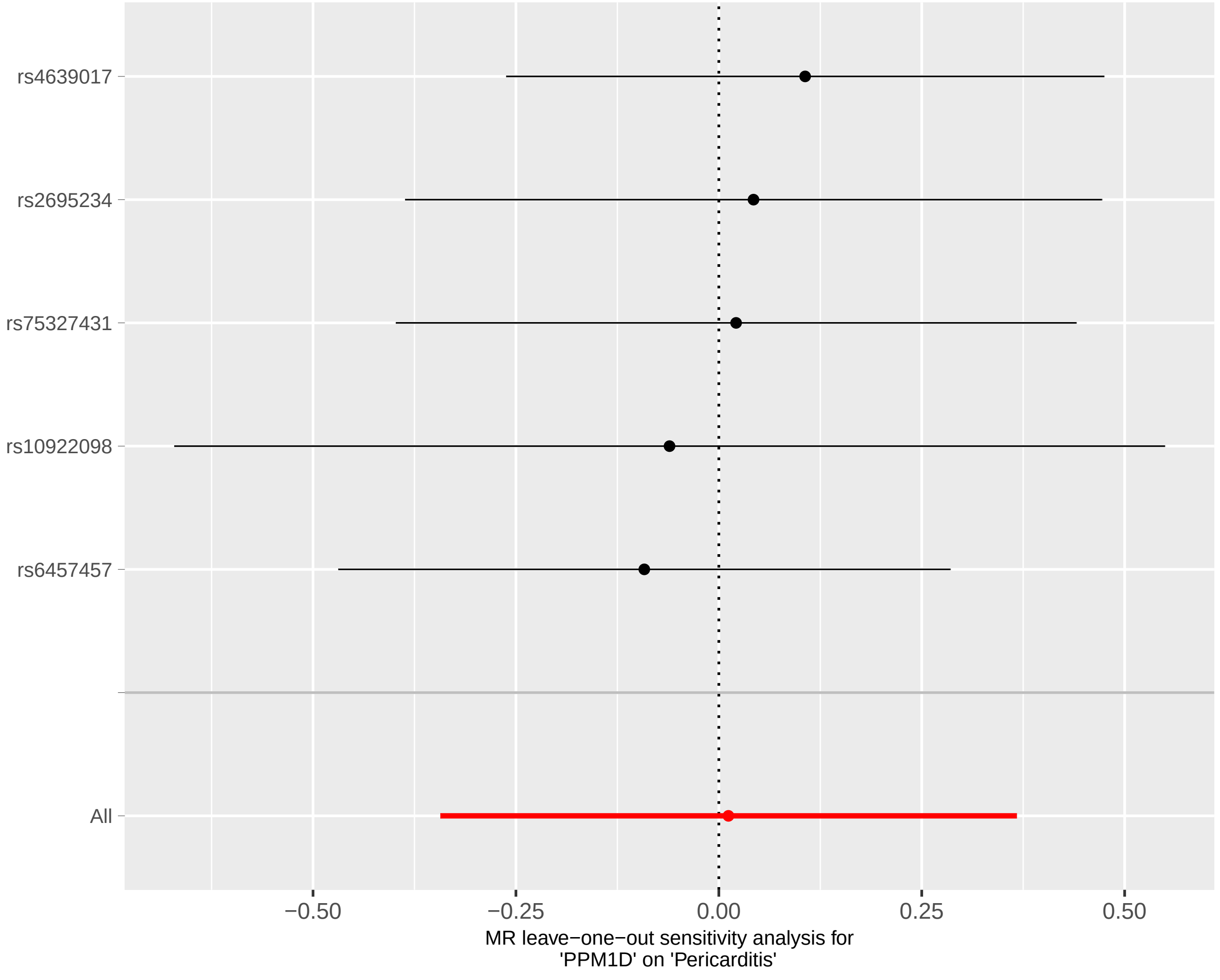
rs4639017
rs2695234
rs75327431
rs10922098
rs6457457
All
−0.50
−0.25
0.00
0.25
0.50
MR leave−one−out sensitivity analysis for
'PPM1D' on 'Pericarditis'

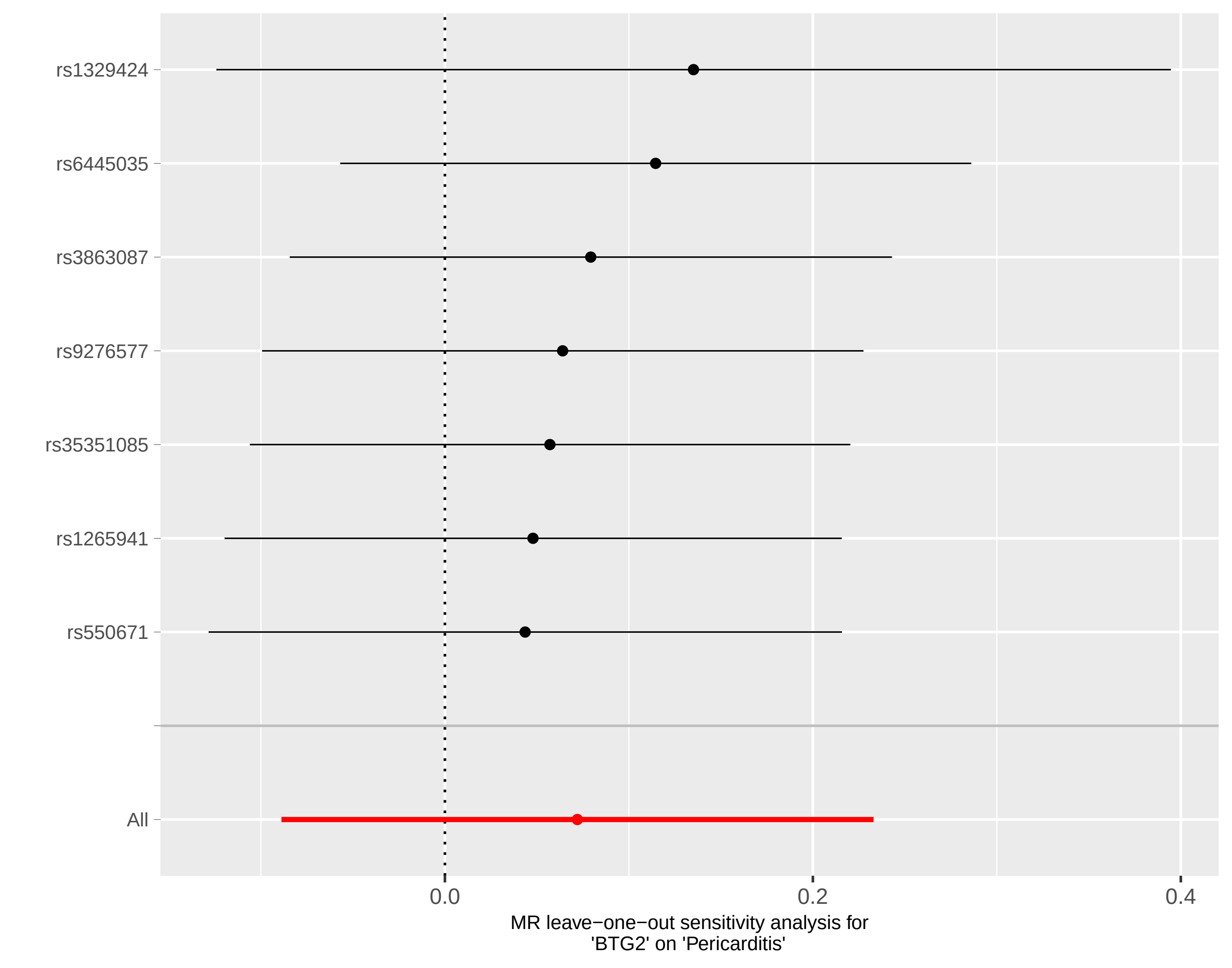

rs1329424
rs6445035
rs3863087
rs9276577
rs35351085
rs1265941
rs550671
All
0.0
0.2
0.4
MR leave−one−out sensitivity analysis for
'BTG2' on 'Pericarditis'

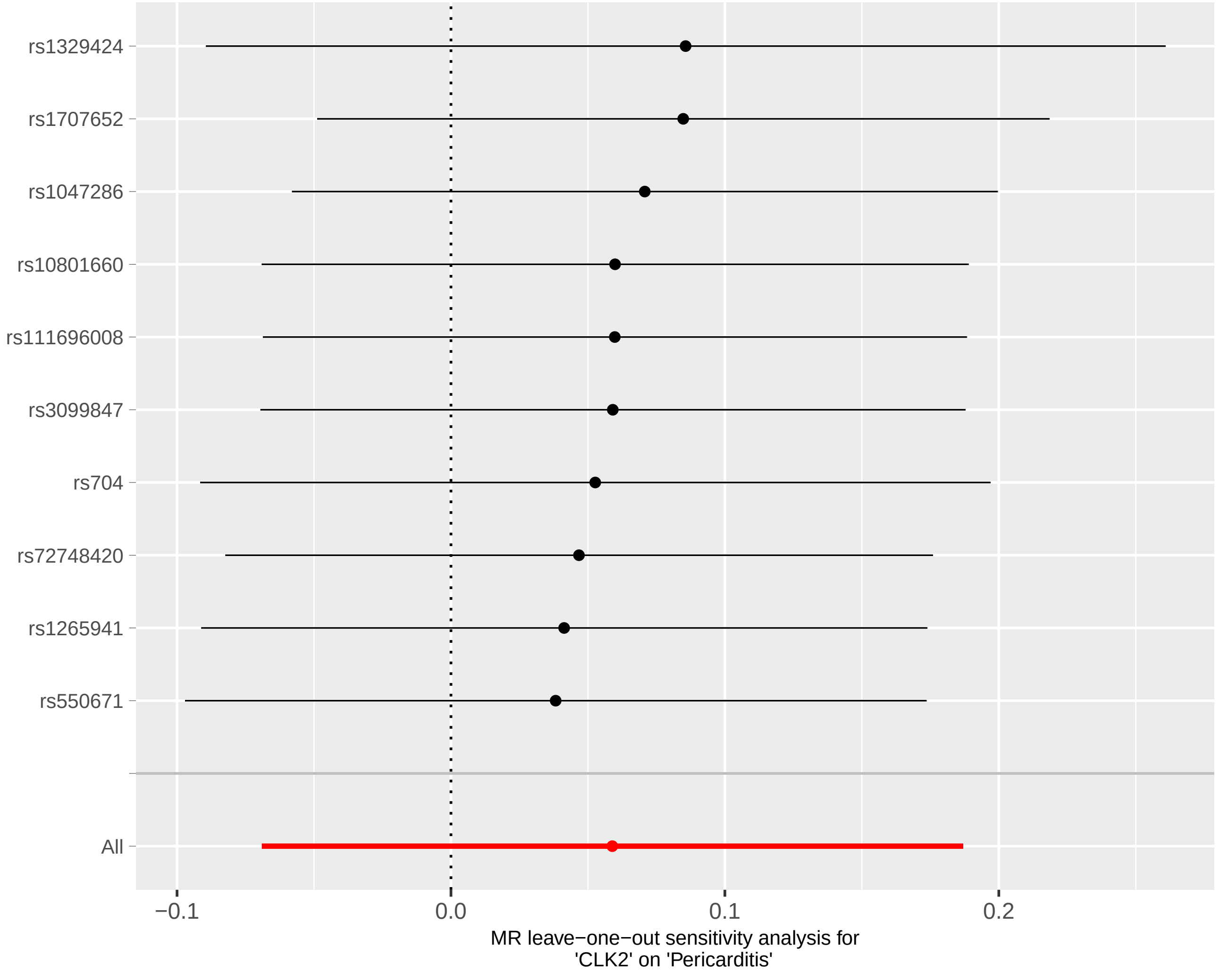
rs1329424
rs1707652
rs1047286
rs10801660
rs111696008
rs3099847
rs704
rs72748420
rs1265941
rs550671
All
−0.1
0.0
0.1
0.2
MR leave−one−out sensitivity analysis for
'CLK2' on 'Pericarditis'

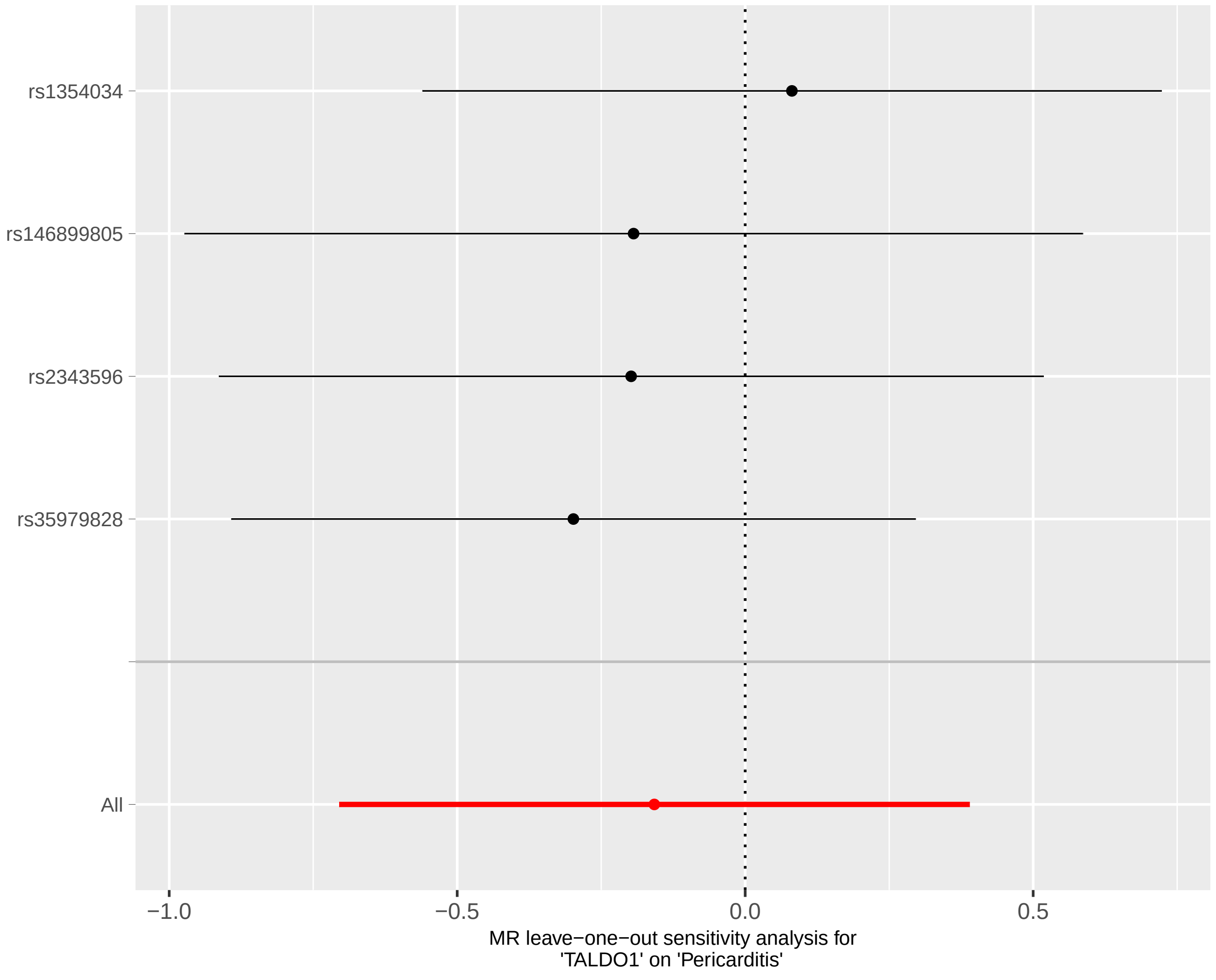
rs1354034
rs146899805
rs2343596
rs35979828
All
−1.0
−0.5
0.0
0.5
MR leave−one−out sensitivity analysis for
'TALDO1' on 'Pericarditis'

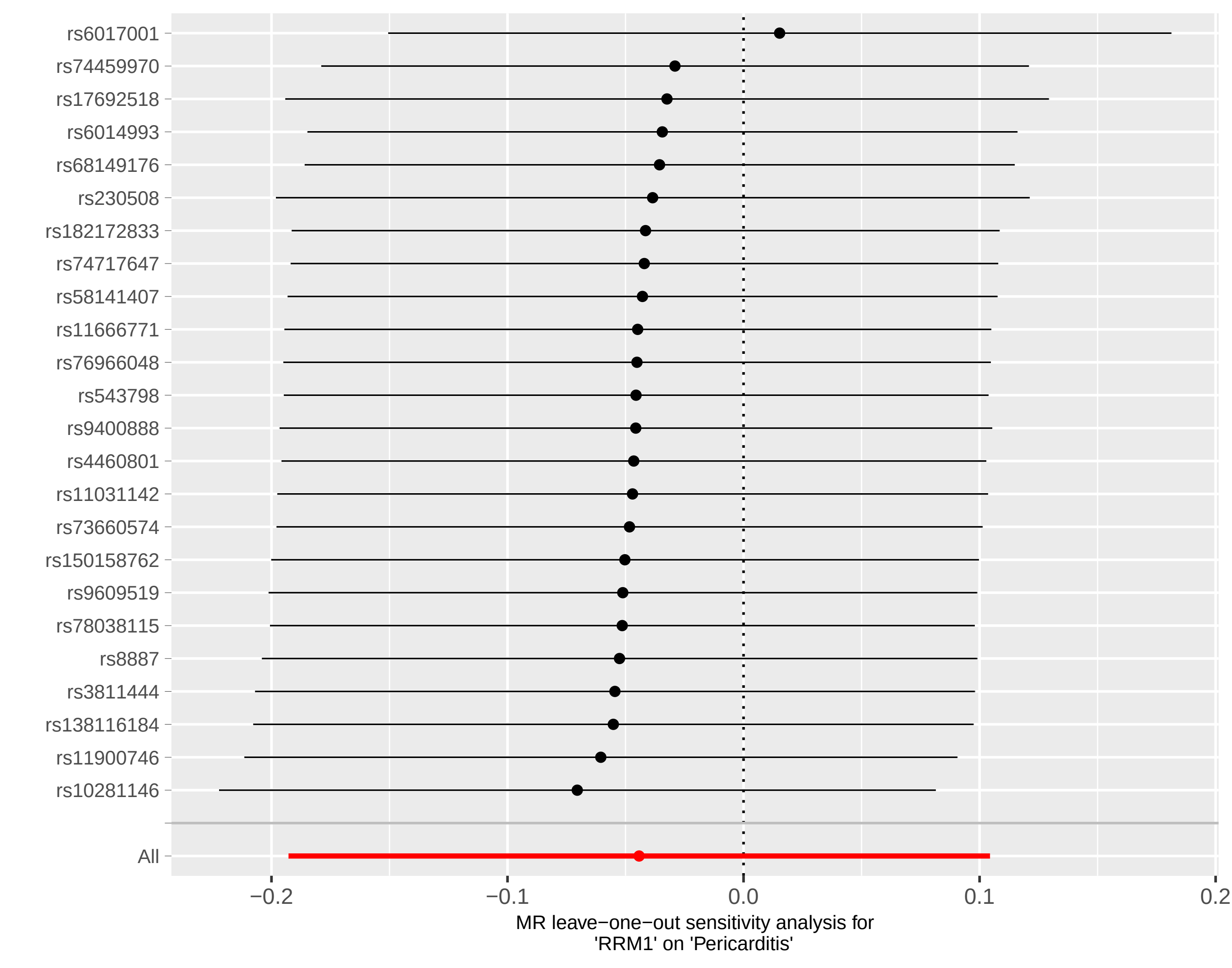

rs6017001
rs74459970
rs17692518
rs6014993
rs68149176
rs230508
rs182172833
rs74717647
rs58141407
rs11666771
rs76966048
rs543798
rs9400888
rs4460801
rs11031142
rs73660574
rs150158762
rs9609519
rs78038115
rs8887
rs3811444
rs138116184
rs11900746
rs10281146
All
−0.2
−0.1
0.0
0.1
0.2
MR leave−one−out sensitivity analysis for
'RRM1' on 'Pericarditis'

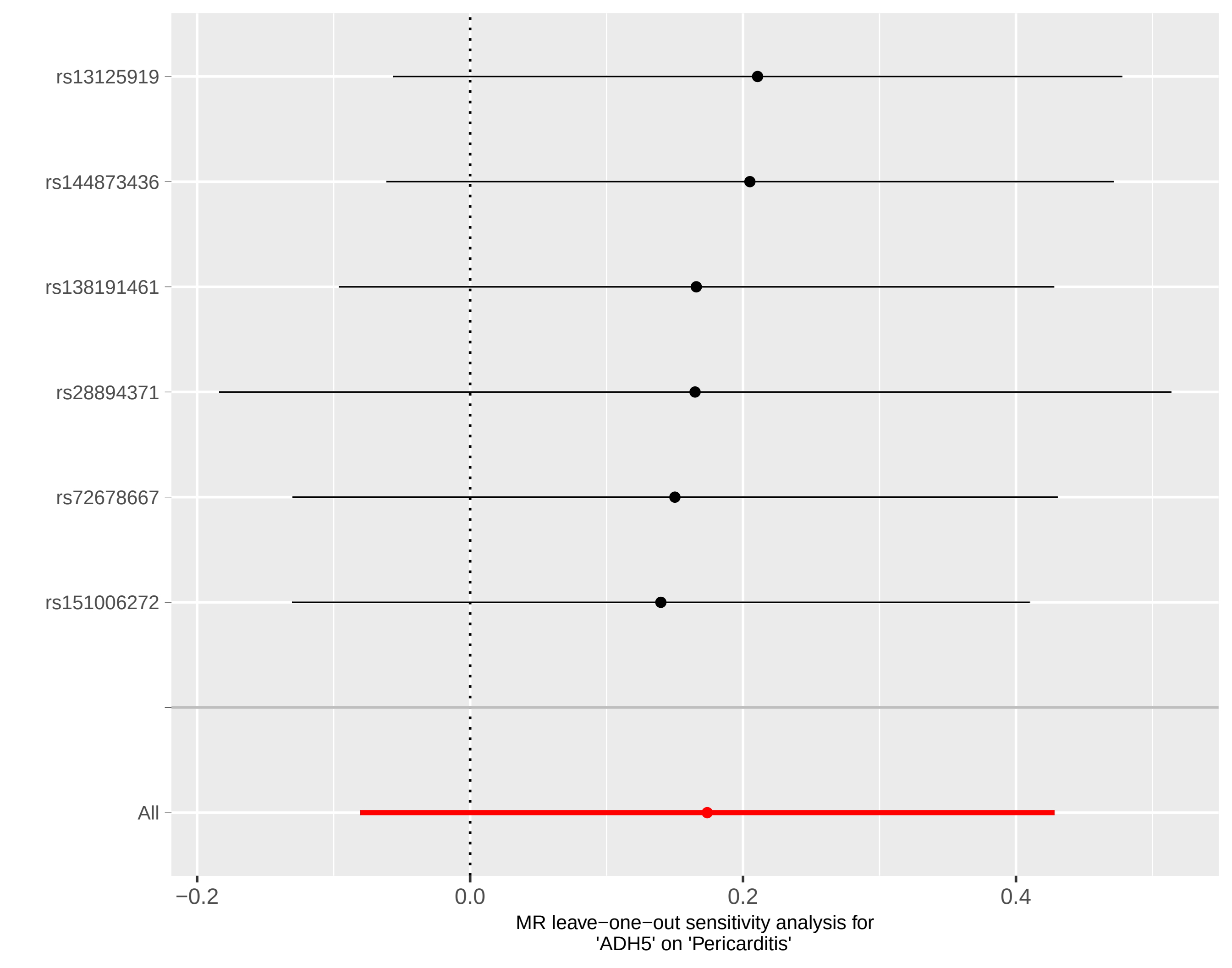
rs13125919
rs144873436
rs138191461
rs28894371
rs72678667
rs151006272
All
−0.2
0.0
0.2
0.4
MR leave−one−out sensitivity analysis for
'ADH5' on 'Pericarditis'

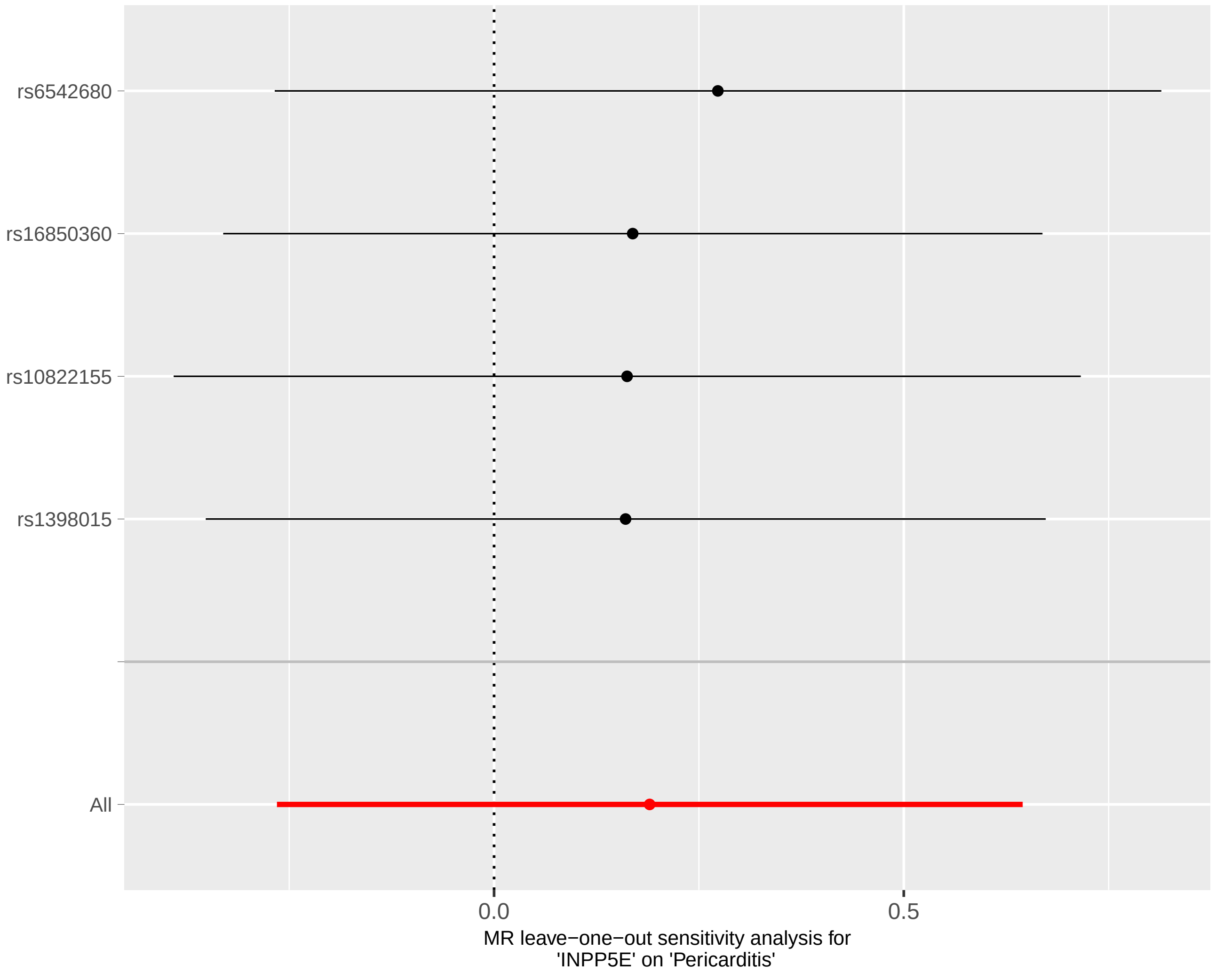
rs6542680
rs16850360
rs10822155
rs1398015
All
0.0
0.5
MR leave−one−out sensitivity analysis for
'INPP5E' on 'Pericarditis'

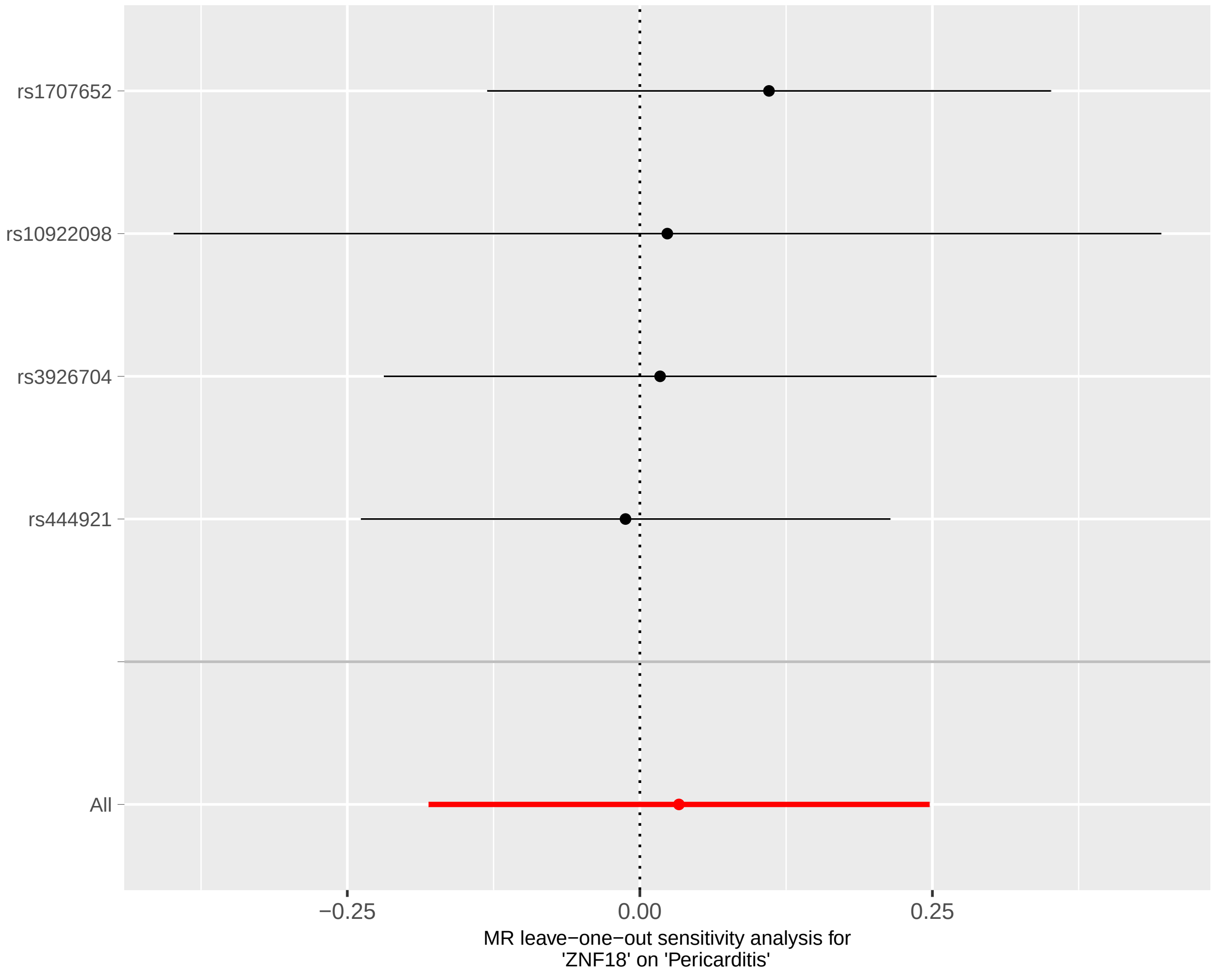

rs1707652
rs10922098
rs3926704
rs444921
All
−0.25
0.00
0.25
MR leave−one−out sensitivity analysis for
'ZNF18' on 'Pericarditis'

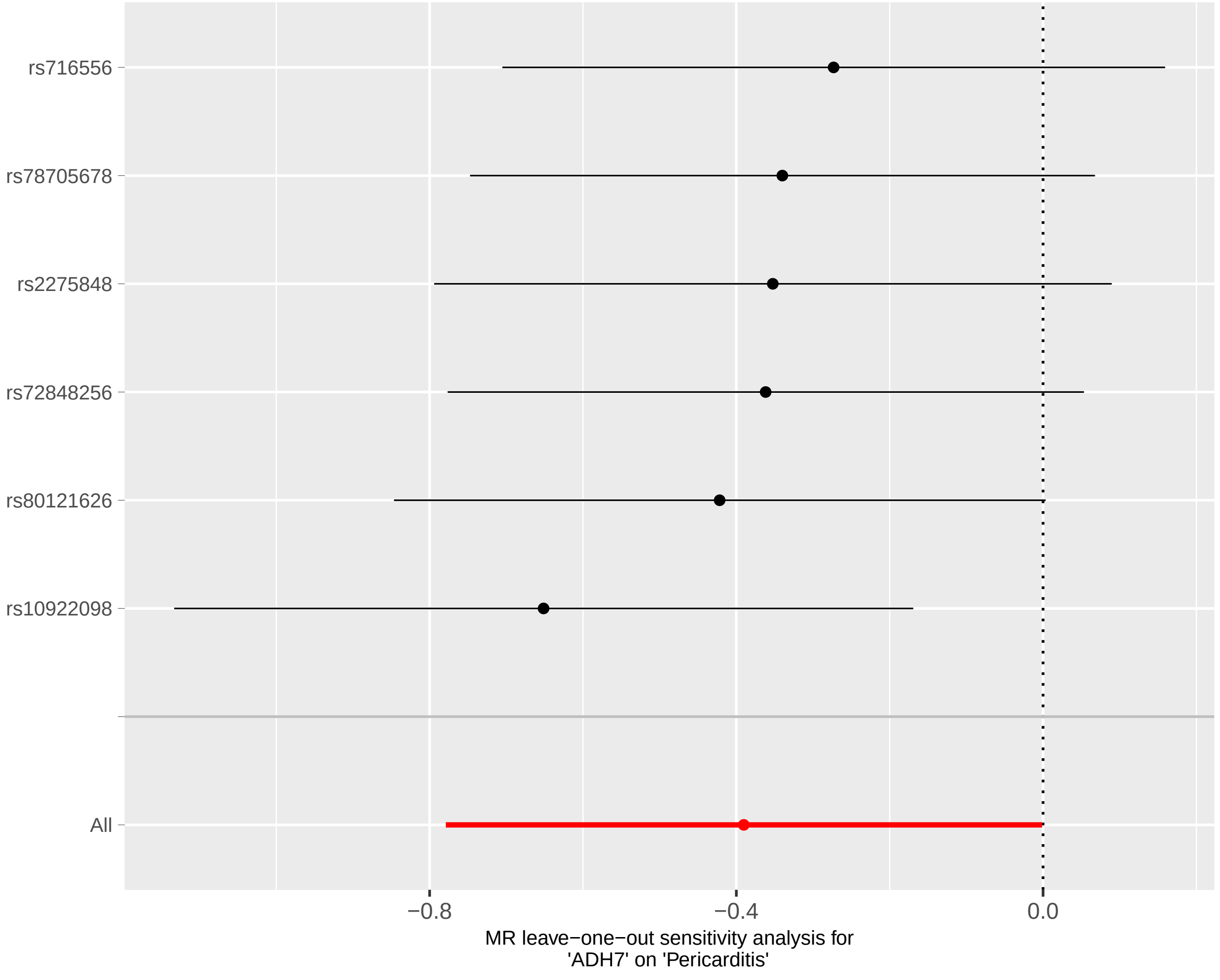
rs716556
rs78705678
rs2275848
rs72848256
rs80121626
rs10922098
All
−0.8
−0.4
0.0
MR leave−one−out sensitivity analysis for
'ADH7' on 'Pericarditis'

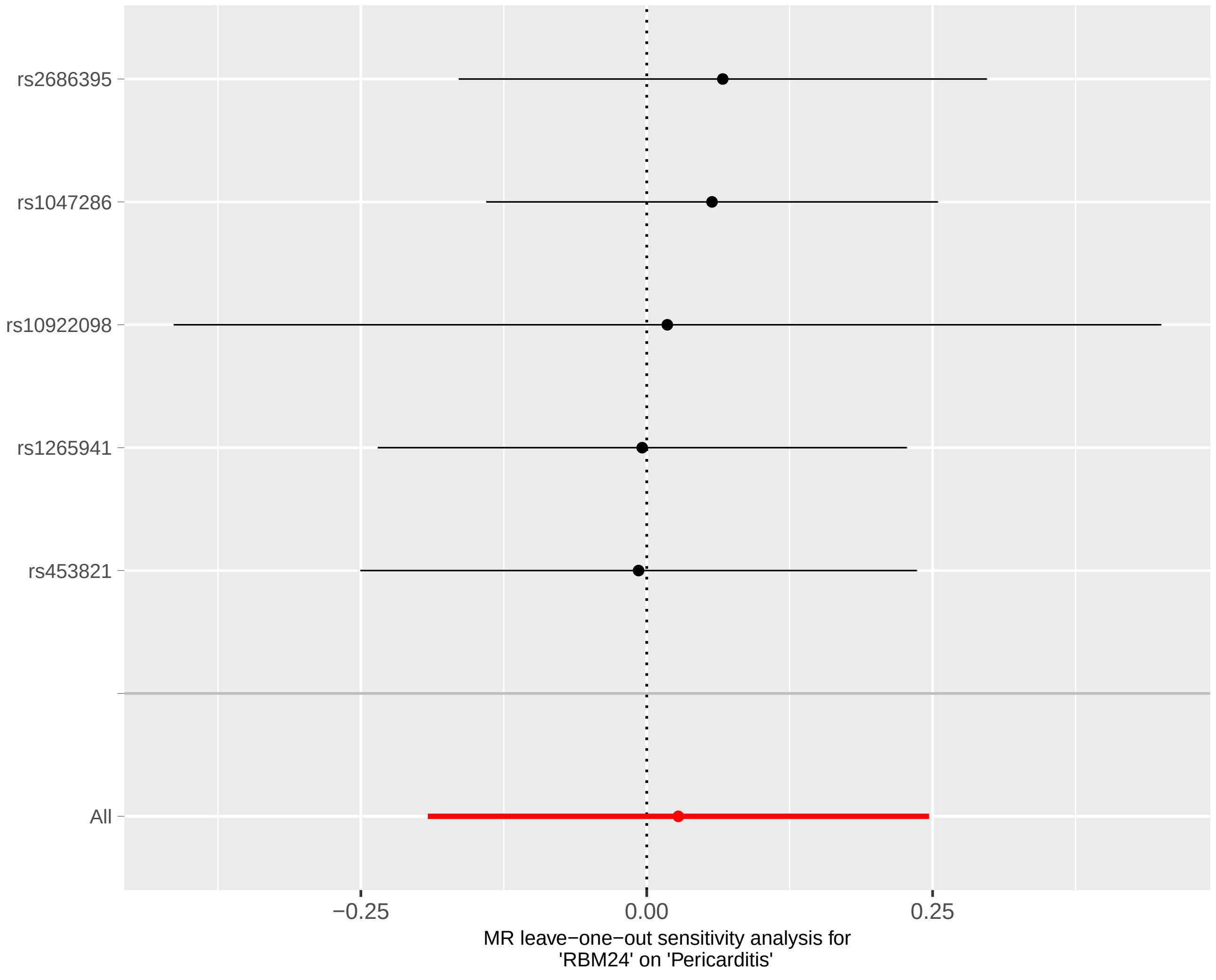
rs2686395
rs1047286
rs10922098
rs1265941
rs453821
All
−0.25
0.00
0.25
MR leave−one−out sensitivity analysis for
'RBM24' on 'Pericarditis'

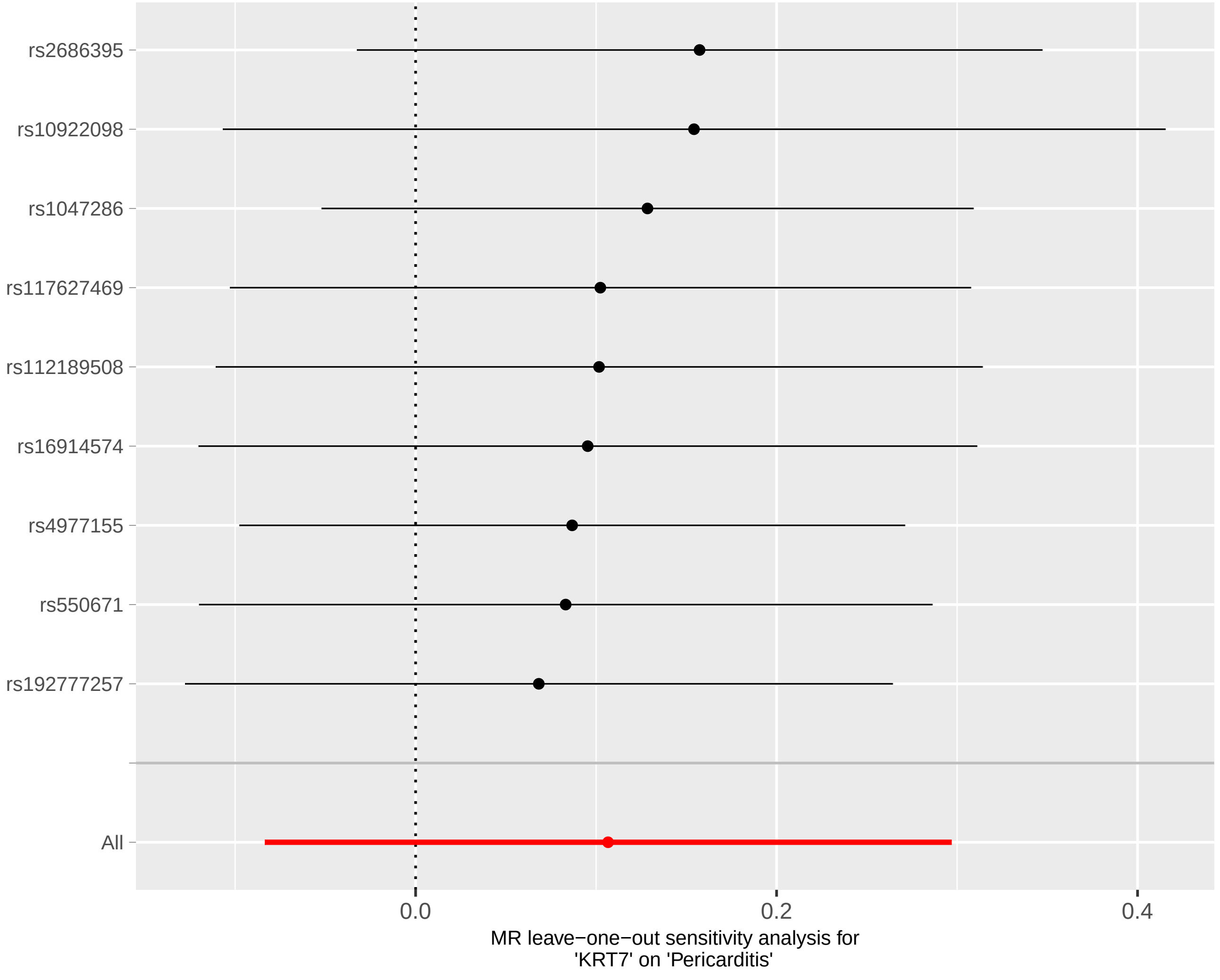

rs2686395
rs10922098
rs1047286
rs117627469
rs112189508
rs16914574
rs4977155
rs550671
rs192777257
All
0.0
0.2
0.4
MR leave−one−out sensitivity analysis for
'KRT7' on 'Pericarditis'

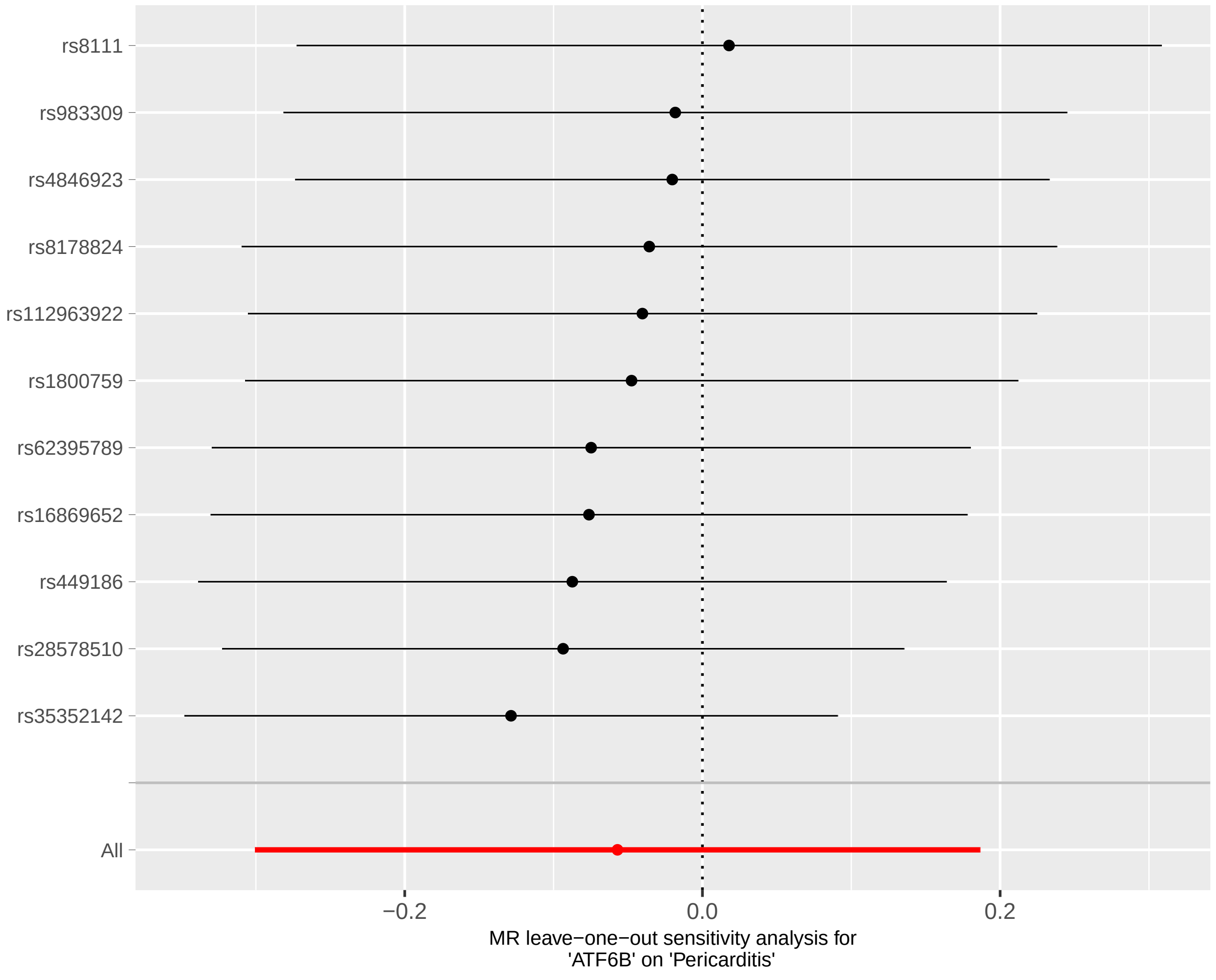
rs8111
rs983309
rs4846923
rs8178824
rs112963922
rs1800759
rs62395789
rs16869652
rs449186
rs28578510
rs35352142
All
−0.2
0.0
0.2
MR leave−one−out sensitivity analysis for
'ATF6B' on 'Pericarditis'

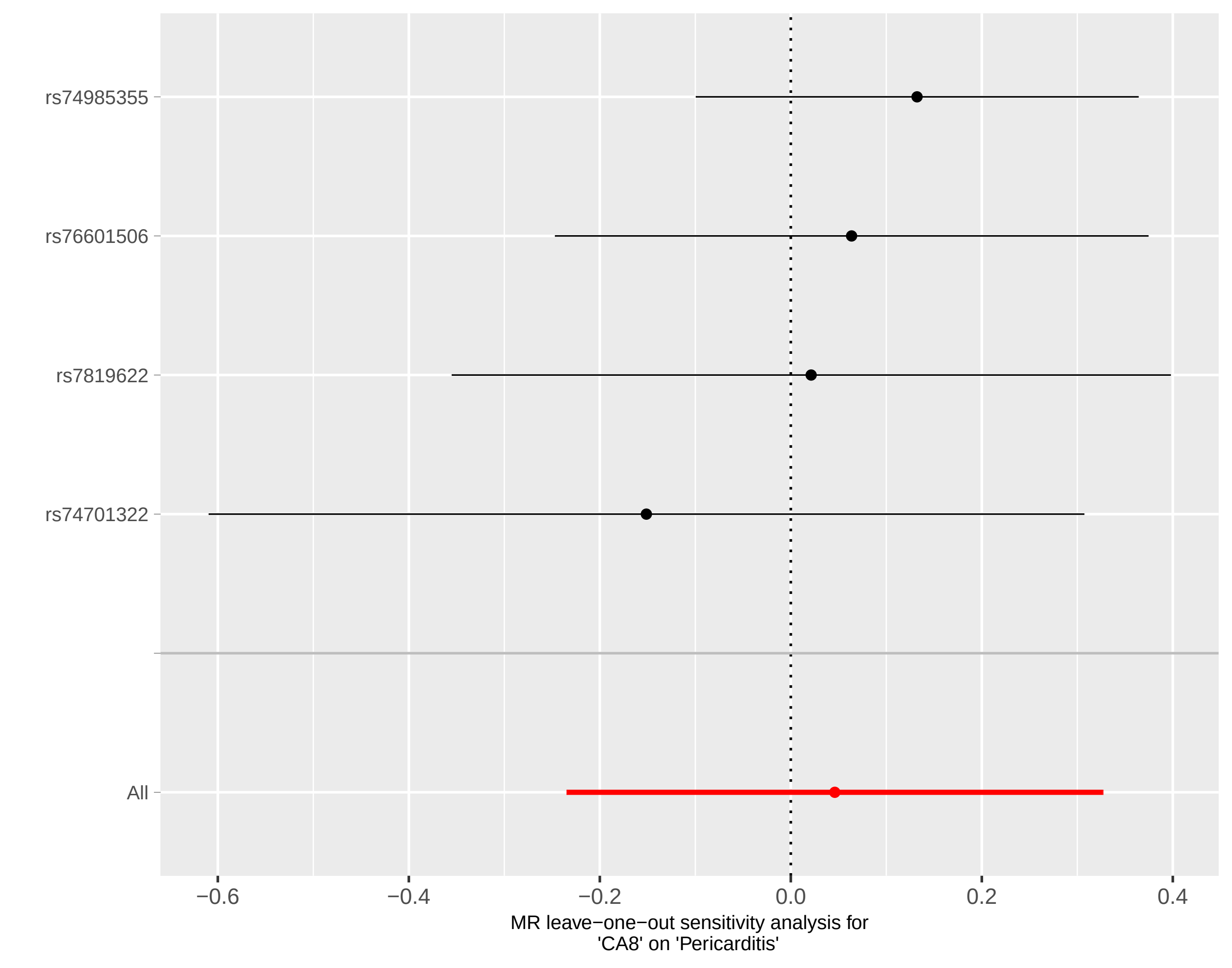

rs74985355
rs76601506
rs7819622
rs74701322
All
−0.6
−0.4
−0.2
0.0
0.2
0.4
MR leave−one−out sensitivity analysis for
'CA8' on 'Pericarditis'

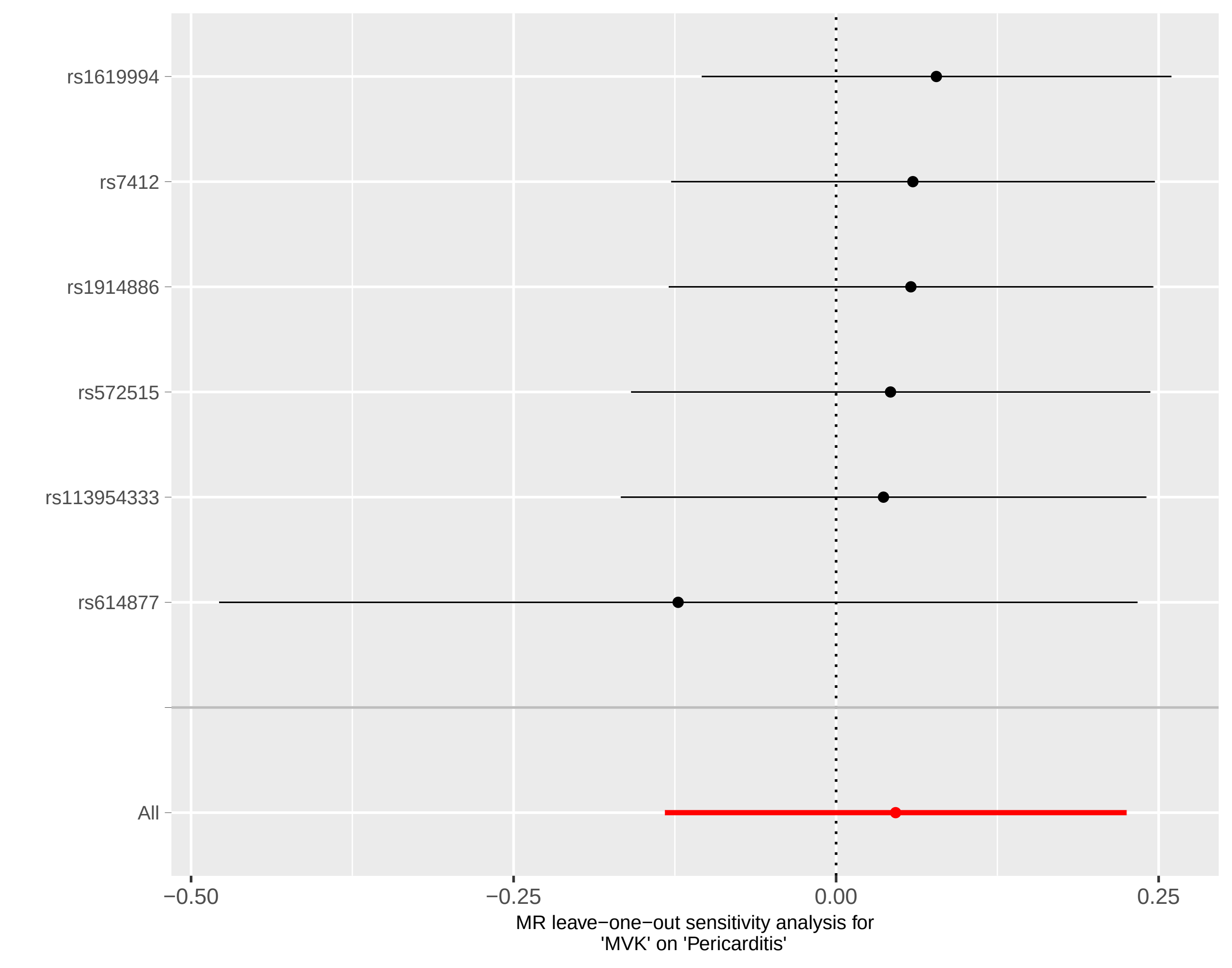

rs1619994
rs7412
rs1914886
rs572515
rs113954333
rs614877
All
−0.50
−0.25
0.00
0.25
MR leave−one−out sensitivity analysis for
'MVK' on 'Pericarditis'

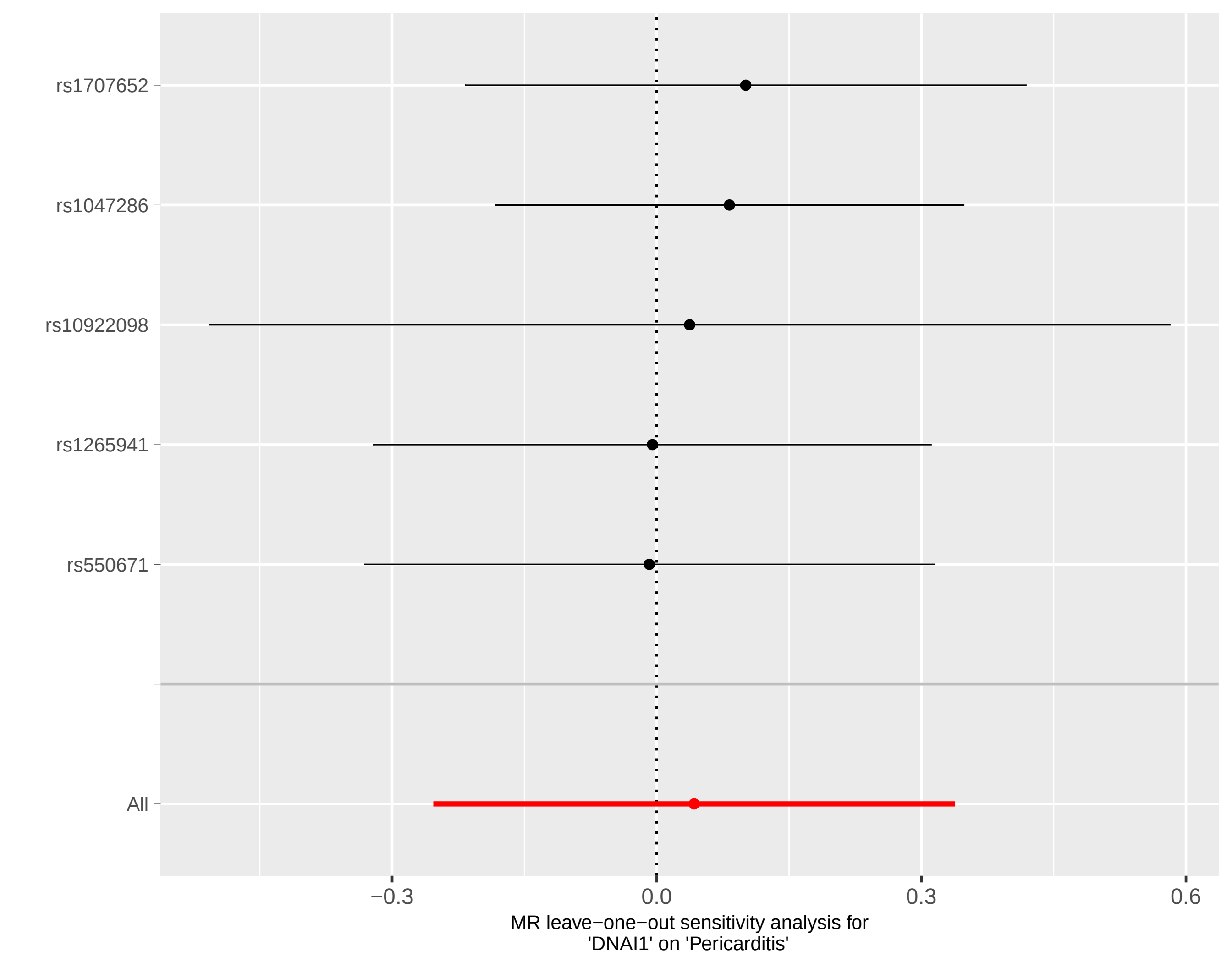

rs1707652
rs1047286
rs10922098
rs1265941
rs550671
All
−0.3
0.0
0.3
0.6
MR leave−one−out sensitivity analysis for
'DNAI1' on 'Pericarditis'

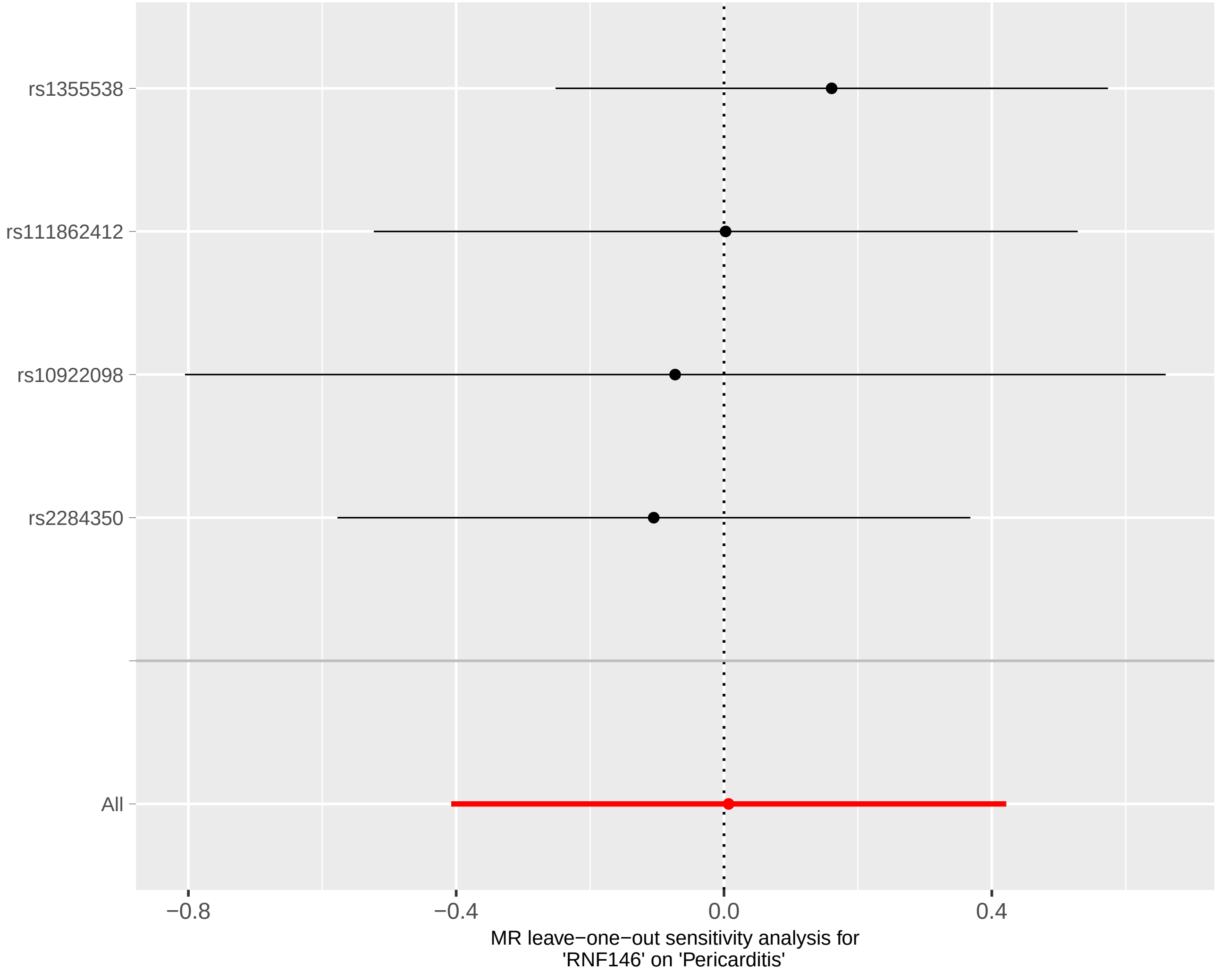

rs1355538
rs111862412
rs10922098
rs2284350
All
−0.8
−0.4
0.0
0.4
MR leave−one−out sensitivity analysis for
'RNF146' on 'Pericarditis'

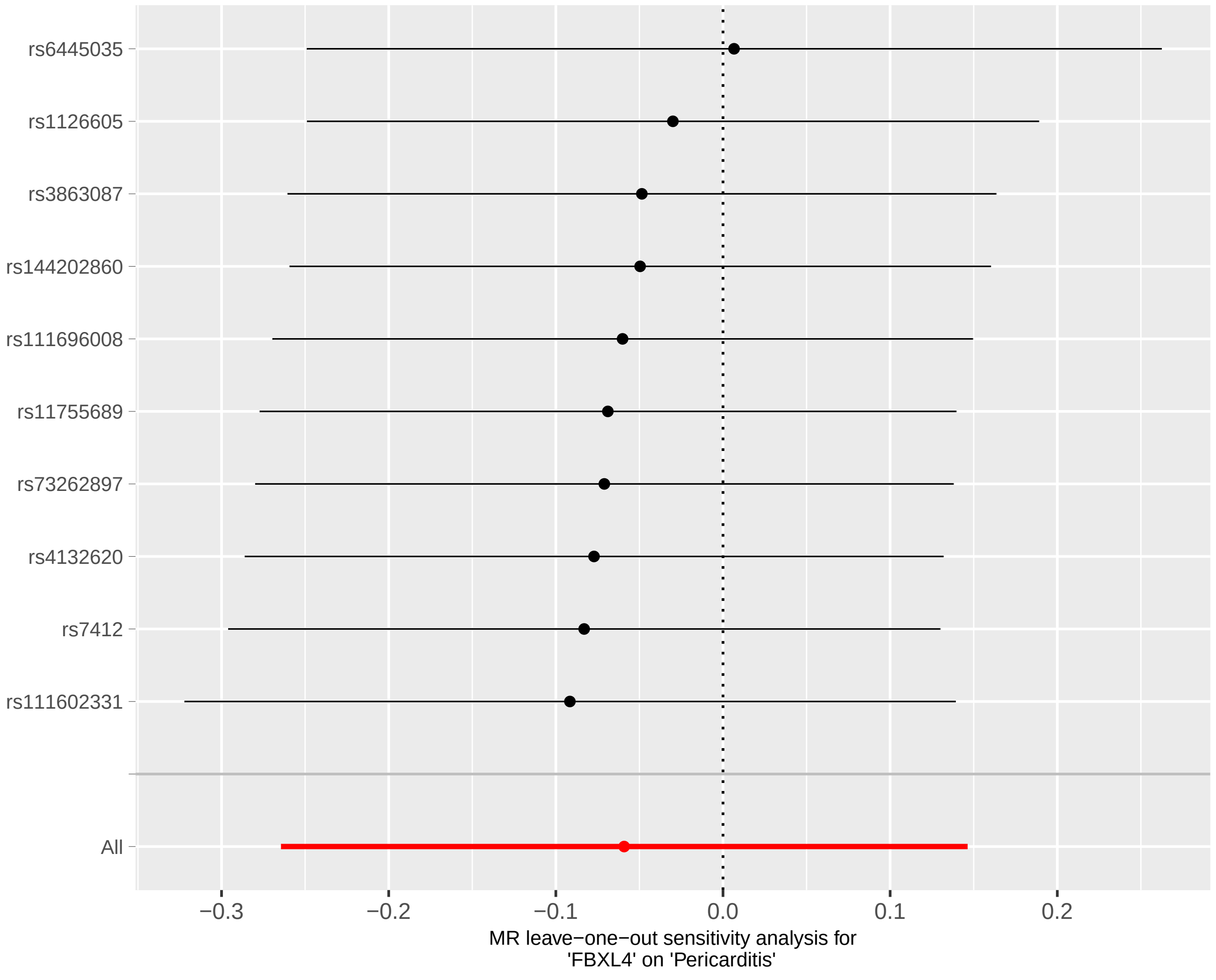

rs6445035
rs1126605
rs3863087
rs144202860
rs111696008
rs11755689
rs73262897
rs4132620
rs7412
rs111602331
All
−0.3
−0.2
−0.1
0.0
0.1
0.2
MR leave−one−out sensitivity analysis for
'FBXL4' on 'Pericarditis'

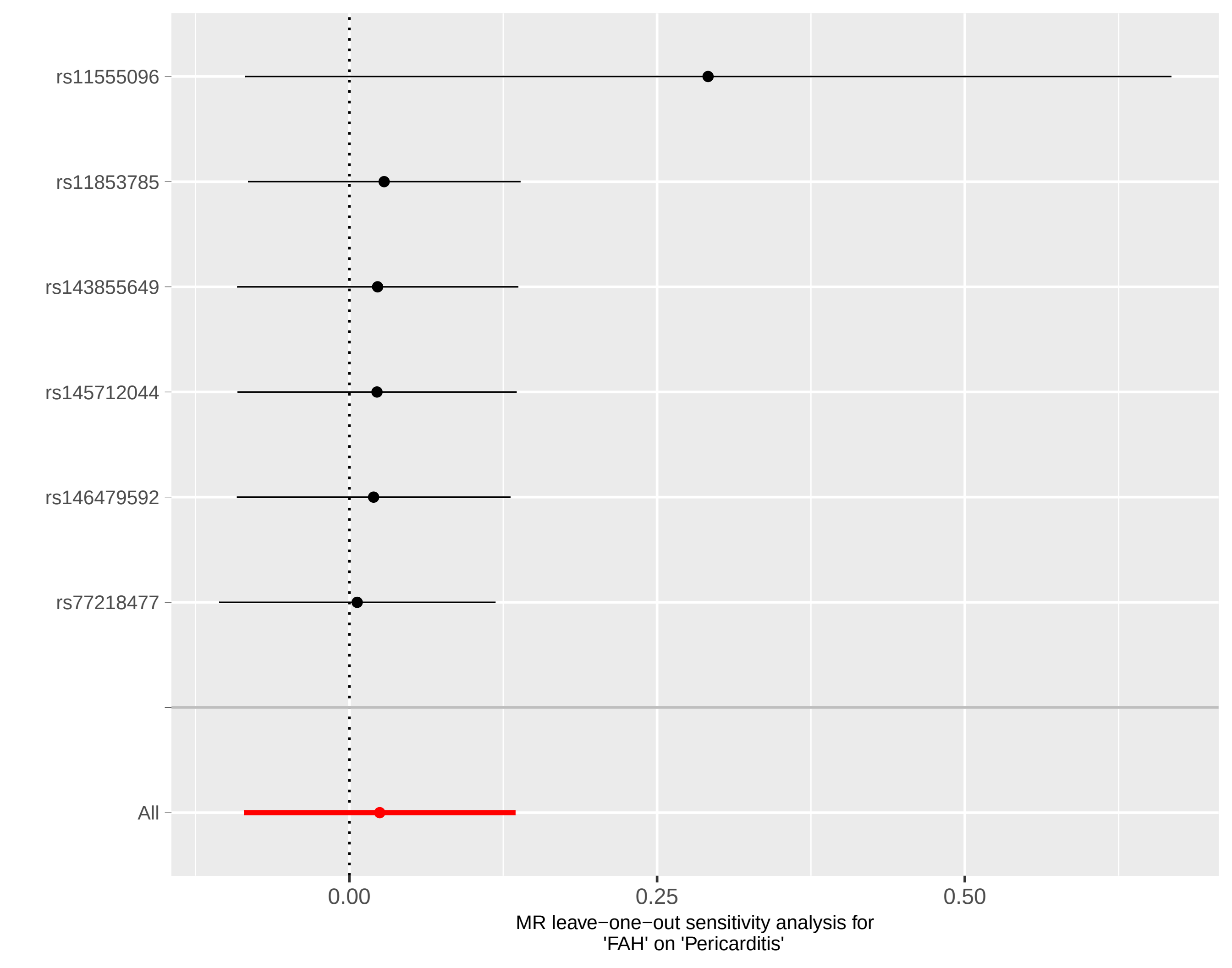
rs11555096
rs11853785
rs143855649
rs145712044
rs146479592
rs77218477
All
0.00
0.25
0.50
MR leave−one−out sensitivity analysis for
'FAH' on 'Pericarditis'

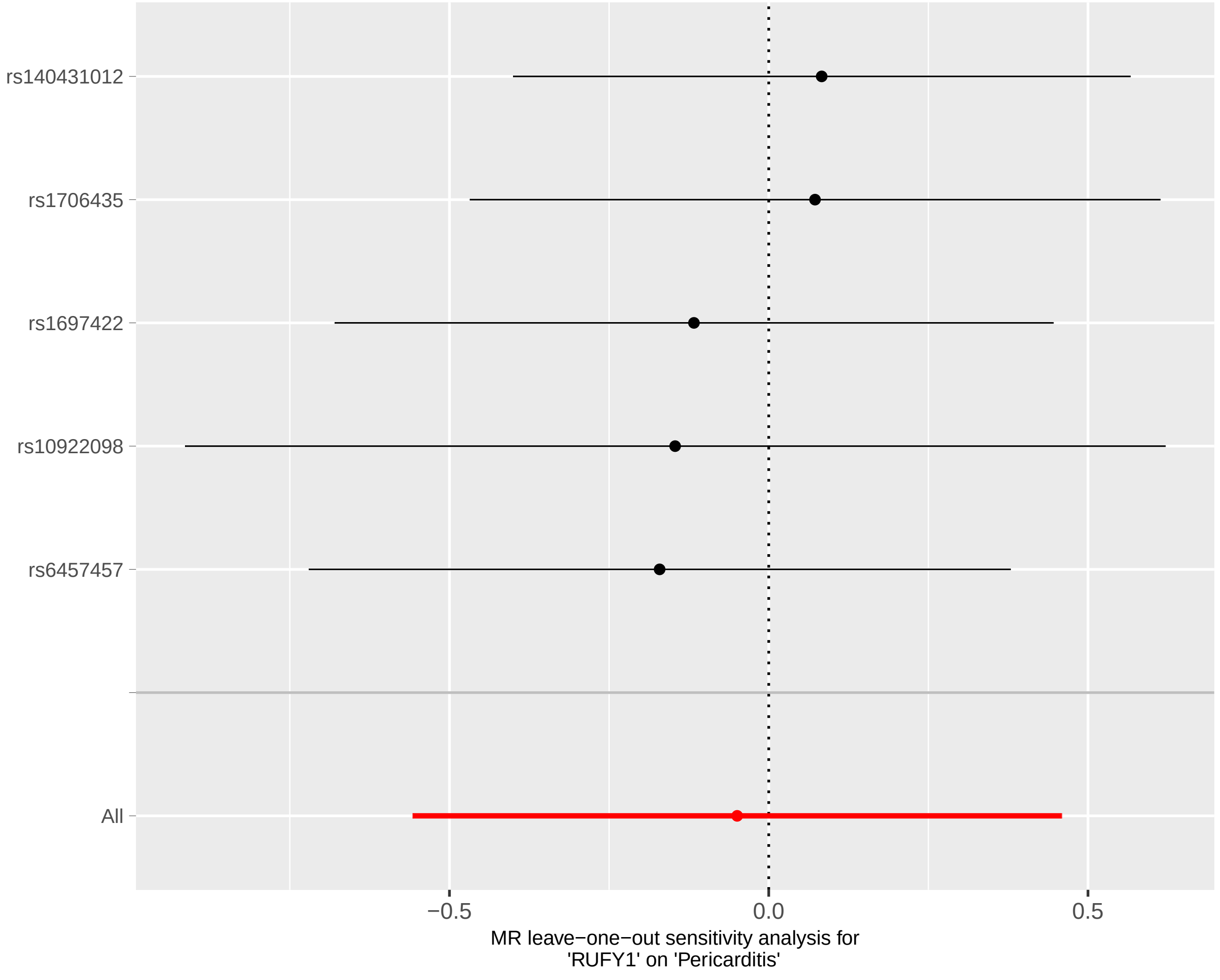

rs140431012
rs1706435
rs1697422
rs10922098
rs6457457
All
−0.5
0.0
0.5
MR leave−one−out sensitivity analysis for
'RUFY1' on 'Pericarditis'

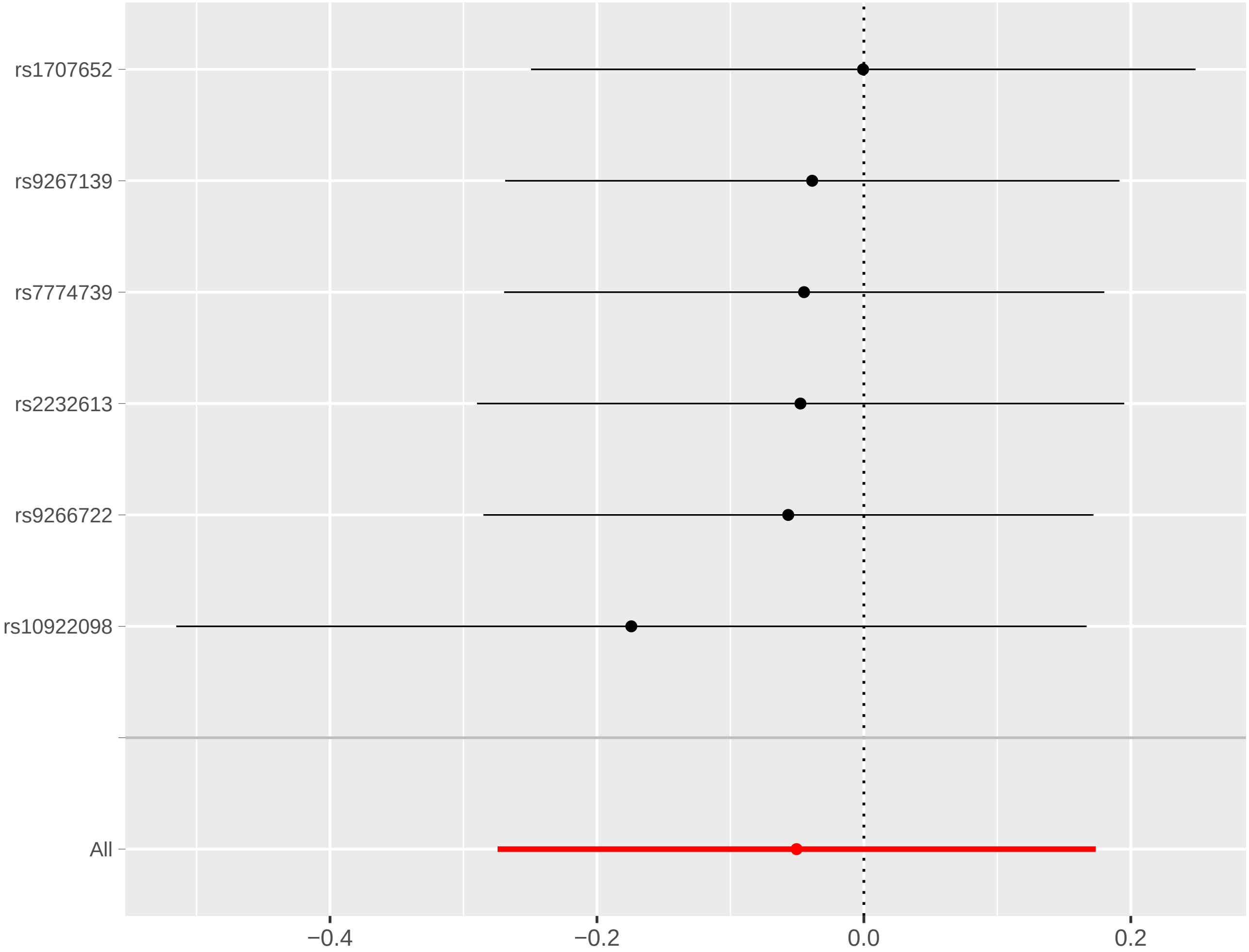

MR leave−one−out sensitivity analysis for
'HNRNPC' on 'Pericarditis'

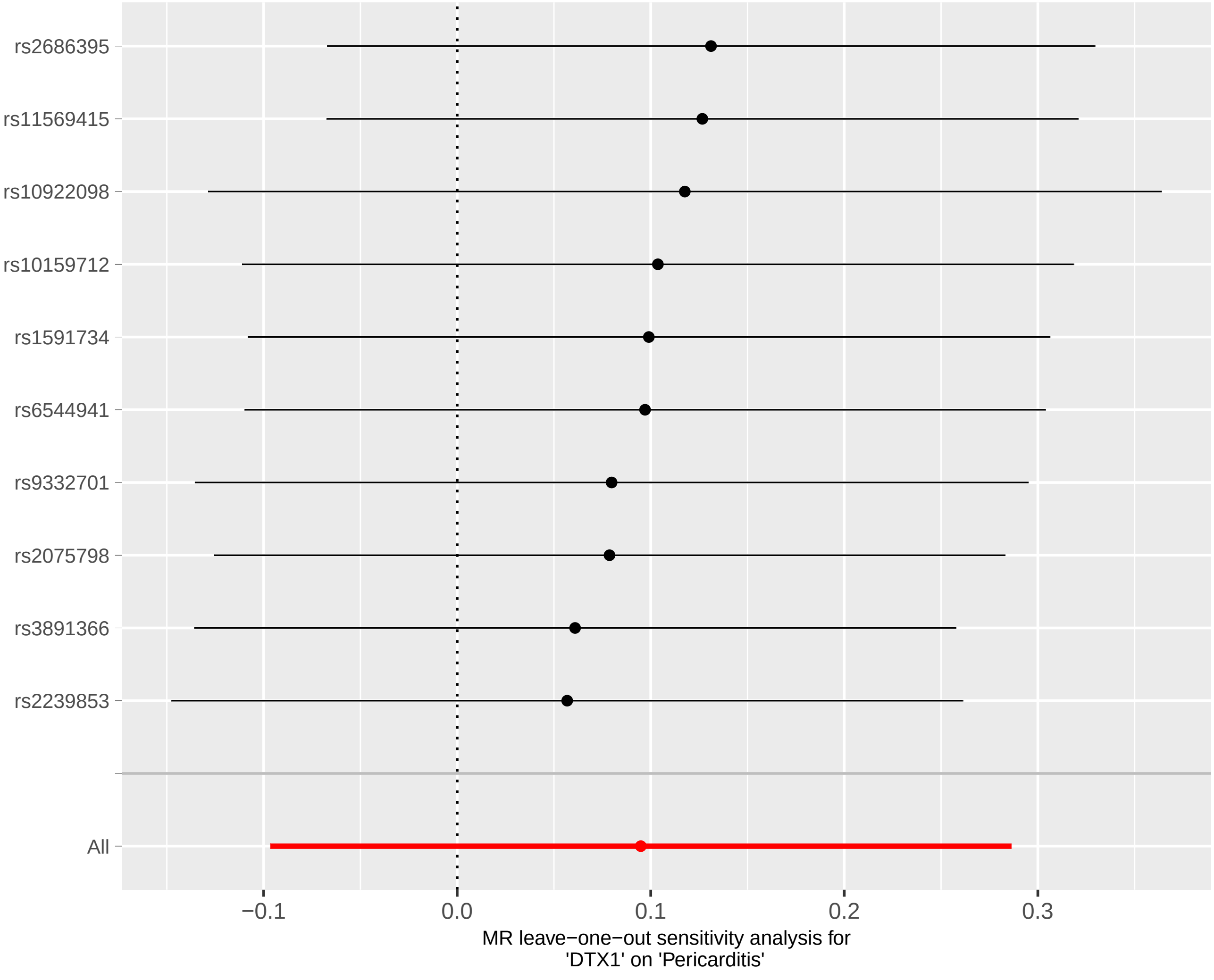
rs2686395
rs11569415
rs10922098
rs10159712
rs1591734
rs6544941
rs9332701
rs2075798
rs3891366
rs2239853
All
−0.1
0.0
0.1
0.2
0.3
MR leave−one−out sensitivity analysis for
'DTX1' on 'Pericarditis'

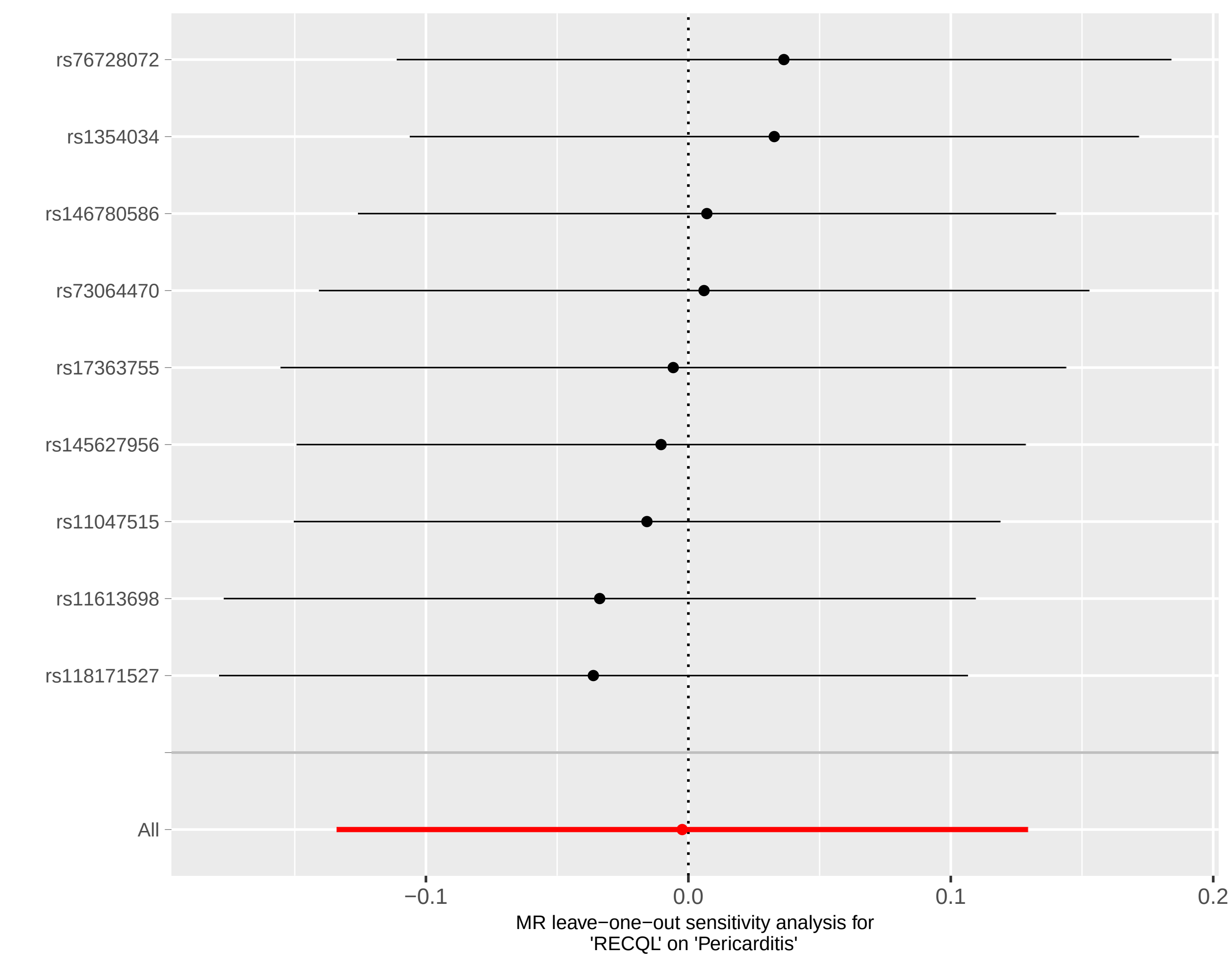

rs76728072
rs1354034
rs146780586
rs73064470
rs17363755
rs145627956
rs11047515
rs11613698
rs118171527
All
−0.1
0.0
0.1
0.2
MR leave−one−out sensitivity analysis for
'RECQL' on 'Pericarditis'

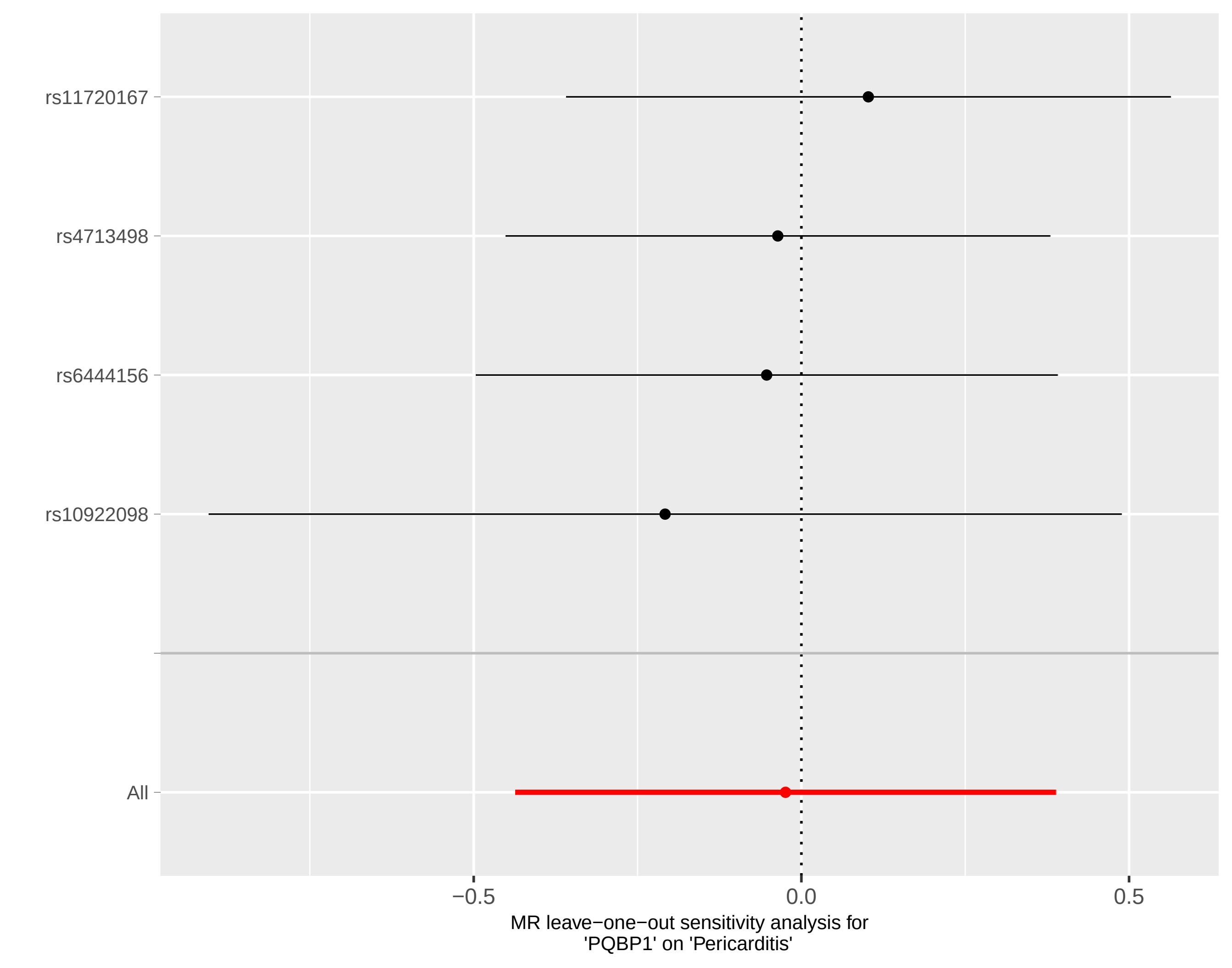
rs11720167
rs4713498
rs6444156
rs10922098
All
−0.5
0.0
0.5
MR leave−one−out sensitivity analysis for
'PQBP1' on 'Pericarditis'

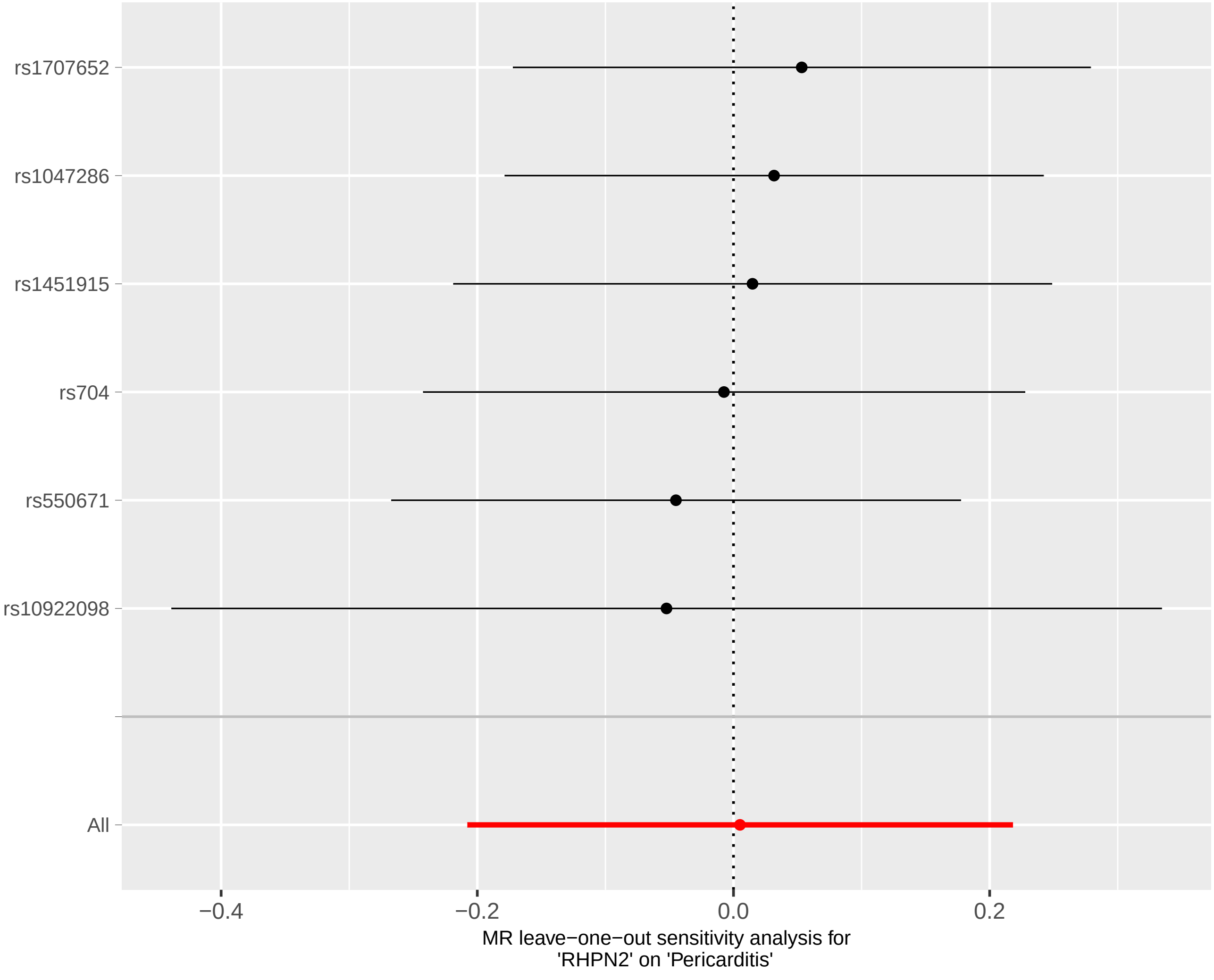

rs1707652
rs1047286
rs1451915
rs704
rs550671
rs10922098
All
−0.4
−0.2
0.0
0.2
MR leave−one−out sensitivity analysis for
'RHPN2' on 'Pericarditis'

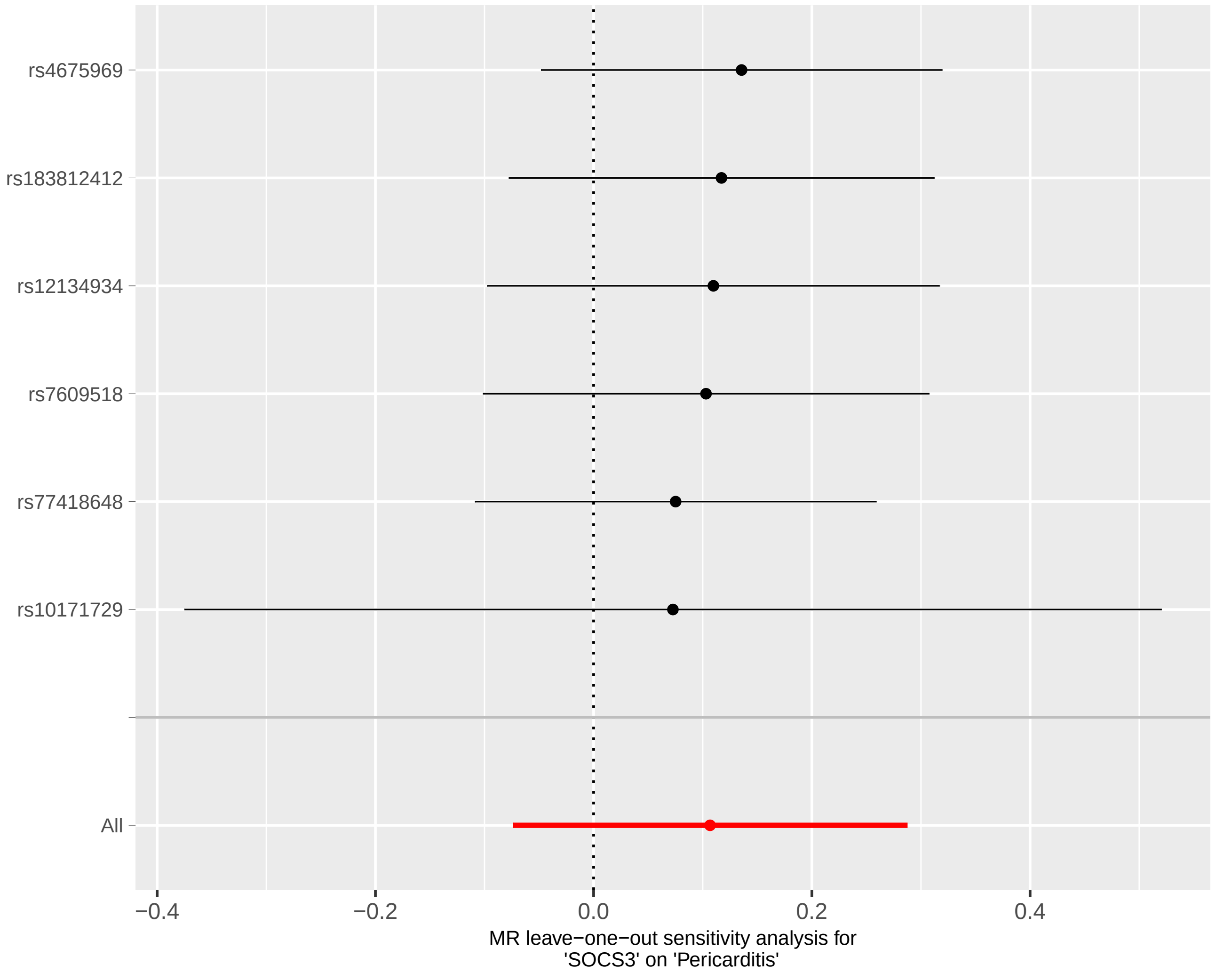
rs4675969
rs183812412
rs12134934
rs7609518
rs77418648
rs10171729
All
−0.4
−0.2
0.0
0.2
0.4
MR leave−one−out sensitivity analysis for
'SOCS3' on 'Pericarditis'

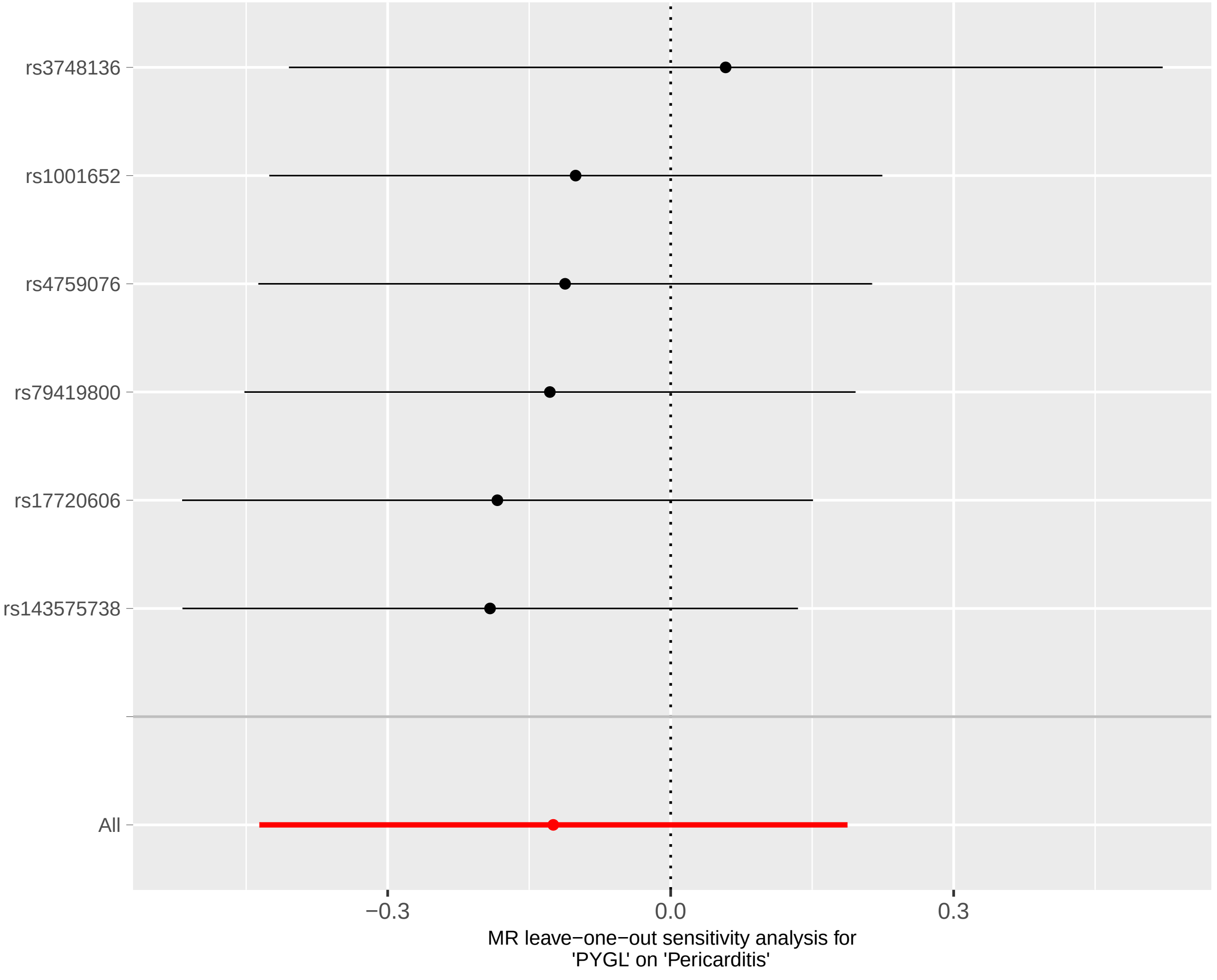
rs3748136
rs1001652
rs4759076
rs79419800
rs17720606
rs143575738
All
−0.3
0.0
0.3
MR leave−one−out sensitivity analysis for
'PYGL' on 'Pericarditis'

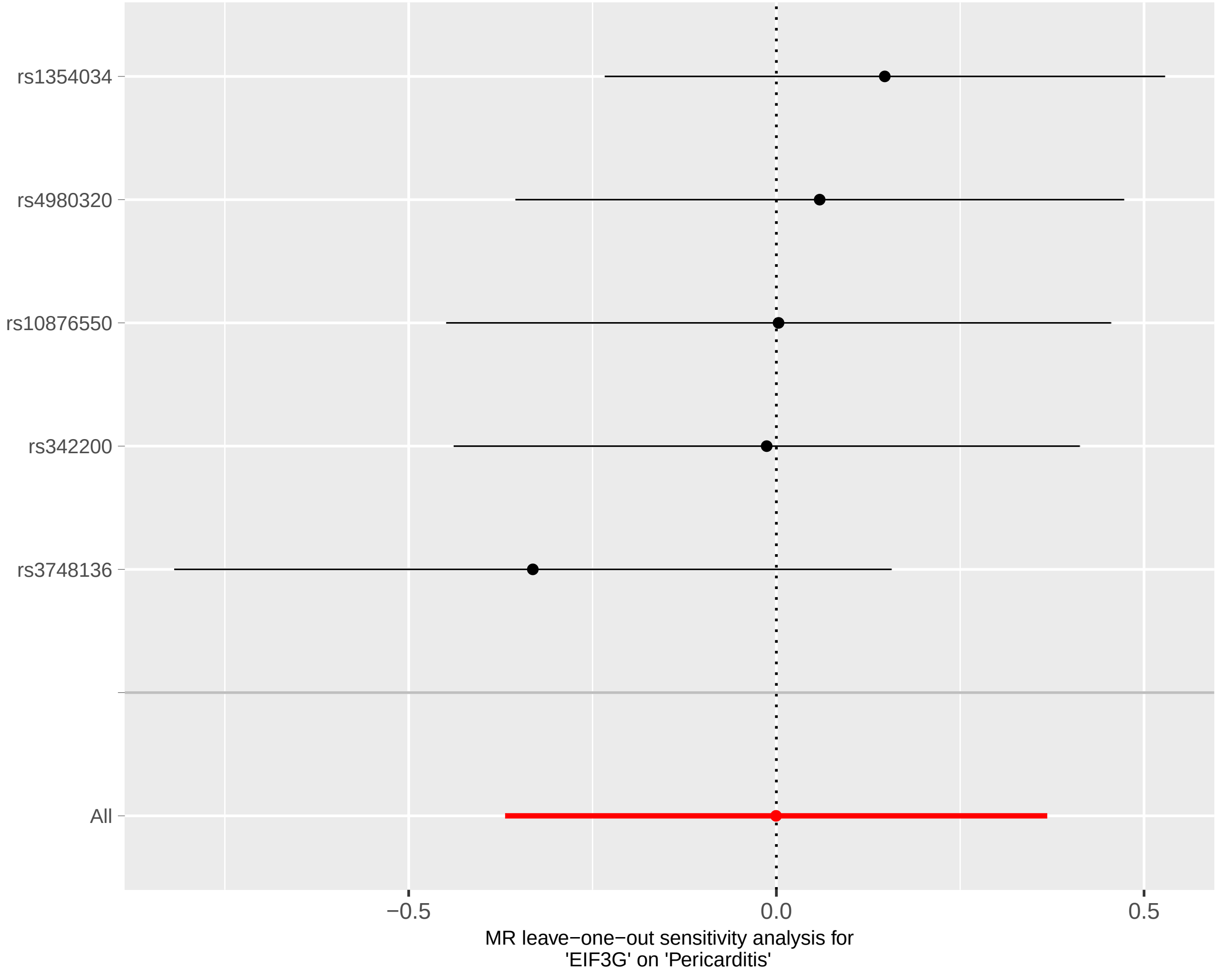
rs1354034
rs4980320
rs10876550
rs342200
rs3748136
All
−0.5
0.0
0.5
MR leave−one−out sensitivity analysis for
'EIF3G' on 'Pericarditis'

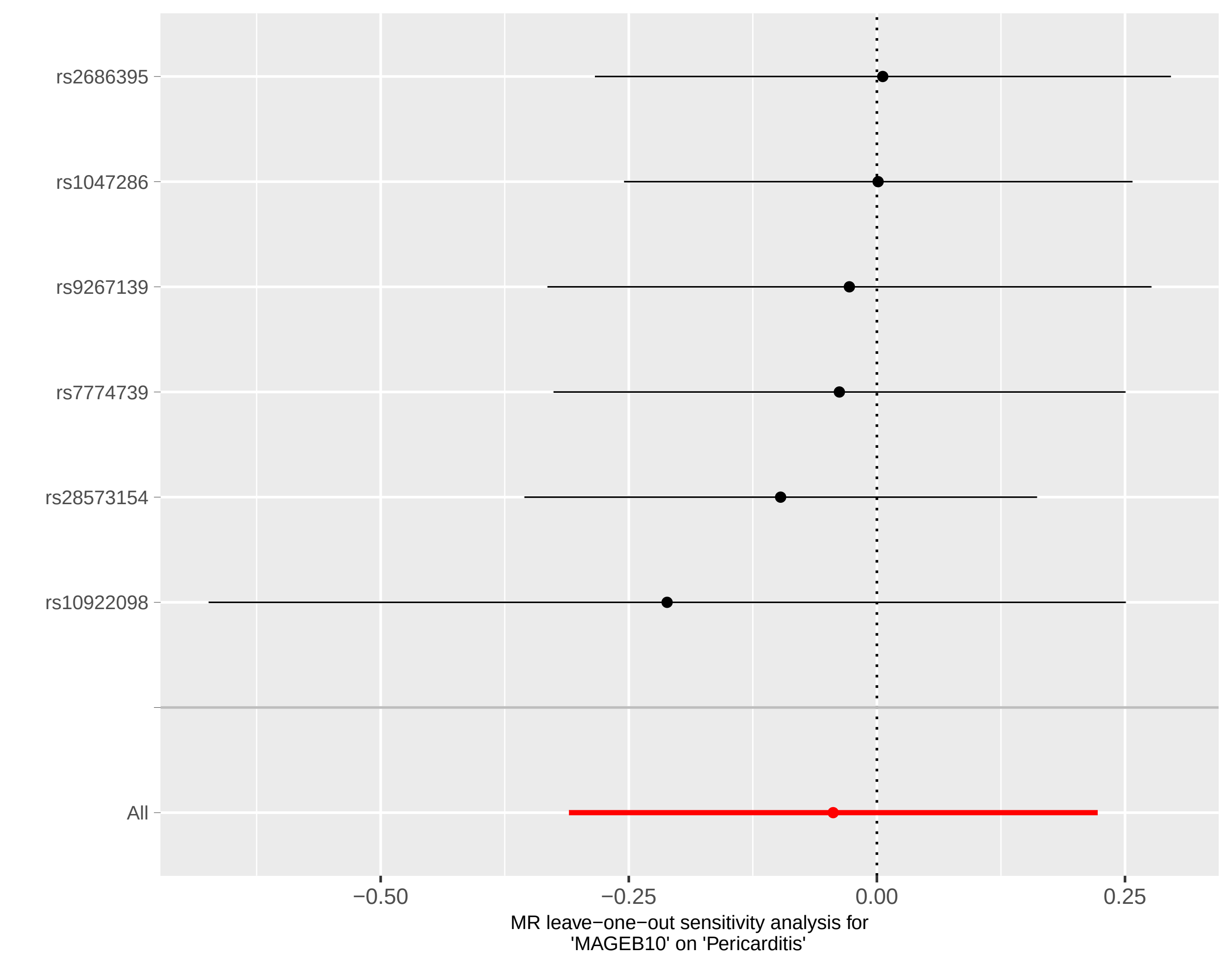
rs2686395
rs1047286
rs9267139
rs7774739
rs28573154
rs10922098
All
−0.50
−0.25
0.00
0.25
MR leave−one−out sensitivity analysis for
'MAGEB10' on 'Pericarditis'

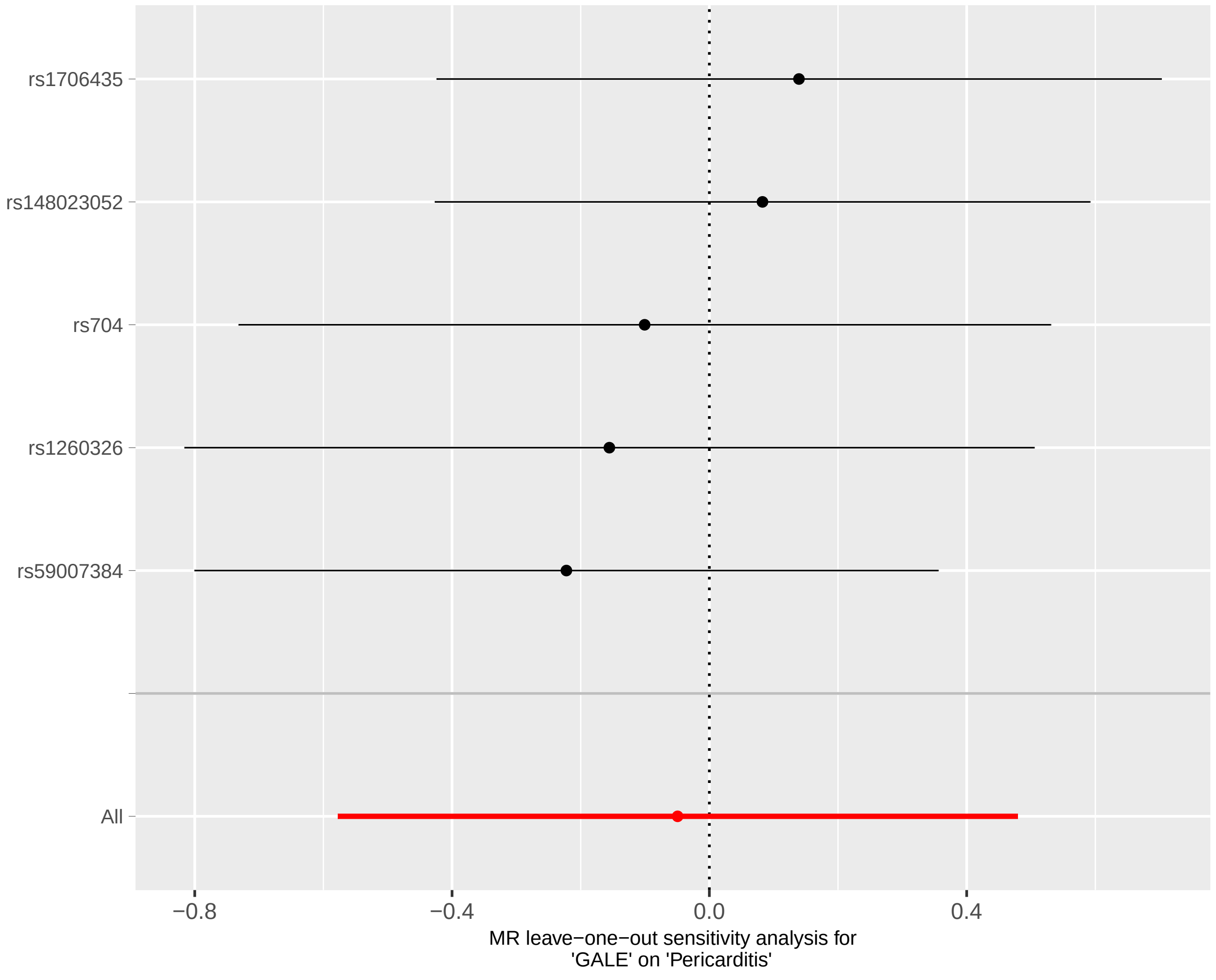

rs1706435
rs148023052
rs704
rs1260326
rs59007384
All
−0.8
−0.4
0.0
0.4
MR leave−one−out sensitivity analysis for
'GALE' on 'Pericarditis'

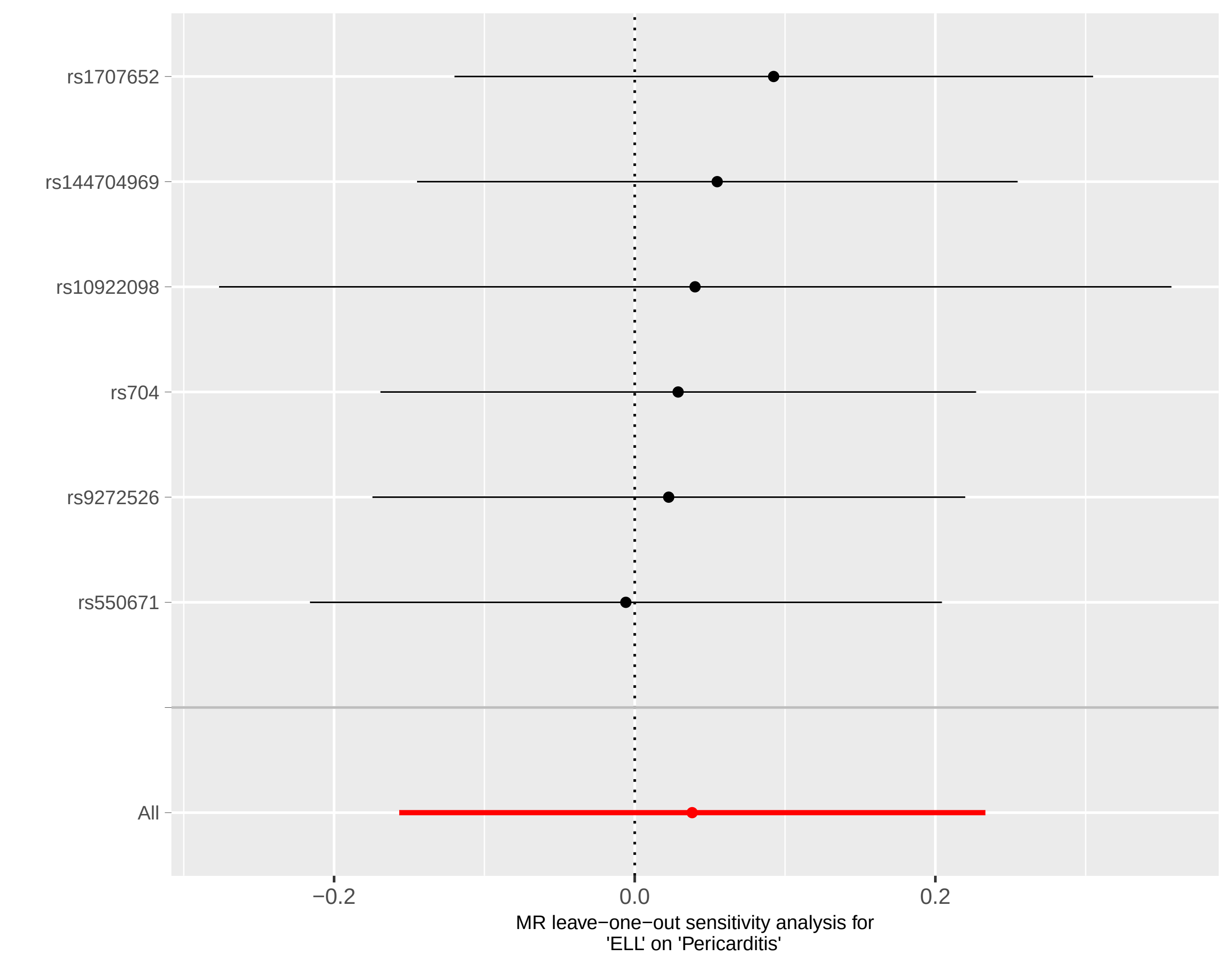
rs1707652
rs144704969
rs10922098
rs704
rs9272526
rs550671
All
−0.2
0.0
0.2
MR leave−one−out sensitivity analysis for
'ELL' on 'Pericarditis'

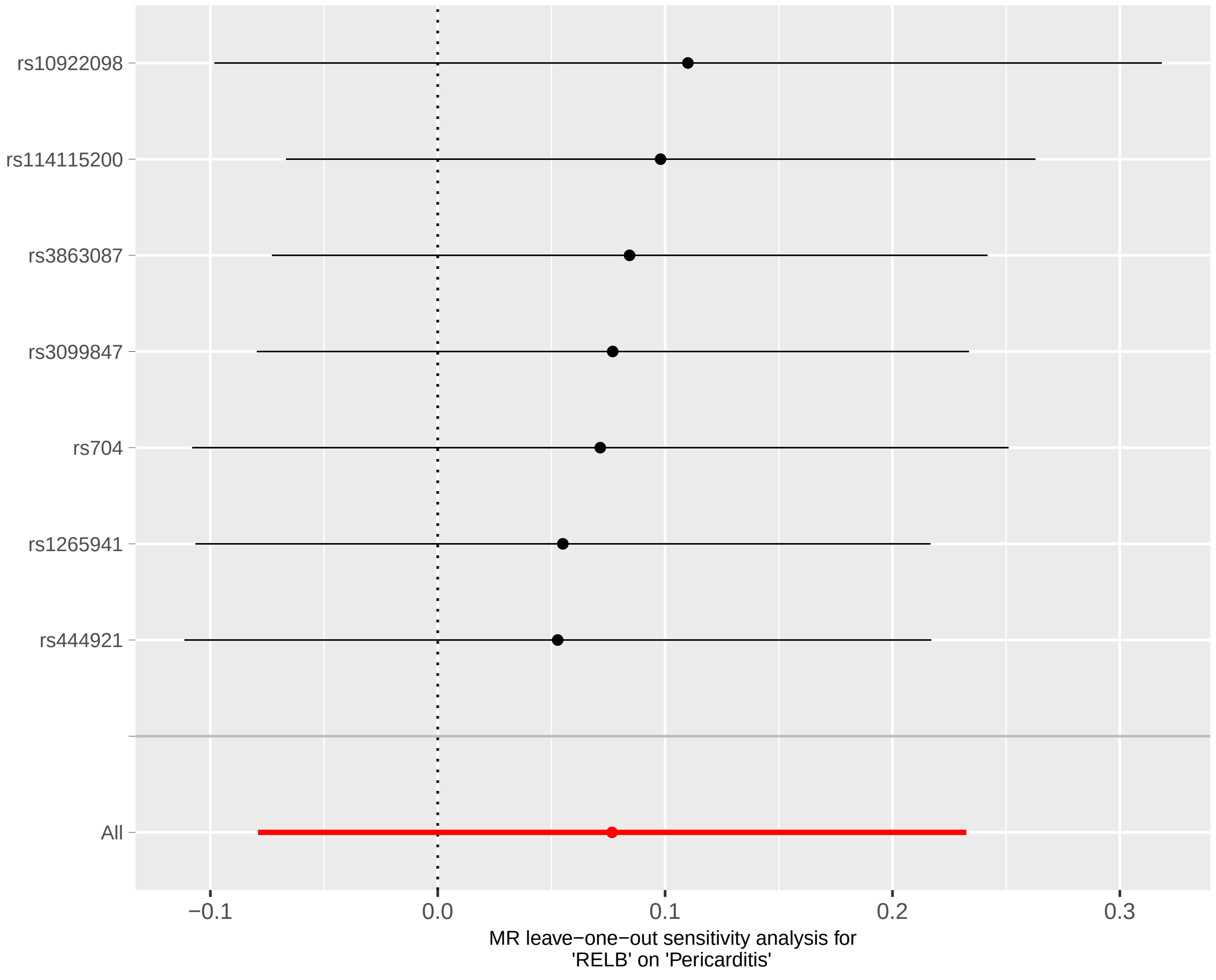

rs10922098
rs114115200
rs3863087
rs3099847
rs704
rs1265941
rs444921
All
−0.1
0.0
0.1
0.2
0.3
MR leave−one−out sensitivity analysis for
'RELB' on 'Pericarditis'

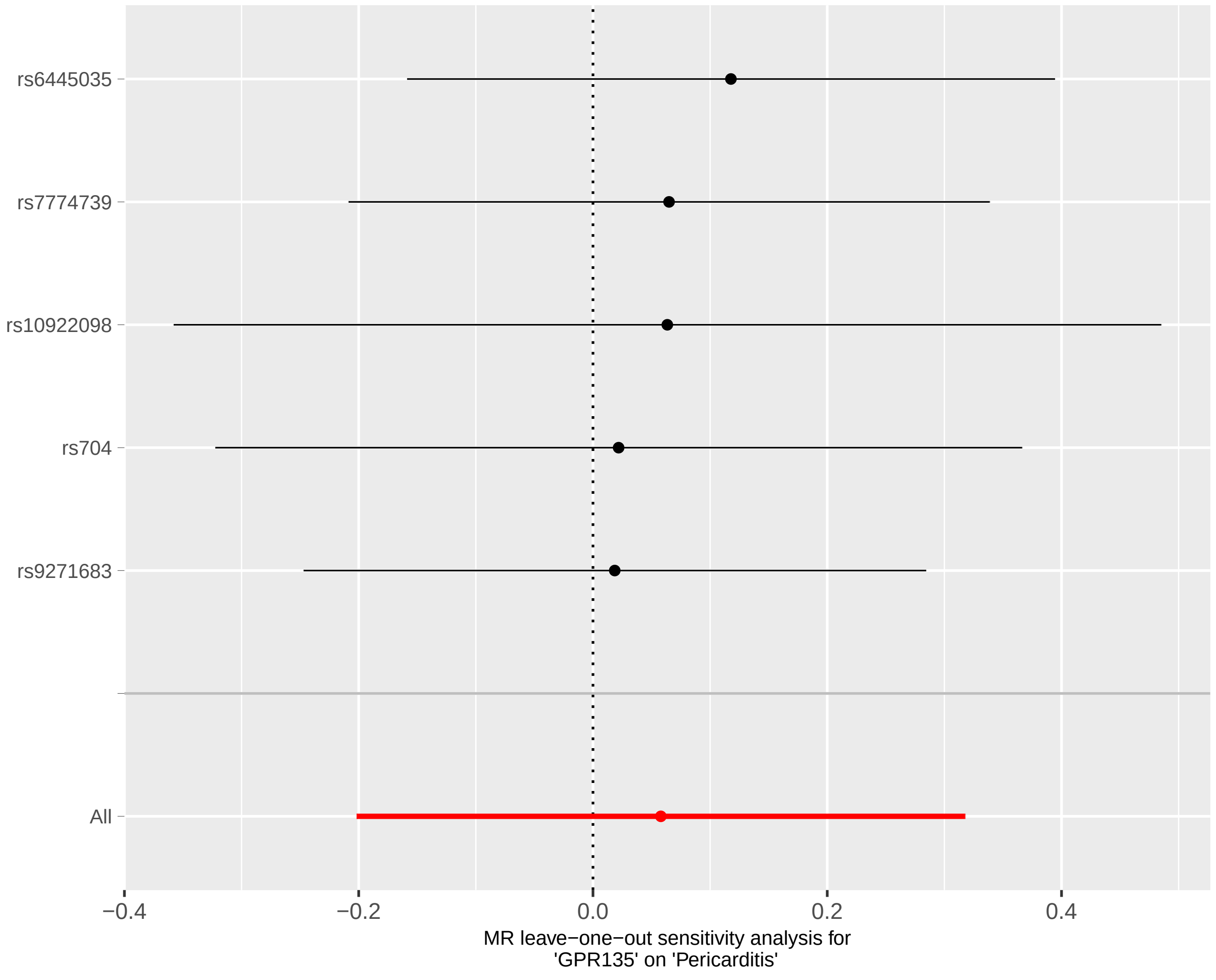
rs6445035
rs7774739
rs10922098
rs704
rs9271683
All
−0.4
−0.2
0.0
0.2
0.4
MR leave−one−out sensitivity analysis for
'GPR135' on 'Pericarditis'

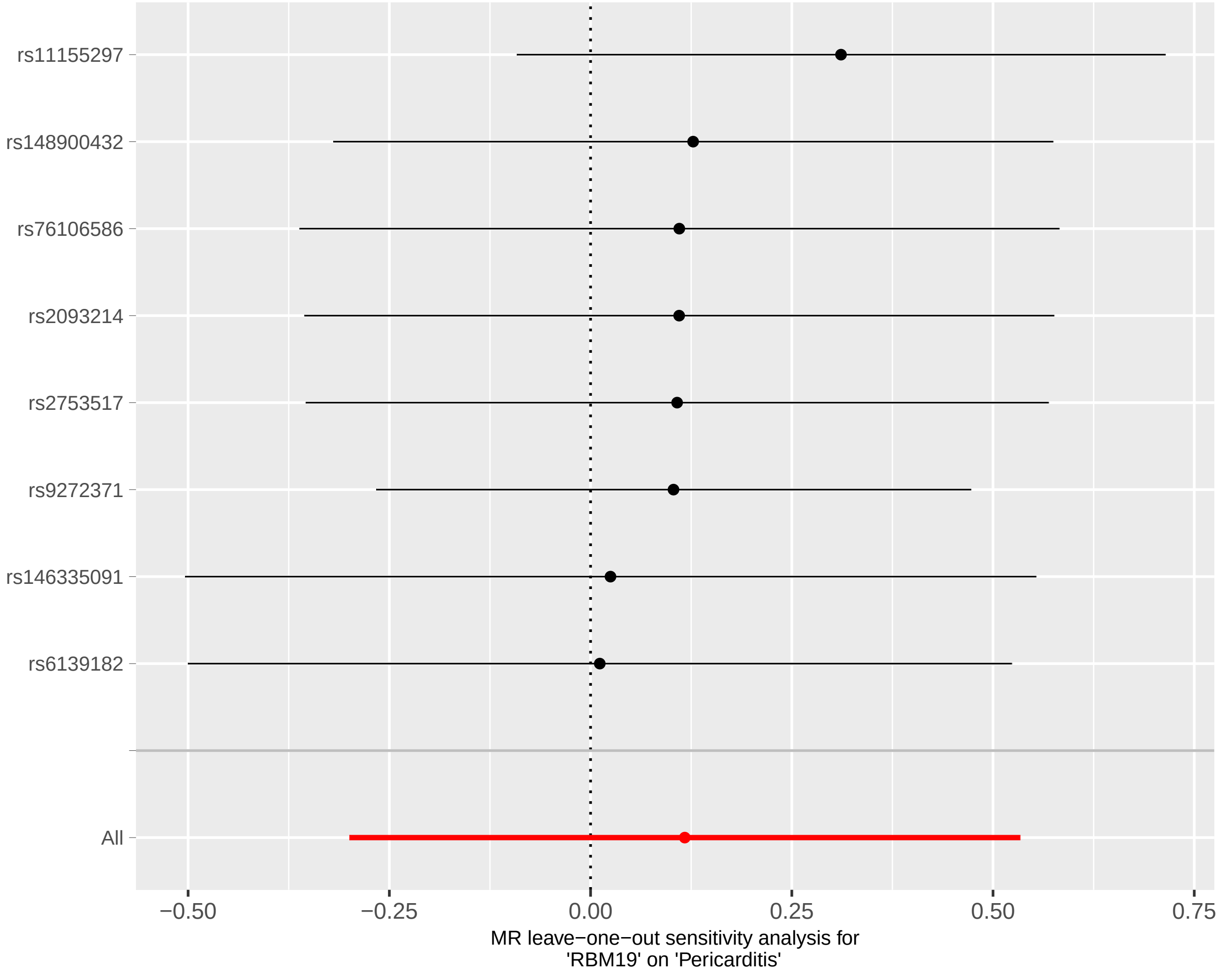

rs11155297
rs148900432
rs76106586
rs2093214
rs2753517
rs9272371
rs146335091
rs6139182
All
−0.50
−0.25
0.00
0.25
0.50
0.75
MR leave−one−out sensitivity analysis for
'RBM19' on 'Pericarditis'

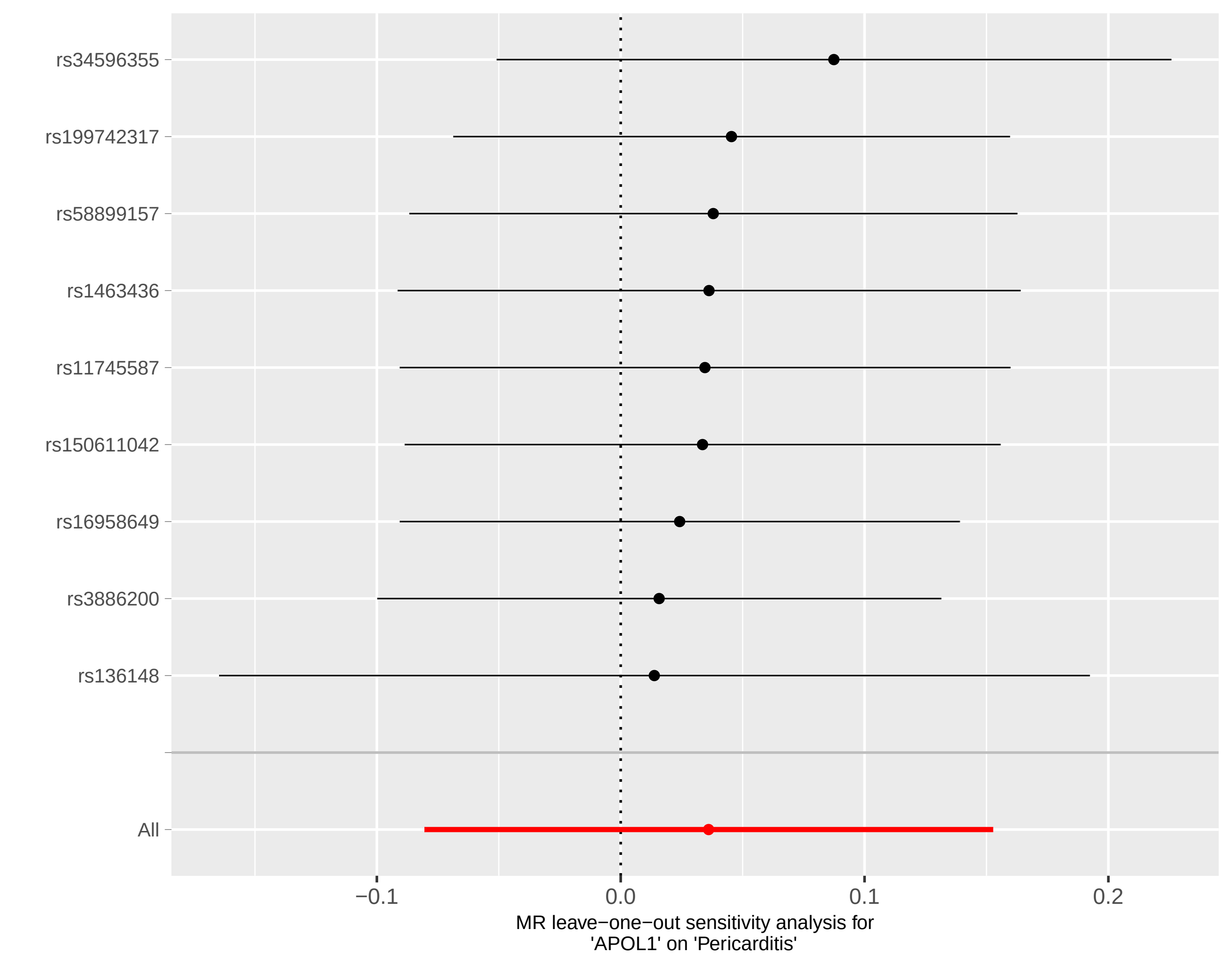

rs34596355
rs199742317
rs58899157
rs1463436
rs11745587
rs150611042
rs16958649
rs3886200
rs136148
All
−0.1
0.0
0.1
0.2
MR leave−one−out sensitivity analysis for
'APOL1' on 'Pericarditis'

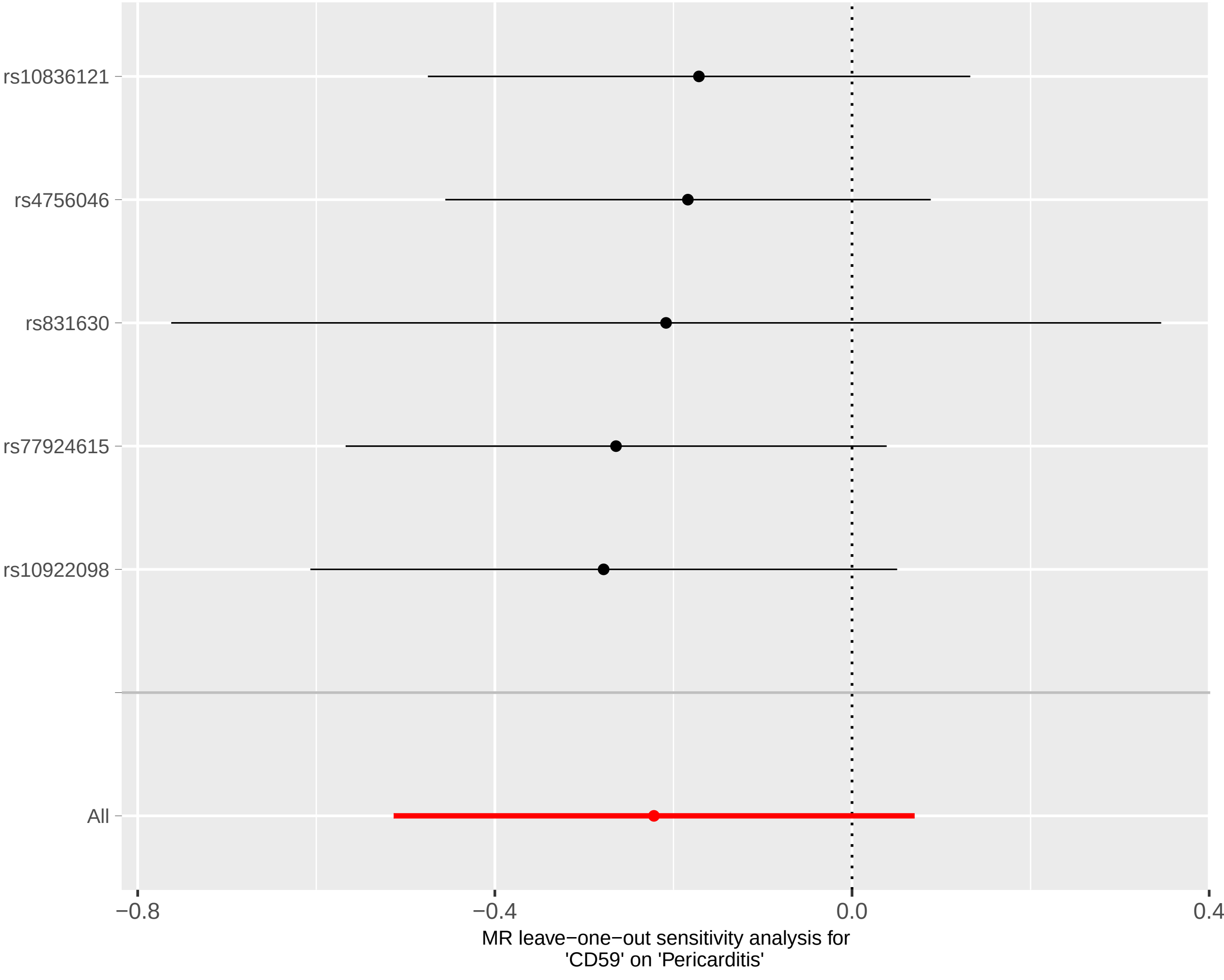
rs10836121
rs4756046
rs831630
rs77924615
rs10922098
All
−0.8
−0.4
0.0
0.4
MR leave−one−out sensitivity analysis for
'CD59' on 'Pericarditis'

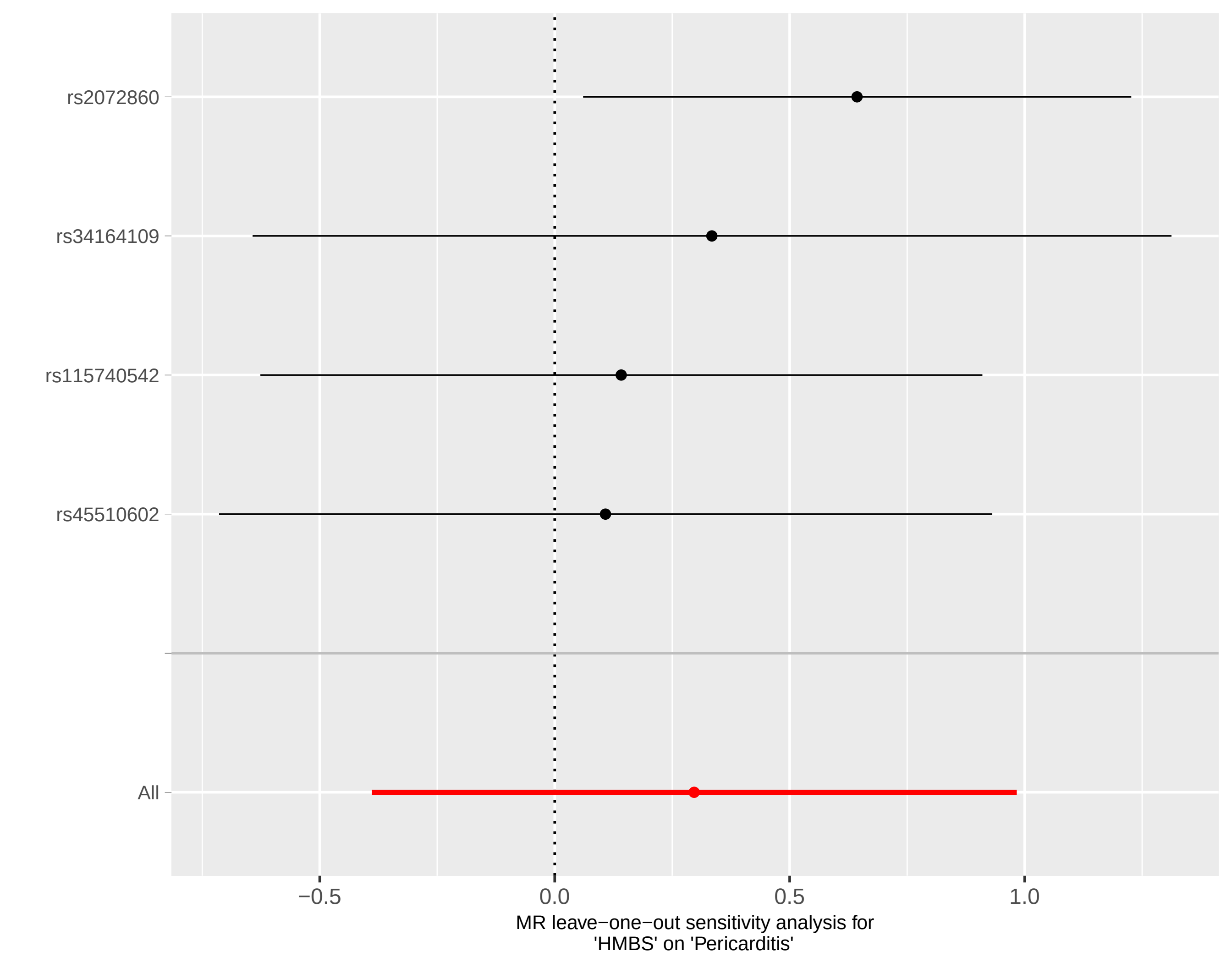
rs2072860
rs34164109
rs115740542
rs45510602
All
−0.5
0.0
0.5
1.0
MR leave−one−out sensitivity analysis for
'HMBS' on 'Pericarditis'

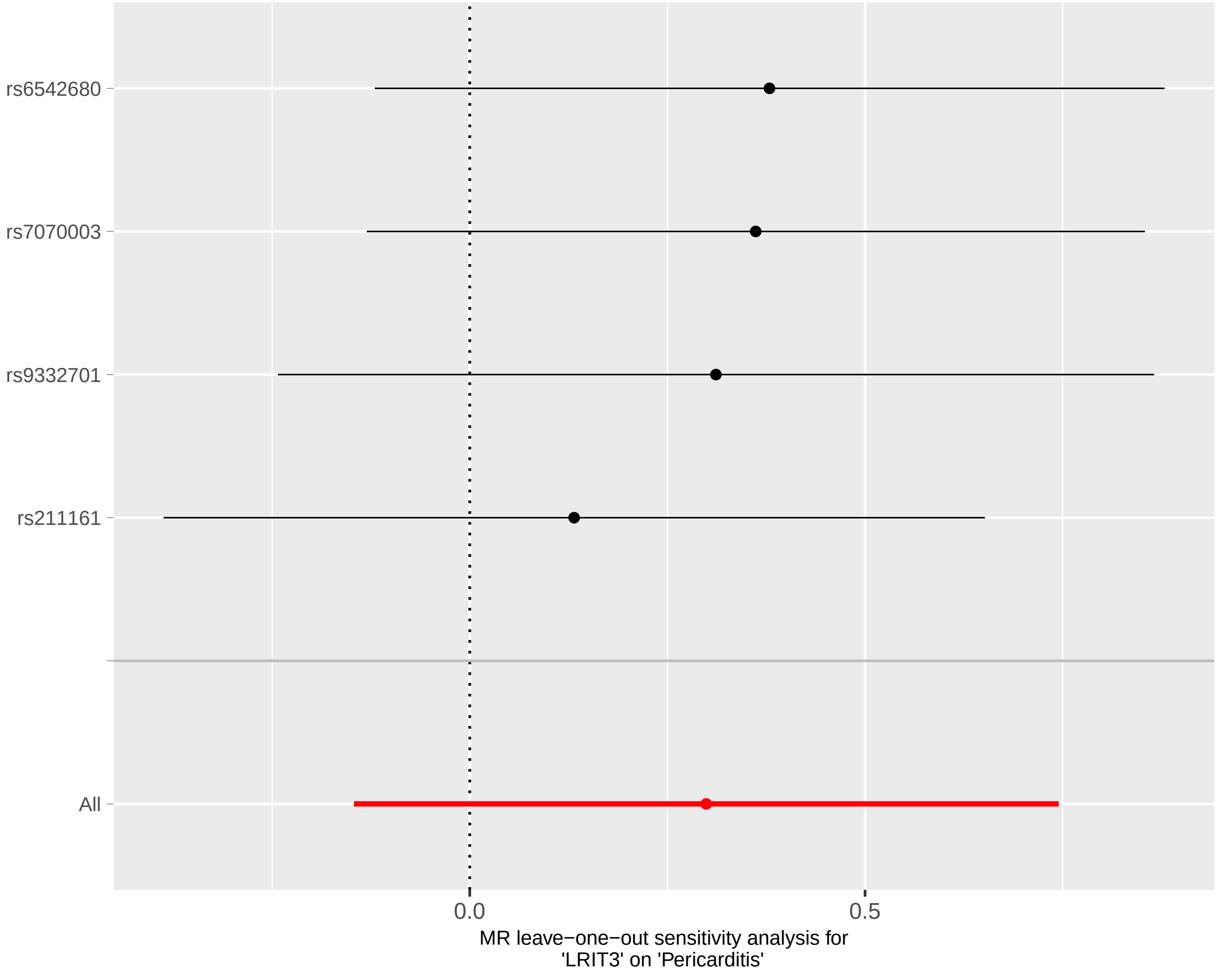
rs6542680
rs7070003
rs9332701
rs211161
All
0.0
0.5
MR leave−one−out sensitivity analysis for
'LRIT3' on 'Pericarditis'

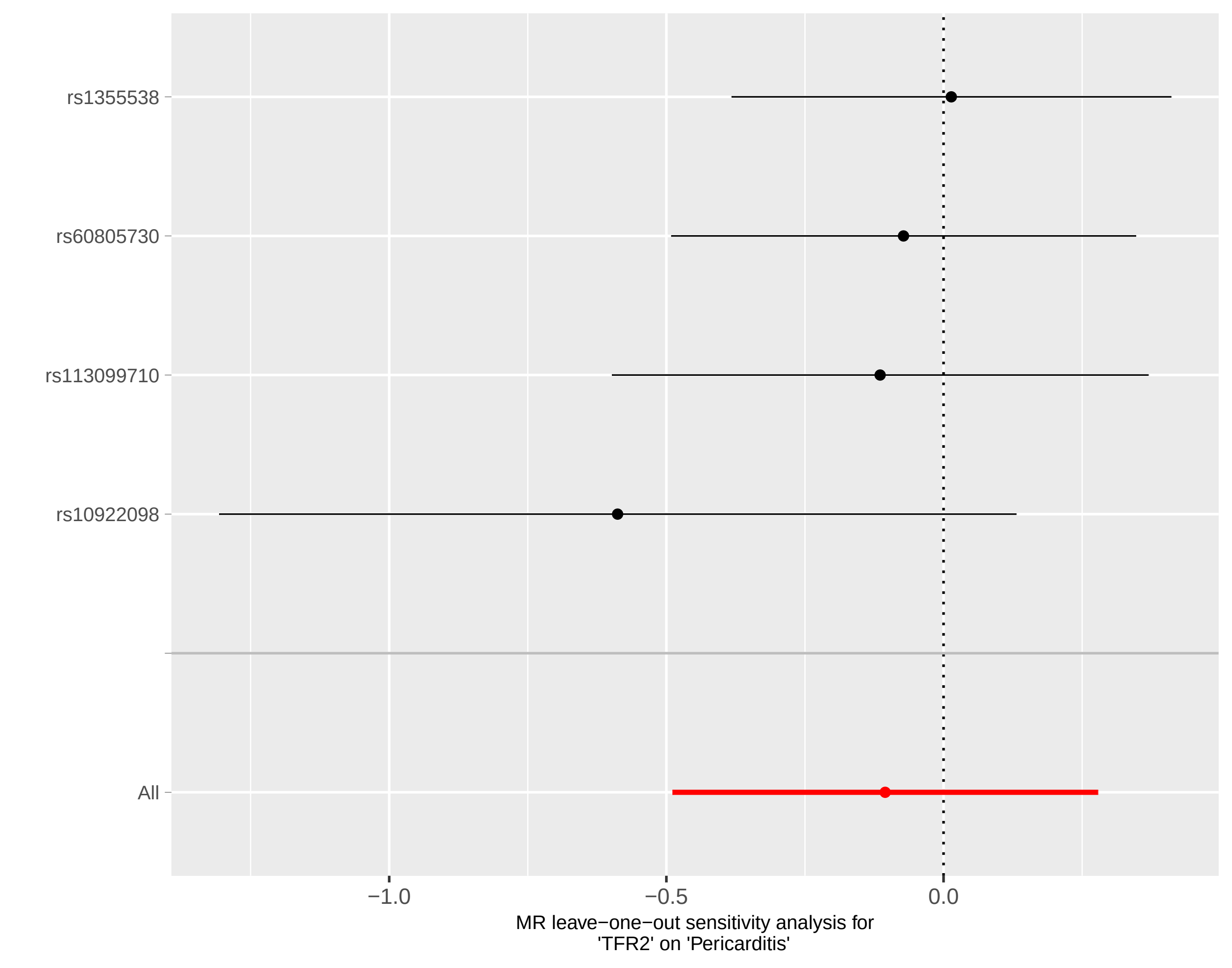
rs1355538
rs60805730
rs113099710
rs10922098
All
−1.0
−0.5
0.0
MR leave−one−out sensitivity analysis for
'TFR2' on 'Pericarditis'

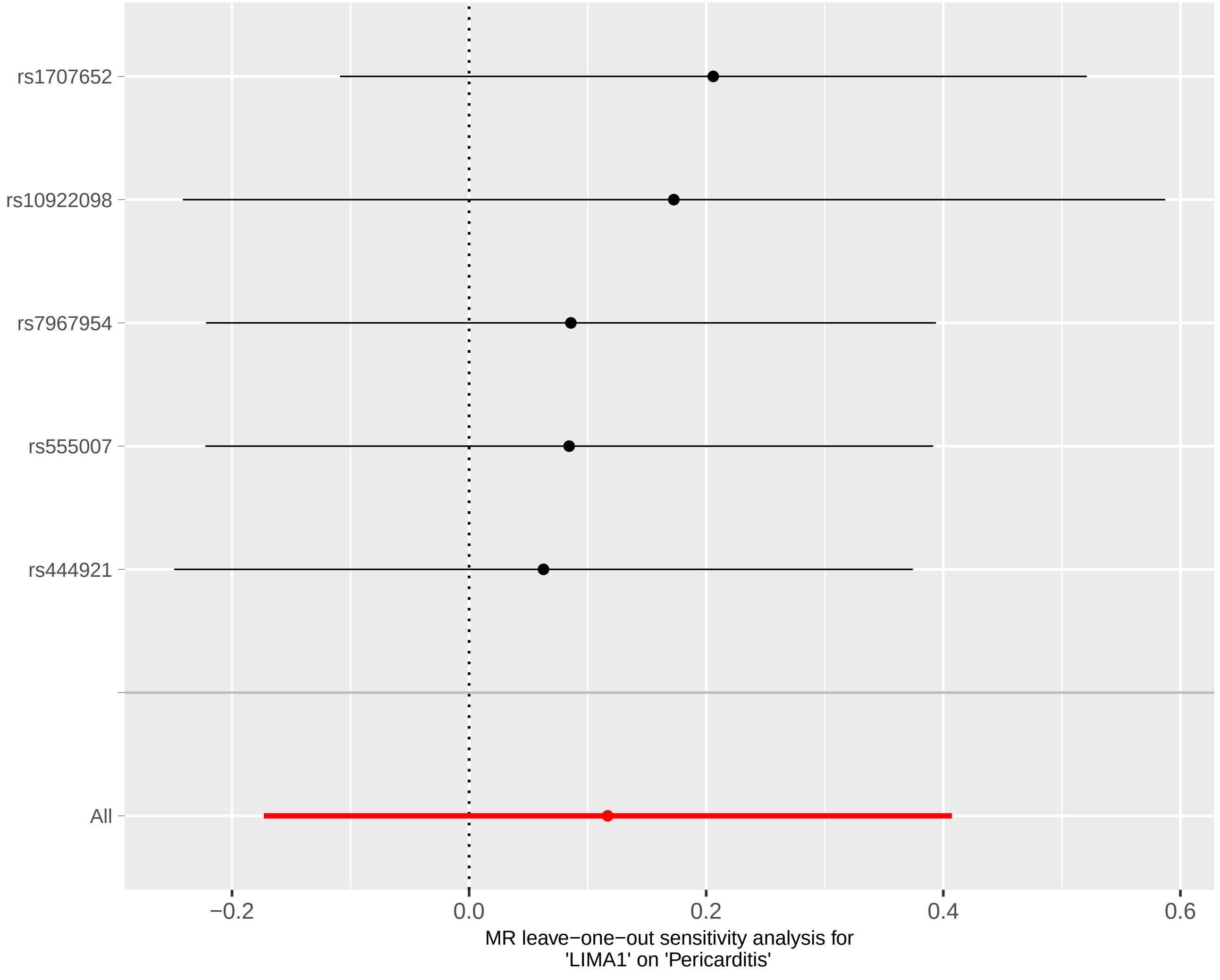

rs1707652
rs10922098
rs7967954
rs555007
rs444921
All
−0.2
0.0
0.2
0.4
0.6
MR leave−one−out sensitivity analysis for
'LIMA1' on 'Pericarditis'

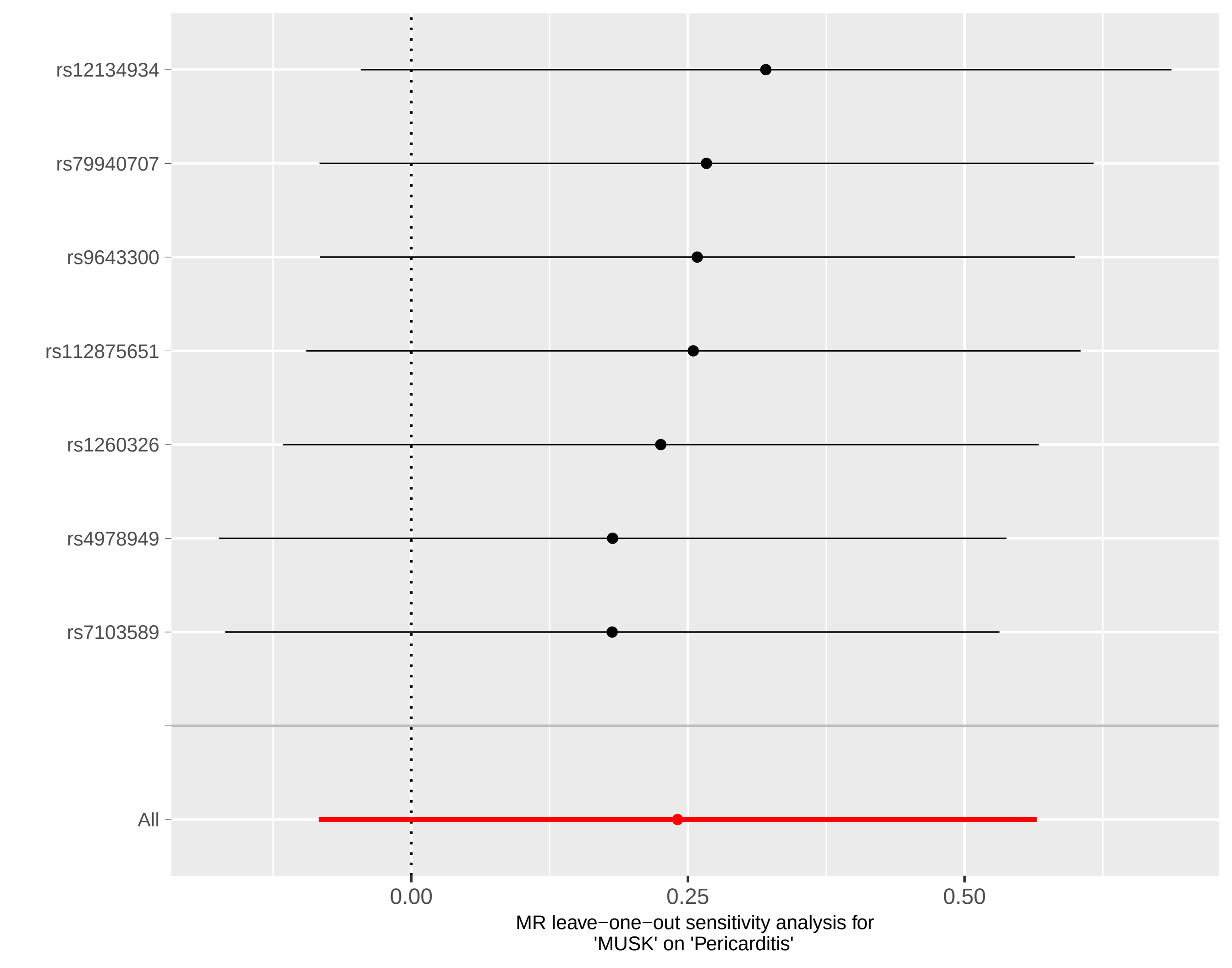
rs12134934
rs79940707
rs9643300
rs112875651
rs1260326
rs4978949
rs7103589
All
0.00
0.25
0.50
MR leave−one−out sensitivity analysis for
'MUSK' on 'Pericarditis'

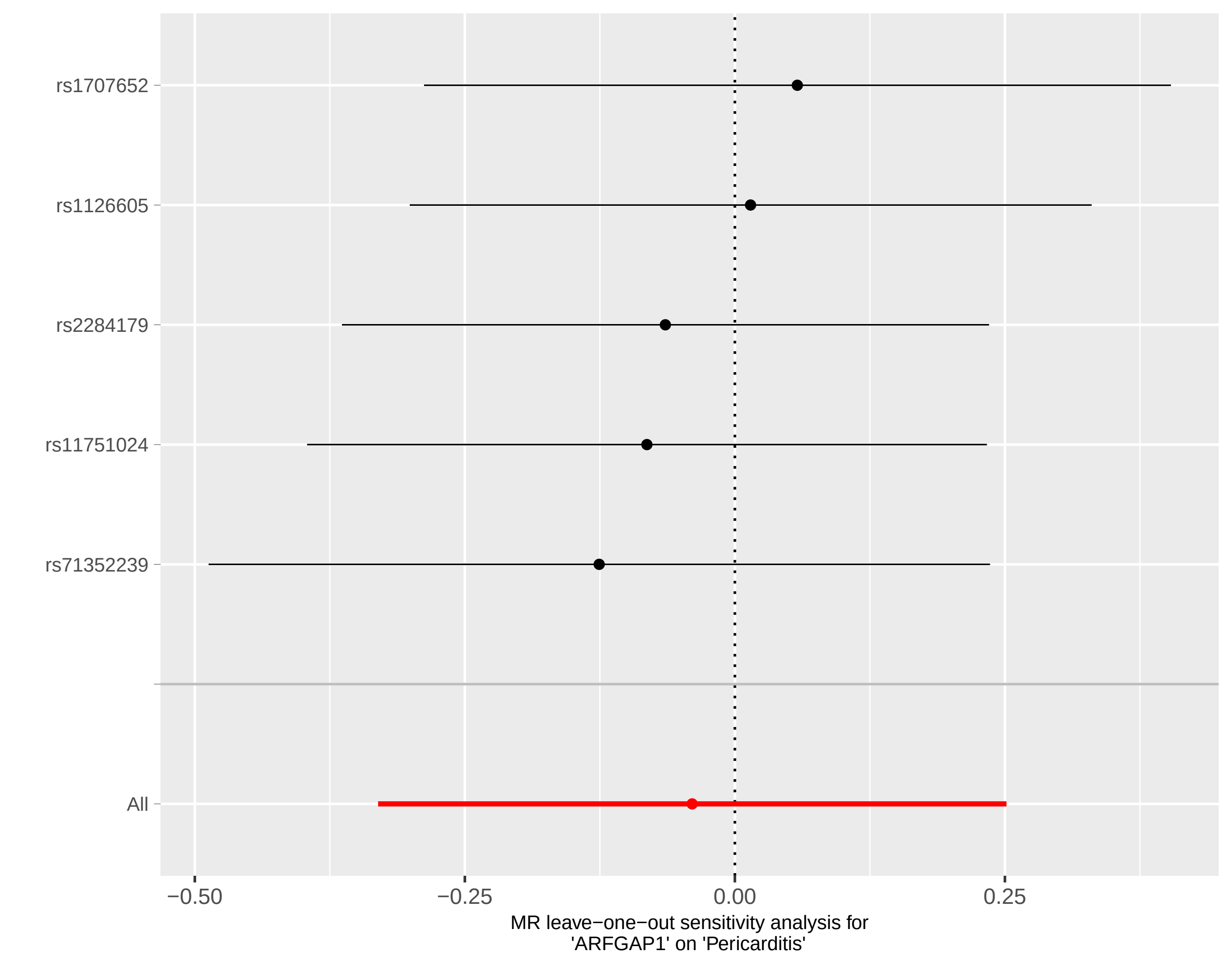
rs1707652
rs1126605
rs2284179
rs11751024
rs71352239
All
−0.50
−0.25
0.00
0.25
MR leave−one−out sensitivity analysis for
'ARFGAP1' on 'Pericarditis'

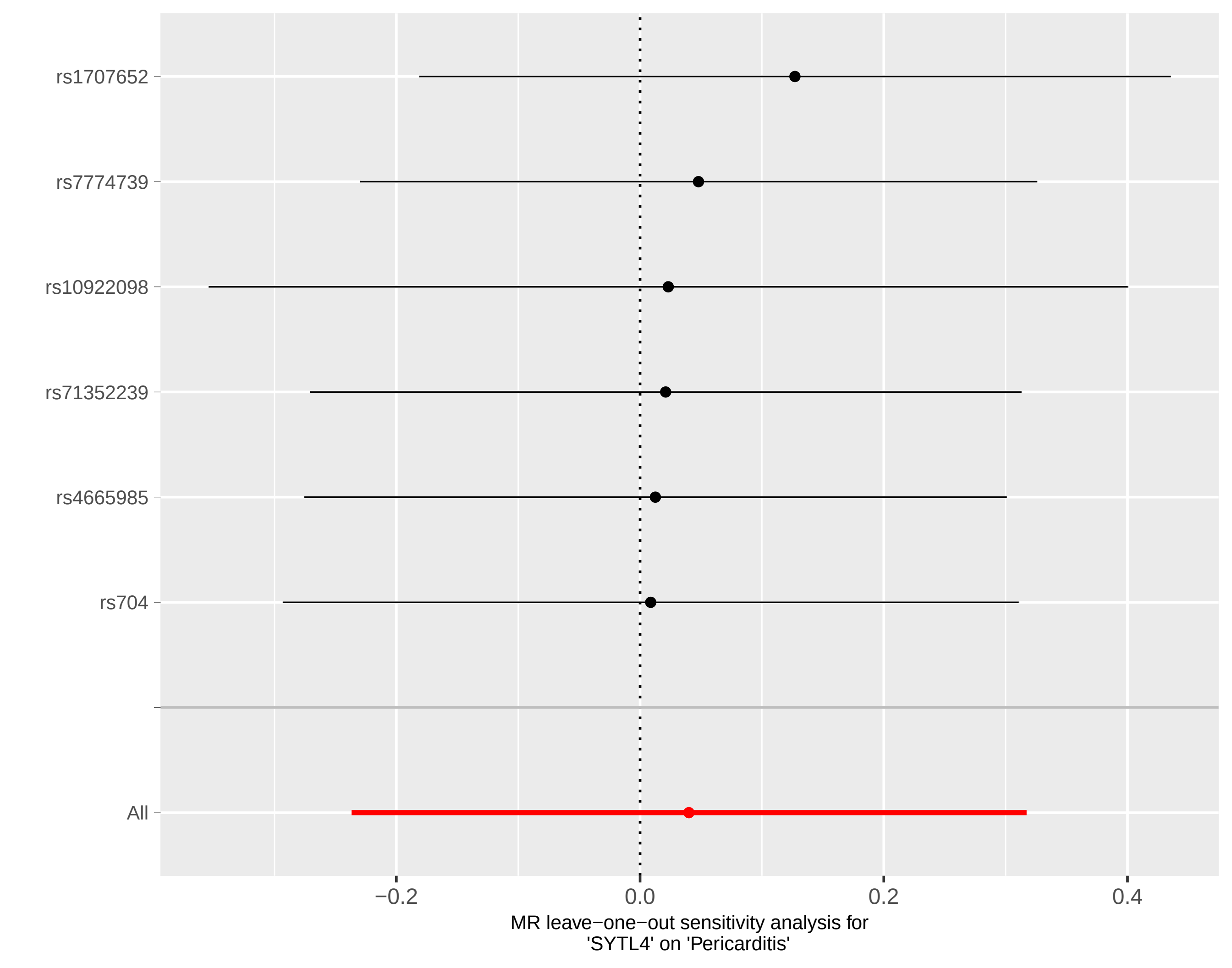

rs1707652
rs7774739
rs10922098
rs71352239
rs4665985
rs704
All
−0.2
0.0
0.2
0.4
MR leave−one−out sensitivity analysis for
'SYTL4' on 'Pericarditis'

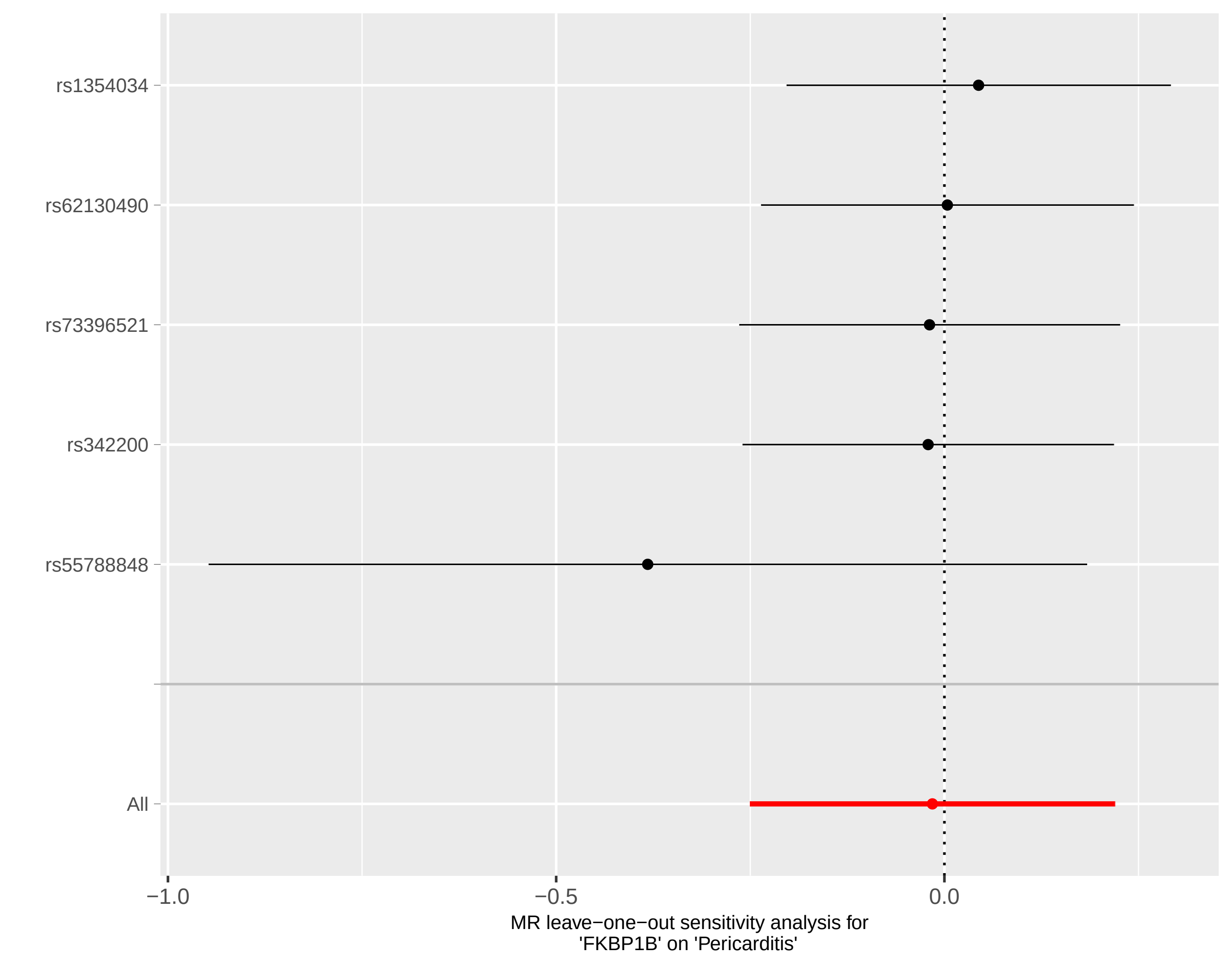
rs1354034
rs62130490
rs73396521
rs342200
rs55788848
All
−1.0
−0.5
0.0
MR leave−one−out sensitivity analysis for
'FKBP1B' on 'Pericarditis'

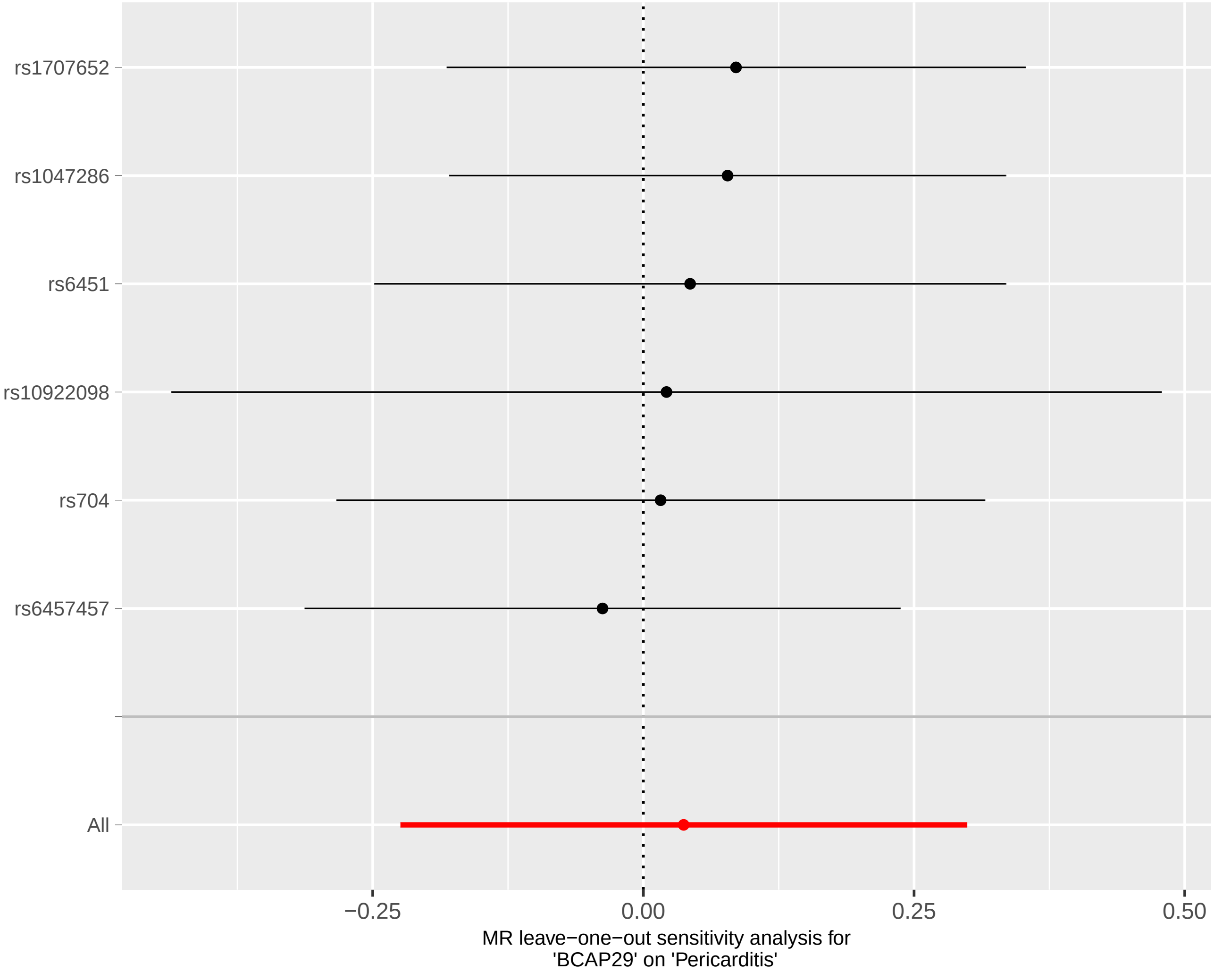
rs1707652
rs1047286
rs6451
rs10922098
rs704
rs6457457
All
−0.25
0.00
0.25
0.50
MR leave−one−out sensitivity analysis for
'BCAP29' on 'Pericarditis'

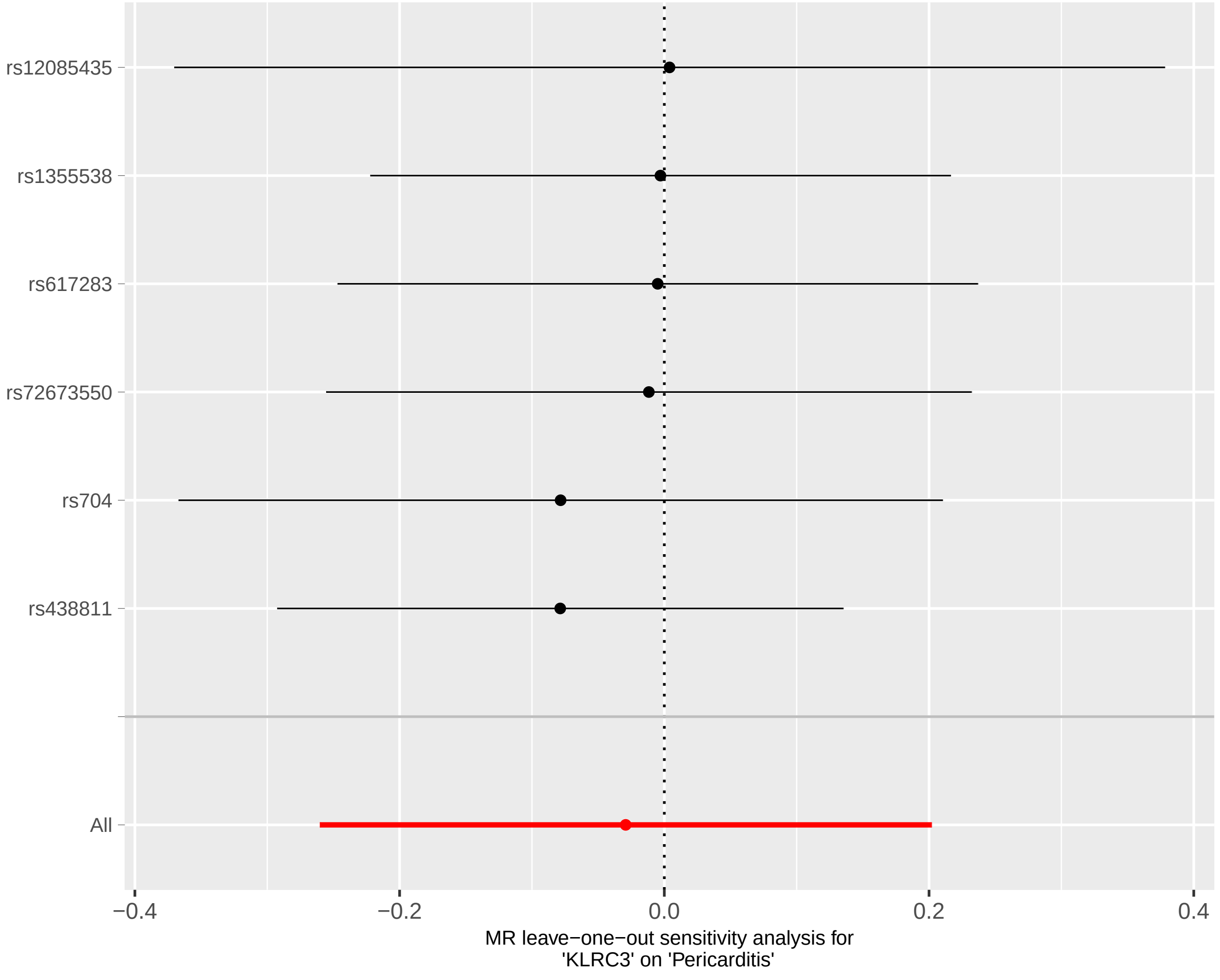
rs12085435
rs1355538
rs617283
rs72673550
rs704
rs438811
All
−0.4
−0.2
0.0
0.2
0.4
MR leave−one−out sensitivity analysis for
'KLRC3' on 'Pericarditis'

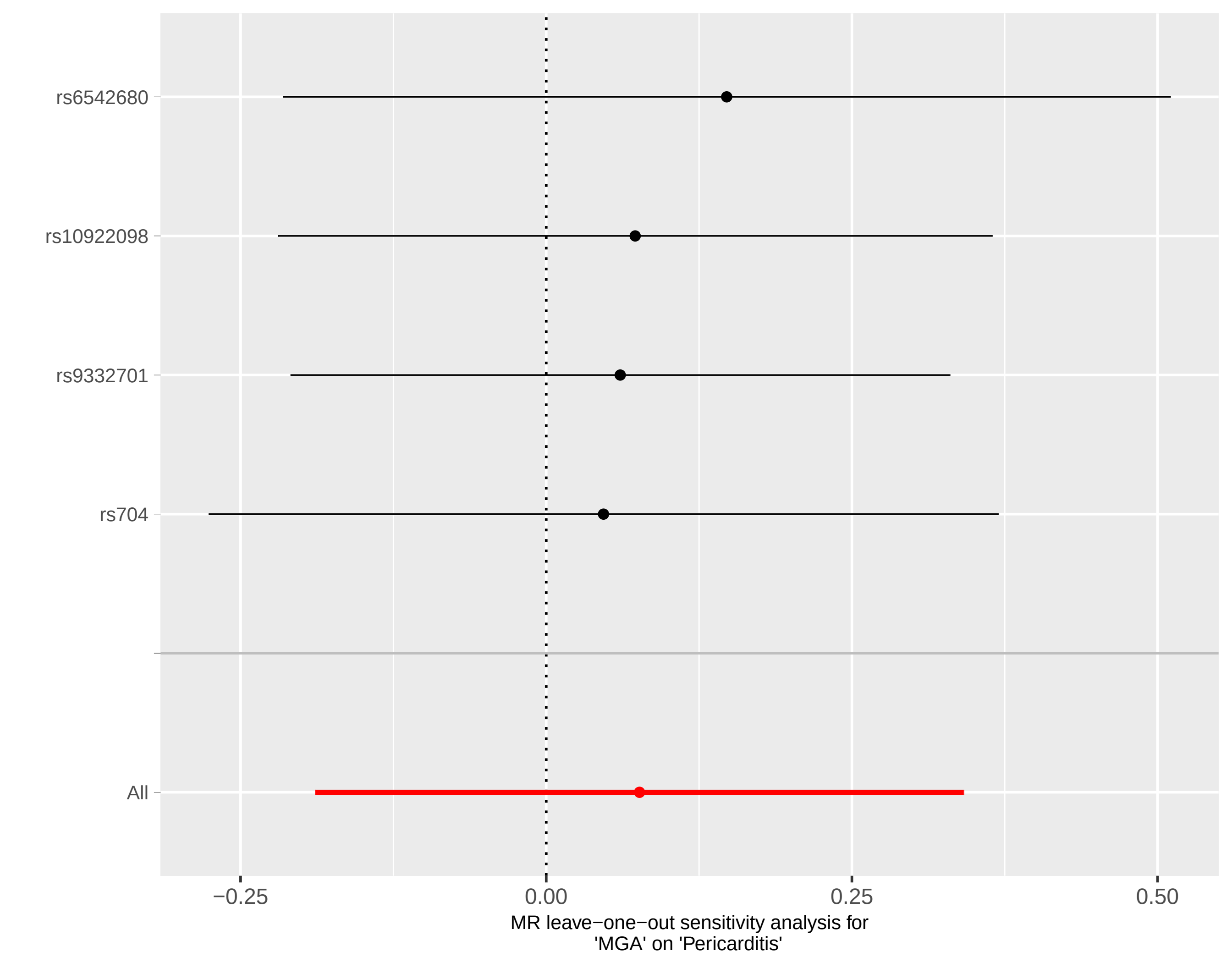
rs6542680
rs10922098
rs9332701
rs704
All
−0.25
0.00
0.25
0.50
MR leave−one−out sensitivity analysis for
'MGA' on 'Pericarditis'

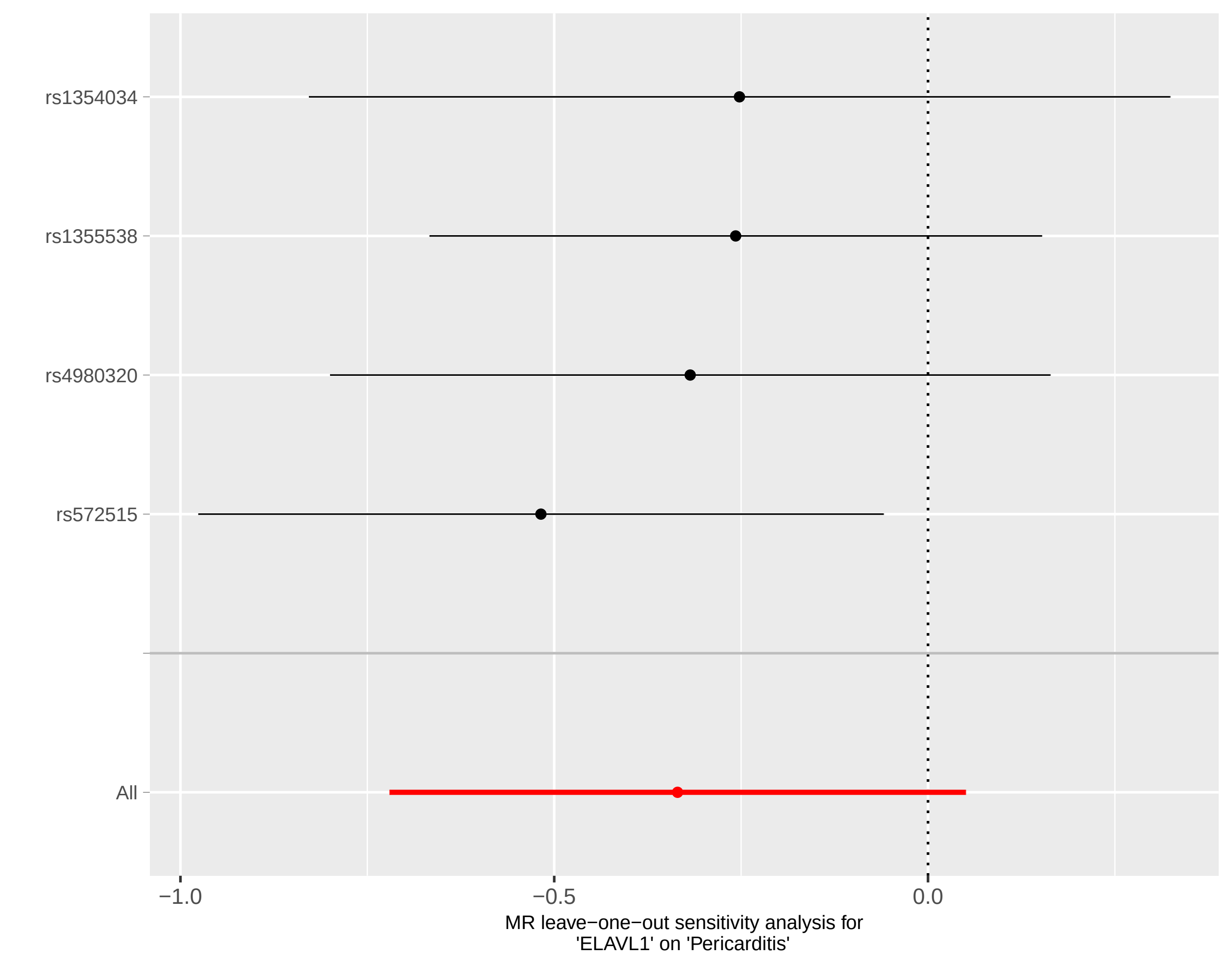
rs1354034
rs1355538
rs4980320
rs572515
All
−1.0
−0.5
0.0
MR leave−one−out sensitivity analysis for
'ELAVL1' on 'Pericarditis'

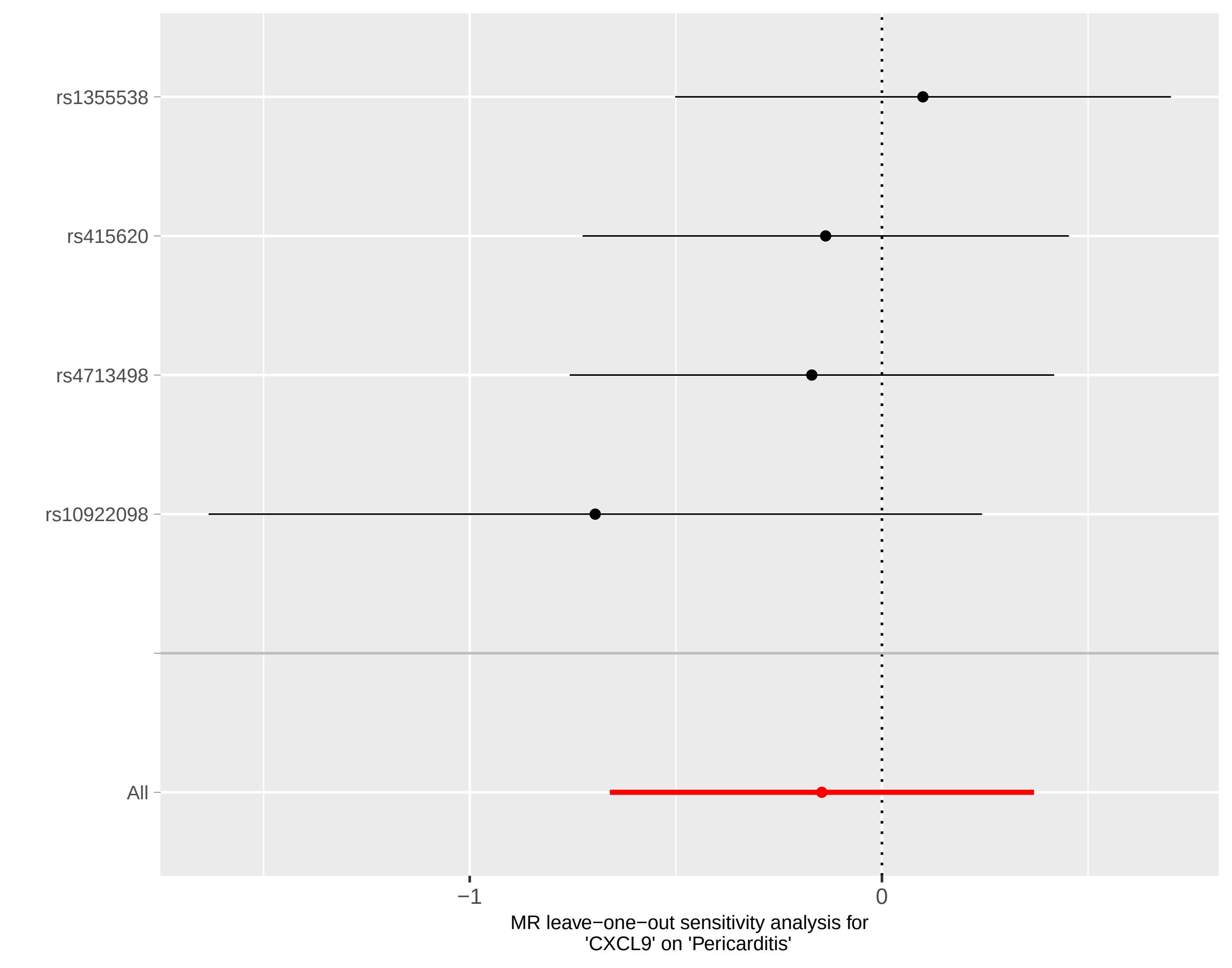
rs1355538
rs415620
rs4713498
rs10922098
All
−1
0
MR leave−one−out sensitivity analysis for
'CXCL9' on 'Pericarditis'

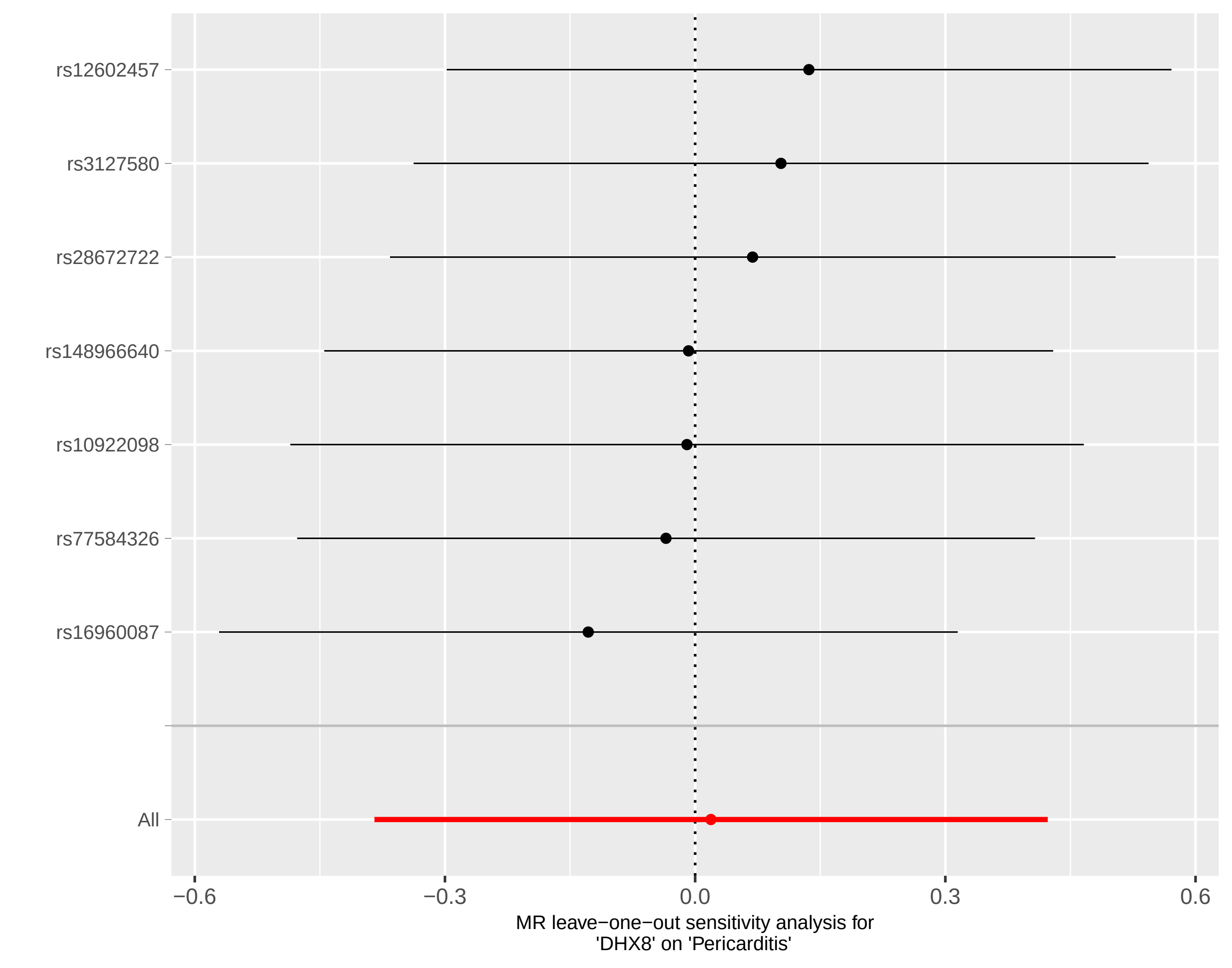

rs12602457
rs3127580
rs28672722
rs148966640
rs10922098
rs77584326
rs16960087
All
−0.6
−0.3
0.0
0.3
0.6
MR leave−one−out sensitivity analysis for
'DHX8' on 'Pericarditis'

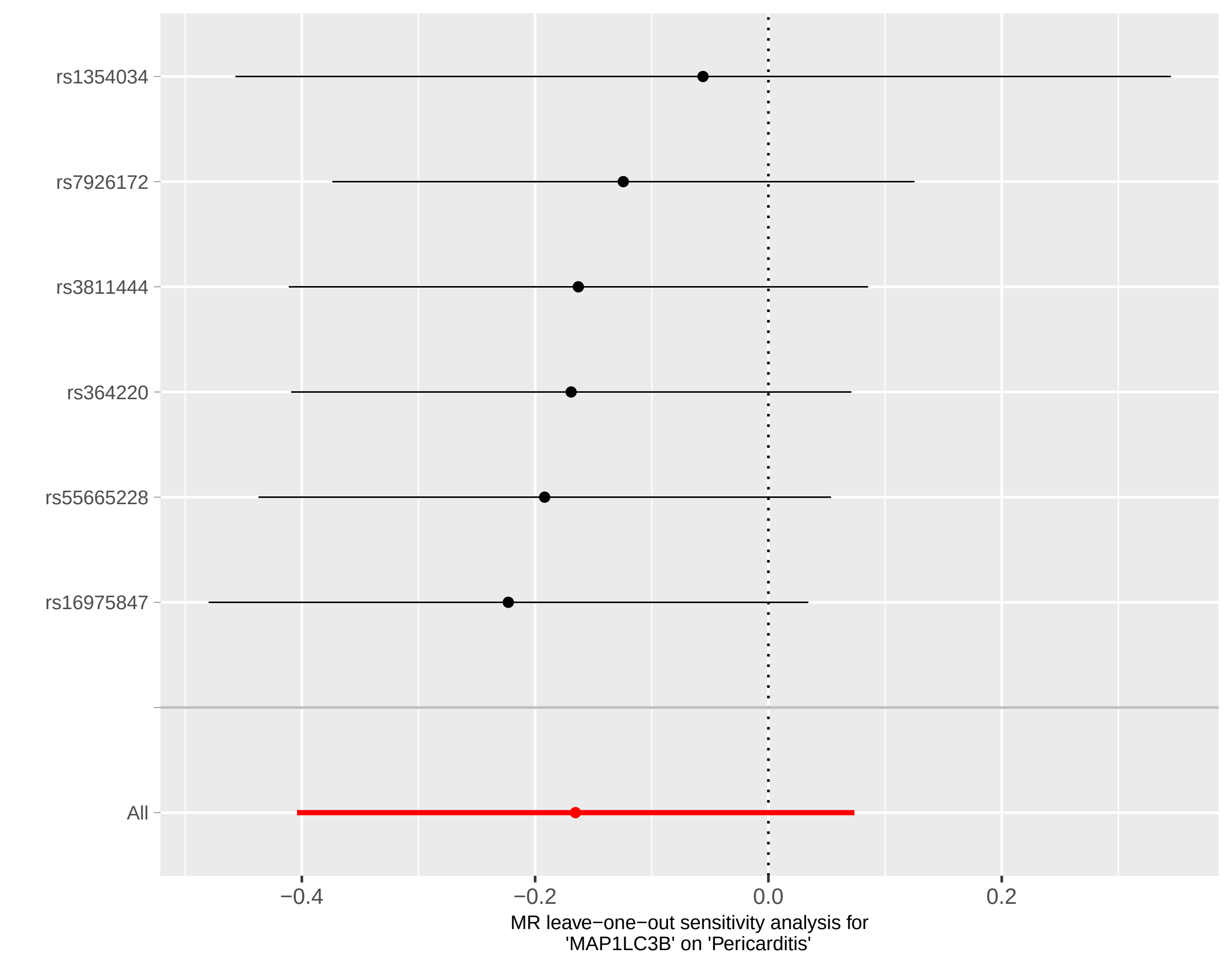

rs1354034
rs7926172
rs3811444
rs364220
rs55665228
rs16975847
All
−0.4
−0.2
0.0
0.2
MR leave−one−out sensitivity analysis for
'MAP1LC3B' on 'Pericarditis'

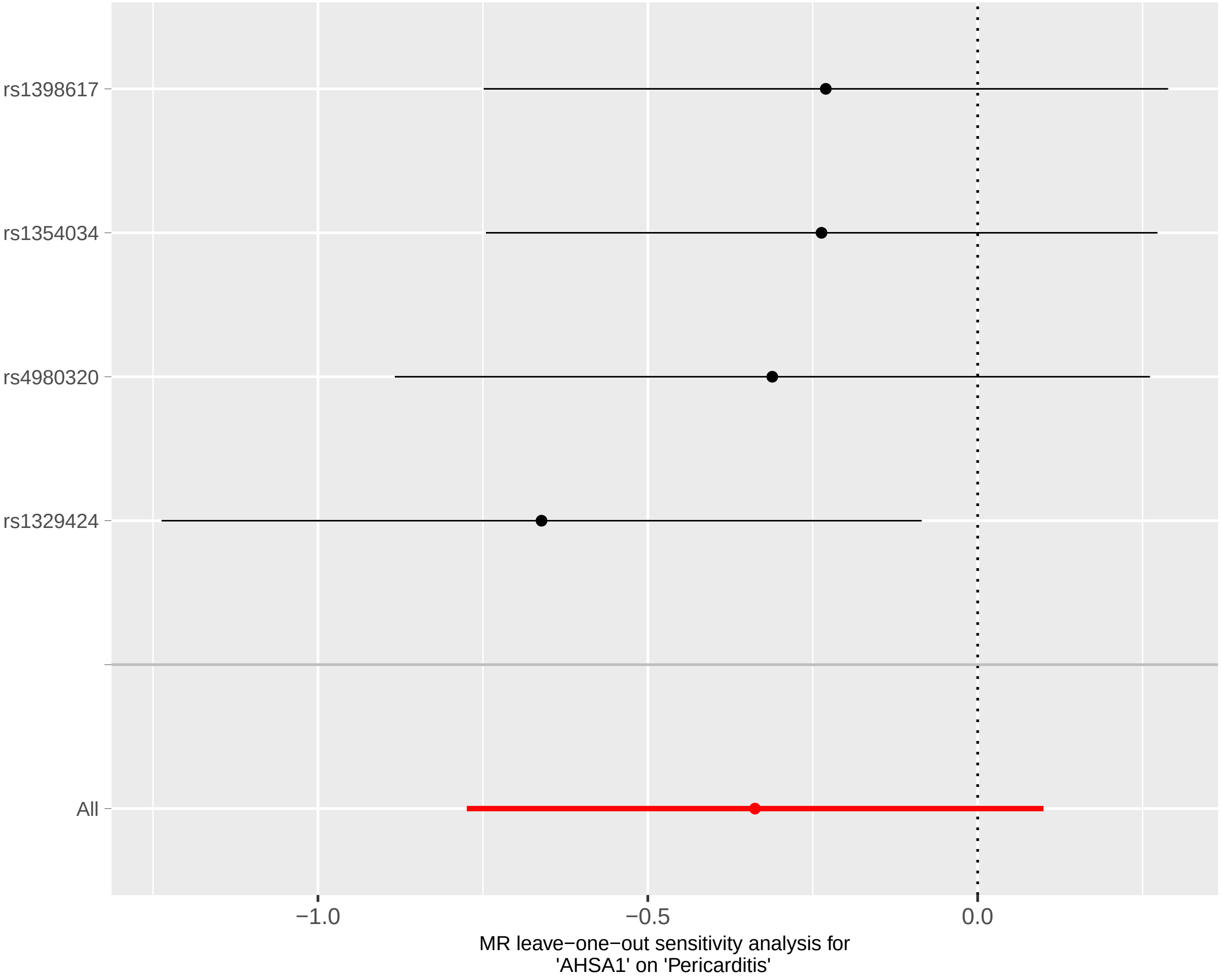
rs1398617
rs1354034
rs4980320
rs1329424
All
−1.0
−0.5
0.0
MR leave−one−out sensitivity analysis for
'AHSA1' on 'Pericarditis'

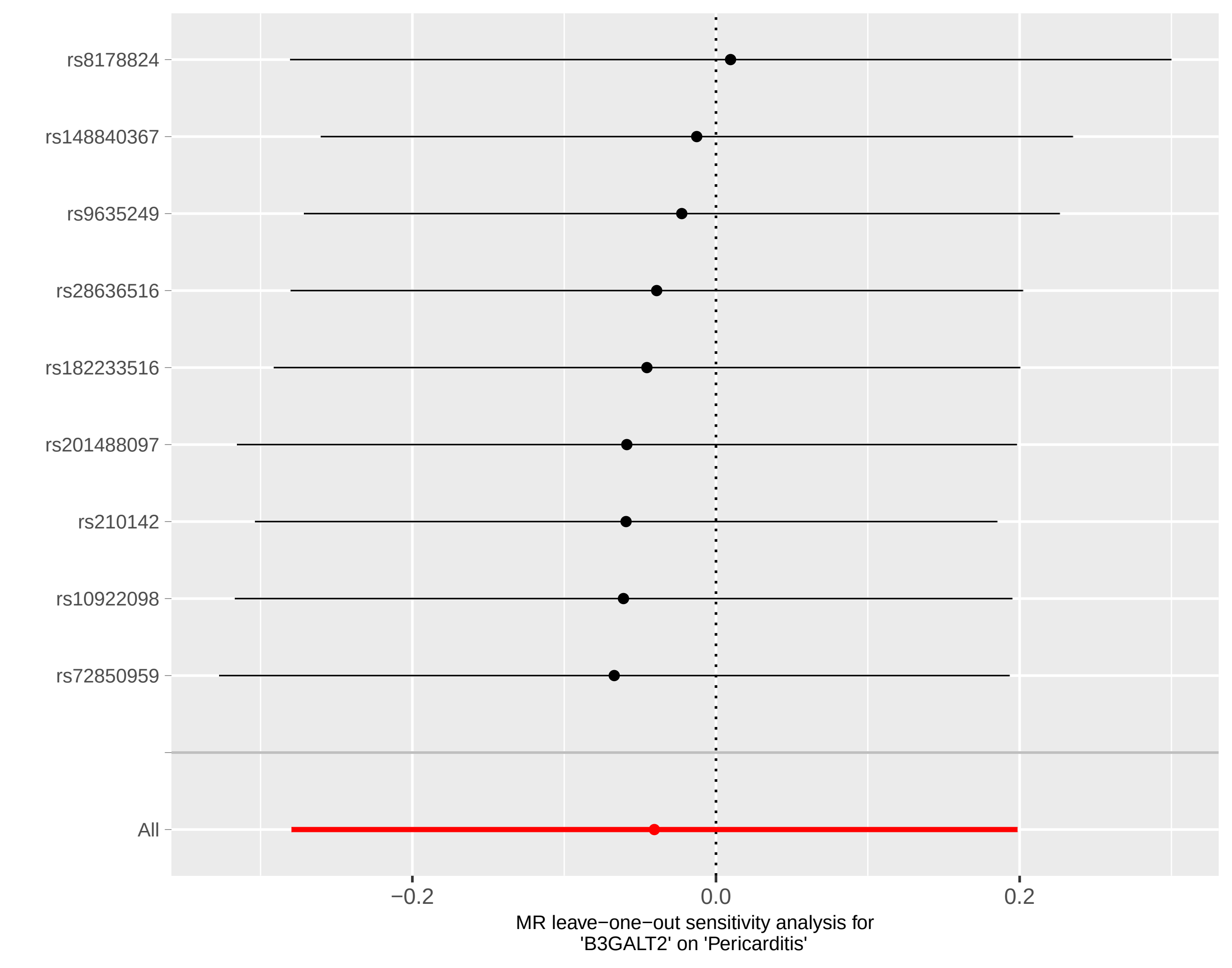
rs8178824
rs148840367
rs9635249
rs28636516
rs182233516
rs201488097
rs210142
rs10922098
rs72850959
All
−0.2
0.0
0.2
MR leave−one−out sensitivity analysis for
'B3GALT2' on 'Pericarditis'

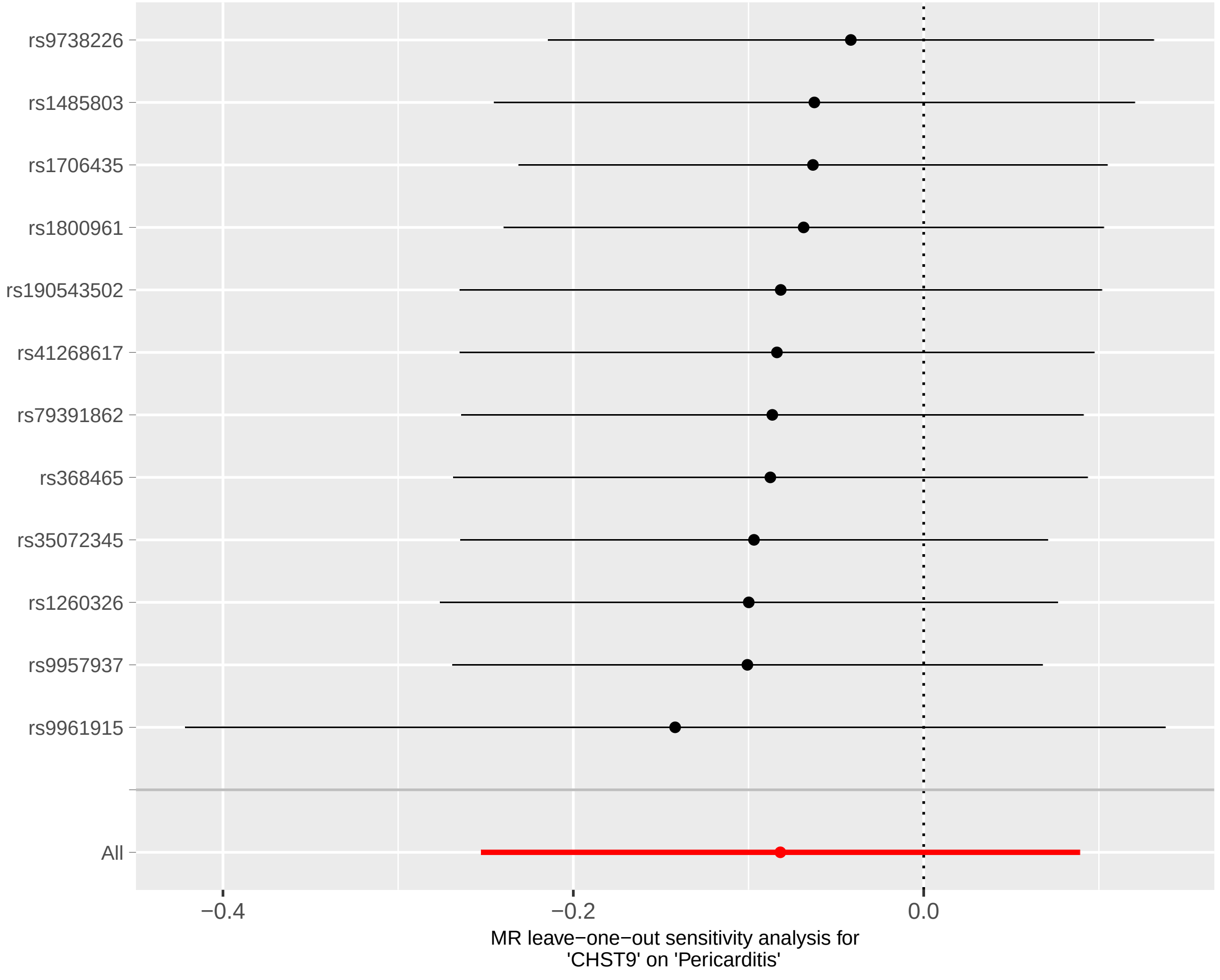

rs9738226
rs1485803
rs1706435
rs1800961
rs190543502
rs41268617
rs79391862
rs368465
rs35072345
rs1260326
rs9957937
rs9961915
All
−0.4
−0.2
0.0
MR leave−one−out sensitivity analysis for
'CHST9' on 'Pericarditis'

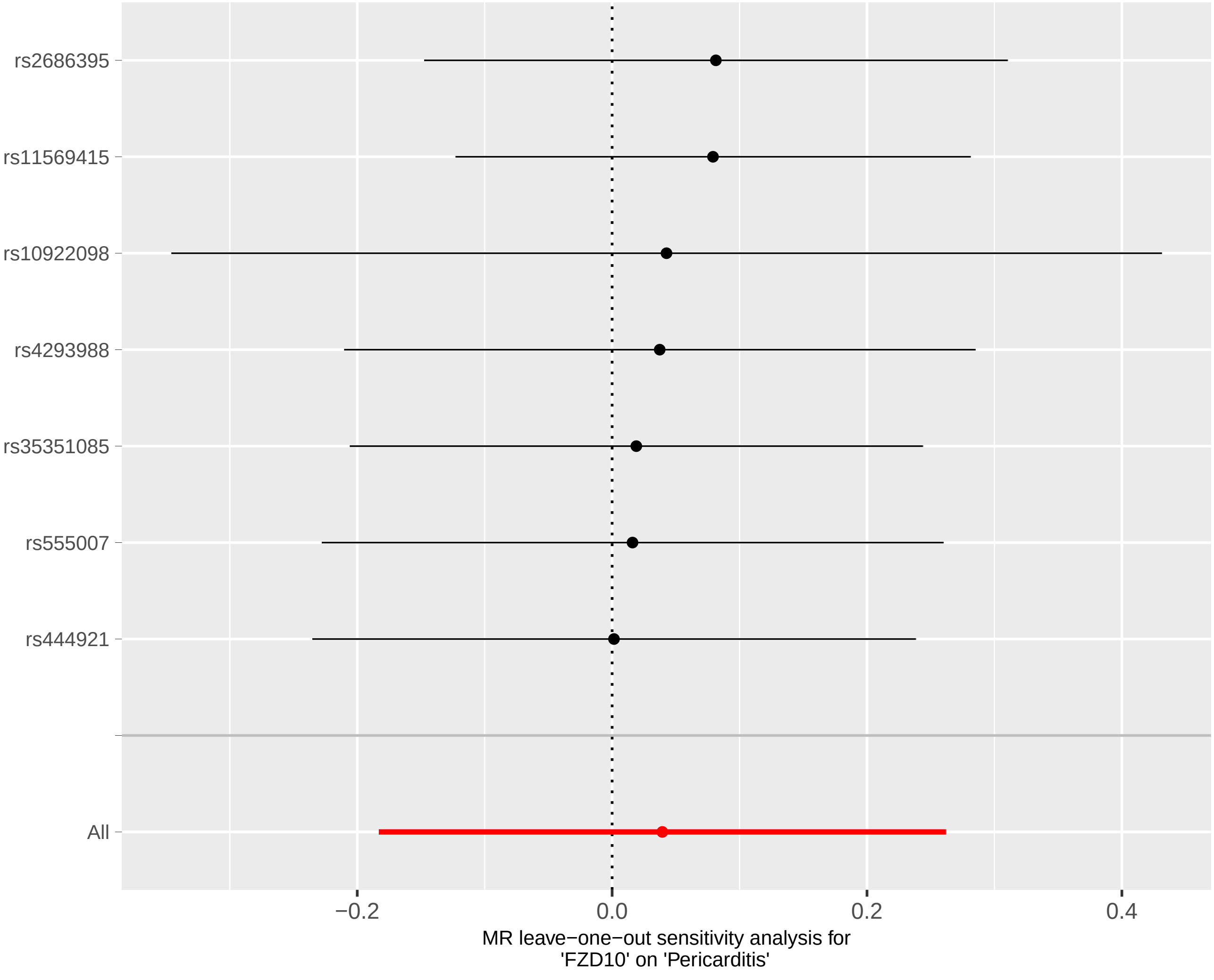
rs2686395
rs11569415
rs10922098
rs4293988
rs35351085
rs555007
rs444921
All
−0.2
0.0
0.2
0.4
MR leave−one−out sensitivity analysis for
'FZD10' on 'Pericarditis'

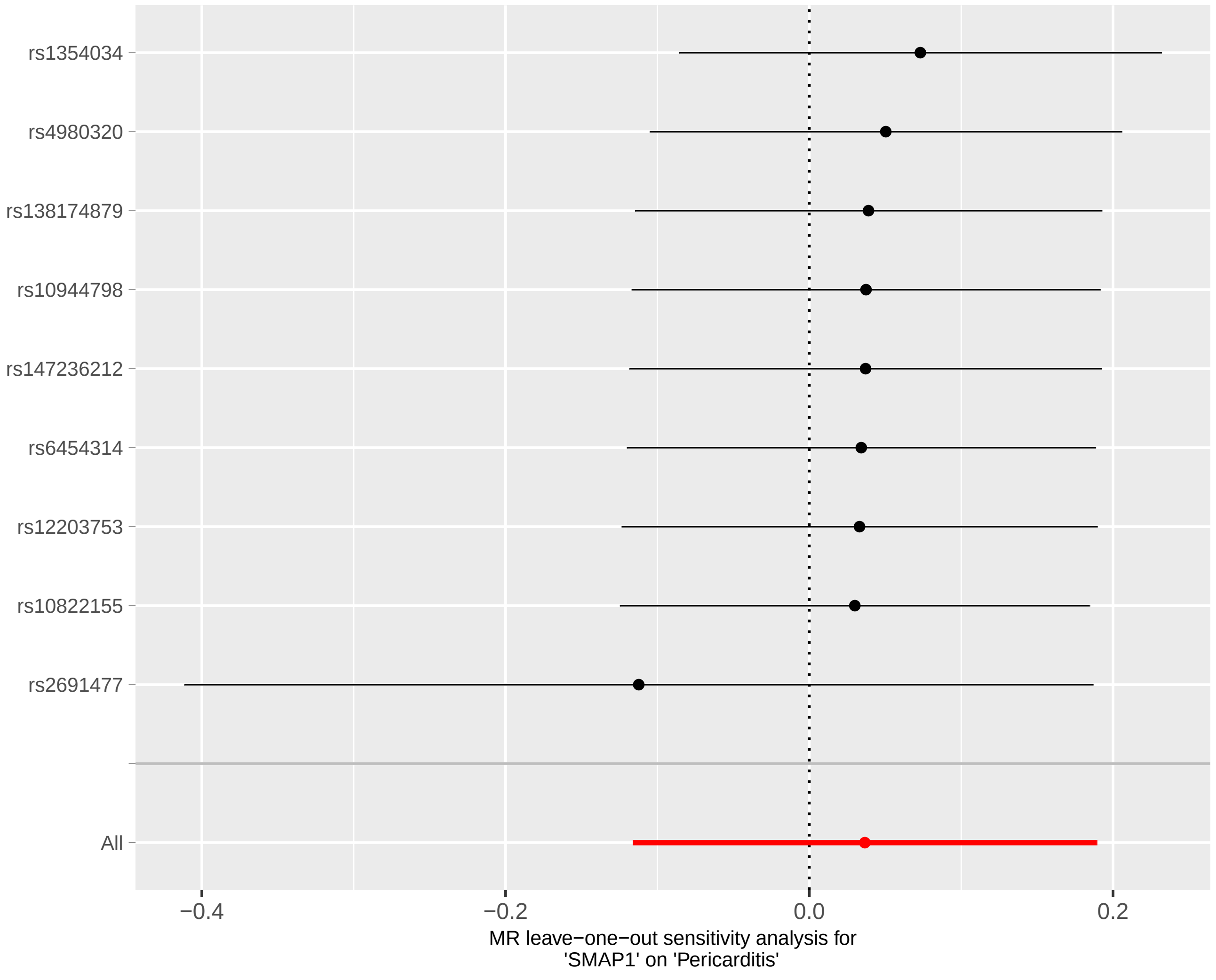
rs1354034
rs4980320
rs138174879
rs10944798
rs147236212
rs6454314
rs12203753
rs10822155
rs2691477
All
−0.4
−0.2
0.0
0.2
MR leave−one−out sensitivity analysis for
'SMAP1' on 'Pericarditis'

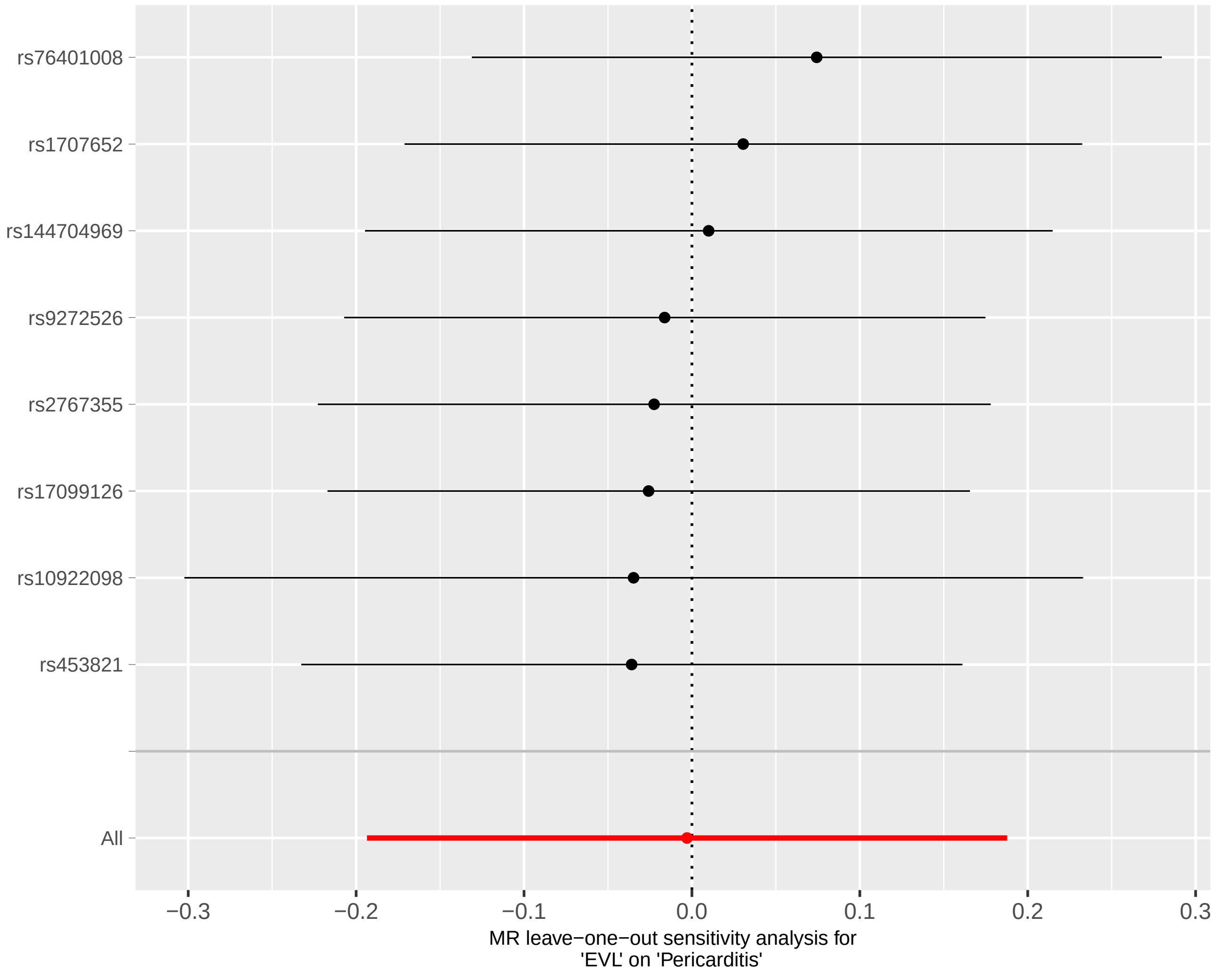

rs76401008
rs1707652
rs144704969
rs9272526
rs2767355
rs17099126
rs10922098
rs453821
All
−0.3
−0.2
−0.1
0.0
0.1
0.2
0.3
MR leave−one−out sensitivity analysis for
'EVL' on 'Pericarditis'

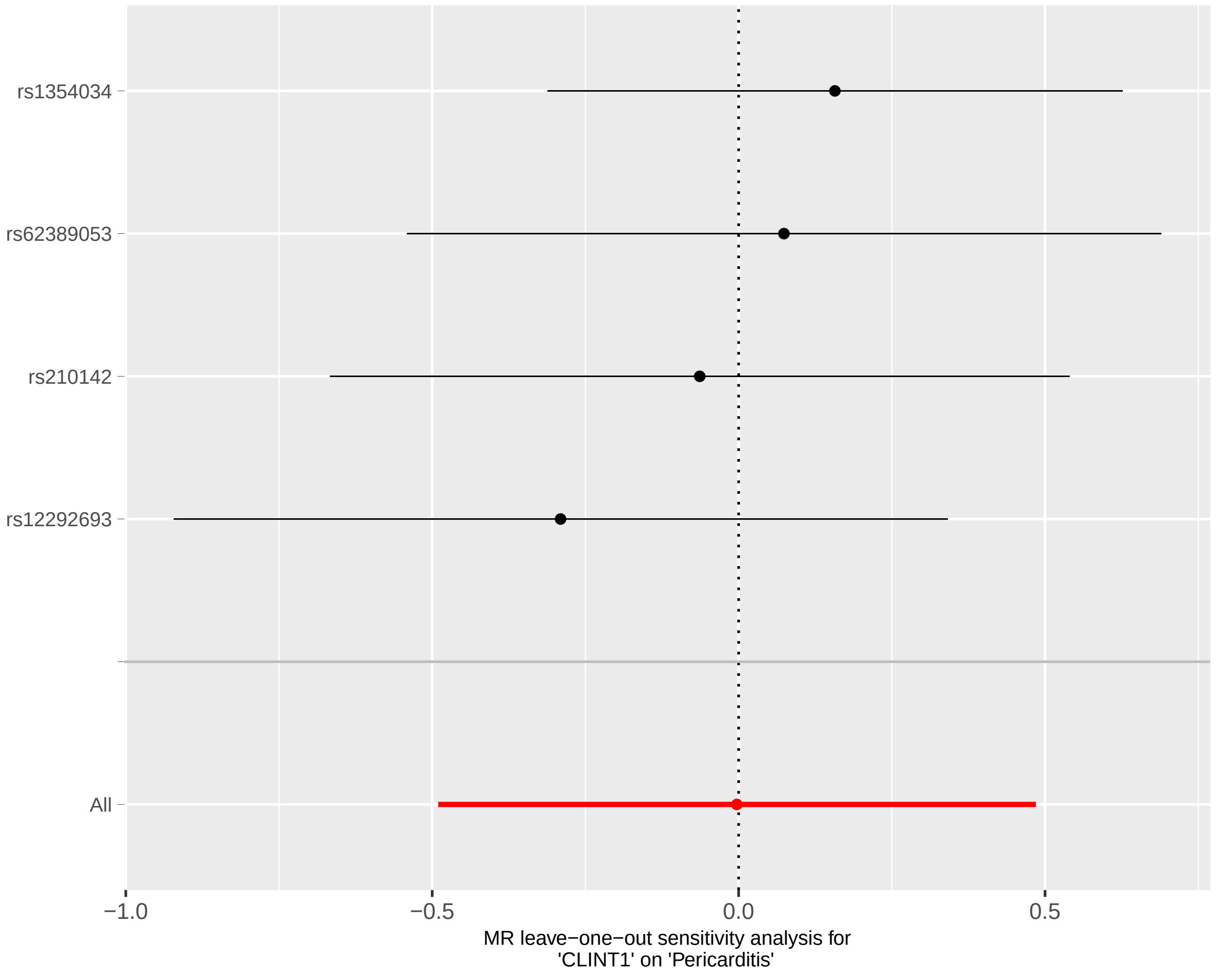
rs1354034
rs62389053
rs210142
rs12292693
All
−1.0
−0.5
0.0
0.5
MR leave−one−out sensitivity analysis for
'CLINT1' on 'Pericarditis'

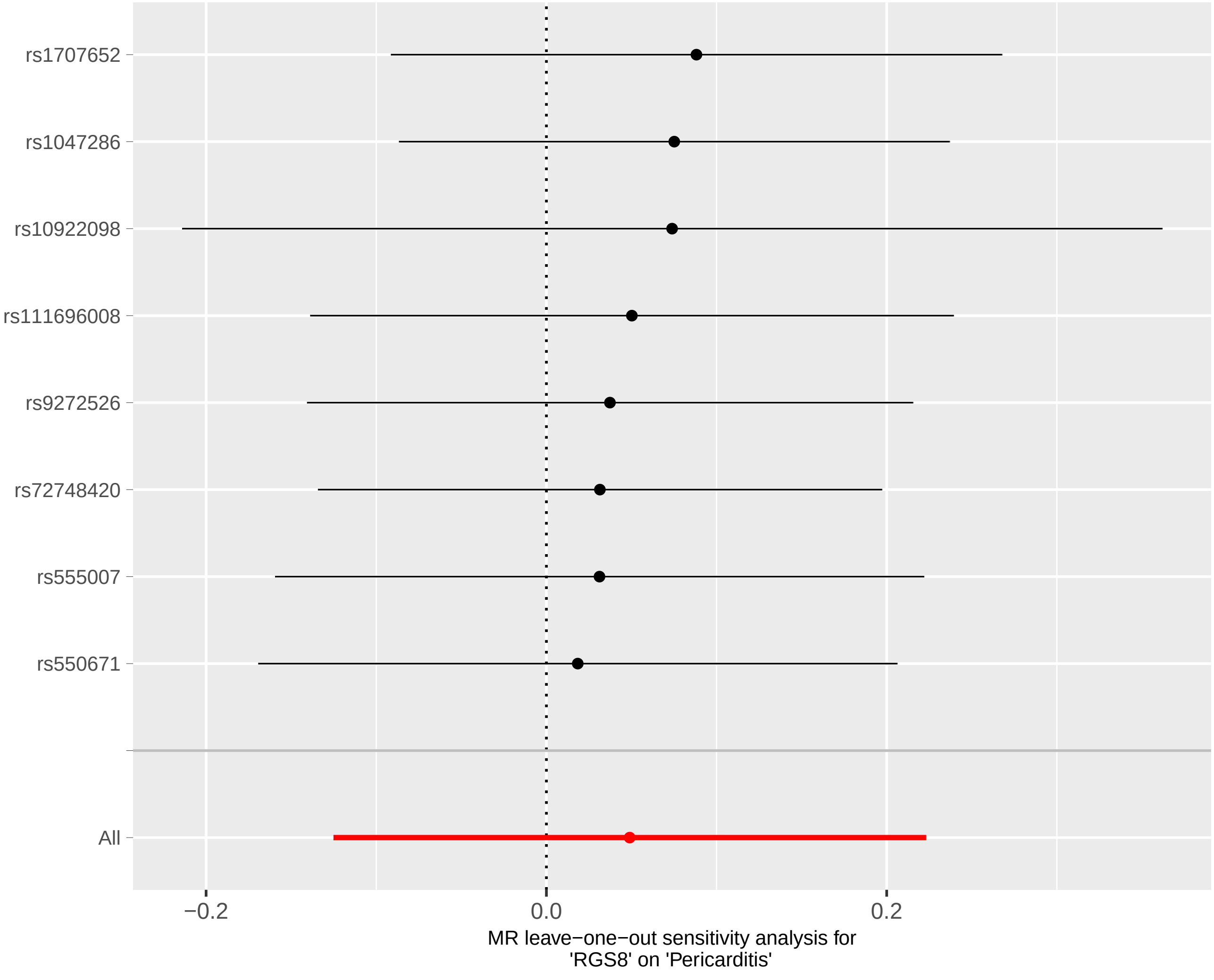

rs1707652
rs1047286
rs10922098
rs111696008
rs9272526
rs72748420
rs555007
rs550671
All
−0.2
0.0
0.2
MR leave−one−out sensitivity analysis for
'RGS8' on 'Pericarditis'

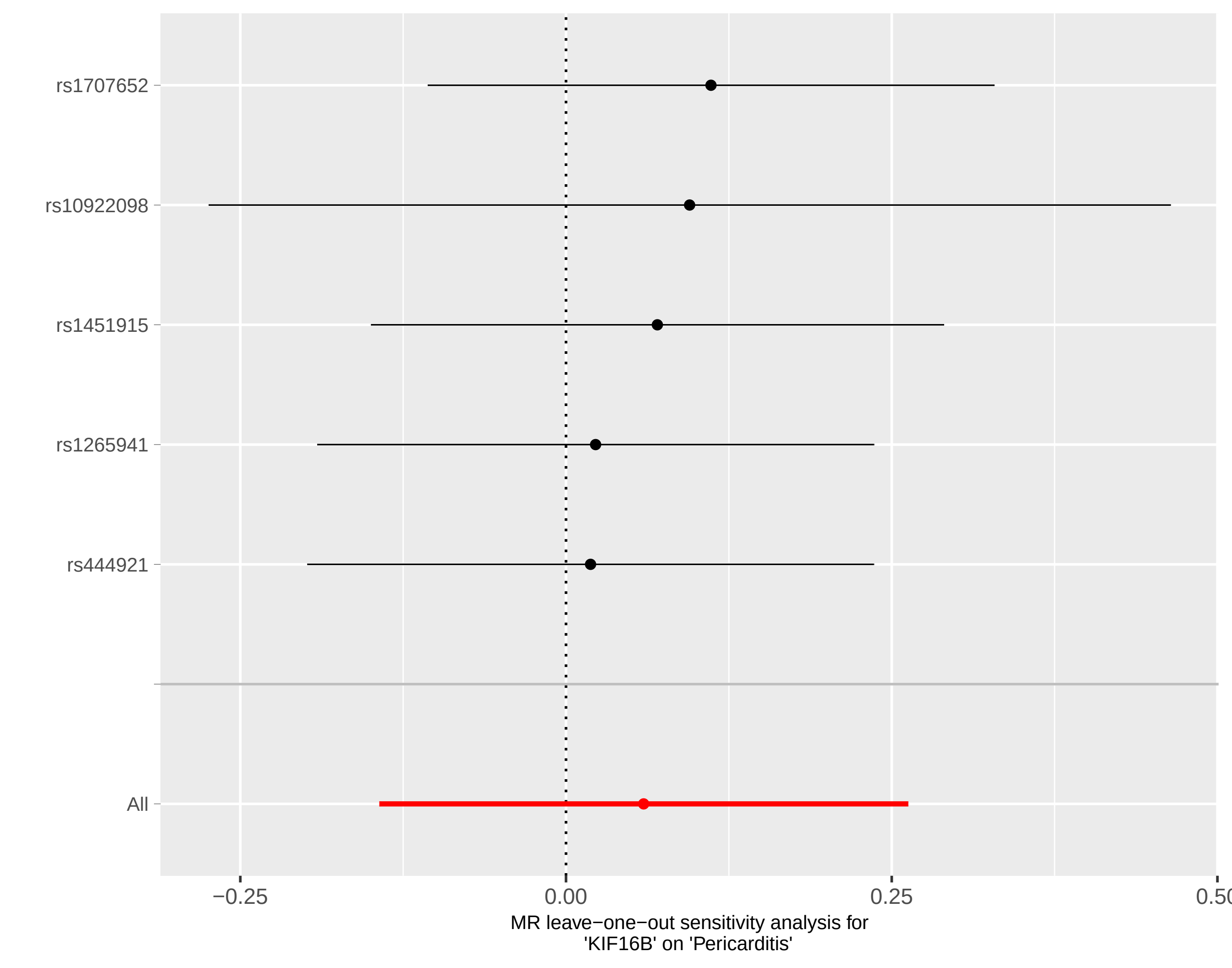
rs1707652
rs10922098
rs1451915
rs1265941
rs444921
All
−0.25
0.00
0.25
0.50
MR leave−one−out sensitivity analysis for
'KIF16B' on 'Pericarditis'

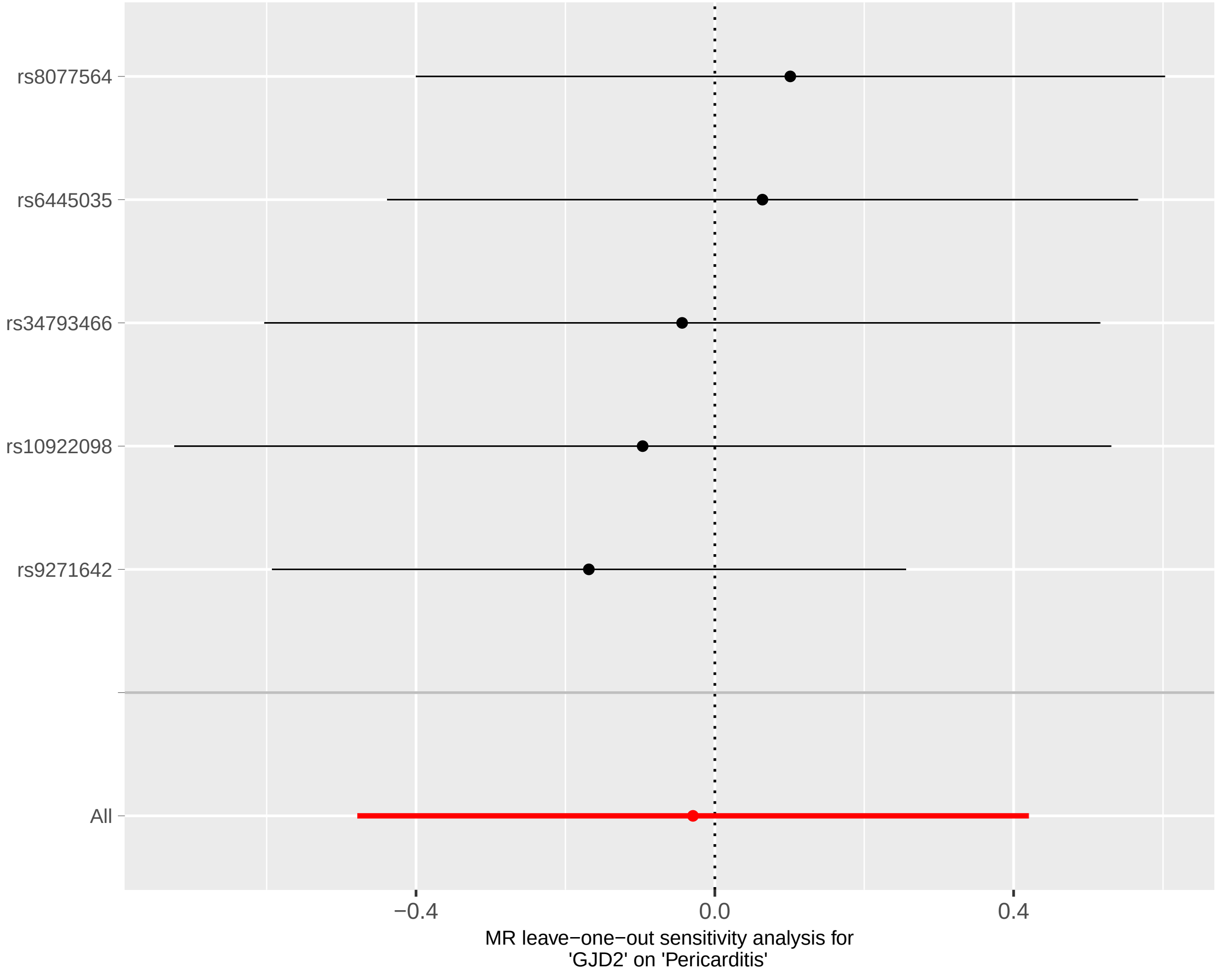

rs8077564
rs6445035
rs34793466
rs10922098
rs9271642
All
−0.4
0.0
0.4
MR leave−one−out sensitivity analysis for
'GJD2' on 'Pericarditis'

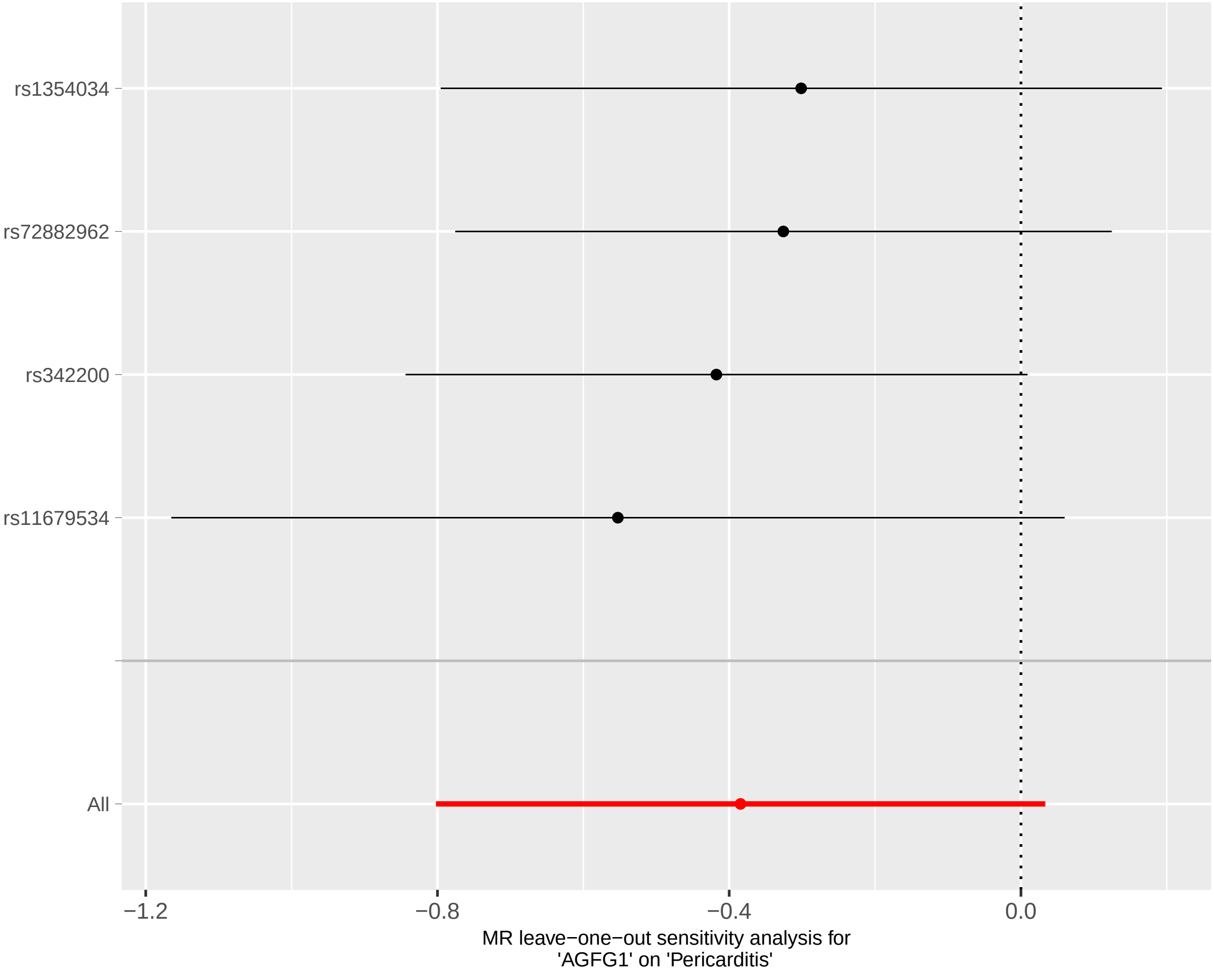
rs1354034
rs72882962
rs342200
rs11679534
All
−1.2
−0.8
−0.4
0.0
MR leave−one−out sensitivity analysis for
'AGFG1' on 'Pericarditis'

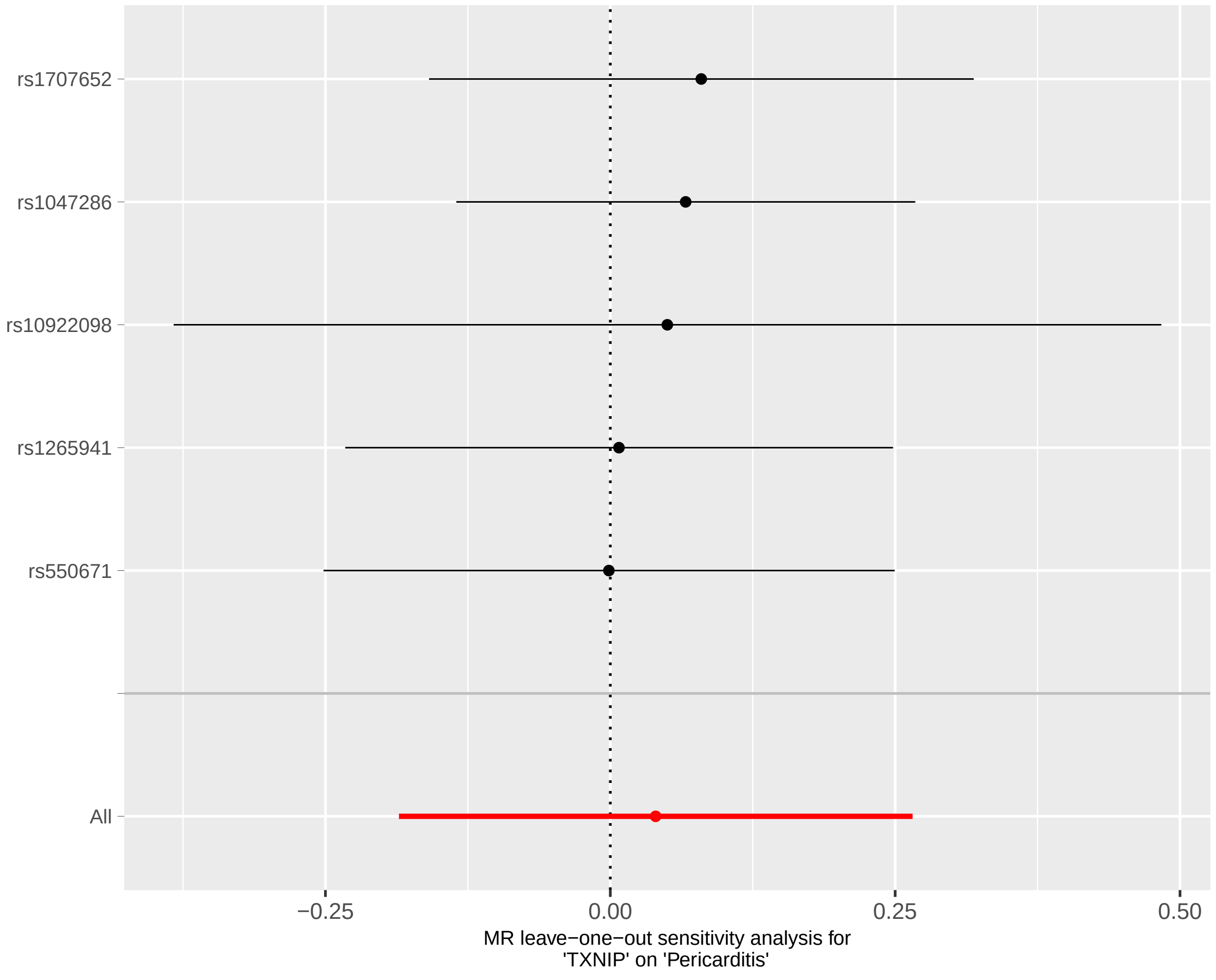

rs1707652
rs1047286
rs10922098
rs1265941
rs550671
All
−0.25
0.00
0.25
0.50
MR leave−one−out sensitivity analysis for
'TXNIP' on 'Pericarditis'

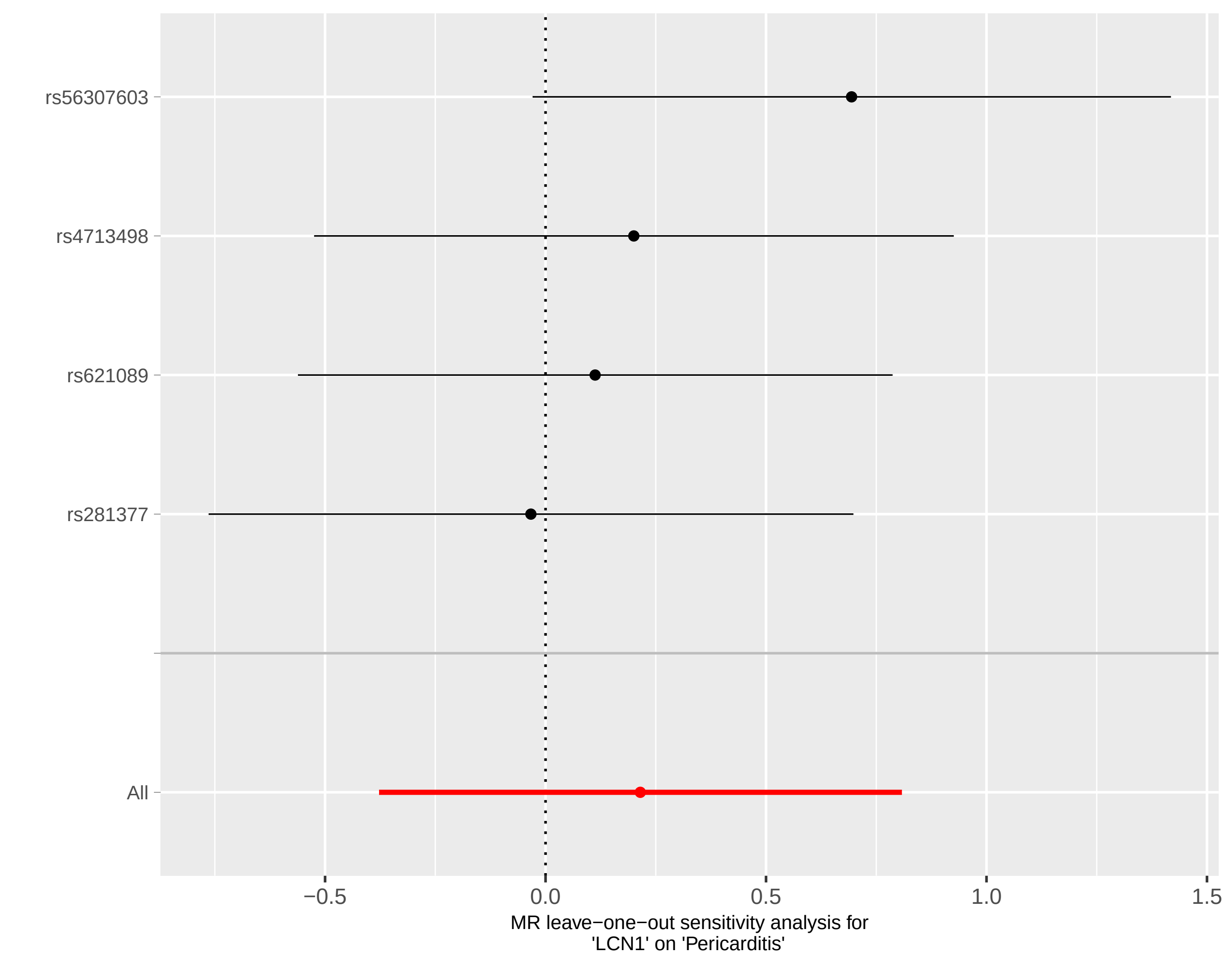

rs56307603
rs4713498
rs621089
rs281377
All
−0.5
0.0
0.5
1.0
1.5
MR leave−one−out sensitivity analysis for
'LCN1' on 'Pericarditis'

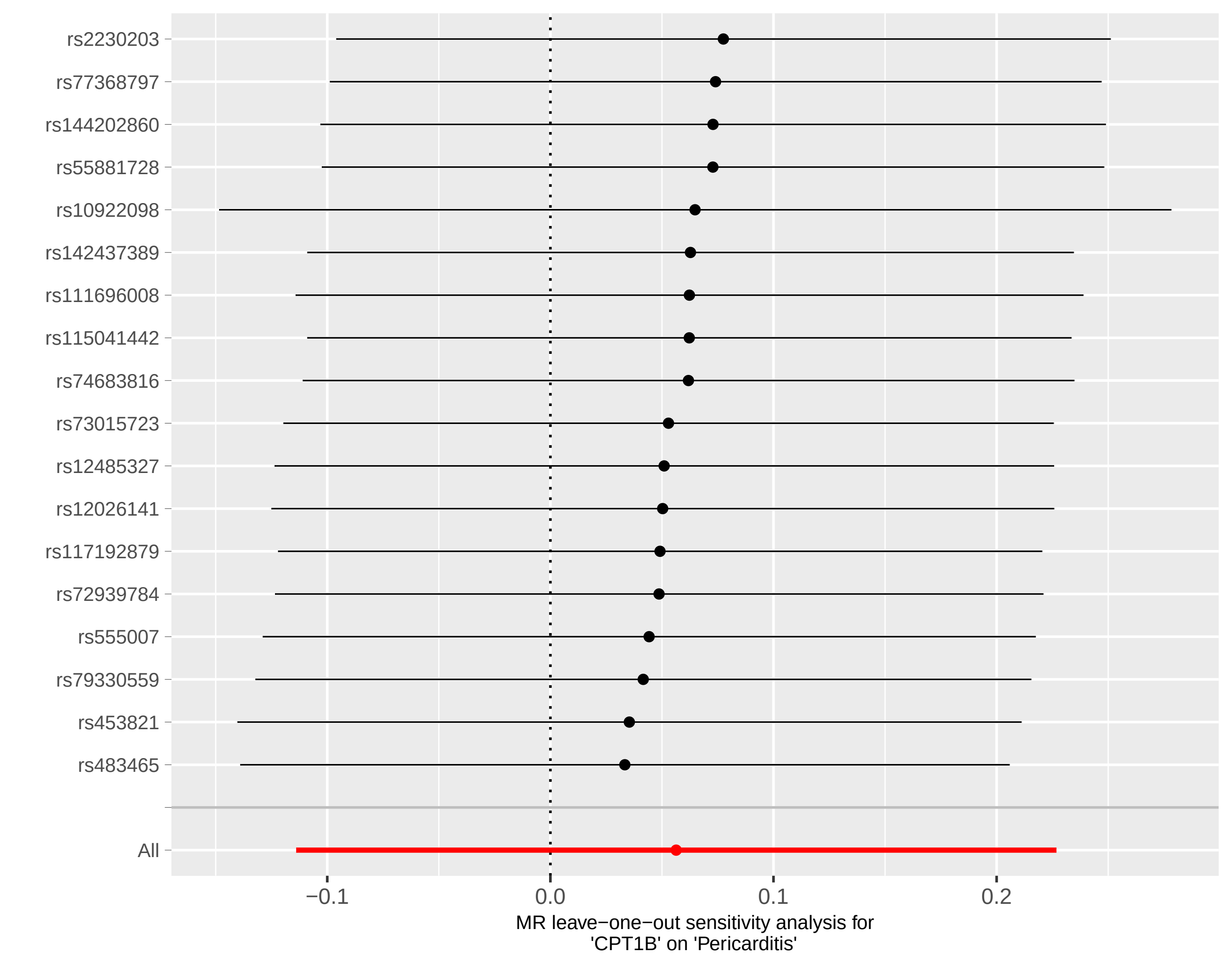

rs2230203
rs77368797
rs144202860
rs55881728
rs10922098
rs142437389
rs111696008
rs115041442
rs74683816
rs73015723
rs12485327
rs12026141
rs117192879
rs72939784
rs555007
rs79330559
rs453821
rs483465
All
−0.1
0.0
0.1
0.2
MR leave−one−out sensitivity analysis for
'CPT1B' on 'Pericarditis'

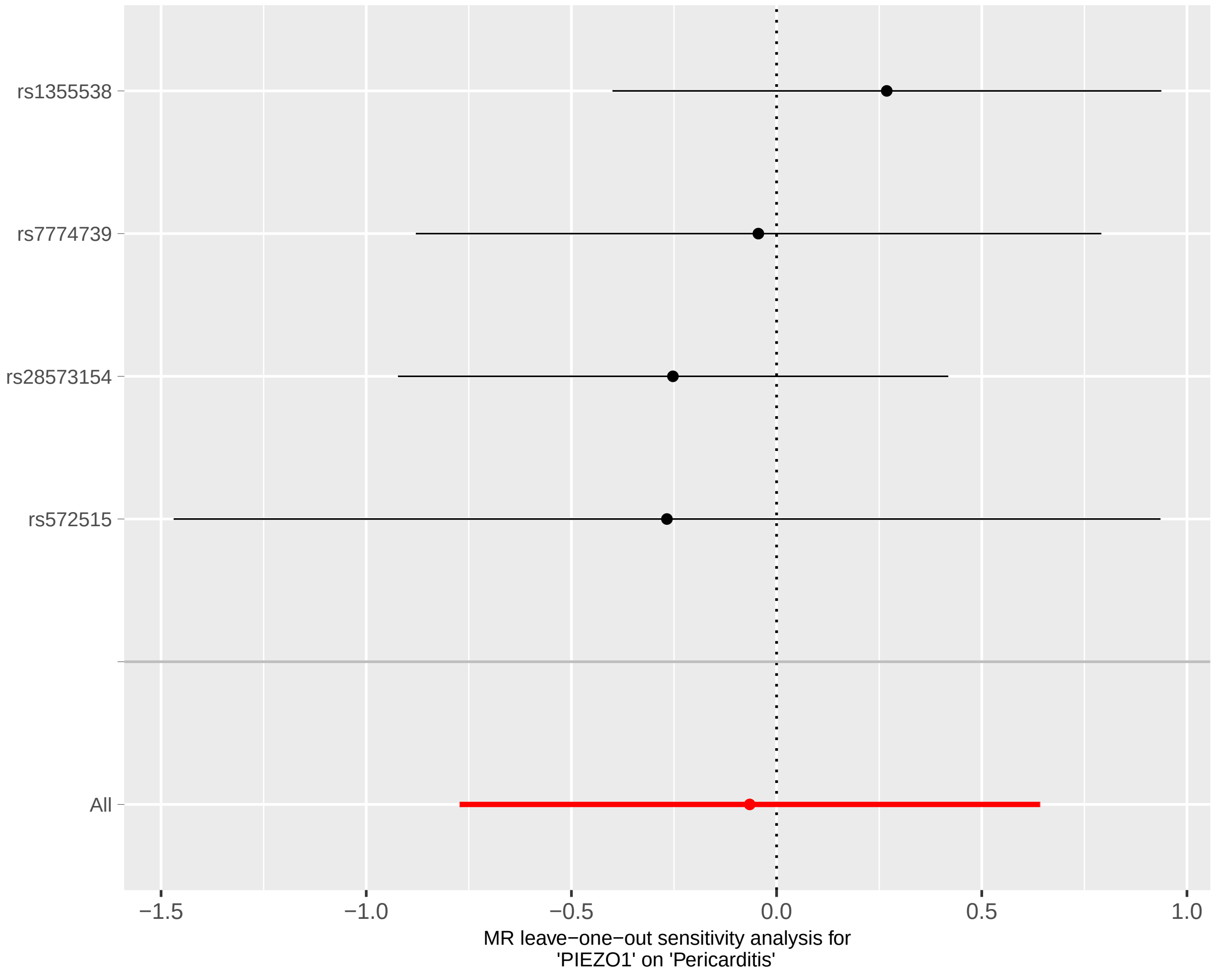
rs1355538
rs7774739
rs28573154
rs572515
All
−1.5
−1.0
−0.5
0.0
0.5
1.0
MR leave−one−out sensitivity analysis for
'PIEZO1' on 'Pericarditis'

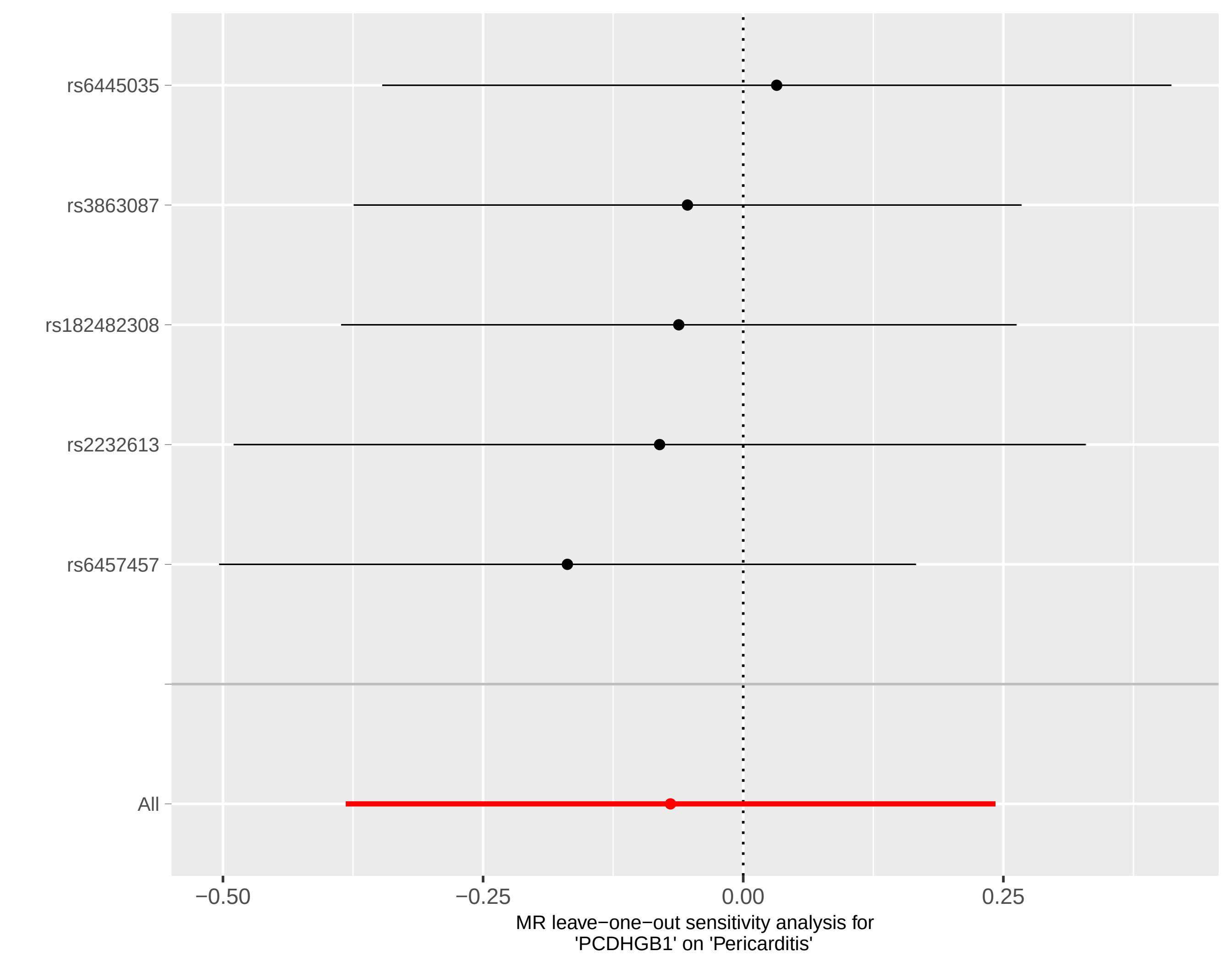
rs6445035
rs3863087
rs182482308
rs2232613
rs6457457
All
−0.50
−0.25
0.00
0.25
MR leave−one−out sensitivity analysis for
'PCDHGB1' on 'Pericarditis'

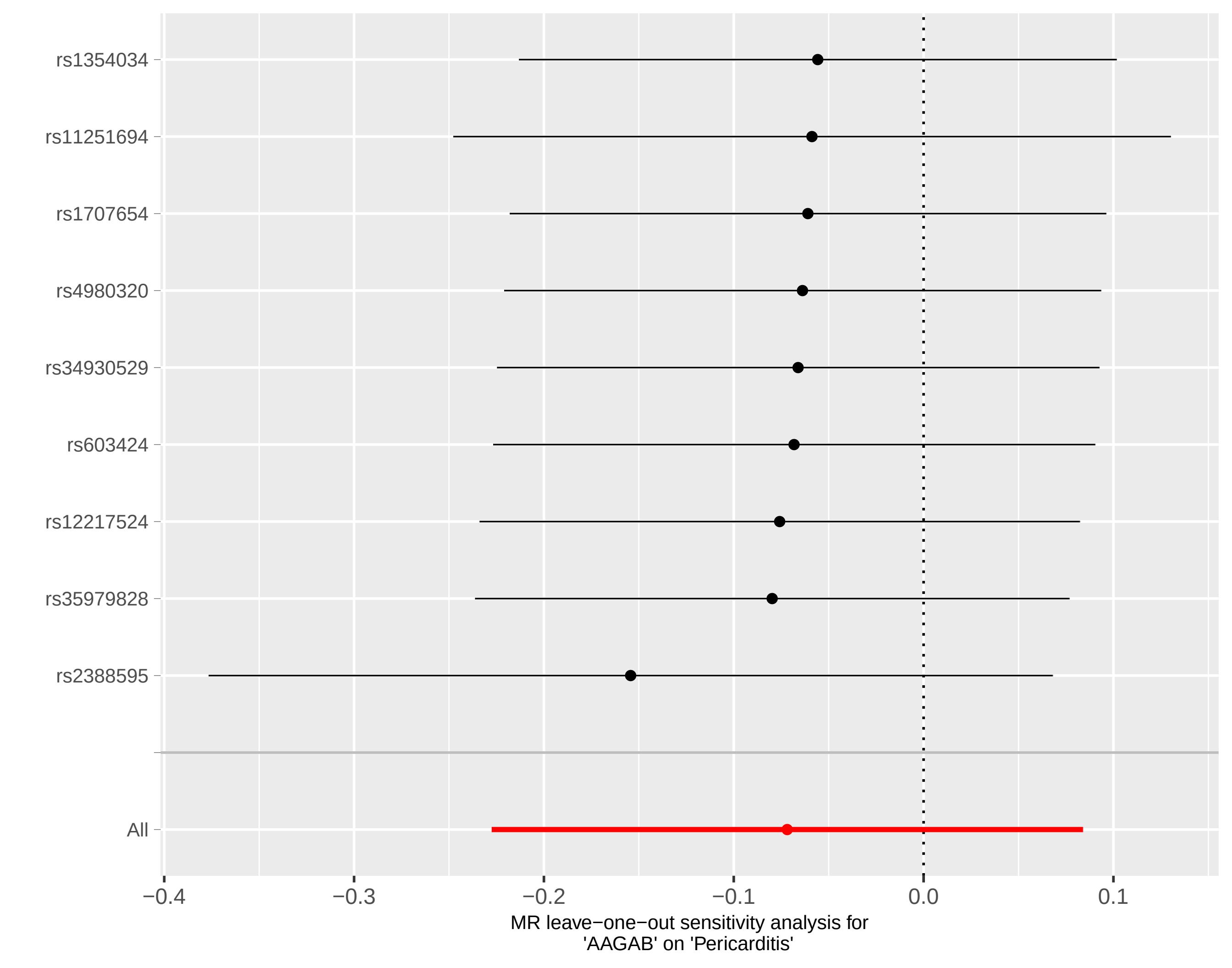
rs1354034
rs11251694
rs1707654
rs4980320
rs34930529
rs603424
rs12217524
rs35979828
rs2388595
All
−0.4
−0.3
−0.2
−0.1
0.0
0.1
MR leave−one−out sensitivity analysis for
'AAGAB' on 'Pericarditis'

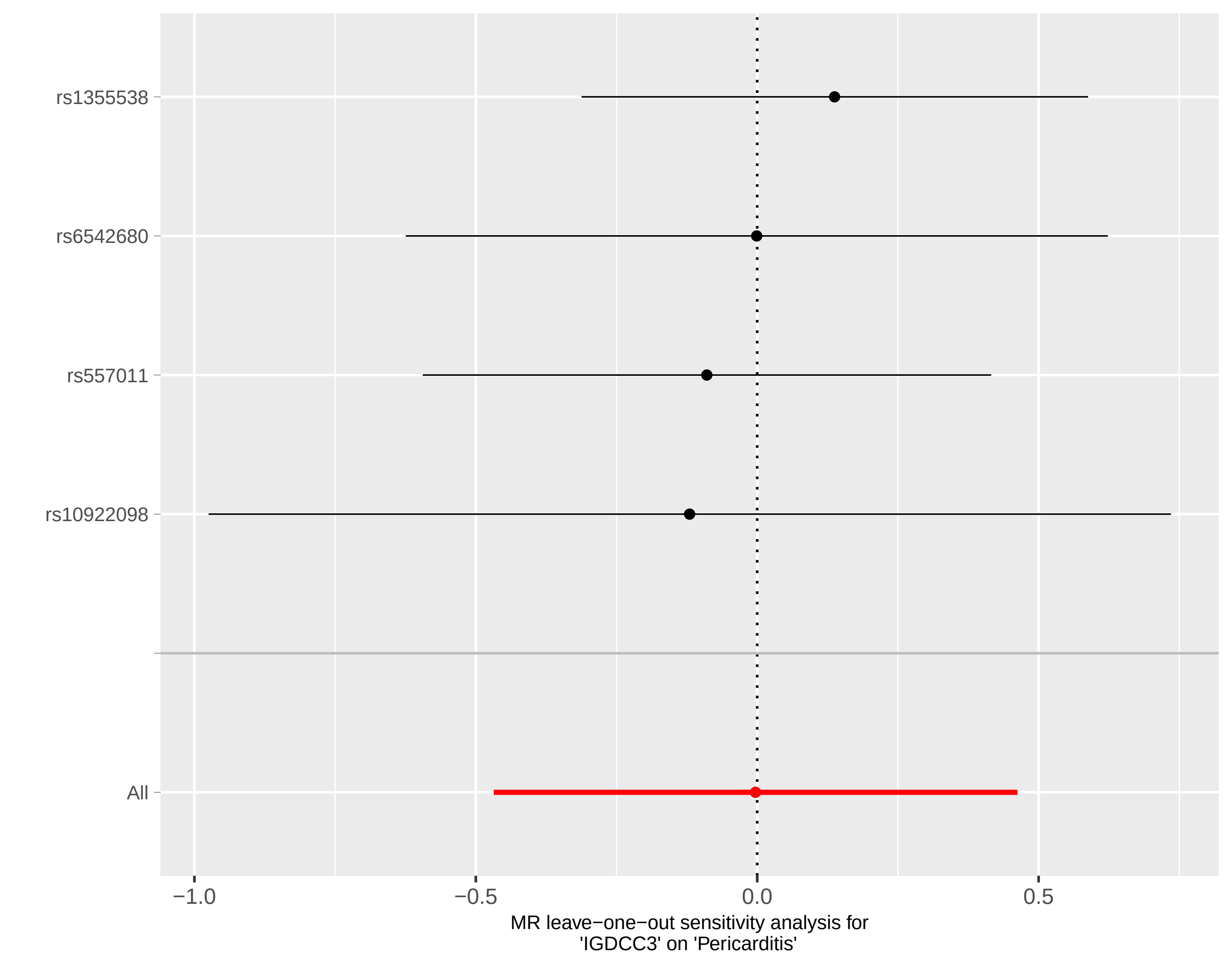
rs1355538
rs6542680
rs557011
rs10922098
All
−1.0
−0.5
0.0
0.5
MR leave−one−out sensitivity analysis for
'IGDCC3' on 'Pericarditis'

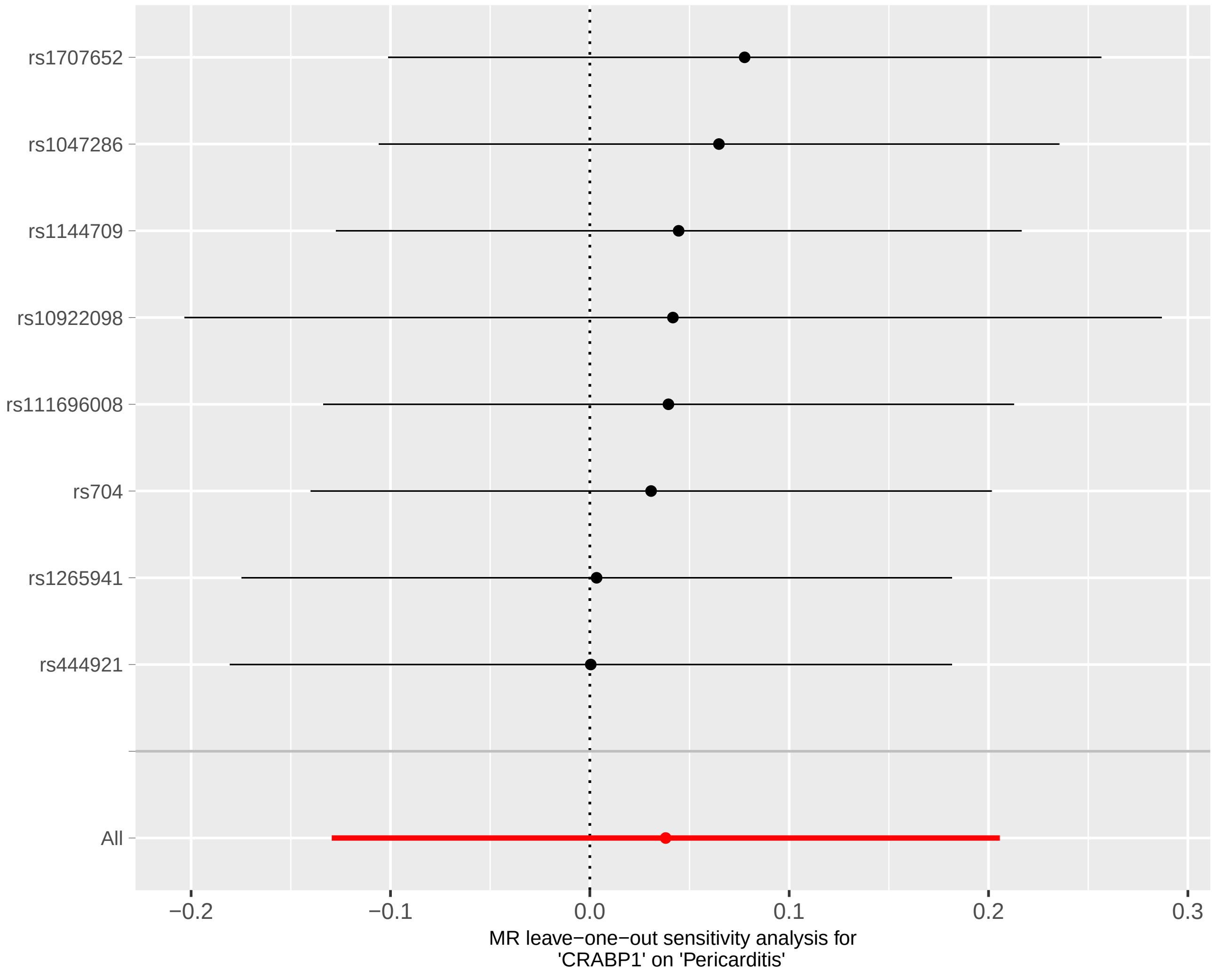

rs1707652
rs1047286
rs1144709
rs10922098
rs111696008
rs704
rs1265941
rs444921
All
−0.2
−0.1
0.0
0.1
0.2
0.3
MR leave−one−out sensitivity analysis for
'CRABP1' on 'Pericarditis'

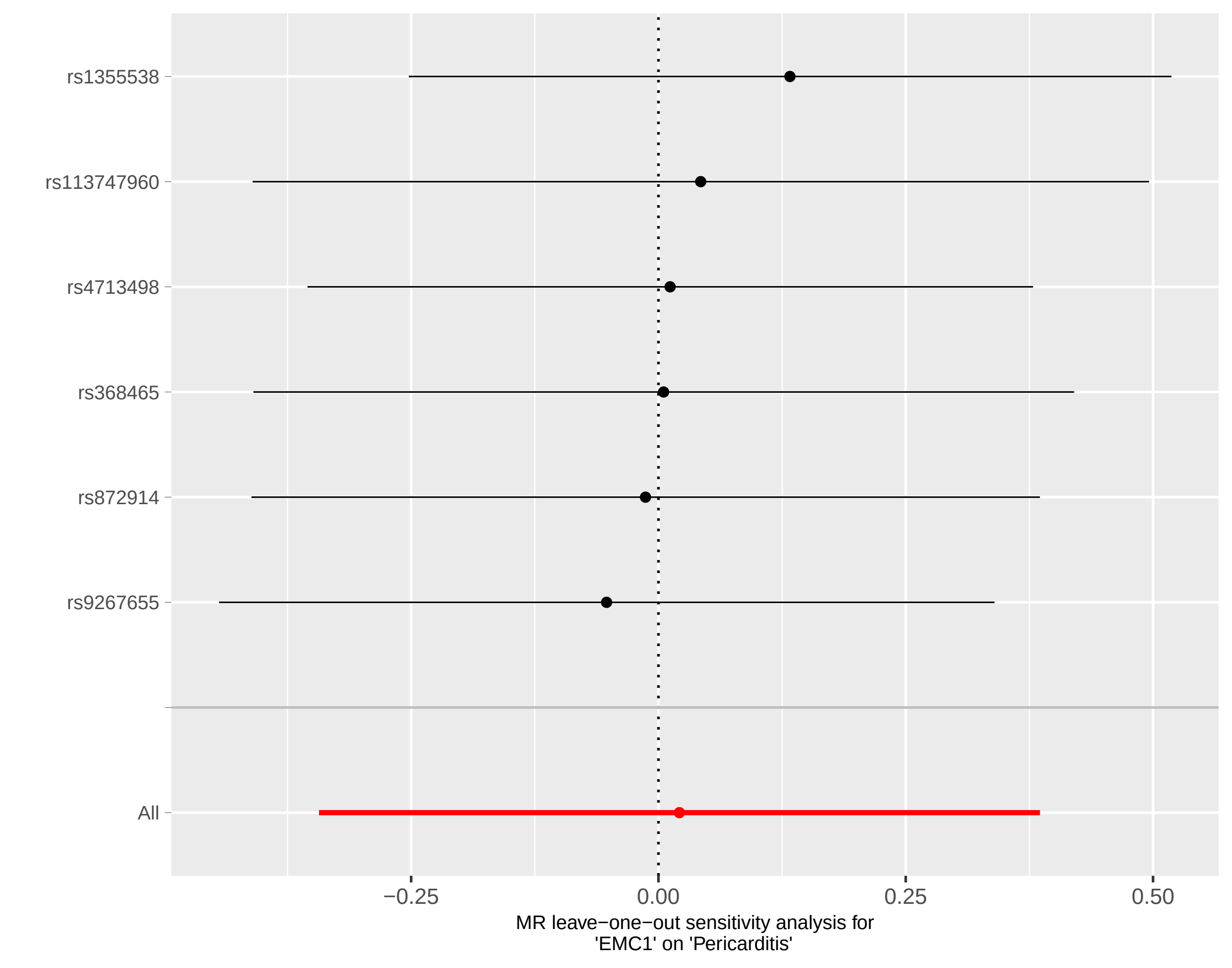
rs1355538
rs113747960
rs4713498
rs368465
rs872914
rs9267655
All
−0.25
0.00
0.25
0.50
MR leave−one−out sensitivity analysis for
'EMC1' on 'Pericarditis'

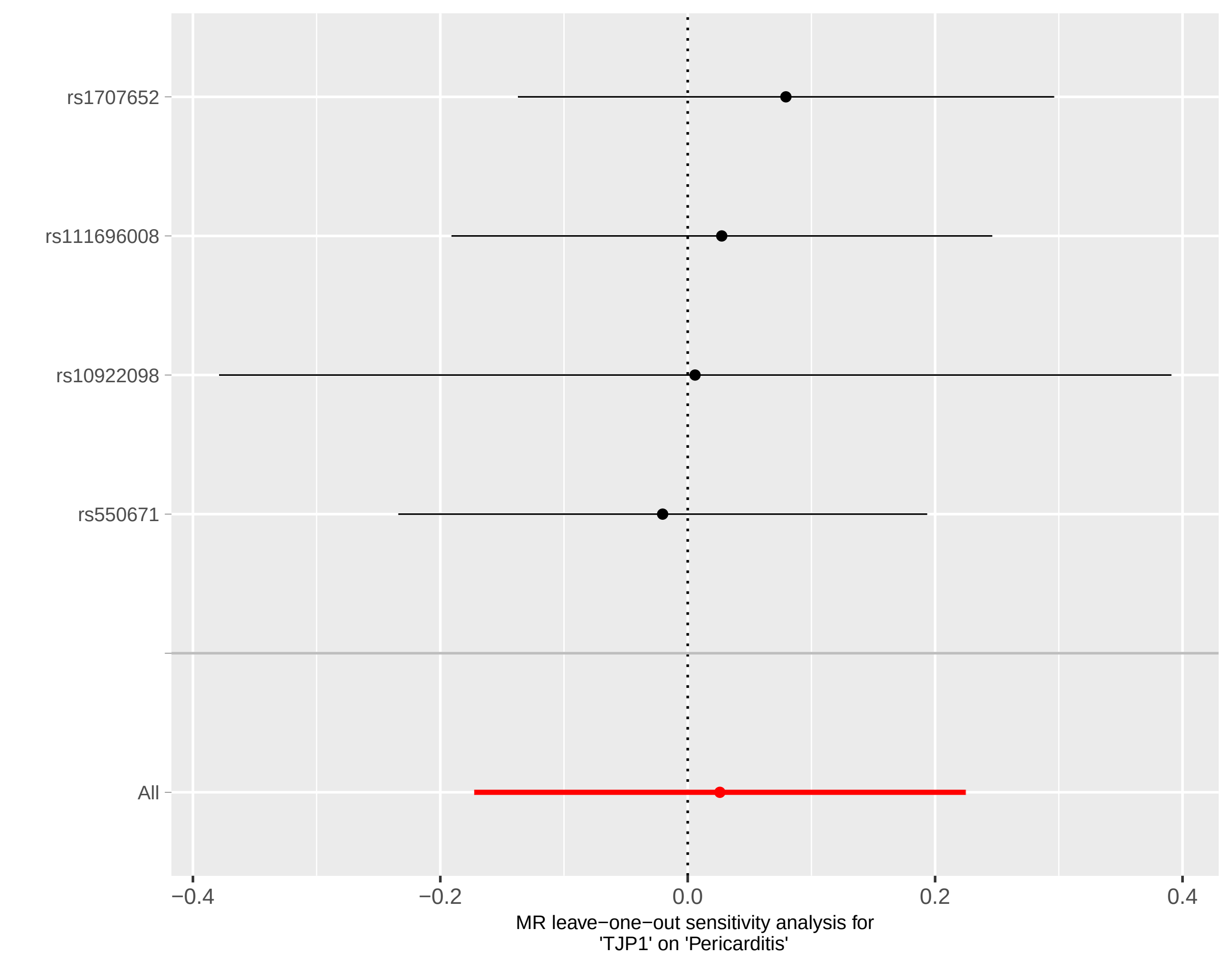

rs1707652
rs111696008
rs10922098
rs550671
All
−0.4
−0.2
0.0
0.2
0.4
MR leave−one−out sensitivity analysis for
'TJP1' on 'Pericarditis'

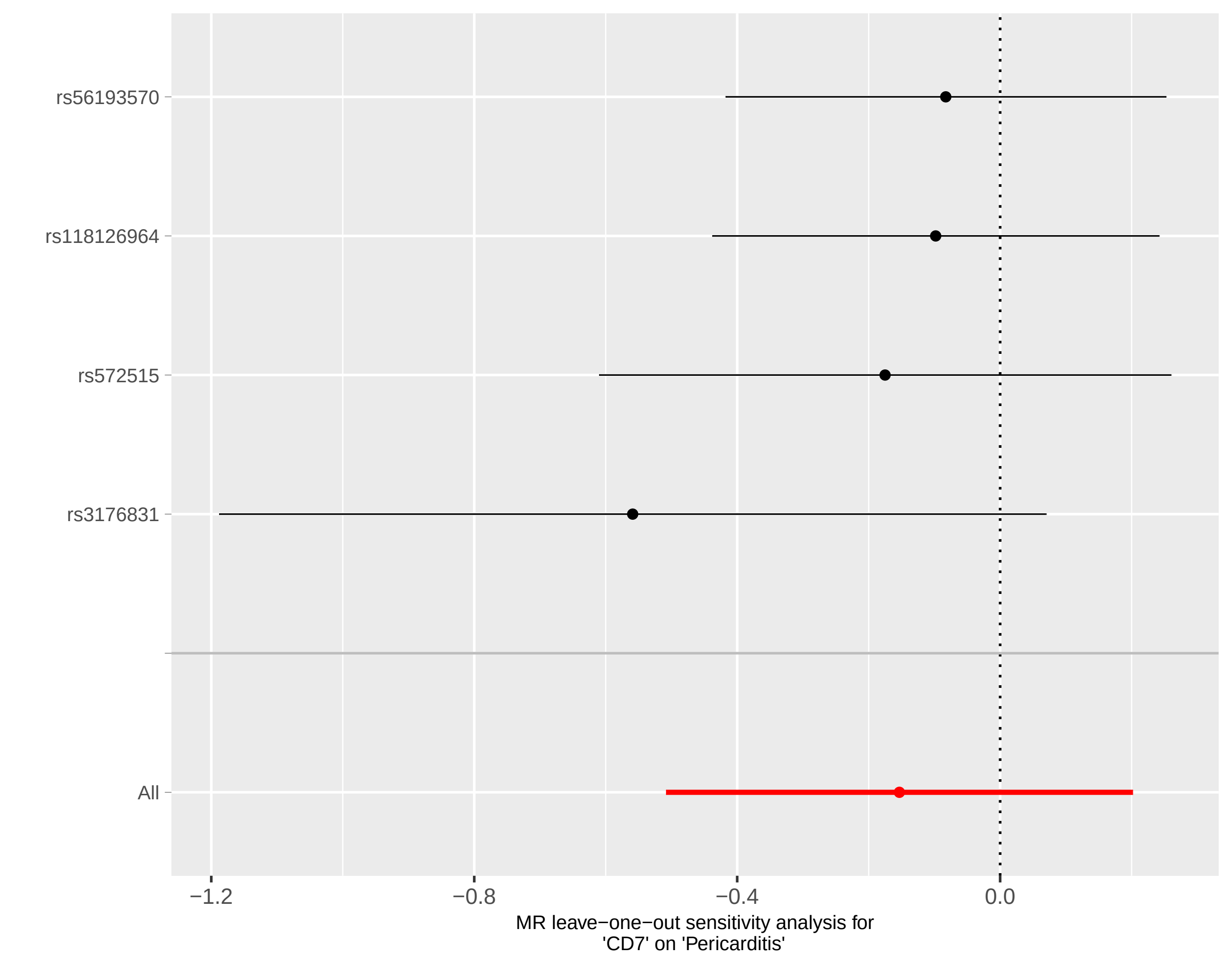

rs56193570
rs118126964
rs572515
rs3176831
All
−1.2
−0.8
−0.4
0.0
MR leave−one−out sensitivity analysis for
'CD7' on 'Pericarditis'

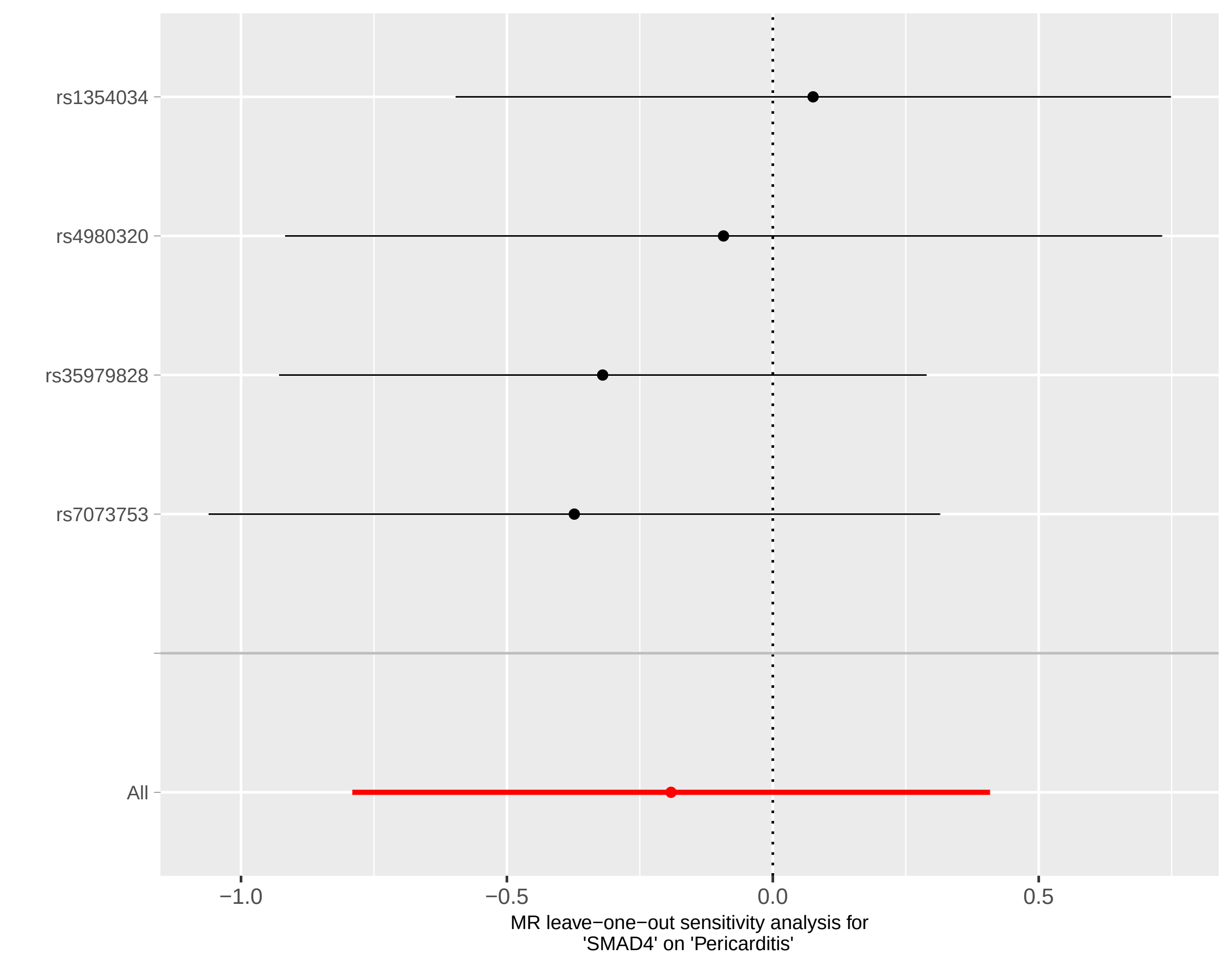

rs1354034
rs4980320
rs35979828
rs7073753
All
−1.0
−0.5
0.0
0.5
MR leave−one−out sensitivity analysis for
'SMAD4' on 'Pericarditis'

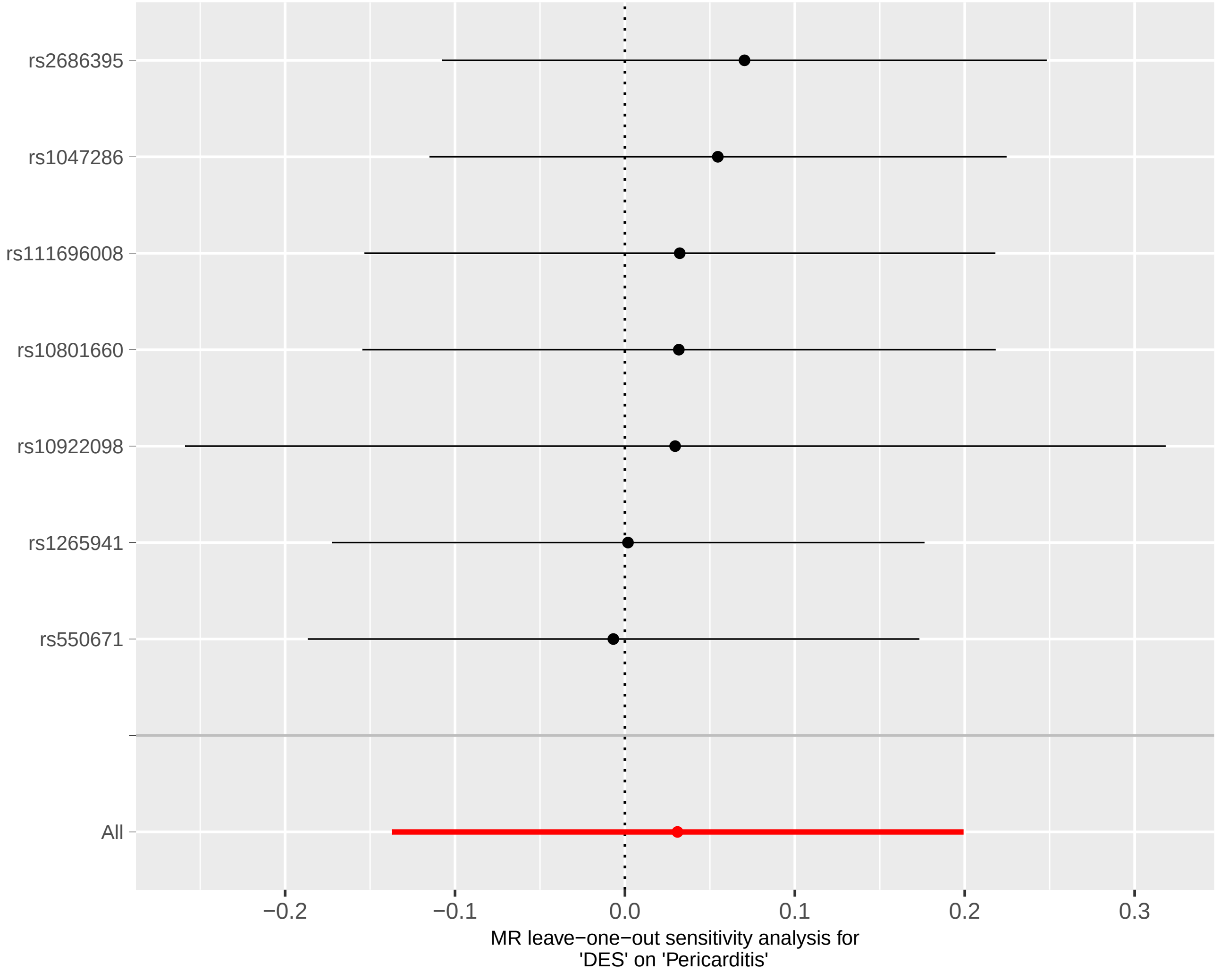
rs2686395
rs1047286
rs111696008
rs10801660
rs10922098
rs1265941
rs550671
All
−0.2
−0.1
0.0
0.1
0.2
0.3
MR leave−one−out sensitivity analysis for
'DES' on 'Pericarditis'

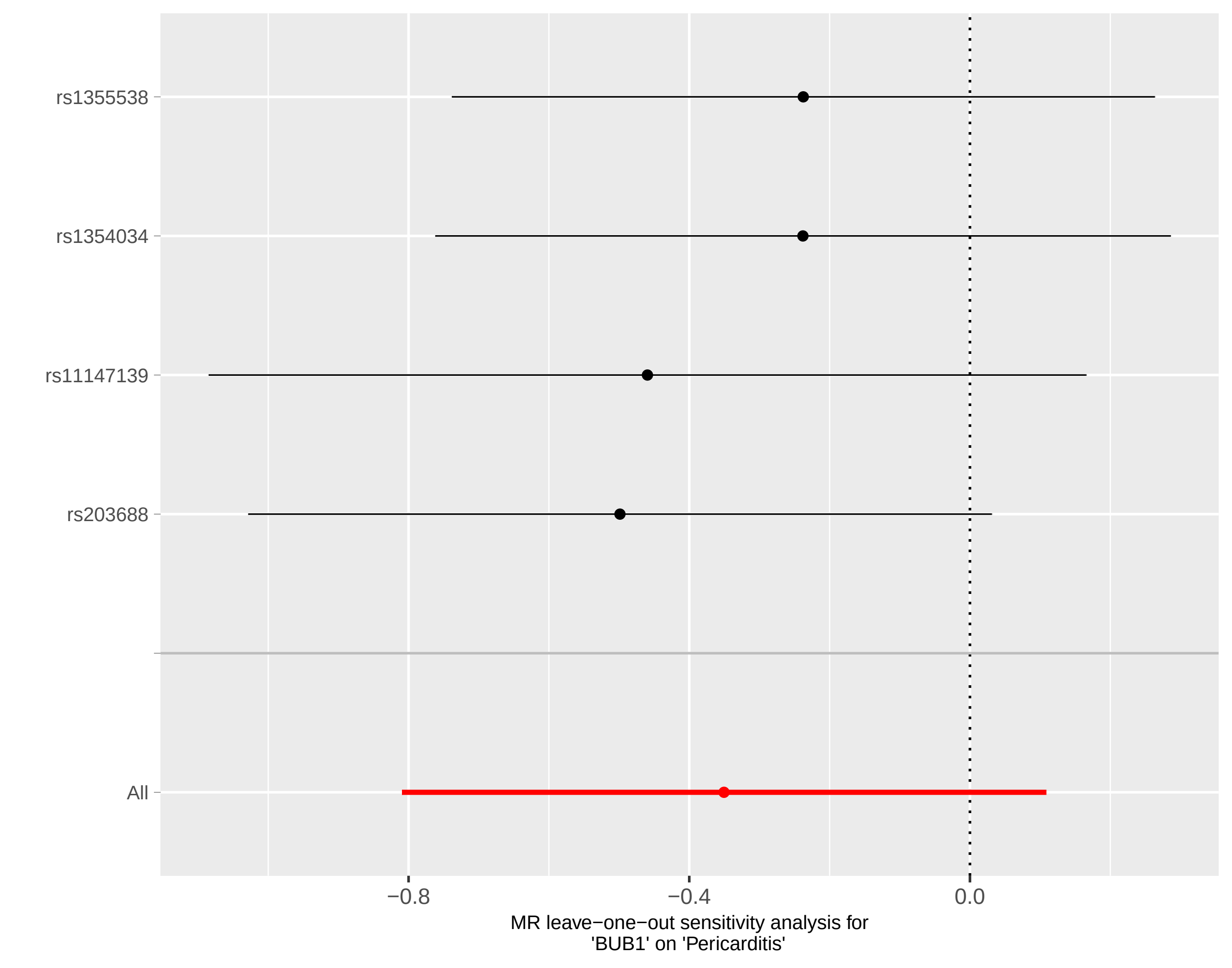
rs1355538
rs1354034
rs11147139
rs203688
All
−0.8
−0.4
0.0
MR leave−one−out sensitivity analysis for
'BUB1' on 'Pericarditis'

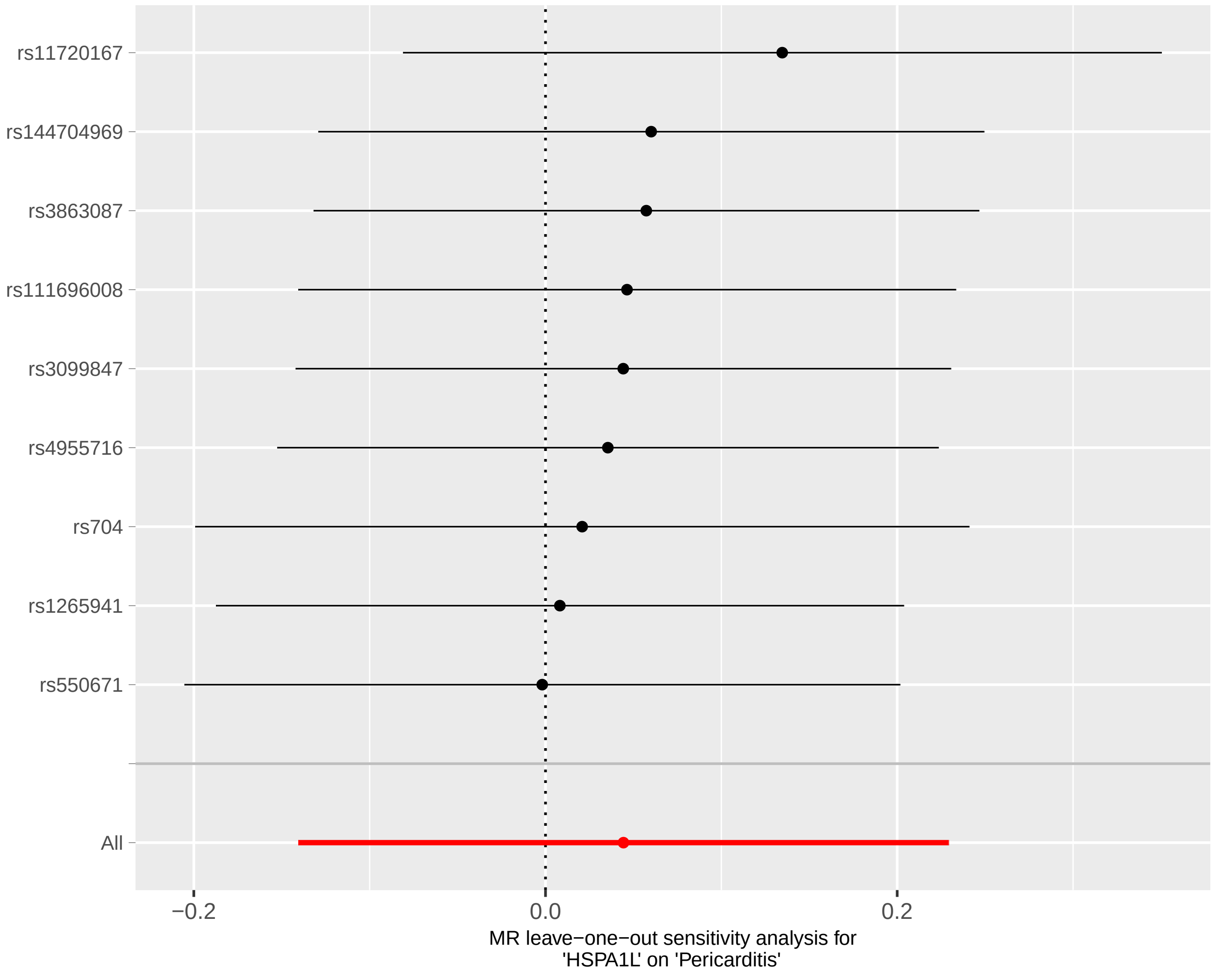
rs11720167
rs144704969
rs3863087
rs111696008
rs3099847
rs4955716
rs704
rs1265941
rs550671
All
−0.2
0.0
0.2
MR leave−one−out sensitivity analysis for
'HSPA1L' on 'Pericarditis'

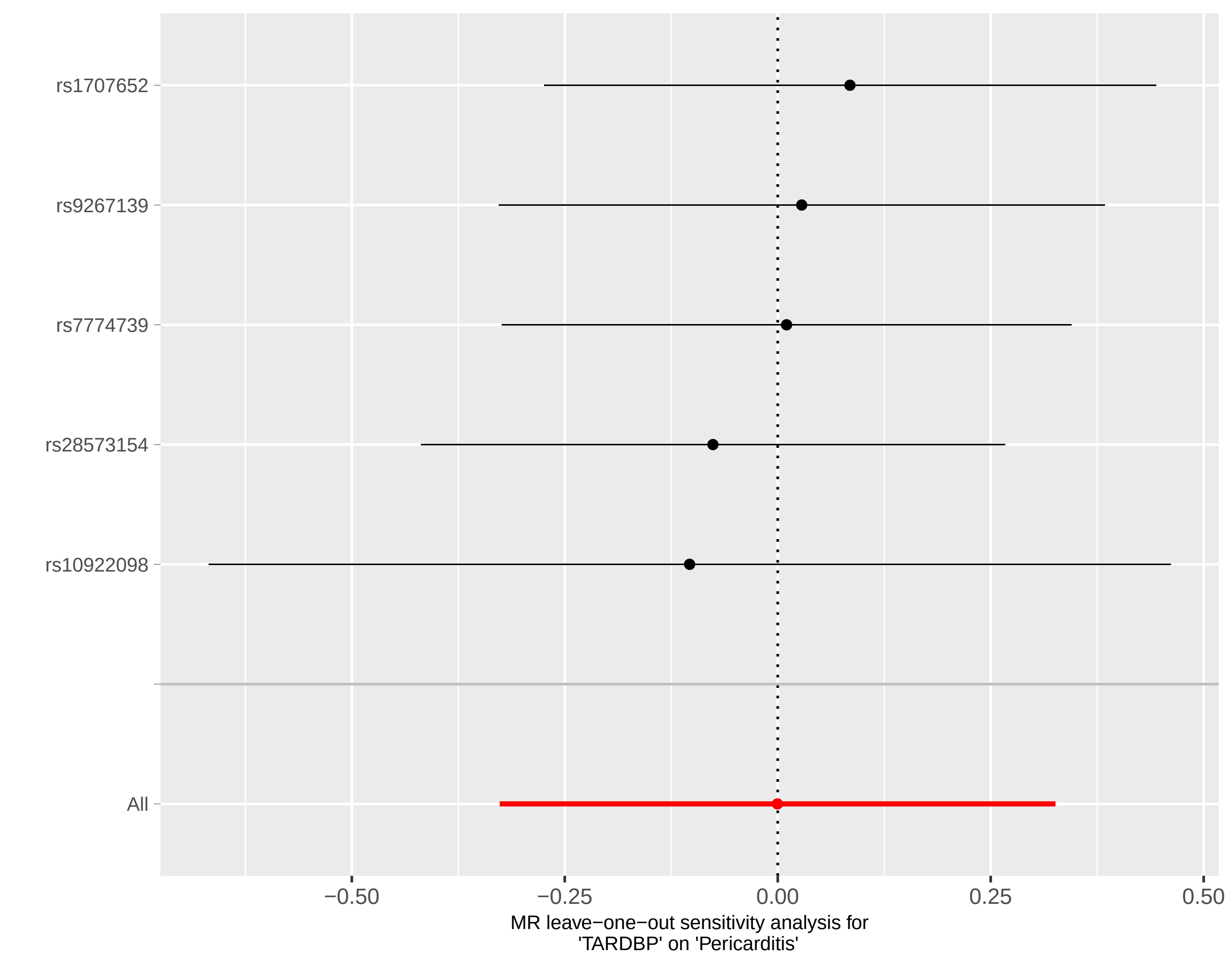

rs1707652
rs9267139
rs7774739
rs28573154
rs10922098
All
−0.50
−0.25
0.00
0.25
0.50
MR leave−one−out sensitivity analysis for
'TARDBP' on 'Pericarditis'

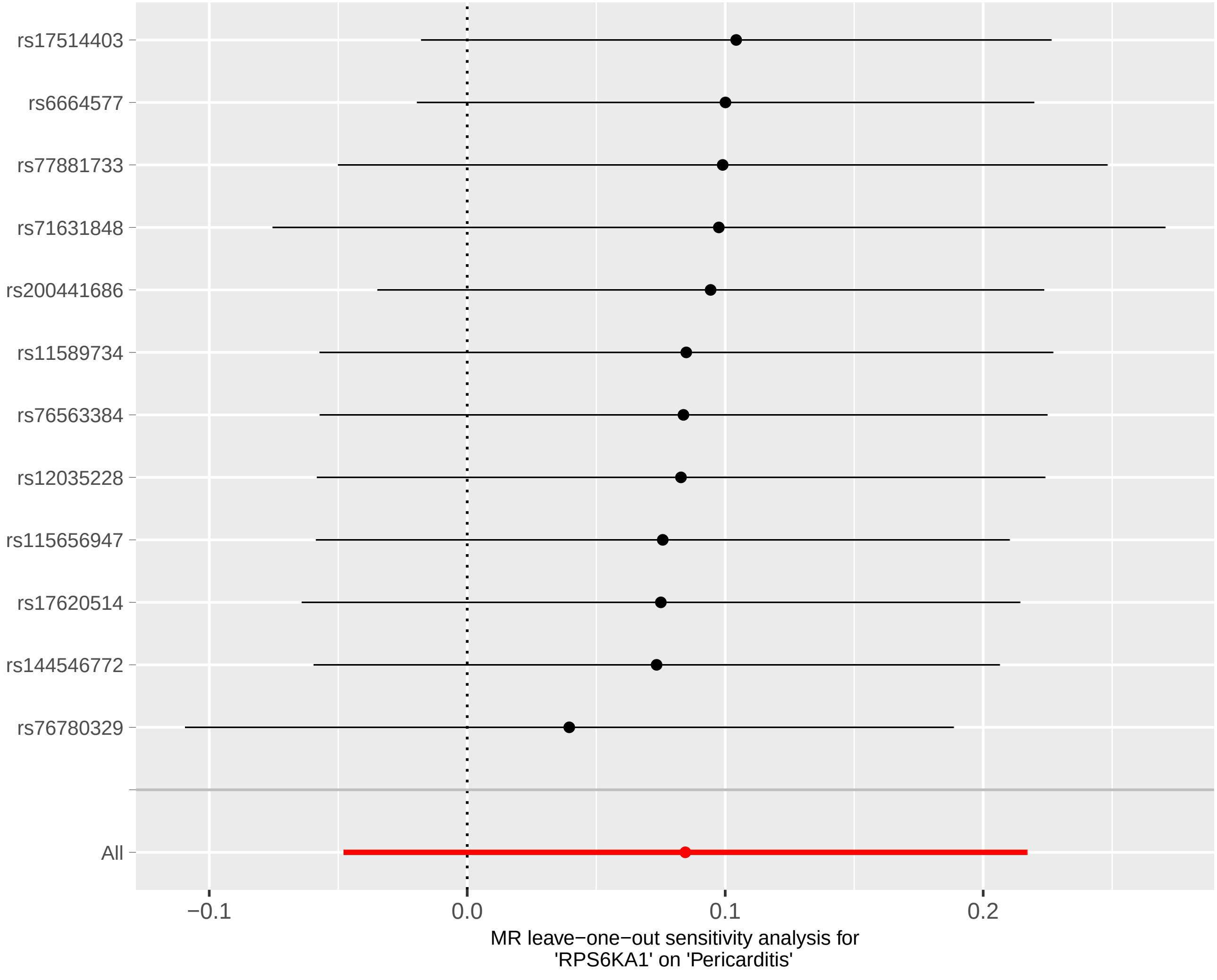

rs17514403
rs6664577
rs77881733
rs71631848
rs200441686
rs11589734
rs76563384
rs12035228
rs115656947
rs17620514
rs144546772
rs76780329
All
−0.1
0.0
0.1
0.2
MR leave−one−out sensitivity analysis for
'RPS6KA1' on 'Pericarditis'

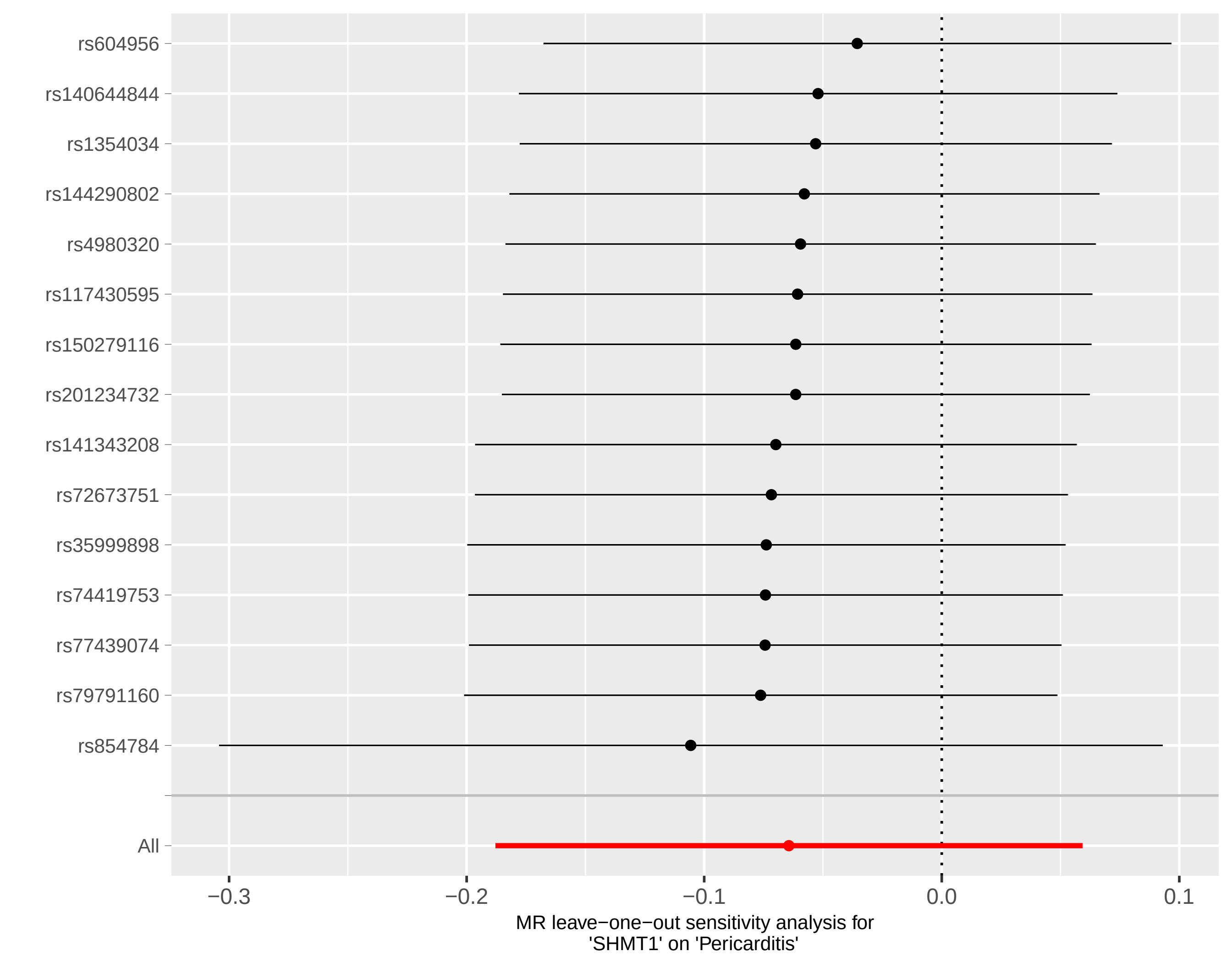
rs604956
rs140644844
rs1354034
rs144290802
rs4980320
rs117430595
rs150279116
rs201234732
rs141343208
rs72673751
rs35999898
rs74419753
rs77439074
rs79791160
rs854784
All
−0.3
−0.2
−0.1
0.0
0.1
MR leave−one−out sensitivity analysis for
'SHMT1' on 'Pericarditis'

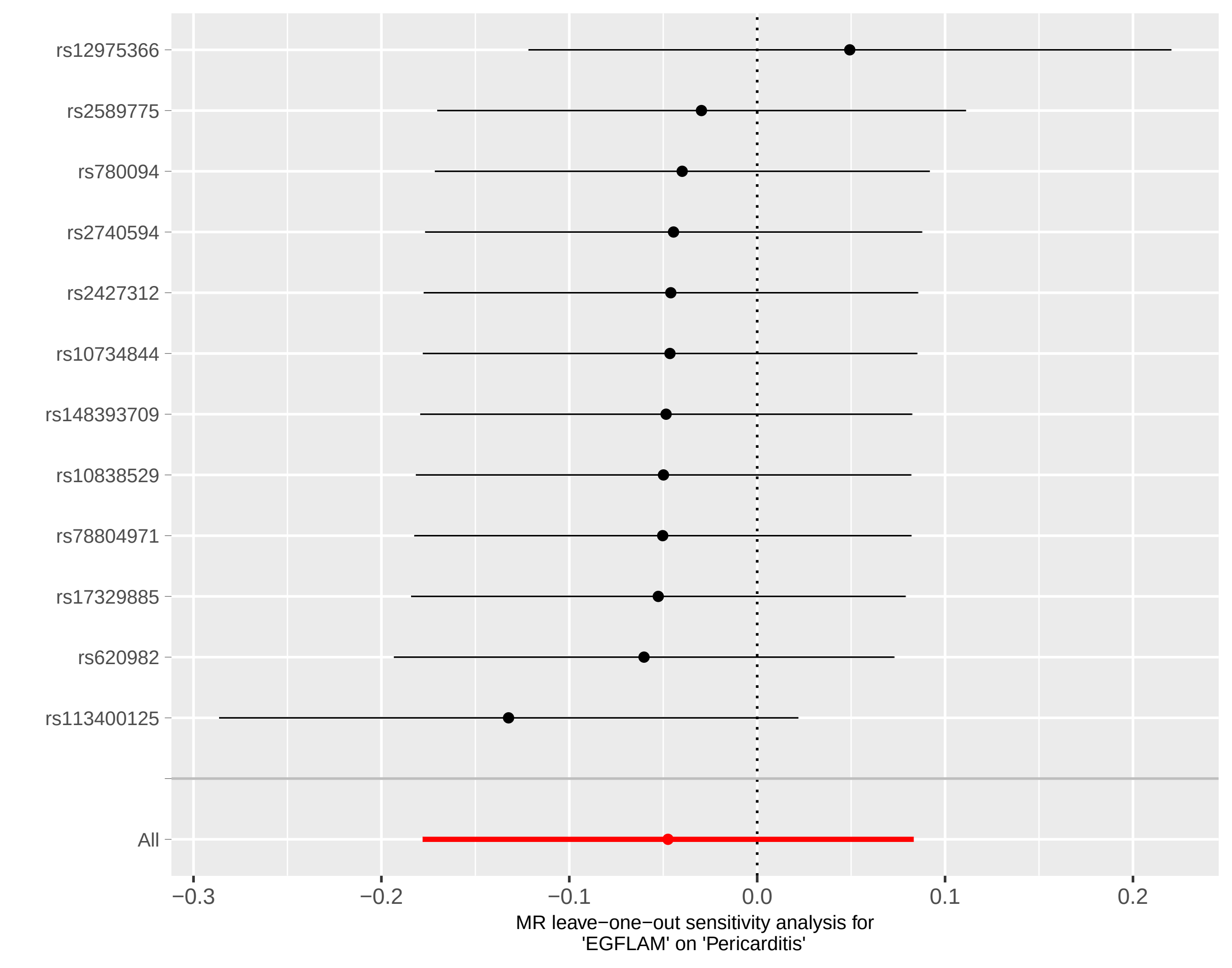
rs12975366
rs2589775
rs780094
rs2740594
rs2427312
rs10734844
rs148393709
rs10838529
rs78804971
rs17329885
rs620982
rs113400125
All
−0.3
−0.2
−0.1
0.0
0.1
0.2
MR leave−one−out sensitivity analysis for
'EGFLAM' on 'Pericarditis'

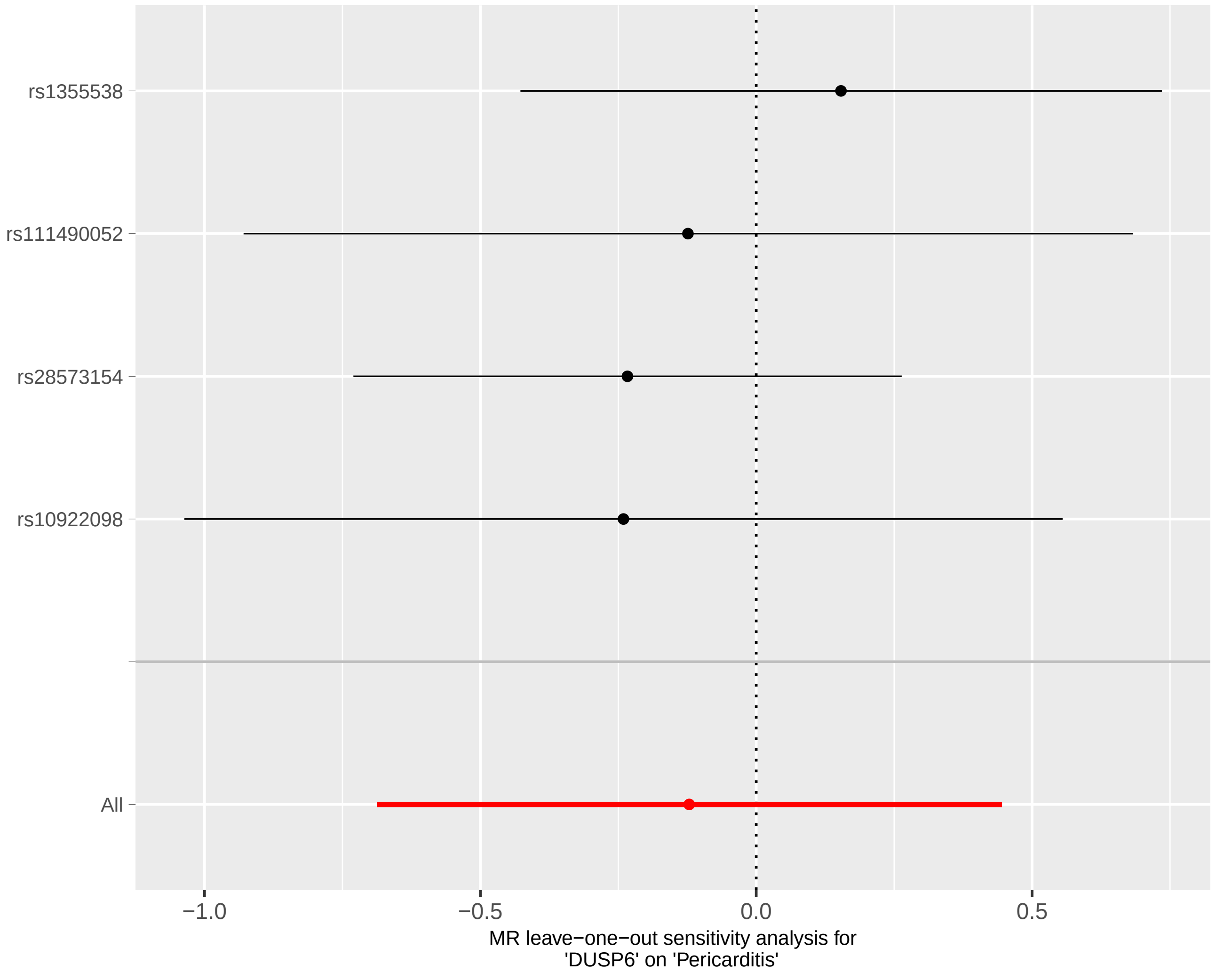

rs1355538
rs111490052
rs28573154
rs10922098
All
−1.0
−0.5
0.0
0.5
MR leave−one−out sensitivity analysis for
'DUSP6' on 'Pericarditis'

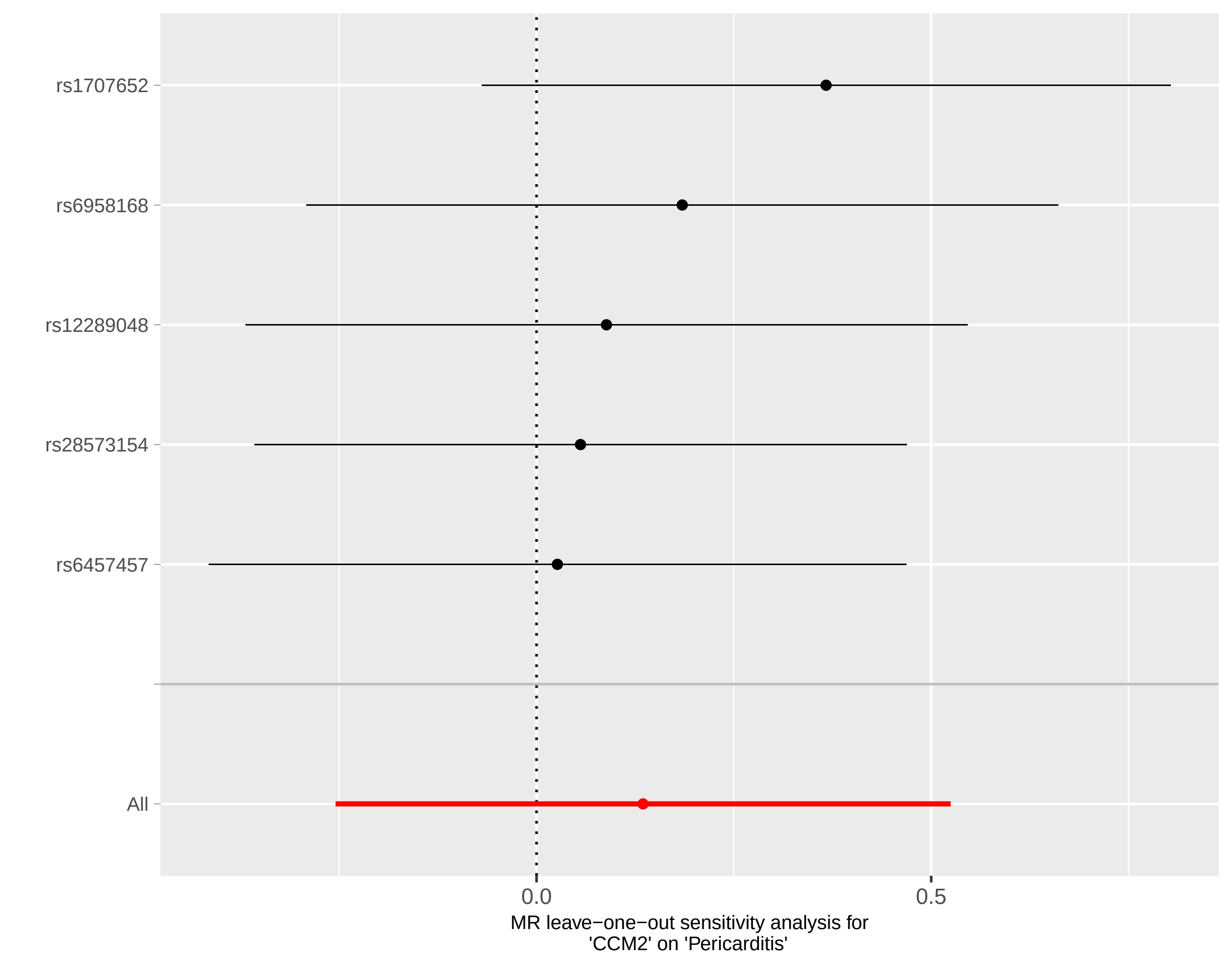
rs1707652
rs6958168
rs12289048
rs28573154
rs6457457
All
0.0
0.5
MR leave−one−out sensitivity analysis for
'CCM2' on 'Pericarditis'

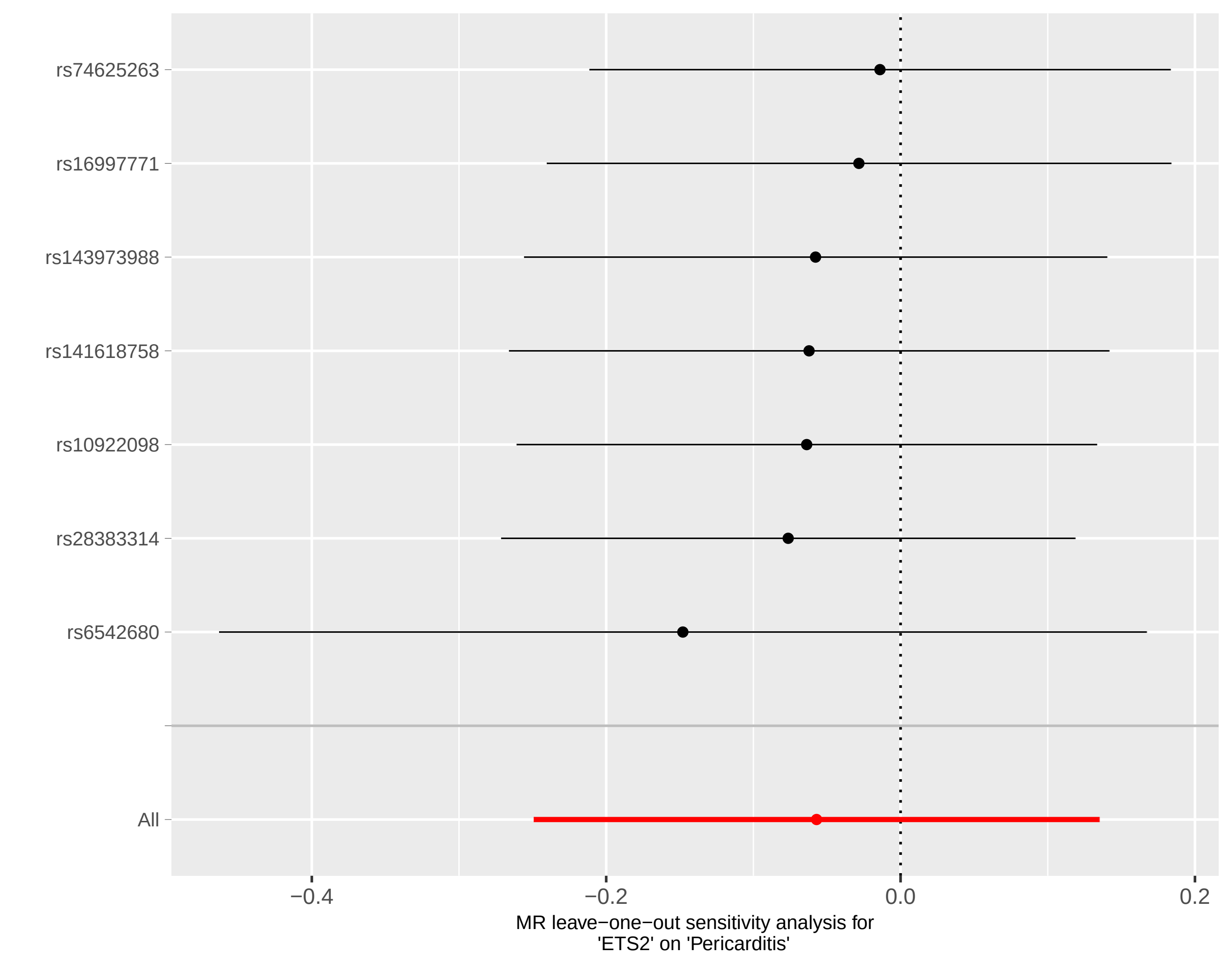
rs74625263
rs16997771
rs143973988
rs141618758
rs10922098
rs28383314
rs6542680
All
−0.4
−0.2
0.0
0.2
MR leave−one−out sensitivity analysis for
'ETS2' on 'Pericarditis'

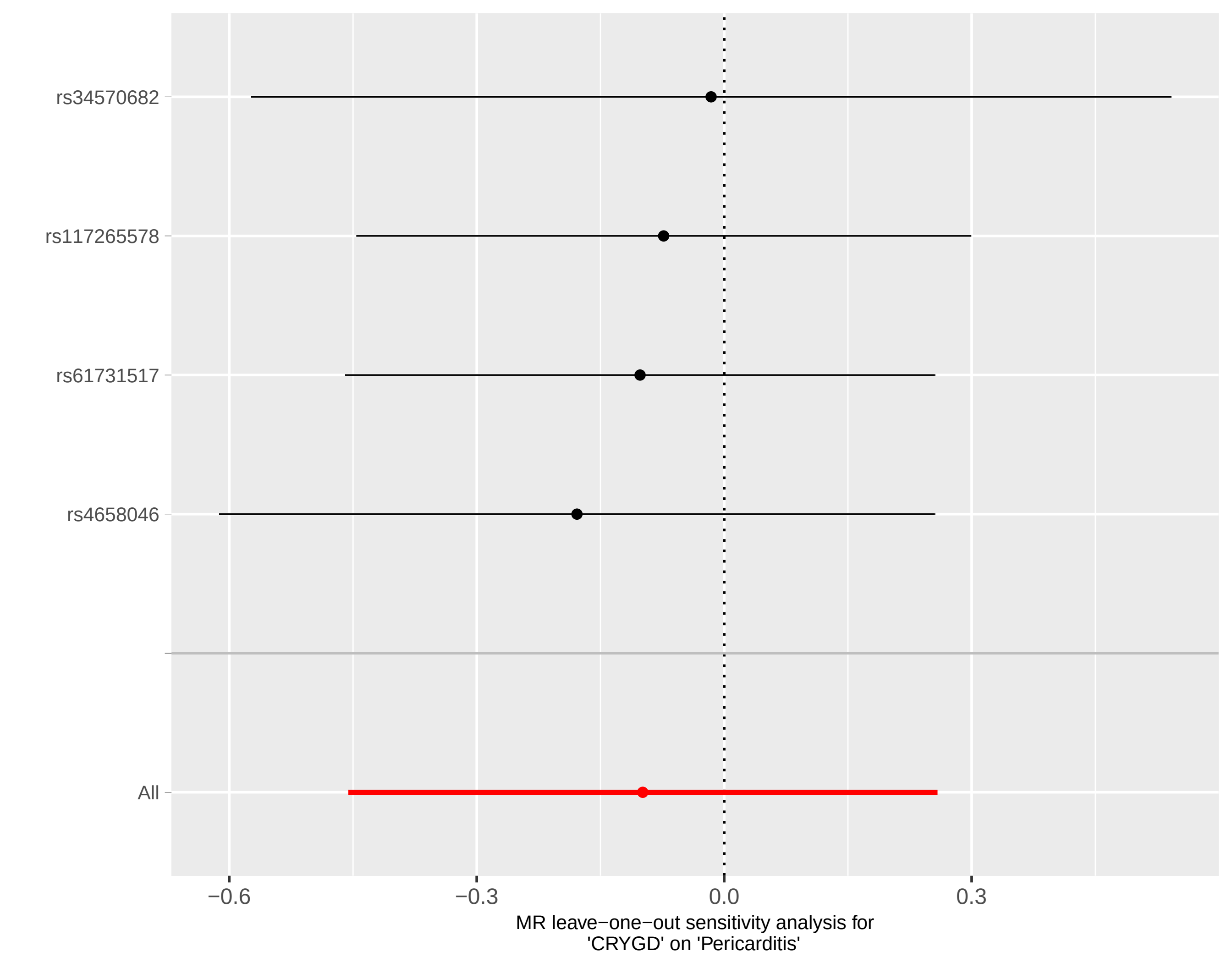
rs34570682
rs117265578
rs61731517
rs4658046
All
−0.6
−0.3
0.0
0.3
MR leave−one−out sensitivity analysis for
'CRYGD' on 'Pericarditis'

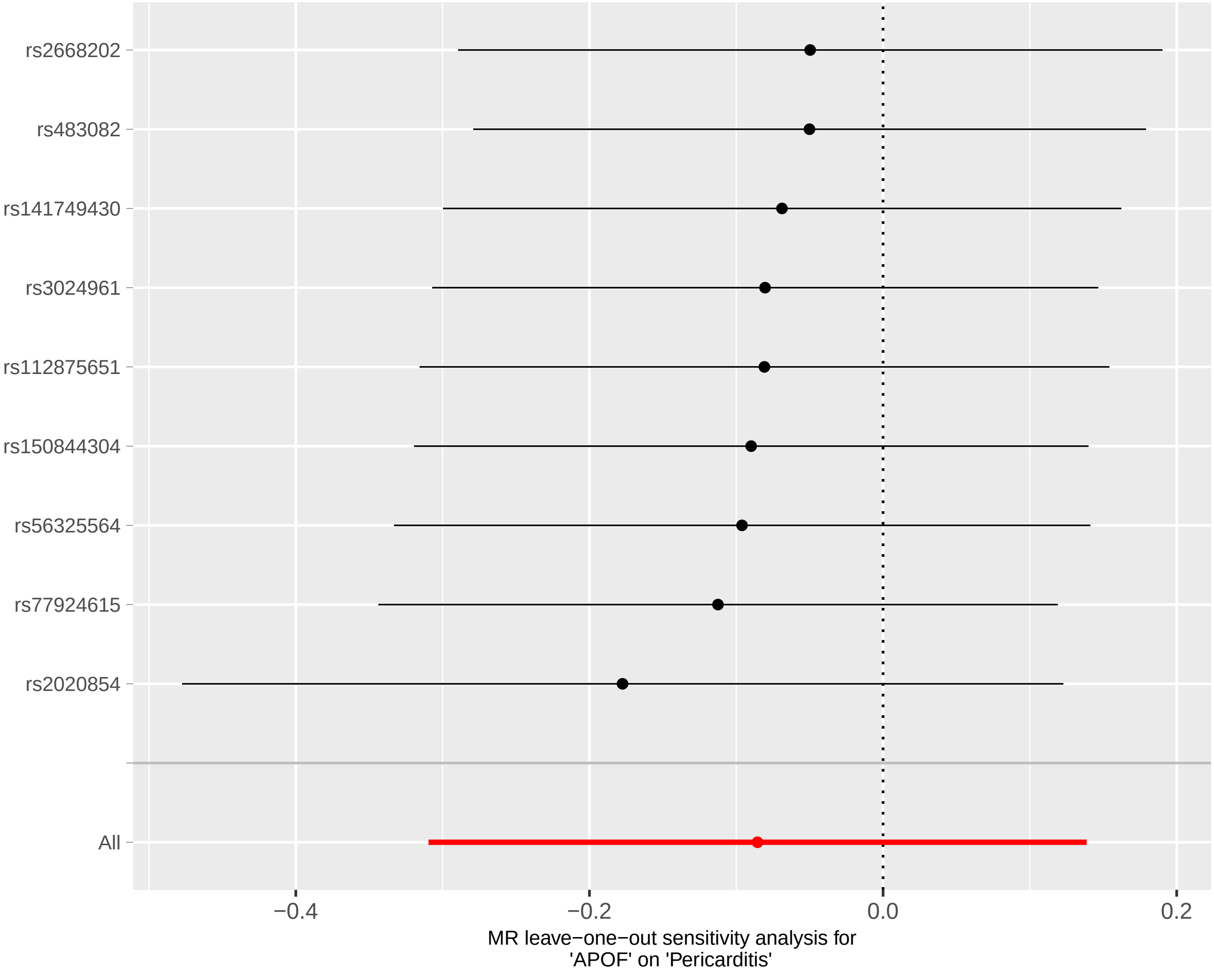
rs2668202
rs483082
rs141749430
rs3024961
rs112875651
rs150844304
rs56325564
rs77924615
rs2020854
All
−0.4
−0.2
0.0
0.2
MR leave−one−out sensitivity analysis for
'APOF' on 'Pericarditis'

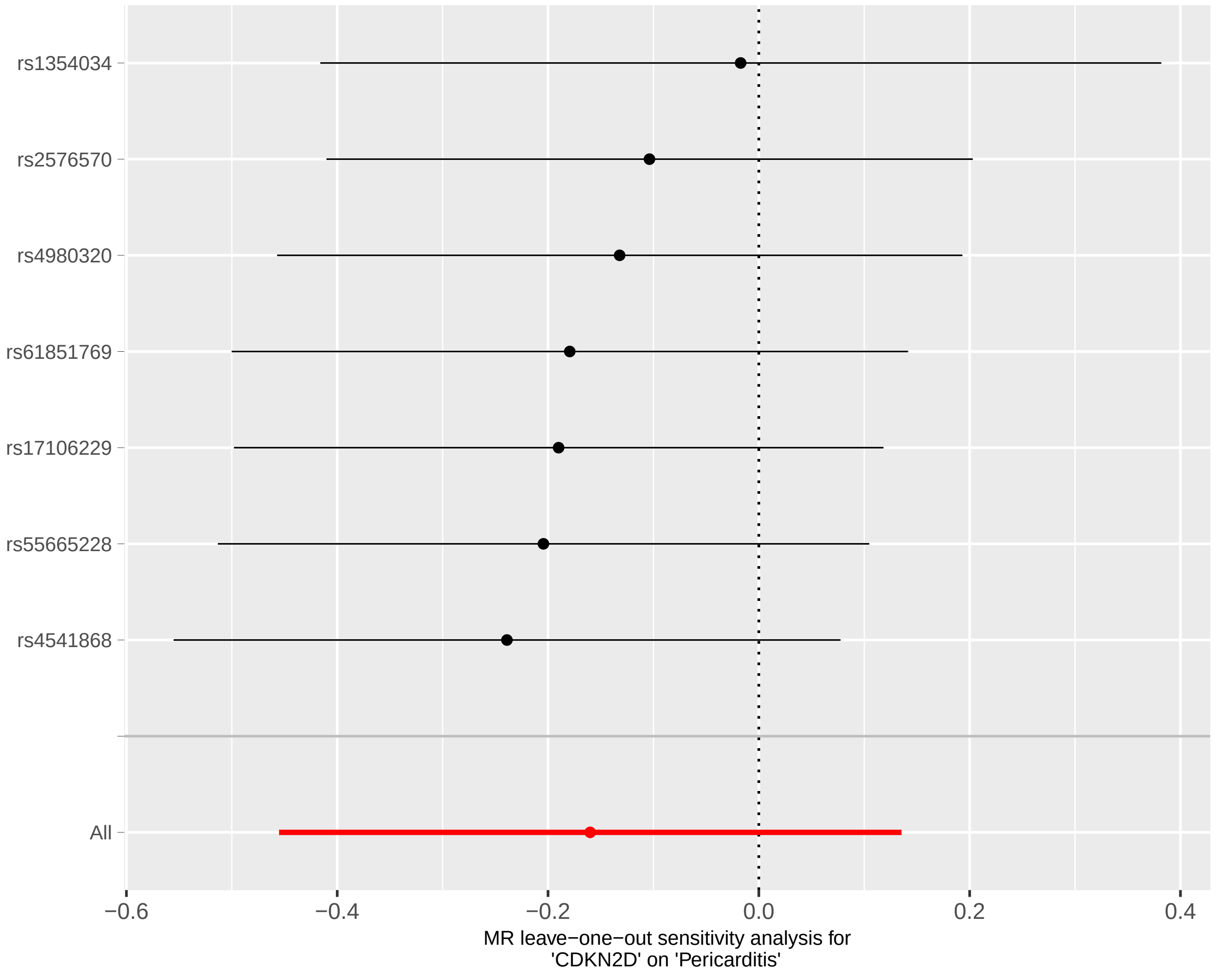

rs1354034
rs2576570
rs4980320
rs61851769
rs17106229
rs55665228
rs4541868
All
−0.6
−0.4
−0.2
0.0
0.2
0.4
MR leave−one−out sensitivity analysis for
'CDKN2D' on 'Pericarditis'

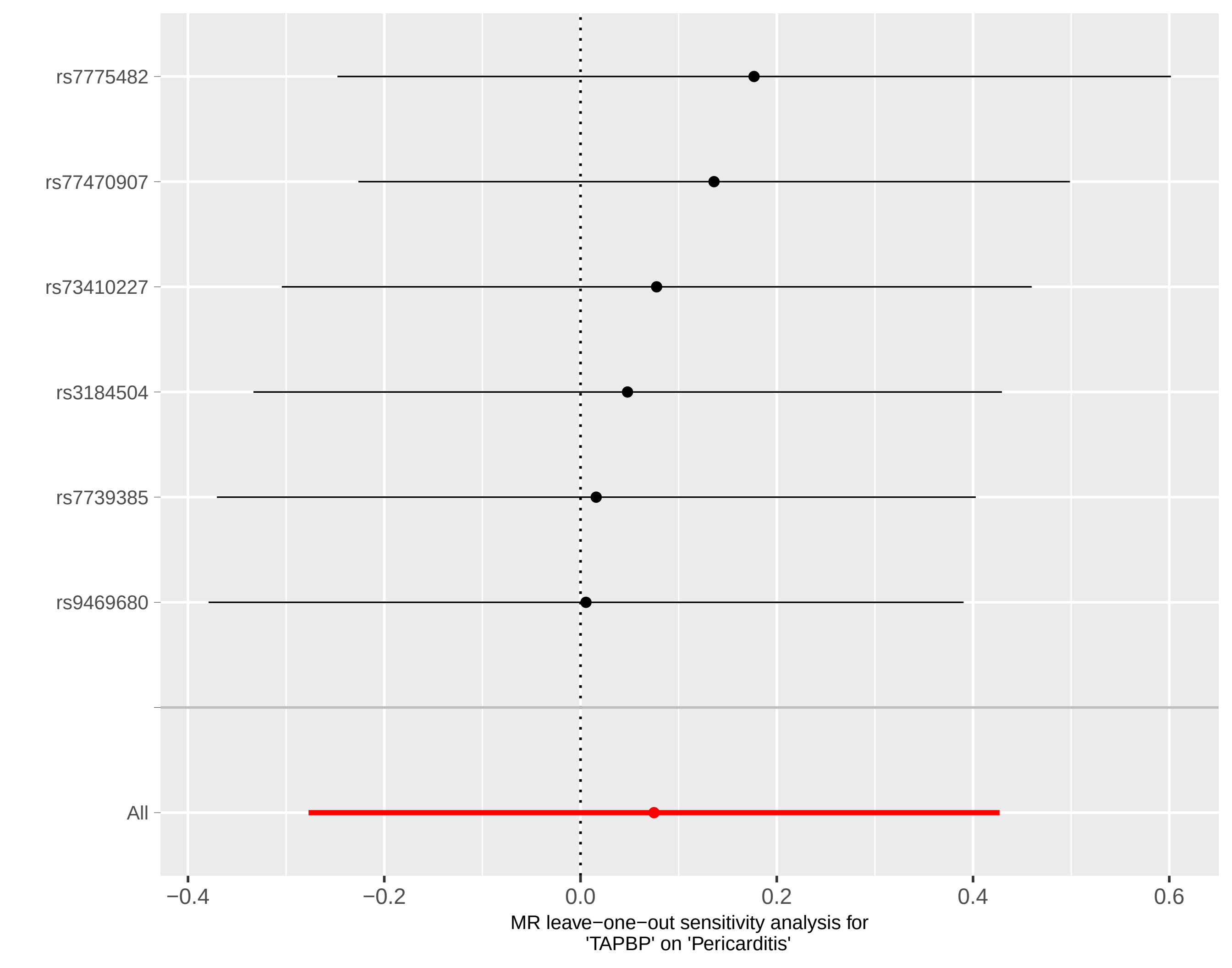
rs7775482
rs77470907
rs73410227
rs3184504
rs7739385
rs9469680
All
−0.4
−0.2
0.0
0.2
0.4
0.6
MR leave−one−out sensitivity analysis for
'TAPBP' on 'Pericarditis'

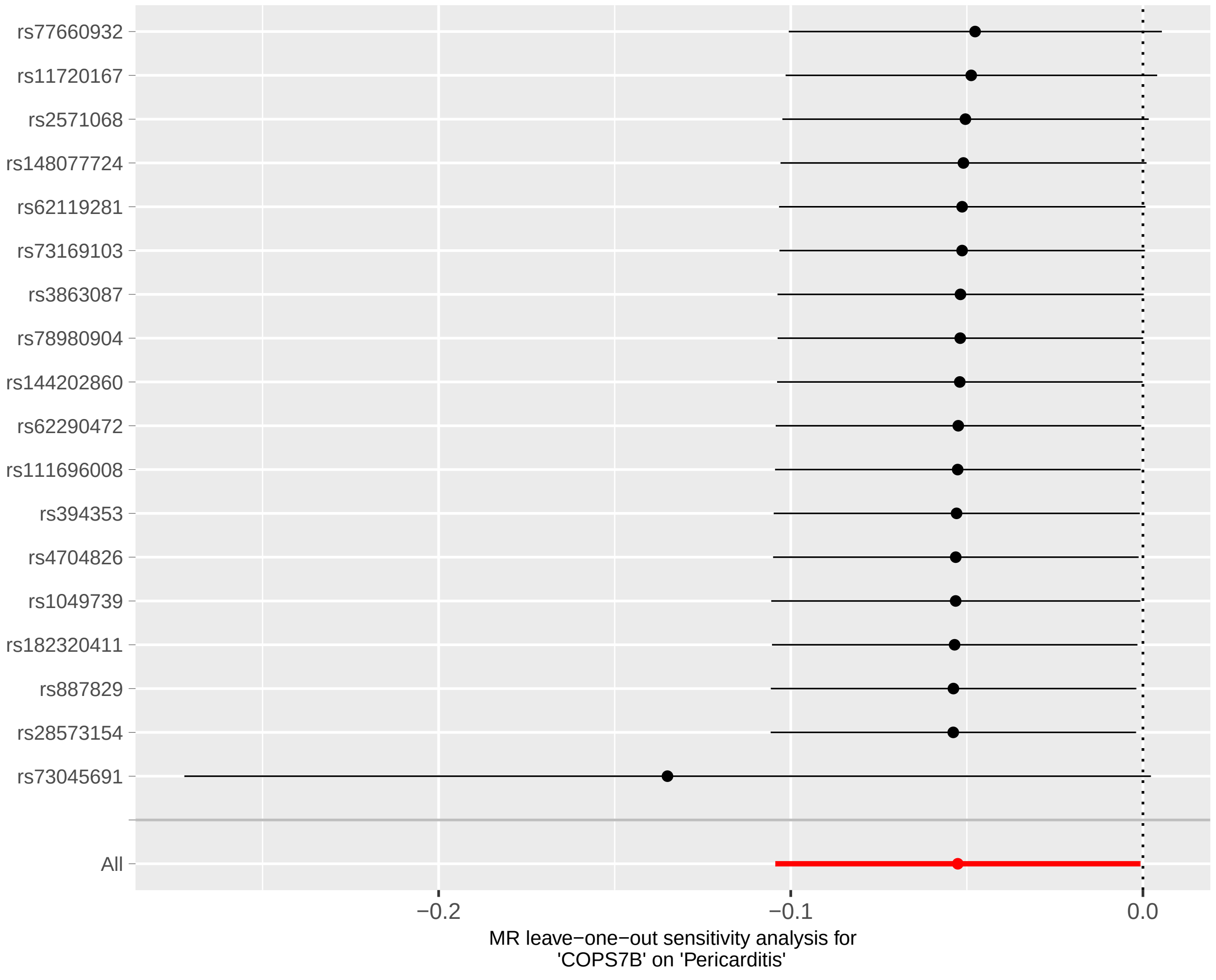
rs77660932
rs11720167
rs2571068
rs148077724
rs62119281
rs73169103
rs3863087
rs78980904
rs144202860
rs62290472
rs111696008
rs394353
rs4704826
rs1049739
rs182320411
rs887829
rs28573154
rs73045691
All
−0.2
−0.1
0.0
MR leave−one−out sensitivity analysis for
'COPS7B' on 'Pericarditis'

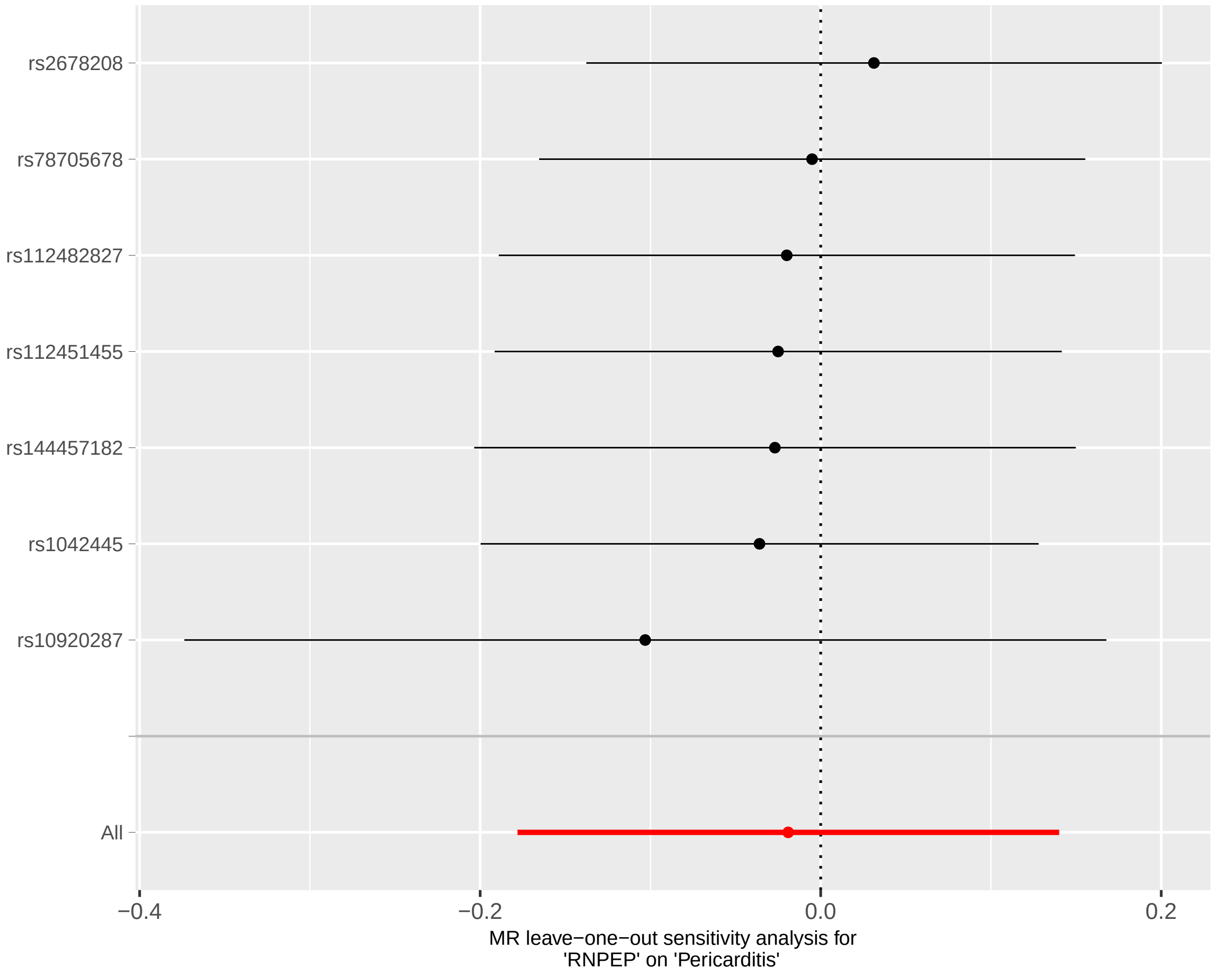

rs2678208
rs78705678
rs112482827
rs112451455
rs144457182
rs1042445
rs10920287
All
−0.4
−0.2
0.0
0.2
MR leave−one−out sensitivity analysis for
'RNPEP' on 'Pericarditis'

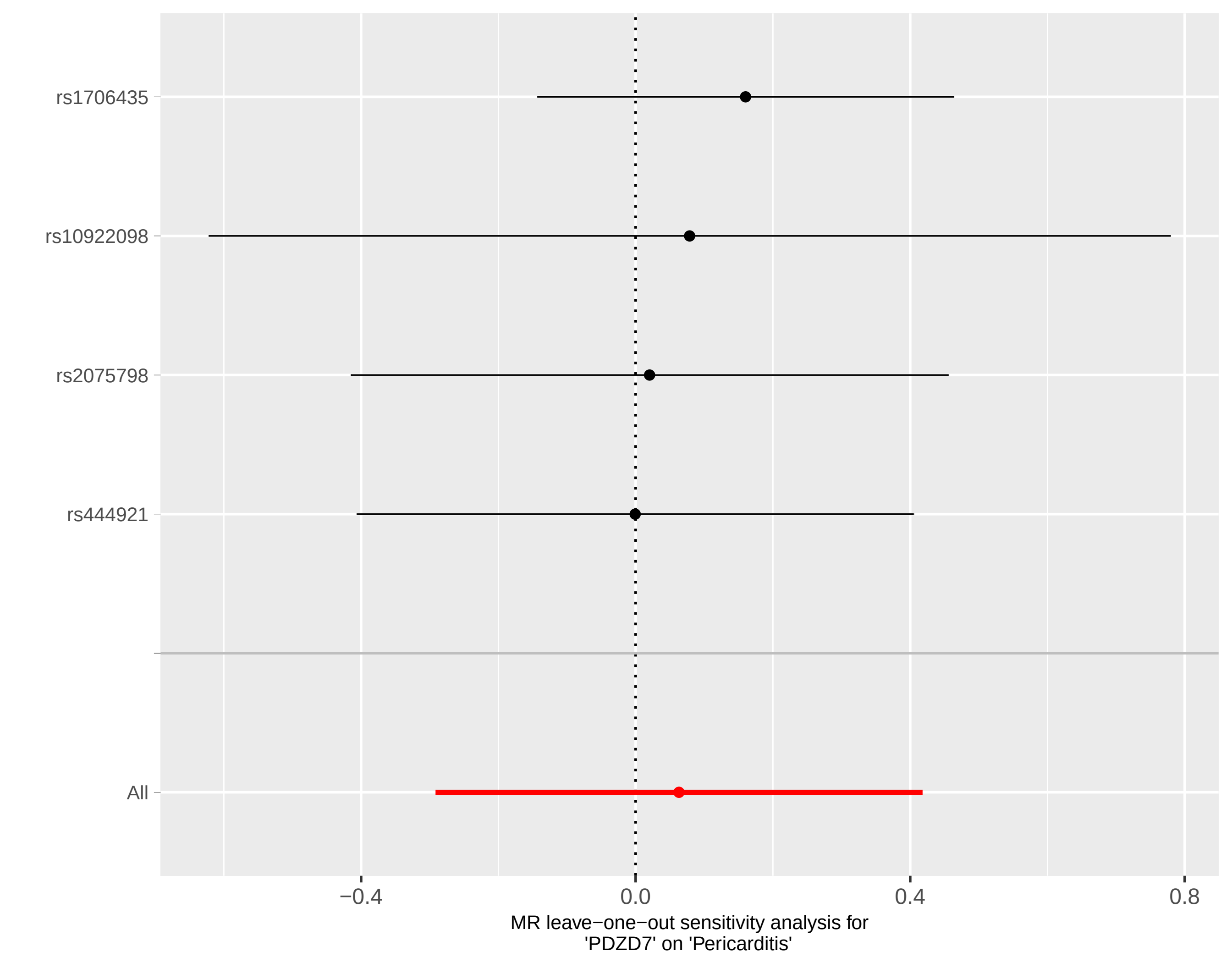
rs1706435
rs10922098
rs2075798
rs444921
All
−0.4
0.0
0.4
0.8
MR leave−one−out sensitivity analysis for
'PDZD7' on 'Pericarditis'

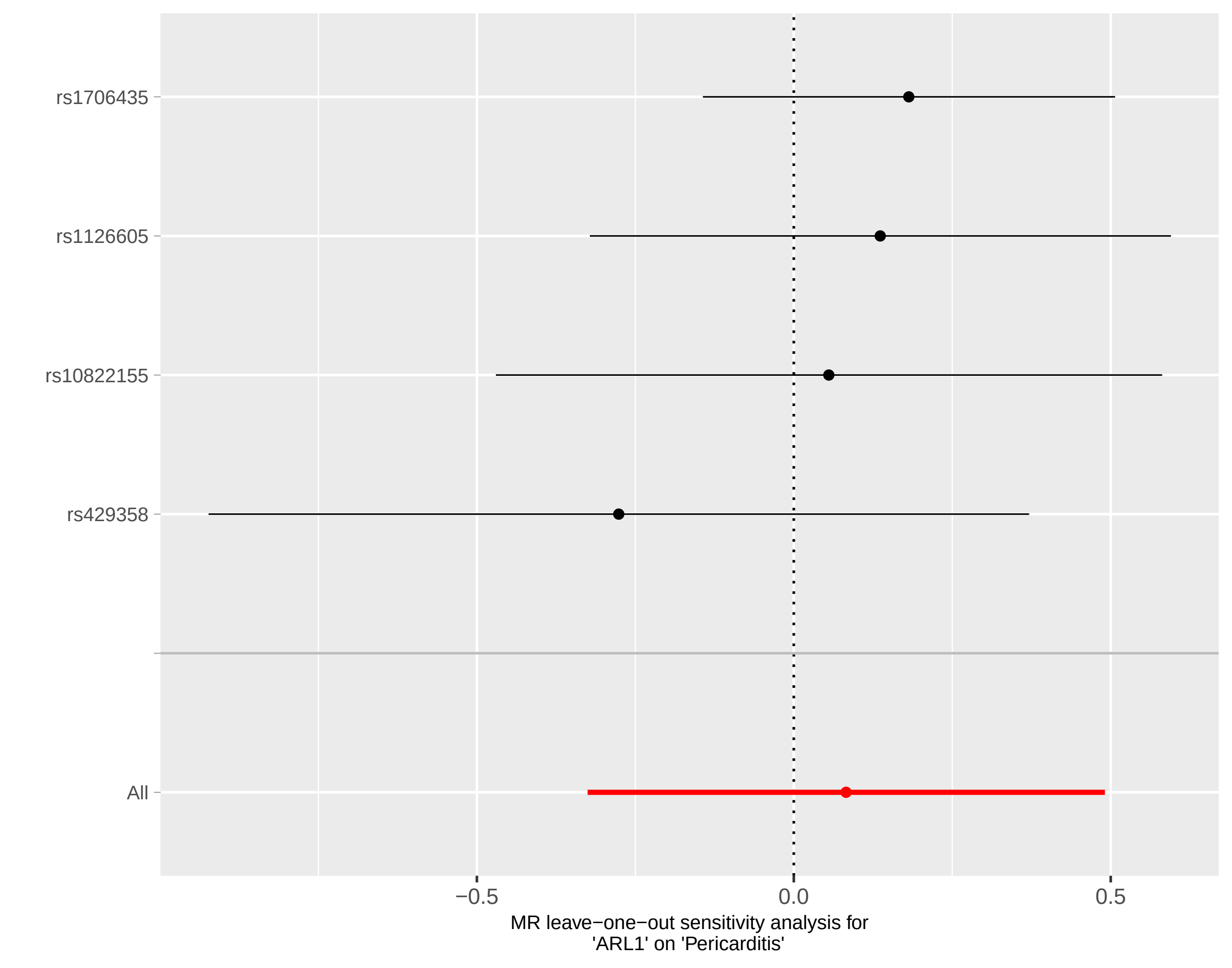
rs1706435
rs1126605
rs10822155
rs429358
All
−0.5
0.0
0.5
MR leave−one−out sensitivity analysis for
'ARL1' on 'Pericarditis'

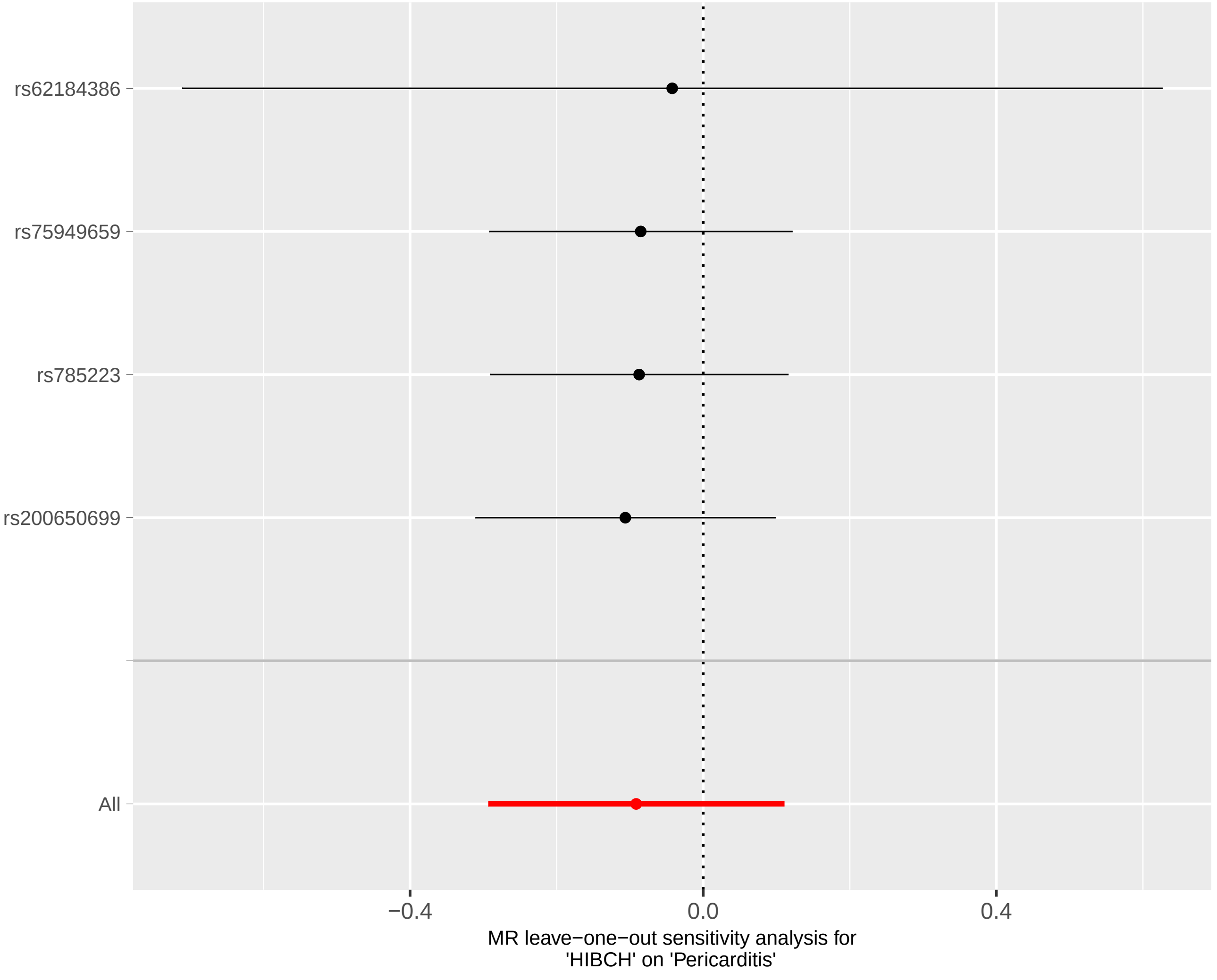
rs62184386
rs75949659
rs785223
rs200650699
All
−0.4
0.0
0.4
MR leave−one−out sensitivity analysis for
'HIBCH' on 'Pericarditis'

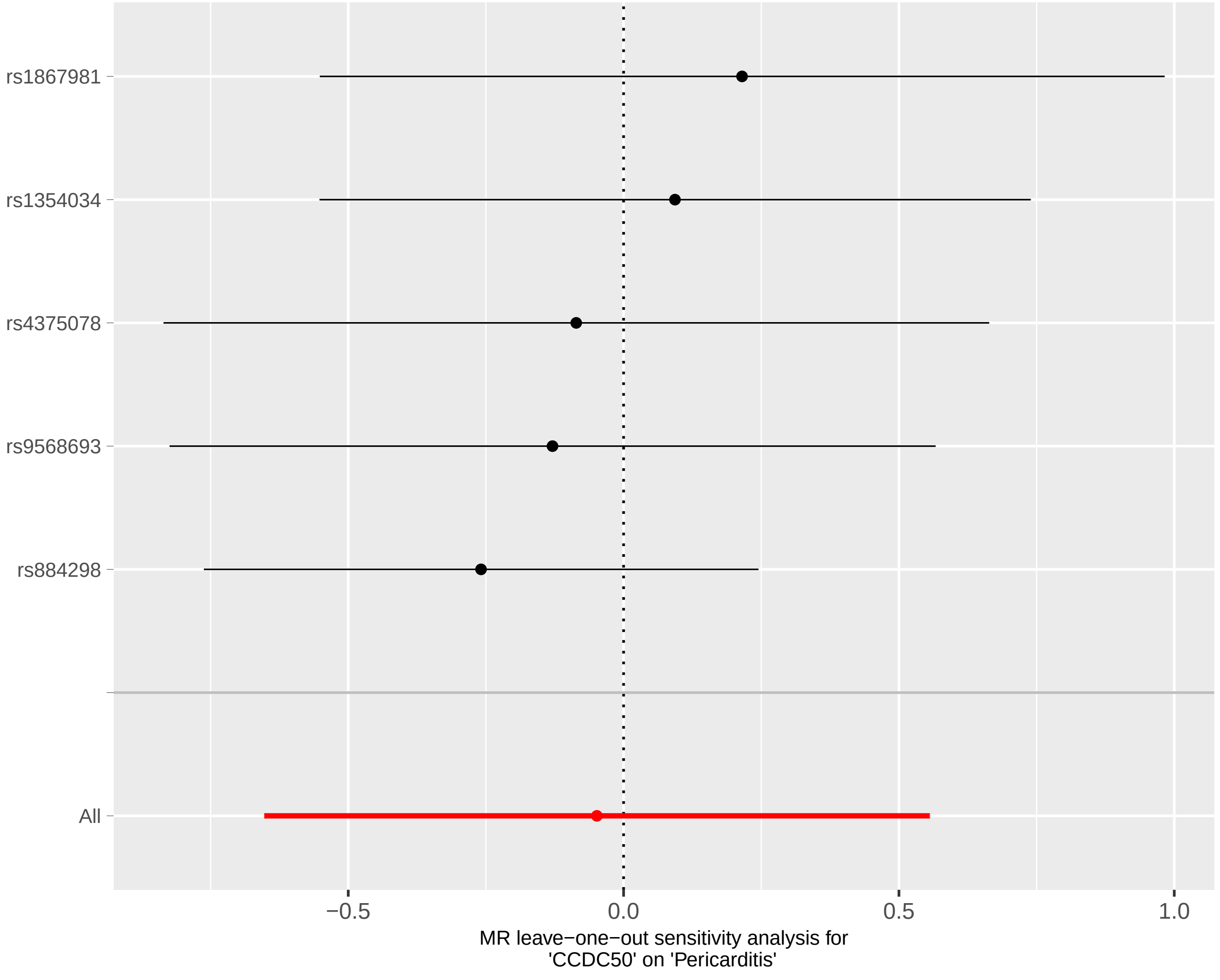
rs1867981
rs1354034
rs4375078
rs9568693
rs884298
All
−0.5
0.0
0.5
1.0
MR leave−one−out sensitivity analysis for
'CCDC50' on 'Pericarditis'

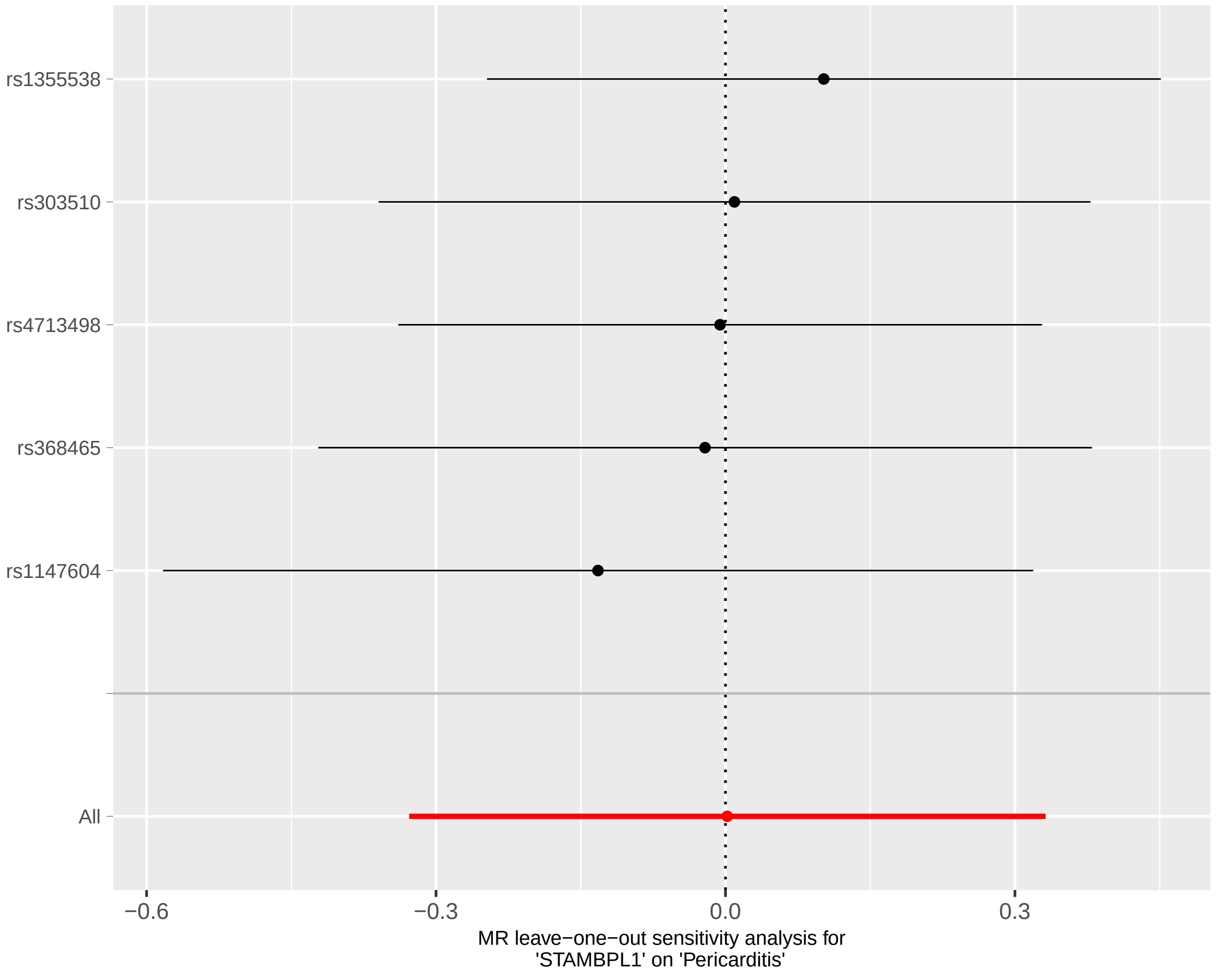
rs1355538
rs303510
rs4713498
rs368465
rs1147604
All
−0.6
−0.3
0.0
0.3
MR leave−one−out sensitivity analysis for
'STAMBPL1' on 'Pericarditis'

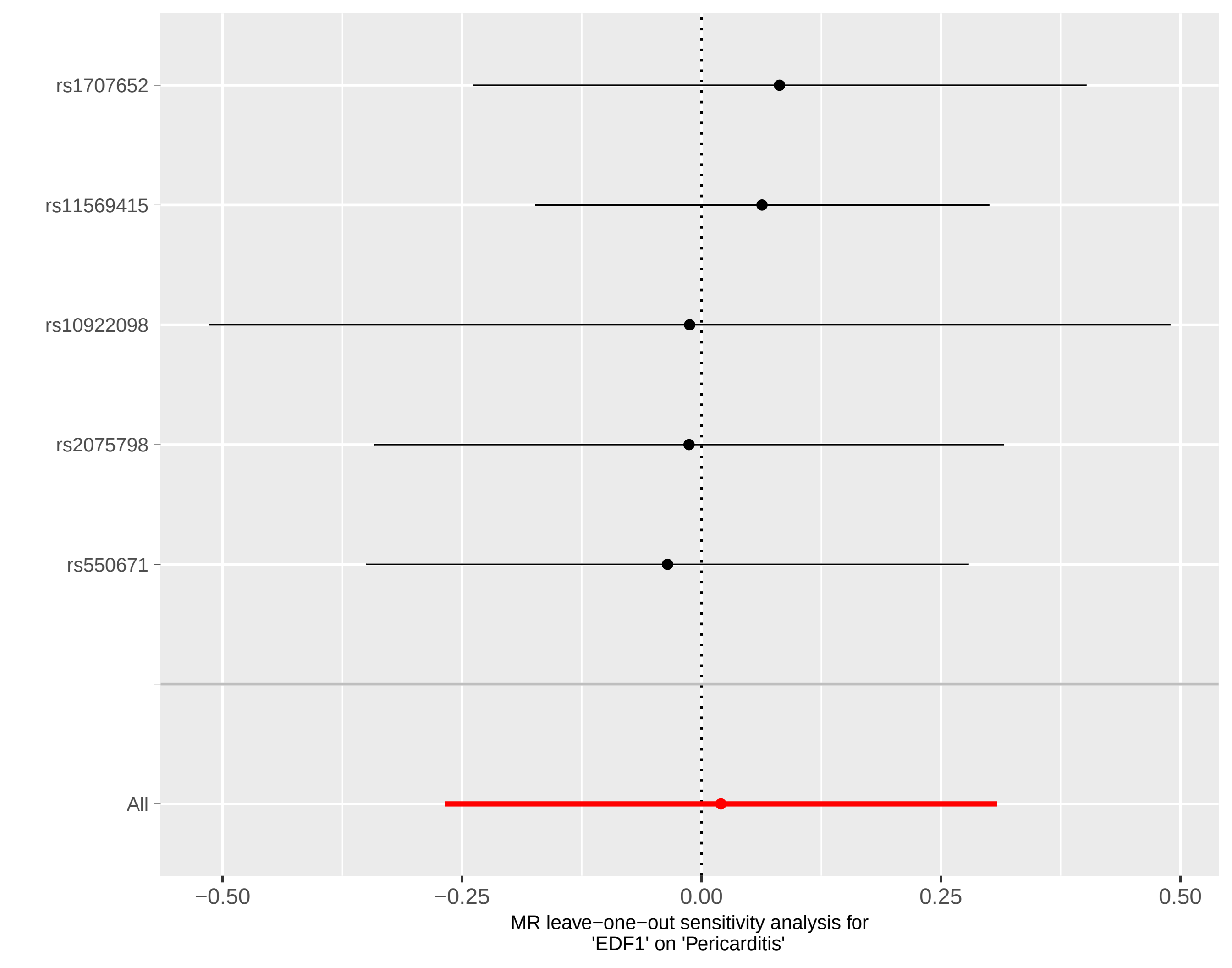
rs1707652
rs11569415
rs10922098
rs2075798
rs550671
All
−0.50
−0.25
0.00
0.25
0.50
MR leave−one−out sensitivity analysis for
'EDF1' on 'Pericarditis'

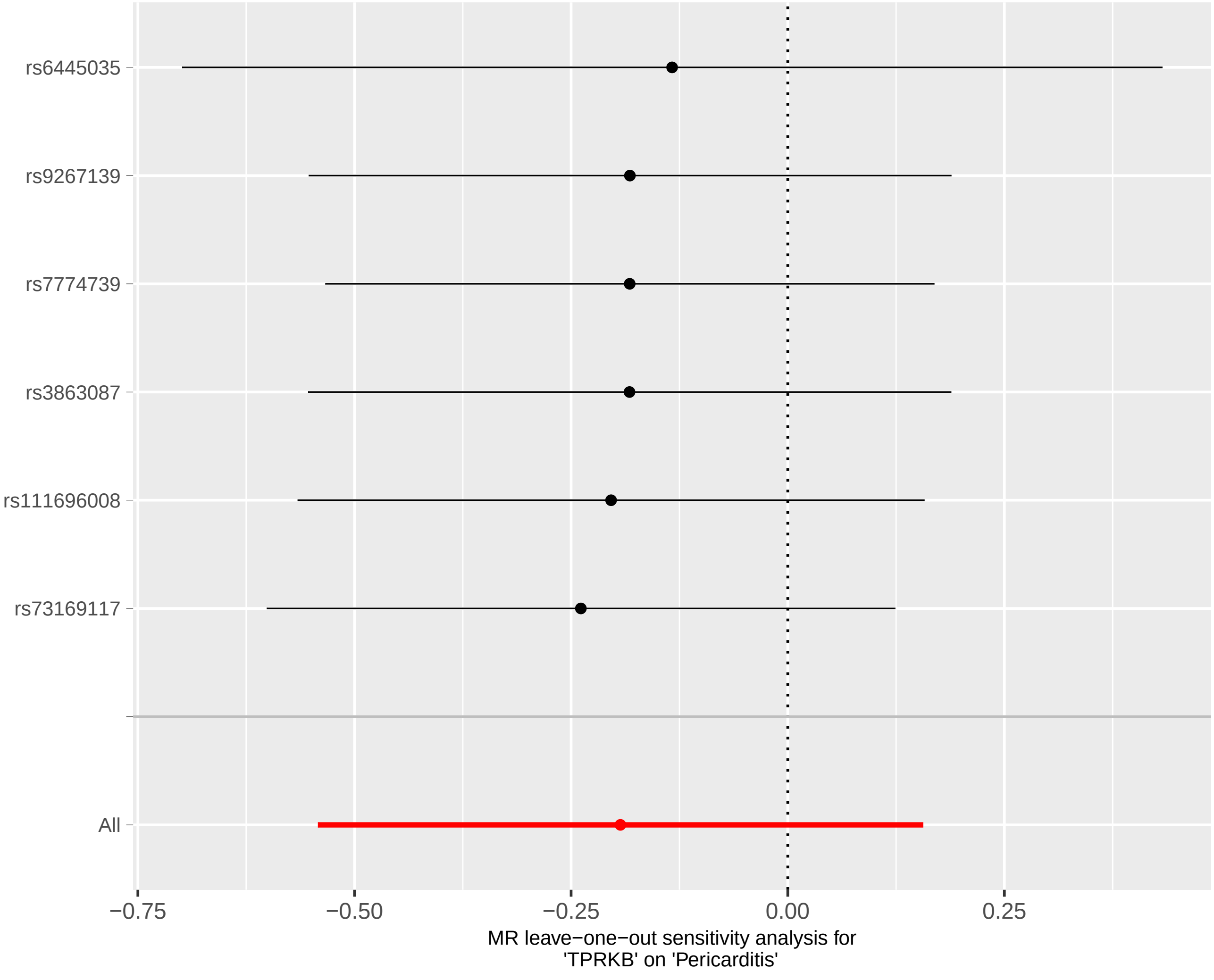
rs6445035
rs9267139
rs7774739
rs3863087
rs111696008
rs73169117
All
−0.75
−0.50
−0.25
0.00
0.25
MR leave−one−out sensitivity analysis for
'TPRKB' on 'Pericarditis'

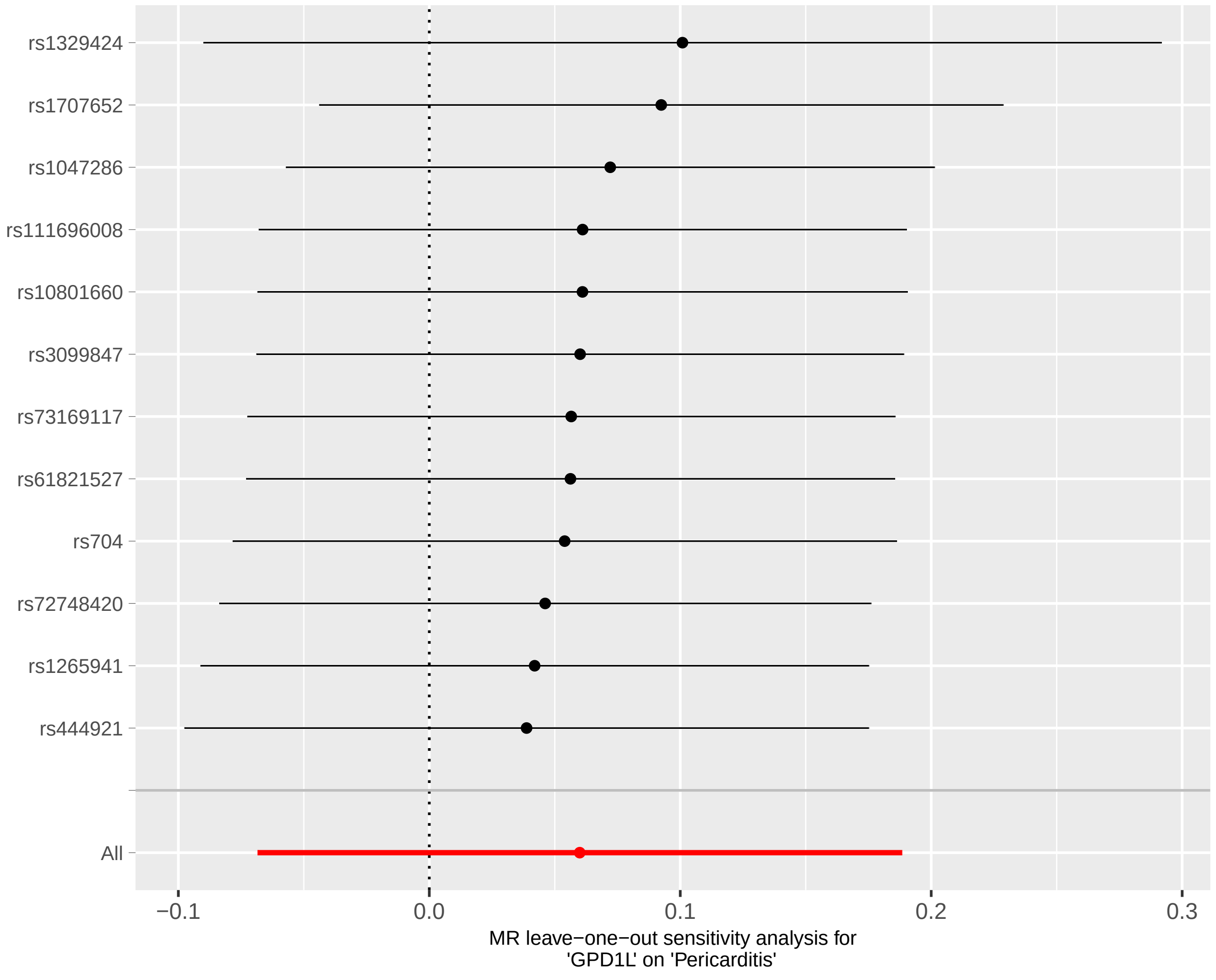

rs1329424
rs1707652
rs1047286
rs111696008
rs10801660
rs3099847
rs73169117
rs61821527
rs704
rs72748420
rs1265941
rs444921
All
−0.1
0.0
0.1
0.2
0.3
MR leave−one−out sensitivity analysis for
'GPD1L' on 'Pericarditis'

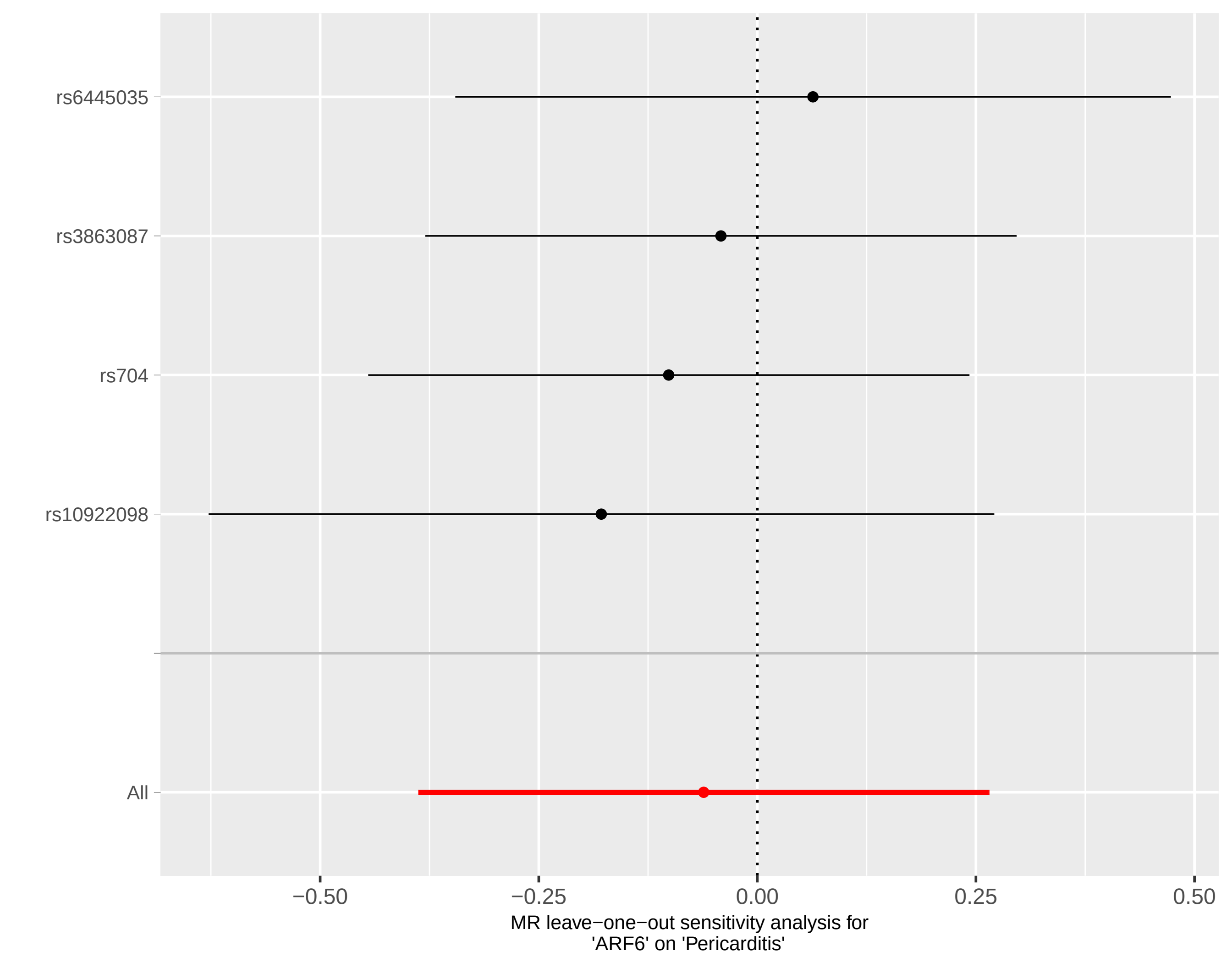
rs6445035
rs3863087
rs704
rs10922098
All
−0.50
−0.25
0.00
0.25
0.50
MR leave−one−out sensitivity analysis for
'ARF6' on 'Pericarditis'

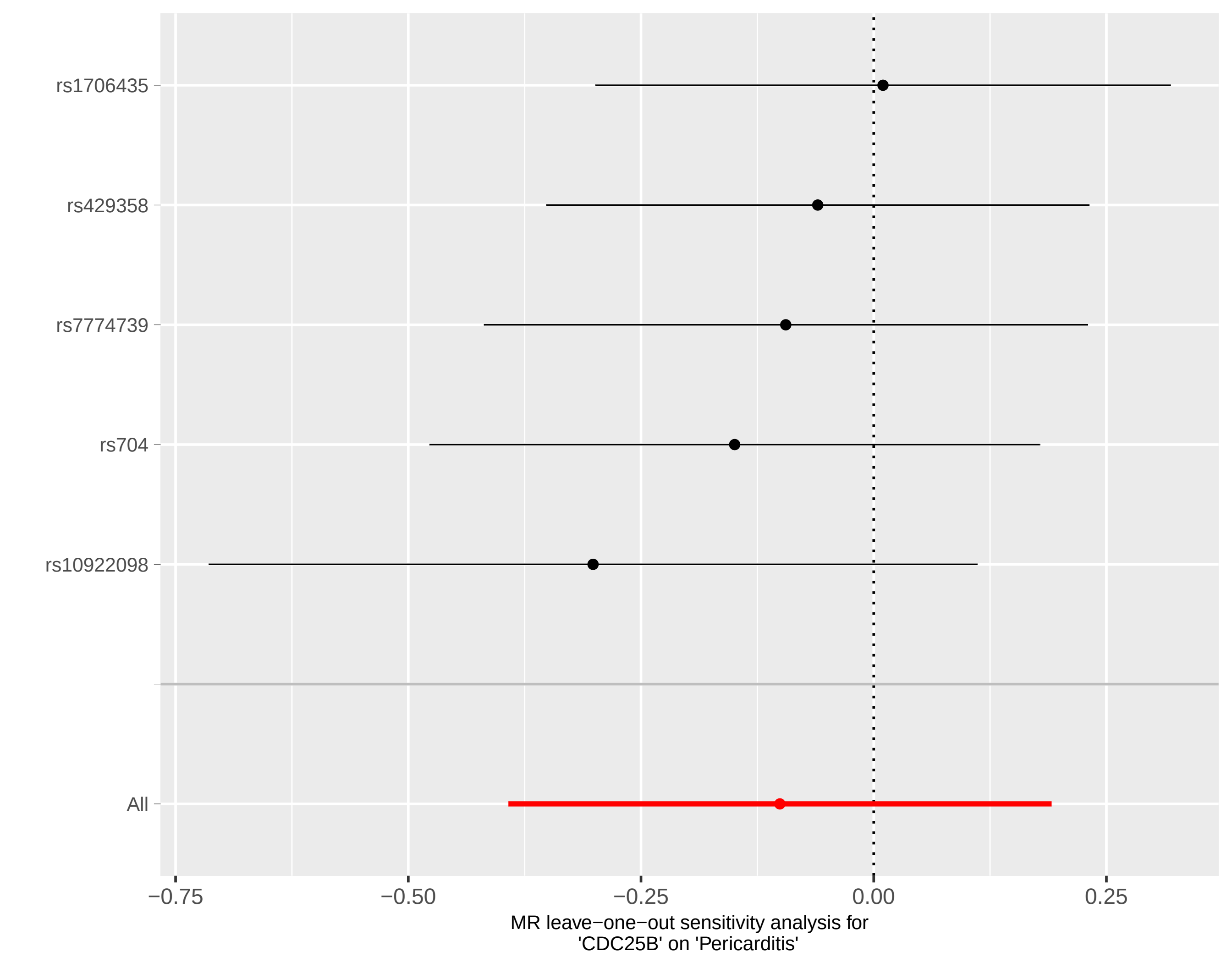
rs1706435
rs429358
rs7774739
rs704
rs10922098
All
−0.75
−0.50
−0.25
0.00
0.25
MR leave−one−out sensitivity analysis for
'CDC25B' on 'Pericarditis'

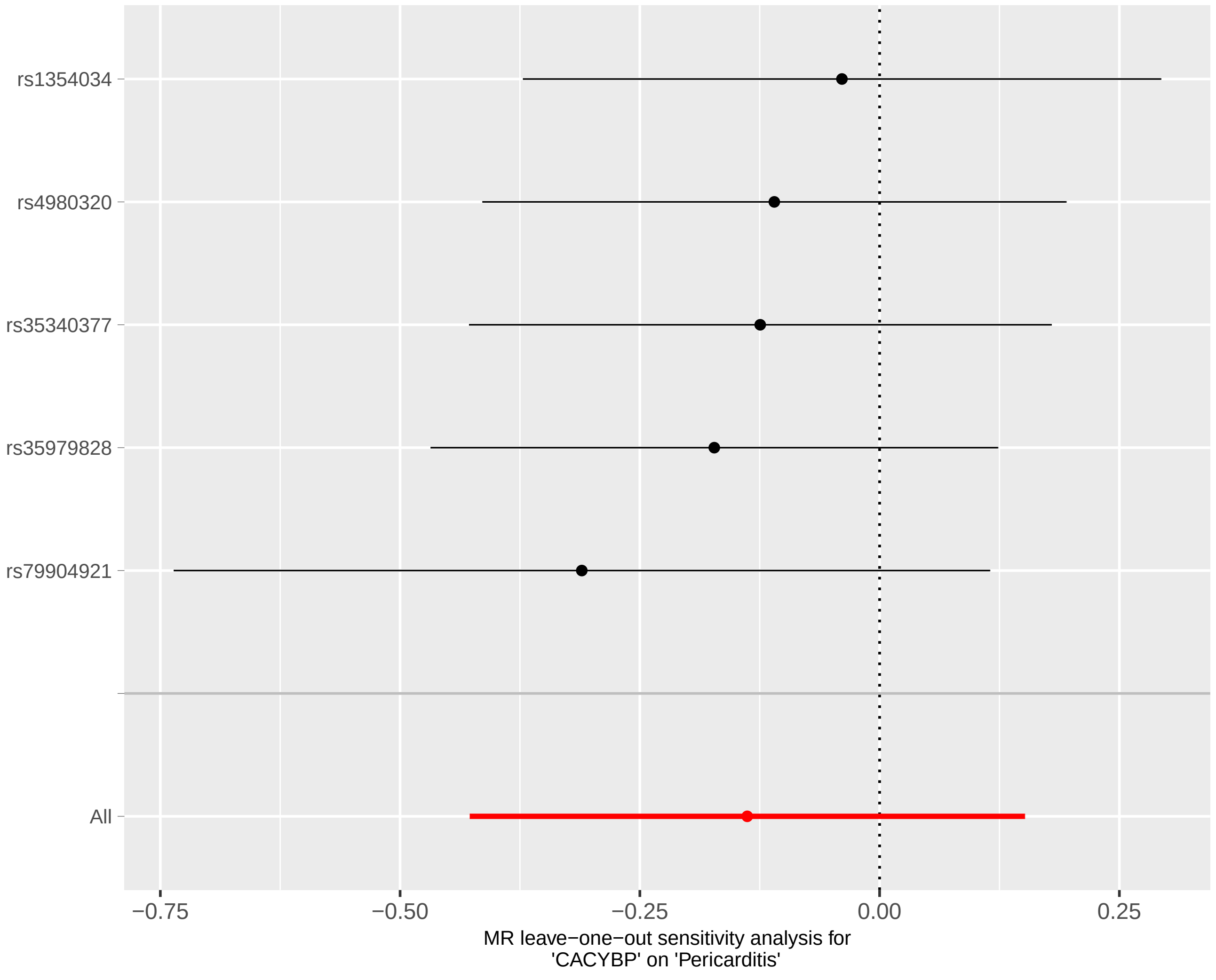

rs1354034
rs4980320
rs35340377
rs35979828
rs79904921
All
−0.75
−0.50
−0.25
0.00
0.25
MR leave−one−out sensitivity analysis for
'CACYBP' on 'Pericarditis'

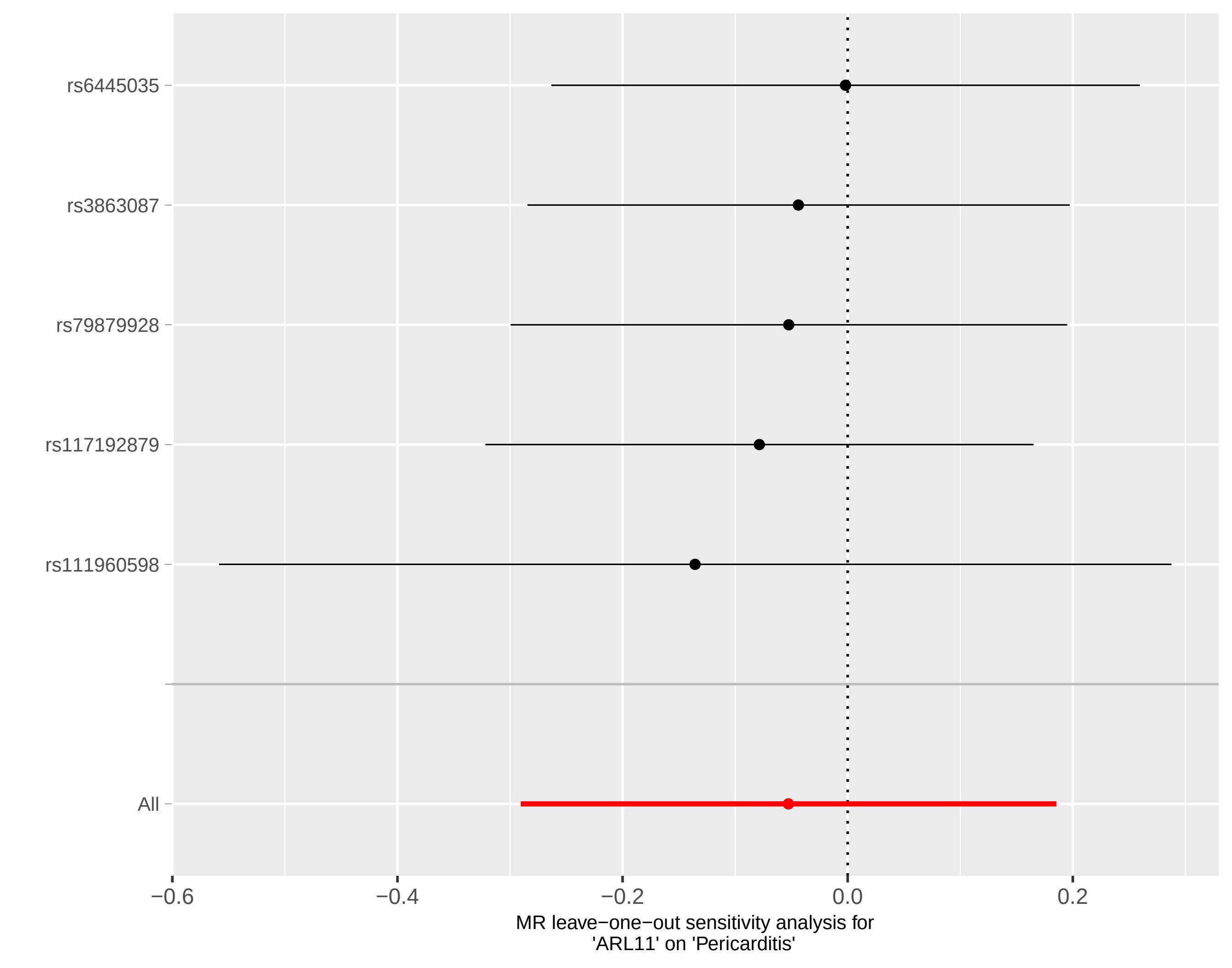
rs6445035
rs3863087
rs79879928
rs117192879
rs111960598
All
−0.6
−0.4
−0.2
0.0
0.2
MR leave−one−out sensitivity analysis for
'ARL11' on 'Pericarditis'

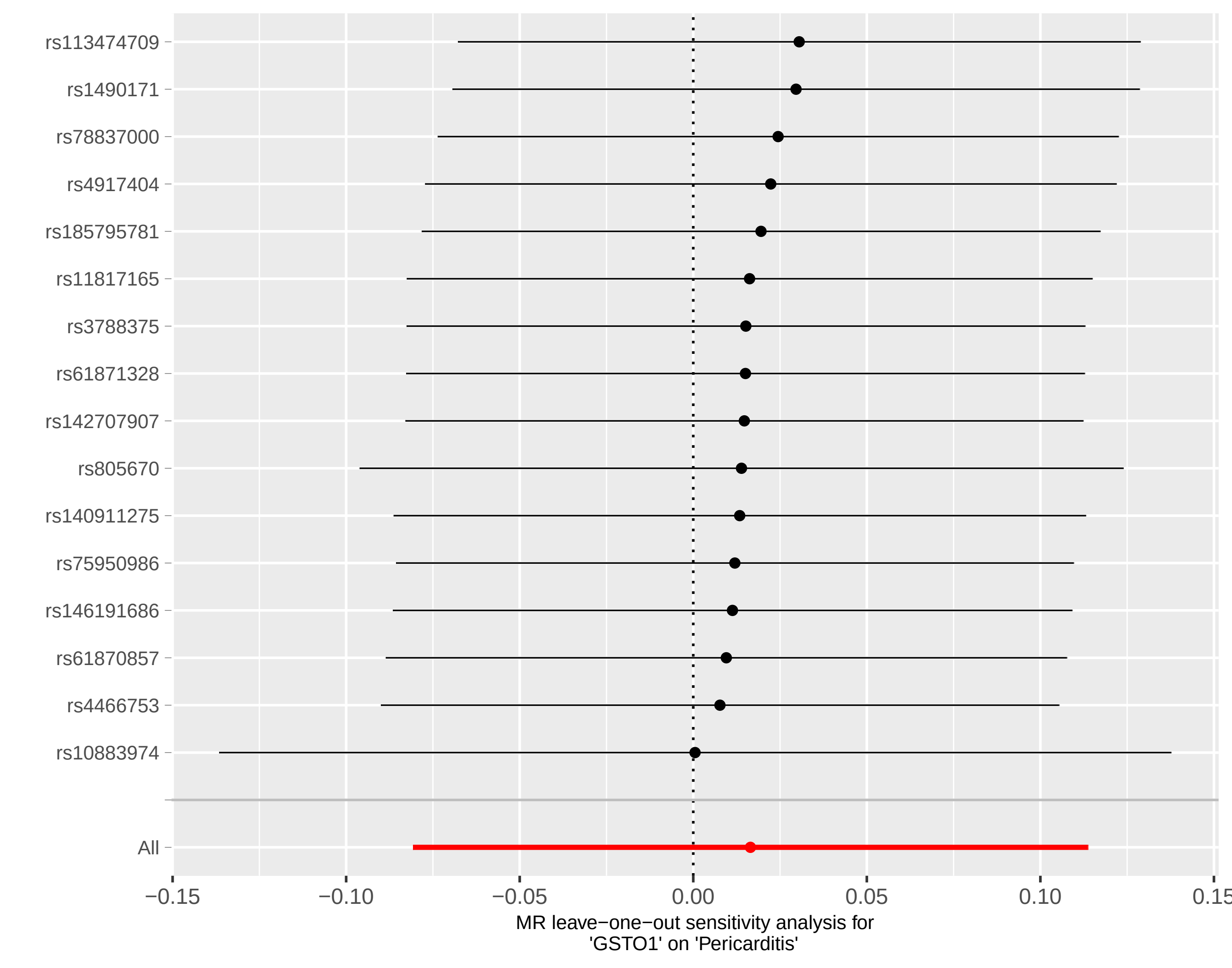
rs113474709
rs1490171
rs78837000
rs4917404
rs185795781
rs11817165
rs3788375
rs61871328
rs142707907
rs805670
rs140911275
rs75950986
rs146191686
rs61870857
rs4466753
rs10883974
All
−0.15
−0.10
−0.05
0.00
0.05
0.10
0.15
MR leave−one−out sensitivity analysis for
'GSTO1' on 'Pericarditis'

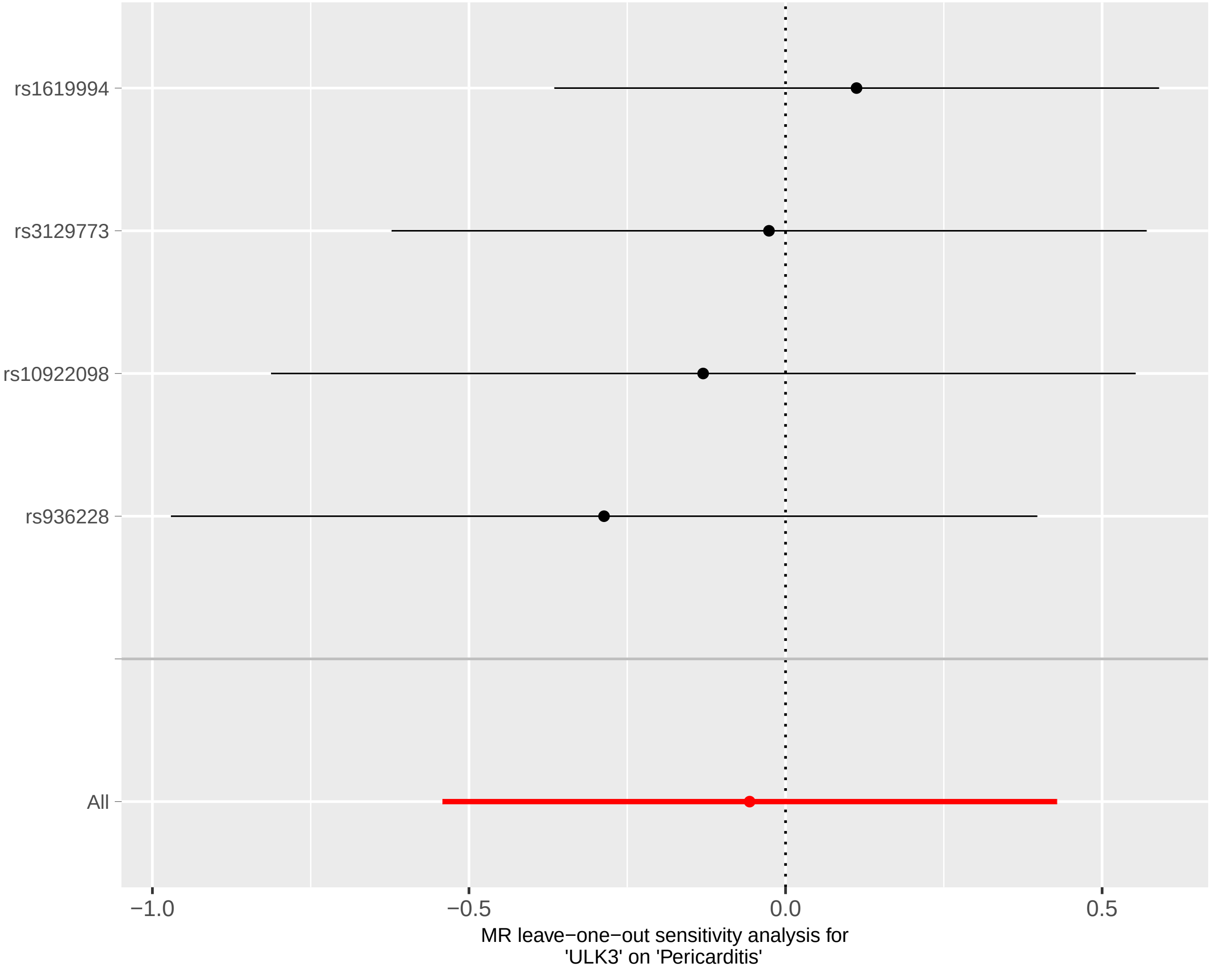

rs1619994
rs3129773
rs10922098
rs936228
All
−1.0
−0.5
0.0
0.5
MR leave−one−out sensitivity analysis for
'ULK3' on 'Pericarditis'

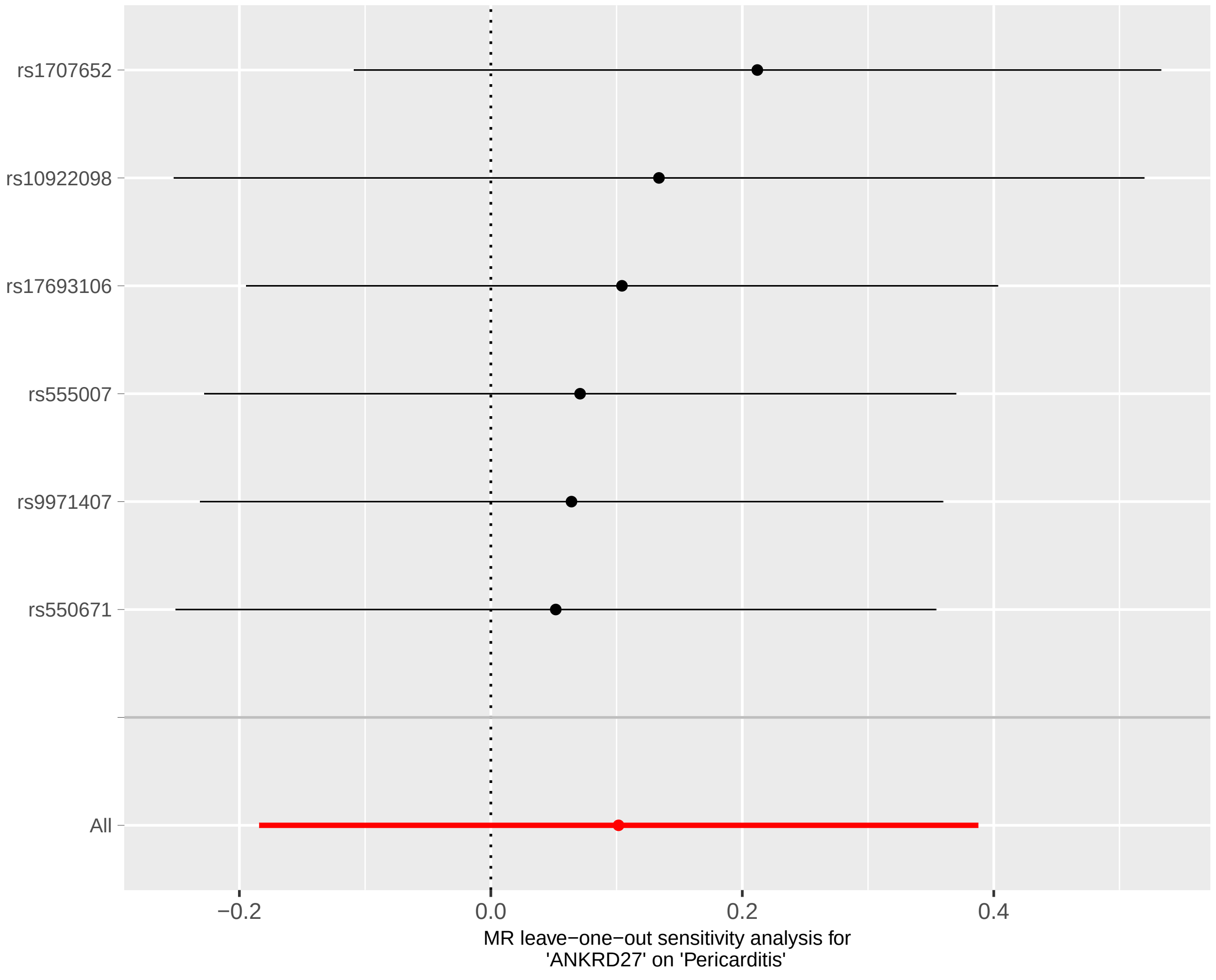
rs1707652
rs10922098
rs17693106
rs555007
rs9971407
rs550671
All
−0.2
0.0
0.2
0.4
MR leave−one−out sensitivity analysis for
'ANKRD27' on 'Pericarditis'

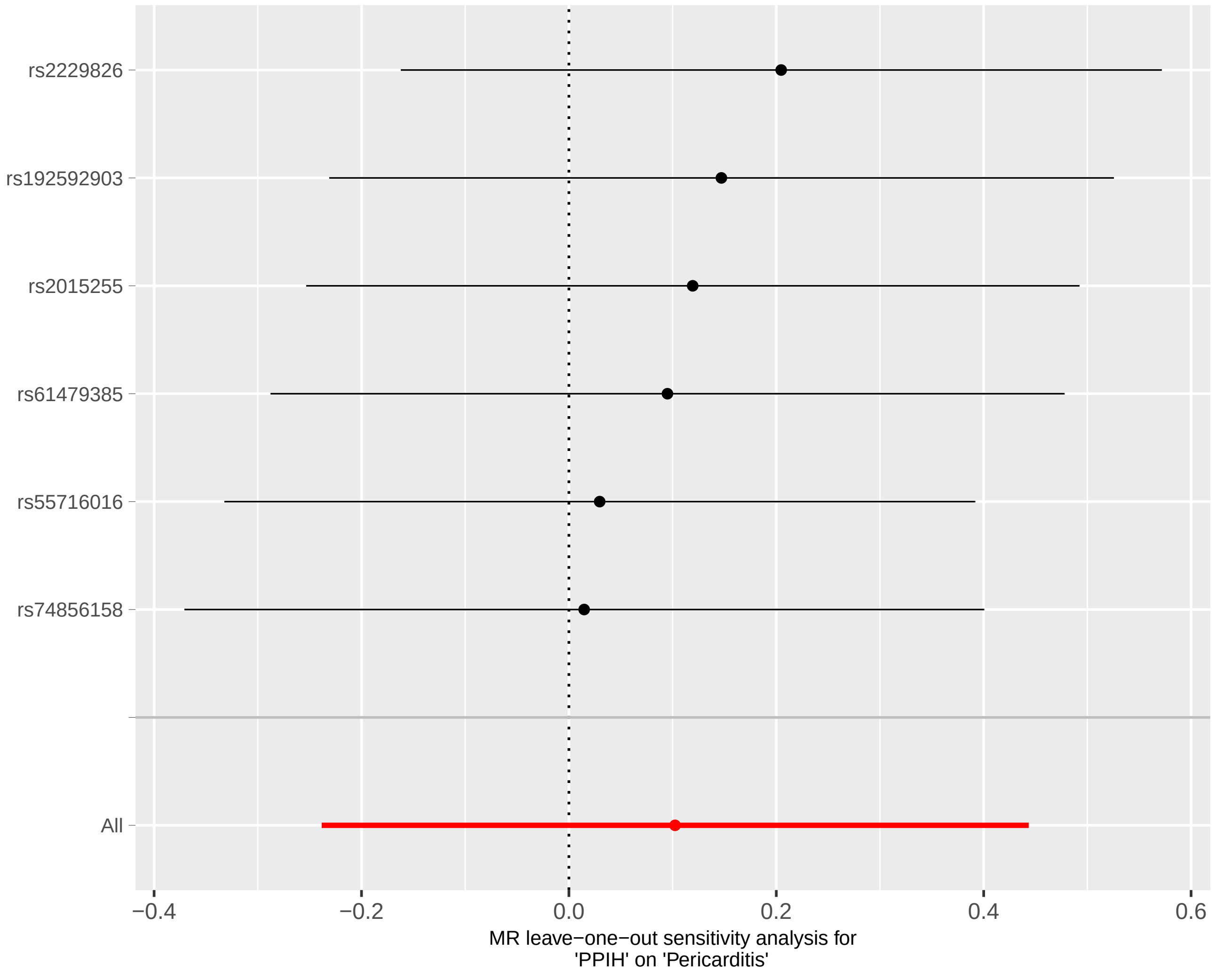

rs2229826
rs192592903
rs2015255
rs61479385
rs55716016
rs74856158
All
−0.4
−0.2
0.0
0.2
0.4
0.6
MR leave−one−out sensitivity analysis for
'PPIH' on 'Pericarditis'

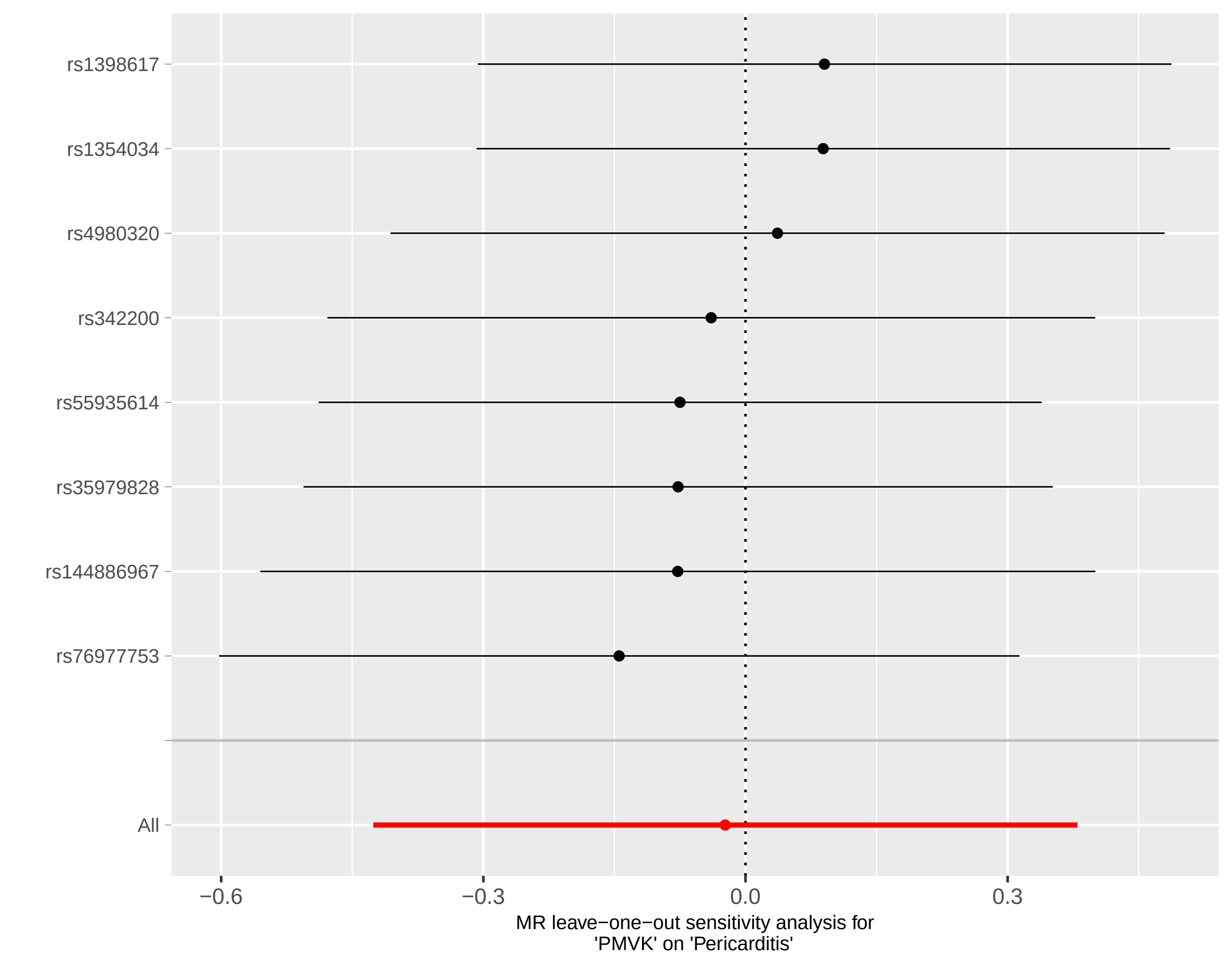
rs1398617
rs1354034
rs4980320
rs342200
rs55935614
rs35979828
rs144886967
rs76977753
All
−0.6
−0.3
0.0
0.3
MR leave−one−out sensitivity analysis for
'PMVK' on 'Pericarditis'

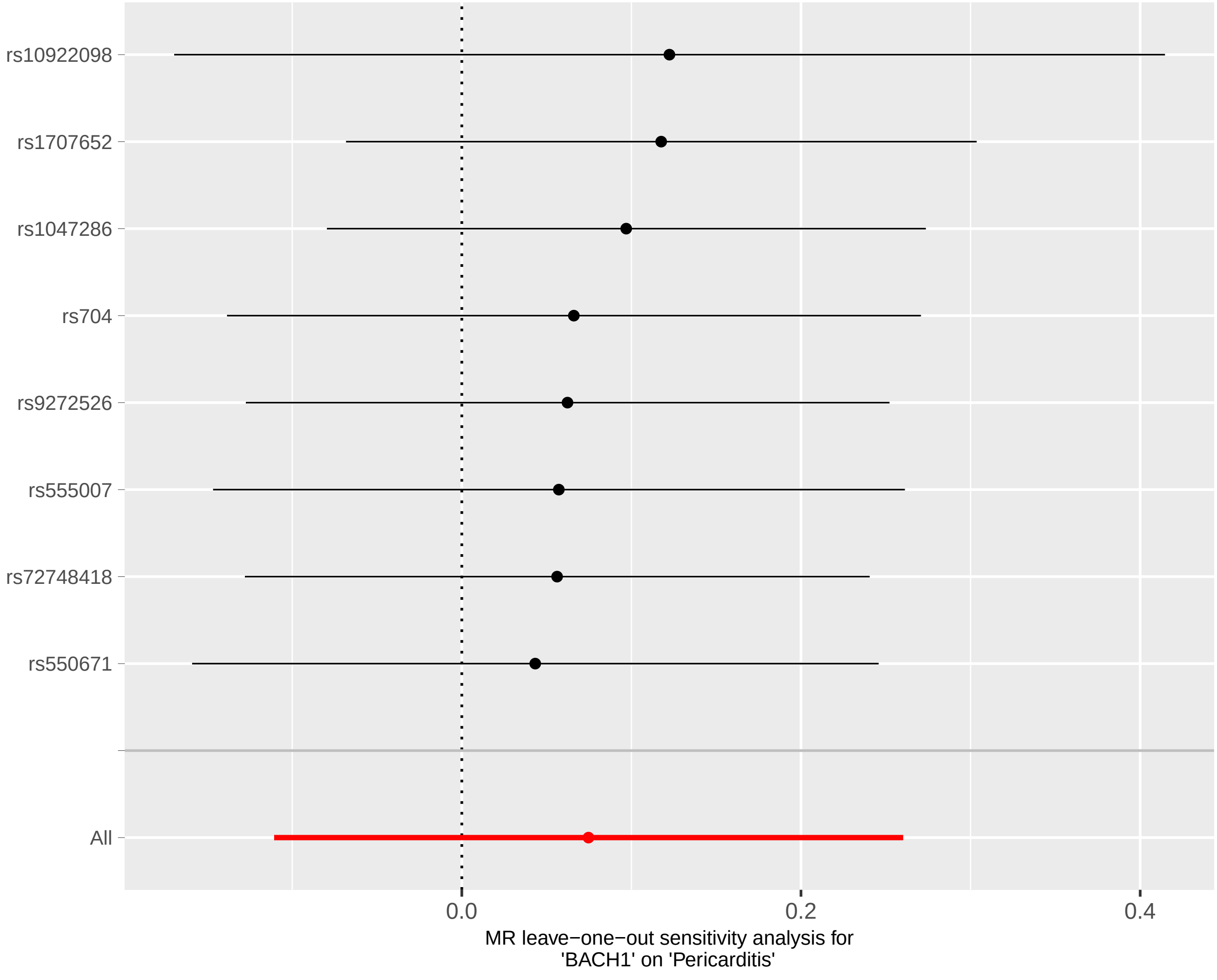
rs10922098
rs1707652
rs1047286
rs704
rs9272526
rs555007
rs72748418
rs550671
All
0.0
0.2
0.4
MR leave−one−out sensitivity analysis for
'BACH1' on 'Pericarditis'

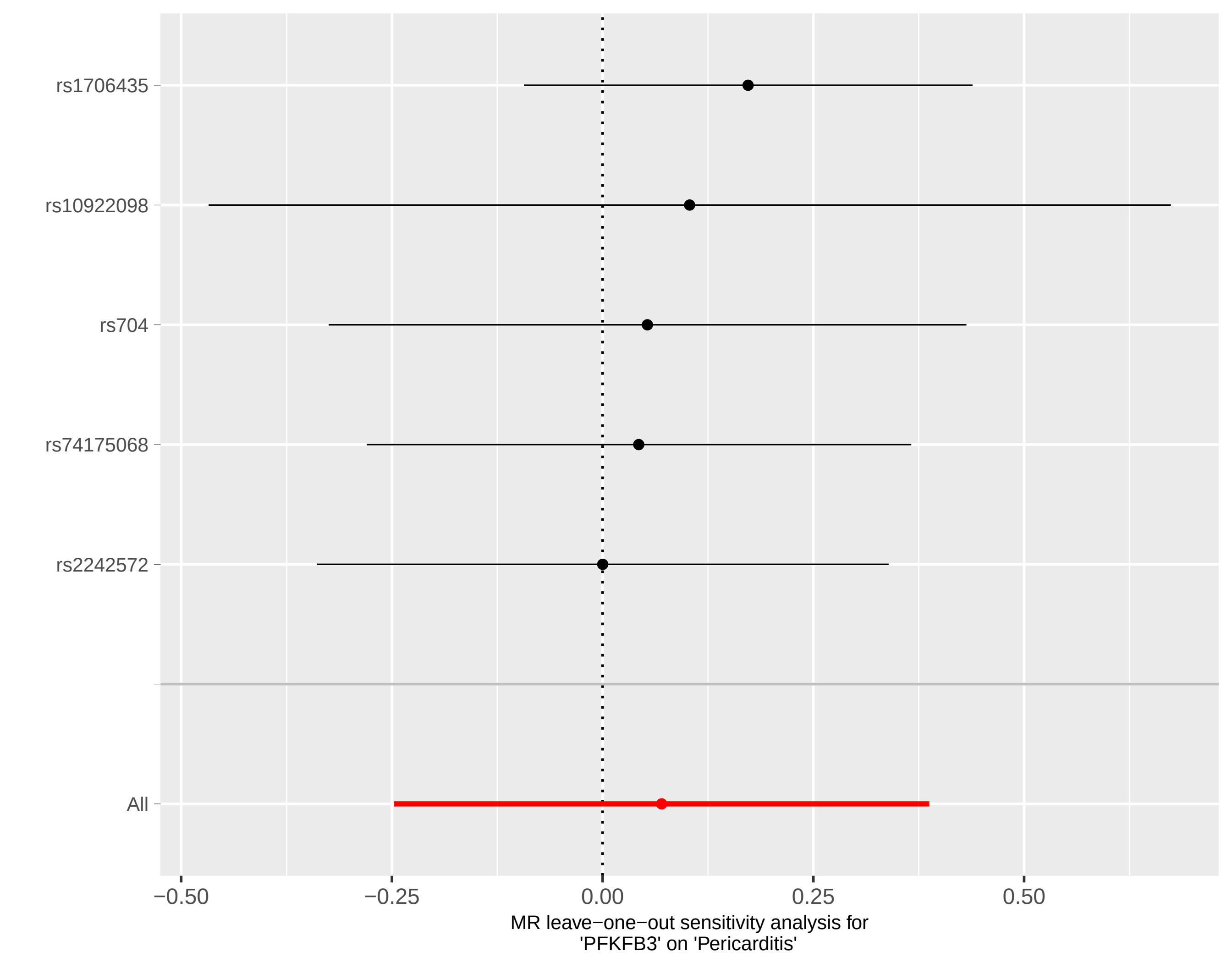
rs1706435
rs10922098
rs704
rs74175068
rs2242572
All
−0.50
−0.25
0.00
0.25
0.50
MR leave−one−out sensitivity analysis for
'PFKFB3' on 'Pericarditis'

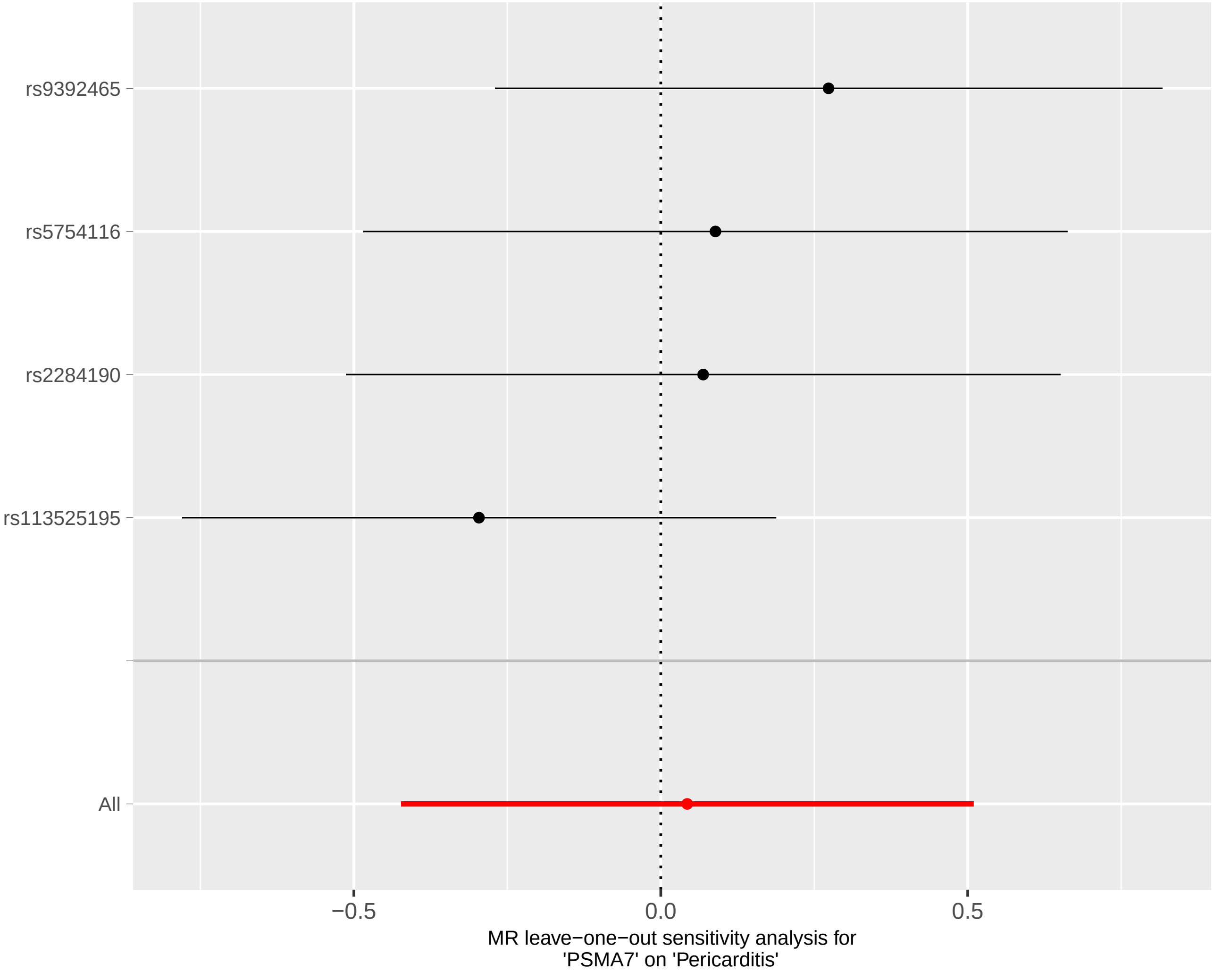
rs9392465
rs5754116
rs2284190
rs113525195
All
−0.5
0.0
0.5
MR leave−one−out sensitivity analysis for
'PSMA7' on 'Pericarditis'

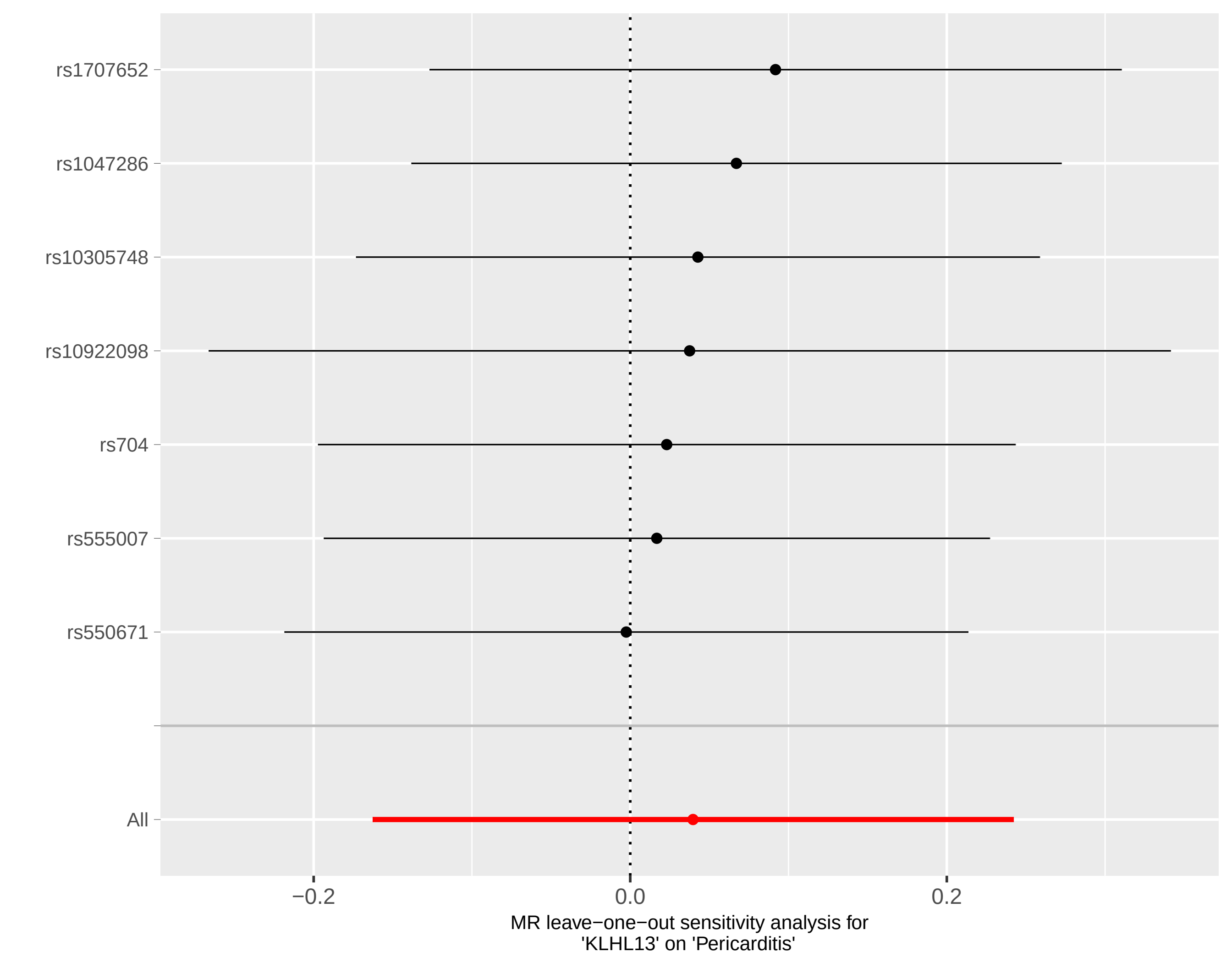

rs1707652
rs1047286
rs10305748
rs10922098
rs704
rs555007
rs550671
All
−0.2
0.0
0.2
MR leave−one−out sensitivity analysis for
'KLHL13' on 'Pericarditis'

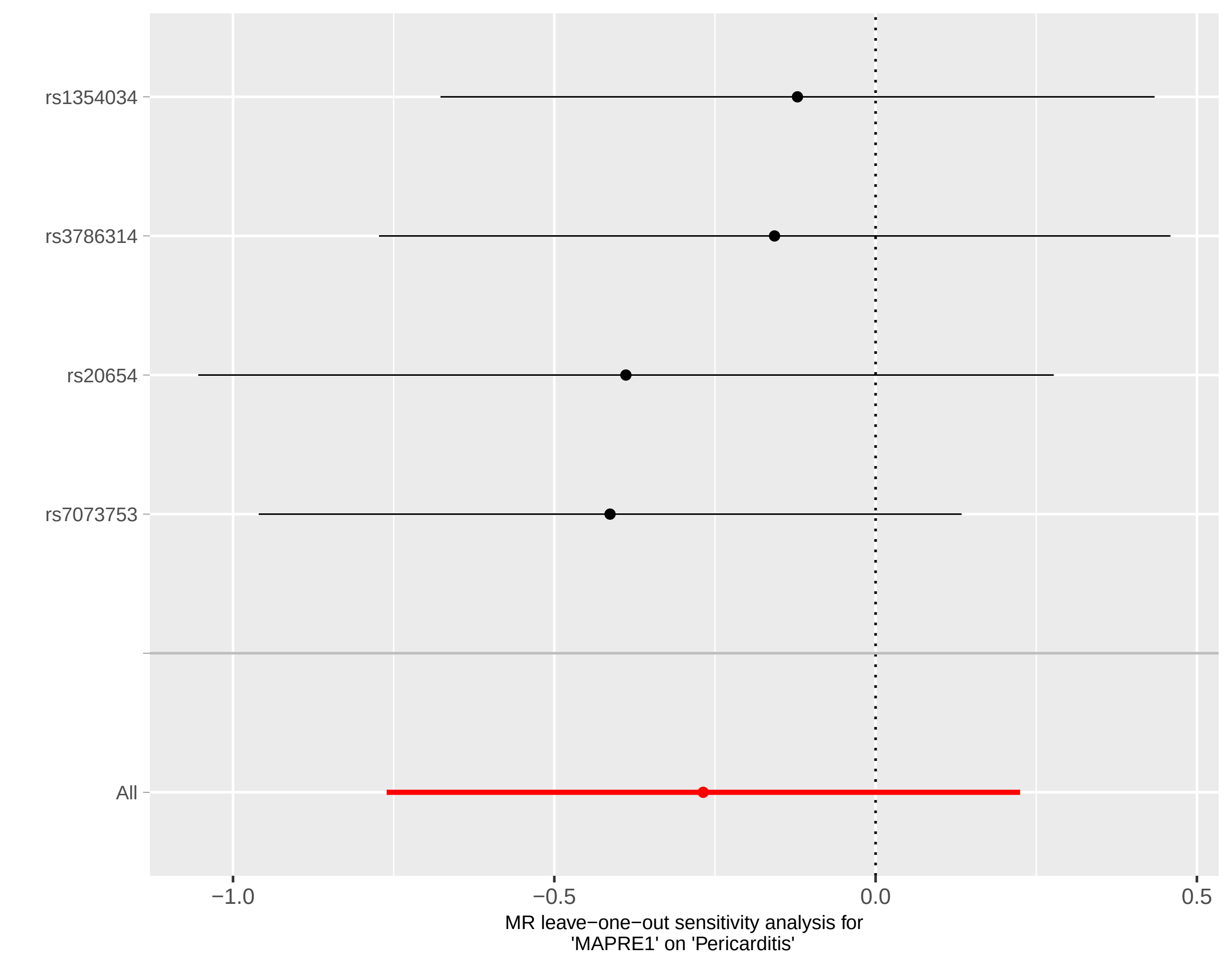
rs1354034
rs3786314
rs20654
rs7073753
All
−1.0
−0.5
0.0
0.5
MR leave−one−out sensitivity analysis for
'MAPRE1' on 'Pericarditis'

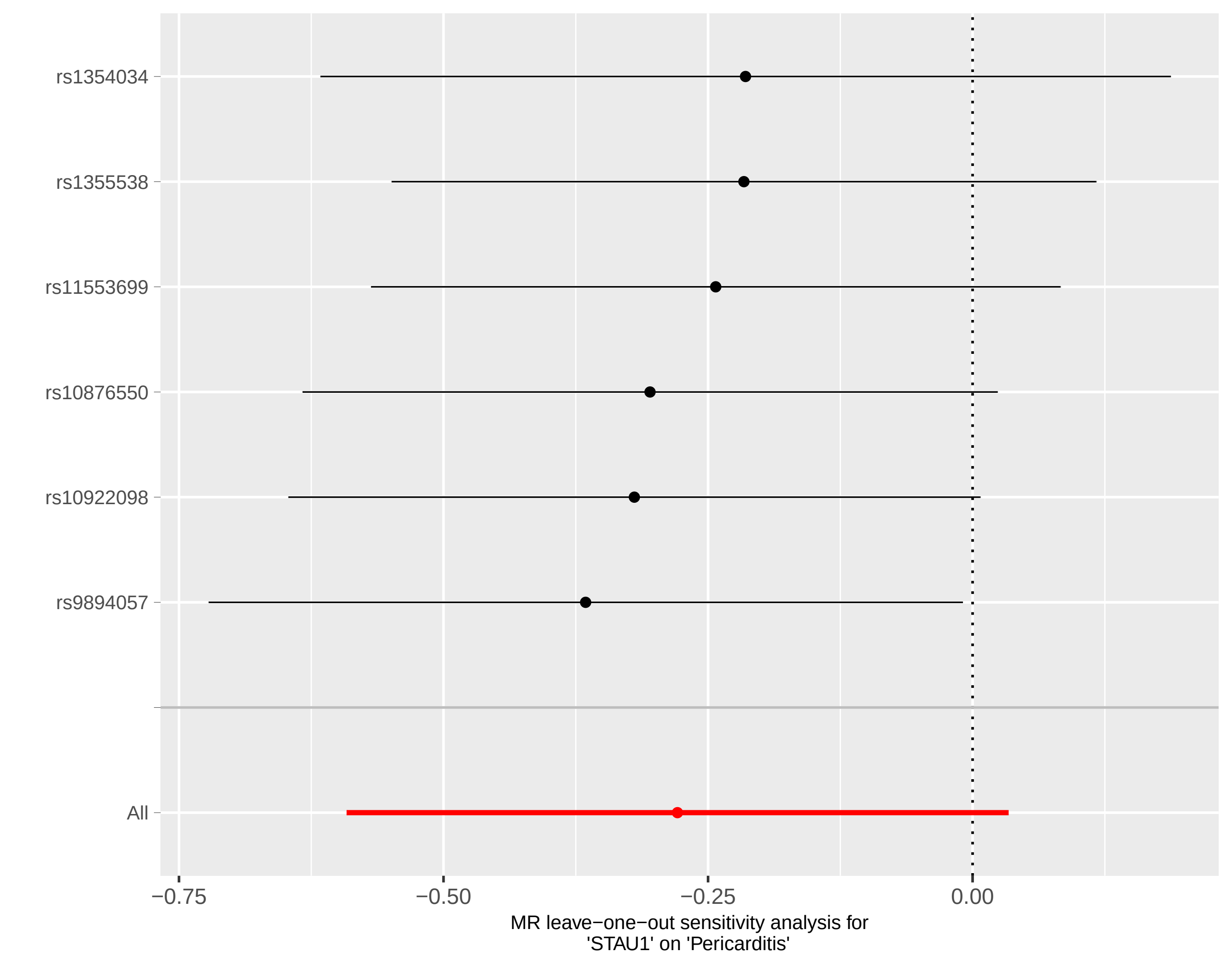

rs1354034
rs1355538
rs11553699
rs10876550
rs10922098
rs9894057
All
−0.75
−0.50
−0.25
0.00
MR leave−one−out sensitivity analysis for
'STAU1' on 'Pericarditis'

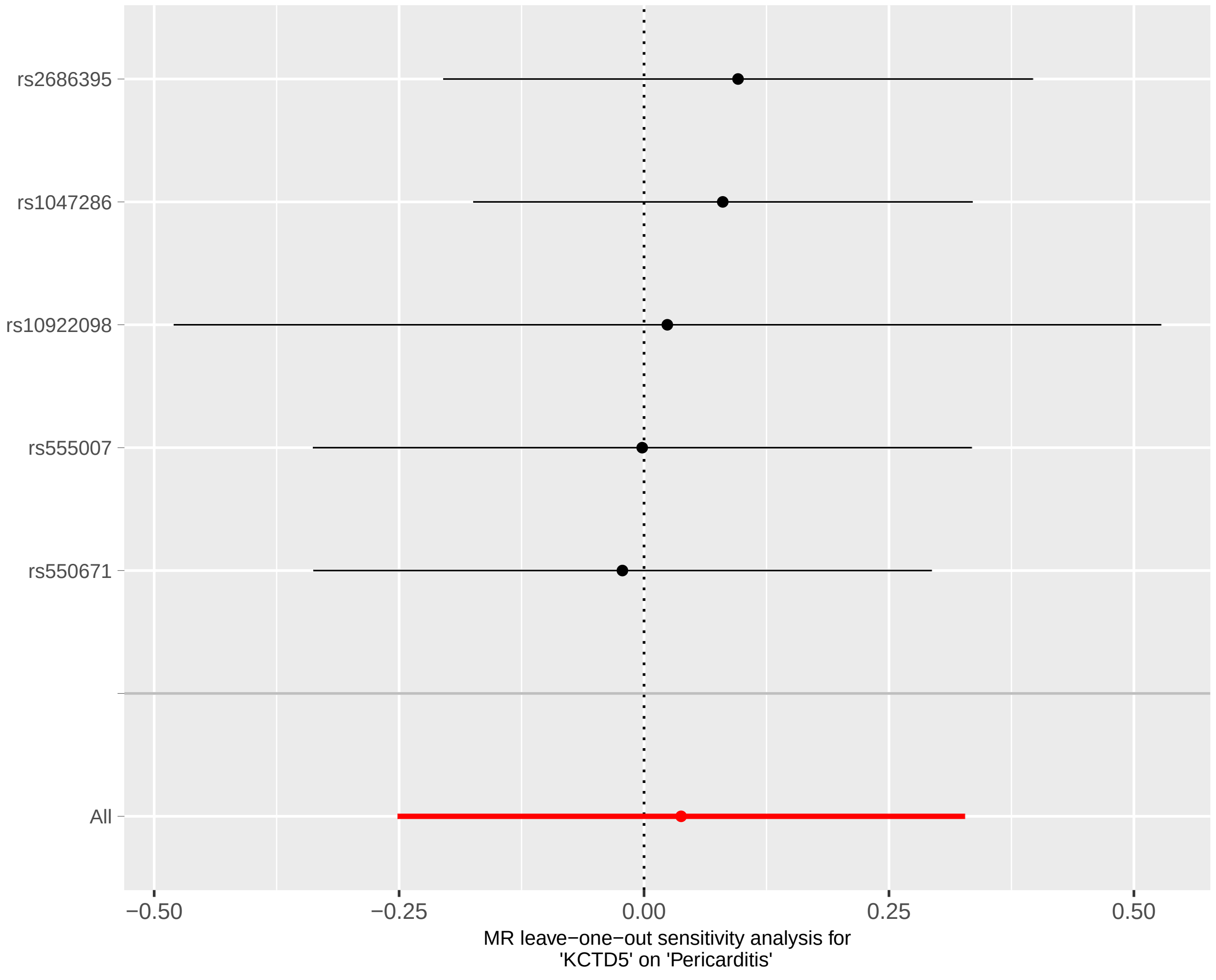

rs2686395
rs1047286
rs10922098
rs555007
rs550671
All
−0.50
−0.25
0.00
0.25
0.50
MR leave−one−out sensitivity analysis for
'KCTD5' on 'Pericarditis'

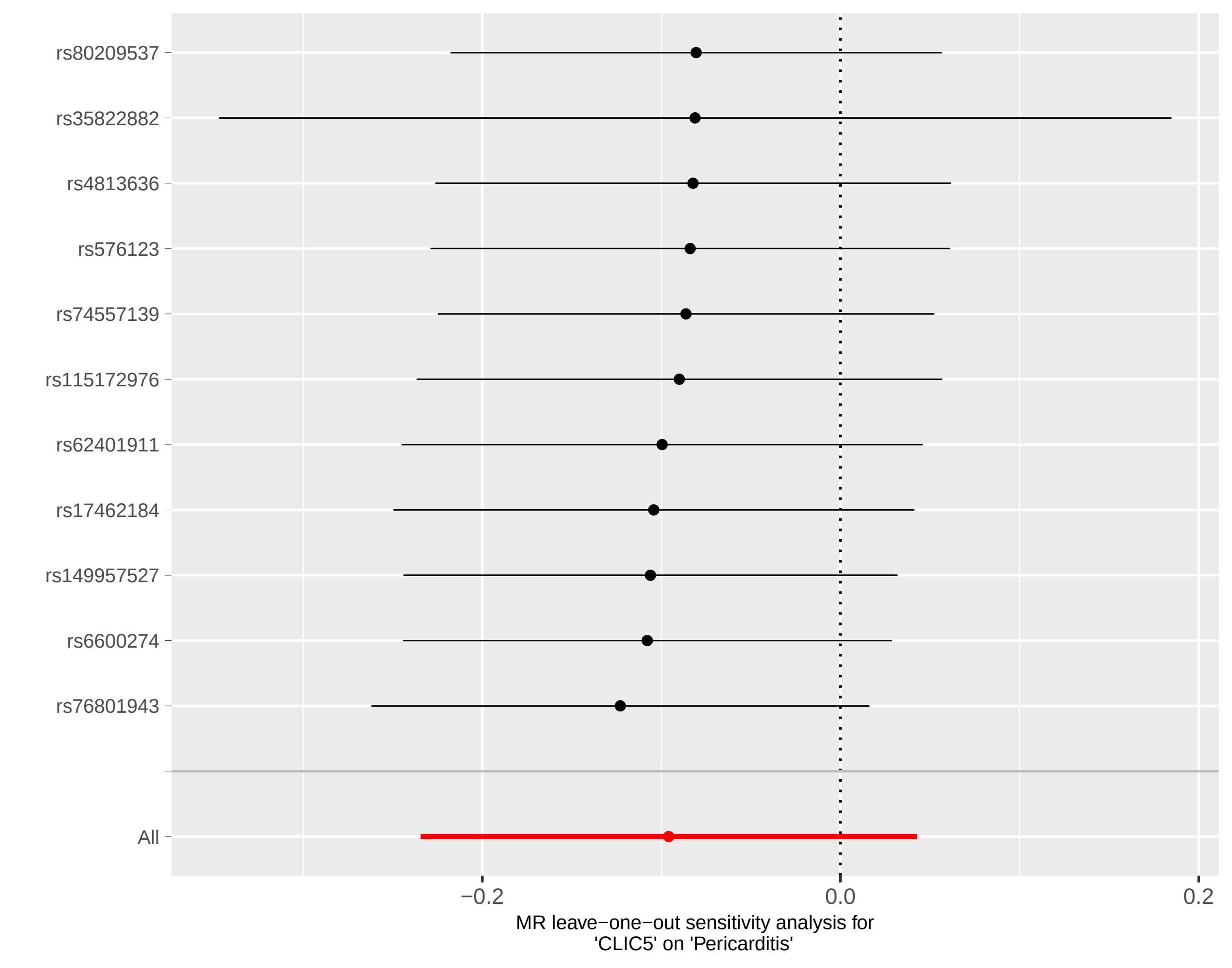
rs80209537
rs35822882
rs4813636
rs576123
rs74557139
rs115172976
rs62401911
rs17462184
rs149957527
rs6600274
rs76801943
All
−0.2
0.0
0.2
MR leave−one−out sensitivity analysis for
'CLIC5' on 'Pericarditis'

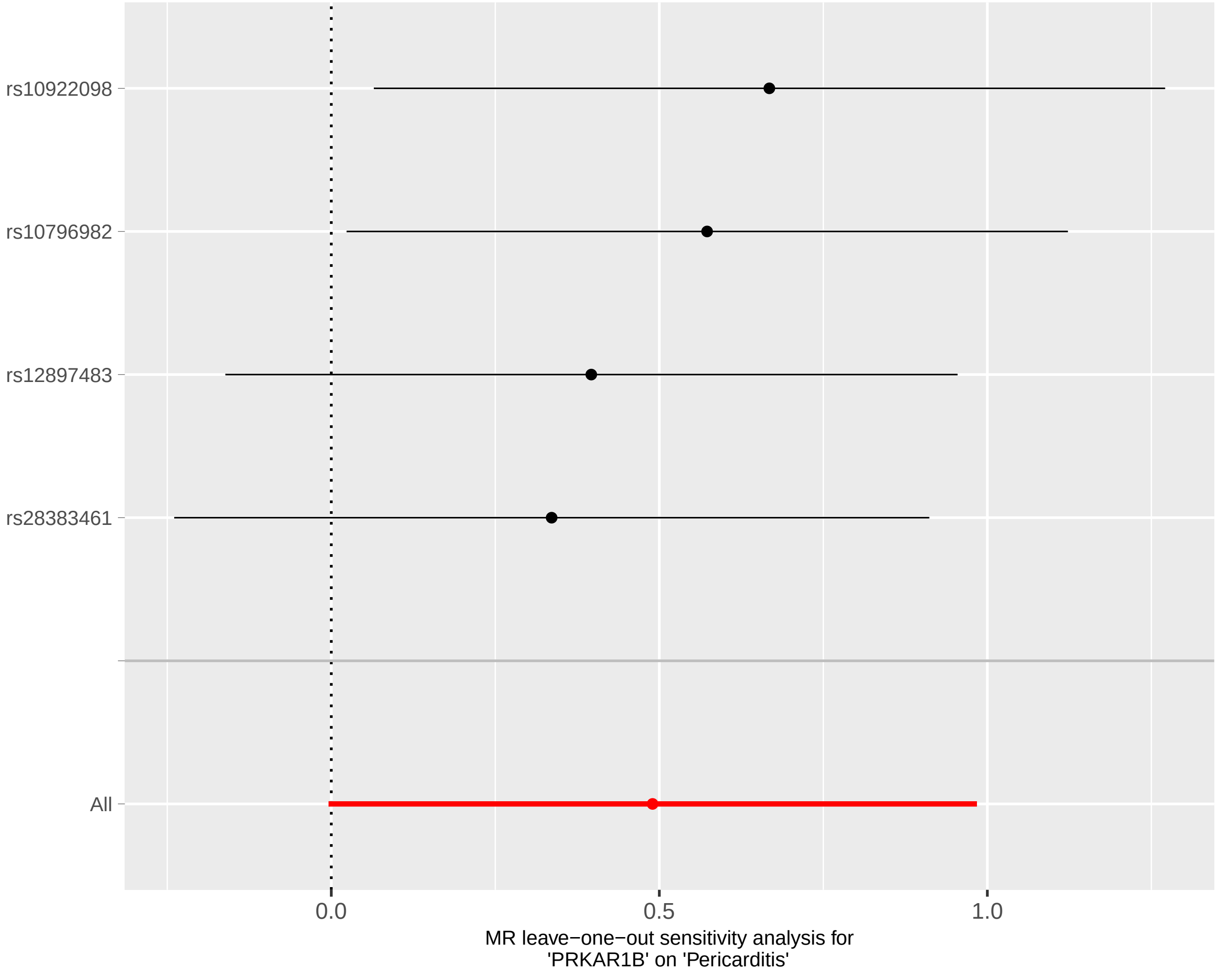

rs10922098
rs10796982
rs12897483
rs28383461
All
0.0
0.5
1.0
MR leave−one−out sensitivity analysis for
'PRKAR1B' on 'Pericarditis'

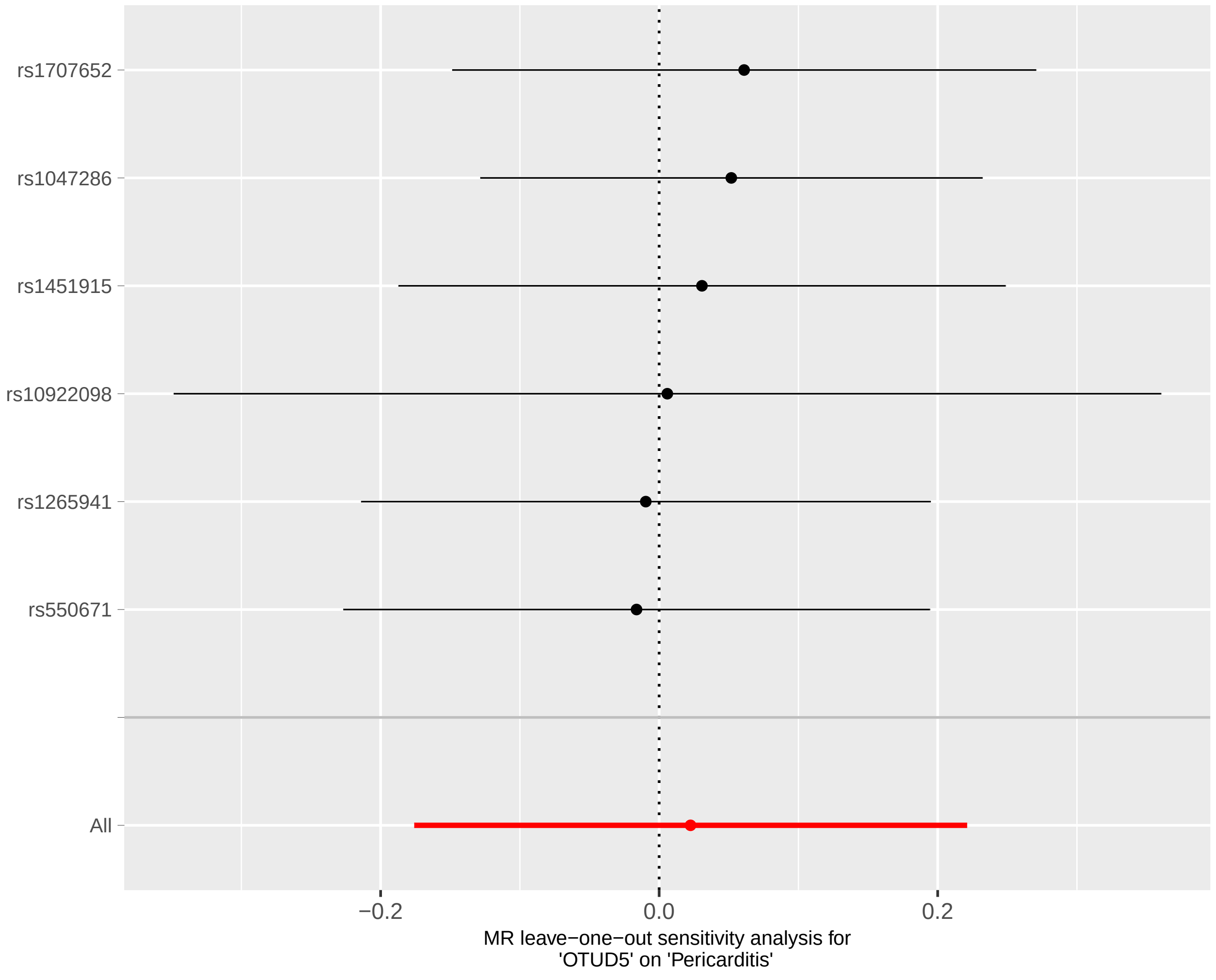
rs1707652
rs1047286
rs1451915
rs10922098
rs1265941
rs550671
All
−0.2
0.0
0.2
MR leave−one−out sensitivity analysis for
'OTUD5' on 'Pericarditis'

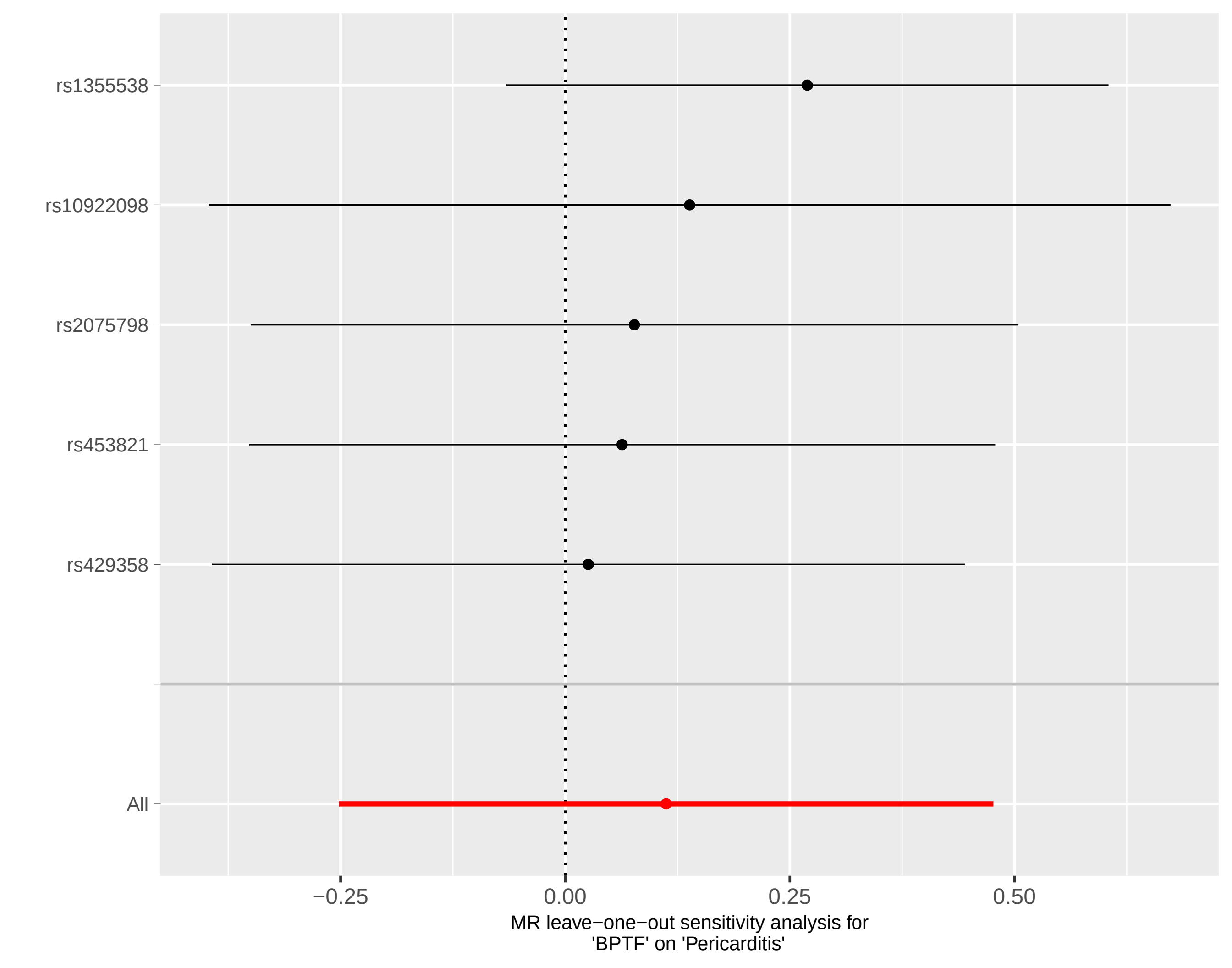
rs1355538
rs10922098
rs2075798
rs453821
rs429358
All
−0.25
0.00
0.25
0.50
MR leave−one−out sensitivity analysis for
'BPTF' on 'Pericarditis'

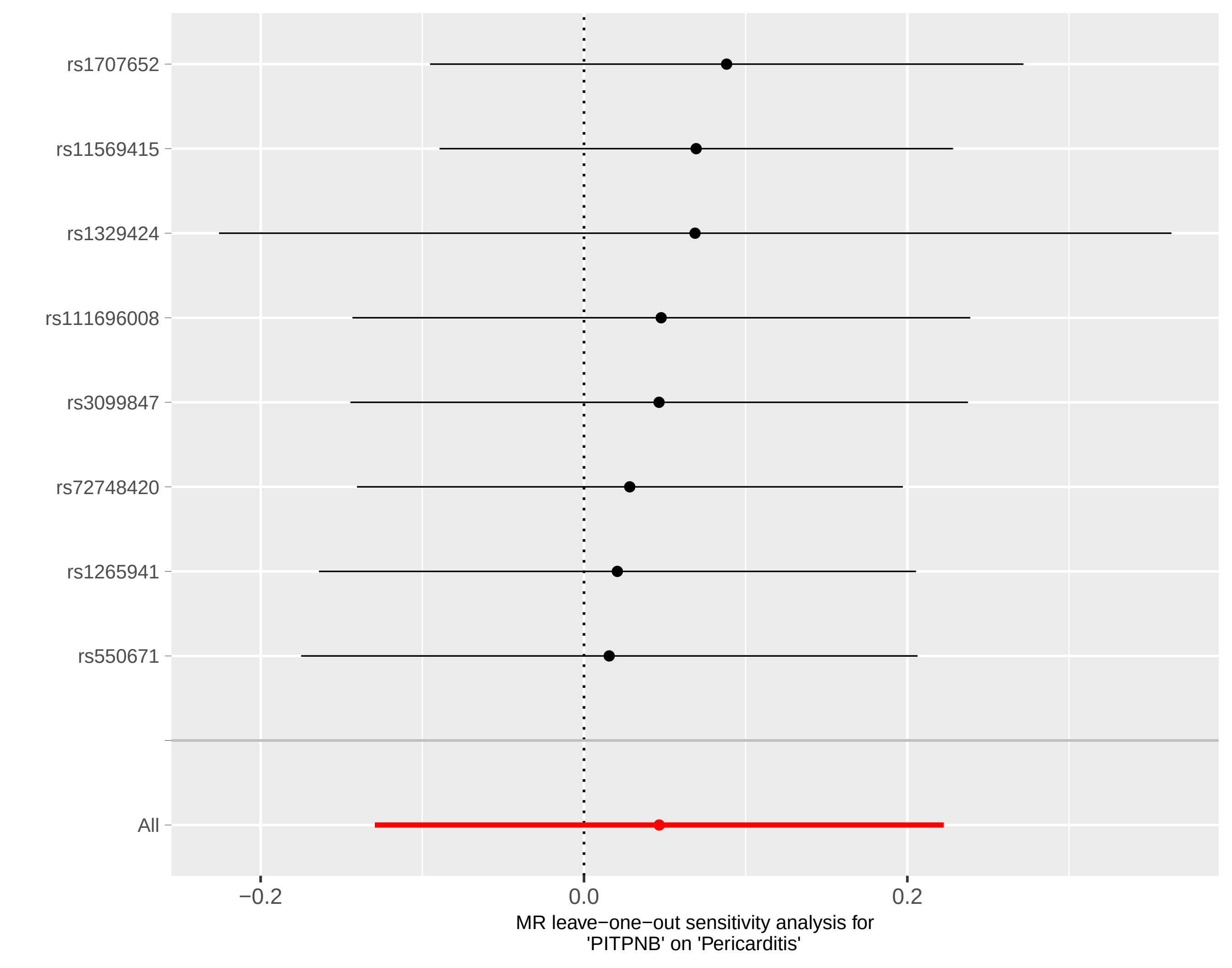
rs1707652
rs11569415
rs1329424
rs111696008
rs3099847
rs72748420
rs1265941
rs550671
All
−0.2
0.0
0.2
MR leave−one−out sensitivity analysis for
'PITPNB' on 'Pericarditis'

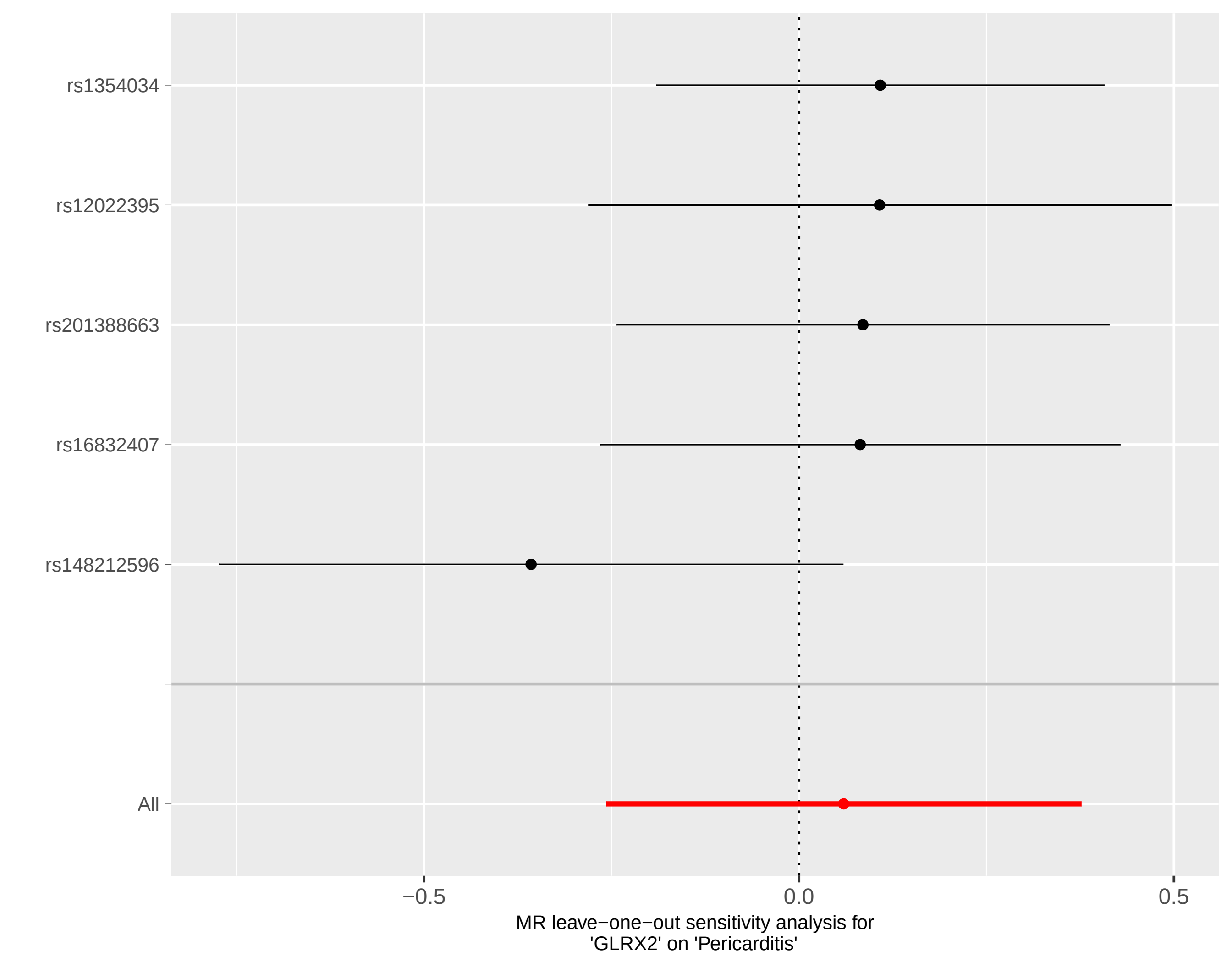
rs1354034
rs12022395
rs201388663
rs16832407
rs148212596
All
−0.5
0.0
0.5
MR leave−one−out sensitivity analysis for
'GLRX2' on 'Pericarditis'

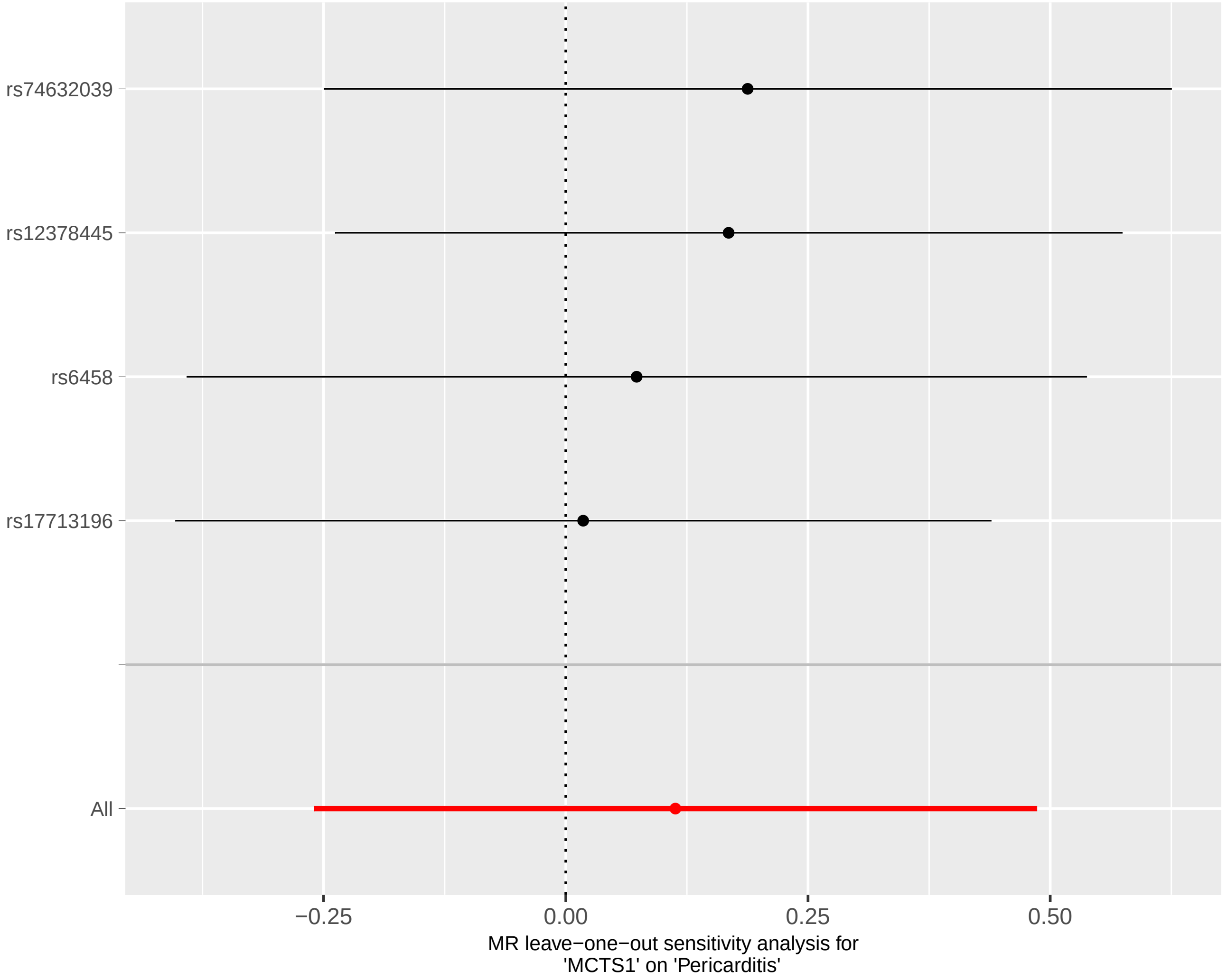

rs74632039
rs12378445
rs6458
rs17713196
All
−0.25
0.00
0.25
0.50
MR leave−one−out sensitivity analysis for
'MCTS1' on 'Pericarditis'

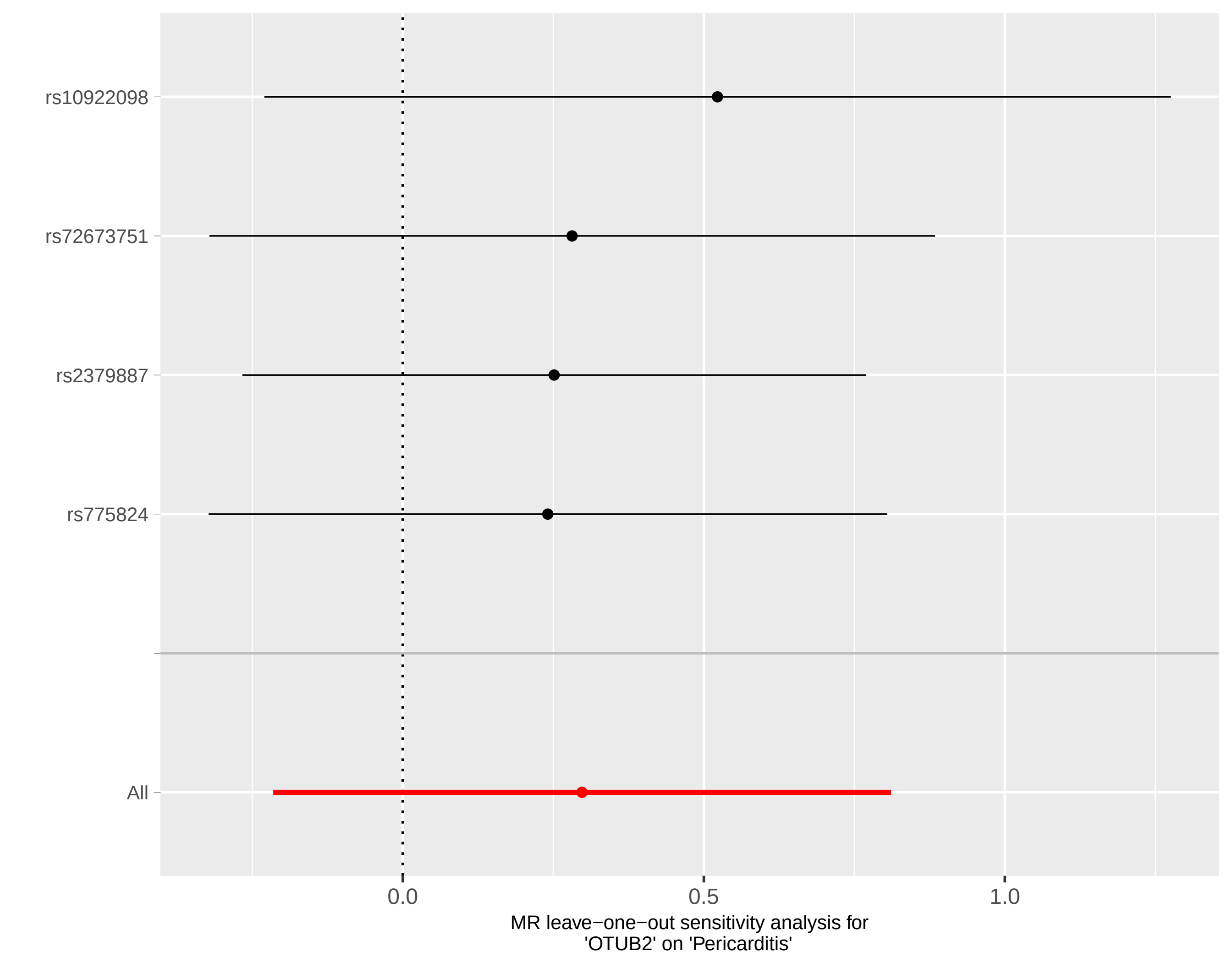
rs10922098
rs72673751
rs2379887
rs775824
All
0.0
0.5
1.0
MR leave−one−out sensitivity analysis for
'OTUB2' on 'Pericarditis'

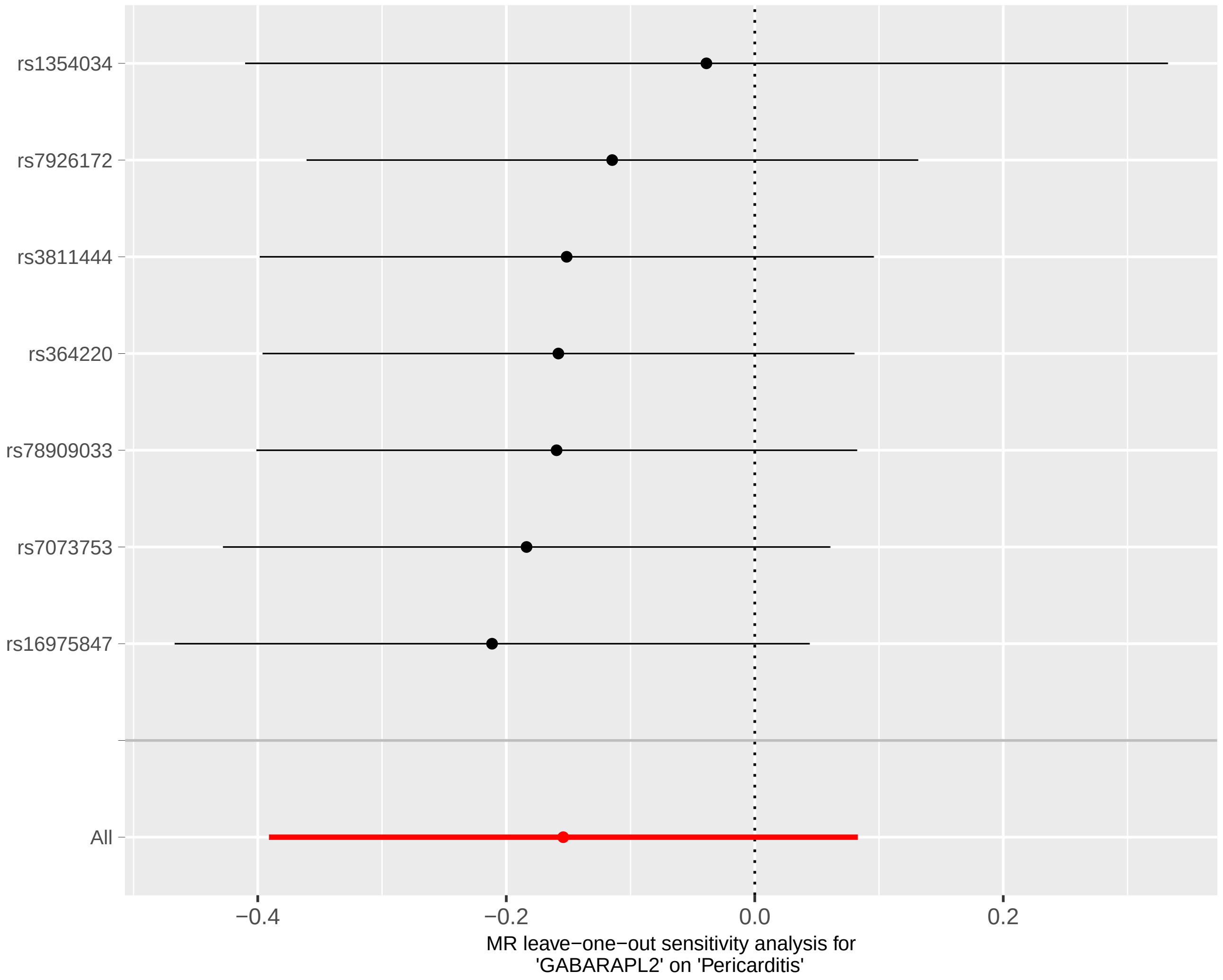

rs1354034
rs7926172
rs3811444
rs364220
rs78909033
rs7073753
rs16975847
All
−0.4
−0.2
0.0
0.2
MR leave−one−out sensitivity analysis for
'GABARAPL2' on 'Pericarditis'

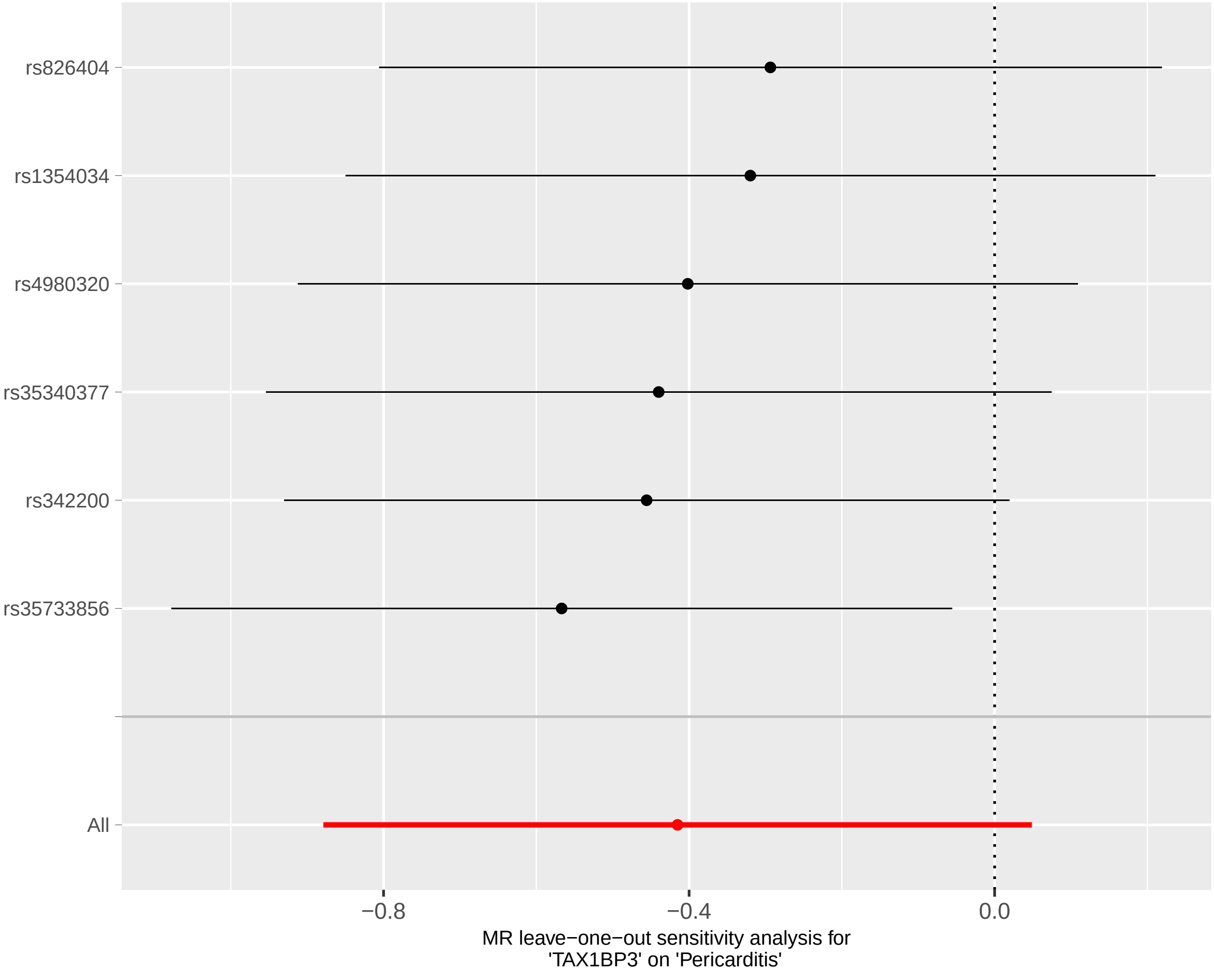
rs826404
rs1354034
rs4980320
rs35340377
rs342200
rs35733856
All
−0.8
−0.4
0.0
MR leave−one−out sensitivity analysis for
'TAX1BP3' on 'Pericarditis'

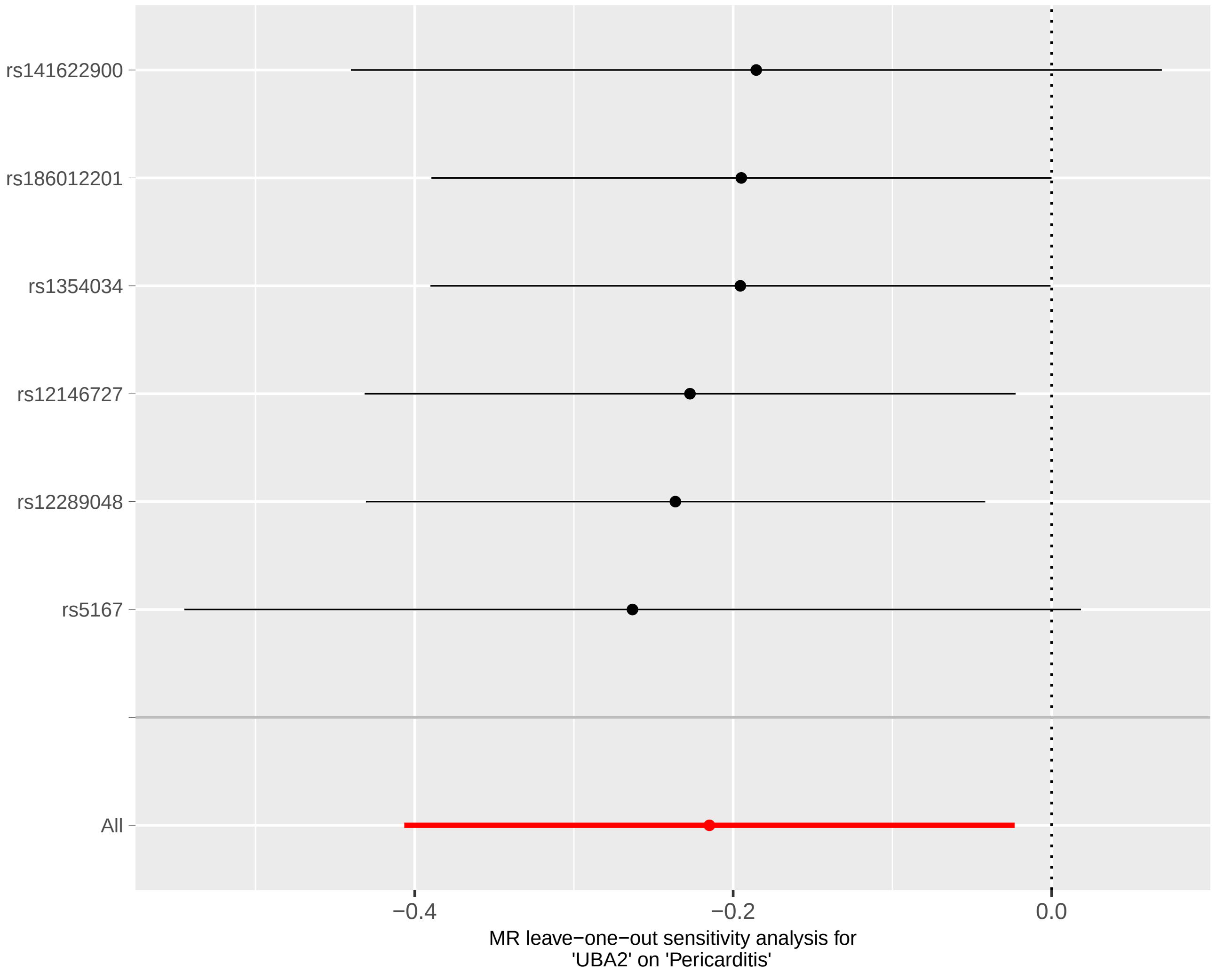
rs141622900
rs186012201
rs1354034
rs12146727
rs12289048
rs5167
All
−0.4
−0.2
0.0
MR leave−one−out sensitivity analysis for
'UBA2' on 'Pericarditis'

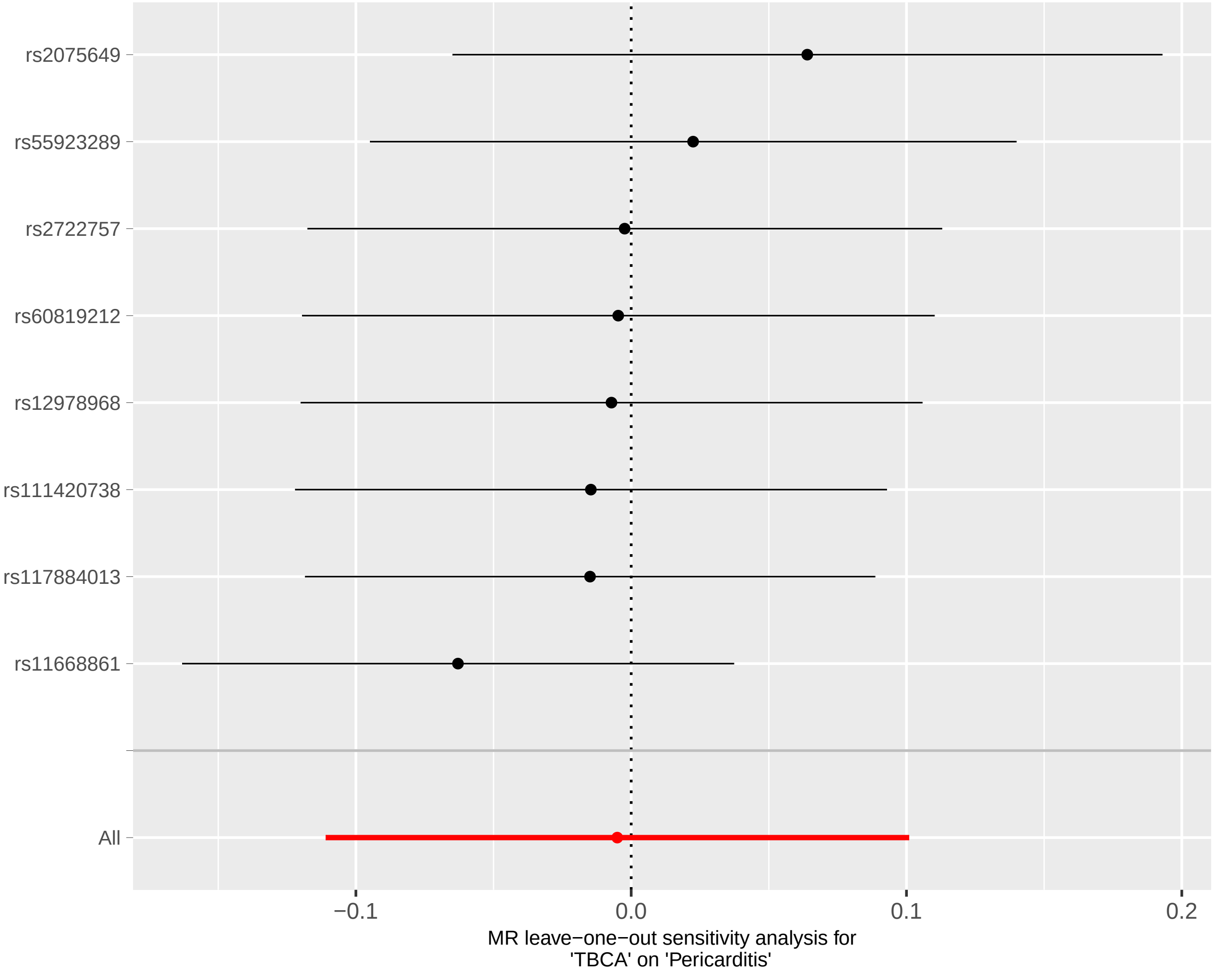

rs2075649
rs55923289
rs2722757
rs60819212
rs12978968
rs111420738
rs117884013
rs11668861
All
−0.1
0.0
0.1
0.2
MR leave−one−out sensitivity analysis for
'TBCA' on 'Pericarditis'

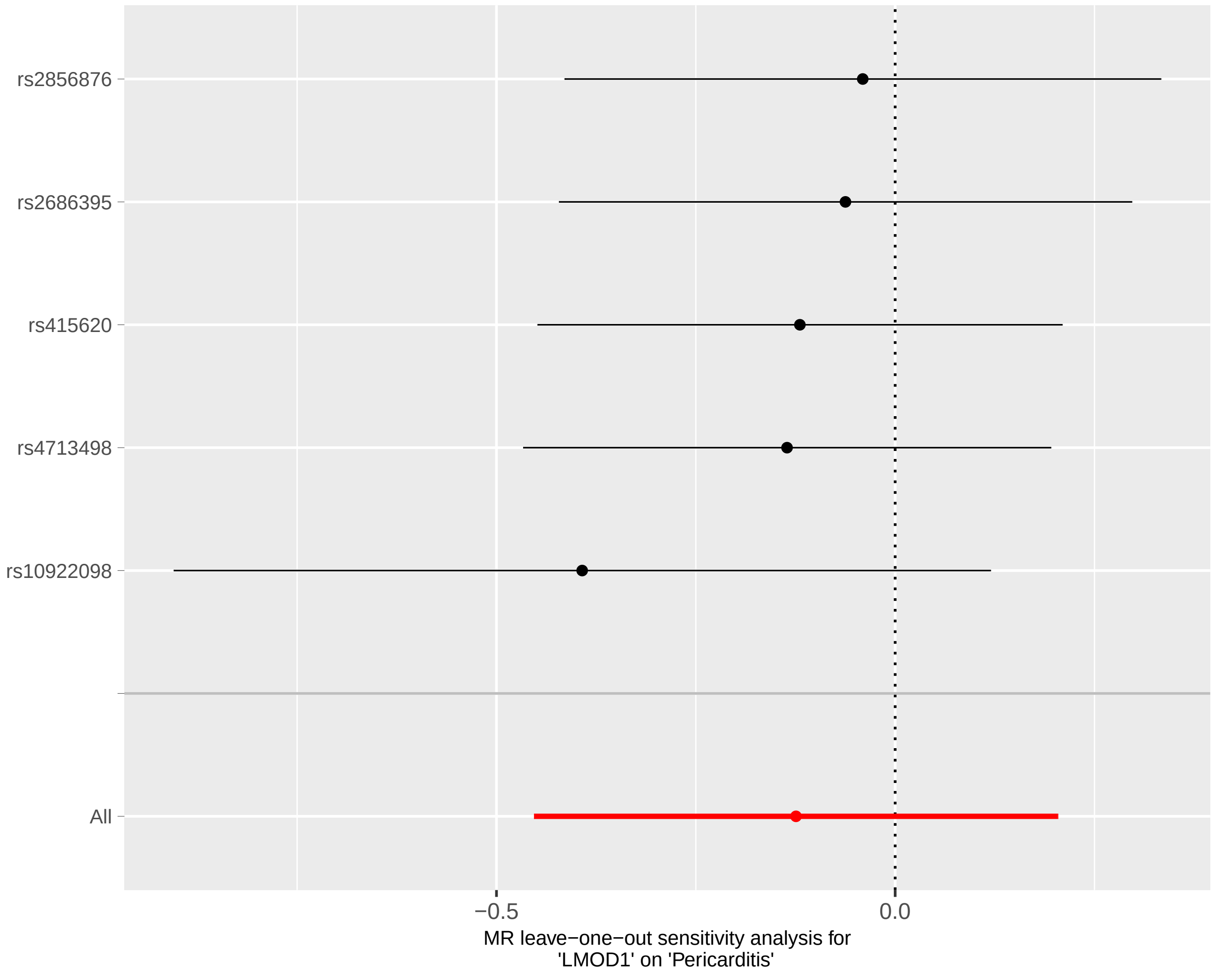
rs2856876
rs2686395
rs415620
rs4713498
rs10922098
All
−0.5
0.0
MR leave−one−out sensitivity analysis for
'LMOD1' on 'Pericarditis'

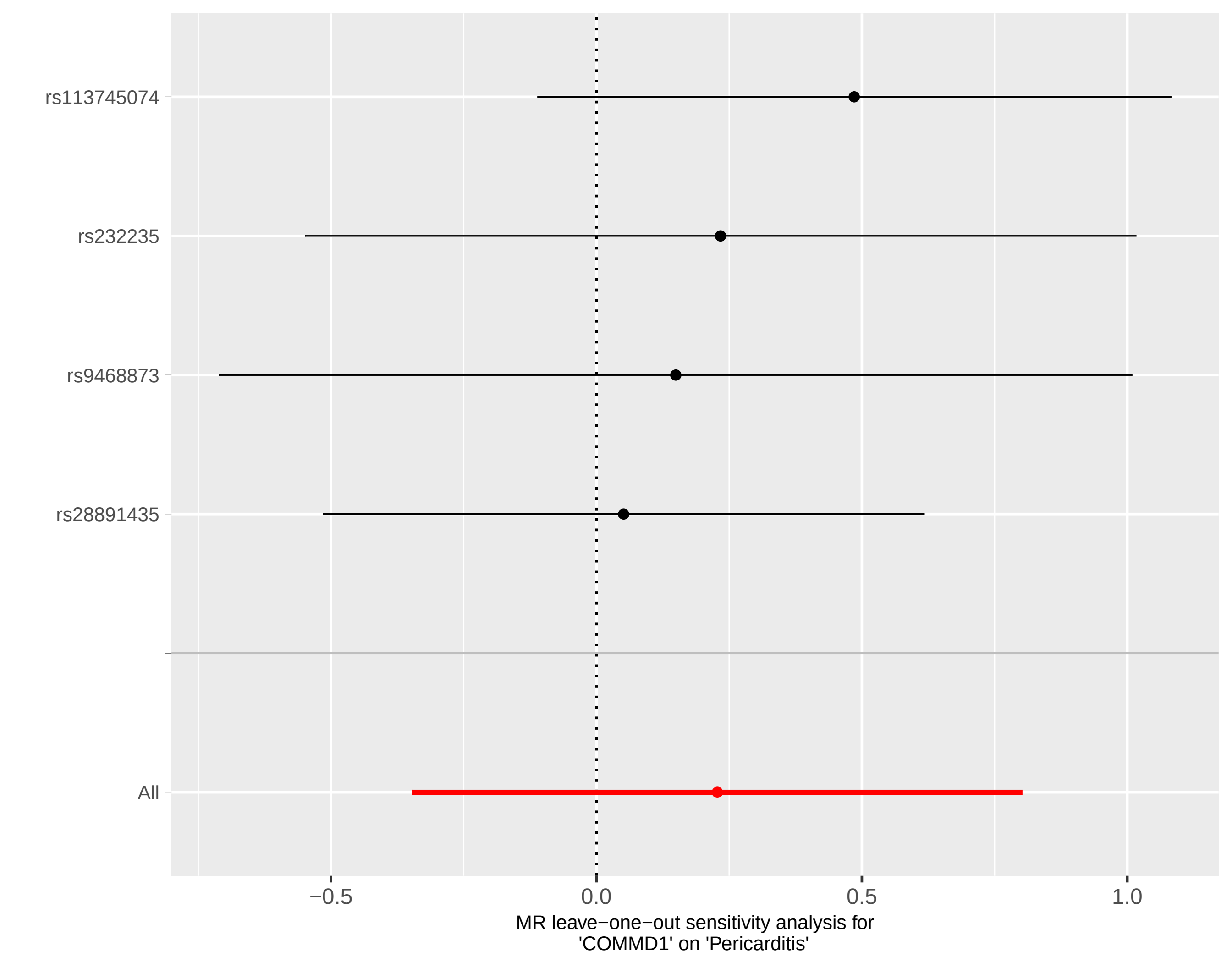
rs113745074
rs232235
rs9468873
rs28891435
All
−0.5
0.0
0.5
1.0
MR leave−one−out sensitivity analysis for
'COMMD1' on 'Pericarditis'

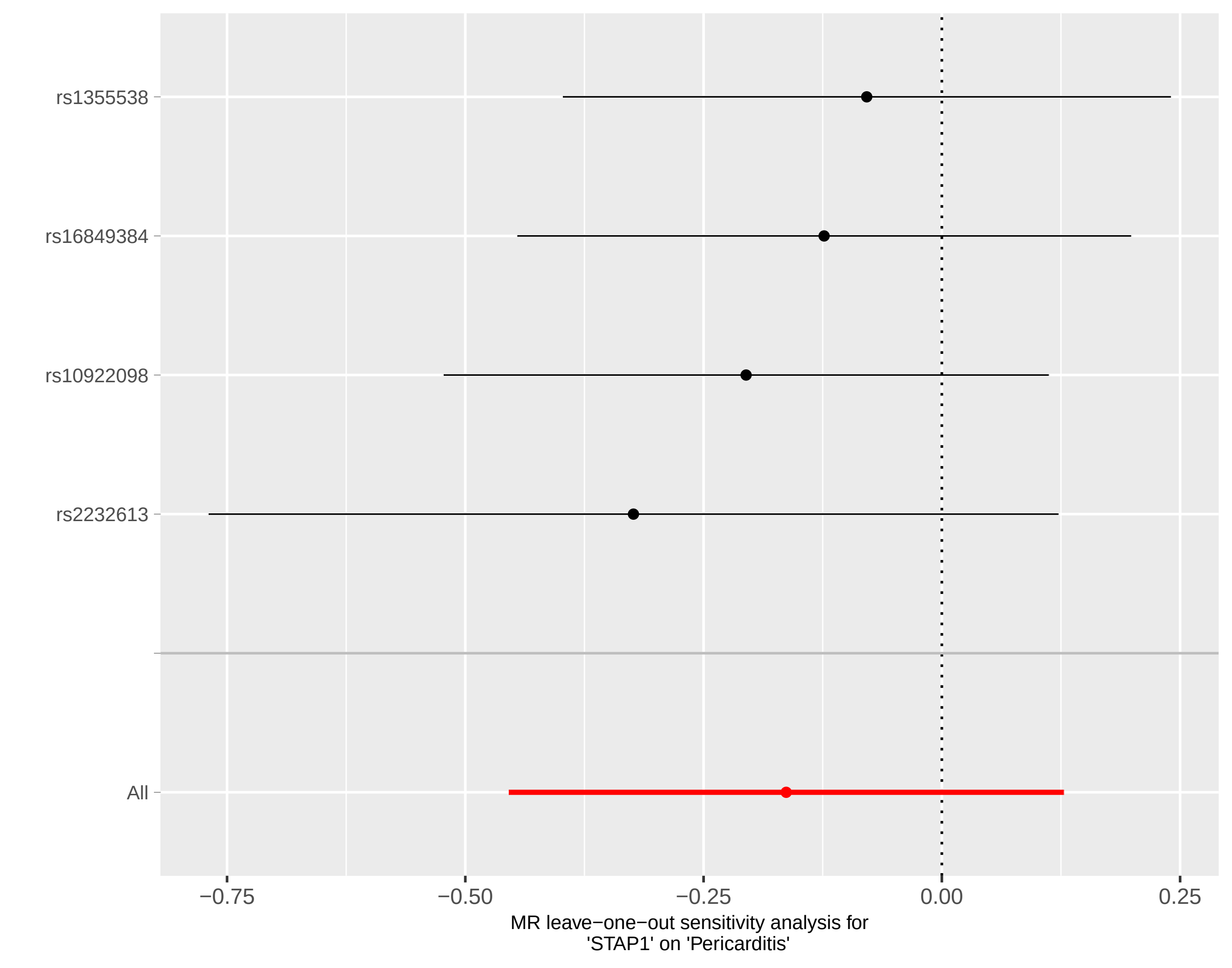
rs1355538
rs16849384
rs10922098
rs2232613
All
−0.75
−0.50
−0.25
0.00
0.25
MR leave−one−out sensitivity analysis for
'STAP1' on 'Pericarditis'

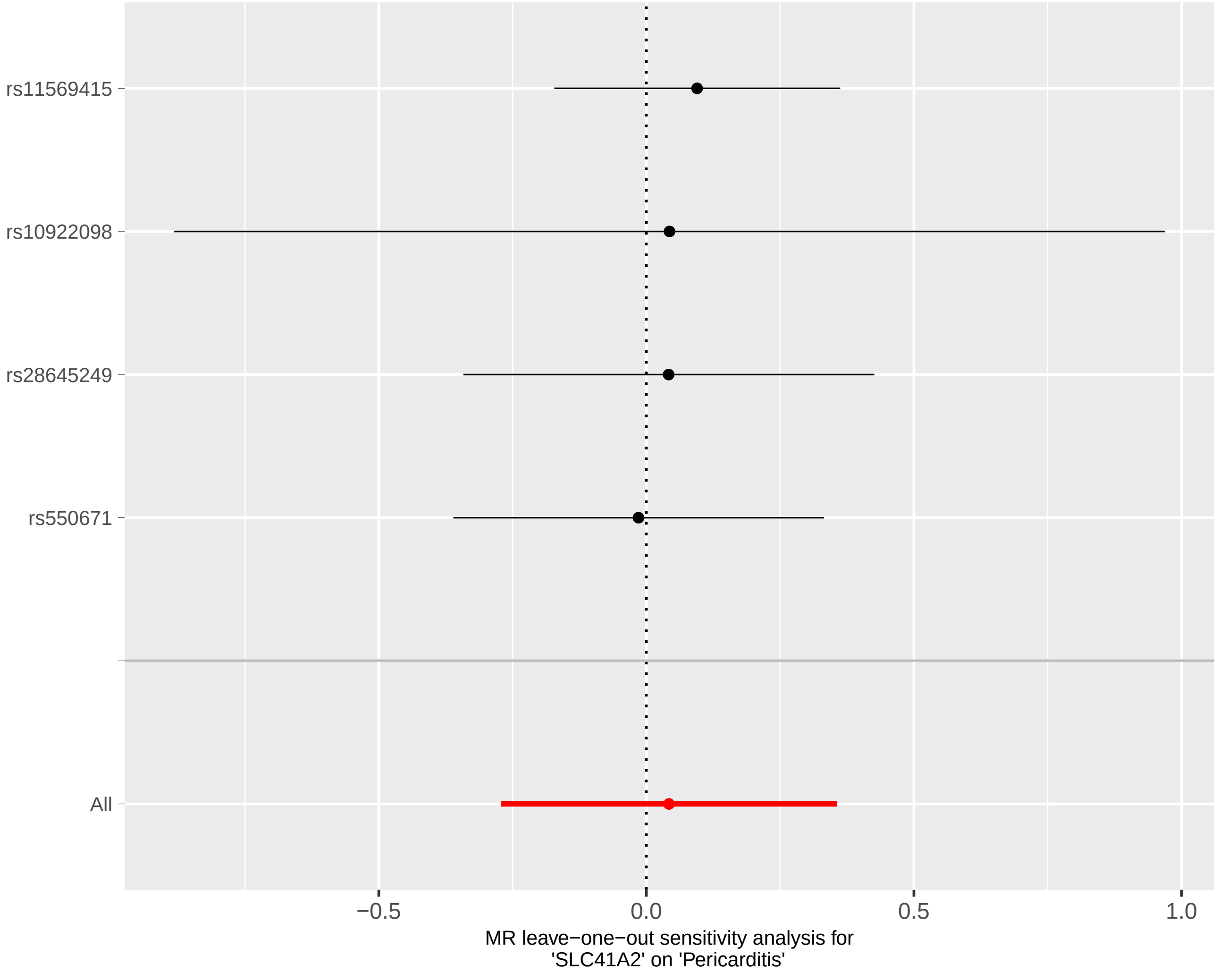

rs11569415
rs10922098
rs28645249
rs550671
All
−0.5
0.0
0.5
1.0
MR leave−one−out sensitivity analysis for
'SLC41A2' on 'Pericarditis'

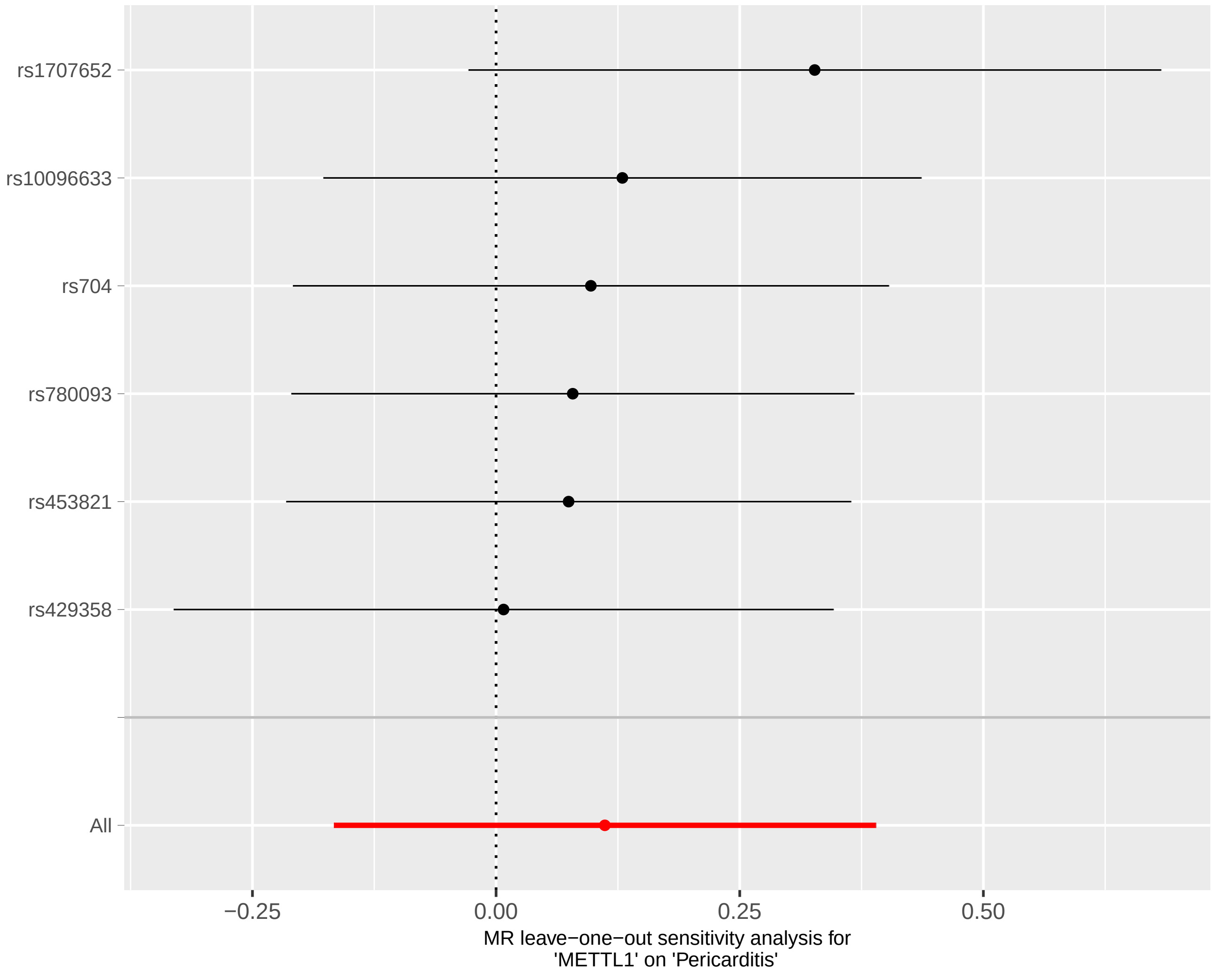

rs1707652
rs10096633
rs704
rs780093
rs453821
rs429358
All
−0.25
0.00
0.25
0.50
MR leave−one−out sensitivity analysis for
'METTL1' on 'Pericarditis'

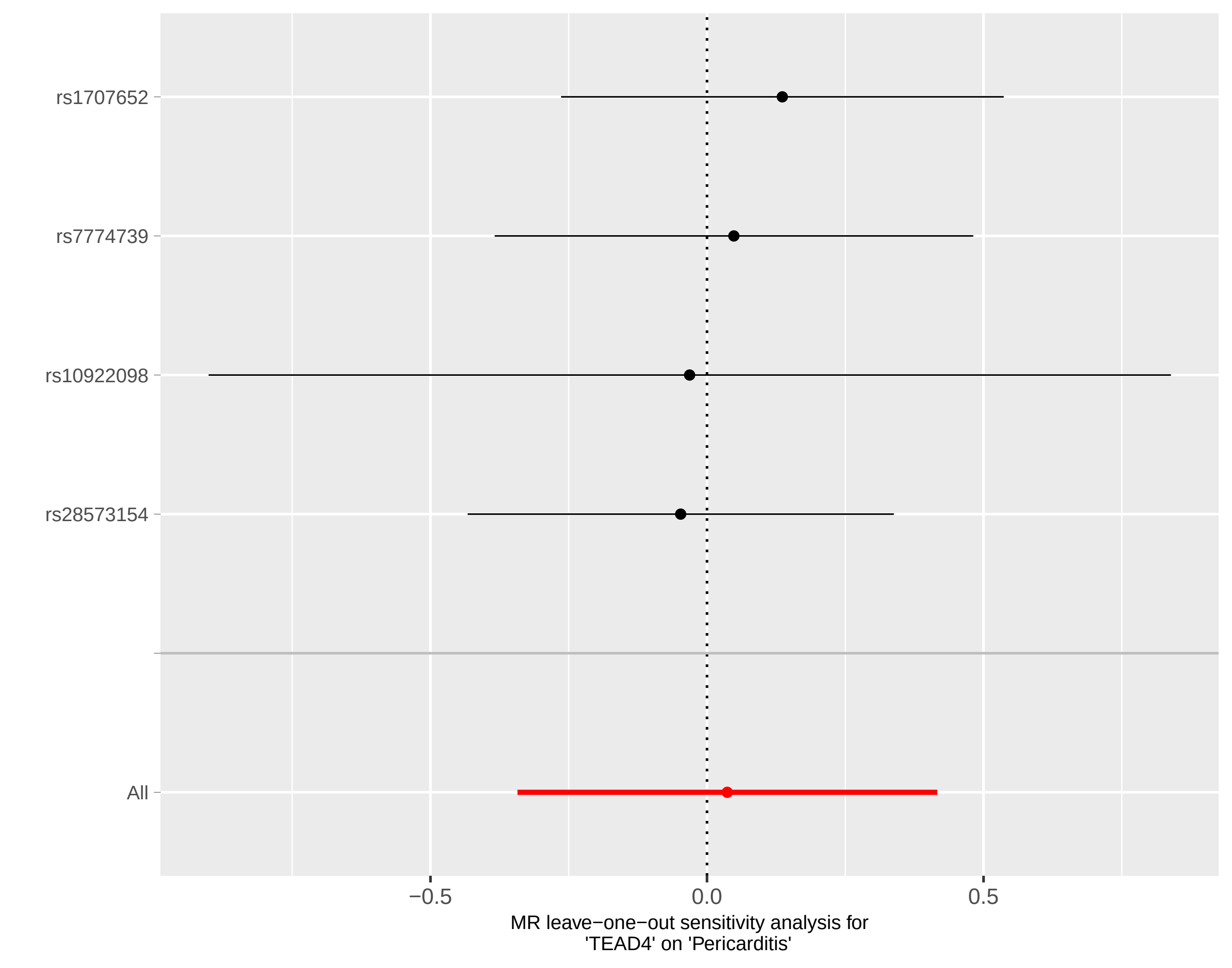
rs1707652
rs7774739
rs10922098
rs28573154
All
−0.5
0.0
0.5
MR leave−one−out sensitivity analysis for
'TEAD4' on 'Pericarditis'

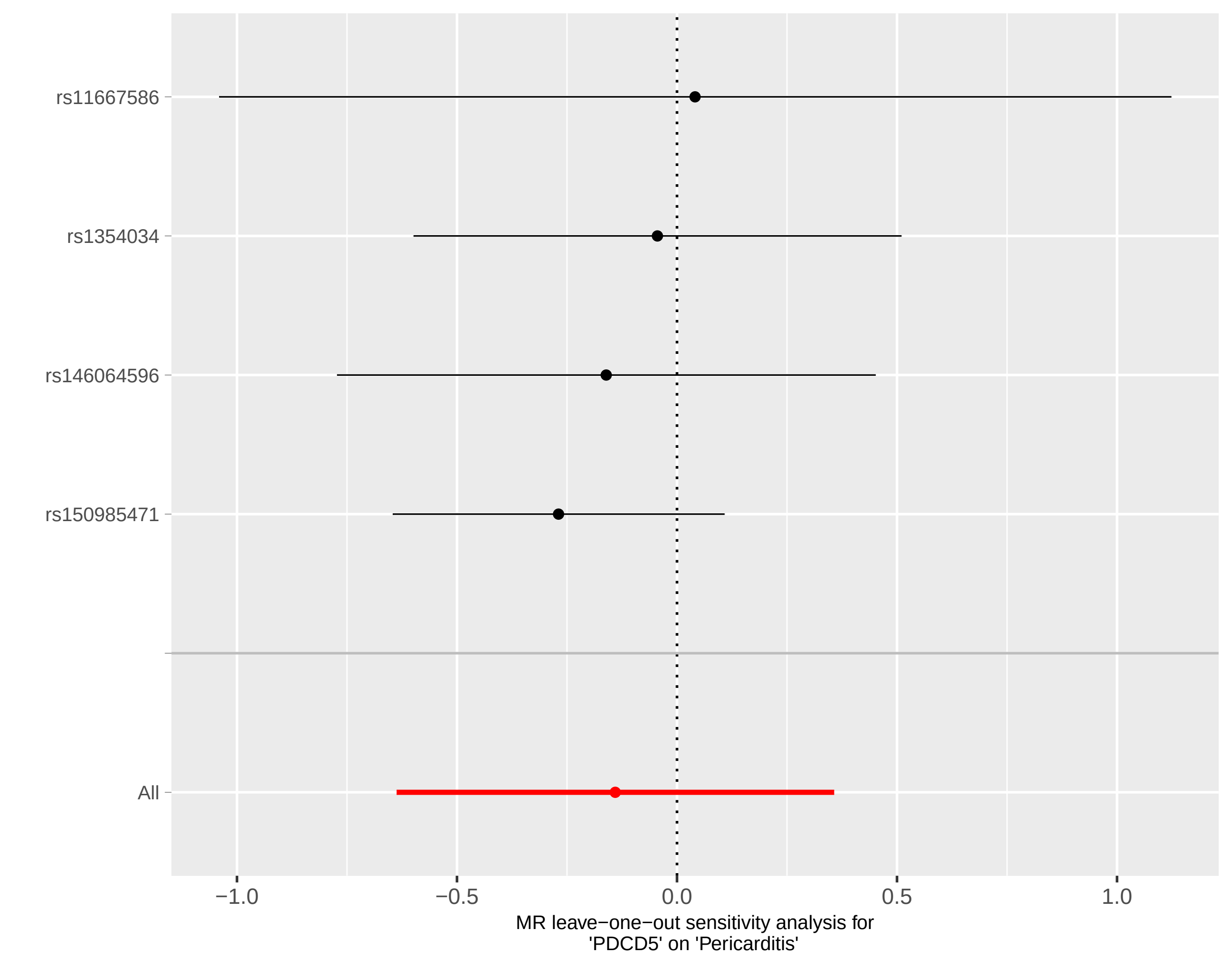
rs11667586
rs1354034
rs146064596
rs150985471
All
−1.0
−0.5
0.0
0.5
1.0
MR leave−one−out sensitivity analysis for
'PDCD5' on 'Pericarditis'

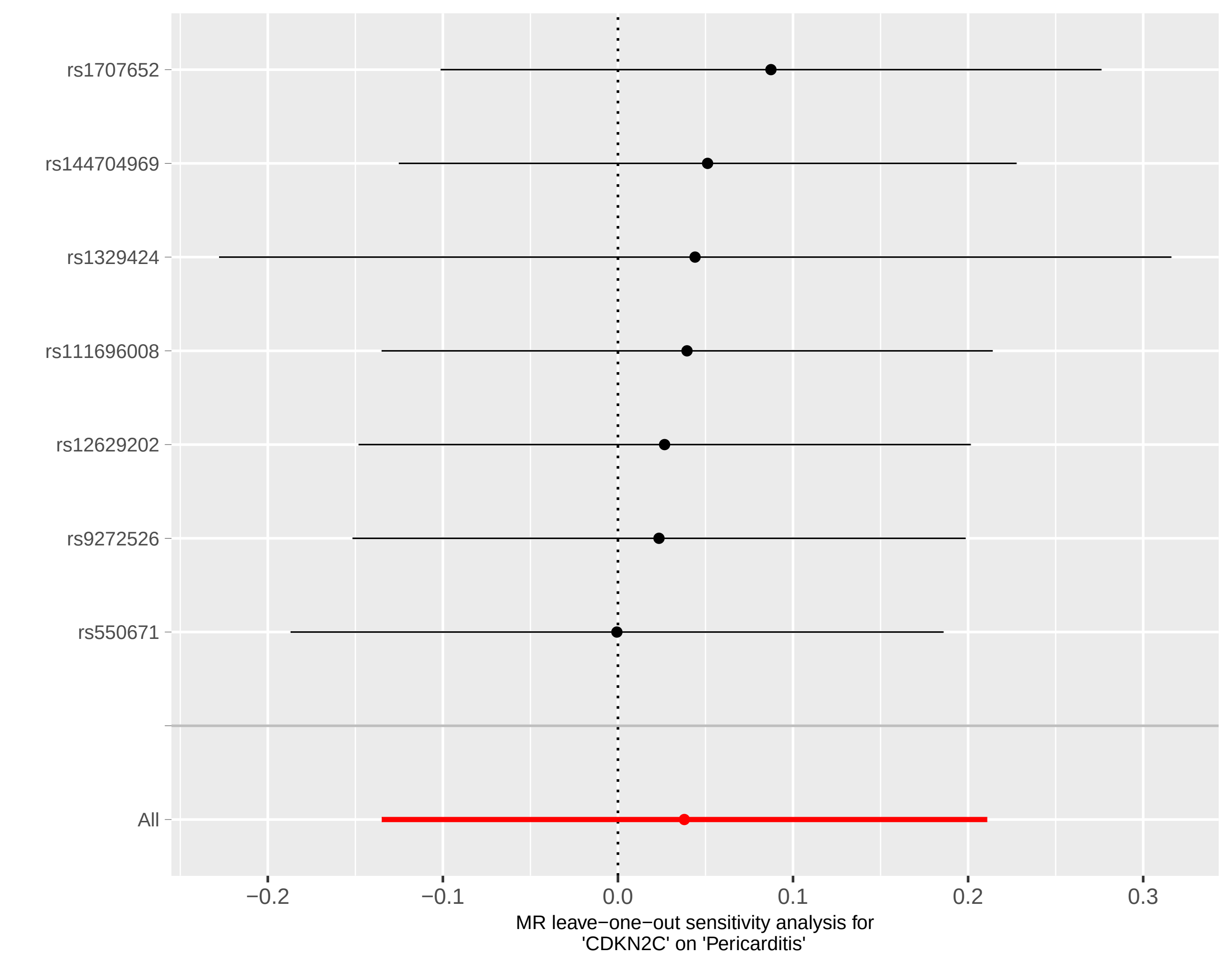

rs1707652
rs144704969
rs1329424
rs111696008
rs12629202
rs9272526
rs550671
All
−0.2
−0.1
0.0
0.1
0.2
0.3
MR leave−one−out sensitivity analysis for
'CDKN2C' on 'Pericarditis'

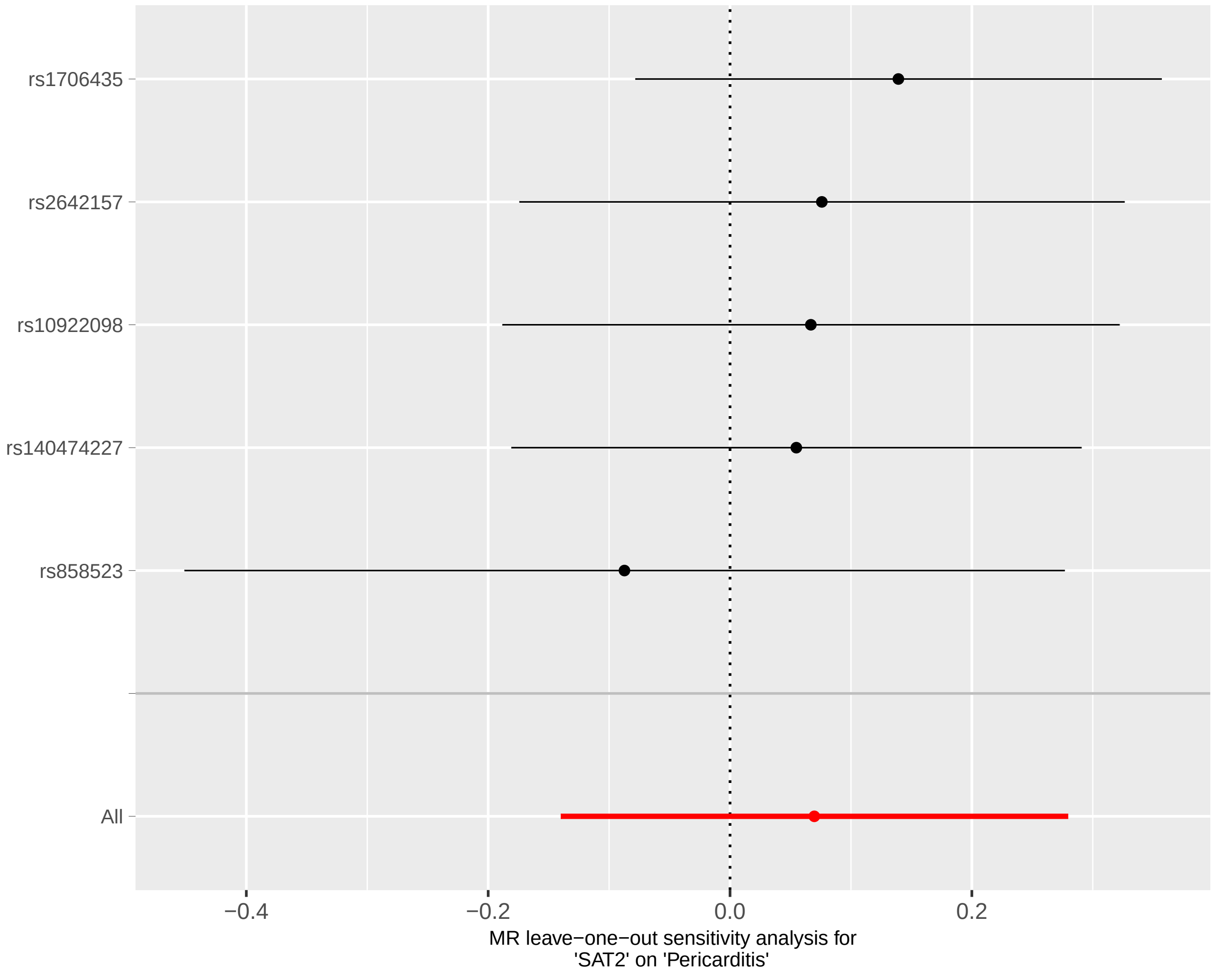
rs1706435
rs2642157
rs10922098
rs140474227
rs858523
All
−0.4
−0.2
0.0
0.2
MR leave−one−out sensitivity analysis for
'SAT2' on 'Pericarditis'

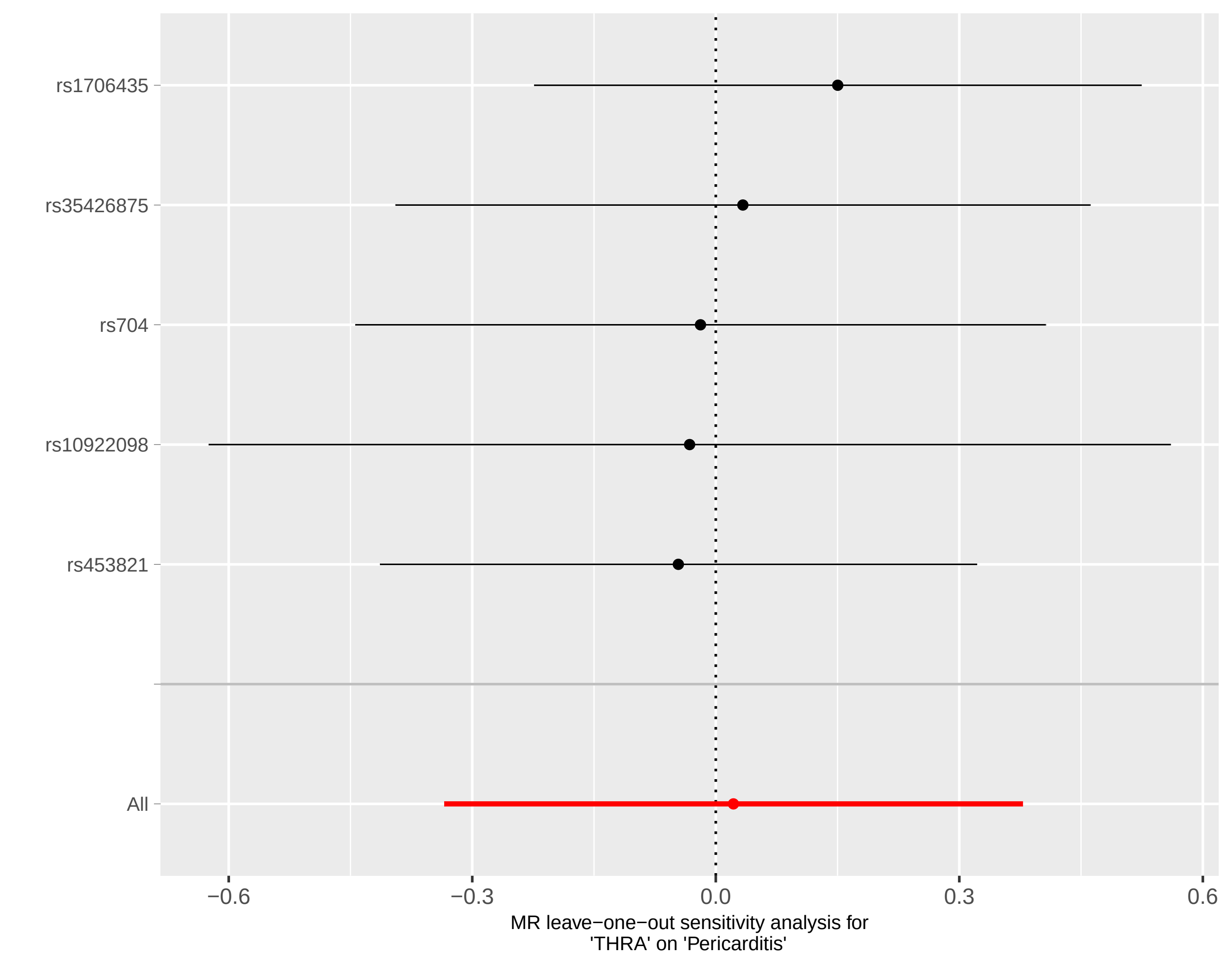
rs1706435
rs35426875
rs704
rs10922098
rs453821
All
−0.6
−0.3
0.0
0.3
0.6
MR leave−one−out sensitivity analysis for
'THRA' on 'Pericarditis'

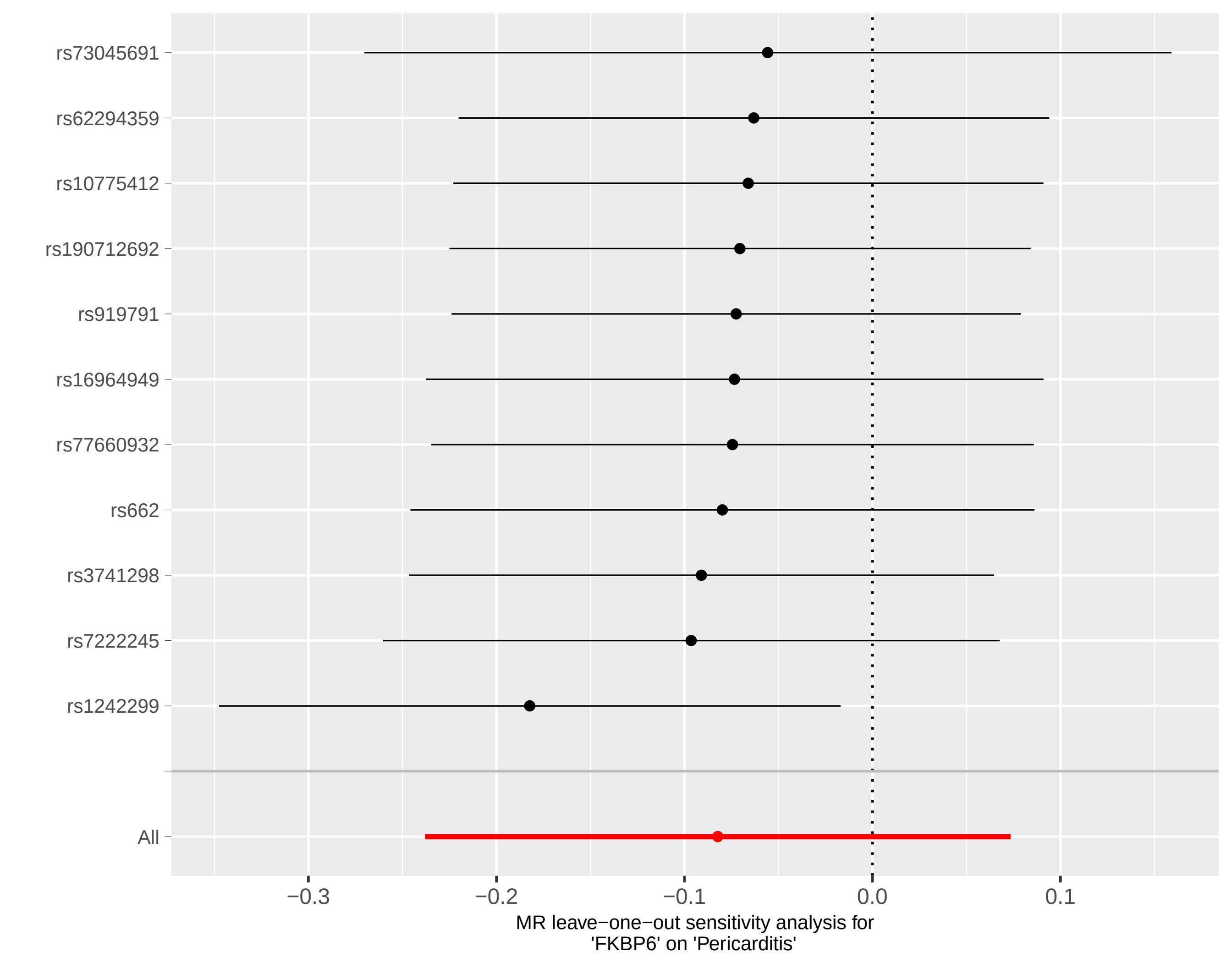
rs73045691
rs62294359
rs10775412
rs190712692
rs919791
rs16964949
rs77660932
rs662
rs3741298
rs7222245
rs1242299
All
−0.3
−0.2
−0.1
0.0
0.1
MR leave−one−out sensitivity analysis for
'FKBP6' on 'Pericarditis'

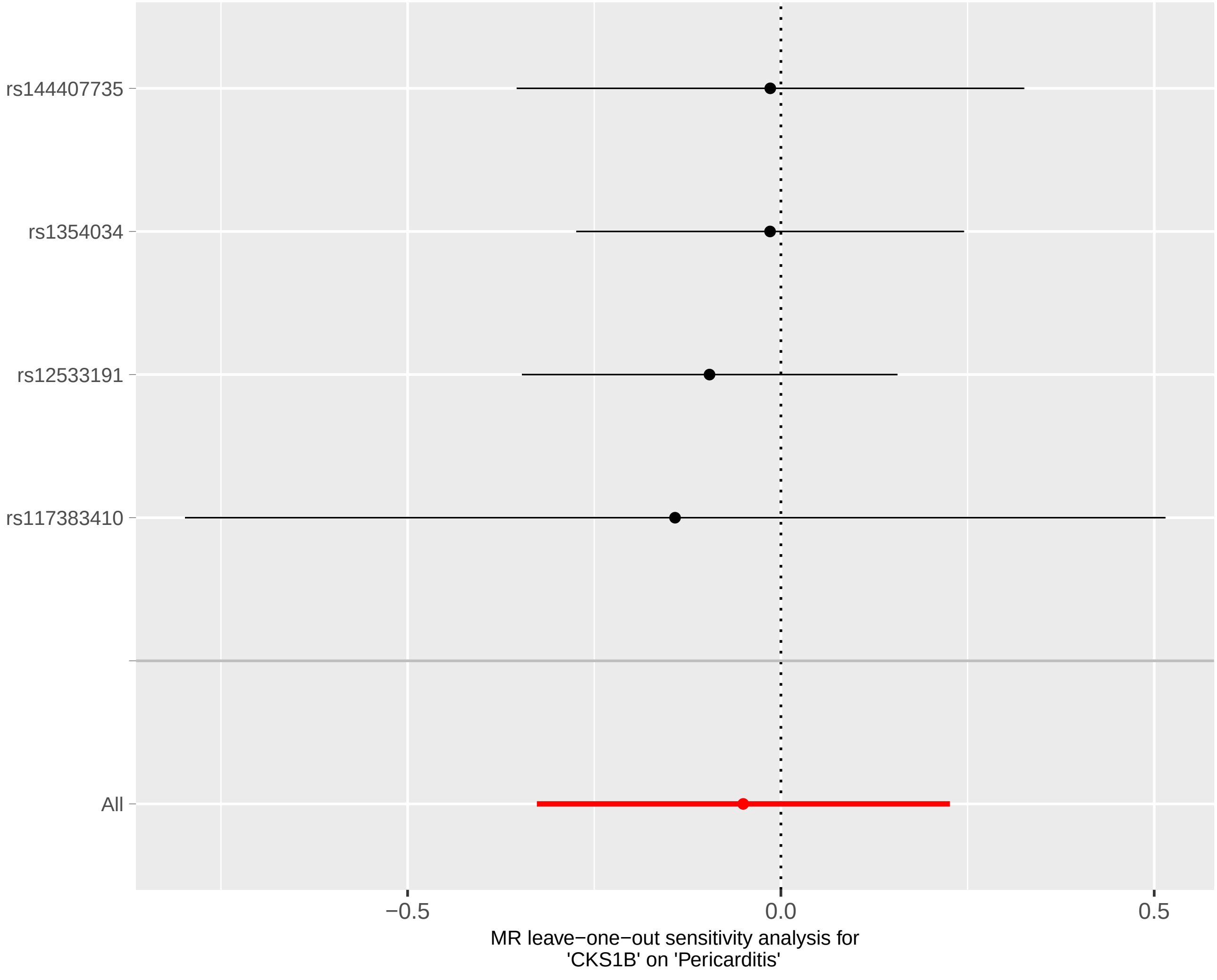
rs144407735
rs1354034
rs12533191
rs117383410
All
−0.5
0.0
0.5
MR leave−one−out sensitivity analysis for
'CKS1B' on 'Pericarditis'

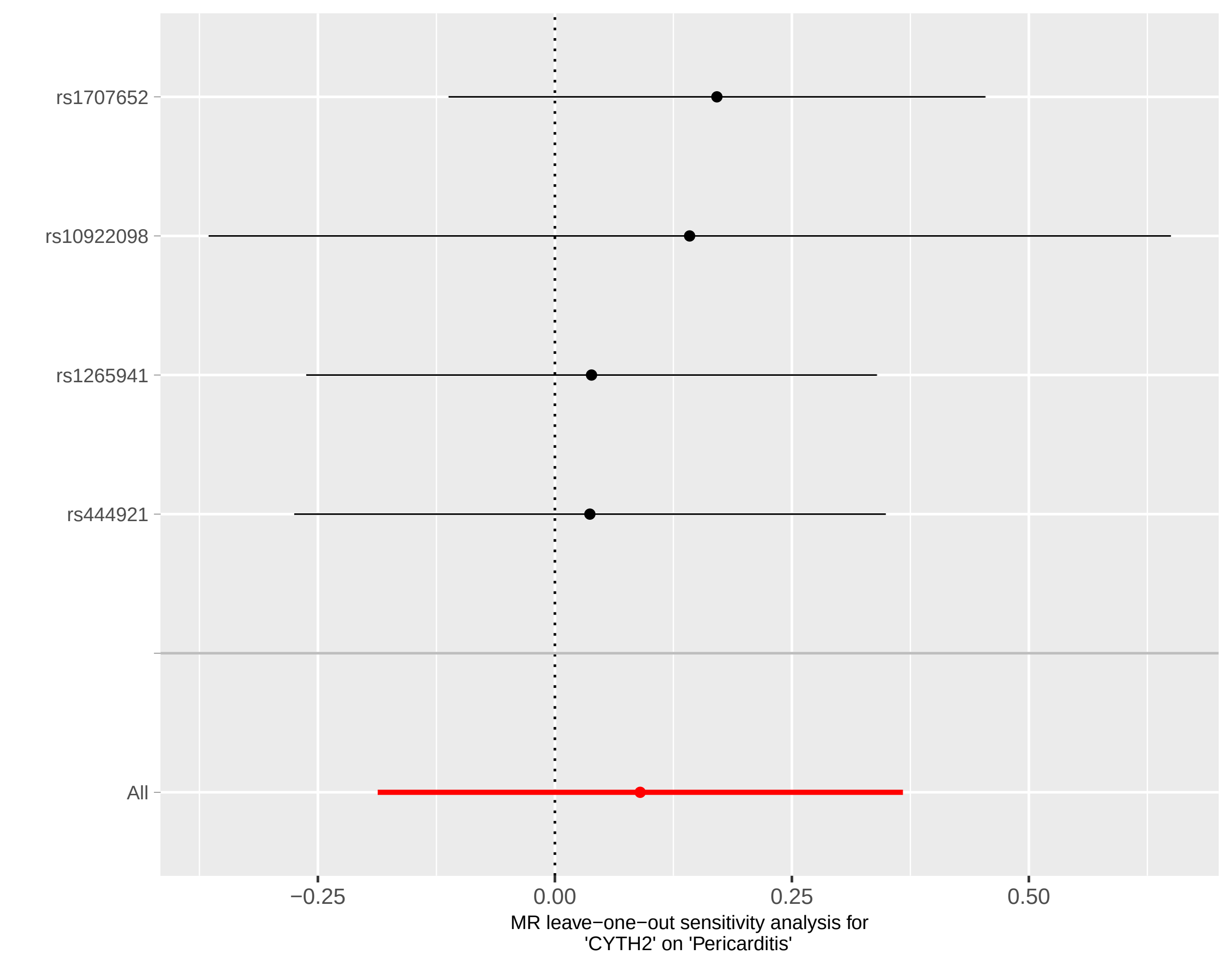
rs1707652
rs10922098
rs1265941
rs444921
All
−0.25
0.00
0.25
0.50
MR leave−one−out sensitivity analysis for
'CYTH2' on 'Pericarditis'

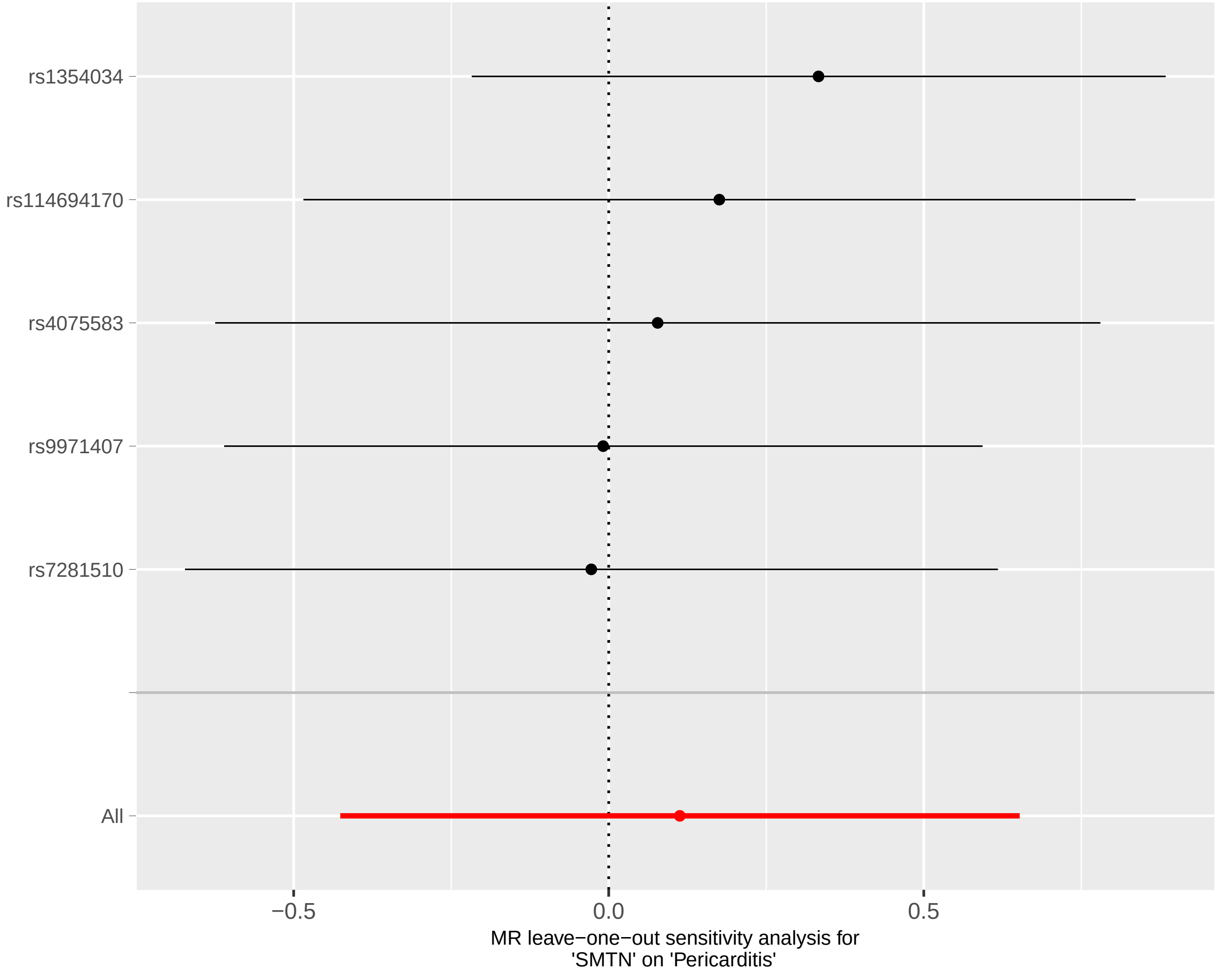

rs1354034
rs114694170
rs4075583
rs9971407
rs7281510
All
−0.5
0.0
0.5
MR leave−one−out sensitivity analysis for
'SMTN' on 'Pericarditis'

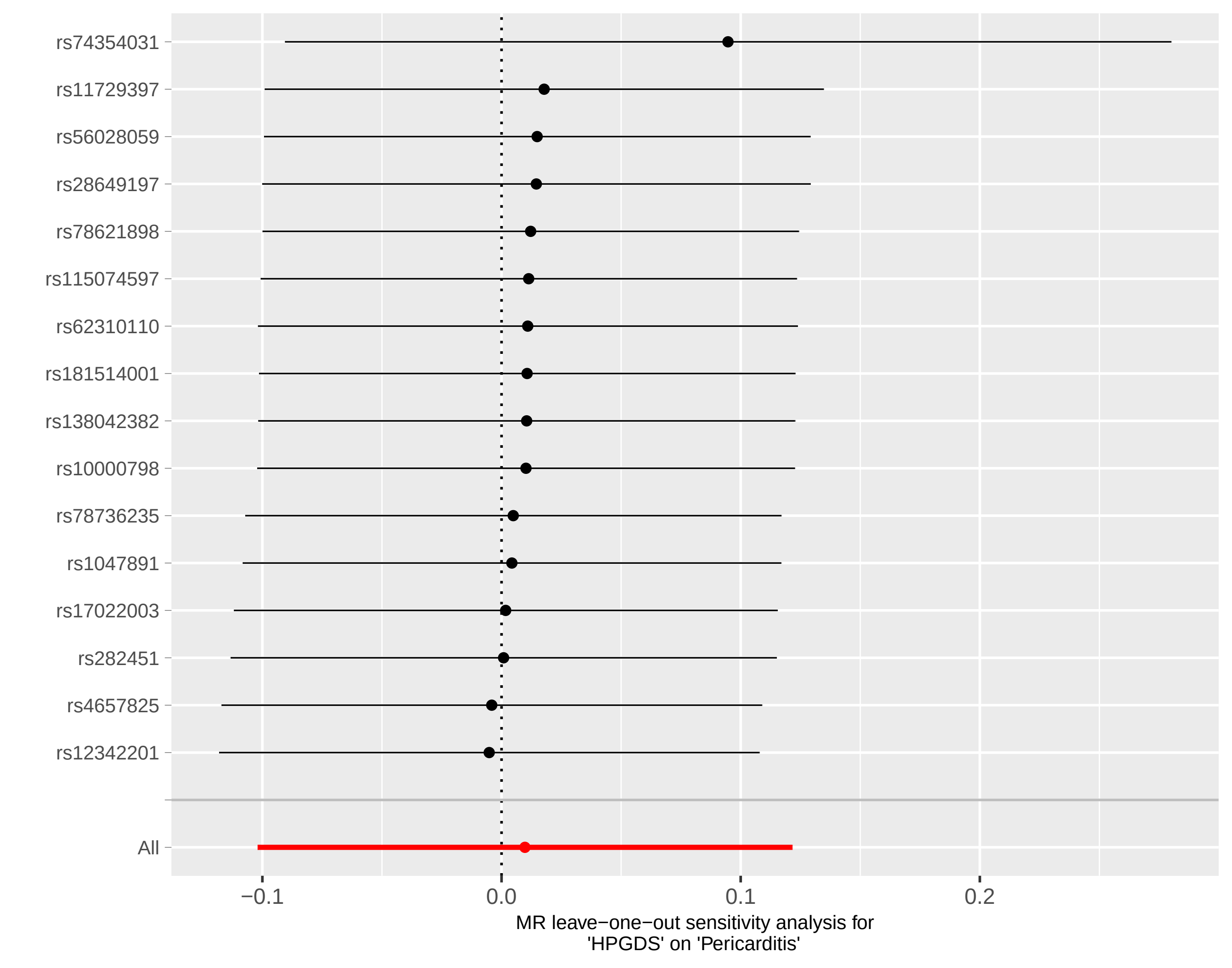
rs74354031
rs11729397
rs56028059
rs28649197
rs78621898
rs115074597
rs62310110
rs181514001
rs138042382
rs10000798
rs78736235
rs1047891
rs17022003
rs282451
rs4657825
rs12342201
All
−0.1
0.0
0.1
0.2
MR leave−one−out sensitivity analysis for
'HPGDS' on 'Pericarditis'

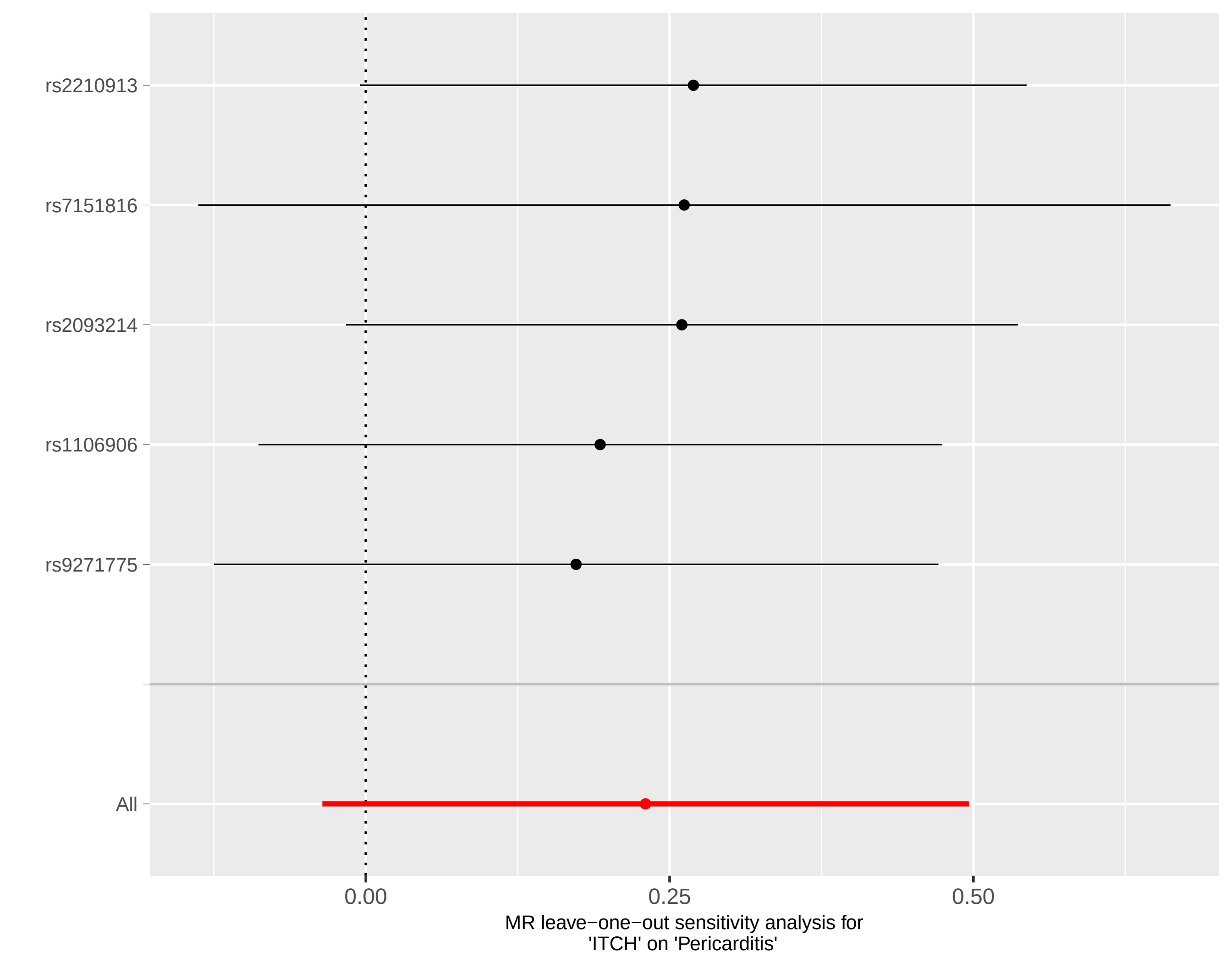

rs2210913
rs7151816
rs2093214
rs1106906
rs9271775
All
0.00
0.25
0.50
MR leave−one−out sensitivity analysis for
'ITCH' on 'Pericarditis'

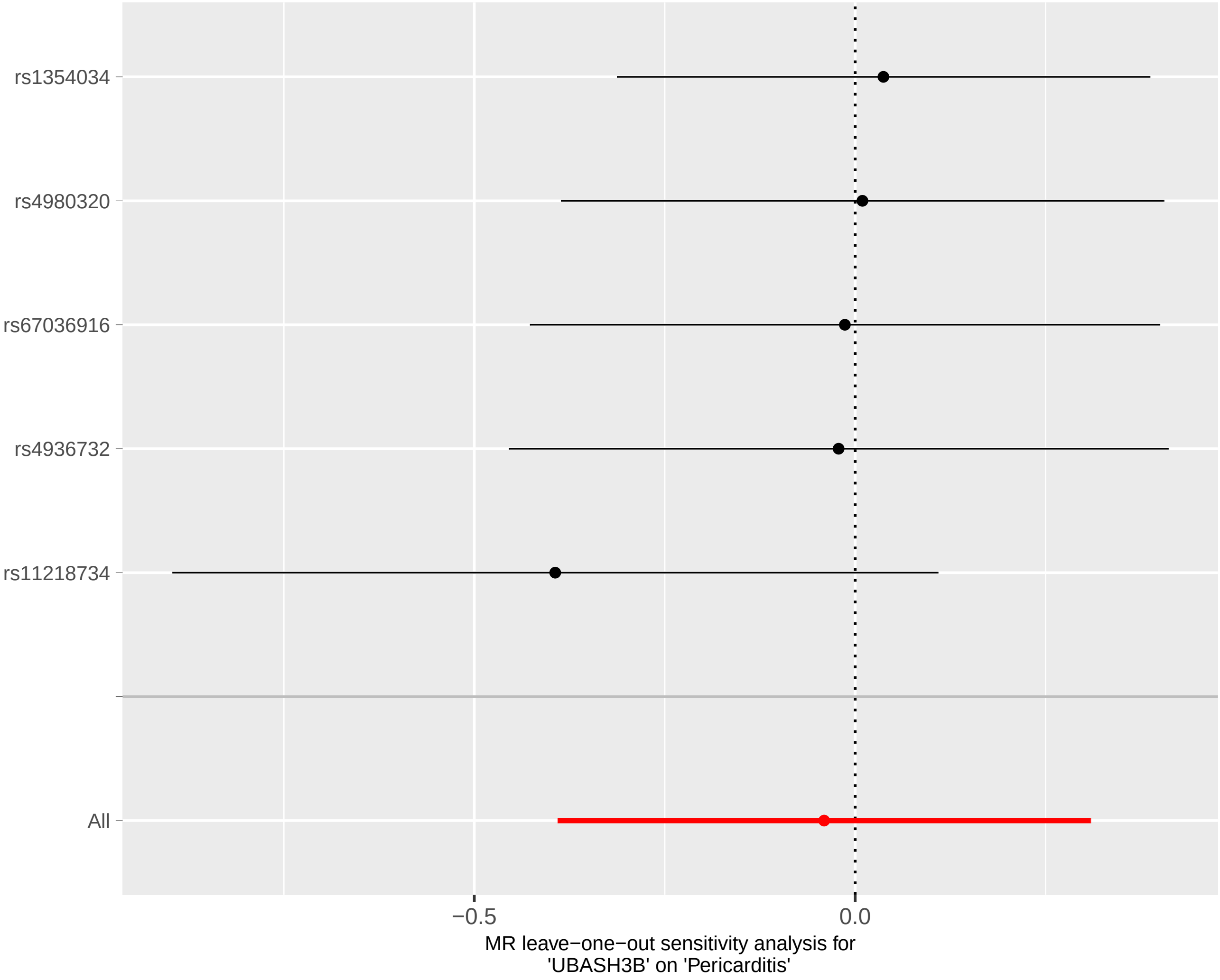
rs1354034
rs4980320
rs67036916
rs4936732
rs11218734
All
−0.5
0.0
MR leave−one−out sensitivity analysis for
'UBASH3B' on 'Pericarditis'

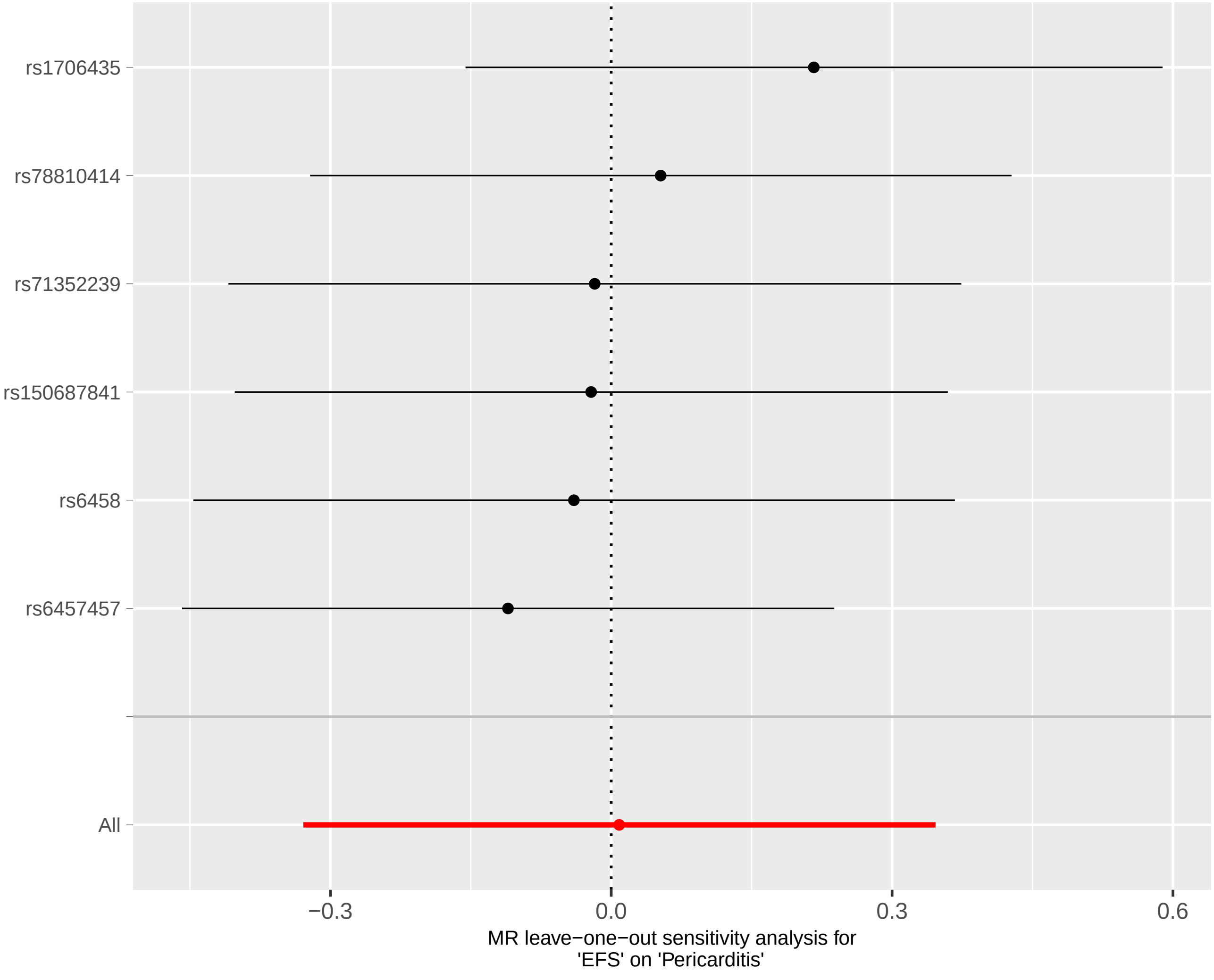
rs1706435
rs78810414
rs71352239
rs150687841
rs6458
rs6457457
All
−0.3
0.0
0.3
0.6
MR leave−one−out sensitivity analysis for
'EFS' on 'Pericarditis'

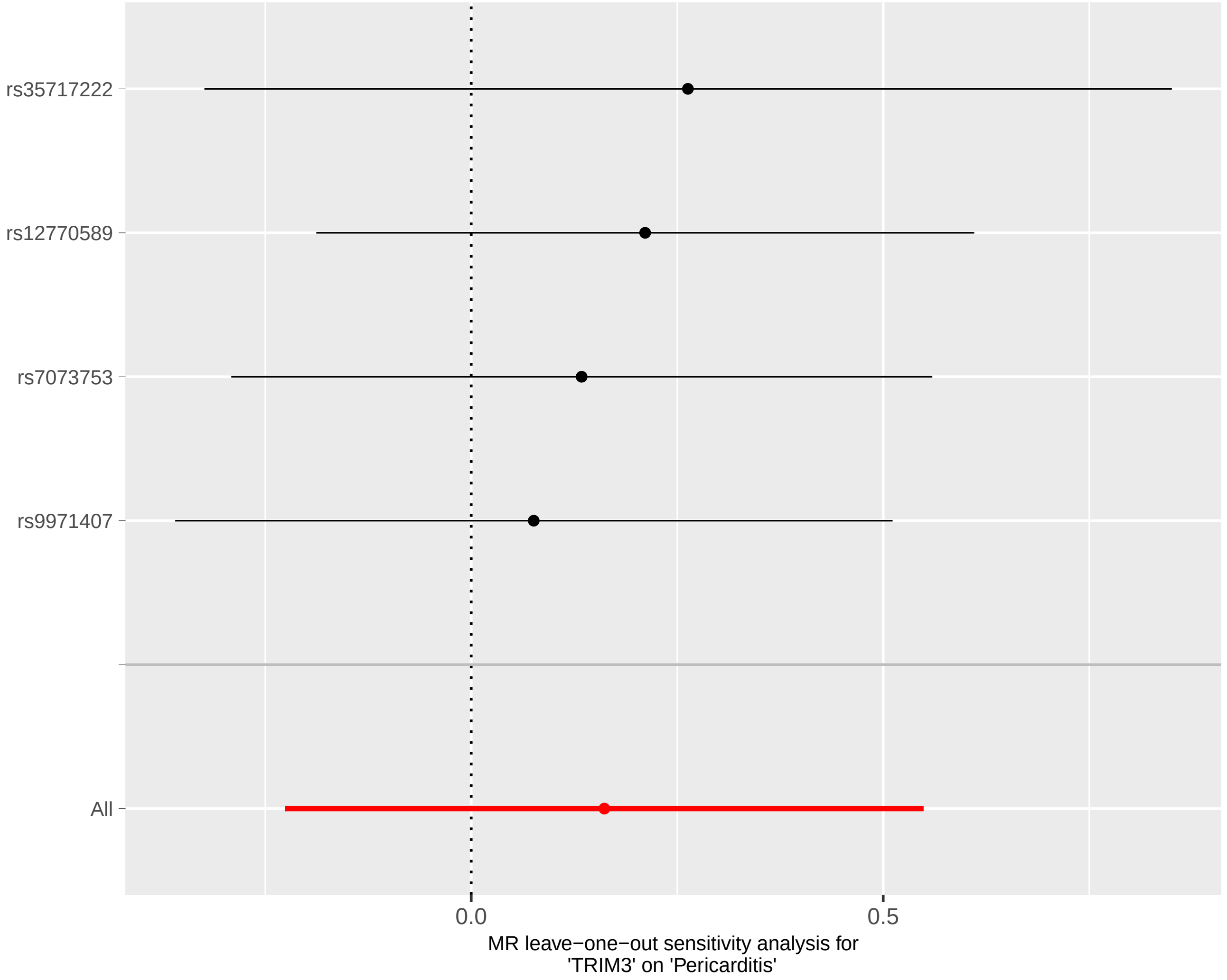

rs35717222
rs12770589
rs7073753
rs9971407
All
0.0
0.5
MR leave−one−out sensitivity analysis for
'TRIM3' on 'Pericarditis'

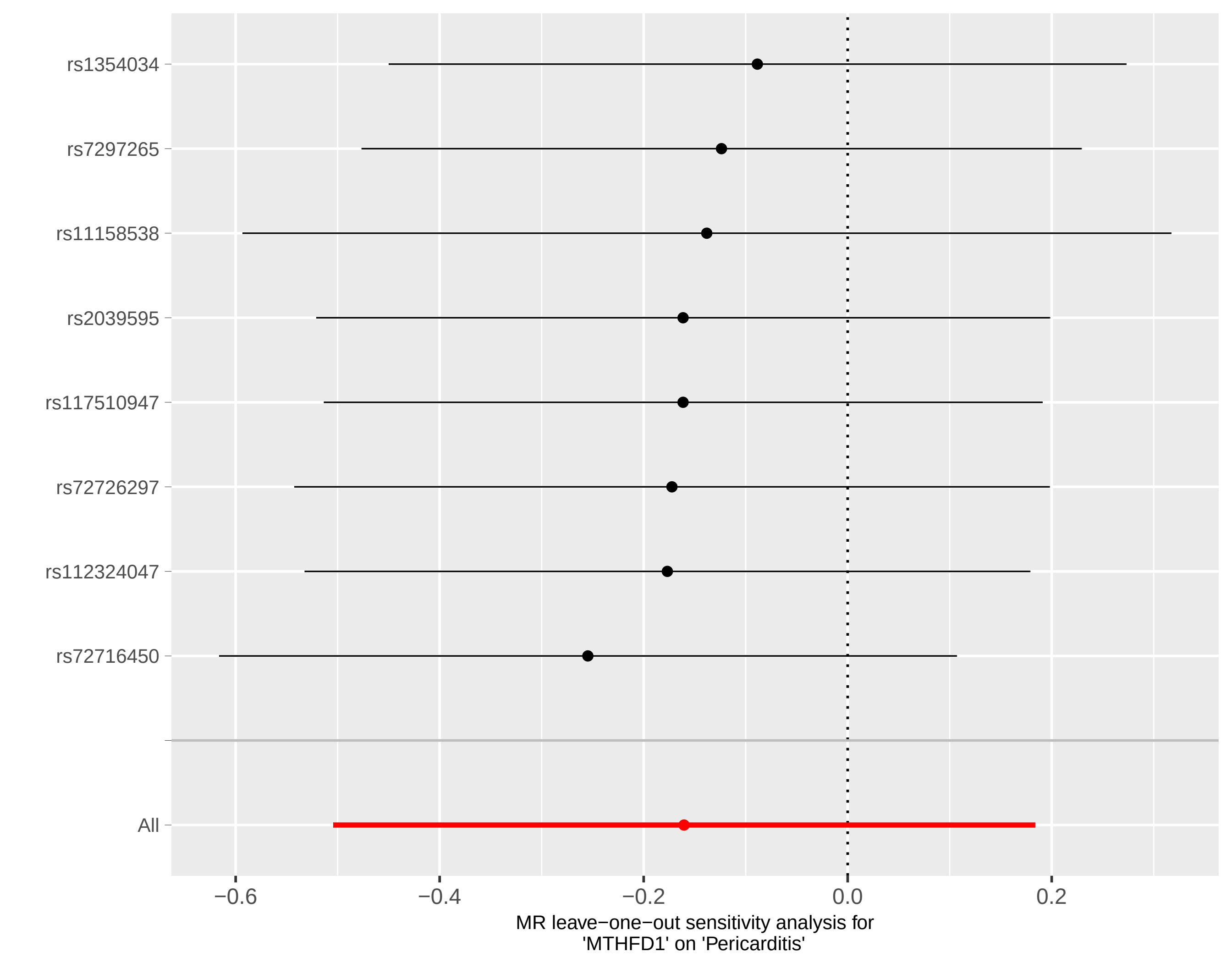
rs1354034
rs7297265
rs11158538
rs2039595
rs117510947
rs72726297
rs112324047
rs72716450
All
−0.6
−0.4
−0.2
0.0
0.2
MR leave−one−out sensitivity analysis for
'MTHFD1' on 'Pericarditis'

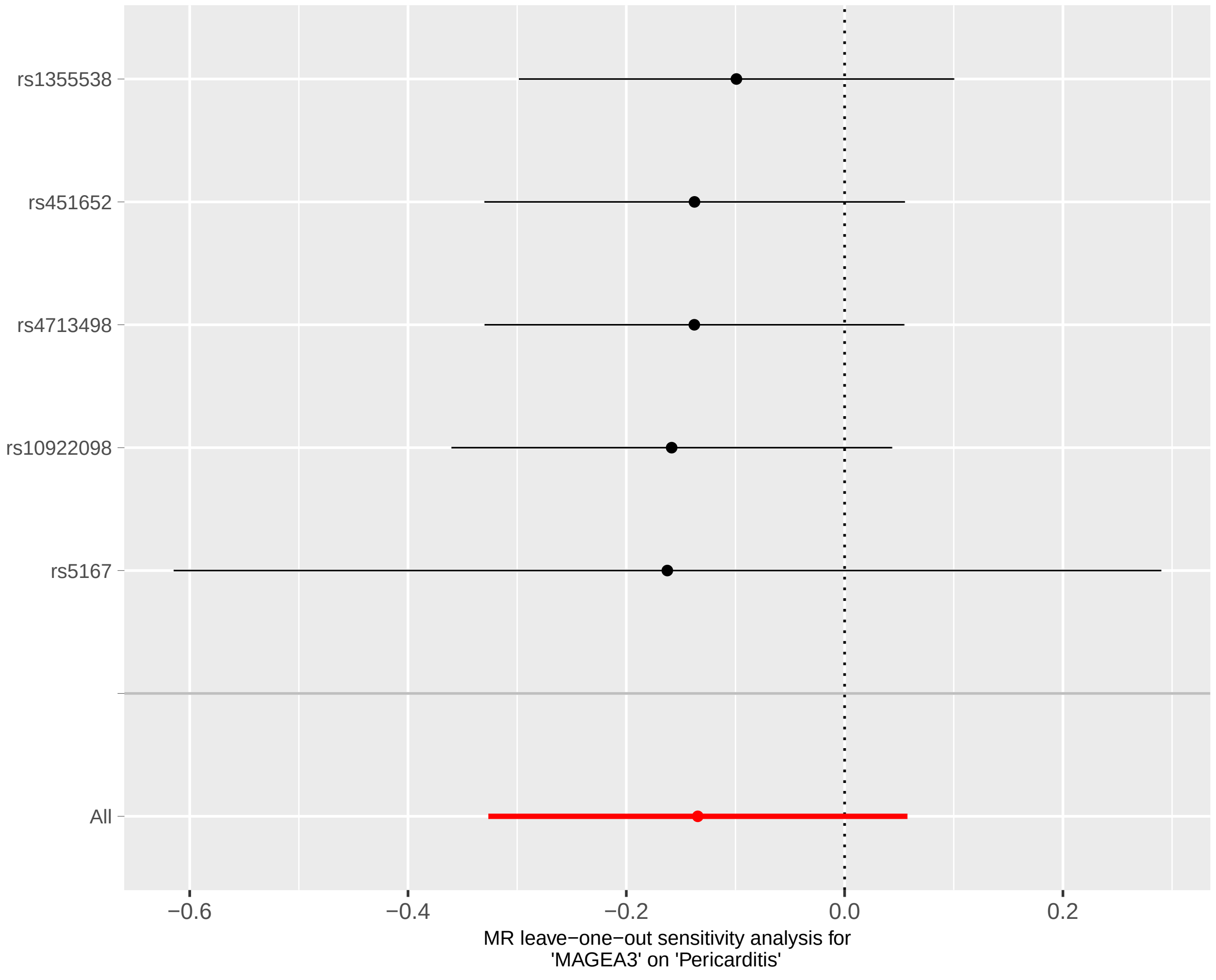
rs1355538
rs451652
rs4713498
rs10922098
rs5167
All
−0.6
−0.4
−0.2
0.0
0.2
MR leave−one−out sensitivity analysis for
'MAGEA3' on 'Pericarditis'

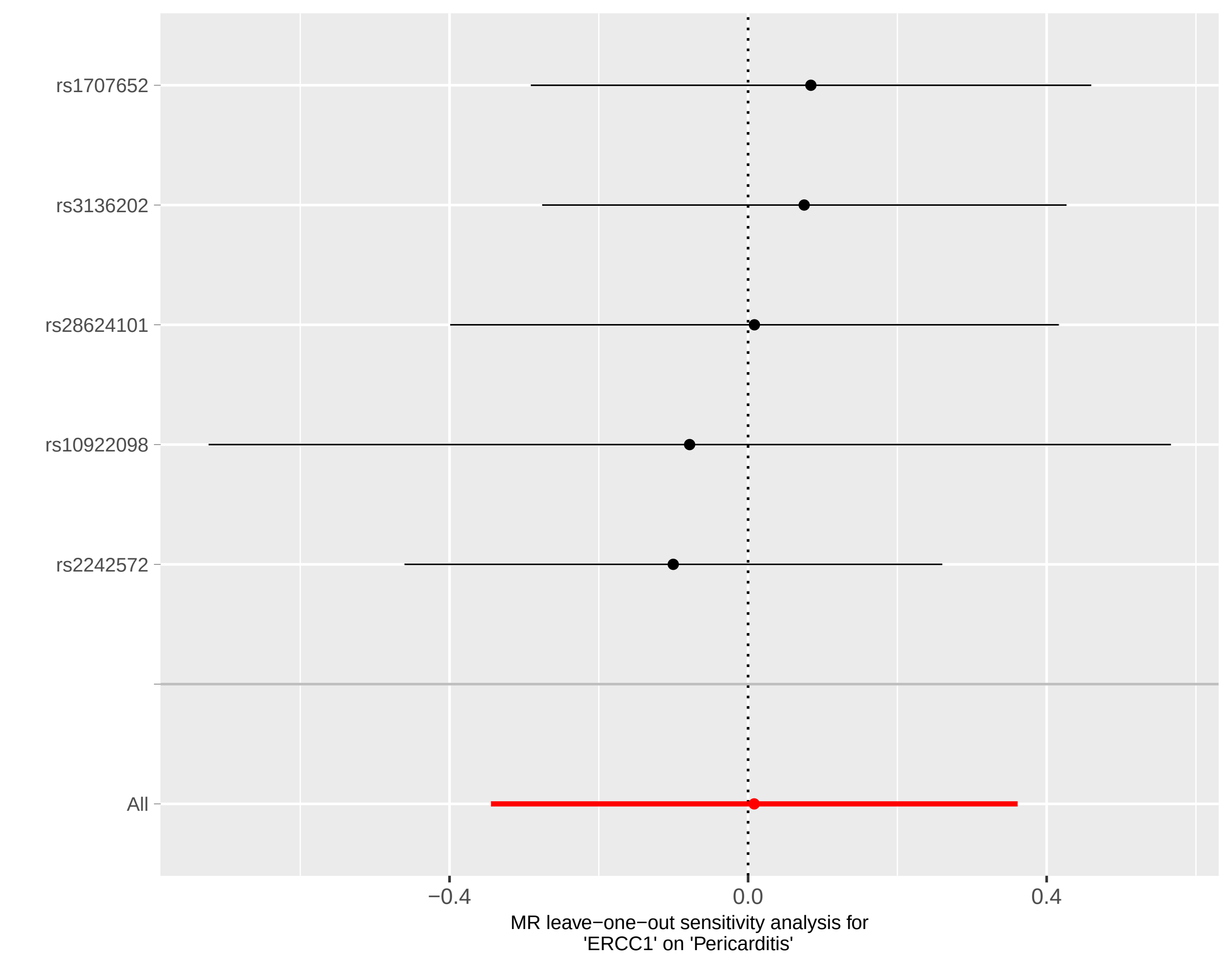
rs1707652
rs3136202
rs28624101
rs10922098
rs2242572
All
−0.4
0.0
0.4
MR leave−one−out sensitivity analysis for
'ERCC1' on 'Pericarditis'

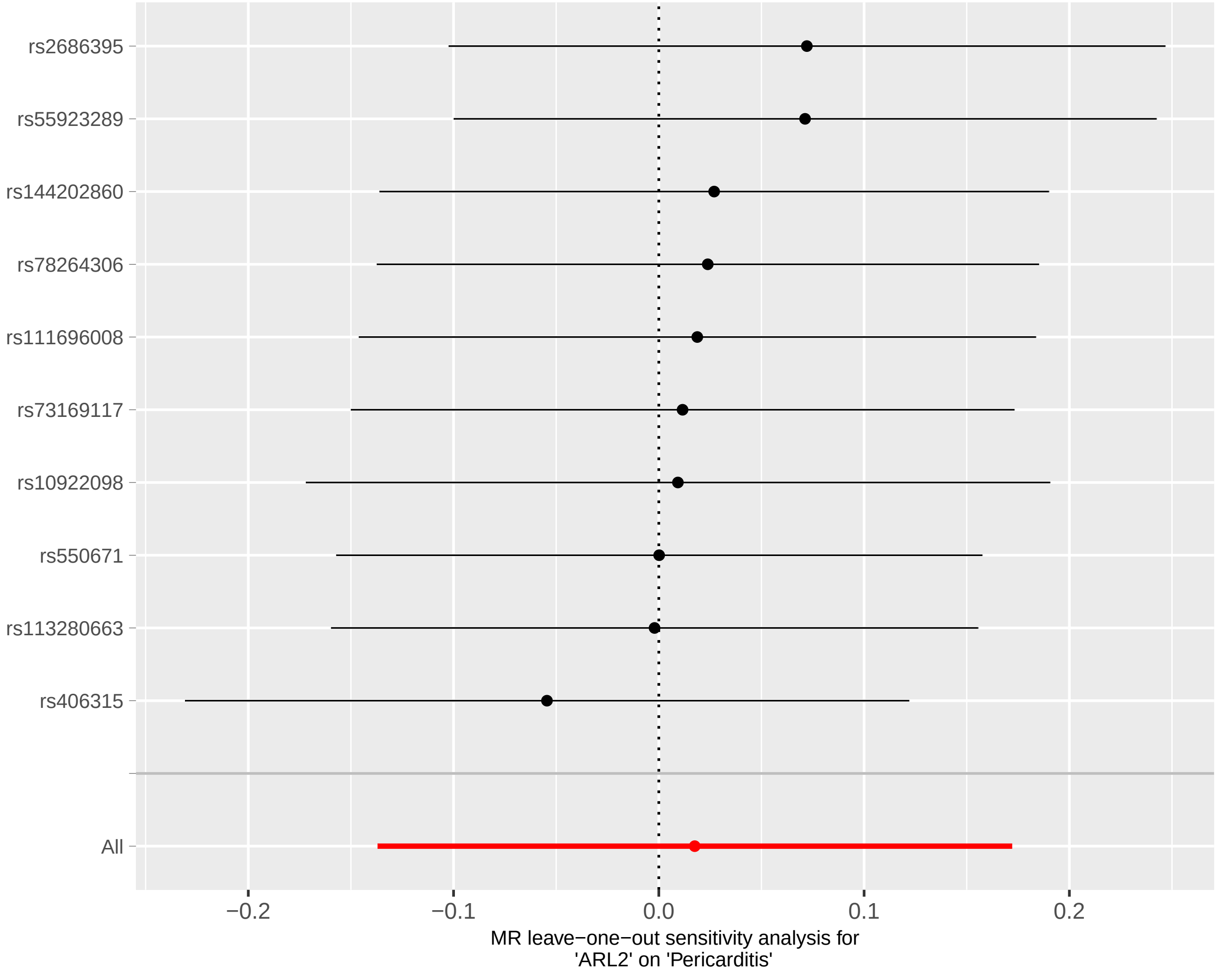
rs2686395
rs55923289
rs144202860
rs78264306
rs111696008
rs73169117
rs10922098
rs550671
rs113280663
rs406315
All
−0.2
−0.1
0.0
0.1
0.2
MR leave−one−out sensitivity analysis for
'ARL2' on 'Pericarditis'

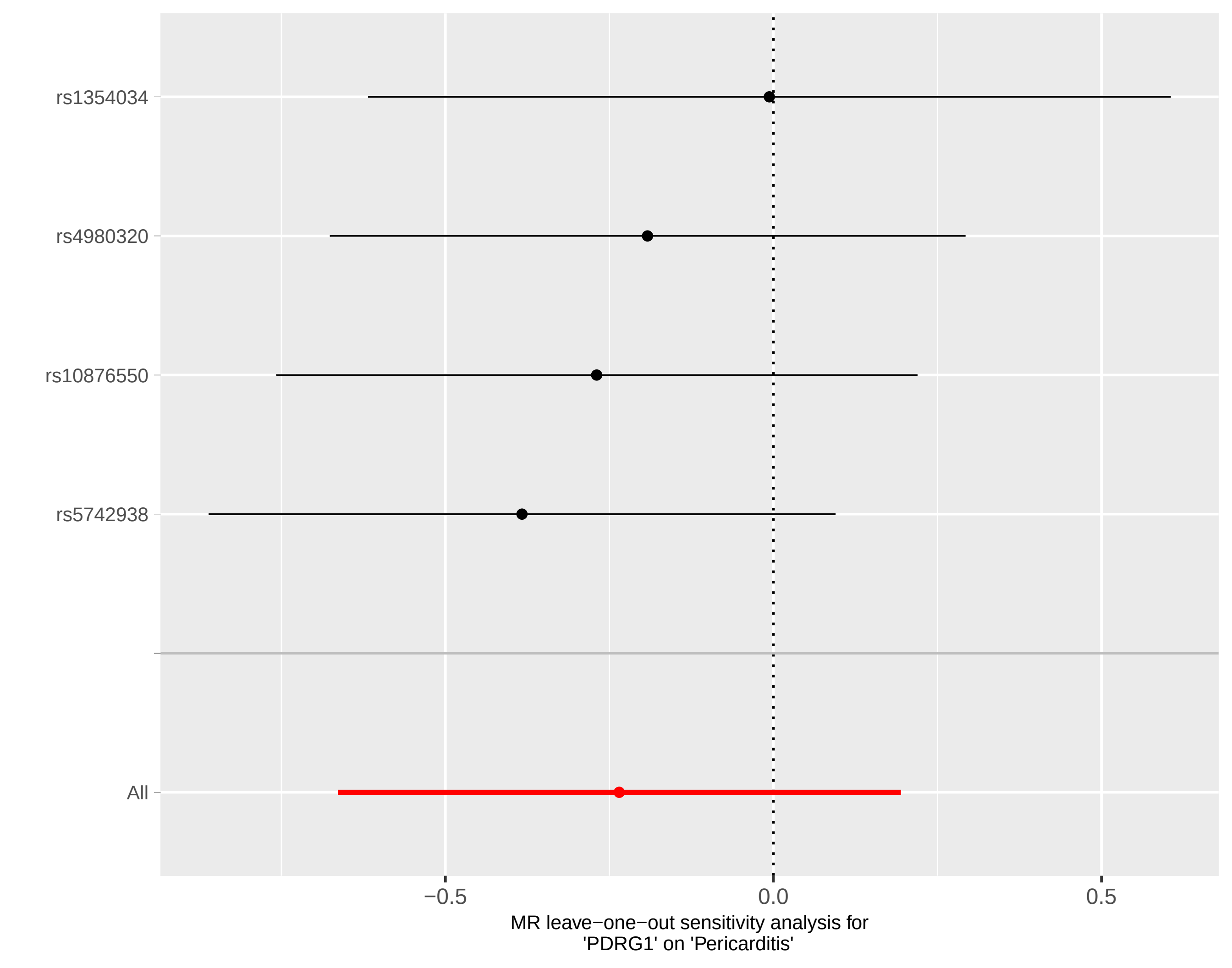
rs1354034
rs4980320
rs10876550
rs5742938
All
−0.5
0.0
0.5
MR leave−one−out sensitivity analysis for
'PDRG1' on 'Pericarditis'

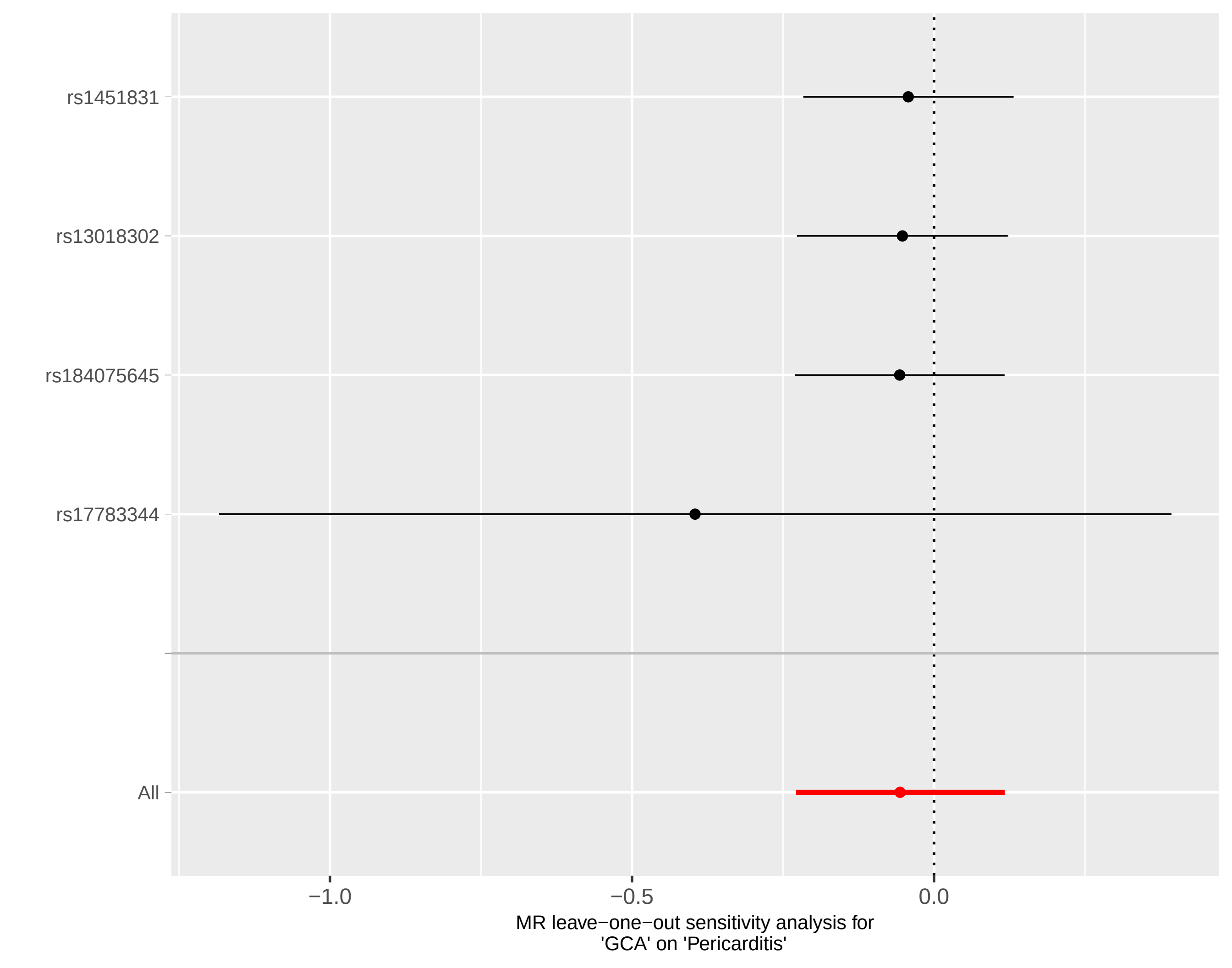
rs1451831
rs13018302
rs184075645
rs17783344
All
−1.0
−0.5
0.0
MR leave−one−out sensitivity analysis for
'GCA' on 'Pericarditis'

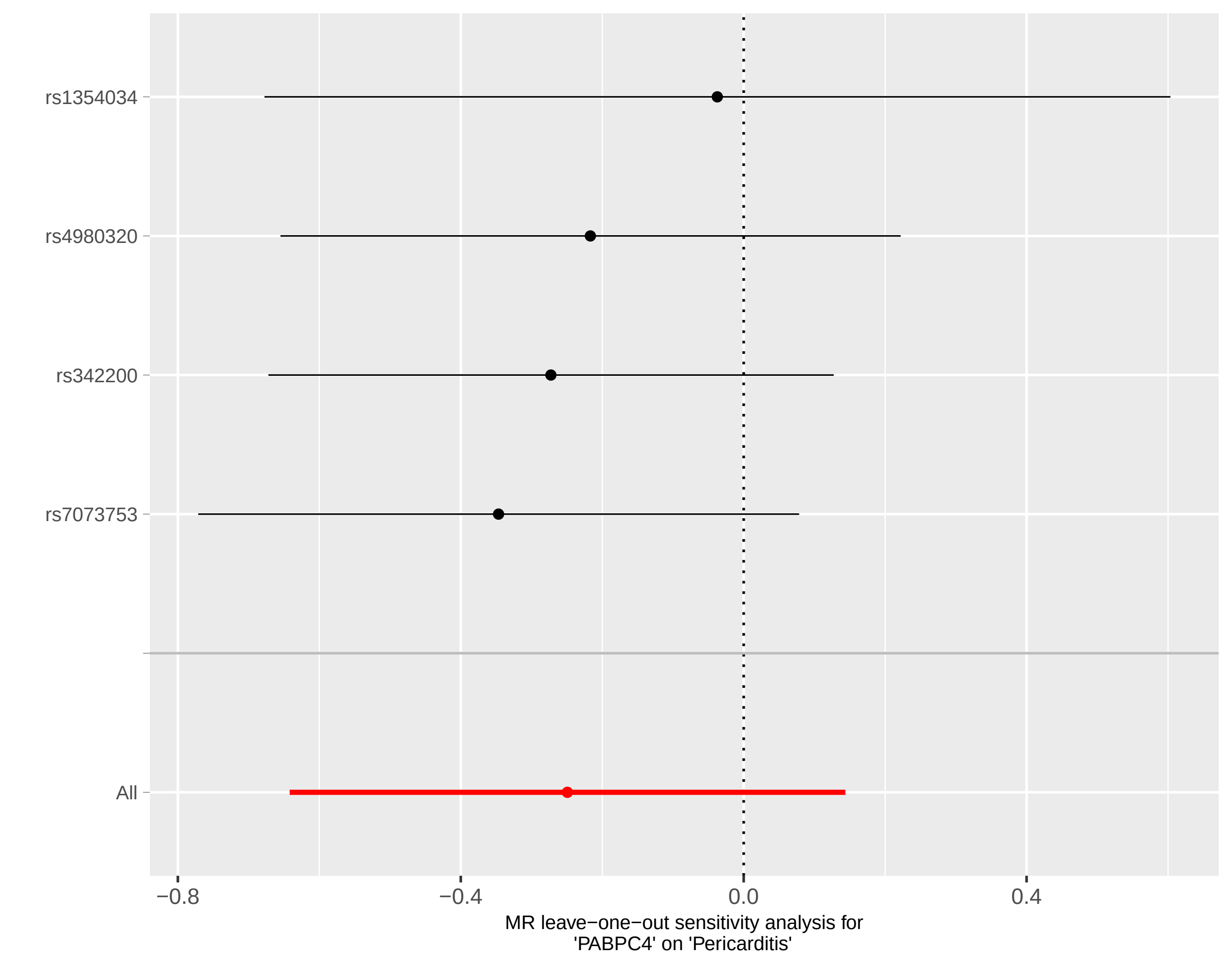
rs1354034
rs4980320
rs342200
rs7073753
All
−0.8
−0.4
0.0
0.4
MR leave−one−out sensitivity analysis for
'PABPC4' on 'Pericarditis'

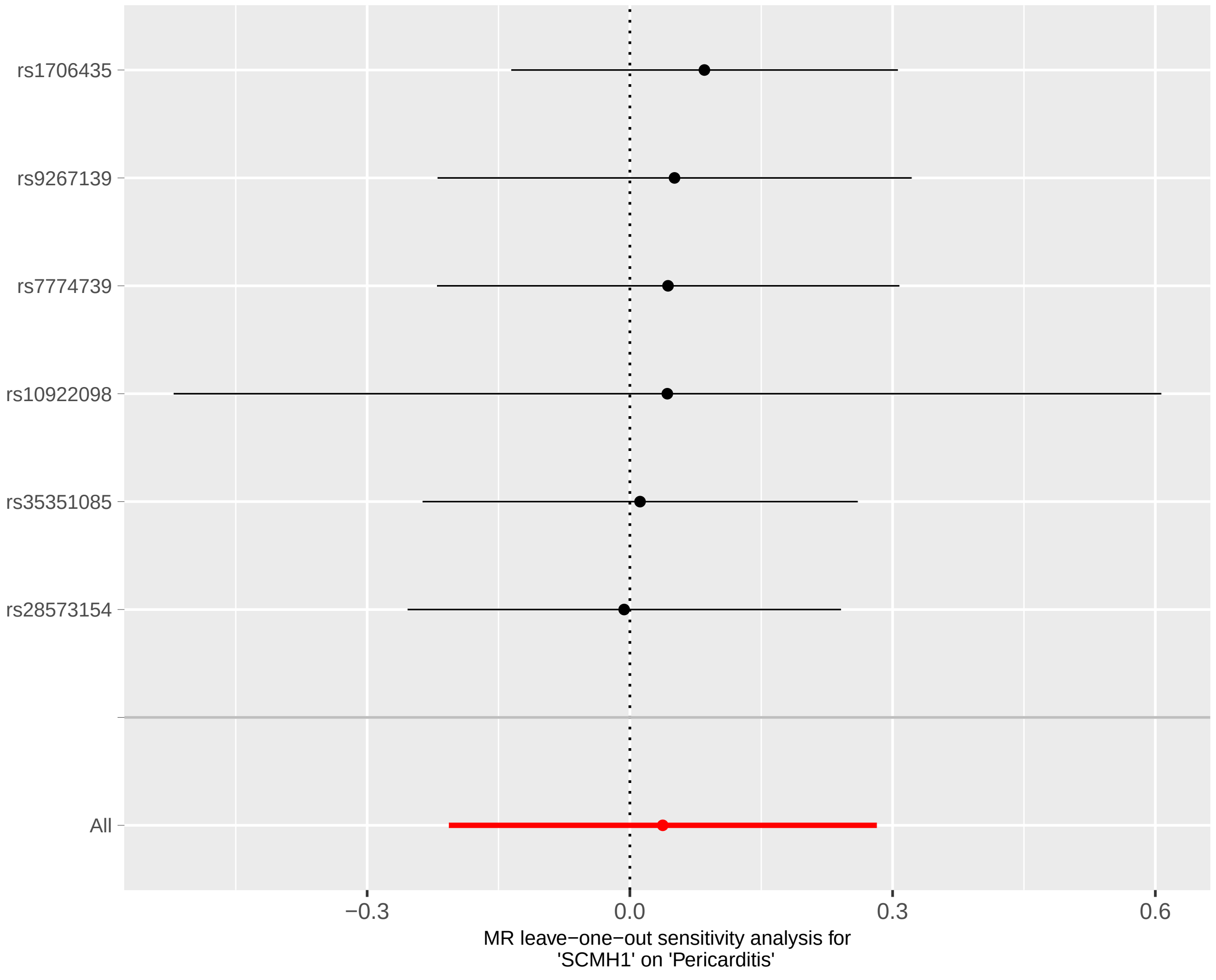
rs1706435
rs9267139
rs7774739
rs10922098
rs35351085
rs28573154
All
−0.3
0.0
0.3
0.6
MR leave−one−out sensitivity analysis for
'SCMH1' on 'Pericarditis'

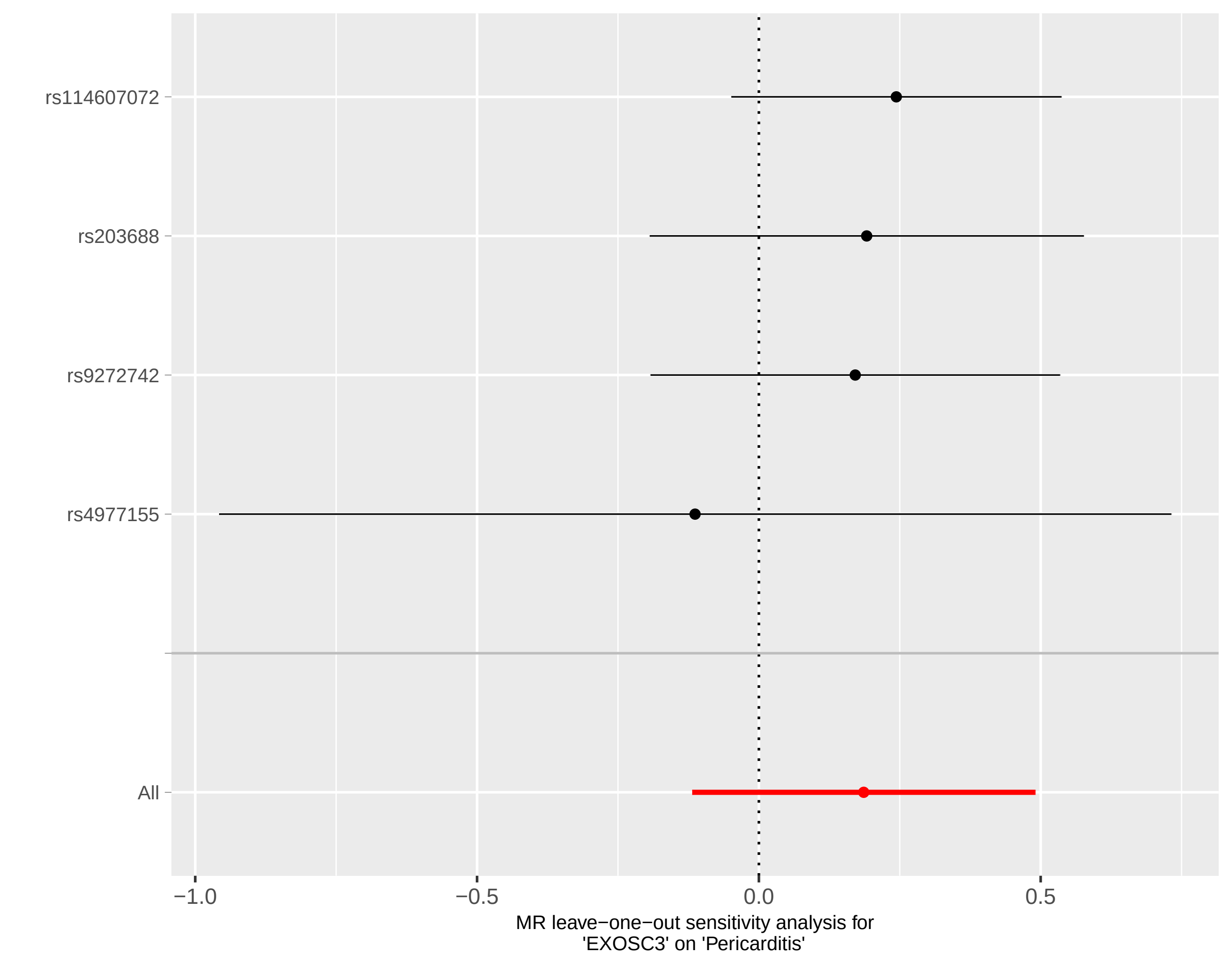
rs114607072
rs203688
rs9272742
rs4977155
All
−1.0
−0.5
0.0
0.5
MR leave−one−out sensitivity analysis for
'EXOSC3' on 'Pericarditis'

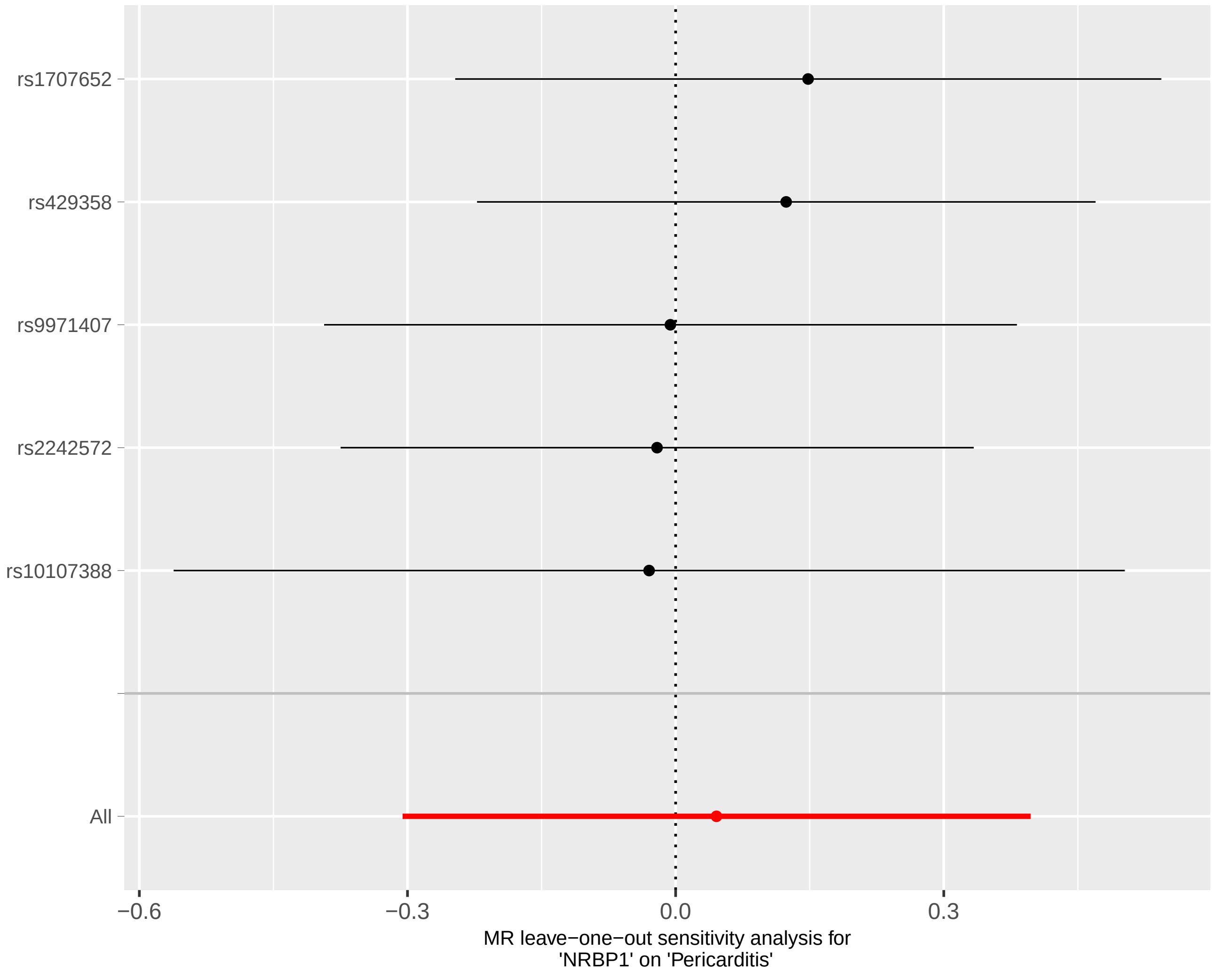
rs1707652
rs429358
rs9971407
rs2242572
rs10107388
All
−0.6
−0.3
0.0
0.3
MR leave−one−out sensitivity analysis for
'NRBP1' on 'Pericarditis'

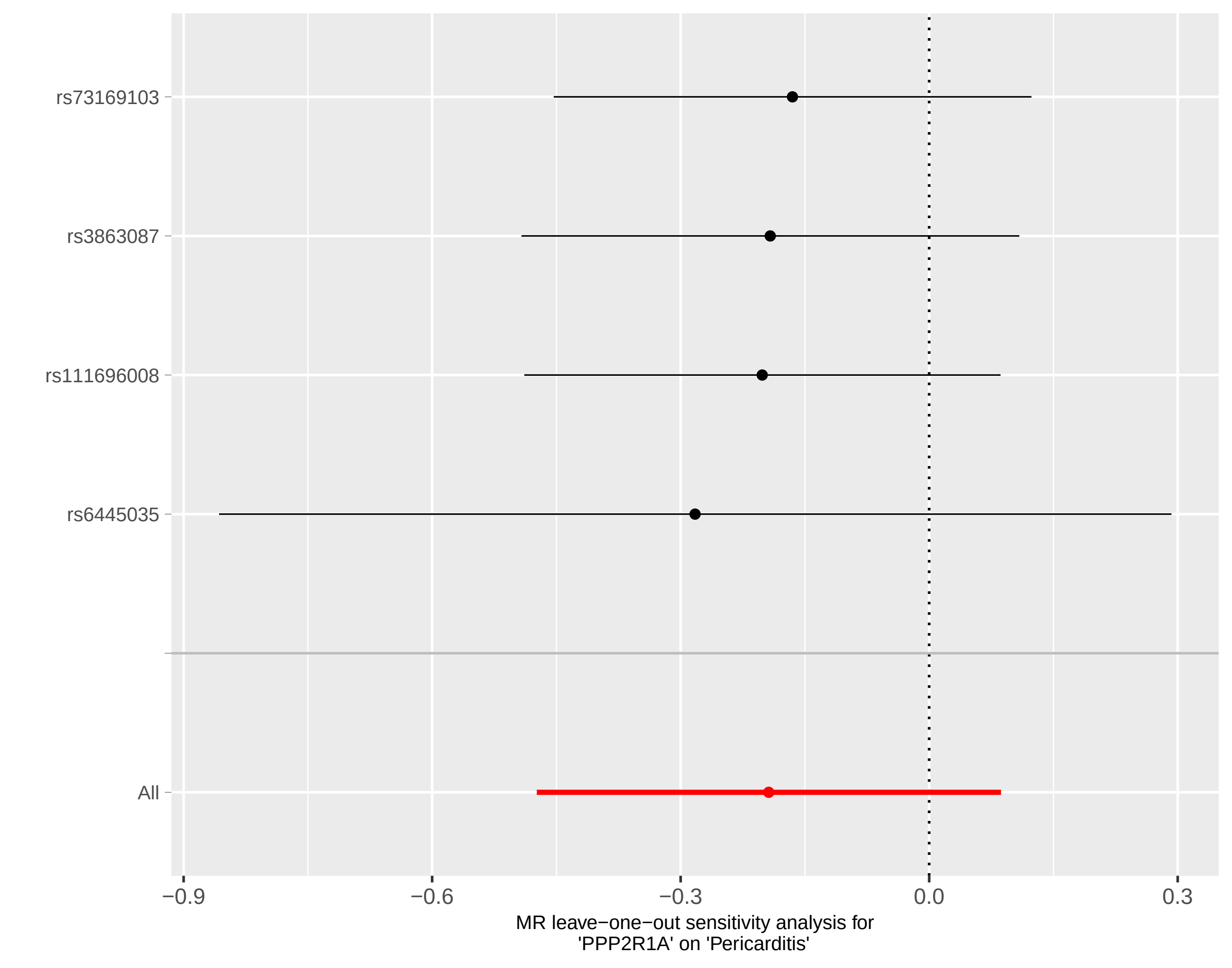

rs73169103
rs3863087
rs111696008
rs6445035
All
−0.9
−0.6
−0.3
0.0
0.3
MR leave−one−out sensitivity analysis for
'PPP2R1A' on 'Pericarditis'

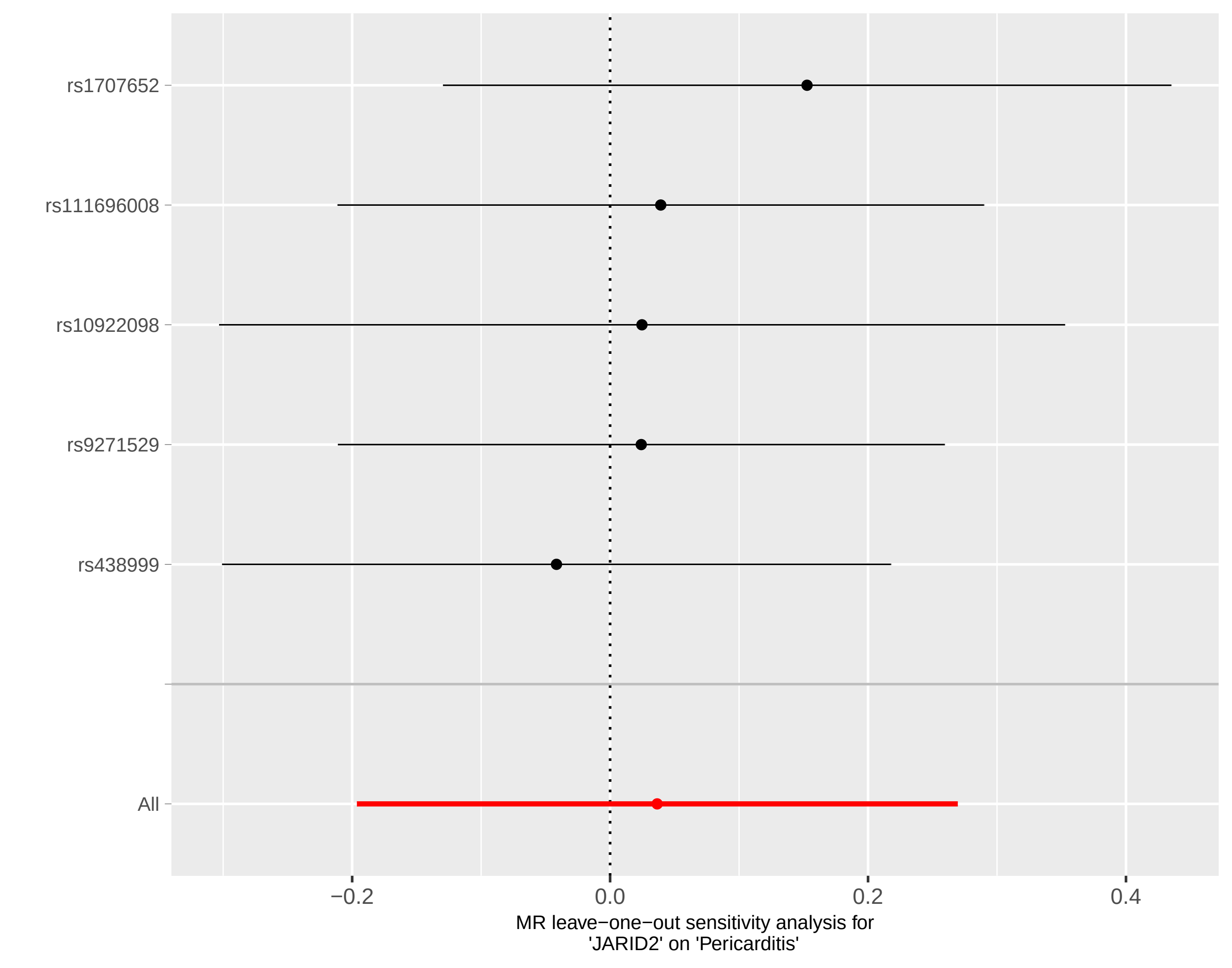
rs1707652
rs111696008
rs10922098
rs9271529
rs438999
All
−0.2
0.0
0.2
0.4
MR leave−one−out sensitivity analysis for
'JARID2' on 'Pericarditis'

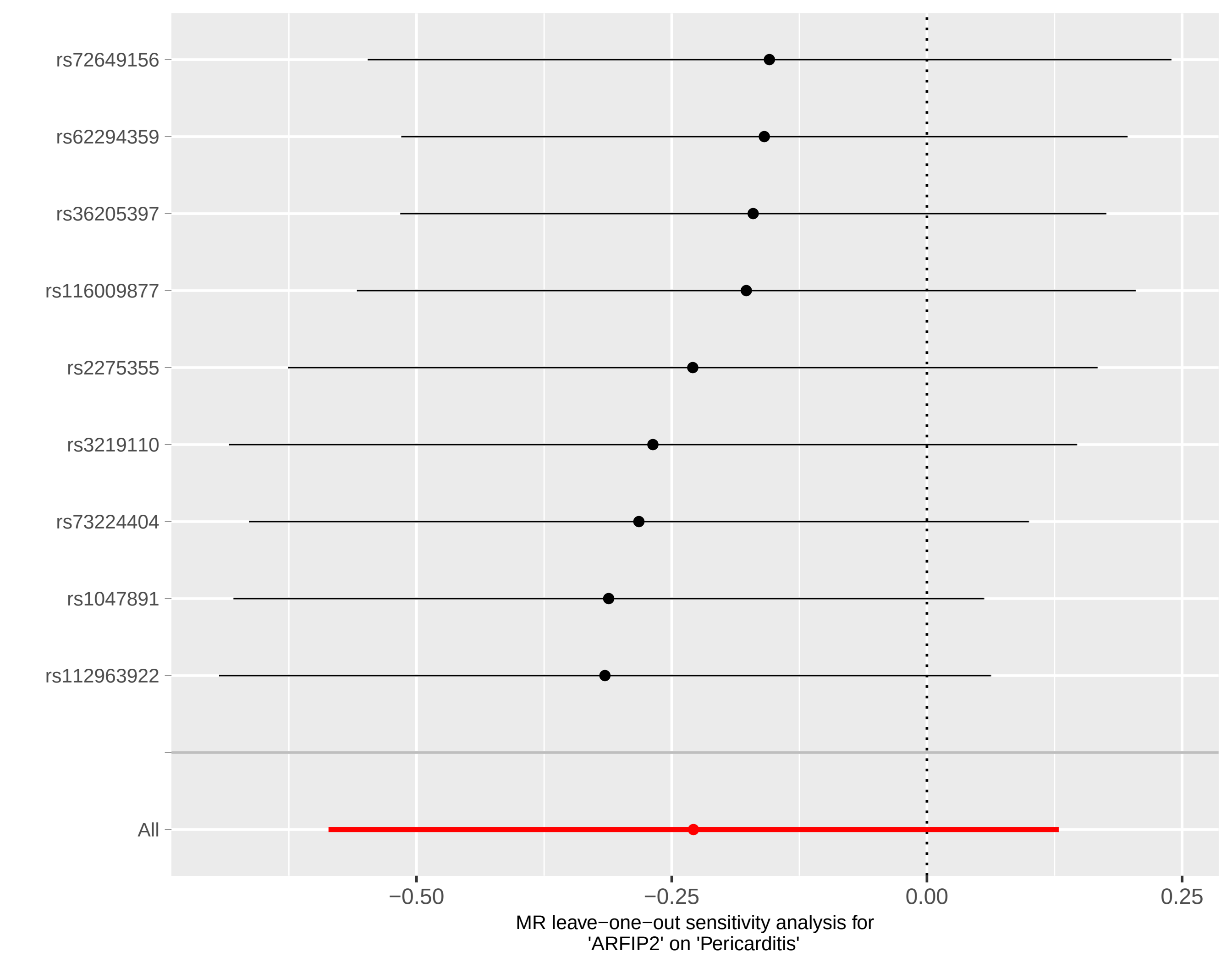
rs72649156
rs62294359
rs36205397
rs116009877
rs2275355
rs3219110
rs73224404
rs1047891
rs112963922
All
−0.50
−0.25
0.00
0.25
MR leave−one−out sensitivity analysis for
'ARFIP2' on 'Pericarditis'

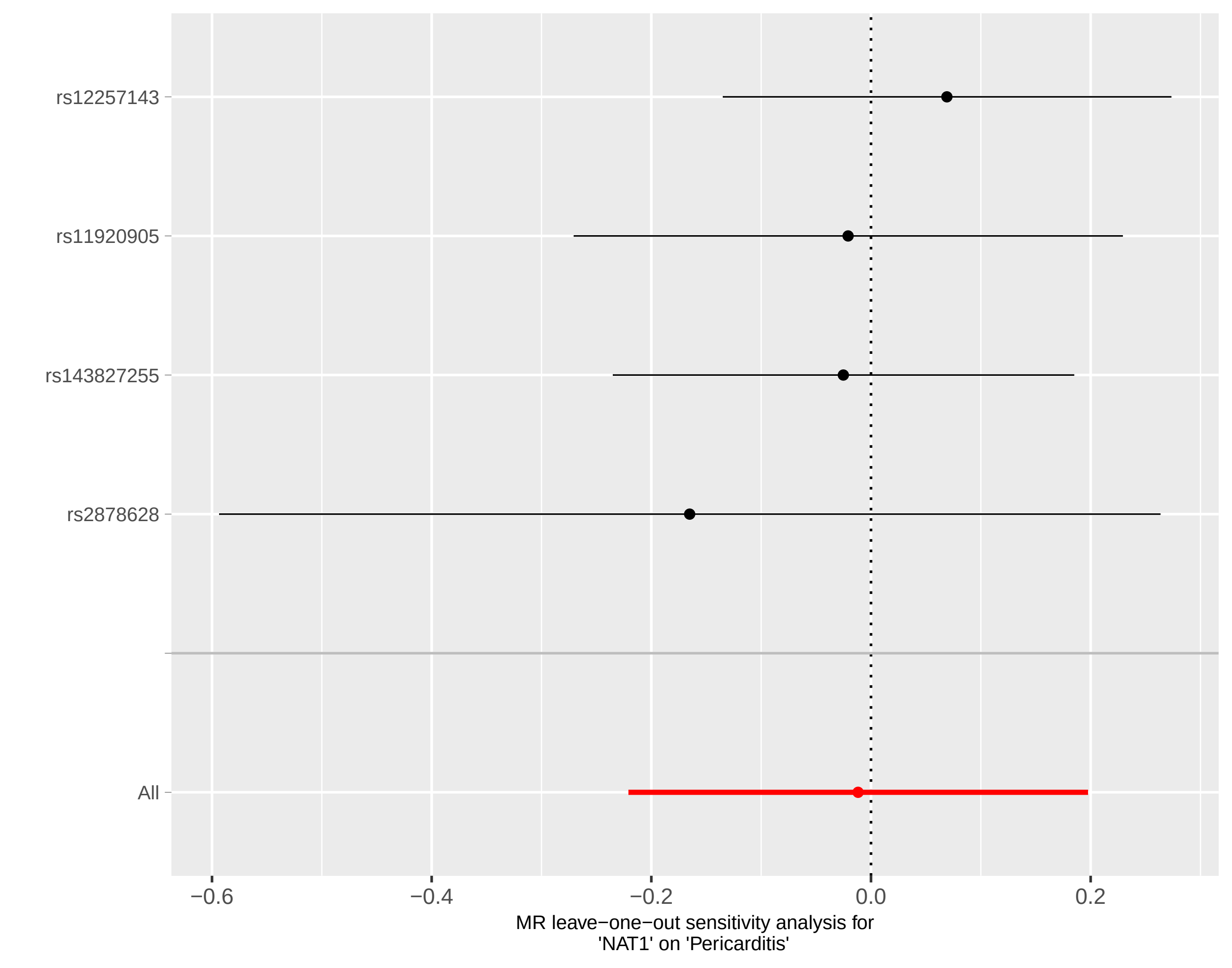

rs12257143
rs11920905
rs143827255
rs2878628
All
−0.6
−0.4
−0.2
0.0
0.2
MR leave−one−out sensitivity analysis for
'NAT1' on 'Pericarditis'

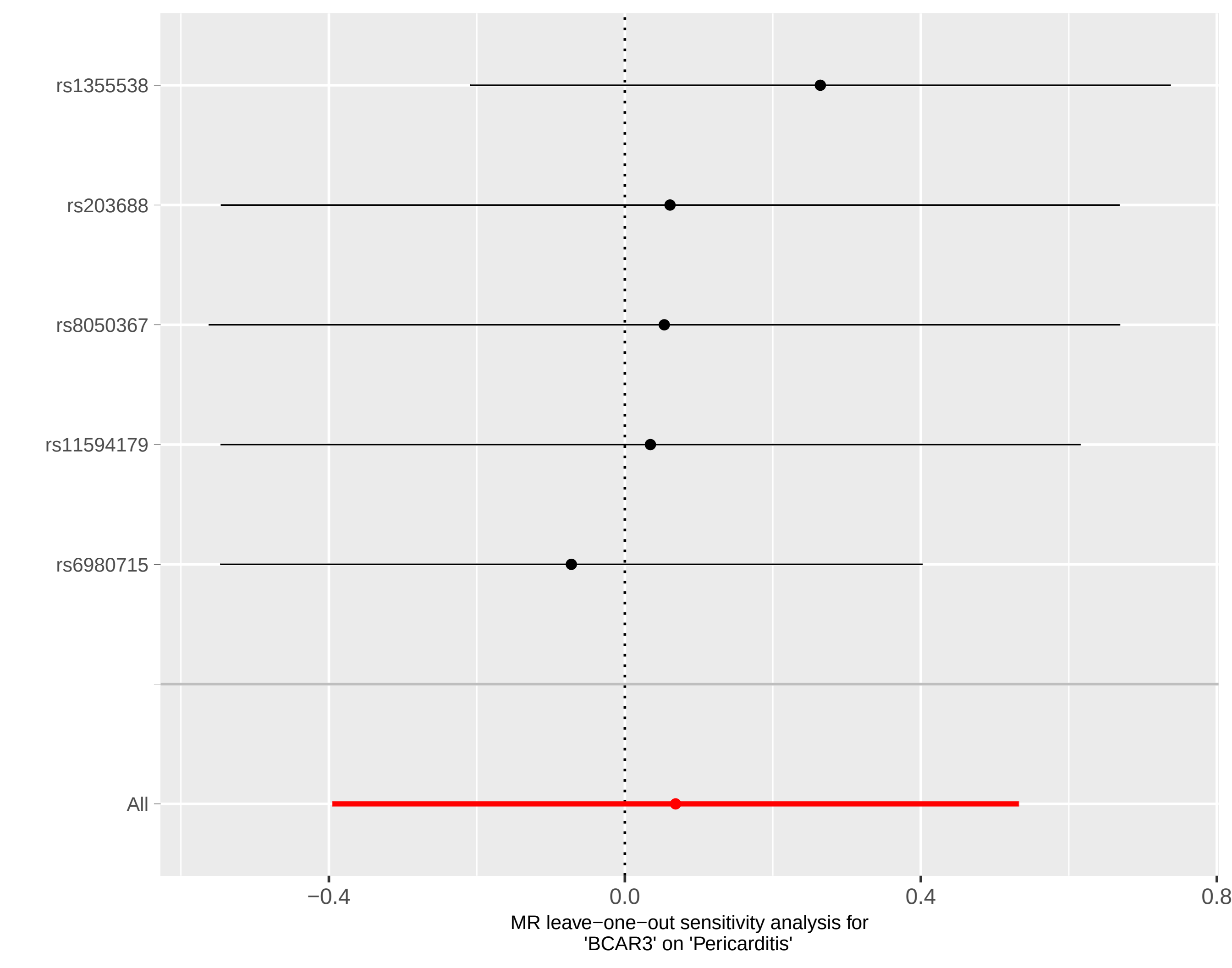

rs1355538
rs203688
rs8050367
rs11594179
rs6980715
All
−0.4
0.0
0.4
0.8
MR leave−one−out sensitivity analysis for
'BCAR3' on 'Pericarditis'

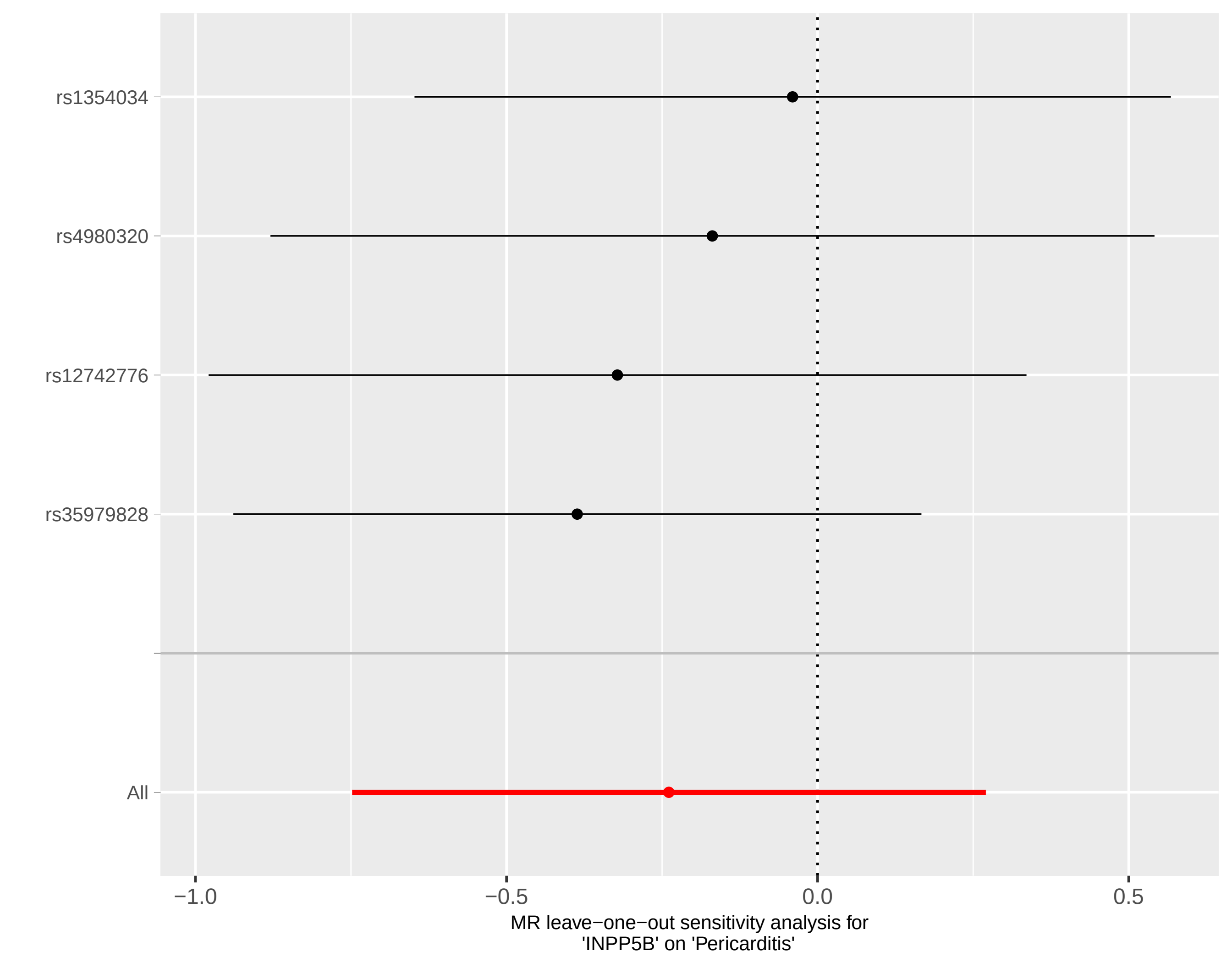

rs1354034
rs4980320
rs12742776
rs35979828
All
−1.0
−0.5
0.0
0.5
MR leave−one−out sensitivity analysis for
'INPP5B' on 'Pericarditis'

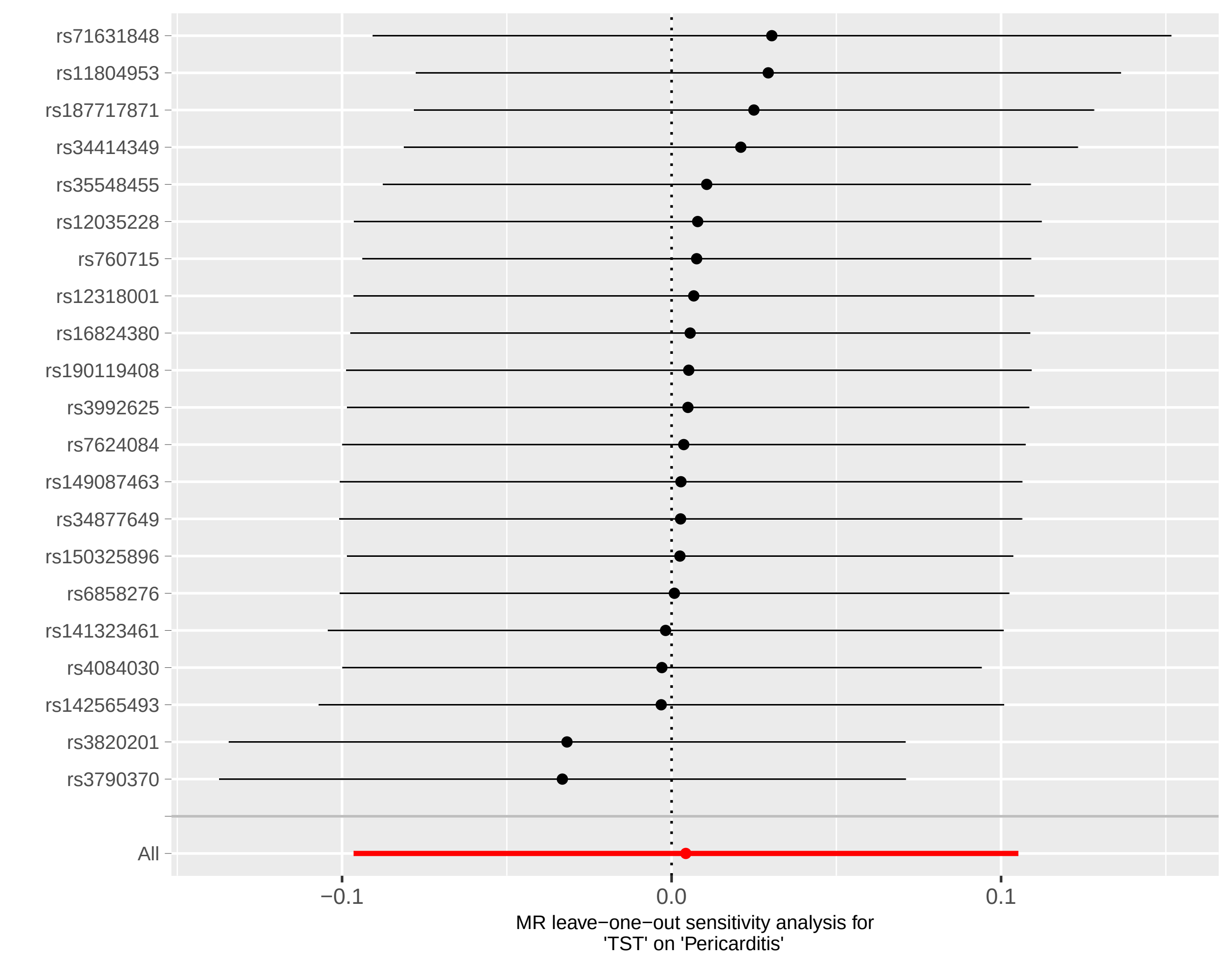
rs71631848
rs11804953
rs187717871
rs34414349
rs35548455
rs12035228
rs760715
rs12318001
rs16824380
rs190119408
rs3992625
rs7624084
rs149087463
rs34877649
rs150325896
rs6858276
rs141323461
rs4084030
rs142565493
rs3820201
rs3790370
All
−0.1
0.0
0.1
MR leave−one−out sensitivity analysis for
'TST' on 'Pericarditis'

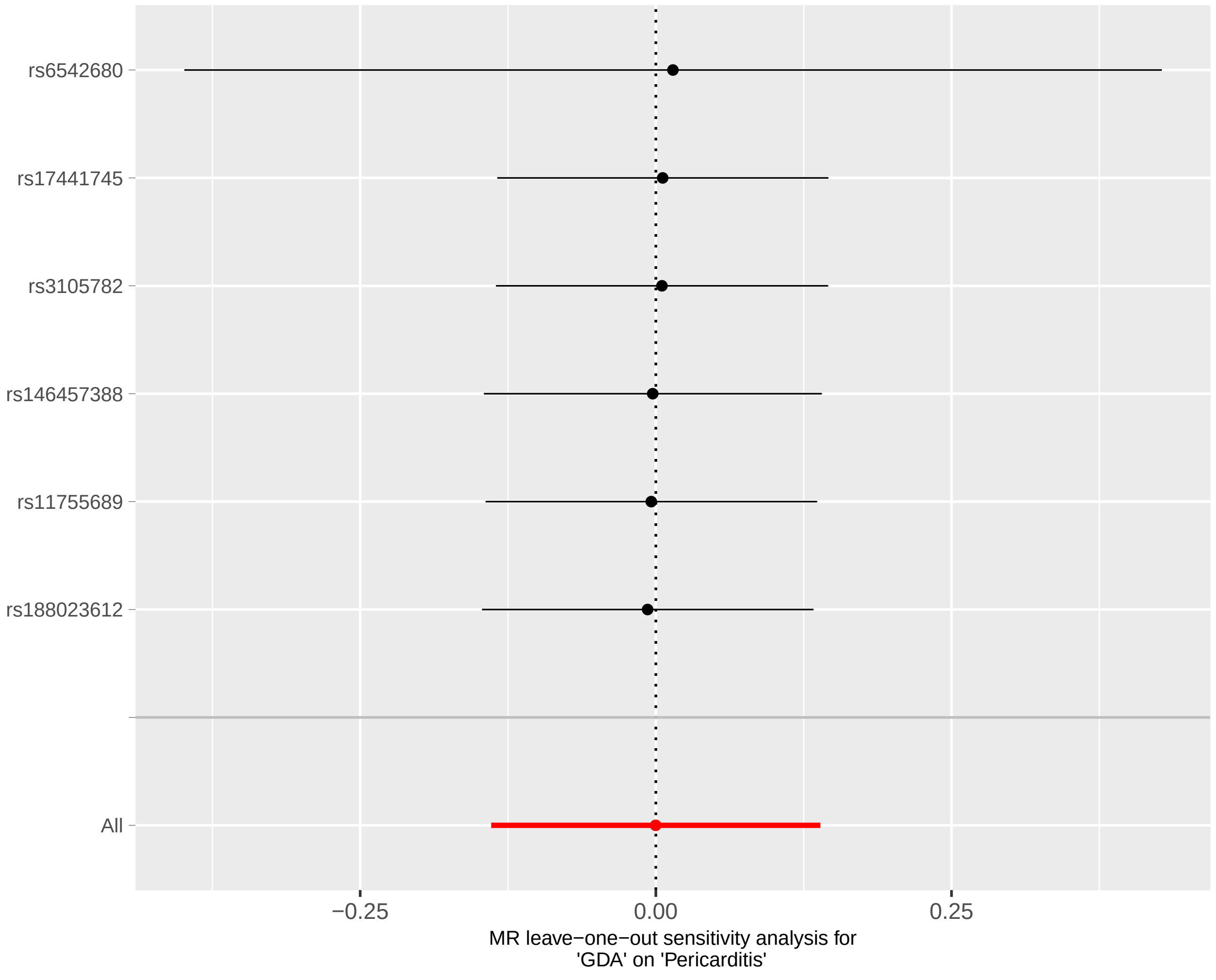
rs6542680
rs17441745
rs3105782
rs146457388
rs11755689
rs188023612
All
−0.25
0.00
0.25
MR leave−one−out sensitivity analysis for
'GDA' on 'Pericarditis'

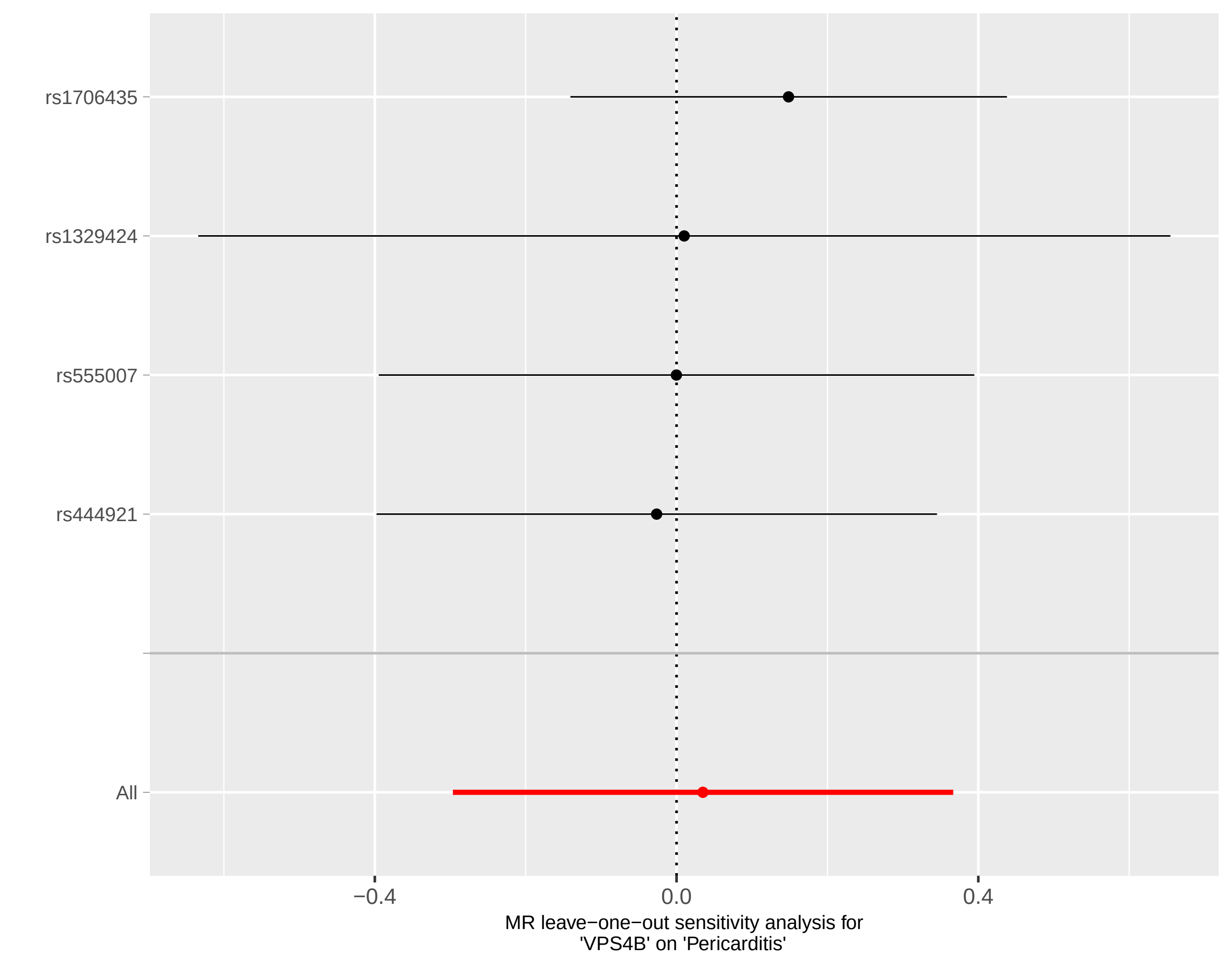
rs1706435
rs1329424
rs555007
rs444921
All
−0.4
0.0
0.4
MR leave−one−out sensitivity analysis for
'VPS4B' on 'Pericarditis'

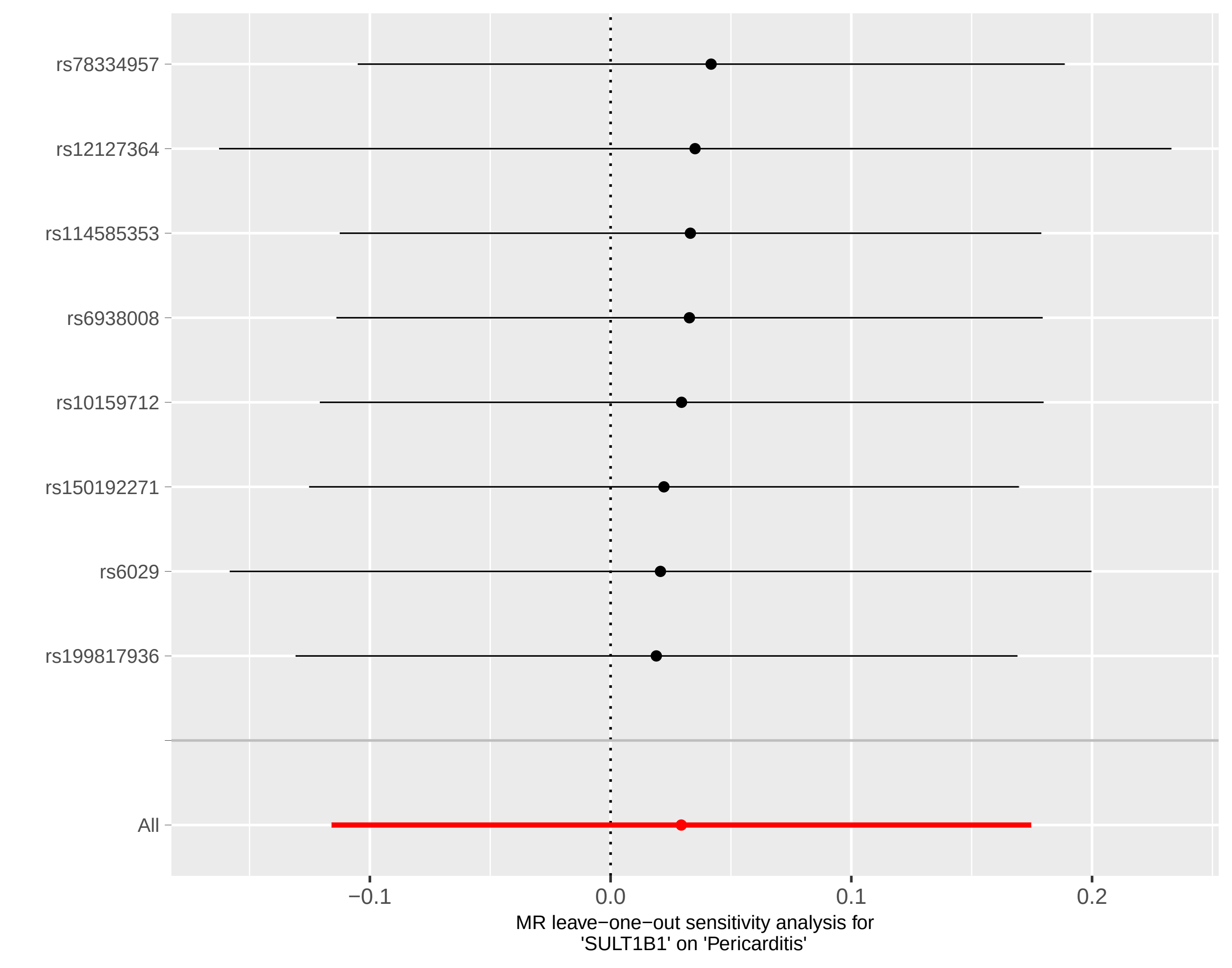
rs78334957
rs12127364
rs114585353
rs6938008
rs10159712
rs150192271
rs6029
rs199817936
All
−0.1
0.0
0.1
0.2
MR leave−one−out sensitivity analysis for
'SULT1B1' on 'Pericarditis'

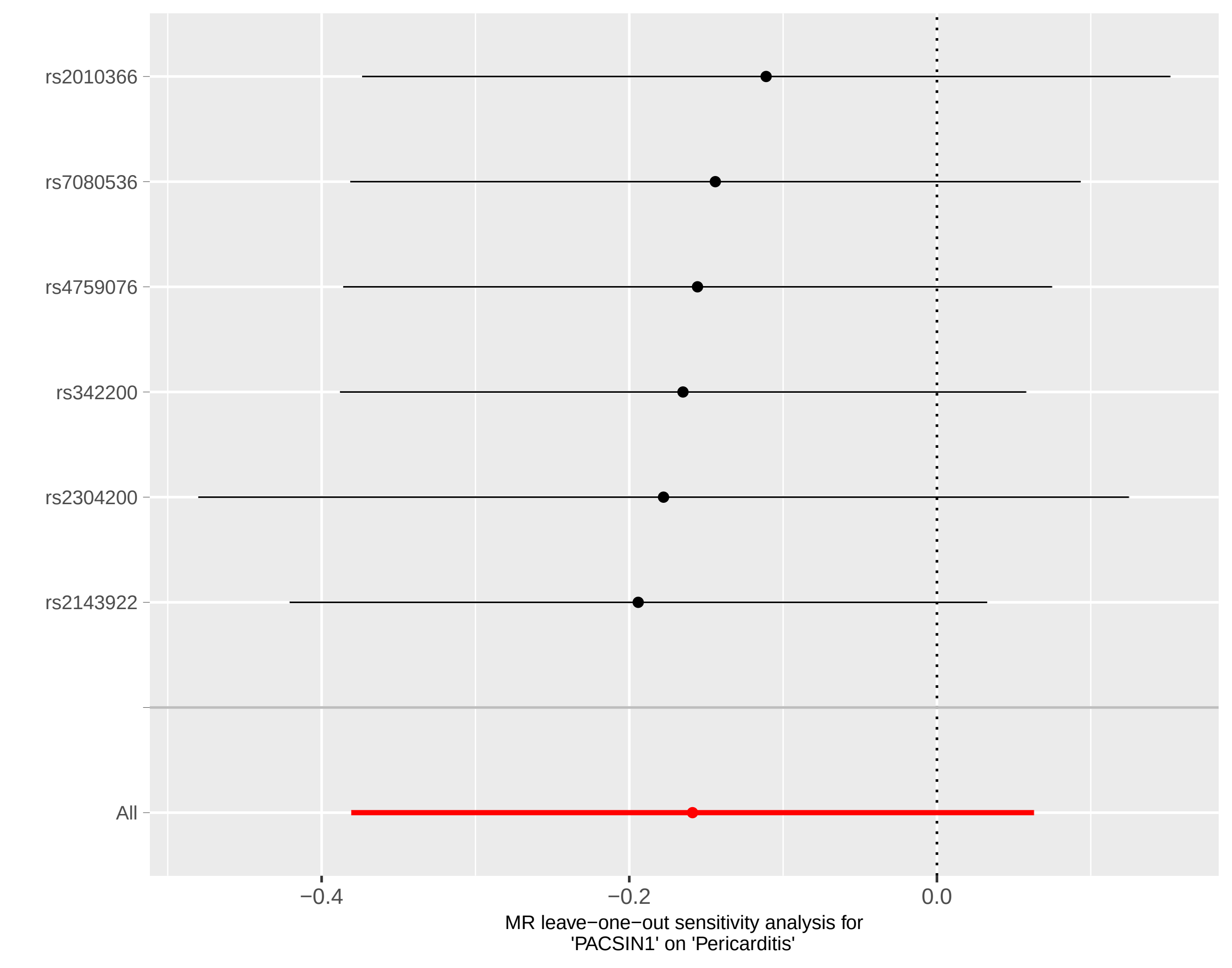
rs2010366
rs7080536
rs4759076
rs342200
rs2304200
rs2143922
All
−0.4
−0.2
0.0
MR leave−one−out sensitivity analysis for
'PACSIN1' on 'Pericarditis'

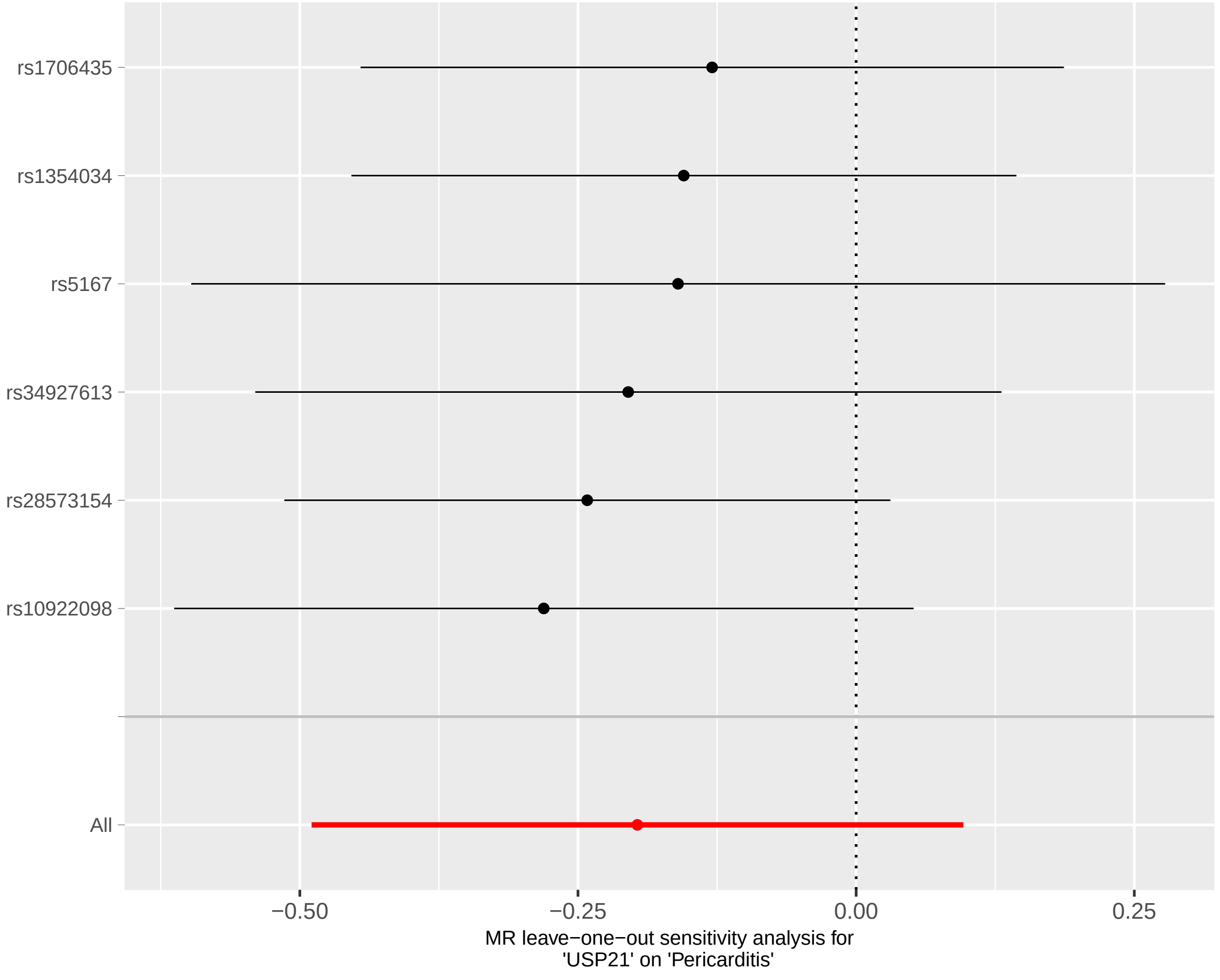

rs1706435
rs1354034
rs5167
rs34927613
rs28573154
rs10922098
All
−0.50
−0.25
0.00
0.25
MR leave−one−out sensitivity analysis for
'USP21' on 'Pericarditis'

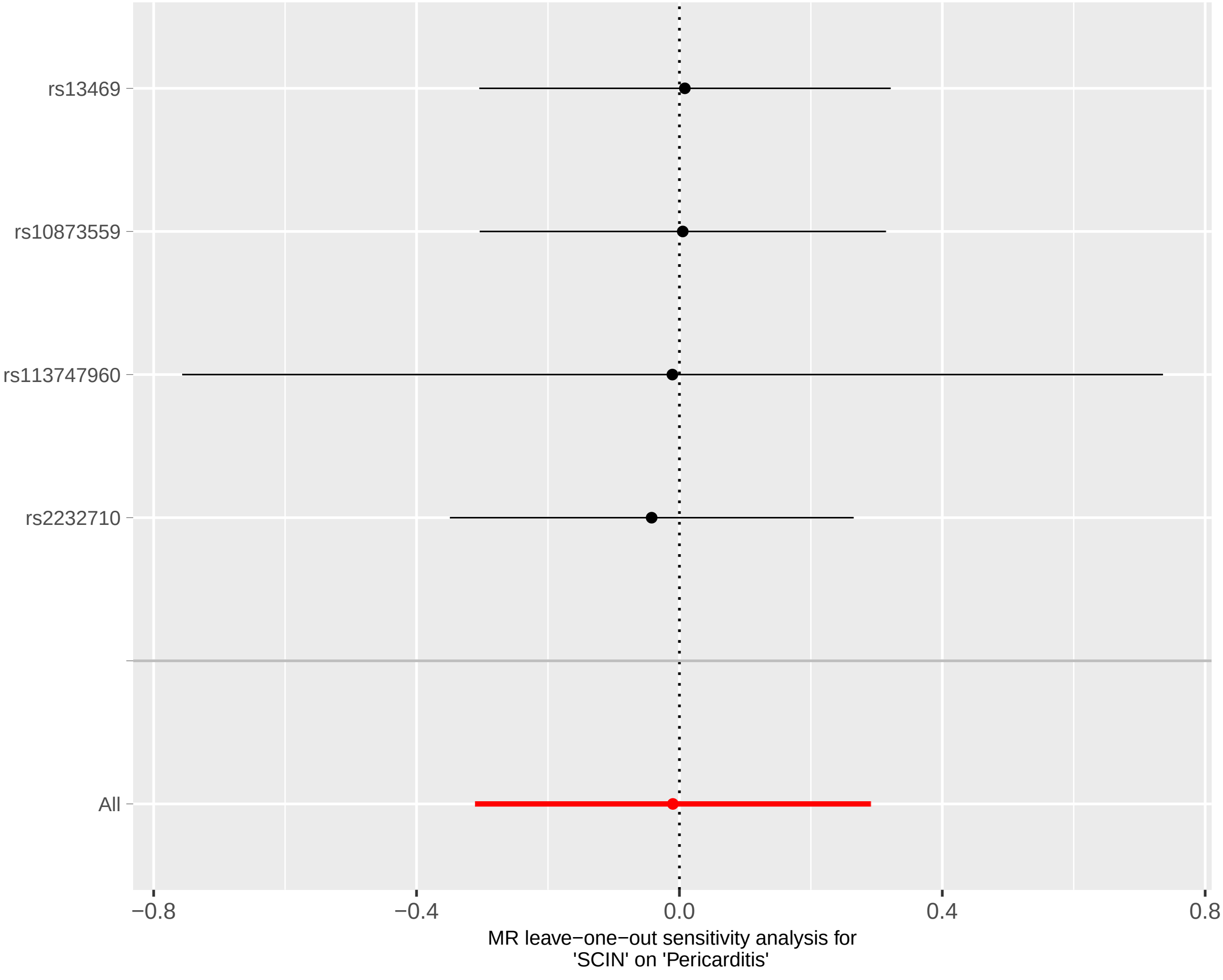

rs13469
rs10873559
rs113747960
rs2232710
All
−0.8
−0.4
0.0
0.4
0.8
MR leave−one−out sensitivity analysis for
'SCIN' on 'Pericarditis'

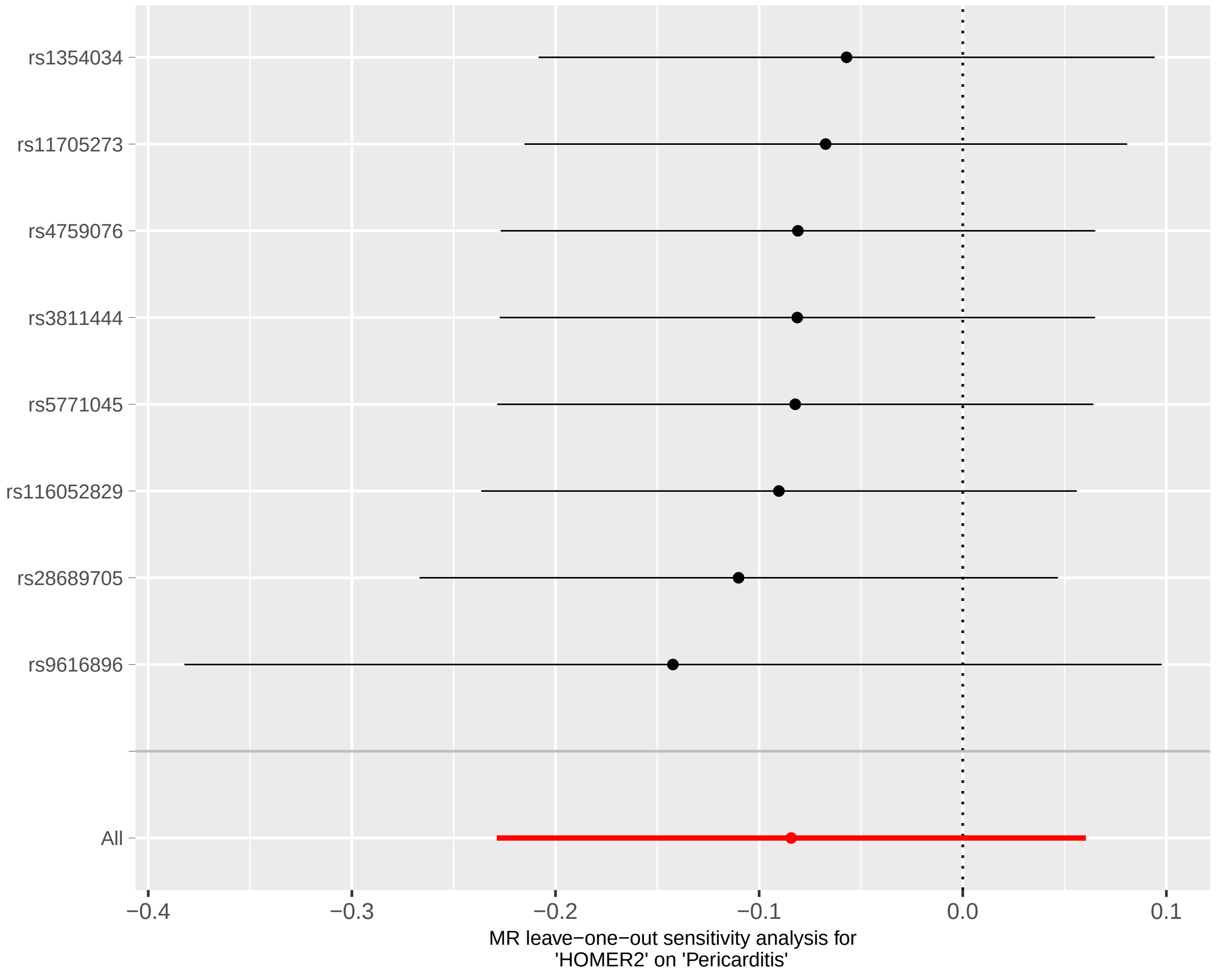

rs1354034
rs11705273
rs4759076
rs3811444
rs5771045
rs116052829
rs28689705
rs9616896
All
−0.4
−0.3
−0.2
−0.1
0.0
0.1
MR leave−one−out sensitivity analysis for
'HOMER2' on 'Pericarditis'

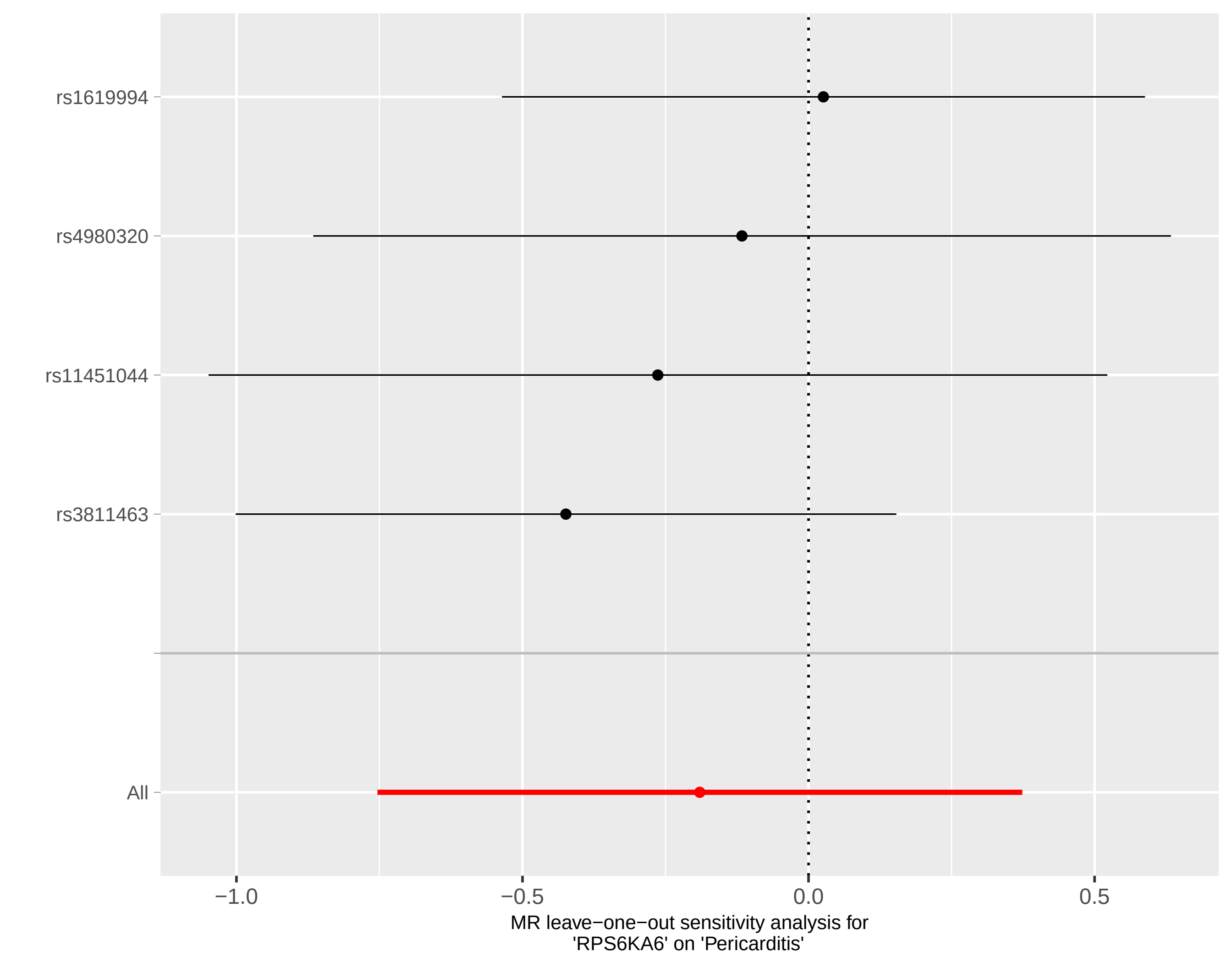
rs1619994
rs4980320
rs11451044
rs3811463
All
−1.0
−0.5
0.0
0.5
MR leave−one−out sensitivity analysis for
'RPS6KA6' on 'Pericarditis'

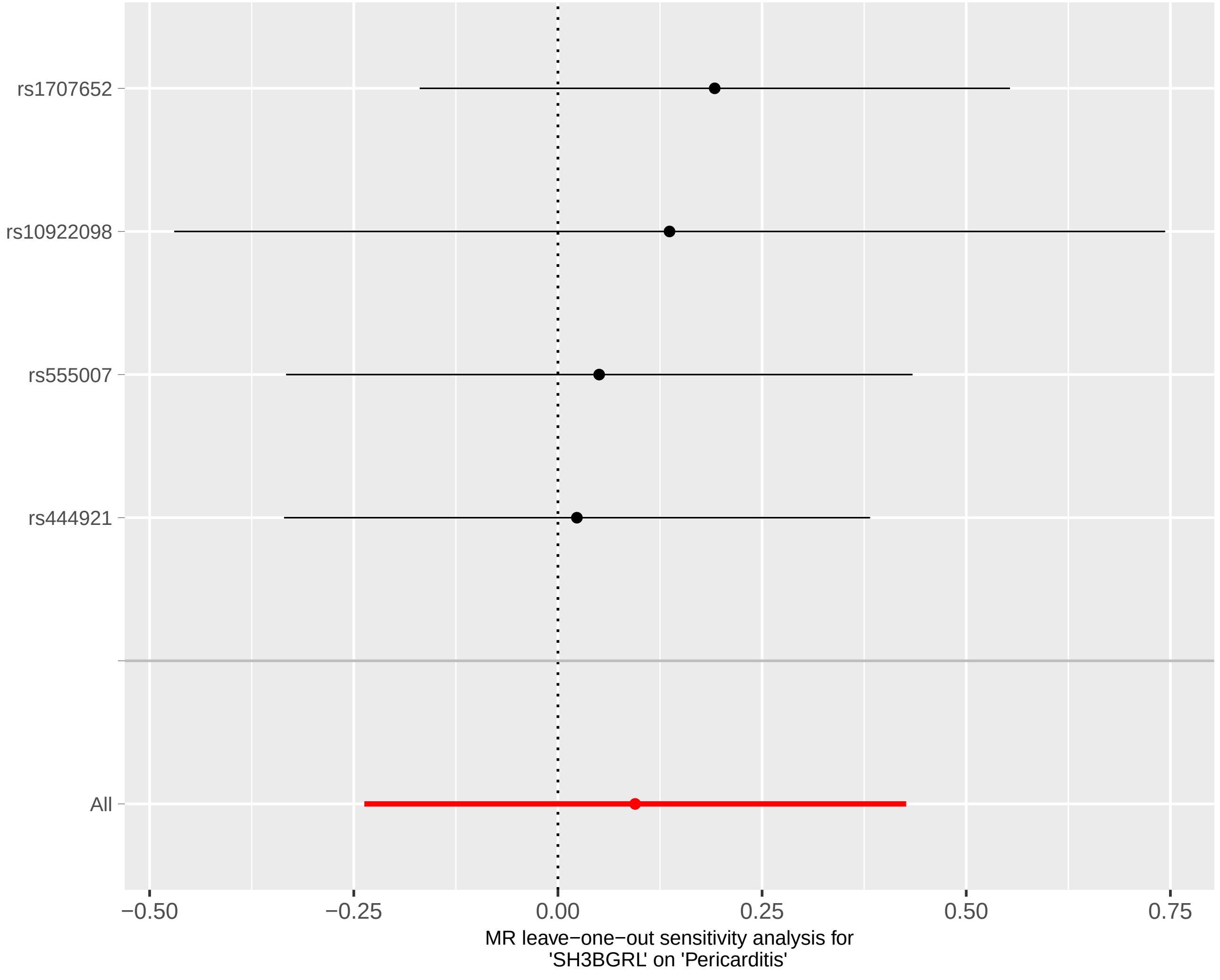

rs1707652
rs10922098
rs555007
rs444921
All
−0.50
−0.25
0.00
0.25
0.50
0.75
MR leave−one−out sensitivity analysis for
'SH3BGRL' on 'Pericarditis'

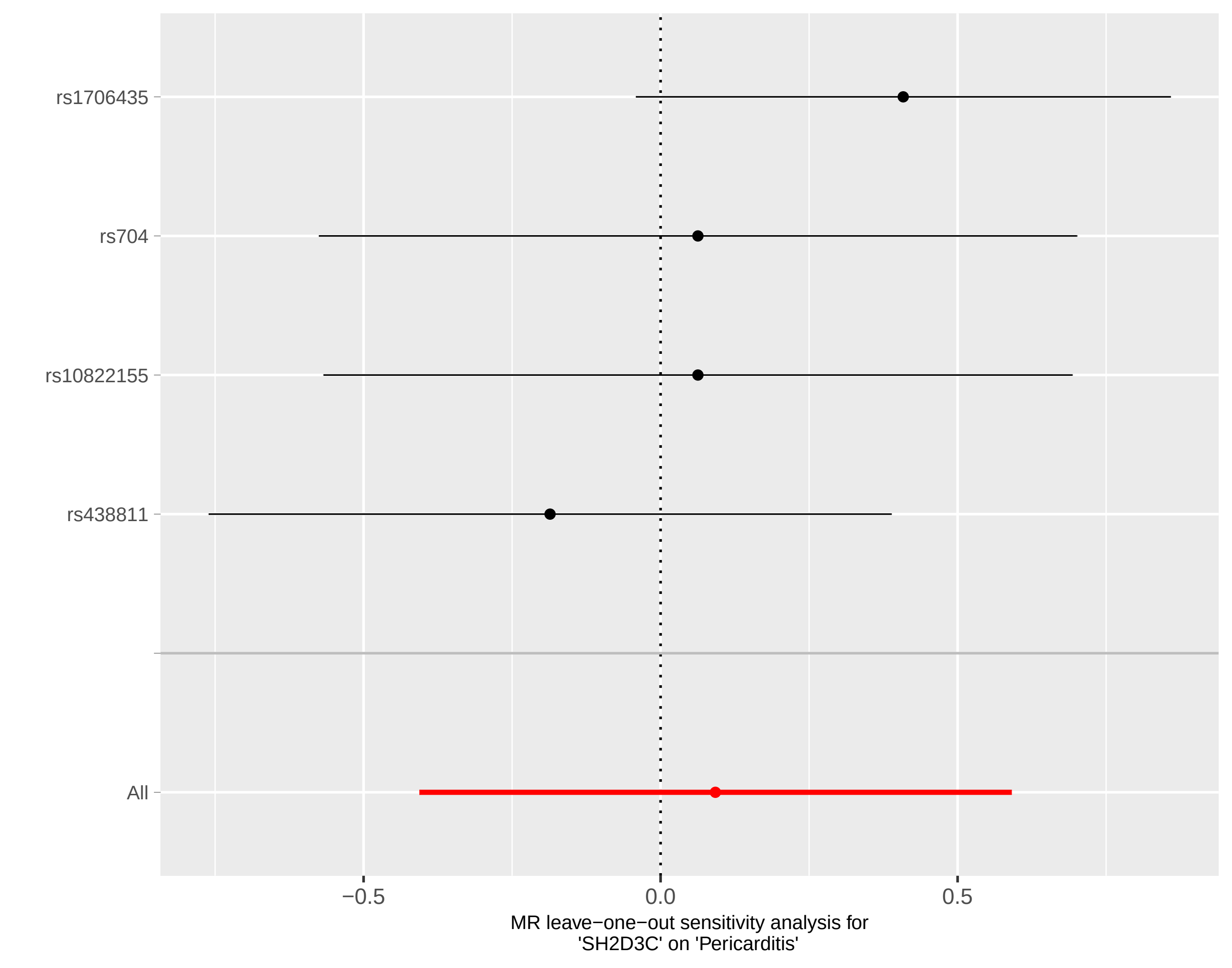
rs1706435
rs704
rs10822155
rs438811
All
−0.5
0.0
0.5
MR leave−one−out sensitivity analysis for
'SH2D3C' on 'Pericarditis'

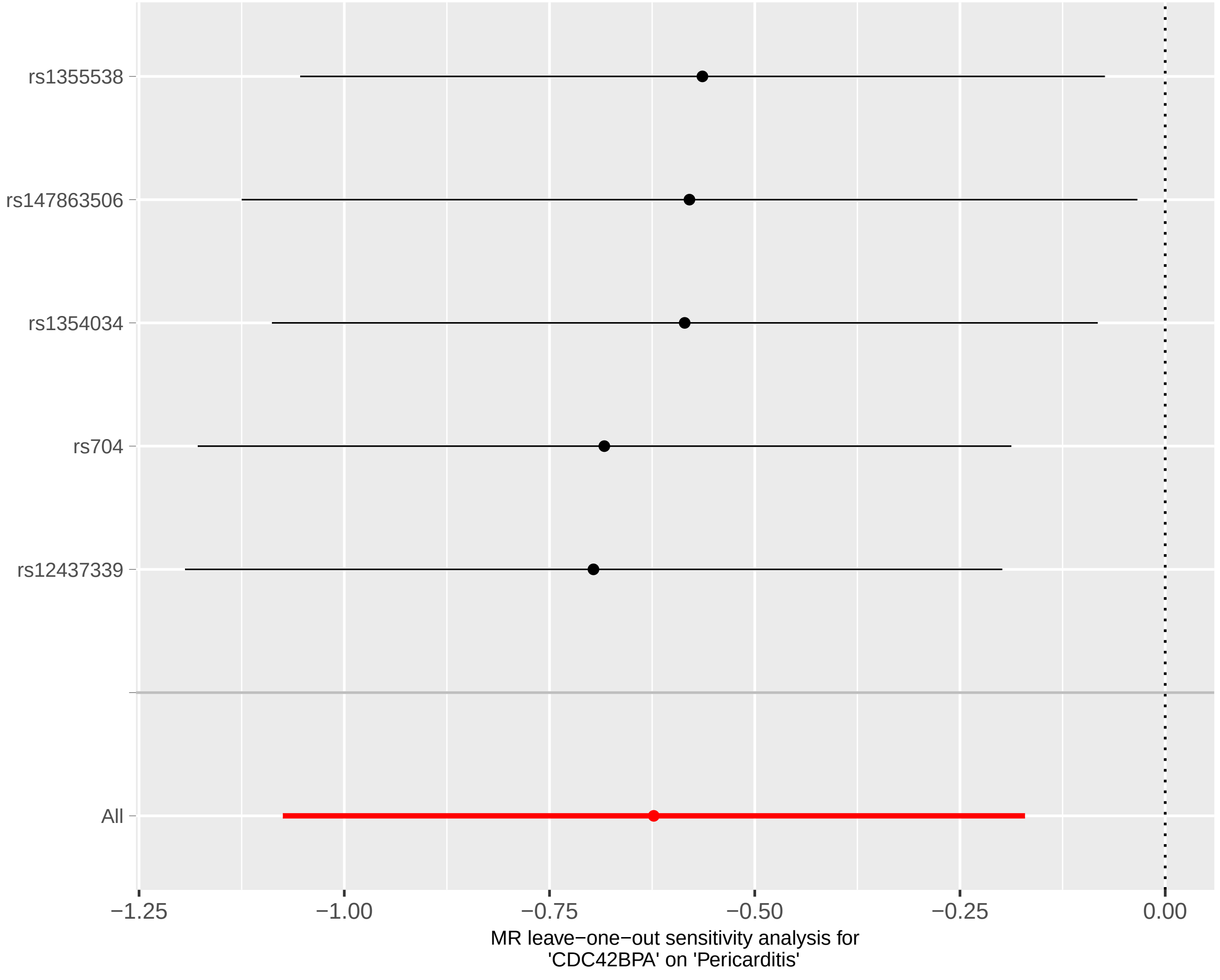
rs1355538
rs147863506
rs1354034
rs704
rs12437339
All
−1.25
−1.00
−0.75
−0.50
−0.25
0.00
MR leave−one−out sensitivity analysis for
'CDC42BPA' on 'Pericarditis'

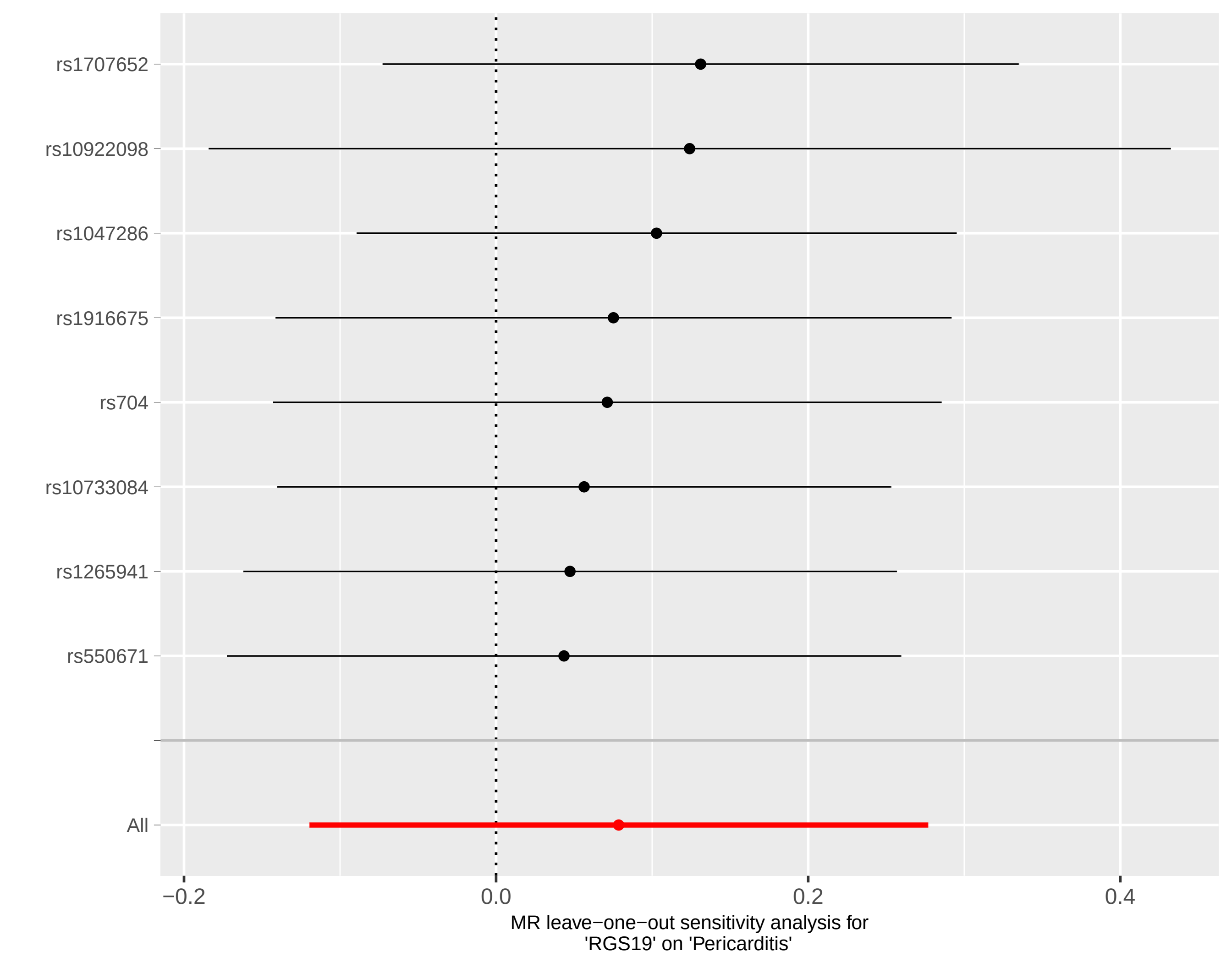

rs1707652
rs10922098
rs1047286
rs1916675
rs704
rs10733084
rs1265941
rs550671
All
−0.2
0.0
0.2
0.4
MR leave−one−out sensitivity analysis for
'RGS19' on 'Pericarditis'

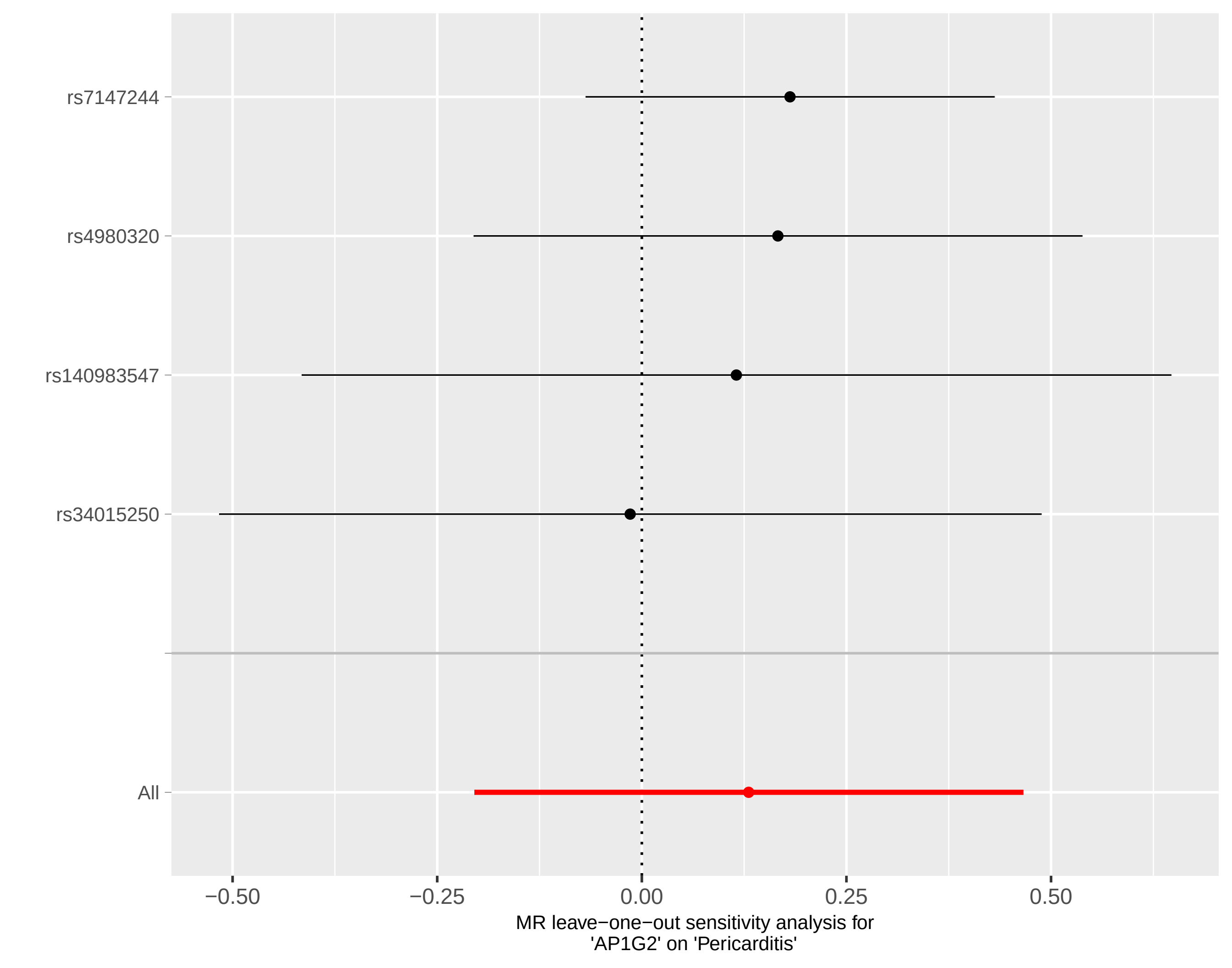
rs7147244
rs4980320
rs140983547
rs34015250
All
−0.50
−0.25
0.00
0.25
0.50
MR leave−one−out sensitivity analysis for
'AP1G2' on 'Pericarditis'

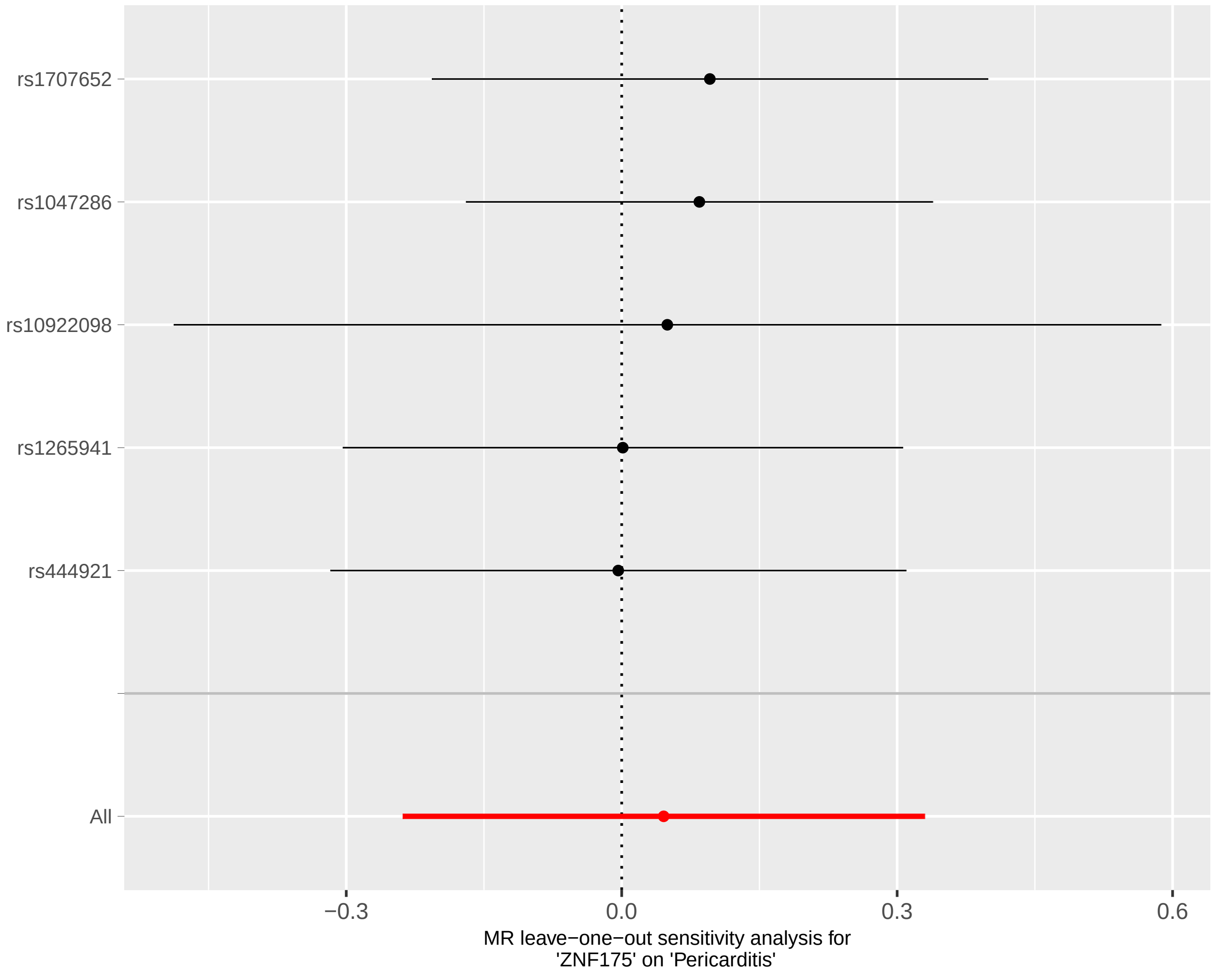
rs1707652
rs1047286
rs10922098
rs1265941
rs444921
All
−0.3
0.0
0.3
0.6
MR leave−one−out sensitivity analysis for
'ZNF175' on 'Pericarditis'

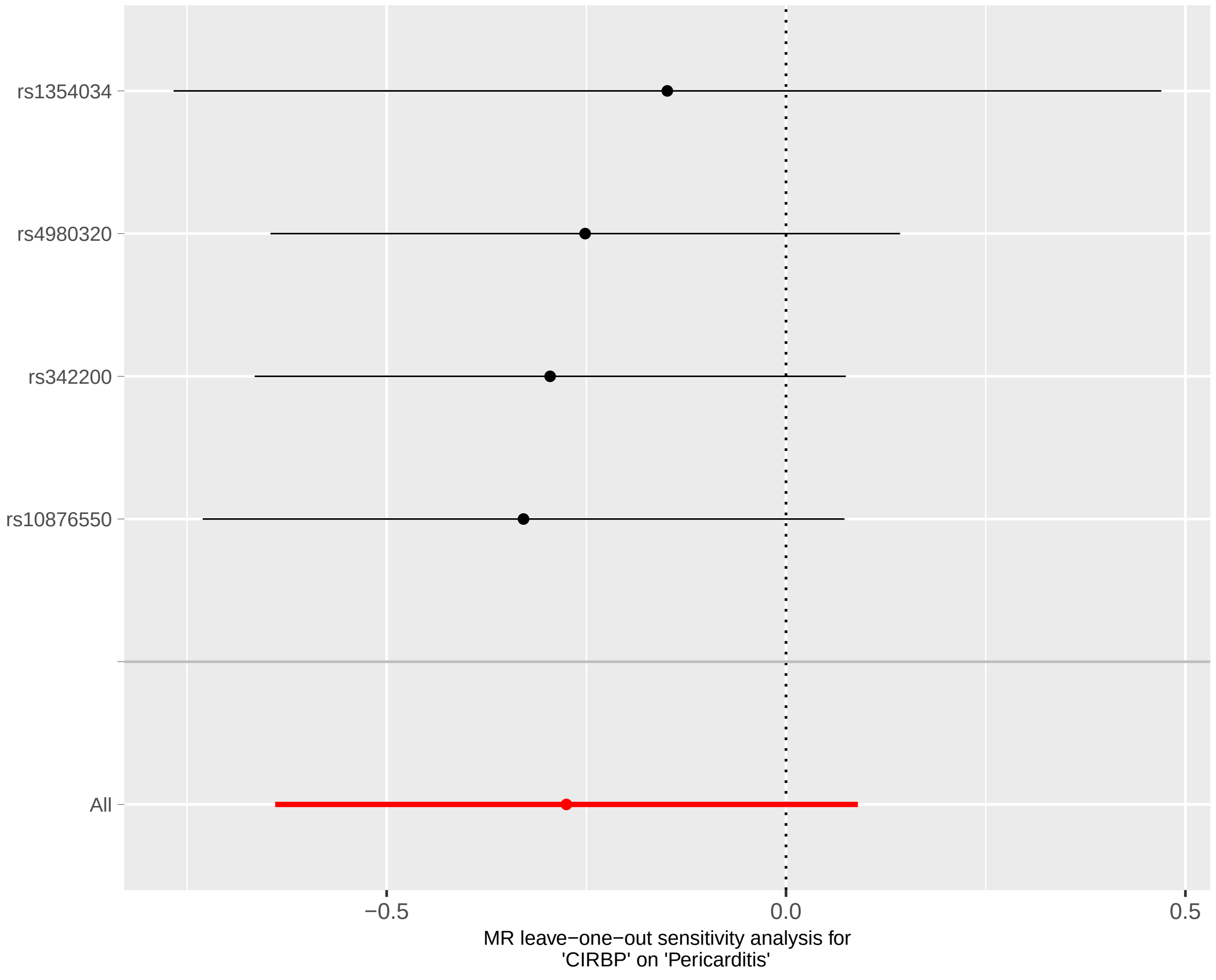
rs1354034
rs4980320
rs342200
rs10876550
All
−0.5
0.0
0.5
MR leave−one−out sensitivity analysis for
'CIRBP' on 'Pericarditis'

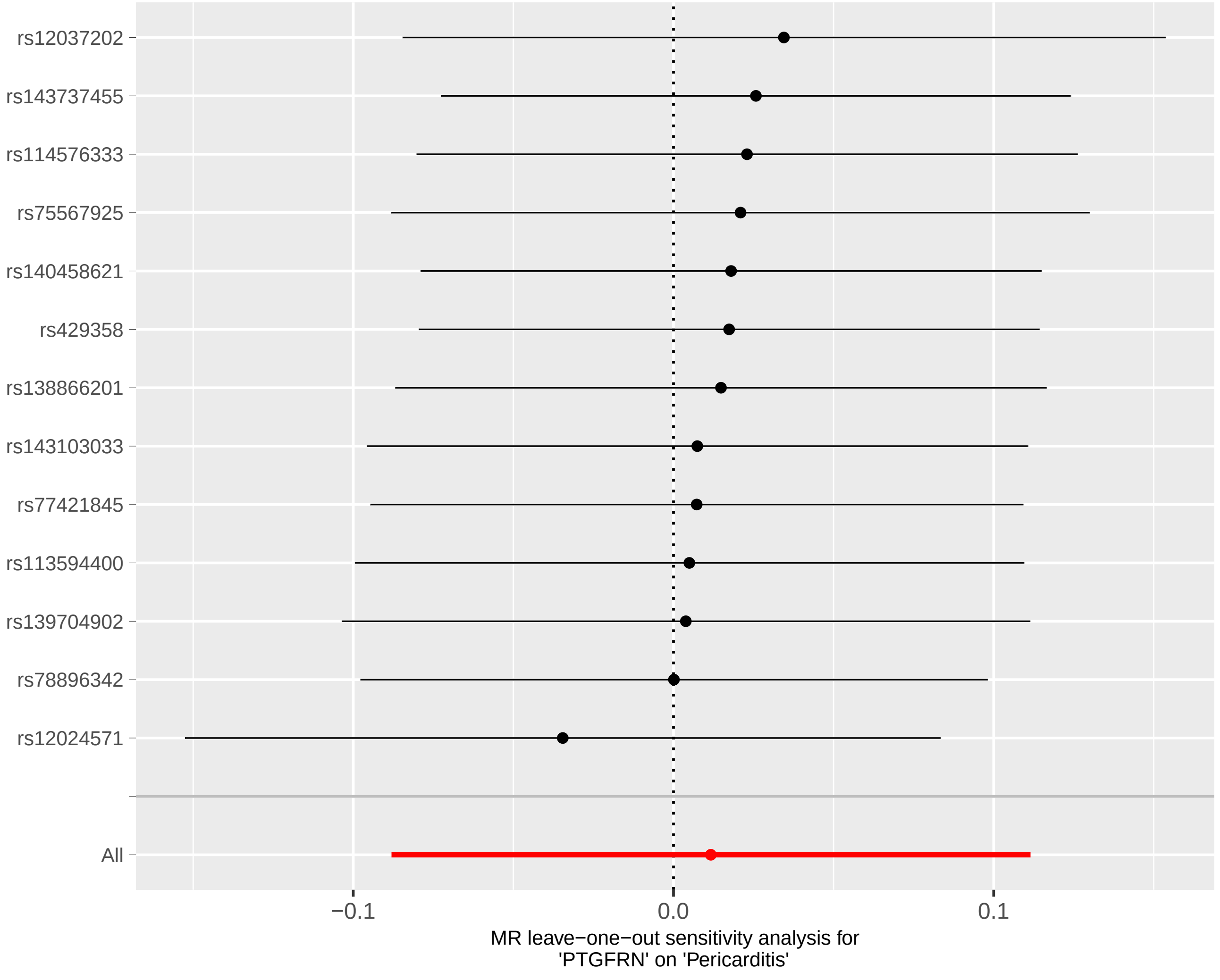
rs12037202
rs143737455
rs114576333
rs75567925
rs140458621
rs429358
rs138866201
rs143103033
rs77421845
rs113594400
rs139704902
rs78896342
rs12024571
All
−0.1
0.0
0.1
MR leave−one−out sensitivity analysis for
'PTGFRN' on 'Pericarditis'

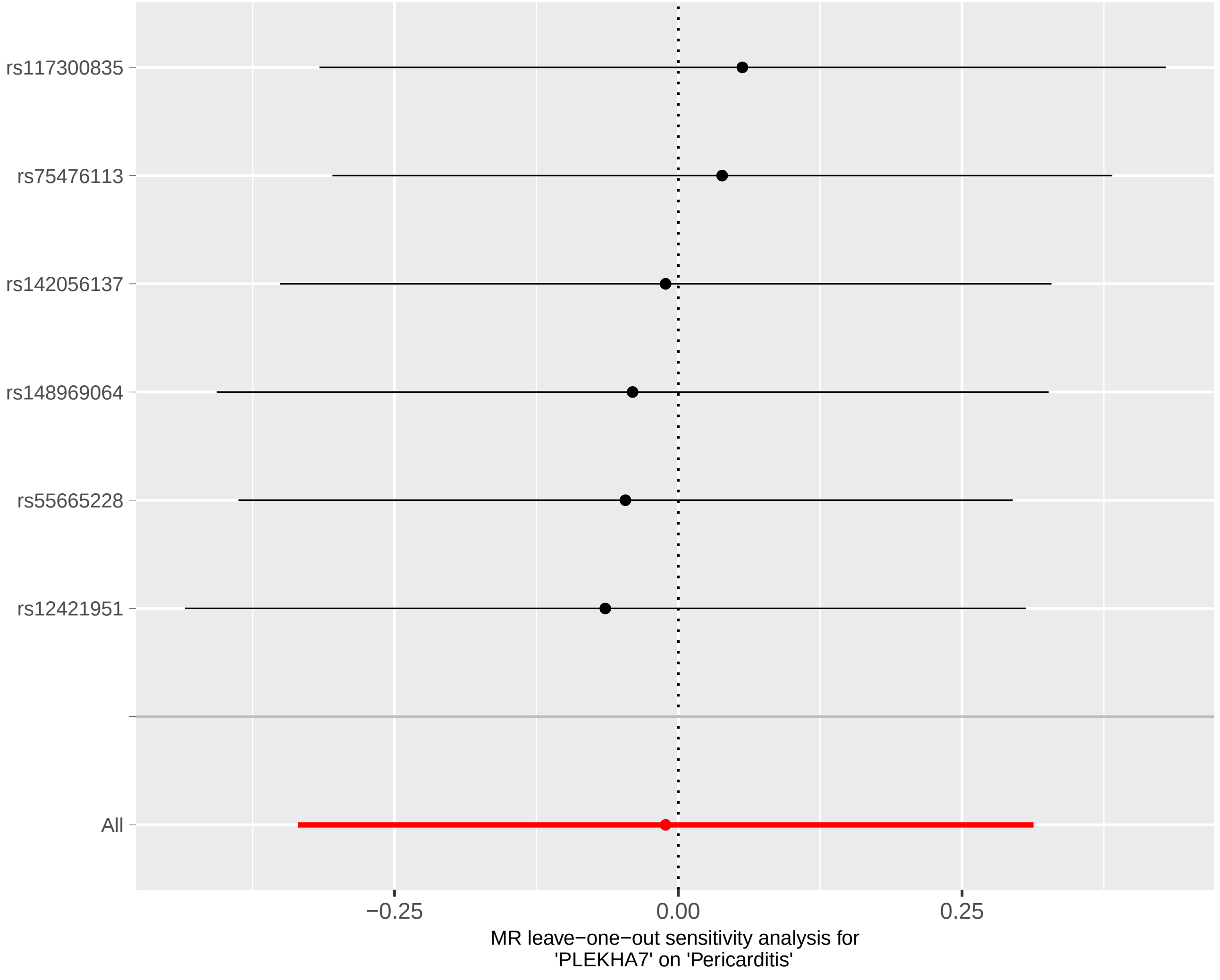

rs117300835
rs75476113
rs142056137
rs148969064
rs55665228
rs12421951
All
−0.25
0.00
0.25
MR leave−one−out sensitivity analysis for
'PLEKHA7' on 'Pericarditis'

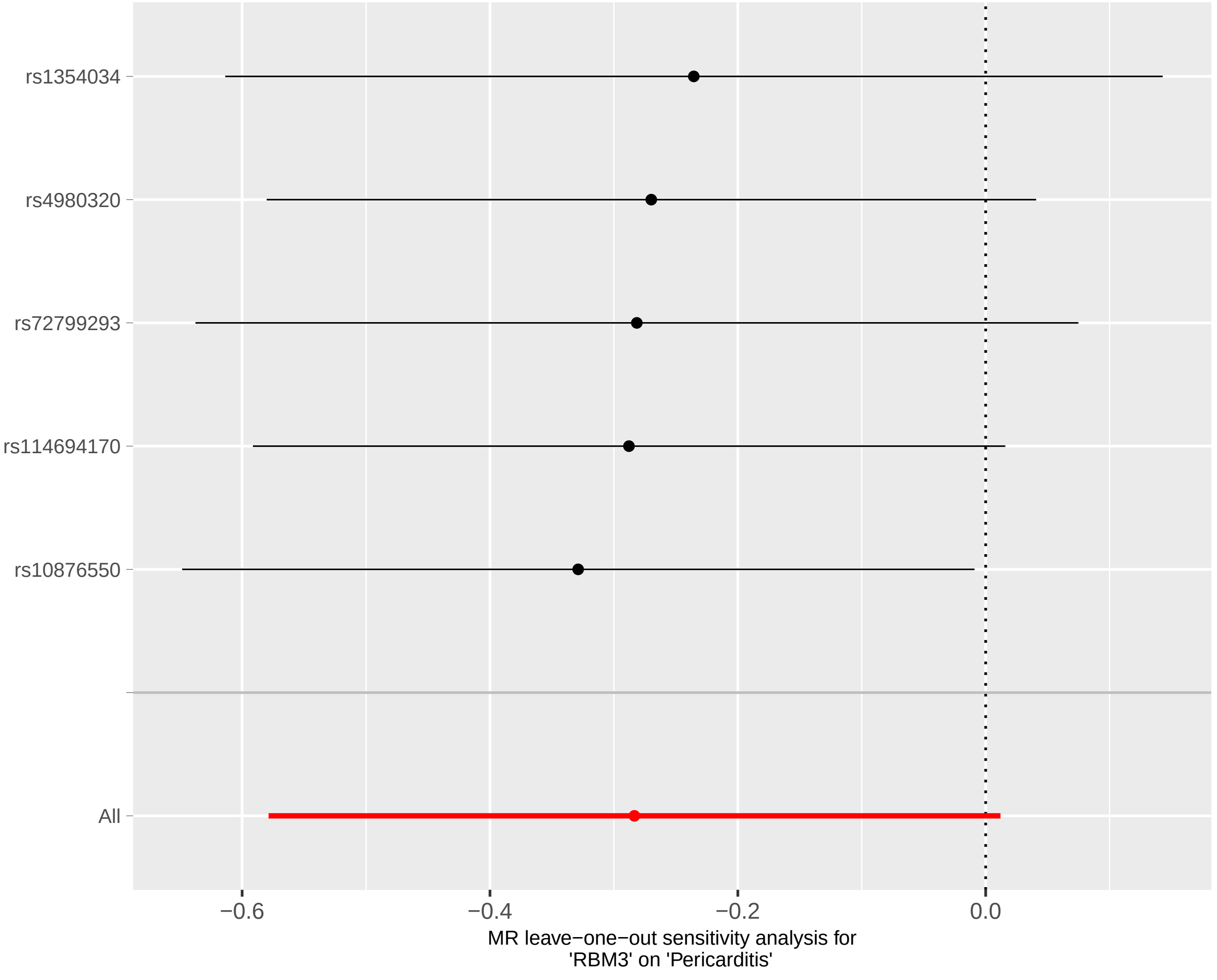

rs1354034
rs4980320
rs72799293
rs114694170
rs10876550
All
−0.6
−0.4
−0.2
0.0
MR leave−one−out sensitivity analysis for
'RBM3' on 'Pericarditis'

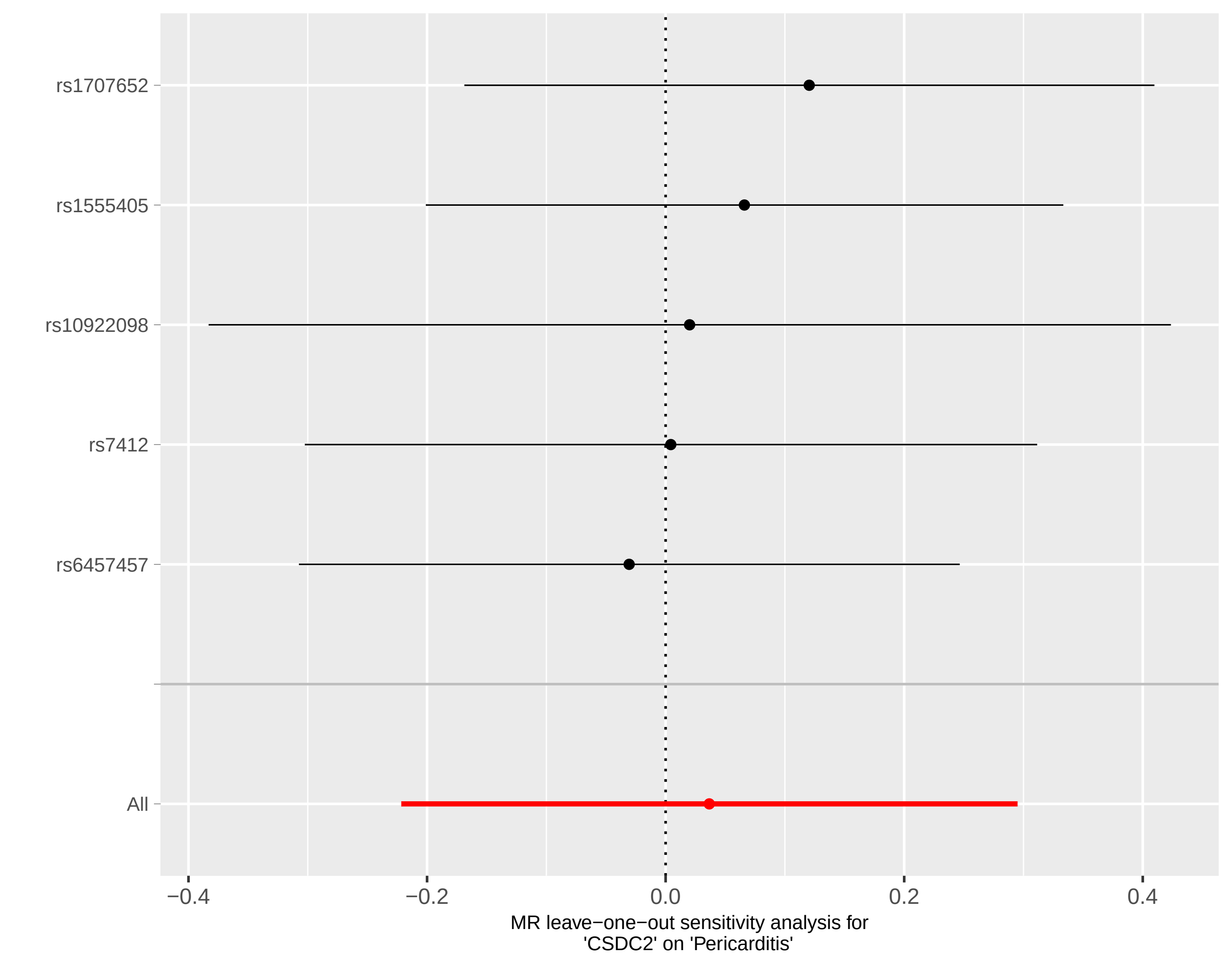

rs1707652
rs1555405
rs10922098
rs7412
rs6457457
All
−0.4
−0.2
0.0
0.2
0.4
MR leave−one−out sensitivity analysis for
'CSDC2' on 'Pericarditis'

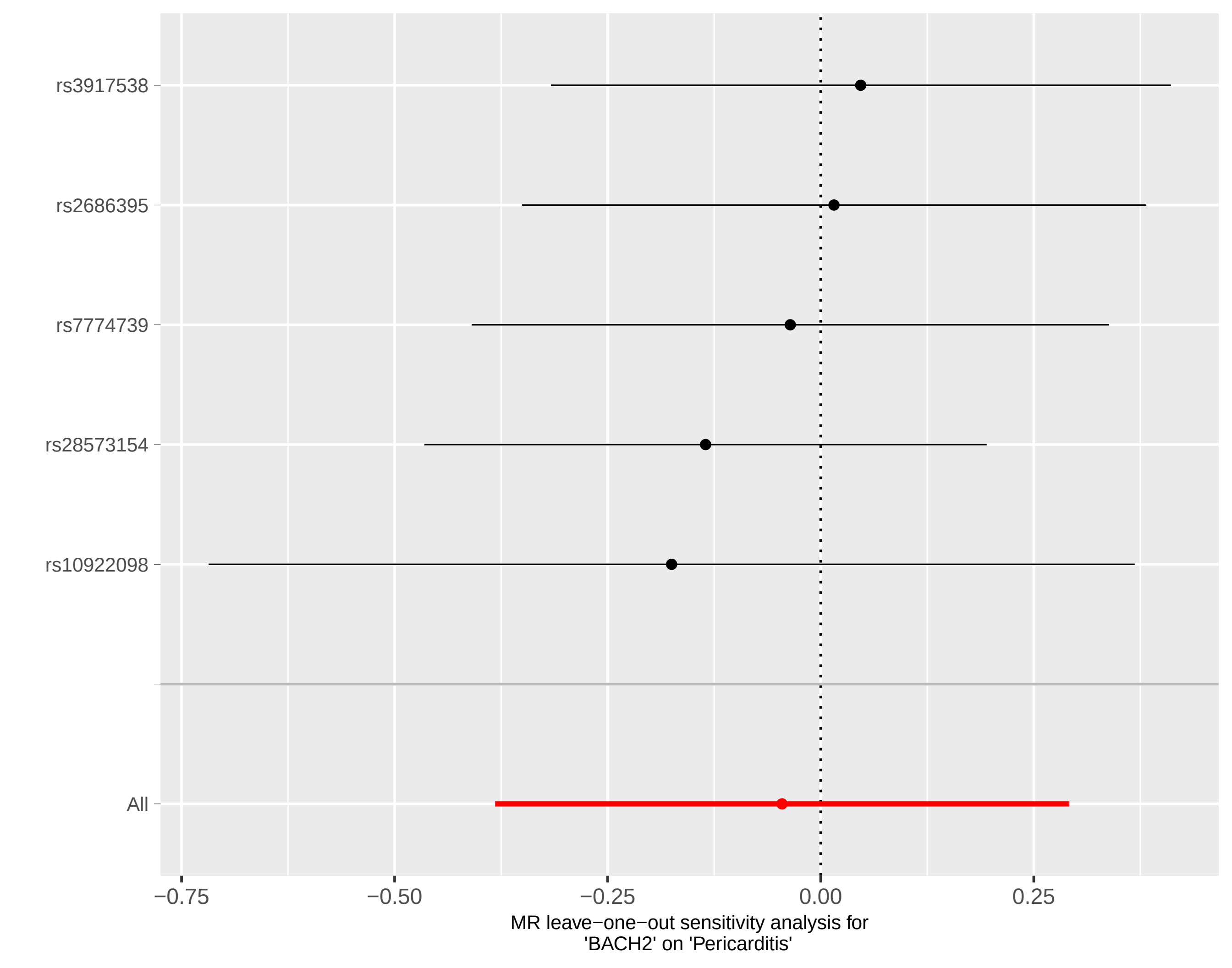

rs3917538
rs2686395
rs7774739
rs28573154
rs10922098
All
−0.75
−0.50
−0.25
0.00
0.25
MR leave−one−out sensitivity analysis for
'BACH2' on 'Pericarditis'

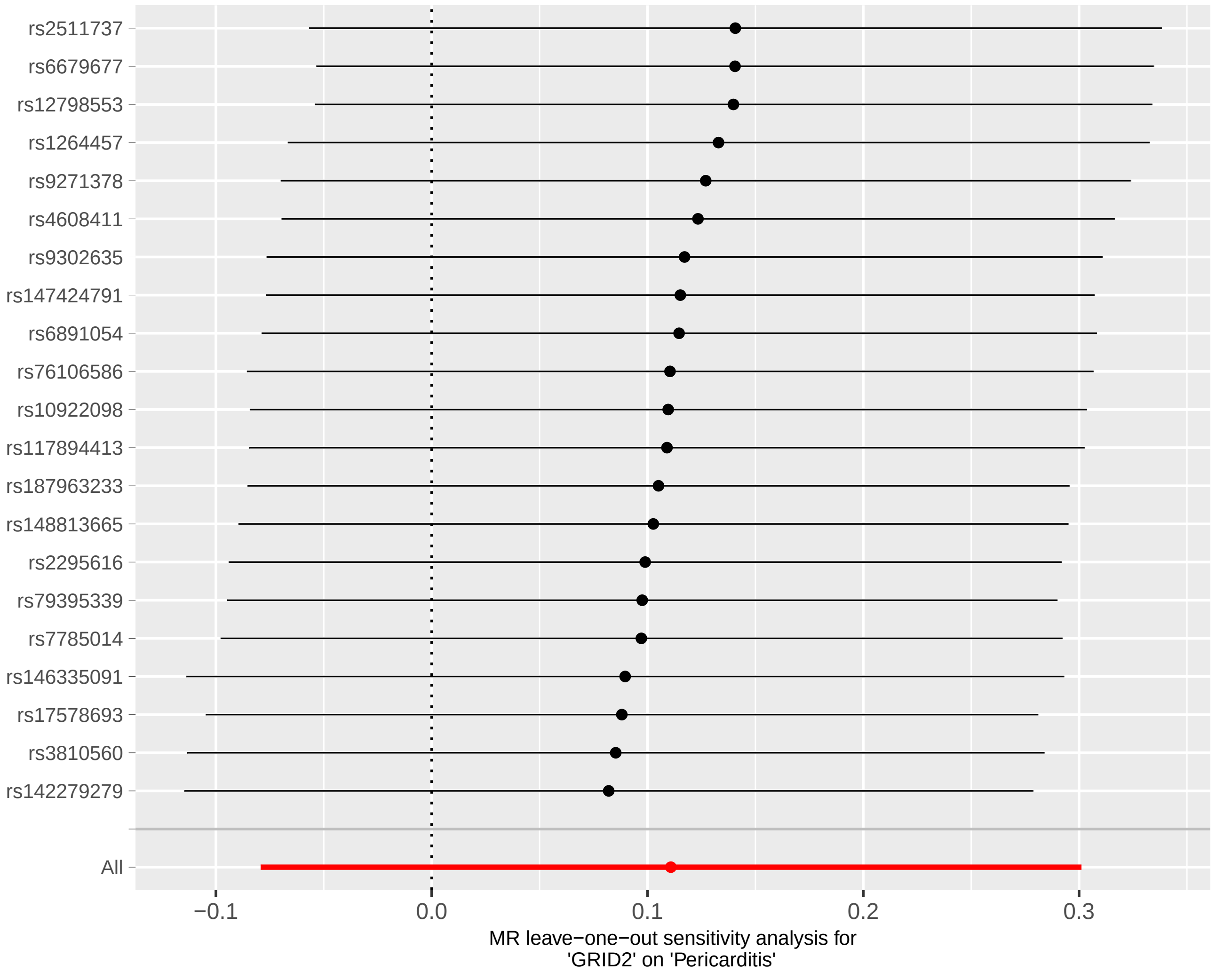

rs2511737
rs6679677
rs12798553
rs1264457
rs9271378
rs4608411
rs9302635
rs147424791
rs6891054
rs76106586
rs10922098
rs117894413
rs187963233
rs148813665
rs2295616
rs79395339
rs7785014
rs146335091
rs17578693
rs3810560
rs142279279
All
−0.1
0.0
0.1
0.2
0.3
MR leave−one−out sensitivity analysis for
'GRID2' on 'Pericarditis'

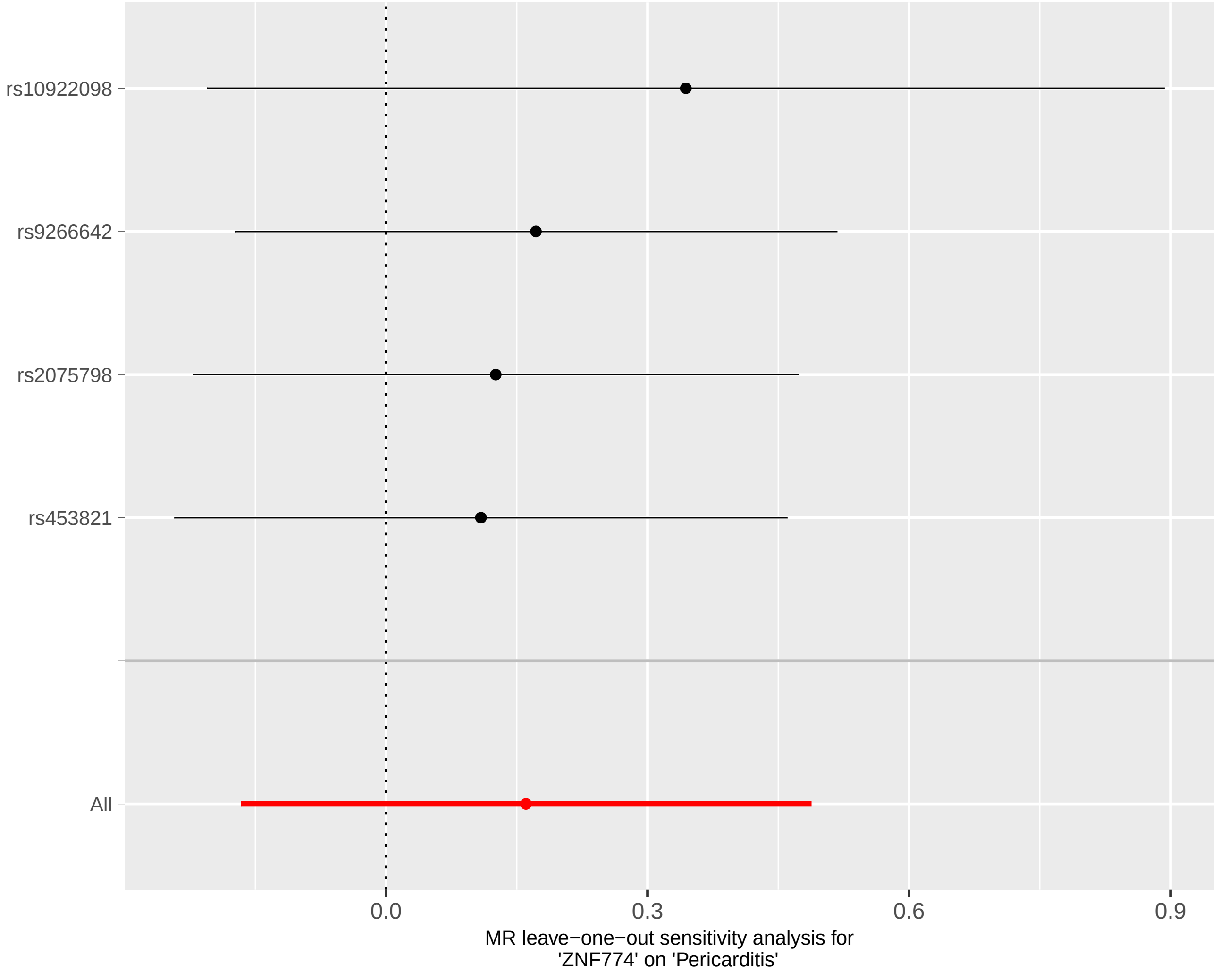

rs10922098
rs9266642
rs2075798
rs453821
All
0.0
0.3
0.6
0.9
MR leave−one−out sensitivity analysis for
'ZNF774' on 'Pericarditis'

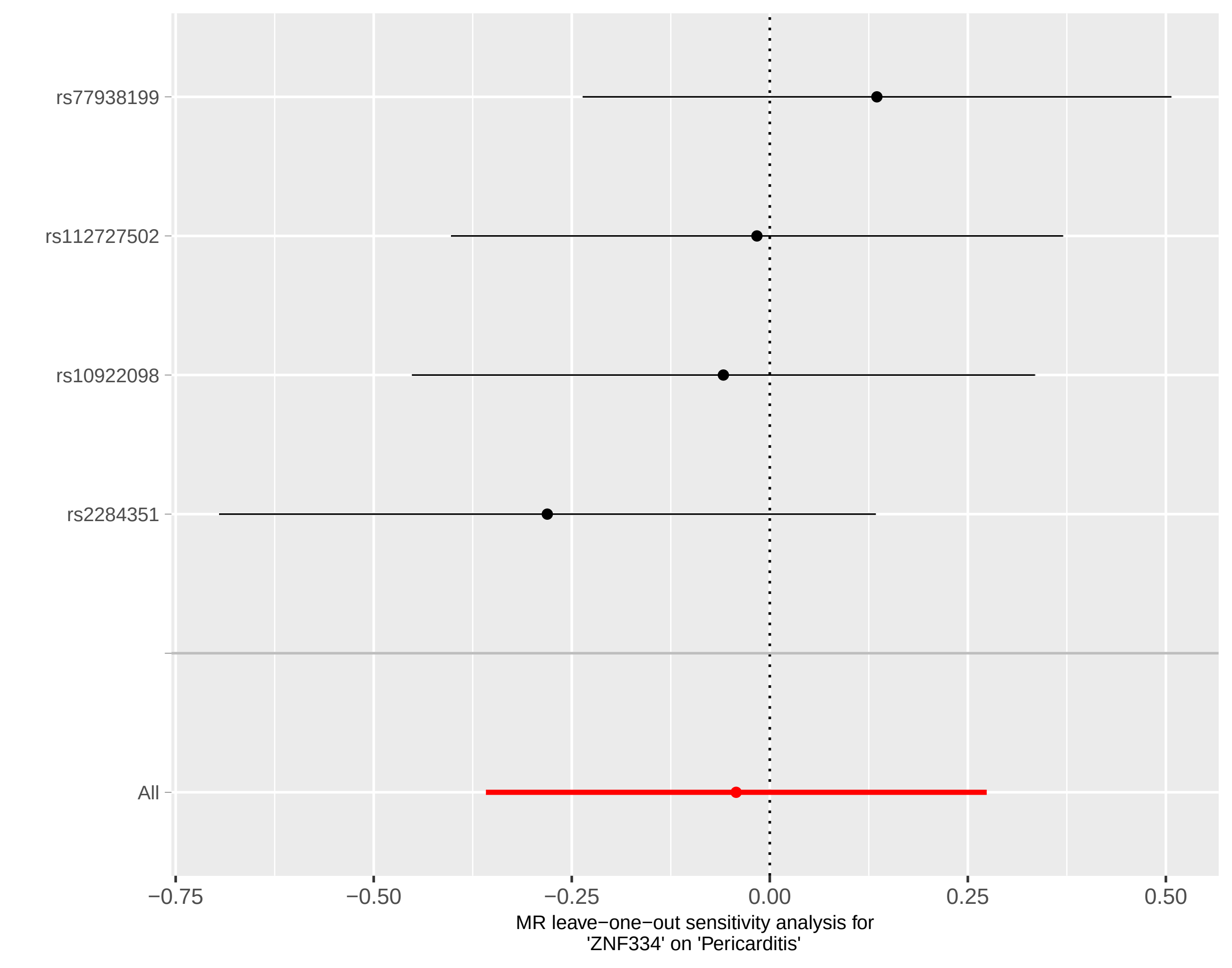

rs77938199
rs112727502
rs10922098
rs2284351
All
−0.75
−0.50
−0.25
0.00
0.25
0.50
MR leave−one−out sensitivity analysis for
'ZNF334' on 'Pericarditis'

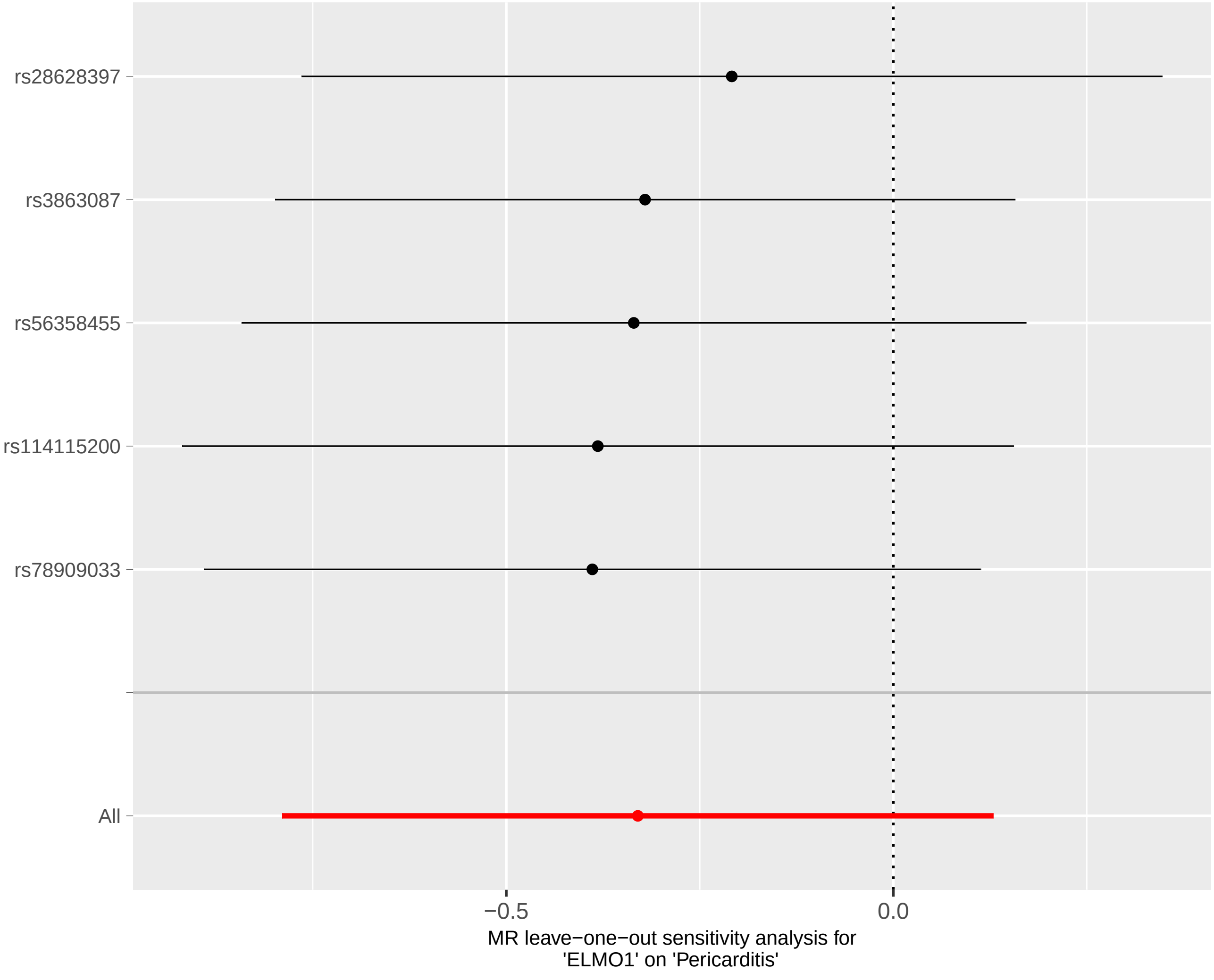
rs28628397
rs3863087
rs56358455
rs114115200
rs78909033
All
−0.5
0.0
MR leave−one−out sensitivity analysis for
'ELMO1' on 'Pericarditis'

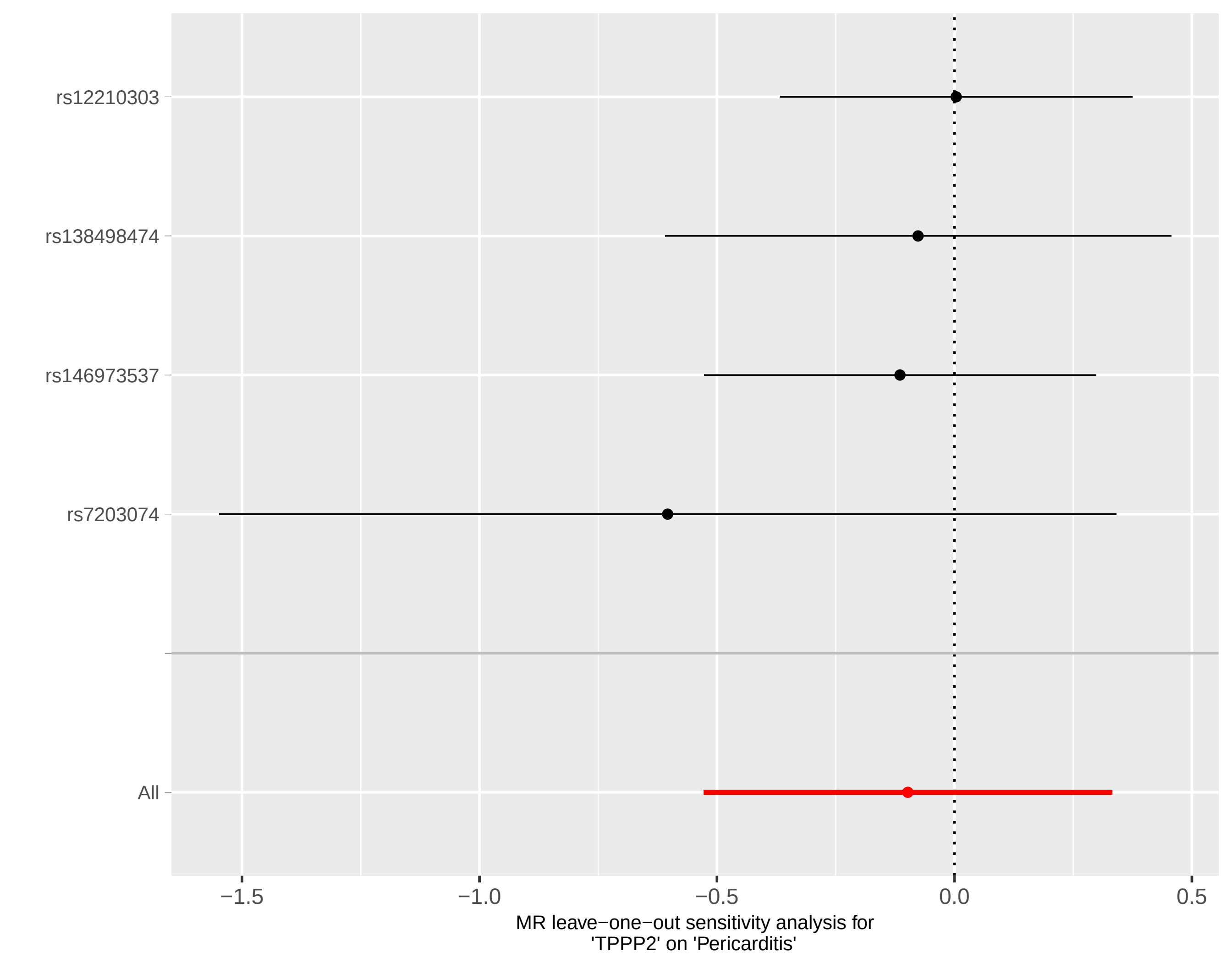
rs12210303
rs138498474
rs146973537
rs7203074
All
−1.5
−1.0
−0.5
0.0
0.5
MR leave−one−out sensitivity analysis for
'TPPP2' on 'Pericarditis'

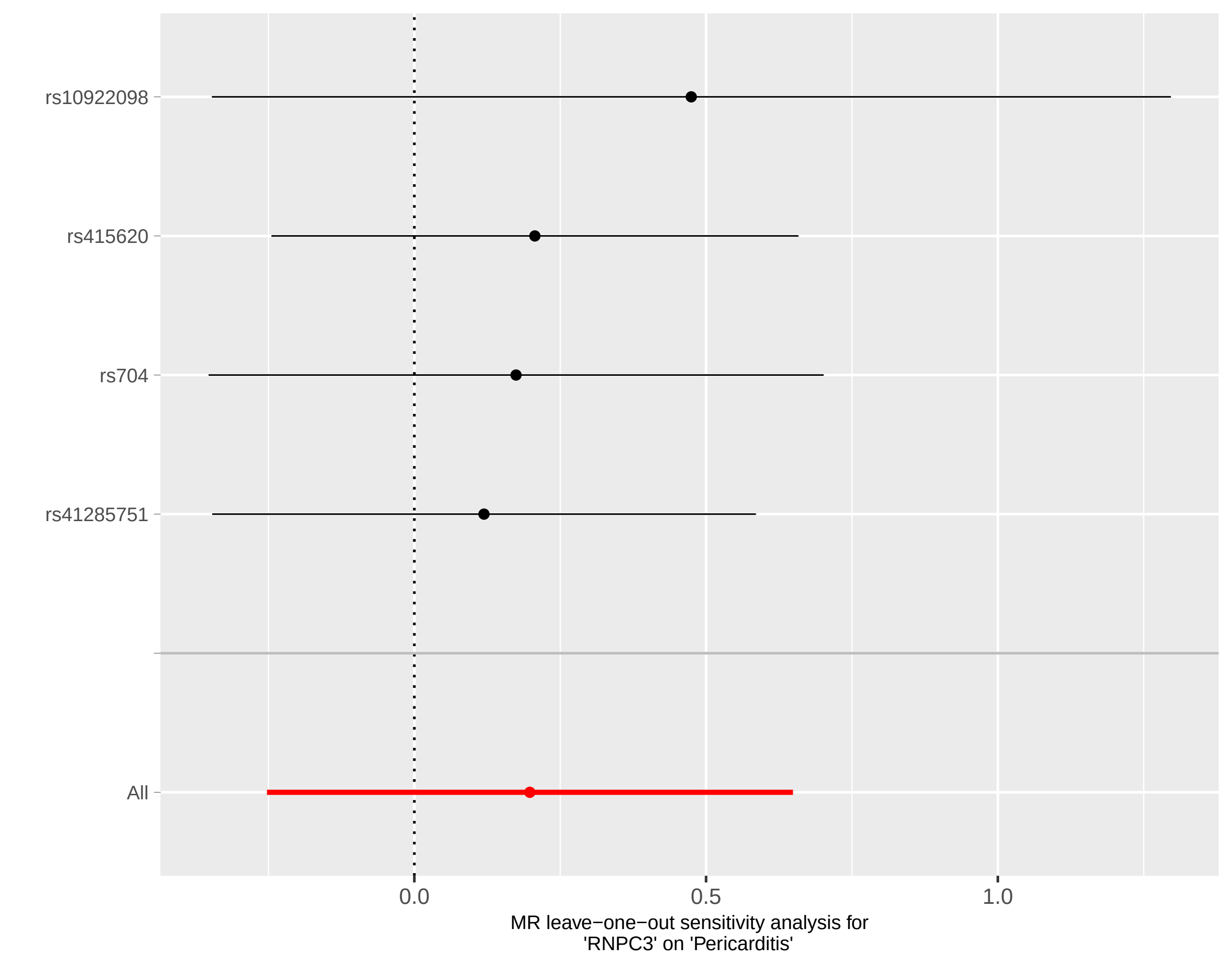
rs10922098
rs415620
rs704
rs41285751
All
0.0
0.5
1.0
MR leave−one−out sensitivity analysis for
'RNPC3' on 'Pericarditis'

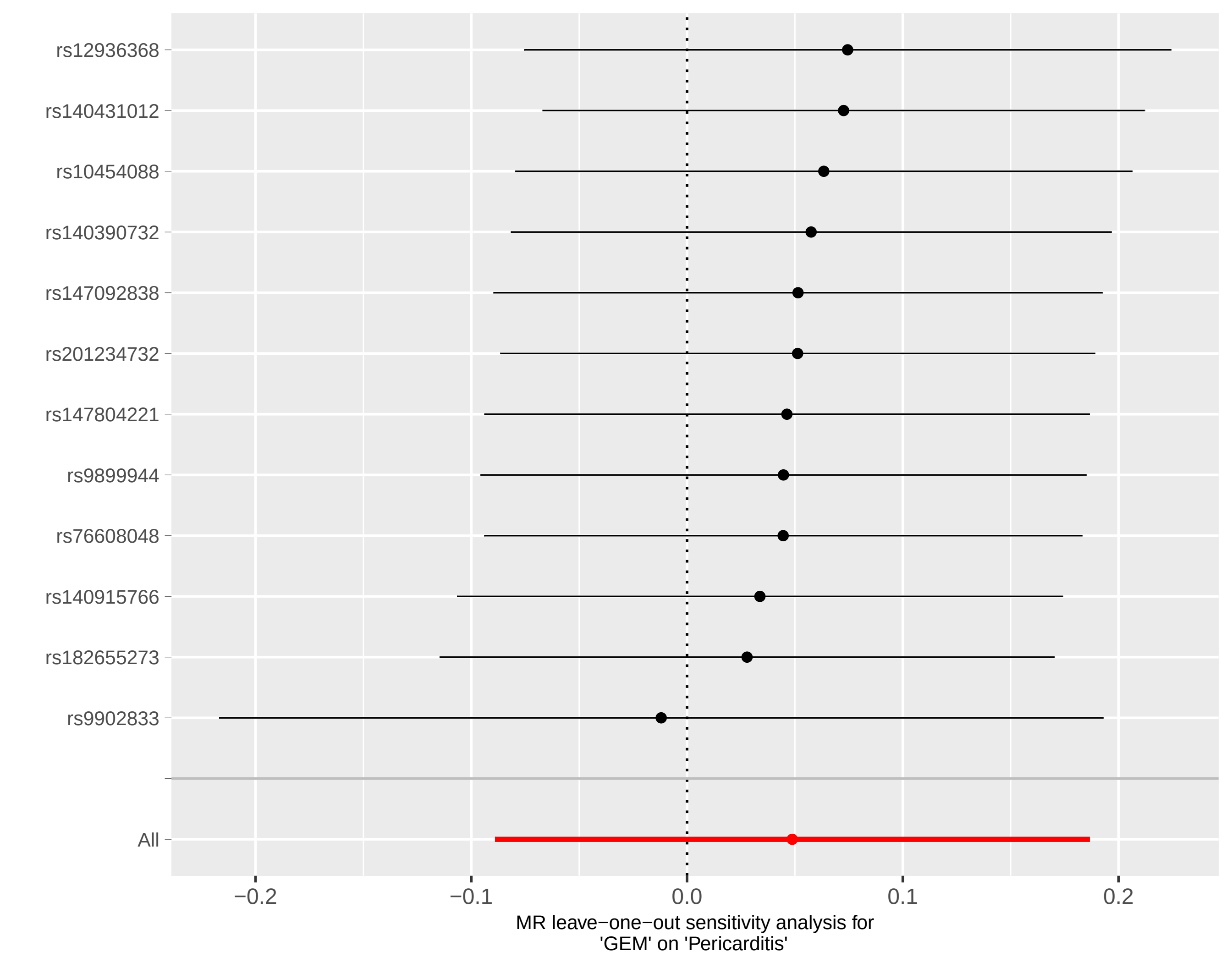
rs12936368
rs140431012
rs10454088
rs140390732
rs147092838
rs201234732
rs147804221
rs9899944
rs76608048
rs140915766
rs182655273
rs9902833
All
−0.2
−0.1
0.0
0.1
0.2
MR leave−one−out sensitivity analysis for
'GEM' on 'Pericarditis'

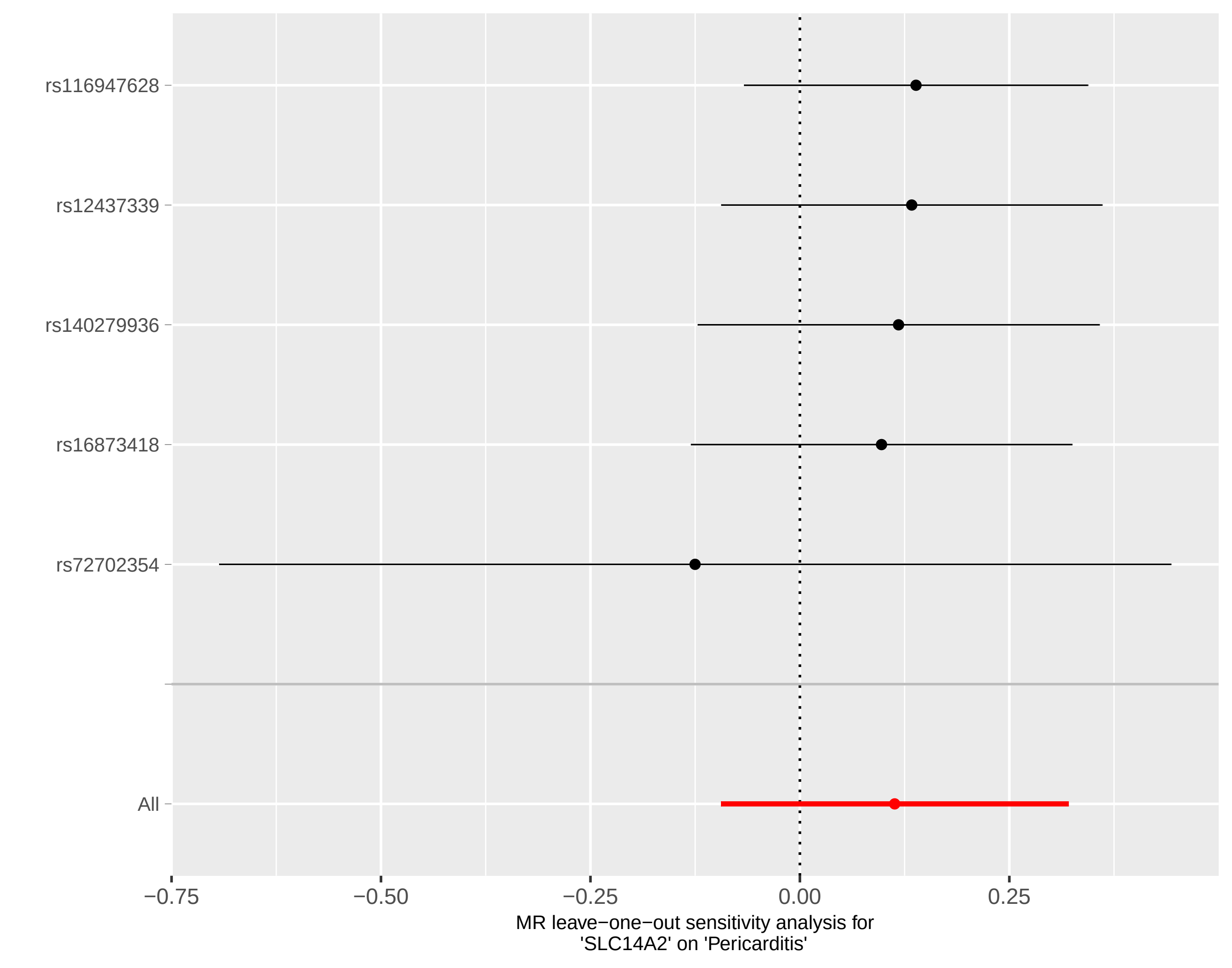

rs116947628
rs12437339
rs140279936
rs16873418
rs72702354
All
−0.75
−0.50
−0.25
0.00
0.25
MR leave−one−out sensitivity analysis for
'SLC14A2' on 'Pericarditis'

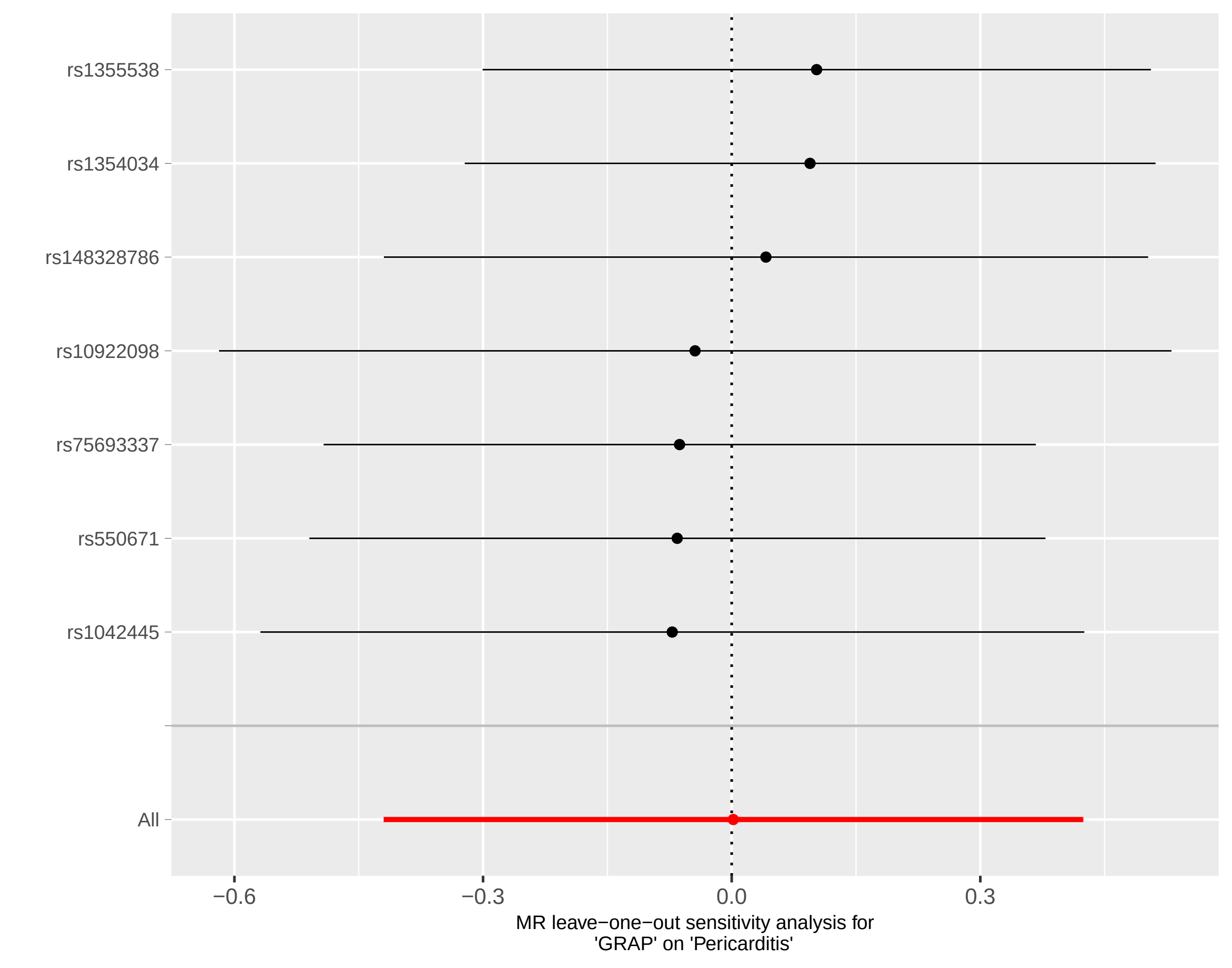
rs1355538
rs1354034
rs148328786
rs10922098
rs75693337
rs550671
rs1042445
All
−0.6
−0.3
0.0
0.3
MR leave−one−out sensitivity analysis for
'GRAP' on 'Pericarditis'

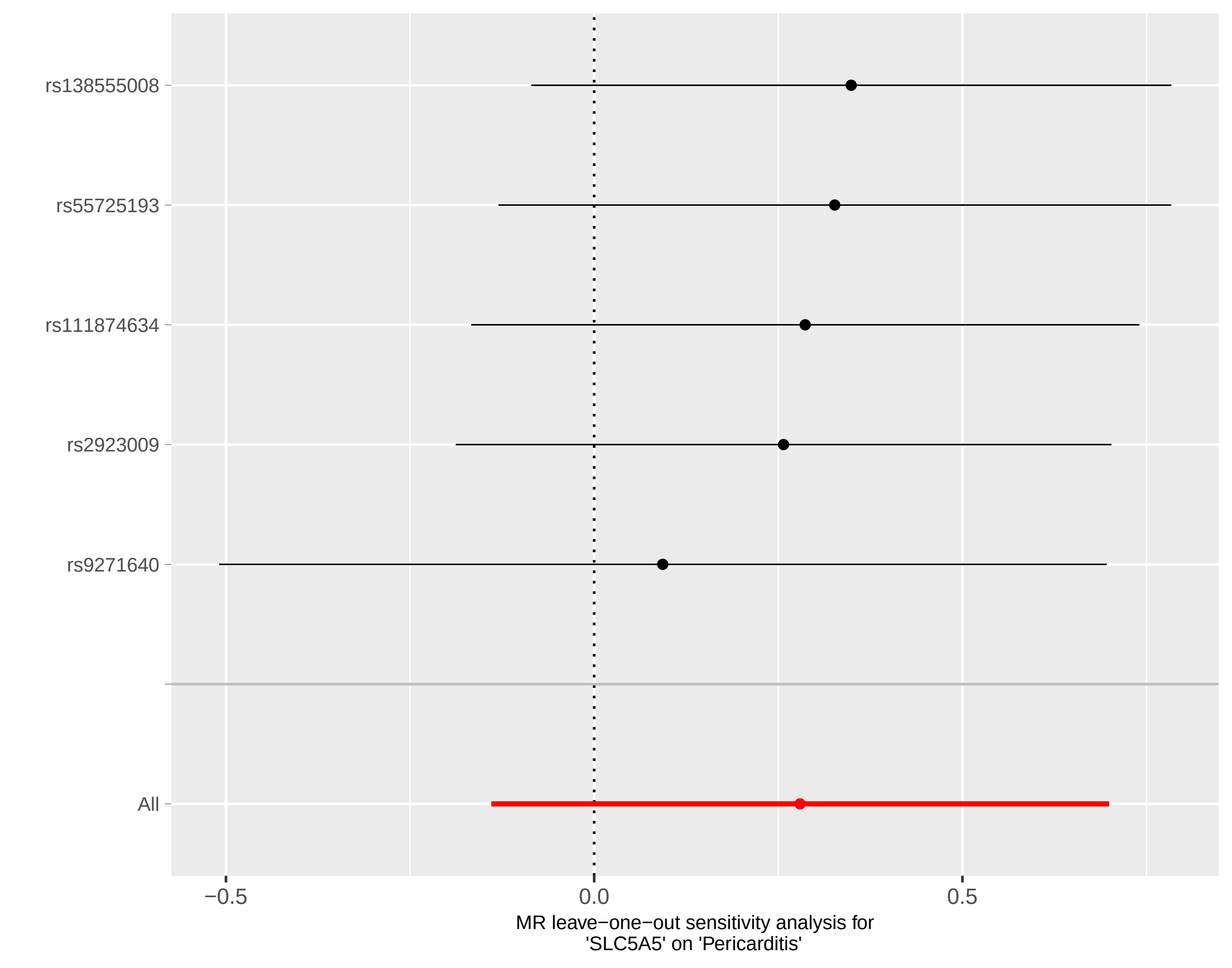

rs138555008
rs55725193
rs111874634
rs2923009
rs9271640
All
−0.5
0.0
0.5
MR leave−one−out sensitivity analysis for
'SLC5A5' on 'Pericarditis'

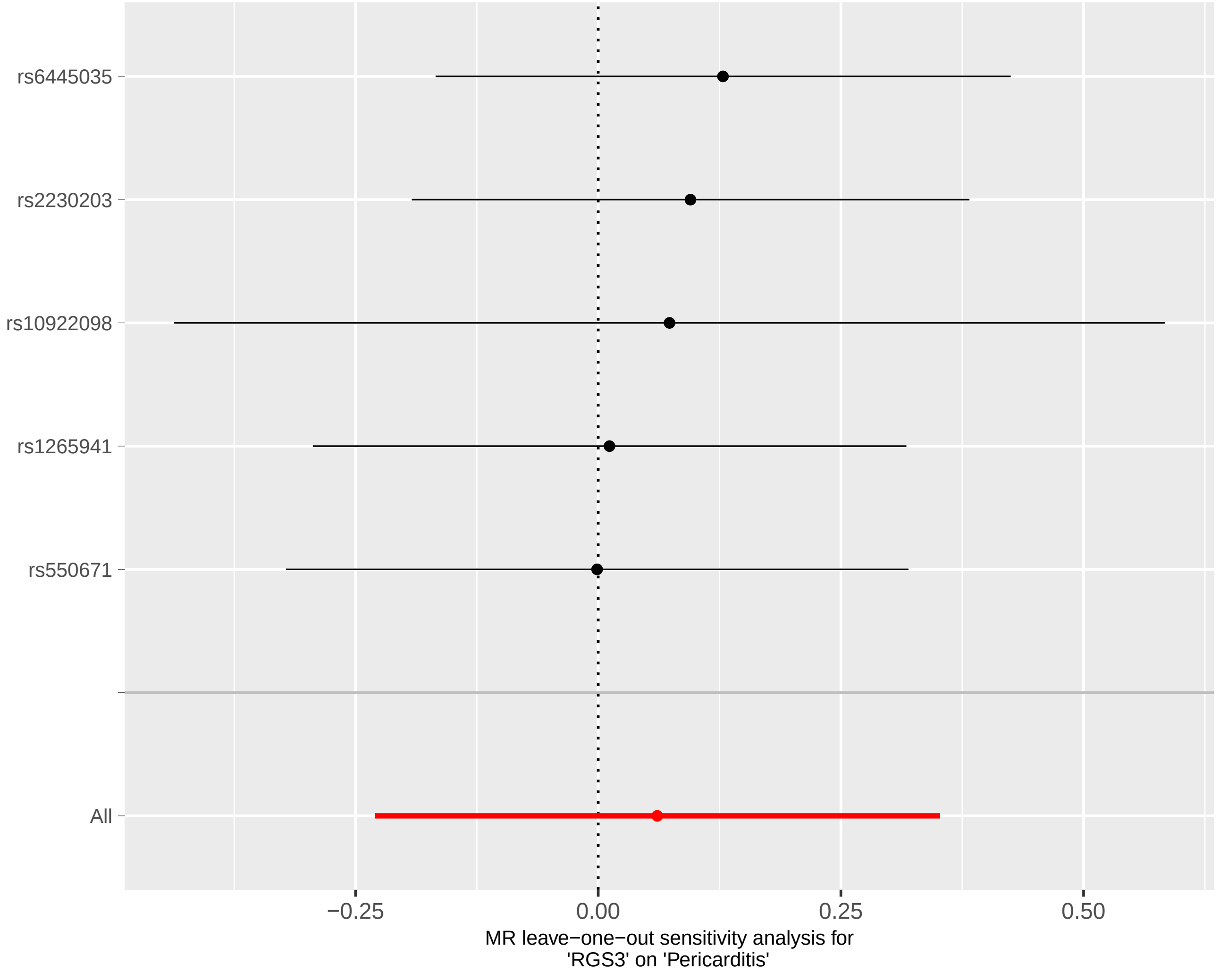
rs6445035
rs2230203
rs10922098
rs1265941
rs550671
All
−0.25
0.00
0.25
0.50
MR leave−one−out sensitivity analysis for
'RGS3' on 'Pericarditis'

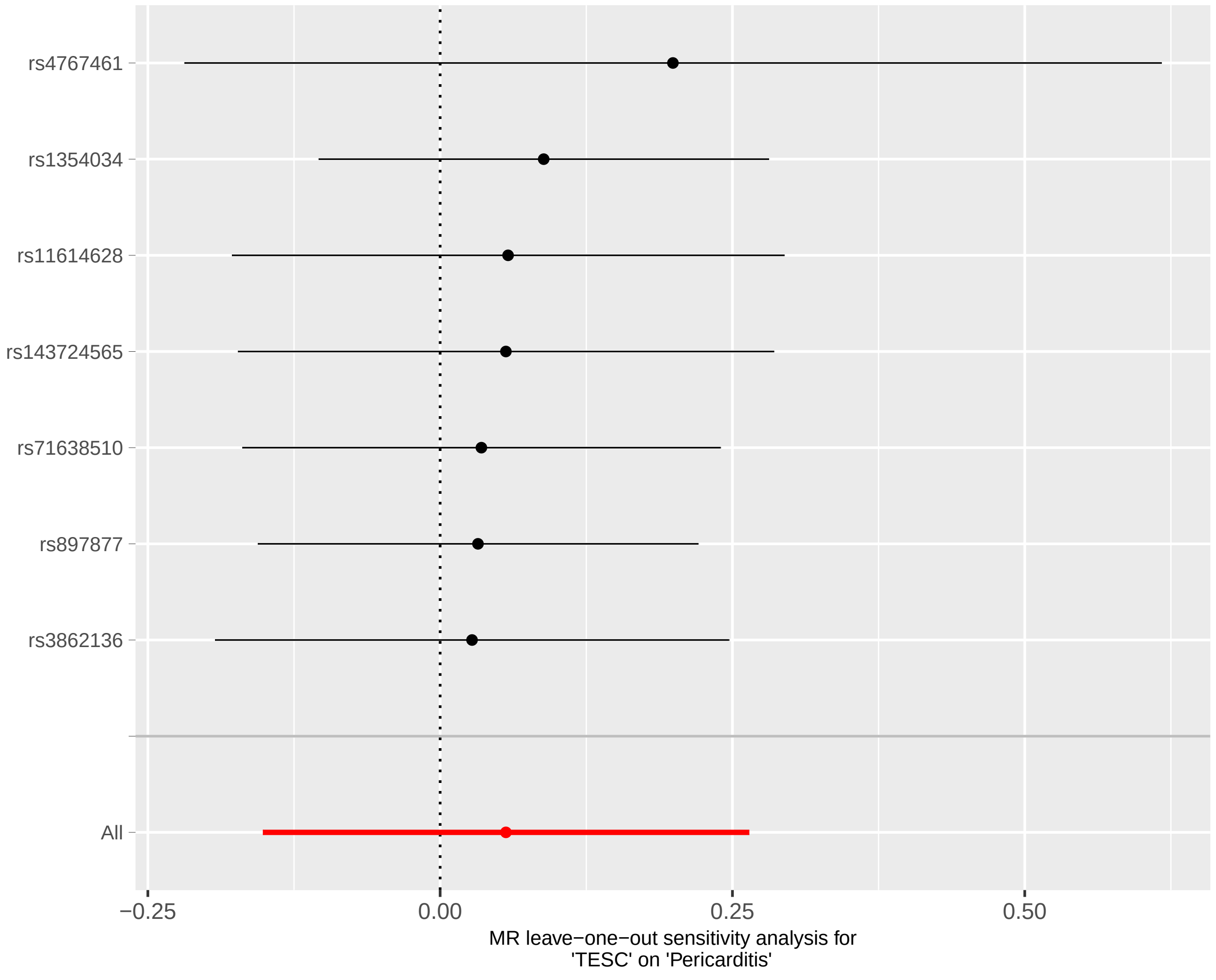
rs4767461
rs1354034
rs11614628
rs143724565
rs71638510
rs897877
rs3862136
All
−0.25
0.00
0.25
0.50
MR leave−one−out sensitivity analysis for
'TESC' on 'Pericarditis'

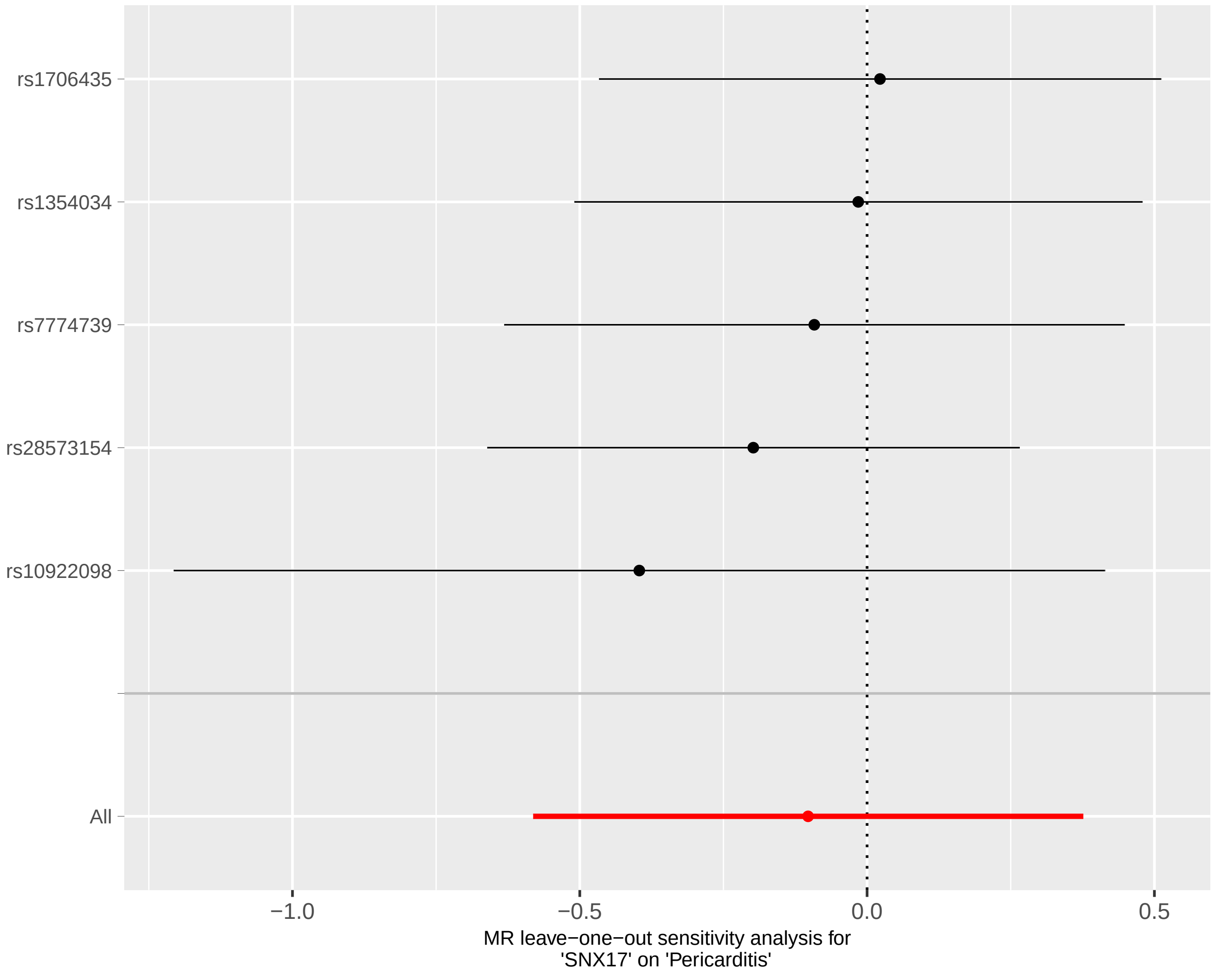

rs1706435
rs1354034
rs7774739
rs28573154
rs10922098
All
−1.0
−0.5
0.0
0.5
MR leave−one−out sensitivity analysis for
'SNX17' on 'Pericarditis'

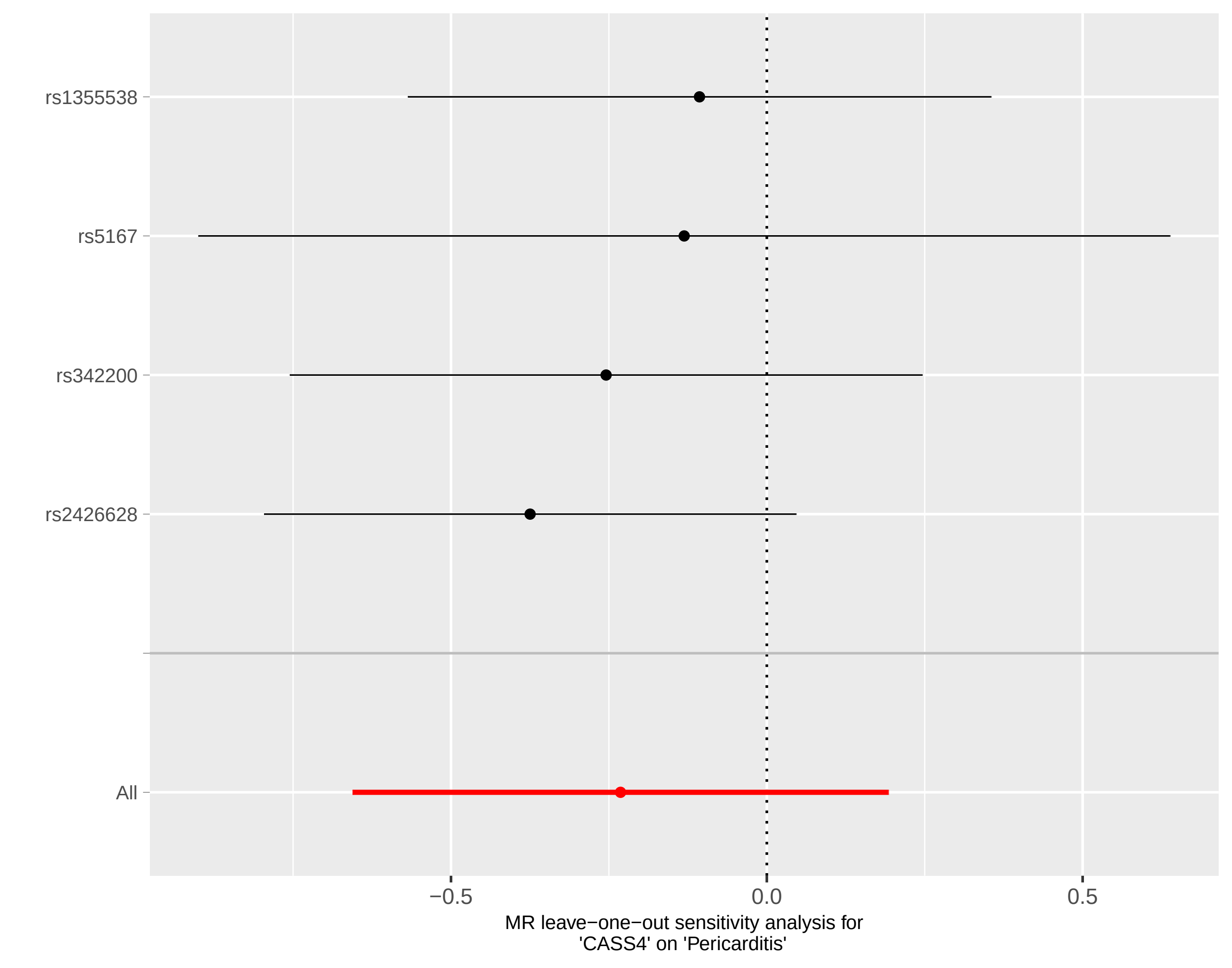
rs1355538
rs5167
rs342200
rs2426628
All
−0.5
0.0
0.5
MR leave−one−out sensitivity analysis for
'CASS4' on 'Pericarditis'

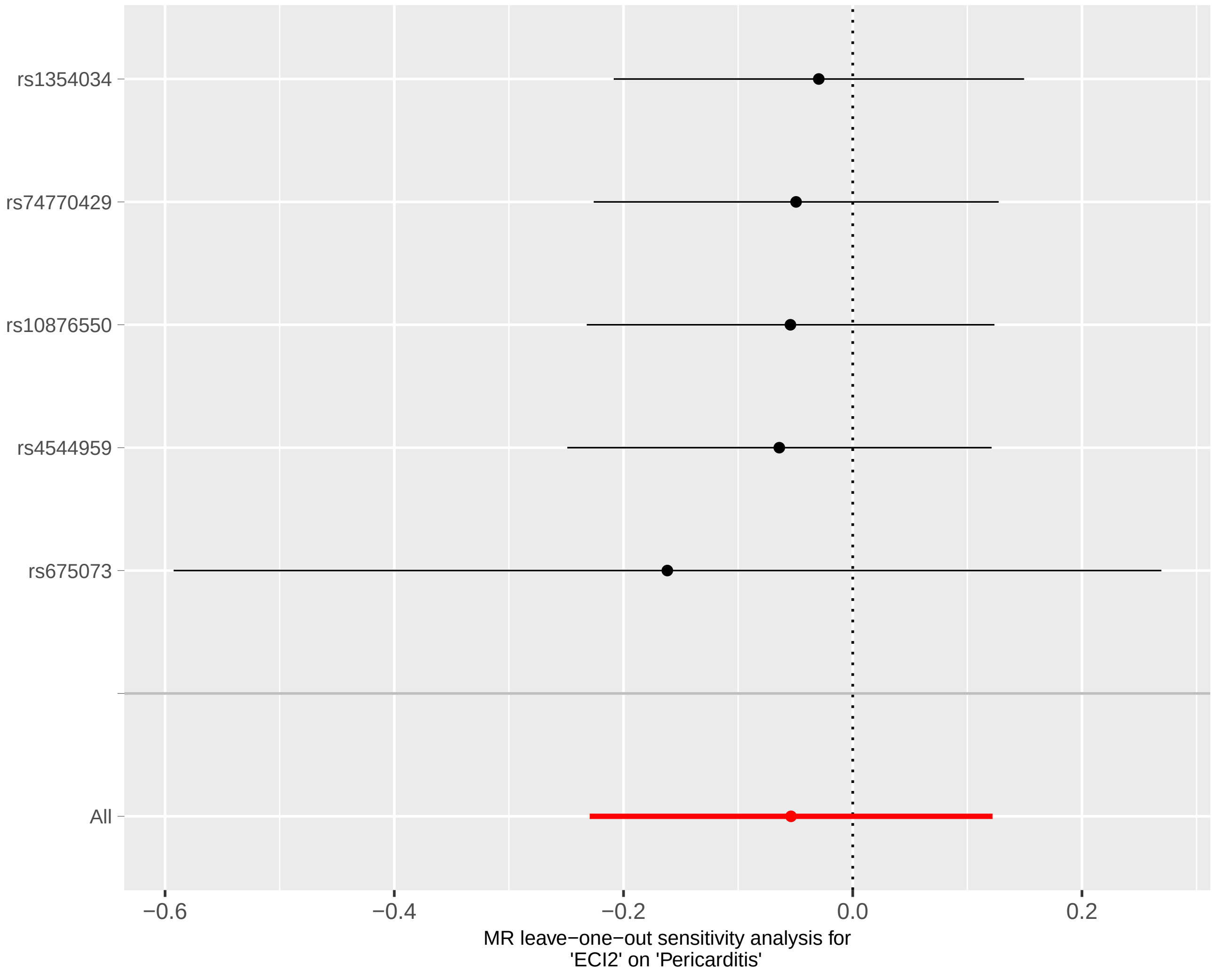

rs1354034
rs74770429
rs10876550
rs4544959
rs675073
All
−0.6
−0.4
−0.2
0.0
0.2
MR leave−one−out sensitivity analysis for
'ECI2' on 'Pericarditis'

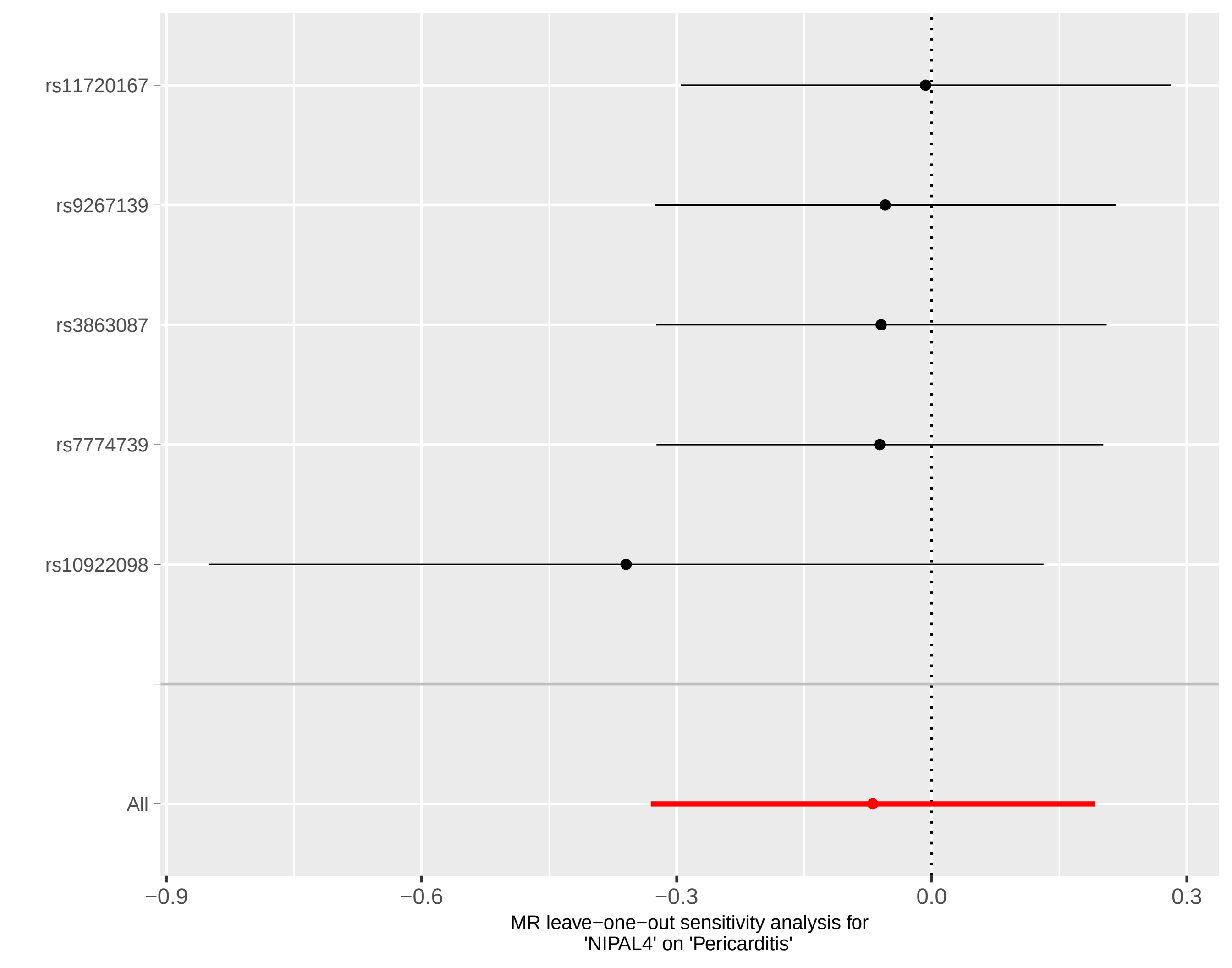

rs11720167
rs9267139
rs3863087
rs7774739
rs10922098
All
−0.9
−0.6
−0.3
0.0
0.3
MR leave−one−out sensitivity analysis for
'NIPAL4' on 'Pericarditis'

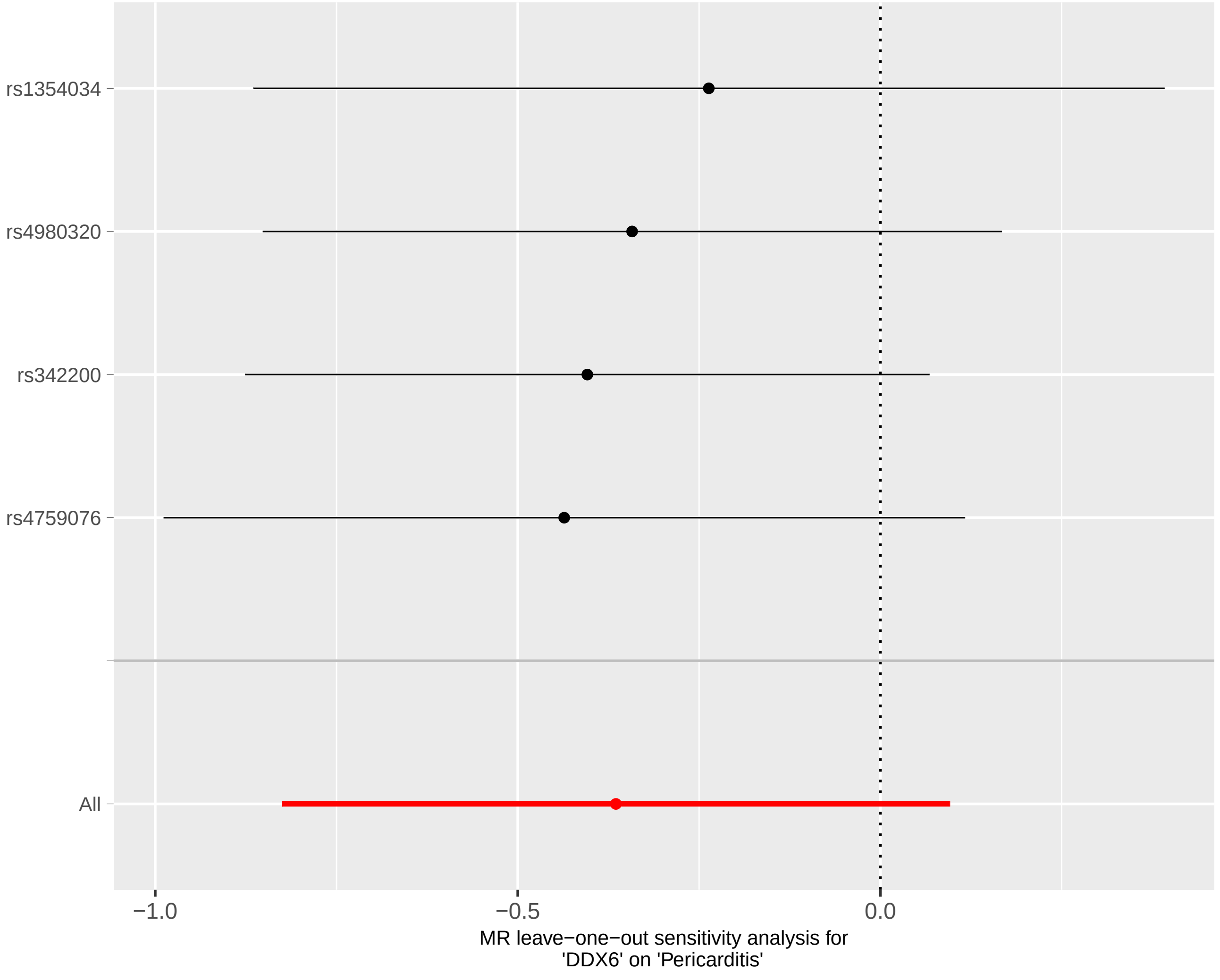
rs1354034
rs4980320
rs342200
rs4759076
All
−1.0
−0.5
0.0
MR leave−one−out sensitivity analysis for
'DDX6' on 'Pericarditis'

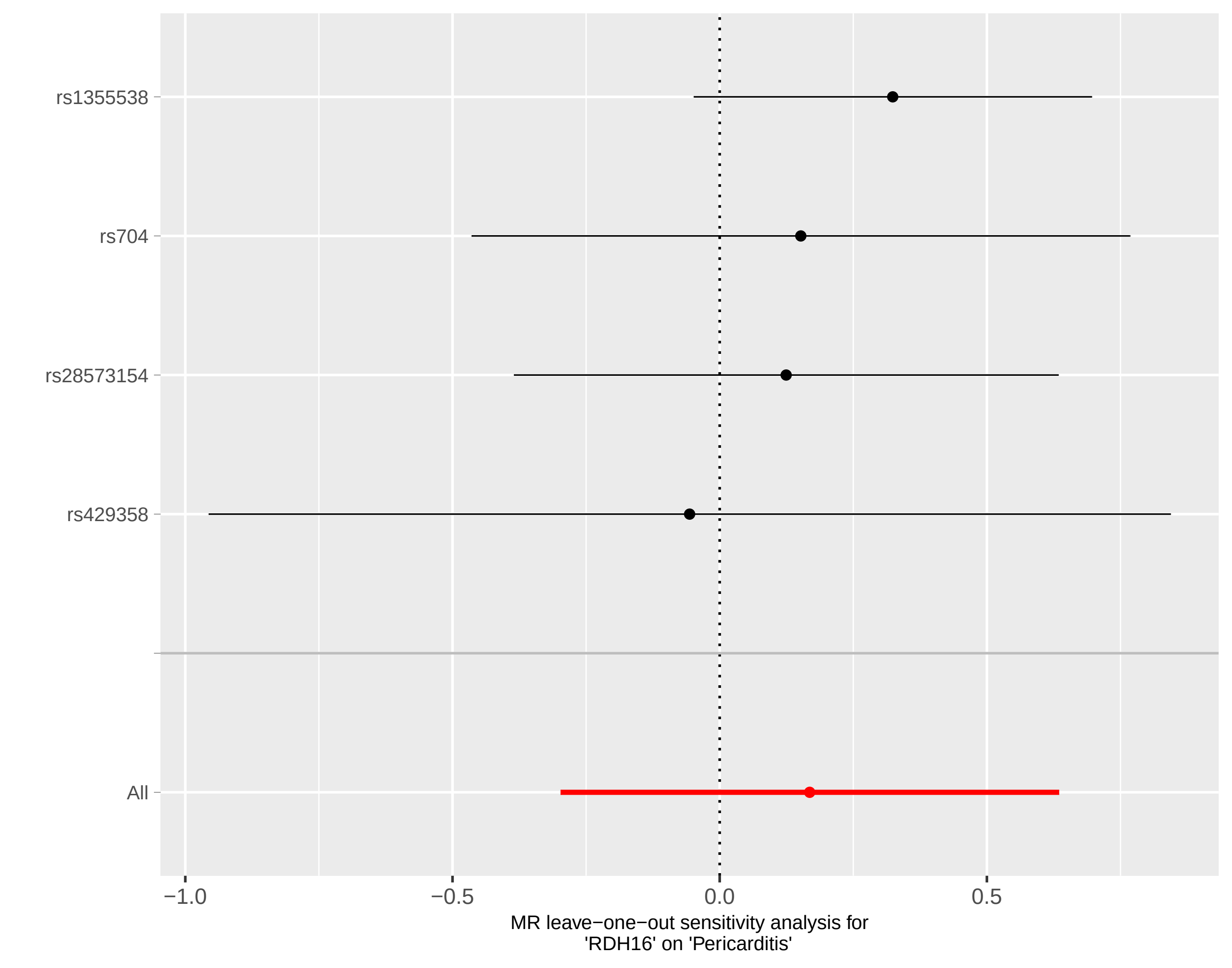
rs1355538
rs704
rs28573154
rs429358
All
−1.0
−0.5
0.0
0.5
MR leave−one−out sensitivity analysis for
'RDH16' on 'Pericarditis'

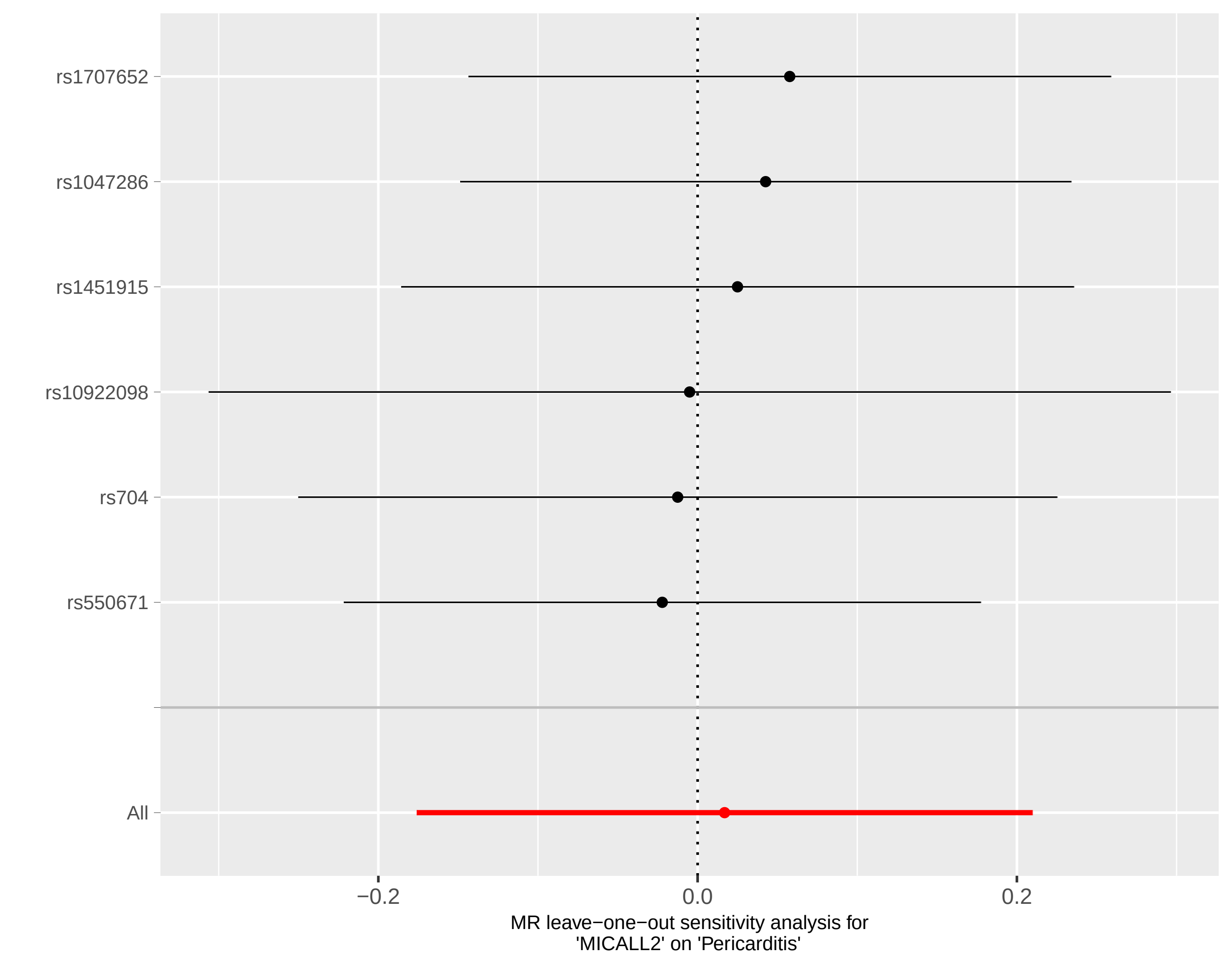
rs1707652
rs1047286
rs1451915
rs10922098
rs704
rs550671
All
−0.2
0.0
0.2
MR leave−one−out sensitivity analysis for
'MICALL2' on 'Pericarditis'

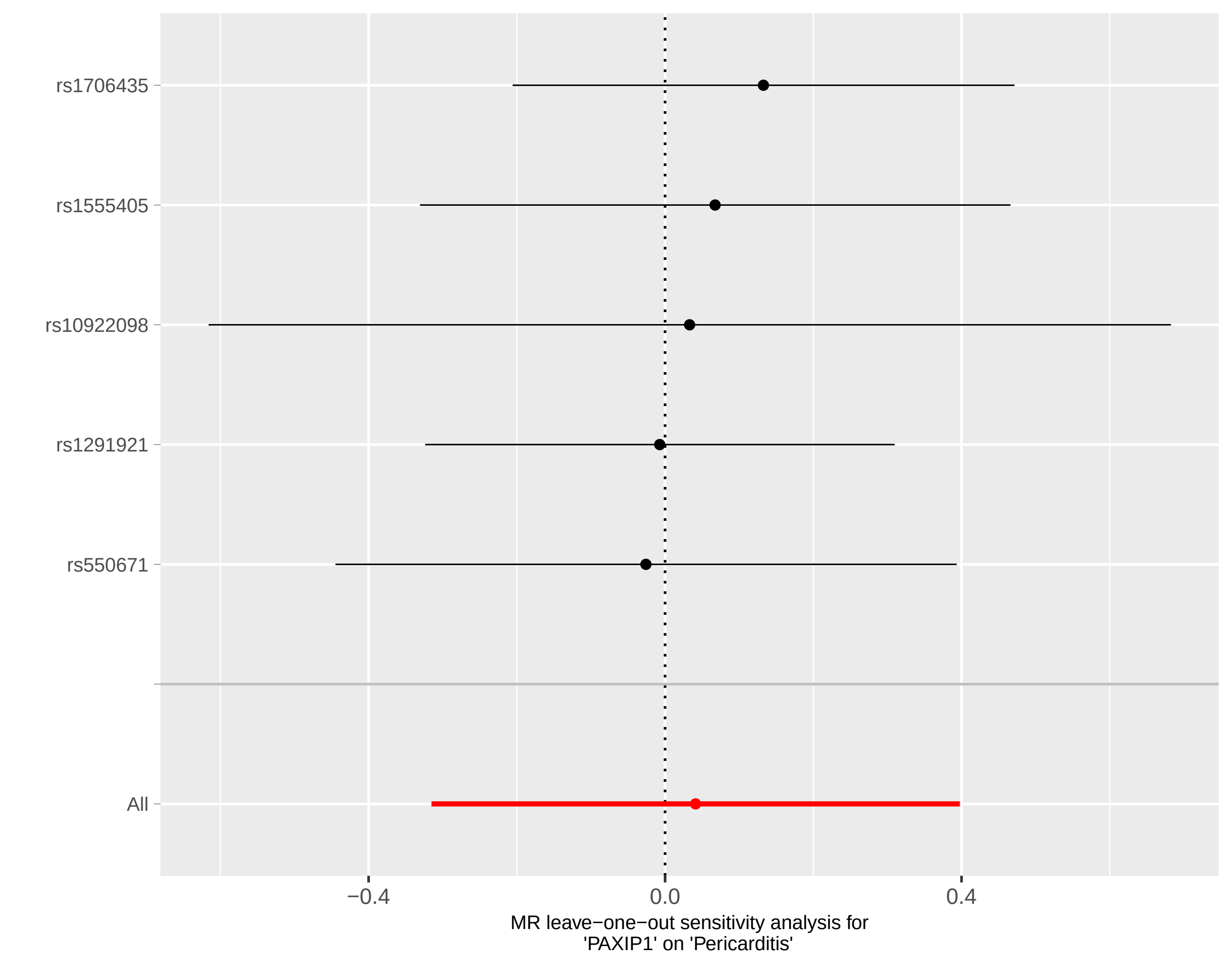
rs1706435
rs1555405
rs10922098
rs1291921
rs550671
All
−0.4
0.0
0.4
MR leave−one−out sensitivity analysis for
'PAXIP1' on 'Pericarditis'

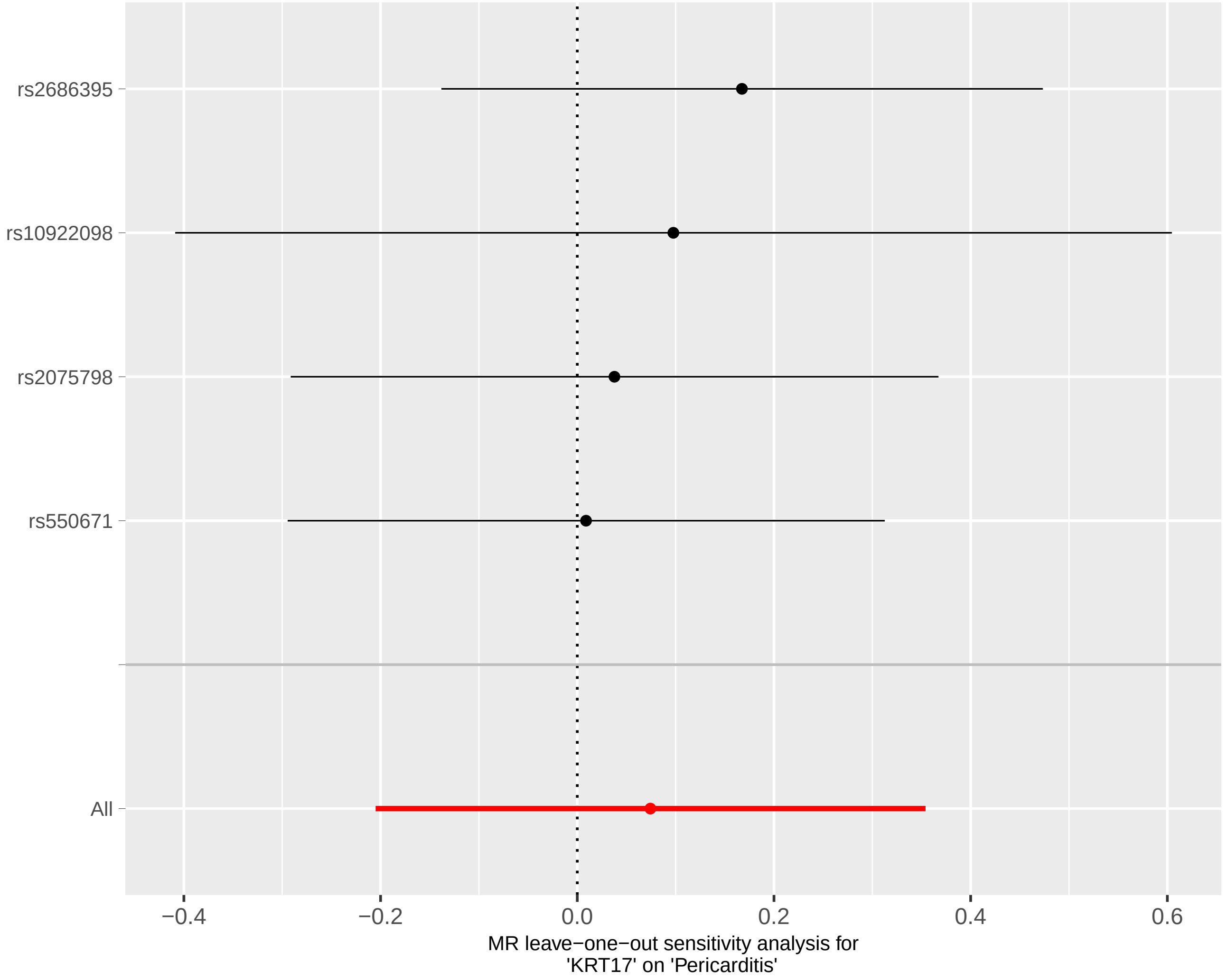
rs2686395
rs10922098
rs2075798
rs550671
All
−0.4
−0.2
0.0
0.2
0.4
0.6
MR leave−one−out sensitivity analysis for
'KRT17' on 'Pericarditis'

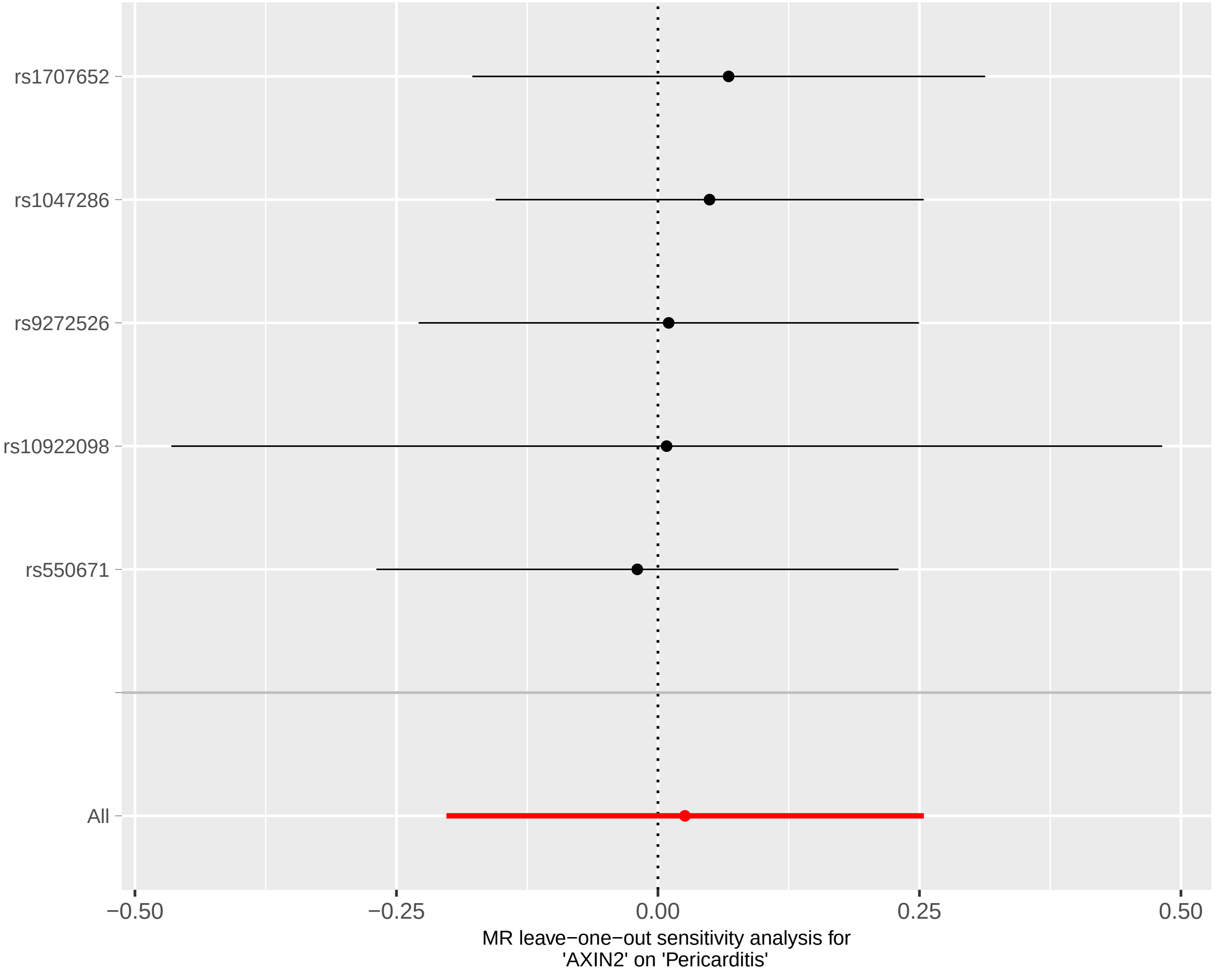
rs1707652
rs1047286
rs9272526
rs10922098
rs550671
All
−0.50
−0.25
0.00
0.25
0.50
MR leave−one−out sensitivity analysis for
'AXIN2' on 'Pericarditis'

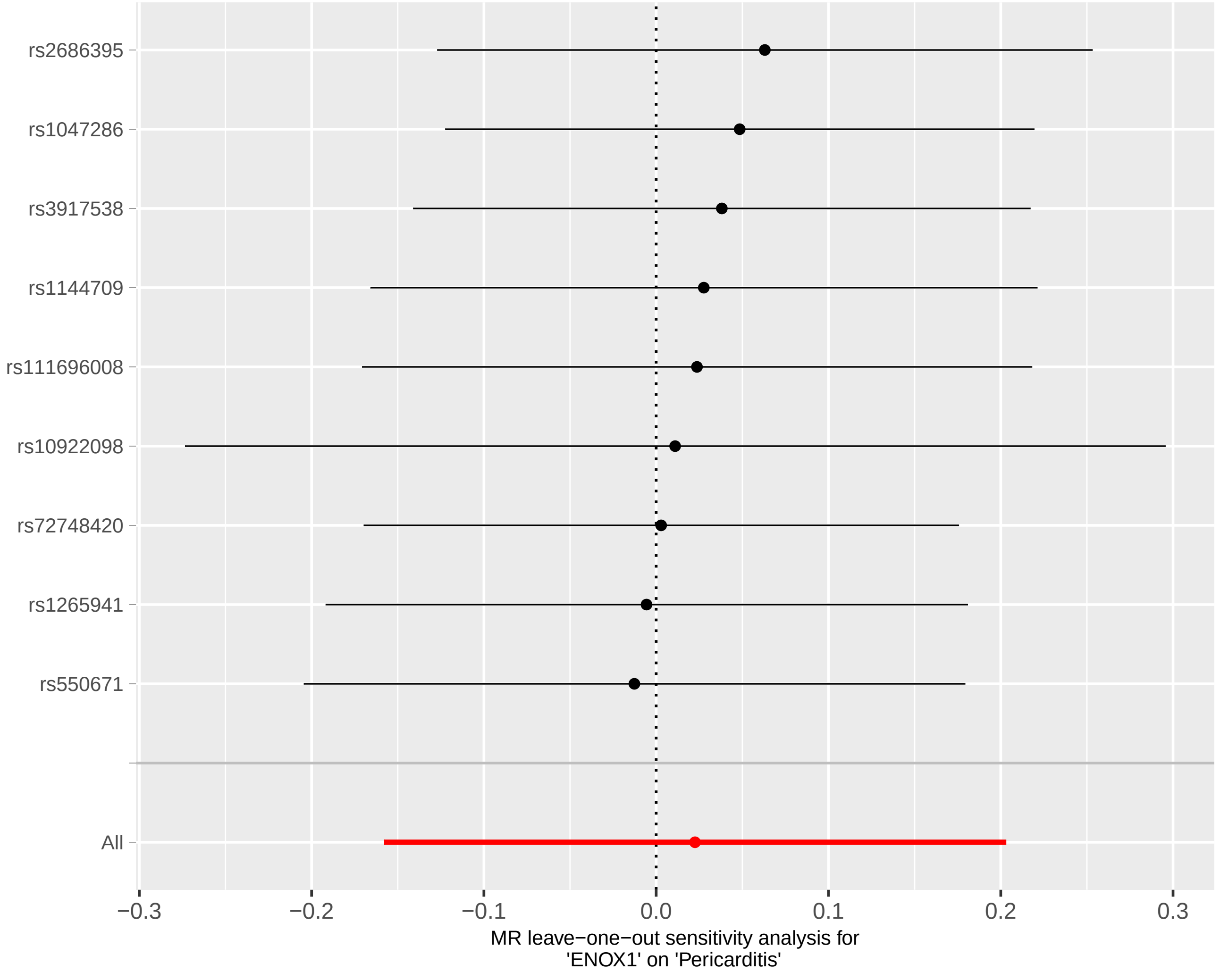

rs2686395
rs1047286
rs3917538
rs1144709
rs111696008
rs10922098
rs72748420
rs1265941
rs550671
All
−0.3
−0.2
−0.1
0.0
0.1
0.2
0.3
MR leave−one−out sensitivity analysis for
'ENOX1' on 'Pericarditis'

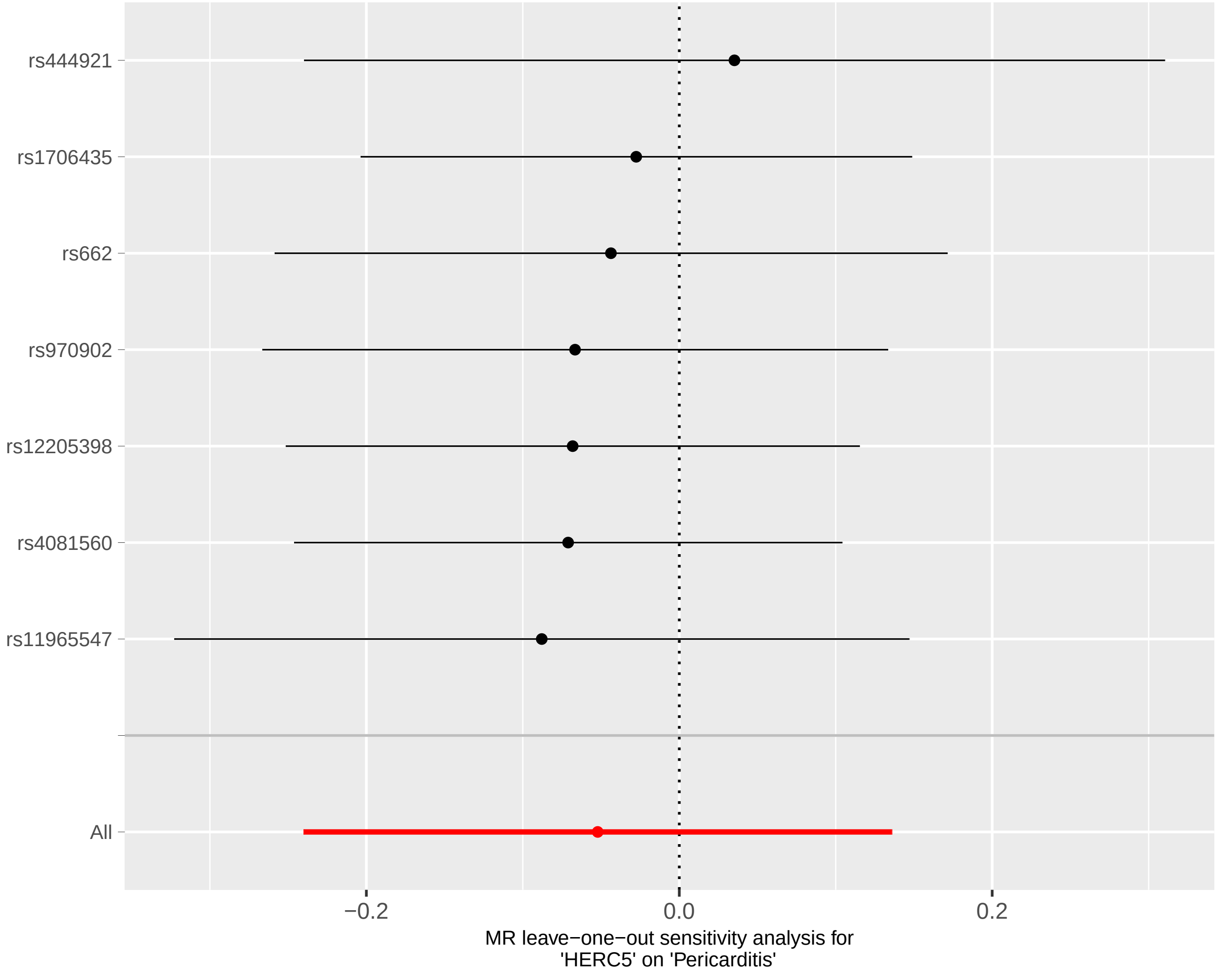
rs444921
rs1706435
rs662
rs970902
rs12205398
rs4081560
rs11965547
All
−0.2
0.0
0.2
MR leave−one−out sensitivity analysis for
'HERC5' on 'Pericarditis'

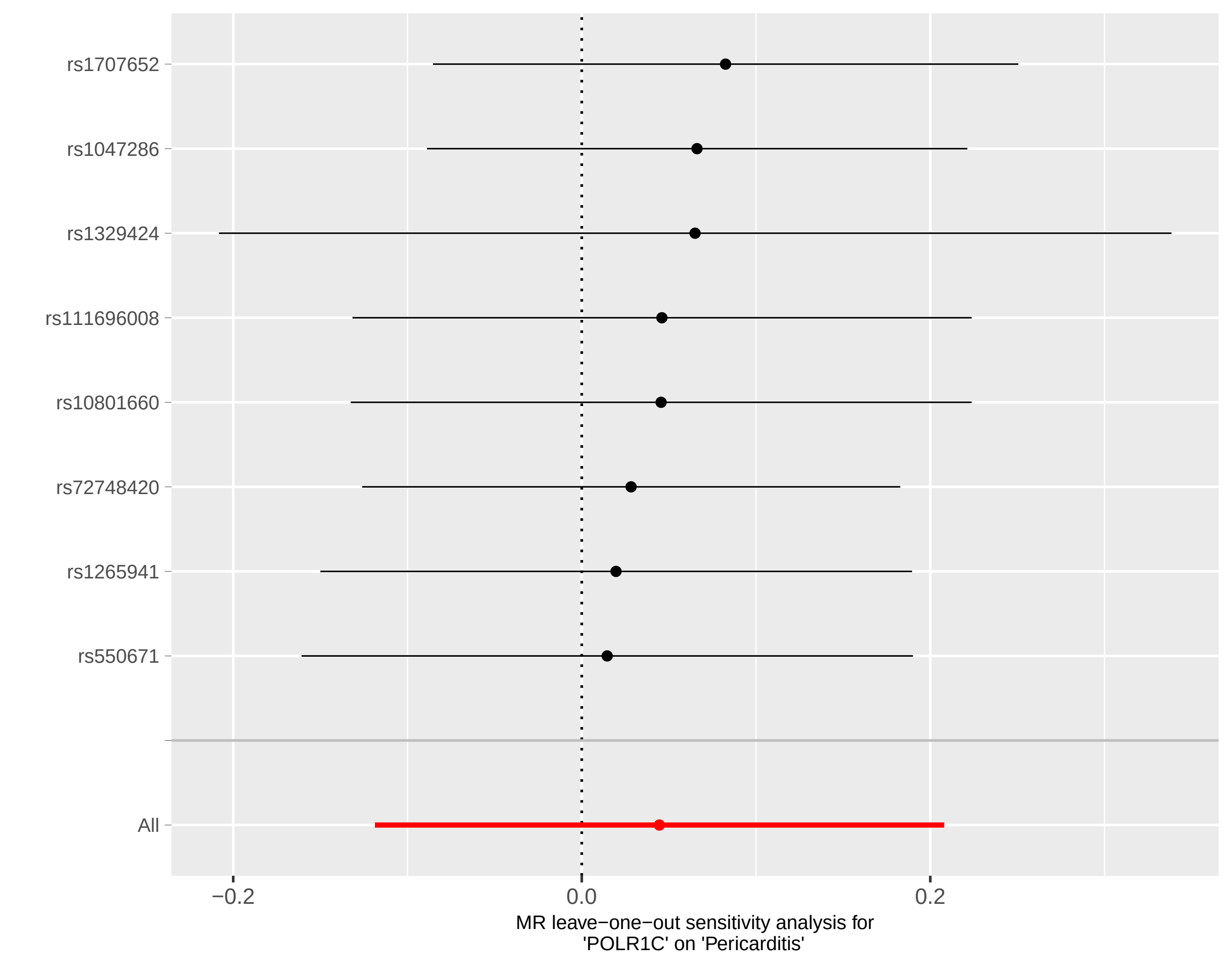
rs1707652
rs1047286
rs1329424
rs111696008
rs10801660
rs72748420
rs1265941
rs550671
All
−0.2
0.0
0.2
MR leave−one−out sensitivity analysis for
'POLR1C' on 'Pericarditis'

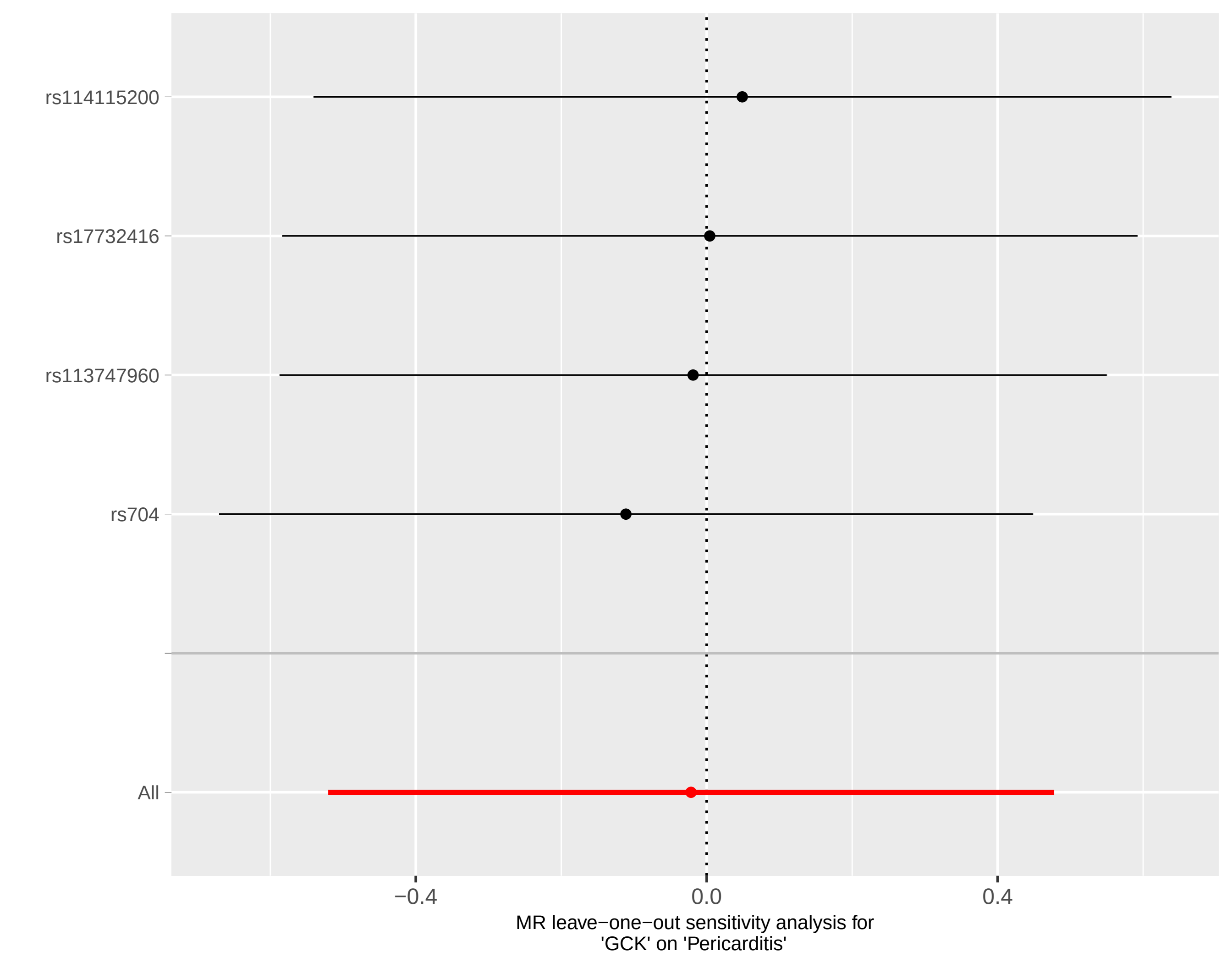
rs114115200
rs17732416
rs113747960
rs704
All
−0.4
0.0
0.4
MR leave−one−out sensitivity analysis for
'GCK' on 'Pericarditis'

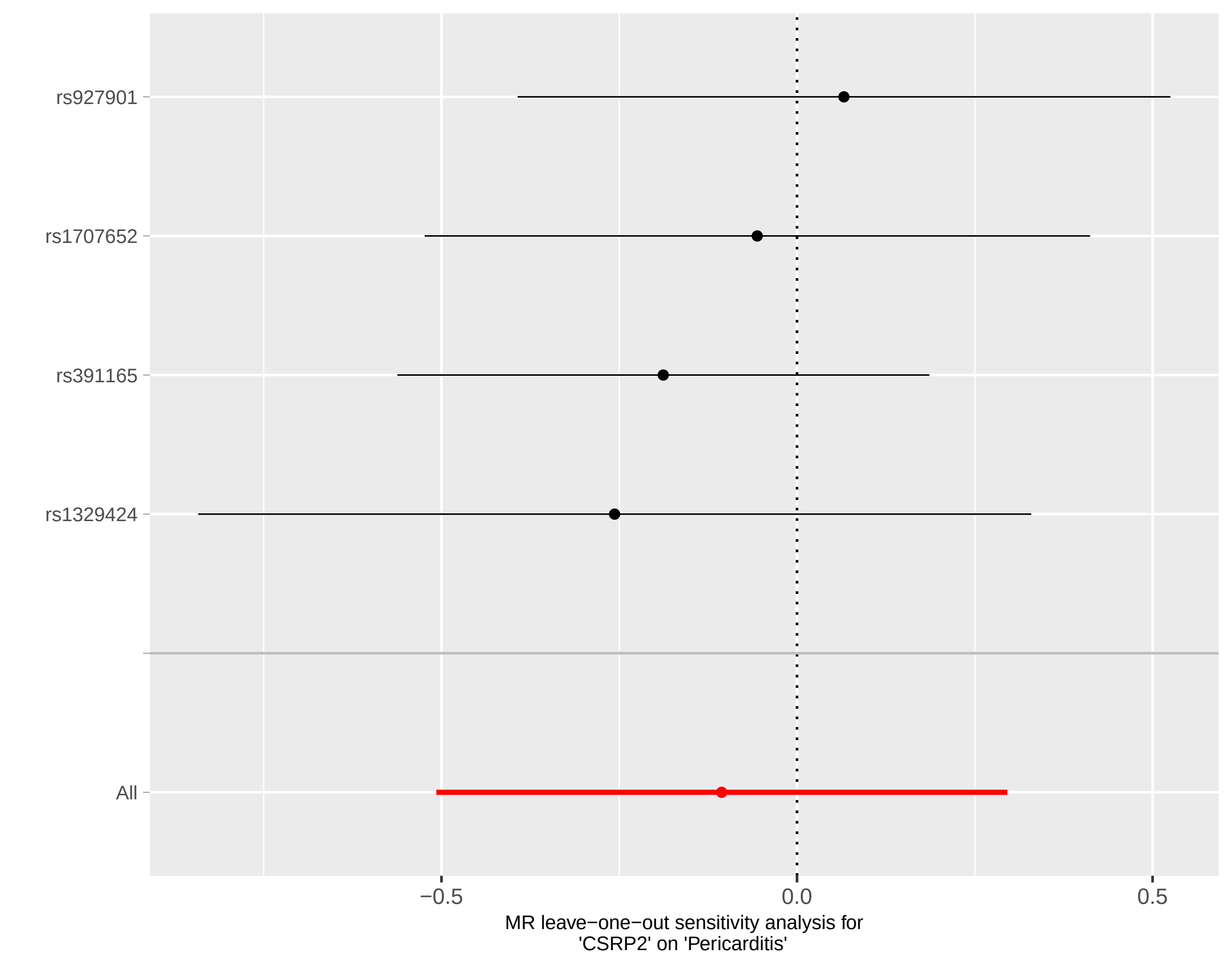

rs927901
rs1707652
rs391165
rs1329424
All
−0.5
0.0
0.5
MR leave−one−out sensitivity analysis for
'CSRP2' on 'Pericarditis'

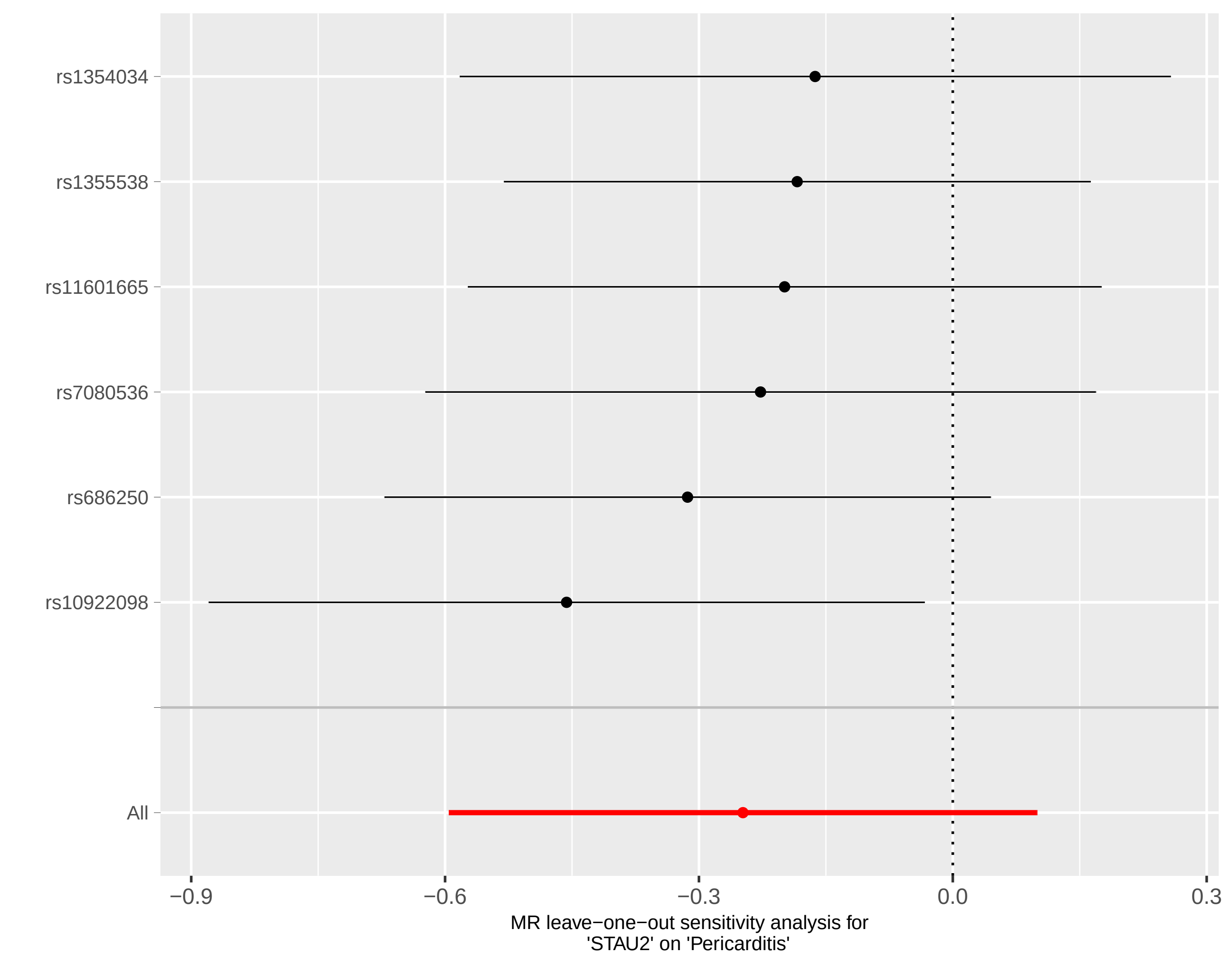

rs1354034
rs1355538
rs11601665
rs7080536
rs686250
rs10922098
All
−0.9
−0.6
−0.3
0.0
0.3
MR leave−one−out sensitivity analysis for
'STAU2' on 'Pericarditis'

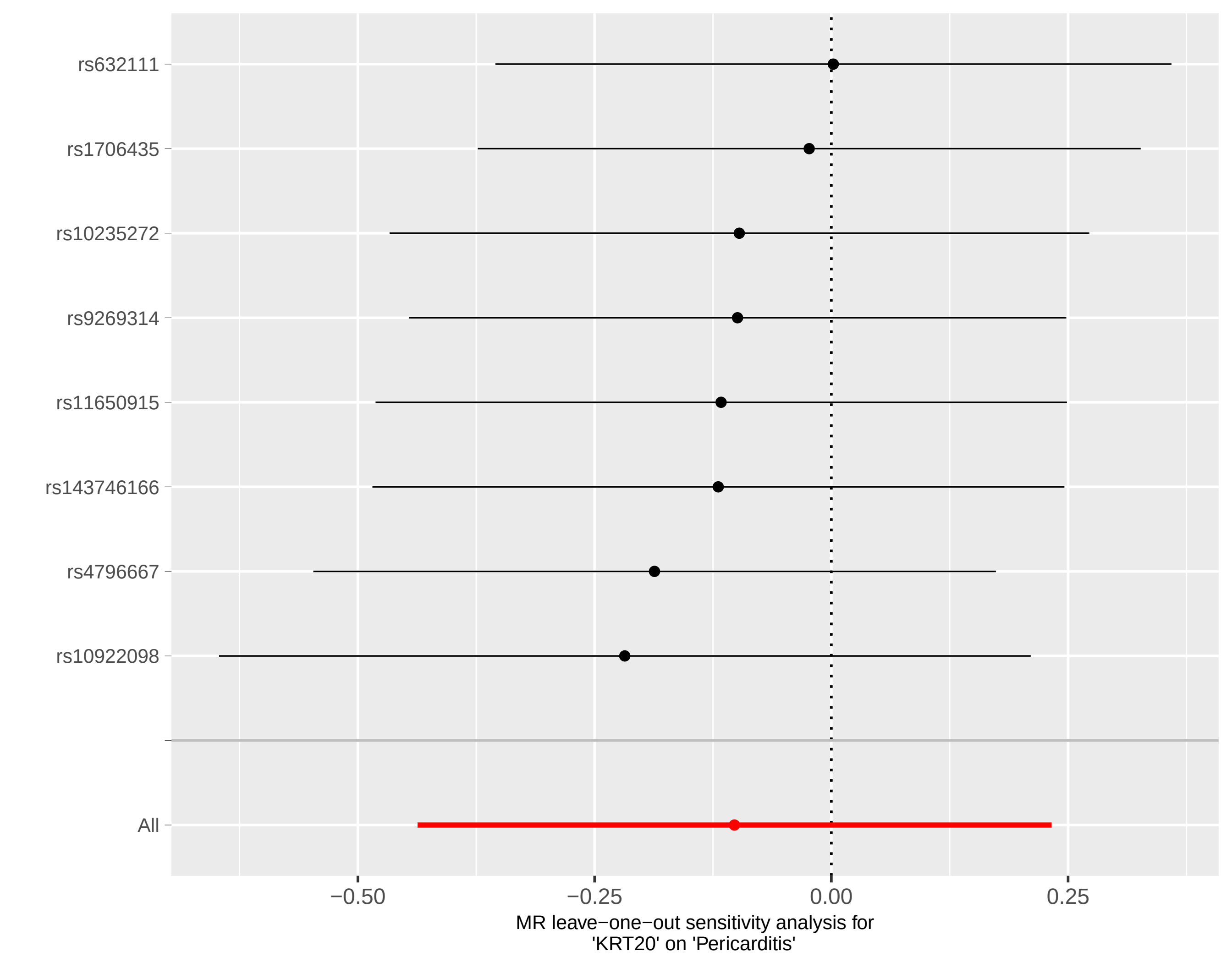
rs632111
rs1706435
rs10235272
rs9269314
rs11650915
rs143746166
rs4796667
rs10922098
All
−0.50
−0.25
0.00
0.25
MR leave−one−out sensitivity analysis for
'KRT20' on 'Pericarditis'

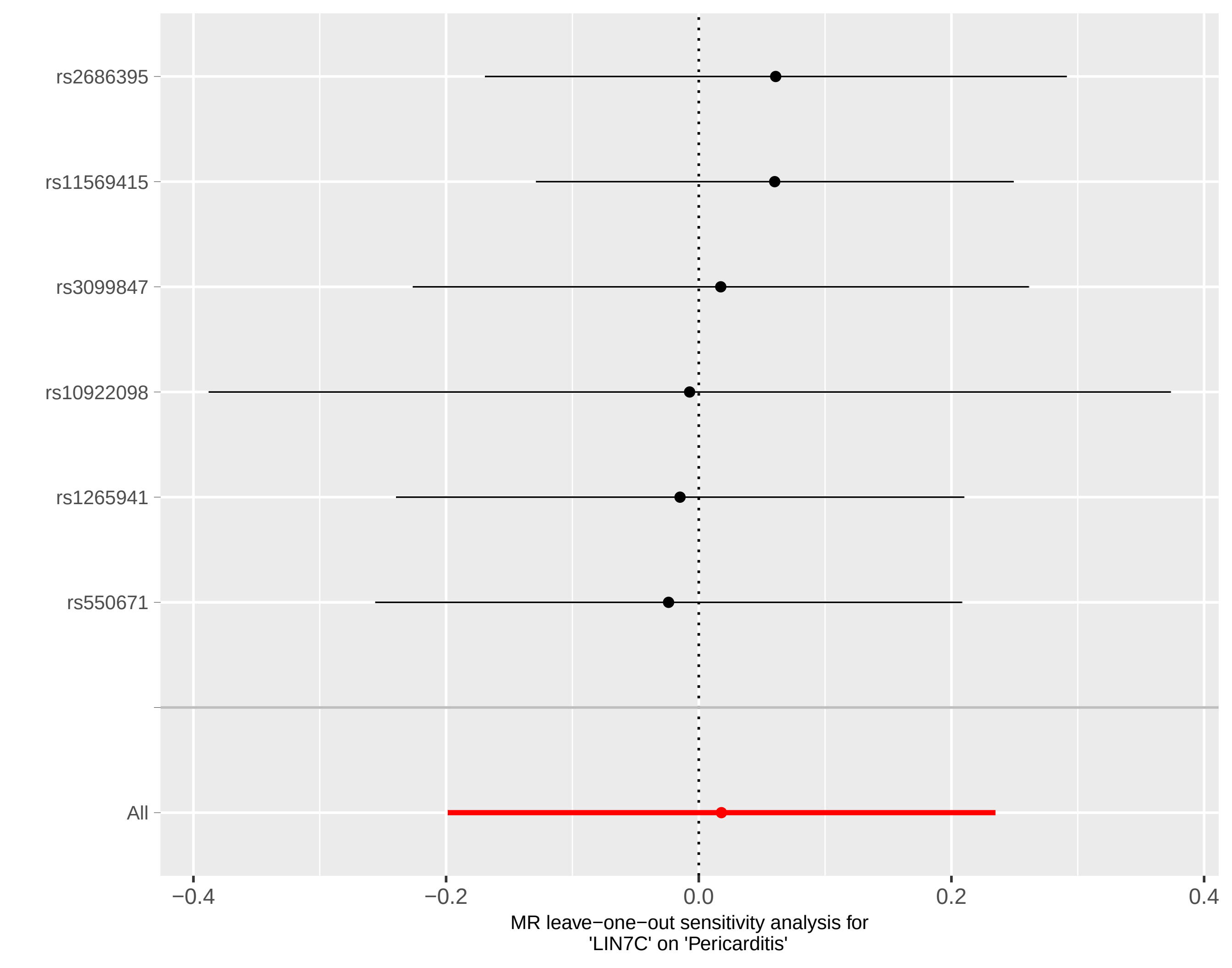

rs2686395
rs11569415
rs3099847
rs10922098
rs1265941
rs550671
All
−0.4
−0.2
0.0
0.2
0.4
MR leave−one−out sensitivity analysis for
'LIN7C' on 'Pericarditis'

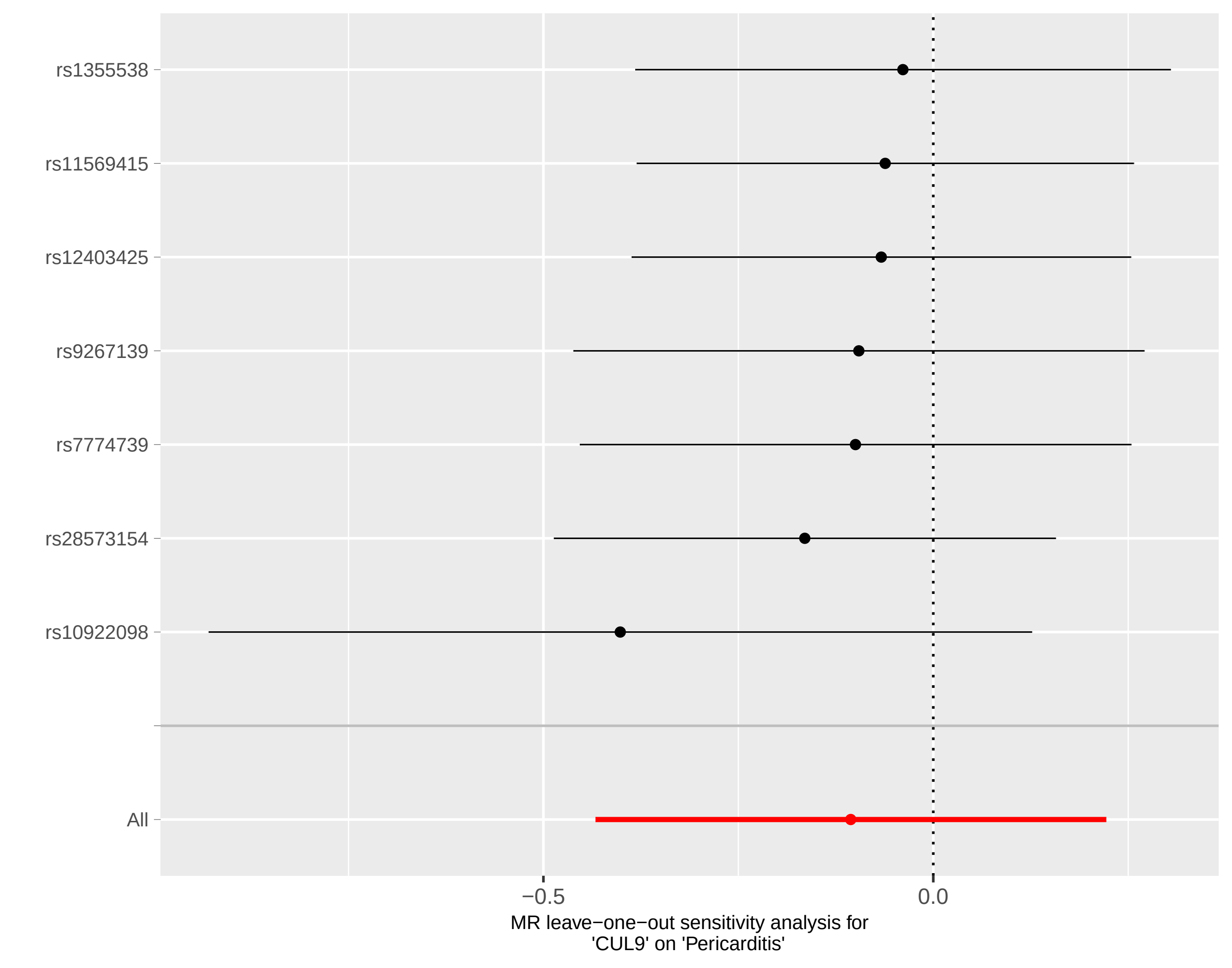

rs1355538
rs11569415
rs12403425
rs9267139
rs7774739
rs28573154
rs10922098
All
−0.5
0.0
MR leave−one−out sensitivity analysis for
'CUL9' on 'Pericarditis'

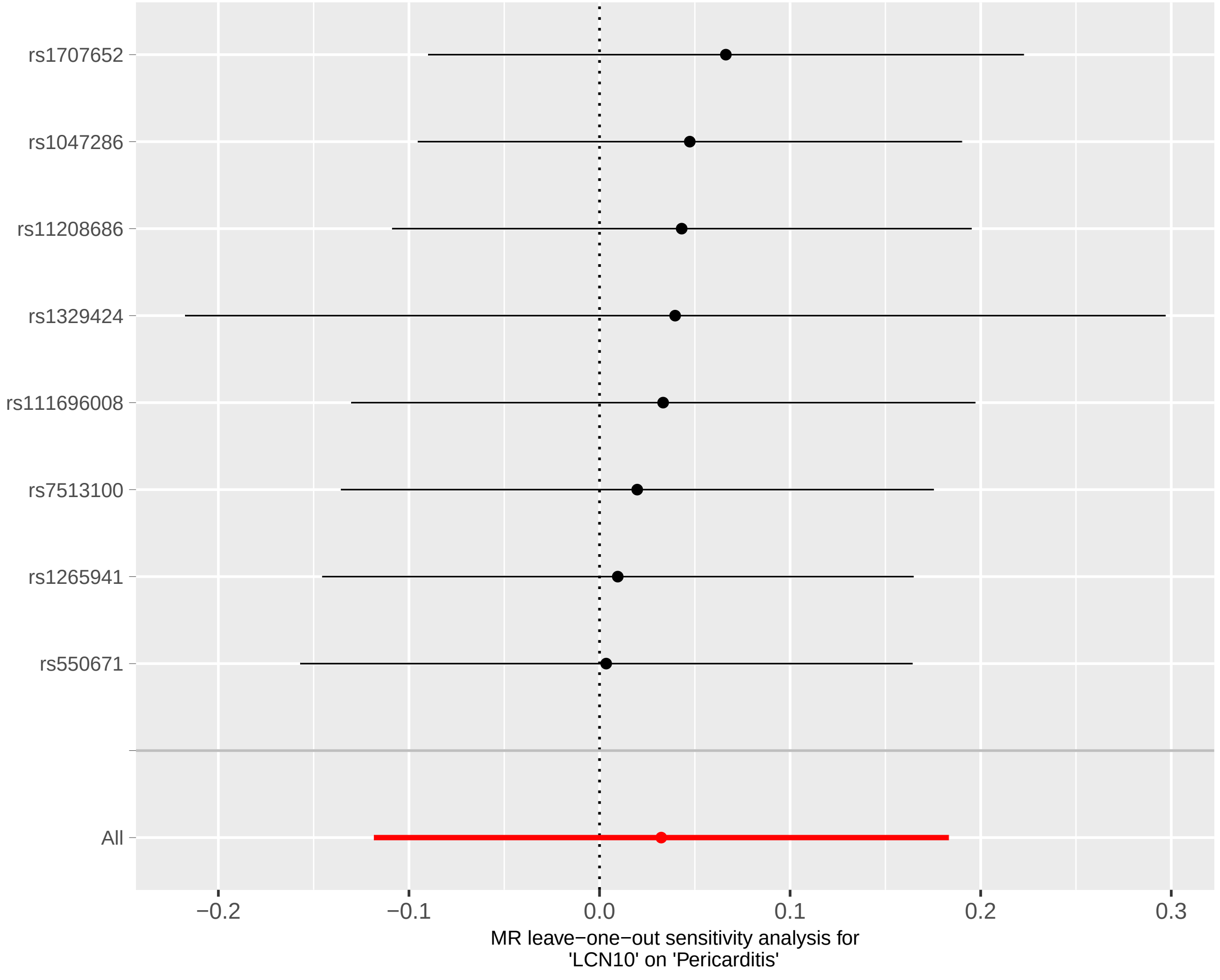

rs1707652
rs1047286
rs11208686
rs1329424
rs111696008
rs7513100
rs1265941
rs550671
All
−0.2
−0.1
0.0
0.1
0.2
0.3
MR leave−one−out sensitivity analysis for
'LCN10' on 'Pericarditis'

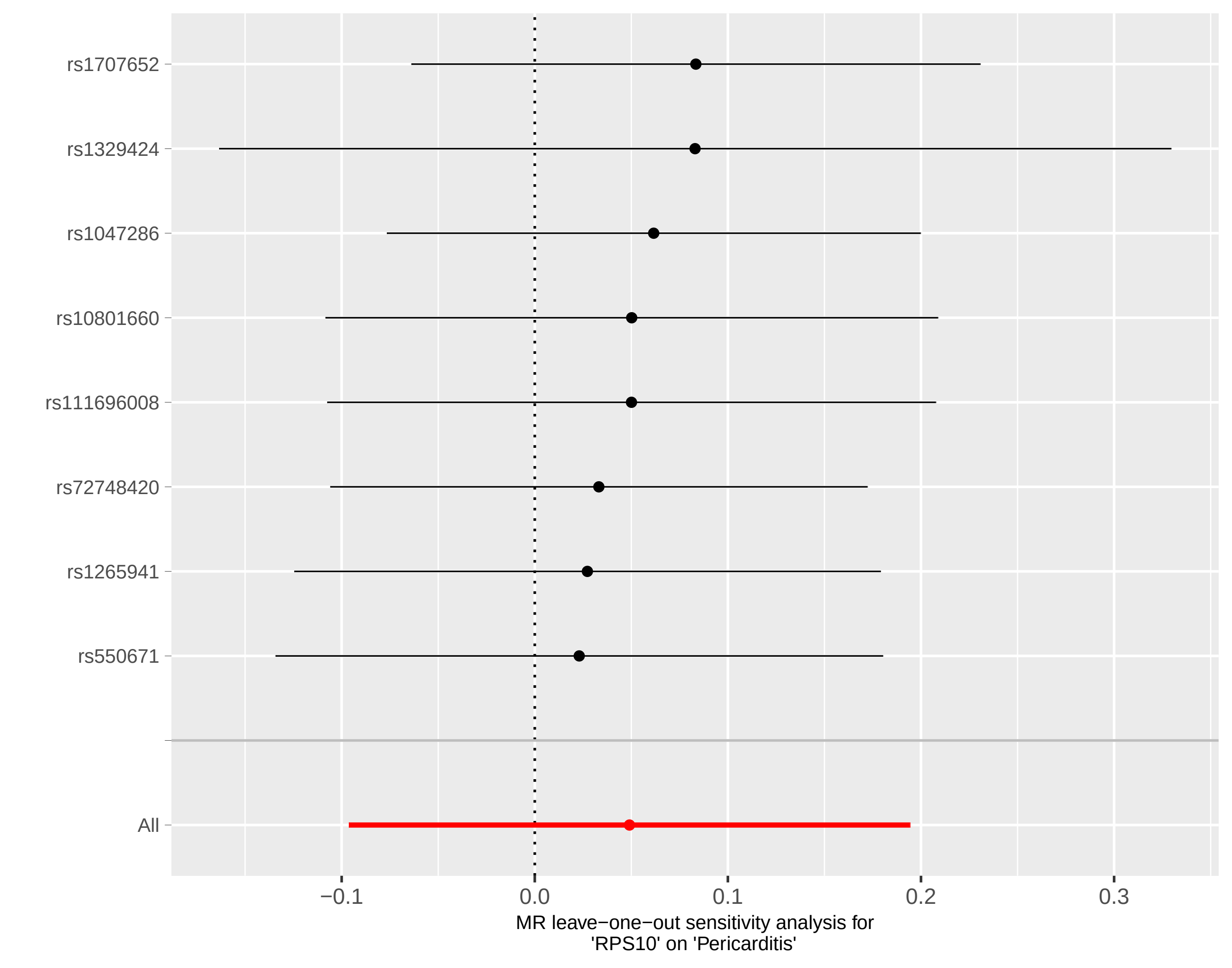

rs1707652
rs1329424
rs1047286
rs10801660
rs111696008
rs72748420
rs1265941
rs550671
All
−0.1
0.0
0.1
0.2
0.3
MR leave−one−out sensitivity analysis for
'RPS10' on 'Pericarditis'

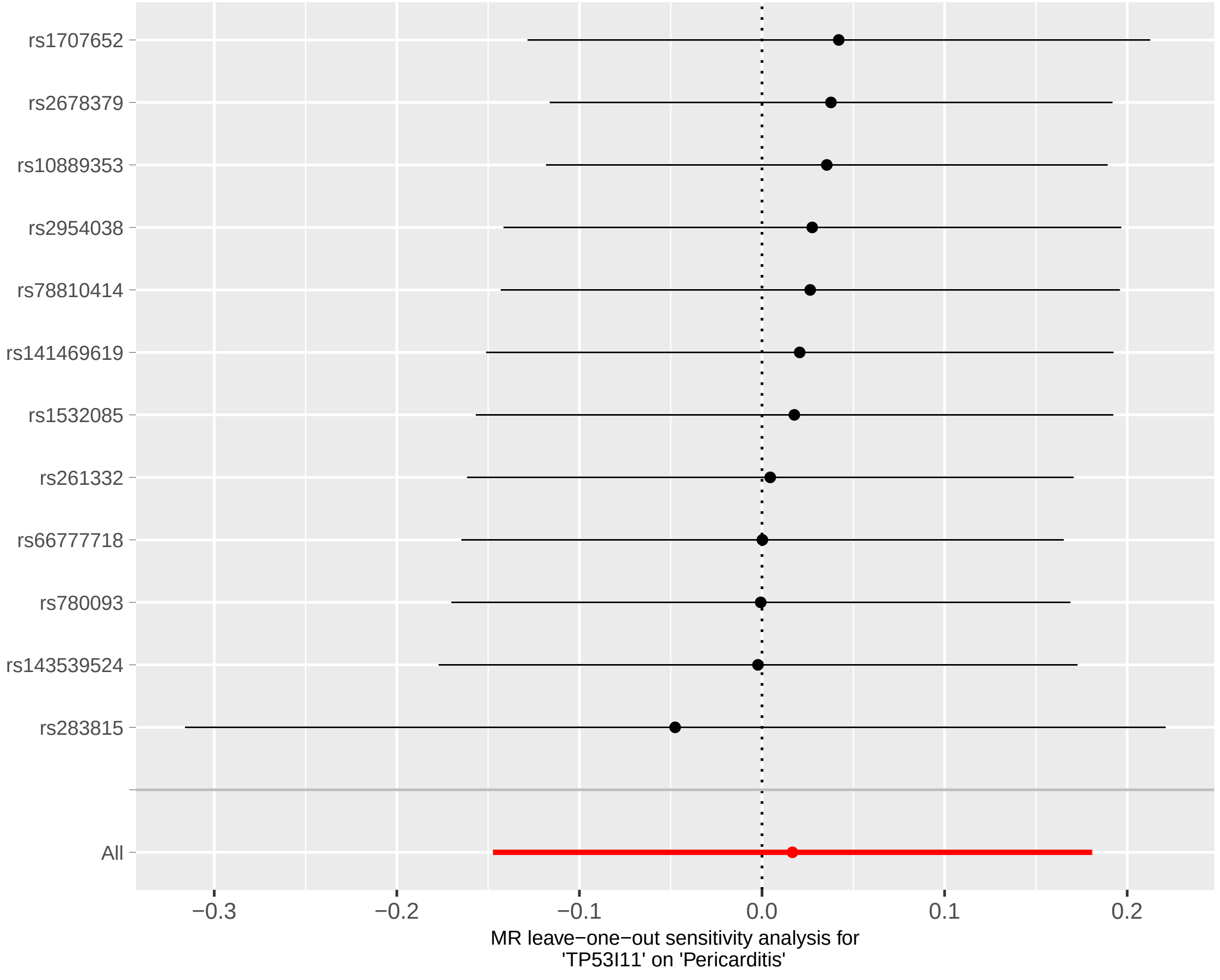
rs1707652
rs2678379
rs10889353
rs2954038
rs78810414
rs141469619
rs1532085
rs261332
rs66777718
rs780093
rs143539524
rs283815
All
−0.3
−0.2
−0.1
0.0
0.1
0.2
MR leave−one−out sensitivity analysis for
'TP53I11' on 'Pericarditis'

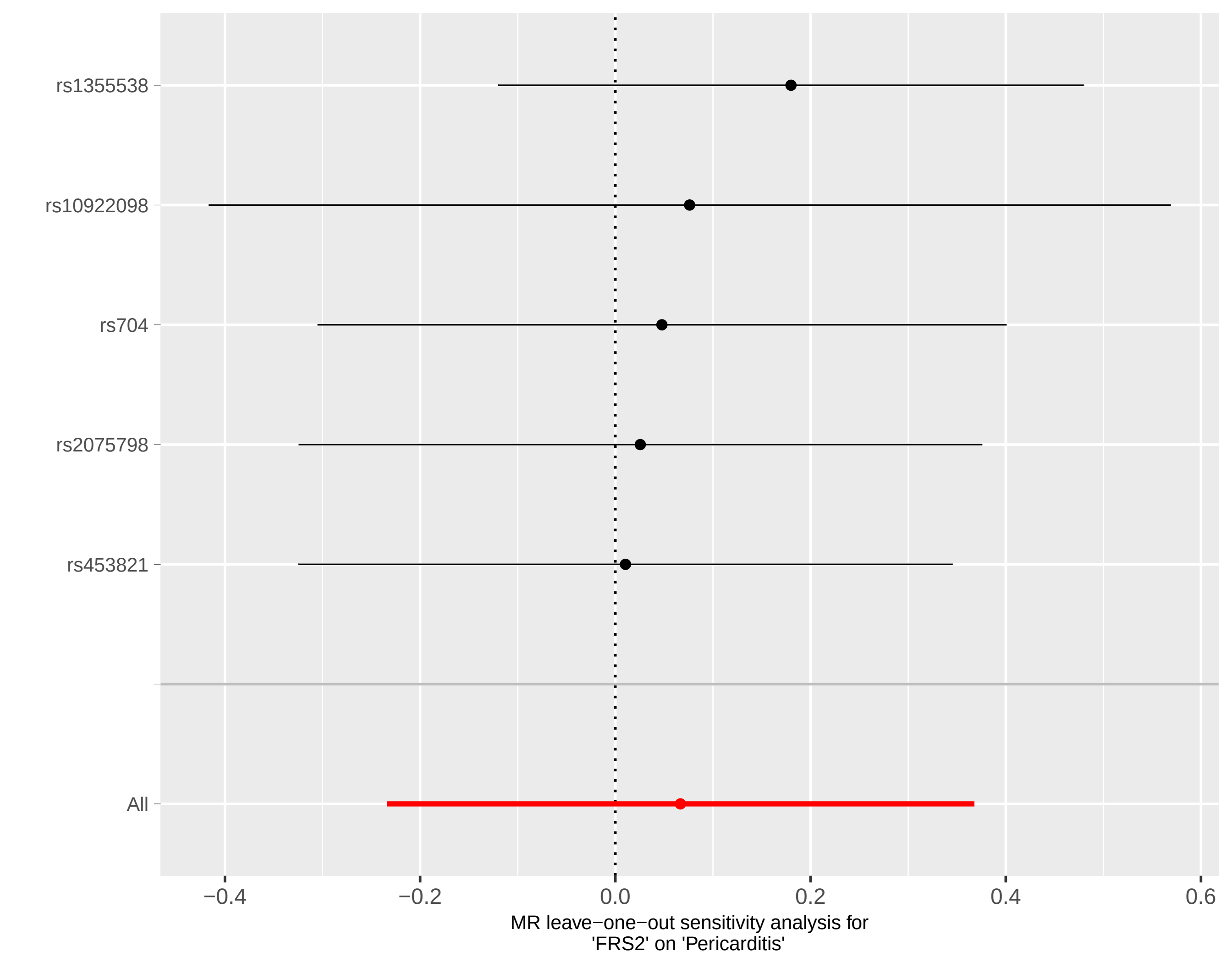
rs1355538
rs10922098
rs704
rs2075798
rs453821
All
−0.4
−0.2
0.0
0.2
0.4
0.6
MR leave−one−out sensitivity analysis for
'FRS2' on 'Pericarditis'

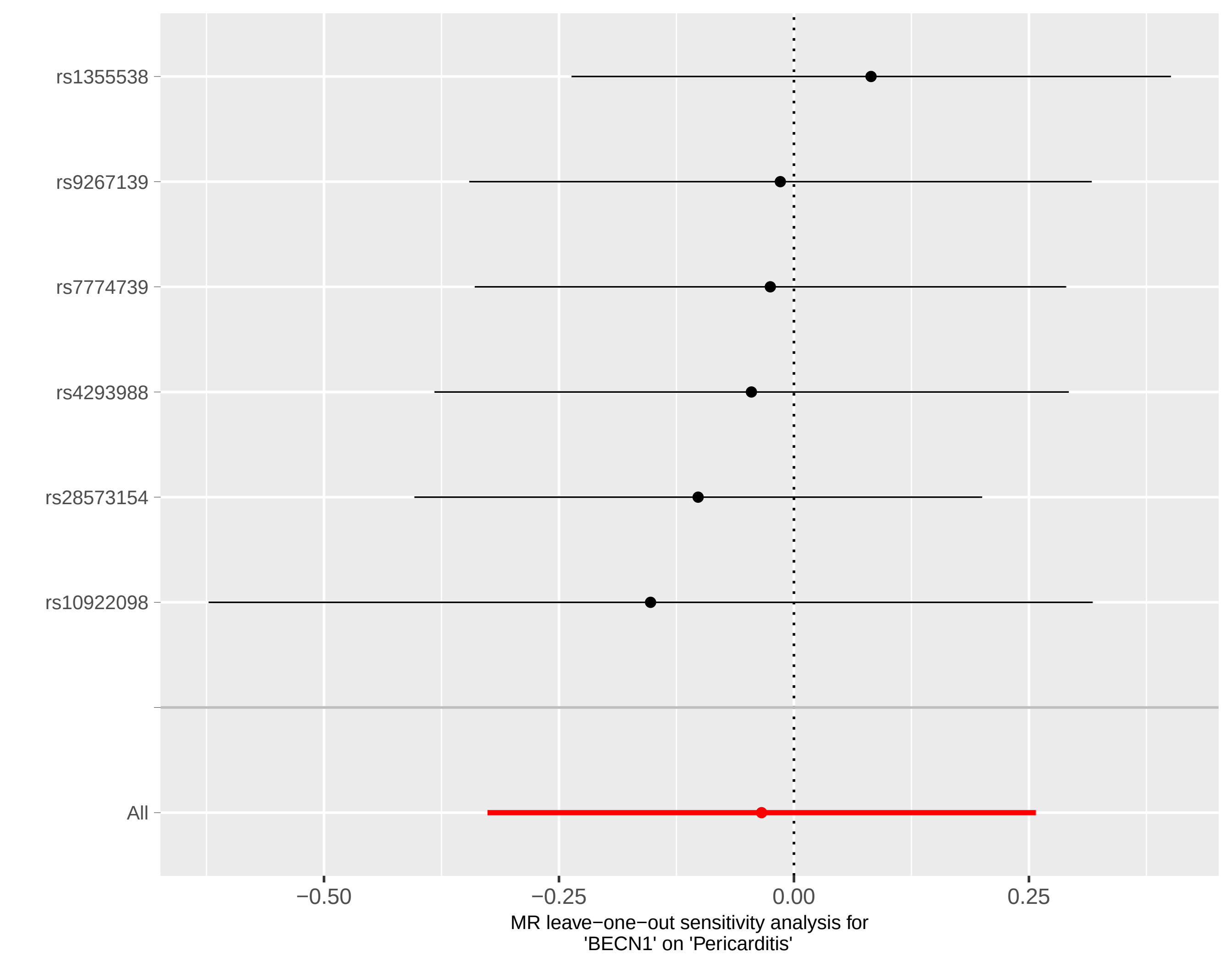

rs1355538
rs9267139
rs7774739
rs4293988
rs28573154
rs10922098
All
−0.50
−0.25
0.00
0.25
MR leave−one−out sensitivity analysis for
'BECN1' on 'Pericarditis'

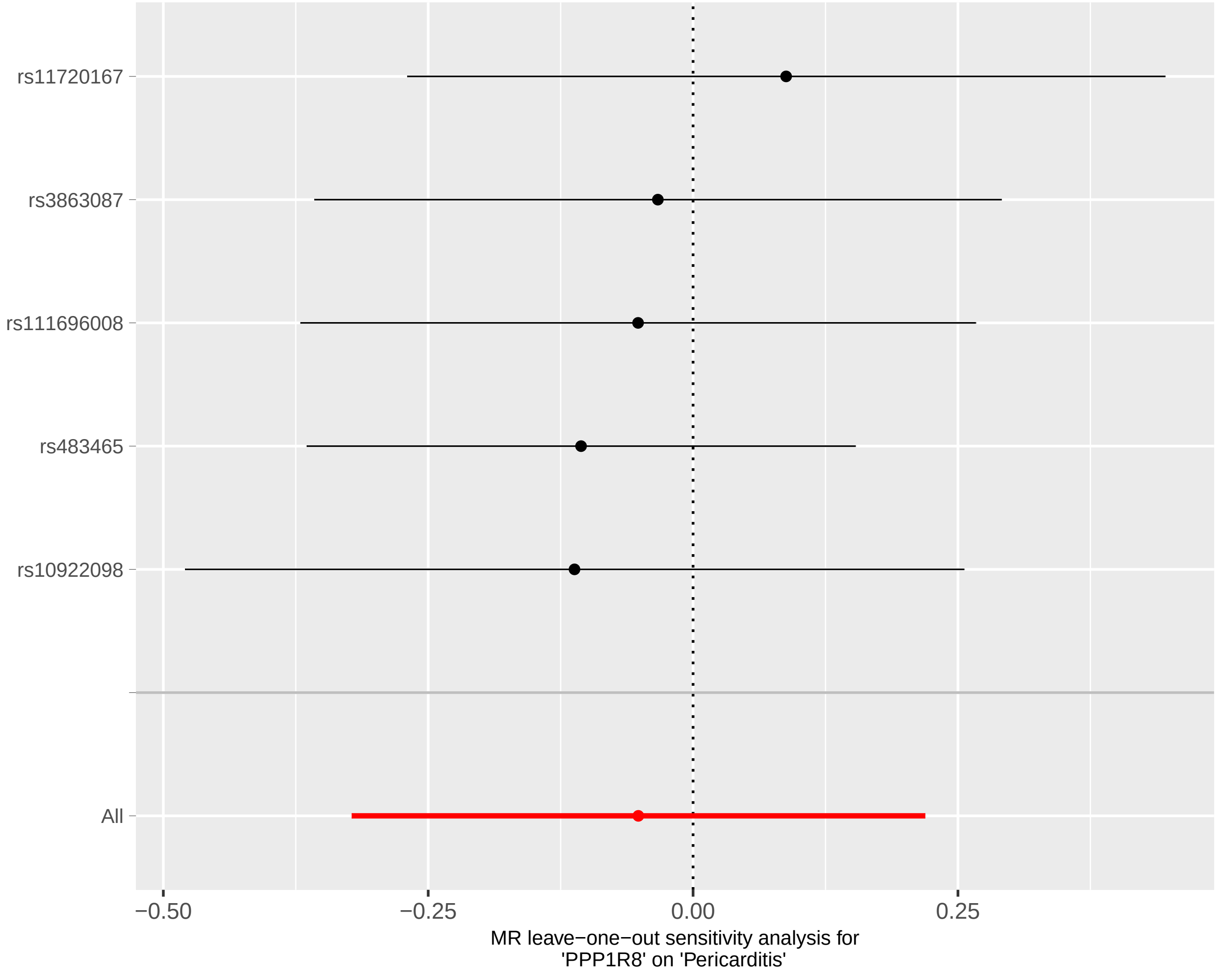
rs11720167
rs3863087
rs111696008
rs483465
rs10922098
All
−0.50
−0.25
0.00
0.25
MR leave−one−out sensitivity analysis for
'PPP1R8' on 'Pericarditis'

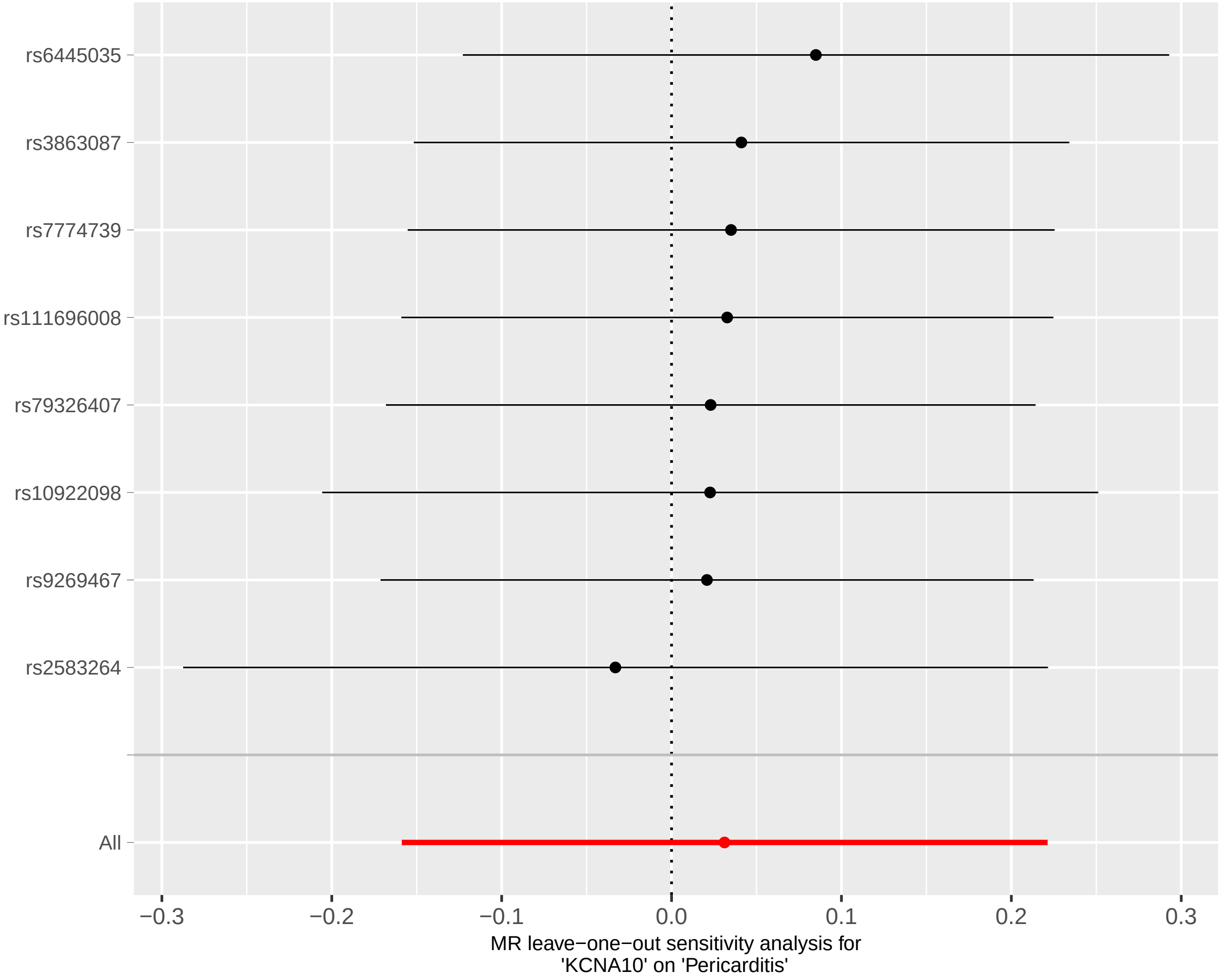

rs6445035
rs3863087
rs7774739
rs111696008
rs79326407
rs10922098
rs9269467
rs2583264
All
−0.3
−0.2
−0.1
0.0
0.1
0.2
0.3
MR leave−one−out sensitivity analysis for
'KCNA10' on 'Pericarditis'

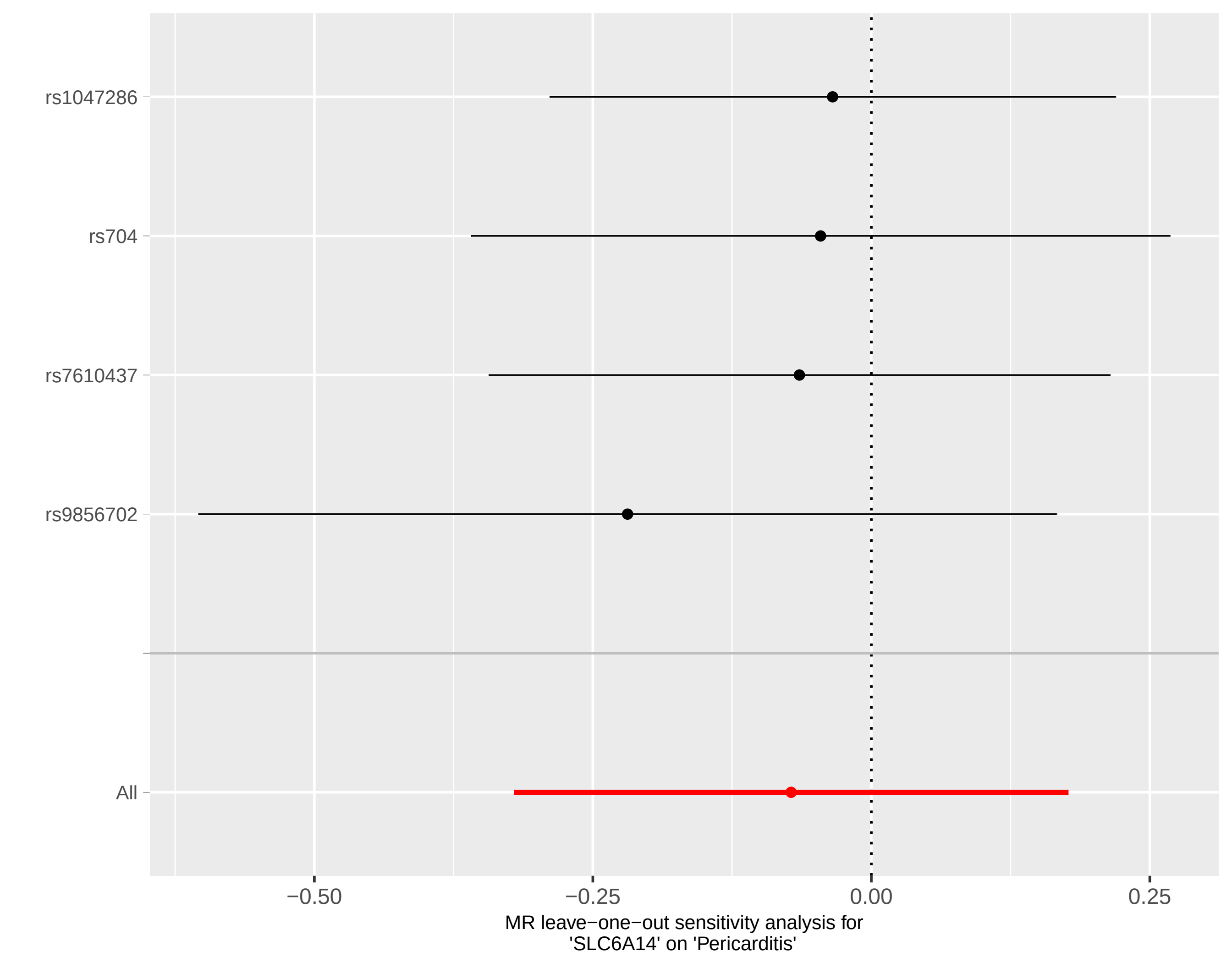

rs1047286
rs704
rs7610437
rs9856702
All
−0.50
−0.25
0.00
0.25
MR leave−one−out sensitivity analysis for
'SLC6A14' on 'Pericarditis'

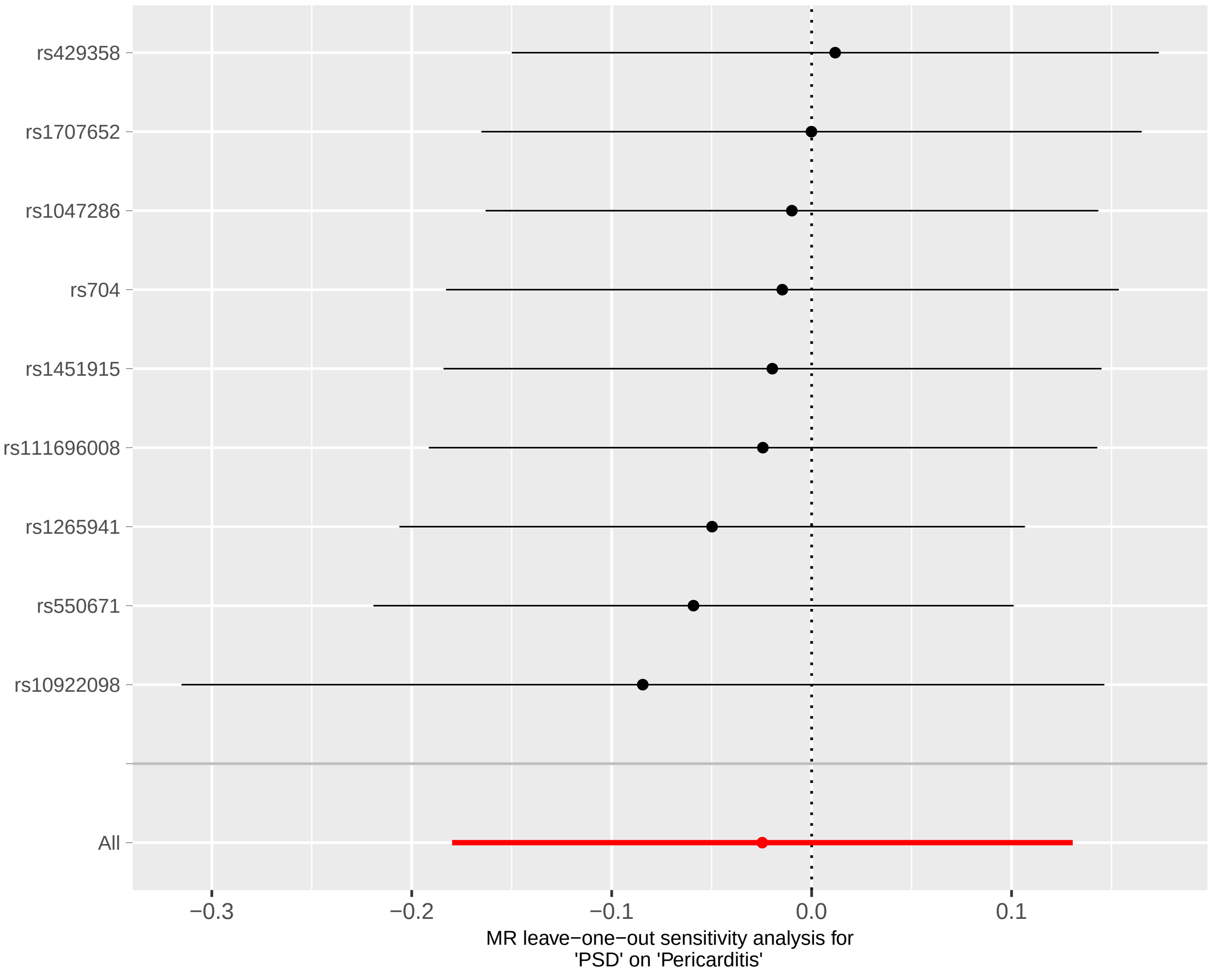
rs429358
rs1707652
rs1047286
rs704
rs1451915
rs111696008
rs1265941
rs550671
rs10922098
All
−0.3
−0.2
−0.1
0.0
0.1
MR leave−one−out sensitivity analysis for
'PSD' on 'Pericarditis'

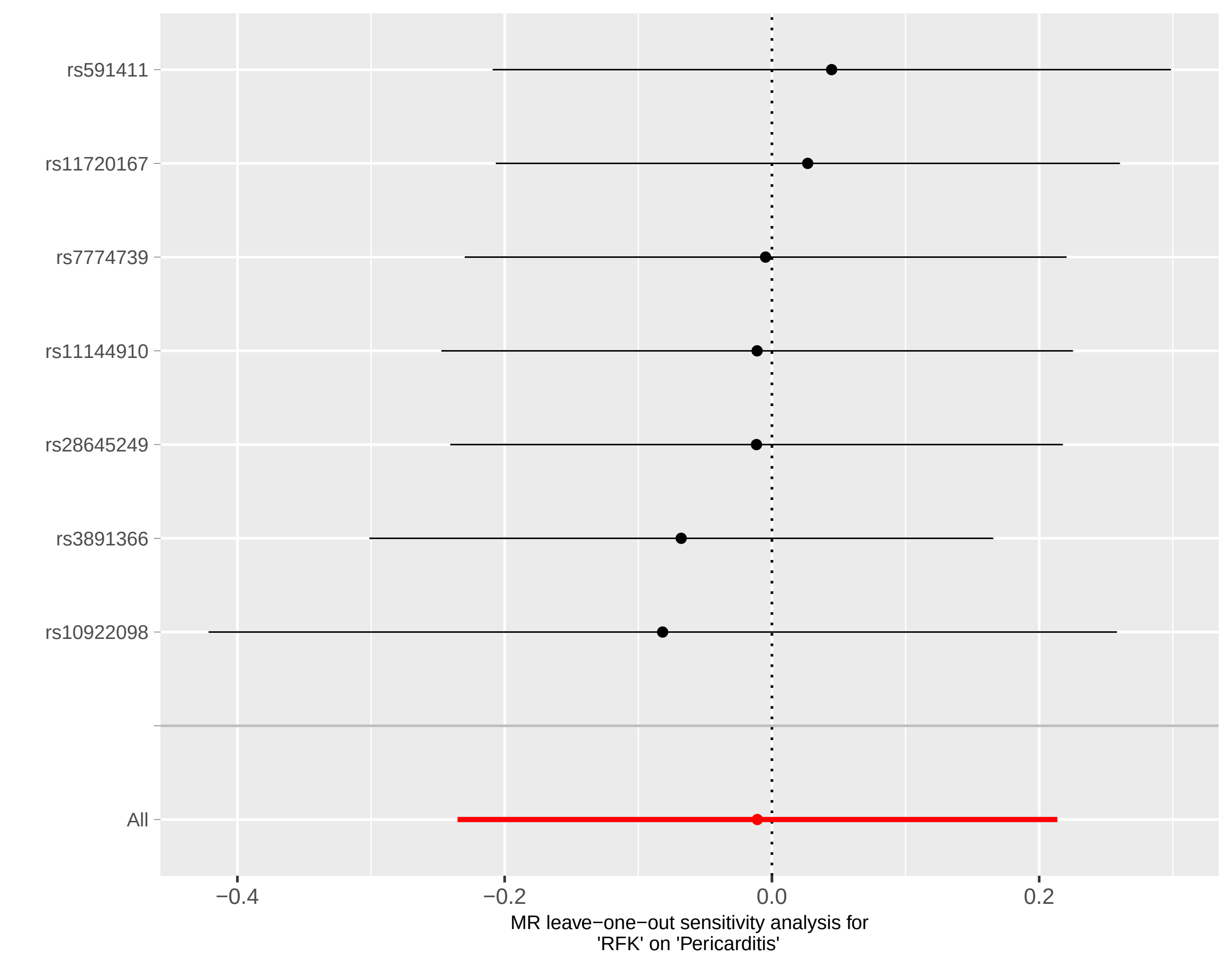
rs591411
rs11720167
rs7774739
rs11144910
rs28645249
rs3891366
rs10922098
All
−0.4
−0.2
0.0
0.2
MR leave−one−out sensitivity analysis for
'RFK' on 'Pericarditis'

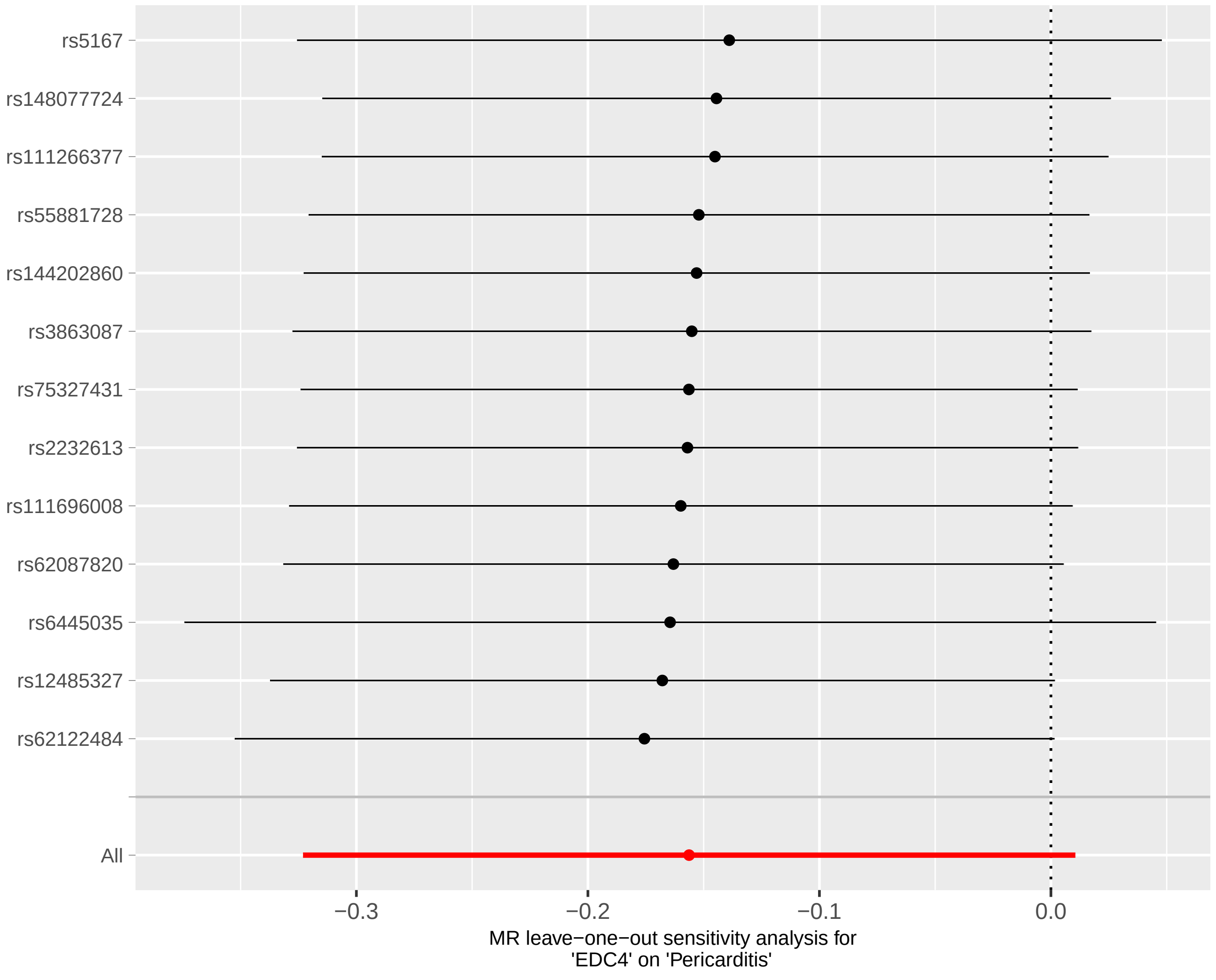
rs5167
rs148077724
rs111266377
rs55881728
rs144202860
rs3863087
rs75327431
rs2232613
rs111696008
rs62087820
rs6445035
rs12485327
rs62122484
All
−0.3
−0.2
−0.1
0.0
MR leave−one−out sensitivity analysis for
'EDC4' on 'Pericarditis'

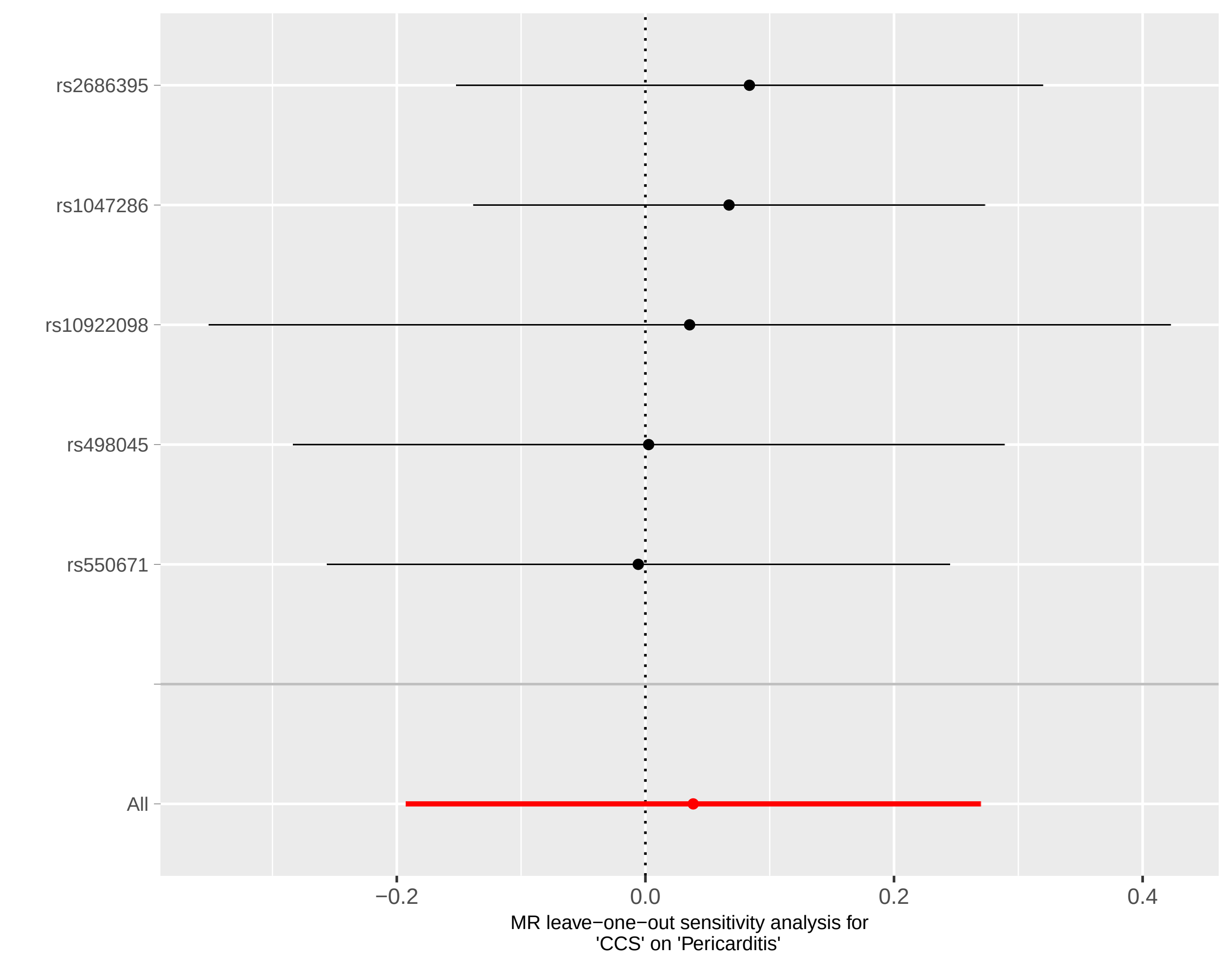

rs2686395
rs1047286
rs10922098
rs498045
rs550671
All
−0.2
0.0
0.2
0.4
MR leave−one−out sensitivity analysis for
'CCS' on 'Pericarditis'

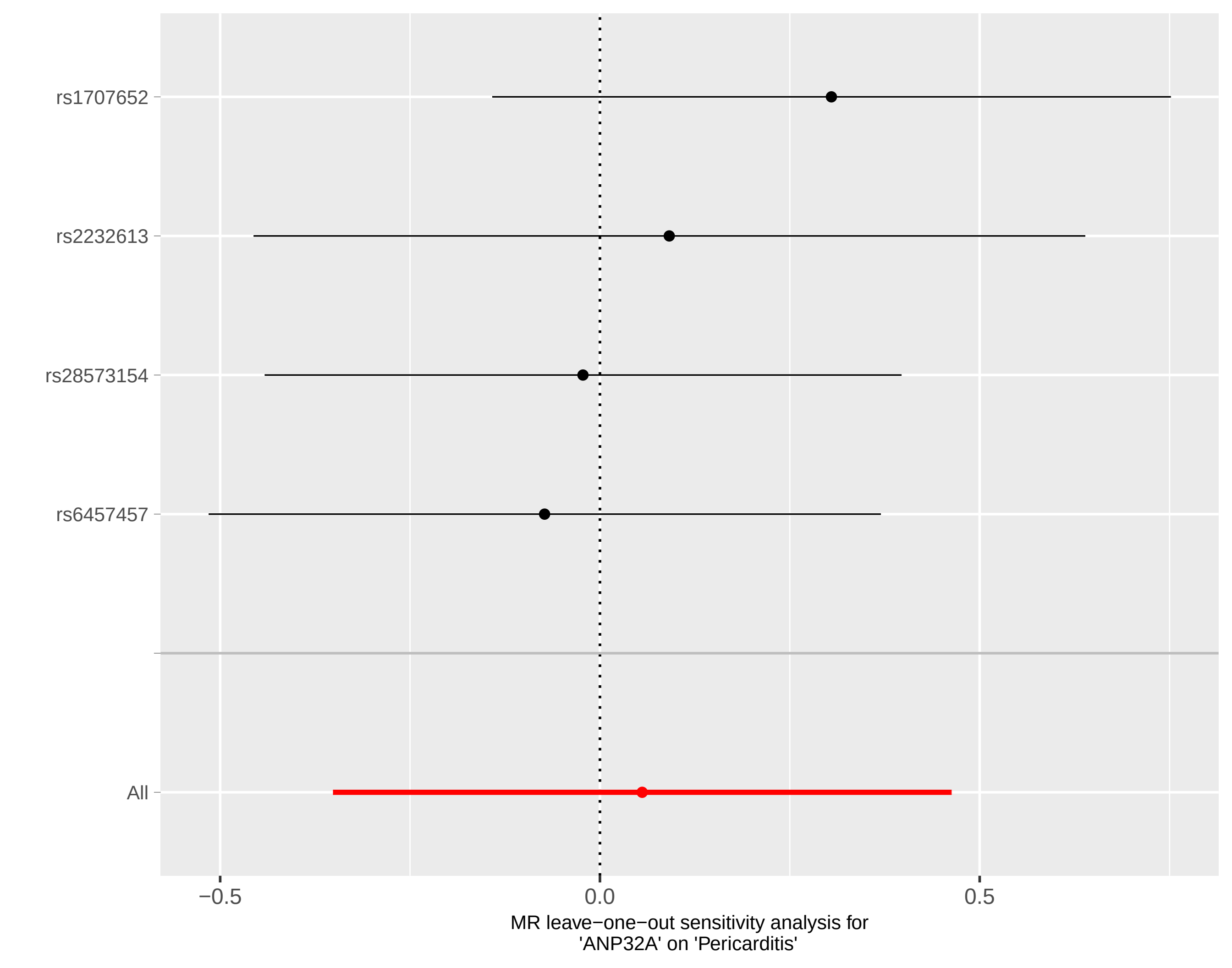
rs1707652
rs2232613
rs28573154
rs6457457
All
−0.5
0.0
0.5
MR leave−one−out sensitivity analysis for
'ANP32A' on 'Pericarditis'

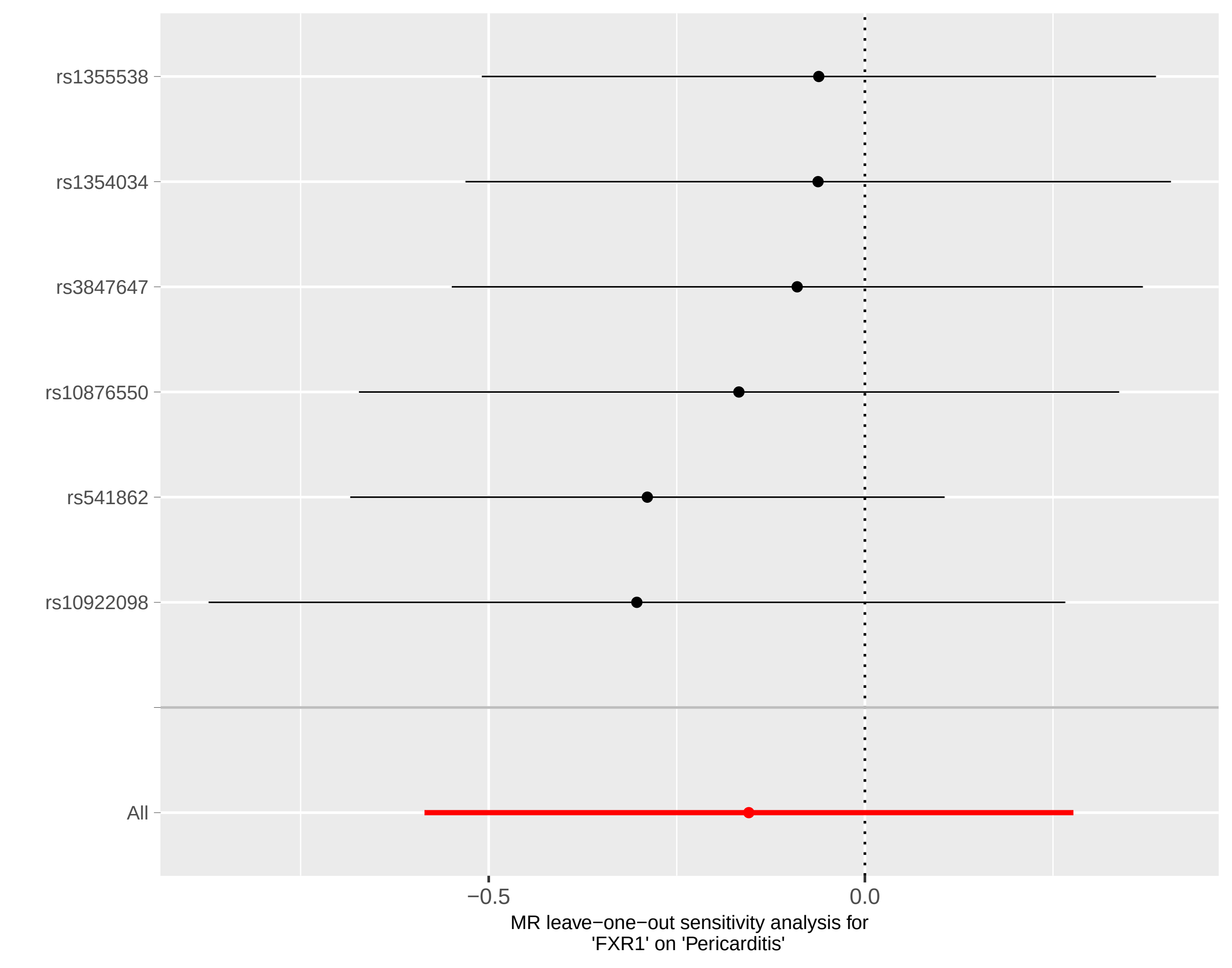
rs1355538
rs1354034
rs3847647
rs10876550
rs541862
rs10922098
All
−0.5
0.0
MR leave−one−out sensitivity analysis for
'FXR1' on 'Pericarditis'

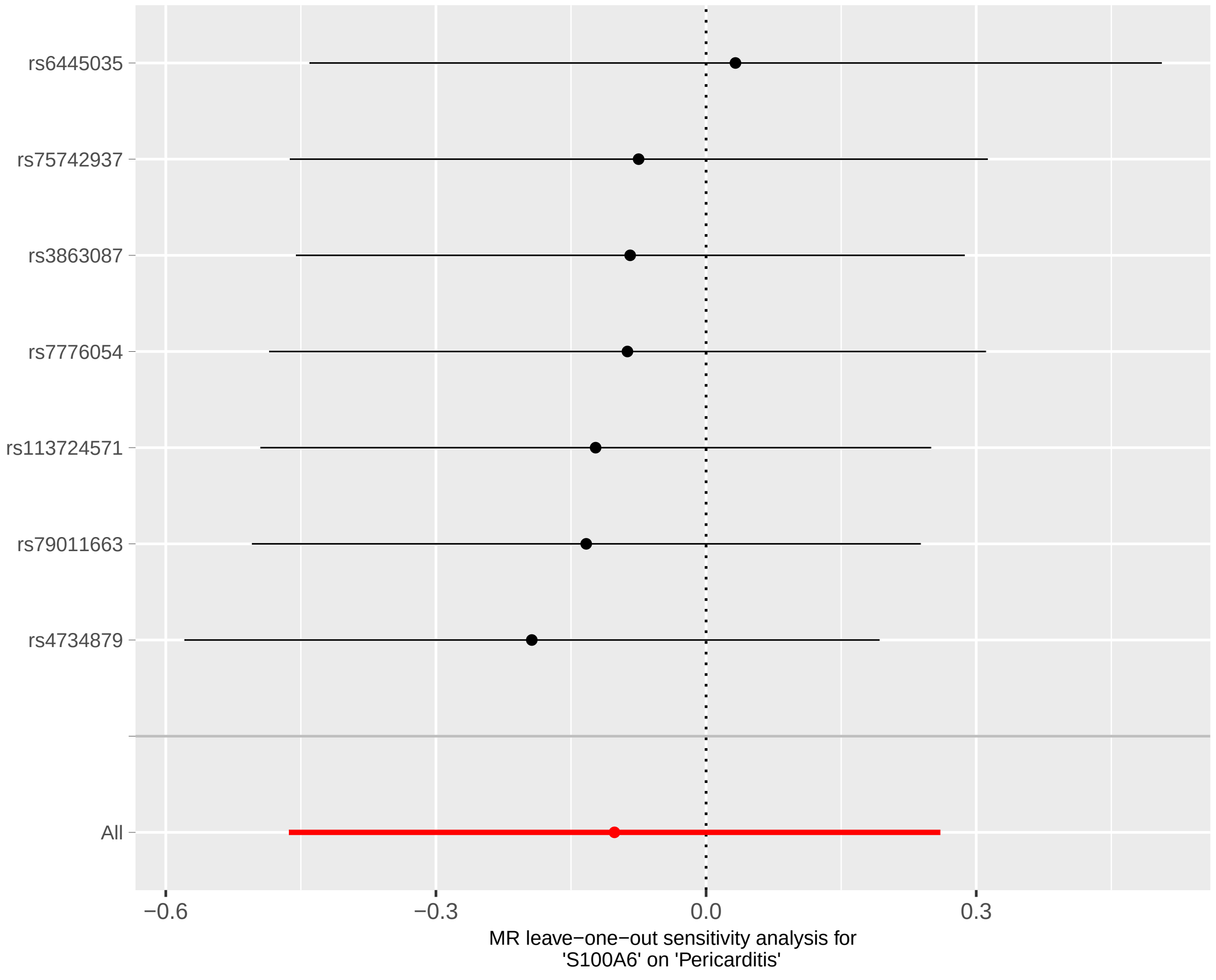
rs6445035
rs75742937
rs3863087
rs7776054
rs113724571
rs79011663
rs4734879
All
−0.6
−0.3
0.0
0.3
MR leave−one−out sensitivity analysis for
'S100A6' on 'Pericarditis'

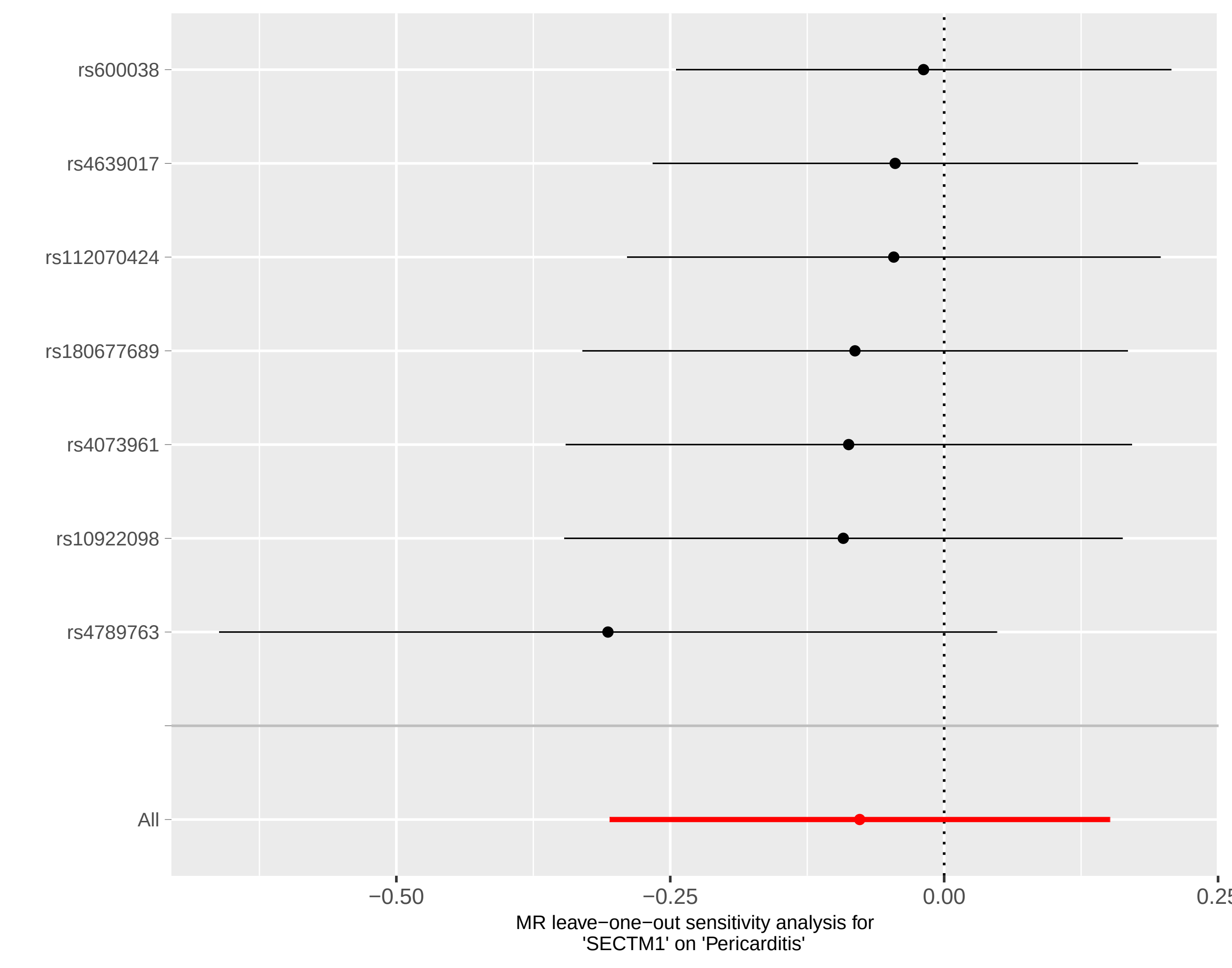
rs600038
rs4639017
rs112070424
rs180677689
rs4073961
rs10922098
rs4789763
All
−0.50
−0.25
0.00
0.25
MR leave−one−out sensitivity analysis for
'SECTM1' on 'Pericarditis'

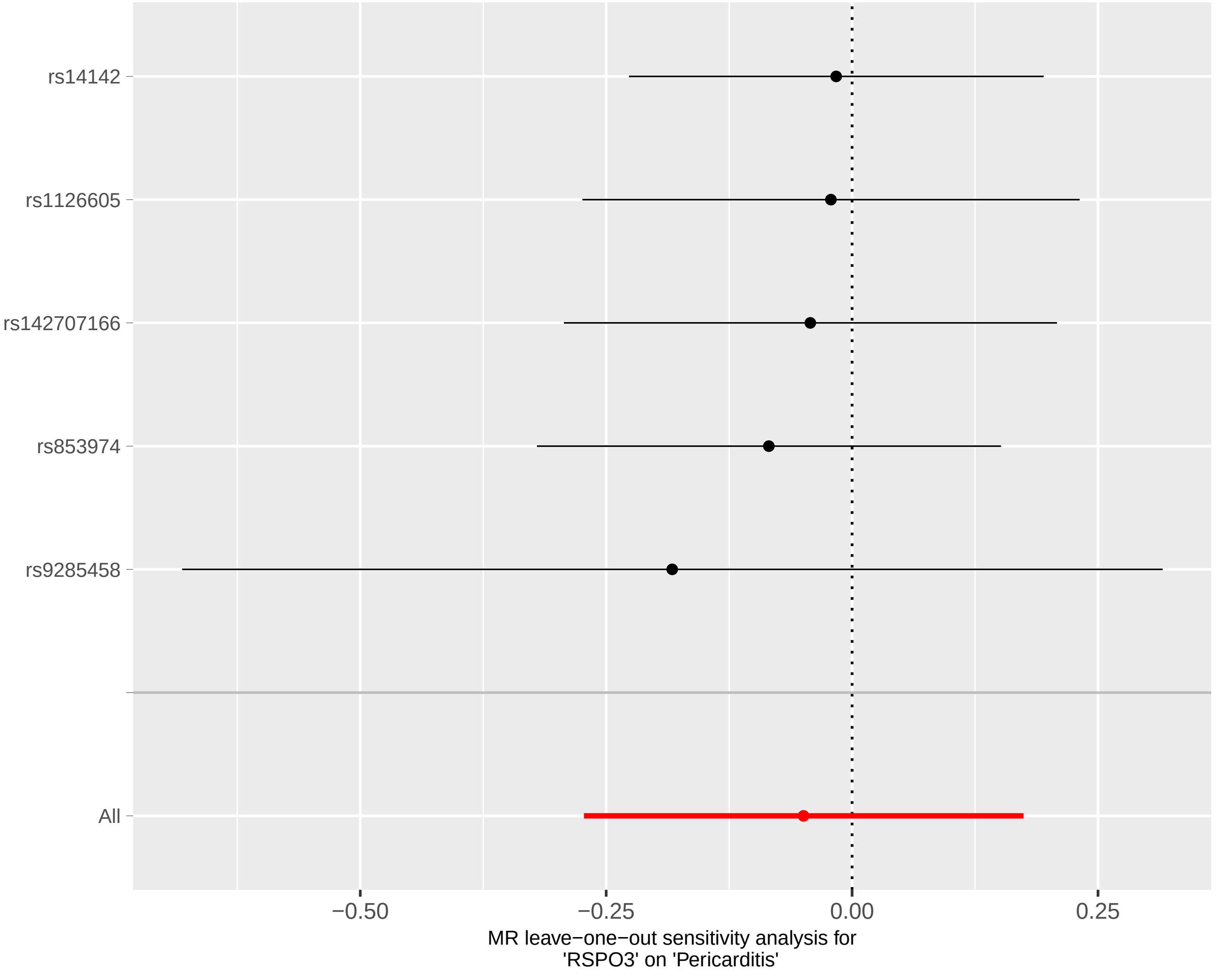
rs14142
rs1126605
rs142707166
rs853974
rs9285458
All
−0.50
−0.25
0.00
0.25
MR leave−one−out sensitivity analysis for
'RSPO3' on 'Pericarditis'

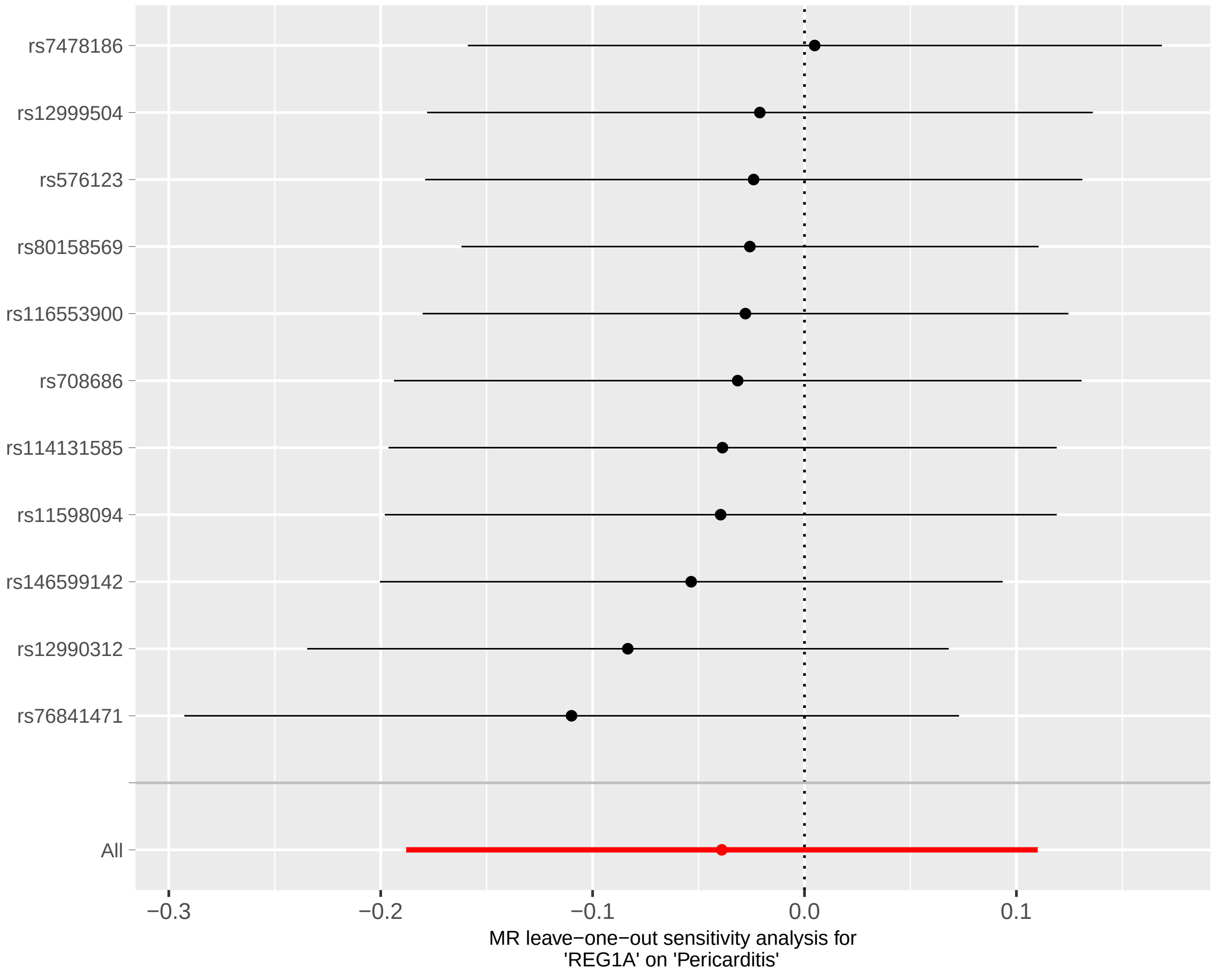

rs7478186
rs12999504
rs576123
rs80158569
rs116553900
rs708686
rs114131585
rs11598094
rs146599142
rs12990312
rs76841471
All
−0.3
−0.2
−0.1
0.0
0.1
MR leave−one−out sensitivity analysis for
'REG1A' on 'Pericarditis'

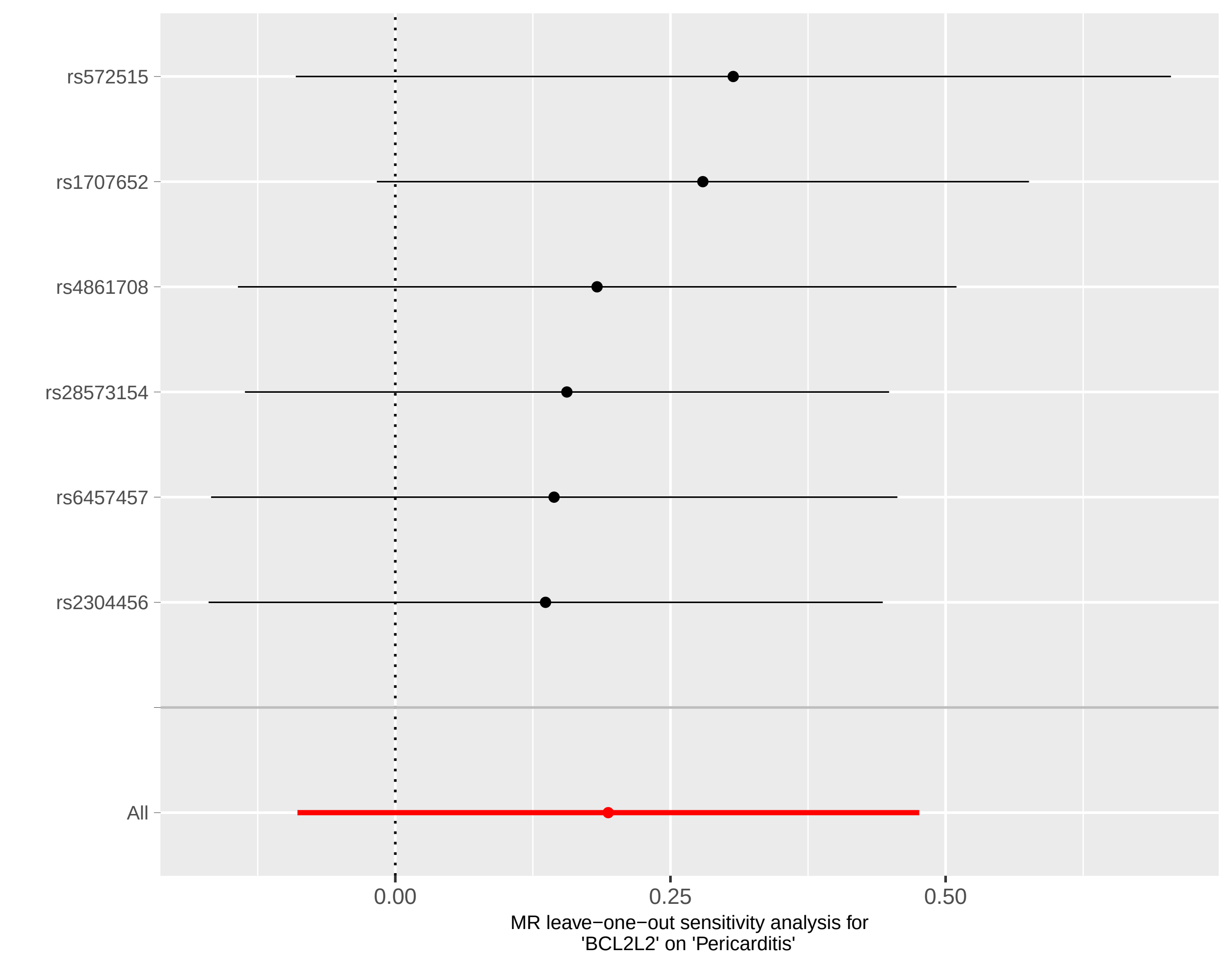

rs572515
rs1707652
rs4861708
rs28573154
rs6457457
rs2304456
All
0.00
0.25
0.50
MR leave−one−out sensitivity analysis for
'BCL2L2' on 'Pericarditis'

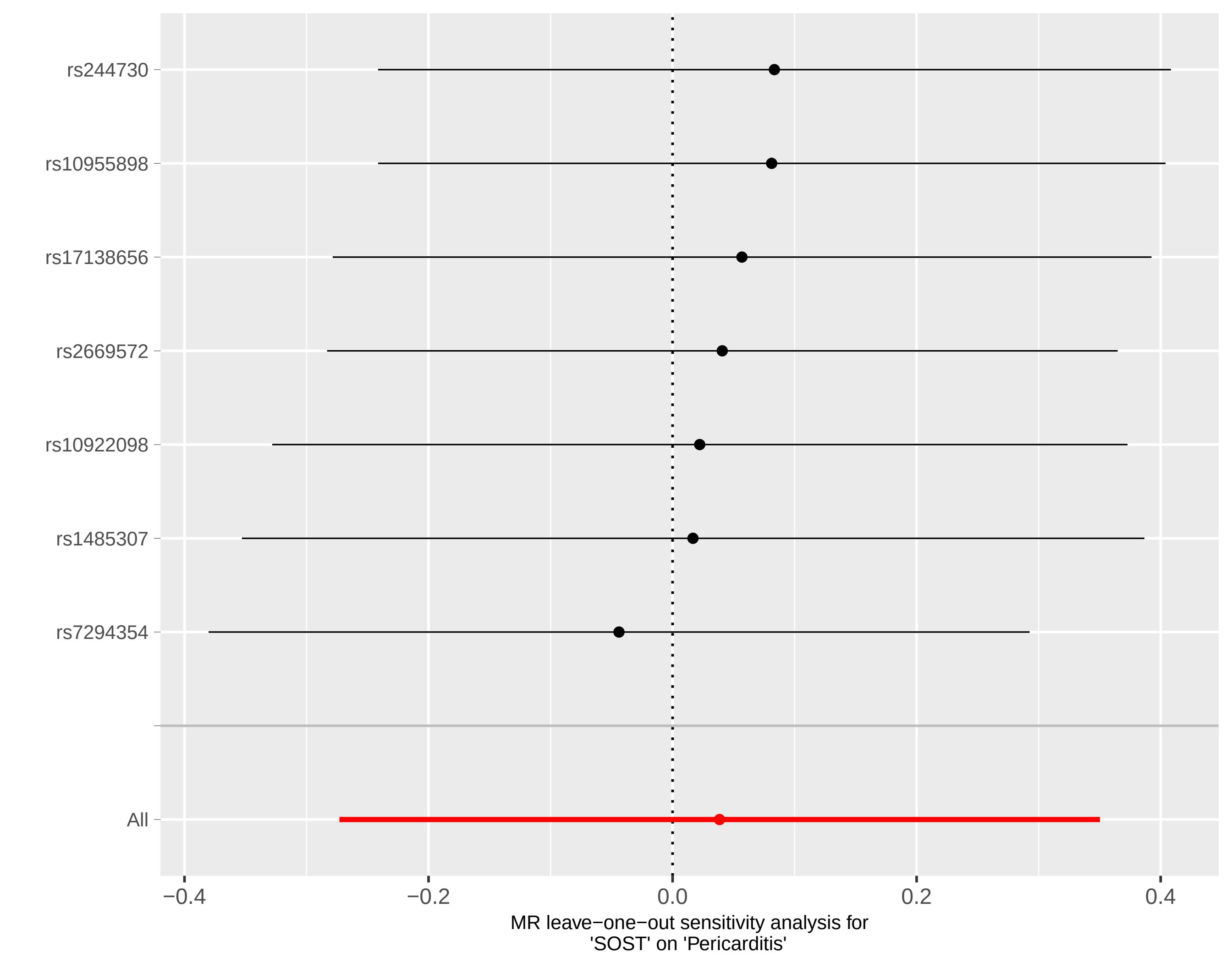
rs244730
rs10955898
rs17138656
rs2669572
rs10922098
rs1485307
rs7294354
All
−0.4
−0.2
0.0
0.2
0.4
MR leave−one−out sensitivity analysis for
'SOST' on 'Pericarditis'

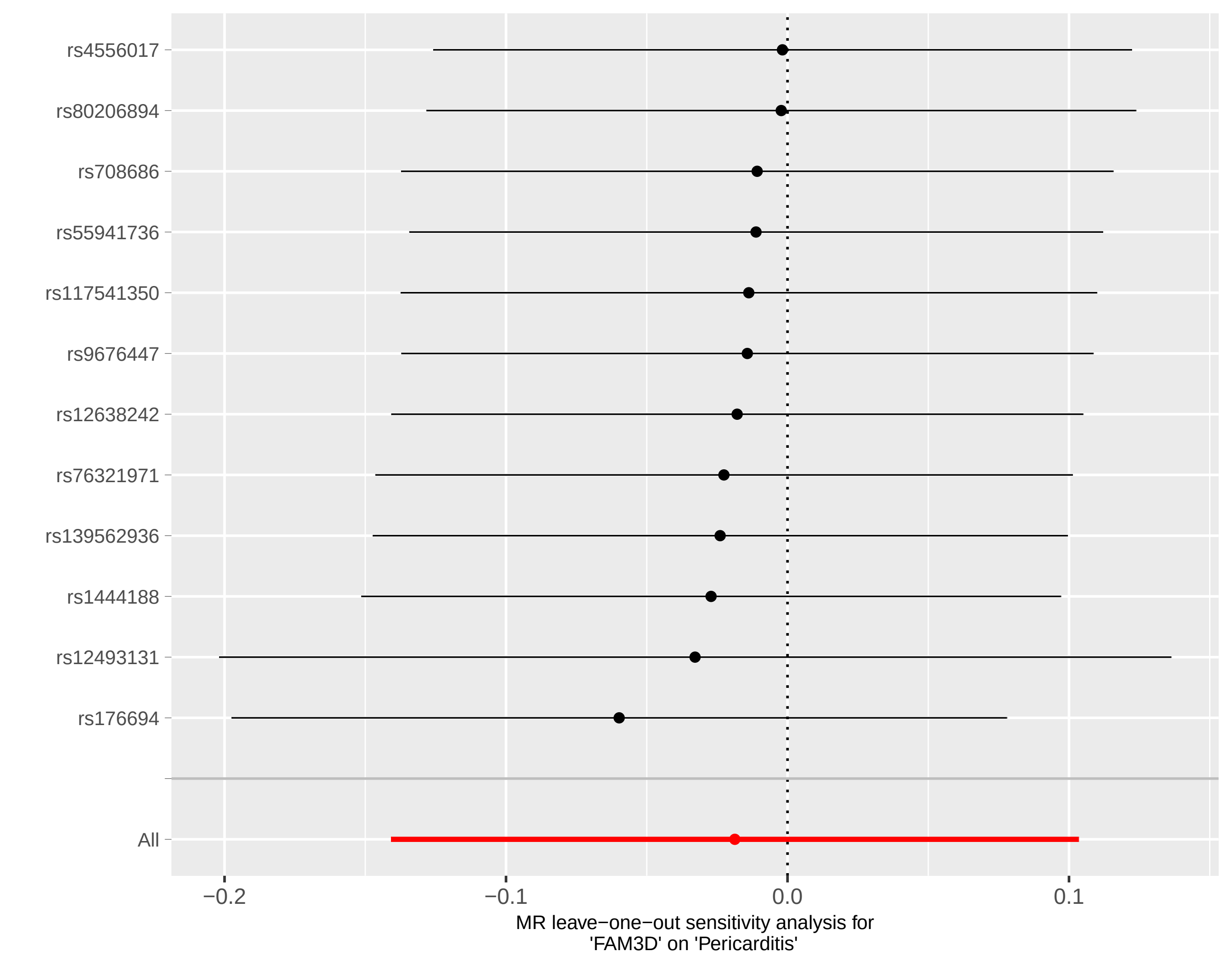
rs4556017
rs80206894
rs708686
rs55941736
rs117541350
rs9676447
rs12638242
rs76321971
rs139562936
rs1444188
rs12493131
rs176694
All
−0.2
−0.1
0.0
0.1
MR leave−one−out sensitivity analysis for
'FAM3D' on 'Pericarditis'

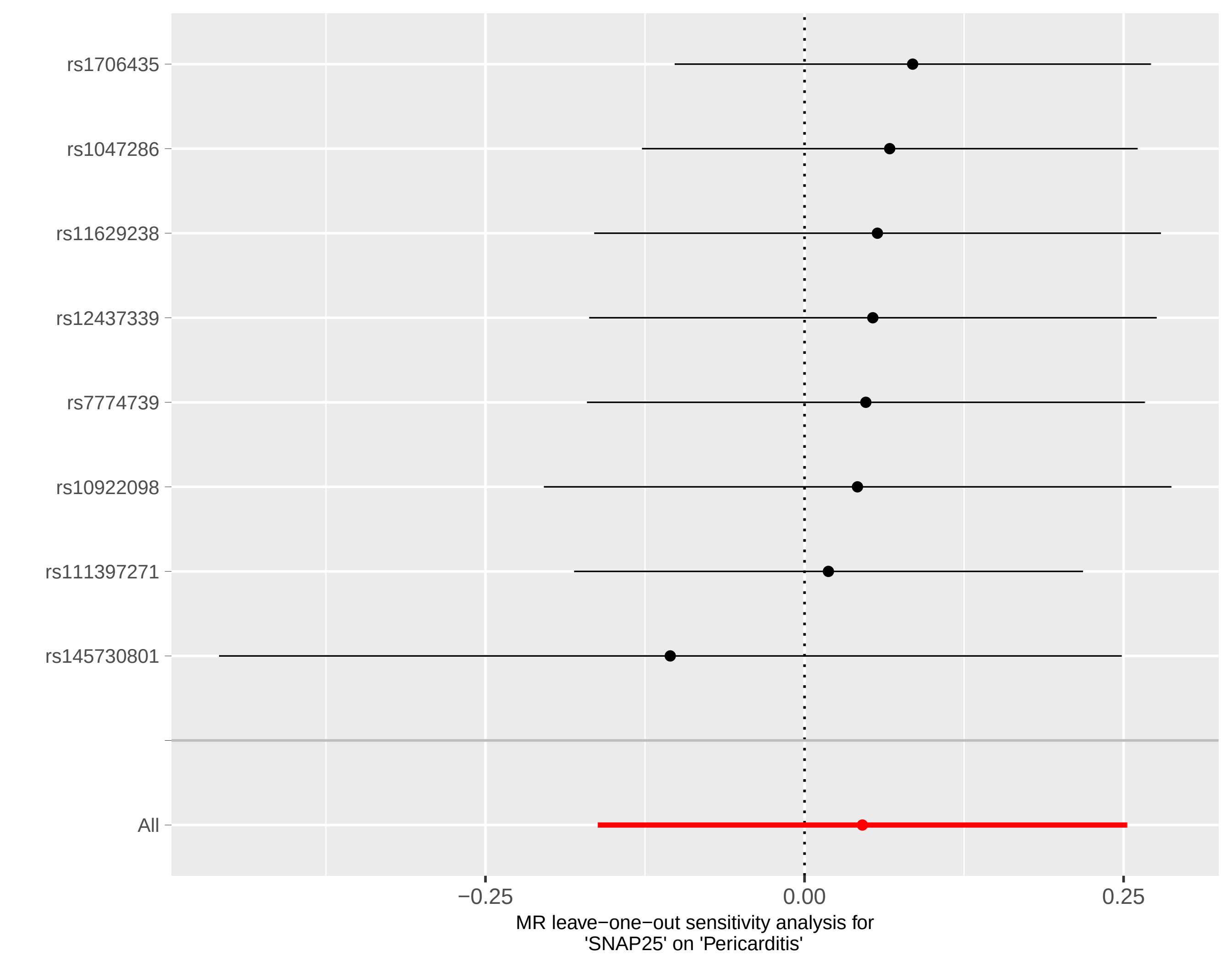
rs1706435
rs1047286
rs11629238
rs12437339
rs7774739
rs10922098
rs111397271
rs145730801
All
−0.25
0.00
0.25
MR leave−one−out sensitivity analysis for
'SNAP25' on 'Pericarditis'

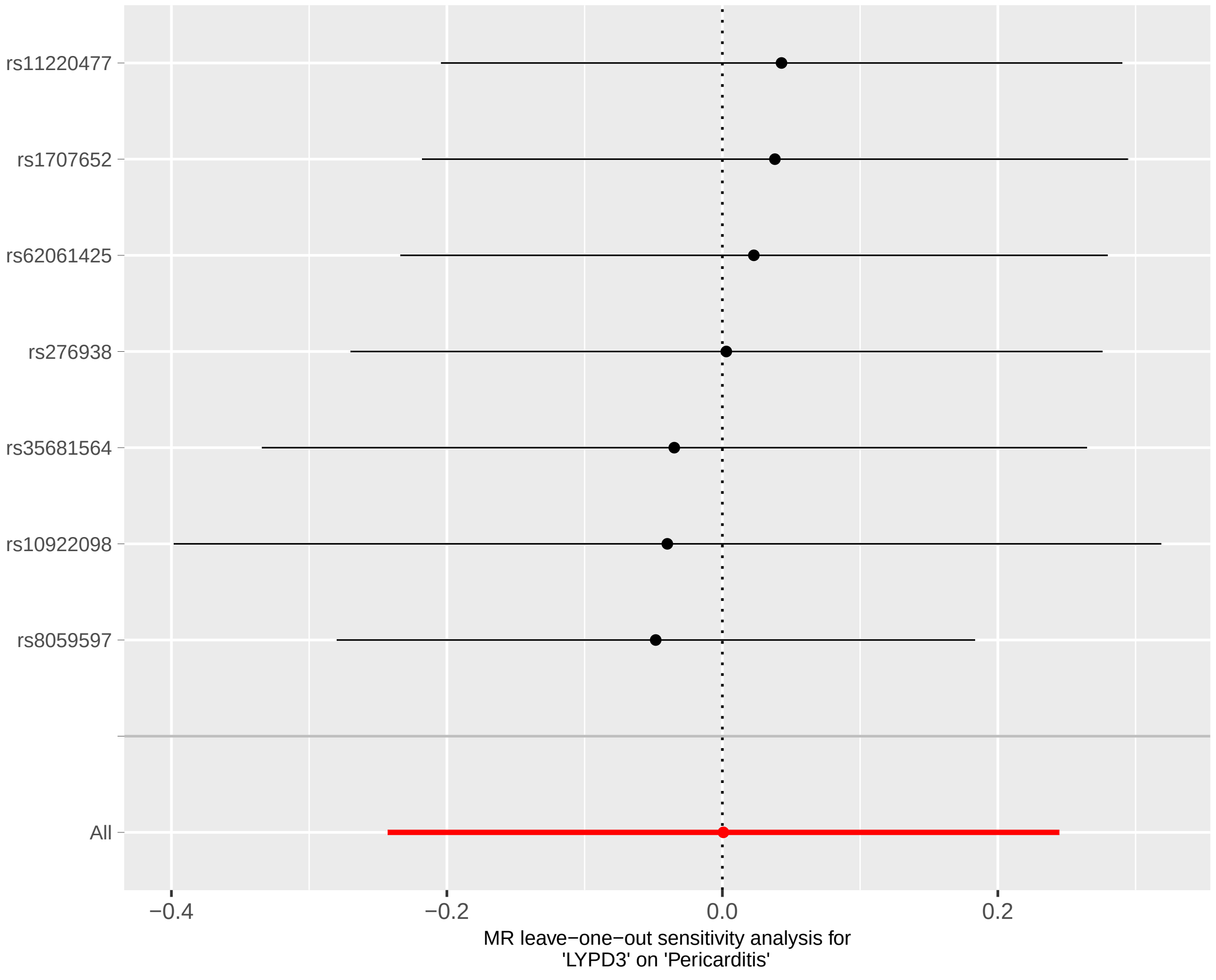
rs11220477
rs1707652
rs62061425
rs276938
rs35681564
rs10922098
rs8059597
All
−0.4
−0.2
0.0
0.2
MR leave−one−out sensitivity analysis for
'LYPD3' on 'Pericarditis'

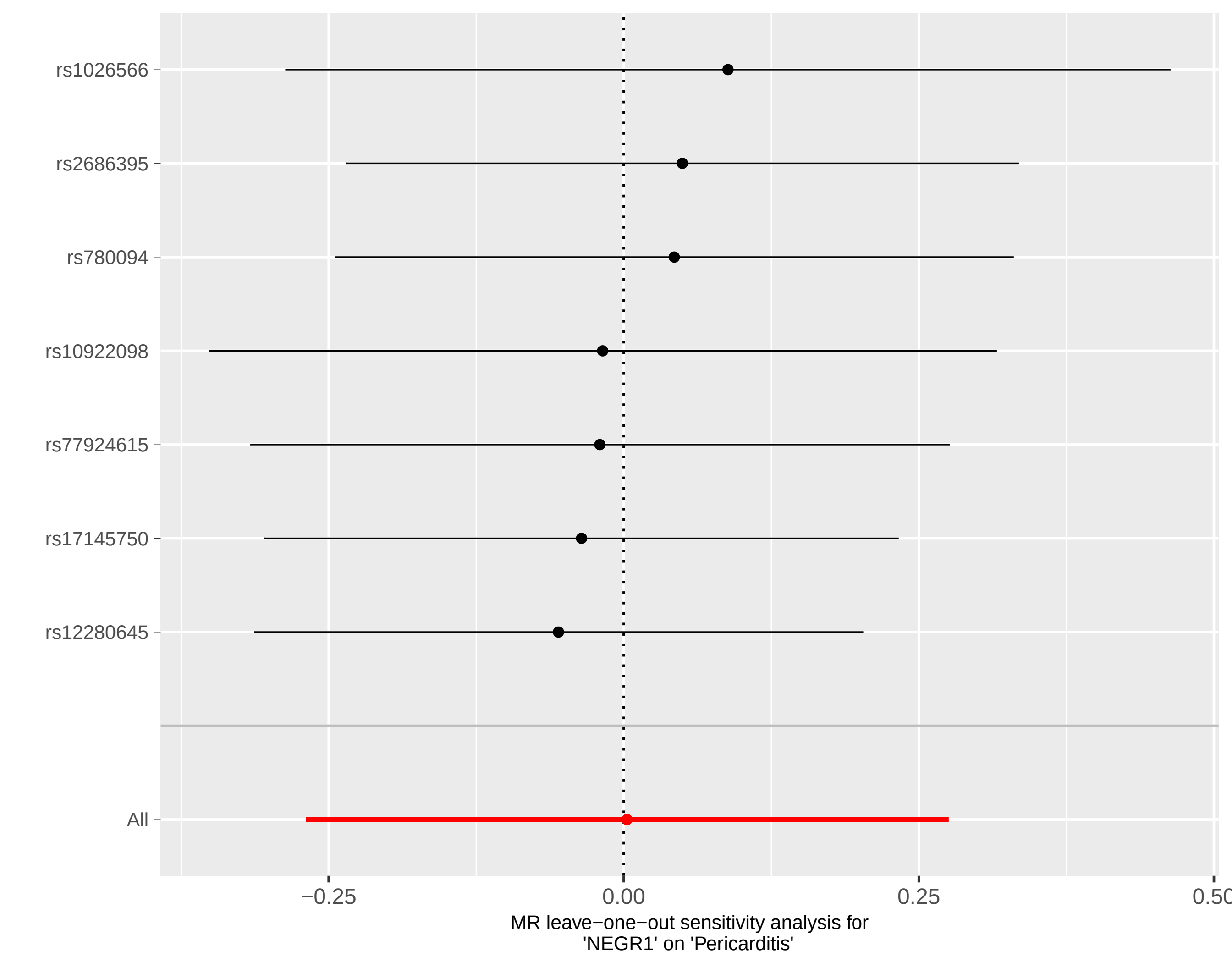
rs1026566
rs2686395
rs780094
rs10922098
rs77924615
rs17145750
rs12280645
All
−0.25
0.00
0.25
0.50
MR leave−one−out sensitivity analysis for
'NEGR1' on 'Pericarditis'

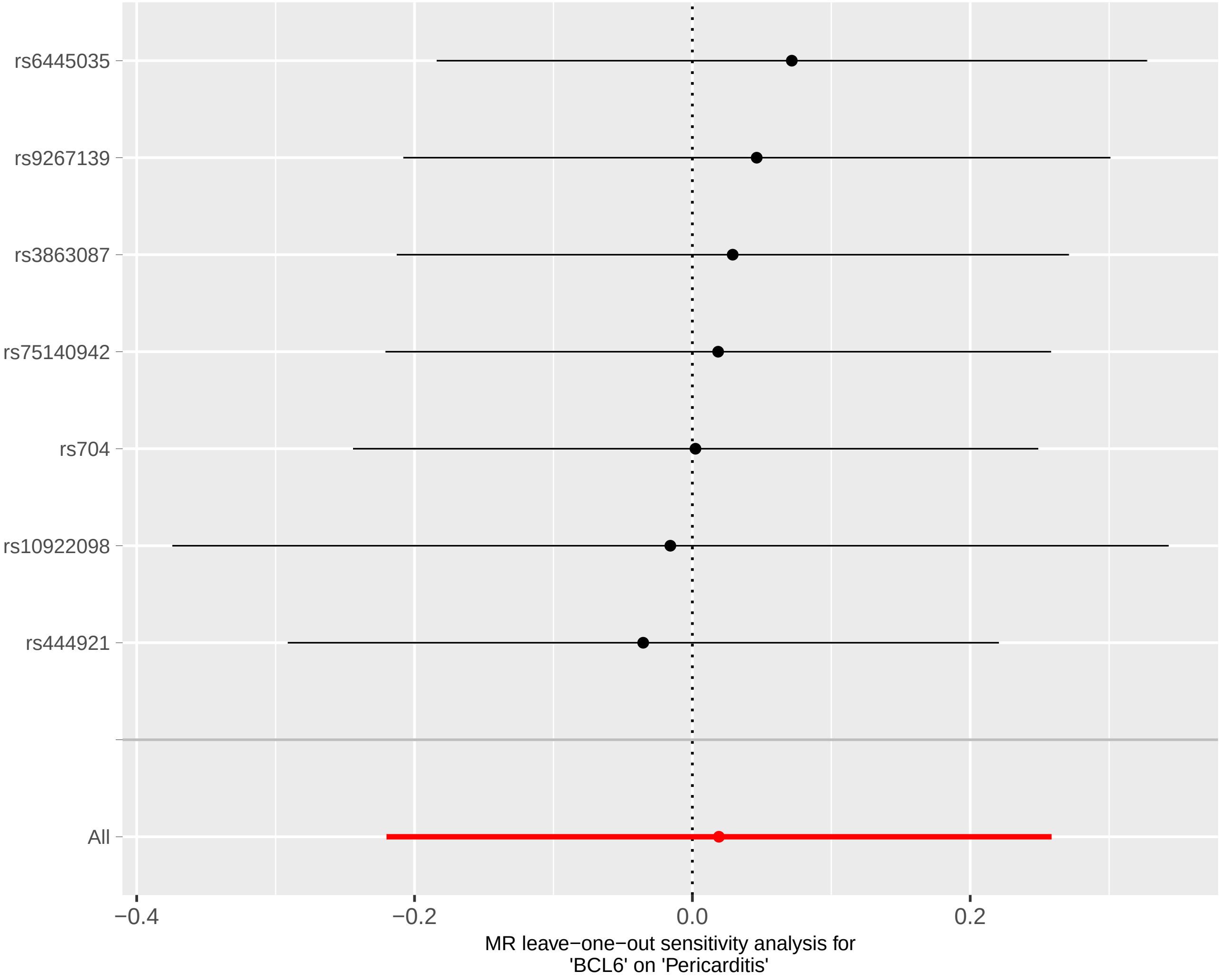
rs6445035
rs9267139
rs3863087
rs75140942
rs704
rs10922098
rs444921
All
−0.4
−0.2
0.0
0.2
MR leave−one−out sensitivity analysis for
'BCL6' on 'Pericarditis'

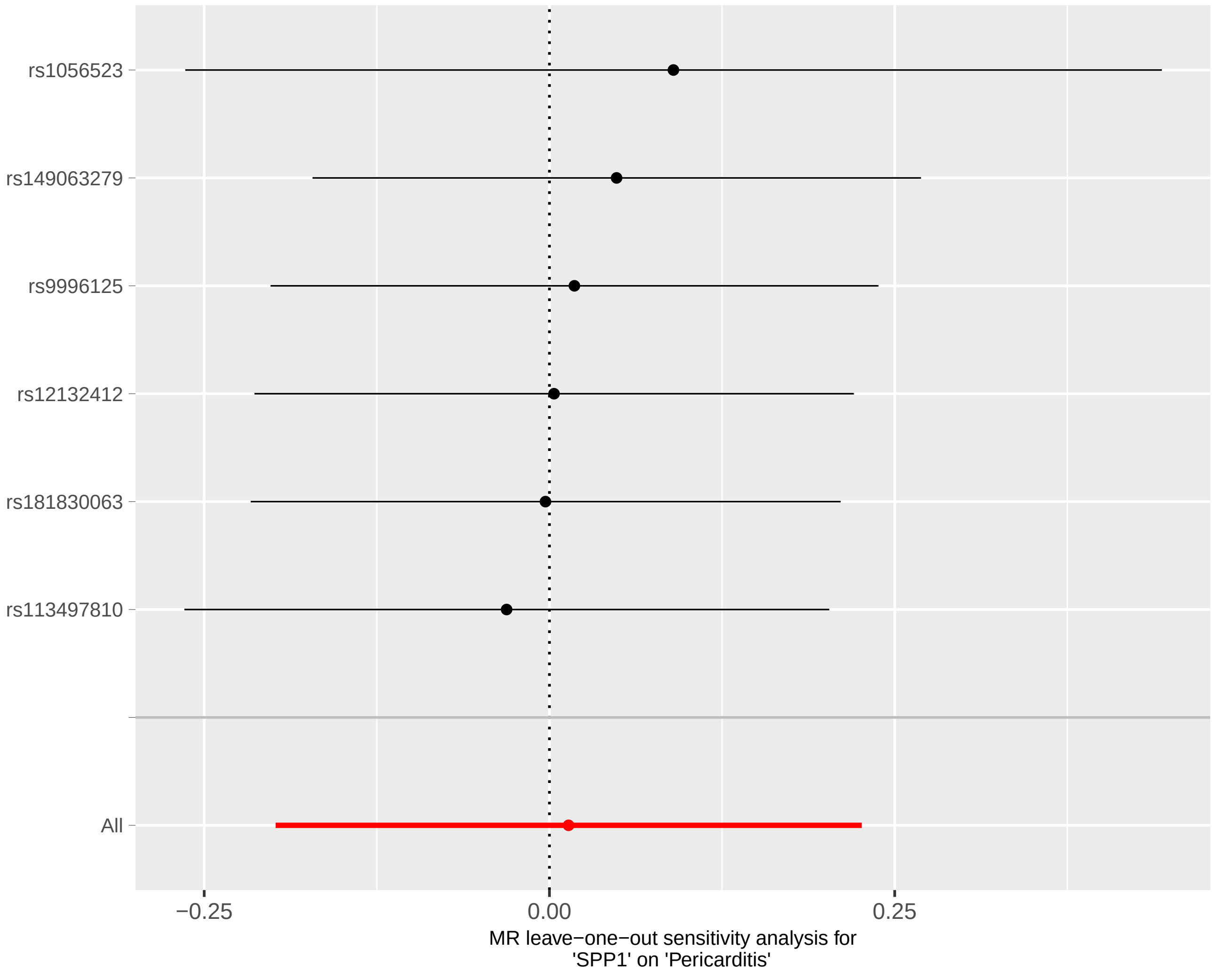
rs1056523
rs149063279
rs9996125
rs12132412
rs181830063
rs113497810
All
−0.25
0.00
0.25
MR leave−one−out sensitivity analysis for
'SPP1' on 'Pericarditis'

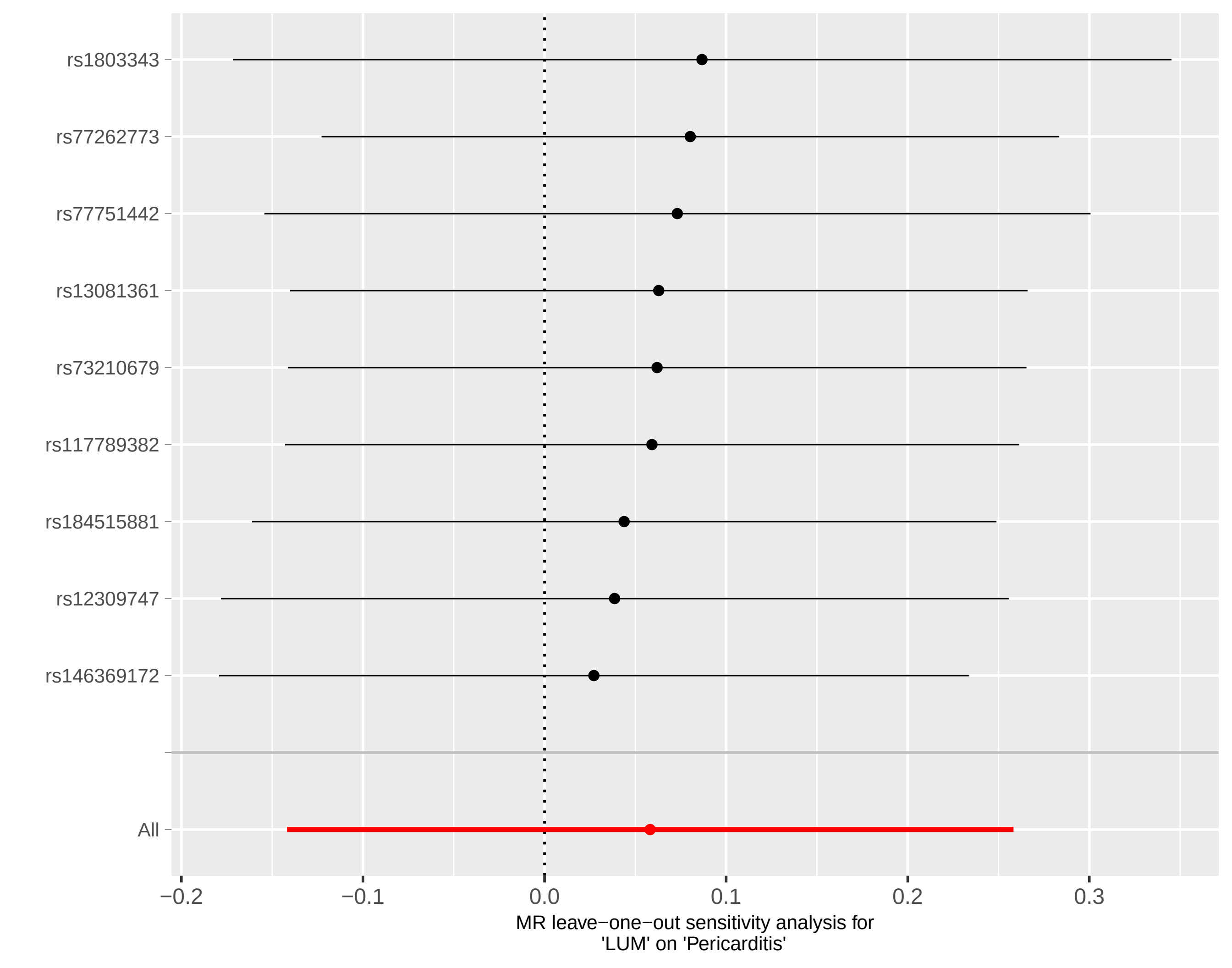
rs1803343
rs77262773
rs77751442
rs13081361
rs73210679
rs117789382
rs184515881
rs12309747
rs146369172
All
−0.2
−0.1
0.0
0.1
0.2
0.3
MR leave−one−out sensitivity analysis for
'LUM' on 'Pericarditis'

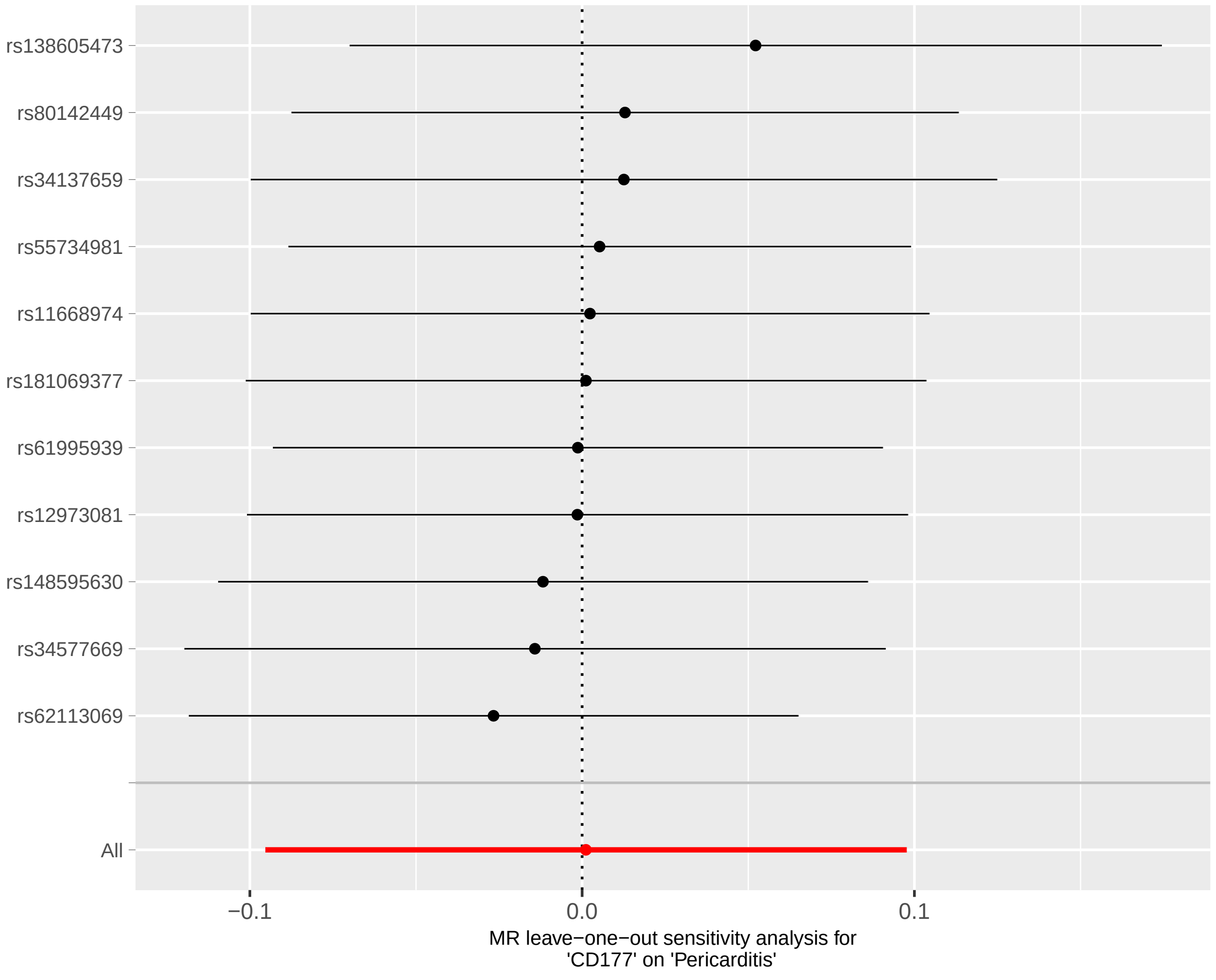
rs138605473
rs80142449
rs34137659
rs55734981
rs11668974
rs181069377
rs61995939
rs12973081
rs148595630
rs34577669
rs62113069
All
−0.1
0.0
0.1
MR leave−one−out sensitivity analysis for
'CD177' on 'Pericarditis'

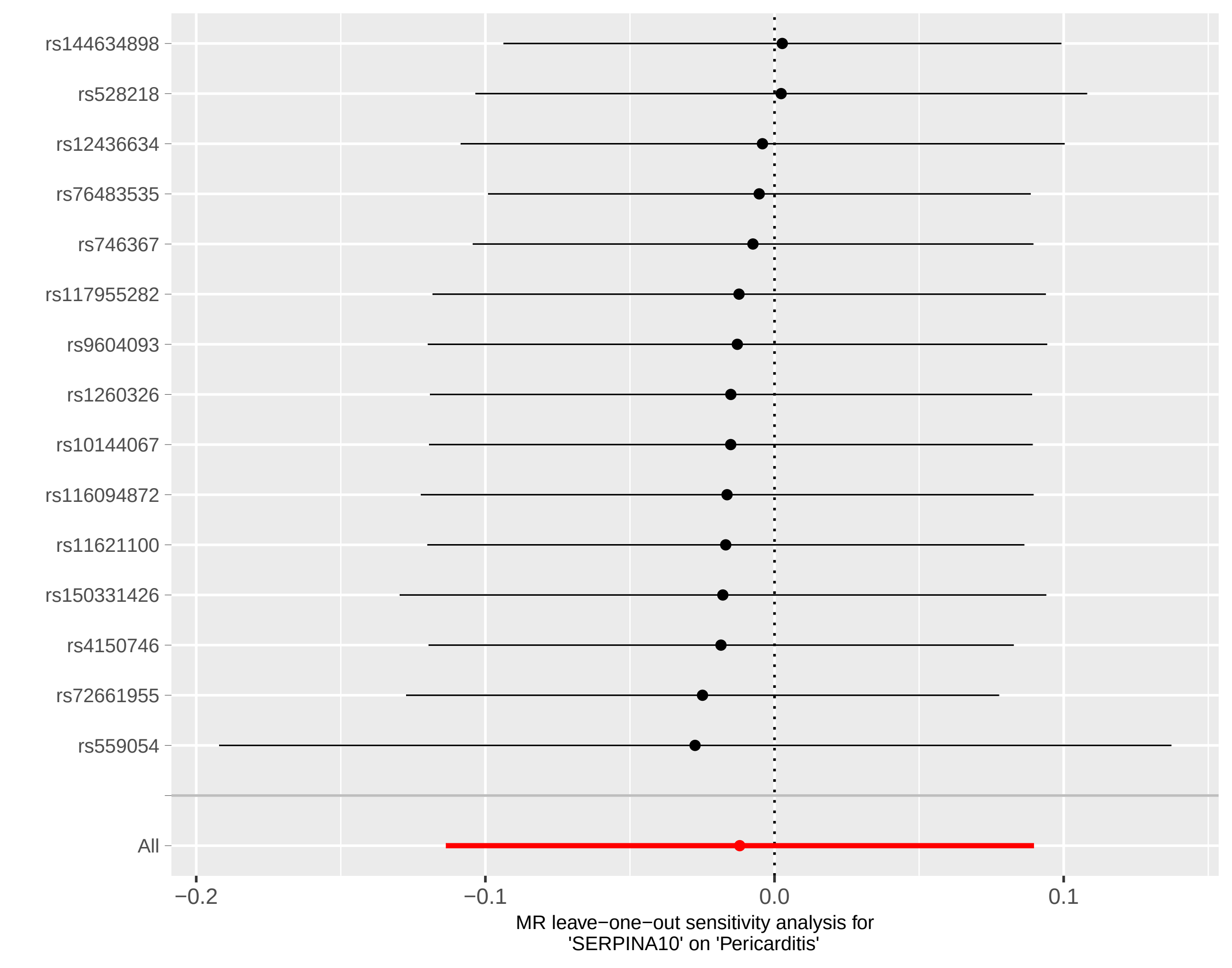
rs144634898
rs528218
rs12436634
rs76483535
rs746367
rs117955282
rs9604093
rs1260326
rs10144067
rs116094872
rs11621100
rs150331426
rs4150746
rs72661955
rs559054
All
−0.2
−0.1
0.0
0.1
MR leave−one−out sensitivity analysis for
'SERPINA10' on 'Pericarditis'

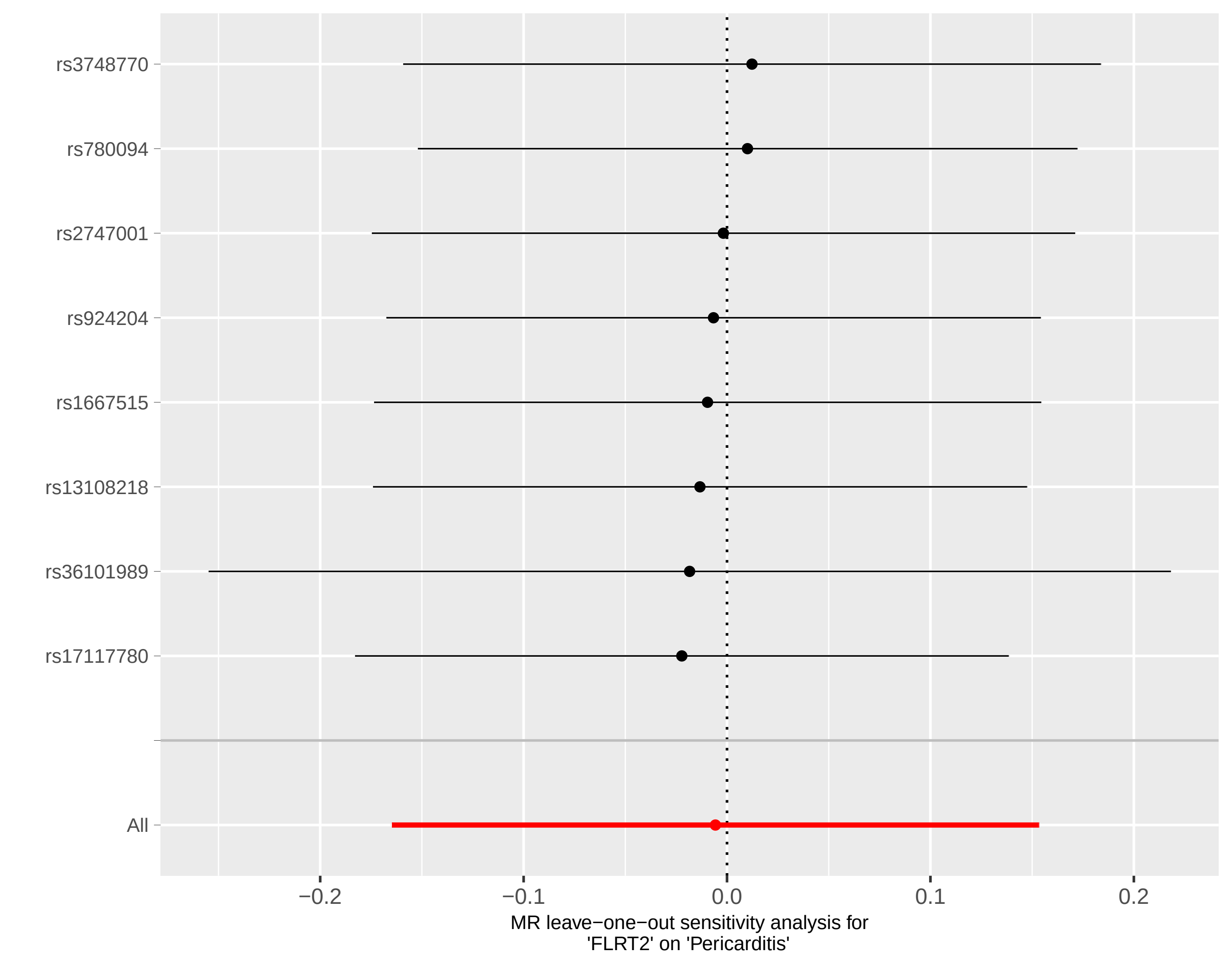
rs3748770
rs780094
rs2747001
rs924204
rs1667515
rs13108218
rs36101989
rs17117780
All
−0.2
−0.1
0.0
0.1
0.2
MR leave−one−out sensitivity analysis for
'FLRT2' on 'Pericarditis'

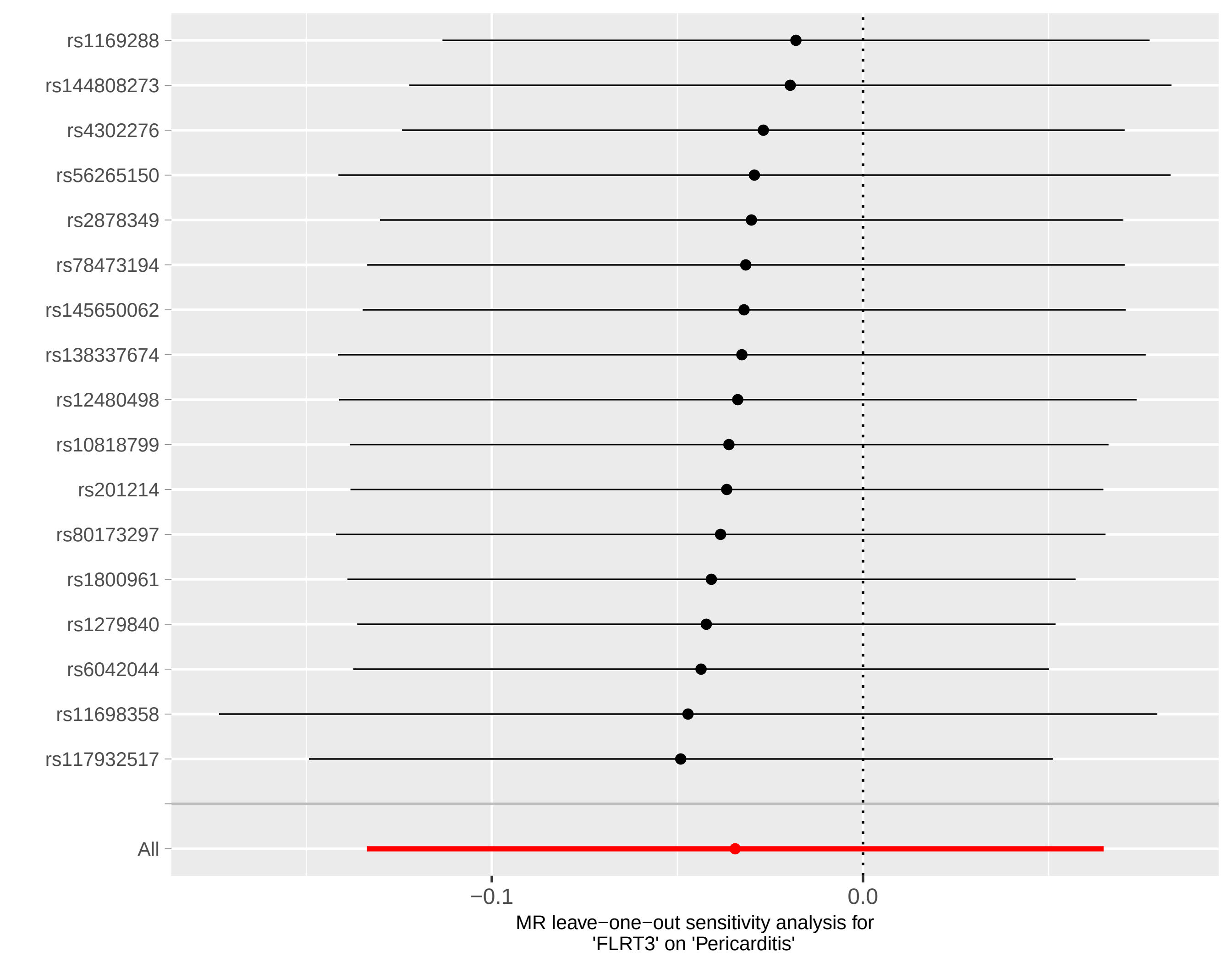

rs1169288
rs144808273
rs4302276
rs56265150
rs2878349
rs78473194
rs145650062
rs138337674
rs12480498
rs10818799
rs201214
rs80173297
rs1800961
rs1279840
rs6042044
rs11698358
rs117932517
All
−0.1
0.0
MR leave−one−out sensitivity analysis for
'FLRT3' on 'Pericarditis'

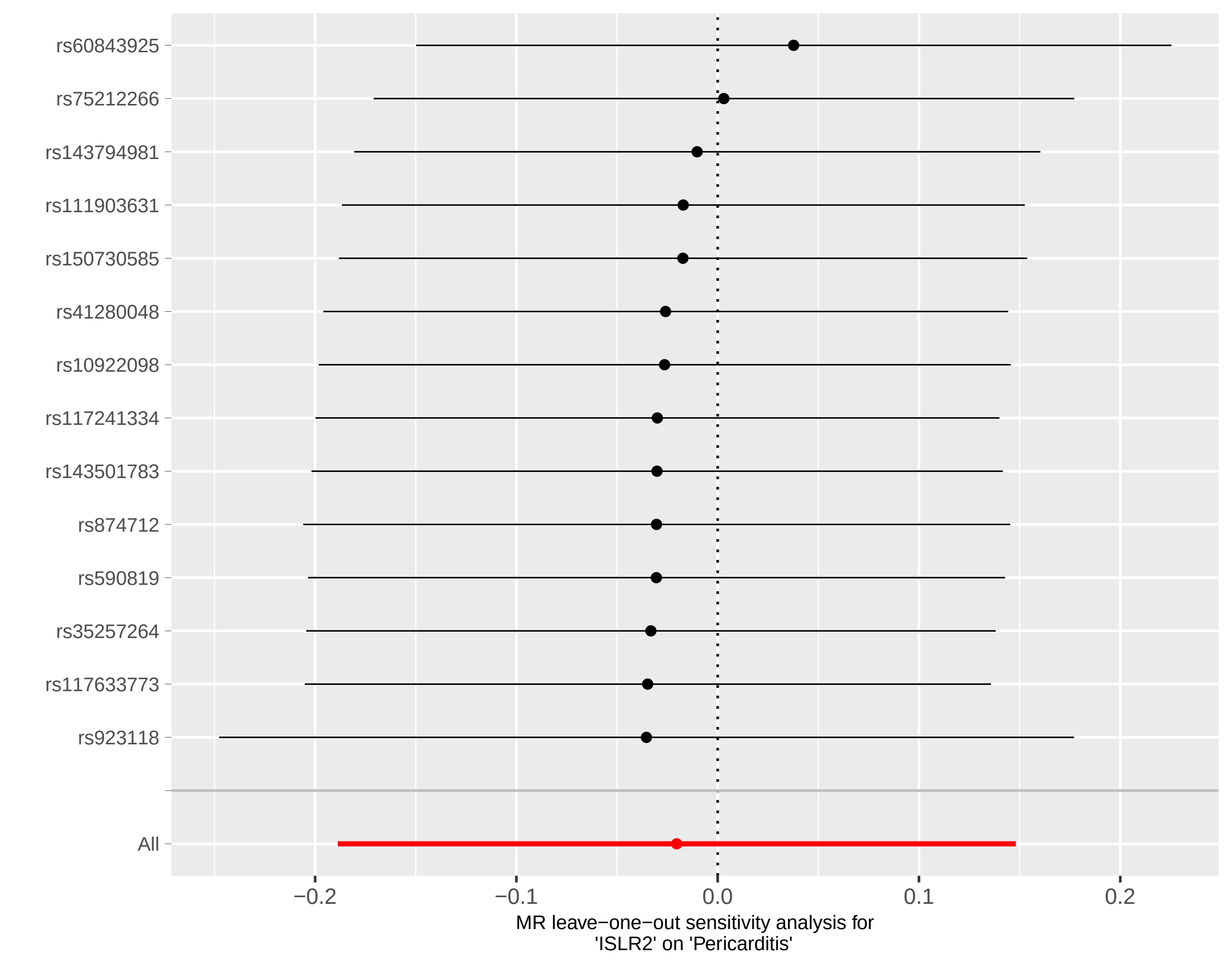

rs60843925
rs75212266
rs143794981
rs111903631
rs150730585
rs41280048
rs10922098
rs117241334
rs143501783
rs874712
rs590819
rs35257264
rs117633773
rs923118
All
−0.2
−0.1
0.0
0.1
0.2
MR leave−one−out sensitivity analysis for
'ISLR2' on 'Pericarditis'

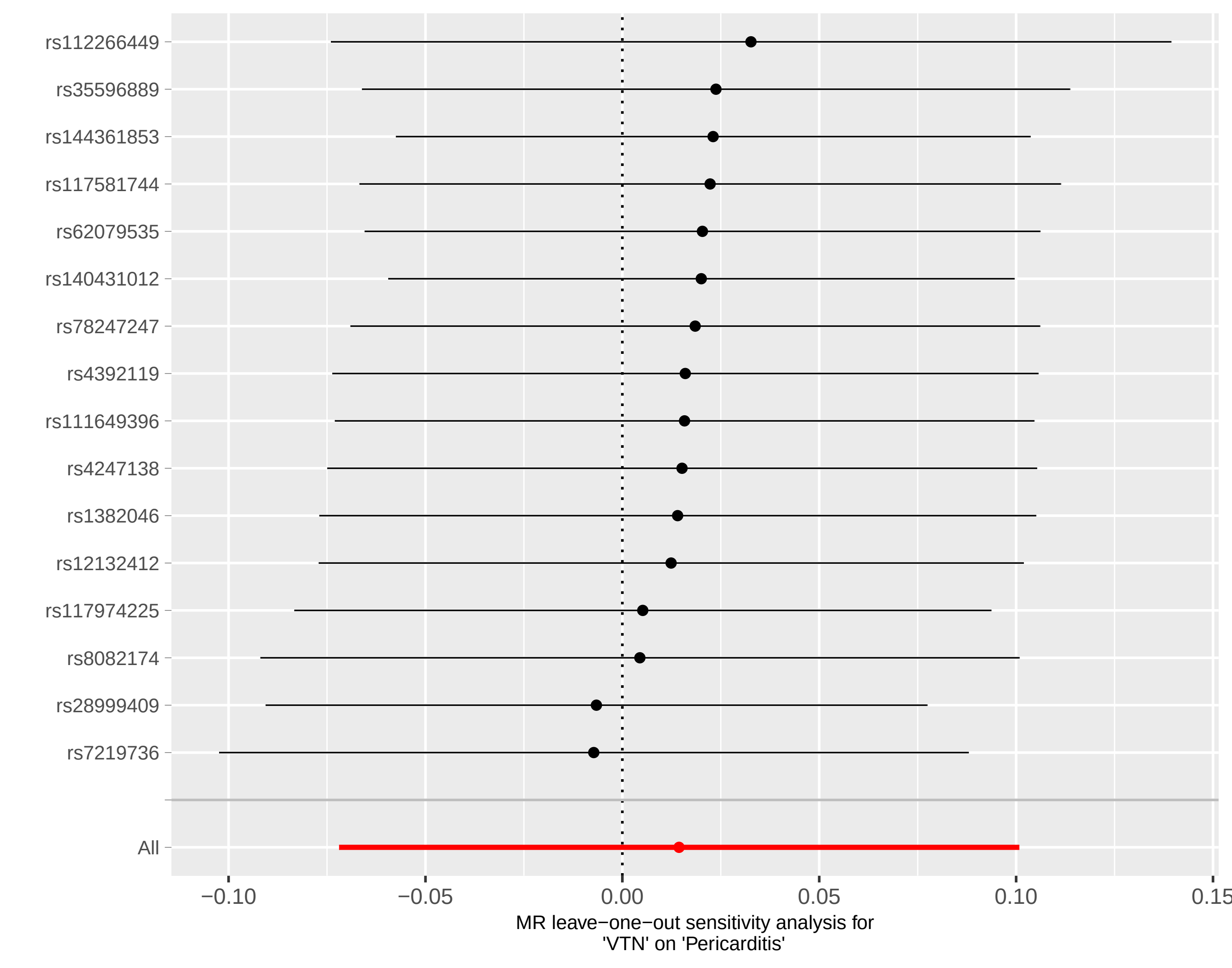

rs112266449
rs35596889
rs144361853
rs117581744
rs62079535
rs140431012
rs78247247
rs4392119
rs111649396
rs4247138
rs1382046
rs12132412
rs117974225
rs8082174
rs28999409
rs7219736
All
−0.10
−0.05
0.00
0.05
0.10
0.15
MR leave−one−out sensitivity analysis for
'VTN' on 'Pericarditis'

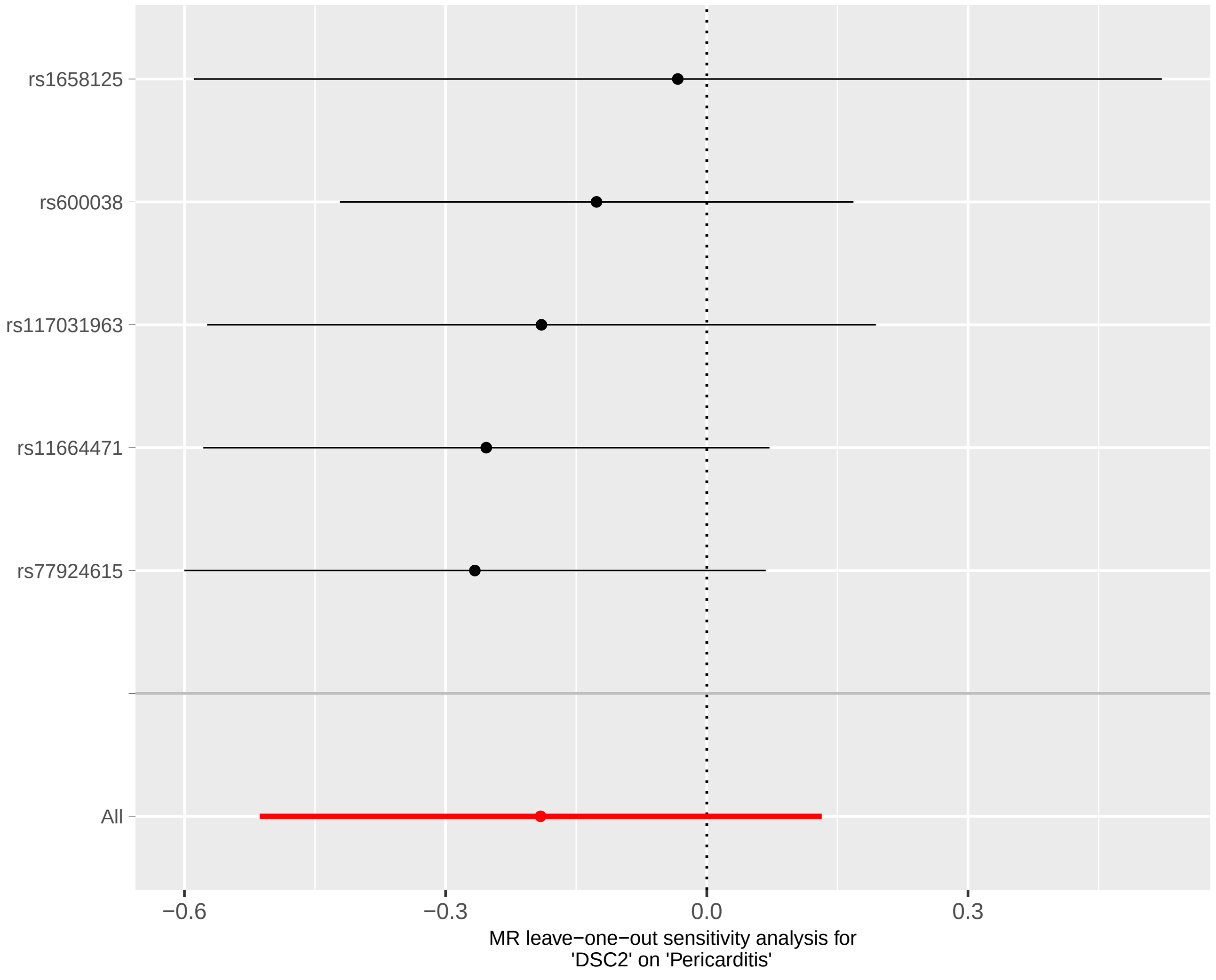
rs1658125
rs600038
rs117031963
rs11664471
rs77924615
All
−0.6
−0.3
0.0
0.3
MR leave−one−out sensitivity analysis for
'DSC2' on 'Pericarditis'

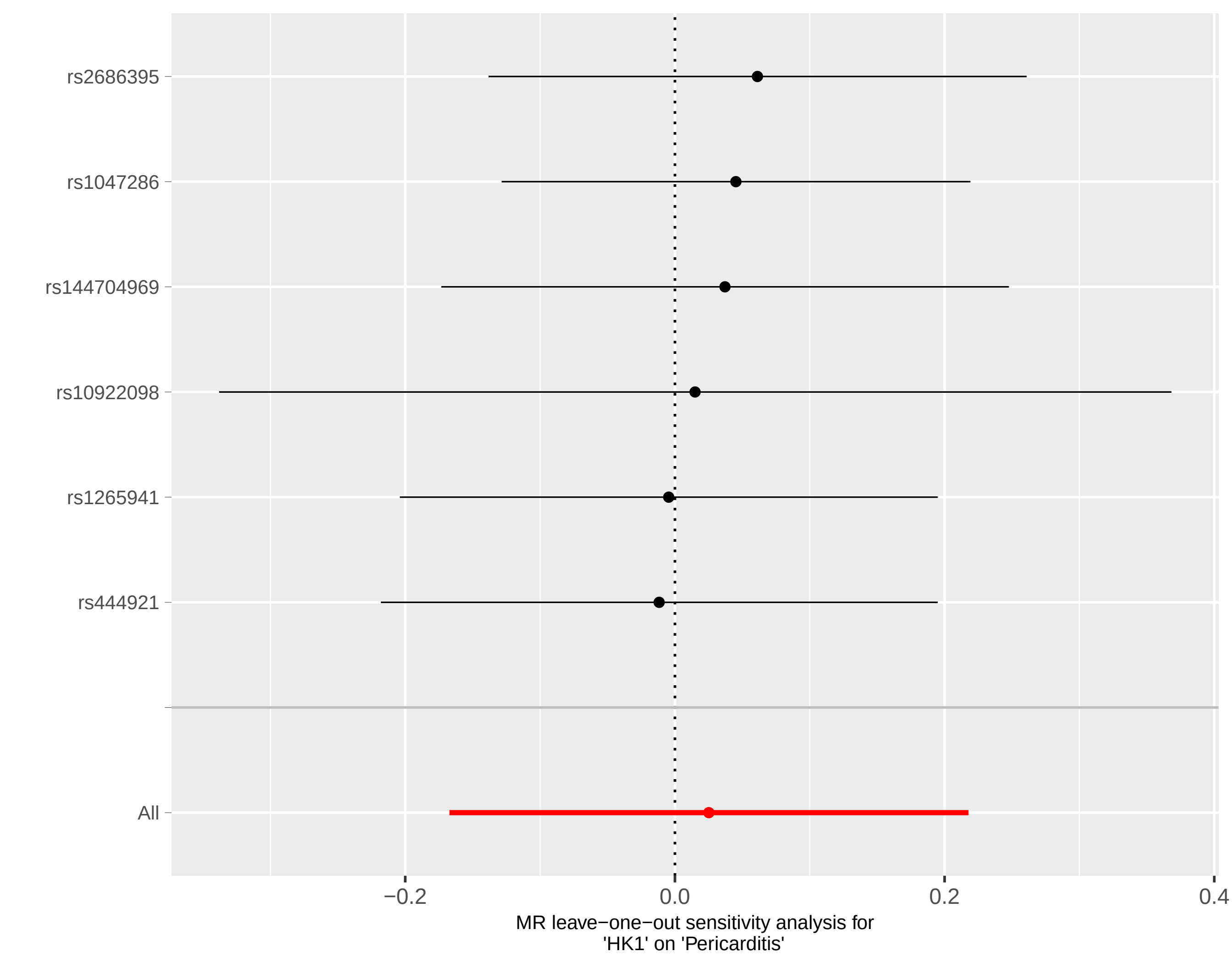
rs2686395
rs1047286
rs144704969
rs10922098
rs1265941
rs444921
All
−0.2
0.0
0.2
0.4
MR leave−one−out sensitivity analysis for
'HK1' on 'Pericarditis'

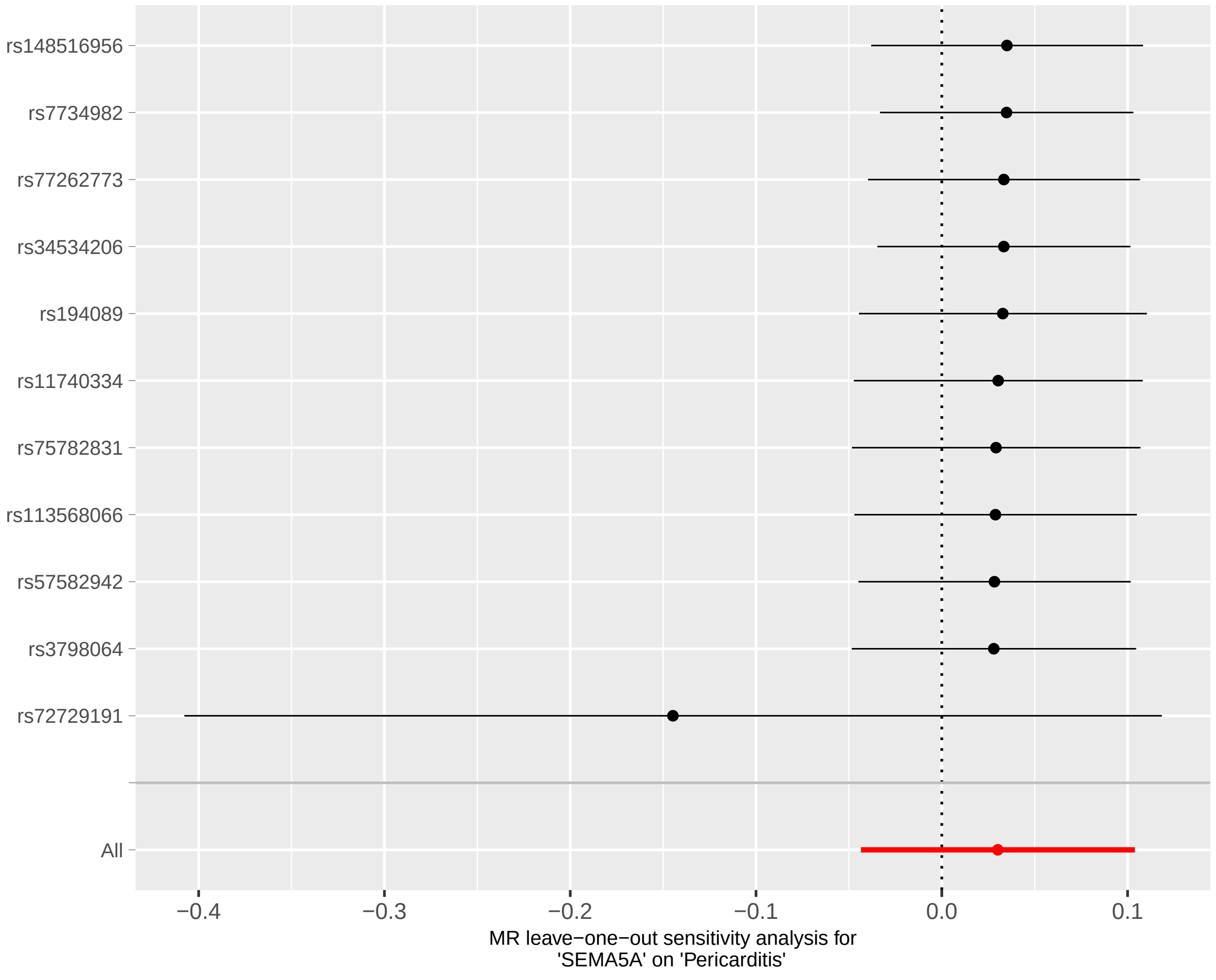

rs148516956
rs7734982
rs77262773
rs34534206
rs194089
rs11740334
rs75782831
rs113568066
rs57582942
rs3798064
rs72729191
All
−0.4
−0.3
−0.2
−0.1
0.0
0.1
MR leave−one−out sensitivity analysis for
'SEMA5A' on 'Pericarditis'

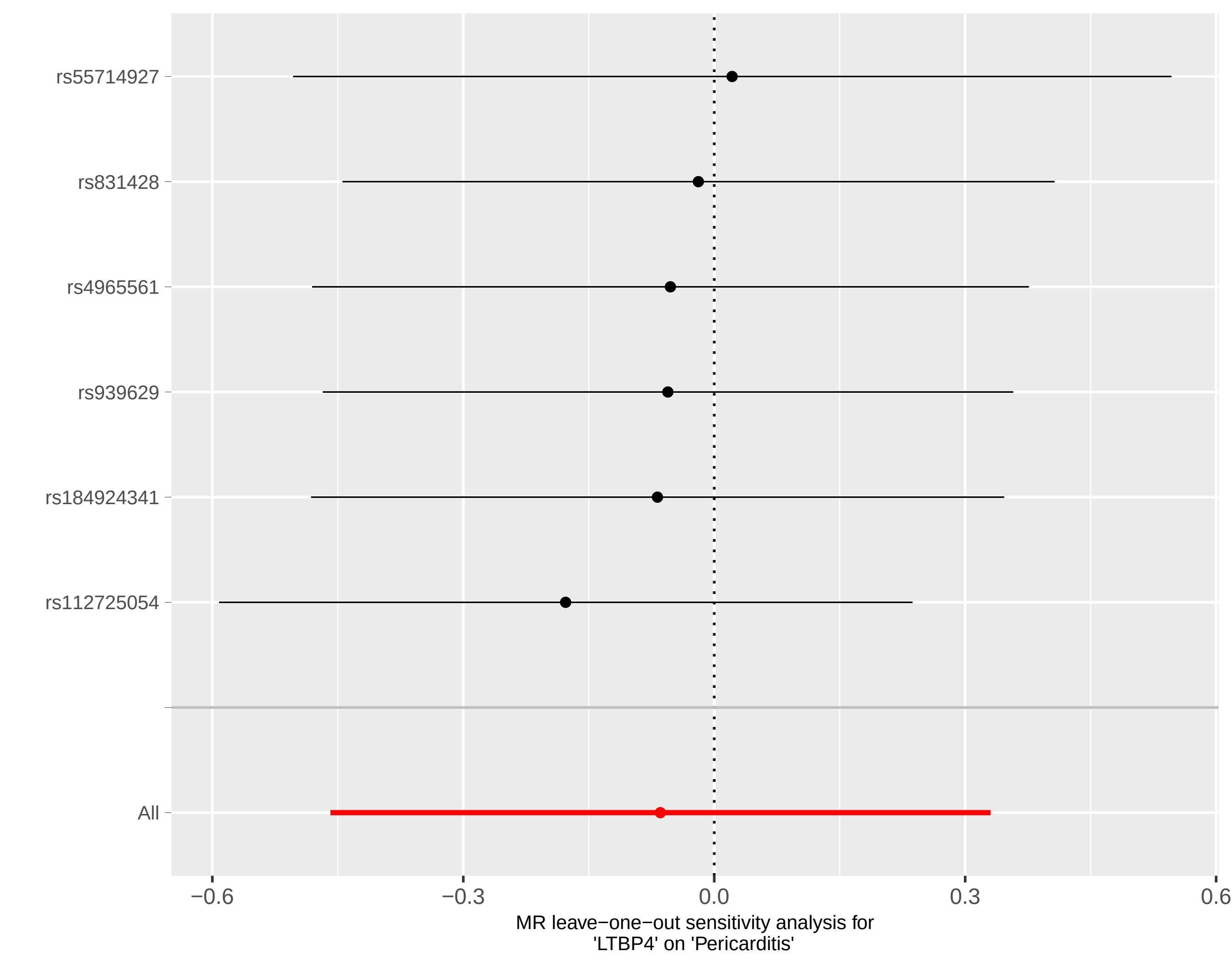

rs55714927
rs831428
rs4965561
rs939629
rs184924341
rs112725054
All
−0.6
−0.3
0.0
0.3
0.6
MR leave−one−out sensitivity analysis for
'LTBP4' on 'Pericarditis'

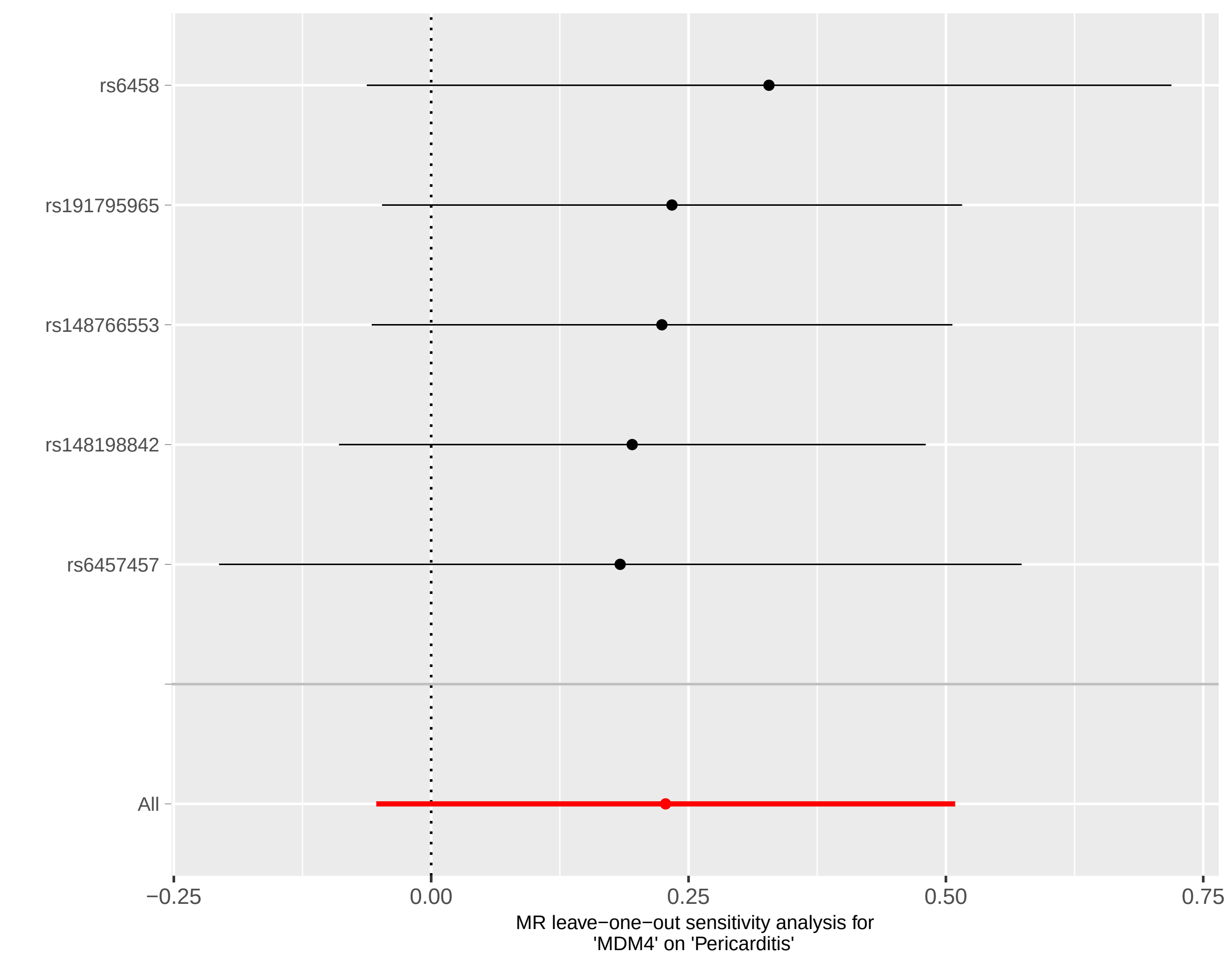

rs6458
rs191795965
rs148766553
rs148198842
rs6457457
All
−0.25
0.00
0.25
0.50
0.75
MR leave−one−out sensitivity analysis for
'MDM4' on 'Pericarditis'

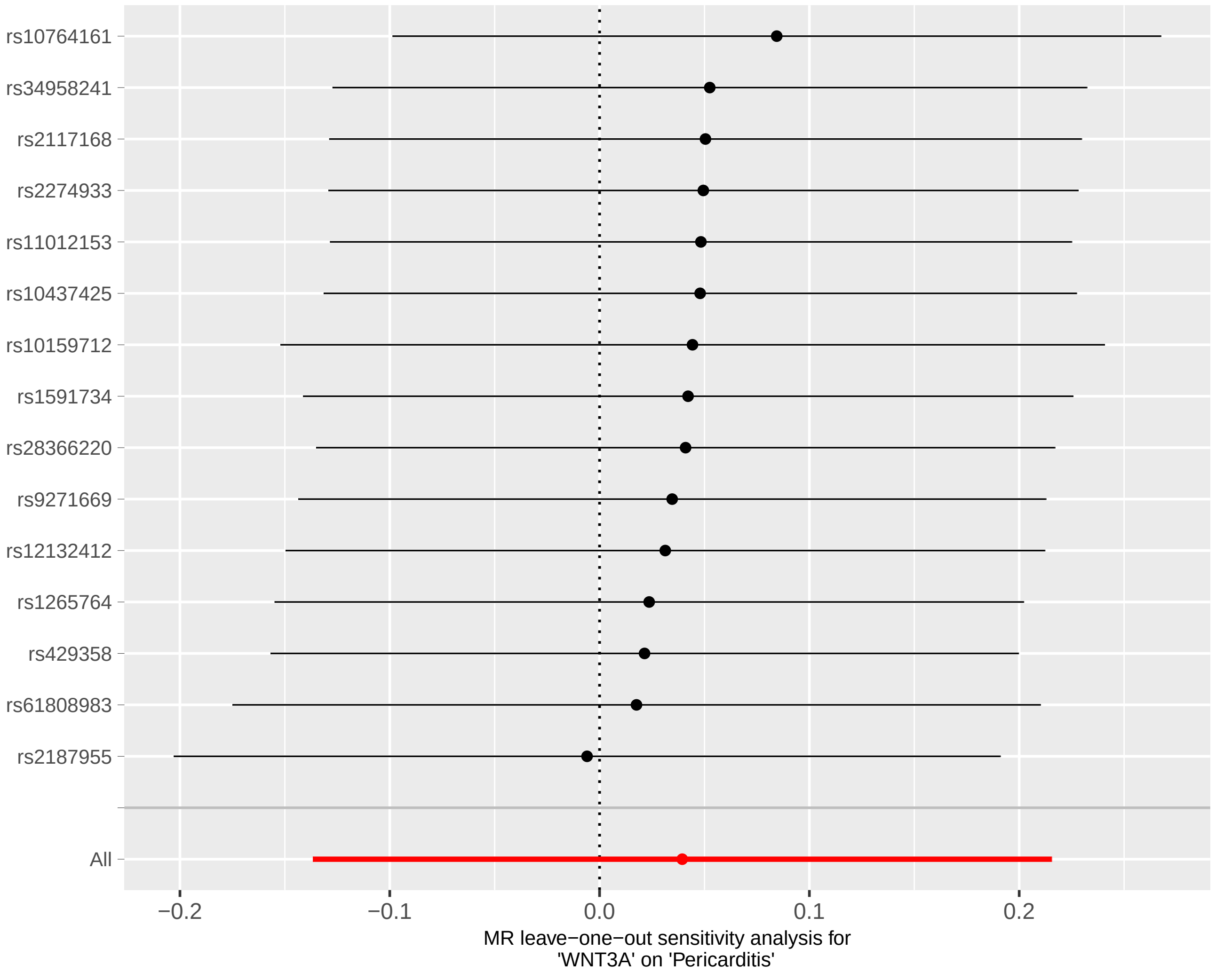

rs10764161
rs34958241
rs2117168
rs2274933
rs11012153
rs10437425
rs10159712
rs1591734
rs28366220
rs9271669
rs12132412
rs1265764
rs429358
rs61808983
rs2187955
All
−0.2
−0.1
0.0
0.1
0.2
MR leave−one−out sensitivity analysis for
'WNT3A' on 'Pericarditis'

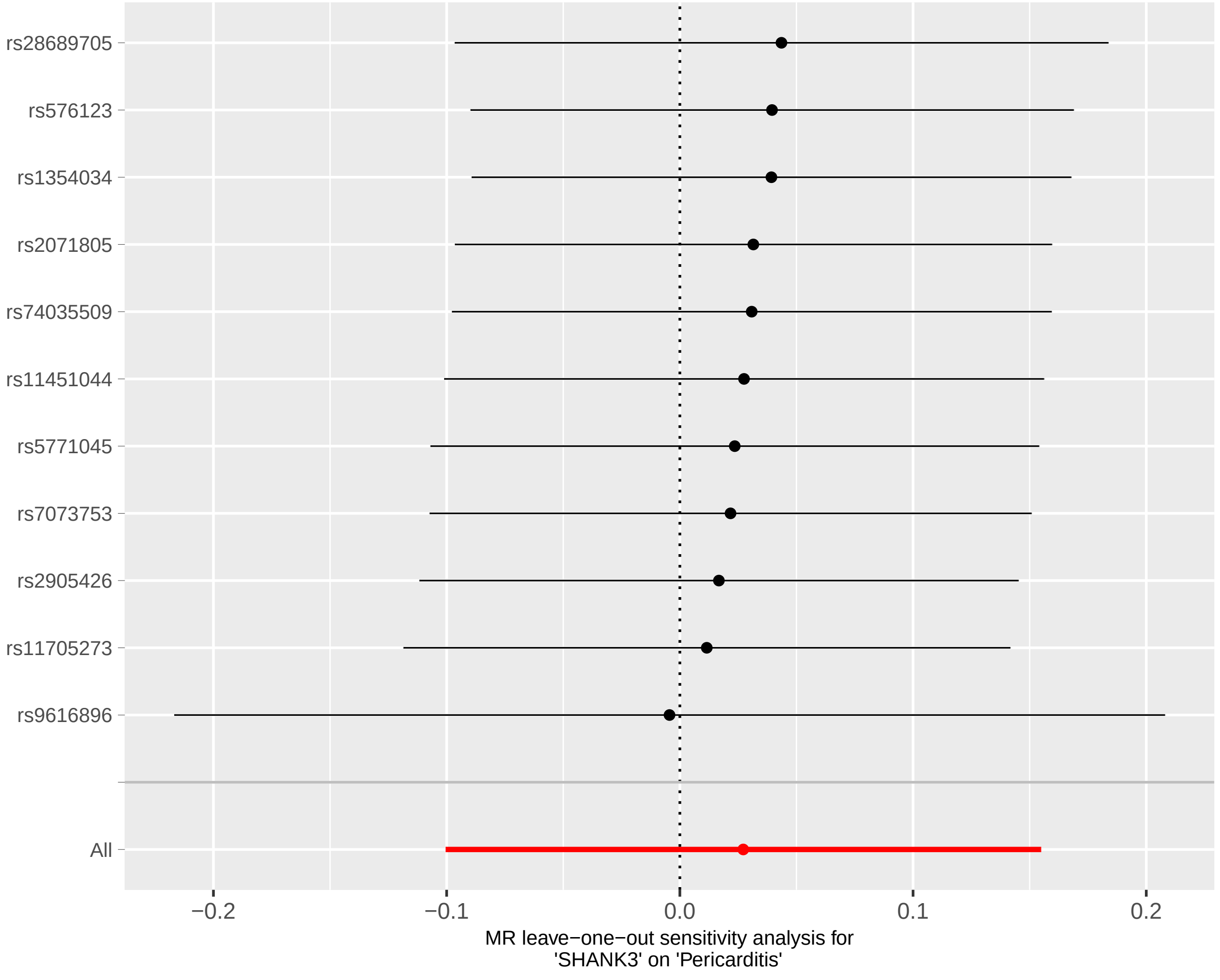
rs28689705
rs576123
rs1354034
rs2071805
rs74035509
rs11451044
rs5771045
rs7073753
rs2905426
rs11705273
rs9616896
All
−0.2
−0.1
0.0
0.1
0.2
MR leave−one−out sensitivity analysis for
'SHANK3' on 'Pericarditis'

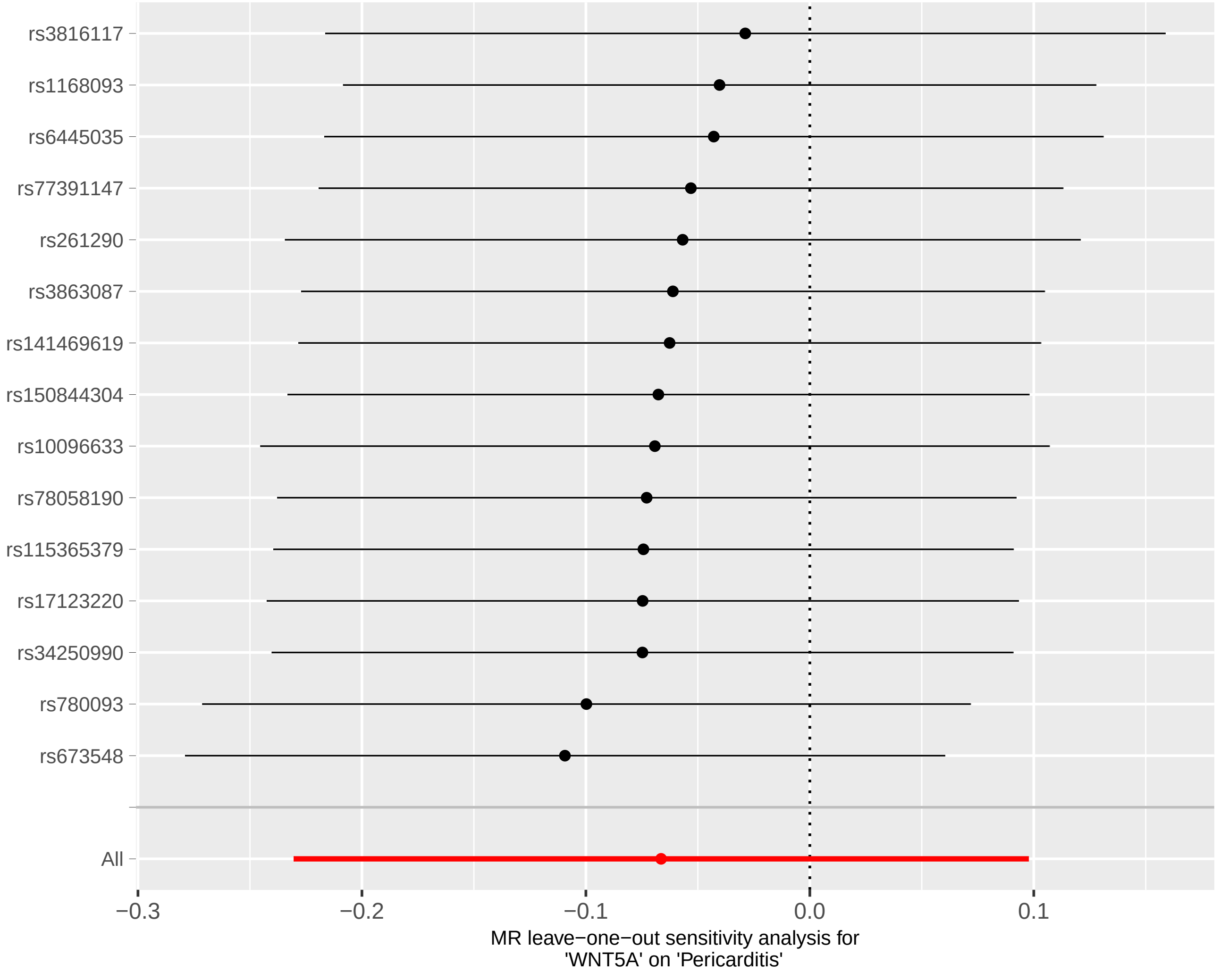

rs3816117
rs1168093
rs6445035
rs77391147
rs261290
rs3863087
rs141469619
rs150844304
rs10096633
rs78058190
rs115365379
rs17123220
rs34250990
rs780093
rs673548
All
−0.3
−0.2
−0.1
0.0
0.1
MR leave−one−out sensitivity analysis for
'WNT5A' on 'Pericarditis'

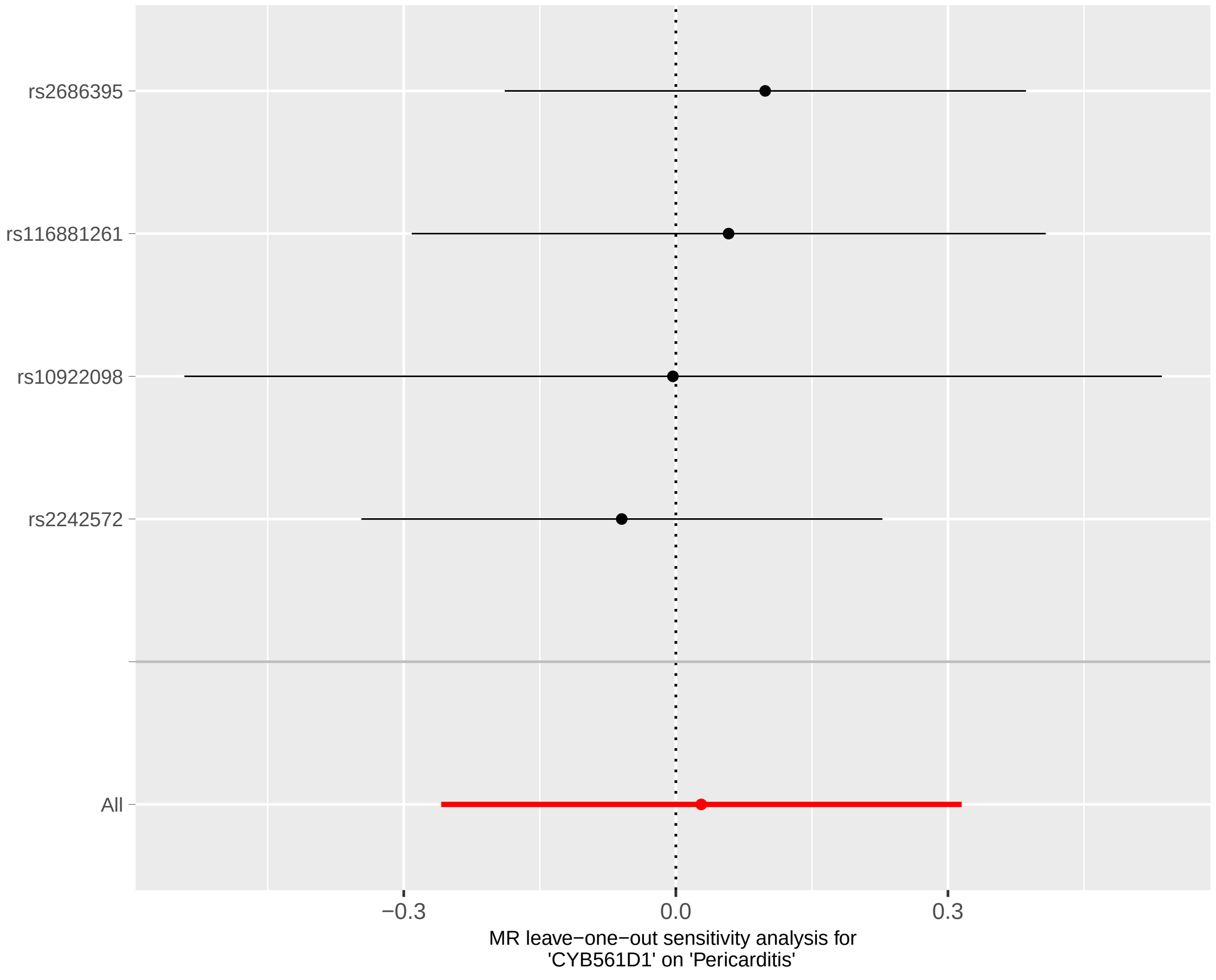
rs2686395
rs116881261
rs10922098
rs2242572
All
−0.3
0.0
0.3
MR leave−one−out sensitivity analysis for
'CYB561D1' on 'Pericarditis'

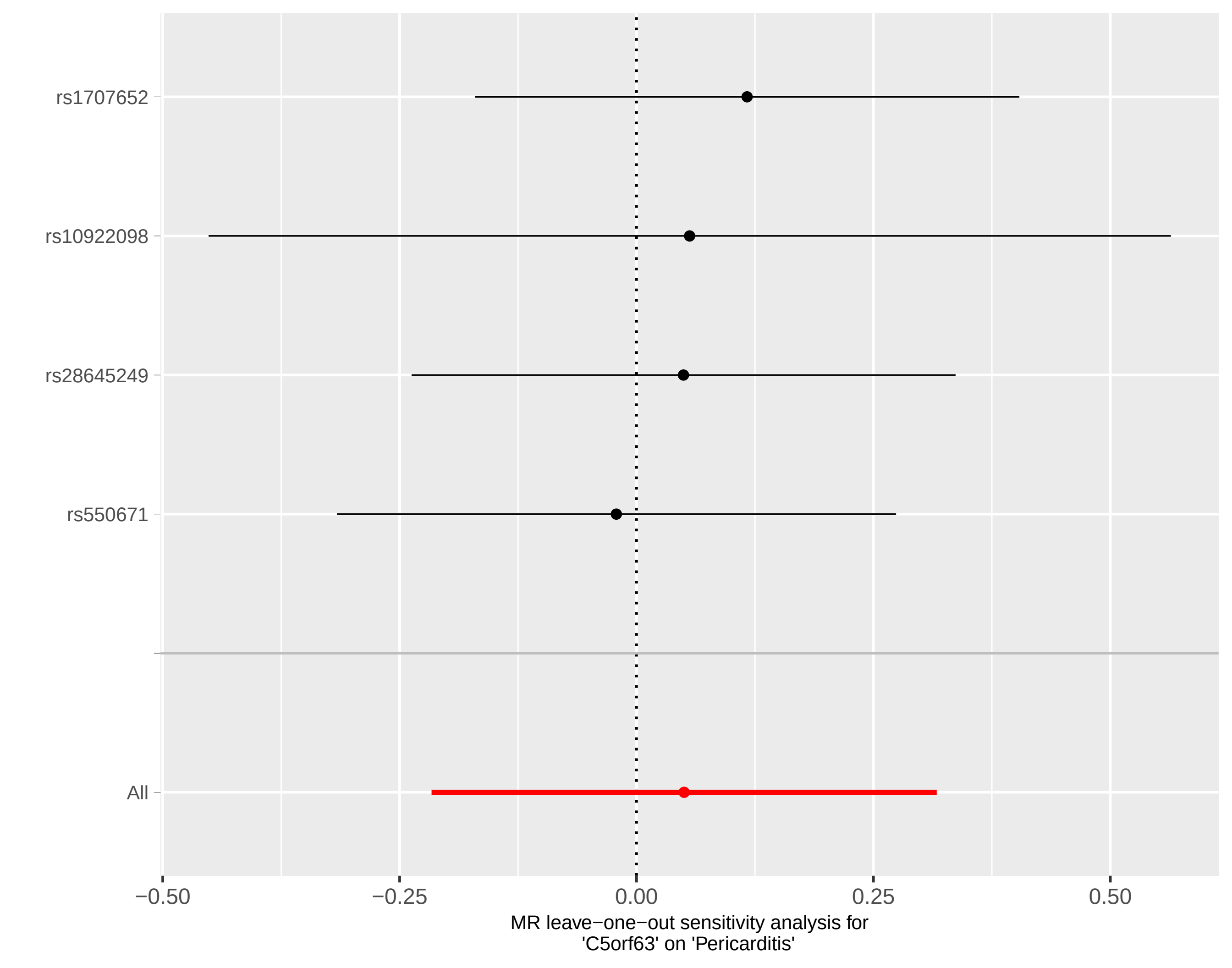
rs1707652
rs10922098
rs28645249
rs550671
All
−0.50
−0.25
0.00
0.25
0.50
MR leave−one−out sensitivity analysis for
'C5orf63' on 'Pericarditis'

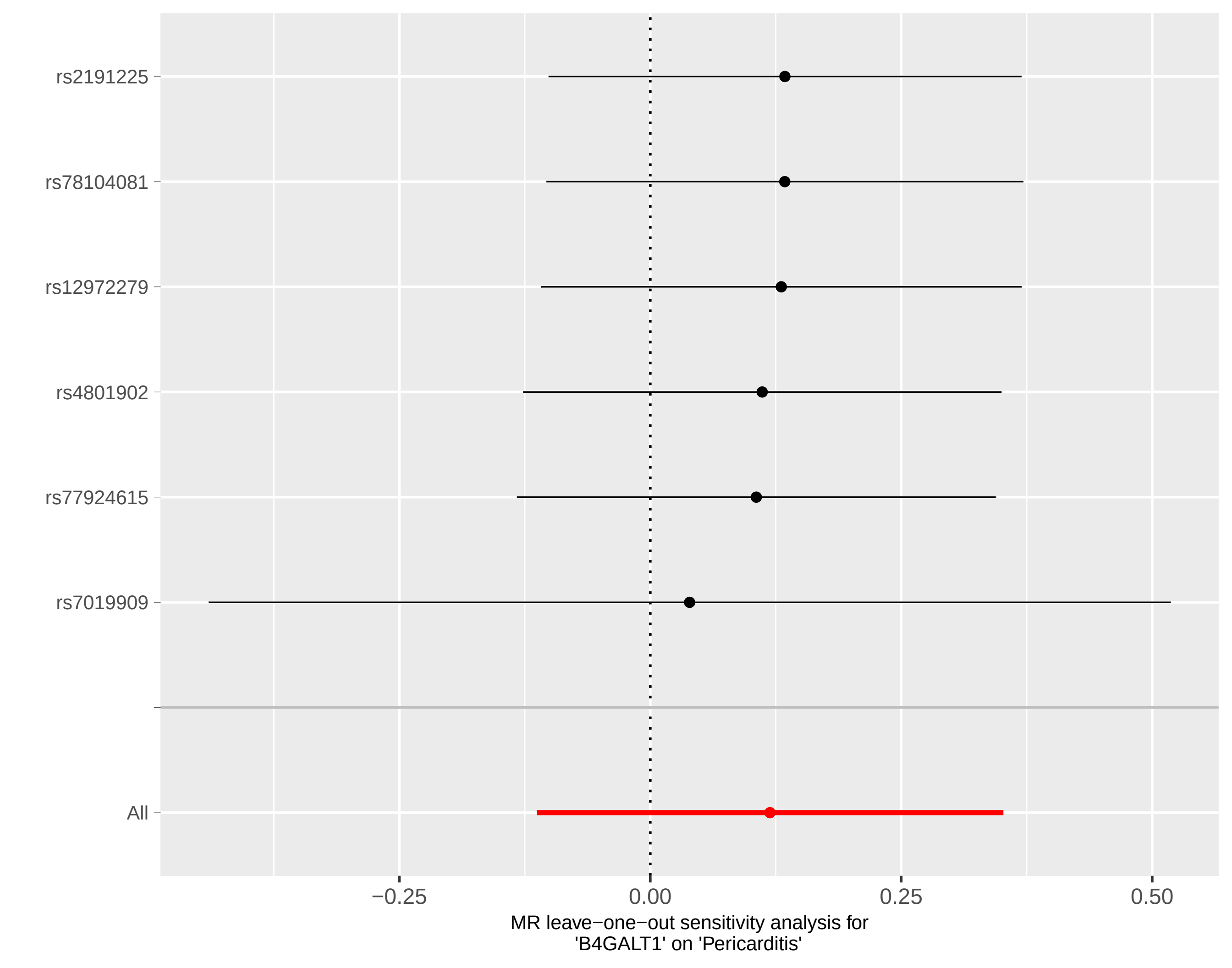
rs2191225
rs78104081
rs12972279
rs4801902
rs77924615
rs7019909
All
−0.25
0.00
0.25
0.50
MR leave−one−out sensitivity analysis for
'B4GALT1' on 'Pericarditis'

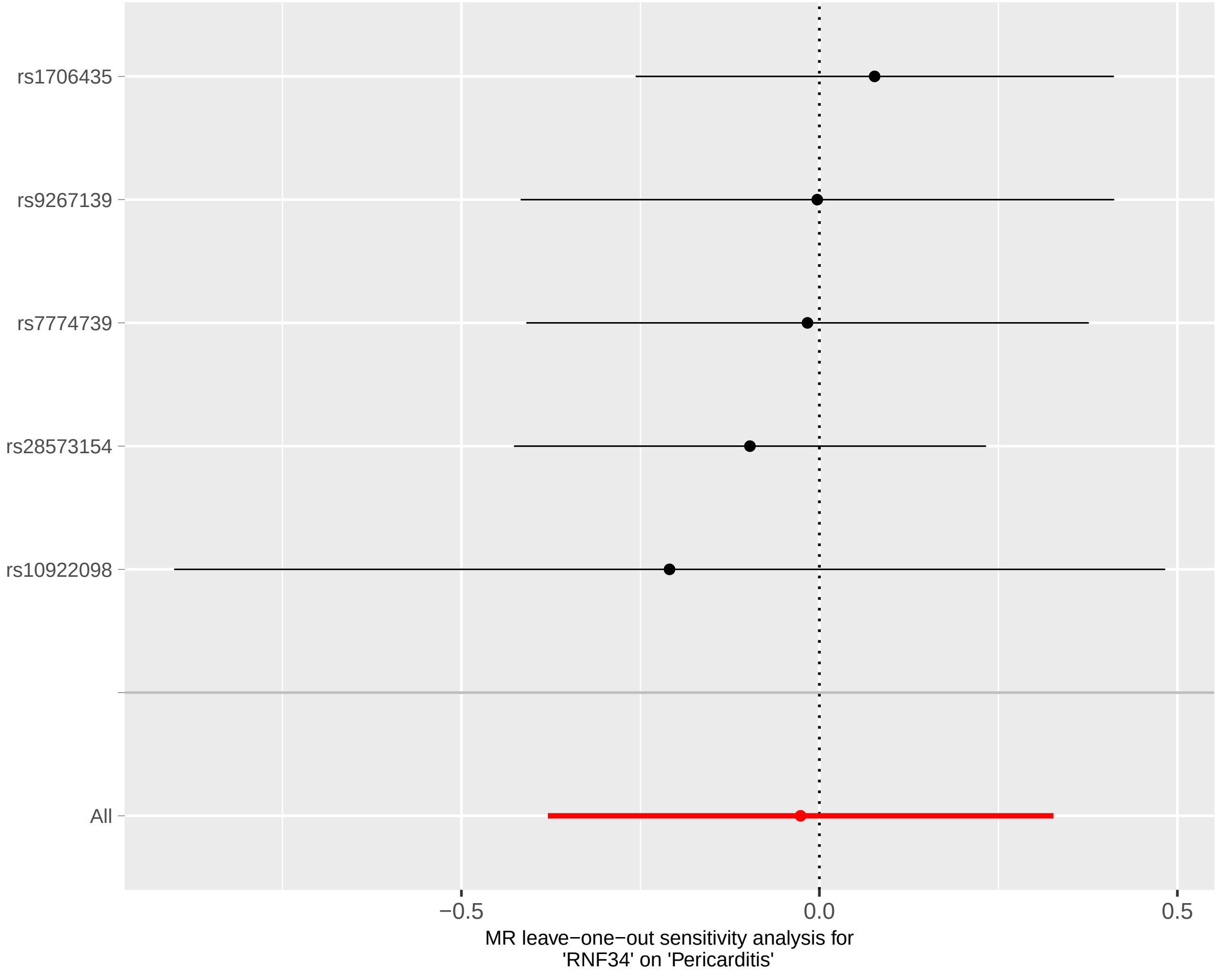
rs1706435
rs9267139
rs7774739
rs28573154
rs10922098
All
−0.5
0.0
0.5
MR leave−one−out sensitivity analysis for
'RNF34' on 'Pericarditis'

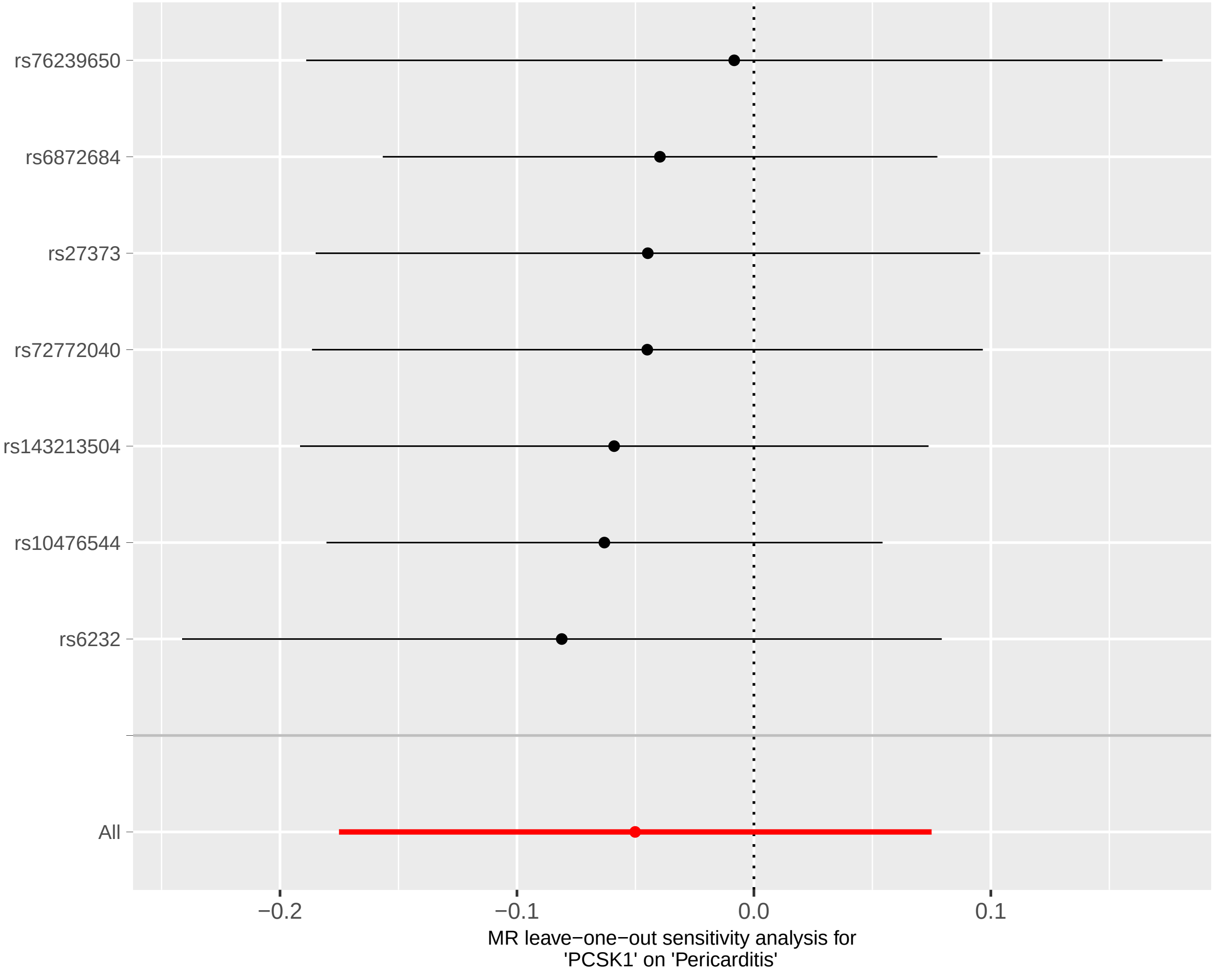
rs76239650
rs6872684
rs27373
rs72772040
rs143213504
rs10476544
rs6232
All
−0.2
−0.1
0.0
0.1
MR leave−one−out sensitivity analysis for
'PCSK1' on 'Pericarditis'

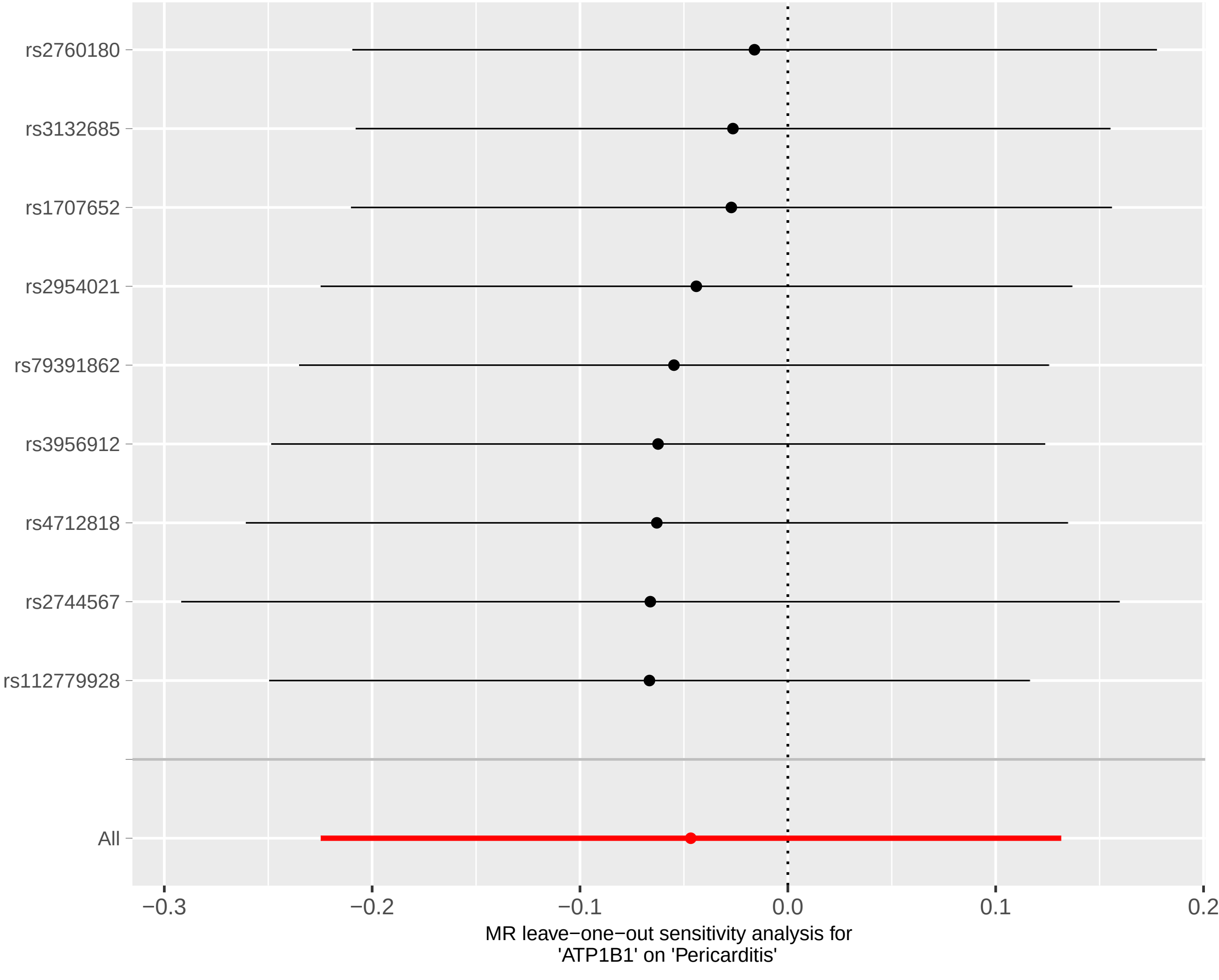

rs2760180
rs3132685
rs1707652
rs2954021
rs79391862
rs3956912
rs4712818
rs2744567
rs112779928
All
−0.3
−0.2
−0.1
0.0
0.1
0.2
MR leave−one−out sensitivity analysis for
'ATP1B1' on 'Pericarditis'

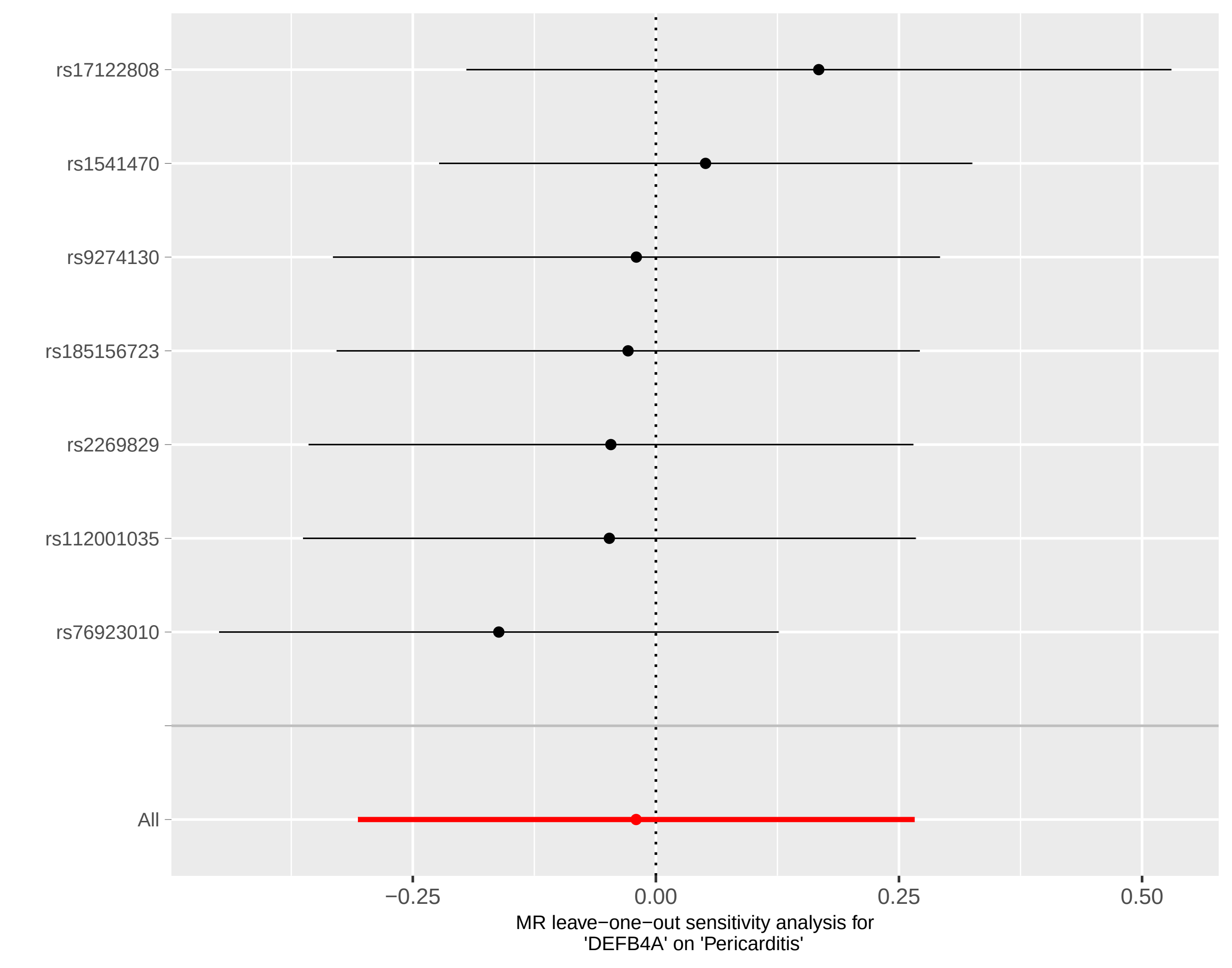

rs17122808
rs1541470
rs9274130
rs185156723
rs2269829
rs112001035
rs76923010
All
−0.25
0.00
0.25
0.50
MR leave−one−out sensitivity analysis for
'DEFB4A' on 'Pericarditis'

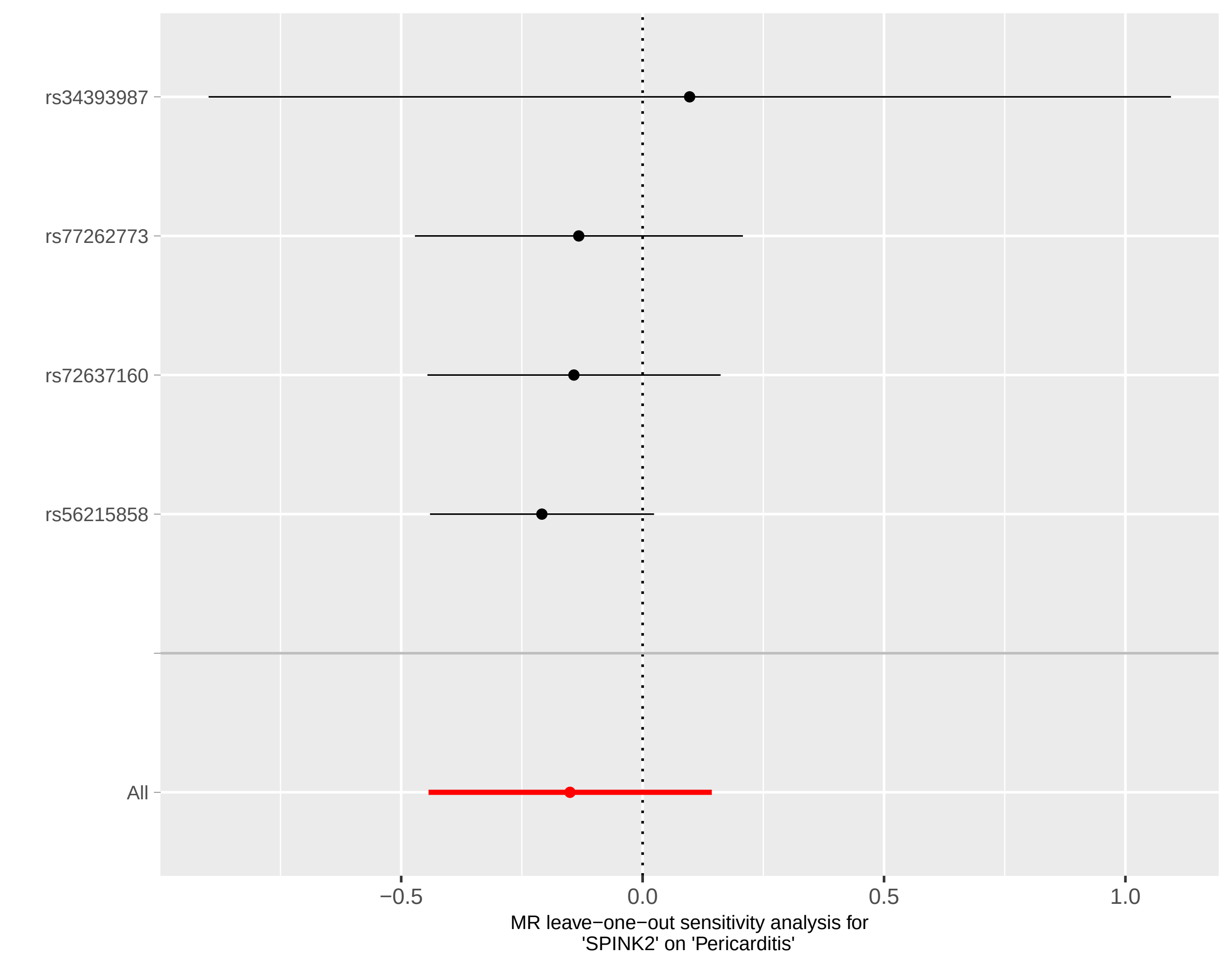
rs34393987
rs77262773
rs72637160
rs56215858
All
−0.5
0.0
0.5
1.0
MR leave−one−out sensitivity analysis for
'SPINK2' on 'Pericarditis'

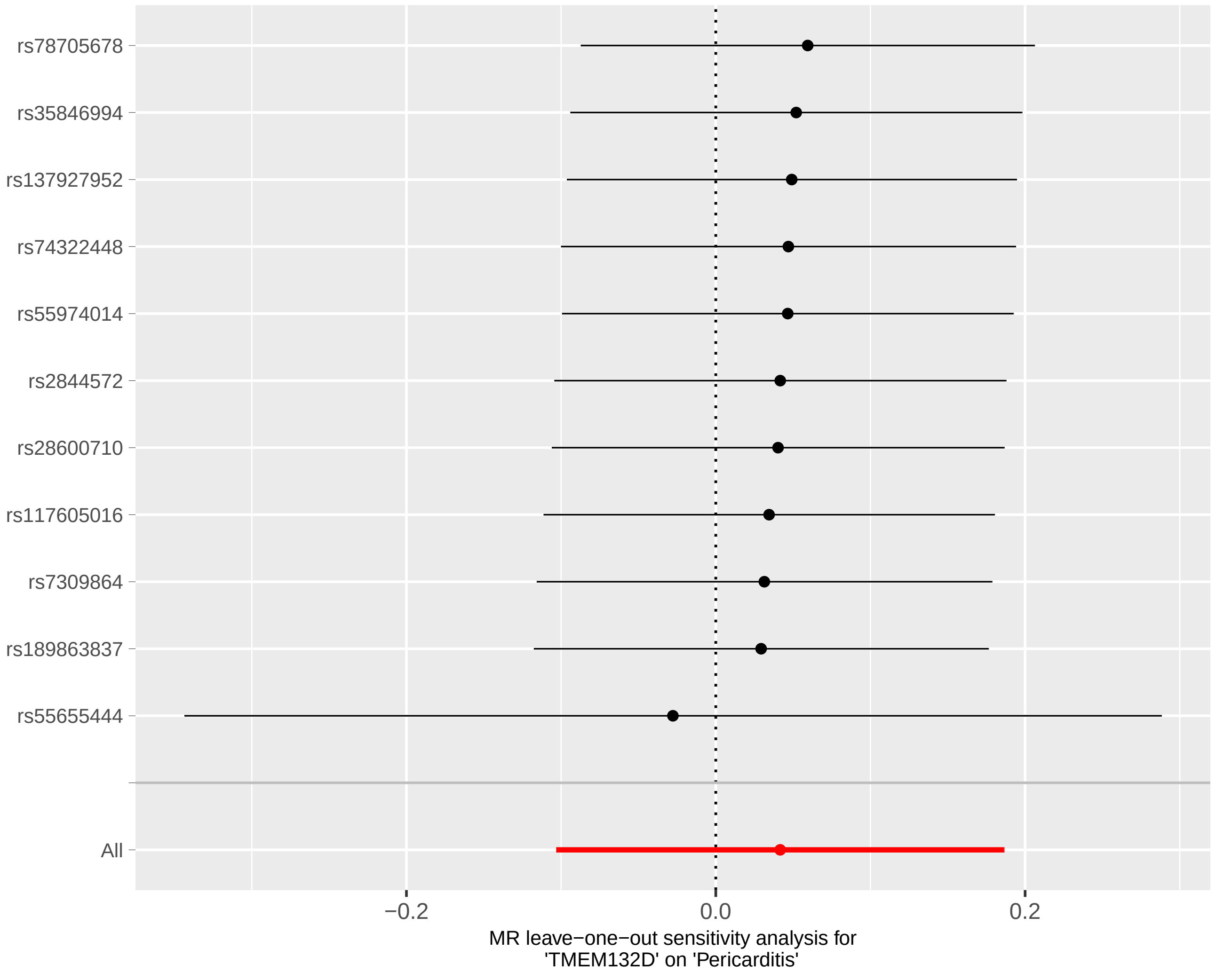

rs78705678
rs35846994
rs137927952
rs74322448
rs55974014
rs2844572
rs28600710
rs117605016
rs7309864
rs189863837
rs55655444
All
−0.2
0.0
0.2
MR leave−one−out sensitivity analysis for
'TMEM132D' on 'Pericarditis'

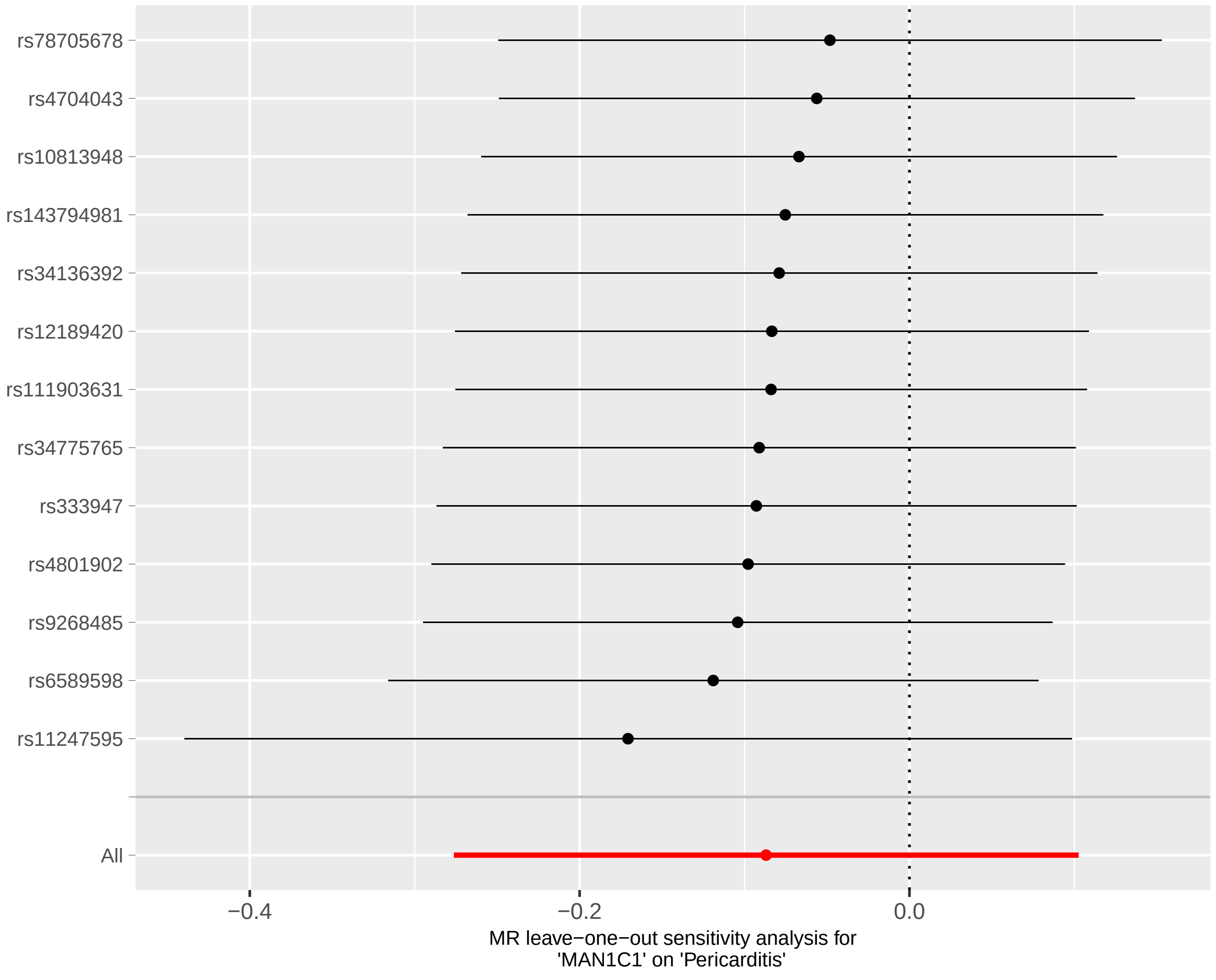
rs78705678
rs4704043
rs10813948
rs143794981
rs34136392
rs12189420
rs111903631
rs34775765
rs333947
rs4801902
rs9268485
rs6589598
rs11247595
All
−0.4
−0.2
0.0
MR leave−one−out sensitivity analysis for
'MAN1C1' on 'Pericarditis'

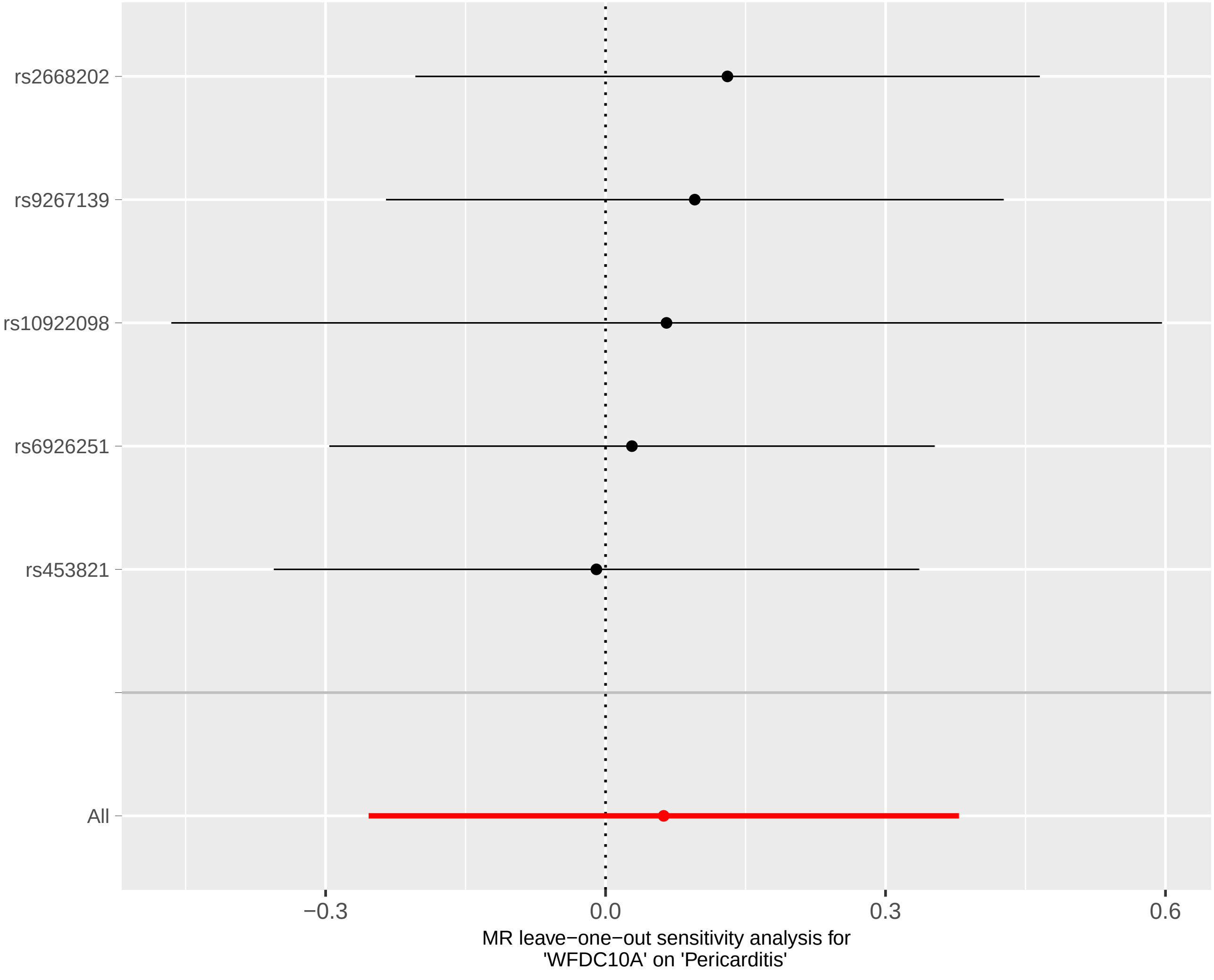

rs2668202
rs9267139
rs10922098
rs6926251
rs453821
All
−0.3
0.0
0.3
0.6
MR leave−one−out sensitivity analysis for
'WFDC10A' on 'Pericarditis'

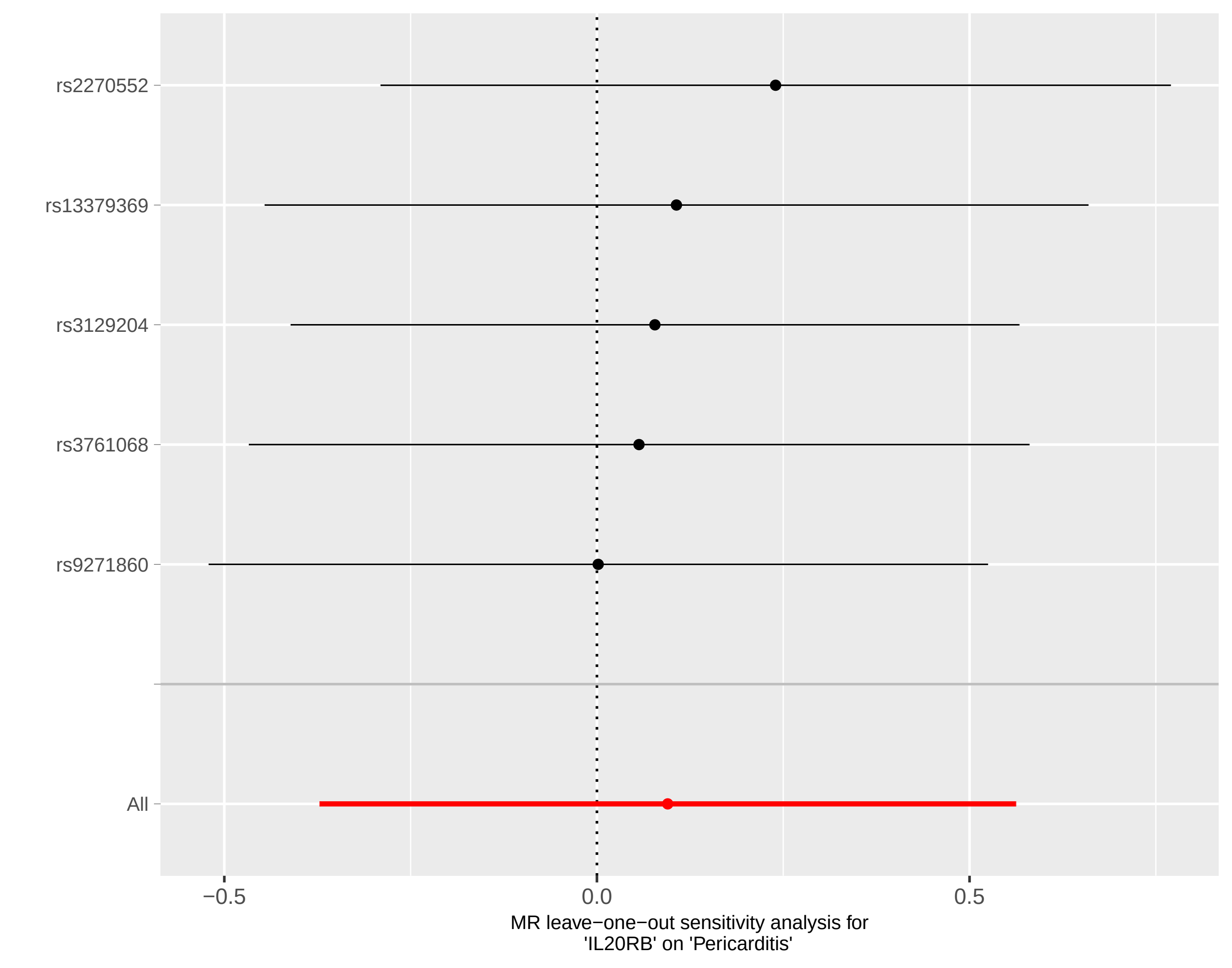

rs2270552
rs13379369
rs3129204
rs3761068
rs9271860
All
−0.5
0.0
0.5
MR leave−one−out sensitivity analysis for
'IL20RB' on 'Pericarditis'

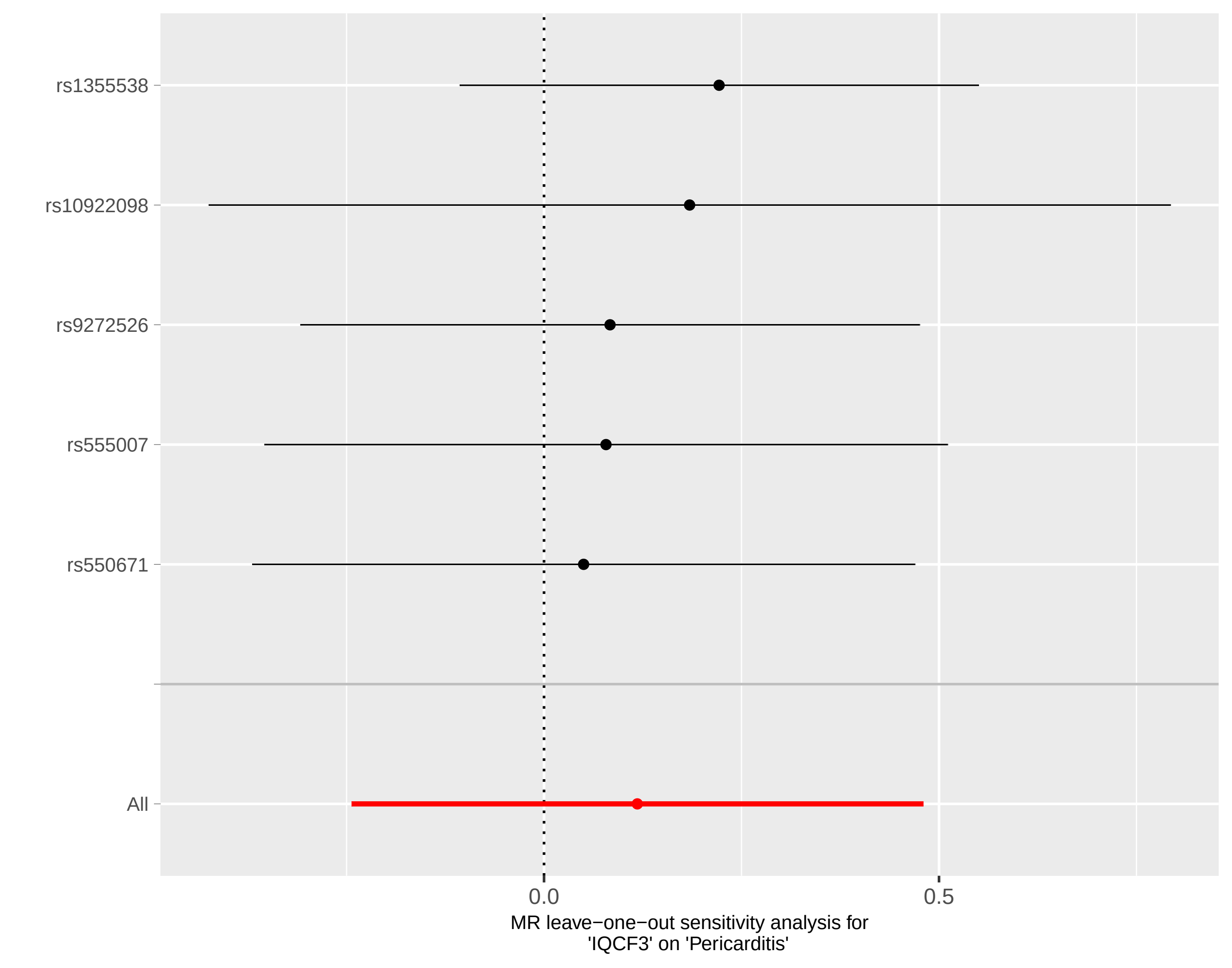
rs1355538
rs10922098
rs9272526
rs555007
rs550671
All
0.0
0.5
MR leave−one−out sensitivity analysis for
'IQCF3' on 'Pericarditis'

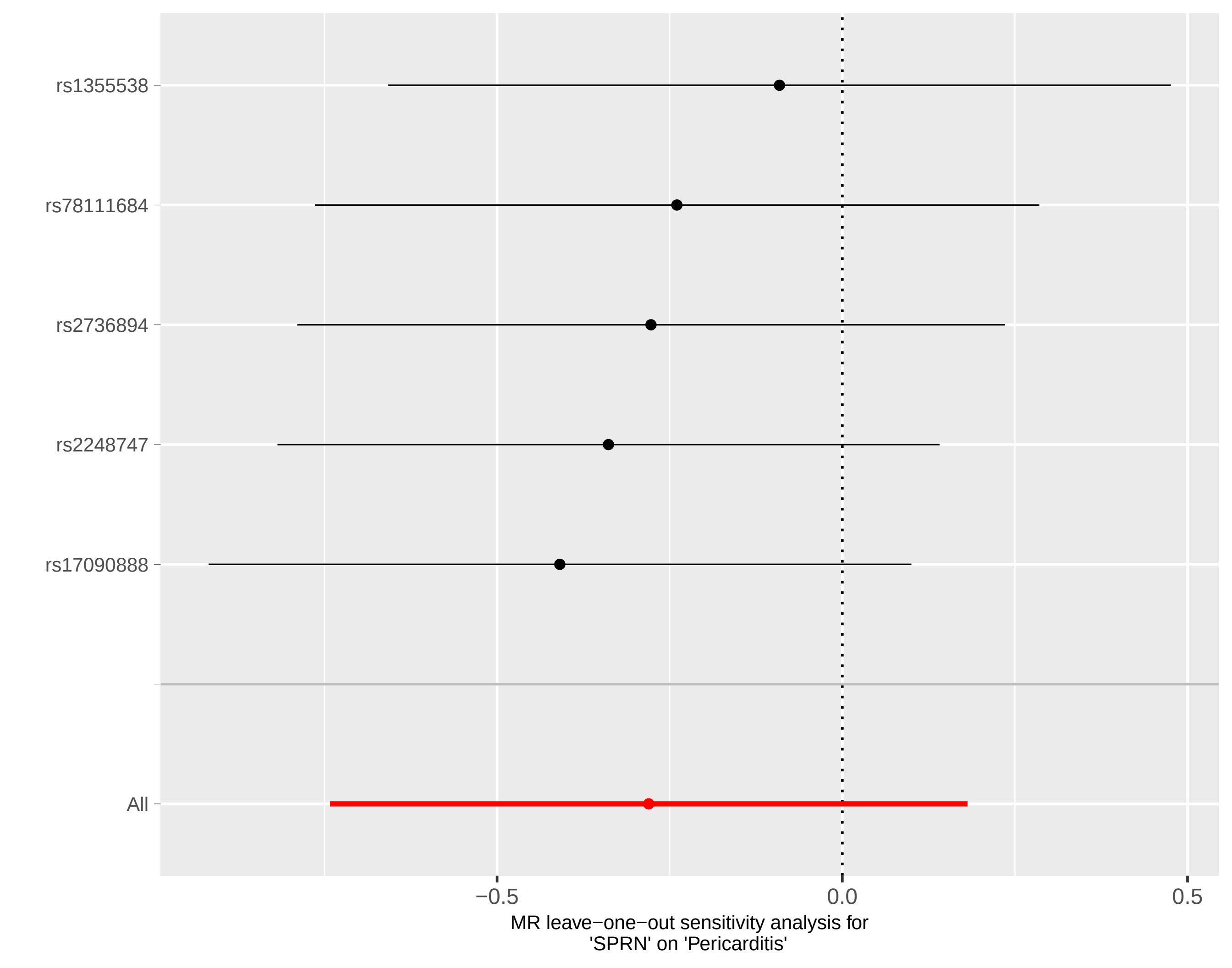
rs1355538
rs78111684
rs2736894
rs2248747
rs17090888
All
−0.5
0.0
0.5
MR leave−one−out sensitivity analysis for
'SPRN' on 'Pericarditis'

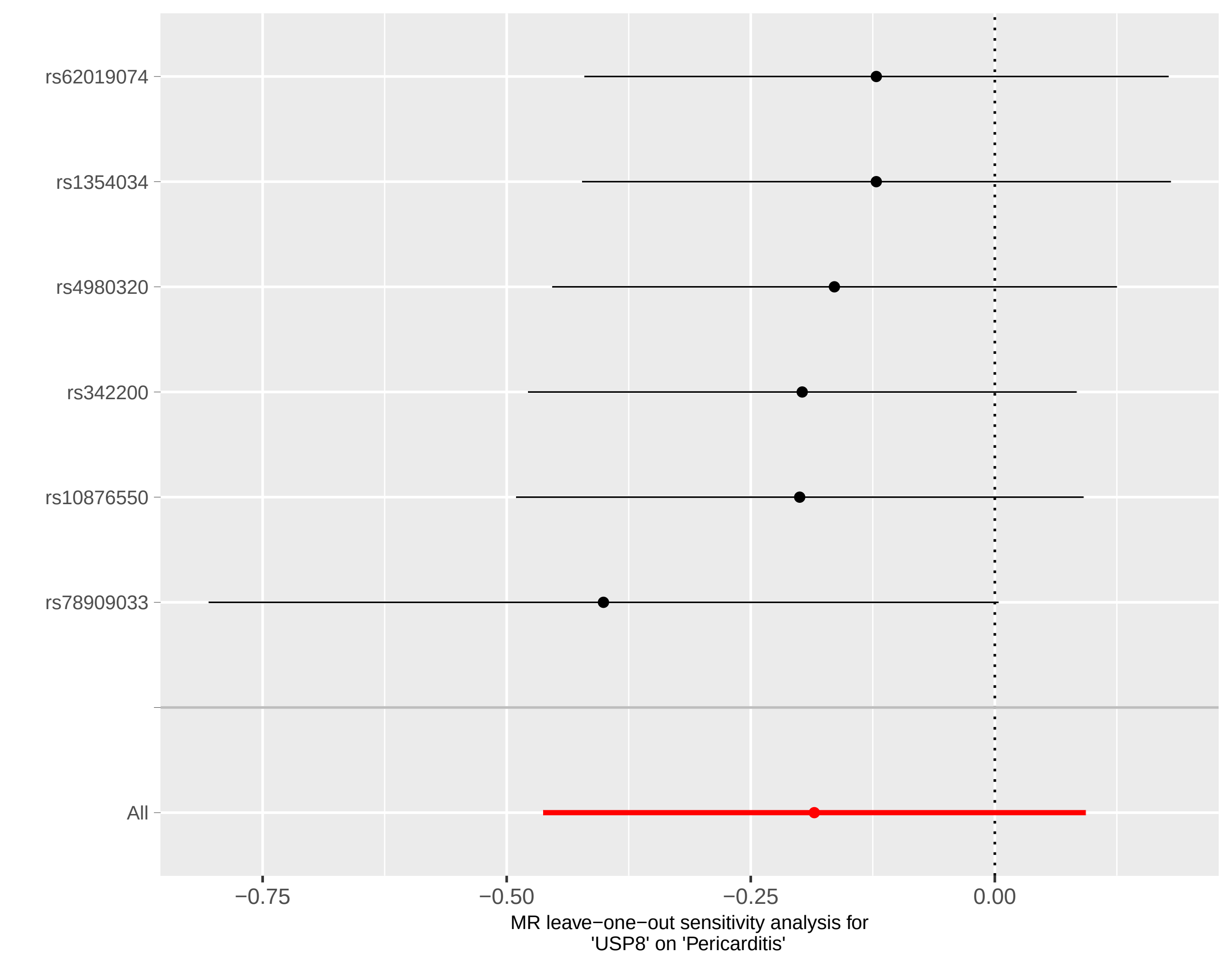

rs62019074
rs1354034
rs4980320
rs342200
rs10876550
rs78909033
All
−0.75
−0.50
−0.25
0.00
MR leave−one−out sensitivity analysis for
'USP8' on 'Pericarditis'

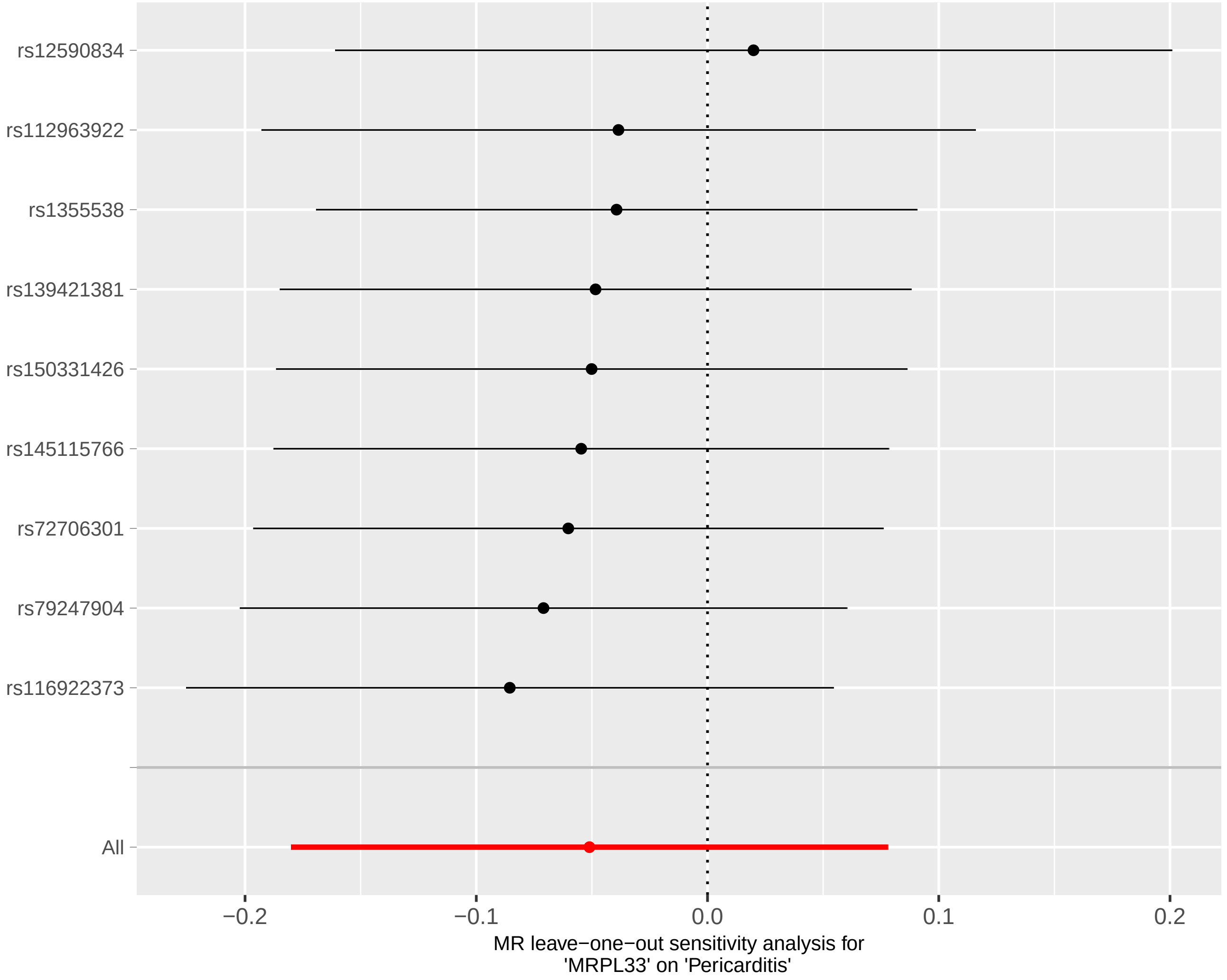

rs12590834
rs112963922
rs1355538
rs139421381
rs150331426
rs145115766
rs72706301
rs79247904
rs116922373
All
−0.2
−0.1
0.0
0.1
0.2
MR leave−one−out sensitivity analysis for
'MRPL33' on 'Pericarditis'

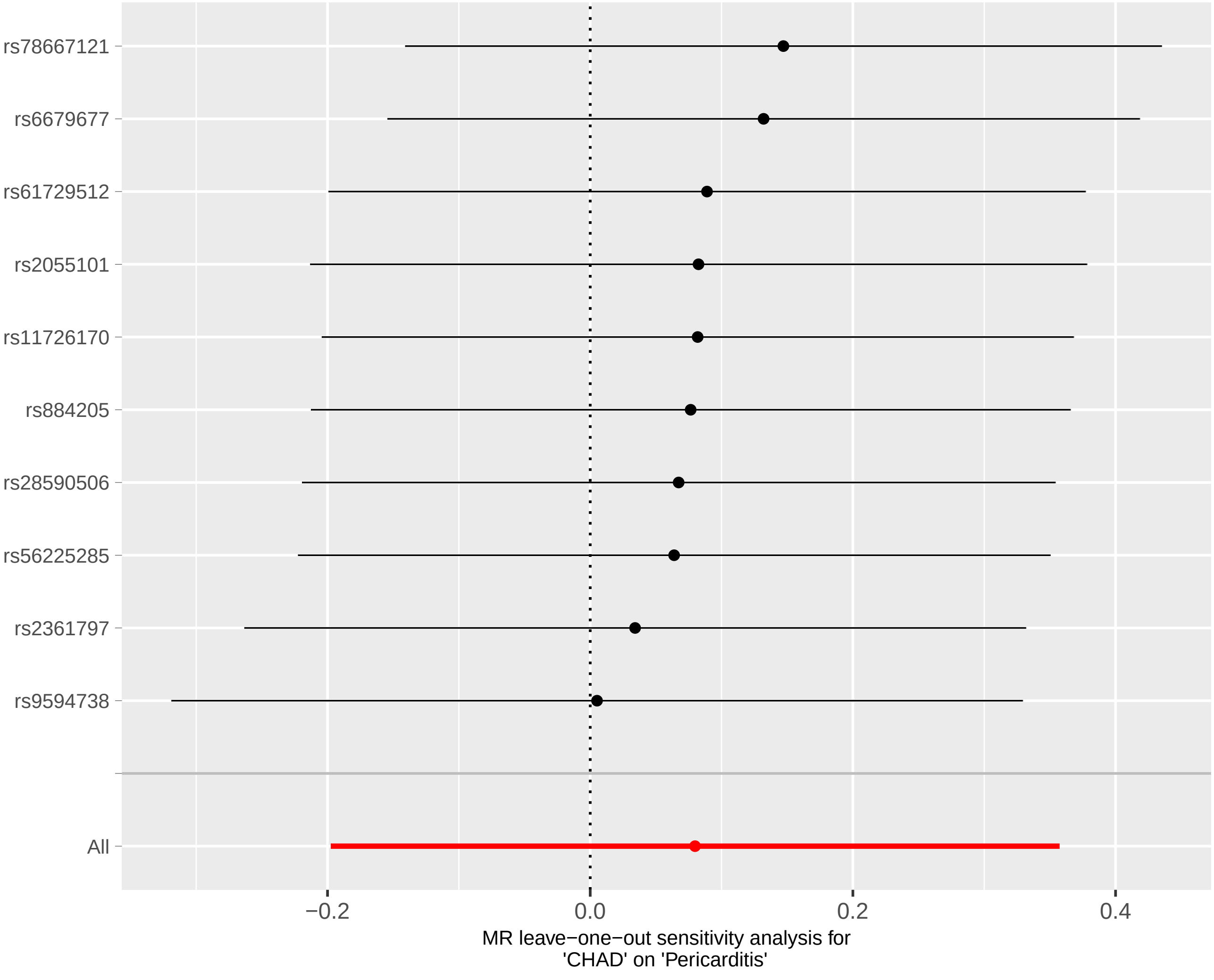
rs78667121
rs6679677
rs61729512
rs2055101
rs11726170
rs884205
rs28590506
rs56225285
rs2361797
rs9594738
All
−0.2
0.0
0.2
0.4
MR leave−one−out sensitivity analysis for
'CHAD' on 'Pericarditis'

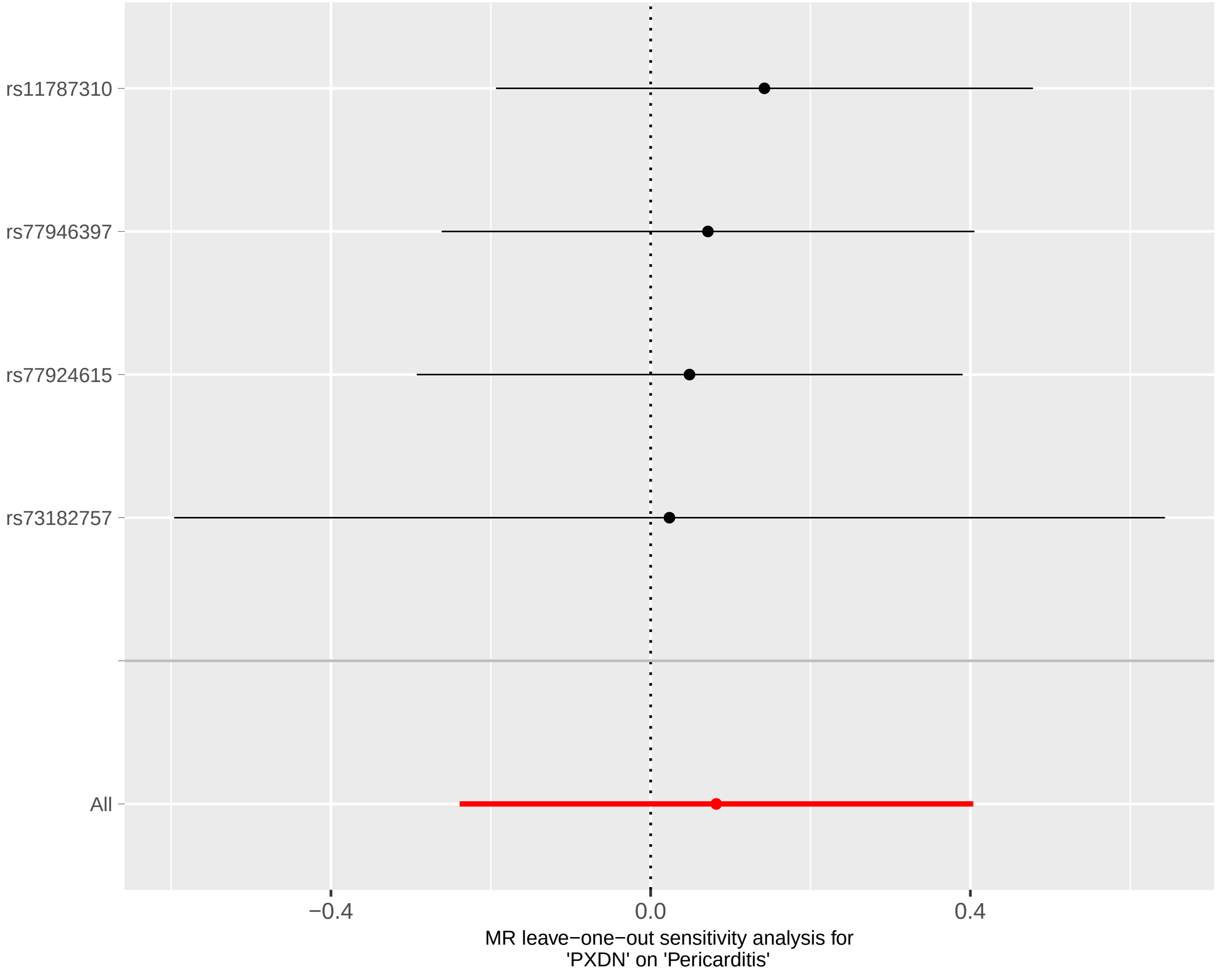

rs11787310
rs77946397
rs77924615
rs73182757
All
−0.4
0.0
0.4
MR leave−one−out sensitivity analysis for
'PXDN' on 'Pericarditis'

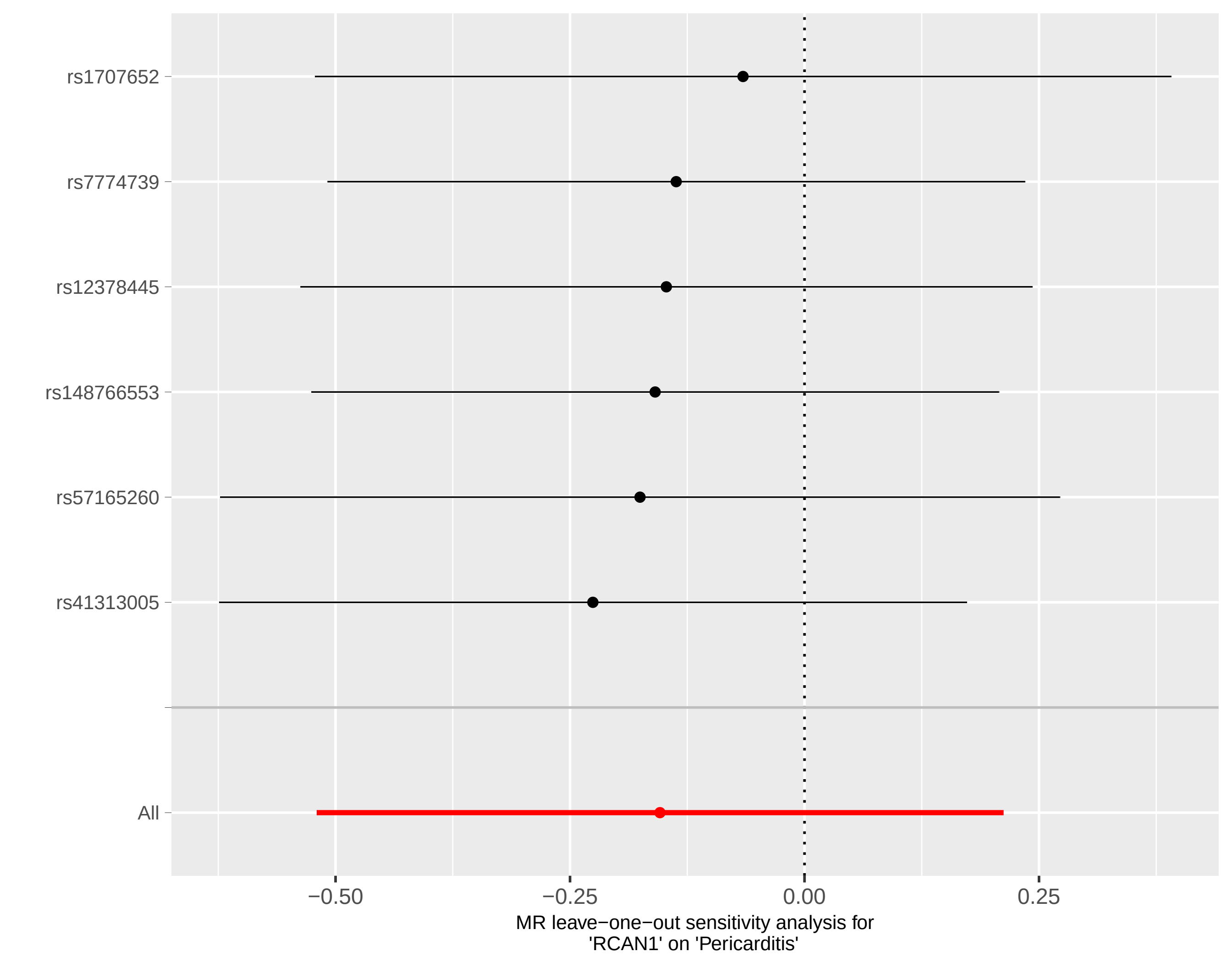
rs1707652
rs7774739
rs12378445
rs148766553
rs57165260
rs41313005
All
−0.50
−0.25
0.00
0.25
MR leave−one−out sensitivity analysis for
'RCAN1' on 'Pericarditis'

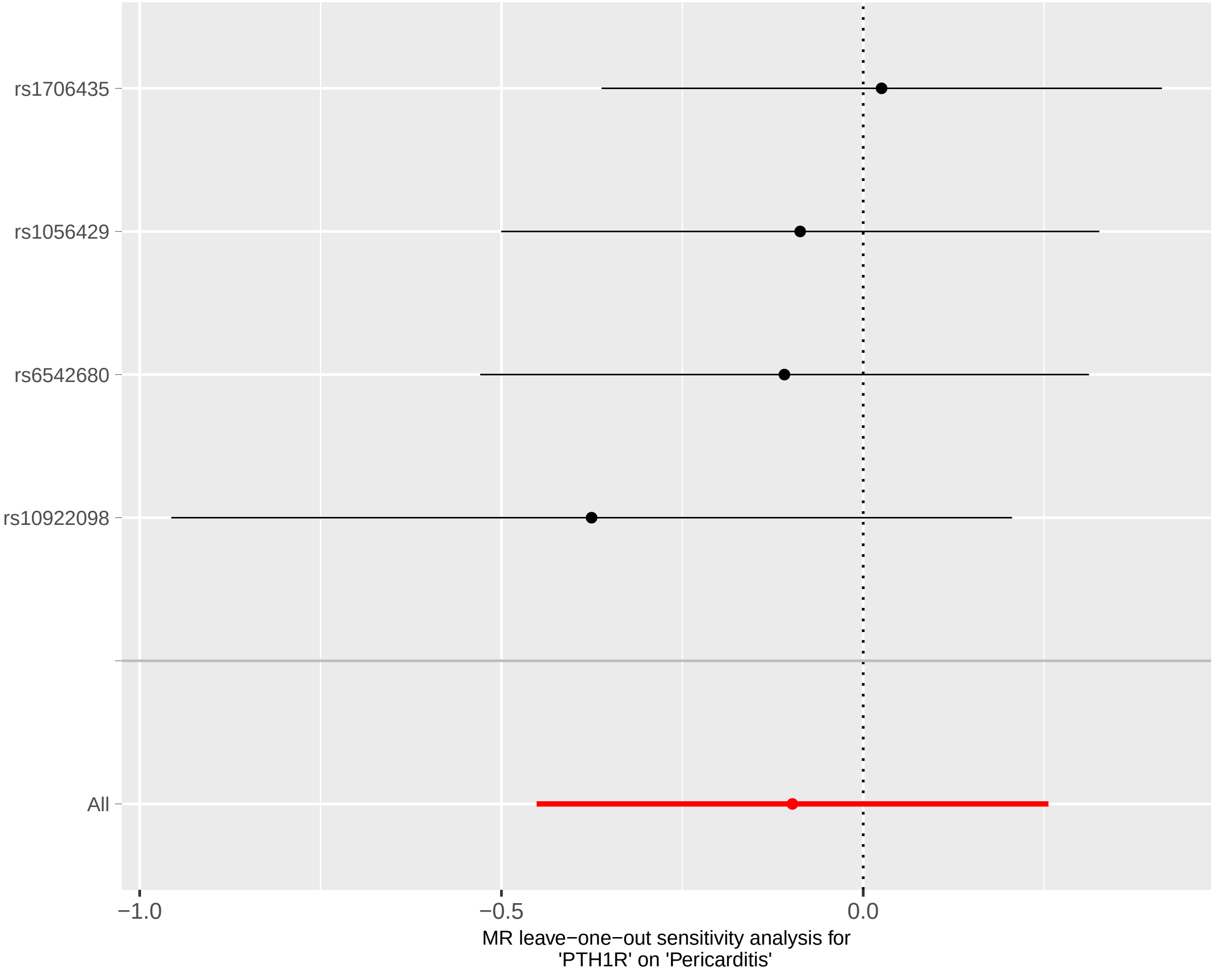
rs1706435
rs1056429
rs6542680
rs10922098
All
−1.0
−0.5
0.0
MR leave−one−out sensitivity analysis for
'PTH1R' on 'Pericarditis'

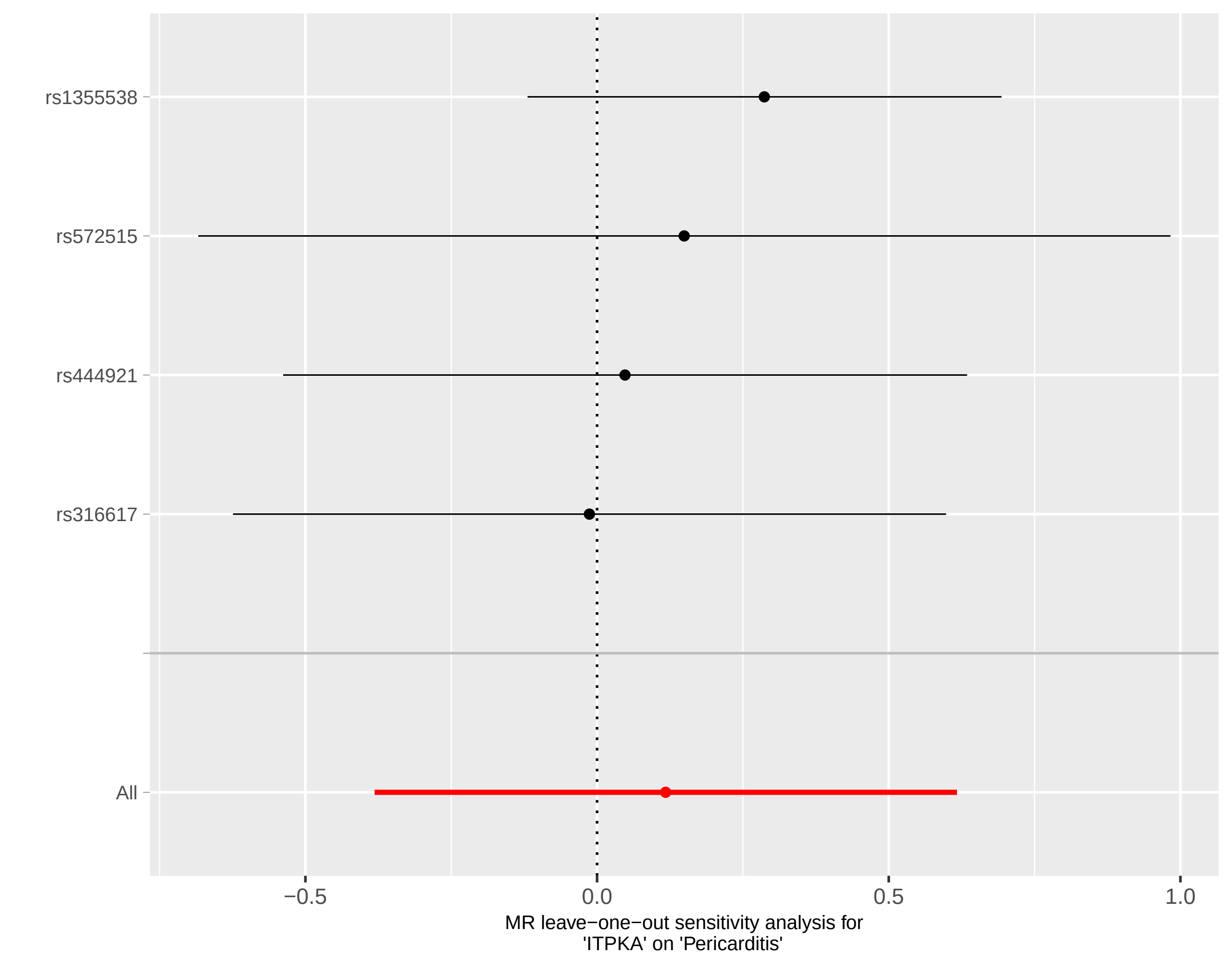
rs1355538
rs572515
rs444921
rs316617
All
−0.5
0.0
0.5
1.0
MR leave−one−out sensitivity analysis for
'ITPKA' on 'Pericarditis'

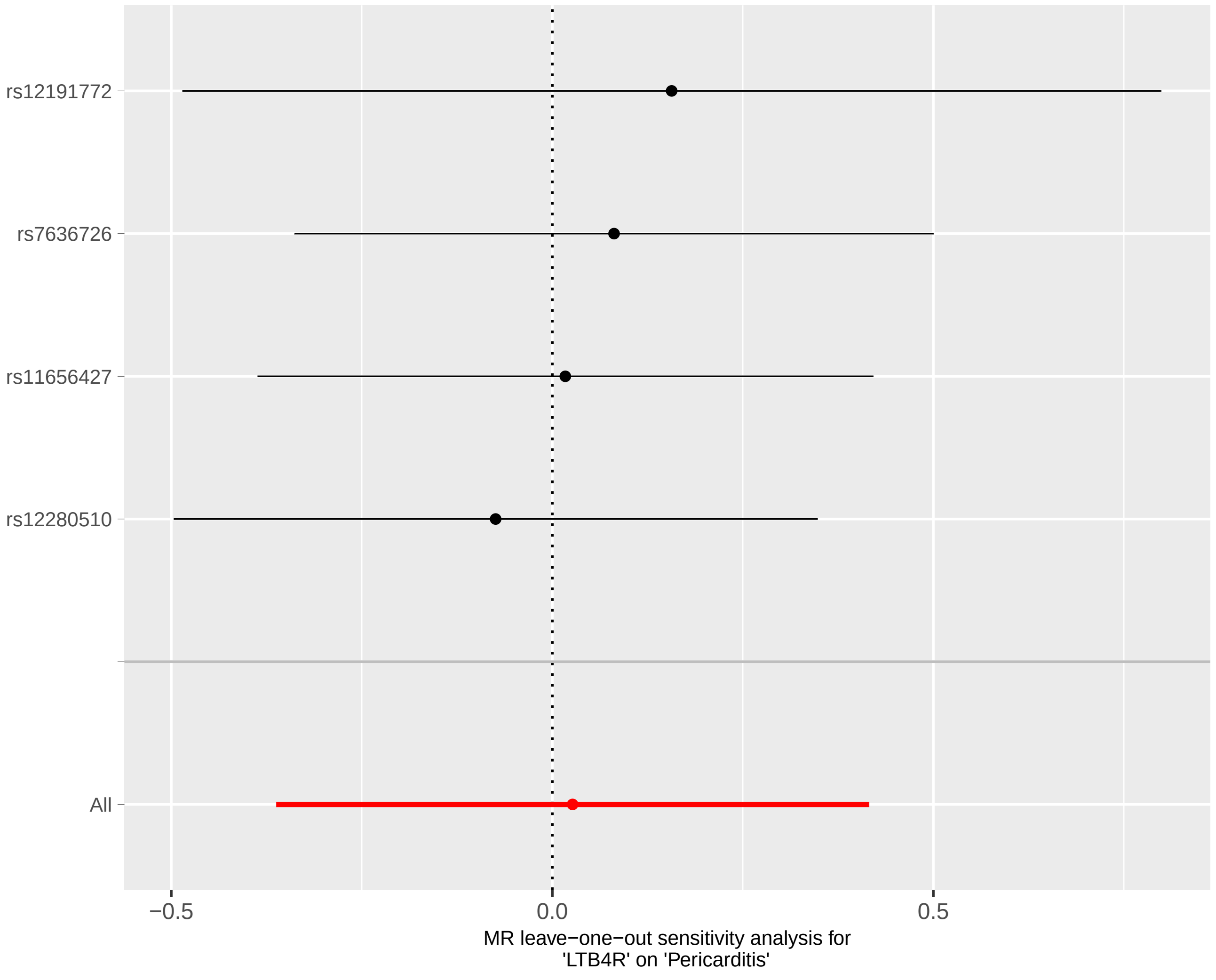
rs12191772
rs7636726
rs11656427
rs12280510
All
−0.5
0.0
0.5
MR leave−one−out sensitivity analysis for
'LTB4R' on 'Pericarditis'

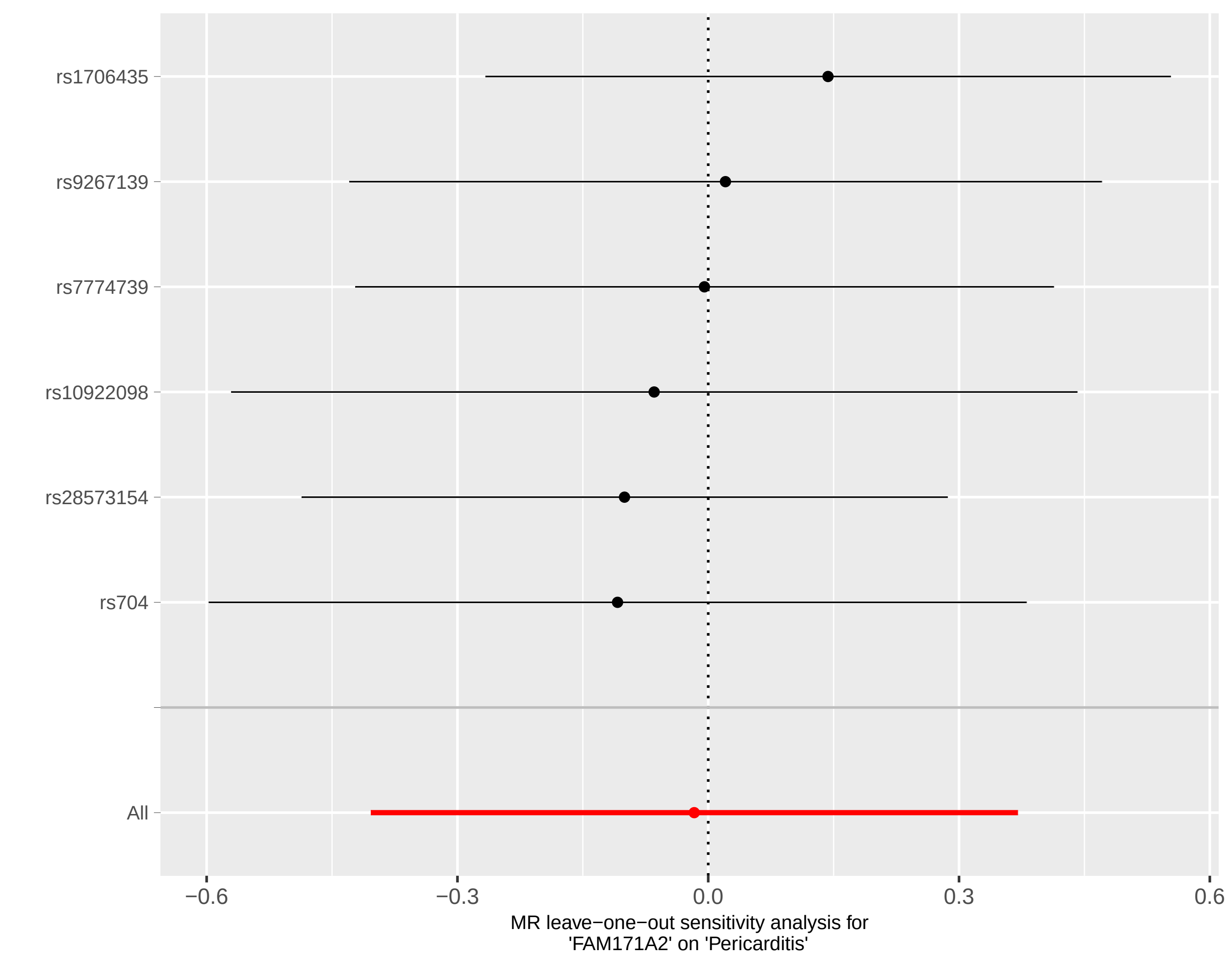
rs1706435
rs9267139
rs7774739
rs10922098
rs28573154
rs704
All
−0.6
−0.3
0.0
0.3
0.6
MR leave−one−out sensitivity analysis for
'FAM171A2' on 'Pericarditis'

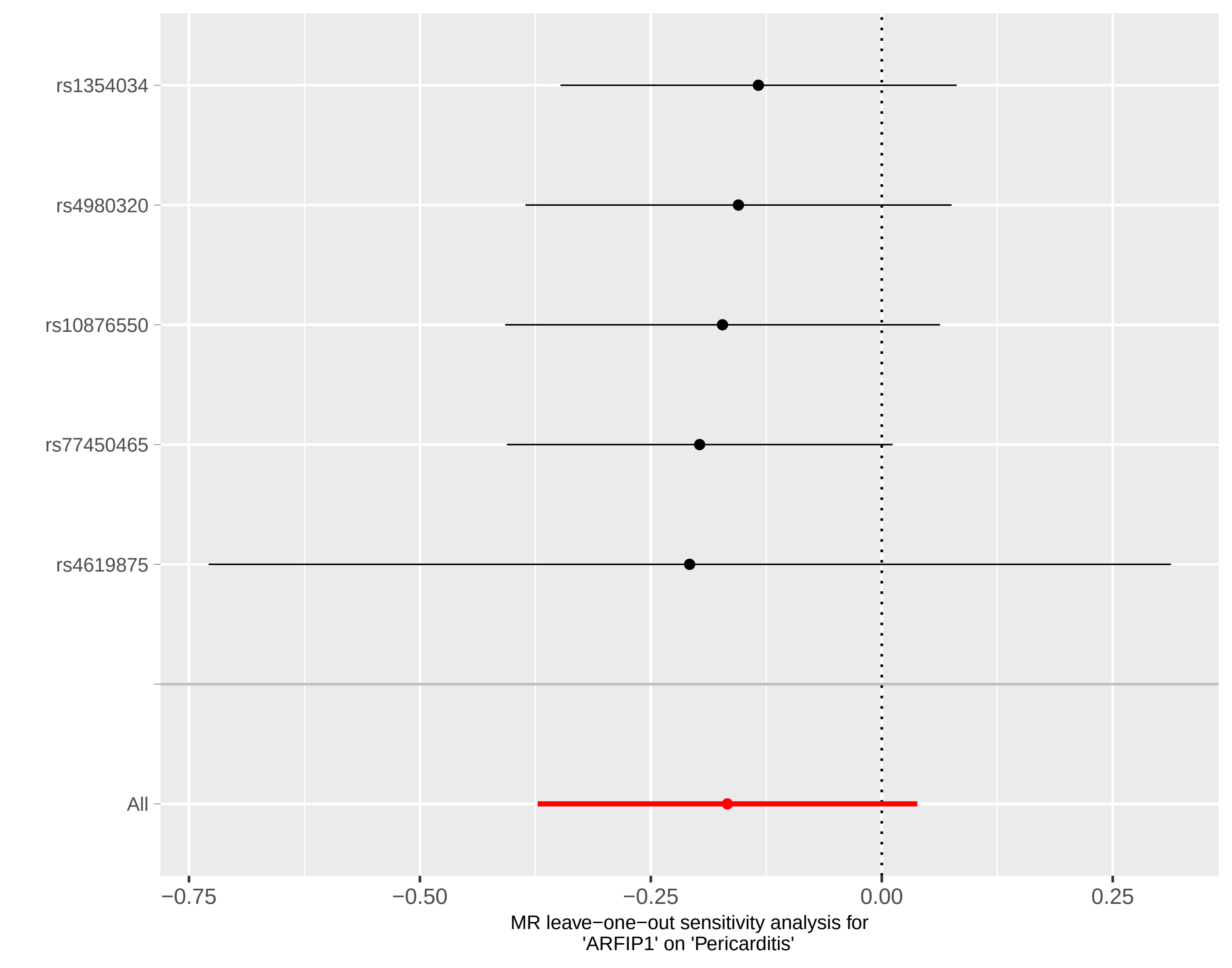
rs1354034
rs4980320
rs10876550
rs77450465
rs4619875
All
−0.75
−0.50
−0.25
0.00
0.25
MR leave−one−out sensitivity analysis for
'ARFIP1' on 'Pericarditis'

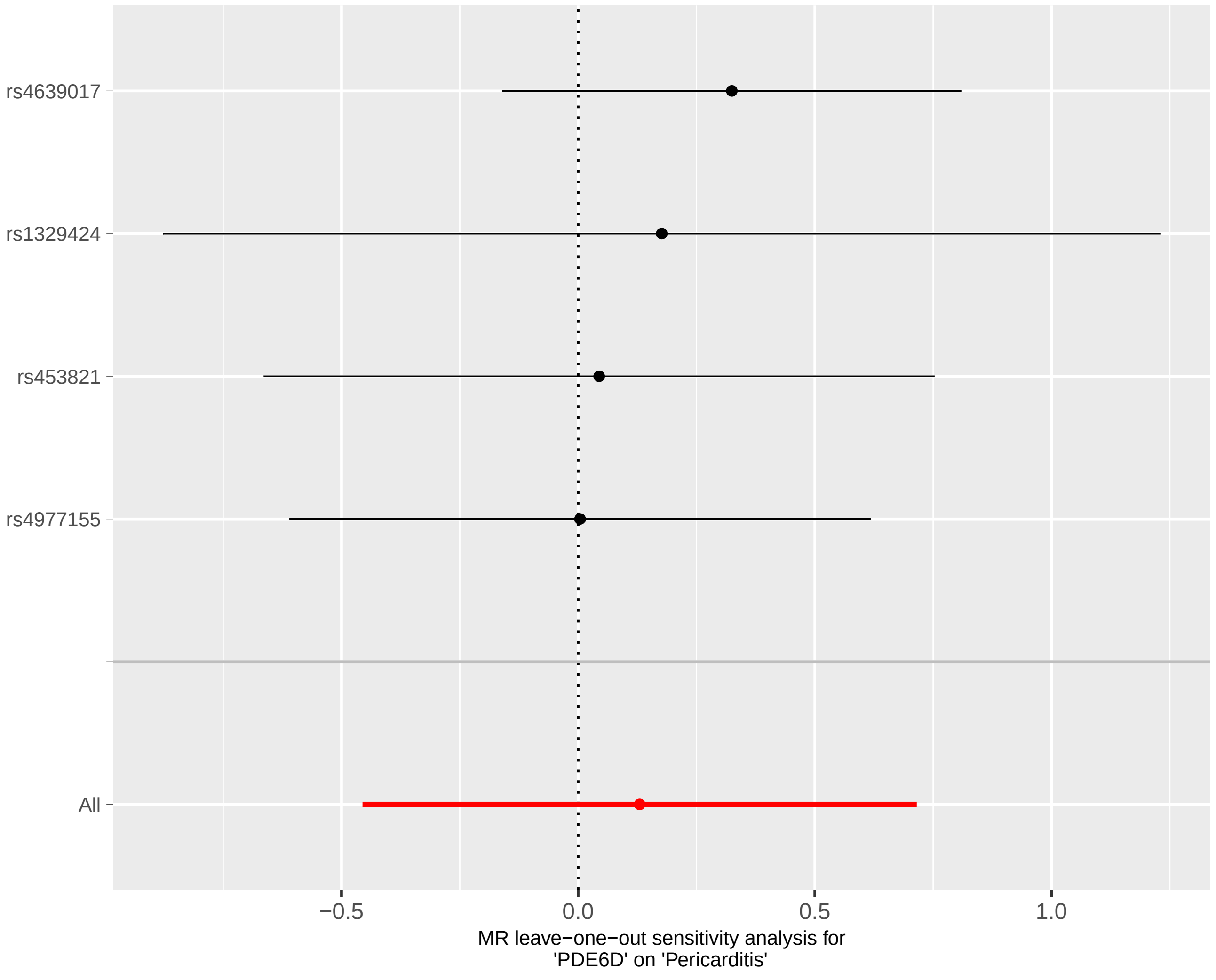

rs4639017
rs1329424
rs453821
rs4977155
All
−0.5
0.0
0.5
1.0
MR leave−one−out sensitivity analysis for
'PDE6D' on 'Pericarditis'

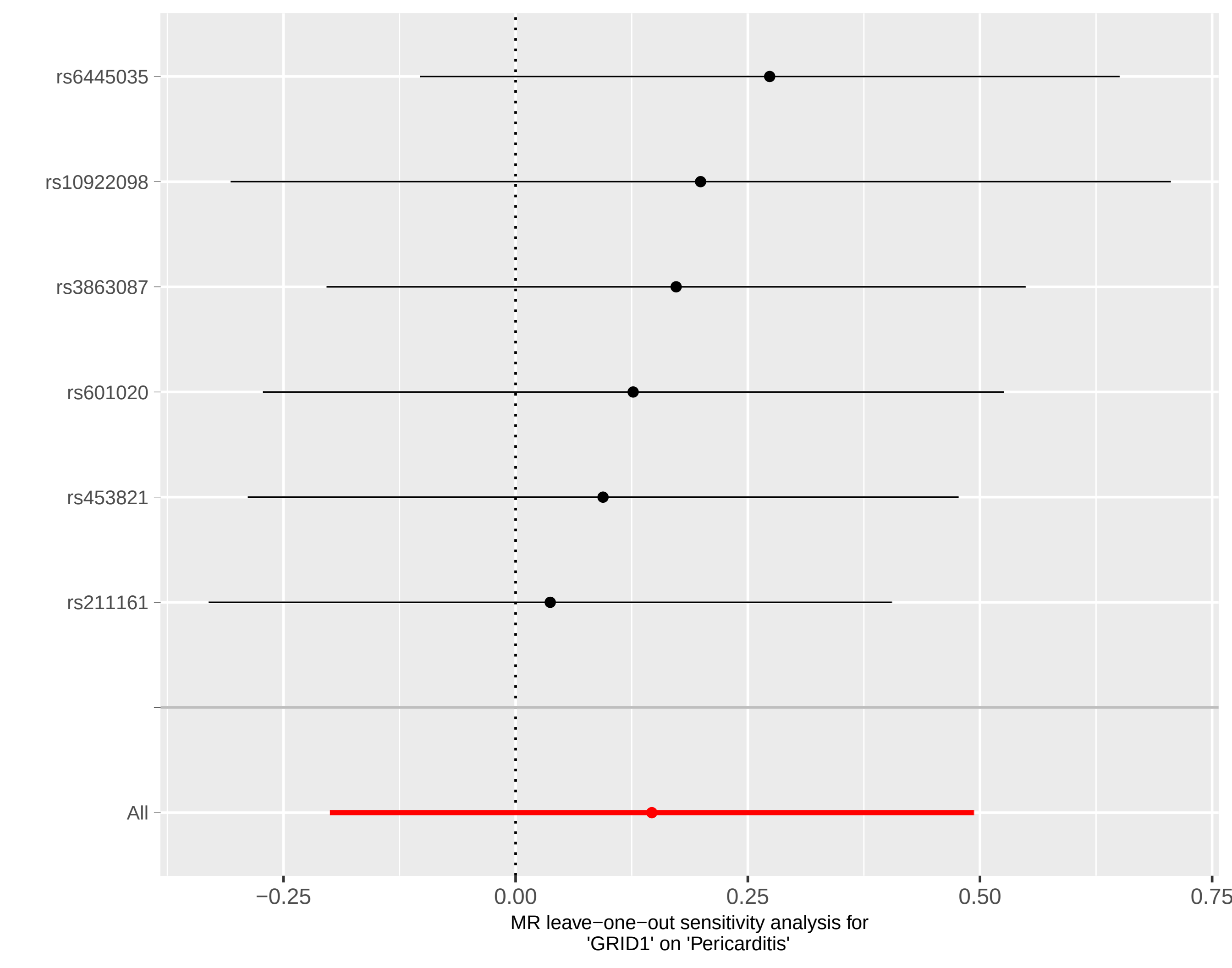

rs6445035
rs10922098
rs3863087
rs601020
rs453821
rs211161
All
−0.25
0.00
0.25
0.50
0.75
MR leave−one−out sensitivity analysis for
'GRID1' on 'Pericarditis'

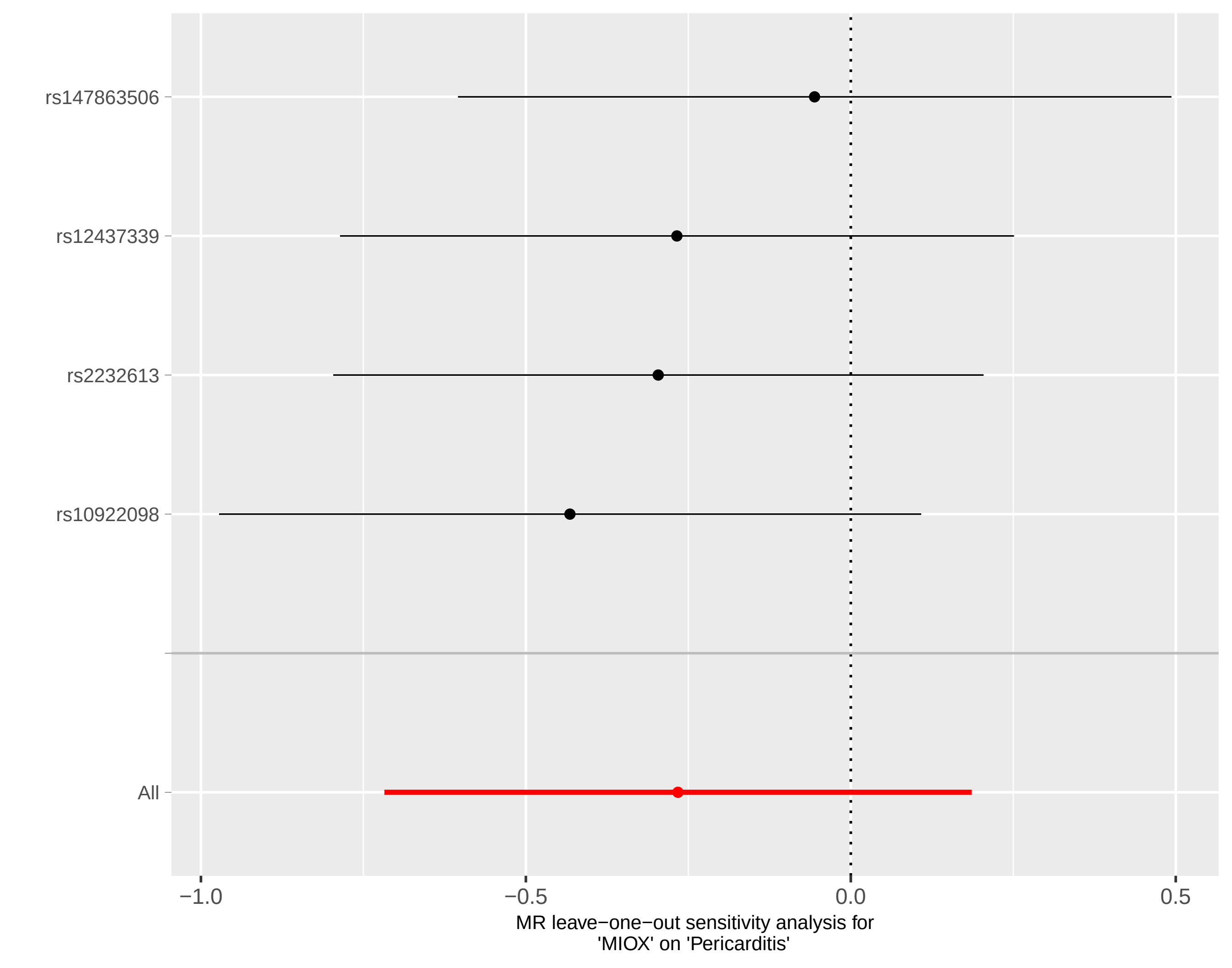

rs147863506
rs12437339
rs2232613
rs10922098
All
−1.0
−0.5
0.0
0.5
MR leave−one−out sensitivity analysis for
'MIOX' on 'Pericarditis'

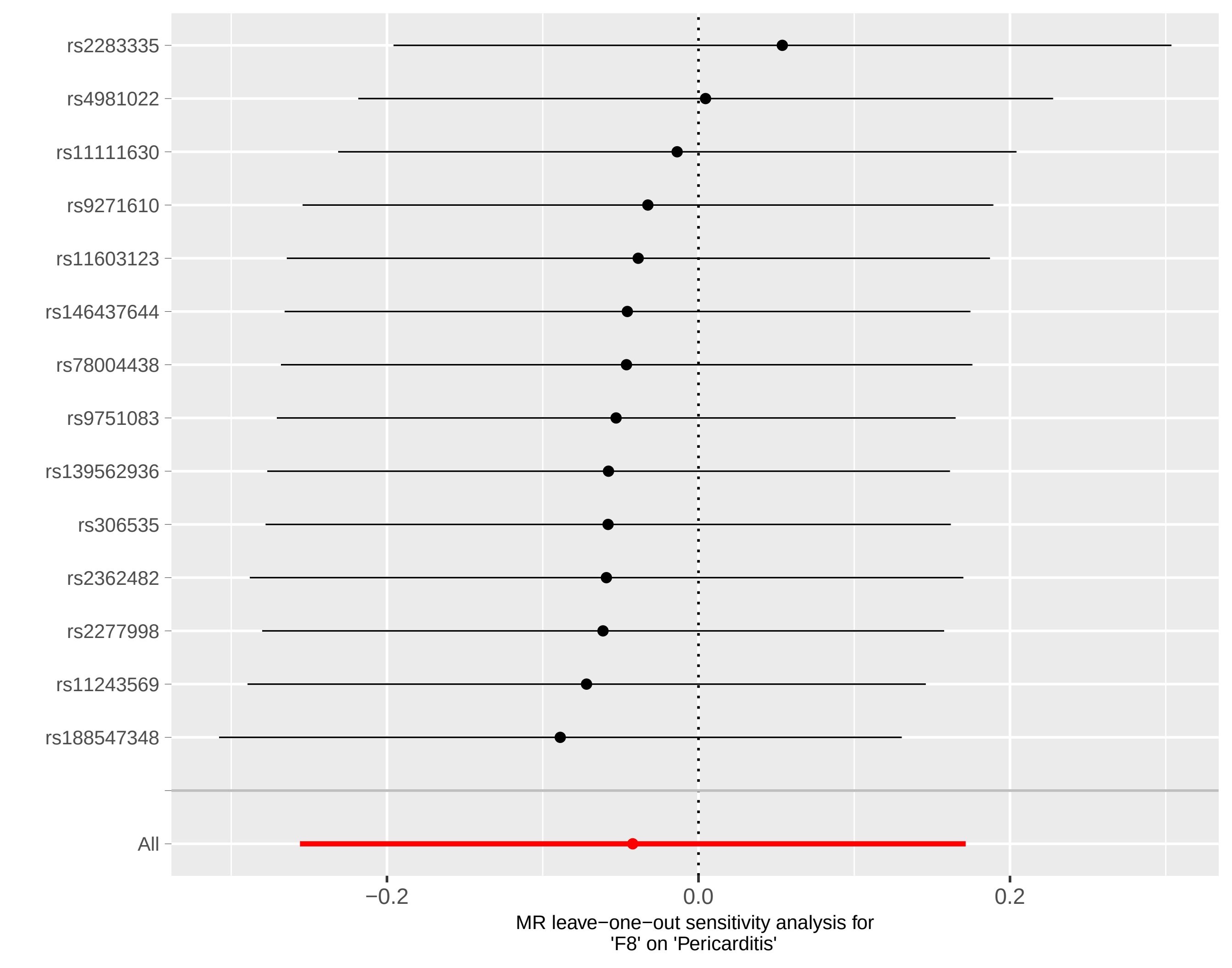
rs2283335
rs4981022
rs11111630
rs9271610
rs11603123
rs146437644
rs78004438
rs9751083
rs139562936
rs306535
rs2362482
rs2277998
rs11243569
rs188547348
All
−0.2
0.0
0.2
MR leave−one−out sensitivity analysis for
'F8' on 'Pericarditis'

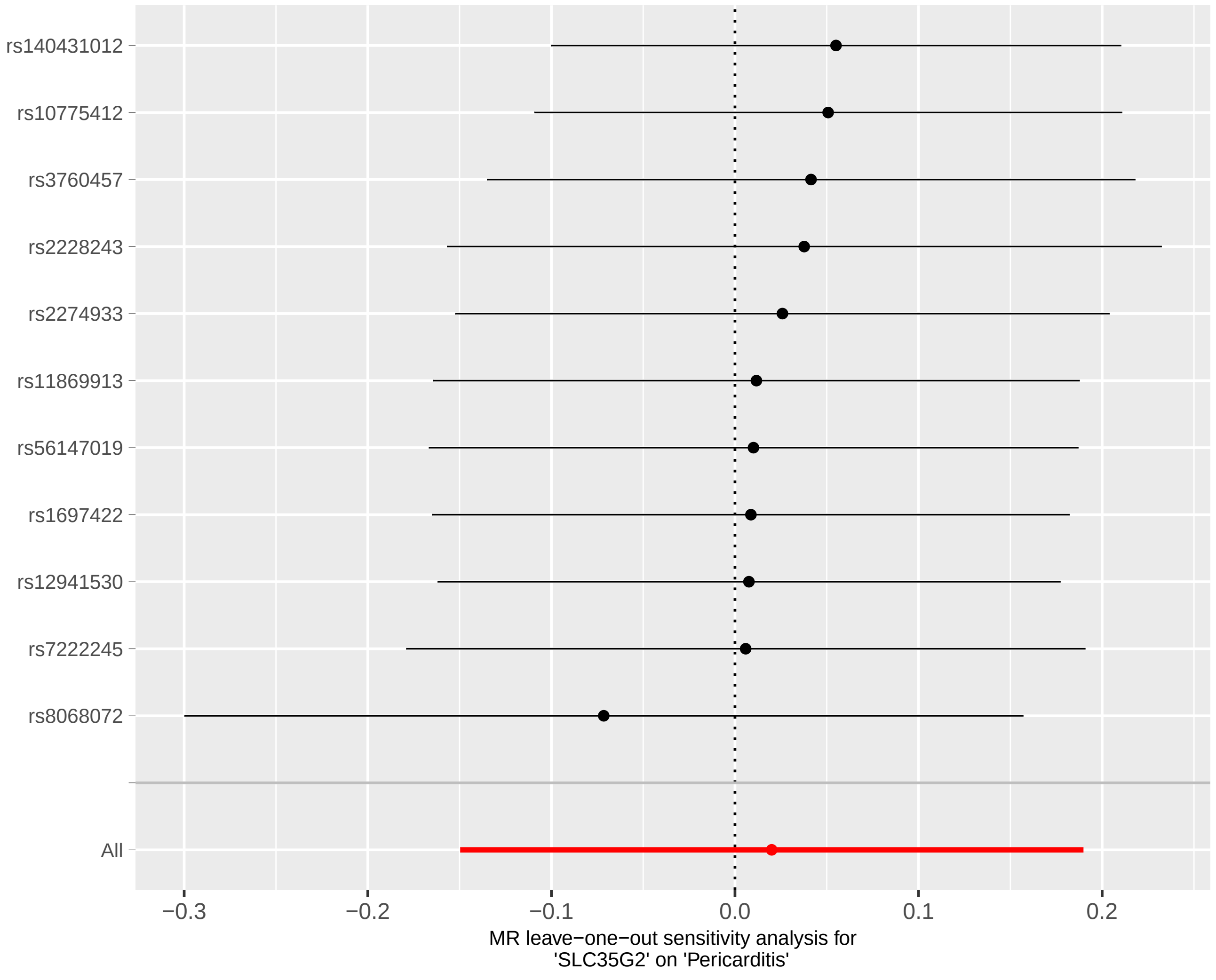
rs140431012
rs10775412
rs3760457
rs2228243
rs2274933
rs11869913
rs56147019
rs1697422
rs12941530
rs7222245
rs8068072
All
−0.3
−0.2
−0.1
0.0
0.1
0.2
MR leave−one−out sensitivity analysis for
'SLC35G2' on 'Pericarditis'

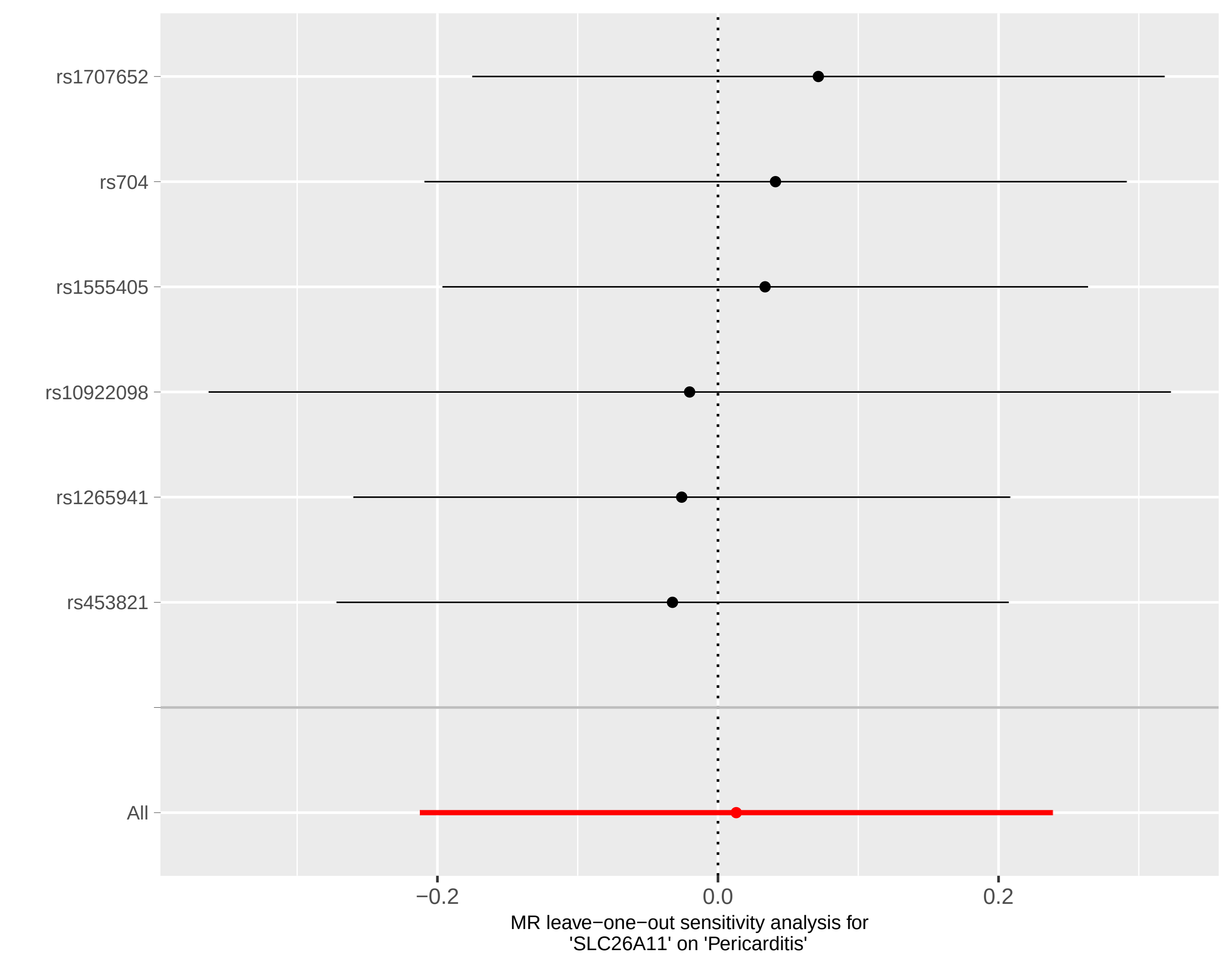

rs1707652
rs704
rs1555405
rs10922098
rs1265941
rs453821
All
−0.2
0.0
0.2
MR leave−one−out sensitivity analysis for
'SLC26A11' on 'Pericarditis'

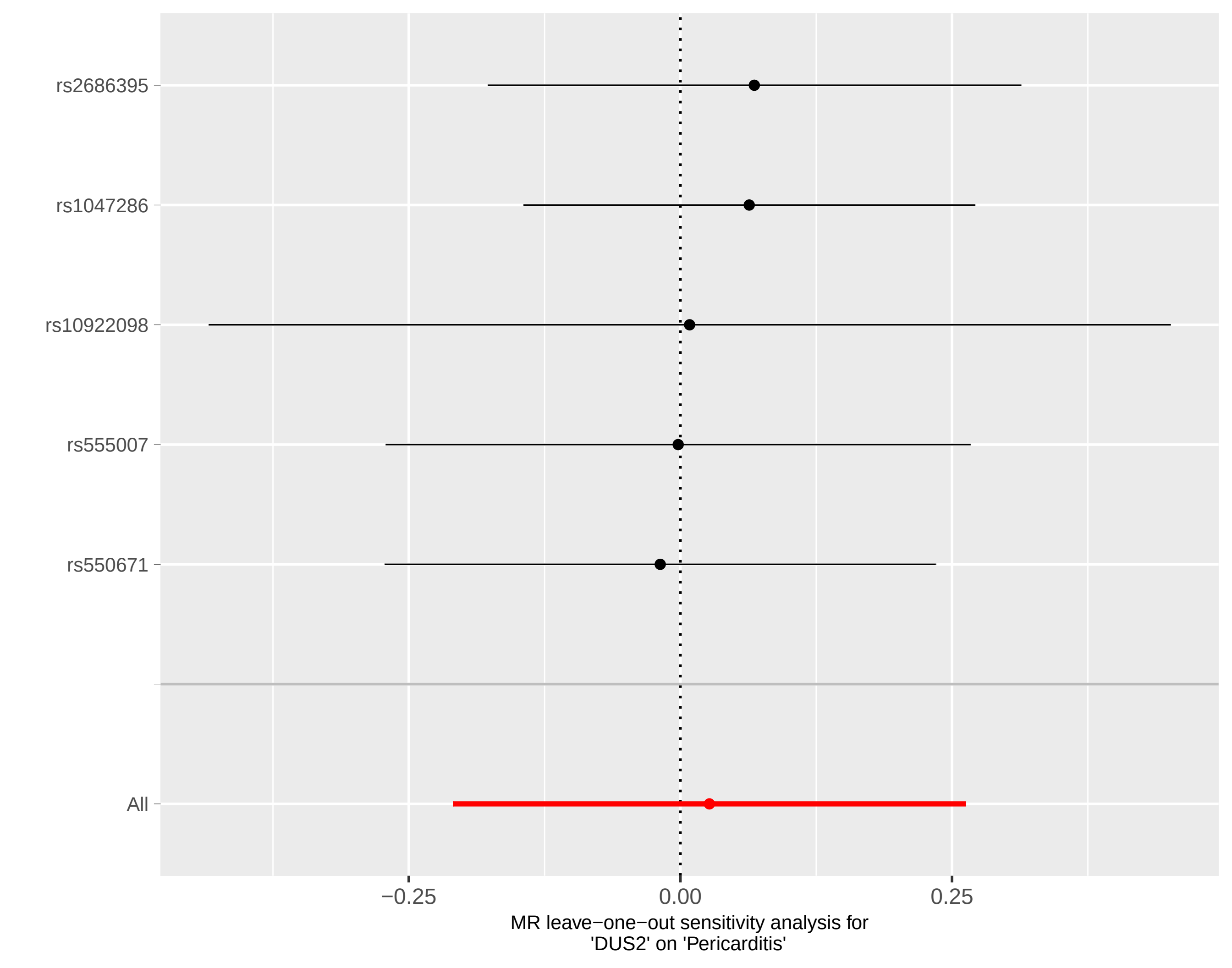
rs2686395
rs1047286
rs10922098
rs555007
rs550671
All
−0.25
0.00
0.25
MR leave−one−out sensitivity analysis for
'DUS2' on 'Pericarditis'

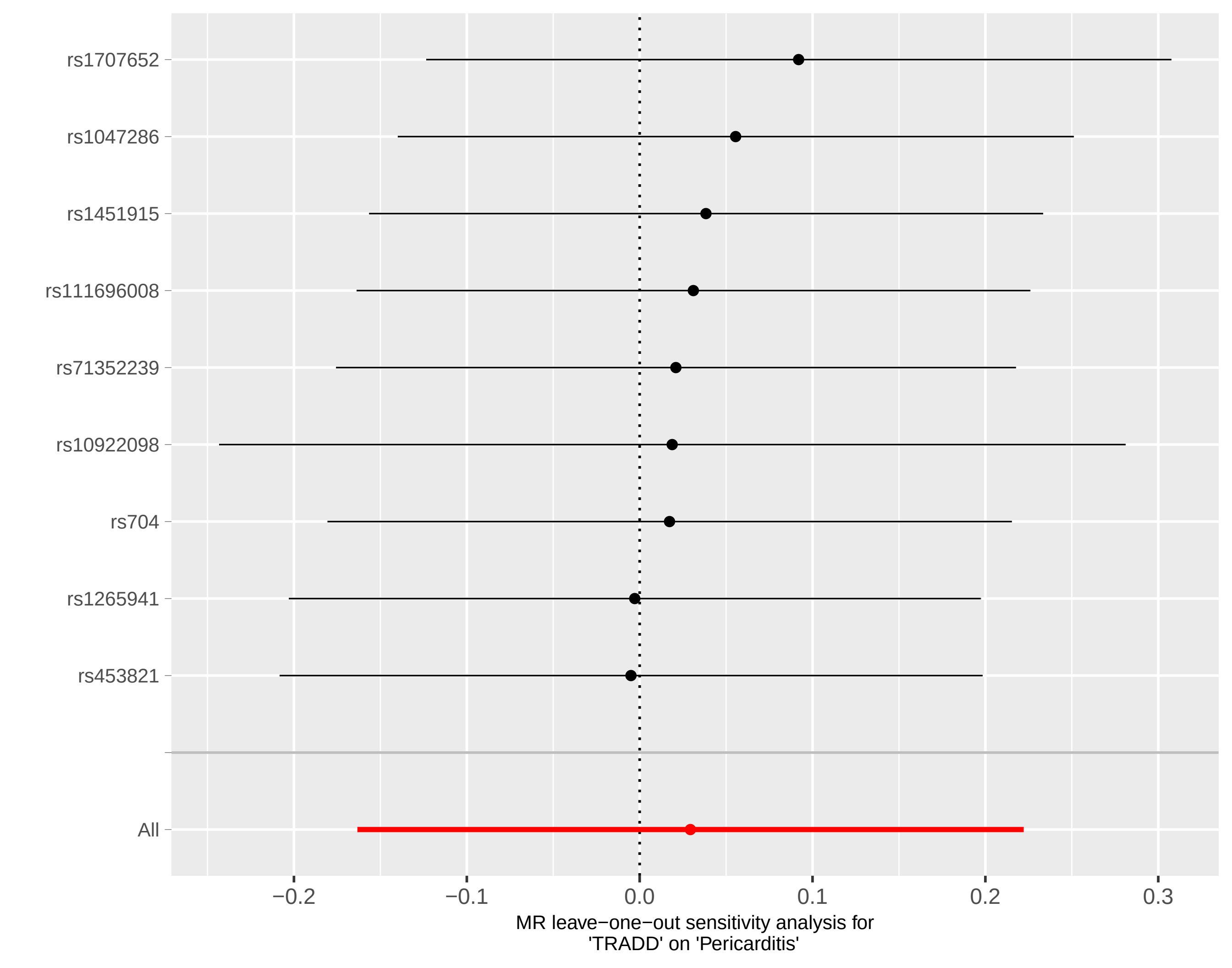
rs1707652
rs1047286
rs1451915
rs111696008
rs71352239
rs10922098
rs704
rs1265941
rs453821
All
−0.2
−0.1
0.0
0.1
0.2
0.3
MR leave−one−out sensitivity analysis for
'TRADD' on 'Pericarditis'

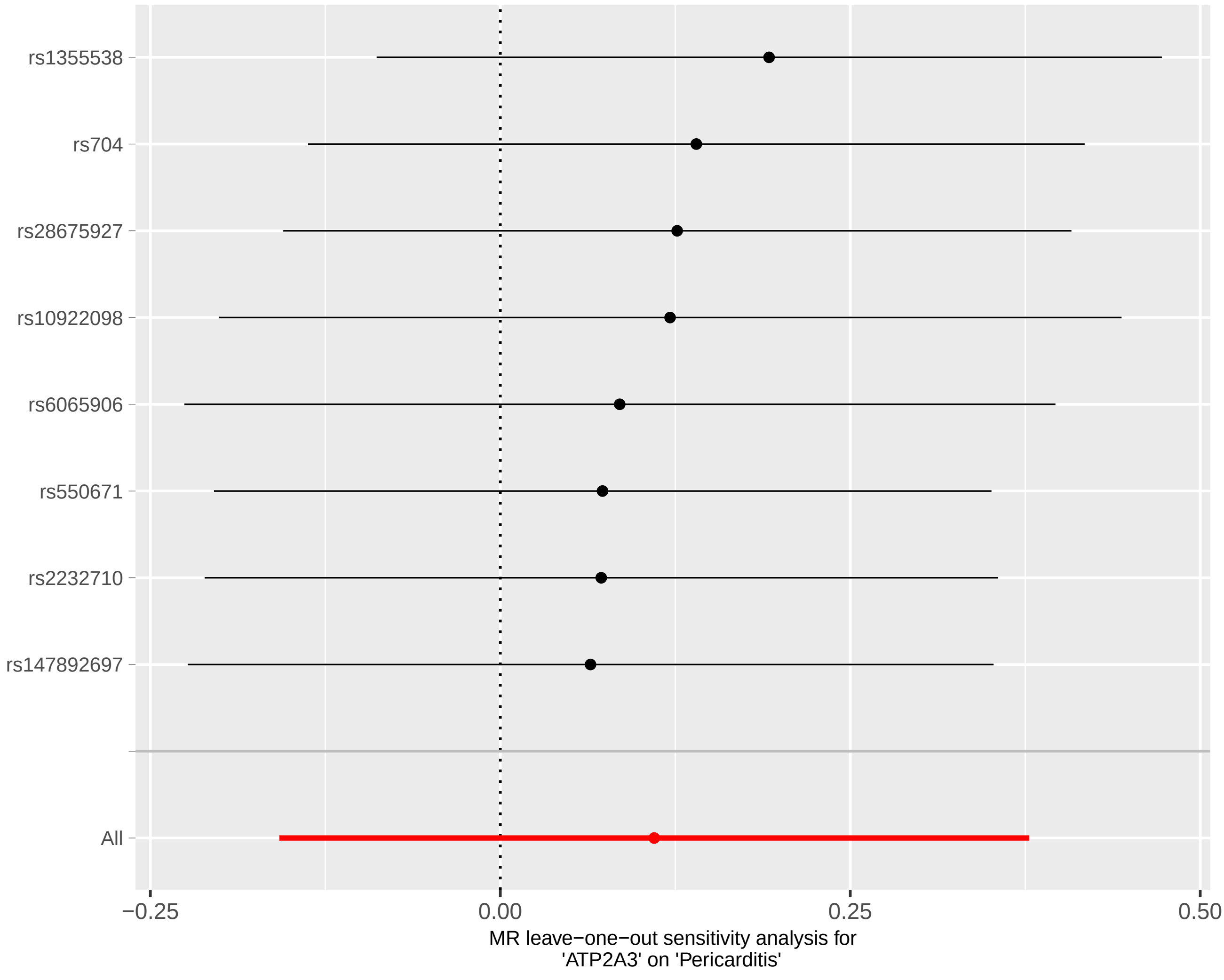

rs1355538
rs704
rs28675927
rs10922098
rs6065906
rs550671
rs2232710
rs147892697
All
−0.25
0.00
0.25
0.50
MR leave−one−out sensitivity analysis for
'ATP2A3' on 'Pericarditis'

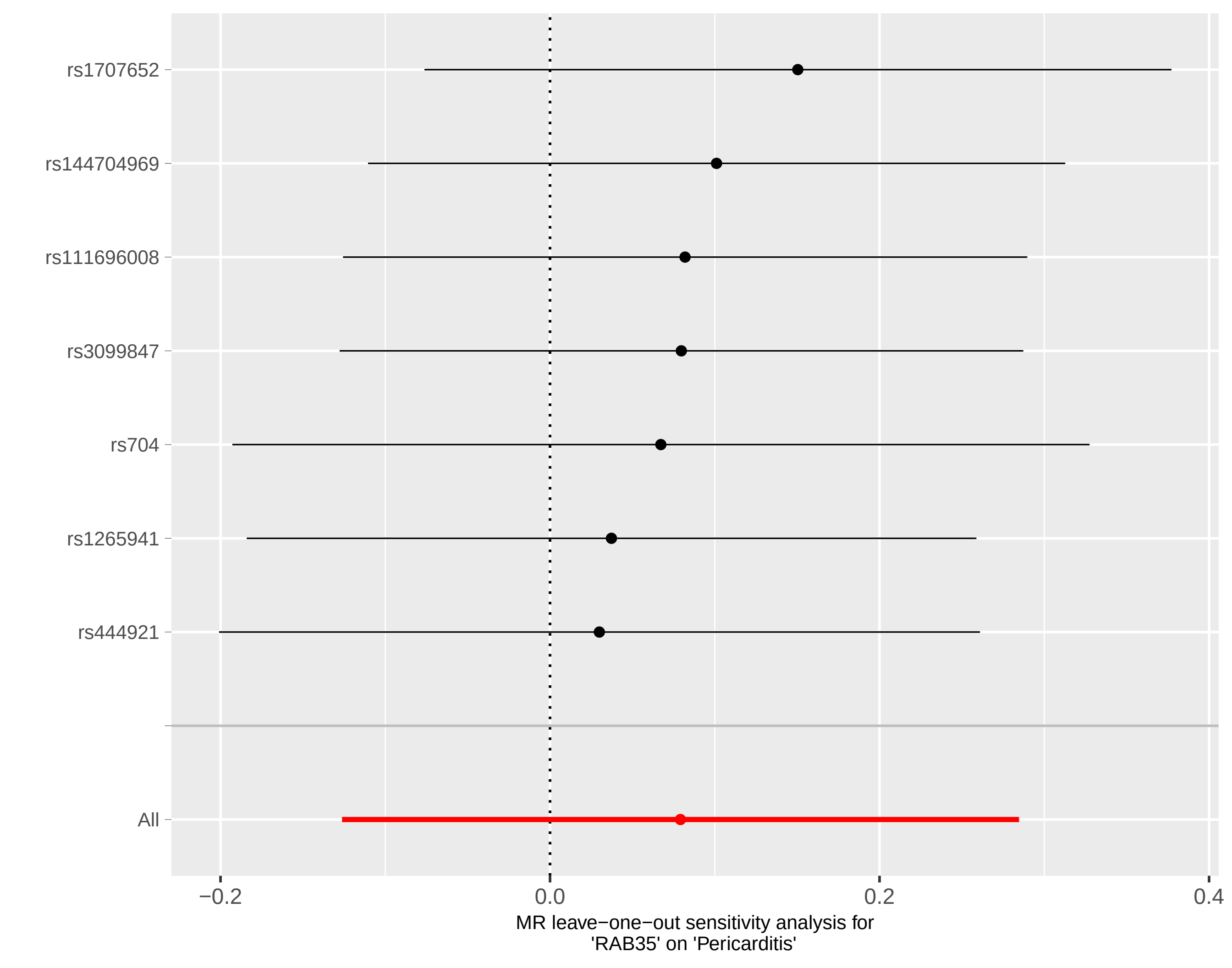
rs1707652
rs144704969
rs111696008
rs3099847
rs704
rs1265941
rs444921
All
−0.2
0.0
0.2
0.4
MR leave−one−out sensitivity analysis for
'RAB35' on 'Pericarditis'

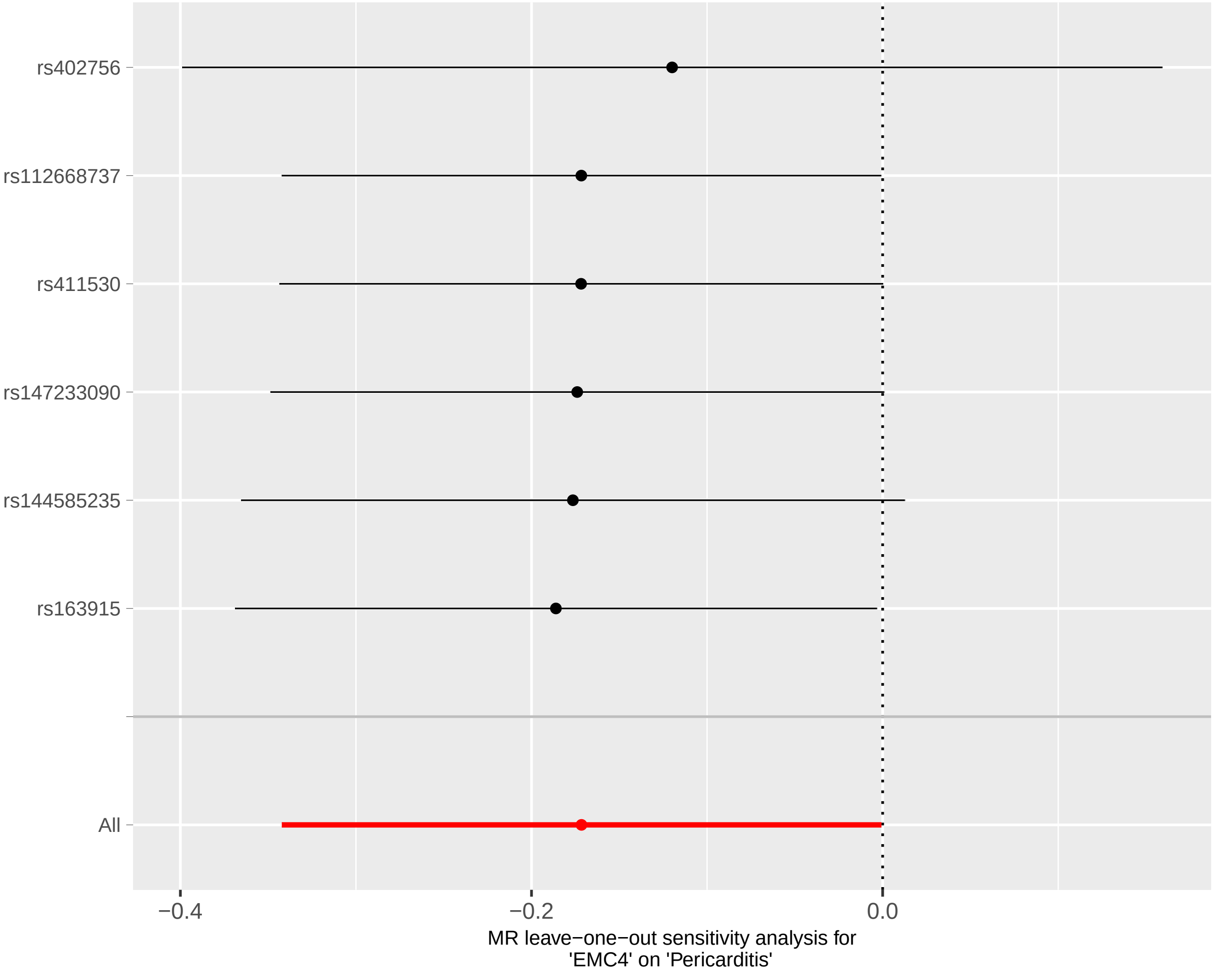
rs402756
rs112668737
rs411530
rs147233090
rs144585235
rs163915
All
−0.4
−0.2
0.0
MR leave−one−out sensitivity analysis for
'EMC4' on 'Pericarditis'

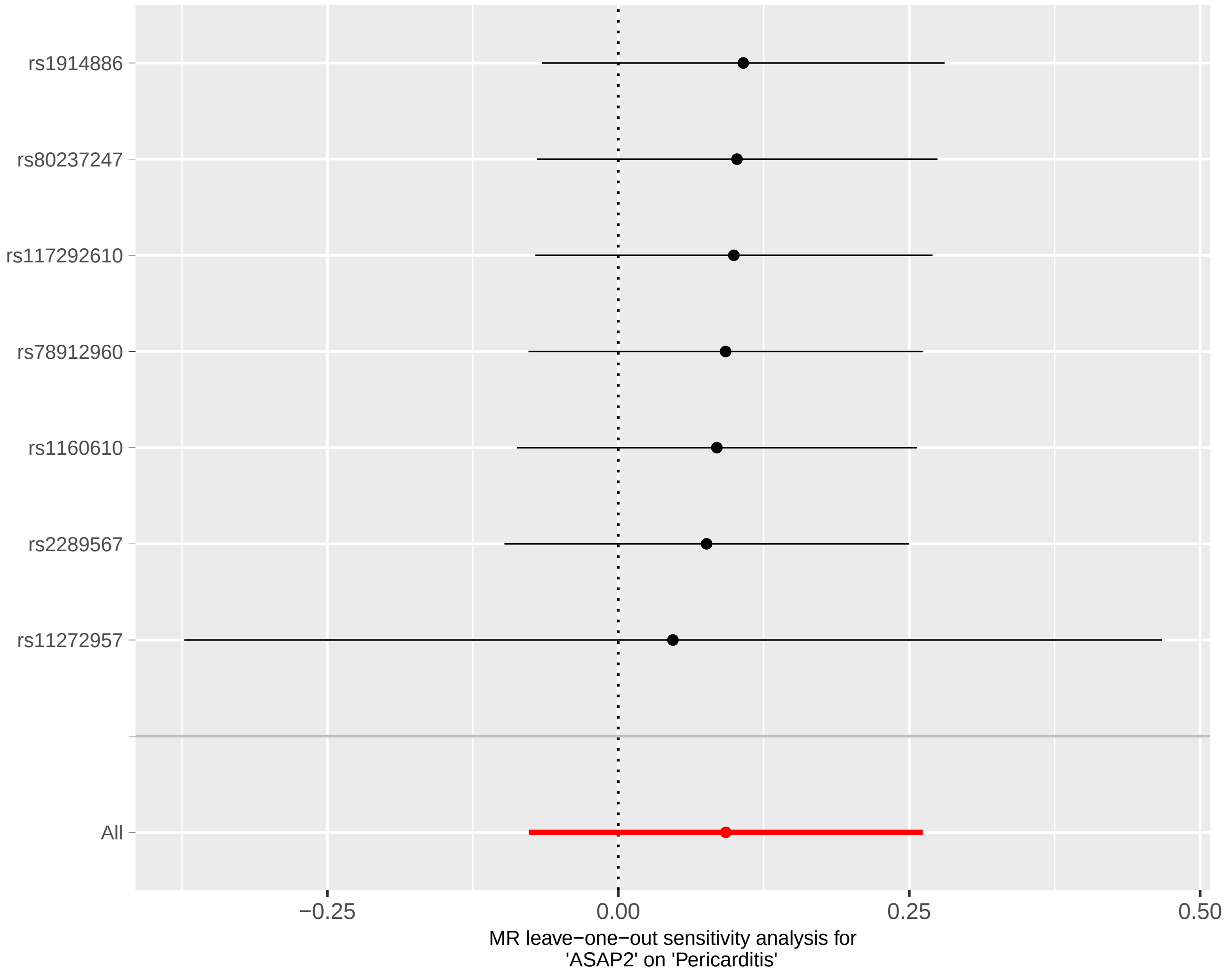
rs1914886
rs80237247
rs117292610
rs78912960
rs1160610
rs2289567
rs11272957
All
−0.25
0.00
0.25
0.50
MR leave−one−out sensitivity analysis for
'ASAP2' on 'Pericarditis'

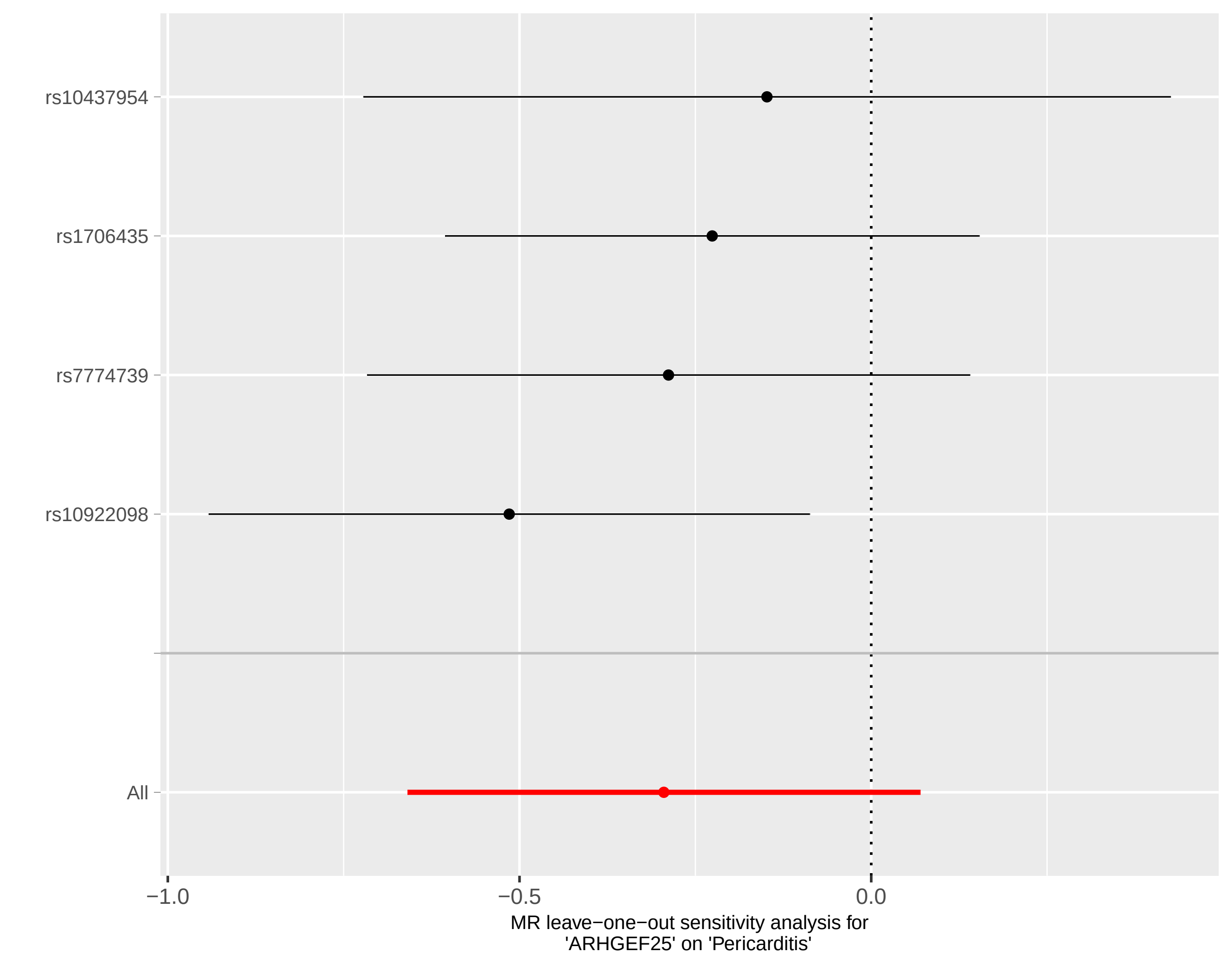
rs10437954
rs1706435
rs7774739
rs10922098
All
−1.0
−0.5
0.0
MR leave−one−out sensitivity analysis for
'ARHGEF25' on 'Pericarditis'

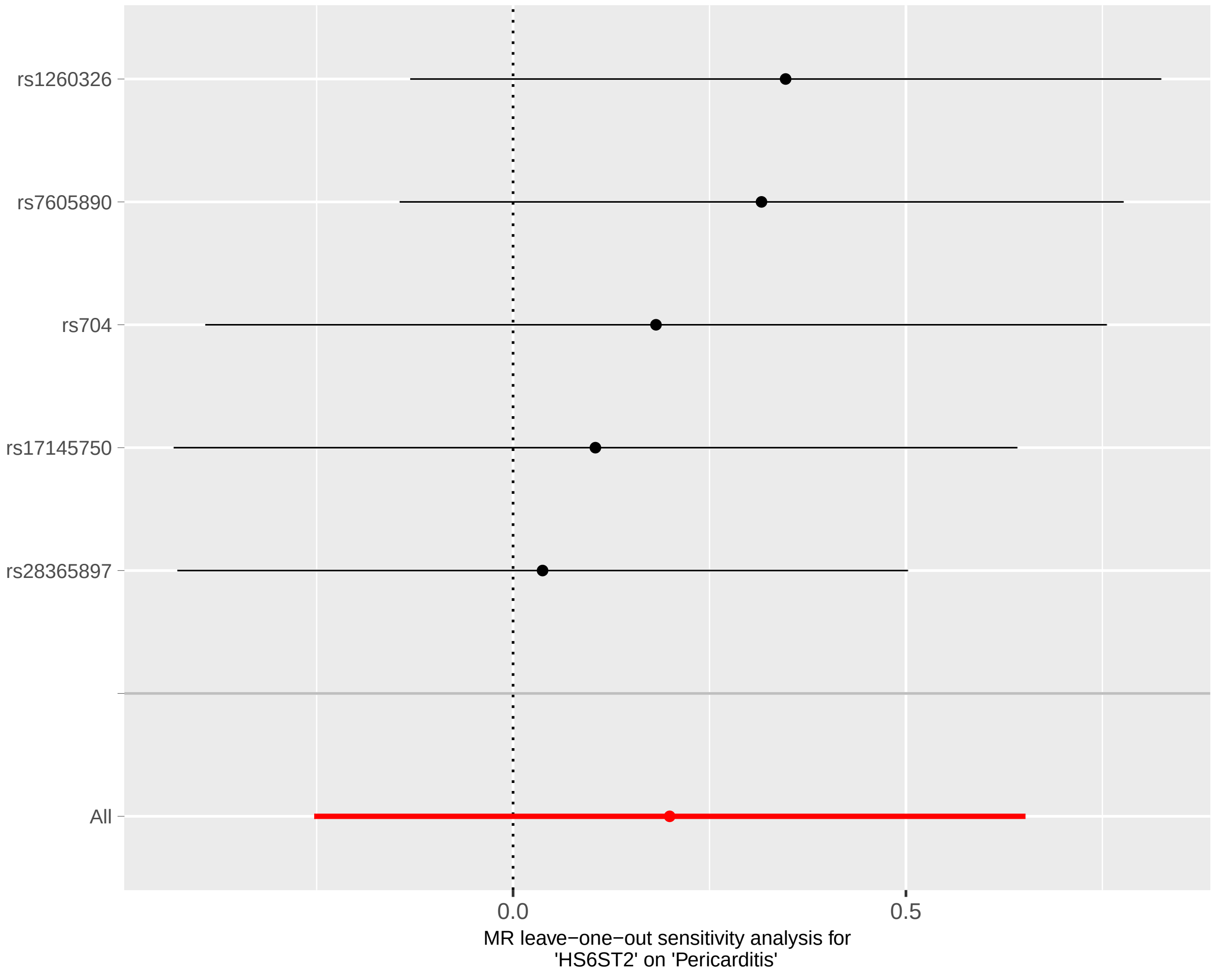

rs1260326
rs7605890
rs704
rs17145750
rs28365897
All
0.0
0.5
MR leave−one−out sensitivity analysis for
'HS6ST2' on 'Pericarditis'

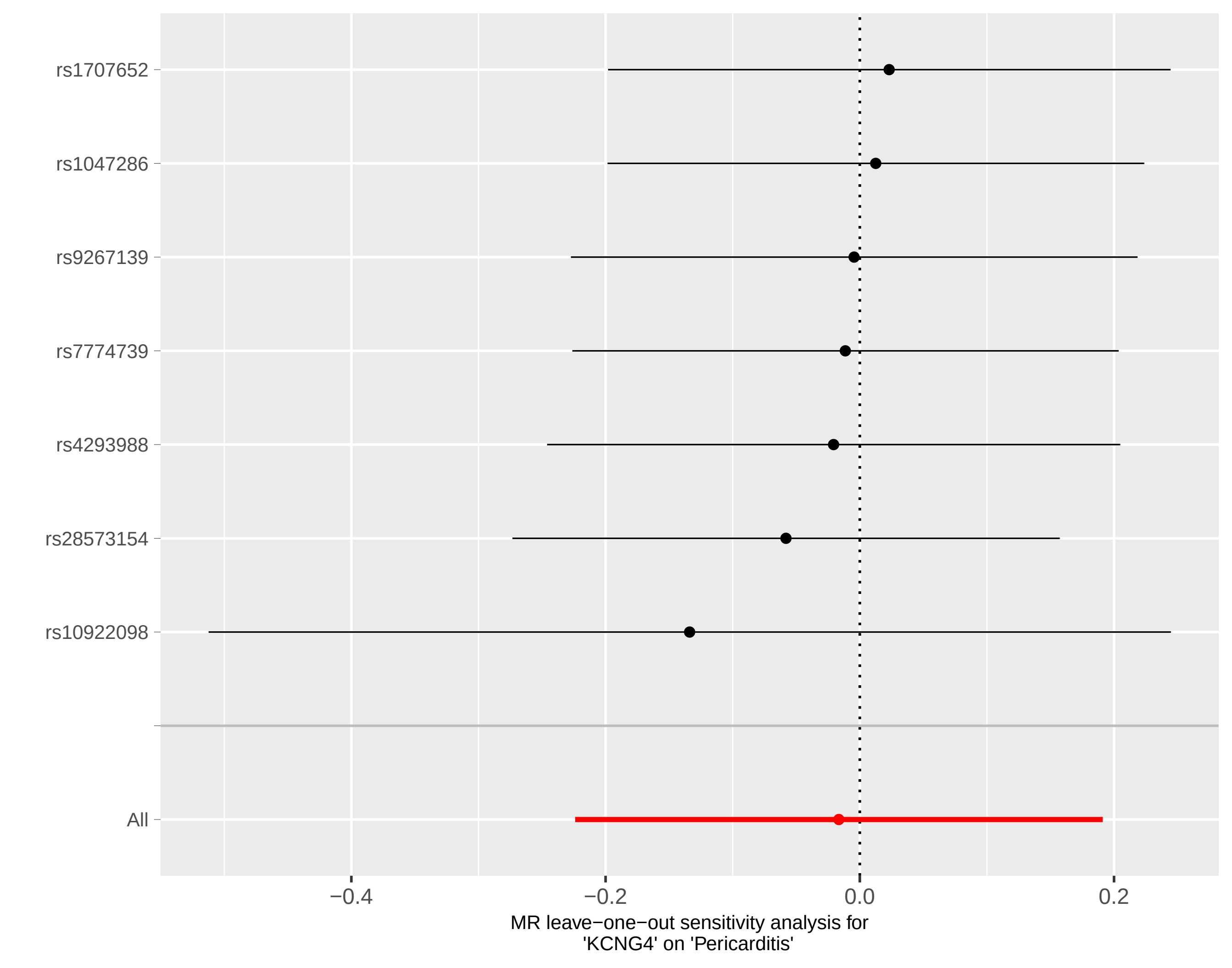

rs1707652
rs1047286
rs9267139
rs7774739
rs4293988
rs28573154
rs10922098
All
−0.4
−0.2
0.0
0.2
MR leave−one−out sensitivity analysis for
'KCNG4' on 'Pericarditis'

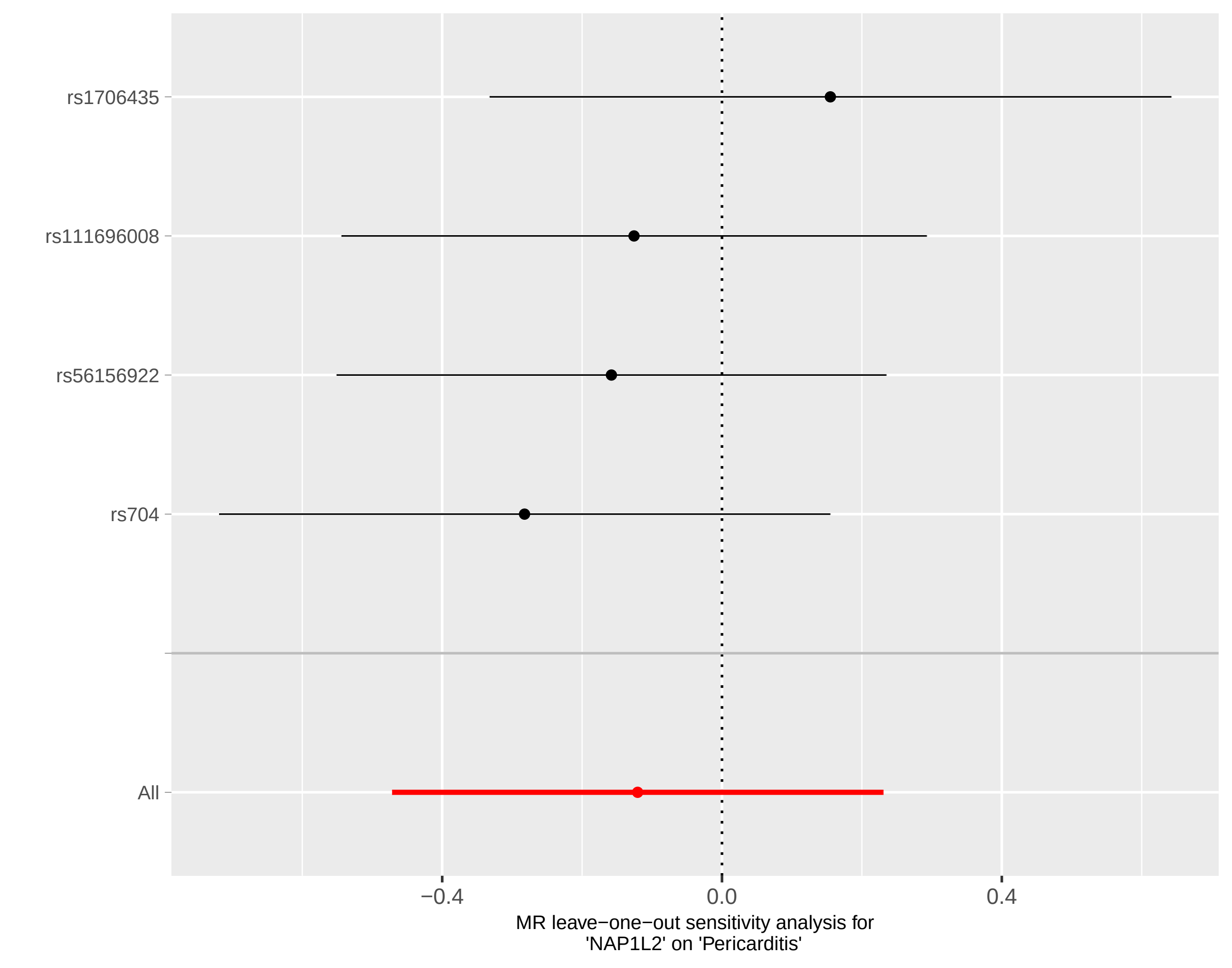
rs1706435
rs111696008
rs56156922
rs704
All
−0.4
0.0
0.4
MR leave−one−out sensitivity analysis for
'NAP1L2' on 'Pericarditis'

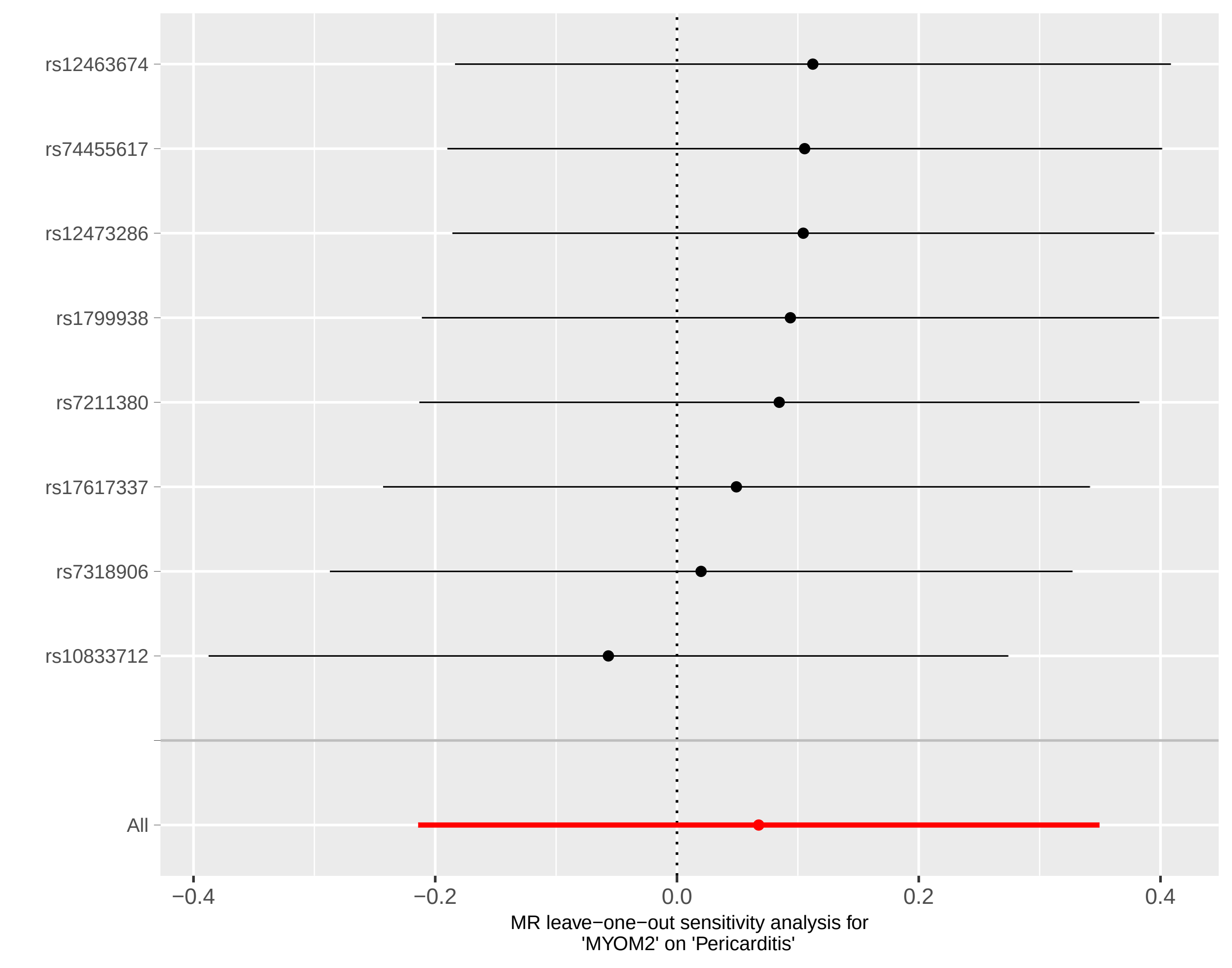
rs12463674
rs74455617
rs12473286
rs1799938
rs7211380
rs17617337
rs7318906
rs10833712
All
−0.4
−0.2
0.0
0.2
0.4
MR leave−one−out sensitivity analysis for
'MYOM2' on 'Pericarditis'

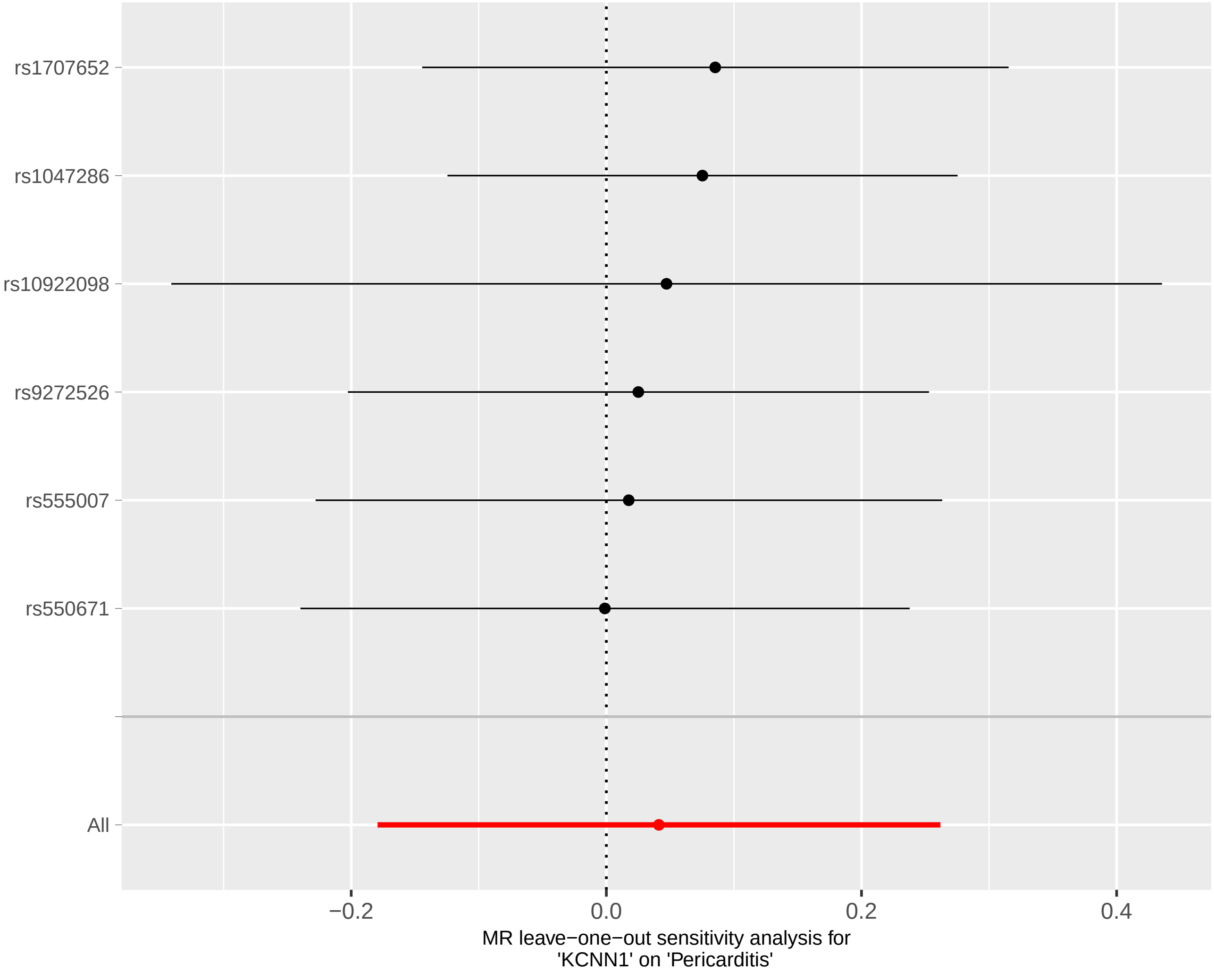
rs1707652
rs1047286
rs10922098
rs9272526
rs555007
rs550671
All
−0.2
0.0
0.2
0.4
MR leave−one−out sensitivity analysis for
'KCNN1' on 'Pericarditis'

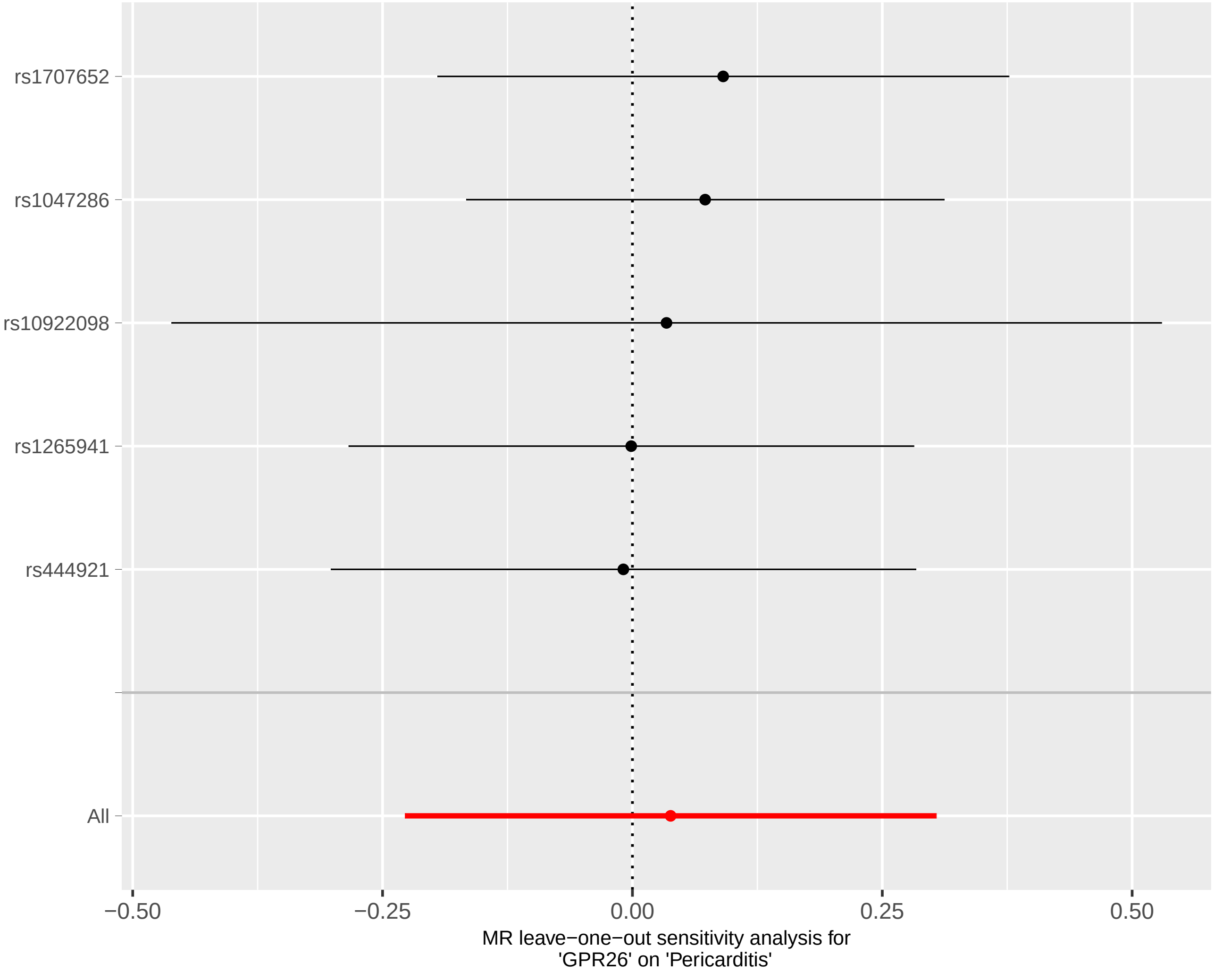
rs1707652
rs1047286
rs10922098
rs1265941
rs444921
All
−0.50
−0.25
0.00
0.25
0.50
MR leave−one−out sensitivity analysis for
'GPR26' on 'Pericarditis'

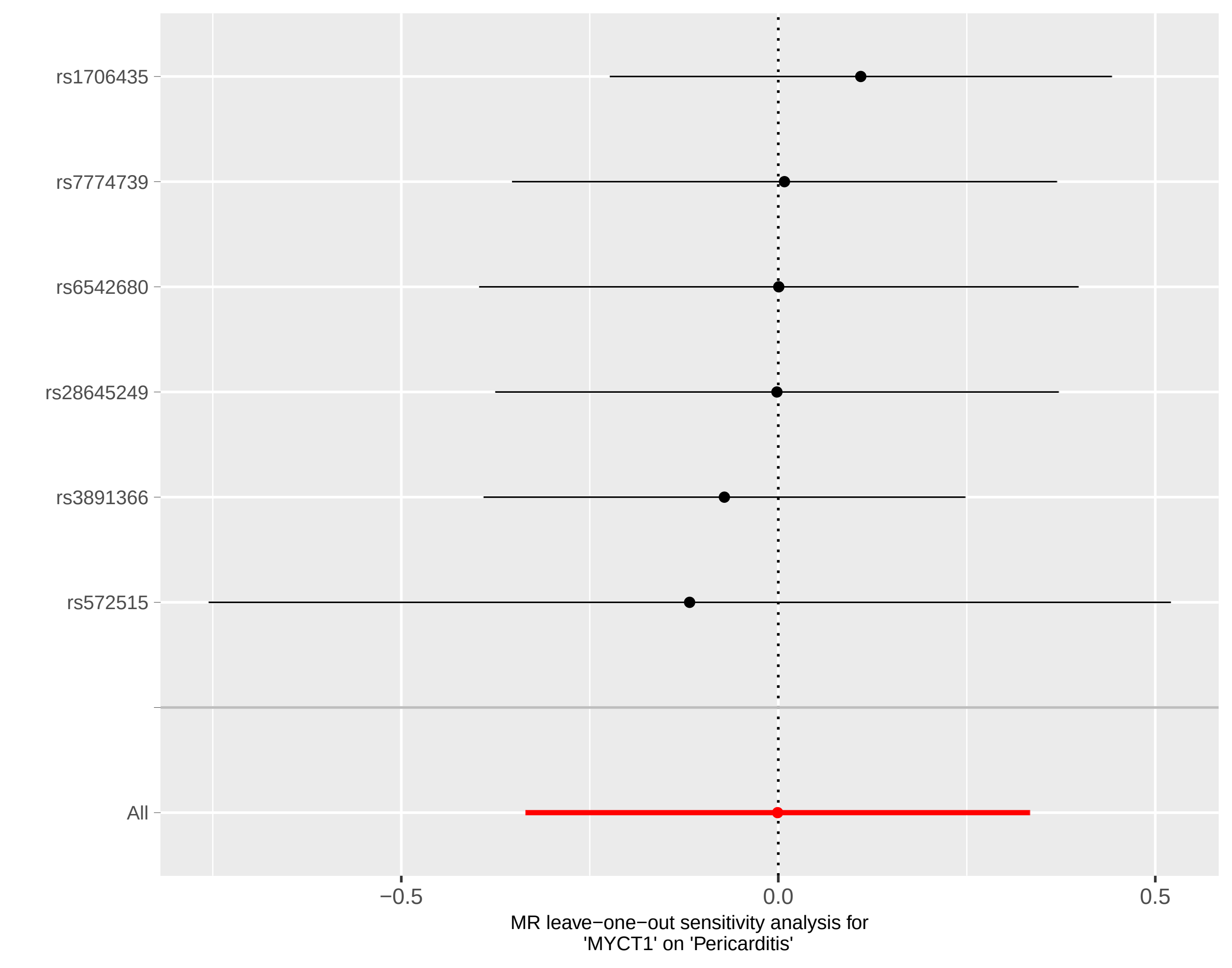

rs1706435
rs7774739
rs6542680
rs28645249
rs3891366
rs572515
All
−0.5
0.0
0.5
MR leave−one−out sensitivity analysis for
'MYCT1' on 'Pericarditis'

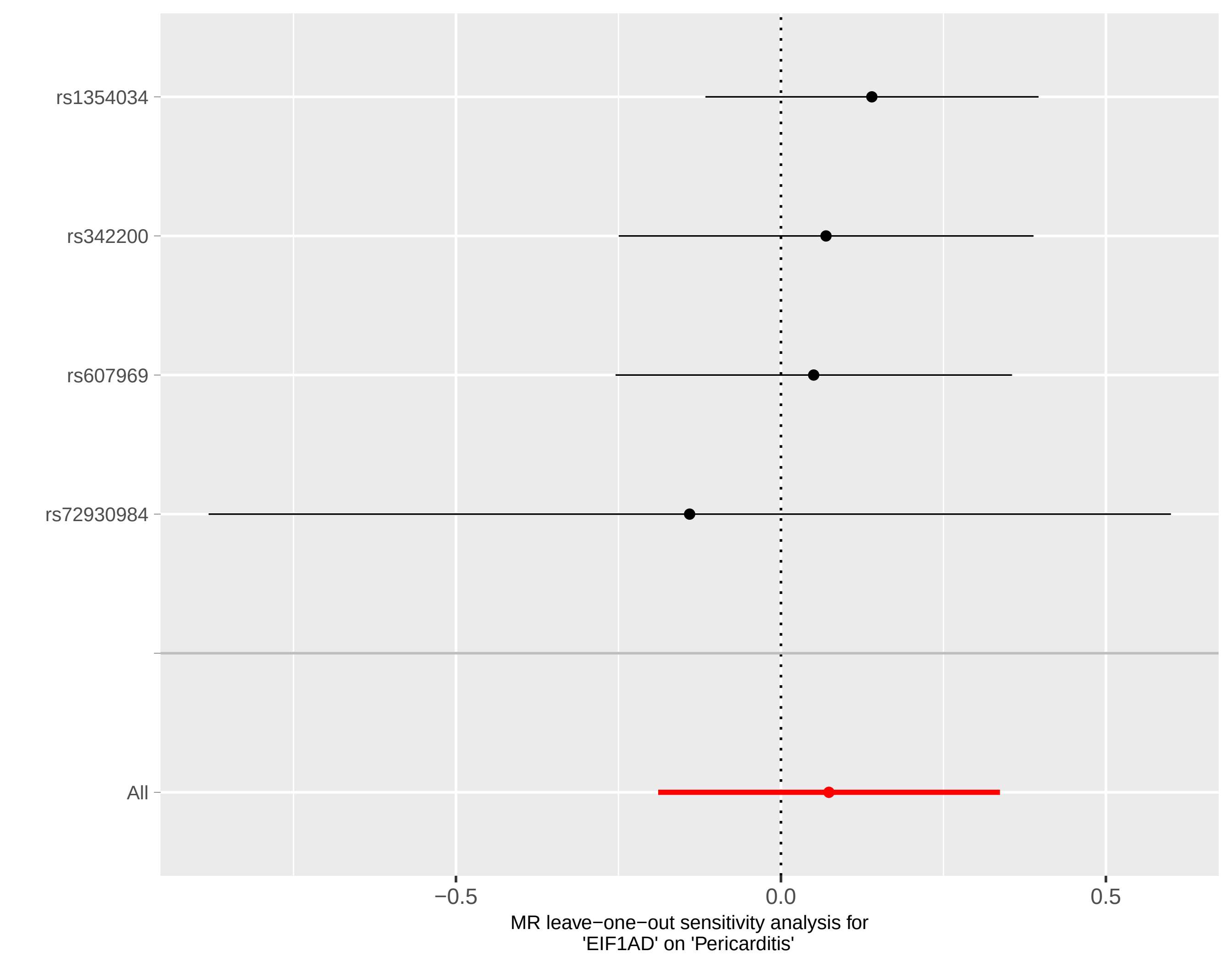

rs1354034
rs342200
rs607969
rs72930984
All
−0.5
0.0
0.5
MR leave−one−out sensitivity analysis for
'EIF1AD' on 'Pericarditis'

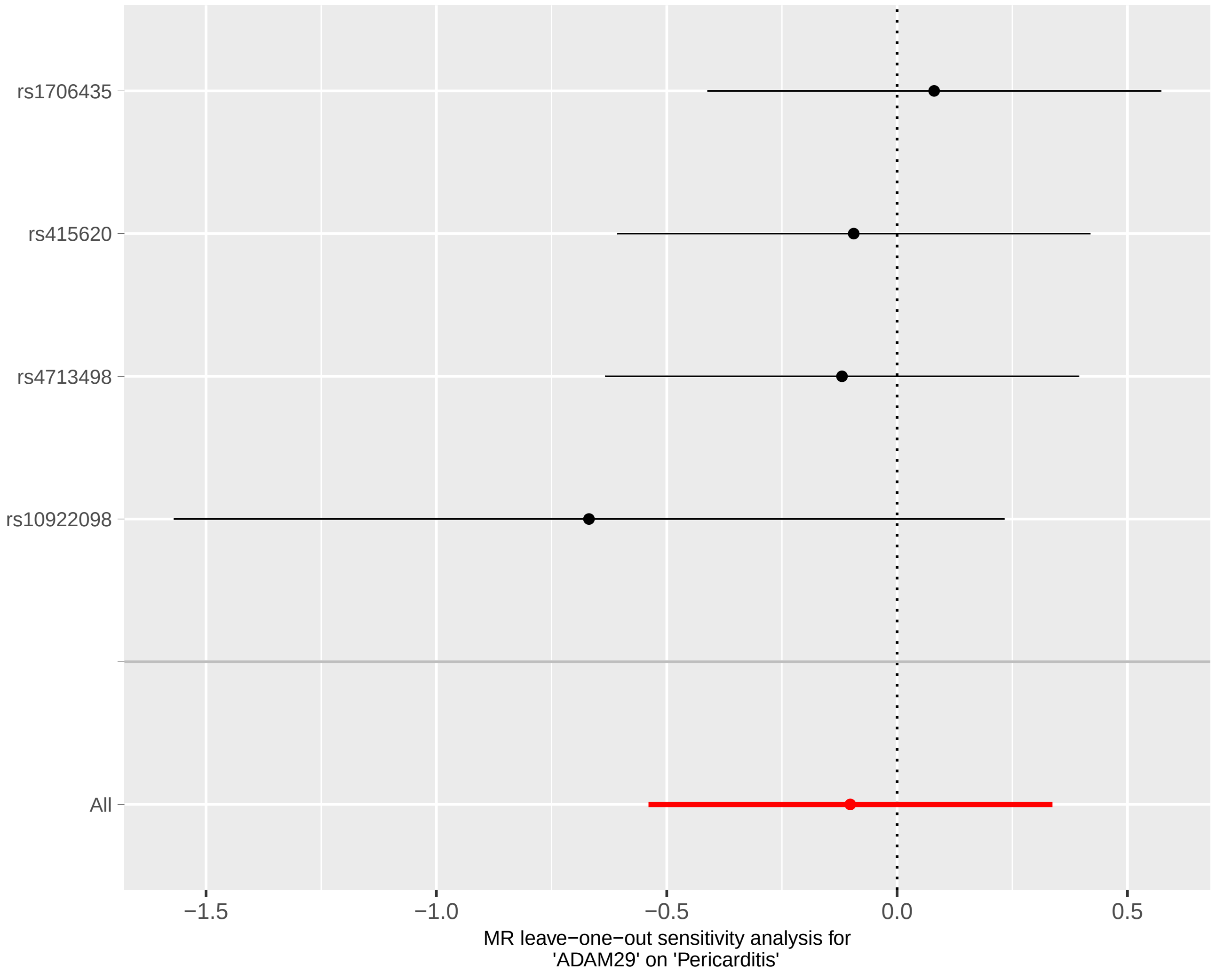
rs1706435
rs415620
rs4713498
rs10922098
All
−1.5
−1.0
−0.5
0.0
0.5
MR leave−one−out sensitivity analysis for
'ADAM29' on 'Pericarditis'

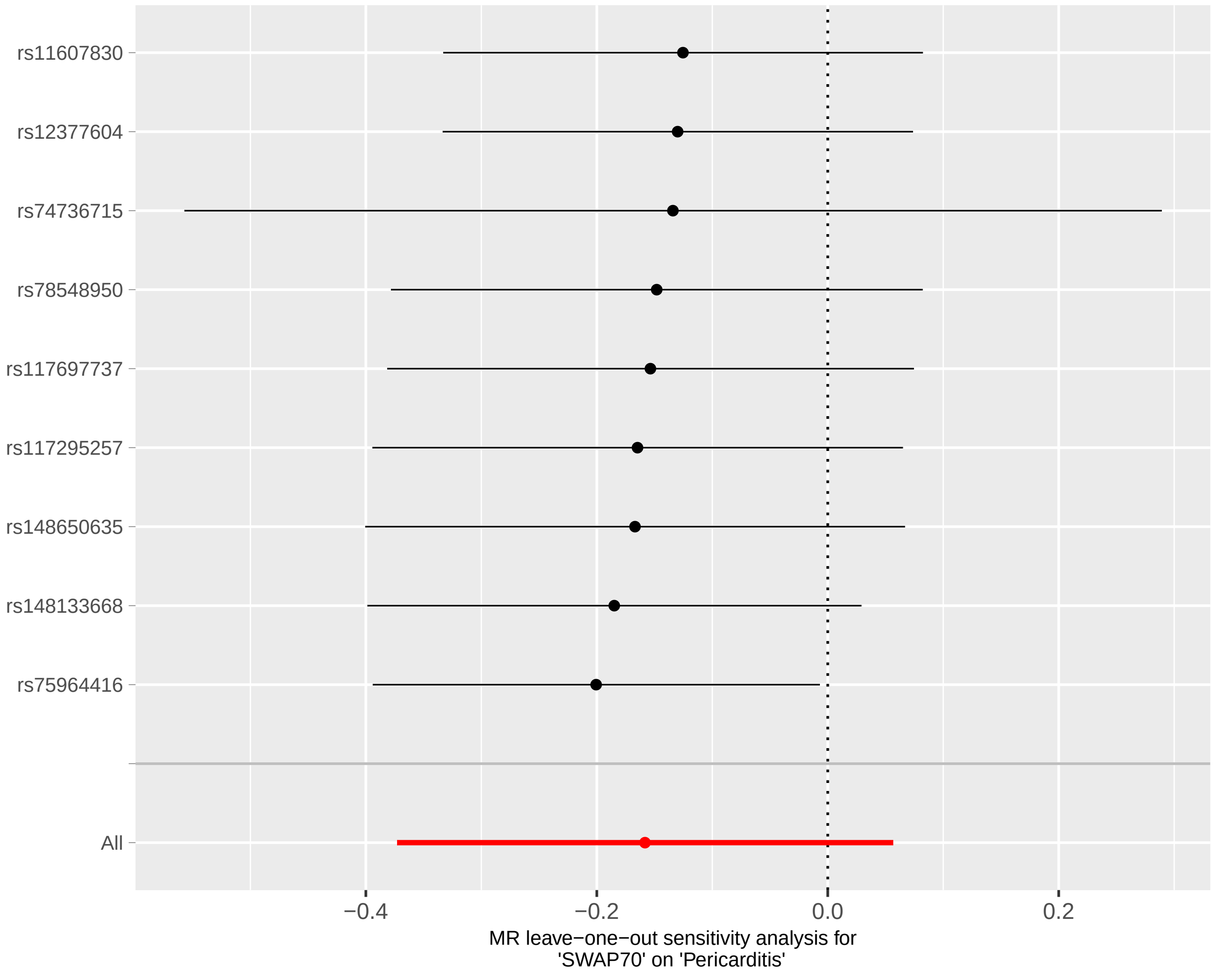

rs11607830
rs12377604
rs74736715
rs78548950
rs117697737
rs117295257
rs148650635
rs148133668
rs75964416
All
−0.4
−0.2
0.0
0.2
MR leave−one−out sensitivity analysis for
'SWAP70' on 'Pericarditis'

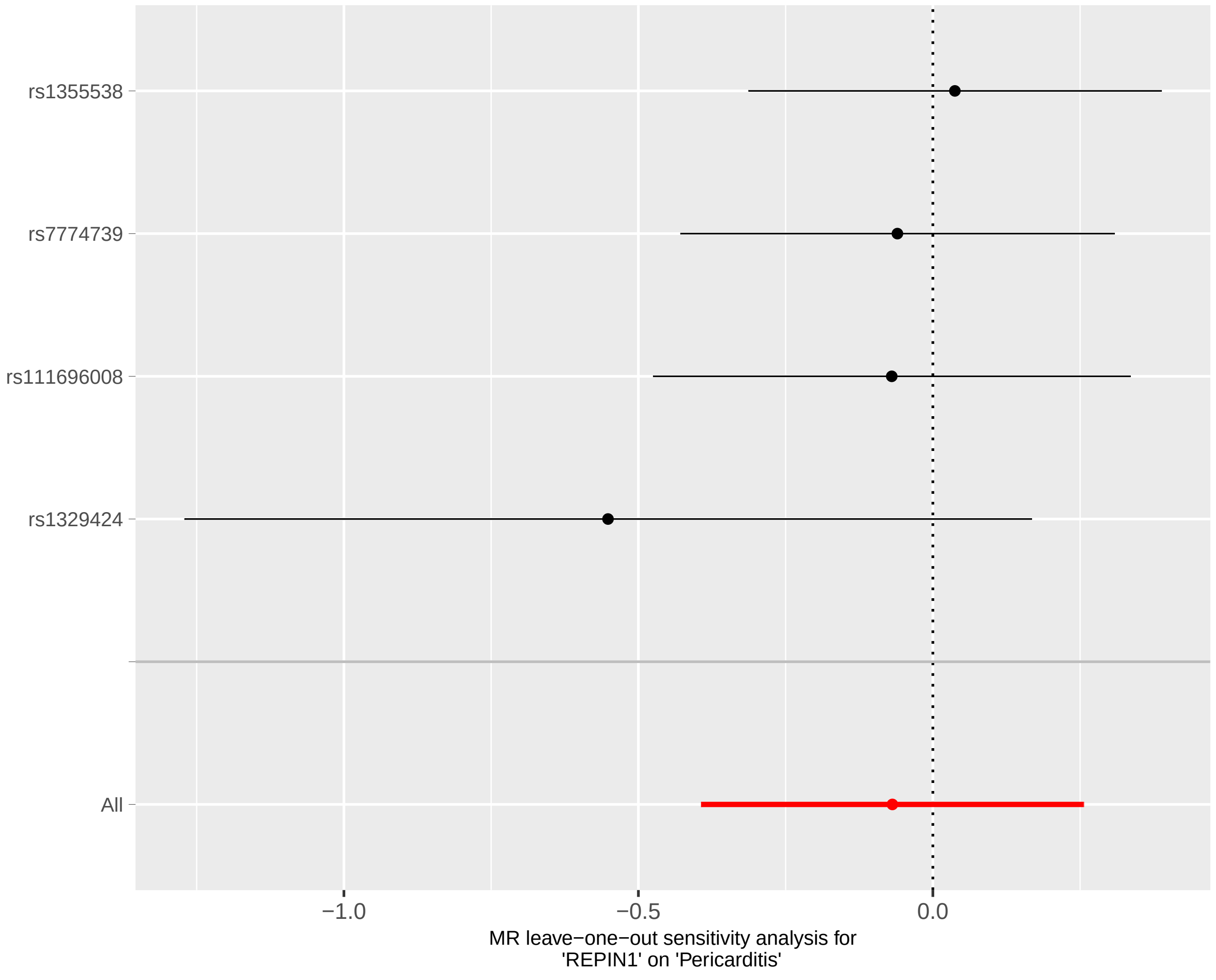

rs1355538
rs7774739
rs111696008
rs1329424
All
−1.0
−0.5
0.0
MR leave−one−out sensitivity analysis for
'REPIN1' on 'Pericarditis'

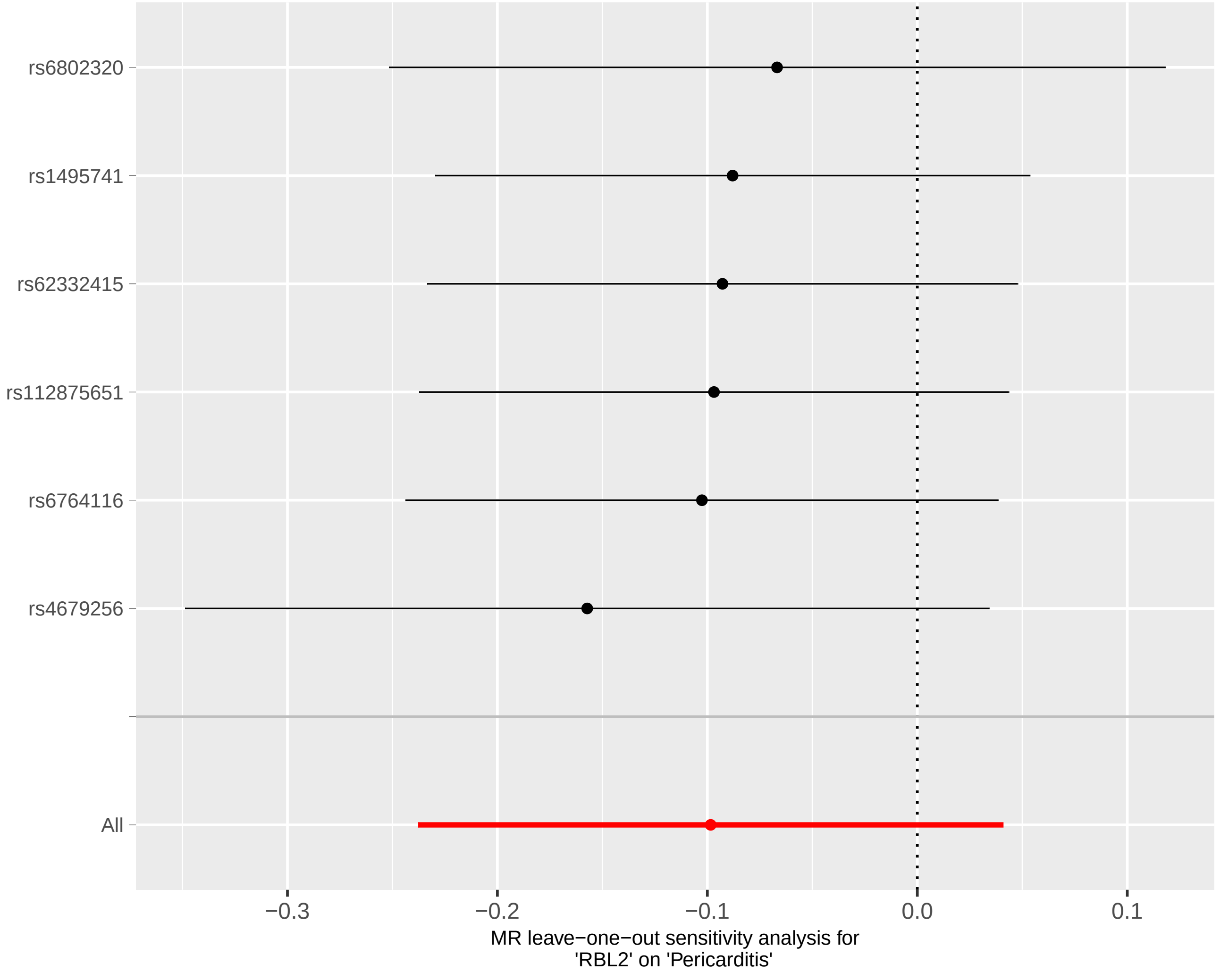
rs6802320
rs1495741
rs62332415
rs112875651
rs6764116
rs4679256
All
−0.3
−0.2
−0.1
0.0
0.1
MR leave−one−out sensitivity analysis for
'RBL2' on 'Pericarditis'

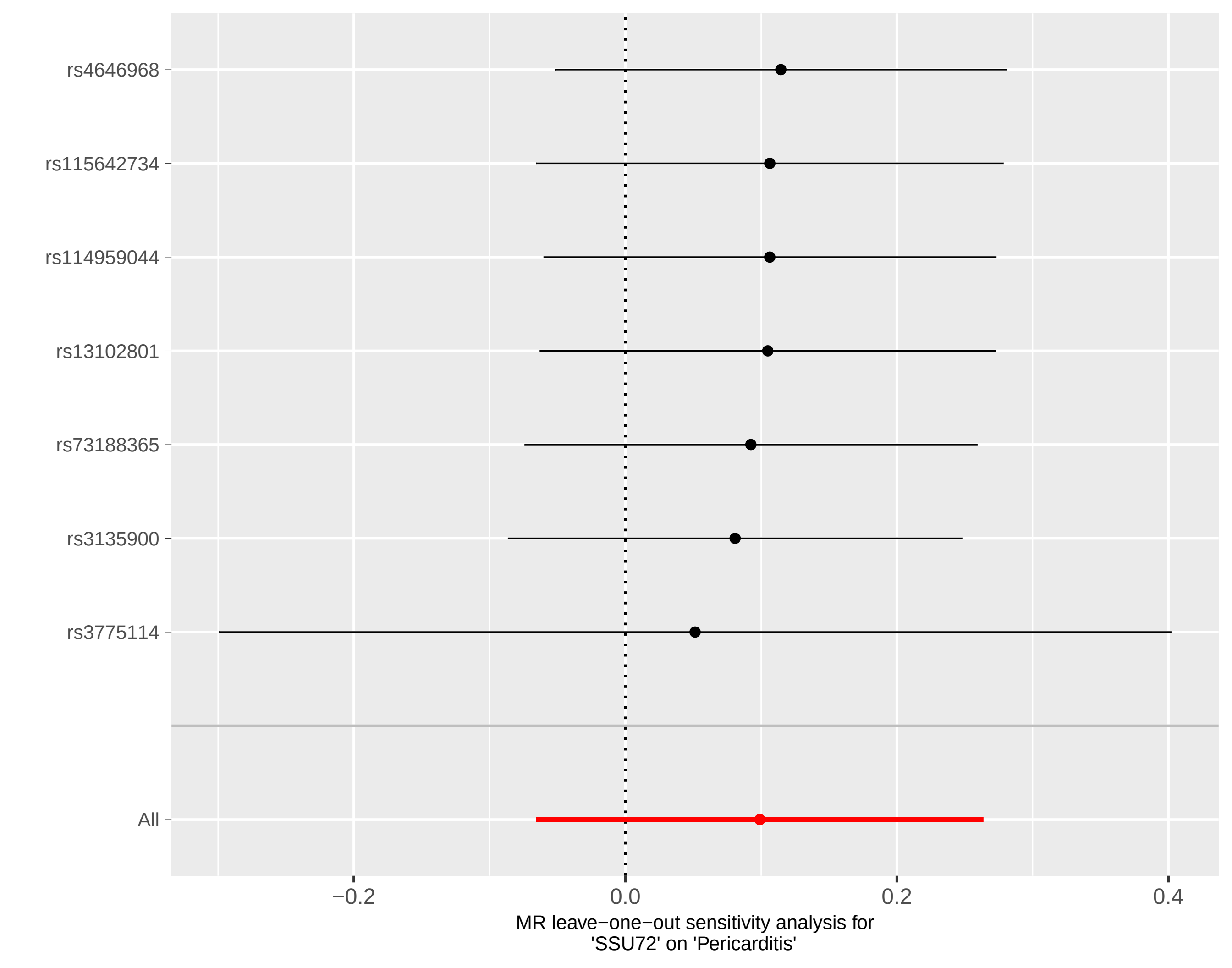

rs4646968
rs115642734
rs114959044
rs13102801
rs73188365
rs3135900
rs3775114
All
−0.2
0.0
0.2
0.4
MR leave−one−out sensitivity analysis for
'SSU72' on 'Pericarditis'

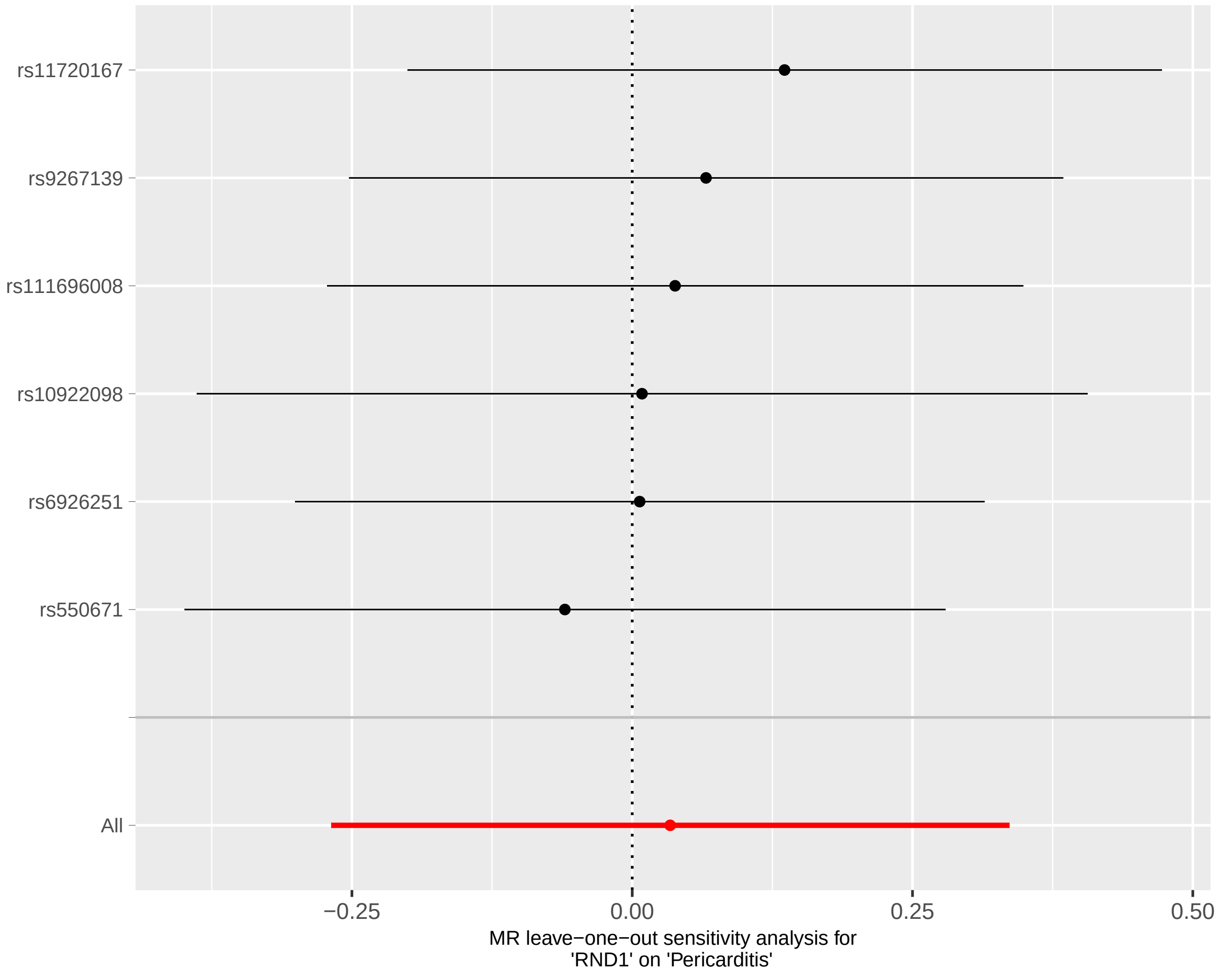

rs11720167
rs9267139
rs111696008
rs10922098
rs6926251
rs550671
All
−0.25
0.00
0.25
0.50
MR leave−one−out sensitivity analysis for
'RND1' on 'Pericarditis'

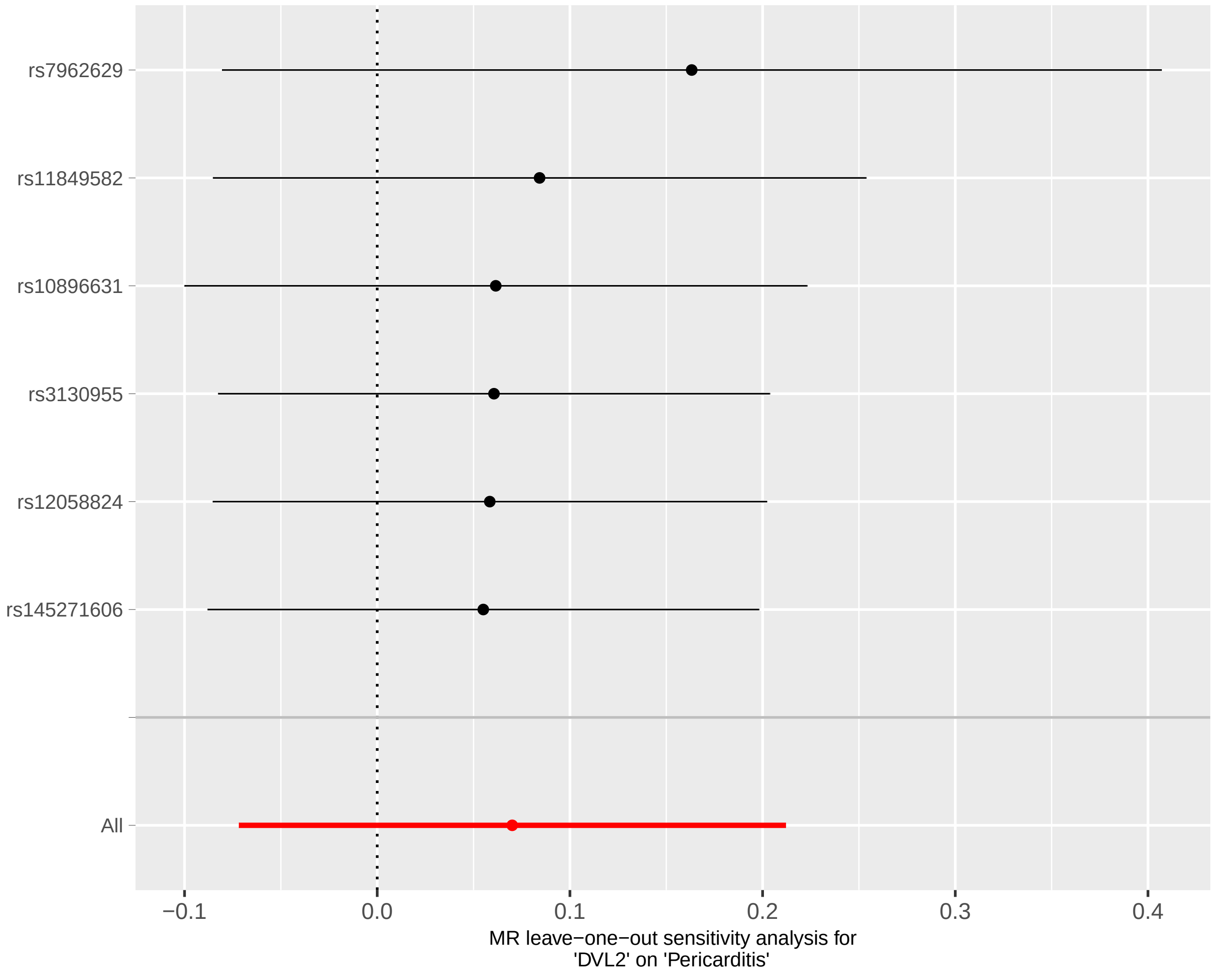
rs7962629
rs11849582
rs10896631
rs3130955
rs12058824
rs145271606
All
−0.1
0.0
0.1
0.2
0.3
0.4
MR leave−one−out sensitivity analysis for
'DVL2' on 'Pericarditis'

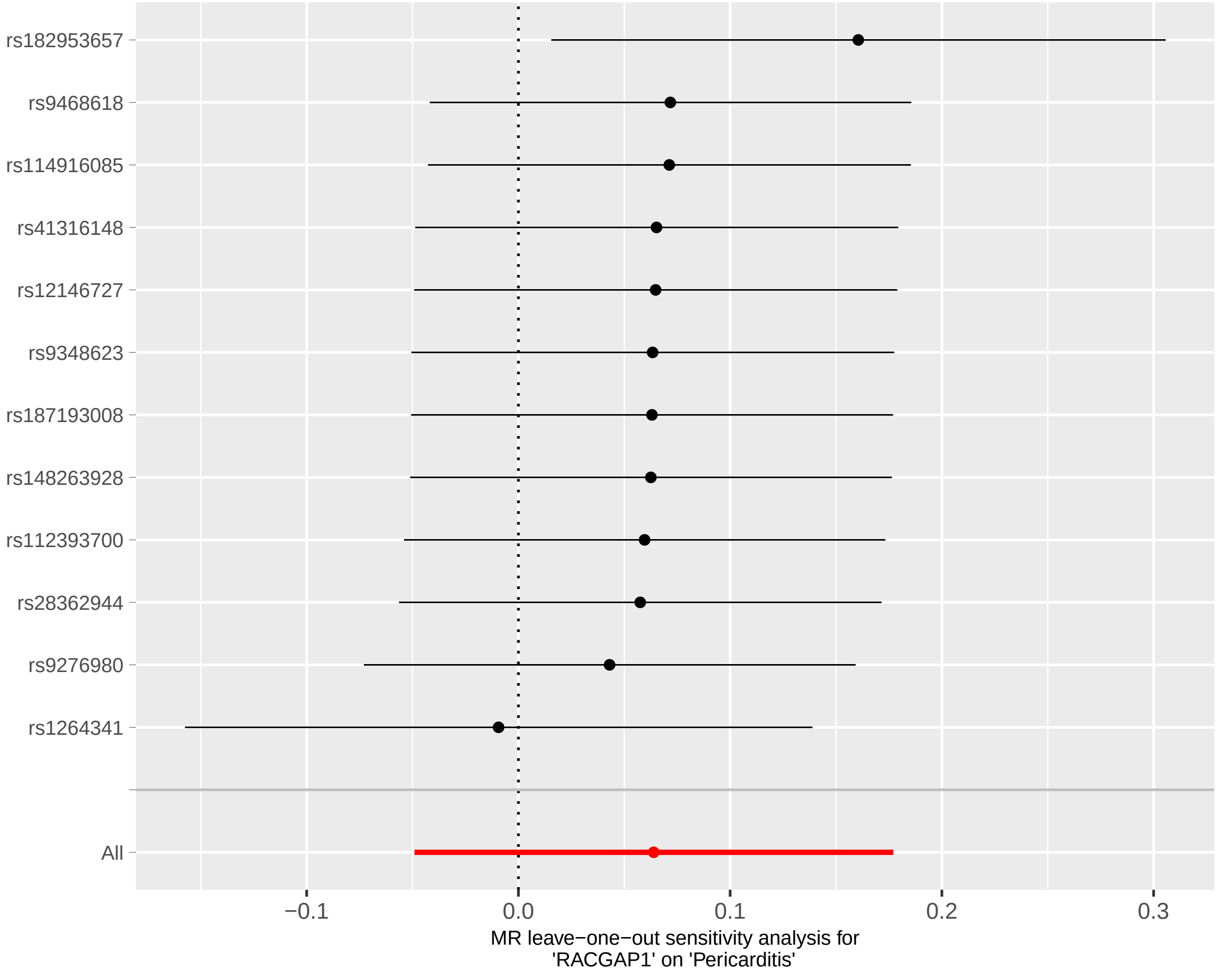

rs182953657
rs9468618
rs114916085
rs41316148
rs12146727
rs9348623
rs187193008
rs148263928
rs112393700
rs28362944
rs9276980
rs1264341
All
−0.1
0.0
0.1
0.2
0.3
MR leave−one−out sensitivity analysis for
'RACGAP1' on 'Pericarditis'

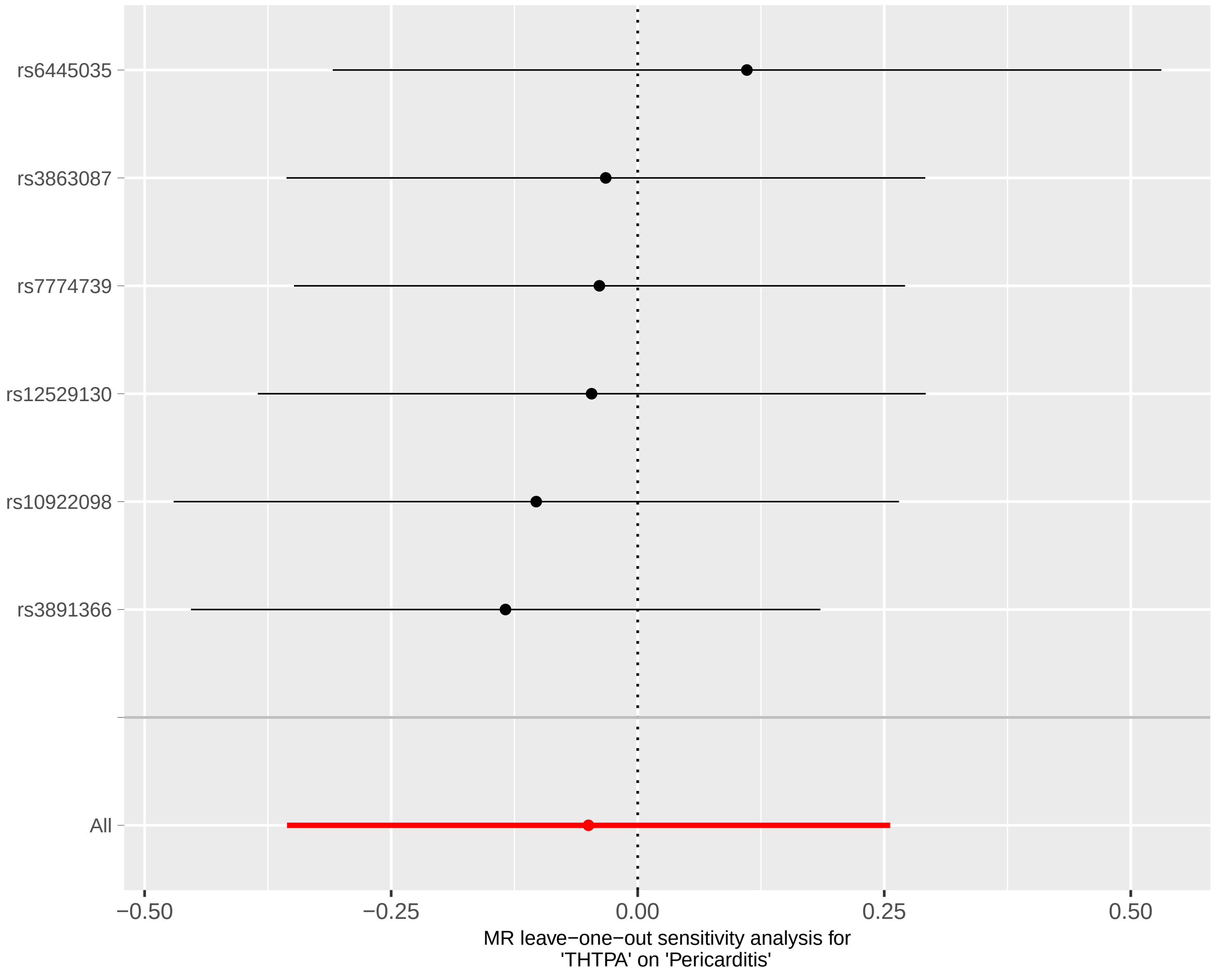

rs6445035
rs3863087
rs7774739
rs12529130
rs10922098
rs3891366
All
−0.50
−0.25
0.00
0.25
0.50
MR leave−one−out sensitivity analysis for
'THTPA' on 'Pericarditis'

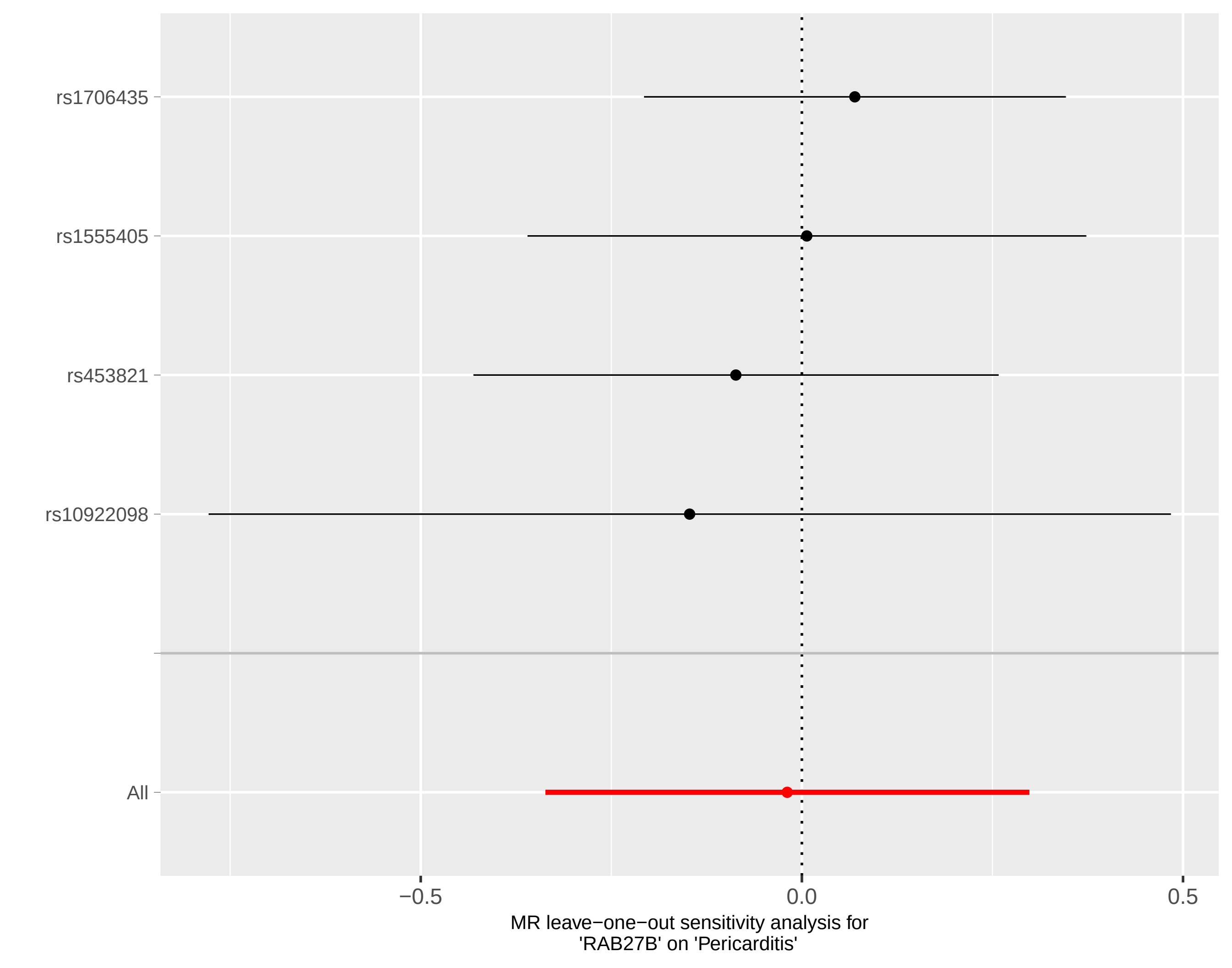
rs1706435
rs1555405
rs453821
rs10922098
All
−0.5
0.0
0.5
MR leave−one−out sensitivity analysis for
'RAB27B' on 'Pericarditis'

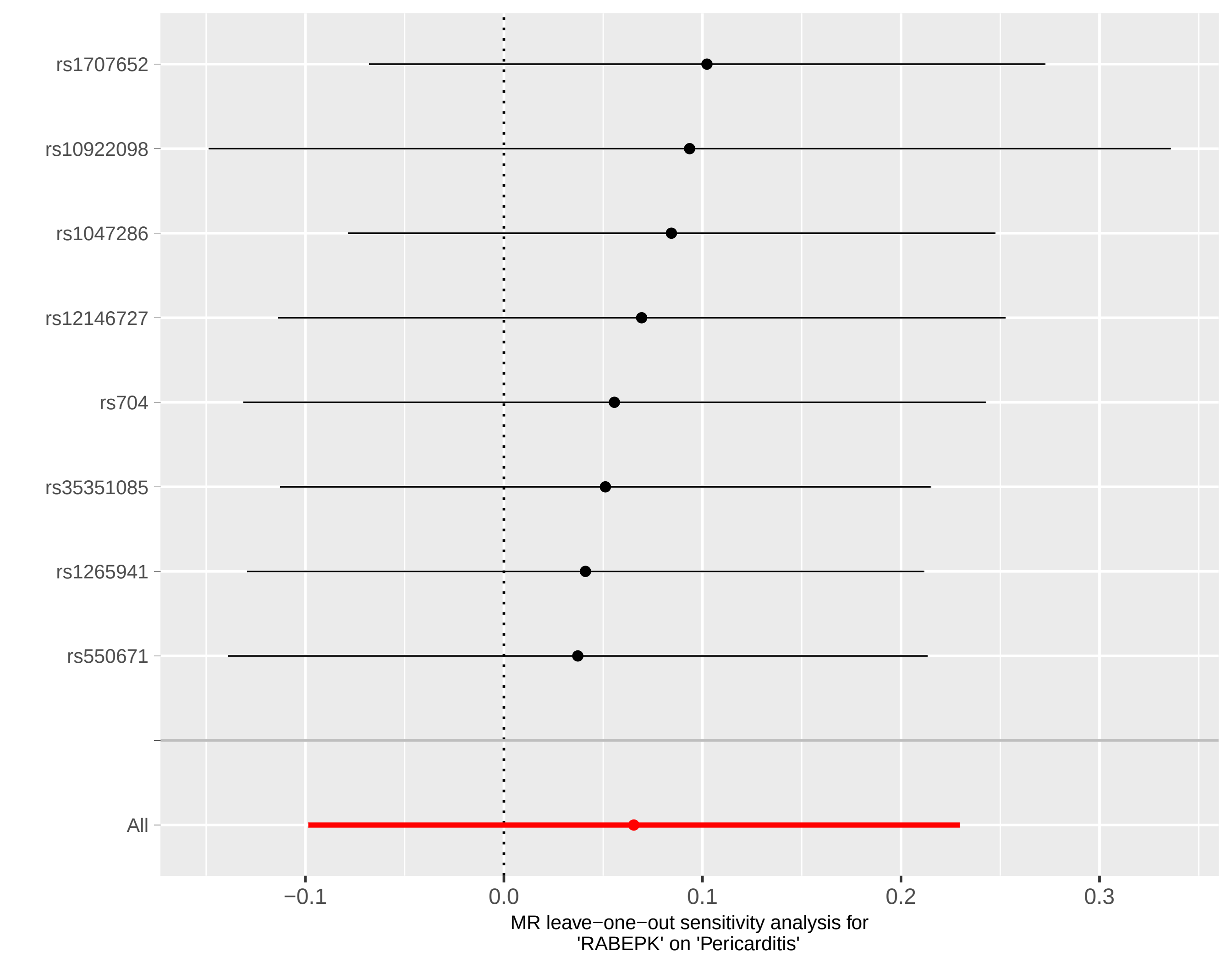
rs1707652
rs10922098
rs1047286
rs12146727
rs704
rs35351085
rs1265941
rs550671
All
−0.1
0.0
0.1
0.2
0.3
MR leave−one−out sensitivity analysis for
'RABEPK' on 'Pericarditis'

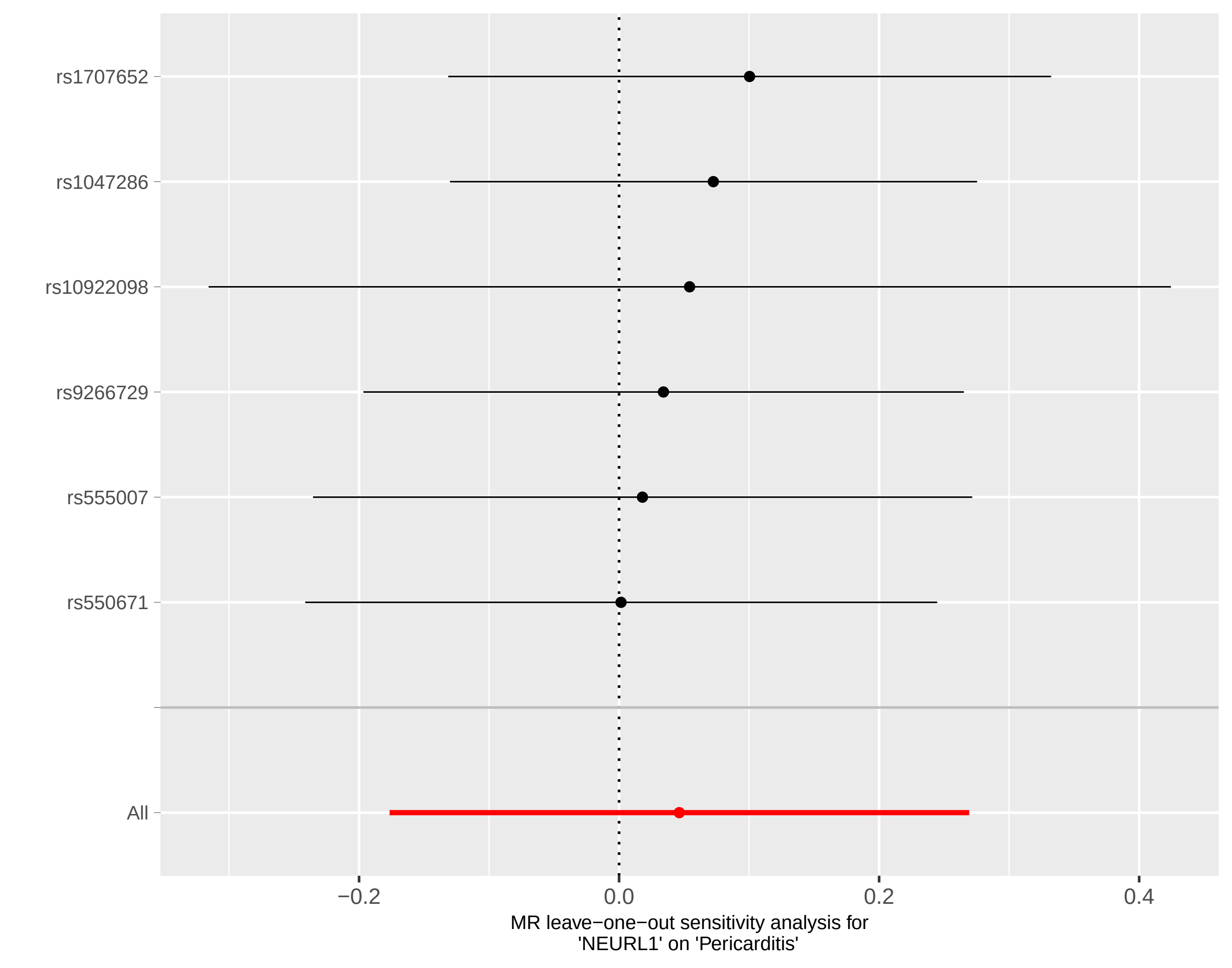

rs1707652
rs1047286
rs10922098
rs9266729
rs555007
rs550671
All
−0.2
0.0
0.2
0.4
MR leave−one−out sensitivity analysis for
'NEURL1' on 'Pericarditis'

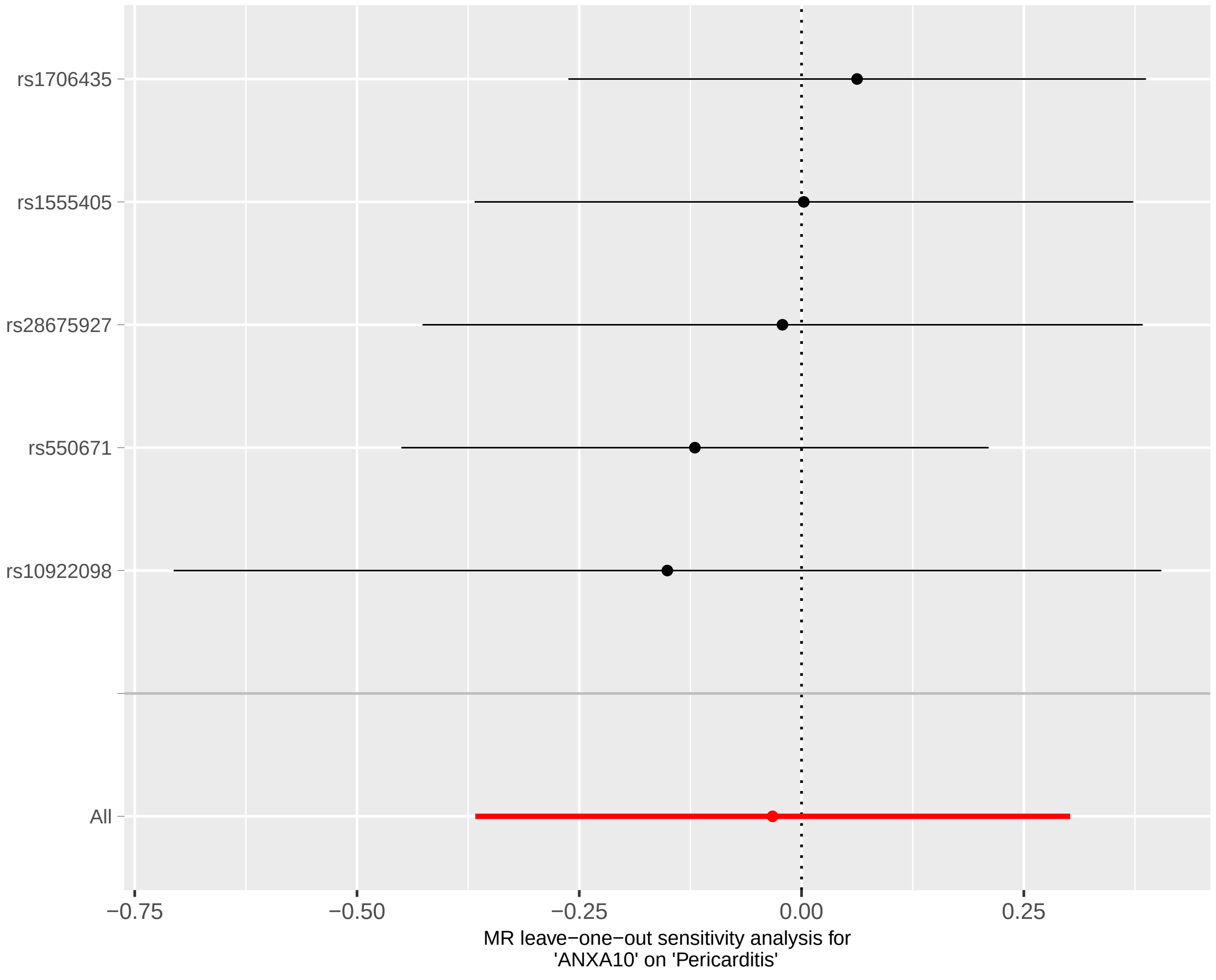
rs1706435
rs1555405
rs28675927
rs550671
rs10922098
All
−0.75
−0.50
−0.25
0.00
0.25
MR leave−one−out sensitivity analysis for
'ANXA10' on 'Pericarditis'

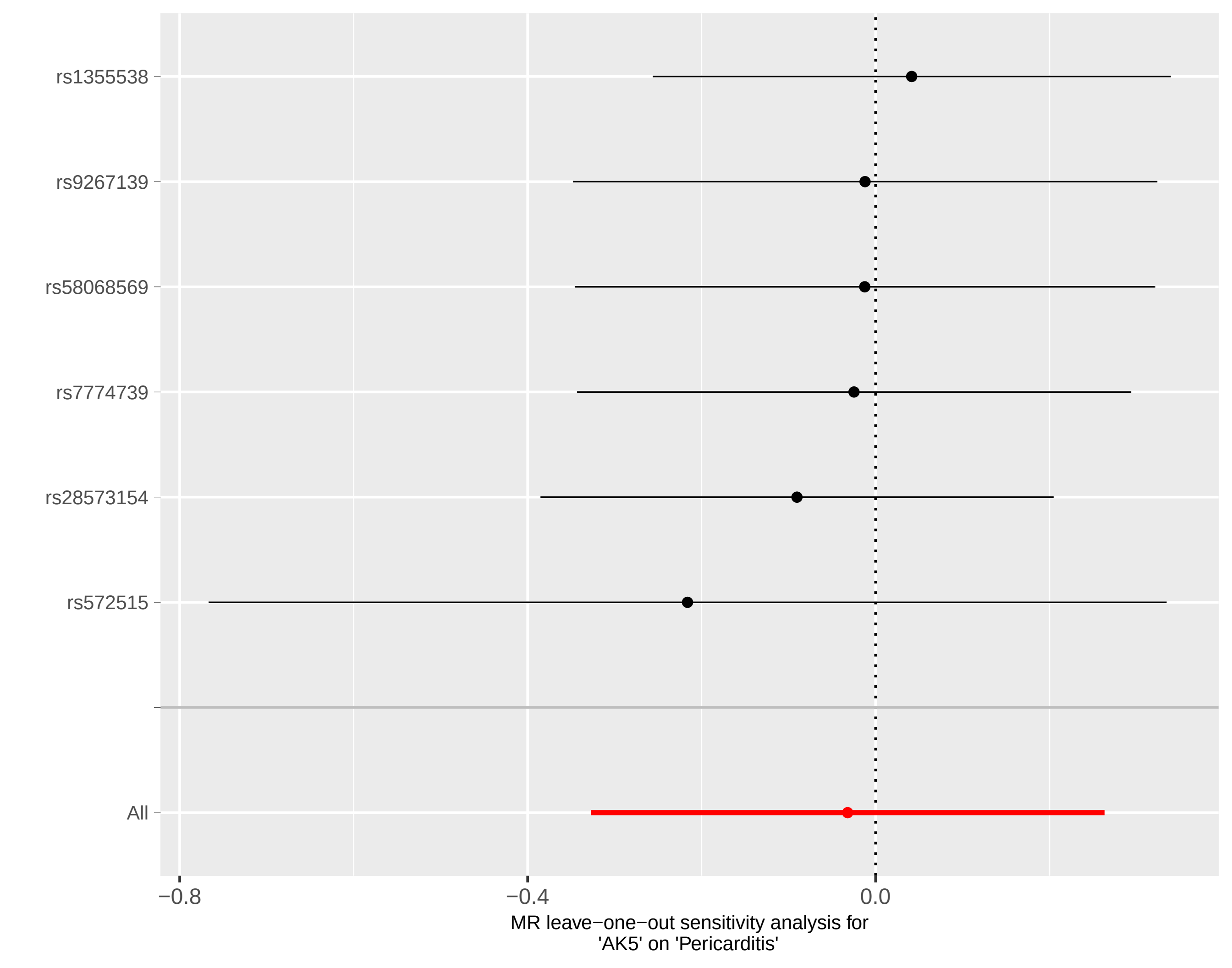
rs1355538
rs9267139
rs58068569
rs7774739
rs28573154
rs572515
All
−0.8
−0.4
0.0
MR leave−one−out sensitivity analysis for
'AK5' on 'Pericarditis'

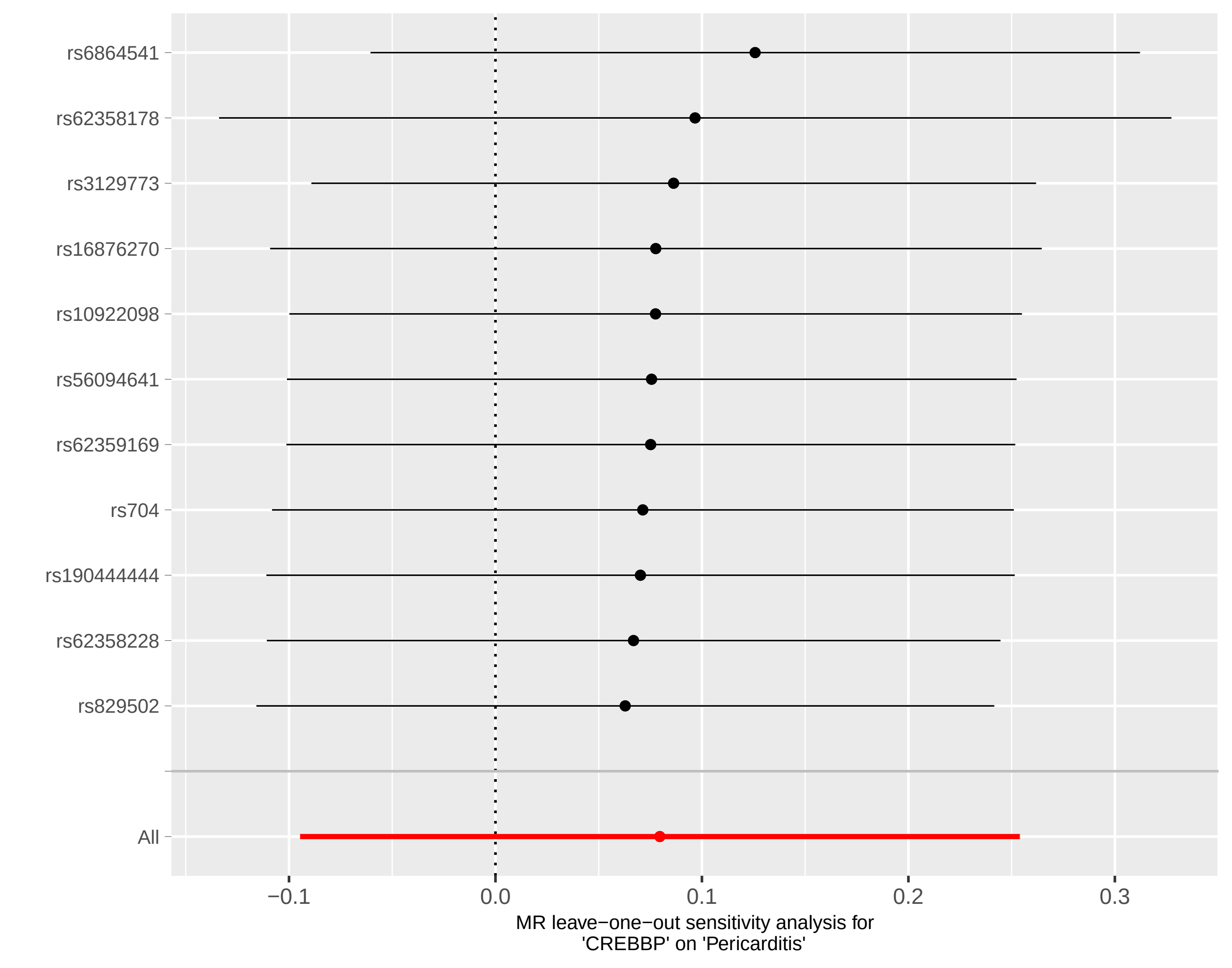

rs6864541
rs62358178
rs3129773
rs16876270
rs10922098
rs56094641
rs62359169
rs704
rs190444444
rs62358228
rs829502
All
−0.1
0.0
0.1
0.2
0.3
MR leave−one−out sensitivity analysis for
'CREBBP' on 'Pericarditis'

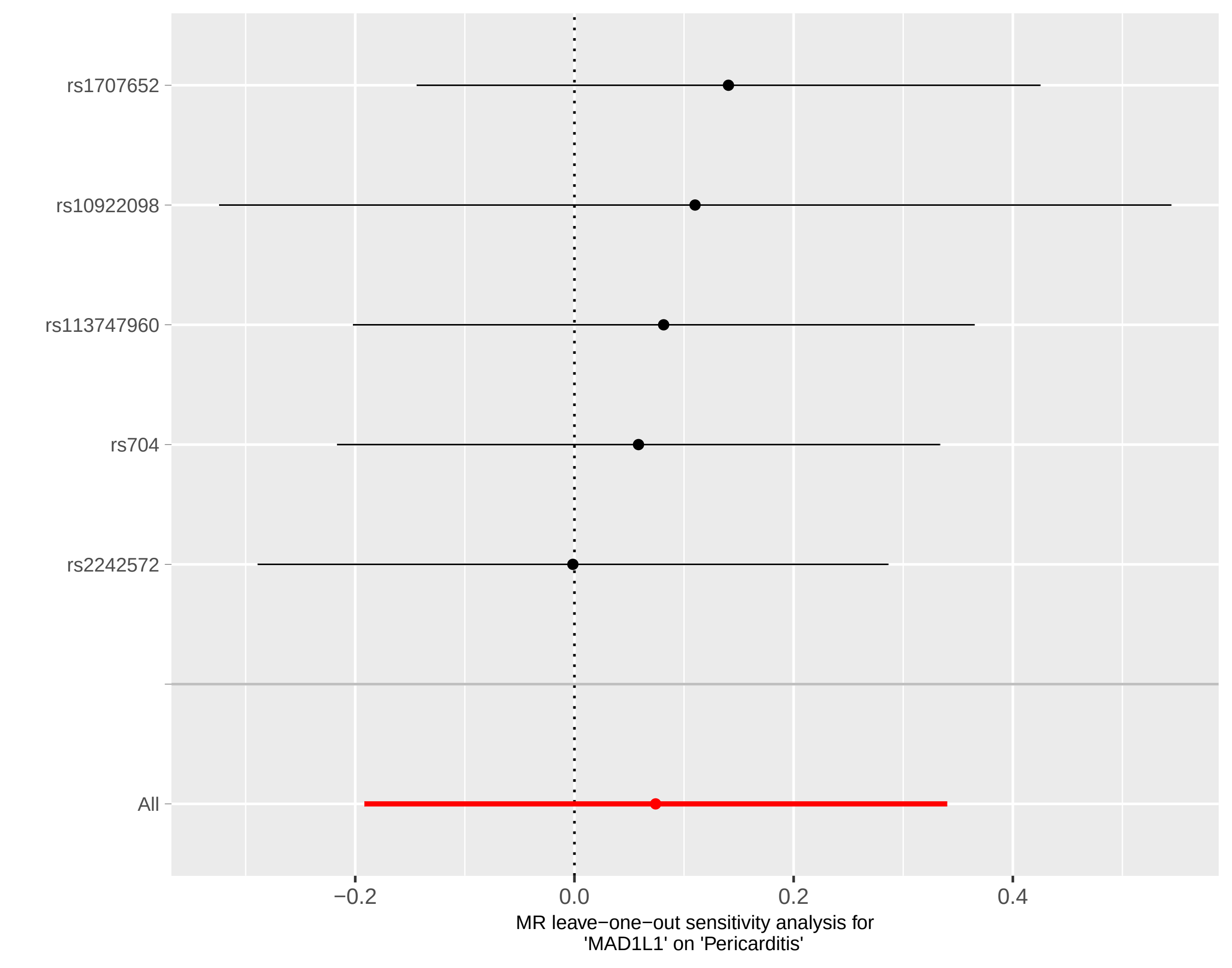
rs1707652
rs10922098
rs113747960
rs704
rs2242572
All
−0.2
0.0
0.2
0.4
MR leave−one−out sensitivity analysis for
'MAD1L1' on 'Pericarditis'

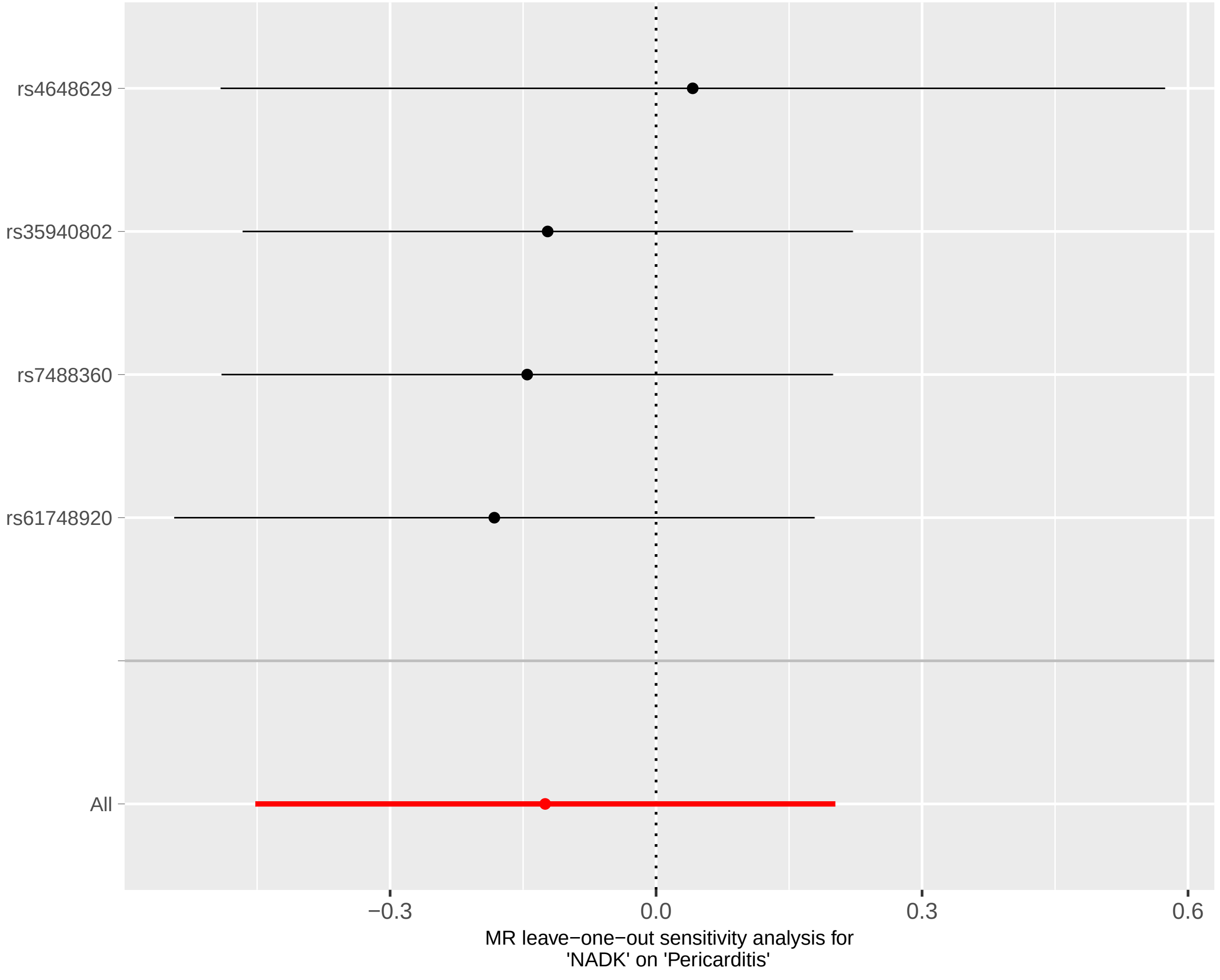
rs4648629
rs35940802
rs7488360
rs61748920
All
−0.3
0.0
0.3
0.6
MR leave−one−out sensitivity analysis for
'NADK' on 'Pericarditis'

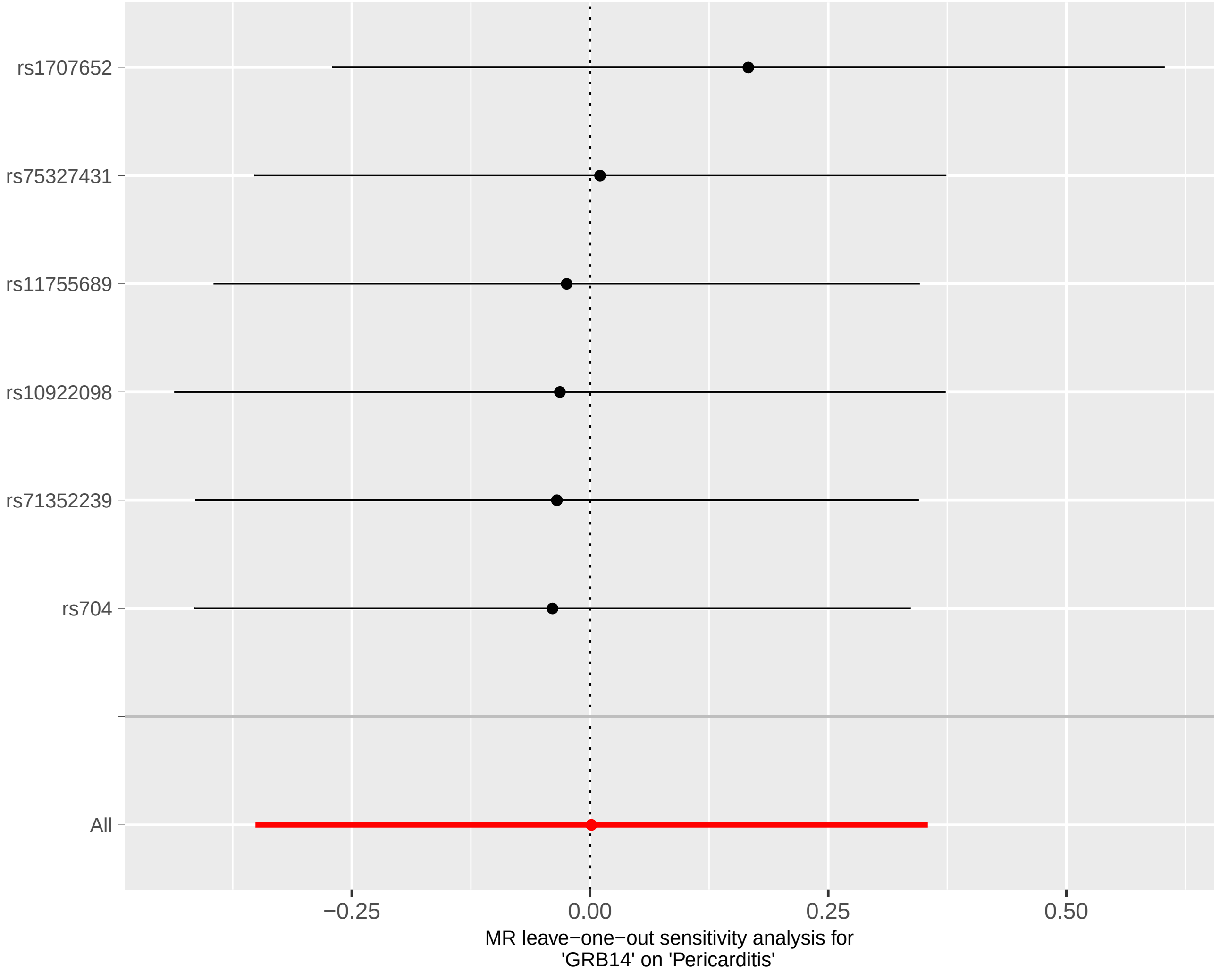
rs1707652
rs75327431
rs11755689
rs10922098
rs71352239
rs704
All
−0.25
0.00
0.25
0.50
MR leave−one−out sensitivity analysis for
'GRB14' on 'Pericarditis'

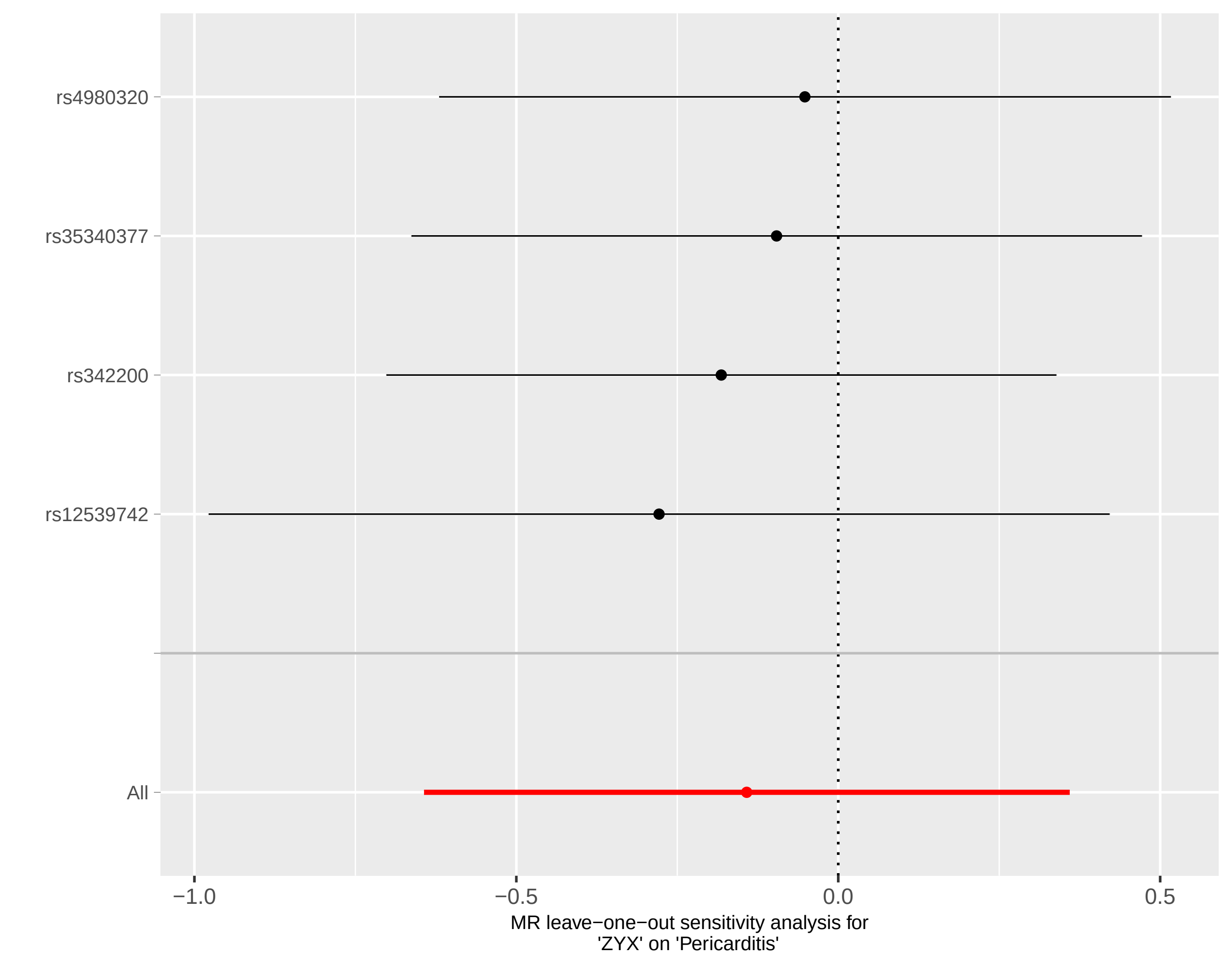
rs4980320
rs35340377
rs342200
rs12539742
All
−1.0
−0.5
0.0
0.5
MR leave−one−out sensitivity analysis for
'ZYX' on 'Pericarditis'

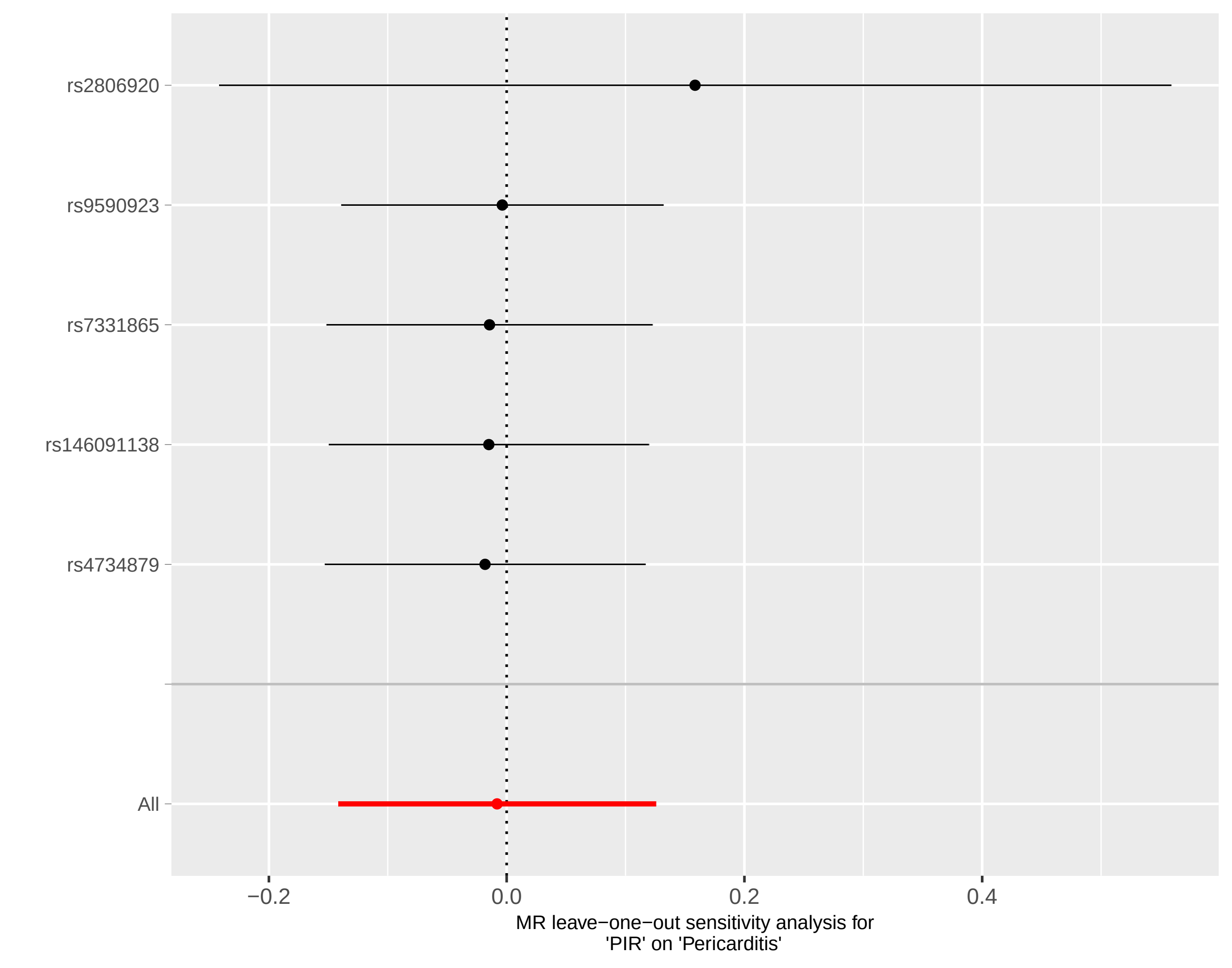

rs2806920
rs9590923
rs7331865
rs146091138
rs4734879
All
−0.2
0.0
0.2
0.4
MR leave−one−out sensitivity analysis for
'PIR' on 'Pericarditis'

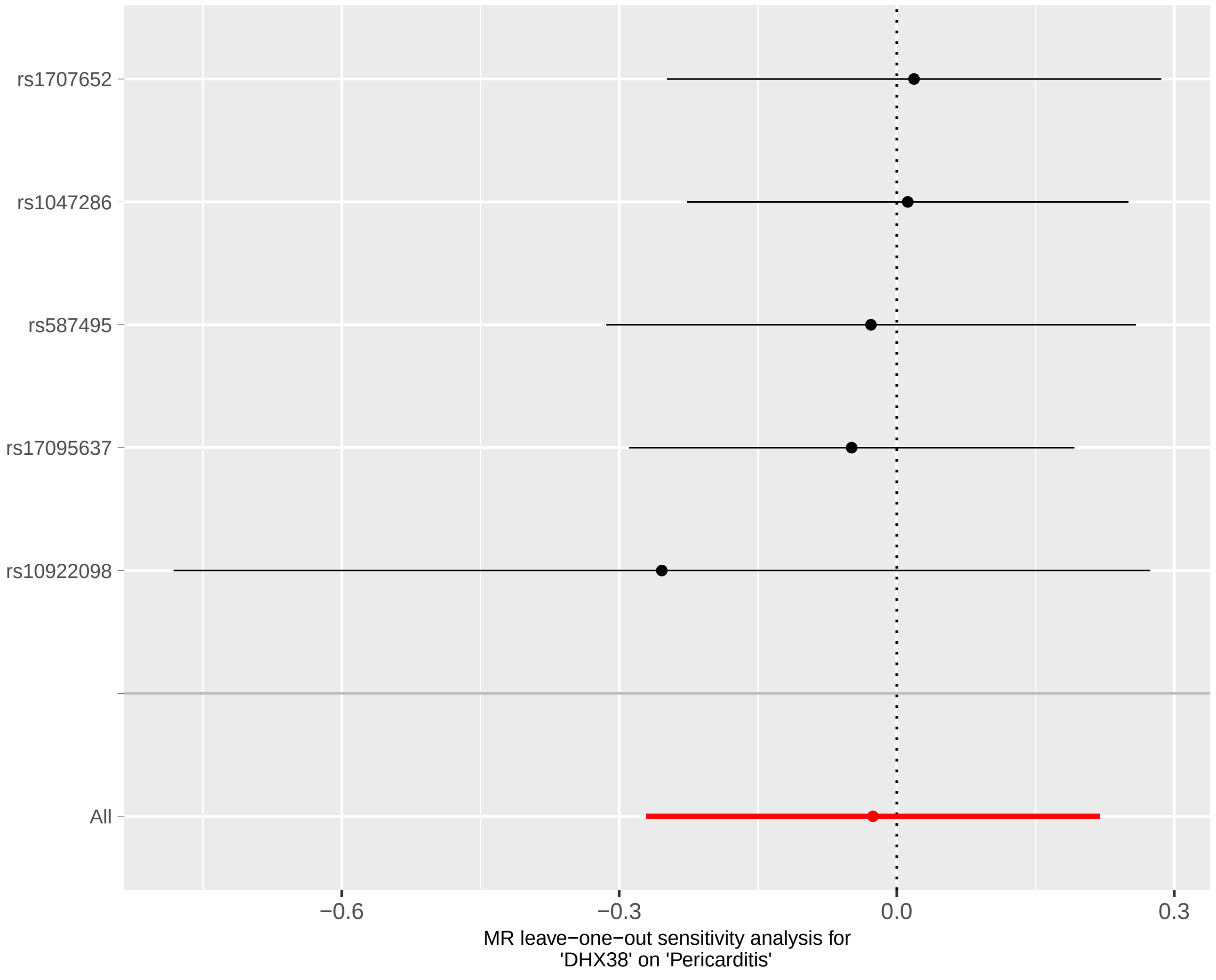

rs1707652
rs1047286
rs587495
rs17095637
rs10922098
All
−0.6
−0.3
0.0
0.3
MR leave−one−out sensitivity analysis for
'DHX38' on 'Pericarditis'

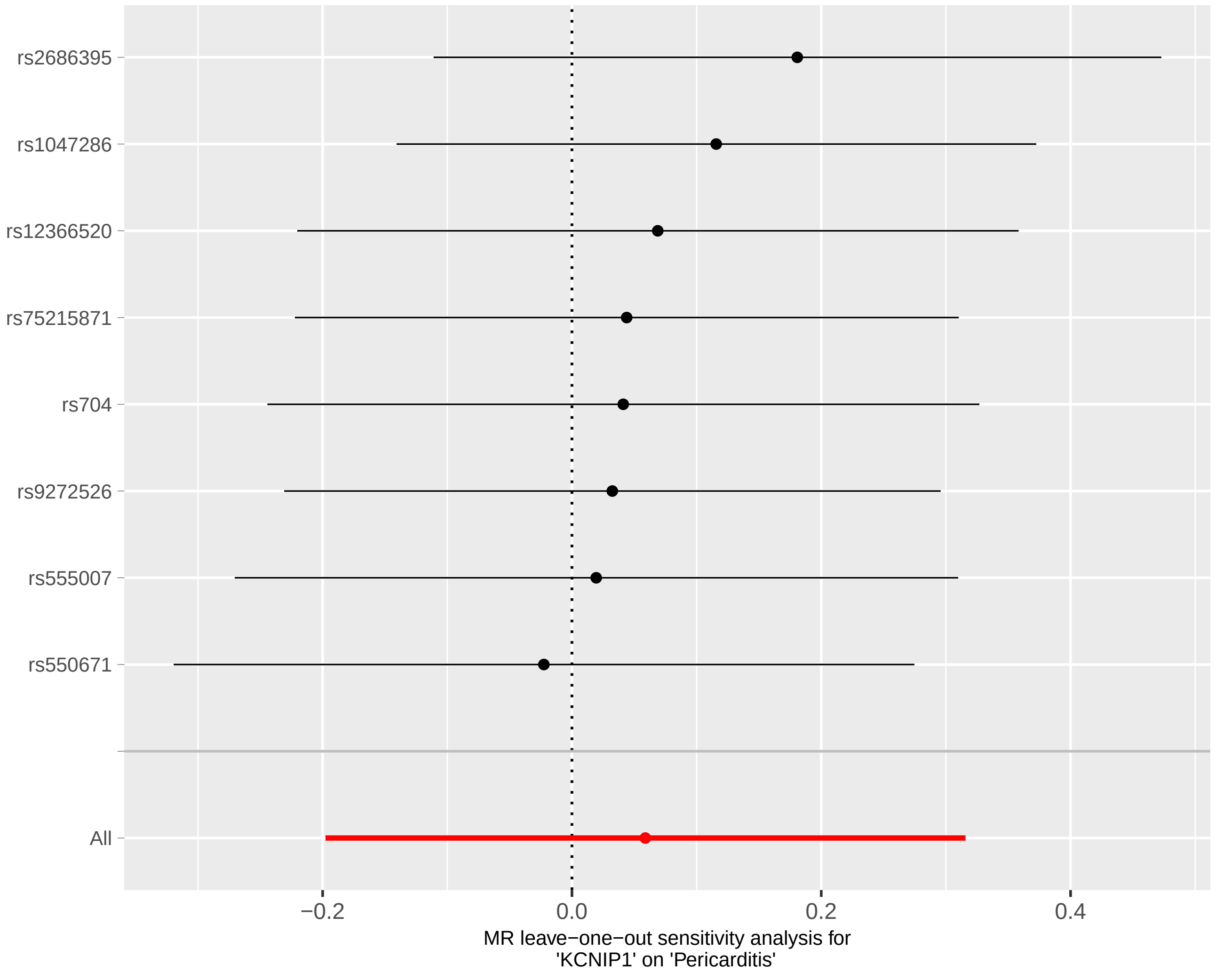
rs2686395
rs1047286
rs12366520
rs75215871
rs704
rs9272526
rs555007
rs550671
All
−0.2
0.0
0.2
0.4
MR leave−one−out sensitivity analysis for
'KCNIP1' on 'Pericarditis'

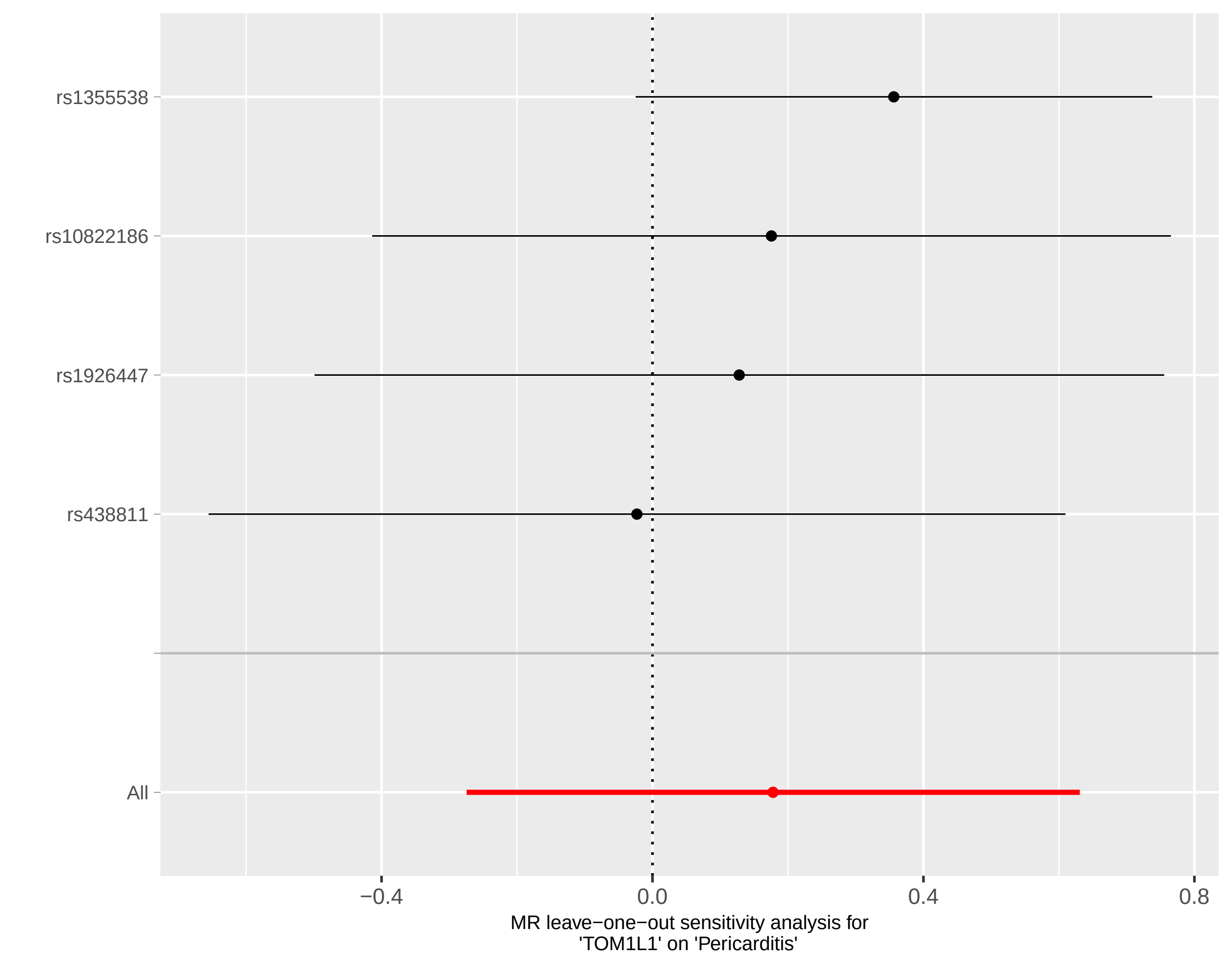

rs1355538
rs10822186
rs1926447
rs438811
All
−0.4
0.0
0.4
0.8
MR leave−one−out sensitivity analysis for
'TOM1L1' on 'Pericarditis'

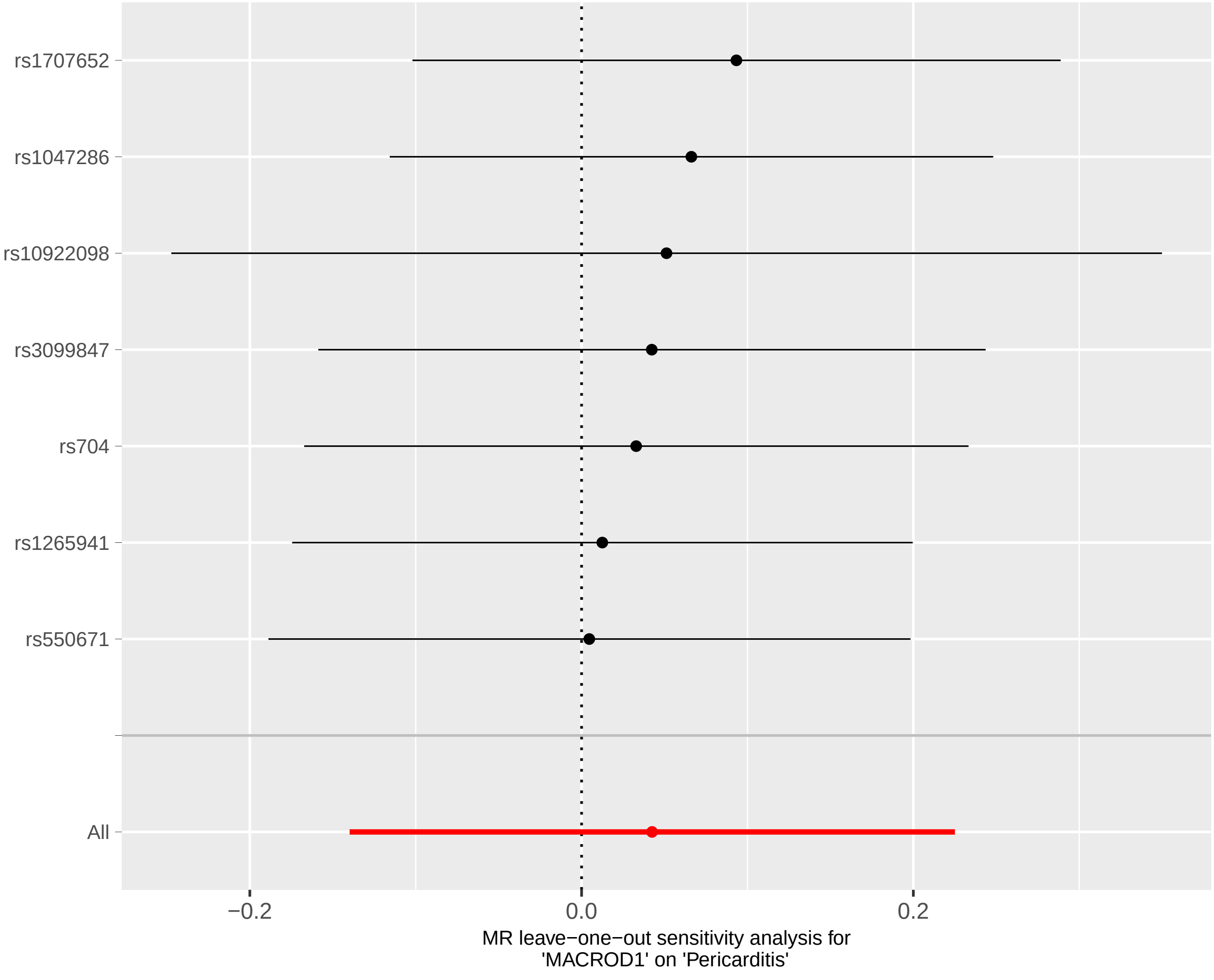

rs1707652
rs1047286
rs10922098
rs3099847
rs704
rs1265941
rs550671
All
−0.2
0.0
0.2
MR leave−one−out sensitivity analysis for
'MACROD1' on 'Pericarditis'

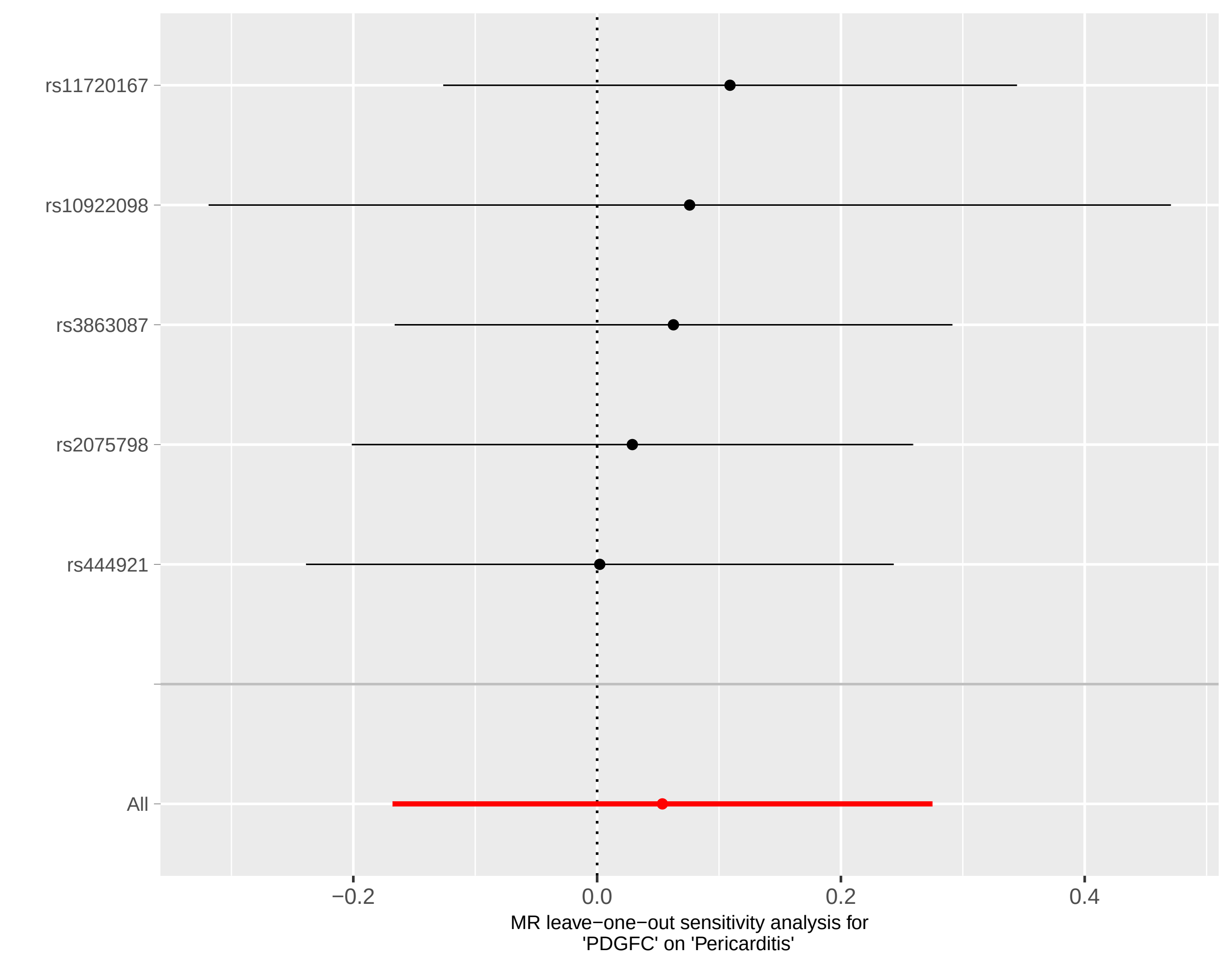
rs11720167
rs10922098
rs3863087
rs2075798
rs444921
All
−0.2
0.0
0.2
0.4
MR leave−one−out sensitivity analysis for
'PDGFC' on 'Pericarditis'

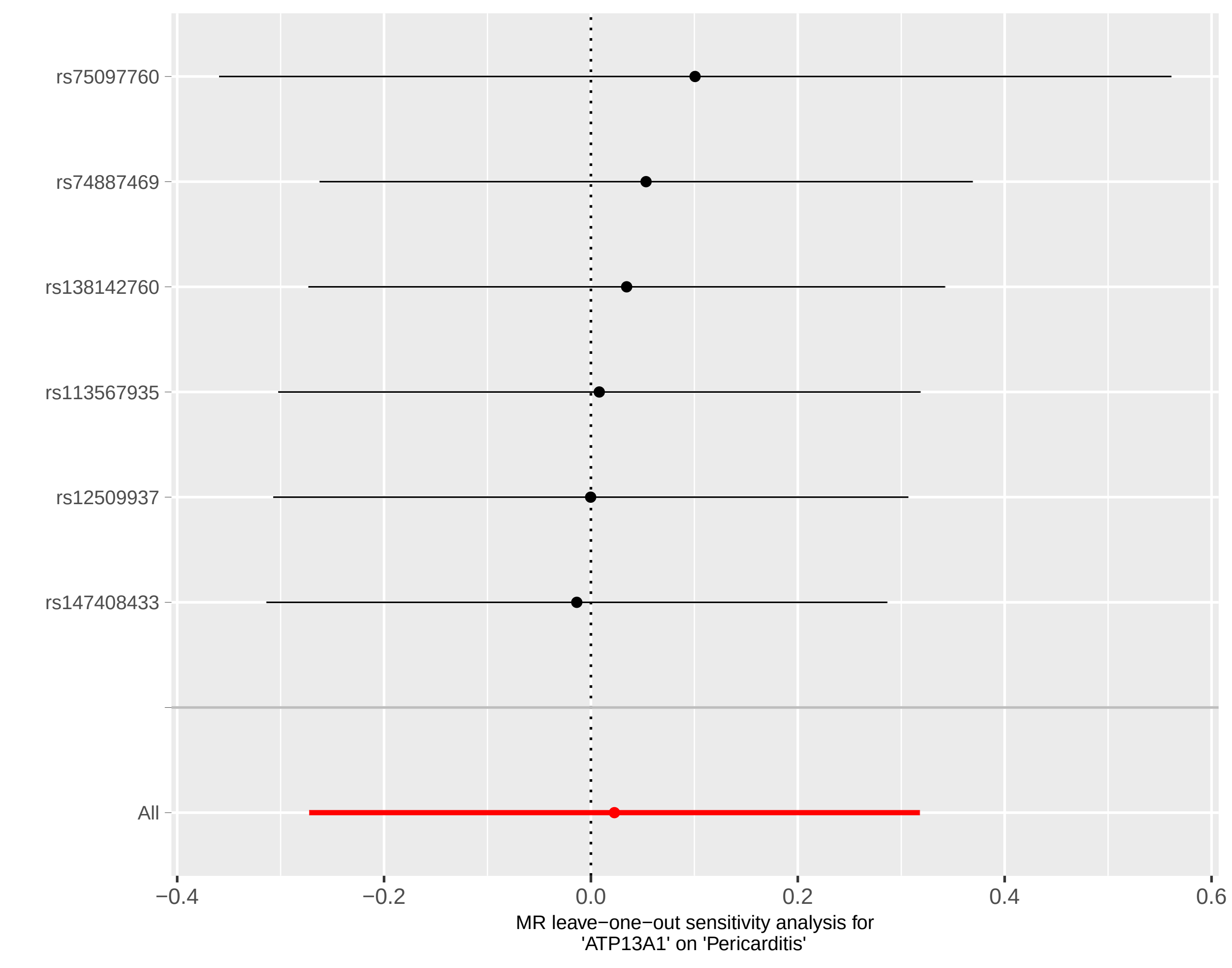

rs75097760
rs74887469
rs138142760
rs113567935
rs12509937
rs147408433
All
−0.4
−0.2
0.0
0.2
0.4
0.6
MR leave−one−out sensitivity analysis for
'ATP13A1' on 'Pericarditis'

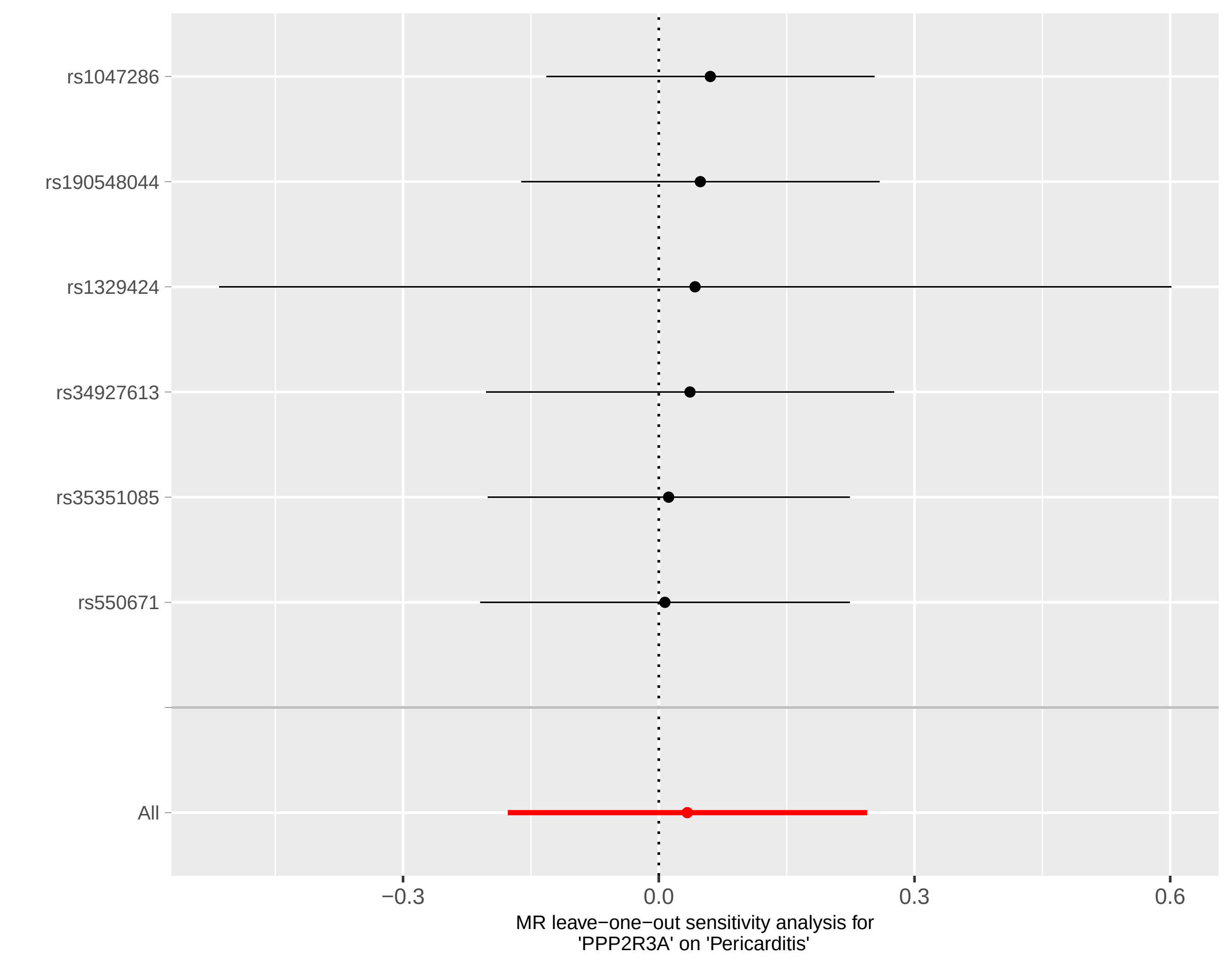
rs1047286
rs190548044
rs1329424
rs34927613
rs35351085
rs550671
All
−0.3
0.0
0.3
0.6
MR leave−one−out sensitivity analysis for
'PPP2R3A' on 'Pericarditis'

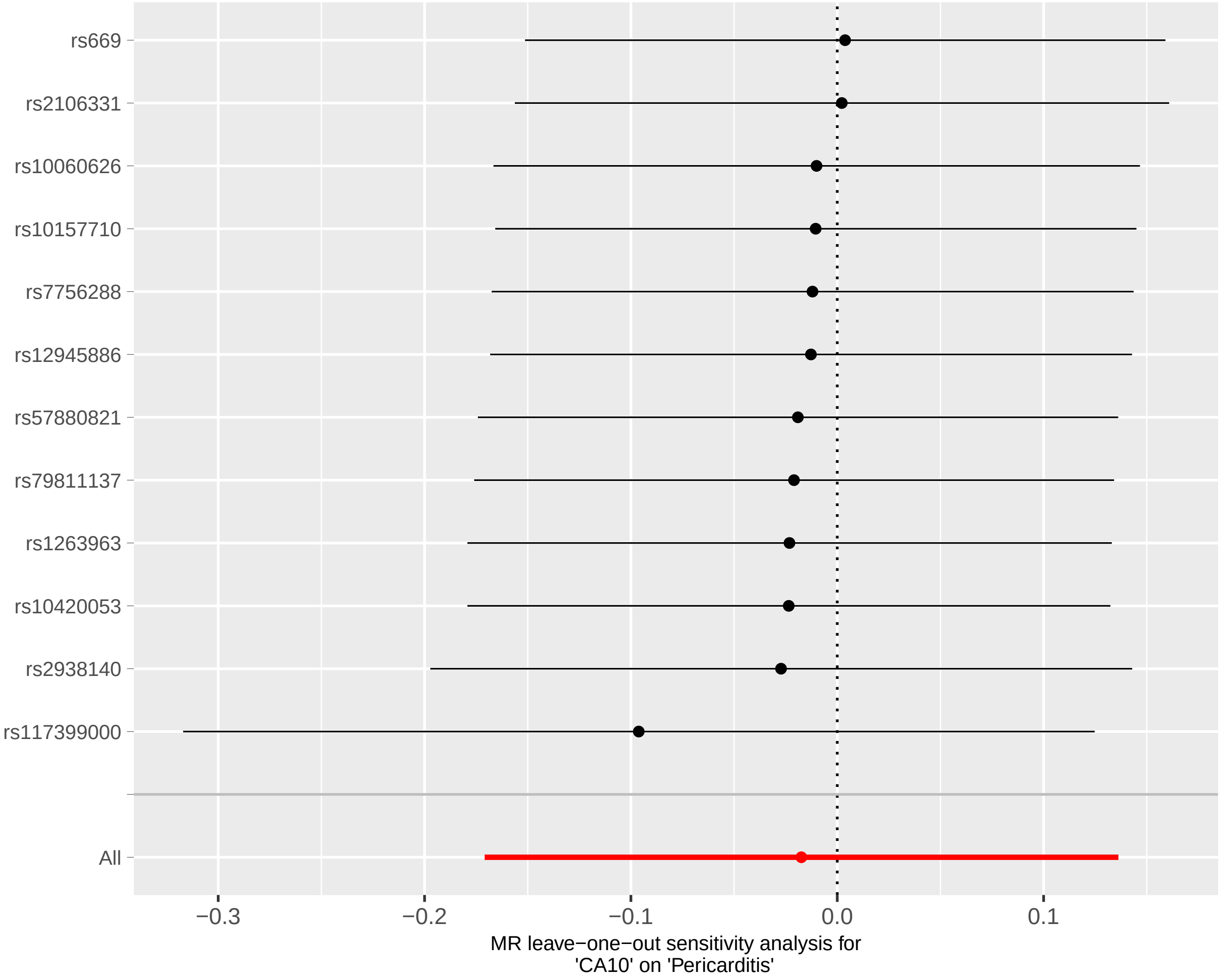

rs669
rs2106331
rs10060626
rs10157710
rs7756288
rs12945886
rs57880821
rs79811137
rs1263963
rs10420053
rs2938140
rs117399000
All
−0.3
−0.2
−0.1
0.0
0.1
MR leave−one−out sensitivity analysis for
'CA10' on 'Pericarditis'

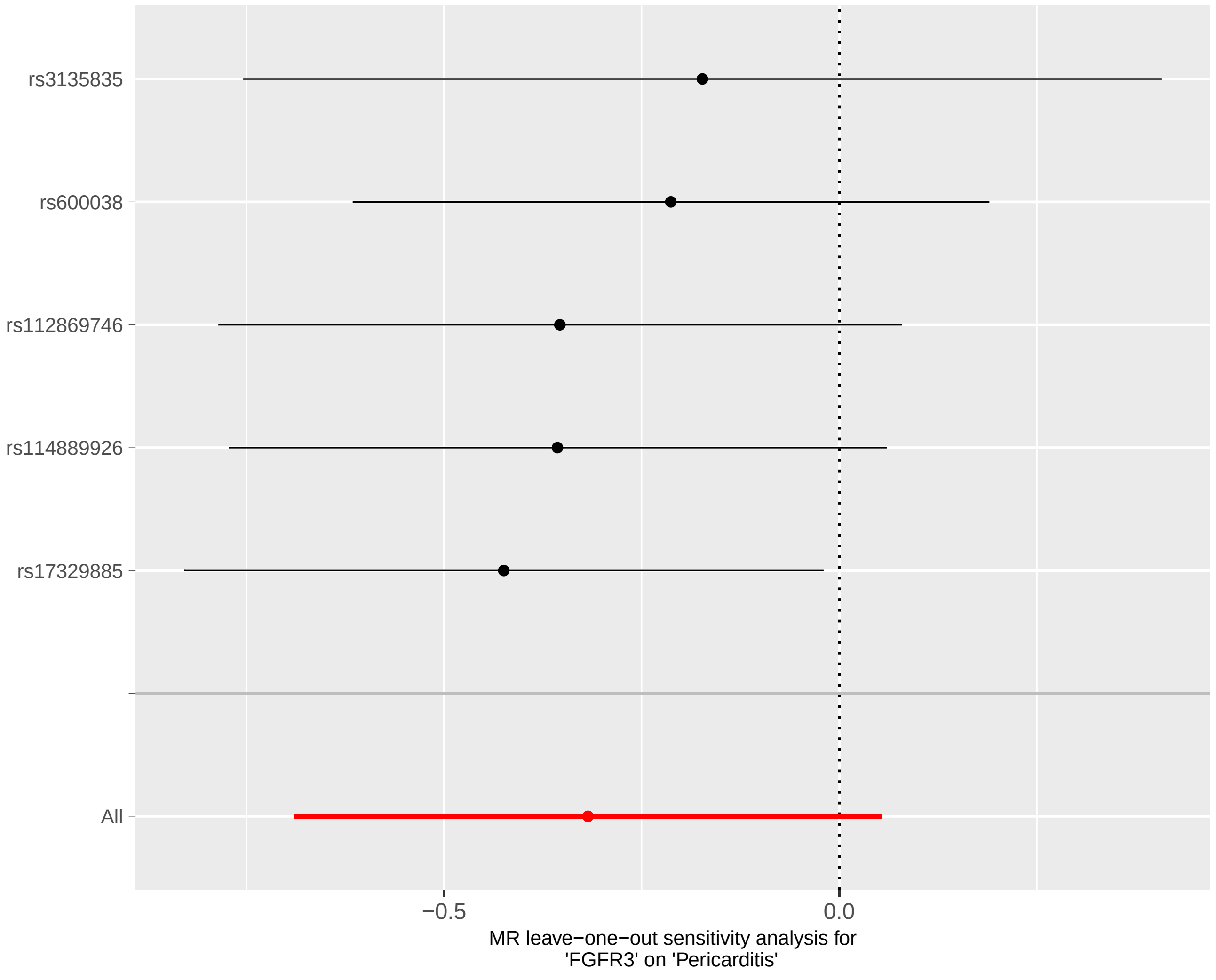
rs3135835
rs600038
rs112869746
rs114889926
rs17329885
All
−0.5
0.0
MR leave−one−out sensitivity analysis for
'FGFR3' on 'Pericarditis'

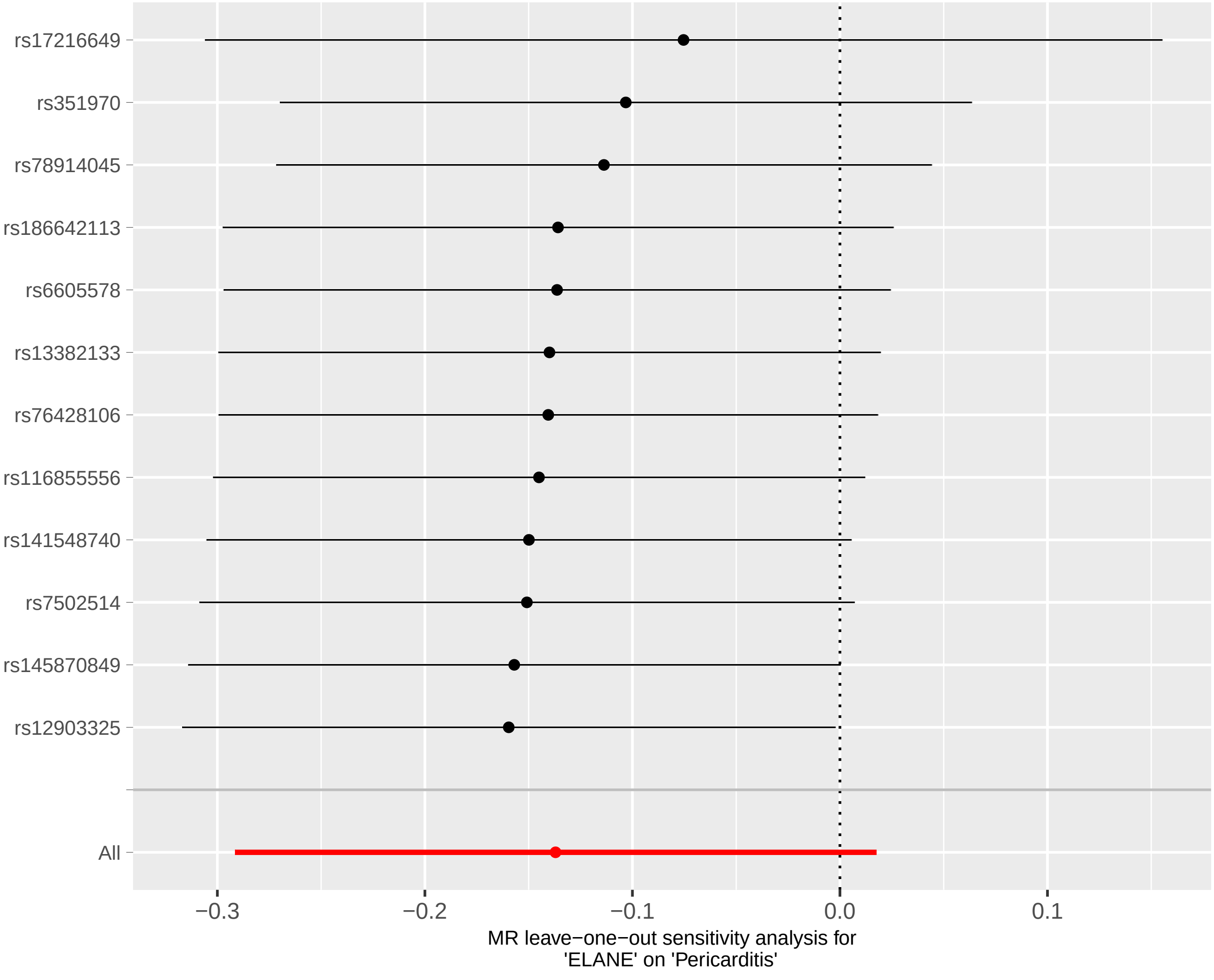
rs17216649
rs351970
rs78914045
rs186642113
rs6605578
rs13382133
rs76428106
rs116855556
rs141548740
rs7502514
rs145870849
rs12903325
All
−0.3
−0.2
−0.1
0.0
0.1
MR leave−one−out sensitivity analysis for
'ELANE' on 'Pericarditis'

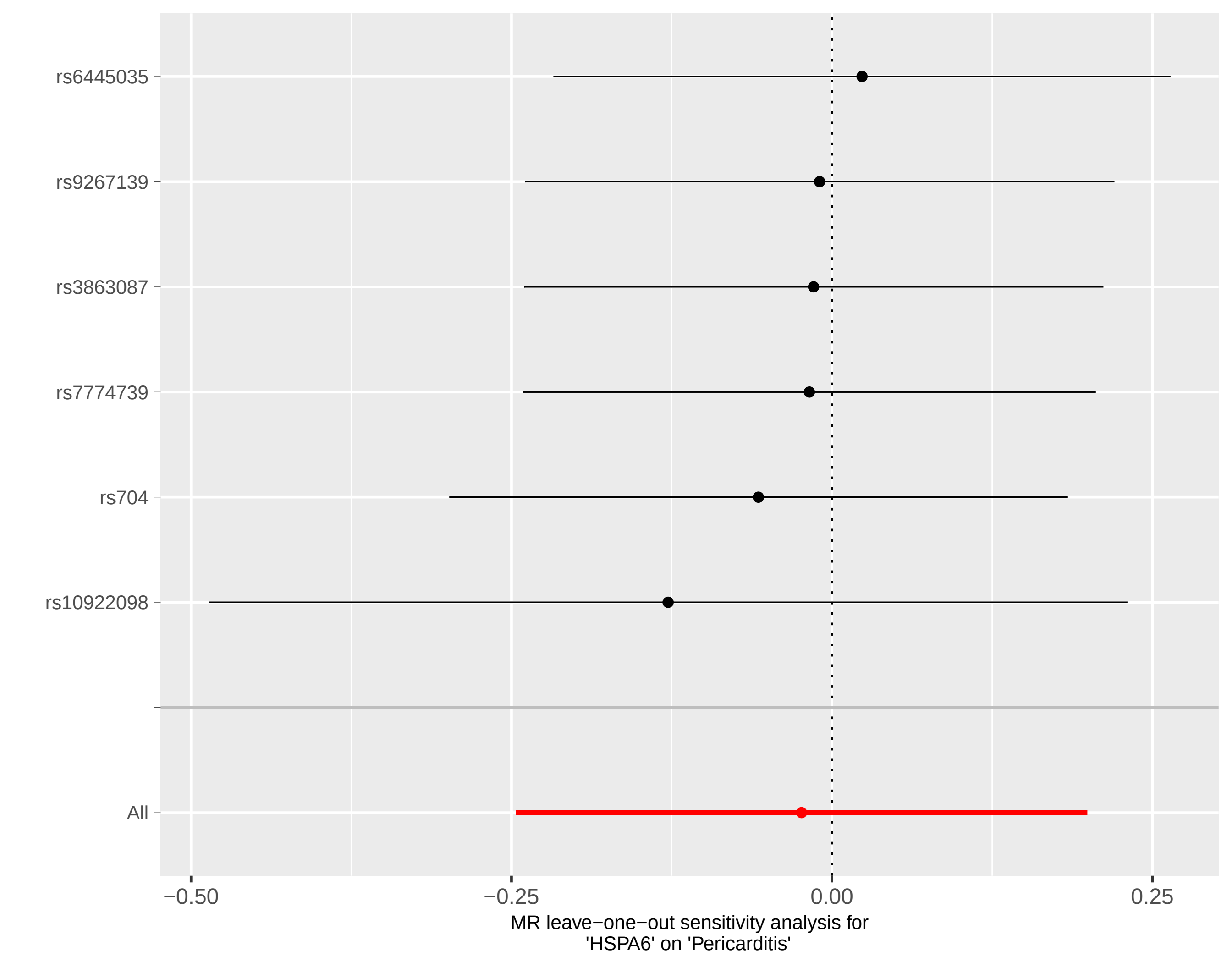

rs6445035
rs9267139
rs3863087
rs7774739
rs704
rs10922098
All
−0.50
−0.25
0.00
0.25
MR leave−one−out sensitivity analysis for
'HSPA6' on 'Pericarditis'

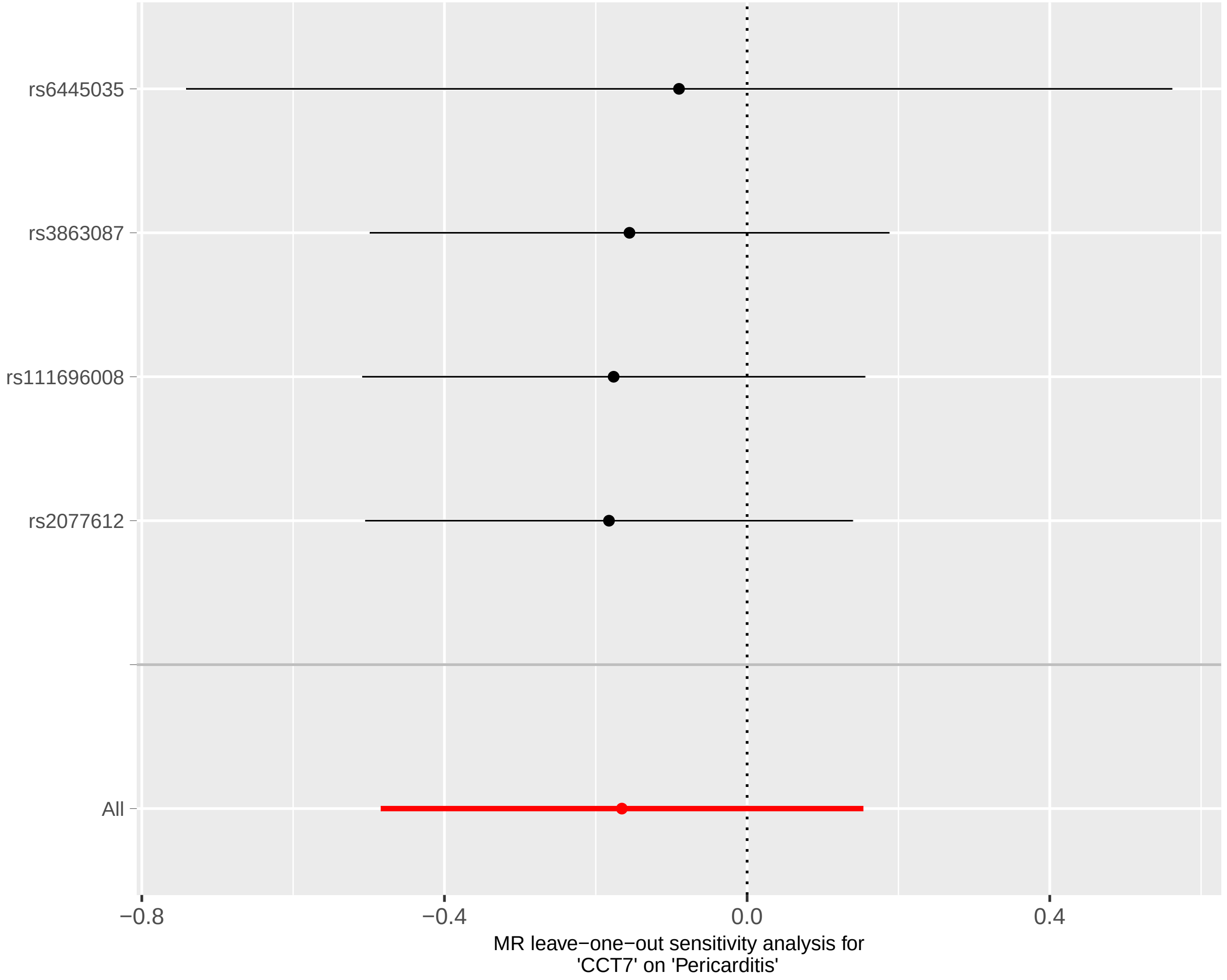

rs6445035
rs3863087
rs111696008
rs2077612
All
−0.8
−0.4
0.0
0.4
MR leave−one−out sensitivity analysis for
'CCT7' on 'Pericarditis'

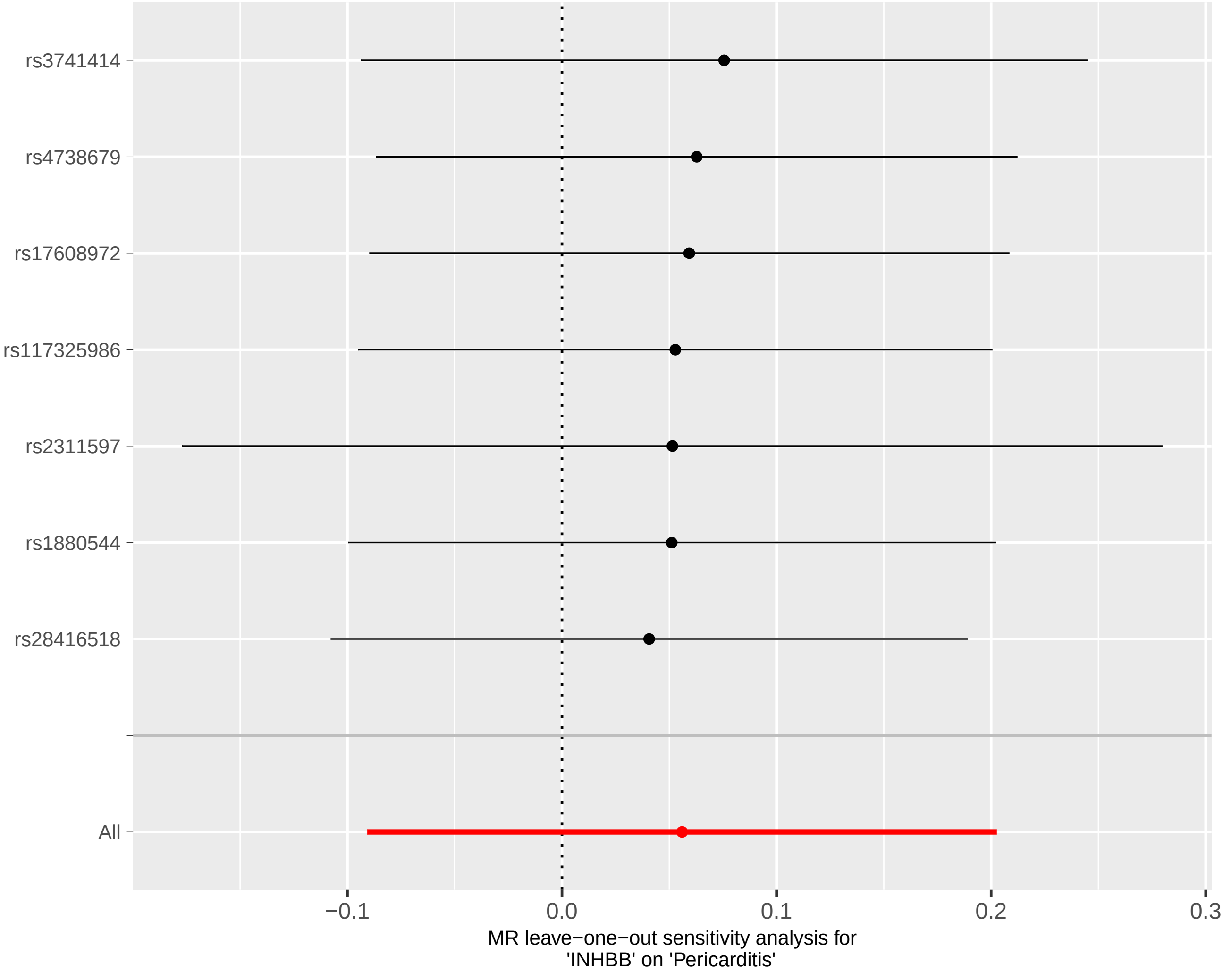

rs3741414
rs4738679
rs17608972
rs117325986
rs2311597
rs1880544
rs28416518
All
−0.1
0.0
0.1
0.2
0.3
MR leave−one−out sensitivity analysis for
'INHBB' on 'Pericarditis'

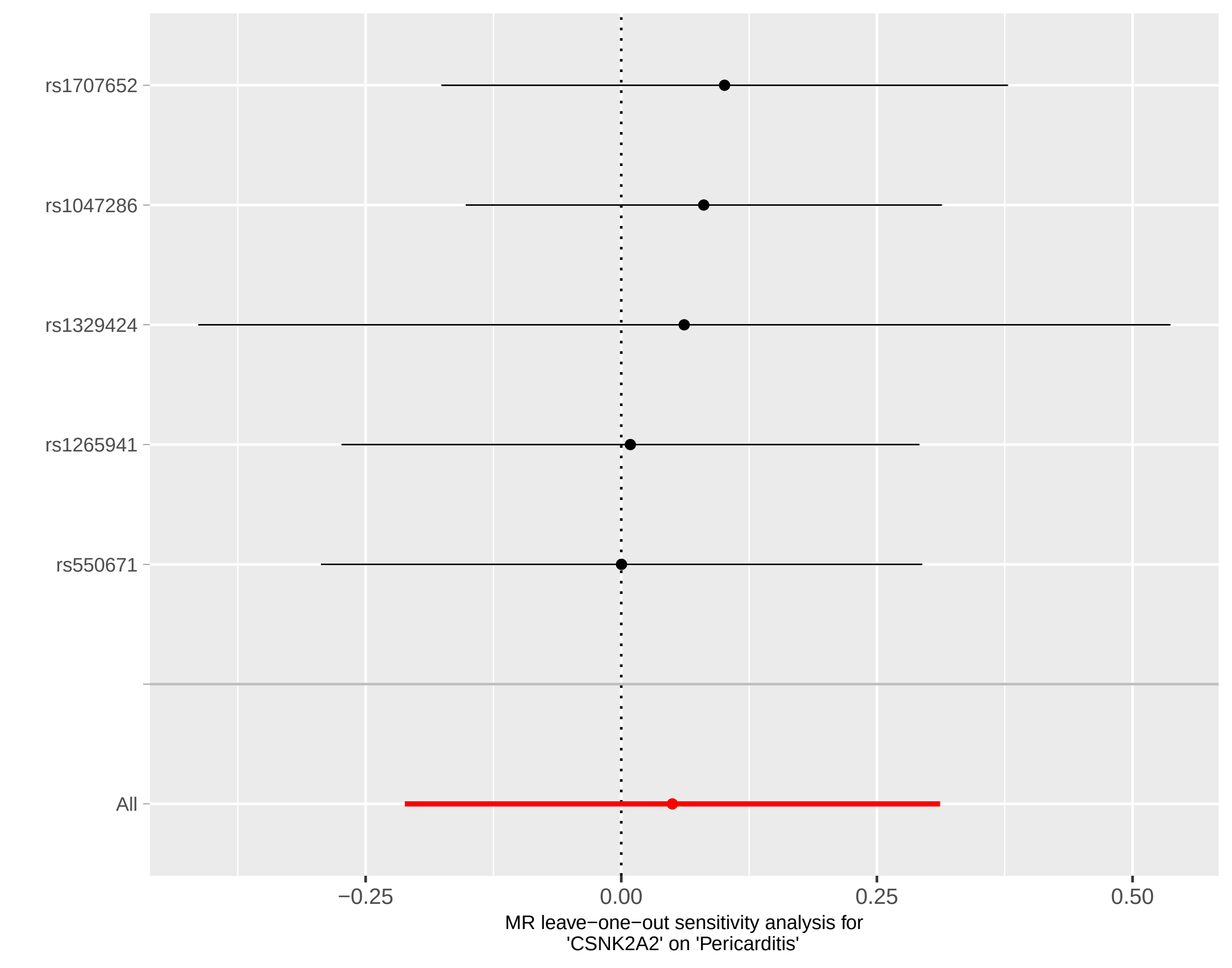

rs1707652
rs1047286
rs1329424
rs1265941
rs550671
All
−0.25
0.00
0.25
0.50
MR leave−one−out sensitivity analysis for
'CSNK2A2' on 'Pericarditis'

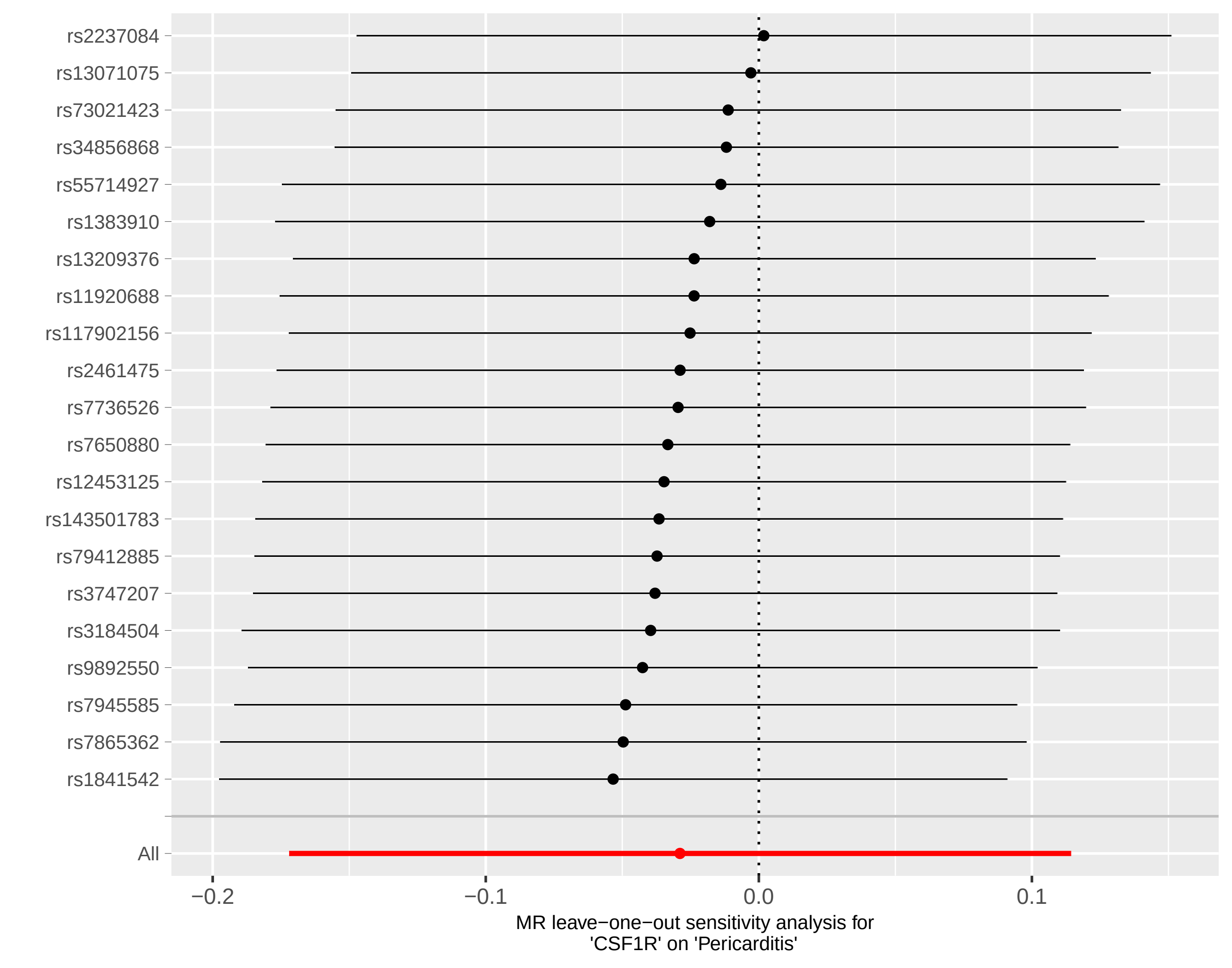
rs2237084
rs13071075
rs73021423
rs34856868
rs55714927
rs1383910
rs13209376
rs11920688
rs117902156
rs2461475
rs7736526
rs7650880
rs12453125
rs143501783
rs79412885
rs3747207
rs3184504
rs9892550
rs7945585
rs7865362
rs1841542
All
−0.2
−0.1
0.0
0.1
MR leave−one−out sensitivity analysis for
'CSF1R' on 'Pericarditis'

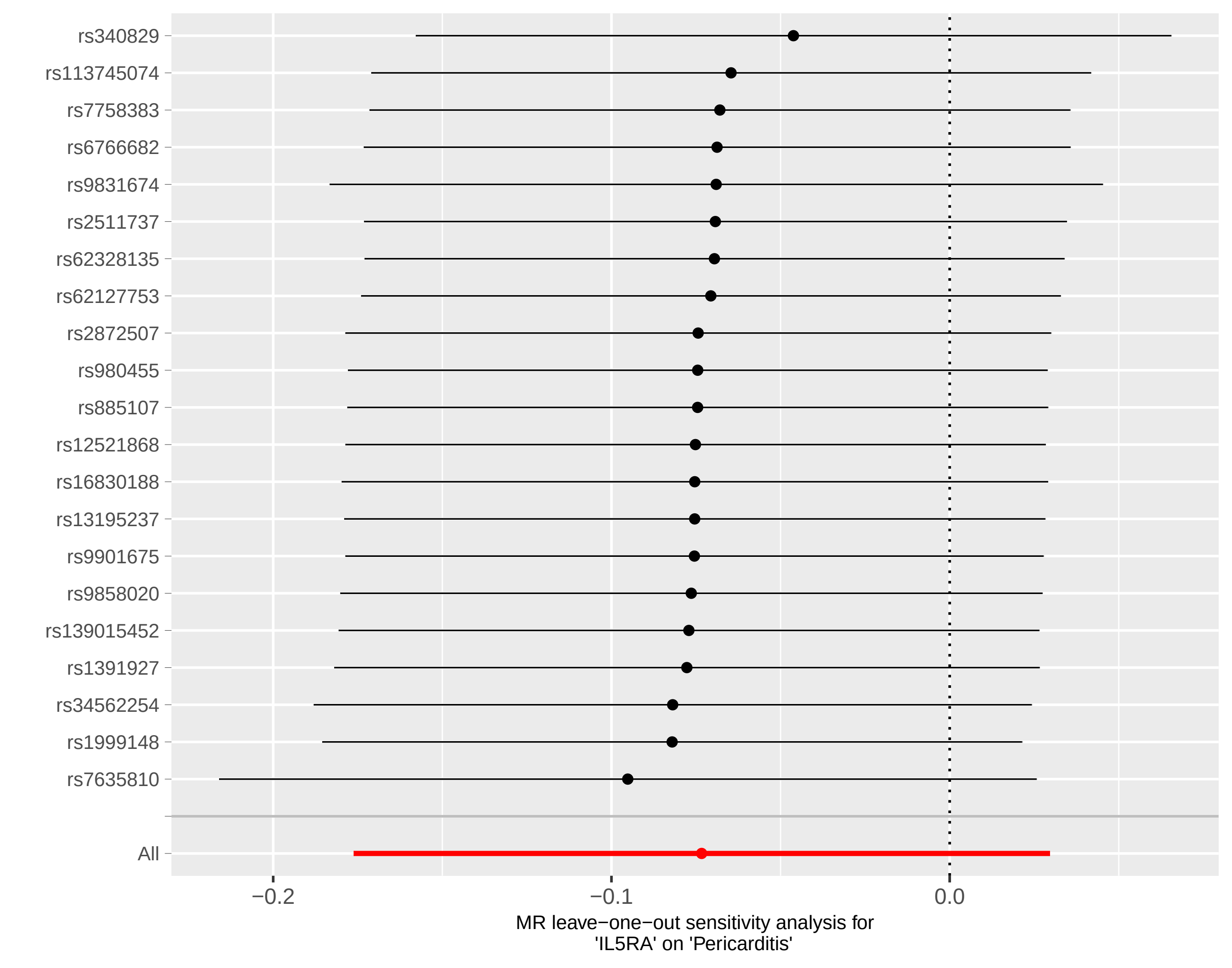
rs340829
rs113745074
rs7758383
rs6766682
rs9831674
rs2511737
rs62328135
rs62127753
rs2872507
rs980455
rs885107
rs12521868
rs16830188
rs13195237
rs9901675
rs9858020
rs139015452
rs1391927
rs34562254
rs1999148
rs7635810
All
−0.2
−0.1
0.0
MR leave−one−out sensitivity analysis for
'IL5RA' on 'Pericarditis'

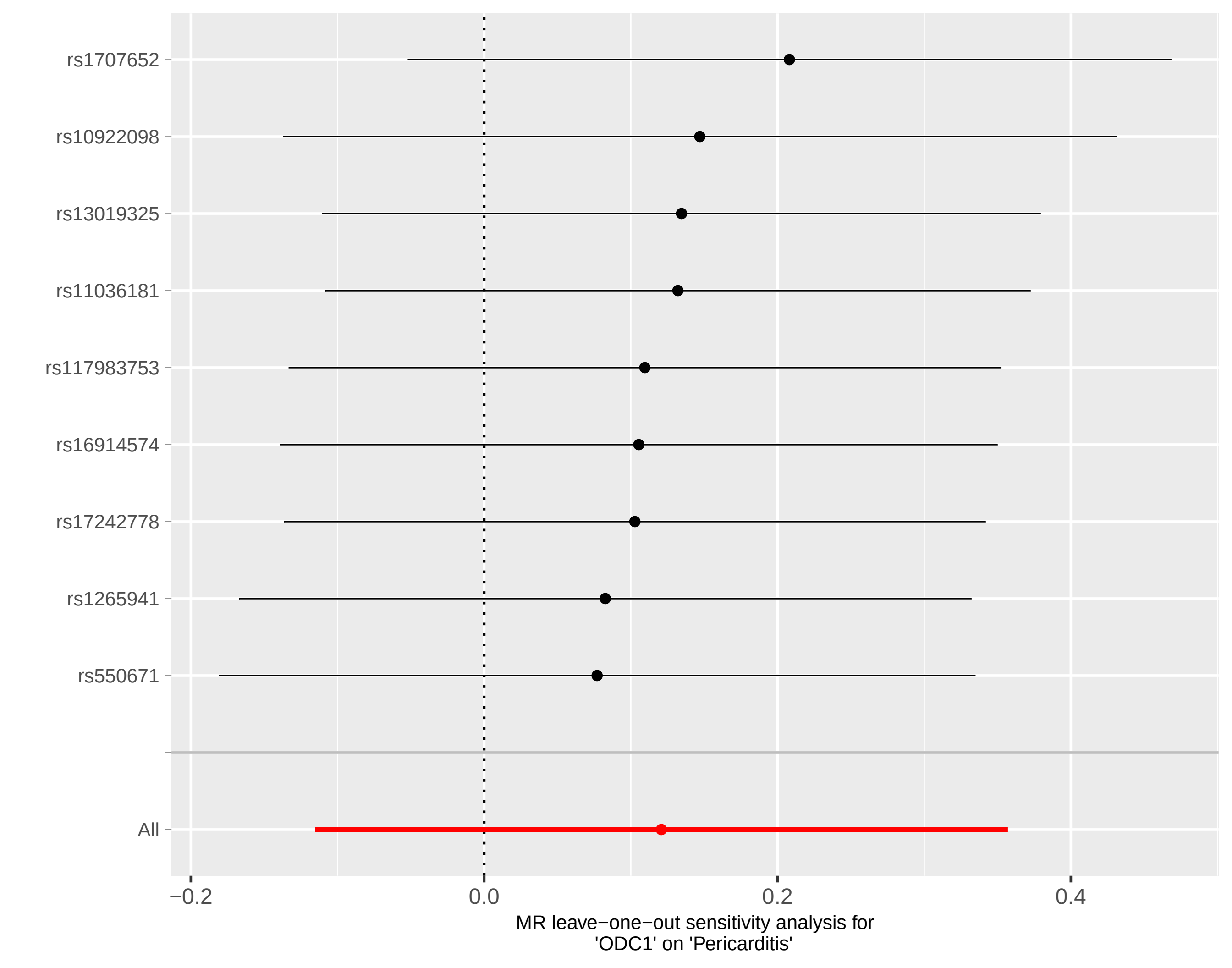

rs1707652
rs10922098
rs13019325
rs11036181
rs117983753
rs16914574
rs17242778
rs1265941
rs550671
All
−0.2
0.0
0.2
0.4
MR leave−one−out sensitivity analysis for
'ODC1' on 'Pericarditis'

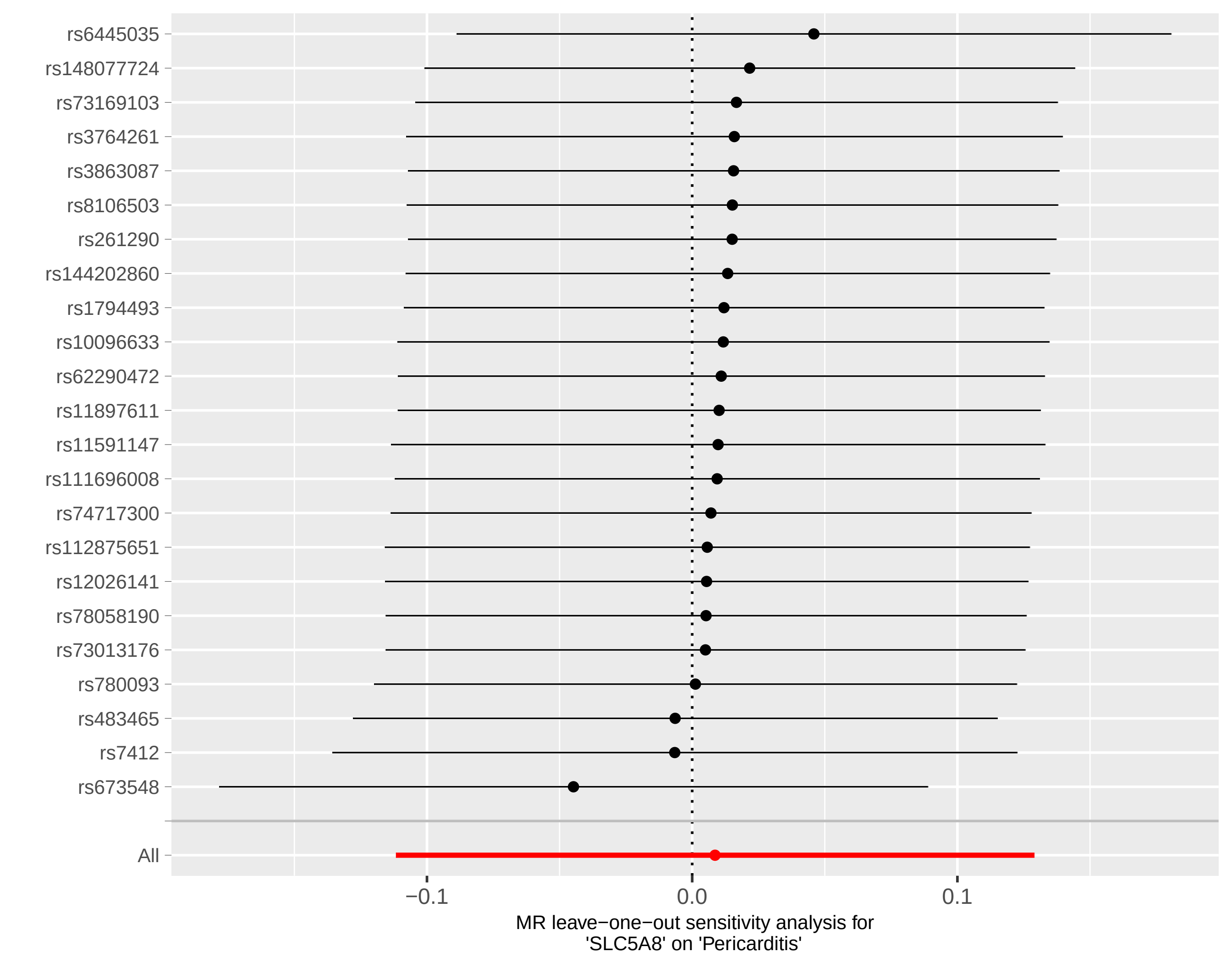
rs6445035
rs148077724
rs73169103
rs3764261
rs3863087
rs8106503
rs261290
rs144202860
rs1794493
rs10096633
rs62290472
rs11897611
rs11591147
rs111696008
rs74717300
rs112875651
rs12026141
rs78058190
rs73013176
rs780093
rs483465
rs7412
rs673548
All
−0.1
0.0
0.1
MR leave−one−out sensitivity analysis for
'SLC5A8' on 'Pericarditis'

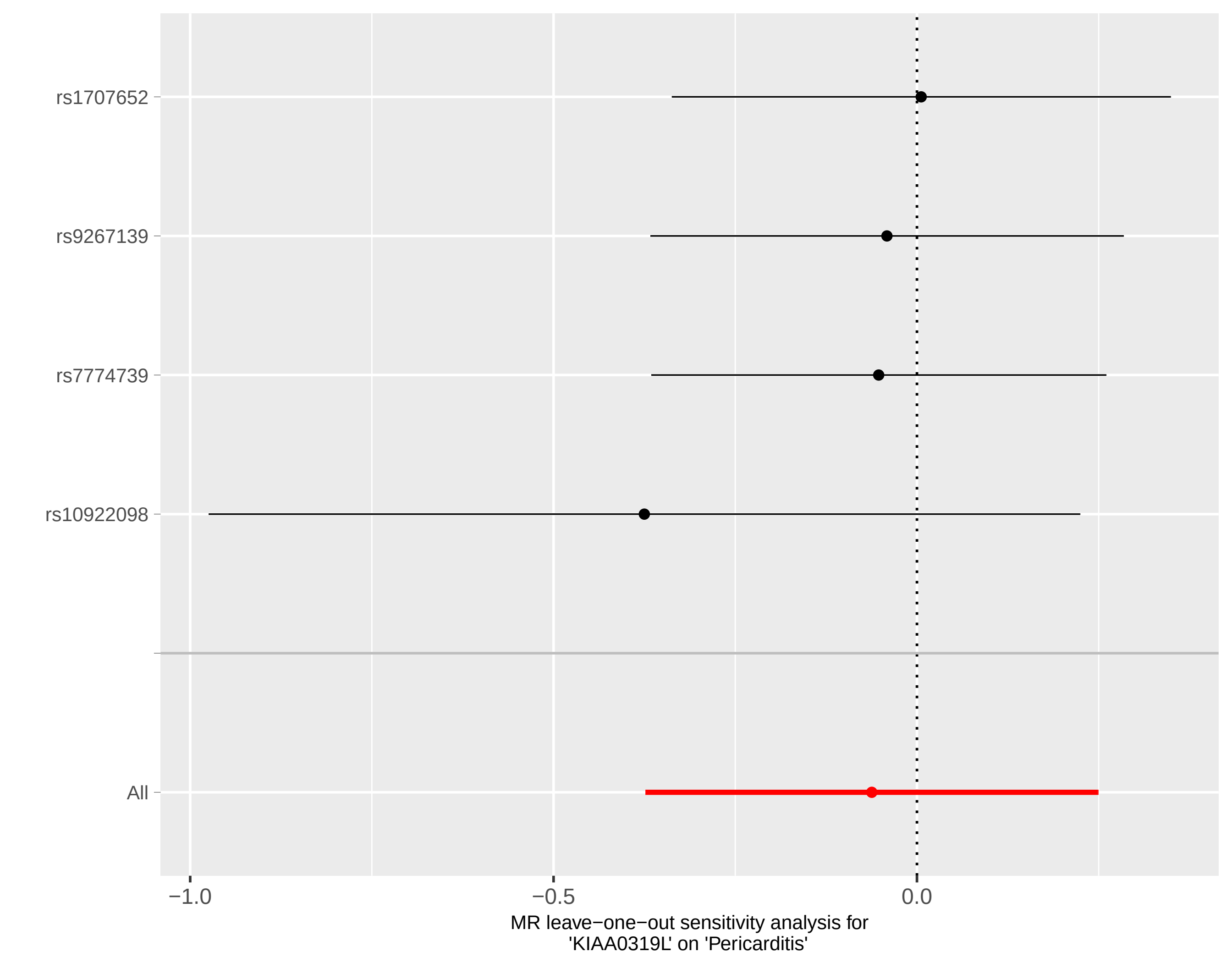
rs1707652
rs9267139
rs7774739
rs10922098
All
−1.0
−0.5
0.0
MR leave−one−out sensitivity analysis for
'KIAA0319L' on 'Pericarditis'

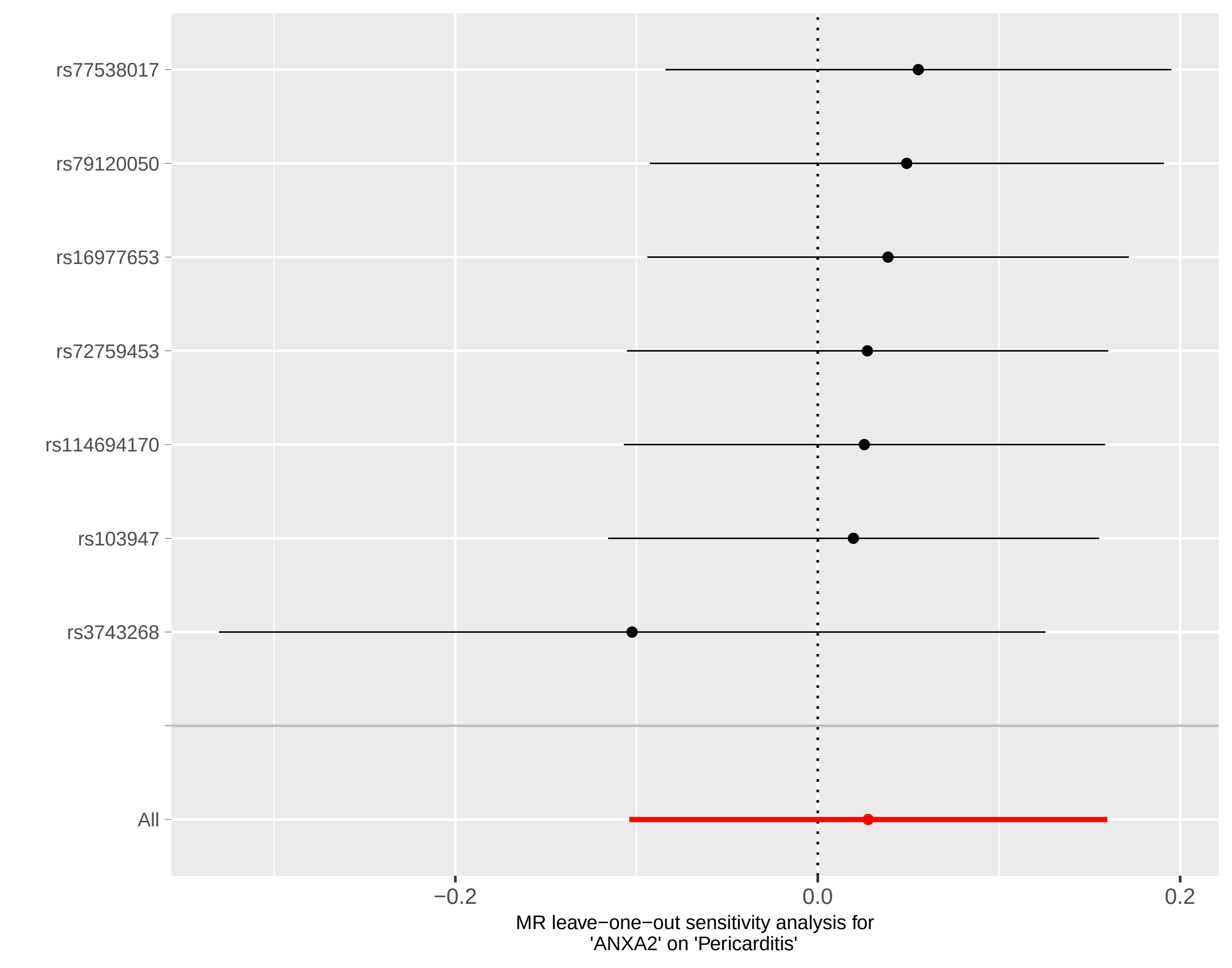
rs77538017
rs79120050
rs16977653
rs72759453
rs114694170
rs103947
rs3743268
All
−0.2
0.0
0.2
MR leave−one−out sensitivity analysis for
'ANXA2' on 'Pericarditis'

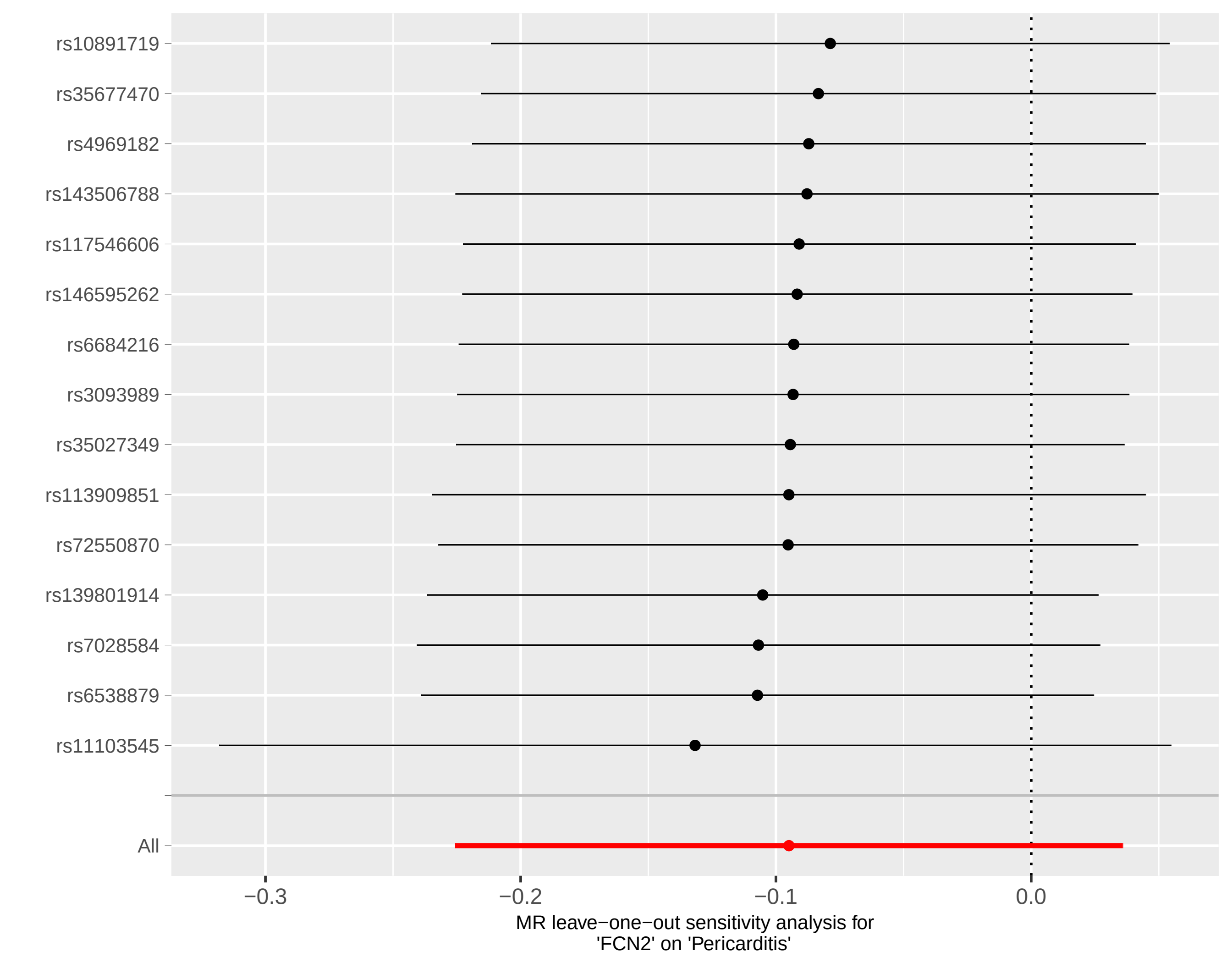
rs10891719
rs35677470
rs4969182
rs143506788
rs117546606
rs146595262
rs6684216
rs3093989
rs35027349
rs113909851
rs72550870
rs139801914
rs7028584
rs6538879
rs11103545
All
−0.3
−0.2
−0.1
0.0
MR leave−one−out sensitivity analysis for
'FCN2' on 'Pericarditis'

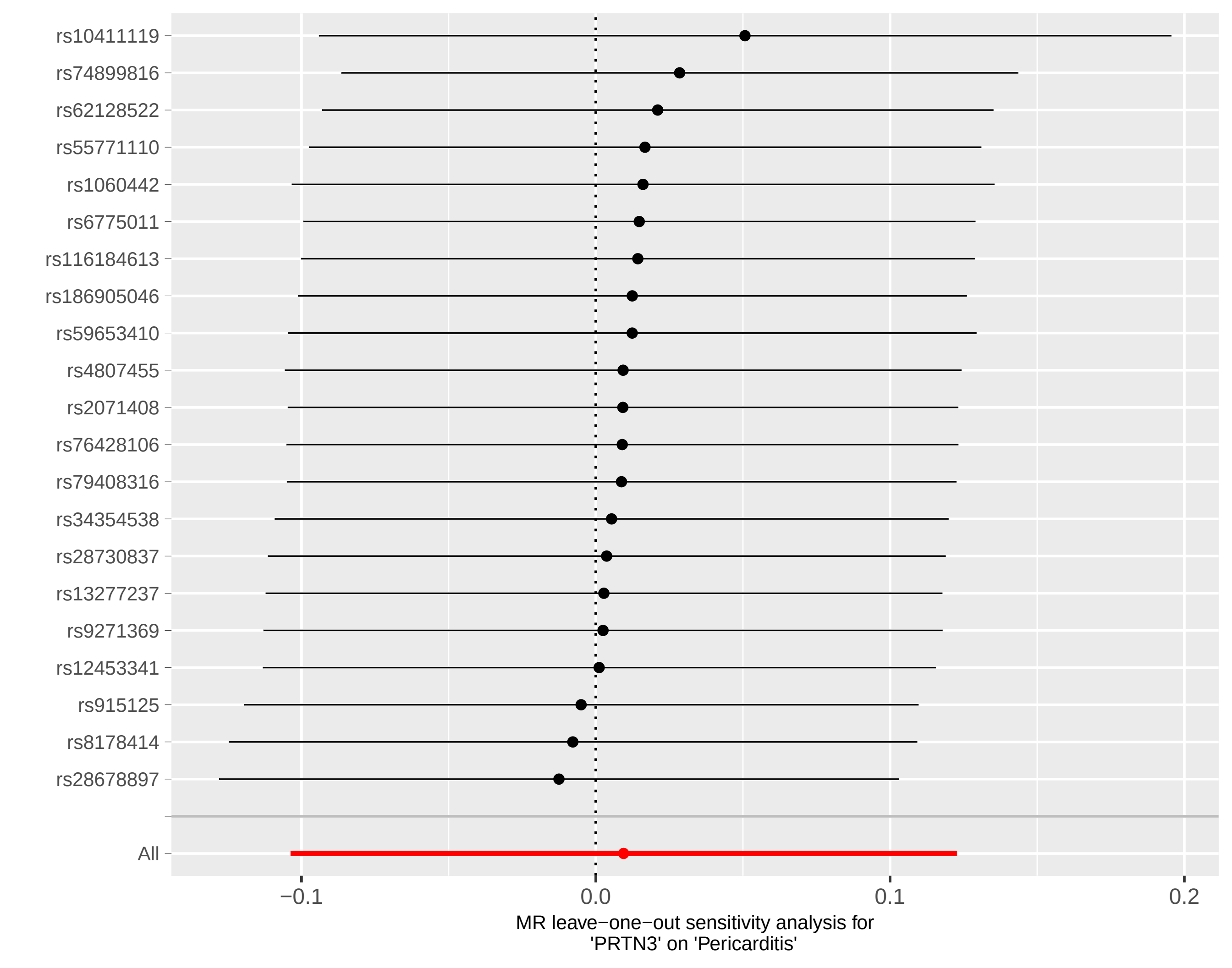
rs10411119
rs74899816
rs62128522
rs55771110
rs1060442
rs6775011
rs116184613
rs186905046
rs59653410
rs4807455
rs2071408
rs76428106
rs79408316
rs34354538
rs28730837
rs13277237
rs9271369
rs12453341
rs915125
rs8178414
rs28678897
All
−0.1
0.0
0.1
0.2
MR leave−one−out sensitivity analysis for
'PRTN3' on 'Pericarditis'

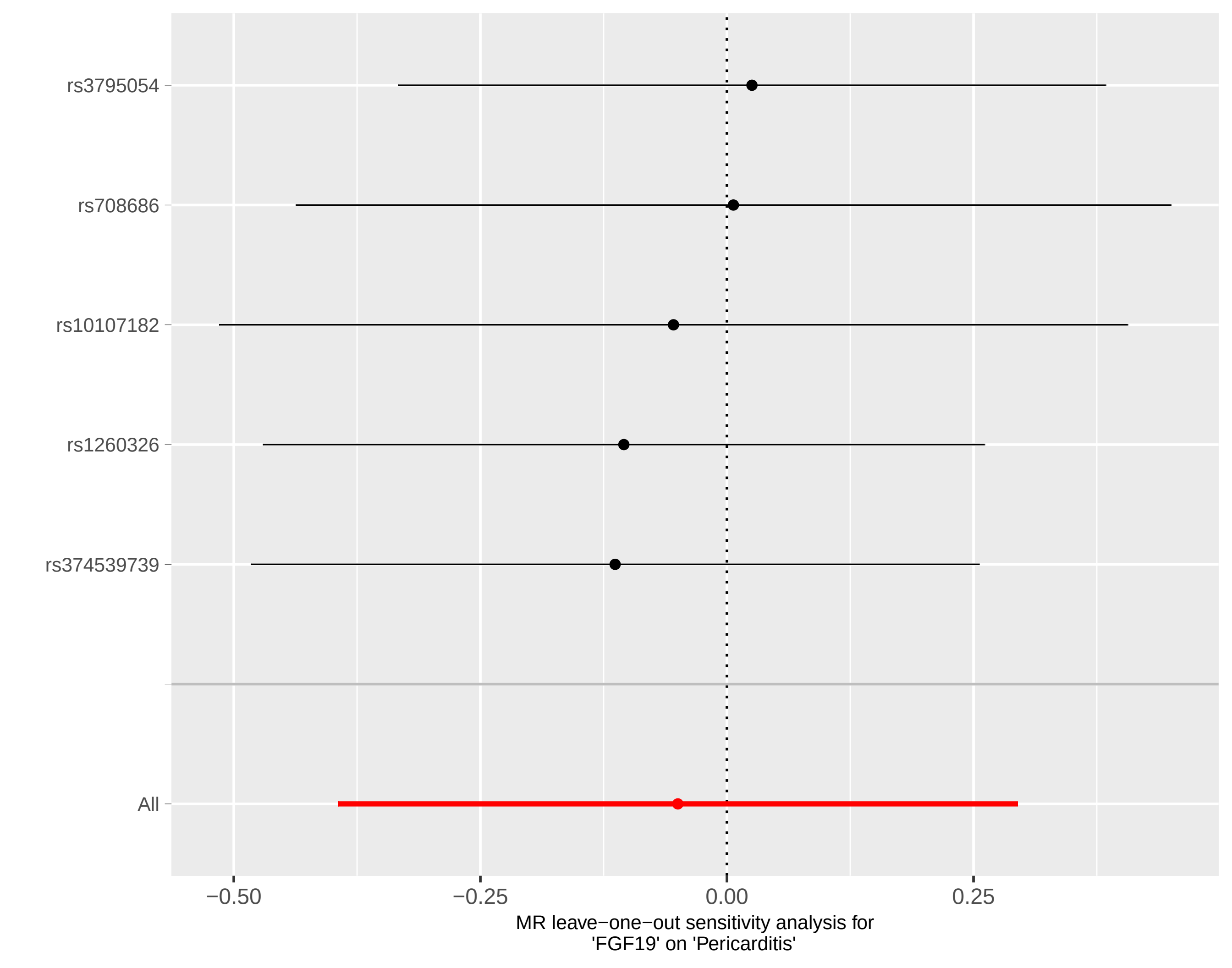
rs3795054
rs708686
rs10107182
rs1260326
rs374539739
All
−0.50
−0.25
0.00
0.25
MR leave−one−out sensitivity analysis for
'FGF19' on 'Pericarditis'

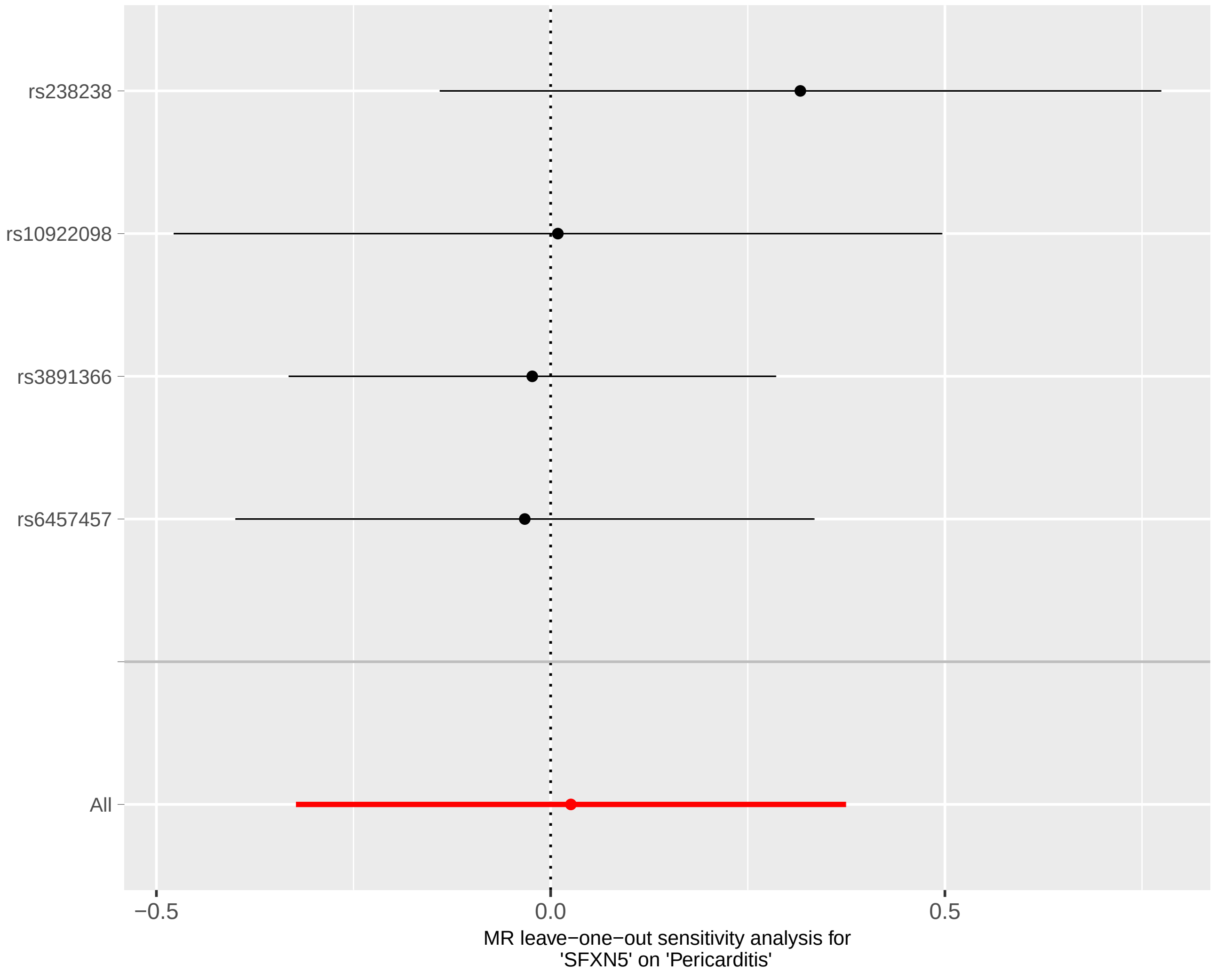
rs238238
rs10922098
rs3891366
rs6457457
All
−0.5
0.0
0.5
MR leave−one−out sensitivity analysis for
'SFXN5' on 'Pericarditis'

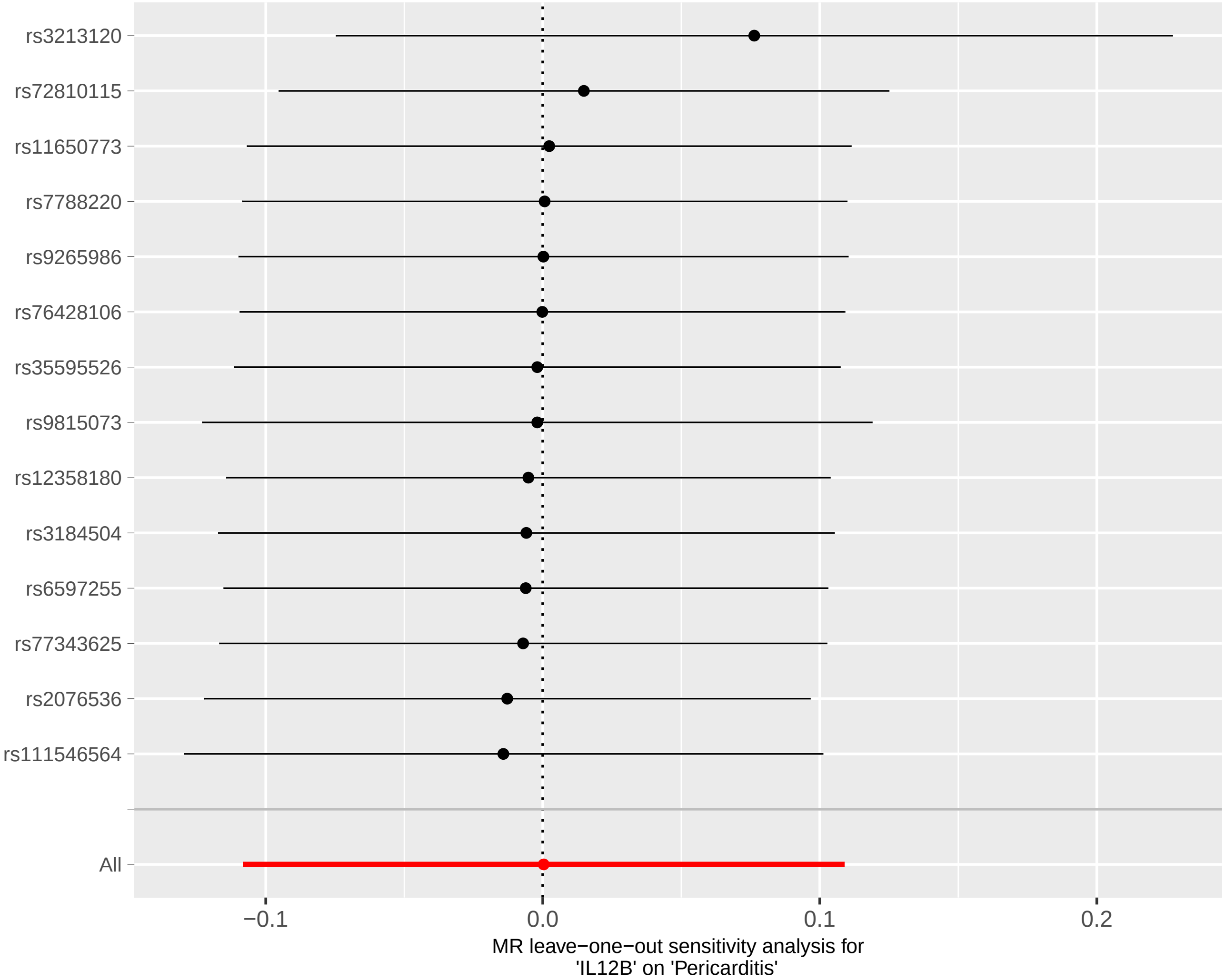
rs3213120
rs72810115
rs11650773
rs7788220
rs9265986
rs76428106
rs35595526
rs9815073
rs12358180
rs3184504
rs6597255
rs77343625
rs2076536
rs111546564
All
−0.1
0.0
0.1
0.2
MR leave−one−out sensitivity analysis for
'IL12B' on 'Pericarditis'

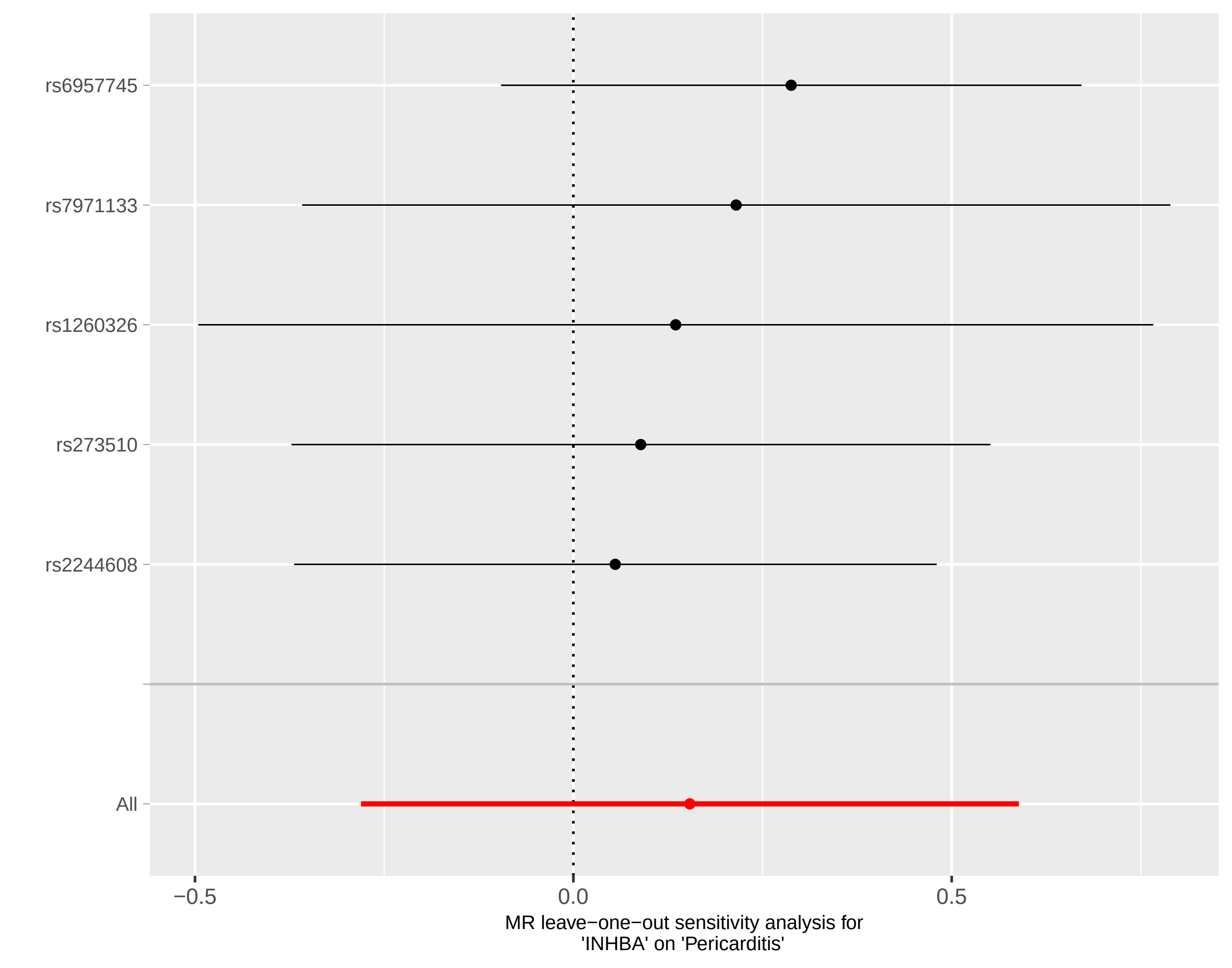

rs6957745
rs7971133
rs1260326
rs273510
rs2244608
All
−0.5
0.0
0.5
MR leave−one−out sensitivity analysis for
'INHBA' on 'Pericarditis'

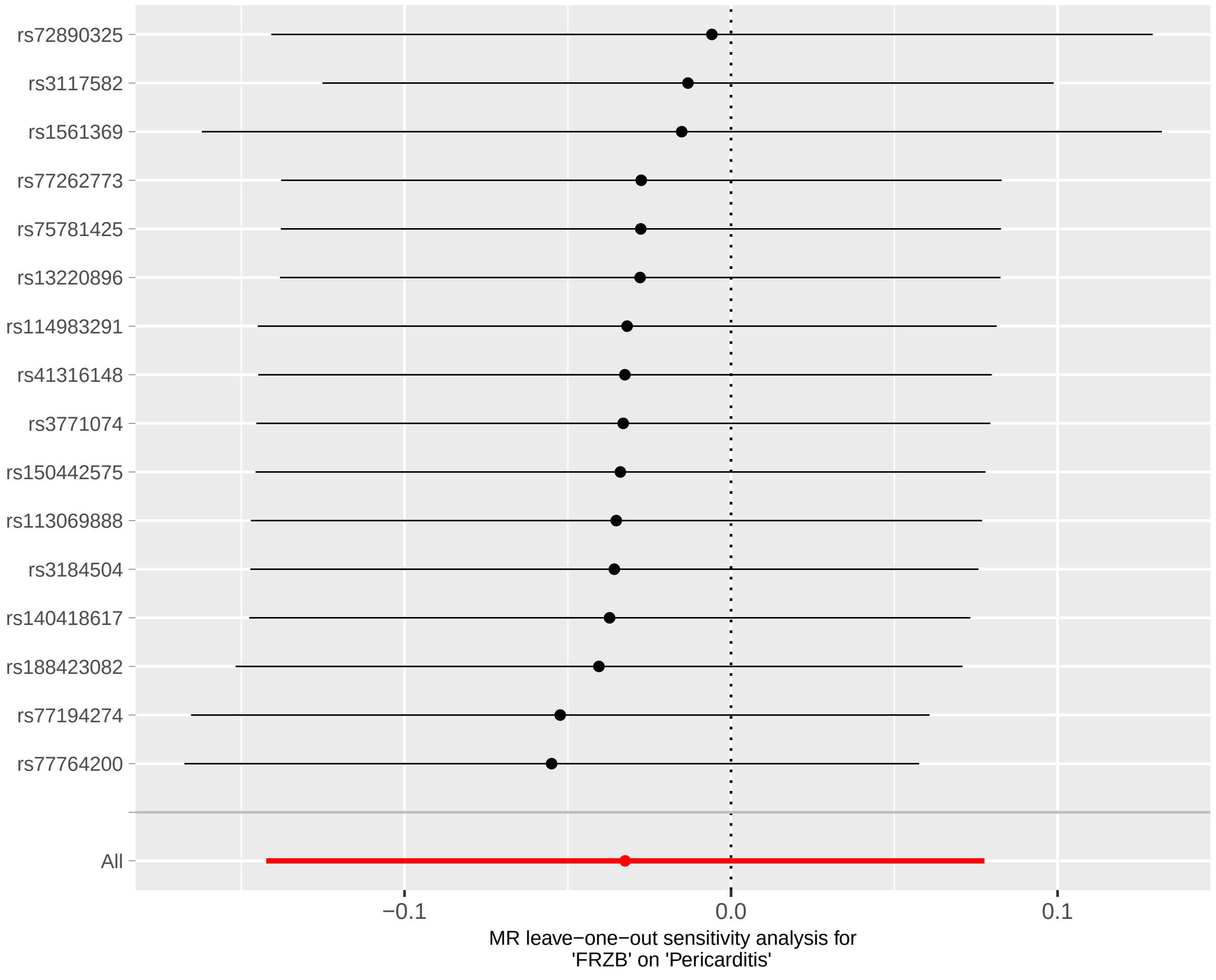
rs72890325
rs3117582
rs1561369
rs77262773
rs75781425
rs13220896
rs114983291
rs41316148
rs3771074
rs150442575
rs113069888
rs3184504
rs140418617
rs188423082
rs77194274
rs77764200
All
−0.1
0.0
0.1
MR leave−one−out sensitivity analysis for
'FRZB' on 'Pericarditis'

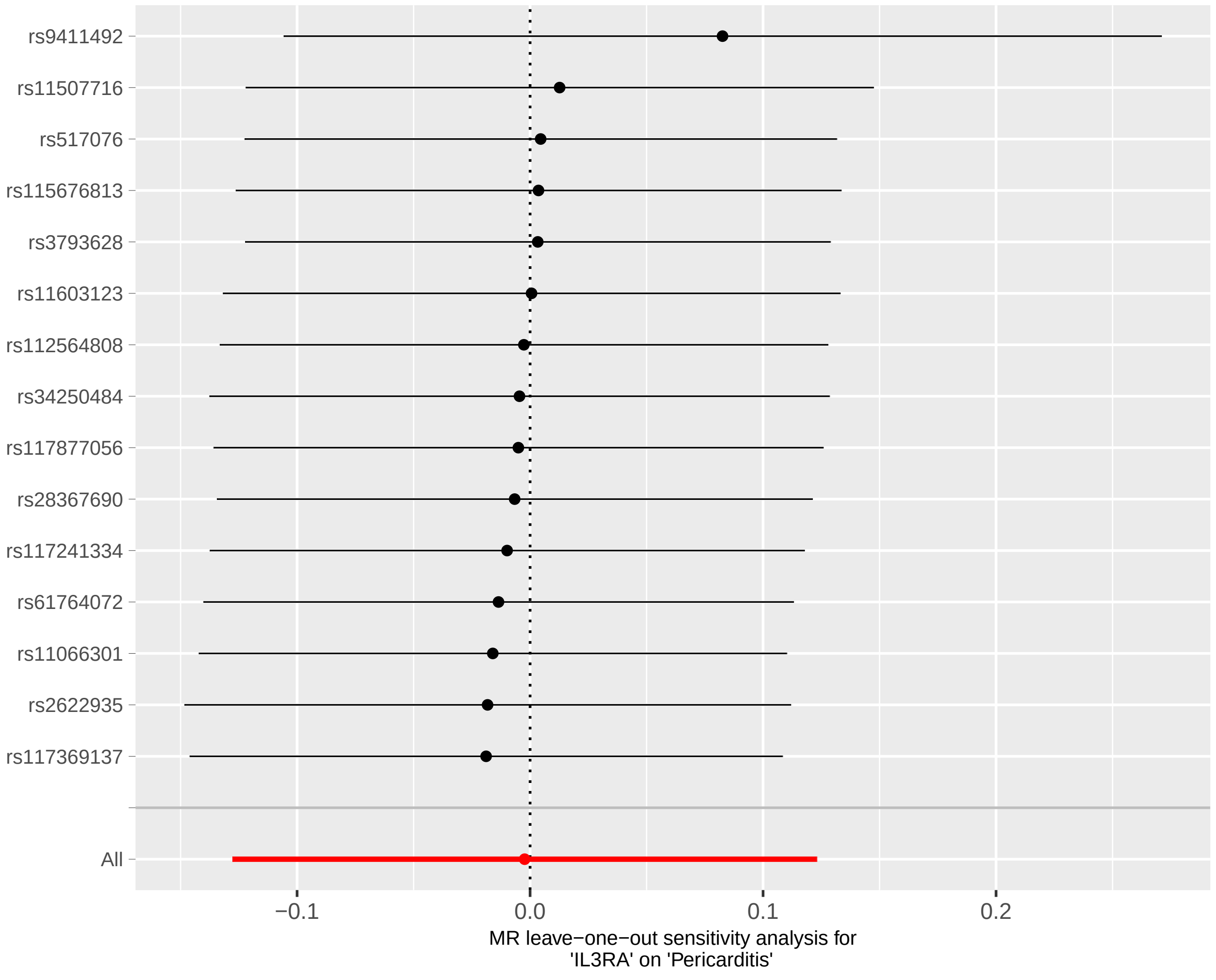
rs9411492
rs11507716
rs517076
rs115676813
rs3793628
rs11603123
rs112564808
rs34250484
rs117877056
rs28367690
rs117241334
rs61764072
rs11066301
rs2622935
rs117369137
All
−0.1
0.0
0.1
0.2
MR leave−one−out sensitivity analysis for
'IL3RA' on 'Pericarditis'

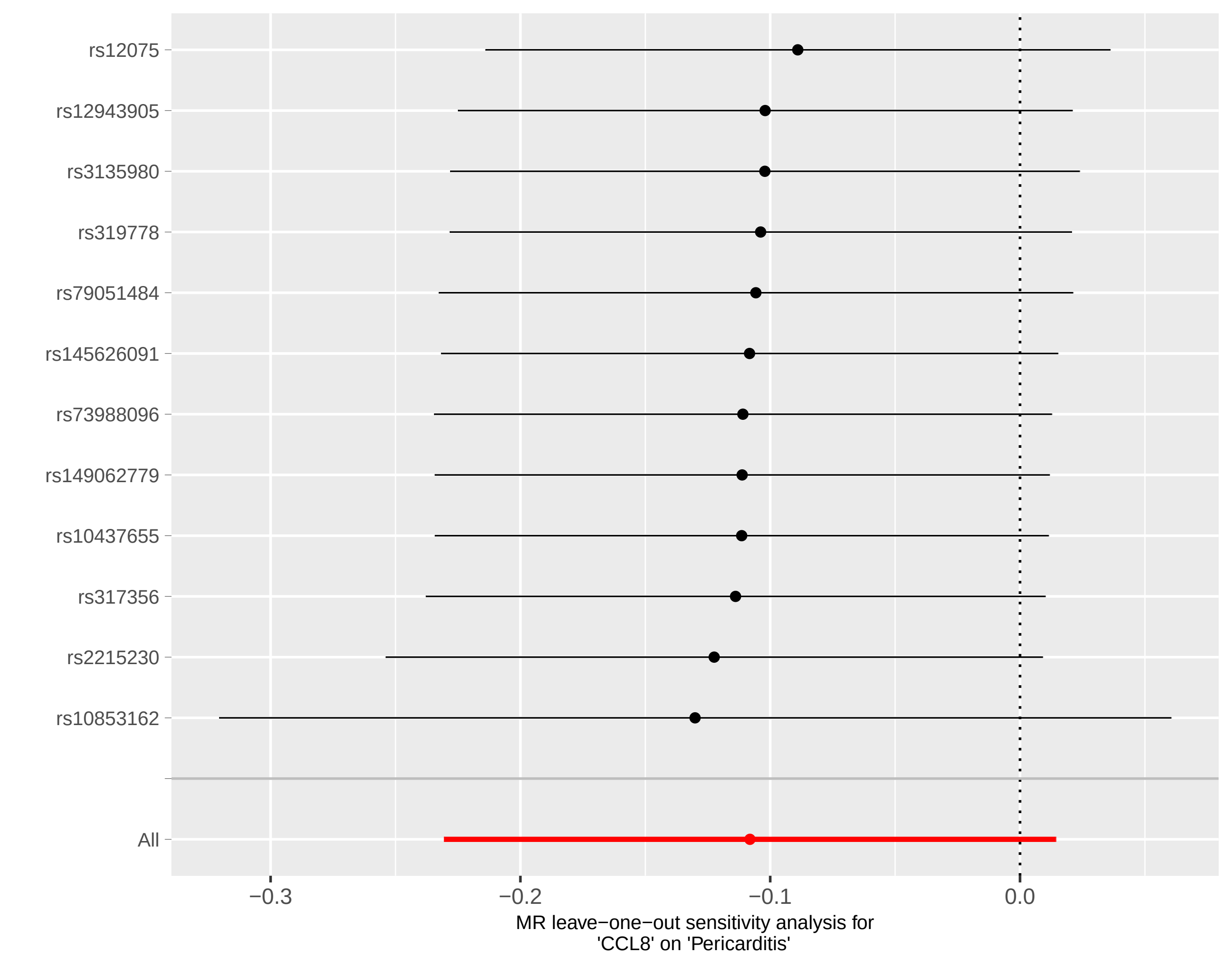
rs12075
rs12943905
rs3135980
rs319778
rs79051484
rs145626091
rs73988096
rs149062779
rs10437655
rs317356
rs2215230
rs10853162
All
−0.3
−0.2
−0.1
0.0
MR leave−one−out sensitivity analysis for
'CCL8' on 'Pericarditis'

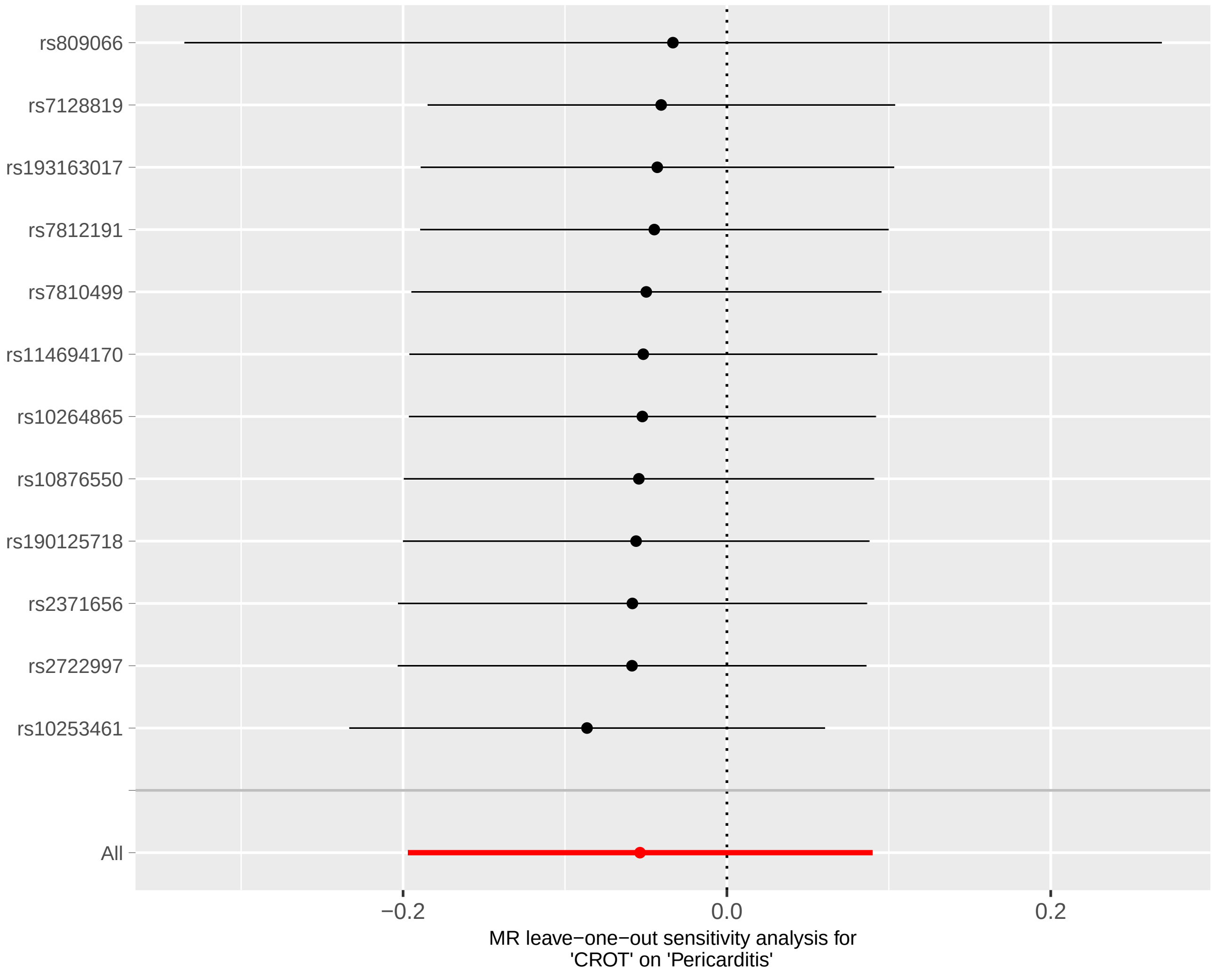
rs809066
rs7128819
rs193163017
rs7812191
rs7810499
rs114694170
rs10264865
rs10876550
rs190125718
rs2371656
rs2722997
rs10253461
All
−0.2
0.0
0.2
MR leave−one−out sensitivity analysis for
'CROT' on 'Pericarditis'

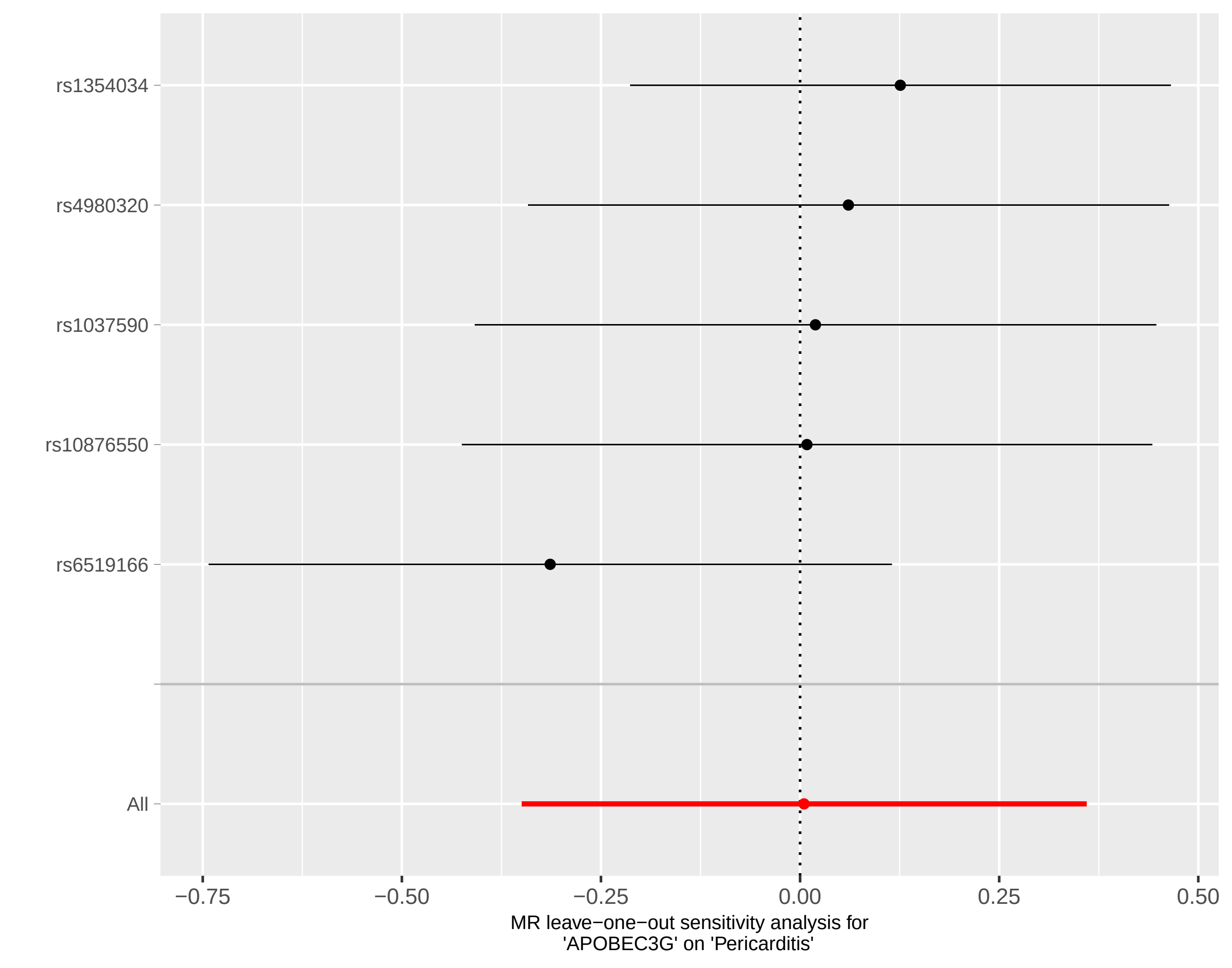

rs1354034
rs4980320
rs1037590
rs10876550
rs6519166
All
−0.75
−0.50
−0.25
0.00
0.25
0.50
MR leave−one−out sensitivity analysis for
'APOBEC3G' on 'Pericarditis'

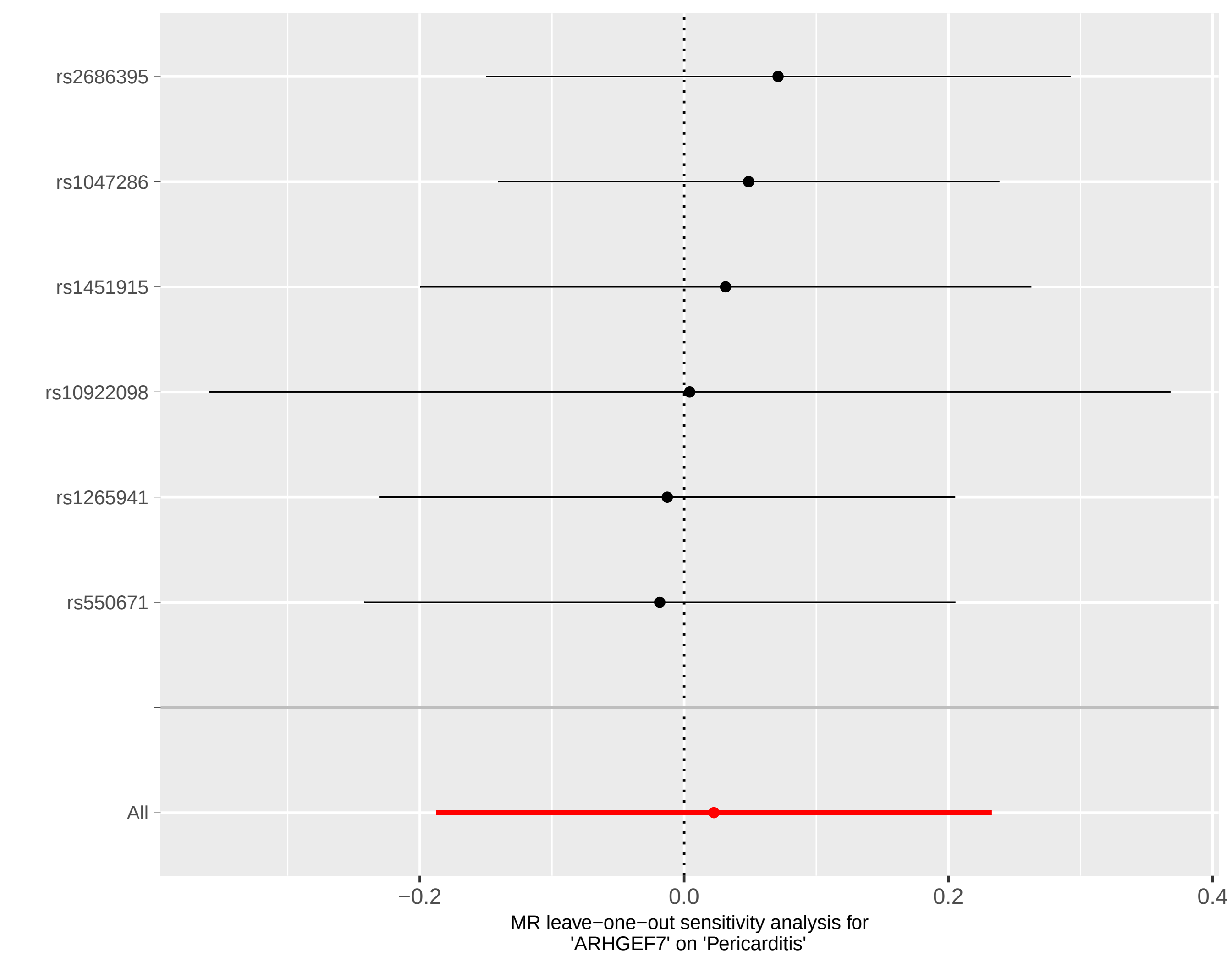
rs2686395
rs1047286
rs1451915
rs10922098
rs1265941
rs550671
All
−0.2
0.0
0.2
0.4
MR leave−one−out sensitivity analysis for
'ARHGEF7' on 'Pericarditis'

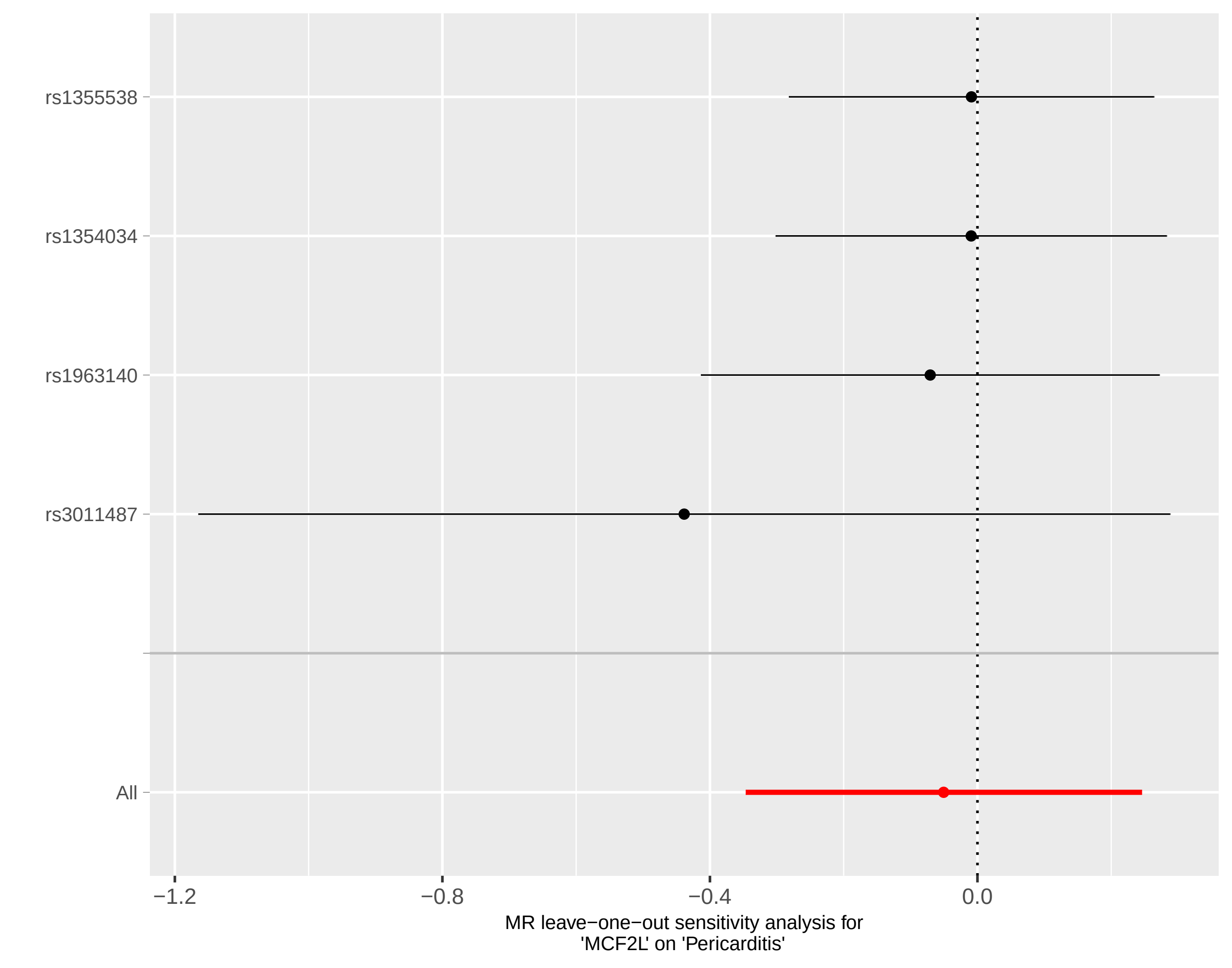
rs1355538
rs1354034
rs1963140
rs3011487
All
−1.2
−0.8
−0.4
0.0
MR leave−one−out sensitivity analysis for
'MCF2L' on 'Pericarditis'

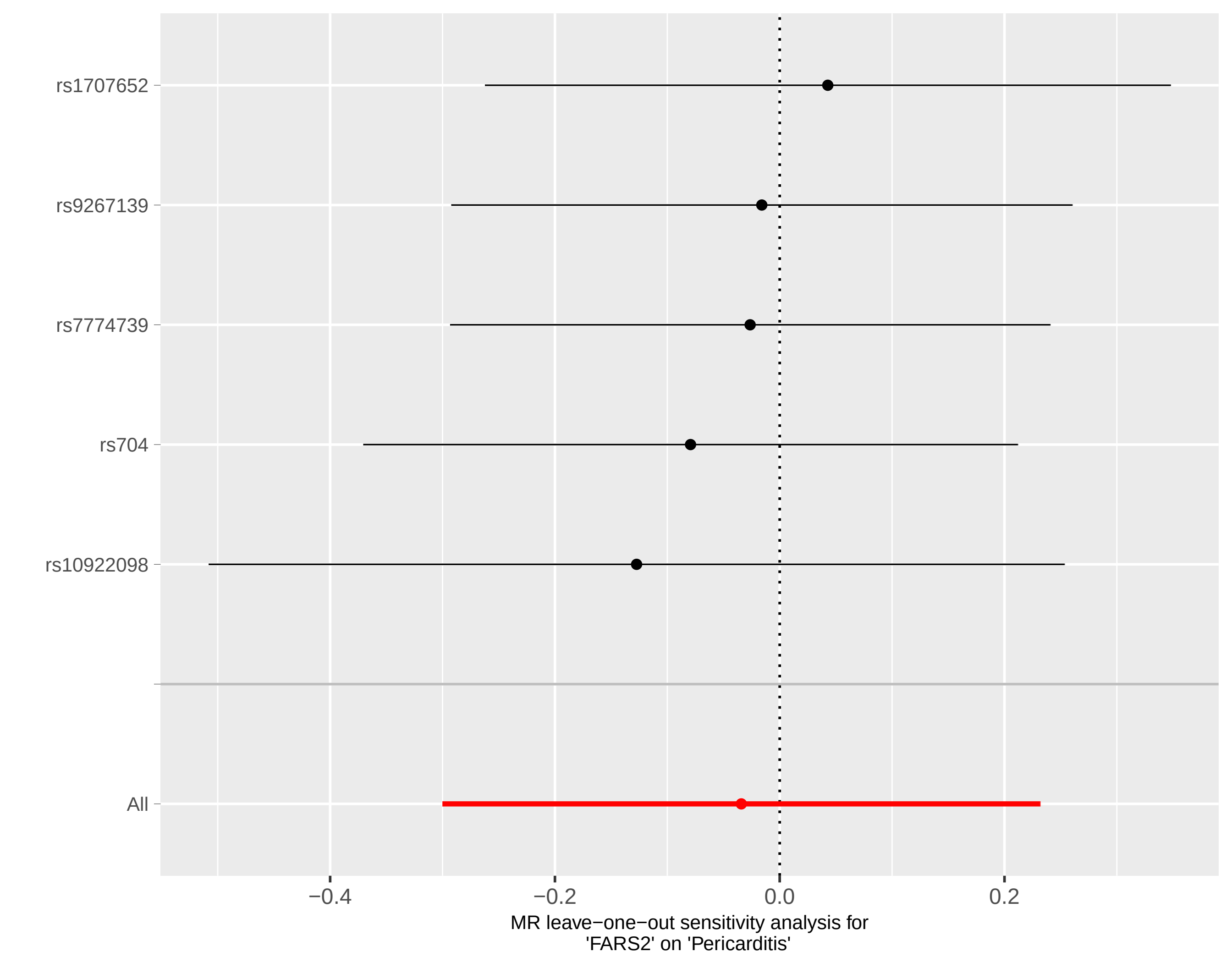
rs1707652
rs9267139
rs7774739
rs704
rs10922098
All
−0.4
−0.2
0.0
0.2
MR leave−one−out sensitivity analysis for
'FARS2' on 'Pericarditis'

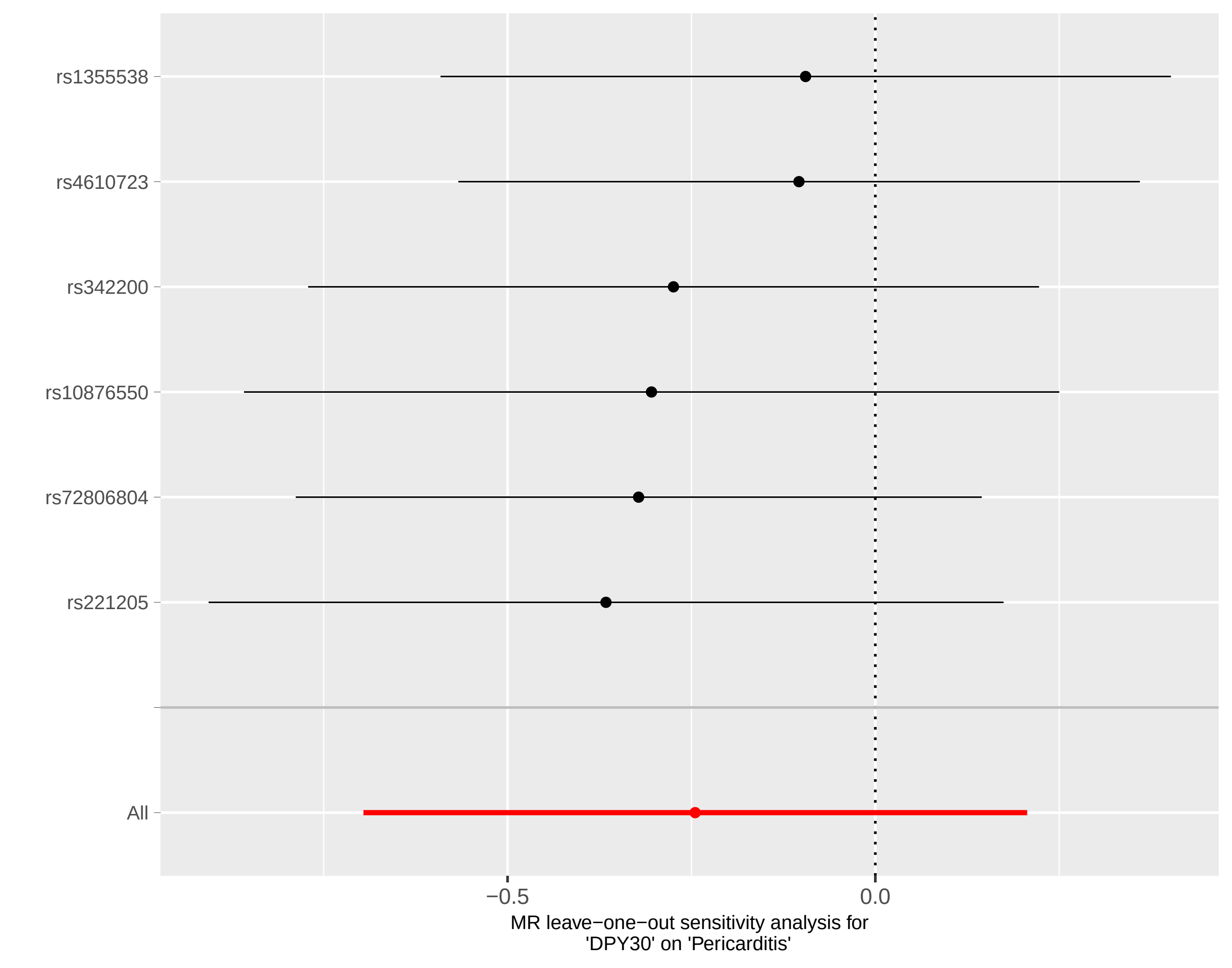

rs1355538
rs4610723
rs342200
rs10876550
rs72806804
rs221205
All
−0.5
0.0
MR leave−one−out sensitivity analysis for
'DPY30' on 'Pericarditis'

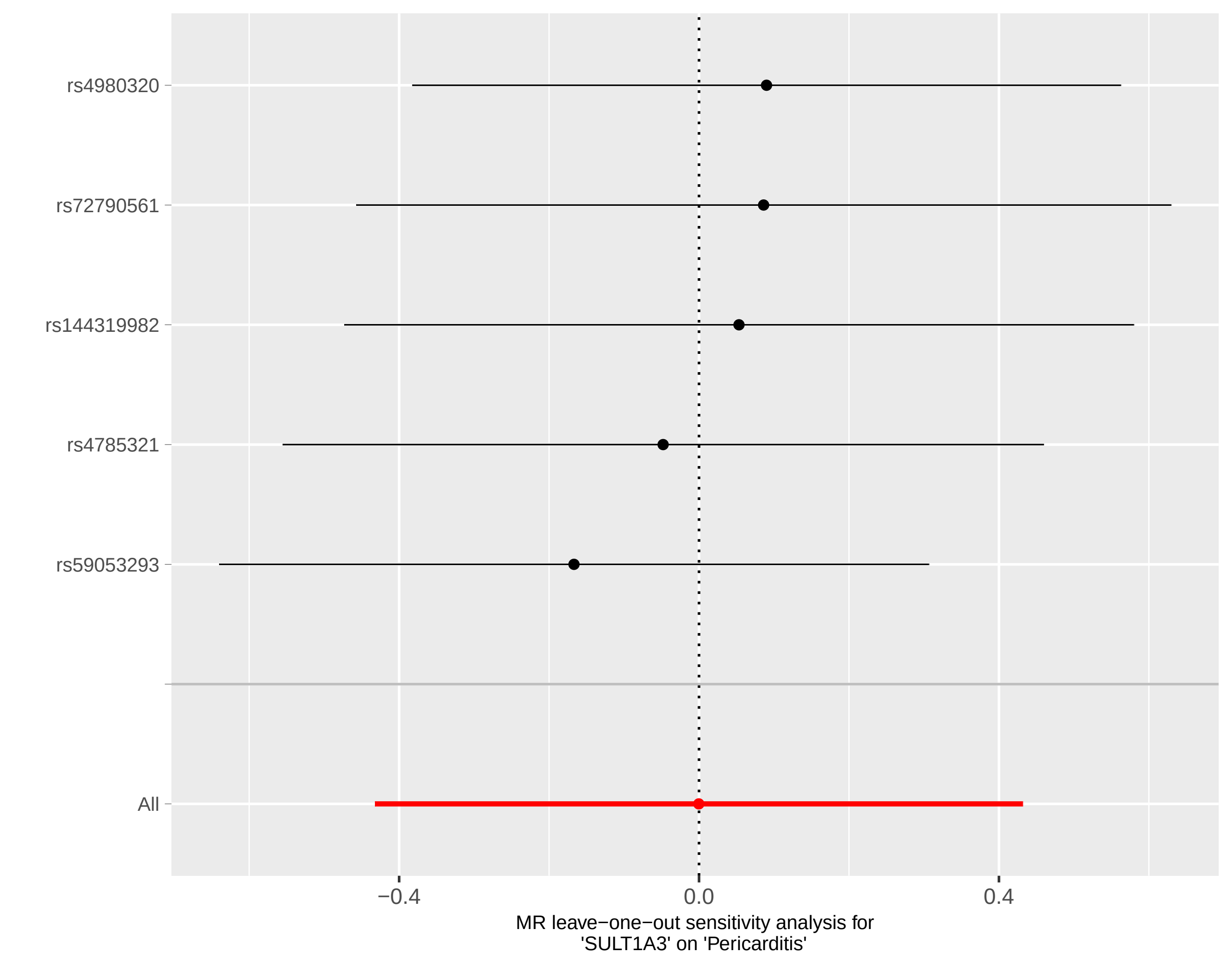
rs4980320
rs72790561
rs144319982
rs4785321
rs59053293
All
−0.4
0.0
0.4
MR leave−one−out sensitivity analysis for
'SULT1A3' on 'Pericarditis'

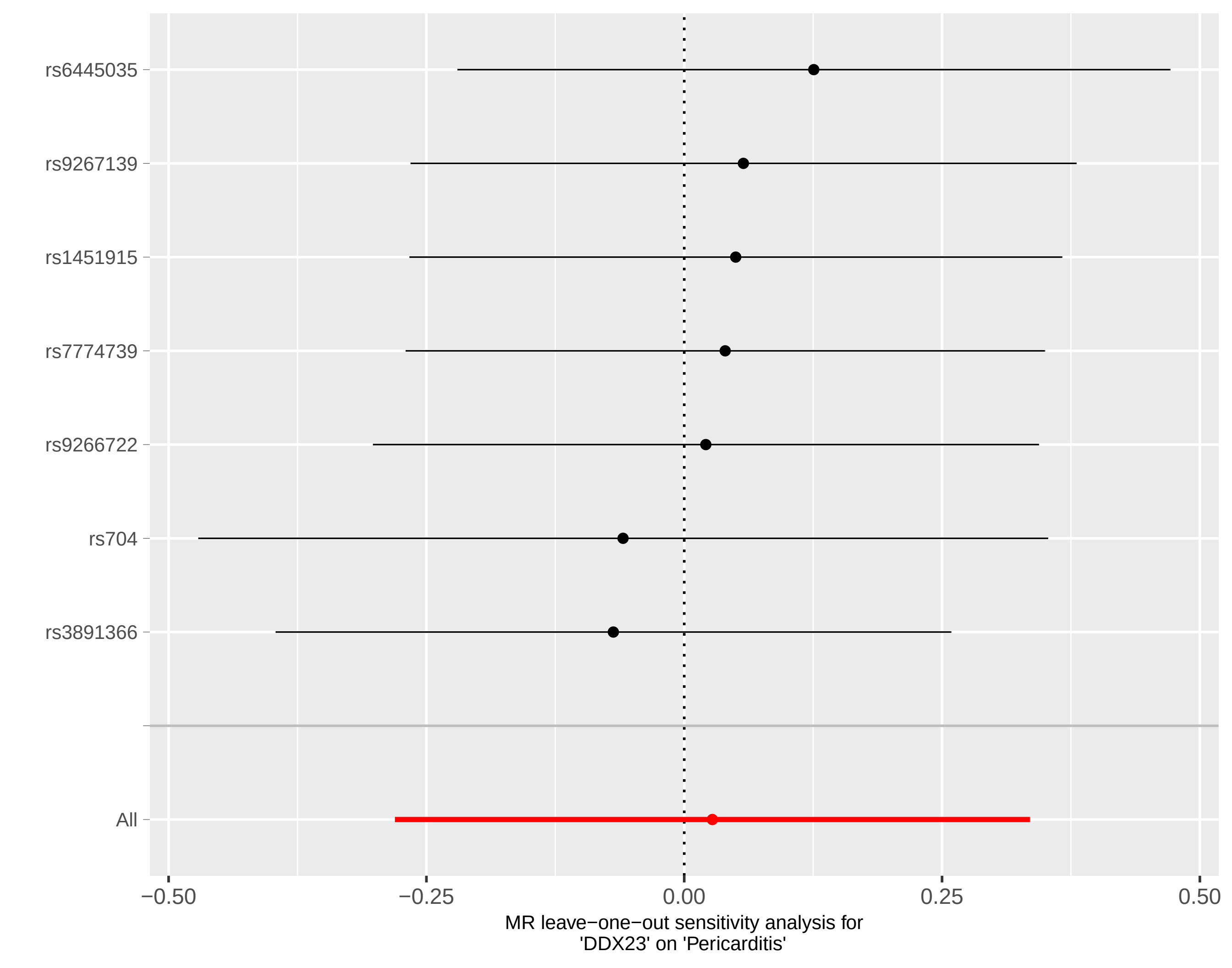
rs6445035
rs9267139
rs1451915
rs7774739
rs9266722
rs704
rs3891366
All
−0.50
−0.25
0.00
0.25
0.50
MR leave−one−out sensitivity analysis for
'DDX23' on 'Pericarditis'

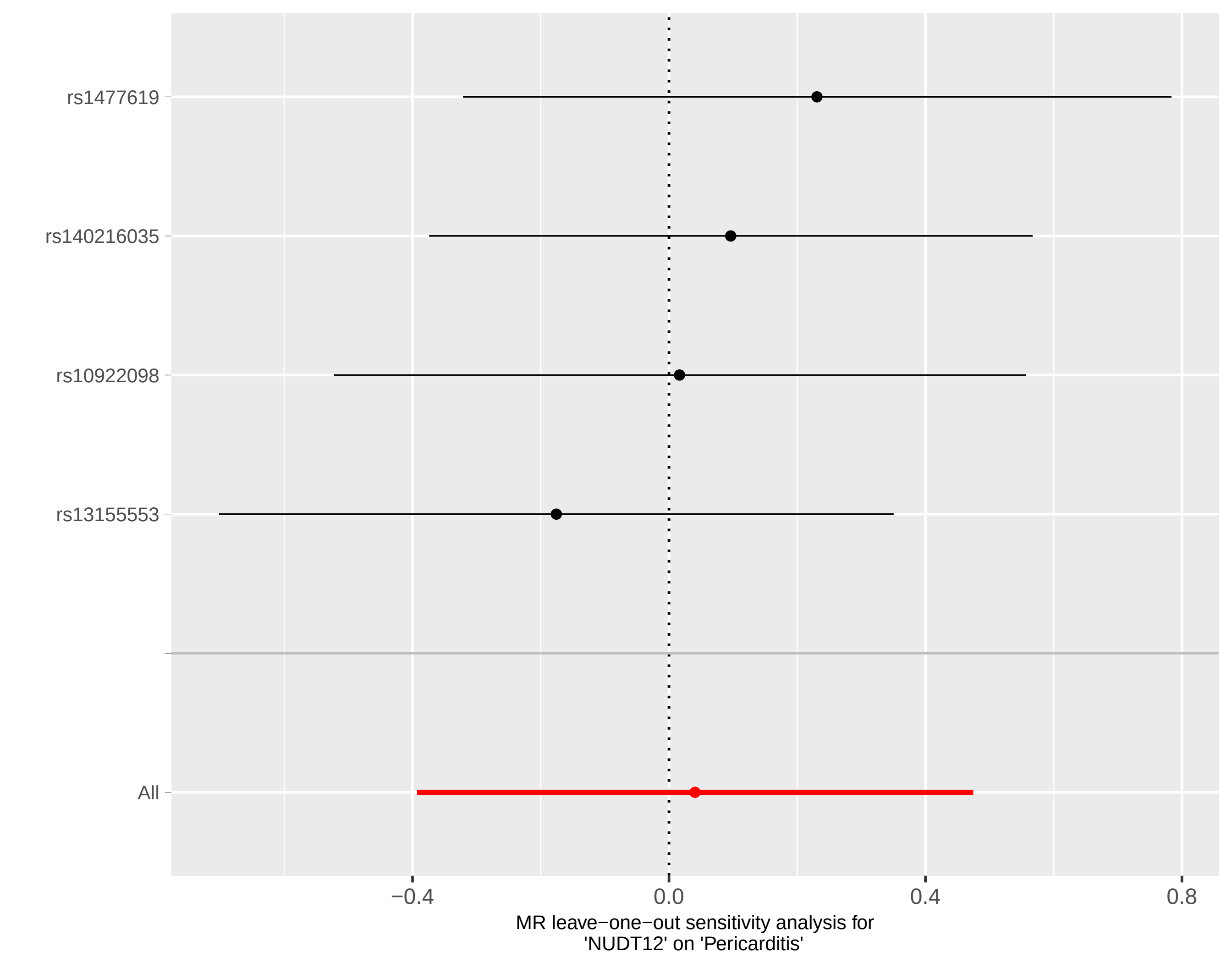
rs1477619
rs140216035
rs10922098
rs13155553
All
−0.4
0.0
0.4
0.8
MR leave−one−out sensitivity analysis for
'NUDT12' on 'Pericarditis'

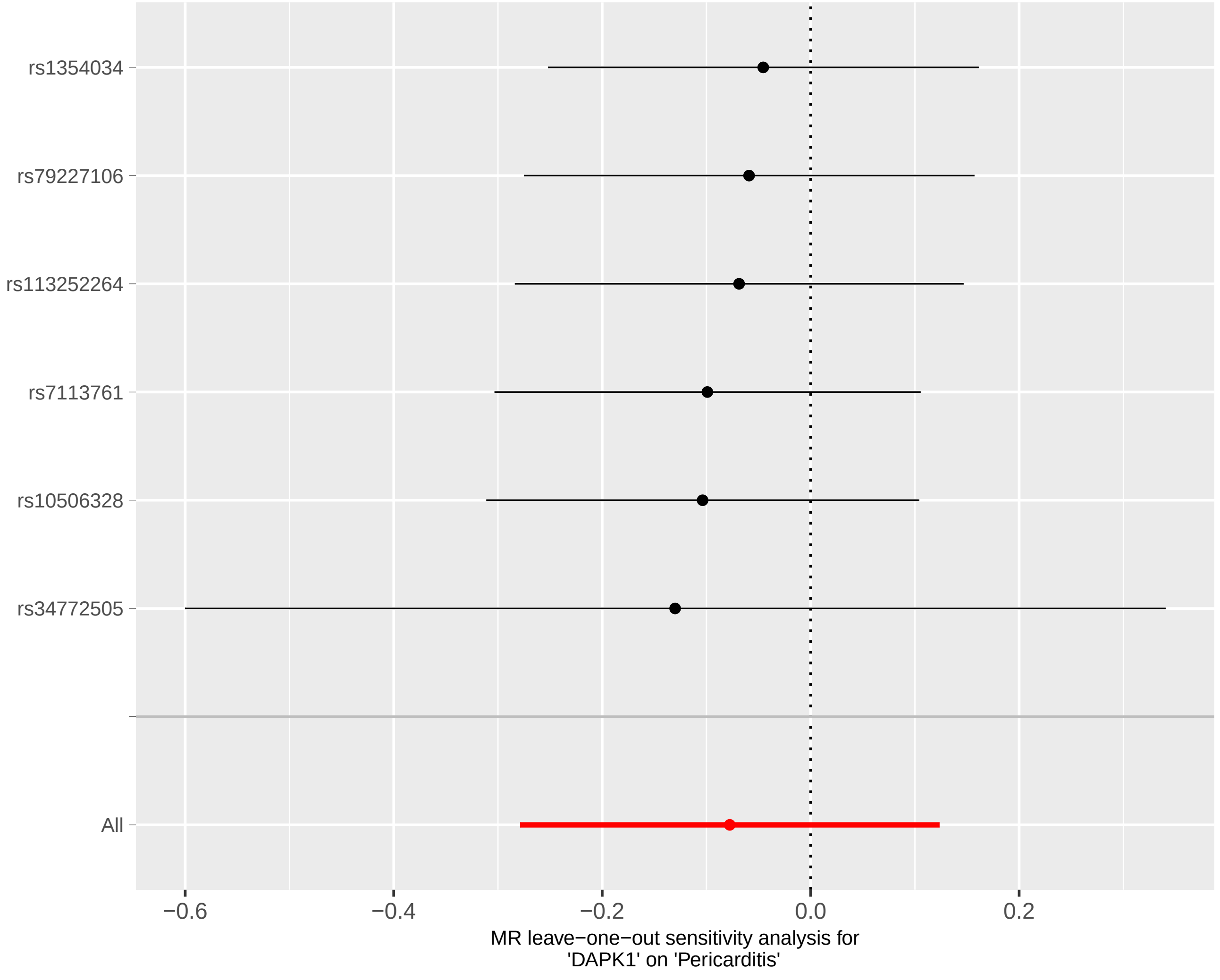
rs1354034
rs79227106
rs113252264
rs7113761
rs10506328
rs34772505
All
−0.6
−0.4
−0.2
0.0
0.2
MR leave−one−out sensitivity analysis for
'DAPK1' on 'Pericarditis'

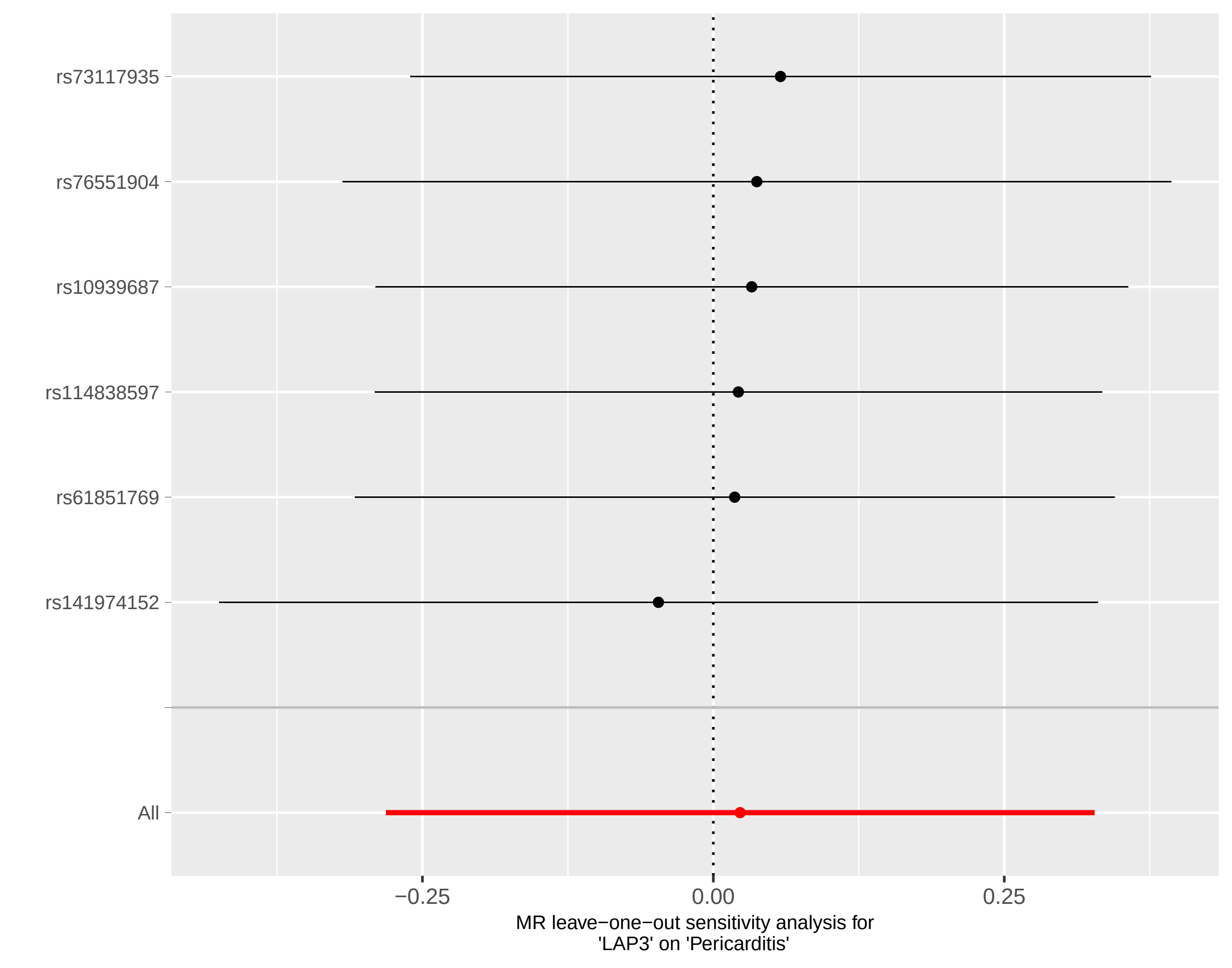
rs73117935
rs76551904
rs10939687
rs114838597
rs61851769
rs141974152
All
−0.25
0.00
0.25
MR leave−one−out sensitivity analysis for
'LAP3' on 'Pericarditis'

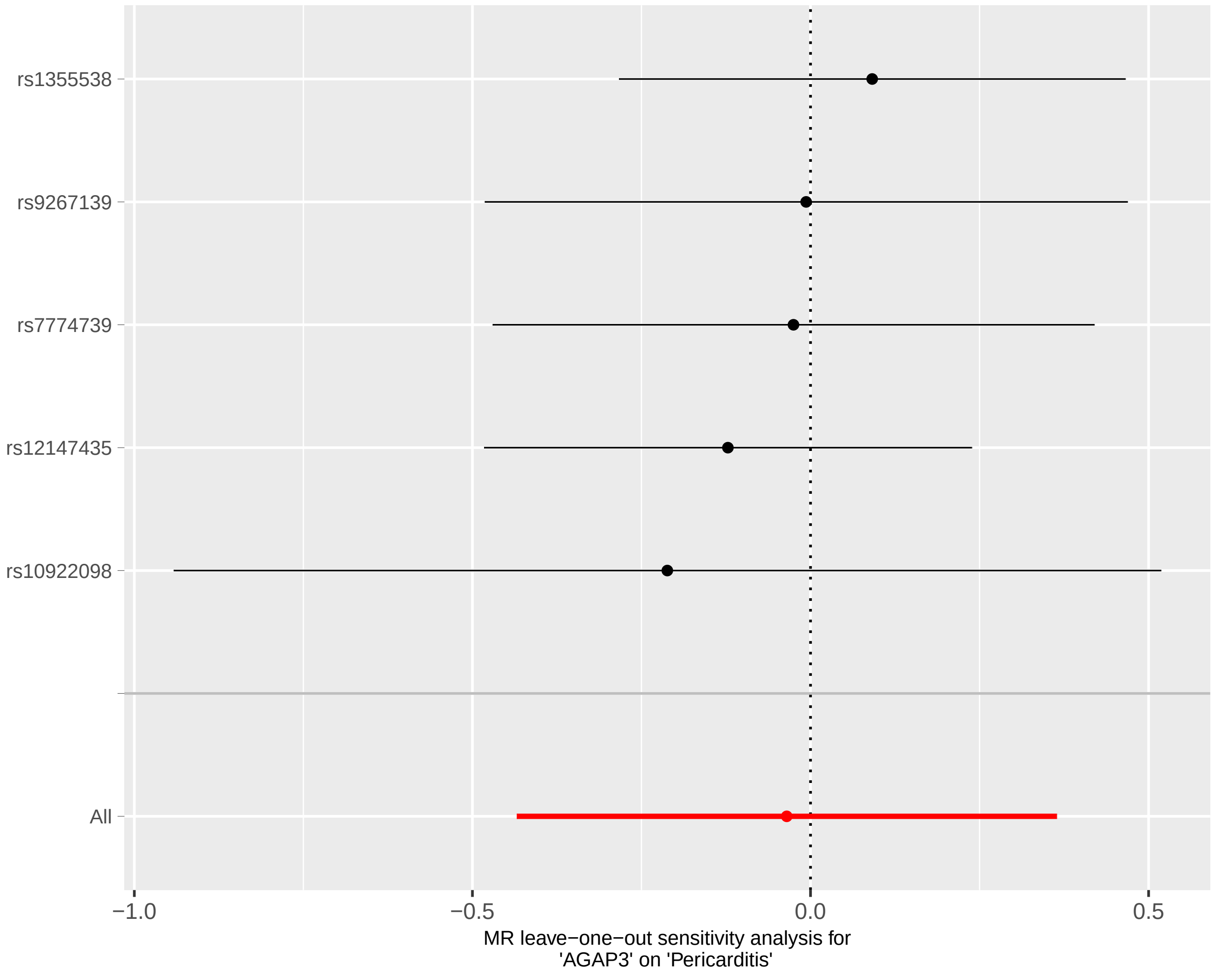

rs1355538
rs9267139
rs7774739
rs12147435
rs10922098
All
−1.0
−0.5
0.0
0.5
MR leave−one−out sensitivity analysis for
'AGAP3' on 'Pericarditis'

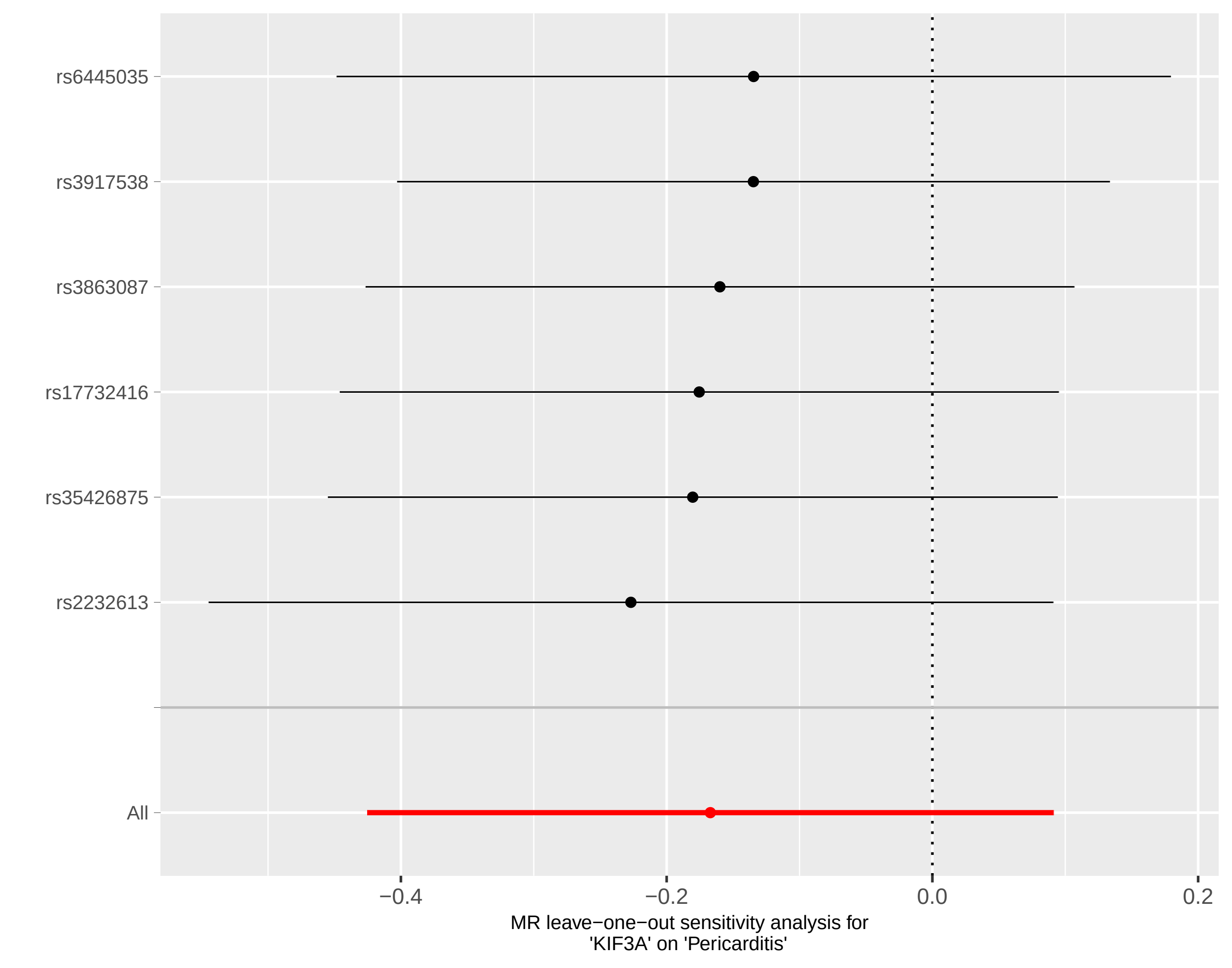

rs6445035
rs3917538
rs3863087
rs17732416
rs35426875
rs2232613
All
−0.4
−0.2
0.0
0.2
MR leave−one−out sensitivity analysis for
'KIF3A' on 'Pericarditis'

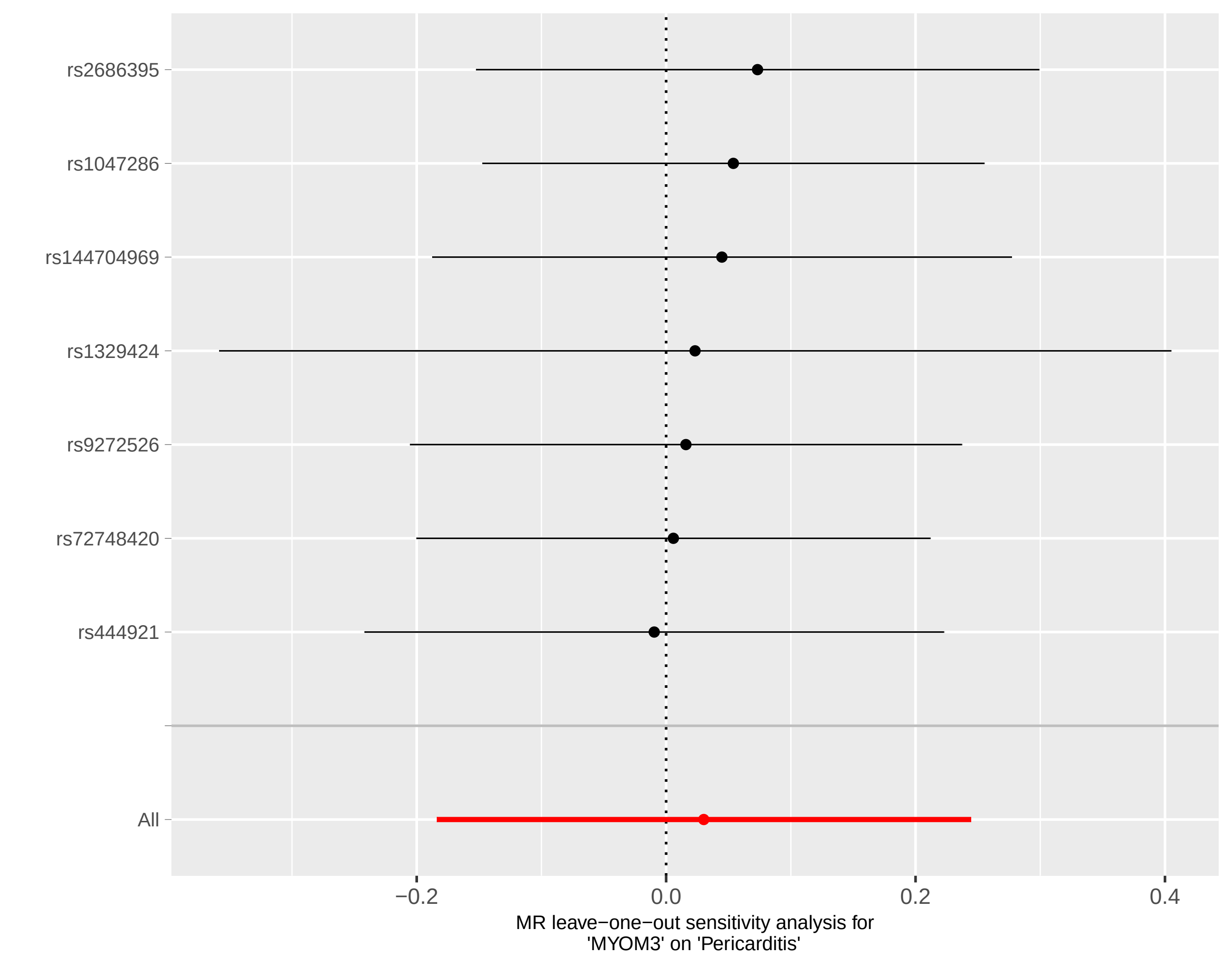
rs2686395
rs1047286
rs144704969
rs1329424
rs9272526
rs72748420
rs444921
All
−0.2
0.0
0.2
0.4
MR leave−one−out sensitivity analysis for
'MYOM3' on 'Pericarditis'

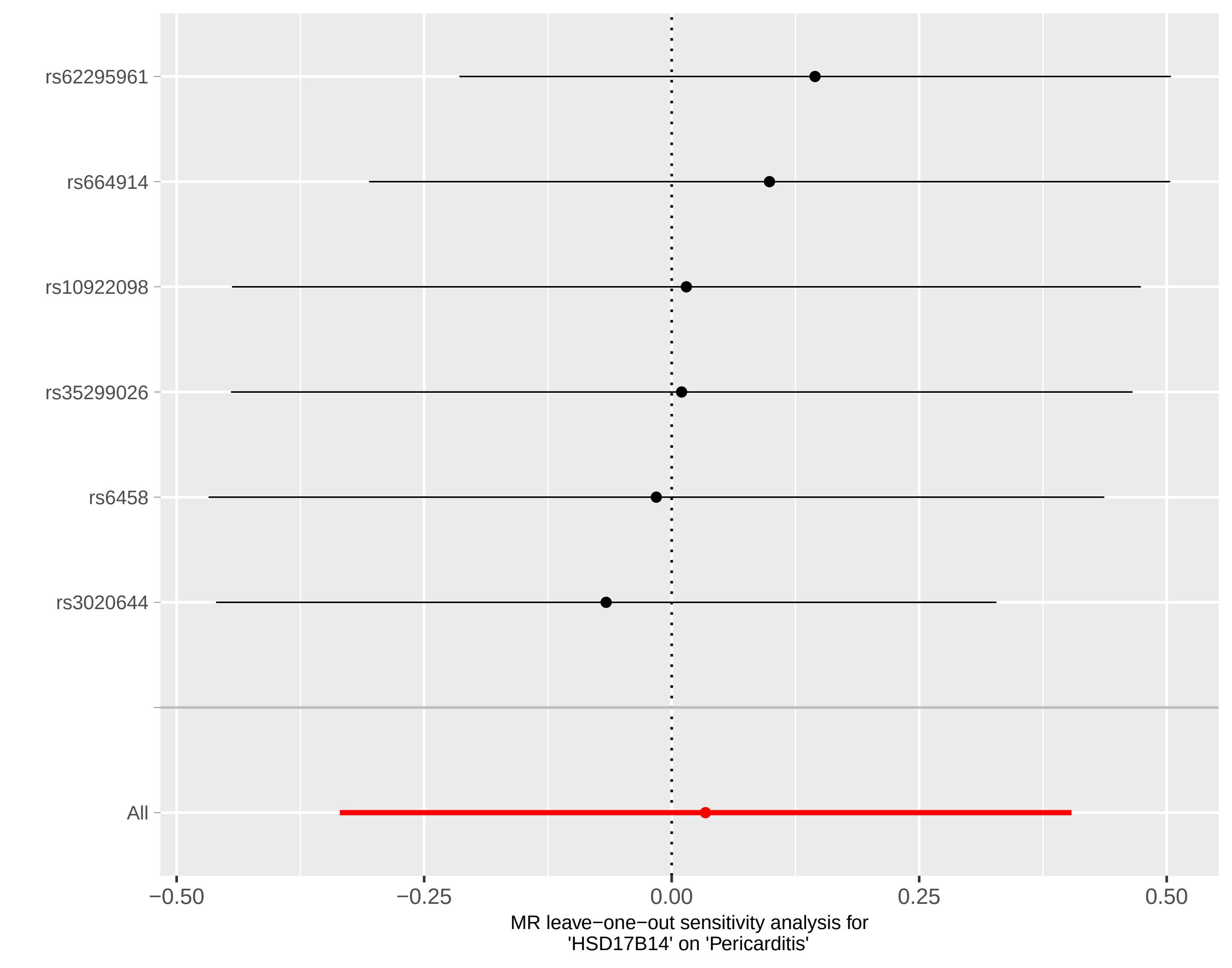

rs62295961
rs664914
rs10922098
rs35299026
rs6458
rs3020644
All
−0.50
−0.25
0.00
0.25
0.50
MR leave−one−out sensitivity analysis for
'HSD17B14' on 'Pericarditis'

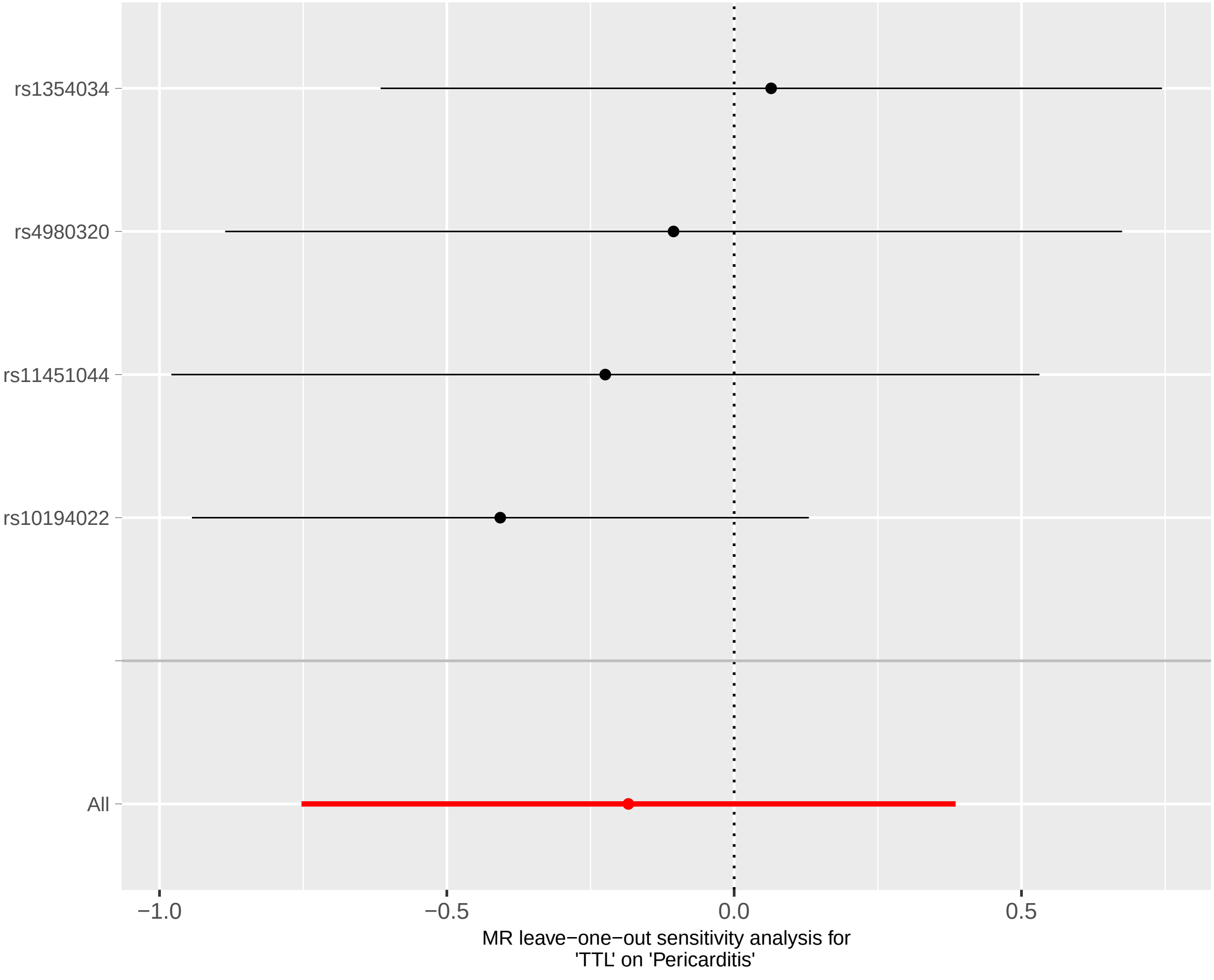
rs1354034
rs4980320
rs11451044
rs10194022
All
−1.0
−0.5
0.0
0.5
MR leave−one−out sensitivity analysis for
'TTL' on 'Pericarditis'

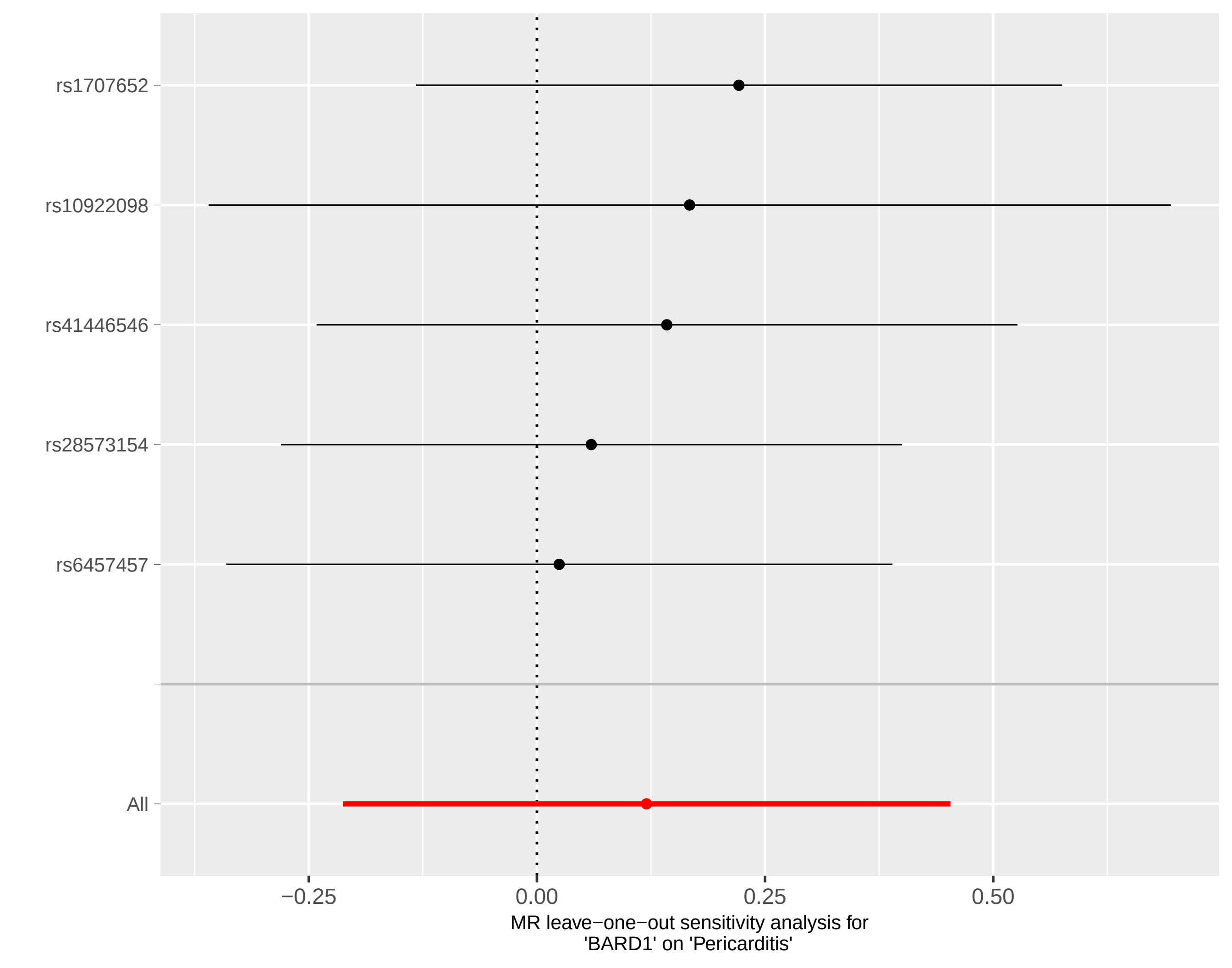
rs1707652
rs10922098
rs41446546
rs28573154
rs6457457
All
−0.25
0.00
0.25
0.50
MR leave−one−out sensitivity analysis for
'BARD1' on 'Pericarditis'

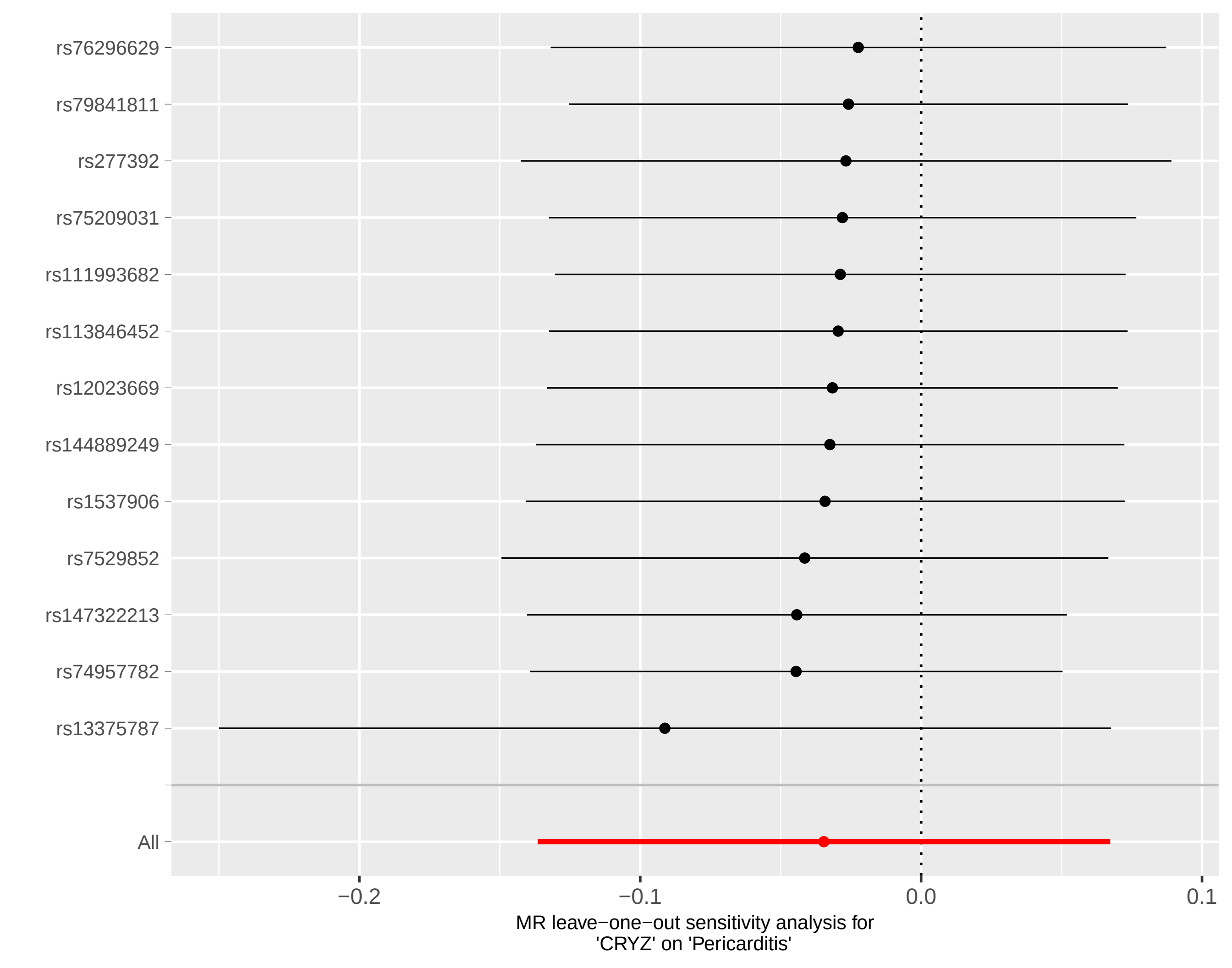
rs76296629
rs79841811
rs277392
rs75209031
rs111993682
rs113846452
rs12023669
rs144889249
rs1537906
rs7529852
rs147322213
rs74957782
rs13375787
All
−0.2
−0.1
0.0
0.1
MR leave−one−out sensitivity analysis for
'CRYZ' on 'Pericarditis'

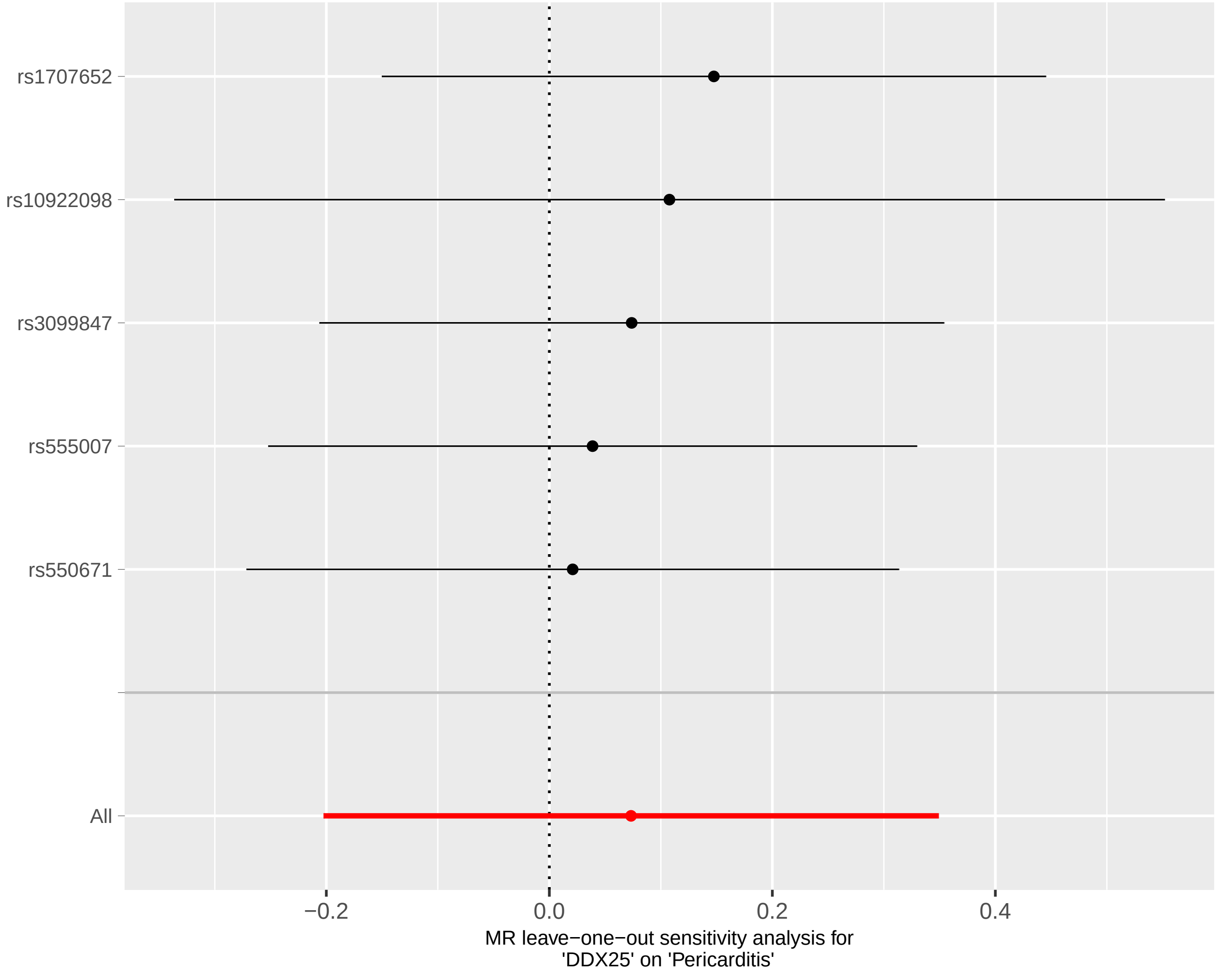
rs1707652
rs10922098
rs3099847
rs555007
rs550671
All
−0.2
0.0
0.2
0.4
MR leave−one−out sensitivity analysis for
'DDX25' on 'Pericarditis'

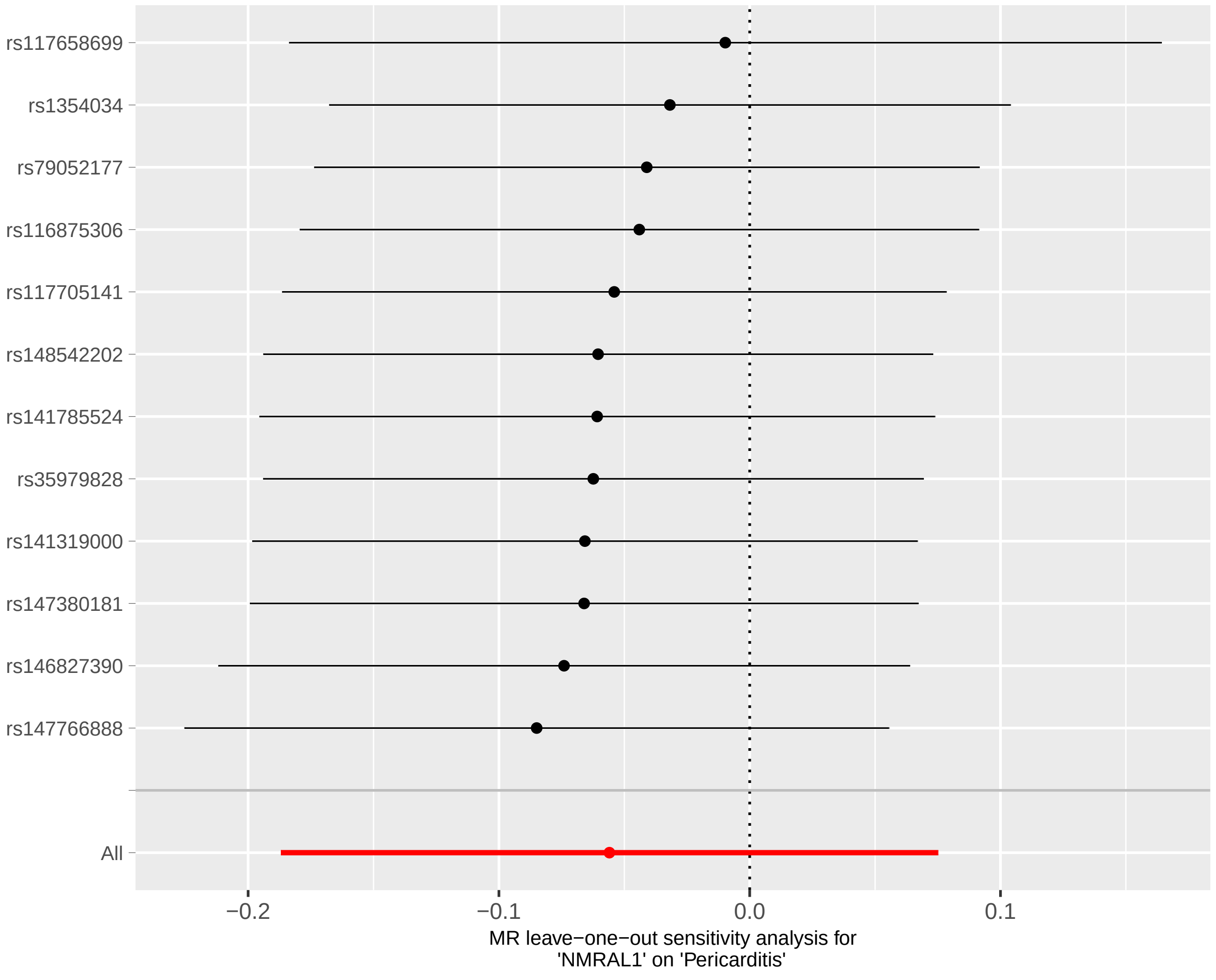

rs117658699
rs1354034
rs79052177
rs116875306
rs117705141
rs148542202
rs141785524
rs35979828
rs141319000
rs147380181
rs146827390
rs147766888
All
−0.2
−0.1
0.0
0.1
MR leave−one−out sensitivity analysis for
'NMRAL1' on 'Pericarditis'

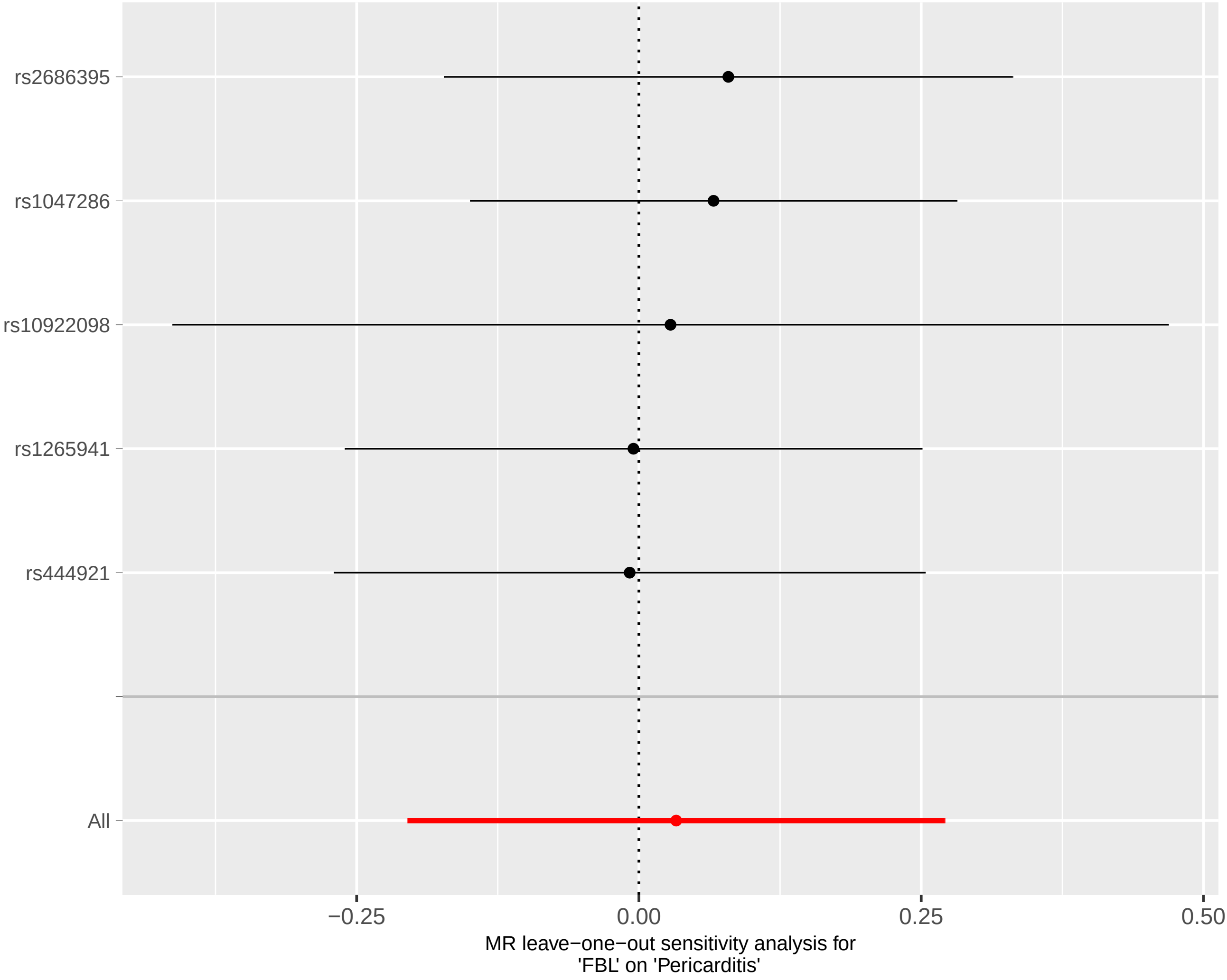

rs2686395
rs1047286
rs10922098
rs1265941
rs444921
All
−0.25
0.00
0.25
0.50
MR leave−one−out sensitivity analysis for
'FBL' on 'Pericarditis'

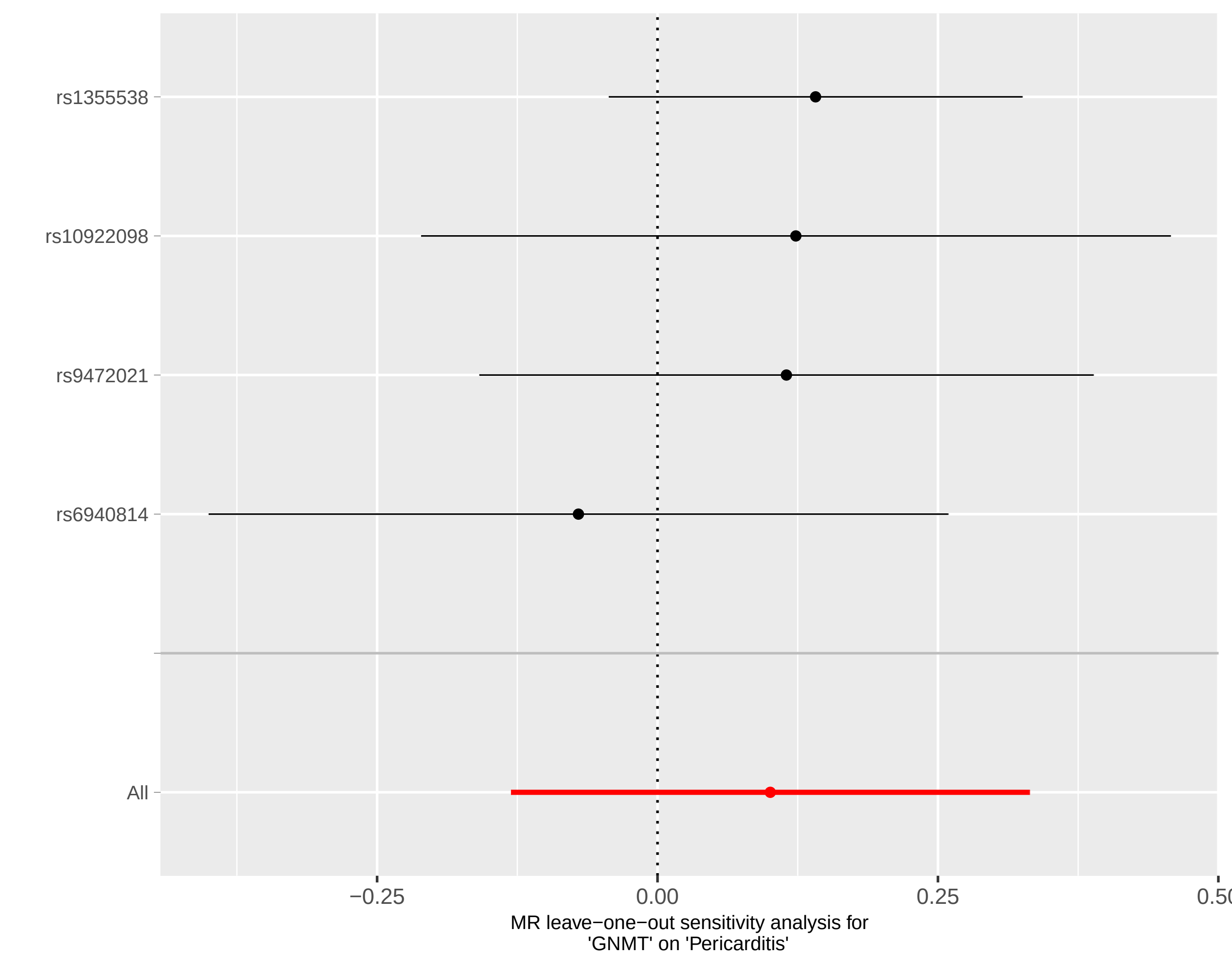
rs1355538
rs10922098
rs9472021
rs6940814
All
−0.25
0.00
0.25
0.50
MR leave−one−out sensitivity analysis for
'GNMT' on 'Pericarditis'

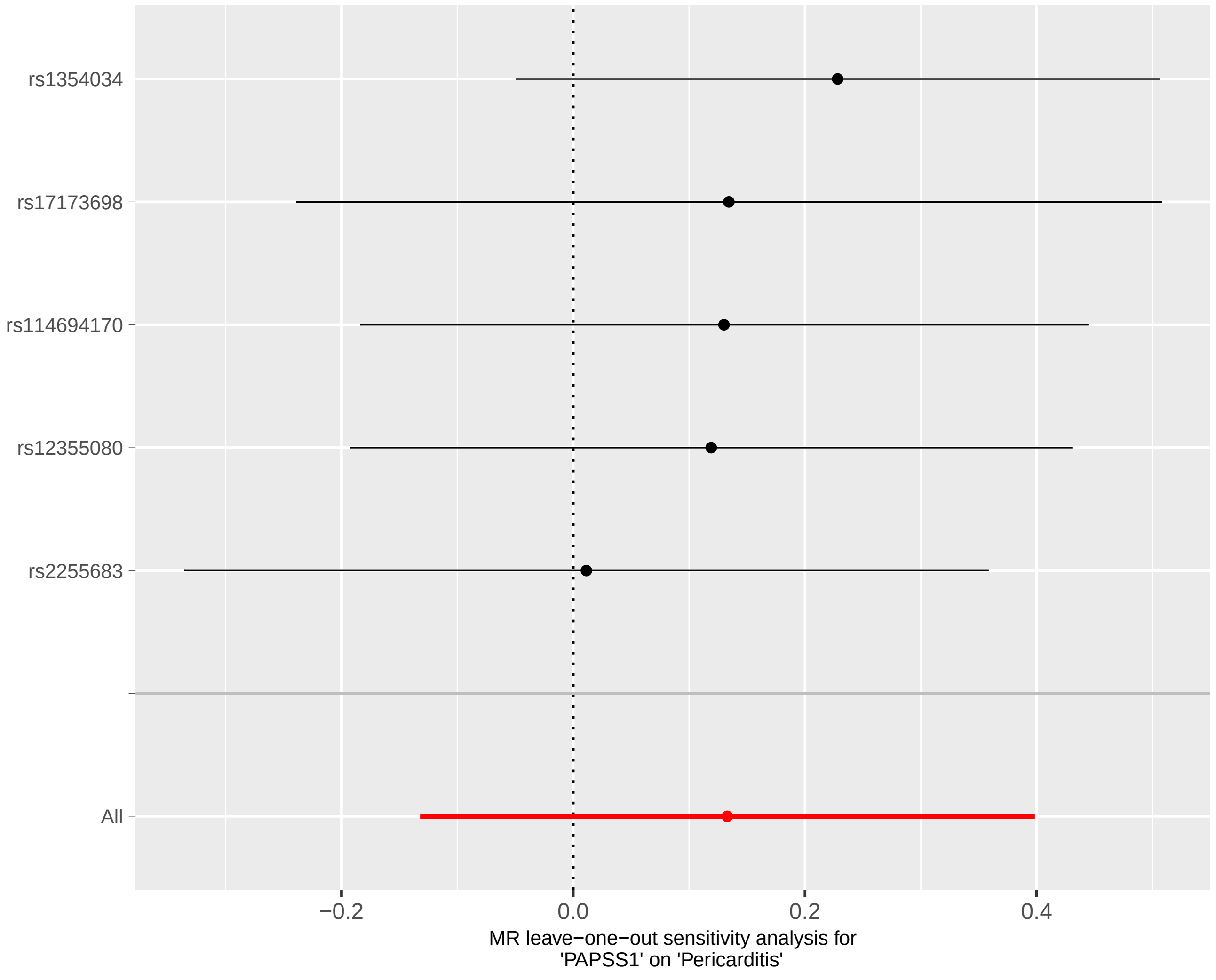
rs1354034
rs17173698
rs114694170
rs12355080
rs2255683
All
−0.2
0.0
0.2
0.4
MR leave−one−out sensitivity analysis for
'PAPSS1' on 'Pericarditis'

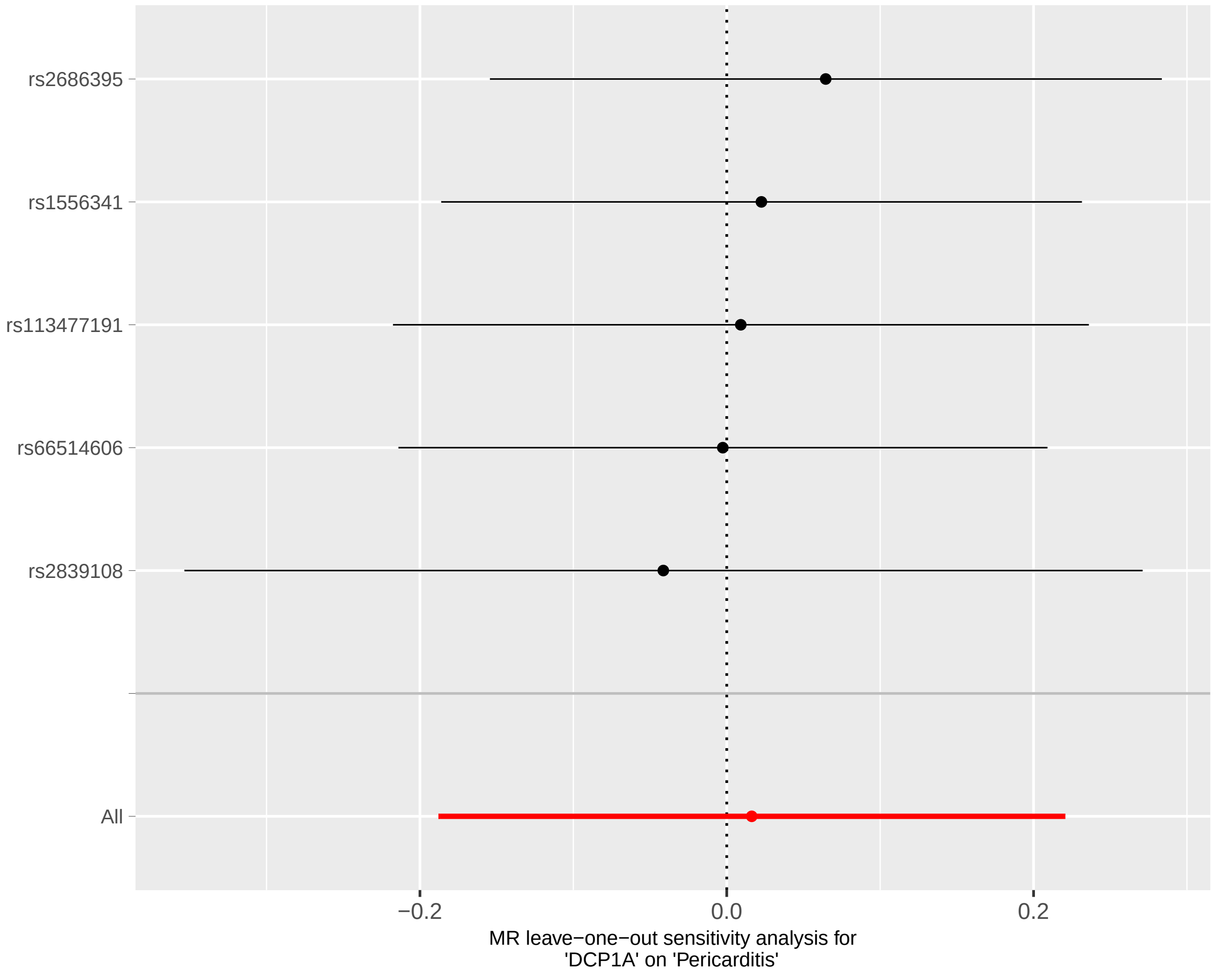
rs2686395
rs1556341
rs113477191
rs66514606
rs2839108
All
−0.2
0.0
0.2
MR leave−one−out sensitivity analysis for
'DCP1A' on 'Pericarditis'

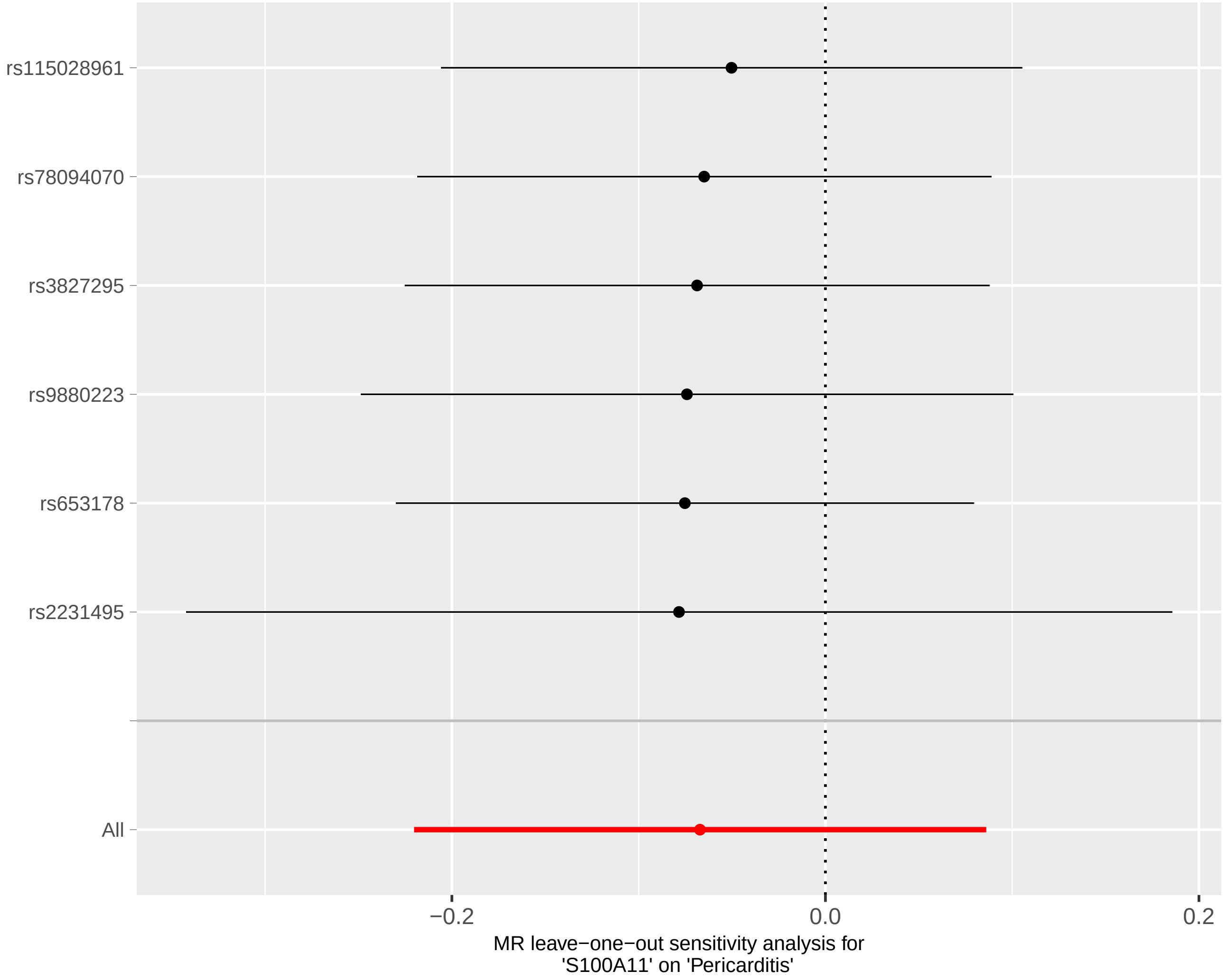

rs115028961
rs78094070
rs3827295
rs9880223
rs653178
rs2231495
All
−0.2
0.0
0.2
MR leave−one−out sensitivity analysis for
'S100A11' on 'Pericarditis'

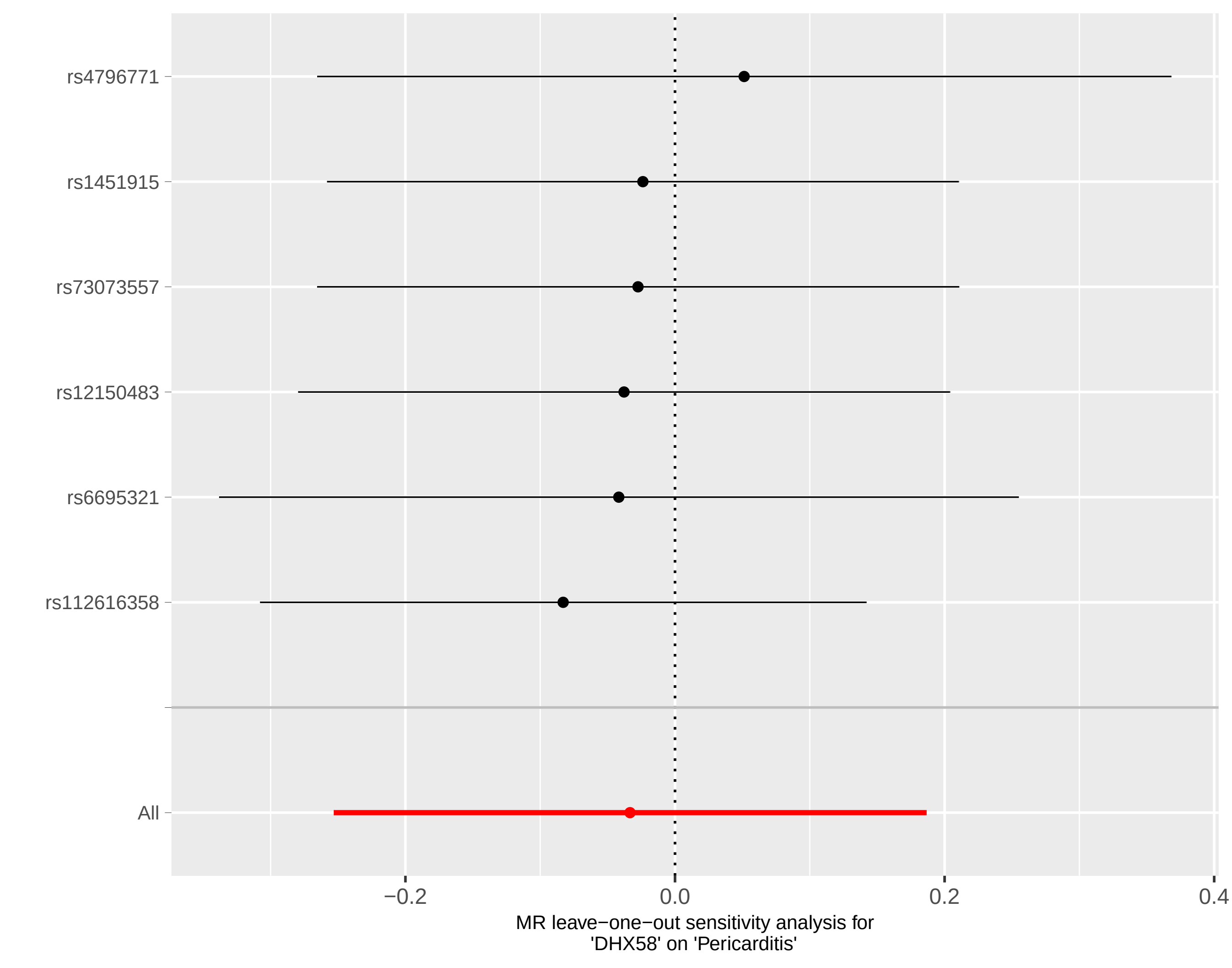
rs4796771
rs1451915
rs73073557
rs12150483
rs6695321
rs112616358
All
−0.2
0.0
0.2
0.4
MR leave−one−out sensitivity analysis for
'DHX58' on 'Pericarditis'

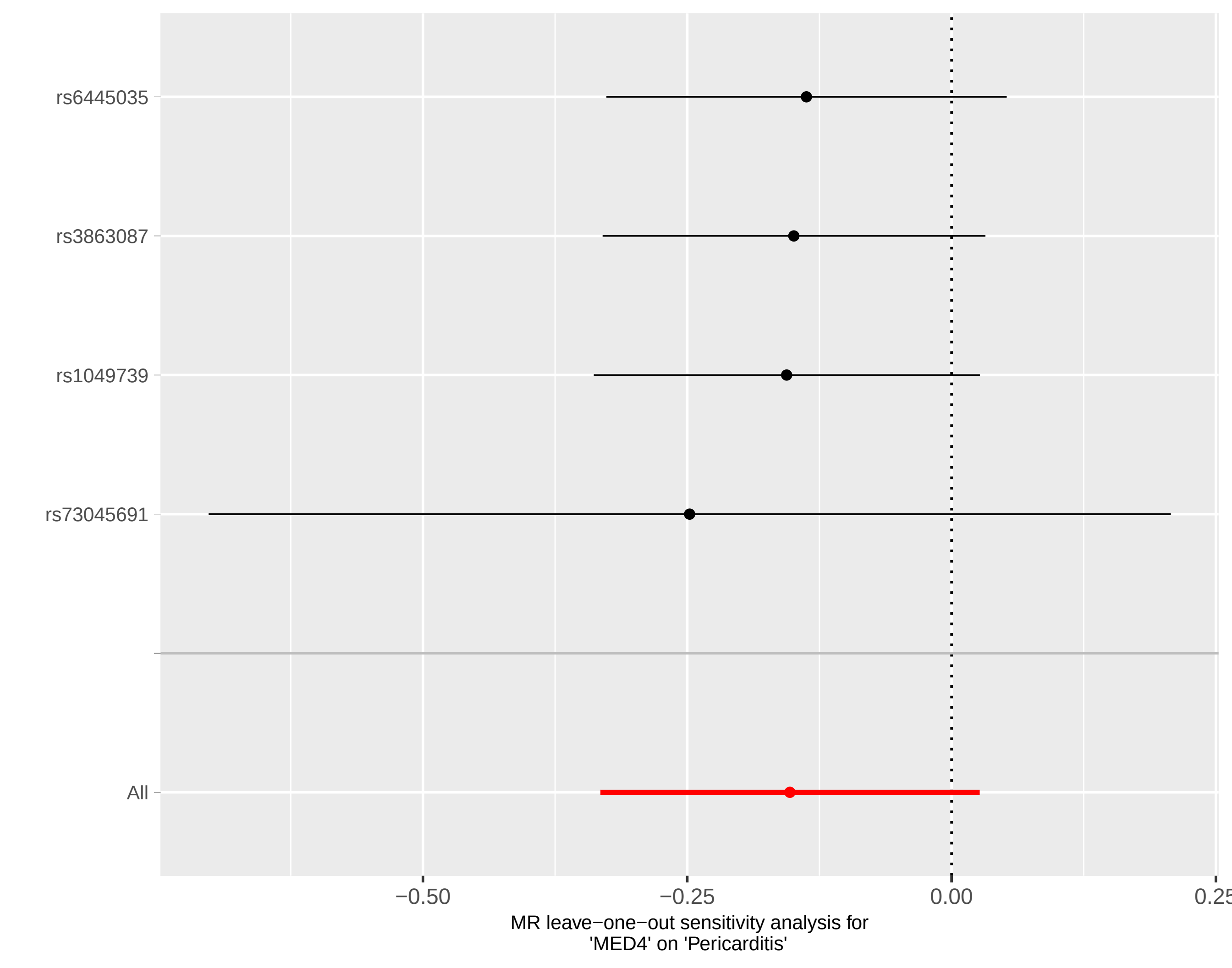
rs6445035
rs3863087
rs1049739
rs73045691
All
−0.50
−0.25
0.00
0.25
MR leave−one−out sensitivity analysis for
'MED4' on 'Pericarditis'

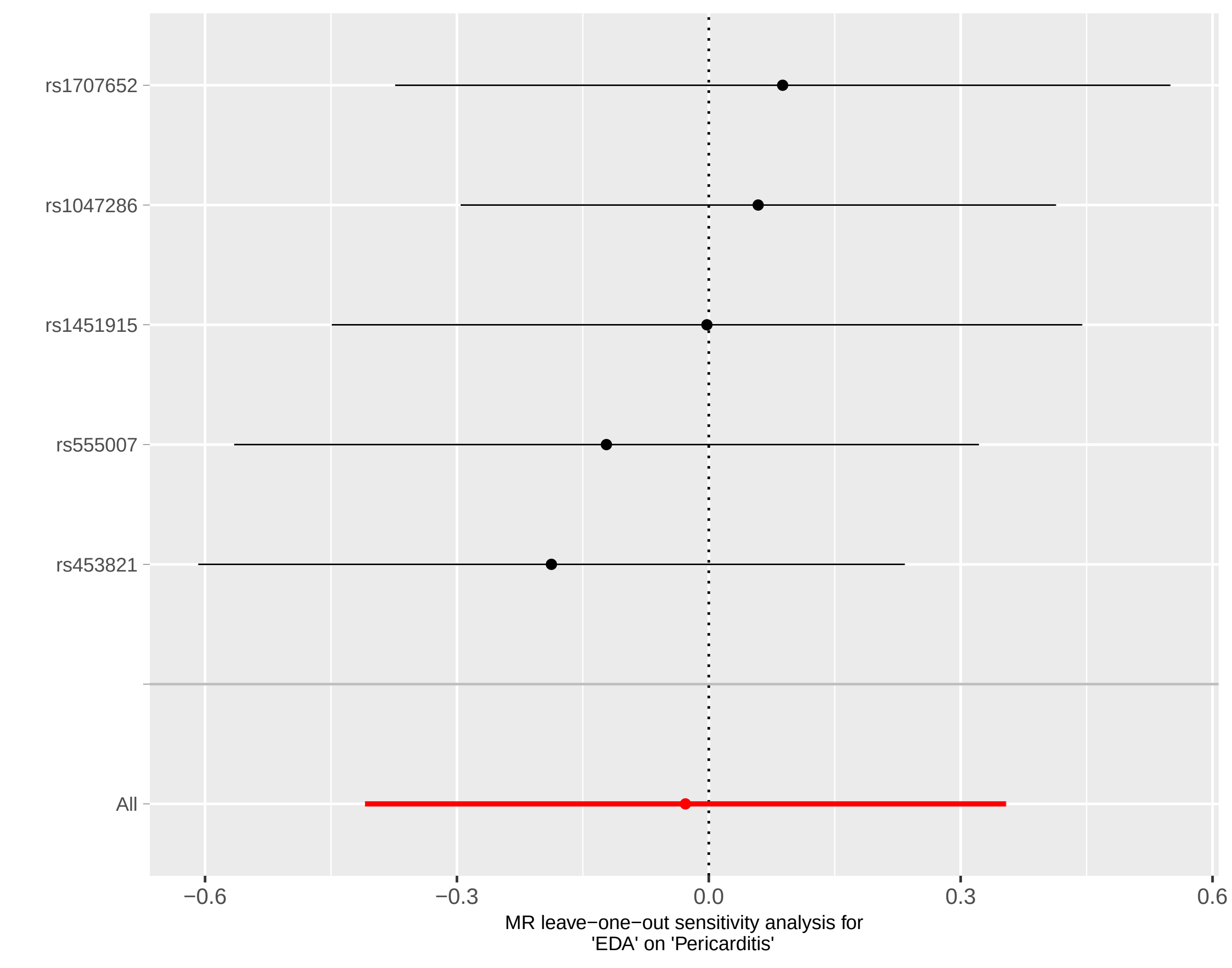

rs1707652
rs1047286
rs1451915
rs555007
rs453821
All
−0.6
−0.3
0.0
0.3
0.6
MR leave−one−out sensitivity analysis for
'EDA' on 'Pericarditis'

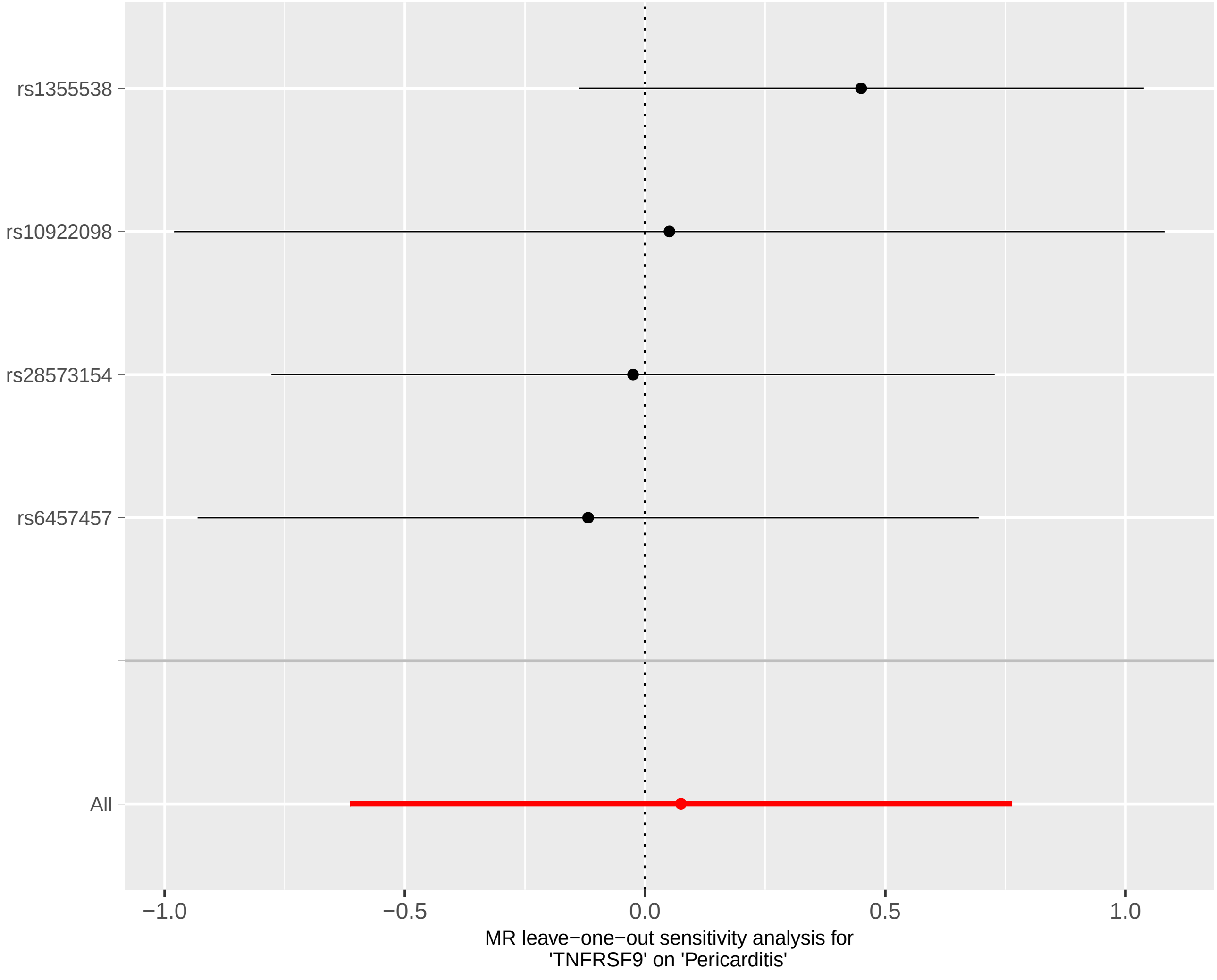
rs1355538
rs10922098
rs28573154
rs6457457
All
−1.0
−0.5
0.0
0.5
1.0
MR leave−one−out sensitivity analysis for
'TNFRSF9' on 'Pericarditis'

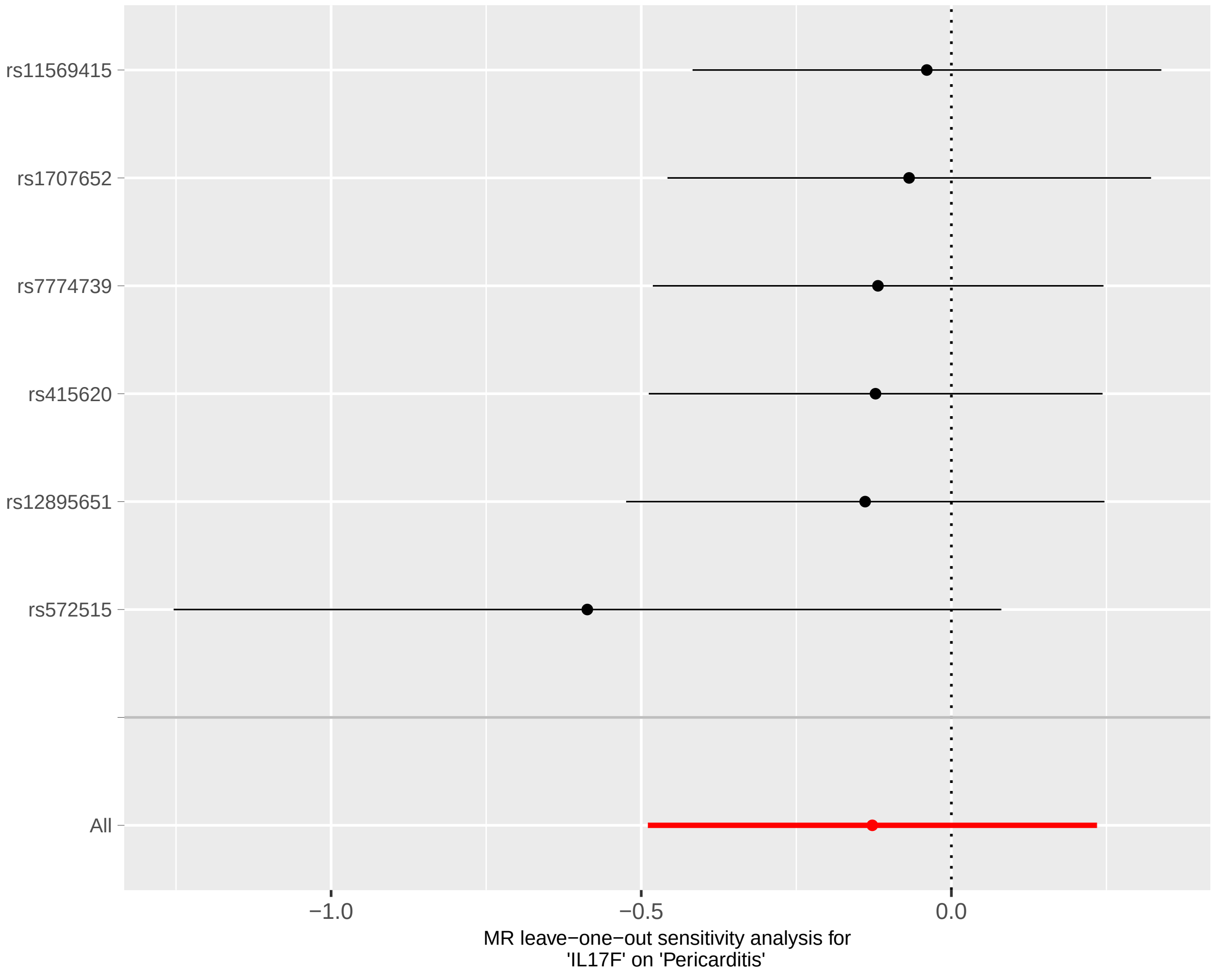

rs11569415
rs1707652
rs7774739
rs415620
rs12895651
rs572515
All
−1.0
−0.5
0.0
MR leave−one−out sensitivity analysis for
'IL17F' on 'Pericarditis'

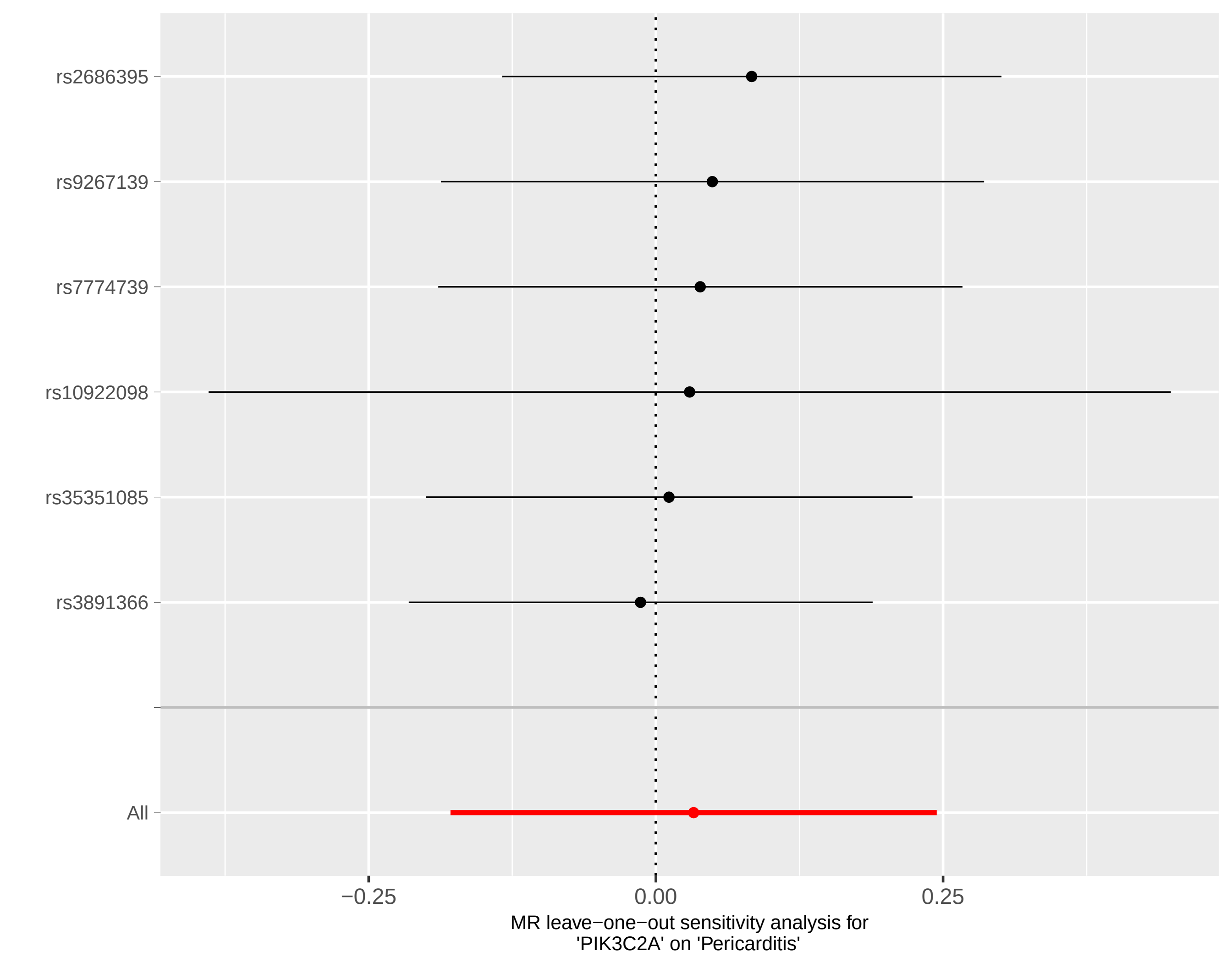
rs2686395
rs9267139
rs7774739
rs10922098
rs35351085
rs3891366
All
−0.25
0.00
0.25
MR leave−one−out sensitivity analysis for
'PIK3C2A' on 'Pericarditis'

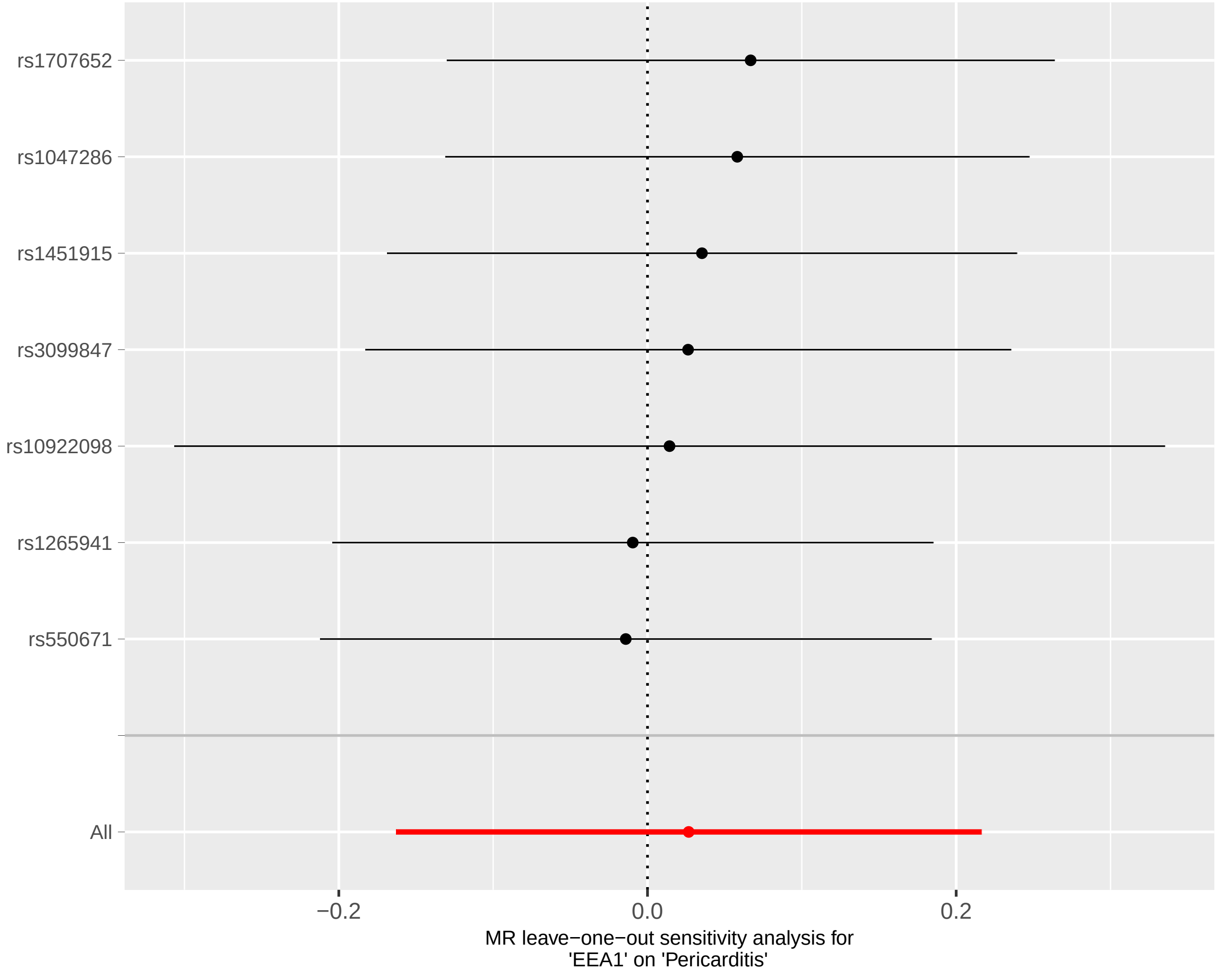
rs1707652
rs1047286
rs1451915
rs3099847
rs10922098
rs1265941
rs550671
All
−0.2
0.0
0.2
MR leave−one−out sensitivity analysis for
'EEA1' on 'Pericarditis'

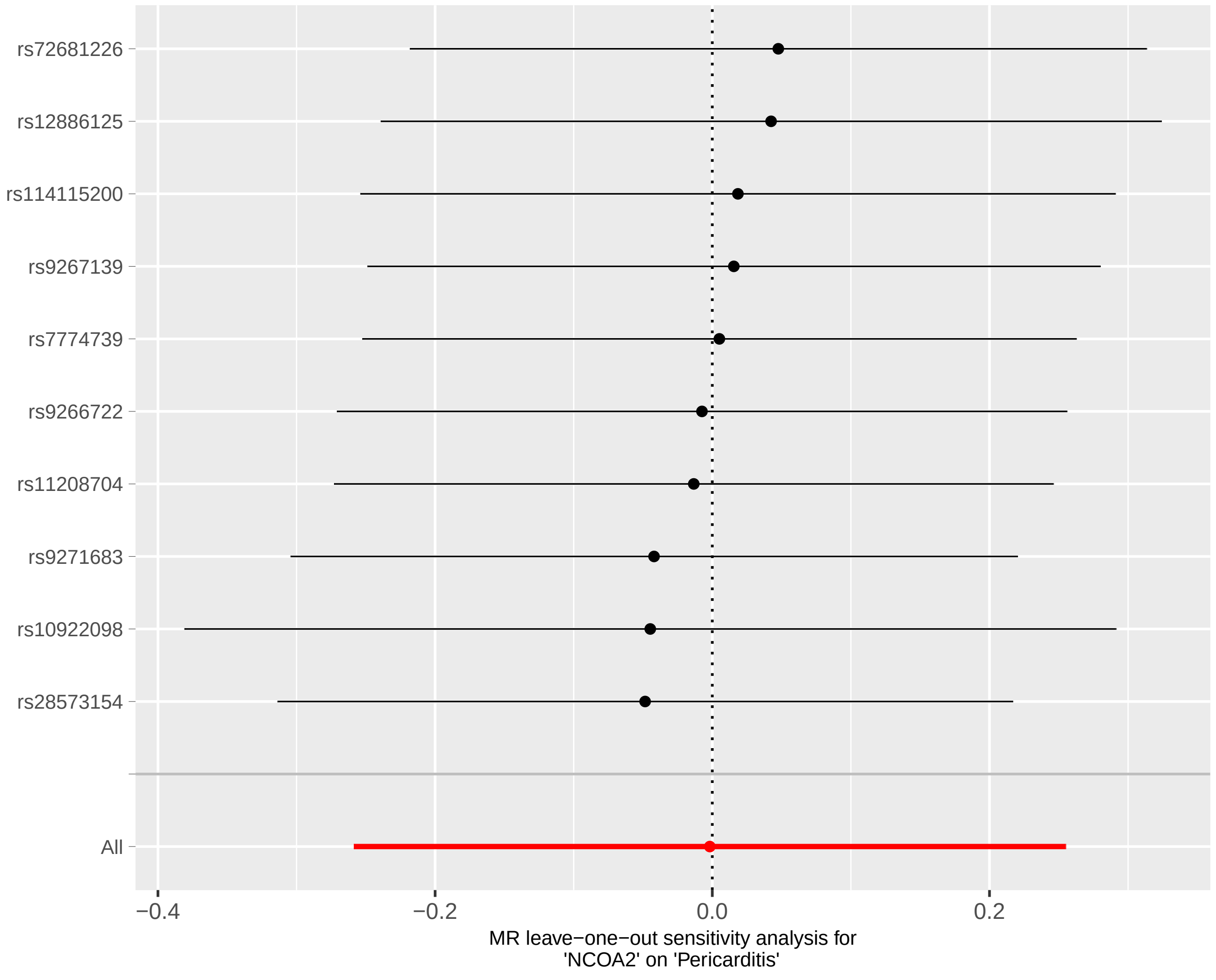
rs72681226
rs12886125
rs114115200
rs9267139
rs7774739
rs9266722
rs11208704
rs9271683
rs10922098
rs28573154
All
−0.4
−0.2
0.0
0.2
MR leave−one−out sensitivity analysis for
'NCOA2' on 'Pericarditis'

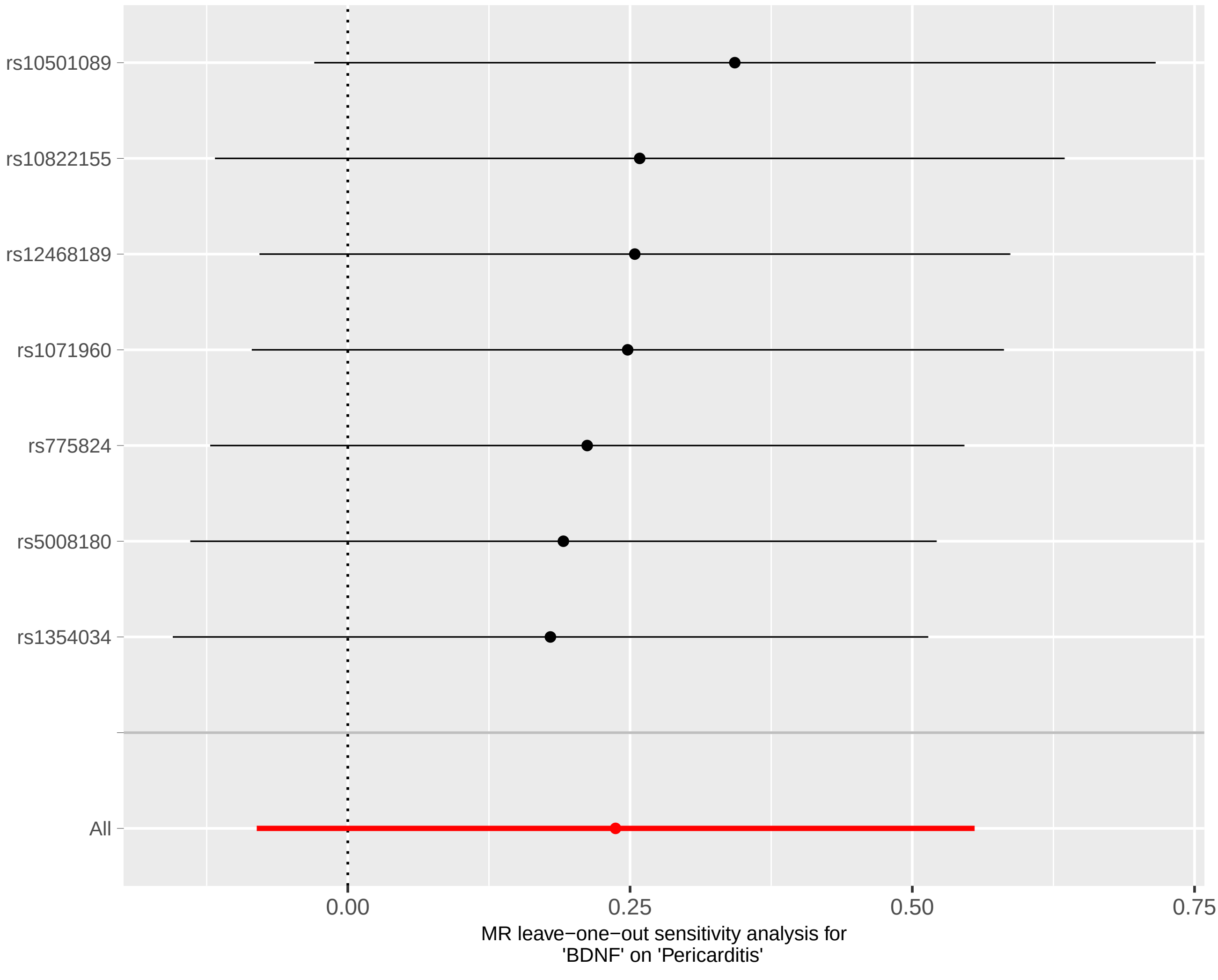
rs10501089
rs10822155
rs12468189
rs1071960
rs775824
rs5008180
rs1354034
All
0.00
0.25
0.50
0.75
MR leave−one−out sensitivity analysis for
'BDNF' on 'Pericarditis'

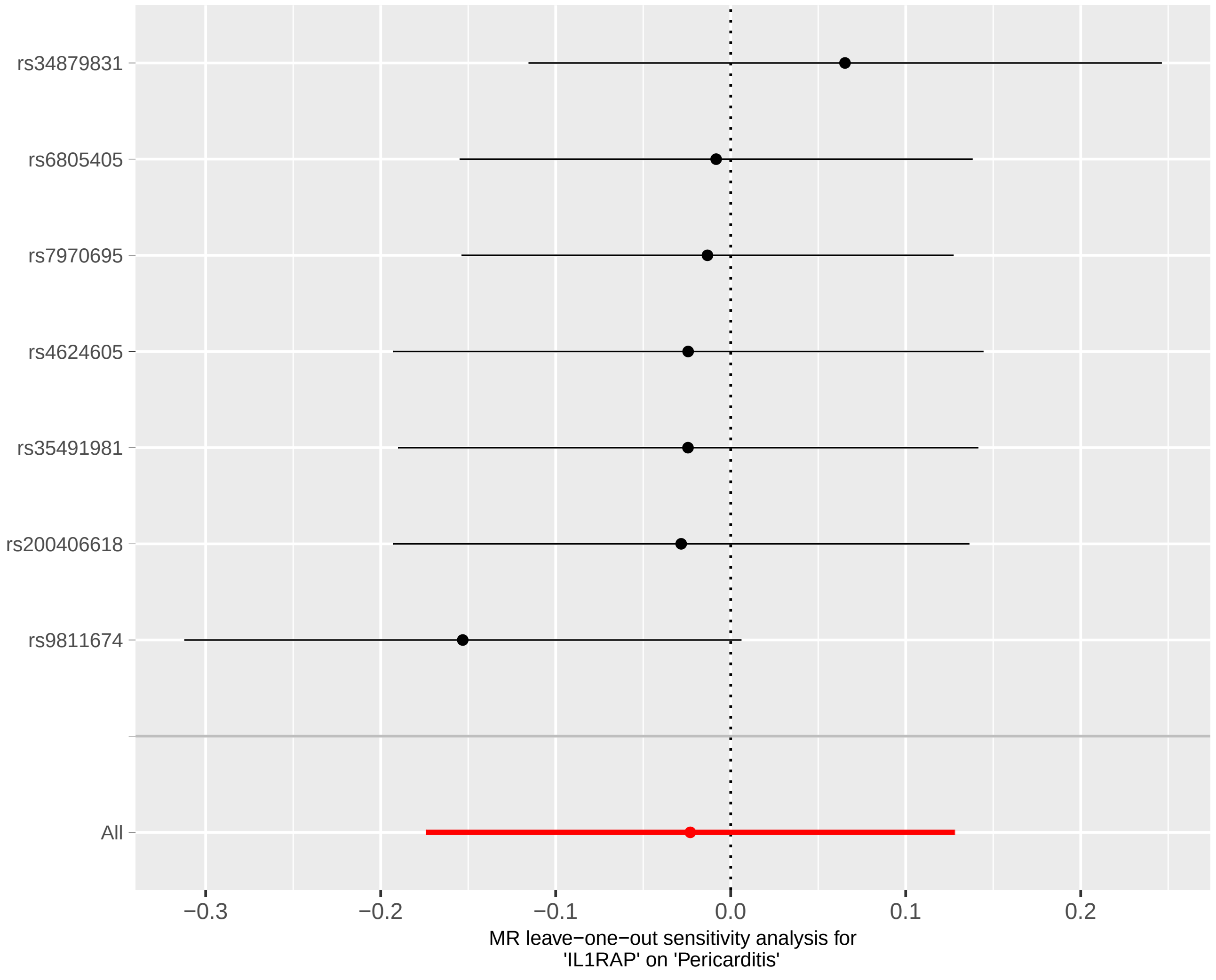

rs34879831
rs6805405
rs7970695
rs4624605
rs35491981
rs200406618
rs9811674
All
−0.3
−0.2
−0.1
0.0
0.1
0.2
MR leave−one−out sensitivity analysis for
'IL1RAP' on 'Pericarditis'

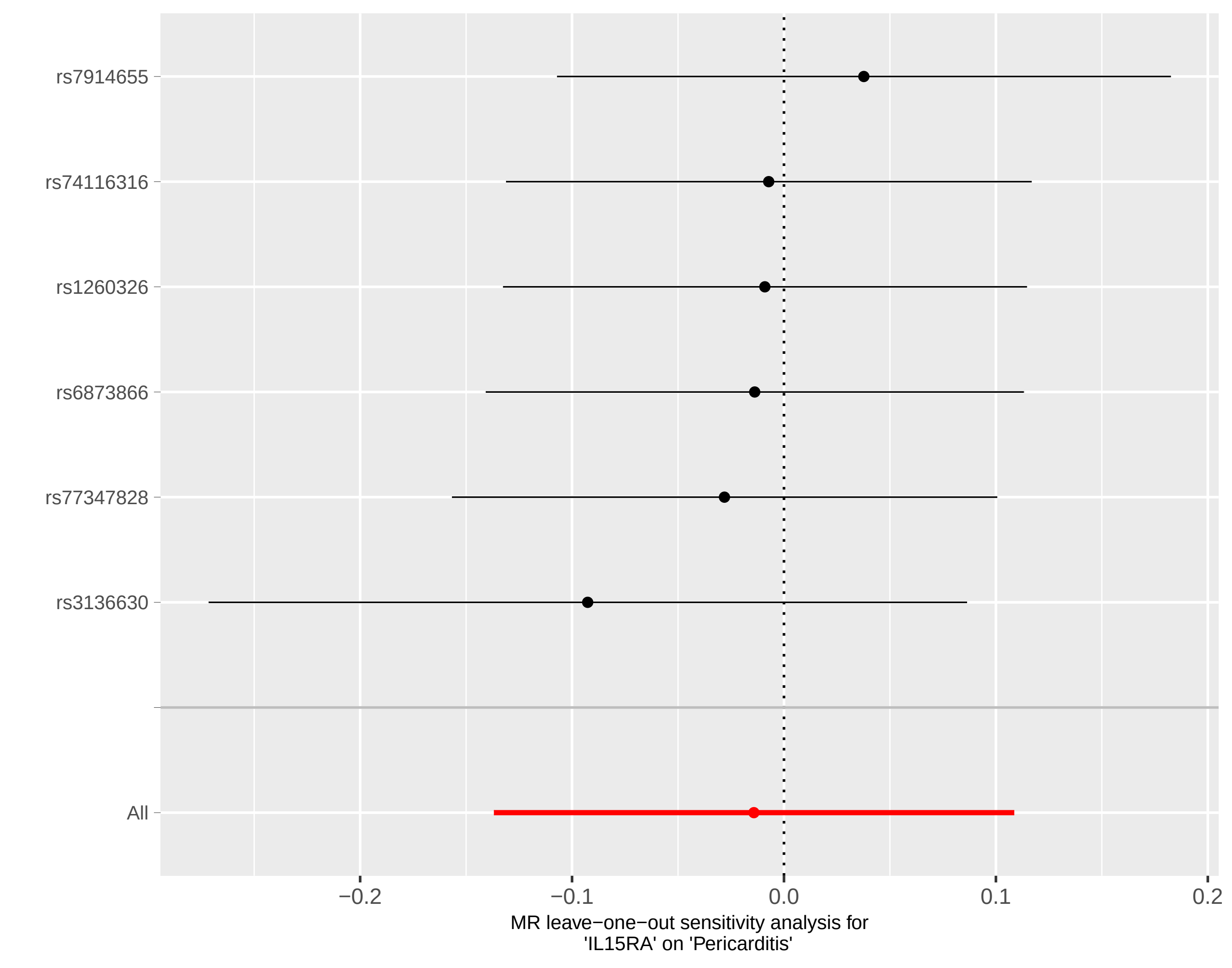
rs7914655
rs74116316
rs1260326
rs6873866
rs77347828
rs3136630
All
−0.2
−0.1
0.0
0.1
0.2
MR leave−one−out sensitivity analysis for
'IL15RA' on 'Pericarditis'

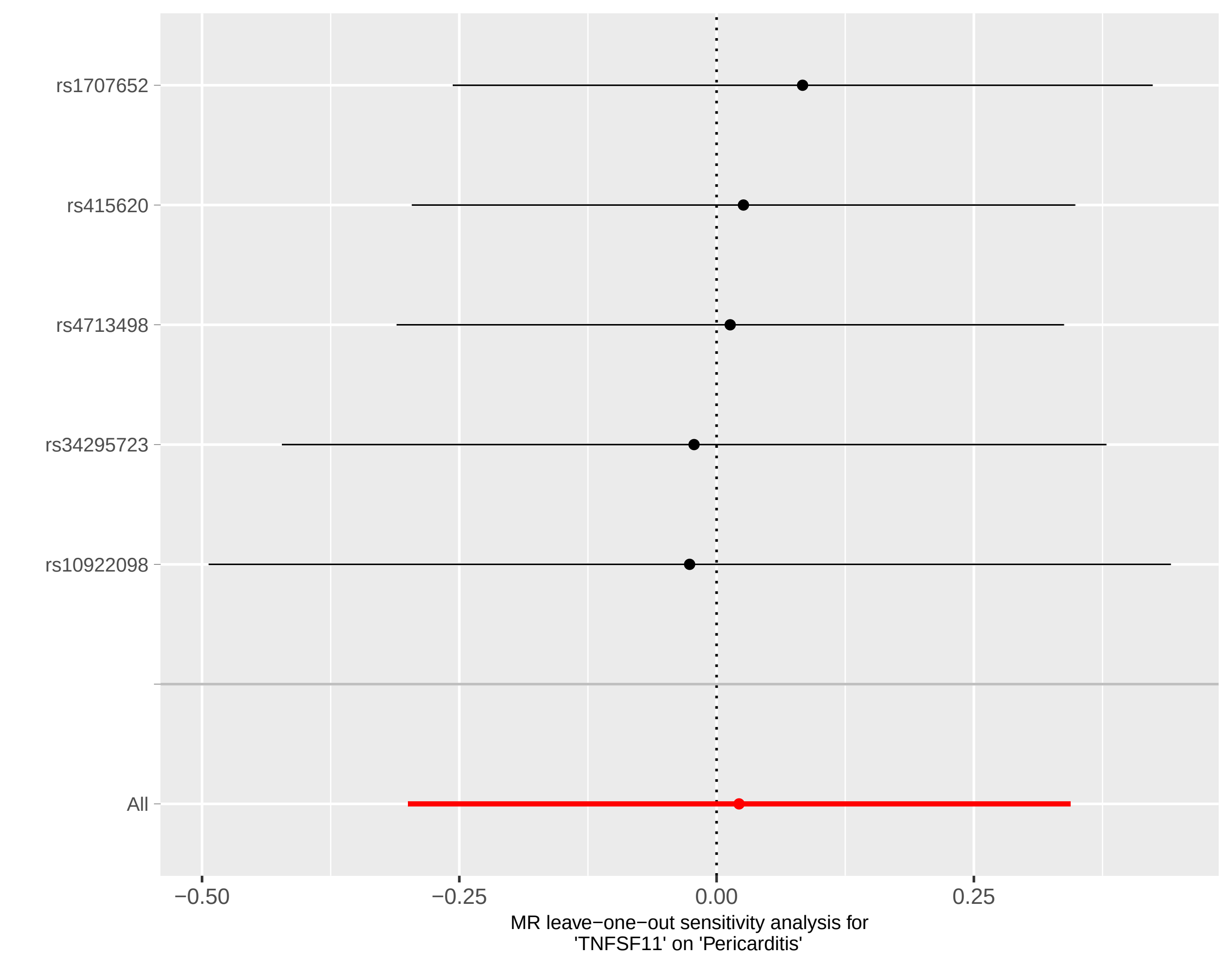
rs1707652
rs415620
rs4713498
rs34295723
rs10922098
All
−0.50
−0.25
0.00
0.25
MR leave−one−out sensitivity analysis for
'TNFSF11' on 'Pericarditis'

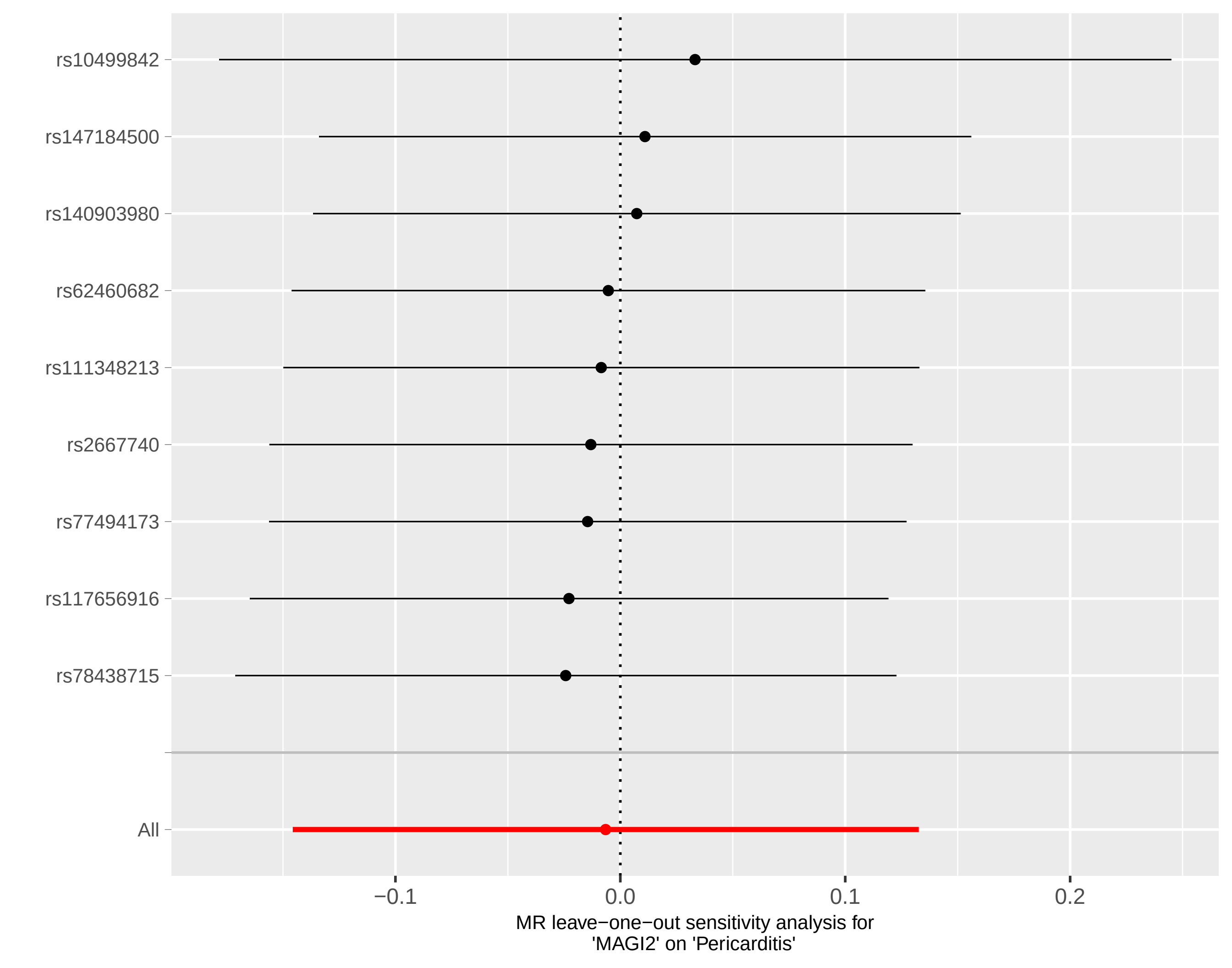

rs10499842
rs147184500
rs140903980
rs62460682
rs111348213
rs2667740
rs77494173
rs117656916
rs78438715
All
−0.1
0.0
0.1
0.2
MR leave−one−out sensitivity analysis for
'MAGI2' on 'Pericarditis'

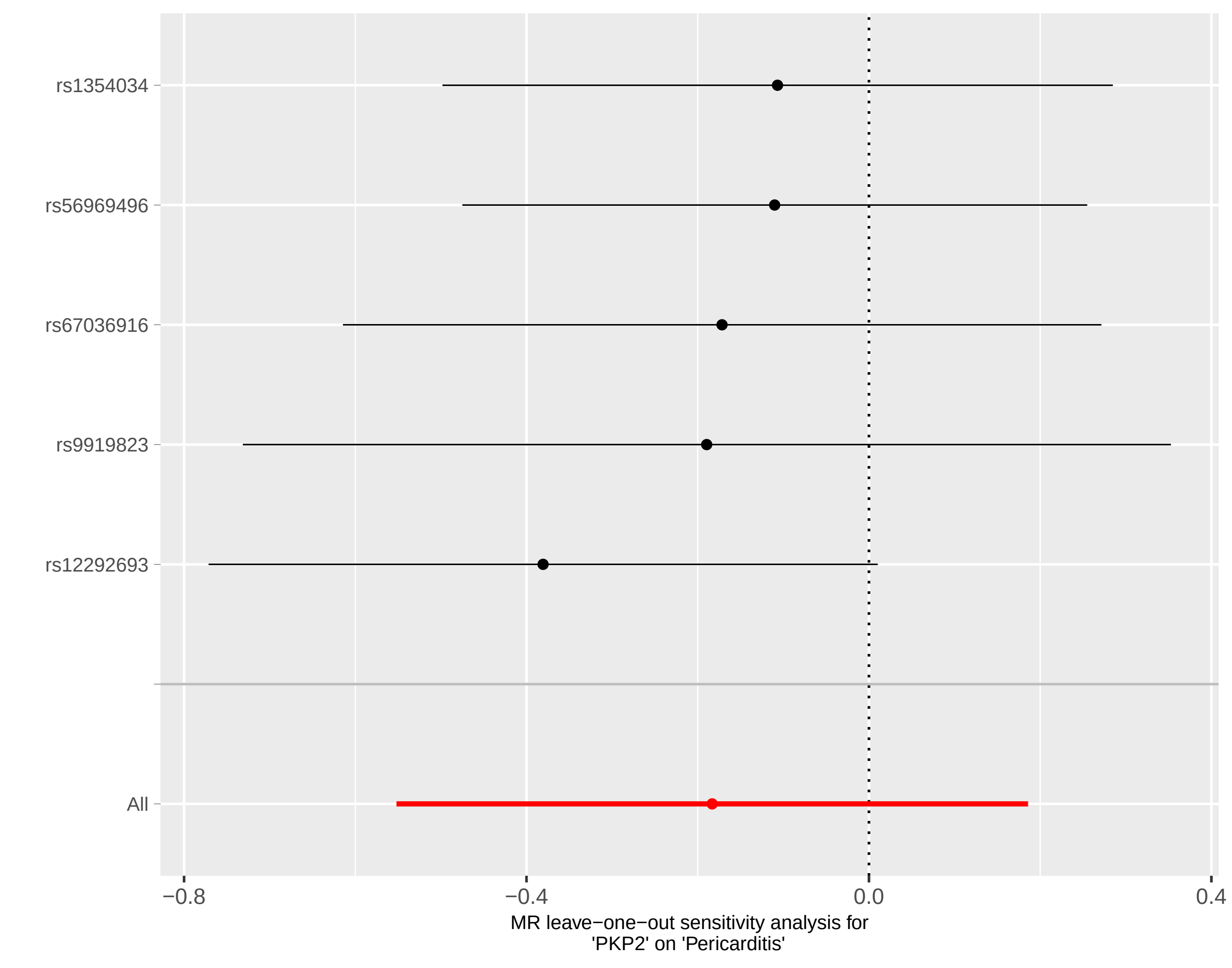
rs1354034
rs56969496
rs67036916
rs9919823
rs12292693
All
−0.8
−0.4
0.0
0.4
MR leave−one−out sensitivity analysis for
'PKP2' on 'Pericarditis'

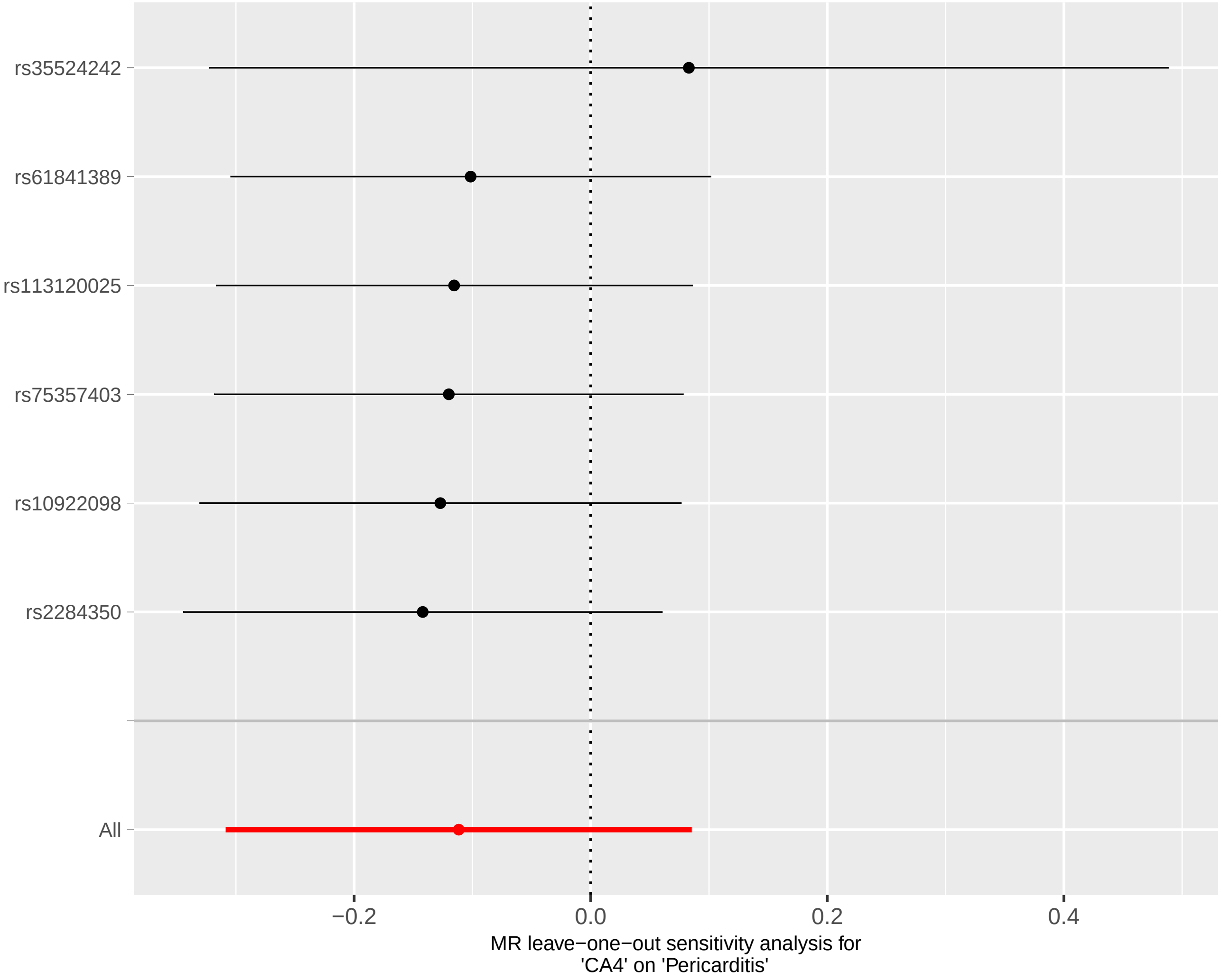
rs35524242
rs61841389
rs113120025
rs75357403
rs10922098
rs2284350
All
−0.2
0.0
0.2
0.4
MR leave−one−out sensitivity analysis for
'CA4' on 'Pericarditis'

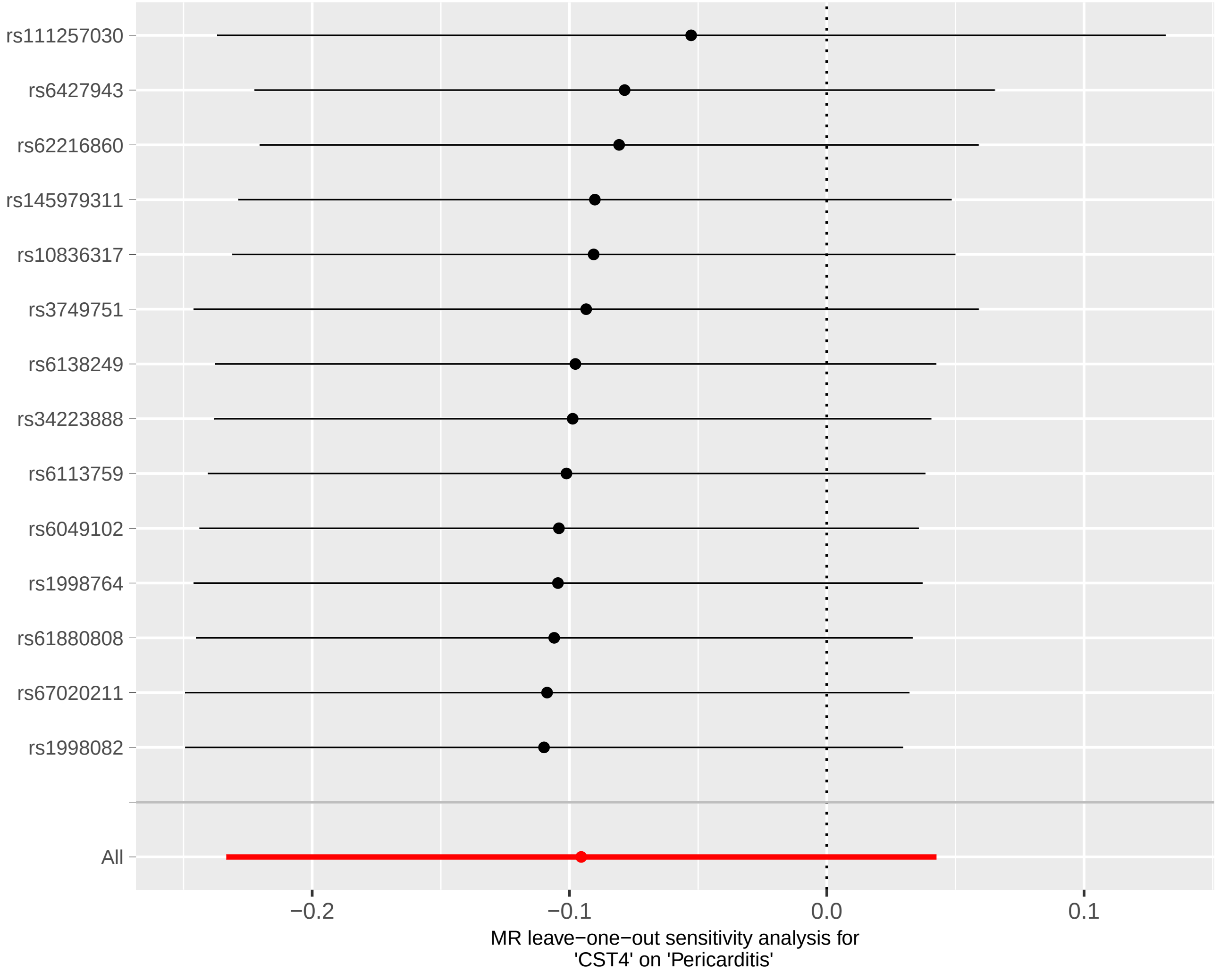
rs111257030
rs6427943
rs62216860
rs145979311
rs10836317
rs3749751
rs6138249
rs34223888
rs6113759
rs6049102
rs1998764
rs61880808
rs67020211
rs1998082
All
−0.2
−0.1
0.0
0.1
MR leave−one−out sensitivity analysis for
'CST4' on 'Pericarditis'

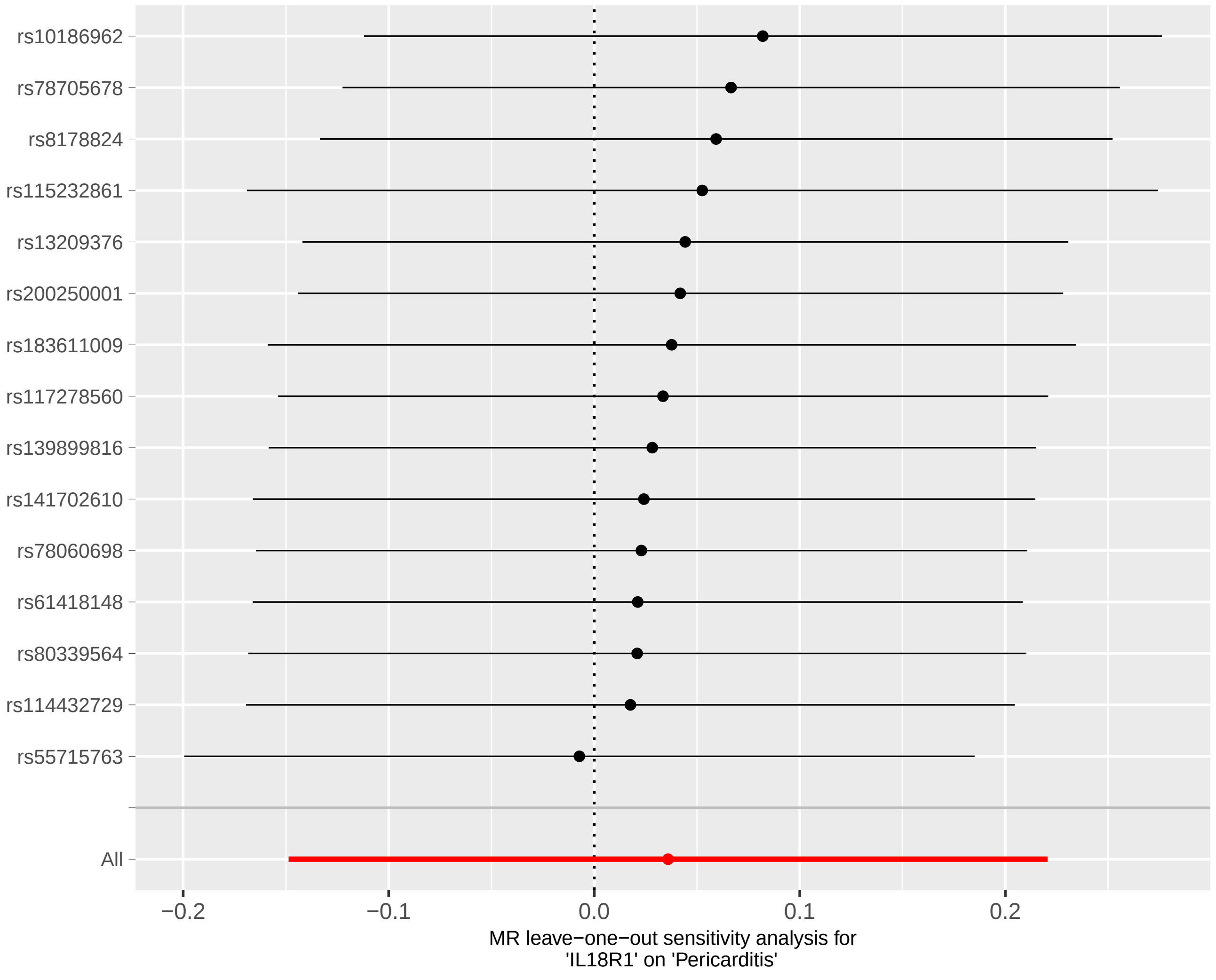
rs10186962
rs78705678
rs8178824
rs115232861
rs13209376
rs200250001
rs183611009
rs117278560
rs139899816
rs141702610
rs78060698
rs61418148
rs80339564
rs114432729
rs55715763
All
−0.2
−0.1
0.0
0.1
0.2
MR leave−one−out sensitivity analysis for
'IL18R1' on 'Pericarditis'

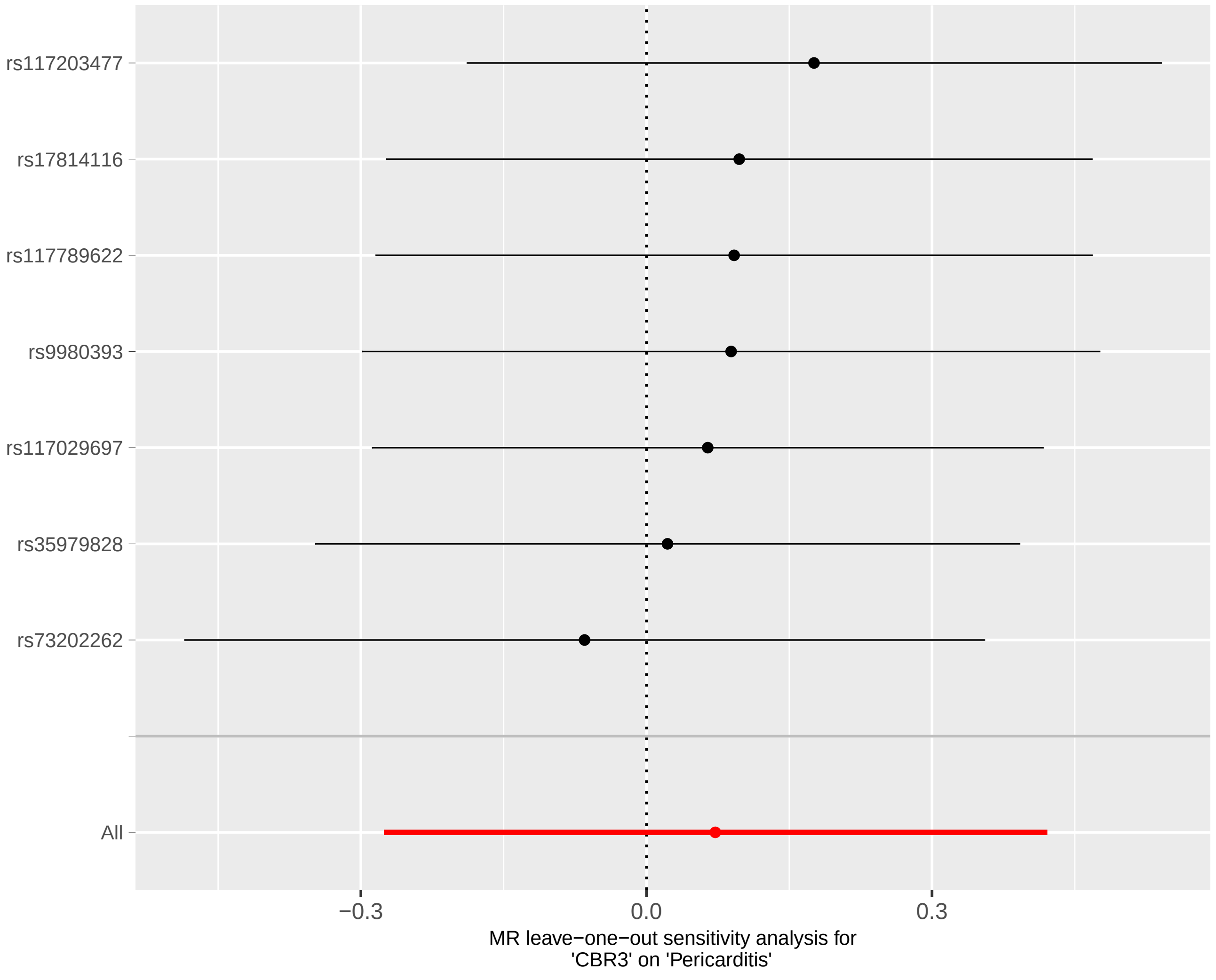

rs117203477
rs17814116
rs117789622
rs9980393
rs117029697
rs35979828
rs73202262
All
−0.3
0.0
0.3
MR leave−one−out sensitivity analysis for
'CBR3' on 'Pericarditis'

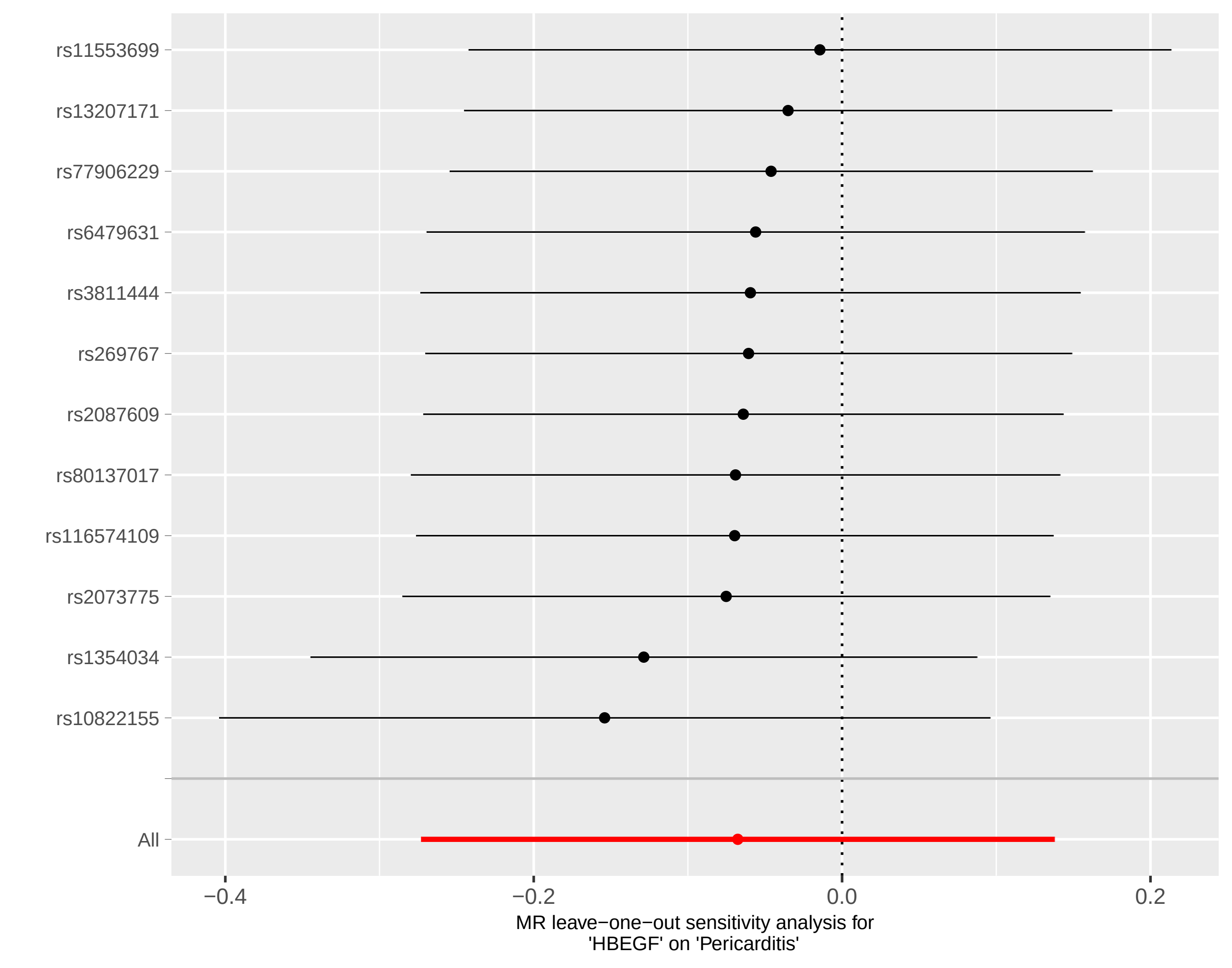
rs11553699
rs13207171
rs77906229
rs6479631
rs3811444
rs269767
rs2087609
rs80137017
rs116574109
rs2073775
rs1354034
rs10822155
All
−0.4
−0.2
0.0
0.2
MR leave−one−out sensitivity analysis for
'HBEGF' on 'Pericarditis'

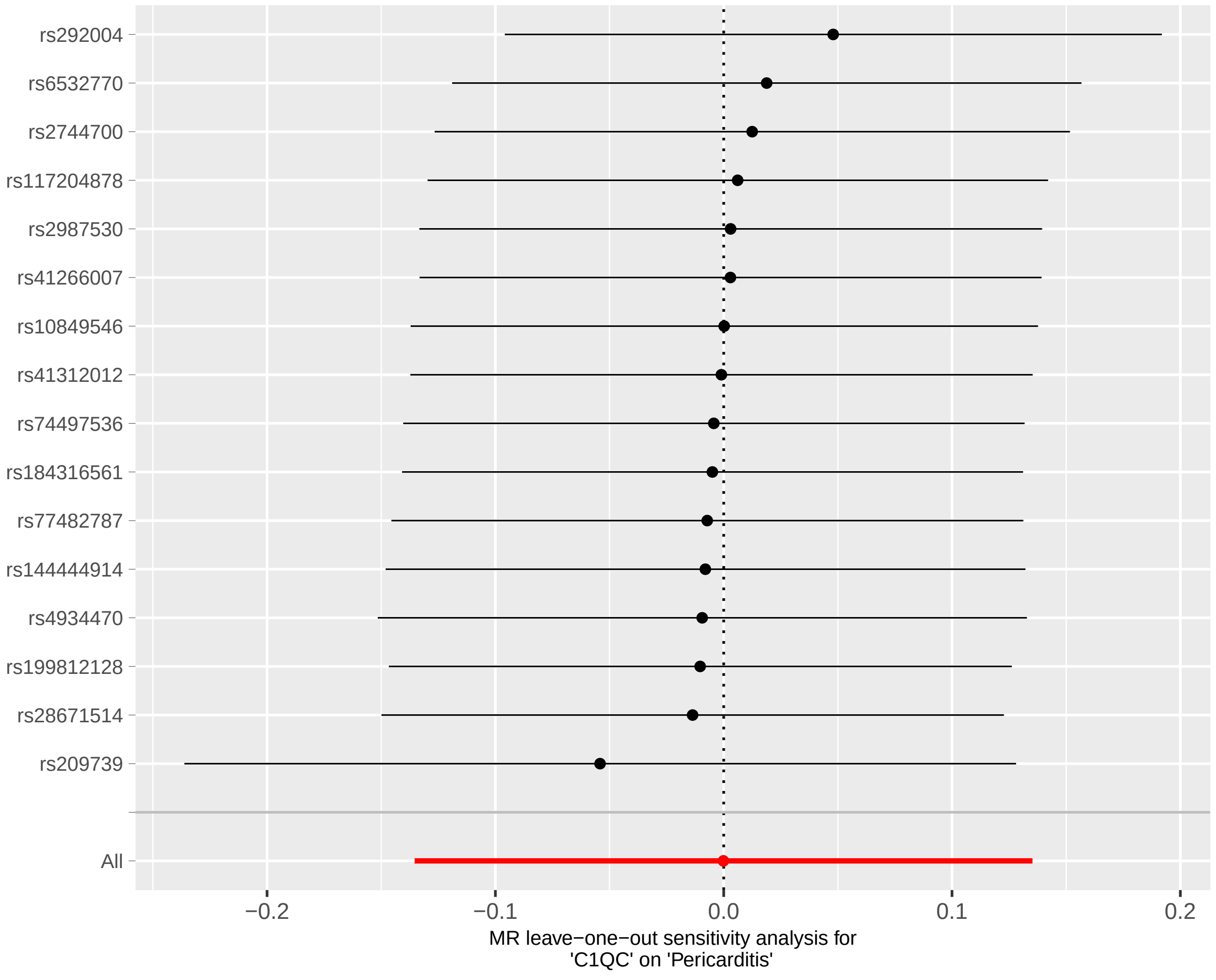

rs292004
rs6532770
rs2744700
rs117204878
rs2987530
rs41266007
rs10849546
rs41312012
rs74497536
rs184316561
rs77482787
rs144444914
rs4934470
rs199812128
rs28671514
rs209739
All
−0.2
−0.1
0.0
0.1
0.2
MR leave−one−out sensitivity analysis for
'C1QC' on 'Pericarditis'

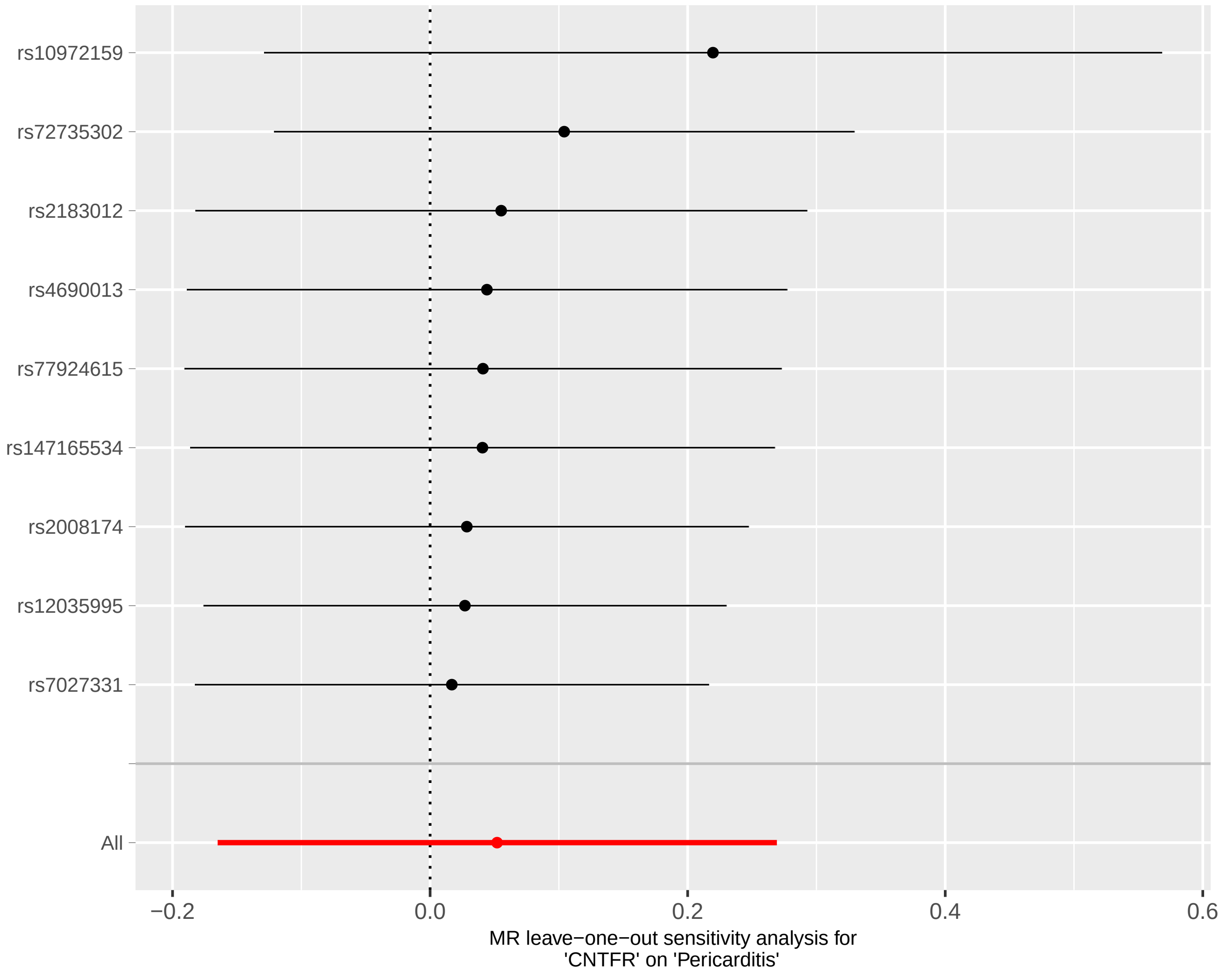
rs10972159
rs72735302
rs2183012
rs4690013
rs77924615
rs147165534
rs2008174
rs12035995
rs7027331
All
−0.2
0.0
0.2
0.4
0.6
MR leave−one−out sensitivity analysis for
'CNTFR' on 'Pericarditis'

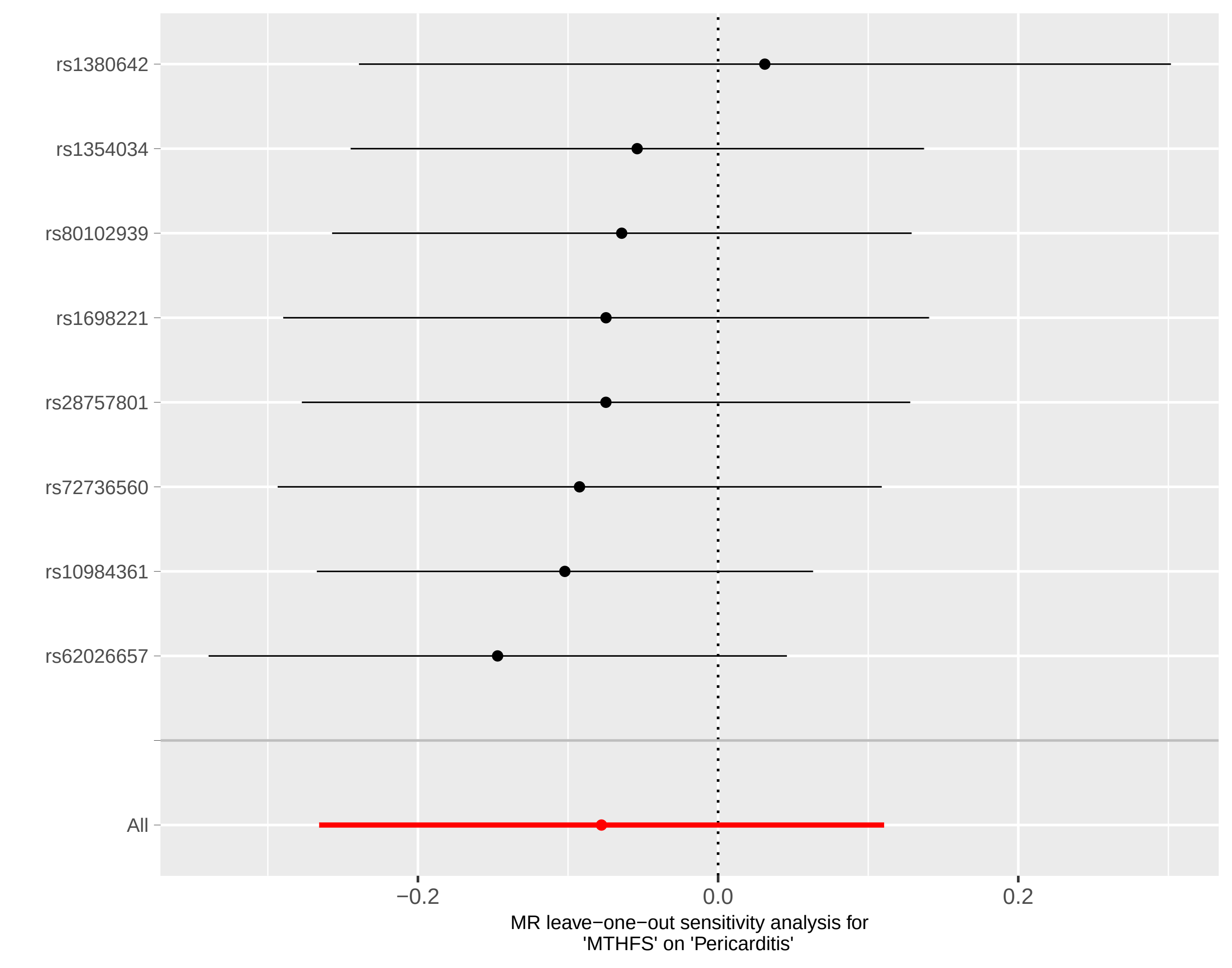
rs1380642
rs1354034
rs80102939
rs1698221
rs28757801
rs72736560
rs10984361
rs62026657
All
−0.2
0.0
0.2
MR leave−one−out sensitivity analysis for
'MTHFS' on 'Pericarditis'

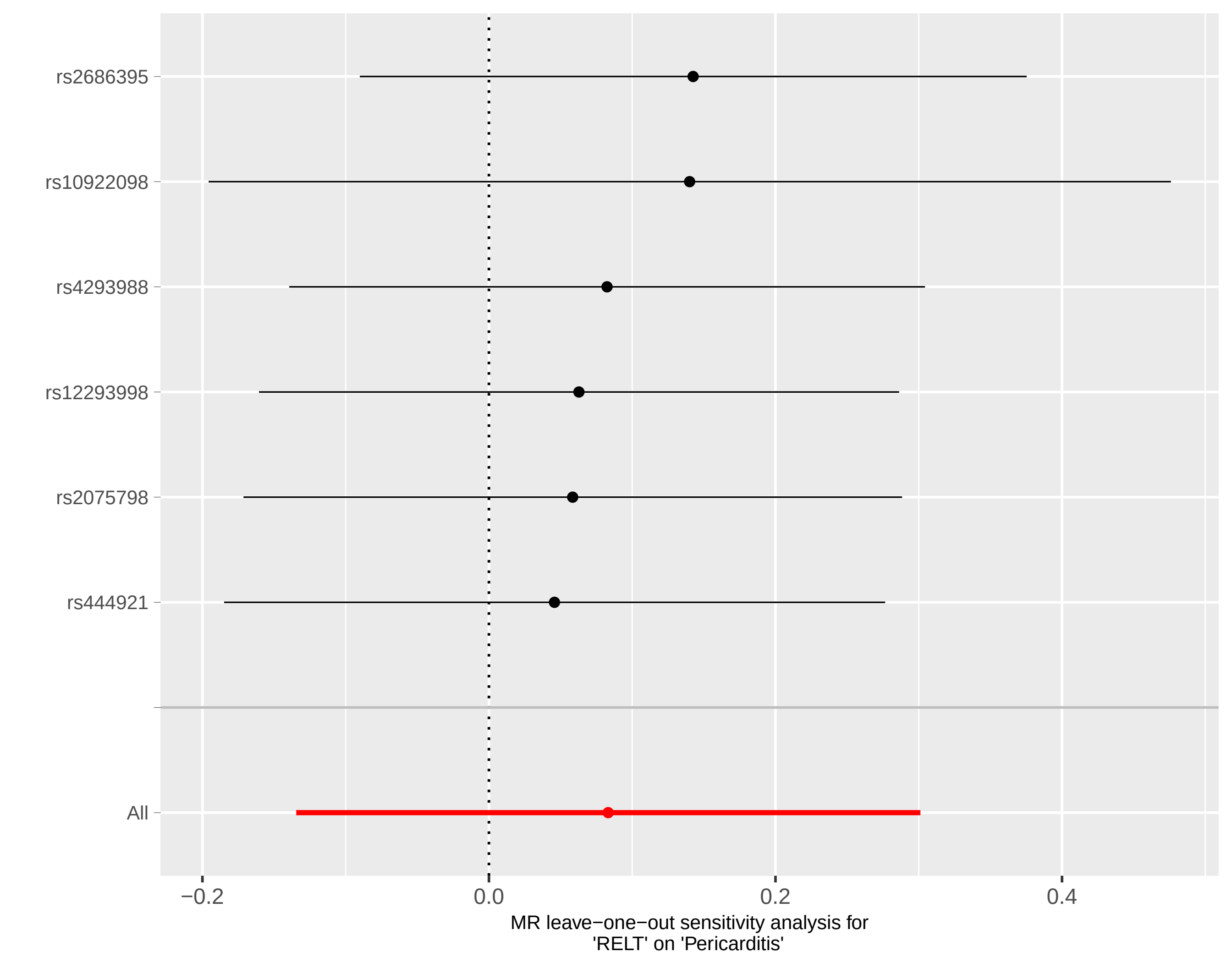
rs2686395
rs10922098
rs4293988
rs12293998
rs2075798
rs444921
All
−0.2
0.0
0.2
0.4
MR leave−one−out sensitivity analysis for
'RELT' on 'Pericarditis'

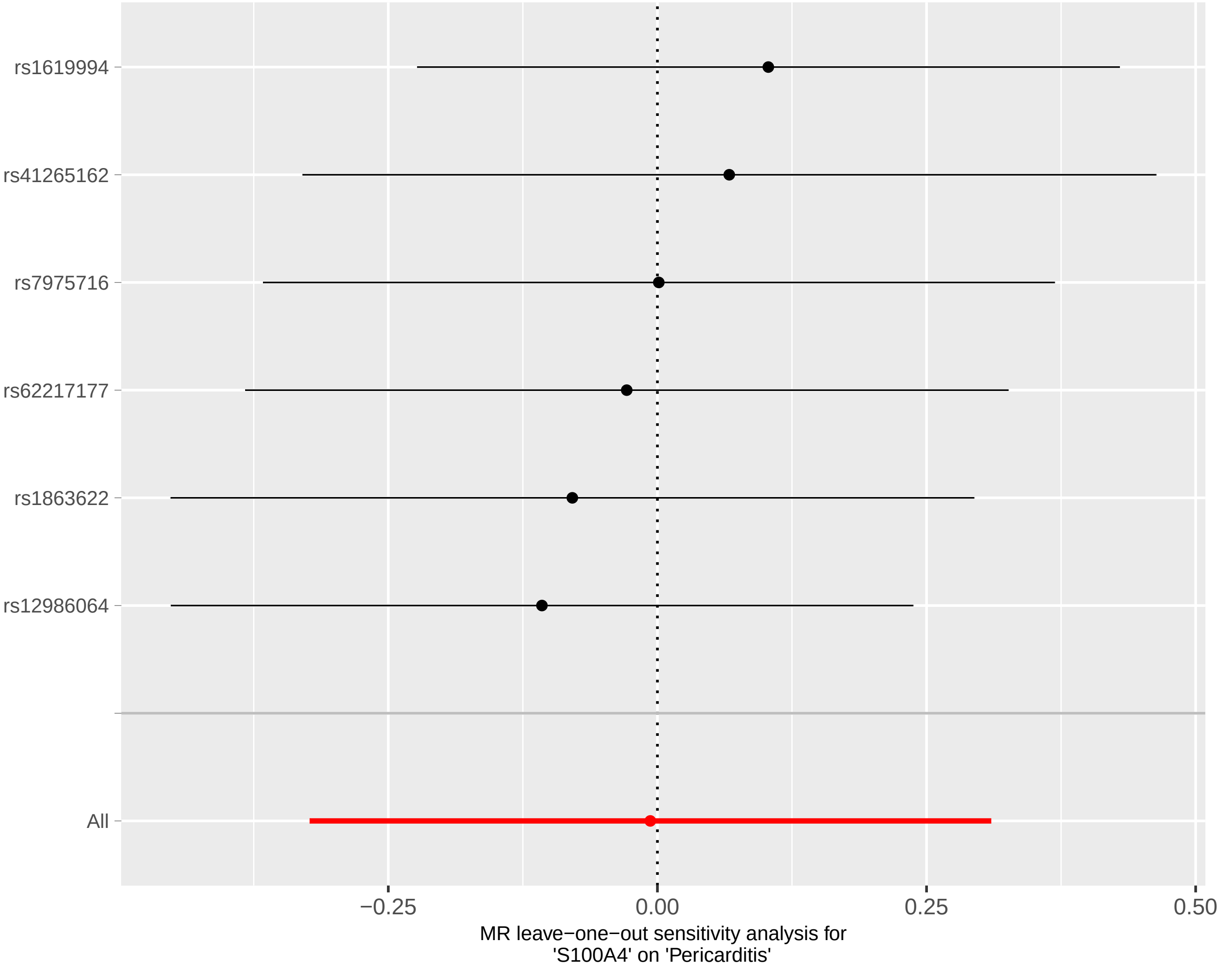
rs1619994
rs41265162
rs7975716
rs62217177
rs1863622
rs12986064
All
−0.25
0.00
0.25
0.50
MR leave−one−out sensitivity analysis for
'S100A4' on 'Pericarditis'

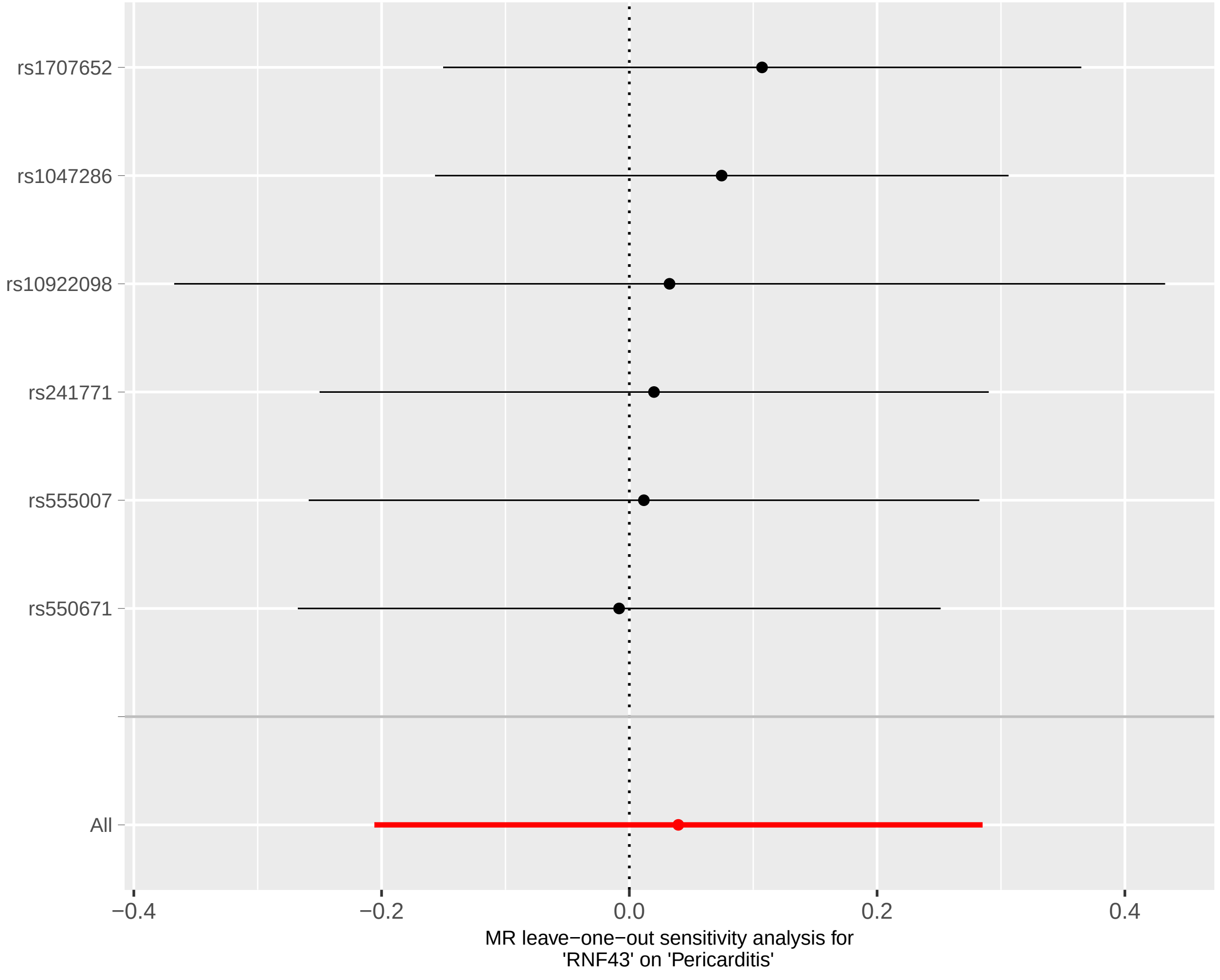

rs1707652
rs1047286
rs10922098
rs241771
rs555007
rs550671
All
−0.4
−0.2
0.0
0.2
0.4
MR leave−one−out sensitivity analysis for
'RNF43' on 'Pericarditis'

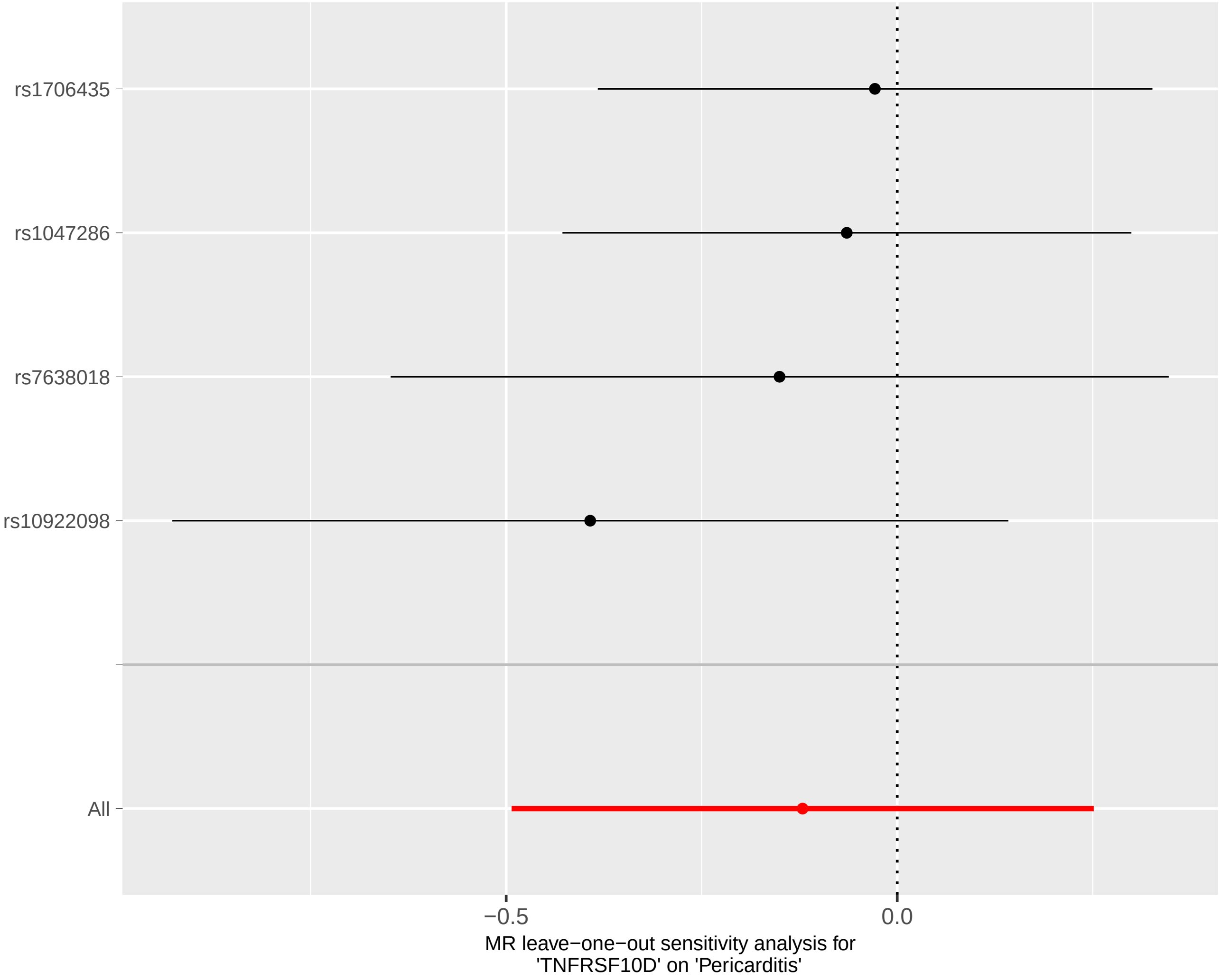
rs1706435
rs1047286
rs7638018
rs10922098
All
−0.5
0.0
MR leave−one−out sensitivity analysis for
'TNFRSF10D' on 'Pericarditis'

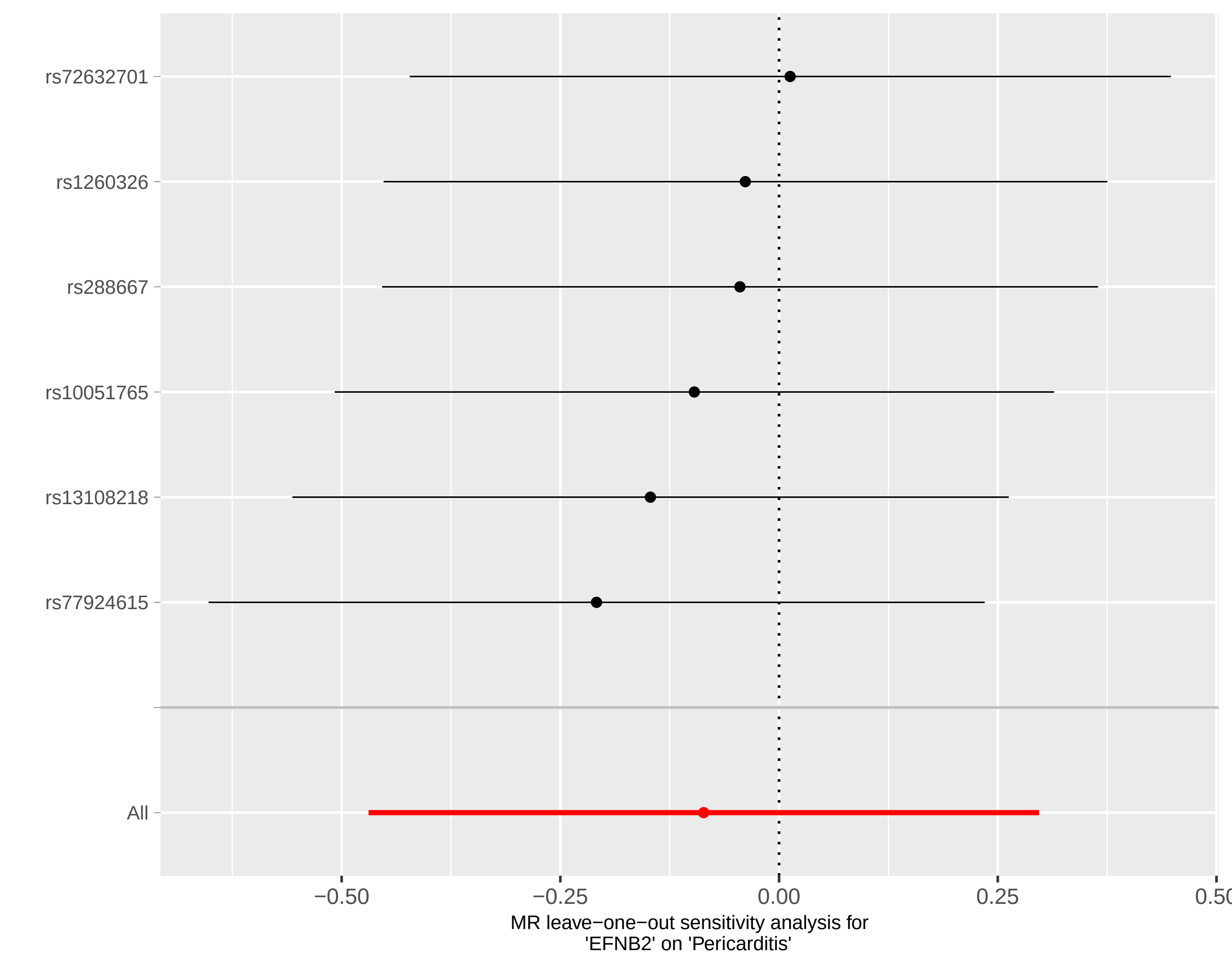
rs72632701
rs1260326
rs288667
rs10051765
rs13108218
rs77924615
All
−0.50
−0.25
0.00
0.25
0.50
MR leave−one−out sensitivity analysis for
'EFNB2' on 'Pericarditis'

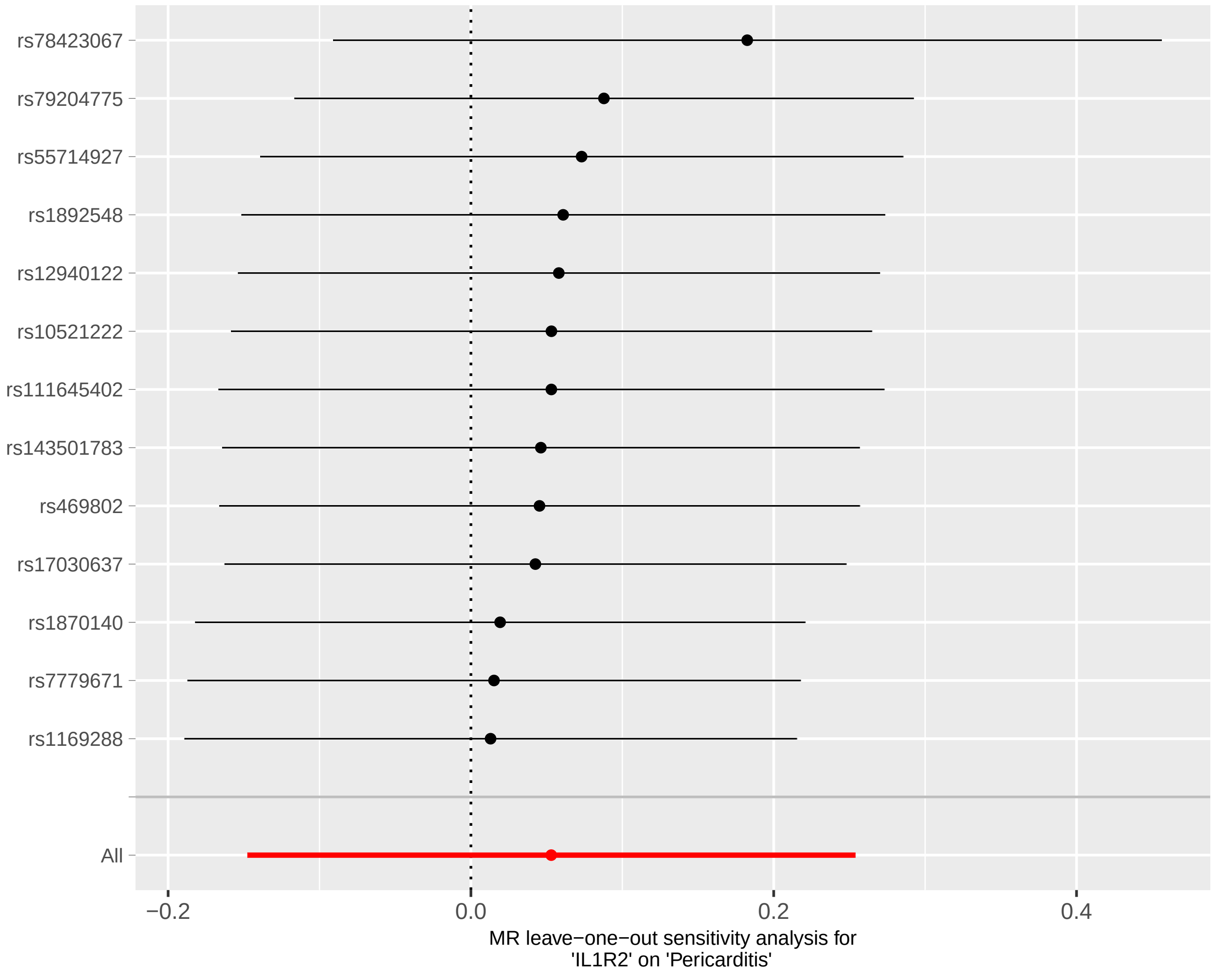

rs78423067
rs79204775
rs55714927
rs1892548
rs12940122
rs10521222
rs111645402
rs143501783
rs469802
rs17030637
rs1870140
rs7779671
rs1169288
All
−0.2
0.0
0.2
0.4
MR leave−one−out sensitivity analysis for
'IL1R2' on 'Pericarditis'

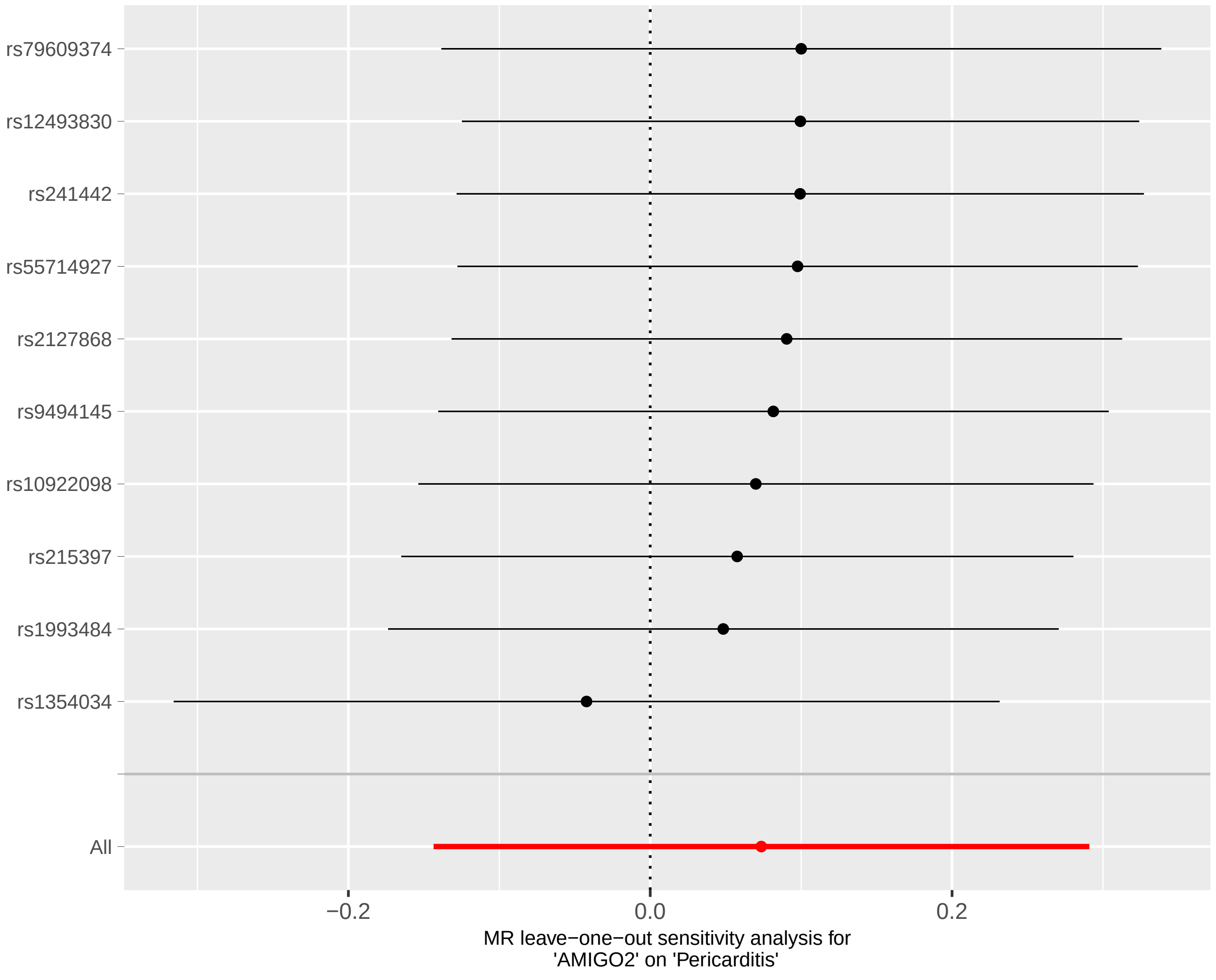

rs79609374
rs12493830
rs241442
rs55714927
rs2127868
rs9494145
rs10922098
rs215397
rs1993484
rs1354034
All
−0.2
0.0
0.2
MR leave−one−out sensitivity analysis for
'AMIGO2' on 'Pericarditis'

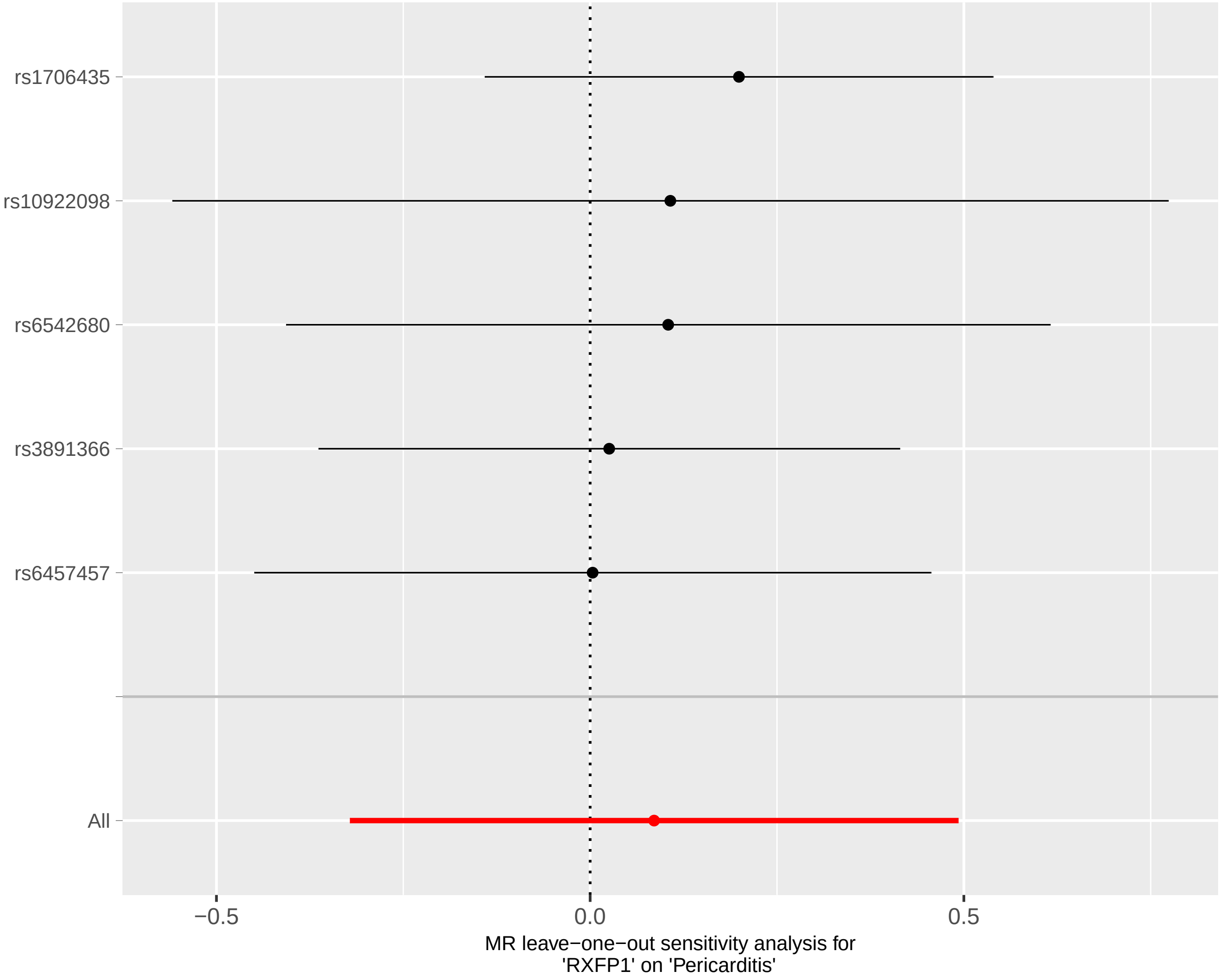
rs1706435
rs10922098
rs6542680
rs3891366
rs6457457
All
−0.5
0.0
0.5
MR leave−one−out sensitivity analysis for
'RXFP1' on 'Pericarditis'

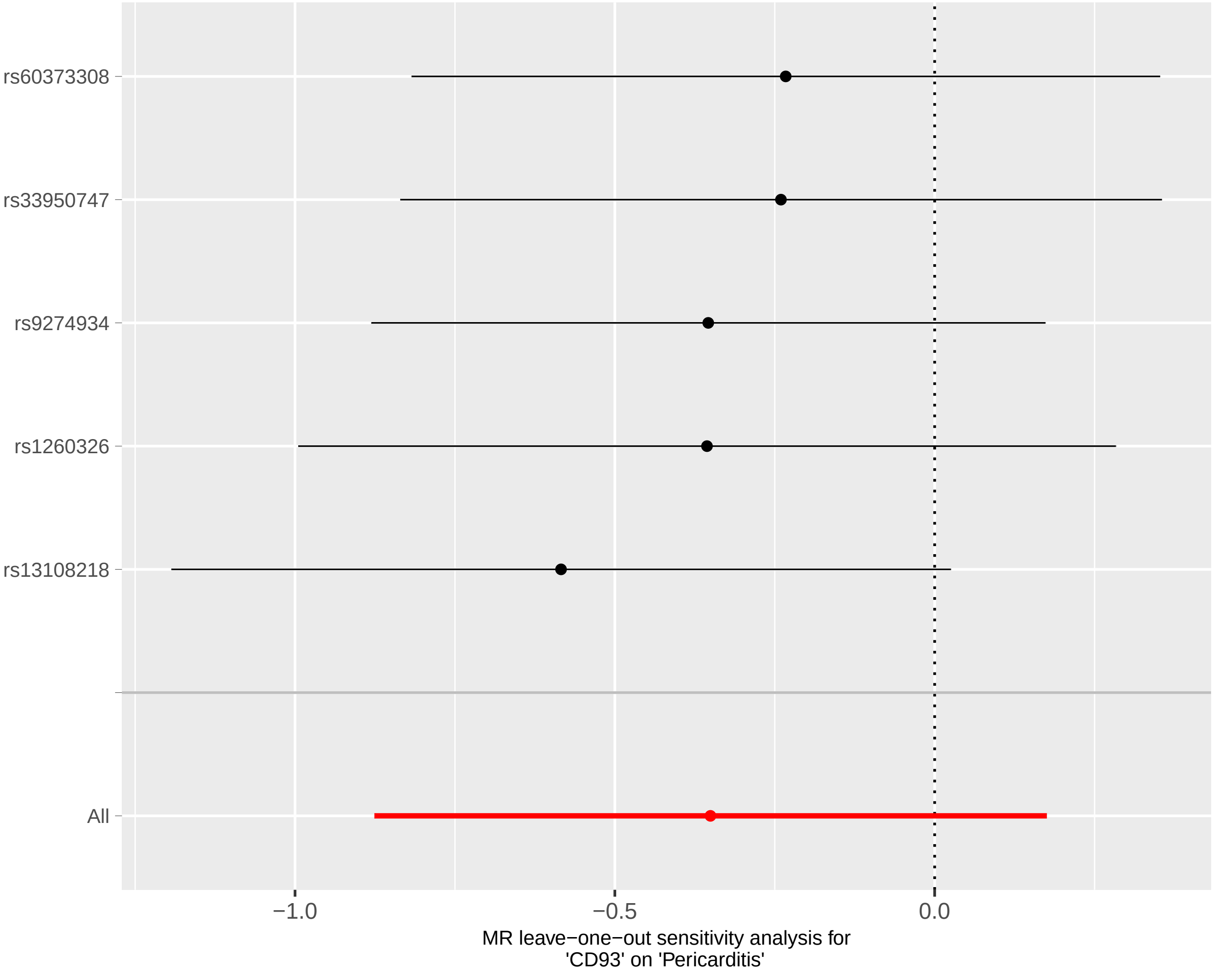

rs60373308
rs33950747
rs9274934
rs1260326
rs13108218
All
−1.0
−0.5
0.0
MR leave−one−out sensitivity analysis for
'CD93' on 'Pericarditis'

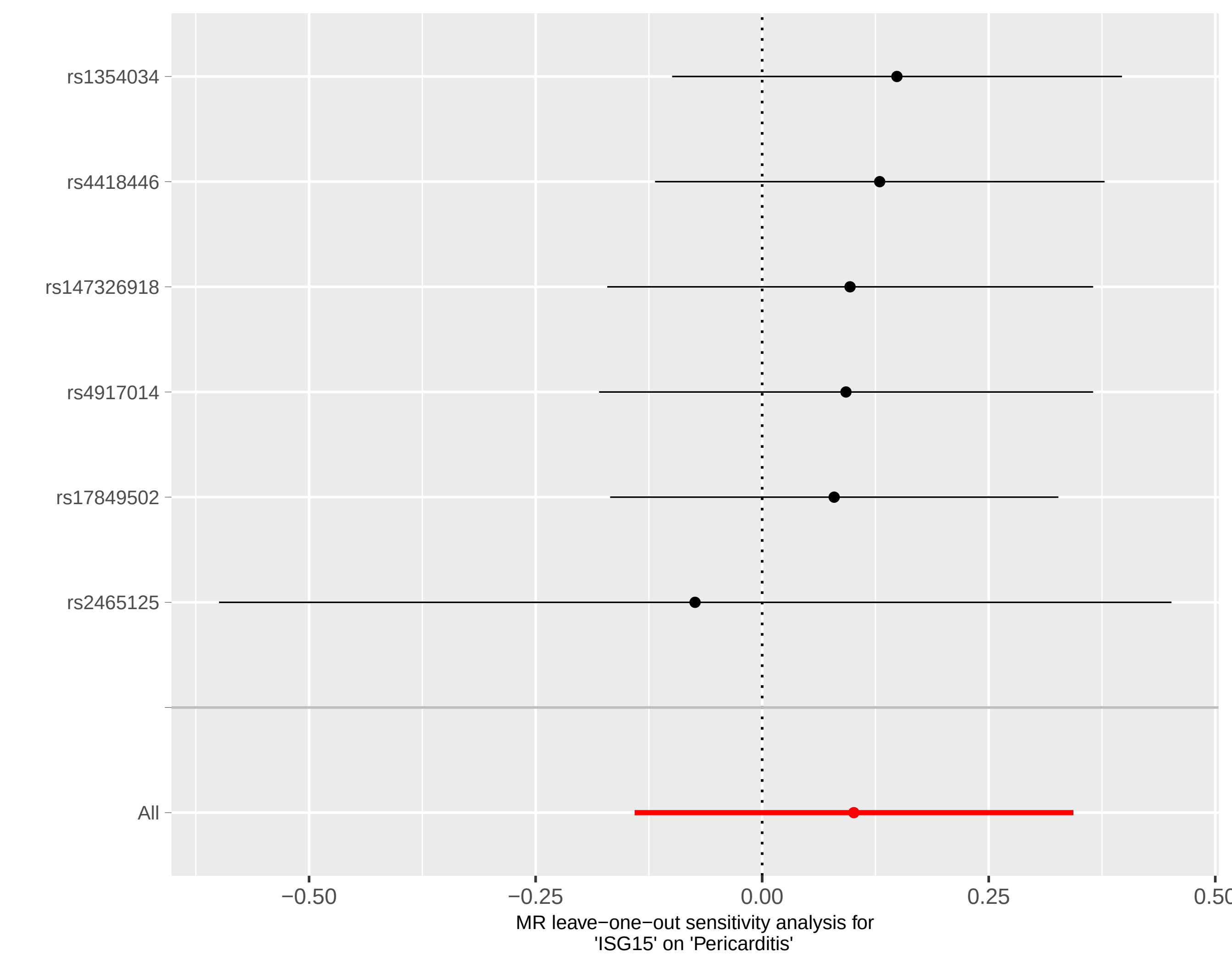
rs1354034
rs4418446
rs147326918
rs4917014
rs17849502
rs2465125
All
−0.50
−0.25
0.00
0.25
0.50
MR leave−one−out sensitivity analysis for
'ISG15' on 'Pericarditis'

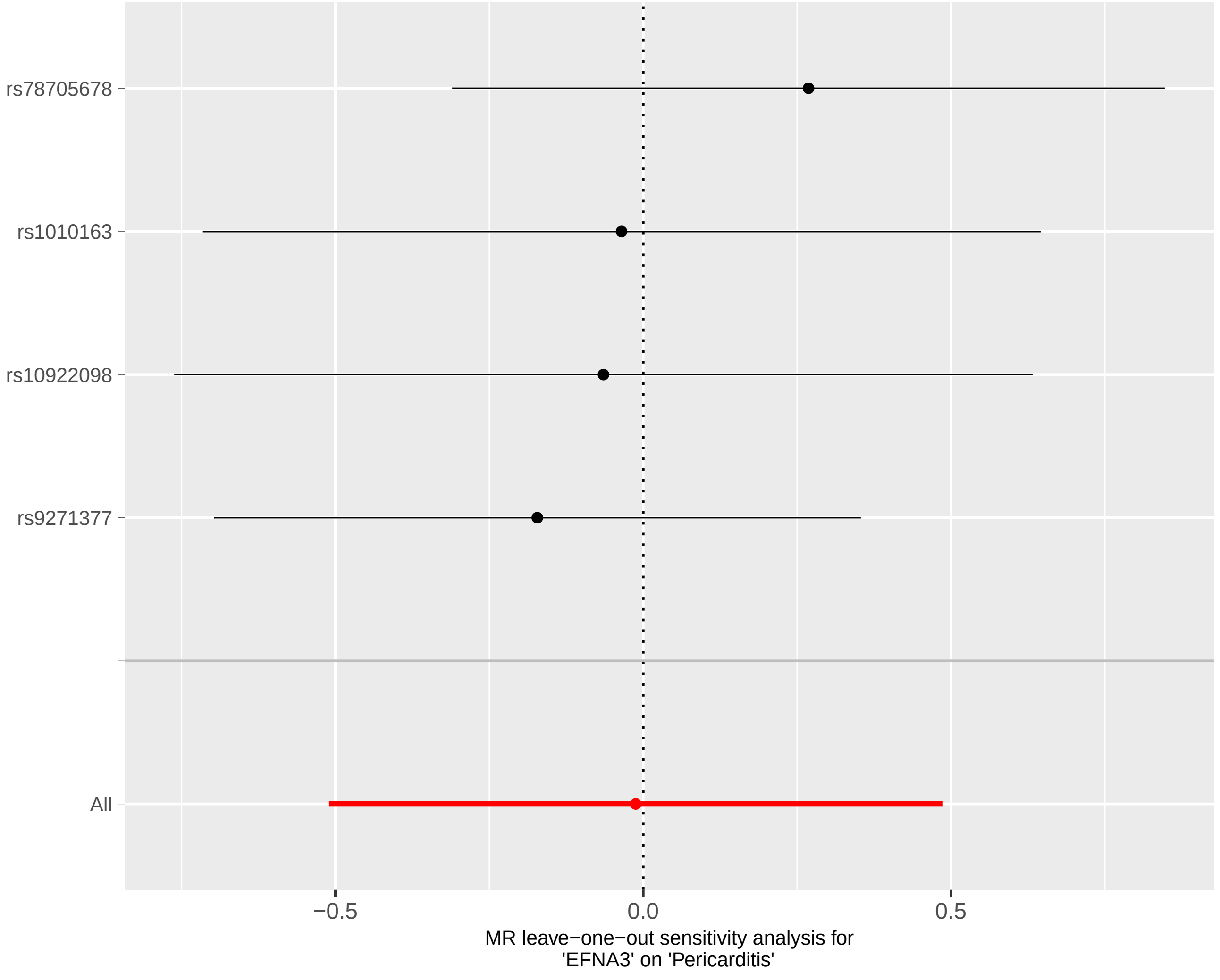
rs78705678
rs1010163
rs10922098
rs9271377
All
−0.5
0.0
0.5
MR leave−one−out sensitivity analysis for
'EFNA3' on 'Pericarditis'

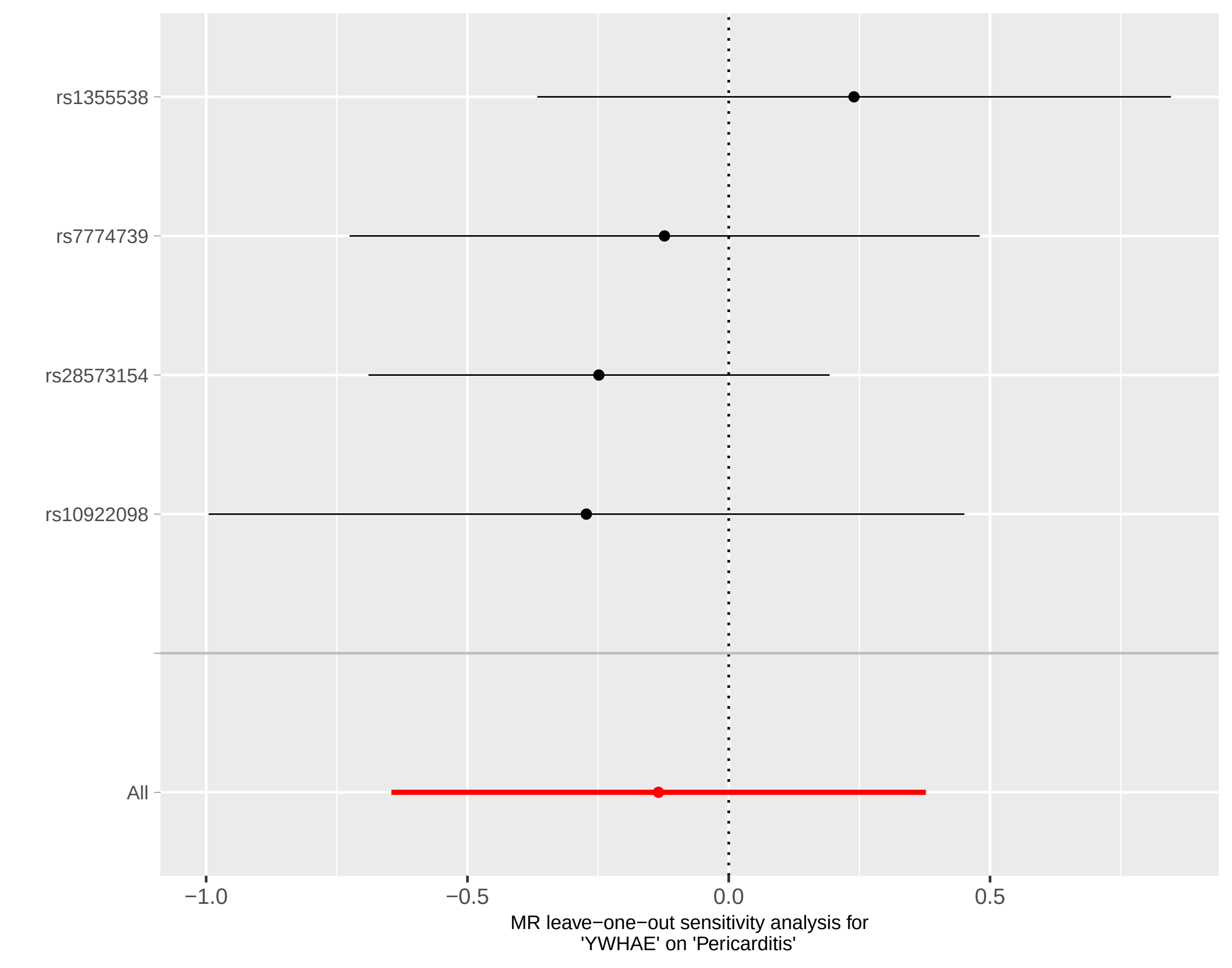

rs1355538
rs7774739
rs28573154
rs10922098
All
−1.0
−0.5
0.0
0.5
MR leave−one−out sensitivity analysis for
'YWHAE' on 'Pericarditis'

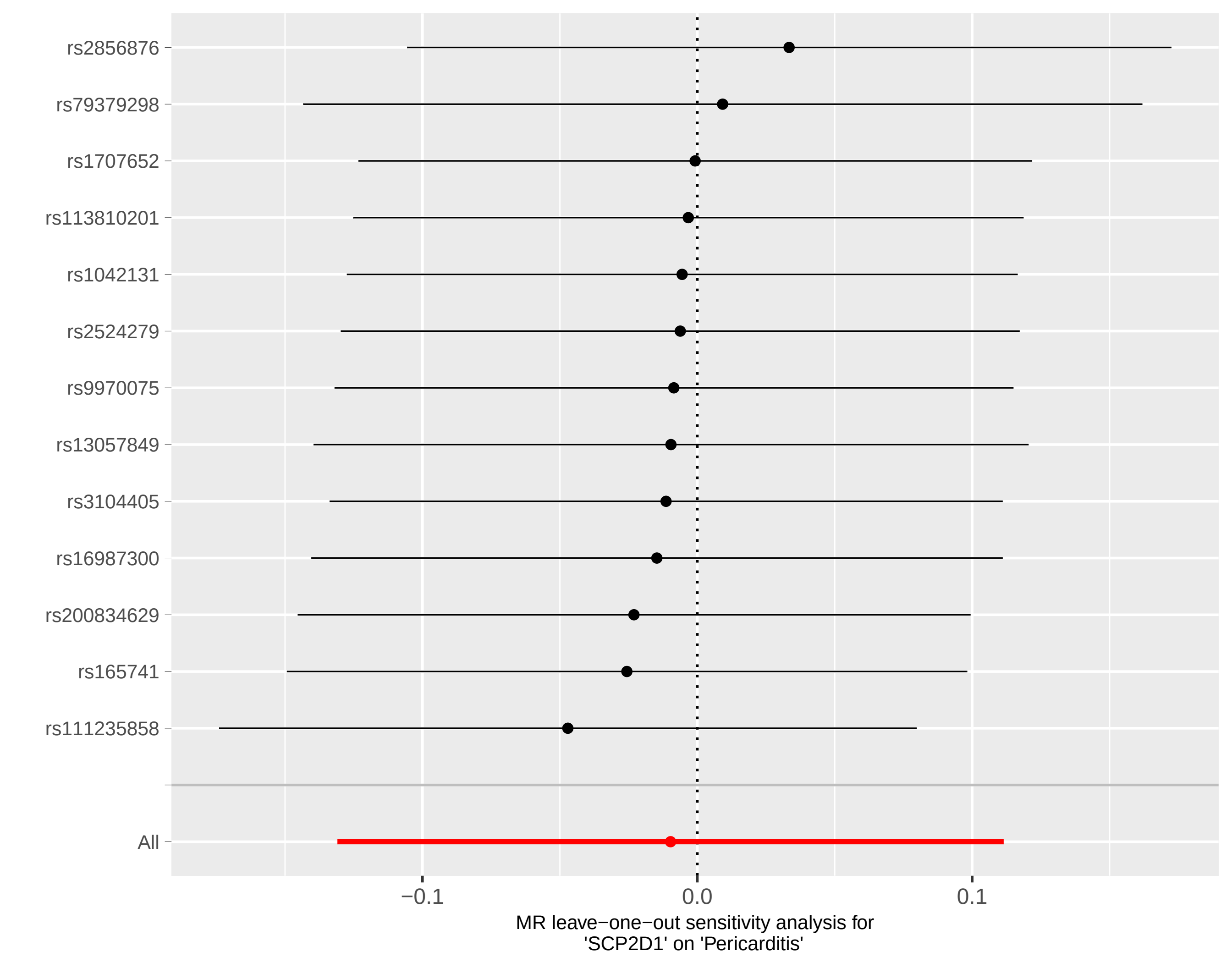
rs2856876
rs79379298
rs1707652
rs113810201
rs1042131
rs2524279
rs9970075
rs13057849
rs3104405
rs16987300
rs200834629
rs165741
rs111235858
All
−0.1
0.0
0.1
MR leave−one−out sensitivity analysis for
'SCP2D1' on 'Pericarditis'

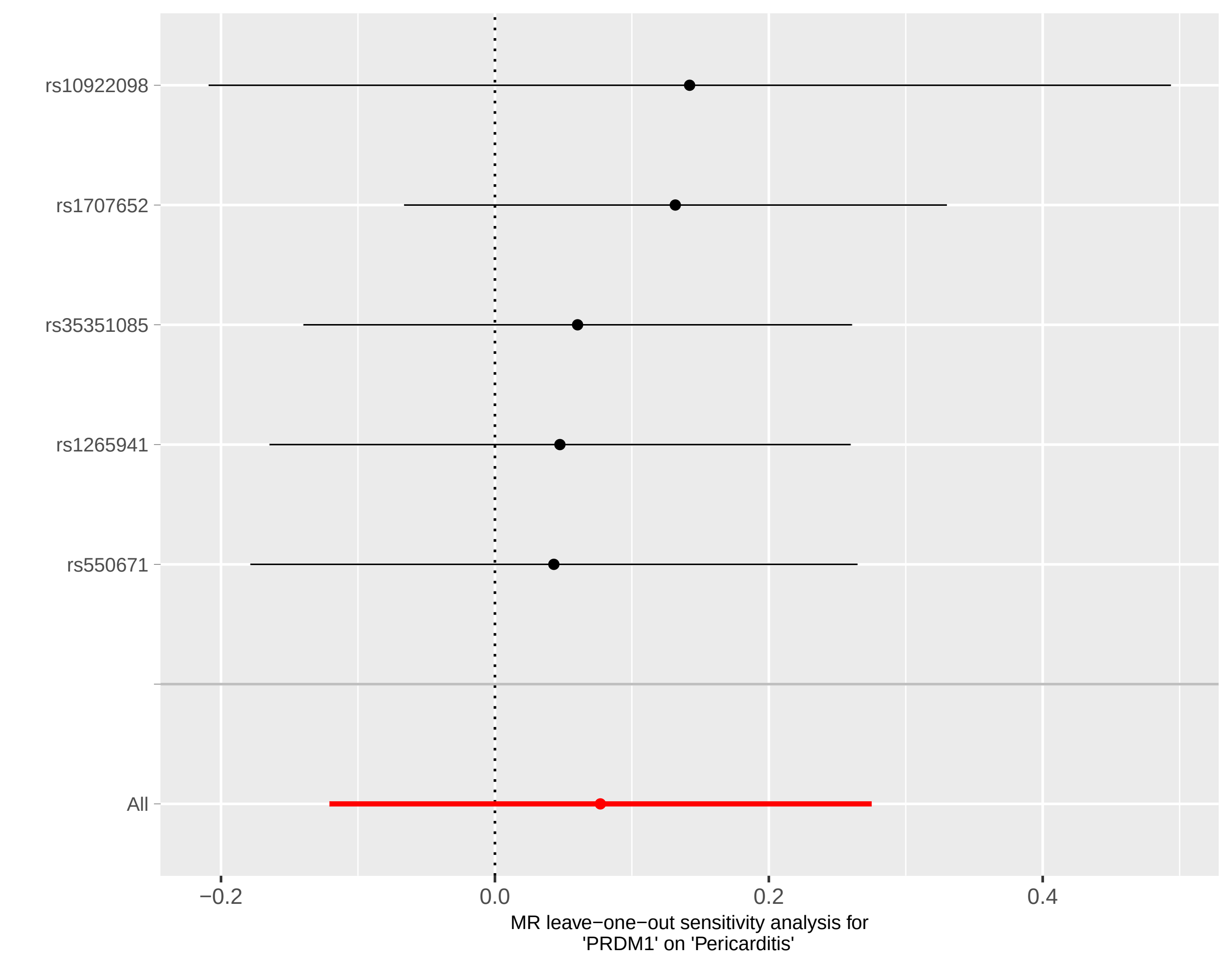

rs10922098
rs1707652
rs35351085
rs1265941
rs550671
All
−0.2
0.0
0.2
0.4
MR leave−one−out sensitivity analysis for
'PRDM1' on 'Pericarditis'

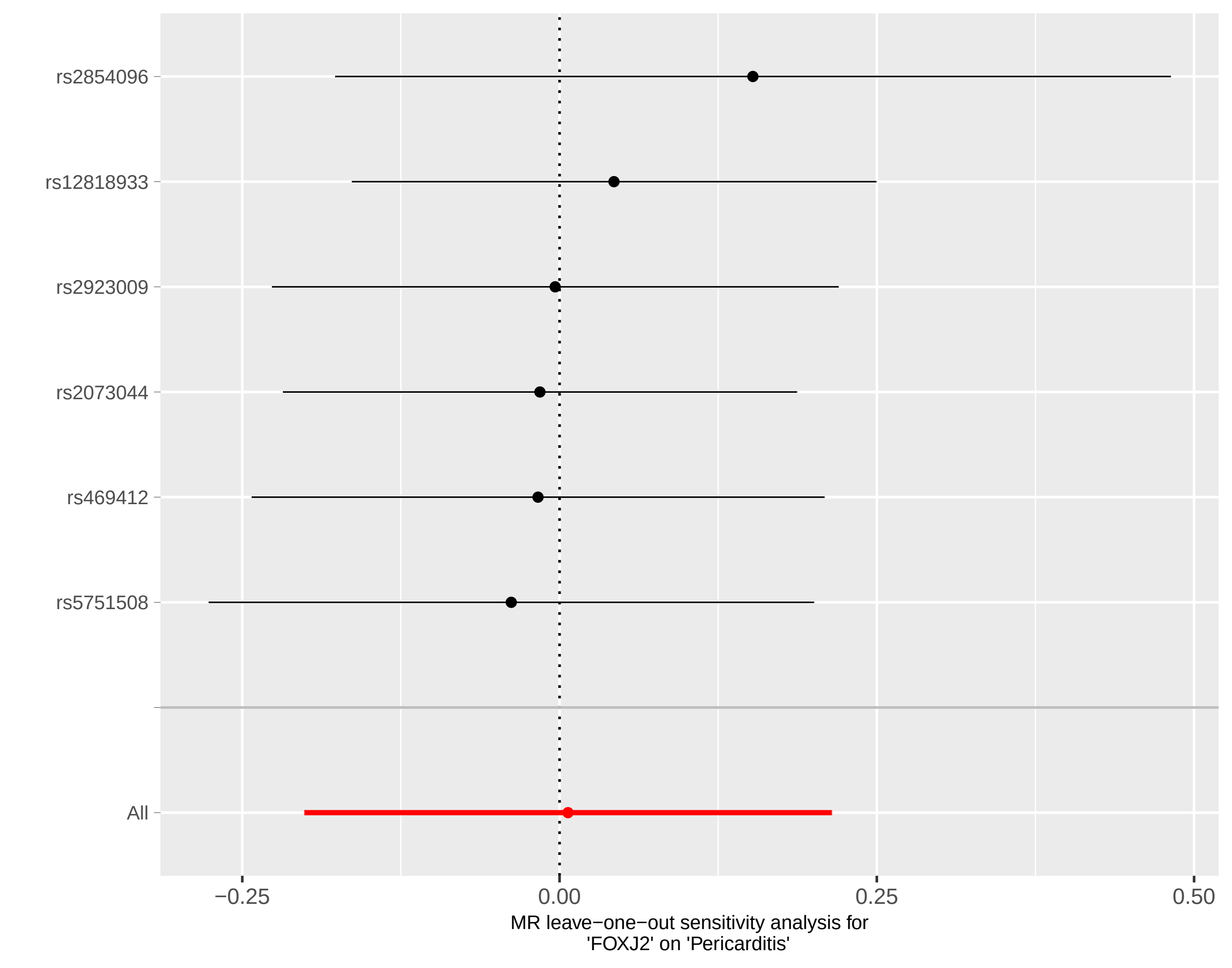
rs2854096
rs12818933
rs2923009
rs2073044
rs469412
rs5751508
All
−0.25
0.00
0.25
0.50
MR leave−one−out sensitivity analysis for
'FOXJ2' on 'Pericarditis'

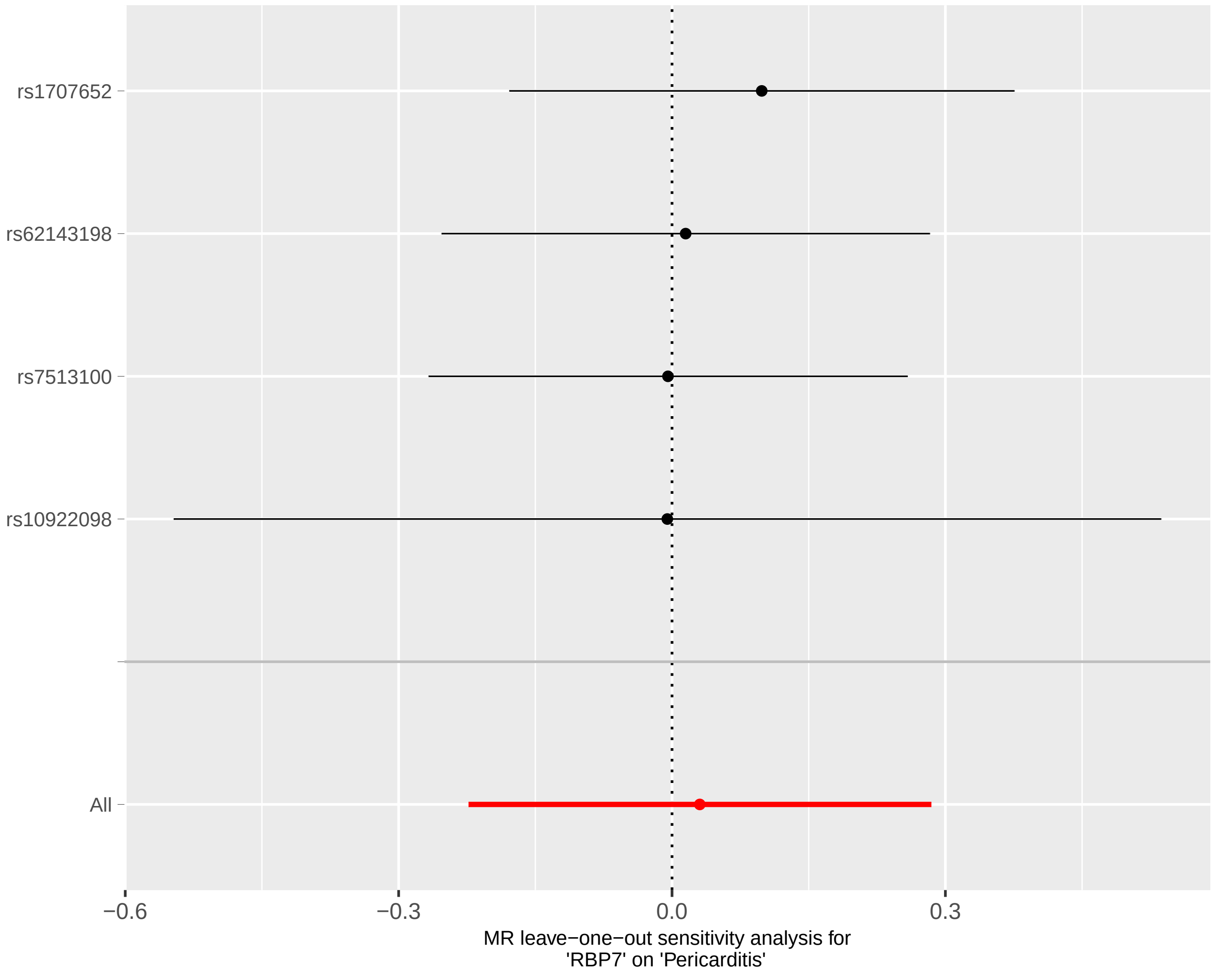
rs1707652
rs62143198
rs7513100
rs10922098
All
−0.6
−0.3
0.0
0.3
MR leave−one−out sensitivity analysis for
'RBP7' on 'Pericarditis'

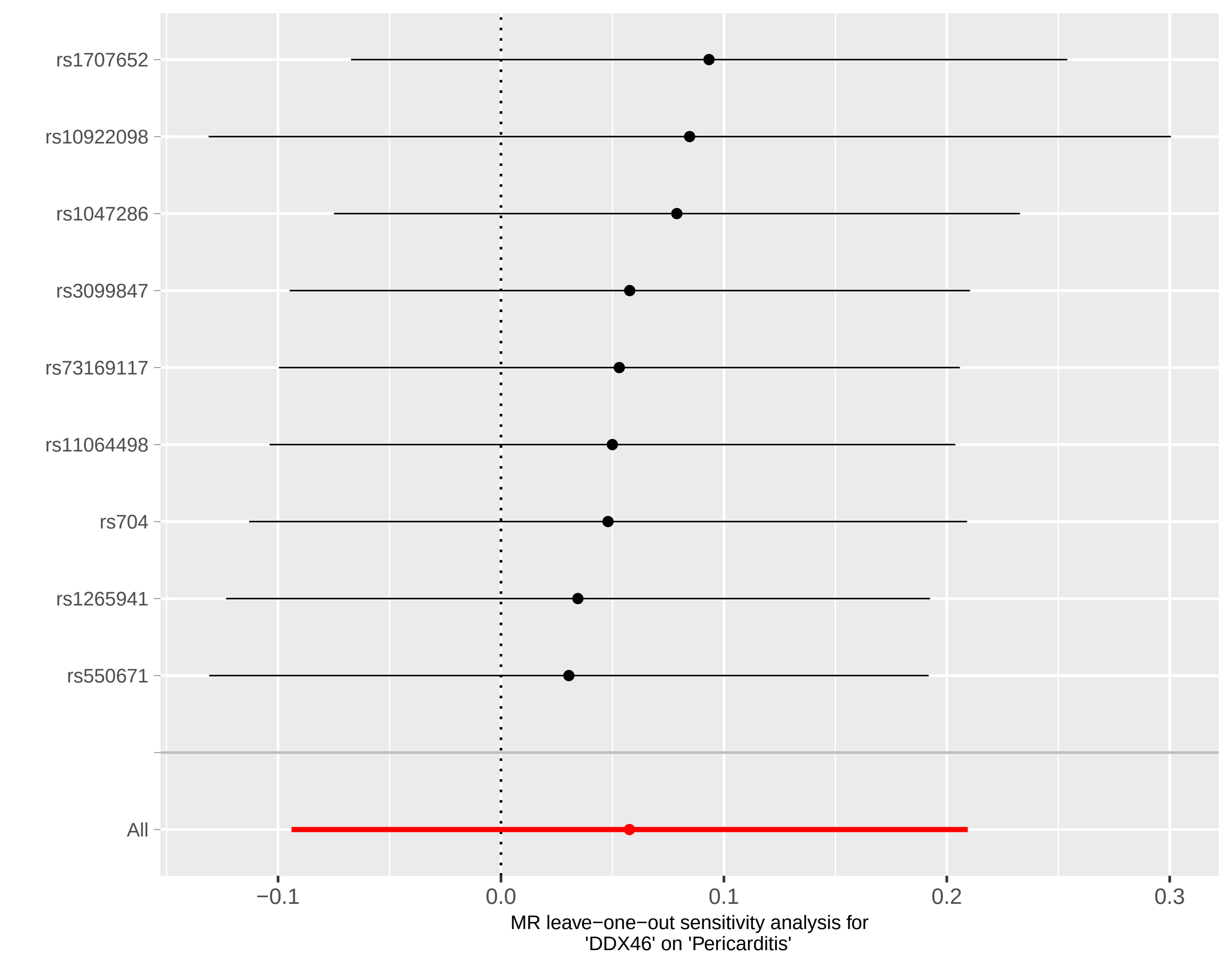

rs1707652
rs10922098
rs1047286
rs3099847
rs73169117
rs11064498
rs704
rs1265941
rs550671
All
−0.1
0.0
0.1
0.2
0.3
MR leave−one−out sensitivity analysis for
'DDX46' on 'Pericarditis'

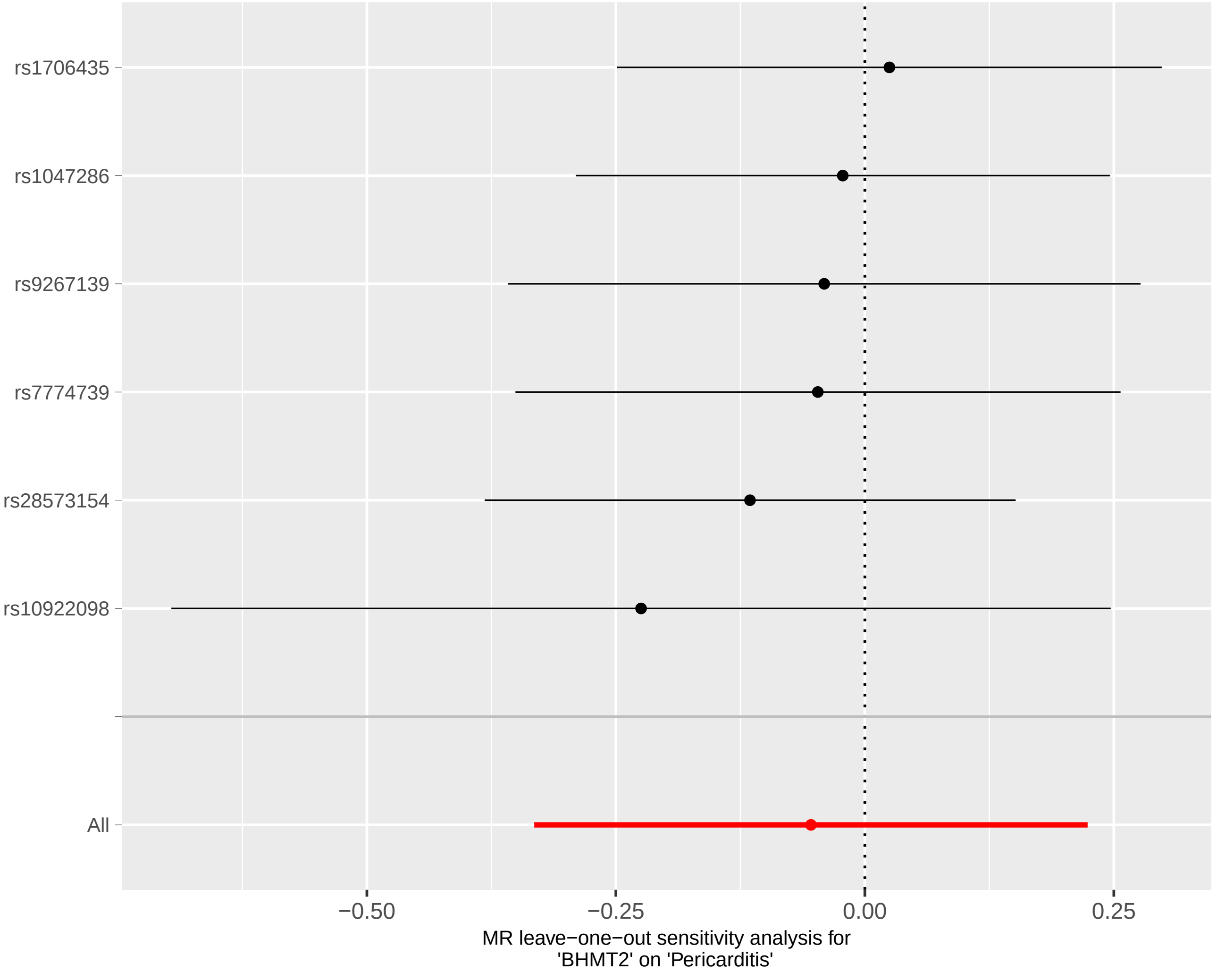
rs1706435
rs1047286
rs9267139
rs7774739
rs28573154
rs10922098
All
−0.50
−0.25
0.00
0.25
MR leave−one−out sensitivity analysis for
'BHMT2' on 'Pericarditis'

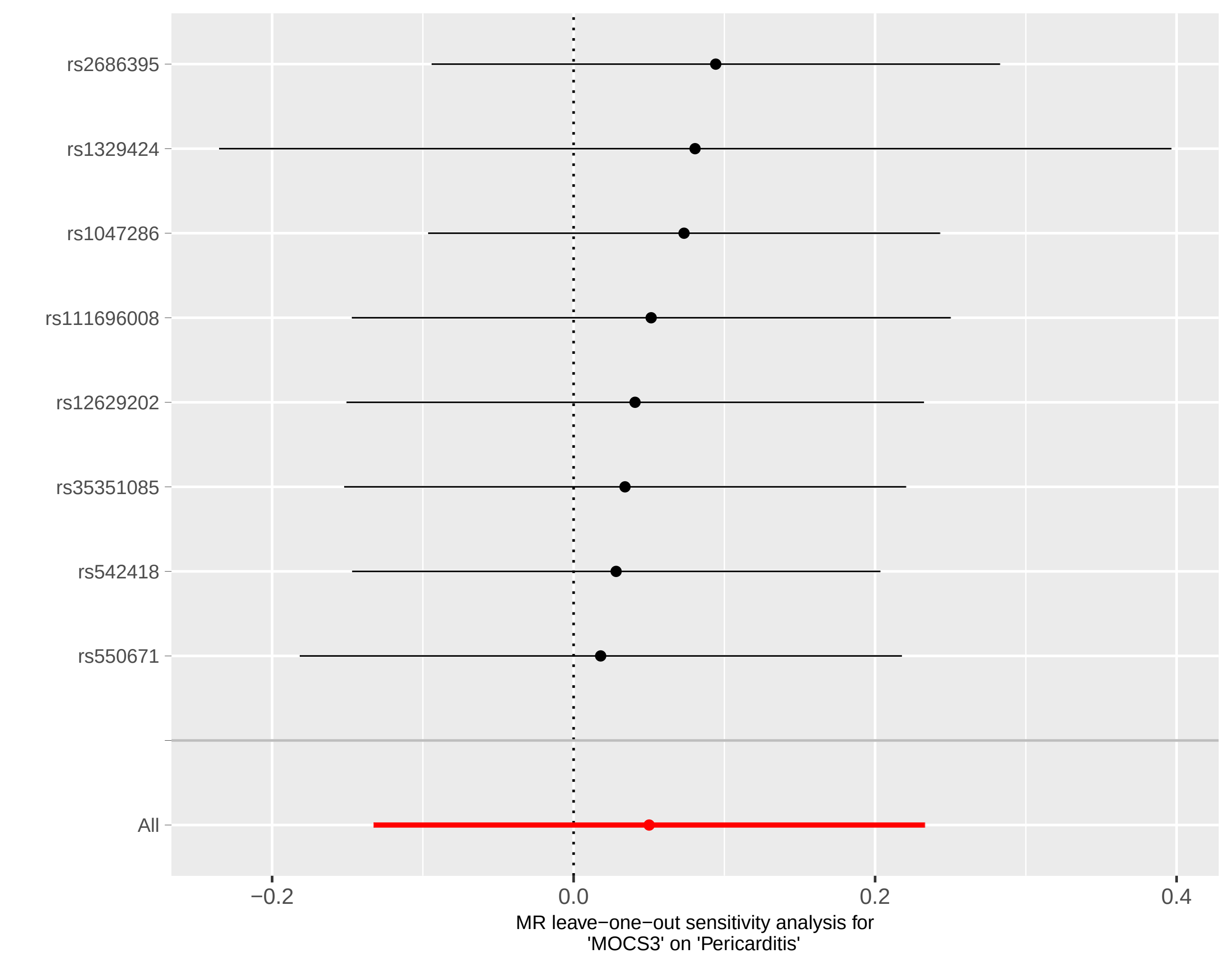

rs2686395
rs1329424
rs1047286
rs111696008
rs12629202
rs35351085
rs542418
rs550671
All
−0.2
0.0
0.2
0.4
MR leave−one−out sensitivity analysis for
'MOCS3' on 'Pericarditis'

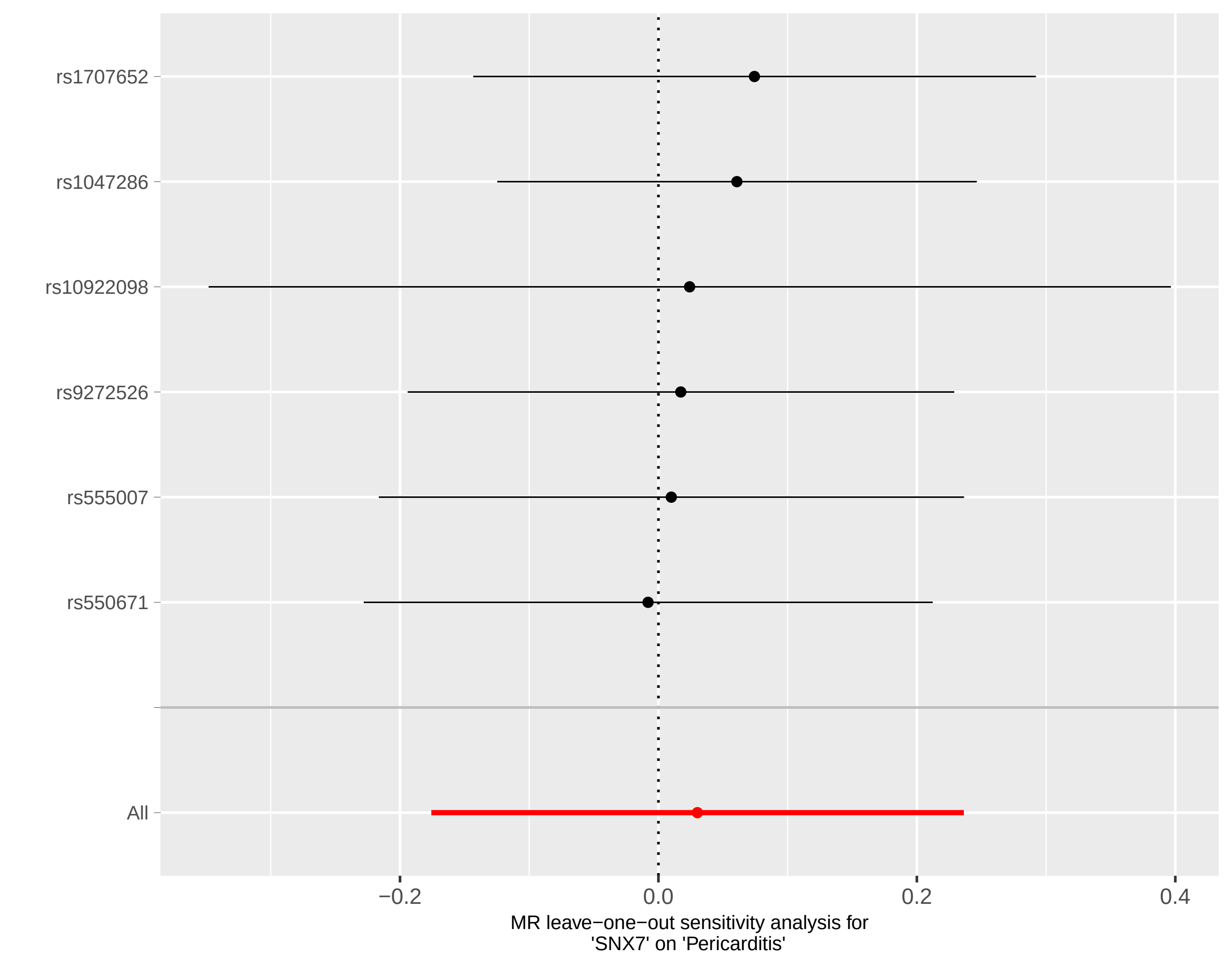
rs1707652
rs1047286
rs10922098
rs9272526
rs555007
rs550671
All
−0.2
0.0
0.2
0.4
MR leave−one−out sensitivity analysis for
'SNX7' on 'Pericarditis'

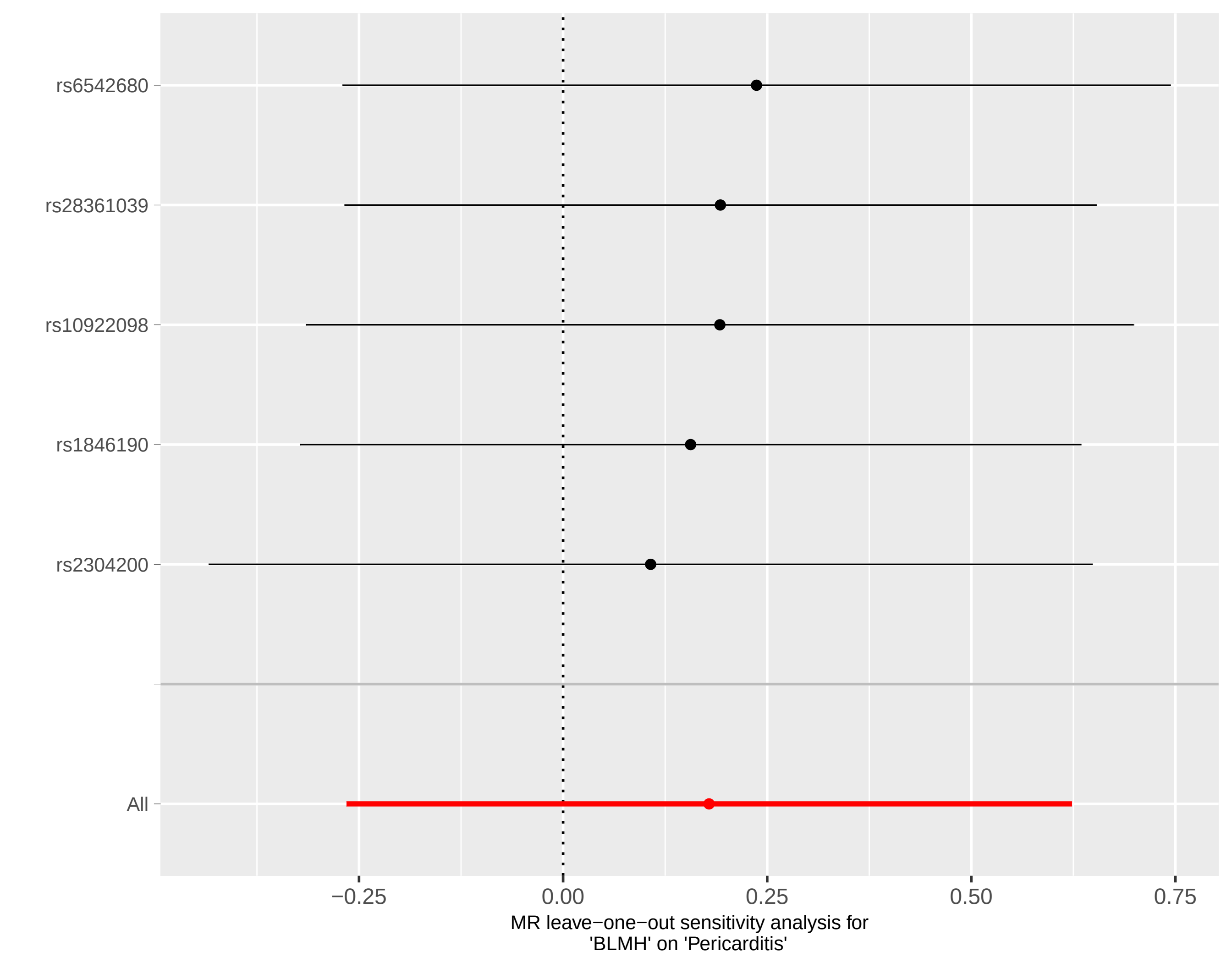
rs6542680
rs28361039
rs10922098
rs1846190
rs2304200
All
−0.25
0.00
0.25
0.50
0.75
MR leave−one−out sensitivity analysis for
'BLMH' on 'Pericarditis'

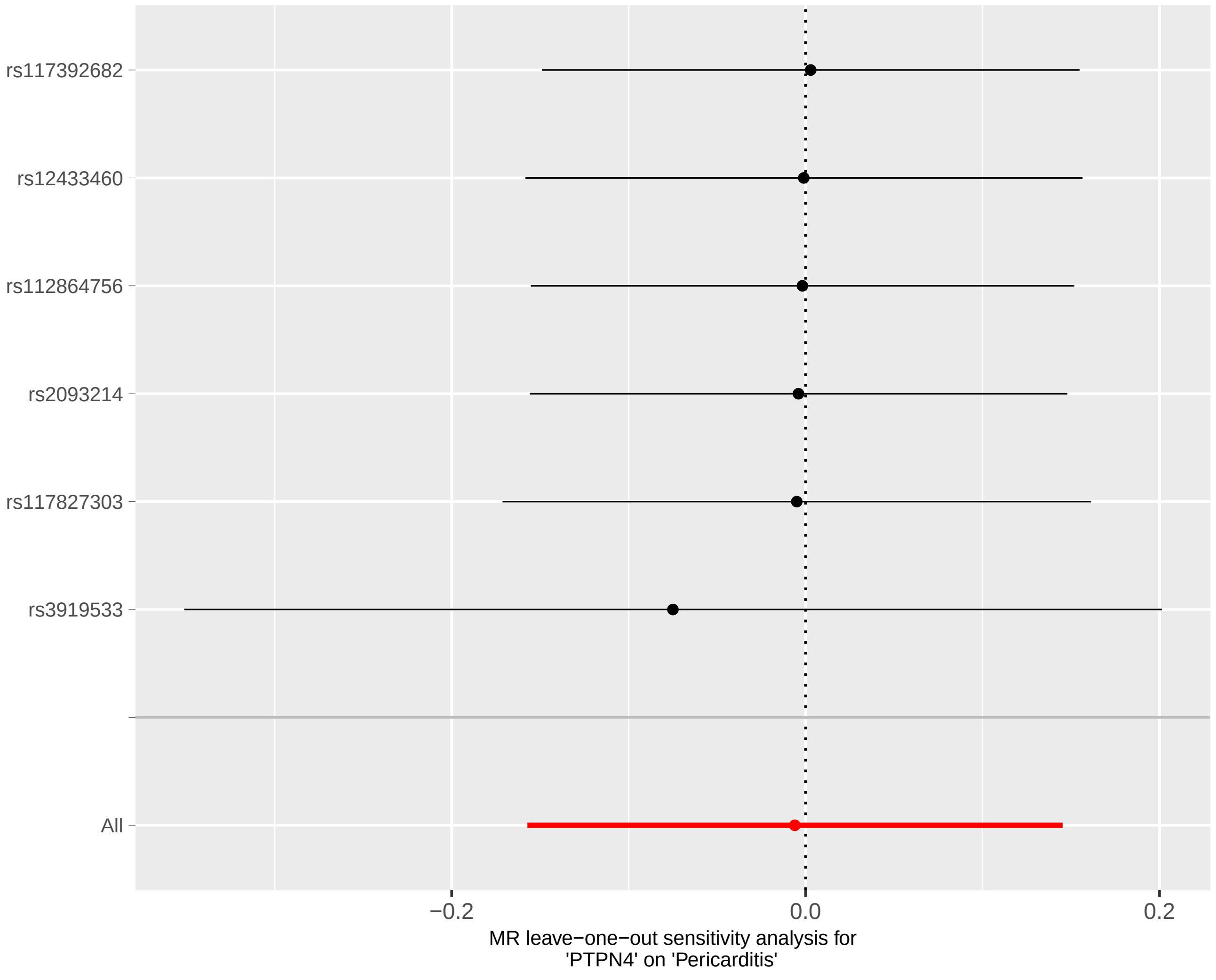
rs117392682
rs12433460
rs112864756
rs2093214
rs117827303
rs3919533
All
−0.2
0.0
0.2
MR leave−one−out sensitivity analysis for
'PTPN4' on 'Pericarditis'

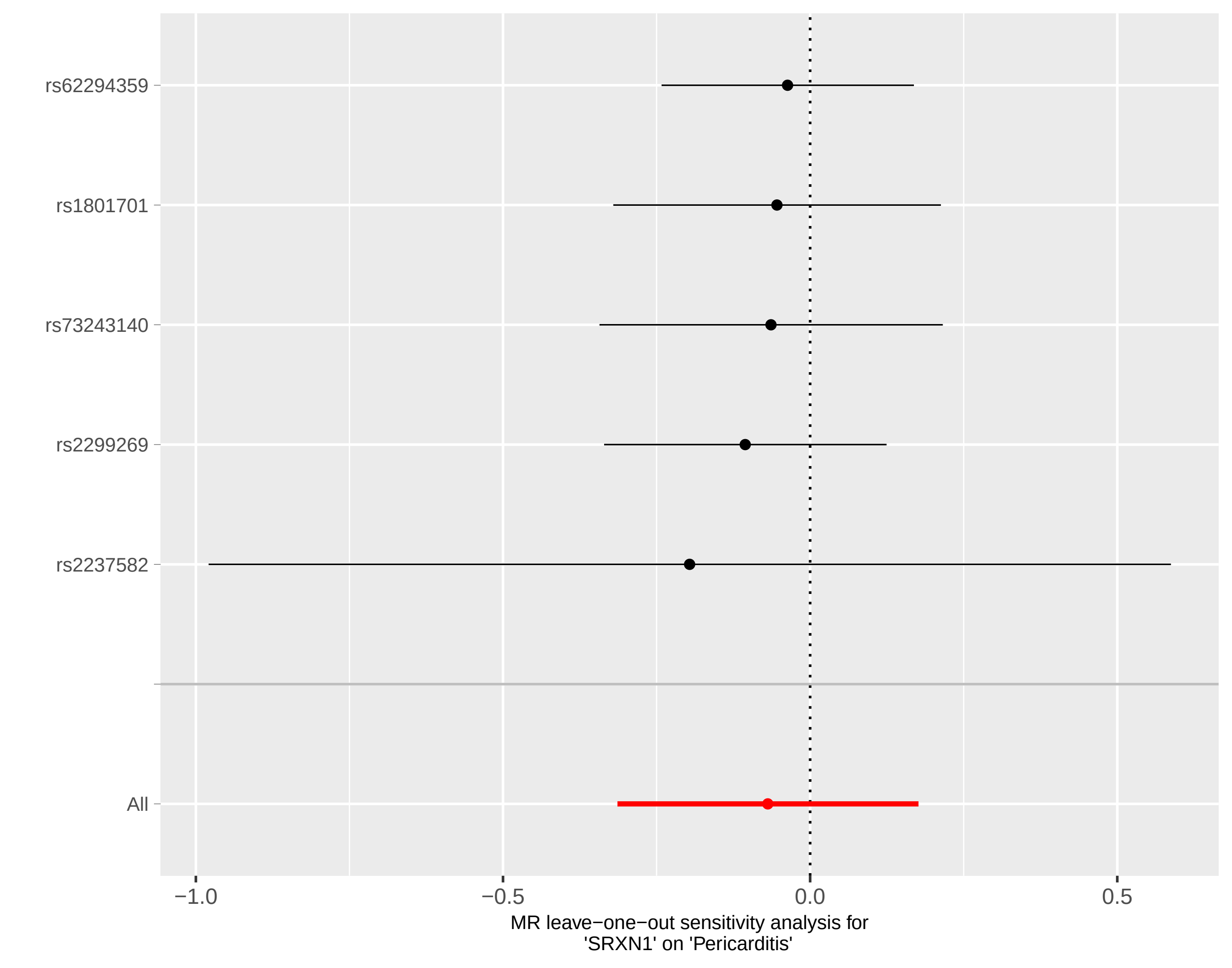
rs62294359
rs1801701
rs73243140
rs2299269
rs2237582
All
−1.0
−0.5
0.0
0.5
MR leave−one−out sensitivity analysis for
'SRXN1' on 'Pericarditis'

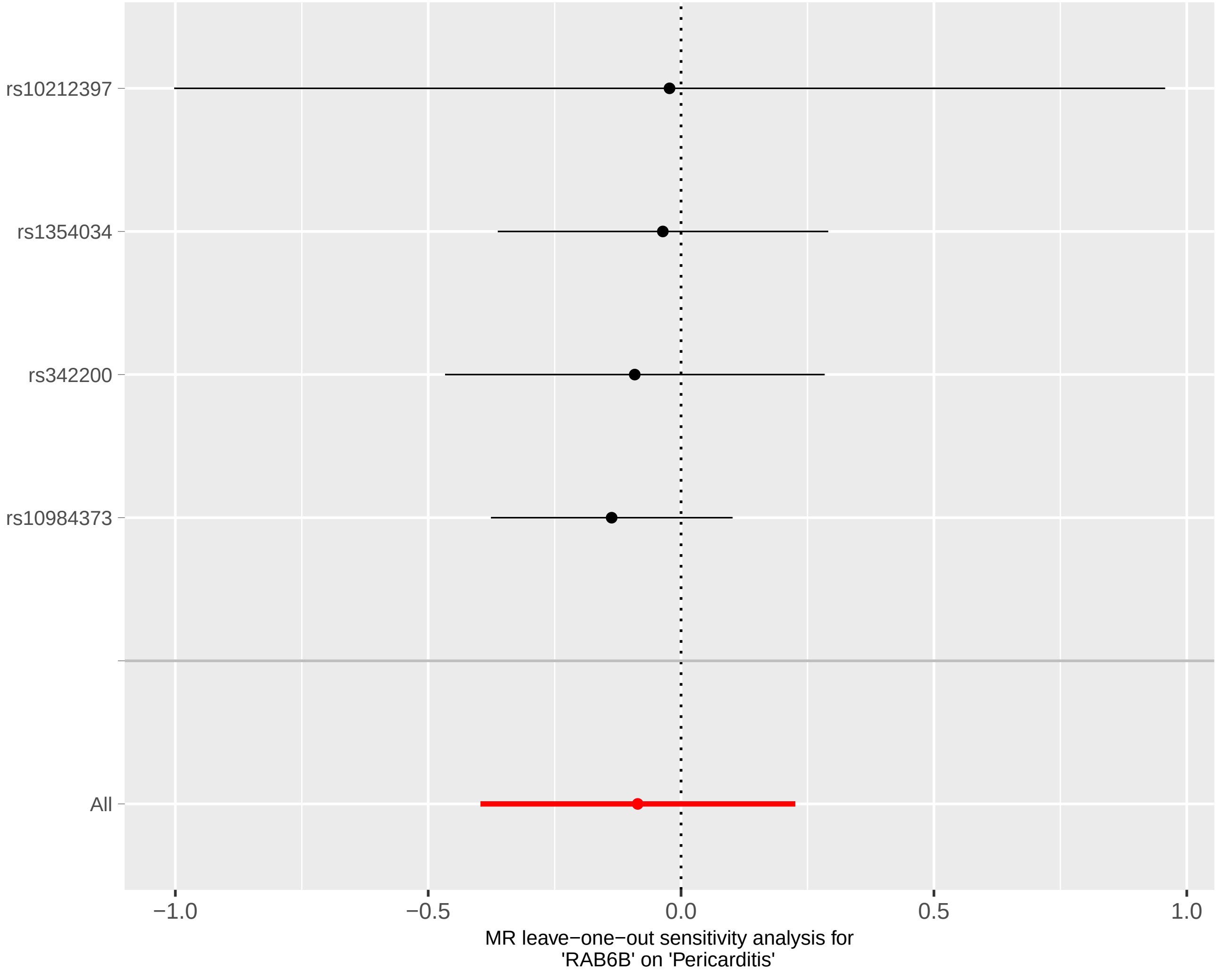
rs10212397
rs1354034
rs342200
rs10984373
All
−1.0
−0.5
0.0
0.5
1.0
MR leave−one−out sensitivity analysis for
'RAB6B' on 'Pericarditis'

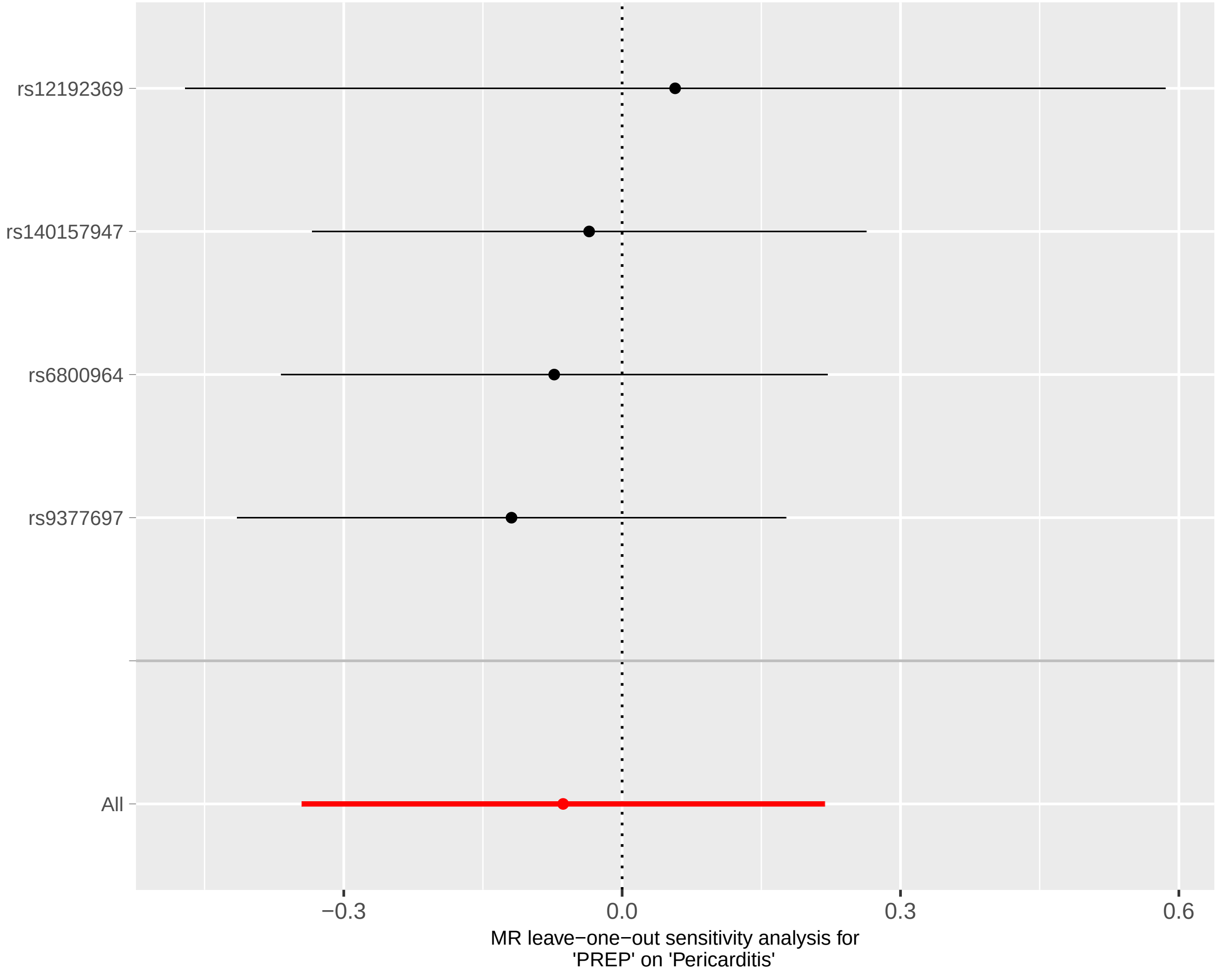
rs12192369
rs140157947
rs6800964
rs9377697
All
−0.3
0.0
0.3
0.6
MR leave−one−out sensitivity analysis for
'PREP' on 'Pericarditis'

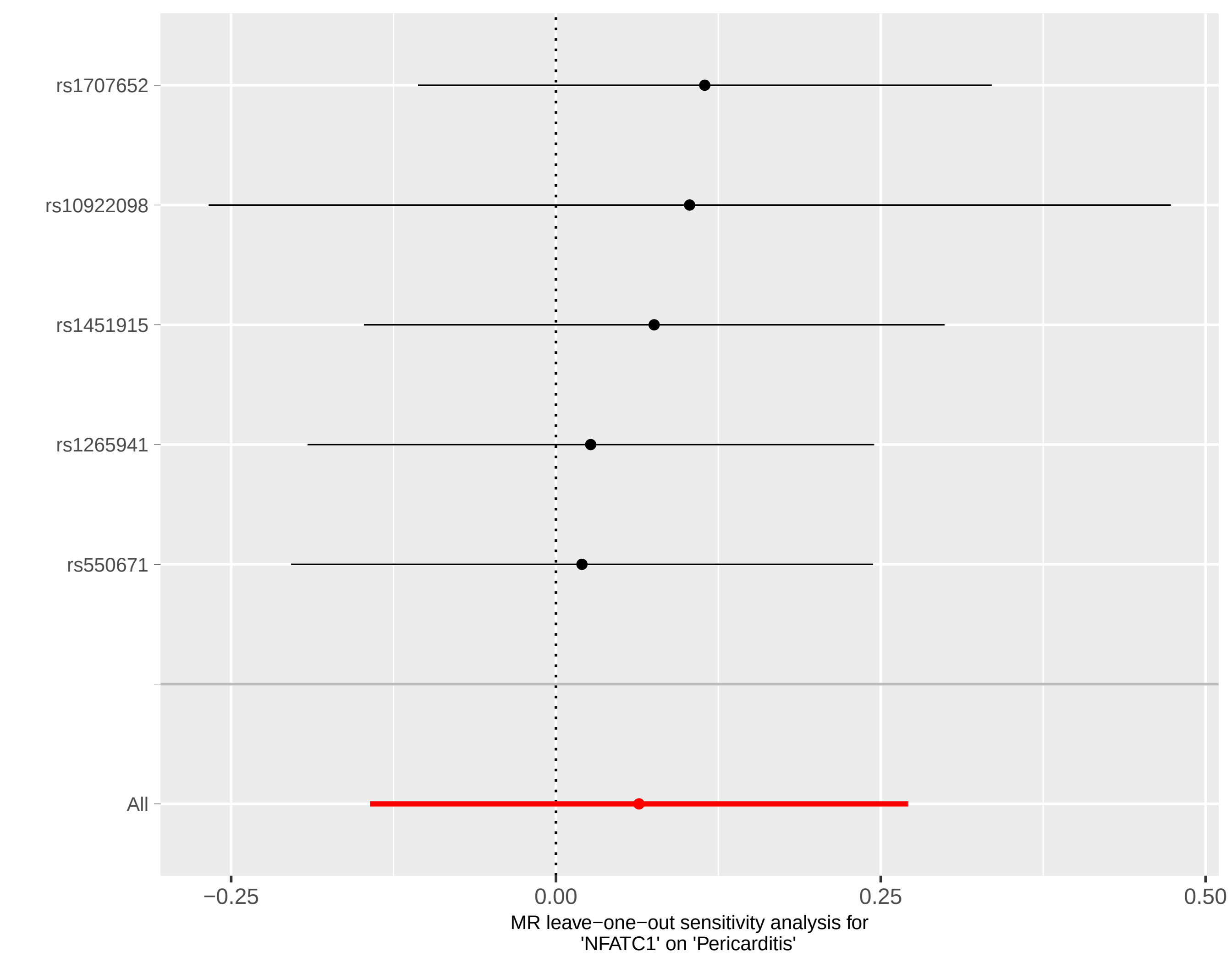
rs1707652
rs10922098
rs1451915
rs1265941
rs550671
All
−0.25
0.00
0.25
0.50
MR leave−one−out sensitivity analysis for
'NFATC1' on 'Pericarditis'

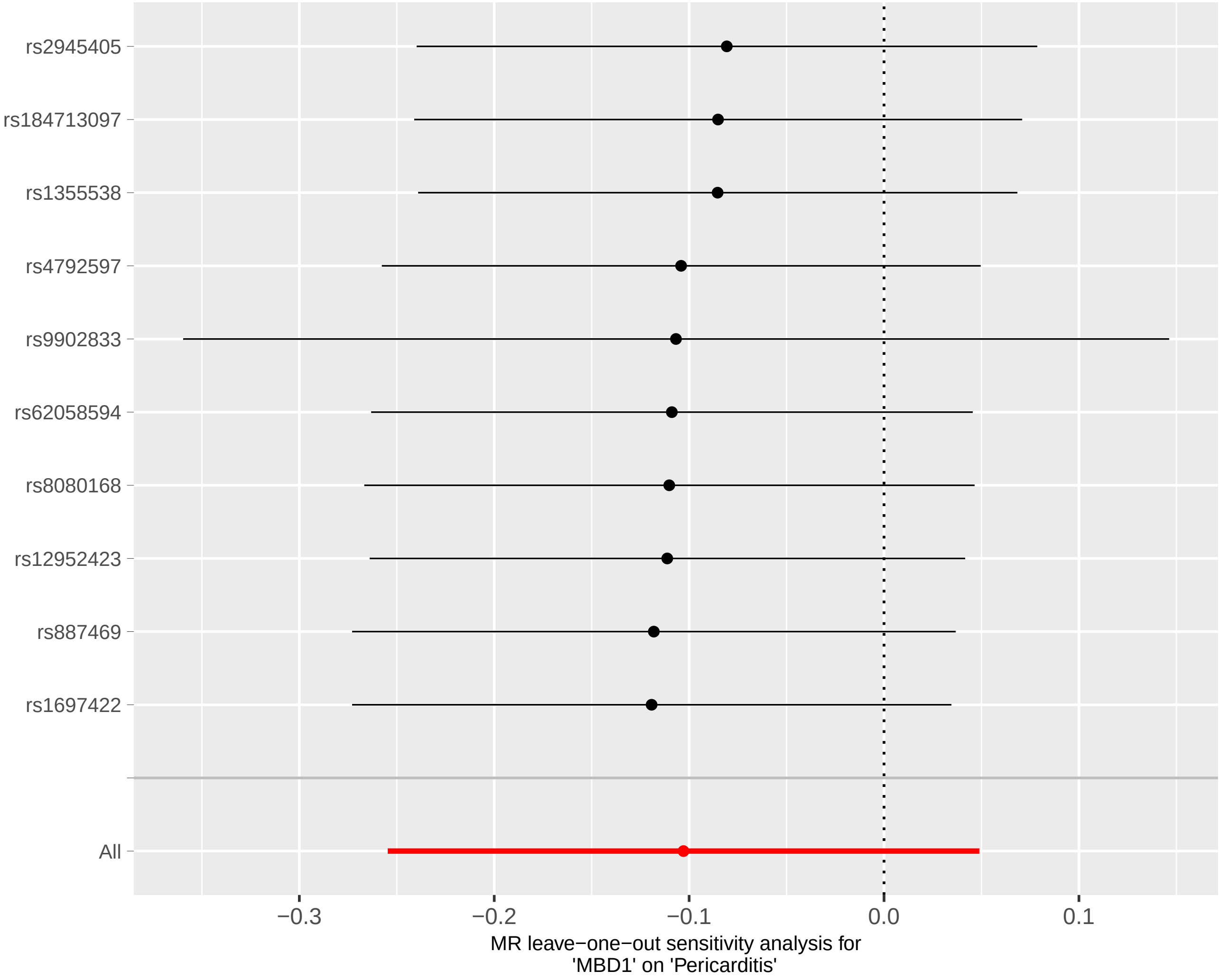
rs2945405
rs184713097
rs1355538
rs4792597
rs9902833
rs62058594
rs8080168
rs12952423
rs887469
rs1697422
All
−0.3
−0.2
−0.1
0.0
0.1
MR leave−one−out sensitivity analysis for
'MBD1' on 'Pericarditis'

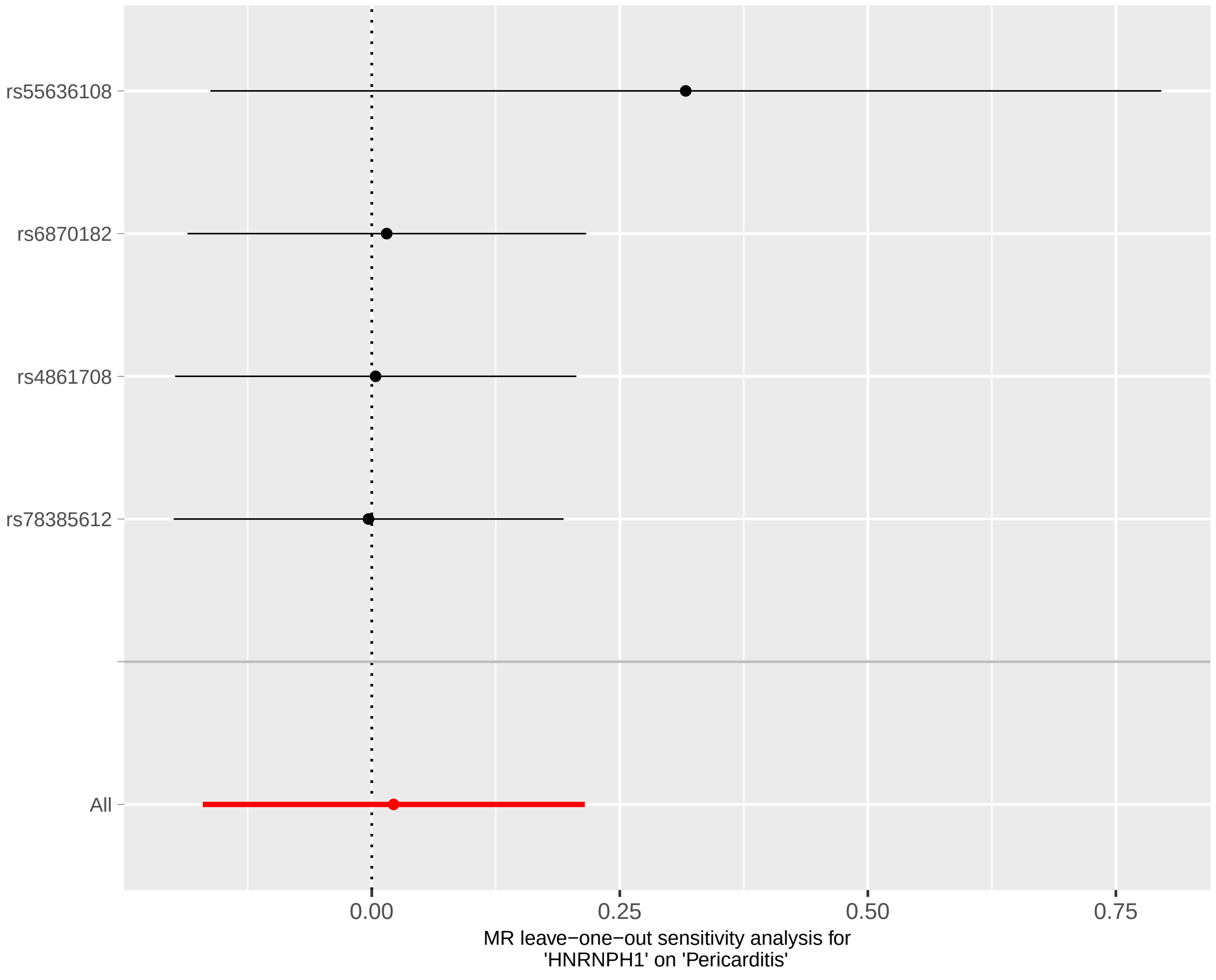
rs55636108
rs6870182
rs4861708
rs78385612
All
0.00
0.25
0.50
0.75
MR leave−one−out sensitivity analysis for
'HNRNPH1' on 'Pericarditis'

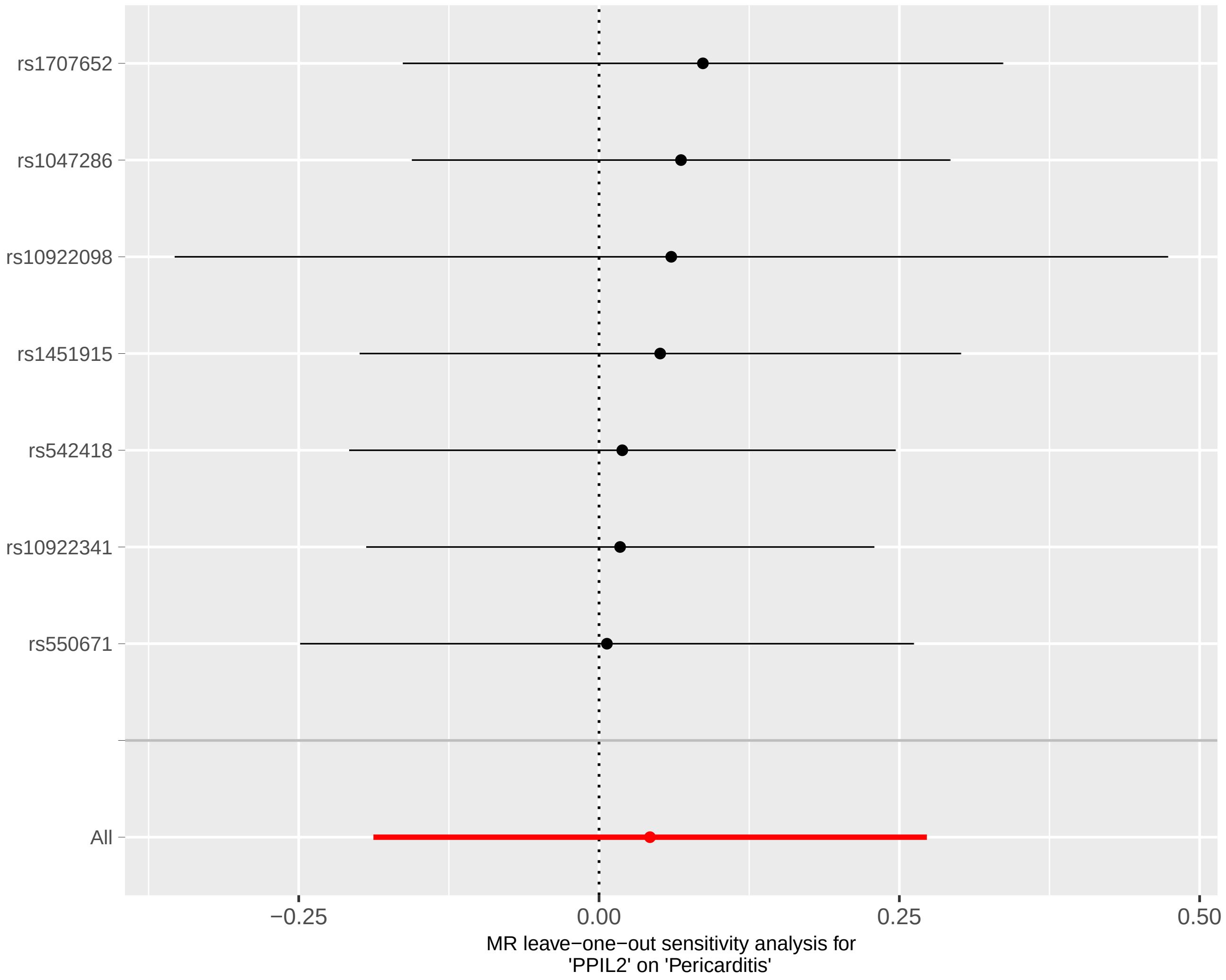
rs1707652
rs1047286
rs10922098
rs1451915
rs542418
rs10922341
rs550671
All
−0.25
0.00
0.25
0.50
MR leave−one−out sensitivity analysis for
'PPIL2' on 'Pericarditis'

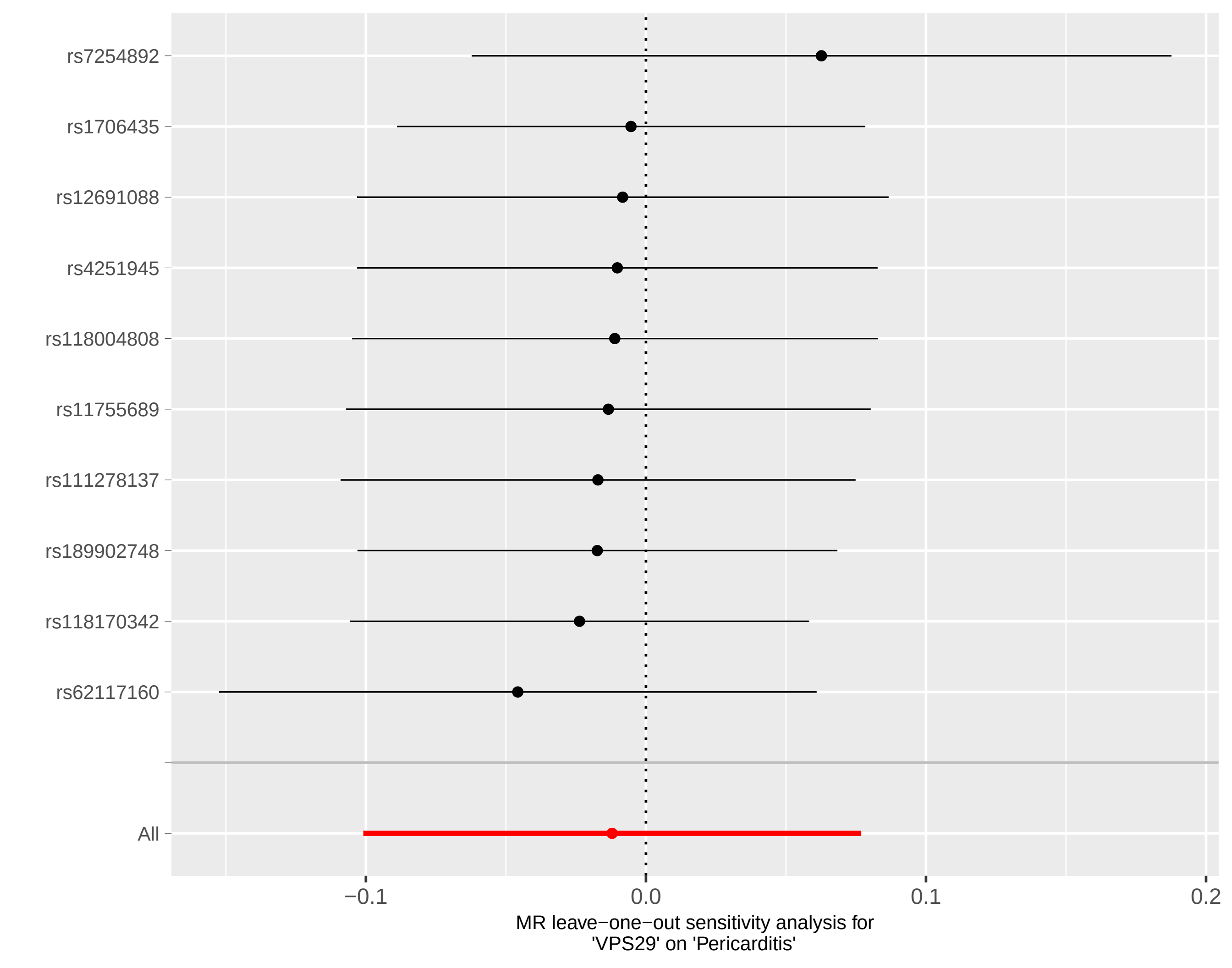
rs7254892
rs1706435
rs12691088
rs4251945
rs118004808
rs11755689
rs111278137
rs189902748
rs118170342
rs62117160
All
−0.1
0.0
0.1
0.2
MR leave−one−out sensitivity analysis for
'VPS29' on 'Pericarditis'

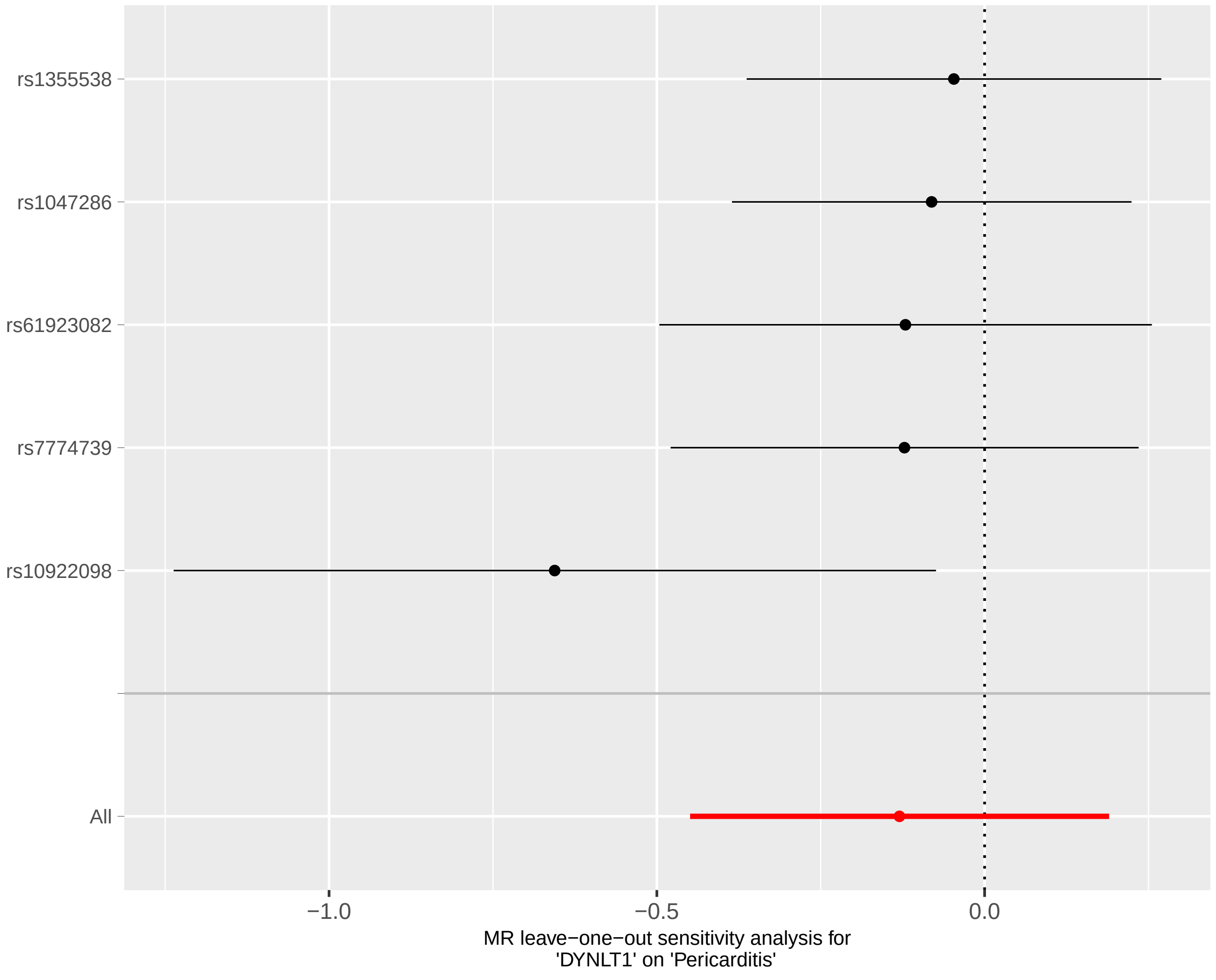

rs1355538
rs1047286
rs61923082
rs7774739
rs10922098
All
−1.0
−0.5
0.0
MR leave−one−out sensitivity analysis for
'DYNLT1' on 'Pericarditis'

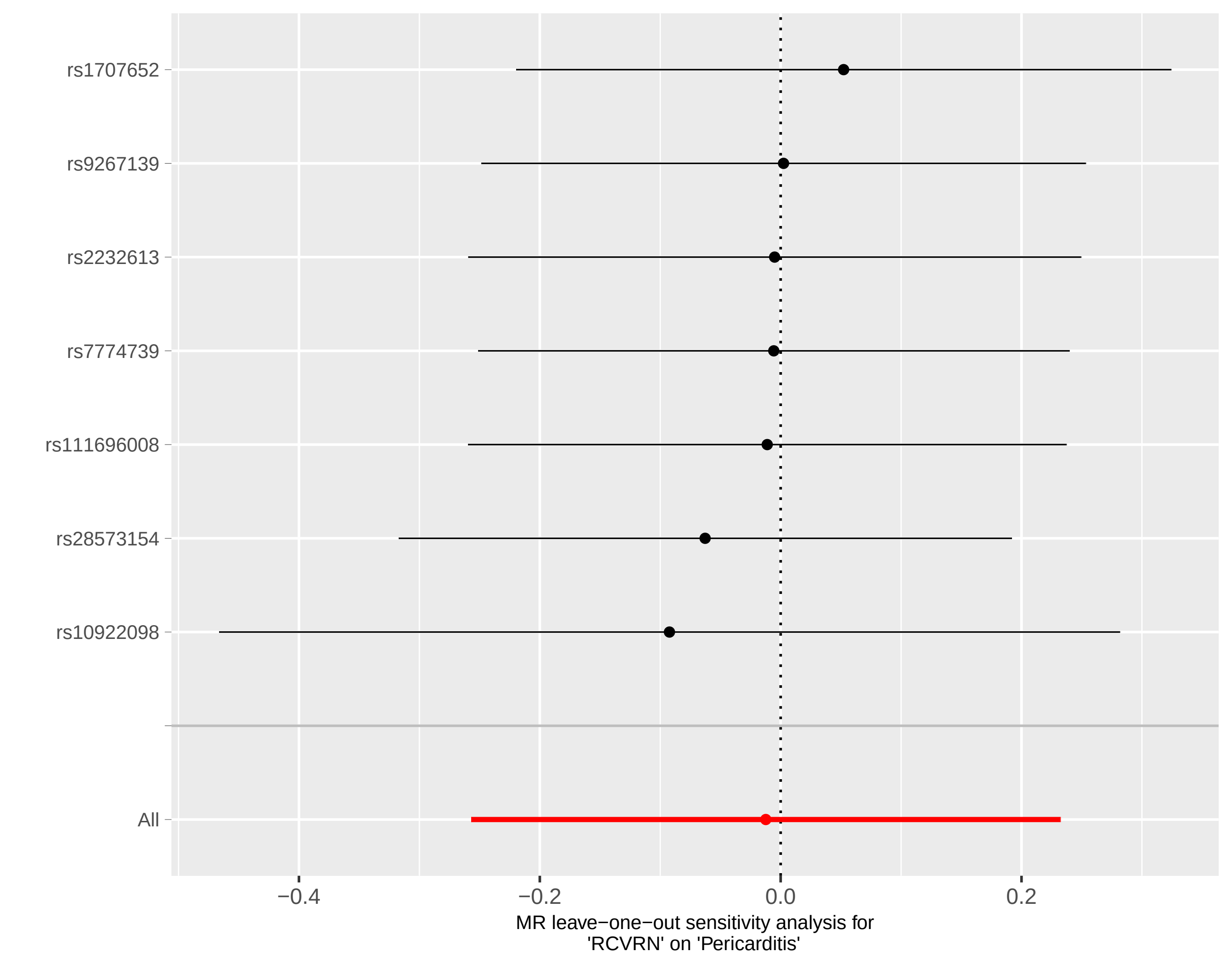
rs1707652
rs9267139
rs2232613
rs7774739
rs111696008
rs28573154
rs10922098
All
−0.4
−0.2
0.0
0.2
MR leave−one−out sensitivity analysis for
'RCVRN' on 'Pericarditis'

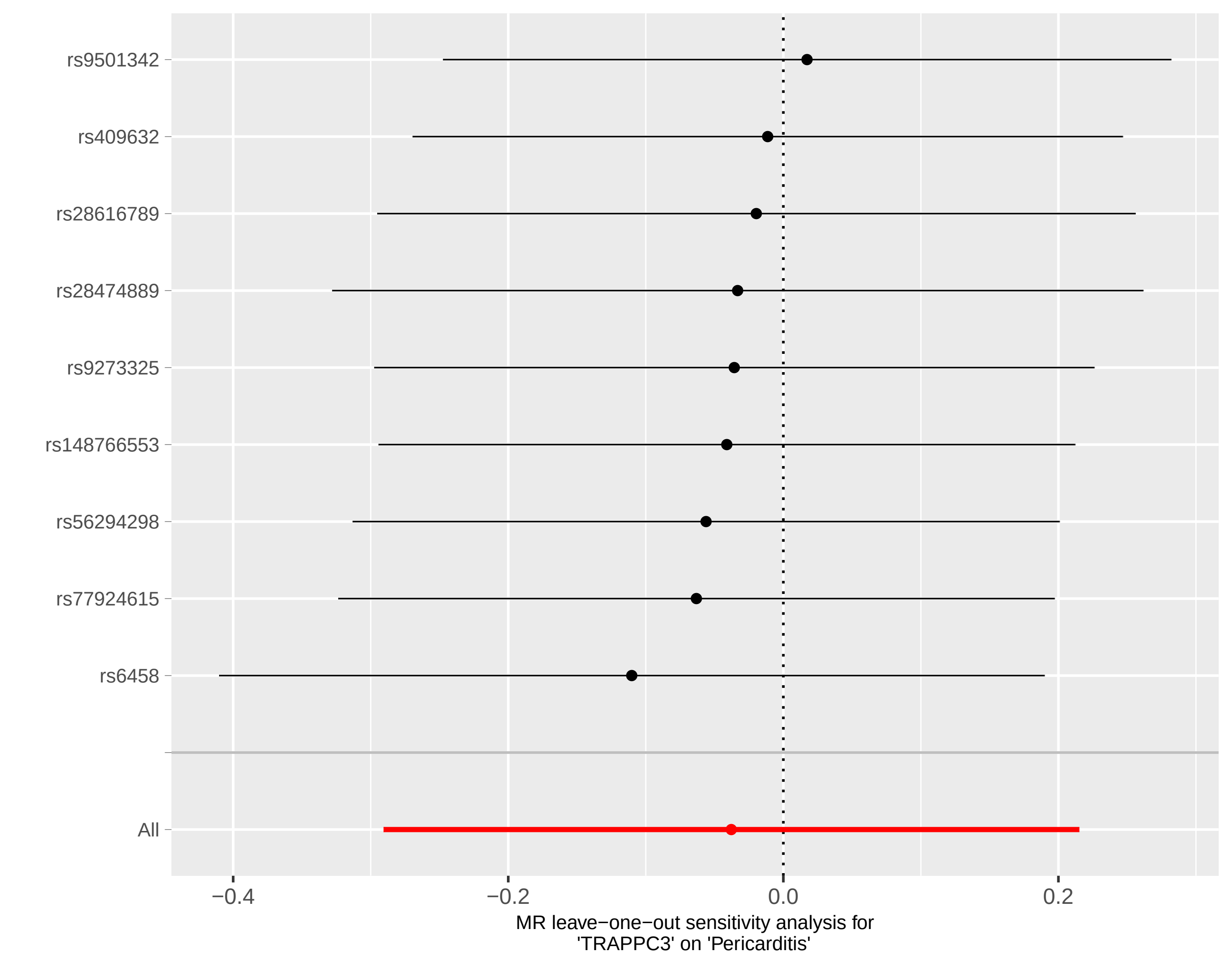

rs9501342
rs409632
rs28616789
rs28474889
rs9273325
rs148766553
rs56294298
rs77924615
rs6458
All
−0.4
−0.2
0.0
0.2
MR leave−one−out sensitivity analysis for
'TRAPPC3' on 'Pericarditis'

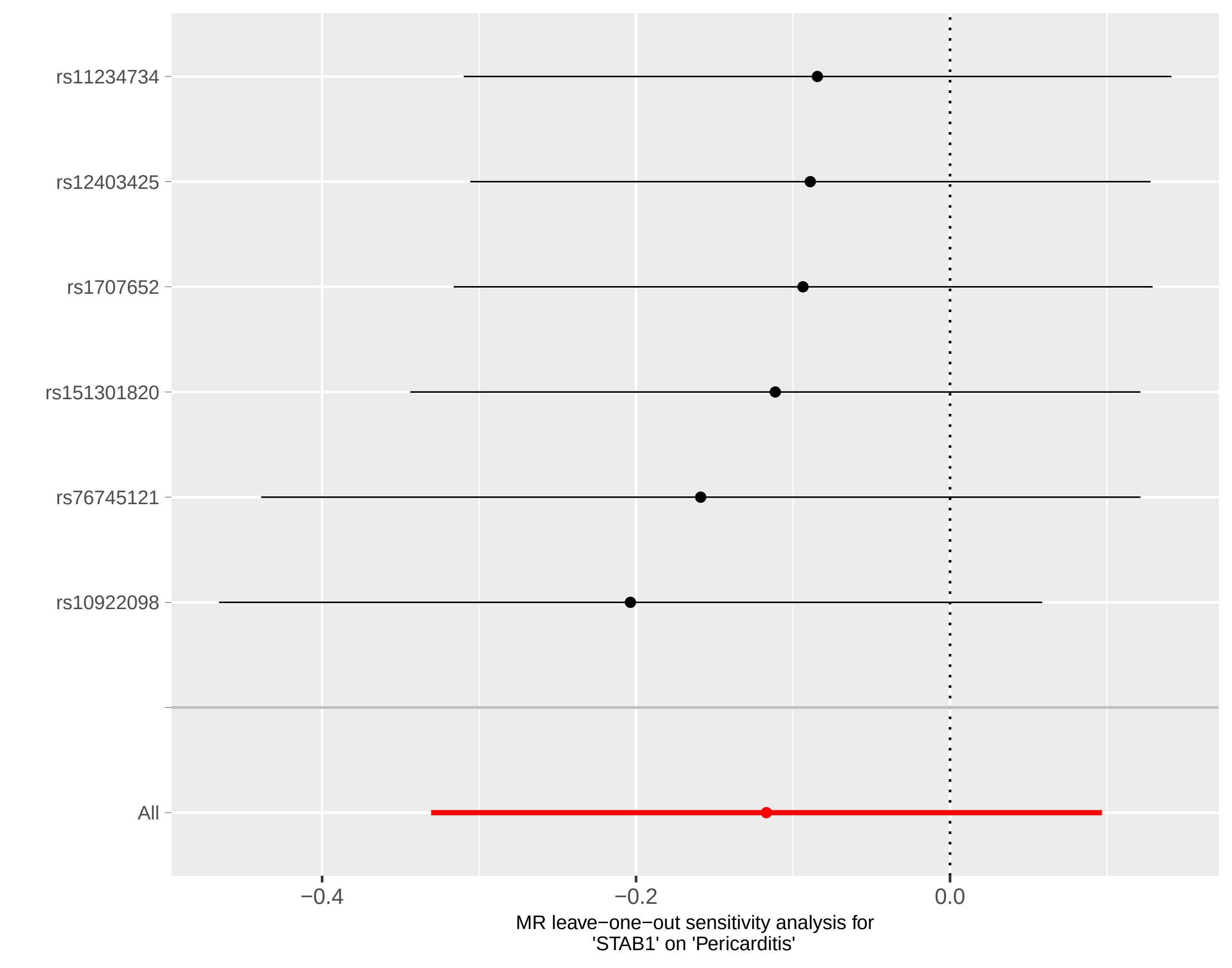
rs11234734
rs12403425
rs1707652
rs151301820
rs76745121
rs10922098
All
−0.4
−0.2
0.0
MR leave−one−out sensitivity analysis for
'STAB1' on 'Pericarditis'

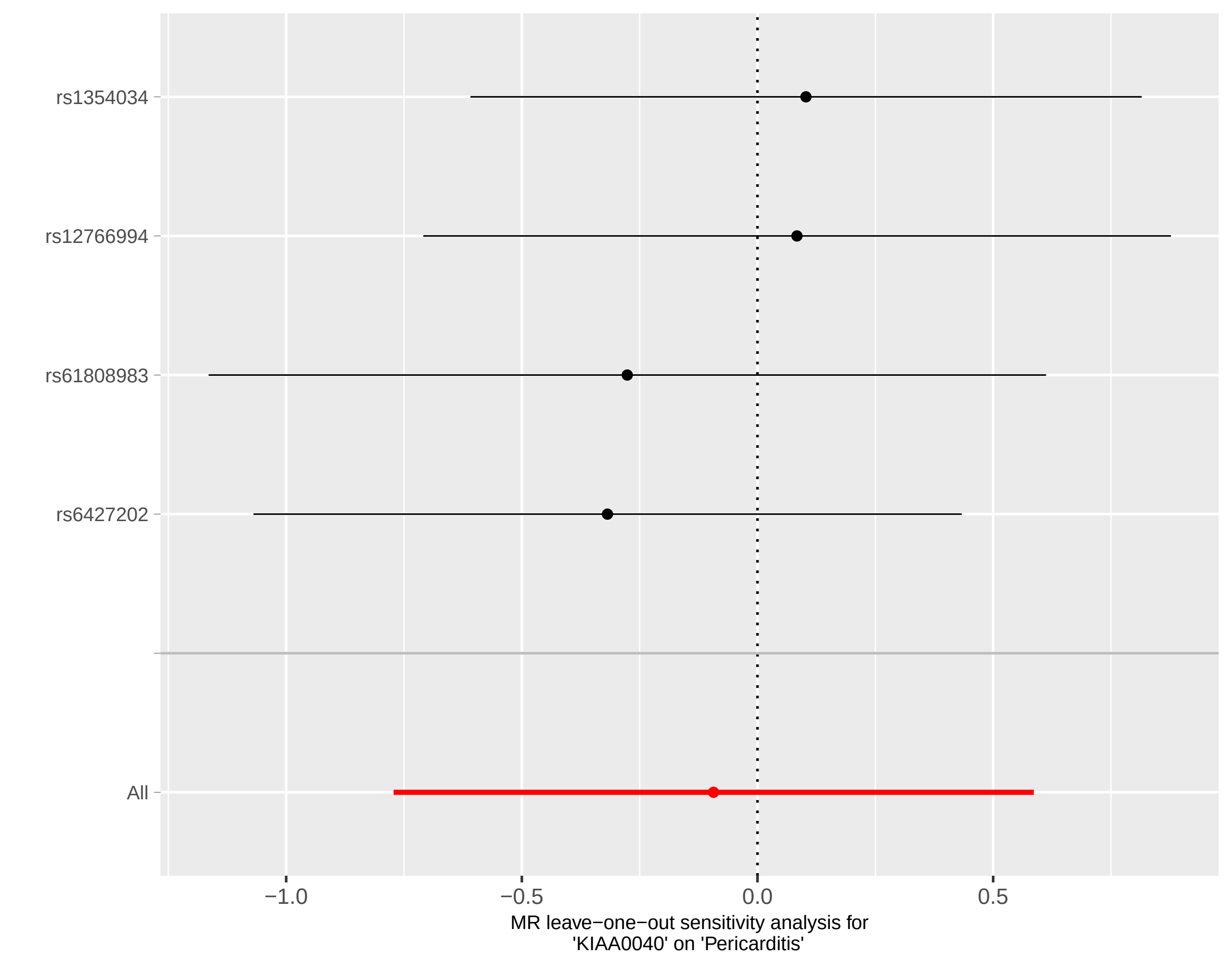
rs1354034
rs12766994
rs61808983
rs6427202
All
−1.0
−0.5
0.0
0.5
MR leave−one−out sensitivity analysis for
'KIAA0040' on 'Pericarditis'

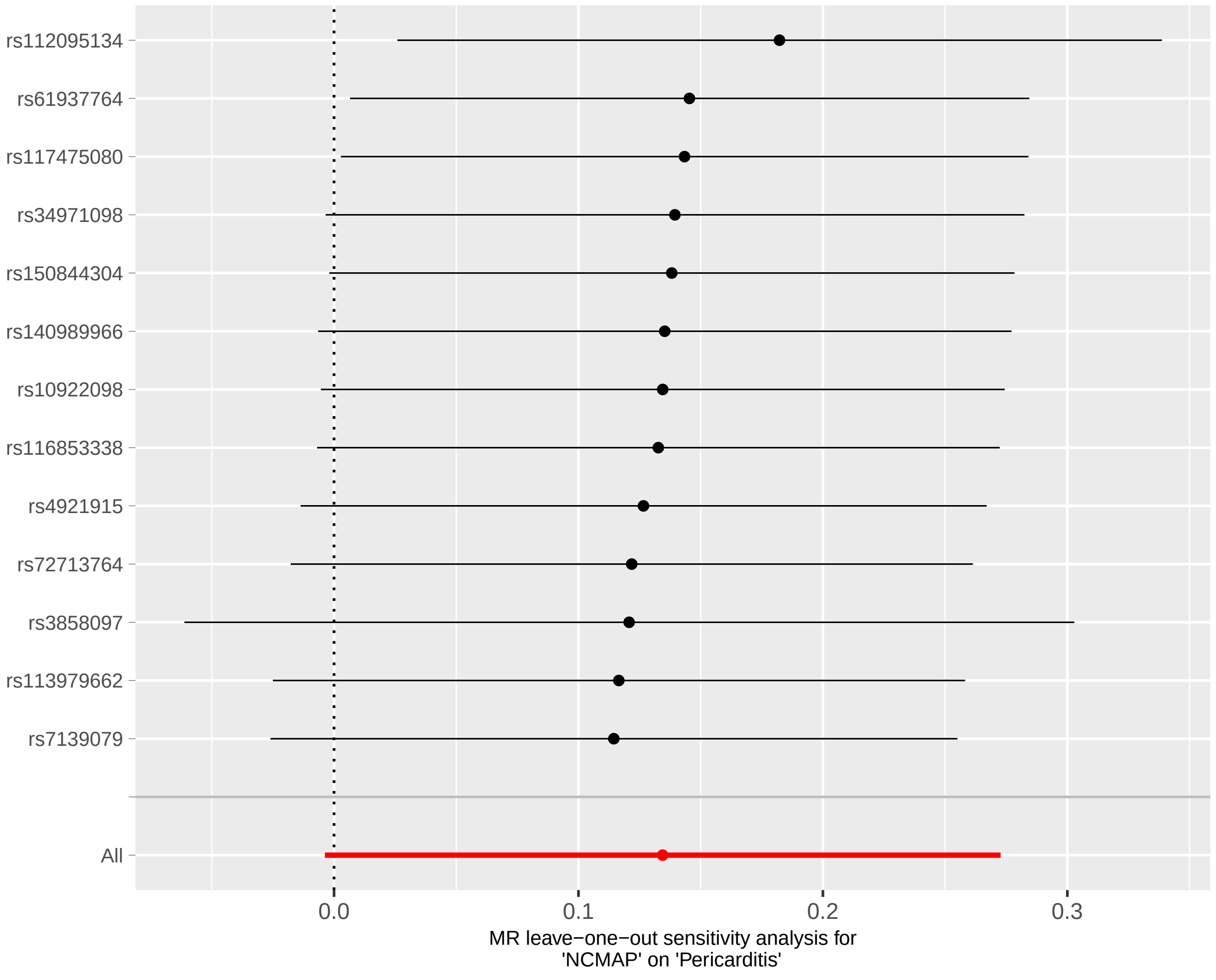
rs112095134
rs61937764
rs117475080
rs34971098
rs150844304
rs140989966
rs10922098
rs116853338
rs4921915
rs72713764
rs3858097
rs113979662
rs7139079
All
0.0
0.1
0.2
0.3
MR leave−one−out sensitivity analysis for
'NCMAP' on 'Pericarditis'

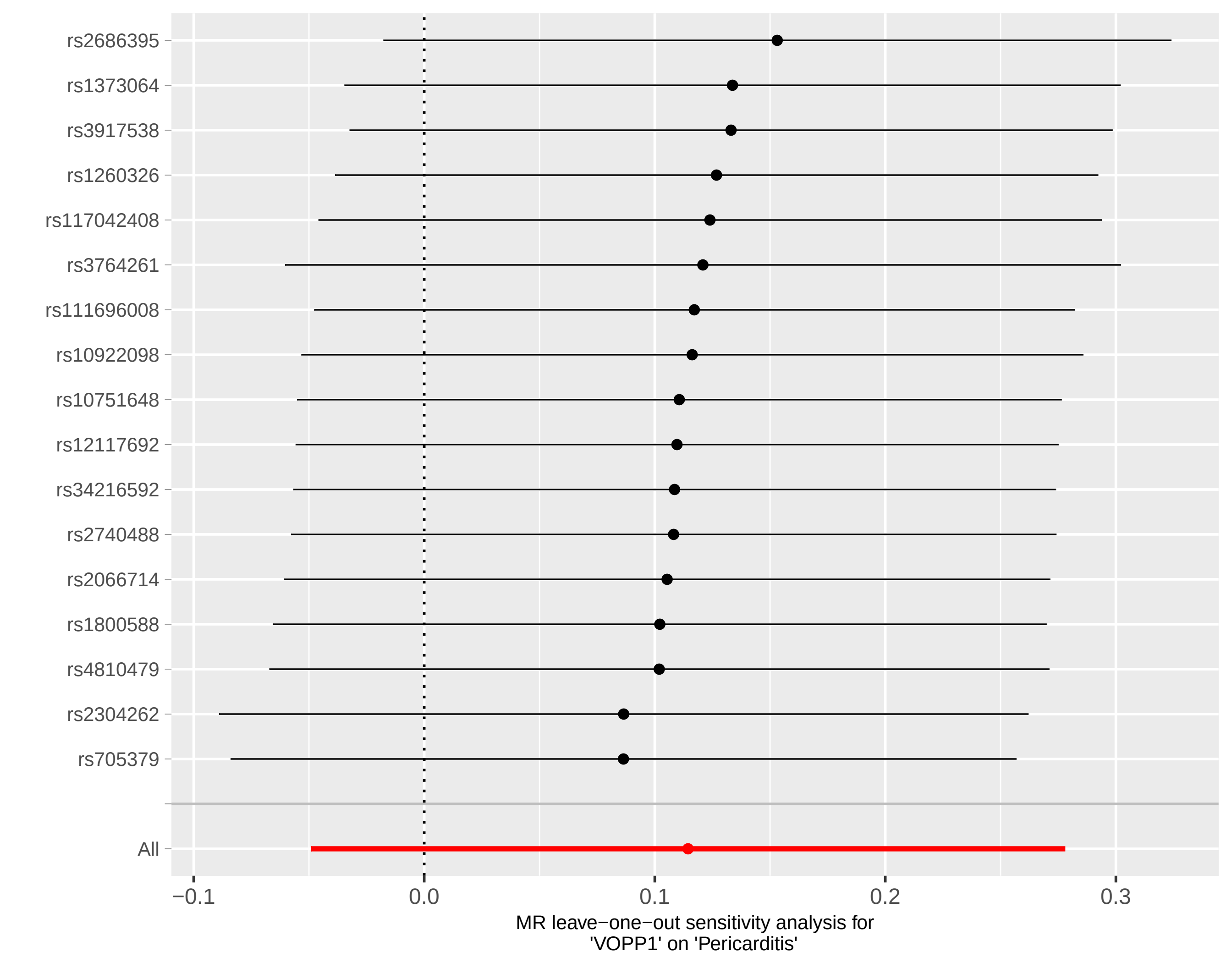
rs2686395
rs1373064
rs3917538
rs1260326
rs117042408
rs3764261
rs111696008
rs10922098
rs10751648
rs12117692
rs34216592
rs2740488
rs2066714
rs1800588
rs4810479
rs2304262
rs705379
All
−0.1
0.0
0.1
0.2
0.3
MR leave−one−out sensitivity analysis for
'VOPP1' on 'Pericarditis'

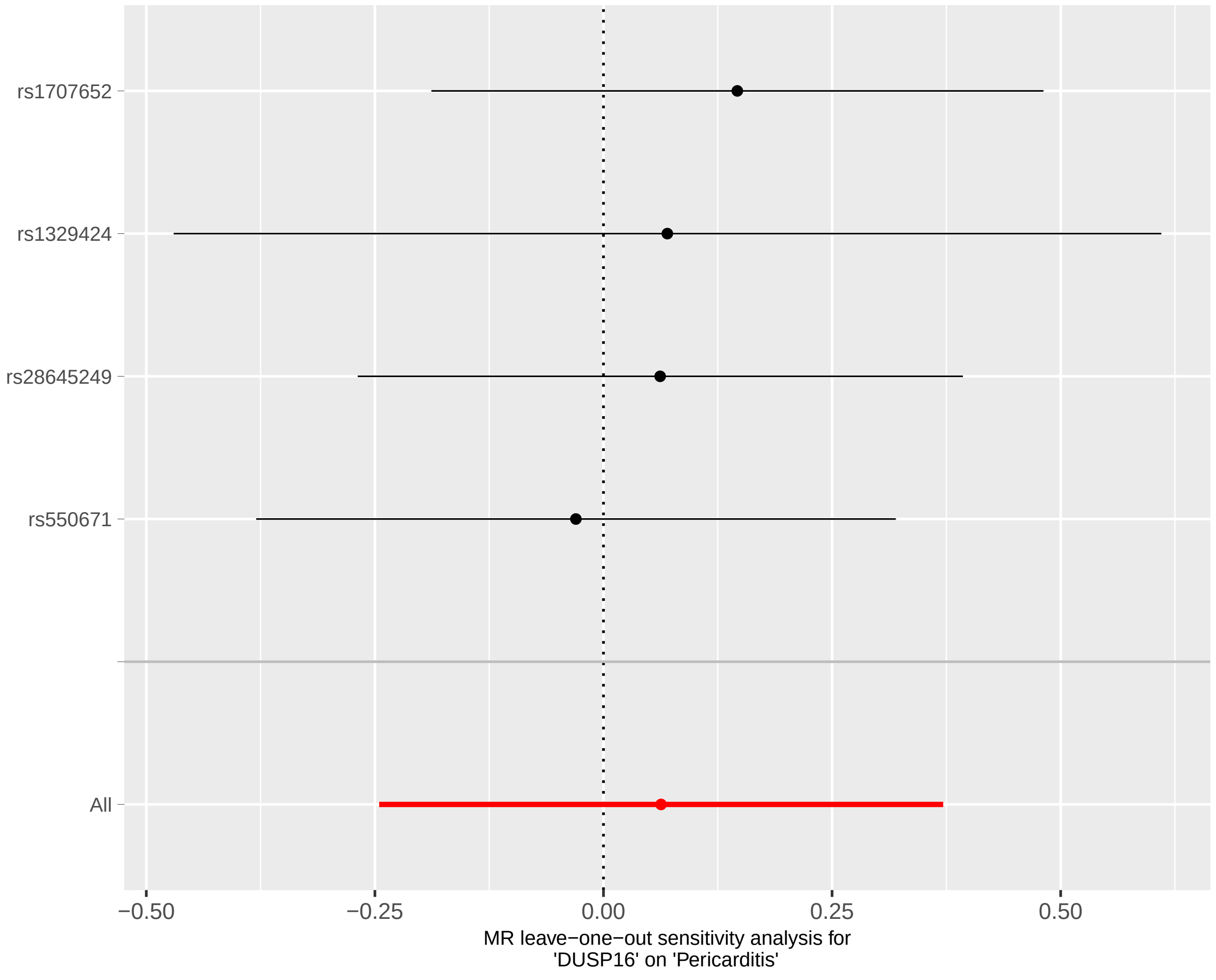
rs1707652
rs1329424
rs28645249
rs550671
All
−0.50
−0.25
0.00
0.25
0.50
MR leave−one−out sensitivity analysis for
'DUSP16' on 'Pericarditis'

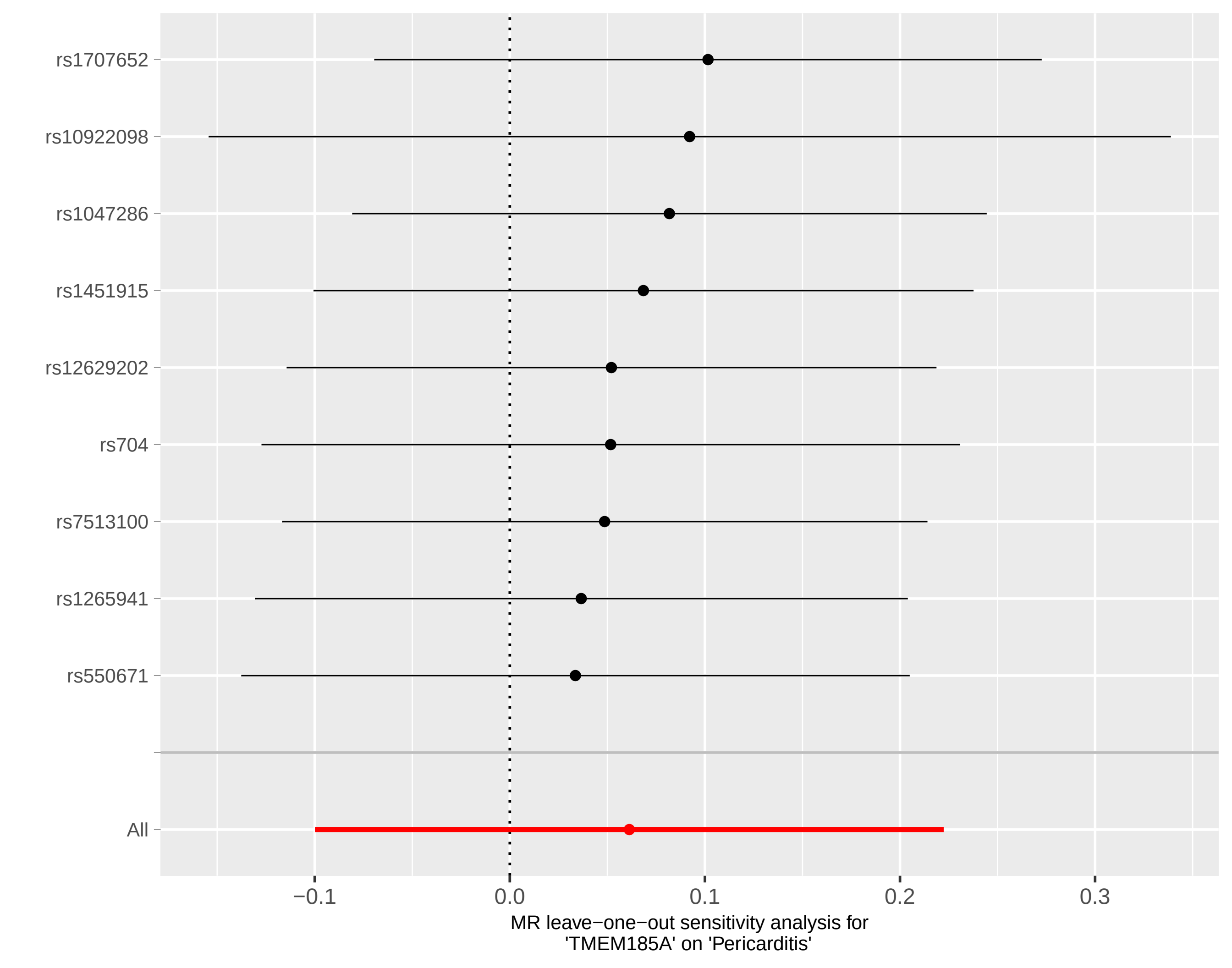
rs1707652
rs10922098
rs1047286
rs1451915
rs12629202
rs704
rs7513100
rs1265941
rs550671
All
−0.1
0.0
0.1
0.2
0.3
MR leave−one−out sensitivity analysis for
'TMEM185A' on 'Pericarditis'

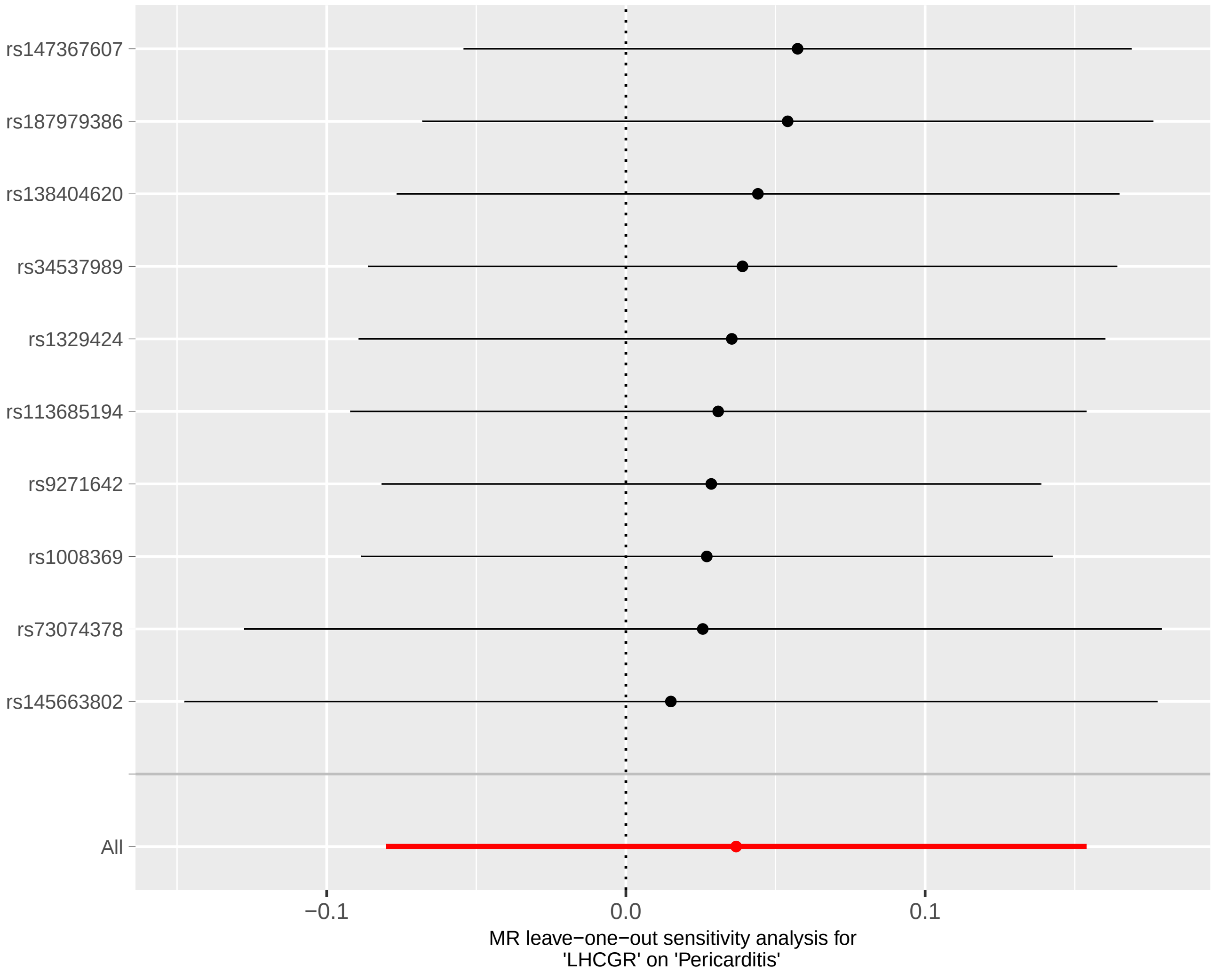
rs147367607
rs187979386
rs138404620
rs34537989
rs1329424
rs113685194
rs9271642
rs1008369
rs73074378
rs145663802
All
−0.1
0.0
0.1
MR leave−one−out sensitivity analysis for
'LHCGR' on 'Pericarditis'

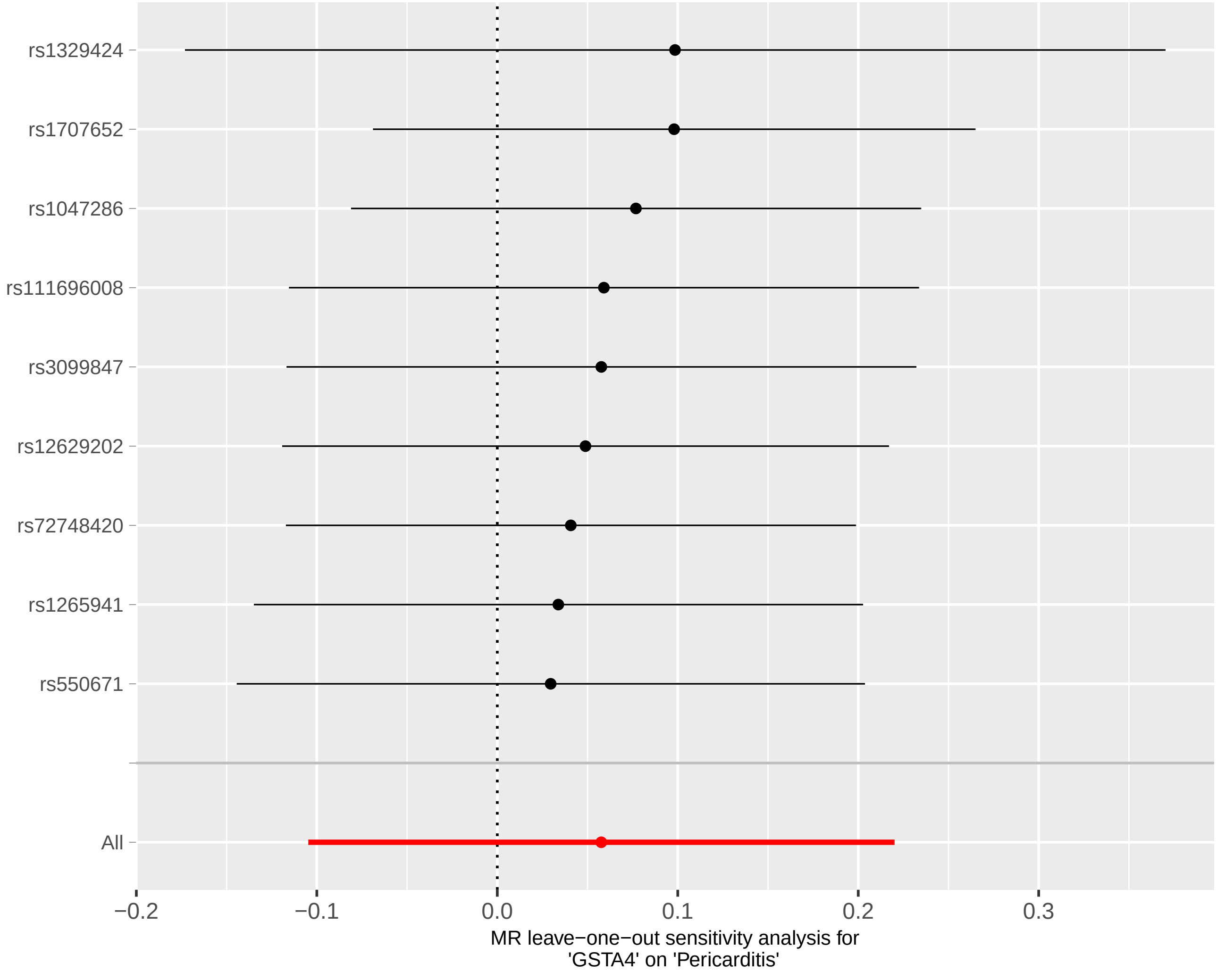

rs1329424
rs1707652
rs1047286
rs111696008
rs3099847
rs12629202
rs72748420
rs1265941
rs550671
All
−0.2
−0.1
0.0
0.1
0.2
0.3
MR leave−one−out sensitivity analysis for
'GSTA4' on 'Pericarditis'

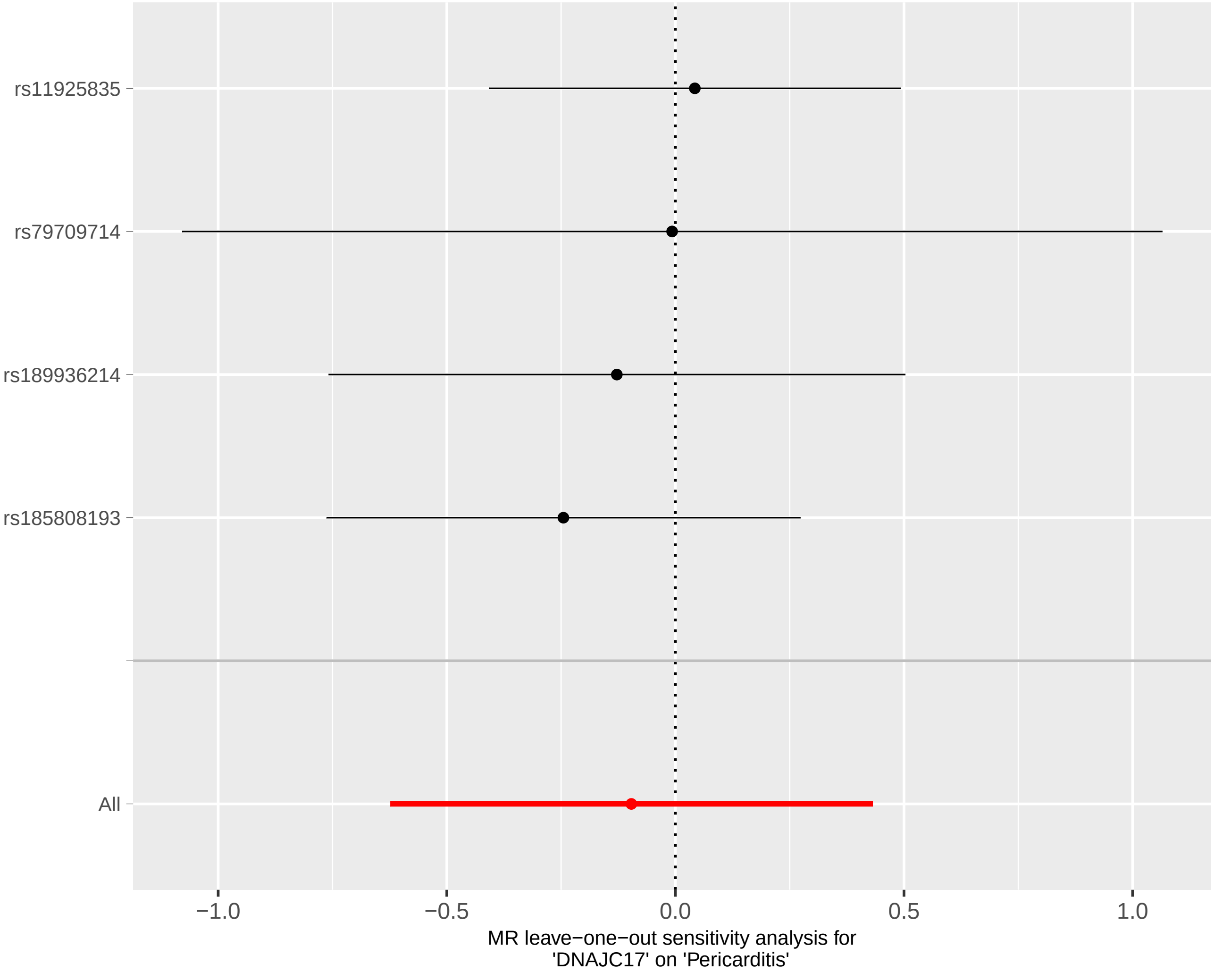

rs11925835
rs79709714
rs189936214
rs185808193
All
−1.0
−0.5
0.0
0.5
1.0
MR leave−one−out sensitivity analysis for
'DNAJC17' on 'Pericarditis'

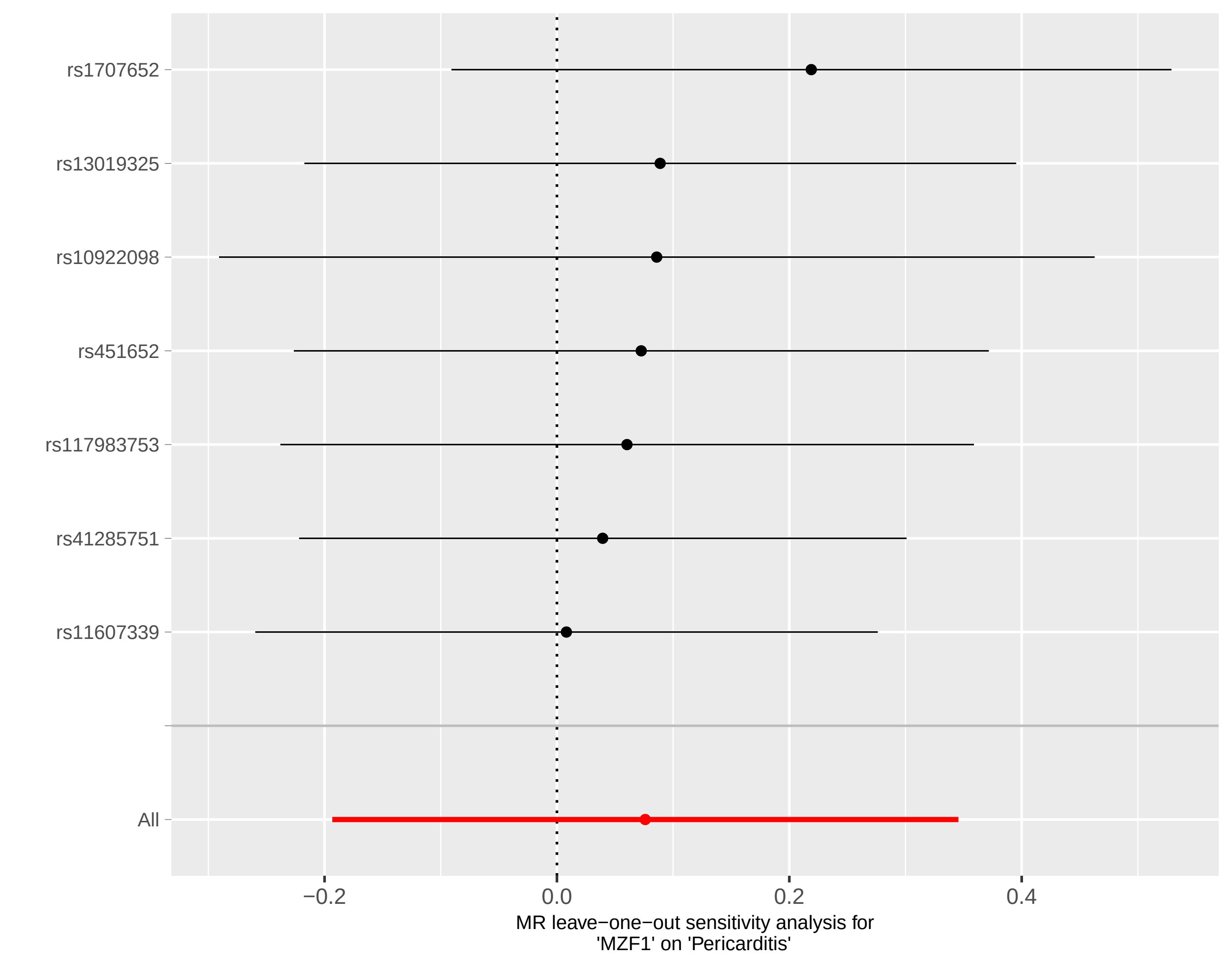
rs1707652
rs13019325
rs10922098
rs451652
rs117983753
rs41285751
rs11607339
All
−0.2
0.0
0.2
0.4
MR leave−one−out sensitivity analysis for
'MZF1' on 'Pericarditis'

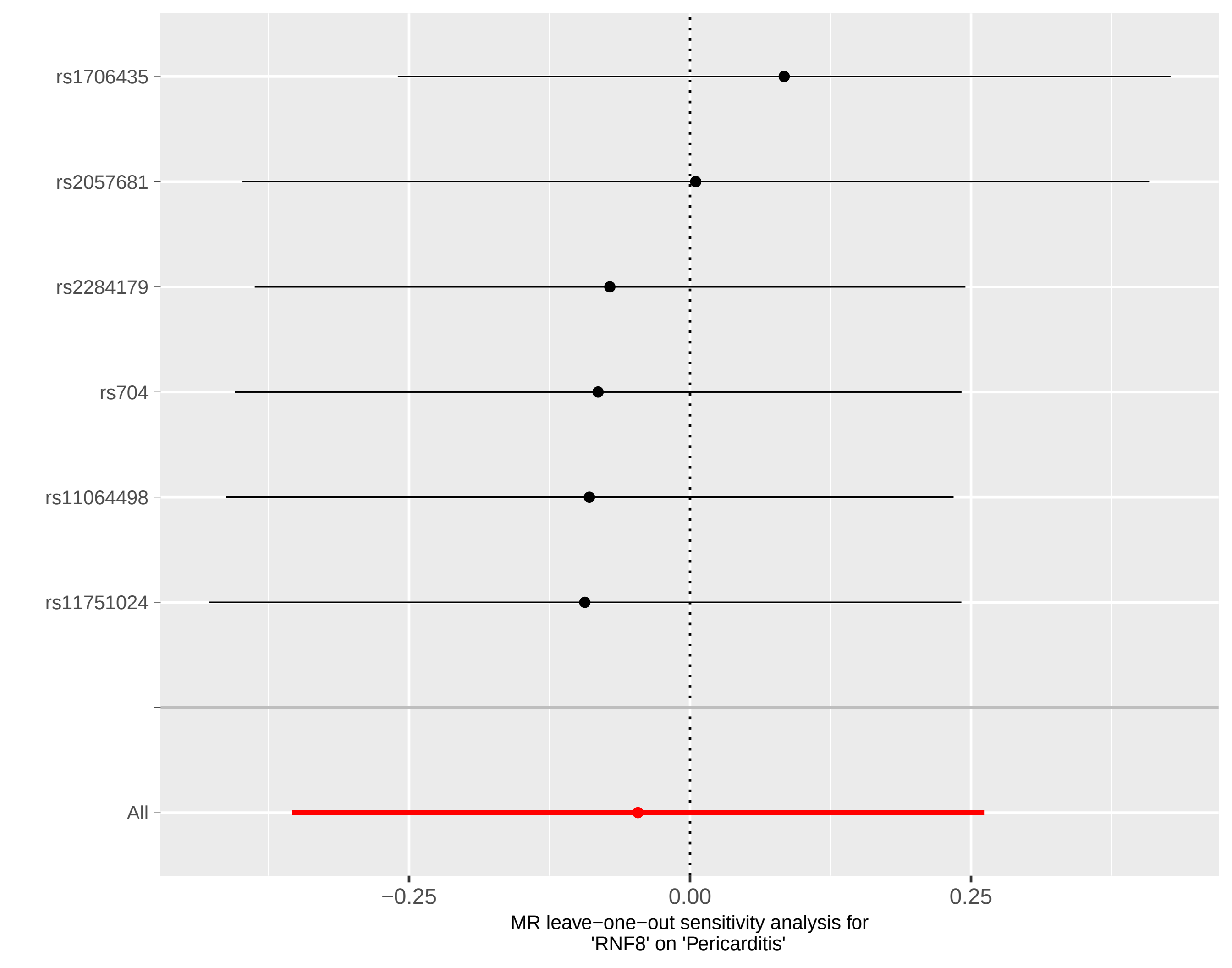
rs1706435
rs2057681
rs2284179
rs704
rs11064498
rs11751024
All
−0.25
0.00
0.25
MR leave−one−out sensitivity analysis for
'RNF8' on 'Pericarditis'

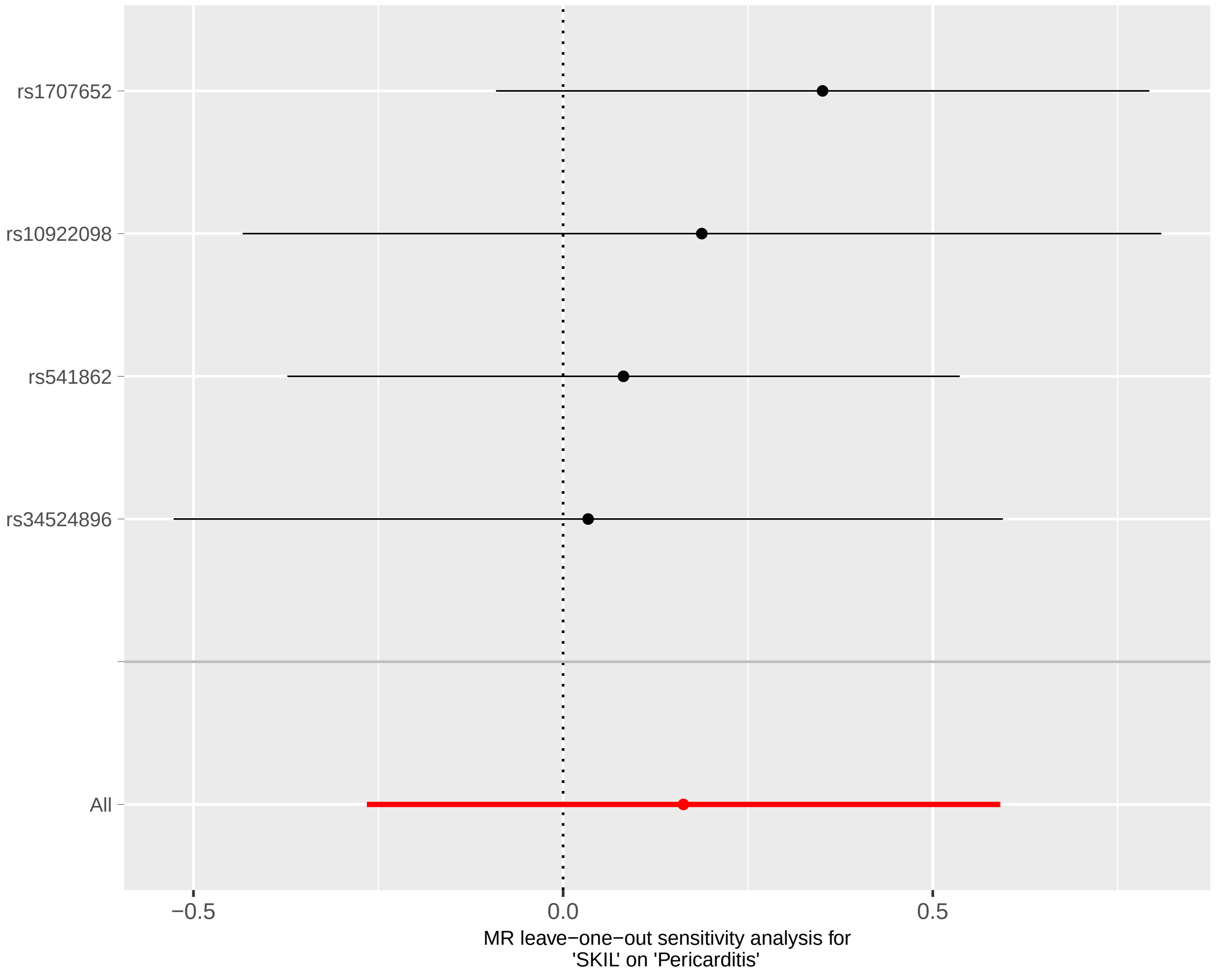

rs1707652
rs10922098
rs541862
rs34524896
All
−0.5
0.0
0.5
MR leave−one−out sensitivity analysis for
'SKIL' on 'Pericarditis'

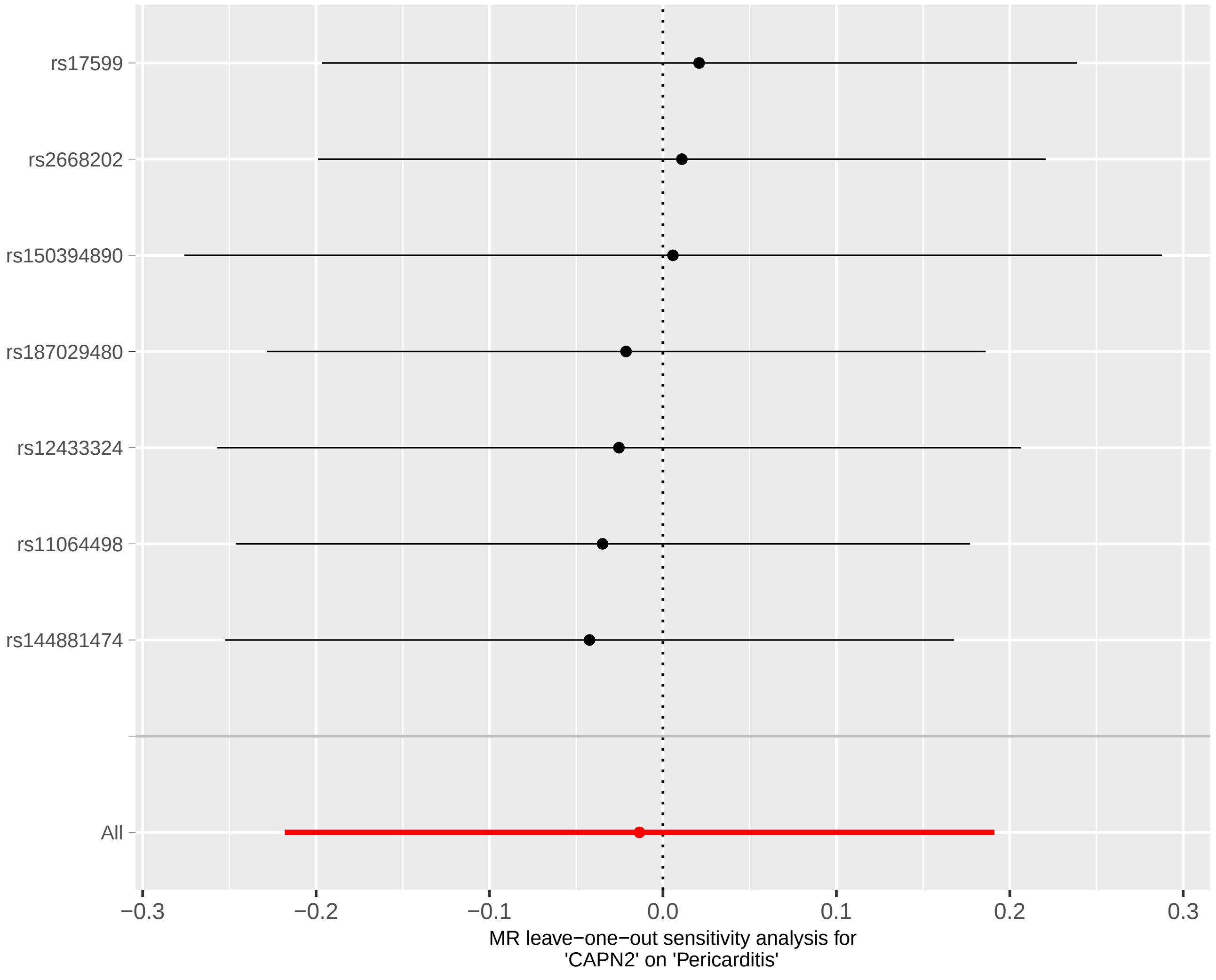
rs17599
rs2668202
rs150394890
rs187029480
rs12433324
rs11064498
rs144881474
All
−0.3
−0.2
−0.1
0.0
0.1
0.2
0.3
MR leave−one−out sensitivity analysis for
'CAPN2' on 'Pericarditis'

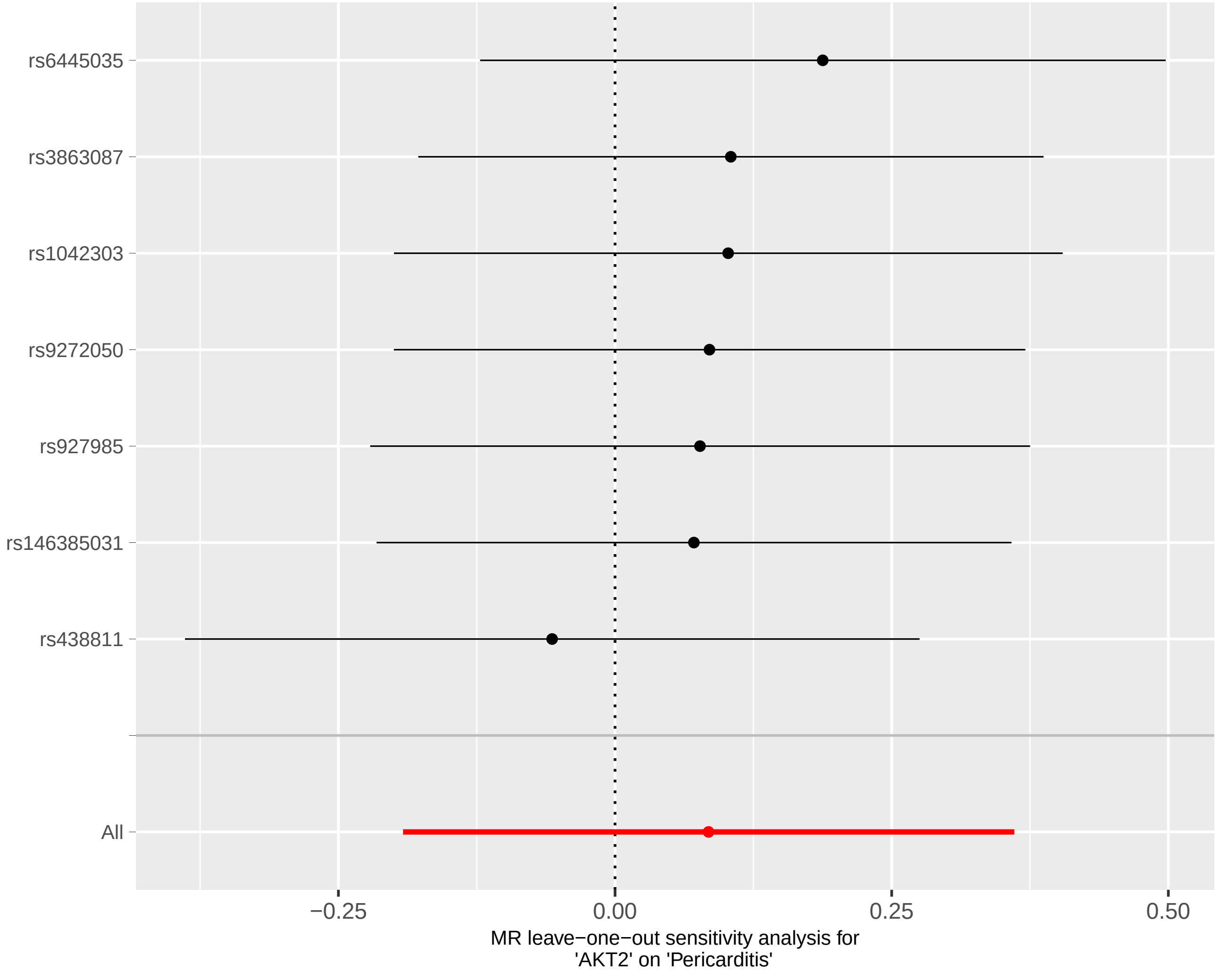
rs6445035
rs3863087
rs1042303
rs9272050
rs927985
rs146385031
rs438811
All
−0.25
0.00
0.25
0.50
MR leave−one−out sensitivity analysis for
'AKT2' on 'Pericarditis'

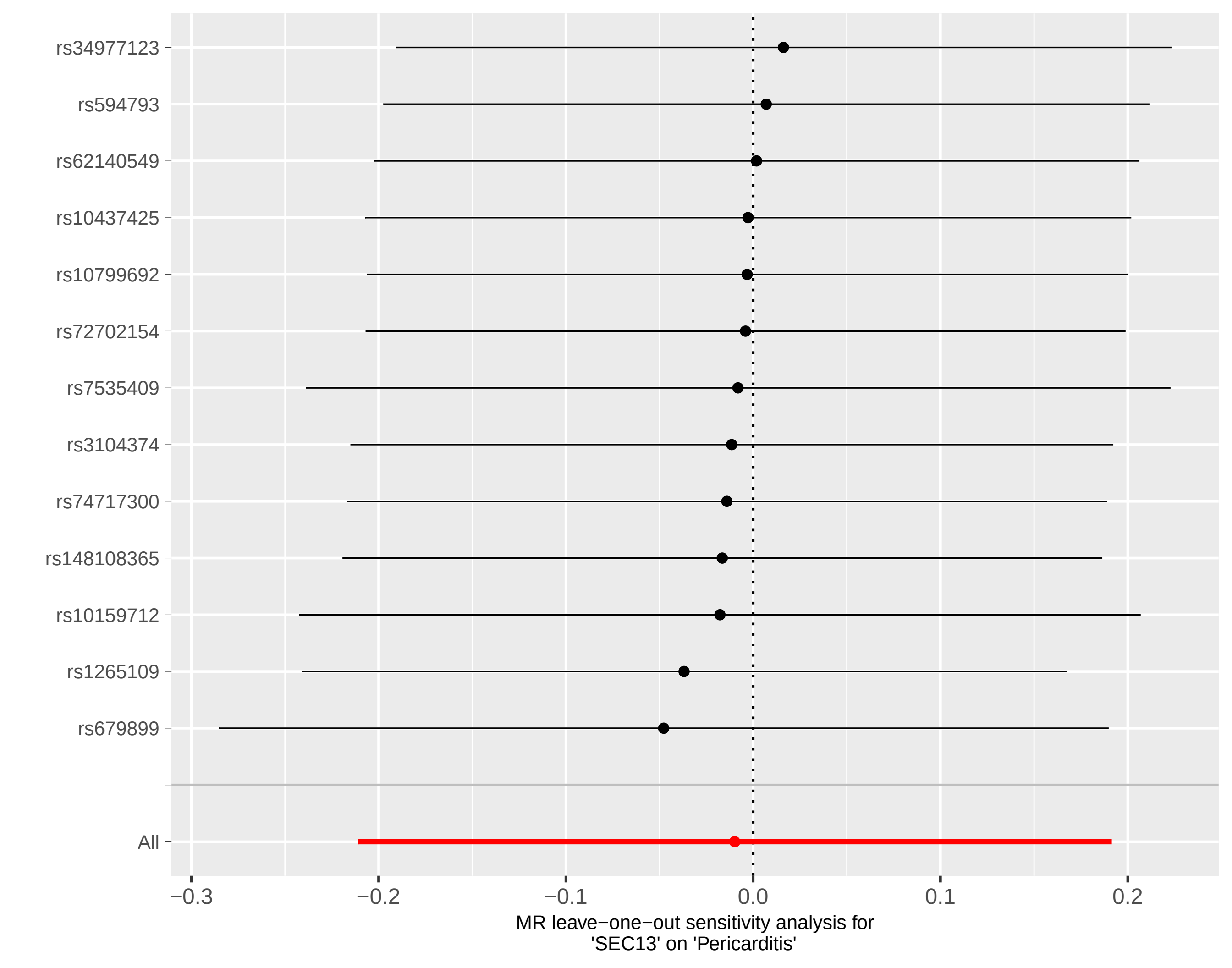

rs34977123
rs594793
rs62140549
rs10437425
rs10799692
rs72702154
rs7535409
rs3104374
rs74717300
rs148108365
rs10159712
rs1265109
rs679899
All
−0.3
−0.2
−0.1
0.0
0.1
0.2
MR leave−one−out sensitivity analysis for
'SEC13' on 'Pericarditis'

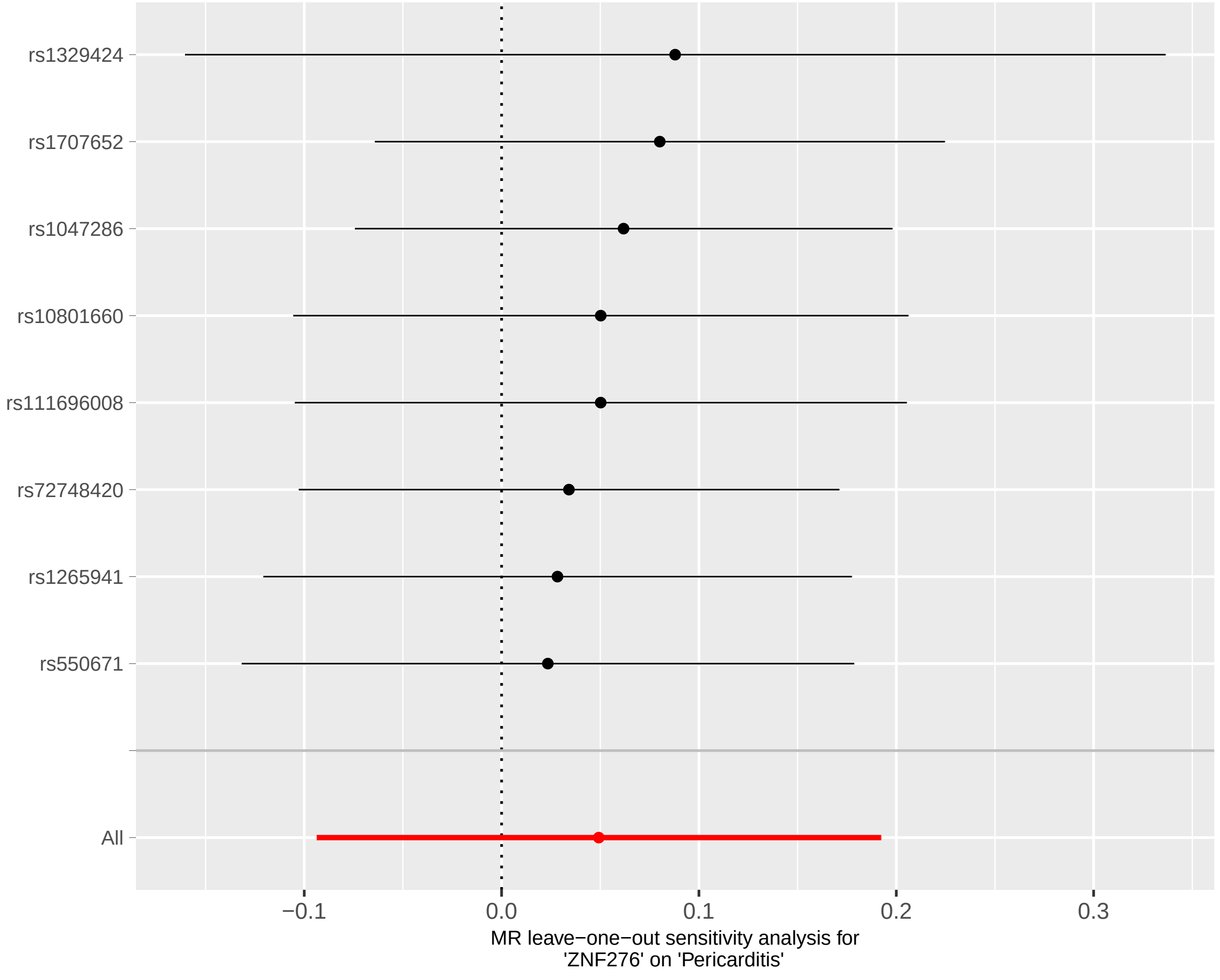
rs1329424
rs1707652
rs1047286
rs10801660
rs111696008
rs72748420
rs1265941
rs550671
All
−0.1
0.0
0.1
0.2
0.3
MR leave−one−out sensitivity analysis for
'ZNF276' on 'Pericarditis'

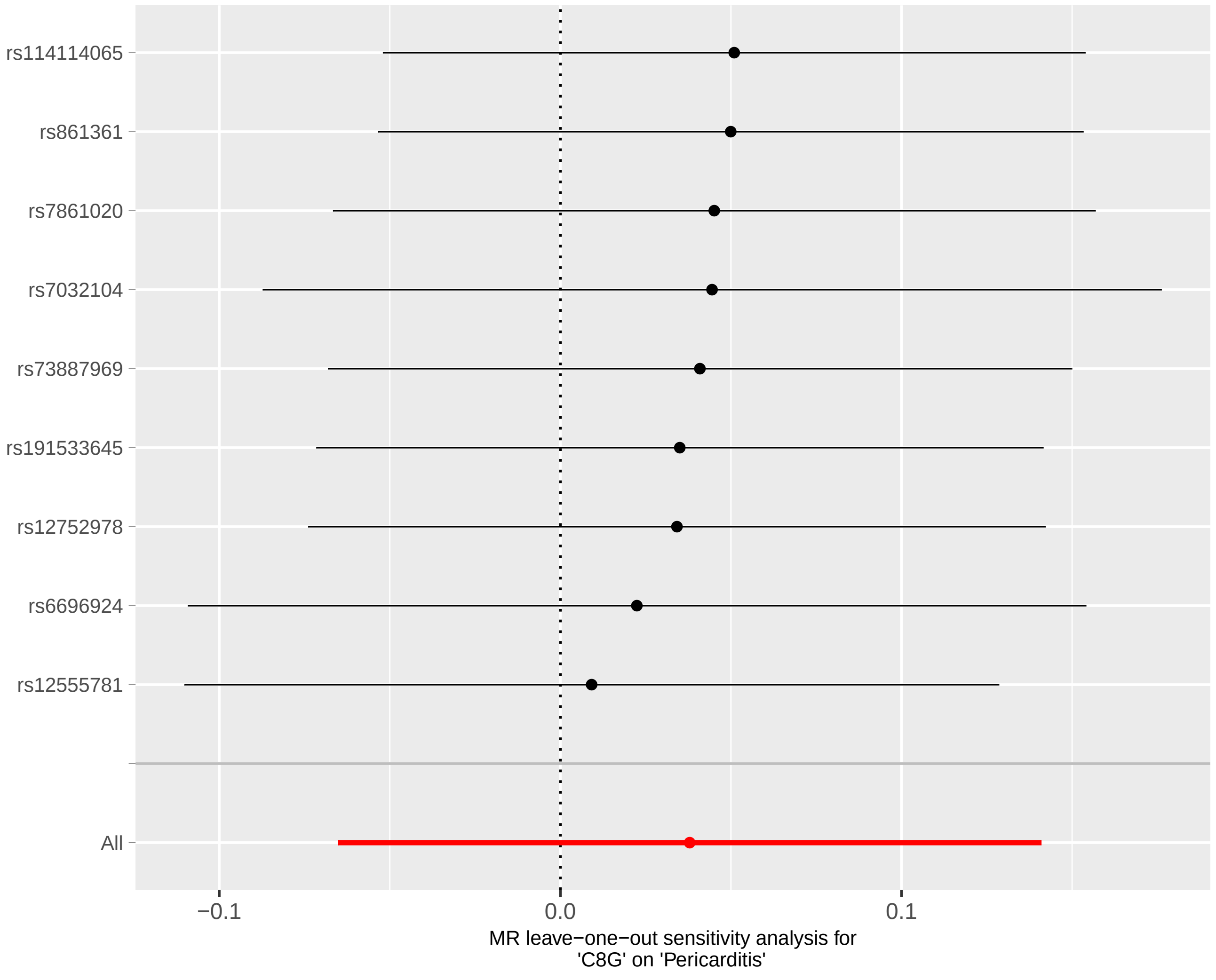

rs114114065
rs861361
rs7861020
rs7032104
rs73887969
rs191533645
rs12752978
rs6696924
rs12555781
All
−0.1
0.0
0.1
MR leave−one−out sensitivity analysis for
'C8G' on 'Pericarditis'

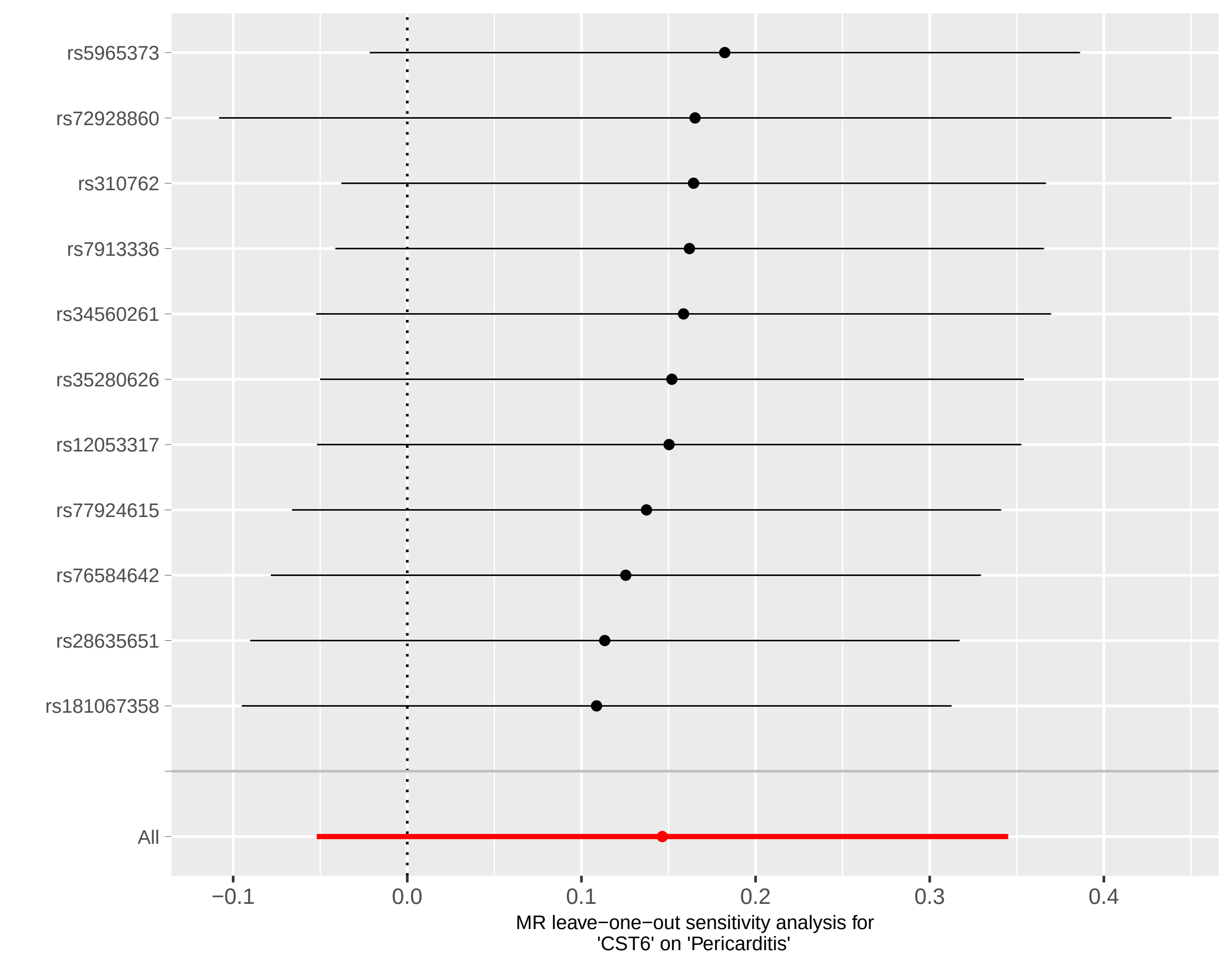
rs5965373
rs72928860
rs310762
rs7913336
rs34560261
rs35280626
rs12053317
rs77924615
rs76584642
rs28635651
rs181067358
All
−0.1
0.0
0.1
0.2
0.3
0.4
MR leave−one−out sensitivity analysis for
'CST6' on 'Pericarditis'

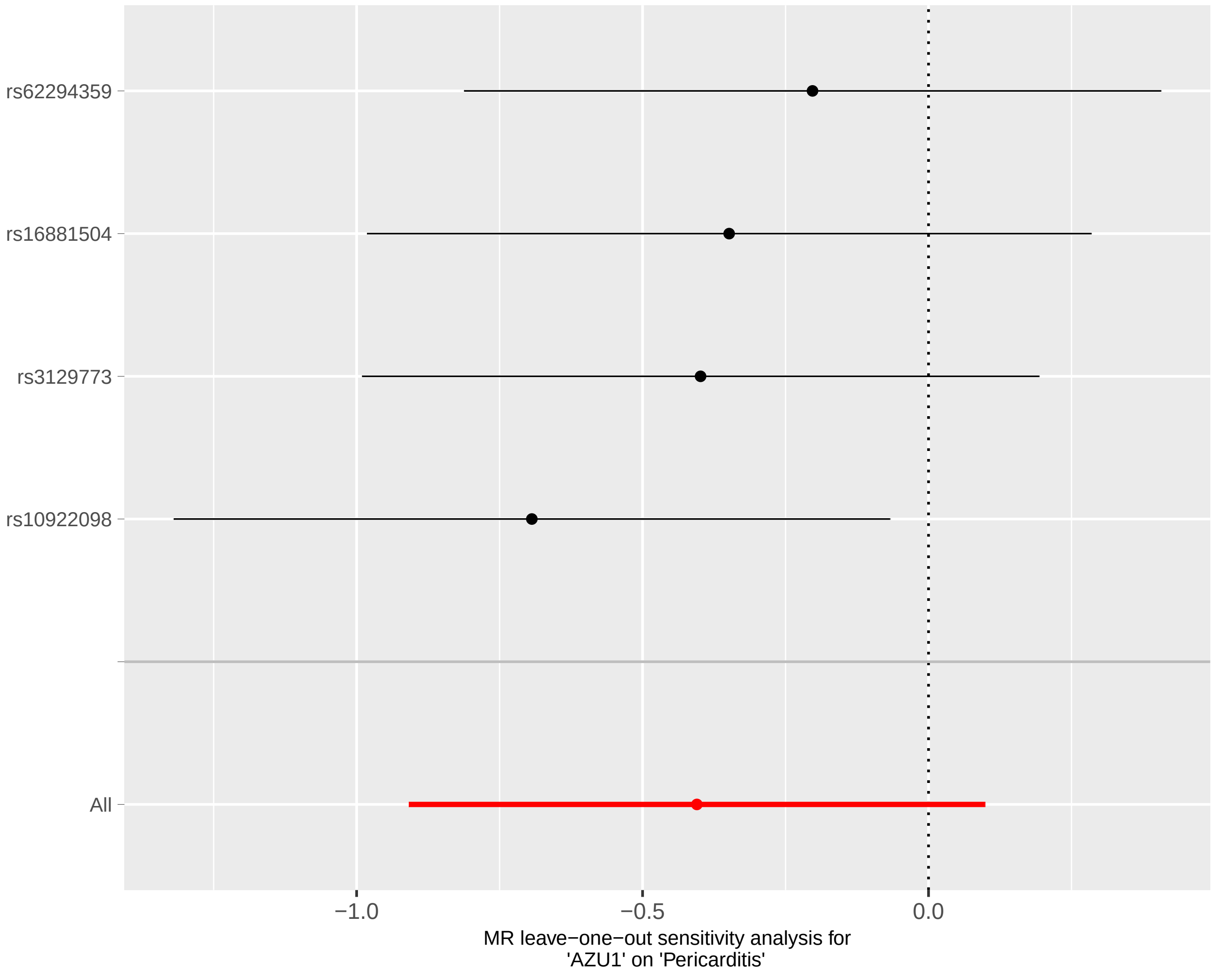

rs62294359
rs16881504
rs3129773
rs10922098
All
−1.0
−0.5
0.0
MR leave−one−out sensitivity analysis for
'AZU1' on 'Pericarditis'

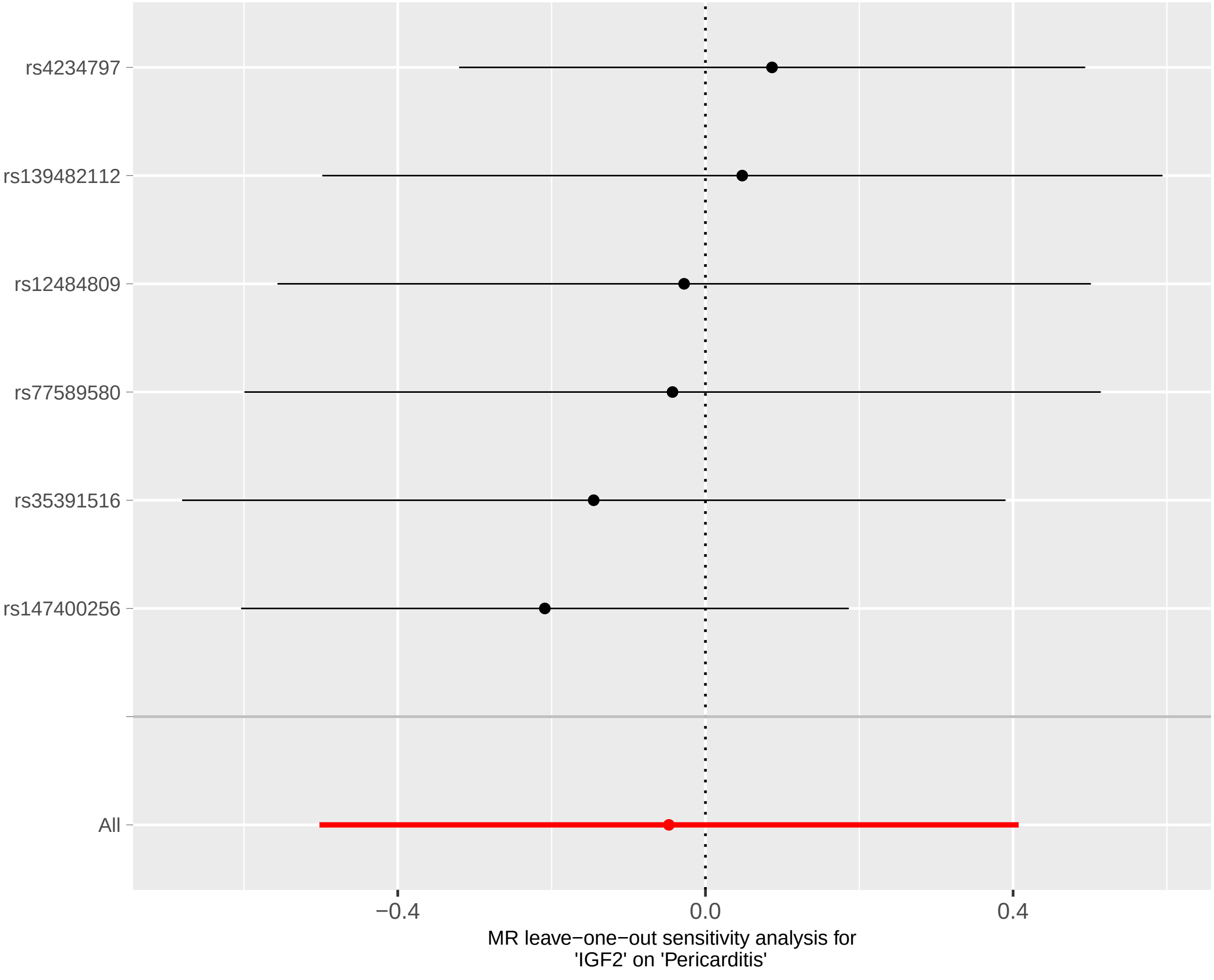

rs4234797
rs139482112
rs12484809
rs77589580
rs35391516
rs147400256
All
−0.4
0.0
0.4
MR leave−one−out sensitivity analysis for
'IGF2' on 'Pericarditis'

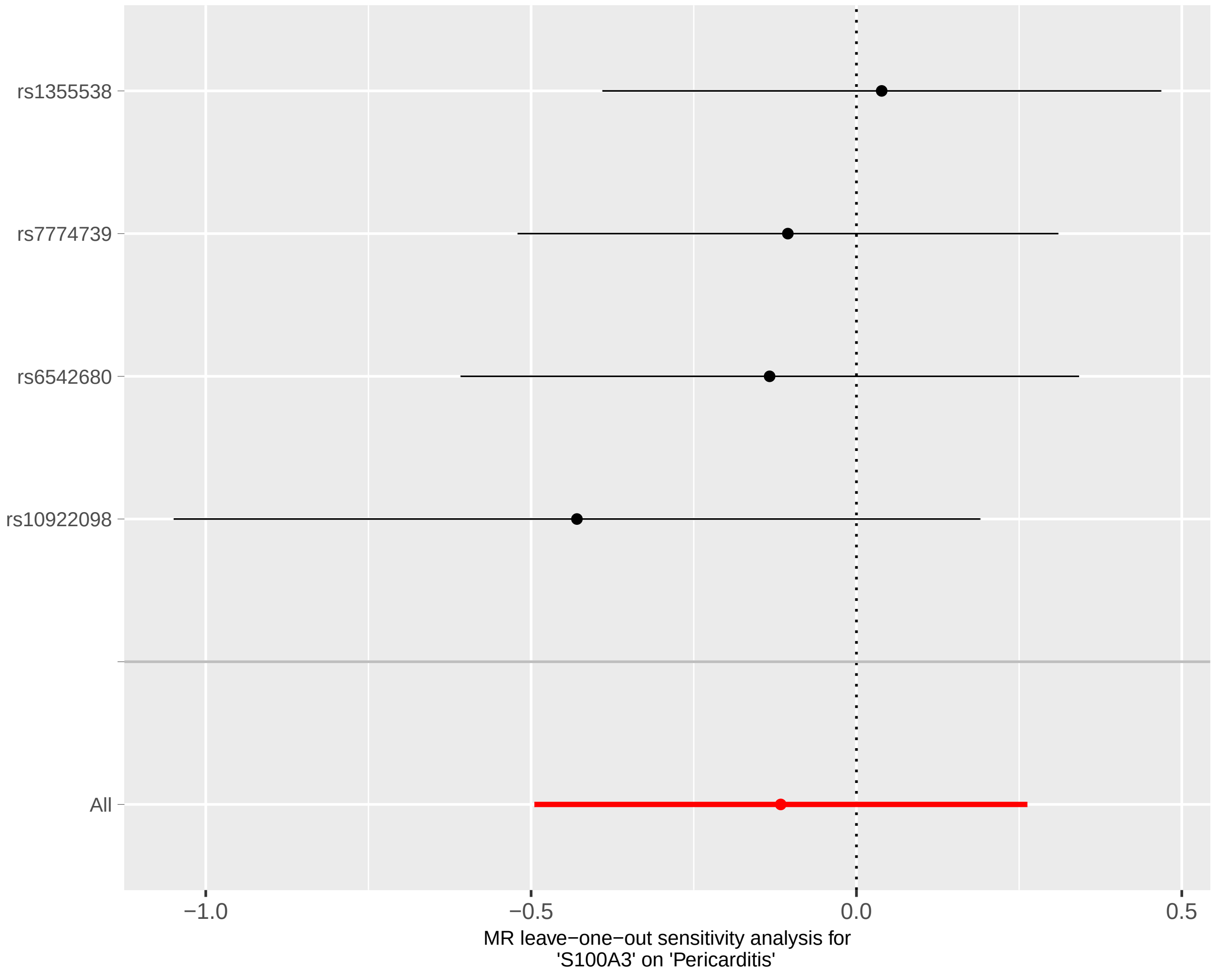

rs1355538
rs7774739
rs6542680
rs10922098
All
−1.0
−0.5
0.0
0.5
MR leave−one−out sensitivity analysis for
'S100A3' on 'Pericarditis'

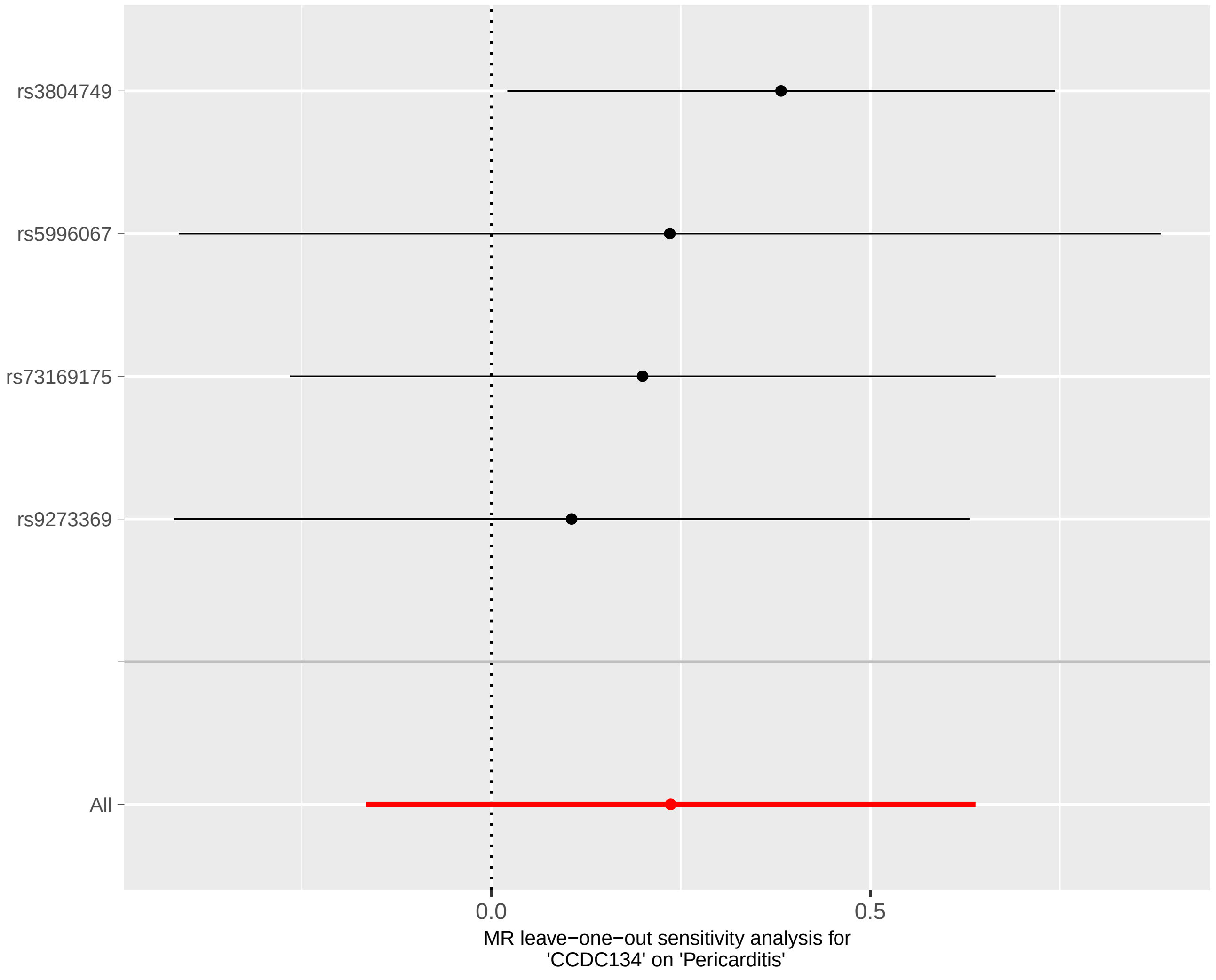

rs3804749
rs5996067
rs73169175
rs9273369
All
0.0
0.5
MR leave−one−out sensitivity analysis for
'CCDC134' on 'Pericarditis'

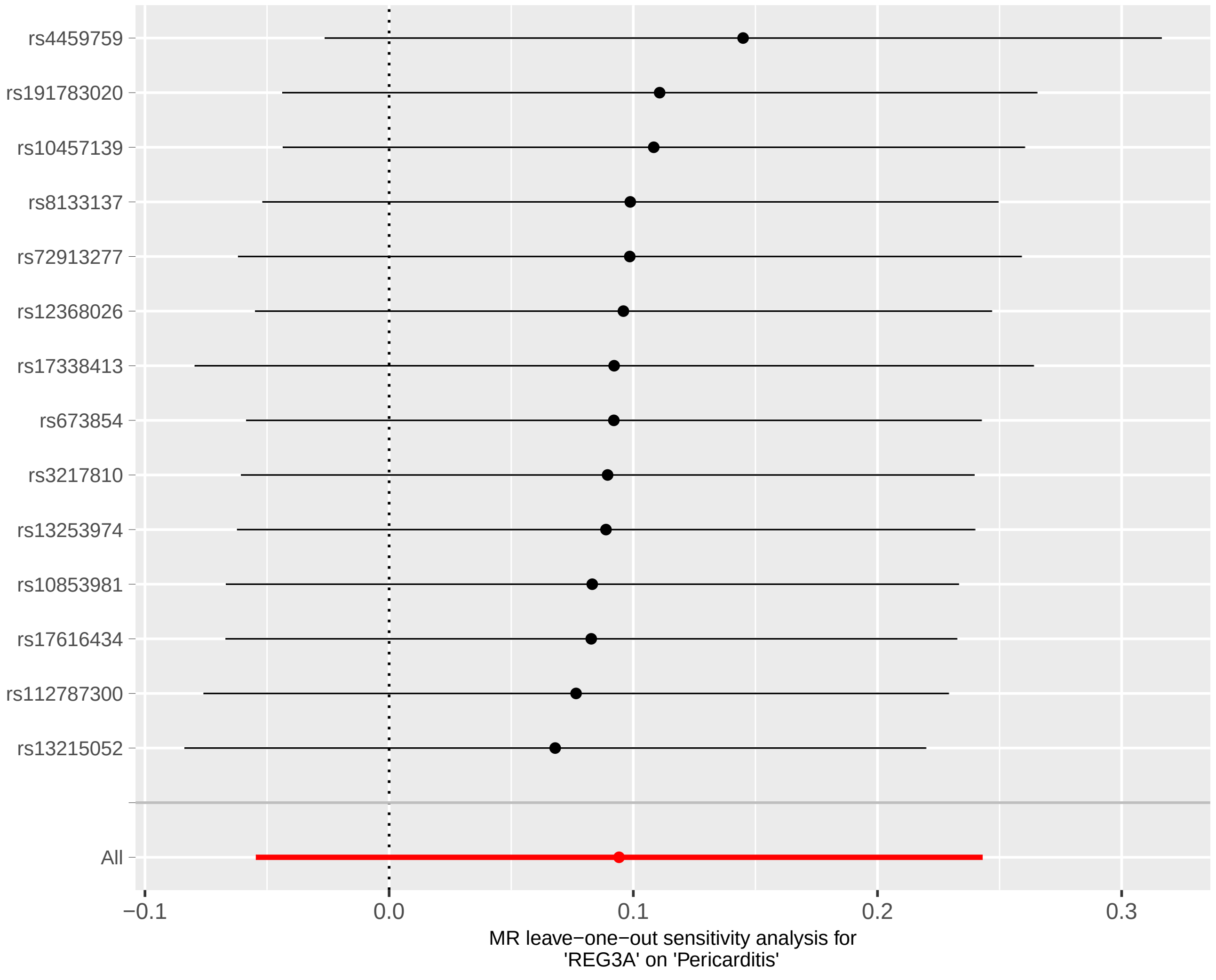

rs4459759
rs191783020
rs10457139
rs8133137
rs72913277
rs12368026
rs17338413
rs673854
rs3217810
rs13253974
rs10853981
rs17616434
rs112787300
rs13215052
All
−0.1
0.0
0.1
0.2
0.3
MR leave−one−out sensitivity analysis for
'REG3A' on 'Pericarditis'

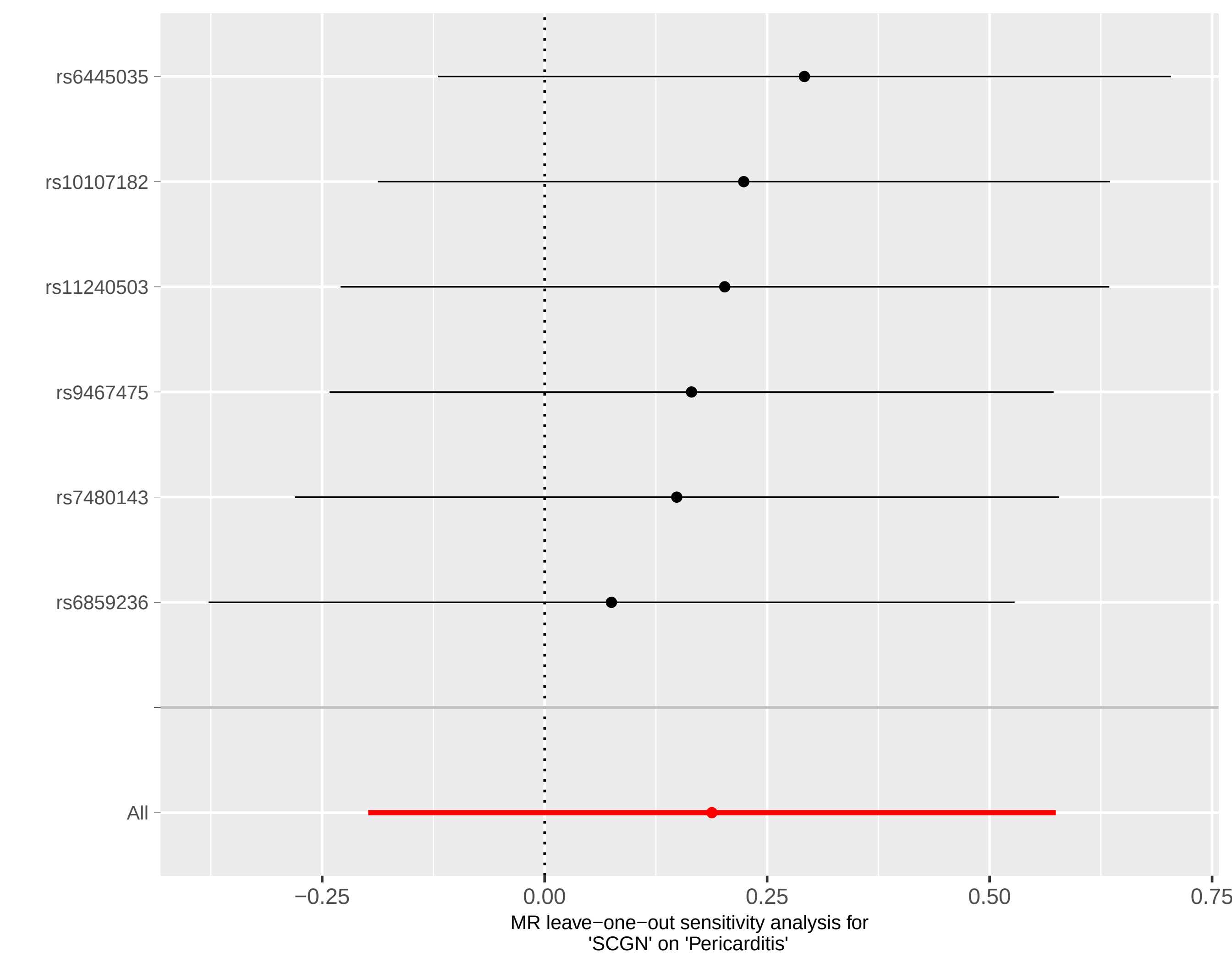
rs6445035
rs10107182
rs11240503
rs9467475
rs7480143
rs6859236
All
−0.25
0.00
0.25
0.50
0.75
MR leave−one−out sensitivity analysis for
'SCGN' on 'Pericarditis'

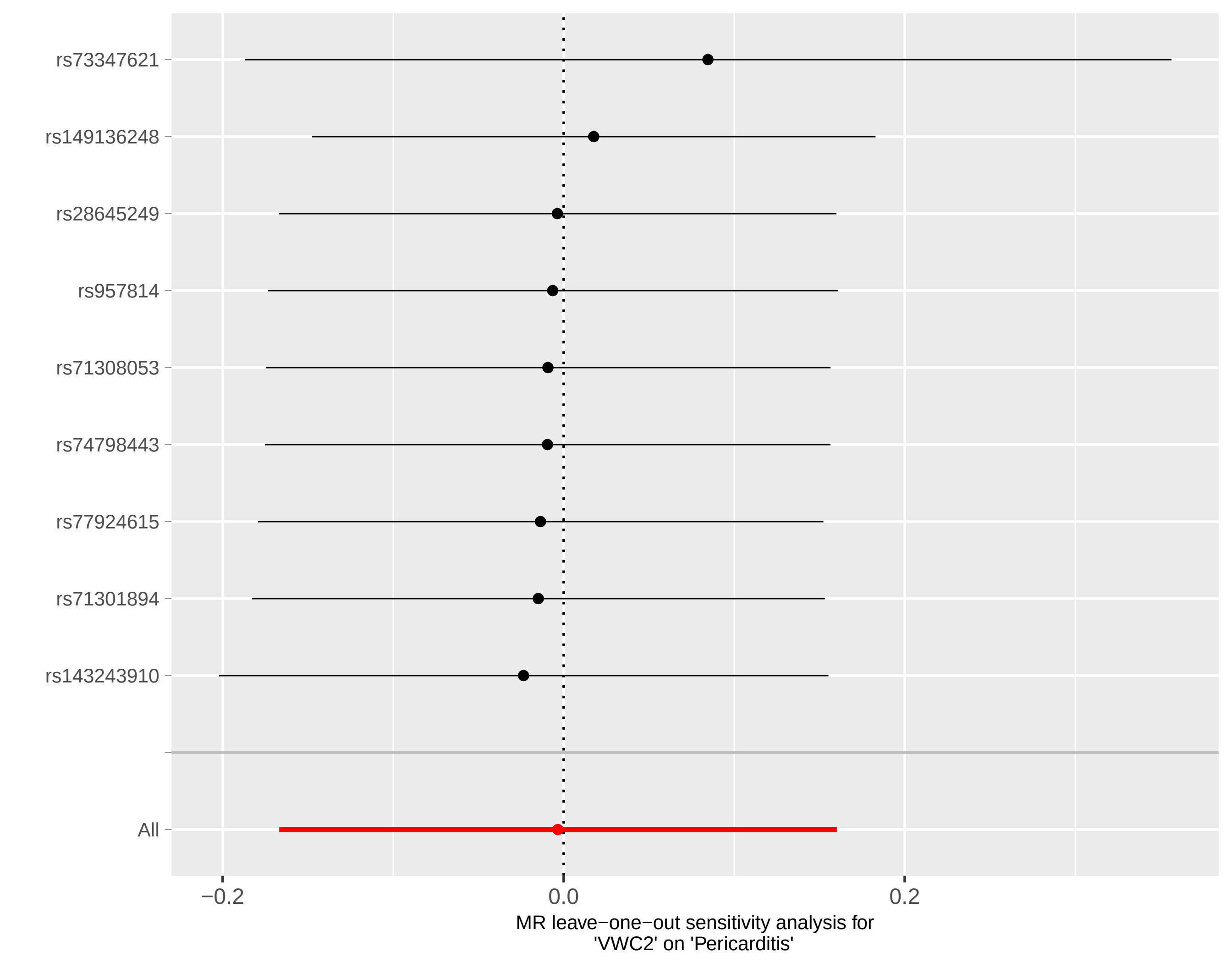
rs73347621
rs149136248
rs28645249
rs957814
rs71308053
rs74798443
rs77924615
rs71301894
rs143243910
All
−0.2
0.0
0.2
MR leave−one−out sensitivity analysis for
'VWC2' on 'Pericarditis'

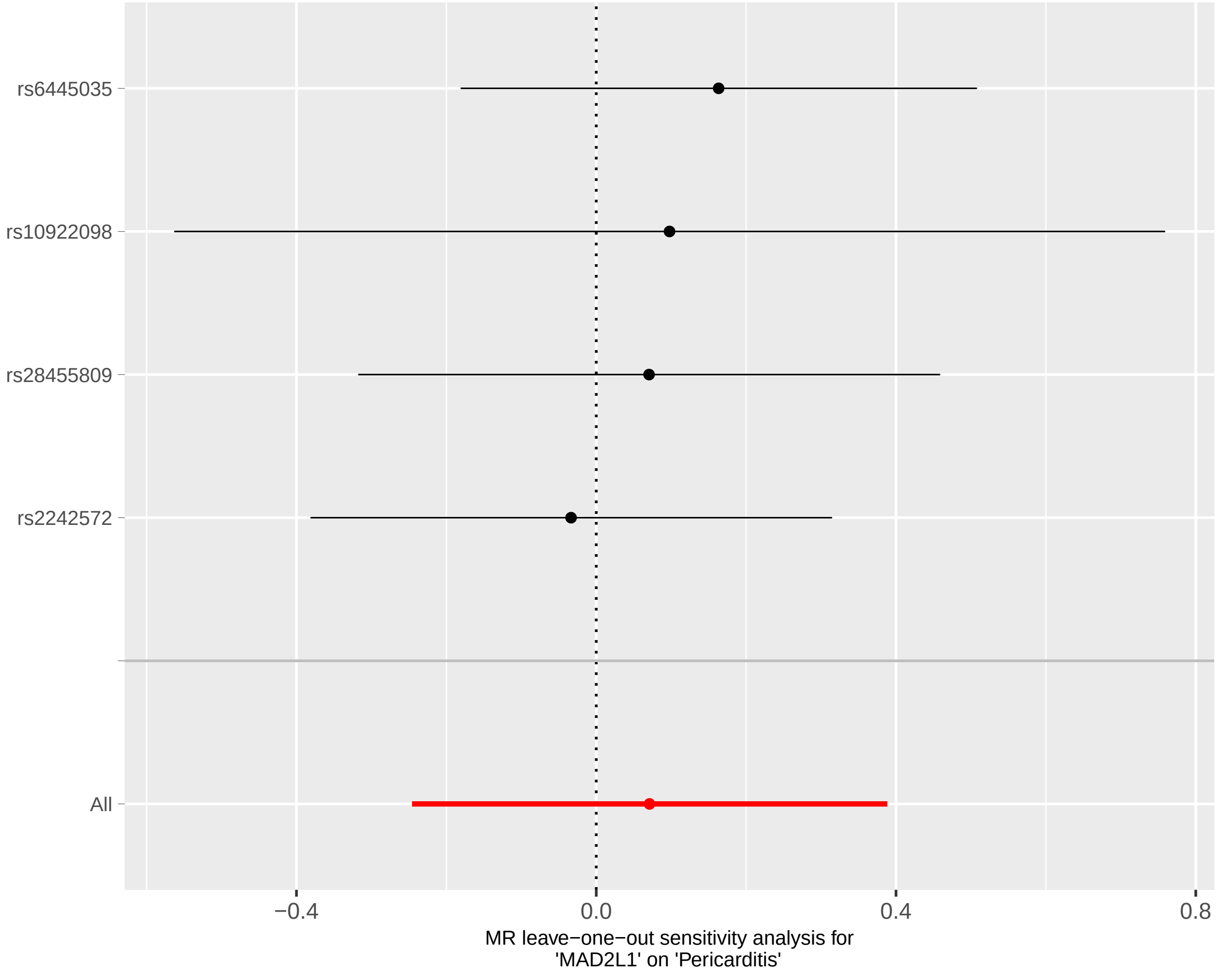
rs6445035
rs10922098
rs28455809
rs2242572
All
−0.4
0.0
0.4
0.8
MR leave−one−out sensitivity analysis for
'MAD2L1' on 'Pericarditis'

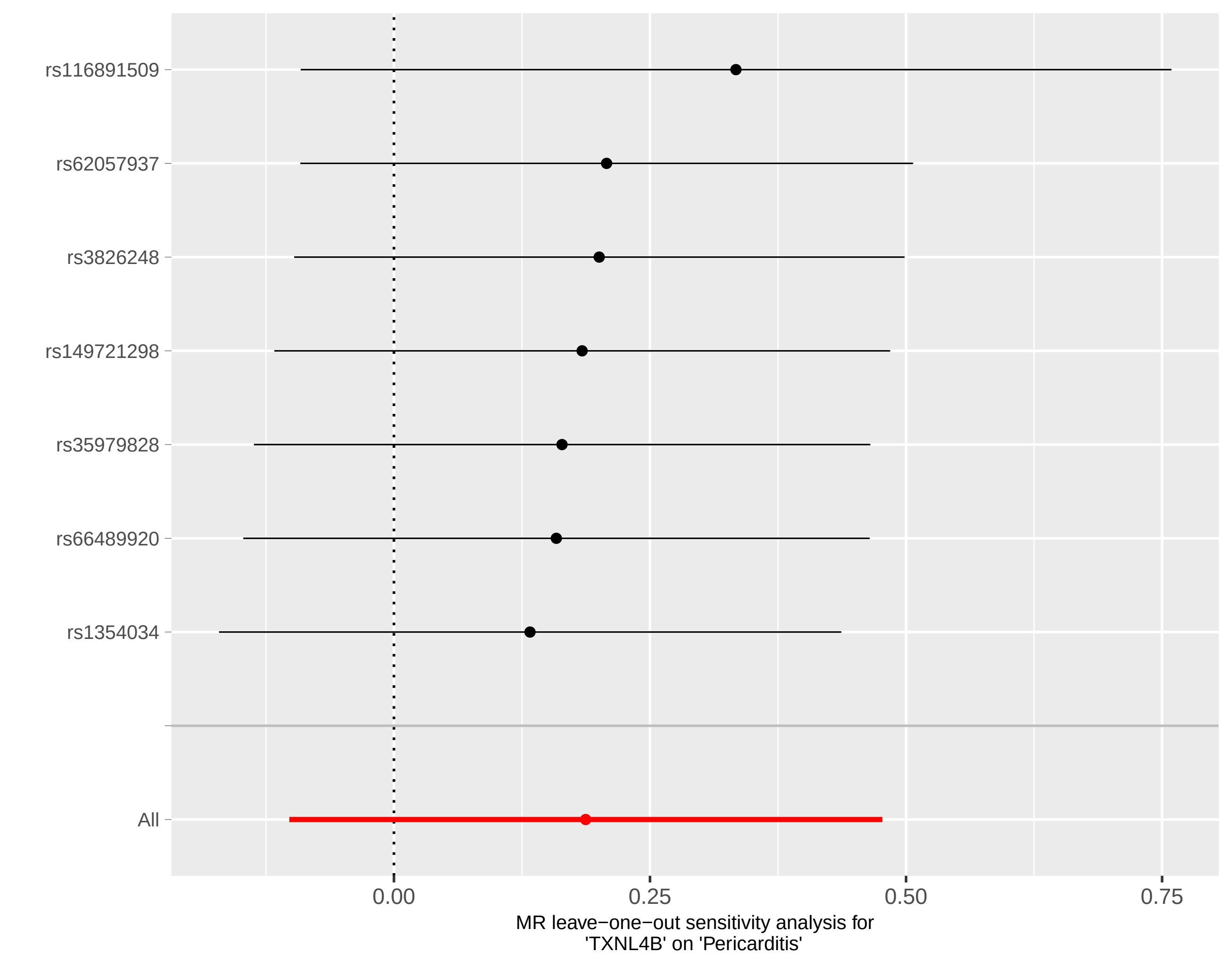

rs116891509
rs62057937
rs3826248
rs149721298
rs35979828
rs66489920
rs1354034
All
0.00
0.25
0.50
0.75
MR leave−one−out sensitivity analysis for
'TXNL4B' on 'Pericarditis'

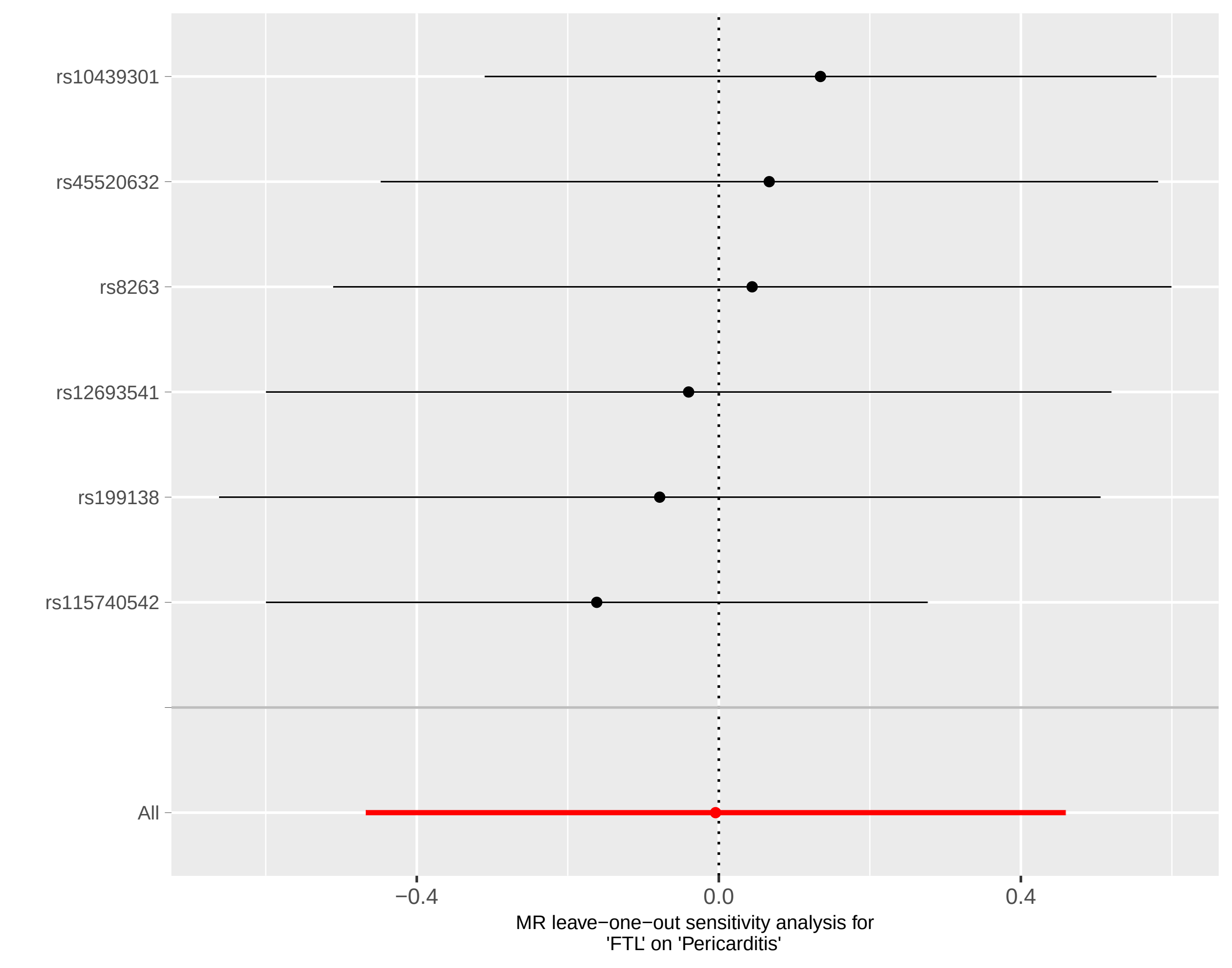
rs10439301
rs45520632
rs8263
rs12693541
rs199138
rs115740542
All
−0.4
0.0
0.4
MR leave−one−out sensitivity analysis for
'FTL' on 'Pericarditis'

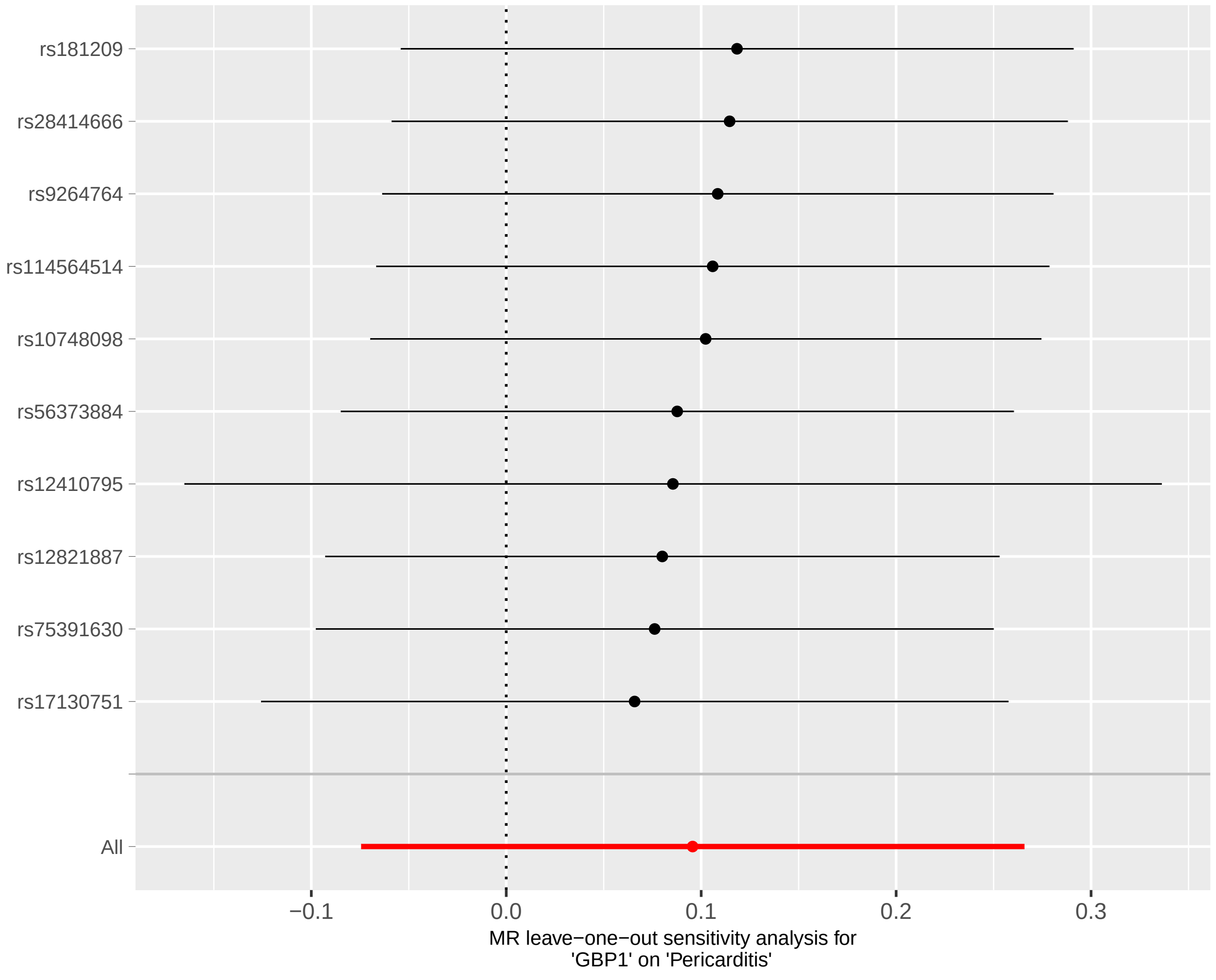
rs181209
rs28414666
rs9264764
rs114564514
rs10748098
rs56373884
rs12410795
rs12821887
rs75391630
rs17130751
All
−0.1
0.0
0.1
0.2
0.3
MR leave−one−out sensitivity analysis for
'GBP1' on 'Pericarditis'

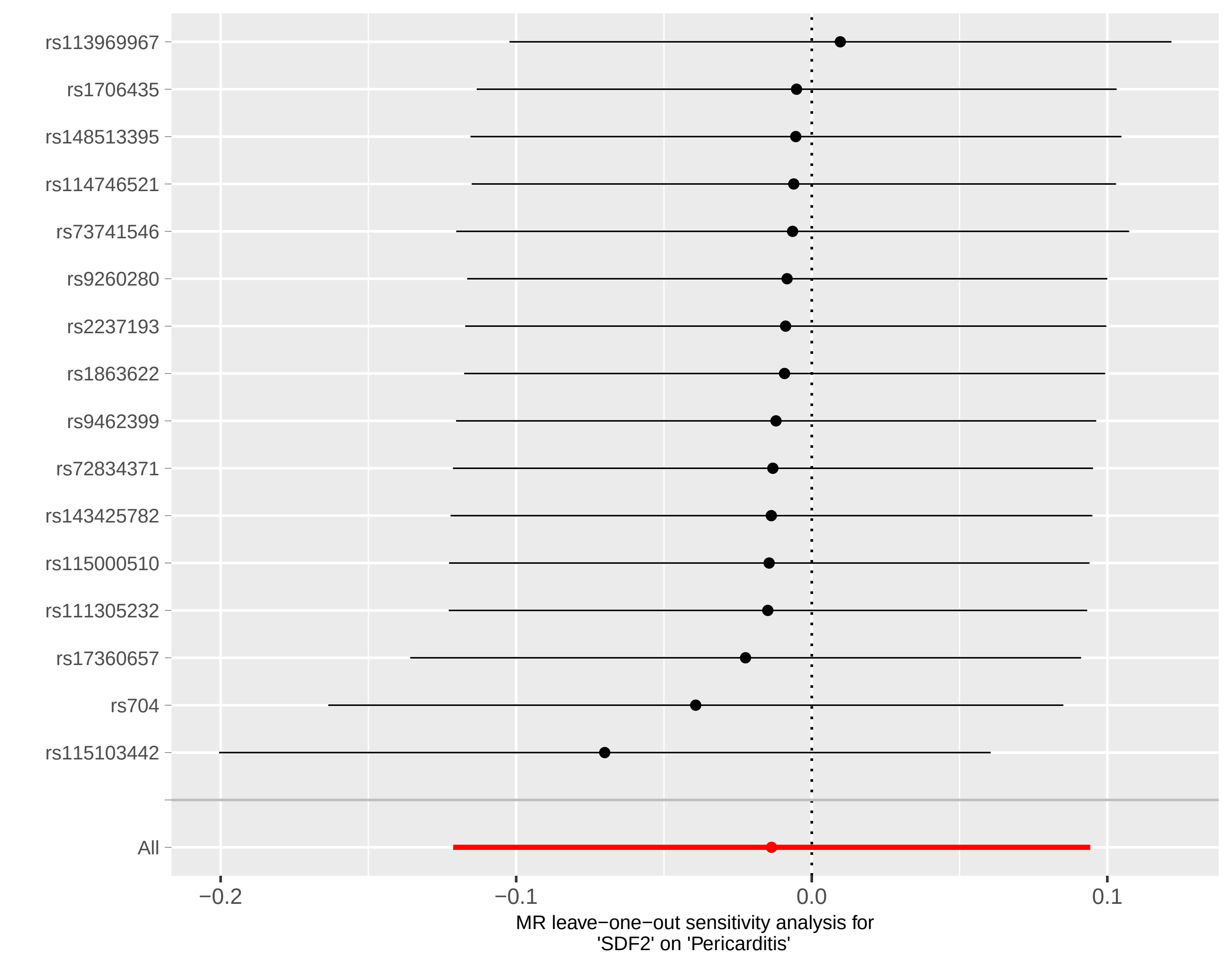

rs113969967
rs1706435
rs148513395
rs114746521
rs73741546
rs9260280
rs2237193
rs1863622
rs9462399
rs72834371
rs143425782
rs115000510
rs111305232
rs17360657
rs704
rs115103442
All
−0.2
−0.1
0.0
0.1
MR leave−one−out sensitivity analysis for
'SDF2' on 'Pericarditis'

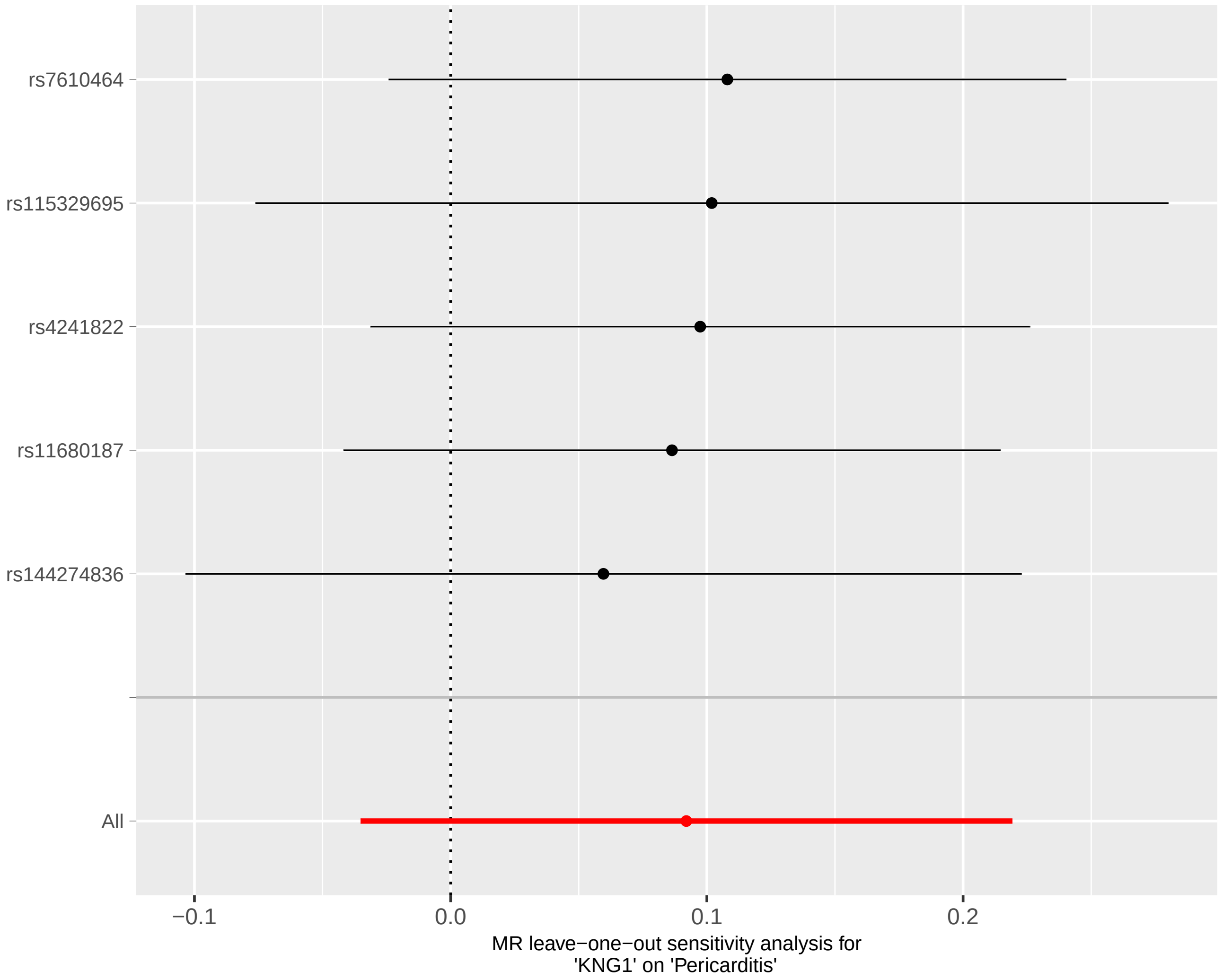
rs7610464
rs115329695
rs4241822
rs11680187
rs144274836
All
−0.1
0.0
0.1
0.2
MR leave−one−out sensitivity analysis for
'KNG1' on 'Pericarditis'

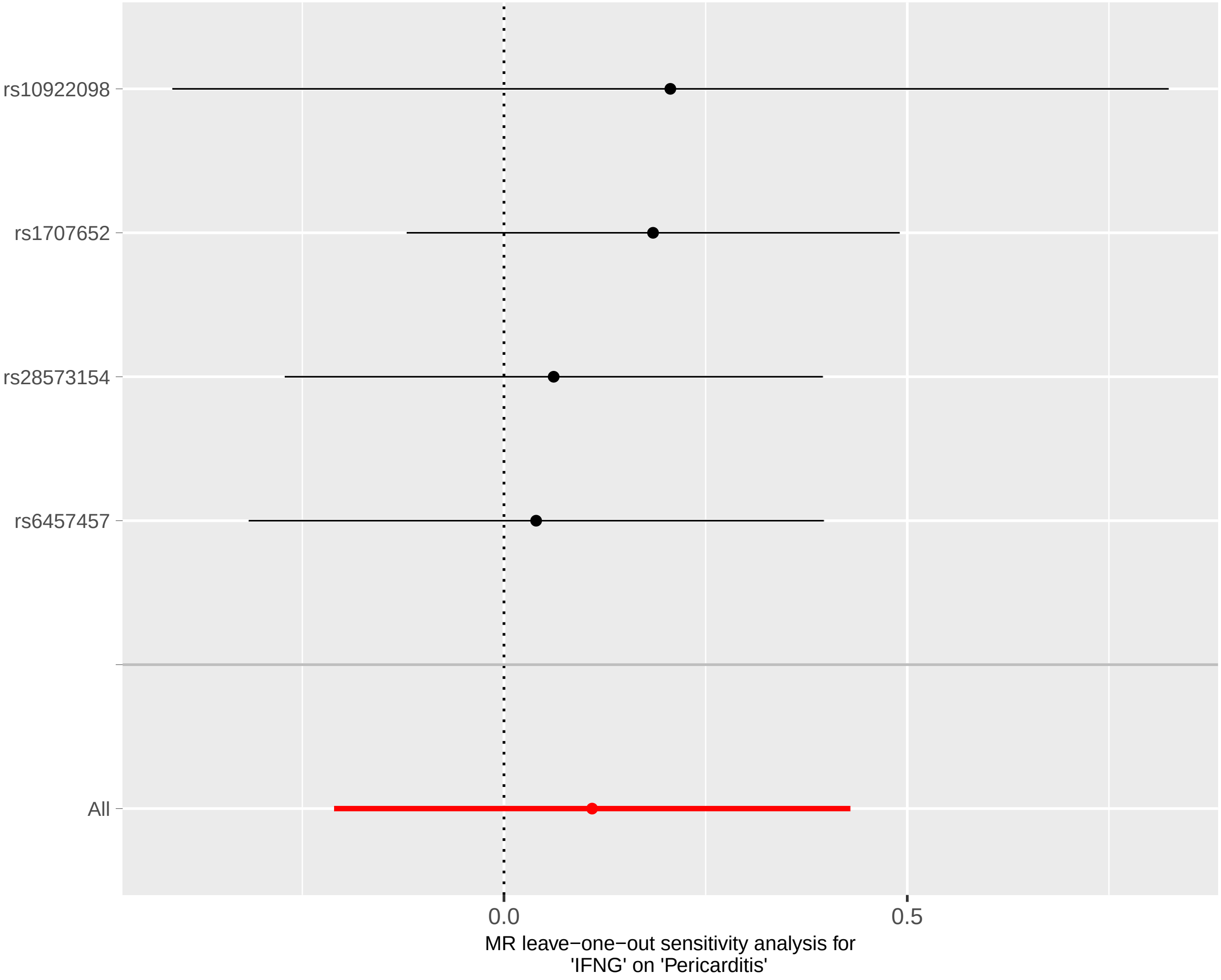

rs10922098
rs1707652
rs28573154
rs6457457
All
0.0
0.5
MR leave−one−out sensitivity analysis for
'IFNG' on 'Pericarditis'

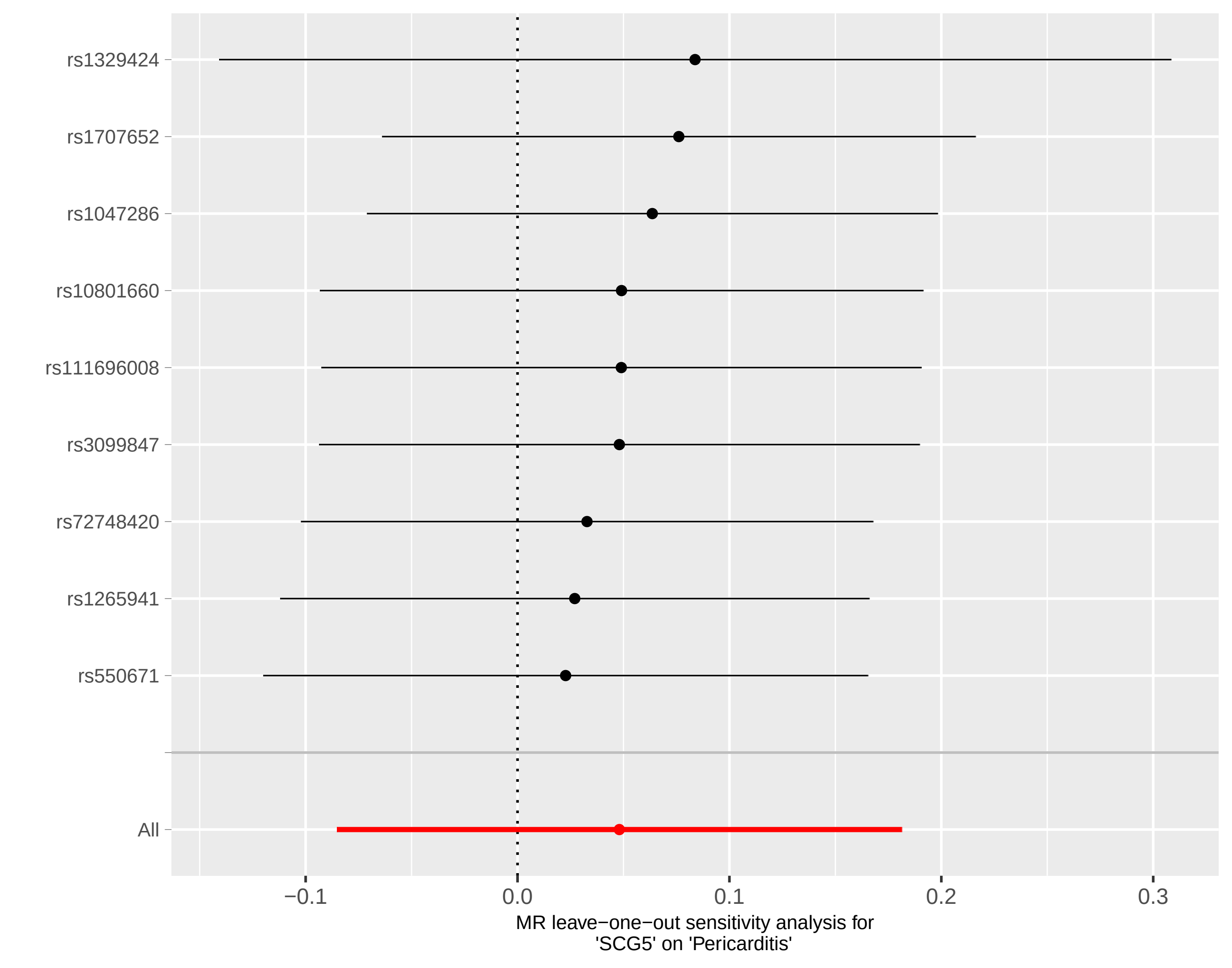

rs1329424
rs1707652
rs1047286
rs10801660
rs111696008
rs3099847
rs72748420
rs1265941
rs550671
All
−0.1
0.0
0.1
0.2
0.3
MR leave−one−out sensitivity analysis for
'SCG5' on 'Pericarditis'

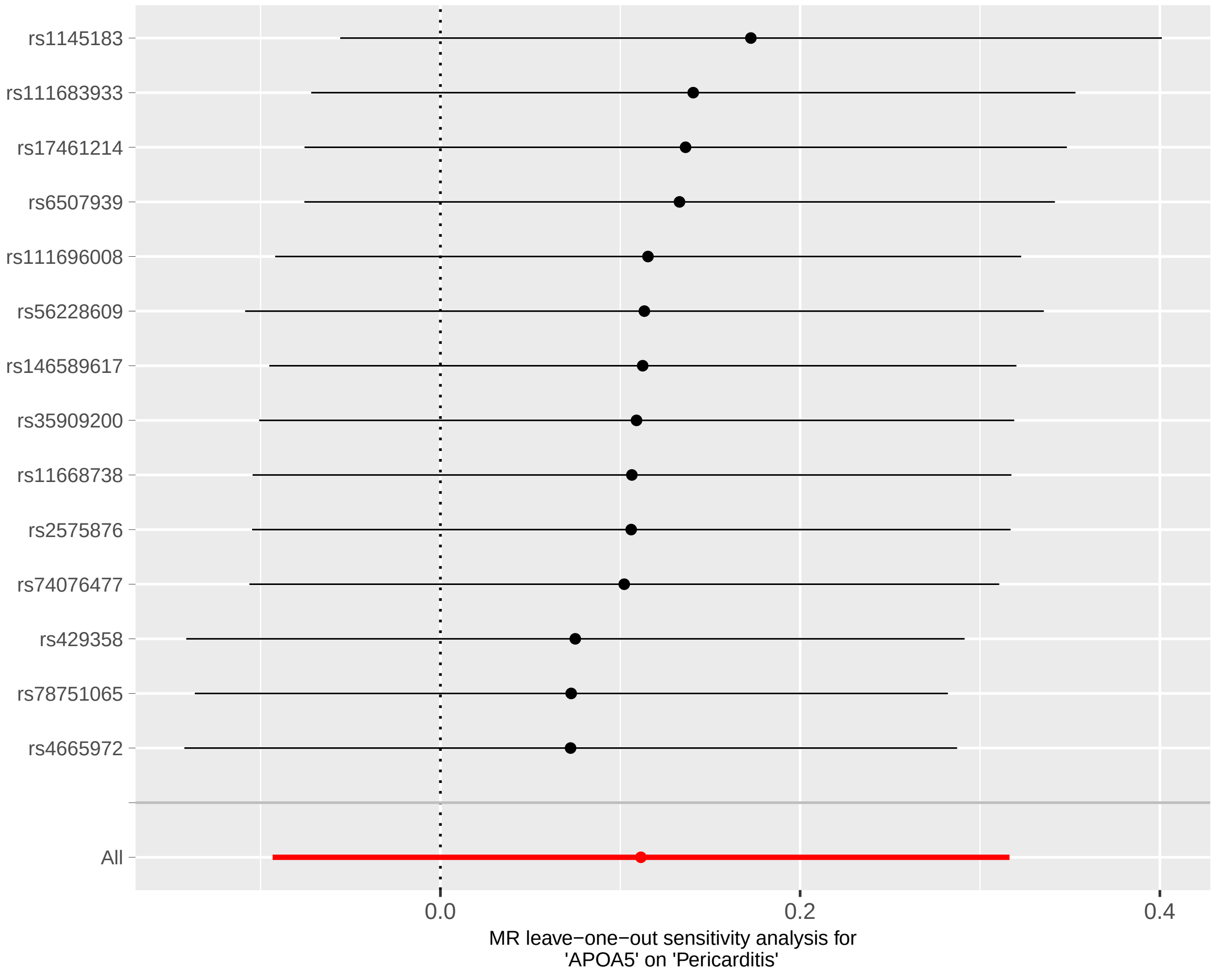

rs1145183
rs111683933
rs17461214
rs6507939
rs111696008
rs56228609
rs146589617
rs35909200
rs11668738
rs2575876
rs74076477
rs429358
rs78751065
rs4665972
All
0.0
0.2
0.4
MR leave−one−out sensitivity analysis for
'APOA5' on 'Pericarditis'

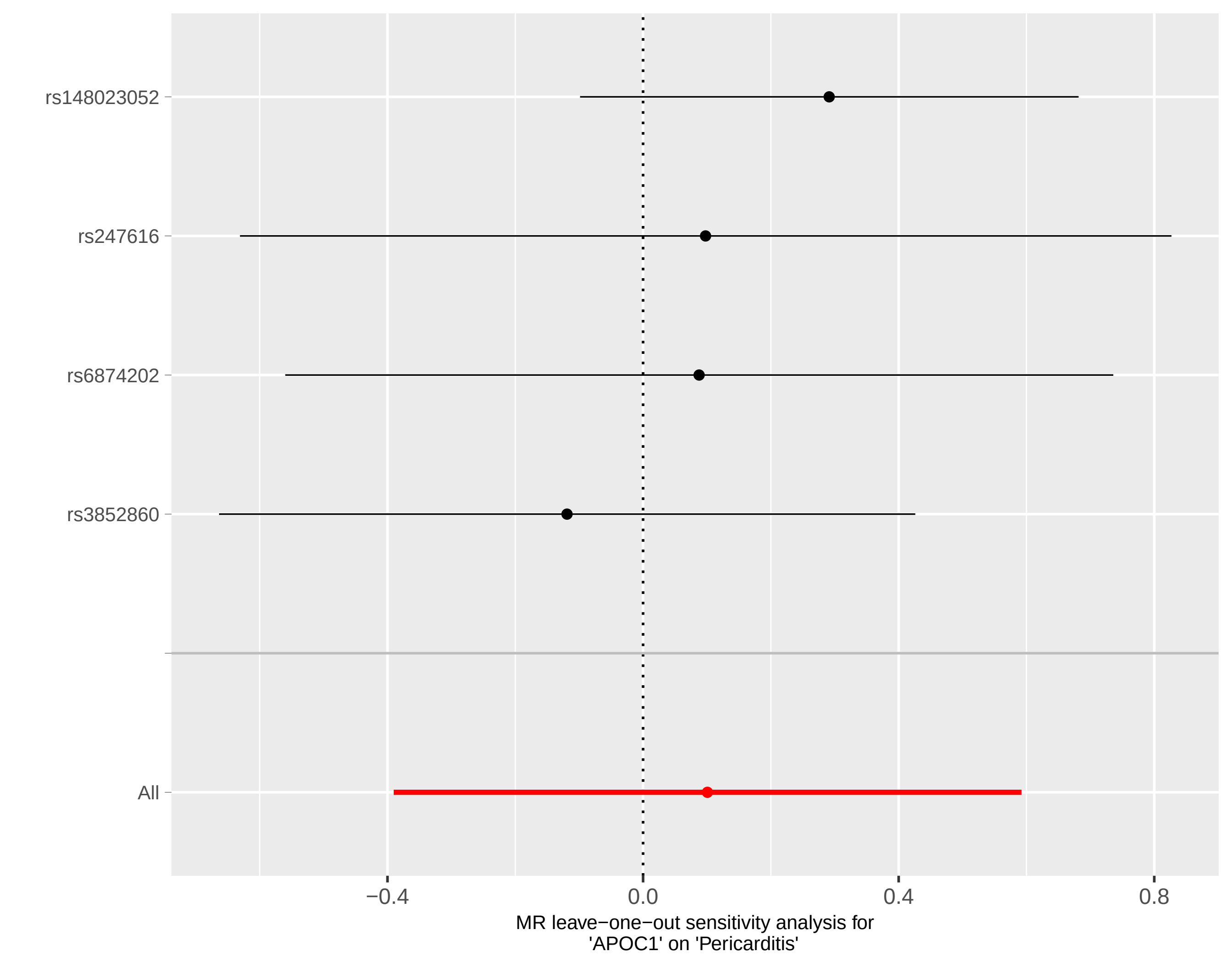
rs148023052
rs247616
rs6874202
rs3852860
All
−0.4
0.0
0.4
0.8
MR leave−one−out sensitivity analysis for
'APOC1' on 'Pericarditis'

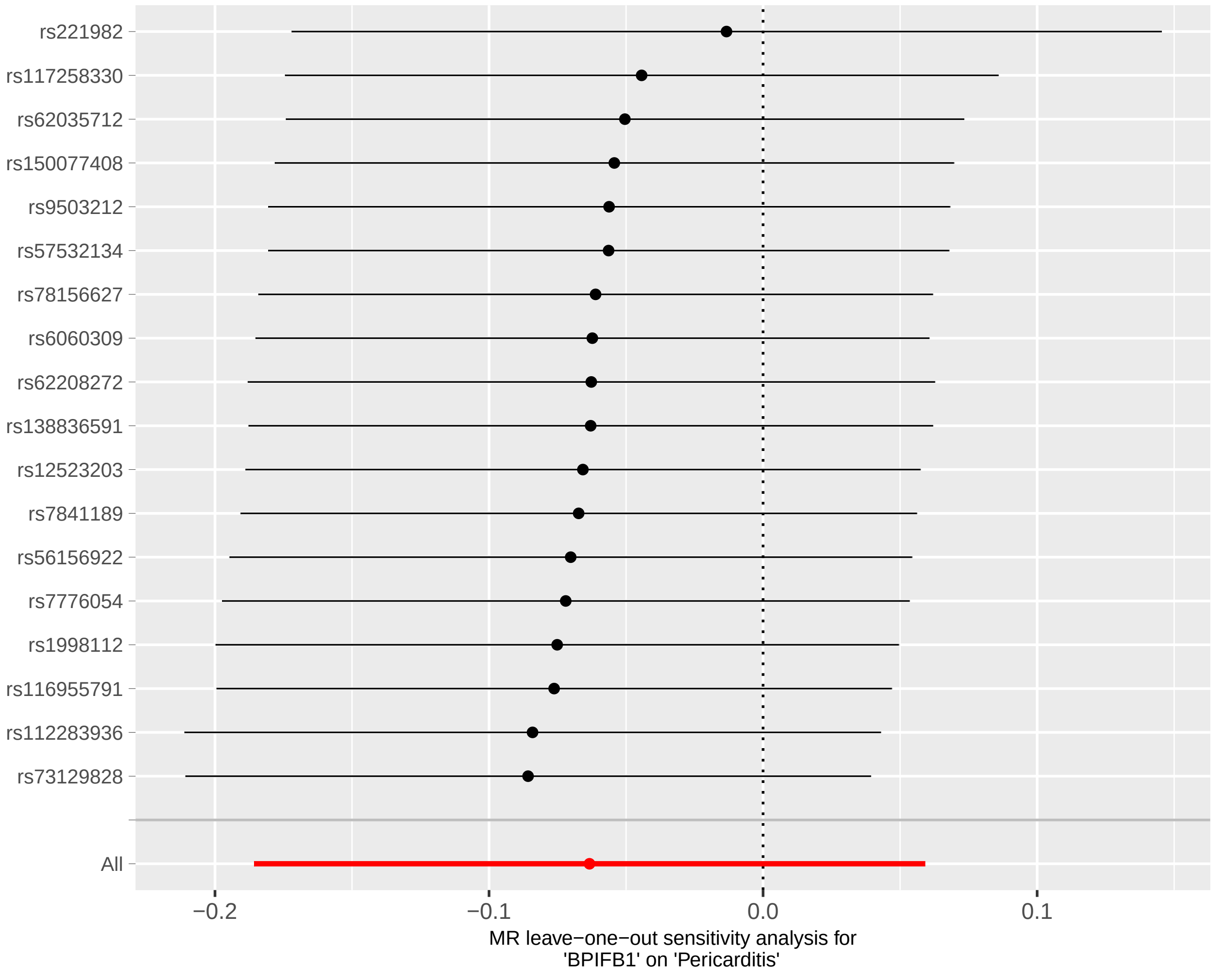
rs221982
rs117258330
rs62035712
rs150077408
rs9503212
rs57532134
rs78156627
rs6060309
rs62208272
rs138836591
rs12523203
rs7841189
rs56156922
rs7776054
rs1998112
rs116955791
rs112283936
rs73129828
All
−0.2
−0.1
0.0
0.1
MR leave−one−out sensitivity analysis for
'BPIFB1' on 'Pericarditis'

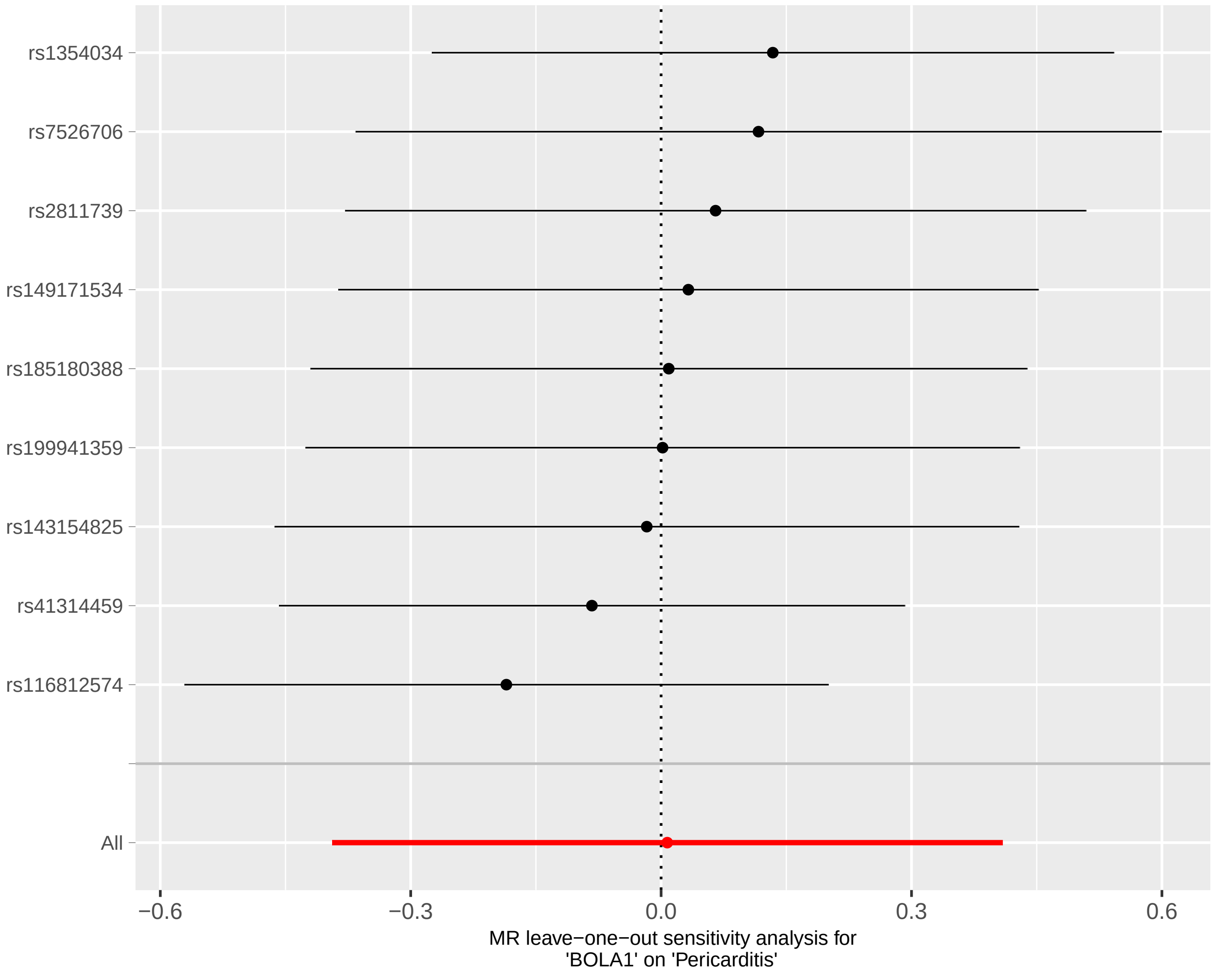
rs1354034
rs7526706
rs2811739
rs149171534
rs185180388
rs199941359
rs143154825
rs41314459
rs116812574
All
−0.6
−0.3
0.0
0.3
0.6
MR leave−one−out sensitivity analysis for
'BOLA1' on 'Pericarditis'

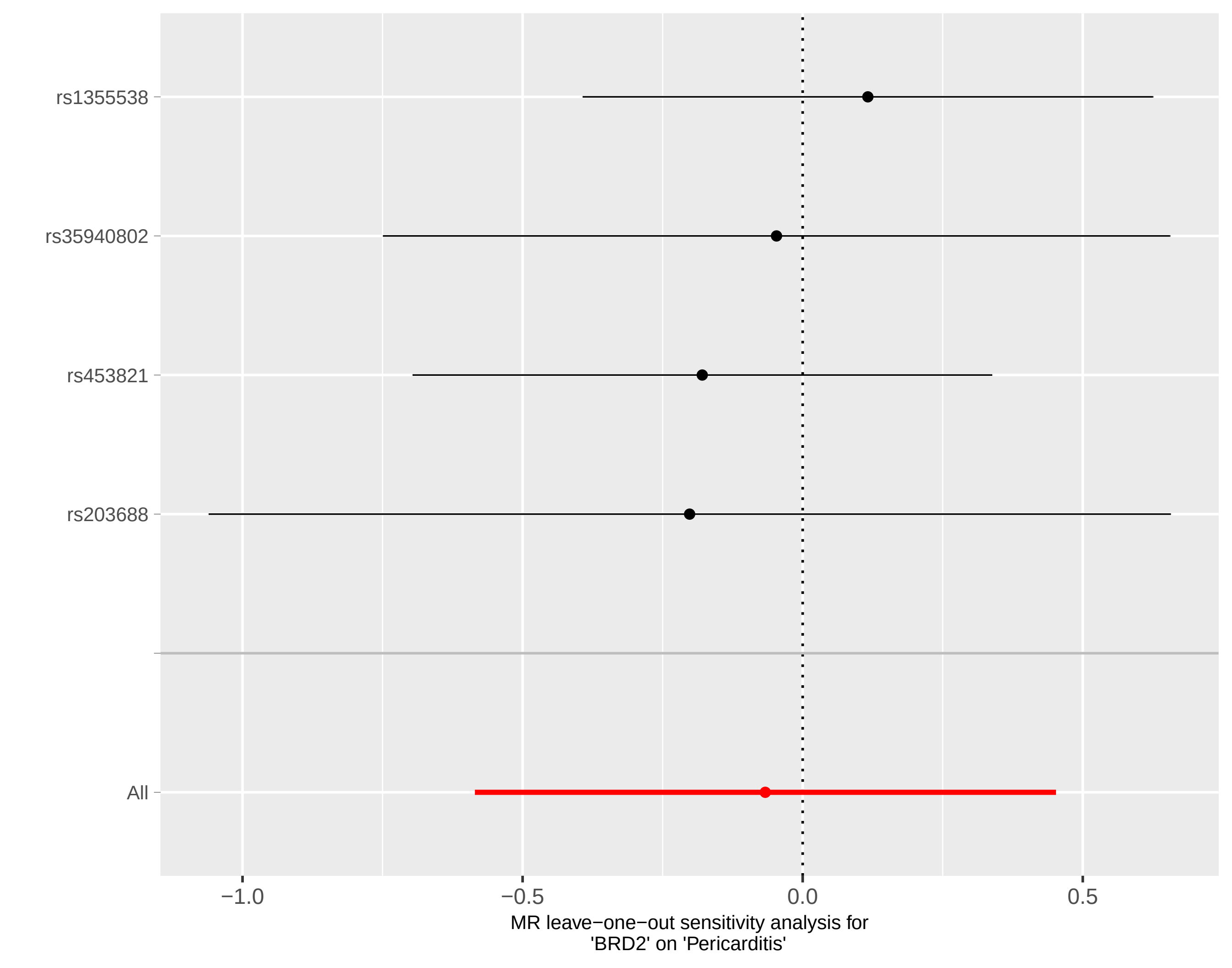
rs1355538
rs35940802
rs453821
rs203688
All
−1.0
−0.5
0.0
0.5
MR leave−one−out sensitivity analysis for
'BRD2' on 'Pericarditis'

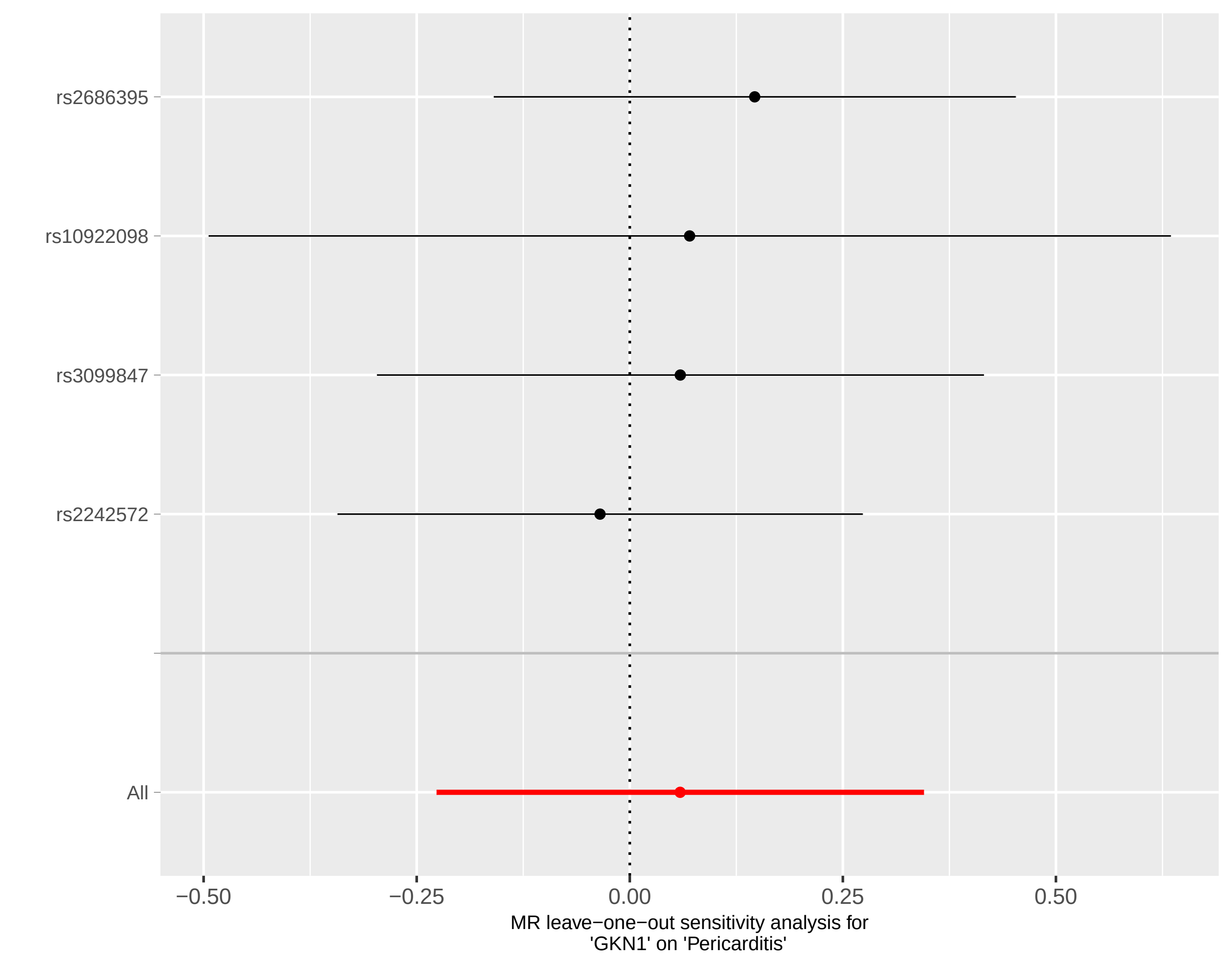
rs2686395
rs10922098
rs3099847
rs2242572
All
−0.50
−0.25
0.00
0.25
0.50
MR leave−one−out sensitivity analysis for
'GKN1' on 'Pericarditis'

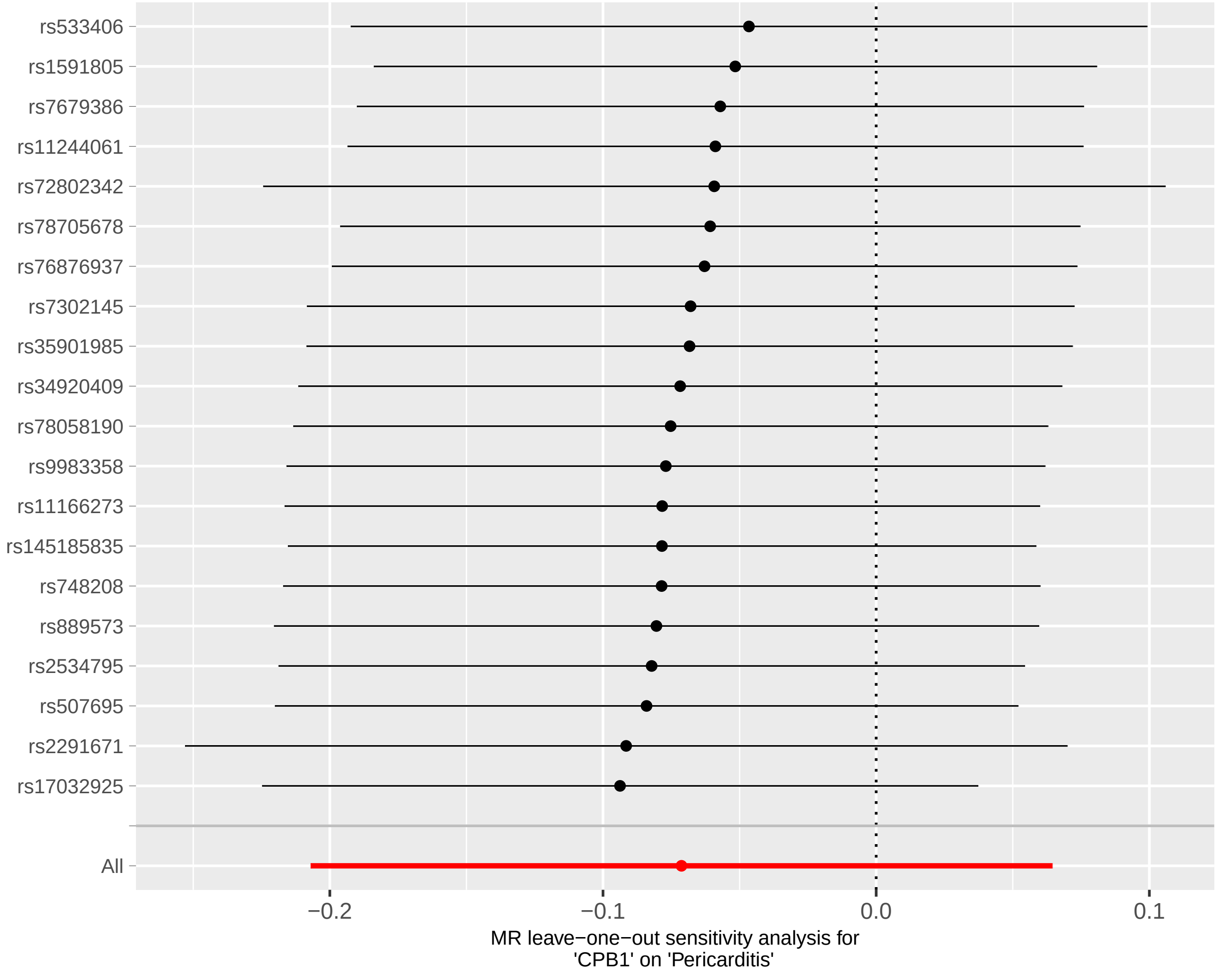

rs533406
rs1591805
rs7679386
rs11244061
rs72802342
rs78705678
rs76876937
rs7302145
rs35901985
rs34920409
rs78058190
rs9983358
rs11166273
rs145185835
rs748208
rs889573
rs2534795
rs507695
rs2291671
rs17032925
All
−0.2
−0.1
0.0
0.1
MR leave−one−out sensitivity analysis for
'CPB1' on 'Pericarditis'

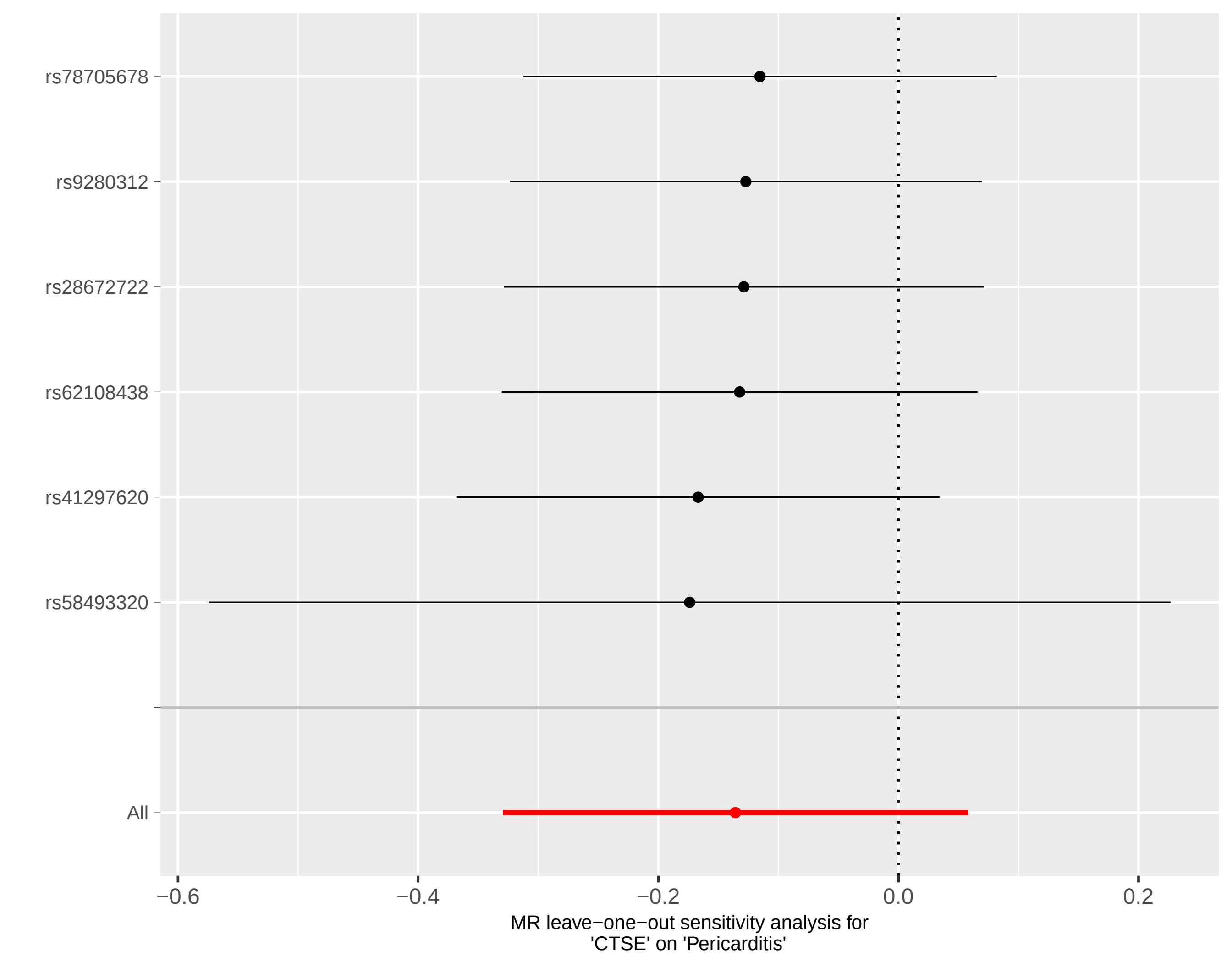

rs78705678
rs9280312
rs28672722
rs62108438
rs41297620
rs58493320
All
−0.6
−0.4
−0.2
0.0
0.2
MR leave−one−out sensitivity analysis for
'CTSE' on 'Pericarditis'

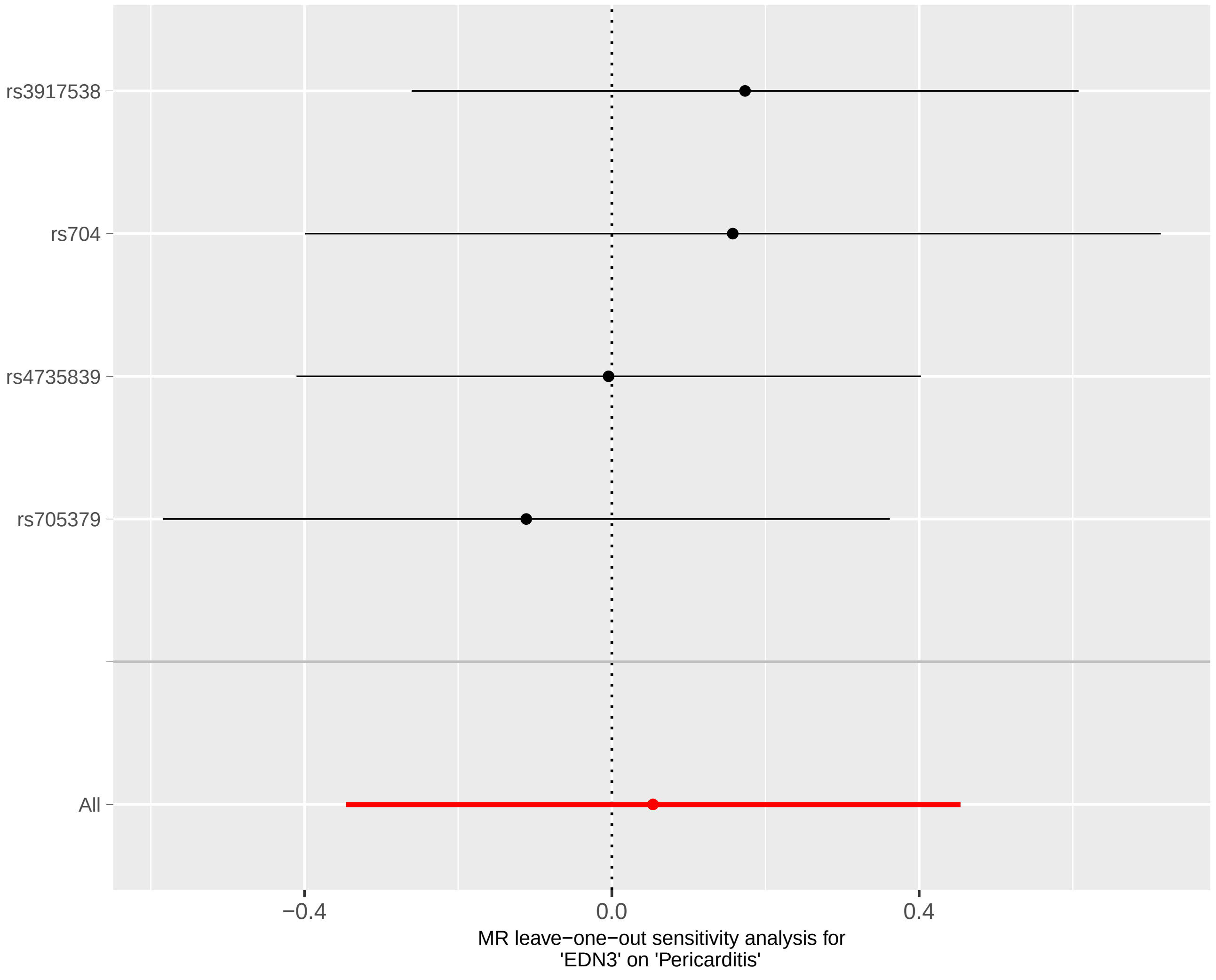

rs3917538
rs704
rs4735839
rs705379
All
−0.4
0.0
0.4
MR leave−one−out sensitivity analysis for
'EDN3' on 'Pericarditis'

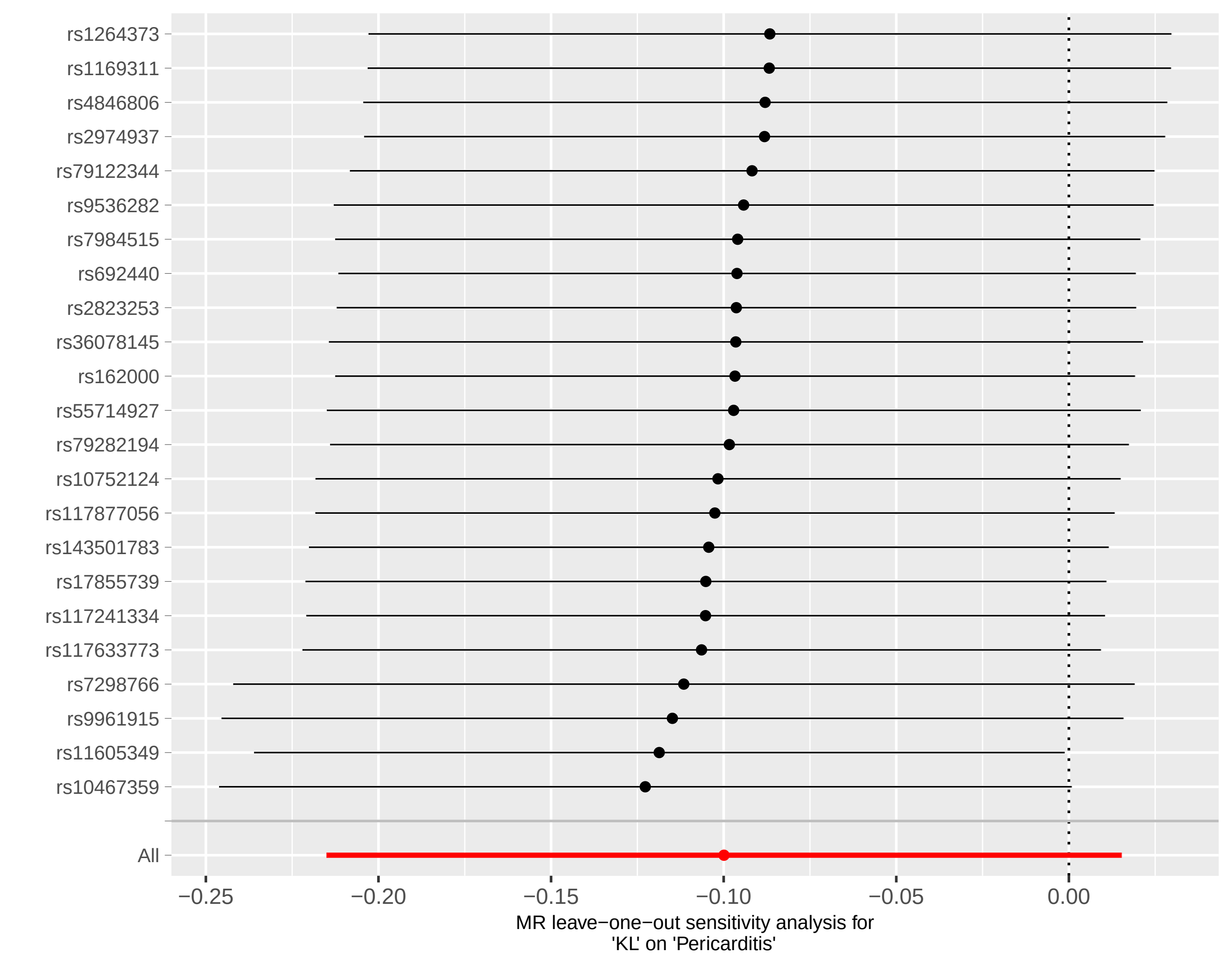
rs1264373
rs1169311
rs4846806
rs2974937
rs79122344
rs9536282
rs7984515
rs692440
rs2823253
rs36078145
rs162000
rs55714927
rs79282194
rs10752124
rs117877056
rs143501783
rs17855739
rs117241334
rs117633773
rs7298766
rs9961915
rs11605349
rs10467359
All
−0.25
−0.20
−0.15
−0.10
−0.05
0.00
MR leave−one−out sensitivity analysis for
'KL' on 'Pericarditis'

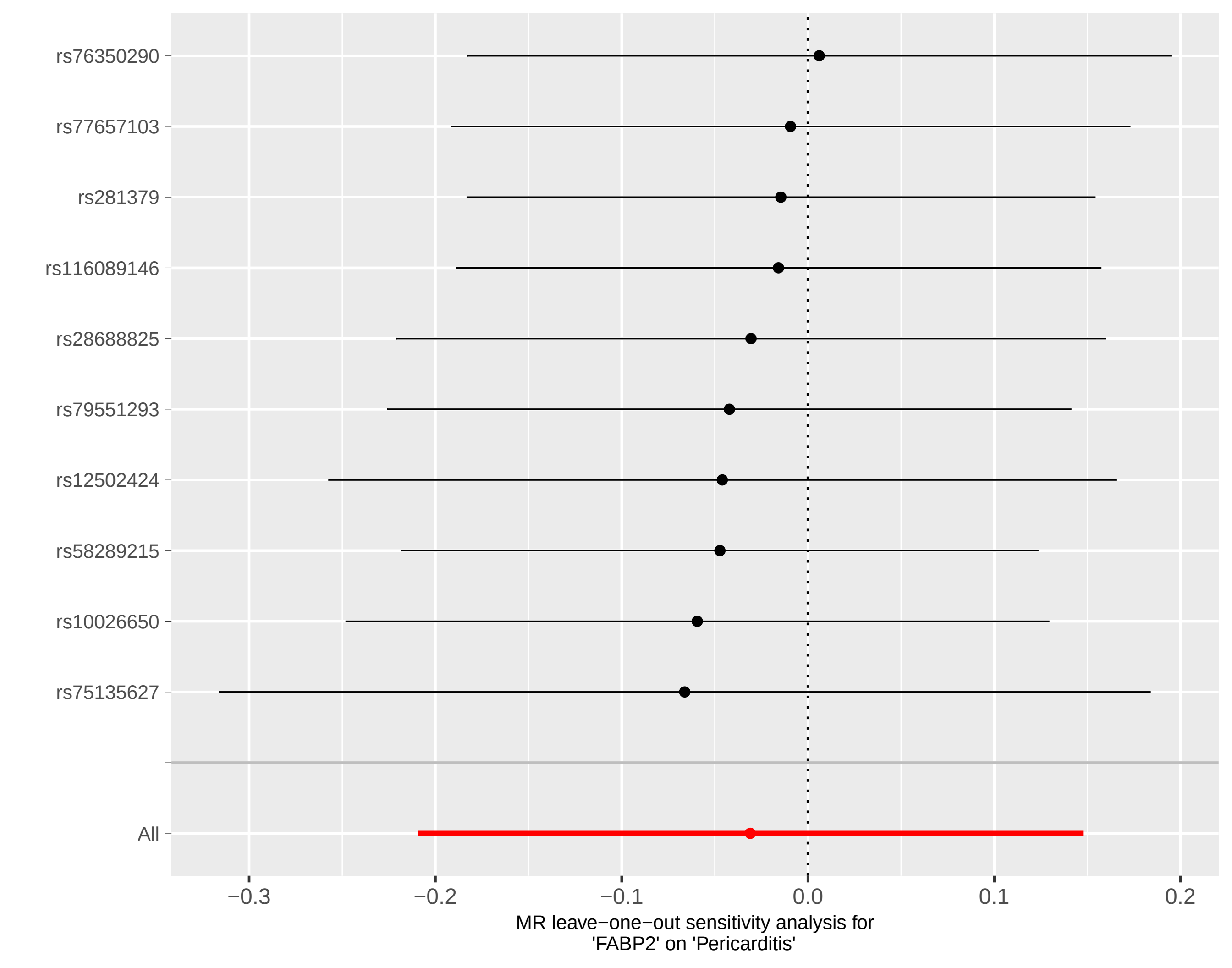

rs76350290
rs77657103
rs281379
rs116089146
rs28688825
rs79551293
rs12502424
rs58289215
rs10026650
rs75135627
All
−0.3
−0.2
−0.1
0.0
0.1
0.2
MR leave−one−out sensitivity analysis for
'FABP2' on 'Pericarditis'

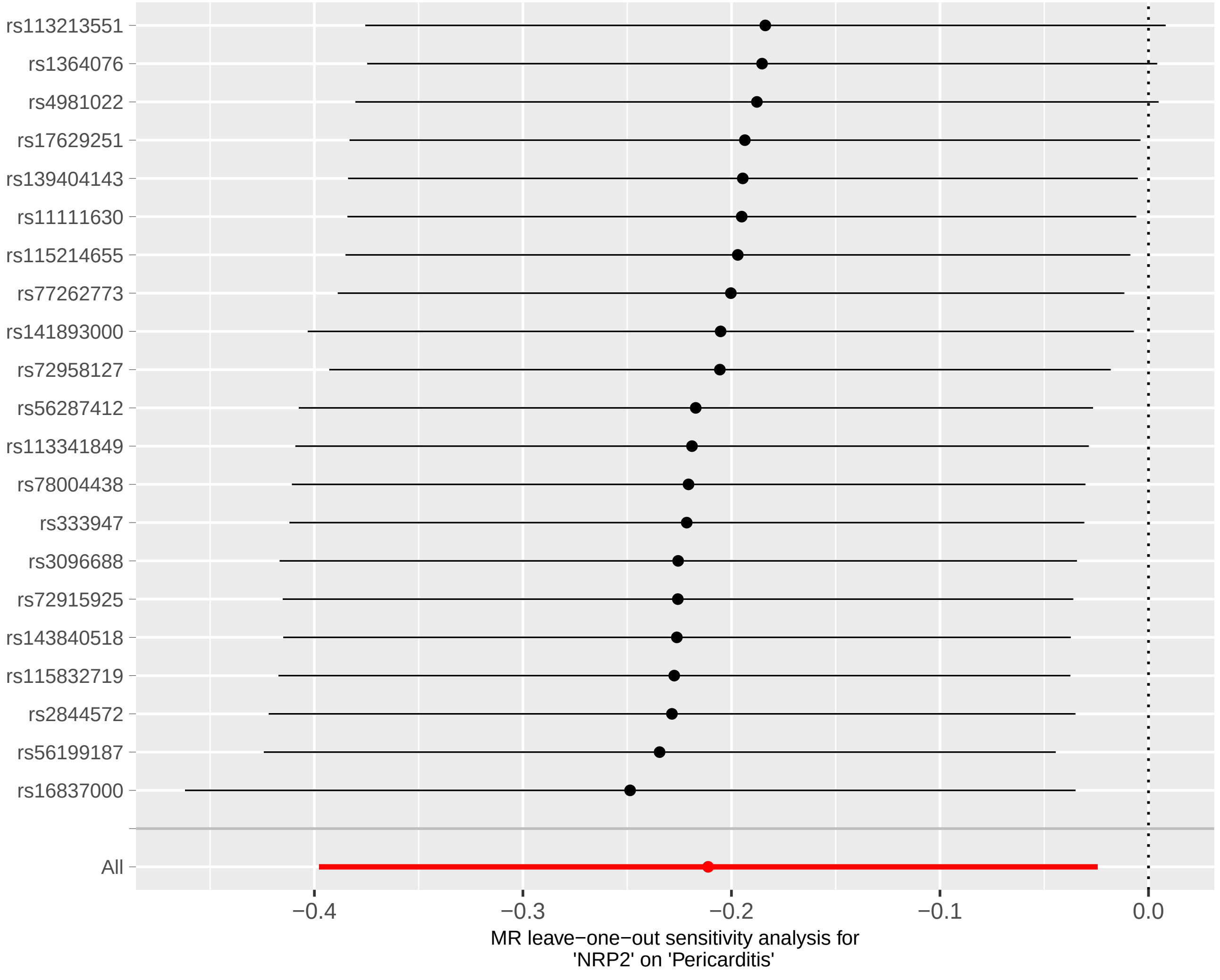

rs113213551
rs1364076
rs4981022
rs17629251
rs139404143
rs11111630
rs115214655
rs77262773
rs141893000
rs72958127
rs56287412
rs113341849
rs78004438
rs333947
rs3096688
rs72915925
rs143840518
rs115832719
rs2844572
rs56199187
rs16837000
All
−0.4
−0.3
−0.2
−0.1
0.0
MR leave−one−out sensitivity analysis for
'NRP2' on 'Pericarditis'

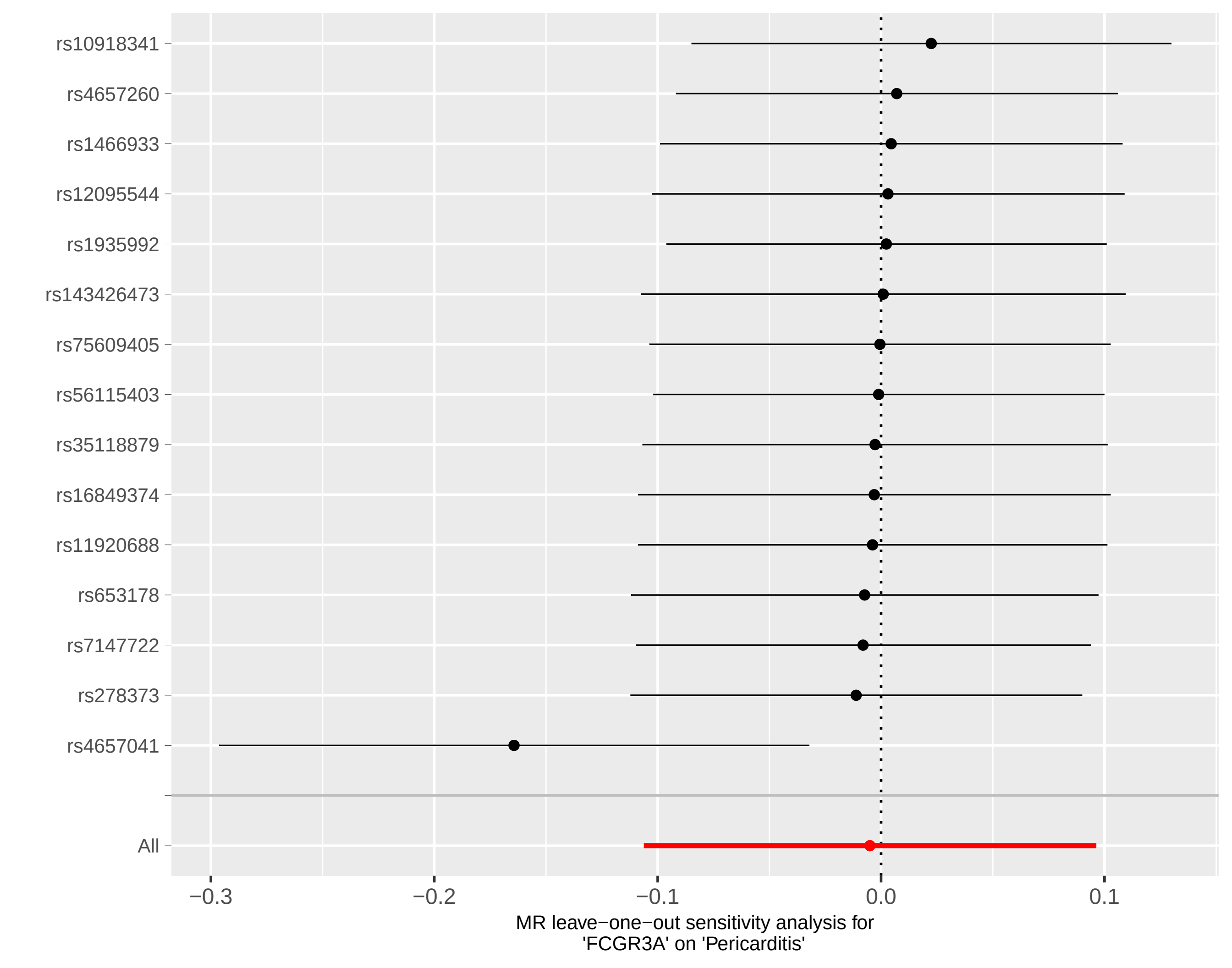

rs10918341
rs4657260
rs1466933
rs12095544
rs1935992
rs143426473
rs75609405
rs56115403
rs35118879
rs16849374
rs11920688
rs653178
rs7147722
rs278373
rs4657041
All
−0.3
−0.2
−0.1
0.0
0.1
MR leave−one−out sensitivity analysis for
'FCGR3A' on 'Pericarditis'

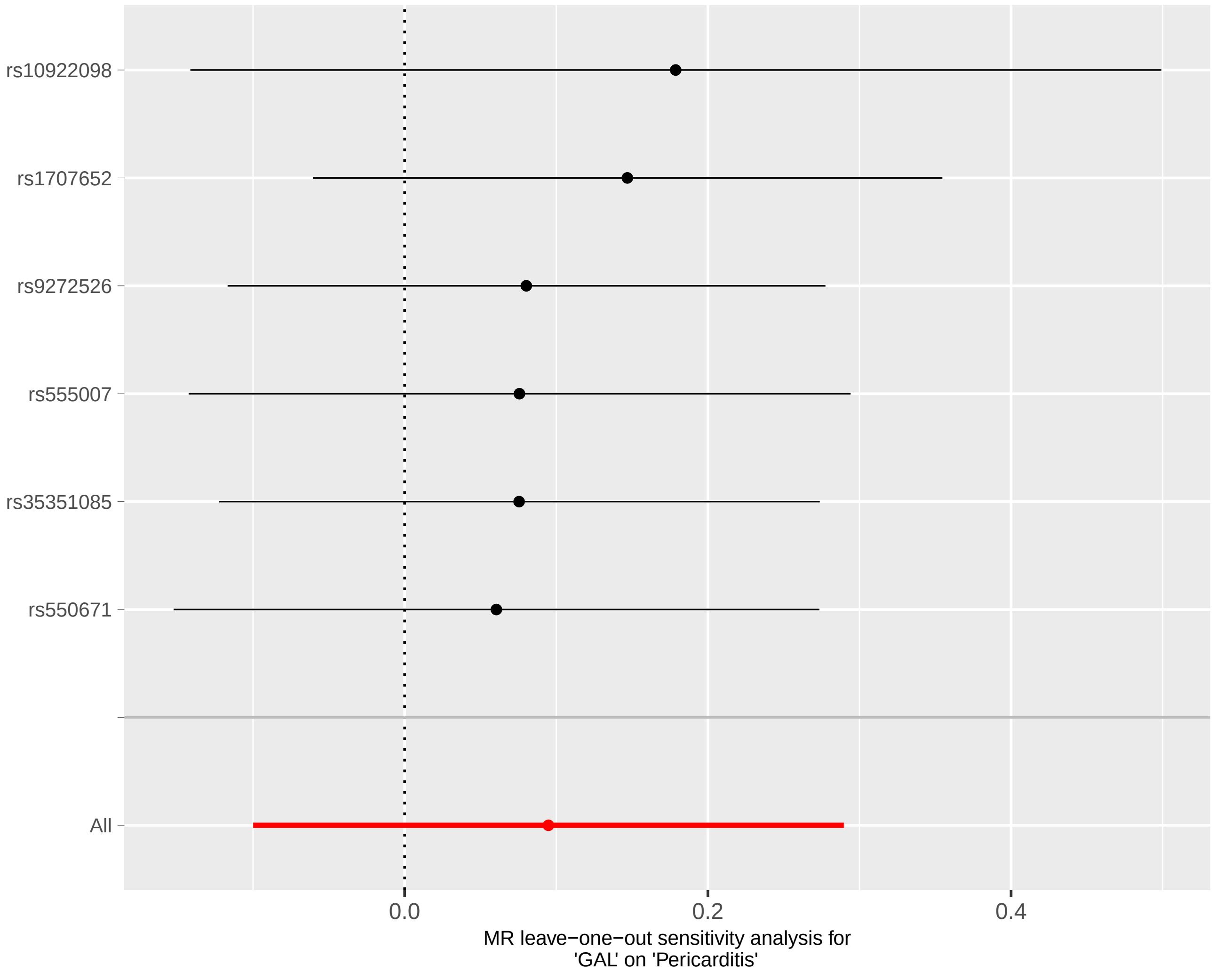

rs10922098
rs1707652
rs9272526
rs555007
rs35351085
rs550671
All
0.0
0.2
0.4
MR leave−one−out sensitivity analysis for
'GAL' on 'Pericarditis'

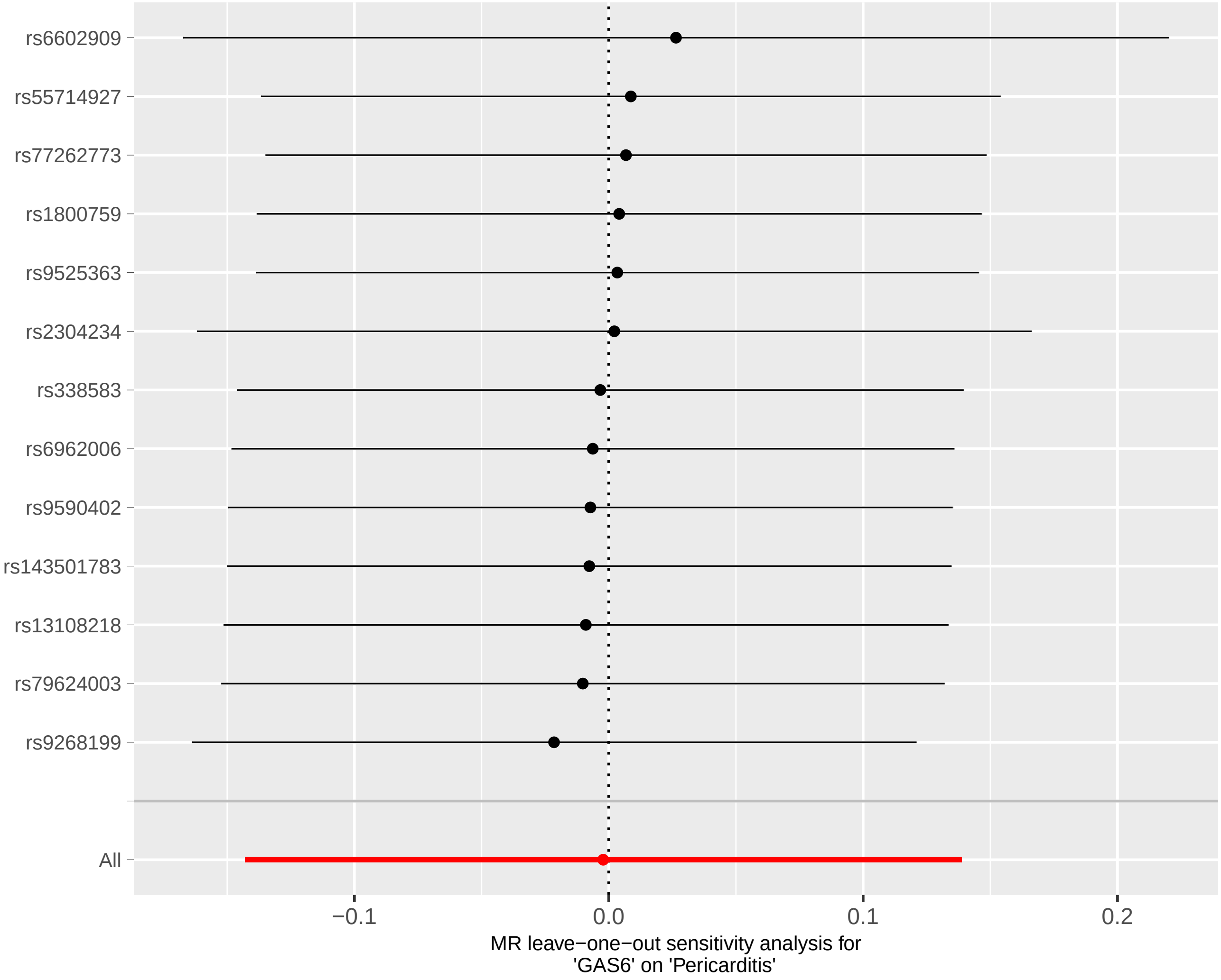
rs6602909
rs55714927
rs77262773
rs1800759
rs9525363
rs2304234
rs338583
rs6962006
rs9590402
rs143501783
rs13108218
rs79624003
rs9268199
All
−0.1
0.0
0.1
0.2
MR leave−one−out sensitivity analysis for
'GAS6' on 'Pericarditis'

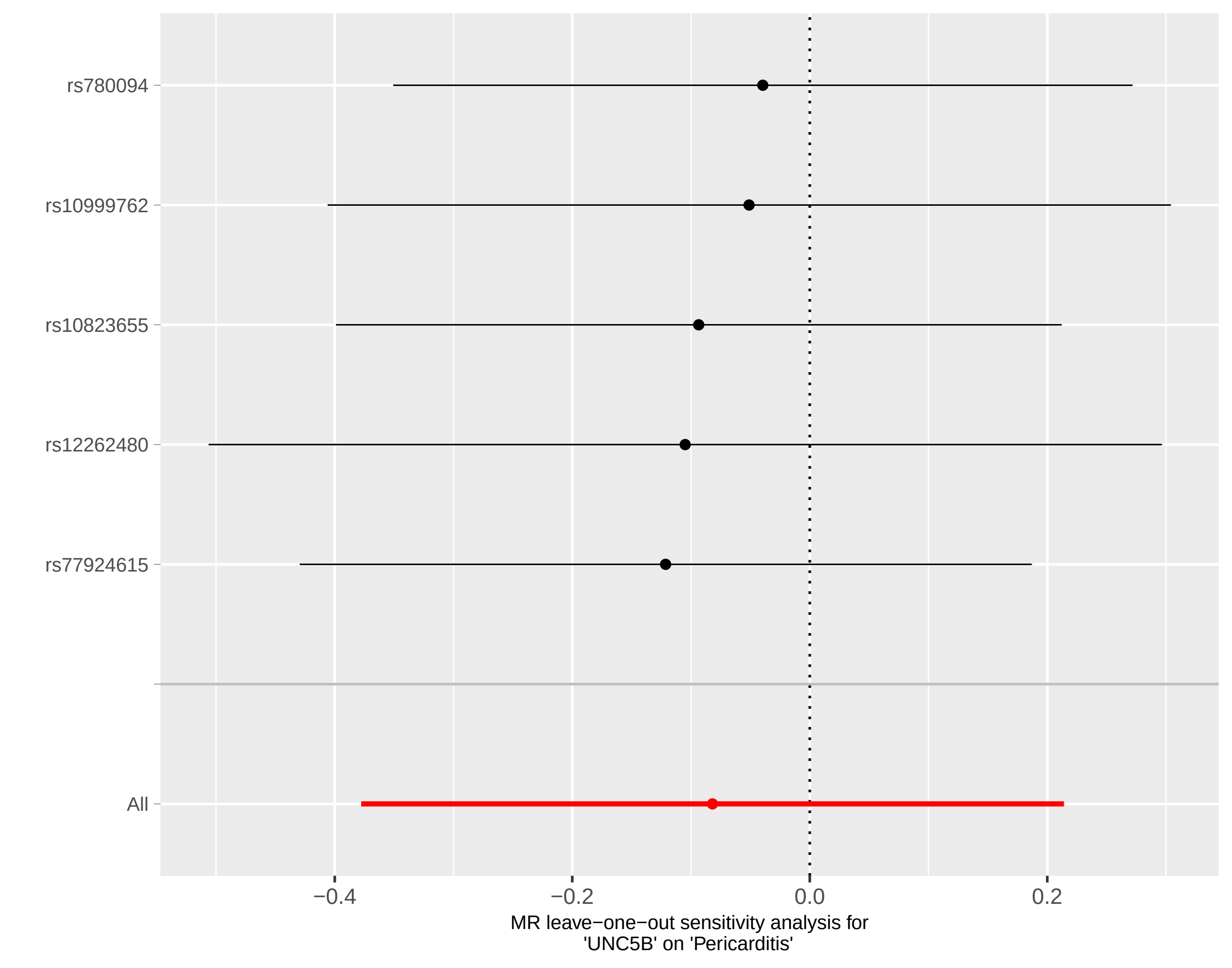
rs780094
rs10999762
rs10823655
rs12262480
rs77924615
All
−0.4
−0.2
0.0
0.2
MR leave−one−out sensitivity analysis for
'UNC5B' on 'Pericarditis'

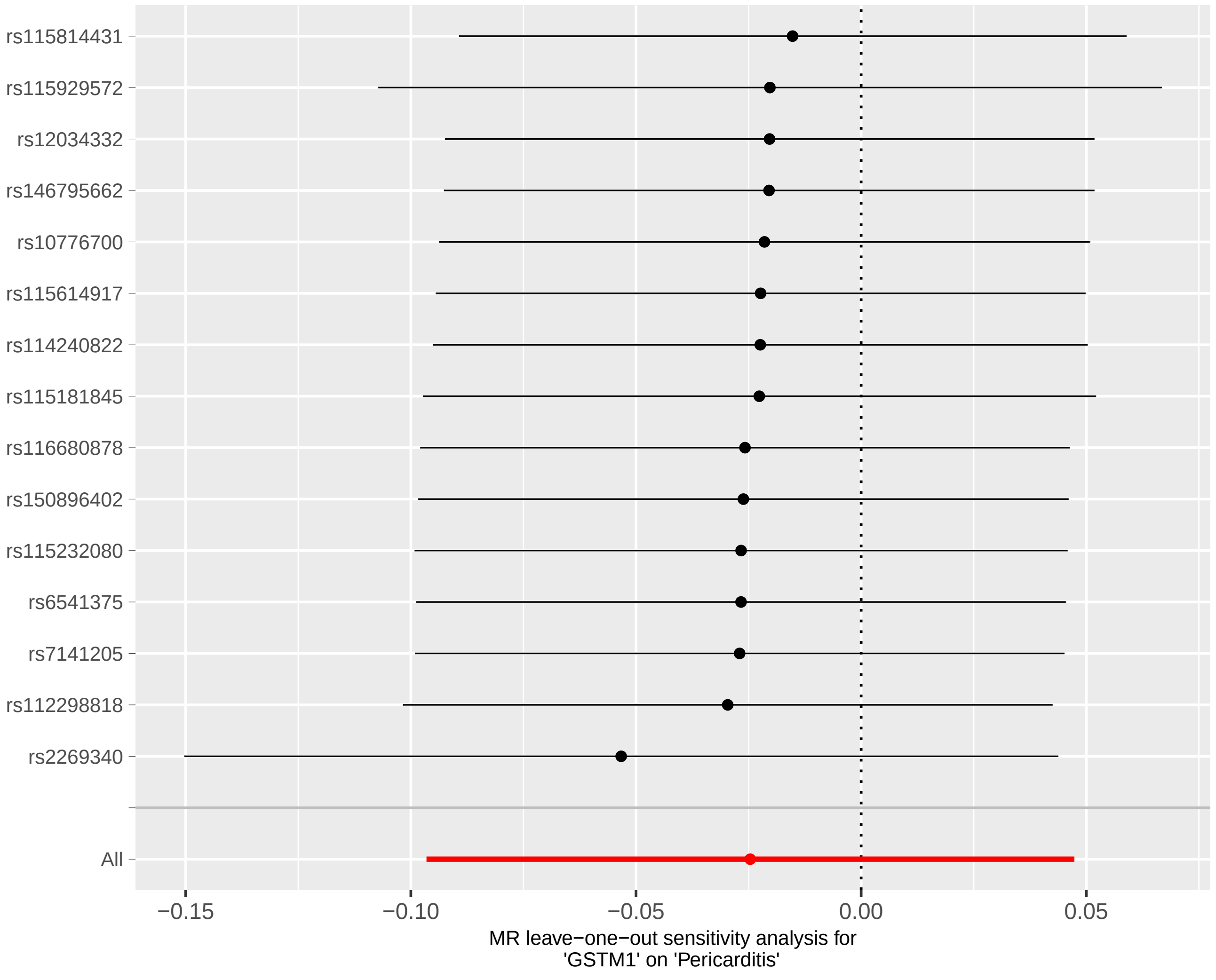
rs115814431
rs115929572
rs12034332
rs146795662
rs10776700
rs115614917
rs114240822
rs115181845
rs116680878
rs150896402
rs115232080
rs6541375
rs7141205
rs112298818
rs2269340
All
−0.15
−0.10
−0.05
0.00
0.05
MR leave−one−out sensitivity analysis for
'GSTM1' on 'Pericarditis'

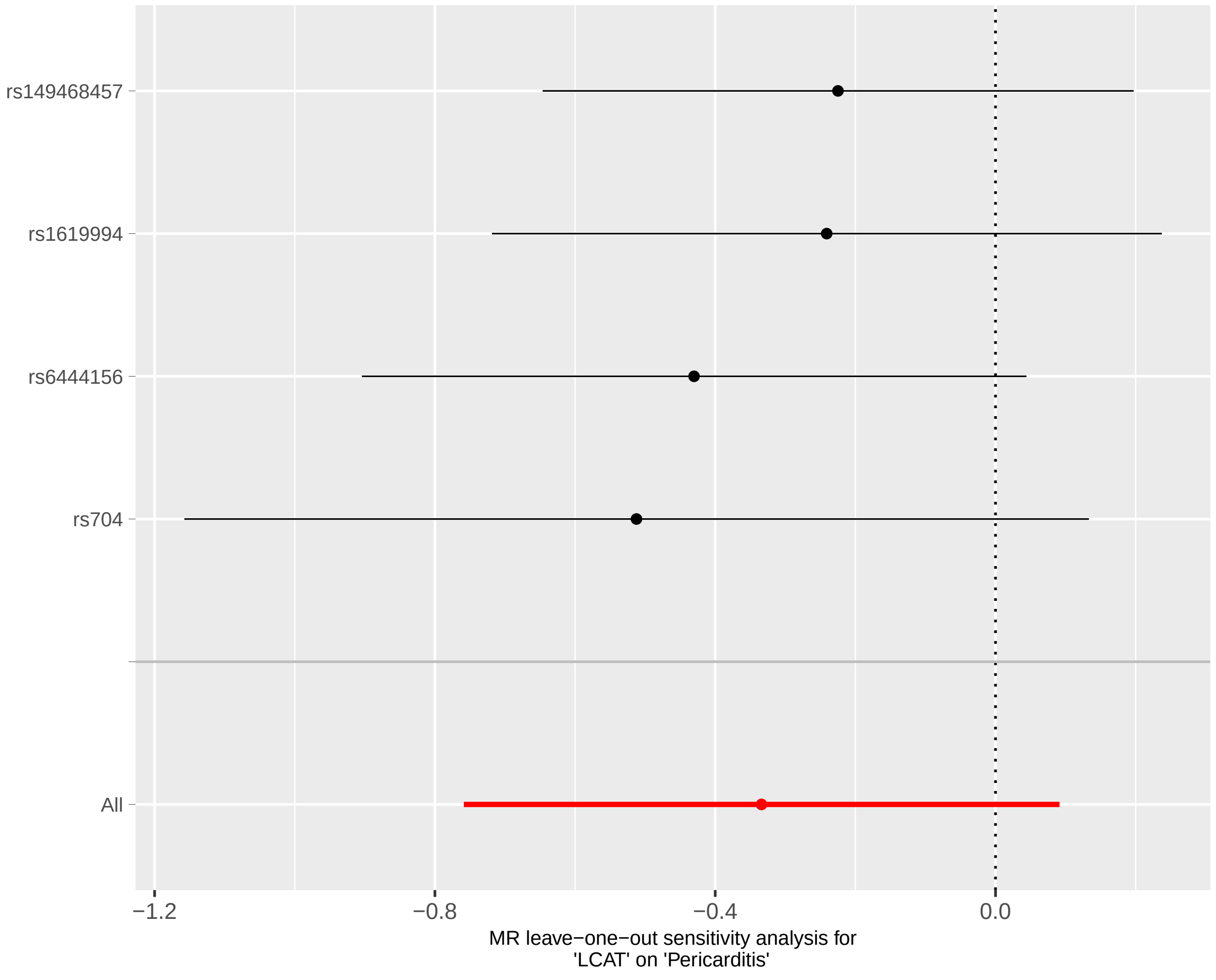
rs149468457
rs1619994
rs6444156
rs704
All
−1.2
−0.8
−0.4
0.0
MR leave−one−out sensitivity analysis for
'LCAT' on 'Pericarditis'

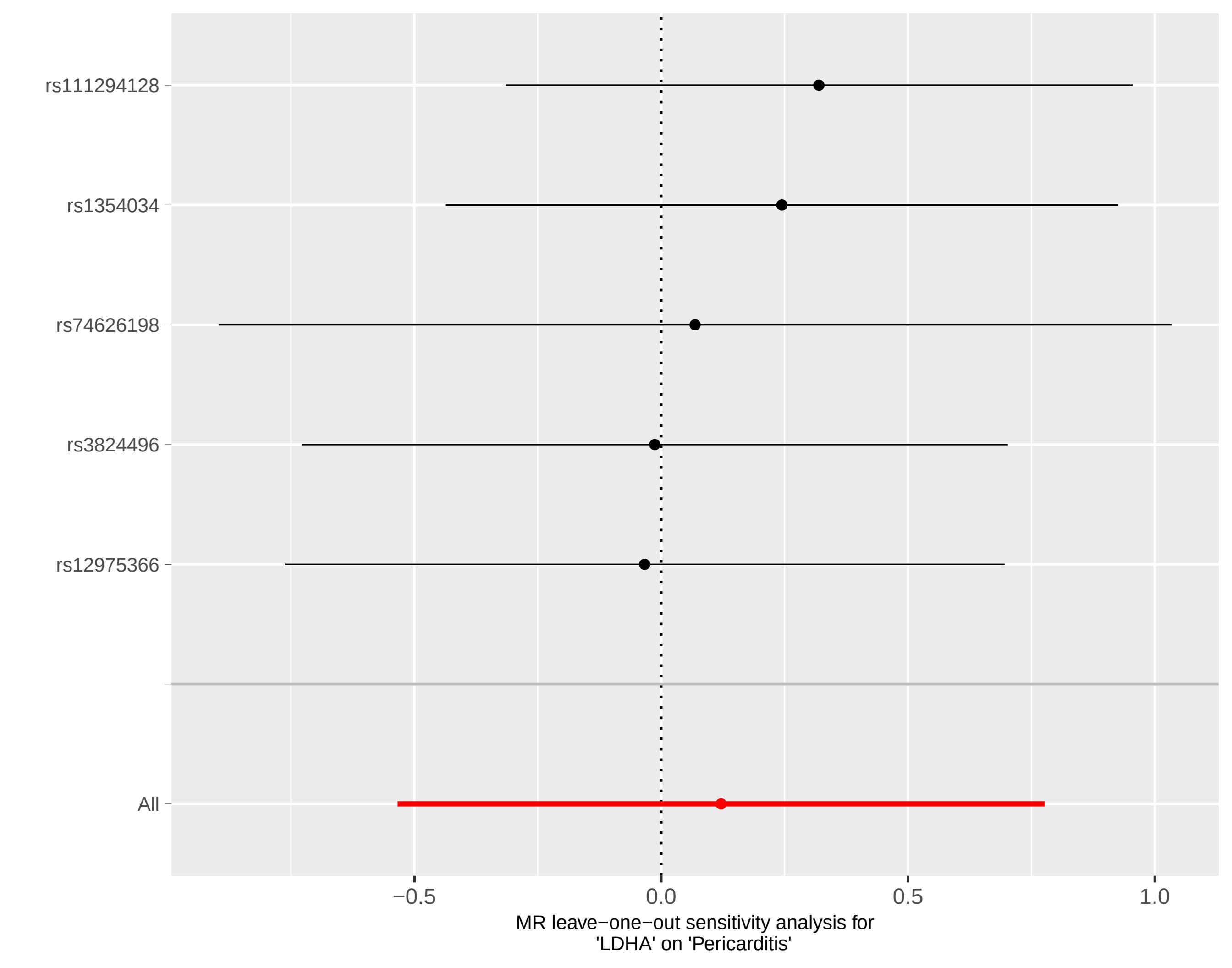
rs111294128
rs1354034
rs74626198
rs3824496
rs12975366
All
−0.5
0.0
0.5
1.0
MR leave−one−out sensitivity analysis for
'LDHA' on 'Pericarditis'

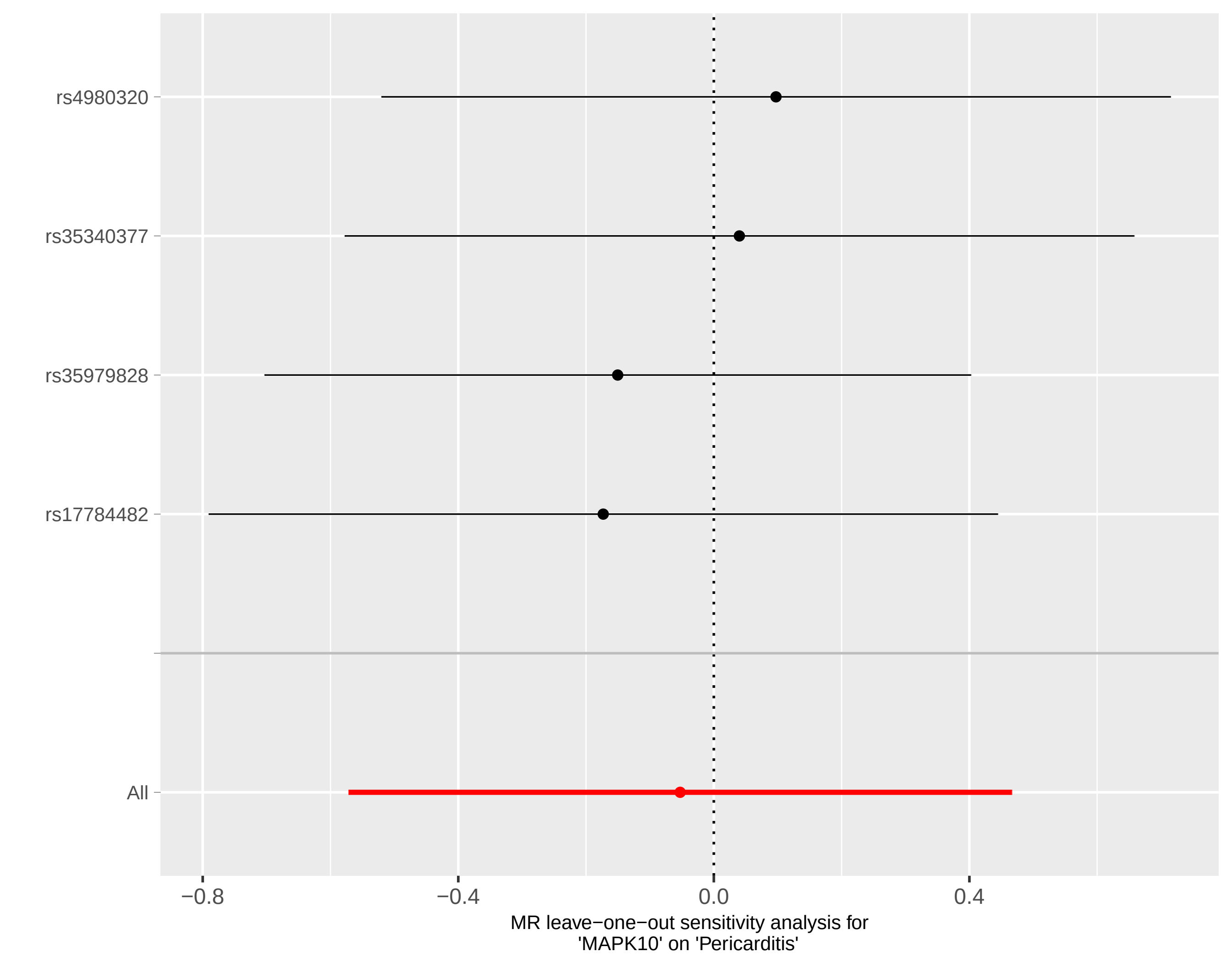
rs4980320
rs35340377
rs35979828
rs17784482
All
−0.8
−0.4
0.0
0.4
MR leave−one−out sensitivity analysis for
'MAPK10' on 'Pericarditis'

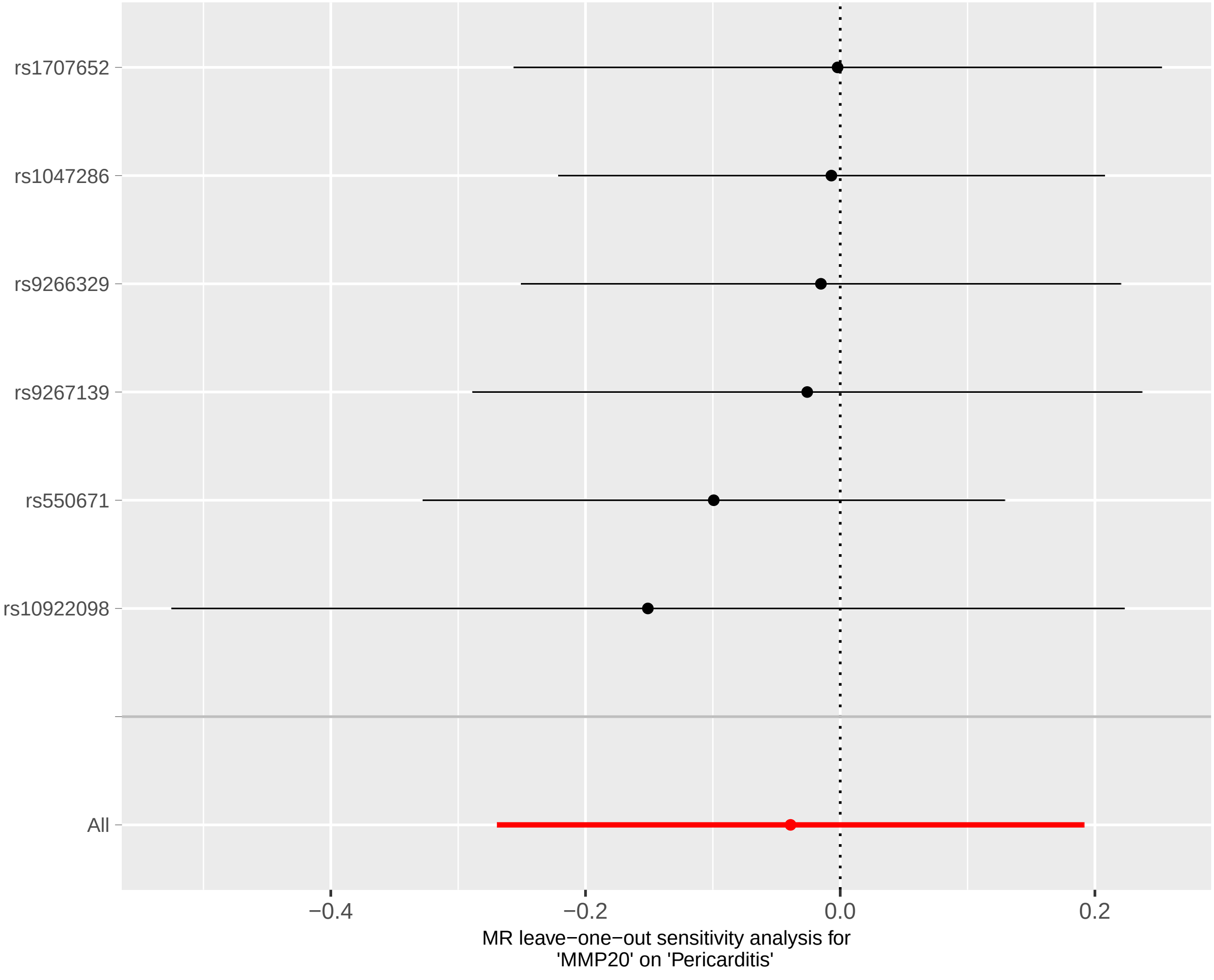

rs1707652
rs1047286
rs9266329
rs9267139
rs550671
rs10922098
All
−0.4
−0.2
0.0
0.2
MR leave−one−out sensitivity analysis for
'MMP20' on 'Pericarditis'

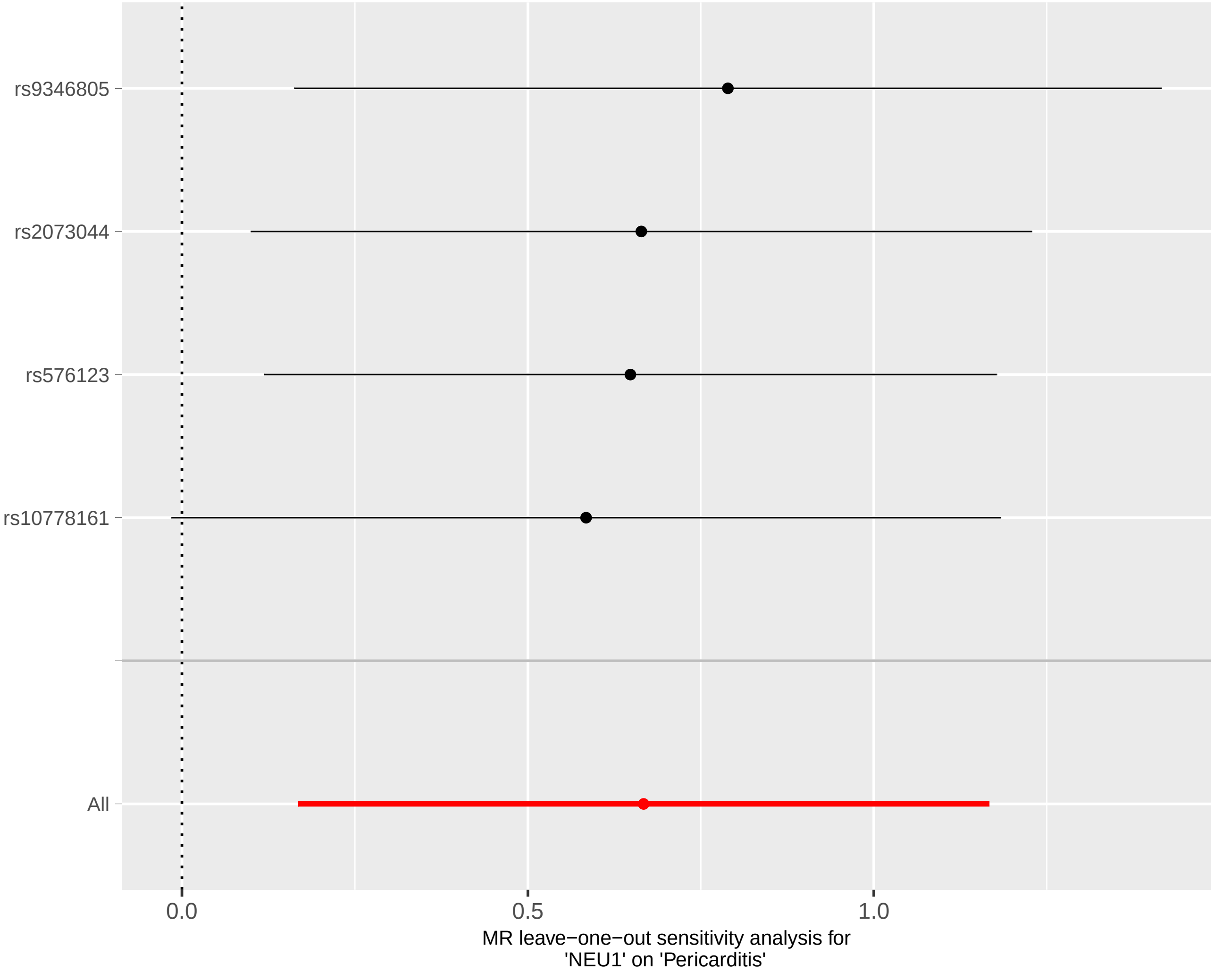

rs9346805
rs2073044
rs576123
rs10778161
All
0.0
0.5
1.0
MR leave−one−out sensitivity analysis for
'NEU1' on 'Pericarditis'

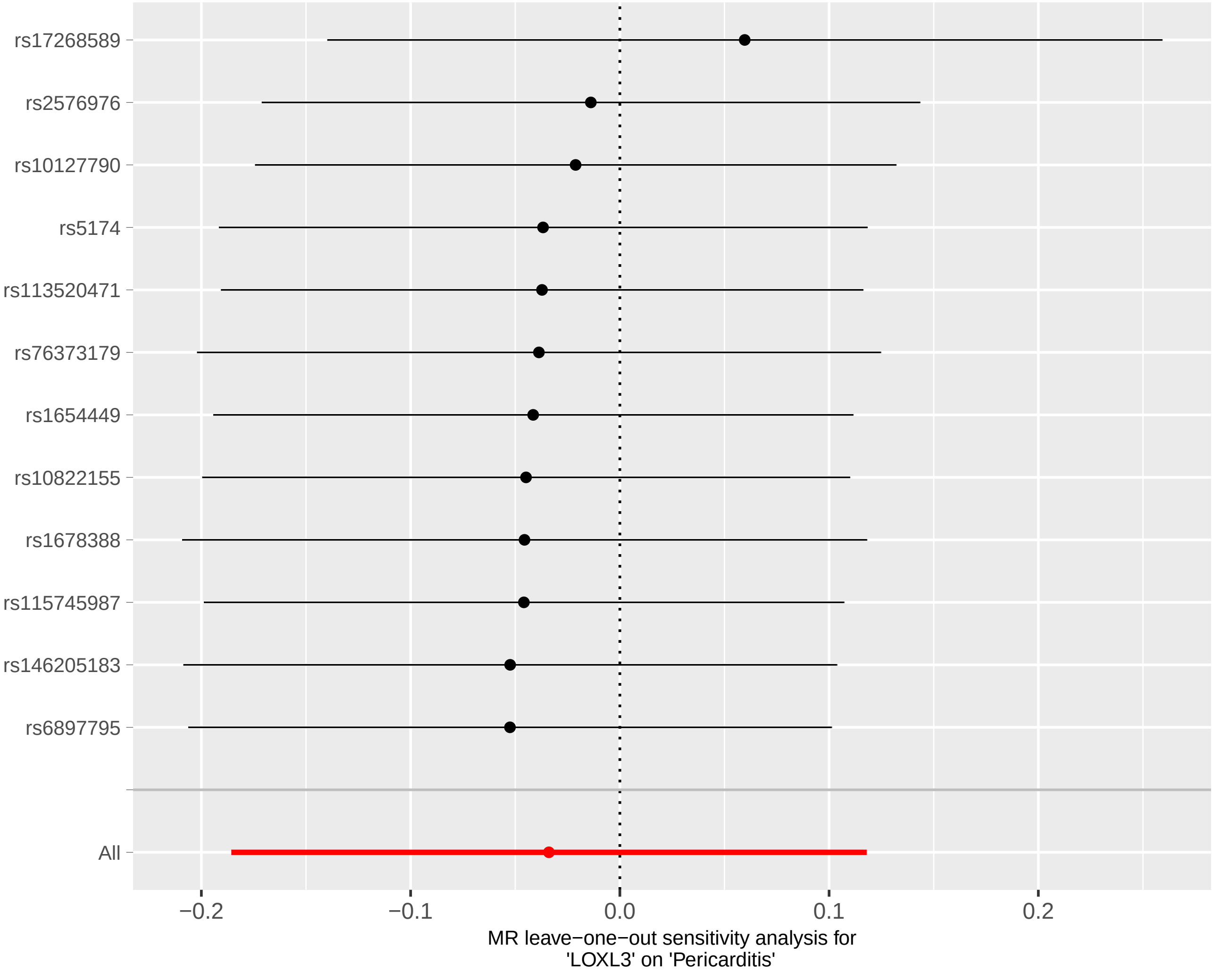

rs17268589
rs2576976
rs10127790
rs5174
rs113520471
rs76373179
rs1654449
rs10822155
rs1678388
rs115745987
rs146205183
rs6897795
All
−0.2
−0.1
0.0
0.1
0.2
MR leave−one−out sensitivity analysis for
'LOXL3' on 'Pericarditis'

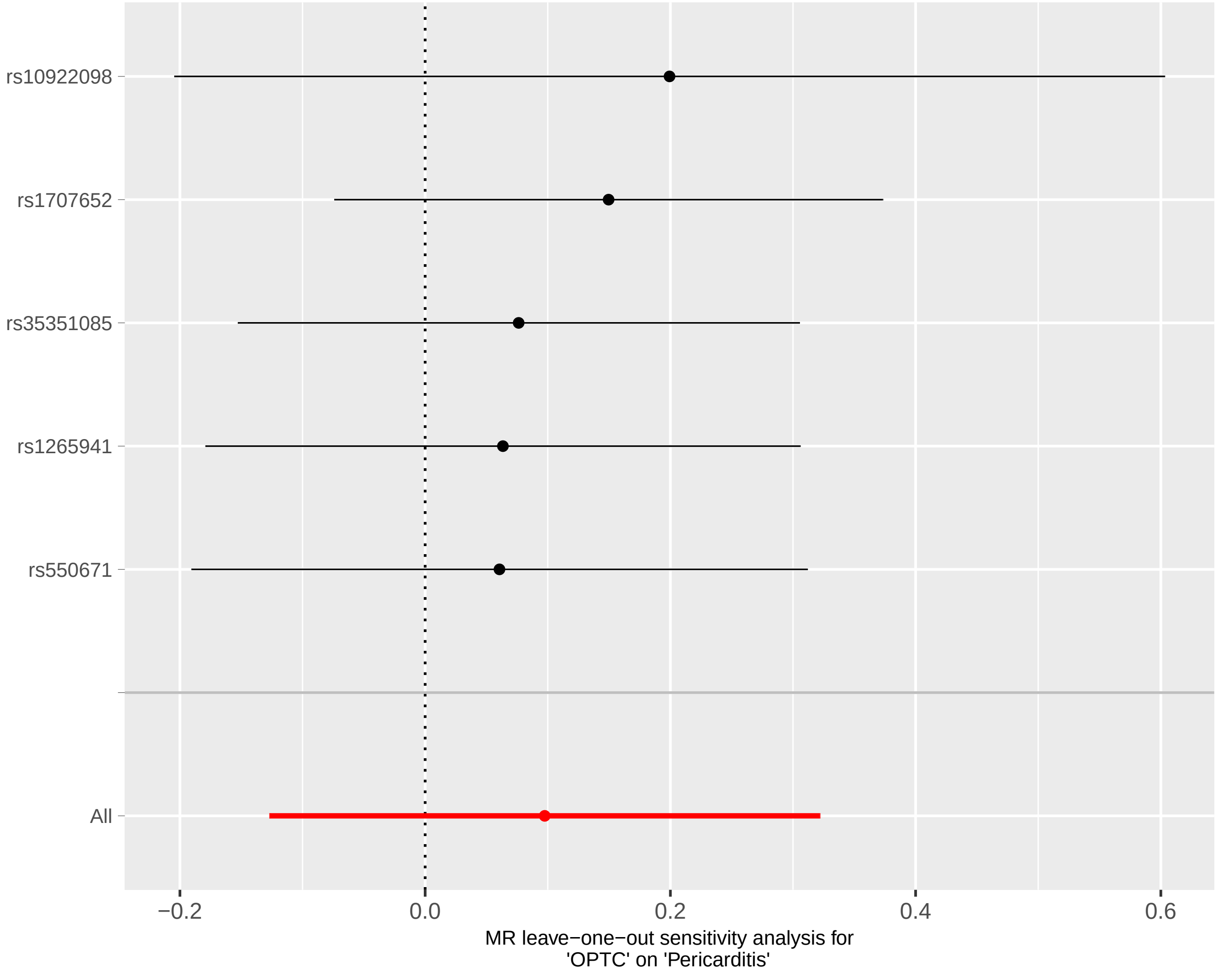
rs10922098
rs1707652
rs35351085
rs1265941
rs550671
All
−0.2
0.0
0.2
0.4
0.6
MR leave−one−out sensitivity analysis for
'OPTC' on 'Pericarditis'

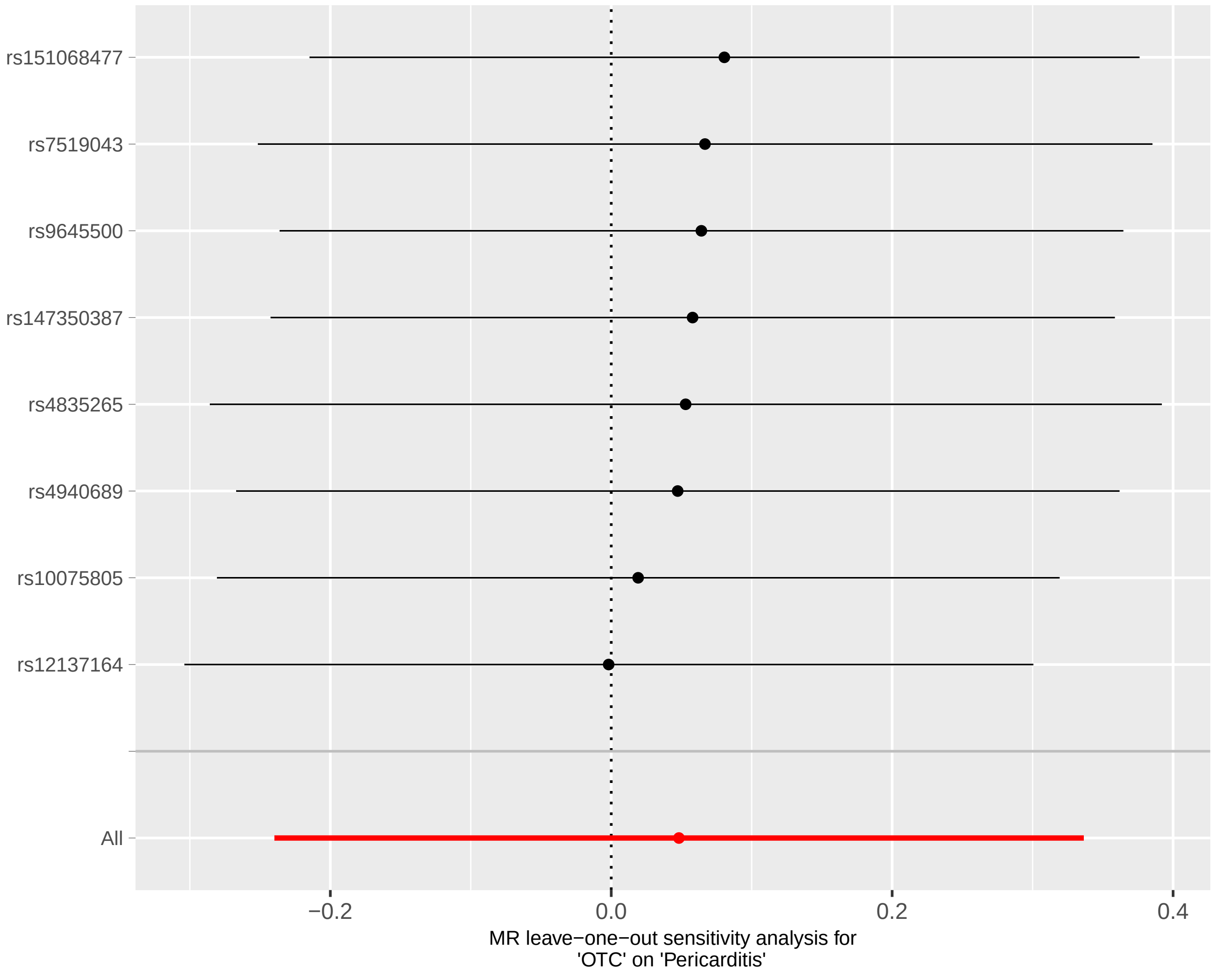

rs151068477
rs7519043
rs9645500
rs147350387
rs4835265
rs4940689
rs10075805
rs12137164
All
−0.2
0.0
0.2
0.4
MR leave−one−out sensitivity analysis for
'OTC' on 'Pericarditis'

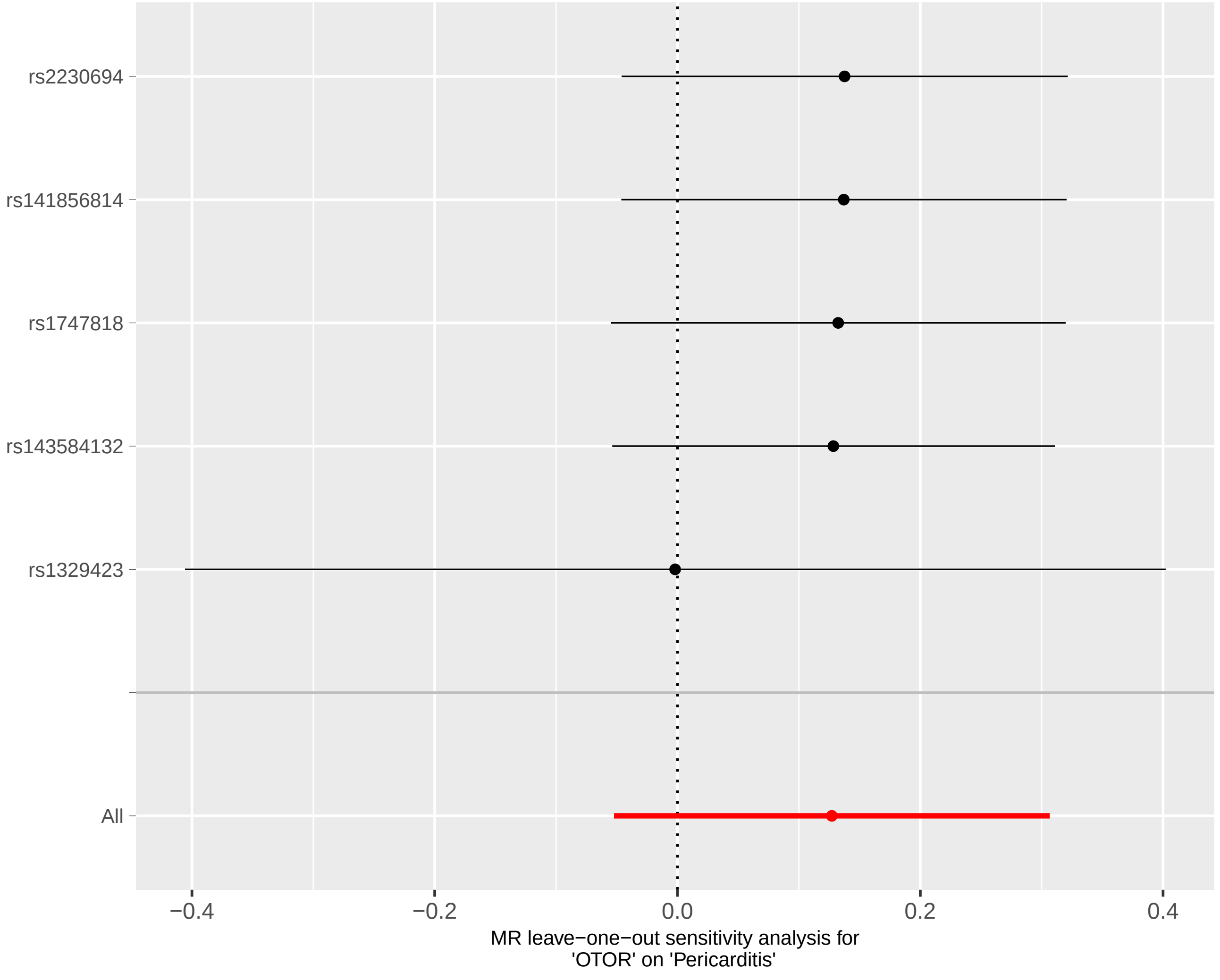
rs2230694
rs141856814
rs1747818
rs143584132
rs1329423
All
−0.4
−0.2
0.0
0.2
0.4
MR leave−one−out sensitivity analysis for
'OTOR' on 'Pericarditis'

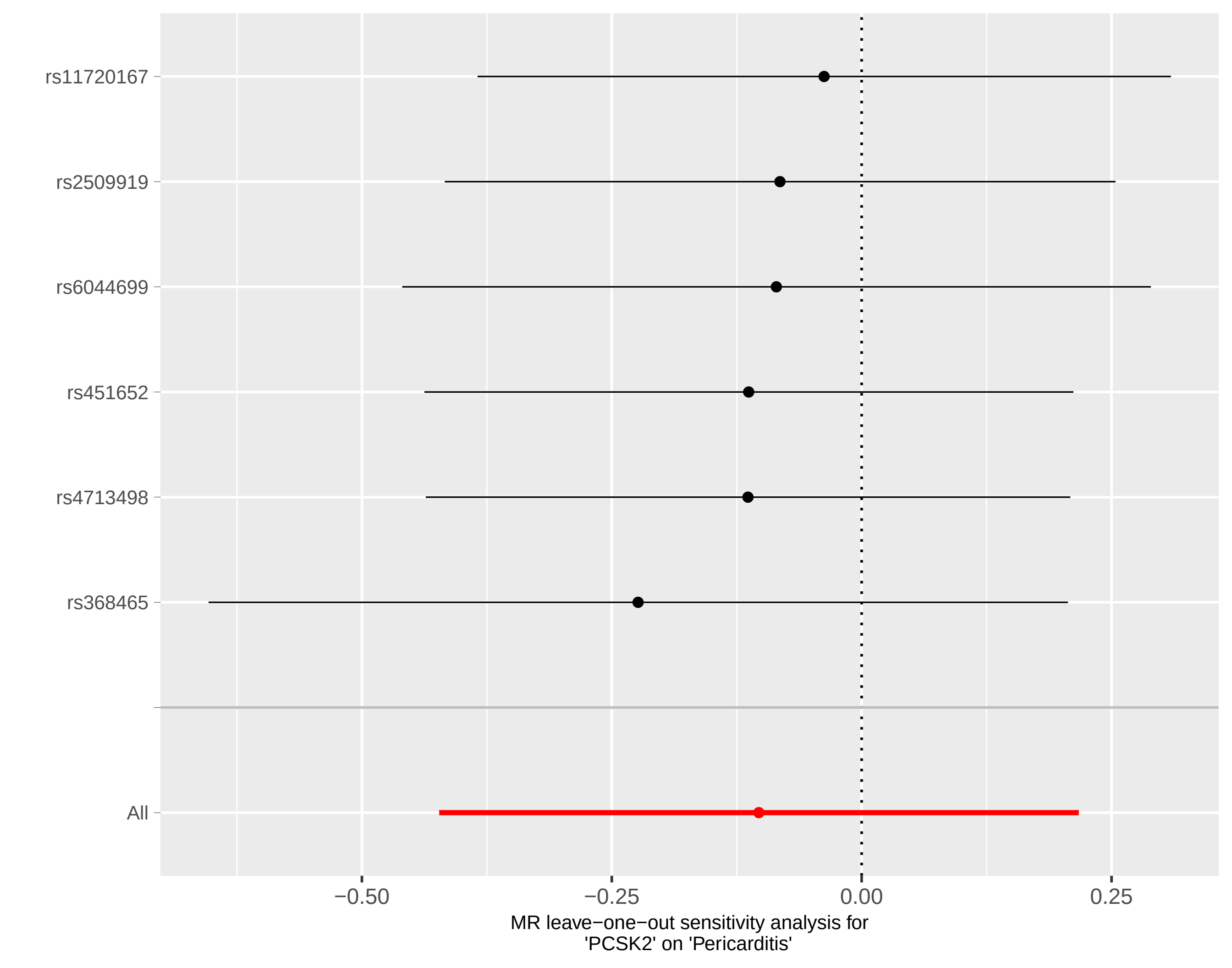
rs11720167
rs2509919
rs6044699
rs451652
rs4713498
rs368465
All
−0.50
−0.25
0.00
0.25
MR leave−one−out sensitivity analysis for
'PCSK2' on 'Pericarditis'

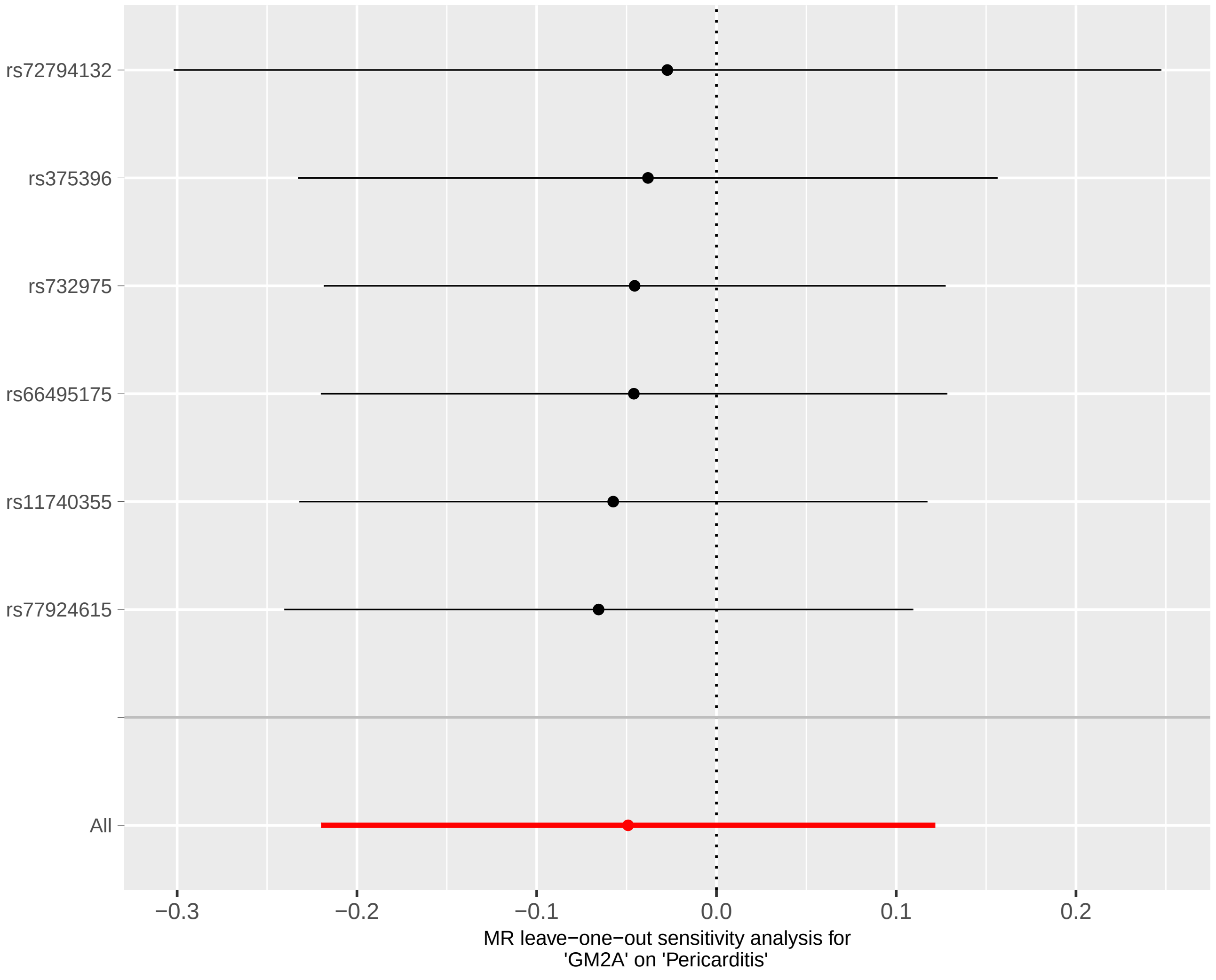
rs72794132
rs375396
rs732975
rs66495175
rs11740355
rs77924615
All
−0.3
−0.2
−0.1
0.0
0.1
0.2
MR leave−one−out sensitivity analysis for
'GM2A' on 'Pericarditis'

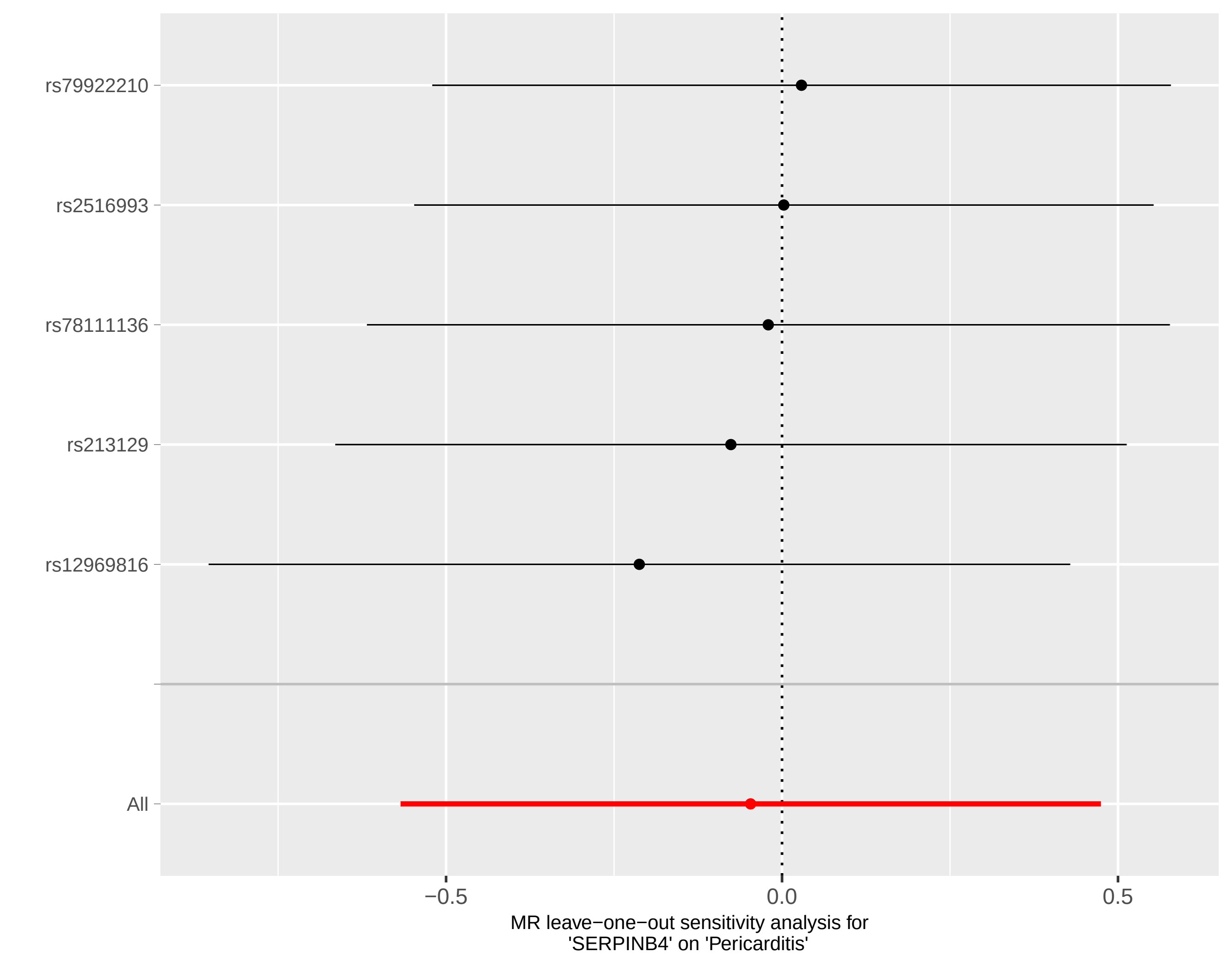

rs79922210
rs2516993
rs78111136
rs213129
rs12969816
All
−0.5
0.0
0.5
MR leave−one−out sensitivity analysis for
'SERPINB4' on 'Pericarditis'

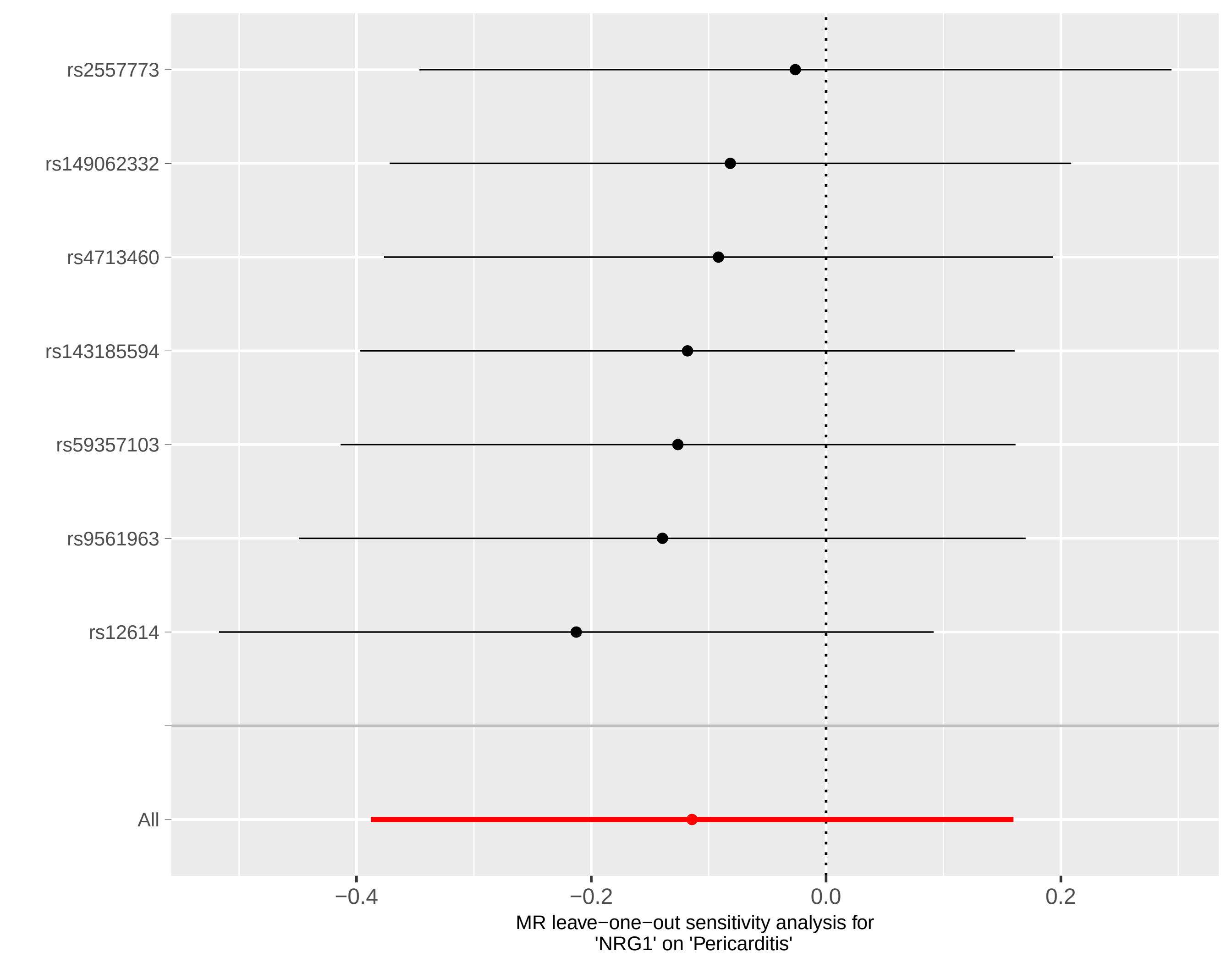
rs2557773
rs149062332
rs4713460
rs143185594
rs59357103
rs9561963
rs12614
All
−0.4
−0.2
0.0
0.2
MR leave−one−out sensitivity analysis for
'NRG1' on 'Pericarditis'

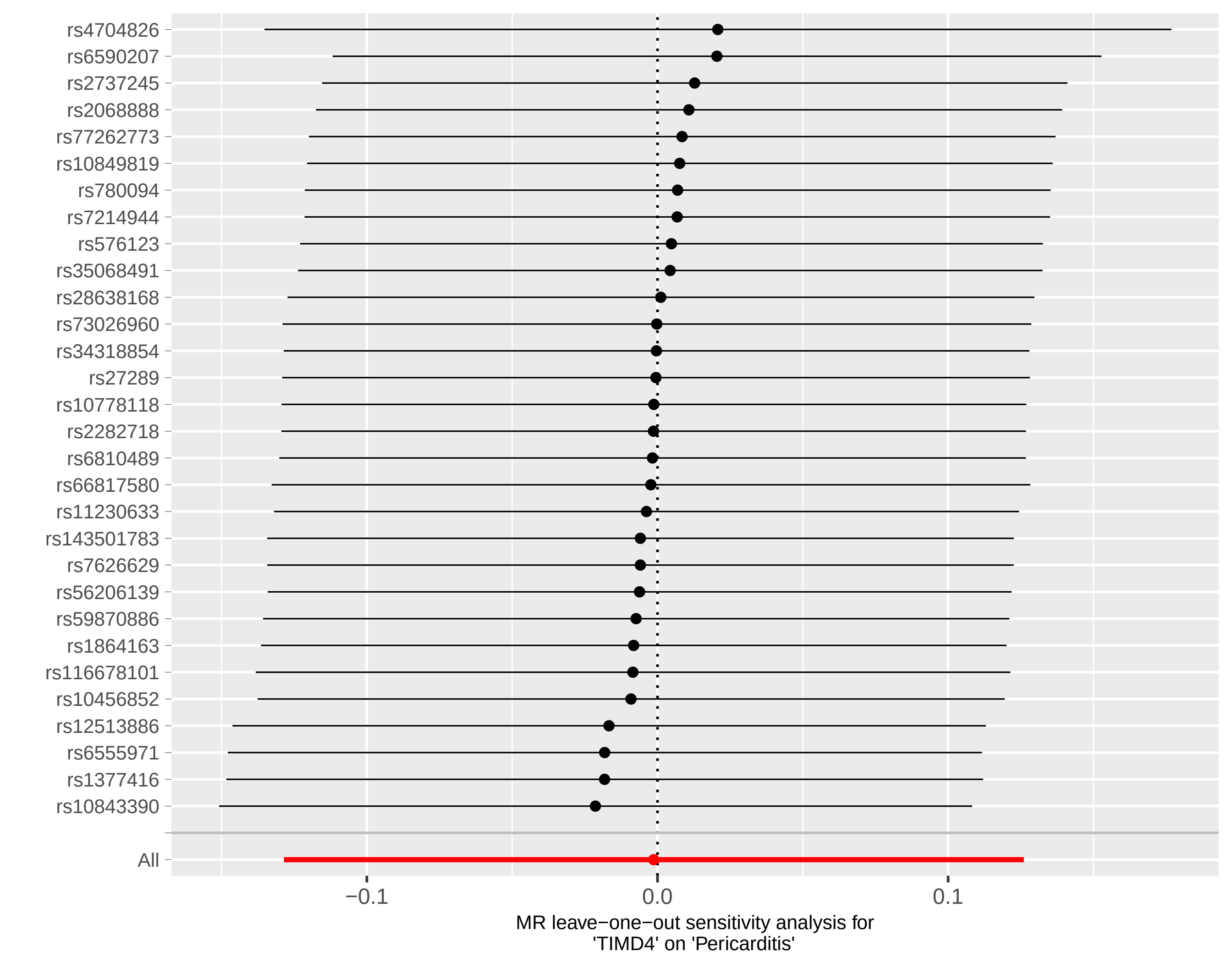
rs4704826
rs6590207
rs2737245
rs2068888
rs77262773
rs10849819
rs780094
rs7214944
rs576123
rs35068491
rs28638168
rs73026960
rs34318854
rs27289
rs10778118
rs2282718
rs6810489
rs66817580
rs11230633
rs143501783
rs7626629
rs56206139
rs59870886
rs1864163
rs116678101
rs10456852
rs12513886
rs6555971
rs1377416
rs10843390
All
−0.1
0.0
0.1
MR leave−one−out sensitivity analysis for
'TIMD4' on 'Pericarditis'

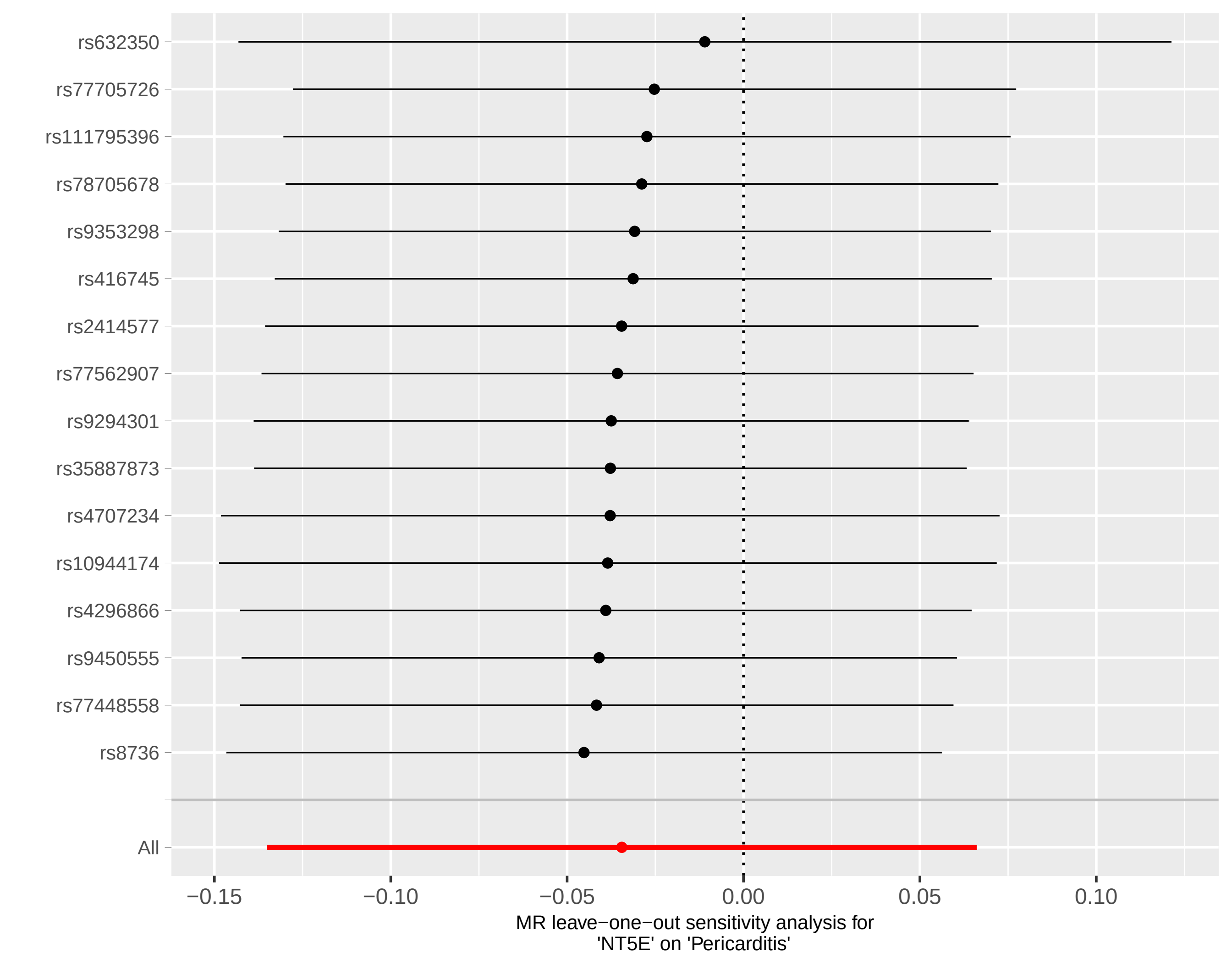
rs632350
rs77705726
rs111795396
rs78705678
rs9353298
rs416745
rs2414577
rs77562907
rs9294301
rs35887873
rs4707234
rs10944174
rs4296866
rs9450555
rs77448558
rs8736
All
−0.15
−0.10
−0.05
0.00
0.05
0.10
MR leave−one−out sensitivity analysis for
'NT5E' on 'Pericarditis'

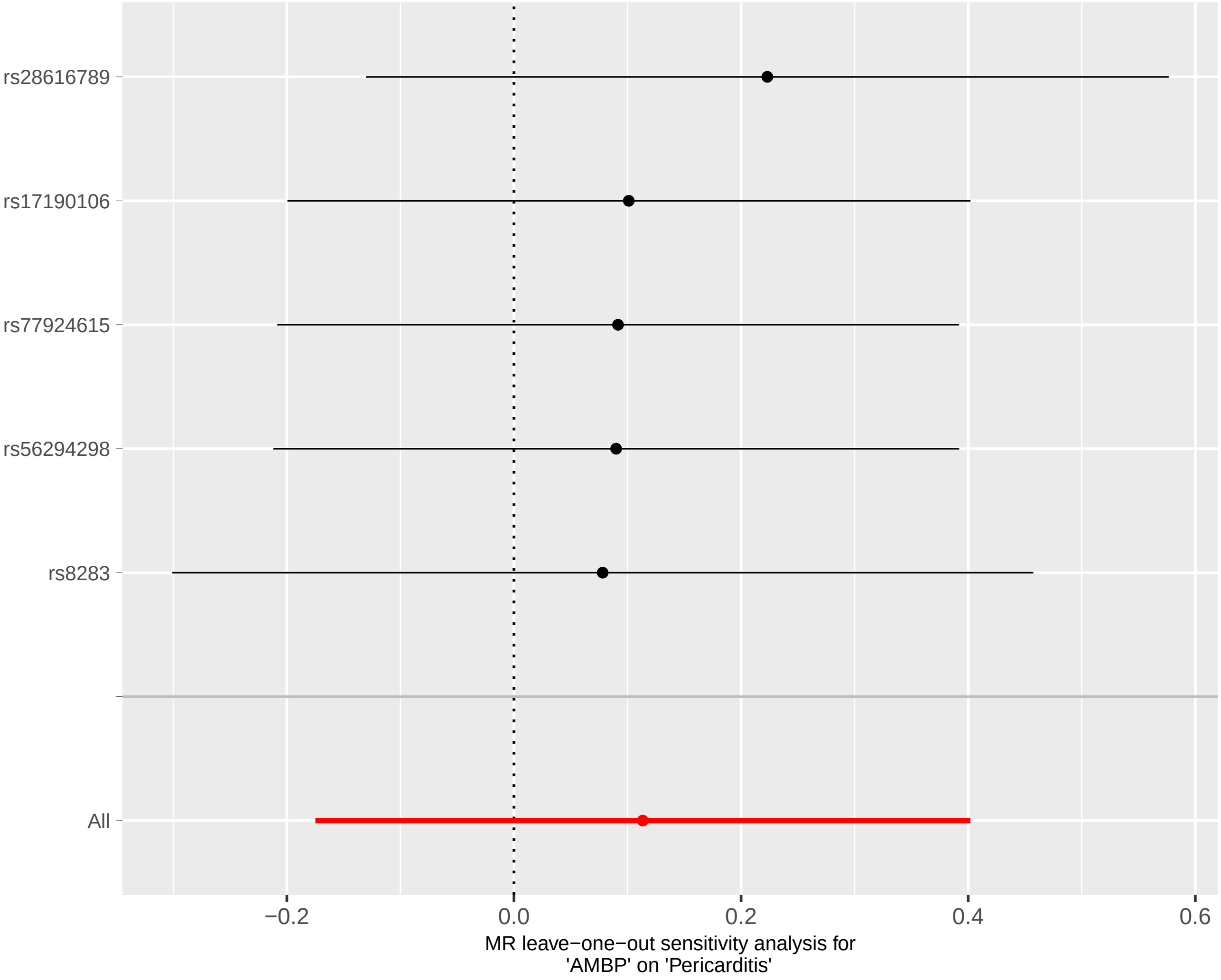
rs28616789
rs17190106
rs77924615
rs56294298
rs8283
All
−0.2
0.0
0.2
0.4
0.6
MR leave−one−out sensitivity analysis for
'AMBP' on 'Pericarditis'

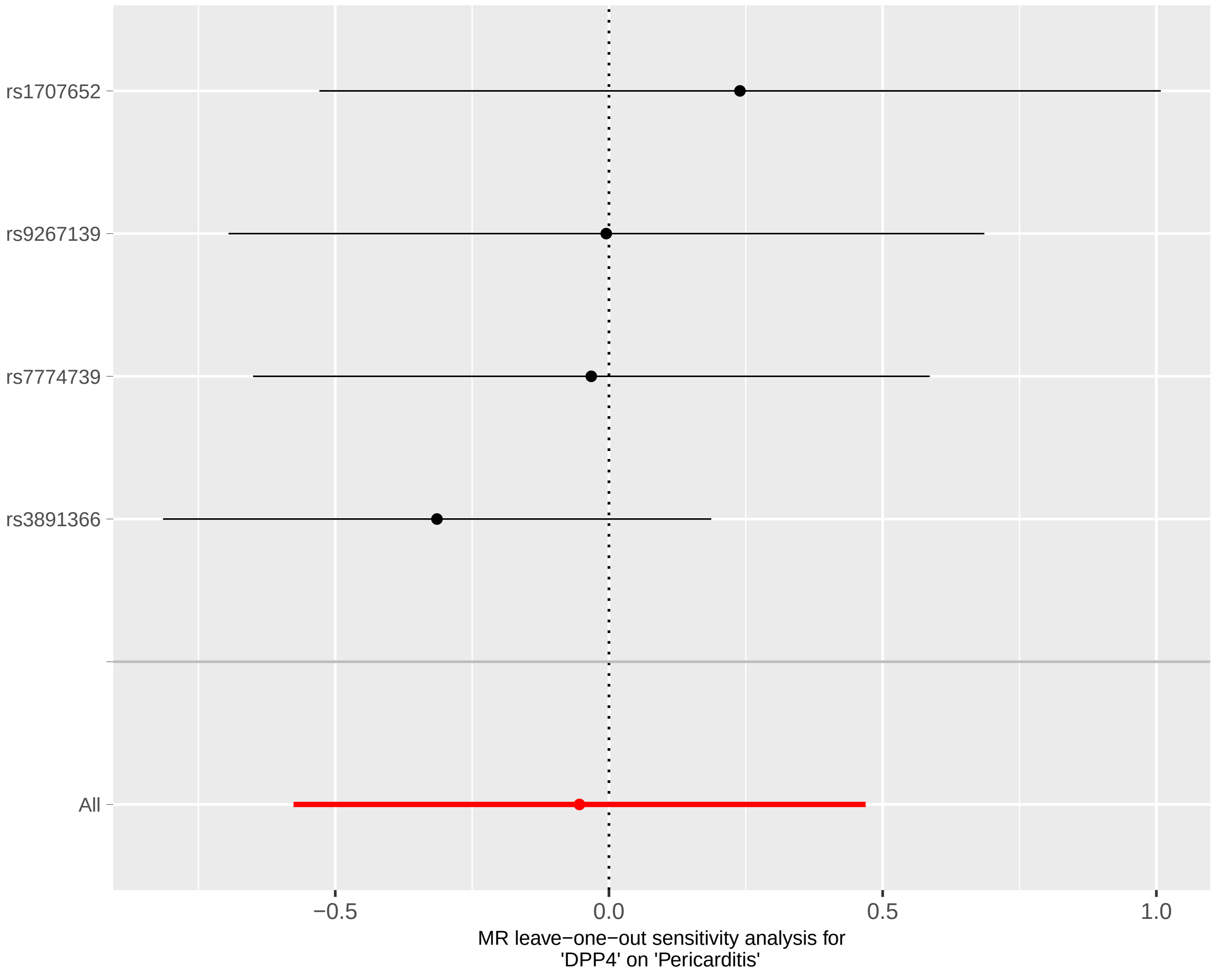
rs1707652
rs9267139
rs7774739
rs3891366
All
−0.5
0.0
0.5
1.0
MR leave−one−out sensitivity analysis for
'DPP4' on 'Pericarditis'

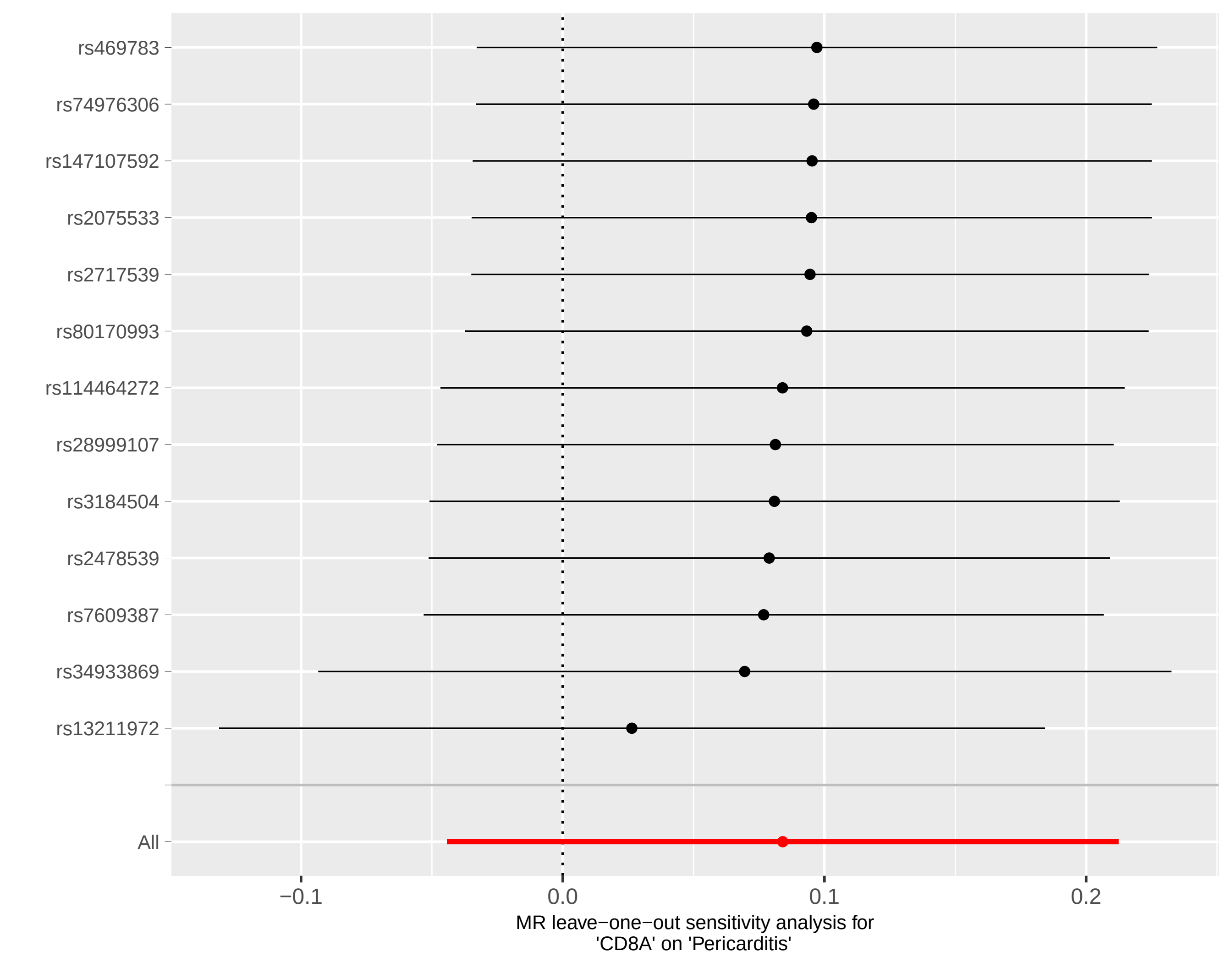

rs469783
rs74976306
rs147107592
rs2075533
rs2717539
rs80170993
rs114464272
rs28999107
rs3184504
rs2478539
rs7609387
rs34933869
rs13211972
All
−0.1
0.0
0.1
0.2
MR leave−one−out sensitivity analysis for
'CD8A' on 'Pericarditis'

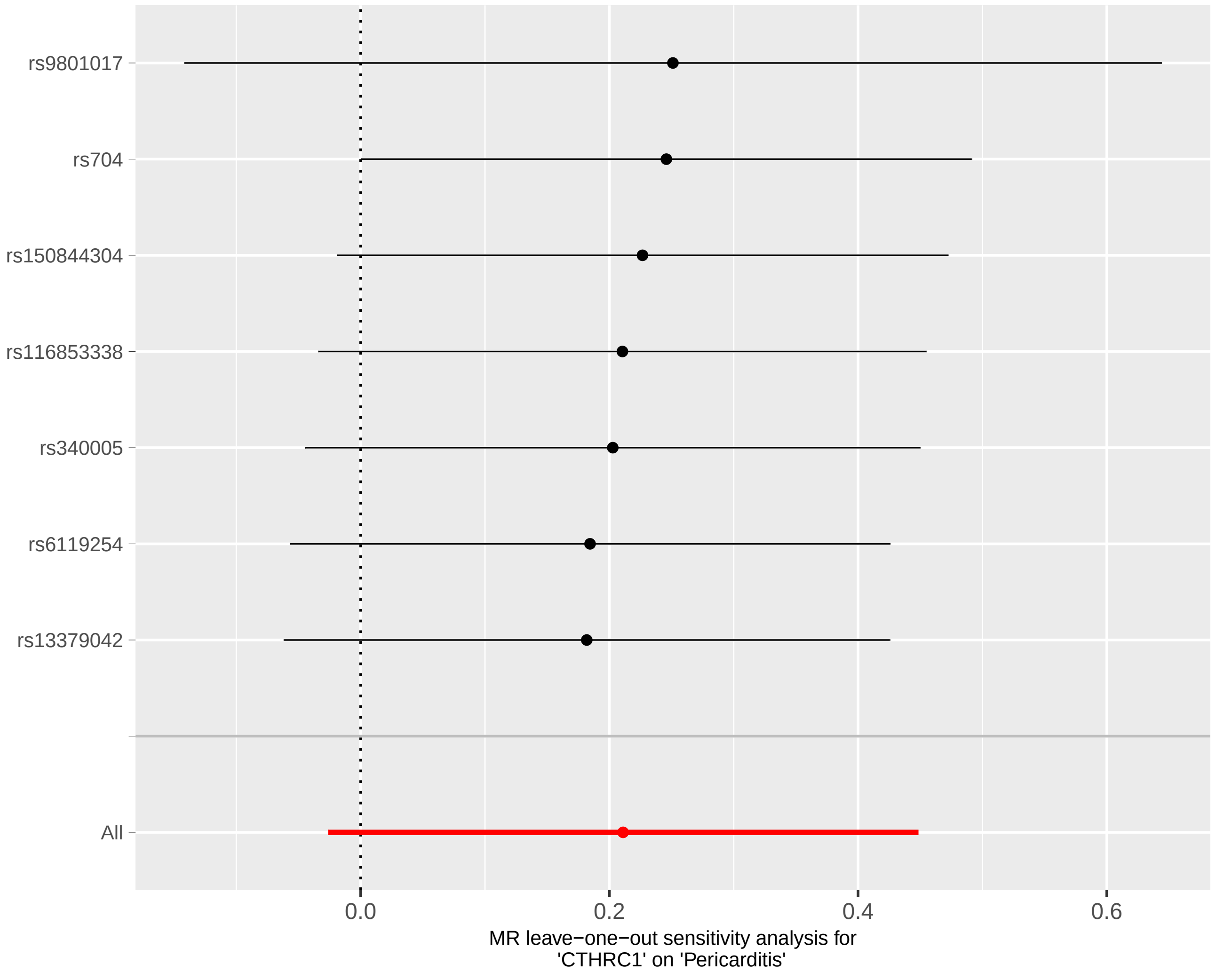
rs9801017
rs704
rs150844304
rs116853338
rs340005
rs6119254
rs13379042
All
0.0
0.2
0.4
0.6
MR leave−one−out sensitivity analysis for
'CTHRC1' on 'Pericarditis'

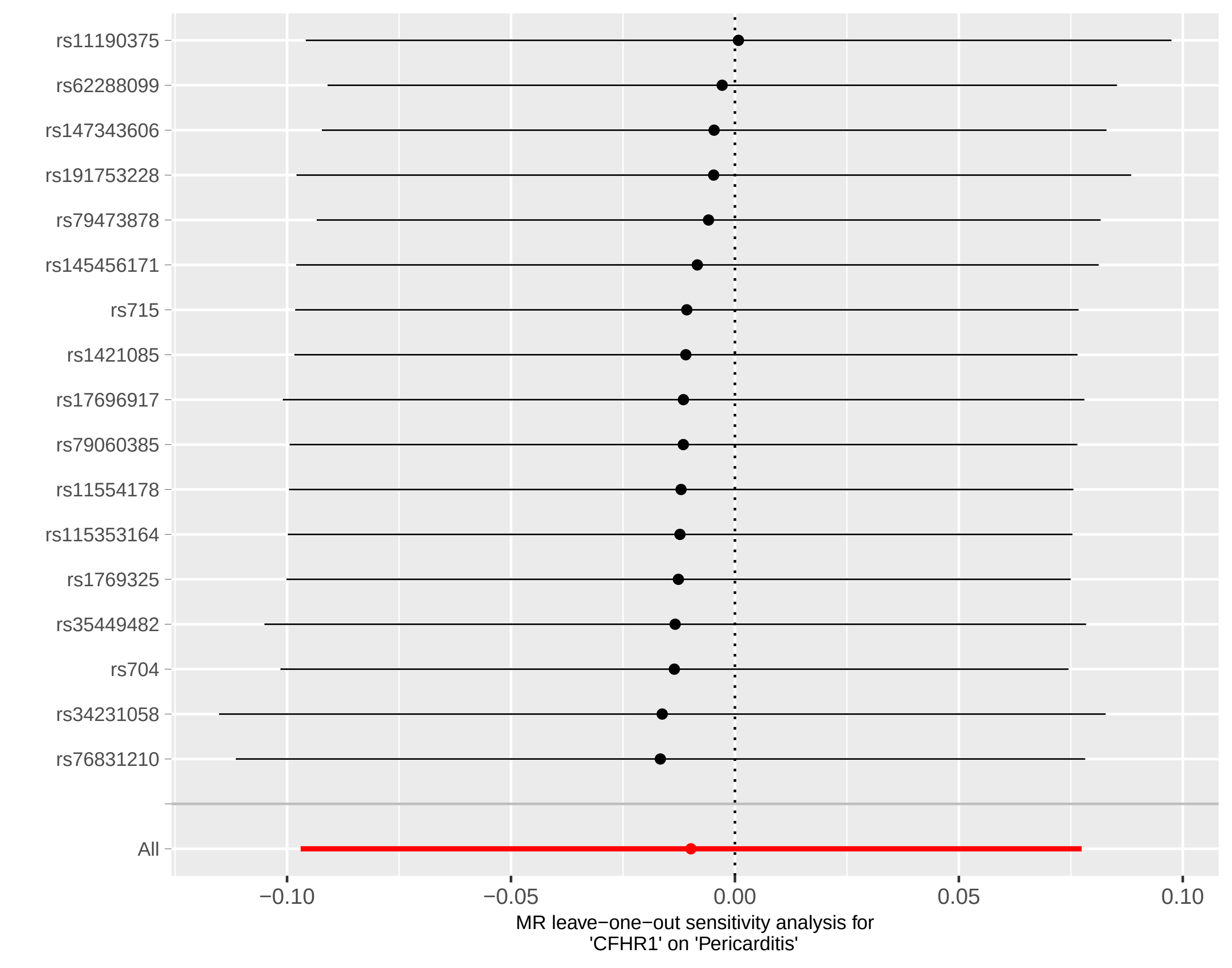

rs11190375
rs62288099
rs147343606
rs191753228
rs79473878
rs145456171
rs715
rs1421085
rs17696917
rs79060385
rs11554178
rs115353164
rs1769325
rs35449482
rs704
rs34231058
rs76831210
All
−0.10
−0.05
0.00
0.05
0.10
MR leave−one−out sensitivity analysis for
'CFHR1' on 'Pericarditis'

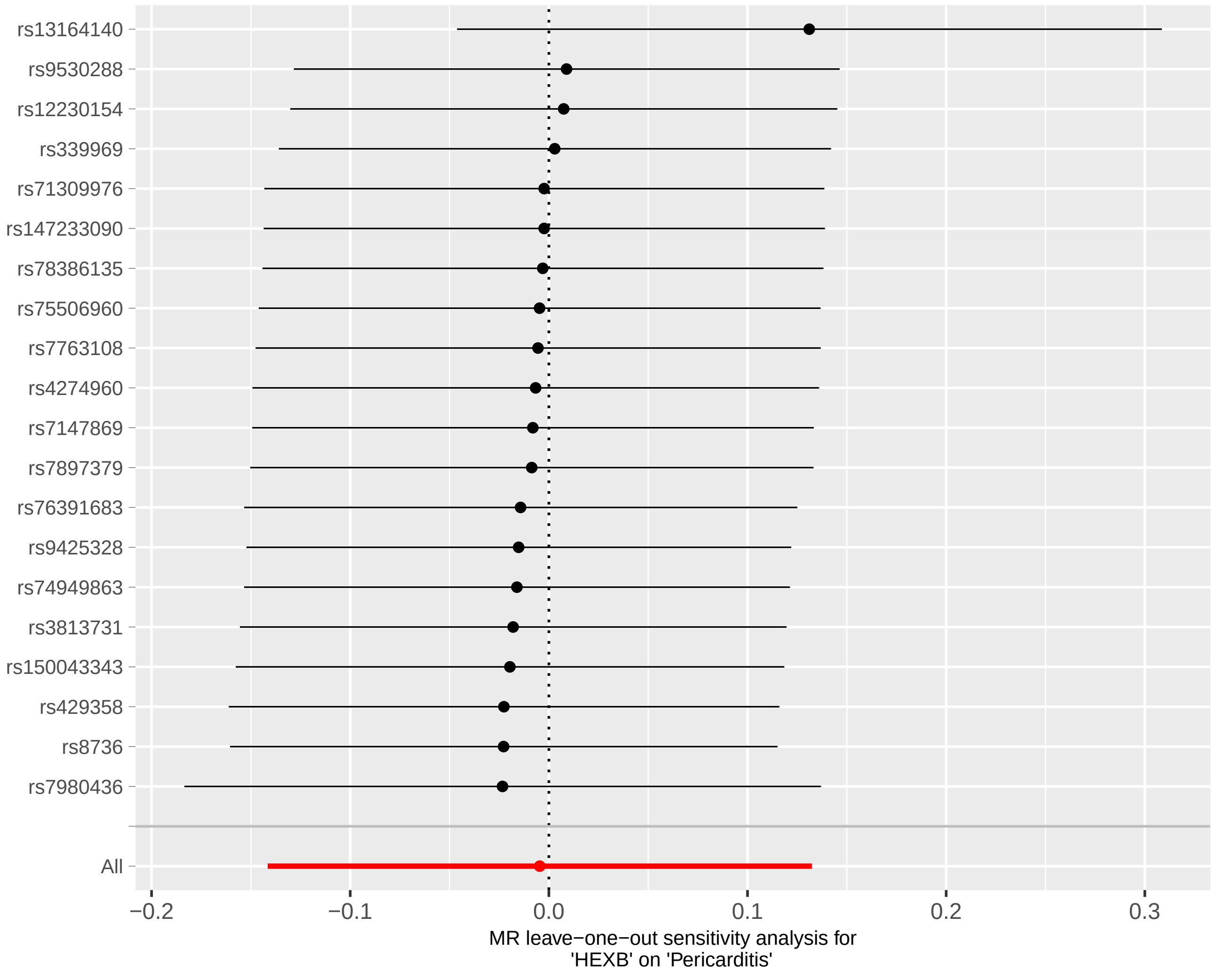

rs13164140
rs9530288
rs12230154
rs339969
rs71309976
rs147233090
rs78386135
rs75506960
rs7763108
rs4274960
rs7147869
rs7897379
rs76391683
rs9425328
rs74949863
rs3813731
rs150043343
rs429358
rs8736
rs7980436
All
−0.2
−0.1
0.0
0.1
0.2
0.3
MR leave−one−out sensitivity analysis for
'HEXB' on 'Pericarditis'

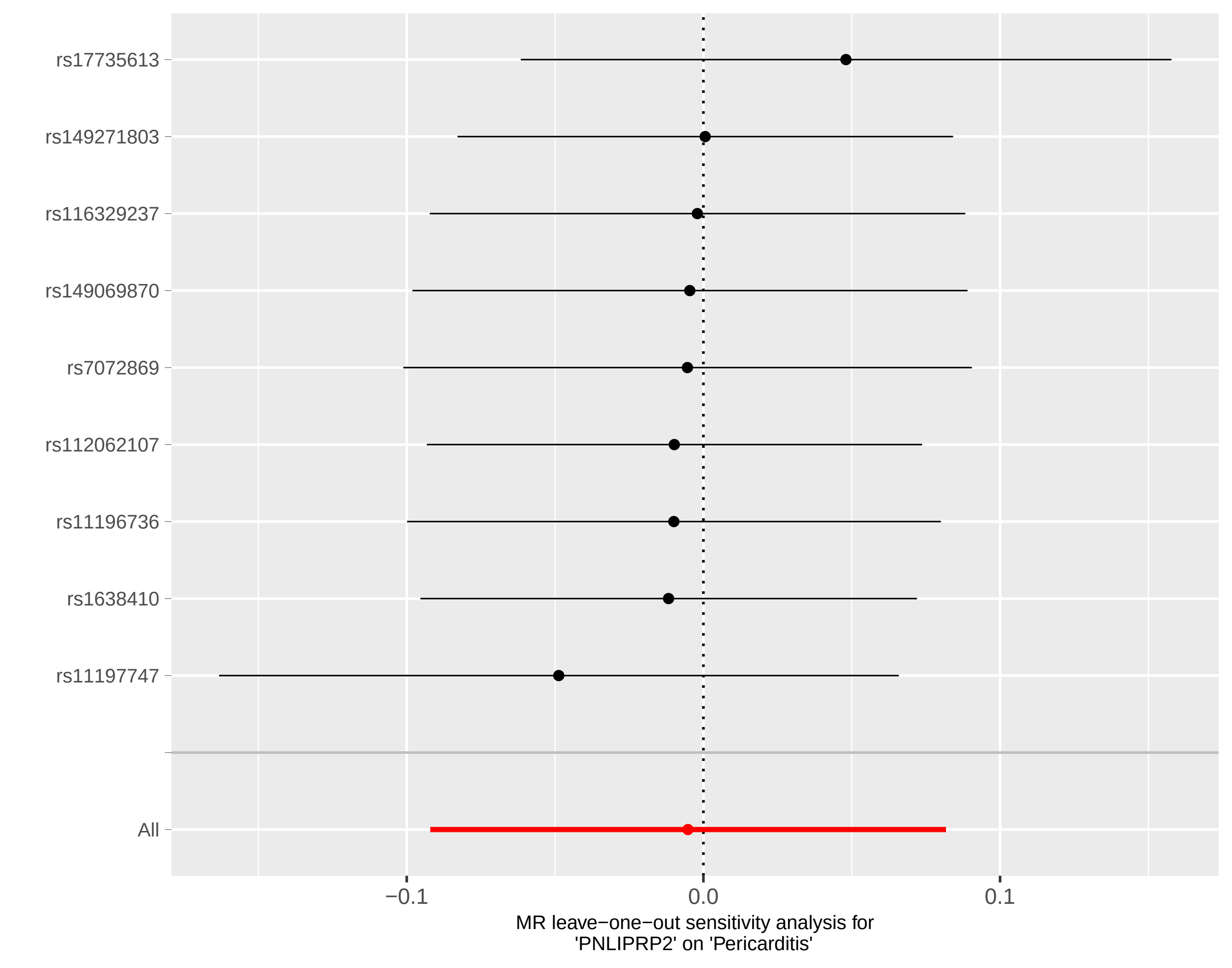
rs17735613
rs149271803
rs116329237
rs149069870
rs7072869
rs112062107
rs11196736
rs1638410
rs11197747
All
−0.1
0.0
0.1
MR leave−one−out sensitivity analysis for
'PNLIPRP2' on 'Pericarditis'

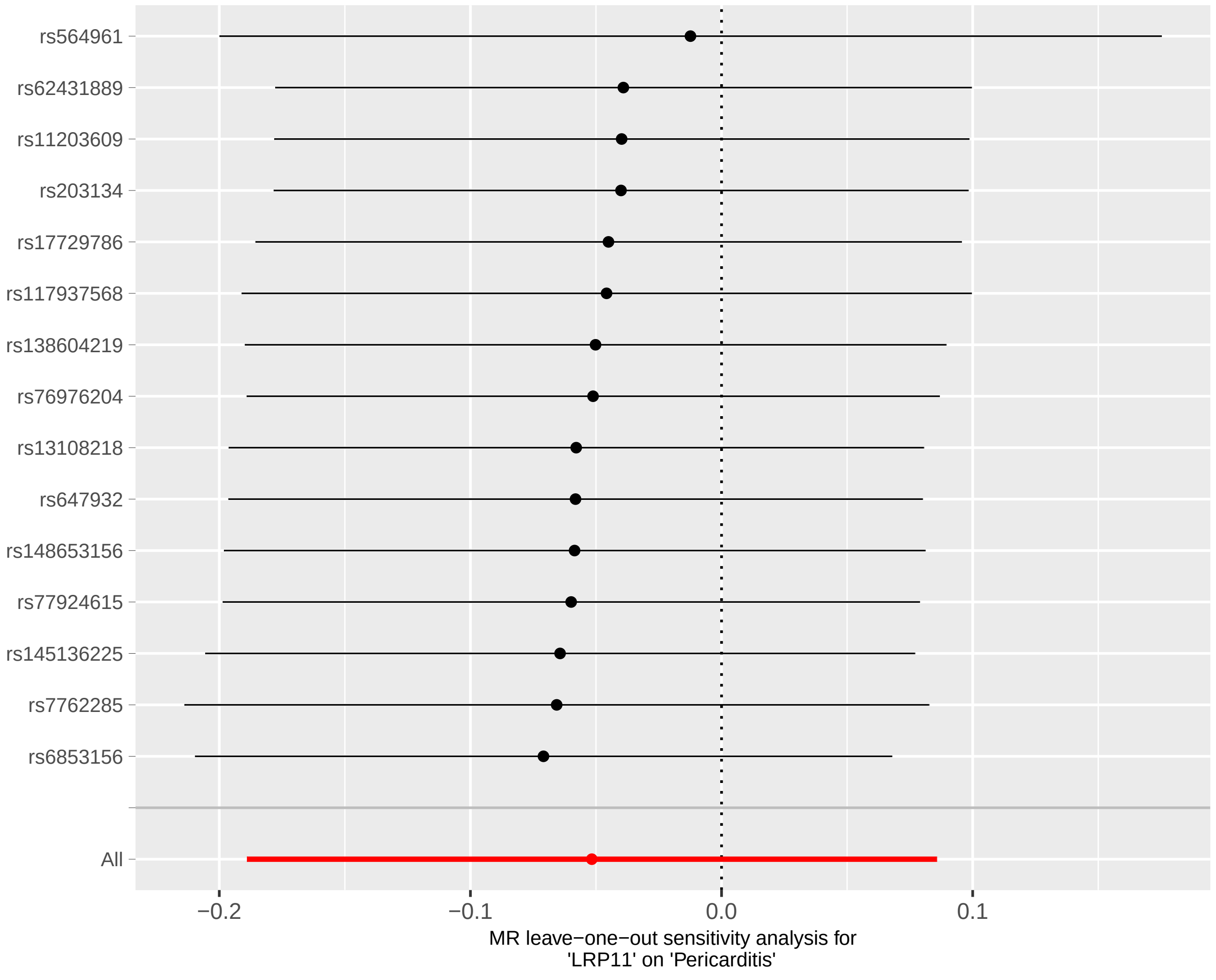

rs564961
rs62431889
rs11203609
rs203134
rs17729786
rs117937568
rs138604219
rs76976204
rs13108218
rs647932
rs148653156
rs77924615
rs145136225
rs7762285
rs6853156
All
−0.2
−0.1
0.0
0.1
MR leave−one−out sensitivity analysis for
'LRP11' on 'Pericarditis'

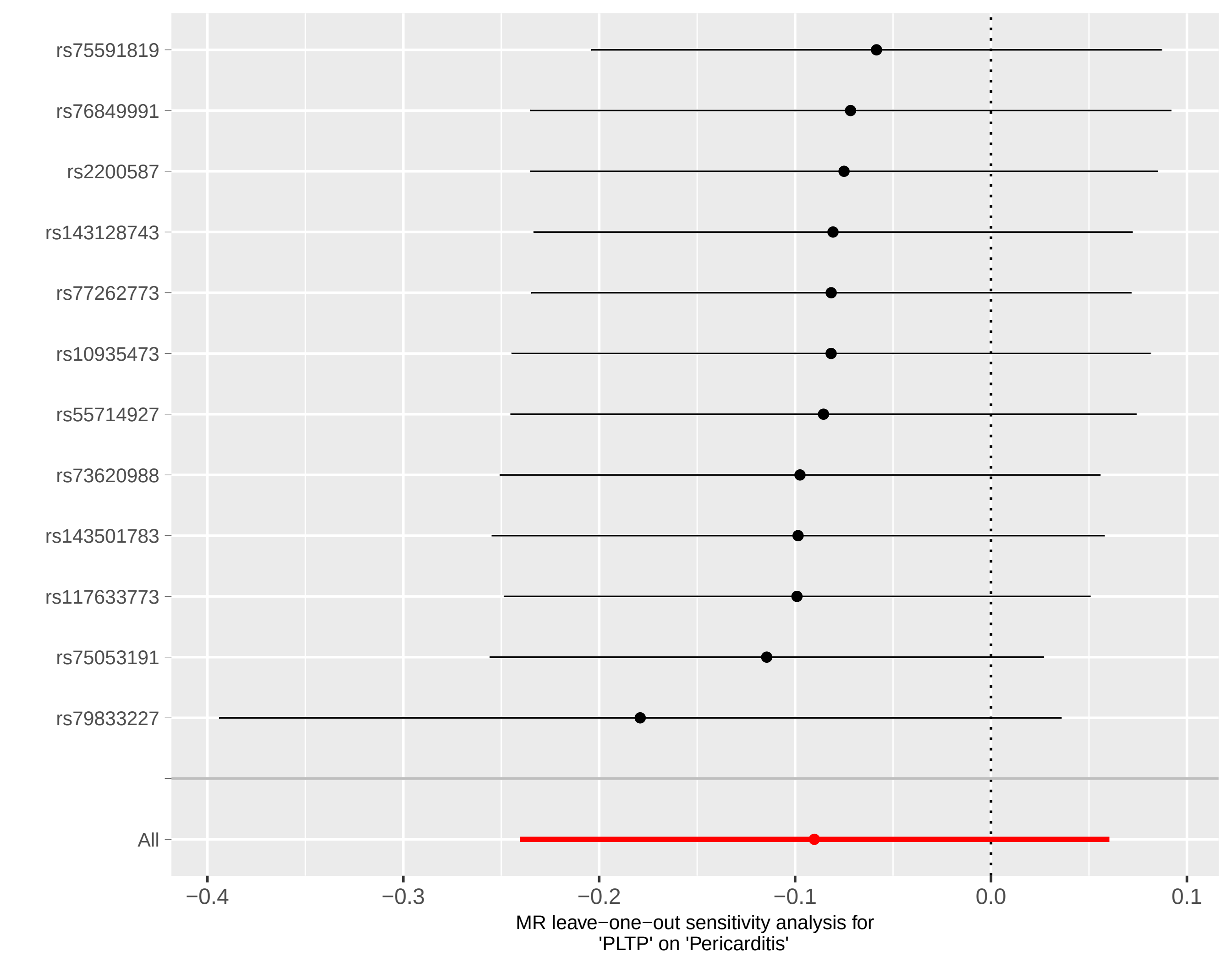
rs75591819
rs76849991
rs2200587
rs143128743
rs77262773
rs10935473
rs55714927
rs73620988
rs143501783
rs117633773
rs75053191
rs79833227
All
−0.4
−0.3
−0.2
−0.1
0.0
0.1
MR leave−one−out sensitivity analysis for
'PLTP' on 'Pericarditis'

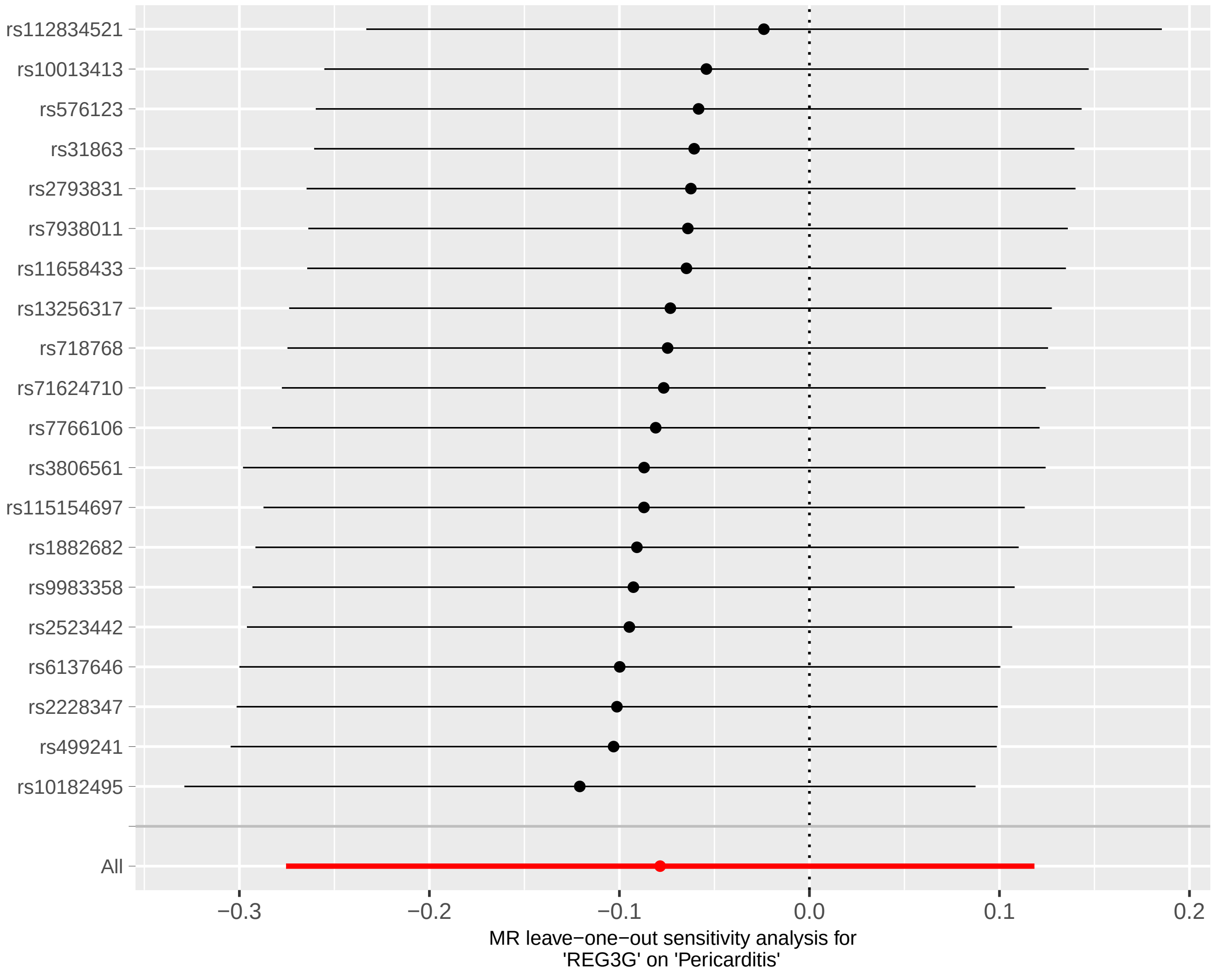
rs112834521
rs10013413
rs576123
rs31863
rs2793831
rs7938011
rs11658433
rs13256317
rs718768
rs71624710
rs7766106
rs3806561
rs115154697
rs1882682
rs9983358
rs2523442
rs6137646
rs2228347
rs499241
rs10182495
All
−0.3
−0.2
−0.1
0.0
0.1
0.2
MR leave−one−out sensitivity analysis for
'REG3G' on 'Pericarditis'

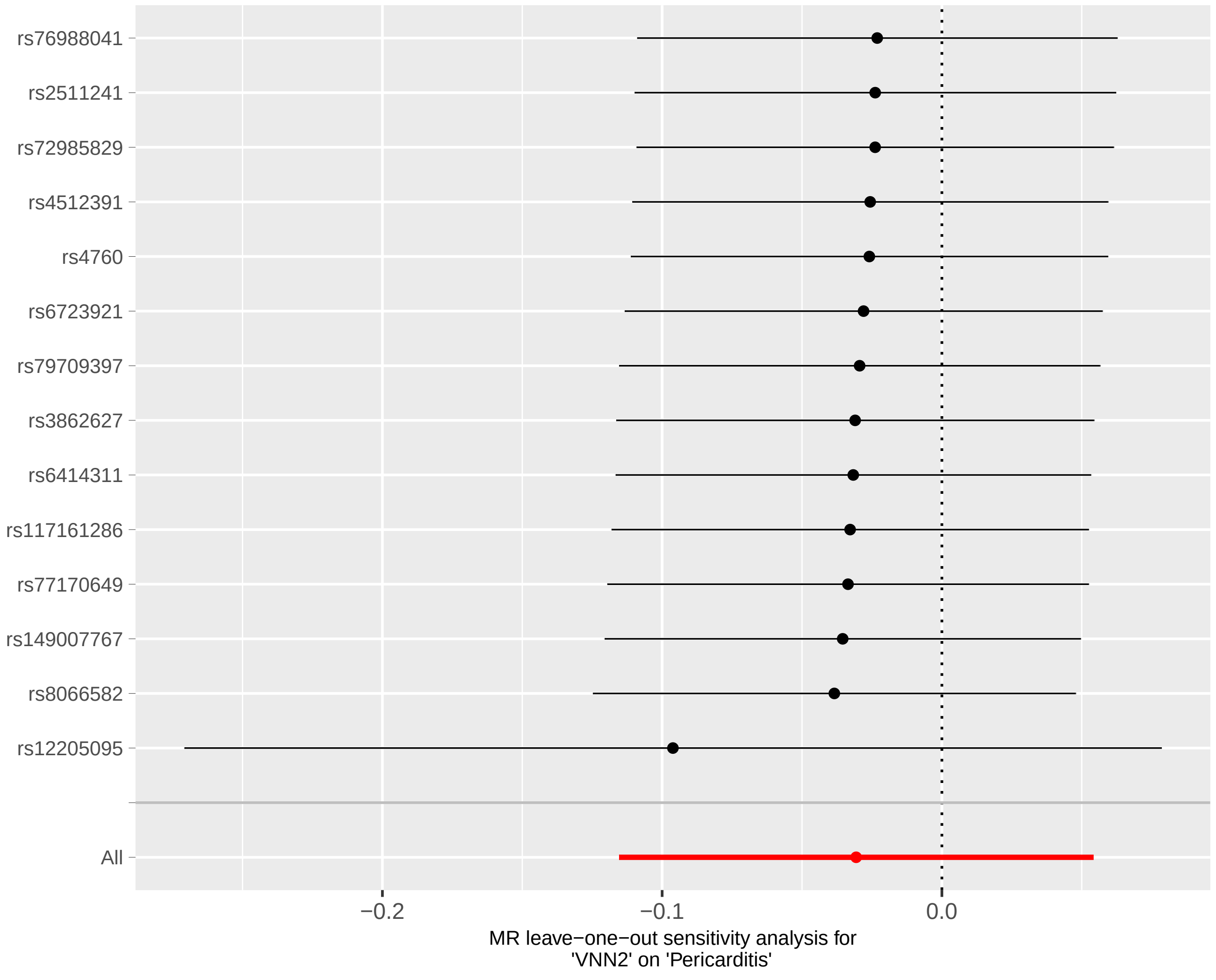

rs76988041
rs2511241
rs72985829
rs4512391
rs4760
rs6723921
rs79709397
rs3862627
rs6414311
rs117161286
rs77170649
rs149007767
rs8066582
rs12205095
All
−0.2
−0.1
0.0
MR leave−one−out sensitivity analysis for
'VNN2' on 'Pericarditis'

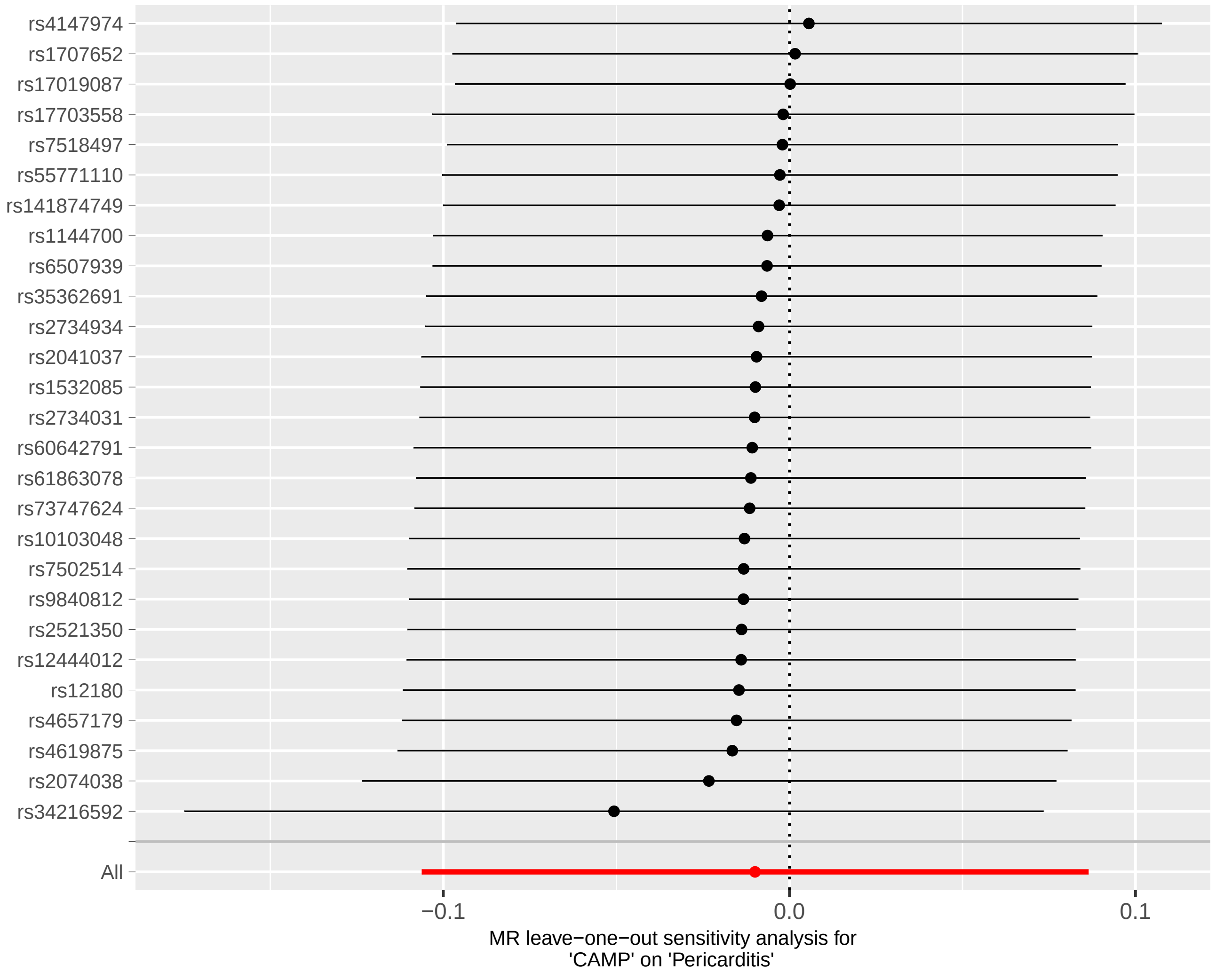

rs4147974
rs1707652
rs17019087
rs17703558
rs7518497
rs55771110
rs141874749
rs1144700
rs6507939
rs35362691
rs2734934
rs2041037
rs1532085
rs2734031
rs60642791
rs61863078
rs73747624
rs10103048
rs7502514
rs9840812
rs2521350
rs12444012
rs12180
rs4657179
rs4619875
rs2074038
rs34216592
All
−0.1
0.0
0.1
MR leave−one−out sensitivity analysis for
'CAMP' on 'Pericarditis'

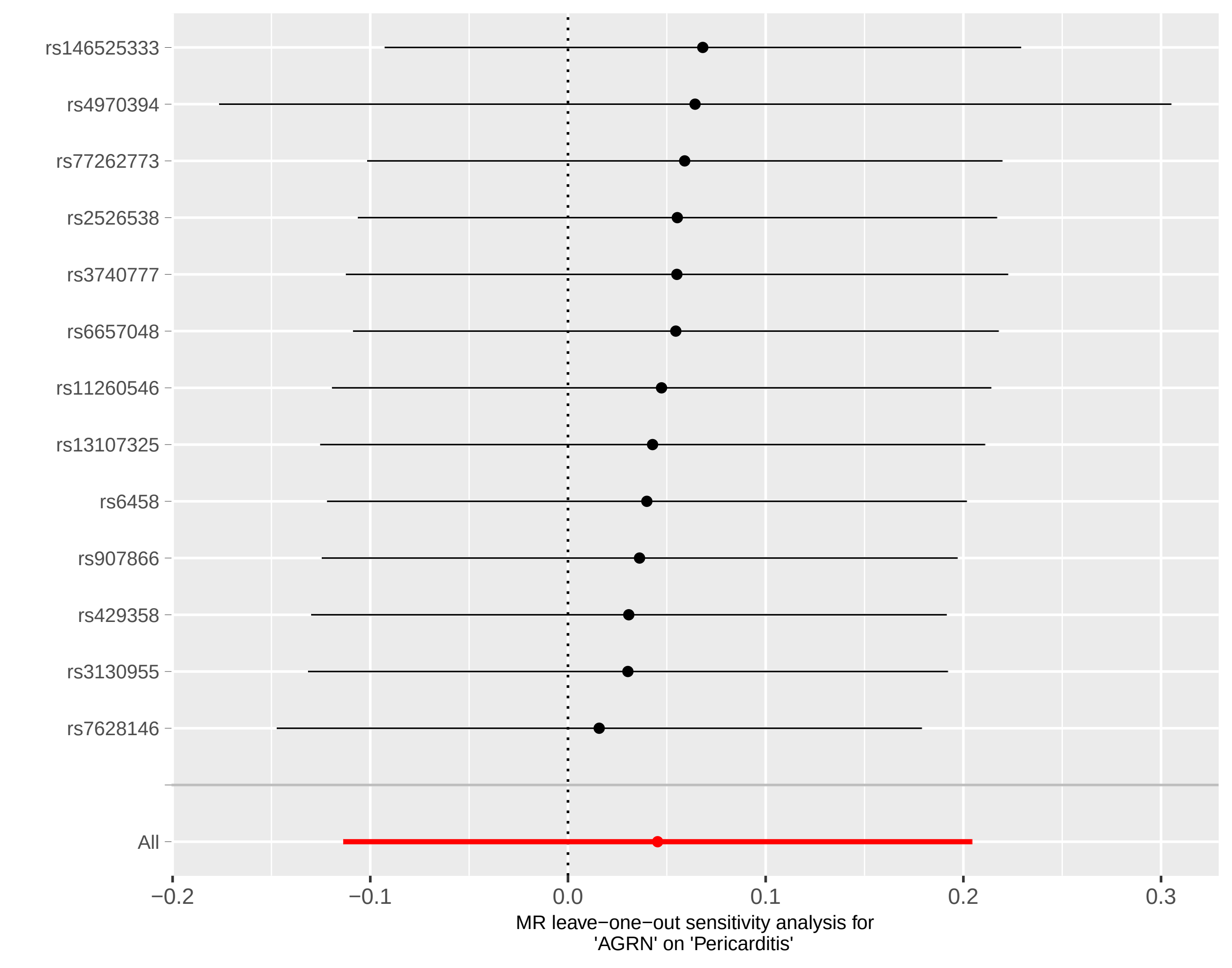

rs146525333
rs4970394
rs77262773
rs2526538
rs3740777
rs6657048
rs11260546
rs13107325
rs6458
rs907866
rs429358
rs3130955
rs7628146
All
−0.2
−0.1
0.0
0.1
0.2
0.3
MR leave−one−out sensitivity analysis for
'AGRN' on 'Pericarditis'

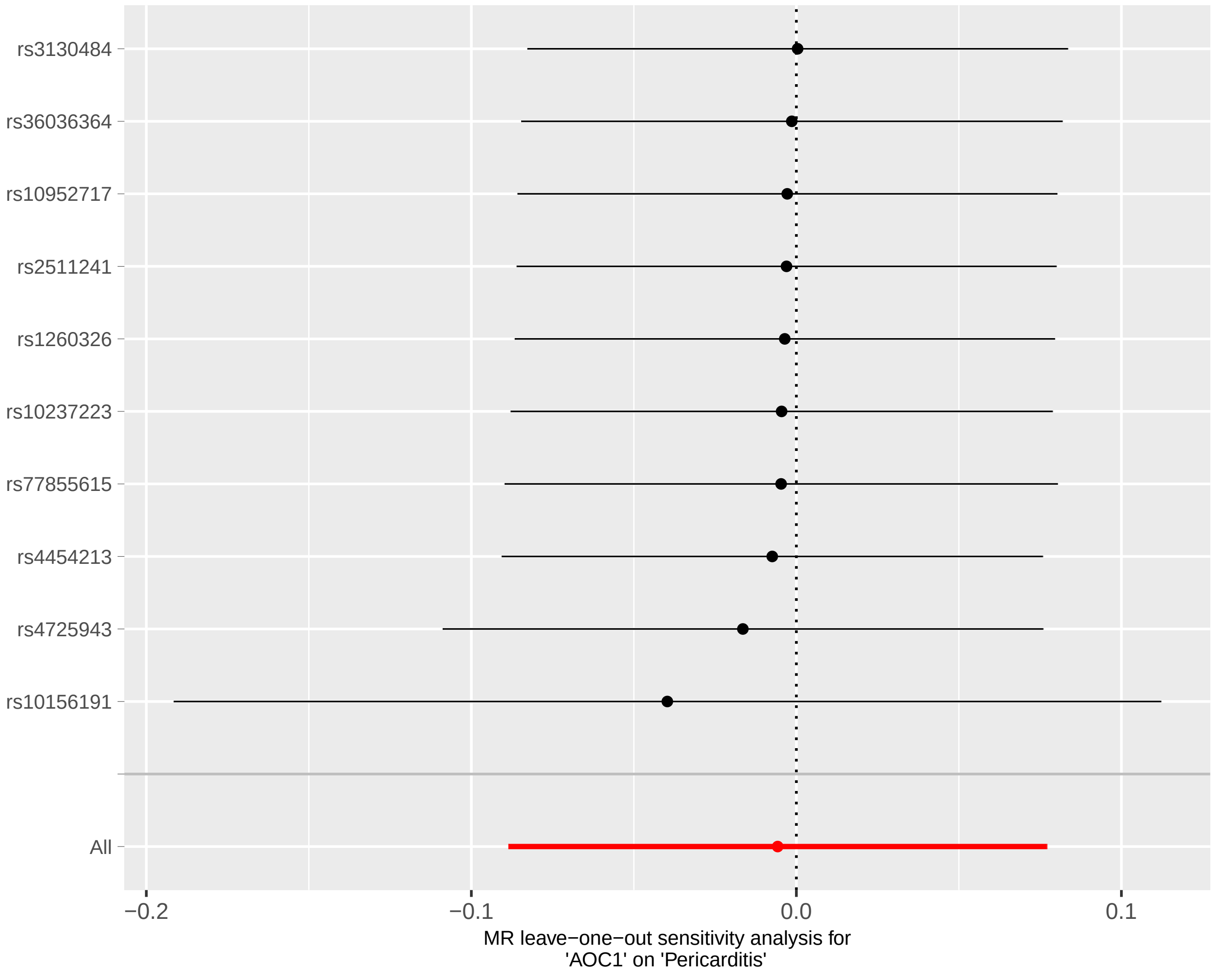

rs3130484
rs36036364
rs10952717
rs2511241
rs1260326
rs10237223
rs77855615
rs4454213
rs4725943
rs10156191
All
−0.2
−0.1
0.0
0.1
MR leave−one−out sensitivity analysis for
'AOC1' on 'Pericarditis'

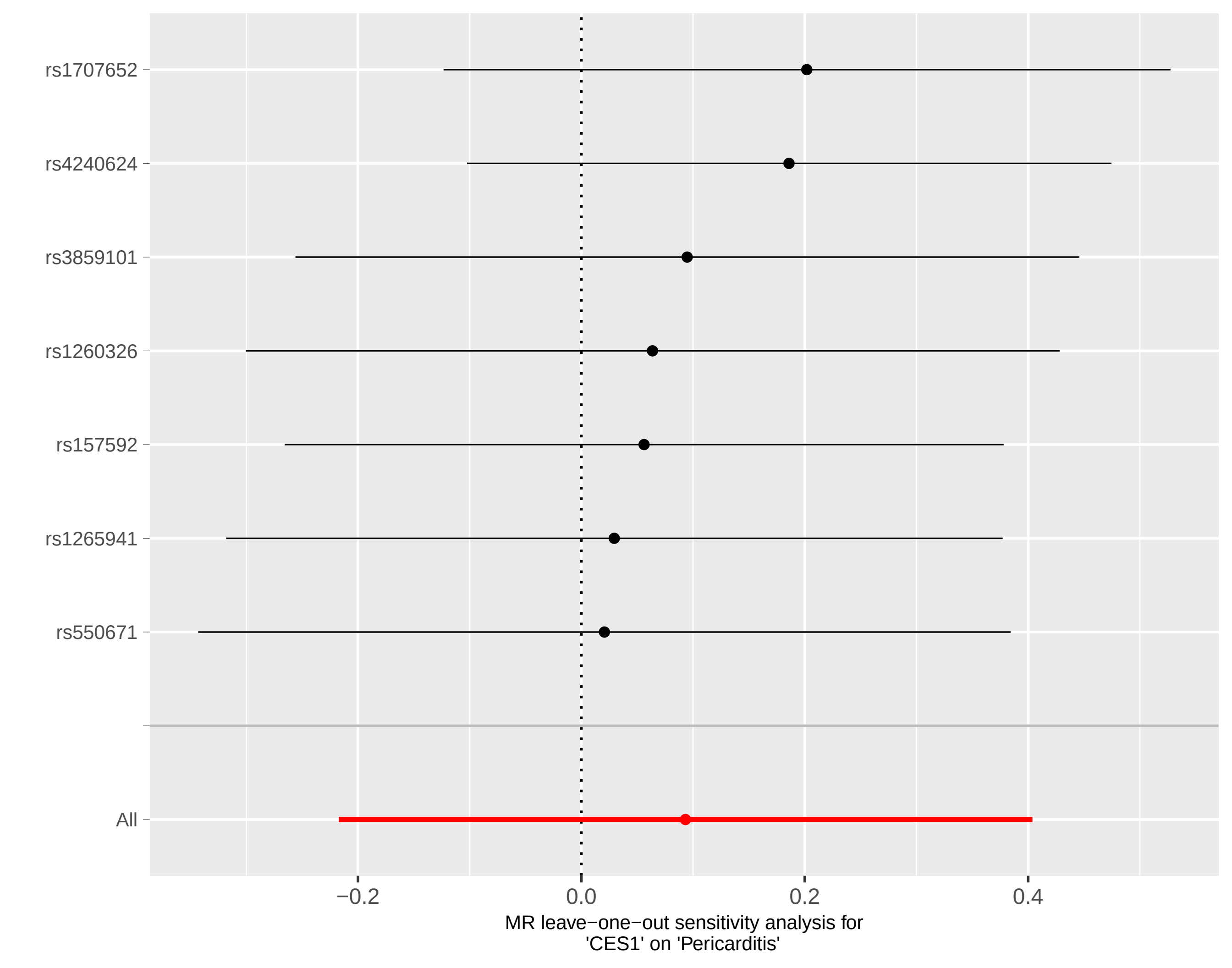

rs1707652
rs4240624
rs3859101
rs1260326
rs157592
rs1265941
rs550671
All
−0.2
0.0
0.2
0.4
MR leave−one−out sensitivity analysis for
'CES1' on 'Pericarditis'

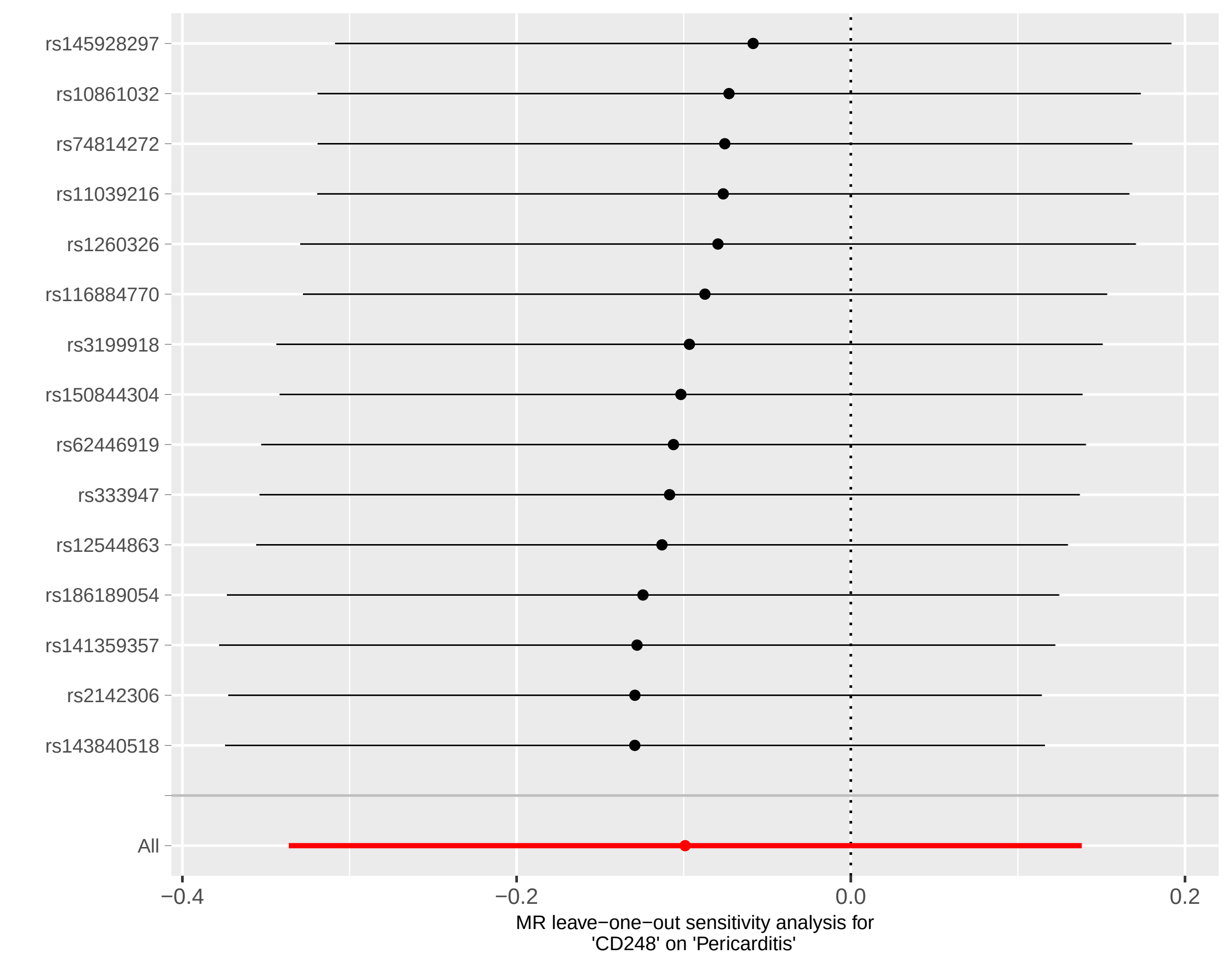
rs145928297
rs10861032
rs74814272
rs11039216
rs1260326
rs116884770
rs3199918
rs150844304
rs62446919
rs333947
rs12544863
rs186189054
rs141359357
rs2142306
rs143840518
All
−0.4
−0.2
0.0
0.2
MR leave−one−out sensitivity analysis for
'CD248' on 'Pericarditis'

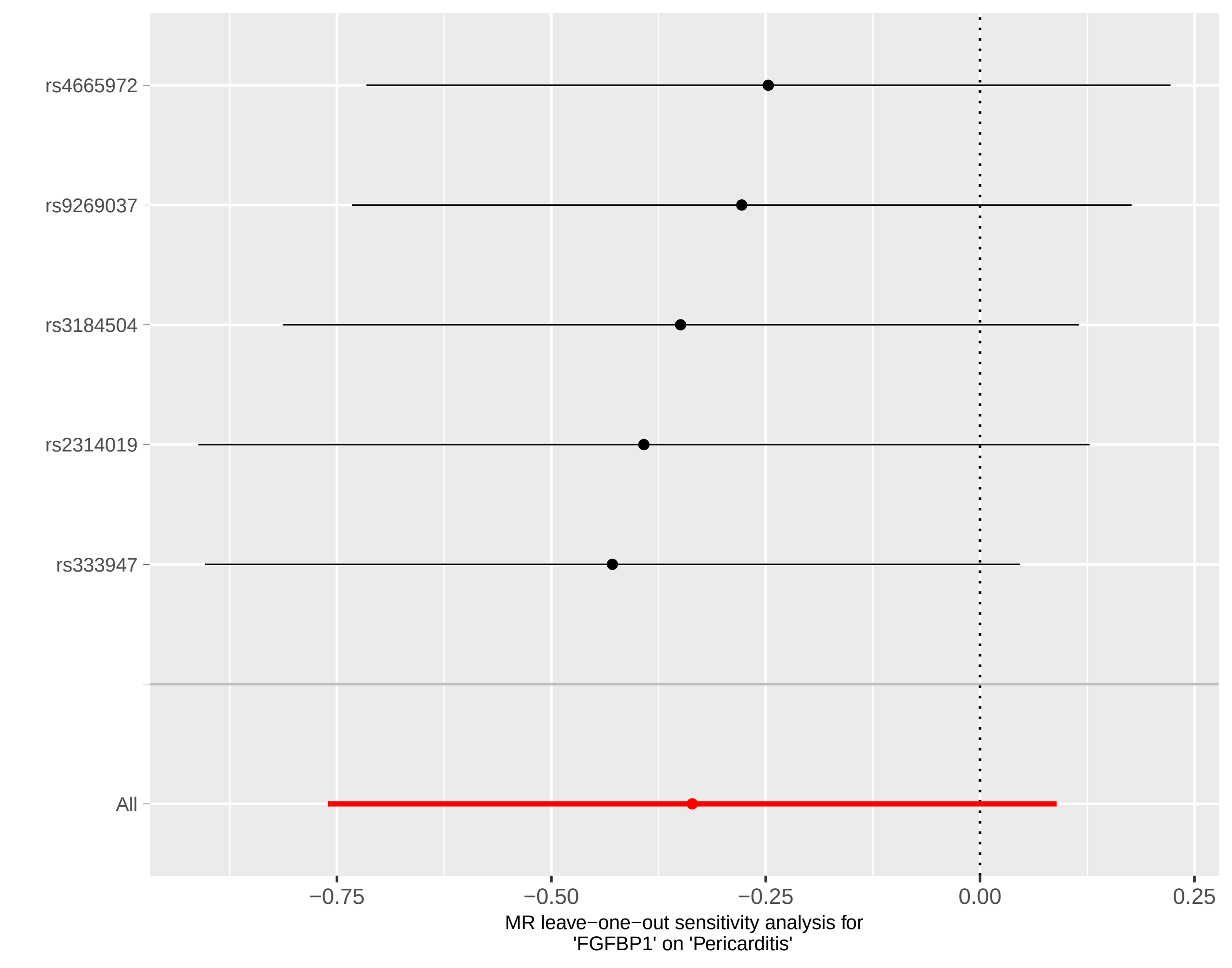
rs4665972
rs9269037
rs3184504
rs2314019
rs333947
All
−0.75
−0.50
−0.25
0.00
0.25
MR leave−one−out sensitivity analysis for
'FGFBP1' on 'Pericarditis'

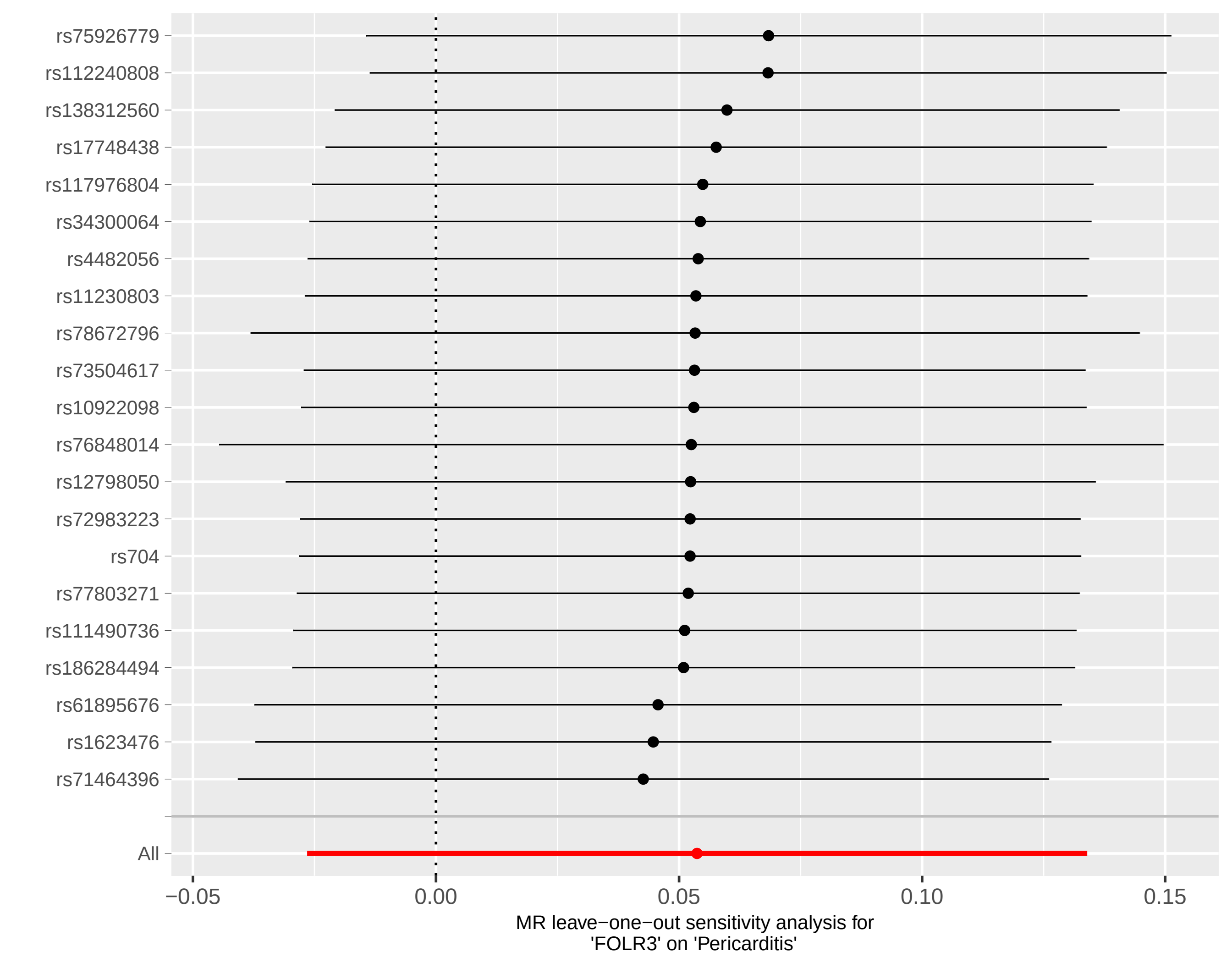
rs75926779
rs112240808
rs138312560
rs17748438
rs117976804
rs34300064
rs4482056
rs11230803
rs78672796
rs73504617
rs10922098
rs76848014
rs12798050
rs72983223
rs704
rs77803271
rs111490736
rs186284494
rs61895676
rs1623476
rs71464396
All
−0.05
0.00
0.05
0.10
0.15
MR leave−one−out sensitivity analysis for
'FOLR3' on 'Pericarditis'

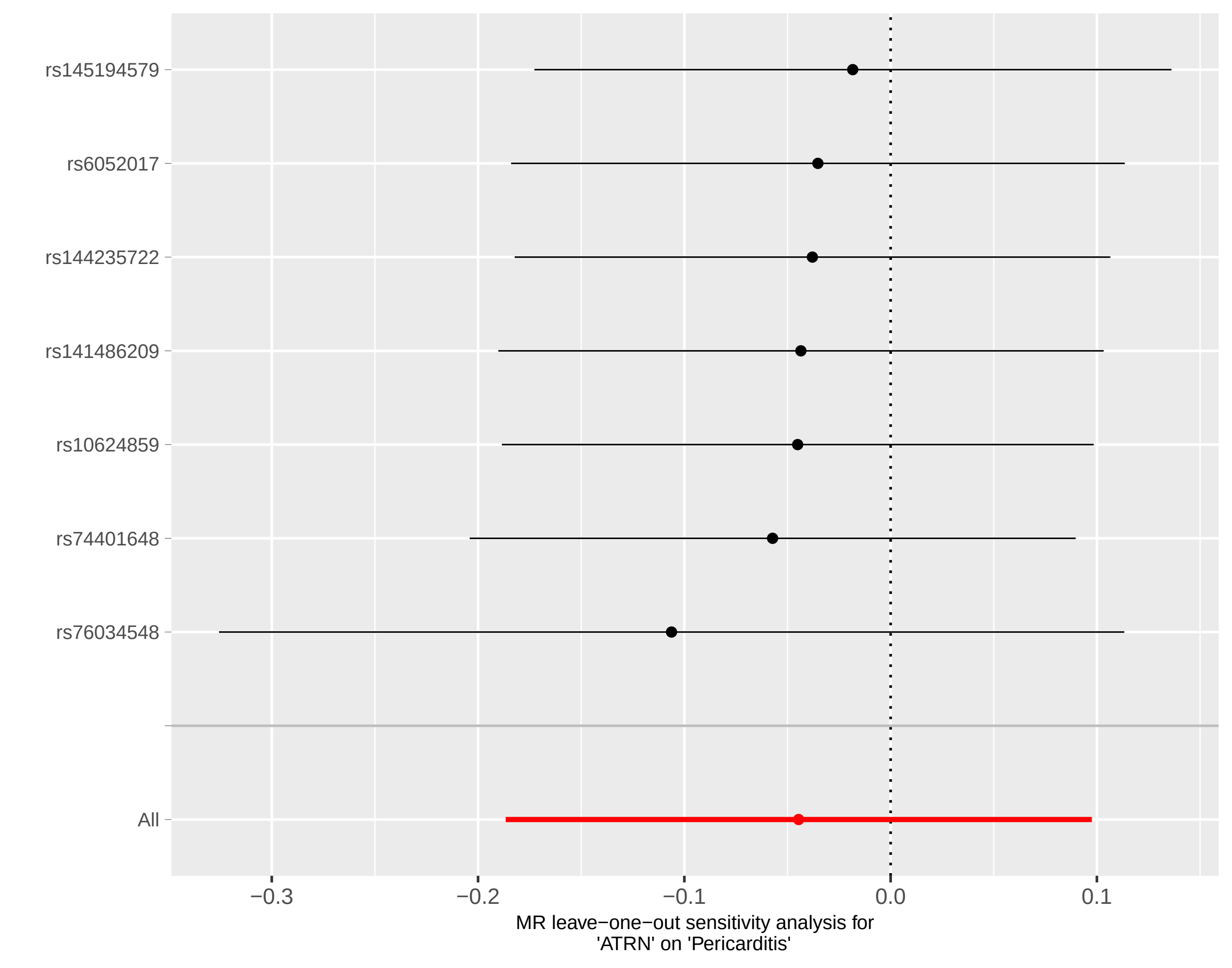
rs145194579
rs6052017
rs144235722
rs141486209
rs10624859
rs74401648
rs76034548
All
−0.3
−0.2
−0.1
0.0
0.1
MR leave−one−out sensitivity analysis for
'ATRN' on 'Pericarditis'

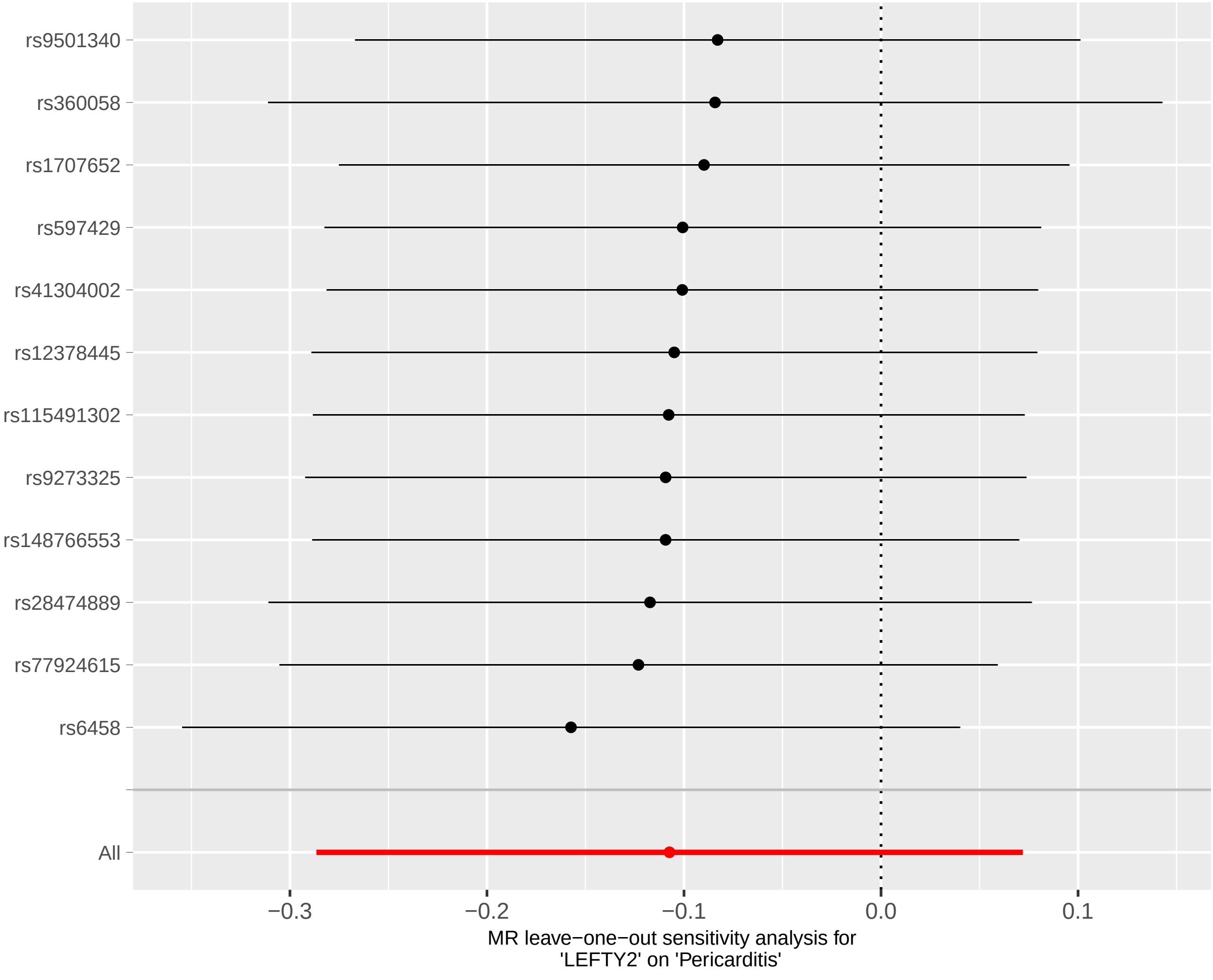

rs9501340
rs360058
rs1707652
rs597429
rs41304002
rs12378445
rs115491302
rs9273325
rs148766553
rs28474889
rs77924615
rs6458
All
−0.3
−0.2
−0.1
0.0
0.1
MR leave−one−out sensitivity analysis for
'LEFTY2' on 'Pericarditis'

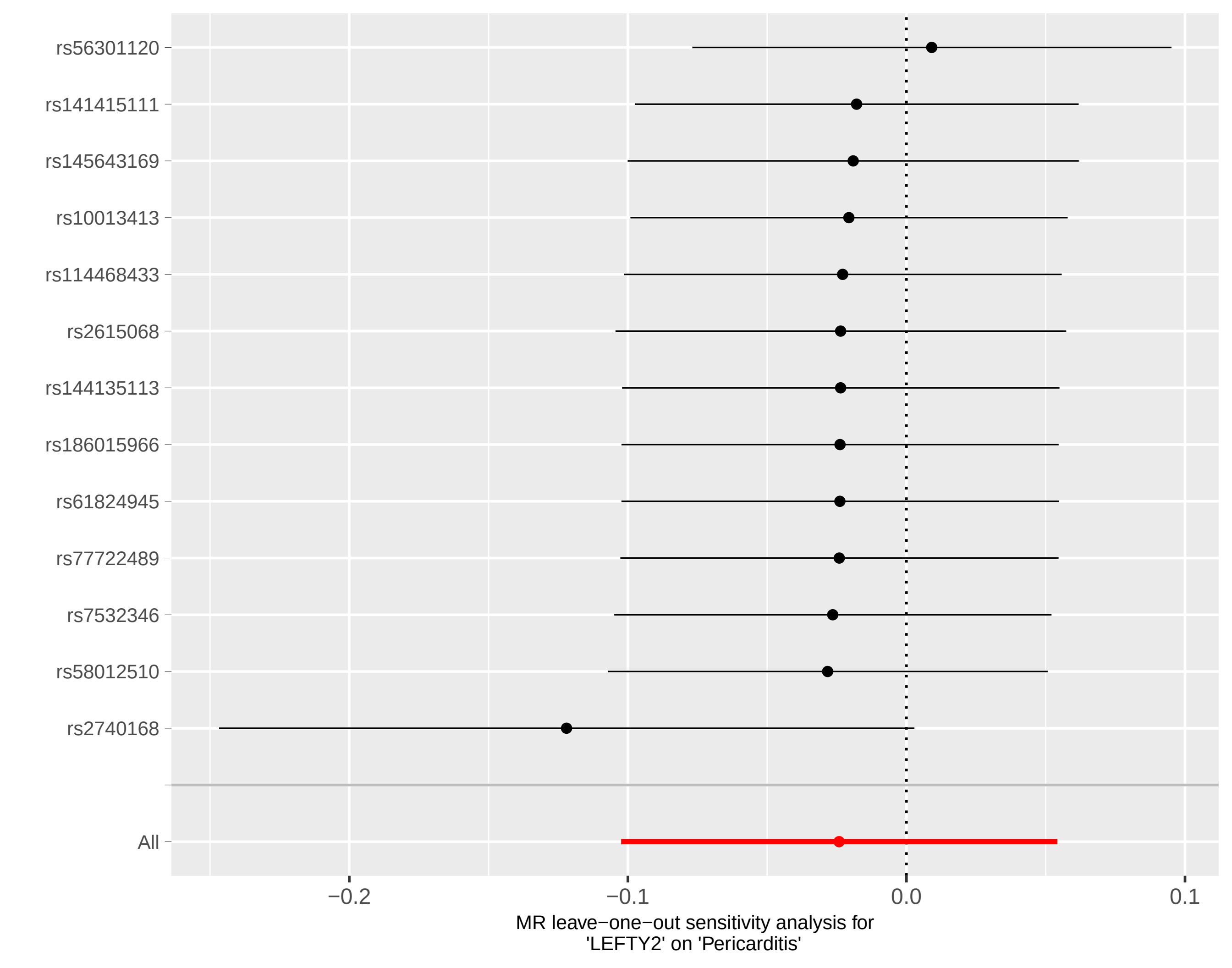

rs56301120
rs141415111
rs145643169
rs10013413
rs114468433
rs2615068
rs144135113
rs186015966
rs61824945
rs77722489
rs7532346
rs58012510
rs2740168
All
−0.2
−0.1
0.0
0.1
MR leave−one−out sensitivity analysis for
'LEFTY2' on 'Pericarditis'

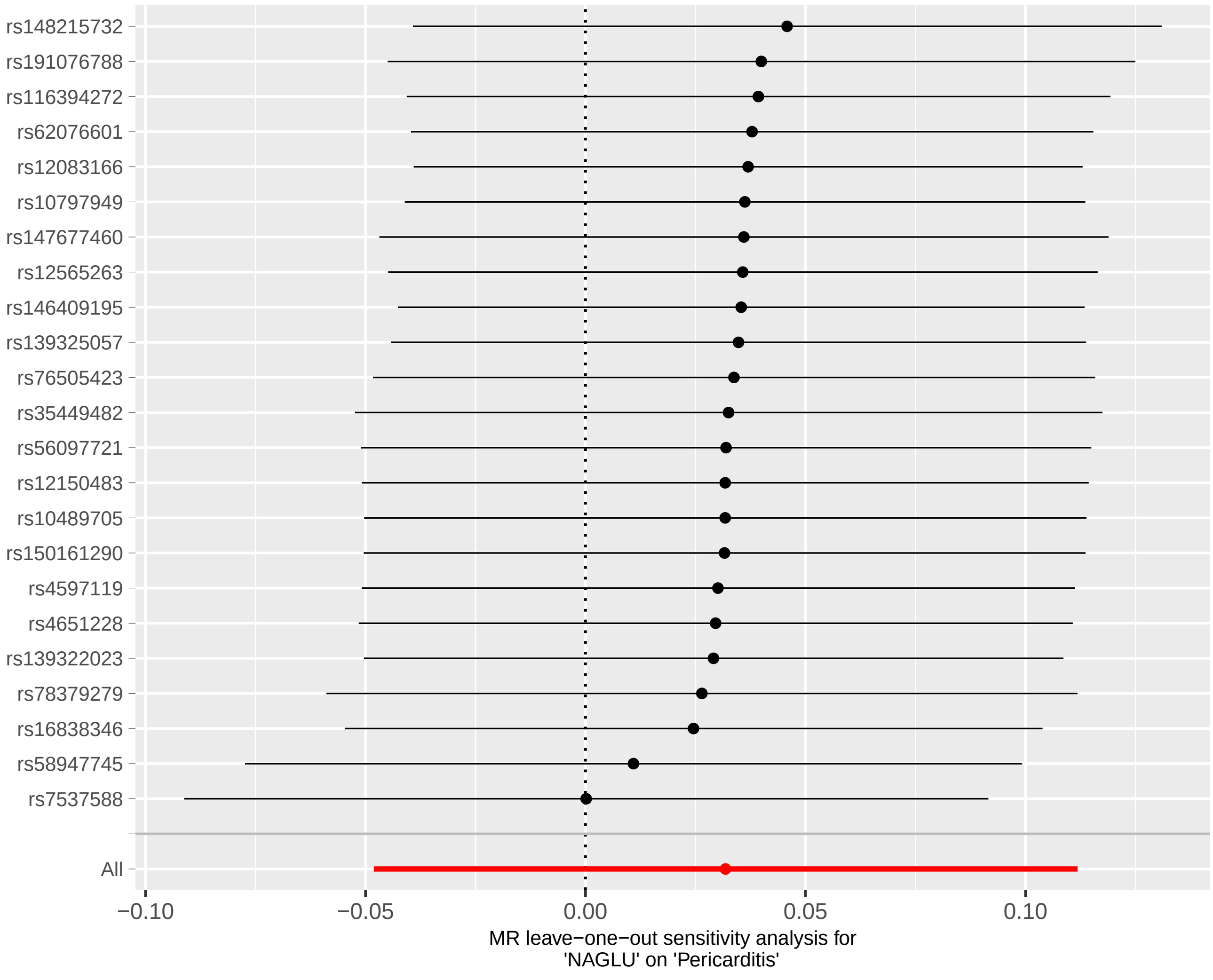

rs148215732
rs191076788
rs116394272
rs62076601
rs12083166
rs10797949
rs147677460
rs12565263
rs146409195
rs139325057
rs76505423
rs35449482
rs56097721
rs12150483
rs10489705
rs150161290
rs4597119
rs4651228
rs139322023
rs78379279
rs16838346
rs58947745
rs7537588
All
−0.10
−0.05
0.00
0.05
0.10
MR leave−one−out sensitivity analysis for
'NAGLU' on 'Pericarditis'

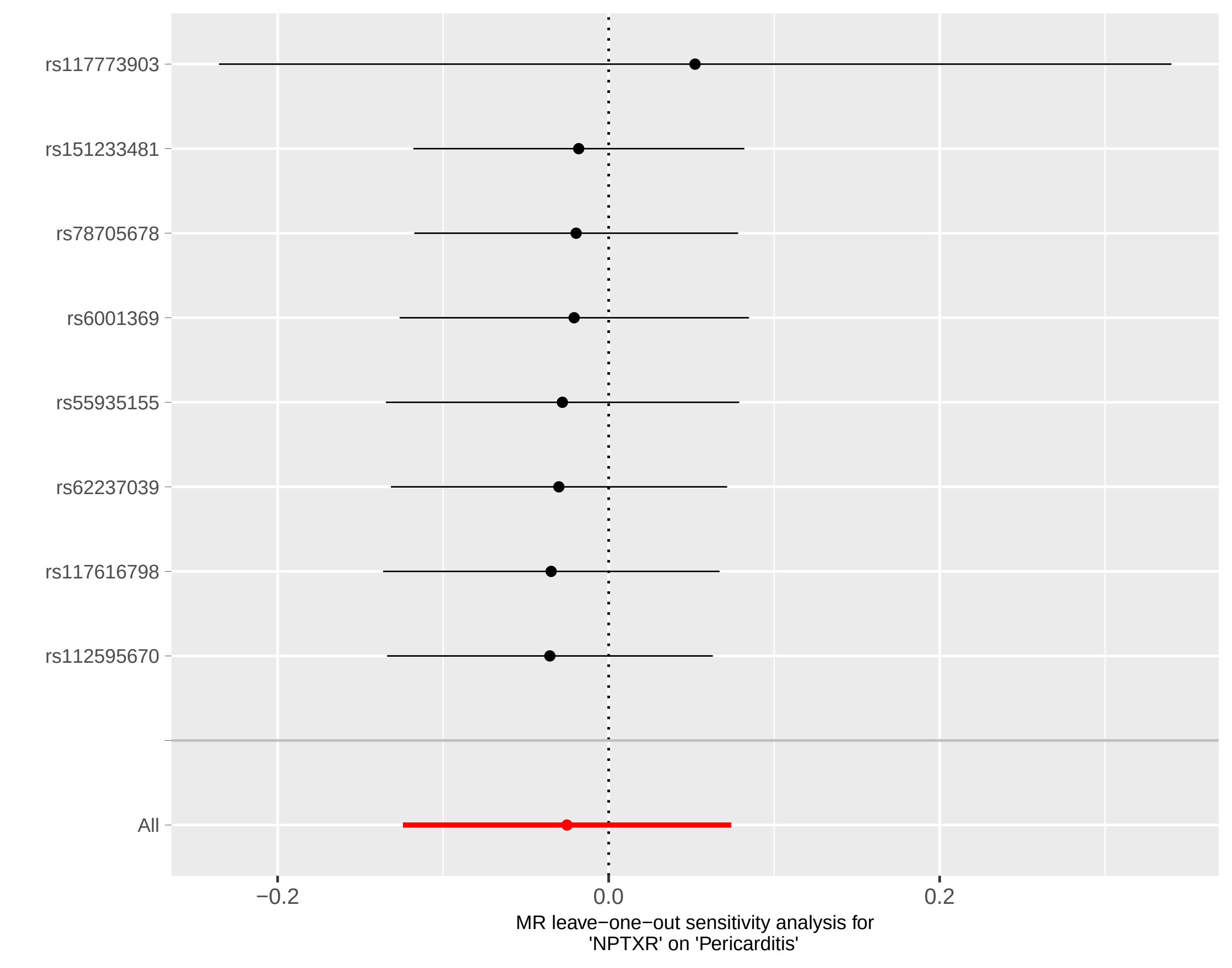

rs117773903
rs151233481
rs78705678
rs6001369
rs55935155
rs62237039
rs117616798
rs112595670
All
−0.2
0.0
0.2
MR leave−one−out sensitivity analysis for
'NPTXR' on 'Pericarditis'

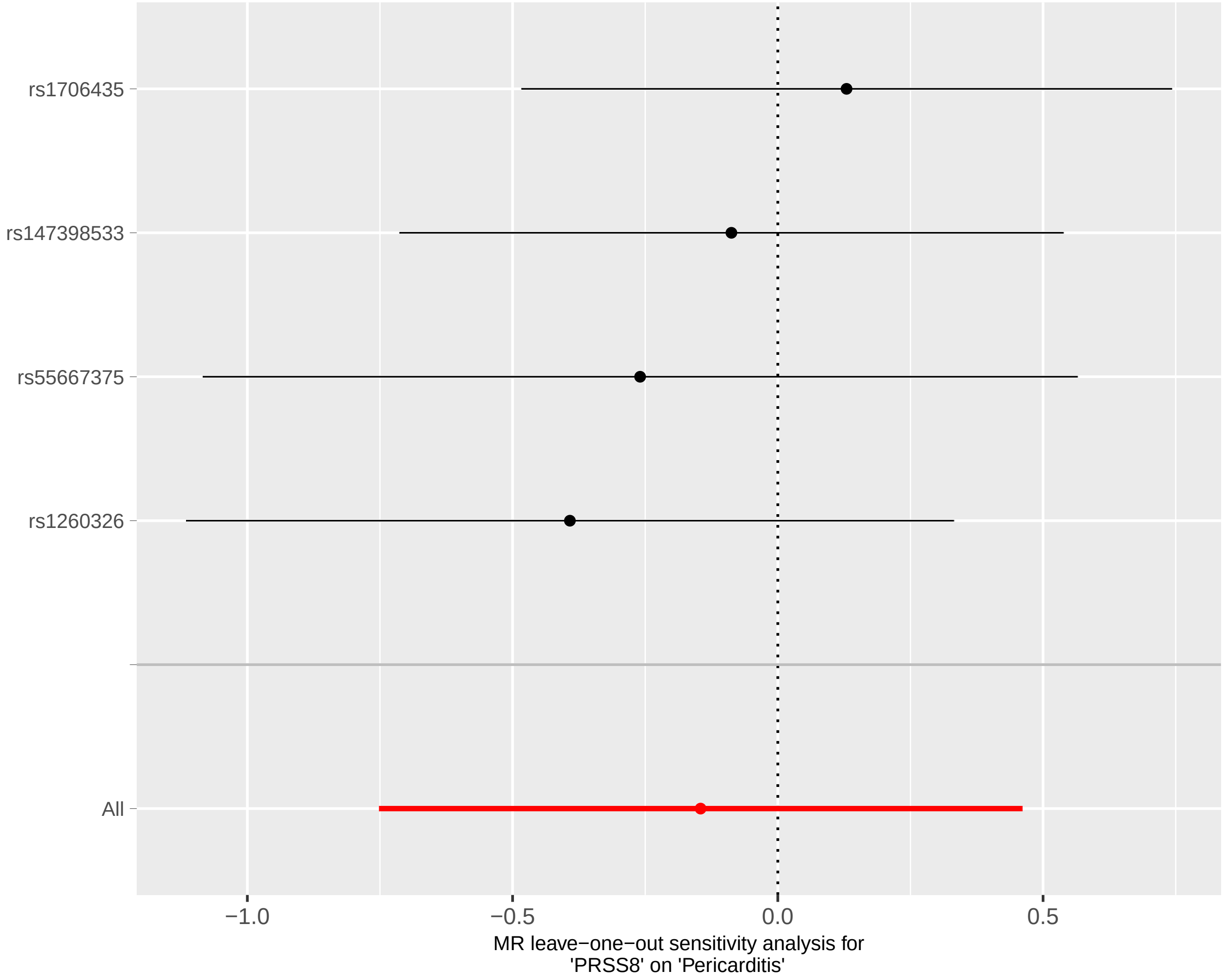
rs1706435
rs147398533
rs55667375
rs1260326
All
−1.0
−0.5
0.0
0.5
MR leave−one−out sensitivity analysis for
'PRSS8' on 'Pericarditis'

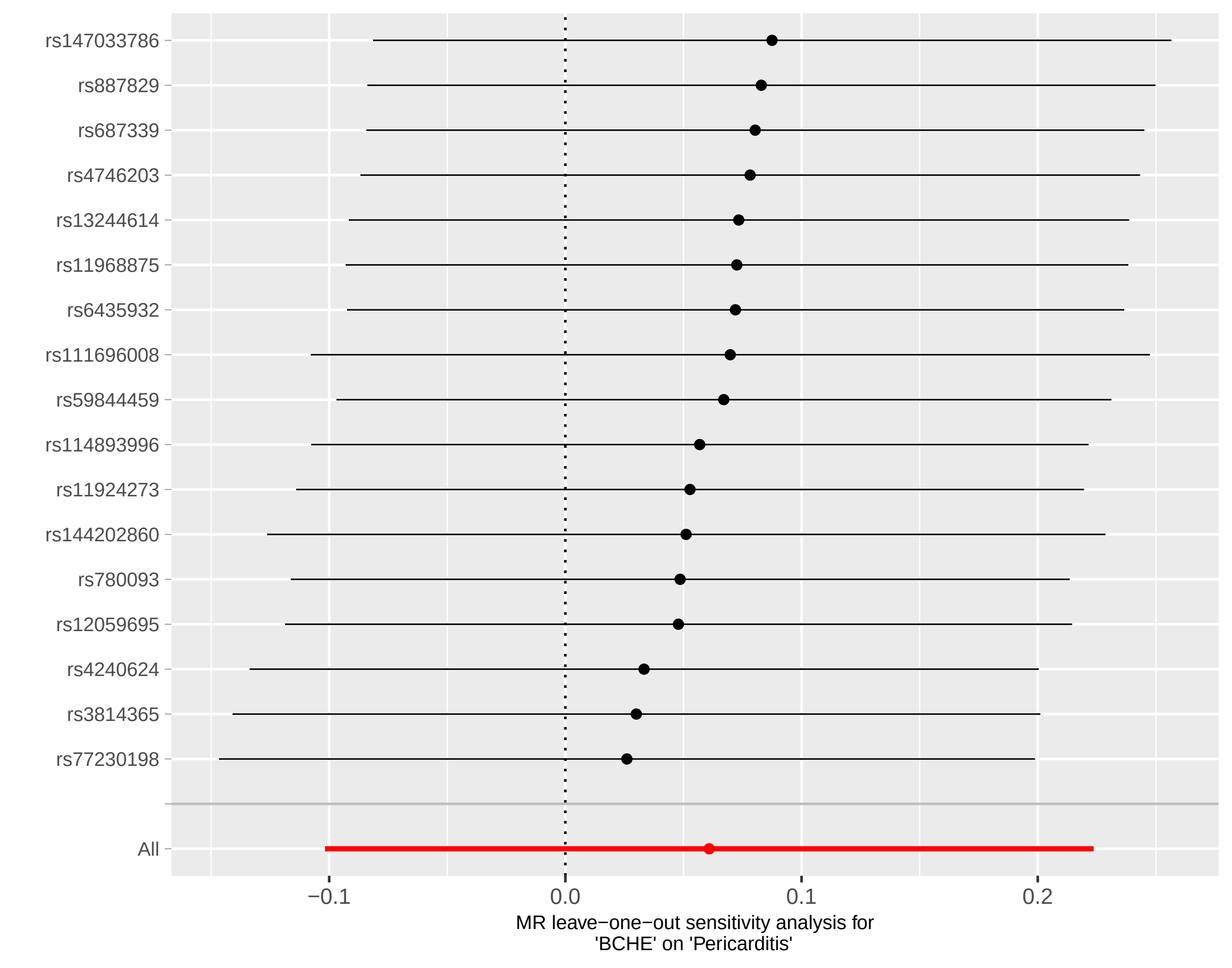
rs147033786
rs887829
rs687339
rs4746203
rs13244614
rs11968875
rs6435932
rs111696008
rs59844459
rs114893996
rs11924273
rs144202860
rs780093
rs12059695
rs4240624
rs3814365
rs77230198
All
−0.1
0.0
0.1
0.2
MR leave−one−out sensitivity analysis for
'BCHE' on 'Pericarditis'

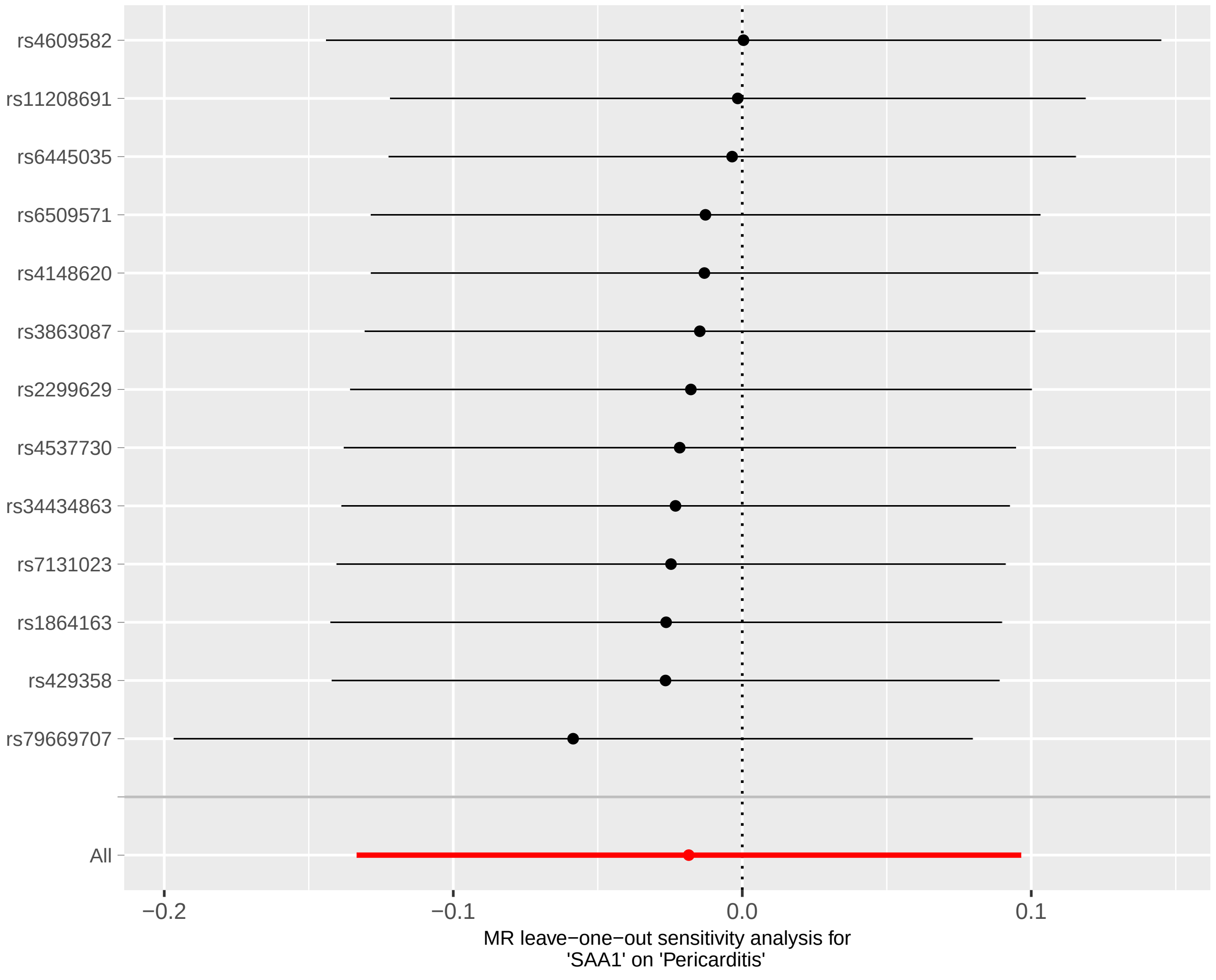
rs4609582
rs11208691
rs6445035
rs6509571
rs4148620
rs3863087
rs2299629
rs4537730
rs34434863
rs7131023
rs1864163
rs429358
rs79669707
All
−0.2
−0.1
0.0
0.1
MR leave−one−out sensitivity analysis for
'SAA1' on 'Pericarditis'

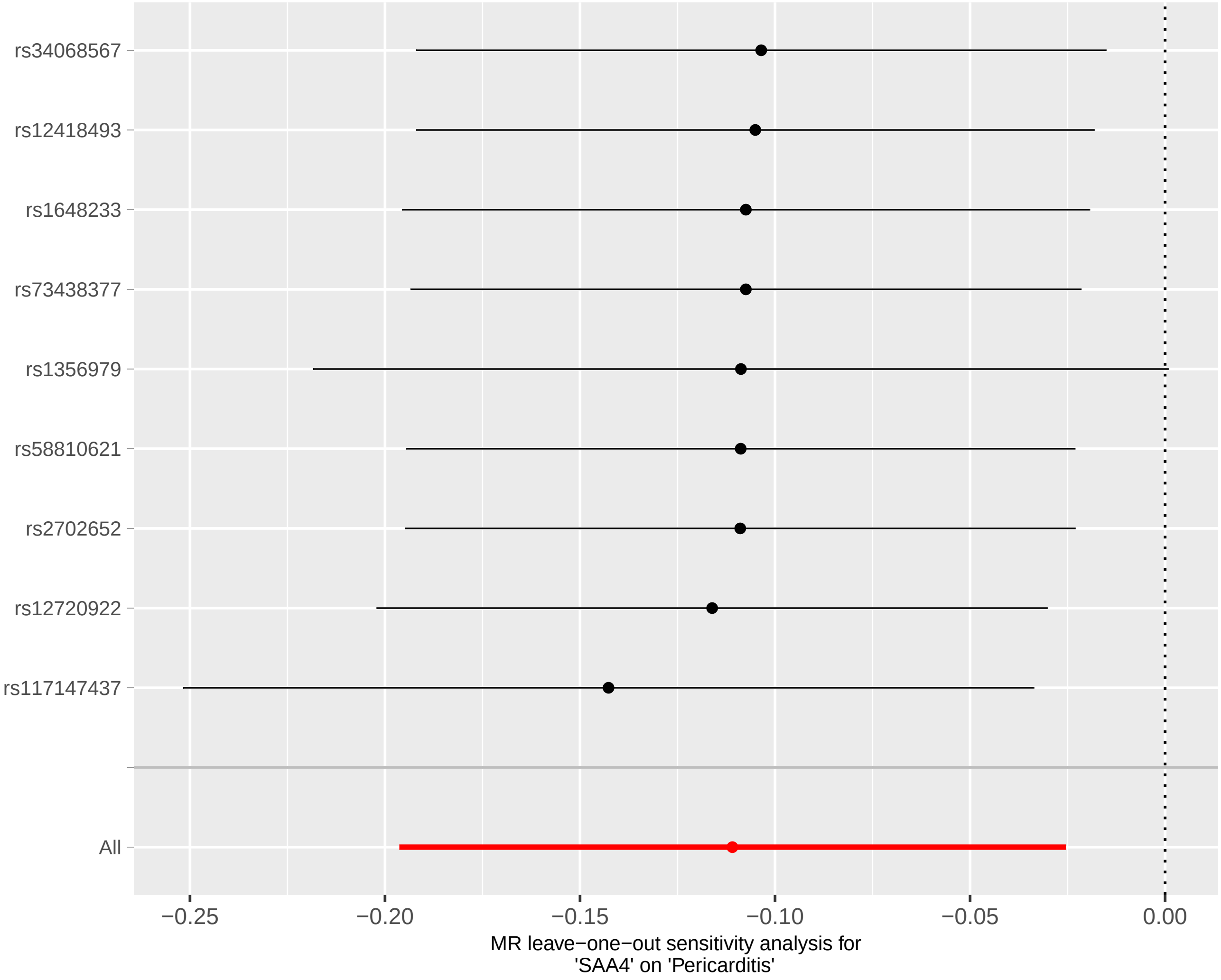
rs34068567
rs12418493
rs1648233
rs73438377
rs1356979
rs58810621
rs2702652
rs12720922
rs117147437
All
−0.25
−0.20
−0.15
−0.10
−0.05
0.00
MR leave−one−out sensitivity analysis for
'SAA4' on 'Pericarditis'

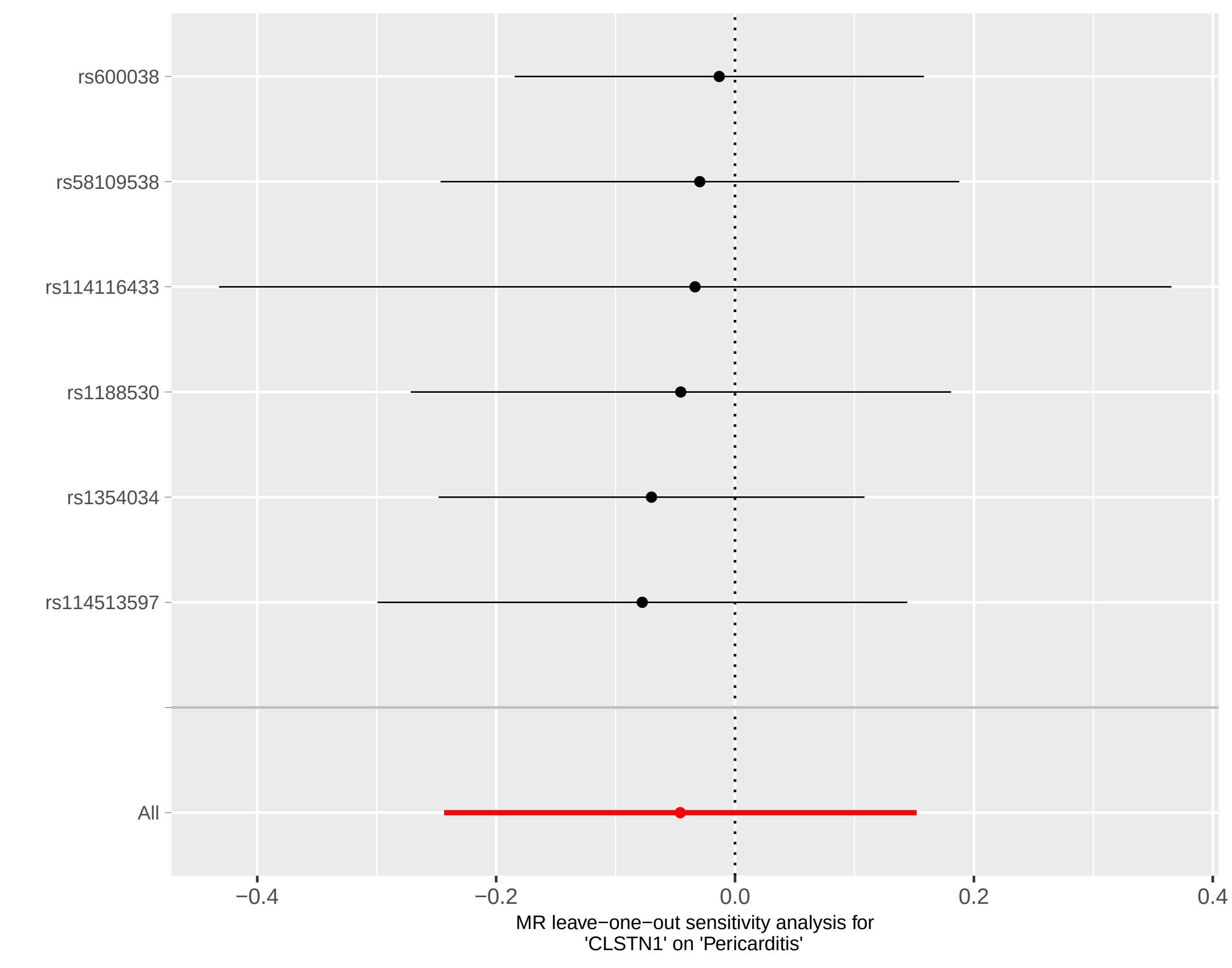

rs600038
rs58109538
rs114116433
rs1188530
rs1354034
rs114513597
All
−0.4
−0.2
0.0
0.2
0.4
MR leave−one−out sensitivity analysis for
'CLSTN1' on 'Pericarditis'

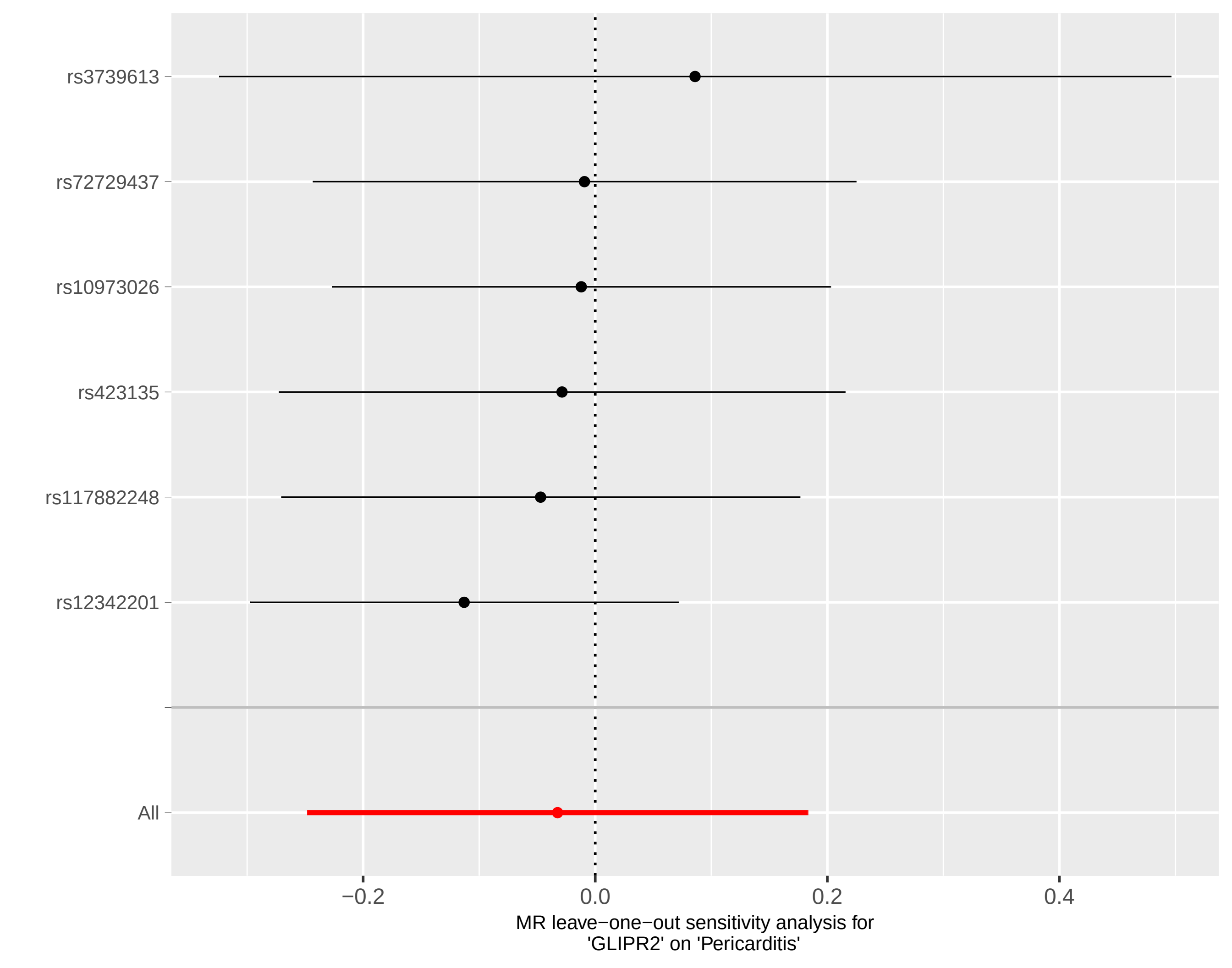
rs3739613
rs72729437
rs10973026
rs423135
rs117882248
rs12342201
All
−0.2
0.0
0.2
0.4
MR leave−one−out sensitivity analysis for
'GLIPR2' on 'Pericarditis'

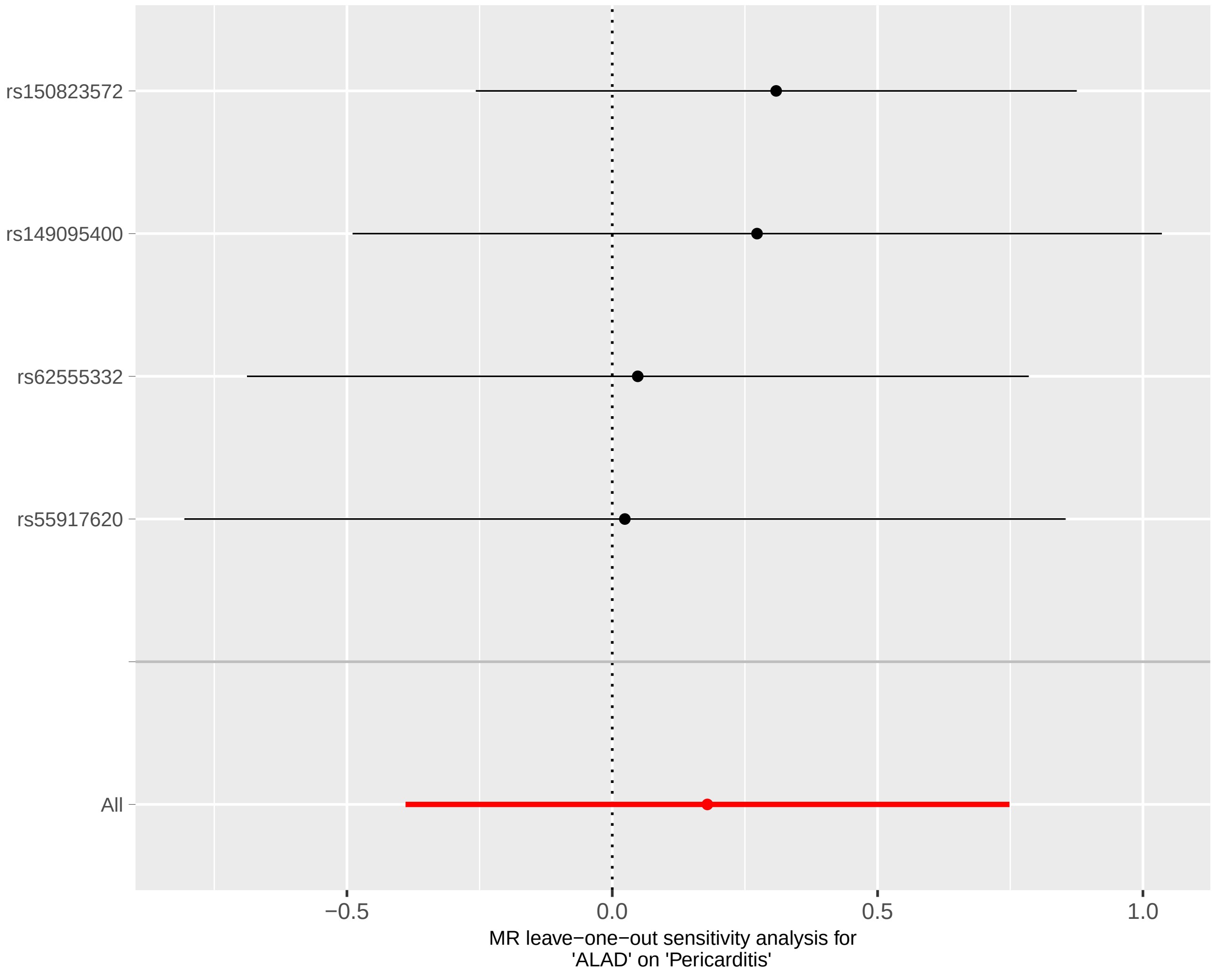

rs150823572
rs149095400
rs62555332
rs55917620
All
−0.5
0.0
0.5
1.0
MR leave−one−out sensitivity analysis for
'ALAD' on 'Pericarditis'

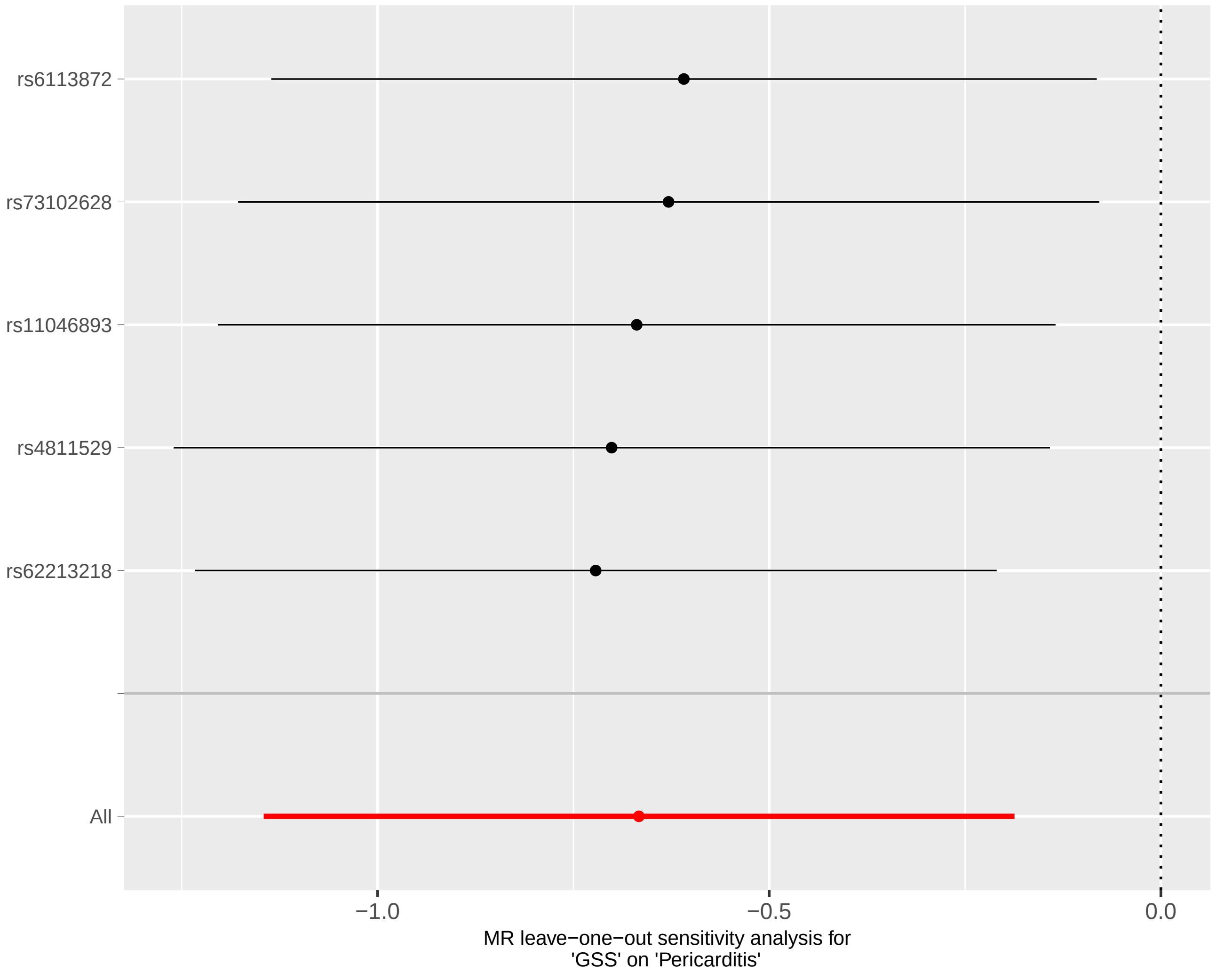

rs6113872
rs73102628
rs11046893
rs4811529
rs62213218
All
−1.0
−0.5
0.0
MR leave−one−out sensitivity analysis for
'GSS' on 'Pericarditis'

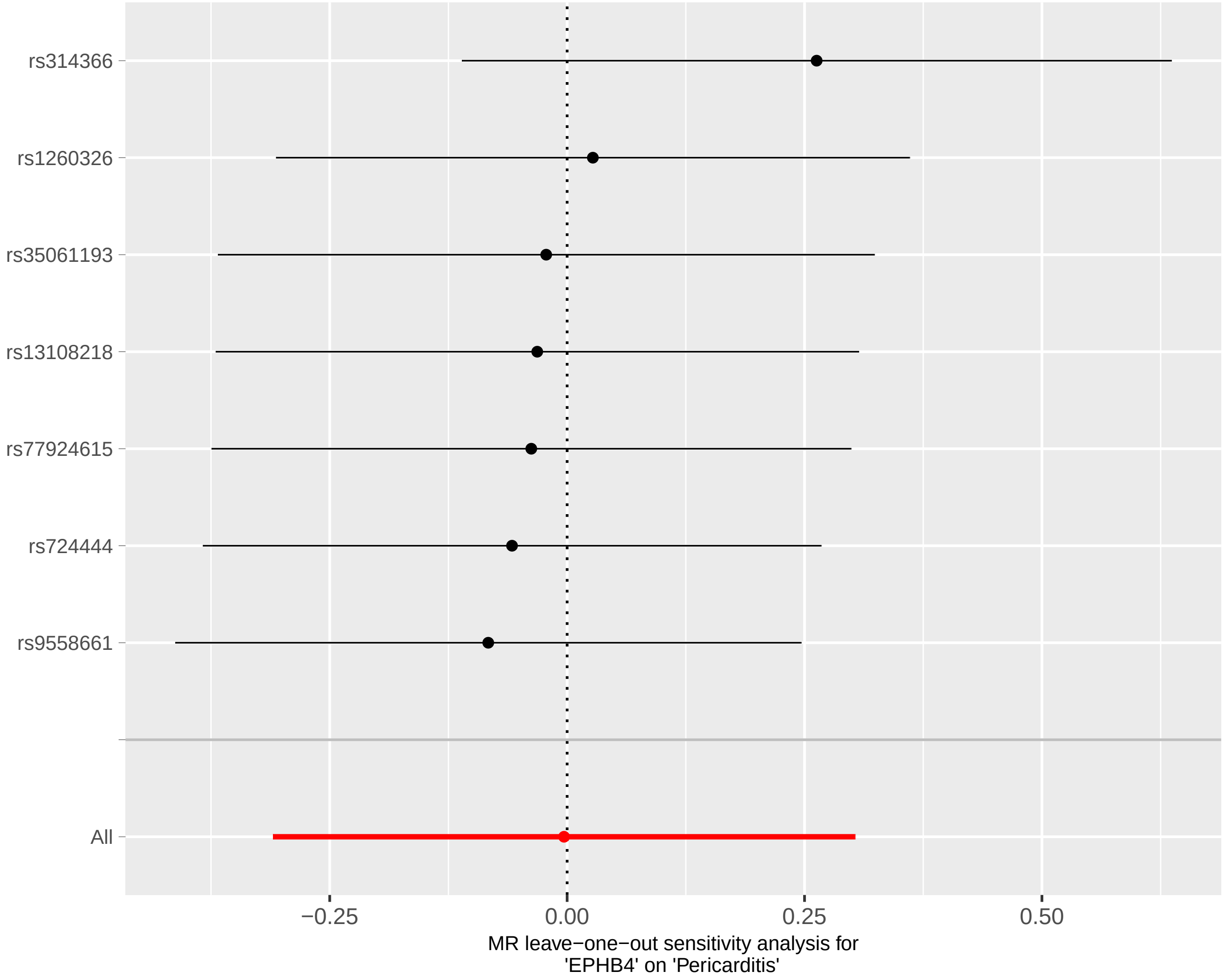
rs314366
rs1260326
rs35061193
rs13108218
rs77924615
rs724444
rs9558661
All
−0.25
0.00
0.25
0.50
MR leave−one−out sensitivity analysis for
'EPHB4' on 'Pericarditis'

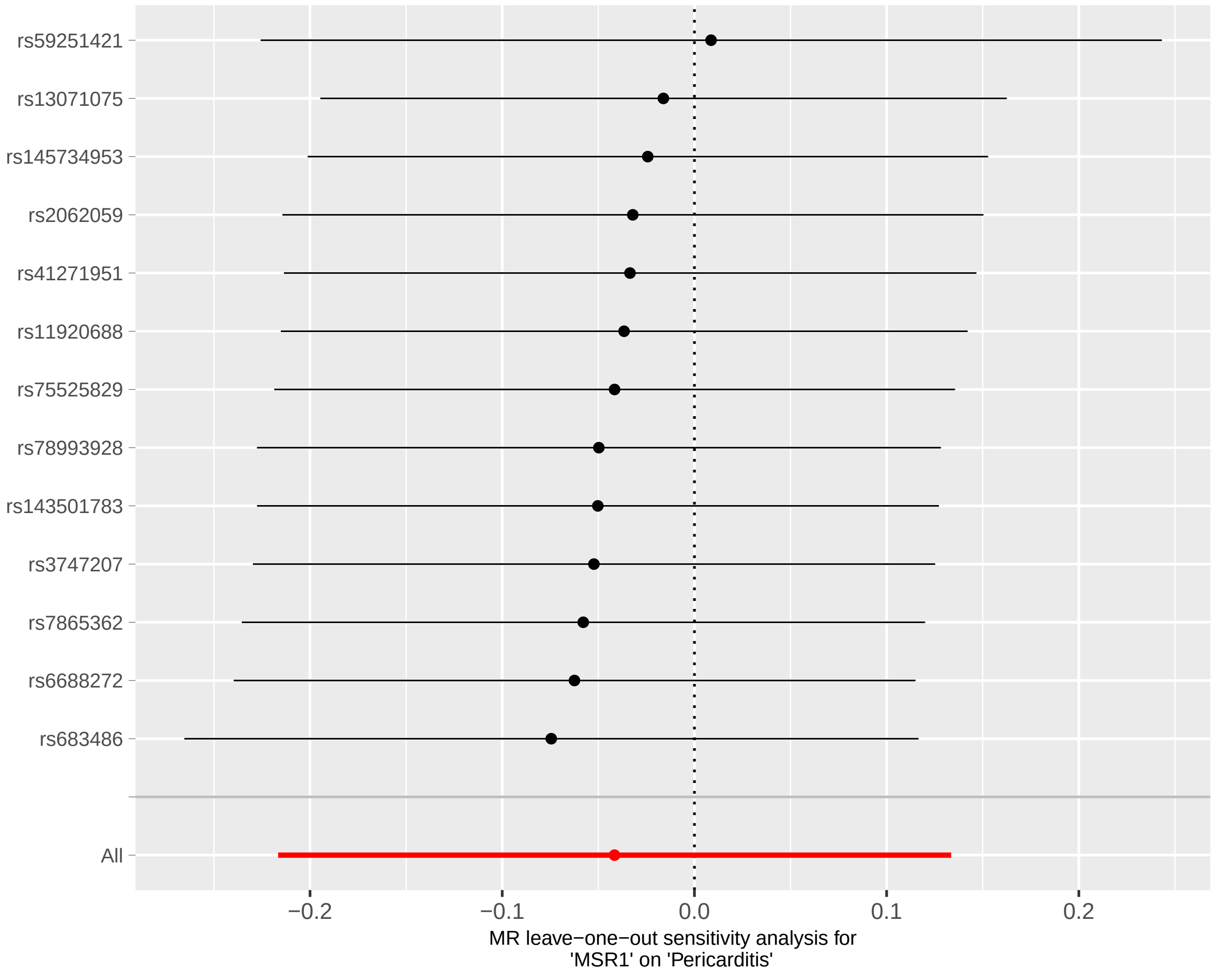
rs59251421
rs13071075
rs145734953
rs2062059
rs41271951
rs11920688
rs75525829
rs78993928
rs143501783
rs3747207
rs7865362
rs6688272
rs683486
All
−0.2
−0.1
0.0
0.1
0.2
MR leave−one−out sensitivity analysis for
'MSR1' on 'Pericarditis'

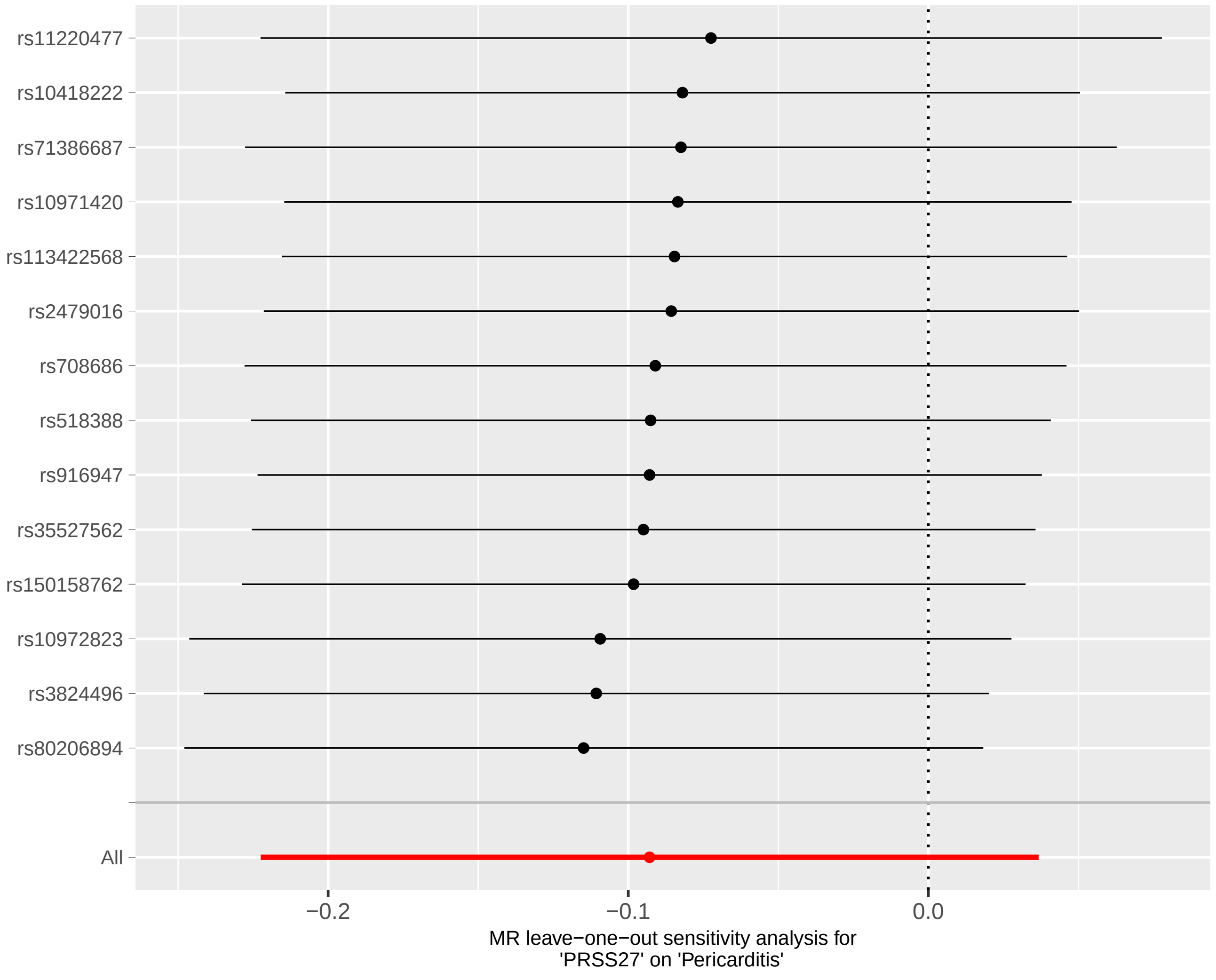
rs11220477
rs10418222
rs71386687
rs10971420
rs113422568
rs2479016
rs708686
rs518388
rs916947
rs35527562
rs150158762
rs10972823
rs3824496
rs80206894
All
−0.2
−0.1
0.0
MR leave−one−out sensitivity analysis for
'PRSS27' on 'Pericarditis'

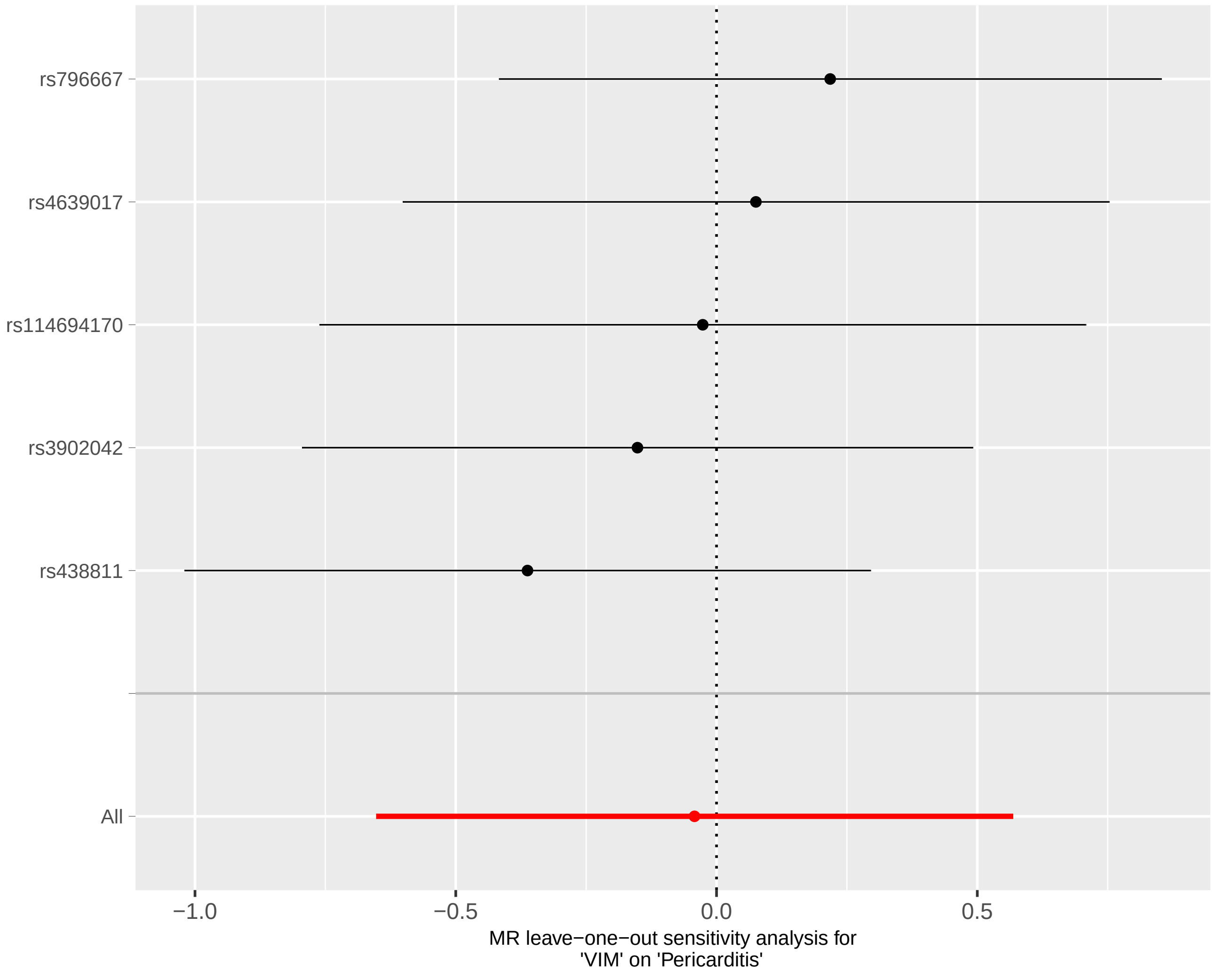
rs796667
rs4639017
rs114694170
rs3902042
rs438811
All
−1.0
−0.5
0.0
0.5
MR leave−one−out sensitivity analysis for
'VIM' on 'Pericarditis'

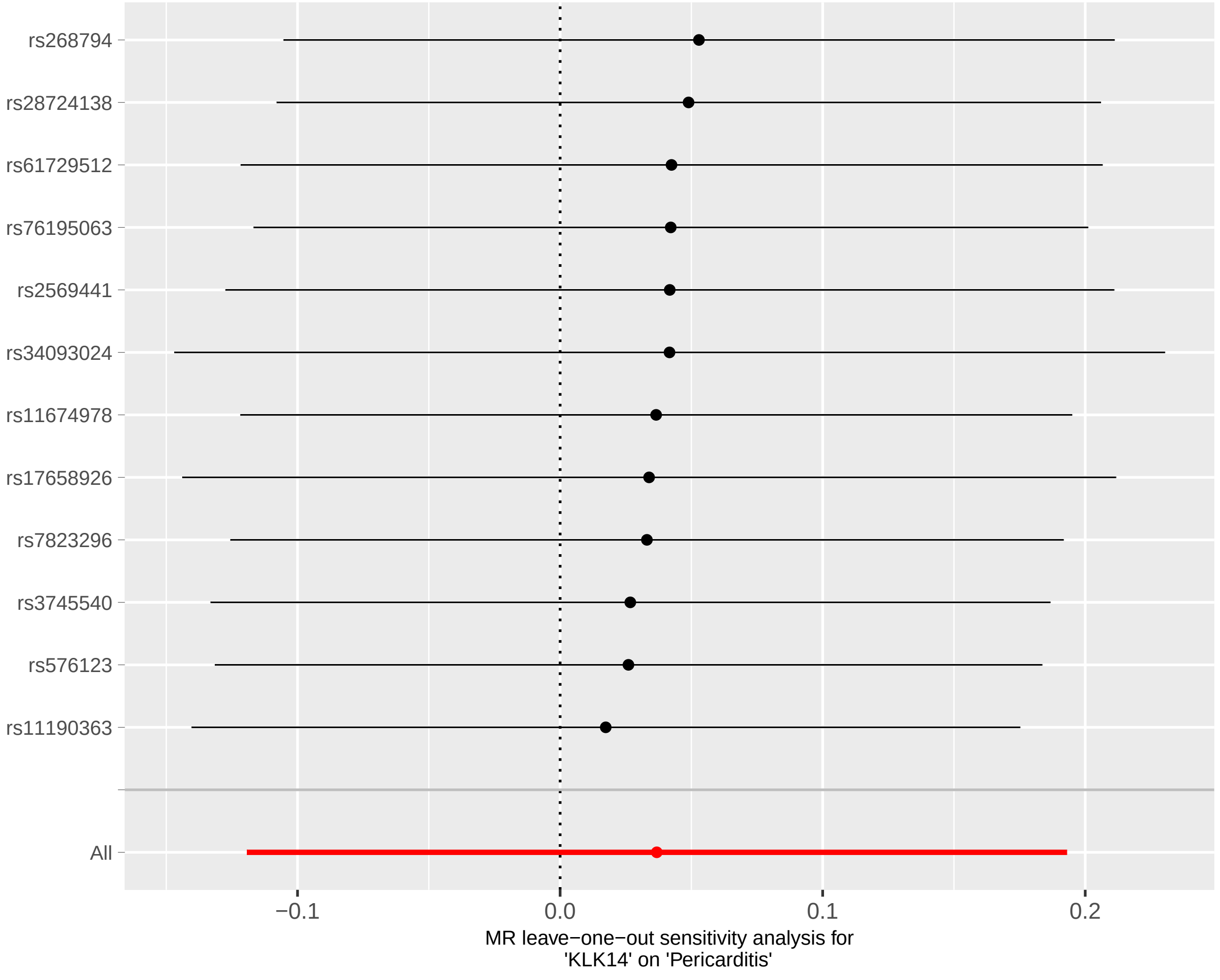
rs268794
rs28724138
rs61729512
rs76195063
rs2569441
rs34093024
rs11674978
rs17658926
rs7823296
rs3745540
rs576123
rs11190363
All
−0.1
0.0
0.1
0.2
MR leave−one−out sensitivity analysis for
'KLK14' on 'Pericarditis'

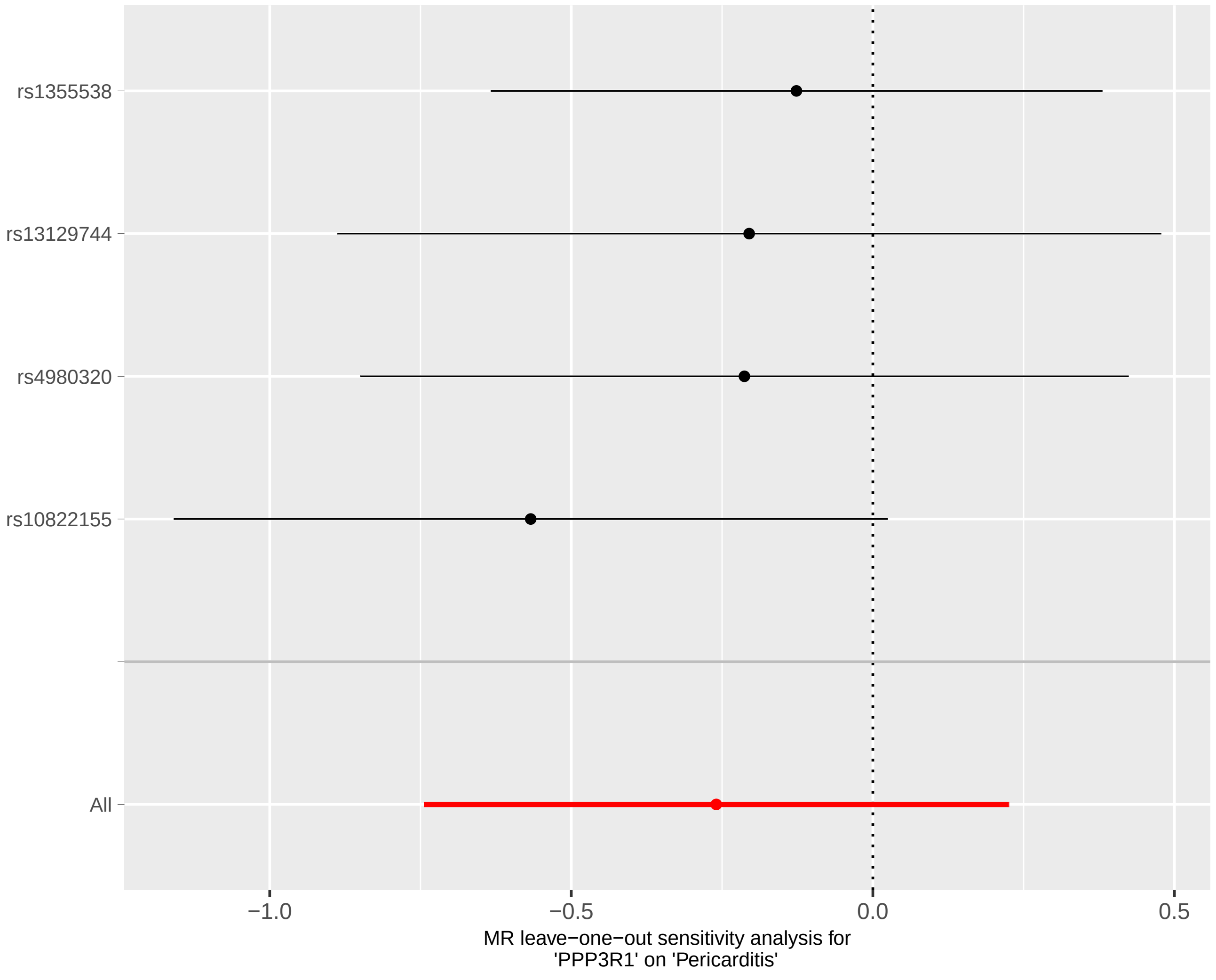

rs1355538
rs13129744
rs4980320
rs10822155
All
−1.0
−0.5
0.0
0.5
MR leave−one−out sensitivity analysis for
'PPP3R1' on 'Pericarditis'

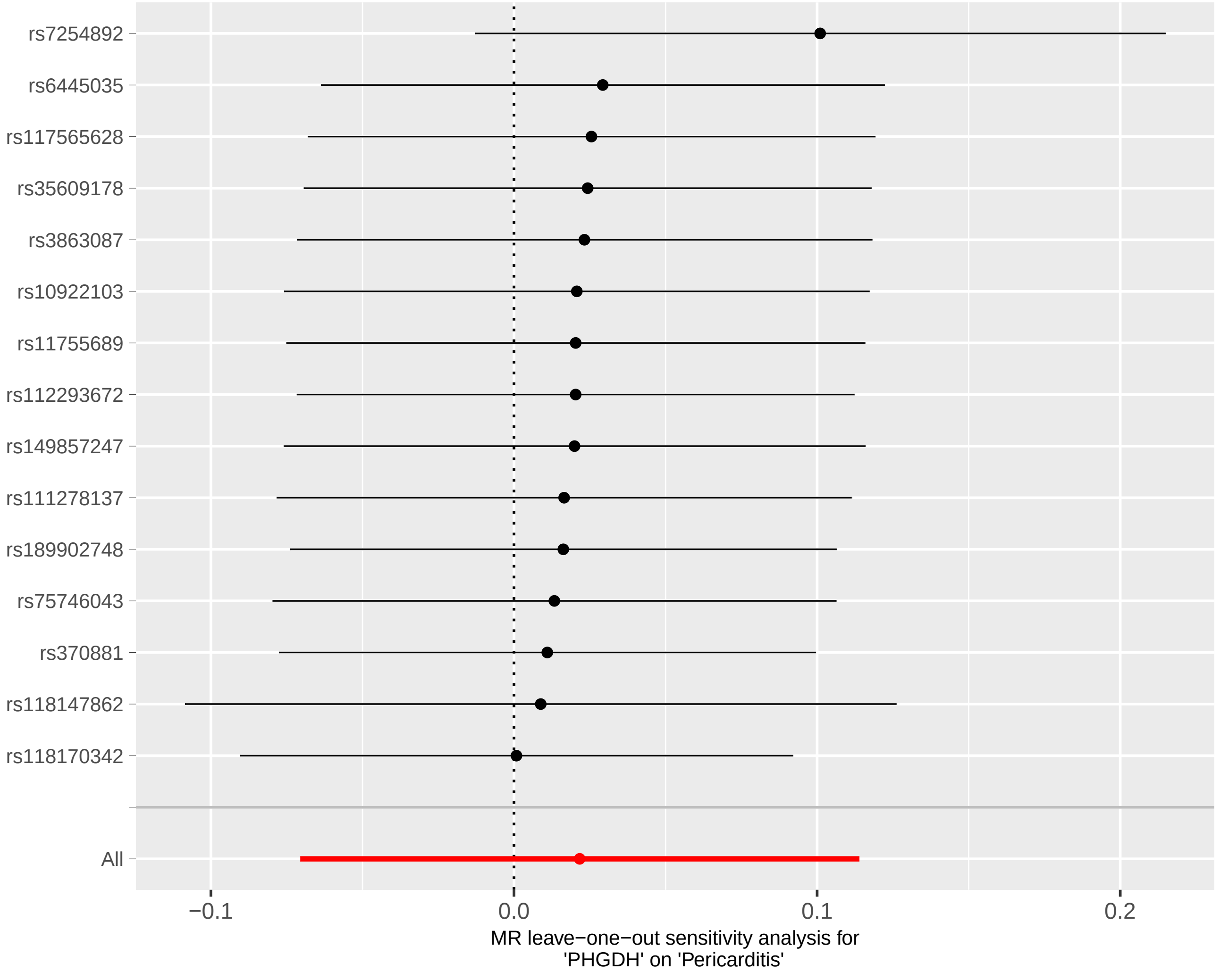

rs7254892
rs6445035
rs117565628
rs35609178
rs3863087
rs10922103
rs11755689
rs112293672
rs149857247
rs111278137
rs189902748
rs75746043
rs370881
rs118147862
rs118170342
All
−0.1
0.0
0.1
0.2
MR leave−one−out sensitivity analysis for
'PHGDH' on 'Pericarditis'

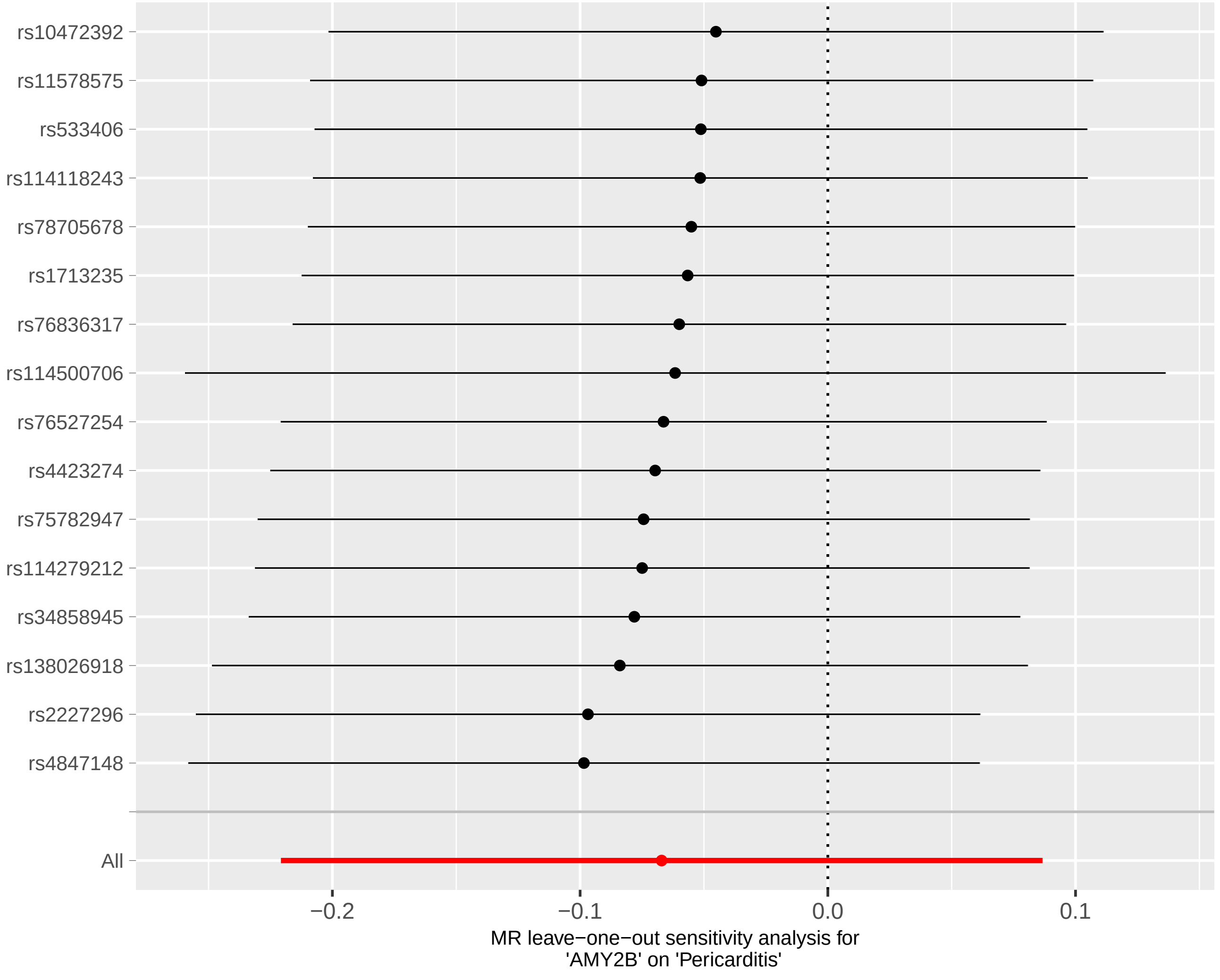

rs10472392
rs11578575
rs533406
rs114118243
rs78705678
rs1713235
rs76836317
rs114500706
rs76527254
rs4423274
rs75782947
rs114279212
rs34858945
rs138026918
rs2227296
rs4847148
All
−0.2
−0.1
0.0
0.1
MR leave−one−out sensitivity analysis for
'AMY2B' on 'Pericarditis'

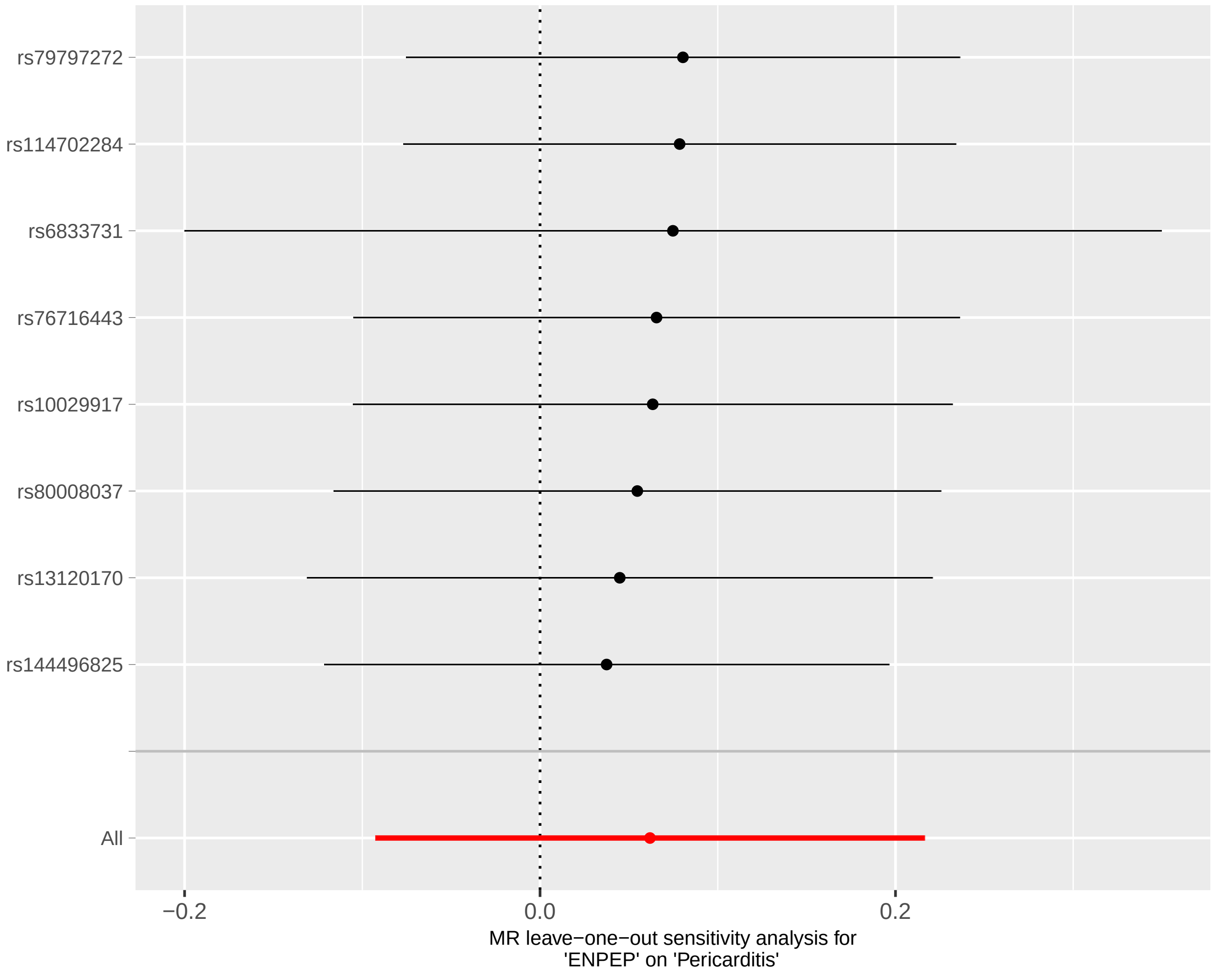
rs79797272
rs114702284
rs6833731
rs76716443
rs10029917
rs80008037
rs13120170
rs144496825
All
−0.2
0.0
0.2
MR leave−one−out sensitivity analysis for
'ENPEP' on 'Pericarditis'

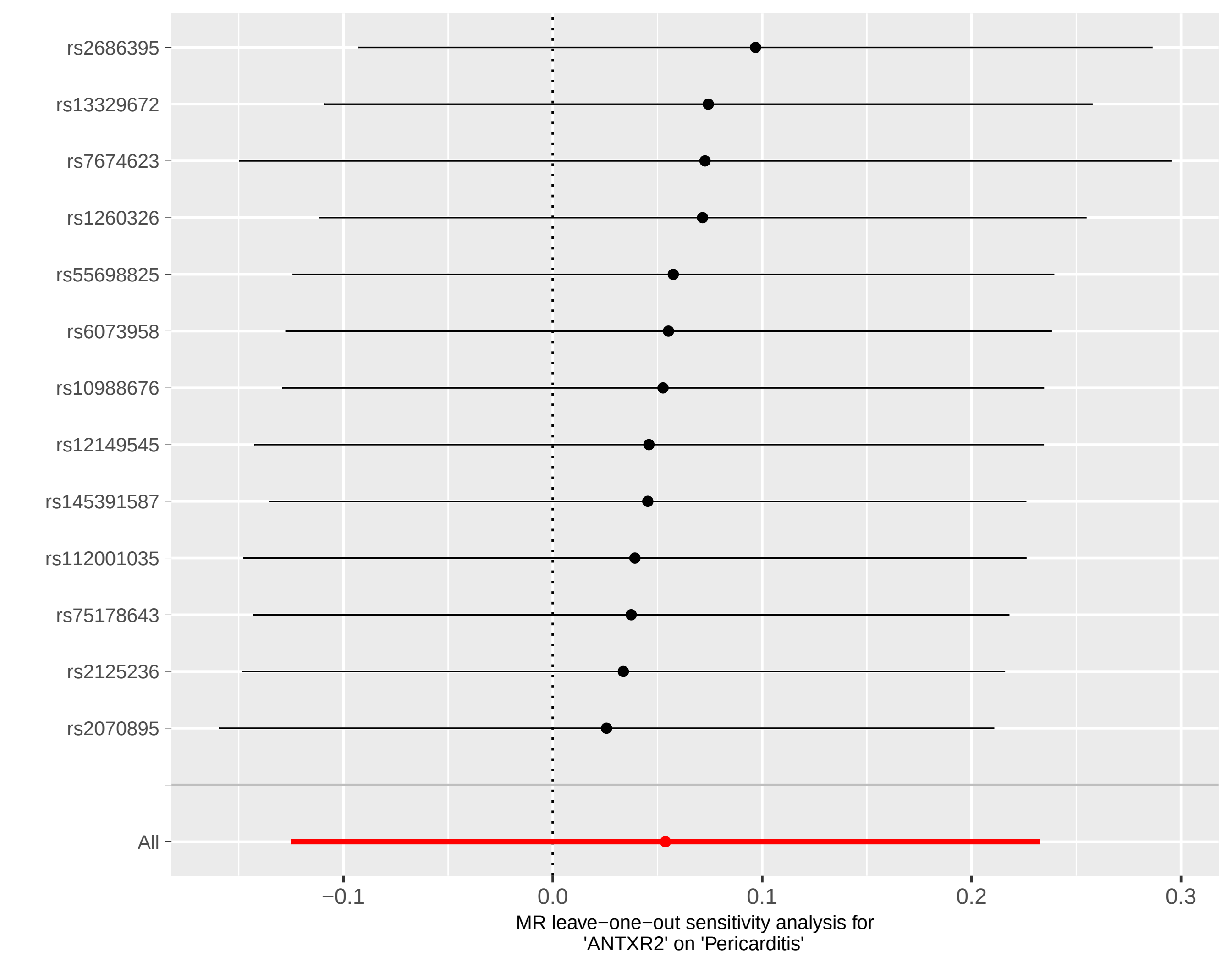
rs2686395
rs13329672
rs7674623
rs1260326
rs55698825
rs6073958
rs10988676
rs12149545
rs145391587
rs112001035
rs75178643
rs2125236
rs2070895
All
−0.1
0.0
0.1
0.2
0.3
MR leave−one−out sensitivity analysis for
'ANTXR2' on 'Pericarditis'

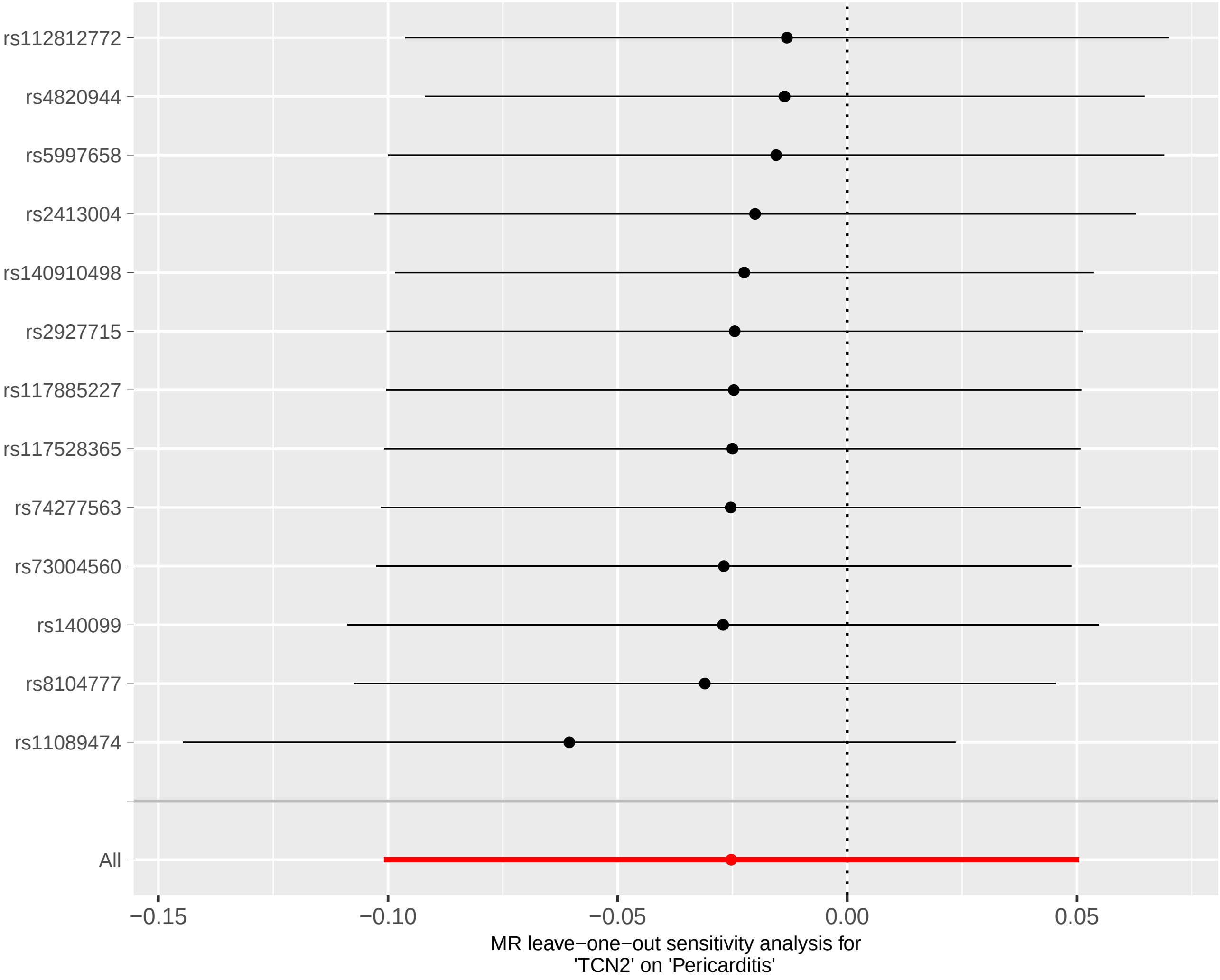
rs112812772
rs4820944
rs5997658
rs2413004
rs140910498
rs2927715
rs117885227
rs117528365
rs74277563
rs73004560
rs140099
rs8104777
rs11089474
All
−0.15
−0.10
−0.05
0.00
0.05
MR leave−one−out sensitivity analysis for
'TCN2' on 'Pericarditis'

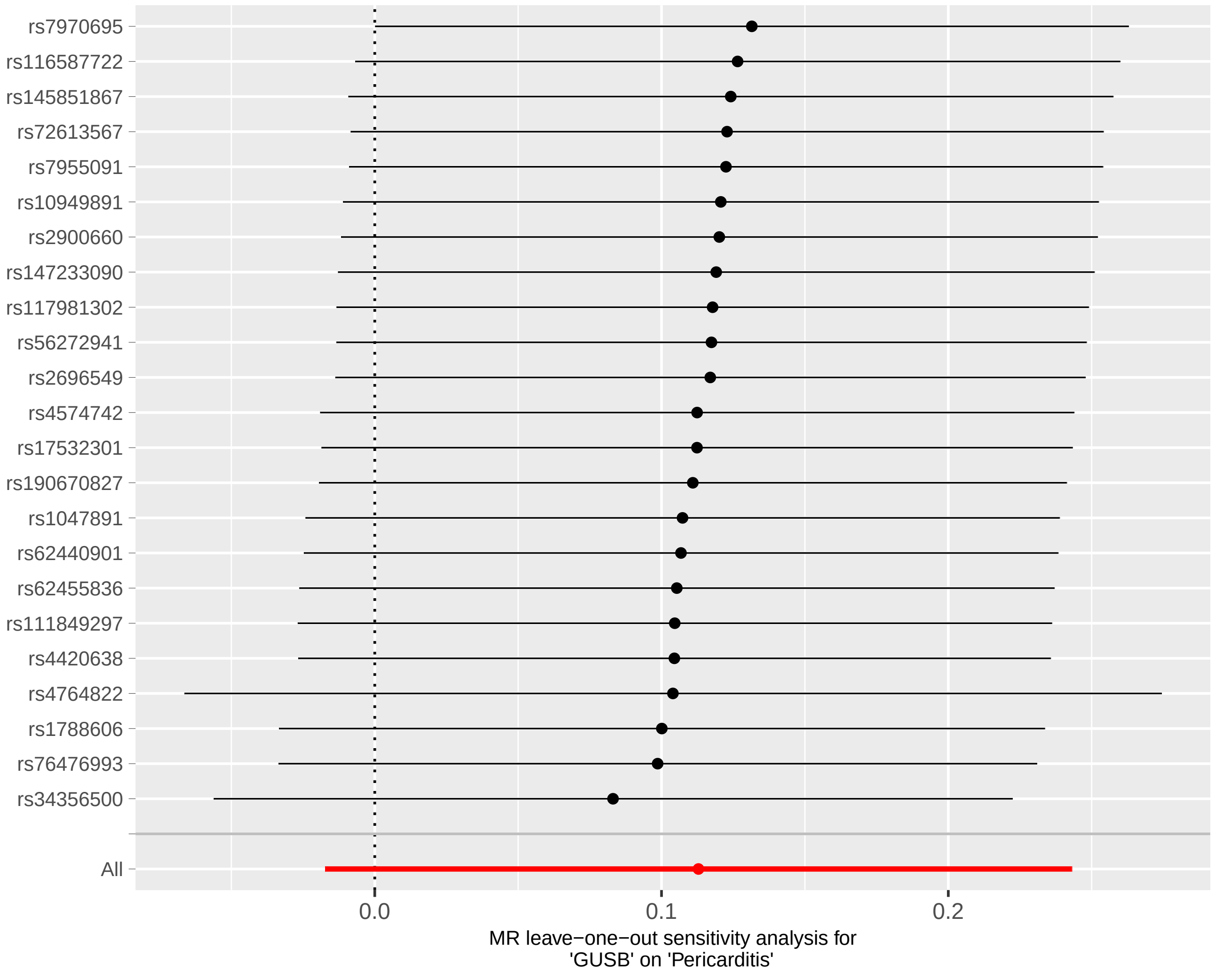
rs7970695
rs116587722
rs145851867
rs72613567
rs7955091
rs10949891
rs2900660
rs147233090
rs117981302
rs56272941
rs2696549
rs4574742
rs17532301
rs190670827
rs1047891
rs62440901
rs62455836
rs111849297
rs4420638
rs4764822
rs1788606
rs76476993
rs34356500
All
0.0
0.1
0.2
MR leave−one−out sensitivity analysis for
'GUSB' on 'Pericarditis'

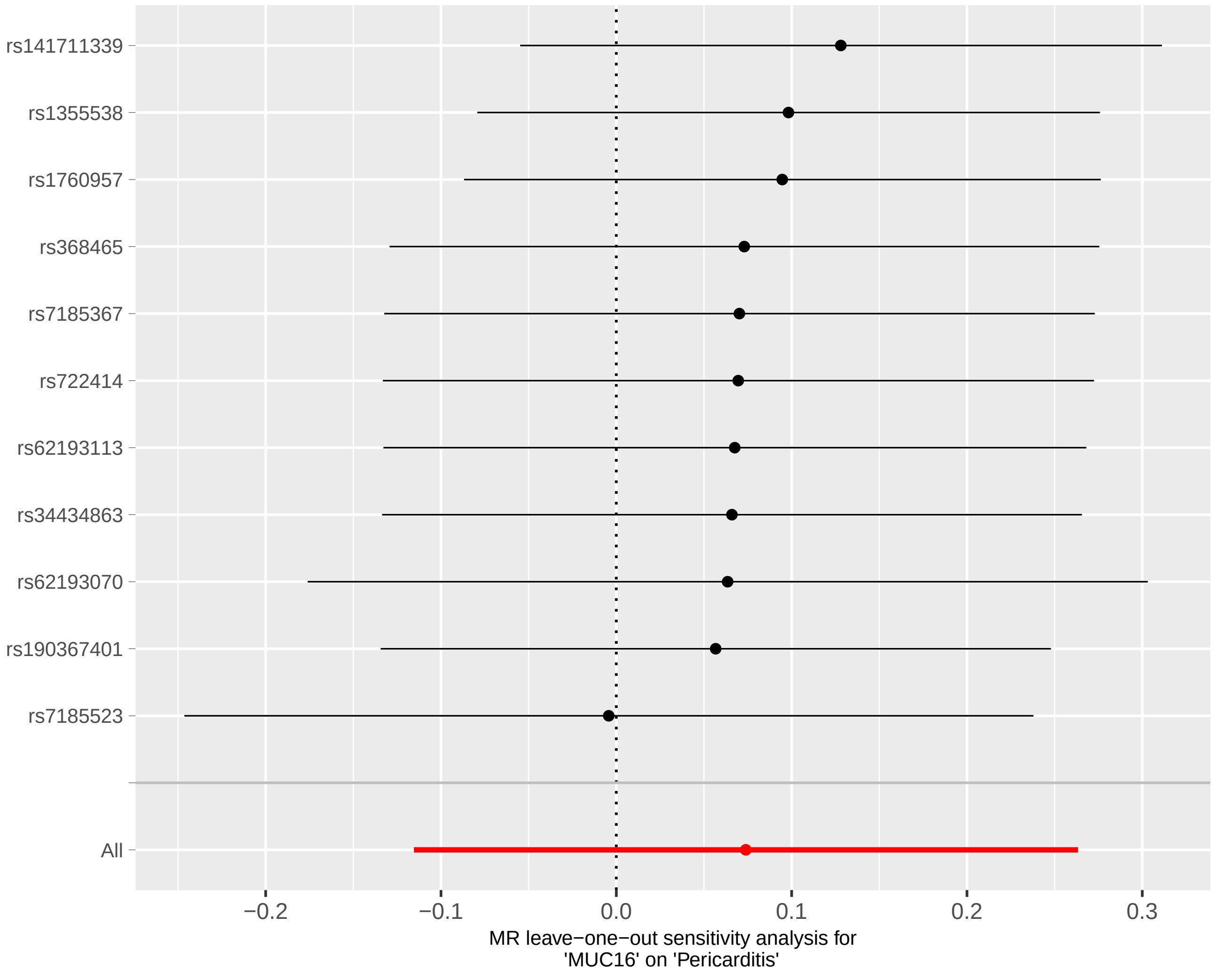

rs141711339
rs1355538
rs1760957
rs368465
rs7185367
rs722414
rs62193113
rs34434863
rs62193070
rs190367401
rs7185523
All
−0.2
−0.1
0.0
0.1
0.2
0.3
MR leave−one−out sensitivity analysis for
'MUC16' on 'Pericarditis'

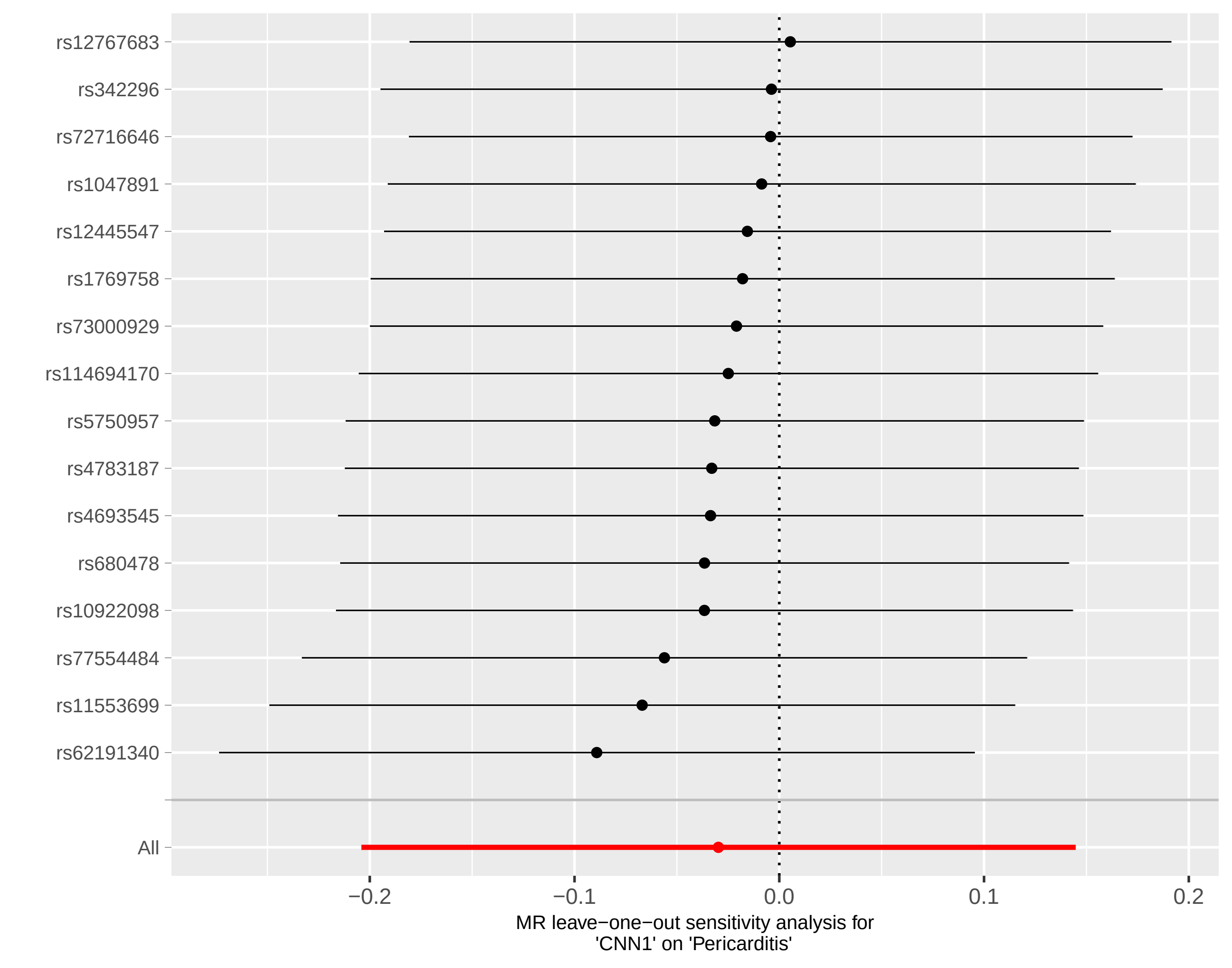

rs12767683
rs342296
rs72716646
rs1047891
rs12445547
rs1769758
rs73000929
rs114694170
rs5750957
rs4783187
rs4693545
rs680478
rs10922098
rs77554484
rs11553699
rs62191340
All
−0.2
−0.1
0.0
0.1
0.2
MR leave−one−out sensitivity analysis for
'CNN1' on 'Pericarditis'

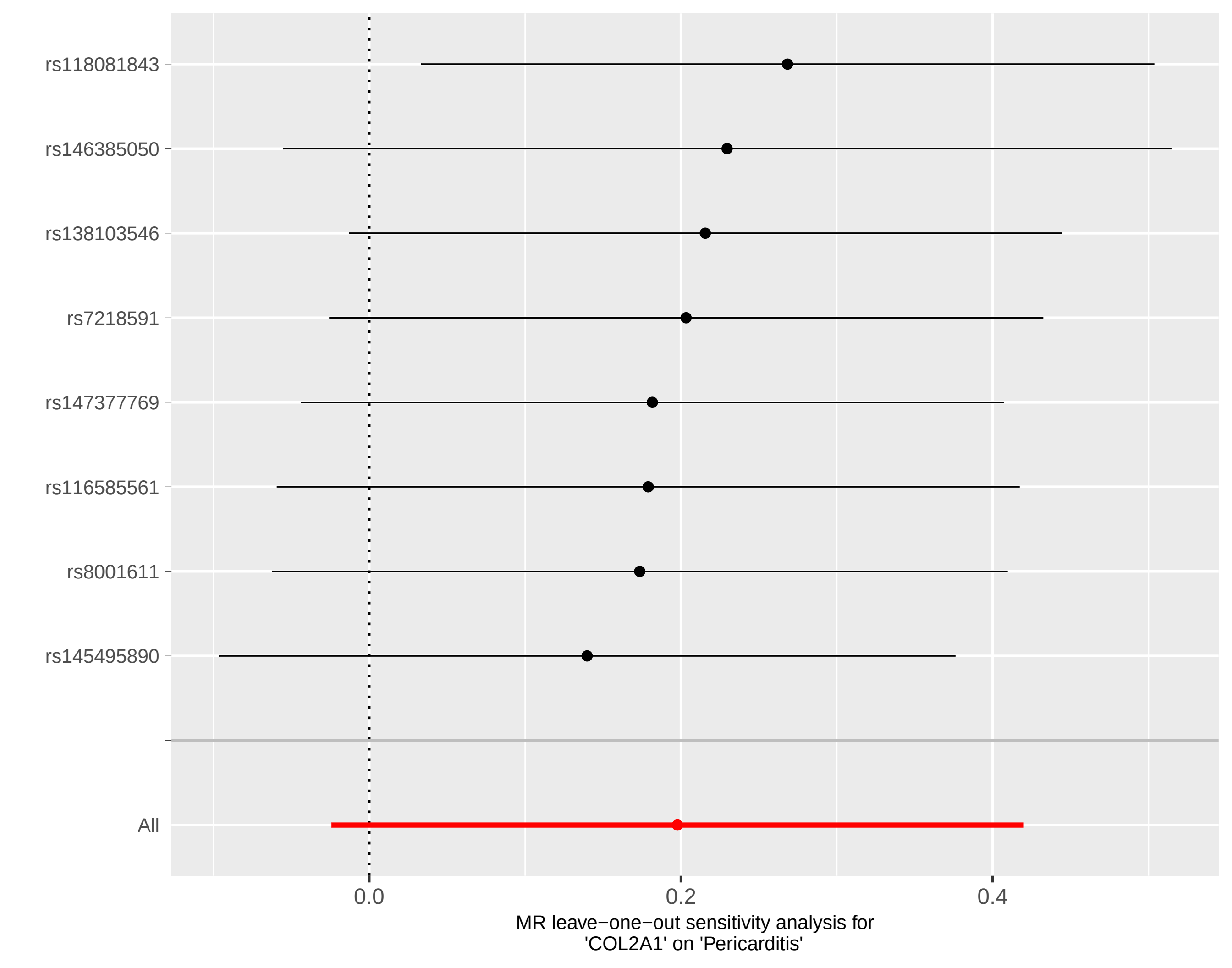

rs118081843
rs146385050
rs138103546
rs7218591
rs147377769
rs116585561
rs8001611
rs145495890
All
0.0
0.2
0.4
MR leave−one−out sensitivity analysis for
'COL2A1' on 'Pericarditis'

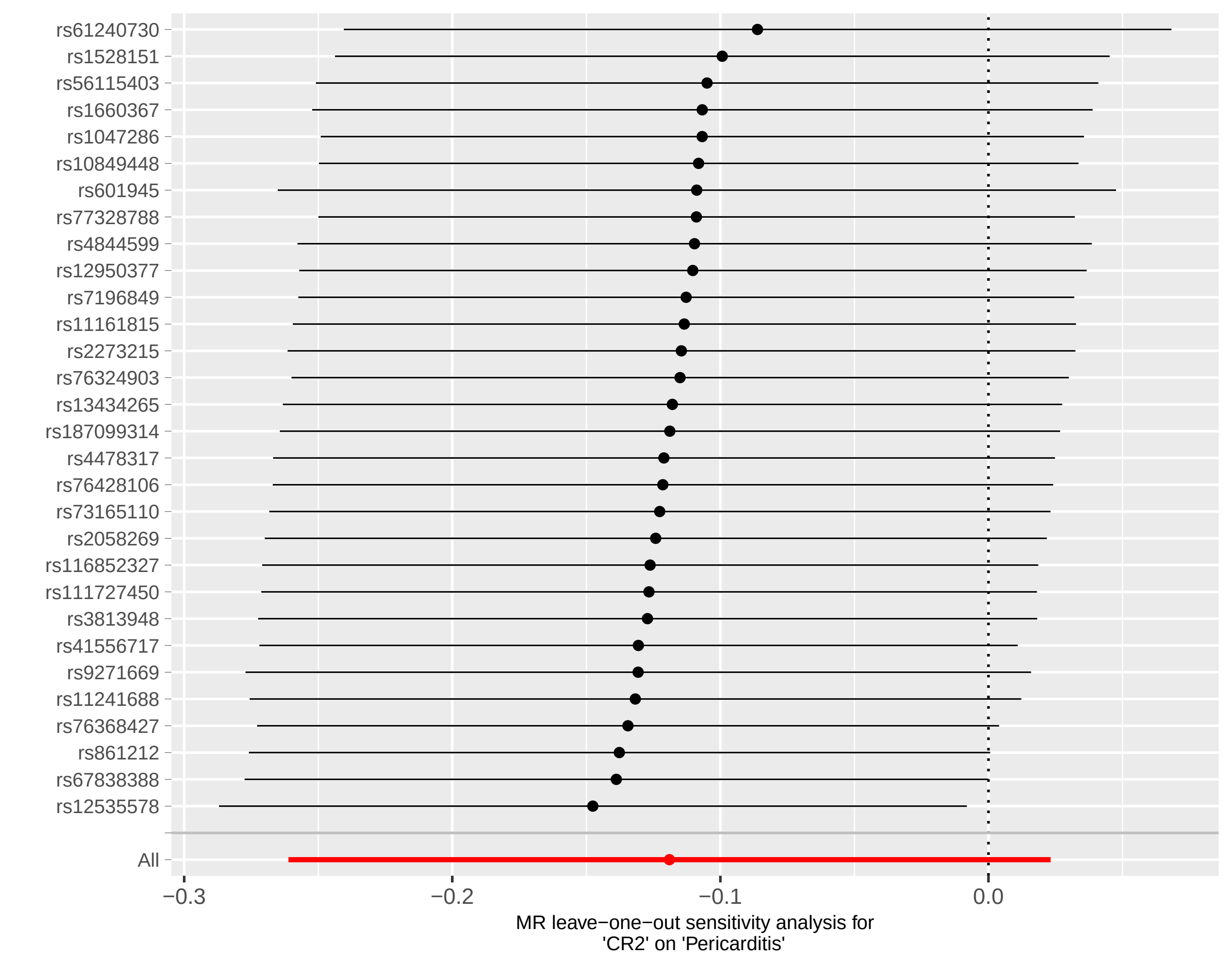

rs61240730
rs1528151
rs56115403
rs1660367
rs1047286
rs10849448
rs601945
rs77328788
rs4844599
rs12950377
rs7196849
rs11161815
rs2273215
rs76324903
rs13434265
rs187099314
rs4478317
rs76428106
rs73165110
rs2058269
rs116852327
rs111727450
rs3813948
rs41556717
rs9271669
rs11241688
rs76368427
rs861212
rs67838388
rs12535578
All
−0.3
−0.2
−0.1
0.0
MR leave−one−out sensitivity analysis for
'CR2' on 'Pericarditis'

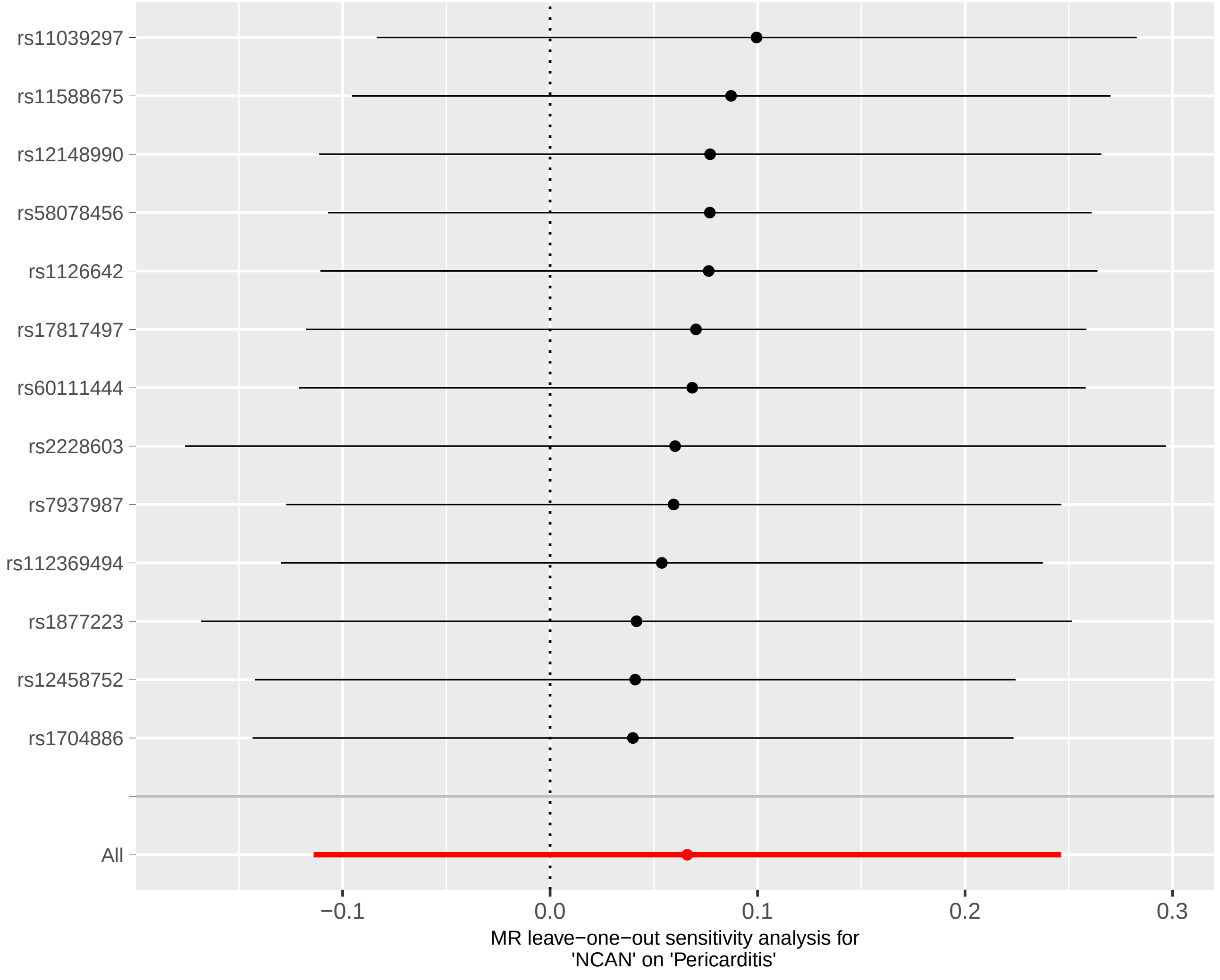

rs11039297
rs11588675
rs12148990
rs58078456
rs1126642
rs17817497
rs60111444
rs2228603
rs7937987
rs112369494
rs1877223
rs12458752
rs1704886
All
−0.1
0.0
0.1
0.2
0.3
MR leave−one−out sensitivity analysis for
'NCAN' on 'Pericarditis'

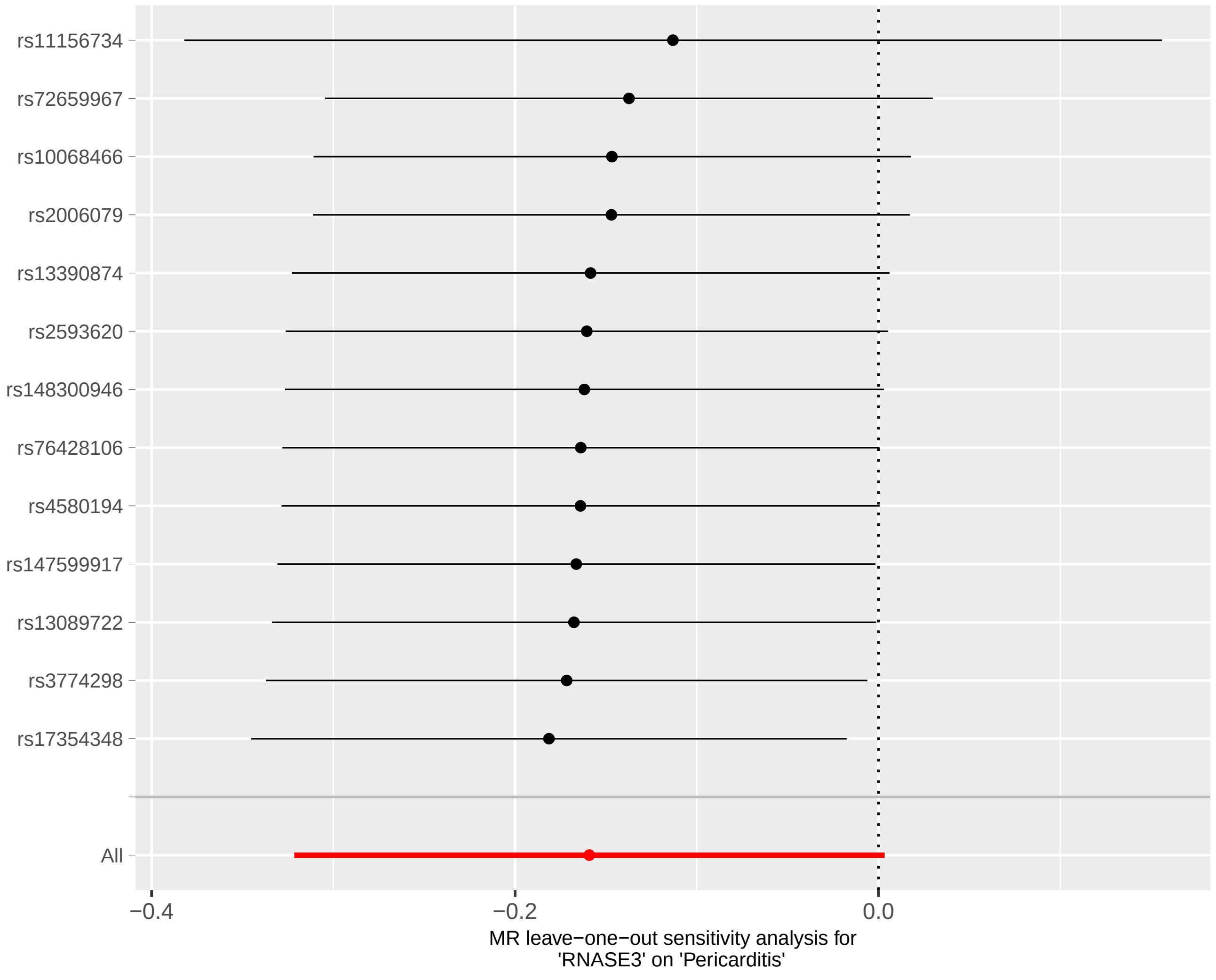

rs11156734
rs72659967
rs10068466
rs2006079
rs13390874
rs2593620
rs148300946
rs76428106
rs4580194
rs147599917
rs13089722
rs3774298
rs17354348
All
−0.4
−0.2
0.0
MR leave−one−out sensitivity analysis for
'RNASE3' on 'Pericarditis'

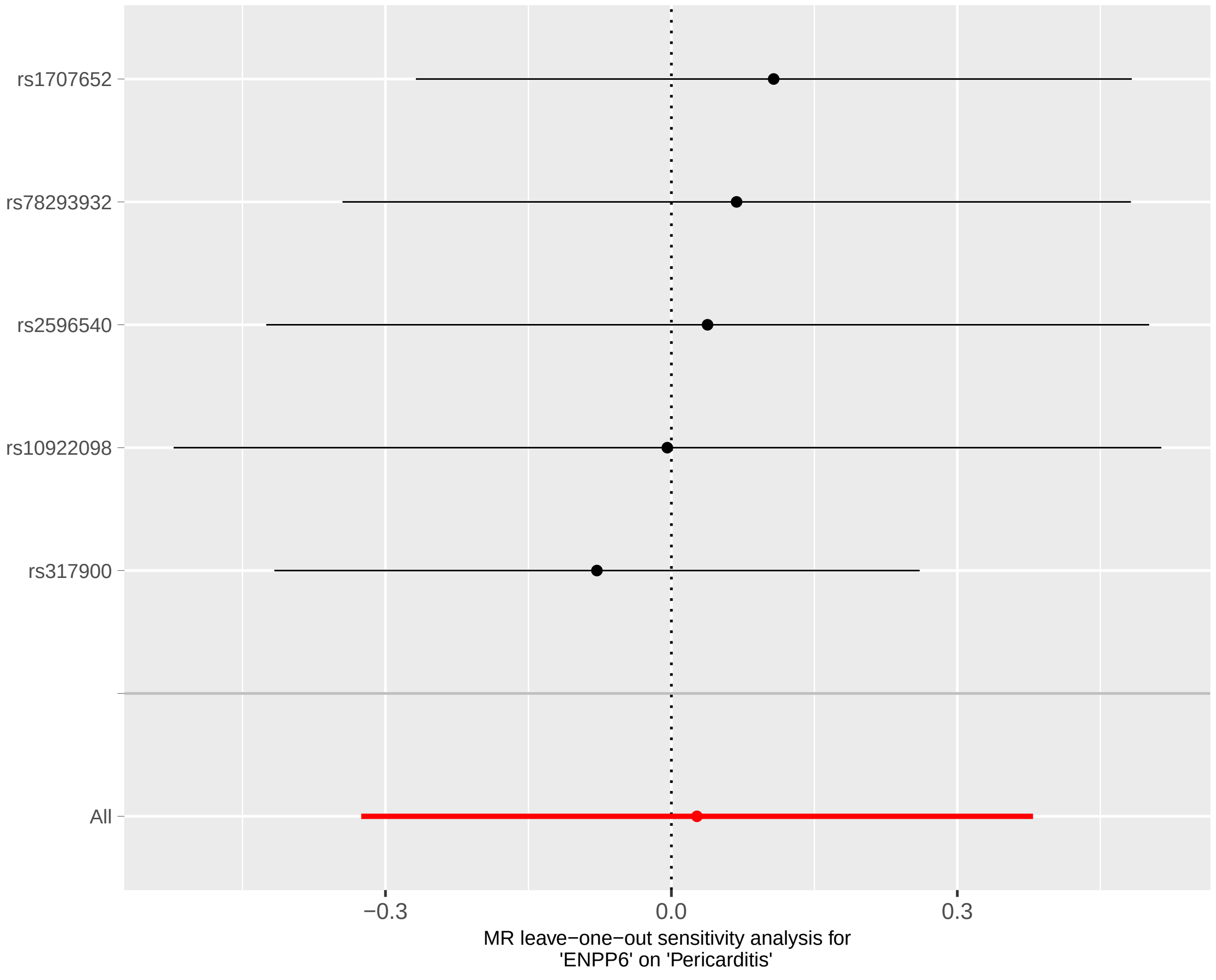

rs1707652
rs78293932
rs2596540
rs10922098
rs317900
All
−0.3
0.0
0.3
MR leave−one−out sensitivity analysis for
'ENPP6' on 'Pericarditis'

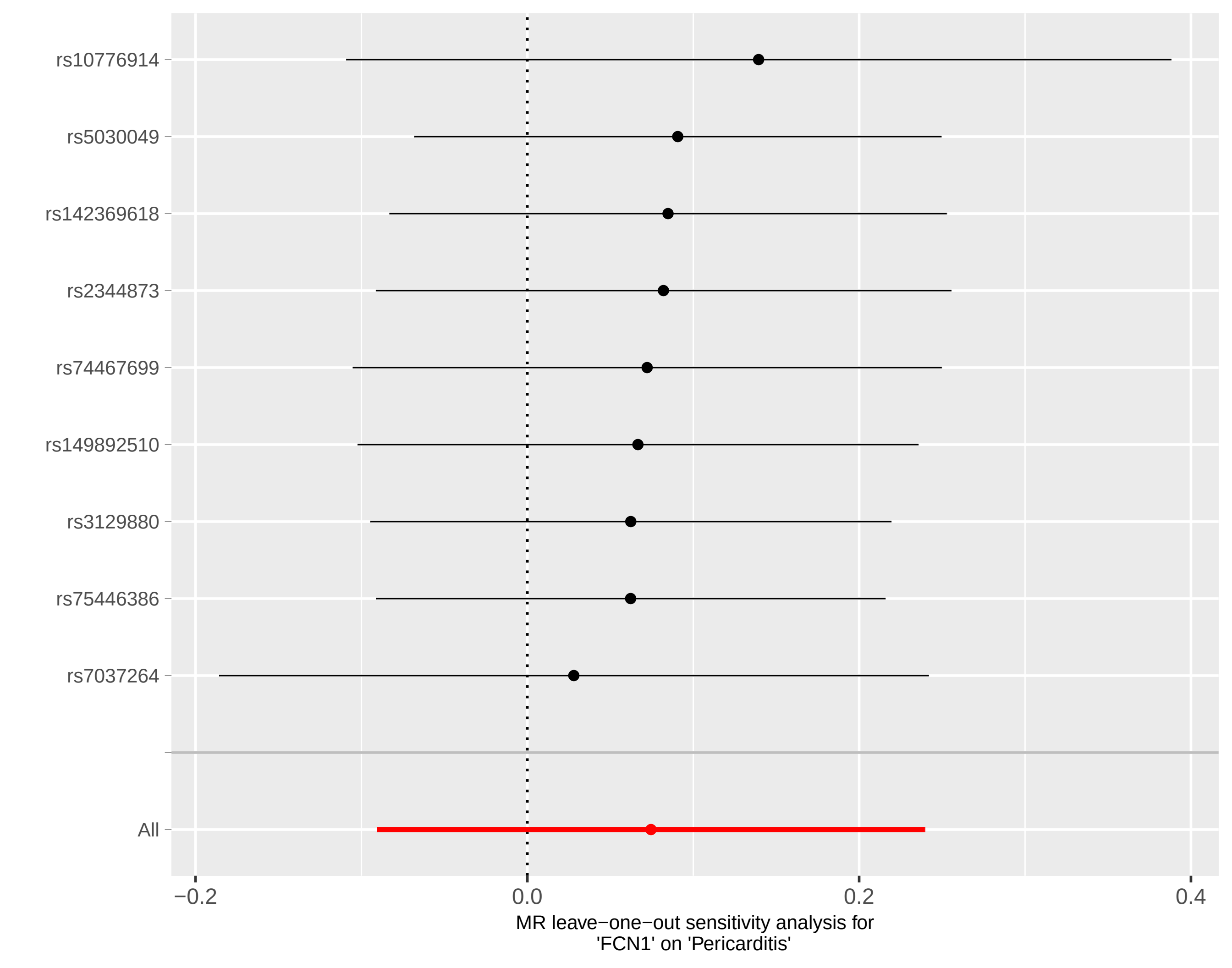
rs10776914
rs5030049
rs142369618
rs2344873
rs74467699
rs149892510
rs3129880
rs75446386
rs7037264
All
−0.2
0.0
0.2
0.4
MR leave−one−out sensitivity analysis for
'FCN1' on 'Pericarditis'

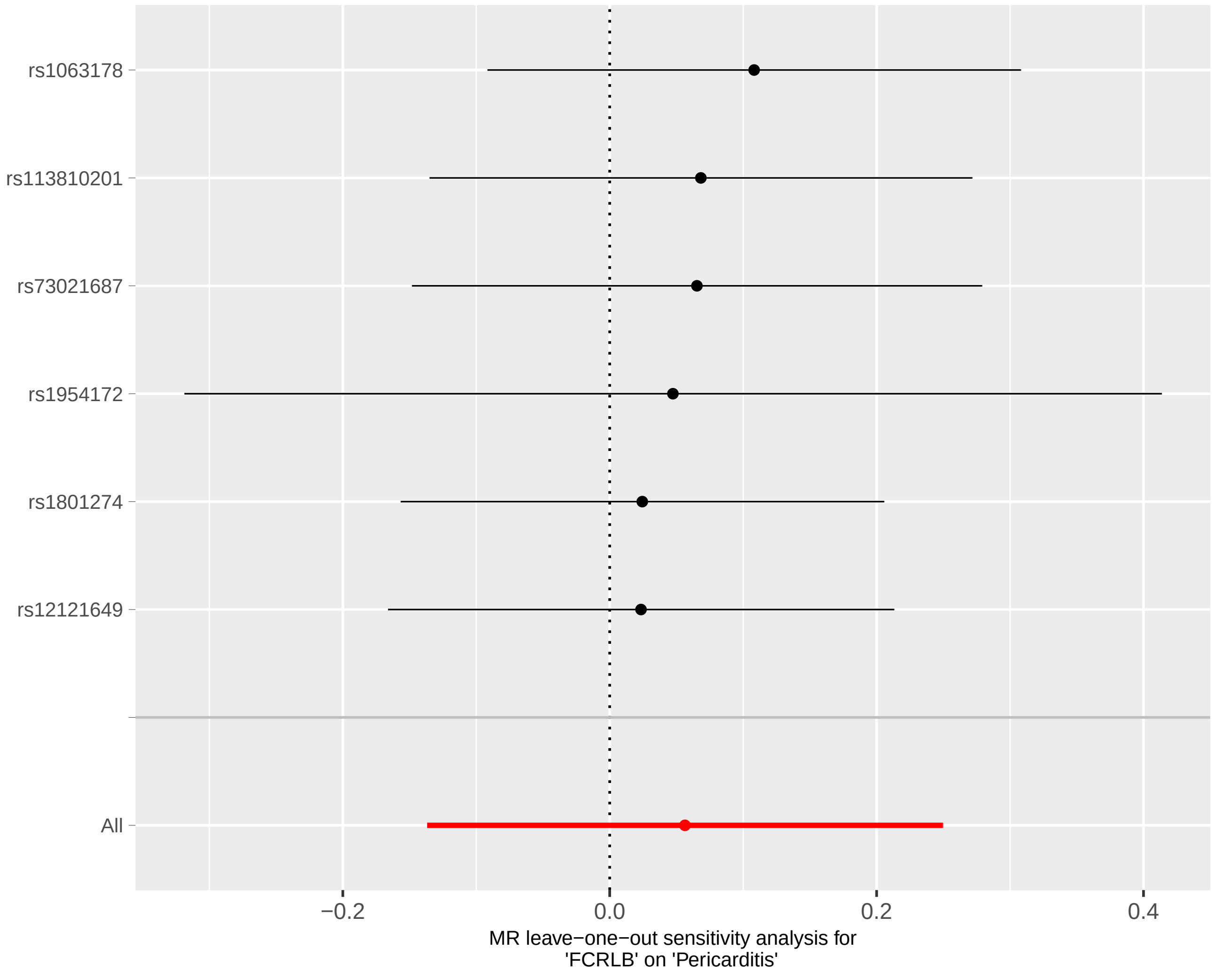

rs1063178
rs113810201
rs73021687
rs1954172
rs1801274
rs12121649
All
−0.2
0.0
0.2
0.4
MR leave−one−out sensitivity analysis for
'FCRLB' on 'Pericarditis'

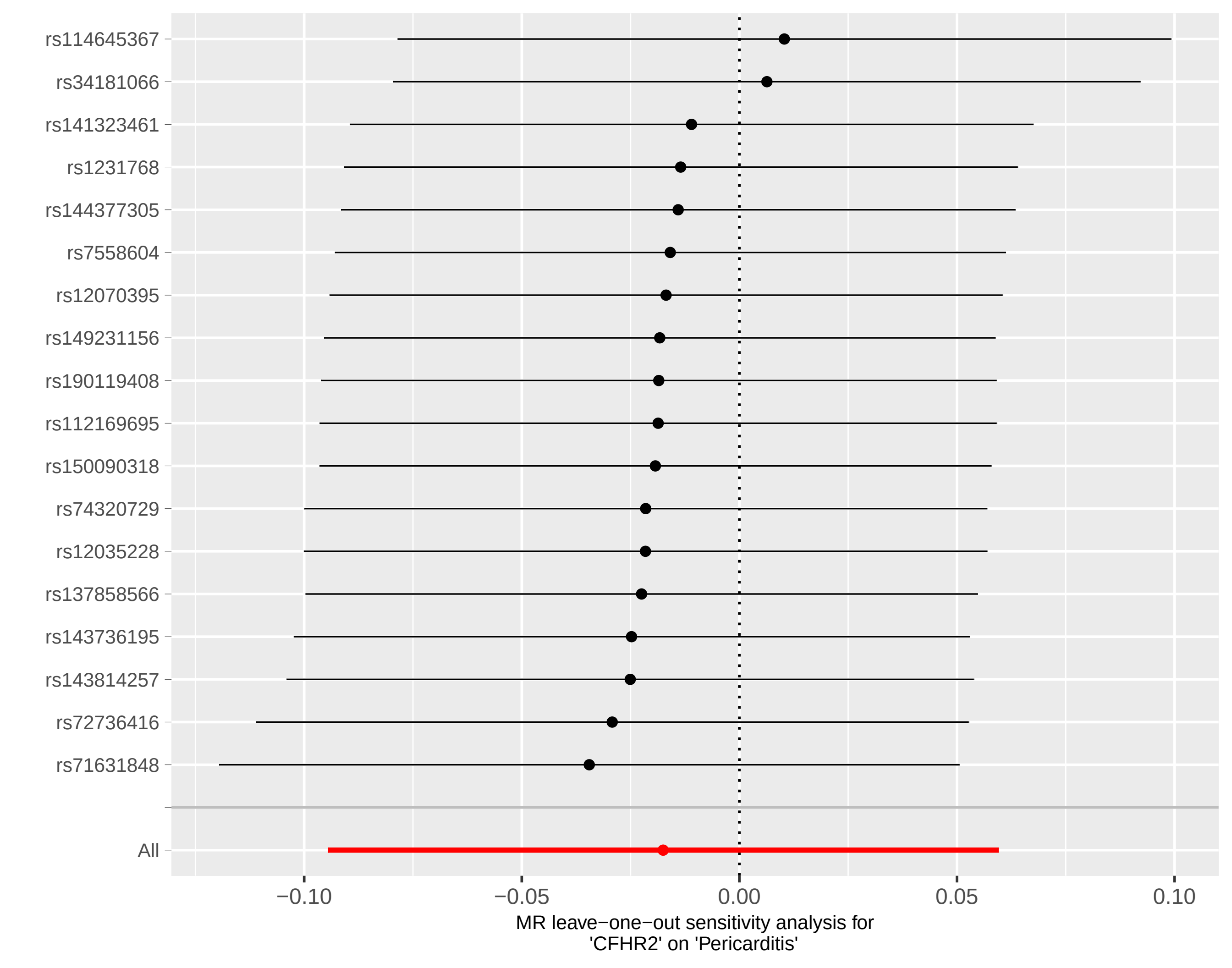
rs114645367
rs34181066
rs141323461
rs1231768
rs144377305
rs7558604
rs12070395
rs149231156
rs190119408
rs112169695
rs150090318
rs74320729
rs12035228
rs137858566
rs143736195
rs143814257
rs72736416
rs71631848
All
−0.10
−0.05
0.00
0.05
0.10
MR leave−one−out sensitivity analysis for
'CFHR2' on 'Pericarditis'

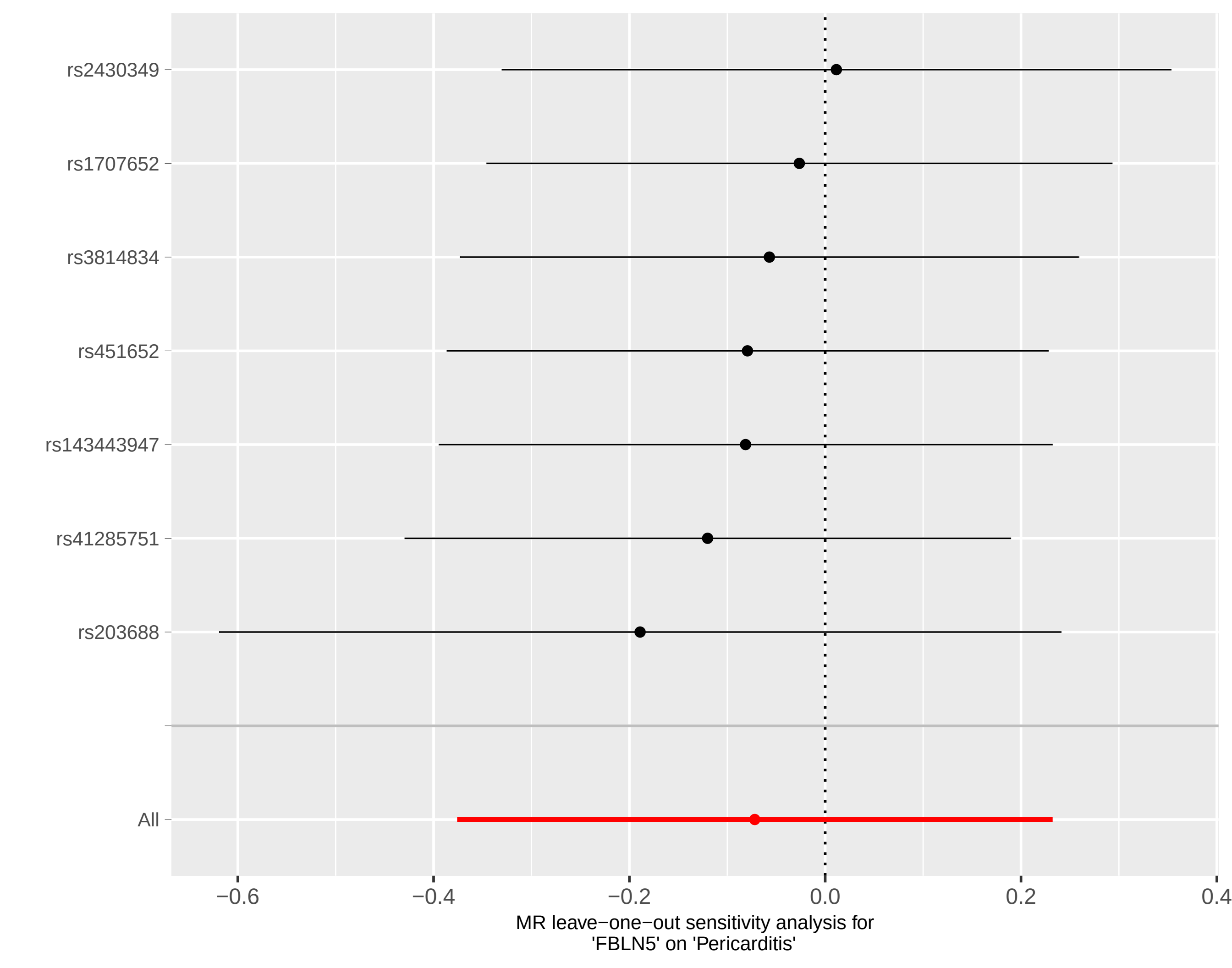

rs2430349
rs1707652
rs3814834
rs451652
rs143443947
rs41285751
rs203688
All
−0.6
−0.4
−0.2
0.0
0.2
0.4
MR leave−one−out sensitivity analysis for
'FBLN5' on 'Pericarditis'

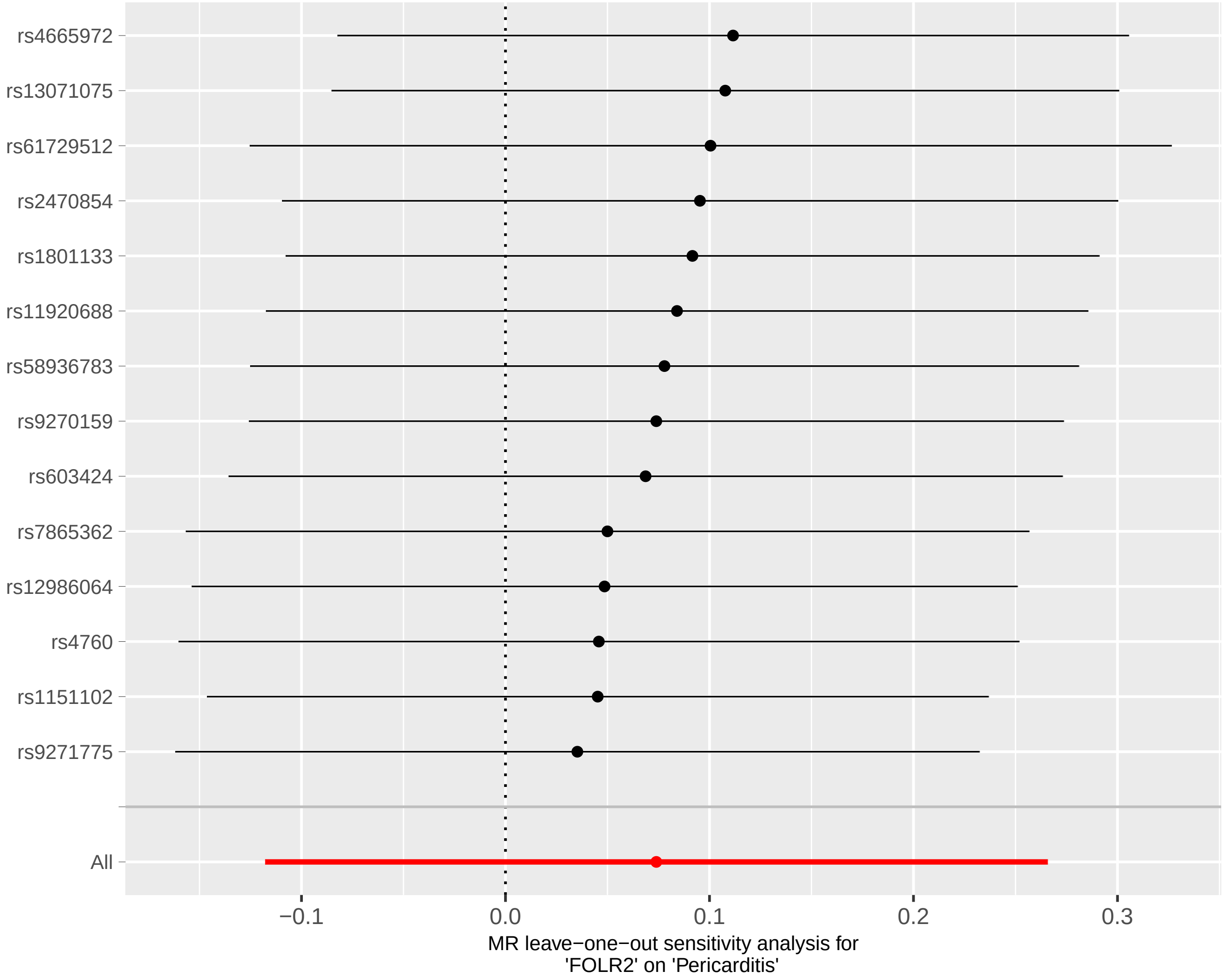
rs4665972
rs13071075
rs61729512
rs2470854
rs1801133
rs11920688
rs58936783
rs9270159
rs603424
rs7865362
rs12986064
rs4760
rs1151102
rs9271775
All
−0.1
0.0
0.1
0.2
0.3
MR leave−one−out sensitivity analysis for
'FOLR2' on 'Pericarditis'

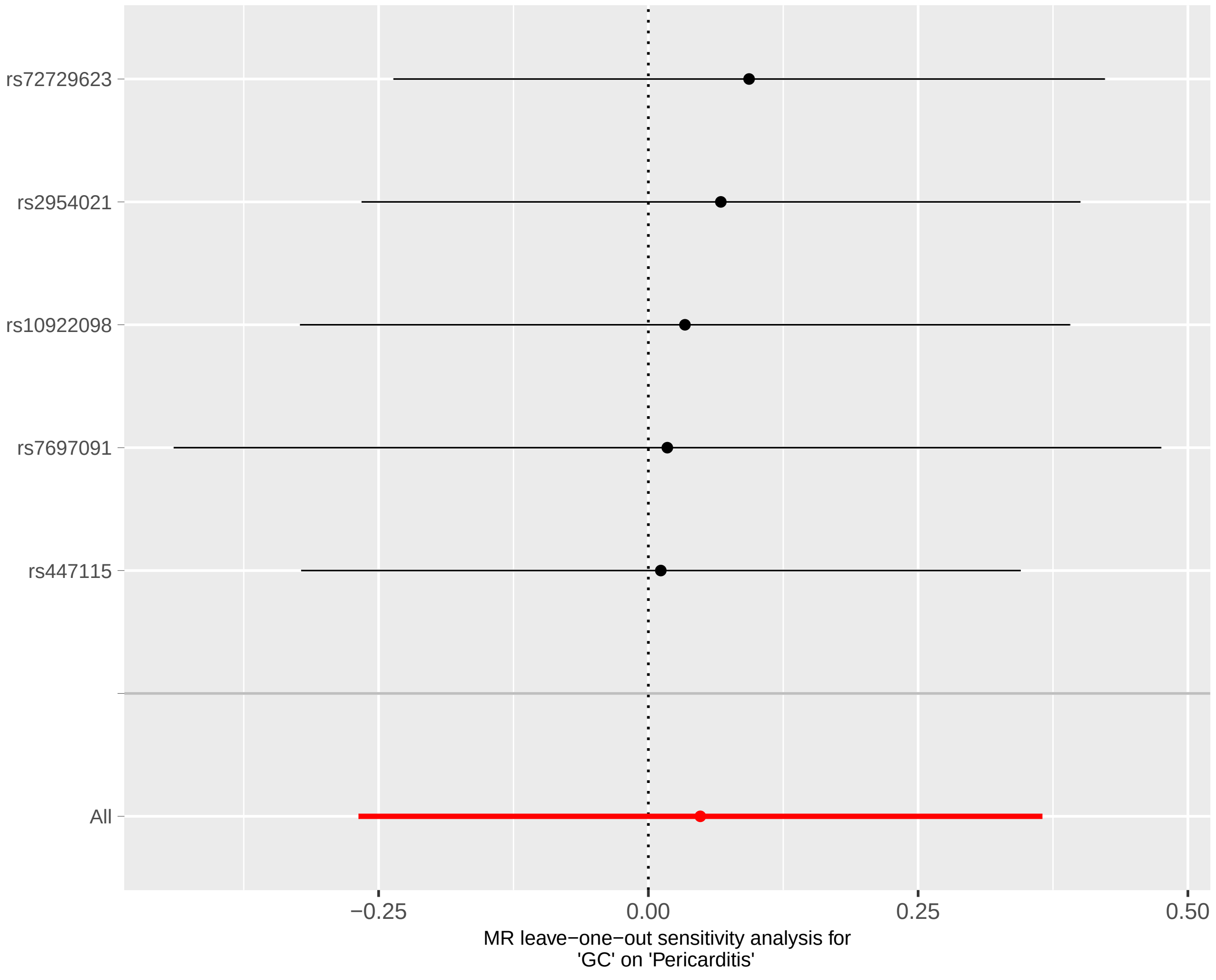

rs72729623
rs2954021
rs10922098
rs7697091
rs447115
All
−0.25
0.00
0.25
0.50
MR leave−one−out sensitivity analysis for
'GC' on 'Pericarditis'

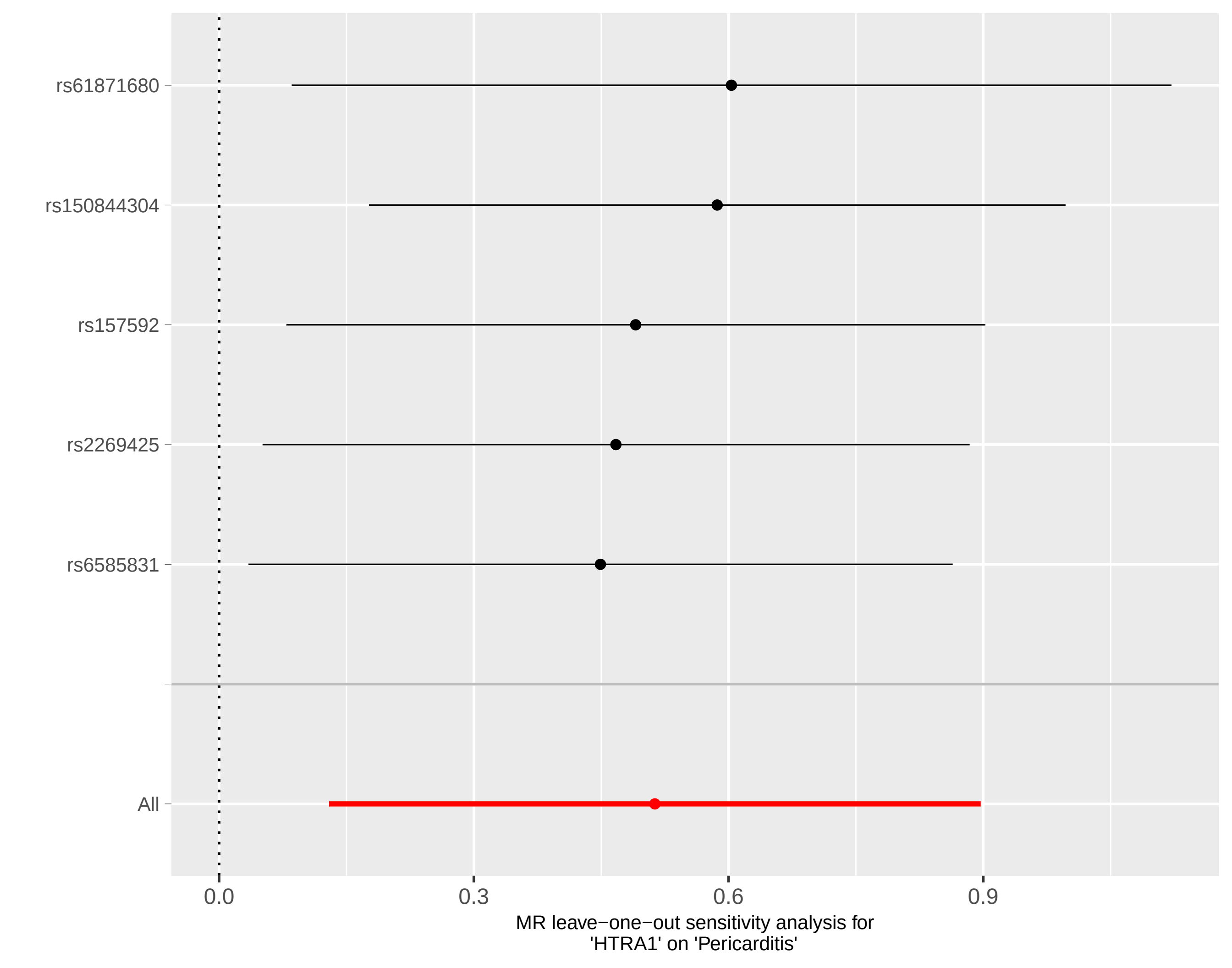

rs61871680
rs150844304
rs157592
rs2269425
rs6585831
All
0.0
0.3
0.6
0.9
MR leave−one−out sensitivity analysis for
'HTRA1' on 'Pericarditis'

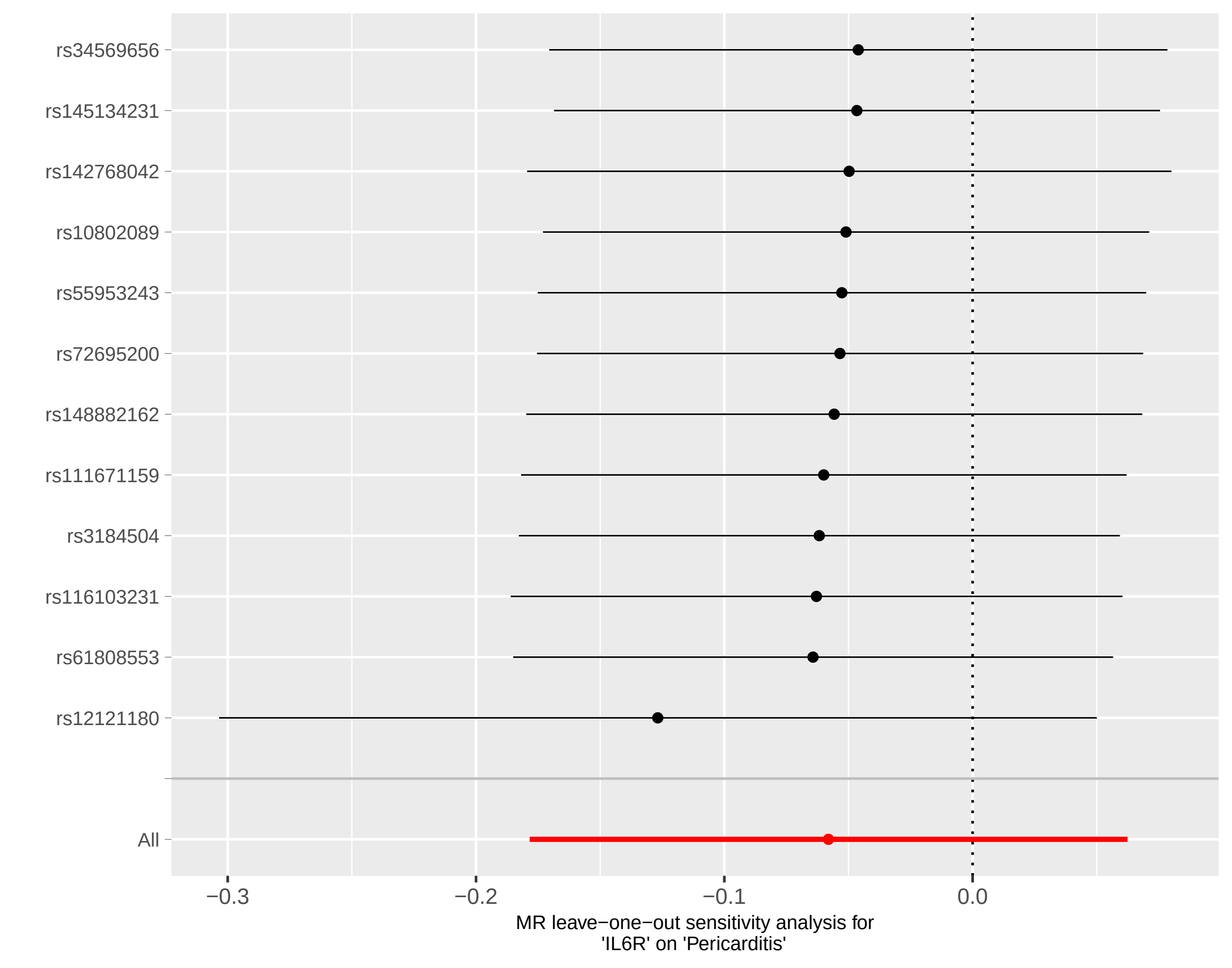
rs34569656
rs145134231
rs142768042
rs10802089
rs55953243
rs72695200
rs148882162
rs111671159
rs3184504
rs116103231
rs61808553
rs12121180
All
−0.3
−0.2
−0.1
0.0
MR leave−one−out sensitivity analysis for
'IL6R' on 'Pericarditis'

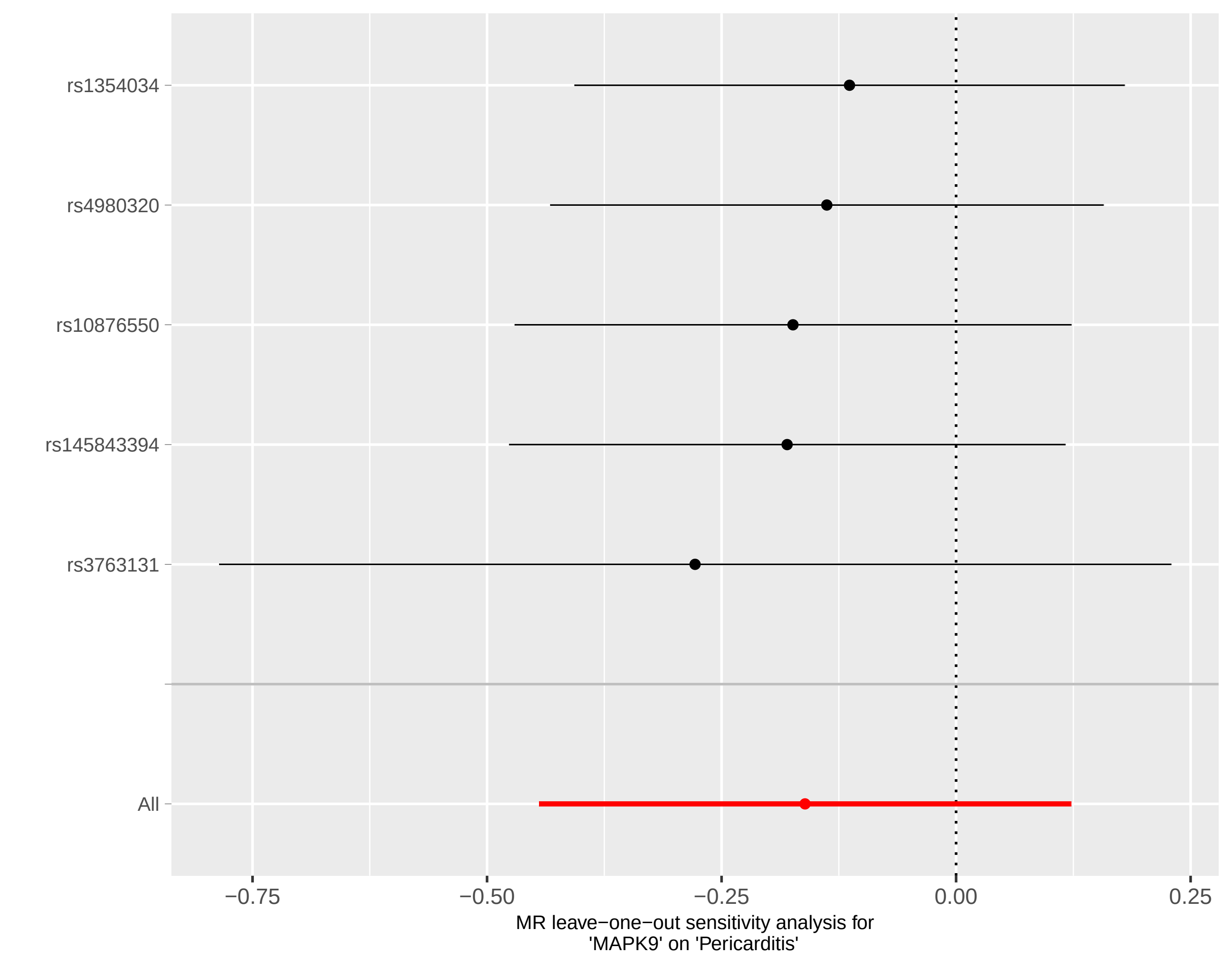

rs1354034
rs4980320
rs10876550
rs145843394
rs3763131
All
−0.75
−0.50
−0.25
0.00
0.25
MR leave−one−out sensitivity analysis for
'MAPK9' on 'Pericarditis'

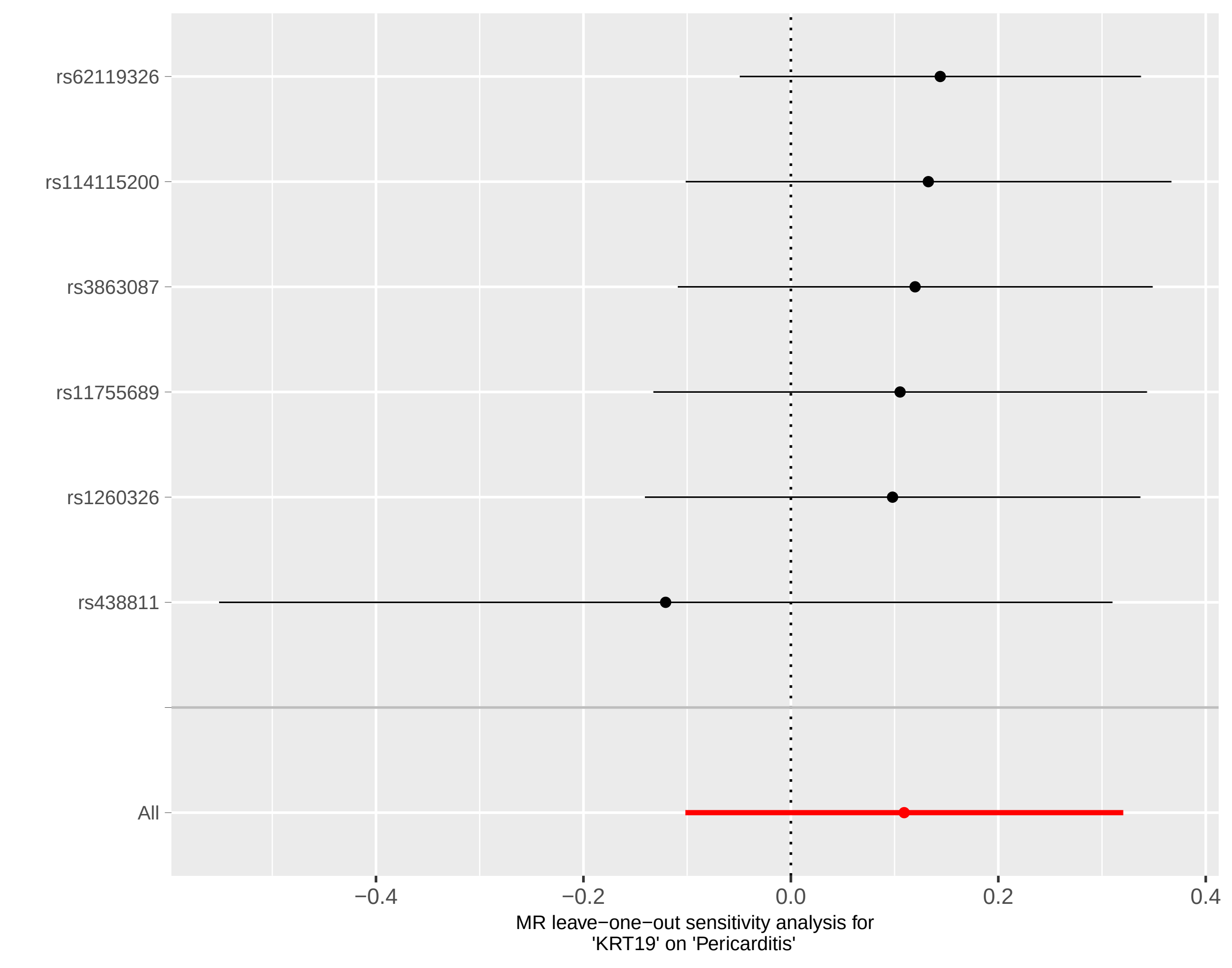

rs62119326
rs114115200
rs3863087
rs11755689
rs1260326
rs438811
All
−0.4
−0.2
0.0
0.2
0.4
MR leave−one−out sensitivity analysis for
'KRT19' on 'Pericarditis'

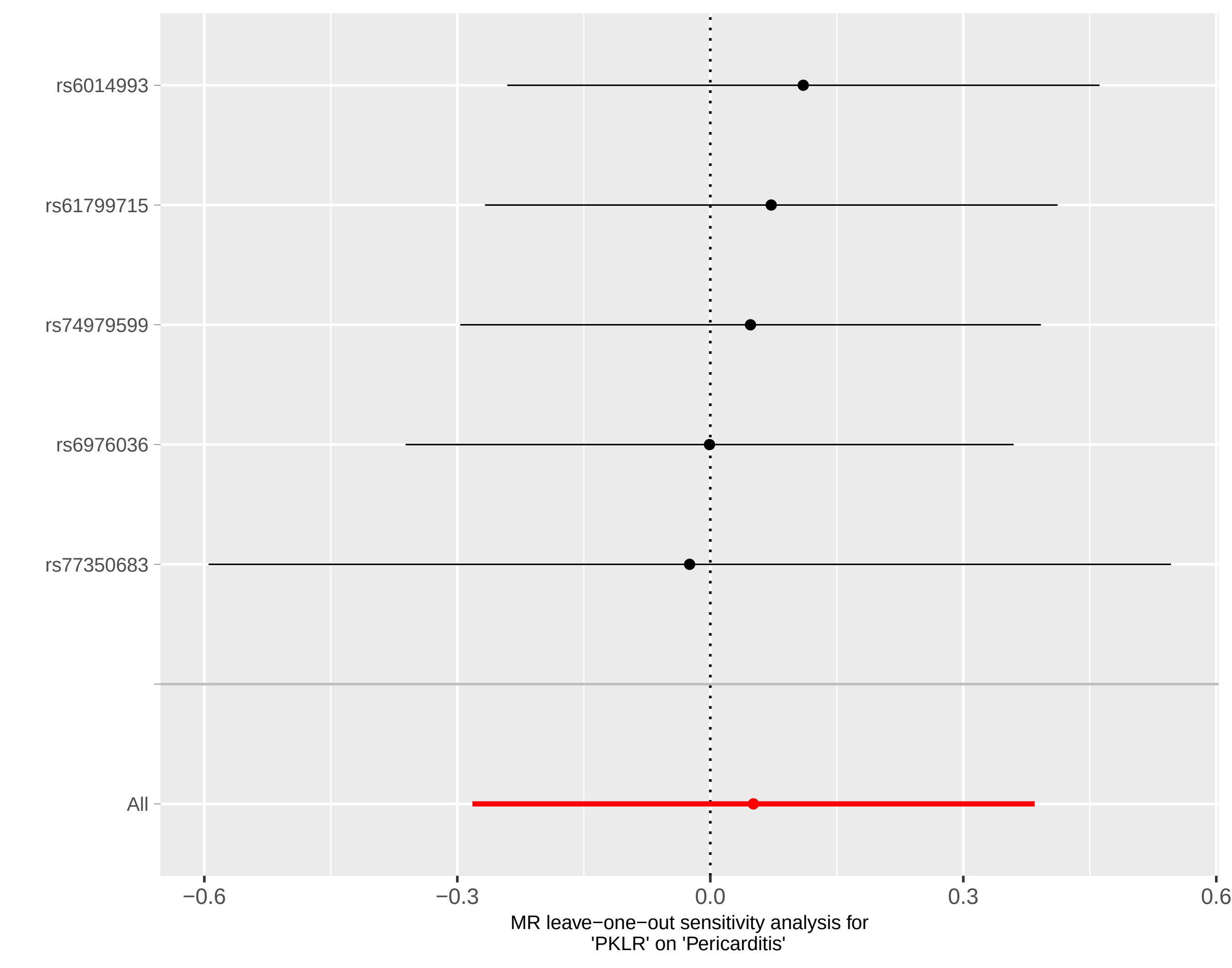
rs6014993
rs61799715
rs74979599
rs6976036
rs77350683
All
−0.6
−0.3
0.0
0.3
0.6
MR leave−one−out sensitivity analysis for
'PKLR' on 'Pericarditis'

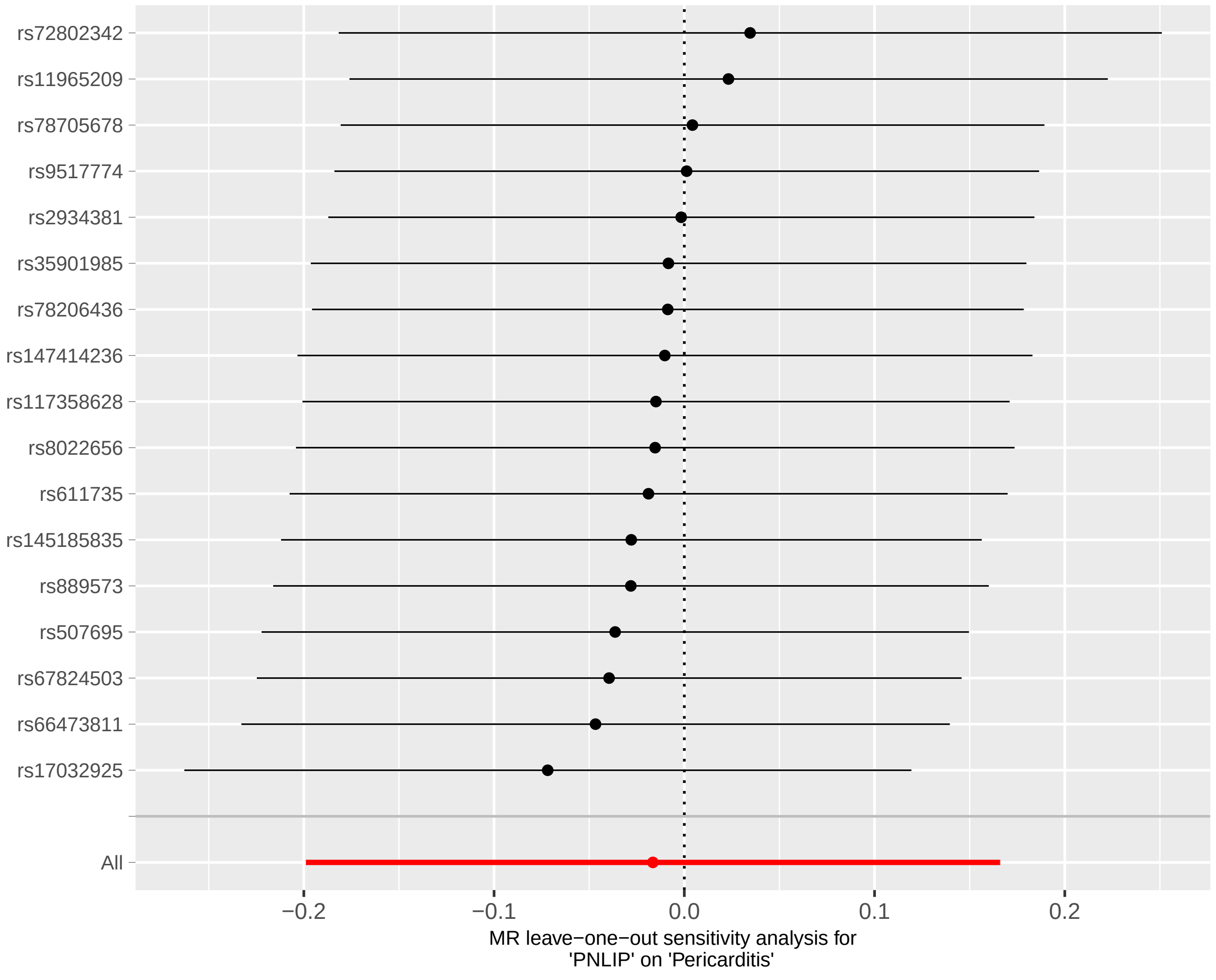
rs72802342
rs11965209
rs78705678
rs9517774
rs2934381
rs35901985
rs78206436
rs147414236
rs117358628
rs8022656
rs611735
rs145185835
rs889573
rs507695
rs67824503
rs66473811
rs17032925
All
−0.2
−0.1
0.0
0.1
0.2
MR leave−one−out sensitivity analysis for
'PNLIP' on 'Pericarditis'

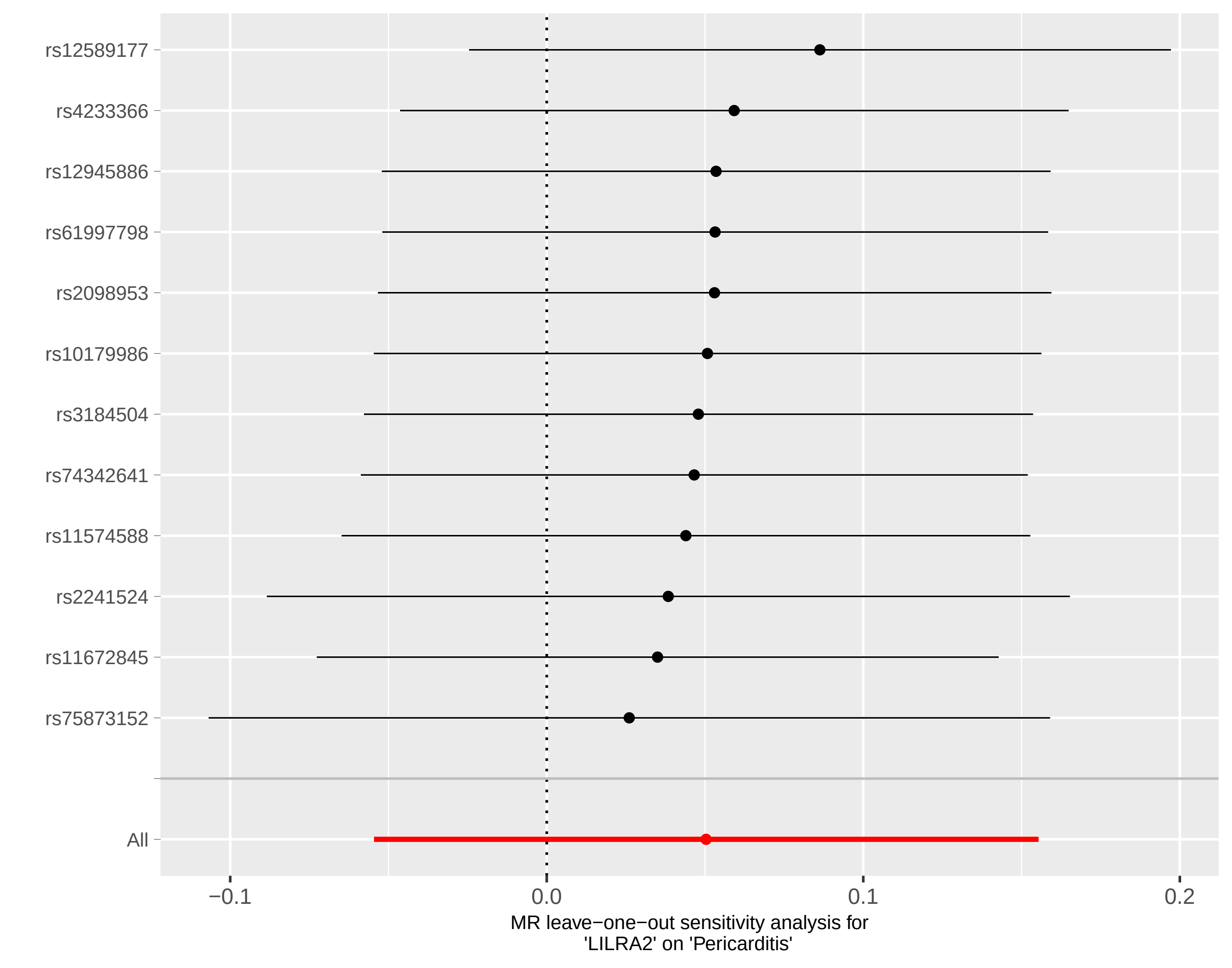

rs12589177
rs4233366
rs12945886
rs61997798
rs2098953
rs10179986
rs3184504
rs74342641
rs11574588
rs2241524
rs11672845
rs75873152
All
−0.1
0.0
0.1
0.2
MR leave−one−out sensitivity analysis for
'LILRA2' on 'Pericarditis'

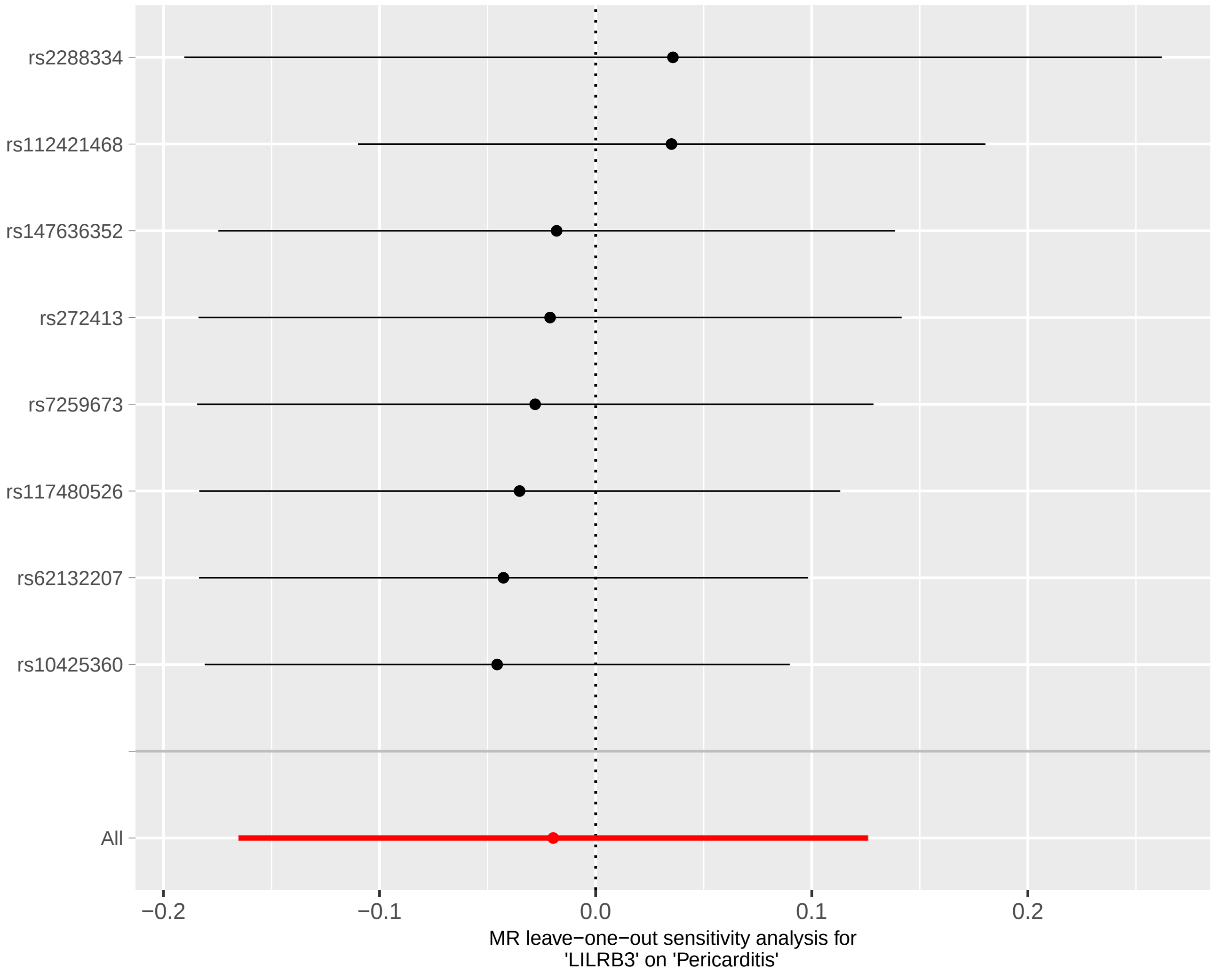

rs2288334
rs112421468
rs147636352
rs272413
rs7259673
rs117480526
rs62132207
rs10425360
All
−0.2
−0.1
0.0
0.1
0.2
MR leave−one−out sensitivity analysis for
'LILRB3' on 'Pericarditis'

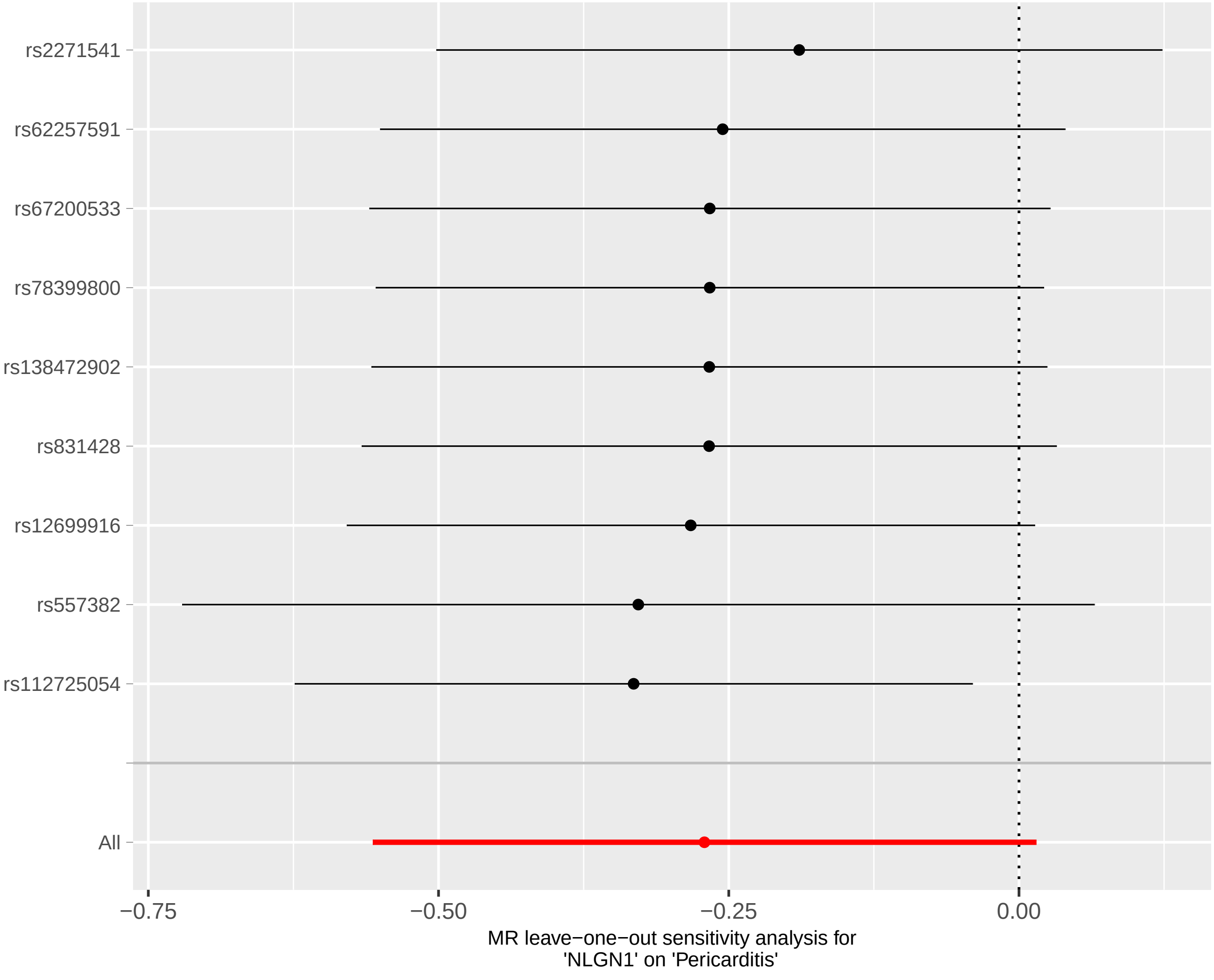
rs2271541
rs62257591
rs67200533
rs78399800
rs138472902
rs831428
rs12699916
rs557382
rs112725054
All
−0.75
−0.50
−0.25
0.00
MR leave−one−out sensitivity analysis for
'NLGN1' on 'Pericarditis'

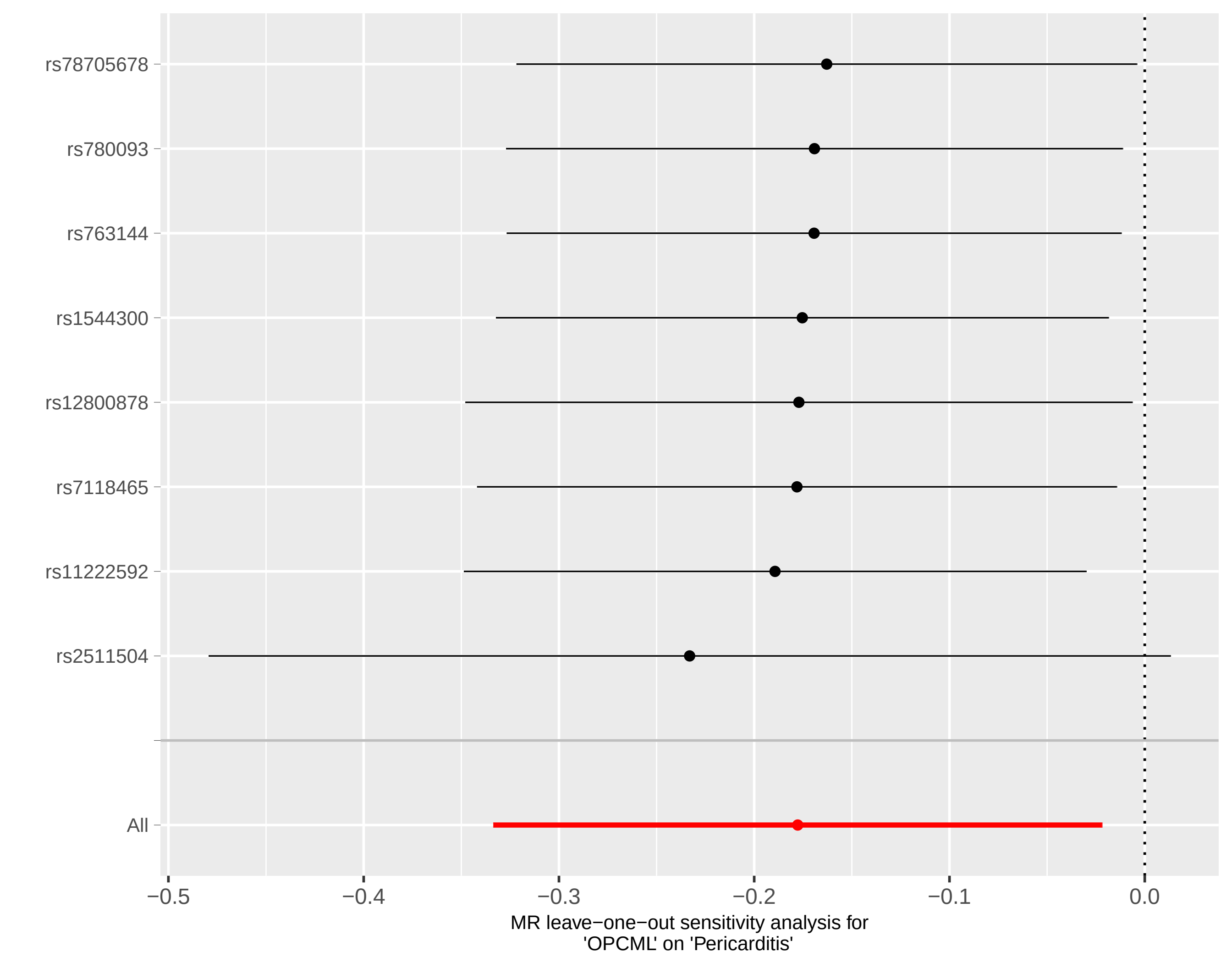
rs78705678
rs780093
rs763144
rs1544300
rs12800878
rs7118465
rs11222592
rs2511504
All
−0.5
−0.4
−0.3
−0.2
−0.1
0.0
MR leave−one−out sensitivity analysis for
'OPCML' on 'Pericarditis'

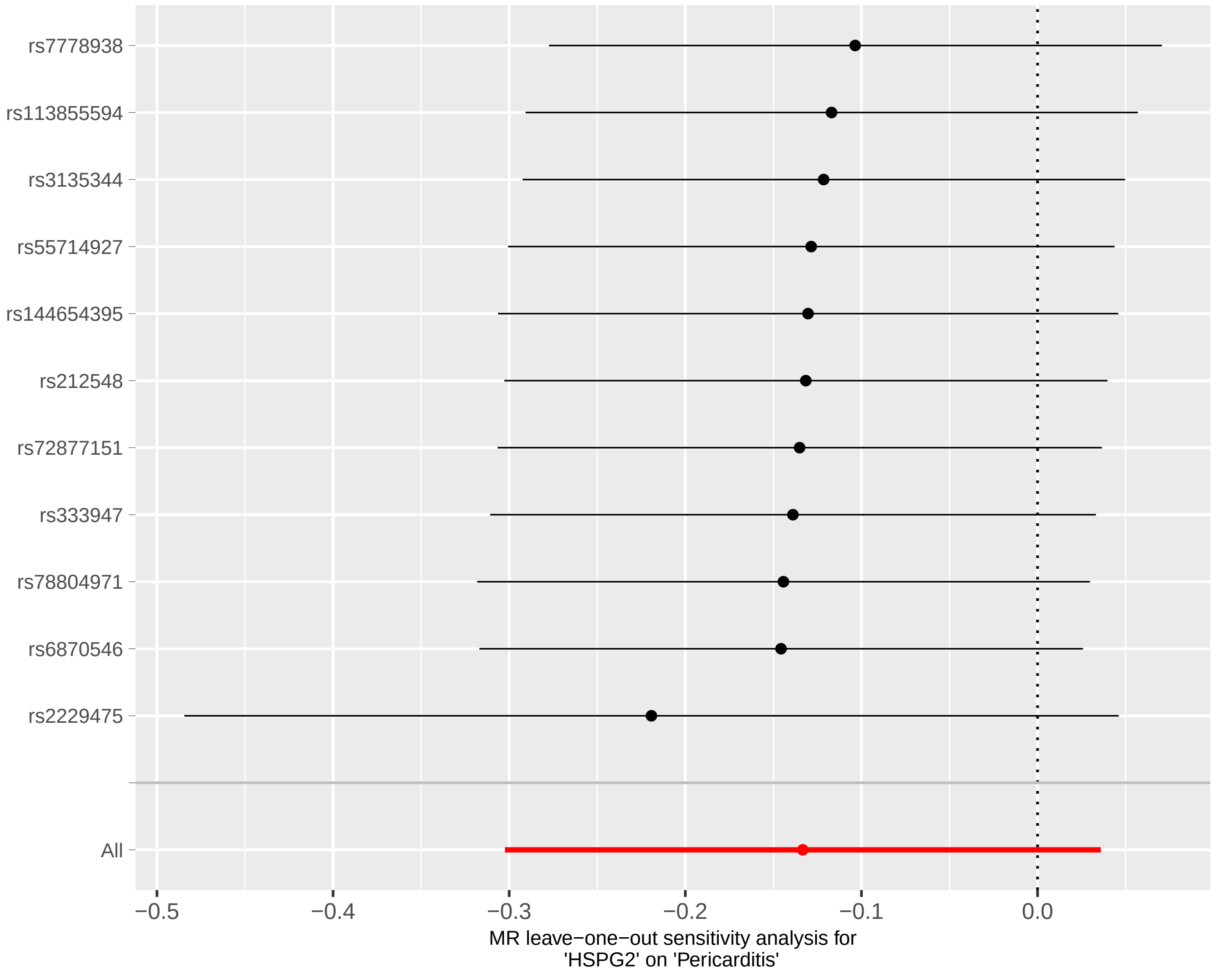
rs7778938
rs113855594
rs3135344
rs55714927
rs144654395
rs212548
rs72877151
rs333947
rs78804971
rs6870546
rs2229475
All
−0.5
−0.4
−0.3
−0.2
−0.1
0.0
MR leave−one−out sensitivity analysis for
'HSPG2' on 'Pericarditis'

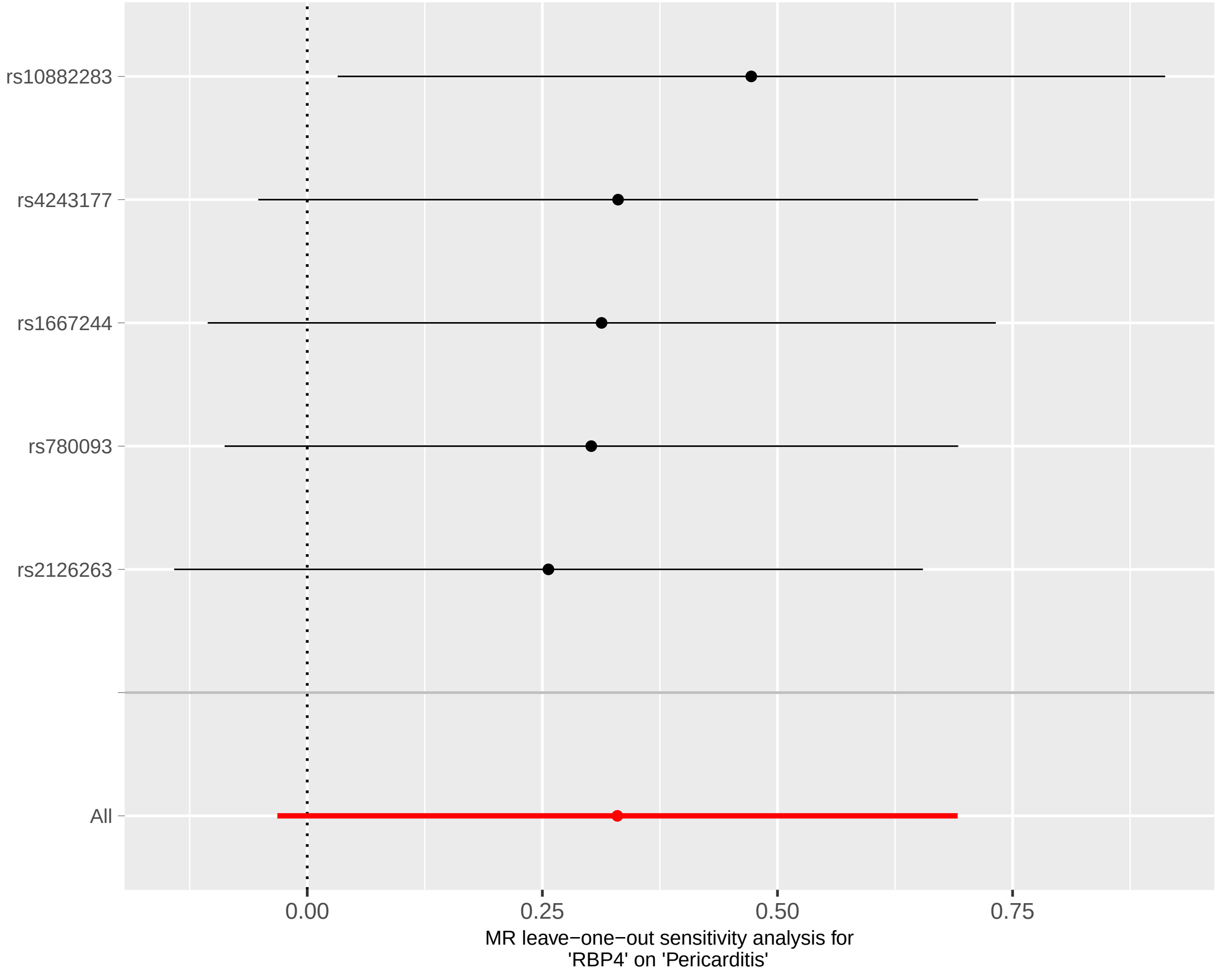

rs10882283
rs4243177
rs1667244
rs780093
rs2126263
All
0.00
0.25
0.50
0.75
MR leave−one−out sensitivity analysis for
'RBP4' on 'Pericarditis'

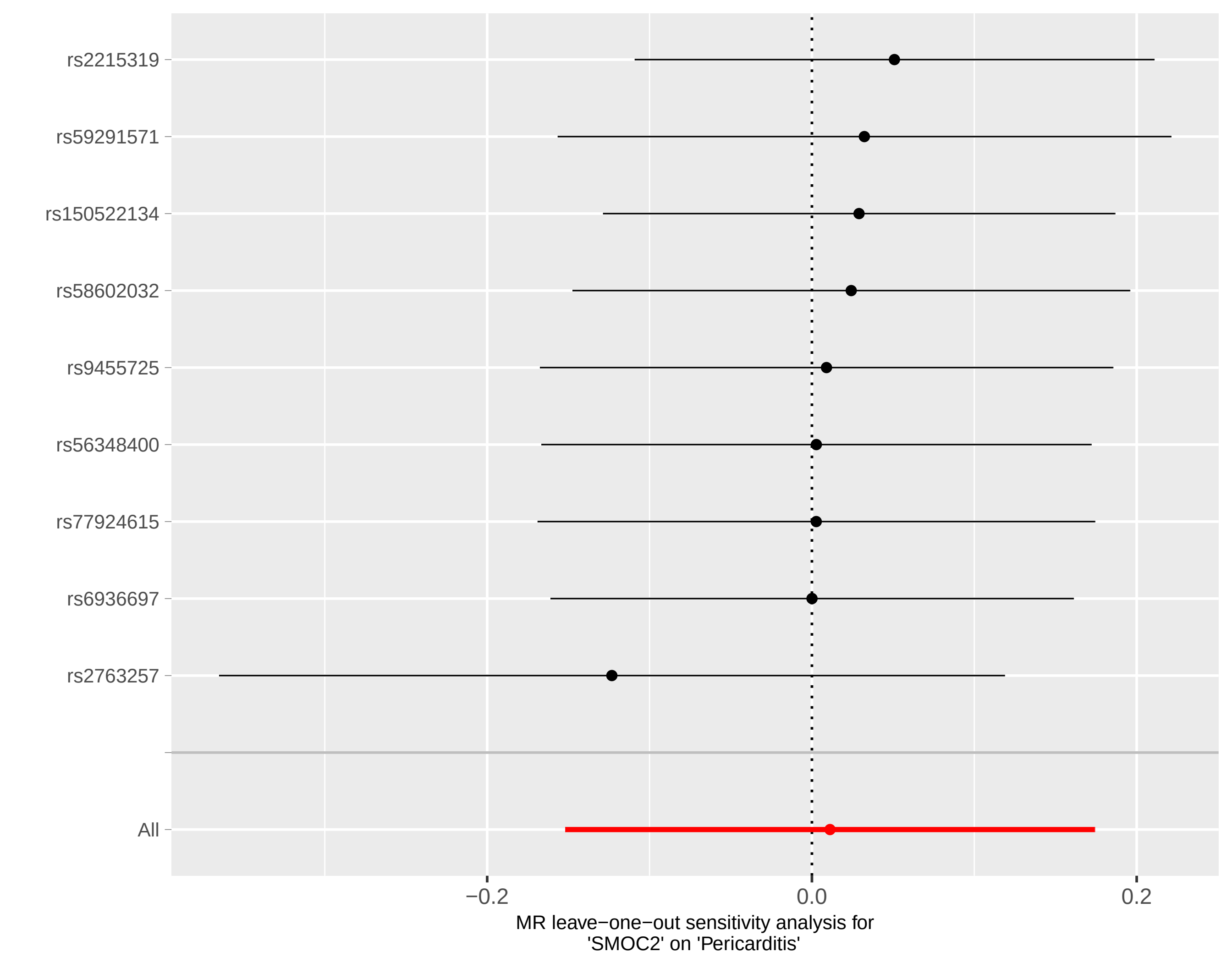
rs2215319
rs59291571
rs150522134
rs58602032
rs9455725
rs56348400
rs77924615
rs6936697
rs2763257
All
−0.2
0.0
0.2
MR leave−one−out sensitivity analysis for
'SMOC2' on 'Pericarditis'

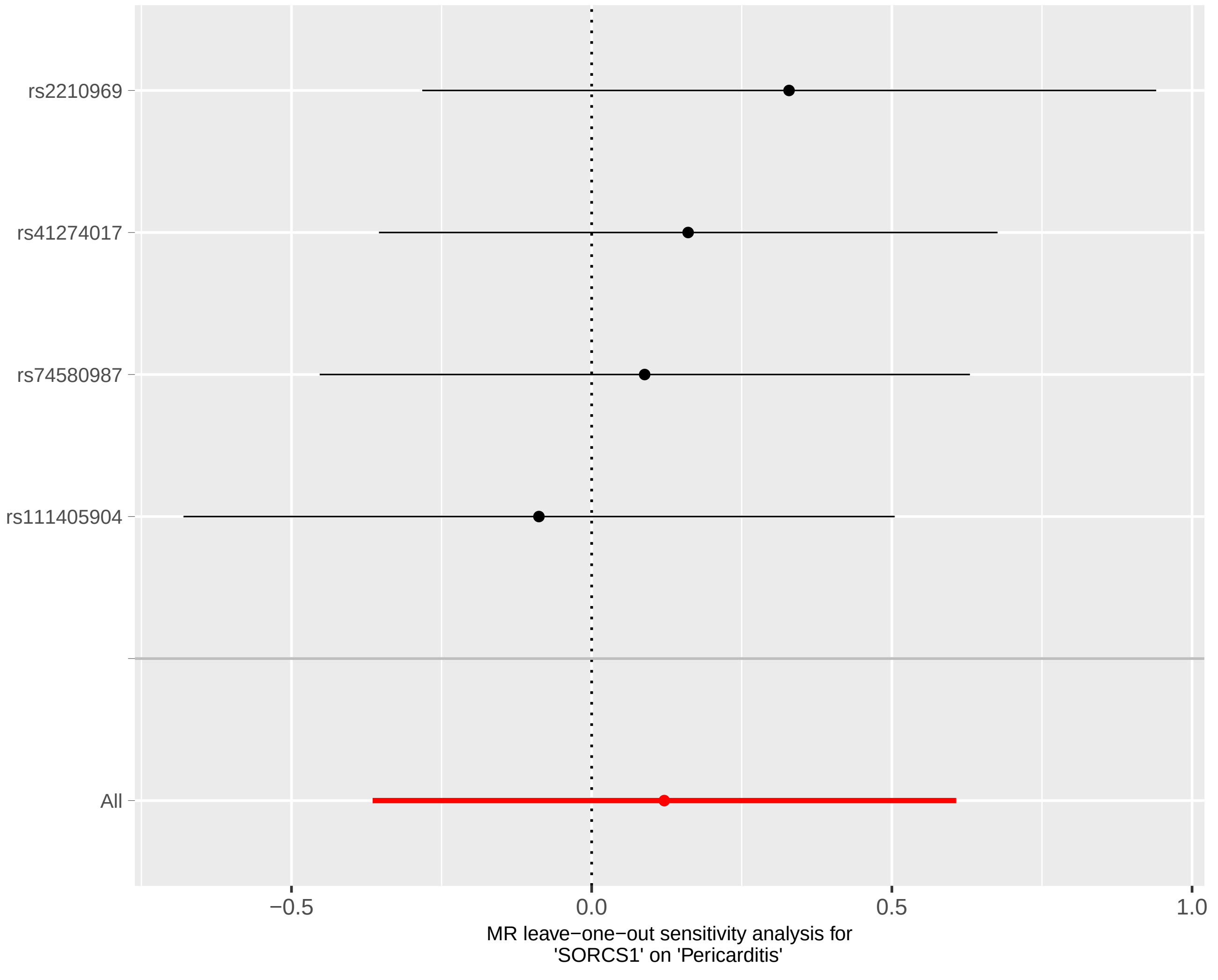
rs2210969
rs41274017
rs74580987
rs111405904
All
−0.5
0.0
0.5
1.0
MR leave−one−out sensitivity analysis for
'SORCS1' on 'Pericarditis'

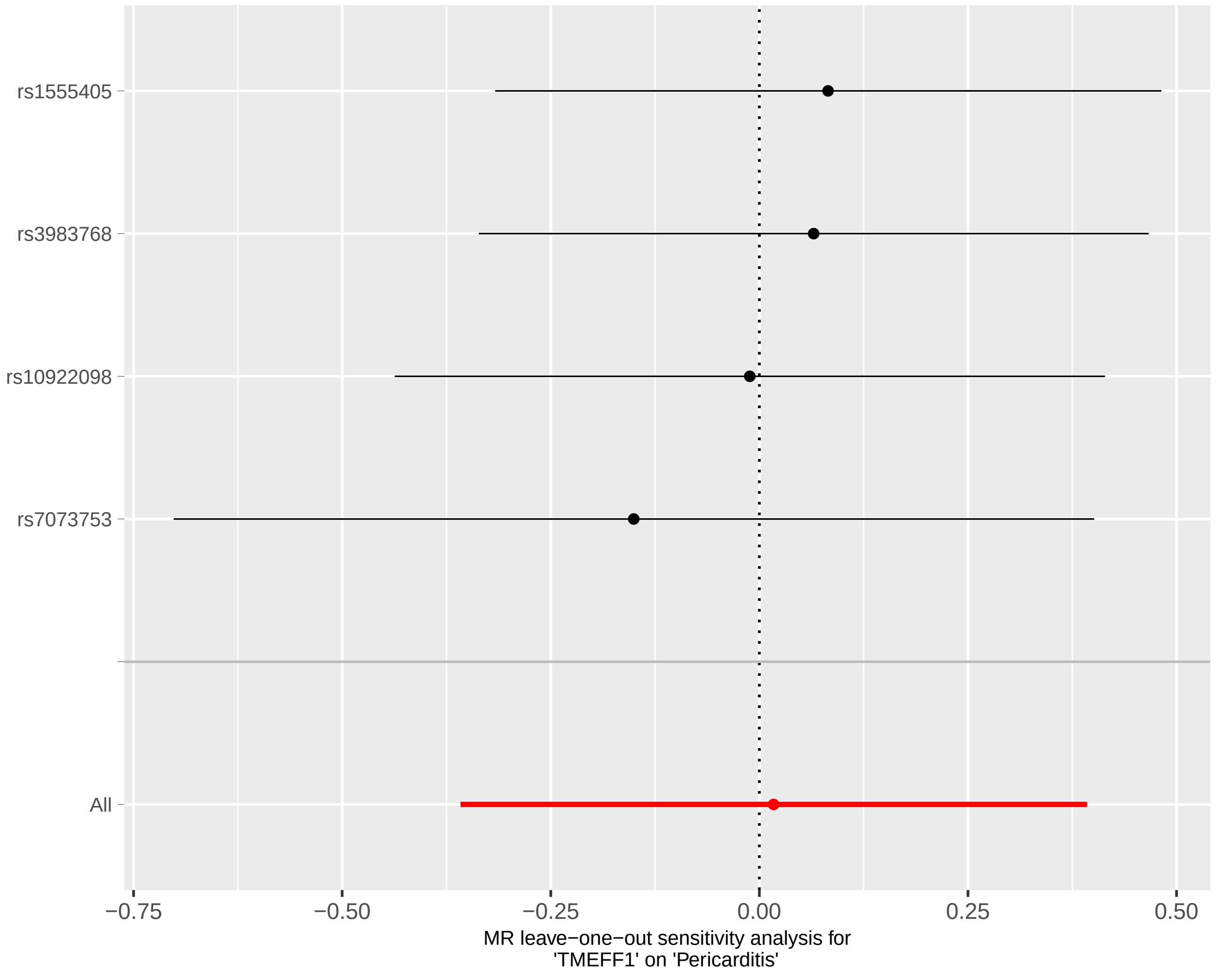
rs1555405
rs3983768
rs10922098
rs7073753
All
−0.75
−0.50
−0.25
0.00
0.25
0.50
MR leave−one−out sensitivity analysis for
'TMEFF1' on 'Pericarditis'

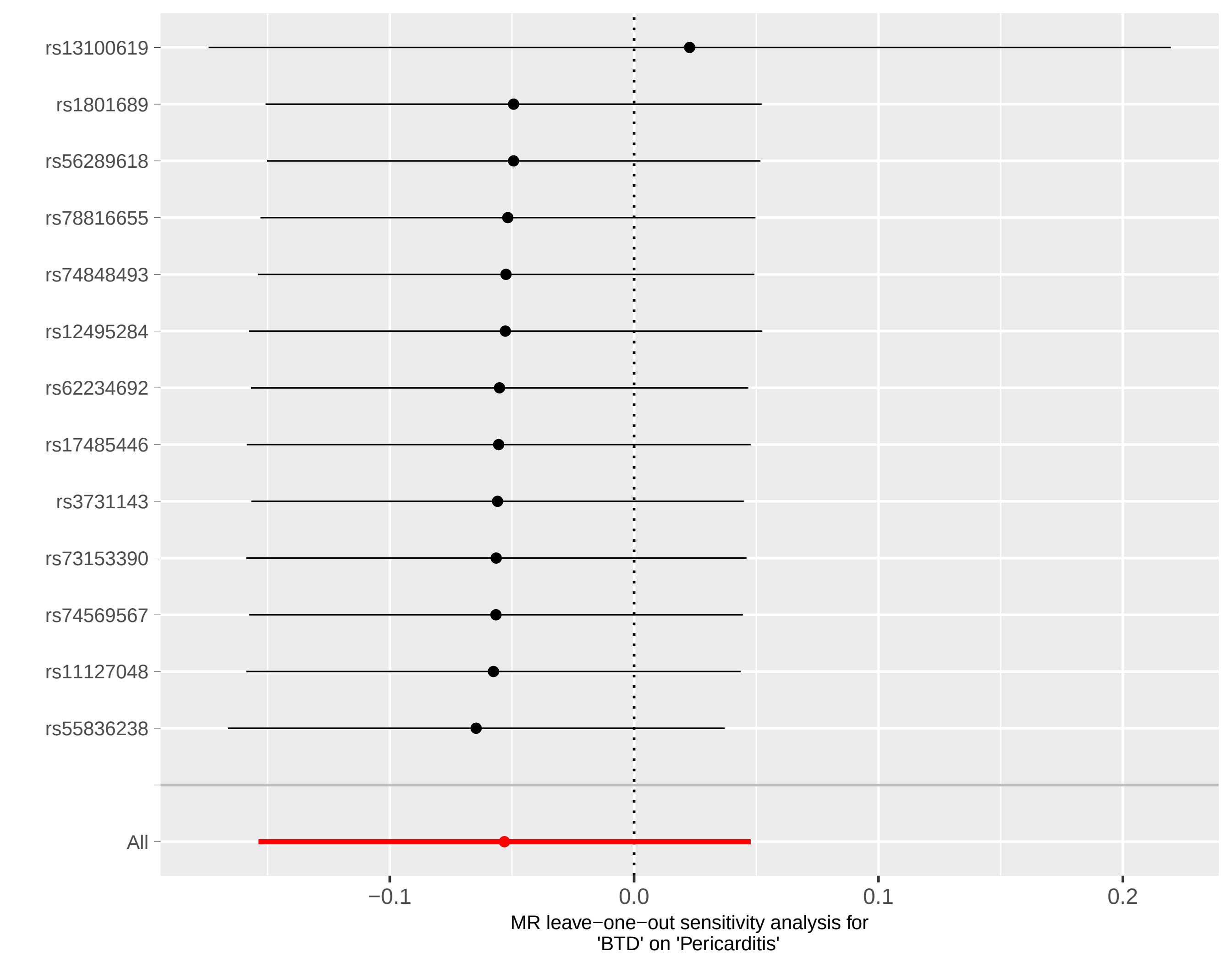
rs13100619
rs1801689
rs56289618
rs78816655
rs74848493
rs12495284
rs62234692
rs17485446
rs3731143
rs73153390
rs74569567
rs11127048
rs55836238
All
−0.1
0.0
0.1
0.2
MR leave−one−out sensitivity analysis for
'BTD' on 'Pericarditis'

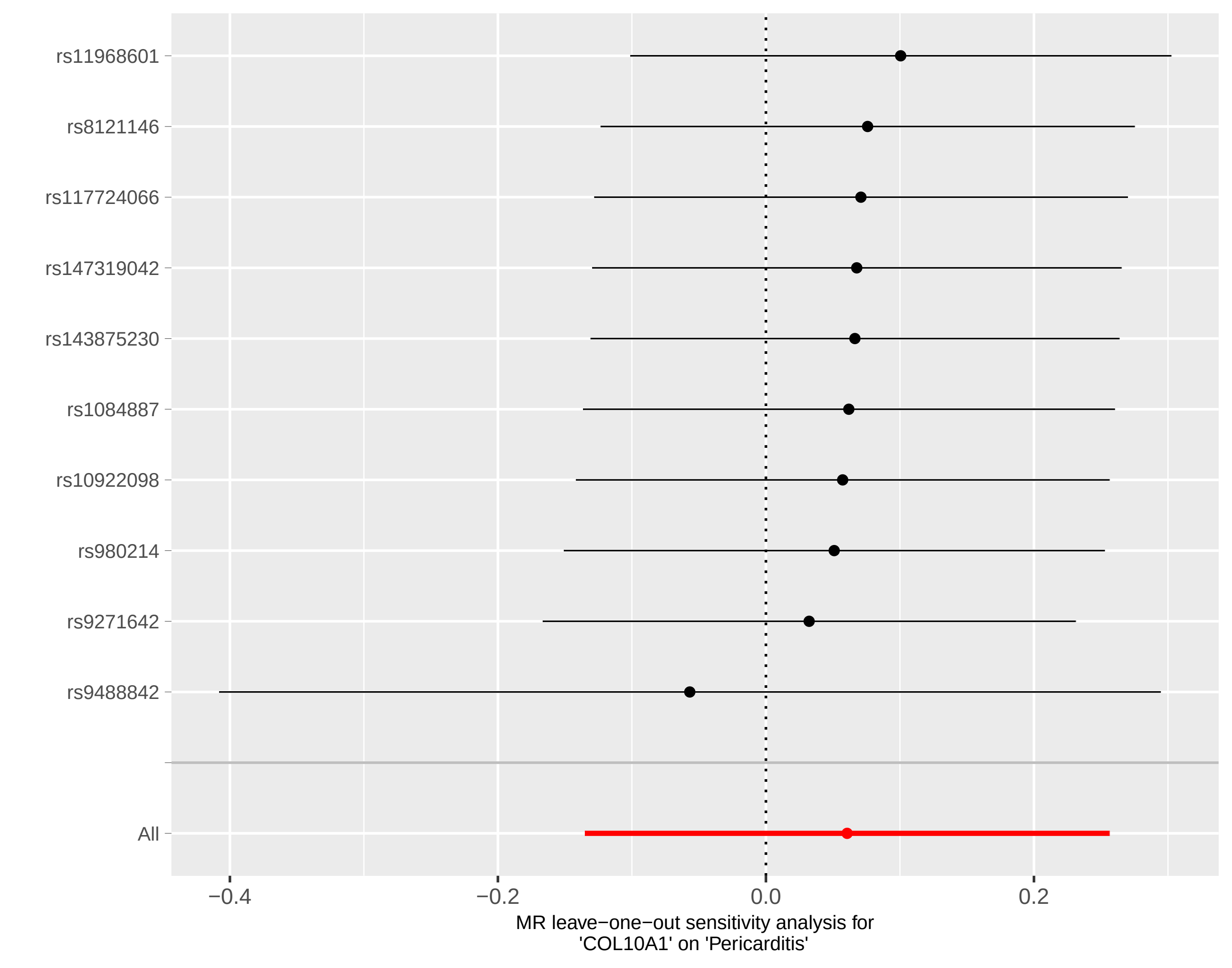
rs11968601
rs8121146
rs117724066
rs147319042
rs143875230
rs1084887
rs10922098
rs980214
rs9271642
rs9488842
All
−0.4
−0.2
0.0
0.2
MR leave−one−out sensitivity analysis for
'COL10A1' on 'Pericarditis'

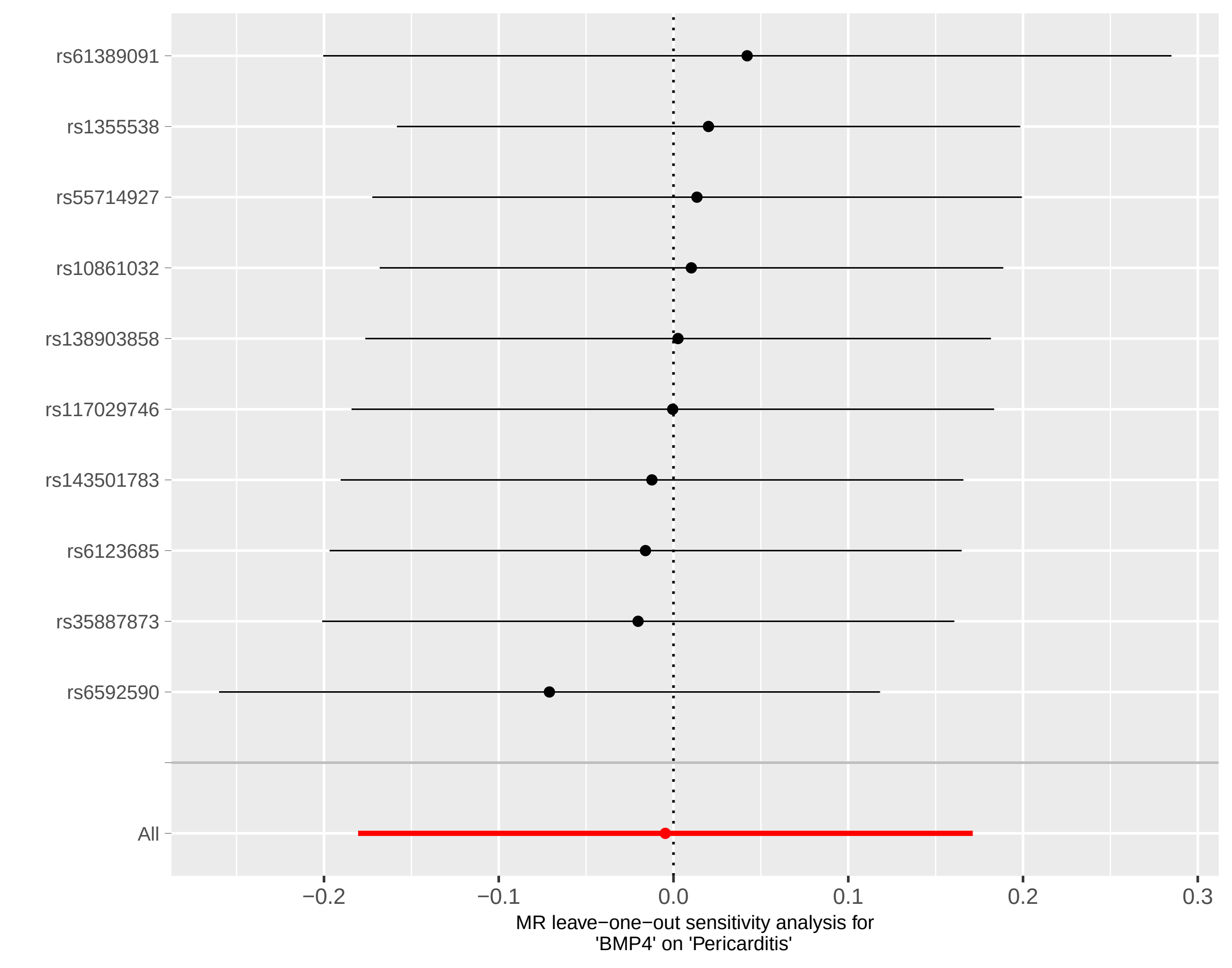
rs61389091
rs1355538
rs55714927
rs10861032
rs138903858
rs117029746
rs143501783
rs6123685
rs35887873
rs6592590
All
−0.2
−0.1
0.0
0.1
0.2
0.3
MR leave−one−out sensitivity analysis for
'BMP4' on 'Pericarditis'

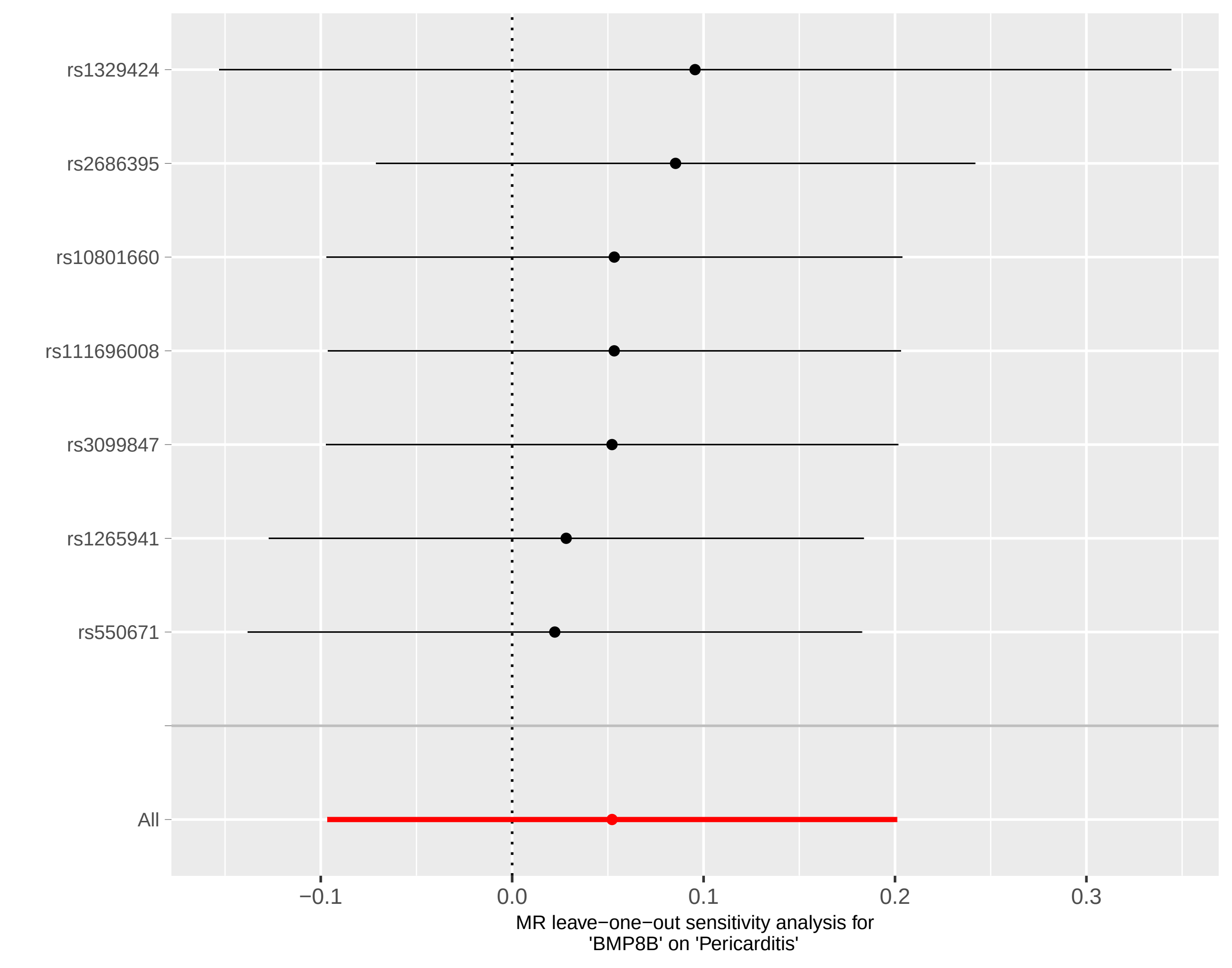

rs1329424
rs2686395
rs10801660
rs111696008
rs3099847
rs1265941
rs550671
All
−0.1
0.0
0.1
0.2
0.3
MR leave−one−out sensitivity analysis for
'BMP8B' on 'Pericarditis'

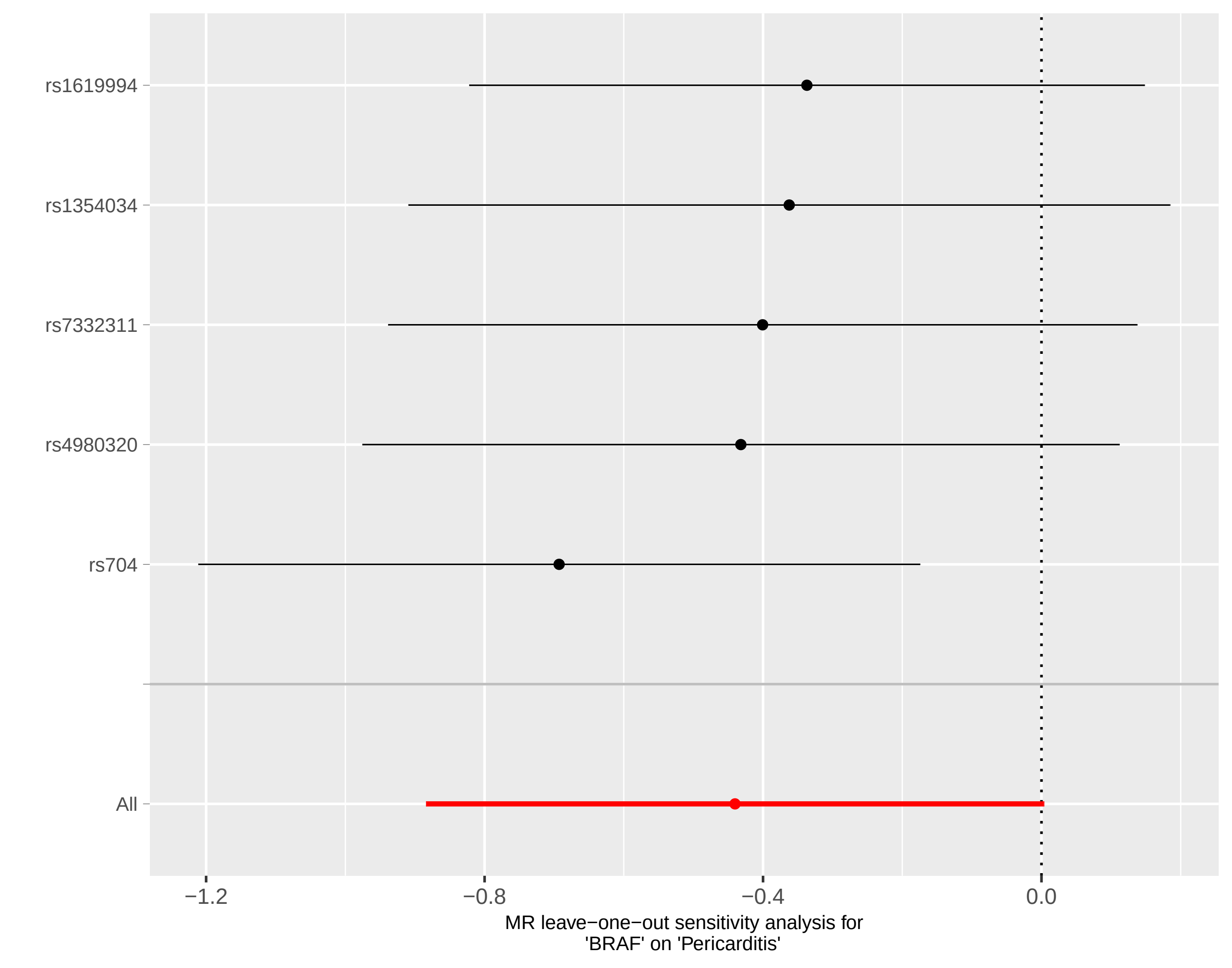
rs1619994
rs1354034
rs7332311
rs4980320
rs704
All
−1.2
−0.8
−0.4
0.0
MR leave−one−out sensitivity analysis for
'BRAF' on 'Pericarditis'

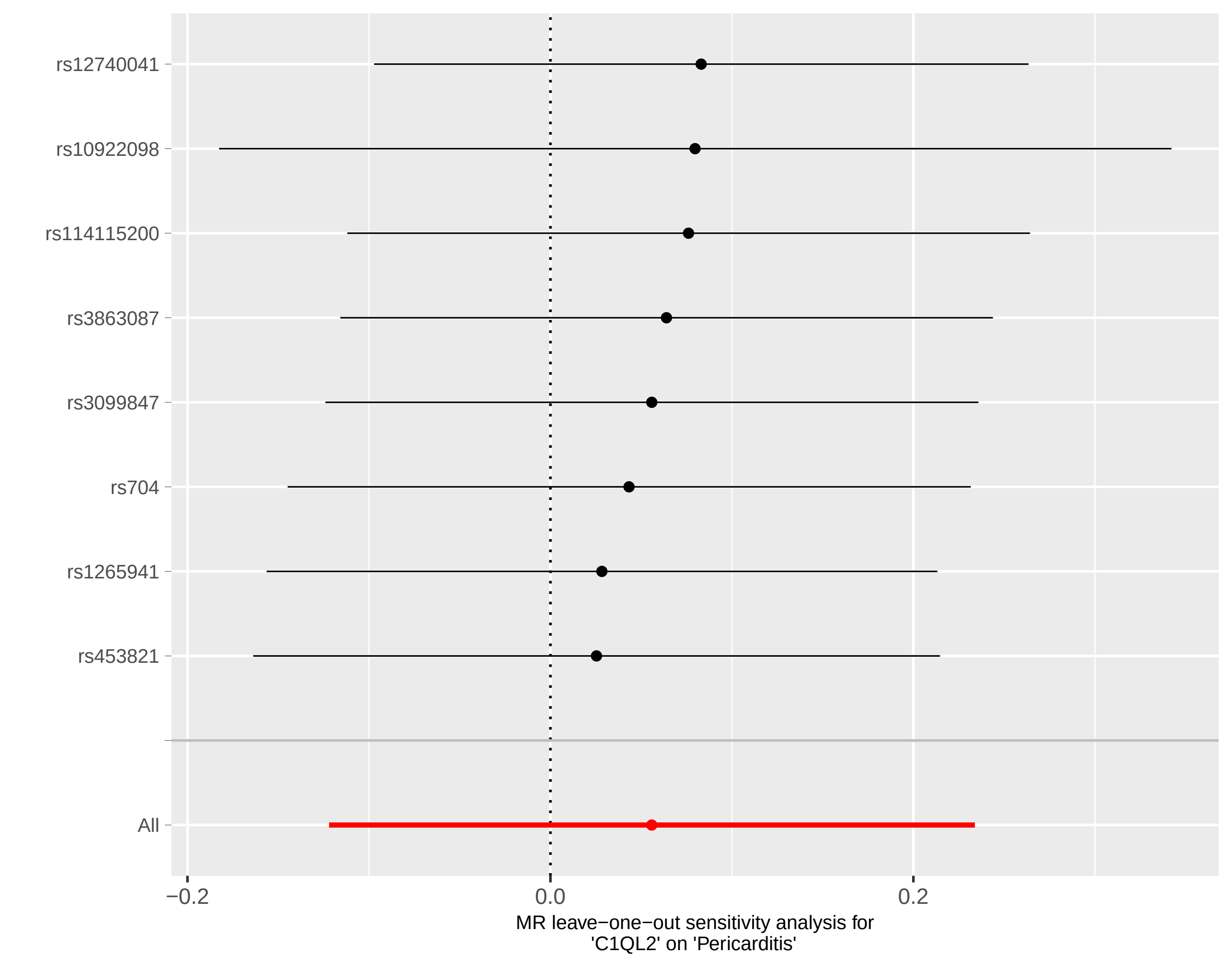
rs12740041
rs10922098
rs114115200
rs3863087
rs3099847
rs704
rs1265941
rs453821
All
−0.2
0.0
0.2
MR leave−one−out sensitivity analysis for
'C1QL2' on 'Pericarditis'

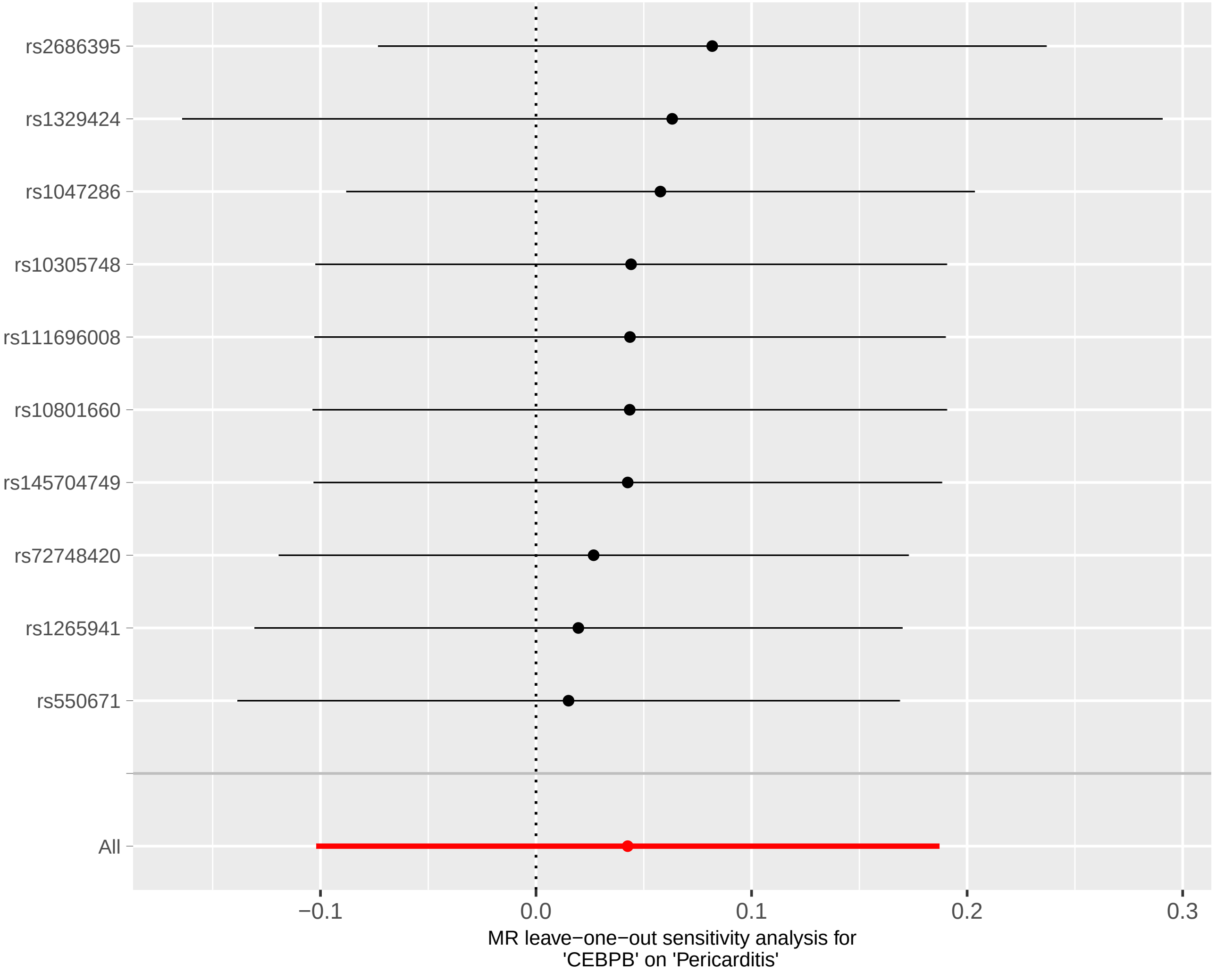
rs2686395
rs1329424
rs1047286
rs10305748
rs111696008
rs10801660
rs145704749
rs72748420
rs1265941
rs550671
All
−0.1
0.0
0.1
0.2
0.3
MR leave−one−out sensitivity analysis for
'CEBPB' on 'Pericarditis'

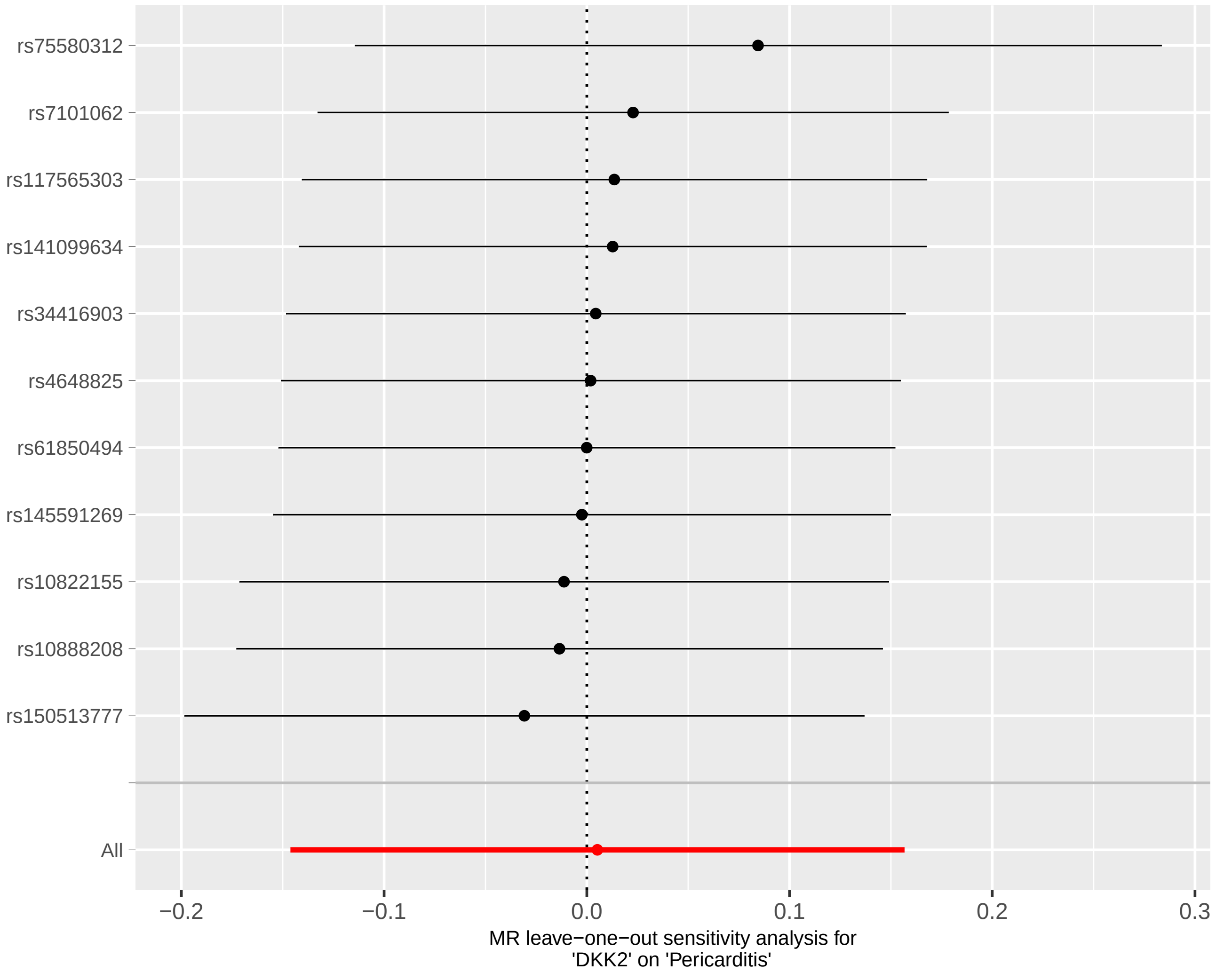
rs75580312
rs7101062
rs117565303
rs141099634
rs34416903
rs4648825
rs61850494
rs145591269
rs10822155
rs10888208
rs150513777
All
−0.2
−0.1
0.0
0.1
0.2
0.3
MR leave−one−out sensitivity analysis for
'DKK2' on 'Pericarditis'

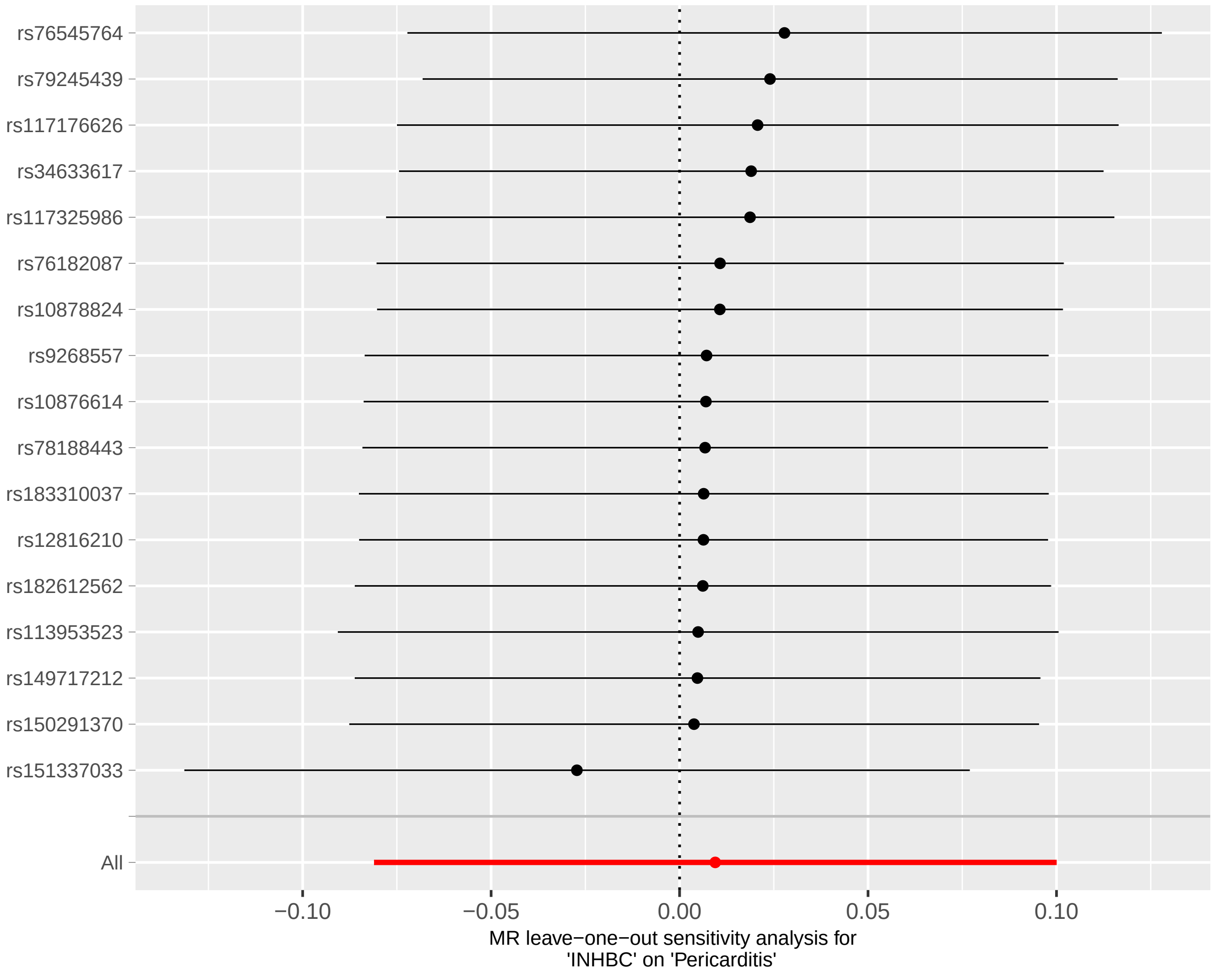
rs76545764
rs79245439
rs117176626
rs34633617
rs117325986
rs76182087
rs10878824
rs9268557
rs10876614
rs78188443
rs183310037
rs12816210
rs182612562
rs113953523
rs149717212
rs150291370
rs151337033
All
−0.10
−0.05
0.00
0.05
0.10
MR leave−one−out sensitivity analysis for
'INHBC' on 'Pericarditis'

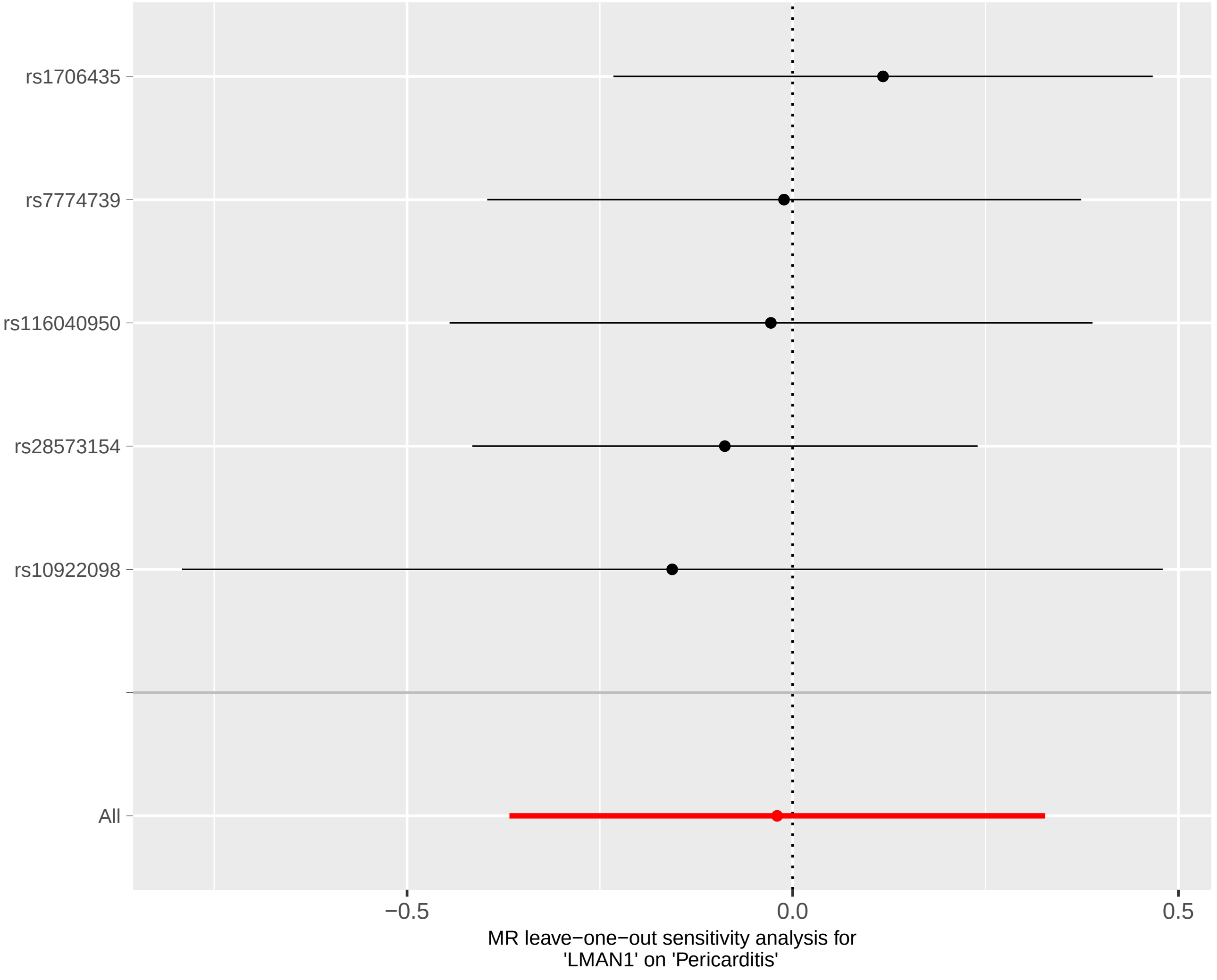

rs1706435
rs7774739
rs116040950
rs28573154
rs10922098
All
−0.5
0.0
0.5
MR leave−one−out sensitivity analysis for
'LMAN1' on 'Pericarditis'

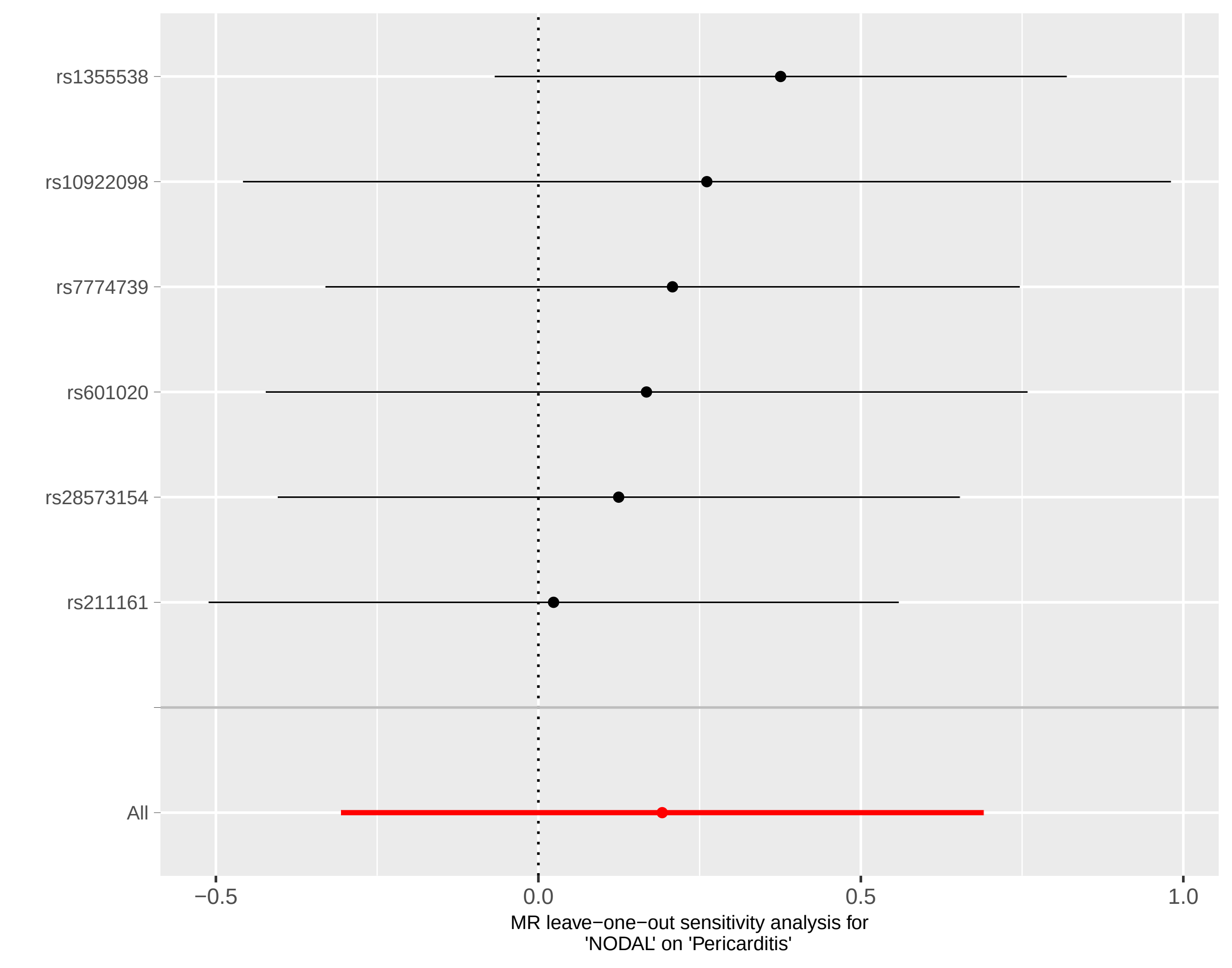
rs1355538
rs10922098
rs7774739
rs601020
rs28573154
rs211161
All
−0.5
0.0
0.5
1.0
MR leave−one−out sensitivity analysis for
'NODAL' on 'Pericarditis'

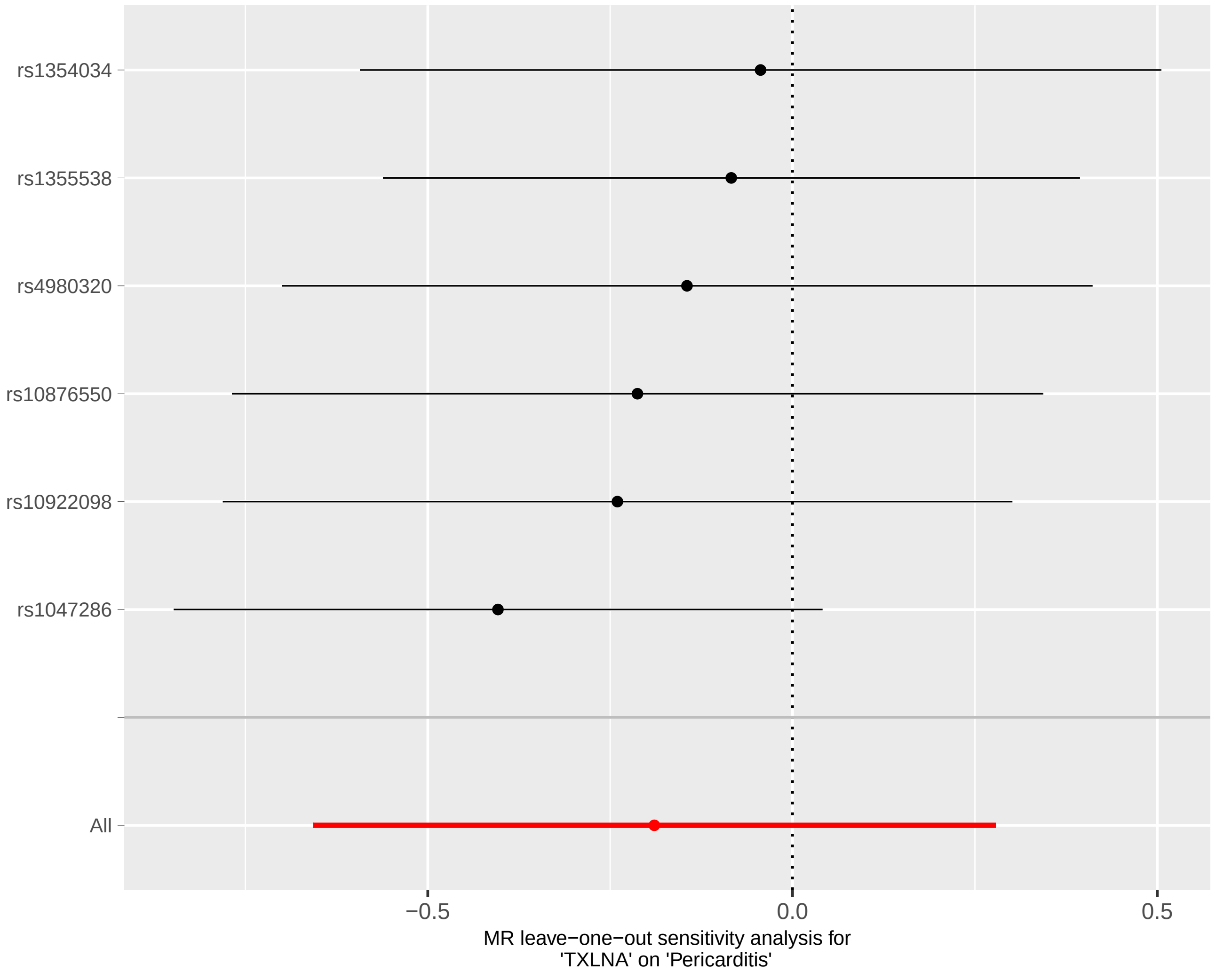
rs1354034
rs1355538
rs4980320
rs10876550
rs10922098
rs1047286
All
−0.5
0.0
0.5
MR leave−one−out sensitivity analysis for
'TXLNA' on 'Pericarditis'

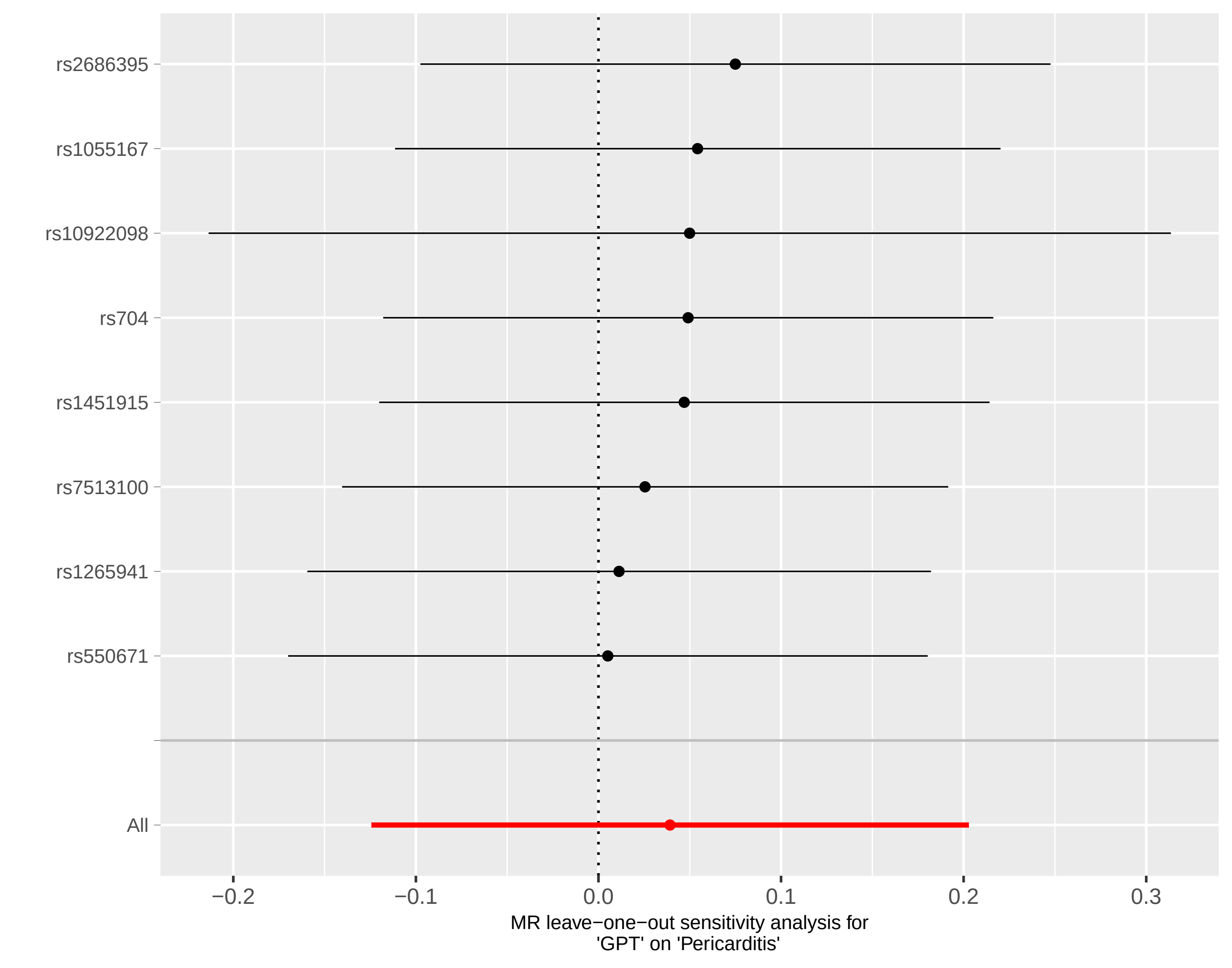

rs2686395
rs1055167
rs10922098
rs704
rs1451915
rs7513100
rs1265941
rs550671
All
−0.2
−0.1
0.0
0.1
0.2
0.3
MR leave−one−out sensitivity analysis for
'GPT' on 'Pericarditis'

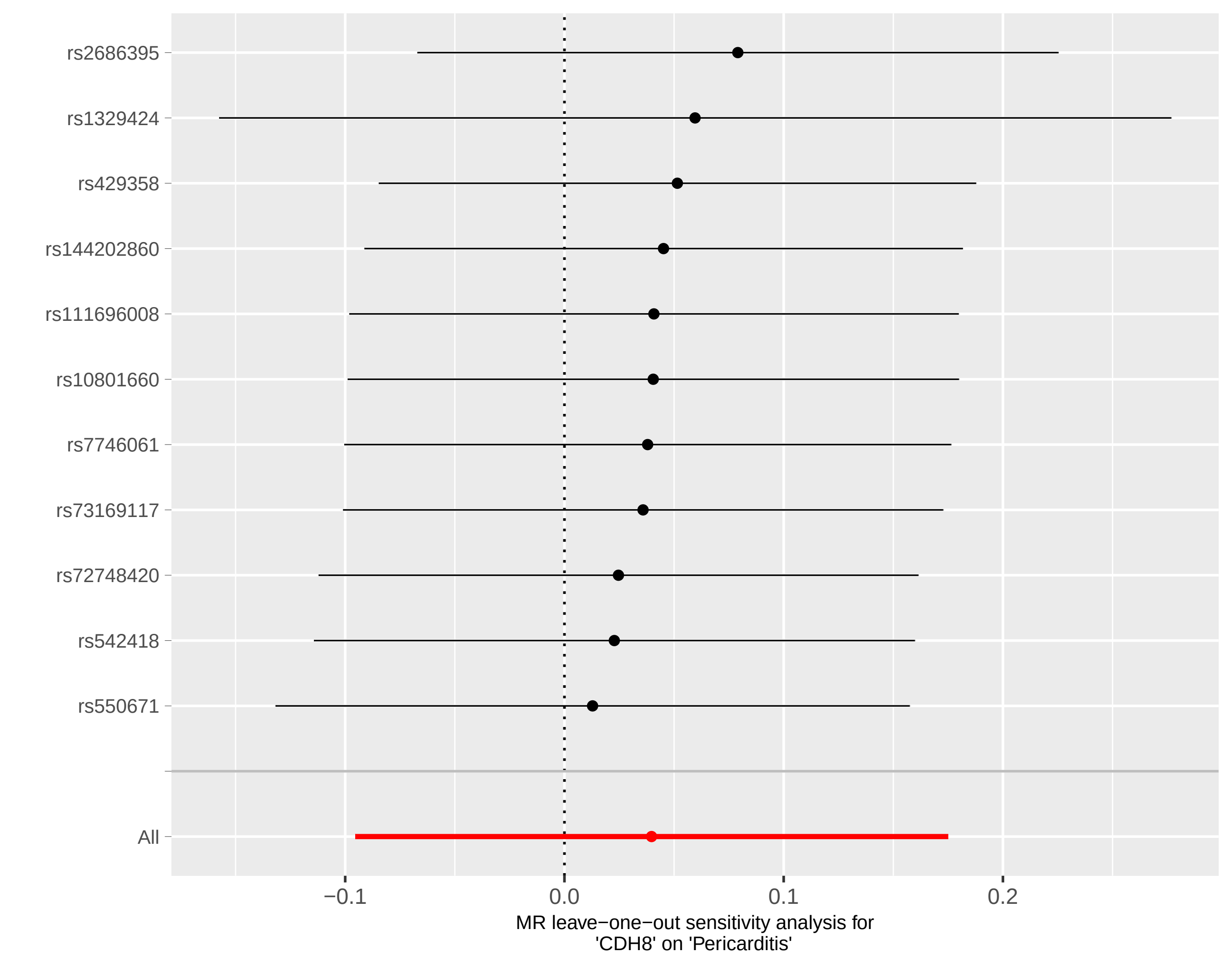

rs2686395
rs1329424
rs429358
rs144202860
rs111696008
rs10801660
rs7746061
rs73169117
rs72748420
rs542418
rs550671
All
−0.1
0.0
0.1
0.2
MR leave−one−out sensitivity analysis for
'CDH8' on 'Pericarditis'

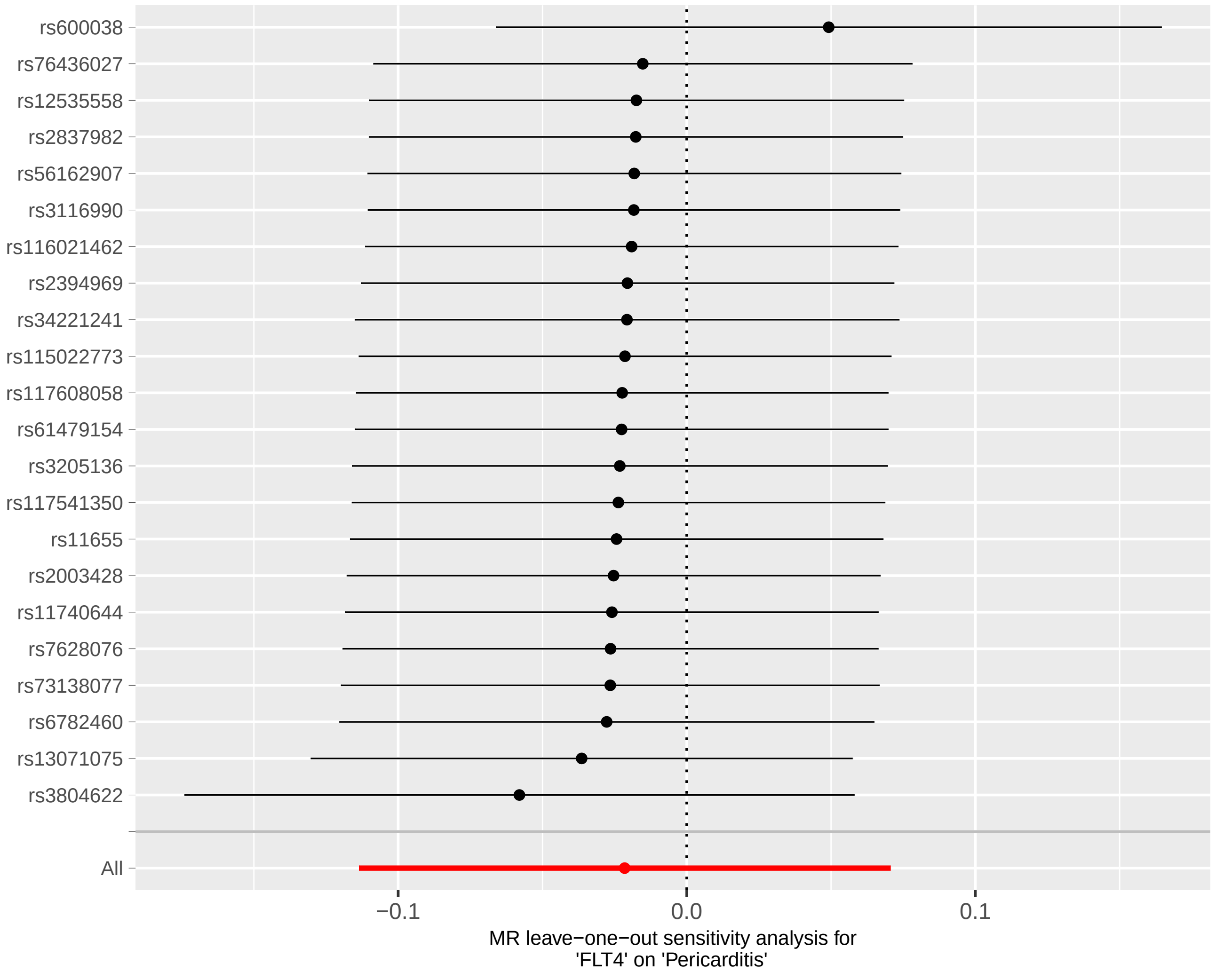
rs600038
rs76436027
rs12535558
rs2837982
rs56162907
rs3116990
rs116021462
rs2394969
rs34221241
rs115022773
rs117608058
rs61479154
rs3205136
rs117541350
rs11655
rs2003428
rs11740644
rs7628076
rs73138077
rs6782460
rs13071075
rs3804622
All
−0.1
0.0
0.1
MR leave−one−out sensitivity analysis for
'FLT4' on 'Pericarditis'

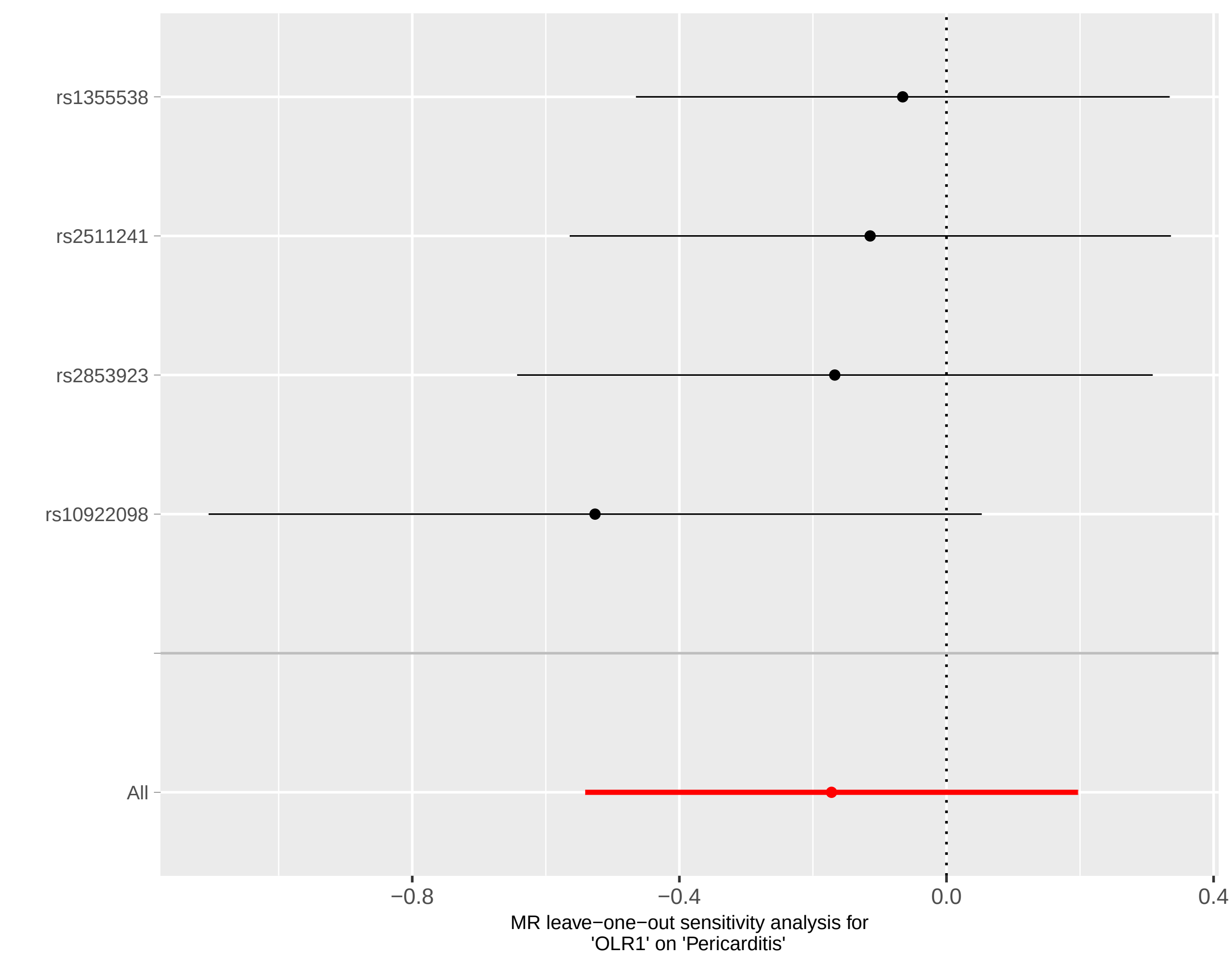
rs1355538
rs2511241
rs2853923
rs10922098
All
−0.8
−0.4
0.0
0.4
MR leave−one−out sensitivity analysis for
'OLR1' on 'Pericarditis'

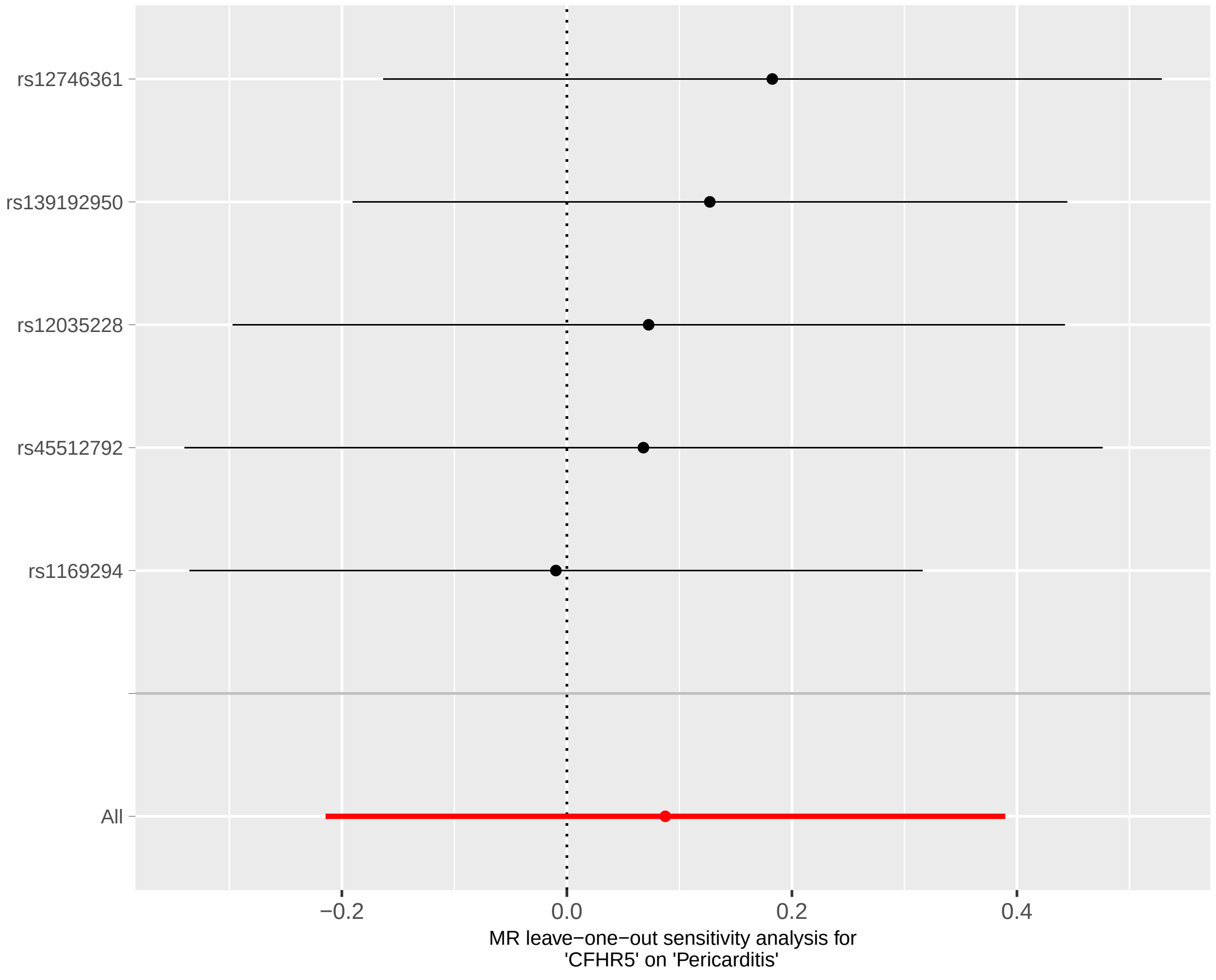

rs12746361
rs139192950
rs12035228
rs45512792
rs1169294
All
−0.2
0.0
0.2
0.4
MR leave−one−out sensitivity analysis for
'CFHR5' on 'Pericarditis'

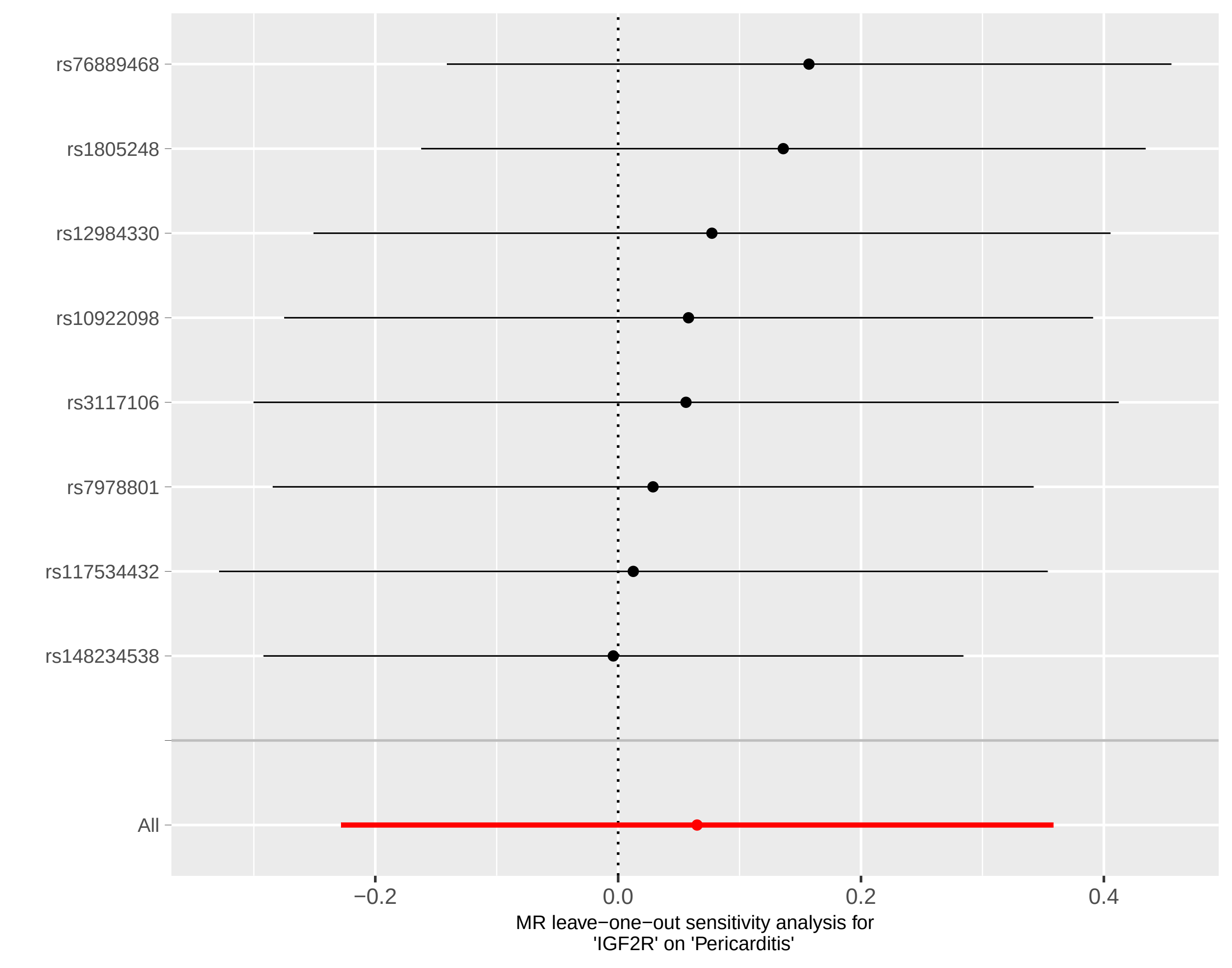
rs76889468
rs1805248
rs12984330
rs10922098
rs3117106
rs7978801
rs117534432
rs148234538
All
−0.2
0.0
0.2
0.4
MR leave−one−out sensitivity analysis for
'IGF2R' on 'Pericarditis'

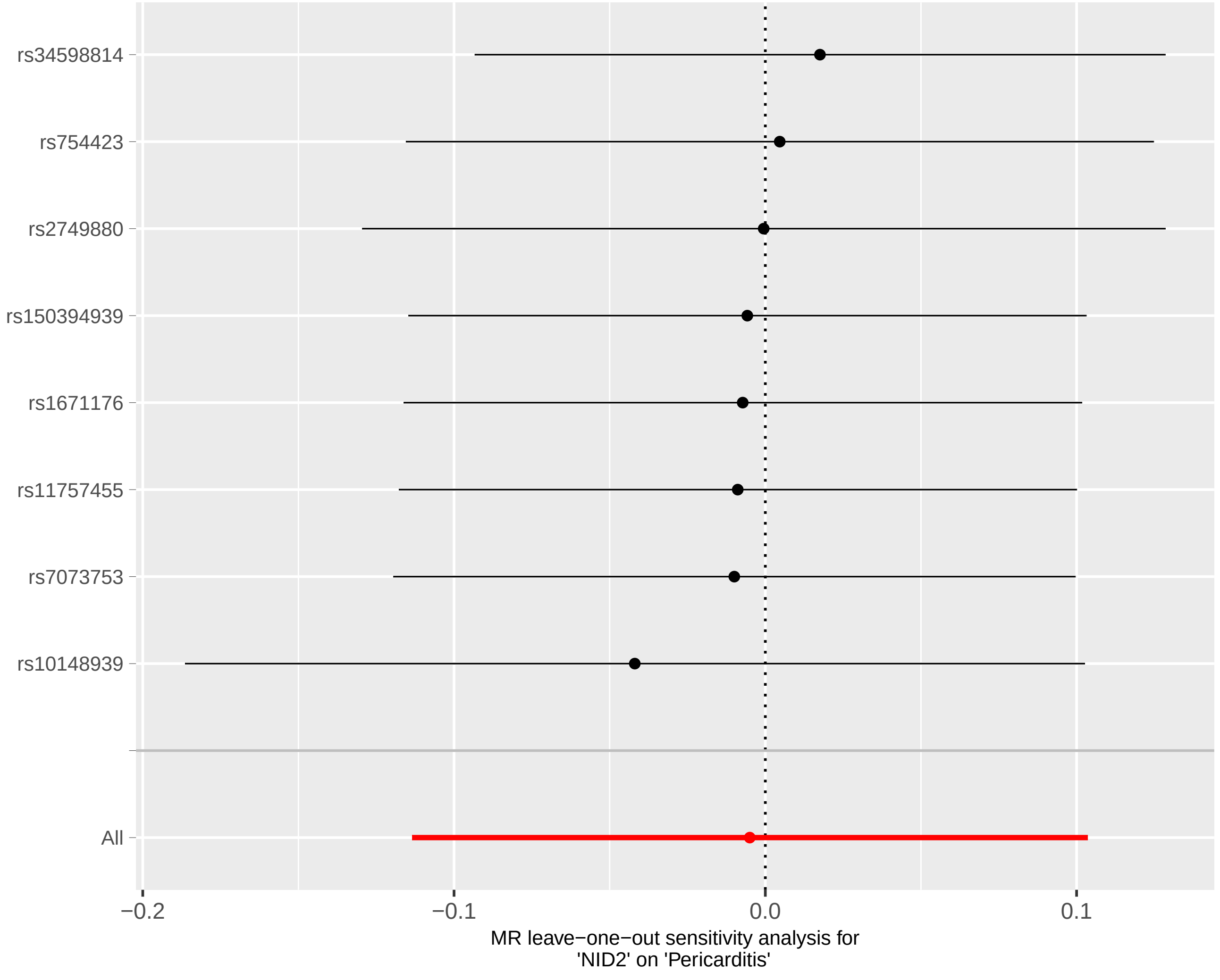
rs34598814
rs754423
rs2749880
rs150394939
rs1671176
rs11757455
rs7073753
rs10148939
All
−0.2
−0.1
0.0
0.1
MR leave−one−out sensitivity analysis for
'NID2' on 'Pericarditis'

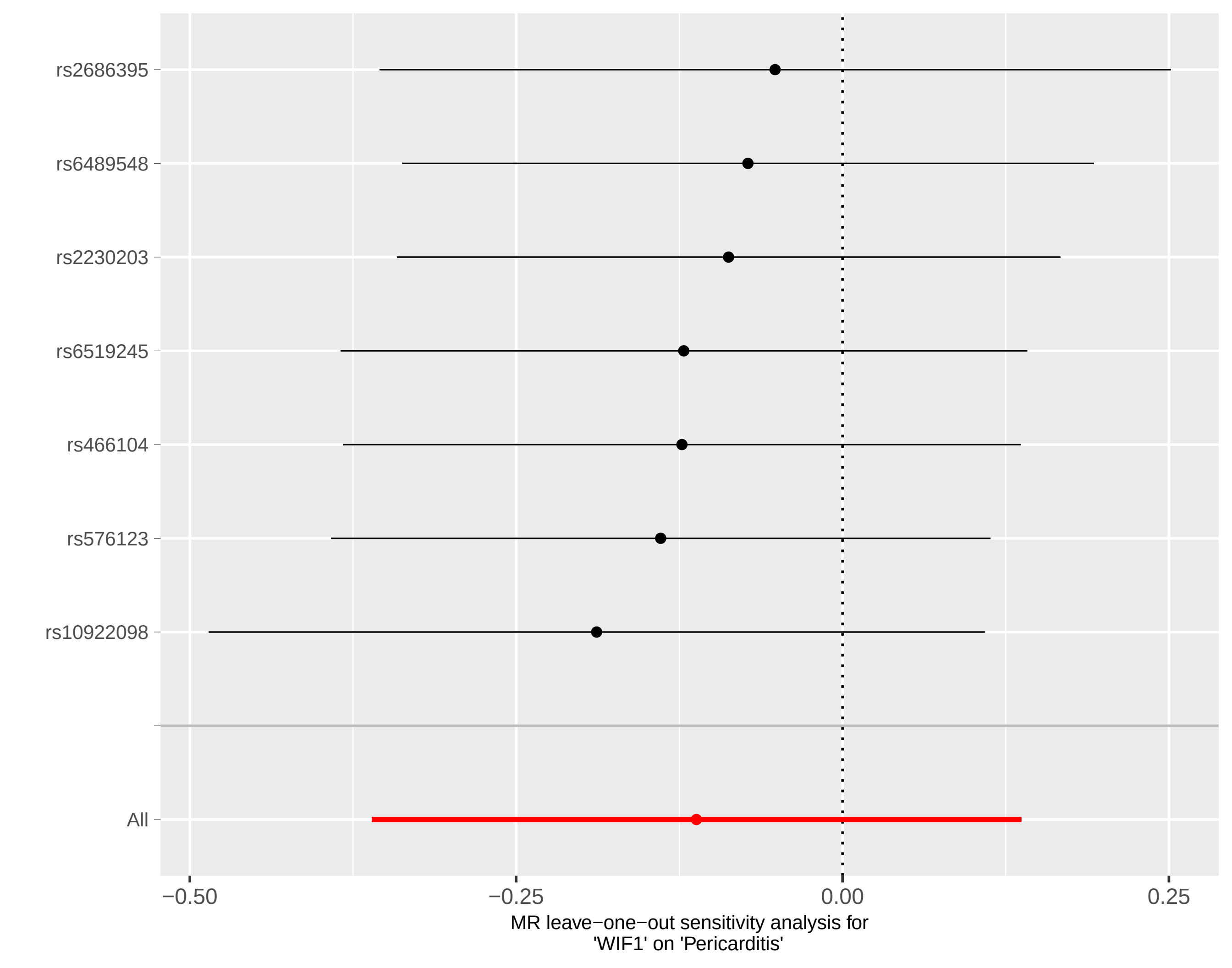

rs2686395
rs6489548
rs2230203
rs6519245
rs466104
rs576123
rs10922098
All
−0.50
−0.25
0.00
0.25
MR leave−one−out sensitivity analysis for
'WIF1' on 'Pericarditis'

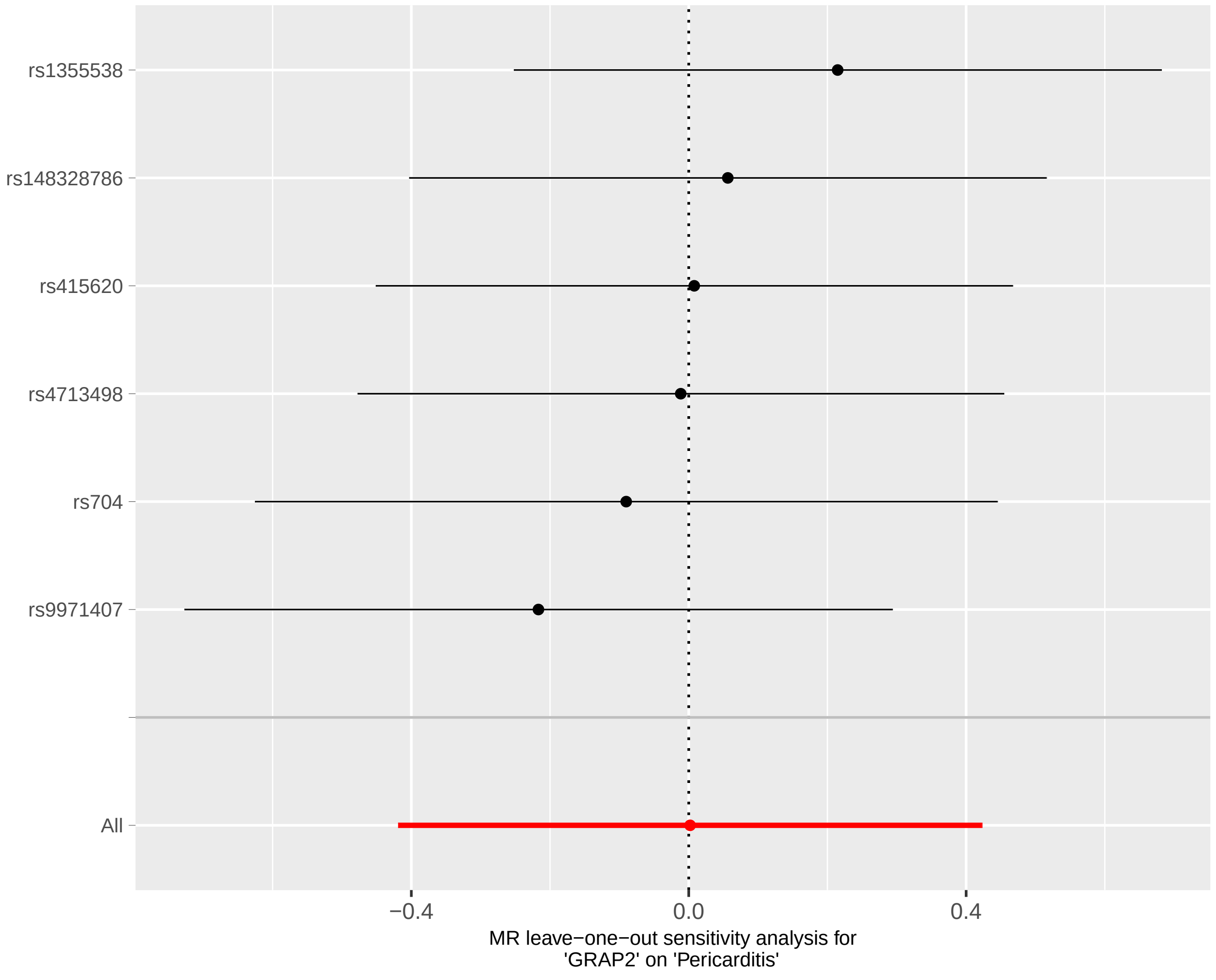
rs1355538
rs148328786
rs415620
rs4713498
rs704
rs9971407
All
−0.4
0.0
0.4
MR leave−one−out sensitivity analysis for
'GRAP2' on 'Pericarditis'

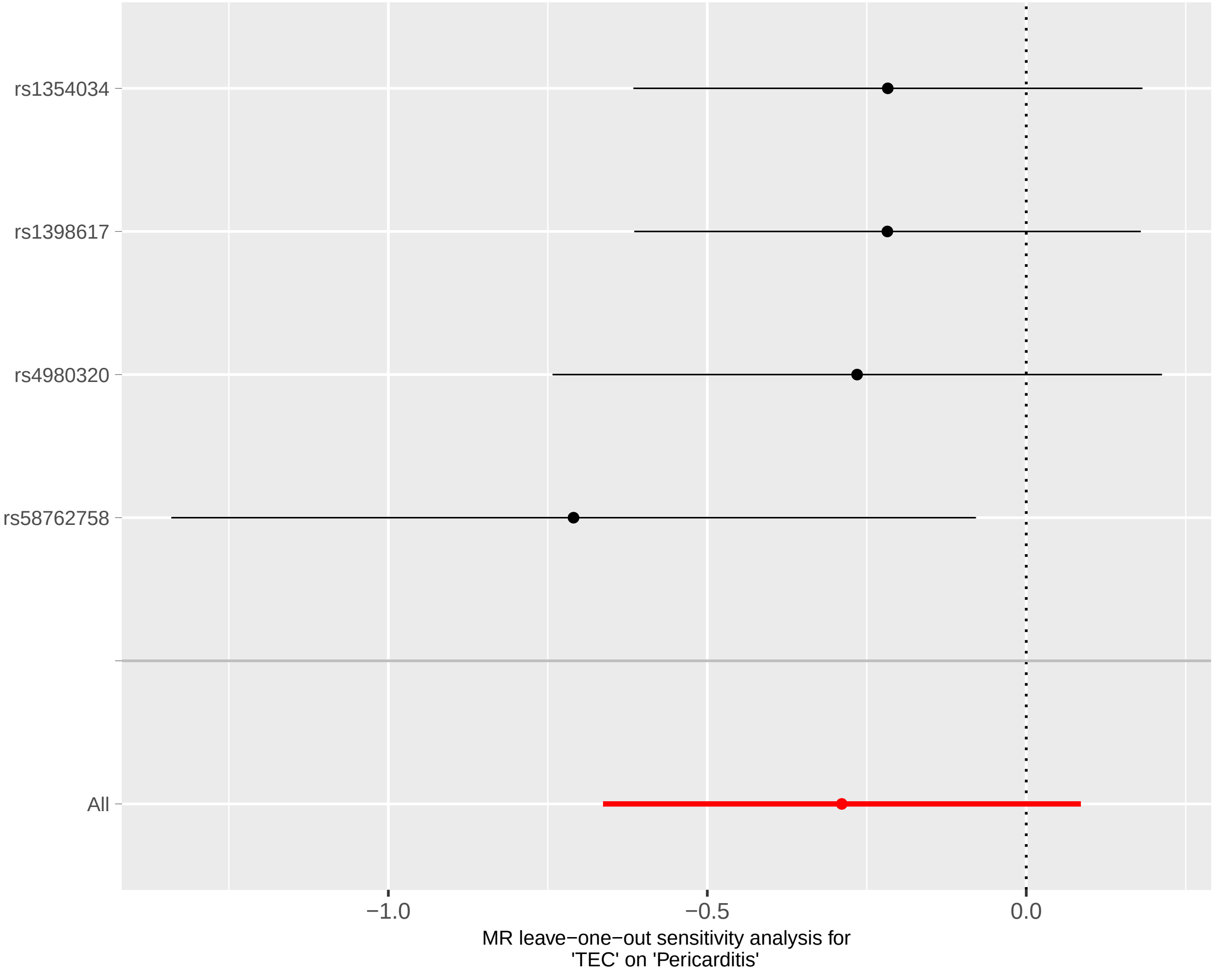

rs1354034
rs1398617
rs4980320
rs58762758
All
−1.0
−0.5
0.0
MR leave−one−out sensitivity analysis for
'TEC' on 'Pericarditis'

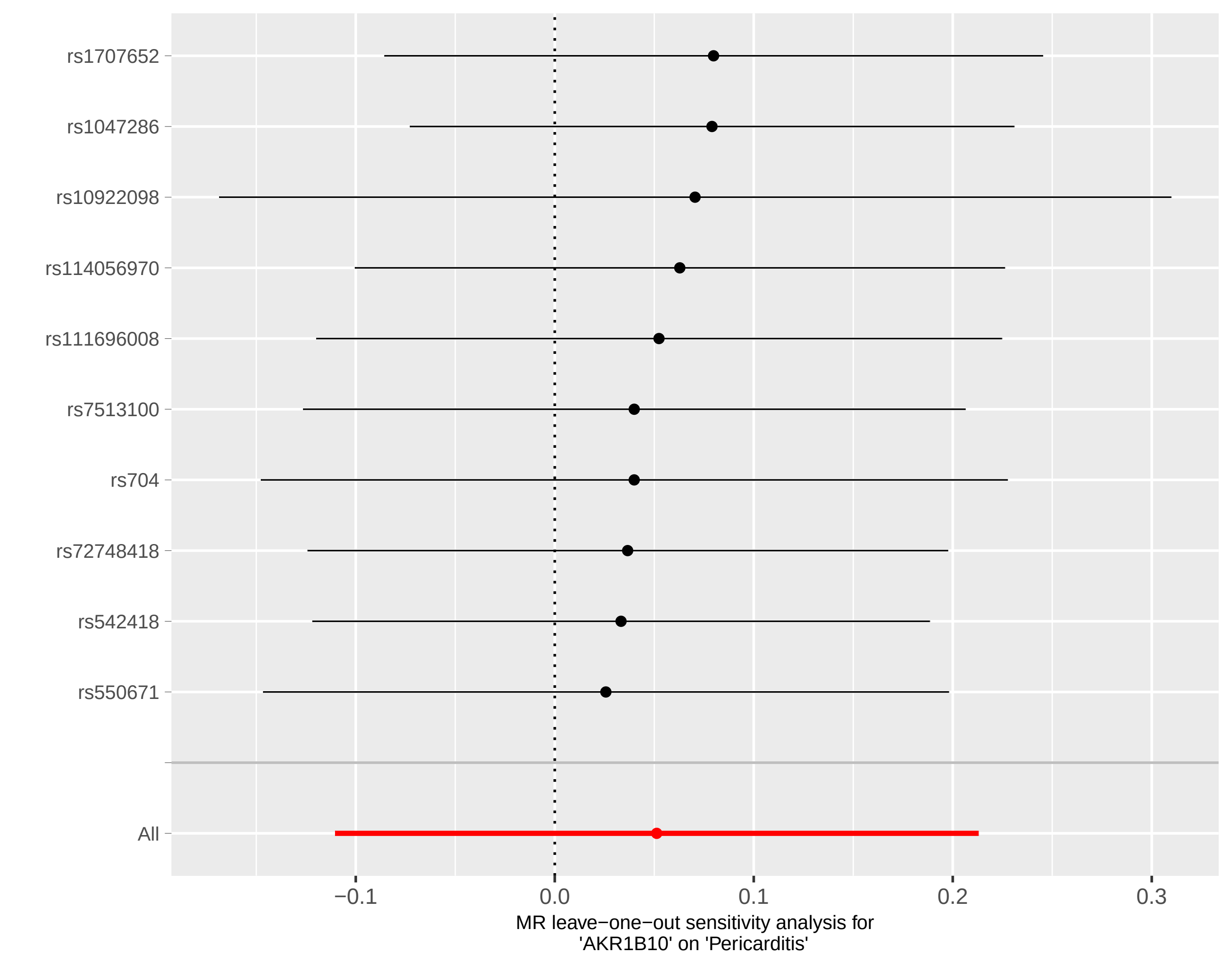
rs1707652
rs1047286
rs10922098
rs114056970
rs111696008
rs7513100
rs704
rs72748418
rs542418
rs550671
All
−0.1
0.0
0.1
0.2
0.3
MR leave−one−out sensitivity analysis for
'AKR1B10' on 'Pericarditis'

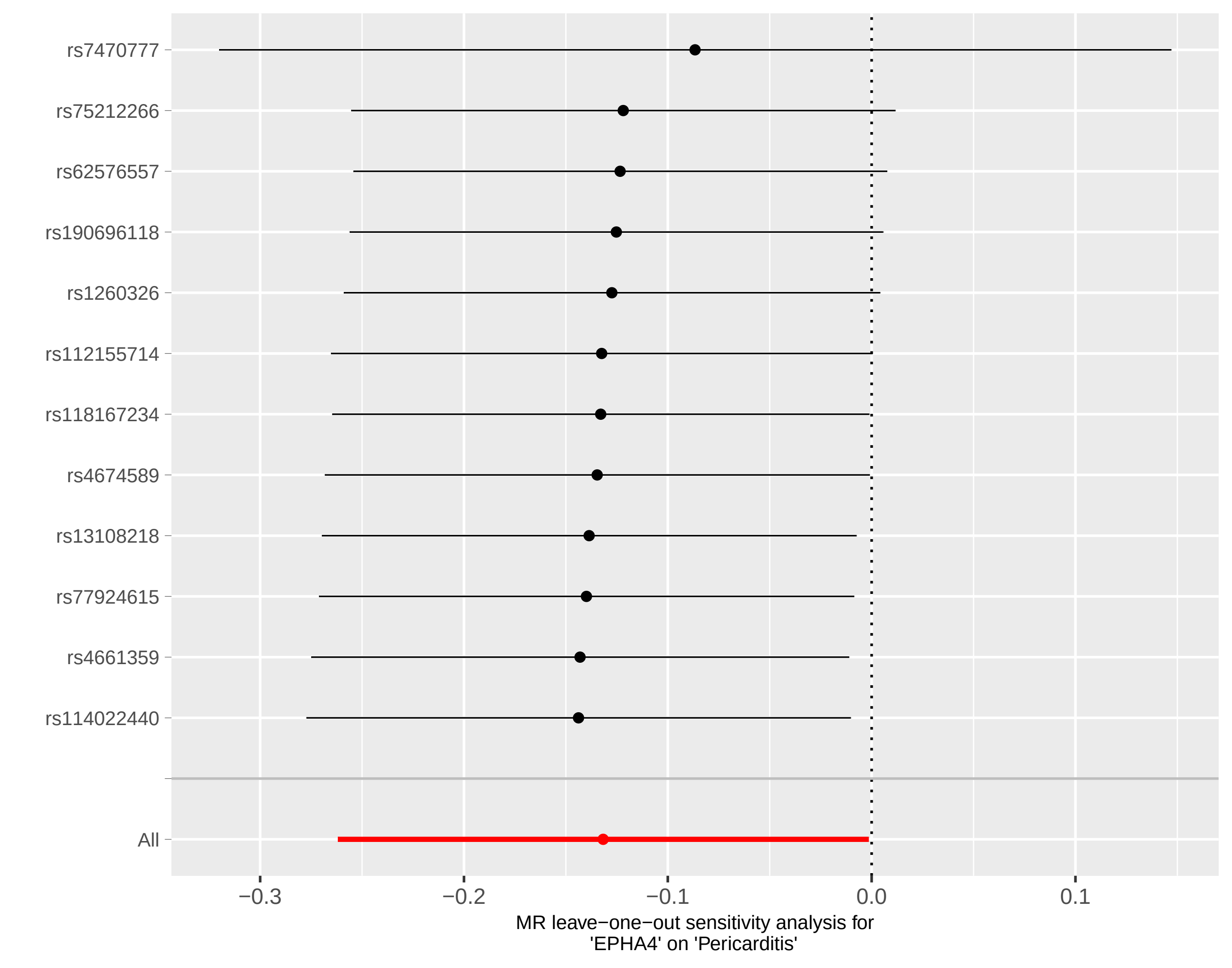

rs7470777
rs75212266
rs62576557
rs190696118
rs1260326
rs112155714
rs118167234
rs4674589
rs13108218
rs77924615
rs4661359
rs114022440
All
−0.3
−0.2
−0.1
0.0
0.1
MR leave−one−out sensitivity analysis for
'EPHA4' on 'Pericarditis'

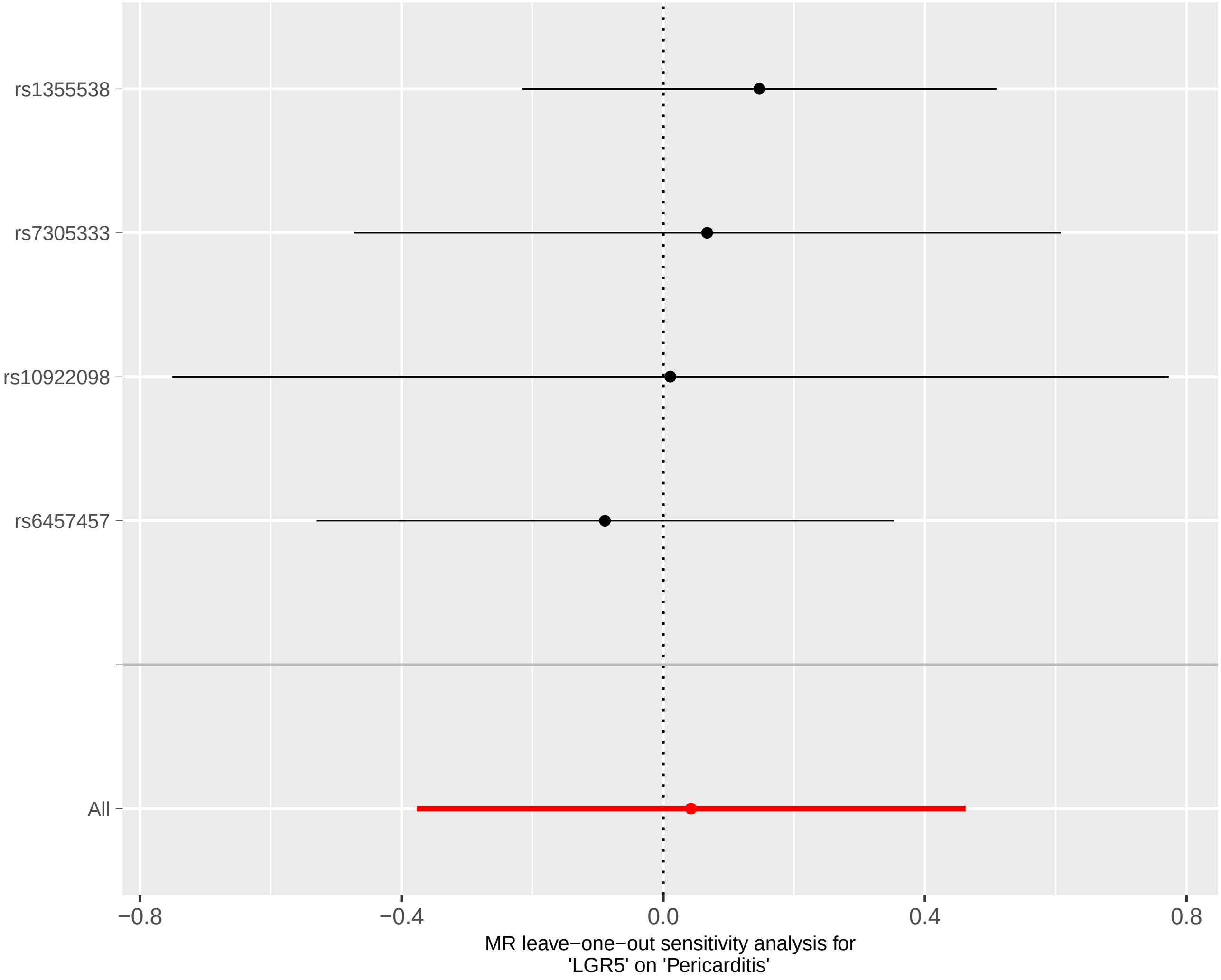
rs1355538
rs7305333
rs10922098
rs6457457
All
−0.8
−0.4
0.0
0.4
0.8
MR leave−one−out sensitivity analysis for
'LGR5' on 'Pericarditis'

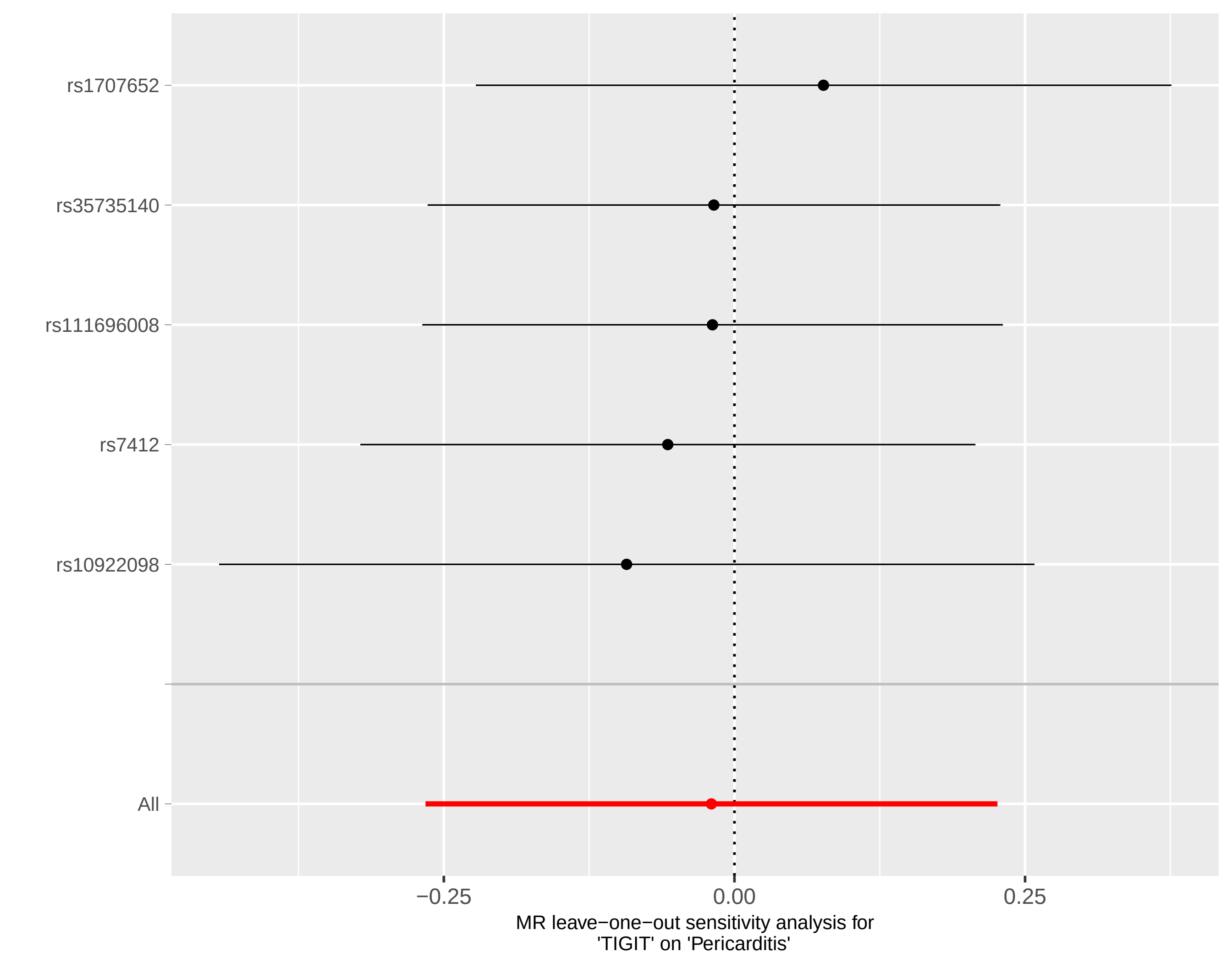
rs1707652
rs35735140
rs111696008
rs7412
rs10922098
All
−0.25
0.00
0.25
MR leave−one−out sensitivity analysis for
'TIGIT' on 'Pericarditis'

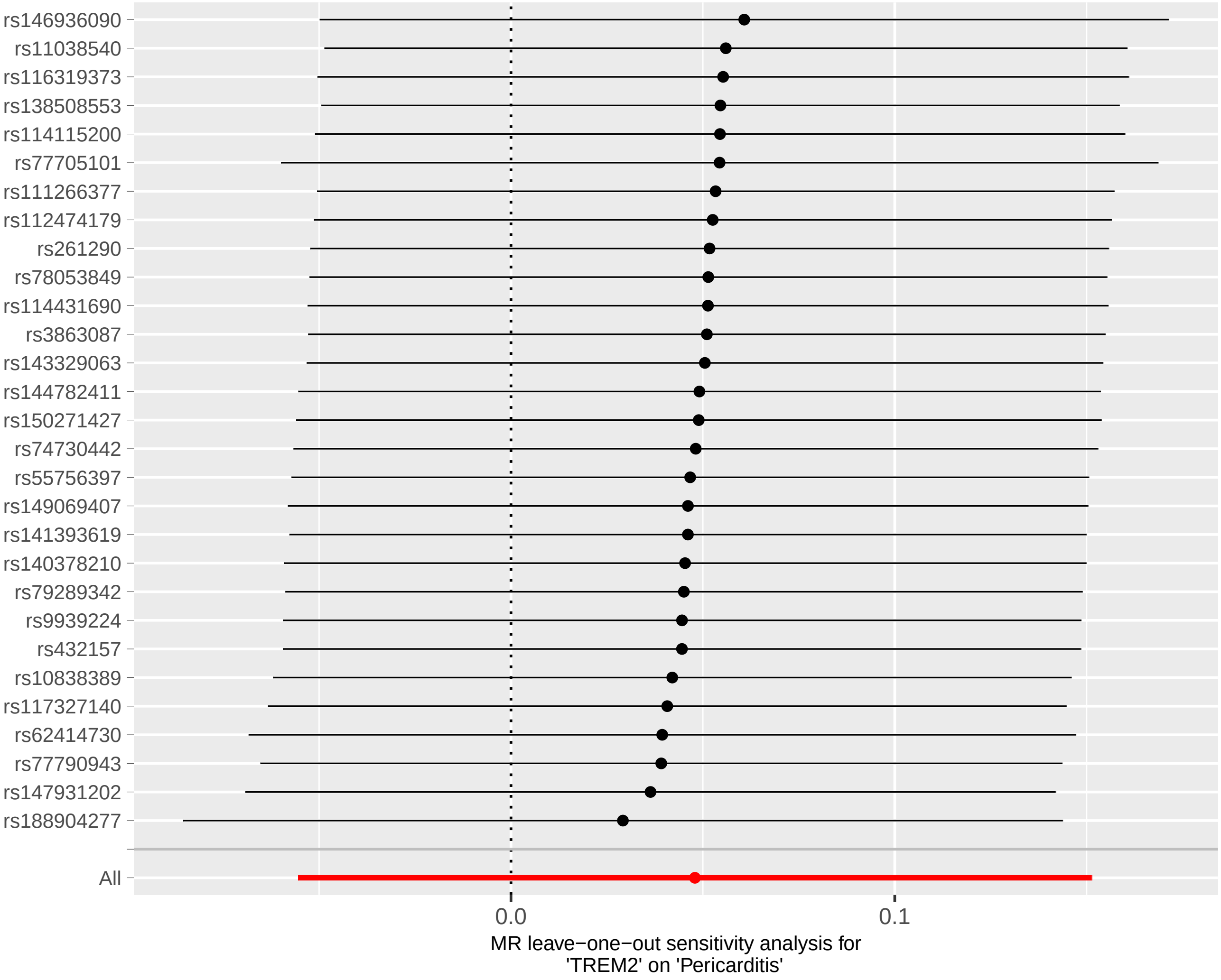
rs146936090
rs11038540
rs116319373
rs138508553
rs114115200
rs77705101
rs111266377
rs112474179
rs261290
rs78053849
rs114431690
rs3863087
rs143329063
rs144782411
rs150271427
rs74730442
rs55756397
rs149069407
rs141393619
rs140378210
rs79289342
rs9939224
rs432157
rs10838389
rs117327140
rs62414730
rs77790943
rs147931202
rs188904277
All
0.0
0.1
MR leave−one−out sensitivity analysis for
'TREM2' on 'Pericarditis'

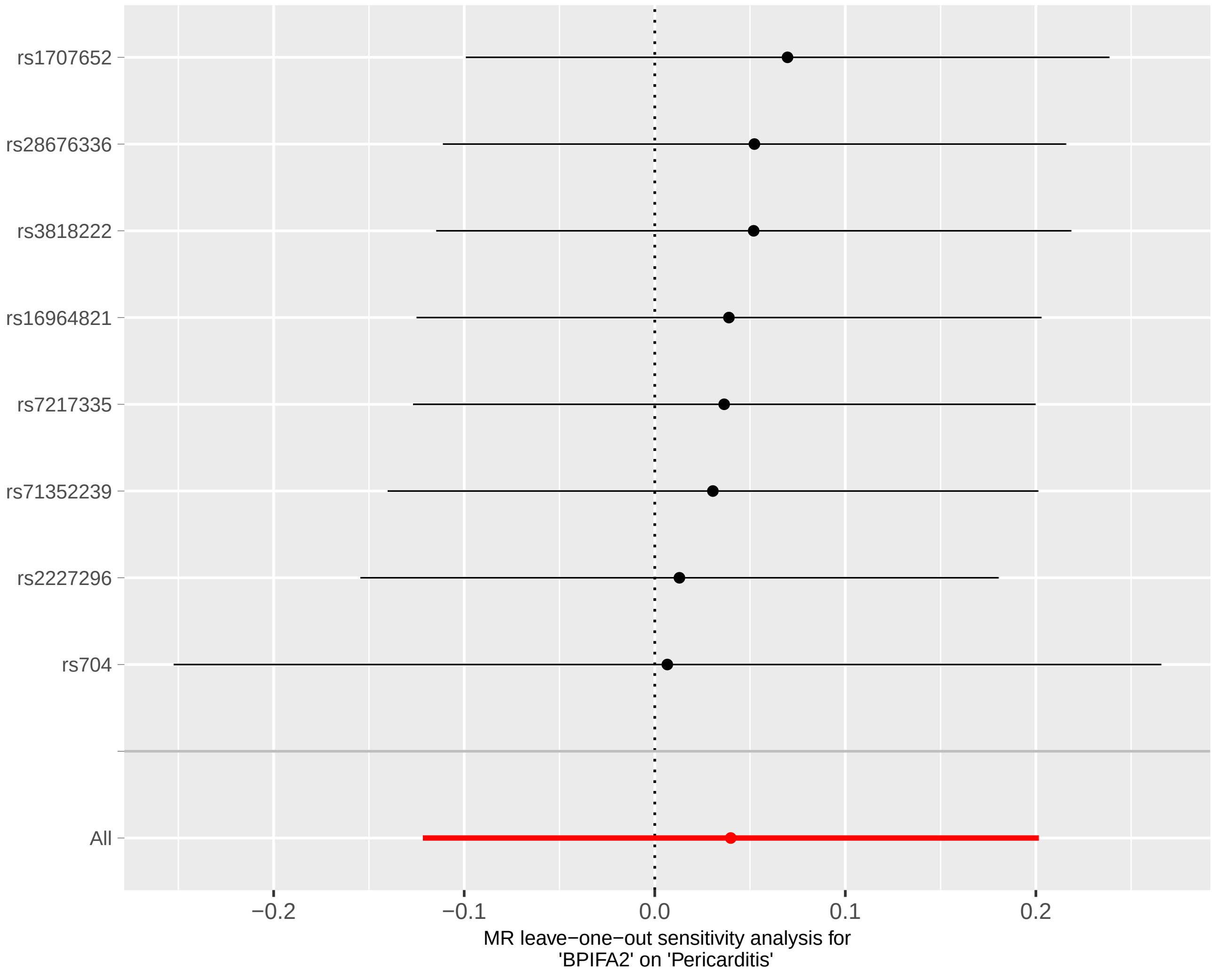

rs1707652
rs28676336
rs3818222
rs16964821
rs7217335
rs71352239
rs2227296
rs704
All
−0.2
−0.1
0.0
0.1
0.2
MR leave−one−out sensitivity analysis for
'BPIFA2' on 'Pericarditis'

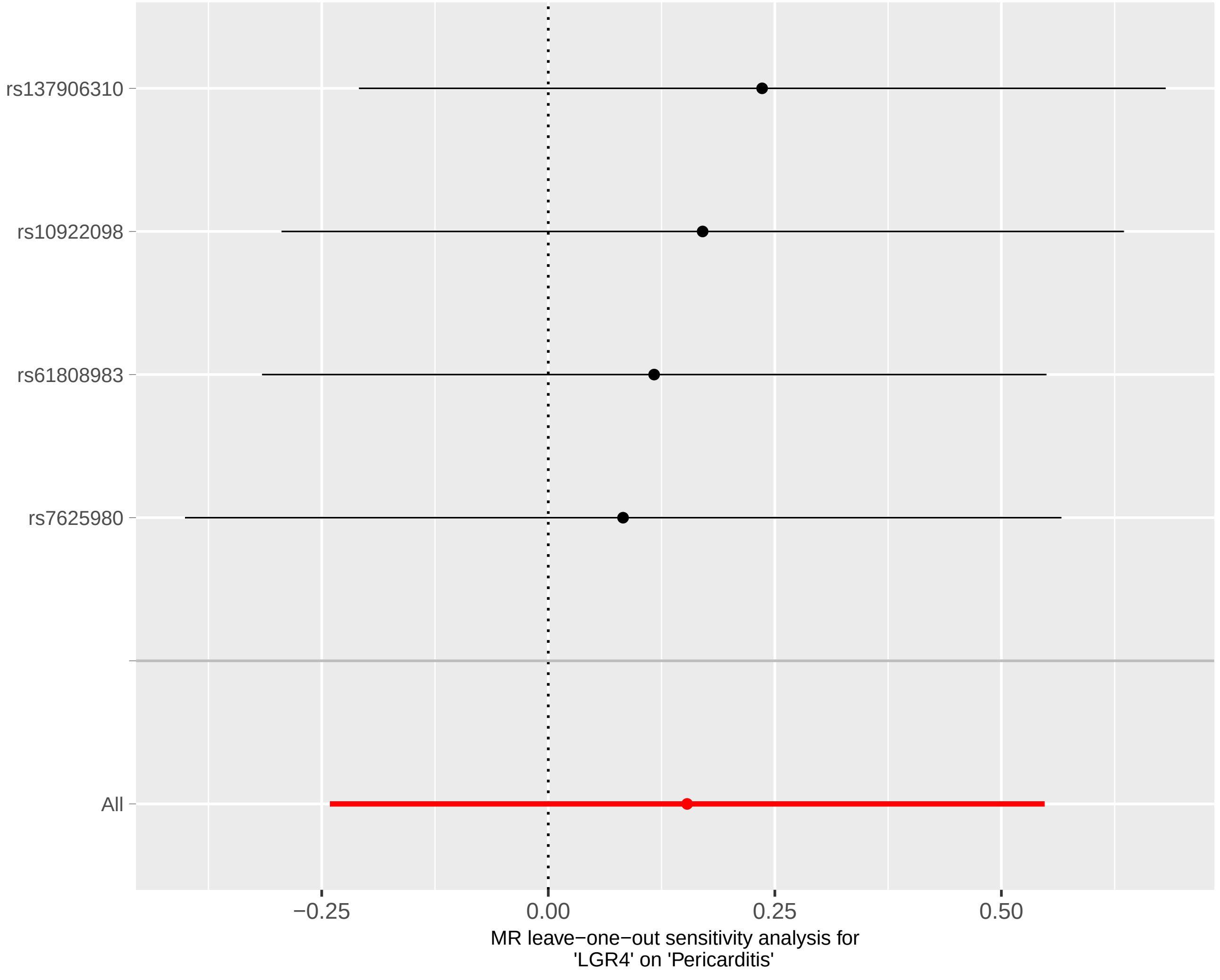
rs137906310
rs10922098
rs61808983
rs7625980
All
−0.25
0.00
0.25
0.50
MR leave−one−out sensitivity analysis for
'LGR4' on 'Pericarditis'

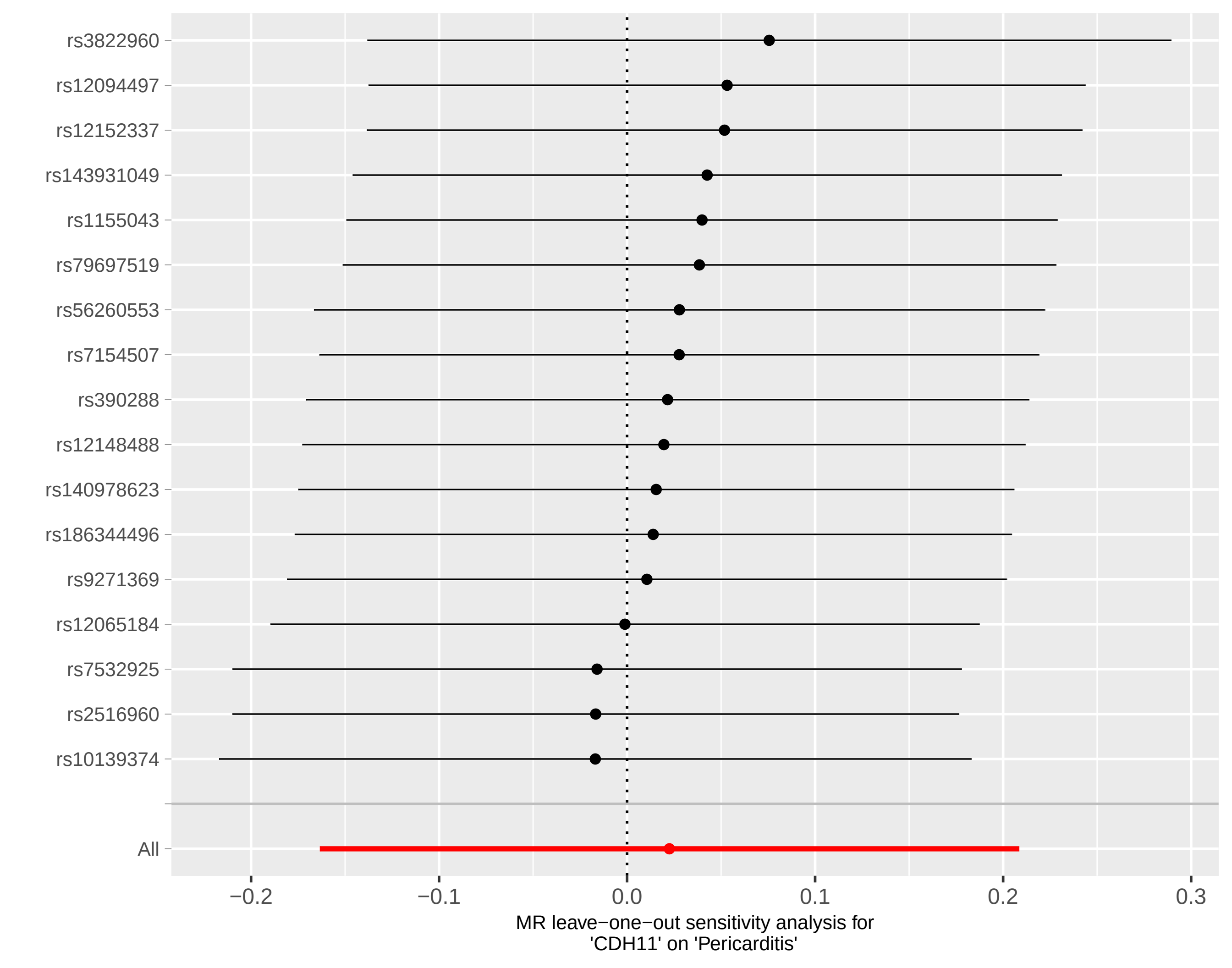

rs3822960
rs12094497
rs12152337
rs143931049
rs1155043
rs79697519
rs56260553
rs7154507
rs390288
rs12148488
rs140978623
rs186344496
rs9271369
rs12065184
rs7532925
rs2516960
rs10139374
All
−0.2
−0.1
0.0
0.1
0.2
0.3
MR leave−one−out sensitivity analysis for
'CDH11' on 'Pericarditis'

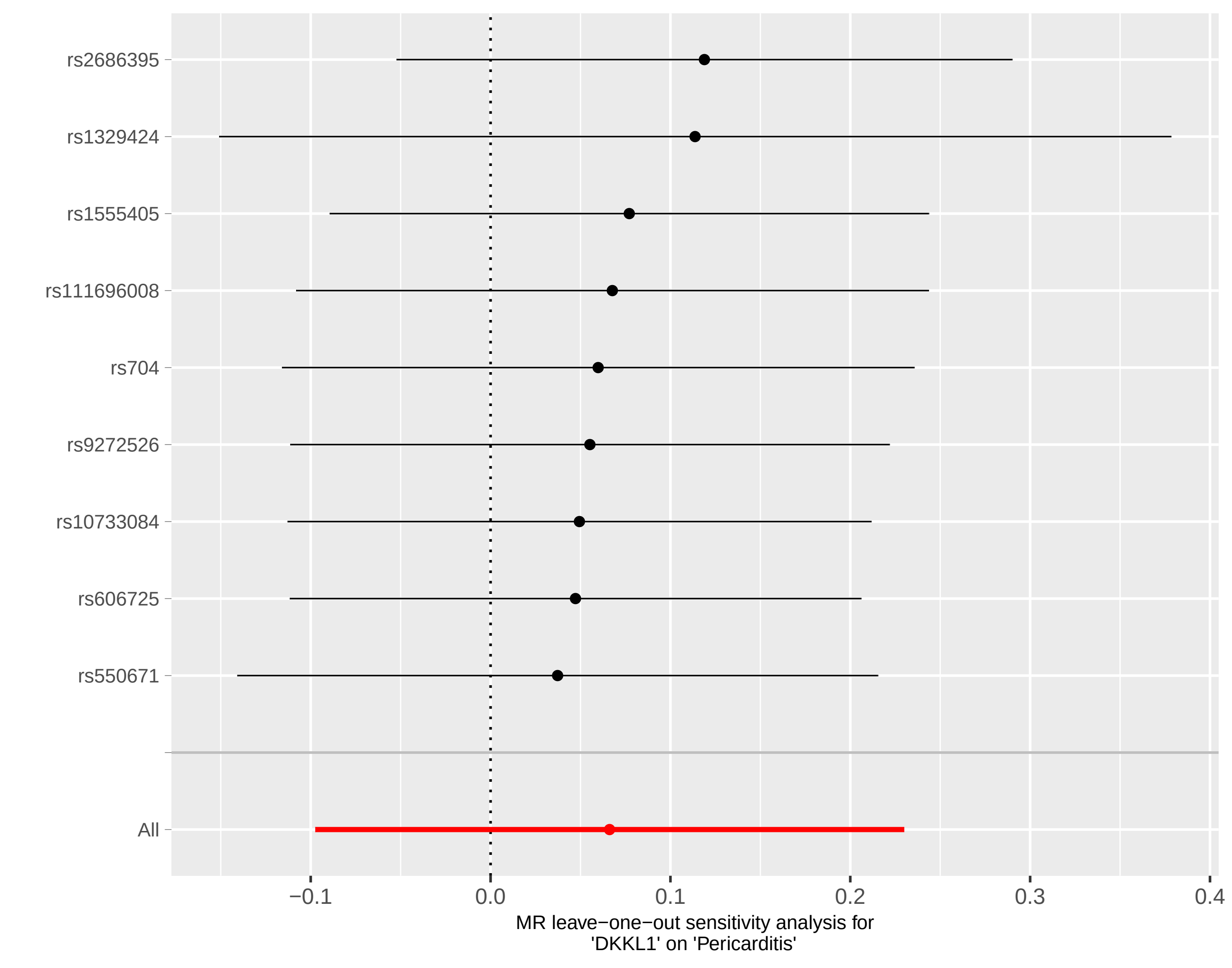
rs2686395
rs1329424
rs1555405
rs111696008
rs704
rs9272526
rs10733084
rs606725
rs550671
All
−0.1
0.0
0.1
0.2
0.3
0.4
MR leave−one−out sensitivity analysis for
'DKKL1' on 'Pericarditis'

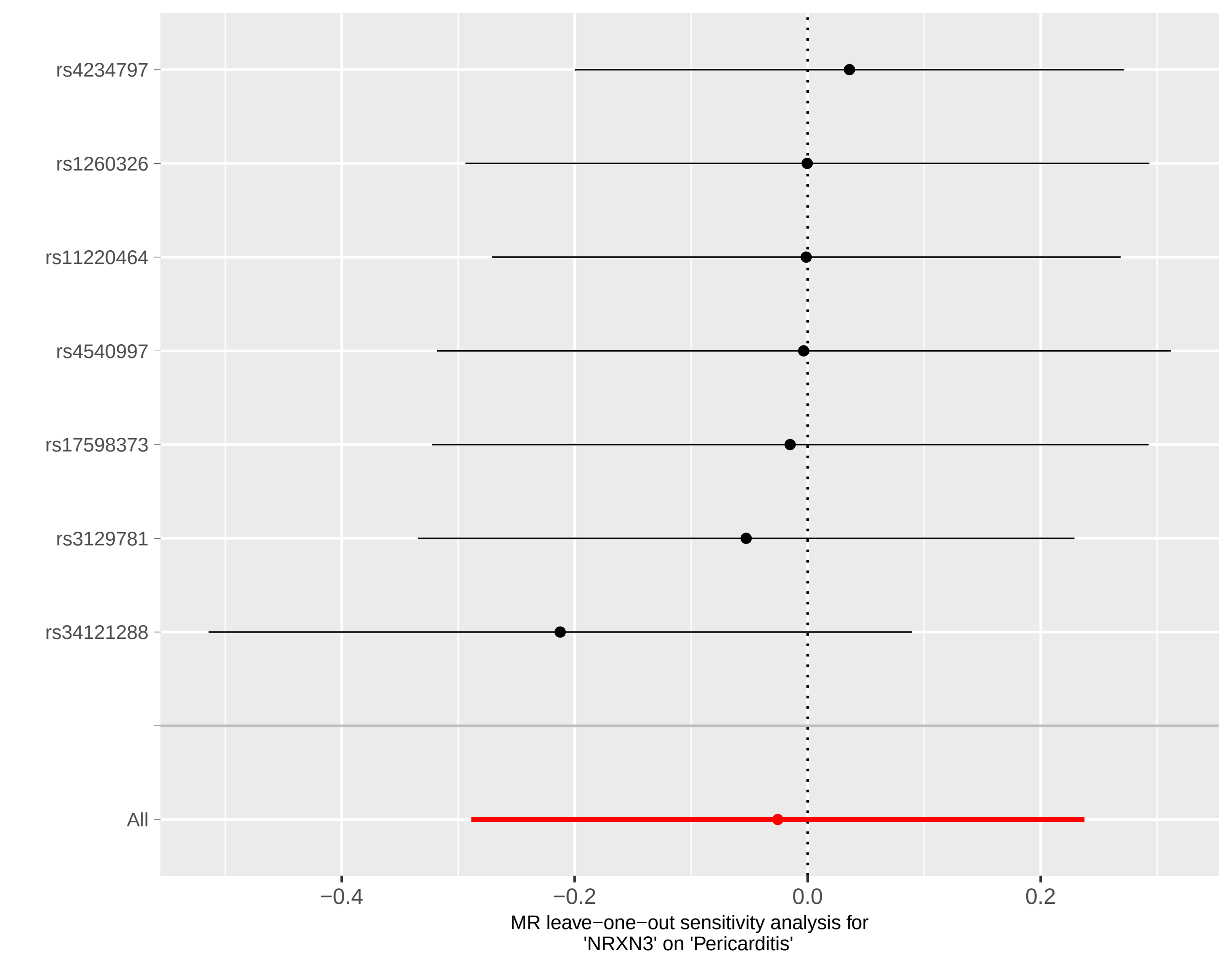

rs4234797
rs1260326
rs11220464
rs4540997
rs17598373
rs3129781
rs34121288
All
−0.4
−0.2
0.0
0.2
MR leave−one−out sensitivity analysis for
'NRXN3' on 'Pericarditis'

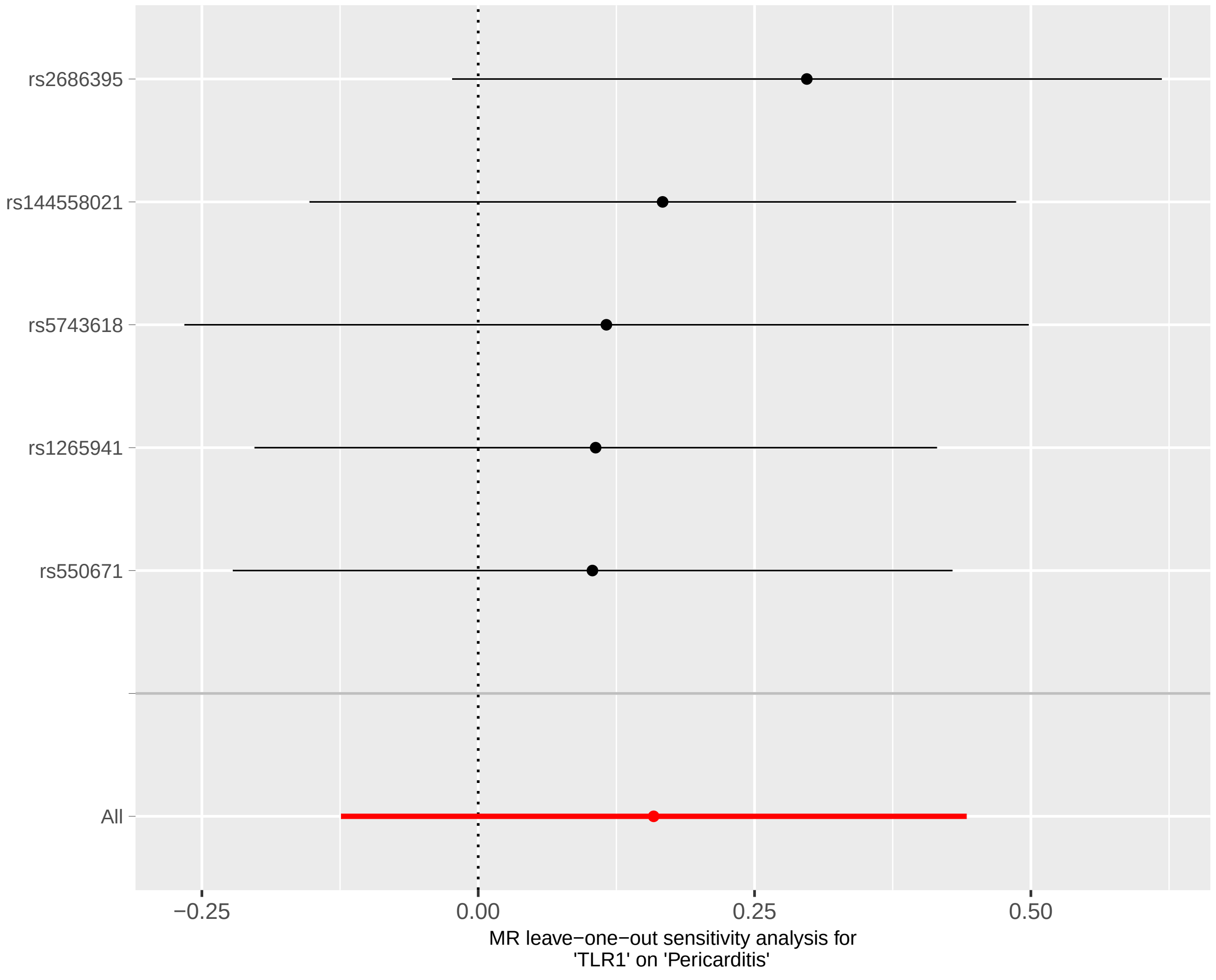
rs2686395
rs144558021
rs5743618
rs1265941
rs550671
All
−0.25
0.00
0.25
0.50
MR leave−one−out sensitivity analysis for
'TLR1' on 'Pericarditis'

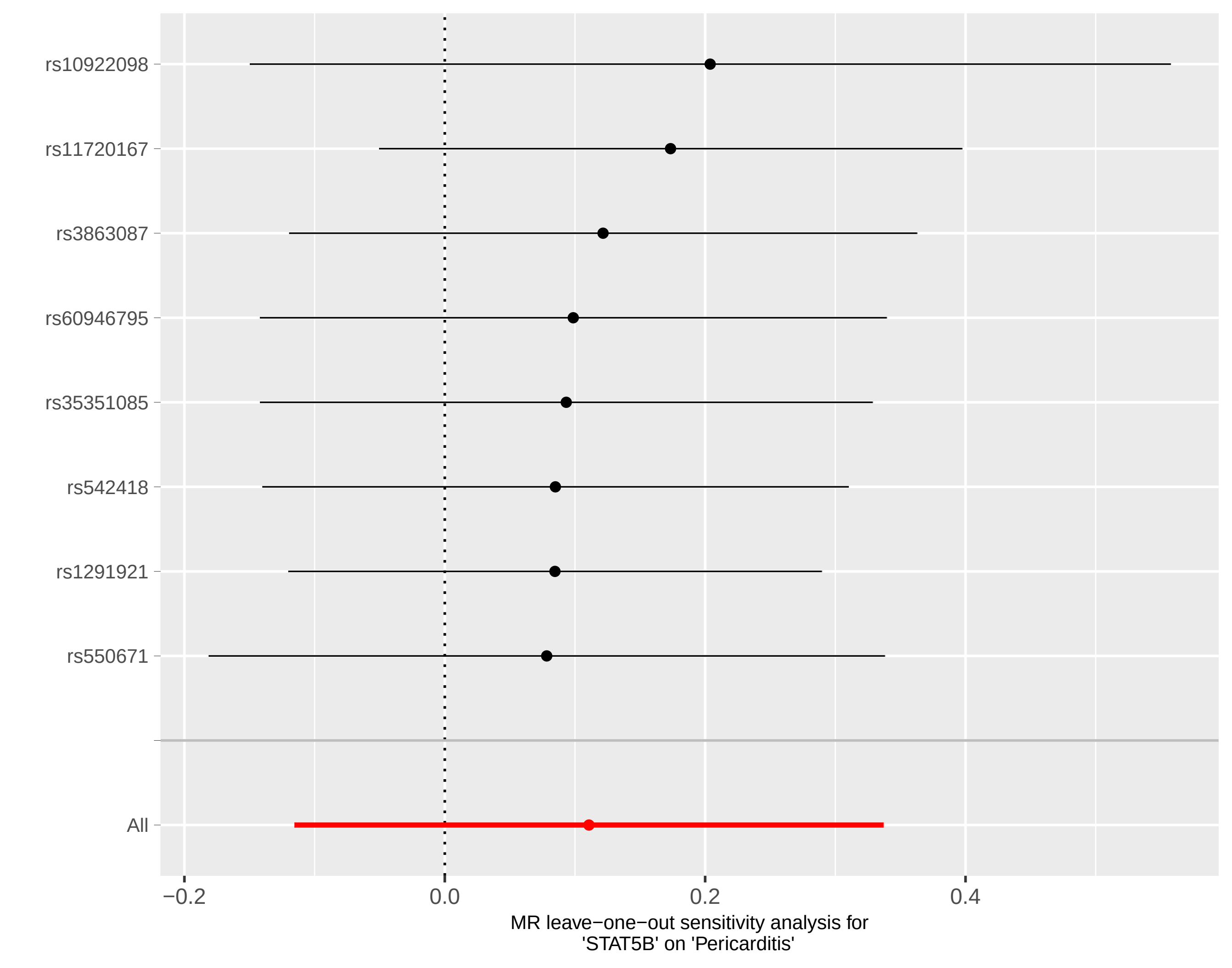

rs10922098
rs11720167
rs3863087
rs60946795
rs35351085
rs542418
rs1291921
rs550671
All
−0.2
0.0
0.2
0.4
MR leave−one−out sensitivity analysis for
'STAT5B' on 'Pericarditis'

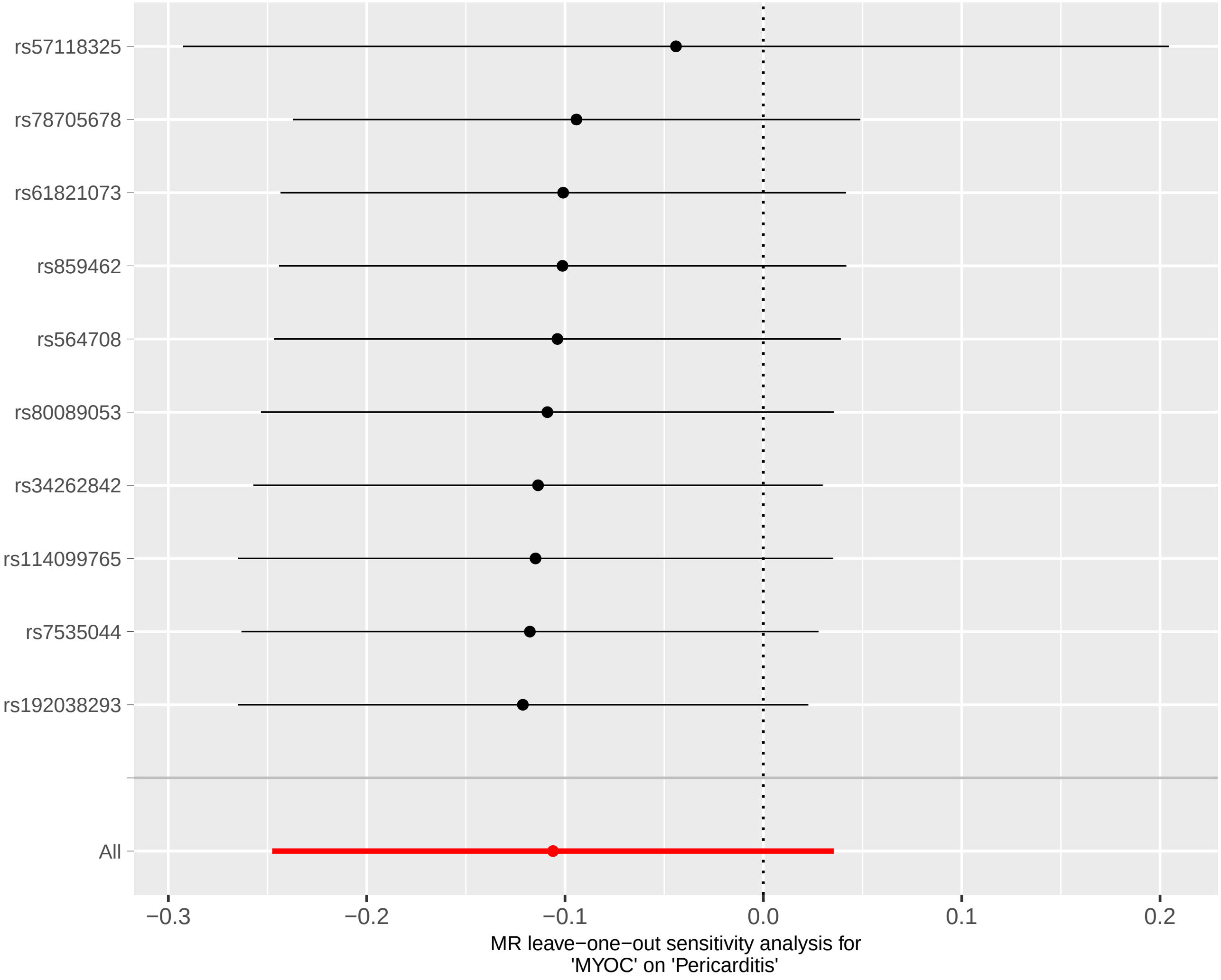

rs57118325
rs78705678
rs61821073
rs859462
rs564708
rs80089053
rs34262842
rs114099765
rs7535044
rs192038293
All
−0.3
−0.2
−0.1
0.0
0.1
0.2
MR leave−one−out sensitivity analysis for
'MYOC' on 'Pericarditis'

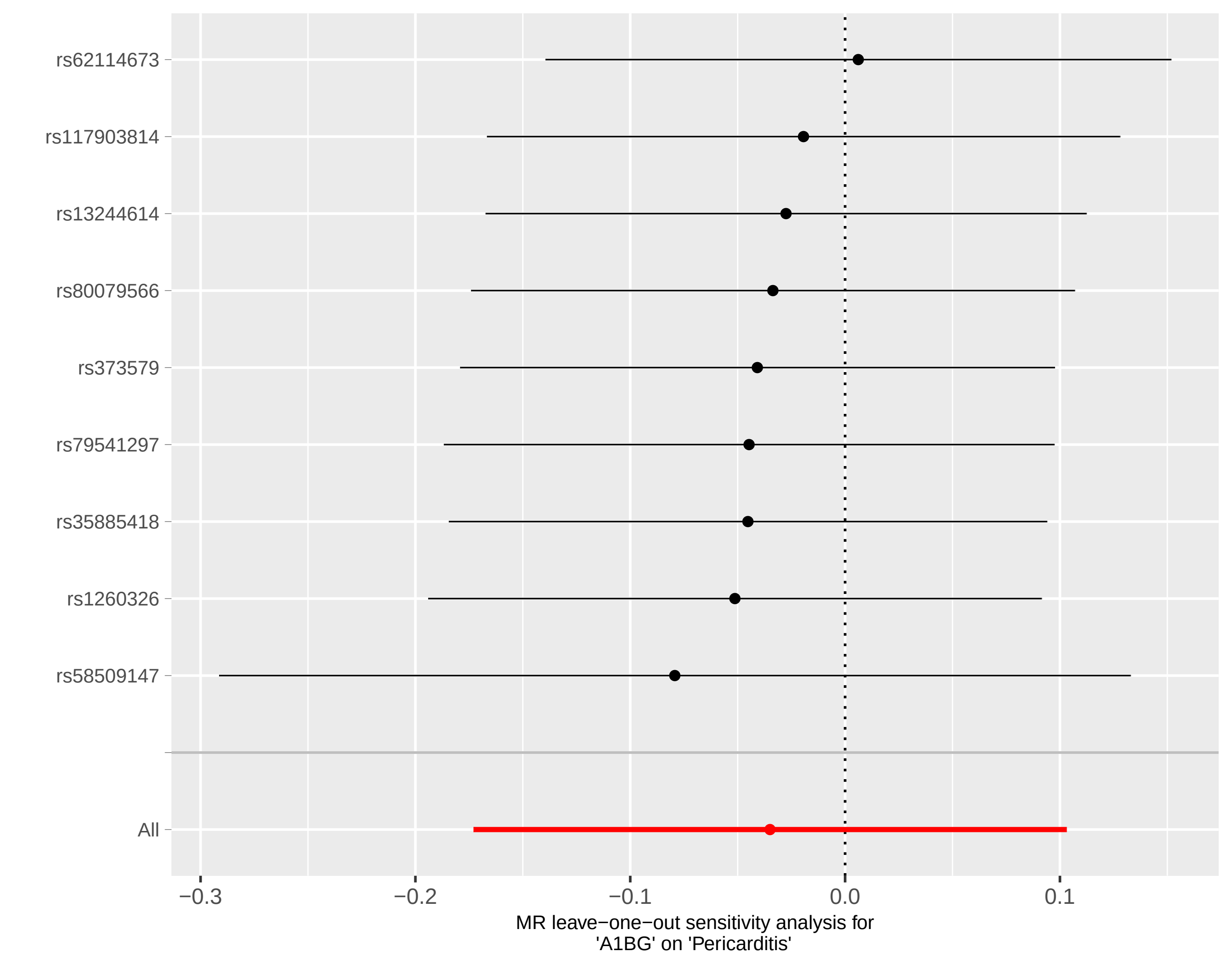

rs62114673
rs117903814
rs13244614
rs80079566
rs373579
rs79541297
rs35885418
rs1260326
rs58509147
All
−0.3
−0.2
−0.1
0.0
0.1
MR leave−one−out sensitivity analysis for
'A1BG' on 'Pericarditis'

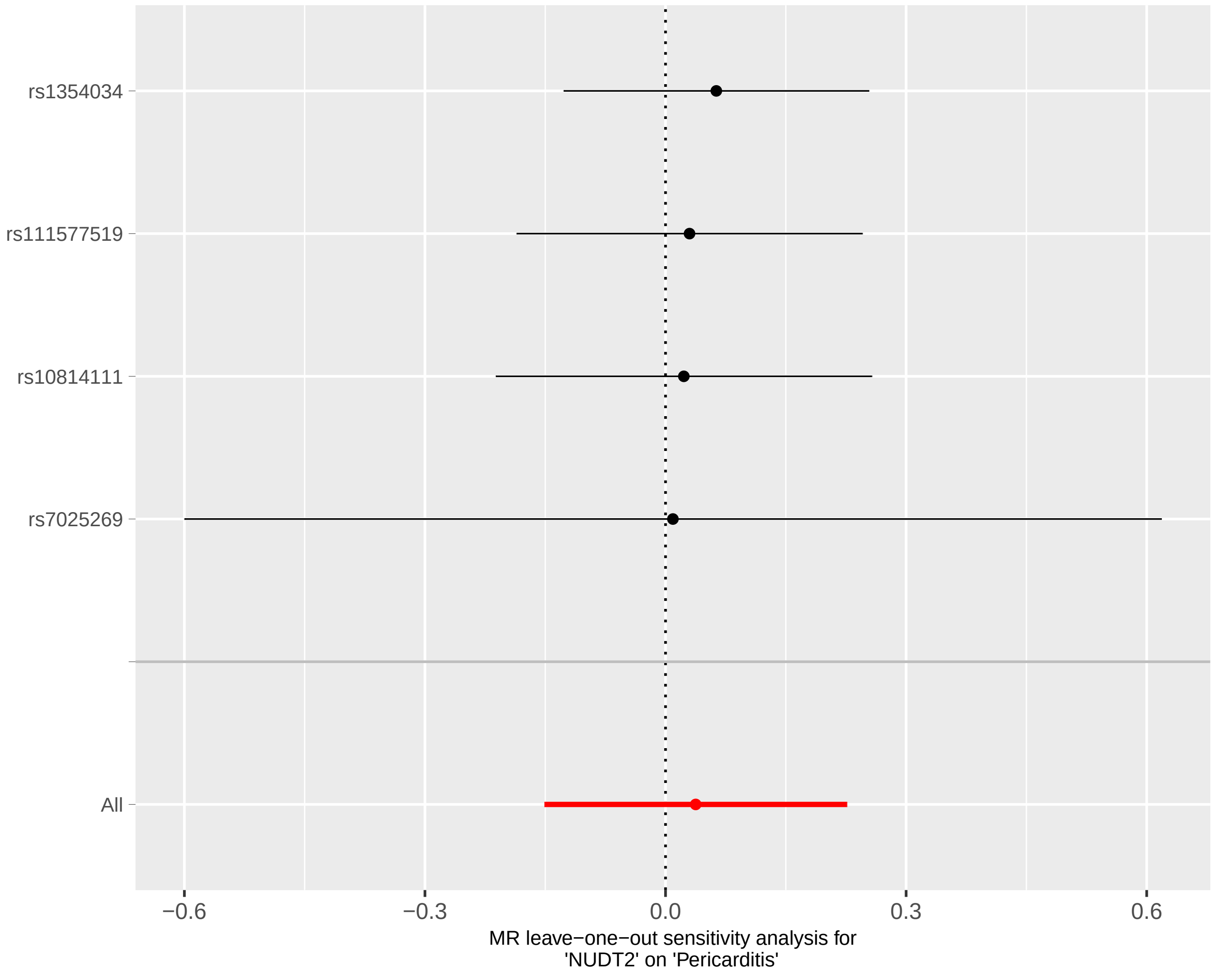
rs1354034
rs111577519
rs10814111
rs7025269
All
−0.6
−0.3
0.0
0.3
0.6
MR leave−one−out sensitivity analysis for
'NUDT2' on 'Pericarditis'

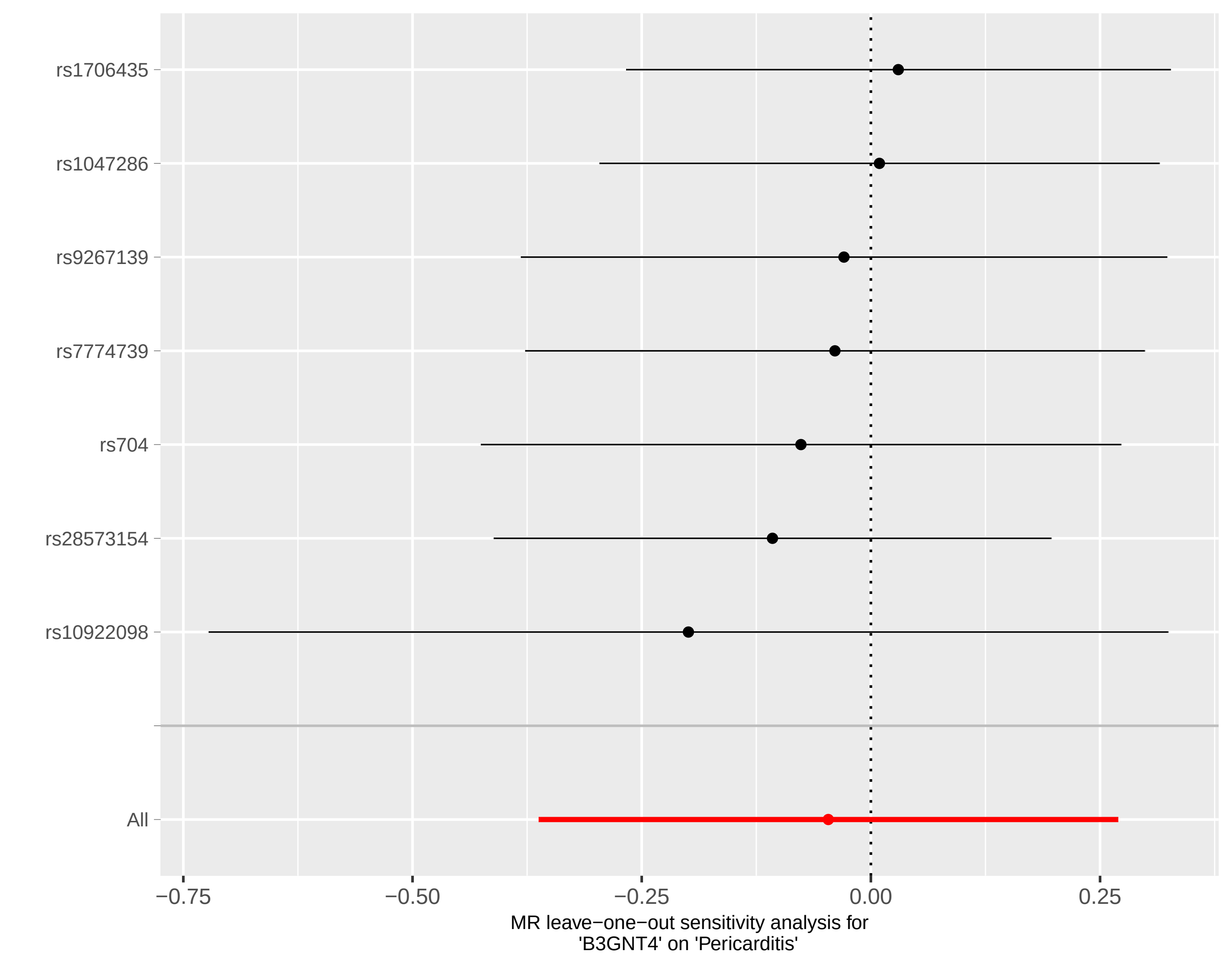

rs1706435
rs1047286
rs9267139
rs7774739
rs704
rs28573154
rs10922098
All
−0.75
−0.50
−0.25
0.00
0.25
MR leave−one−out sensitivity analysis for
'B3GNT4' on 'Pericarditis'

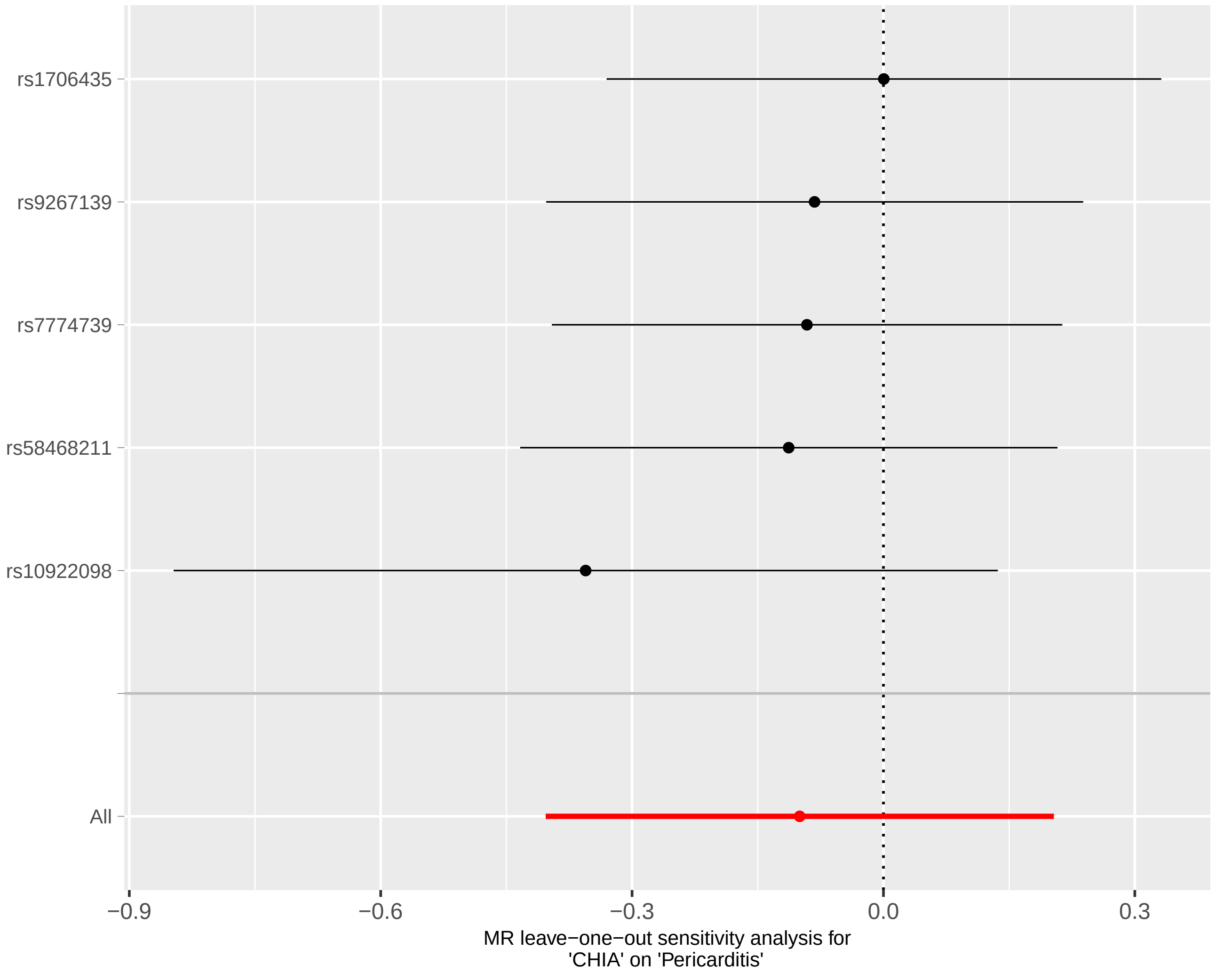

rs1706435
rs9267139
rs7774739
rs58468211
rs10922098
All
−0.9
−0.6
−0.3
0.0
0.3
MR leave−one−out sensitivity analysis for
'CHIA' on 'Pericarditis'

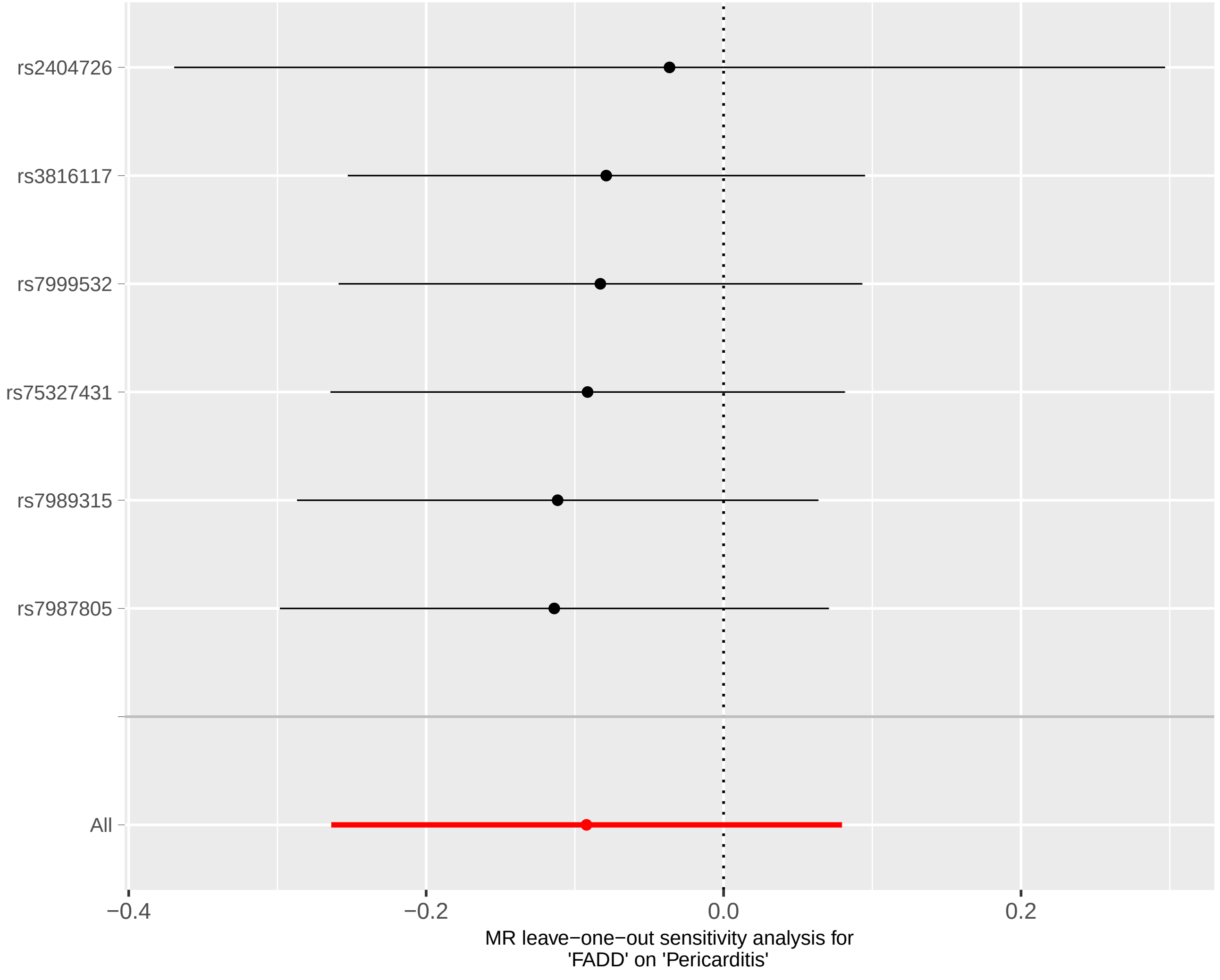
rs2404726
rs3816117
rs7999532
rs75327431
rs7989315
rs7987805
All
−0.4
−0.2
0.0
0.2
MR leave−one−out sensitivity analysis for
'FADD' on 'Pericarditis'

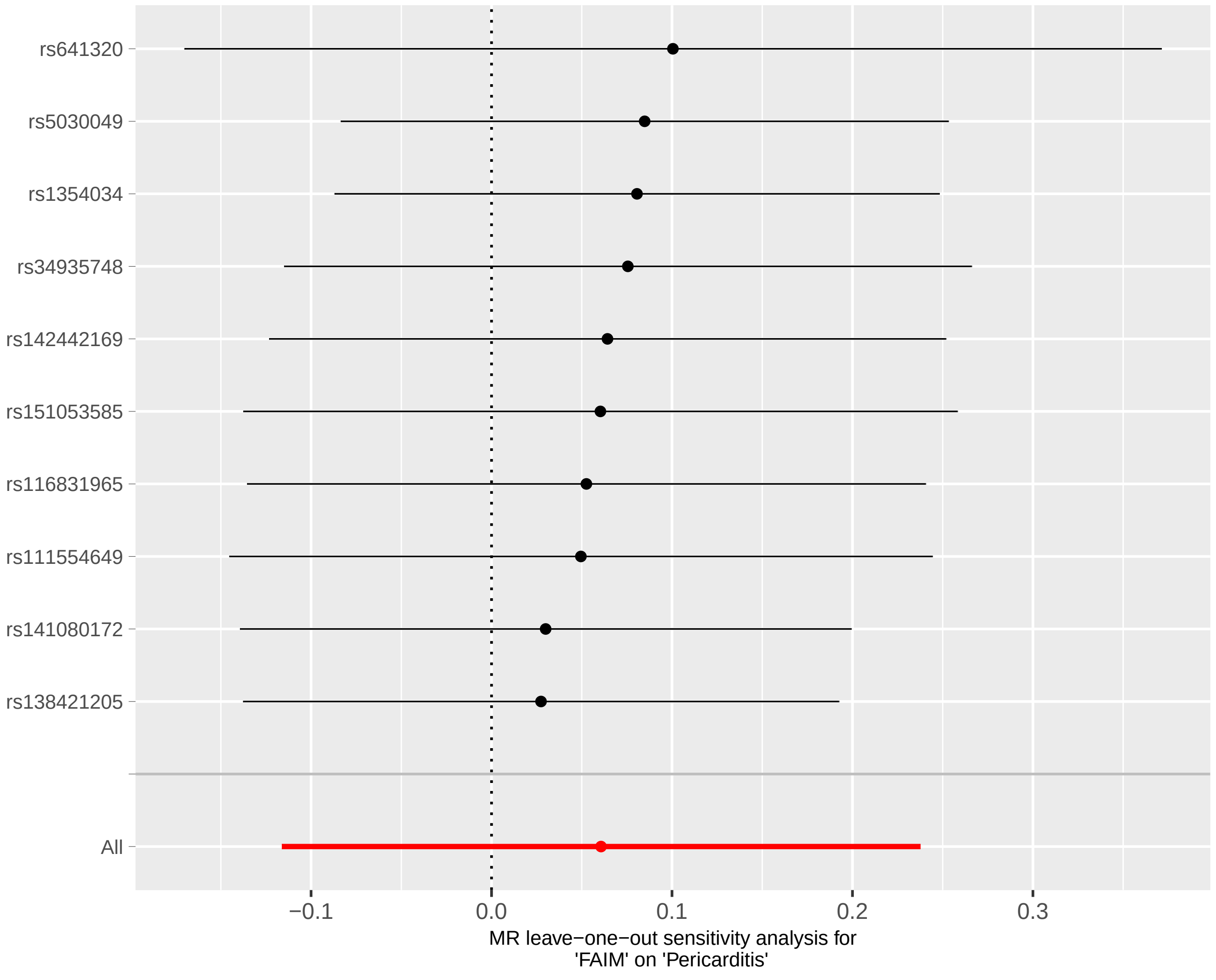
rs641320
rs5030049
rs1354034
rs34935748
rs142442169
rs151053585
rs116831965
rs111554649
rs141080172
rs138421205
All
−0.1
0.0
0.1
0.2
0.3
MR leave−one−out sensitivity analysis for
'FAIM' on 'Pericarditis'

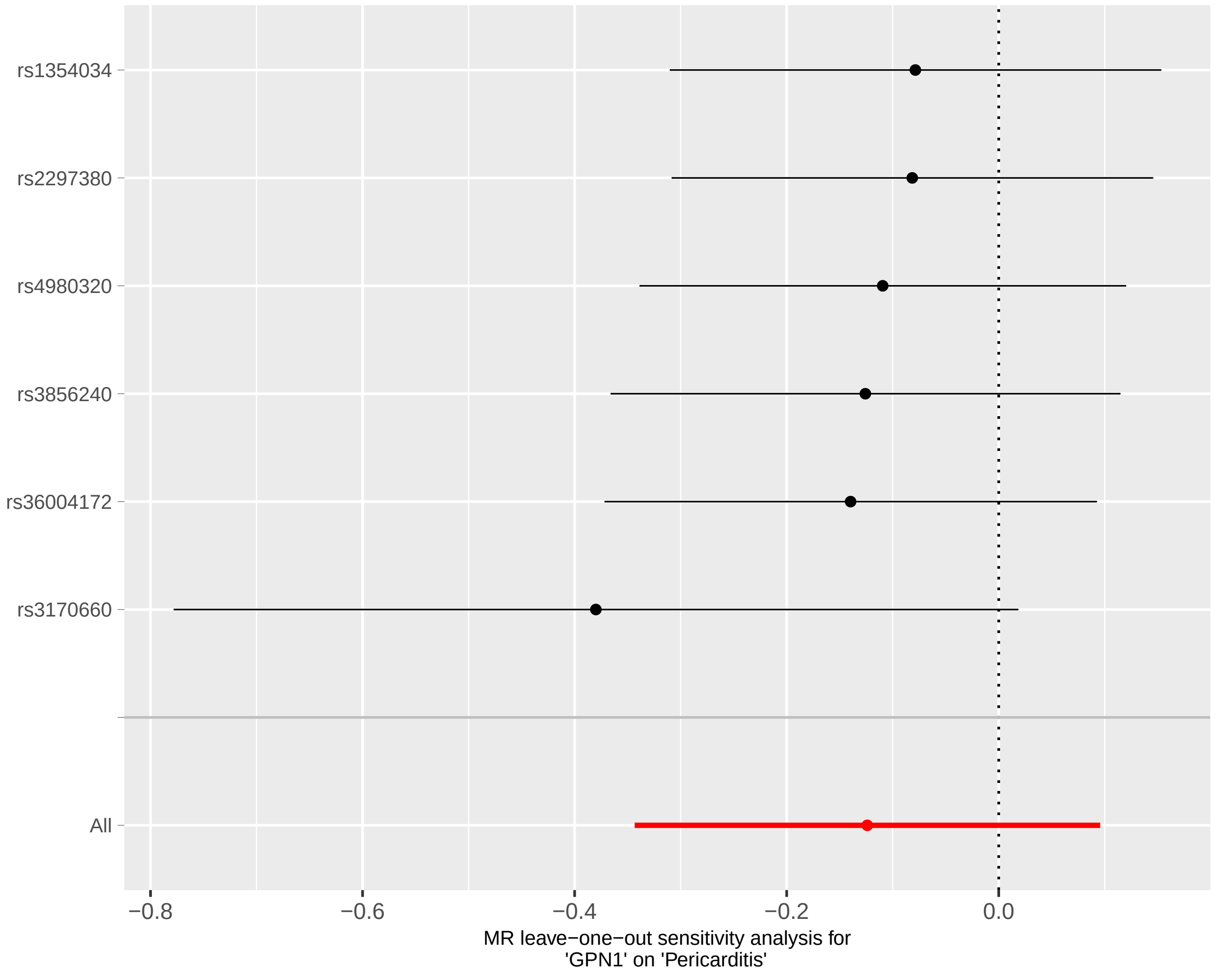

rs1354034
rs2297380
rs4980320
rs3856240
rs36004172
rs3170660
All
−0.8
−0.6
−0.4
−0.2
0.0
MR leave−one−out sensitivity analysis for
'GPN1' on 'Pericarditis'

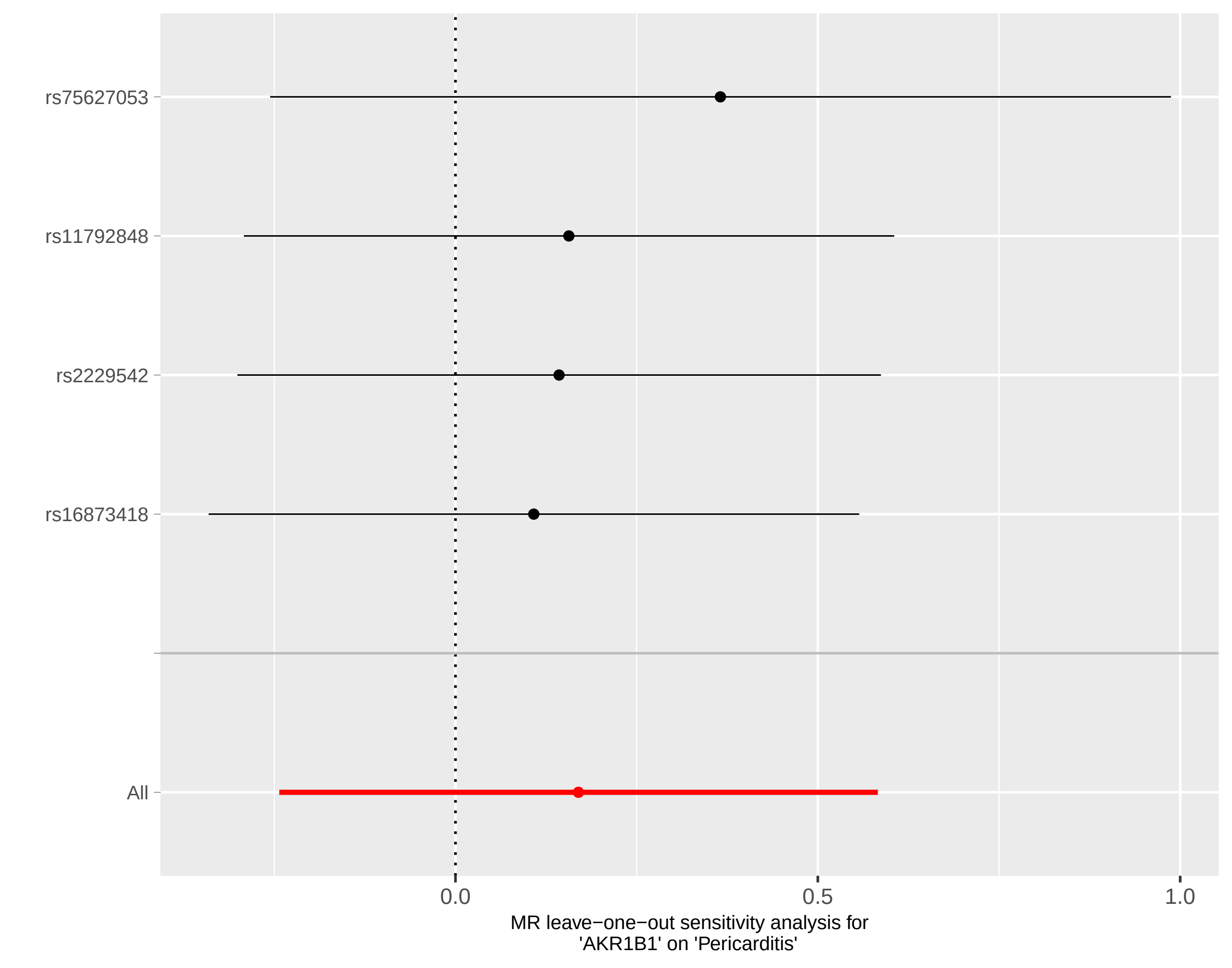

rs75627053
rs11792848
rs2229542
rs16873418
All
0.0
0.5
1.0
MR leave−one−out sensitivity analysis for
'AKR1B1' on 'Pericarditis'

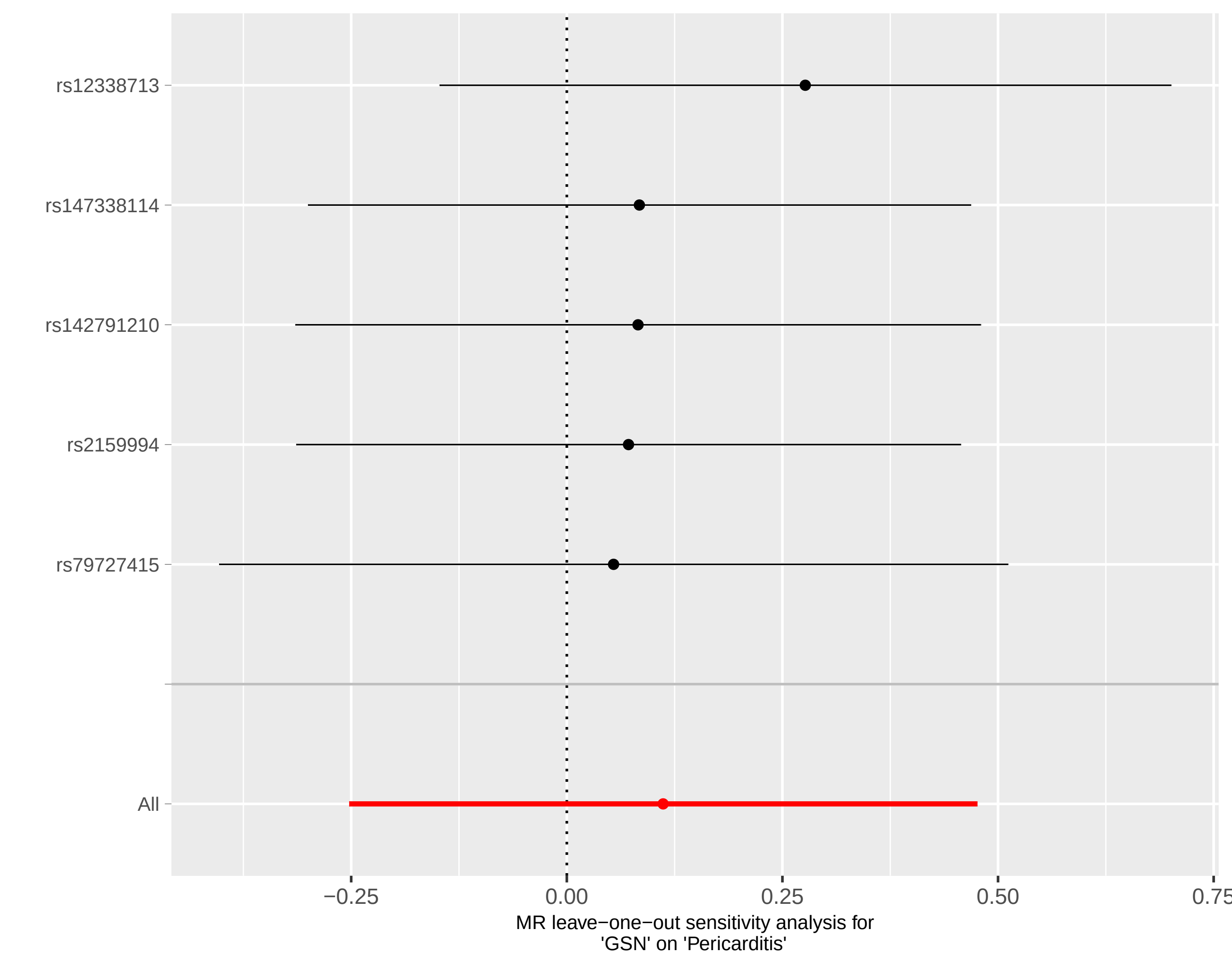
rs12338713
rs147338114
rs142791210
rs2159994
rs79727415
All
−0.25
0.00
0.25
0.50
0.75
MR leave−one−out sensitivity analysis for
'GSN' on 'Pericarditis'

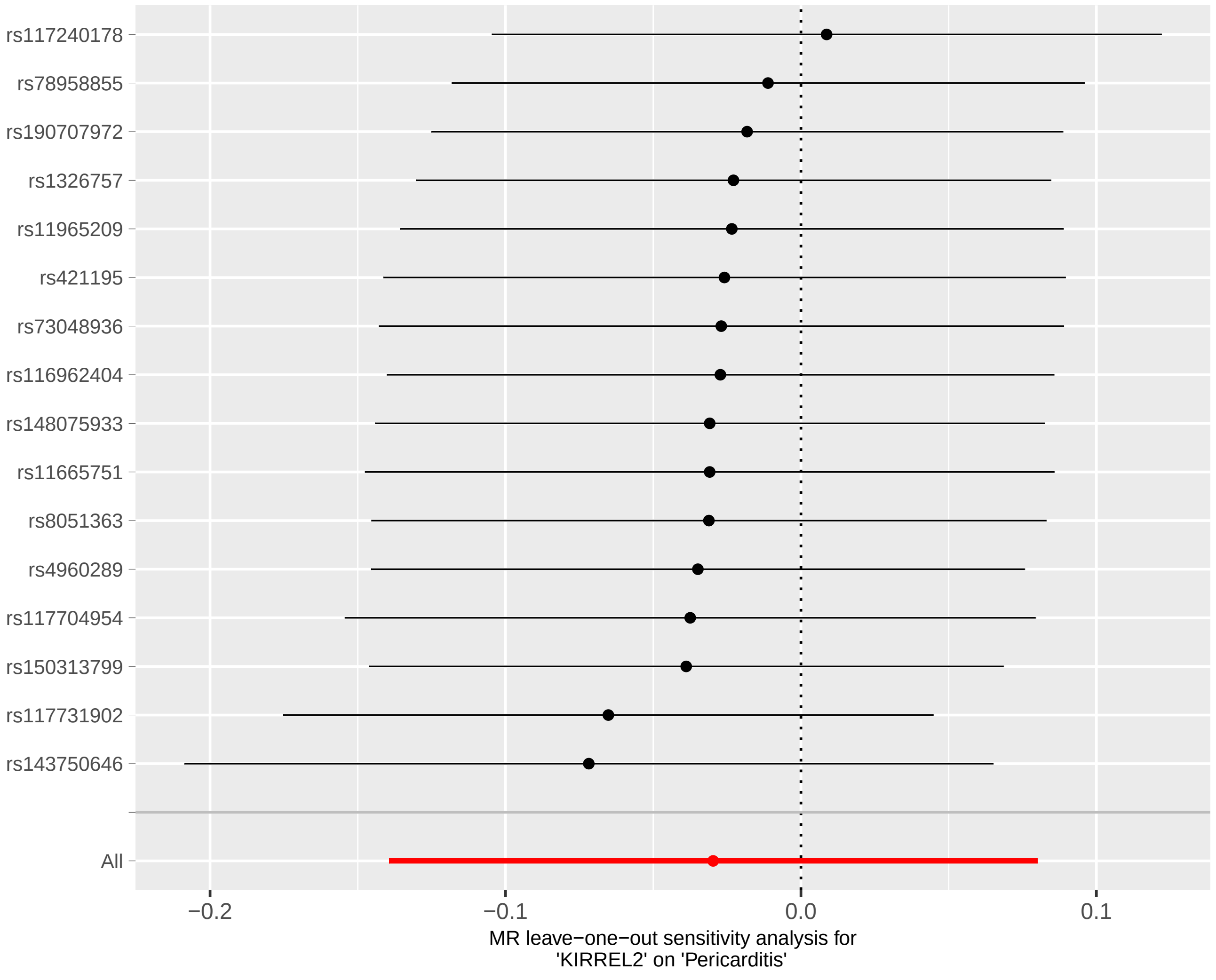

rs117240178
rs78958855
rs190707972
rs1326757
rs11965209
rs421195
rs73048936
rs116962404
rs148075933
rs11665751
rs8051363
rs4960289
rs117704954
rs150313799
rs117731902
rs143750646
All
−0.2
−0.1
0.0
0.1
MR leave−one−out sensitivity analysis for
'KIRREL2' on 'Pericarditis'

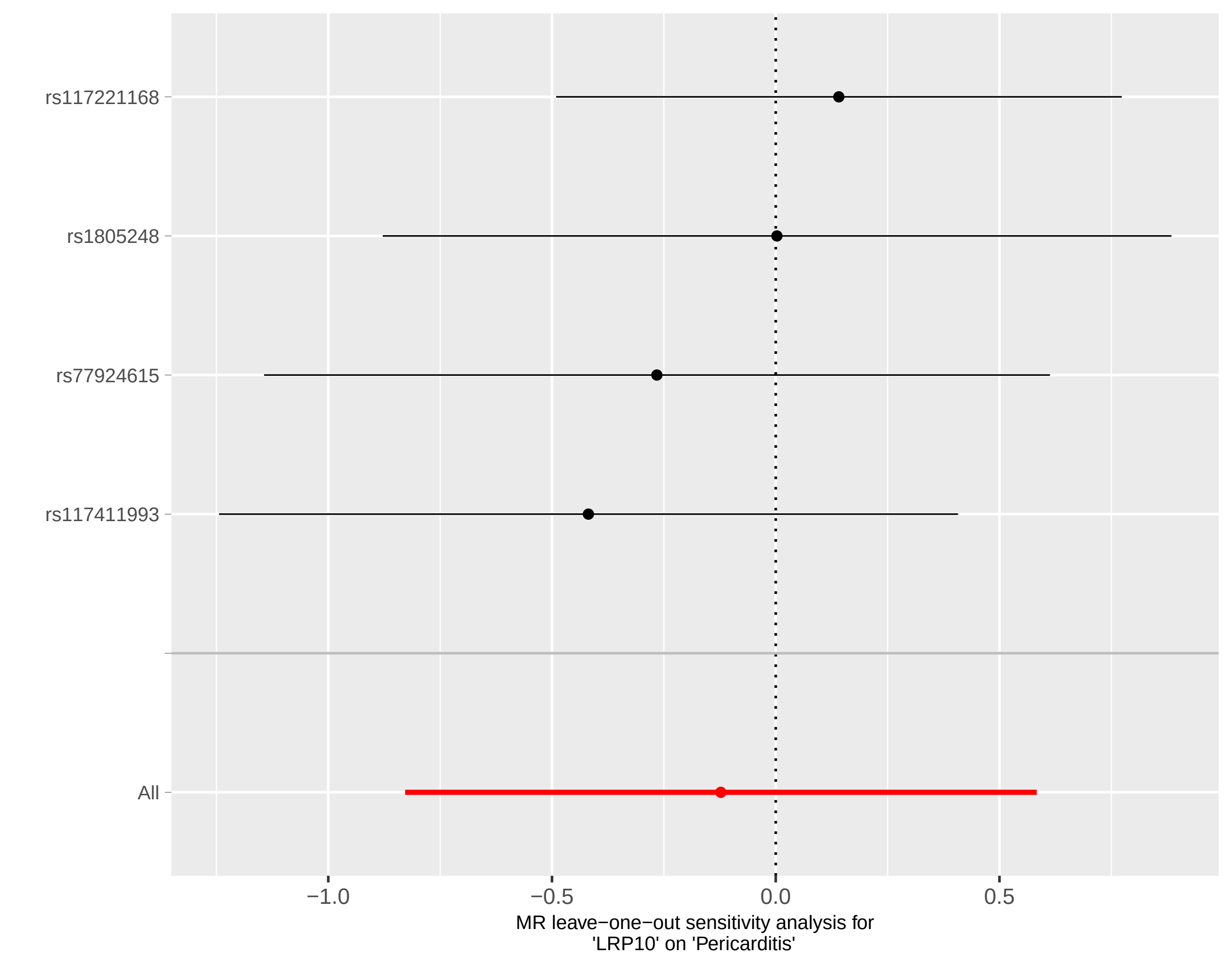
rs117221168
rs1805248
rs77924615
rs117411993
All
−1.0
−0.5
0.0
0.5
MR leave−one−out sensitivity analysis for
'LRP10' on 'Pericarditis'

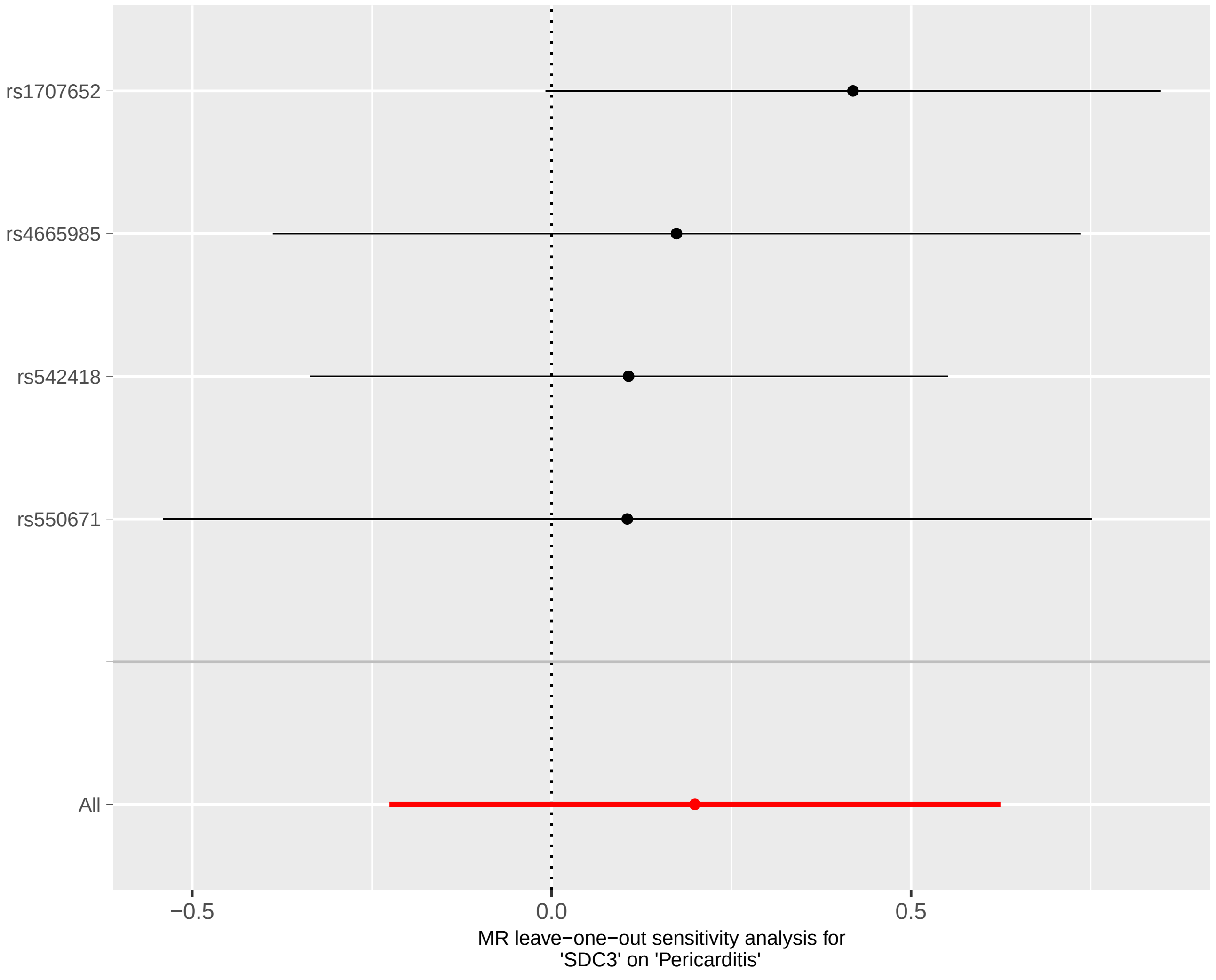

rs1707652
rs4665985
rs542418
rs550671
All
−0.5
0.0
0.5
MR leave−one−out sensitivity analysis for
'SDC3' on 'Pericarditis'

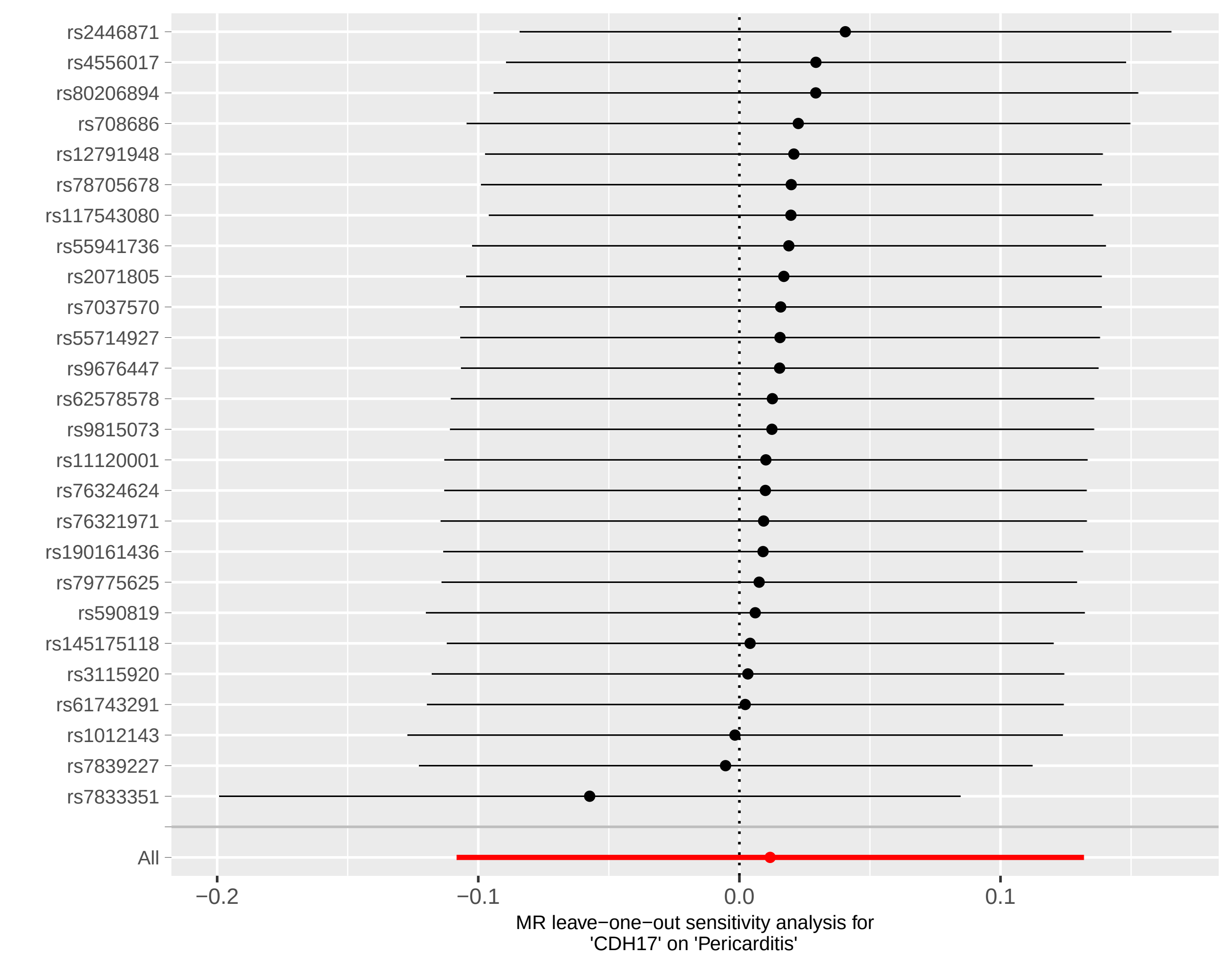
rs2446871
rs4556017
rs80206894
rs708686
rs12791948
rs78705678
rs117543080
rs55941736
rs2071805
rs7037570
rs55714927
rs9676447
rs62578578
rs9815073
rs11120001
rs76324624
rs76321971
rs190161436
rs79775625
rs590819
rs145175118
rs3115920
rs61743291
rs1012143
rs7839227
rs7833351
All
−0.2
−0.1
0.0
0.1
MR leave−one−out sensitivity analysis for
'CDH17' on 'Pericarditis'

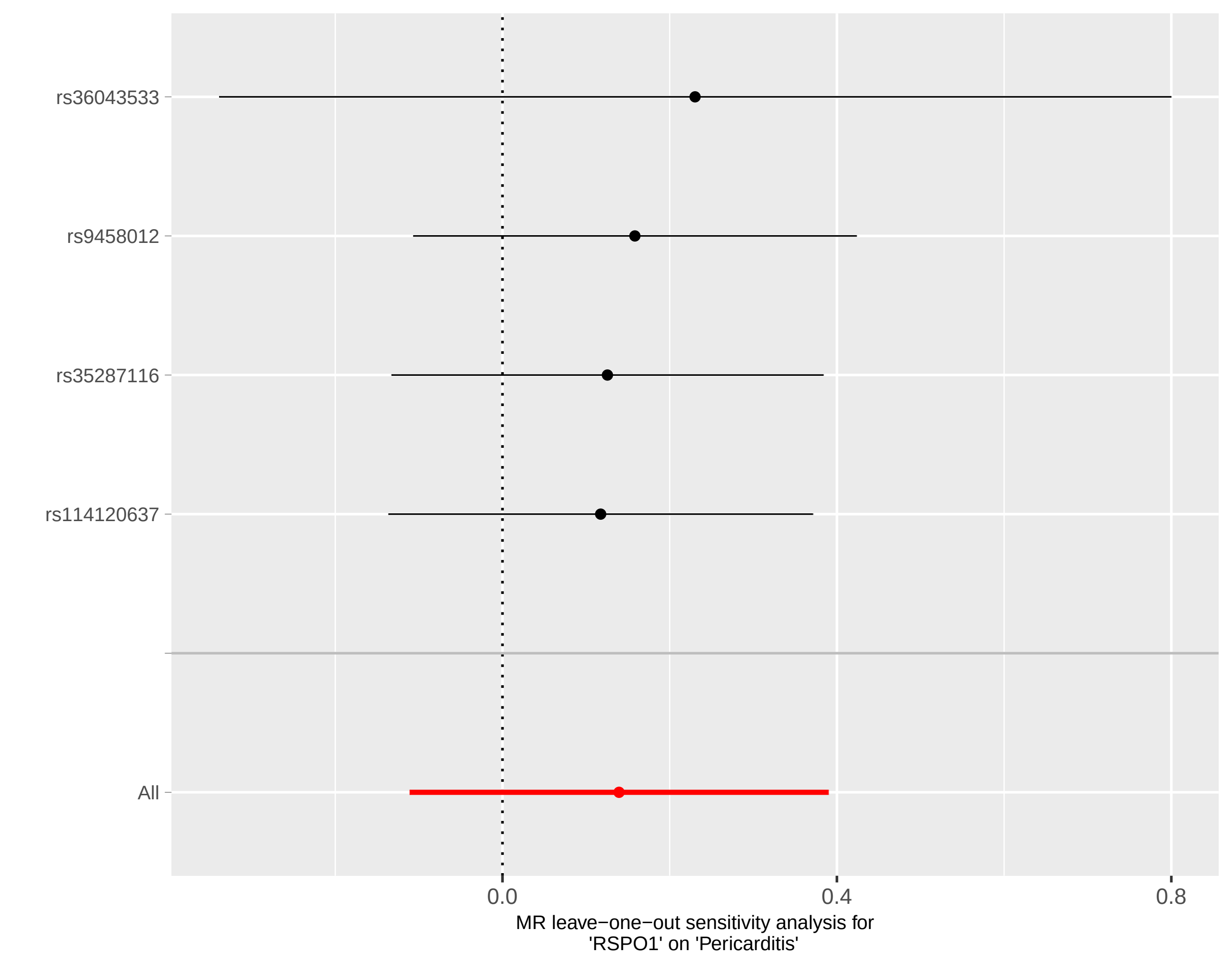
rs36043533
rs9458012
rs35287116
rs114120637
All
0.0
0.4
0.8
MR leave−one−out sensitivity analysis for
'RSPO1' on 'Pericarditis'

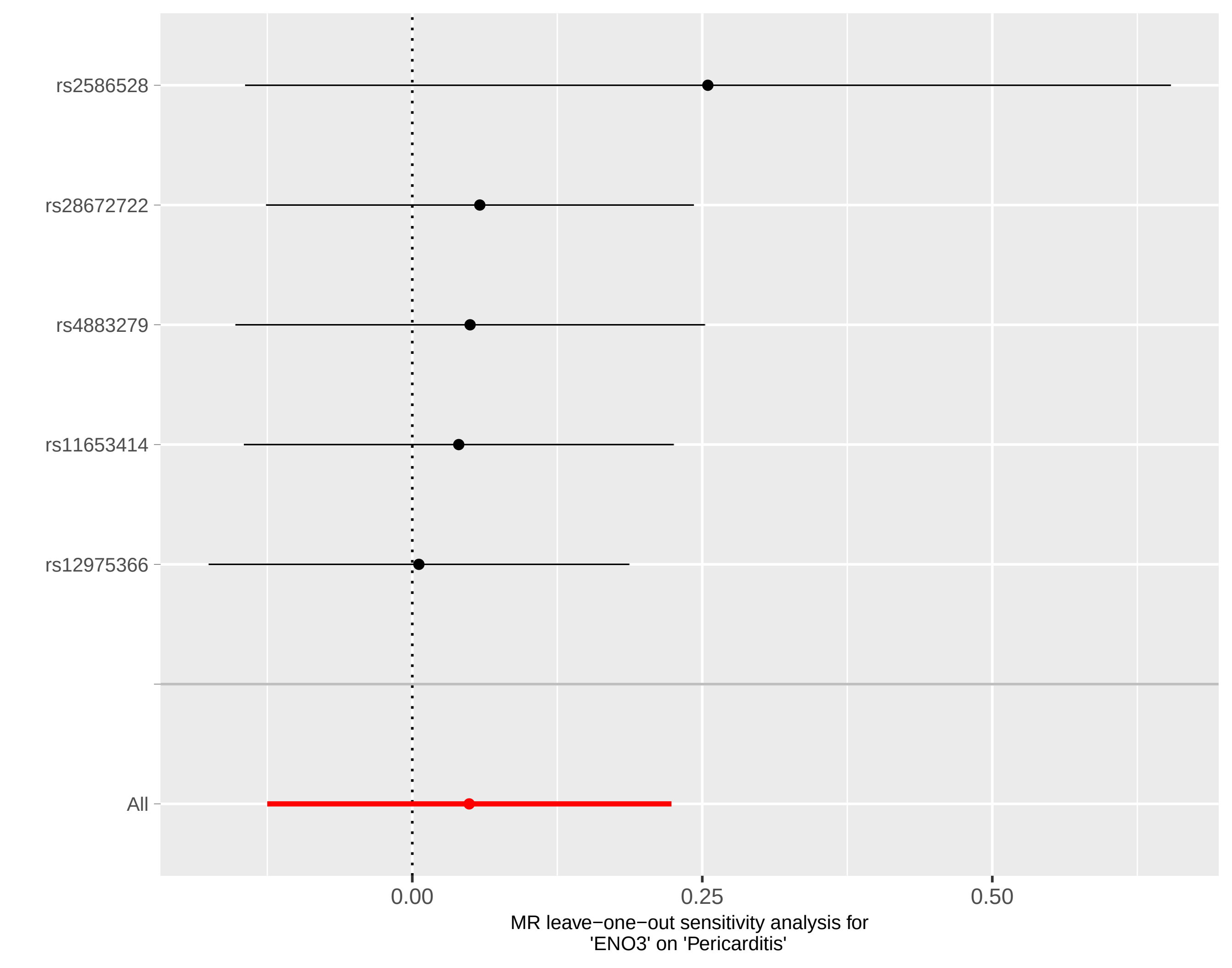

rs2586528
rs28672722
rs4883279
rs11653414
rs12975366
All
0.00
0.25
0.50
MR leave−one−out sensitivity analysis for
'ENO3' on 'Pericarditis'

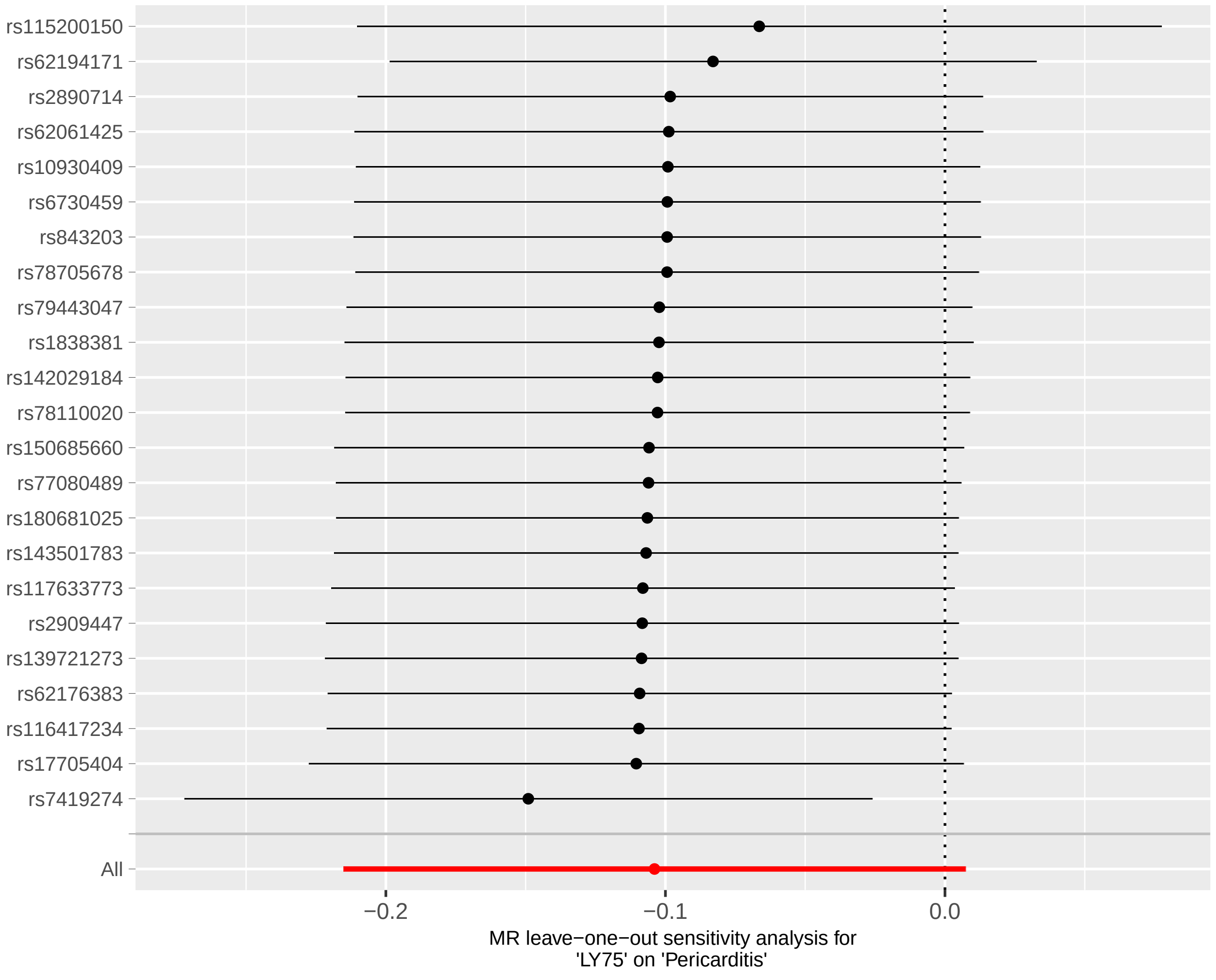
rs115200150
rs62194171
rs2890714
rs62061425
rs10930409
rs6730459
rs843203
rs78705678
rs79443047
rs1838381
rs142029184
rs78110020
rs150685660
rs77080489
rs180681025
rs143501783
rs117633773
rs2909447
rs139721273
rs62176383
rs116417234
rs17705404
rs7419274
All
−0.2
−0.1
0.0
MR leave−one−out sensitivity analysis for
'LY75' on 'Pericarditis'

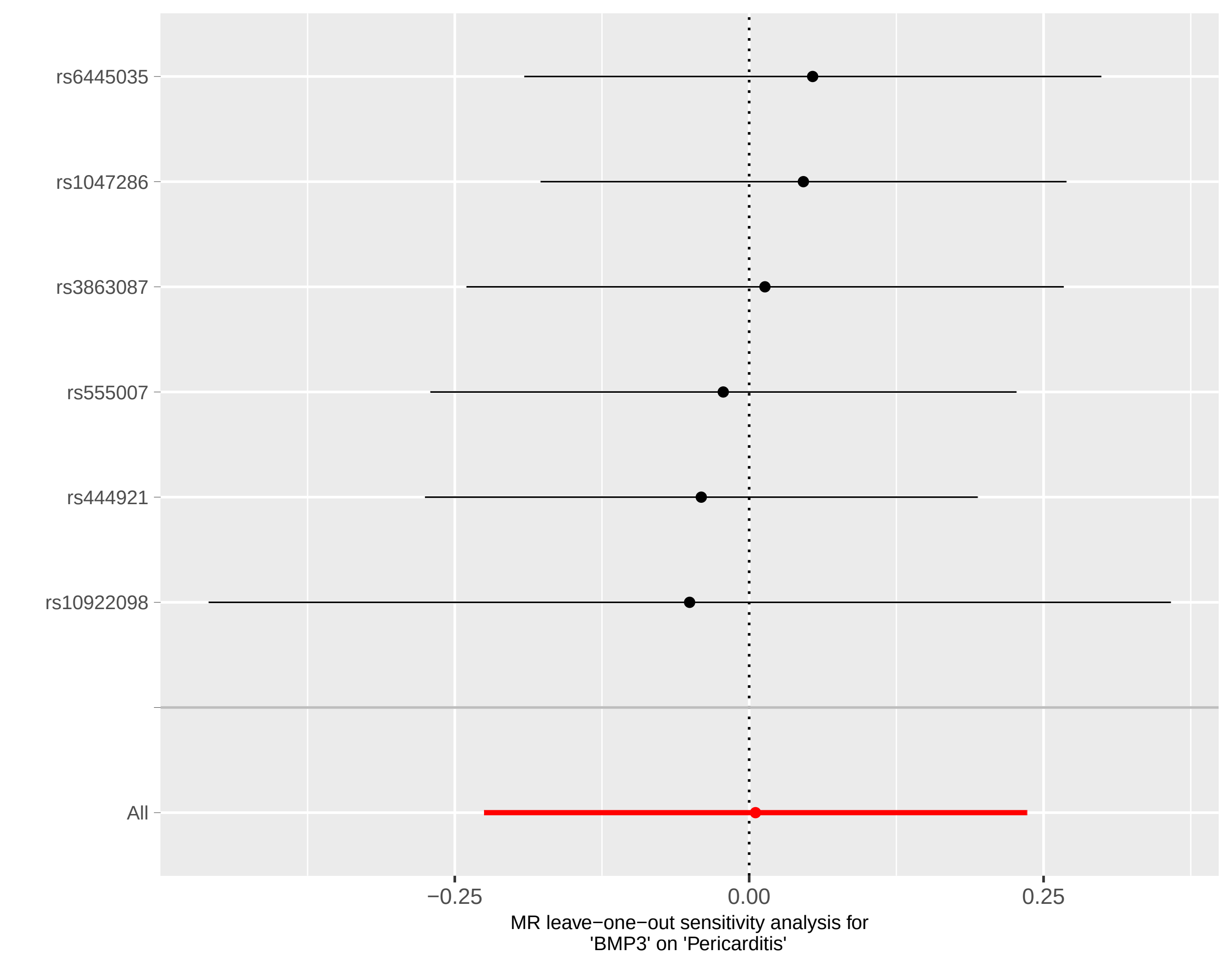

rs6445035
rs1047286
rs3863087
rs555007
rs444921
rs10922098
All
−0.25
0.00
0.25
MR leave−one−out sensitivity analysis for
'BMP3' on 'Pericarditis'

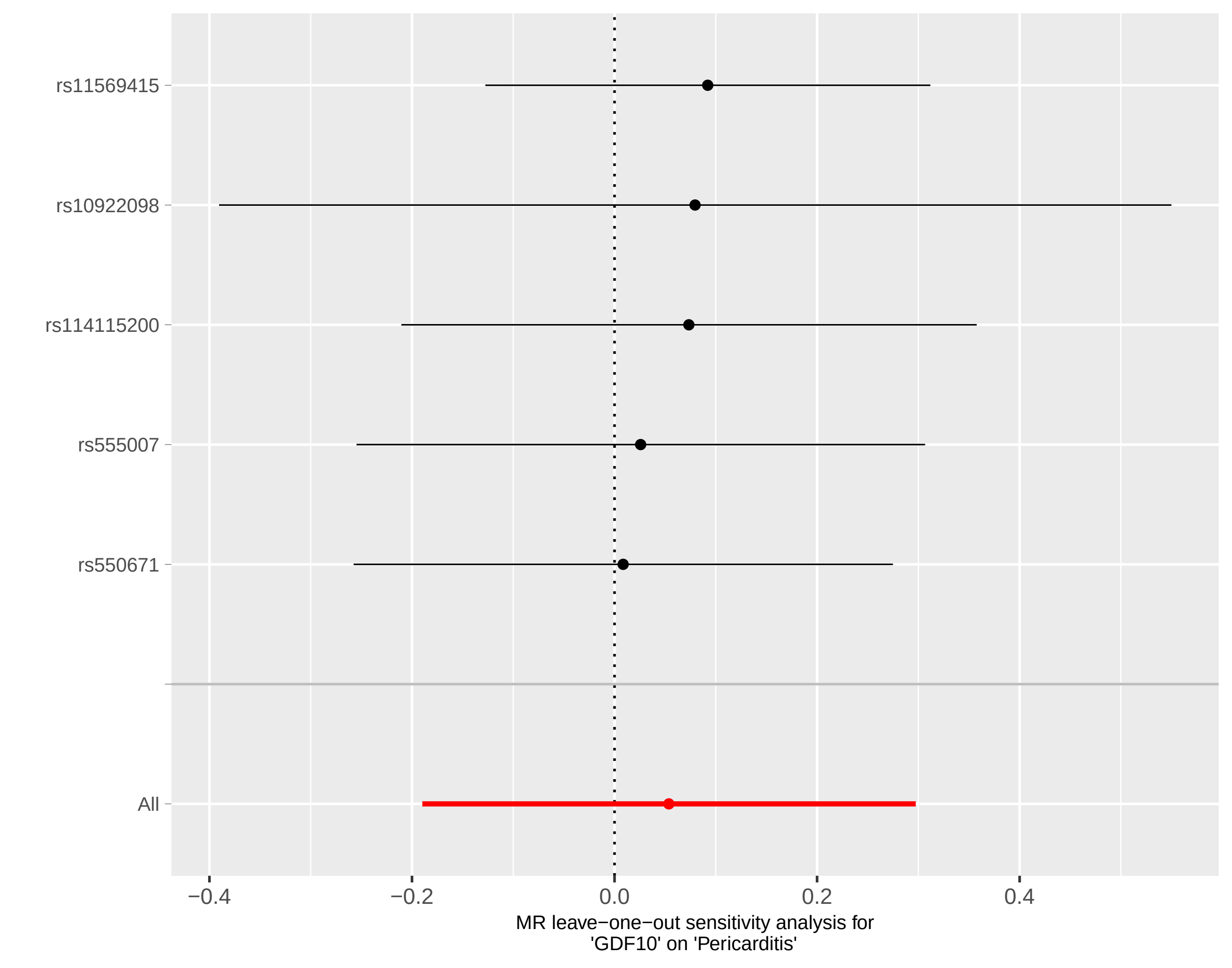
rs11569415
rs10922098
rs114115200
rs555007
rs550671
All
−0.4
−0.2
0.0
0.2
0.4
MR leave−one−out sensitivity analysis for
'GDF10' on 'Pericarditis'

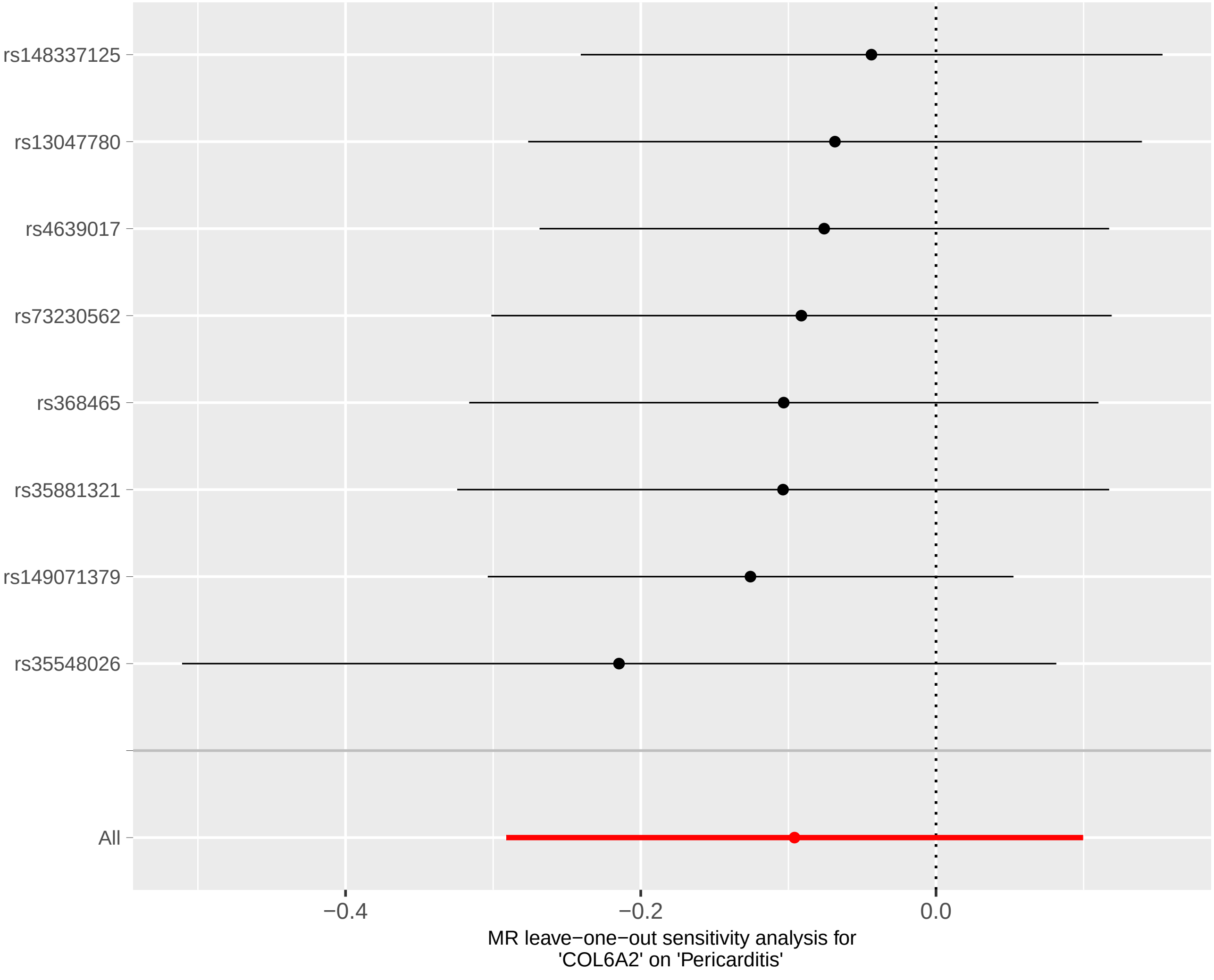
rs148337125
rs13047780
rs4639017
rs73230562
rs368465
rs35881321
rs149071379
rs35548026
All
−0.4
−0.2
0.0
MR leave−one−out sensitivity analysis for
'COL6A2' on 'Pericarditis'

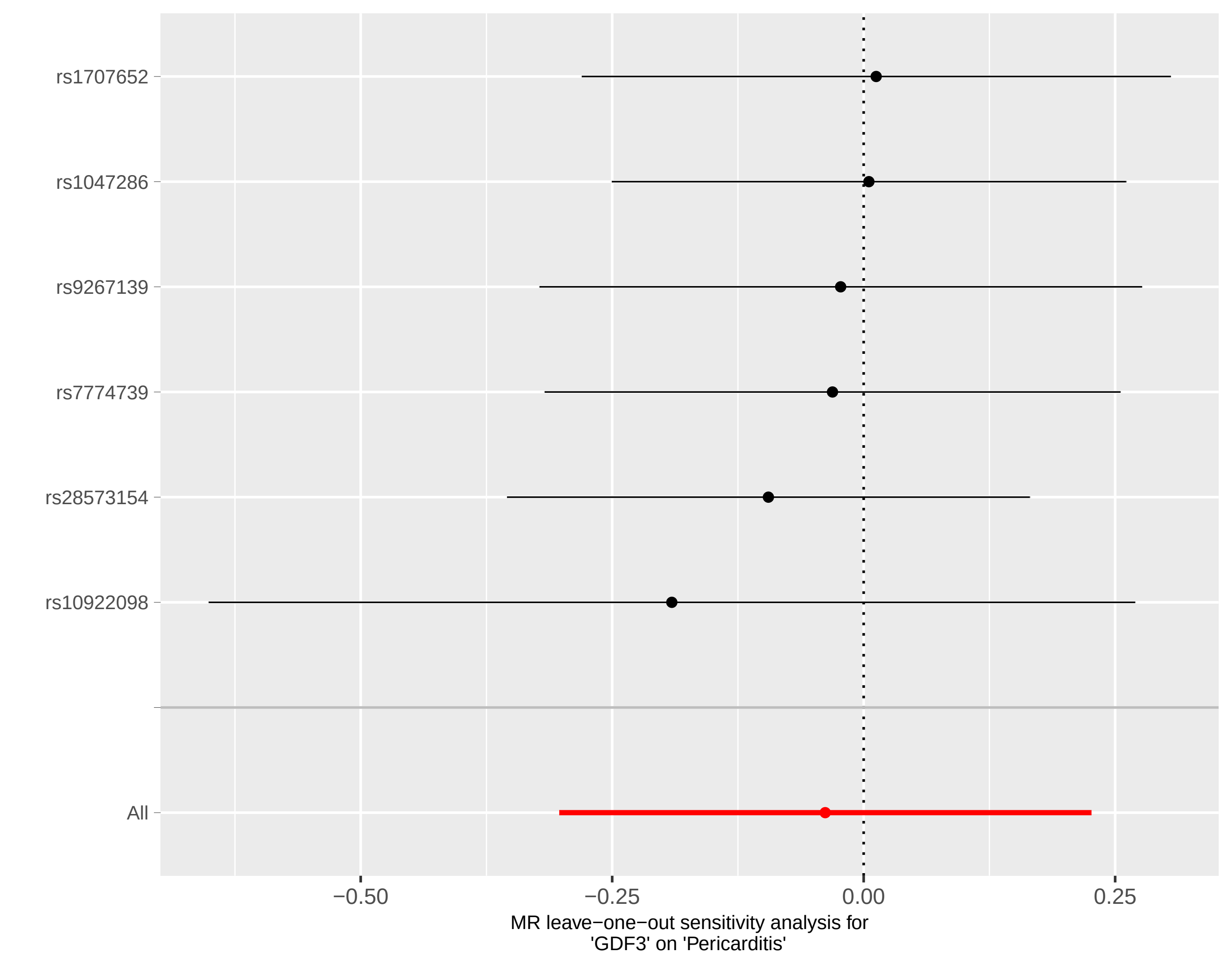
rs1707652
rs1047286
rs9267139
rs7774739
rs28573154
rs10922098
All
−0.50
−0.25
0.00
0.25
MR leave−one−out sensitivity analysis for
'GDF3' on 'Pericarditis'

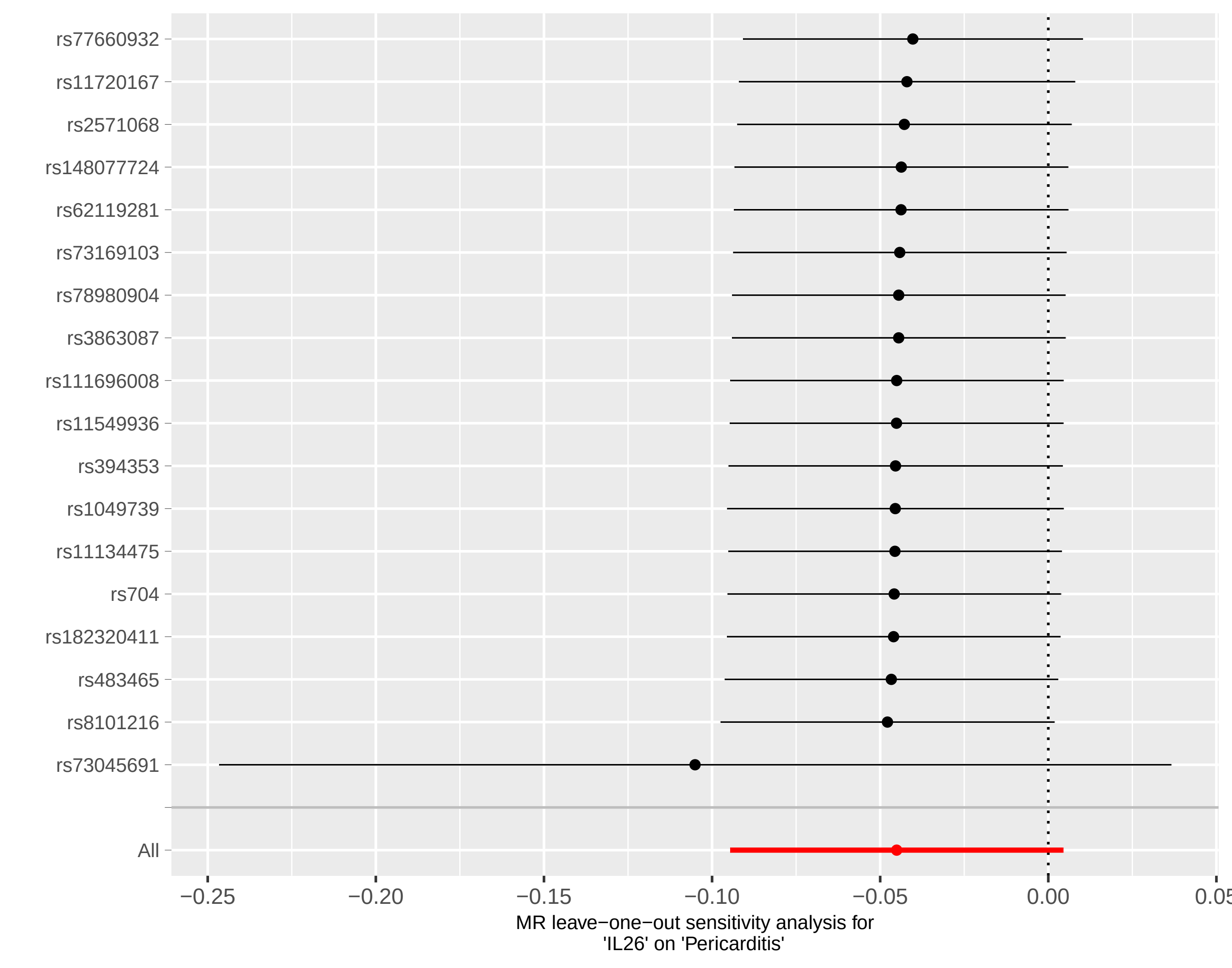

rs77660932
rs11720167
rs2571068
rs148077724
rs62119281
rs73169103
rs78980904
rs3863087
rs111696008
rs11549936
rs394353
rs1049739
rs11134475
rs704
rs182320411
rs483465
rs8101216
rs73045691
All
−0.25
−0.20
−0.15
−0.10
−0.05
0.00
MR leave−one−out sensitivity analysis for
'IL26' on 'Pericarditis'

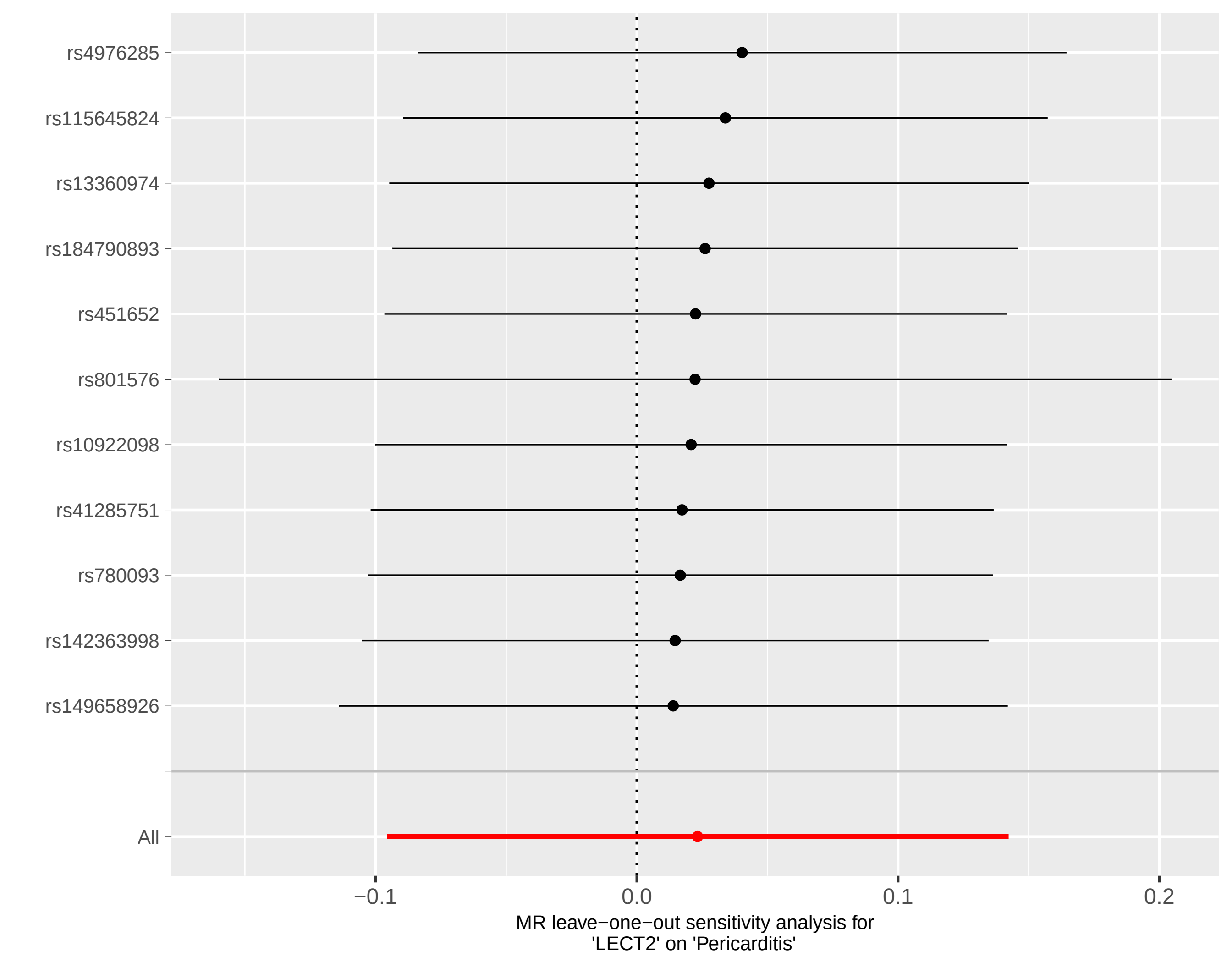

rs4976285
rs115645824
rs13360974
rs184790893
rs451652
rs801576
rs10922098
rs41285751
rs780093
rs142363998
rs149658926
All
−0.1
0.0
0.1
0.2
MR leave−one−out sensitivity analysis for
'LECT2' on 'Pericarditis'

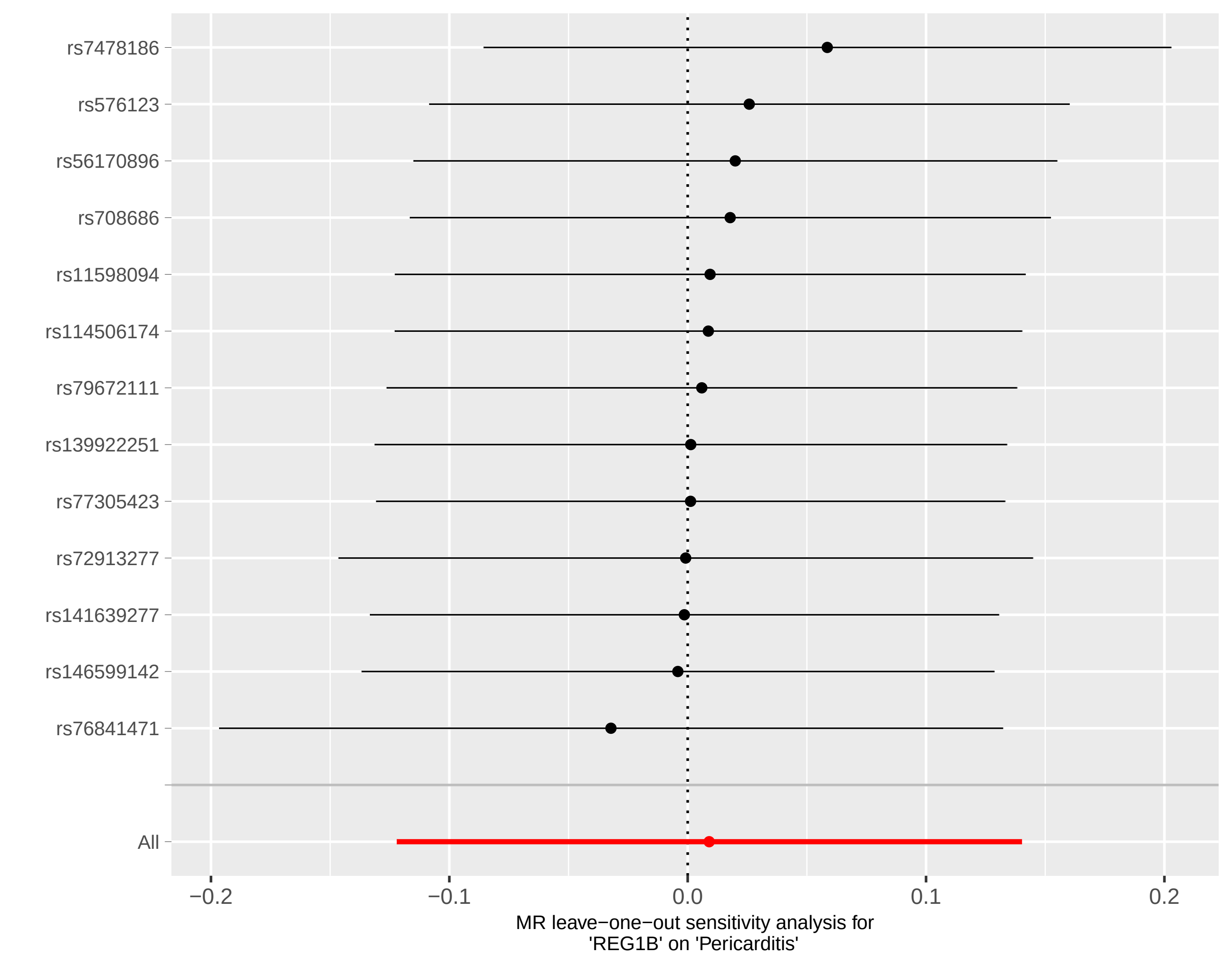
rs7478186
rs576123
rs56170896
rs708686
rs11598094
rs114506174
rs79672111
rs139922251
rs77305423
rs72913277
rs141639277
rs146599142
rs76841471
All
−0.2
−0.1
0.0
0.1
0.2
MR leave−one−out sensitivity analysis for
'REG1B' on 'Pericarditis'

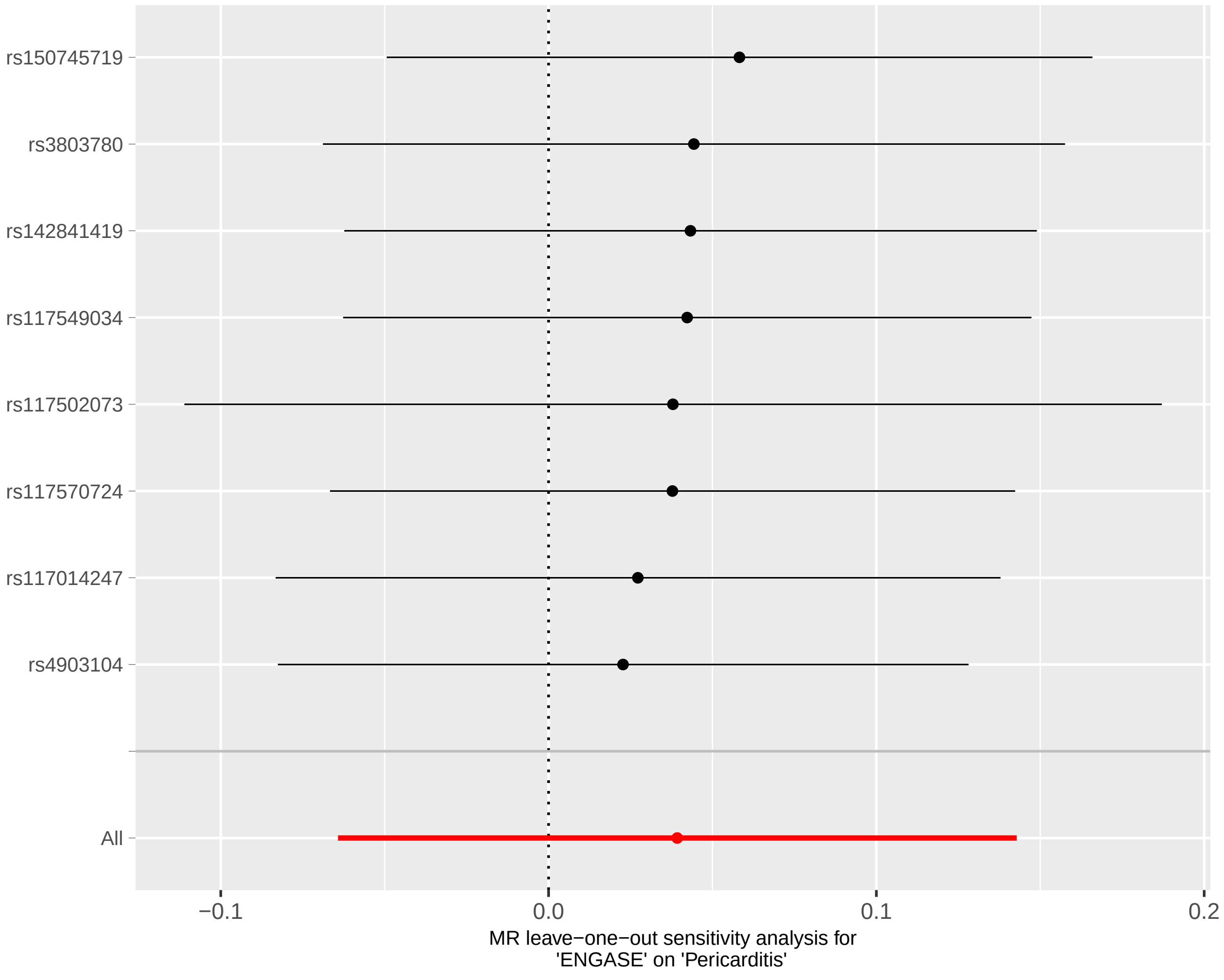
rs150745719
rs3803780
rs142841419
rs117549034
rs117502073
rs117570724
rs117014247
rs4903104
All
−0.1
0.0
0.1
0.2
MR leave−one−out sensitivity analysis for
'ENGASE' on 'Pericarditis'

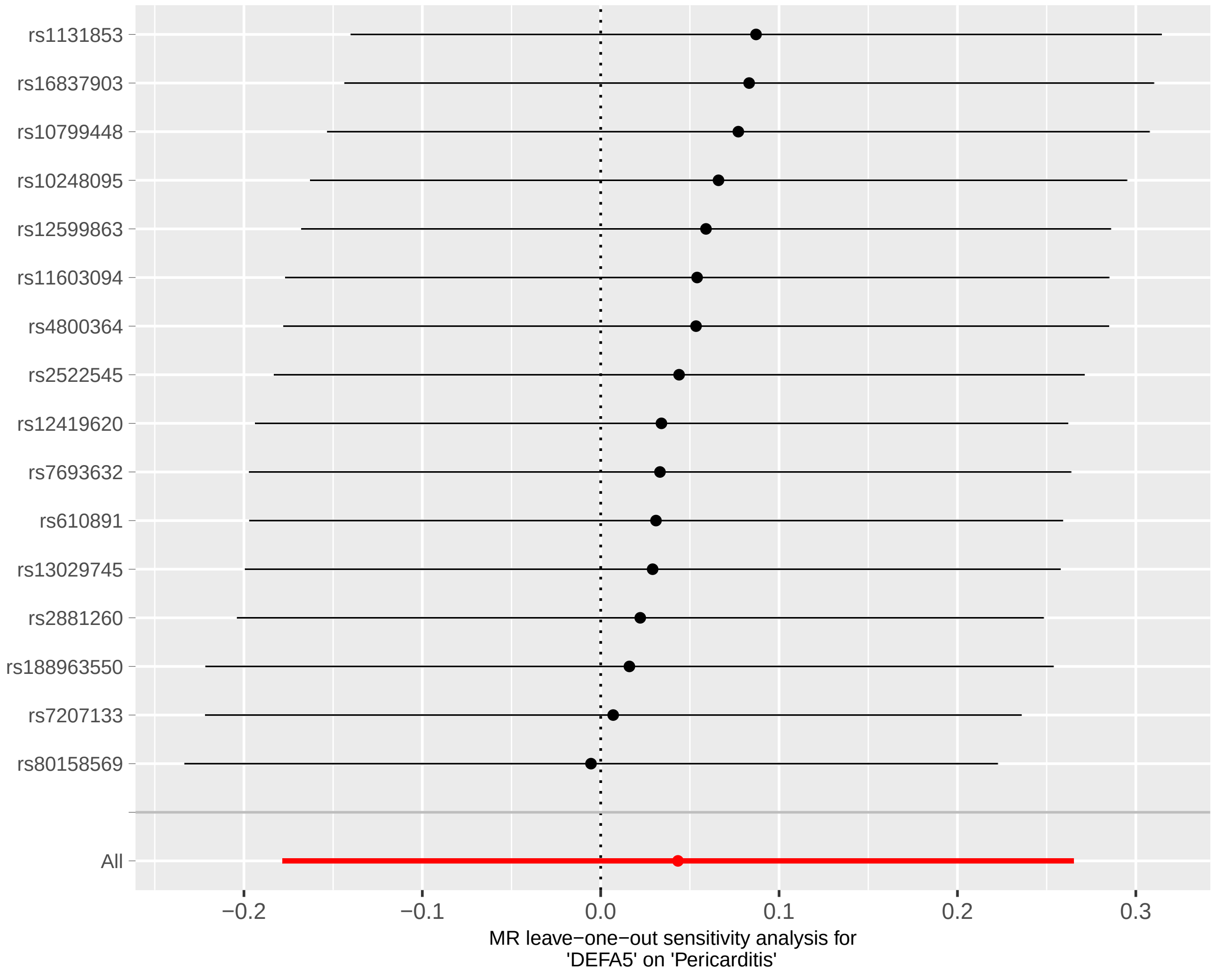
rs1131853
rs16837903
rs10799448
rs10248095
rs12599863
rs11603094
rs4800364
rs2522545
rs12419620
rs7693632
rs610891
rs13029745
rs2881260
rs188963550
rs7207133
rs80158569
All
−0.2
−0.1
0.0
0.1
0.2
0.3
MR leave−one−out sensitivity analysis for
'DEFA5' on 'Pericarditis'

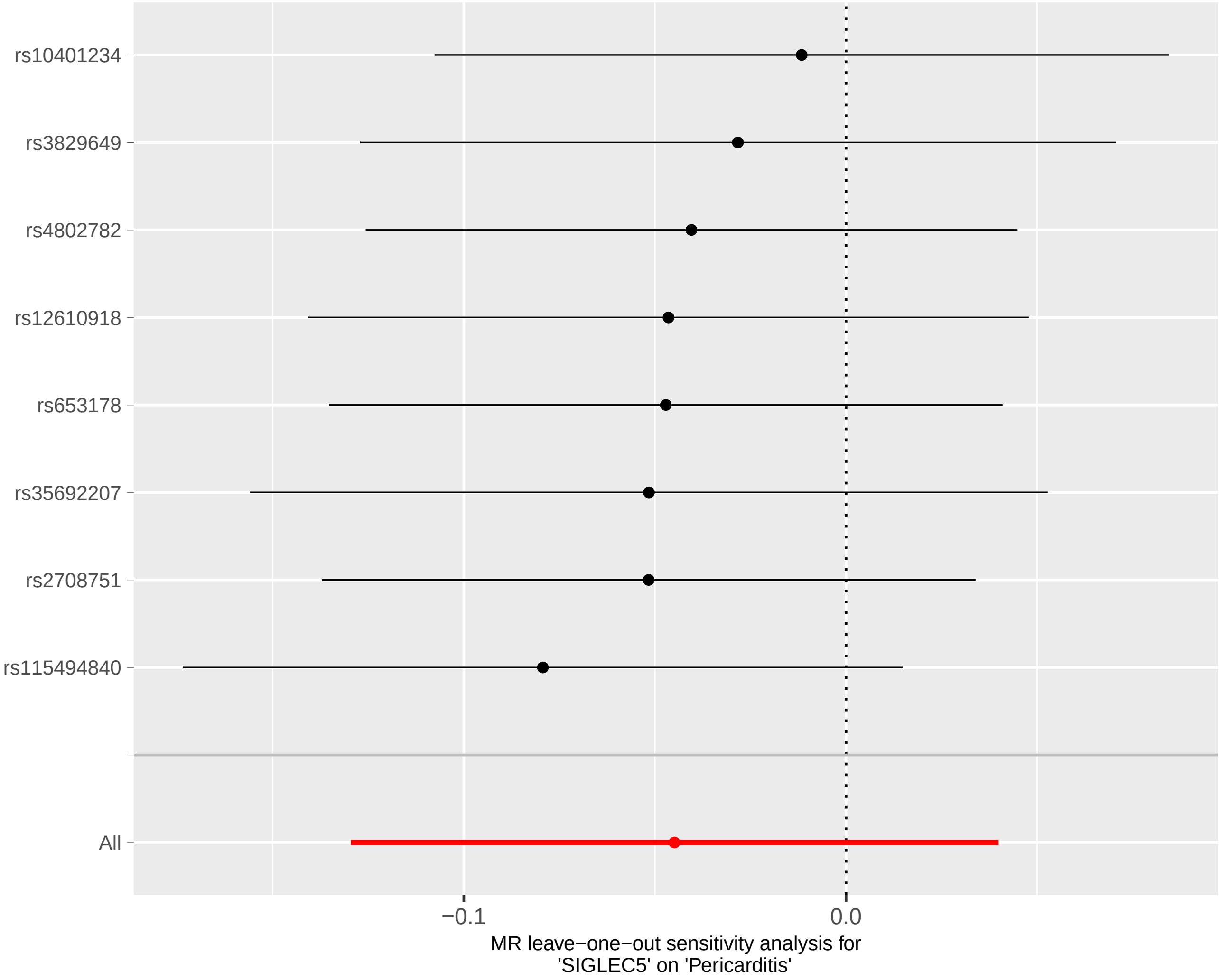
rs10401234
rs3829649
rs4802782
rs12610918
rs653178
rs35692207
rs2708751
rs115494840
All
−0.1
0.0
MR leave−one−out sensitivity analysis for
'SIGLEC5' on 'Pericarditis'

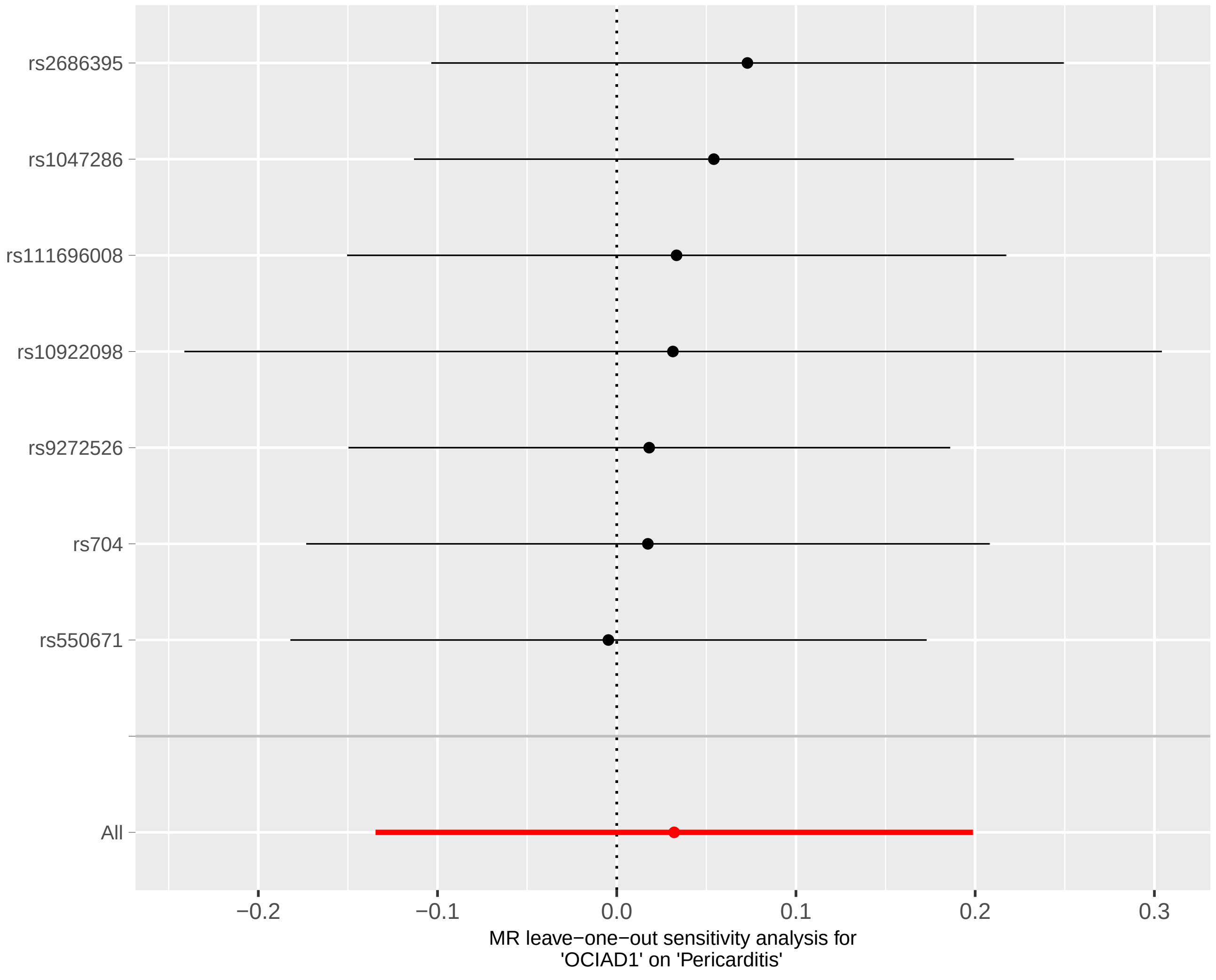

rs2686395
rs1047286
rs111696008
rs10922098
rs9272526
rs704
rs550671
All
−0.2
−0.1
0.0
0.1
0.2
0.3
MR leave−one−out sensitivity analysis for
'OCIAD1' on 'Pericarditis'

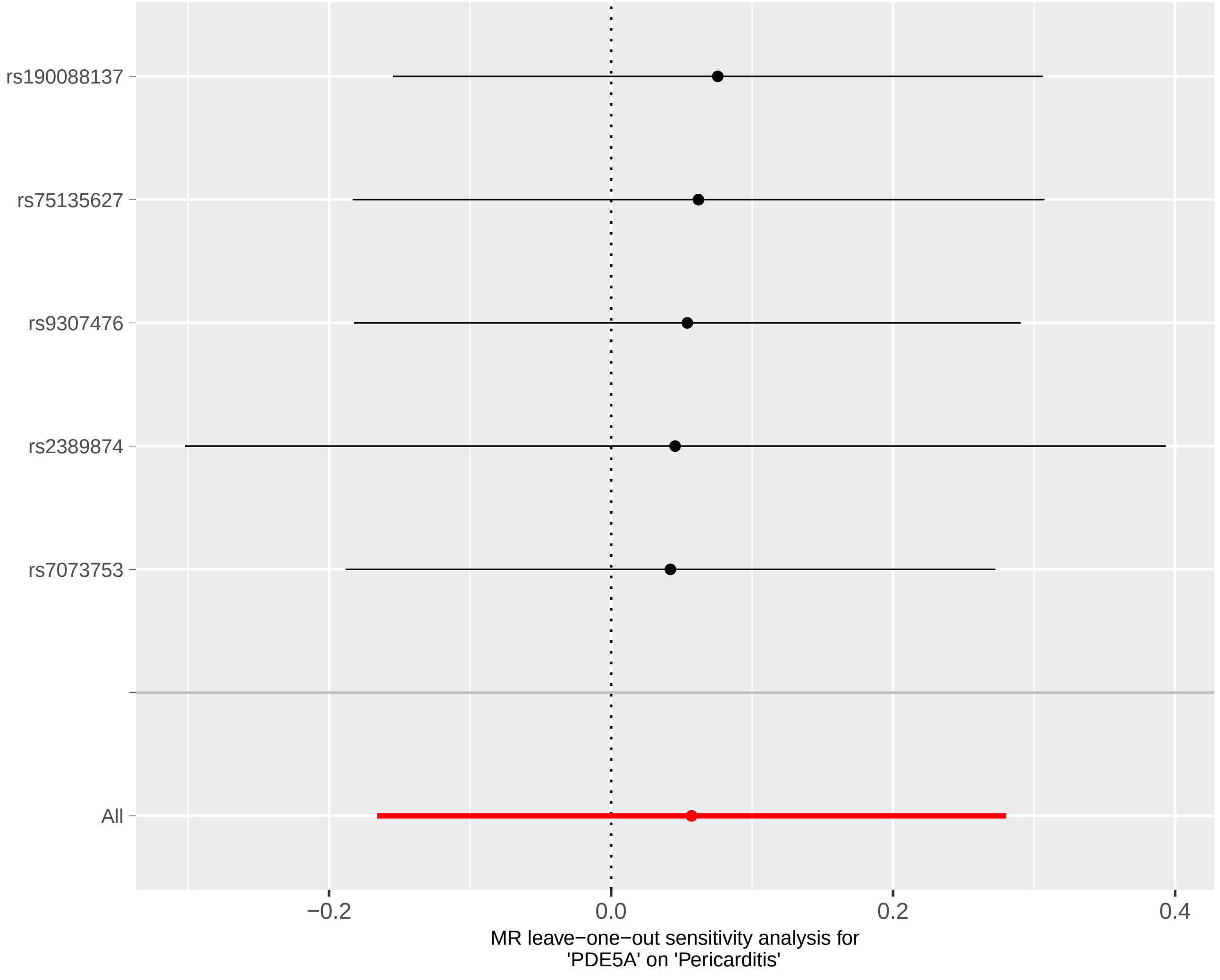
rs190088137
rs75135627
rs9307476
rs2389874
rs7073753
All
−0.2
0.0
0.2
0.4
MR leave−one−out sensitivity analysis for
'PDE5A' on 'Pericarditis'

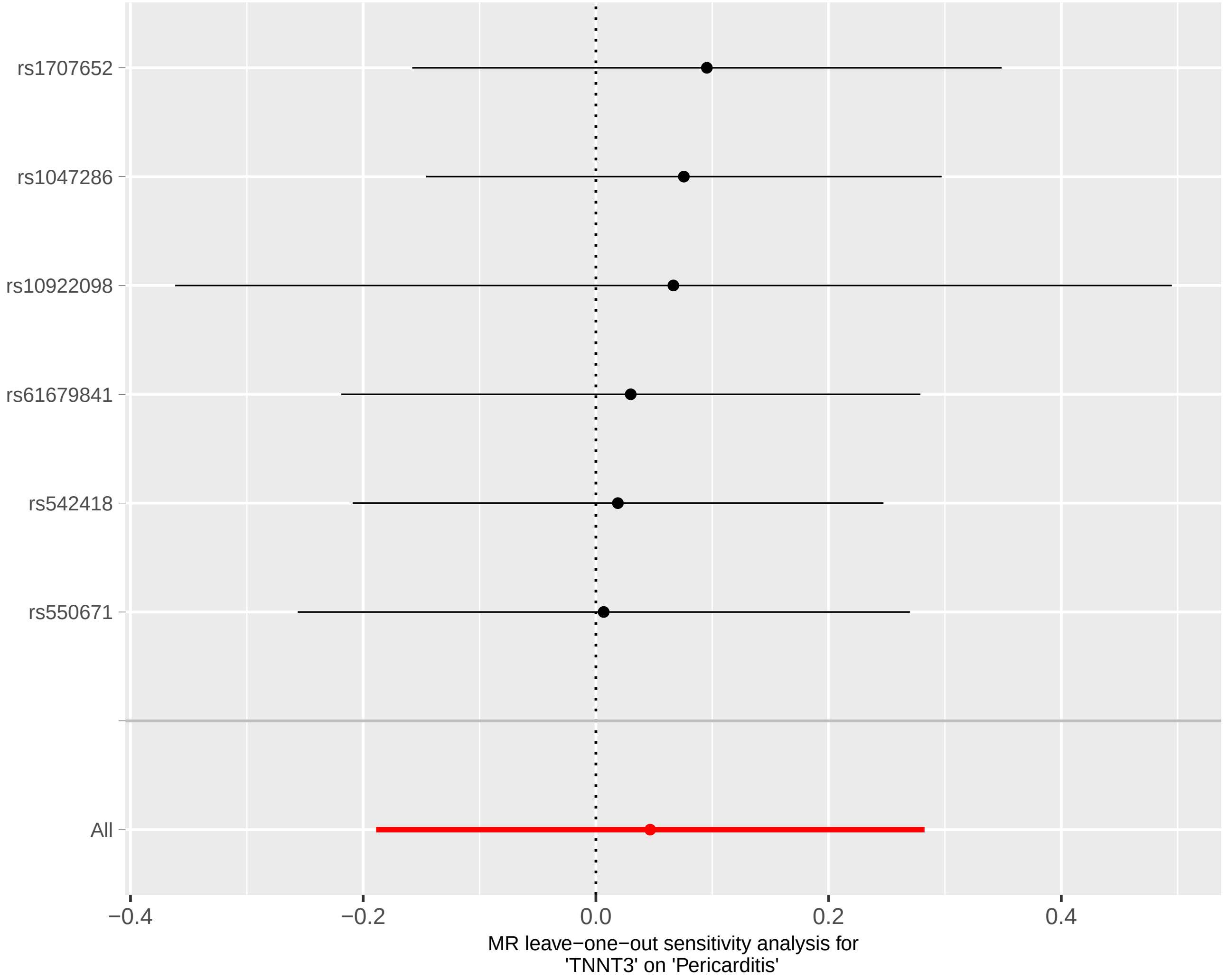
rs1707652
rs1047286
rs10922098
rs61679841
rs542418
rs550671
All
−0.4
−0.2
0.0
0.2
0.4
MR leave−one−out sensitivity analysis for
'TNNT3' on 'Pericarditis'

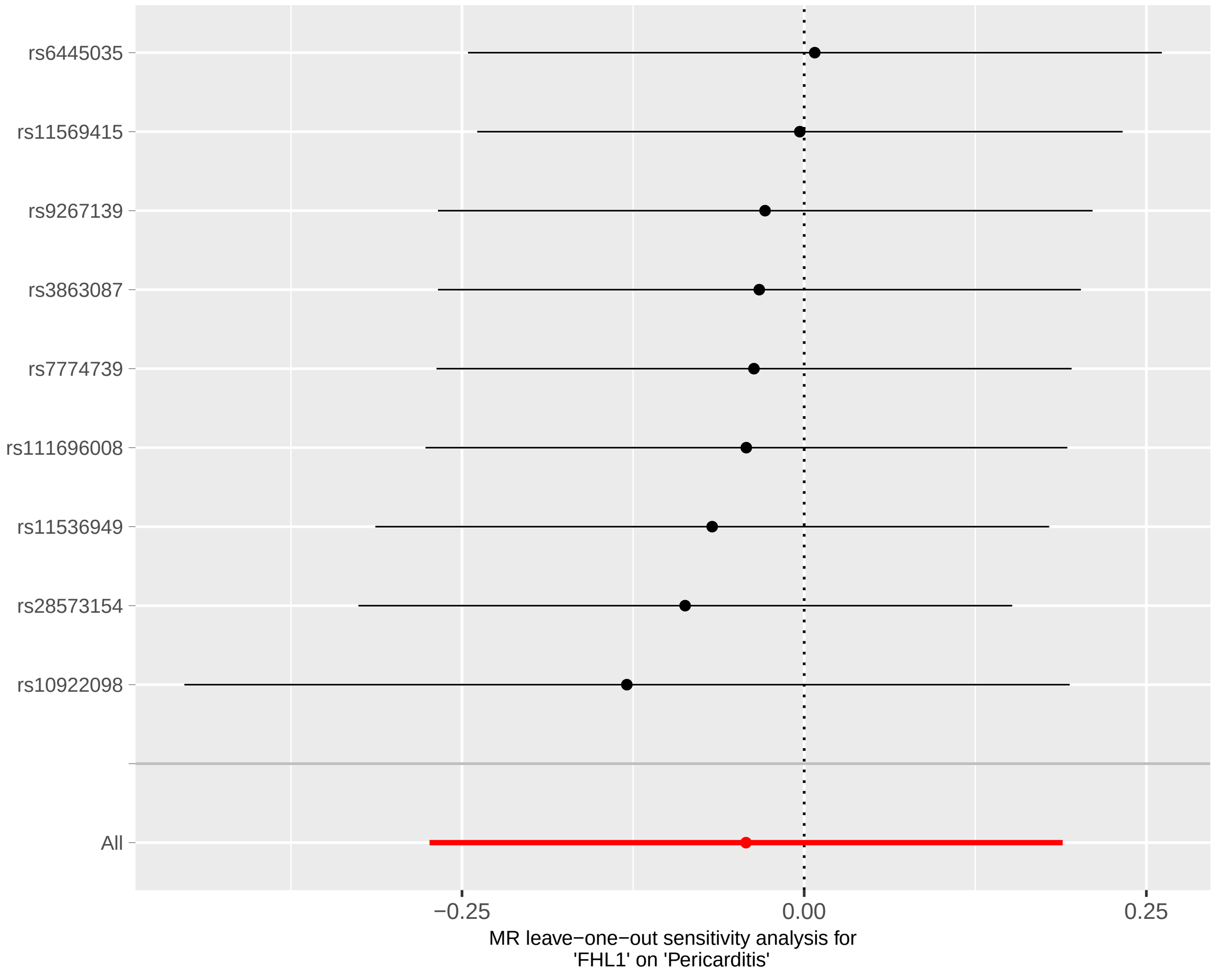
rs6445035
rs11569415
rs9267139
rs3863087
rs7774739
rs111696008
rs11536949
rs28573154
rs10922098
All
−0.25
0.00
0.25
MR leave−one−out sensitivity analysis for
'FHL1' on 'Pericarditis'

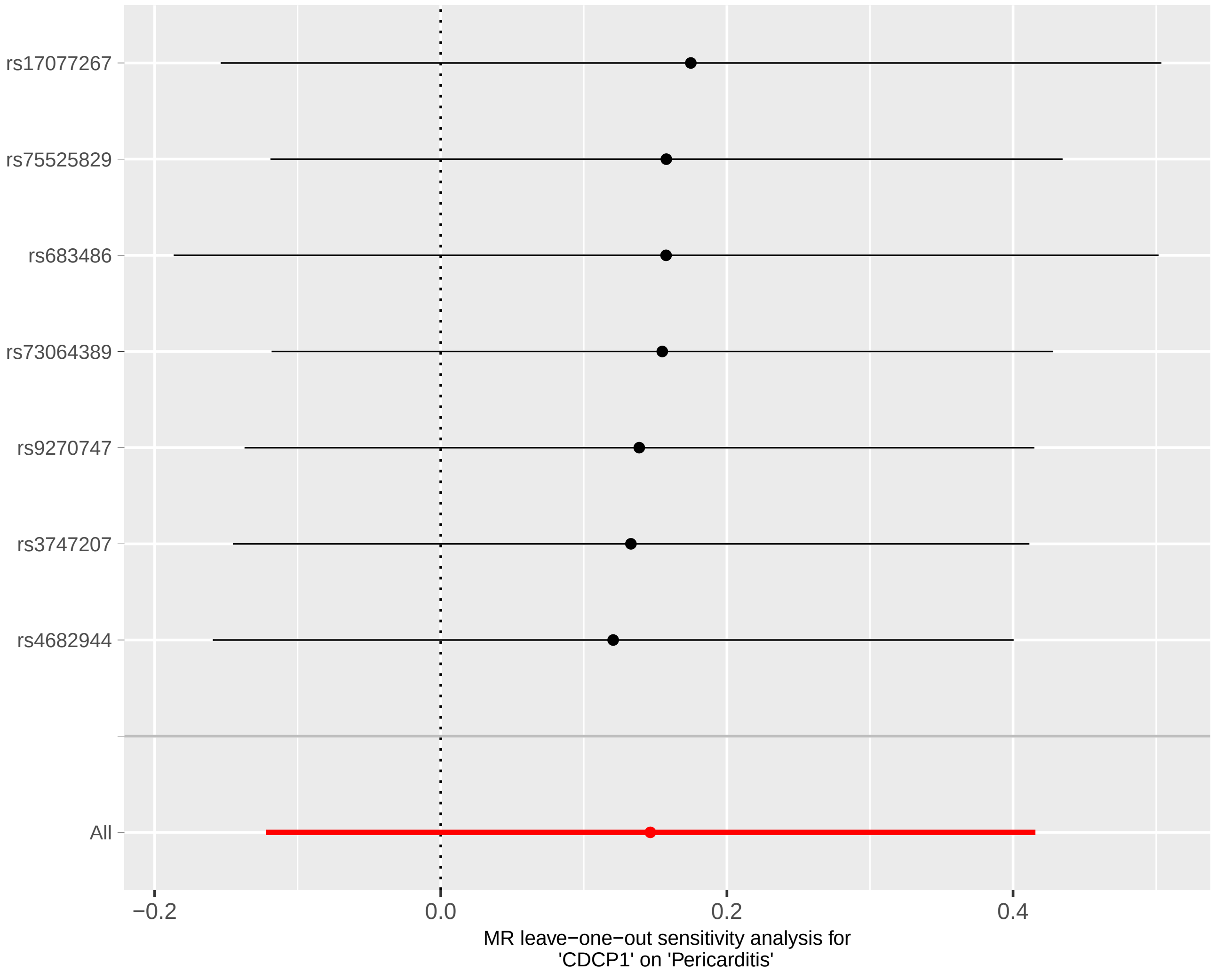
rs17077267
rs75525829
rs683486
rs73064389
rs9270747
rs3747207
rs4682944
All
−0.2
0.0
0.2
0.4
MR leave−one−out sensitivity analysis for
'CDCP1' on 'Pericarditis'

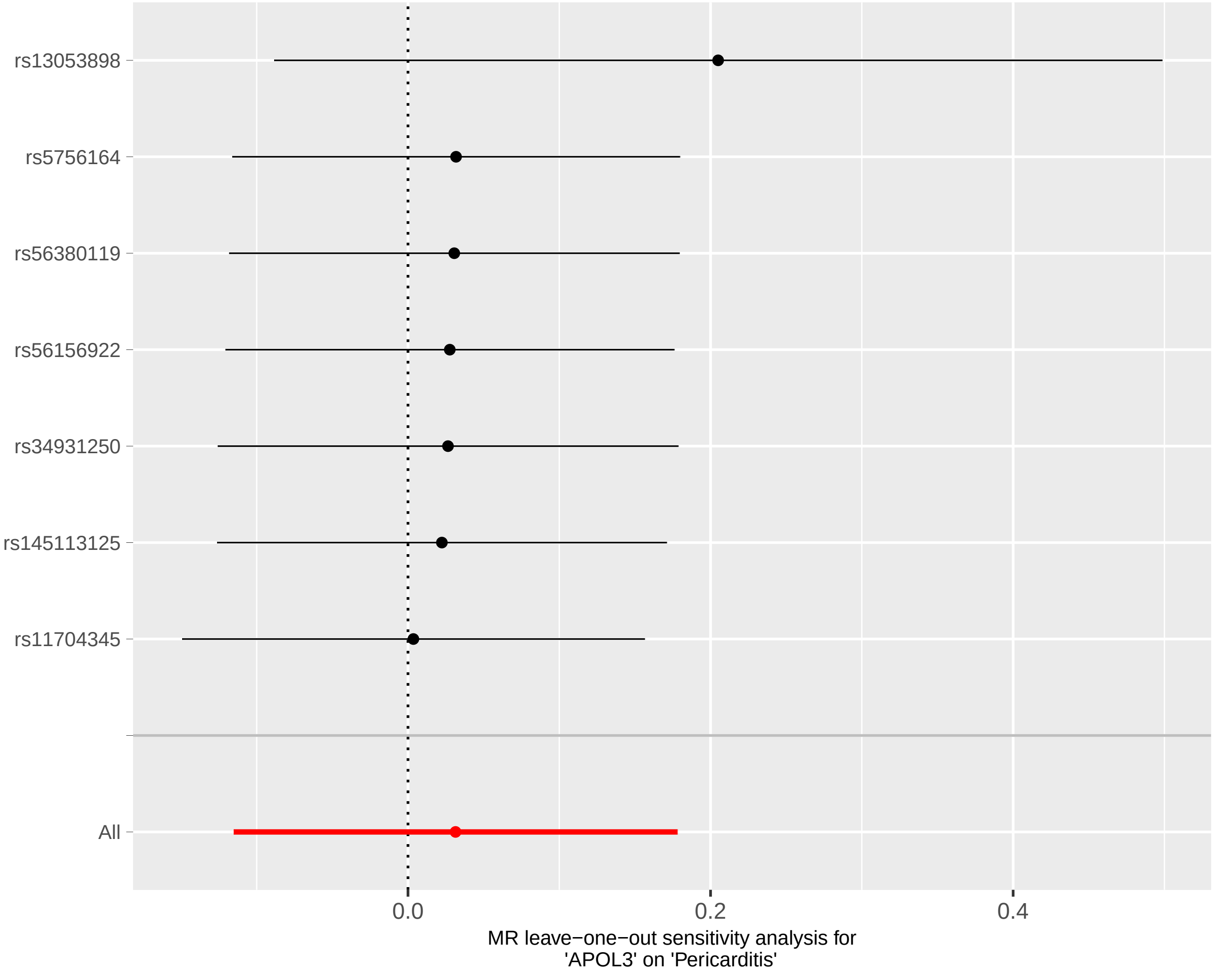

rs13053898
rs5756164
rs56380119
rs56156922
rs34931250
rs145113125
rs11704345
All
0.0
0.2
0.4
MR leave−one−out sensitivity analysis for
'APOL3' on 'Pericarditis'

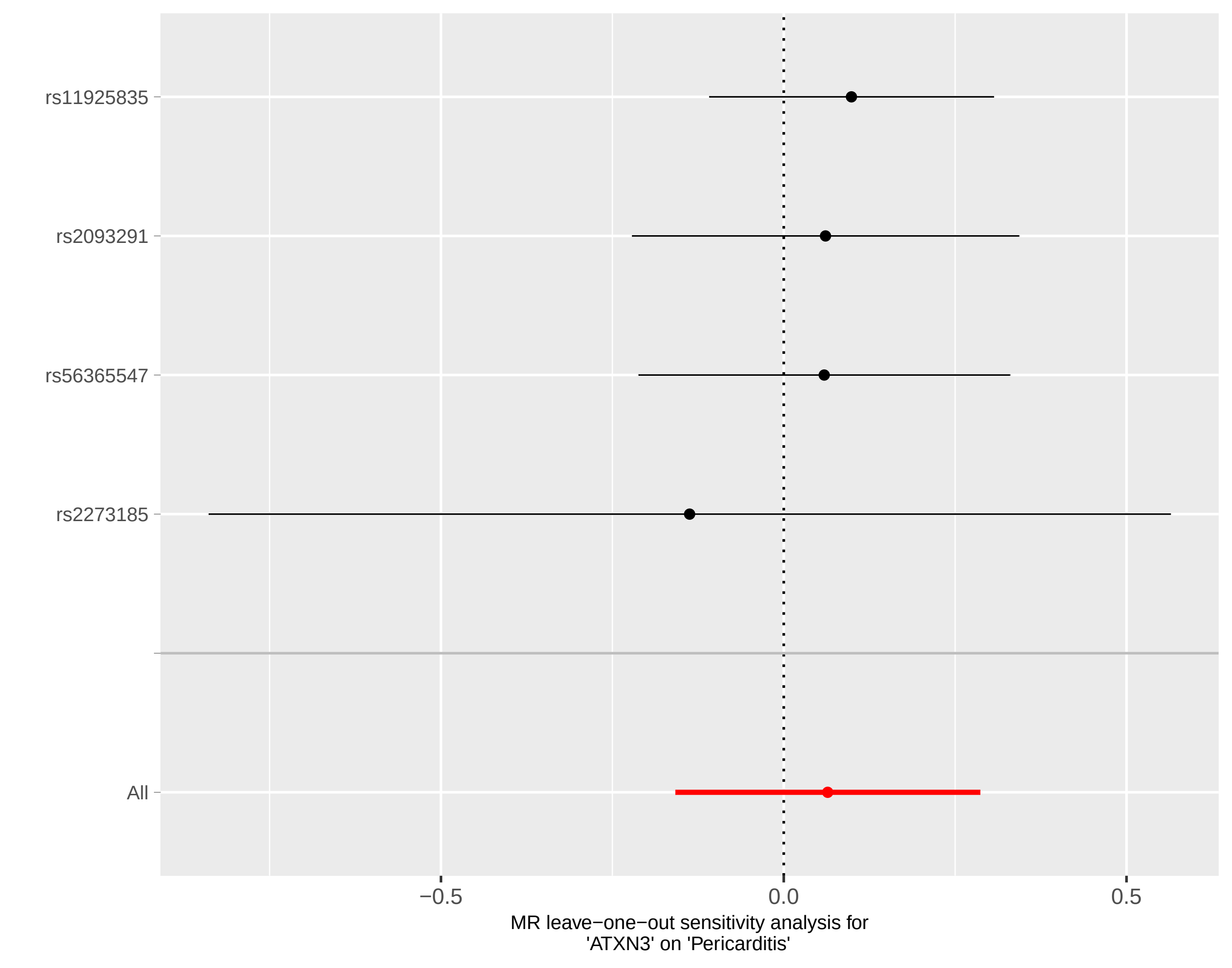

rs11925835
rs2093291
rs56365547
rs2273185
All
−0.5
0.0
0.5
MR leave−one−out sensitivity analysis for
'ATXN3' on 'Pericarditis'

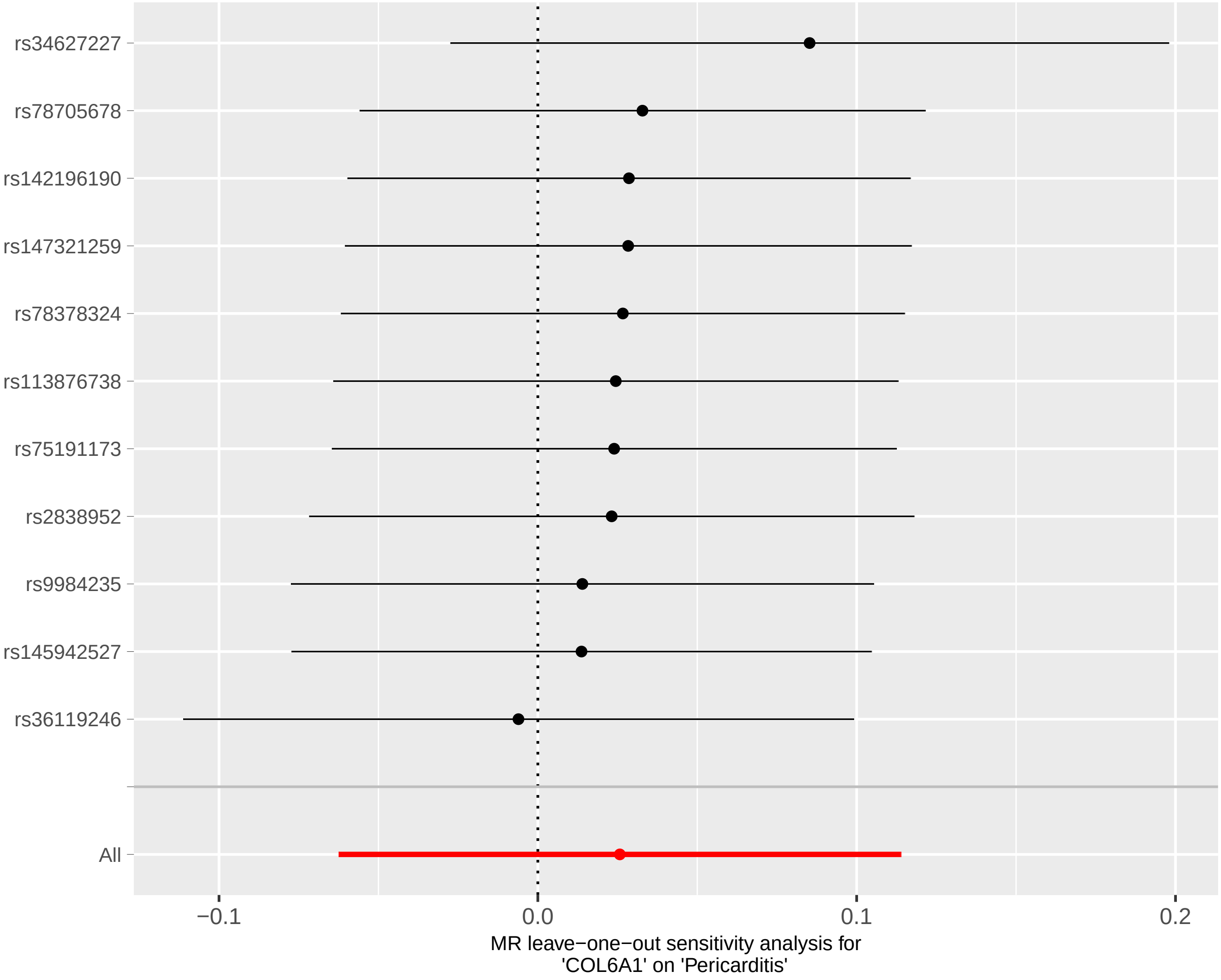

rs34627227
rs78705678
rs142196190
rs147321259
rs78378324
rs113876738
rs75191173
rs2838952
rs9984235
rs145942527
rs36119246
All
−0.1
0.0
0.1
0.2
MR leave−one−out sensitivity analysis for
'COL6A1' on 'Pericarditis'

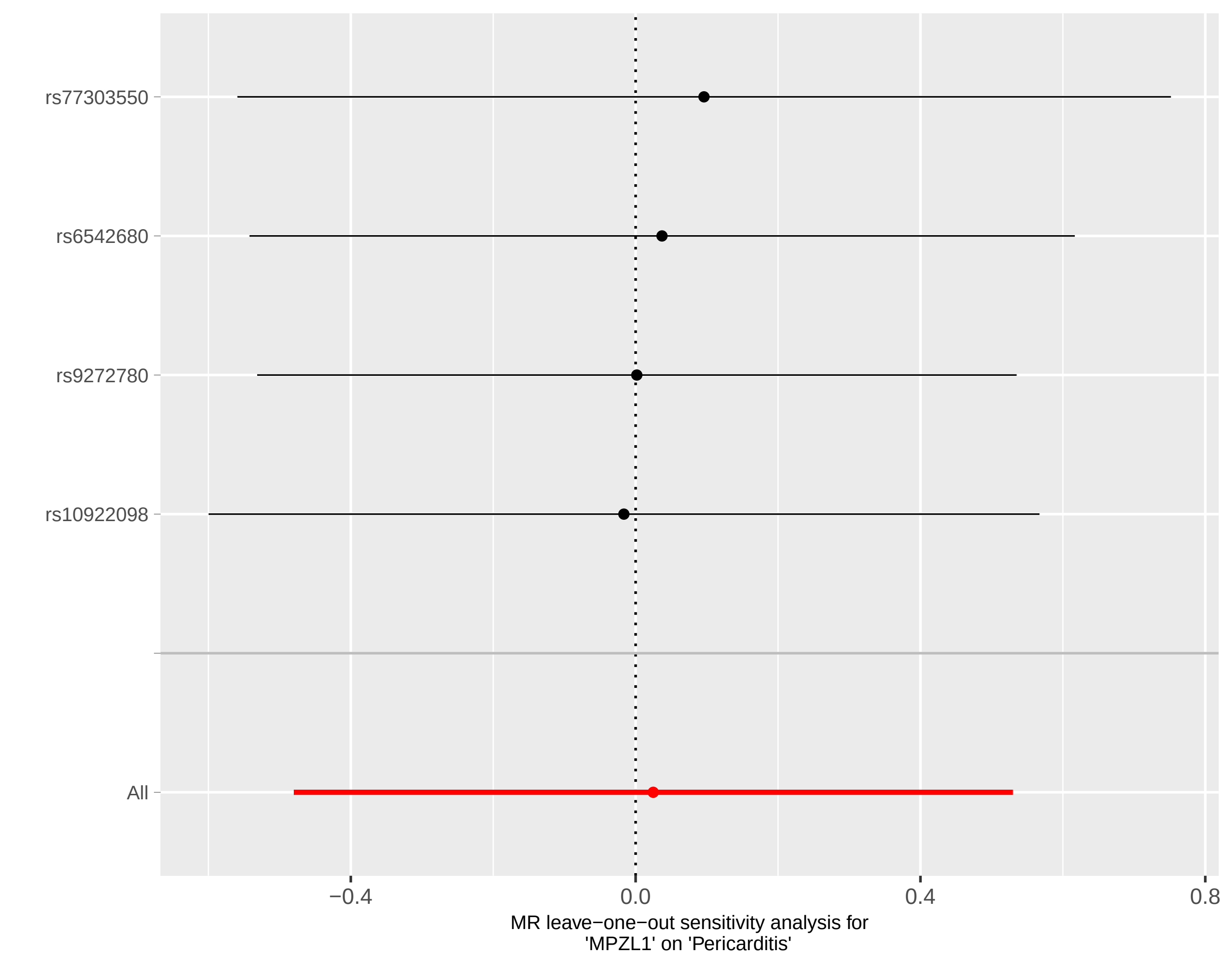

rs77303550
rs6542680
rs9272780
rs10922098
All
−0.4
0.0
0.4
0.8
MR leave−one−out sensitivity analysis for
'MPZL1' on 'Pericarditis'

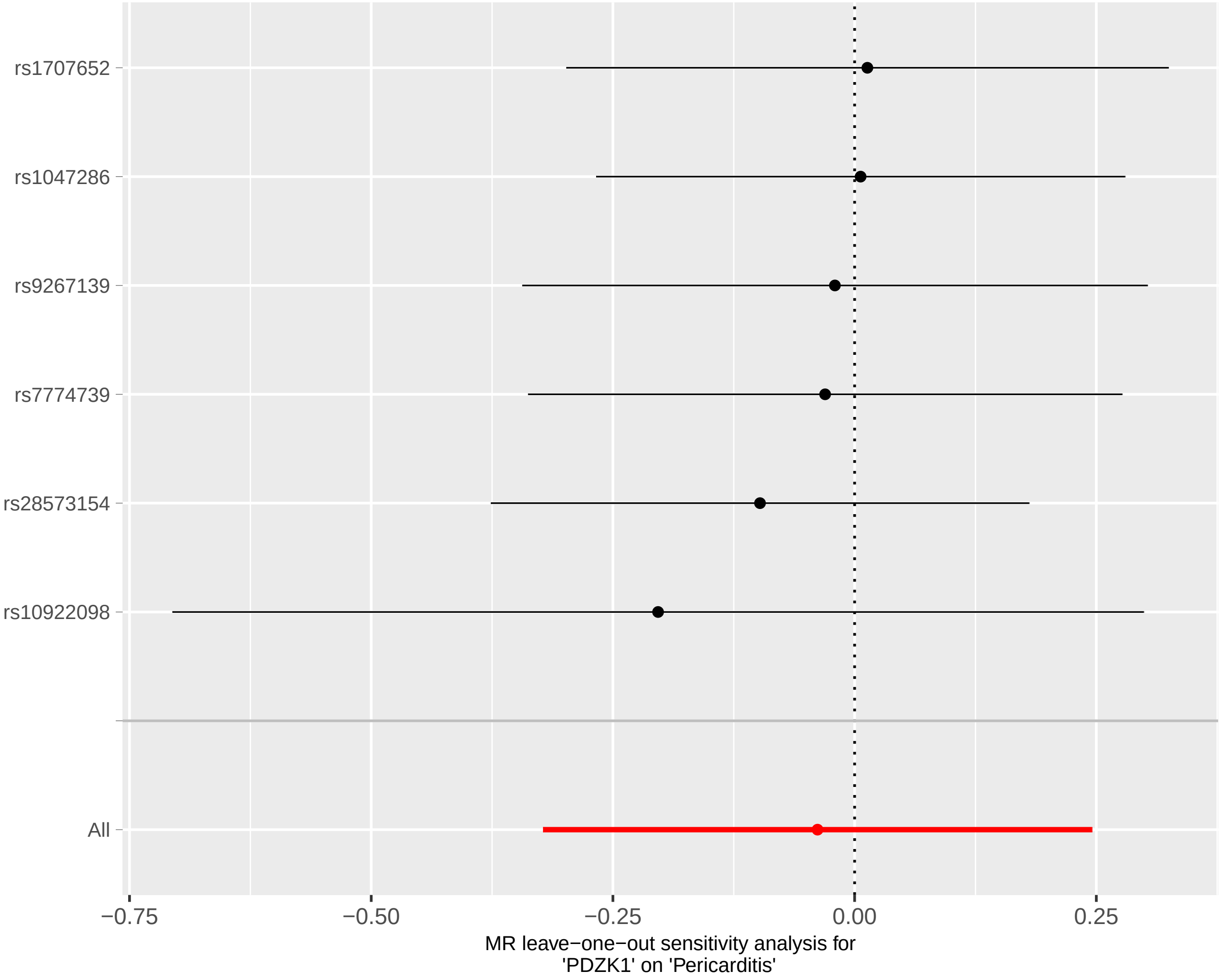
rs1707652
rs1047286
rs9267139
rs7774739
rs28573154
rs10922098
All
−0.75
−0.50
−0.25
0.00
0.25
MR leave−one−out sensitivity analysis for
'PDZK1' on 'Pericarditis'

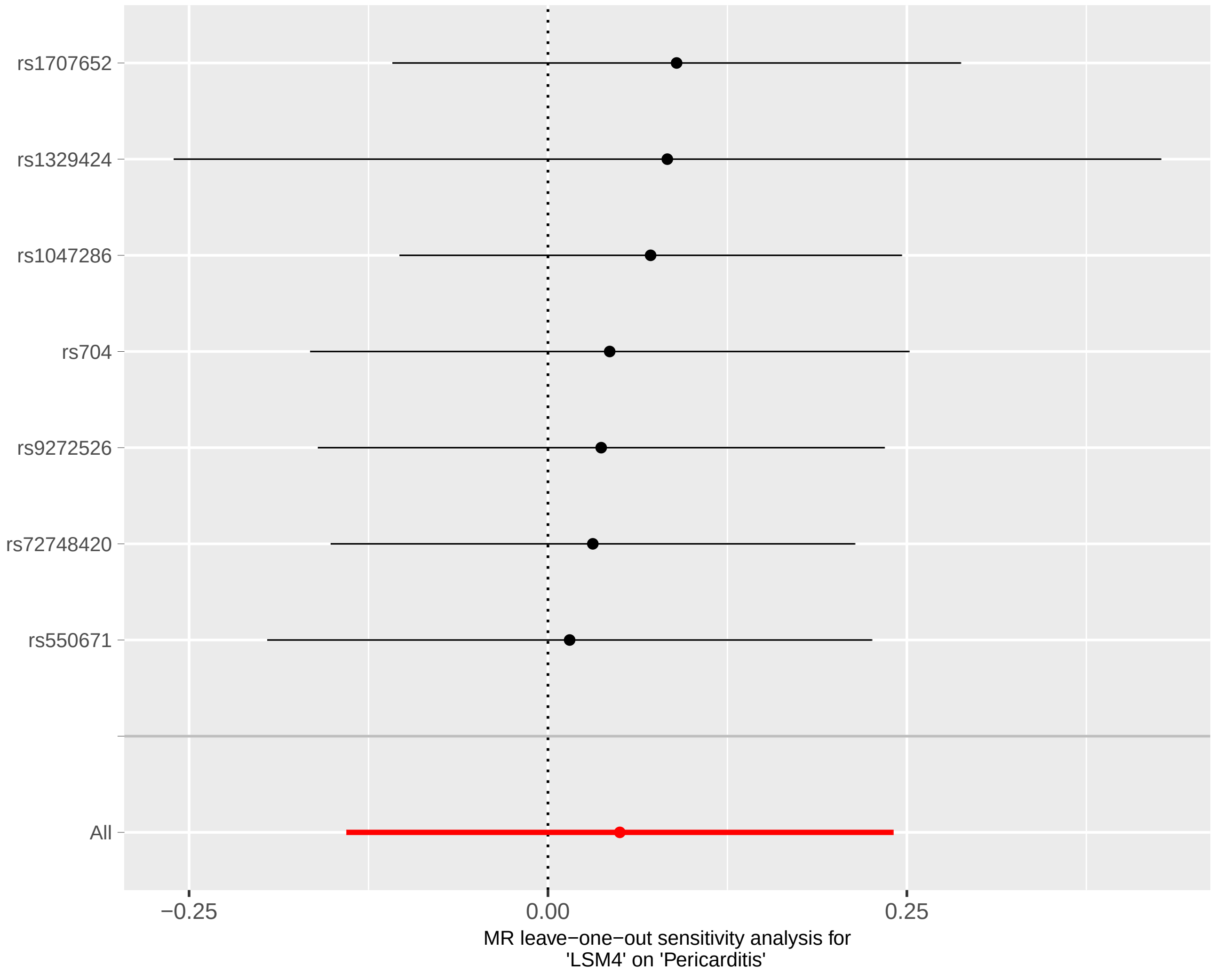
rs1707652
rs1329424
rs1047286
rs704
rs9272526
rs72748420
rs550671
All
−0.25
0.00
0.25
MR leave−one−out sensitivity analysis for
'LSM4' on 'Pericarditis'

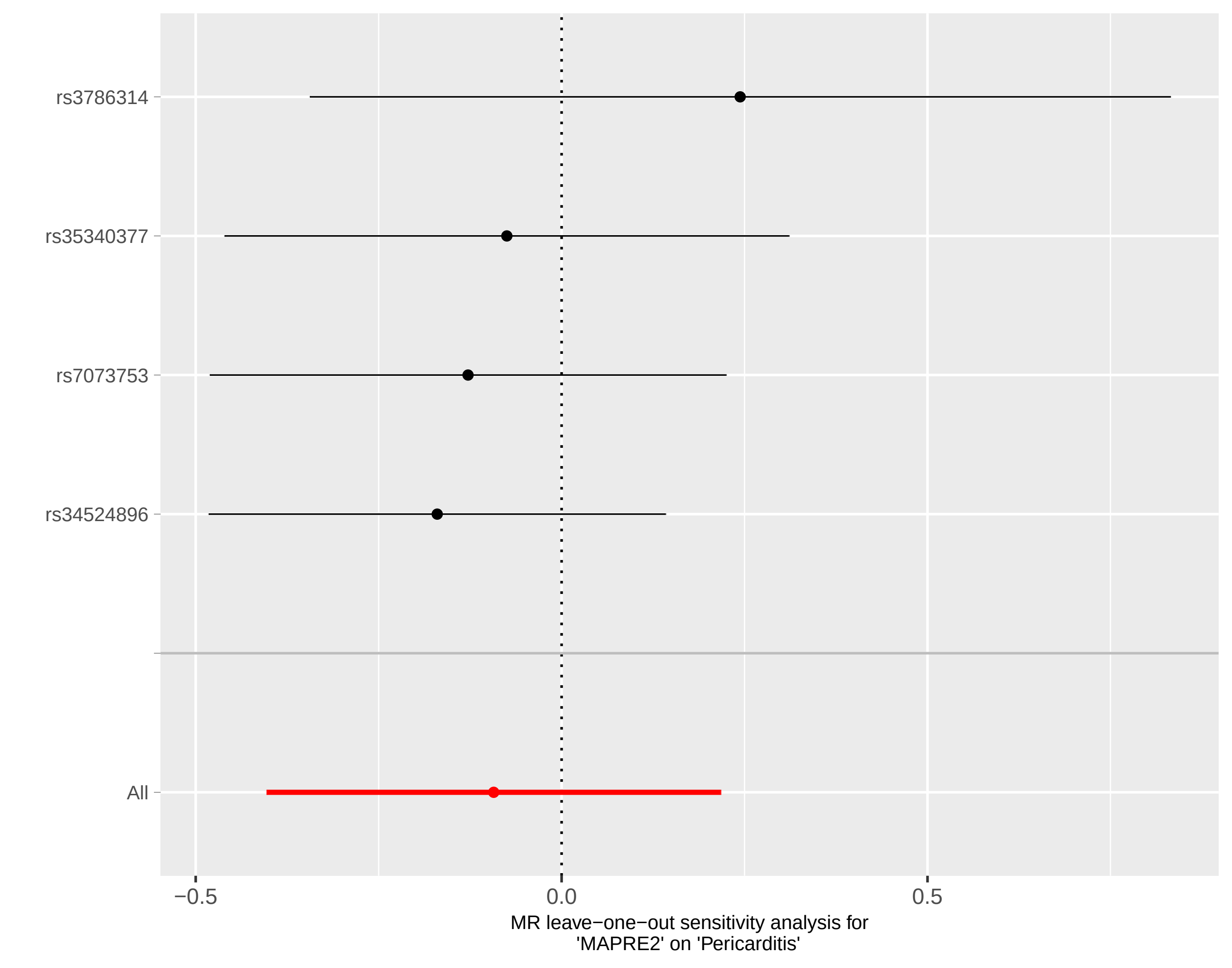

rs3786314
rs35340377
rs7073753
rs34524896
All
−0.5
0.0
0.5
MR leave−one−out sensitivity analysis for
'MAPRE2' on 'Pericarditis'

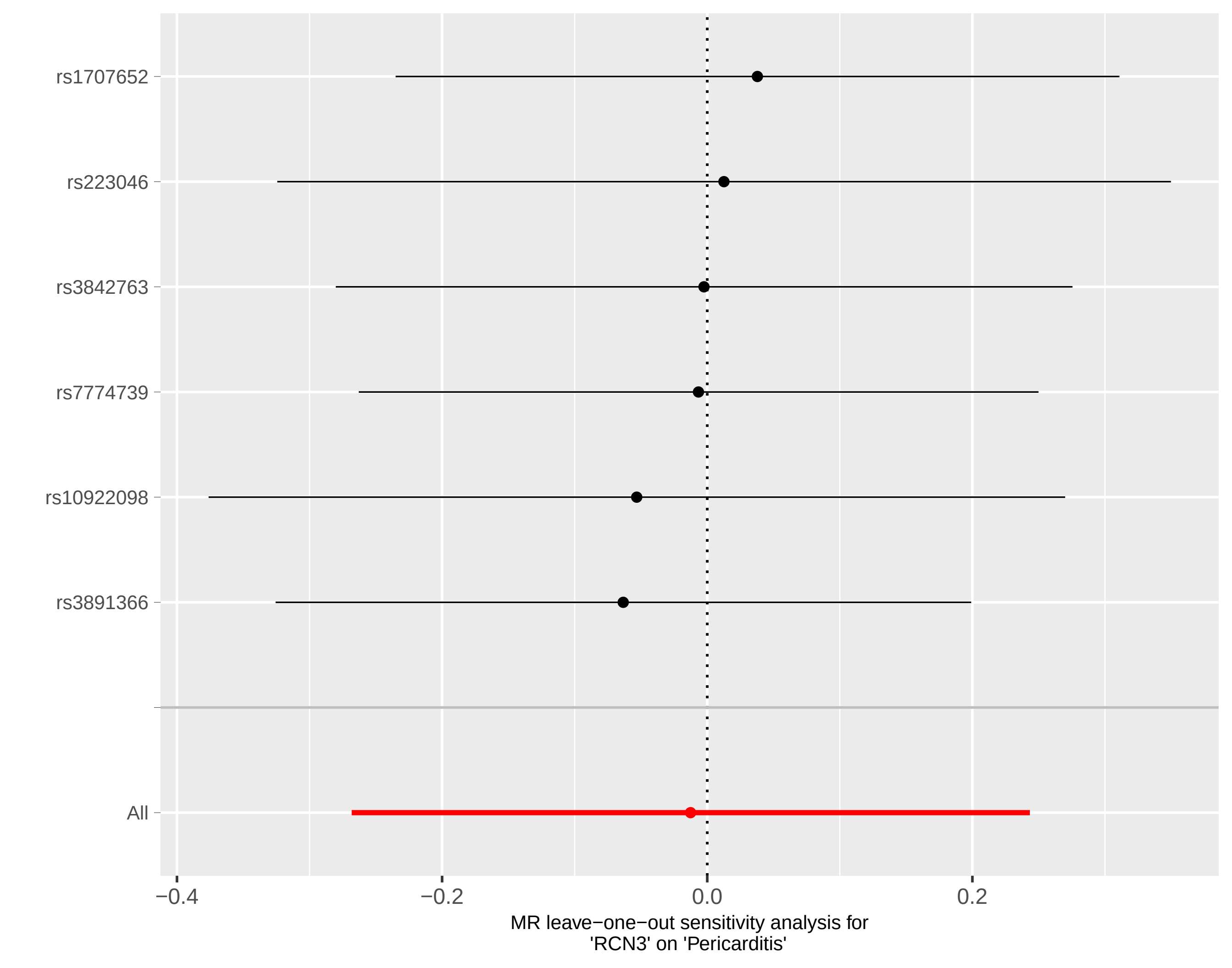

rs1707652
rs223046
rs3842763
rs7774739
rs10922098
rs3891366
All
−0.4
−0.2
0.0
0.2
MR leave−one−out sensitivity analysis for
'RCN3' on 'Pericarditis'

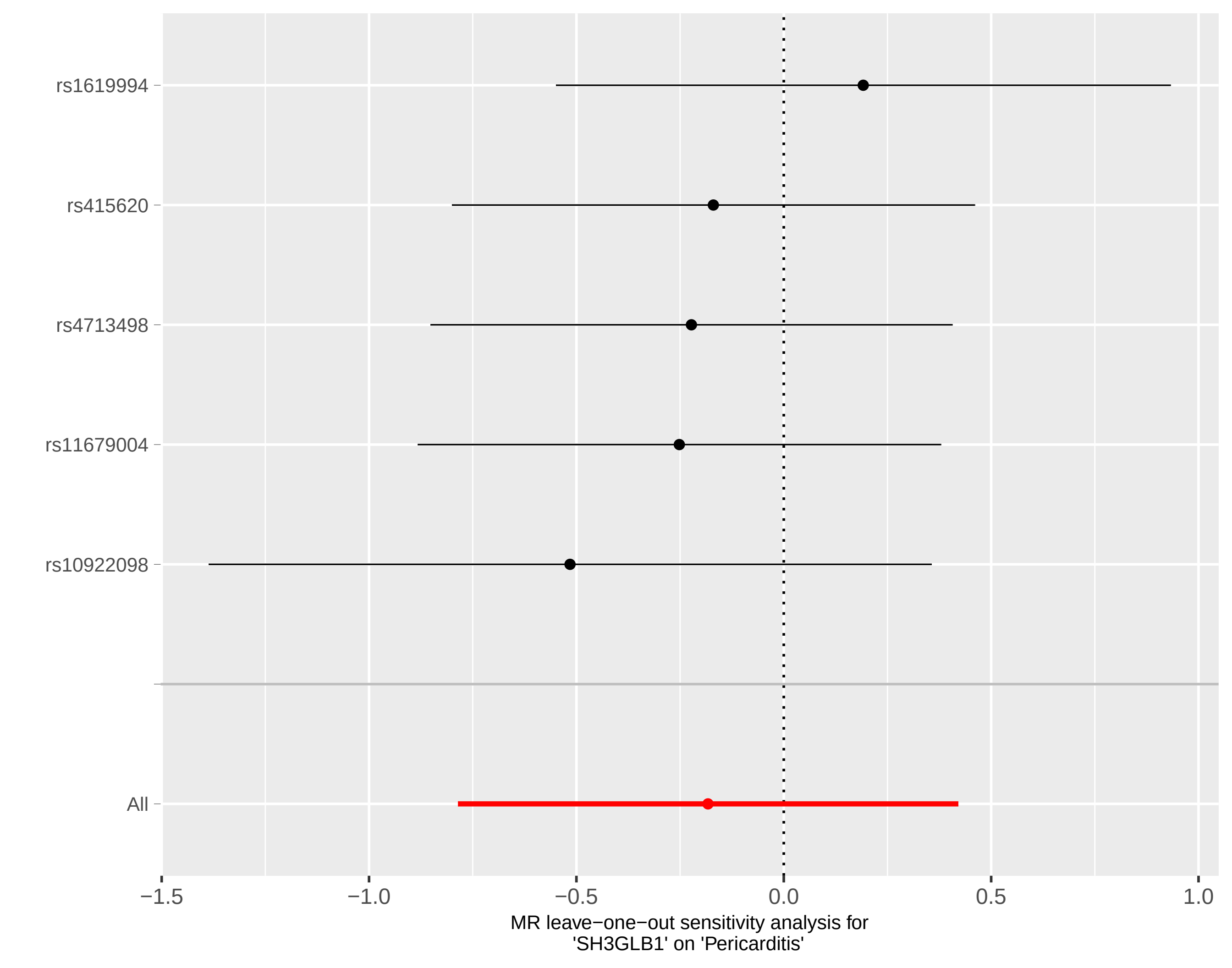

rs1619994
rs415620
rs4713498
rs11679004
rs10922098
All
−1.5
−1.0
−0.5
0.0
0.5
1.0
MR leave−one−out sensitivity analysis for
'SH3GLB1' on 'Pericarditis'

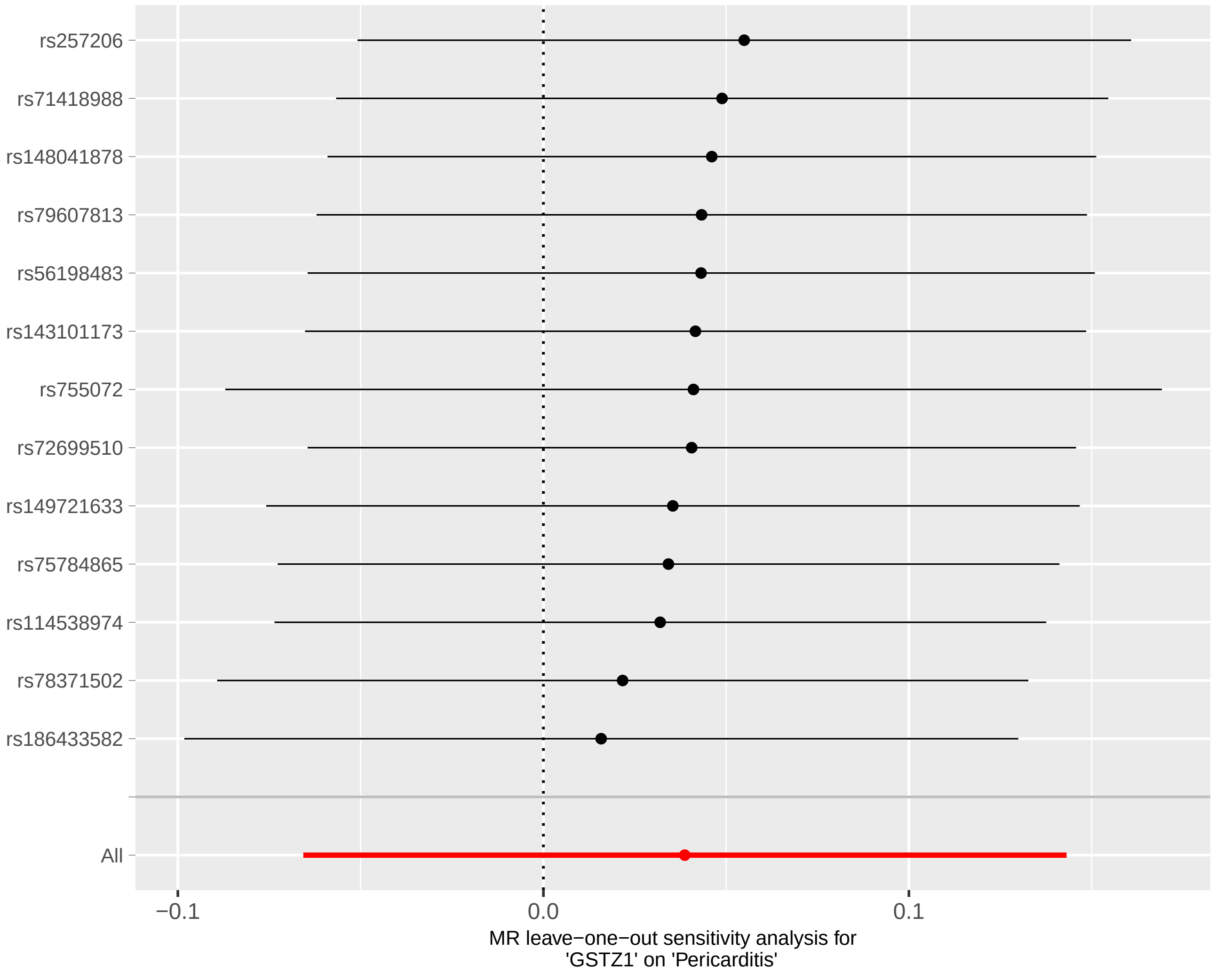
rs257206
rs71418988
rs148041878
rs79607813
rs56198483
rs143101173
rs755072
rs72699510
rs149721633
rs75784865
rs114538974
rs78371502
rs186433582
All
−0.1
0.0
0.1
MR leave−one−out sensitivity analysis for
'GSTZ1' on 'Pericarditis'

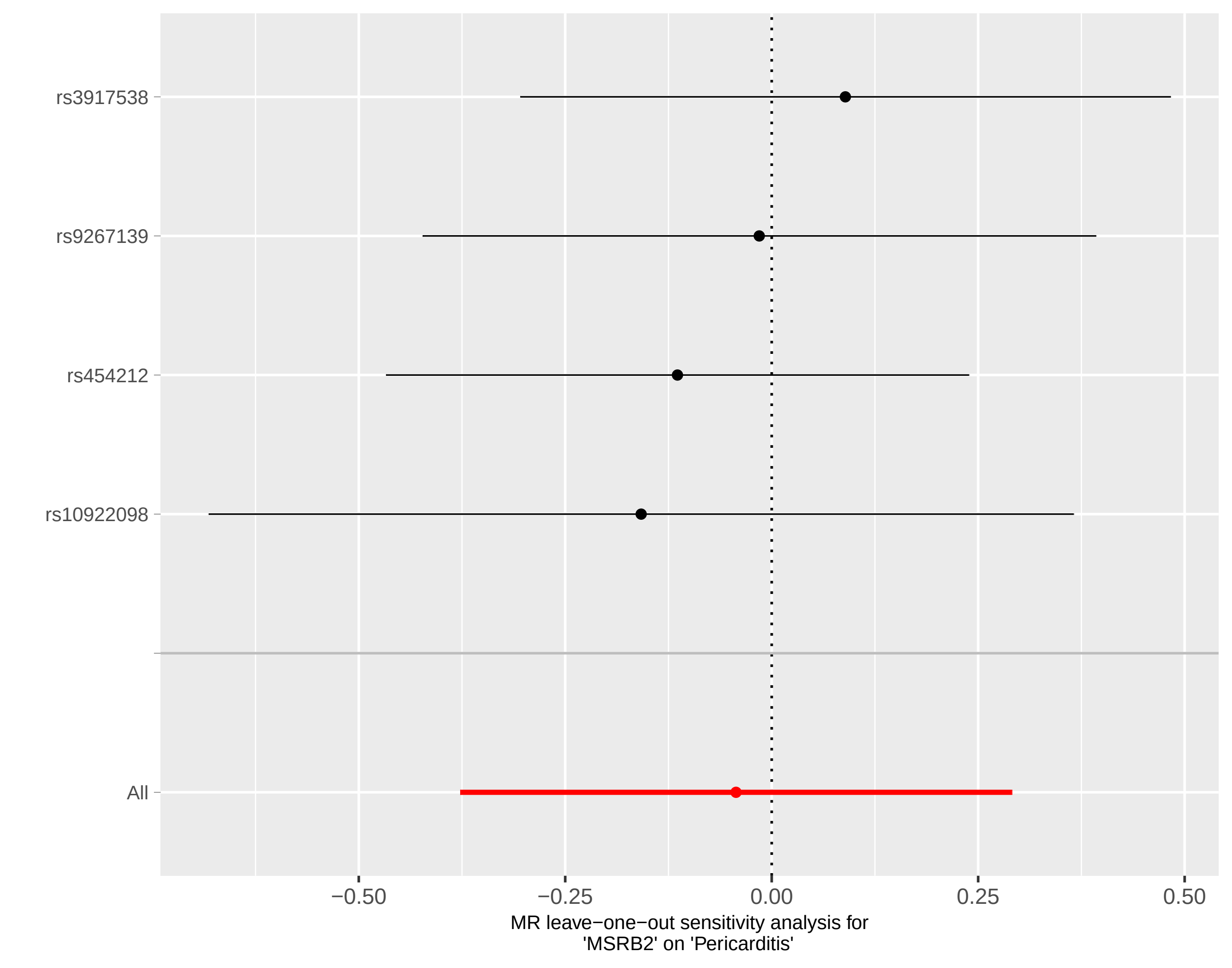

rs3917538
rs9267139
rs454212
rs10922098
All
−0.50
−0.25
0.00
0.25
0.50
MR leave−one−out sensitivity analysis for
'MSRB2' on 'Pericarditis'

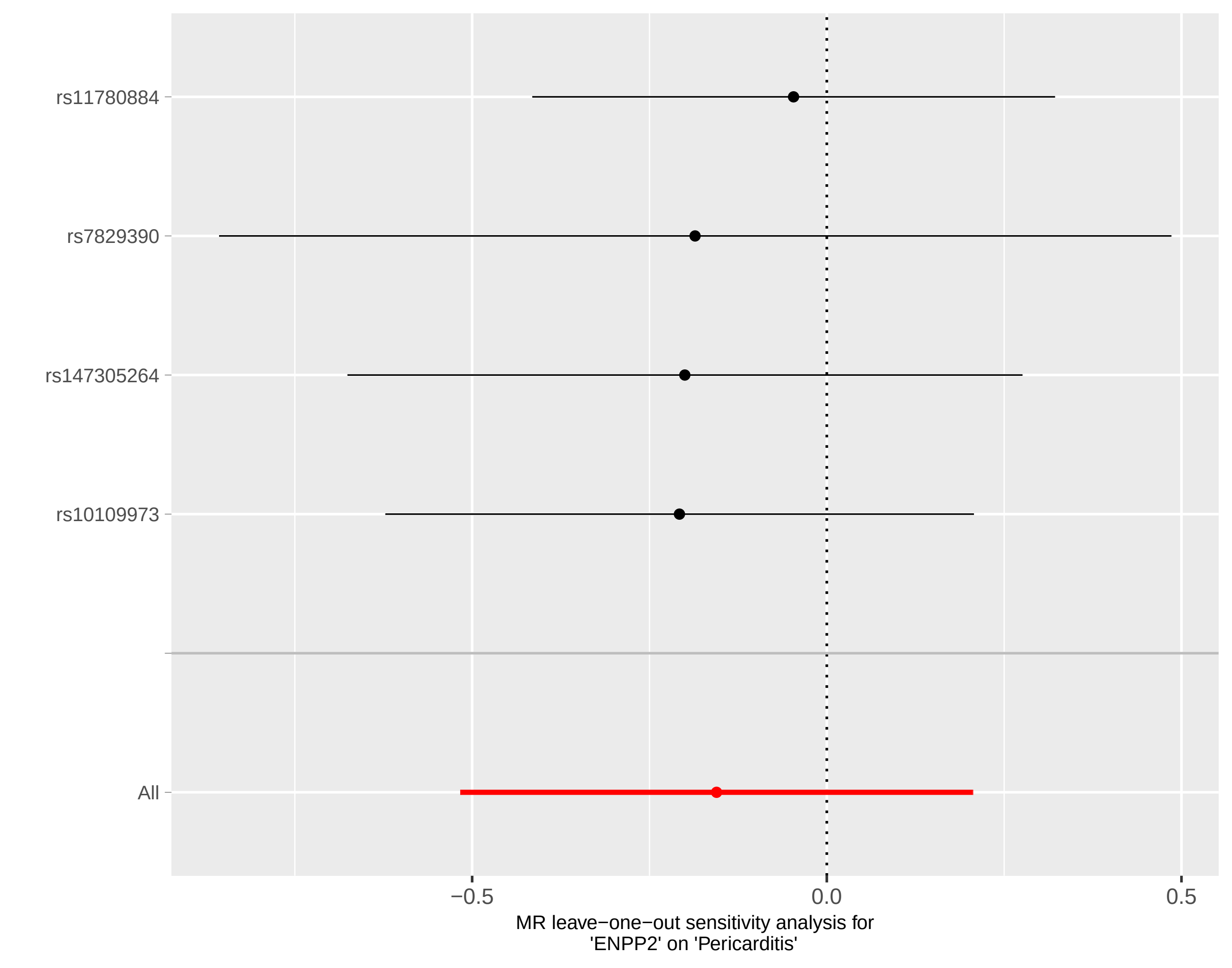
rs11780884
rs7829390
rs147305264
rs10109973
All
−0.5
0.0
0.5
MR leave−one−out sensitivity analysis for
'ENPP2' on 'Pericarditis'

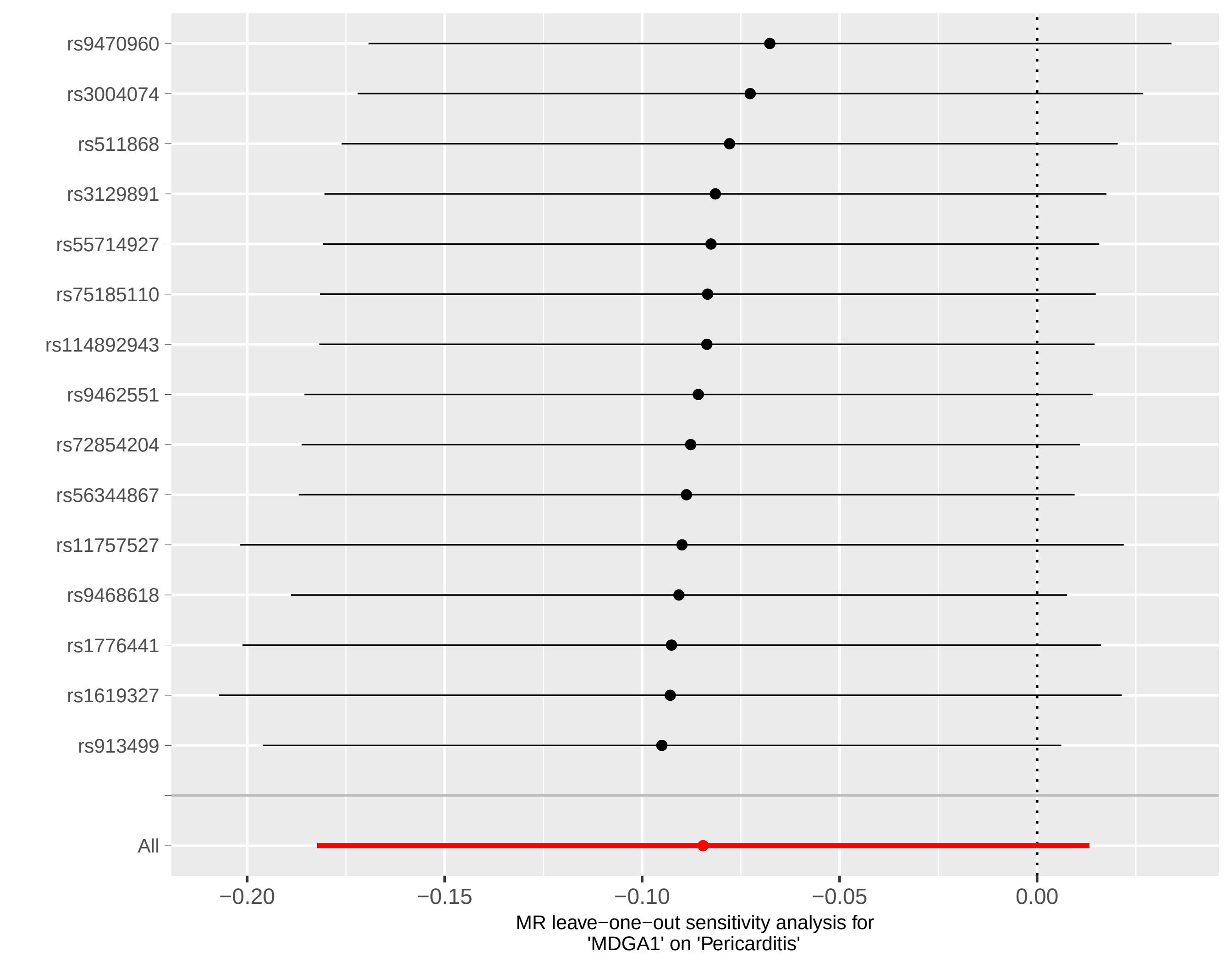
rs9470960
rs3004074
rs511868
rs3129891
rs55714927
rs75185110
rs114892943
rs9462551
rs72854204
rs56344867
rs11757527
rs9468618
rs1776441
rs1619327
rs913499
All
−0.20
−0.15
−0.10
−0.05
0.00
MR leave−one−out sensitivity analysis for
'MDGA1' on 'Pericarditis'

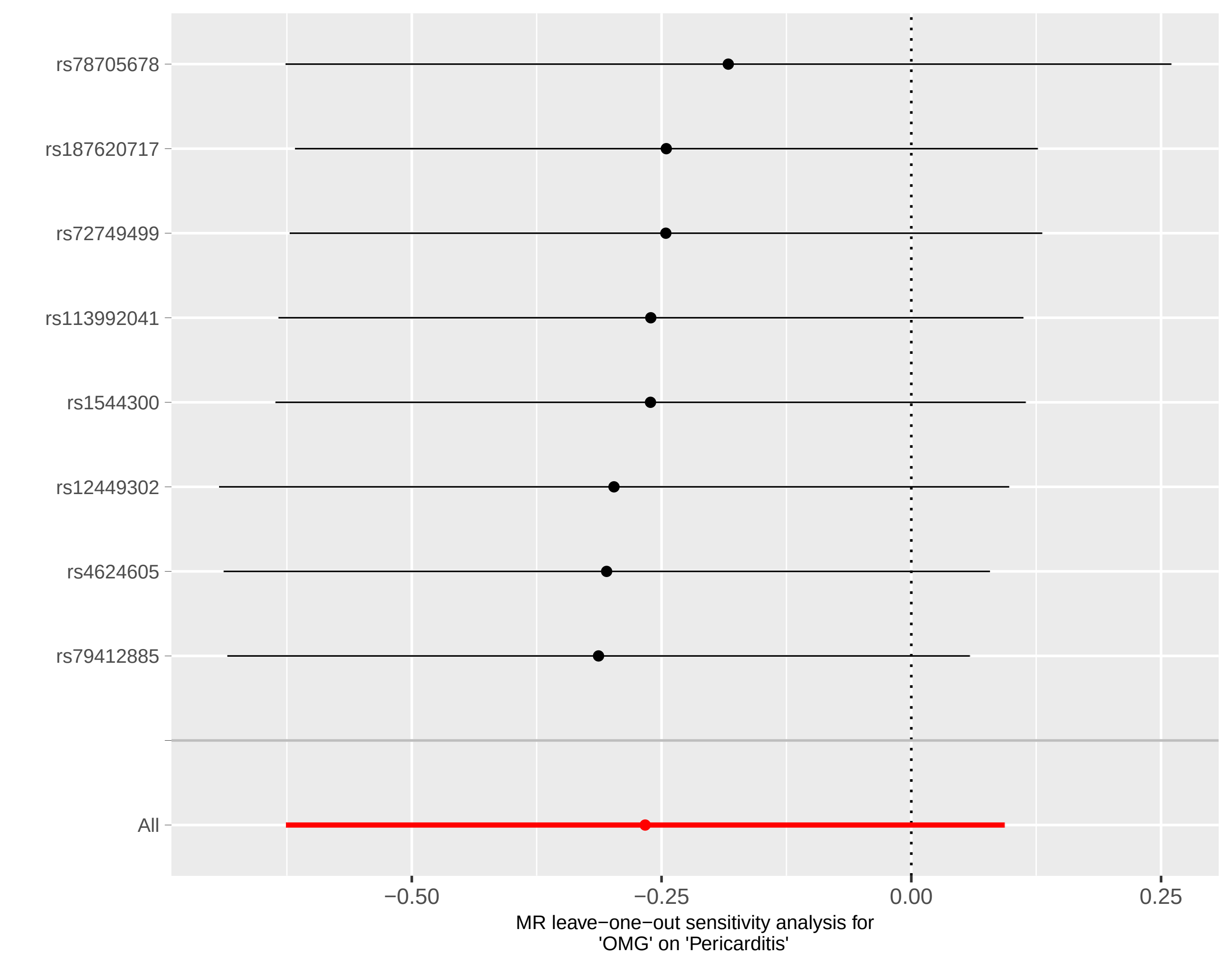

rs78705678
rs187620717
rs72749499
rs113992041
rs1544300
rs12449302
rs4624605
rs79412885
All
−0.50
−0.25
0.00
0.25
MR leave−one−out sensitivity analysis for
'OMG' on 'Pericarditis'

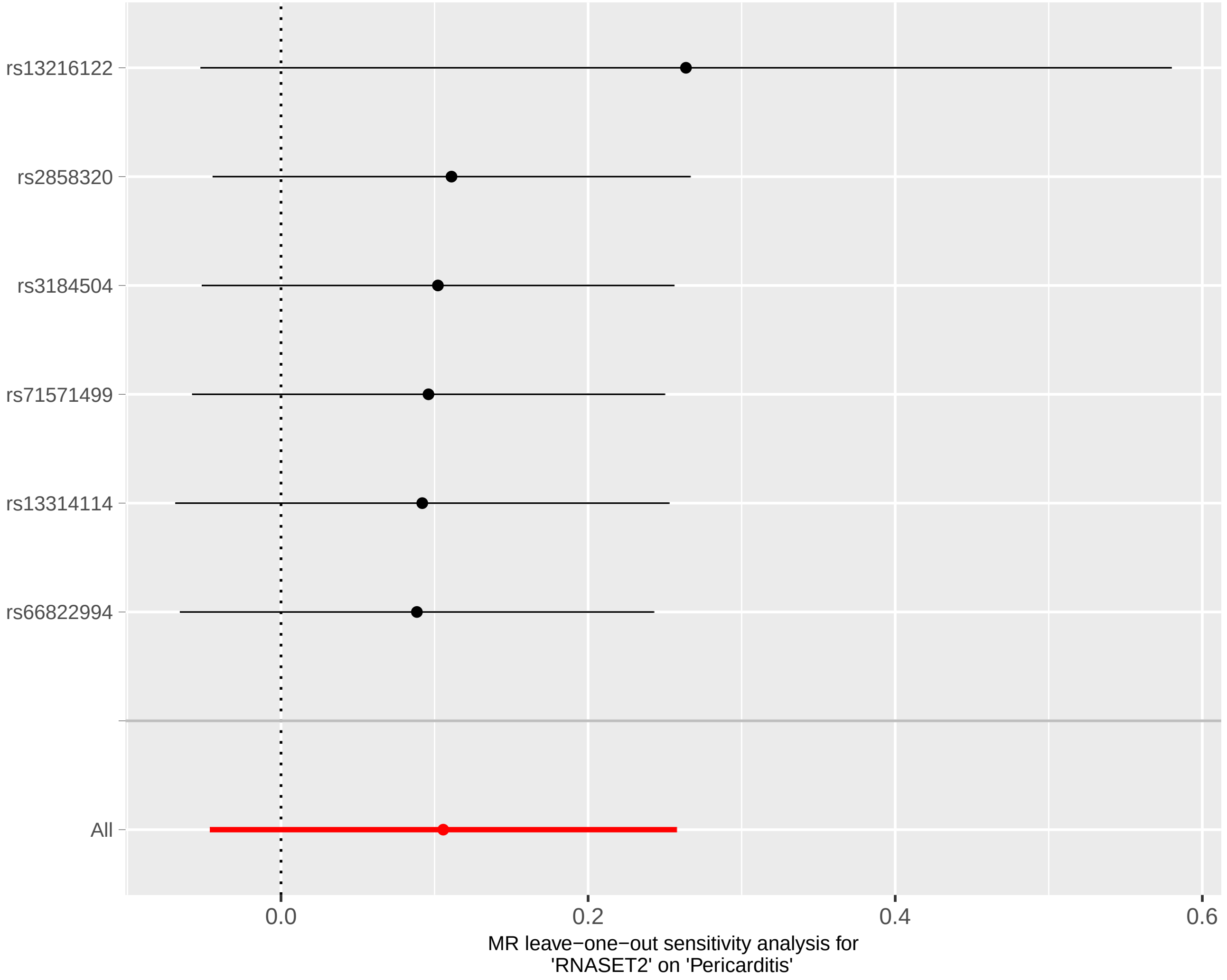

rs13216122
rs2858320
rs3184504
rs71571499
rs13314114
rs66822994
All
0.0
0.2
0.4
0.6
MR leave−one−out sensitivity analysis for
'RNASET2' on 'Pericarditis'

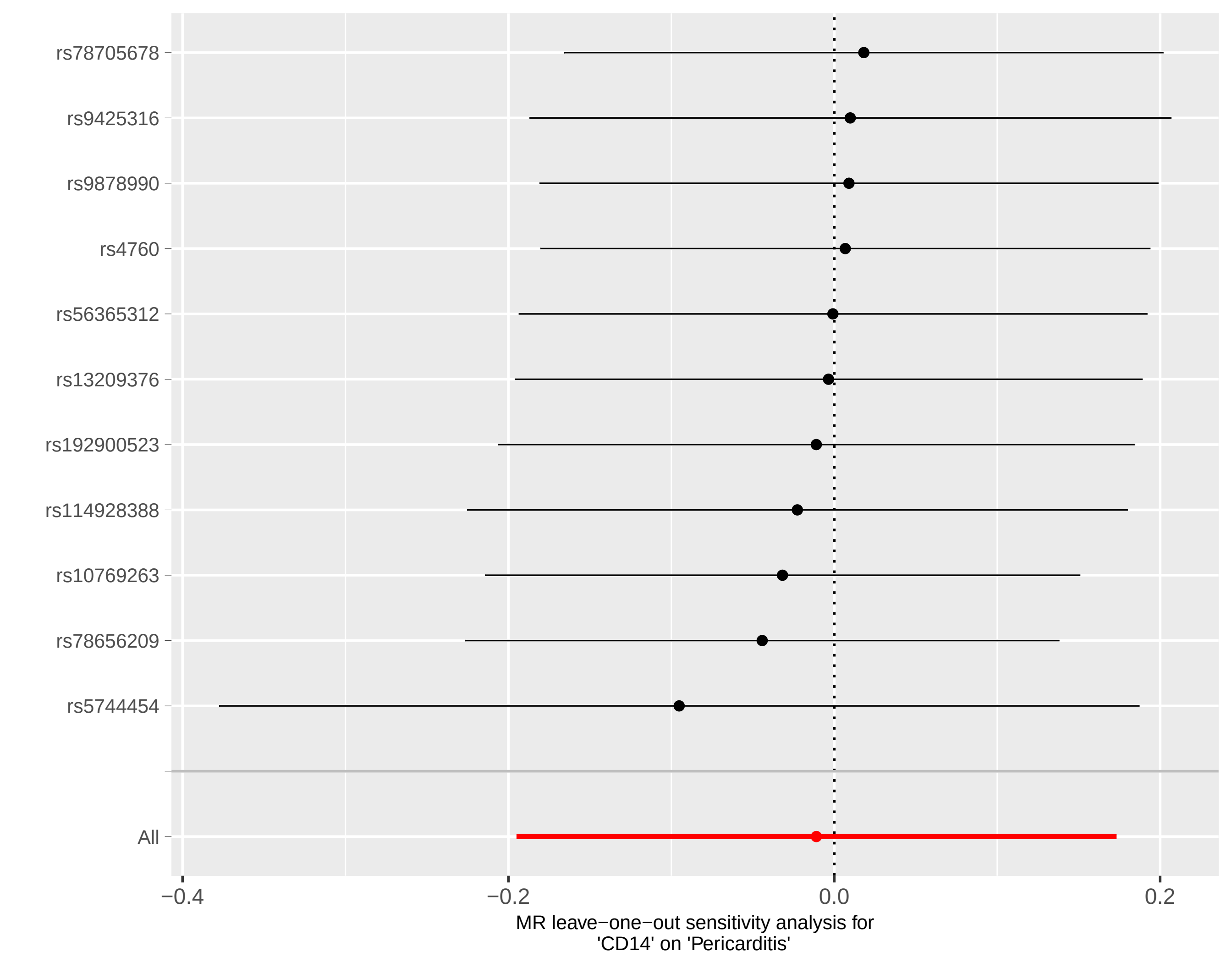

rs78705678
rs9425316
rs9878990
rs4760
rs56365312
rs13209376
rs192900523
rs114928388
rs10769263
rs78656209
rs5744454
All
−0.4
−0.2
0.0
0.2
MR leave−one−out sensitivity analysis for
'CD14' on 'Pericarditis'

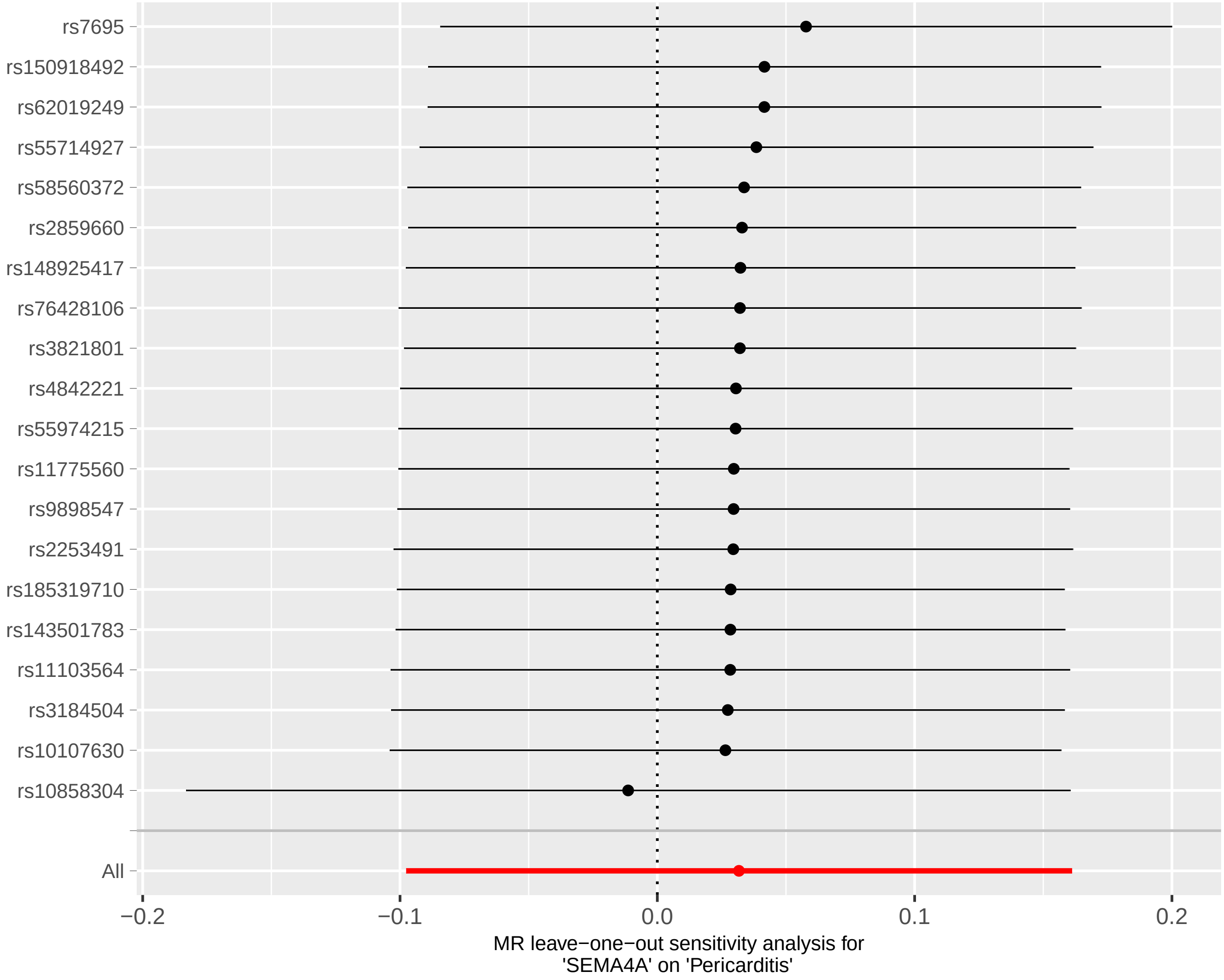
rs7695
rs150918492
rs62019249
rs55714927
rs58560372
rs2859660
rs148925417
rs76428106
rs3821801
rs4842221
rs55974215
rs11775560
rs9898547
rs2253491
rs185319710
rs143501783
rs11103564
rs3184504
rs10107630
rs10858304
All
−0.2
−0.1
0.0
0.1
0.2
MR leave−one−out sensitivity analysis for
'SEMA4A' on 'Pericarditis'

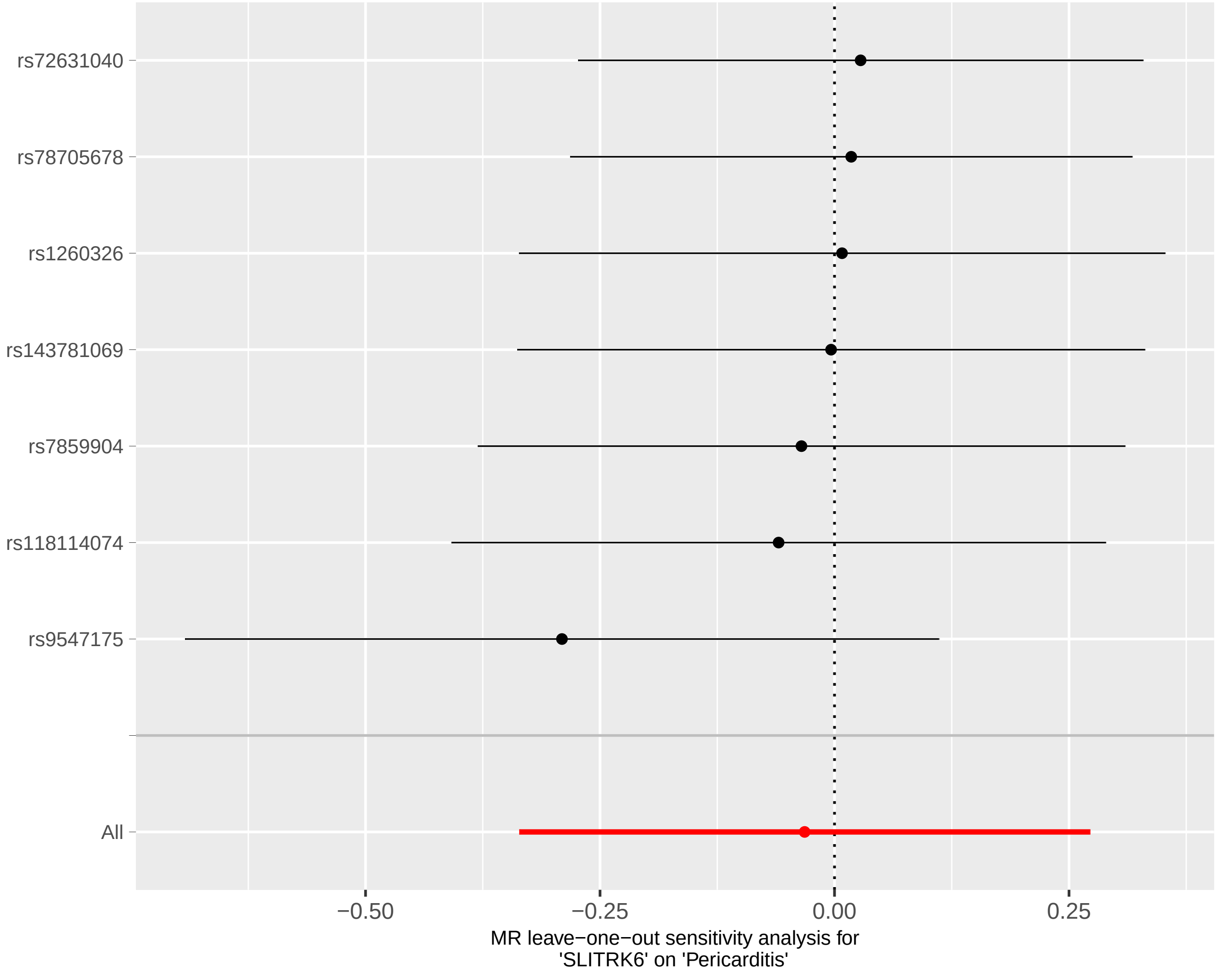

rs72631040
rs78705678
rs1260326
rs143781069
rs7859904
rs118114074
rs9547175
All
−0.50
−0.25
0.00
0.25
MR leave−one−out sensitivity analysis for
'SLITRK6' on 'Pericarditis'

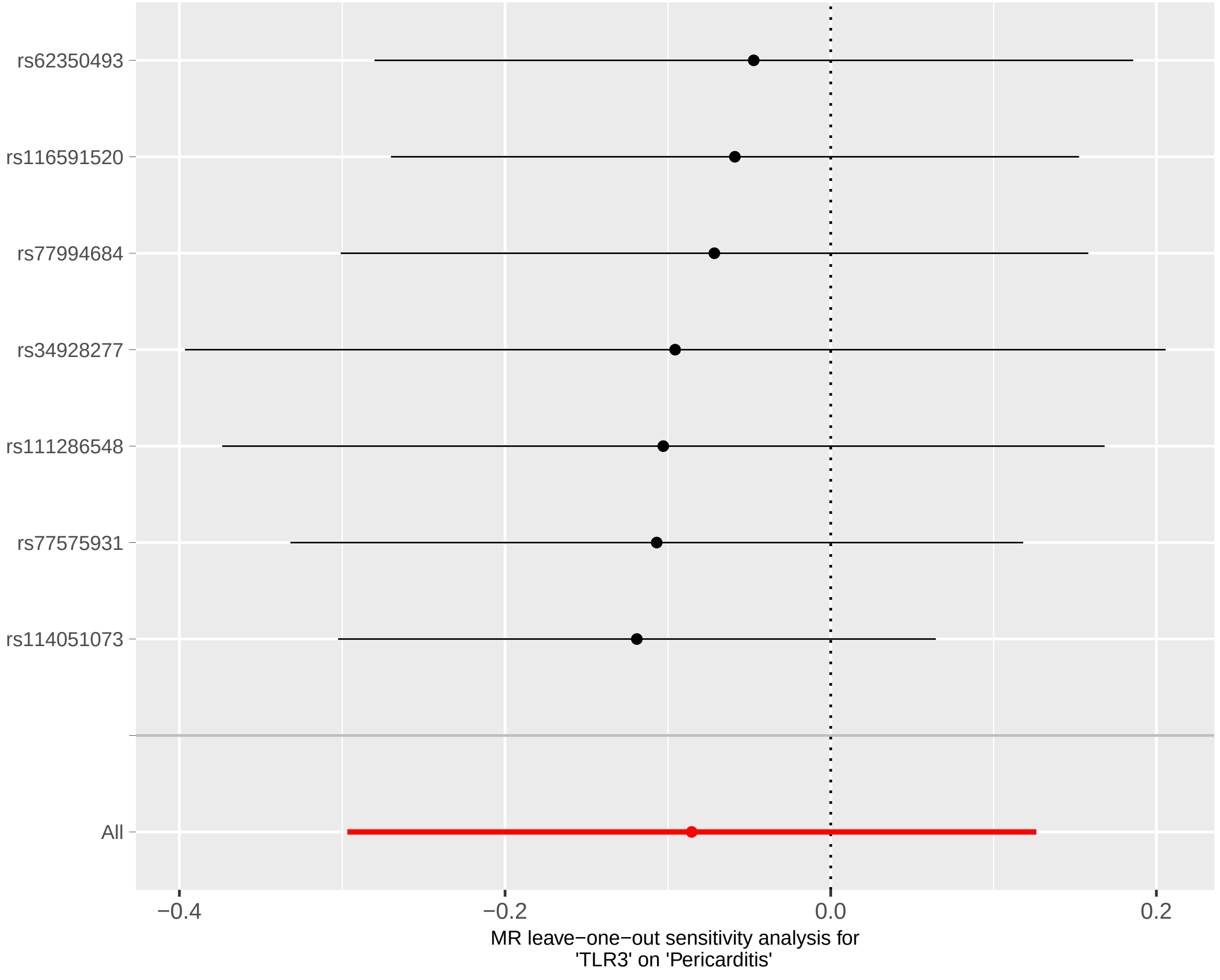

rs62350493
rs116591520
rs77994684
rs34928277
rs111286548
rs77575931
rs114051073
All
−0.4
−0.2
0.0
0.2
MR leave−one−out sensitivity analysis for
'TLR3' on 'Pericarditis'

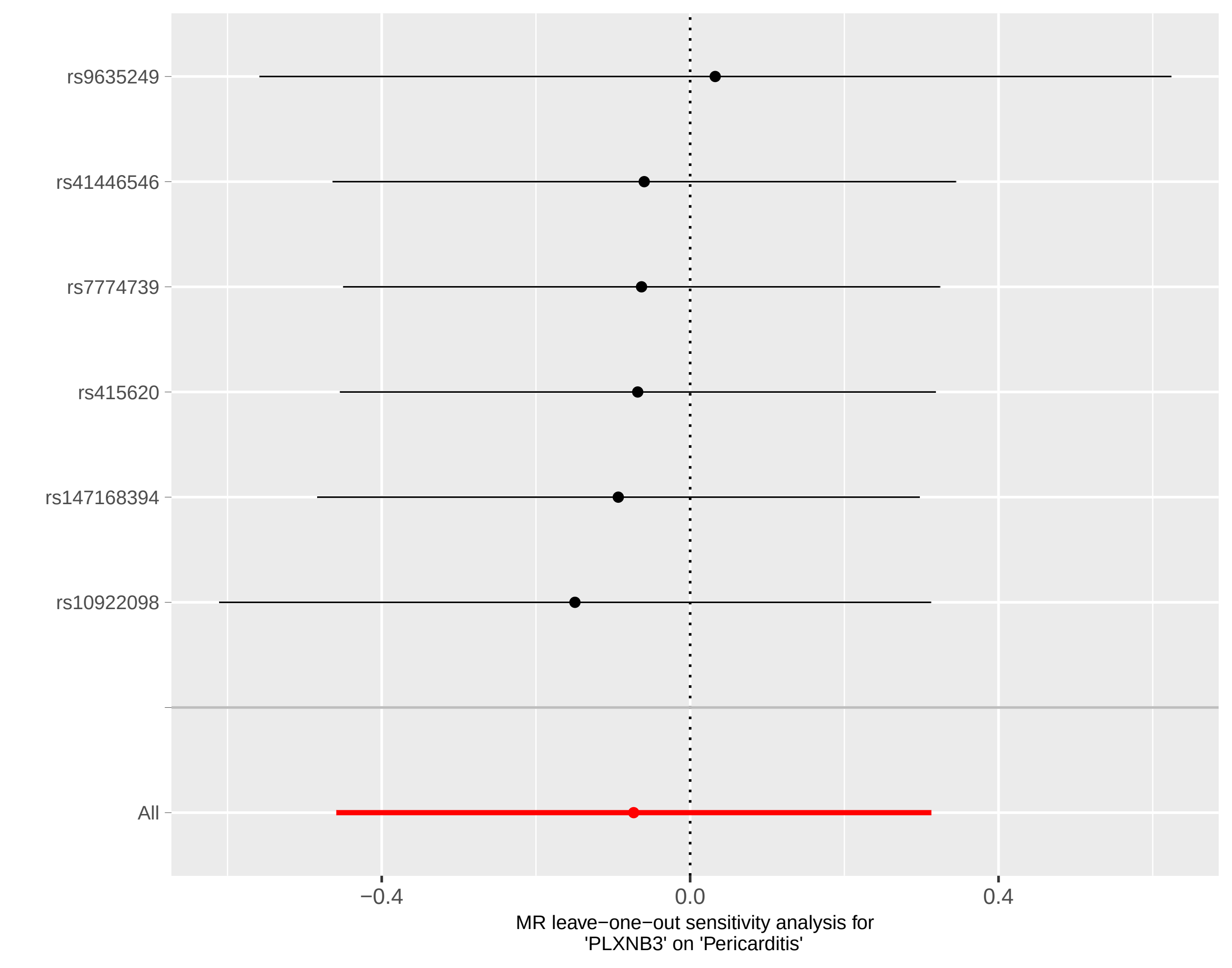

rs9635249
rs41446546
rs7774739
rs415620
rs147168394
rs10922098
All
−0.4
0.0
0.4
MR leave−one−out sensitivity analysis for
'PLXNB3' on 'Pericarditis'

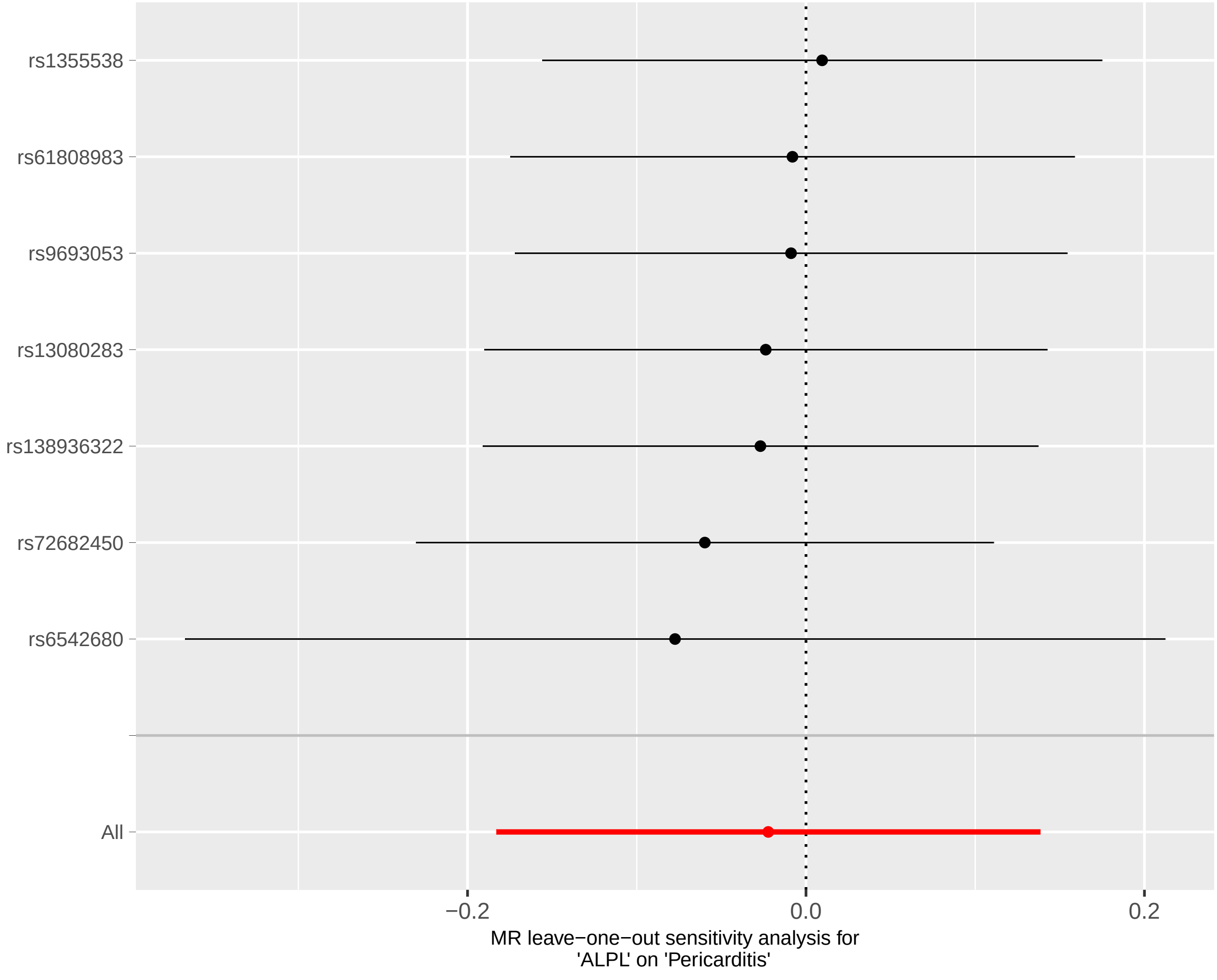
rs1355538
rs61808983
rs9693053
rs13080283
rs138936322
rs72682450
rs6542680
All
−0.2
0.0
0.2
MR leave−one−out sensitivity analysis for
'ALPL' on 'Pericarditis'

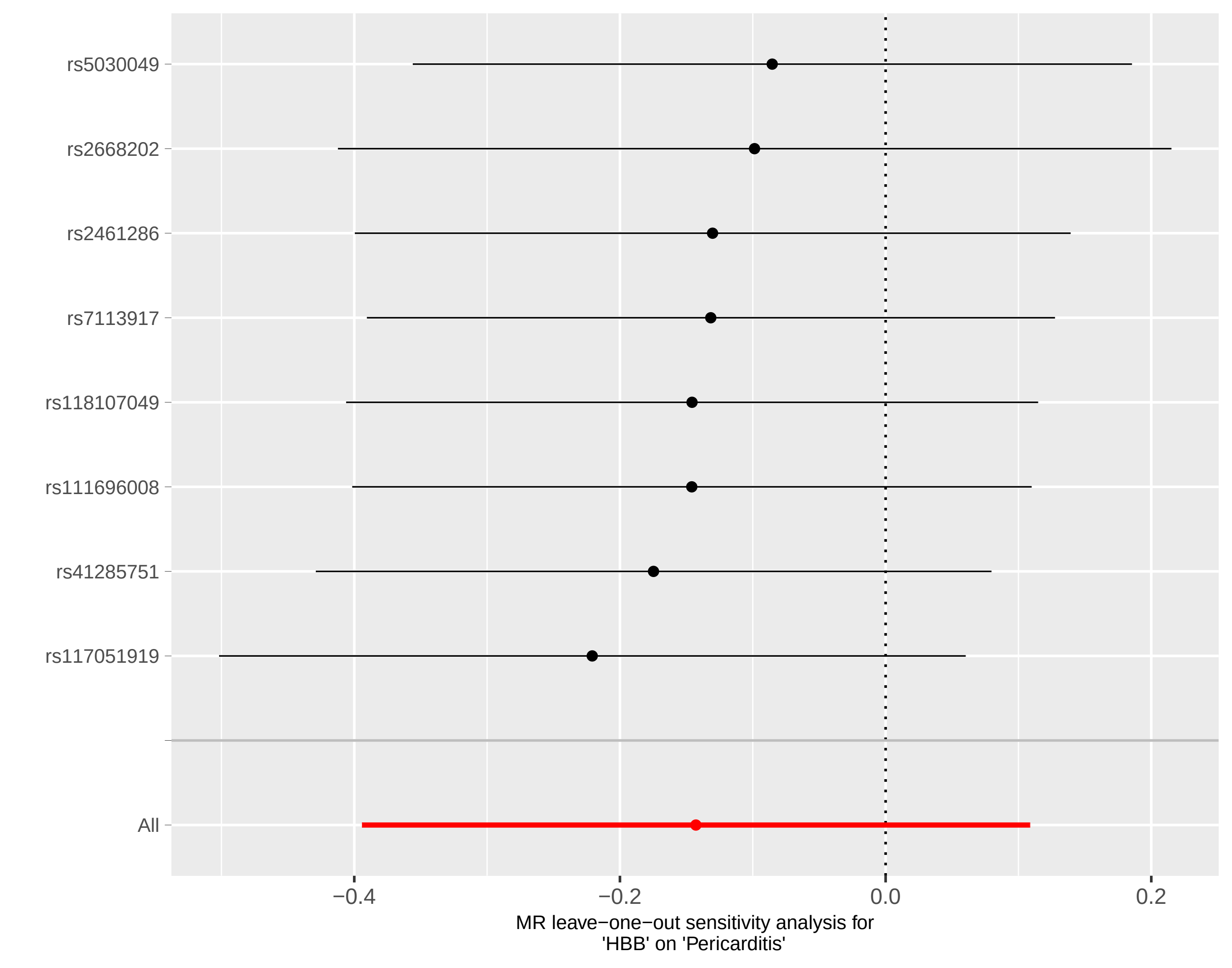
rs5030049
rs2668202
rs2461286
rs7113917
rs118107049
rs111696008
rs41285751
rs117051919
All
−0.4
−0.2
0.0
0.2
MR leave−one−out sensitivity analysis for
'HBB' on 'Pericarditis'

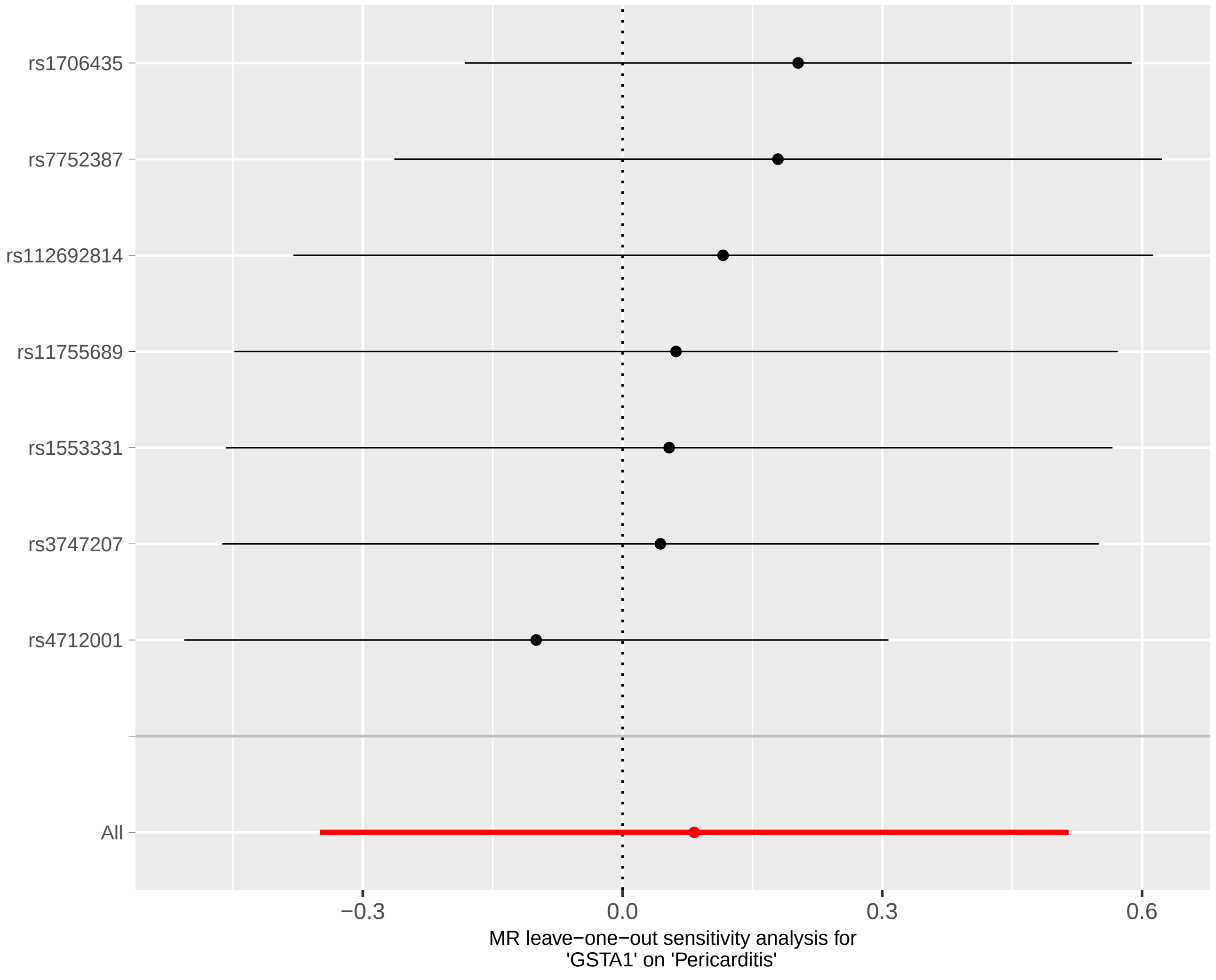
rs1706435
rs7752387
rs112692814
rs11755689
rs1553331
rs3747207
rs4712001
All
−0.3
0.0
0.3
0.6
MR leave−one−out sensitivity analysis for
'GSTA1' on 'Pericarditis'

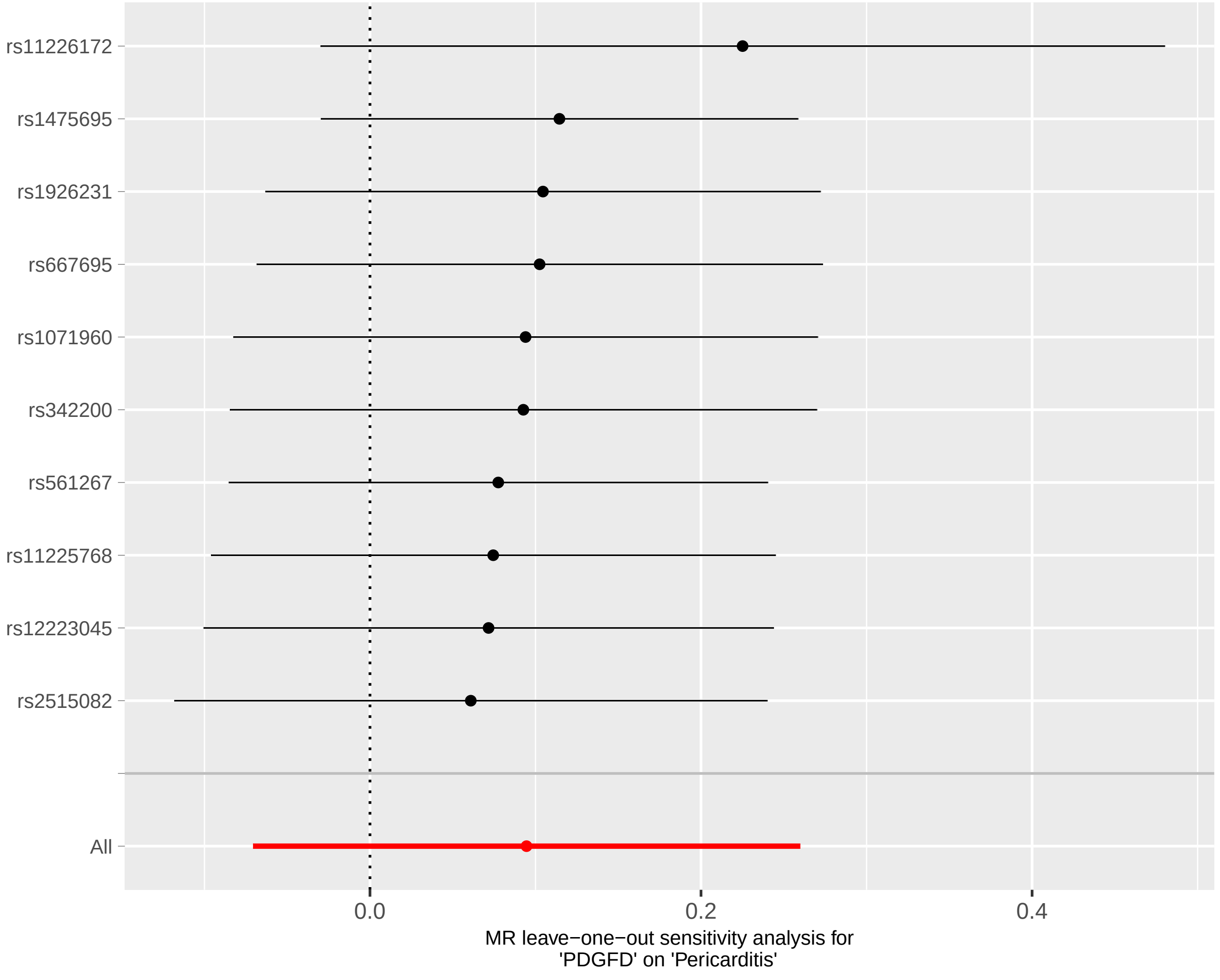

rs11226172
rs1475695
rs1926231
rs667695
rs1071960
rs342200
rs561267
rs11225768
rs12223045
rs2515082
All
0.0
0.2
0.4
MR leave−one−out sensitivity analysis for
'PDGFD' on 'Pericarditis'

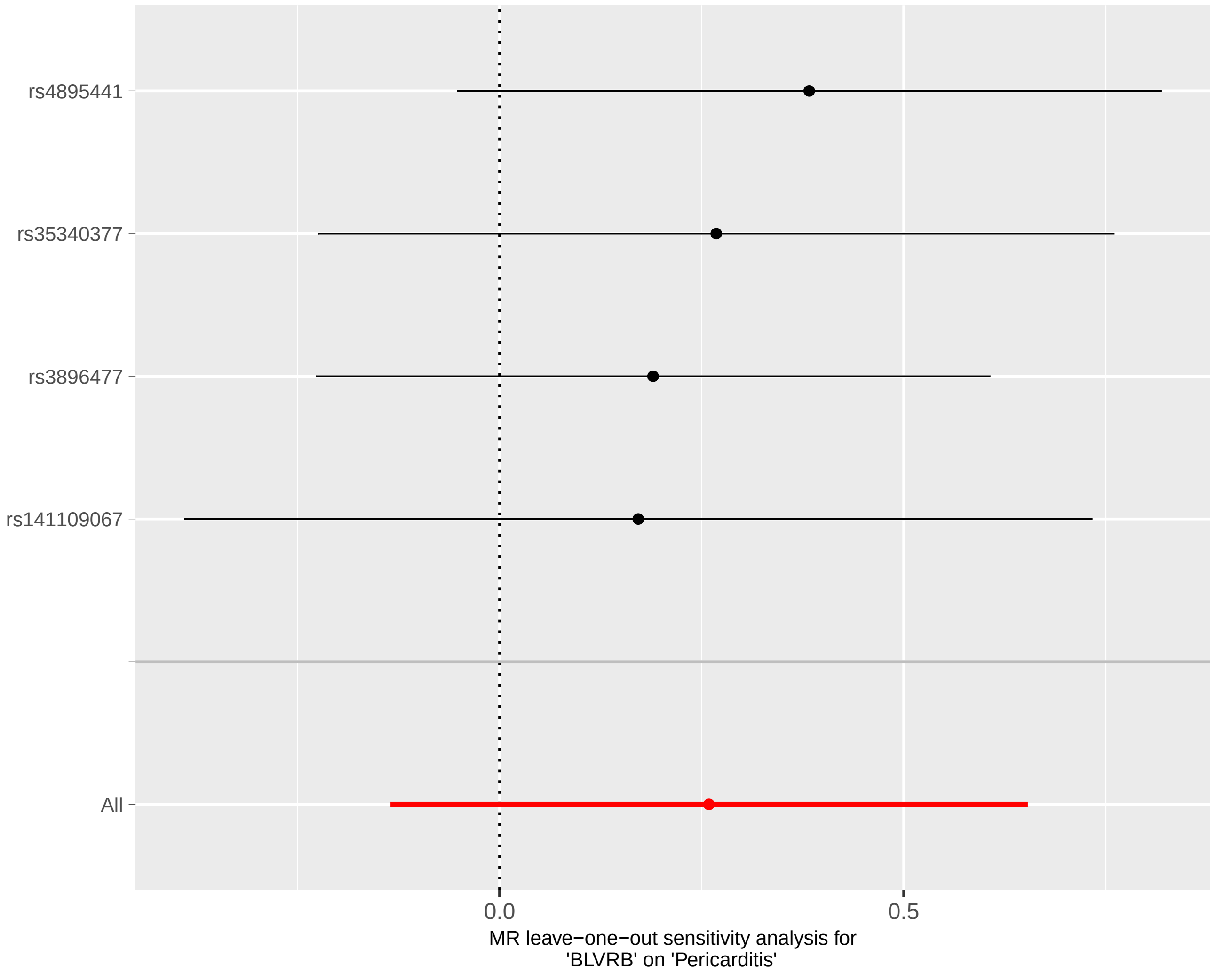
rs4895441
rs35340377
rs3896477
rs141109067
All
0.0
0.5
MR leave−one−out sensitivity analysis for
'BLVRB' on 'Pericarditis'

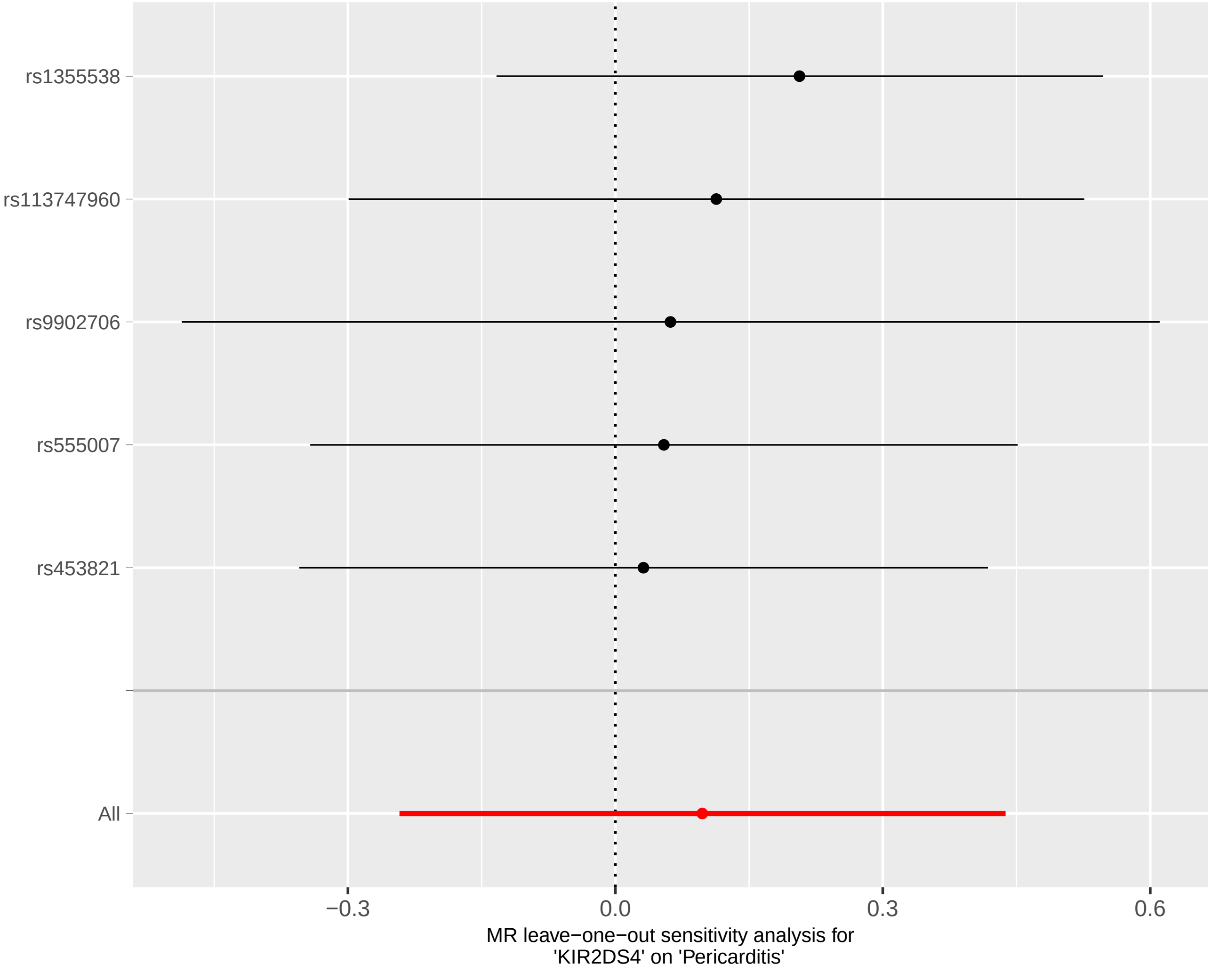
rs1355538
rs113747960
rs9902706
rs555007
rs453821
All
−0.3
0.0
0.3
0.6
MR leave−one−out sensitivity analysis for
'KIR2DS4' on 'Pericarditis'

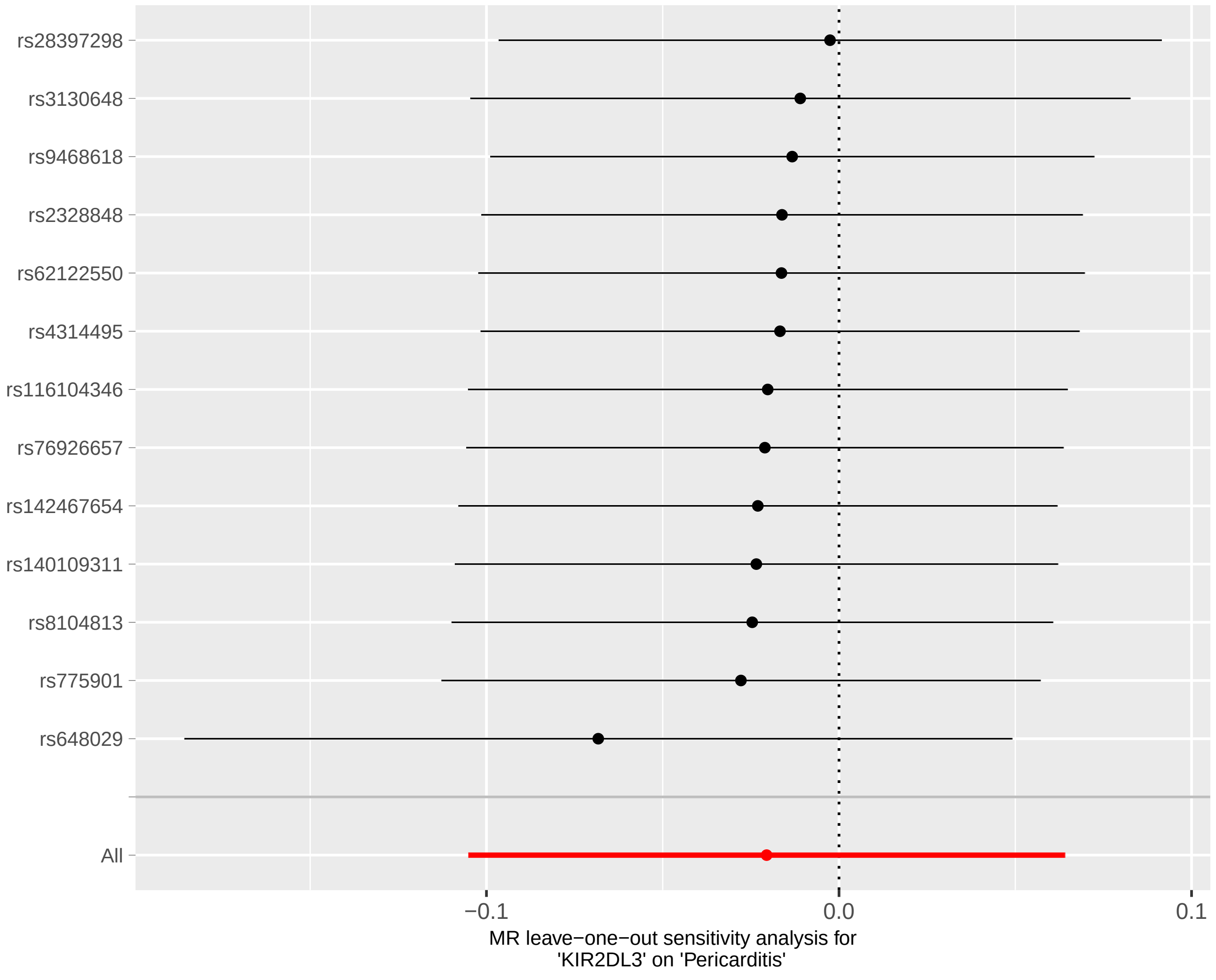
rs28397298
rs3130648
rs9468618
rs2328848
rs62122550
rs4314495
rs116104346
rs76926657
rs142467654
rs140109311
rs8104813
rs775901
rs648029
All
−0.1
0.0
0.1
MR leave−one−out sensitivity analysis for
'KIR2DL3' on 'Pericarditis'

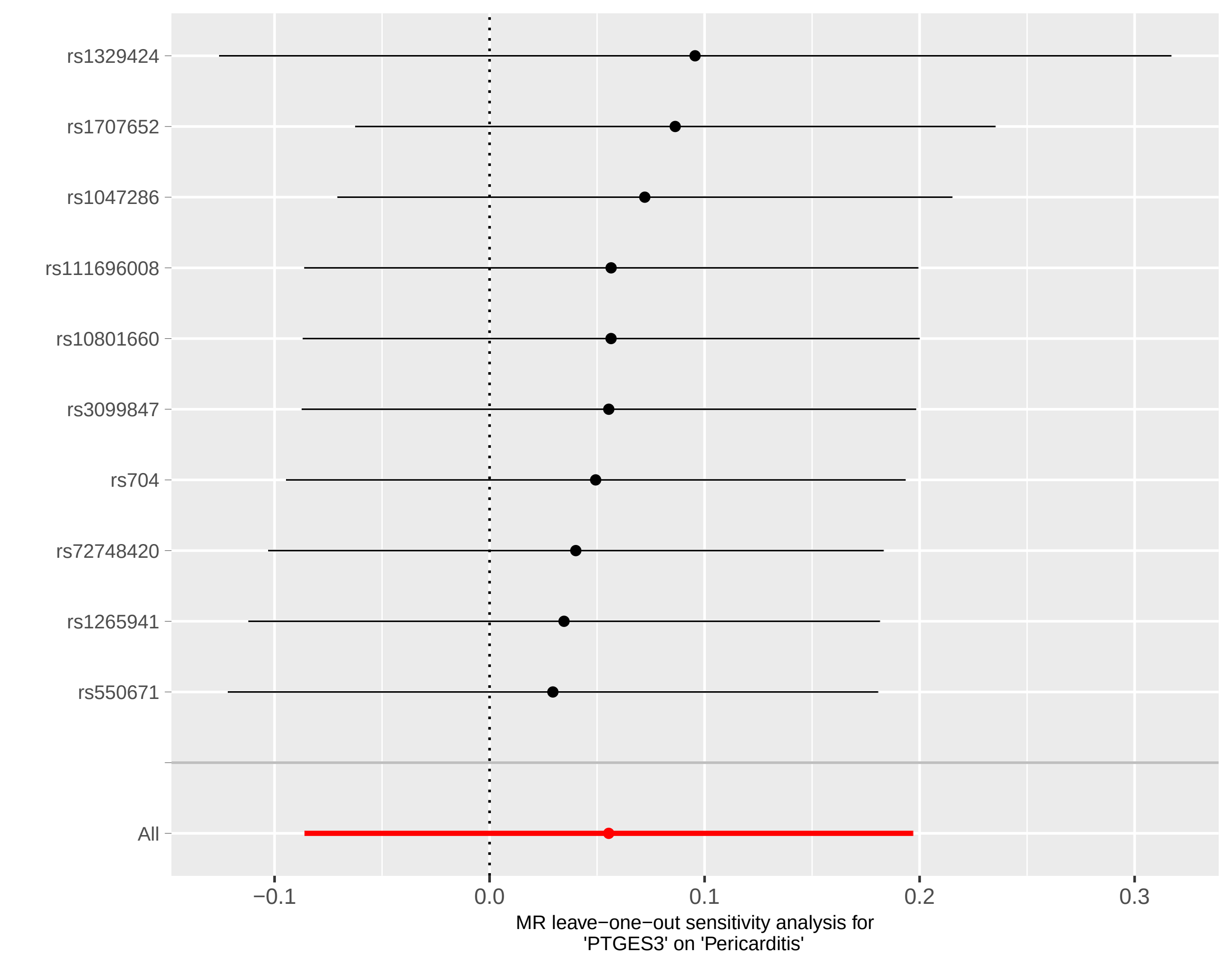
rs1329424
rs1707652
rs1047286
rs111696008
rs10801660
rs3099847
rs704
rs72748420
rs1265941
rs550671
All
−0.1
0.0
0.1
0.2
0.3
MR leave−one−out sensitivity analysis for
'PTGES3' on 'Pericarditis'

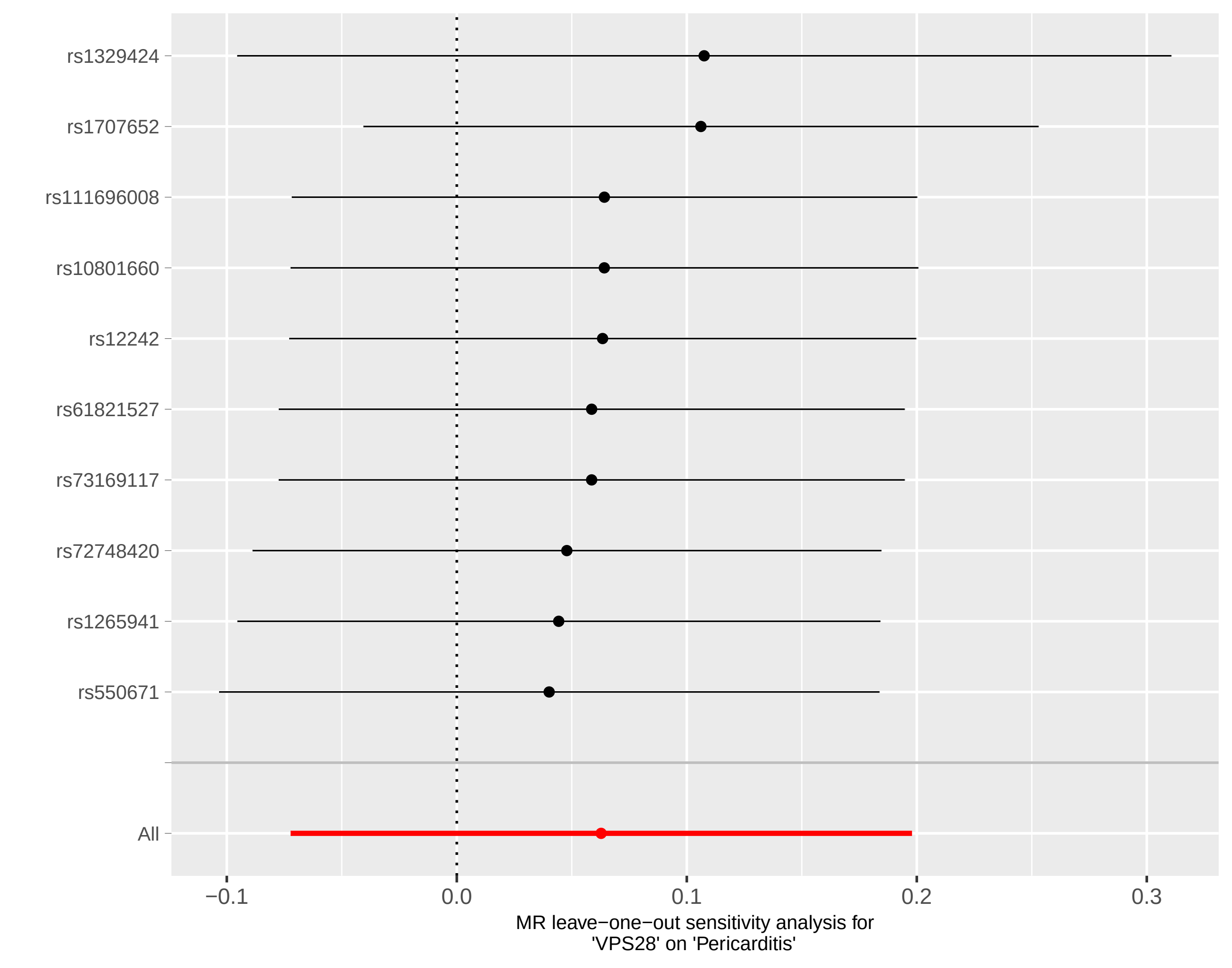

rs1329424
rs1707652
rs111696008
rs10801660
rs12242
rs61821527
rs73169117
rs72748420
rs1265941
rs550671
All
−0.1
0.0
0.1
0.2
0.3
MR leave−one−out sensitivity analysis for
'VPS28' on 'Pericarditis'

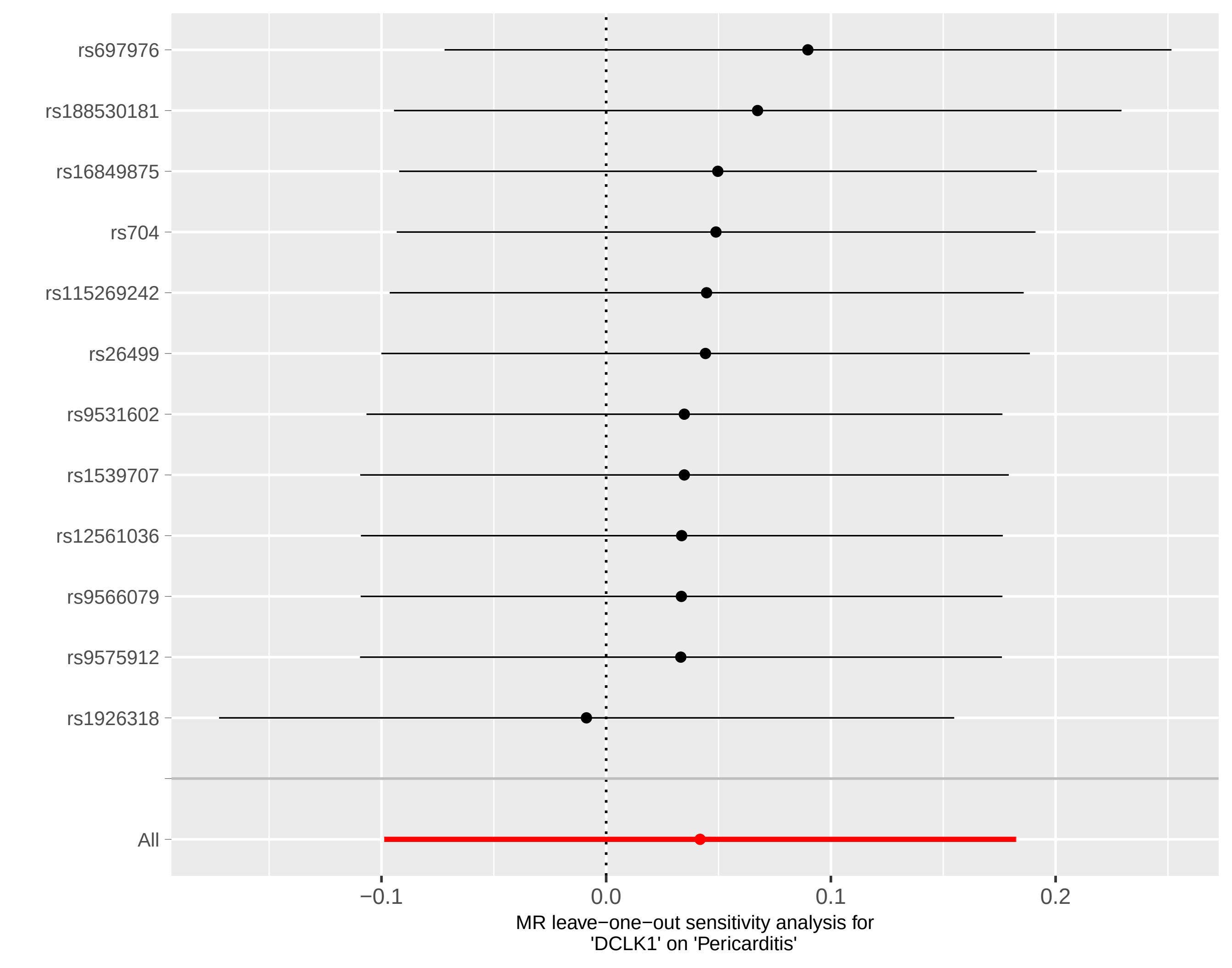
rs697976
rs188530181
rs16849875
rs704
rs115269242
rs26499
rs9531602
rs1539707
rs12561036
rs9566079
rs9575912
rs1926318
All
−0.1
0.0
0.1
0.2
MR leave−one−out sensitivity analysis for
'DCLK1' on 'Pericarditis'

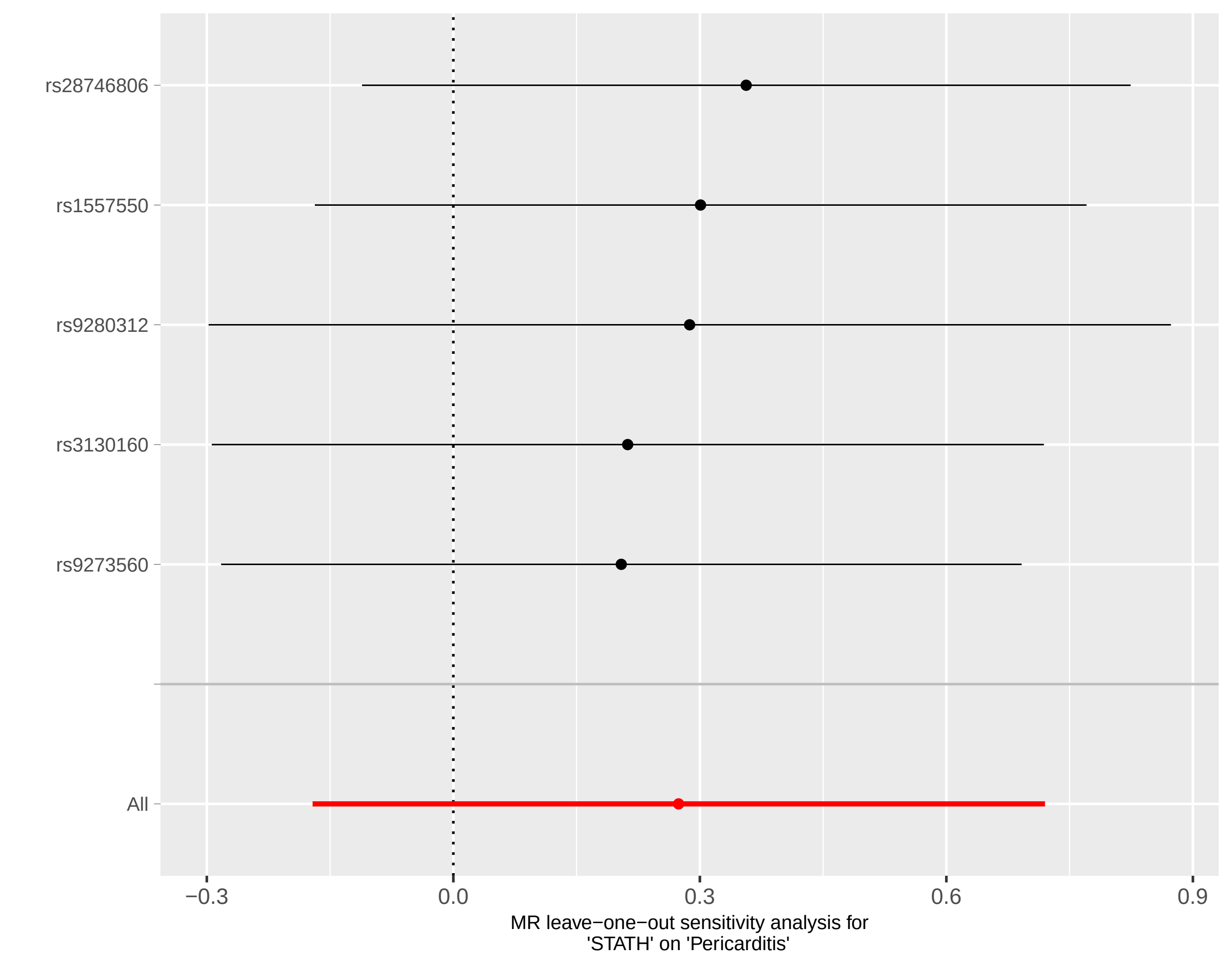
rs28746806
rs1557550
rs9280312
rs3130160
rs9273560
All
−0.3
0.0
0.3
0.6
0.9
MR leave−one−out sensitivity analysis for
'STATH' on 'Pericarditis'

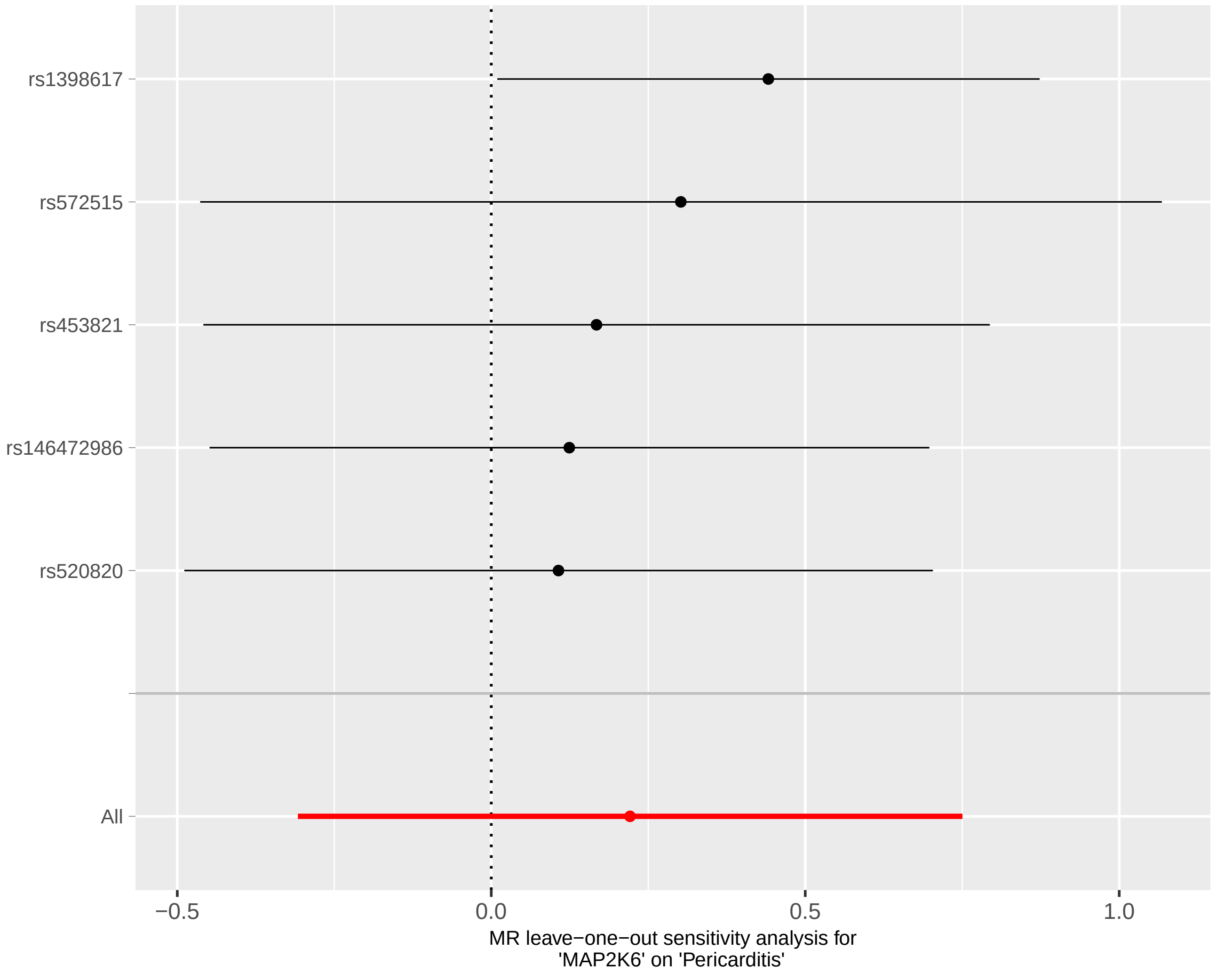
rs1398617
rs572515
rs453821
rs146472986
rs520820
All
−0.5
0.0
0.5
1.0
MR leave−one−out sensitivity analysis for
'MAP2K6' on 'Pericarditis'

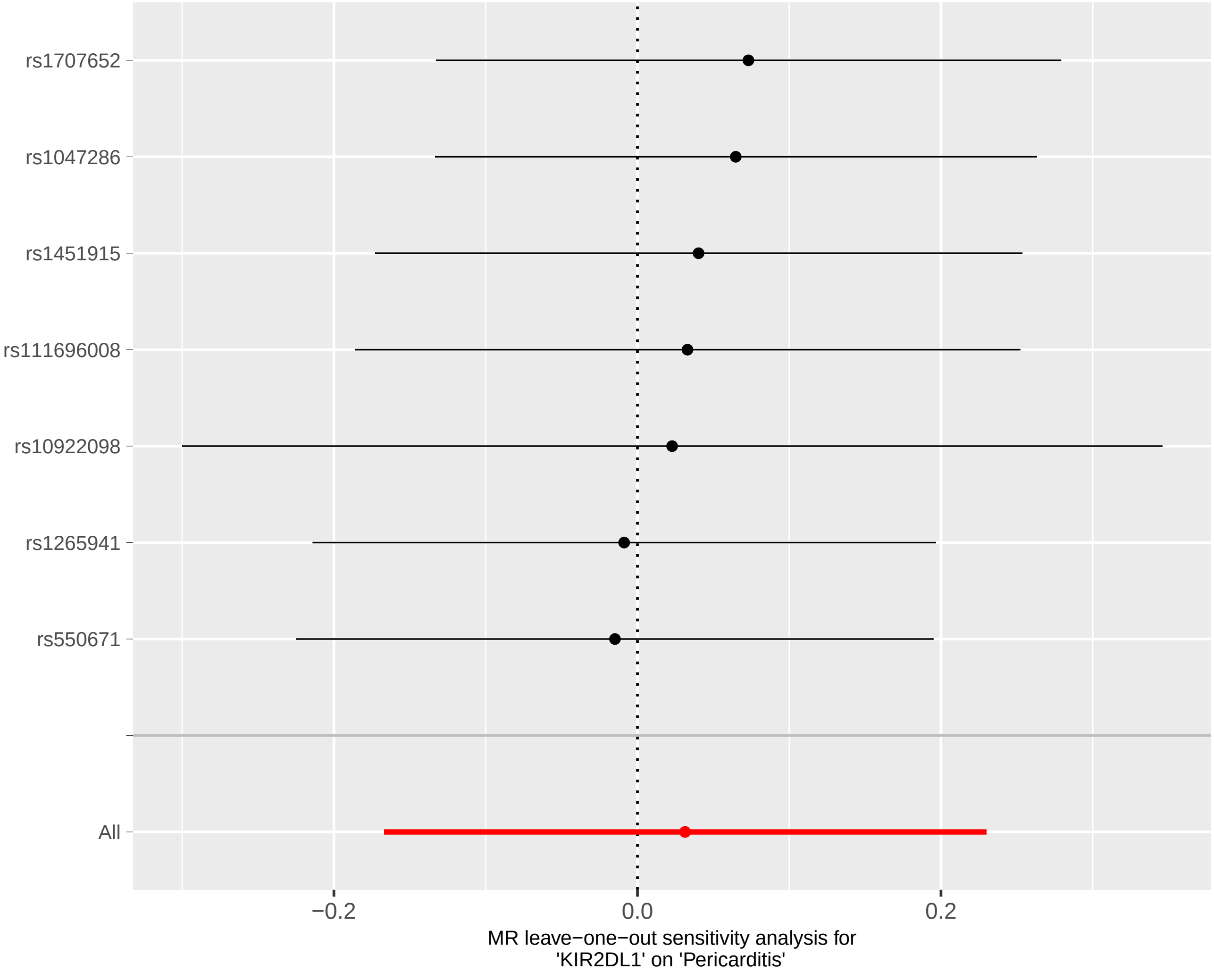
rs1707652
rs1047286
rs1451915
rs111696008
rs10922098
rs1265941
rs550671
All
−0.2
0.0
0.2
MR leave−one−out sensitivity analysis for
'KIR2DL1' on 'Pericarditis'

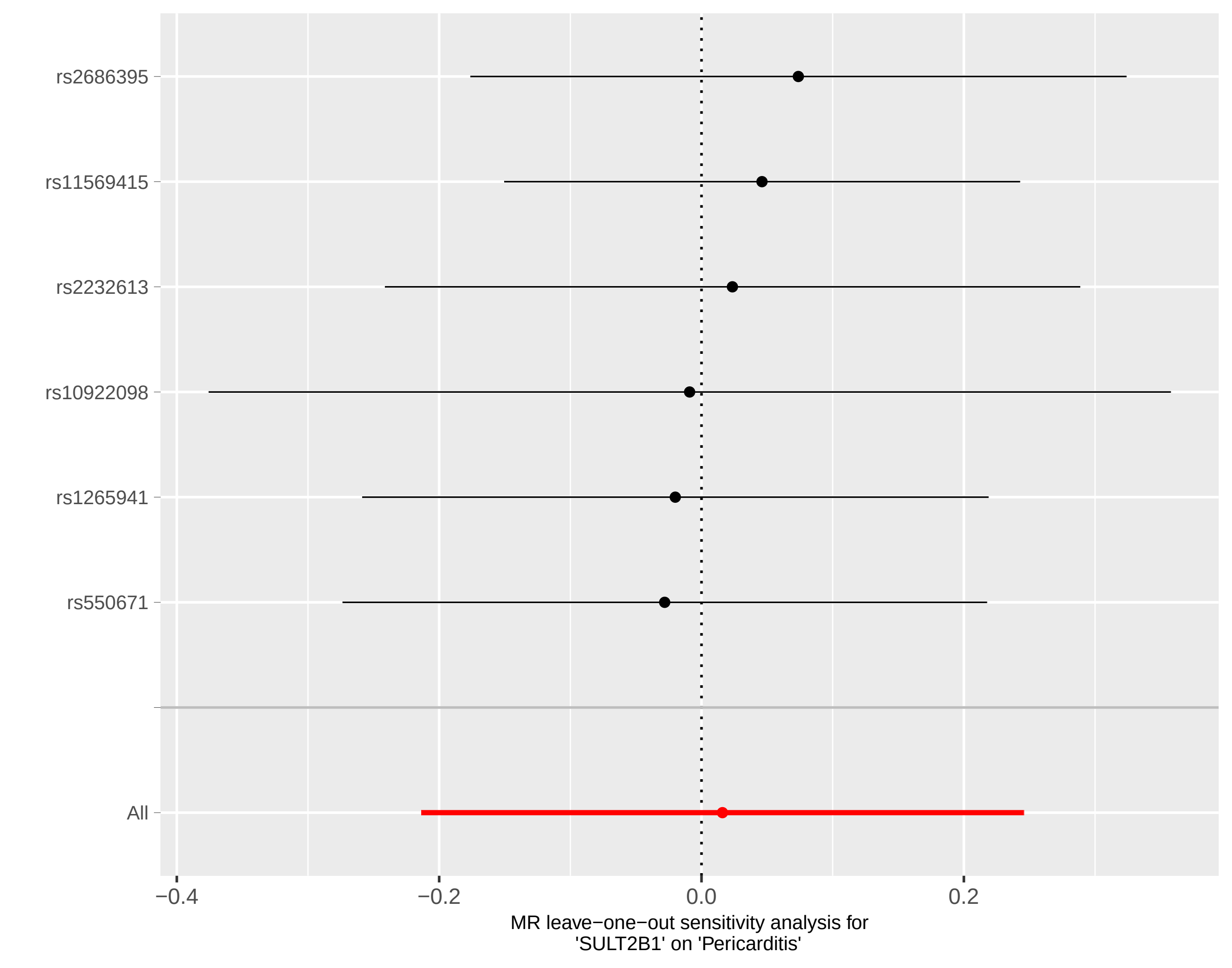
rs2686395
rs11569415
rs2232613
rs10922098
rs1265941
rs550671
All
−0.4
−0.2
0.0
0.2
MR leave−one−out sensitivity analysis for
'SULT2B1' on 'Pericarditis'

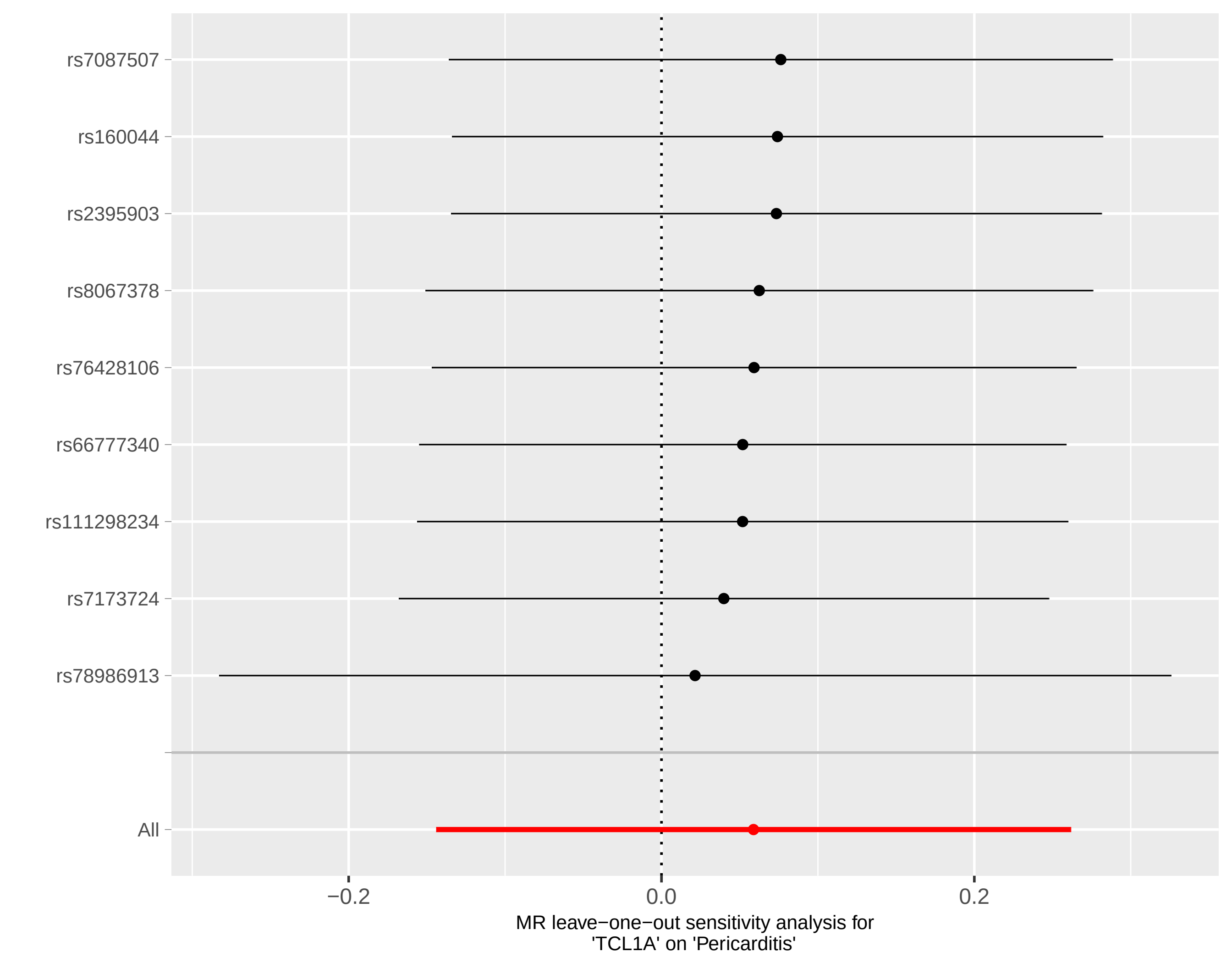
rs7087507
rs160044
rs2395903
rs8067378
rs76428106
rs66777340
rs111298234
rs7173724
rs78986913
All
−0.2
0.0
0.2
MR leave−one−out sensitivity analysis for
'TCL1A' on 'Pericarditis'

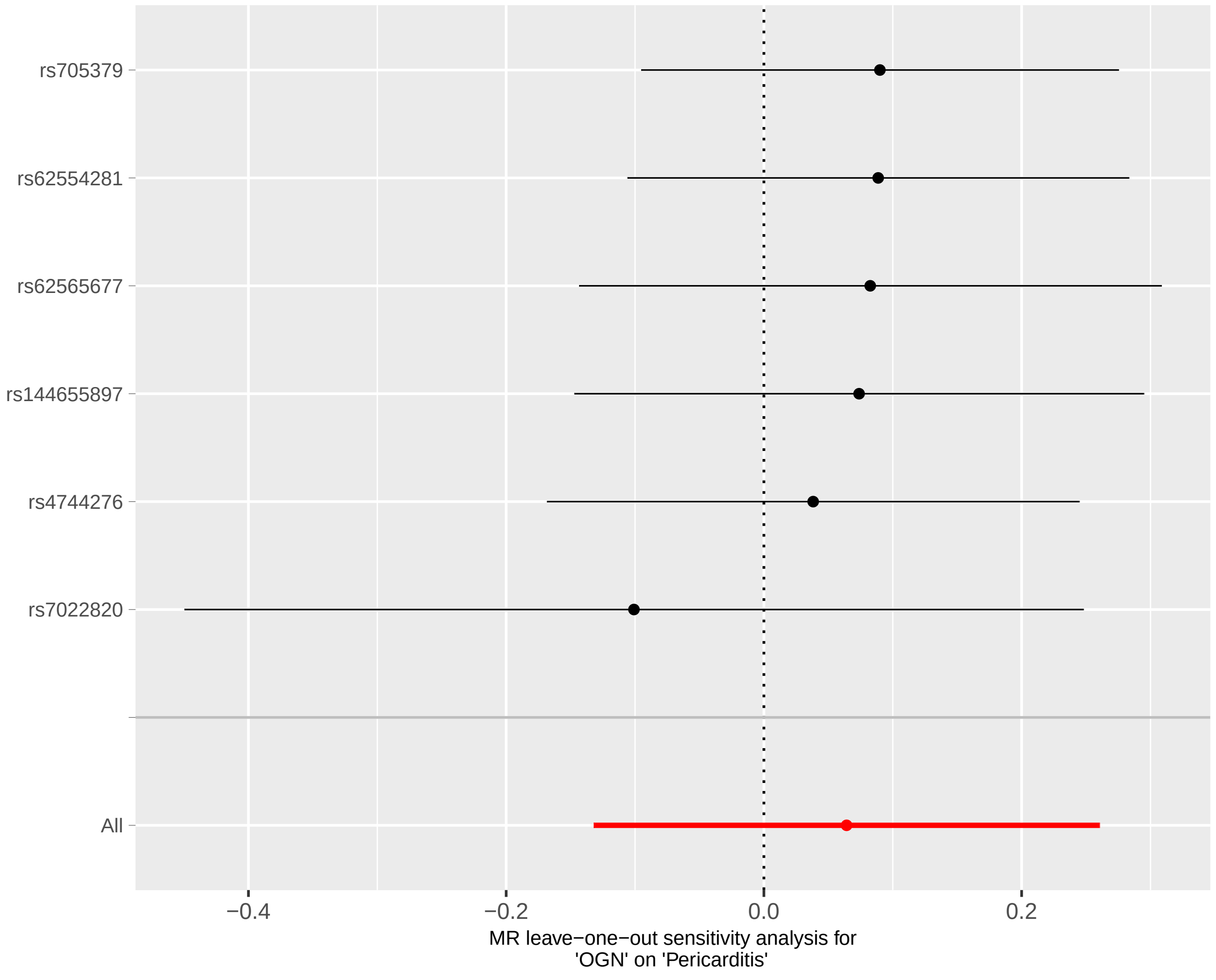
rs705379
rs62554281
rs62565677
rs144655897
rs4744276
rs7022820
All
−0.4
−0.2
0.0
0.2
MR leave−one−out sensitivity analysis for
'OGN' on 'Pericarditis'

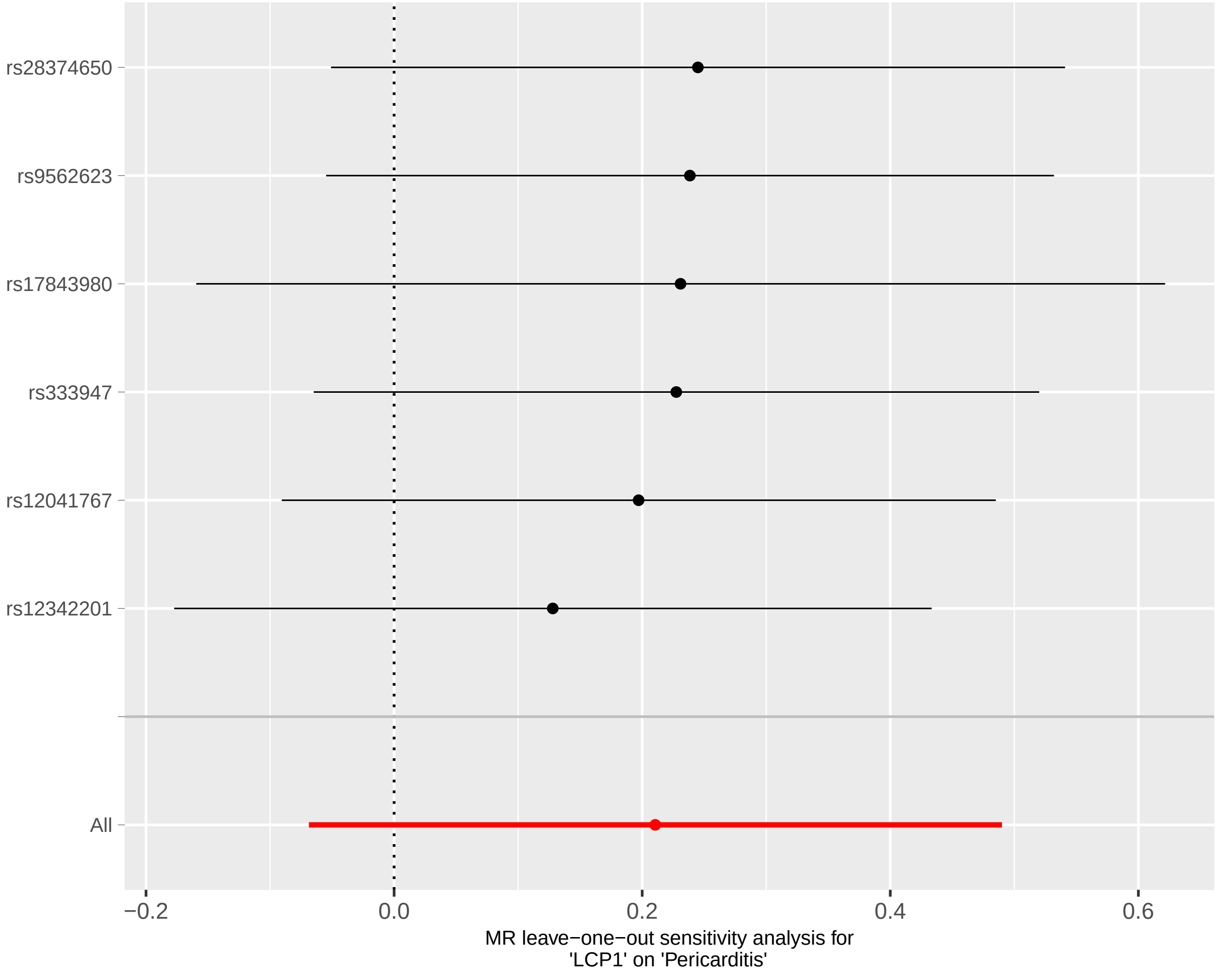
rs28374650
rs9562623
rs17843980
rs333947
rs12041767
rs12342201
All
−0.2
0.0
0.2
0.4
0.6
MR leave−one−out sensitivity analysis for
'LCP1' on 'Pericarditis'

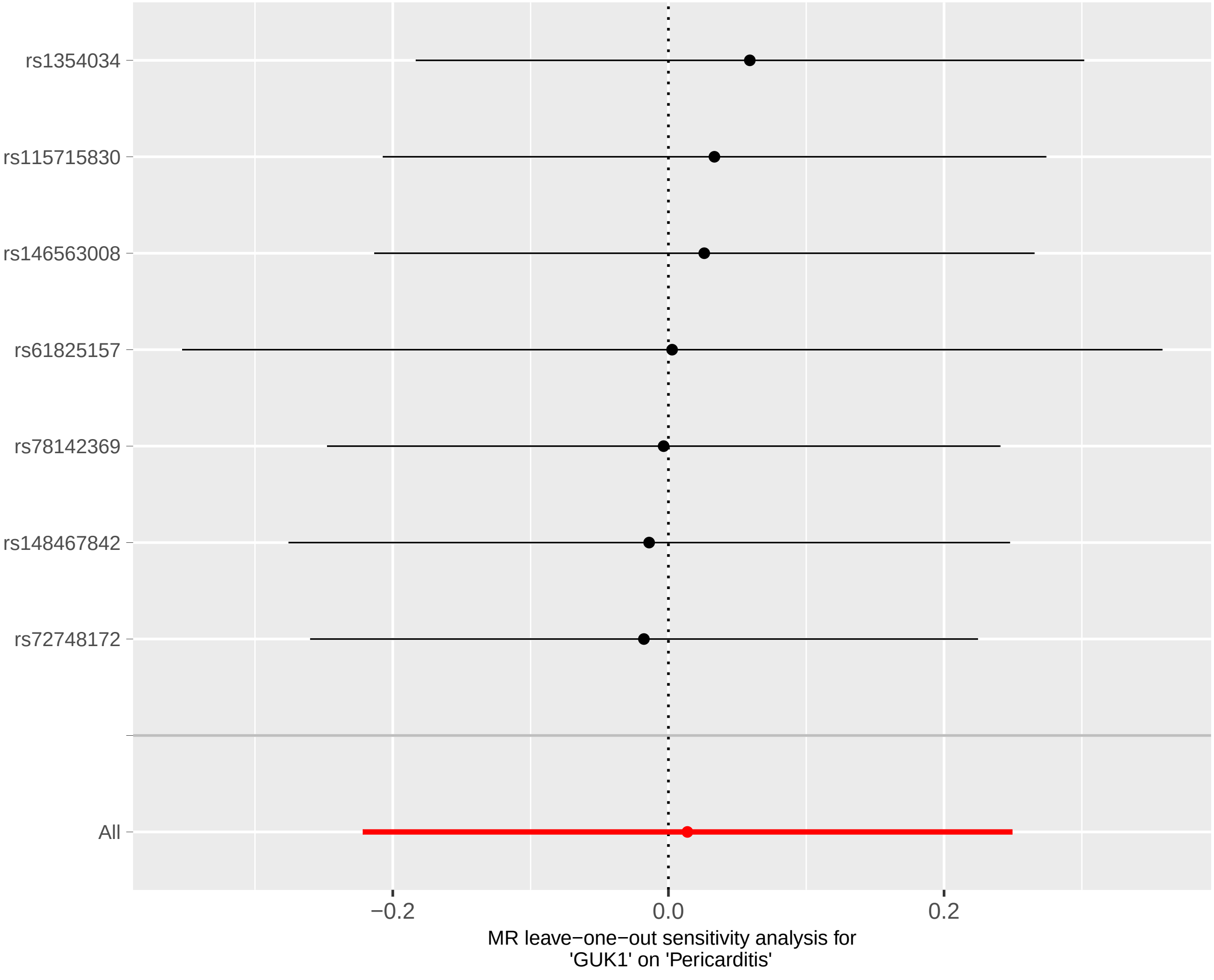
rs1354034
rs115715830
rs146563008
rs61825157
rs78142369
rs148467842
rs72748172
All
−0.2
0.0
0.2
MR leave−one−out sensitivity analysis for
'GUK1' on 'Pericarditis'

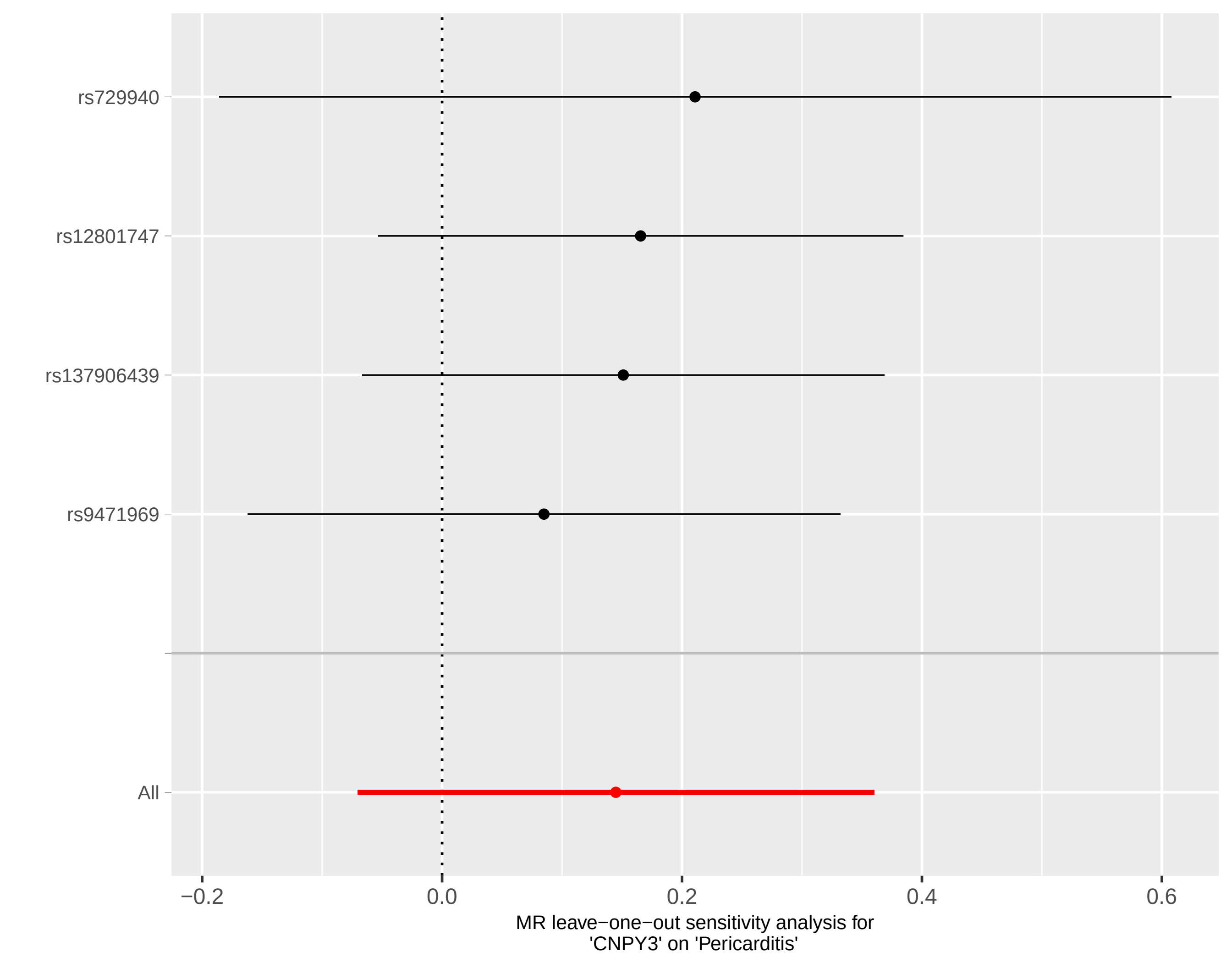
rs729940
rs12801747
rs137906439
rs9471969
All
−0.2
0.0
0.2
0.4
0.6
MR leave−one−out sensitivity analysis for
'CNPY3' on 'Pericarditis'

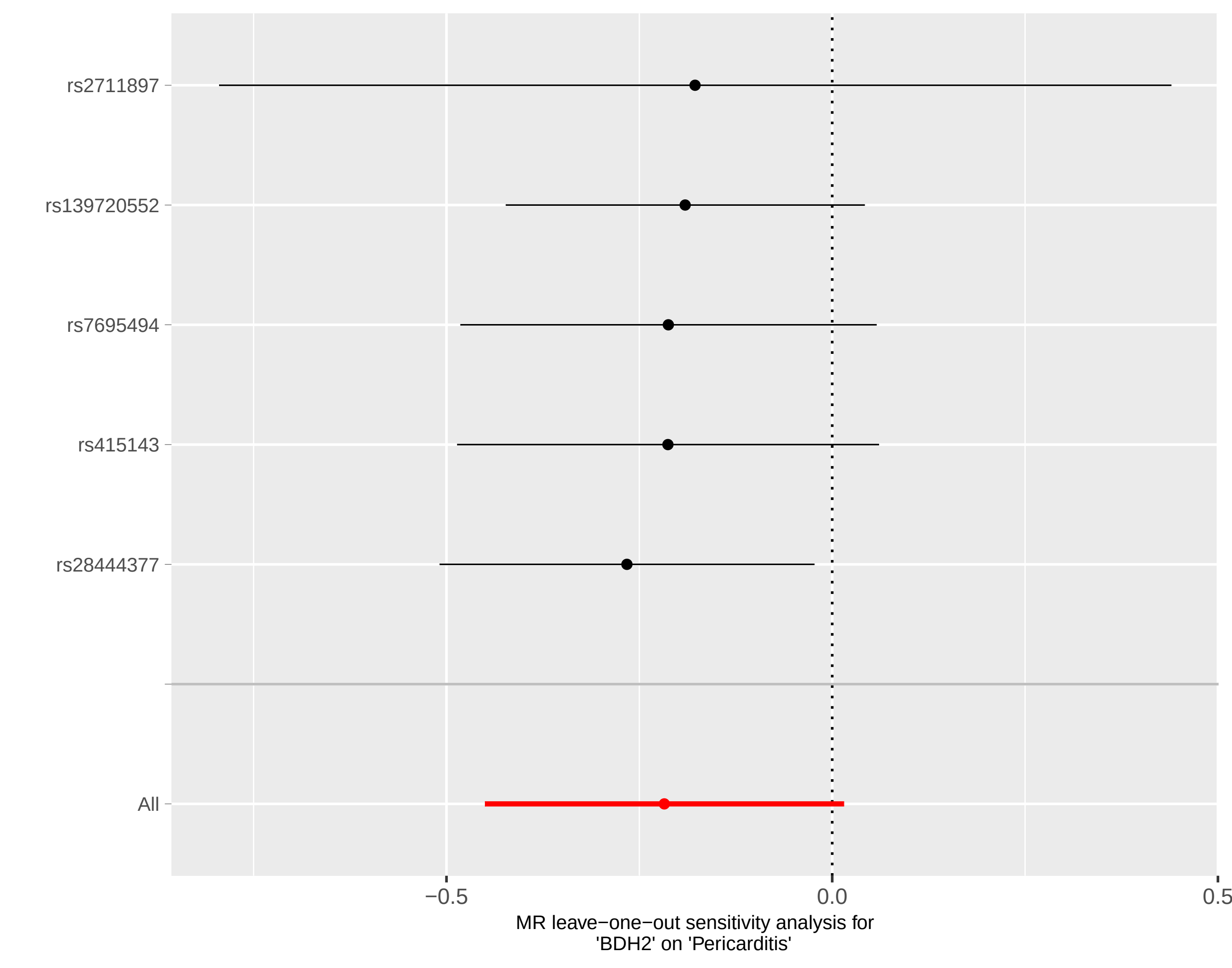
rs2711897
rs139720552
rs7695494
rs415143
rs28444377
All
−0.5
0.0
0.5
MR leave−one−out sensitivity analysis for
'BDH2' on 'Pericarditis'

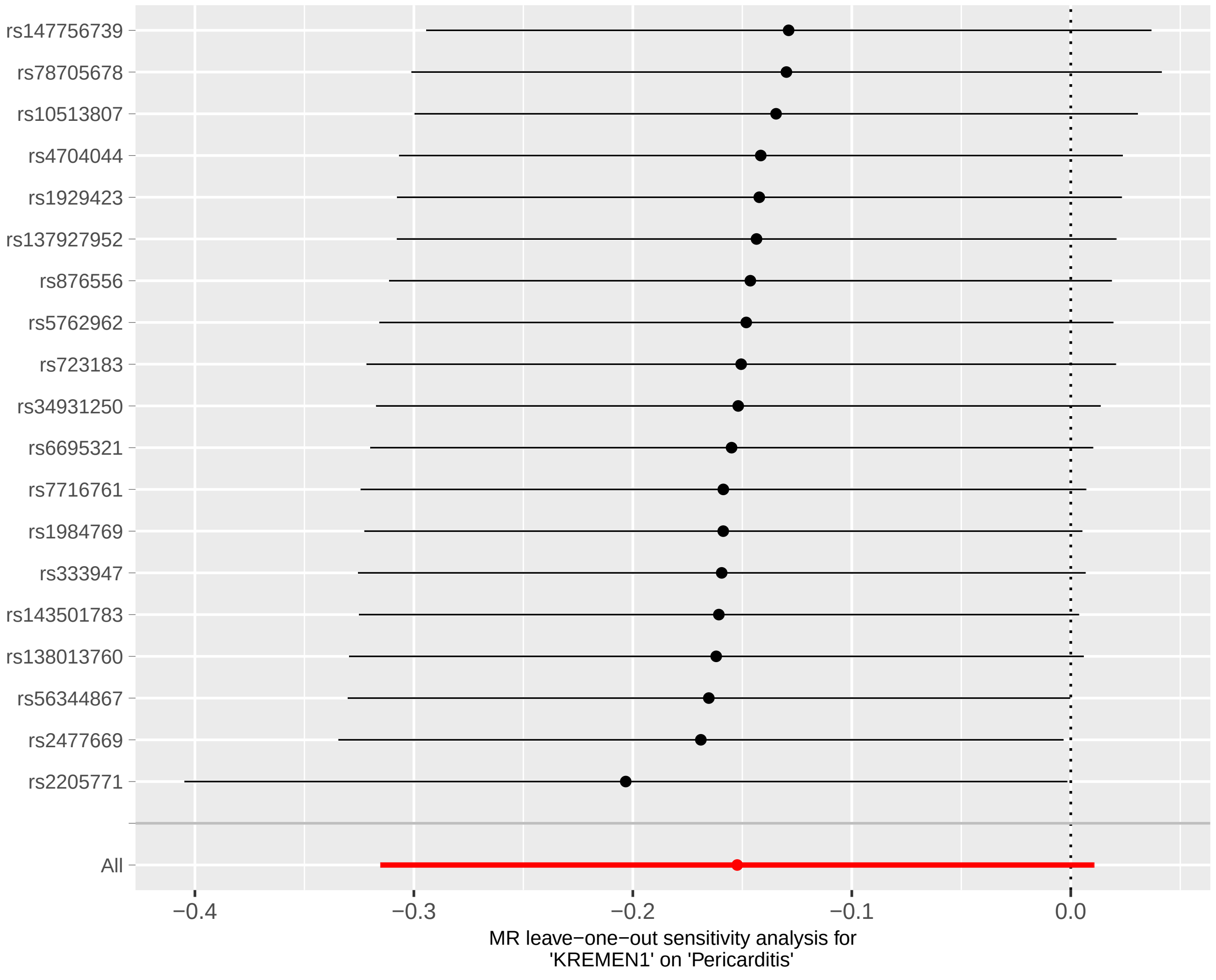

rs147756739
rs78705678
rs10513807
rs4704044
rs1929423
rs137927952
rs876556
rs5762962
rs723183
rs34931250
rs6695321
rs7716761
rs1984769
rs333947
rs143501783
rs138013760
rs56344867
rs2477669
rs2205771
All
−0.4
−0.3
−0.2
−0.1
0.0
MR leave−one−out sensitivity analysis for
'KREMEN1' on 'Pericarditis'

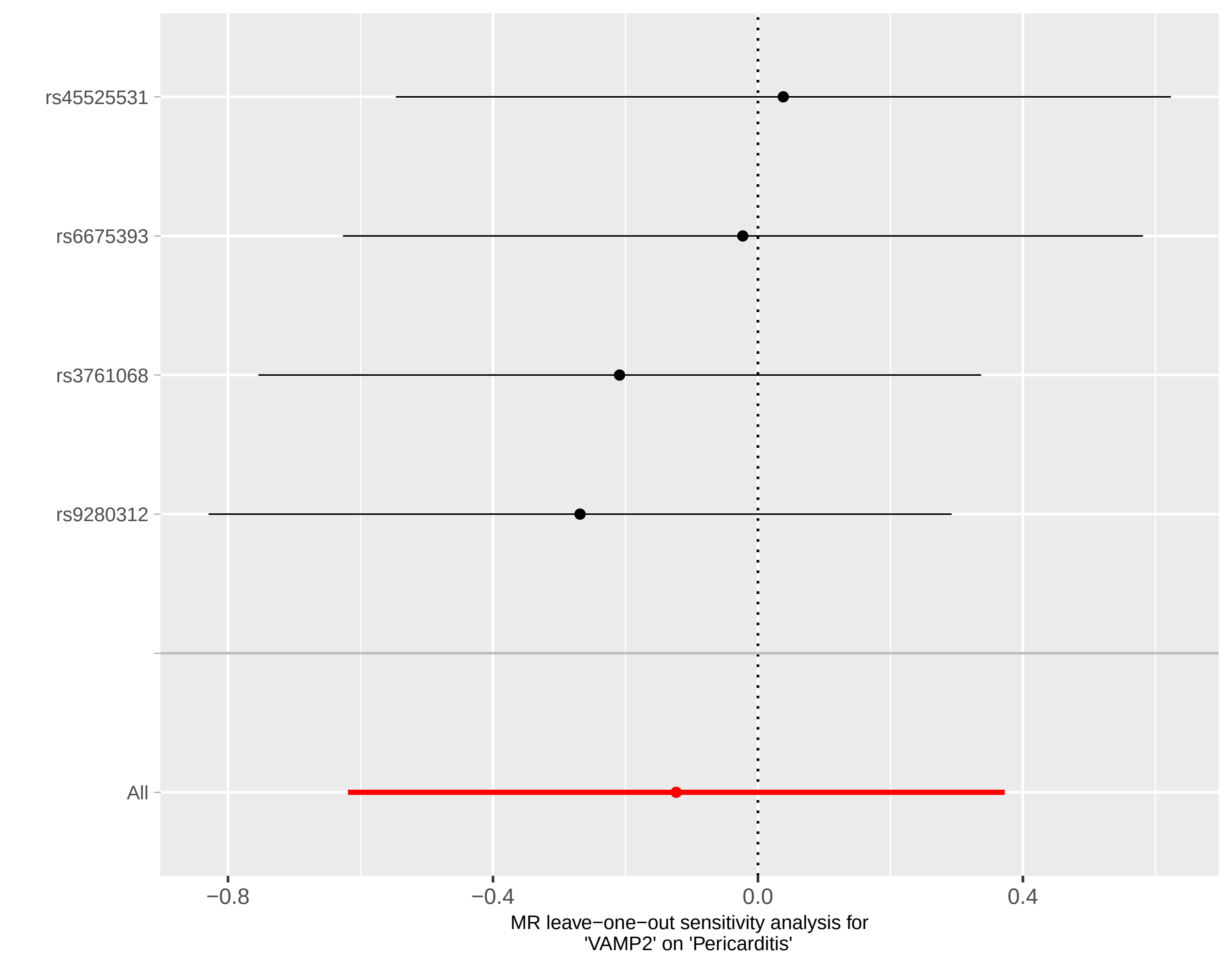
rs45525531
rs6675393
rs3761068
rs9280312
All
−0.8
−0.4
0.0
0.4
MR leave−one−out sensitivity analysis for
'VAMP2' on 'Pericarditis'

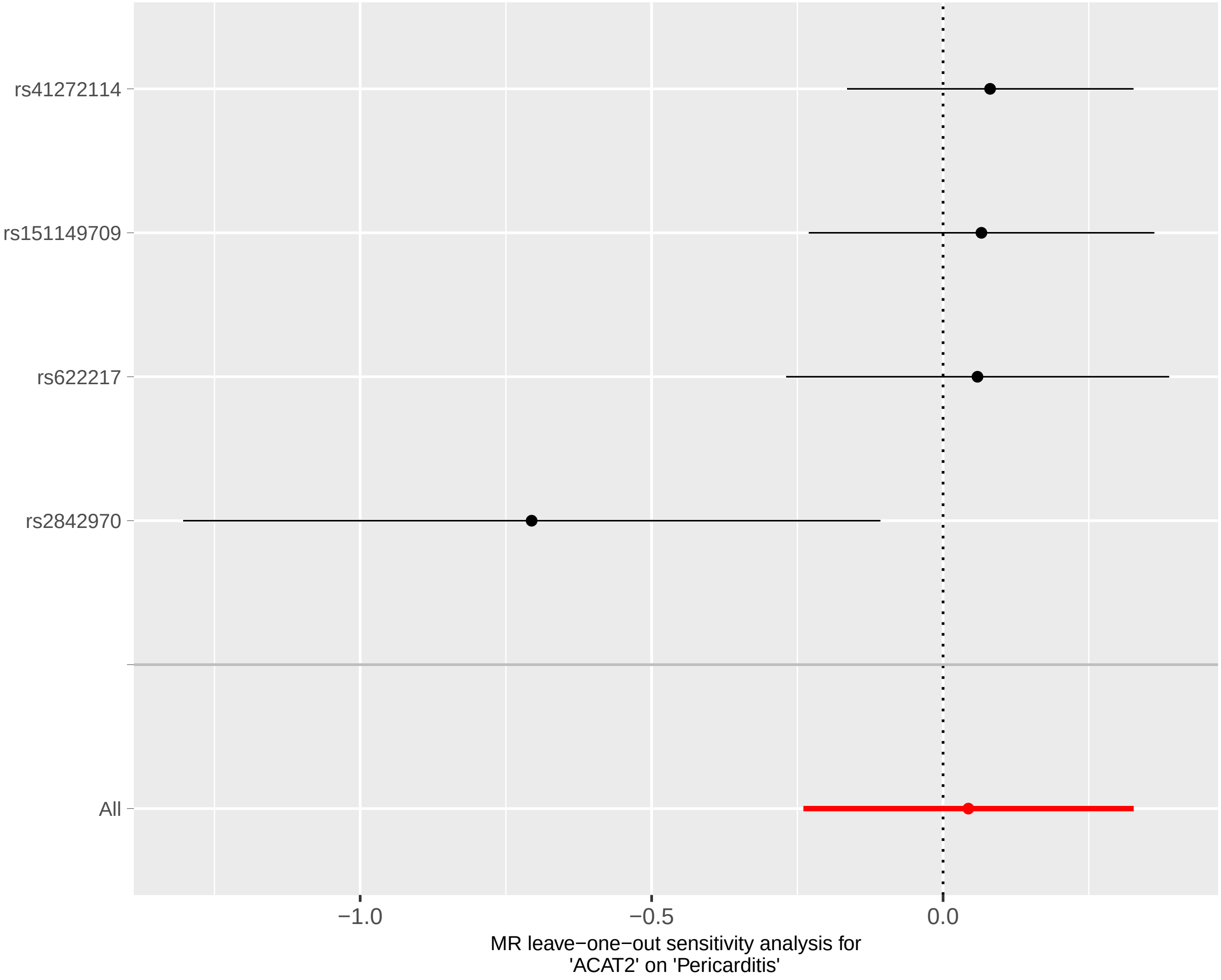

rs41272114
rs151149709
rs622217
rs2842970
All
−1.0
−0.5
0.0
MR leave−one−out sensitivity analysis for
'ACAT2' on 'Pericarditis'

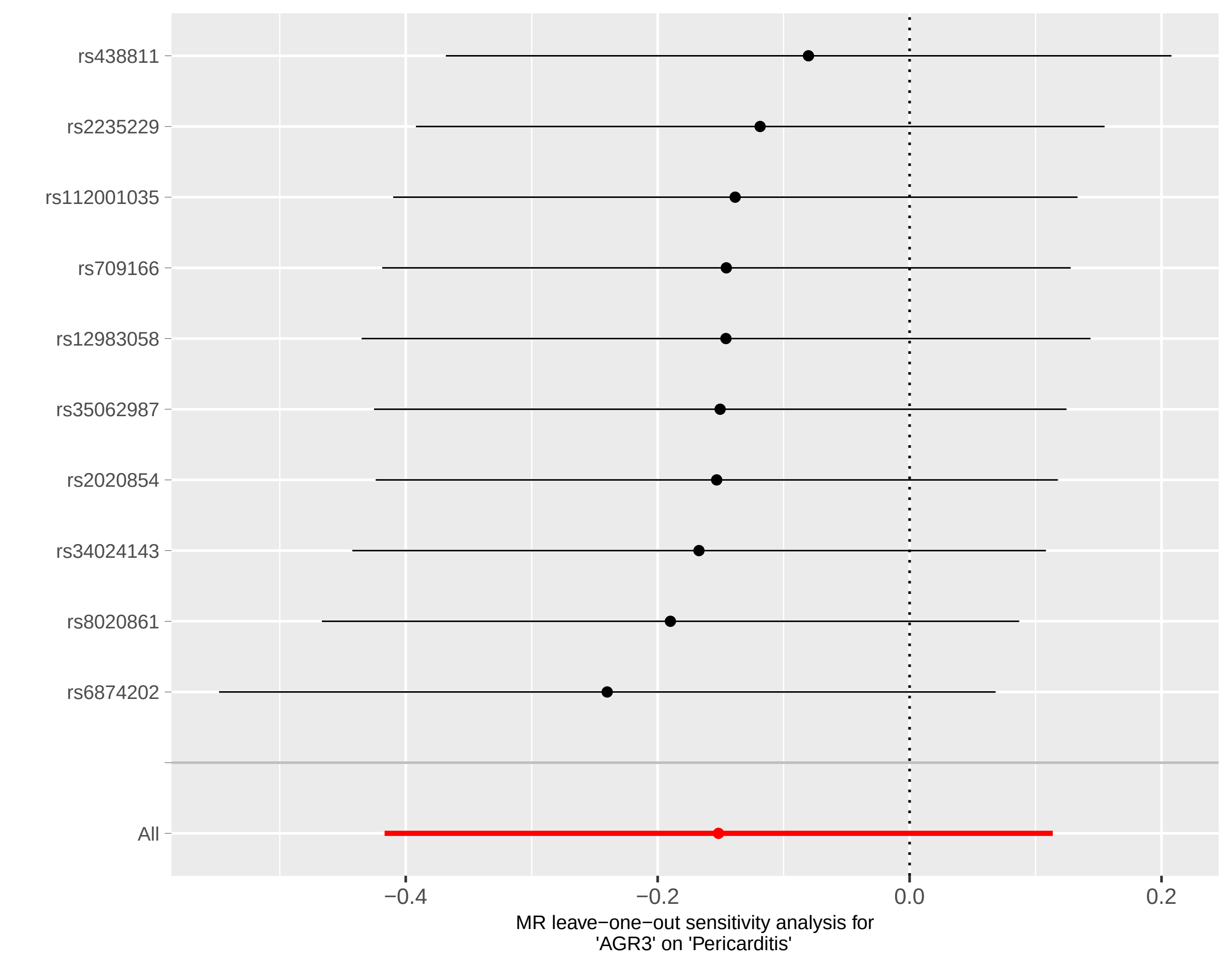

rs438811
rs2235229
rs112001035
rs709166
rs12983058
rs35062987
rs2020854
rs34024143
rs8020861
rs6874202
All
−0.4
−0.2
0.0
0.2
MR leave−one−out sensitivity analysis for
'AGR3' on 'Pericarditis'

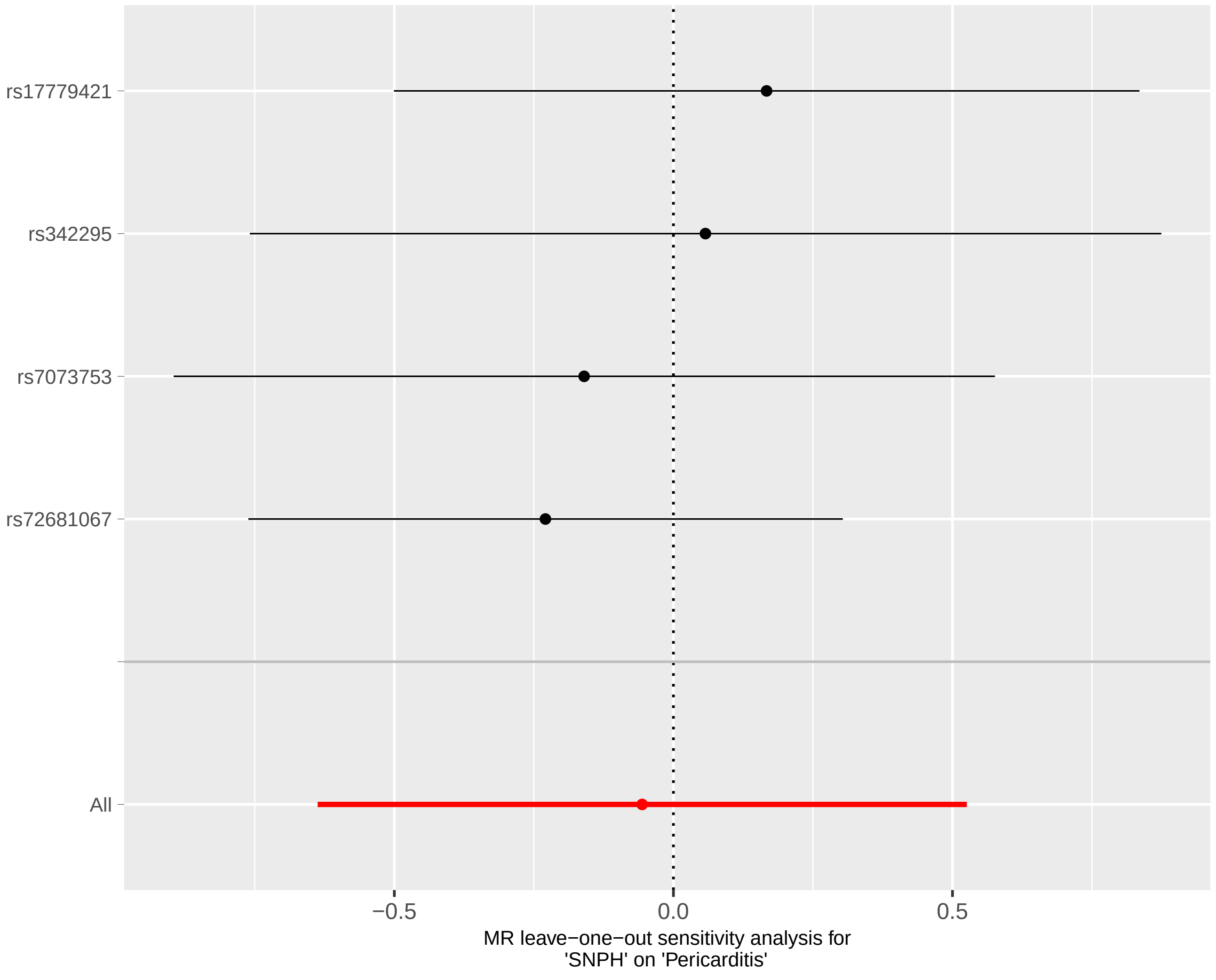

rs17779421
rs342295
rs7073753
rs72681067
All
−0.5
0.0
0.5
MR leave−one−out sensitivity analysis for
'SNPH' on 'Pericarditis'

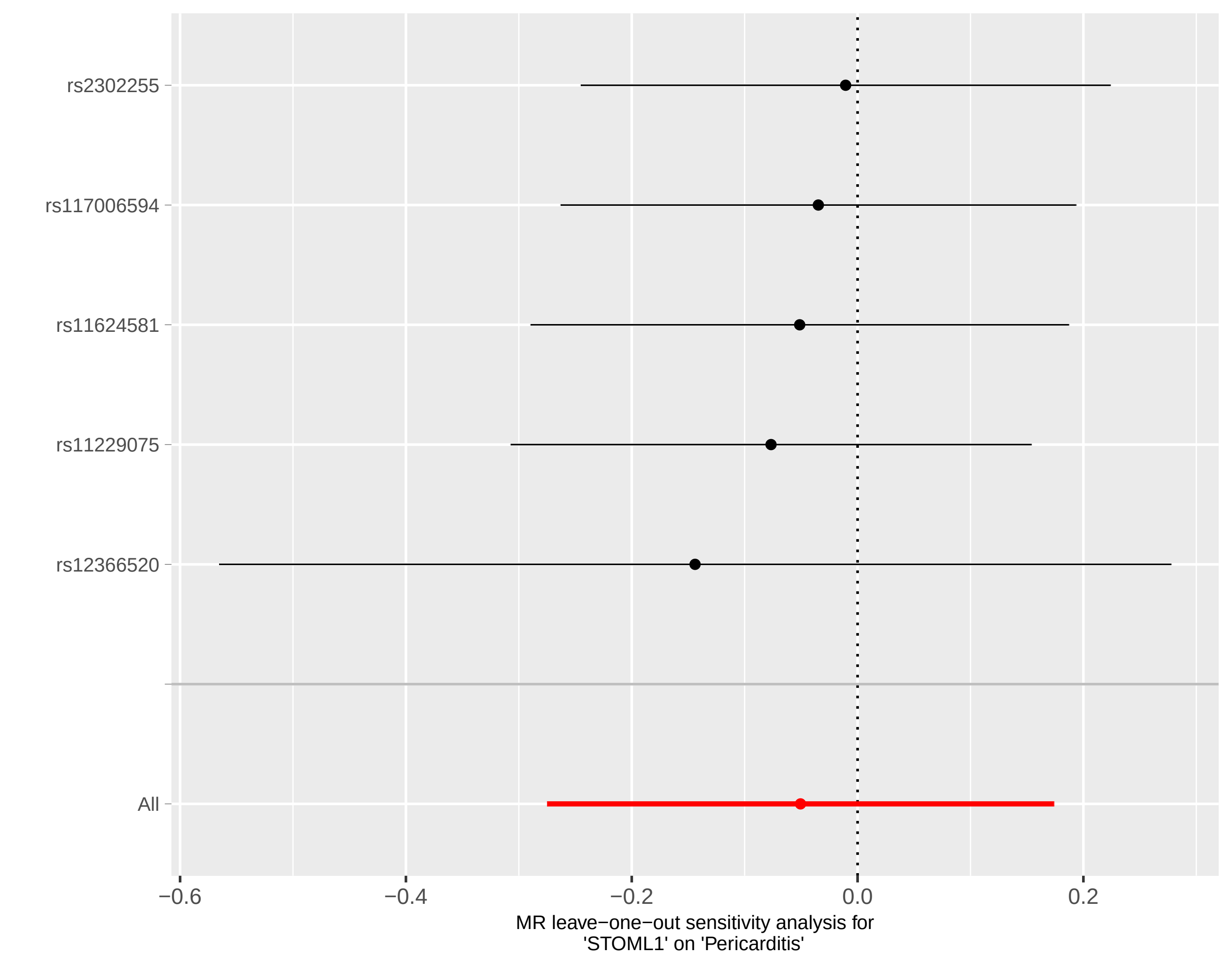

rs2302255
rs117006594
rs11624581
rs11229075
rs12366520
All
−0.6
−0.4
−0.2
0.0
0.2
MR leave−one−out sensitivity analysis for
'STOML1' on 'Pericarditis'

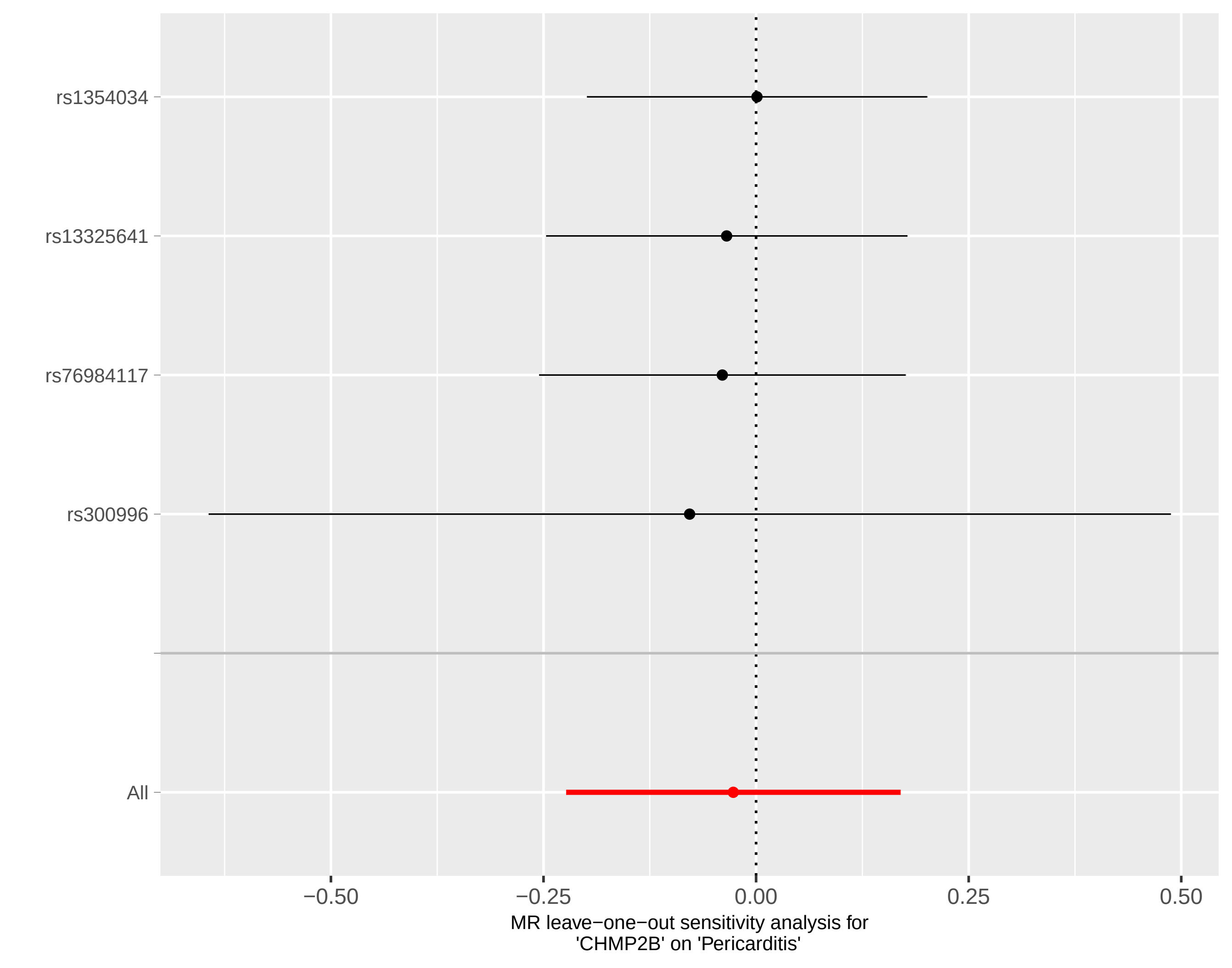
rs1354034
rs13325641
rs76984117
rs300996
All
−0.50
−0.25
0.00
0.25
0.50
MR leave−one−out sensitivity analysis for
'CHMP2B' on 'Pericarditis'

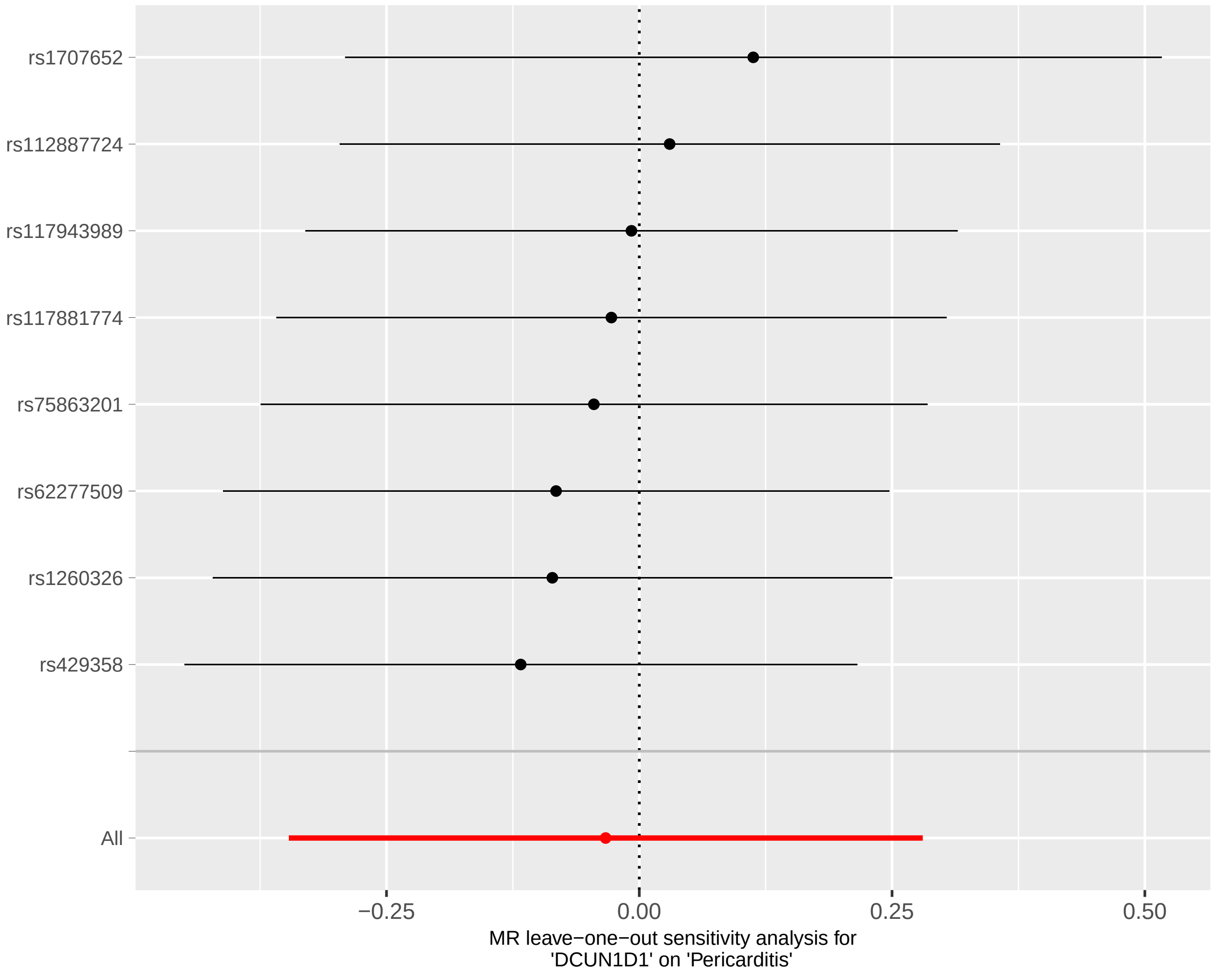

rs1707652
rs112887724
rs117943989
rs117881774
rs75863201
rs62277509
rs1260326
rs429358
All
−0.25
0.00
0.25
0.50
MR leave−one−out sensitivity analysis for
'DCUN1D1' on 'Pericarditis'

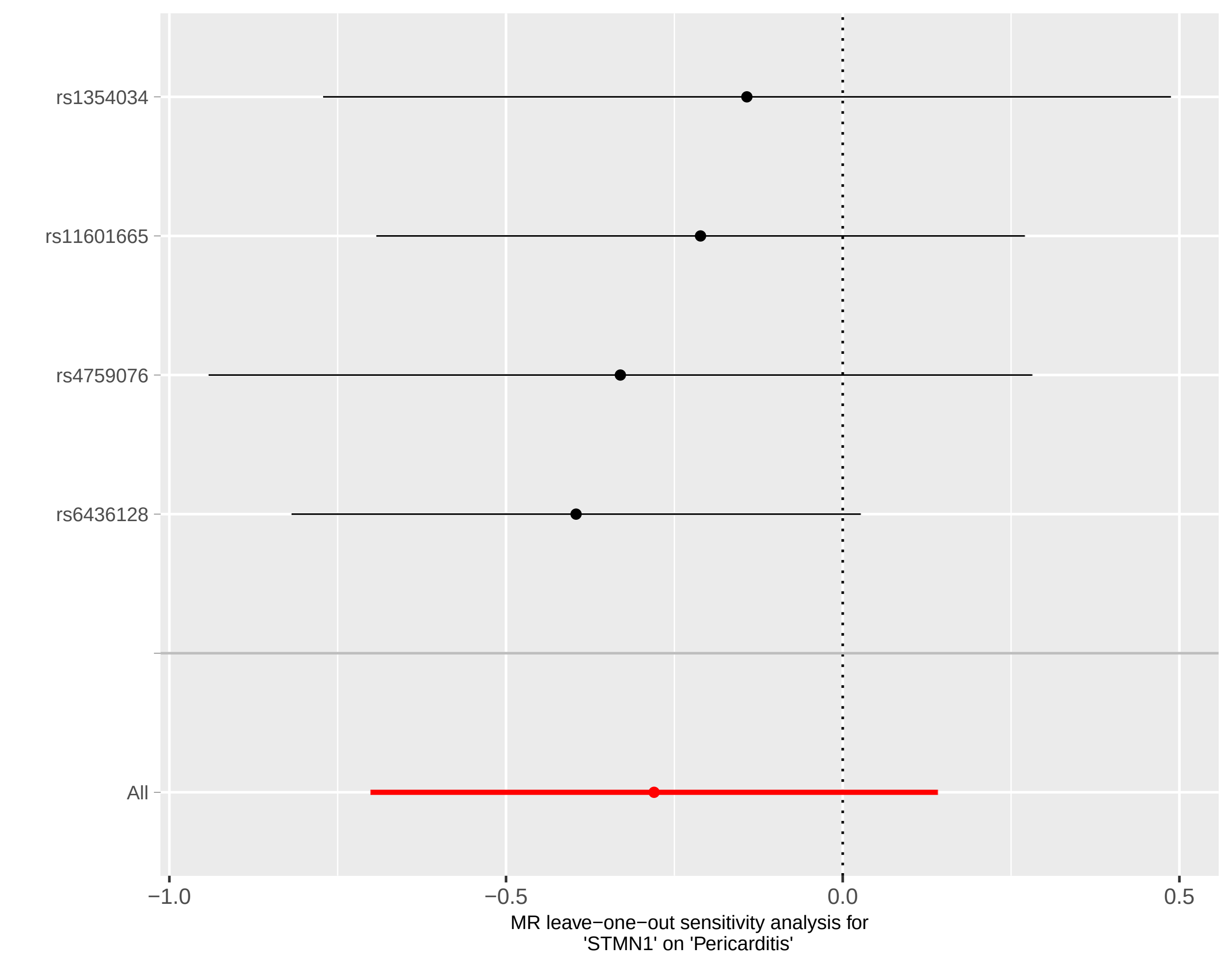

rs1354034
rs11601665
rs4759076
rs6436128
All
−1.0
−0.5
0.0
0.5
MR leave−one−out sensitivity analysis for
'STMN1' on 'Pericarditis'

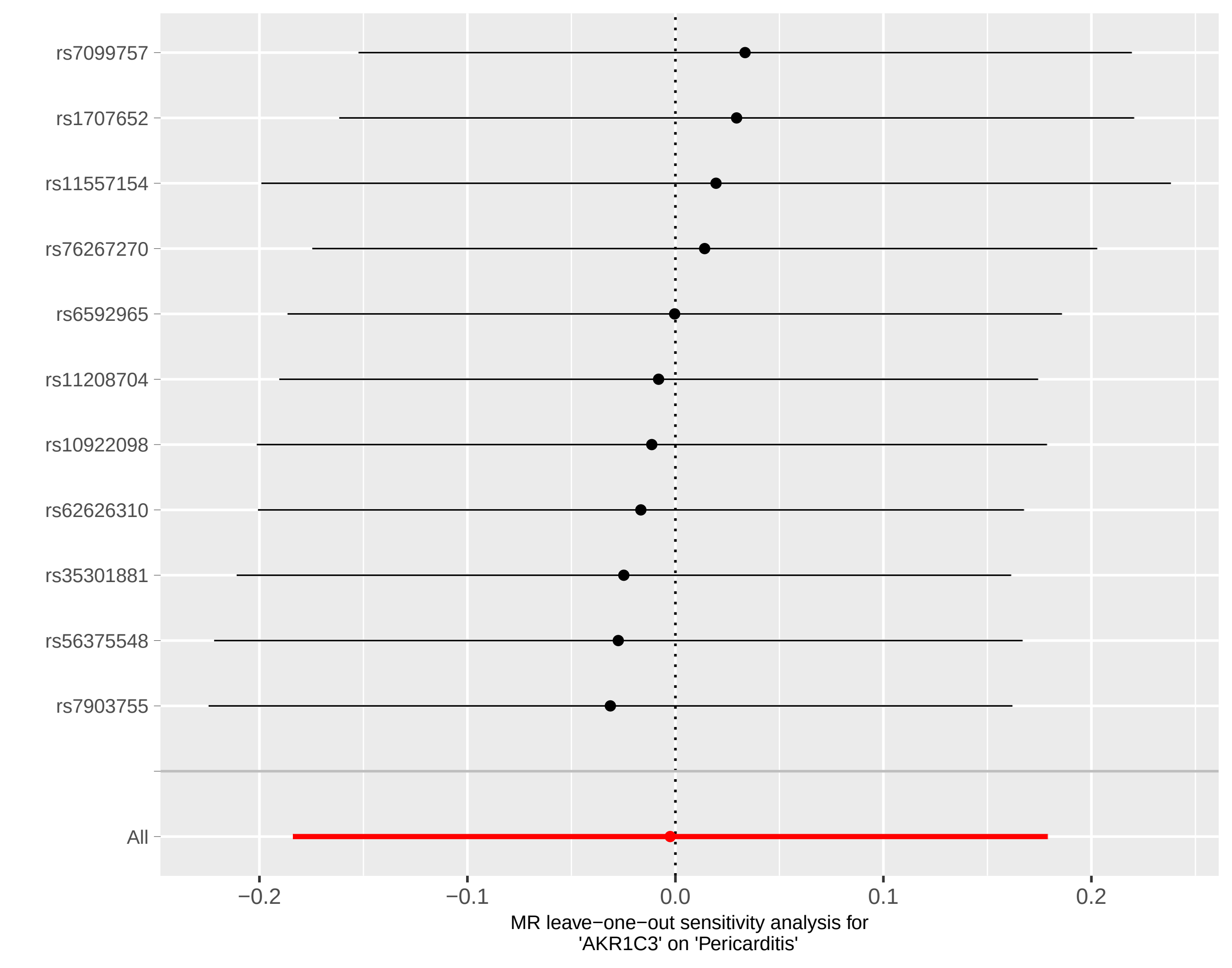
rs7099757
rs1707652
rs11557154
rs76267270
rs6592965
rs11208704
rs10922098
rs62626310
rs35301881
rs56375548
rs7903755
All
−0.2
−0.1
0.0
0.1
0.2
MR leave−one−out sensitivity analysis for
'AKR1C3' on 'Pericarditis'

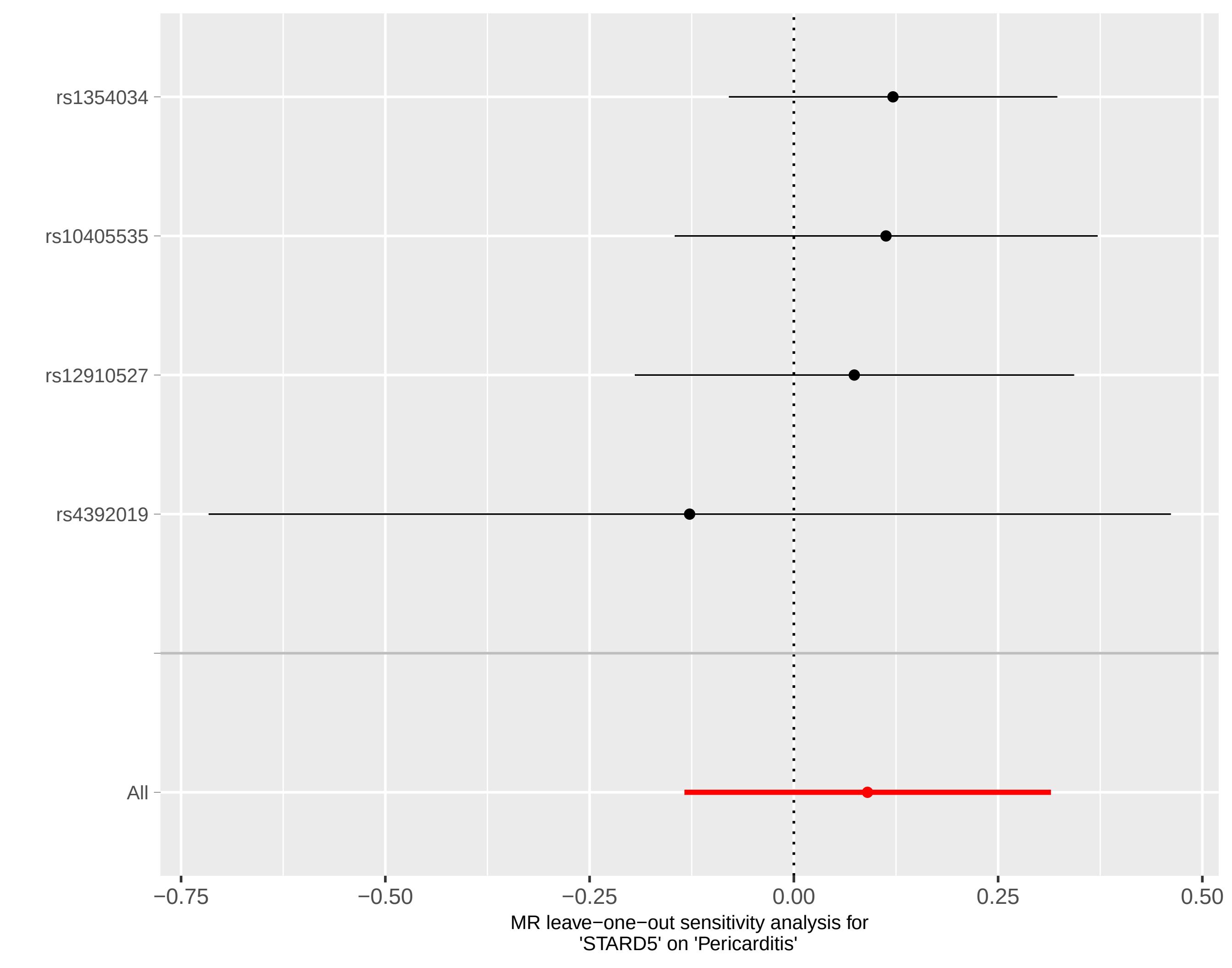
rs1354034
rs10405535
rs12910527
rs4392019
All
−0.75
−0.50
−0.25
0.00
0.25
0.50
MR leave−one−out sensitivity analysis for
'STARD5' on 'Pericarditis'

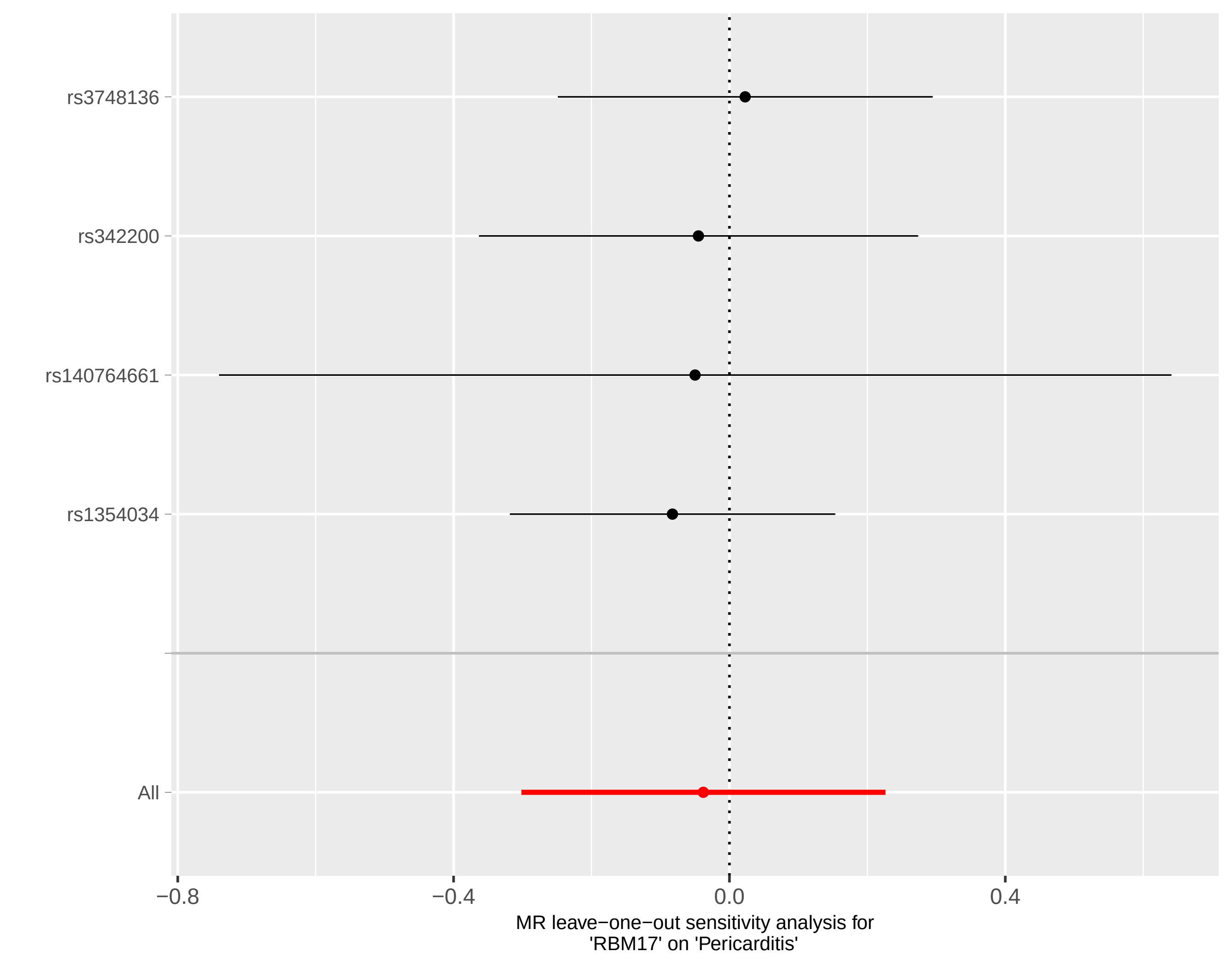

rs3748136
rs342200
rs140764661
rs1354034
All
−0.8
−0.4
0.0
0.4
MR leave−one−out sensitivity analysis for
'RBM17' on 'Pericarditis'

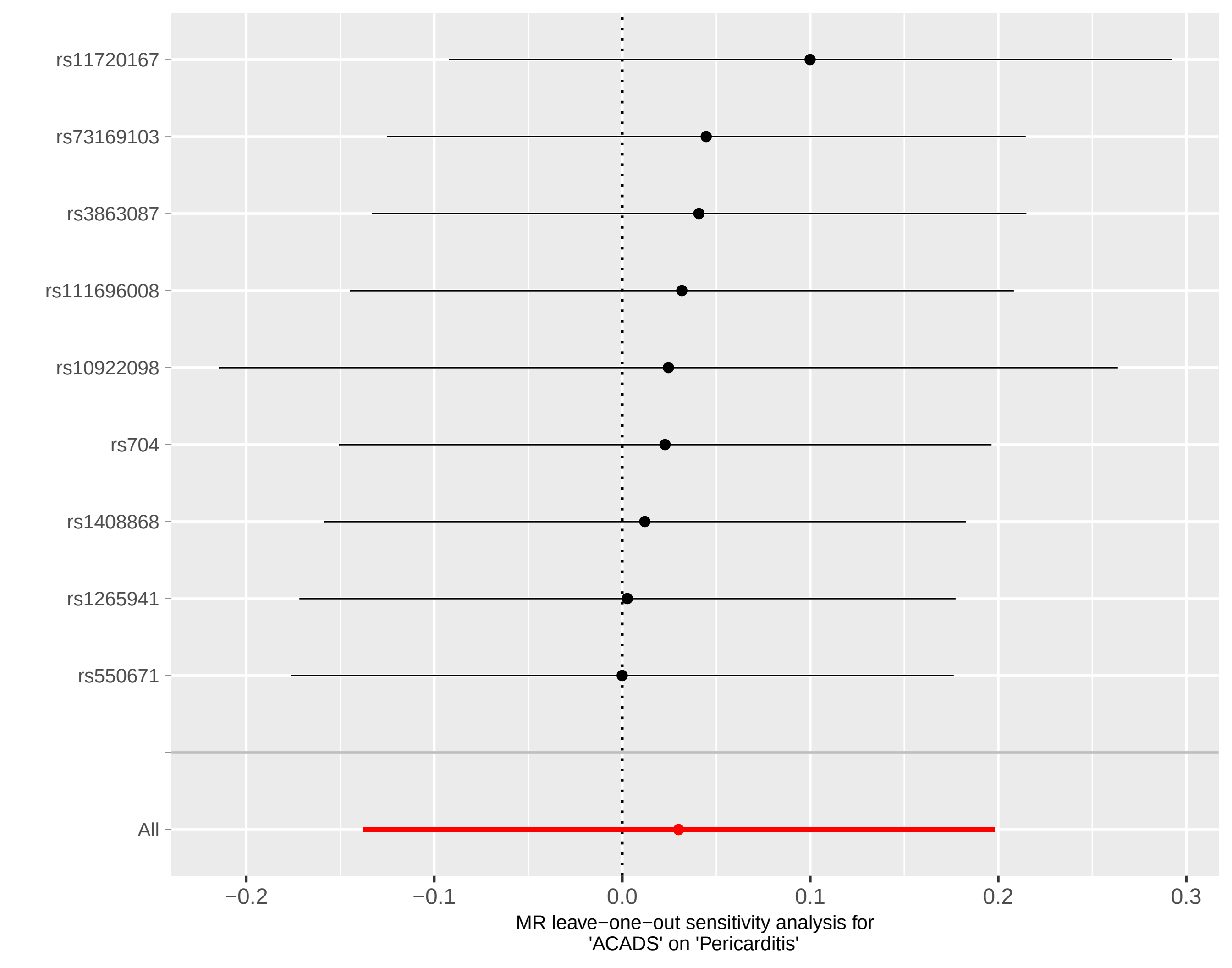
rs11720167
rs73169103
rs3863087
rs111696008
rs10922098
rs704
rs1408868
rs1265941
rs550671
All
−0.2
−0.1
0.0
0.1
0.2
0.3
MR leave−one−out sensitivity analysis for
'ACADS' on 'Pericarditis'

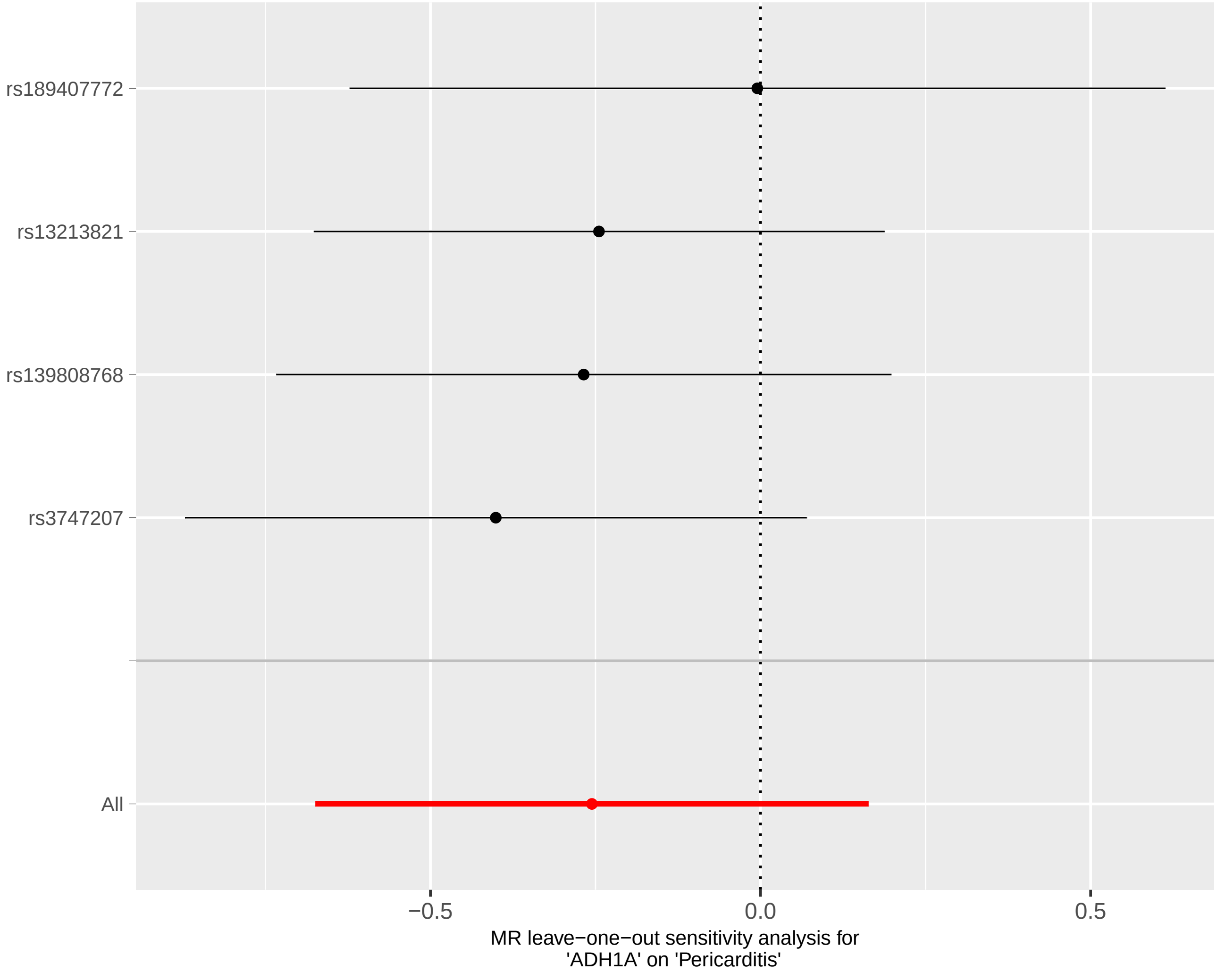
rs189407772
rs13213821
rs139808768
rs3747207
All
−0.5
0.0
0.5
MR leave−one−out sensitivity analysis for
'ADH1A' on 'Pericarditis'

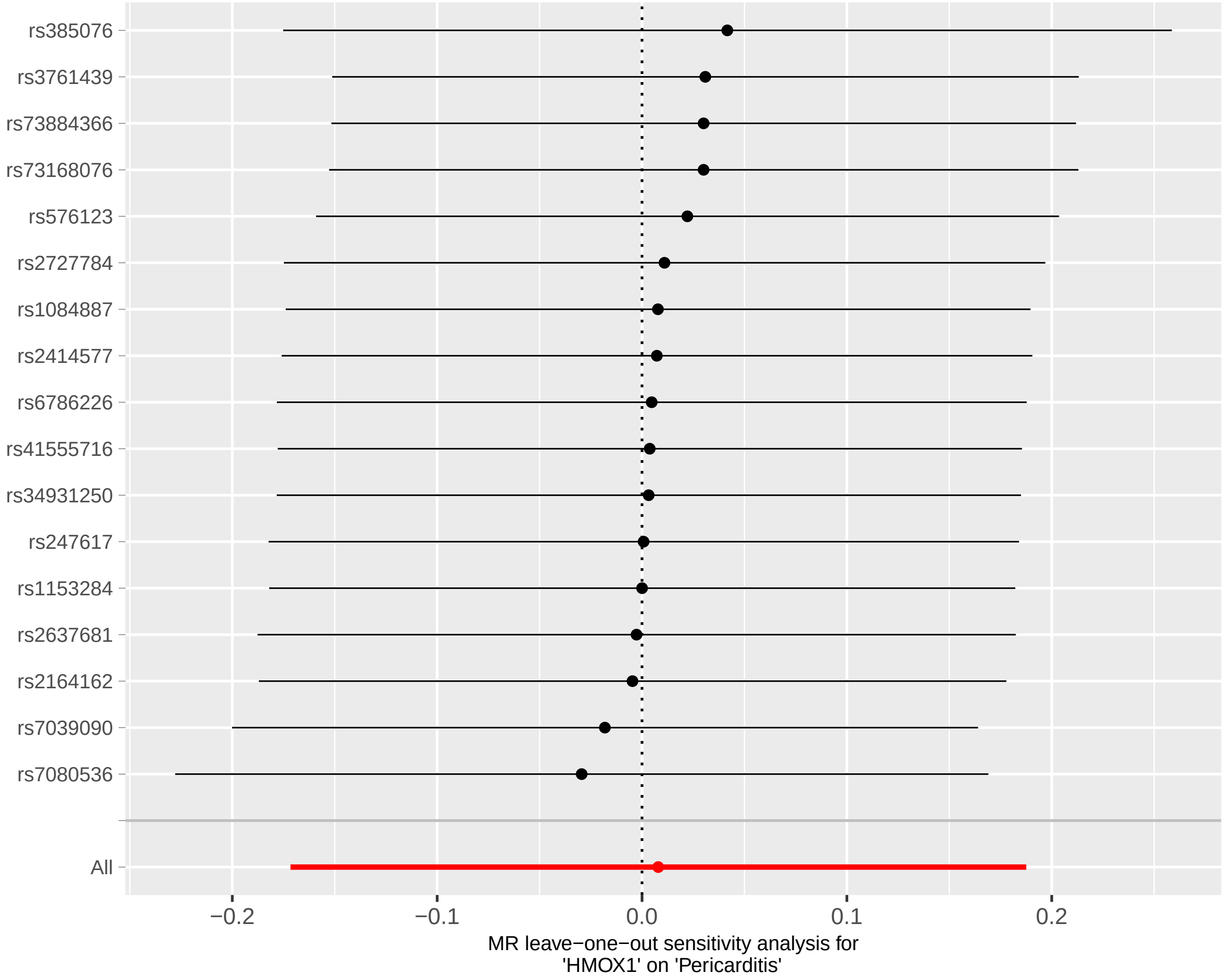
rs385076
rs3761439
rs73884366
rs73168076
rs576123
rs2727784
rs1084887
rs2414577
rs6786226
rs41555716
rs34931250
rs247617
rs1153284
rs2637681
rs2164162
rs7039090
rs7080536
All
−0.2
−0.1
0.0
0.1
0.2
MR leave−one−out sensitivity analysis for
'HMOX1' on 'Pericarditis'

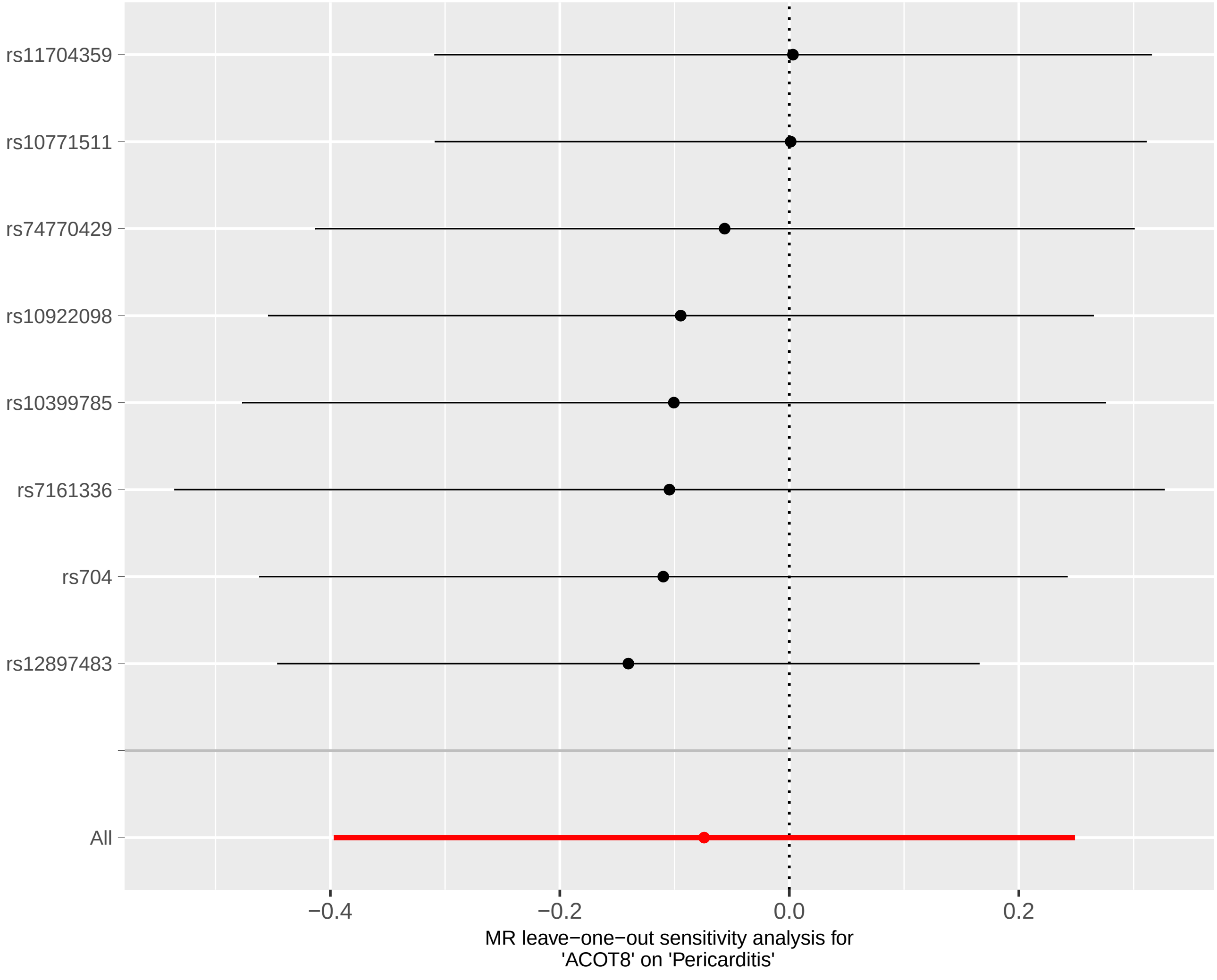

rs11704359
rs10771511
rs74770429
rs10922098
rs10399785
rs7161336
rs704
rs12897483
All
−0.4
−0.2
0.0
0.2
MR leave−one−out sensitivity analysis for
'ACOT8' on 'Pericarditis'

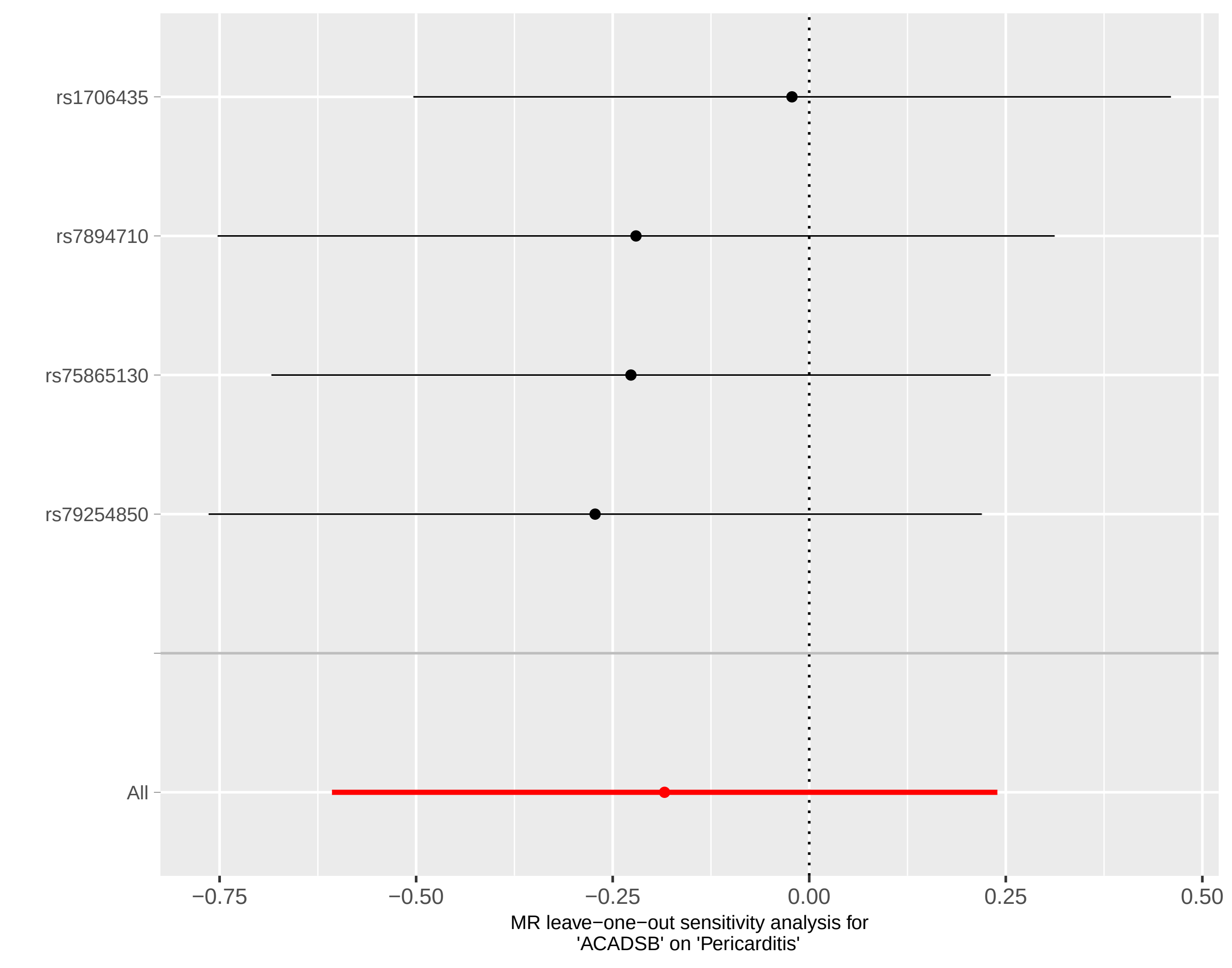
rs1706435
rs7894710
rs75865130
rs79254850
All
−0.75
−0.50
−0.25
0.00
0.25
0.50
MR leave−one−out sensitivity analysis for
'ACADSB' on 'Pericarditis'

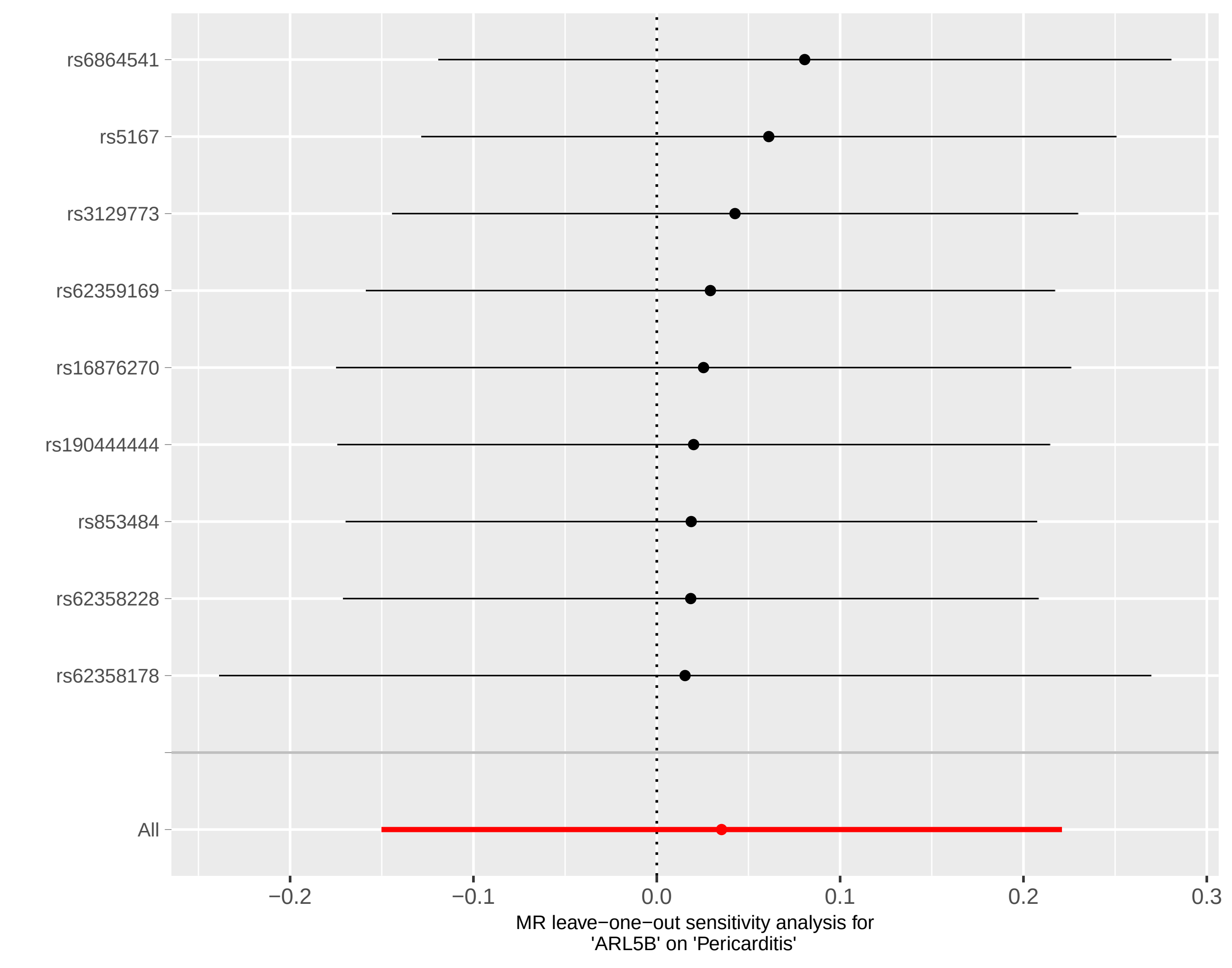
rs6864541
rs5167
rs3129773
rs62359169
rs16876270
rs190444444
rs853484
rs62358228
rs62358178
All
−0.2
−0.1
0.0
0.1
0.2
0.3
MR leave−one−out sensitivity analysis for
'ARL5B' on 'Pericarditis'

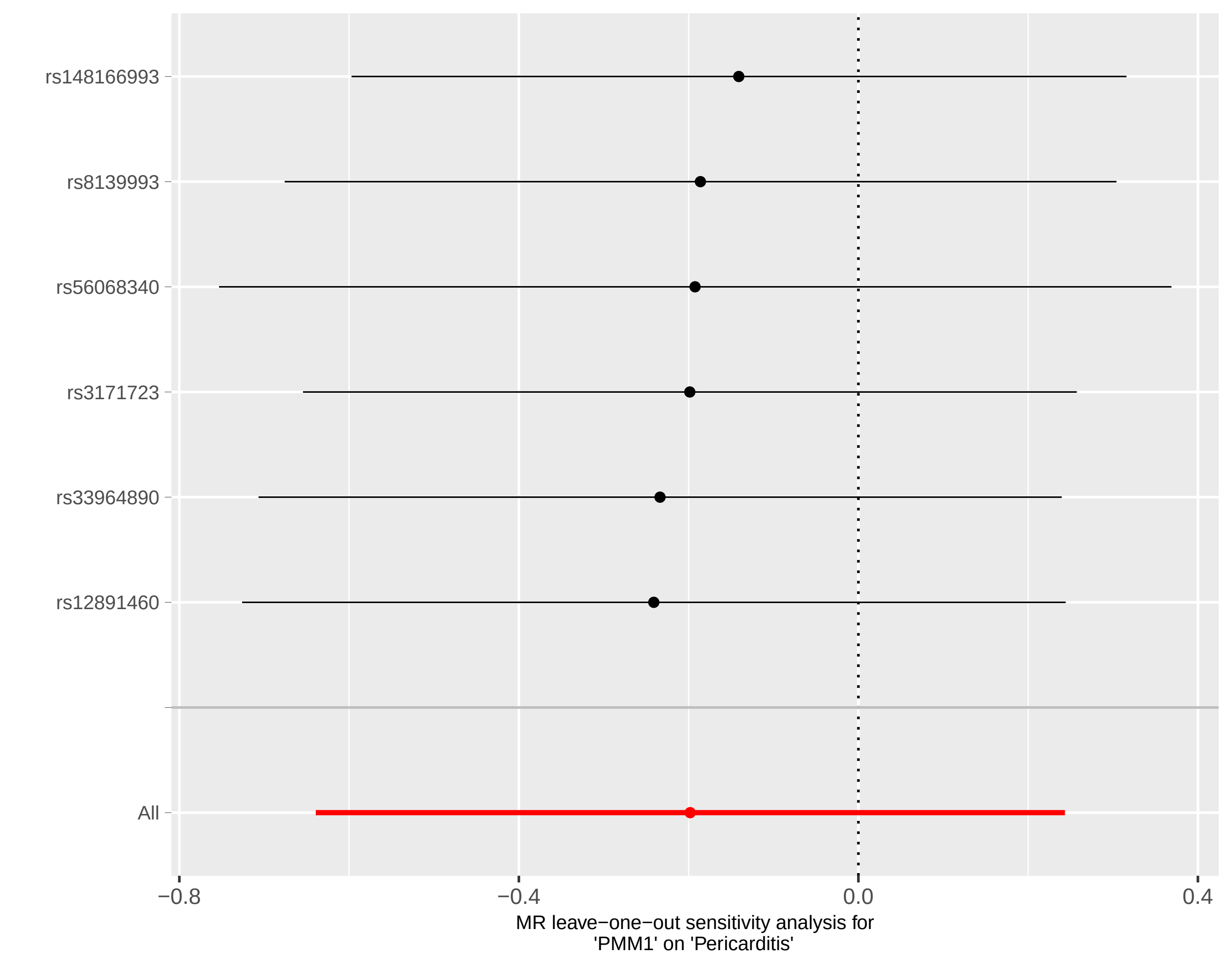
rs148166993
rs8139993
rs56068340
rs3171723
rs33964890
rs12891460
All
−0.8
−0.4
0.0
0.4
MR leave−one−out sensitivity analysis for
'PMM1' on 'Pericarditis'

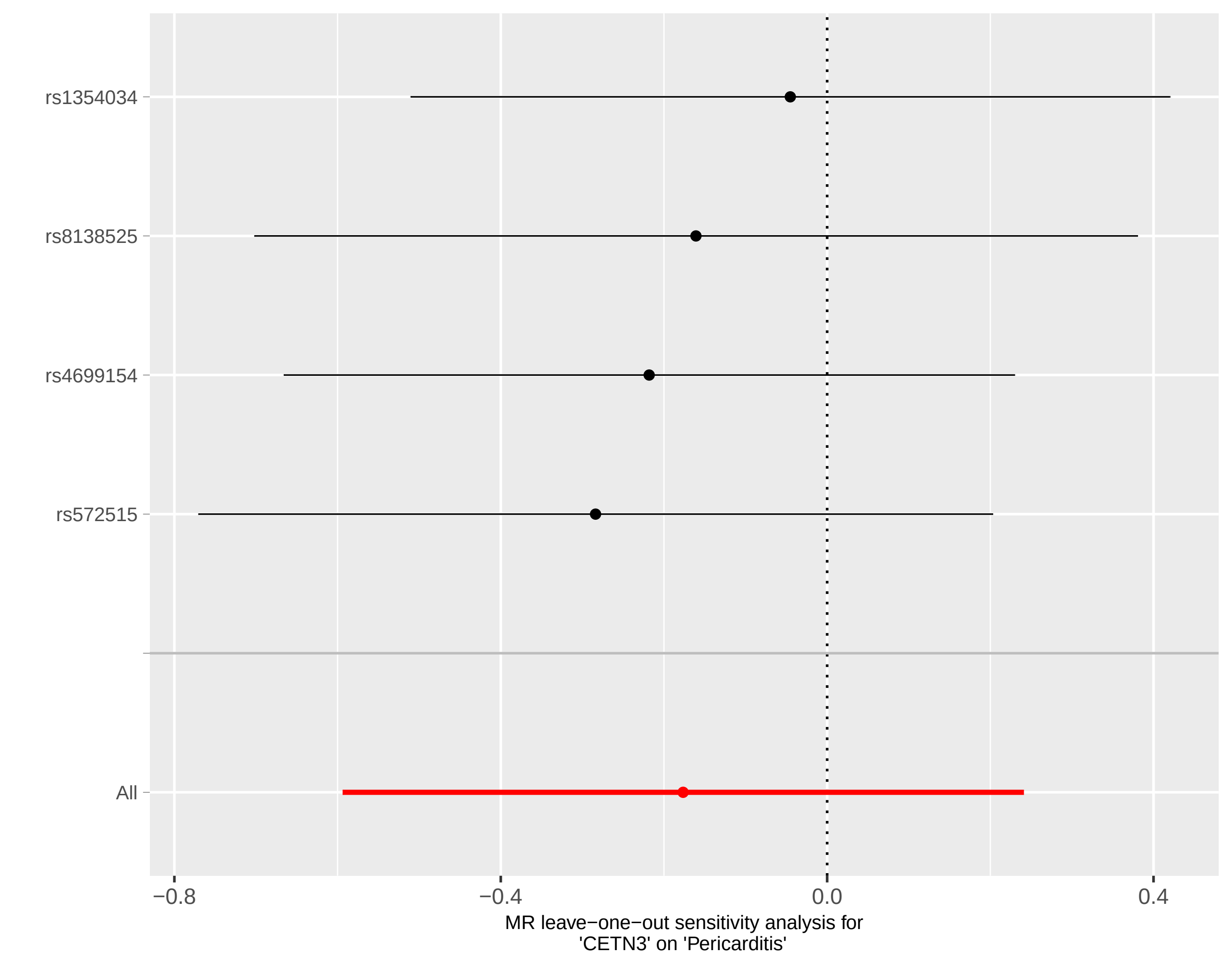
rs1354034
rs8138525
rs4699154
rs572515
All
−0.8
−0.4
0.0
0.4
MR leave−one−out sensitivity analysis for
'CETN3' on 'Pericarditis'

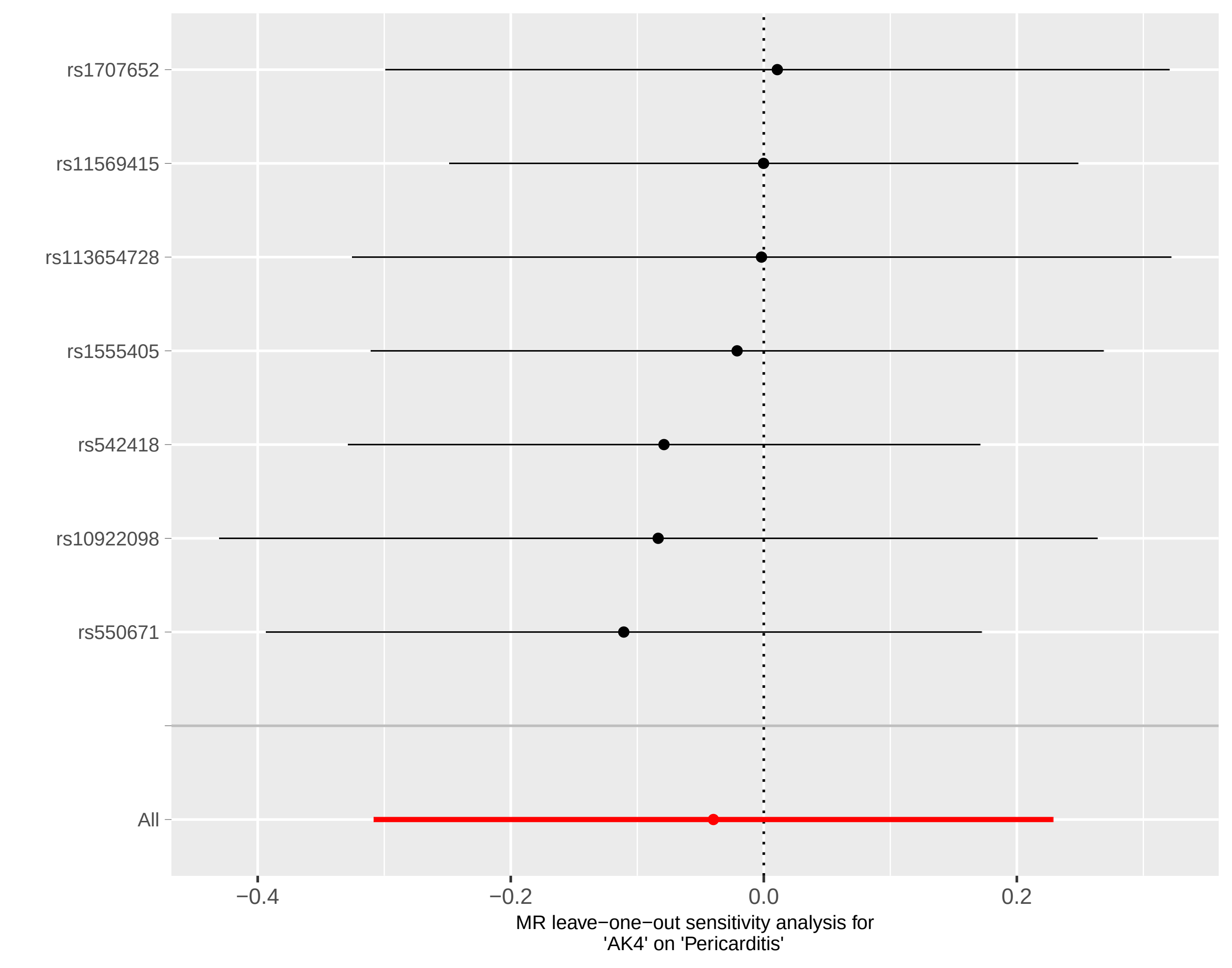
rs1707652
rs11569415
rs113654728
rs1555405
rs542418
rs10922098
rs550671
All
−0.4
−0.2
0.0
0.2
MR leave−one−out sensitivity analysis for
'AK4' on 'Pericarditis'

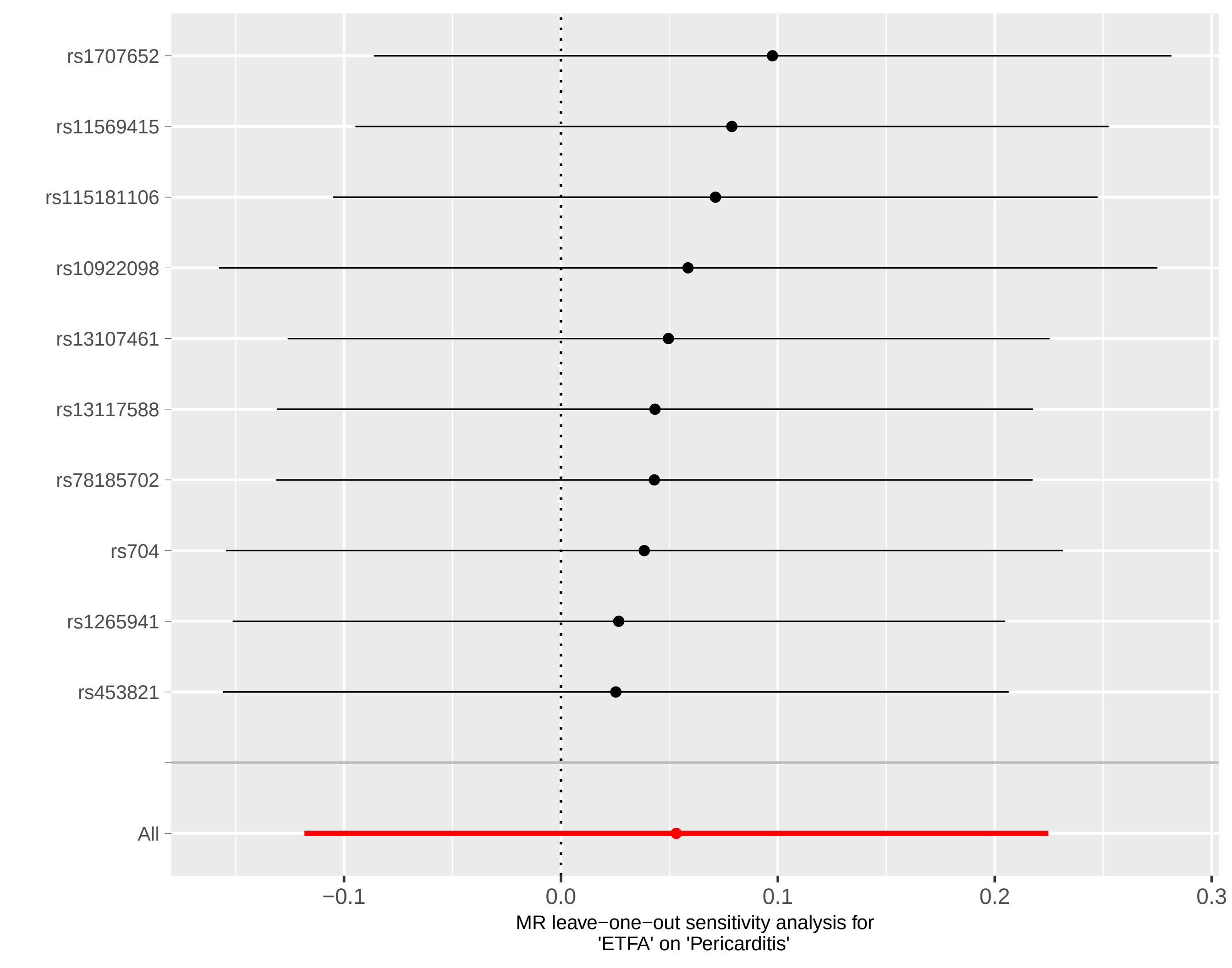
rs1707652
rs11569415
rs115181106
rs10922098
rs13107461
rs13117588
rs78185702
rs704
rs1265941
rs453821
All
−0.1
0.0
0.1
0.2
0.3
MR leave−one−out sensitivity analysis for
'ETFA' on 'Pericarditis'

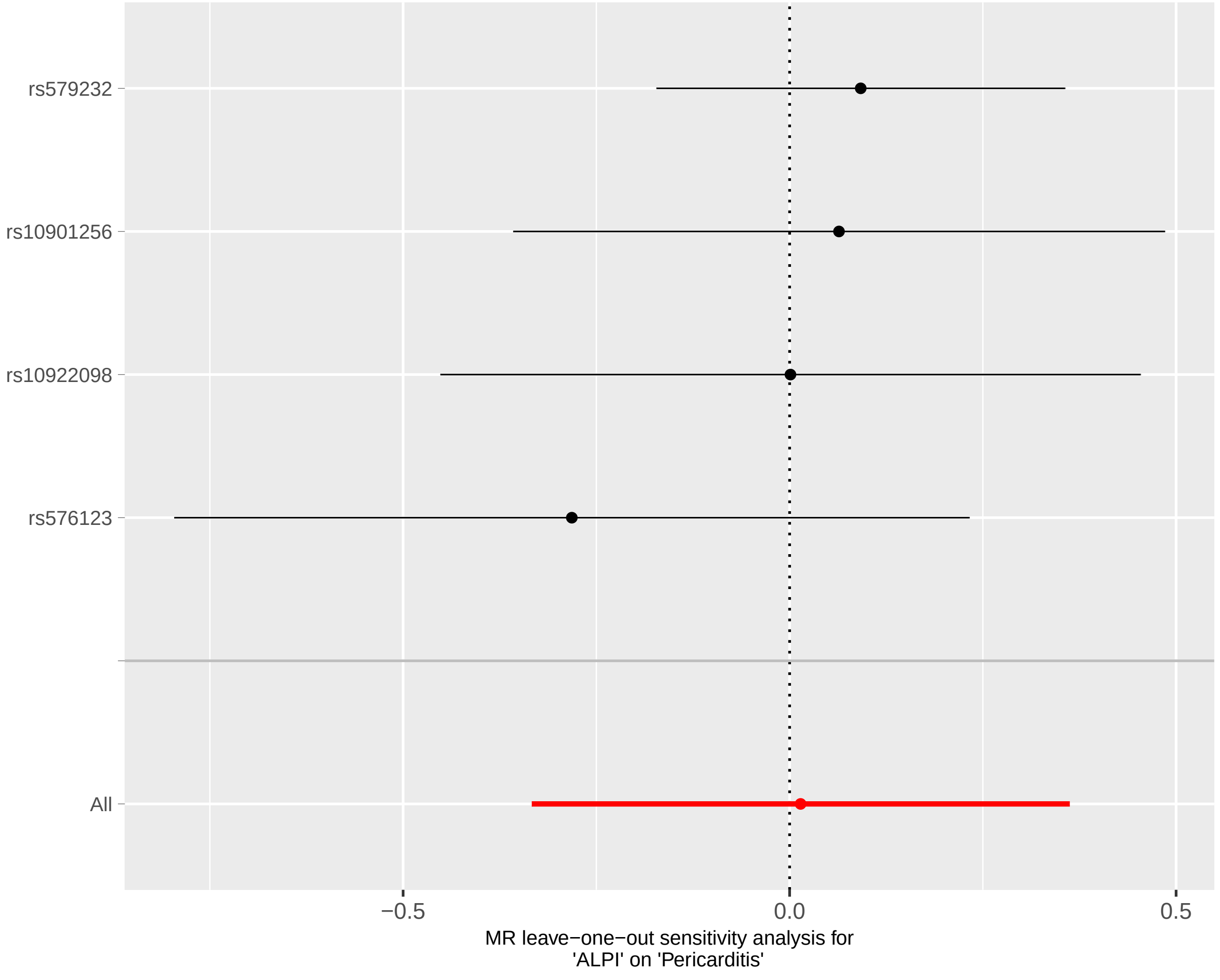
rs579232
rs10901256
rs10922098
rs576123
All
−0.5
0.0
0.5
MR leave−one−out sensitivity analysis for
'ALPI' on 'Pericarditis'

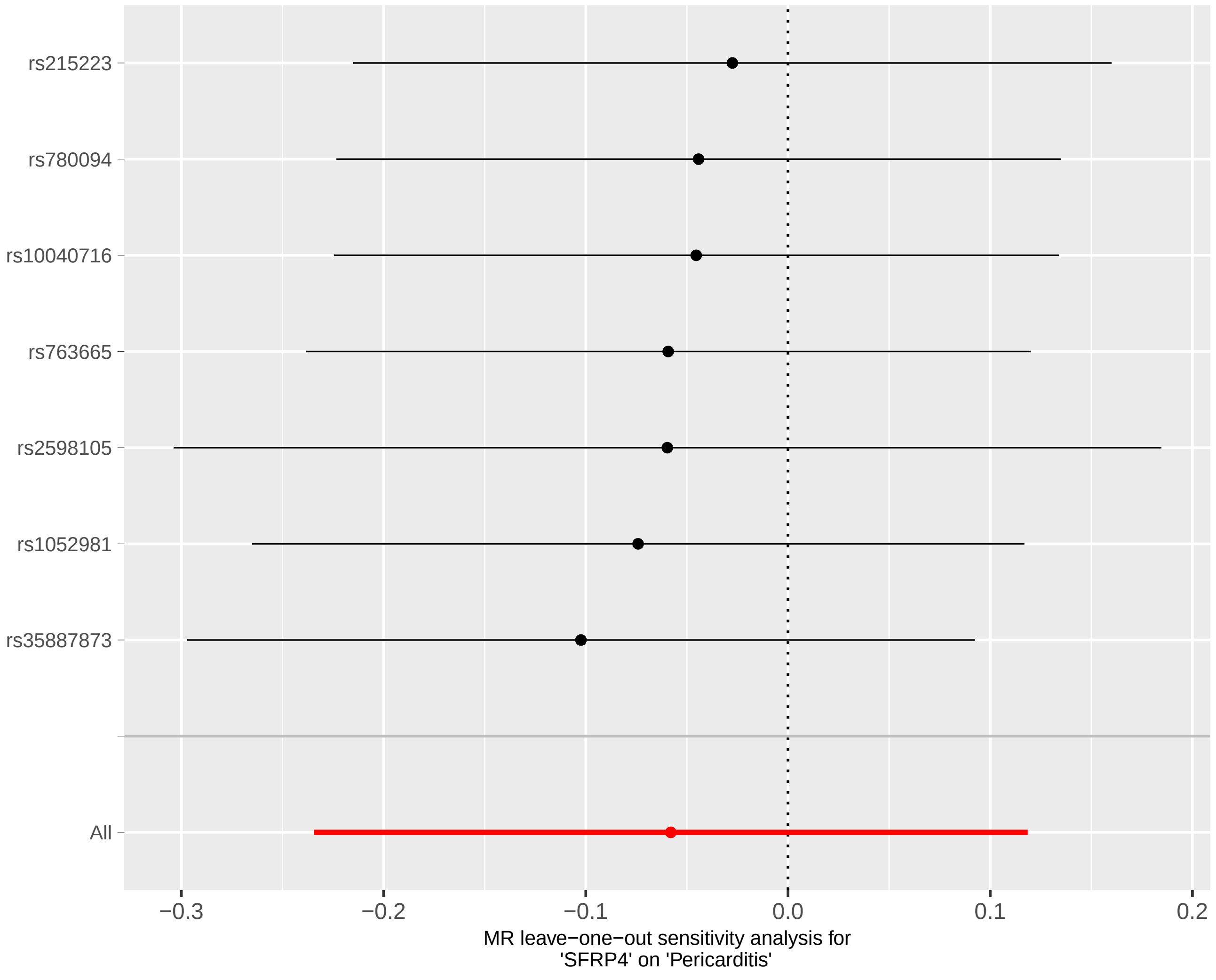
rs215223
rs780094
rs10040716
rs763665
rs2598105
rs1052981
rs35887873
All
−0.3
−0.2
−0.1
0.0
0.1
0.2
MR leave−one−out sensitivity analysis for
'SFRP4' on 'Pericarditis'

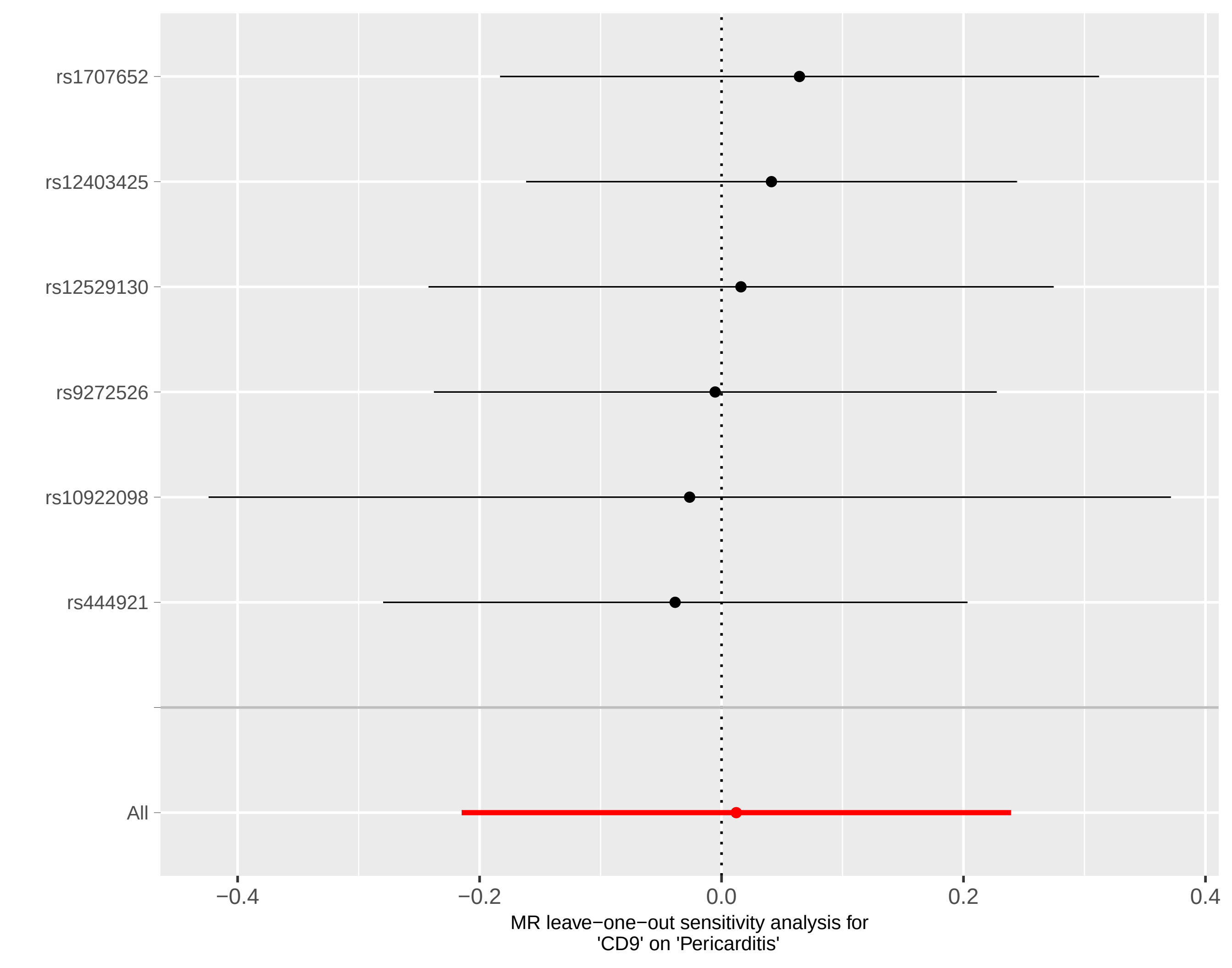

rs1707652
rs12403425
rs12529130
rs9272526
rs10922098
rs444921
All
−0.4
−0.2
0.0
0.2
0.4
MR leave−one−out sensitivity analysis for
'CD9' on 'Pericarditis'

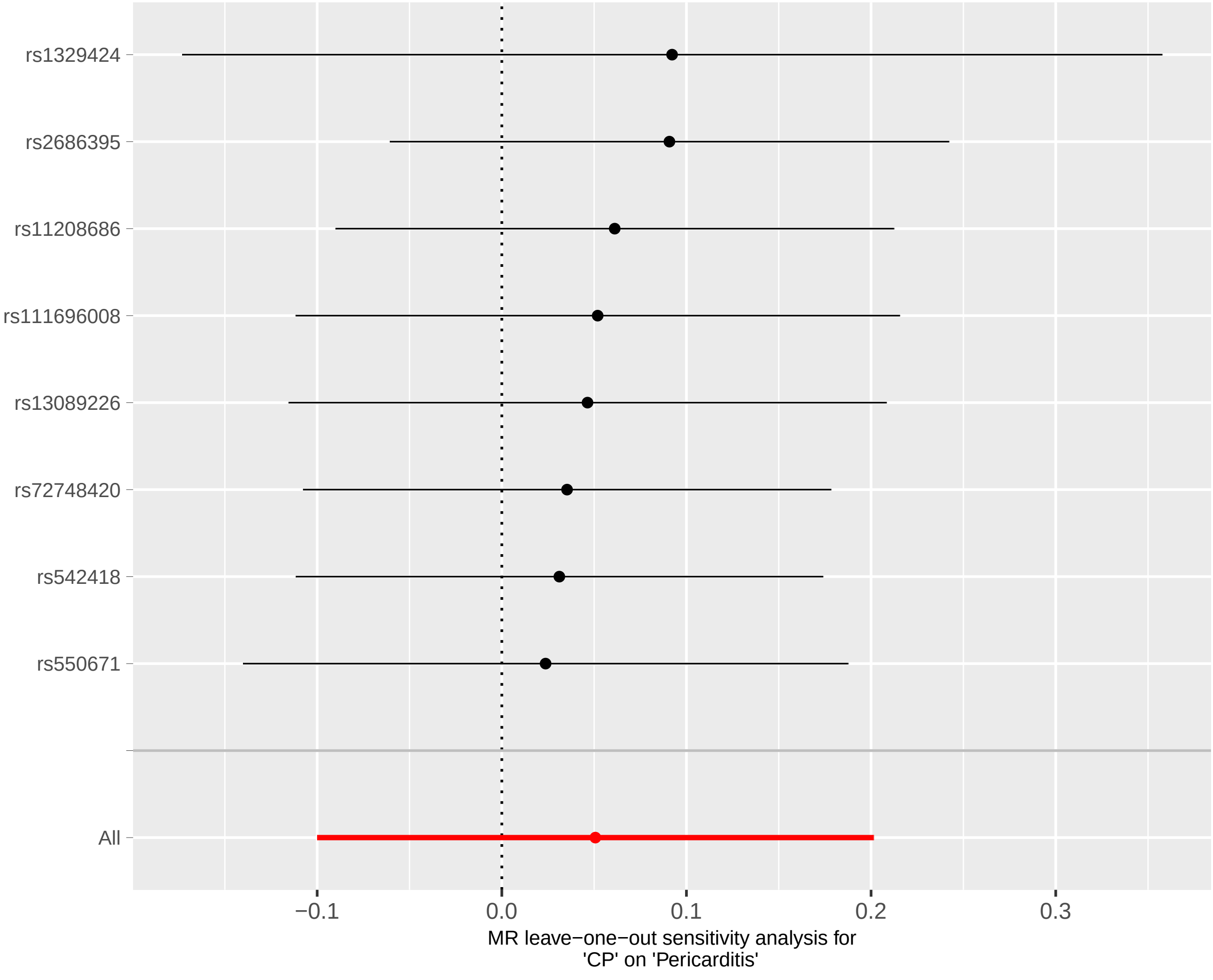

rs1329424
rs2686395
rs11208686
rs111696008
rs13089226
rs72748420
rs542418
rs550671
All
−0.1
0.0
0.1
0.2
0.3
MR leave−one−out sensitivity analysis for
'CP' on 'Pericarditis'

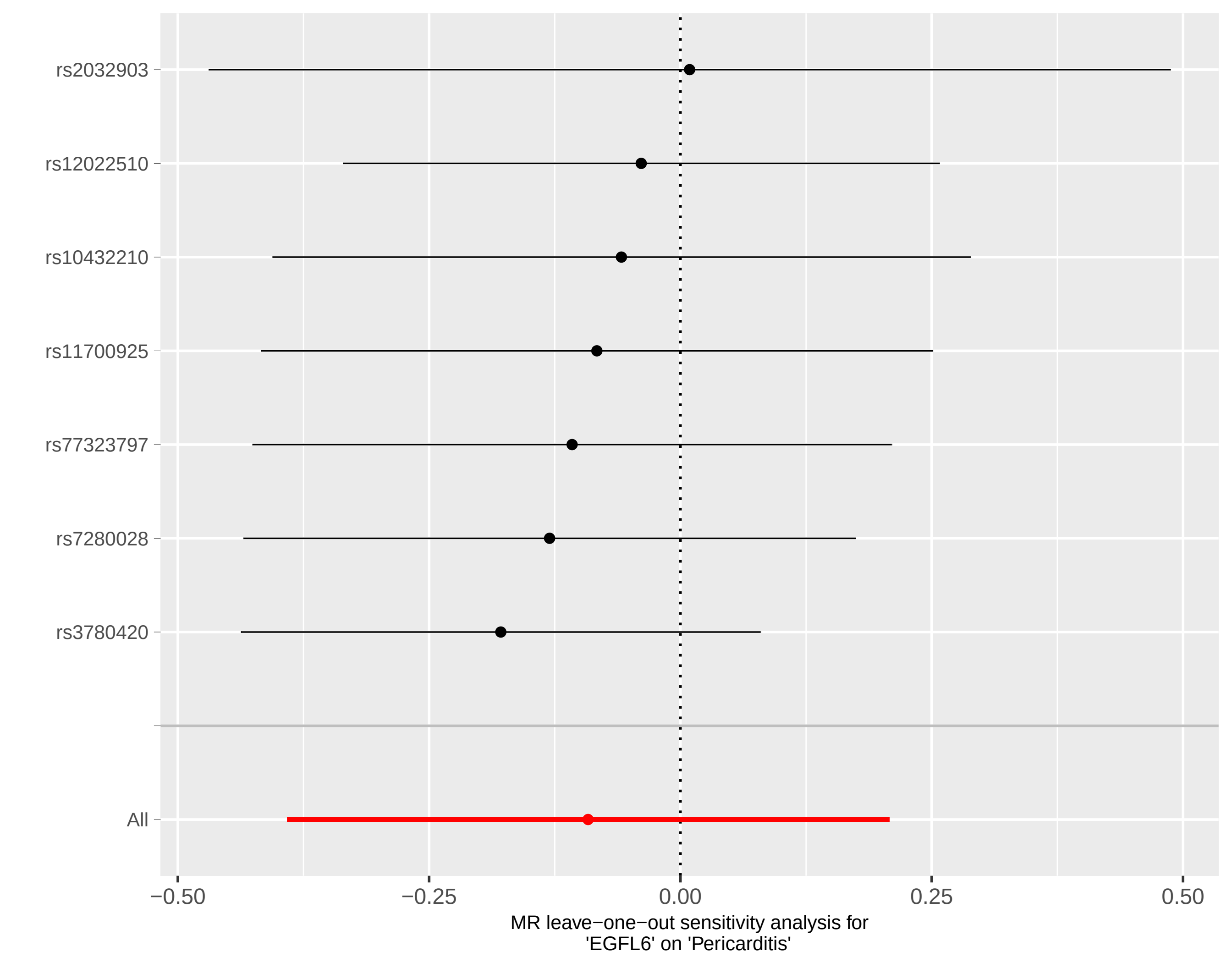
rs2032903
rs12022510
rs10432210
rs11700925
rs77323797
rs7280028
rs3780420
All
−0.50
−0.25
0.00
0.25
0.50
MR leave−one−out sensitivity analysis for
'EGFL6' on 'Pericarditis'

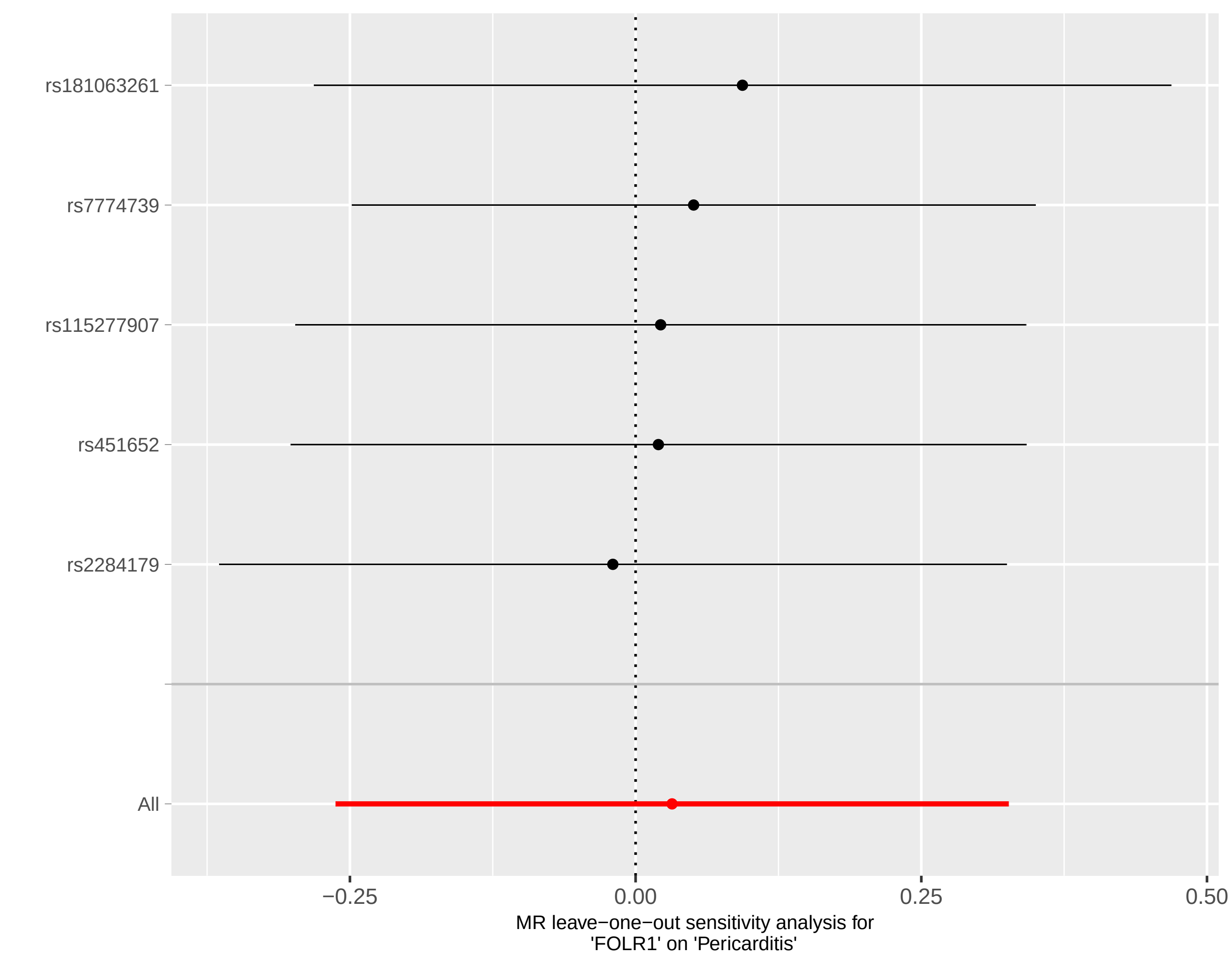
rs181063261
rs7774739
rs115277907
rs451652
rs2284179
All
−0.25
0.00
0.25
0.50
MR leave−one−out sensitivity analysis for
'FOLR1' on 'Pericarditis'

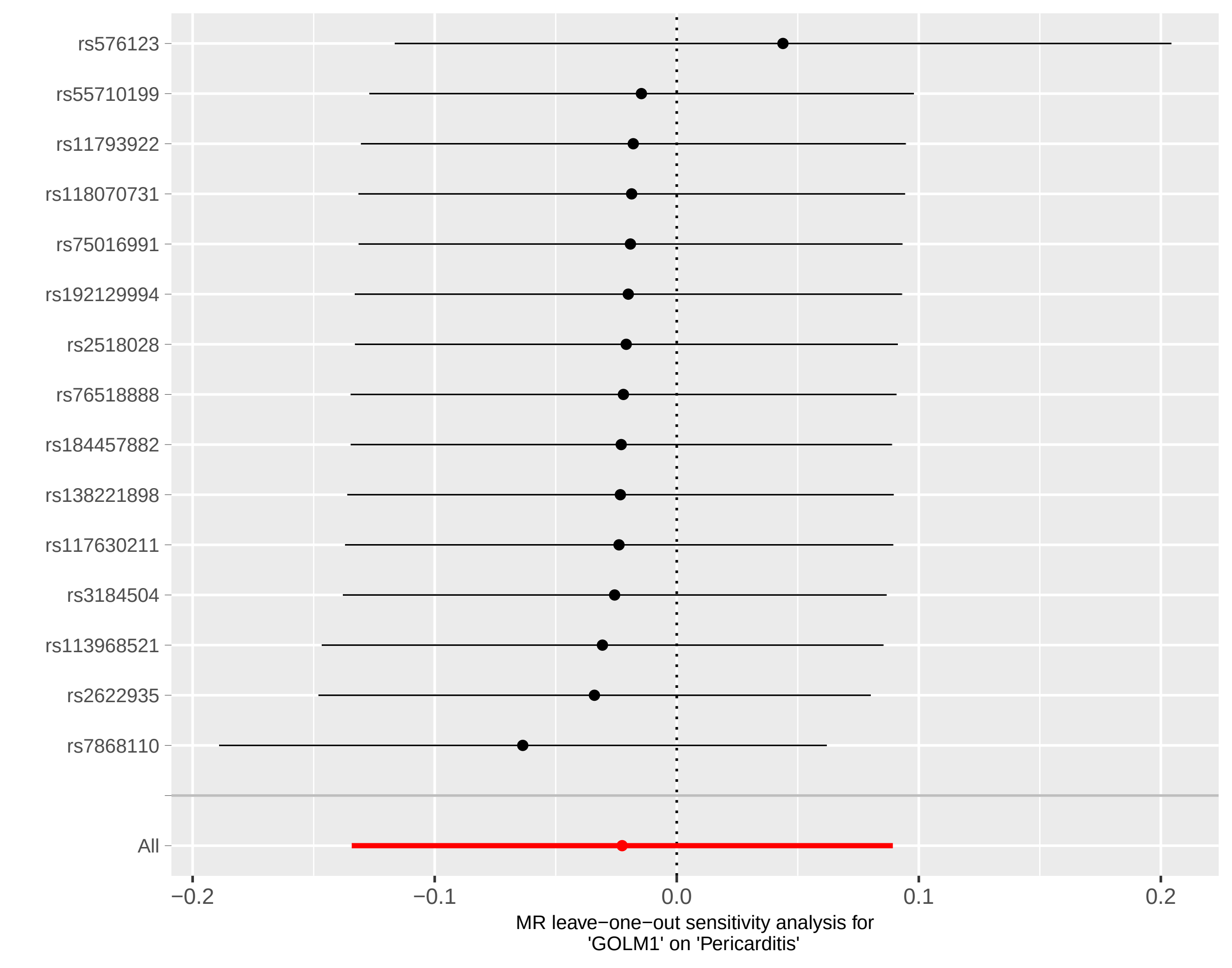
rs576123
rs55710199
rs11793922
rs118070731
rs75016991
rs192129994
rs2518028
rs76518888
rs184457882
rs138221898
rs117630211
rs3184504
rs113968521
rs2622935
rs7868110
All
−0.2
−0.1
0.0
0.1
0.2
MR leave−one−out sensitivity analysis for
'GOLM1' on 'Pericarditis'

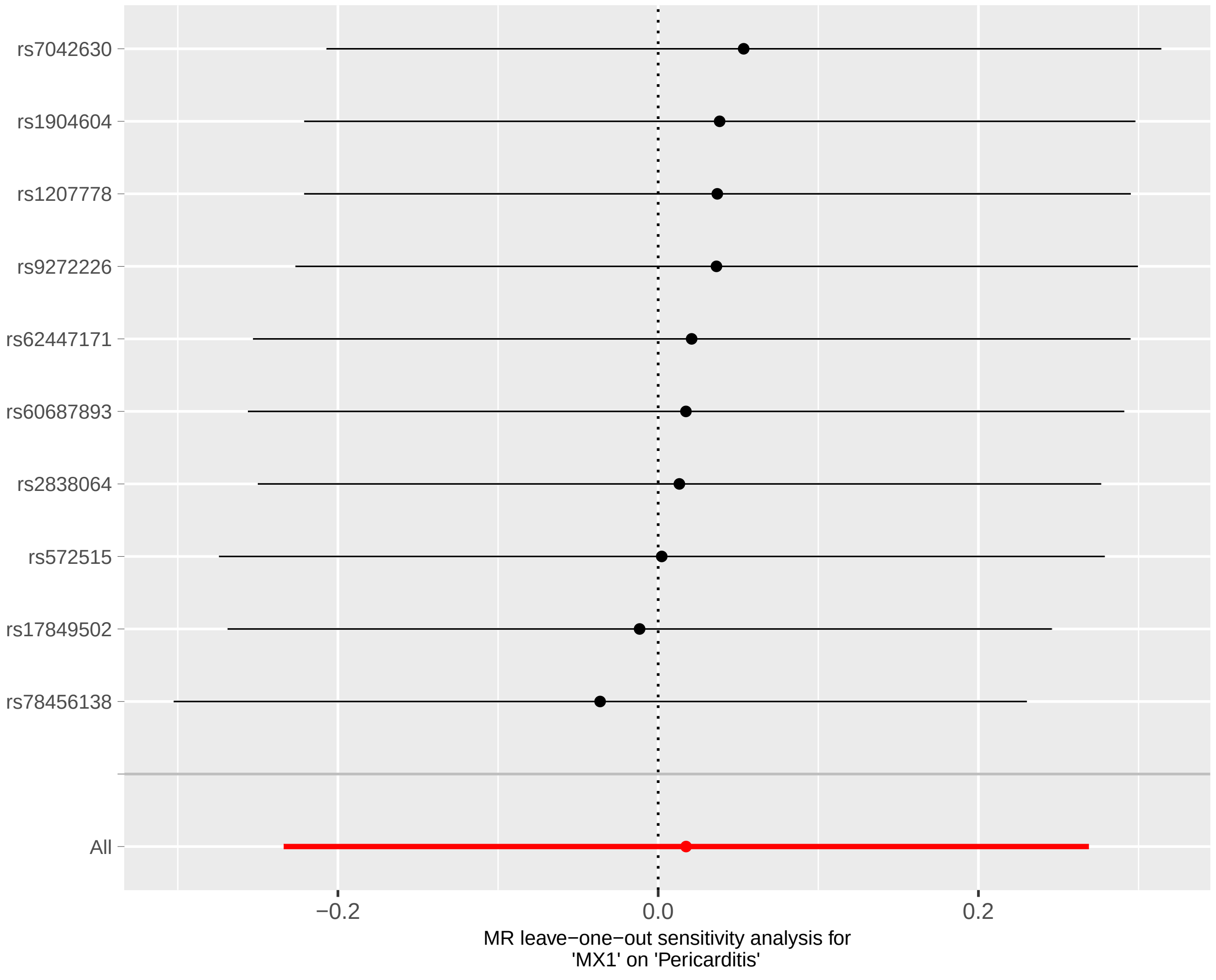

rs7042630
rs1904604
rs1207778
rs9272226
rs62447171
rs60687893
rs2838064
rs572515
rs17849502
rs78456138
All
−0.2
0.0
0.2
MR leave−one−out sensitivity analysis for
'MX1' on 'Pericarditis'

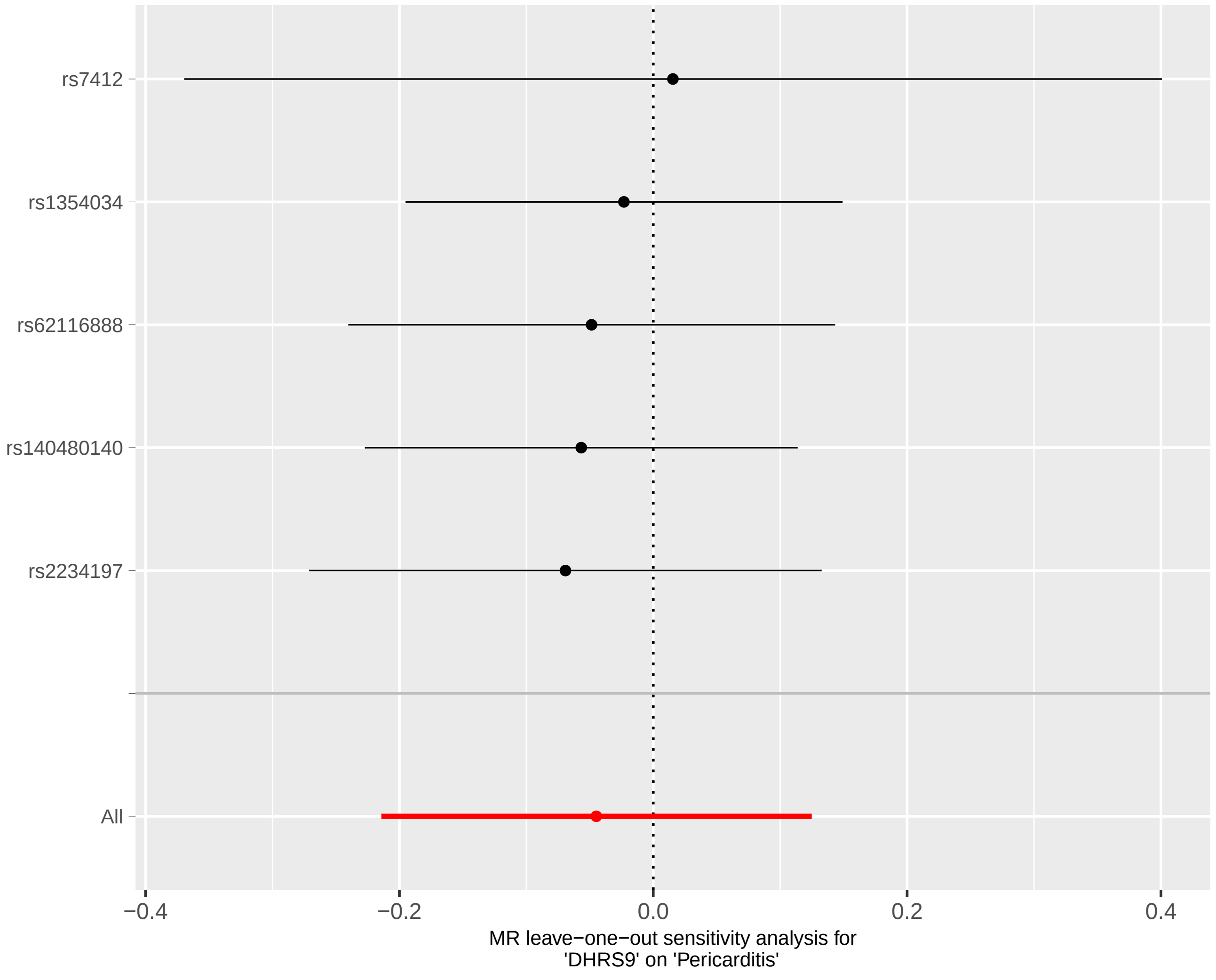
rs7412
rs1354034
rs62116888
rs140480140
rs2234197
All
−0.4
−0.2
0.0
0.2
0.4
MR leave−one−out sensitivity analysis for
'DHRS9' on 'Pericarditis'

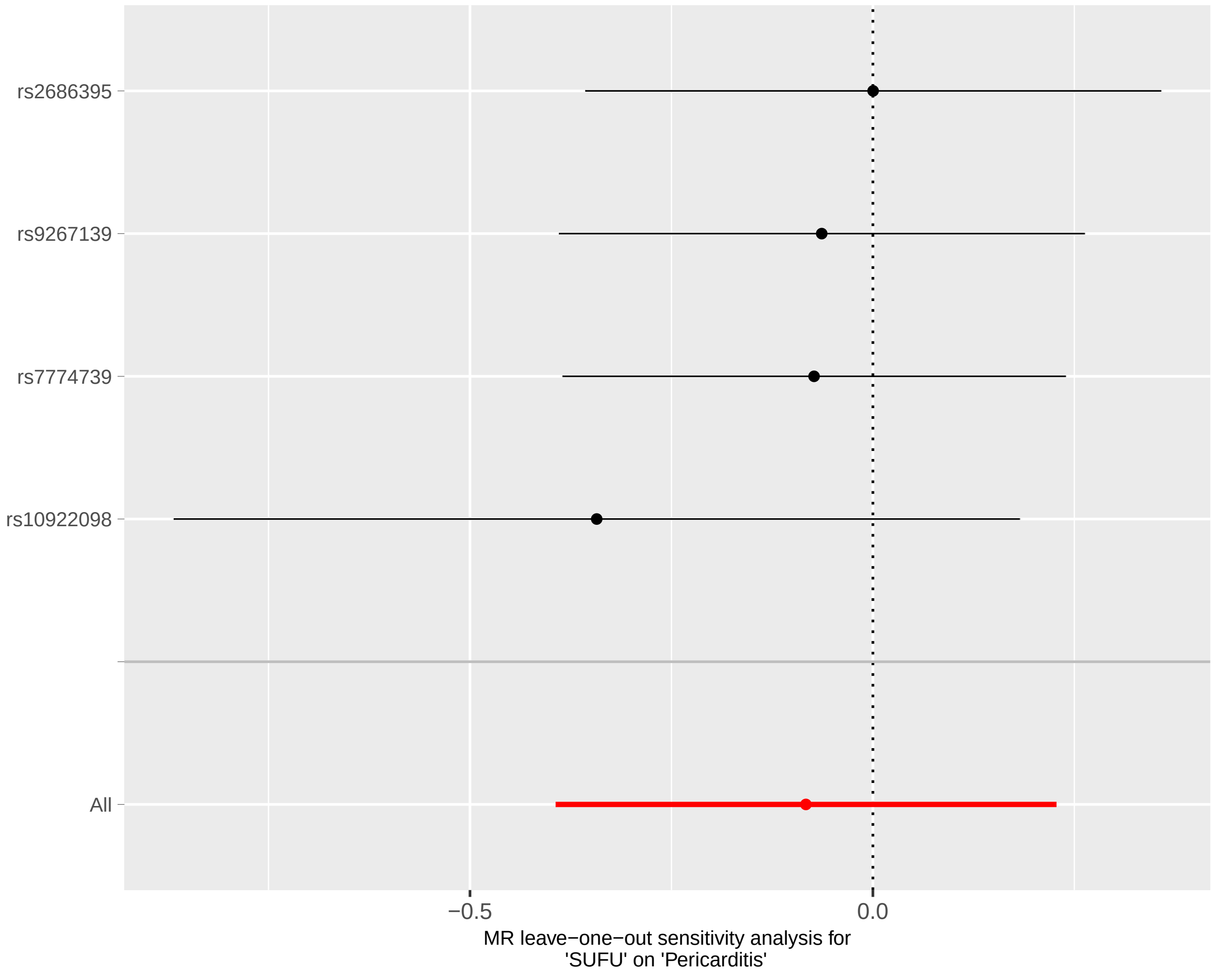
rs2686395
rs9267139
rs7774739
rs10922098
All
−0.5
0.0
MR leave−one−out sensitivity analysis for
'SUFU' on 'Pericarditis'

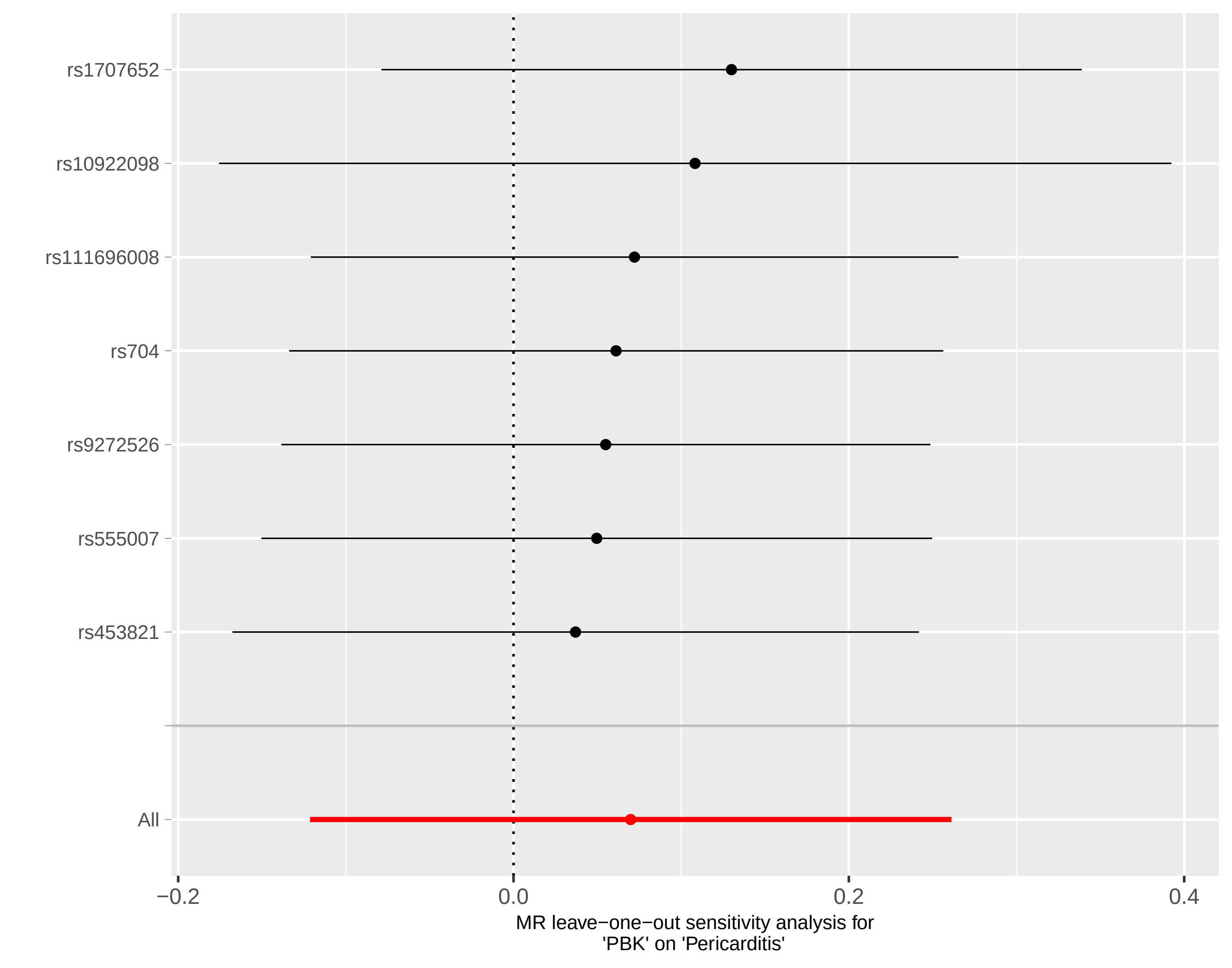

rs1707652
rs10922098
rs111696008
rs704
rs9272526
rs555007
rs453821
All
−0.2
0.0
0.2
0.4
MR leave−one−out sensitivity analysis for
'PBK' on 'Pericarditis'

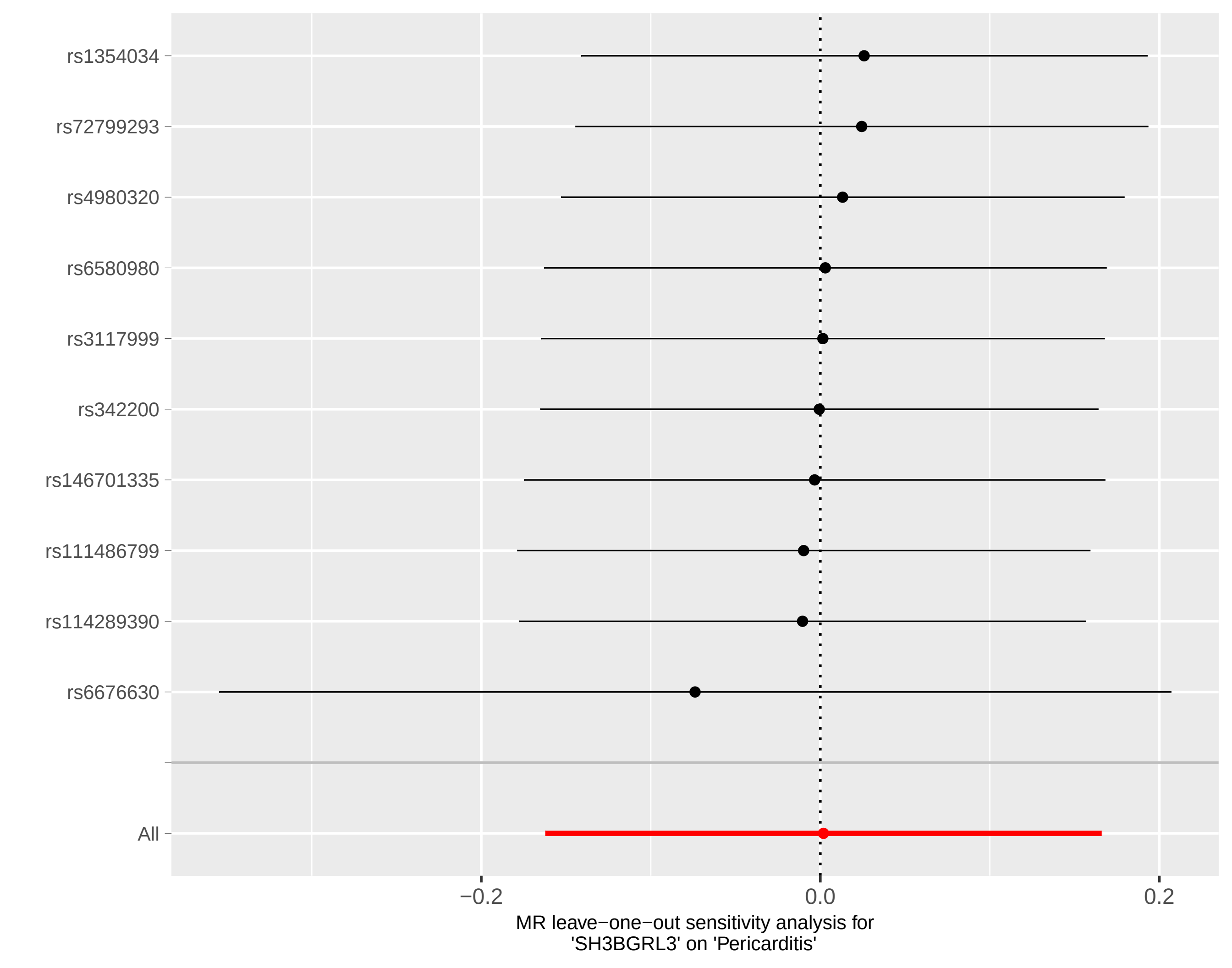
rs1354034
rs72799293
rs4980320
rs6580980
rs3117999
rs342200
rs146701335
rs111486799
rs114289390
rs6676630
All
−0.2
0.0
0.2
MR leave−one−out sensitivity analysis for
'SH3BGRL3' on 'Pericarditis'

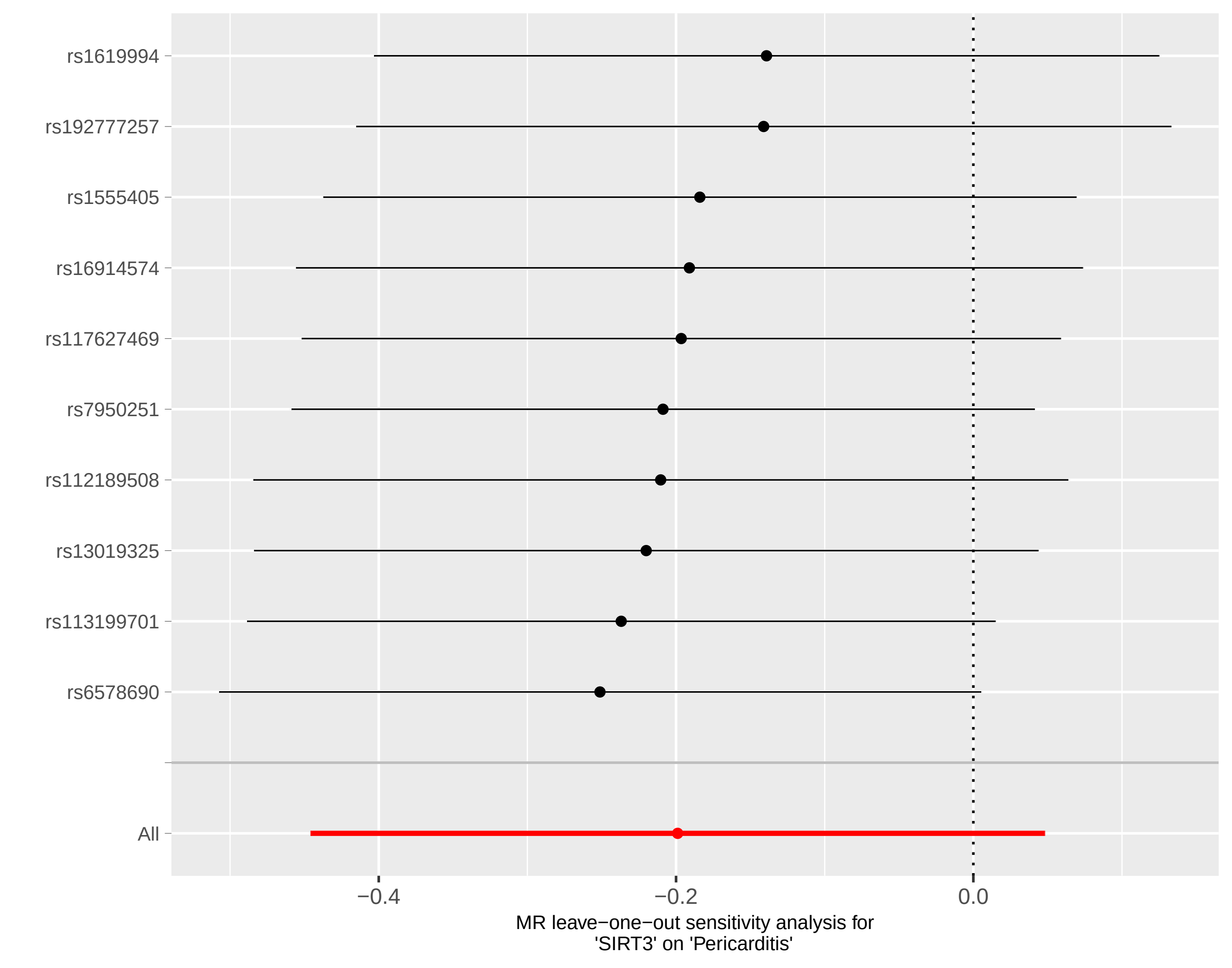
rs1619994
rs192777257
rs1555405
rs16914574
rs117627469
rs7950251
rs112189508
rs13019325
rs113199701
rs6578690
All
−0.4
−0.2
0.0
MR leave−one−out sensitivity analysis for
'SIRT3' on 'Pericarditis'

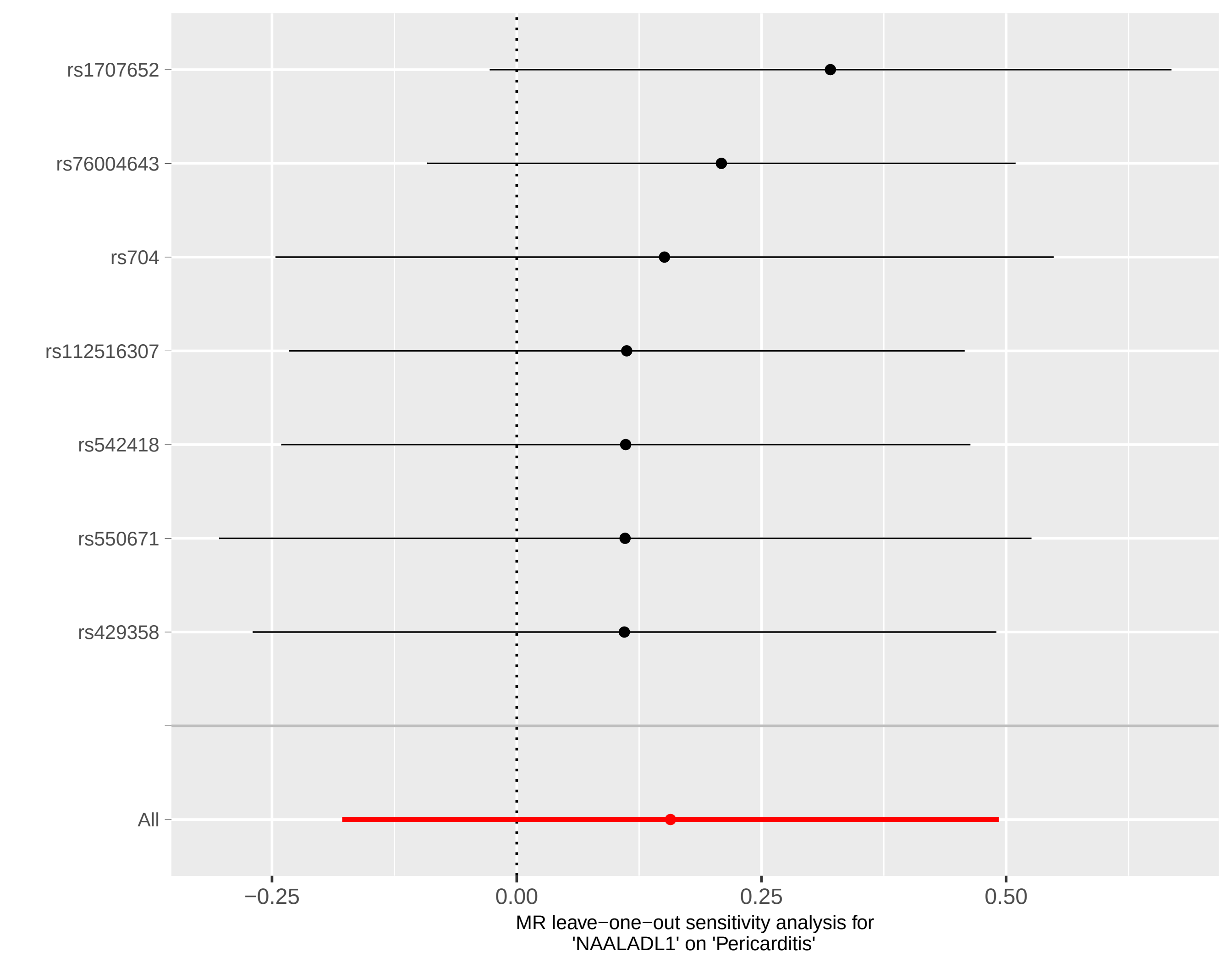
rs1707652
rs76004643
rs704
rs112516307
rs542418
rs550671
rs429358
All
−0.25
0.00
0.25
0.50
MR leave−one−out sensitivity analysis for
'NAALADL1' on 'Pericarditis'

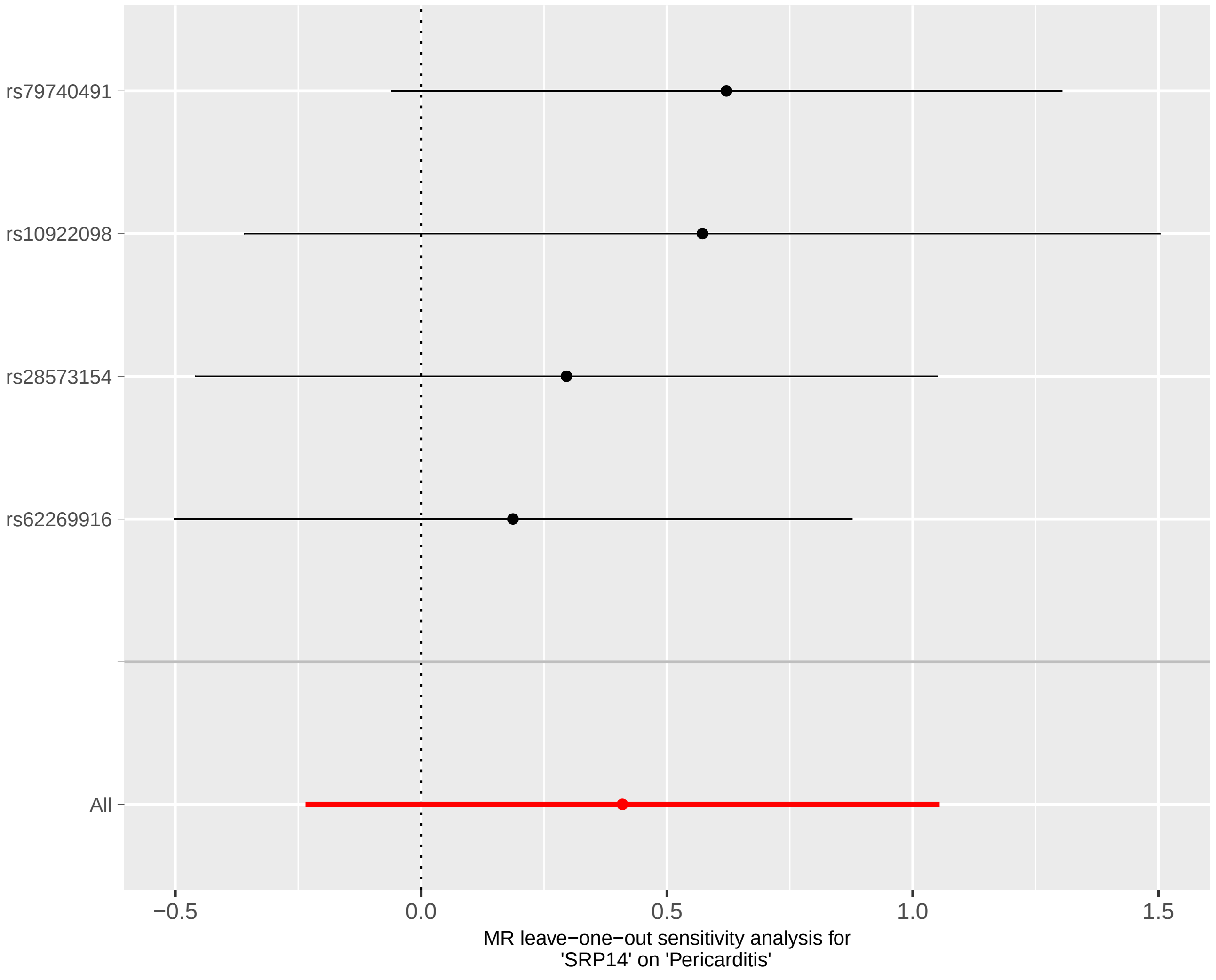
rs79740491
rs10922098
rs28573154
rs62269916
All
−0.5
0.0
0.5
1.0
1.5
MR leave−one−out sensitivity analysis for
'SRP14' on 'Pericarditis'

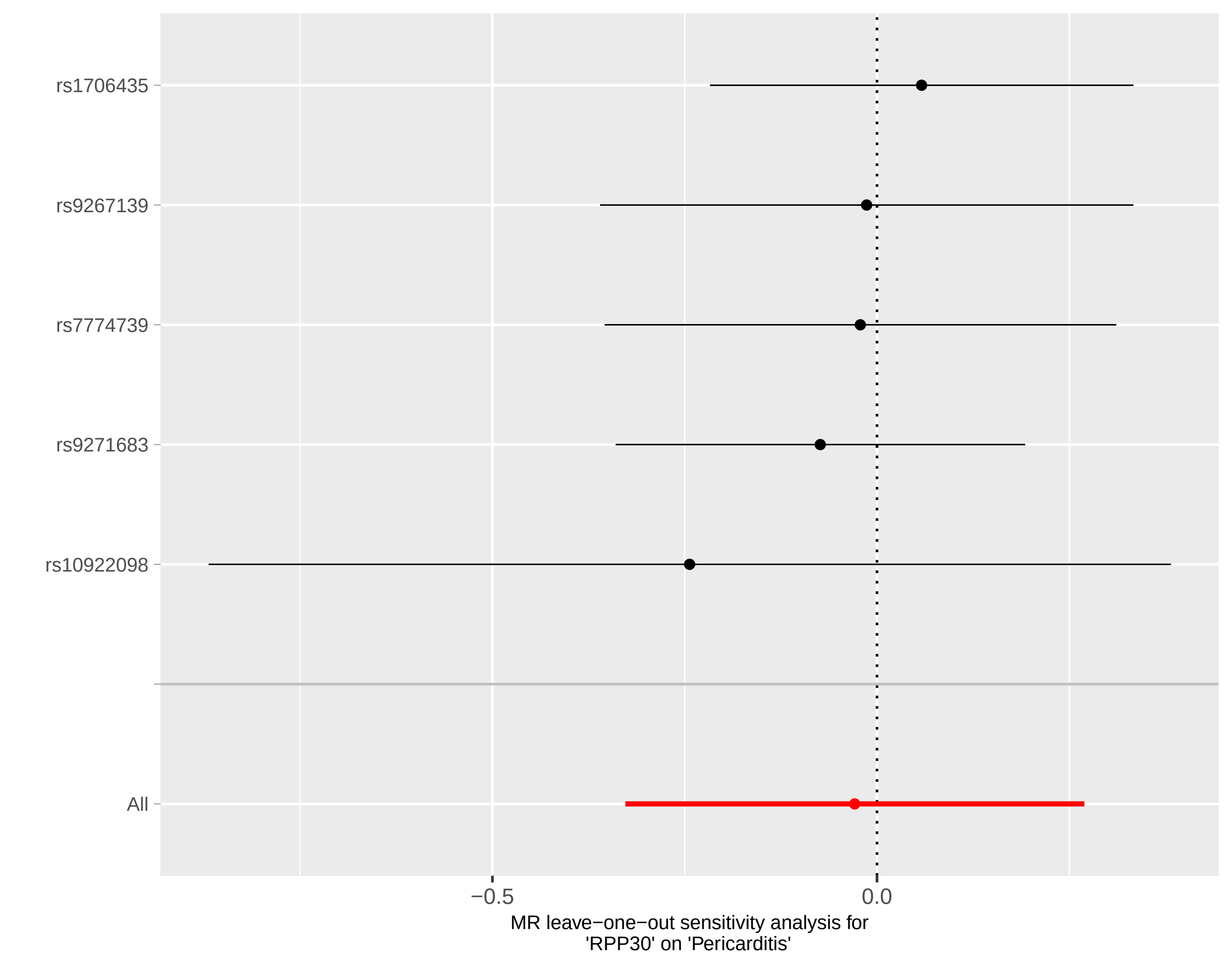
rs1706435
rs9267139
rs7774739
rs9271683
rs10922098
All
−0.5
0.0
MR leave−one−out sensitivity analysis for
'RPP30' on 'Pericarditis'

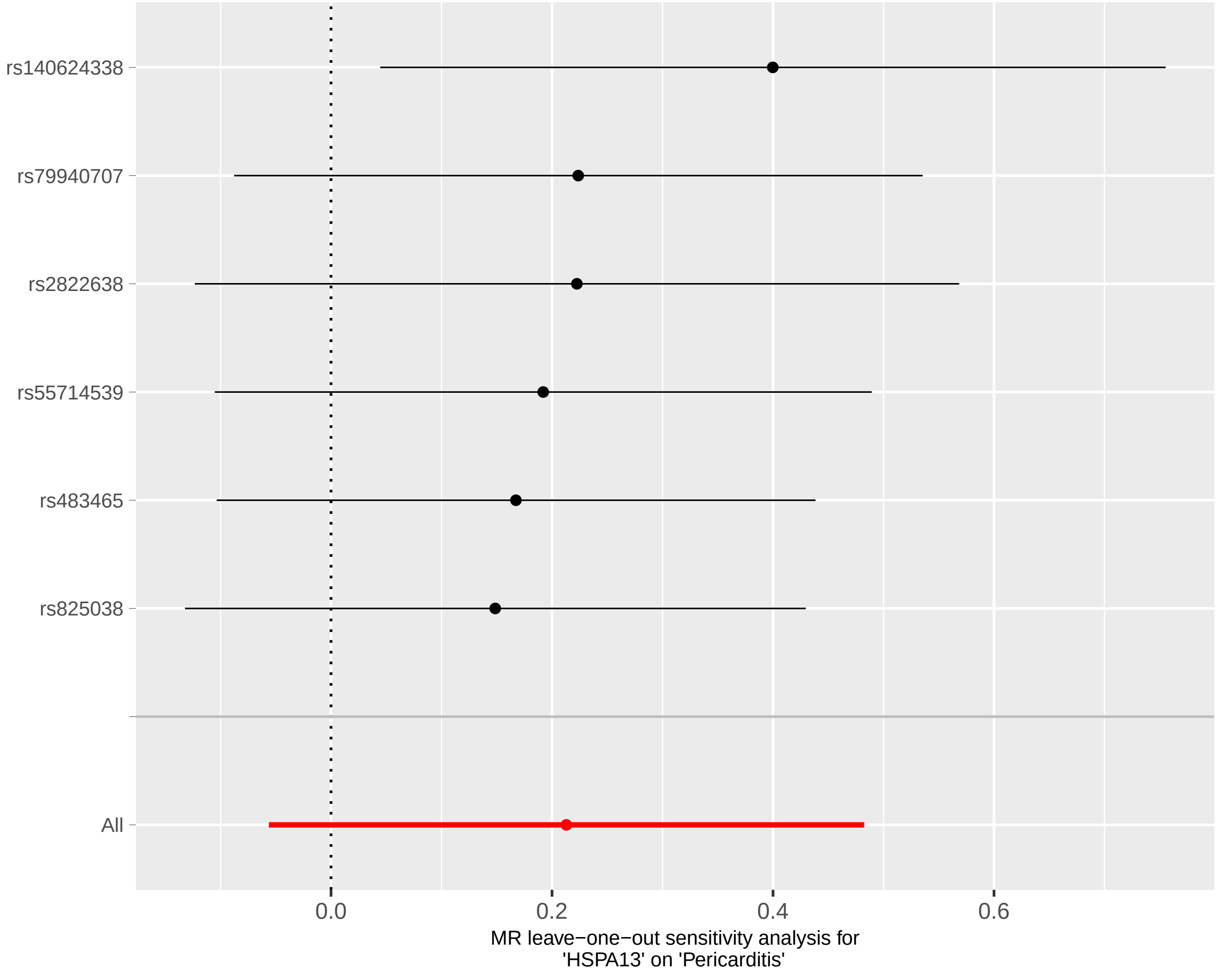

rs140624338
rs79940707
rs2822638
rs55714539
rs483465
rs825038
All
0.0
0.2
0.4
0.6
MR leave−one−out sensitivity analysis for
'HSPA13' on 'Pericarditis'

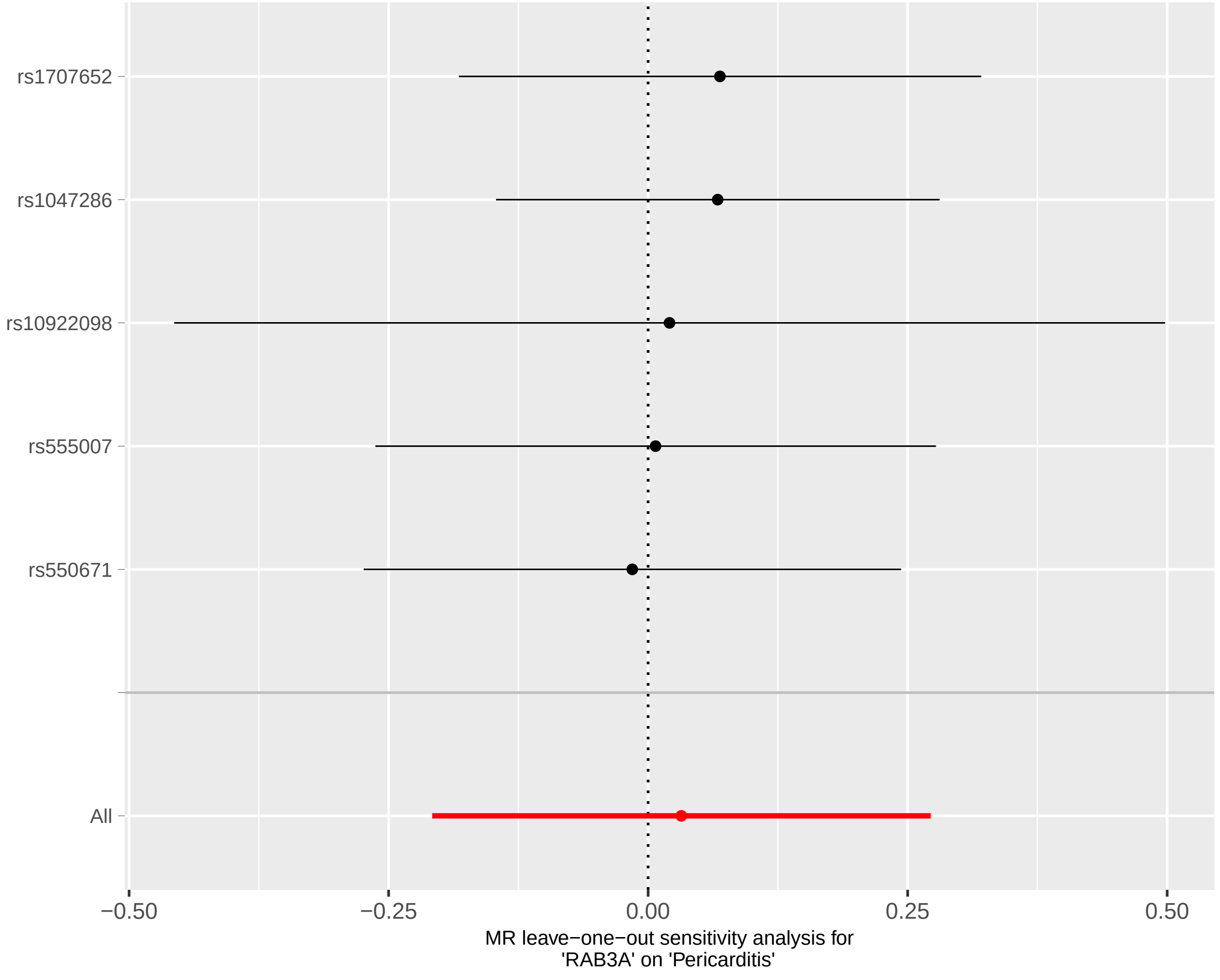

rs1707652
rs1047286
rs10922098
rs555007
rs550671
All
−0.50
−0.25
0.00
0.25
0.50
MR leave−one−out sensitivity analysis for
'RAB3A' on 'Pericarditis'

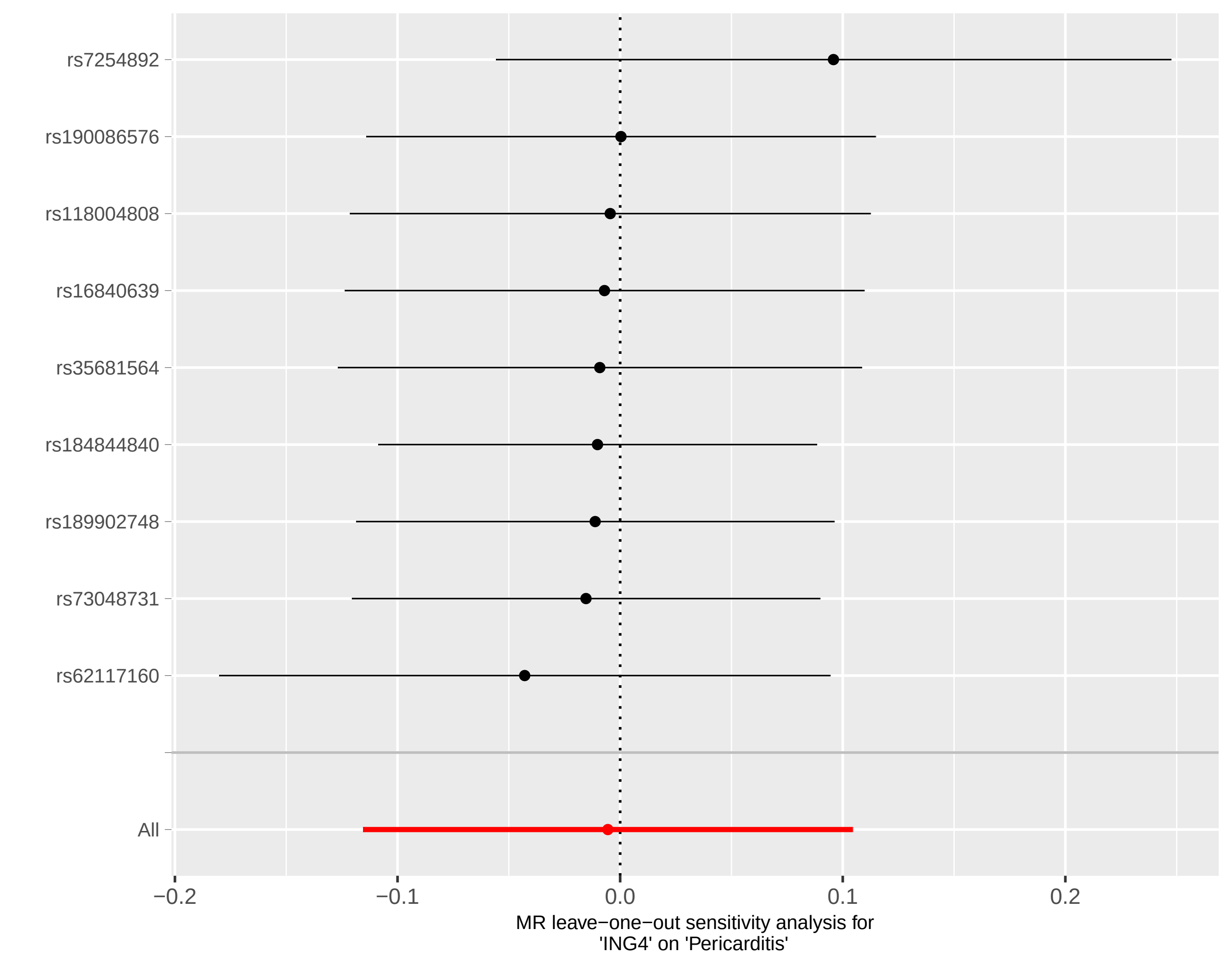
rs7254892
rs190086576
rs118004808
rs16840639
rs35681564
rs184844840
rs189902748
rs73048731
rs62117160
All
−0.2
−0.1
0.0
0.1
0.2
MR leave−one−out sensitivity analysis for
'ING4' on 'Pericarditis'

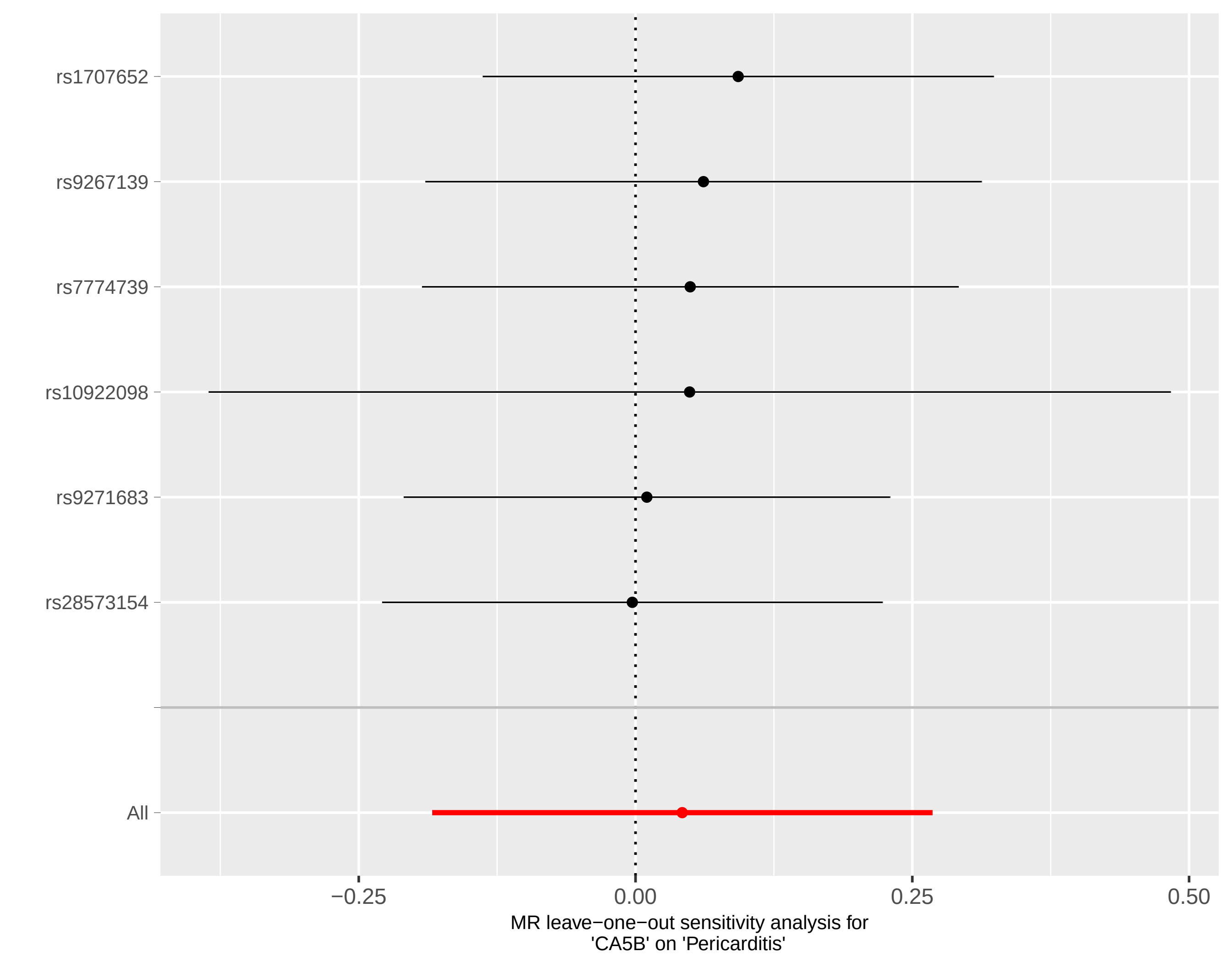
rs1707652
rs9267139
rs7774739
rs10922098
rs9271683
rs28573154
All
−0.25
0.00
0.25
0.50
MR leave−one−out sensitivity analysis for
'CA5B' on 'Pericarditis'

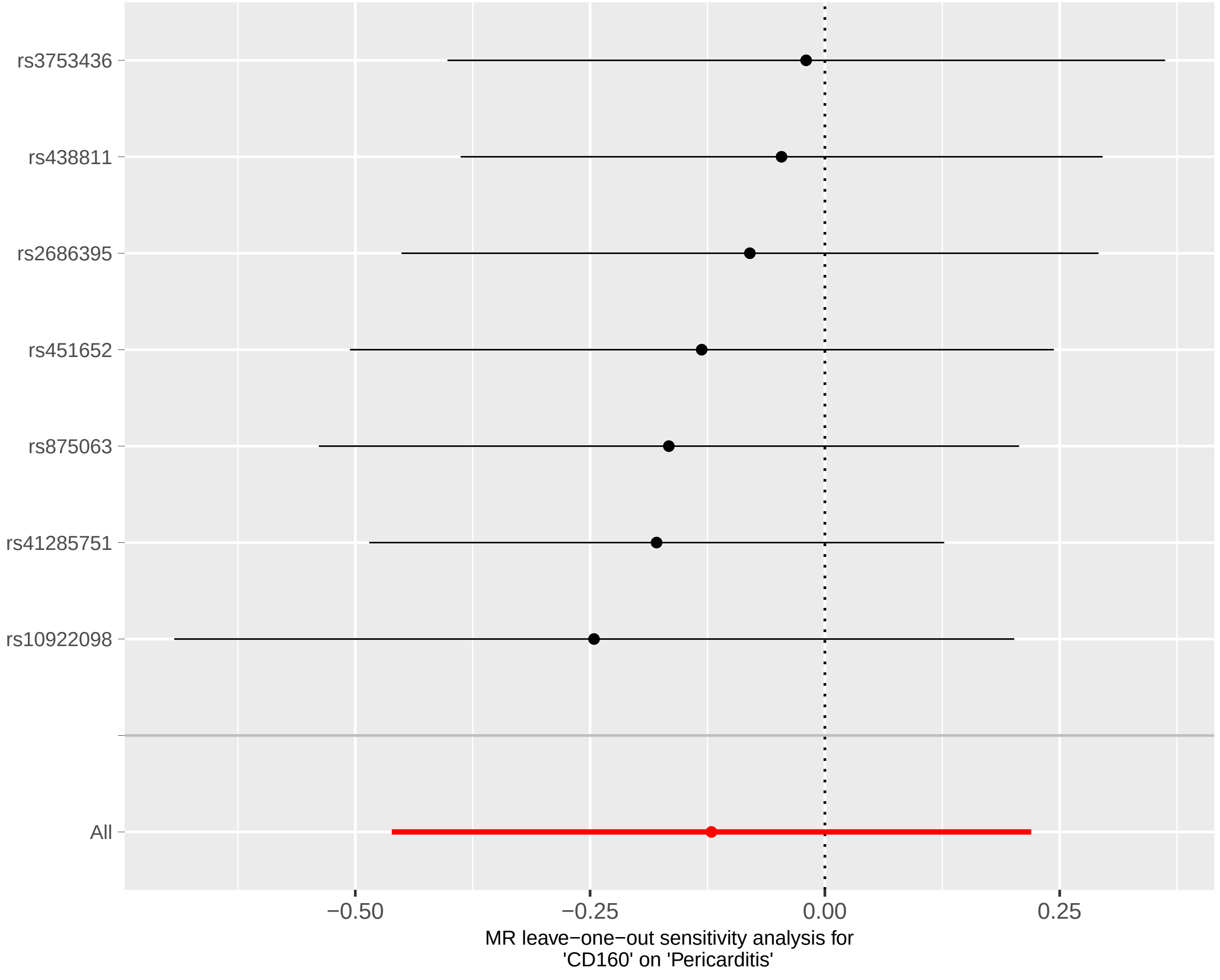
rs3753436
rs438811
rs2686395
rs451652
rs875063
rs41285751
rs10922098
All
−0.50
−0.25
0.00
0.25
MR leave−one−out sensitivity analysis for
'CD160' on 'Pericarditis'

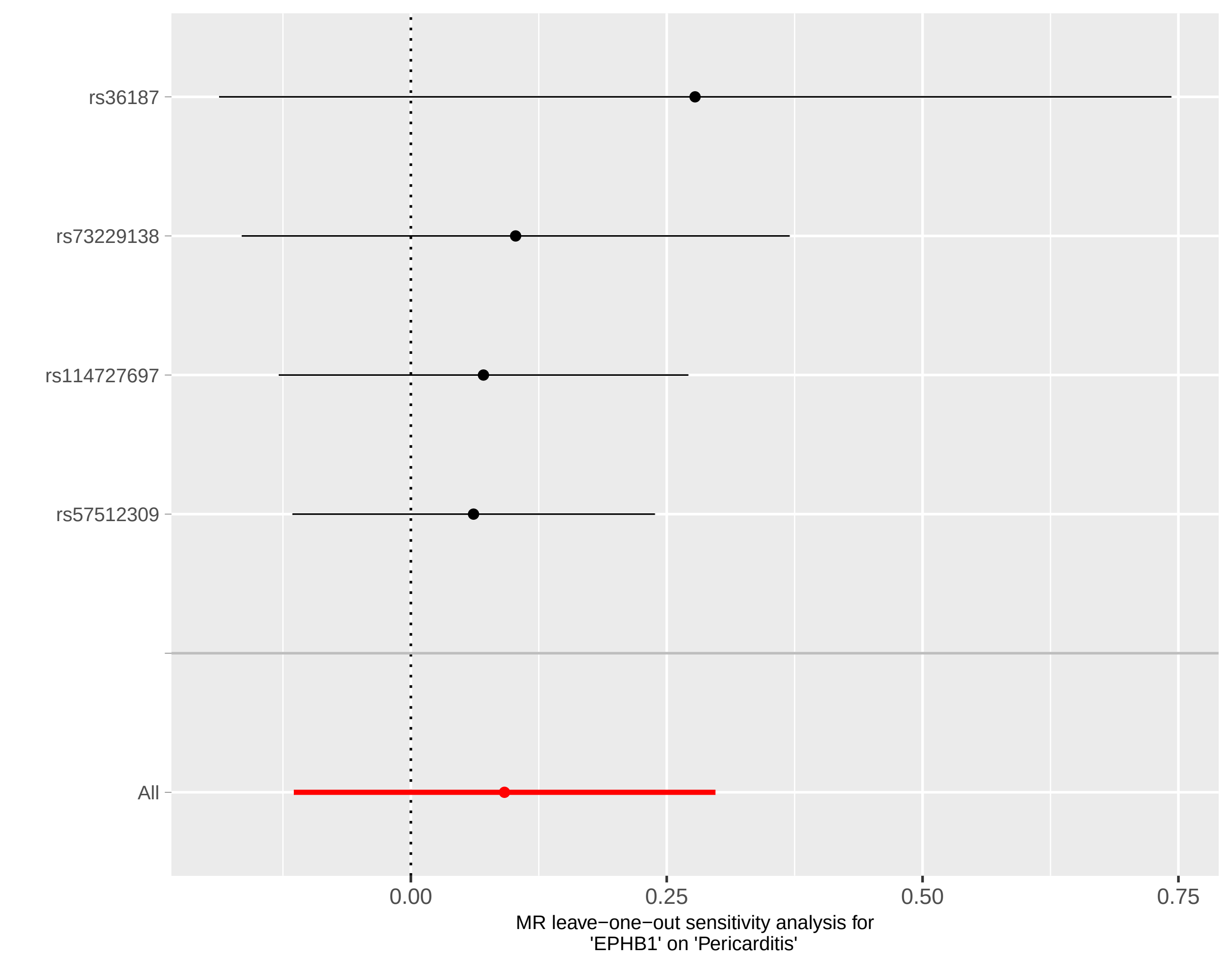

rs36187
rs73229138
rs114727697
rs57512309
All
0.00
0.25
0.50
0.75
MR leave−one−out sensitivity analysis for
'EPHB1' on 'Pericarditis'

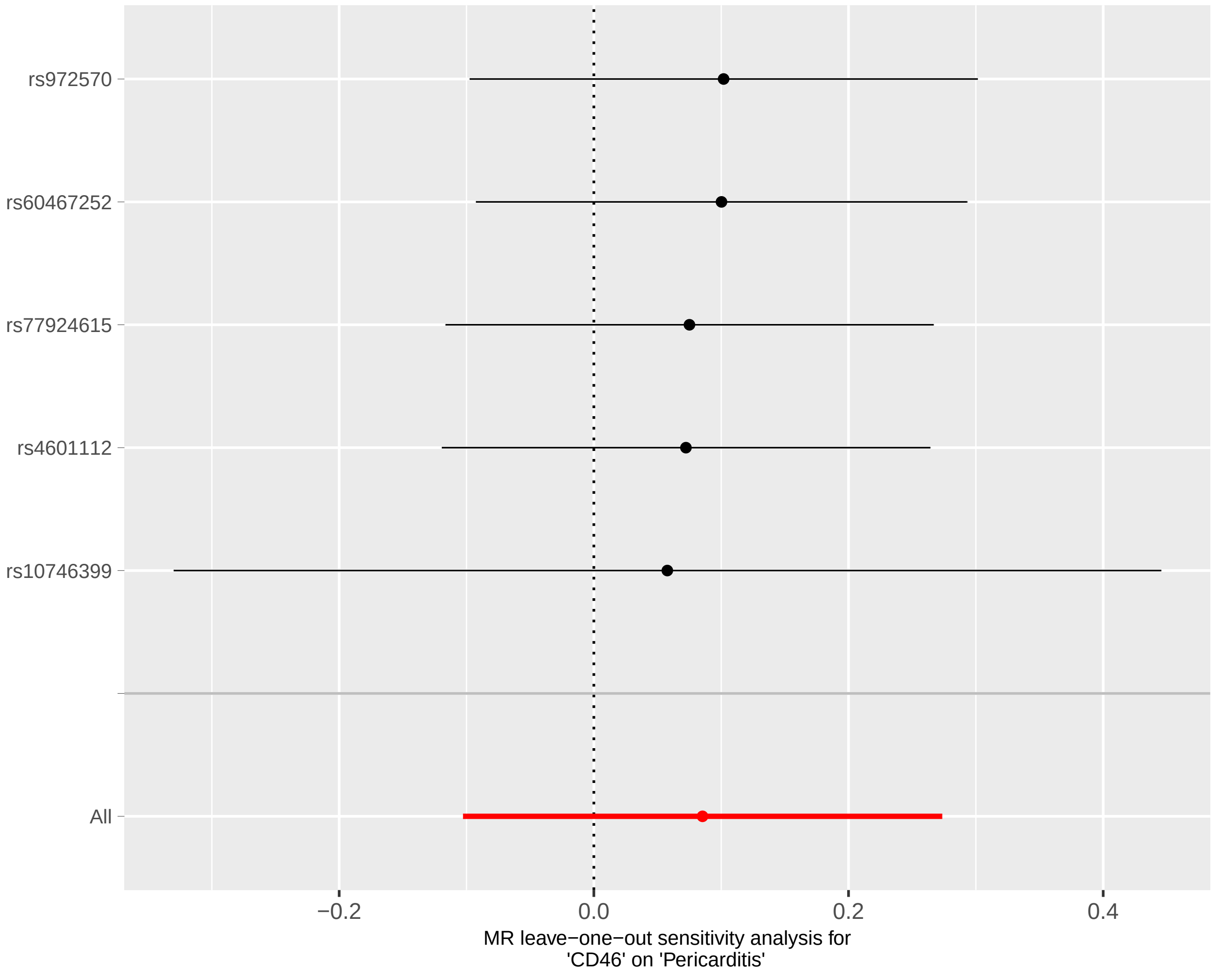

rs972570
rs60467252
rs77924615
rs4601112
rs10746399
All
−0.2
0.0
0.2
0.4
MR leave−one−out sensitivity analysis for
'CD46' on 'Pericarditis'

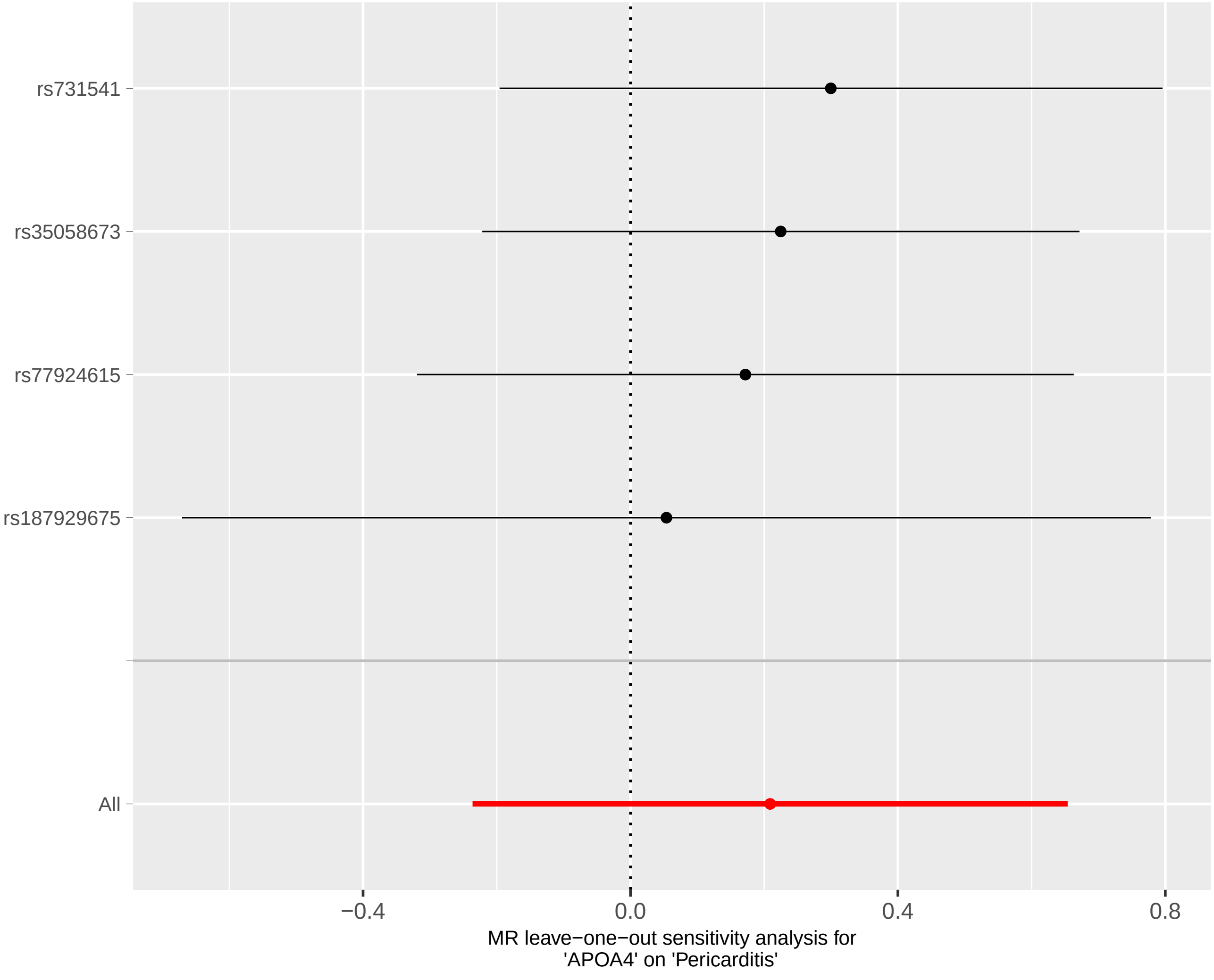

rs731541
rs35058673
rs77924615
rs187929675
All
−0.4
0.0
0.4
0.8
MR leave−one−out sensitivity analysis for
'APOA4' on 'Pericarditis'

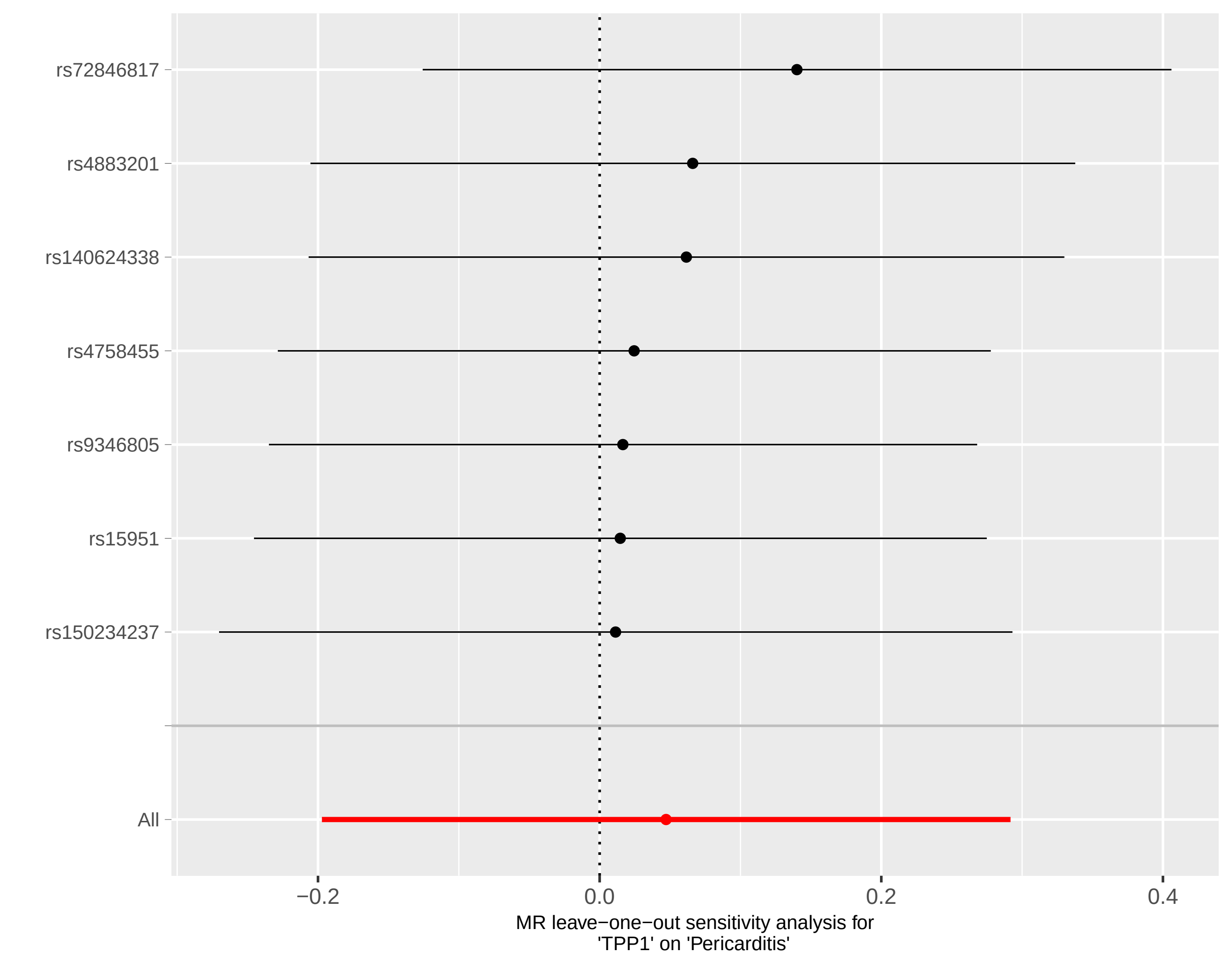

rs72846817
rs4883201
rs140624338
rs4758455
rs9346805
rs15951
rs150234237
All
−0.2
0.0
0.2
0.4
MR leave−one−out sensitivity analysis for
'TPP1' on 'Pericarditis'

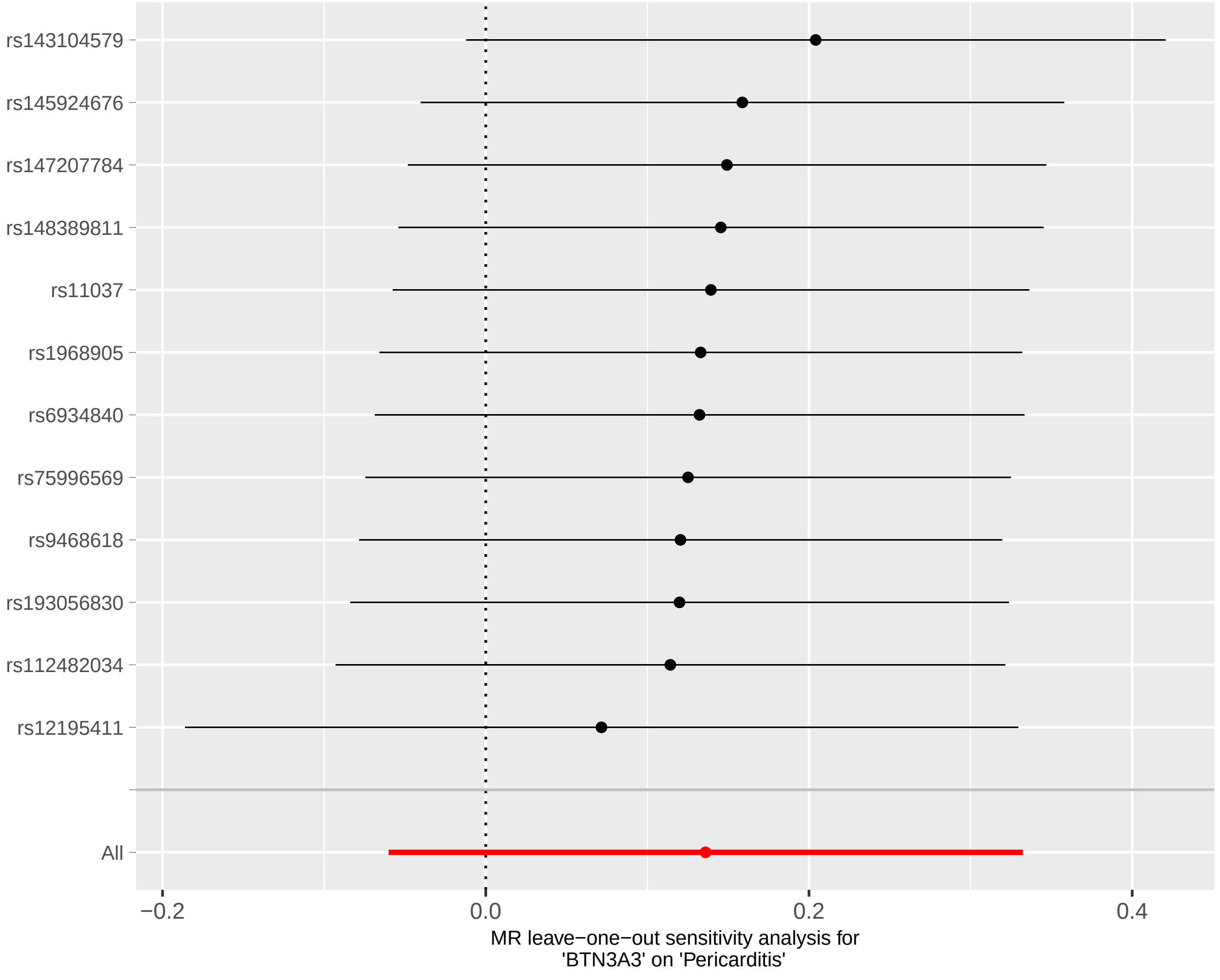

rs143104579
rs145924676
rs147207784
rs148389811
rs11037
rs1968905
rs6934840
rs75996569
rs9468618
rs193056830
rs112482034
rs12195411
All
−0.2
0.0
0.2
0.4
MR leave−one−out sensitivity analysis for
'BTN3A3' on 'Pericarditis'

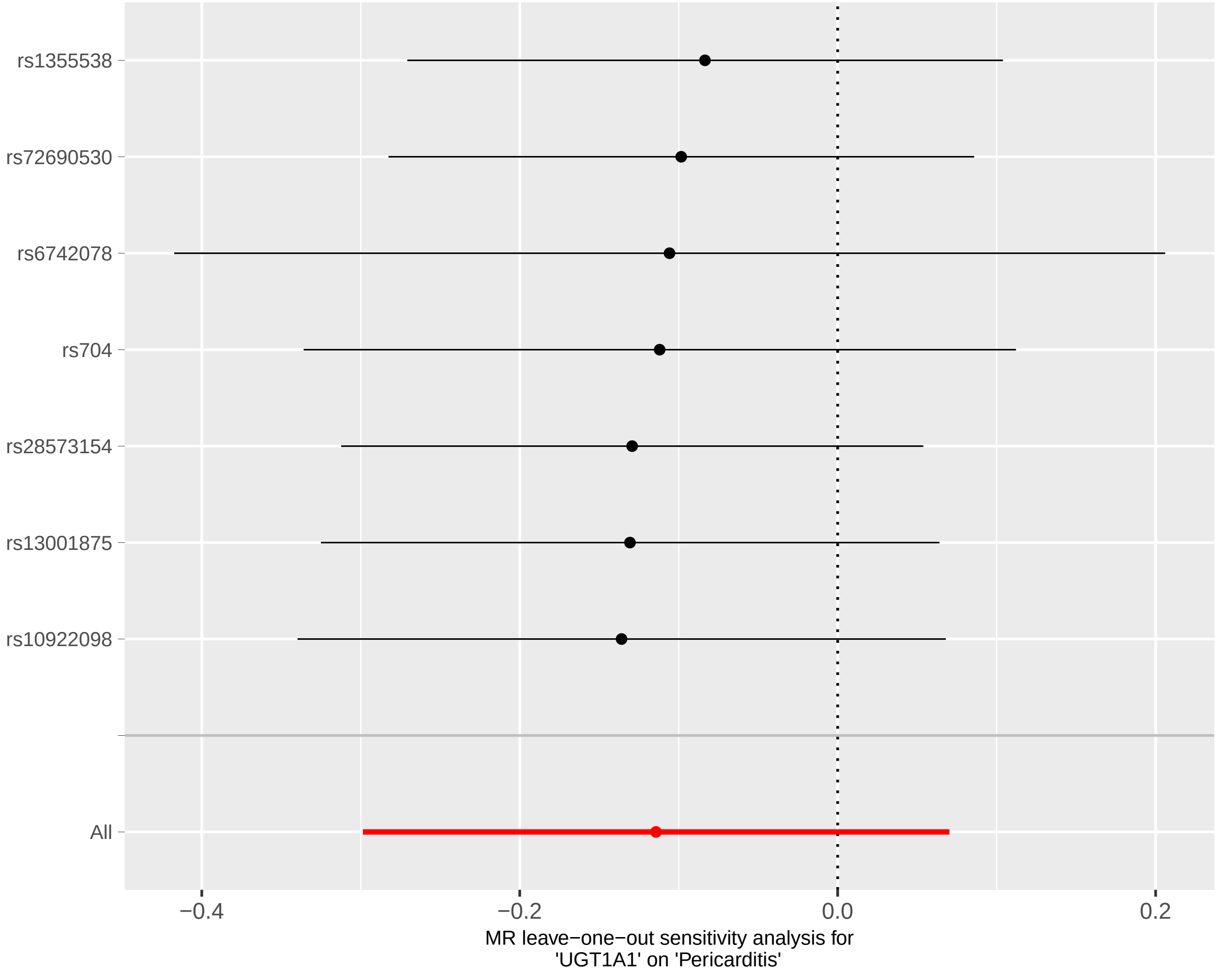
rs1355538
rs72690530
rs6742078
rs704
rs28573154
rs13001875
rs10922098
All
−0.4
−0.2
0.0
0.2
MR leave−one−out sensitivity analysis for
'UGT1A1' on 'Pericarditis'

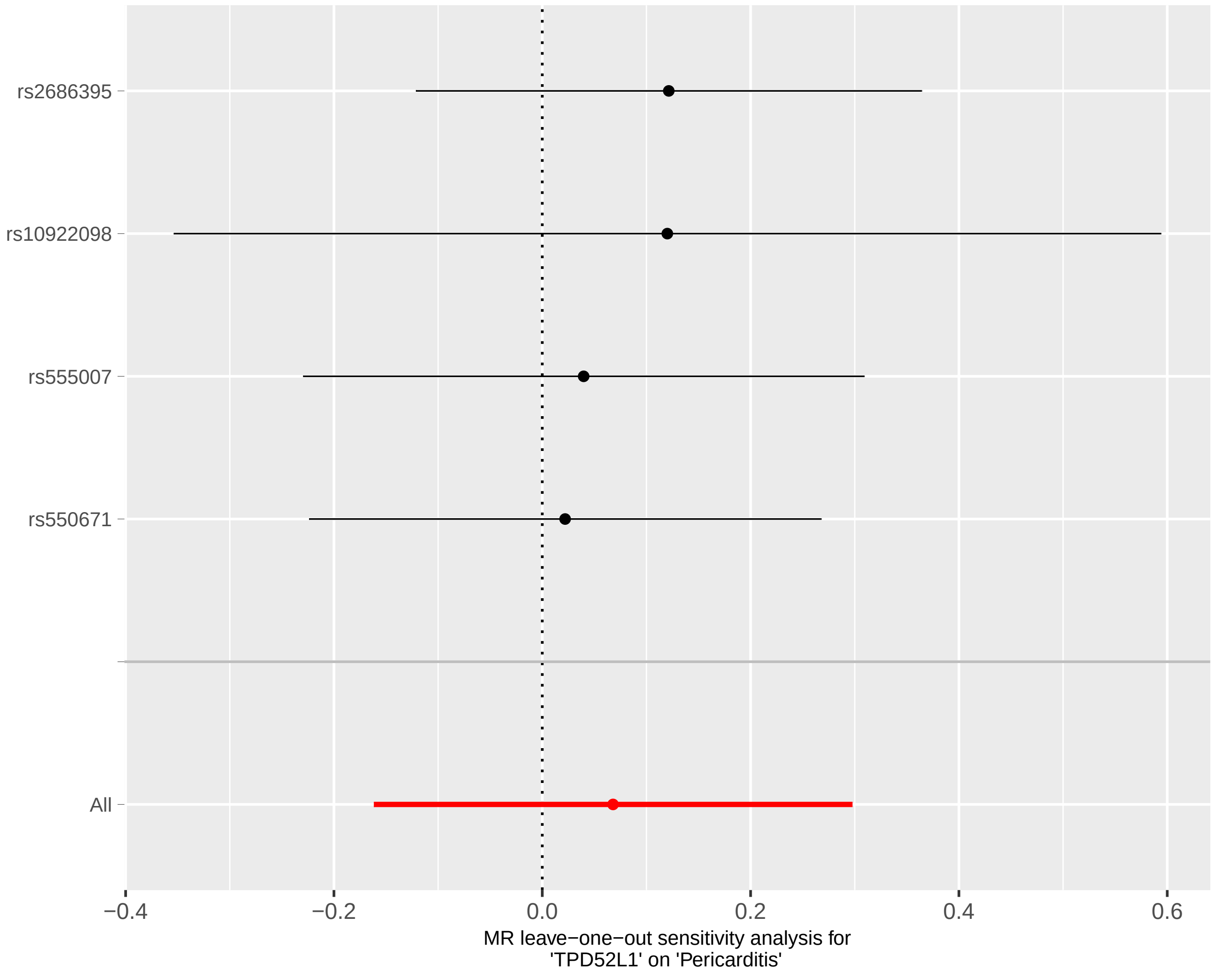

rs2686395
rs10922098
rs555007
rs550671
All
−0.4
−0.2
0.0
0.2
0.4
0.6
MR leave−one−out sensitivity analysis for
'TPD52L1' on 'Pericarditis'

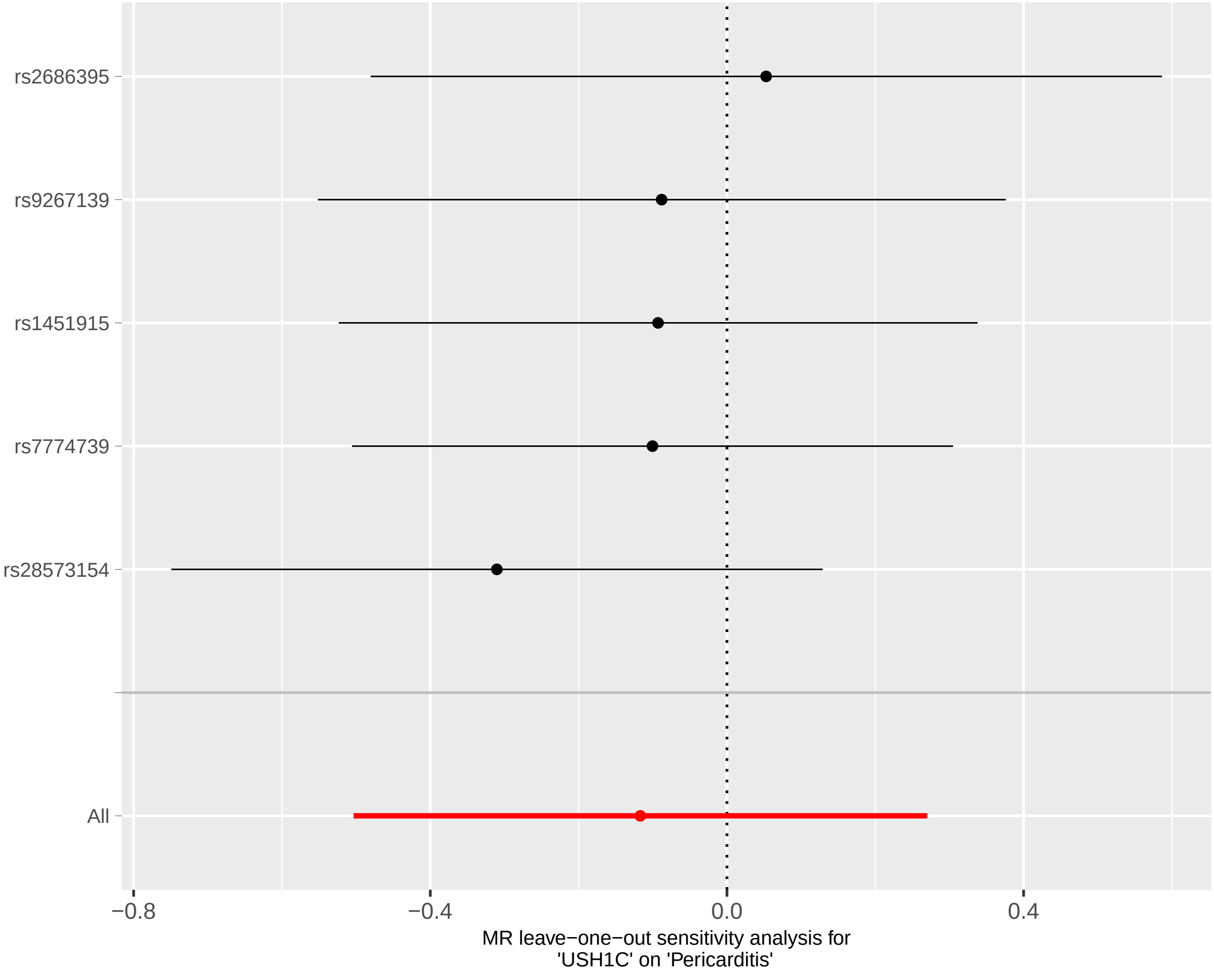
rs2686395
rs9267139
rs1451915
rs7774739
rs28573154
All
−0.8
−0.4
0.0
0.4
MR leave−one−out sensitivity analysis for
'USH1C' on 'Pericarditis'

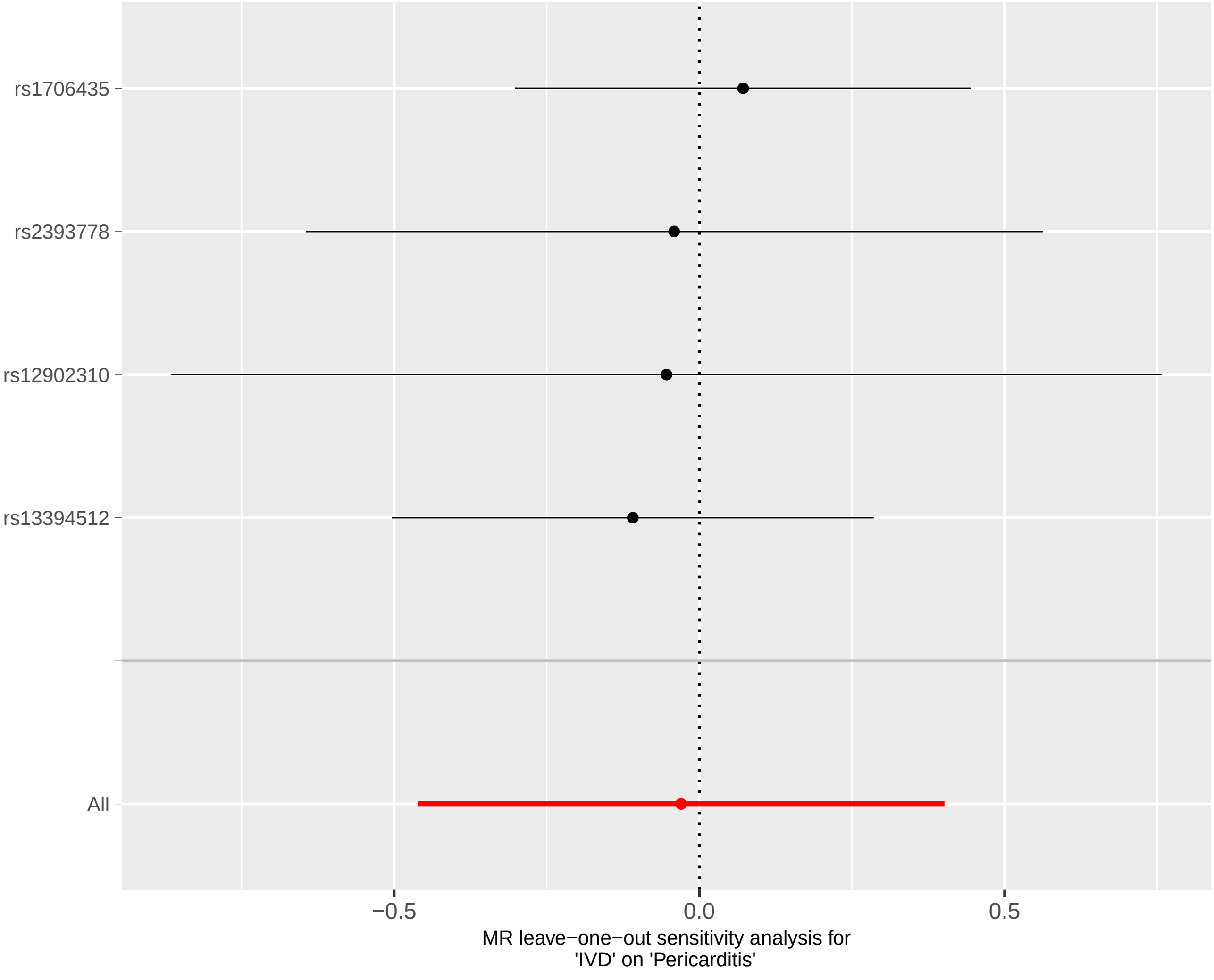

rs1706435
rs2393778
rs12902310
rs13394512
All
−0.5
0.0
0.5
MR leave−one−out sensitivity analysis for
'IVD' on 'Pericarditis'

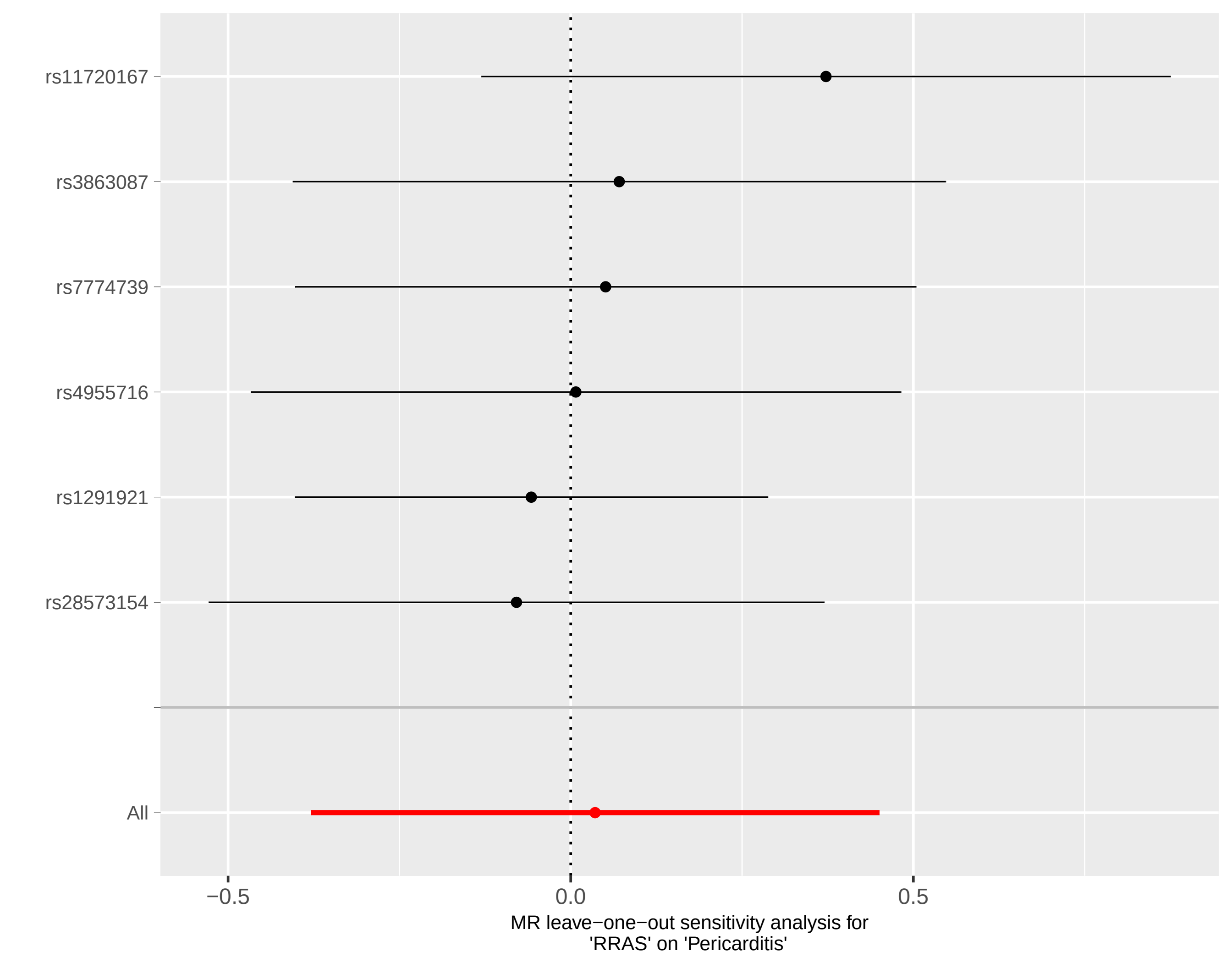

rs11720167
rs3863087
rs7774739
rs4955716
rs1291921
rs28573154
All
−0.5
0.0
0.5
MR leave−one−out sensitivity analysis for
'RRAS' on 'Pericarditis'

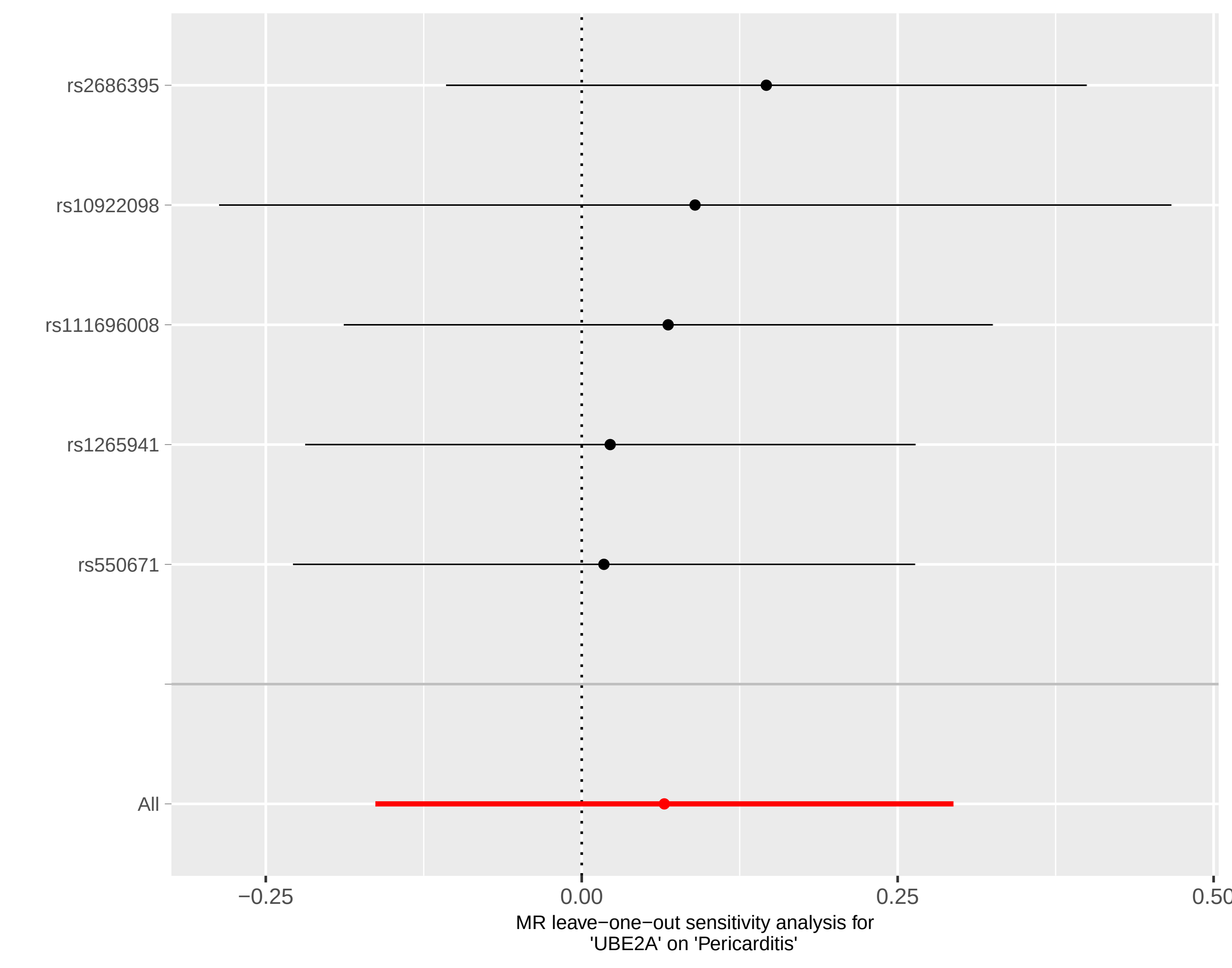

rs2686395
rs10922098
rs111696008
rs1265941
rs550671
All
−0.25
0.00
0.25
0.50
MR leave−one−out sensitivity analysis for
'UBE2A' on 'Pericarditis'

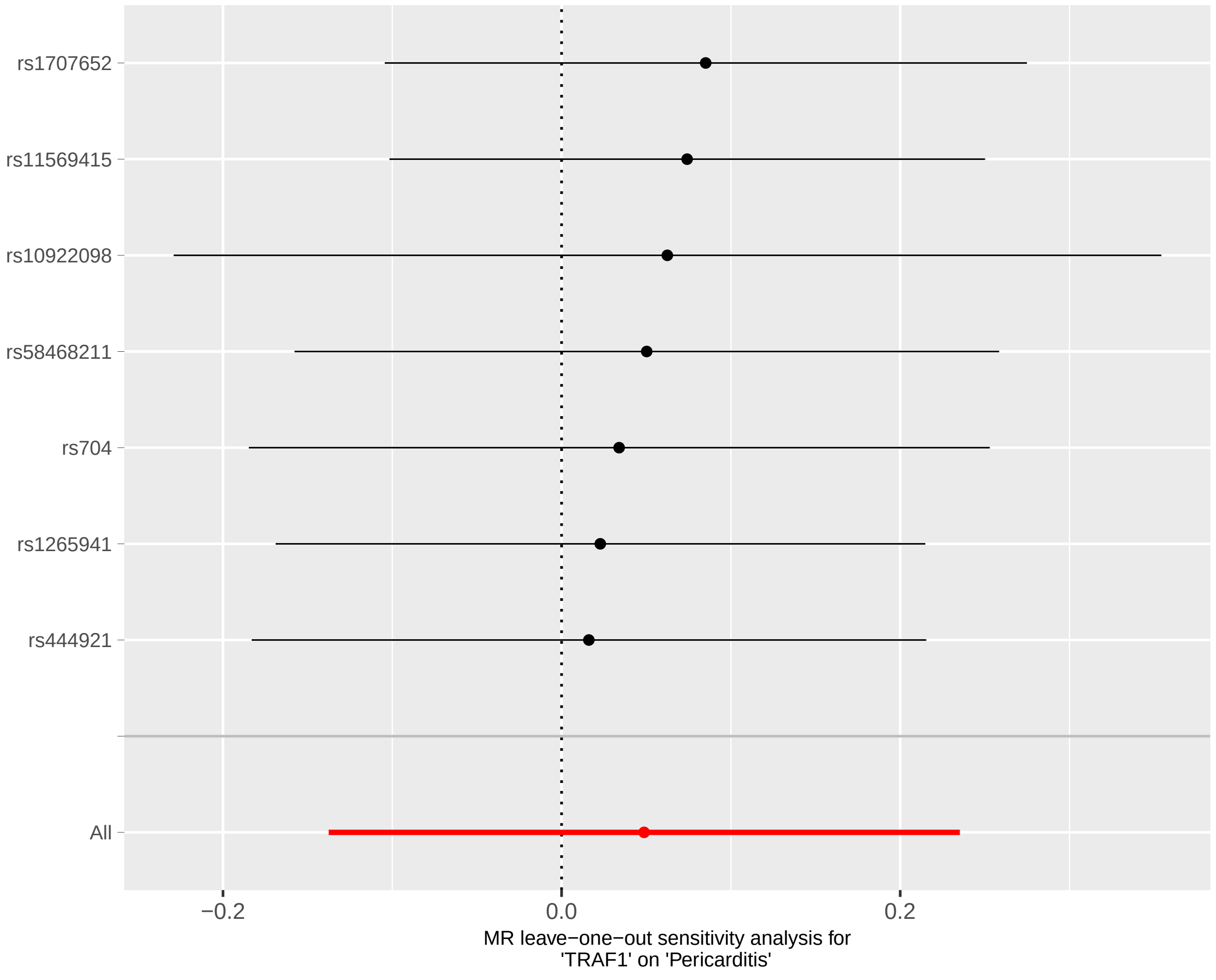
rs1707652
rs11569415
rs10922098
rs58468211
rs704
rs1265941
rs444921
All
−0.2
0.0
0.2
MR leave−one−out sensitivity analysis for
'TRAF1' on 'Pericarditis'

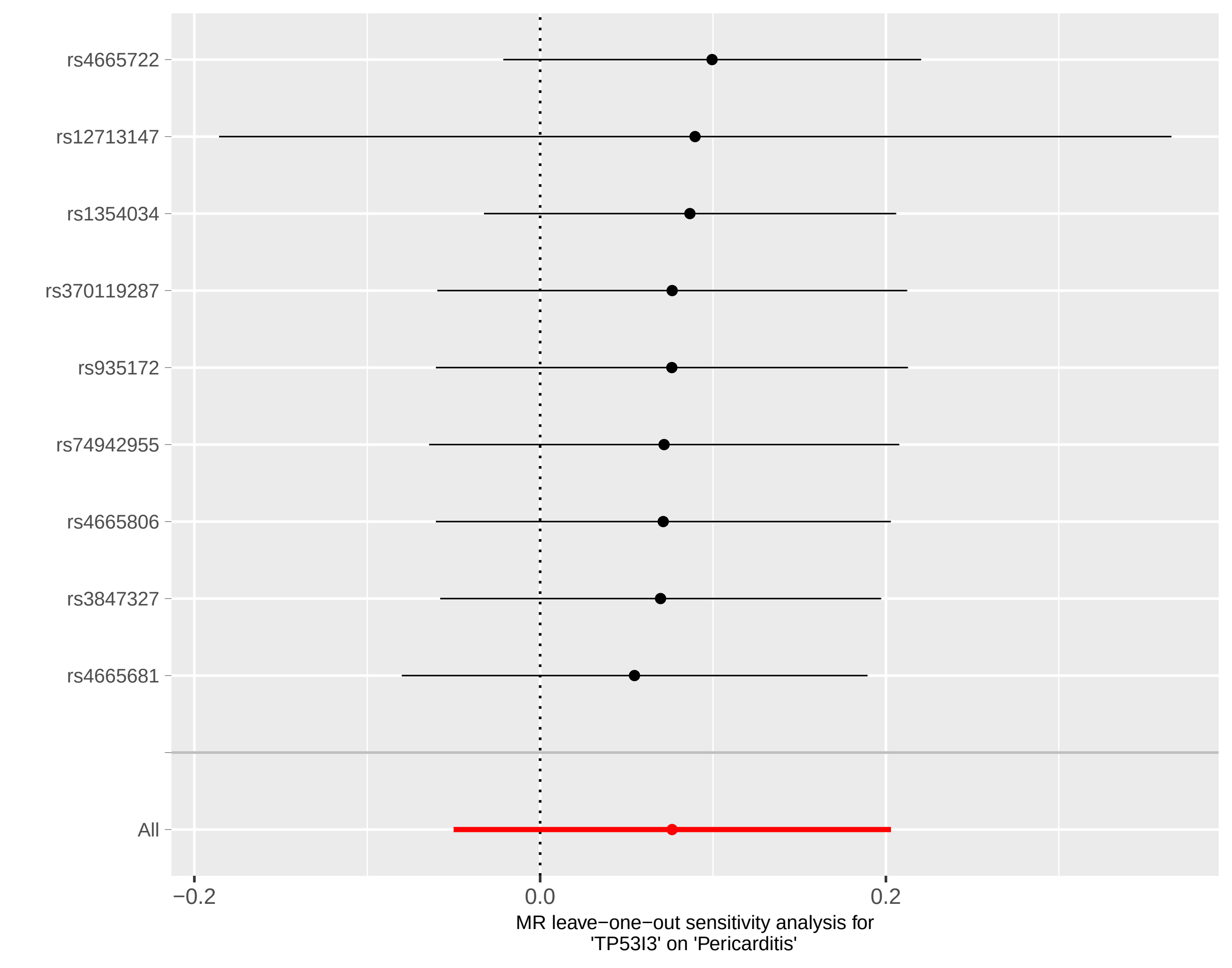

rs4665722
rs12713147
rs1354034
rs370119287
rs935172
rs74942955
rs4665806
rs3847327
rs4665681
All
−0.2
0.0
0.2
MR leave−one−out sensitivity analysis for
'TP53I3' on 'Pericarditis'

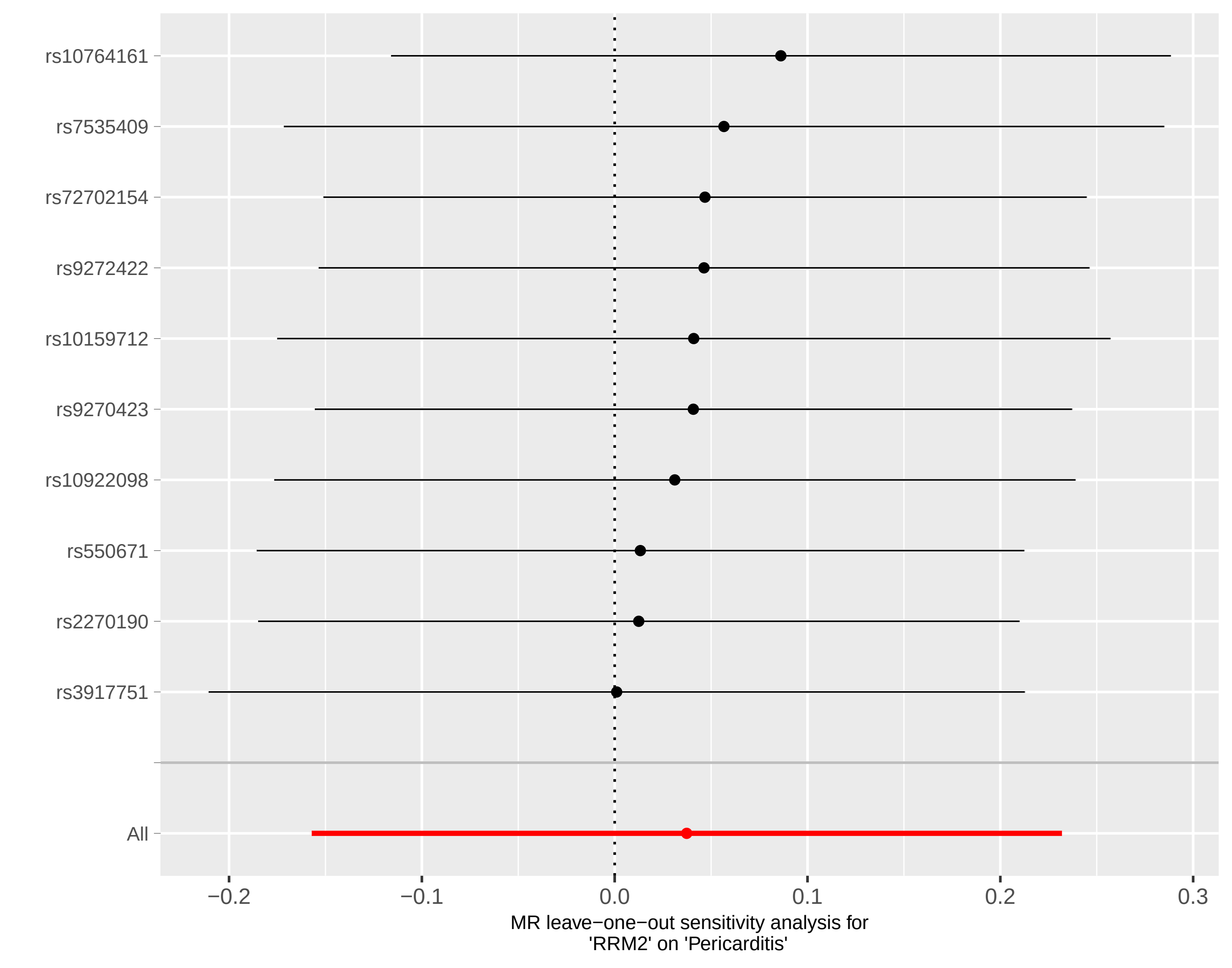
rs10764161
rs7535409
rs72702154
rs9272422
rs10159712
rs9270423
rs10922098
rs550671
rs2270190
rs3917751
All
−0.2
−0.1
0.0
0.1
0.2
0.3
MR leave−one−out sensitivity analysis for
'RRM2' on 'Pericarditis'

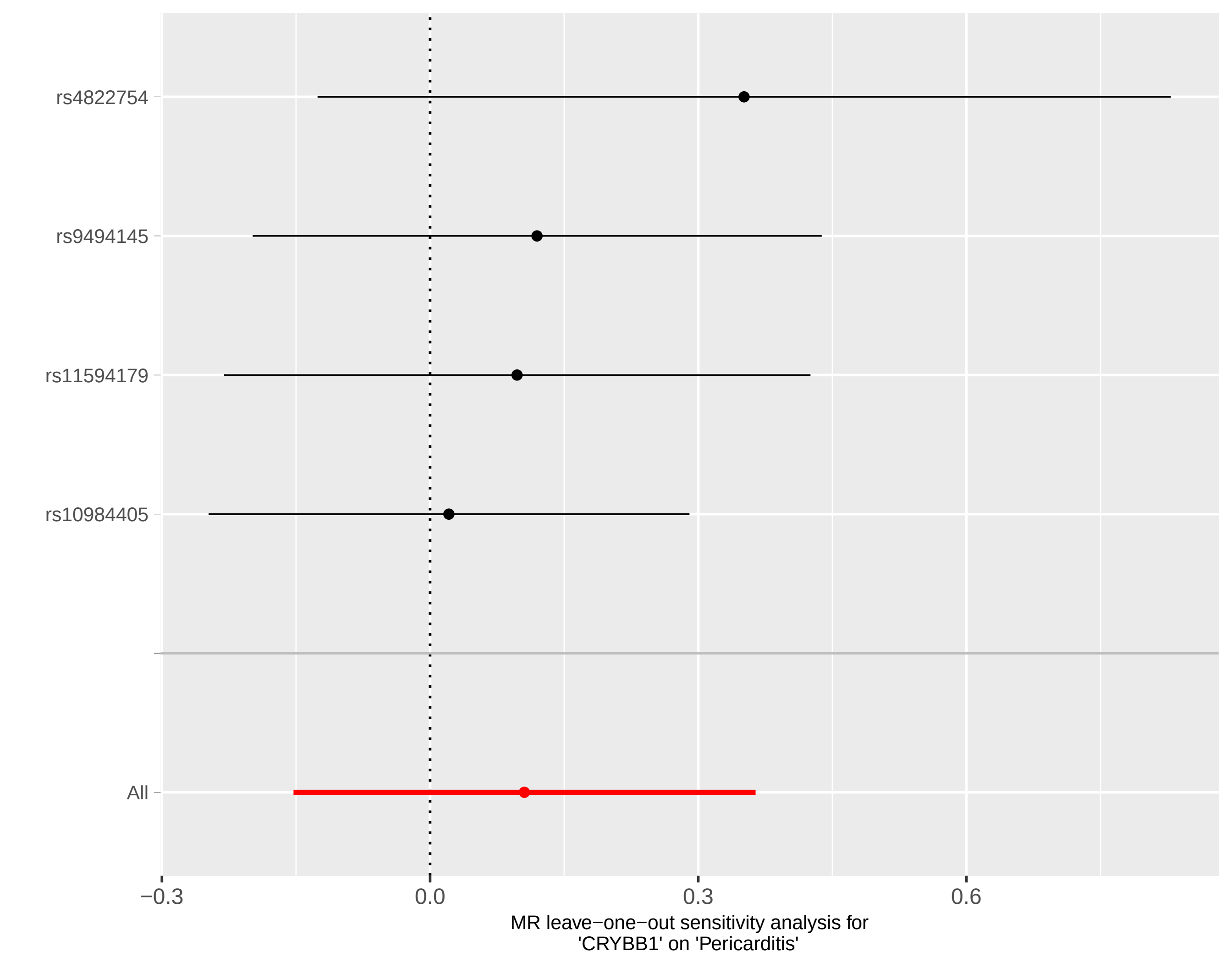

rs4822754
rs9494145
rs11594179
rs10984405
All
−0.3
0.0
0.3
0.6
MR leave−one−out sensitivity analysis for
'CRYBB1' on 'Pericarditis'

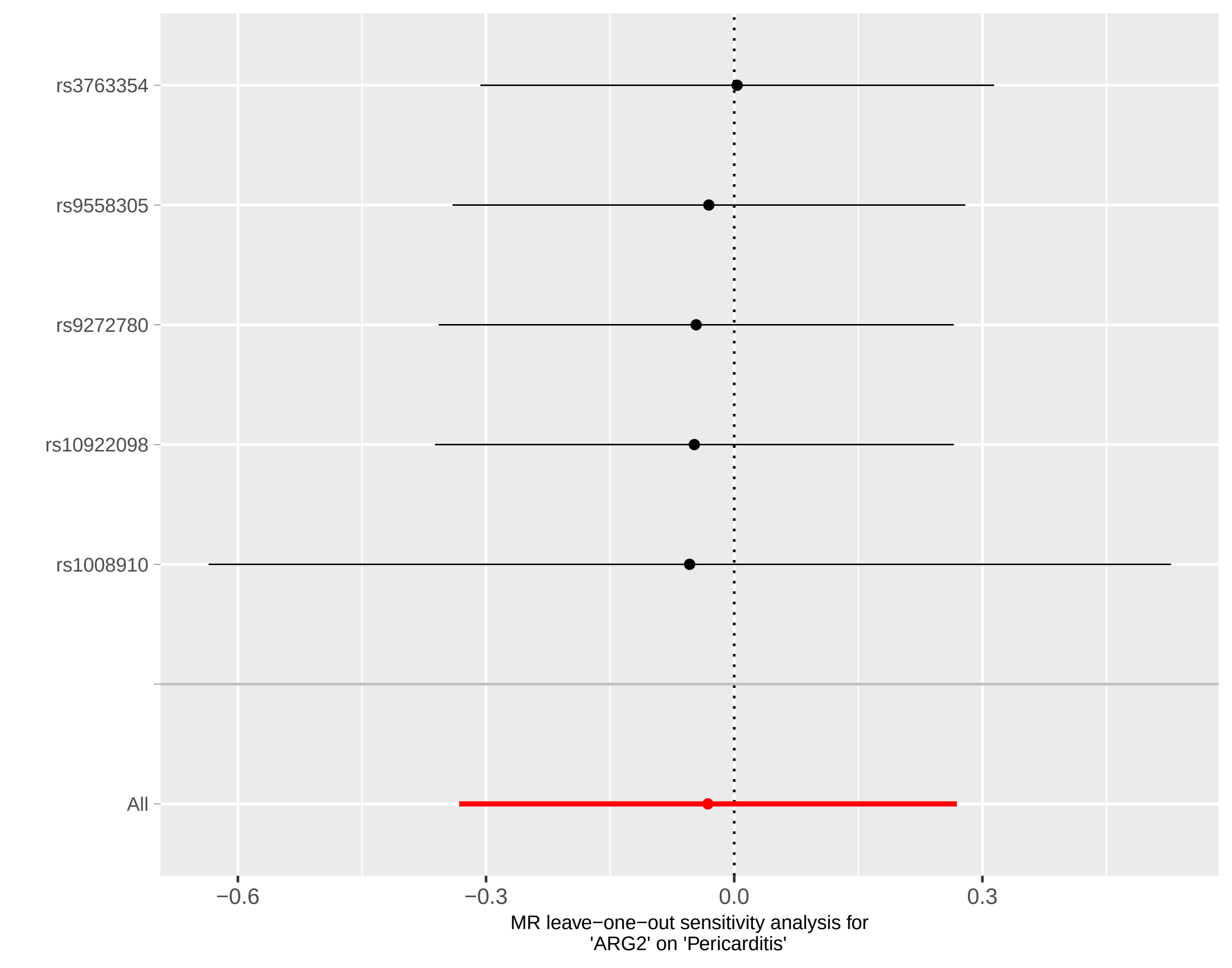

rs3763354
rs9558305
rs9272780
rs10922098
rs1008910
All
−0.6
−0.3
0.0
0.3
MR leave−one−out sensitivity analysis for
'ARG2' on 'Pericarditis'

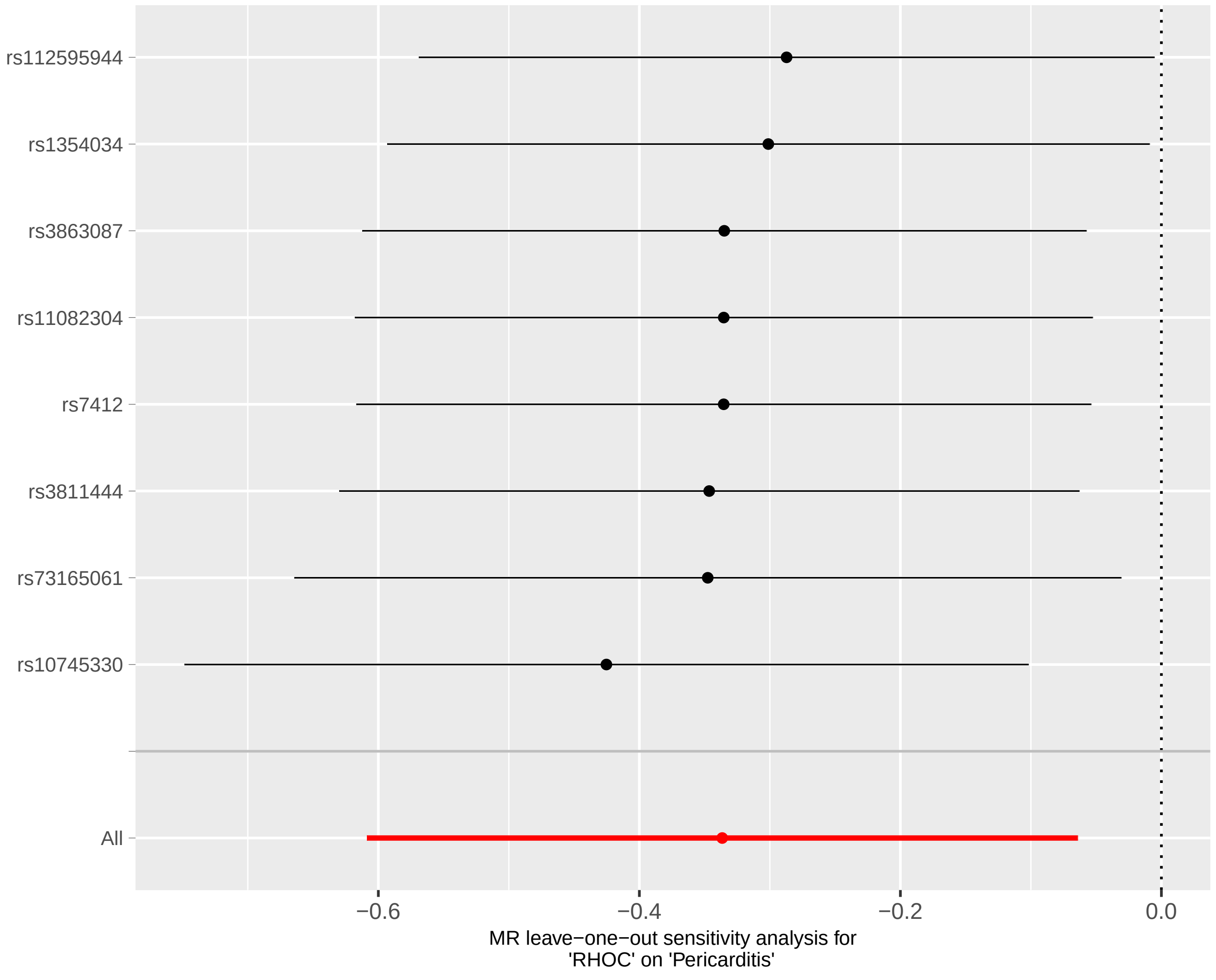

rs112595944
rs1354034
rs3863087
rs11082304
rs7412
rs3811444
rs73165061
rs10745330
All
−0.6
−0.4
−0.2
0.0
MR leave−one−out sensitivity analysis for
'RHOC' on 'Pericarditis'

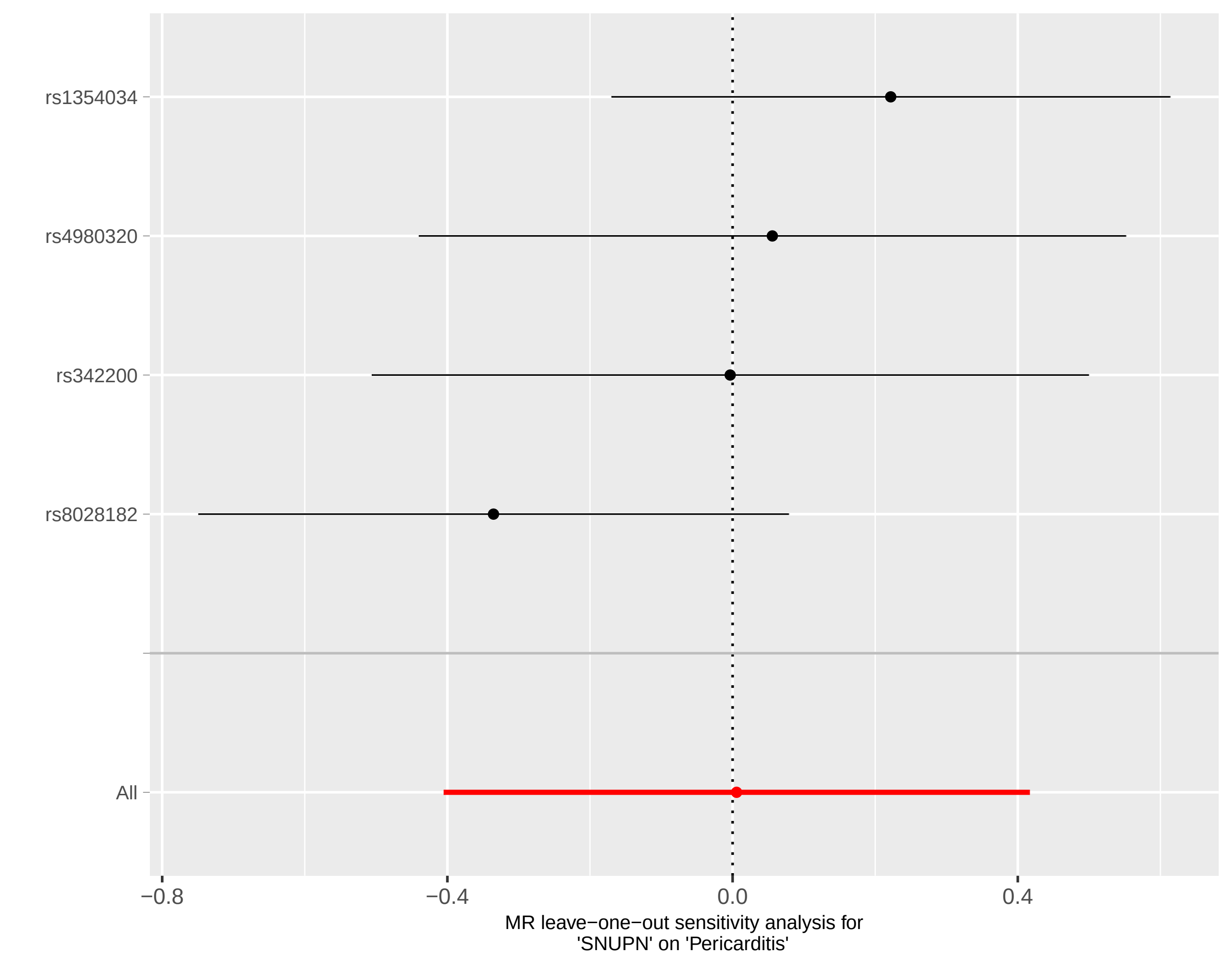

rs1354034
rs4980320
rs342200
rs8028182
All
−0.8
−0.4
0.0
0.4
MR leave−one−out sensitivity analysis for
'SNUPN' on 'Pericarditis'

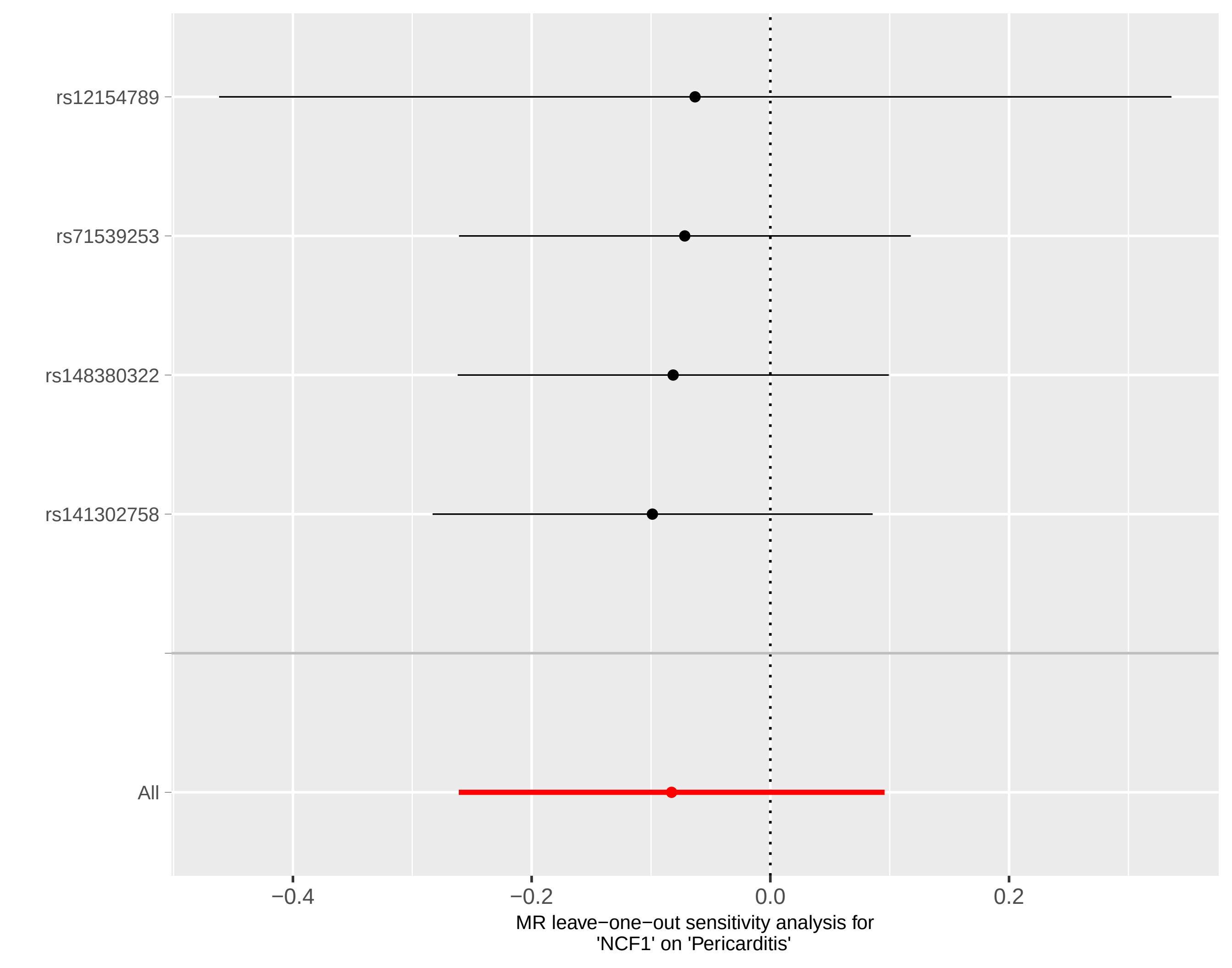
rs12154789
rs71539253
rs148380322
rs141302758
All
−0.4
−0.2
0.0
0.2
MR leave−one−out sensitivity analysis for
'NCF1' on 'Pericarditis'

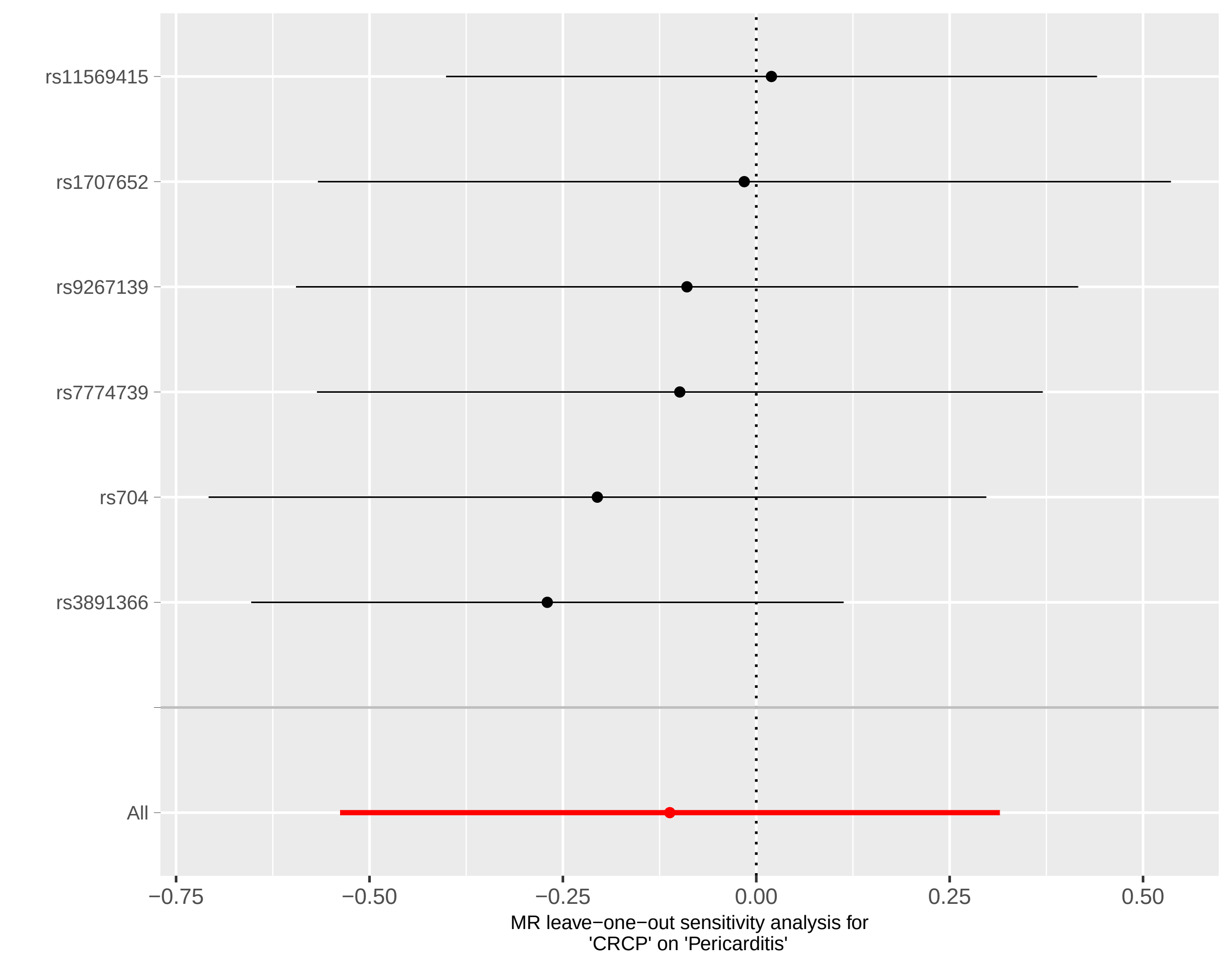

rs11569415
rs1707652
rs9267139
rs7774739
rs704
rs3891366
All
−0.75
−0.50
−0.25
0.00
0.25
0.50
MR leave−one−out sensitivity analysis for
'CRCP' on 'Pericarditis'

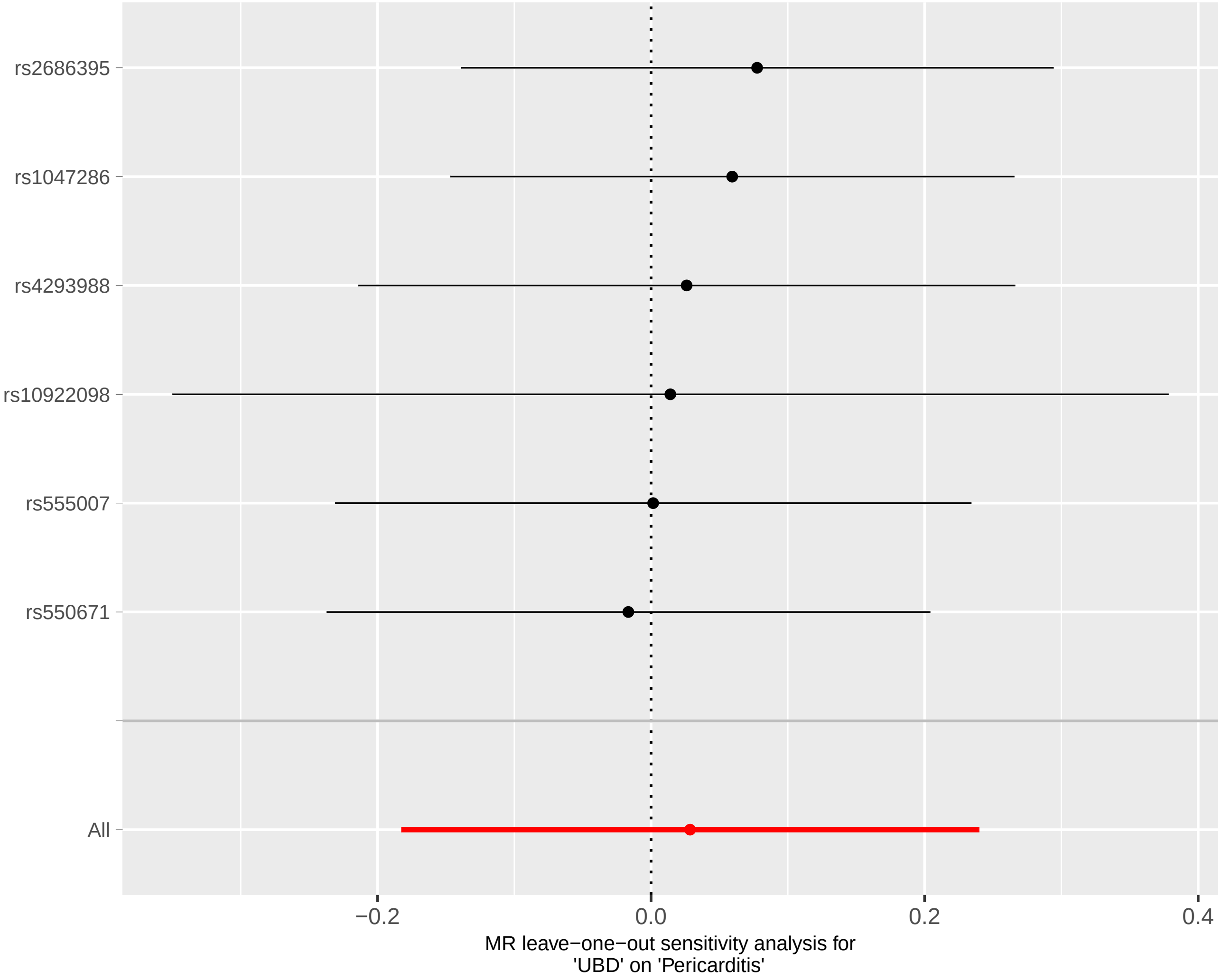
rs2686395
rs1047286
rs4293988
rs10922098
rs555007
rs550671
All
−0.2
0.0
0.2
0.4
MR leave−one−out sensitivity analysis for
'UBD' on 'Pericarditis'

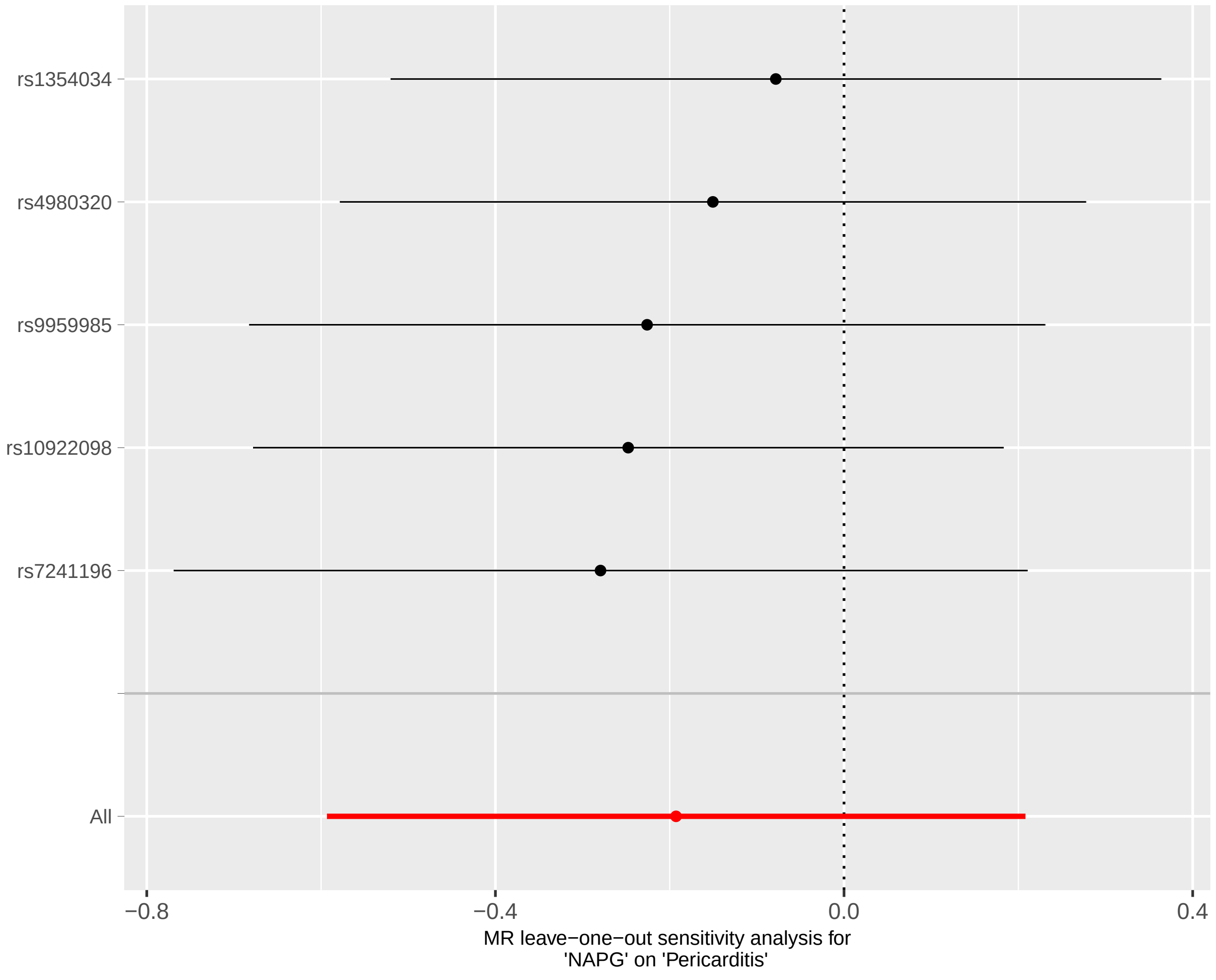

rs1354034
rs4980320
rs9959985
rs10922098
rs7241196
All
−0.8
−0.4
0.0
0.4
MR leave−one−out sensitivity analysis for
'NAPG' on 'Pericarditis'

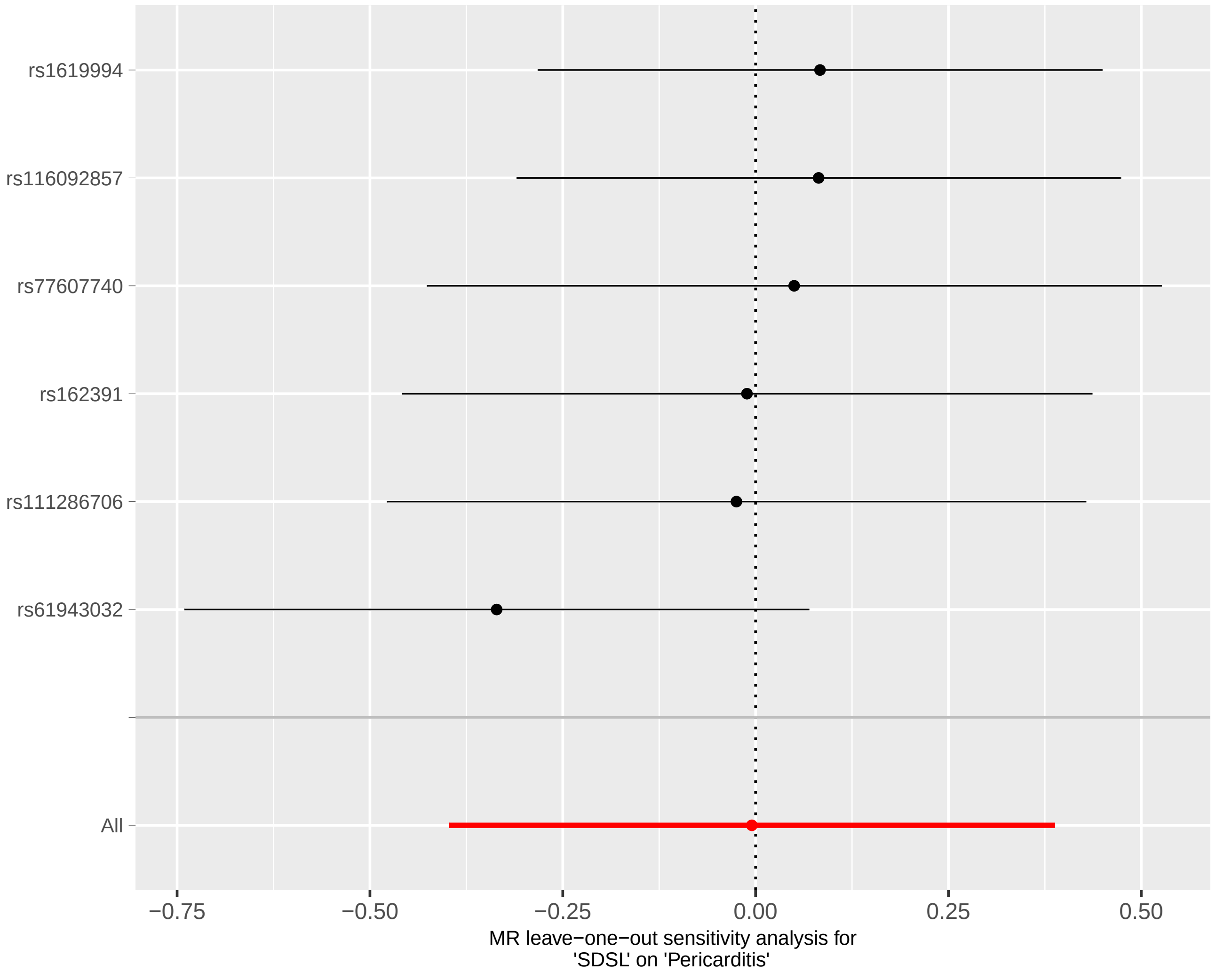
rs1619994
rs116092857
rs77607740
rs162391
rs111286706
rs61943032
All
−0.75
−0.50
−0.25
0.00
0.25
0.50
MR leave−one−out sensitivity analysis for
'SDSL' on 'Pericarditis'

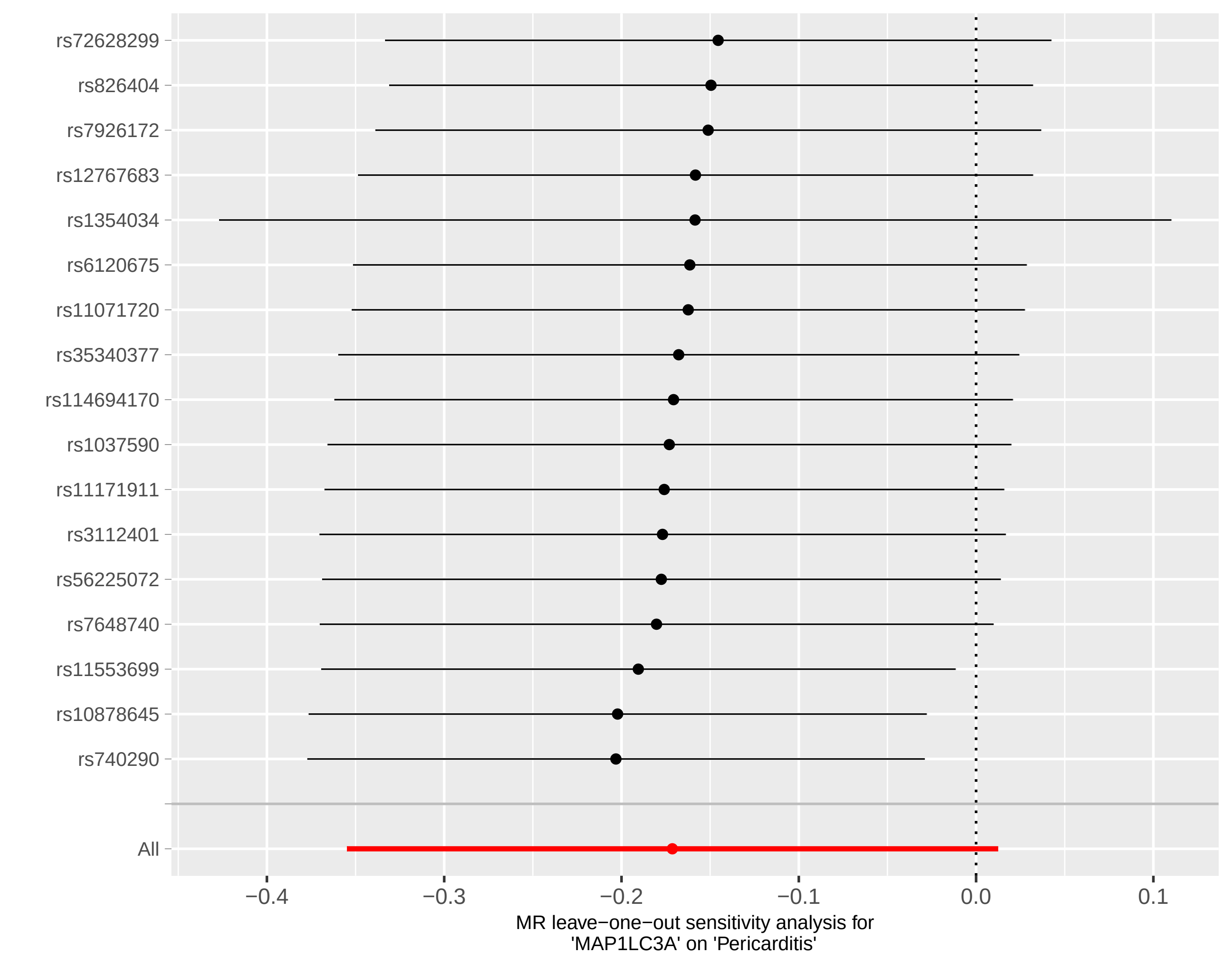
rs72628299
rs826404
rs7926172
rs12767683
rs1354034
rs6120675
rs11071720
rs35340377
rs114694170
rs1037590
rs11171911
rs3112401
rs56225072
rs7648740
rs11553699
rs10878645
rs740290
All
−0.4
−0.3
−0.2
−0.1
0.0
0.1
MR leave−one−out sensitivity analysis for
'MAP1LC3A' on 'Pericarditis'

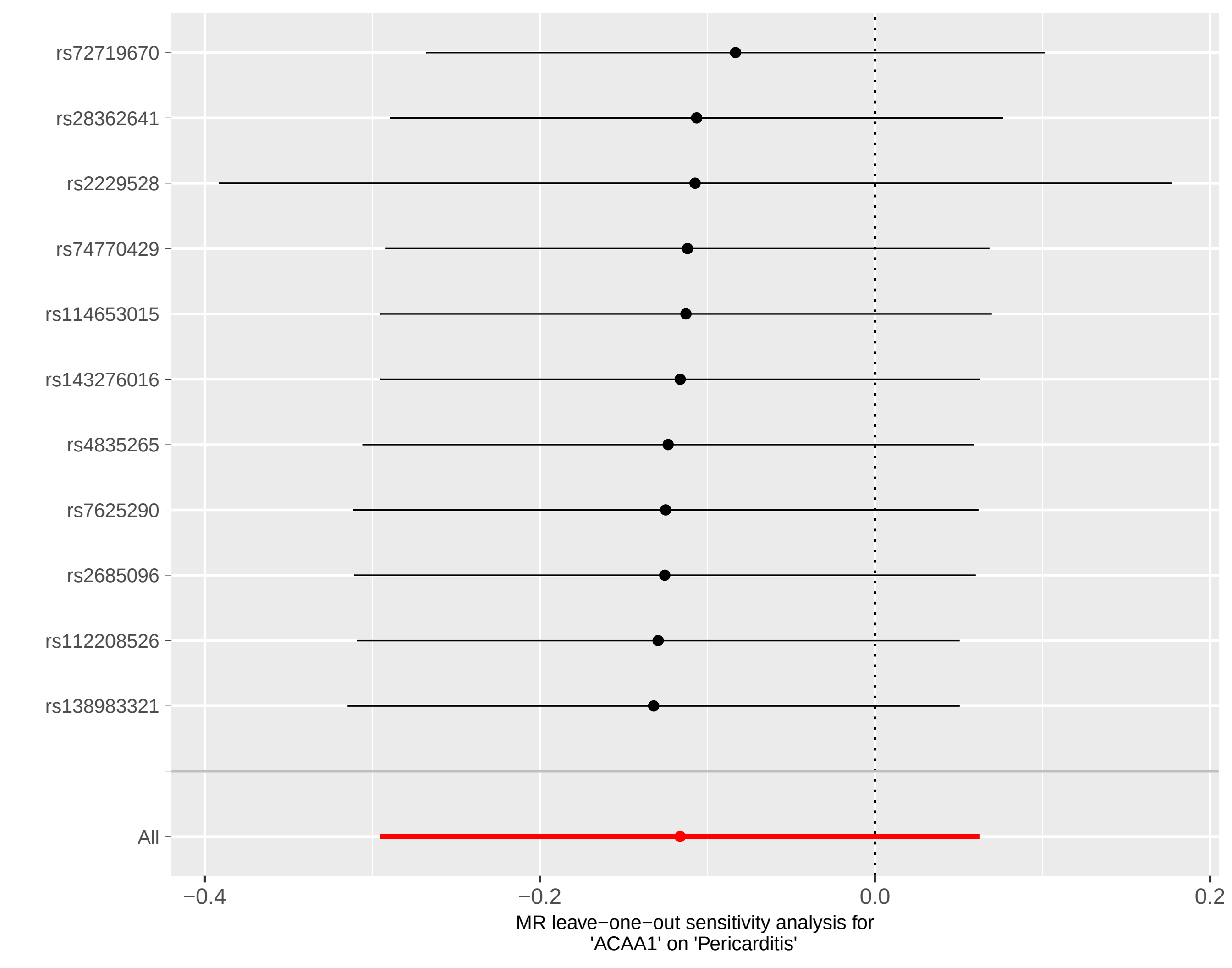
rs72719670
rs28362641
rs2229528
rs74770429
rs114653015
rs143276016
rs4835265
rs7625290
rs2685096
rs112208526
rs138983321
All
−0.4
−0.2
0.0
0.2
MR leave−one−out sensitivity analysis for
'ACAA1' on 'Pericarditis'

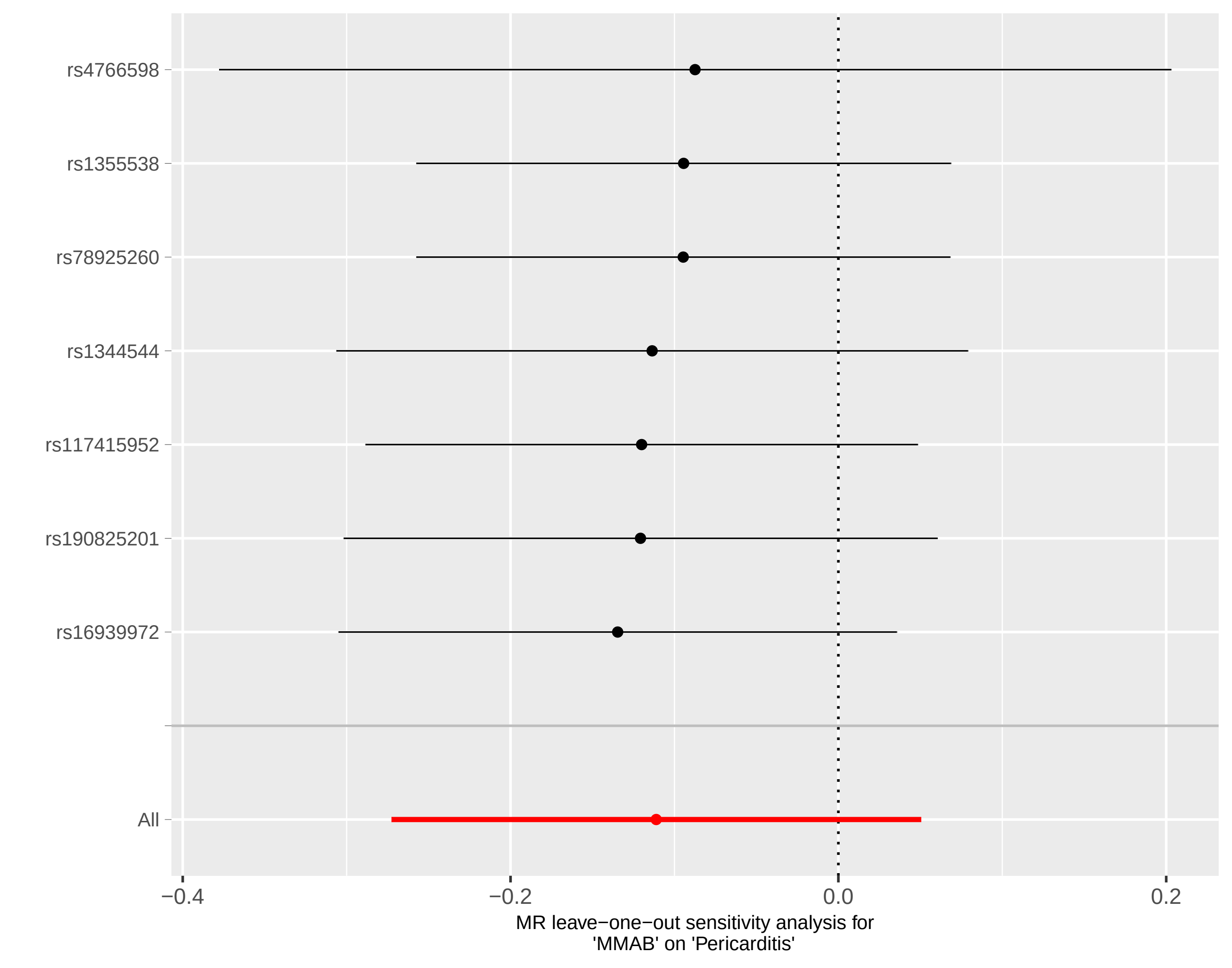
rs4766598
rs1355538
rs78925260
rs1344544
rs117415952
rs190825201
rs16939972
All
−0.4
−0.2
0.0
0.2
MR leave−one−out sensitivity analysis for
'MMAB' on 'Pericarditis'

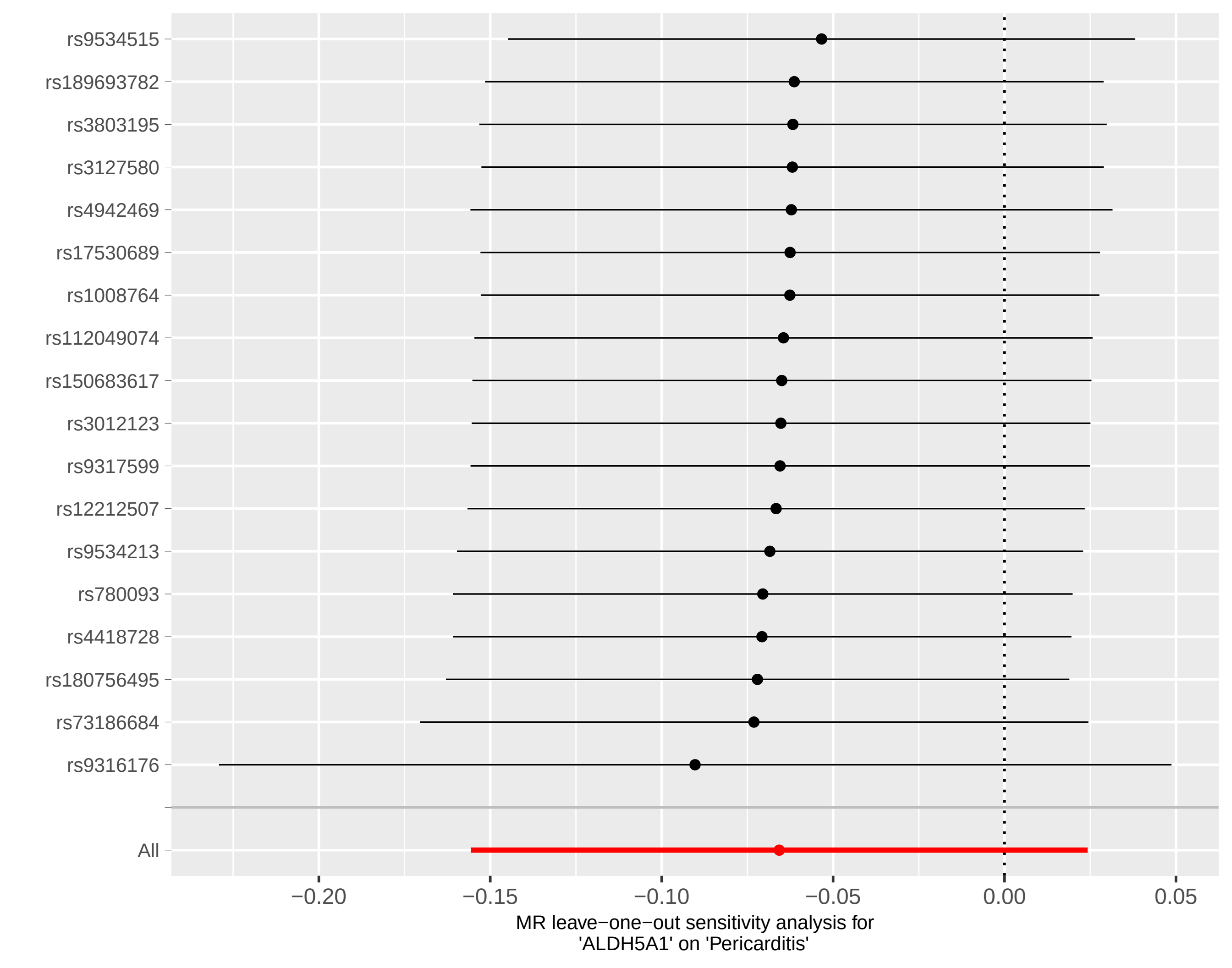
rs9534515
rs189693782
rs3803195
rs3127580
rs4942469
rs17530689
rs1008764
rs112049074
rs150683617
rs3012123
rs9317599
rs12212507
rs9534213
rs780093
rs4418728
rs180756495
rs73186684
rs9316176
All
−0.20
−0.15
−0.10
−0.05
0.00
0.05
MR leave−one−out sensitivity analysis for
'ALDH5A1' on 'Pericarditis'

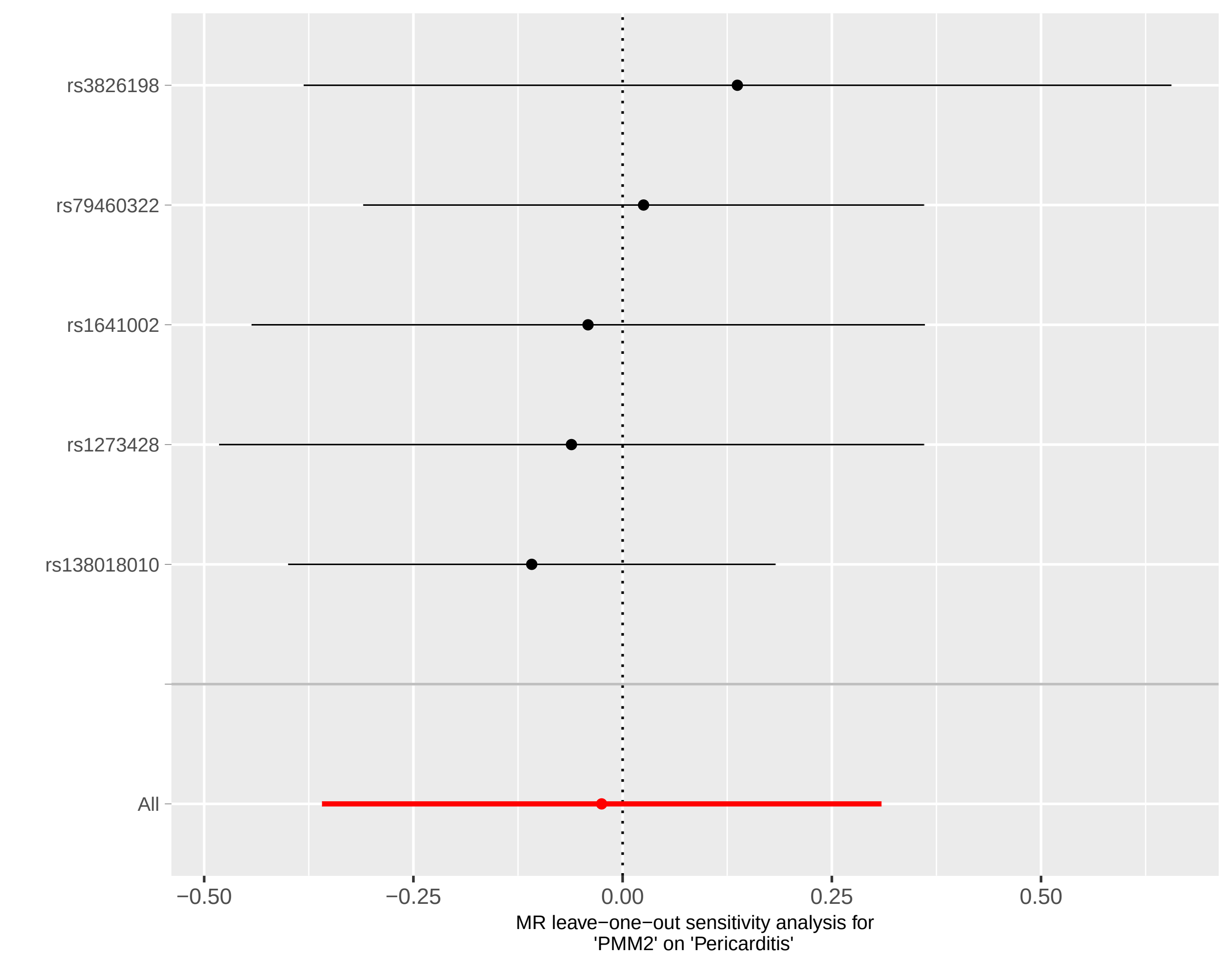
rs3826198
rs79460322
rs1641002
rs1273428
rs138018010
All
−0.50
−0.25
0.00
0.25
0.50
MR leave−one−out sensitivity analysis for
'PMM2' on 'Pericarditis'

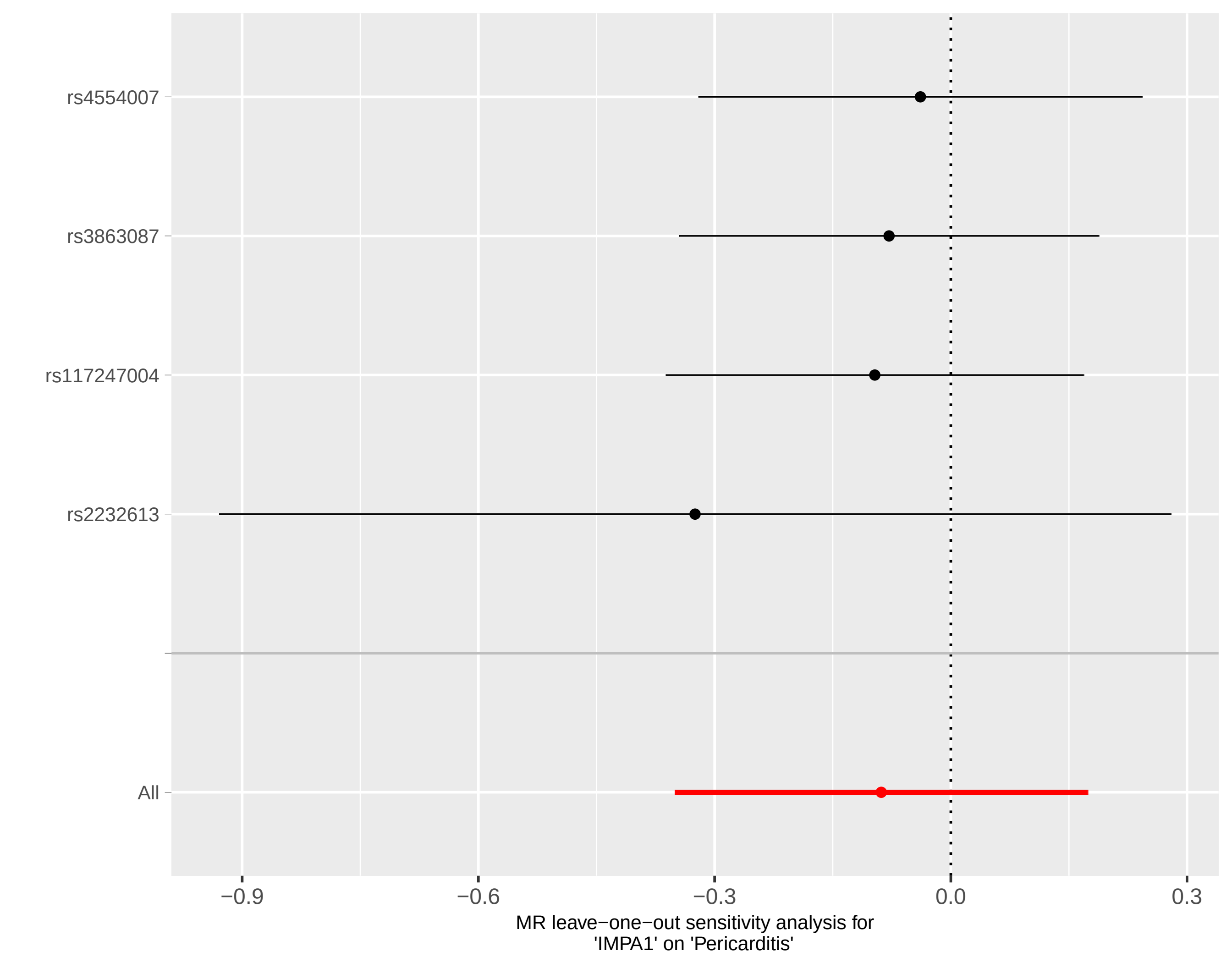
rs4554007
rs3863087
rs117247004
rs2232613
All
−0.9
−0.6
−0.3
0.0
0.3
MR leave−one−out sensitivity analysis for
'IMPA1' on 'Pericarditis'

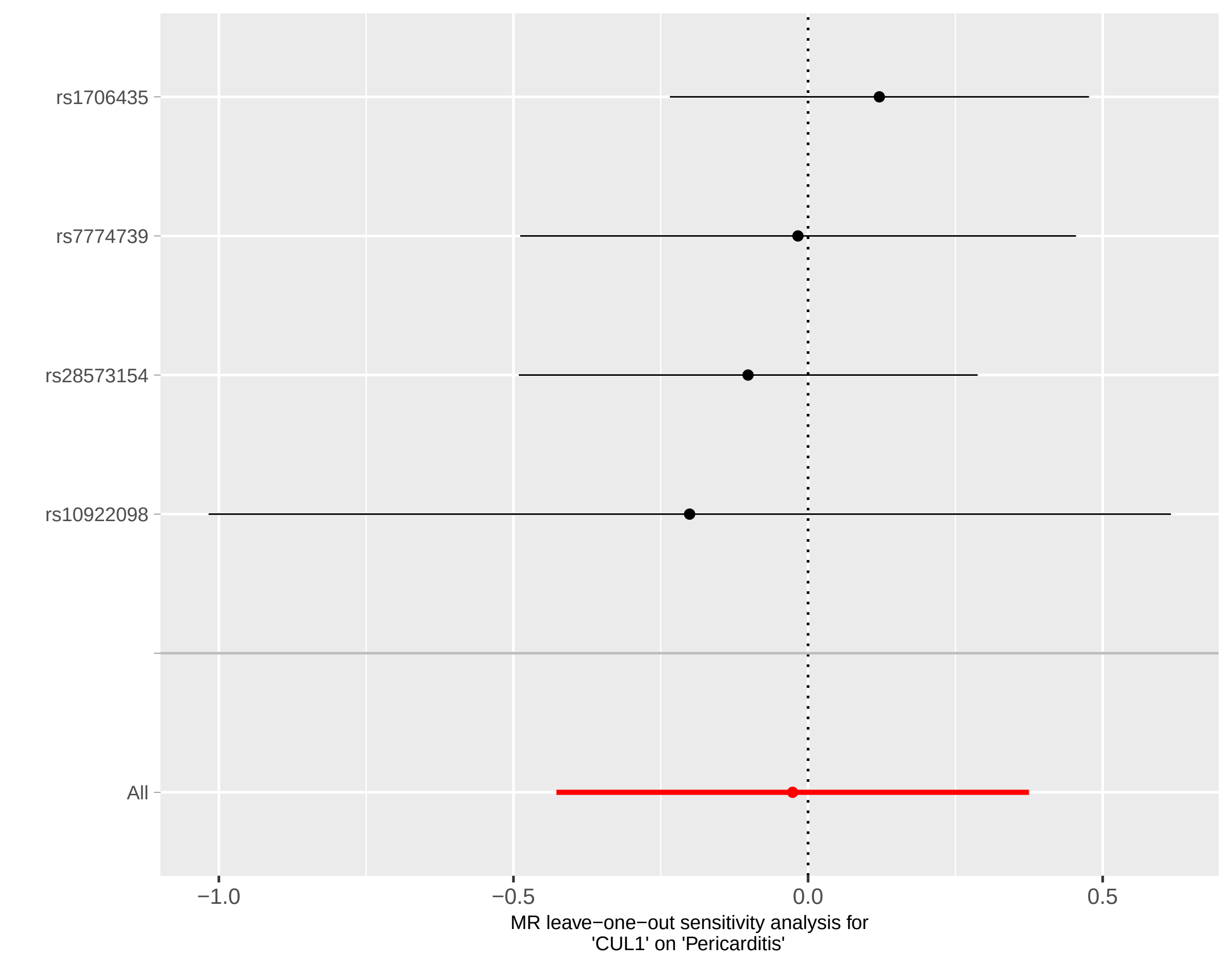

rs1706435
rs7774739
rs28573154
rs10922098
All
−1.0
−0.5
0.0
0.5
MR leave−one−out sensitivity analysis for
'CUL1' on 'Pericarditis'

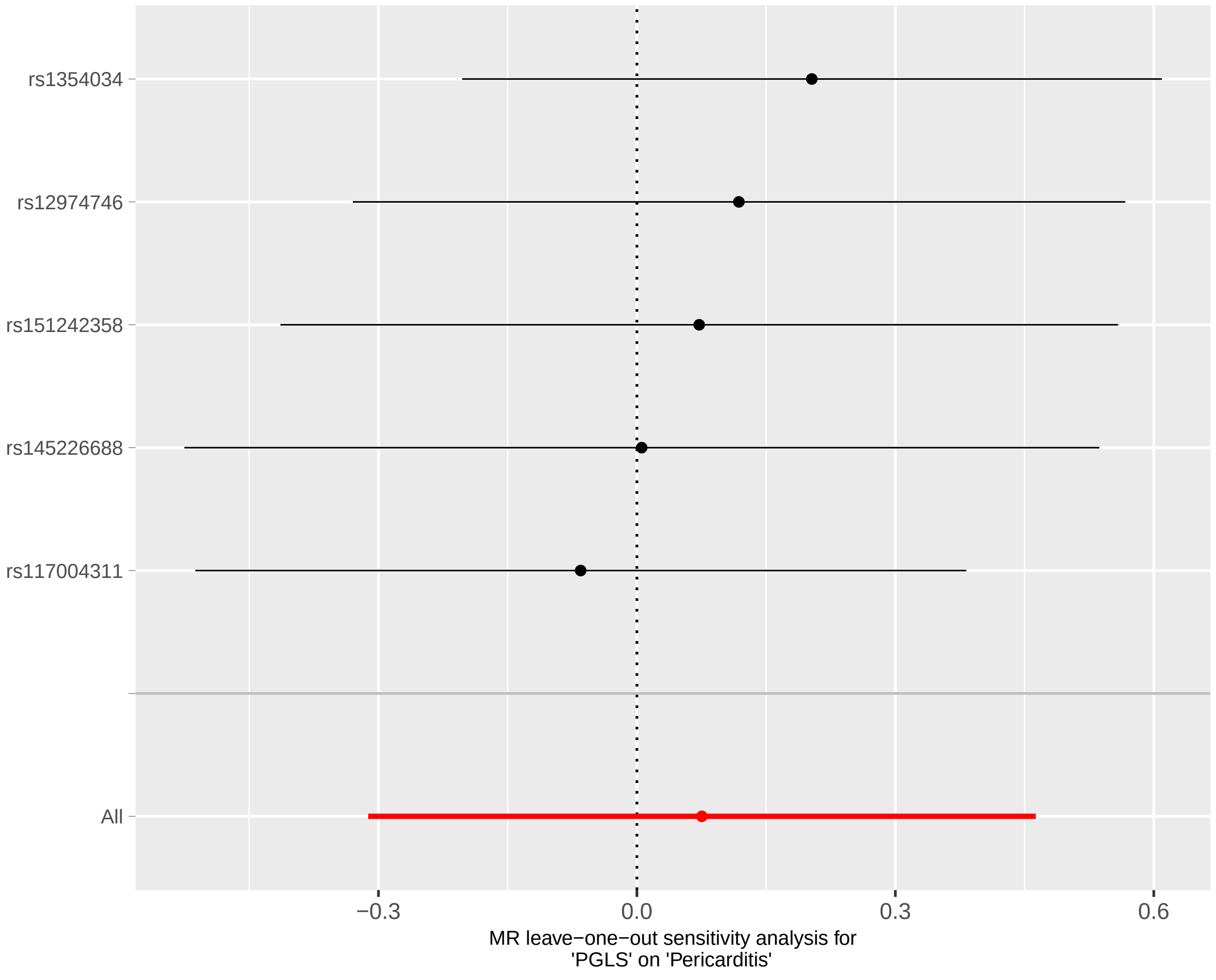

rs1354034
rs12974746
rs151242358
rs145226688
rs117004311
All
−0.3
0.0
0.3
0.6
MR leave−one−out sensitivity analysis for
'PGLS' on 'Pericarditis'

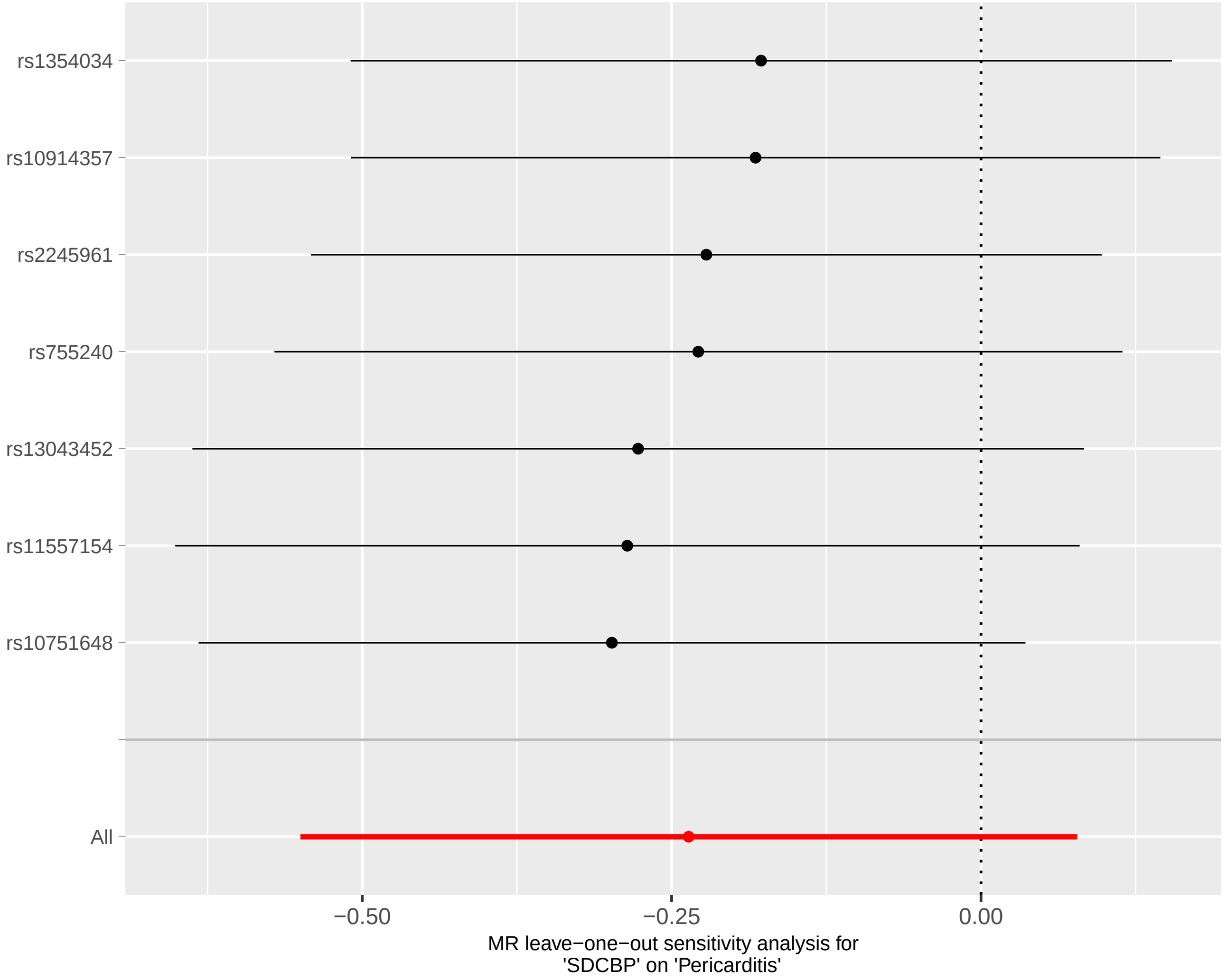
rs1354034
rs10914357
rs2245961
rs755240
rs13043452
rs11557154
rs10751648
All
−0.50
−0.25
0.00
MR leave−one−out sensitivity analysis for
'SDCBP' on 'Pericarditis'

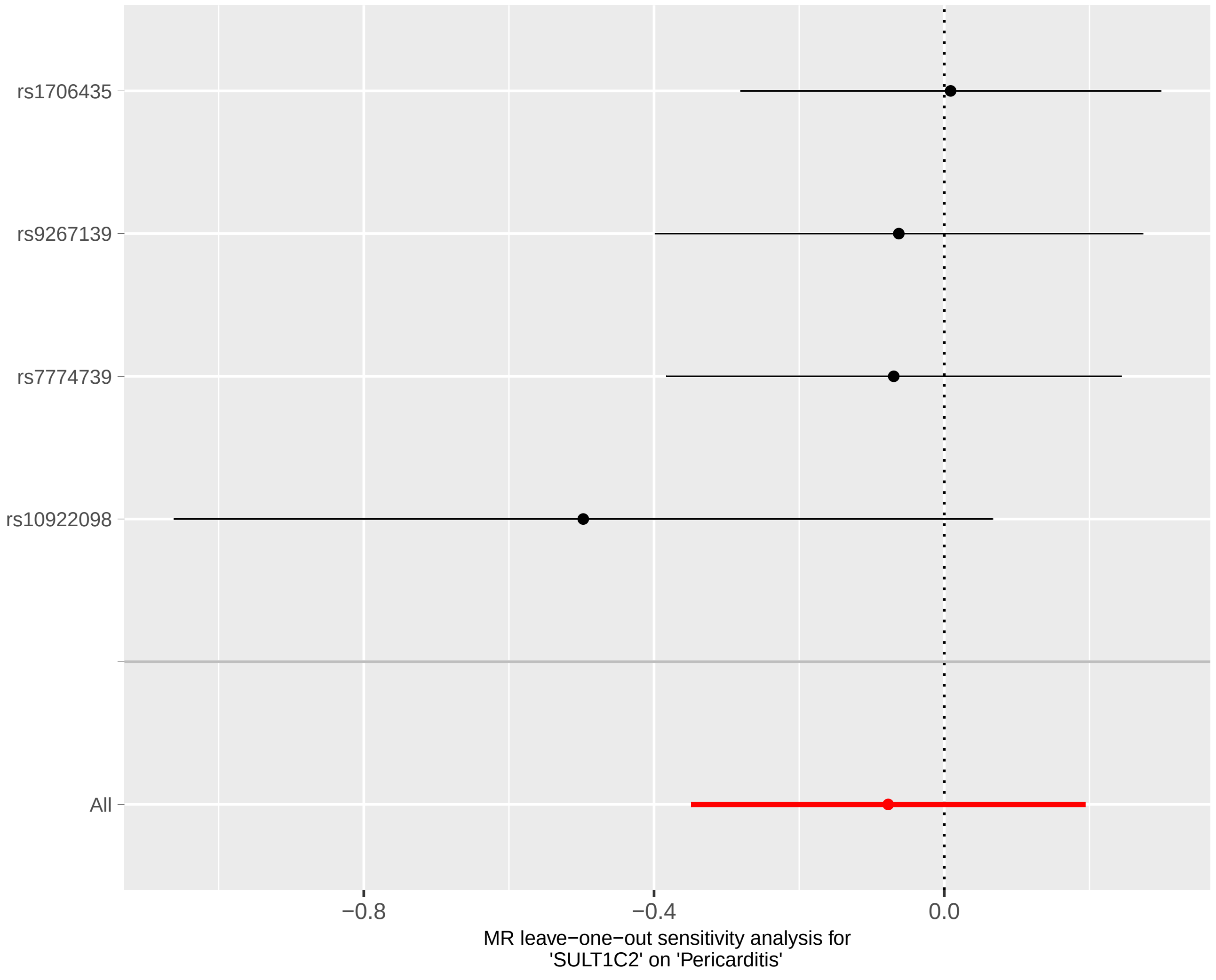
rs1706435
rs9267139
rs7774739
rs10922098
All
−0.8
−0.4
0.0
MR leave−one−out sensitivity analysis for
'SULT1C2' on 'Pericarditis'

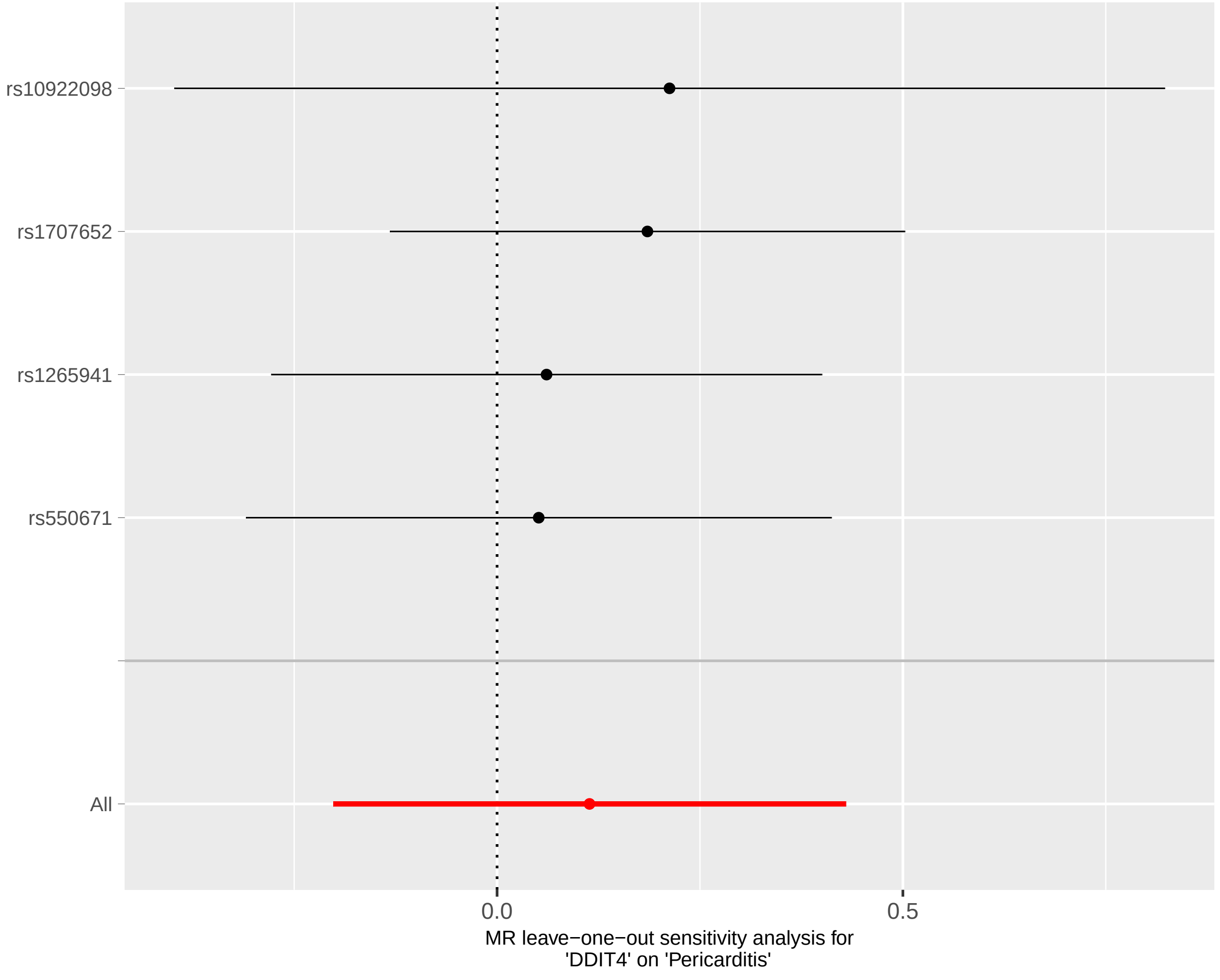
rs10922098
rs1707652
rs1265941
rs550671
All
0.0
0.5
MR leave−one−out sensitivity analysis for
'DDIT4' on 'Pericarditis'

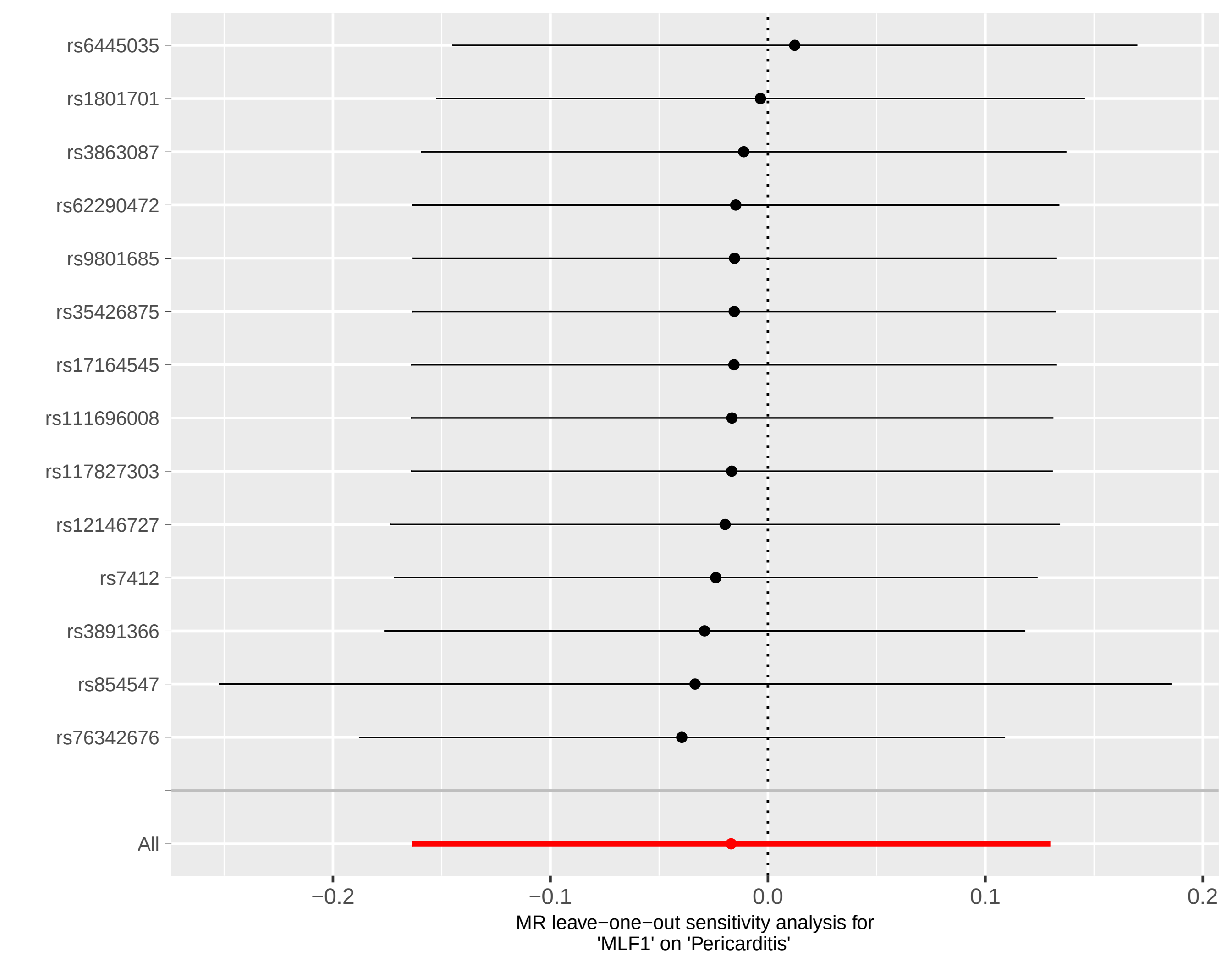
rs6445035
rs1801701
rs3863087
rs62290472
rs9801685
rs35426875
rs17164545
rs111696008
rs117827303
rs12146727
rs7412
rs3891366
rs854547
rs76342676
All
−0.2
−0.1
0.0
0.1
0.2
MR leave−one−out sensitivity analysis for
'MLF1' on 'Pericarditis'

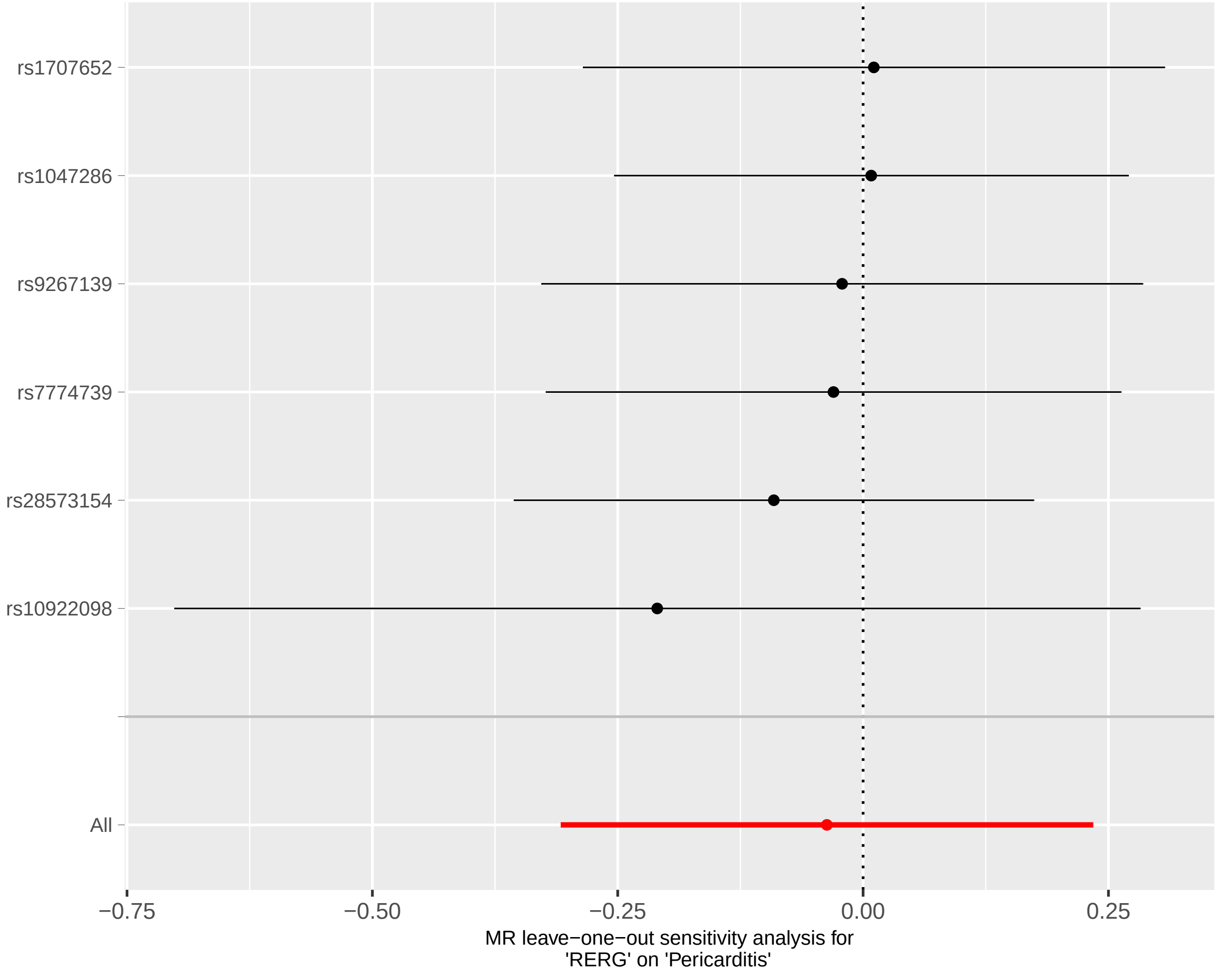
rs1707652
rs1047286
rs9267139
rs7774739
rs28573154
rs10922098
All
−0.75
−0.50
−0.25
0.00
0.25
MR leave−one−out sensitivity analysis for
'RERG' on 'Pericarditis'

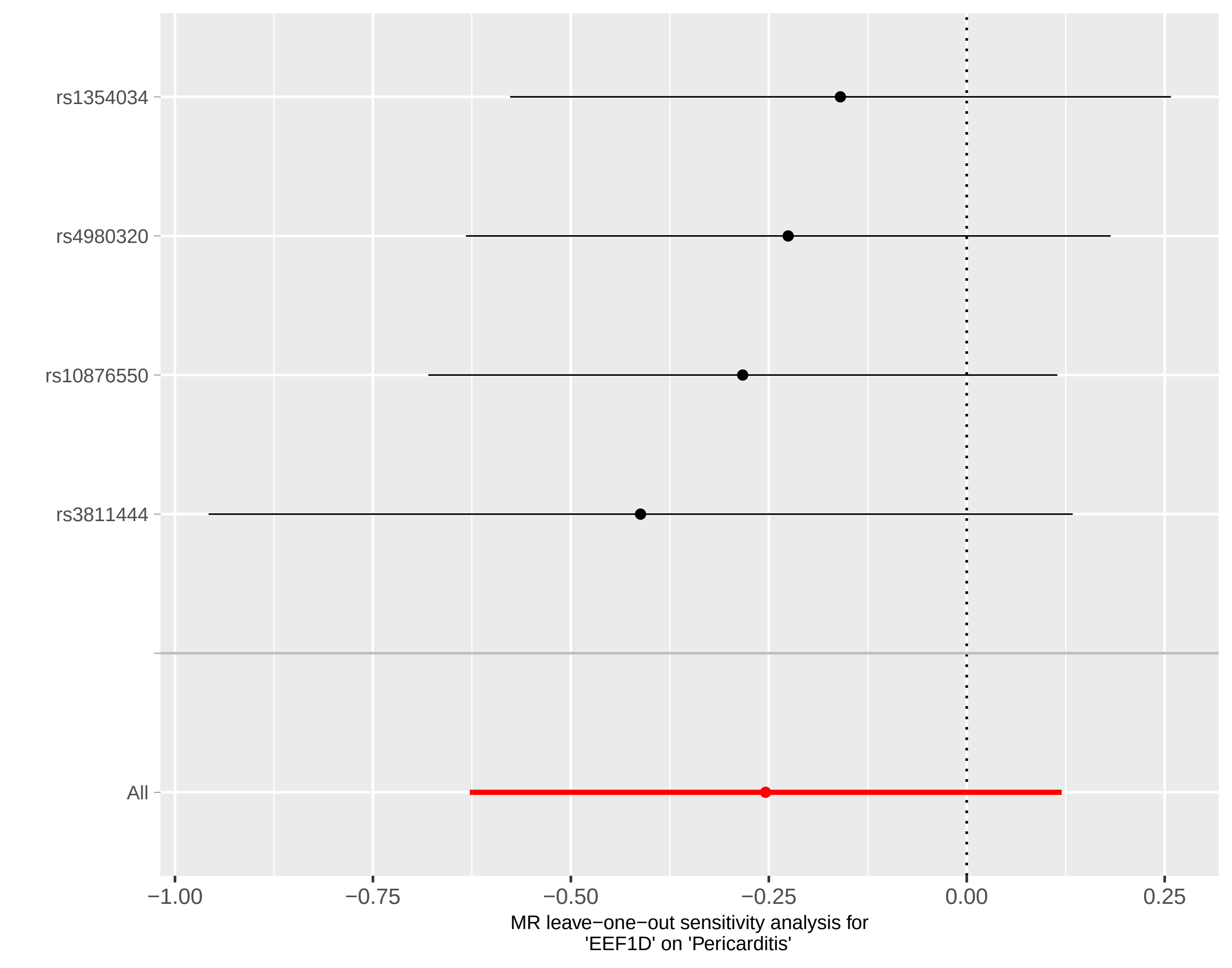
rs1354034
rs4980320
rs10876550
rs3811444
All
−1.00
−0.75
−0.50
−0.25
0.00
0.25
MR leave−one−out sensitivity analysis for
'EEF1D' on 'Pericarditis'

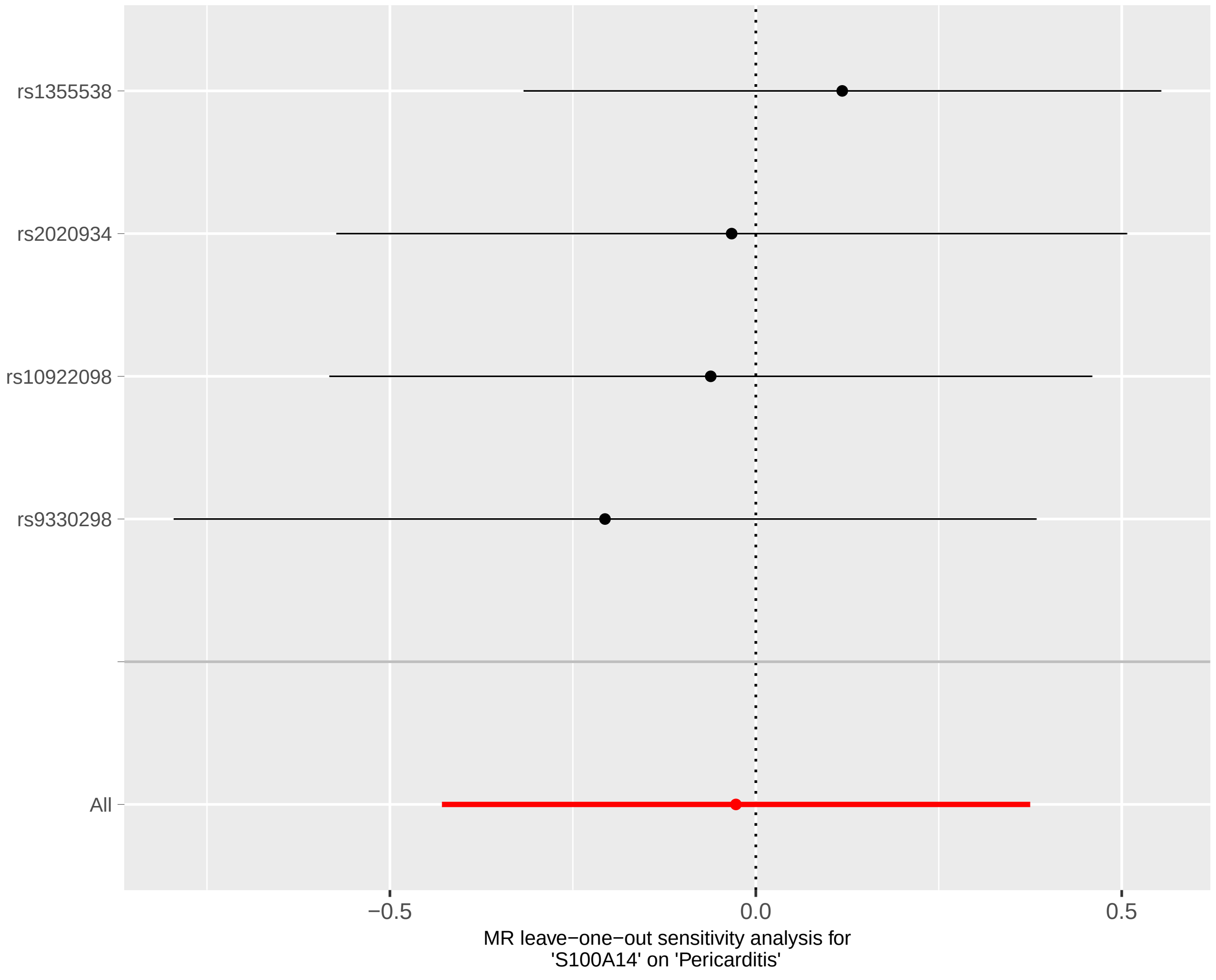
rs1355538
rs2020934
rs10922098
rs9330298
All
−0.5
0.0
0.5
MR leave−one−out sensitivity analysis for
'S100A14' on 'Pericarditis'

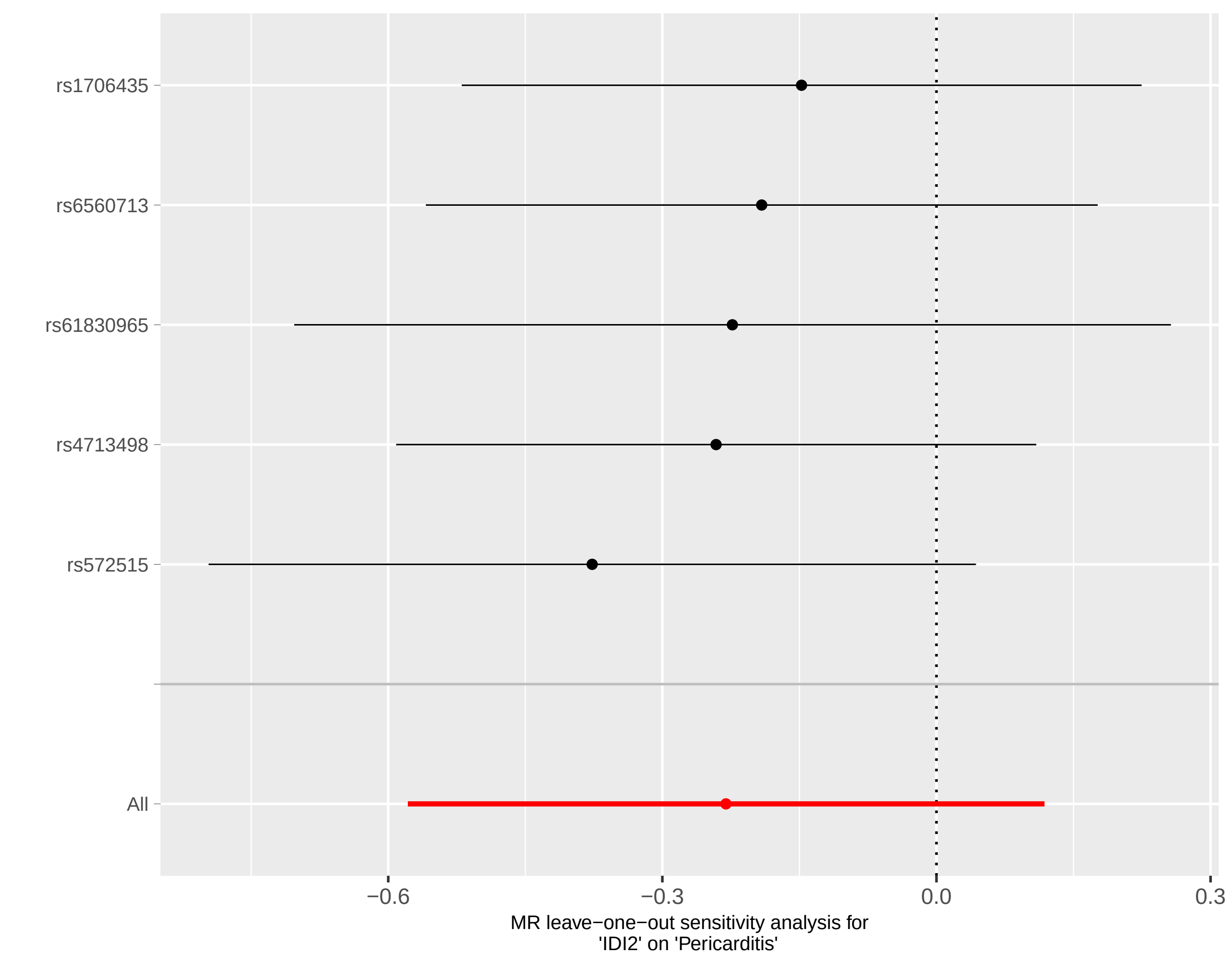
rs1706435
rs6560713
rs61830965
rs4713498
rs572515
All
−0.6
−0.3
0.0
0.3
MR leave−one−out sensitivity analysis for
'IDI2' on 'Pericarditis'

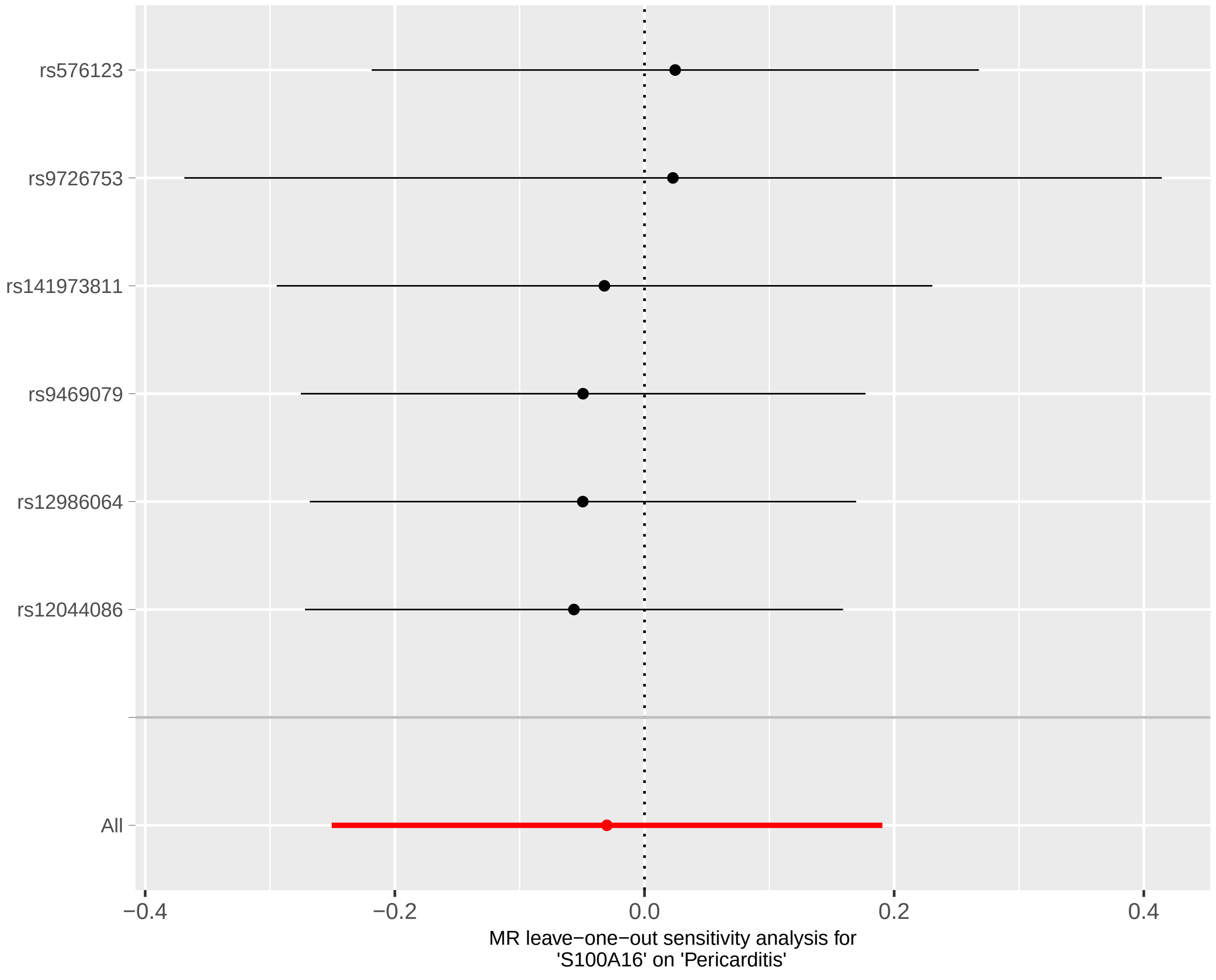
rs576123
rs9726753
rs141973811
rs9469079
rs12986064
rs12044086
All
−0.4
−0.2
0.0
0.2
0.4
MR leave−one−out sensitivity analysis for
'S100A16' on 'Pericarditis'

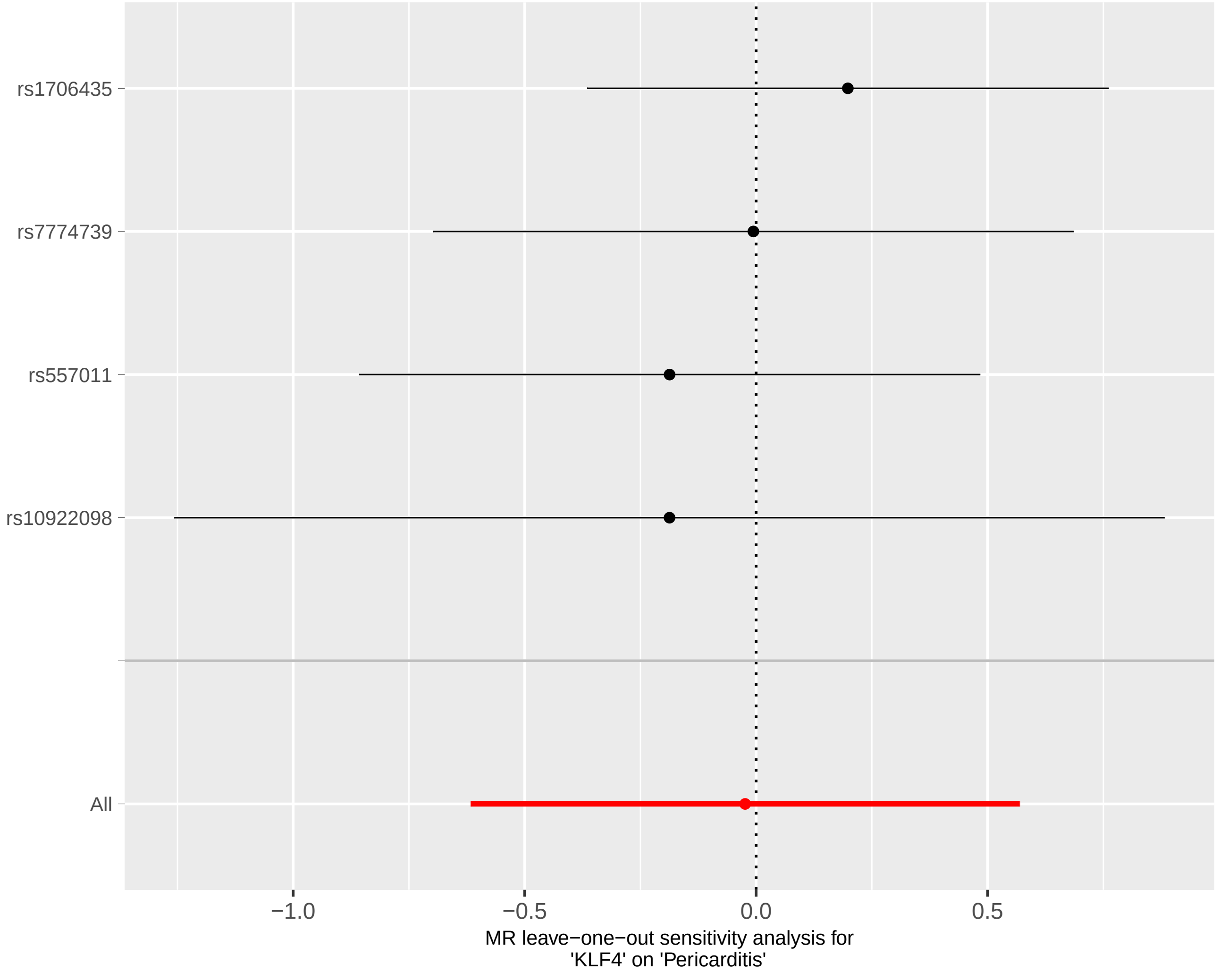

rs1706435
rs7774739
rs557011
rs10922098
All
−1.0
−0.5
0.0
0.5
MR leave−one−out sensitivity analysis for
'KLF4' on 'Pericarditis'

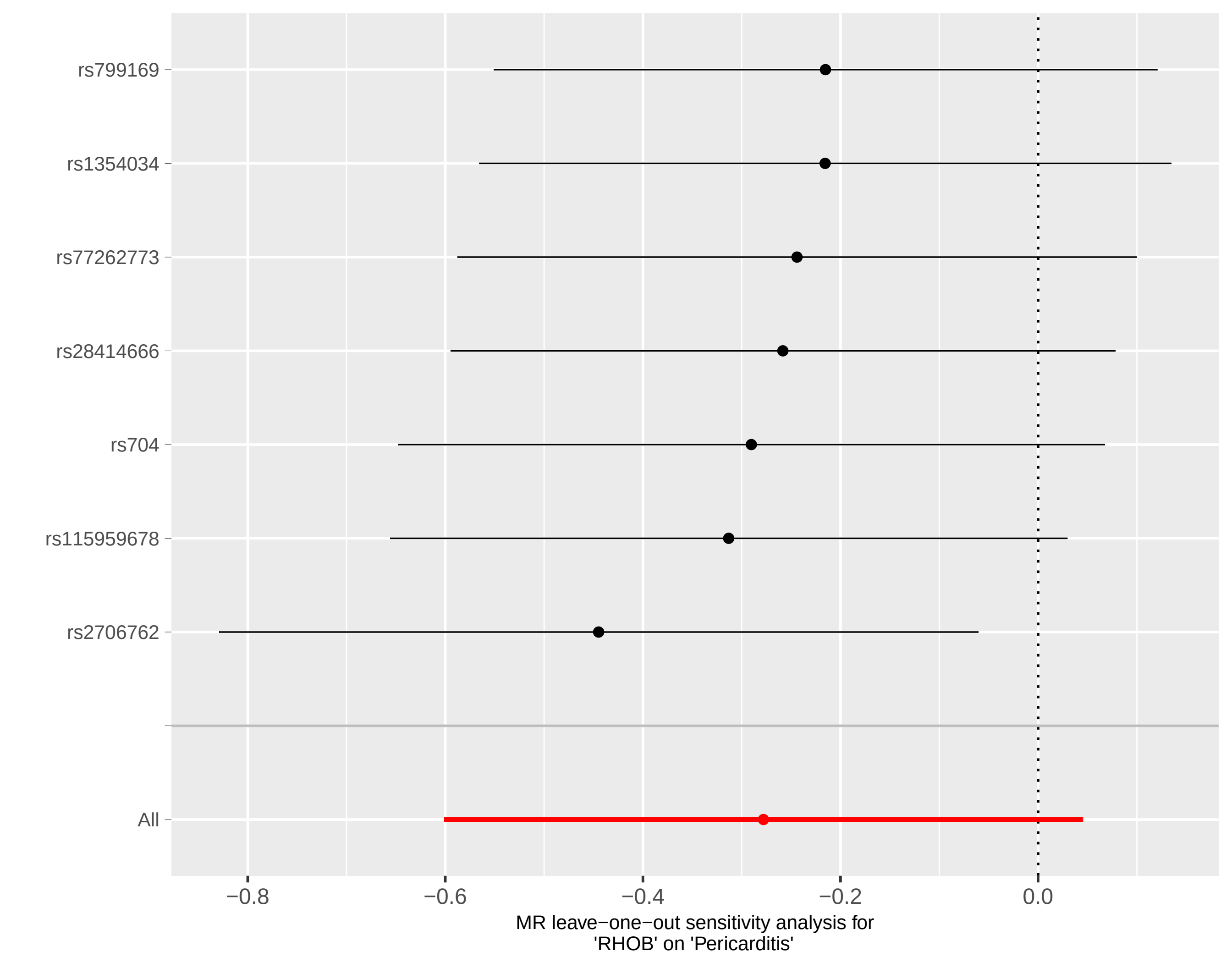
rs799169
rs1354034
rs77262773
rs28414666
rs704
rs115959678
rs2706762
All
−0.8
−0.6
−0.4
−0.2
0.0
MR leave−one−out sensitivity analysis for
'RHOB' on 'Pericarditis'

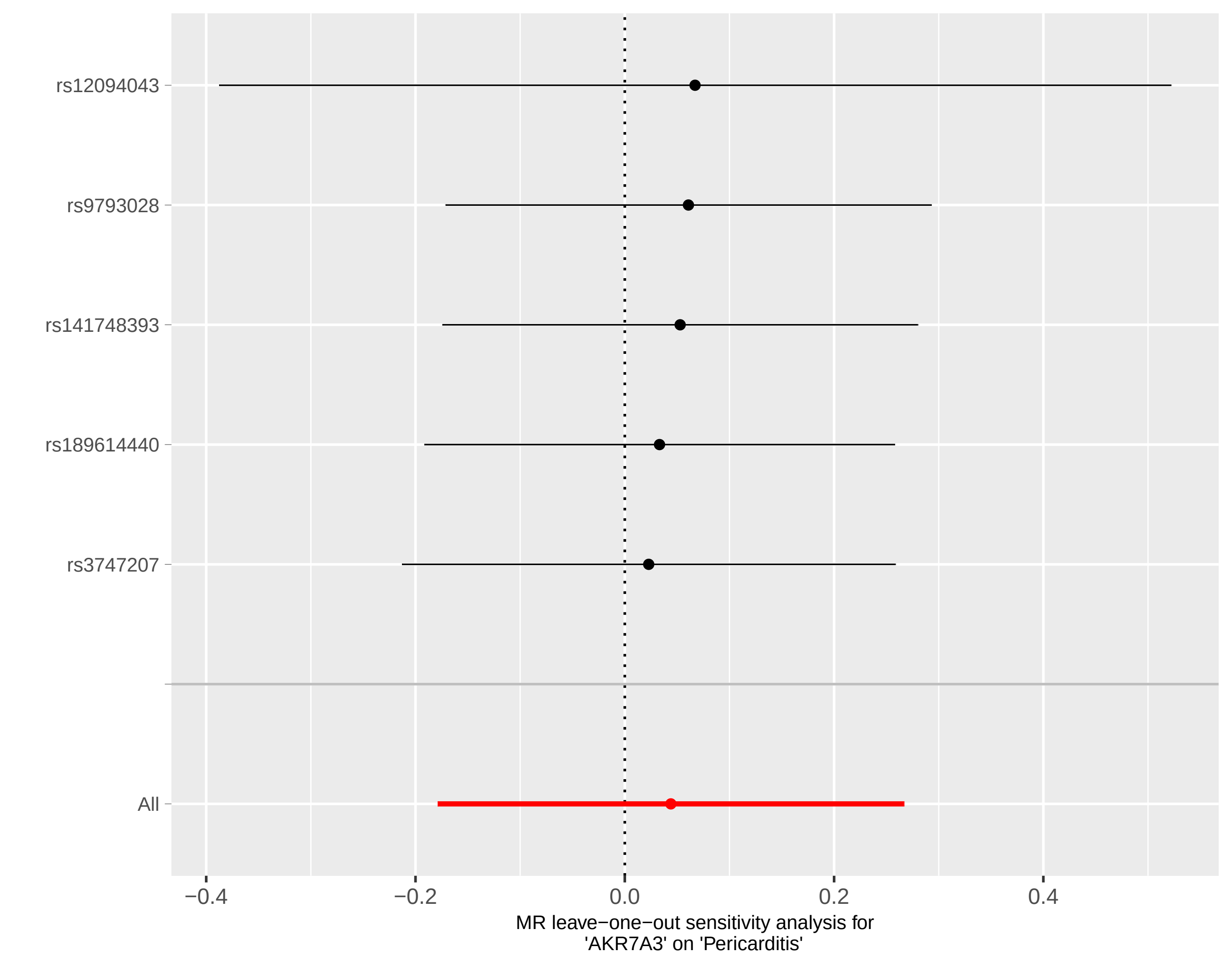

rs12094043
rs9793028
rs141748393
rs189614440
rs3747207
All
−0.4
−0.2
0.0
0.2
0.4
MR leave−one−out sensitivity analysis for
'AKR7A3' on 'Pericarditis'

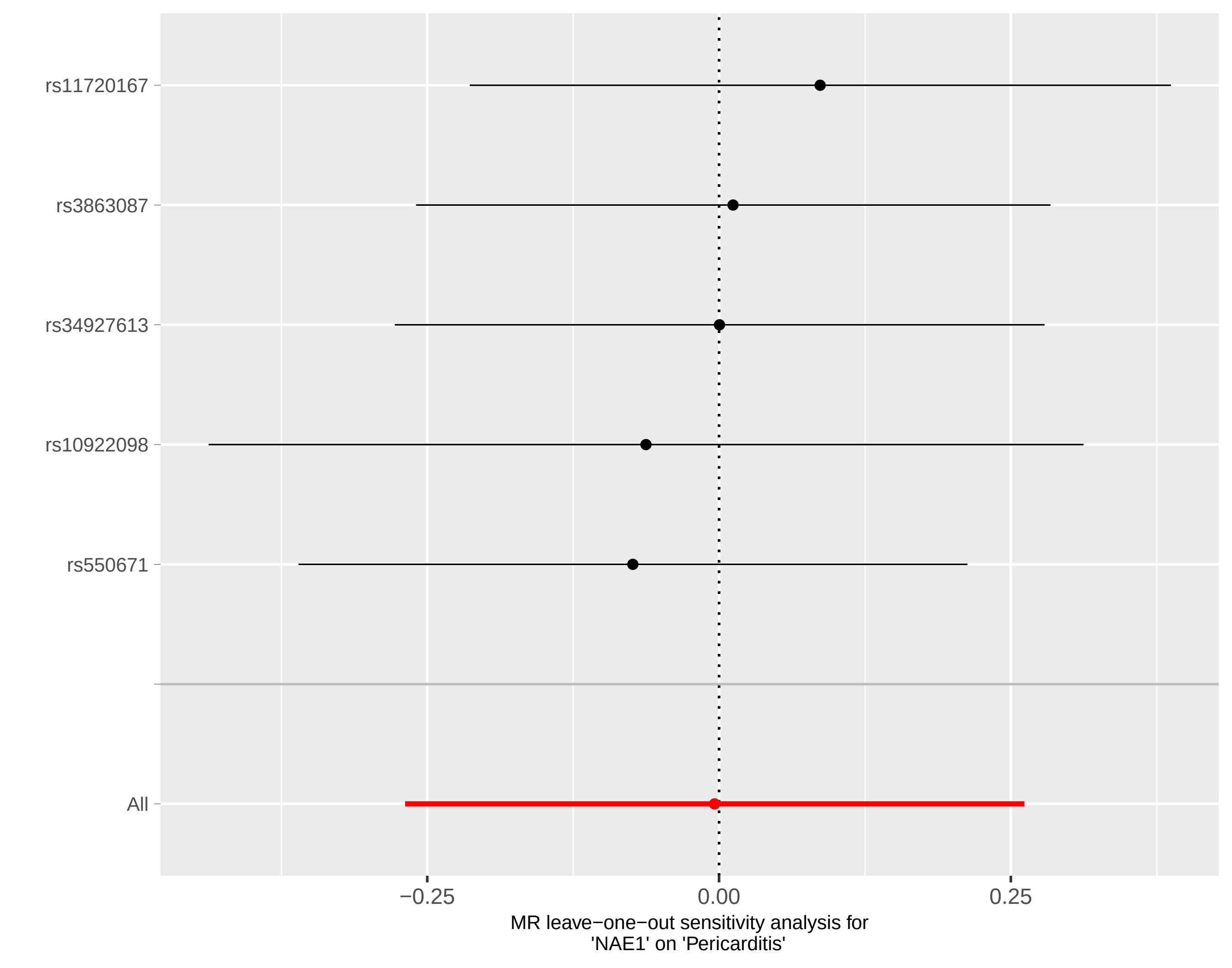
rs11720167
rs3863087
rs34927613
rs10922098
rs550671
All
−0.25
0.00
0.25
MR leave−one−out sensitivity analysis for
'NAE1' on 'Pericarditis'

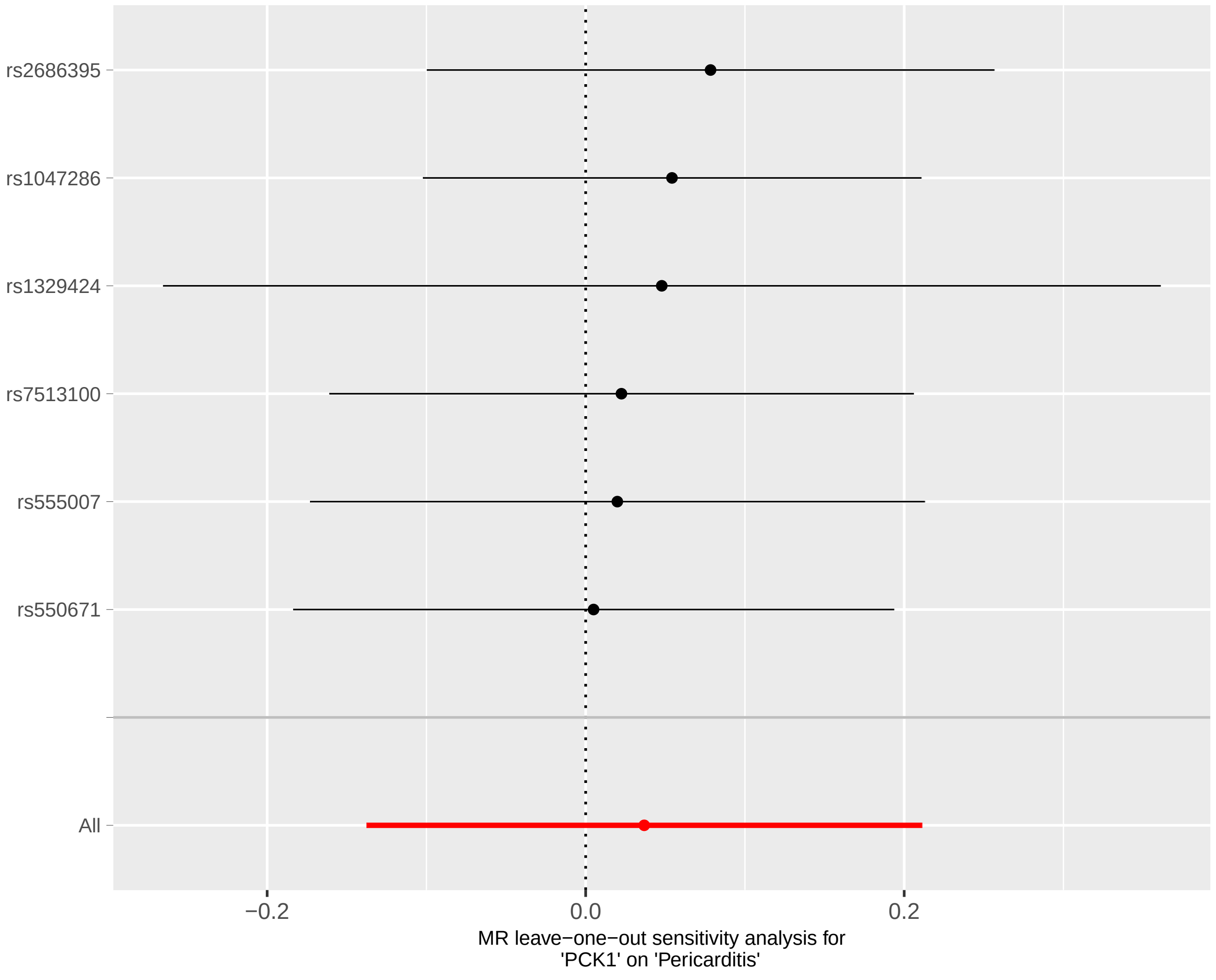
rs2686395
rs1047286
rs1329424
rs7513100
rs555007
rs550671
All
−0.2
0.0
0.2
MR leave−one−out sensitivity analysis for
'PCK1' on 'Pericarditis'

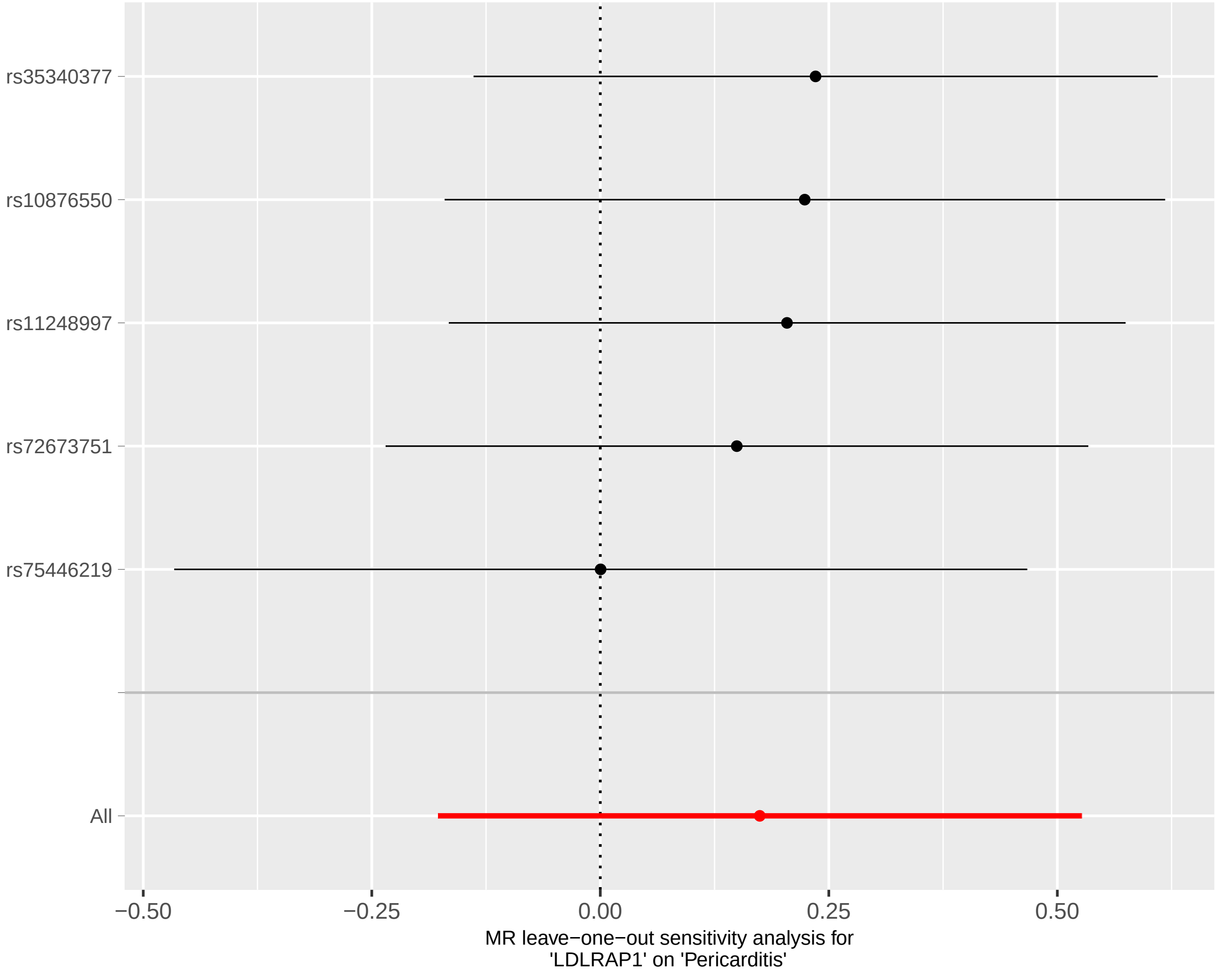
rs35340377
rs10876550
rs11248997
rs72673751
rs75446219
All
−0.50
−0.25
0.00
0.25
0.50
MR leave−one−out sensitivity analysis for
'LDLRAP1' on 'Pericarditis'

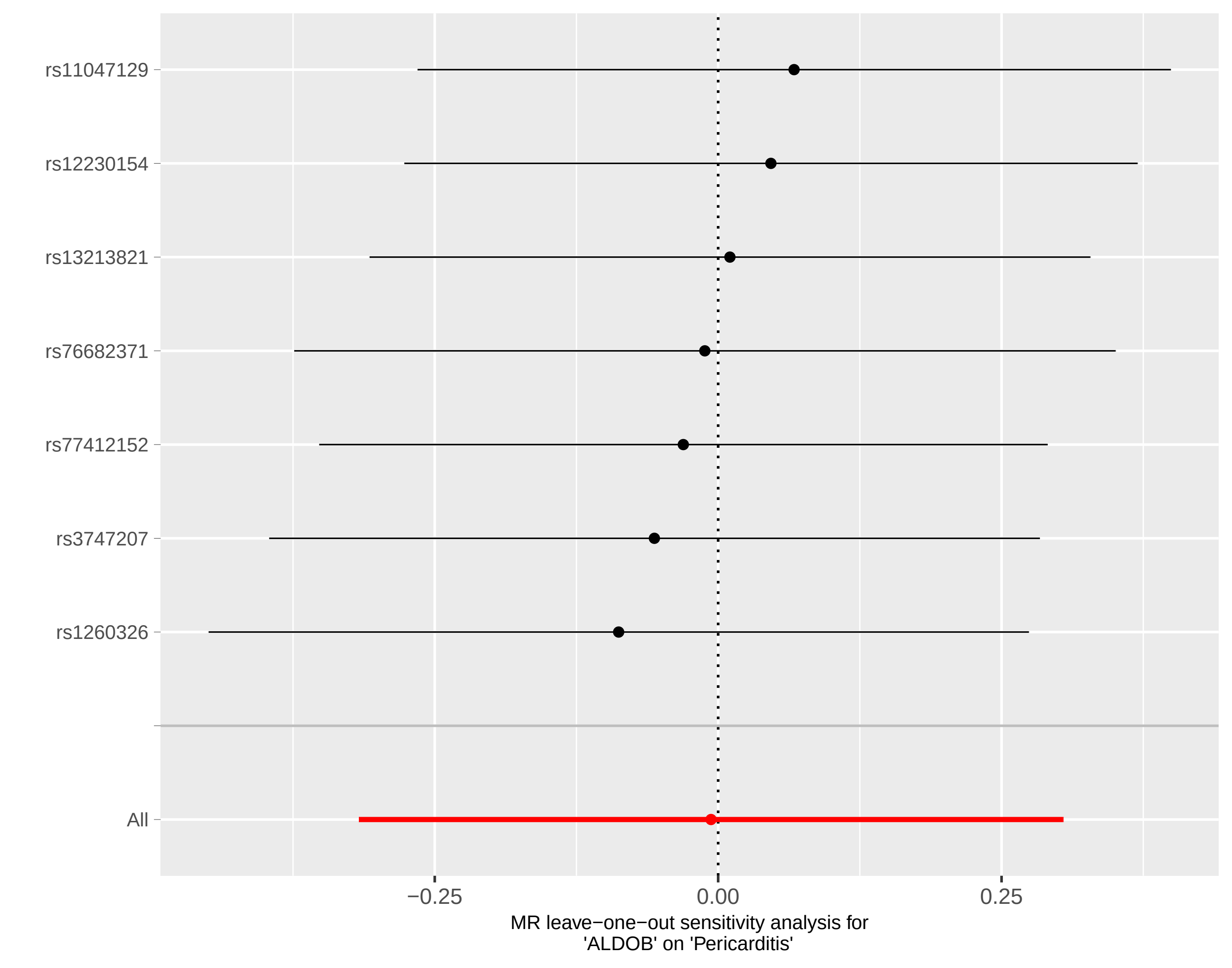
rs11047129
rs12230154
rs13213821
rs76682371
rs77412152
rs3747207
rs1260326
All
−0.25
0.00
0.25
MR leave−one−out sensitivity analysis for
'ALDOB' on 'Pericarditis'

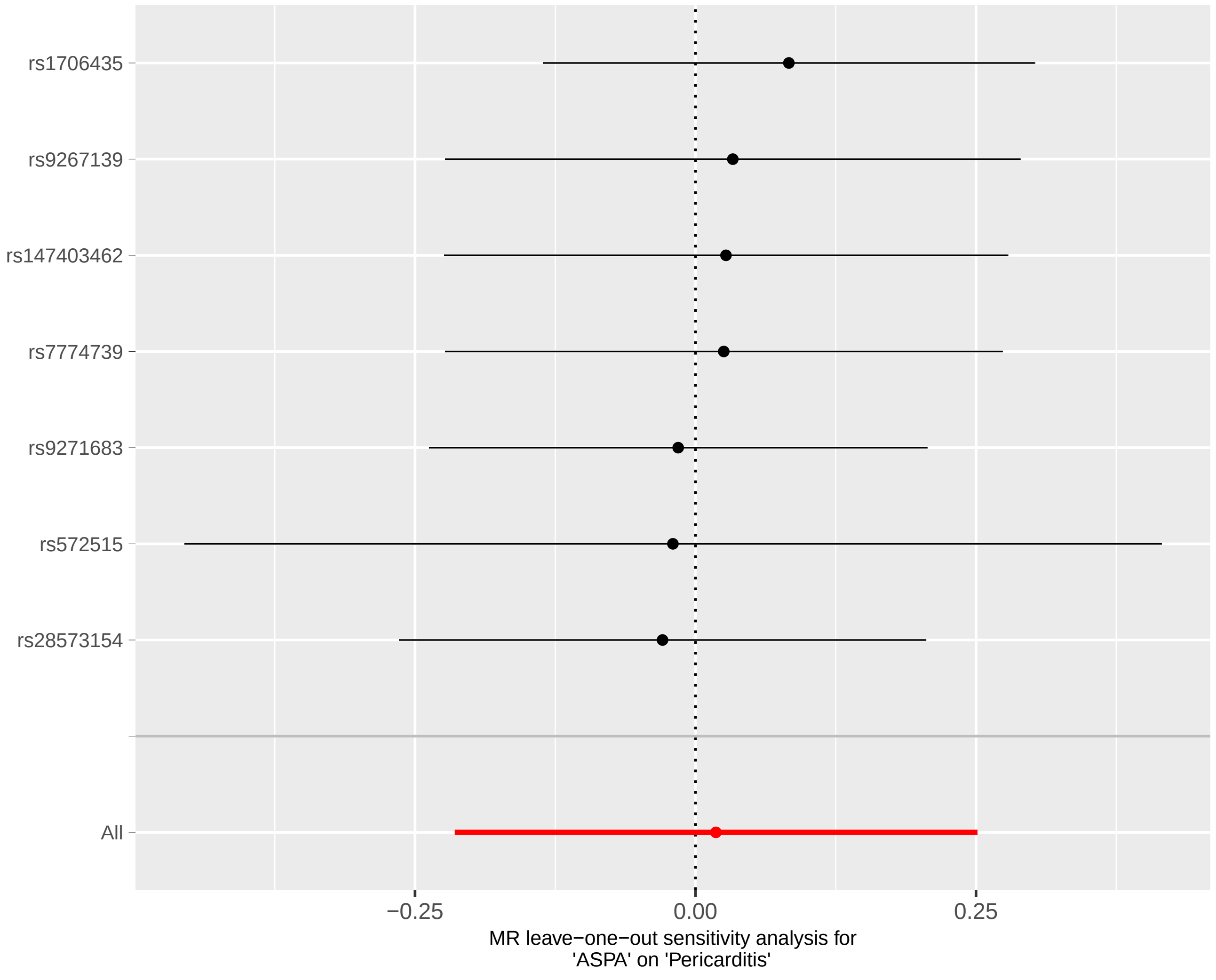
rs1706435
rs9267139
rs147403462
rs7774739
rs9271683
rs572515
rs28573154
All
−0.25
0.00
0.25
MR leave−one−out sensitivity analysis for
'ASPA' on 'Pericarditis'

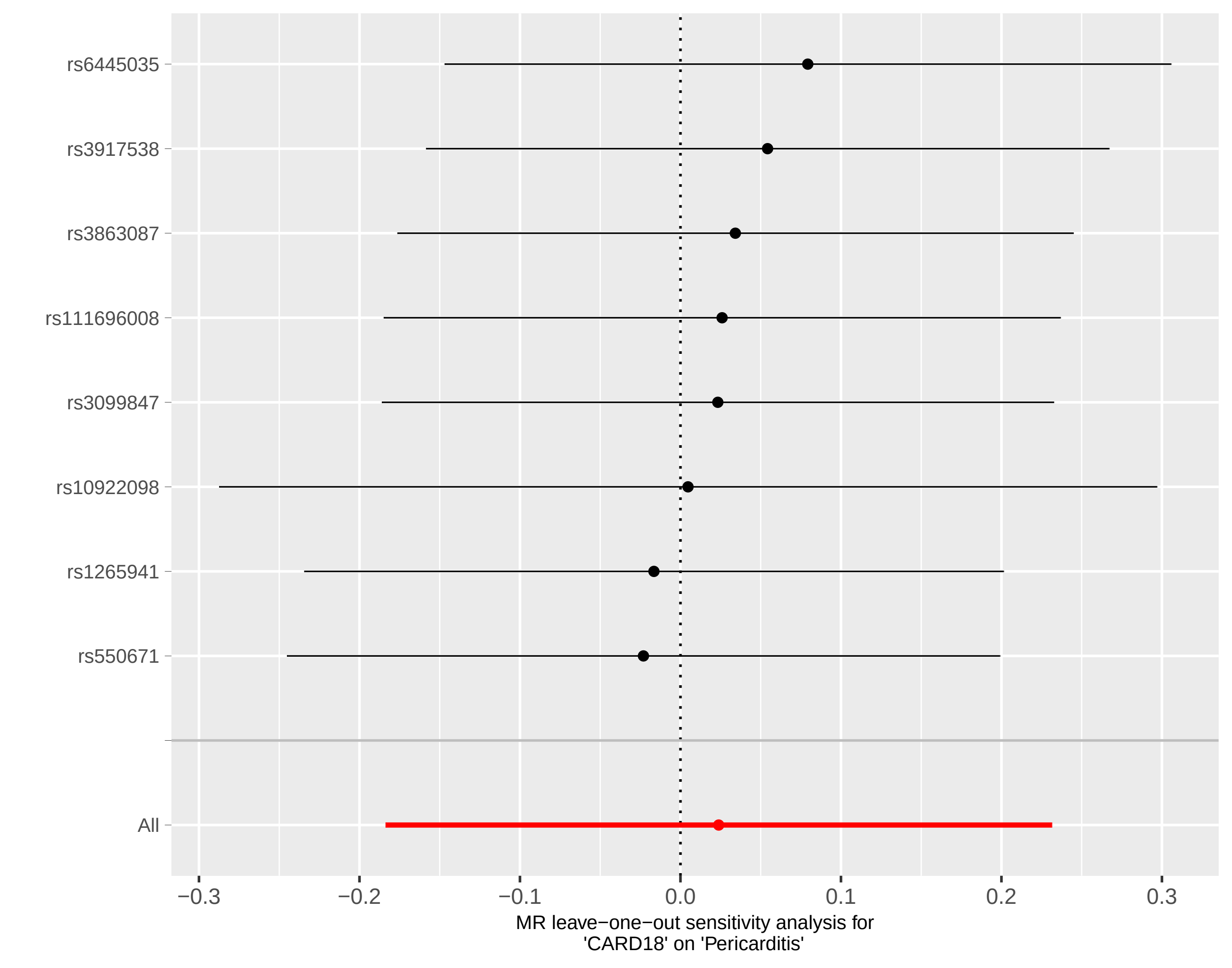

rs6445035
rs3917538
rs3863087
rs111696008
rs3099847
rs10922098
rs1265941
rs550671
All
−0.3
−0.2
−0.1
0.0
0.1
0.2
0.3
MR leave−one−out sensitivity analysis for
'CARD18' on 'Pericarditis'

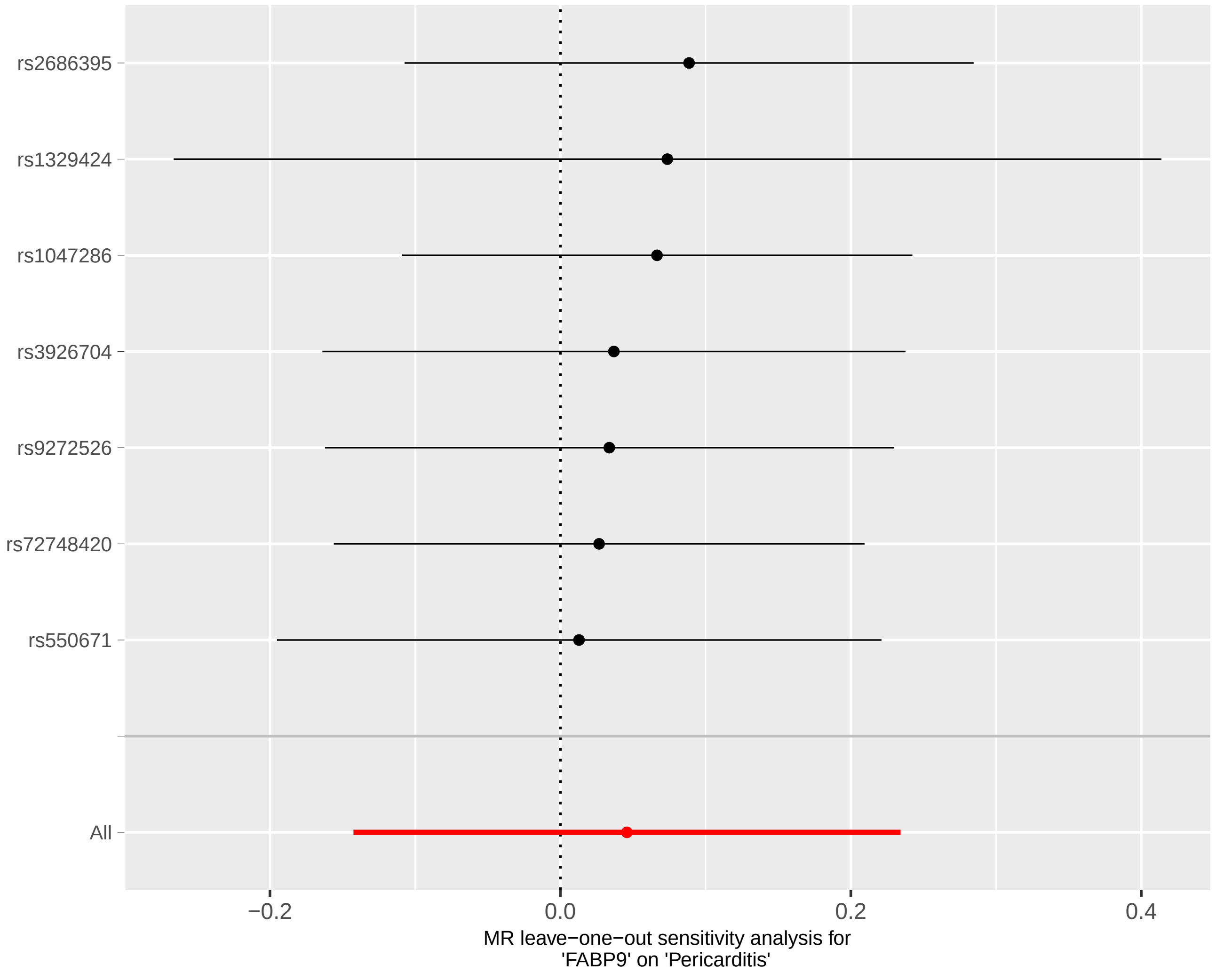

rs2686395
rs1329424
rs1047286
rs3926704
rs9272526
rs72748420
rs550671
All
−0.2
0.0
0.2
0.4
MR leave−one−out sensitivity analysis for
'FABP9' on 'Pericarditis'

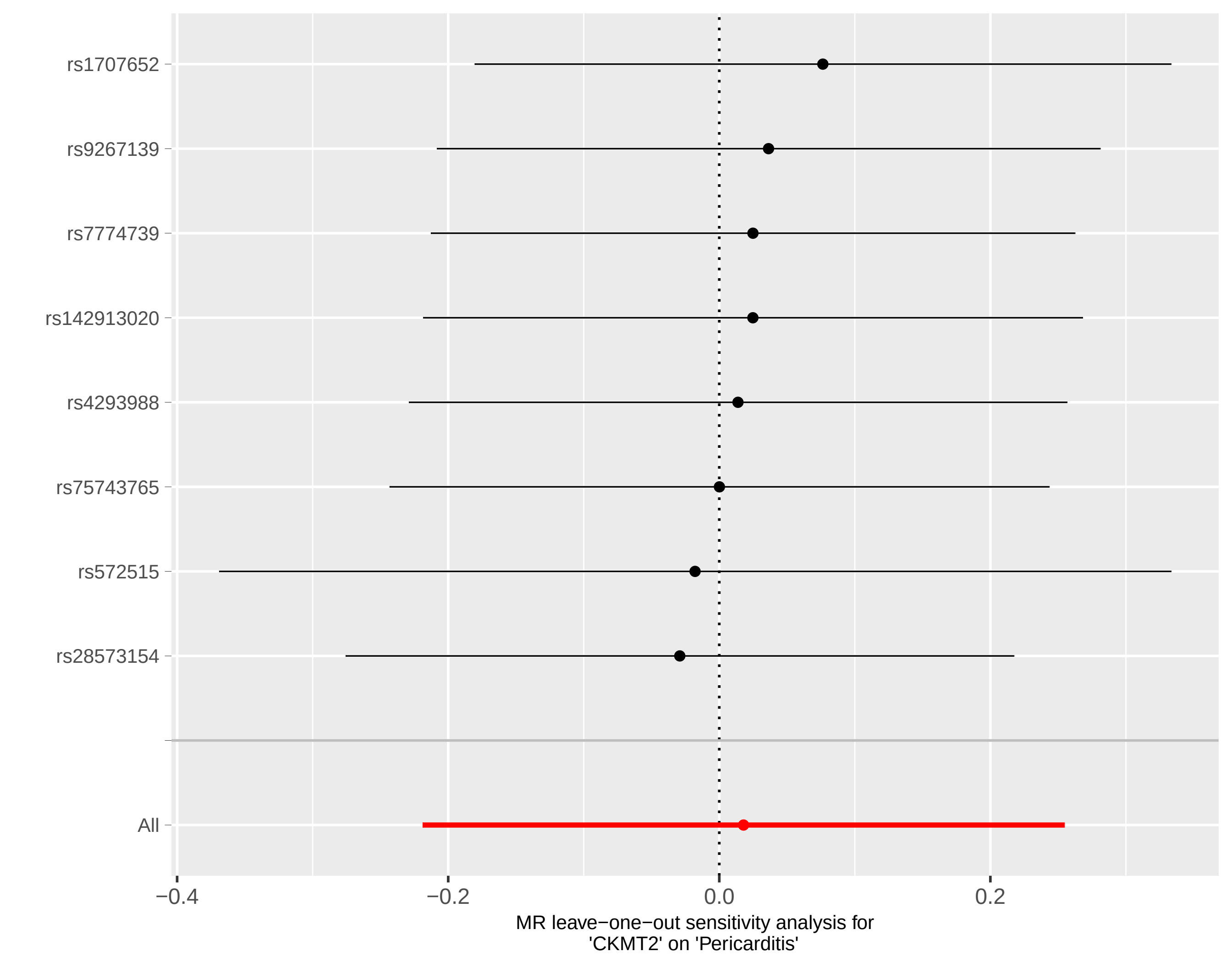
rs1707652
rs9267139
rs7774739
rs142913020
rs4293988
rs75743765
rs572515
rs28573154
All
−0.4
−0.2
0.0
0.2
MR leave−one−out sensitivity analysis for
'CKMT2' on 'Pericarditis'

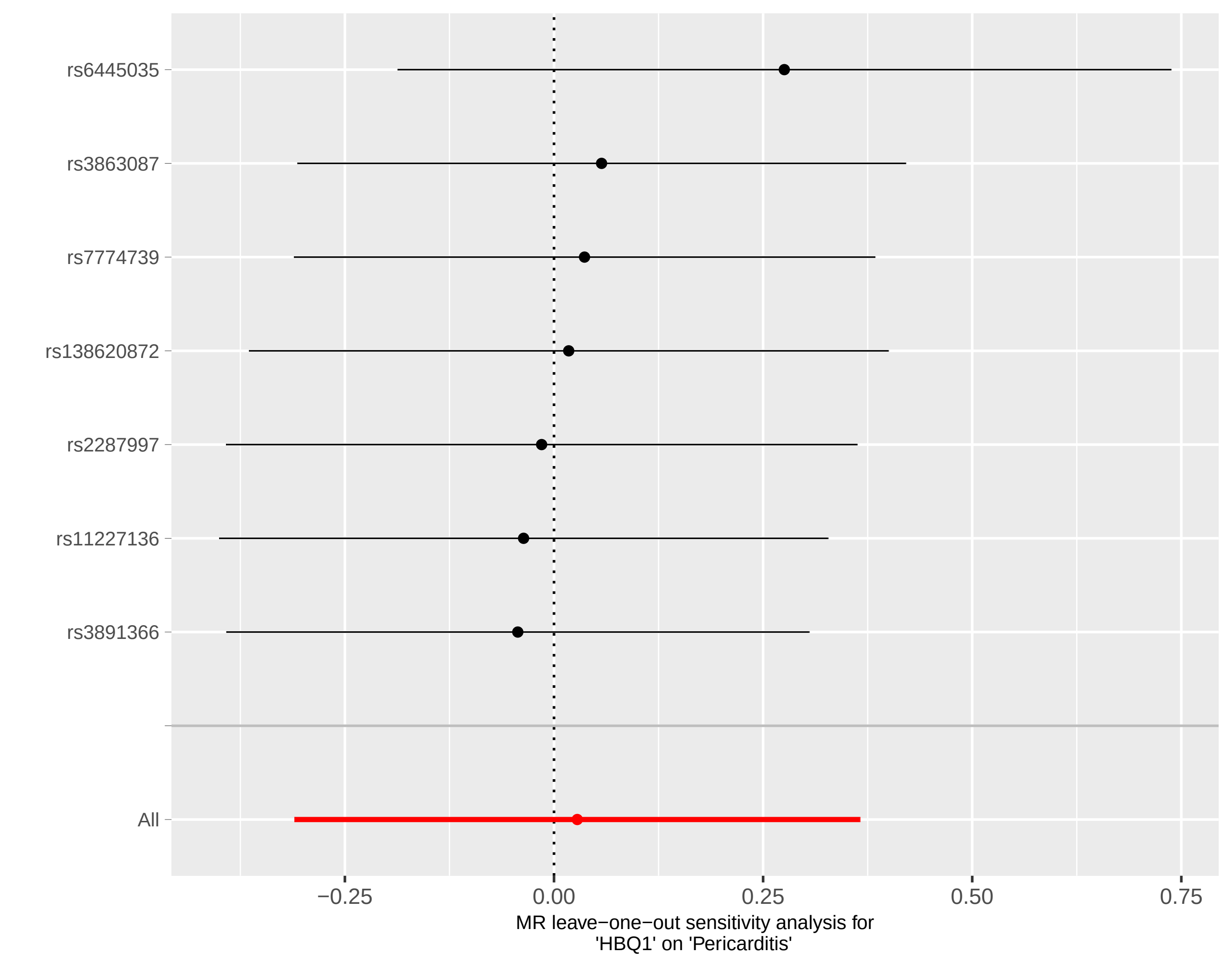
rs6445035
rs3863087
rs7774739
rs138620872
rs2287997
rs11227136
rs3891366
All
−0.25
0.00
0.25
0.50
0.75
MR leave−one−out sensitivity analysis for
'HBQ1' on 'Pericarditis'

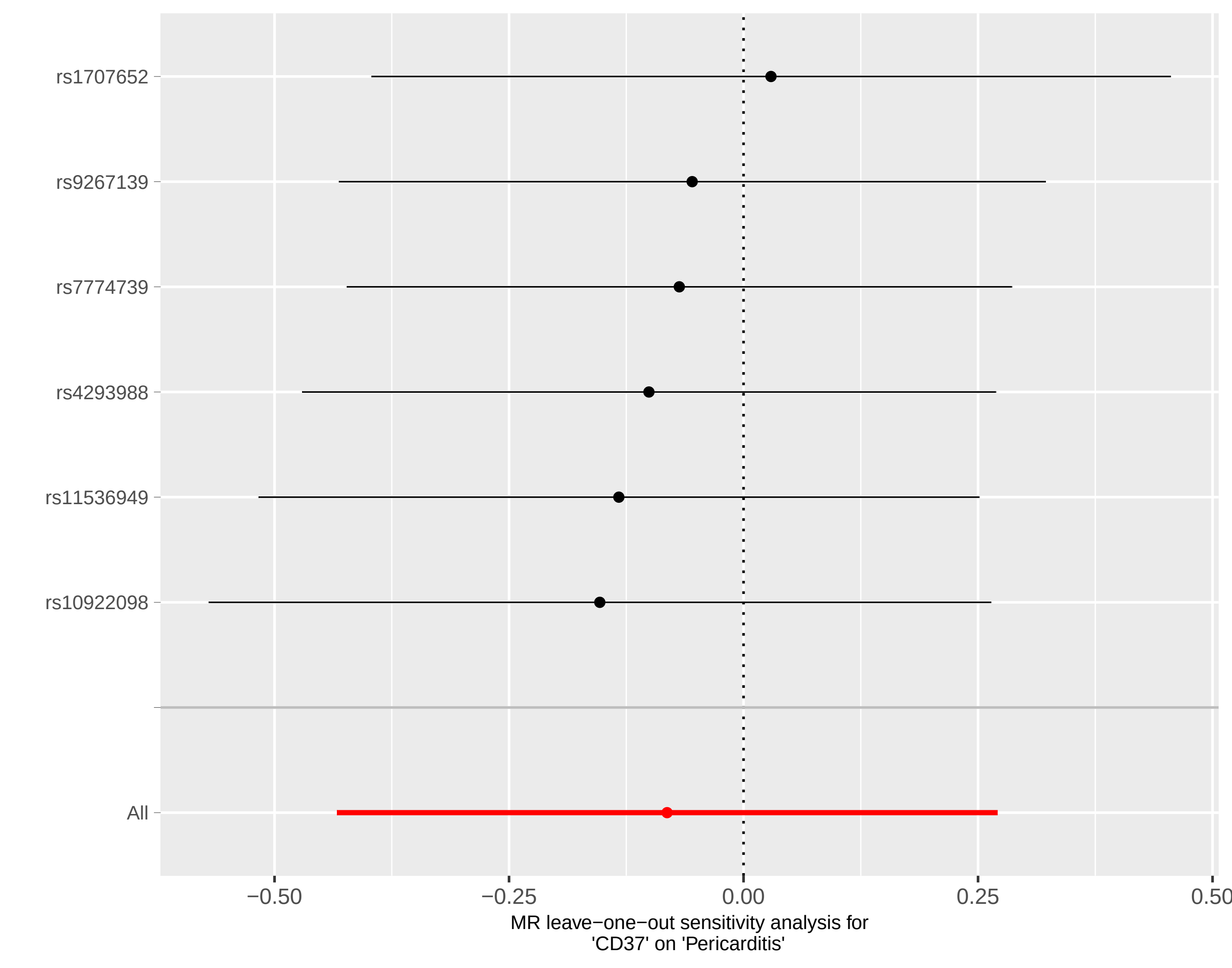

rs1707652
rs9267139
rs7774739
rs4293988
rs11536949
rs10922098
All
−0.50
−0.25
0.00
0.25
0.50
MR leave−one−out sensitivity analysis for
'CD37' on 'Pericarditis'

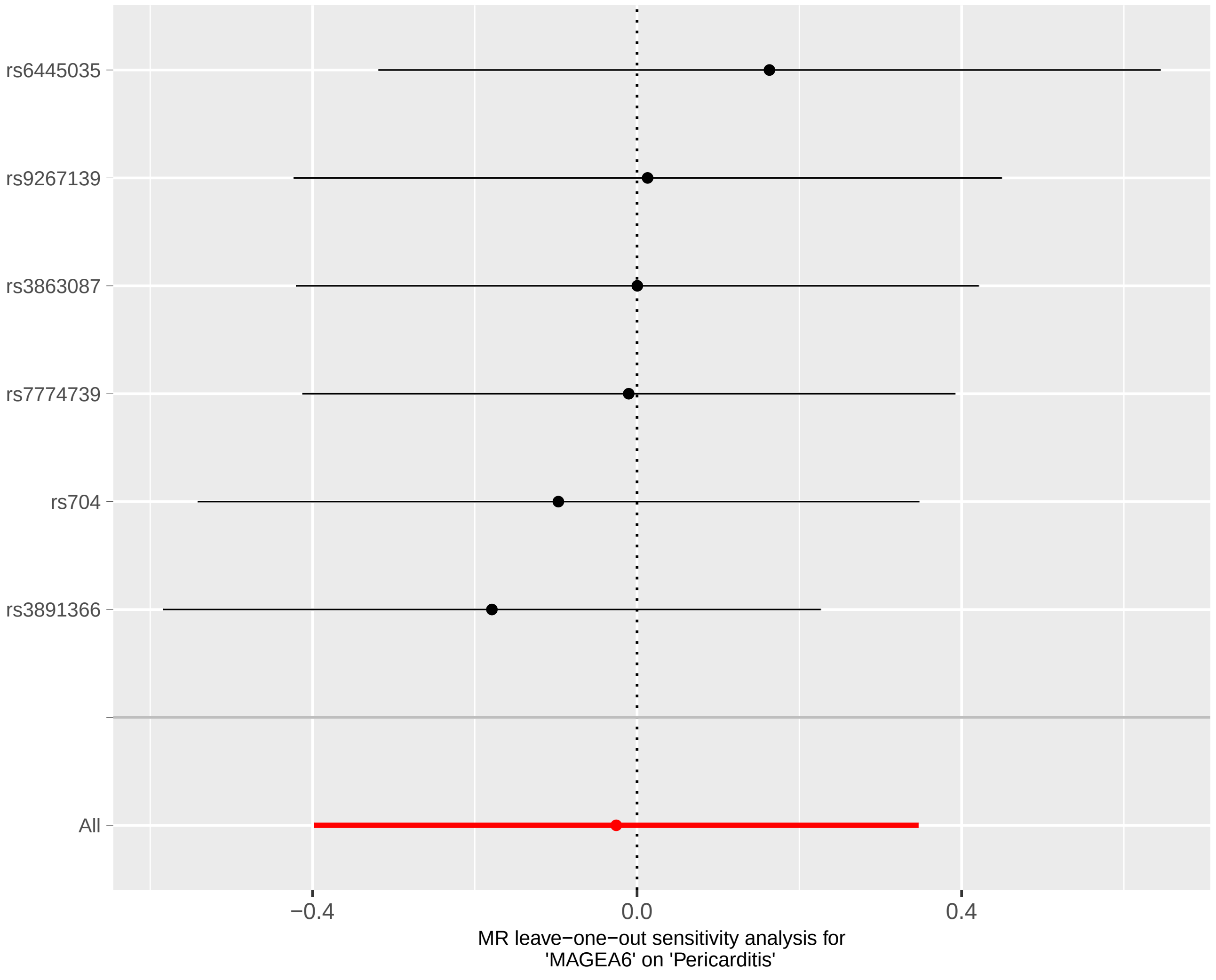
rs6445035
rs9267139
rs3863087
rs7774739
rs704
rs3891366
All
−0.4
0.0
0.4
MR leave−one−out sensitivity analysis for
'MAGEA6' on 'Pericarditis'

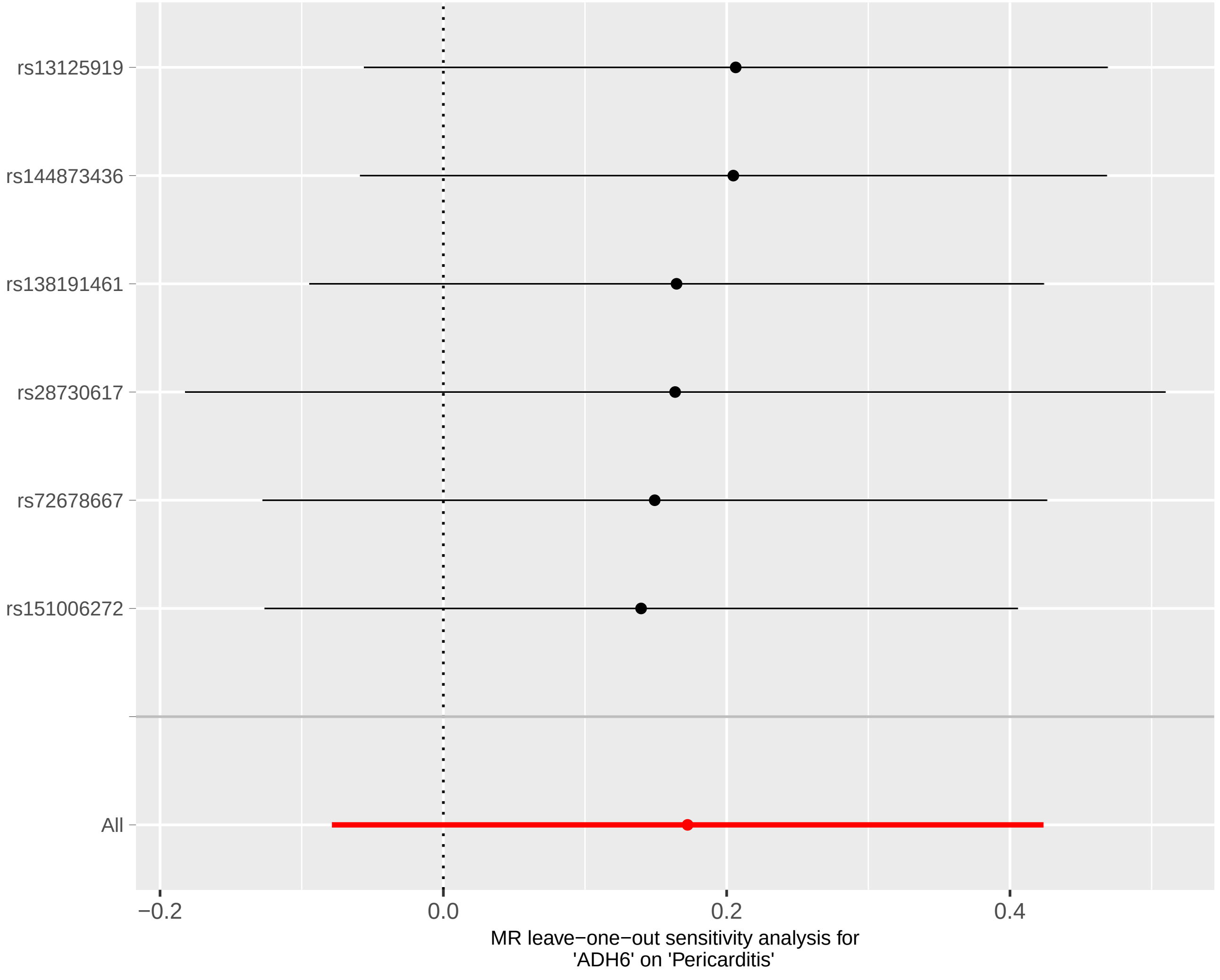

rs13125919
rs144873436
rs138191461
rs28730617
rs72678667
rs151006272
All
−0.2
0.0
0.2
0.4
MR leave−one−out sensitivity analysis for
'ADH6' on 'Pericarditis'

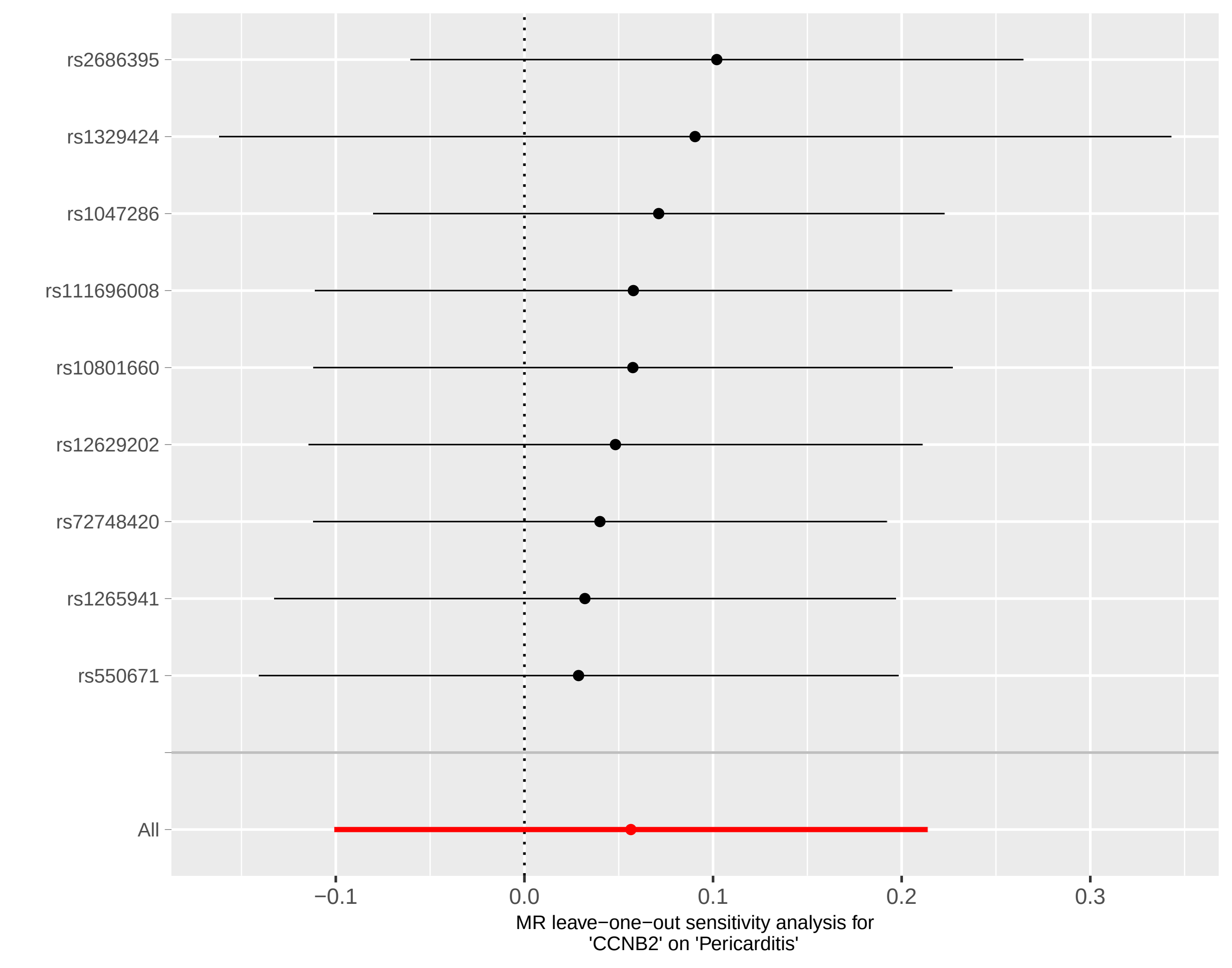
rs2686395
rs1329424
rs1047286
rs111696008
rs10801660
rs12629202
rs72748420
rs1265941
rs550671
All
−0.1
0.0
0.1
0.2
0.3
MR leave−one−out sensitivity analysis for
'CCNB2' on 'Pericarditis'

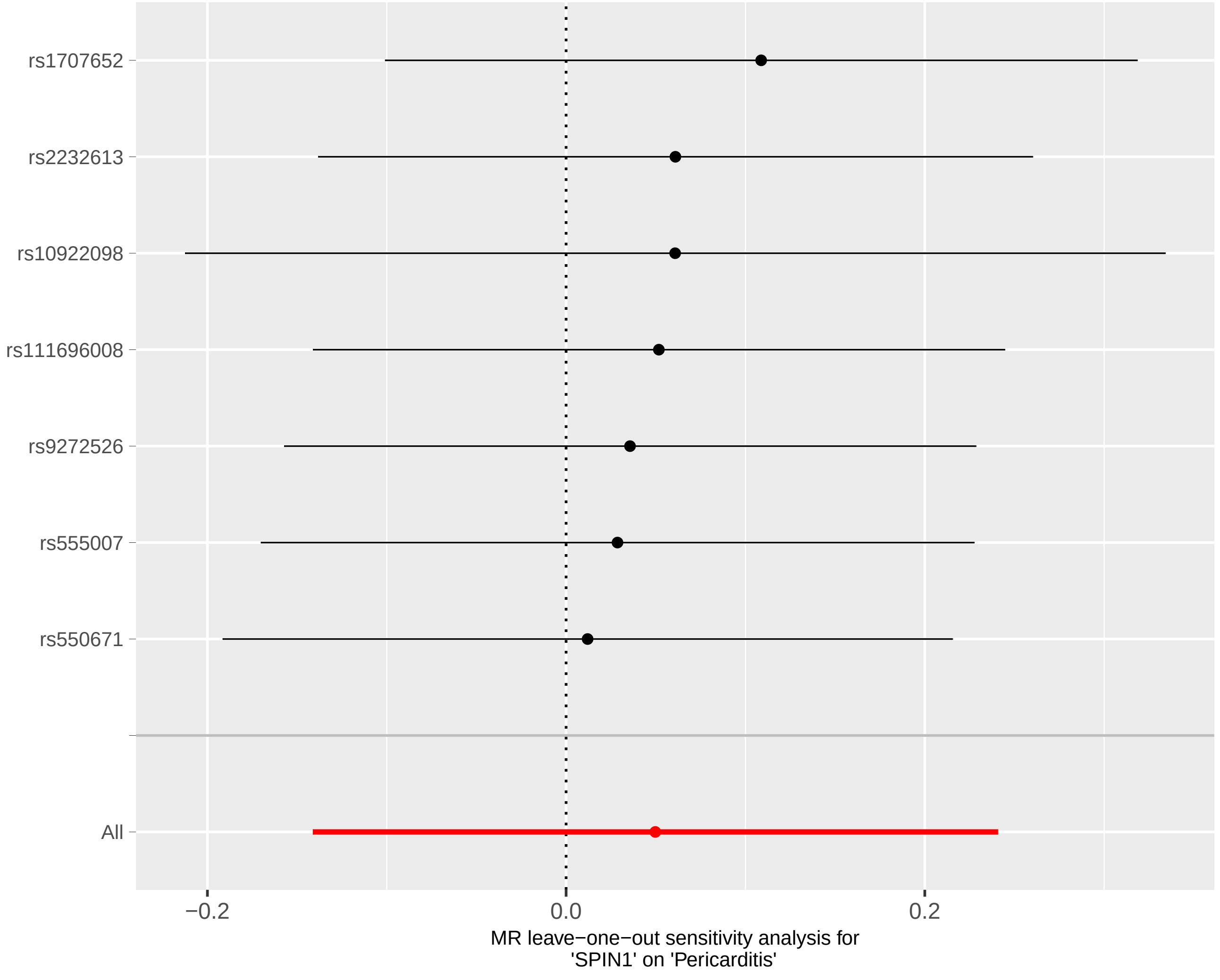

rs1707652
rs2232613
rs10922098
rs111696008
rs9272526
rs555007
rs550671
All
−0.2
0.0
0.2
MR leave−one−out sensitivity analysis for
'SPIN1' on 'Pericarditis'

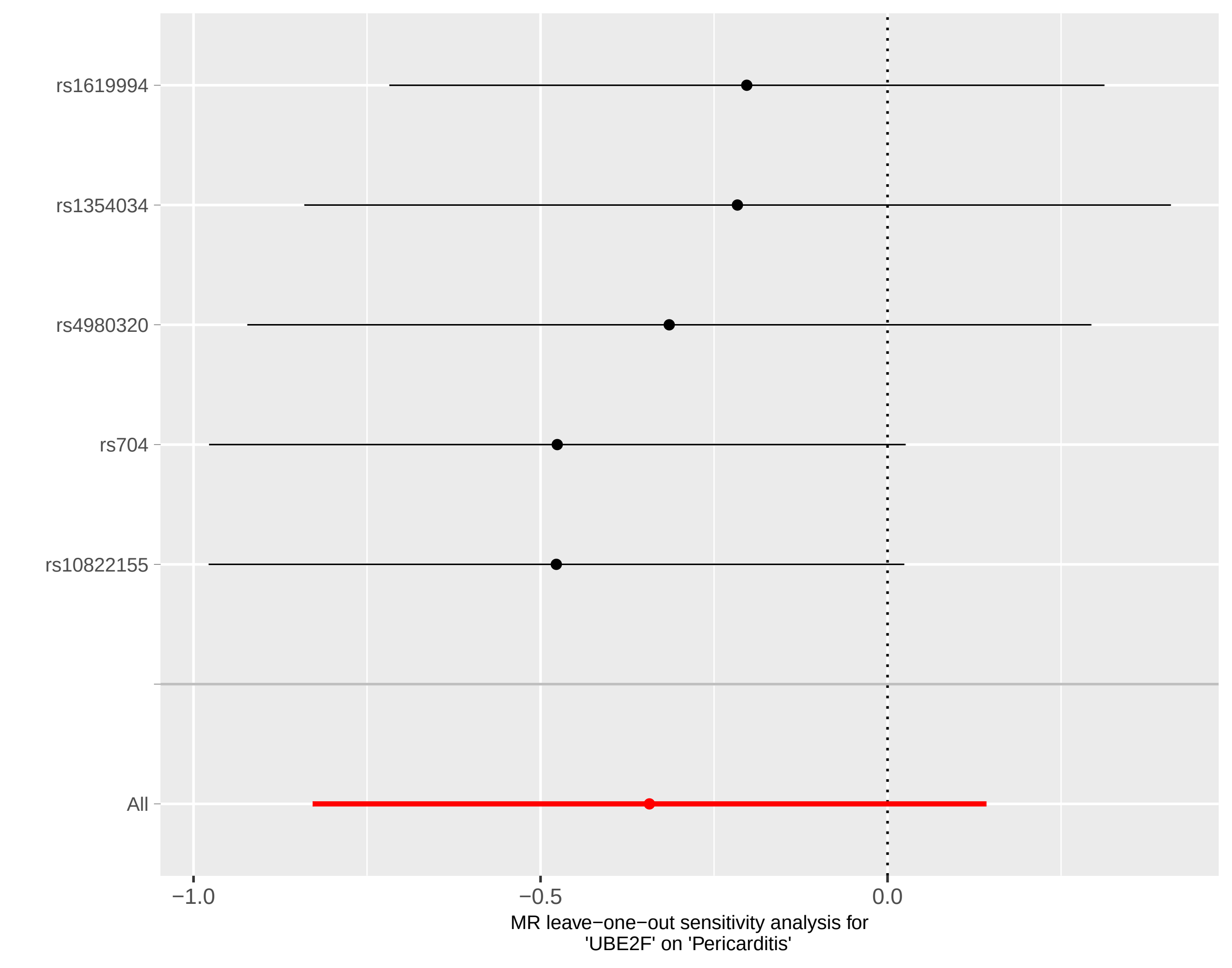

rs1619994
rs1354034
rs4980320
rs704
rs10822155
All
−1.0
−0.5
0.0
MR leave−one−out sensitivity analysis for
'UBE2F' on 'Pericarditis'

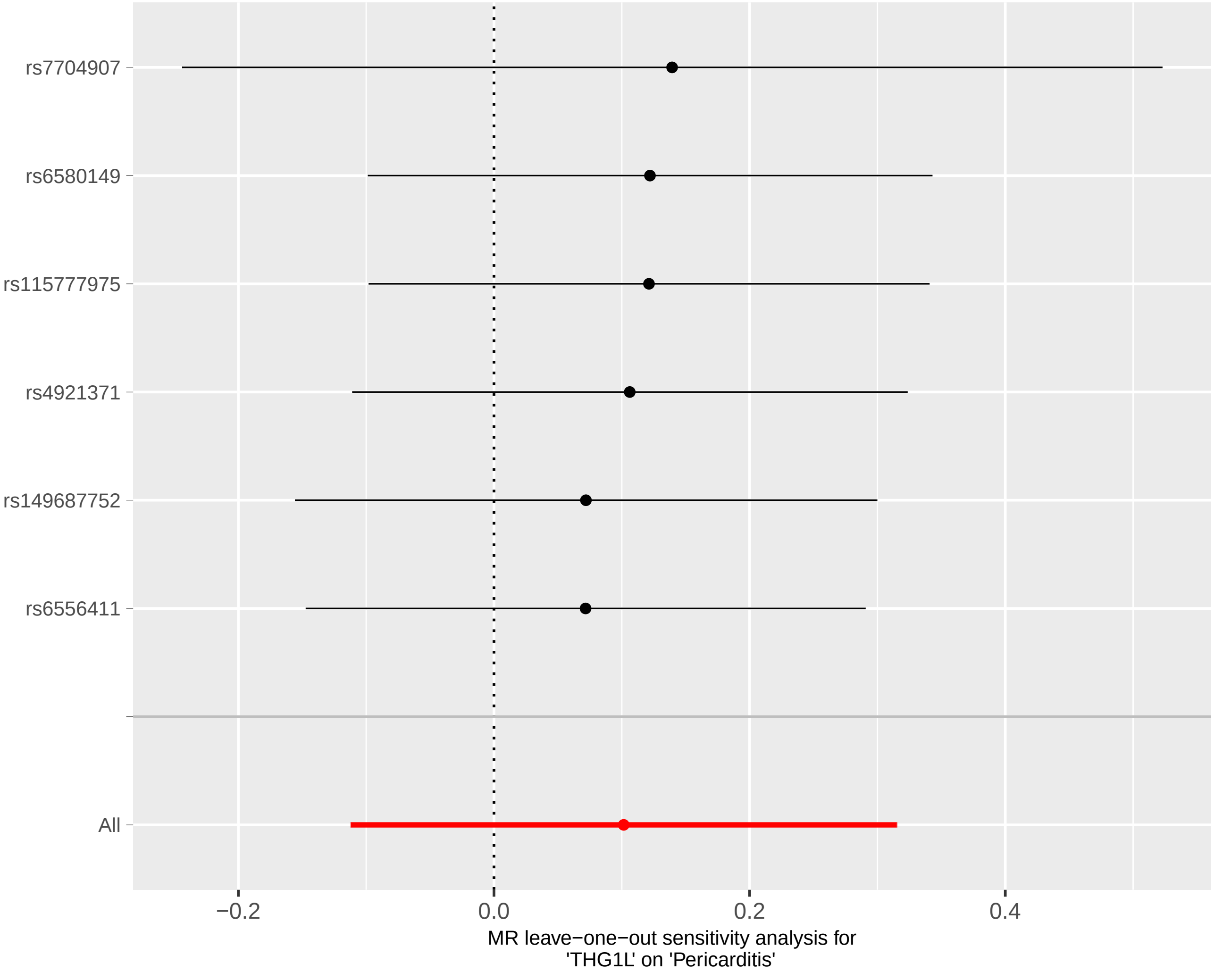
rs7704907
rs6580149
rs115777975
rs4921371
rs149687752
rs6556411
All
−0.2
0.0
0.2
0.4
MR leave−one−out sensitivity analysis for
'THG1L' on 'Pericarditis'

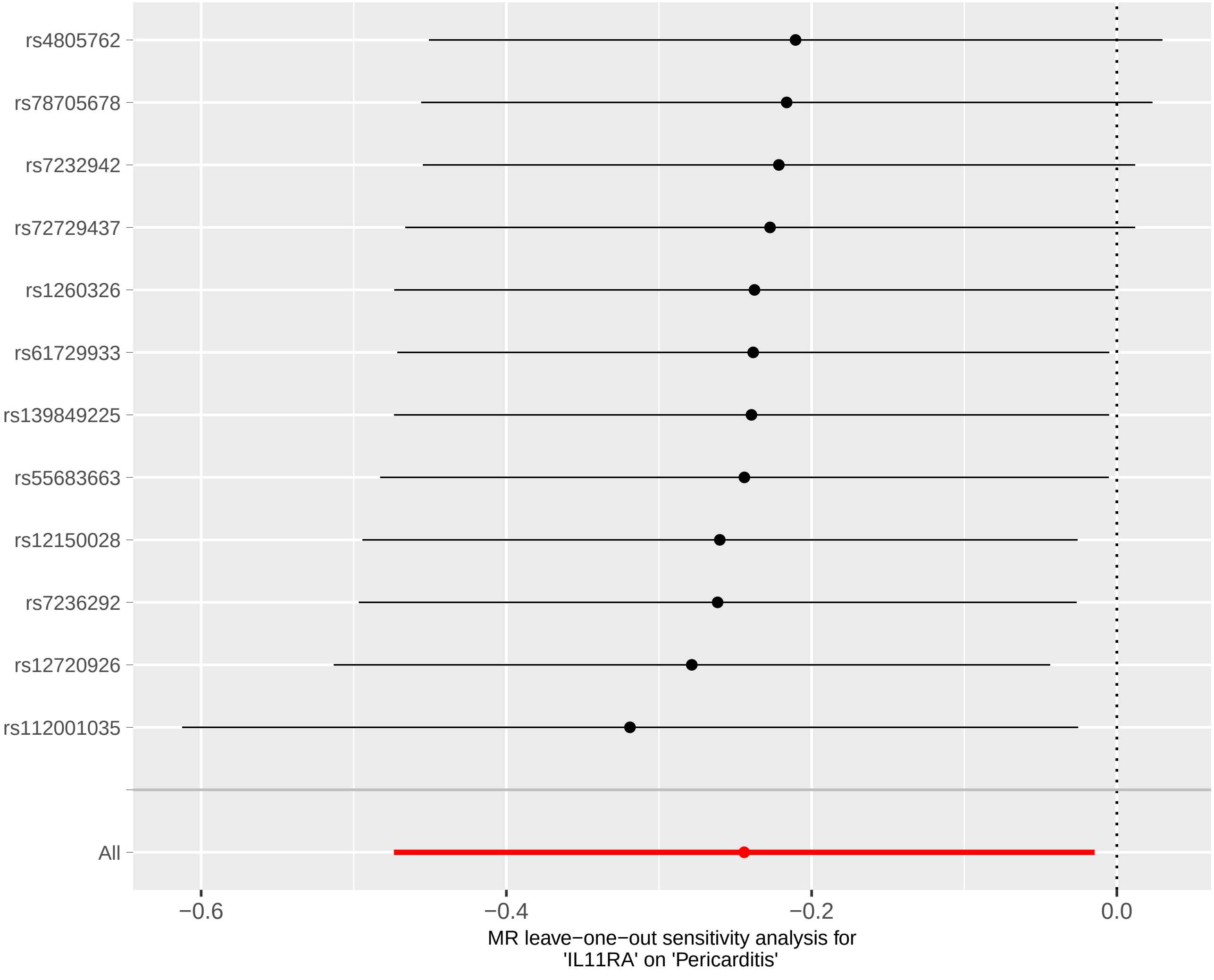
rs4805762
rs78705678
rs7232942
rs72729437
rs1260326
rs61729933
rs139849225
rs55683663
rs12150028
rs7236292
rs12720926
rs112001035
All
−0.6
−0.4
−0.2
0.0
MR leave−one−out sensitivity analysis for
'IL11RA' on 'Pericarditis'

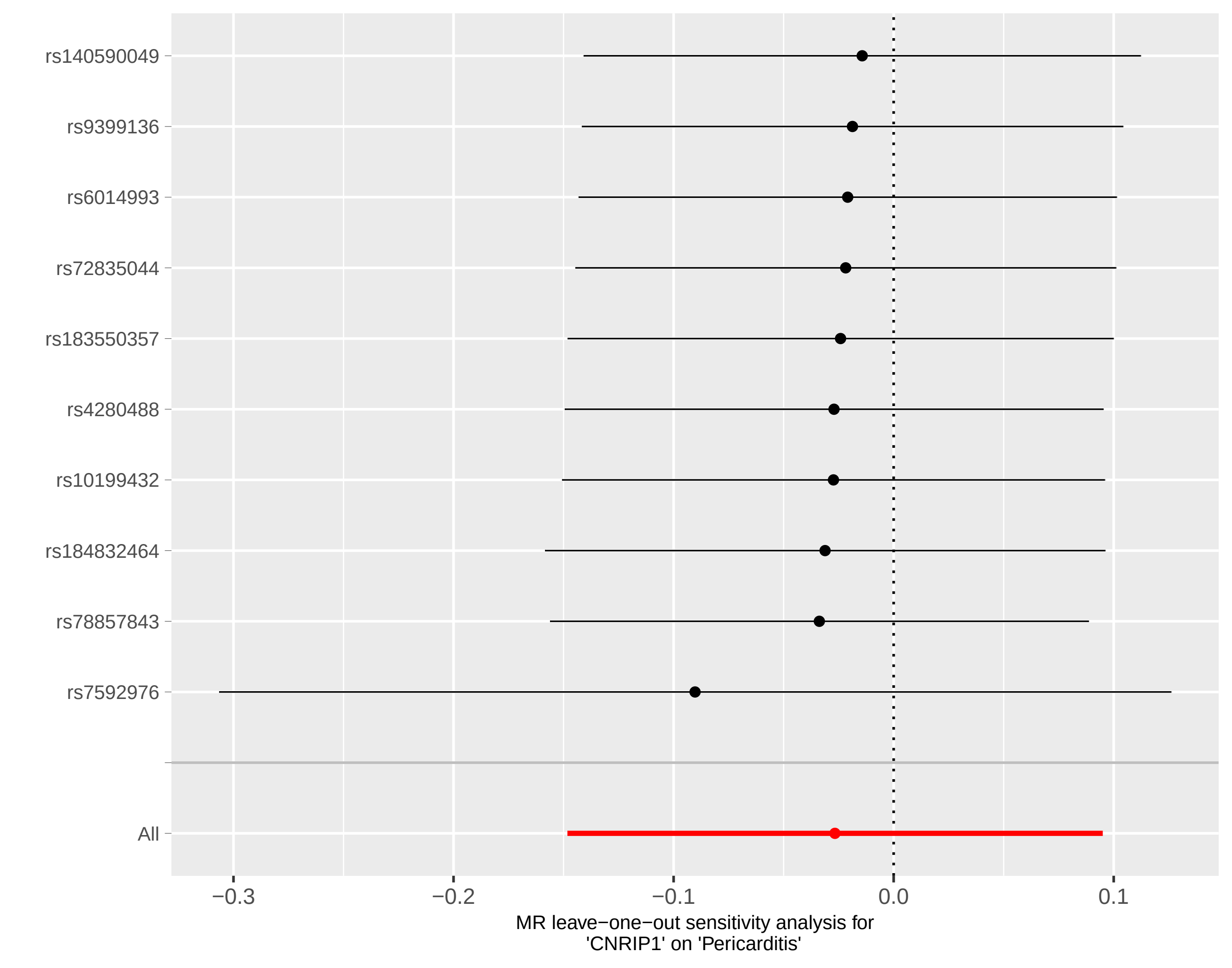
rs140590049
rs9399136
rs6014993
rs72835044
rs183550357
rs4280488
rs10199432
rs184832464
rs78857843
rs7592976
All
−0.3
−0.2
−0.1
0.0
0.1
MR leave−one−out sensitivity analysis for
'CNRIP1' on 'Pericarditis'

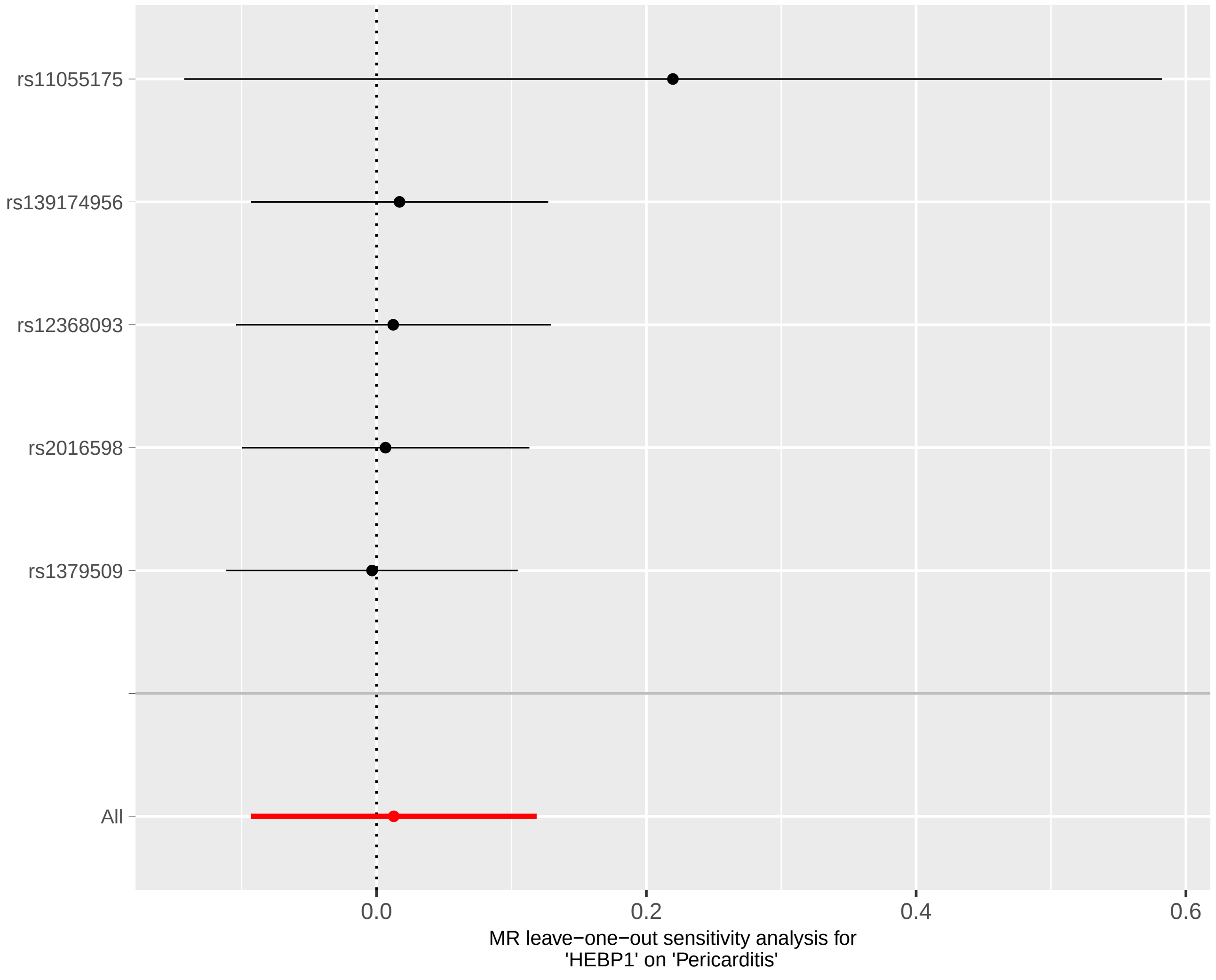

rs11055175
rs139174956
rs12368093
rs2016598
rs1379509
All
0.0
0.2
0.4
0.6
MR leave−one−out sensitivity analysis for
'HEBP1' on 'Pericarditis'

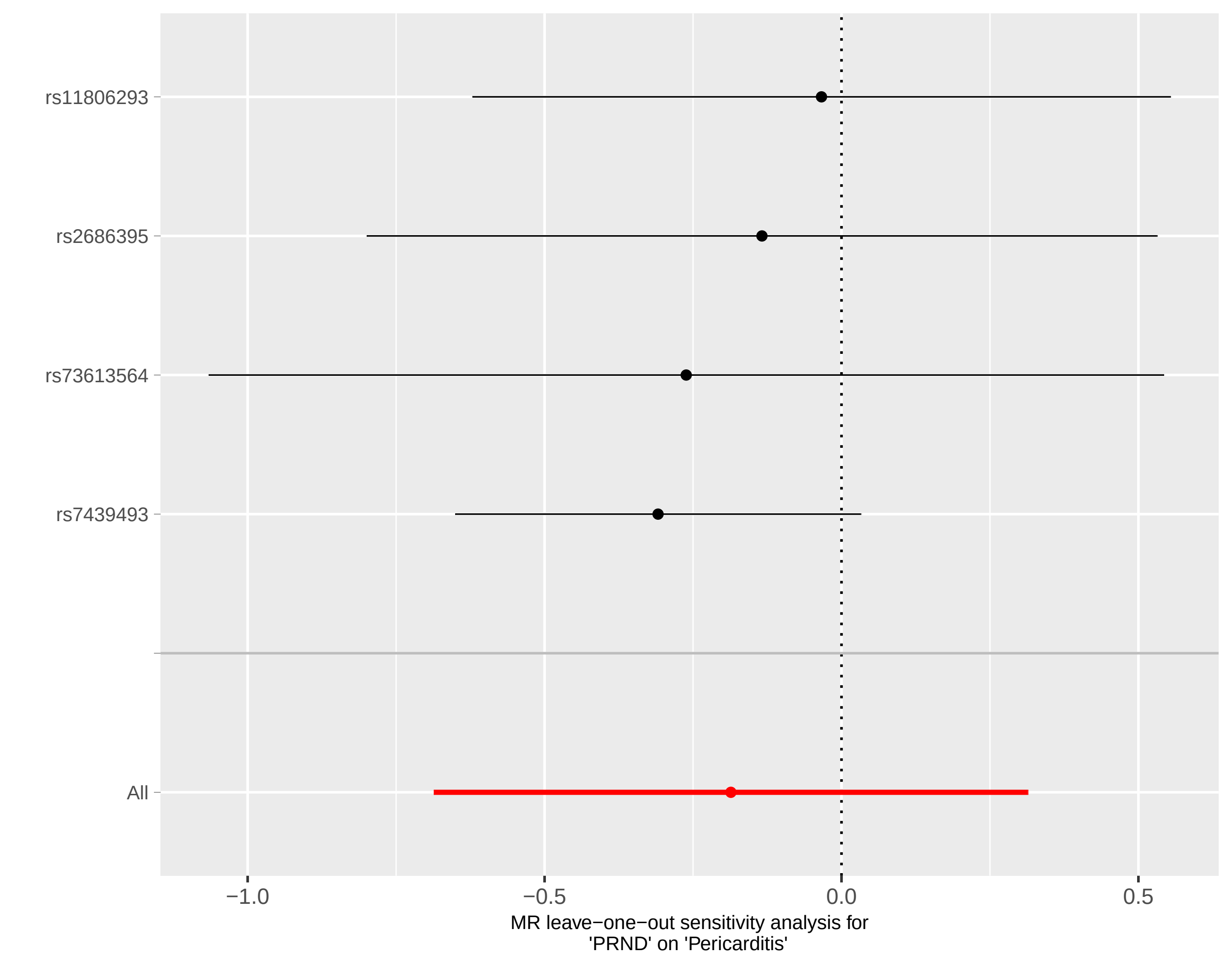

rs11806293
rs2686395
rs73613564
rs7439493
All
−1.0
−0.5
0.0
0.5
MR leave−one−out sensitivity analysis for
'PRND' on 'Pericarditis'

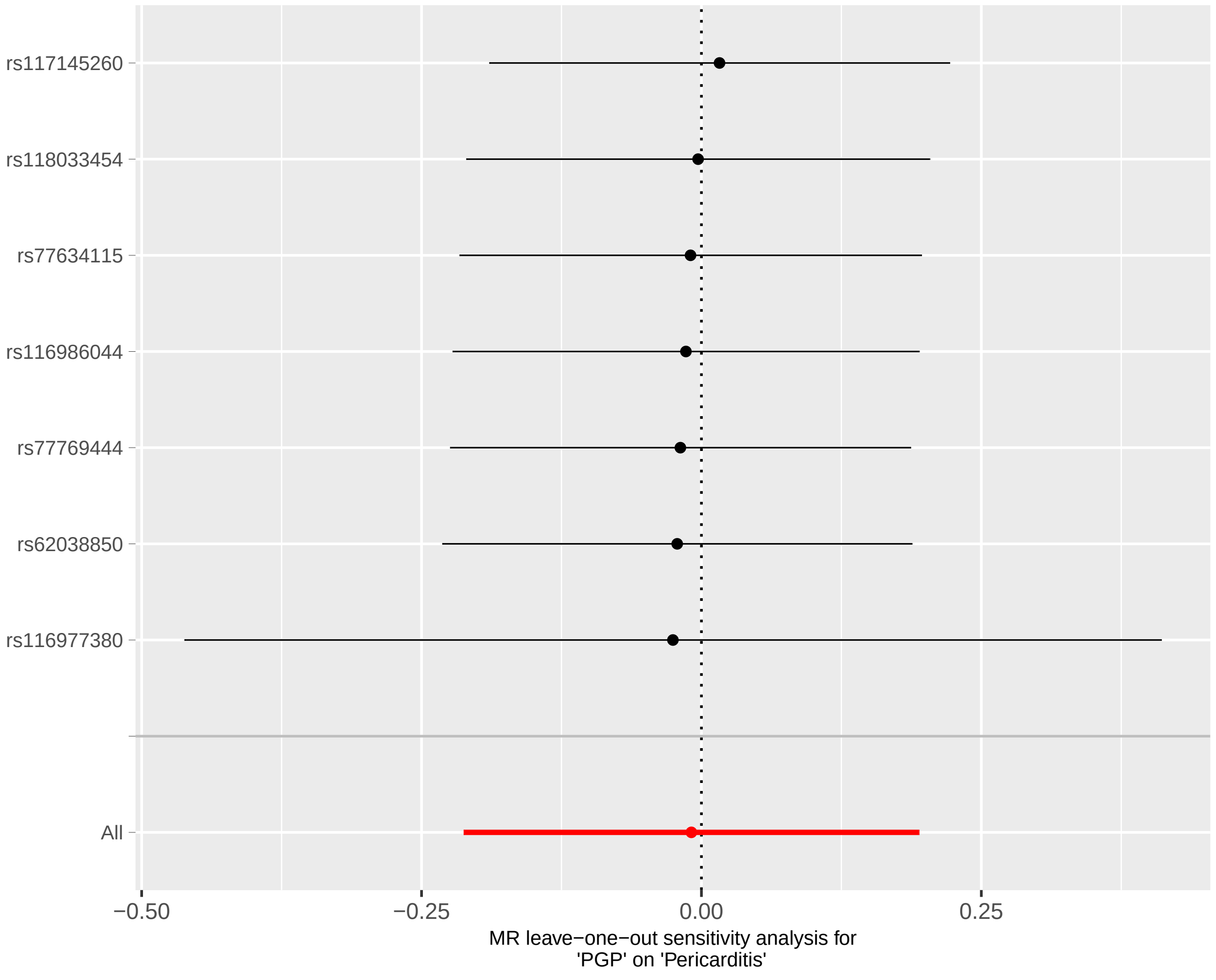
rs117145260
rs118033454
rs77634115
rs116986044
rs77769444
rs62038850
rs116977380
All
−0.50
−0.25
0.00
0.25
MR leave−one−out sensitivity analysis for
'PGP' on 'Pericarditis'

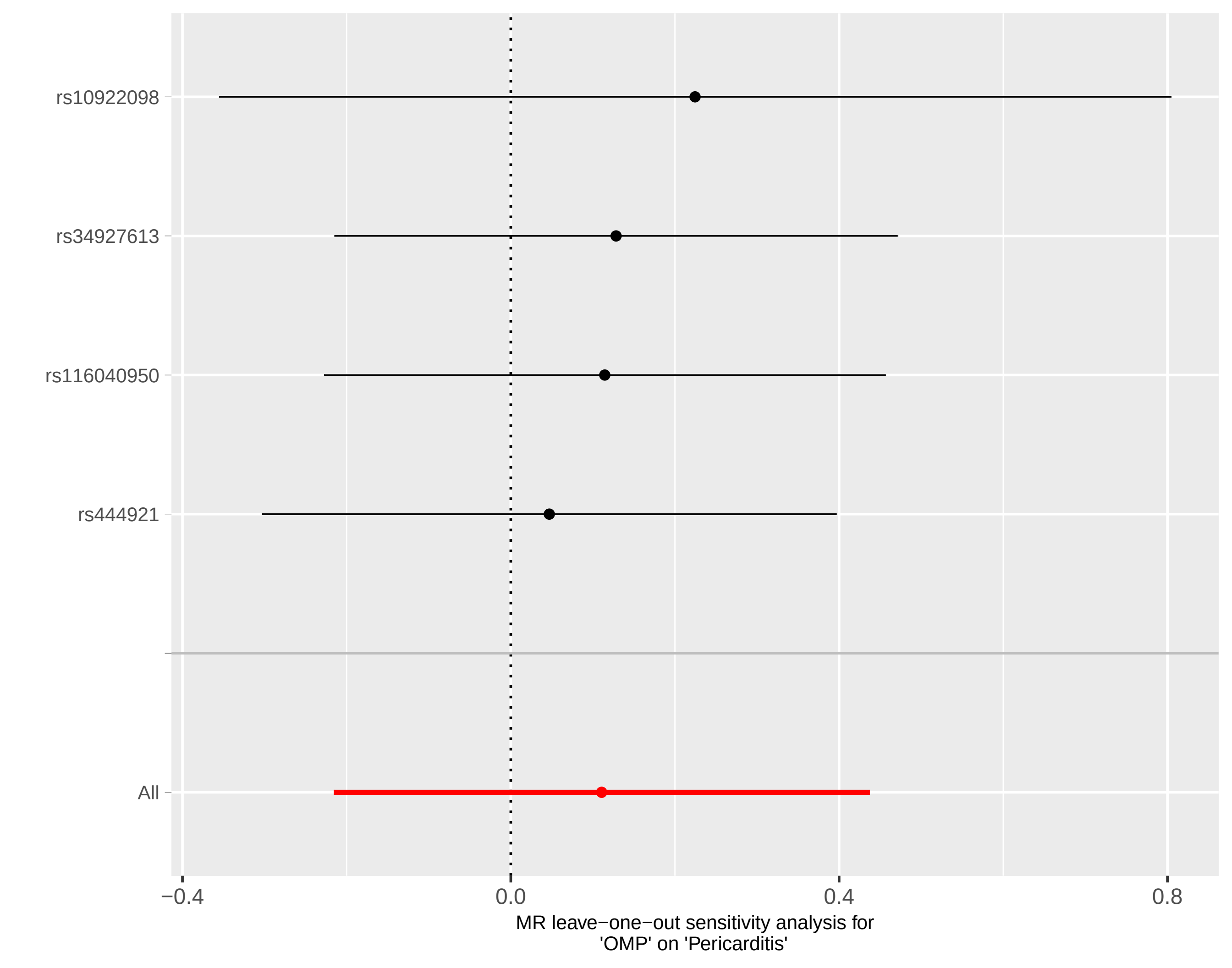
rs10922098
rs34927613
rs116040950
rs444921
All
−0.4
0.0
0.4
0.8
MR leave−one−out sensitivity analysis for
'OMP' on 'Pericarditis'

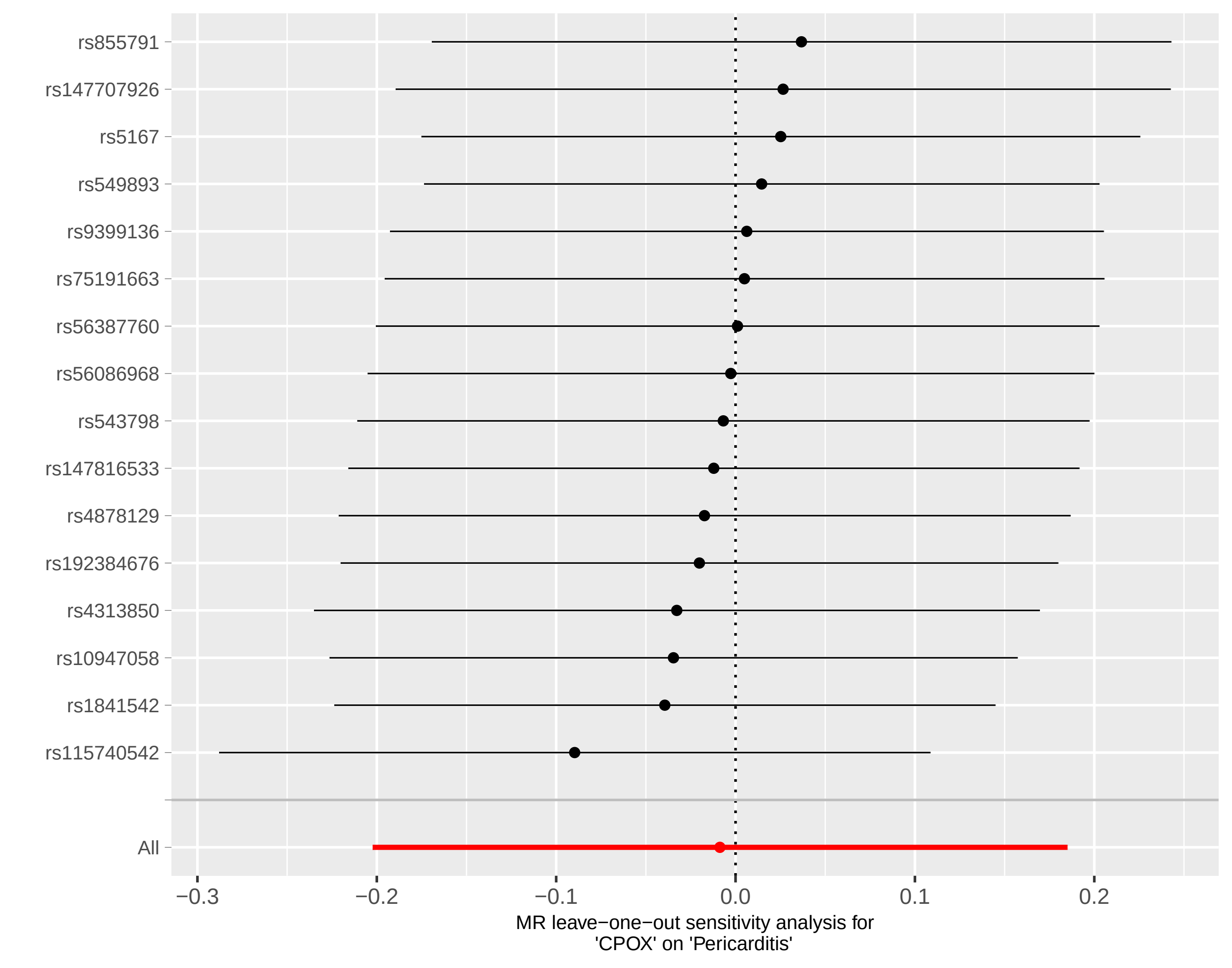

rs855791
rs147707926
rs5167
rs549893
rs9399136
rs75191663
rs56387760
rs56086968
rs543798
rs147816533
rs4878129
rs192384676
rs4313850
rs10947058
rs1841542
rs115740542
All
−0.3
−0.2
−0.1
0.0
0.1
0.2
MR leave−one−out sensitivity analysis for
'CPOX' on 'Pericarditis'

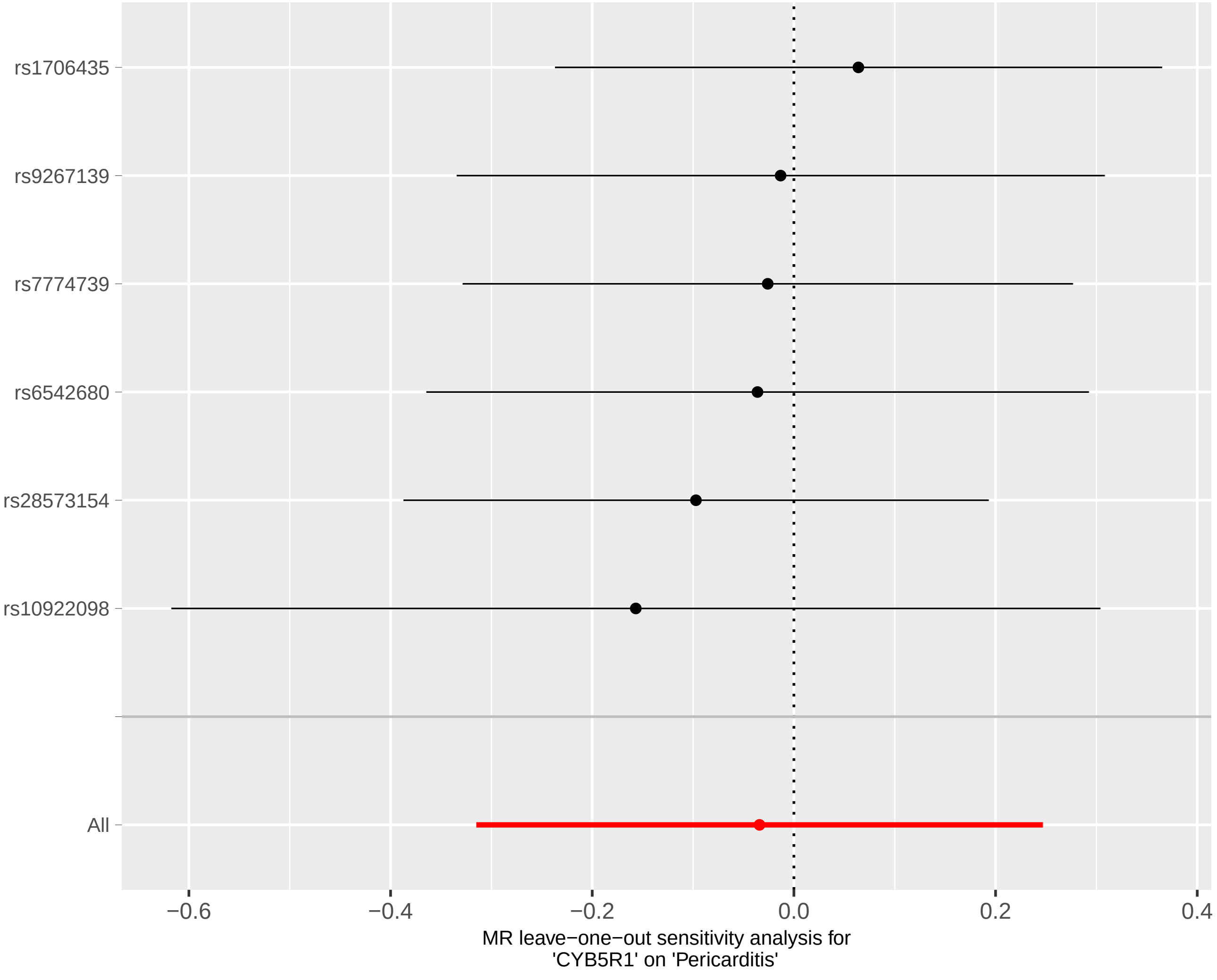

rs1706435
rs9267139
rs7774739
rs6542680
rs28573154
rs10922098
All
−0.6
−0.4
−0.2
0.0
0.2
0.4
MR leave−one−out sensitivity analysis for
'CYB5R1' on 'Pericarditis'

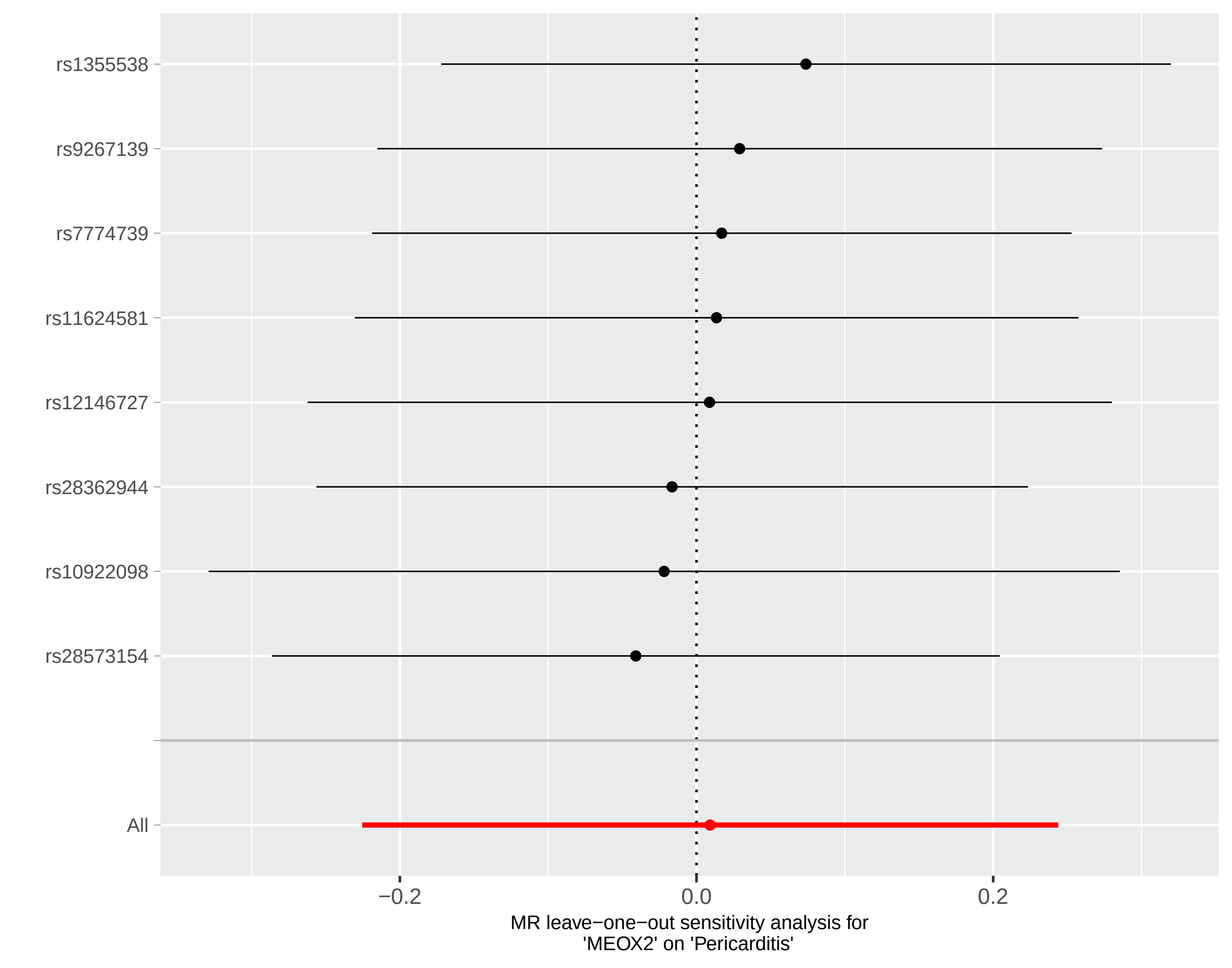
rs1355538
rs9267139
rs7774739
rs11624581
rs12146727
rs28362944
rs10922098
rs28573154
All
−0.2
0.0
0.2
MR leave−one−out sensitivity analysis for
'MEOX2' on 'Pericarditis'

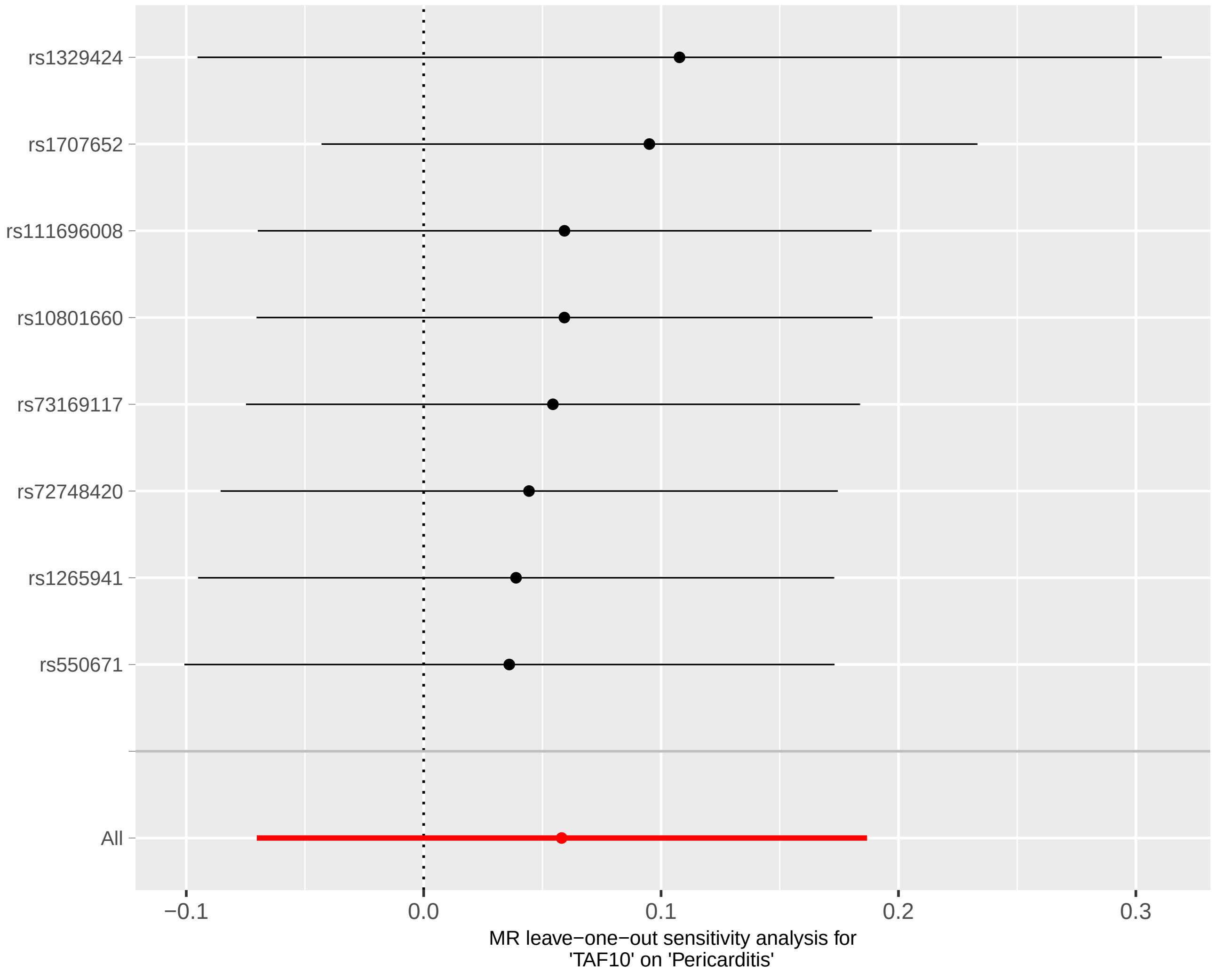
rs1329424
rs1707652
rs111696008
rs10801660
rs73169117
rs72748420
rs1265941
rs550671
All
−0.1
0.0
0.1
0.2
0.3
MR leave−one−out sensitivity analysis for
'TAF10' on 'Pericarditis'

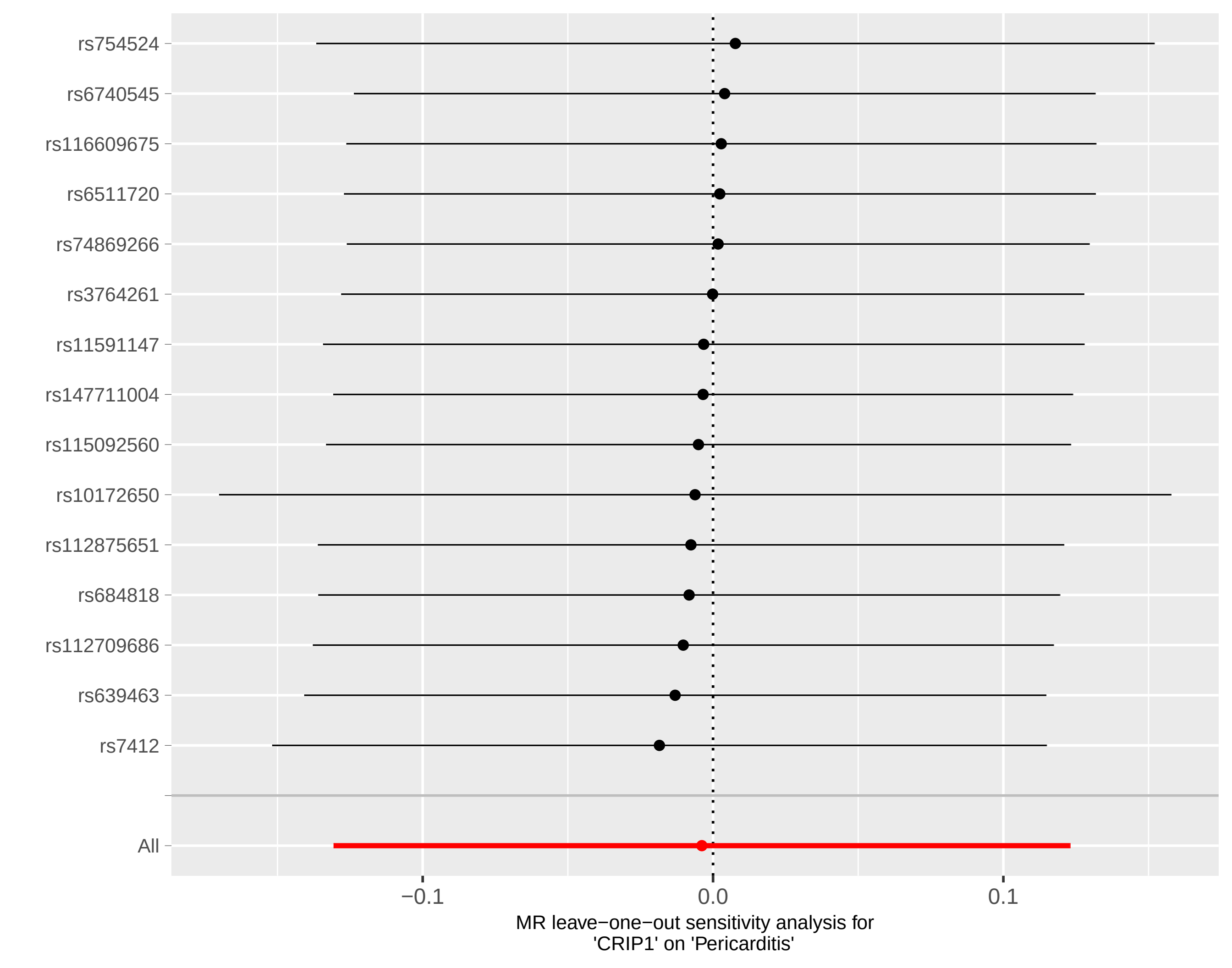
rs754524
rs6740545
rs116609675
rs6511720
rs74869266
rs3764261
rs11591147
rs147711004
rs115092560
rs10172650
rs112875651
rs684818
rs112709686
rs639463
rs7412
All
−0.1
0.0
0.1
MR leave−one−out sensitivity analysis for
'CRIP1' on 'Pericarditis'

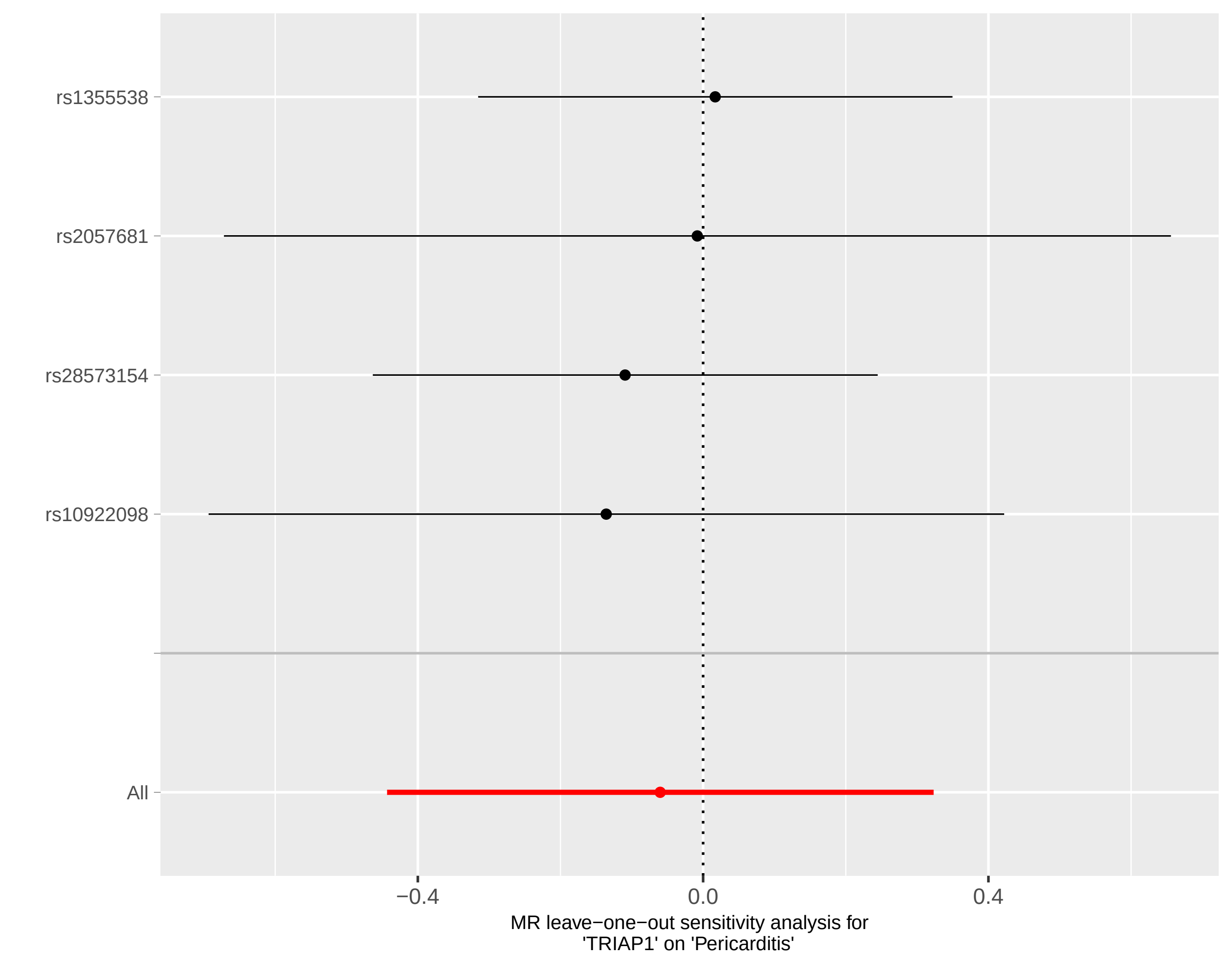
rs1355538
rs2057681
rs28573154
rs10922098
All
−0.4
0.0
0.4
MR leave−one−out sensitivity analysis for
'TRIAP1' on 'Pericarditis'

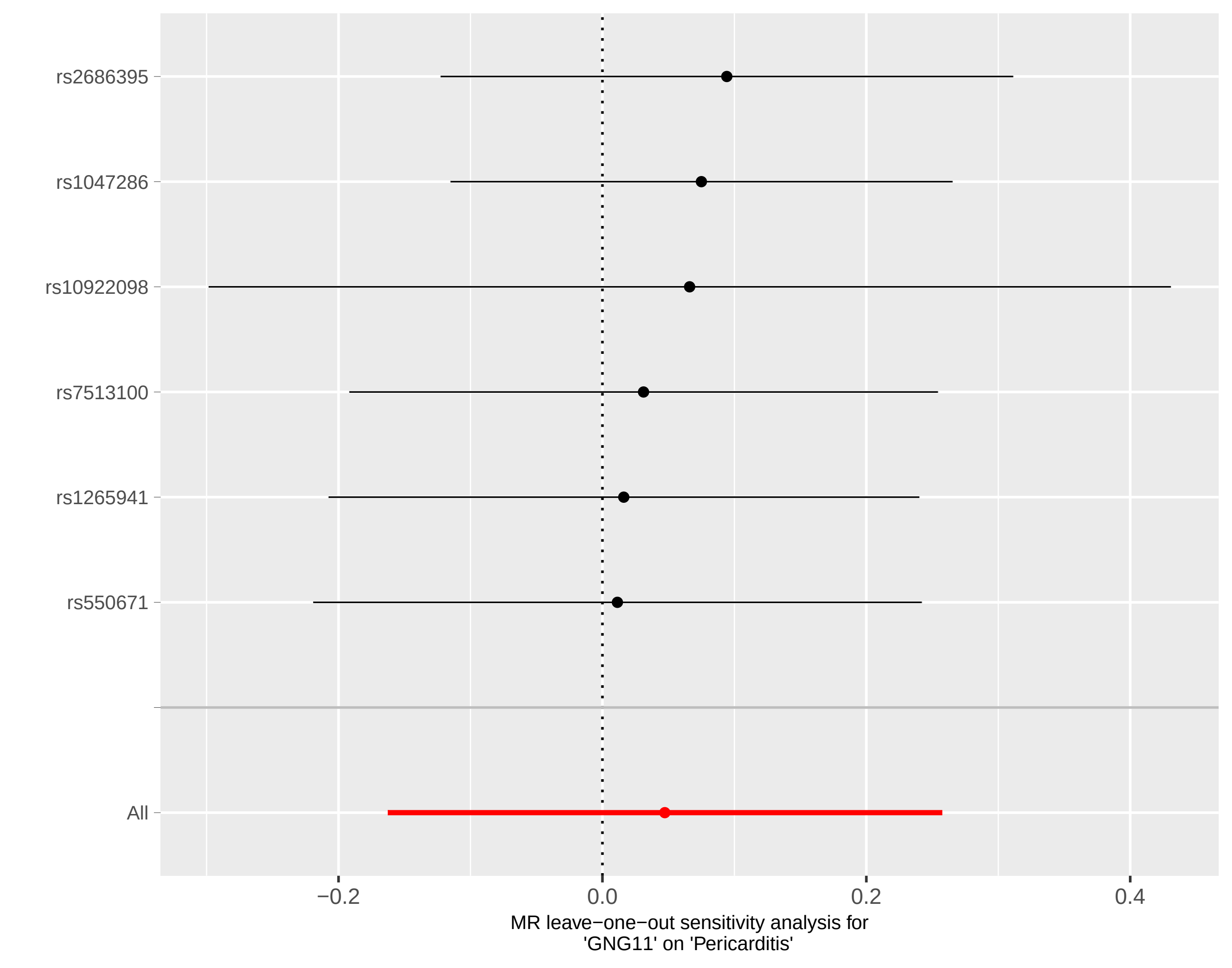
rs2686395
rs1047286
rs10922098
rs7513100
rs1265941
rs550671
All
−0.2
0.0
0.2
0.4
MR leave−one−out sensitivity analysis for
'GNG11' on 'Pericarditis'

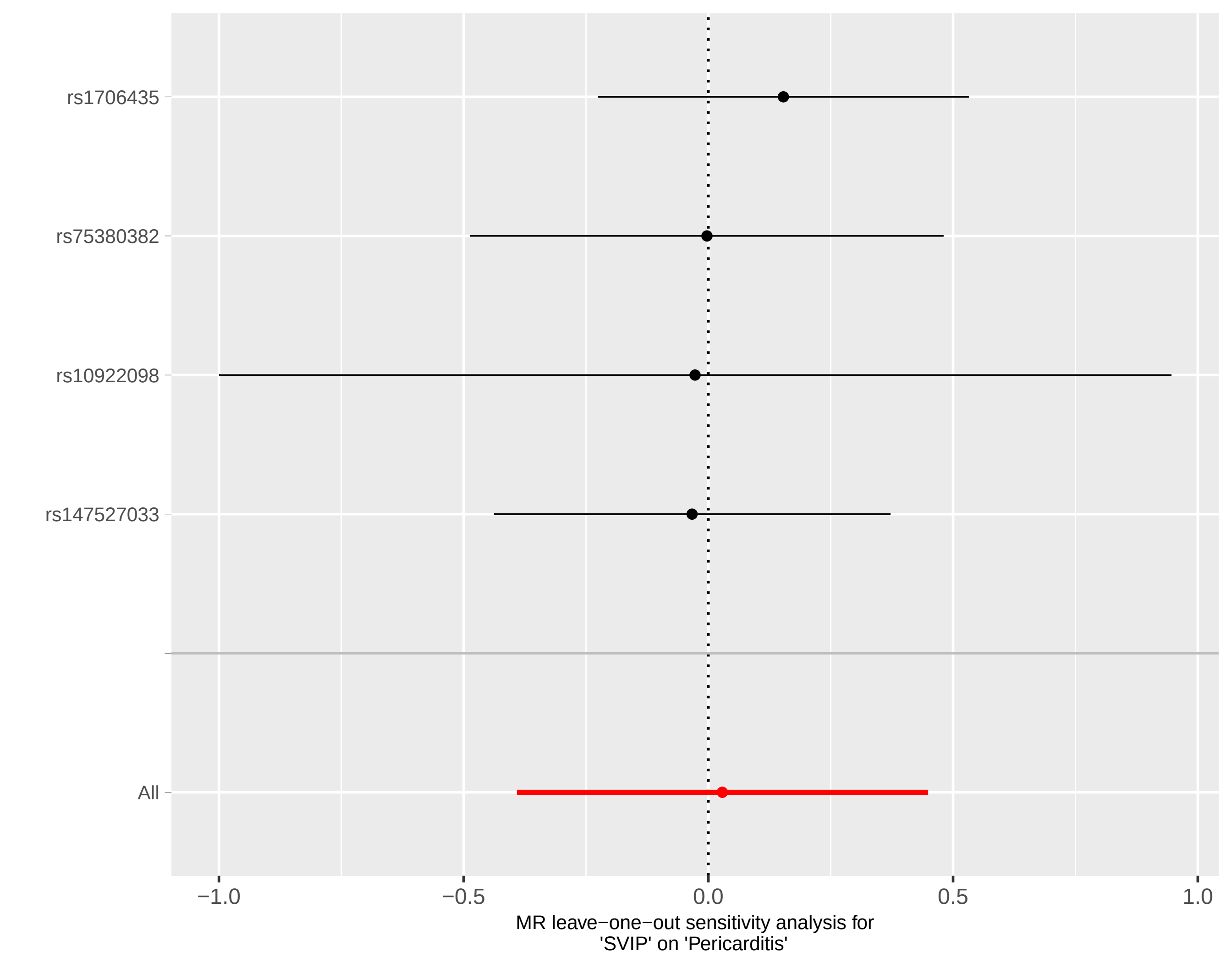
rs1706435
rs75380382
rs10922098
rs147527033
All
−1.0
−0.5
0.0
0.5
1.0
MR leave−one−out sensitivity analysis for
'SVIP' on 'Pericarditis'

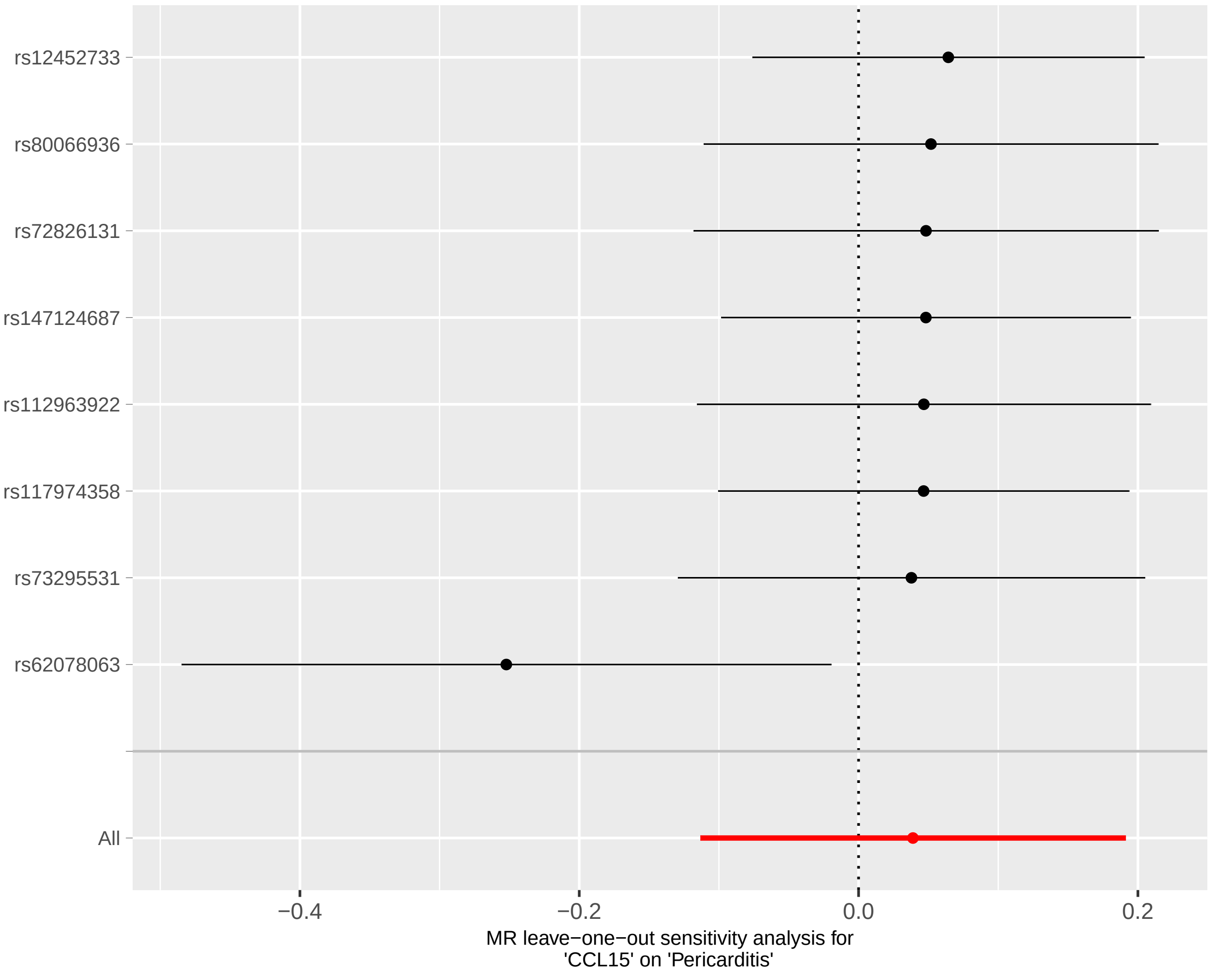

rs12452733
rs80066936
rs72826131
rs147124687
rs112963922
rs117974358
rs73295531
rs62078063
All
−0.4
−0.2
0.0
0.2
MR leave−one−out sensitivity analysis for
'CCL15' on 'Pericarditis'

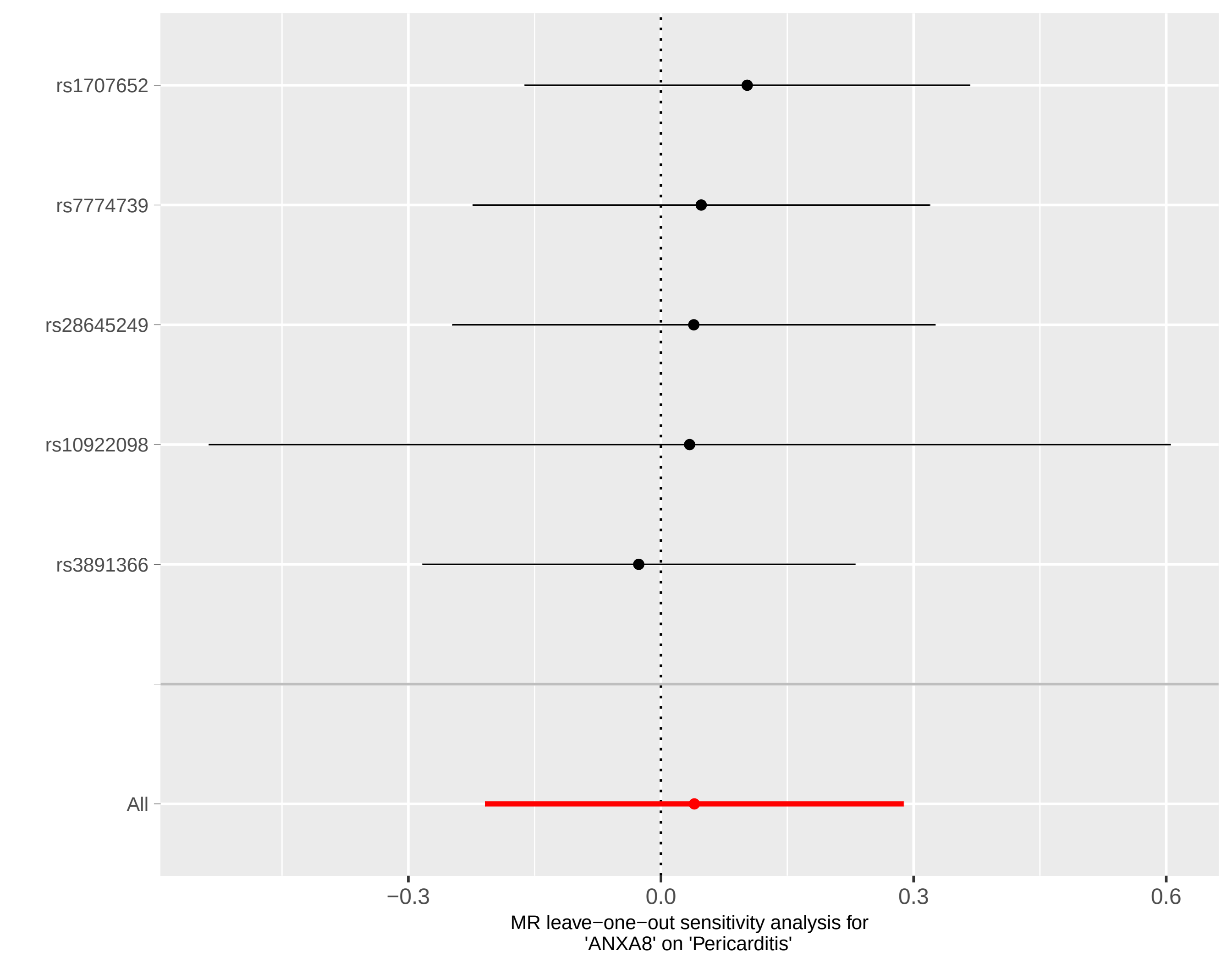
rs1707652
rs7774739
rs28645249
rs10922098
rs3891366
All
−0.3
0.0
0.3
0.6
MR leave−one−out sensitivity analysis for
'ANXA8' on 'Pericarditis'

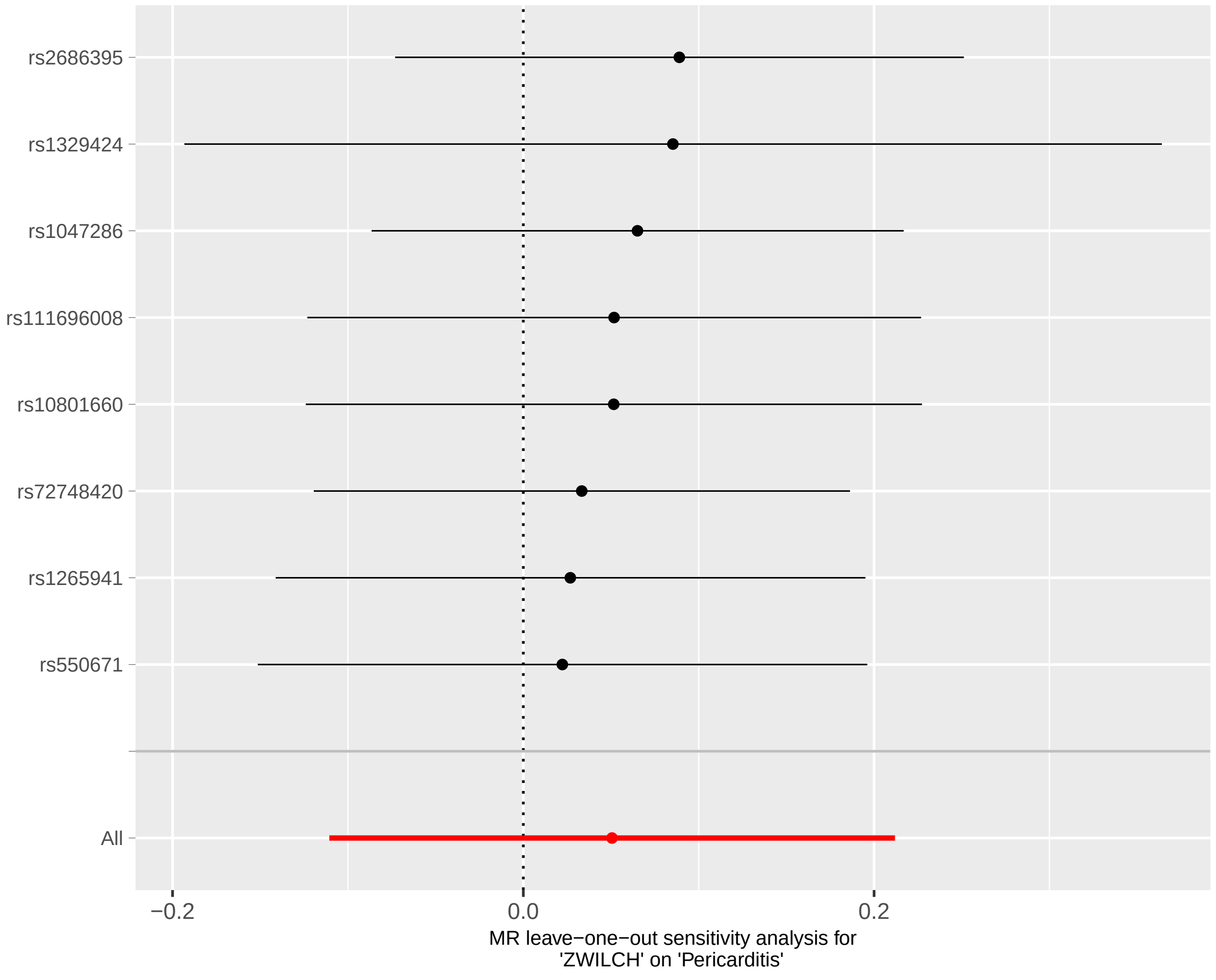
rs2686395
rs1329424
rs1047286
rs111696008
rs10801660
rs72748420
rs1265941
rs550671
All
−0.2
0.0
0.2
MR leave−one−out sensitivity analysis for
'ZWILCH' on 'Pericarditis'

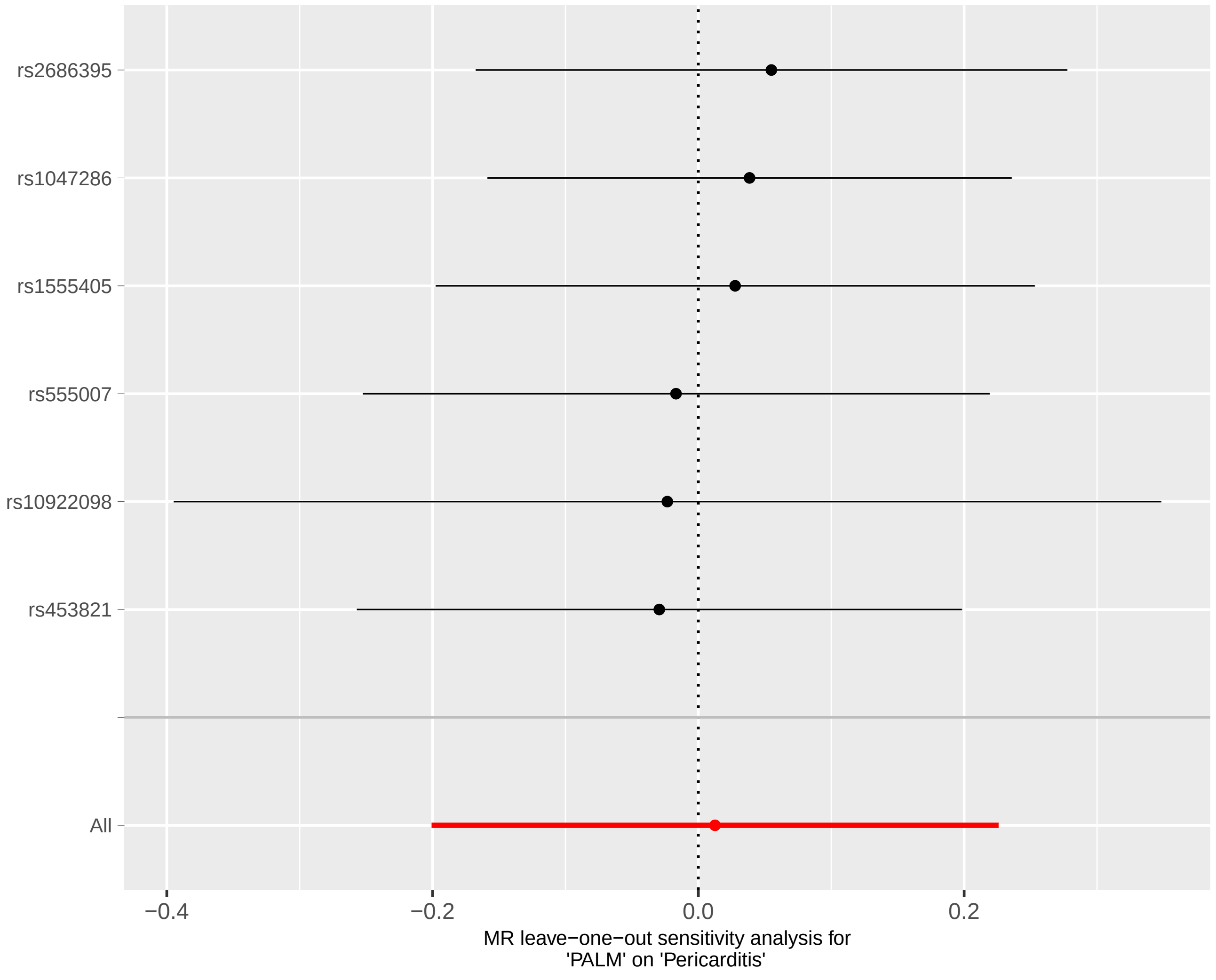

rs2686395
rs1047286
rs1555405
rs555007
rs10922098
rs453821
All
−0.4
−0.2
0.0
0.2
MR leave−one−out sensitivity analysis for
'PALM' on 'Pericarditis'

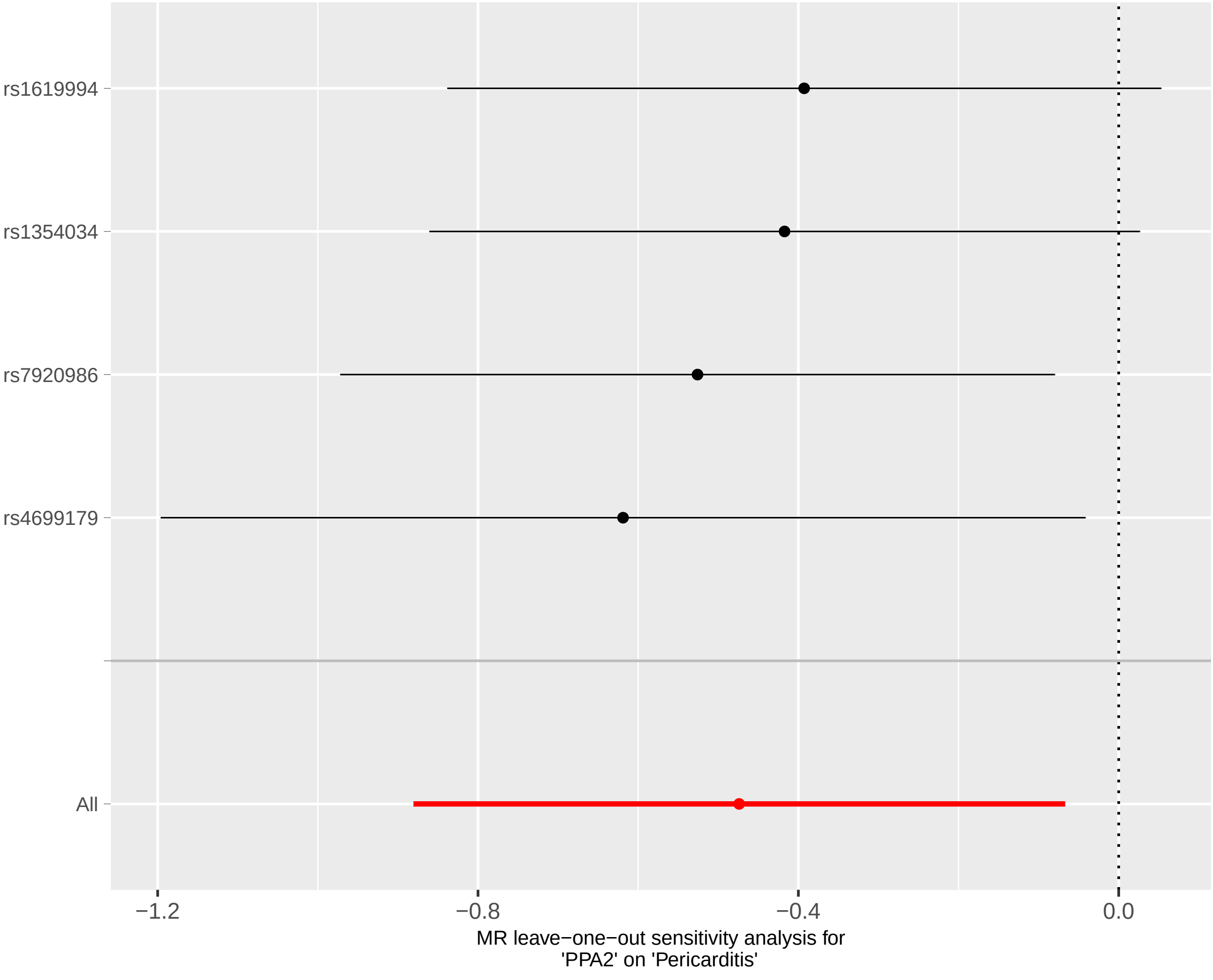
rs1619994
rs1354034
rs7920986
rs4699179
All
−1.2
−0.8
−0.4
0.0
MR leave−one−out sensitivity analysis for
'PPA2' on 'Pericarditis'

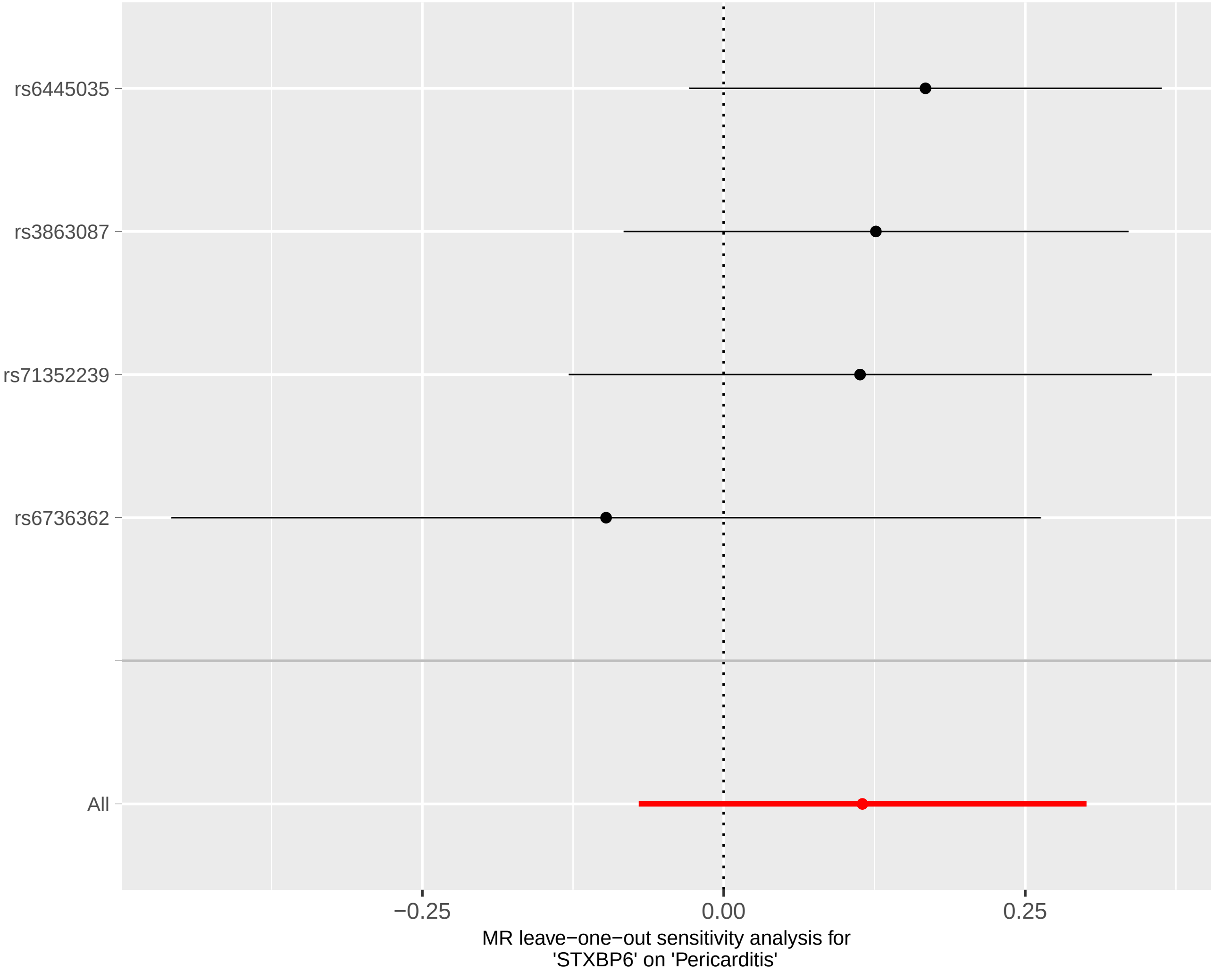
rs6445035
rs3863087
rs71352239
rs6736362
All
−0.25
0.00
0.25
MR leave−one−out sensitivity analysis for
'STXBP6' on 'Pericarditis'

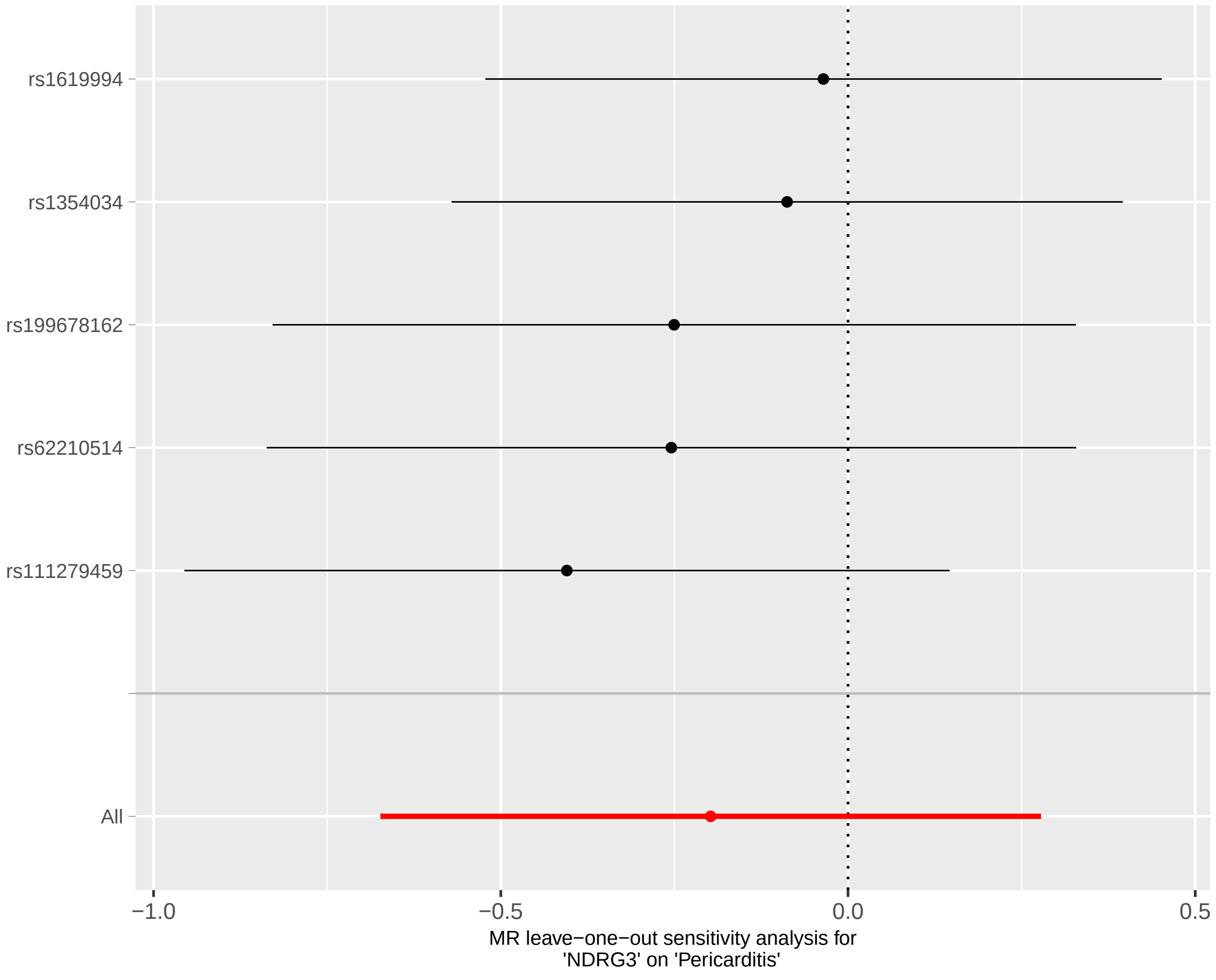
rs1619994
rs1354034
rs199678162
rs62210514
rs111279459
All
−1.0
−0.5
0.0
0.5
MR leave−one−out sensitivity analysis for
'NDRG3' on 'Pericarditis'

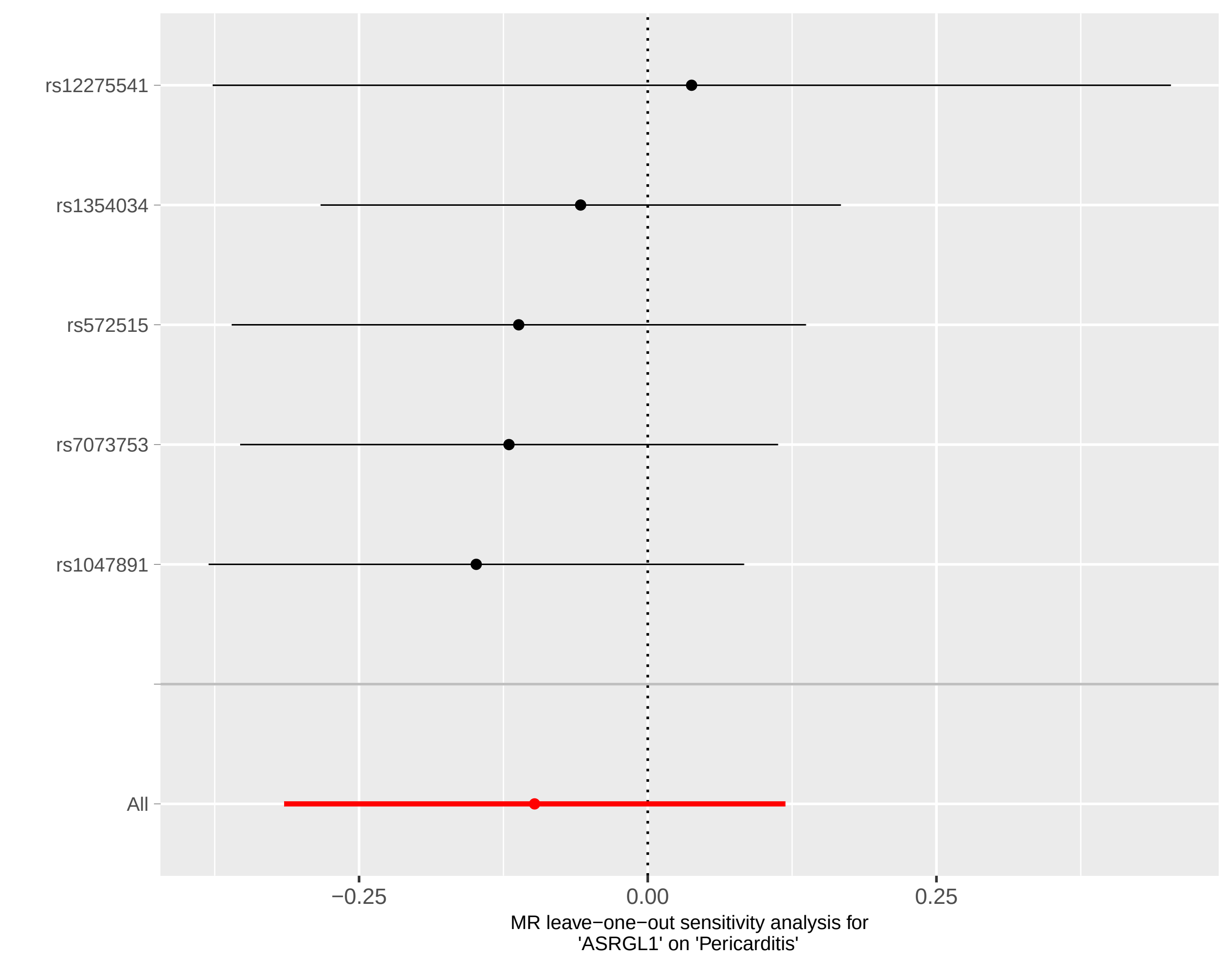
rs12275541
rs1354034
rs572515
rs7073753
rs1047891
All
−0.25
0.00
0.25
MR leave−one−out sensitivity analysis for
'ASRGL1' on 'Pericarditis'

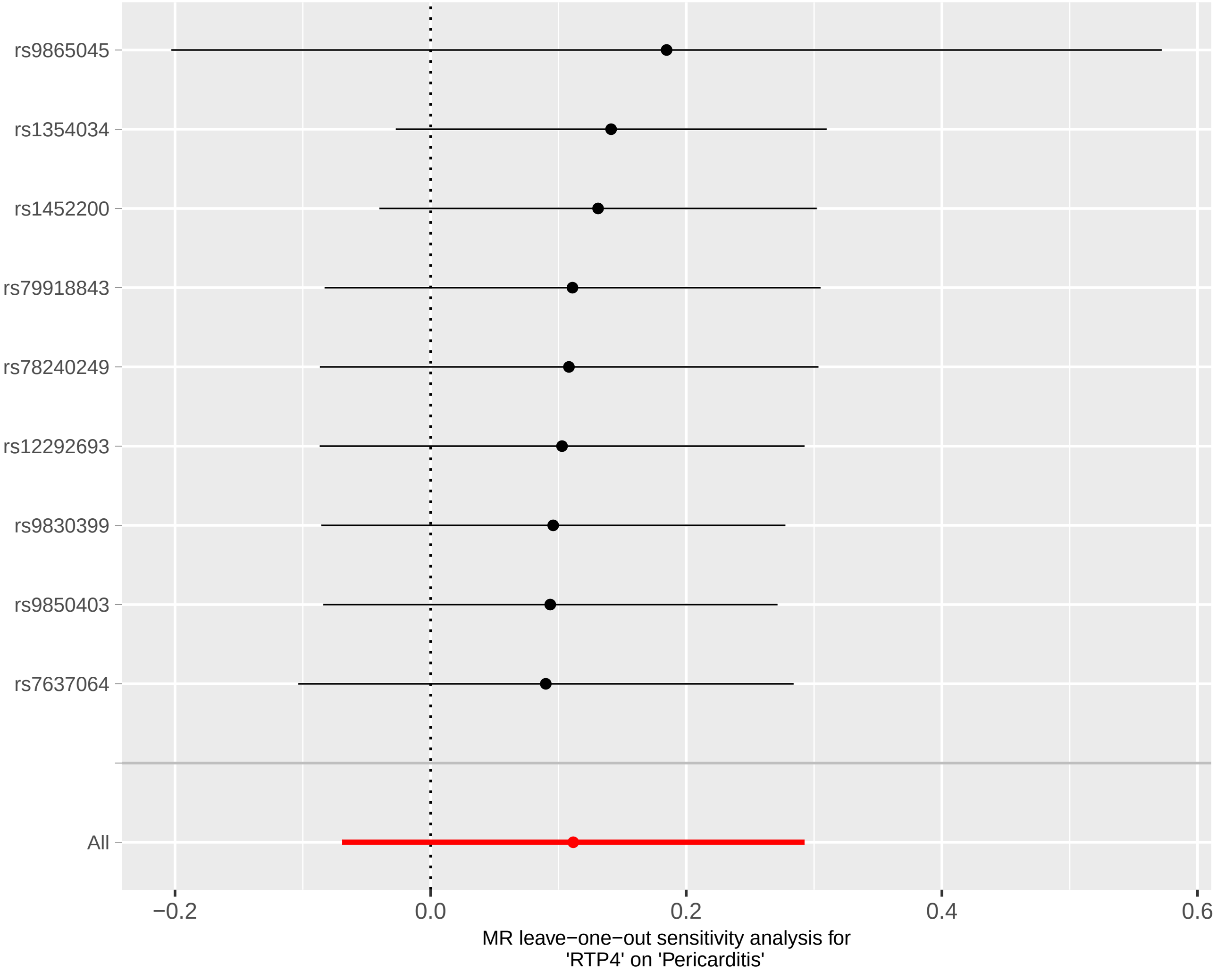
rs9865045
rs1354034
rs1452200
rs79918843
rs78240249
rs12292693
rs9830399
rs9850403
rs7637064
All
−0.2
0.0
0.2
0.4
0.6
MR leave−one−out sensitivity analysis for
'RTP4' on 'Pericarditis'

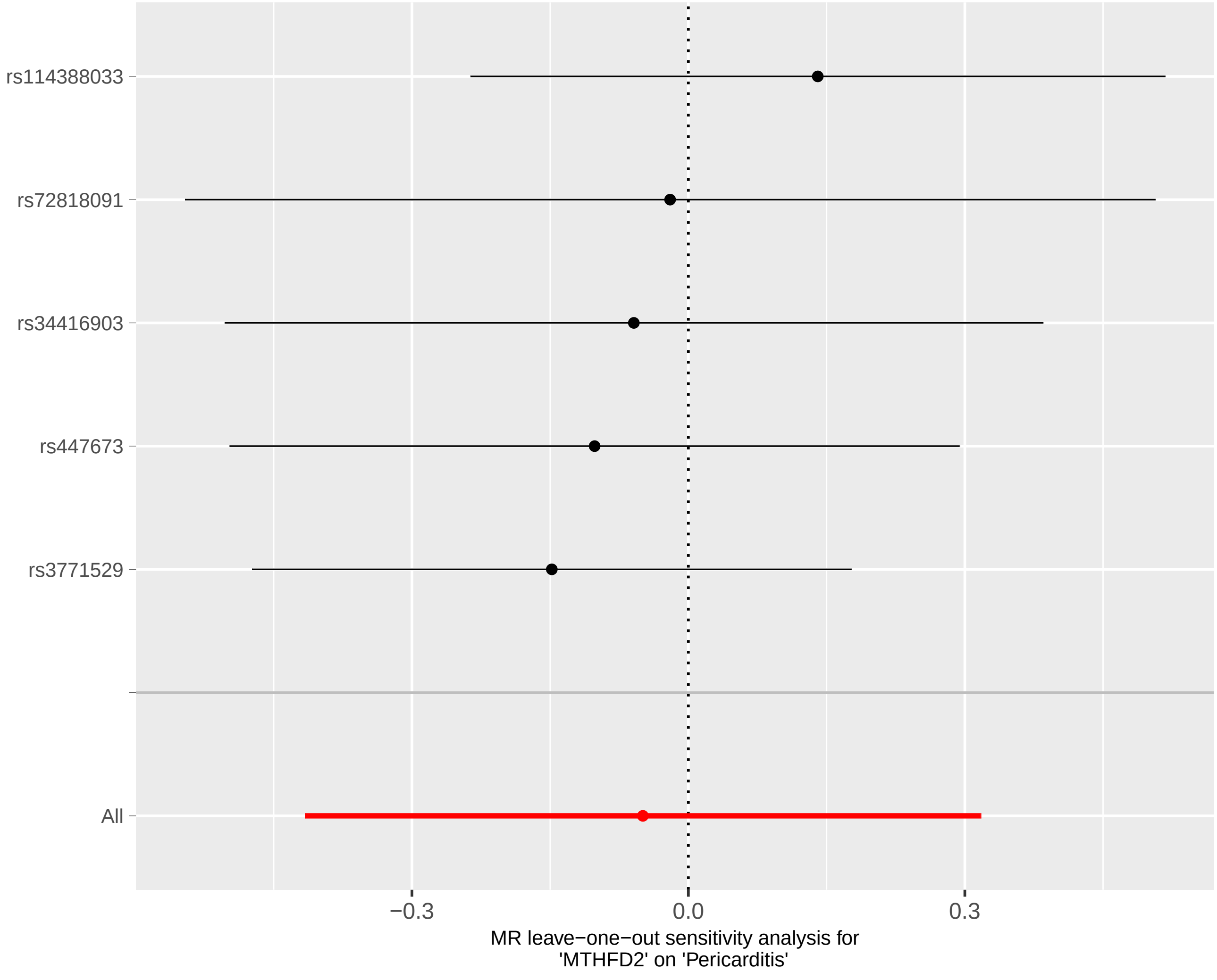

rs114388033
rs72818091
rs34416903
rs447673
rs3771529
All
−0.3
0.0
0.3
MR leave−one−out sensitivity analysis for
'MTHFD2' on 'Pericarditis'

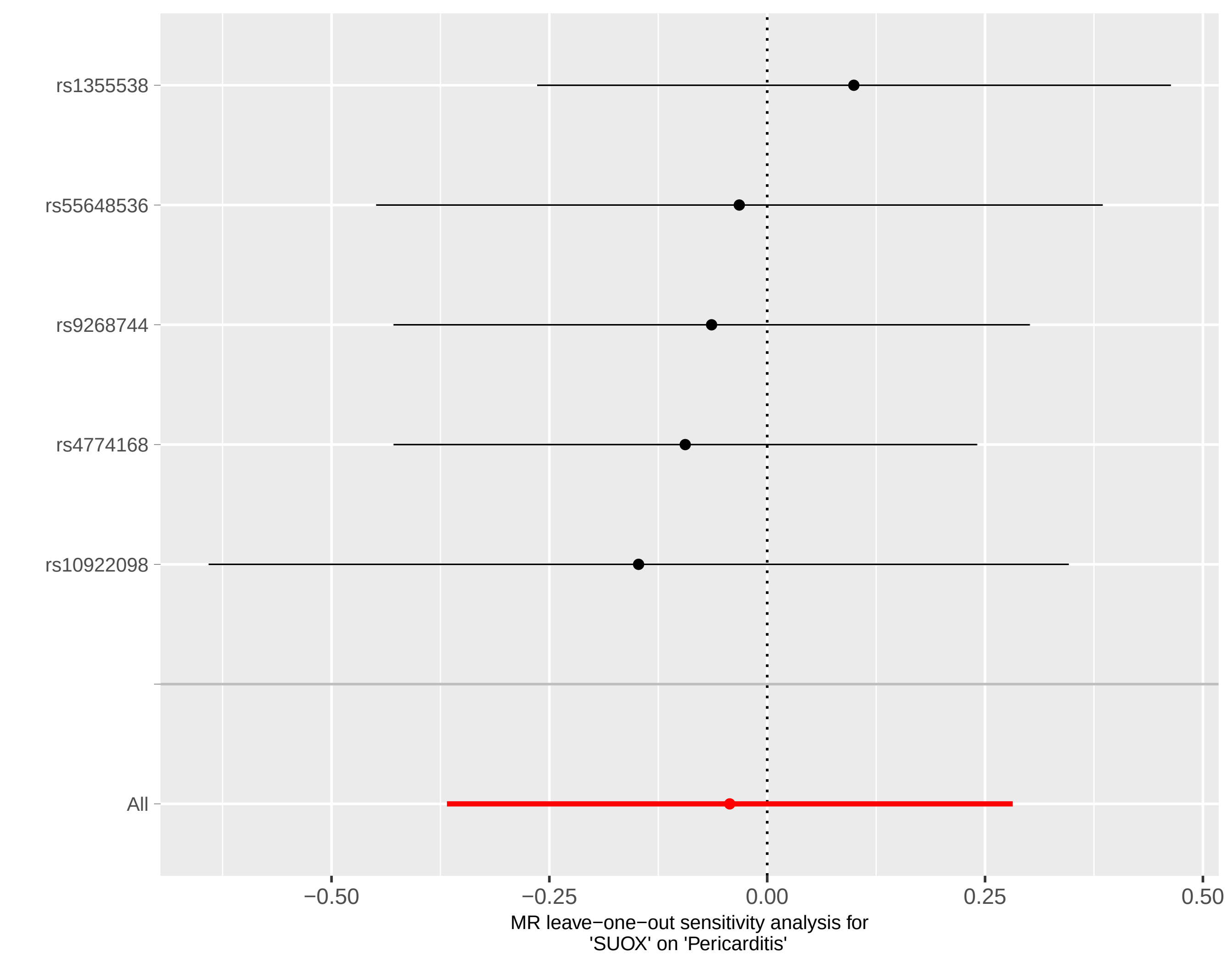
rs1355538
rs55648536
rs9268744
rs4774168
rs10922098
All
−0.50
−0.25
0.00
0.25
0.50
MR leave−one−out sensitivity analysis for
'SUOX' on 'Pericarditis'

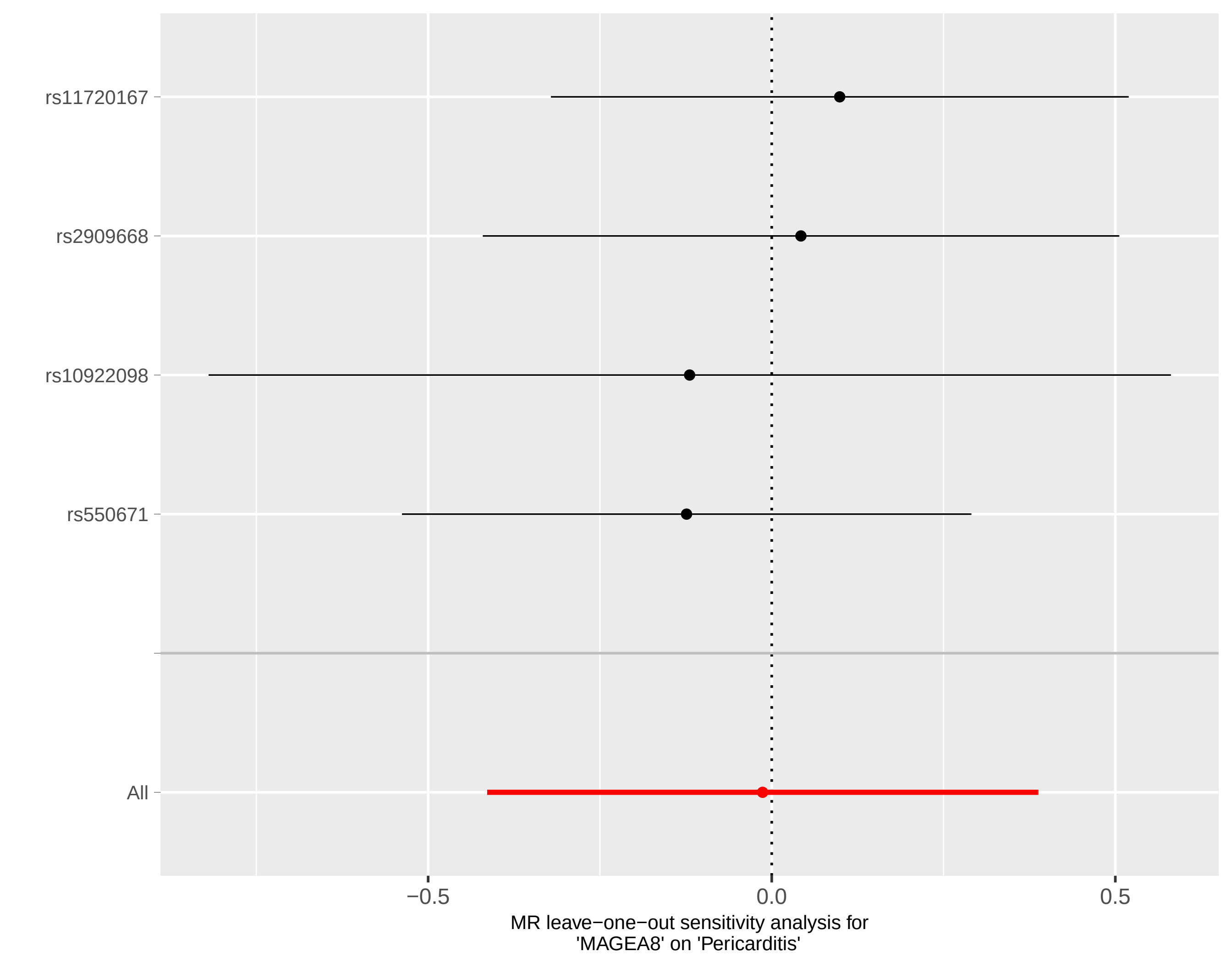
rs11720167
rs2909668
rs10922098
rs550671
All
−0.5
0.0
0.5
MR leave−one−out sensitivity analysis for
'MAGEA8' on 'Pericarditis'

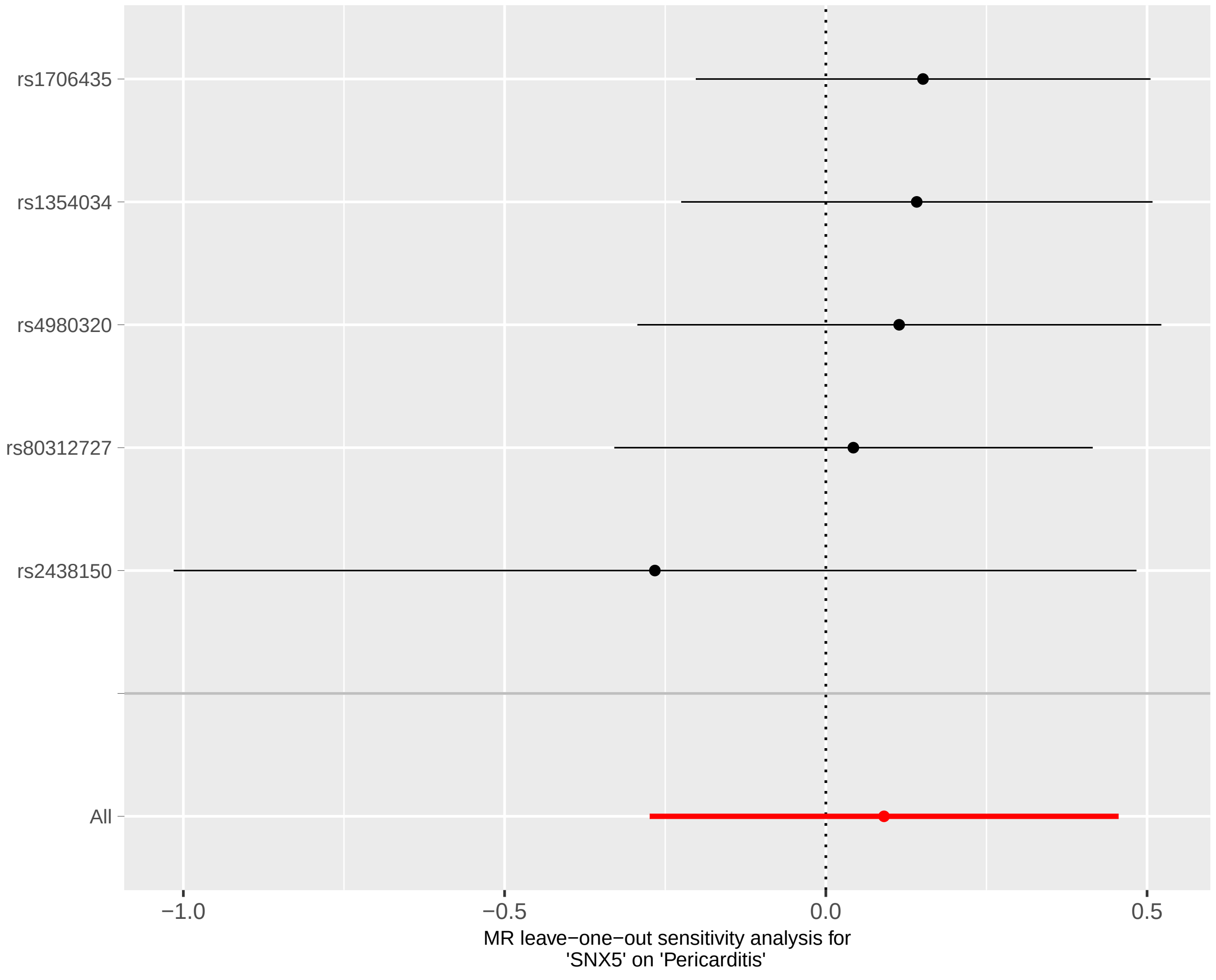

rs1706435
rs1354034
rs4980320
rs80312727
rs2438150
All
−1.0
−0.5
0.0
0.5
MR leave−one−out sensitivity analysis for
'SNX5' on 'Pericarditis'

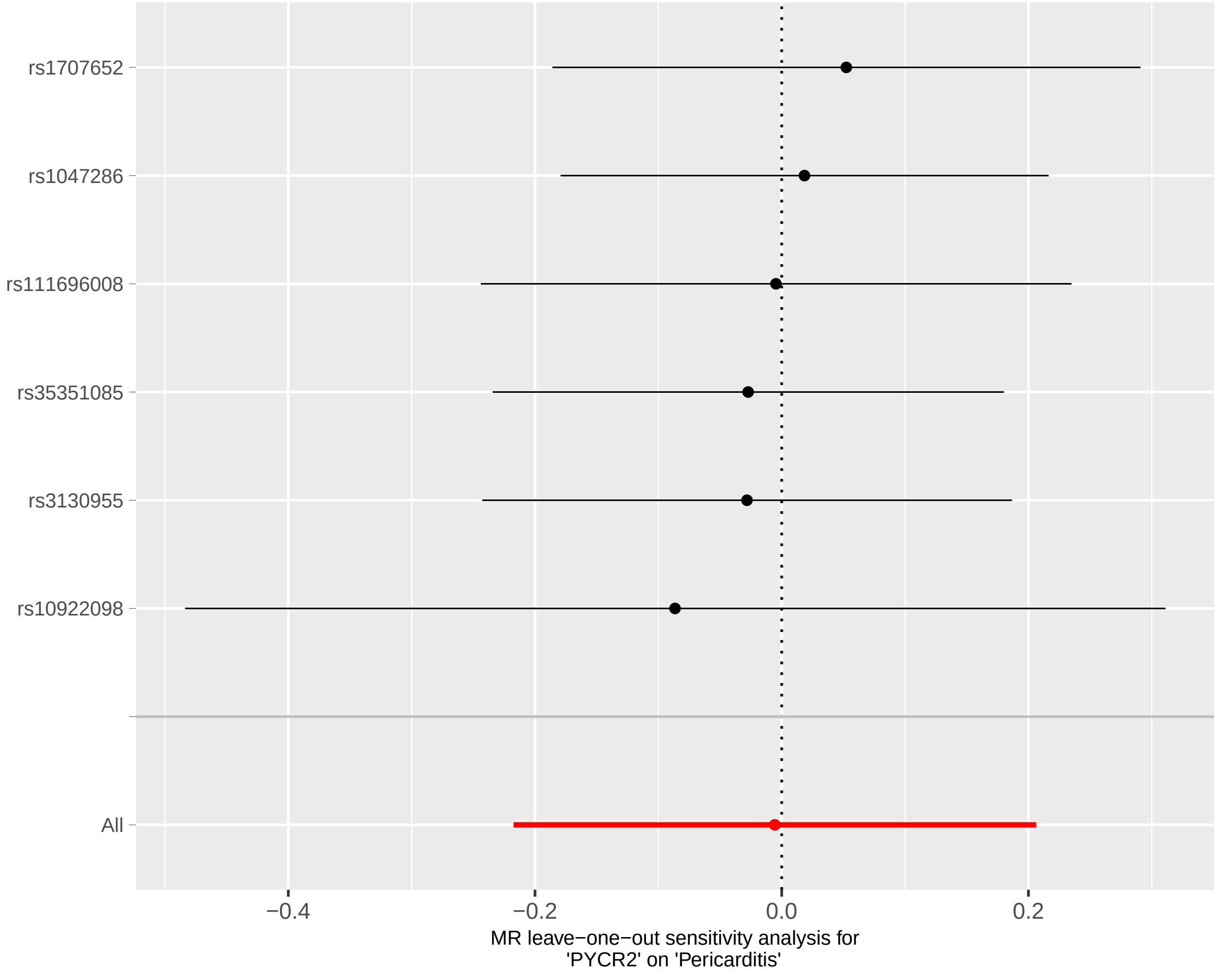

rs1707652
rs1047286
rs111696008
rs35351085
rs3130955
rs10922098
All
−0.4
−0.2
0.0
0.2
MR leave−one−out sensitivity analysis for
'PYCR2' on 'Pericarditis'

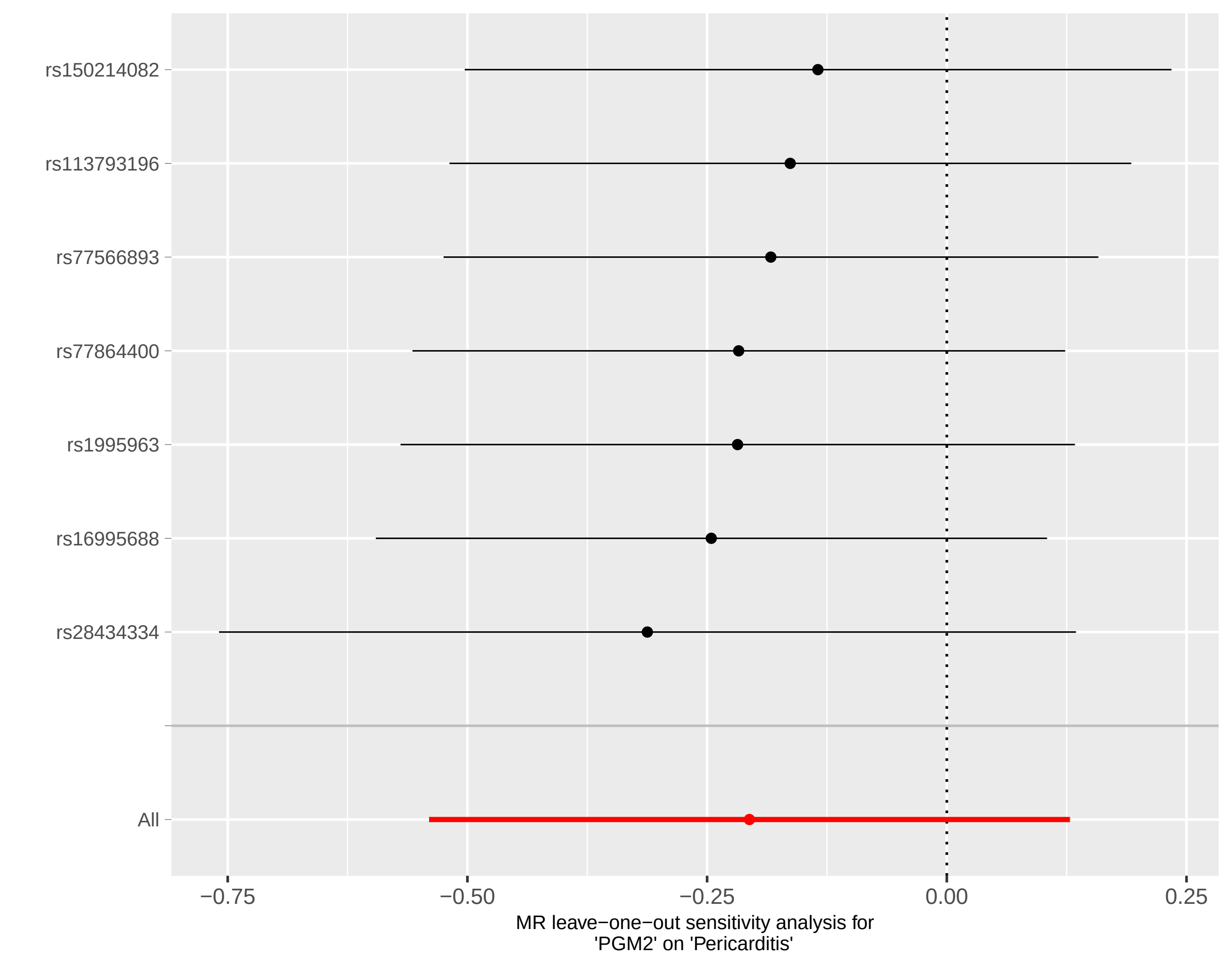
rs150214082
rs113793196
rs77566893
rs77864400
rs1995963
rs16995688
rs28434334
All
−0.75
−0.50
−0.25
0.00
0.25
MR leave−one−out sensitivity analysis for
'PGM2' on 'Pericarditis'

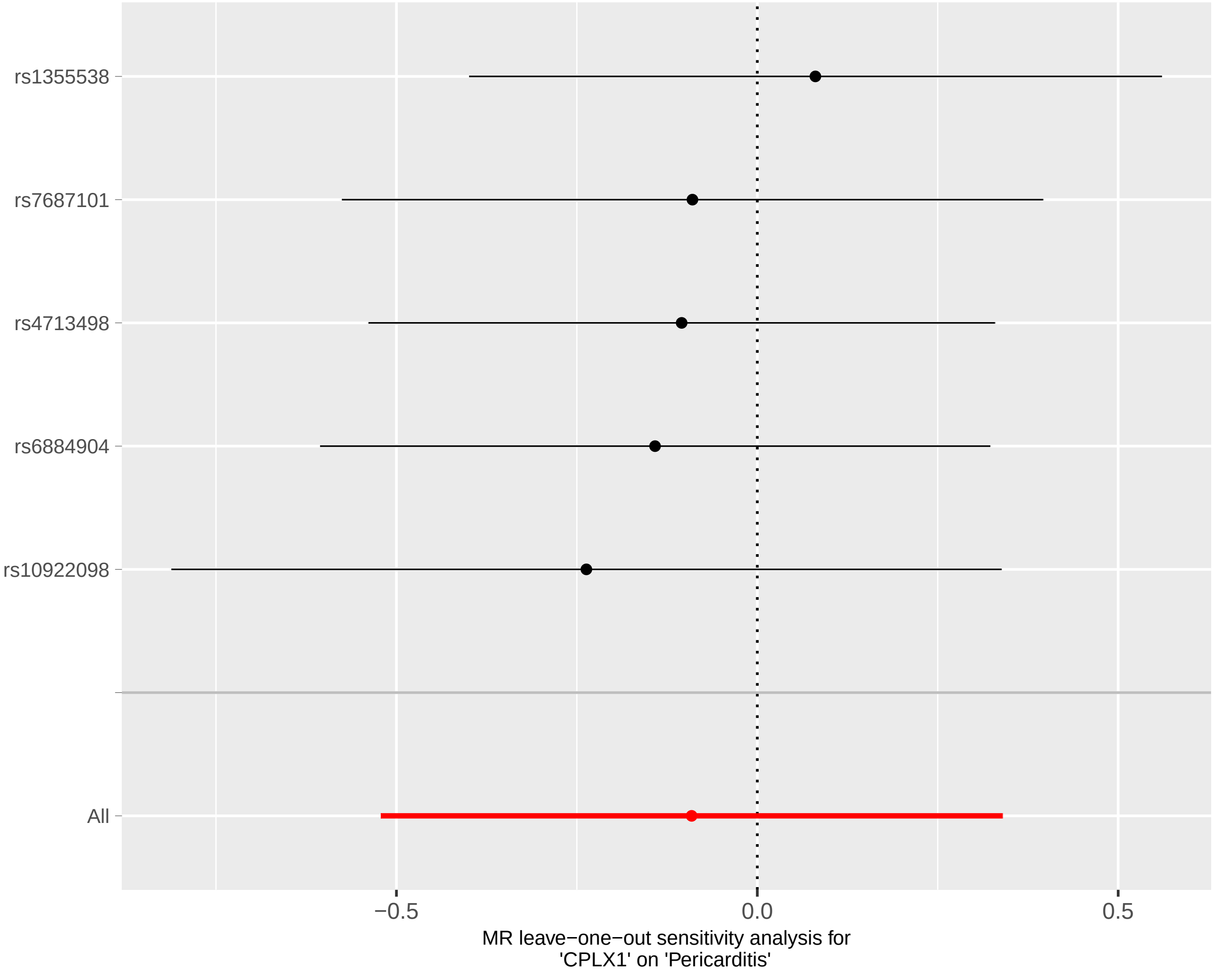
rs1355538
rs7687101
rs4713498
rs6884904
rs10922098
All
−0.5
0.0
0.5
MR leave−one−out sensitivity analysis for
'CPLX1' on 'Pericarditis'

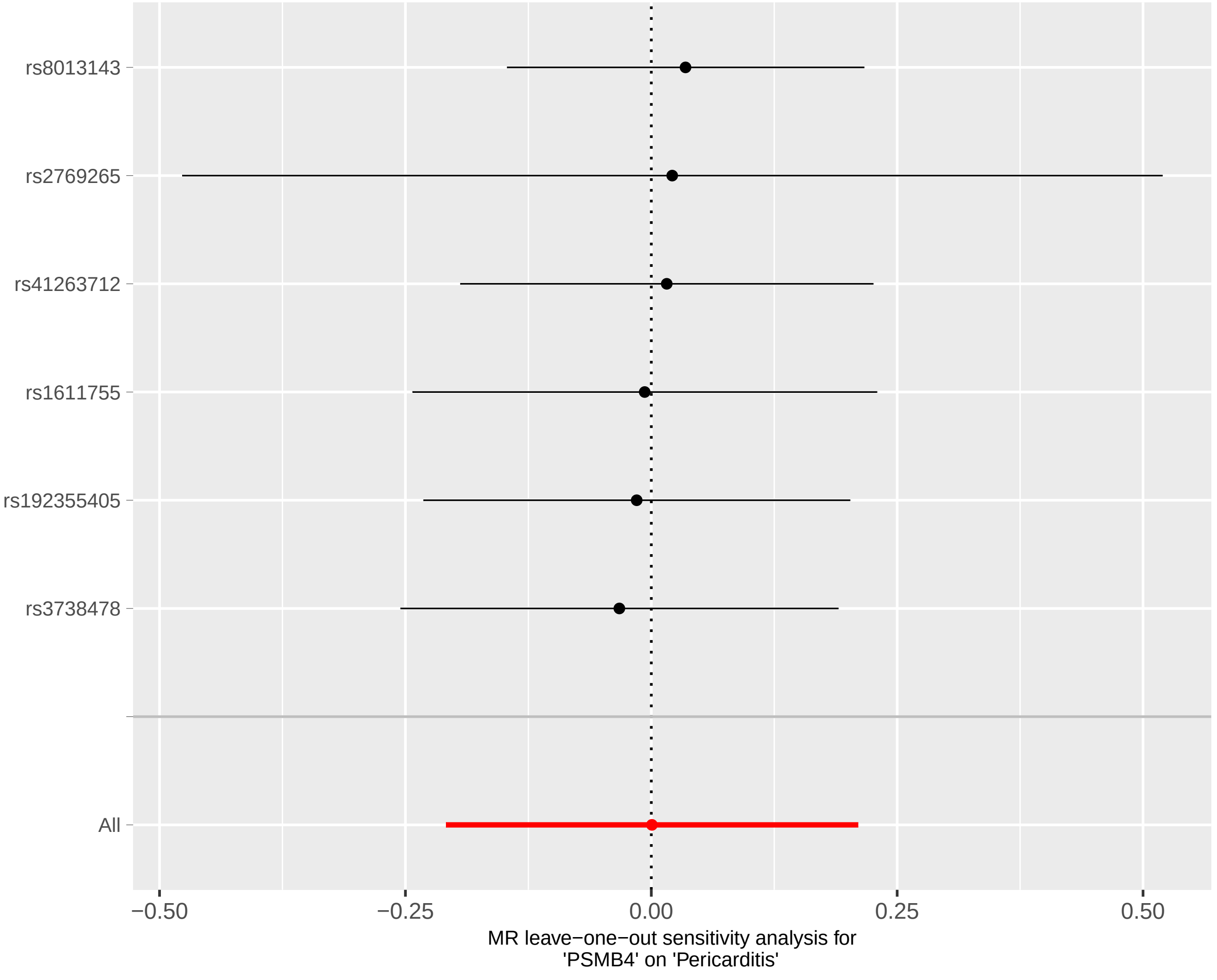
rs8013143
rs2769265
rs41263712
rs1611755
rs192355405
rs3738478
All
−0.50
−0.25
0.00
0.25
0.50
MR leave−one−out sensitivity analysis for
'PSMB4' on 'Pericarditis'

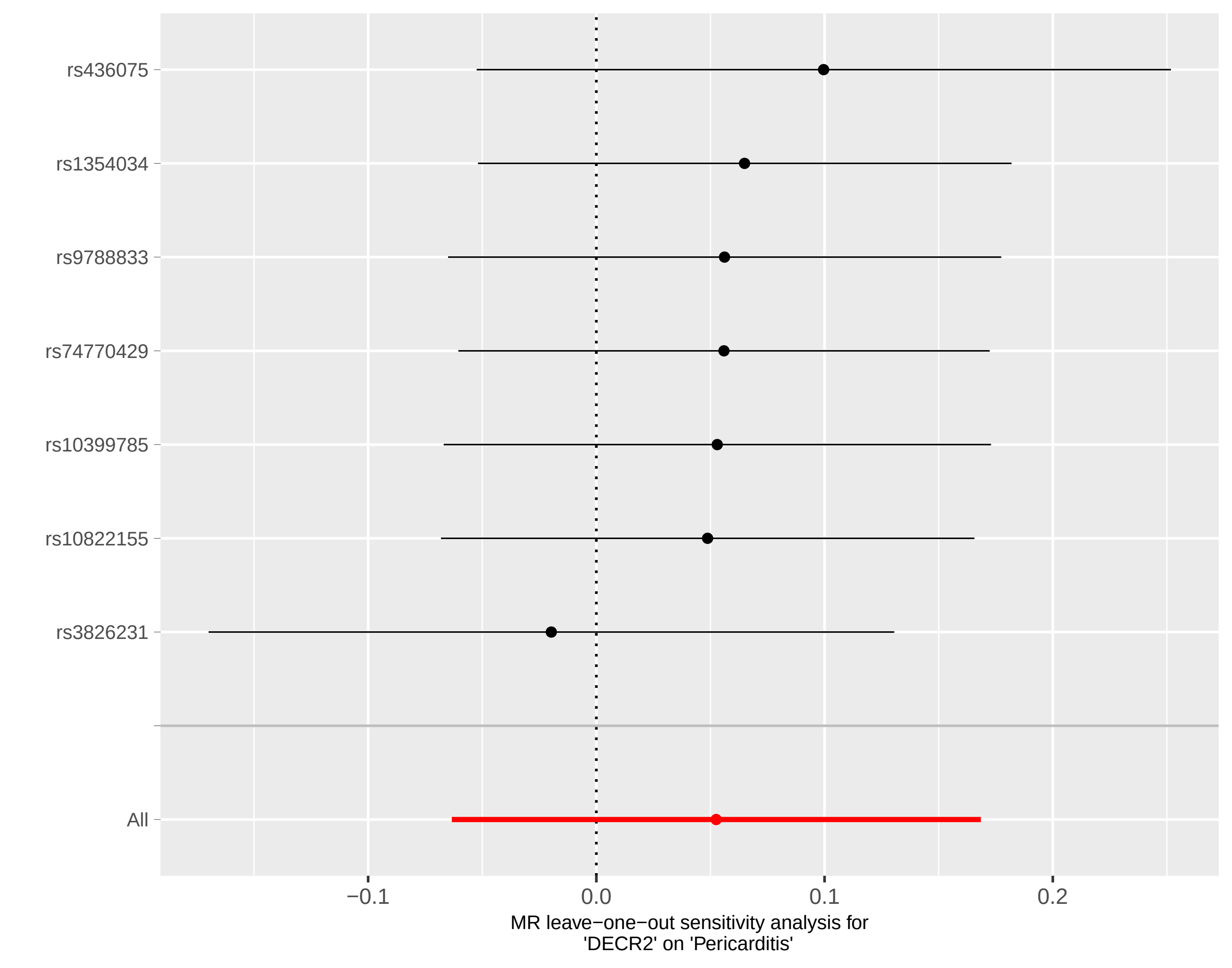
rs436075
rs1354034
rs9788833
rs74770429
rs10399785
rs10822155
rs3826231
All
−0.1
0.0
0.1
0.2
MR leave−one−out sensitivity analysis for
'DECR2' on 'Pericarditis'

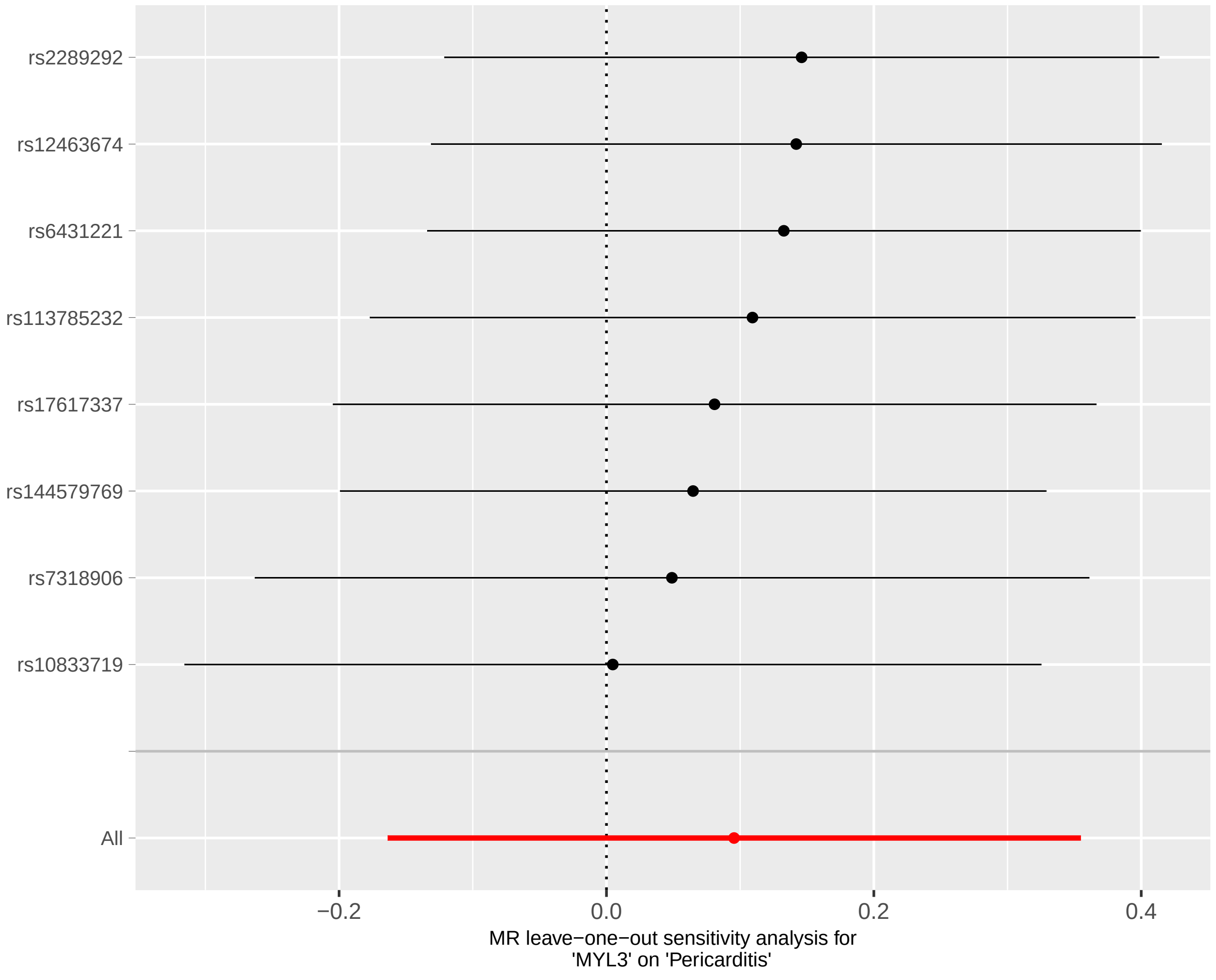
rs2289292
rs12463674
rs6431221
rs113785232
rs17617337
rs144579769
rs7318906
rs10833719
All
−0.2
0.0
0.2
0.4
MR leave−one−out sensitivity analysis for
'MYL3' on 'Pericarditis'

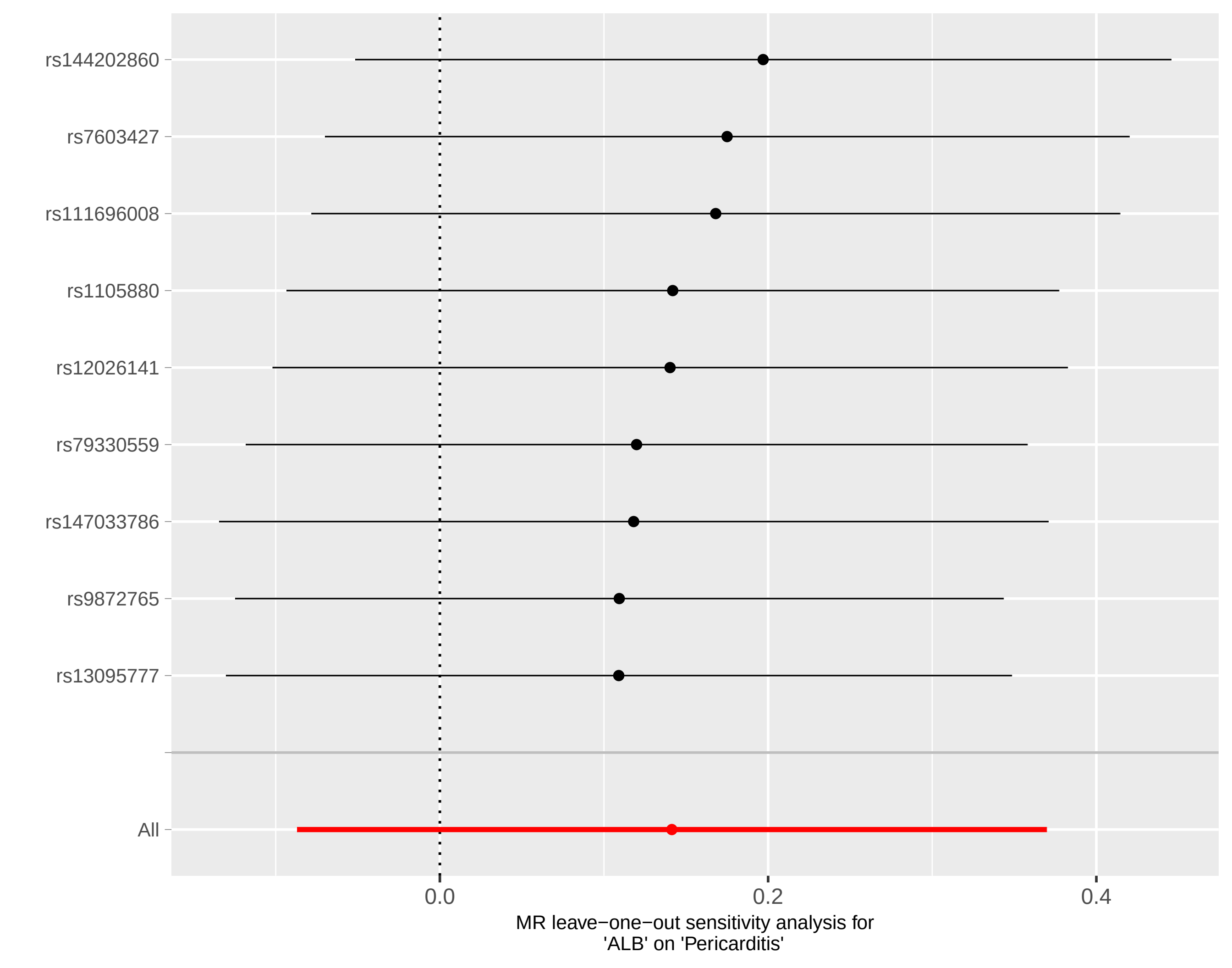

rs144202860
rs7603427
rs111696008
rs1105880
rs12026141
rs79330559
rs147033786
rs9872765
rs13095777
All
0.0
0.2
0.4
MR leave−one−out sensitivity analysis for
'ALB' on 'Pericarditis'

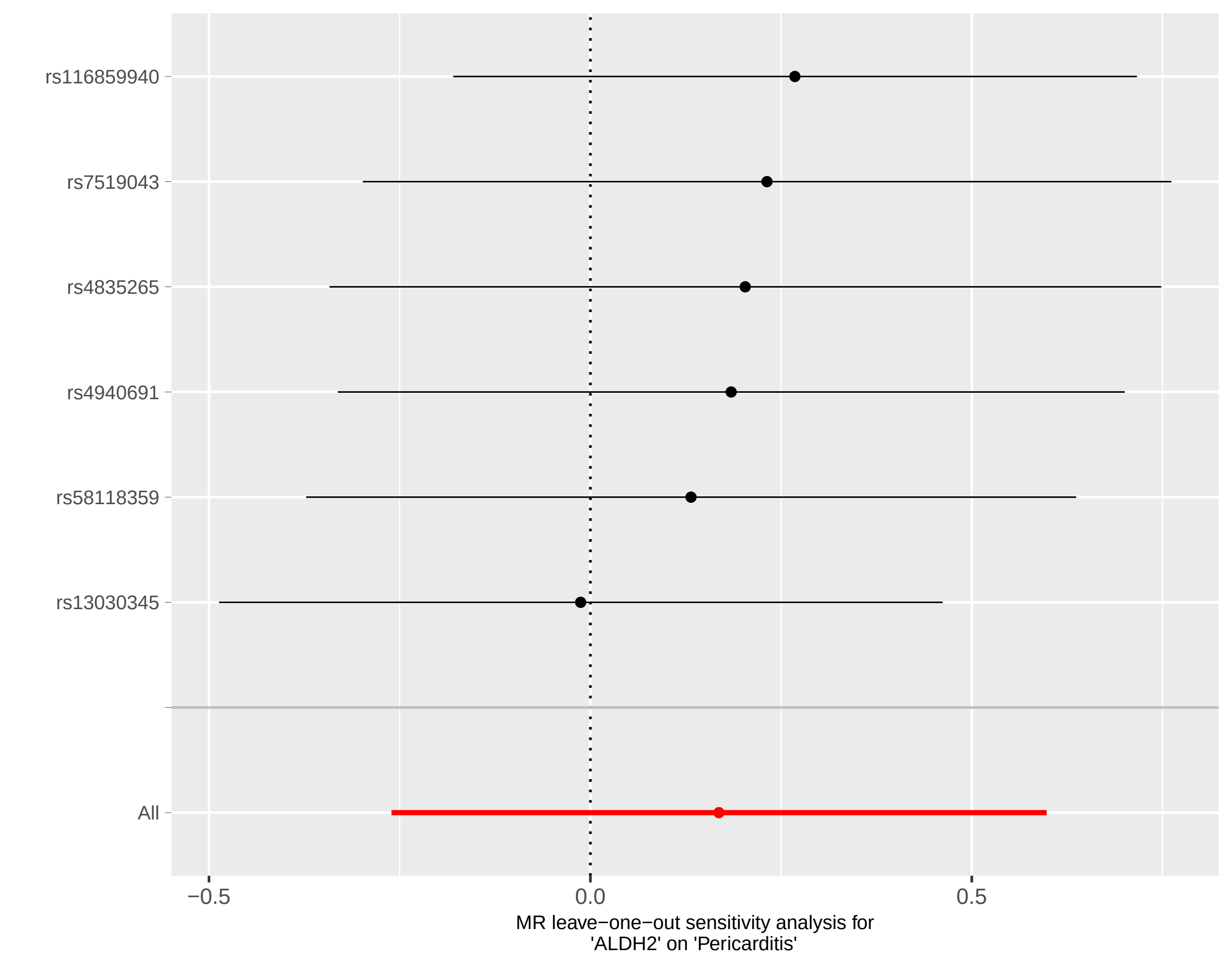

rs116859940
rs7519043
rs4835265
rs4940691
rs58118359
rs13030345
All
−0.5
0.0
0.5
MR leave−one−out sensitivity analysis for
'ALDH2' on 'Pericarditis'

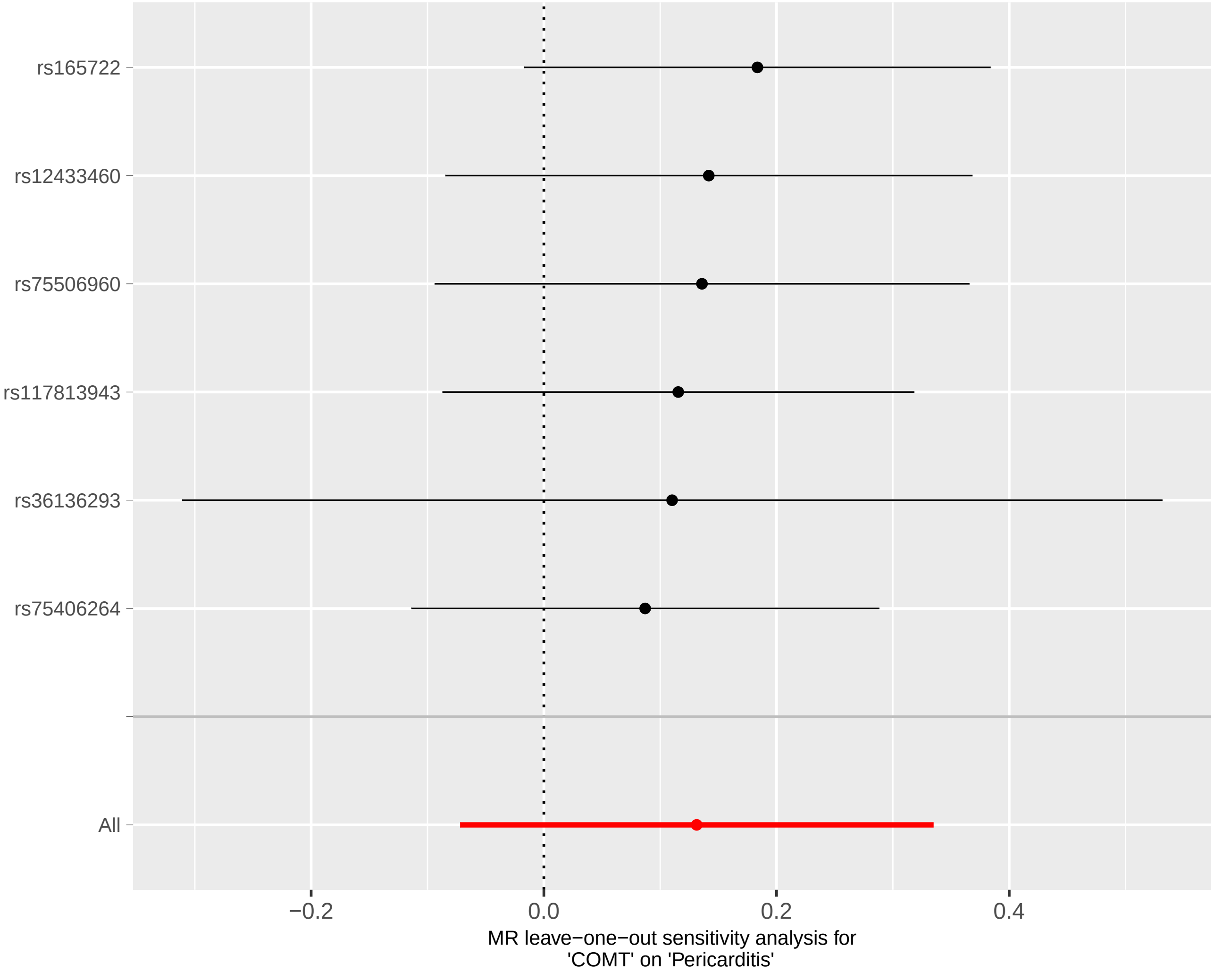
rs165722
rs12433460
rs75506960
rs117813943
rs36136293
rs75406264
All
−0.2
0.0
0.2
0.4
MR leave−one−out sensitivity analysis for
'COMT' on 'Pericarditis'

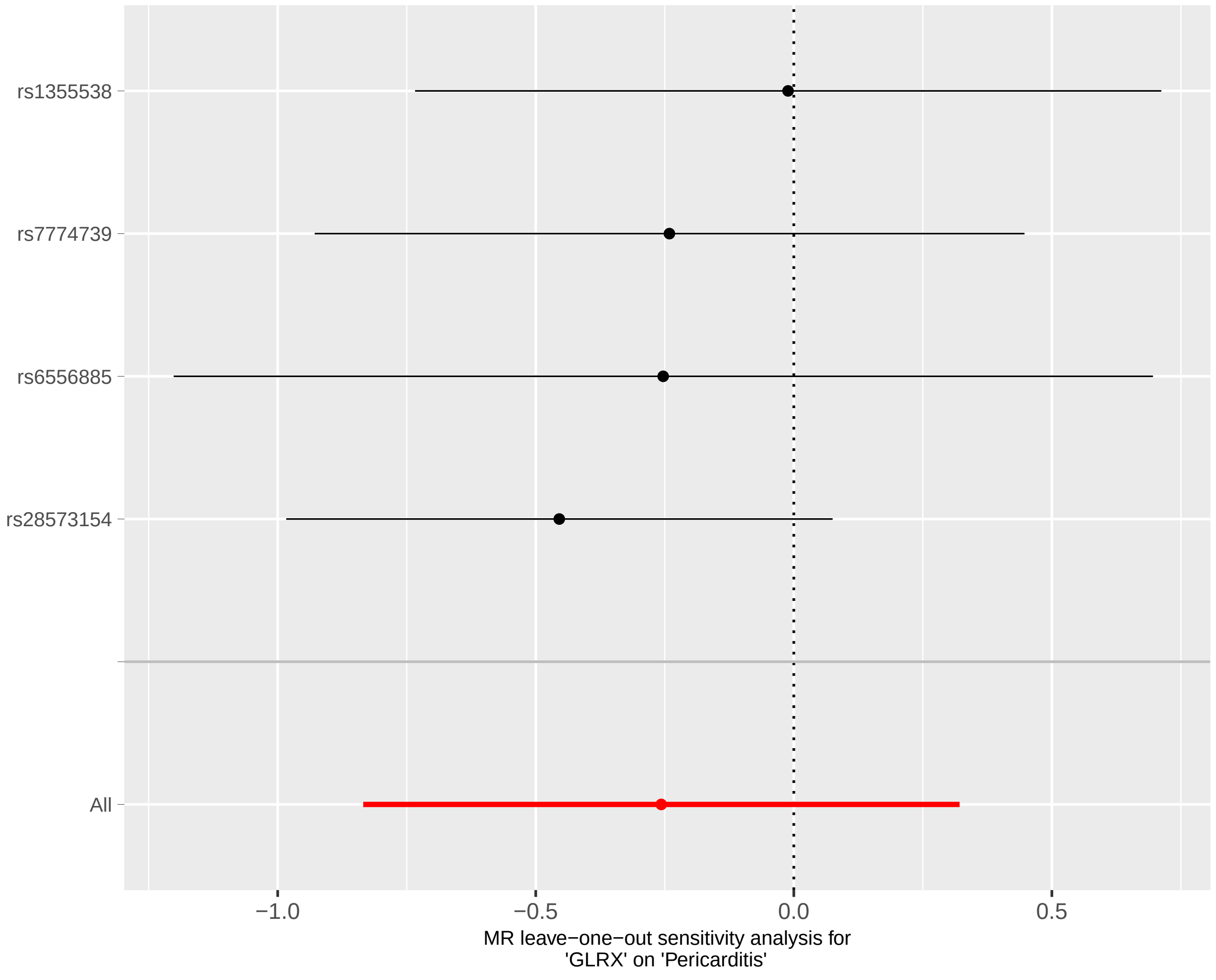
rs1355538
rs7774739
rs6556885
rs28573154
All
−1.0
−0.5
0.0
0.5
MR leave−one−out sensitivity analysis for
'GLRX' on 'Pericarditis'

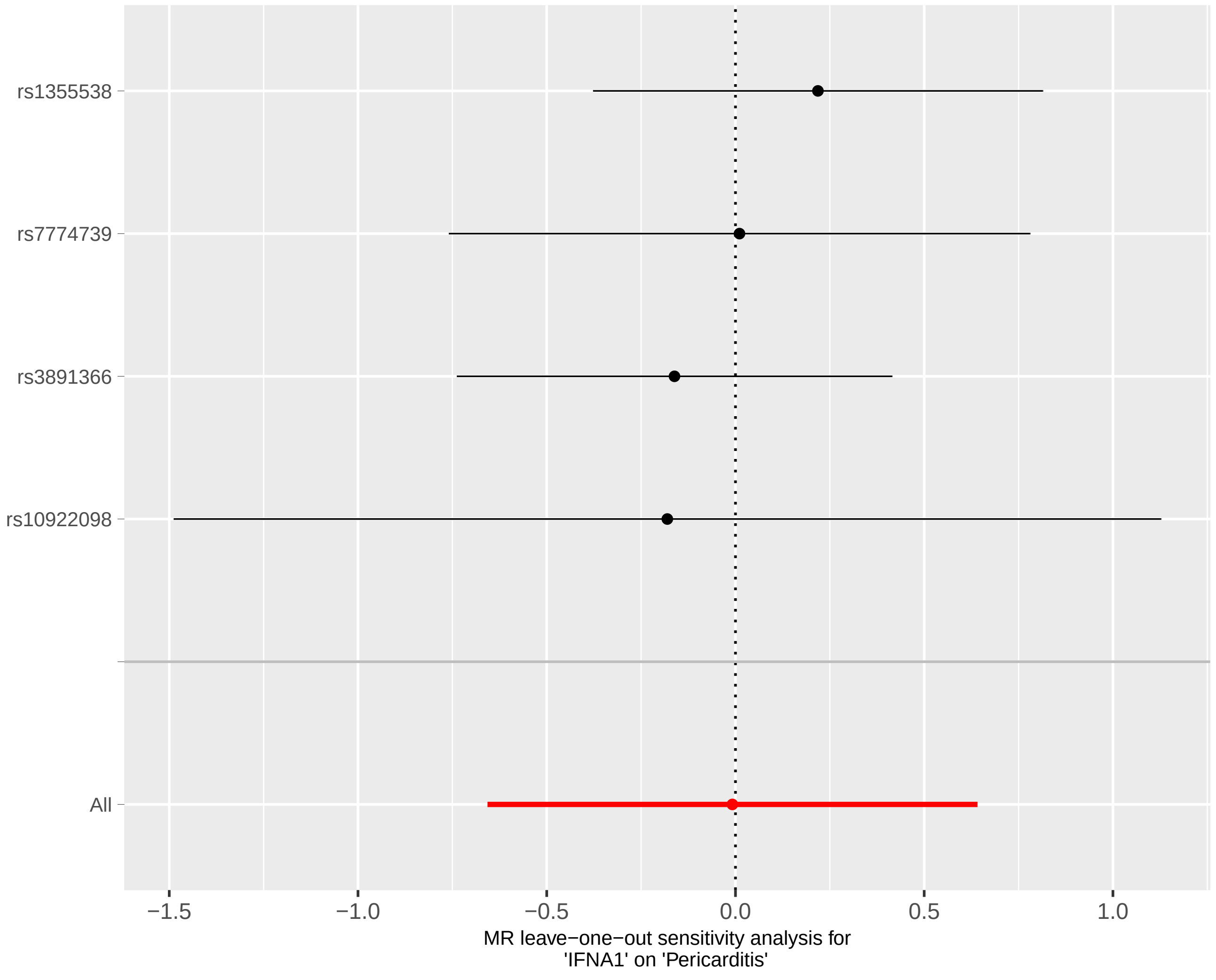
rs1355538
rs7774739
rs3891366
rs10922098
All
−1.5
−1.0
−0.5
0.0
0.5
1.0
MR leave−one−out sensitivity analysis for
'IFNA1' on 'Pericarditis'

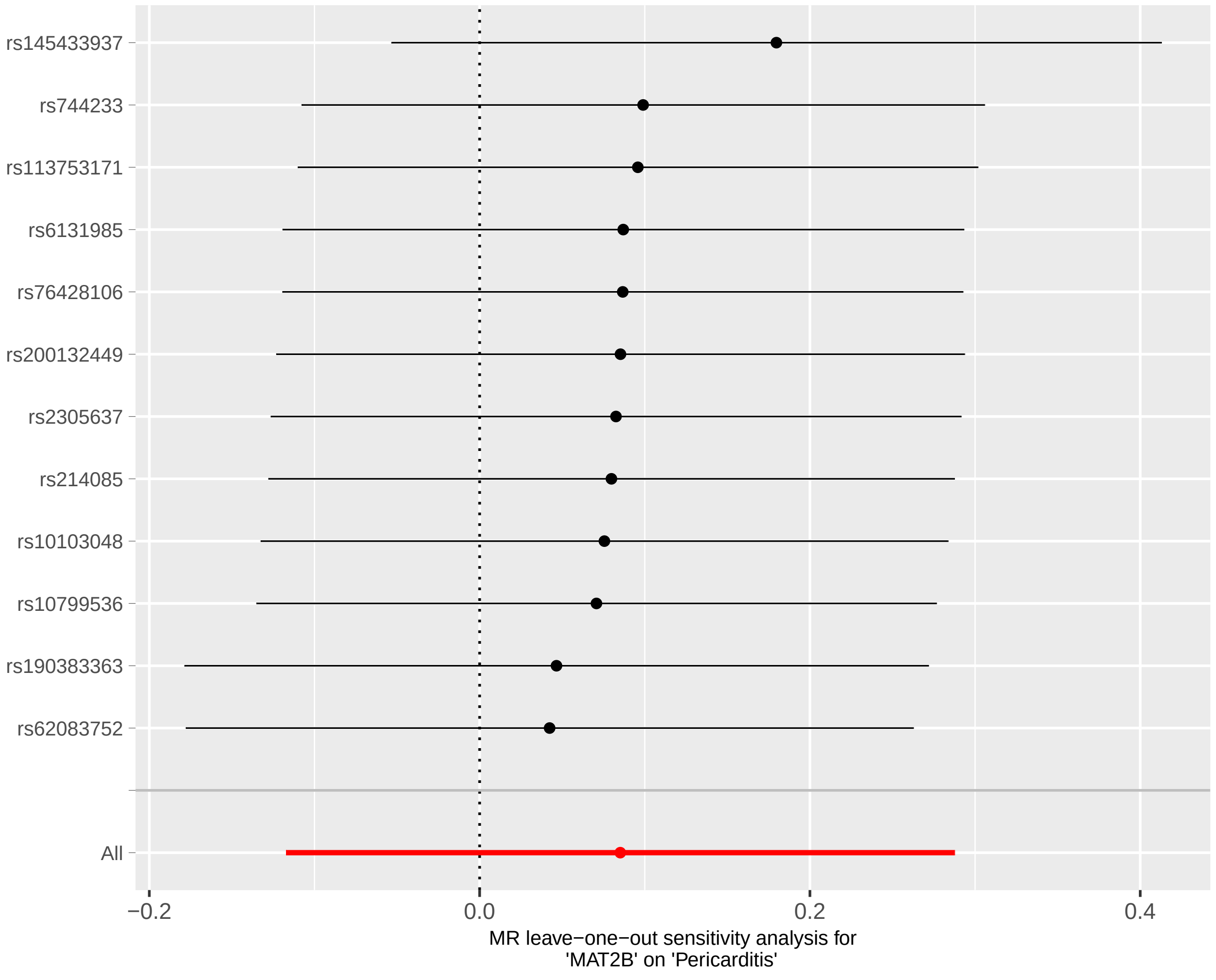

rs145433937
rs744233
rs113753171
rs6131985
rs76428106
rs200132449
rs2305637
rs214085
rs10103048
rs10799536
rs190383363
rs62083752
All
−0.2
0.0
0.2
0.4
MR leave−one−out sensitivity analysis for
'MAT2B' on 'Pericarditis'

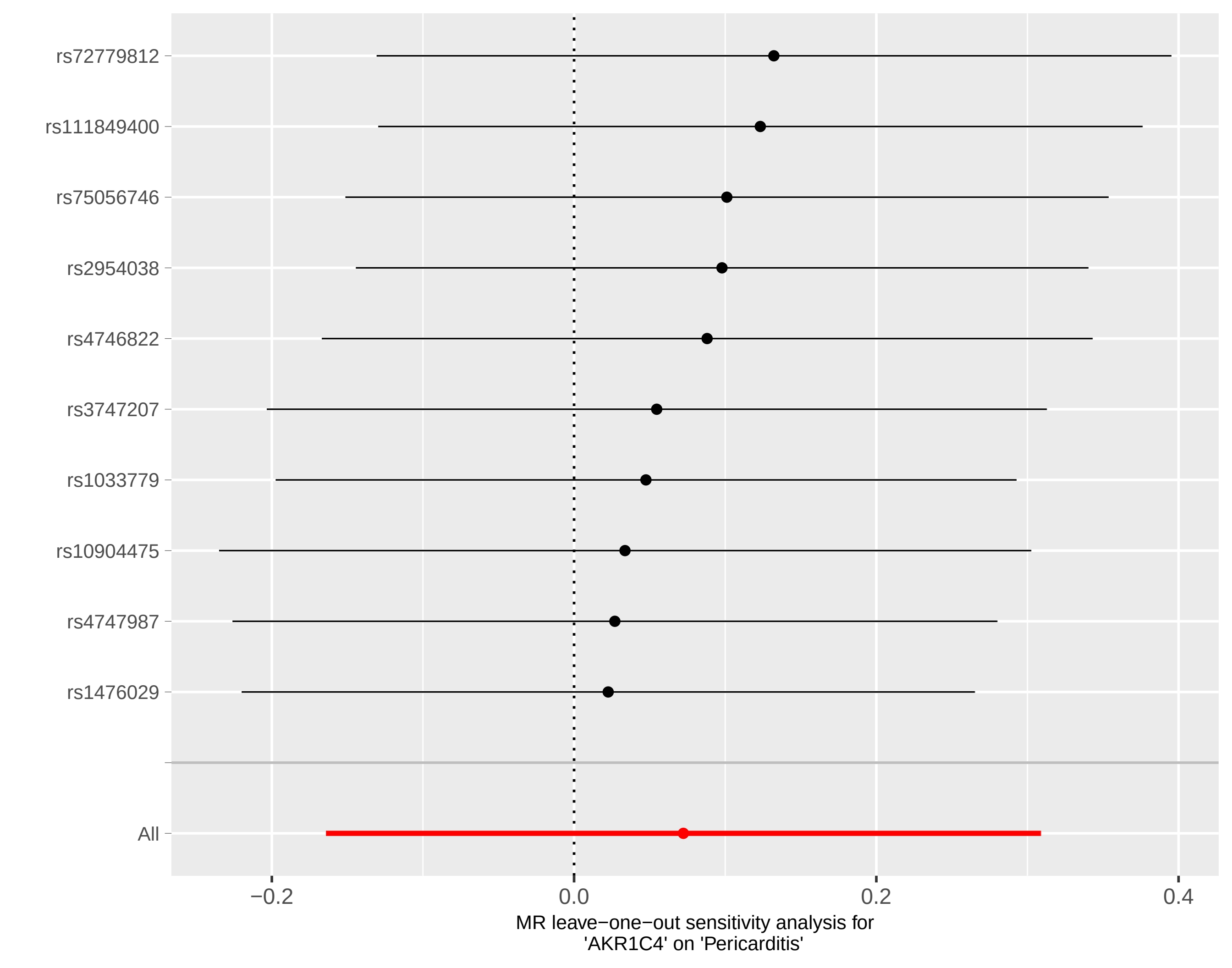

rs72779812
rs111849400
rs75056746
rs2954038
rs4746822
rs3747207
rs1033779
rs10904475
rs4747987
rs1476029
All
−0.2
0.0
0.2
0.4
MR leave−one−out sensitivity analysis for
'AKR1C4' on 'Pericarditis'

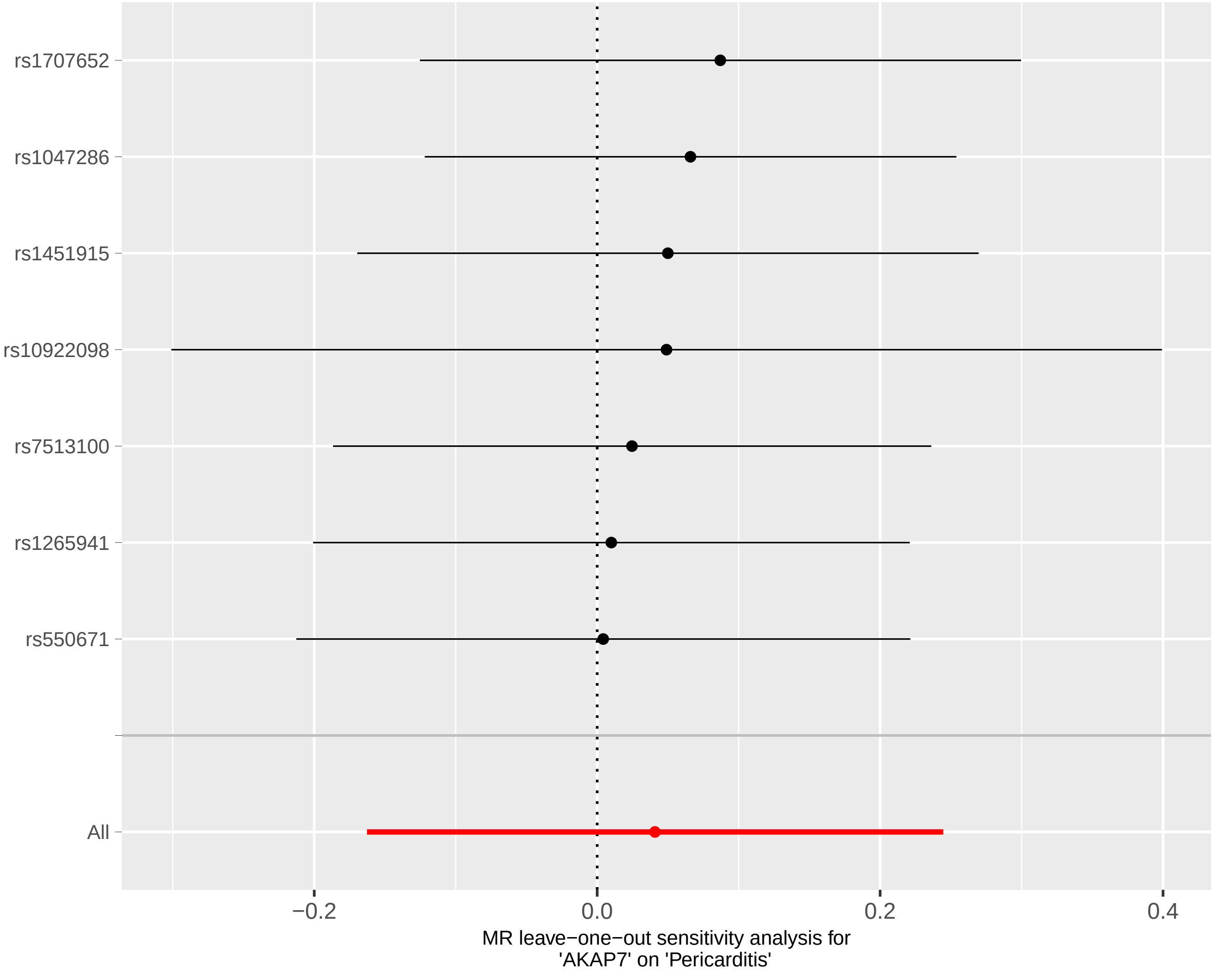

rs1707652
rs1047286
rs1451915
rs10922098
rs7513100
rs1265941
rs550671
All
−0.2
0.0
0.2
0.4
MR leave−one−out sensitivity analysis for
'AKAP7' on 'Pericarditis'

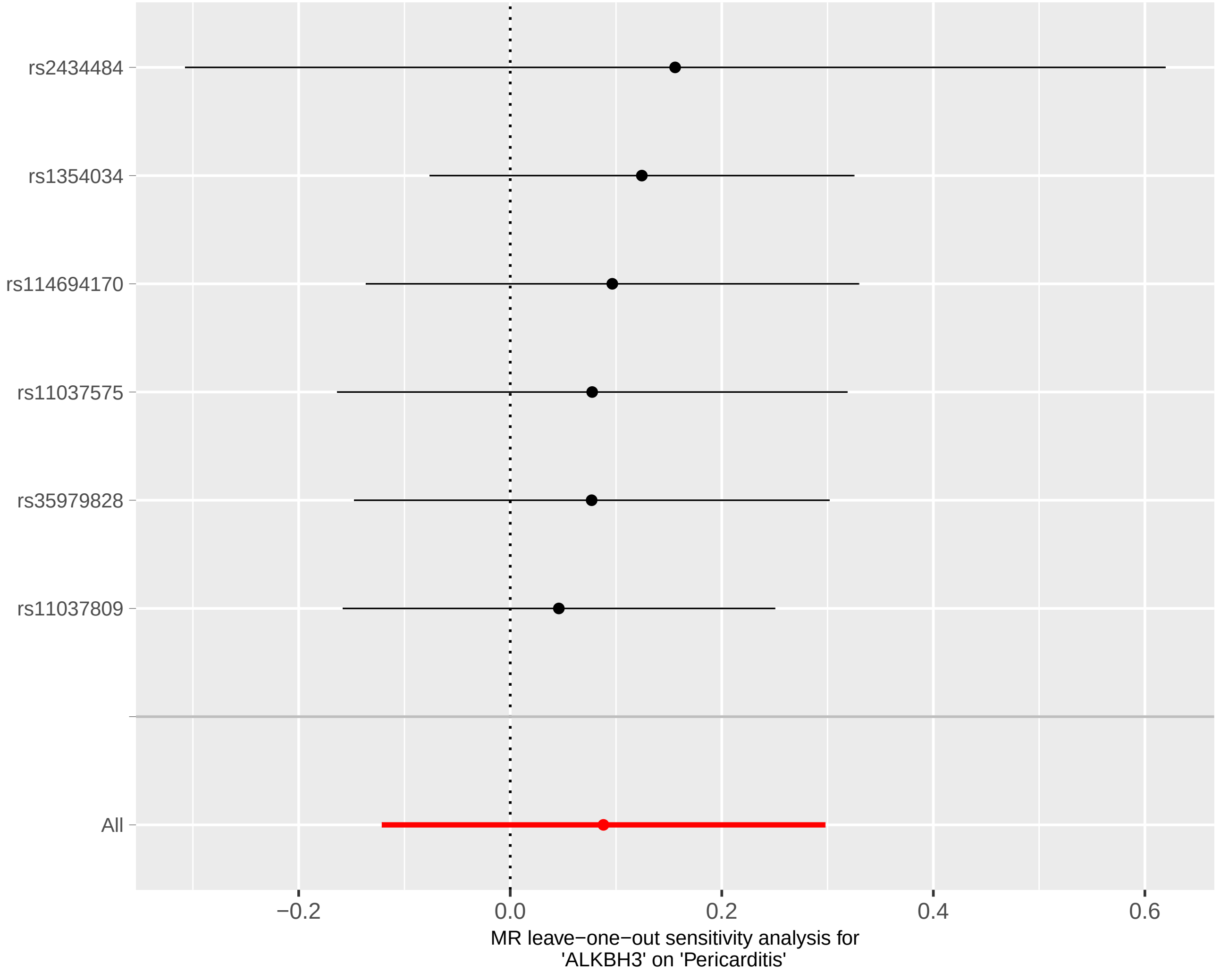
rs2434484
rs1354034
rs114694170
rs11037575
rs35979828
rs11037809
All
−0.2
0.0
0.2
0.4
0.6
MR leave−one−out sensitivity analysis for
'ALKBH3' on 'Pericarditis'

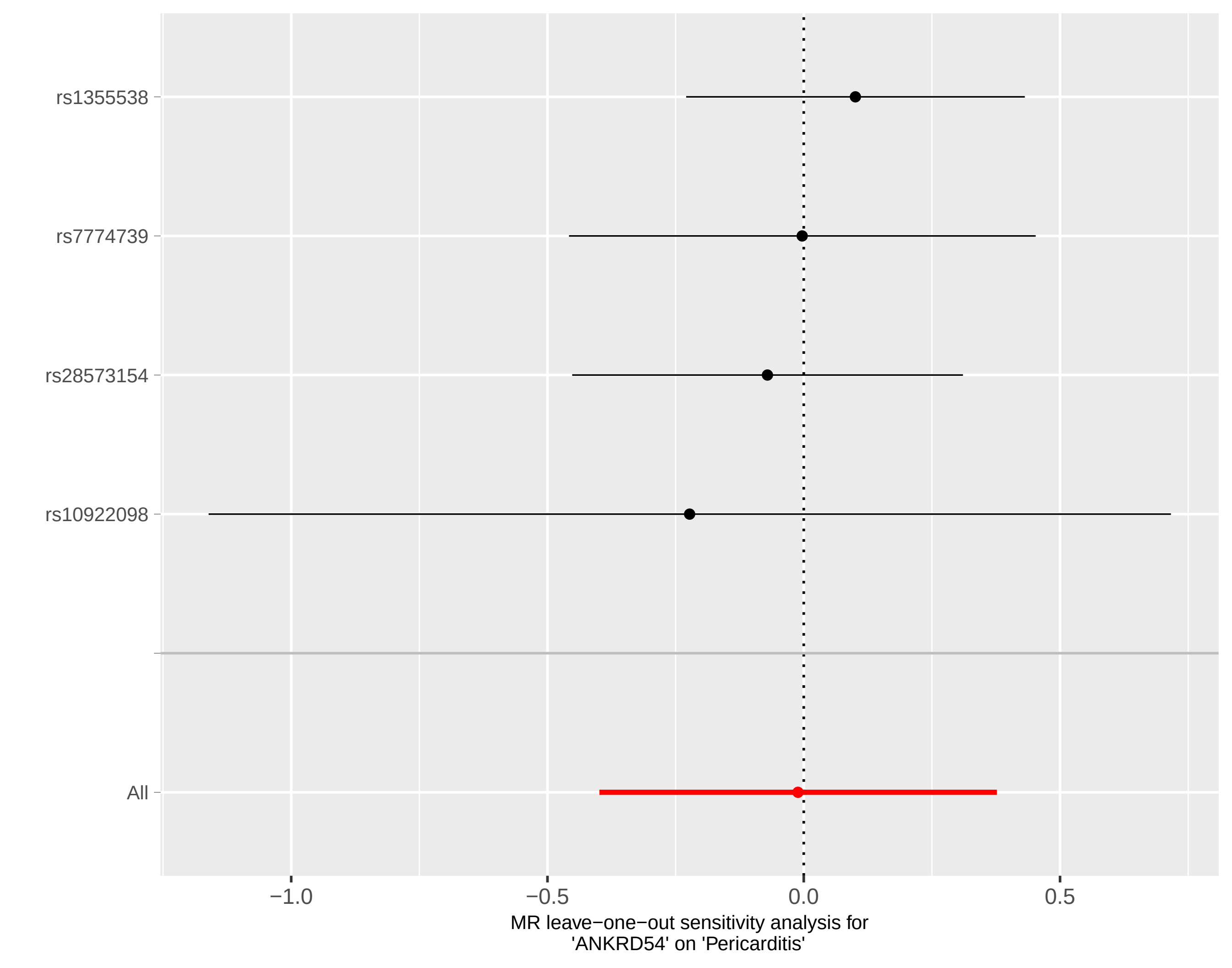

rs1355538
rs7774739
rs28573154
rs10922098
All
−1.0
−0.5
0.0
0.5
MR leave−one−out sensitivity analysis for
'ANKRD54' on 'Pericarditis'

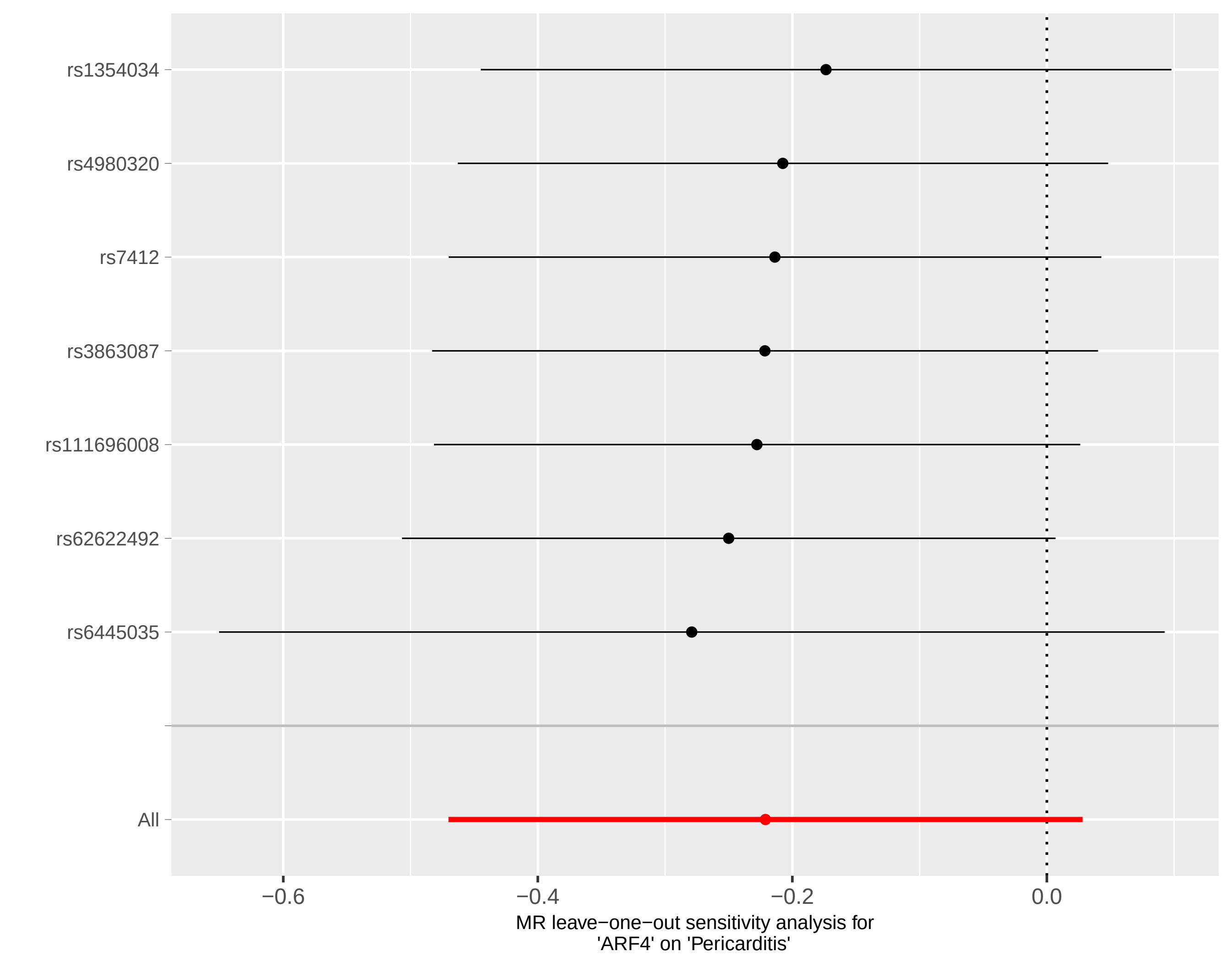
rs1354034
rs4980320
rs7412
rs3863087
rs111696008
rs62622492
rs6445035
All
−0.6
−0.4
−0.2
0.0
MR leave−one−out sensitivity analysis for
'ARF4' on 'Pericarditis'

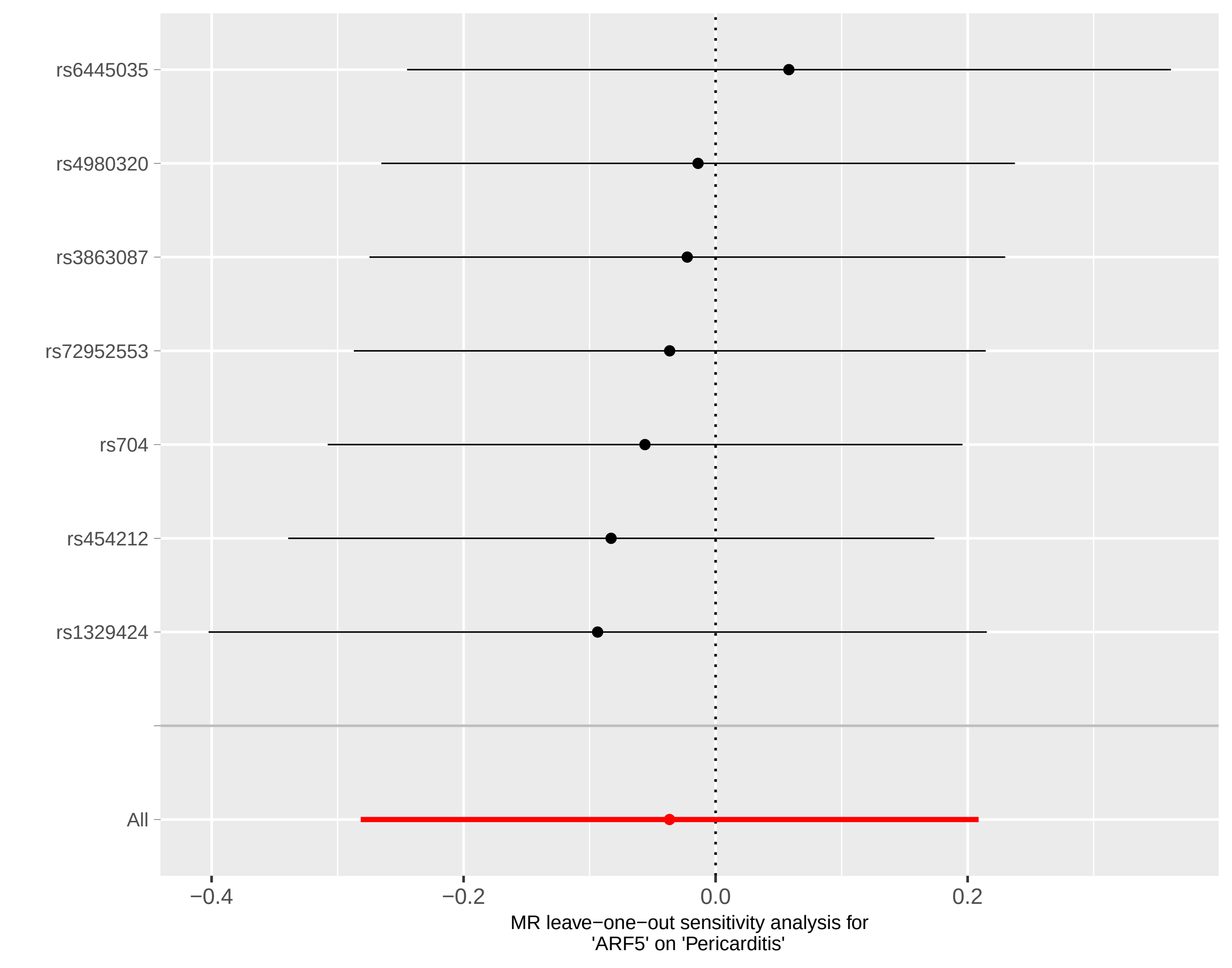
rs6445035
rs4980320
rs3863087
rs72952553
rs704
rs454212
rs1329424
All
−0.4
−0.2
0.0
0.2
MR leave−one−out sensitivity analysis for
'ARF5' on 'Pericarditis'

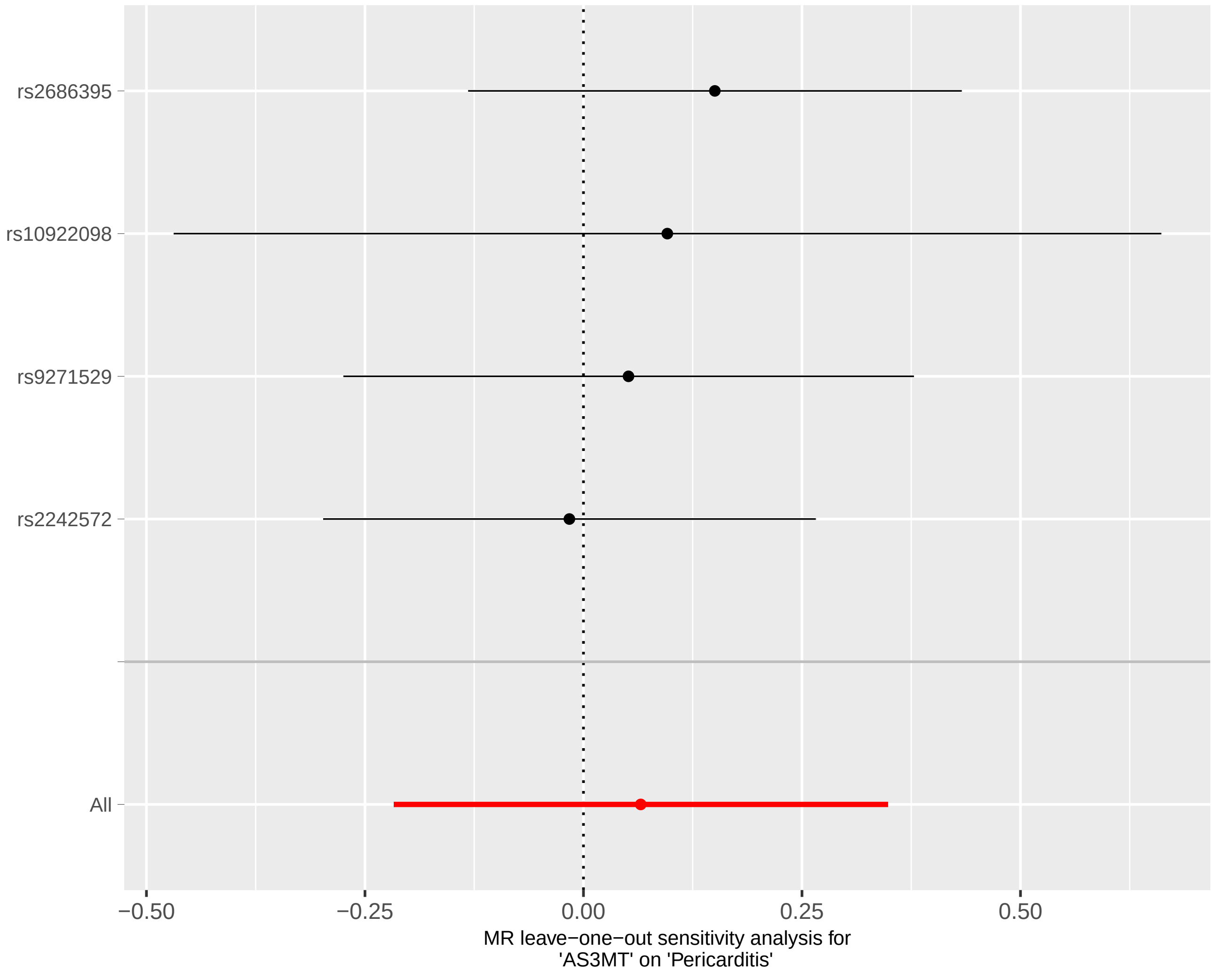
rs2686395
rs10922098
rs9271529
rs2242572
All
−0.50
−0.25
0.00
0.25
0.50
MR leave−one−out sensitivity analysis for
'AS3MT' on 'Pericarditis'

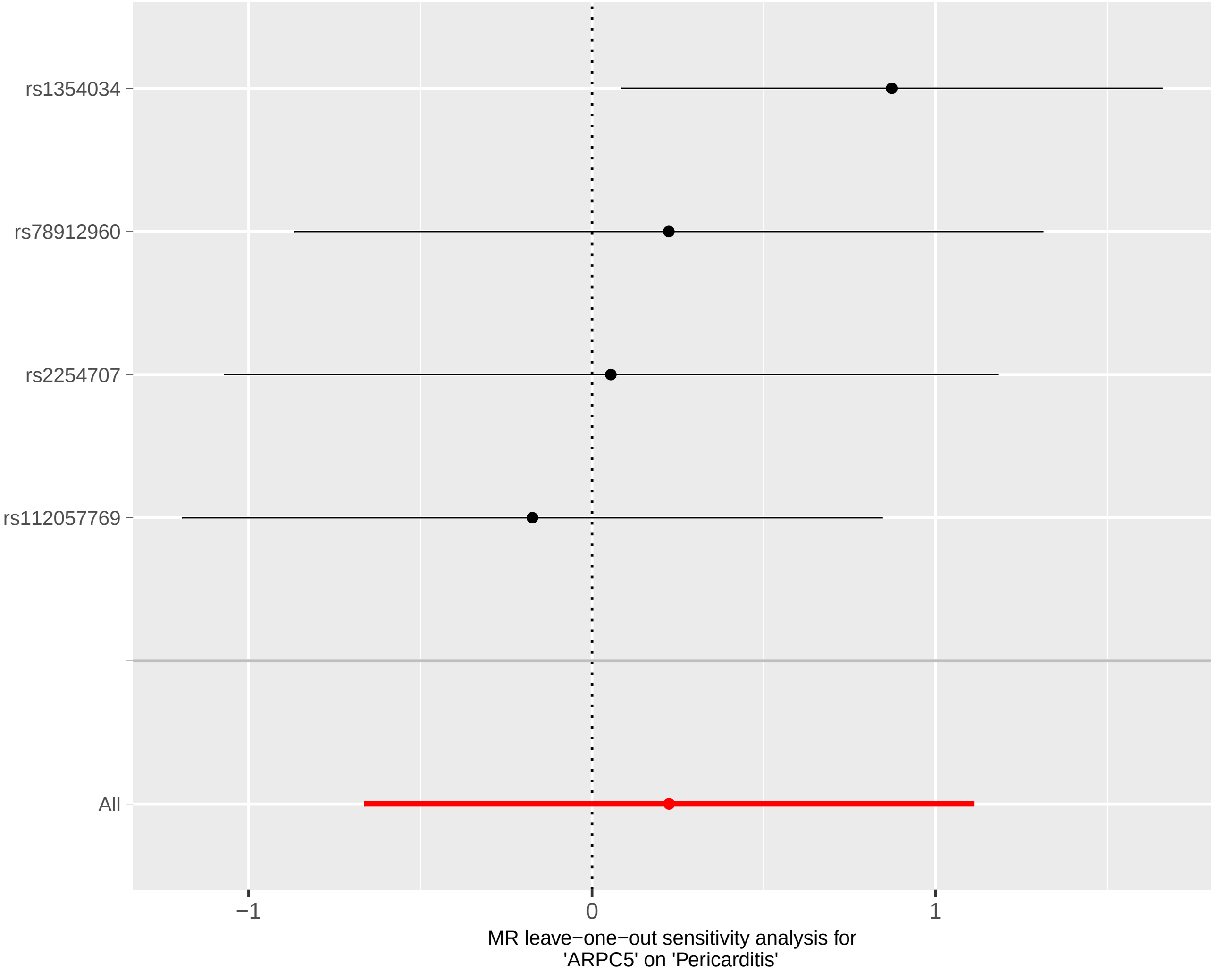
rs1354034
rs78912960
rs2254707
rs112057769
All
−1
0
1
MR leave−one−out sensitivity analysis for
'ARPC5' on 'Pericarditis'

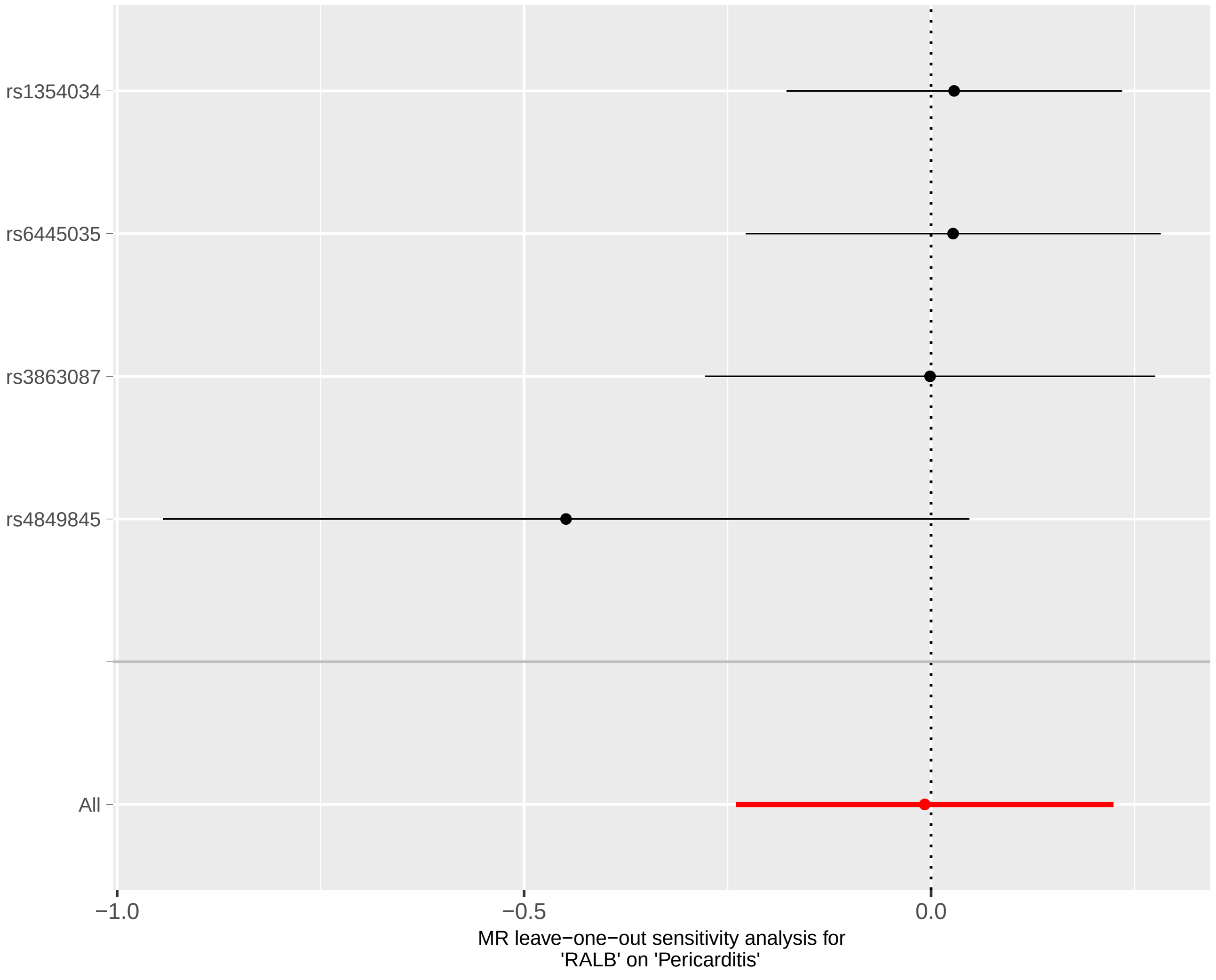
rs1354034
rs6445035
rs3863087
rs4849845
All
−1.0
−0.5
0.0
MR leave−one−out sensitivity analysis for
'RALB' on 'Pericarditis'

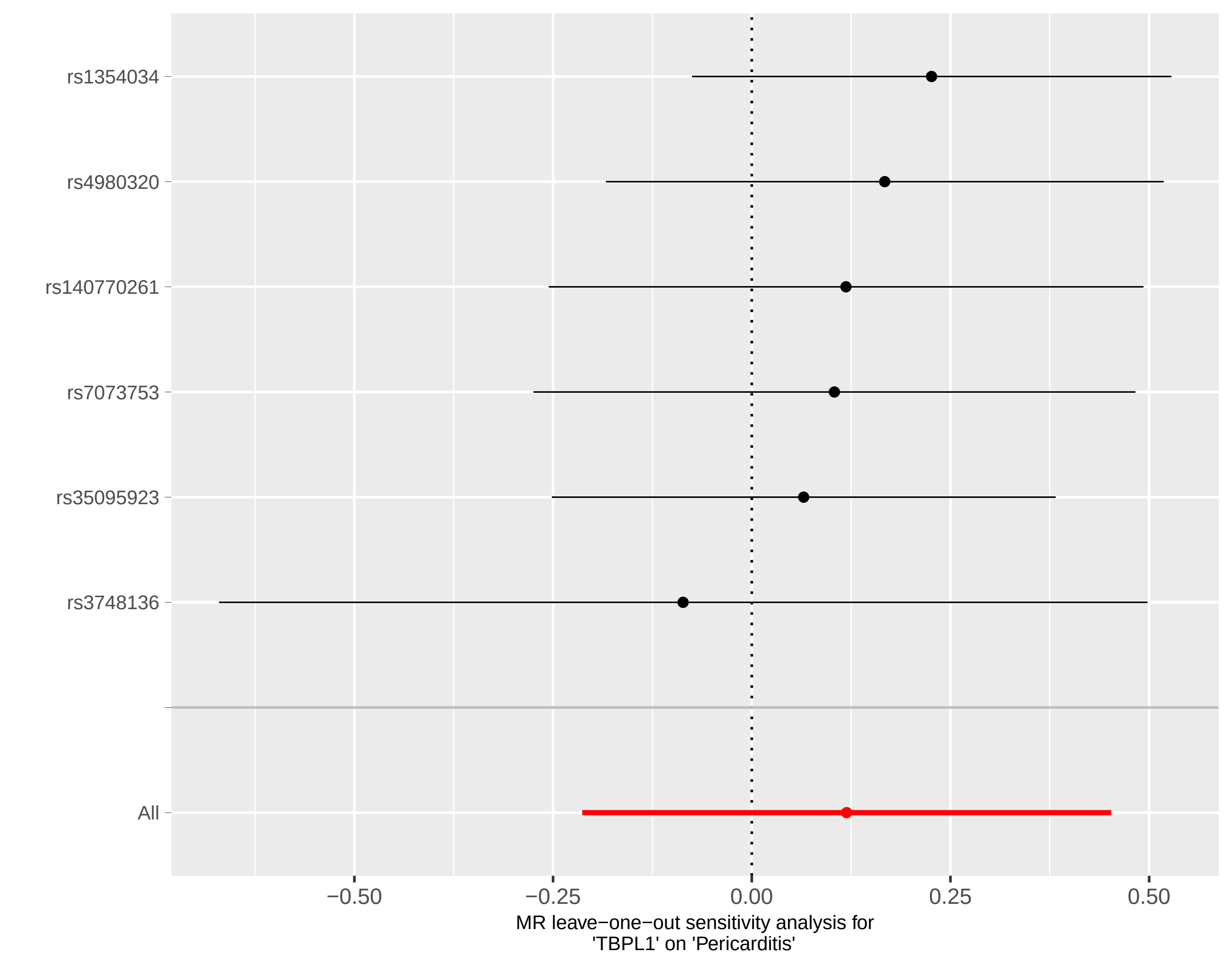
rs1354034
rs4980320
rs140770261
rs7073753
rs35095923
rs3748136
All
−0.50
−0.25
0.00
0.25
0.50
MR leave−one−out sensitivity analysis for
'TBPL1' on 'Pericarditis'

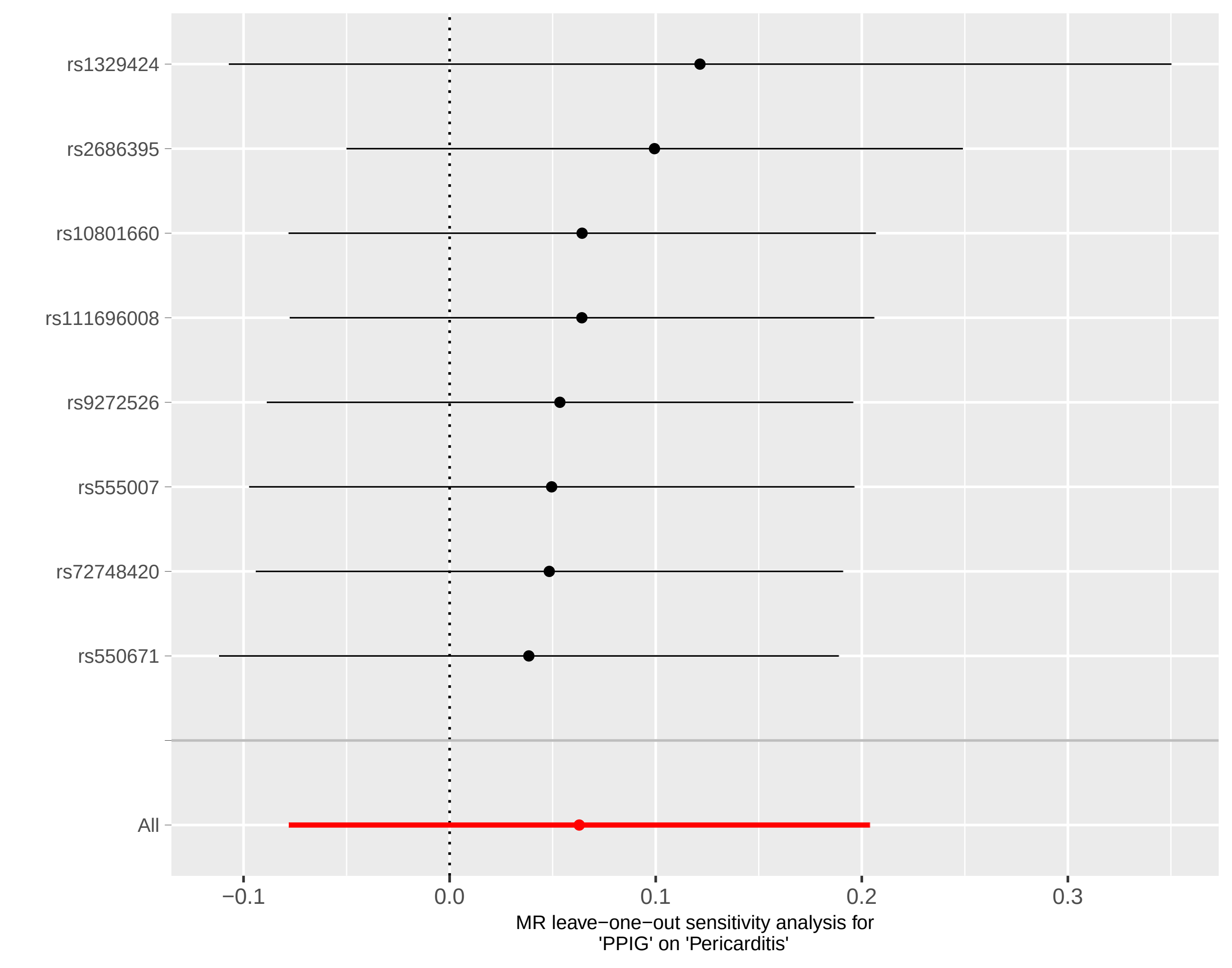

rs1329424
rs2686395
rs10801660
rs111696008
rs9272526
rs555007
rs72748420
rs550671
All
−0.1
0.0
0.1
0.2
0.3
MR leave−one−out sensitivity analysis for
'PPIG' on 'Pericarditis'
