# Supplementary material for: Causal Association Between Plasma Proteins and Pericarditis: A Mendelian Randomization Study With Therapeutic Target Identification
Source: Mediators Inflamm. 2026 Feb 9;2026:4659271. doi: 10.1155/mi/4659271 (PMC12887433; doi:10.1155/mi/4659271)
Supplement: Supplementary file 7 — Supporting Information 7 Figure S3. Funnel plot results of MR analysis between plasmas proteins and Pericarditis [file MI-2026-4659271-s006.pdf]

Figure S3. Funnel plot results of MR analysis between plasmas proteins and Pericarditis

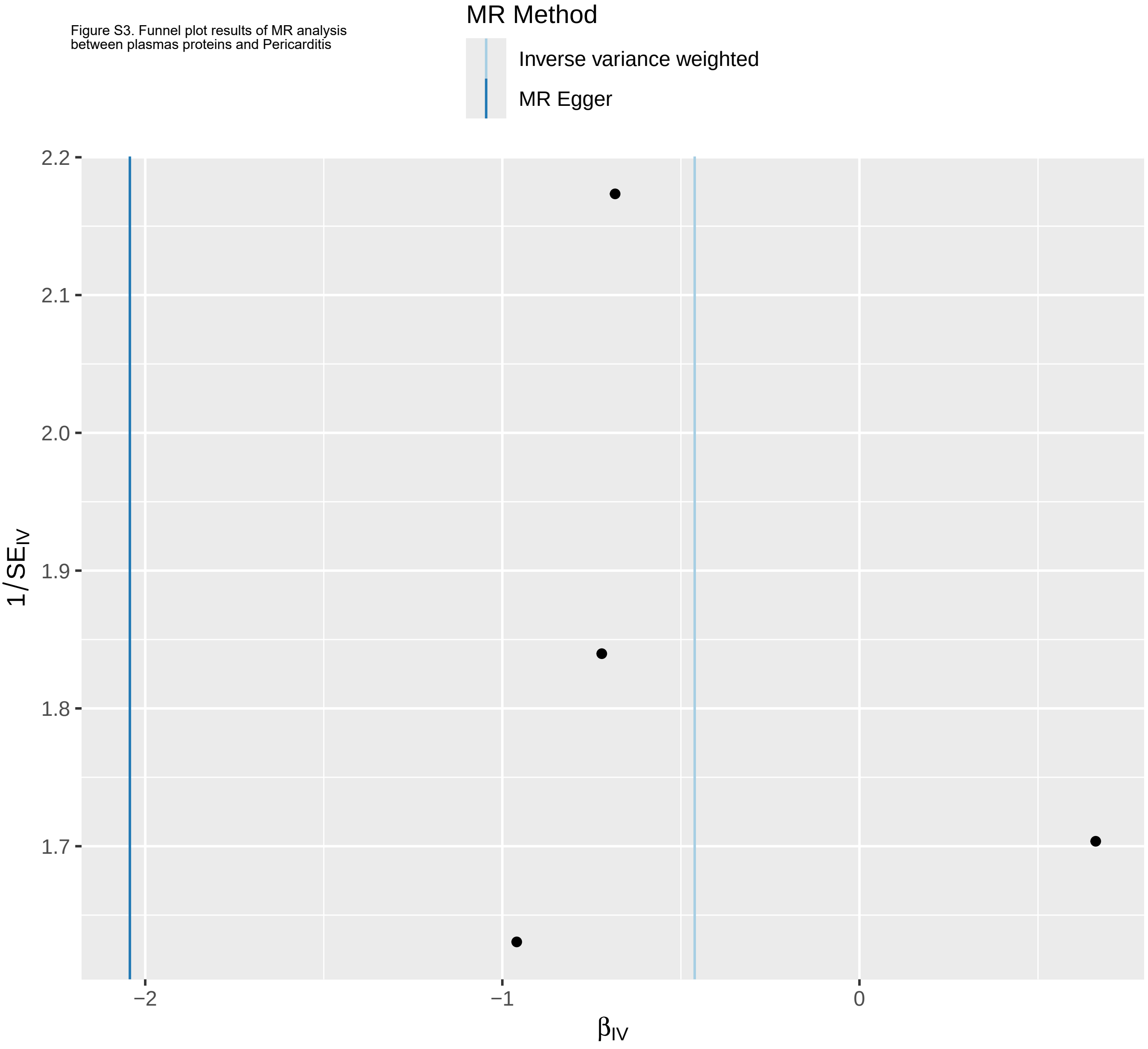

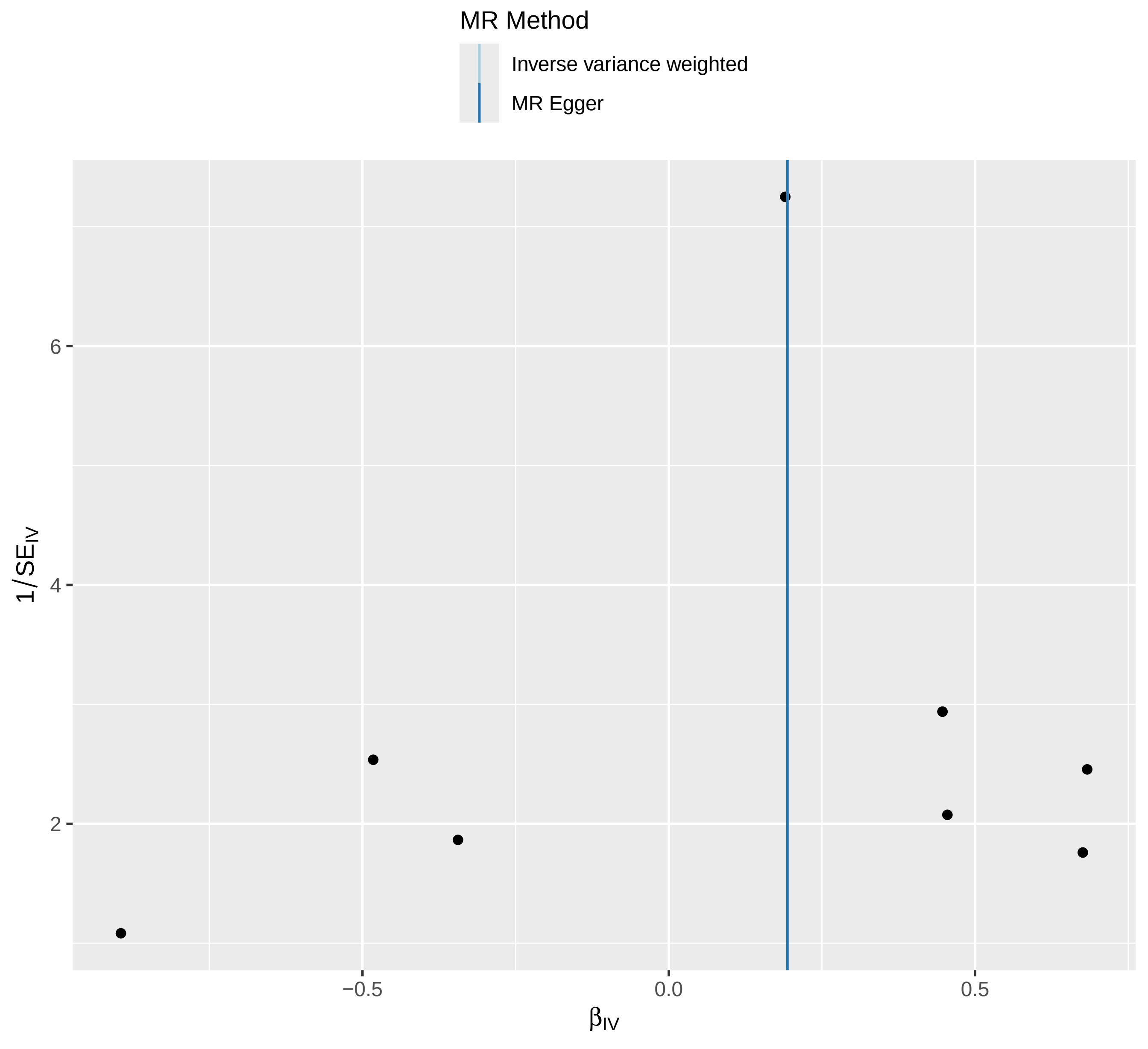
MR Method
Inverse variance weighted
MR Egger
1/SE_IV
6
4
2
−0.5
0.0
0.5
β_IV

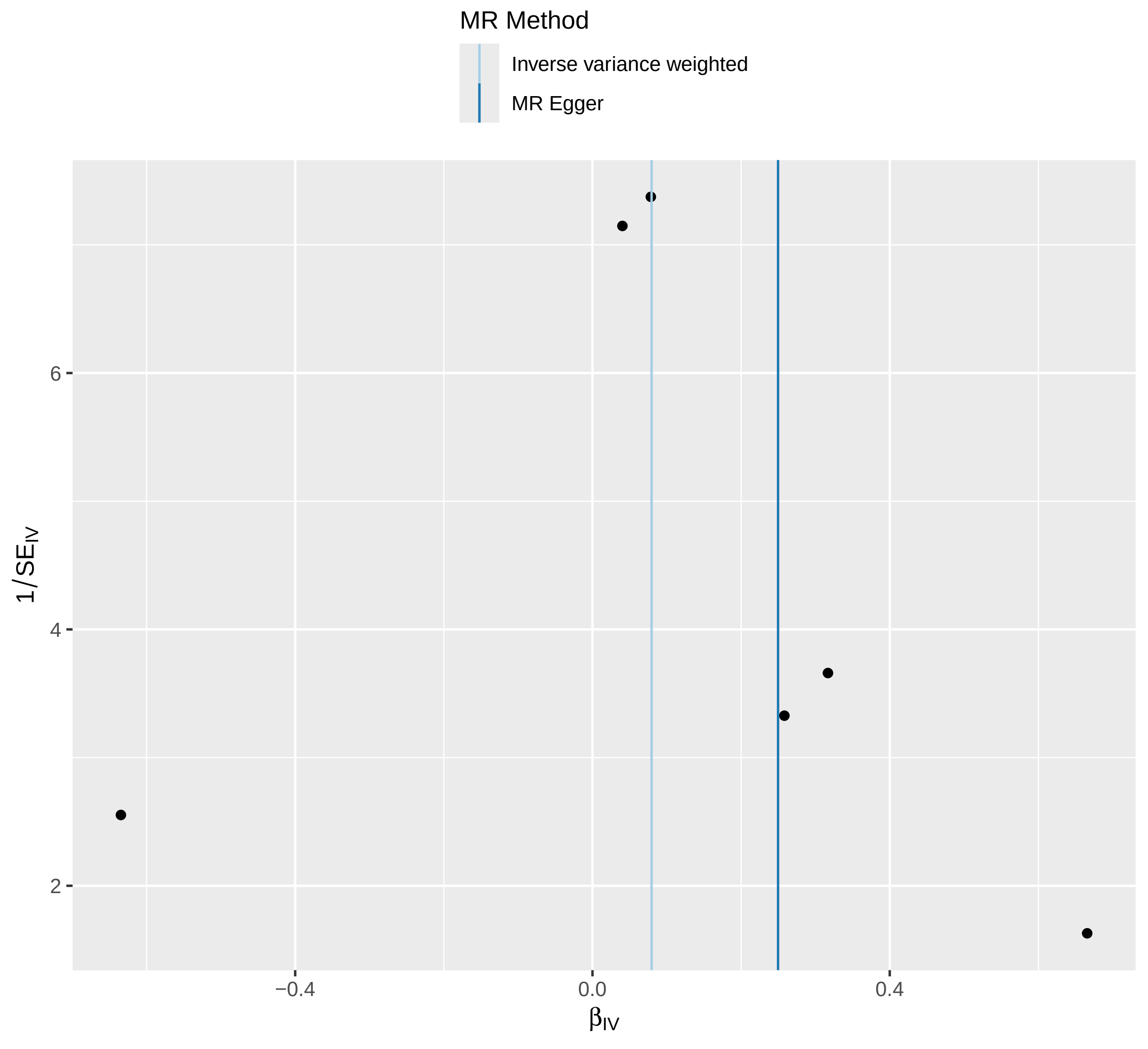

MR Method
Inverse variance weighted
MR Egger
6
4
2
1/SE_IV
−0.4
0.0
0.4
β_IV

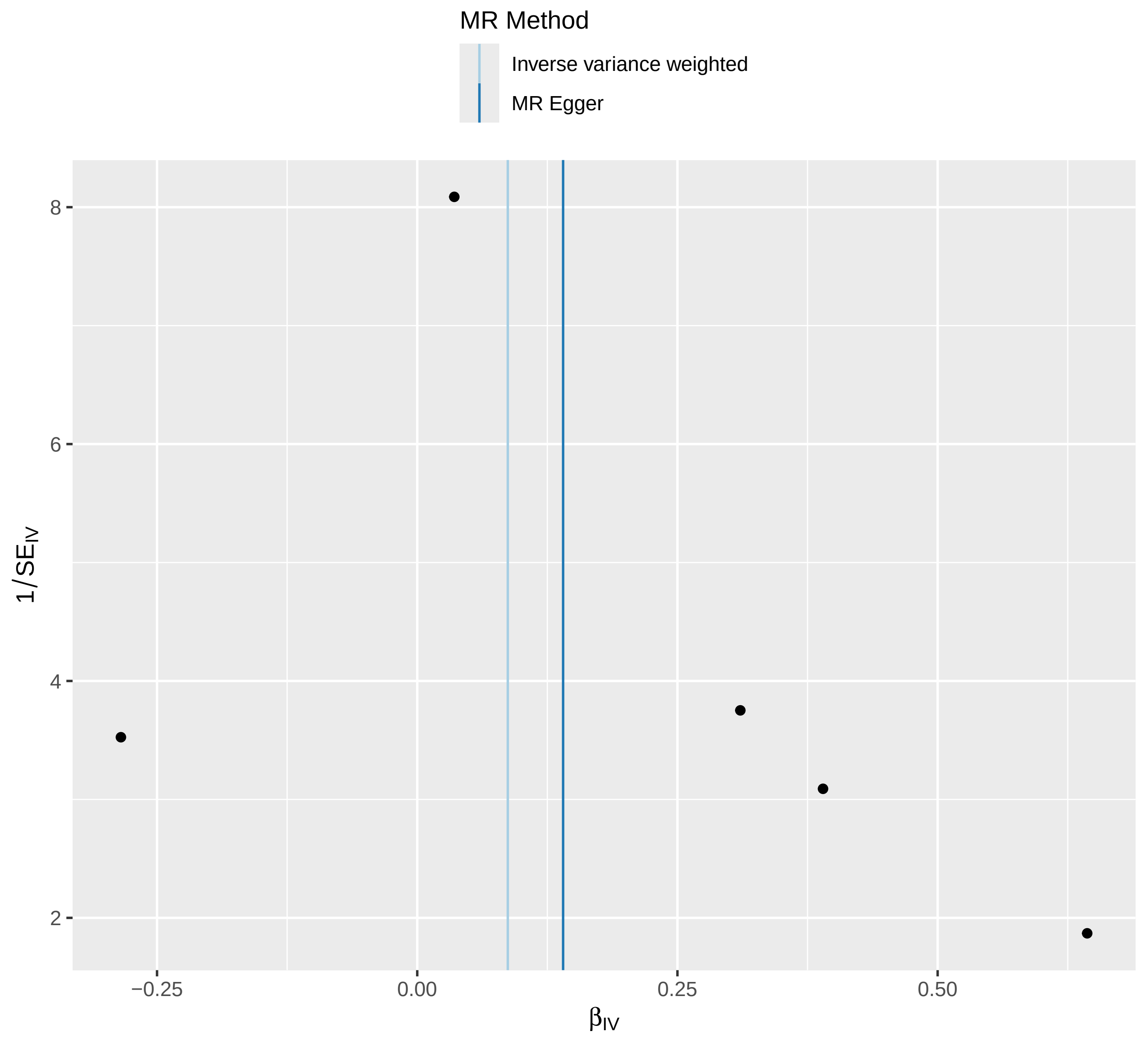
MR Method
Inverse variance weighted
MR Egger
8
6
4
2
−0.25
0.00
0.25
0.50
1/SE_IV
β_IV

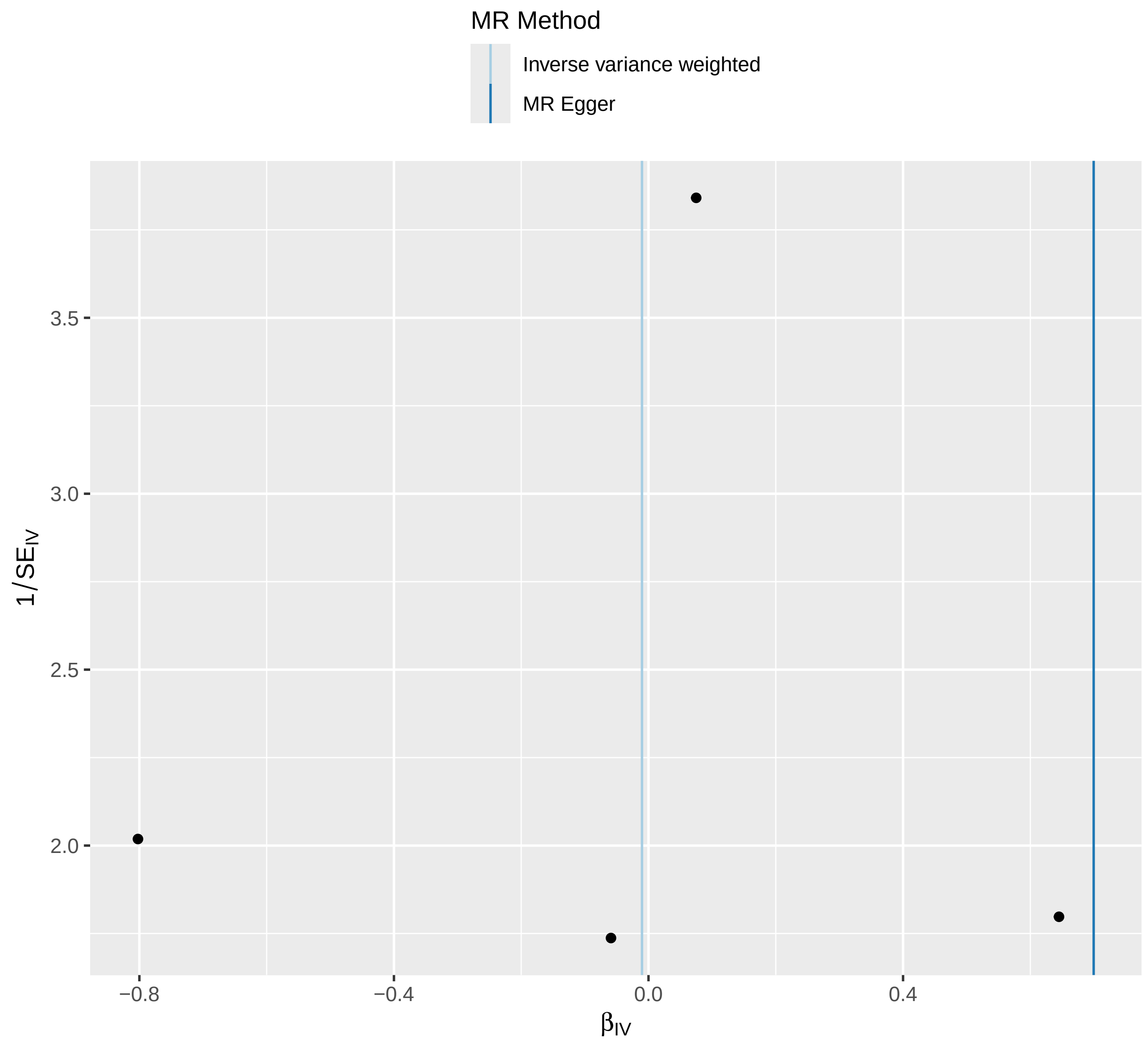
MR Method
Inverse variance weighted
MR Egger
3.5
3.0
2.5
2.0
1/SE_IV
−0.8
−0.4
0.0
0.4
β_IV

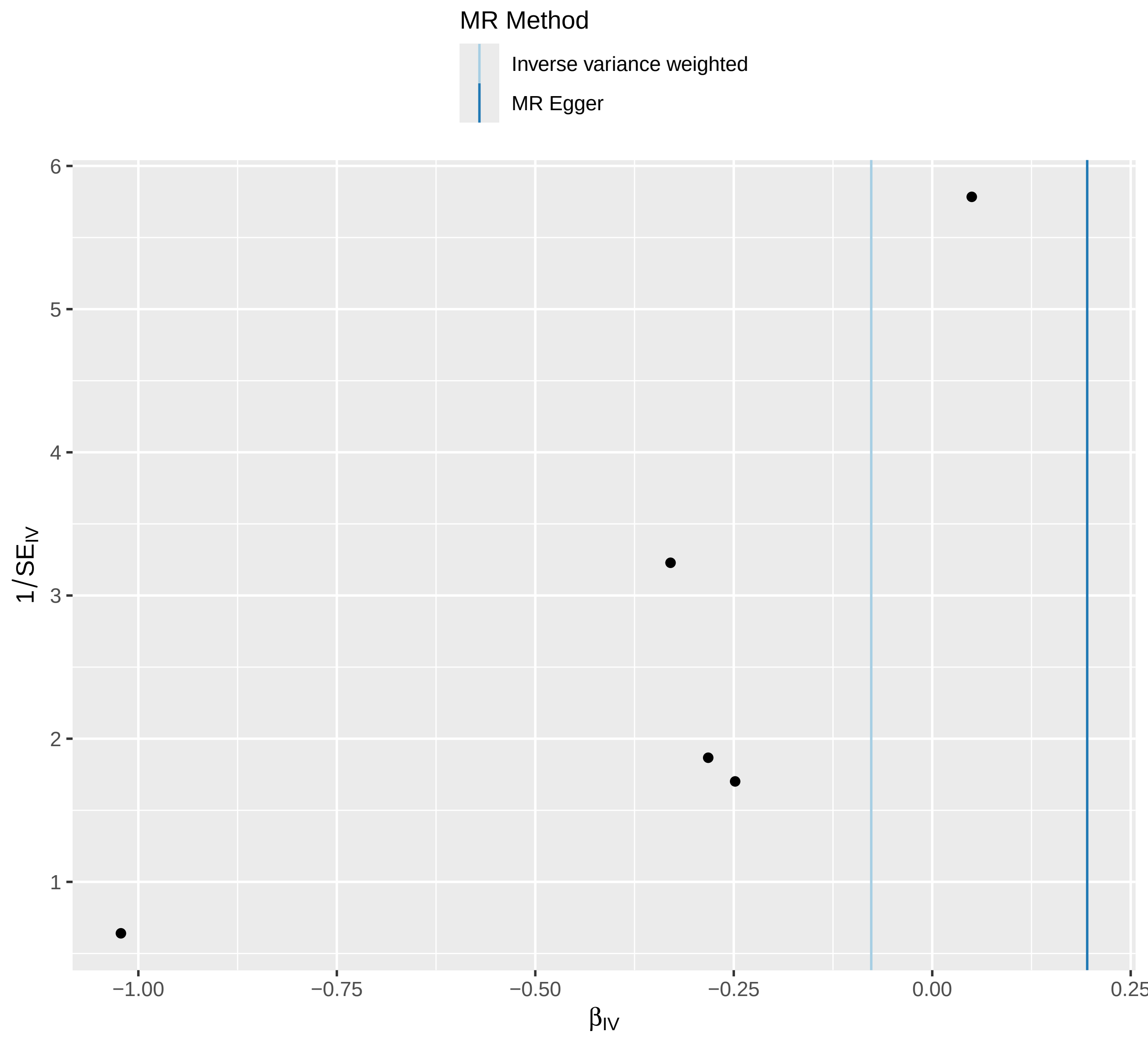
MR Method
Inverse variance weighted
MR Egger
6
5
4
3
2
1
$1/SE_{IV}$
−1.00
−0.75
−0.50
−0.25
0.00
0.25
β_{IV}

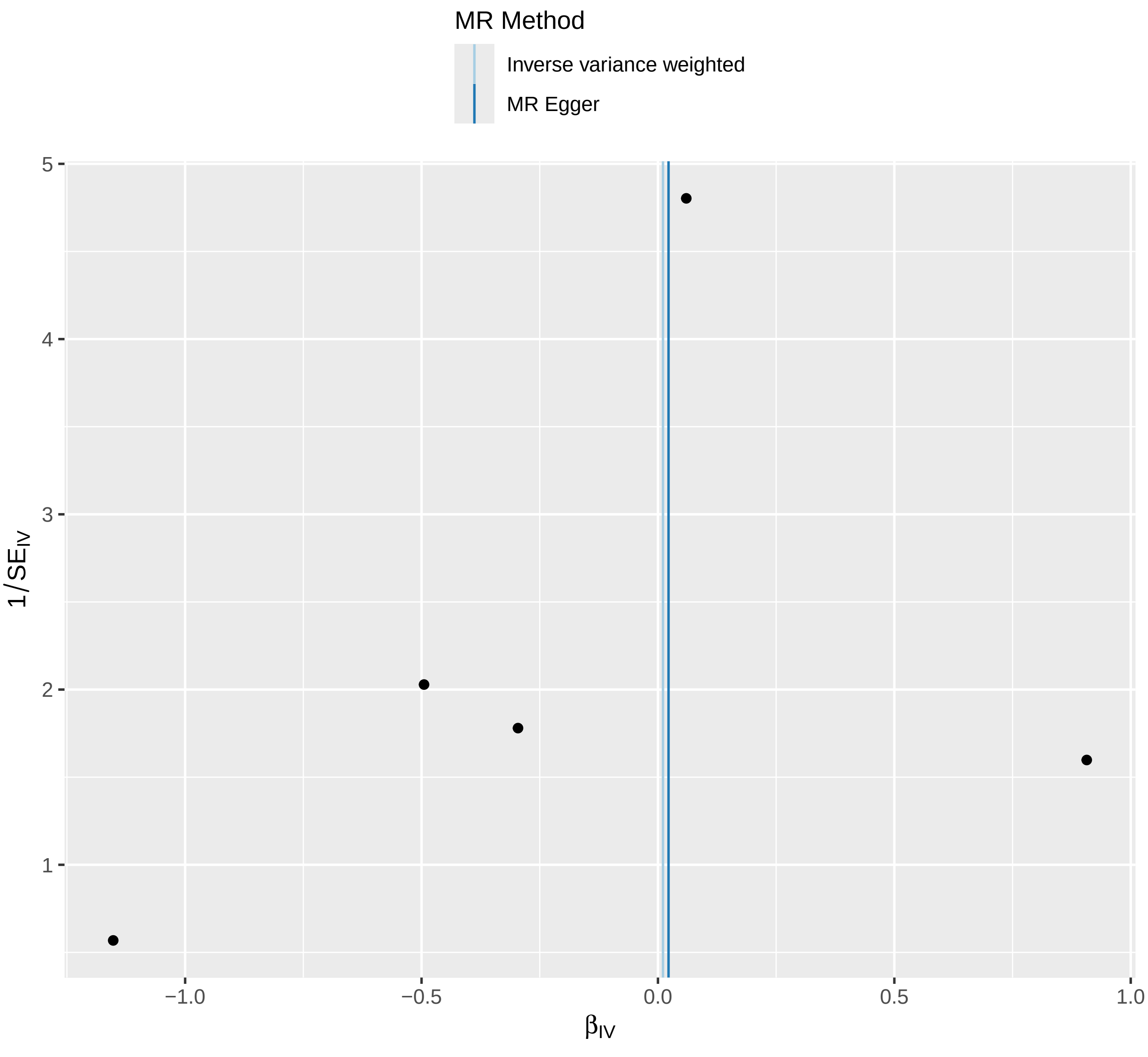

MR Method
Inverse variance weighted
MR Egger
1/SE$_{IV}$
5
4
3
2
1
β$_{IV}$
−1.0
−0.5
0.0
0.5
1.0

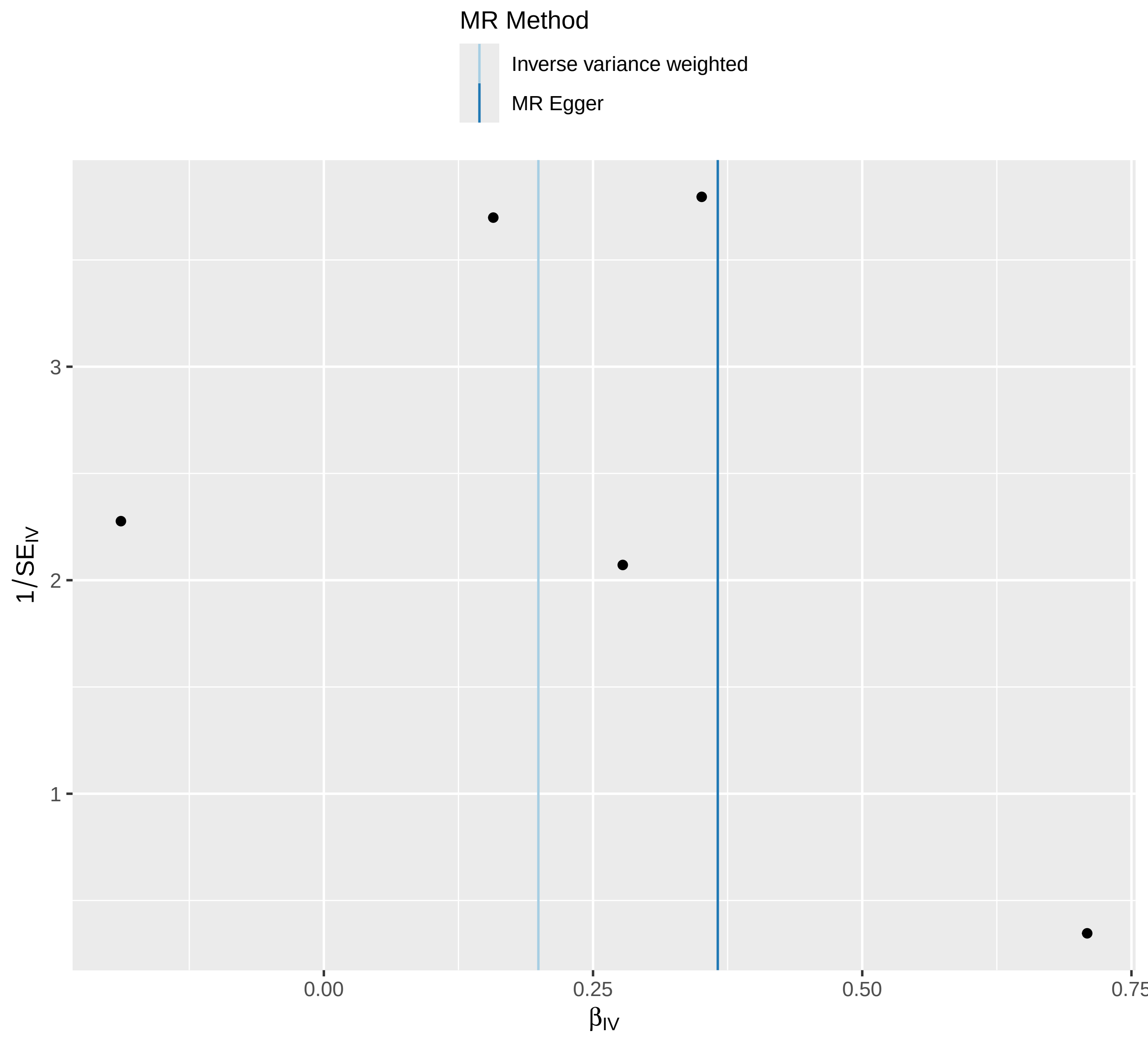
MR Method
Inverse variance weighted
MR Egger
1/SE_{IV}
3
2
1
0.00
0.25
0.50
0.75
β_{IV}

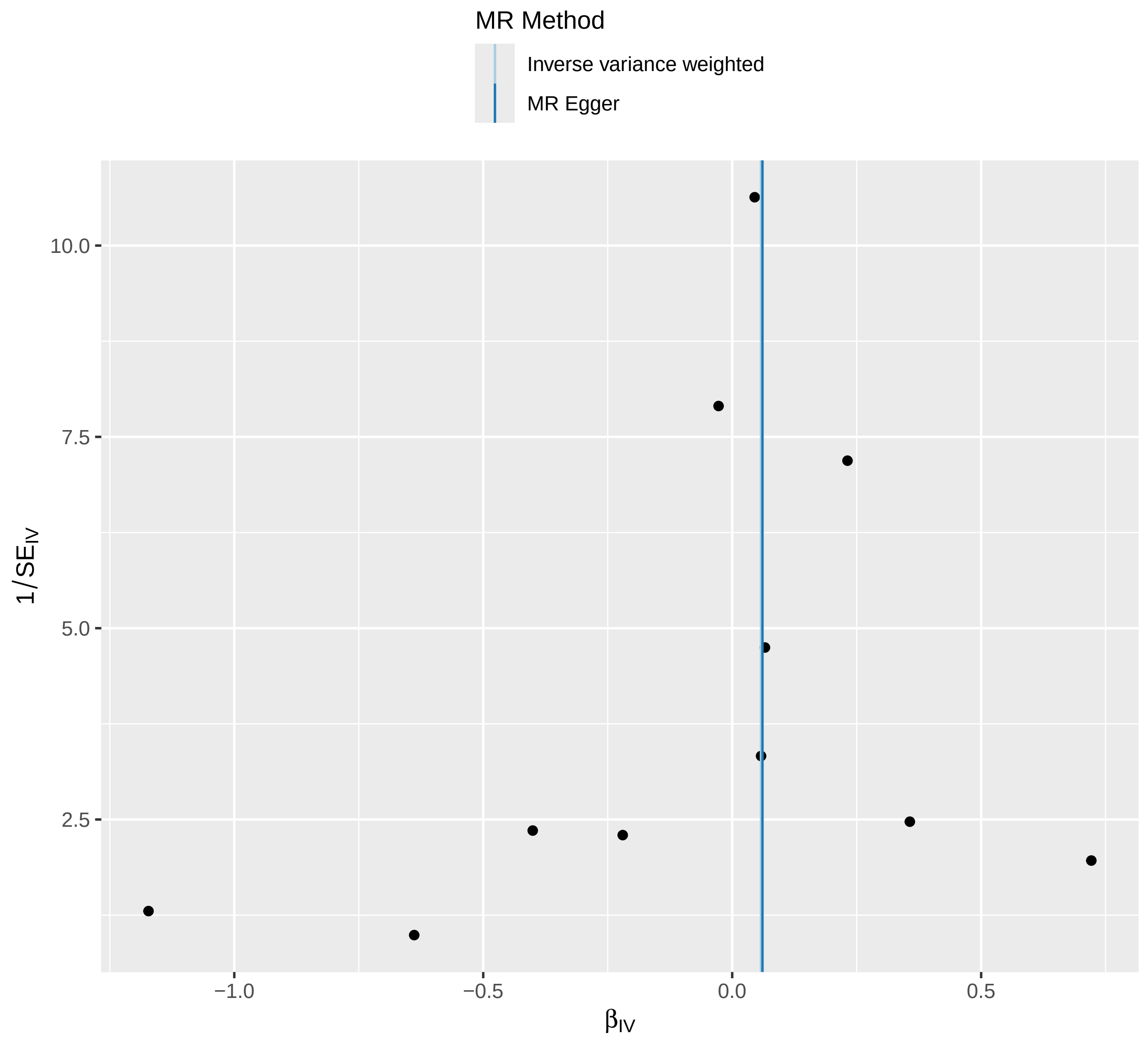
MR Method
Inverse variance weighted
MR Egger
10.0
7.5
5.0
2.5
1/SE_IV
−1.0
−0.5
0.0
0.5
β_IV

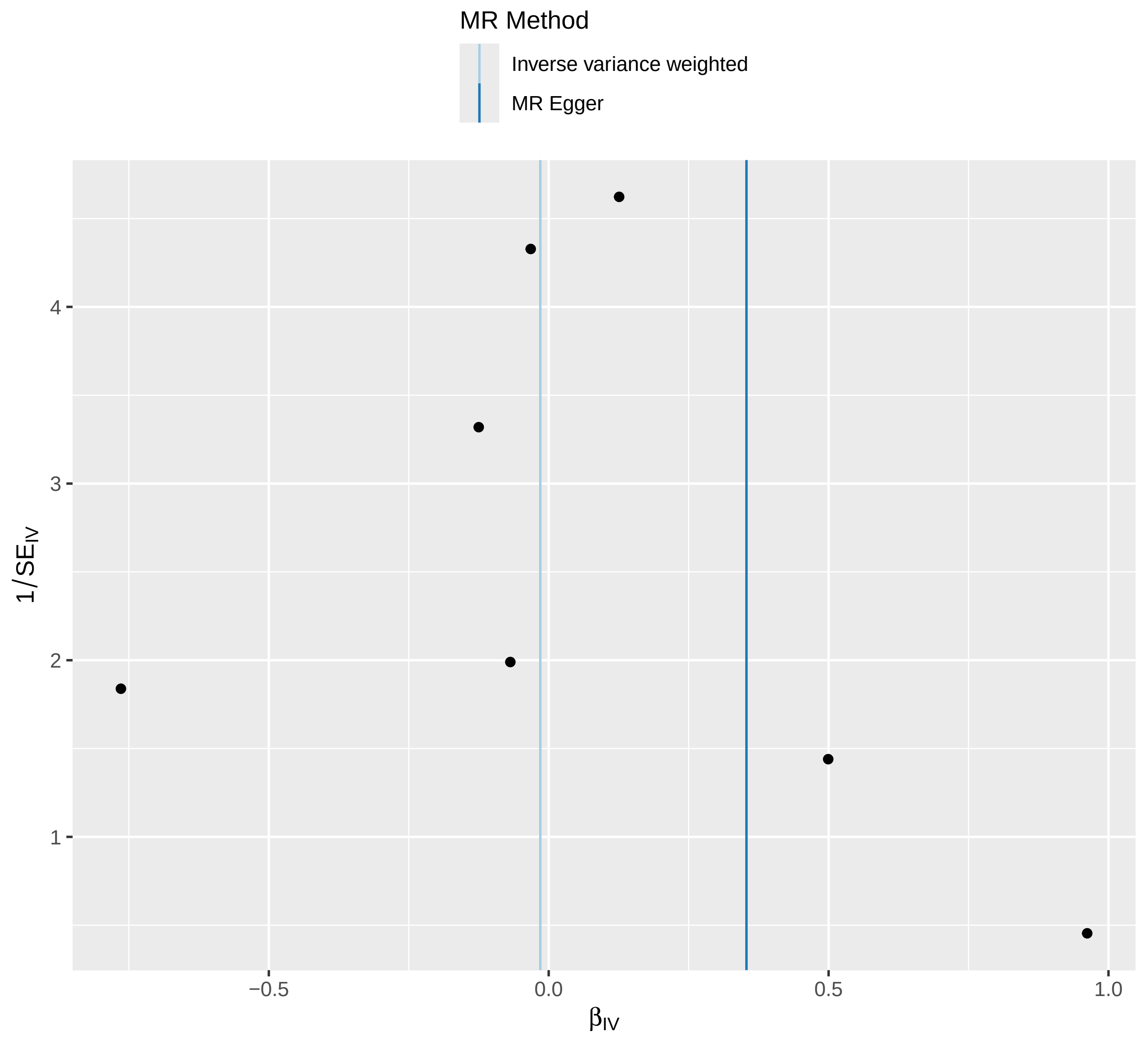

MR Method
Inverse variance weighted
MR Egger
4
3
2
1
1/SE_IV
−0.5
0.0
0.5
1.0
β_IV

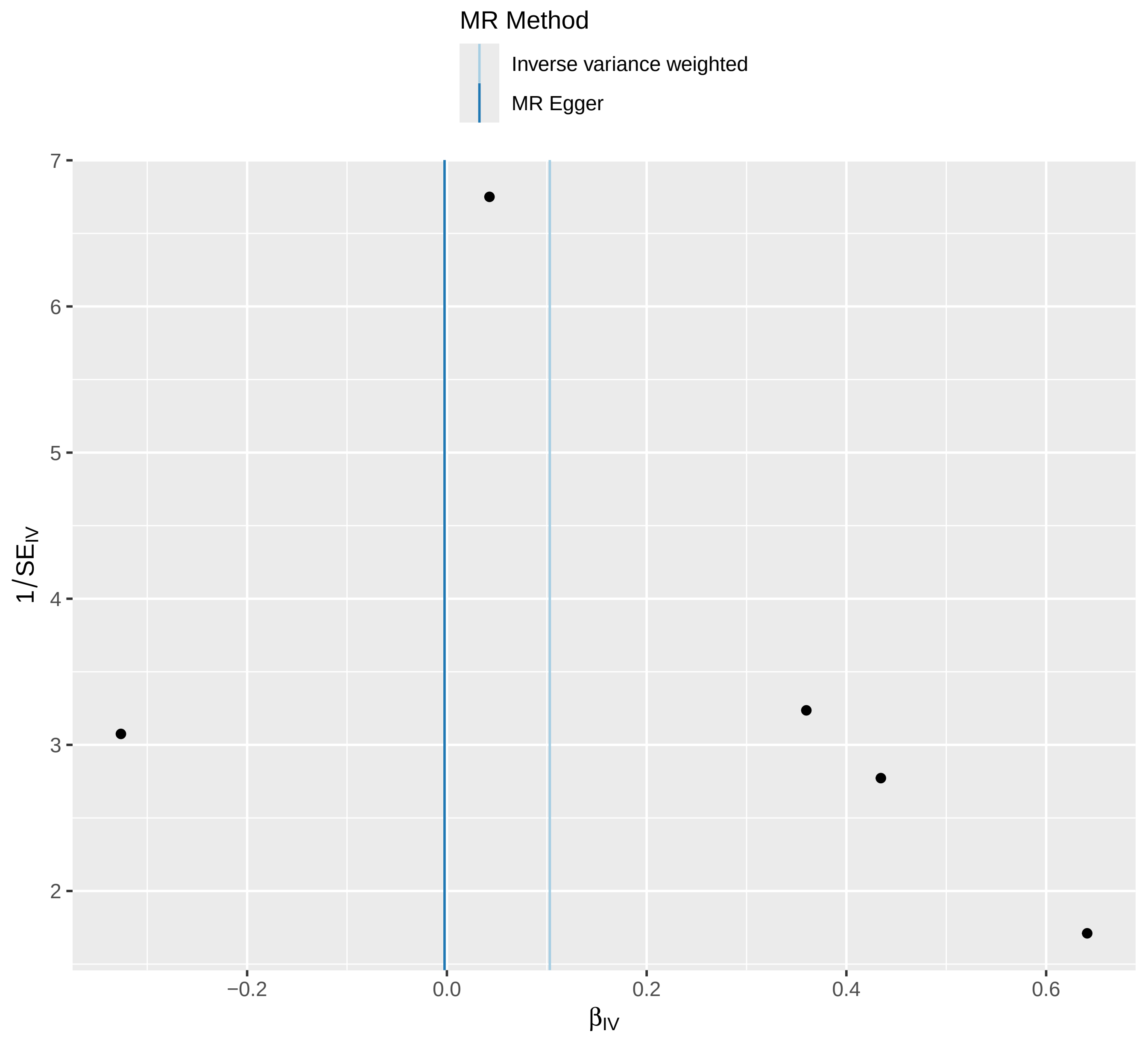

MR Method
Inverse variance weighted
MR Egger
7
6
5
4
3
2
1/SE_IV
−0.2
0.0
0.2
0.4
0.6
β_IV

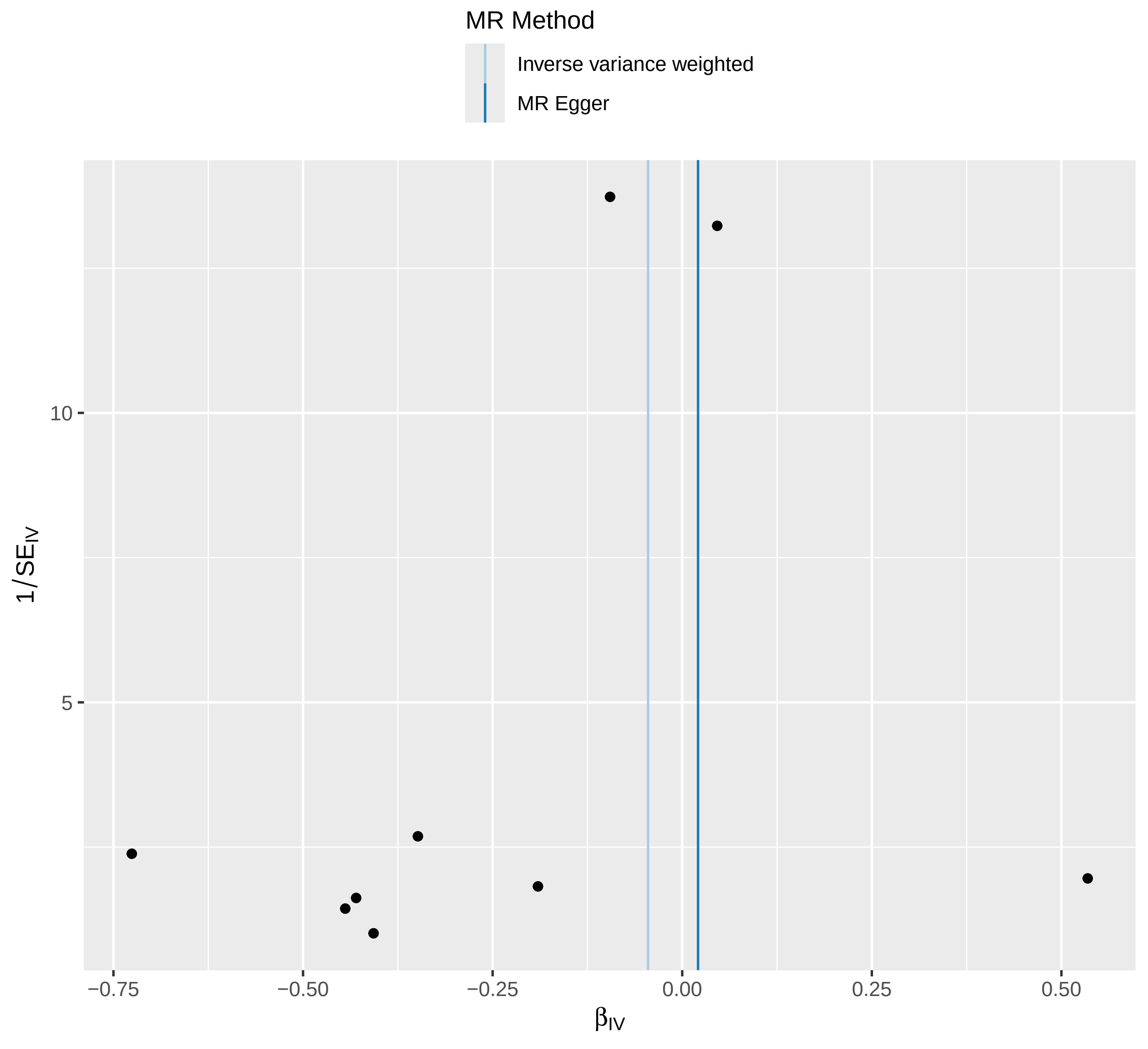
MR Method
Inverse variance weighted
MR Egger
10
5
1/SE_IV
−0.75
−0.50
−0.25
0.00
0.25
0.50
β_IV

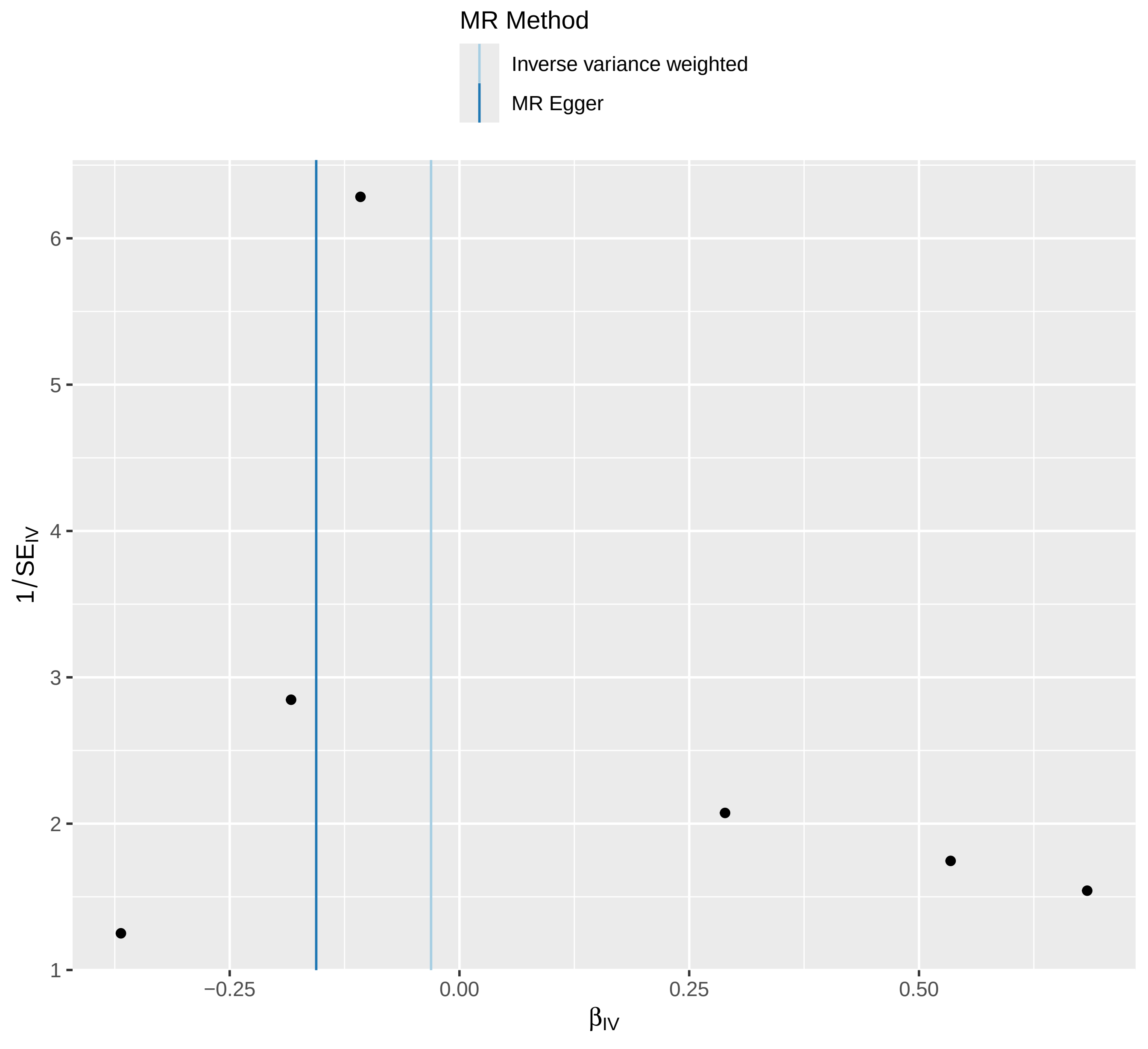

MR Method
Inverse variance weighted
MR Egger
6
5
4
3
2
1
1/SE_IV
−0.25
0.00
0.25
0.50
β_IV

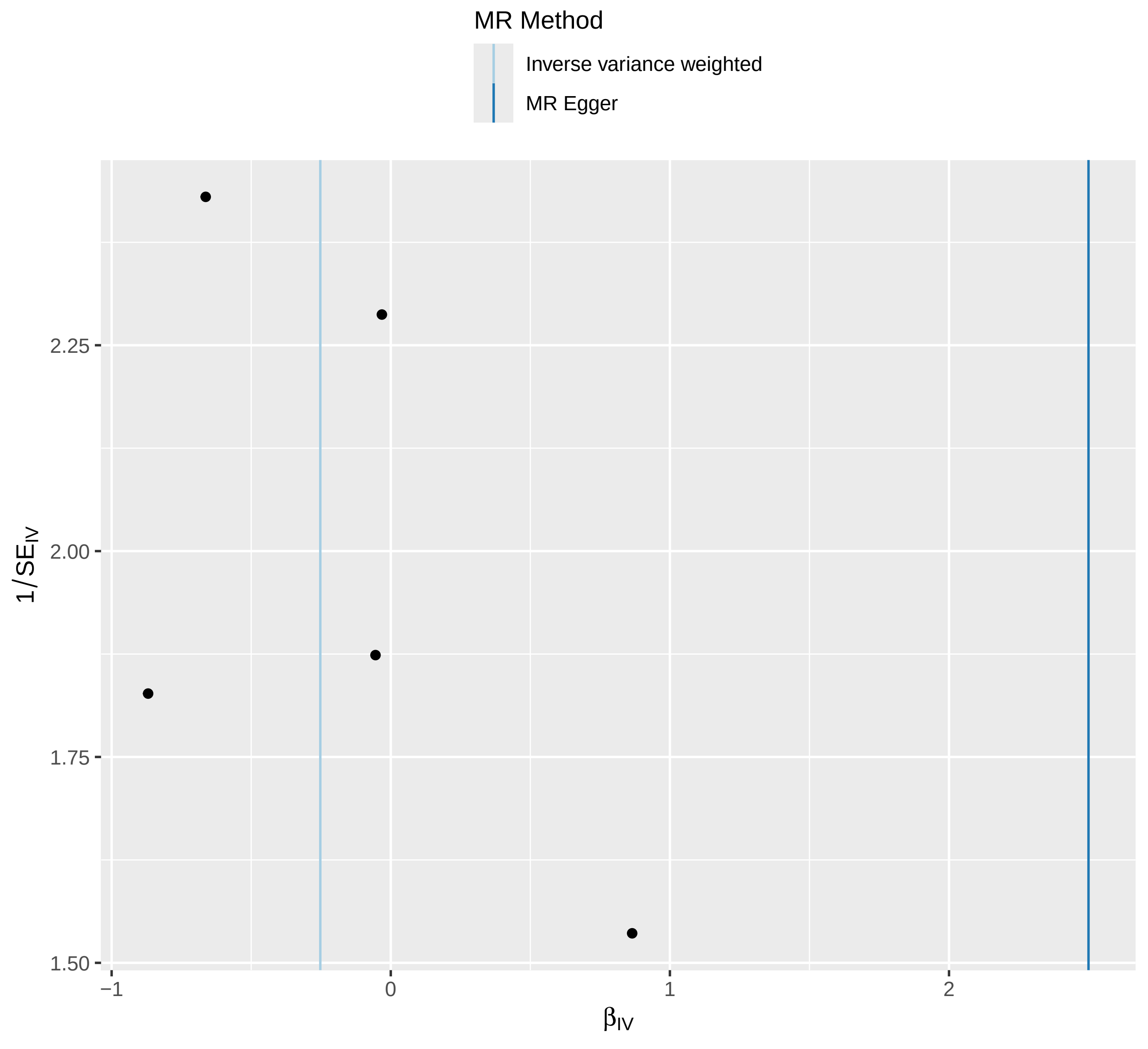

MR Method
Inverse variance weighted
MR Egger
2.25
2.00
1.75
1.50
1/SE$_{IV}$
−1
0
1
2
β$_{IV}$

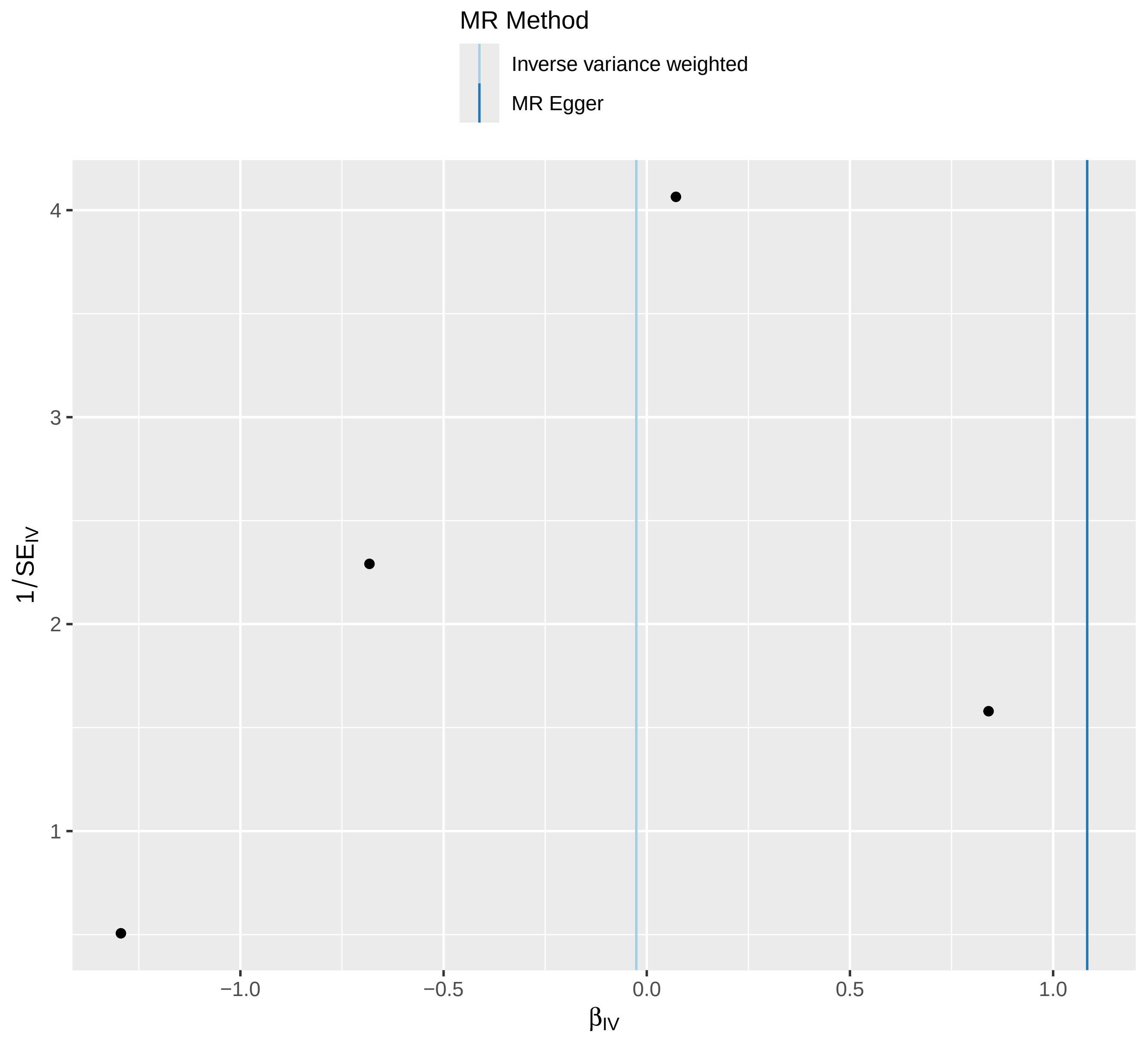
MR Method
Inverse variance weighted
MR Egger
1/SE_IV
4
3
2
1
−1.0
−0.5
0.0
0.5
1.0
β_IV

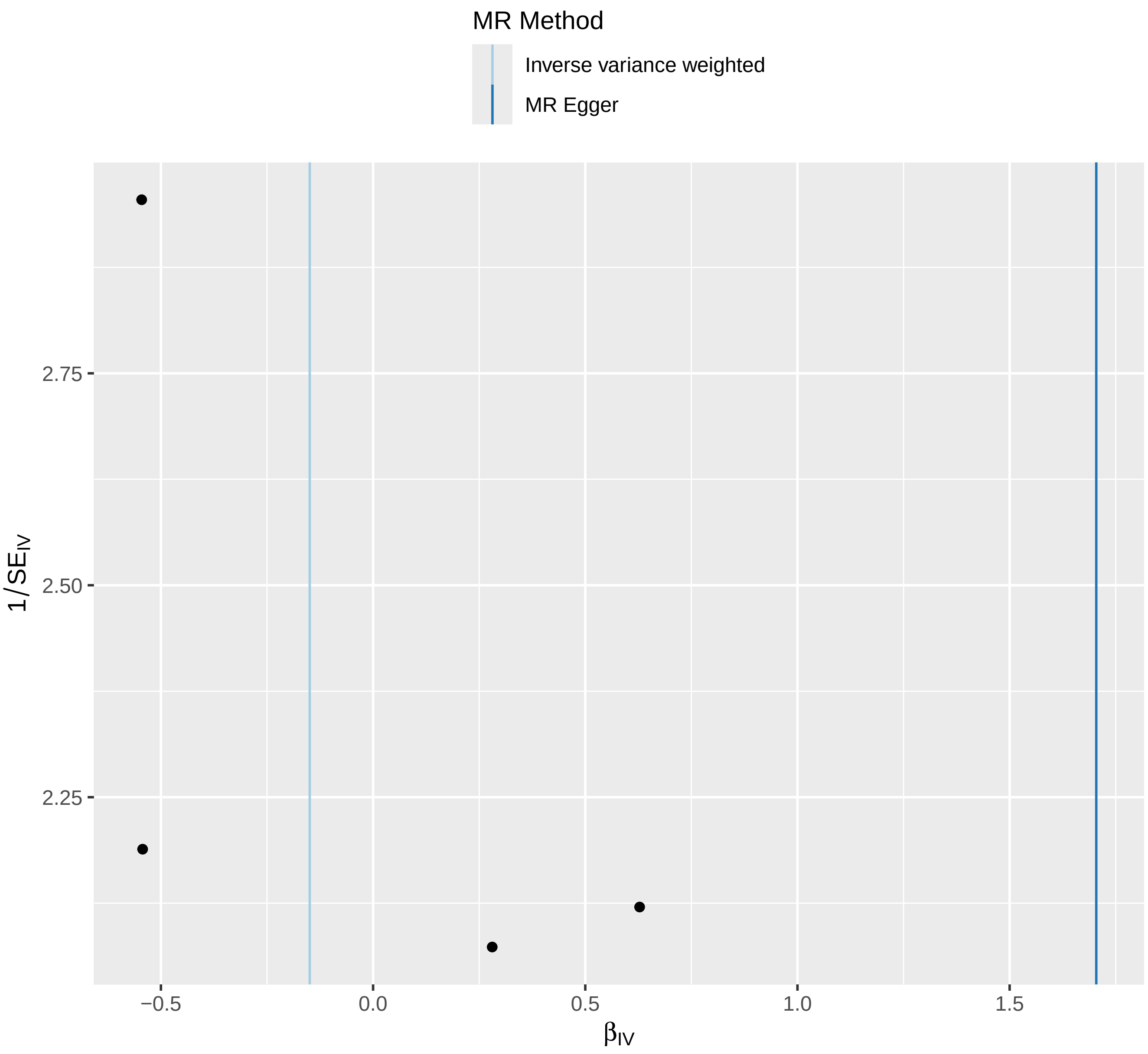
MR Method
Inverse variance weighted
MR Egger
2.75
2.50
2.25
−0.5
0.0
0.5
1.0
1.5
$1/SE_{IV}$
β_{IV}

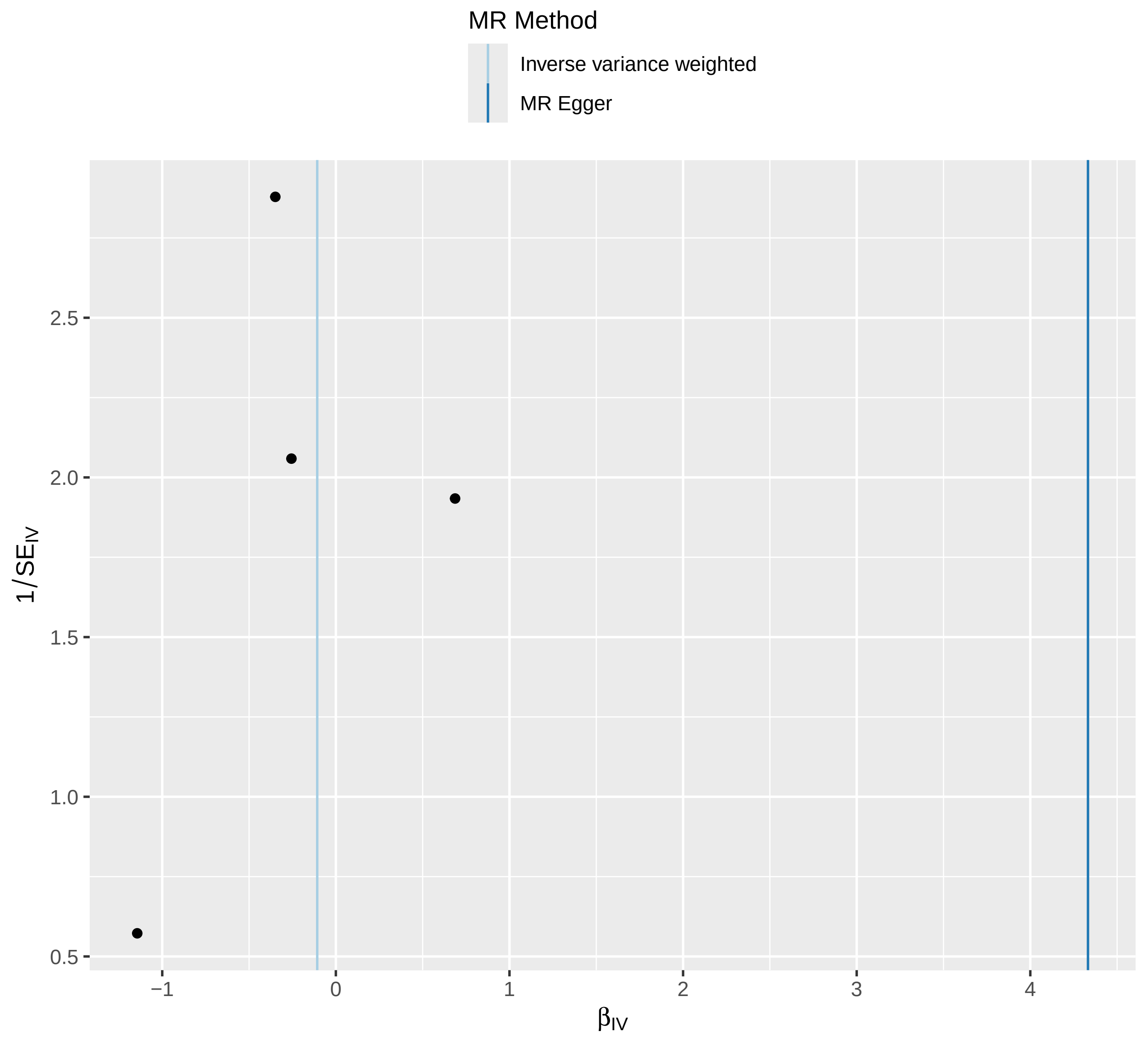
MR Method
Inverse variance weighted
MR Egger
2.5
2.0
1.5
1.0
0.5
1/SE_IV
−1
0
1
2
3
4
β_IV

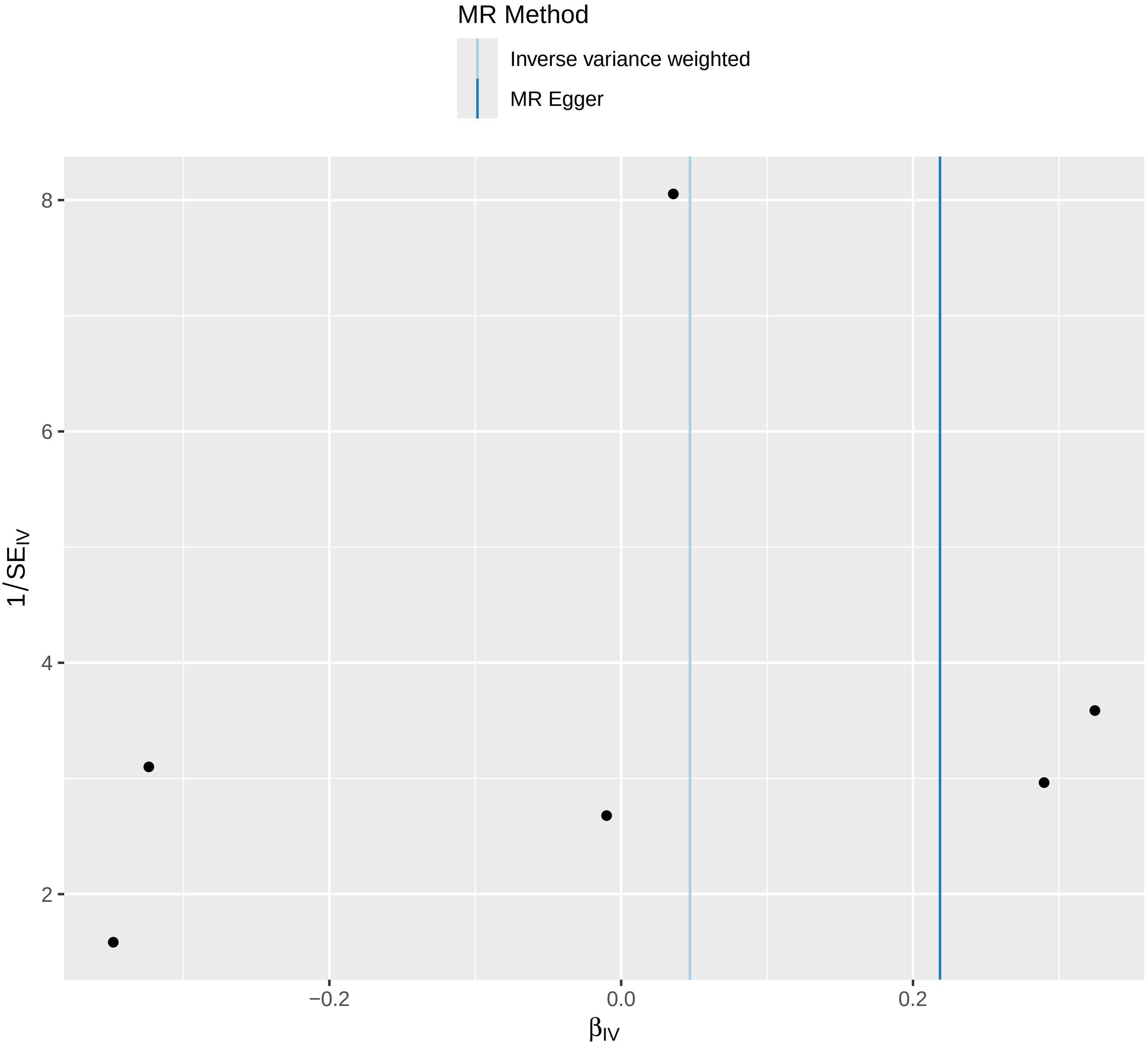

MR Method
Inverse variance weighted
MR Egger
8
6
4
2
1/SE_IV
−0.2
0.0
0.2
β_IV

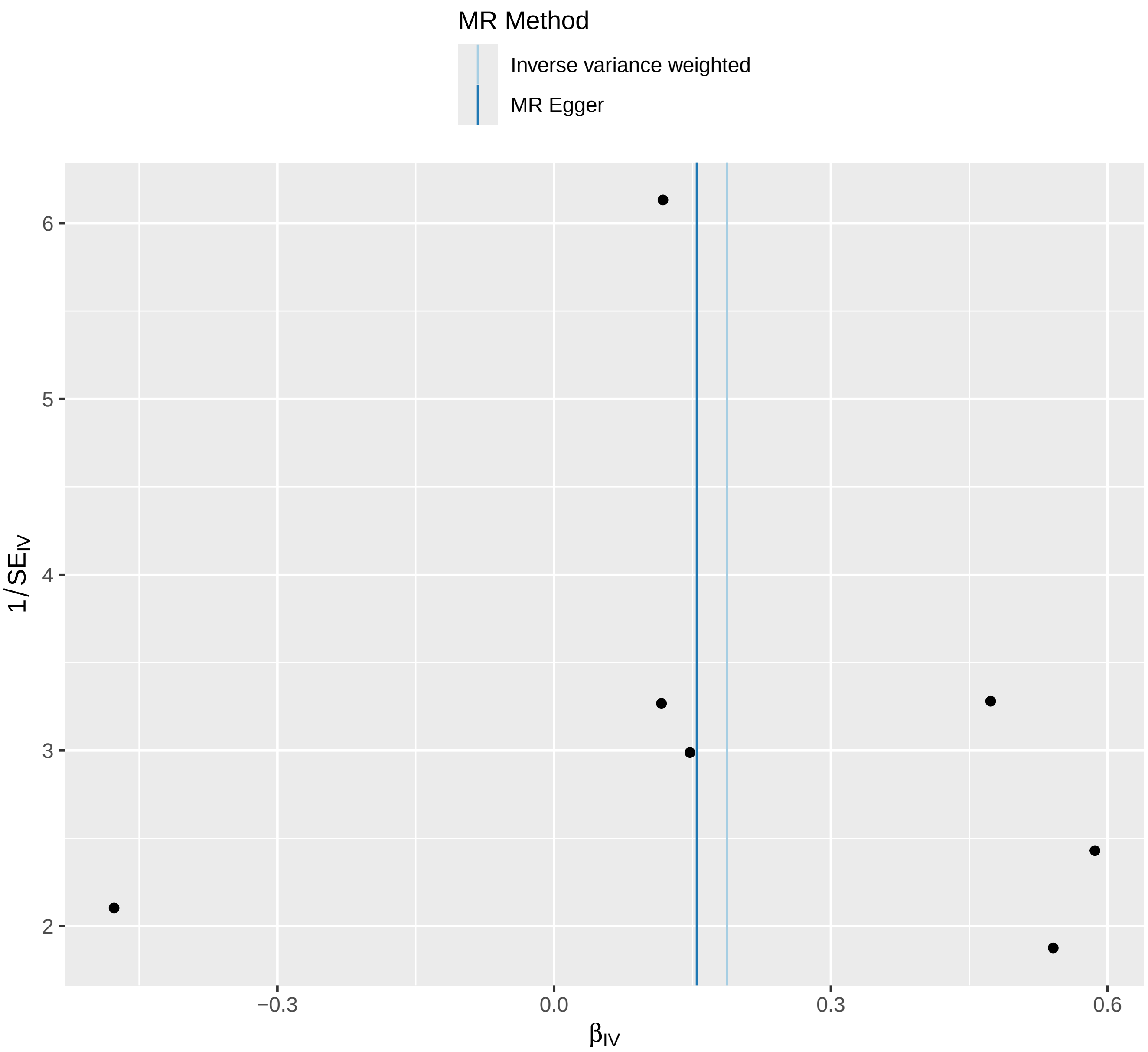

MR Method
Inverse variance weighted
MR Egger
1/SE_IV
6
5
4
3
2
−0.3
0.0
0.3
0.6
β_IV

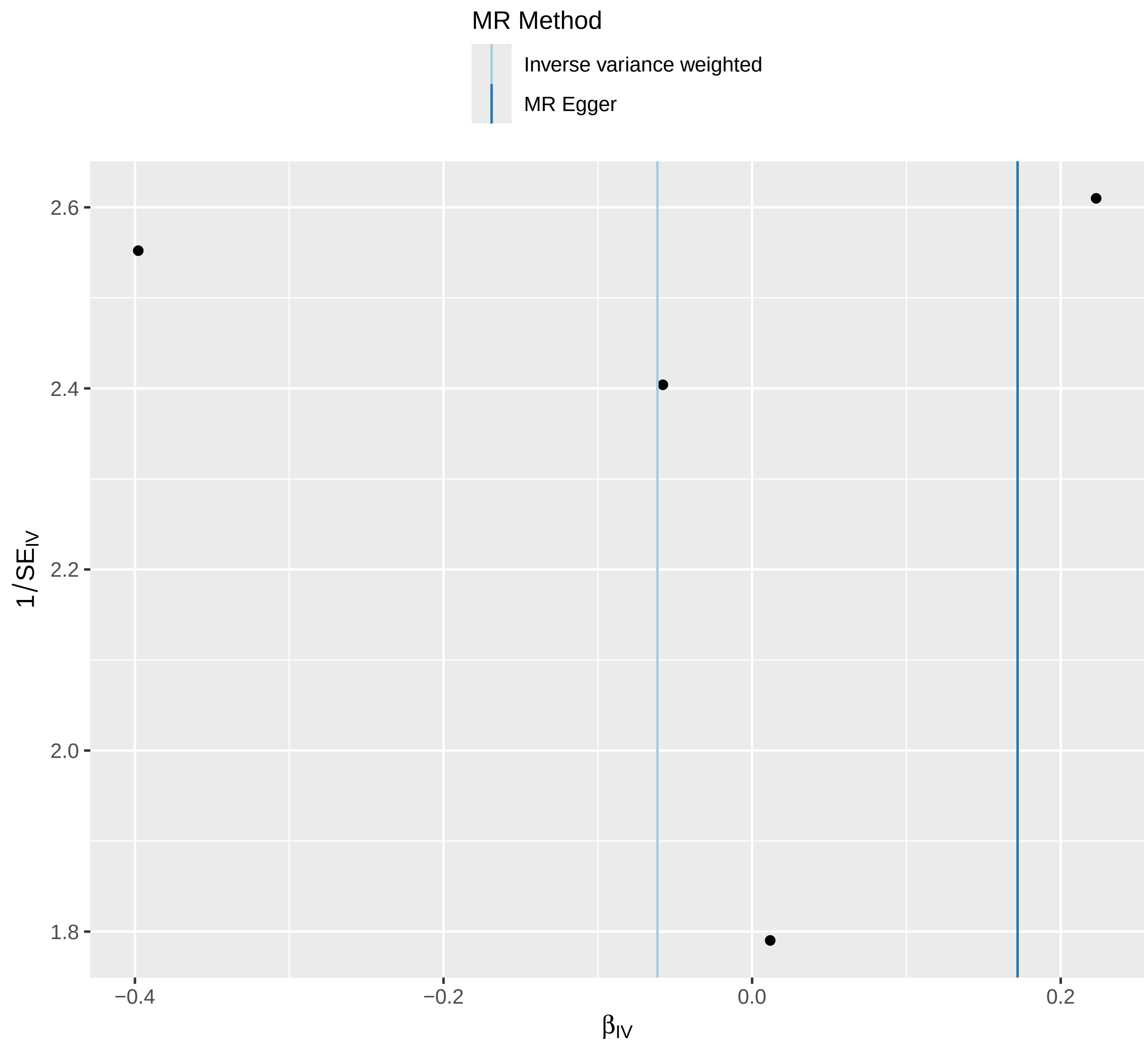
MR Method
Inverse variance weighted
MR Egger
2.6
2.4
2.2
2.0
1.8
$1/SE_{IV}$
−0.4
−0.2
0.0
0.2
β_{IV}

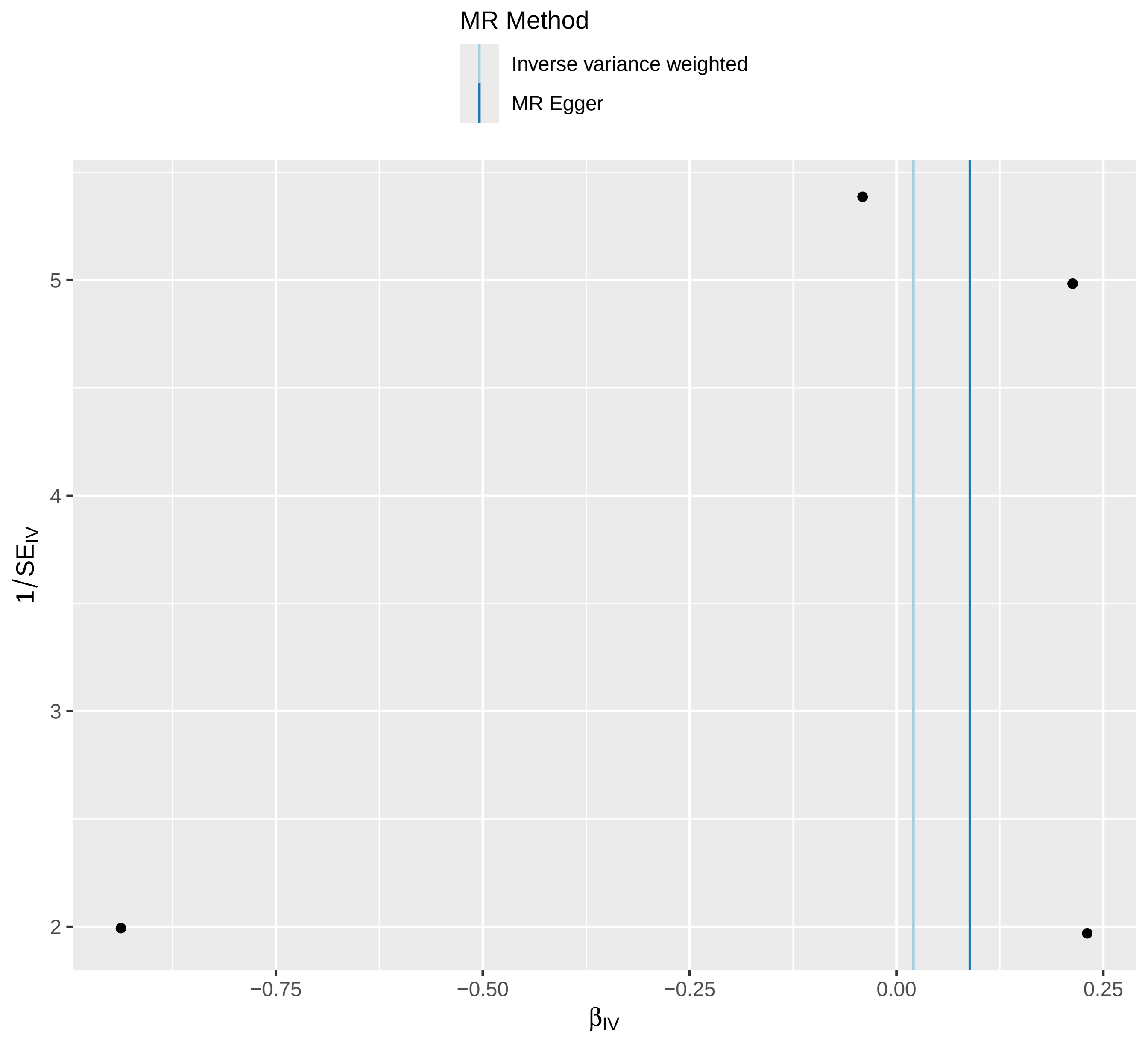
MR Method
Inverse variance weighted
MR Egger
5
4
3
2
1/SE_IV
−0.75
−0.50
−0.25
0.00
0.25
β_IV

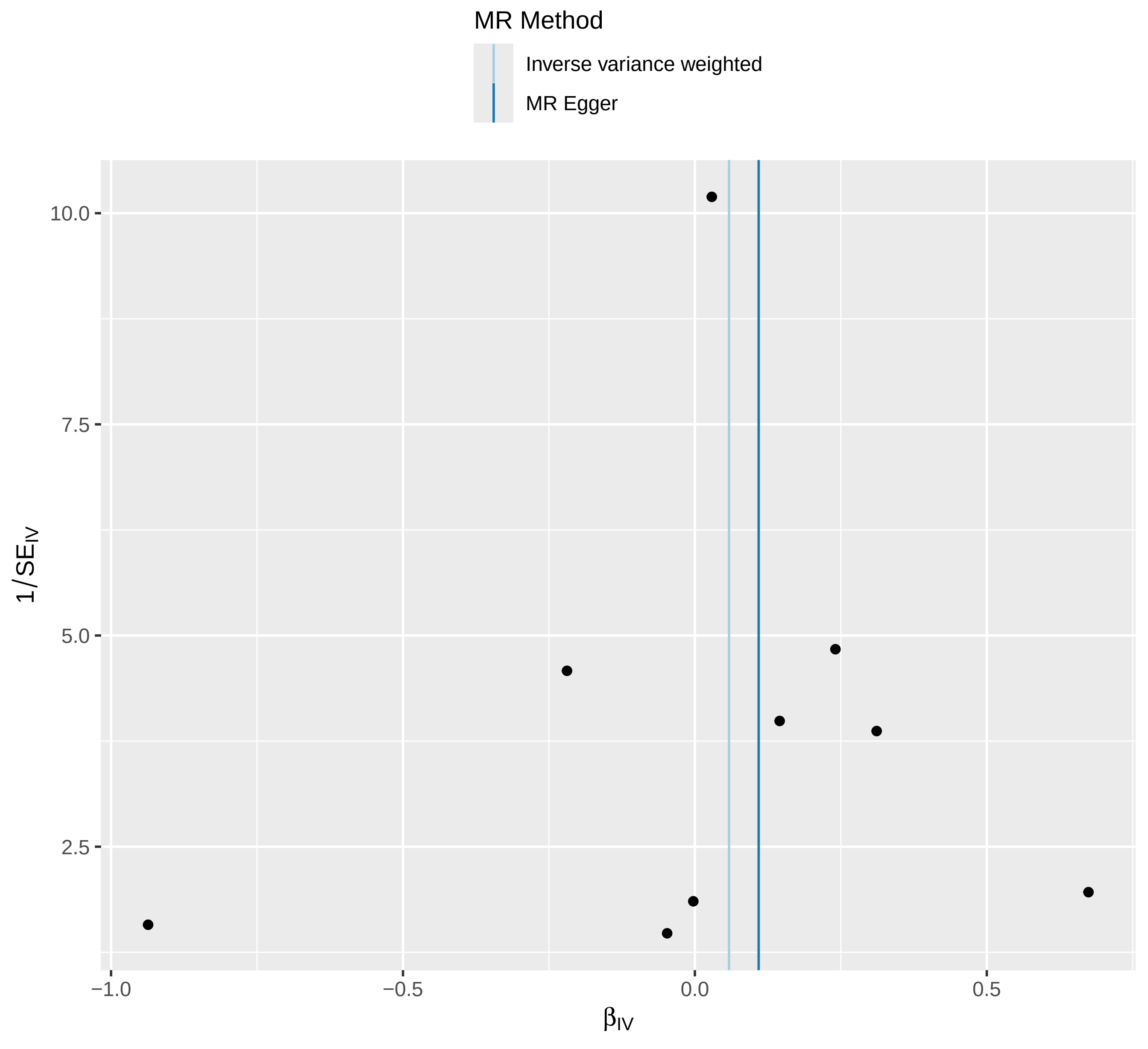

MR Method
Inverse variance weighted
MR Egger
10.0
7.5
5.0
2.5
$1/SE_{IV}$
−1.0
−0.5
0.0
0.5
β_{IV}

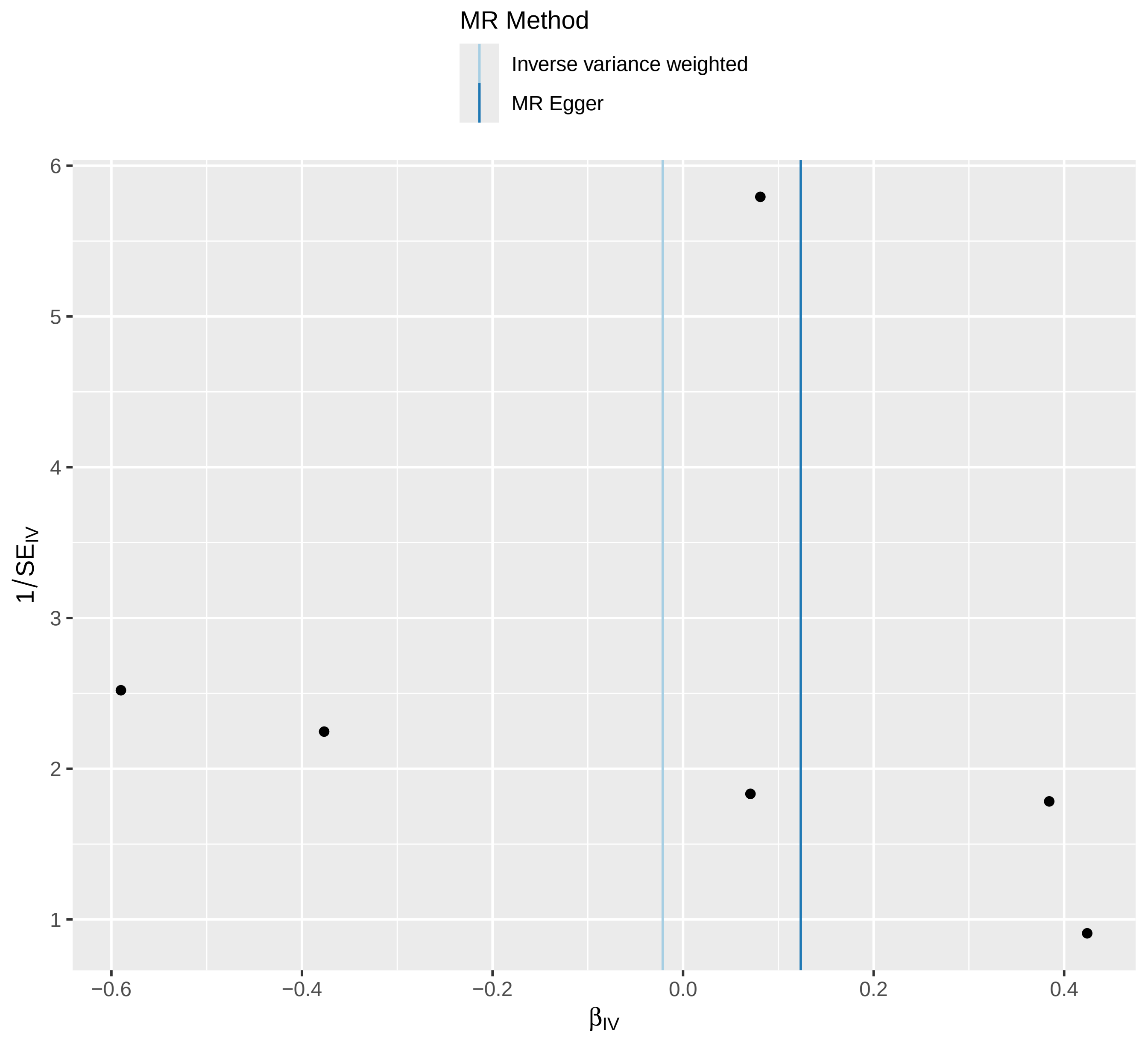
MR Method
Inverse variance weighted
MR Egger
6
5
4
3
2
1
1/SE_IV
−0.6
−0.4
−0.2
0.0
0.2
0.4
β_IV

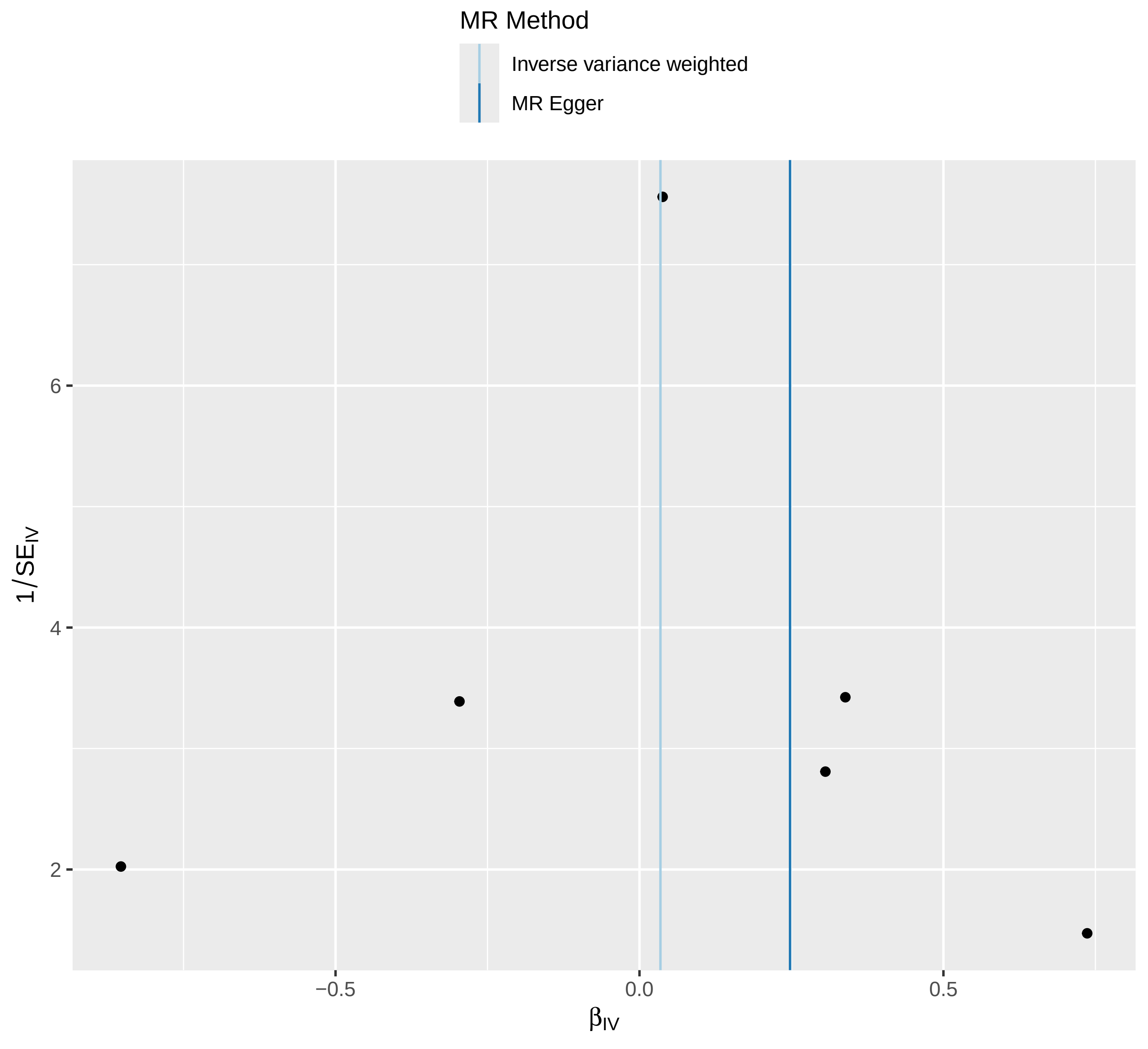

MR Method
Inverse variance weighted
MR Egger
6
4
2
1/SE_IV
−0.5
0.0
0.5
β_IV

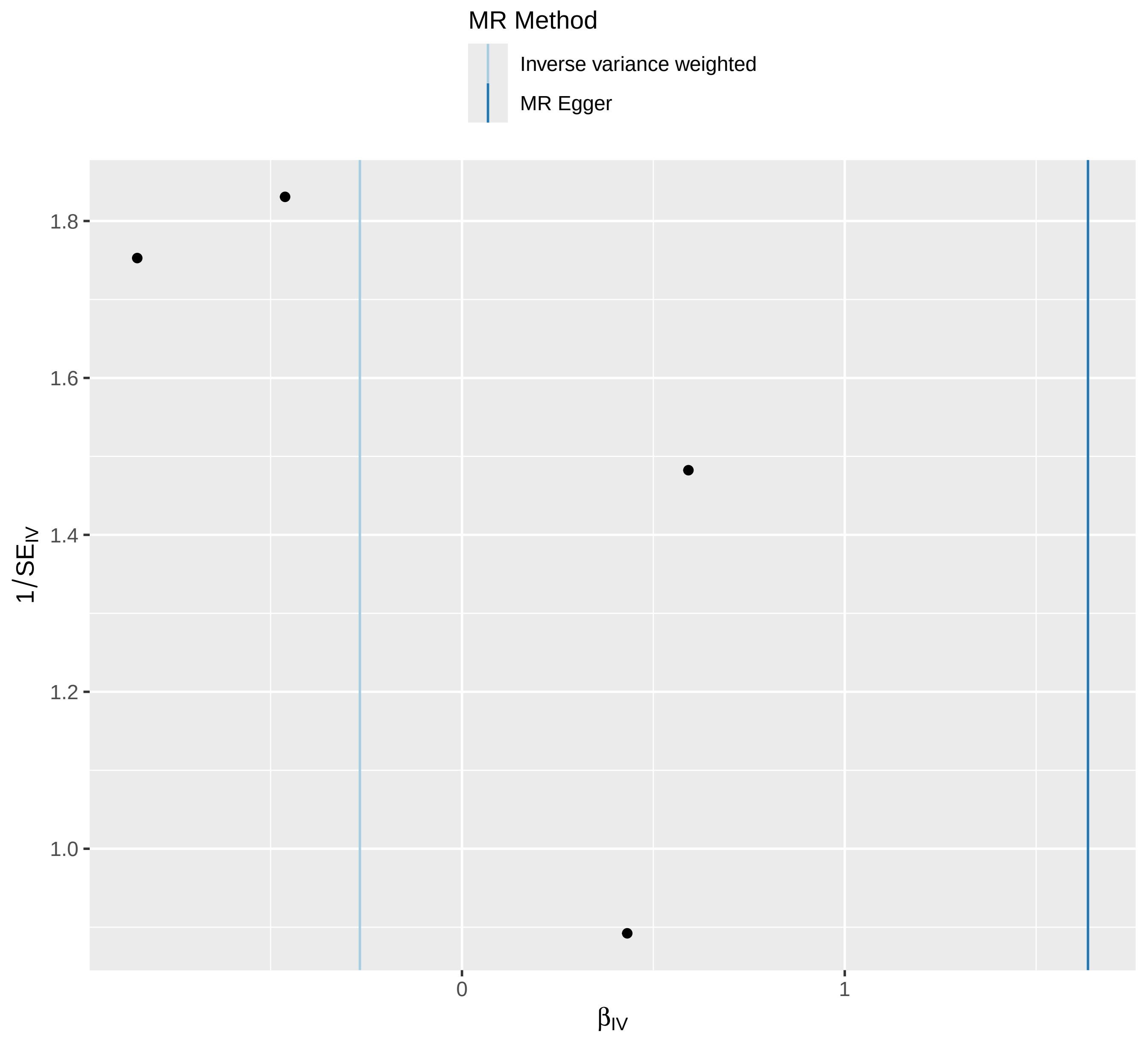
MR Method
Inverse variance weighted
MR Egger
1.8
1.6
1.4
1.2
1.0
1/SE_IV
0
1
β_IV

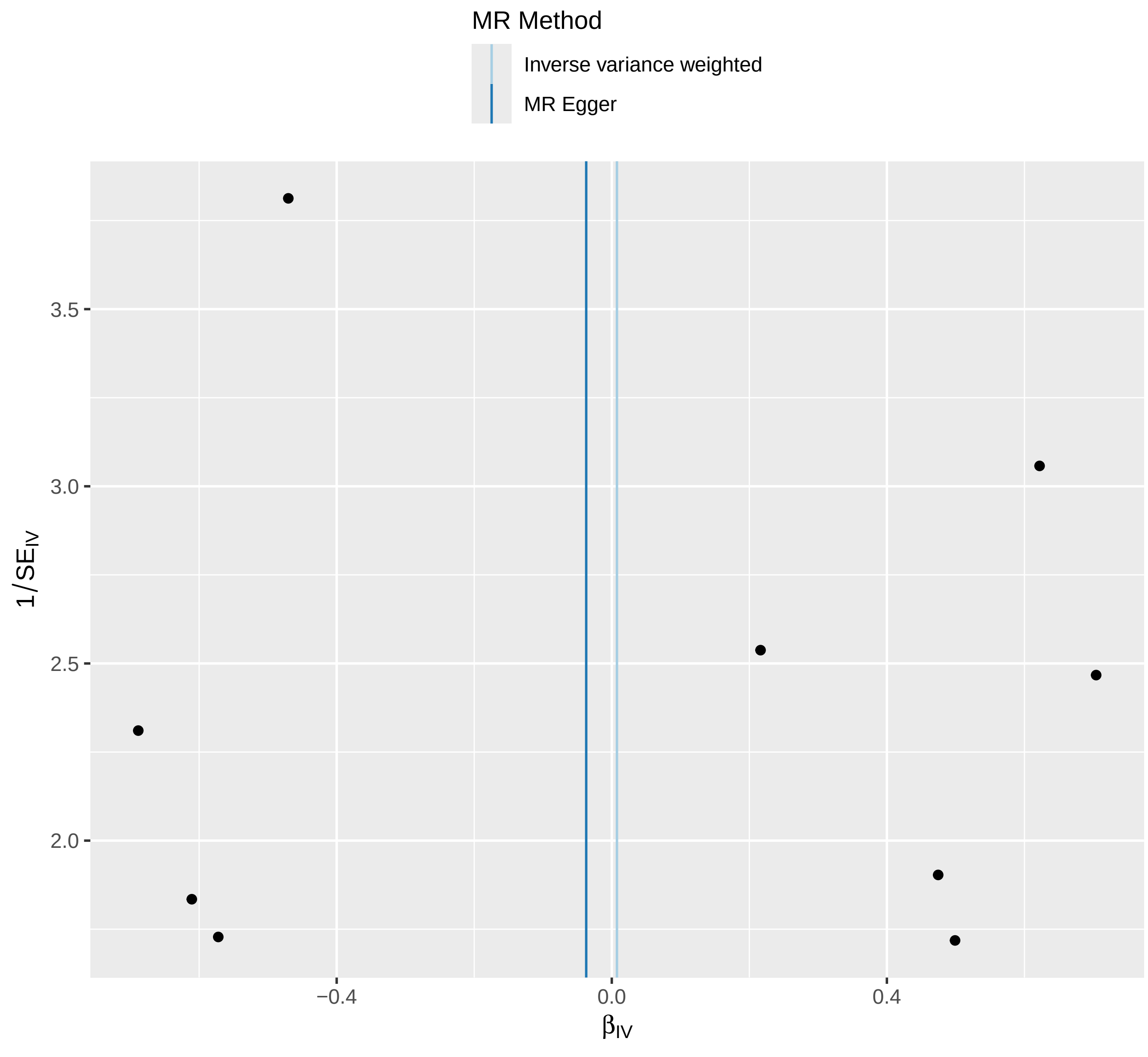
MR Method
Inverse variance weighted
MR Egger
3.5
3.0
2.5
2.0
$1/SE_{IV}$
−0.4
0.0
0.4
β_{IV}

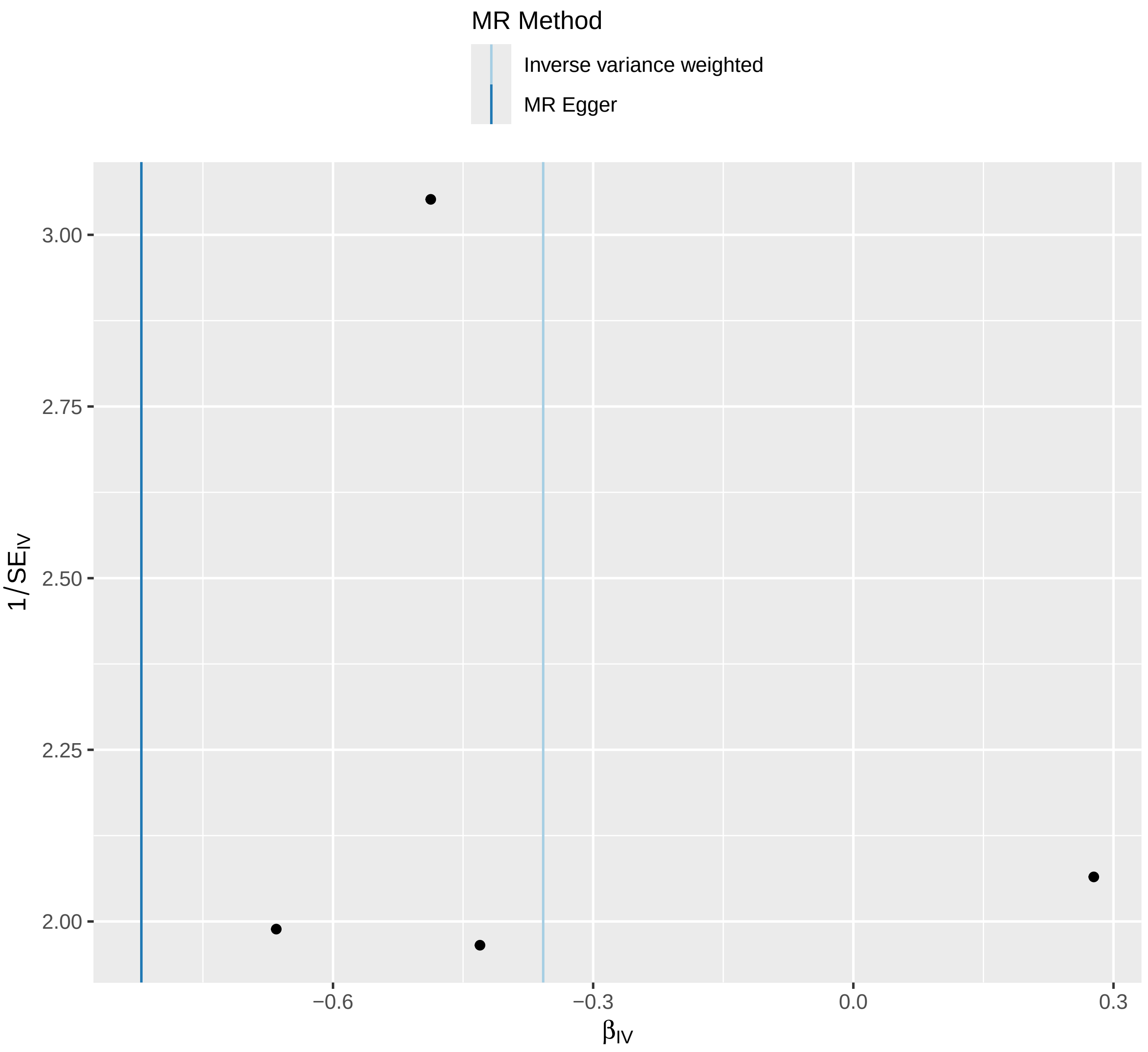

MR Method
Inverse variance weighted
MR Egger
3.00
2.75
2.50
2.25
2.00
1/SE$_{IV}$
−0.6
−0.3
0.0
0.3
β$_{IV}$

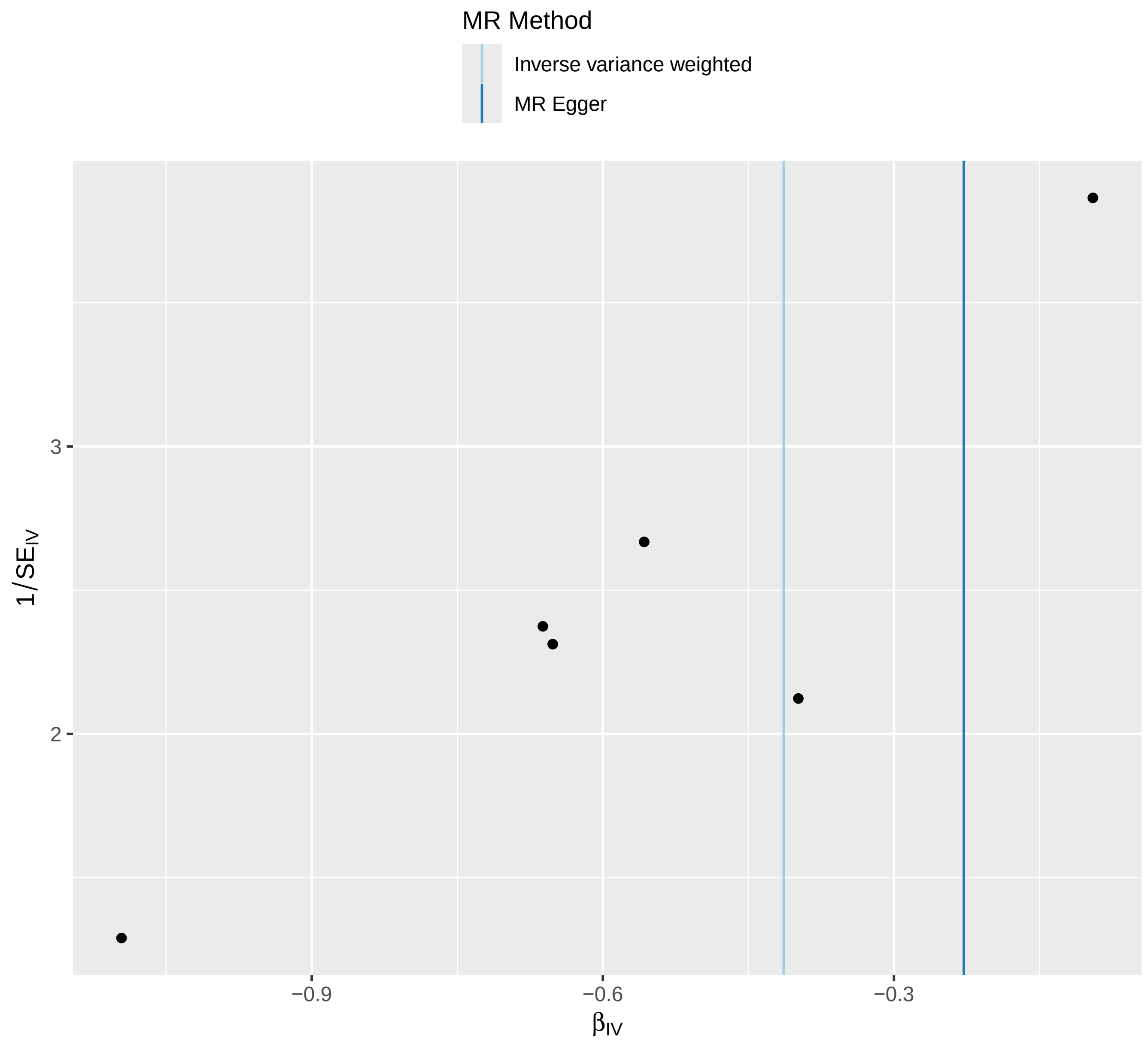

MR Method
Inverse variance weighted
MR Egger
3
2
1/SE_IV
−0.9
−0.6
−0.3
β_IV

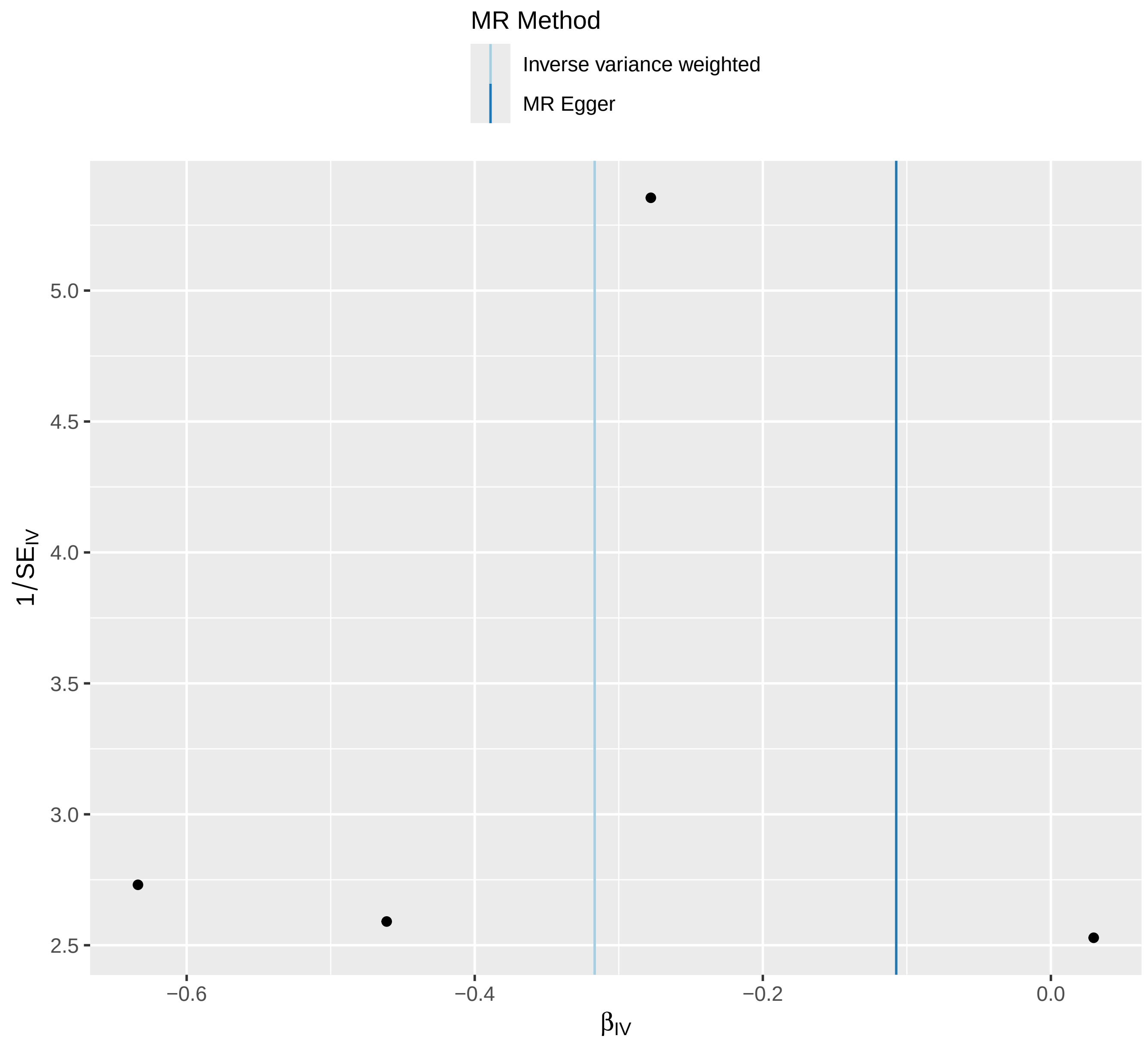
MR Method
Inverse variance weighted
MR Egger
5.0
4.5
4.0
3.5
3.0
2.5
$1/SE_{IV}$
−0.6
−0.4
−0.2
0.0
β_{IV}

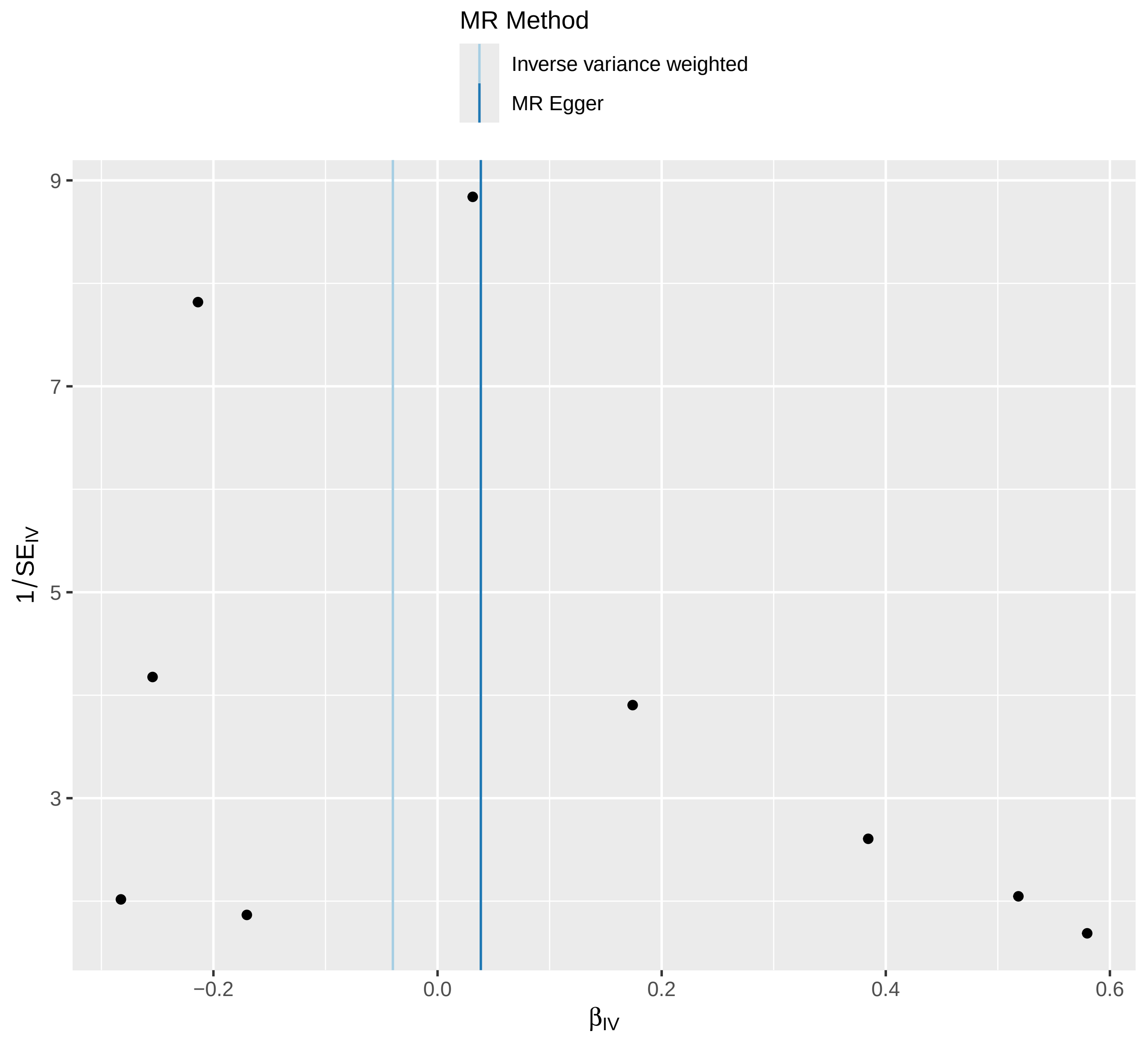

MR Method
Inverse variance weighted
MR Egger
9
7
5
3
1/SE_IV
−0.2
0.0
0.2
0.4
0.6
β_IV

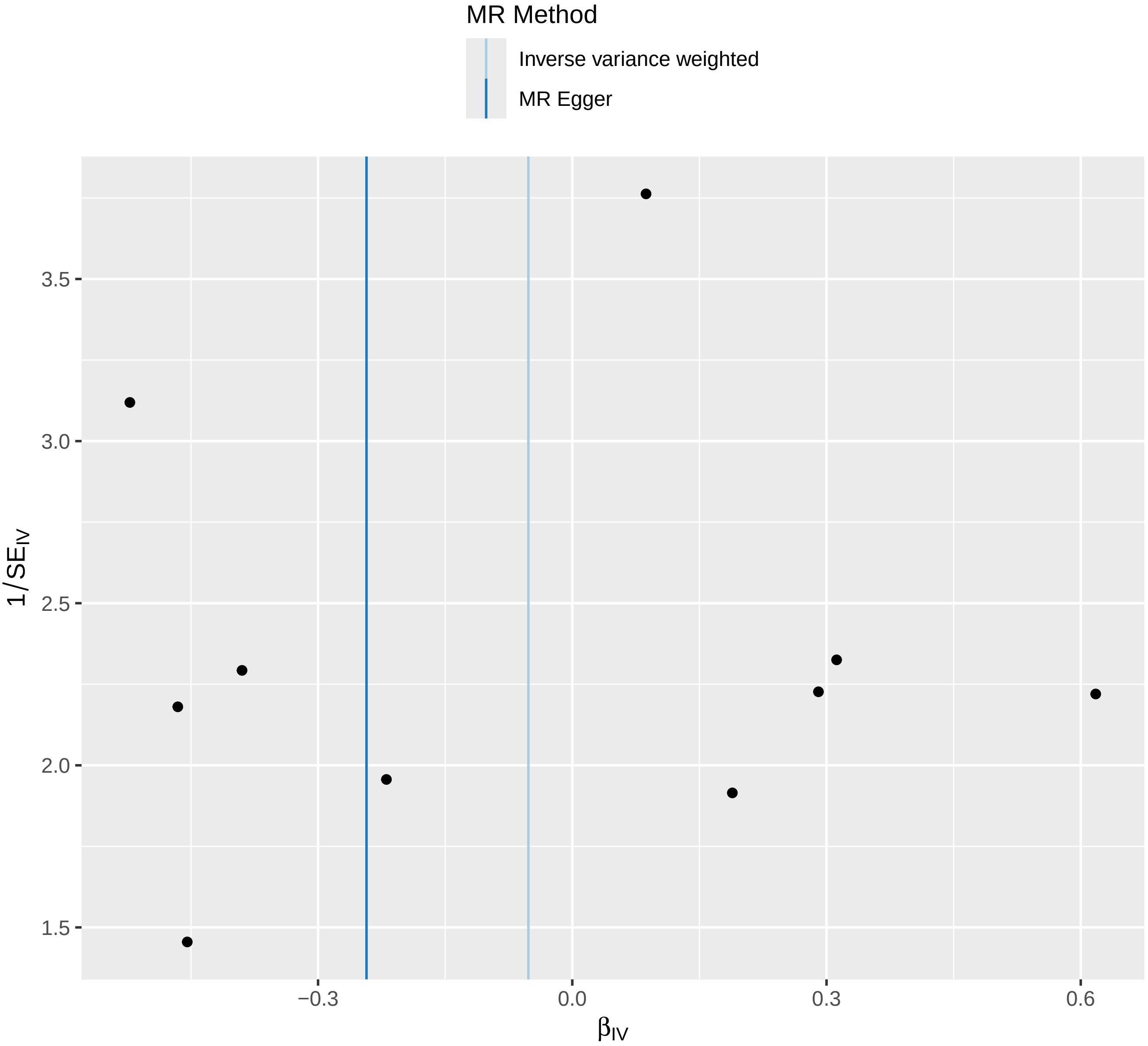
MR Method
Inverse variance weighted
MR Egger
1.5
2.0
2.5
3.0
3.5
$1/SE_{IV}$
−0.3
0.0
0.3
0.6
β_{IV}

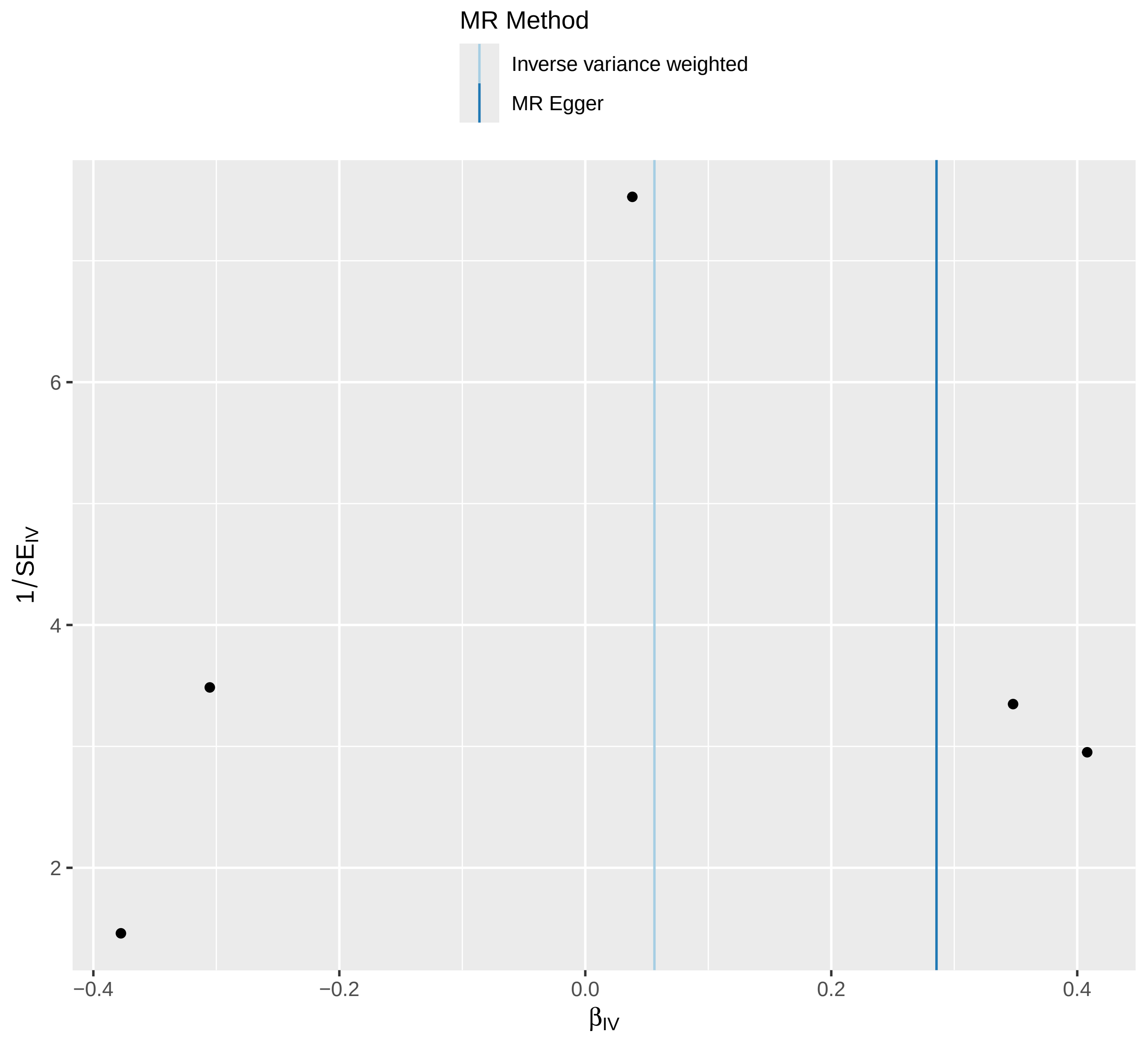

MR Method
Inverse variance weighted
MR Egger
6
4
2
$1/SE_{IV}$
−0.4
−0.2
0.0
0.2
0.4
β_{IV}

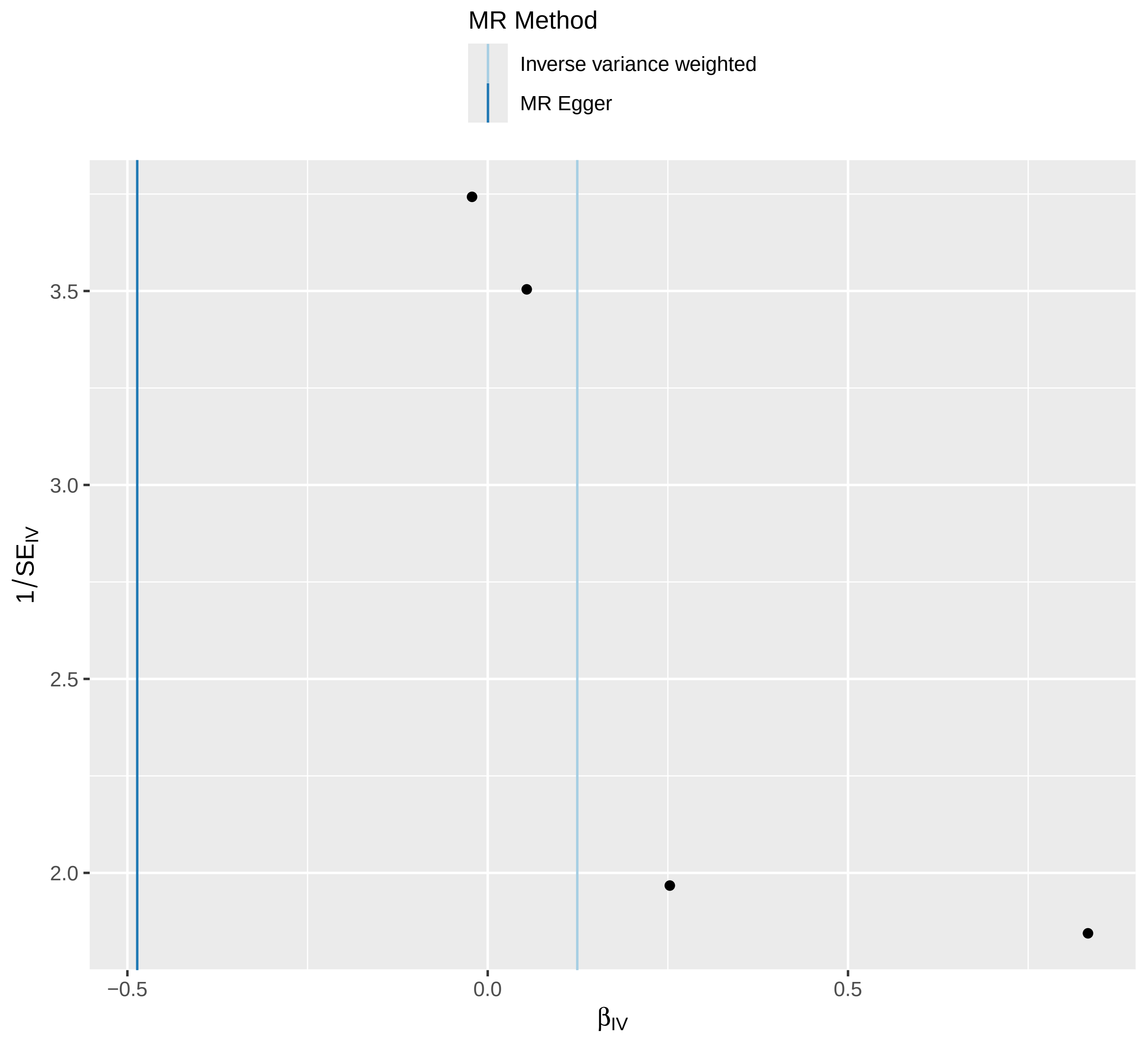

MR Method
Inverse variance weighted
MR Egger
3.5
3.0
2.5
2.0
$1/SE_{IV}$
−0.5
0.0
0.5
β_{IV}

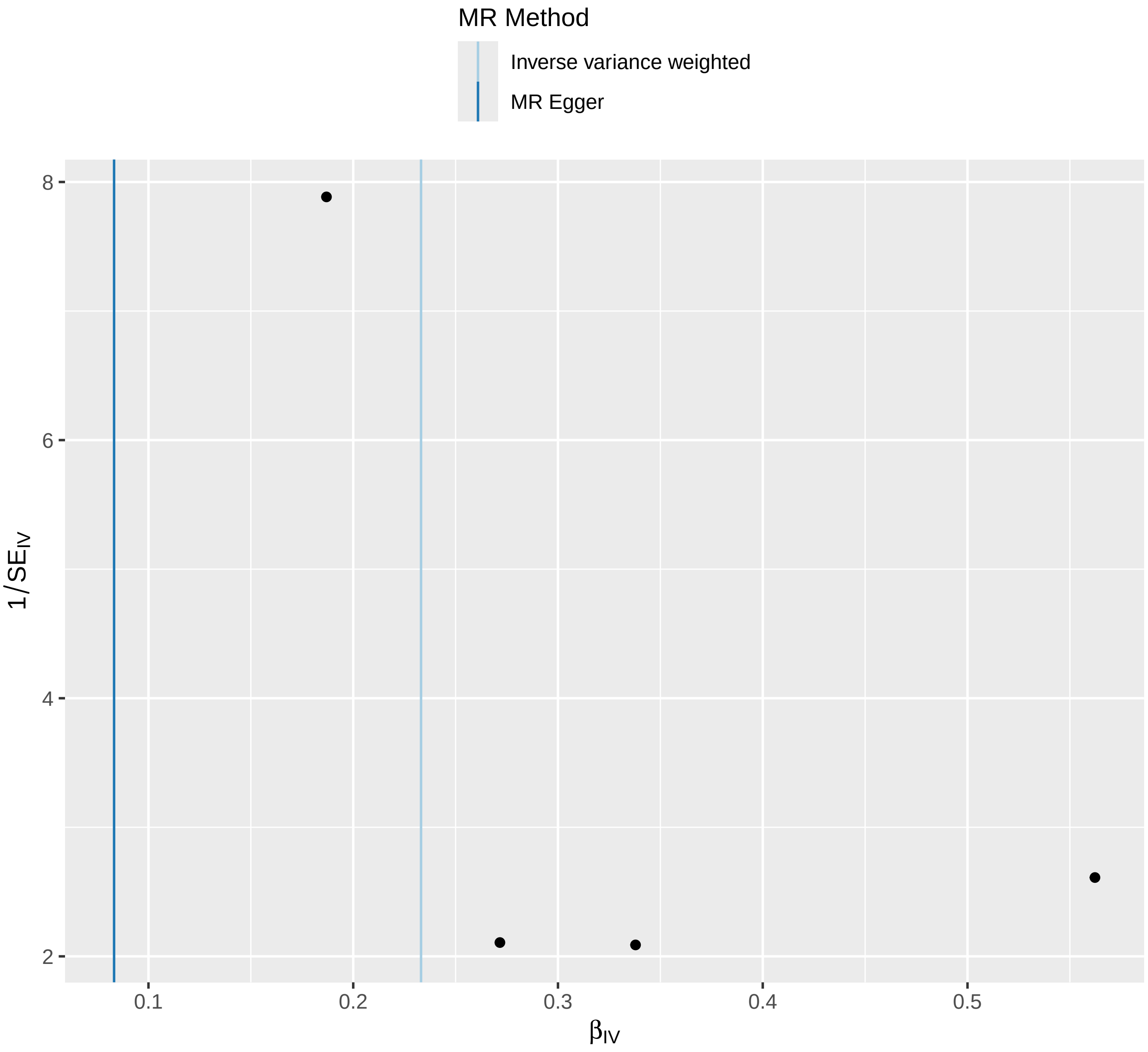
MR Method
Inverse variance weighted
MR Egger
8
6
4
2
0.1
0.2
0.3
0.4
0.5
$1/SE_{IV}$
β_{IV}

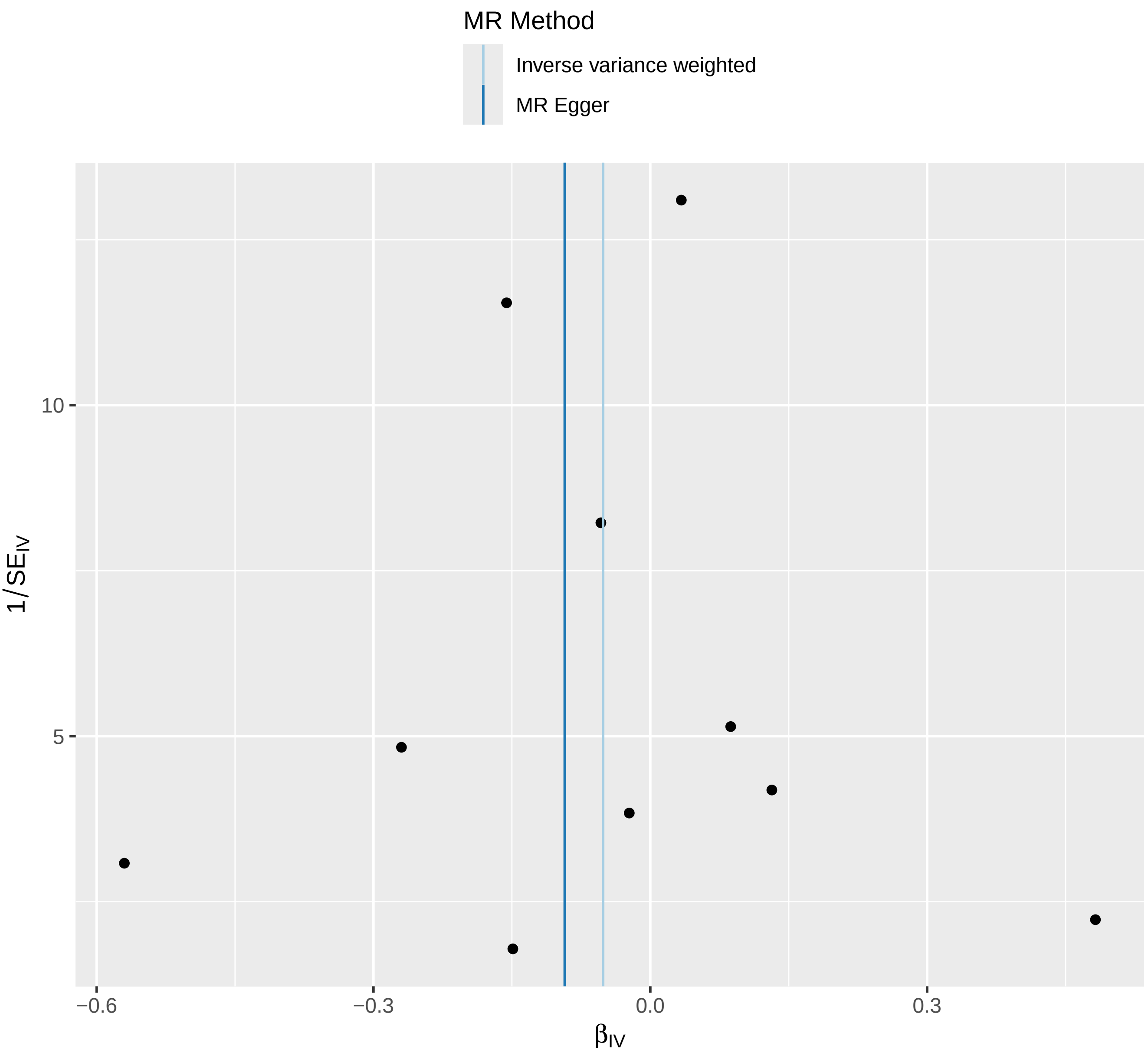

MR Method
Inverse variance weighted
MR Egger
10
5
1/SE_IV
−0.6
−0.3
0.0
0.3
β_IV

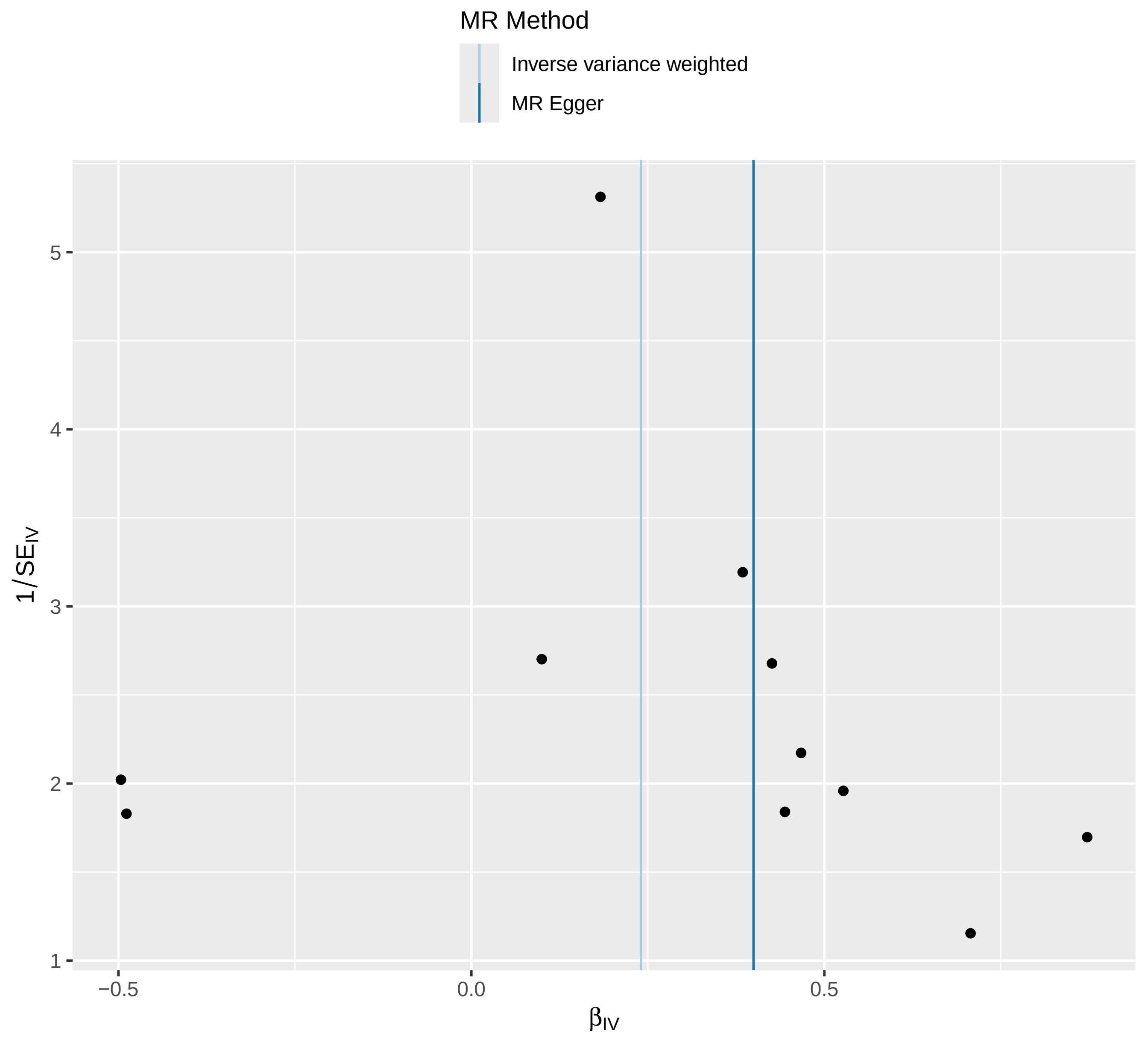
MR Method
Inverse variance weighted
MR Egger
1/SE$_{IV}$
β$_{IV}$
−0.5
0.0
0.5
1
2
3
4
5

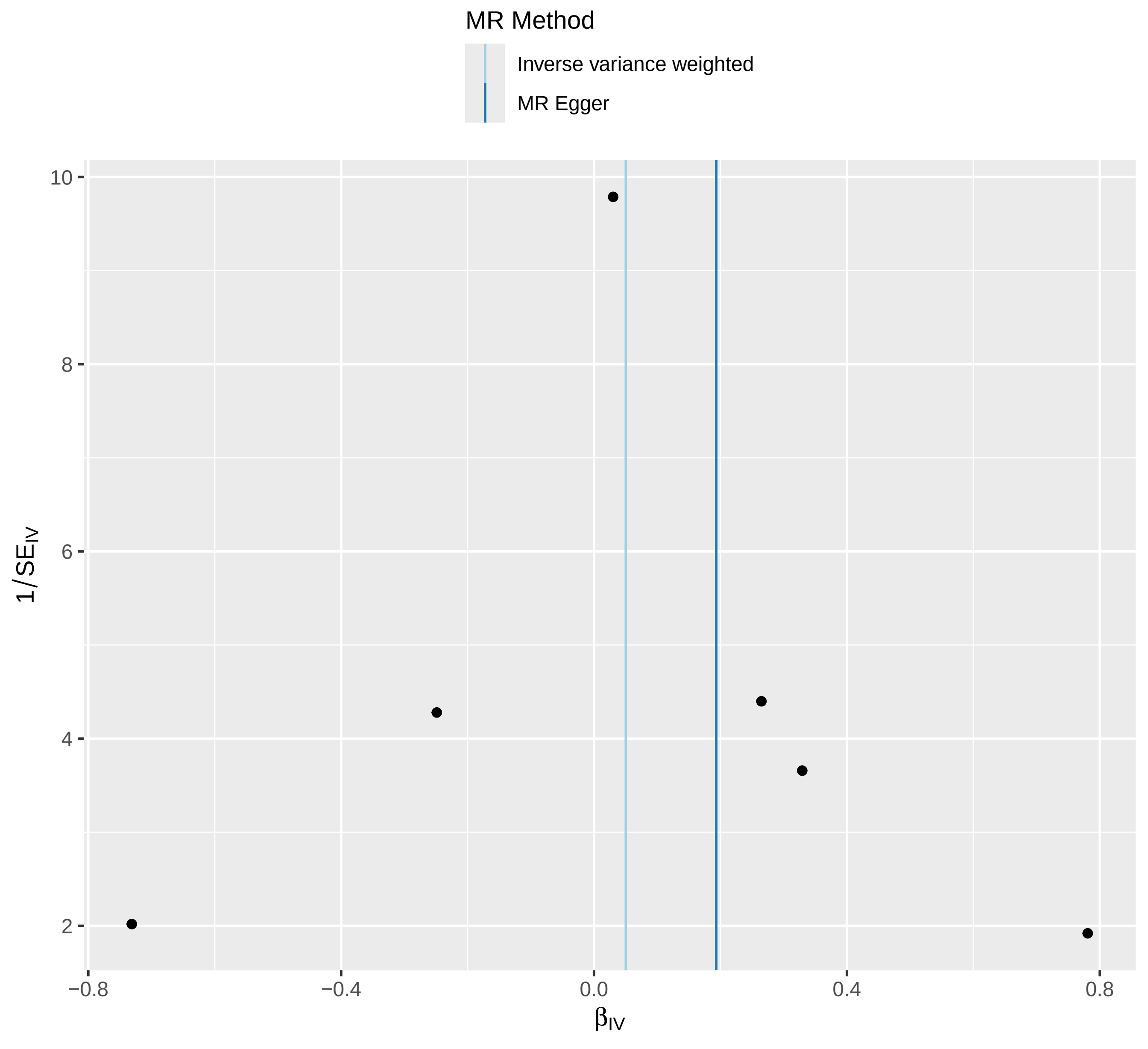
MR Method
Inverse variance weighted
MR Egger
1/SE_IV
β_IV
10
8
6
4
2
−0.8
−0.4
0.0
0.4
0.8

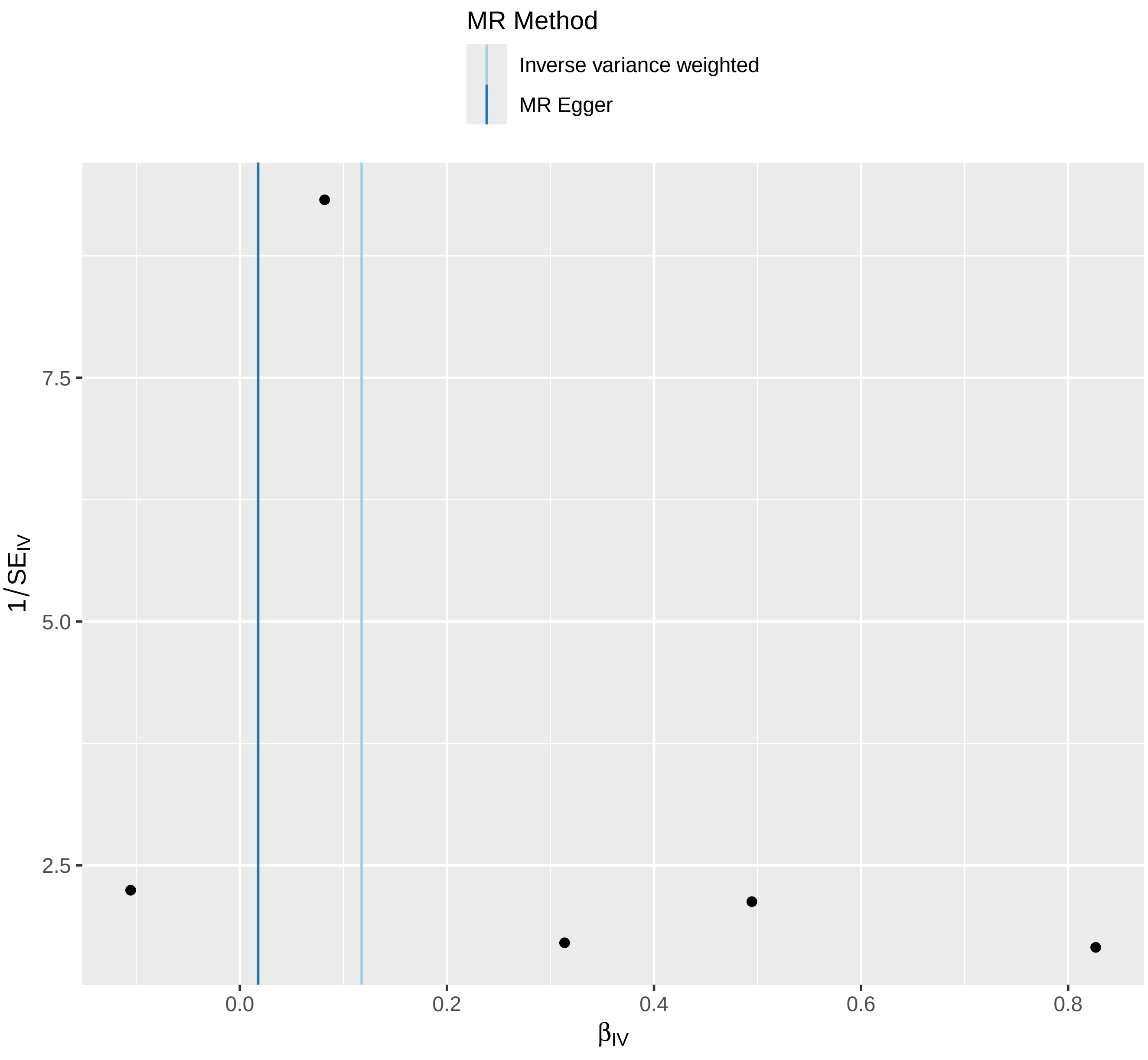

MR Method
Inverse variance weighted
MR Egger
7.5
5.0
2.5
1/SE_IV
0.0
0.2
0.4
0.6
0.8
β_IV

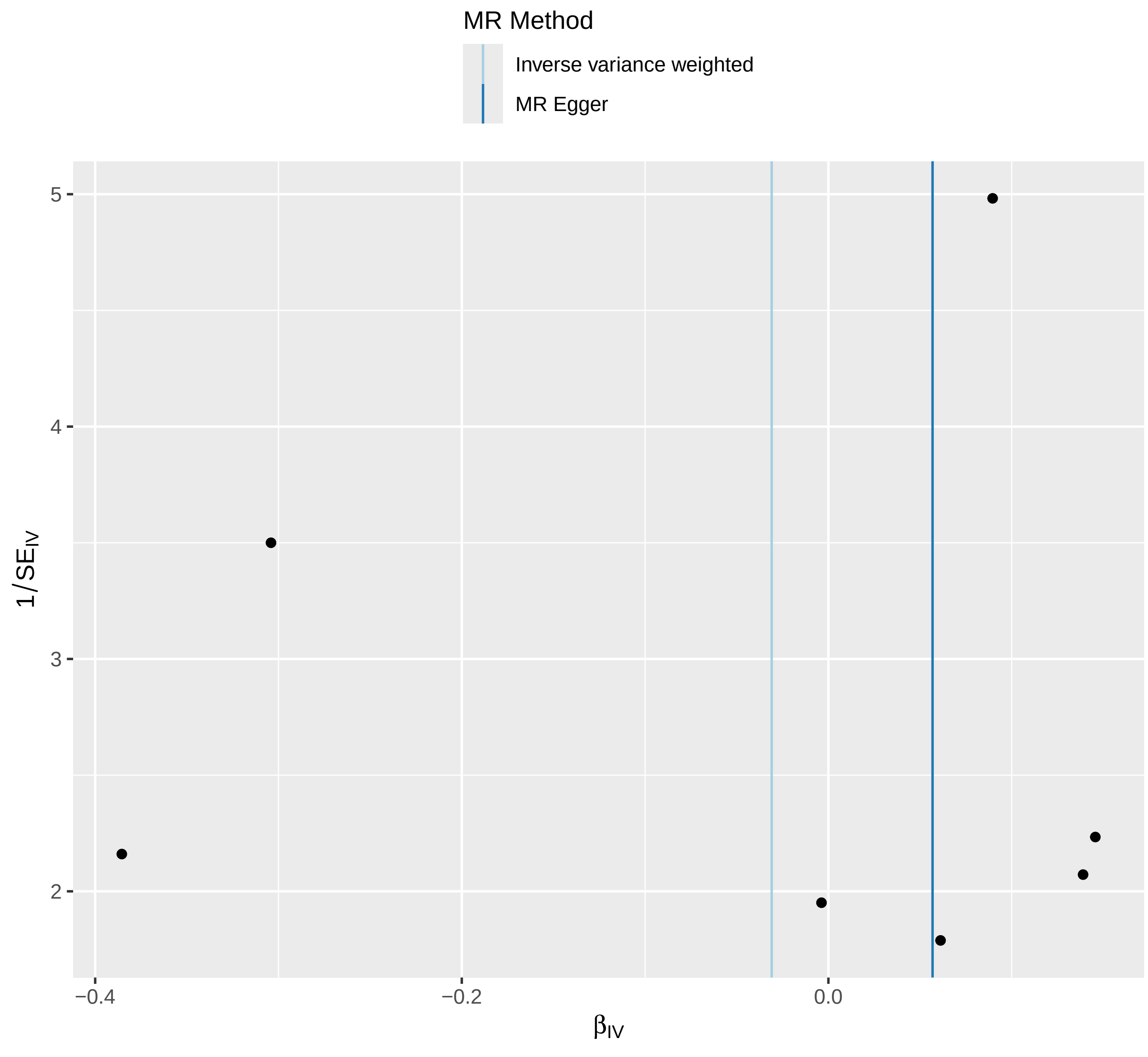

MR Method
Inverse variance weighted
MR Egger
5
4
3
2
1/SE_IV
−0.4
−0.2
0.0
β_IV

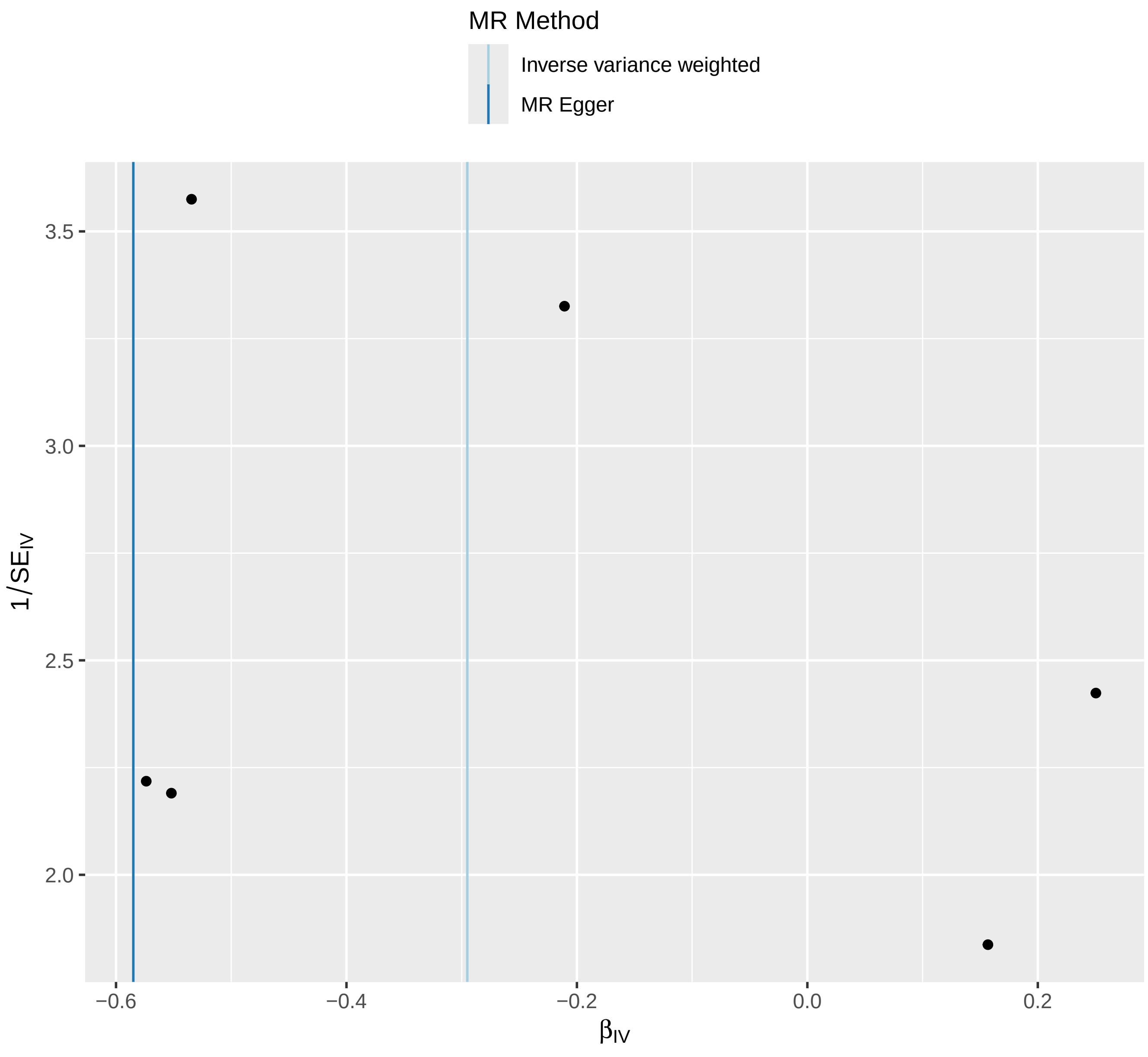

MR Method
Inverse variance weighted
MR Egger
3.5
3.0
2.5
2.0
1/SE$_{IV}$
−0.6
−0.4
−0.2
0.0
0.2
β$_{IV}$

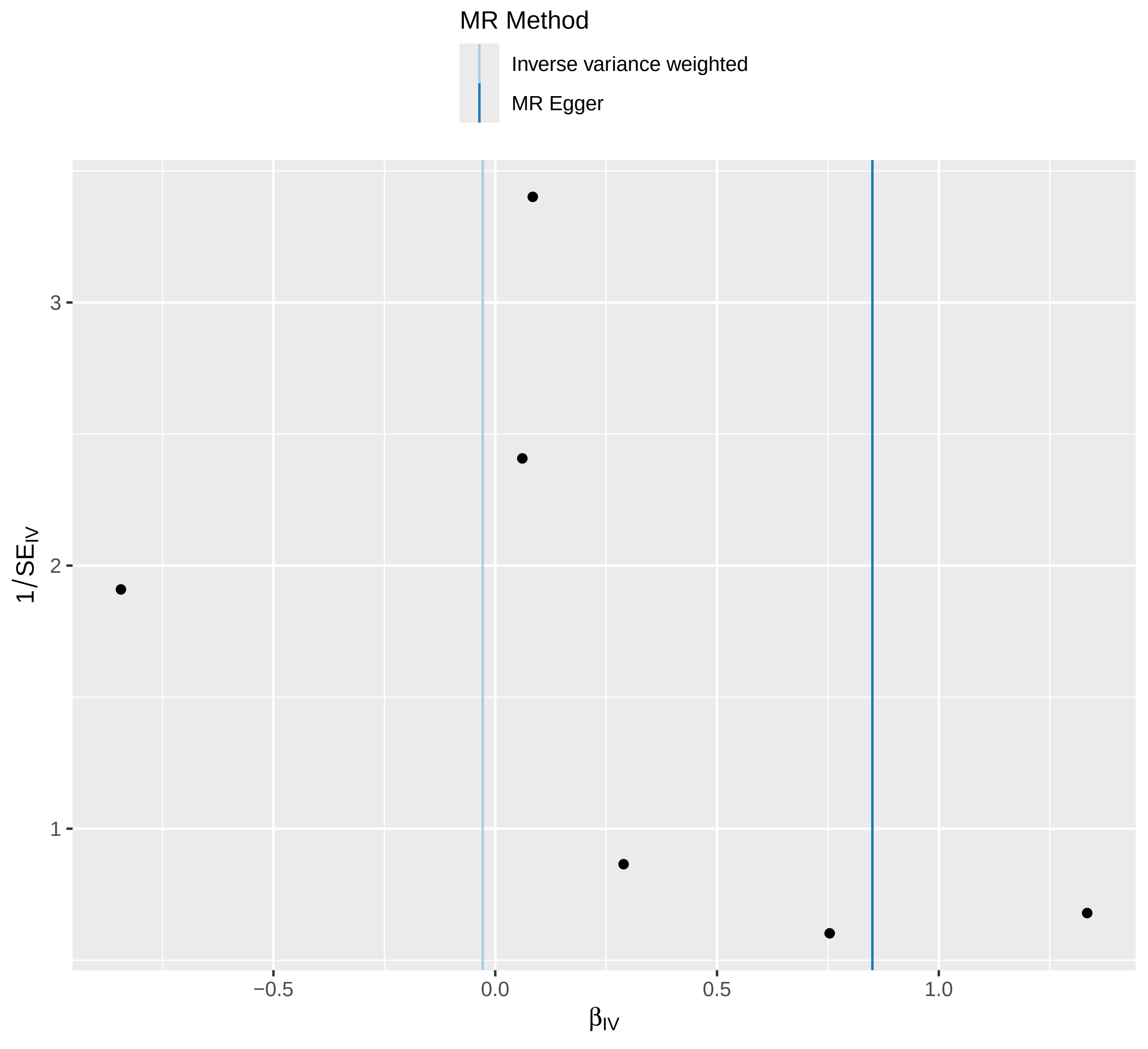
MR Method
Inverse variance weighted
MR Egger
1/SE$_{IV}$
3
2
1
−0.5
0.0
0.5
1.0
β$_{IV}$

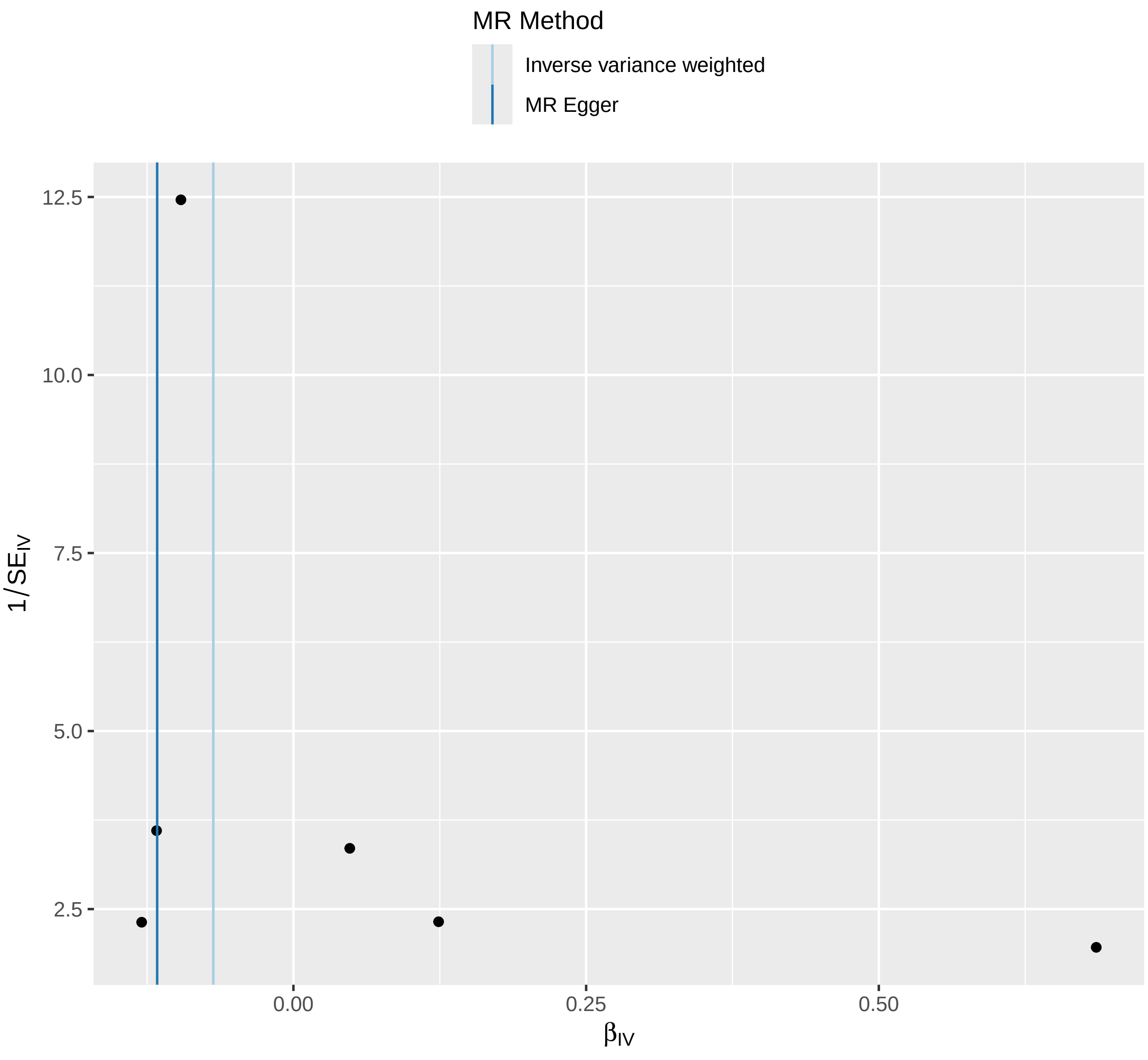
MR Method
Inverse variance weighted
MR Egger
12.5
10.0
7.5
5.0
2.5
1/SE_IV
0.00
0.25
0.50
β_IV

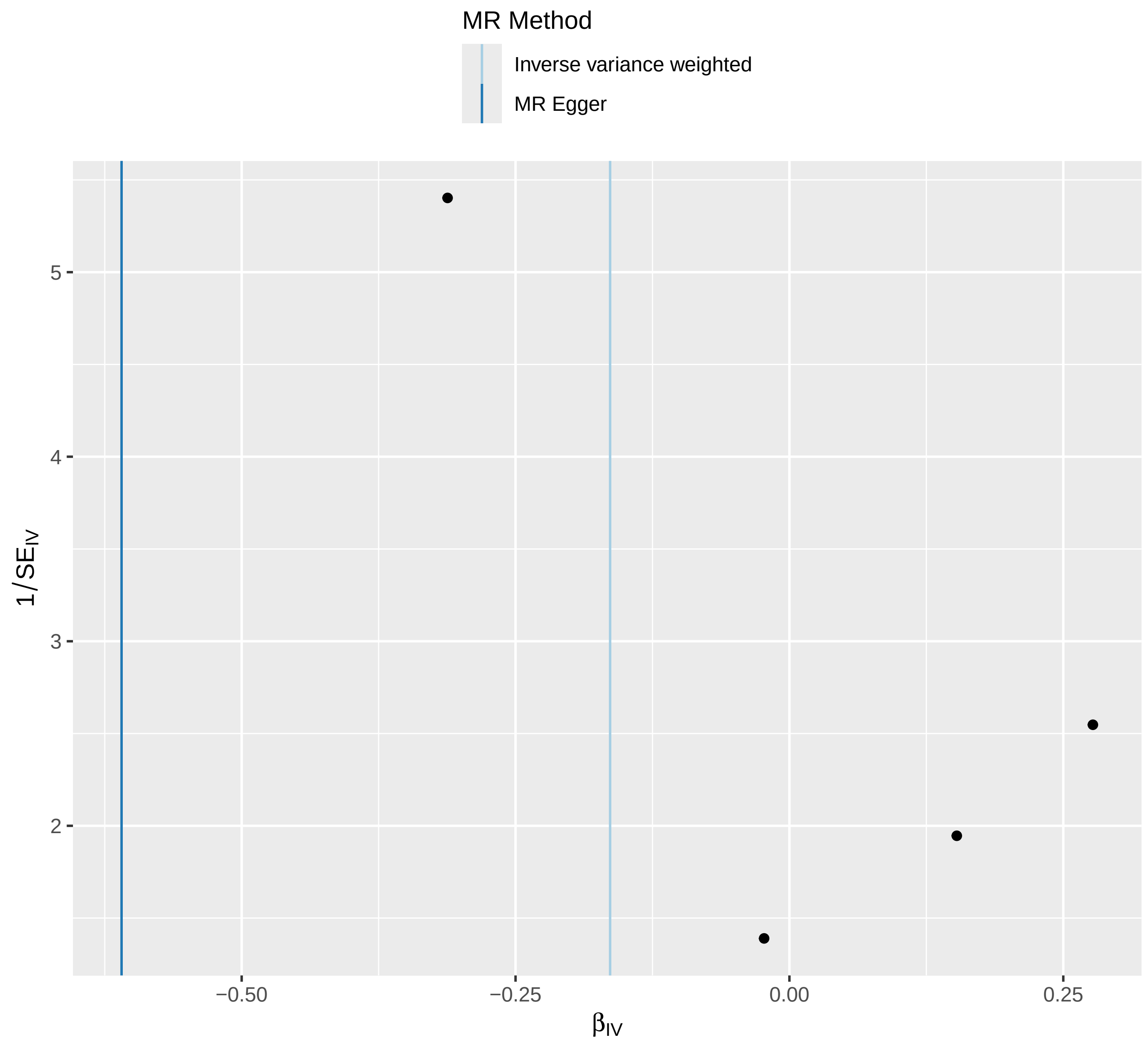

MR Method
Inverse variance weighted
MR Egger
5
4
3
2
1/SE_IV
−0.50
−0.25
0.00
0.25
β_IV

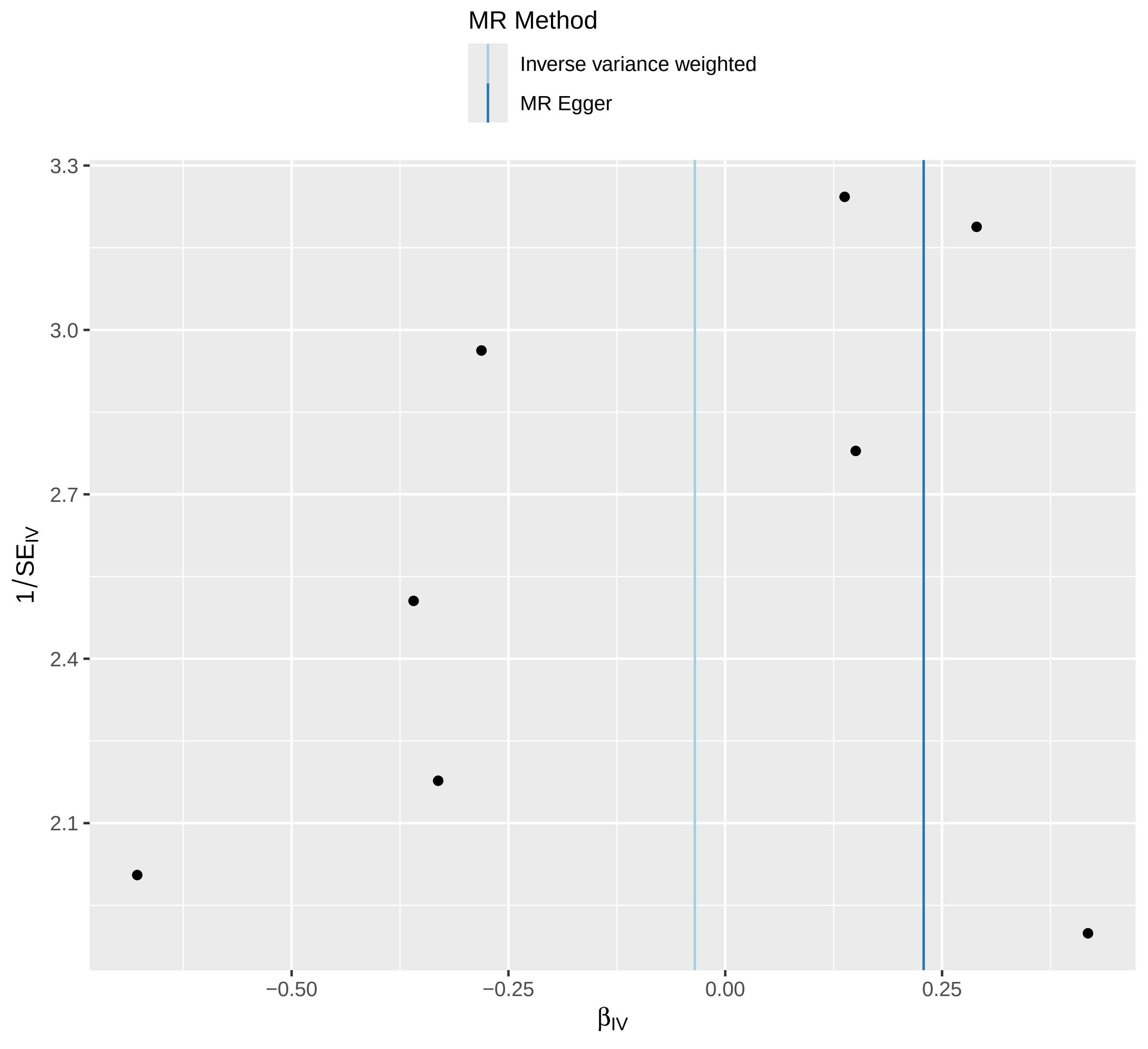

MR Method
Inverse variance weighted
MR Egger
3.3
3.0
2.7
2.4
2.1
1/SE$_{IV}$
−0.50
−0.25
0.00
0.25
β$_{IV}$

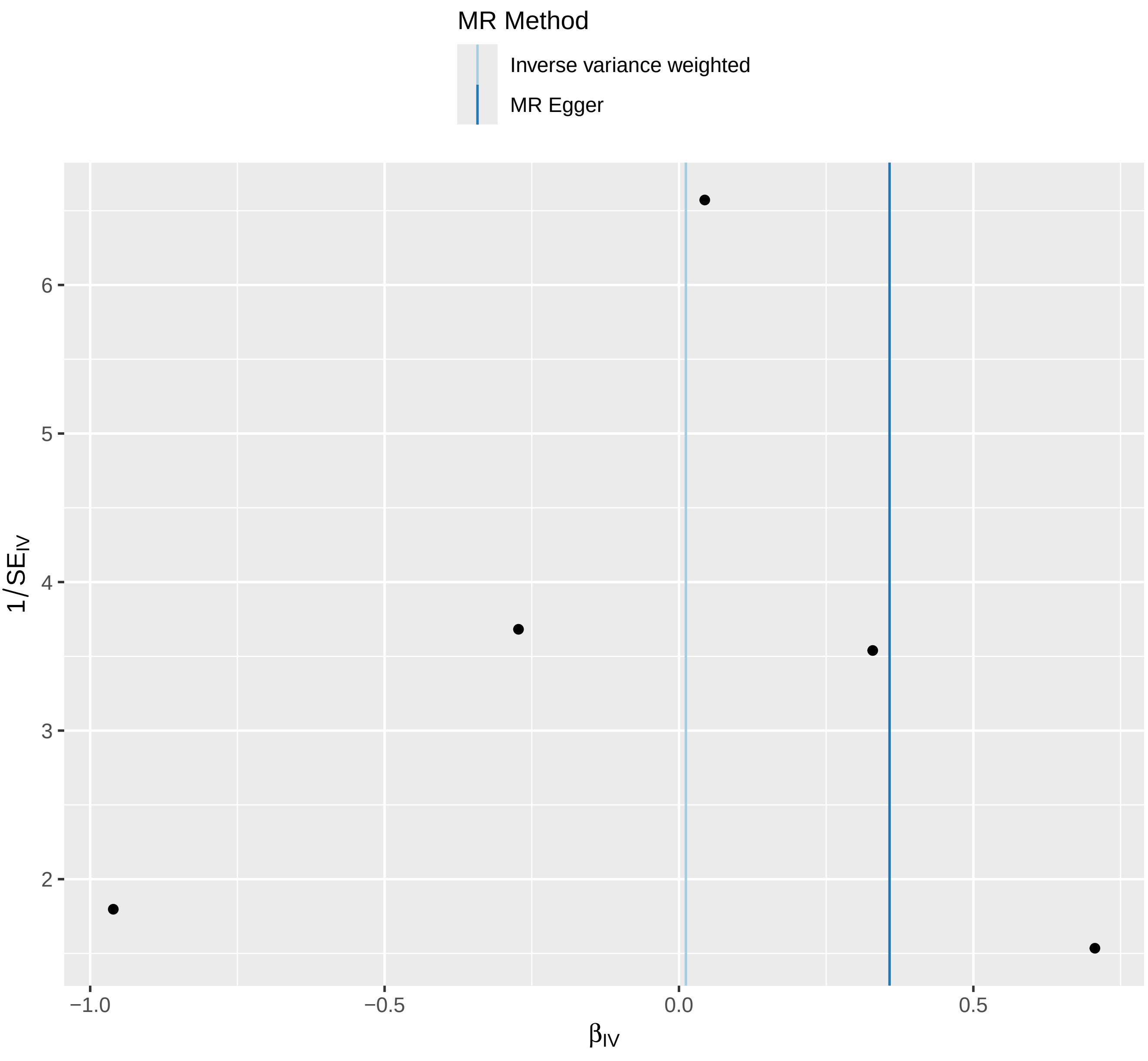

MR Method
Inverse variance weighted
MR Egger
1/SE_IV
6
5
4
3
2
−1.0
−0.5
0.0
0.5
β_IV

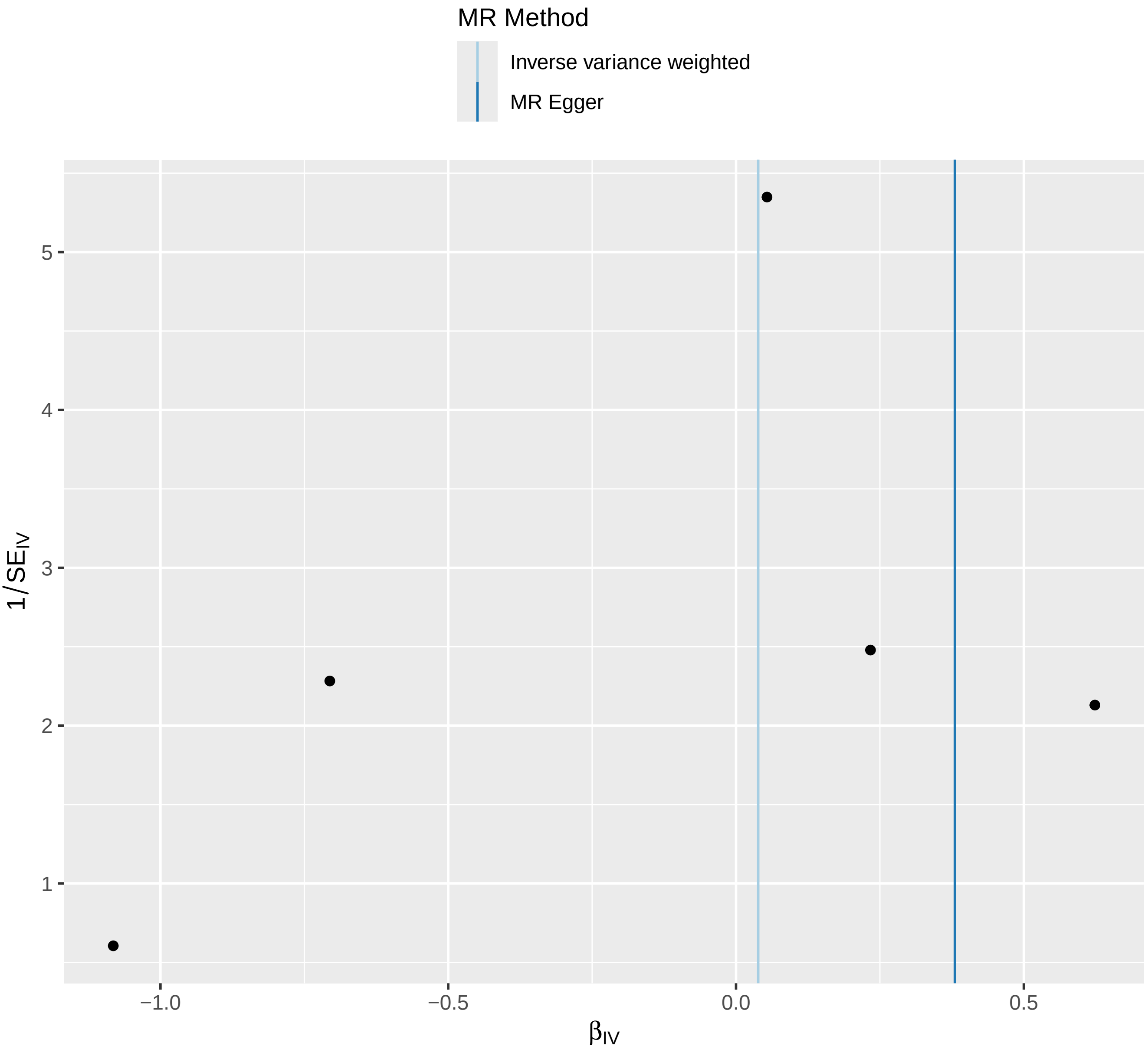

MR Method
Inverse variance weighted
MR Egger
1/SE_IV
5
4
3
2
1
−1.0
−0.5
0.0
0.5
β_IV

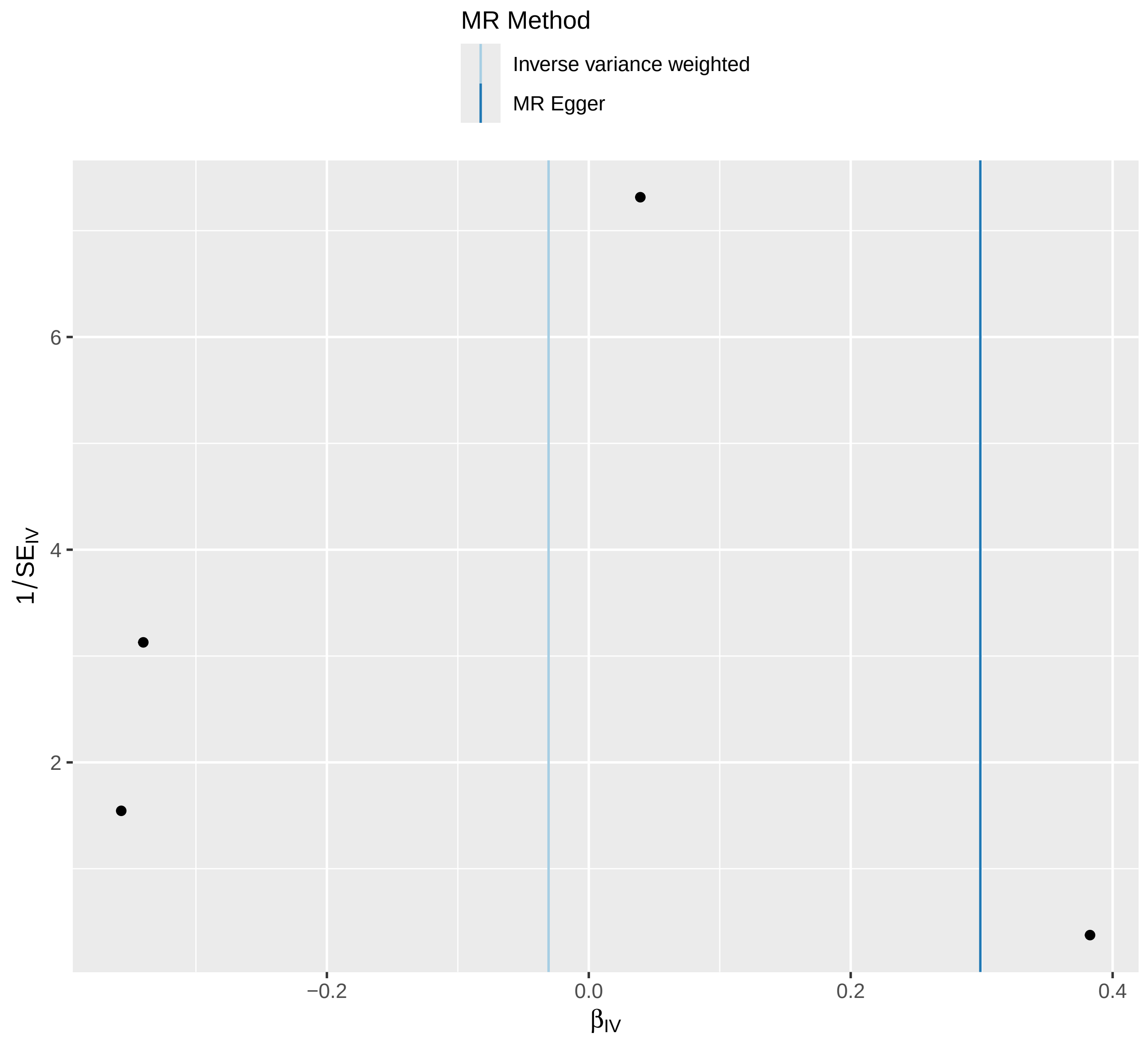
MR Method
Inverse variance weighted
MR Egger
6
4
2
1/SE_IV
−0.2
0.0
0.2
0.4
β_IV

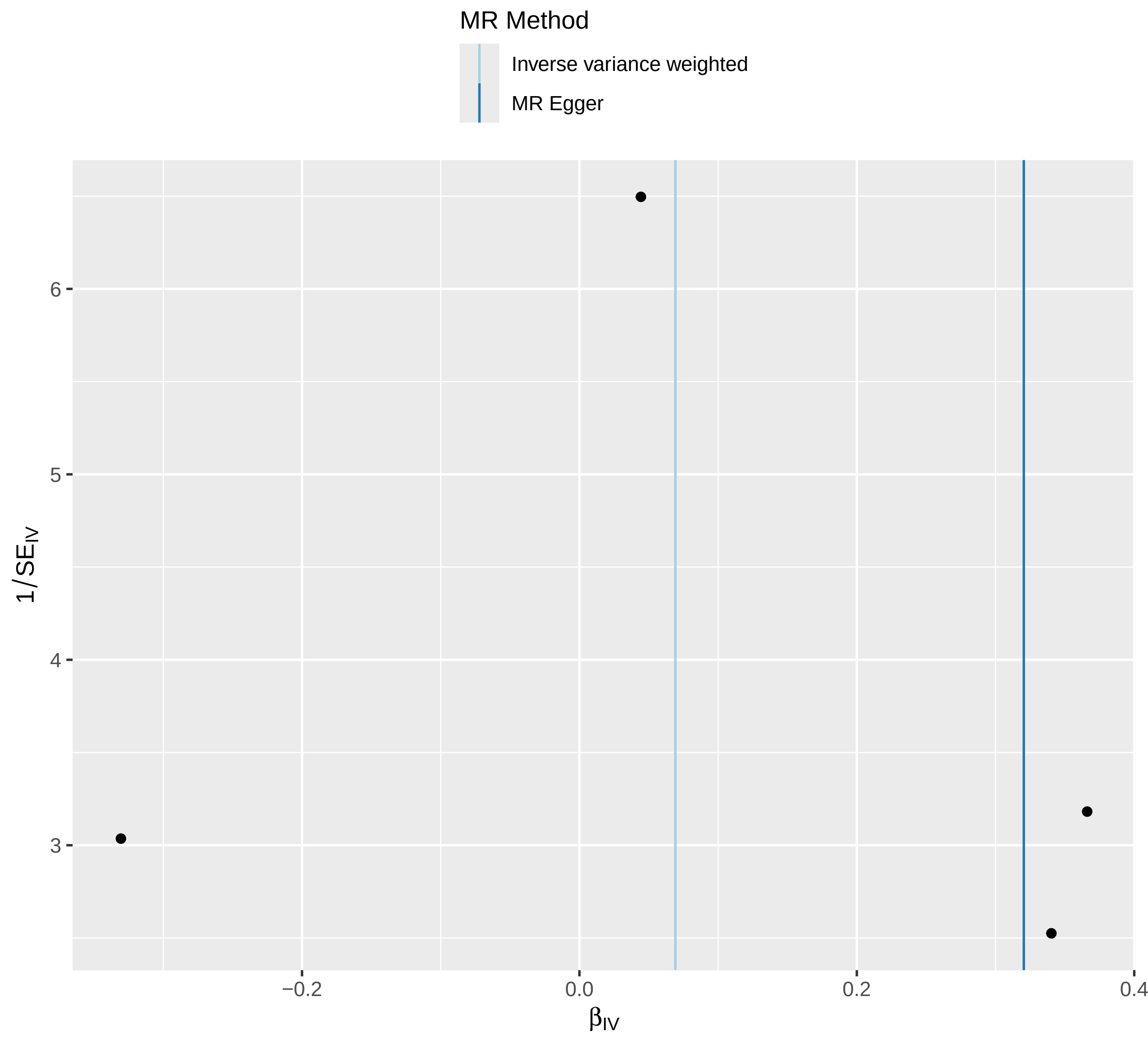
MR Method
Inverse variance weighted
MR Egger
1/SE$_{IV}$
6
5
4
3
−0.2
0.0
0.2
0.4
β$_{IV}$

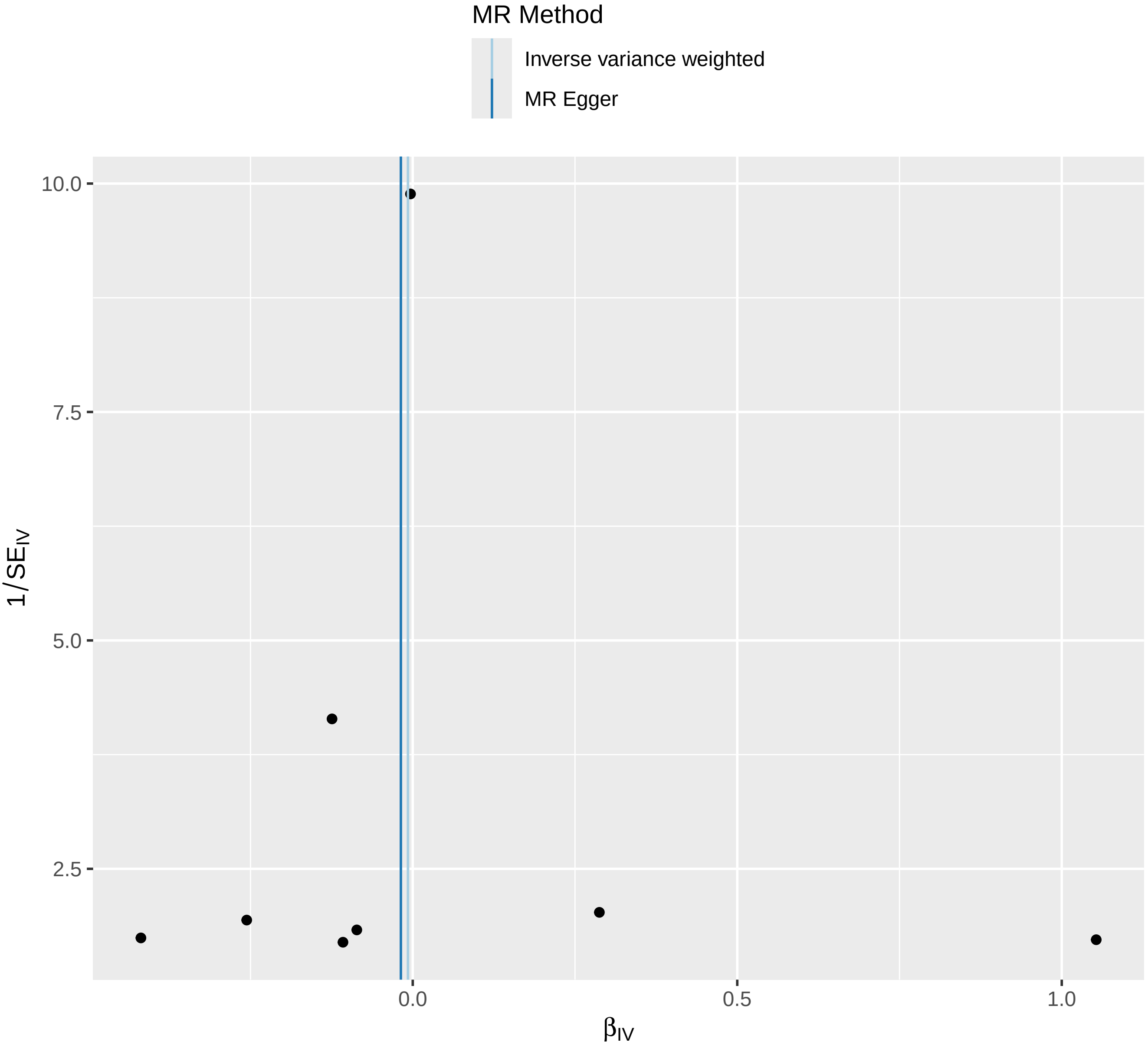

MR Method
Inverse variance weighted
MR Egger
10.0
7.5
5.0
2.5
1/SE$_{IV}$
0.0
0.5
1.0
β$_{IV}$

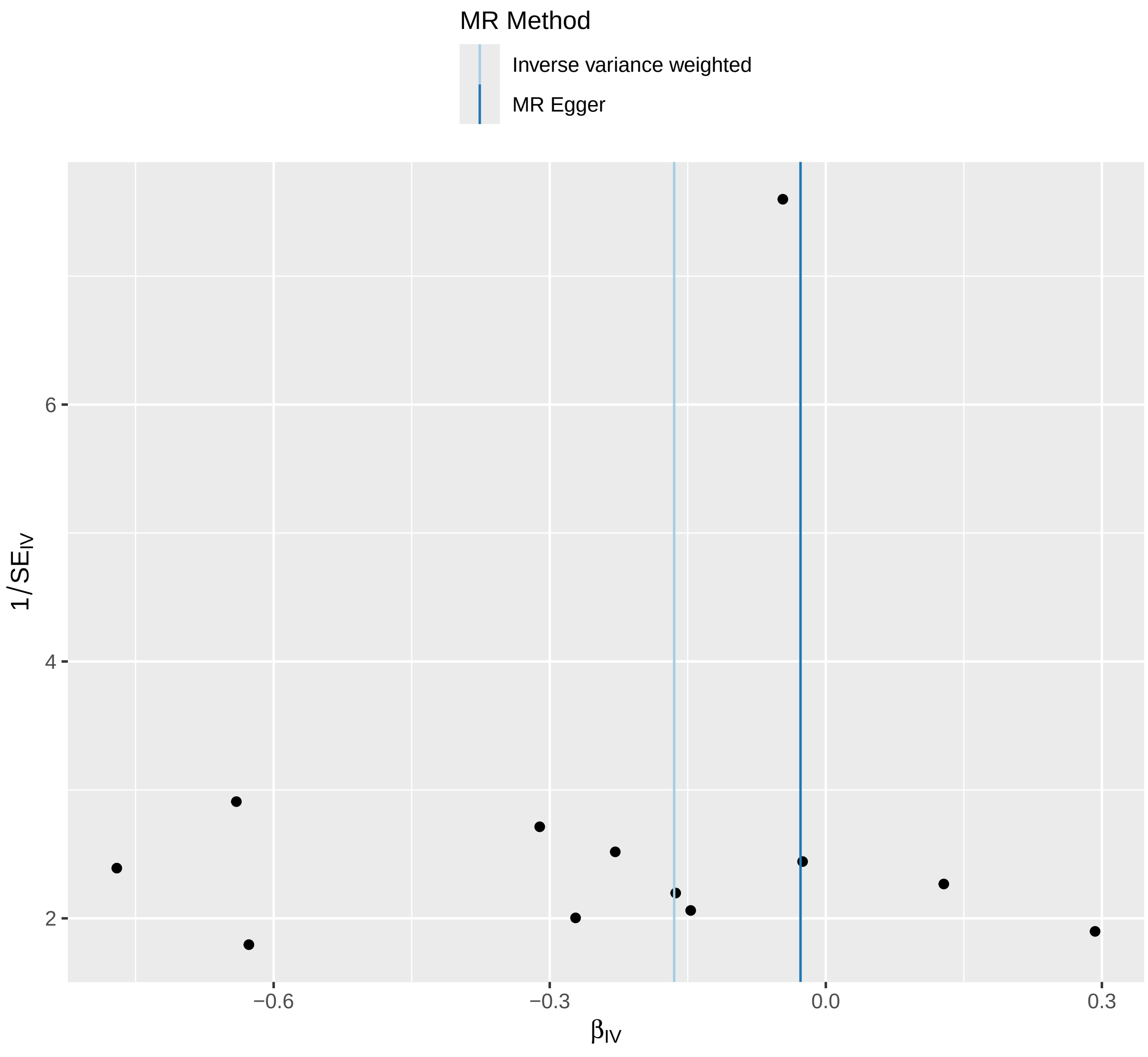

MR Method
Inverse variance weighted
MR Egger
6
4
2
1/SE$_{IV}$
−0.6
−0.3
0.0
0.3
β$_{IV}$

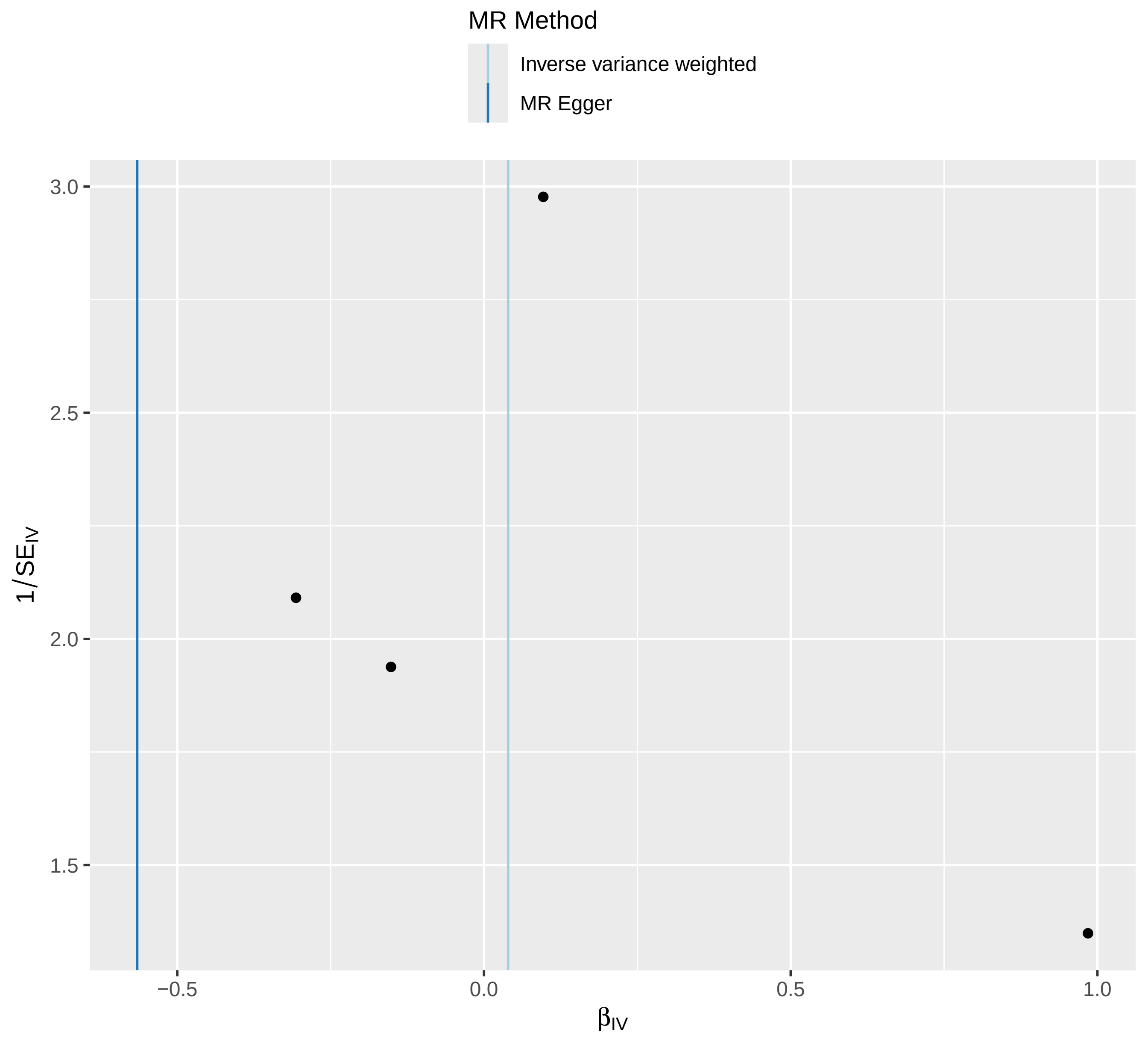
MR Method
Inverse variance weighted
MR Egger
3.0
2.5
2.0
1.5
1/SE$_{IV}$
−0.5
0.0
0.5
1.0
β$_{IV}$

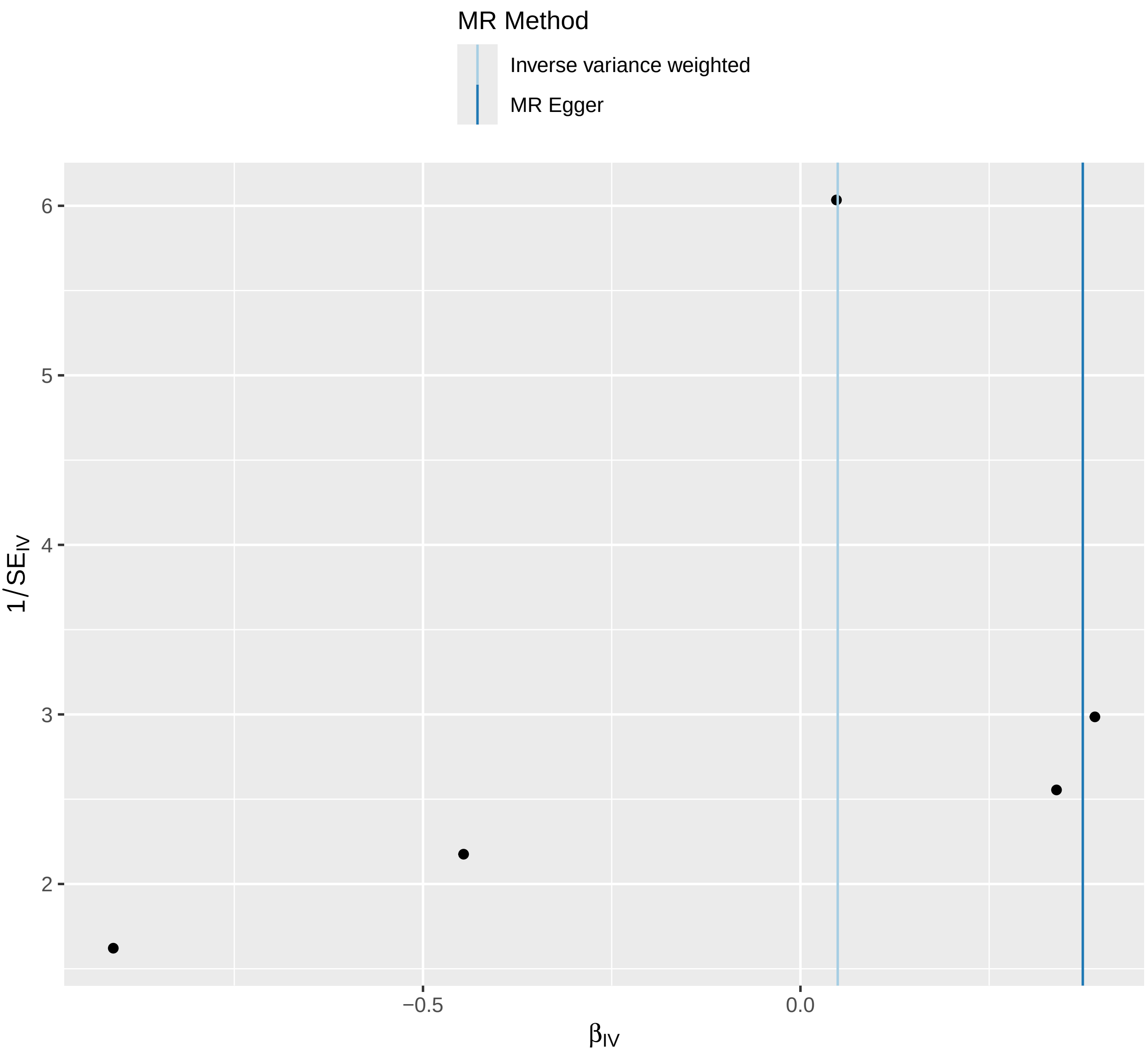
MR Method
Inverse variance weighted
MR Egger
6
5
4
3
2
1/SE_IV
−0.5
0.0
β_IV

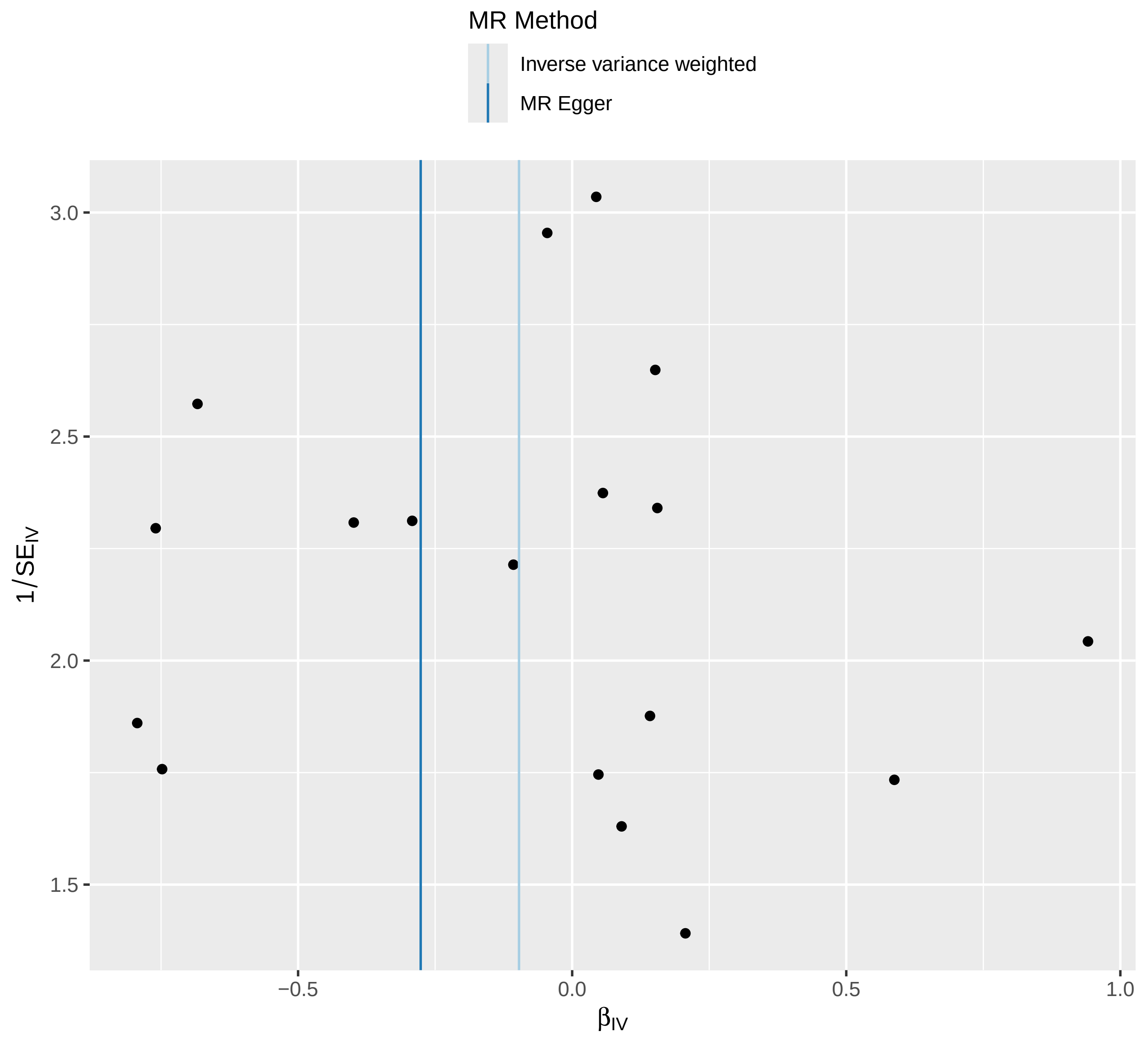

MR Method
Inverse variance weighted
MR Egger
3.0
2.5
2.0
1.5
1/SE_IV
−0.5
0.0
0.5
1.0
β_IV

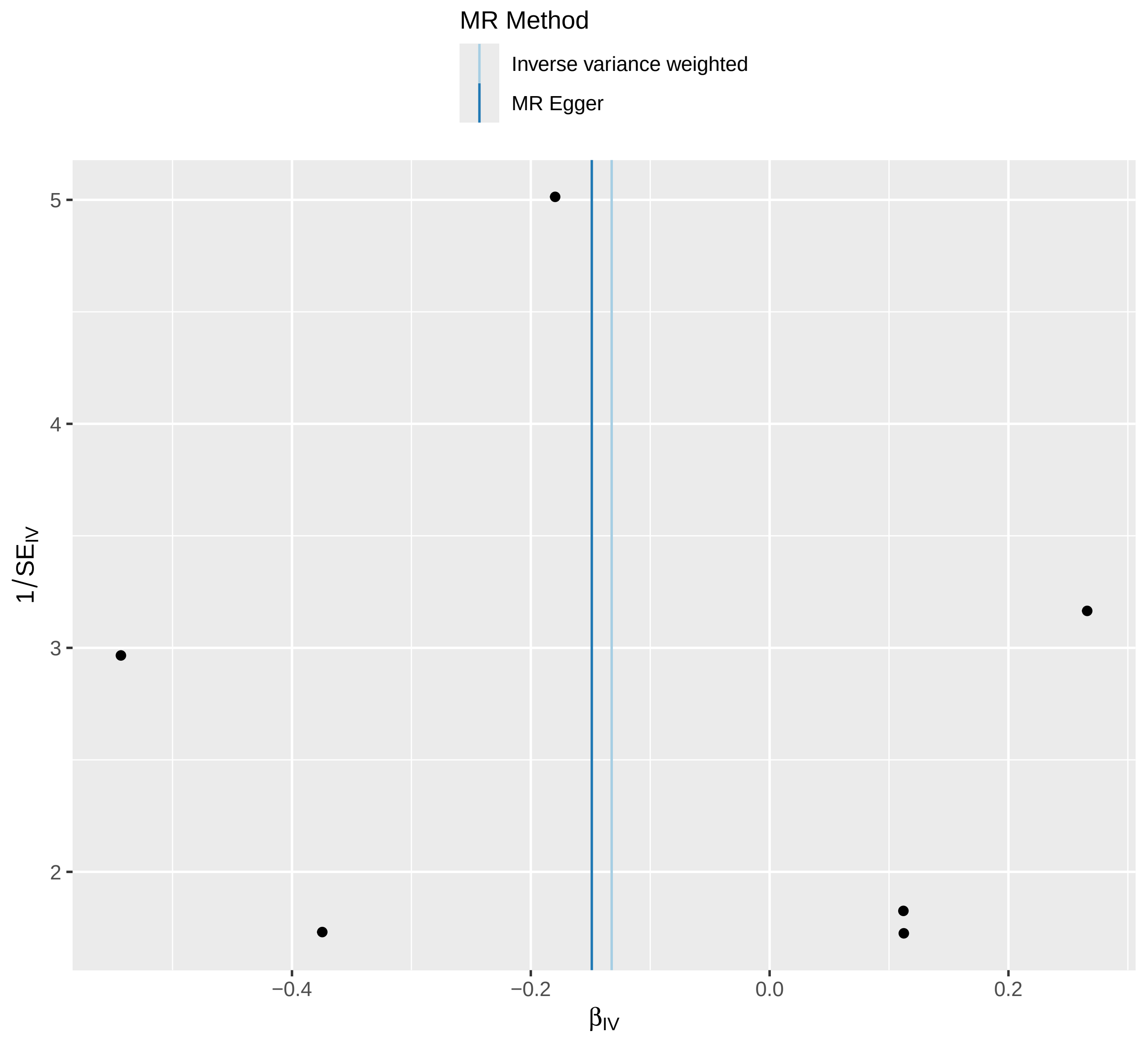
MR Method
Inverse variance weighted
MR Egger
5
4
3
2
1/SE$_{IV}$
−0.4
−0.2
0.0
0.2
β$_{IV}$

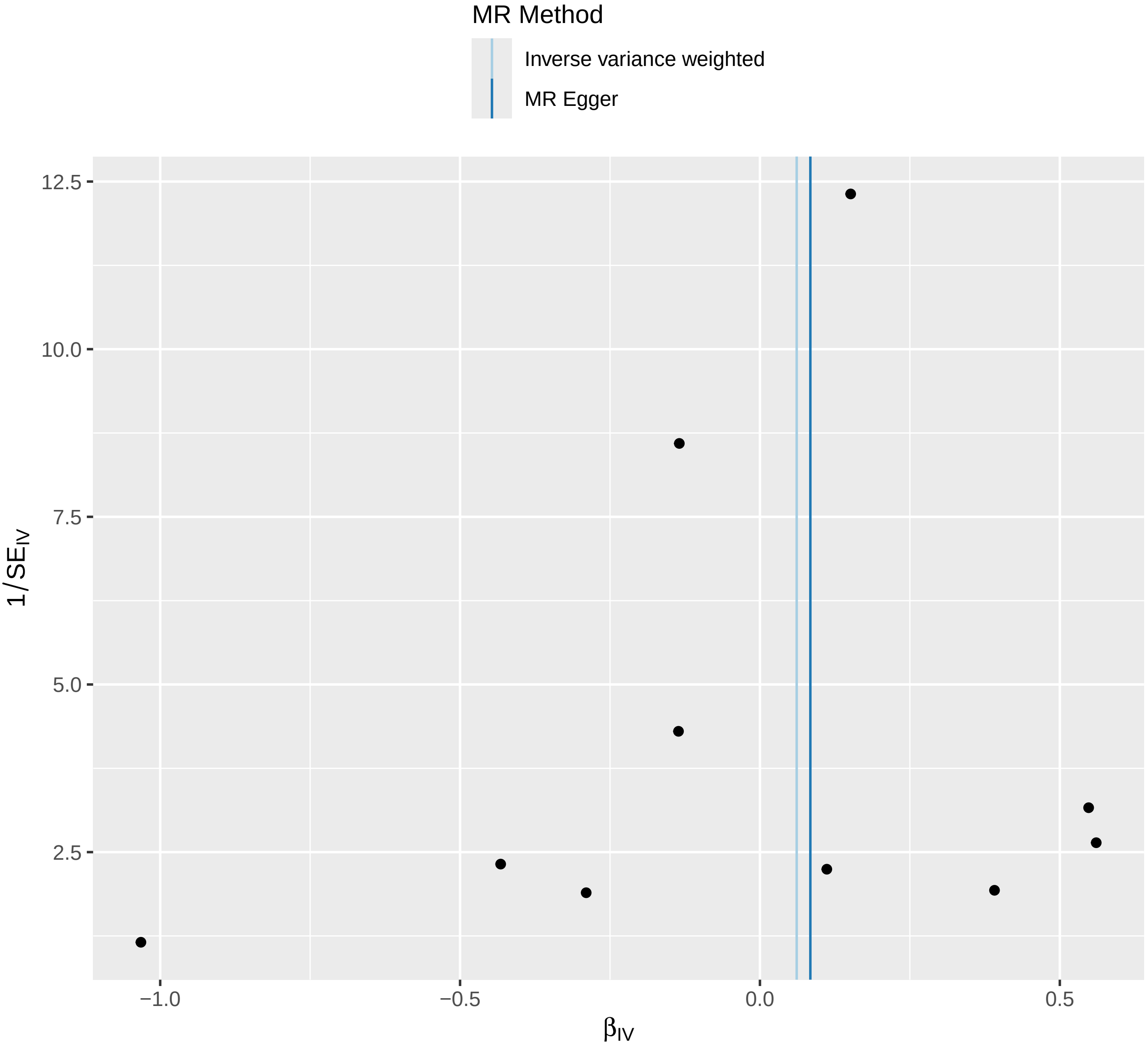
MR Method
Inverse variance weighted
MR Egger
12.5
10.0
7.5
5.0
2.5
$1/SE_{IV}$
−1.0
−0.5
0.0
0.5
β_{IV}

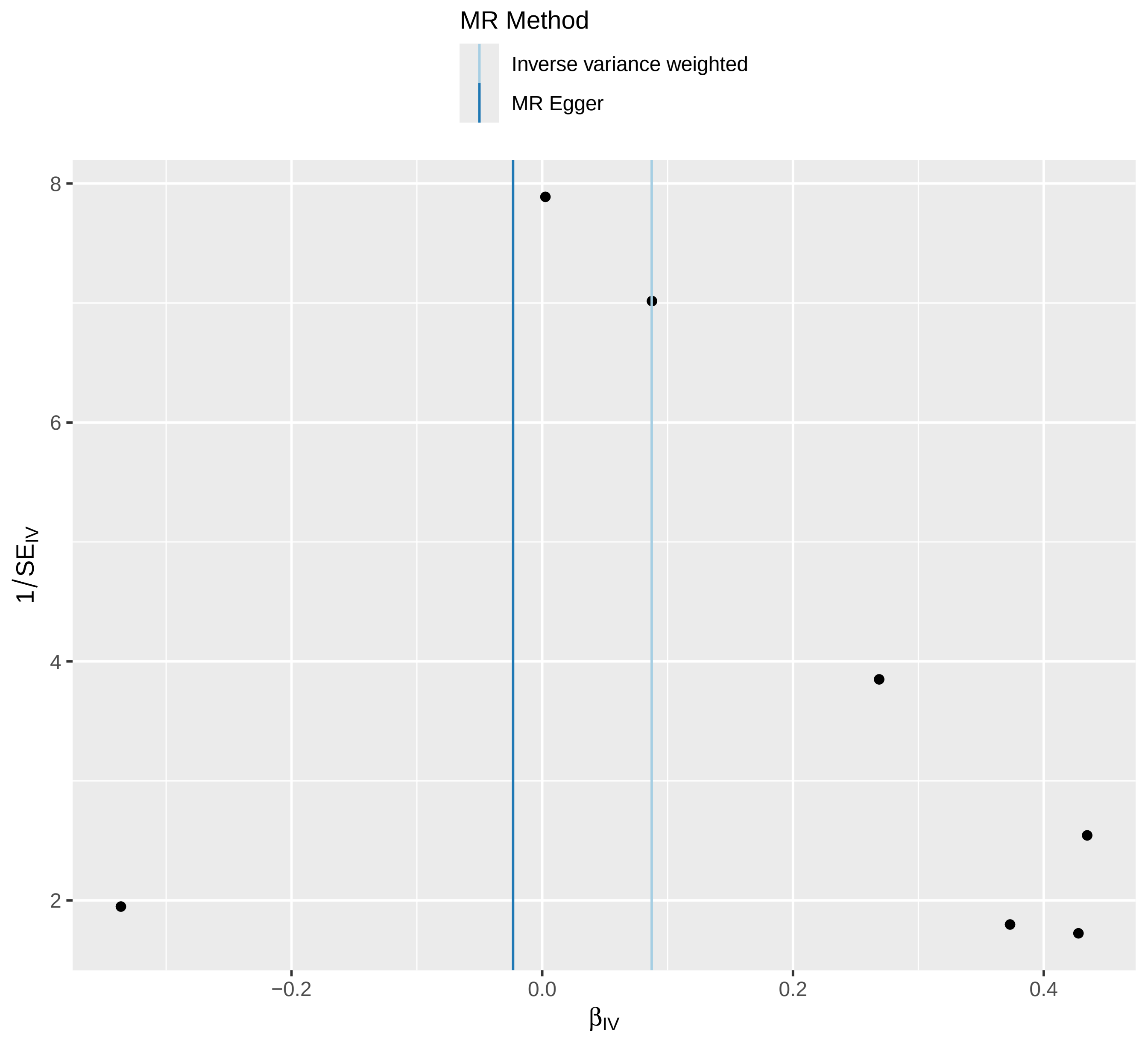

MR Method
Inverse variance weighted
MR Egger
8
6
4
2
−0.2
0.0
0.2
0.4
β_{IV}
$1/SE_{IV}$

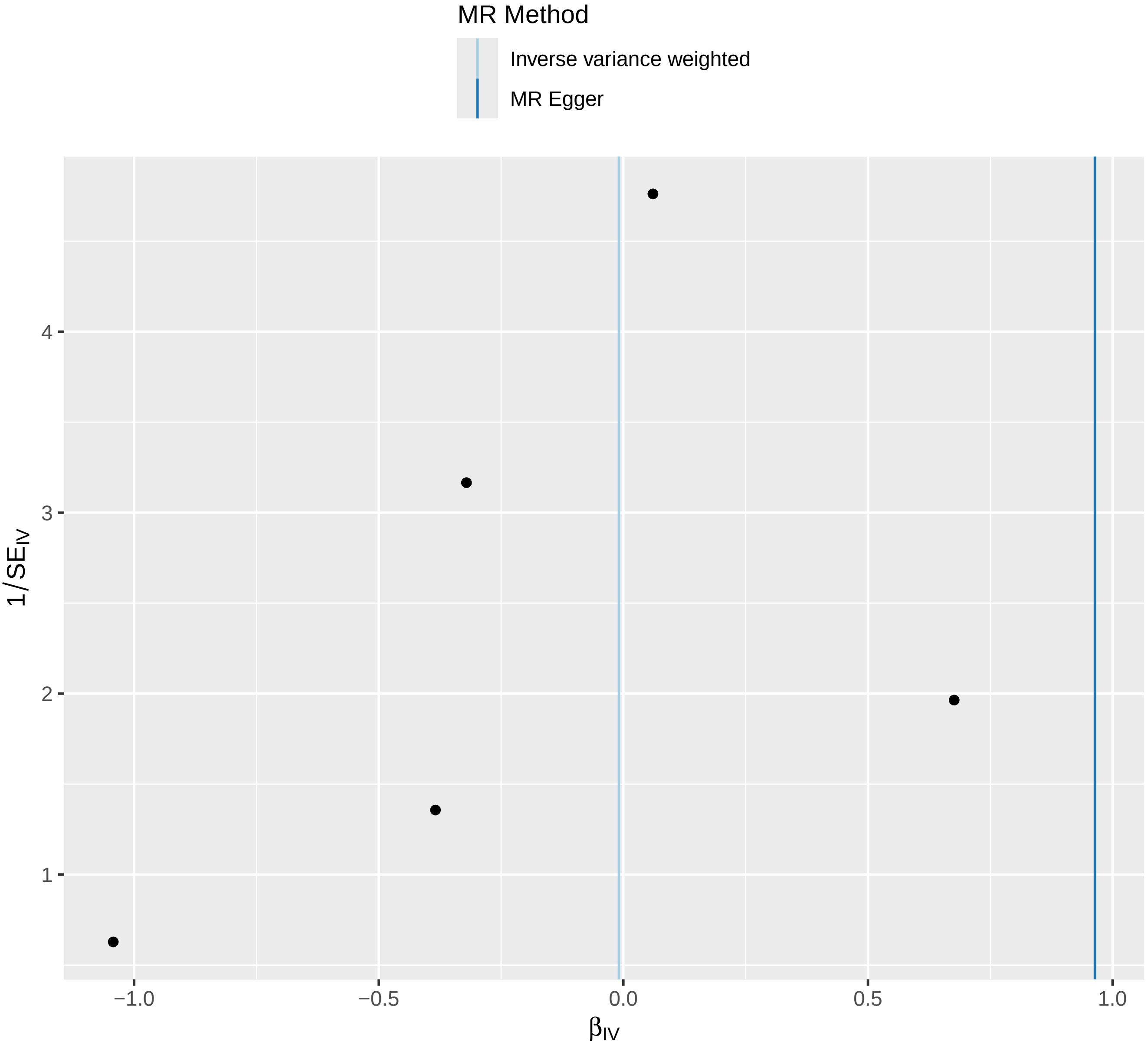

MR Method
Inverse variance weighted
MR Egger
1/SE_IV
β_IV
4
3
2
1
−1.0
−0.5
0.0
0.5
1.0

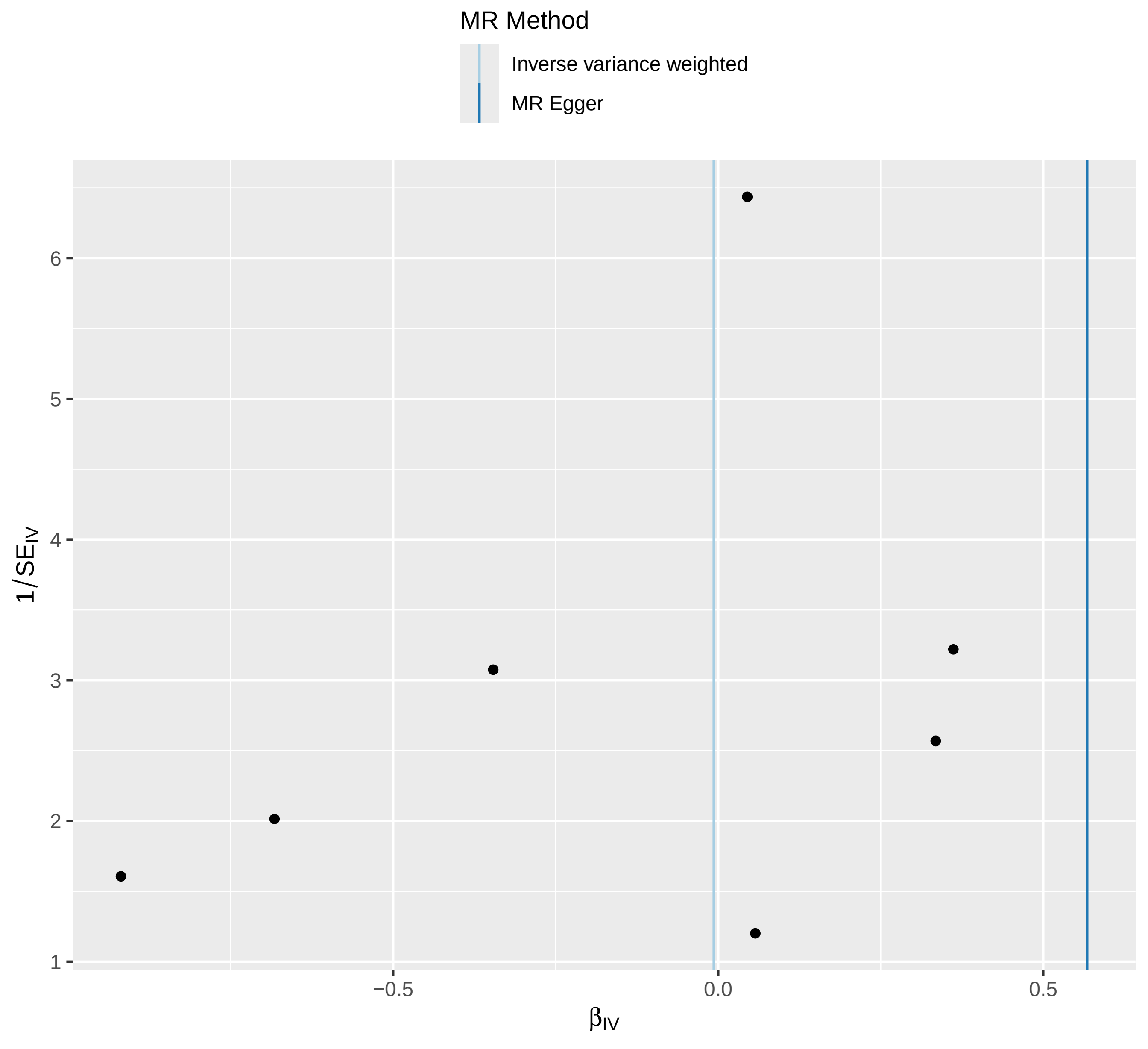

MR Method
Inverse variance weighted
MR Egger
6
5
4
3
2
1
1/SE_IV
−0.5
0.0
0.5
β_IV

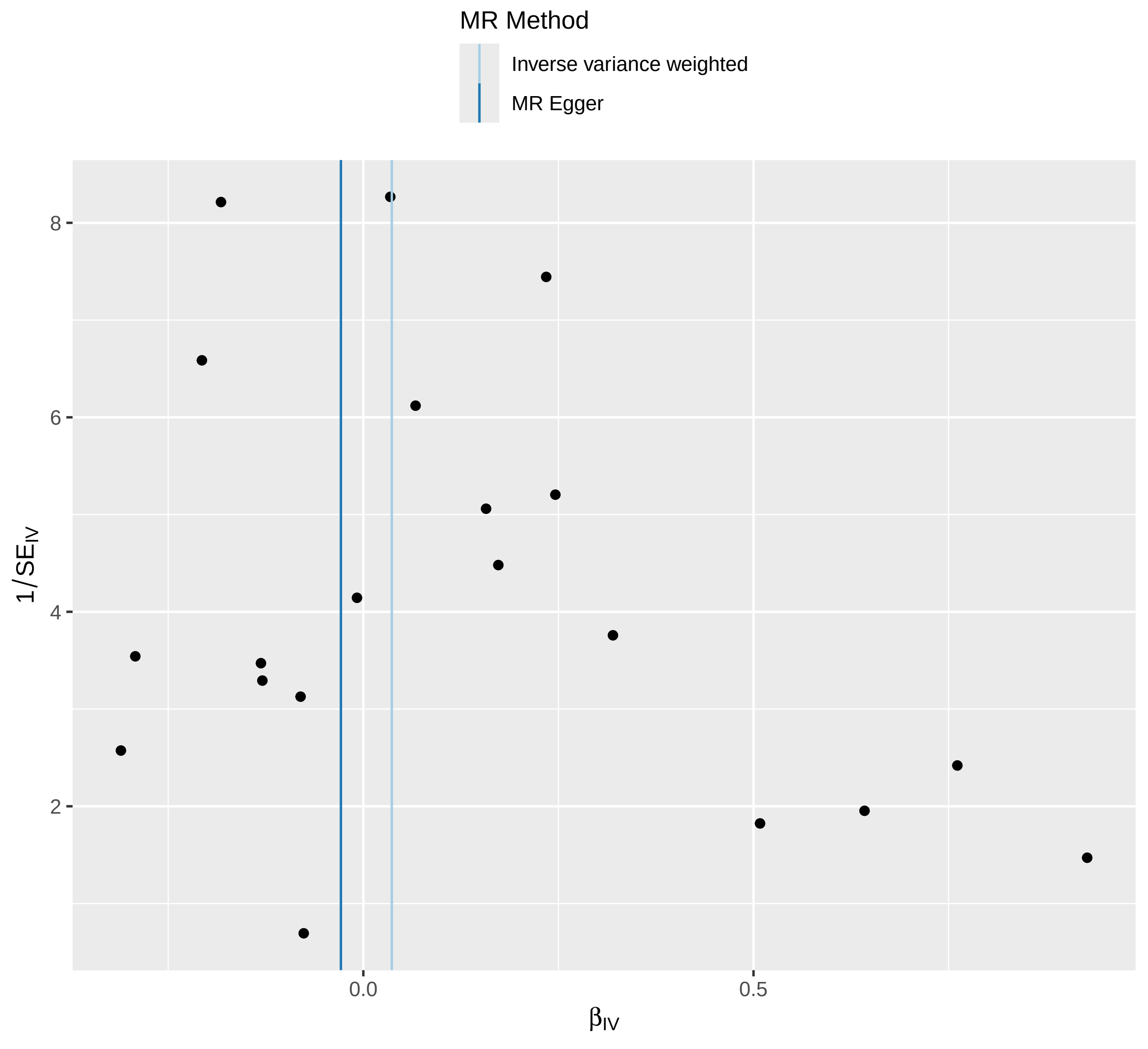

MR Method
Inverse variance weighted
MR Egger
8
6
4
2
1/SE_IV
0.0
0.5
β_IV

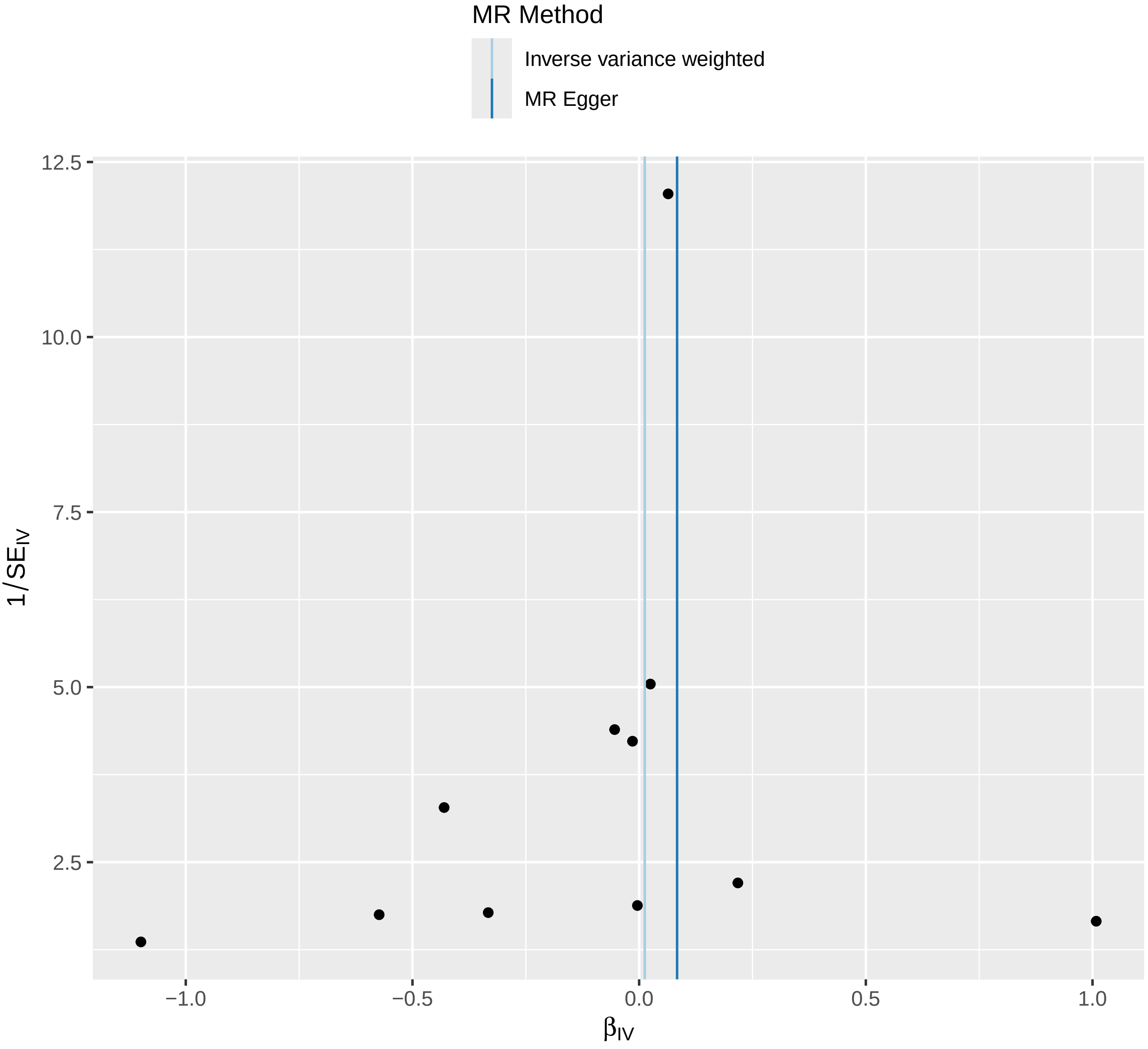

MR Method
Inverse variance weighted
MR Egger
12.5
10.0
7.5
5.0
2.5
1/SE$_{IV}$
−1.0
−0.5
0.0
0.5
1.0
β$_{IV}$

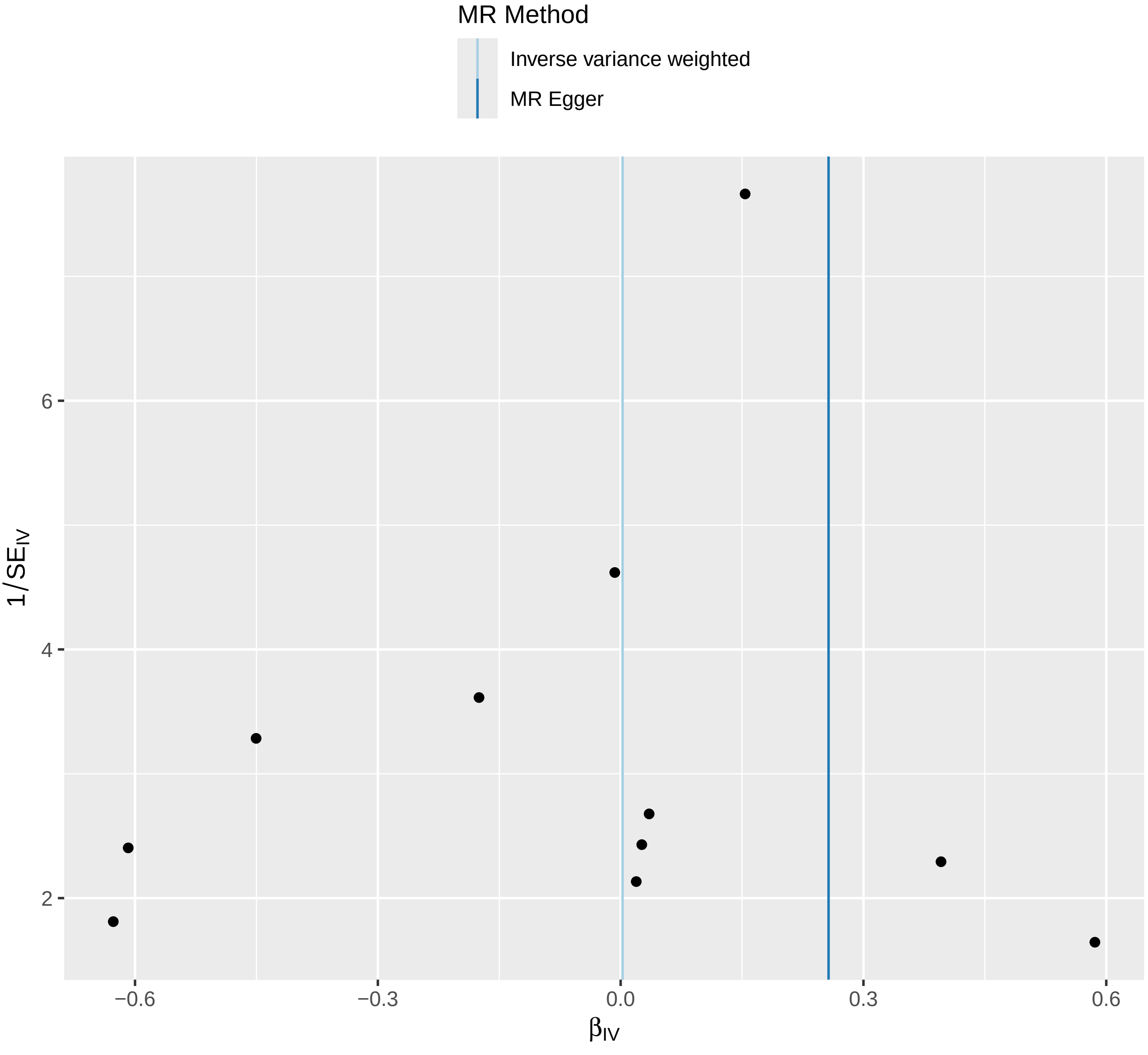
MR Method
Inverse variance weighted
MR Egger
1/SE$_{IV}$
6
4
2
−0.6
−0.3
0.0
0.3
0.6
β$_{IV}$

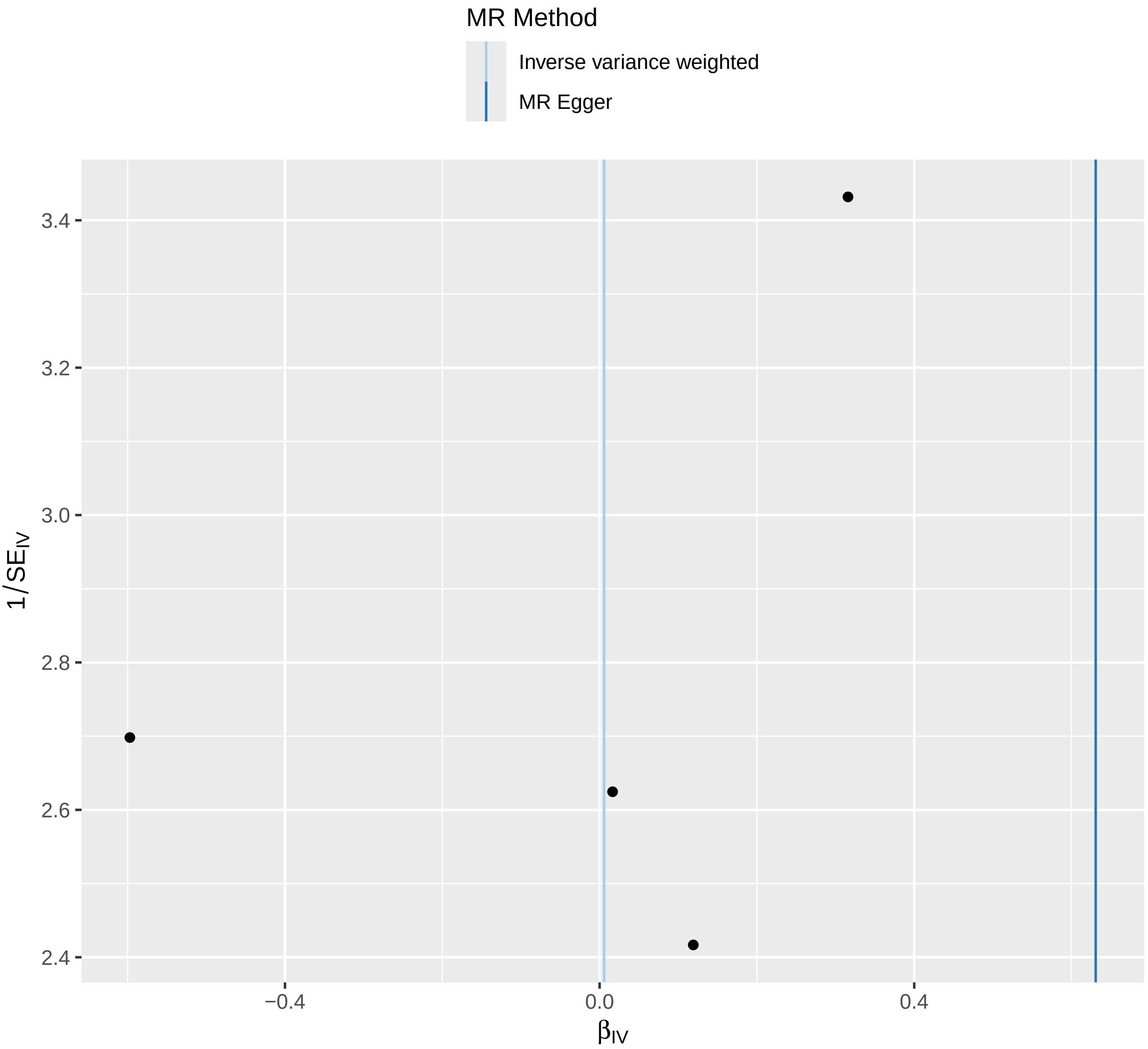
MR Method
Inverse variance weighted
MR Egger
1/SE_IV
β_IV
3.4
3.2
3.0
2.8
2.6
2.4
−0.4
0.0
0.4

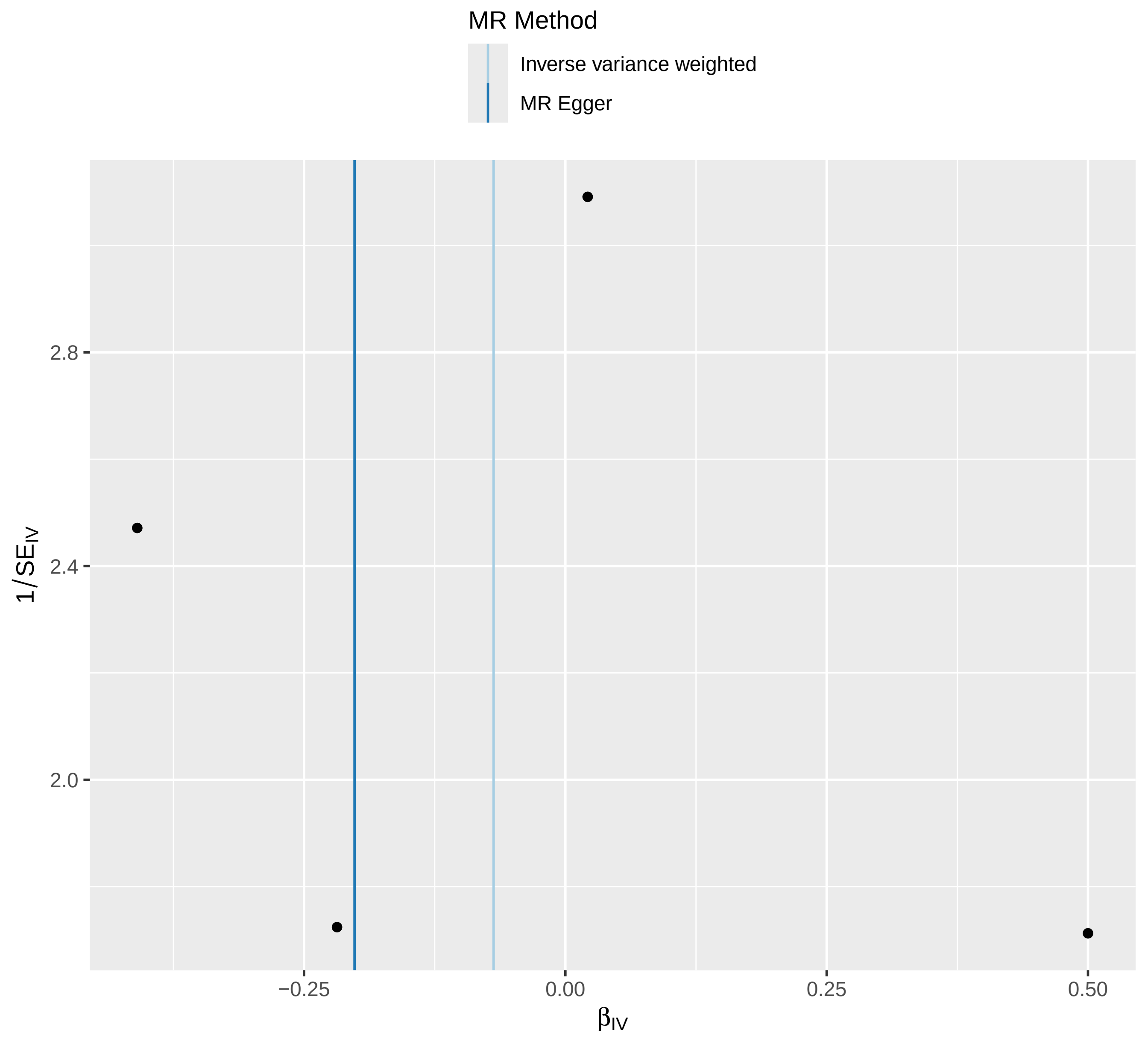

MR Method
Inverse variance weighted
MR Egger
2.8
2.4
2.0
1/SE$_{IV}$
−0.25
0.00
0.25
0.50
β$_{IV}$

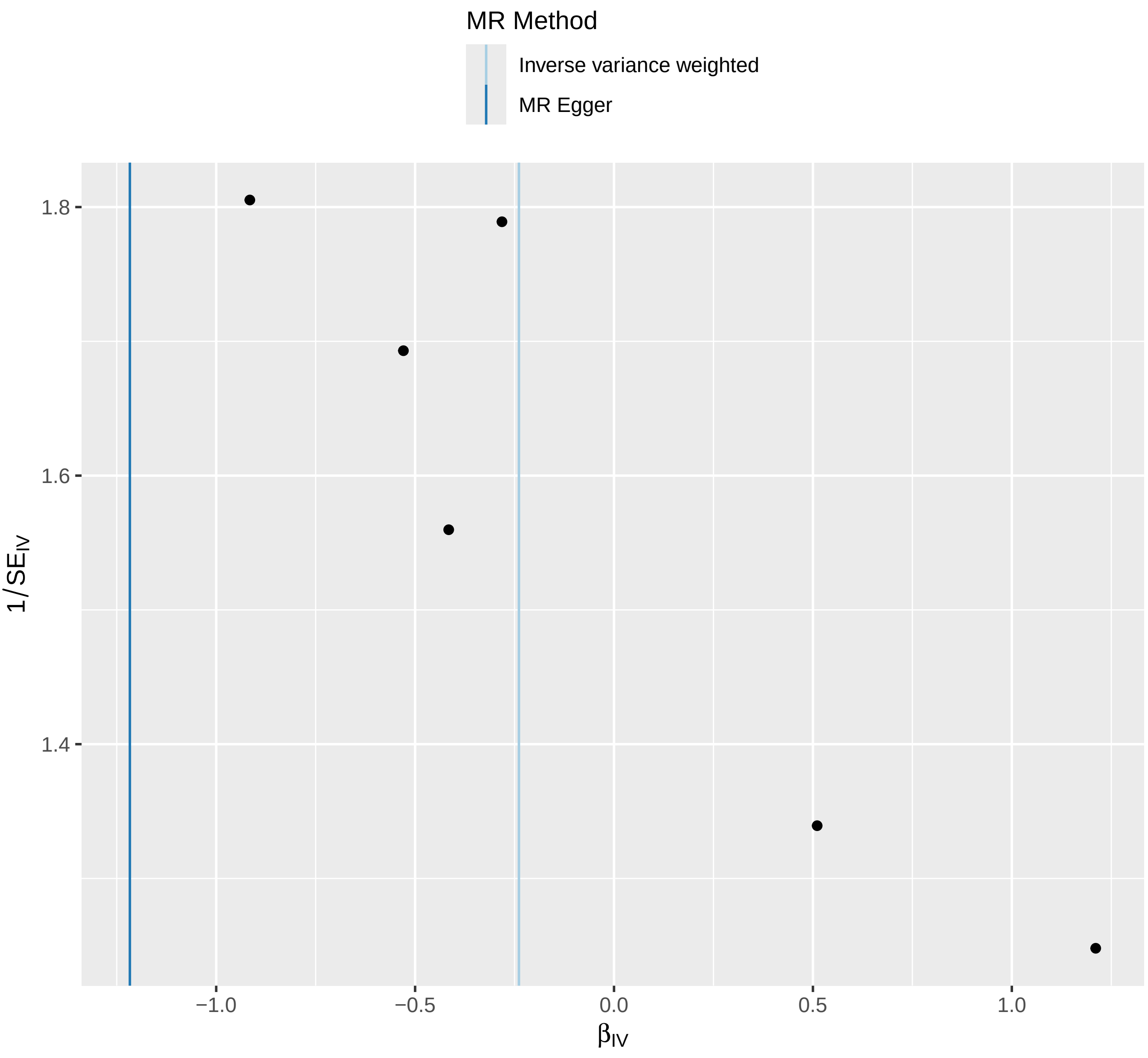
MR Method
Inverse variance weighted
MR Egger
1.8
1.6
1.4
−1.0
−0.5
0.0
0.5
1.0
$1/SE_{IV}$
β_{IV}

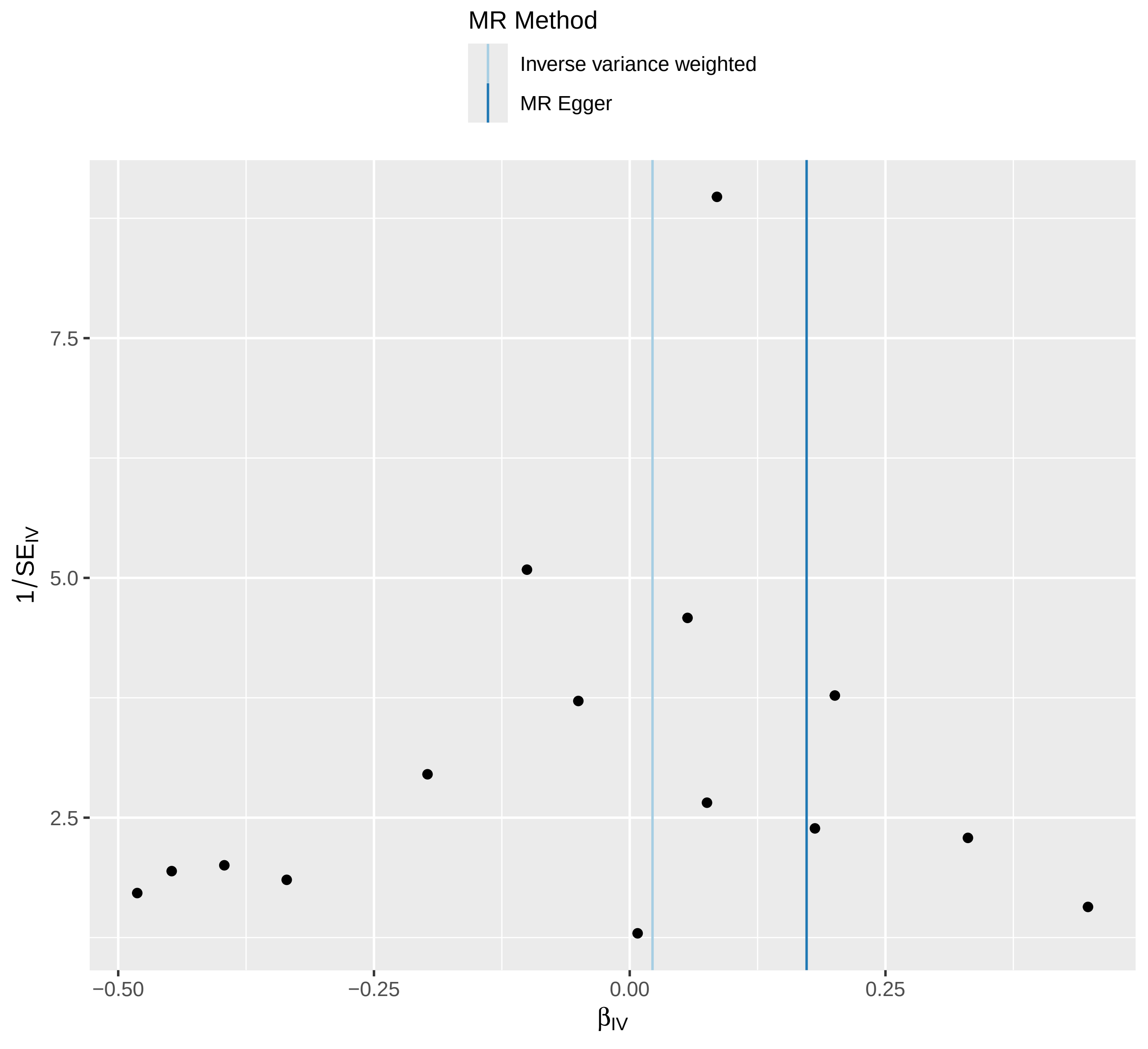
MR Method
Inverse variance weighted
MR Egger
7.5
5.0
2.5
1/SE_IV
−0.50
−0.25
0.00
0.25
β_IV

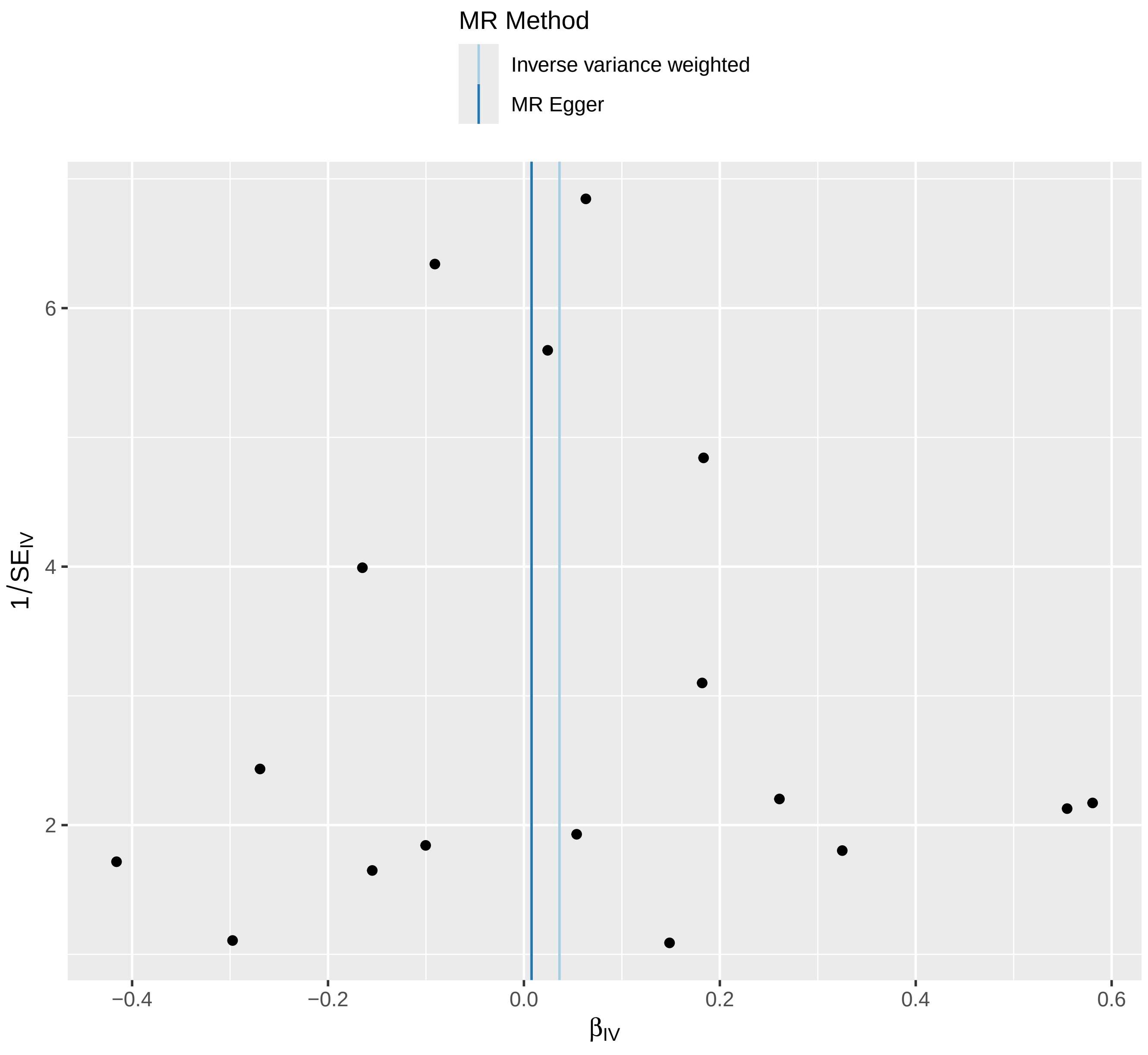

MR Method
Inverse variance weighted
MR Egger
1/SE_IV
6
4
2
β_IV
−0.4
−0.2
0.0
0.2
0.4
0.6

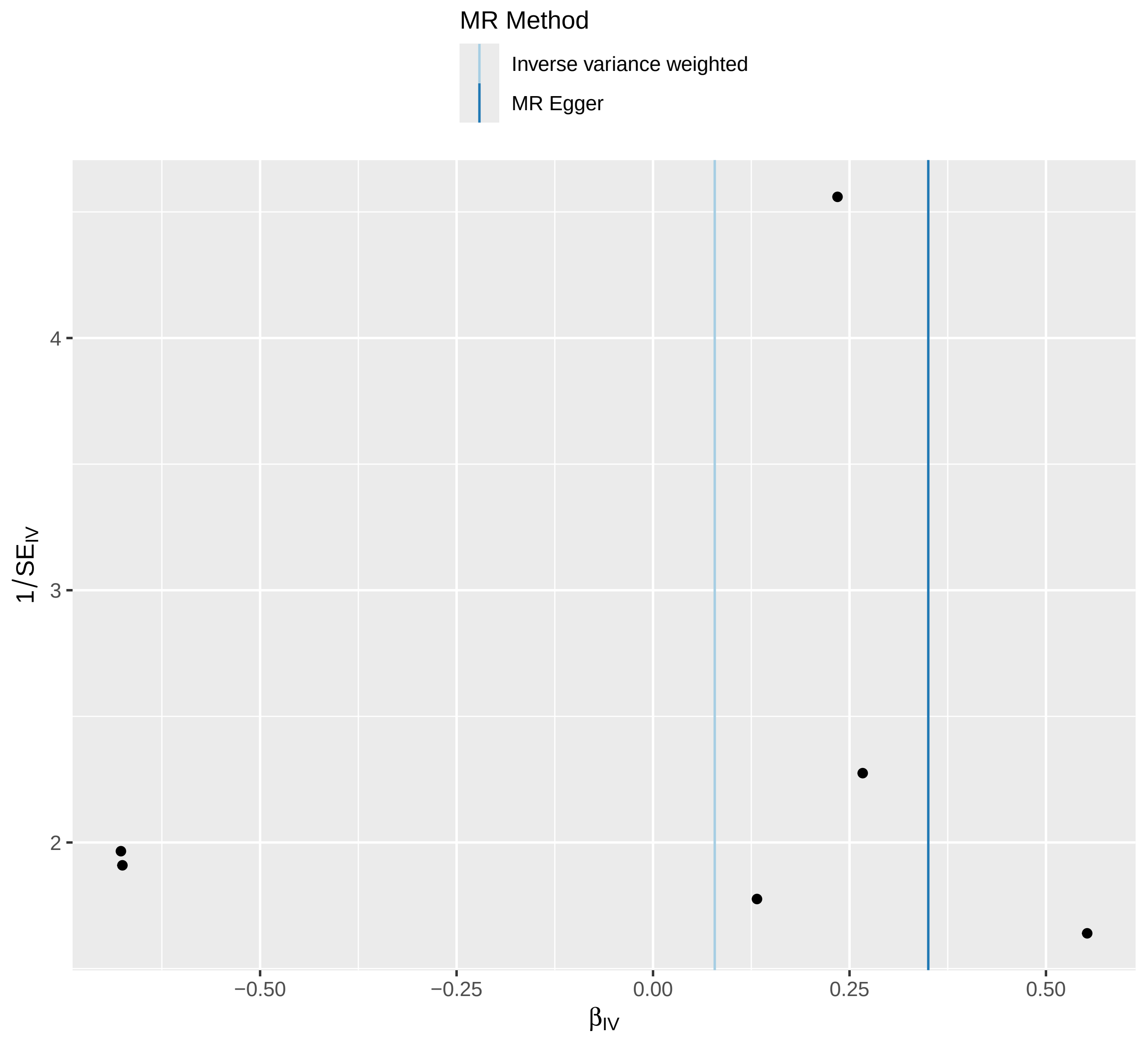
MR Method
Inverse variance weighted
MR Egger
1/SE$_{IV}$
4
3
2
−0.50
−0.25
0.00
0.25
0.50
β$_{IV}$

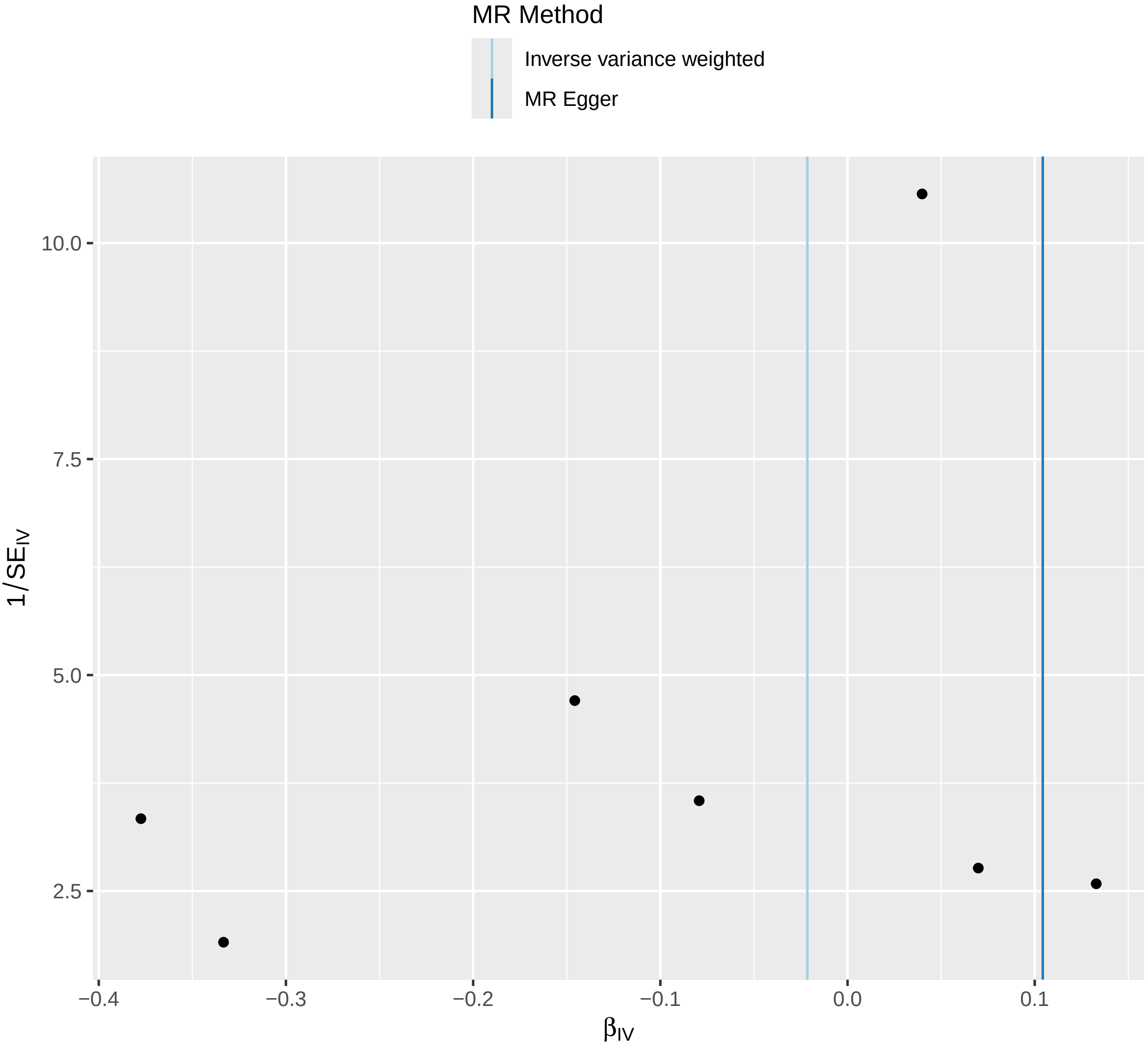
MR Method
Inverse variance weighted
MR Egger
10.0
7.5
5.0
2.5
1/SE_IV
−0.4
−0.3
−0.2
−0.1
0.0
0.1
β_IV

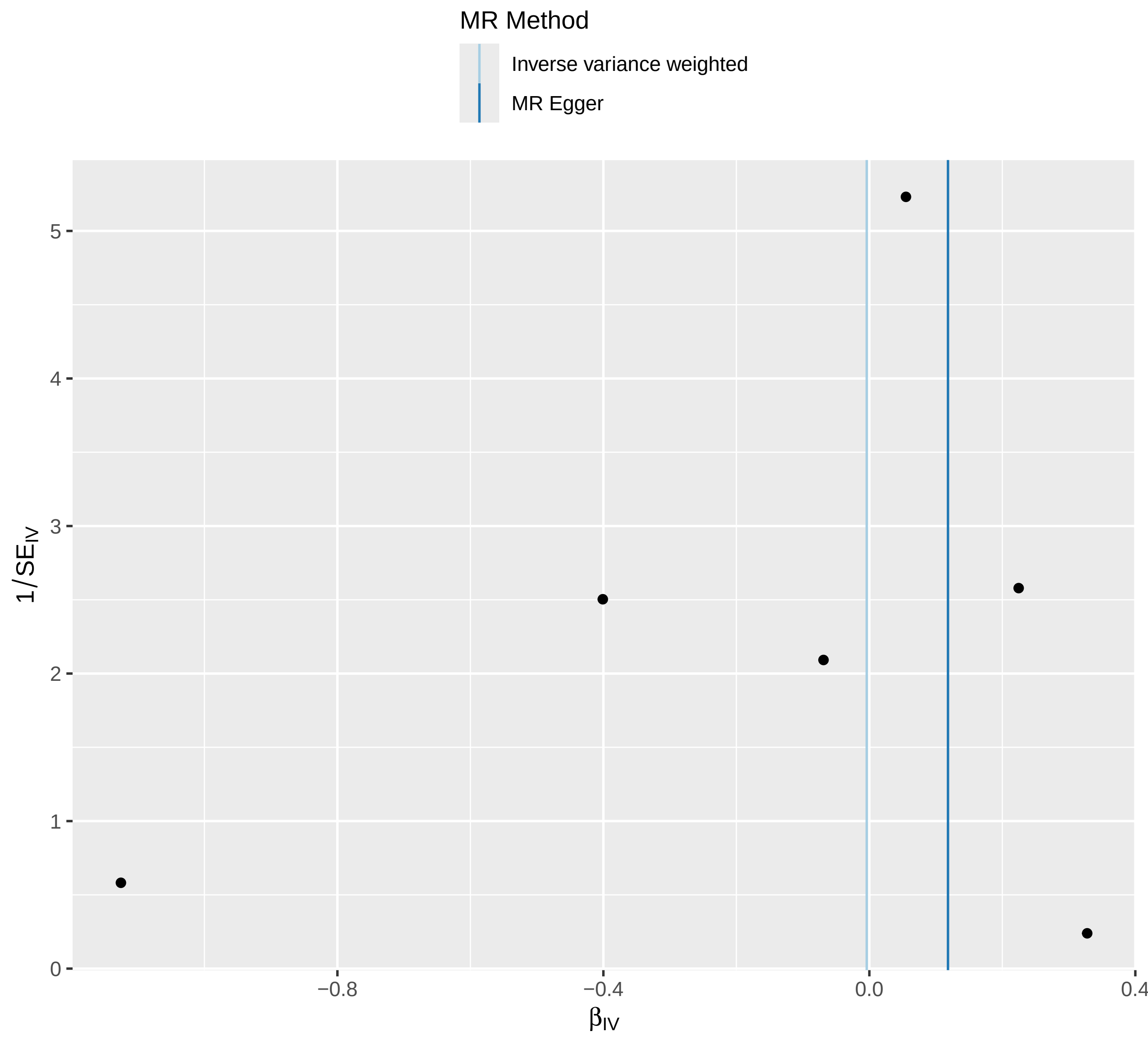

MR Method
Inverse variance weighted
MR Egger
5
4
3
2
1
0
1/SE_IV
−0.8
−0.4
0.0
0.4
β_IV

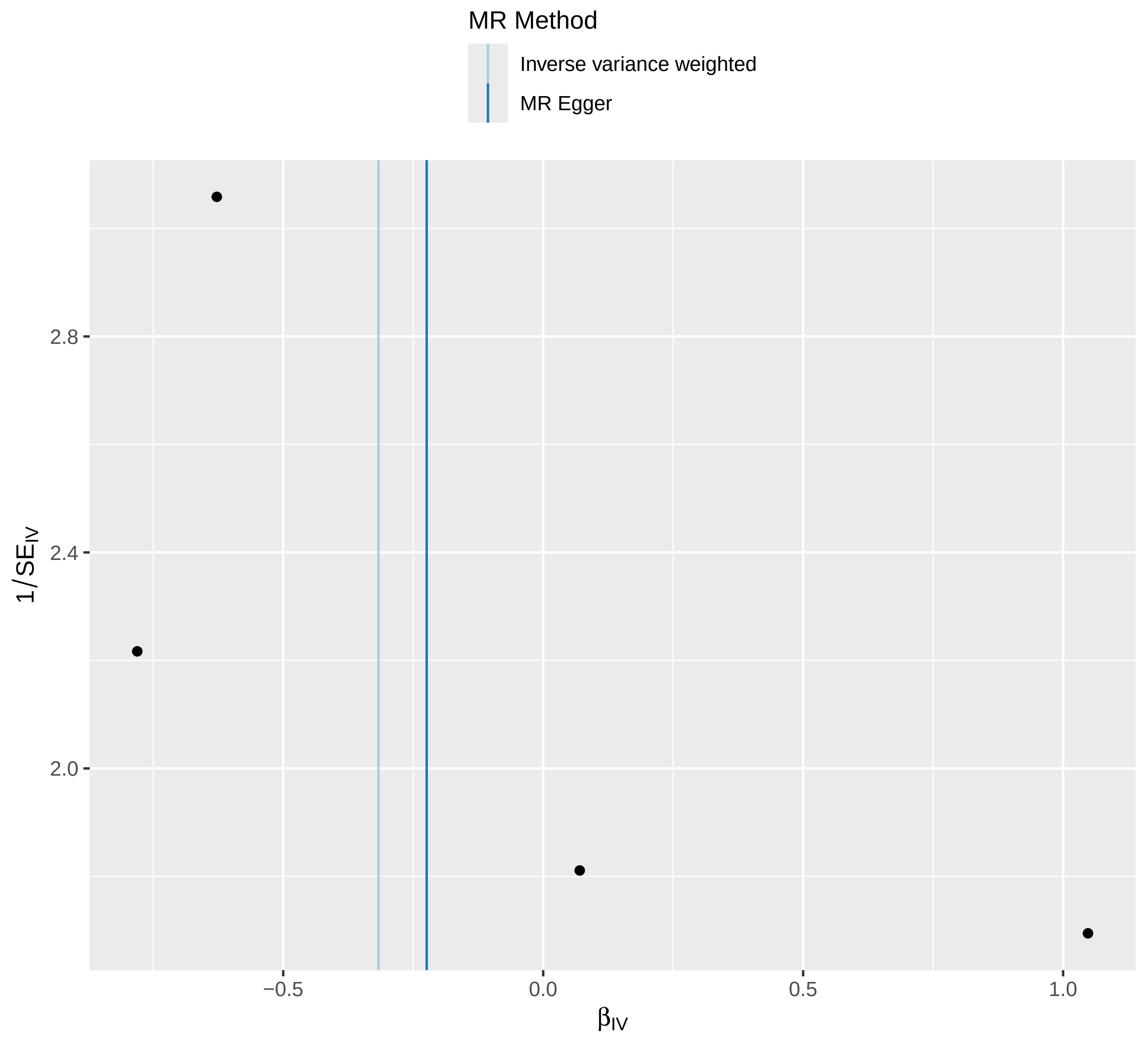

MR Method
Inverse variance weighted
MR Egger
2.8
2.4
2.0
1/SE_IV
−0.5
0.0
0.5
1.0
β_IV

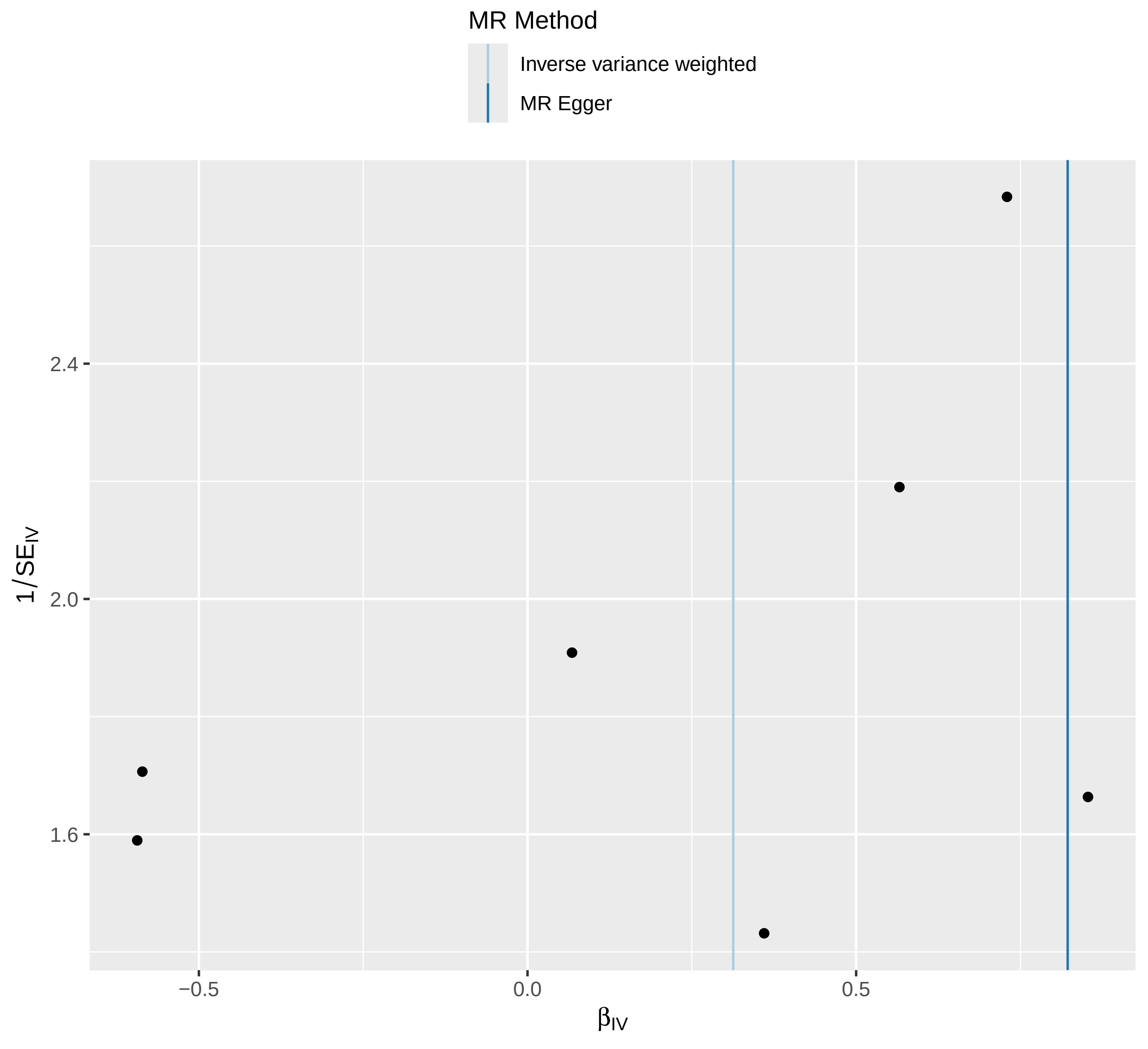
MR Method
Inverse variance weighted
MR Egger
2.4
2.0
1.6
1/SE$_{IV}$
−0.5
0.0
0.5
β$_{IV}$

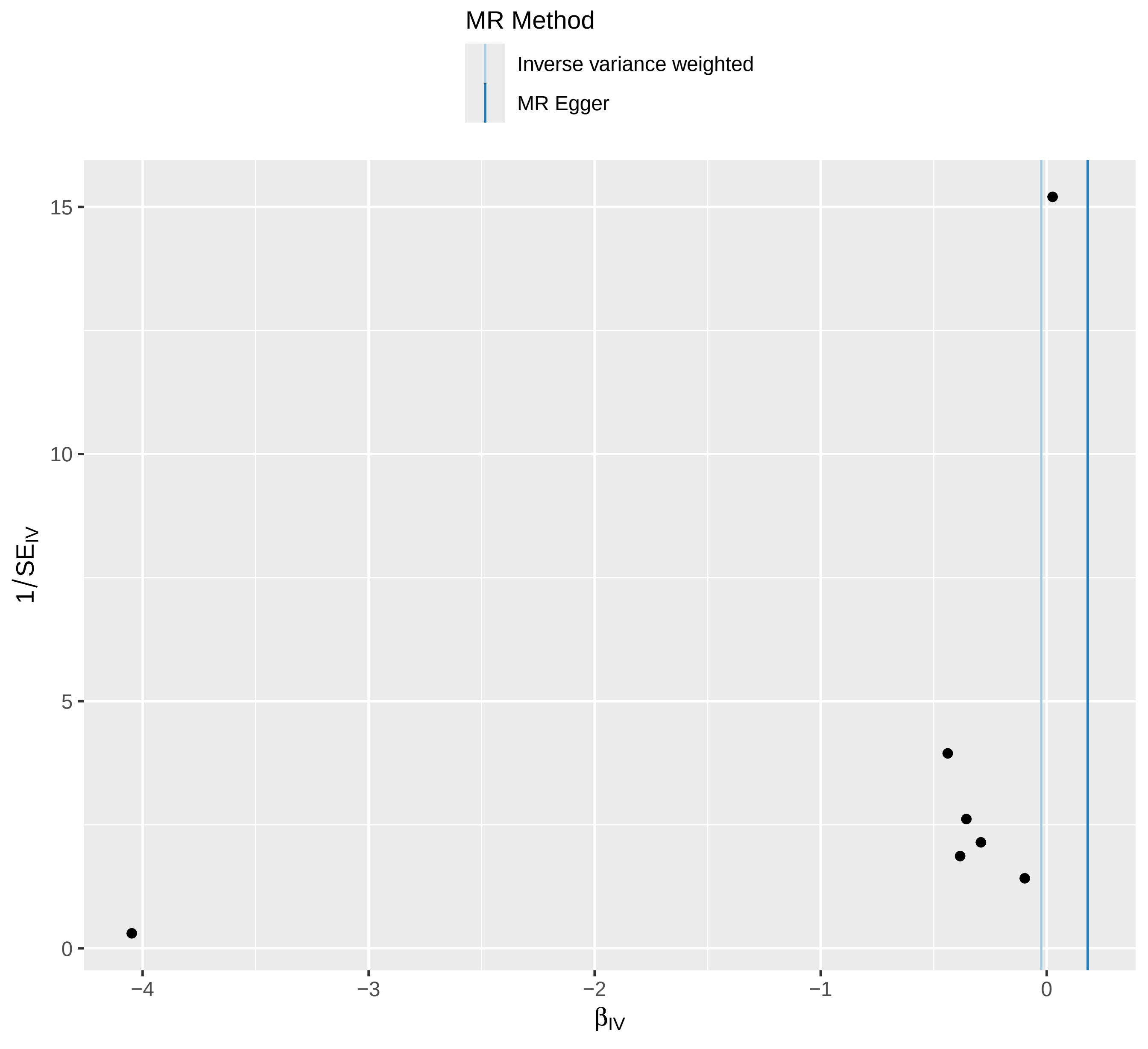

MR Method
Inverse variance weighted
MR Egger
15
10
5
0
1/SE$_{IV}$
−4
−3
−2
−1
0
β$_{IV}$

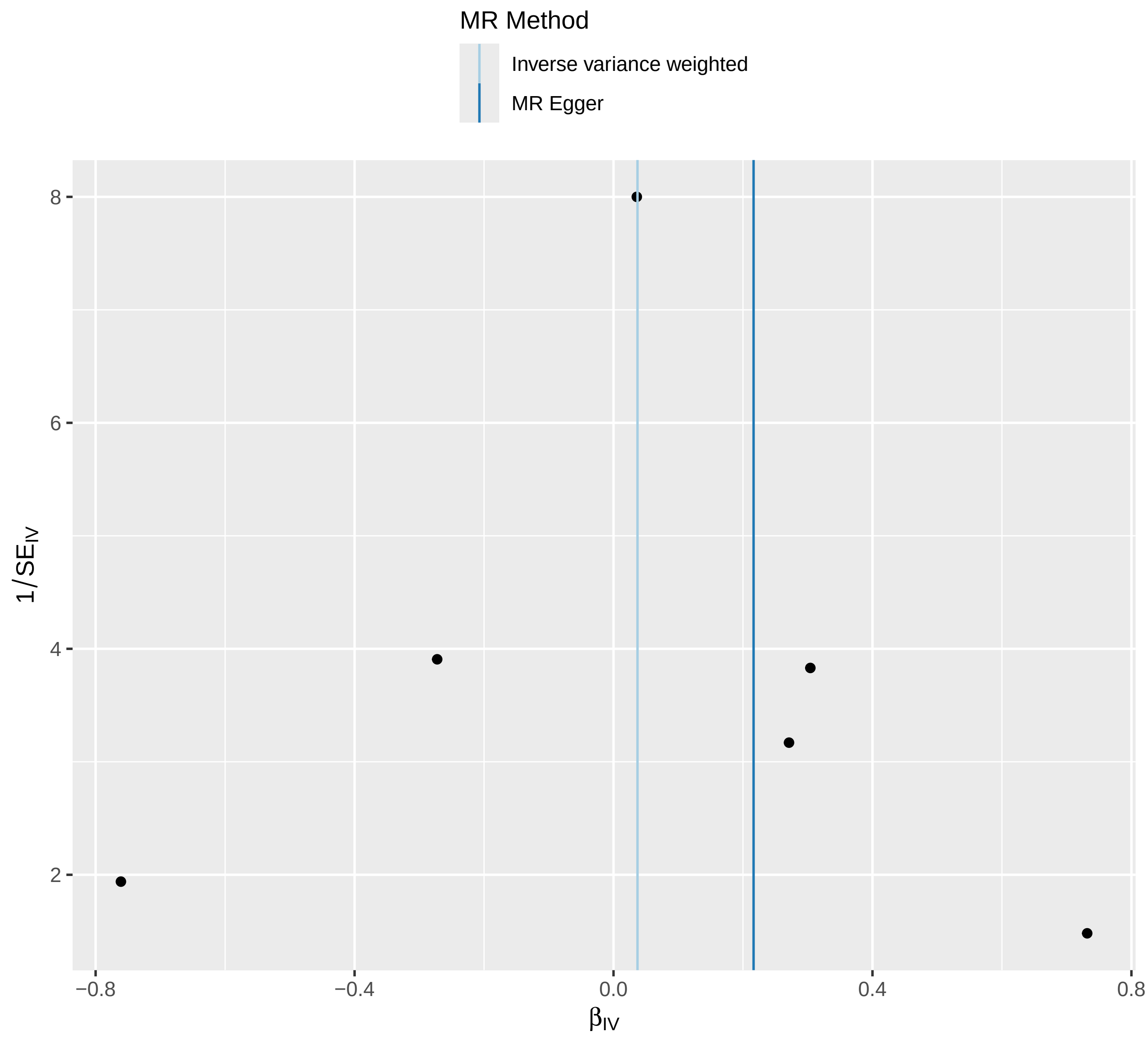

MR Method
Inverse variance weighted
MR Egger
8
6
4
2
1/SE_IV
−0.8
−0.4
0.0
0.4
0.8
β_IV

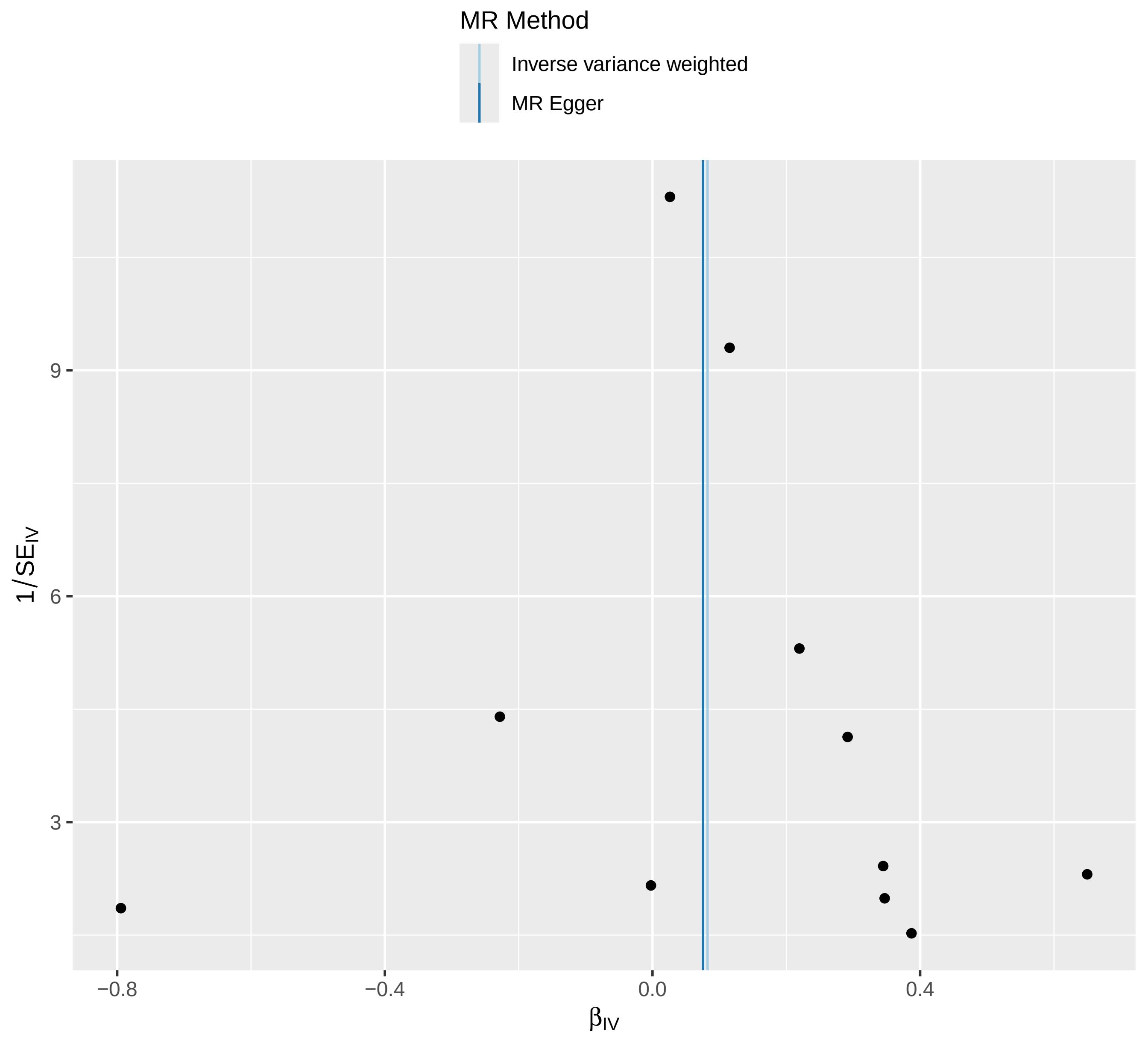
MR Method
Inverse variance weighted
MR Egger
1/SE_IV
9
6
3
−0.8
−0.4
0.0
0.4
β_IV

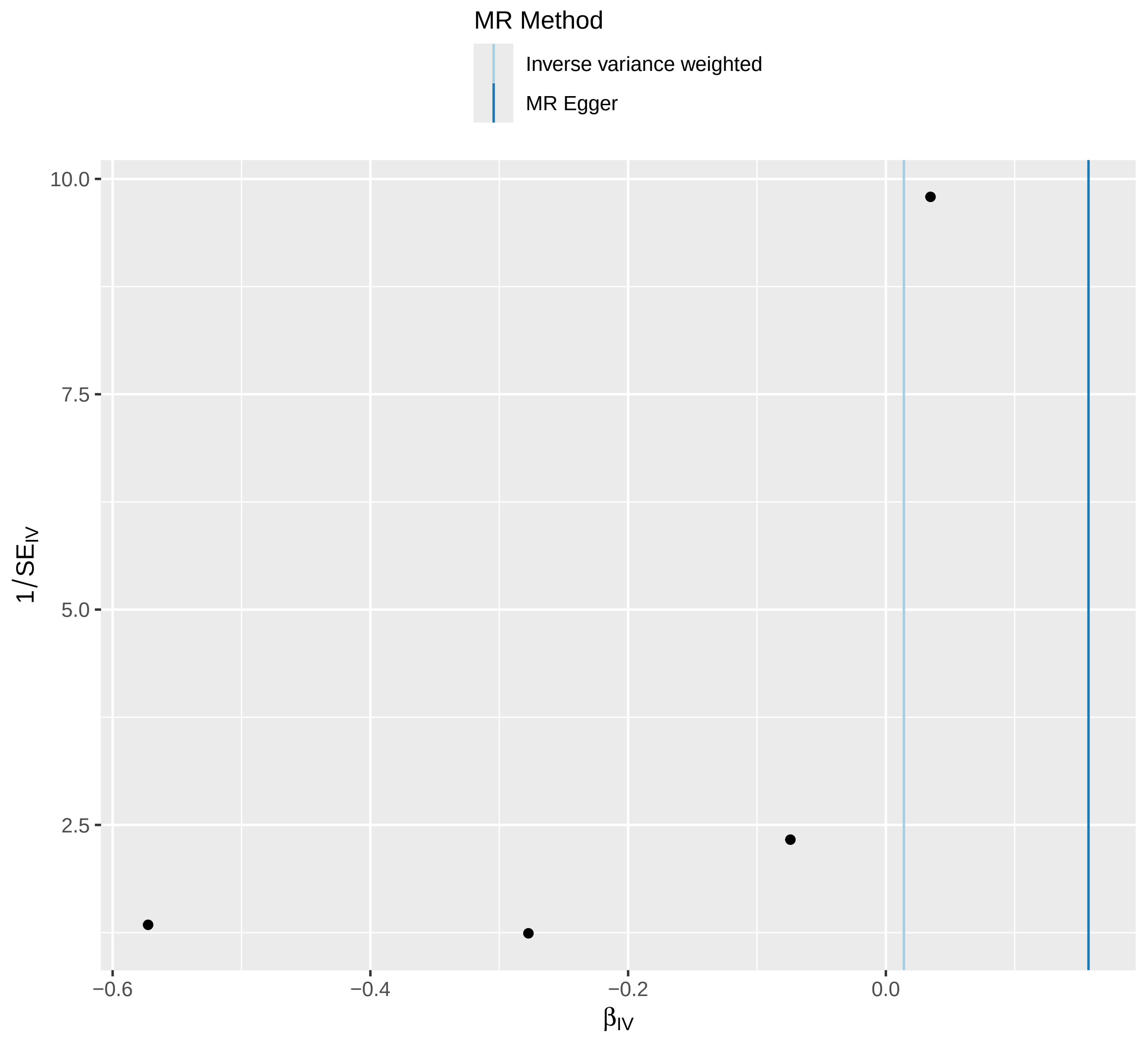

MR Method
Inverse variance weighted
MR Egger
10.0
7.5
5.0
2.5
$1/SE_{IV}$
−0.6
−0.4
−0.2
0.0
β_{IV}

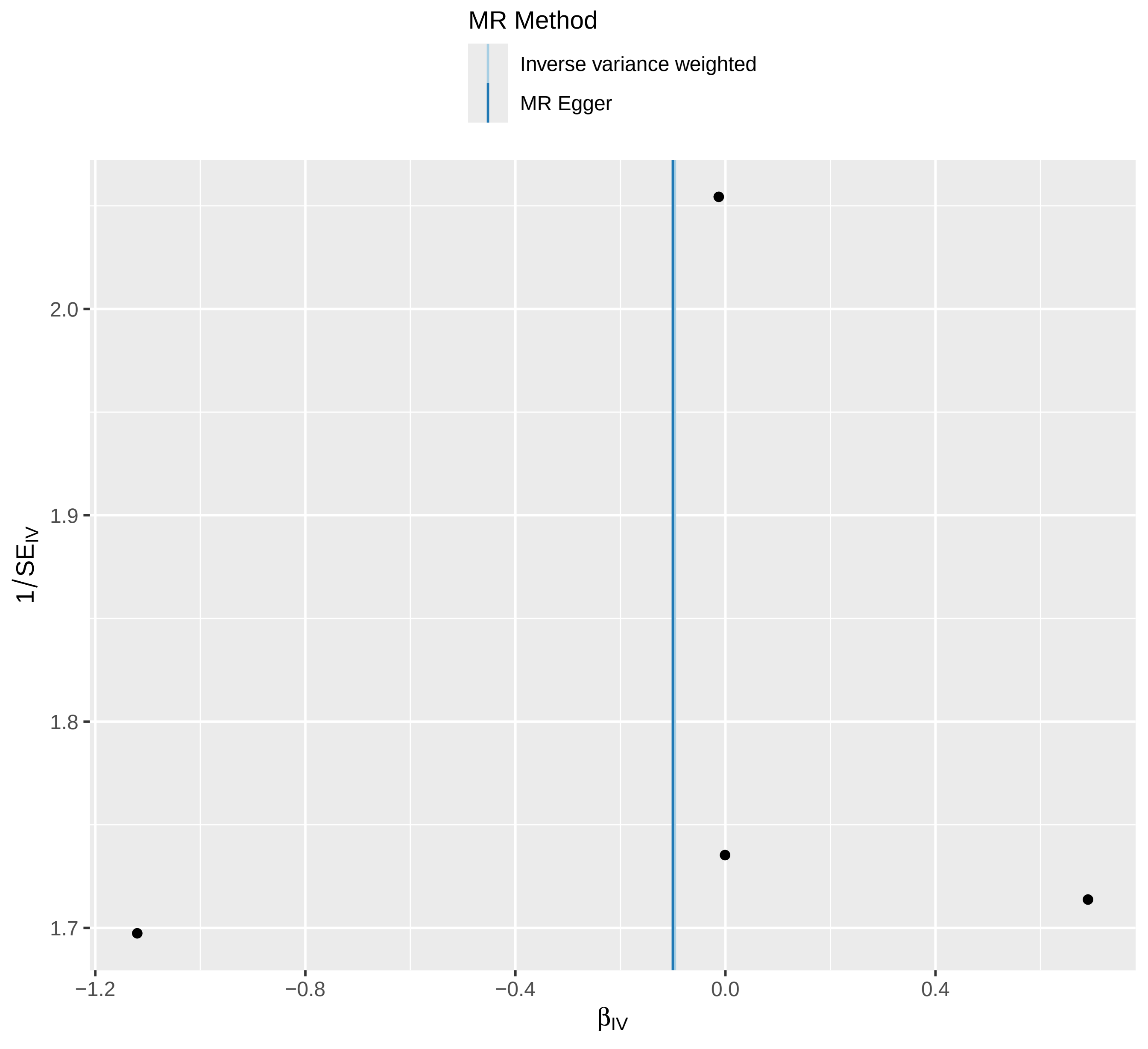

MR Method
Inverse variance weighted
MR Egger
2.0
1.9
1.8
1.7
1/SE_IV
−1.2
−0.8
−0.4
0.0
0.4
β_IV

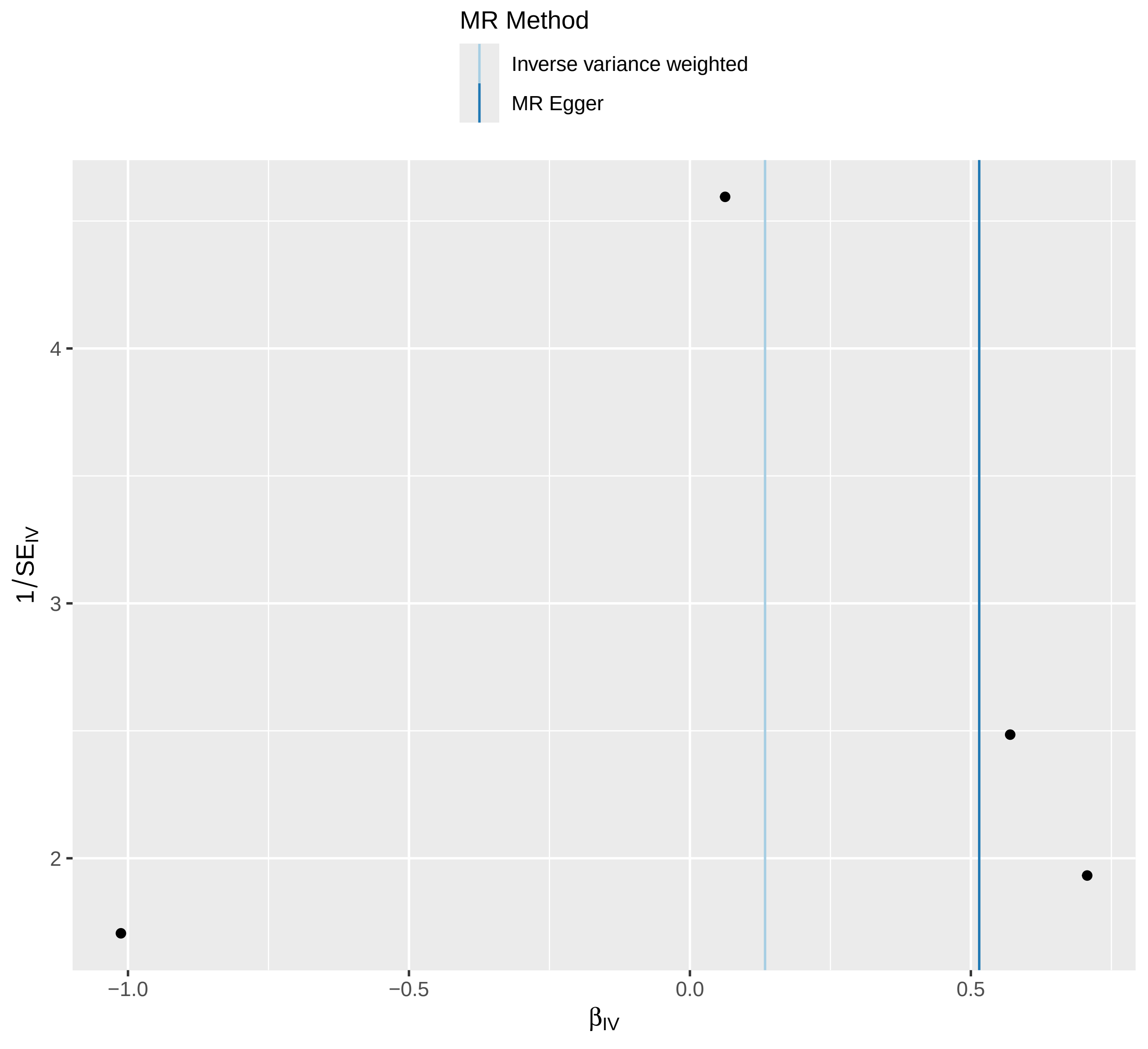

MR Method
Inverse variance weighted
MR Egger
4
3
2
1/SE_IV
−1.0
−0.5
0.0
0.5
β_IV

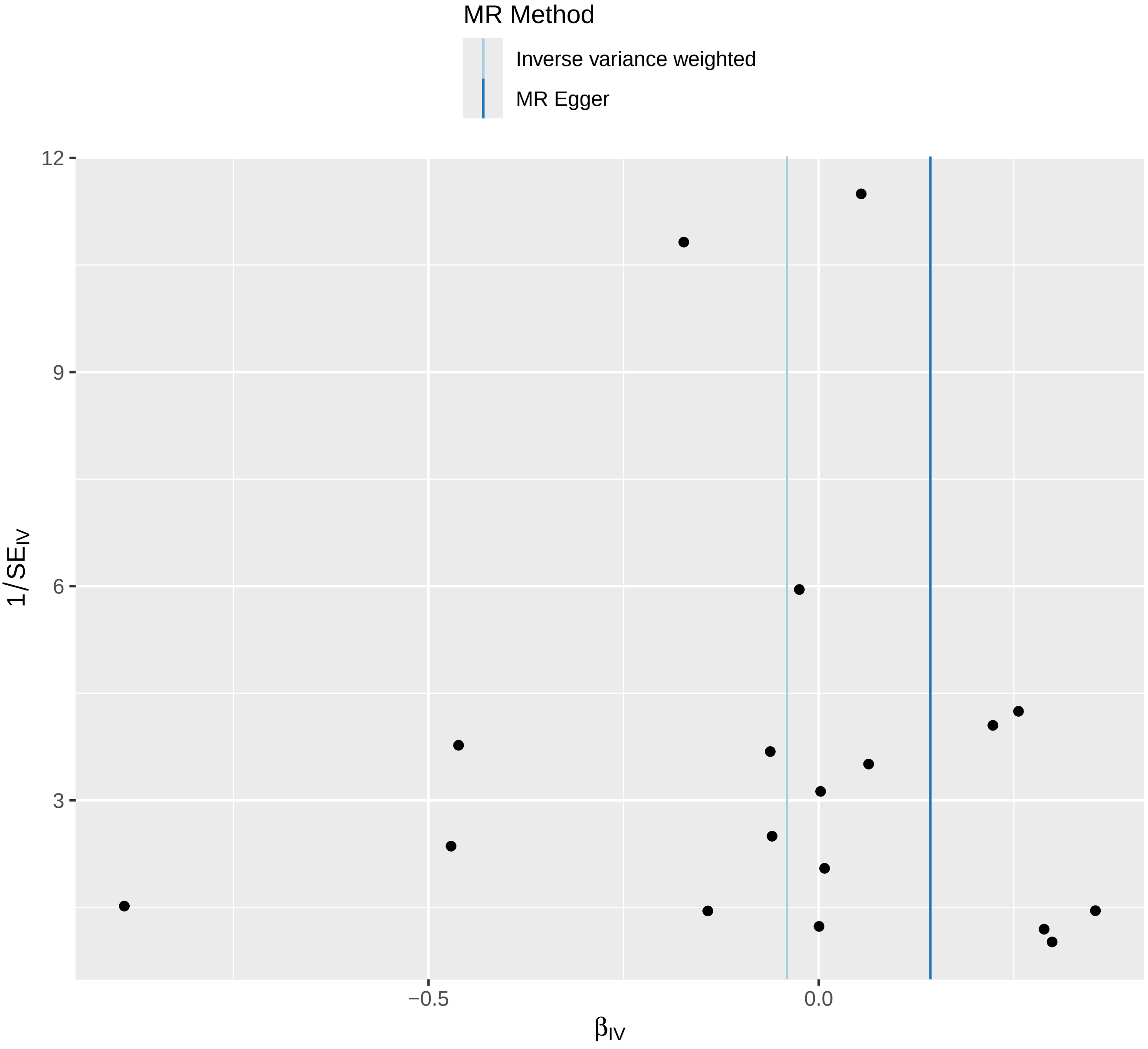

MR Method
Inverse variance weighted
MR Egger
12
9
6
3
−0.5
0.0
1/SE$_{IV}$
β$_{IV}$

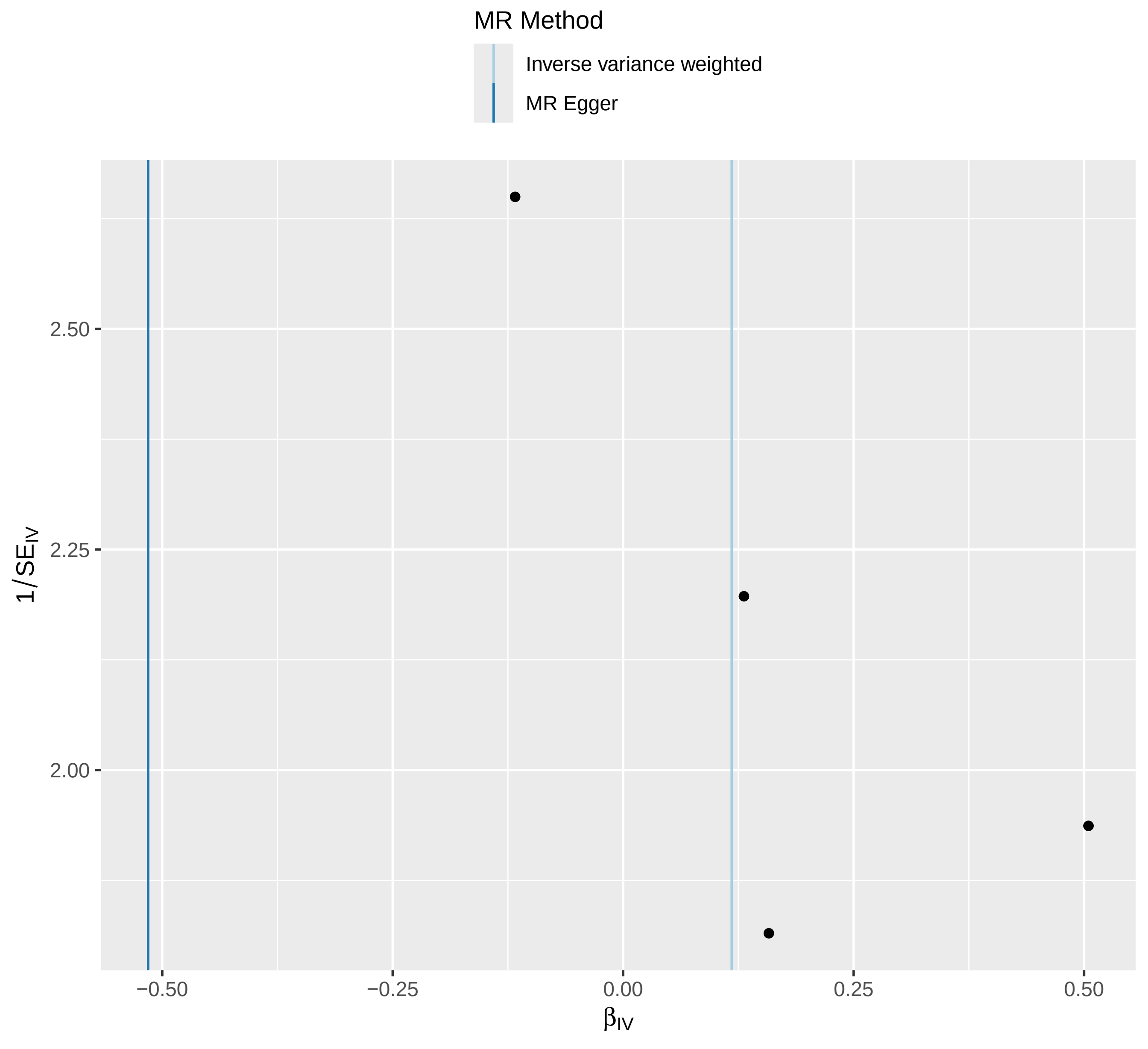

MR Method
Inverse variance weighted
MR Egger
2.50
2.25
2.00
$1/SE_{IV}$
−0.50
−0.25
0.00
0.25
0.50
β_{IV}

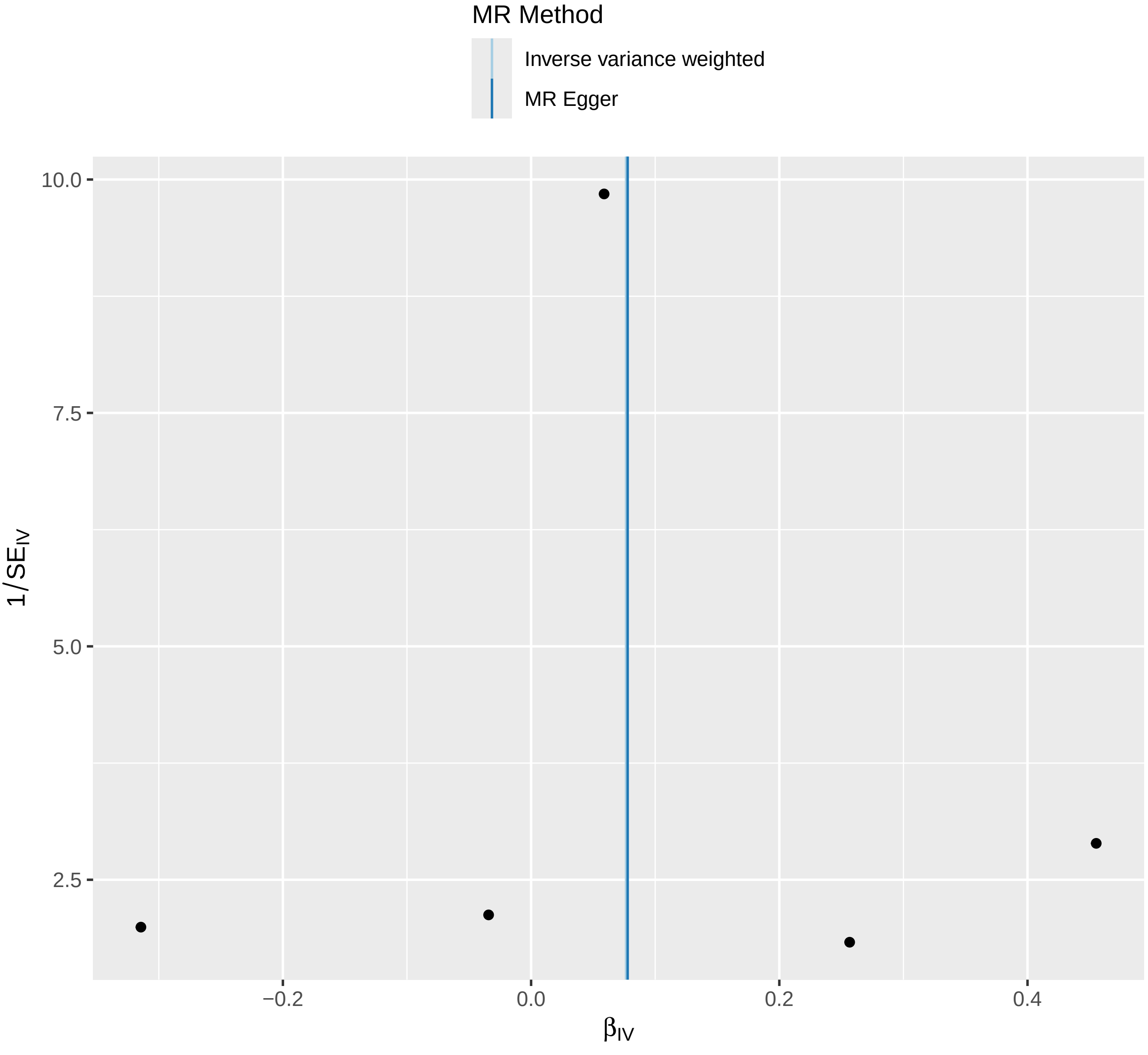

MR Method
Inverse variance weighted
MR Egger
10.0
7.5
5.0
2.5
1/SE_IV
−0.2
0.0
0.2
0.4
β_IV

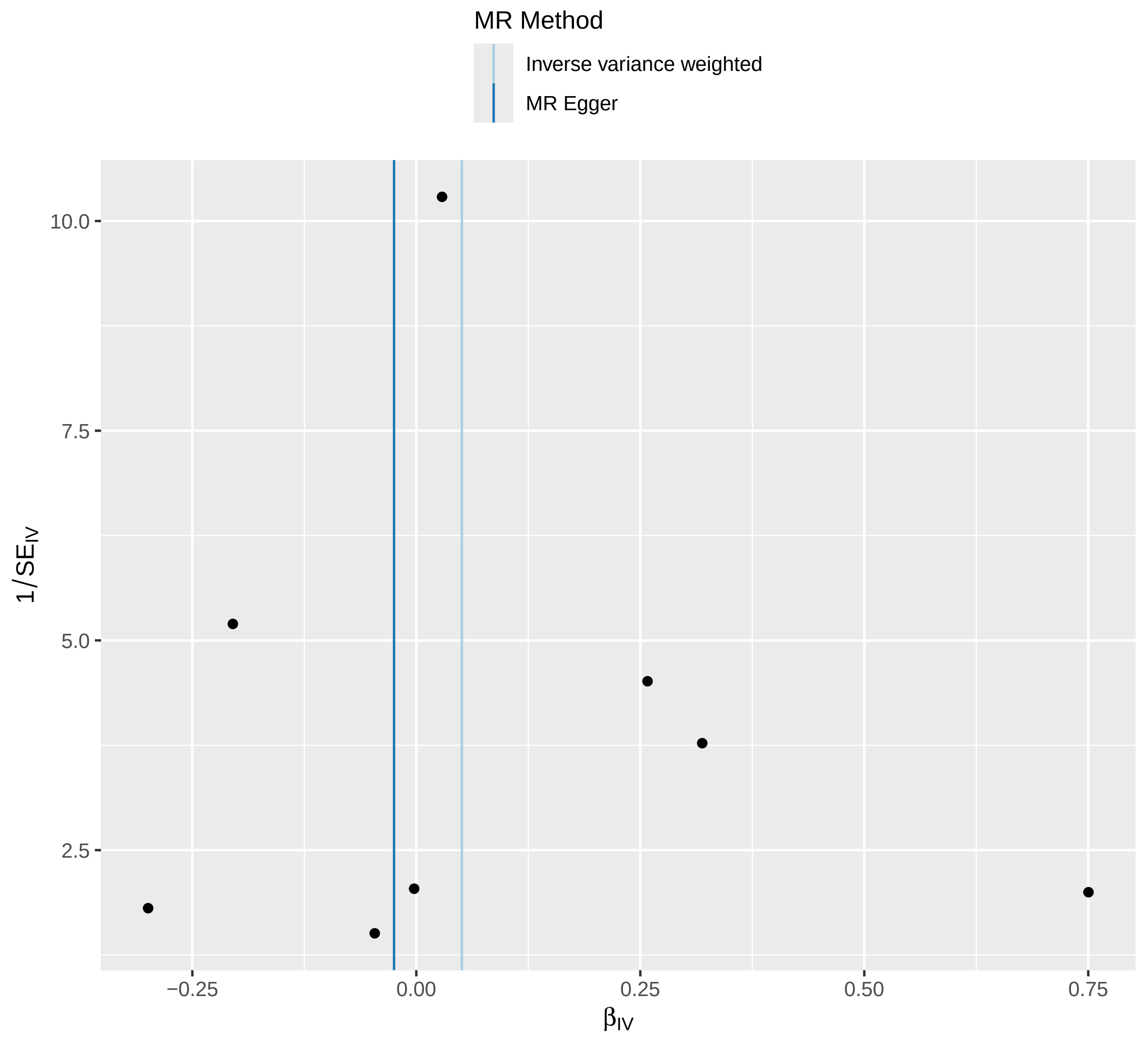

MR Method
Inverse variance weighted
MR Egger
10.0
7.5
5.0
2.5
−0.25
0.00
0.25
0.50
0.75
$1/SE_{IV}$
β_{IV}

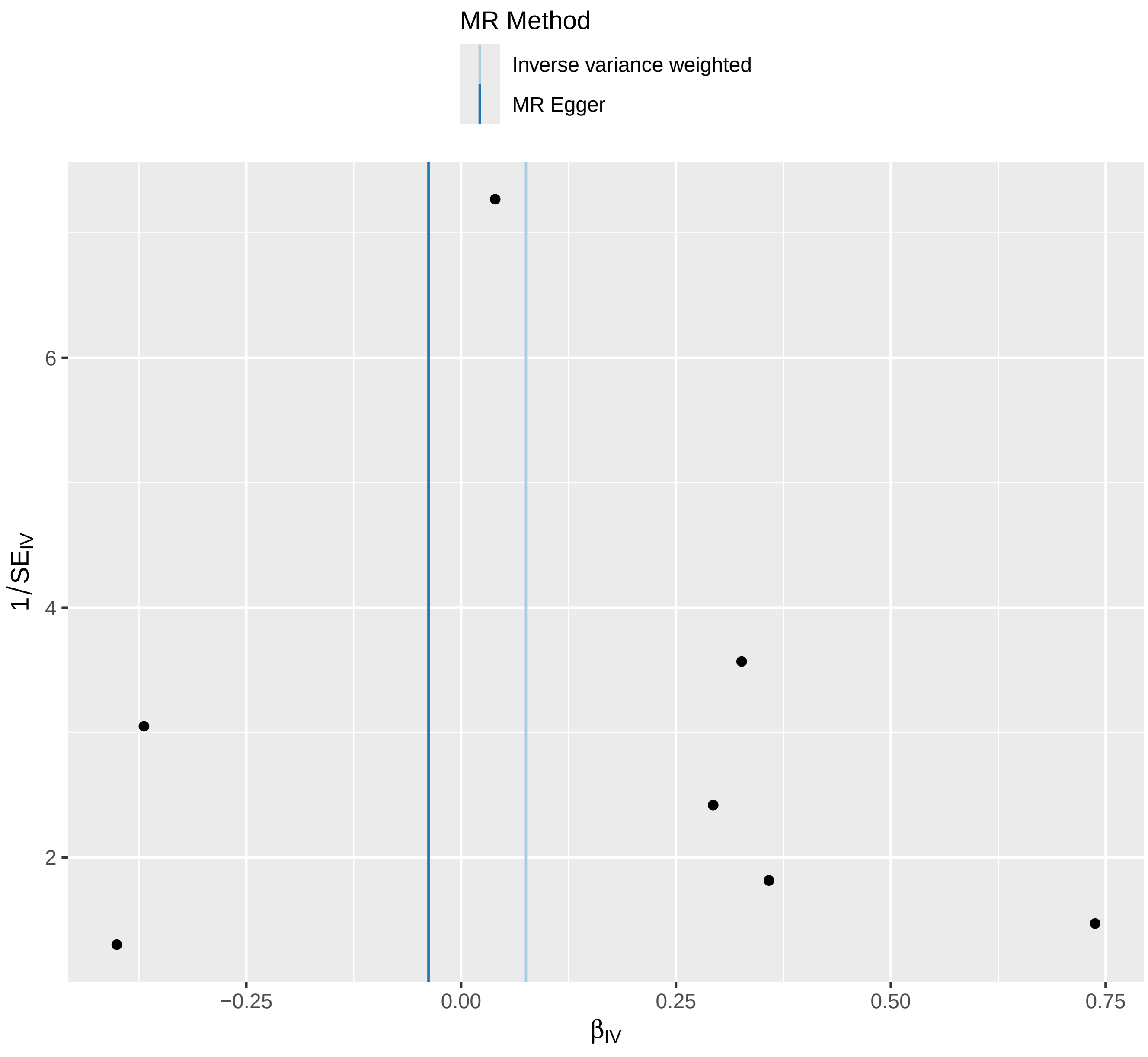

MR Method
Inverse variance weighted
MR Egger
6
4
2
1/SE_IV
−0.25
0.00
0.25
0.50
0.75
β_IV

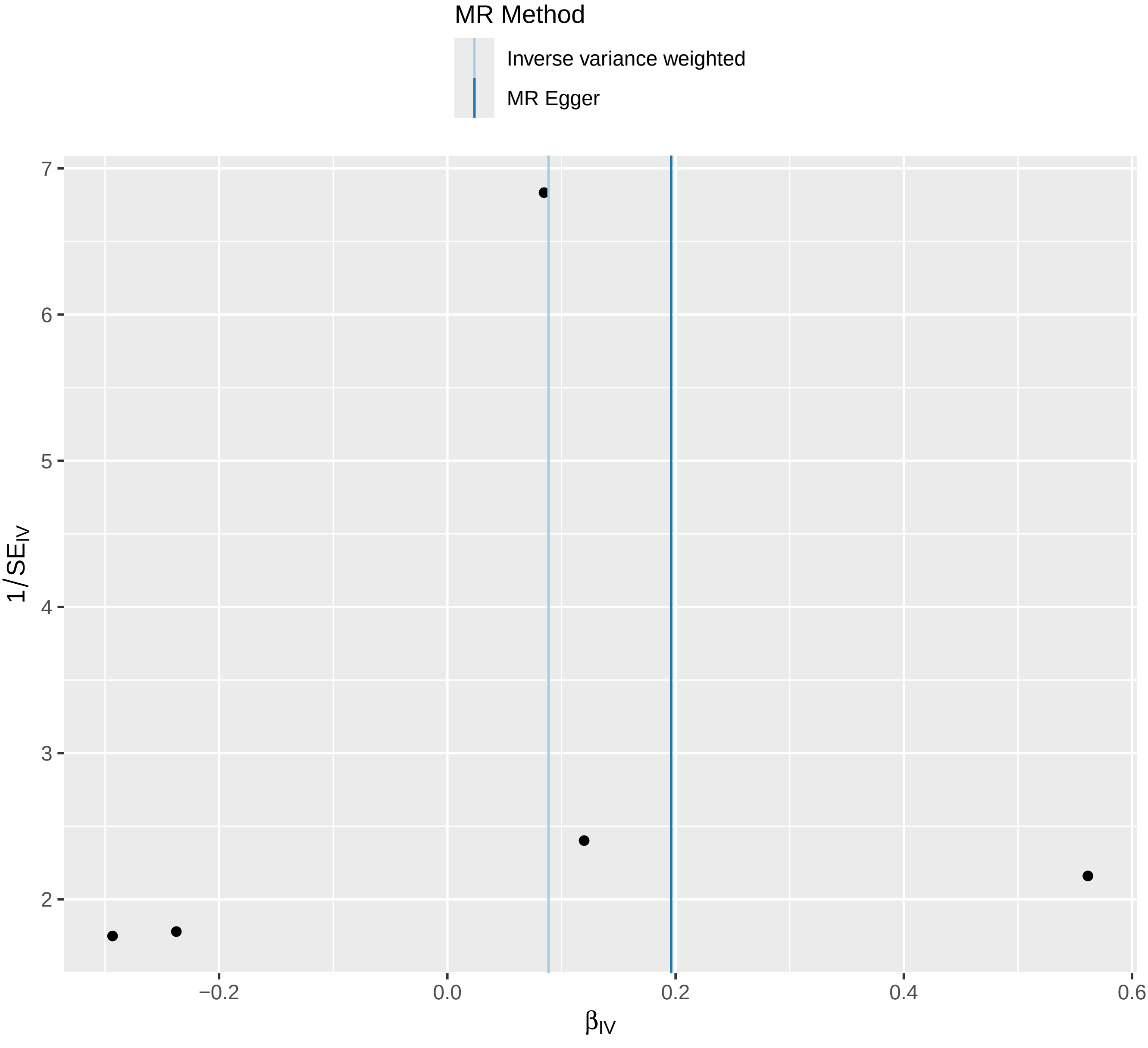
MR Method
Inverse variance weighted
MR Egger
7
6
5
4
3
2
1/SE$_{IV}$
−0.2
0.0
0.2
0.4
0.6
β$_{IV}$

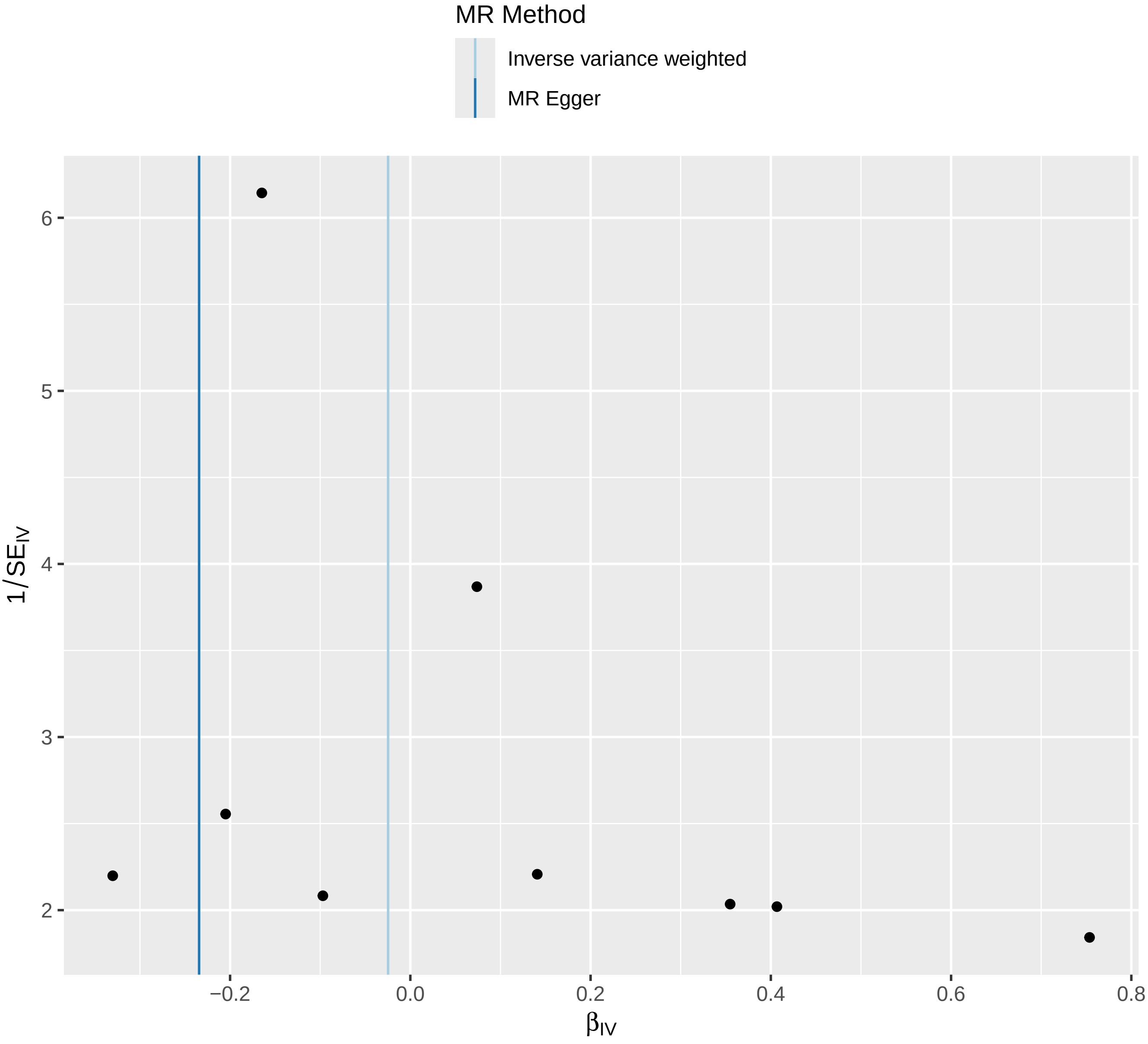
MR Method
Inverse variance weighted
MR Egger
1/SE_IV
β_IV
6
5
4
3
2
−0.2
0.0
0.2
0.4
0.6
0.8

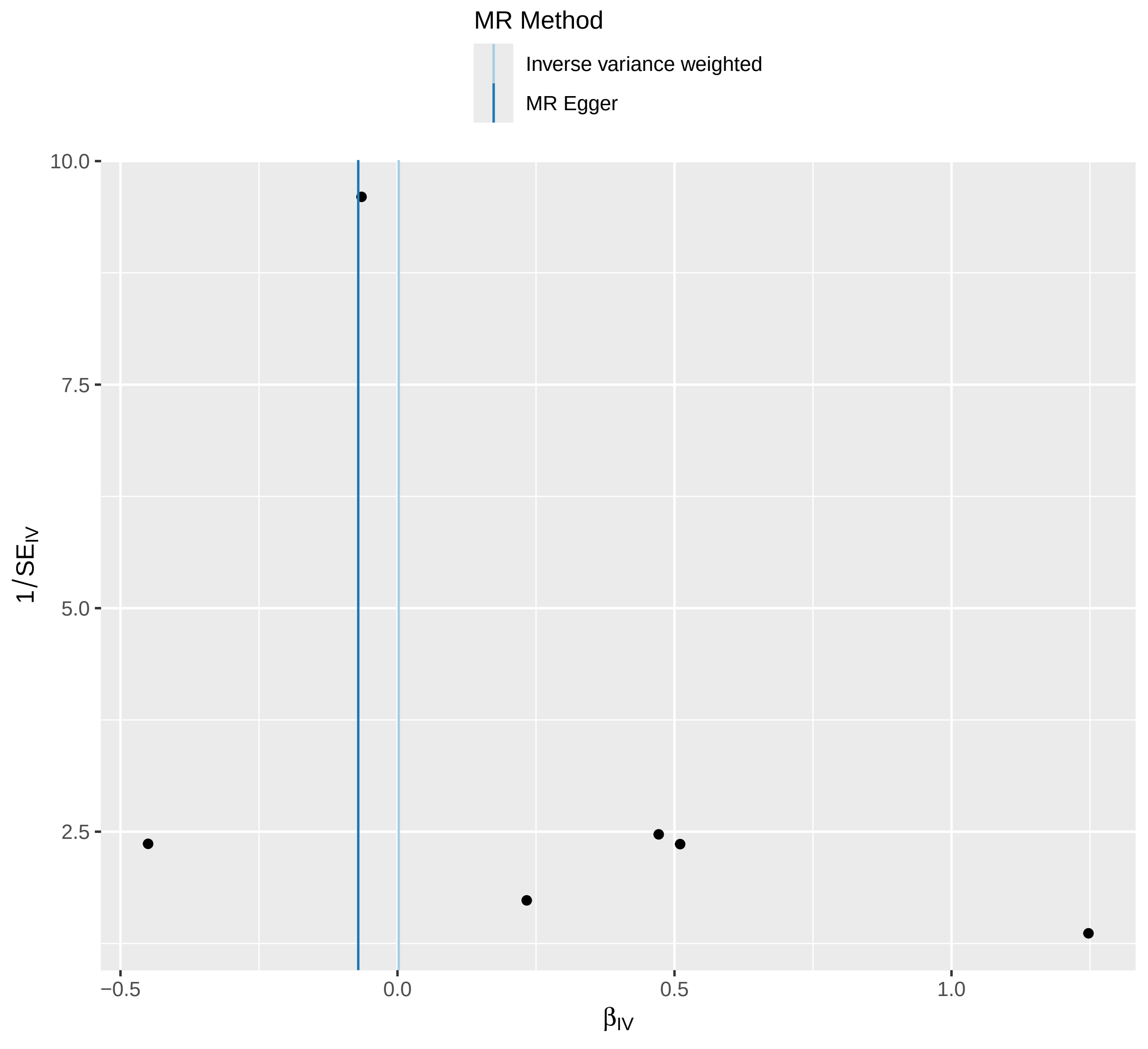

MR Method
Inverse variance weighted
MR Egger
10.0
7.5
5.0
2.5
1/SE_IV
−0.5
0.0
0.5
1.0
β_IV

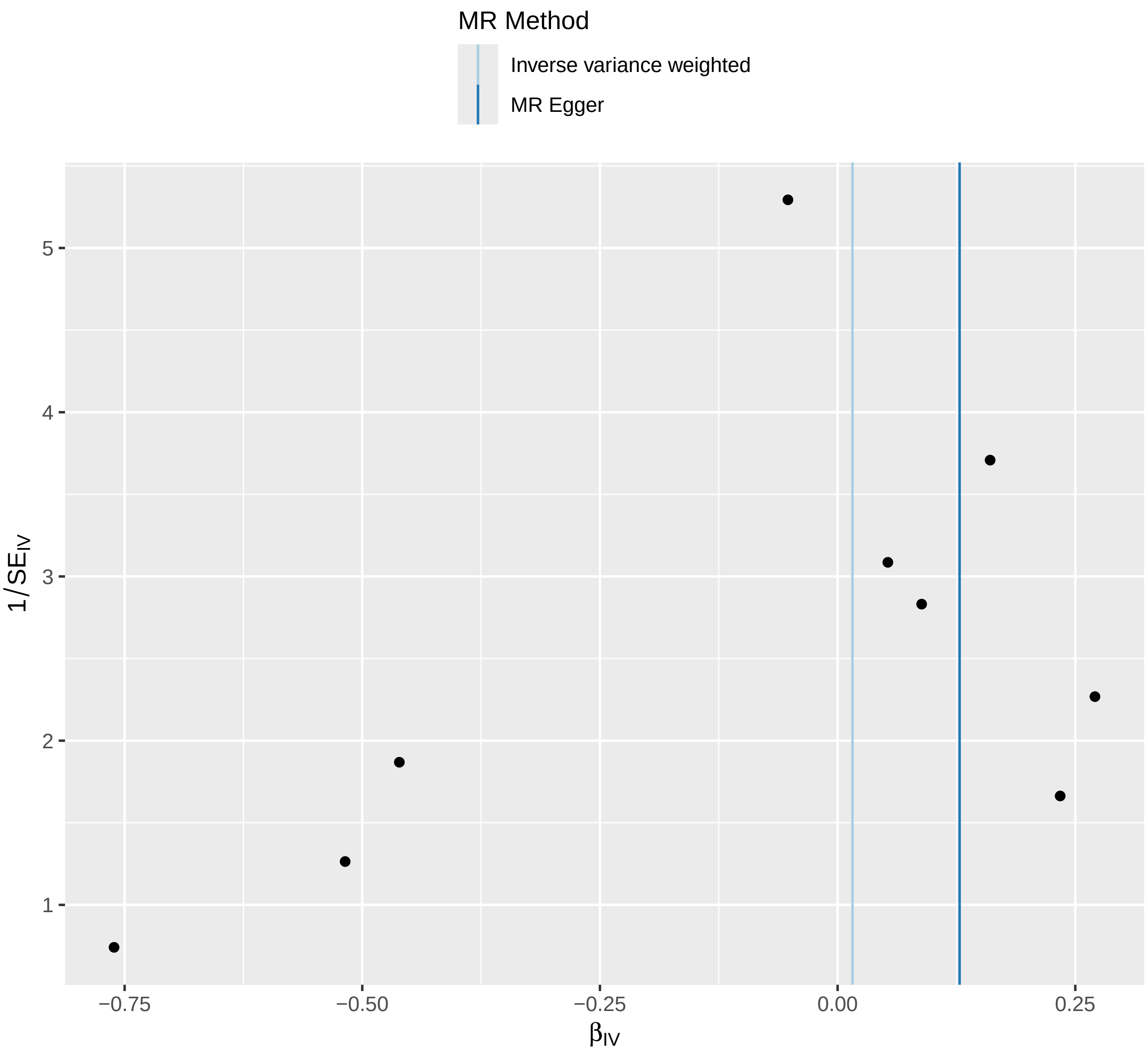

MR Method
Inverse variance weighted
MR Egger
5
4
3
2
1
1/SE_IV
−0.75
−0.50
−0.25
0.00
0.25
β_IV

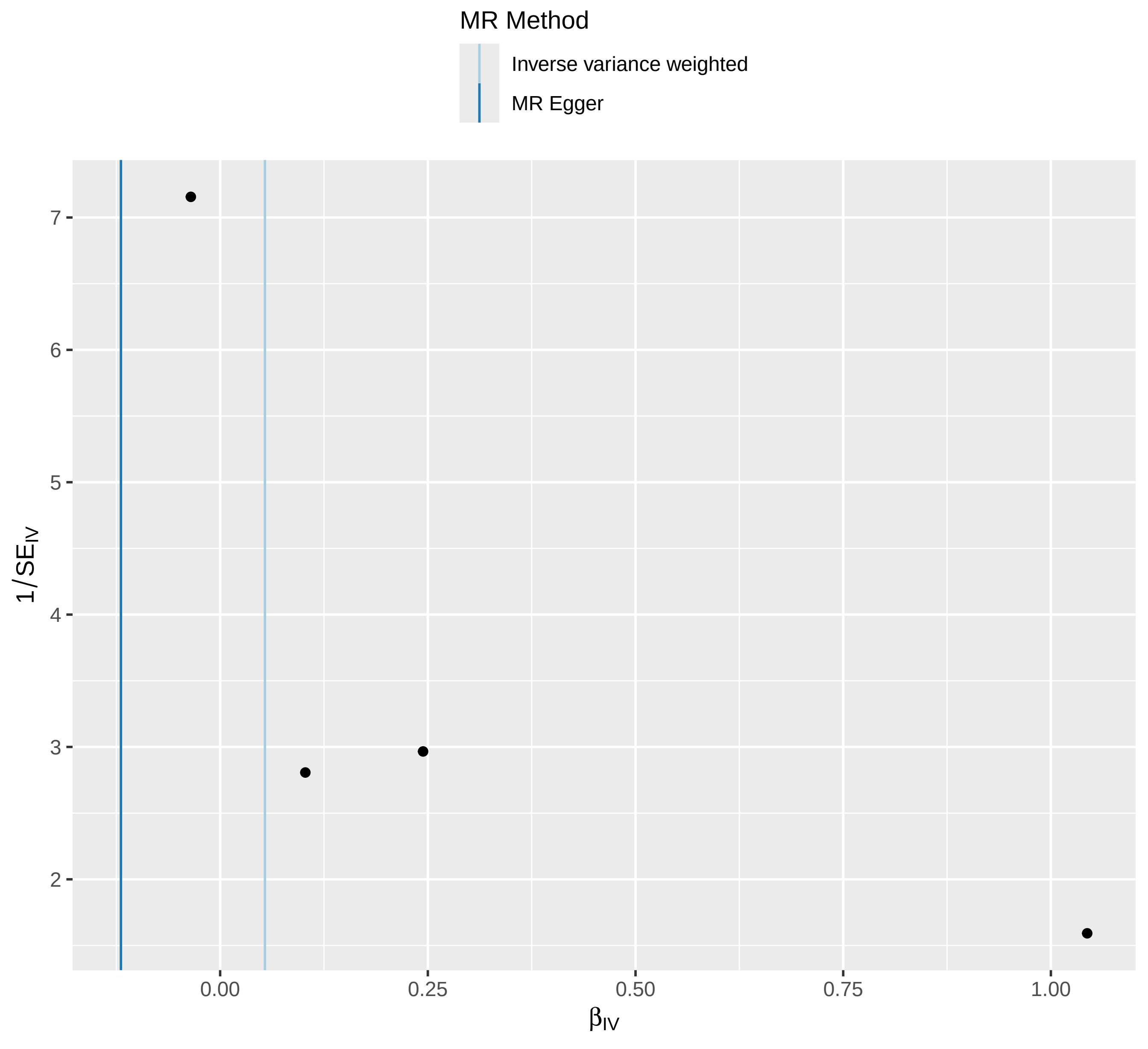
MR Method
Inverse variance weighted
MR Egger
7
6
5
4
3
2
1/SE$_{IV}$
0.00
0.25
0.50
0.75
1.00
β$_{IV}$

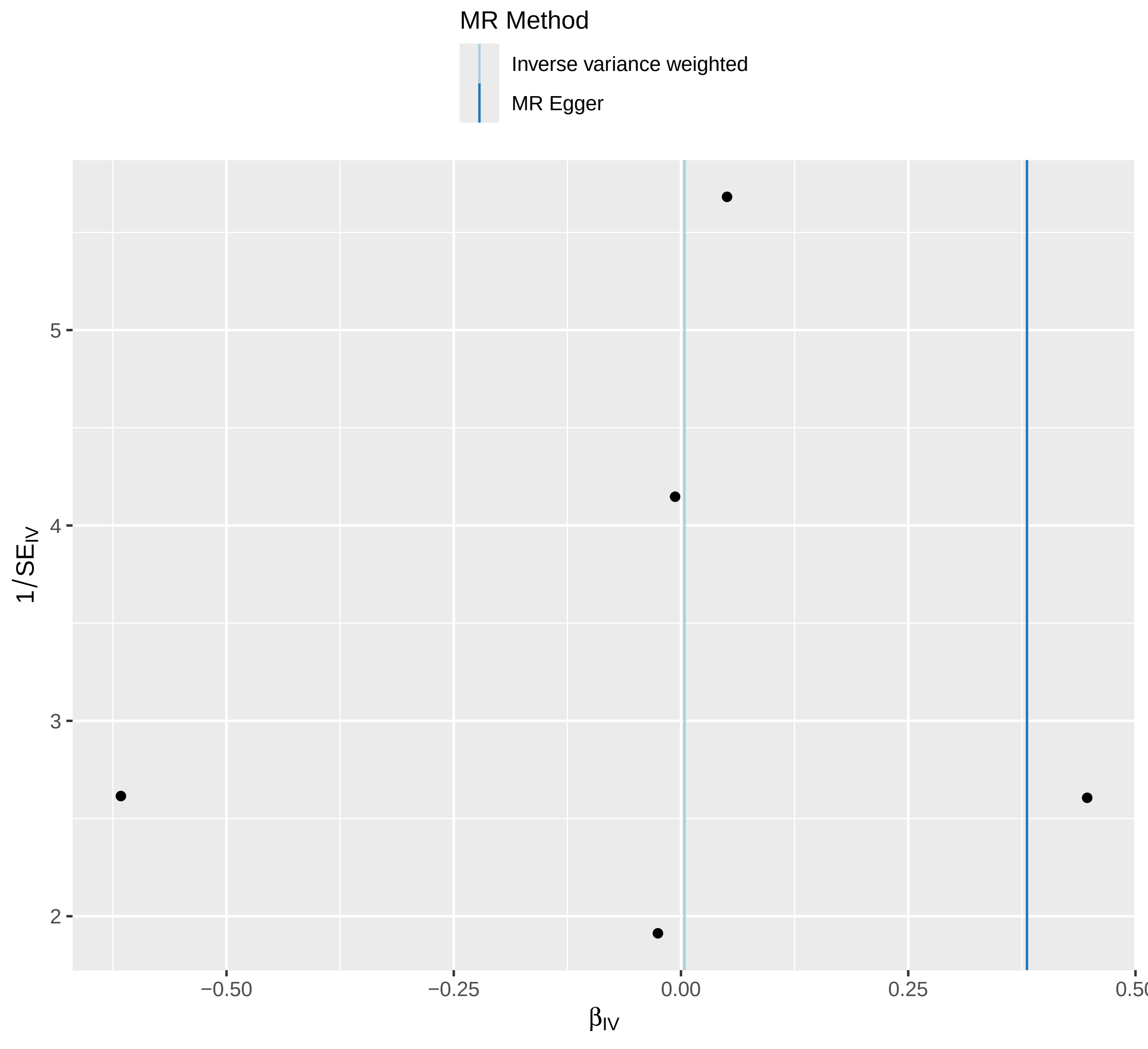

MR Method
Inverse variance weighted
MR Egger
5
4
3
2
1/SE$_{IV}$
−0.50
−0.25
0.00
0.25
0.50
β$_{IV}$

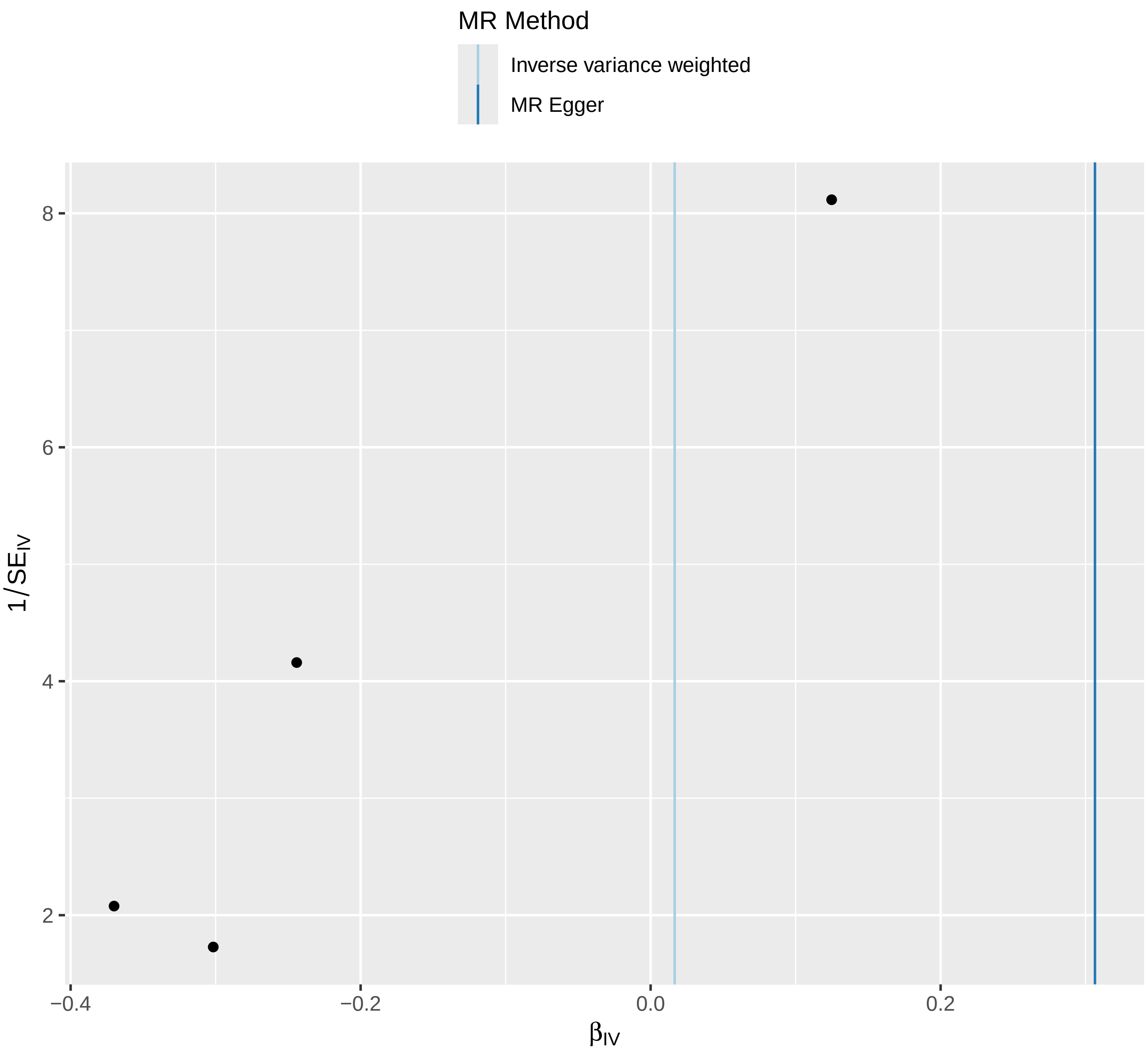

MR Method
Inverse variance weighted
MR Egger
8
6
4
2
1/SE$_{IV}$
−0.4
−0.2
0.0
0.2
β$_{IV}$

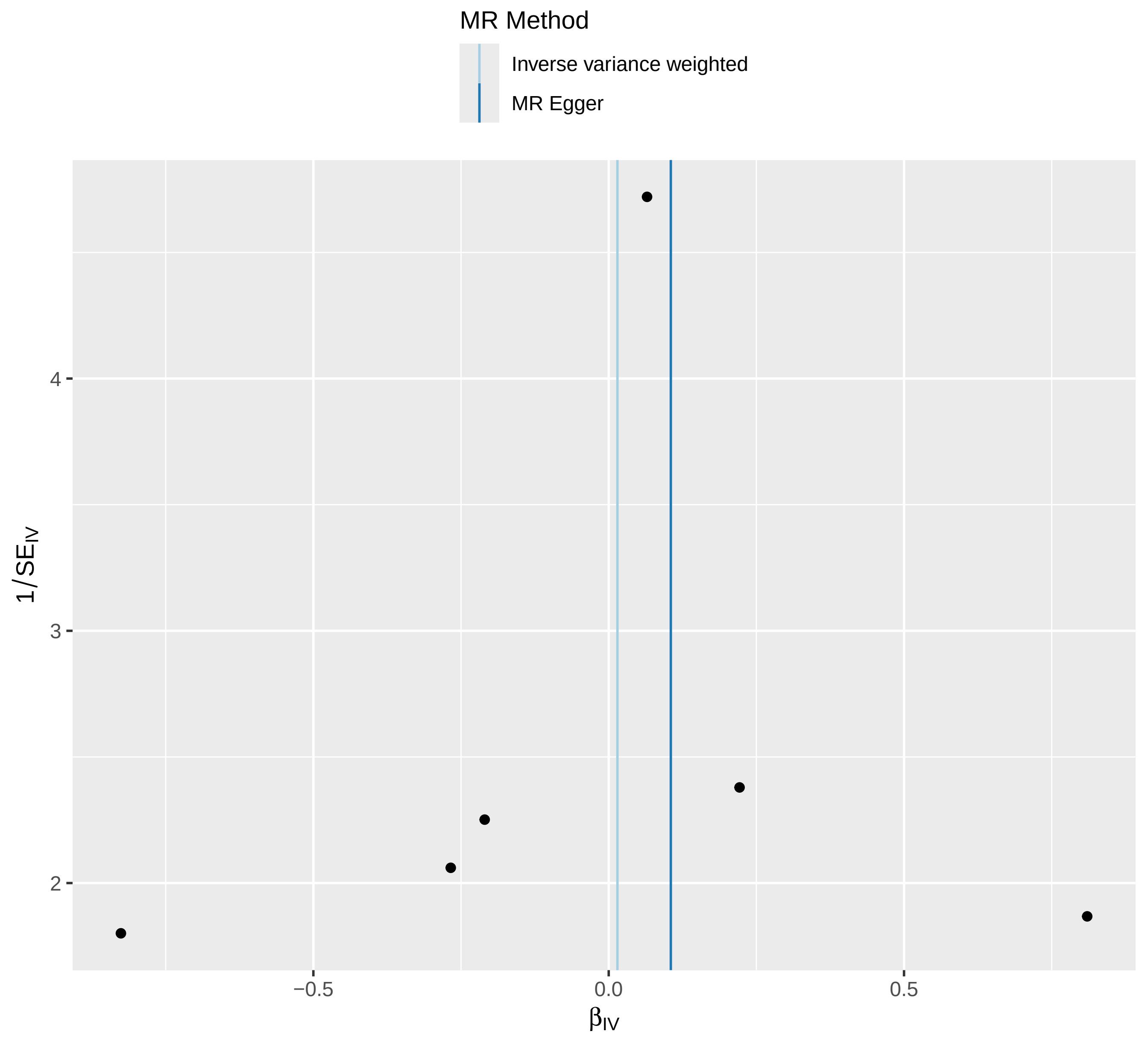
MR Method
Inverse variance weighted
MR Egger
1/SE_IV
4
3
2
−0.5
0.0
0.5
β_IV

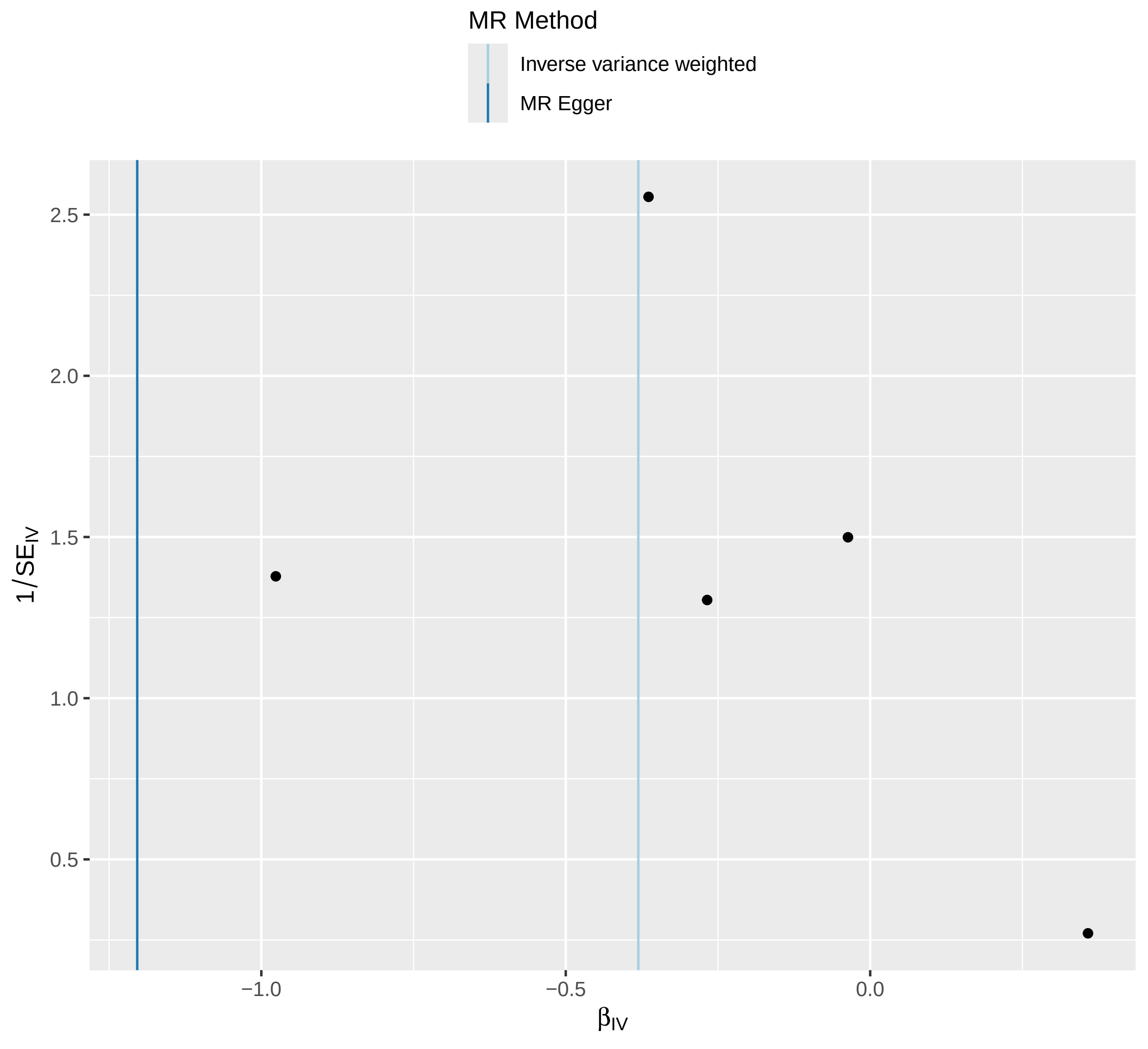

MR Method
Inverse variance weighted
MR Egger
2.5
2.0
1.5
1.0
0.5
1/SE_IV
−1.0
−0.5
0.0
β_IV

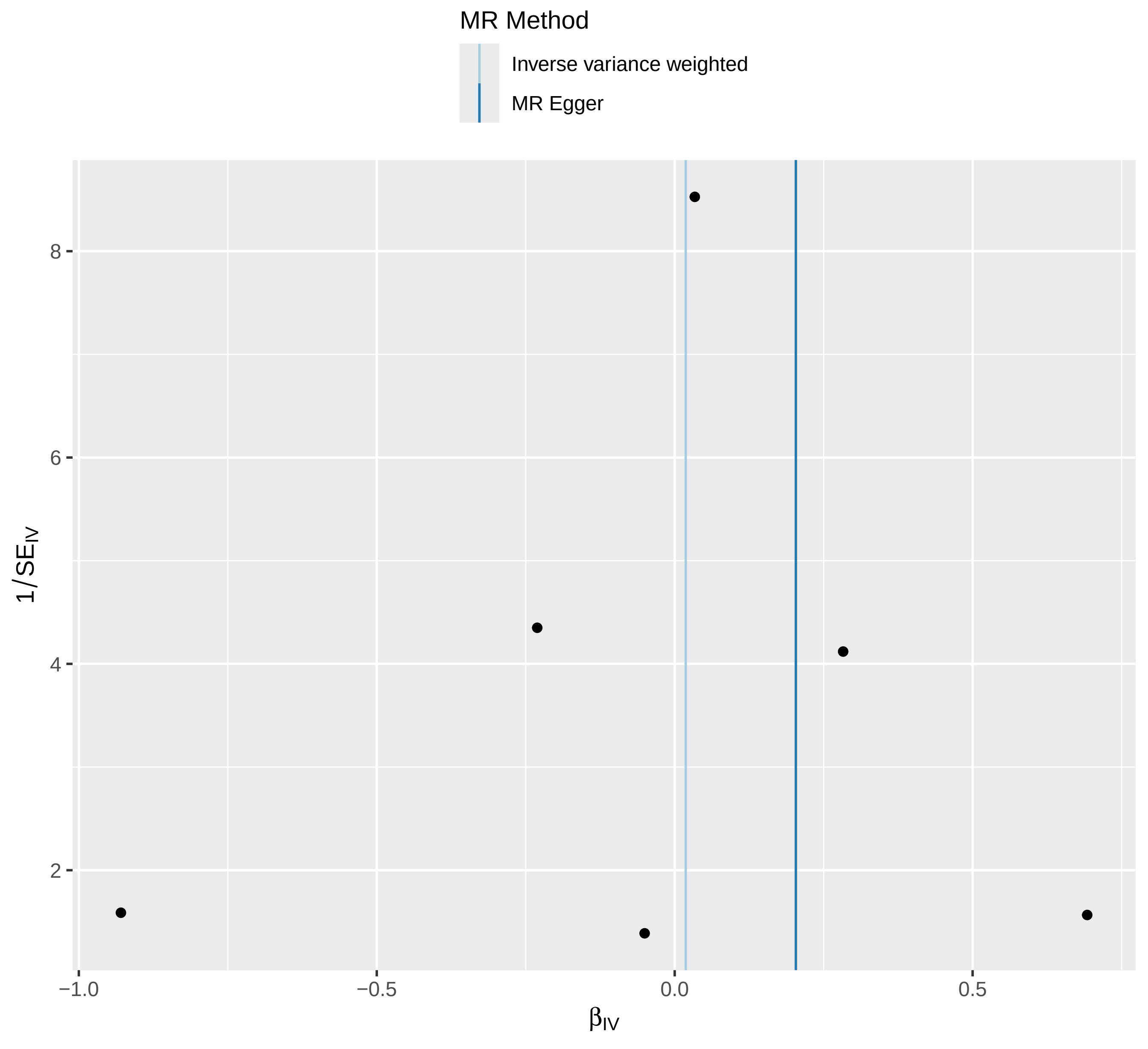

MR Method
Inverse variance weighted
MR Egger
8
6
4
2
1/SE_IV
−1.0
−0.5
0.0
0.5
β_IV

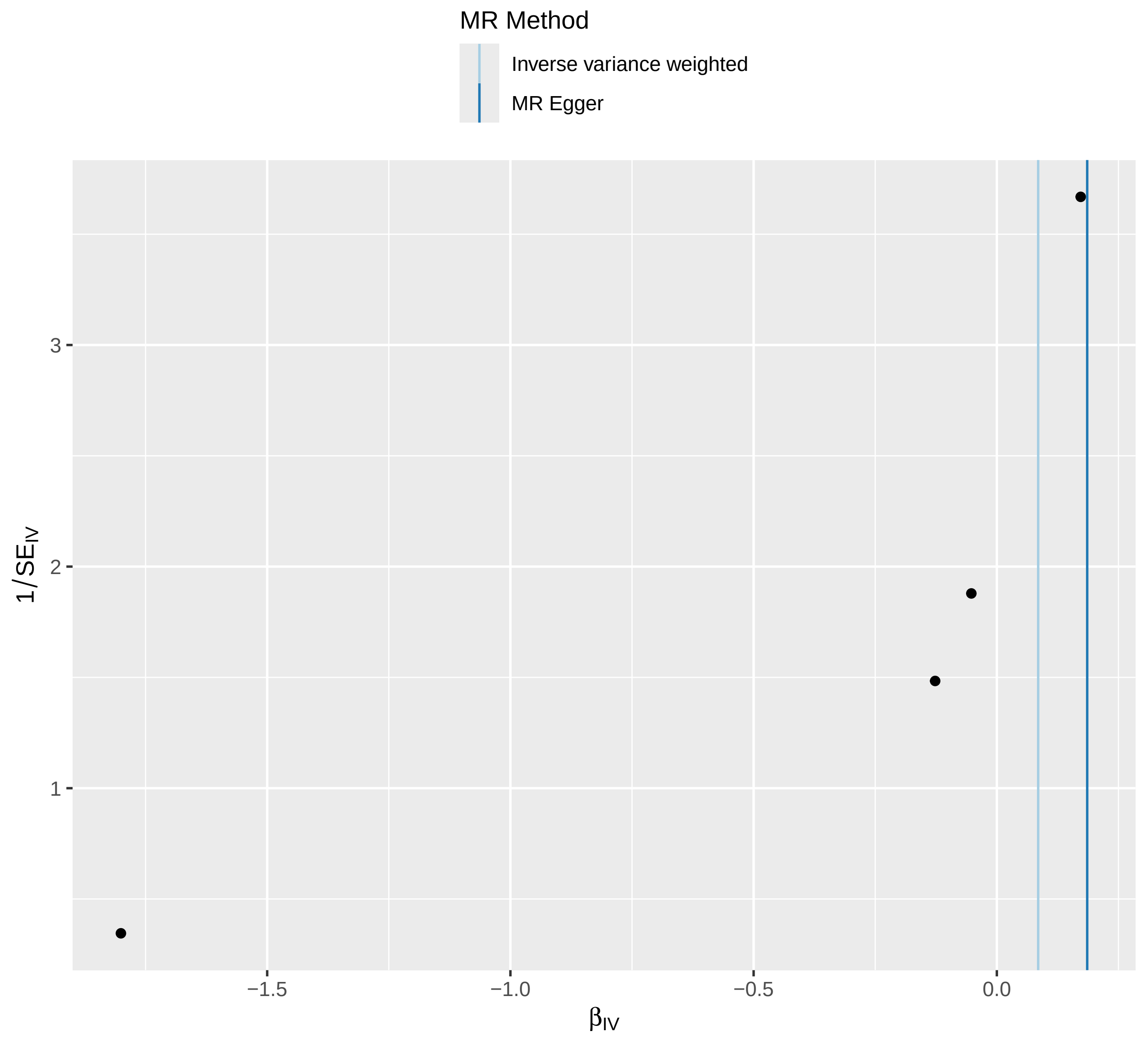
MR Method
Inverse variance weighted
MR Egger
3
2
1
−1.5
−1.0
−0.5
0.0
1/SE_IV
β_IV

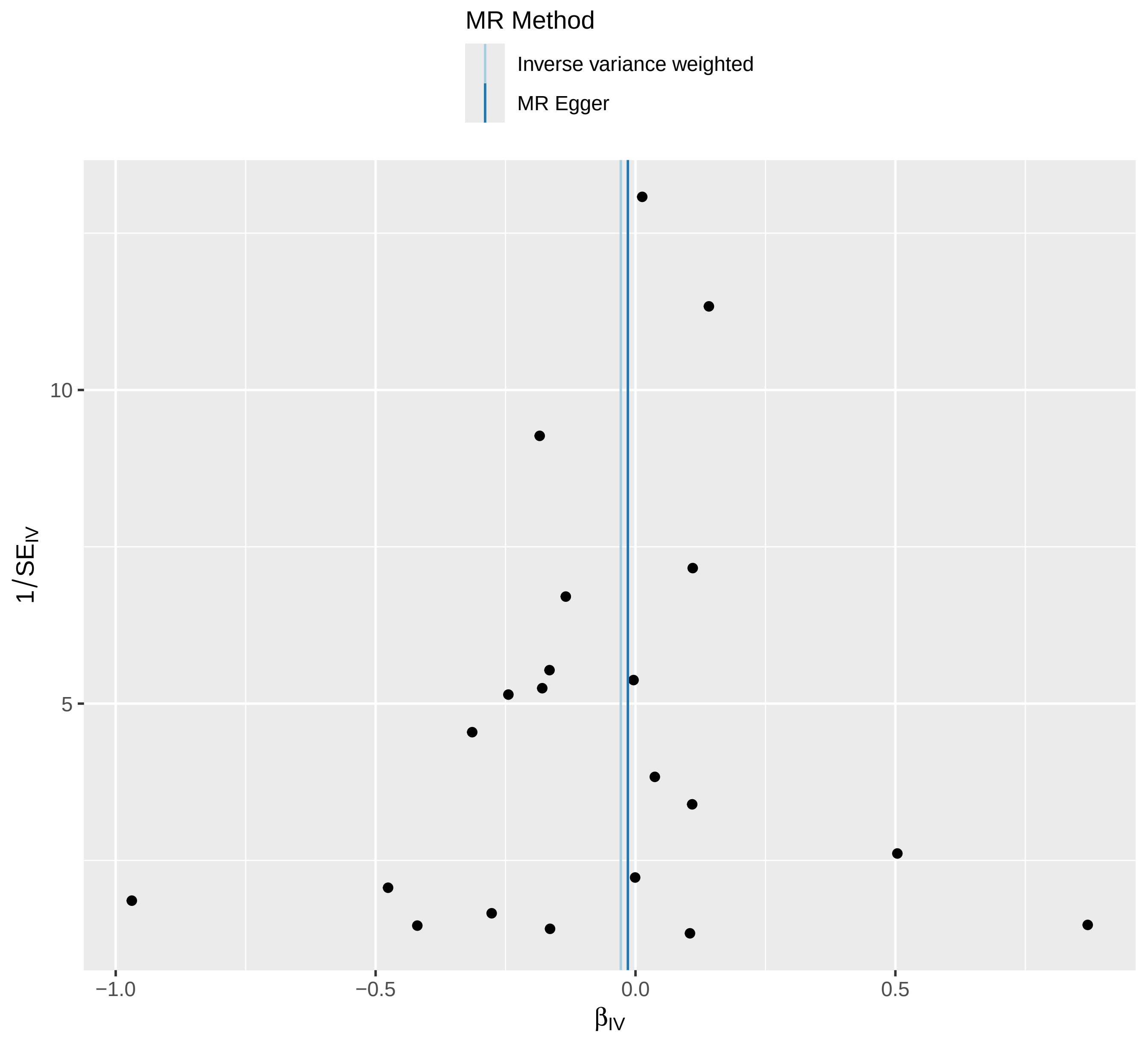

MR Method
Inverse variance weighted
MR Egger
10
5
1/SE_IV
−1.0
−0.5
0.0
0.5
β_IV

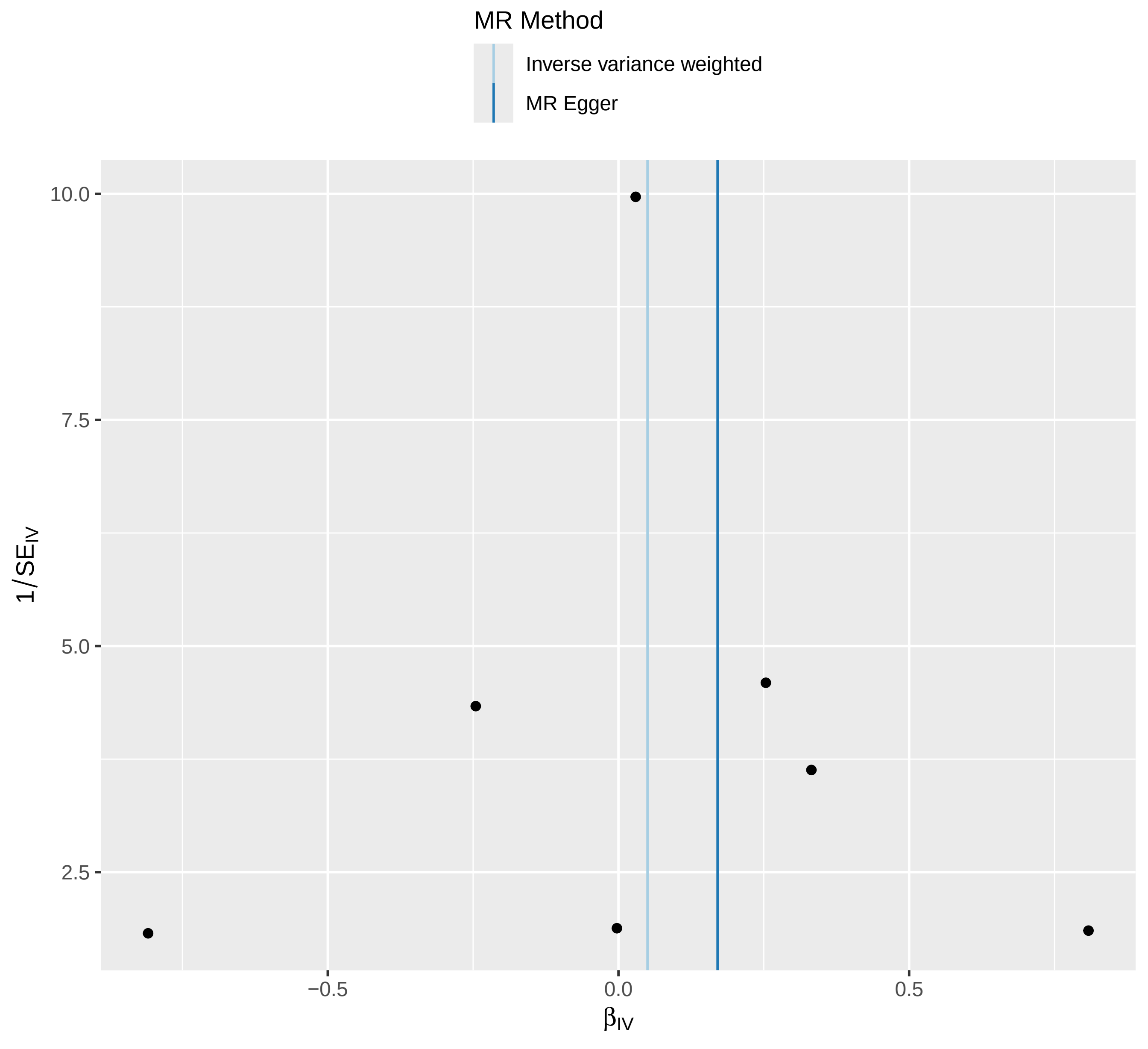

MR Method
Inverse variance weighted
MR Egger
10.0
7.5
5.0
2.5
1/SE$_{IV}$
−0.5
0.0
0.5
β$_{IV}$

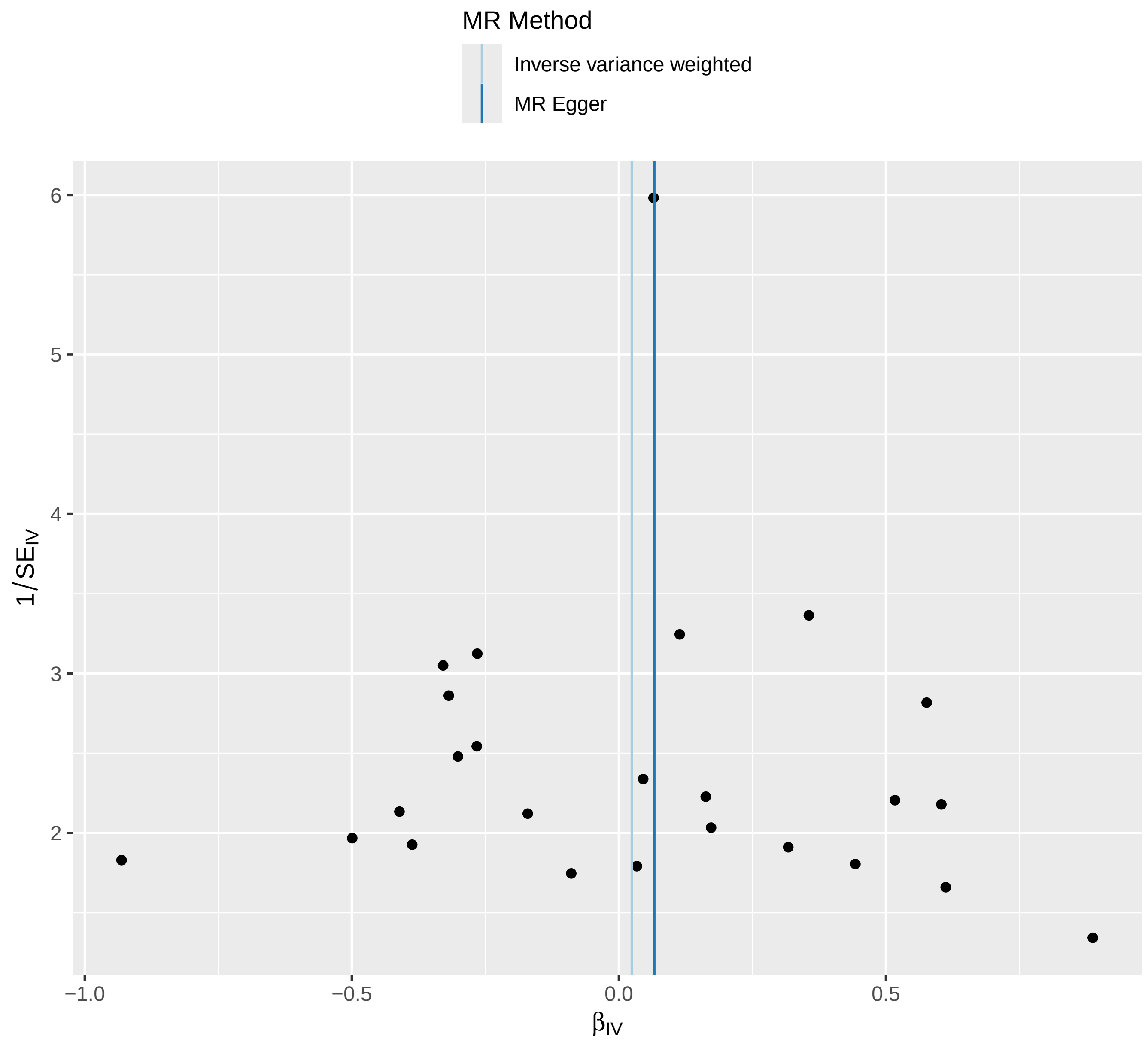

MR Method
Inverse variance weighted
MR Egger
1/SE_IV
β_IV
6
5
4
3
2
−1.0
−0.5
0.0
0.5

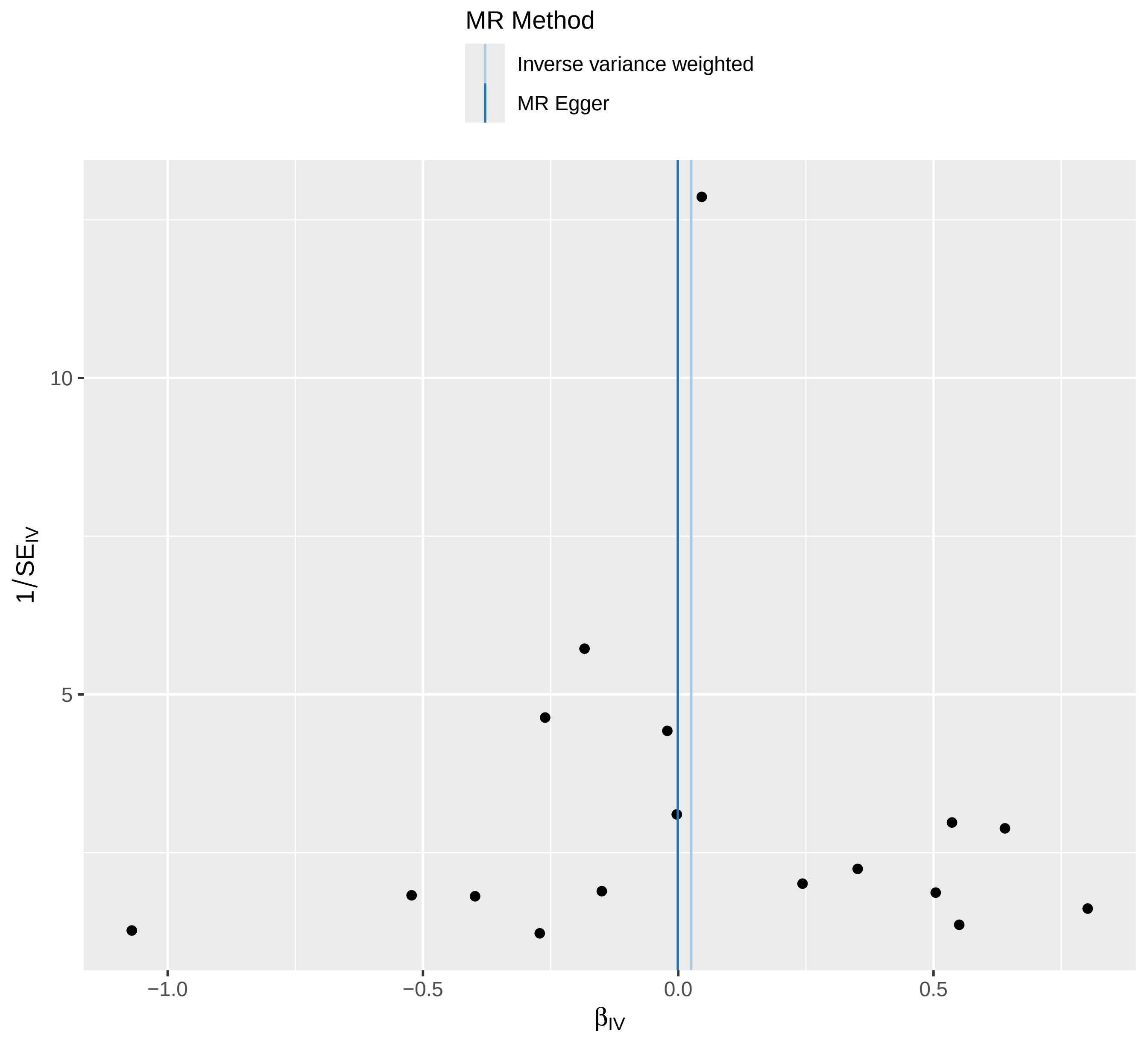
MR Method
Inverse variance weighted
MR Egger
10
5
1/SE_{IV}
−1.0
−0.5
0.0
0.5
β_{IV}

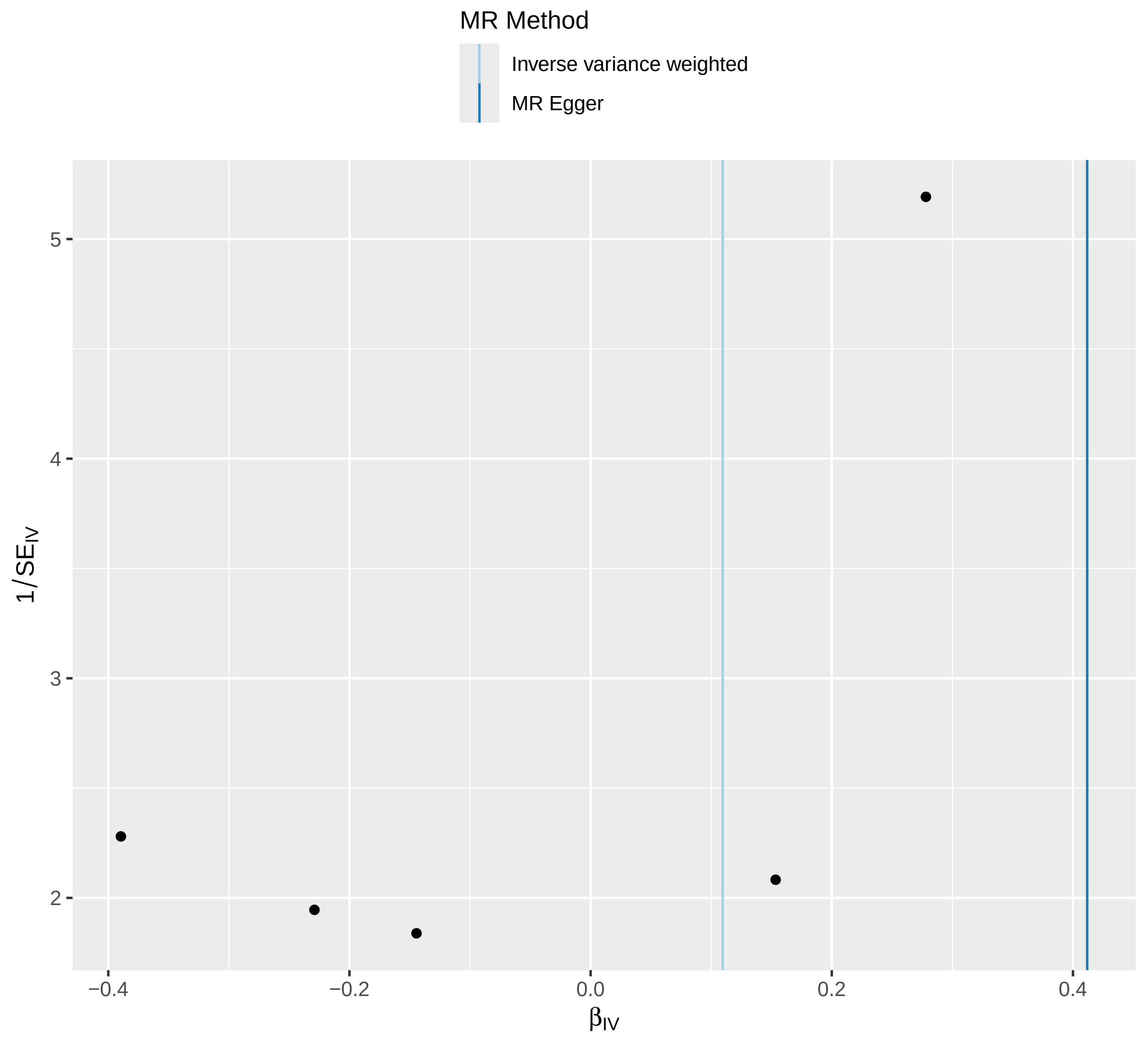

MR Method
Inverse variance weighted
MR Egger
5
4
3
2
1/SE_IV
−0.4
−0.2
0.0
0.2
0.4
β_IV

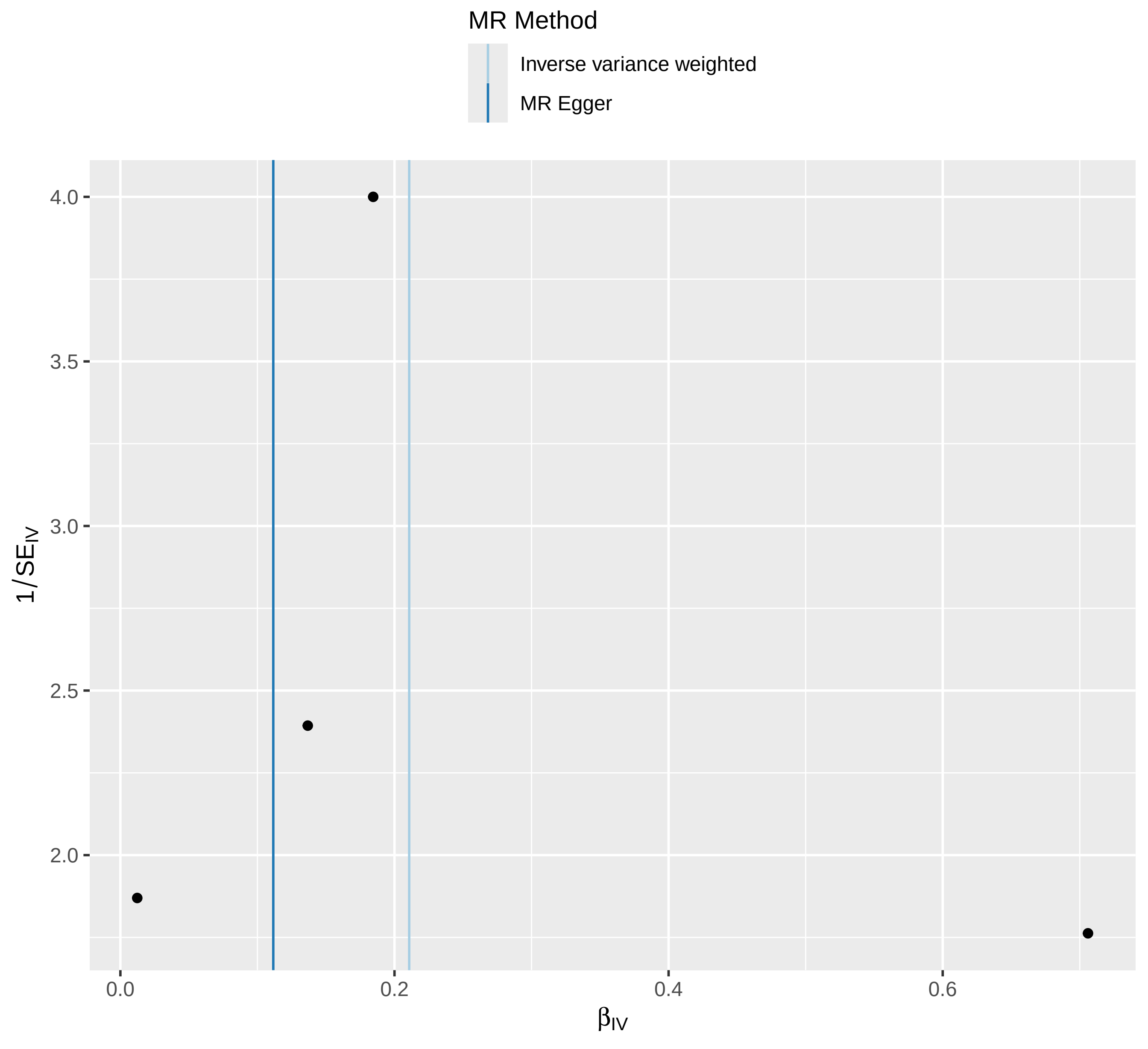
MR Method
Inverse variance weighted
MR Egger
4.0
3.5
3.0
2.5
2.0
$1/SE_{IV}$
0.0
0.2
0.4
0.6
β_{IV}

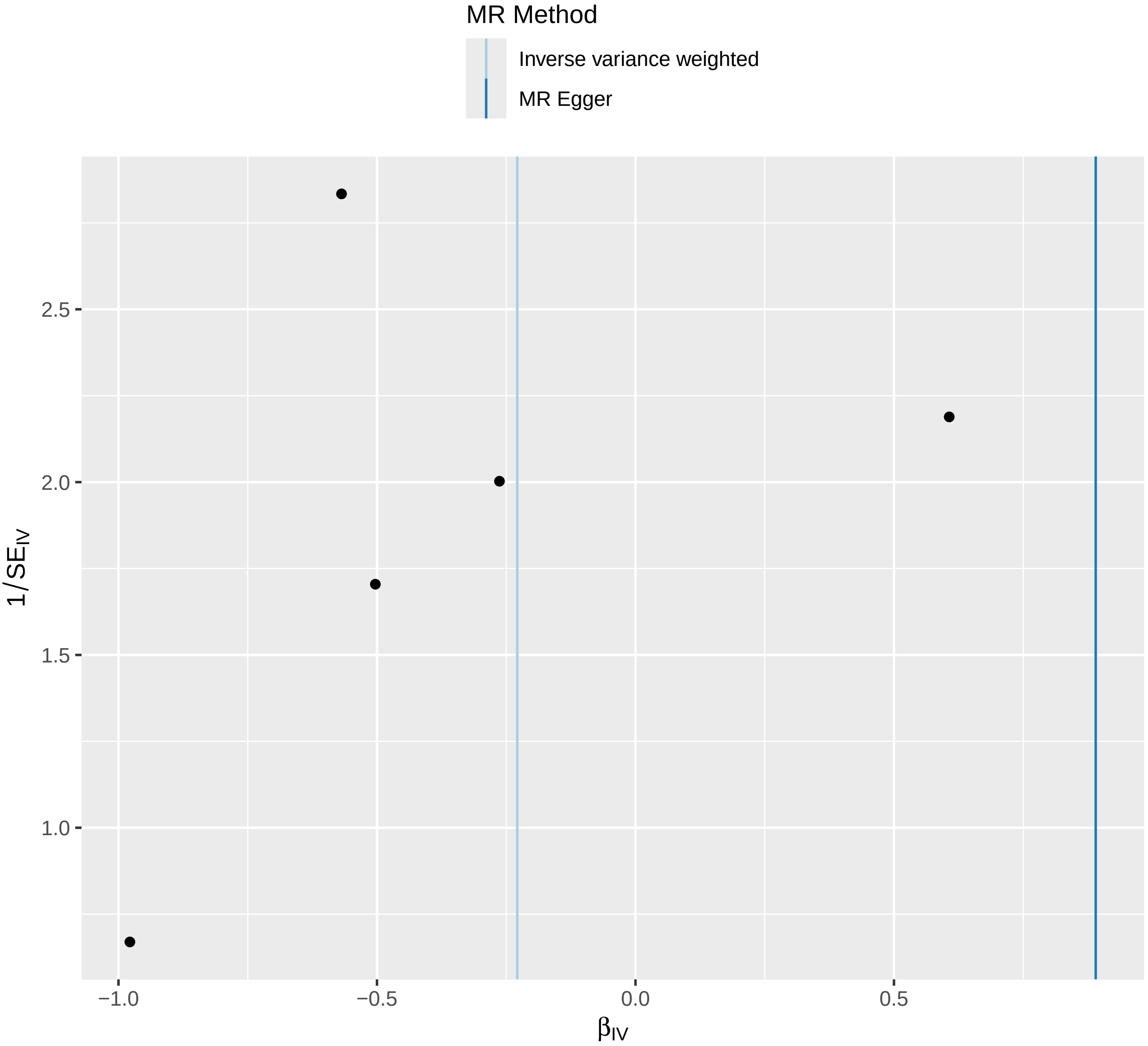

MR Method
Inverse variance weighted
MR Egger
2.5
2.0
1.5
1.0
1/SE_IV
−1.0
−0.5
0.0
0.5
β_IV

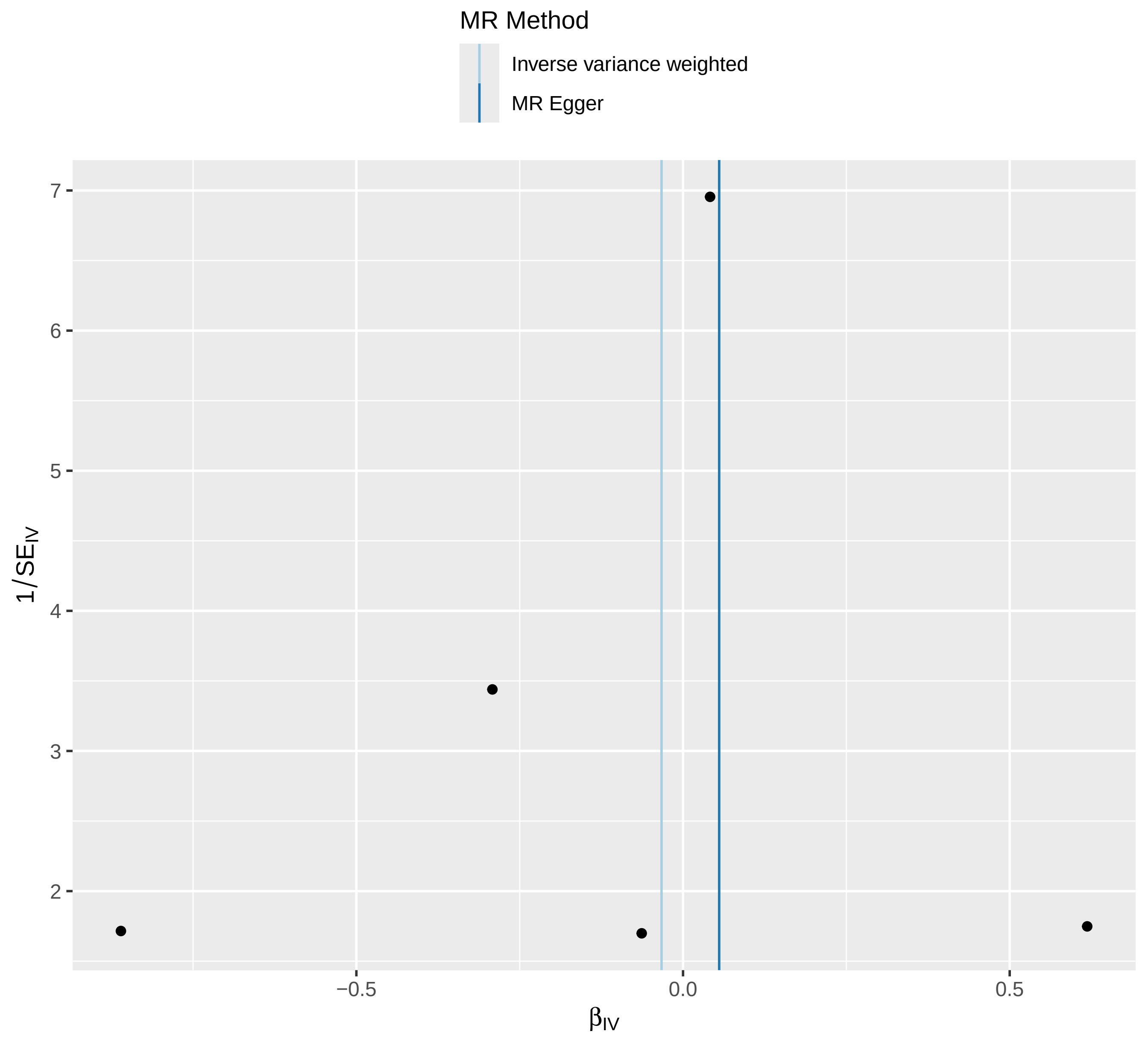

MR Method
Inverse variance weighted
MR Egger
1/SE_IV
7
6
5
4
3
2
−0.5
0.0
0.5
β_IV

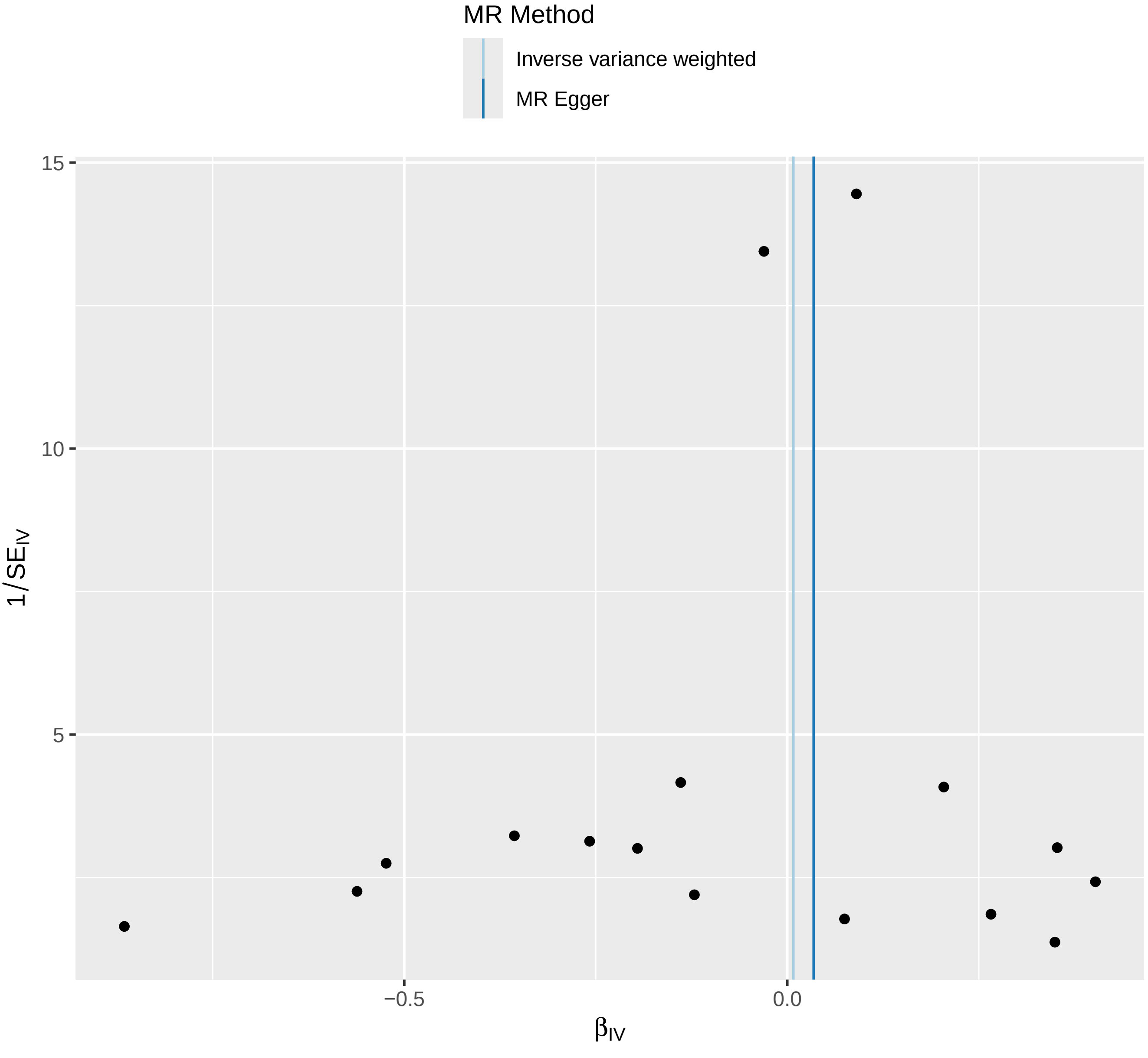
MR Method
Inverse variance weighted
MR Egger
15
10
5
1/SE$_{IV}$
−0.5
0.0
β$_{IV}$

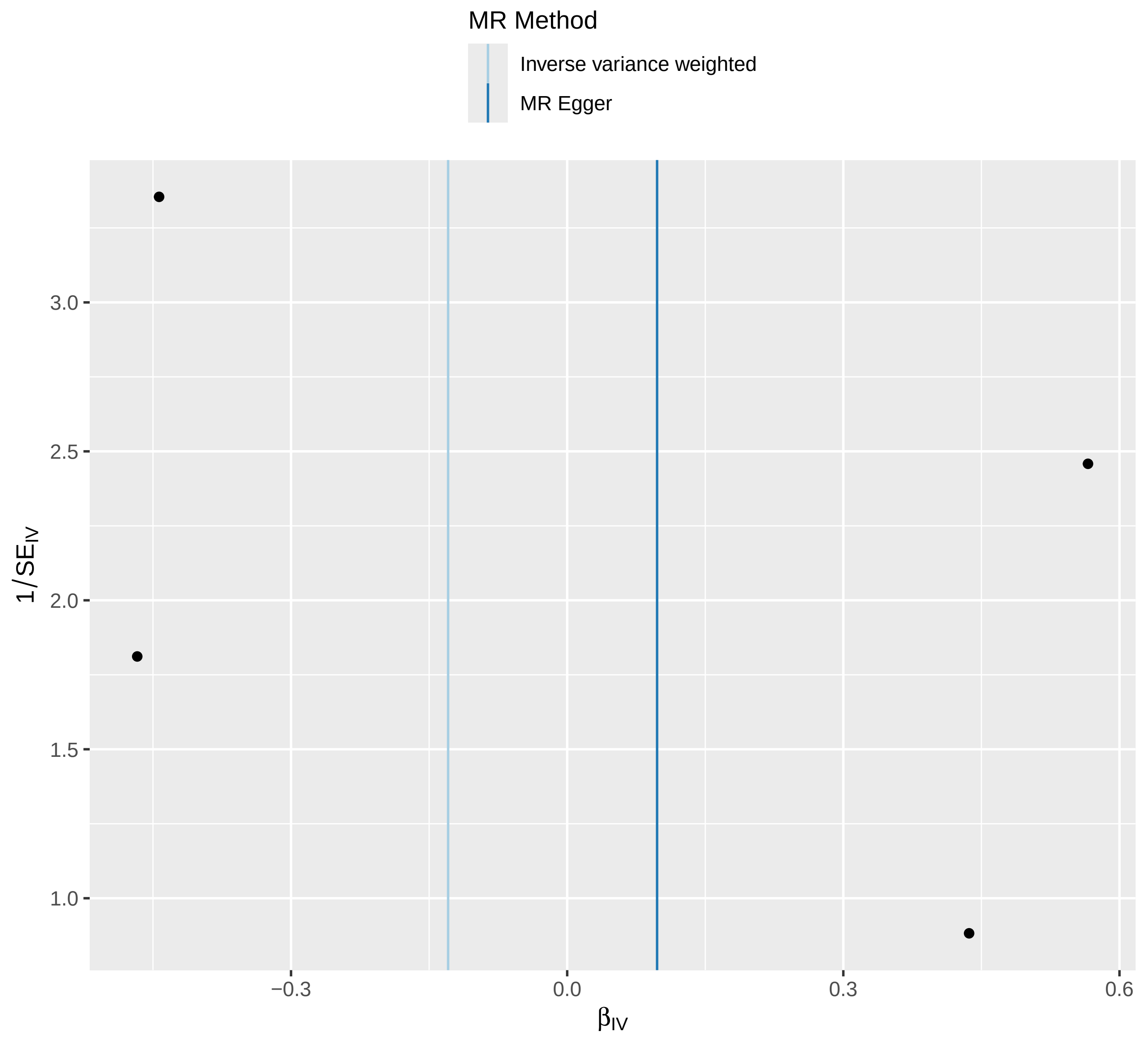
MR Method
Inverse variance weighted
MR Egger
3.0
2.5
2.0
1.5
1.0
$1/SE_{IV}$
−0.3
0.0
0.3
0.6
β_{IV}

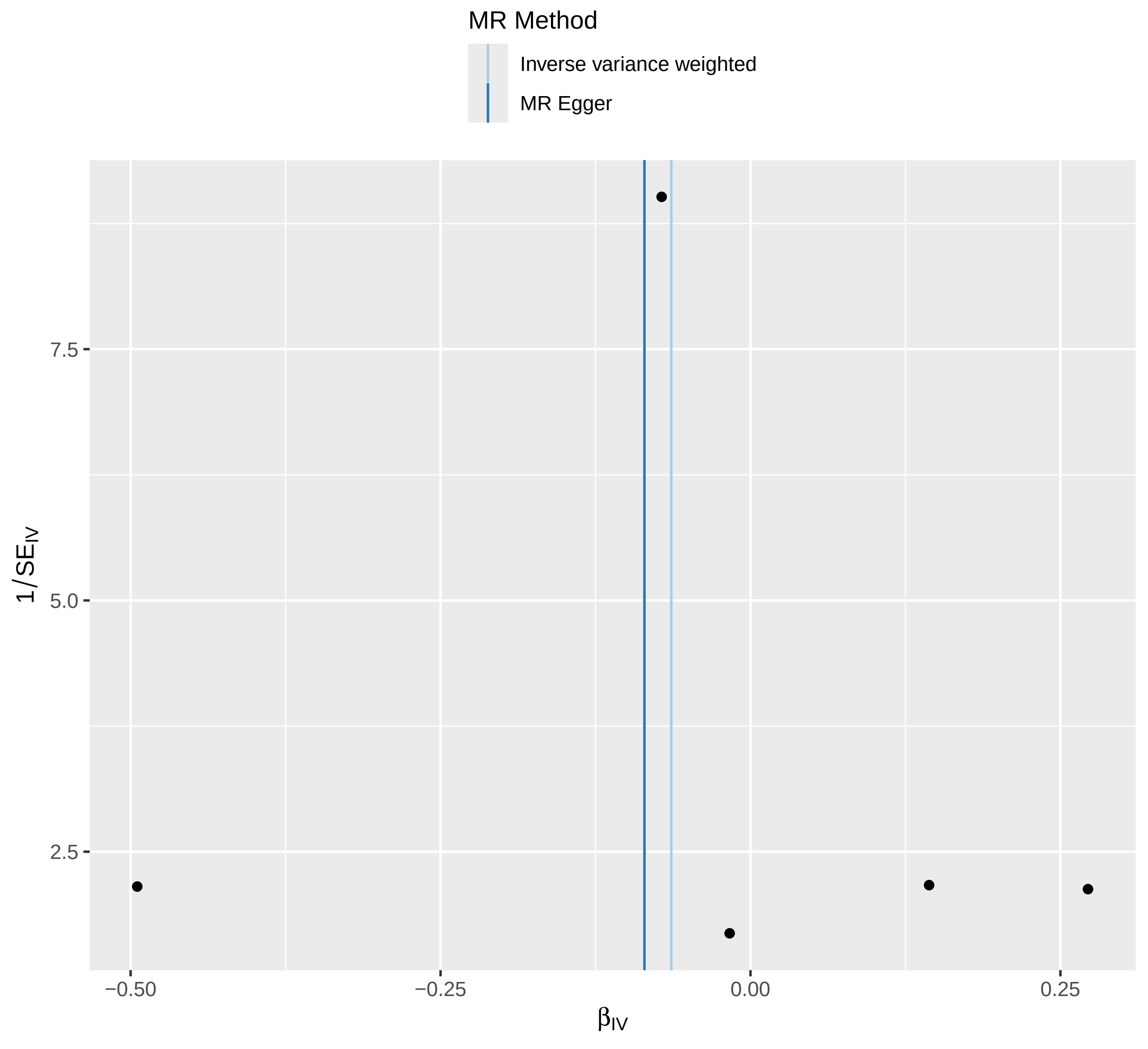

MR Method
Inverse variance weighted
MR Egger
1/SE_IV
7.5
5.0
2.5
−0.50
−0.25
0.00
0.25
β_IV

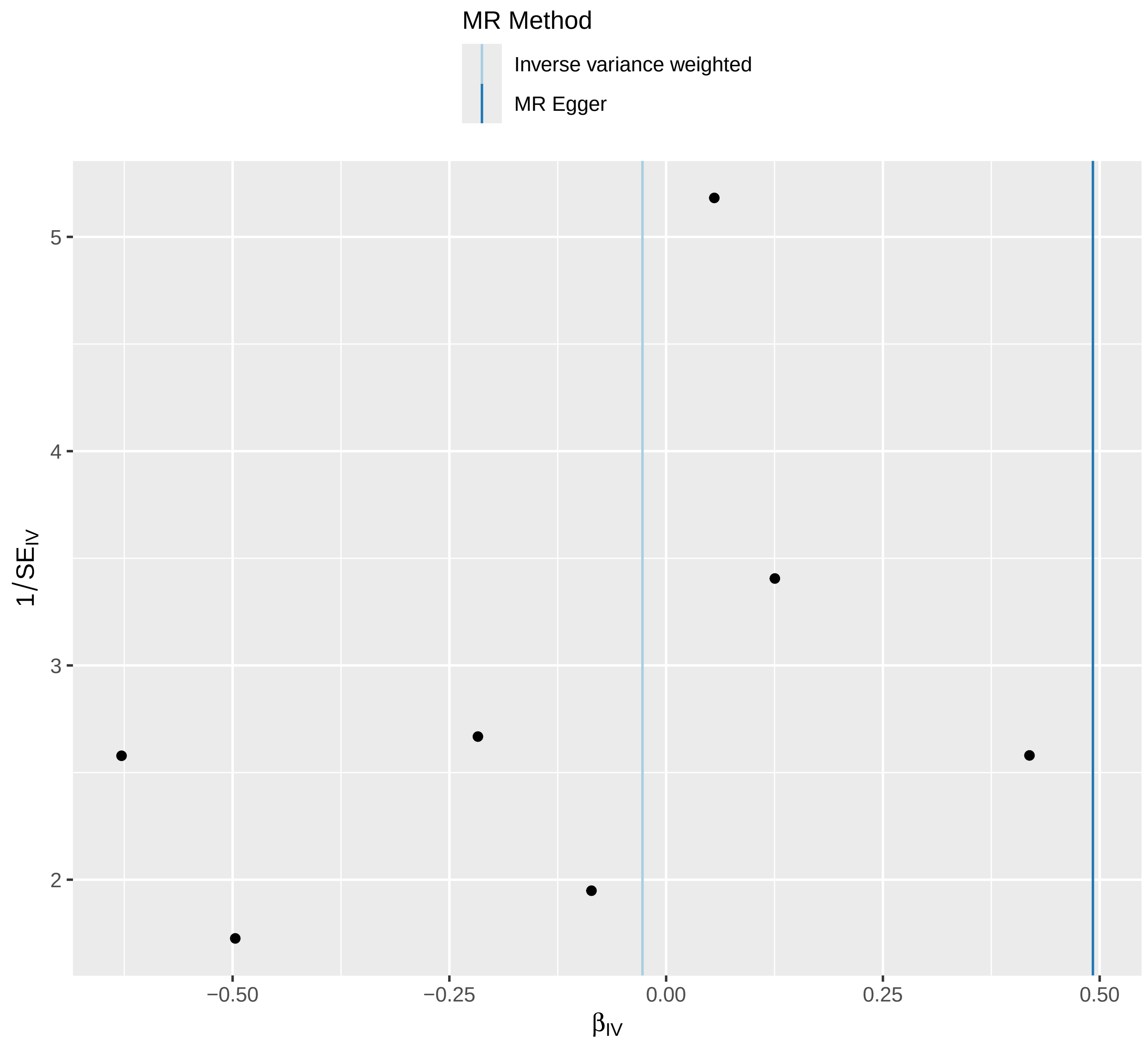
MR Method
Inverse variance weighted
MR Egger
5
4
3
2
1/SE$_{IV}$
−0.50
−0.25
0.00
0.25
0.50
β$_{IV}$

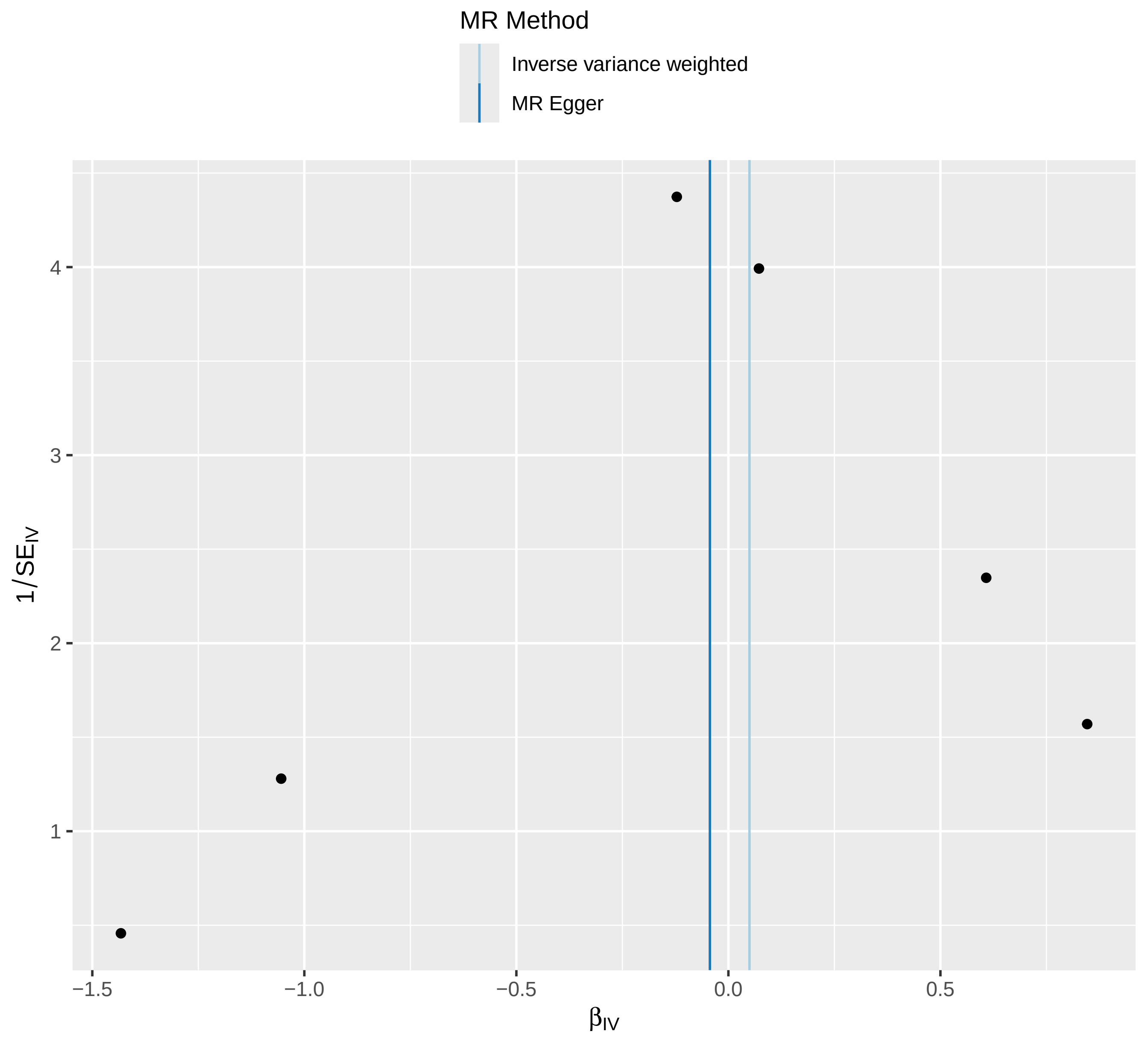

MR Method
Inverse variance weighted
MR Egger
1/SE_IV
4
3
2
1
−1.5
−1.0
−0.5
0.0
0.5
β_IV

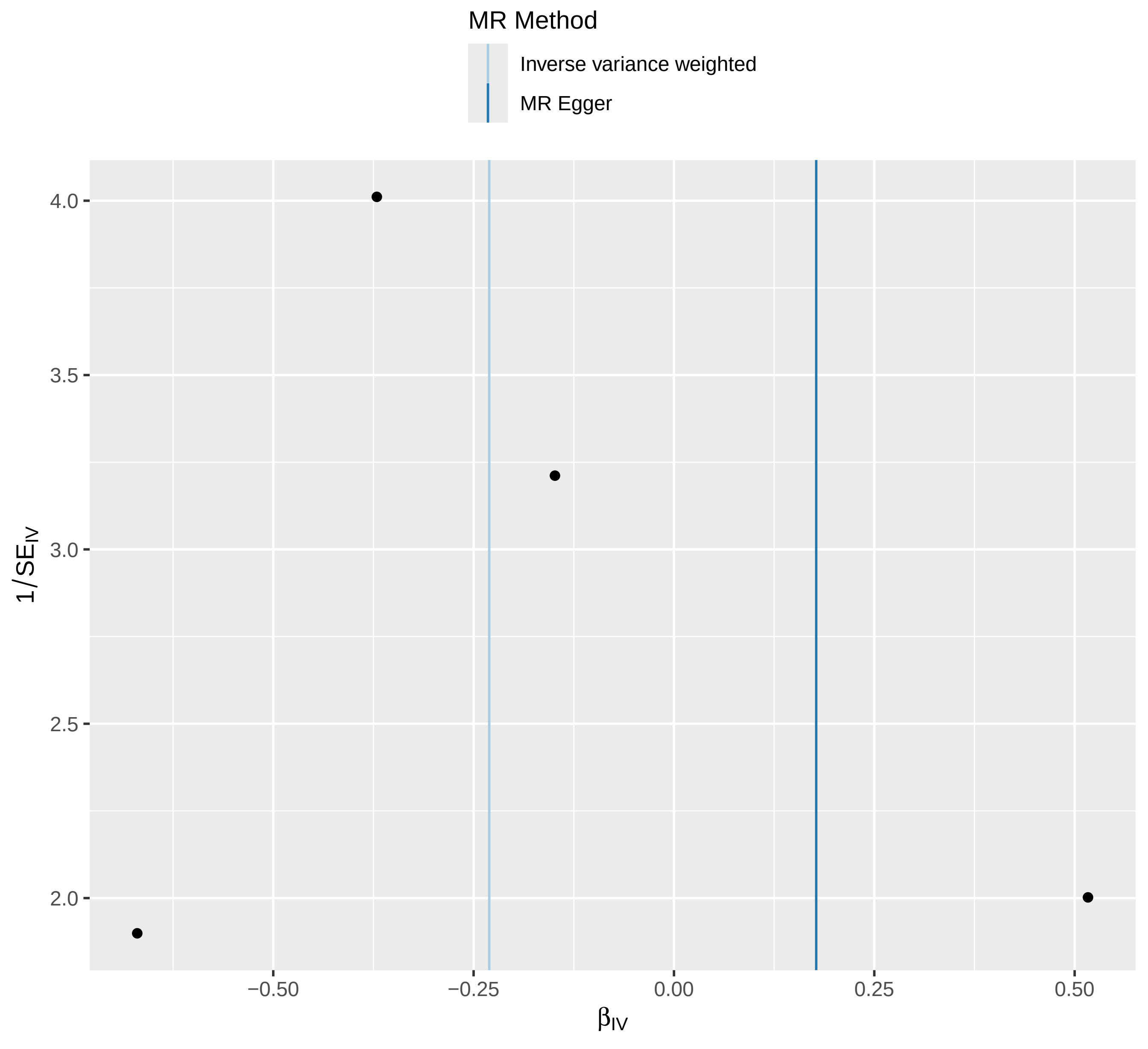

MR Method
Inverse variance weighted
MR Egger
4.0
3.5
3.0
2.5
2.0
1/SE_IV
−0.50
−0.25
0.00
0.25
0.50
β_IV

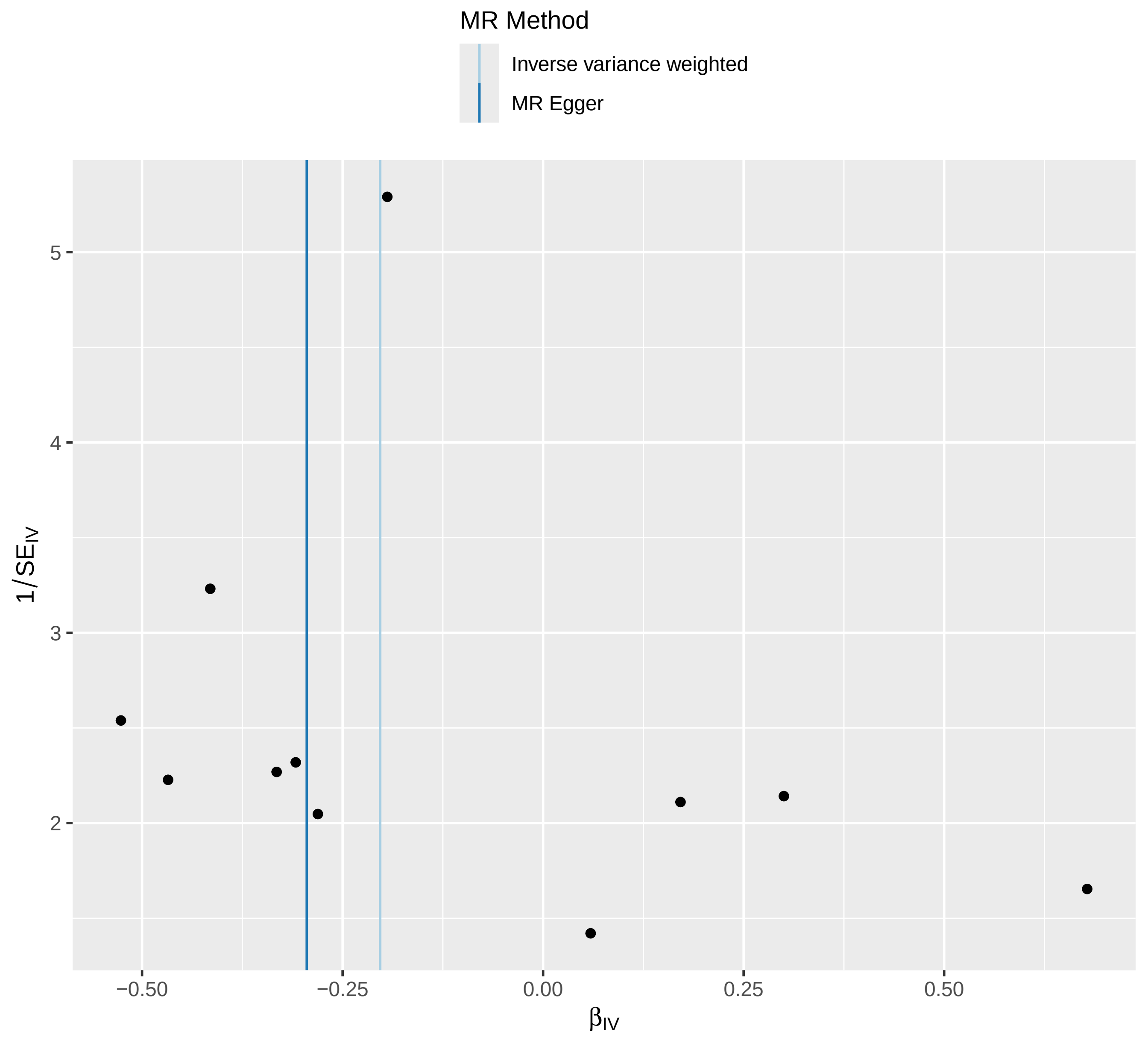

MR Method
Inverse variance weighted
MR Egger
1/SE_IV
5
4
3
2
−0.50
−0.25
0.00
0.25
0.50
β_IV

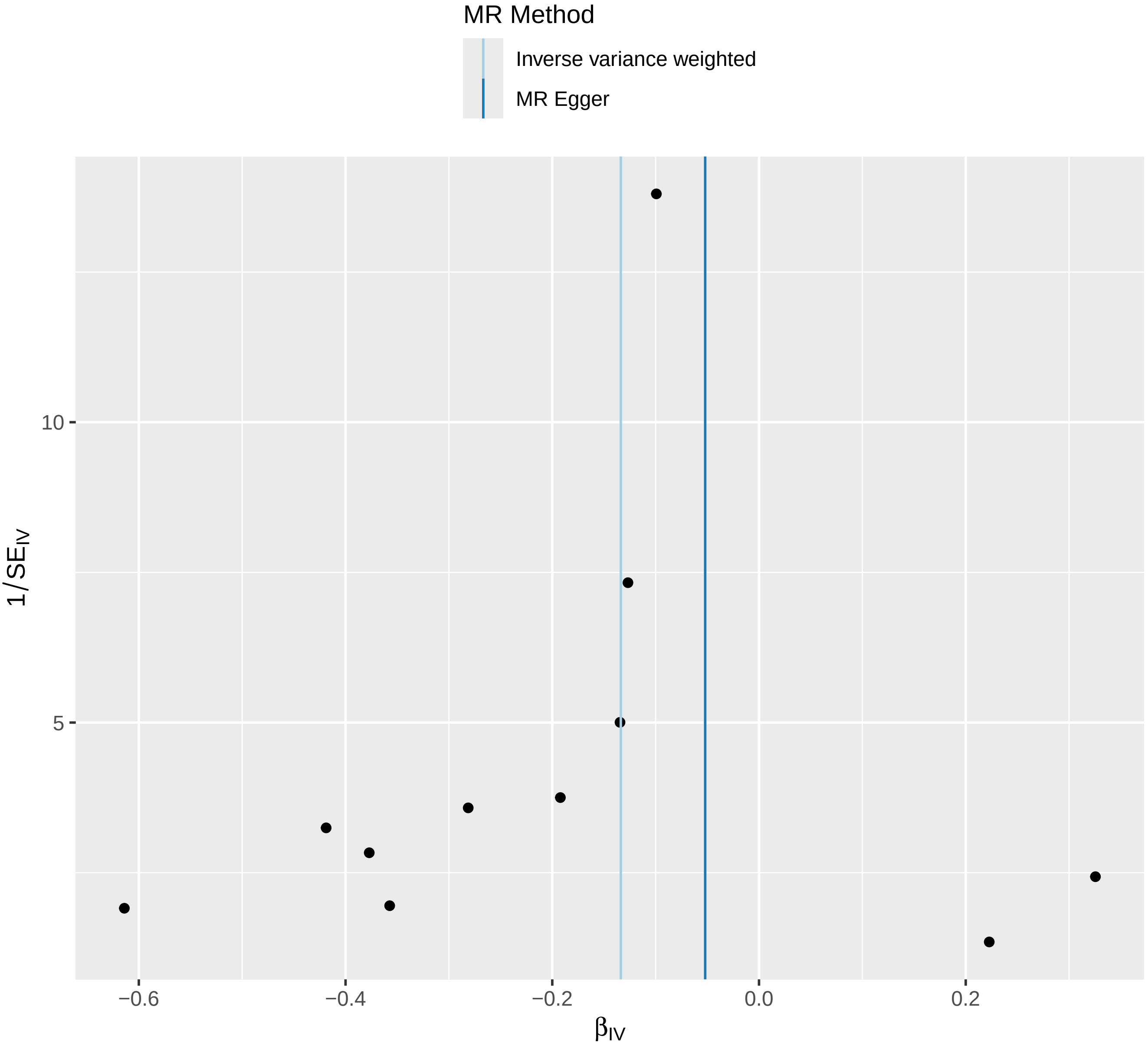
MR Method
Inverse variance weighted
MR Egger
10
5
$1/SE_{IV}$
−0.6
−0.4
−0.2
0.0
0.2
β_{IV}

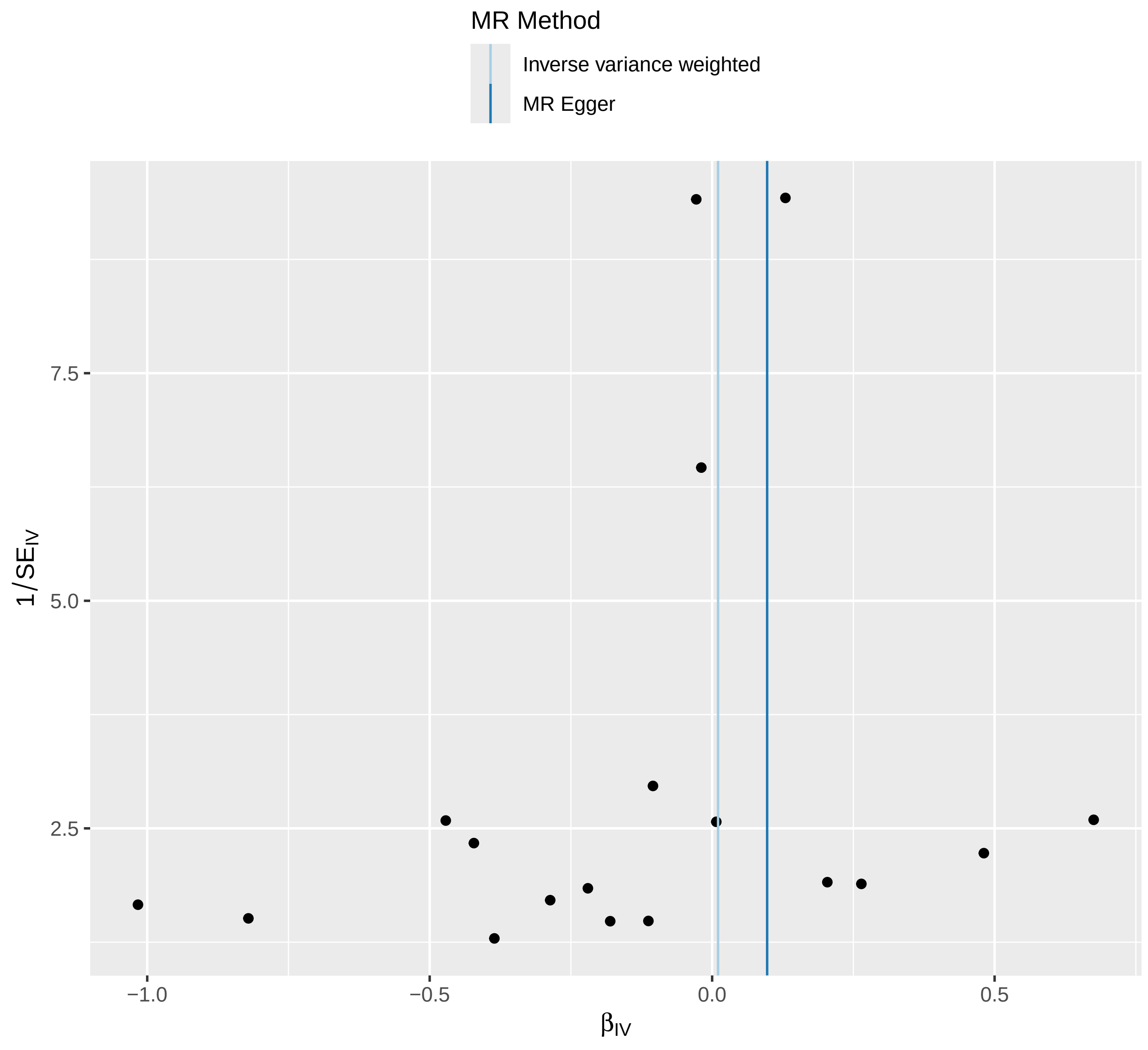
MR Method
Inverse variance weighted
MR Egger
7.5
5.0
2.5
1/SE$_{IV}$
−1.0
−0.5
0.0
0.5
β$_{IV}$

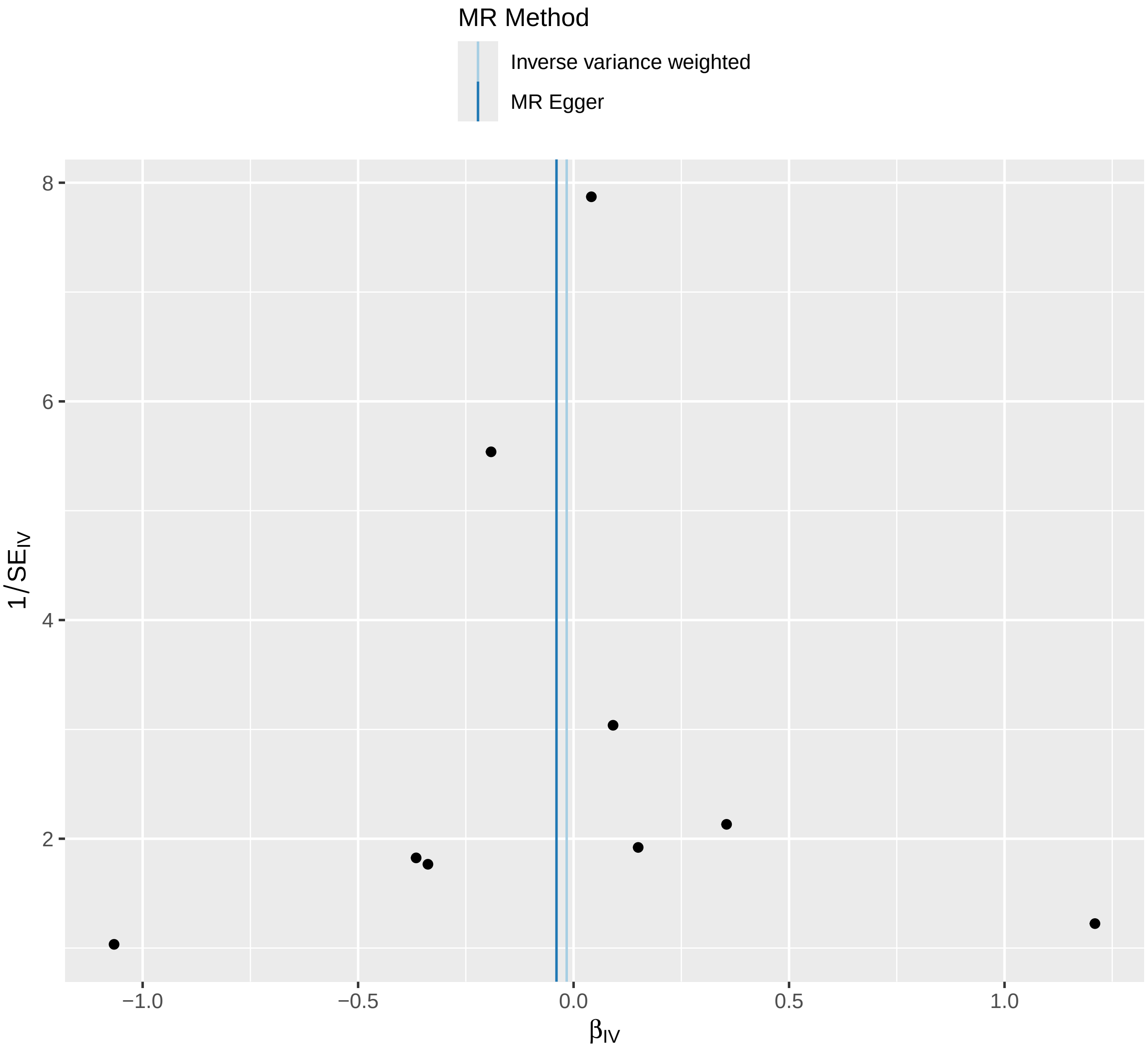

MR Method
Inverse variance weighted
MR Egger
8
6
4
2
$1/SE_{IV}$
−1.0
−0.5
0.0
0.5
1.0
β_{IV}

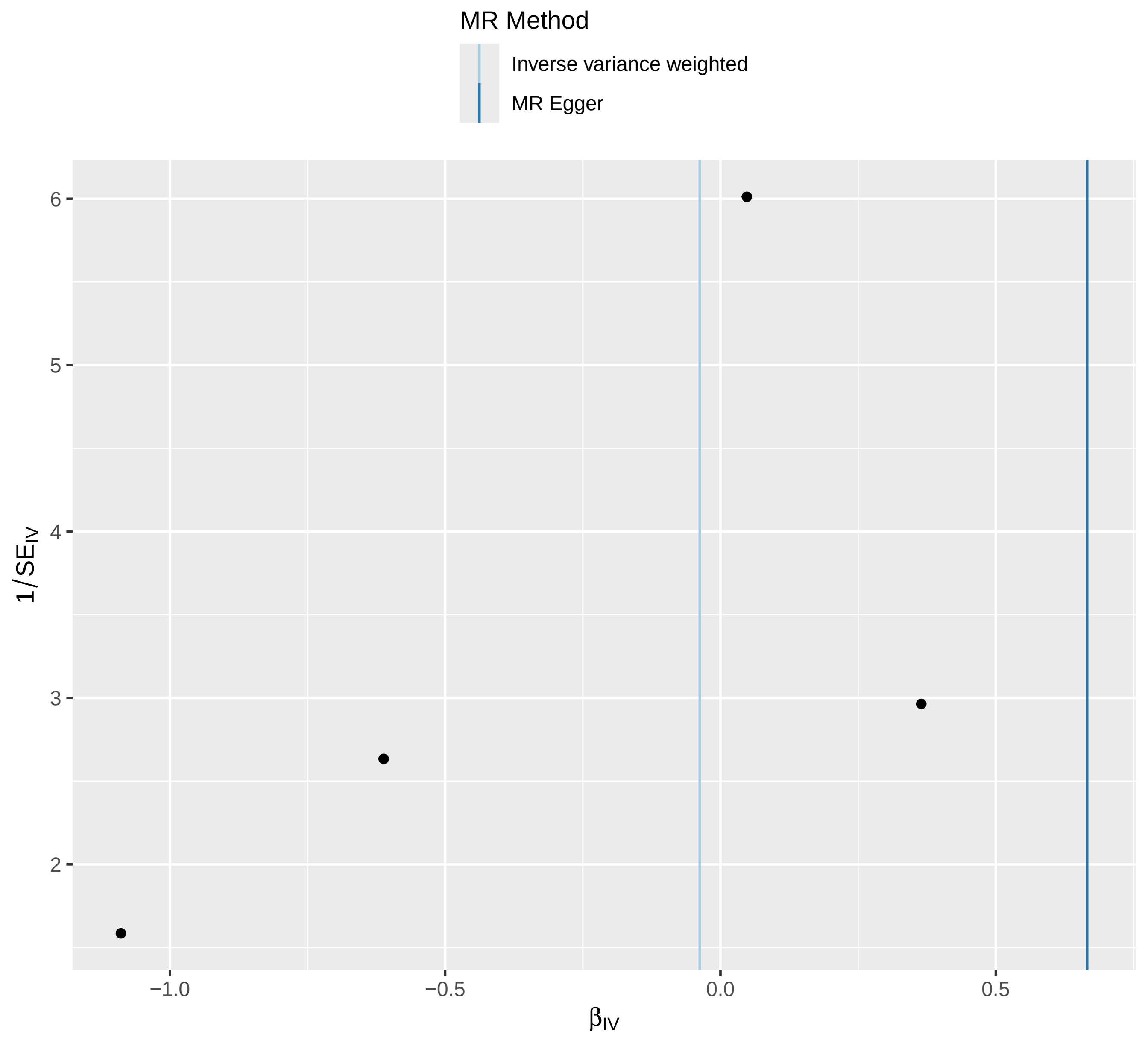
MR Method
Inverse variance weighted
MR Egger
6
5
4
3
2
1/SE_IV
−1.0
−0.5
0.0
0.5
β_IV

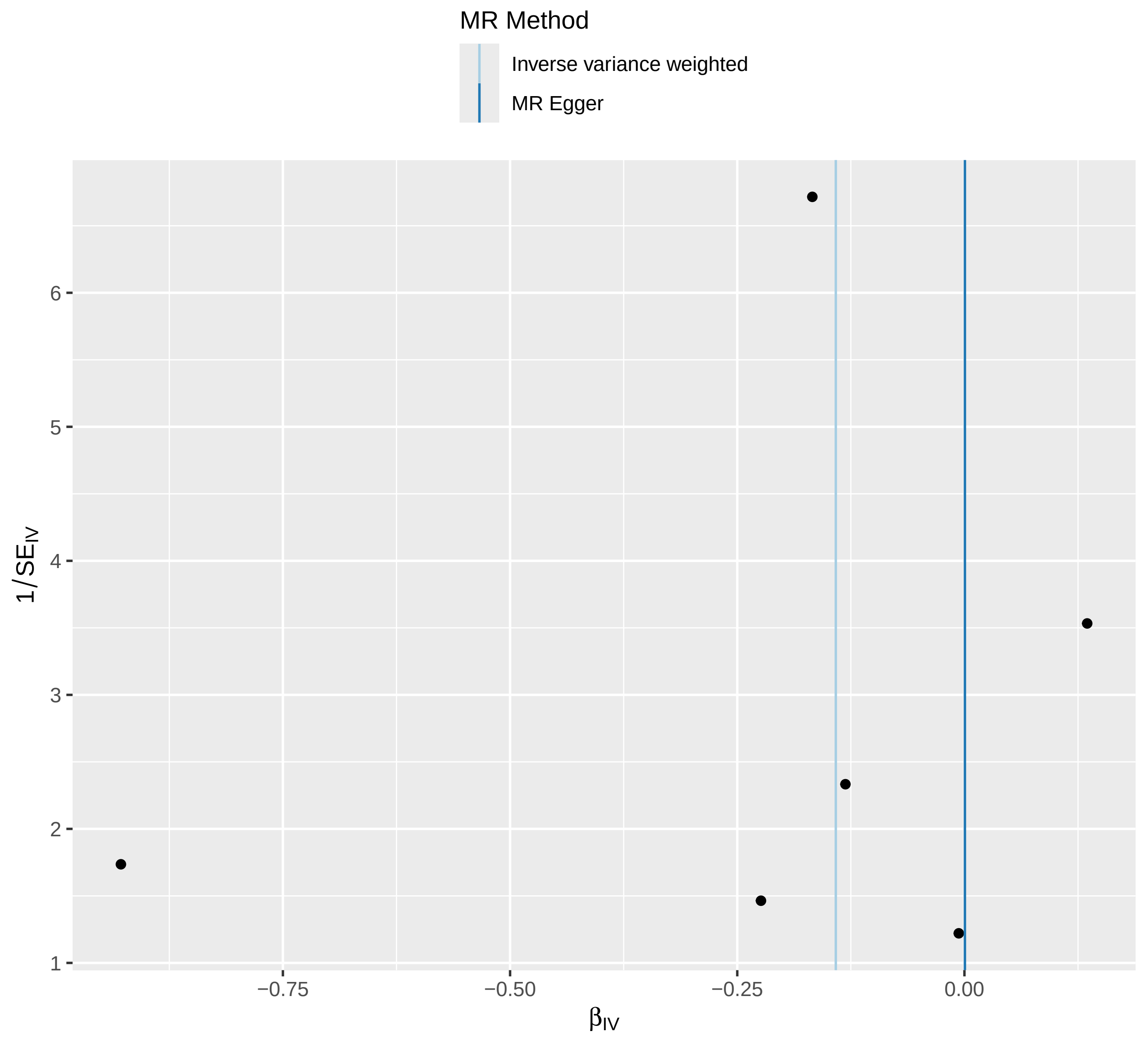

MR Method
Inverse variance weighted
MR Egger
6
5
4
3
2
1
1/SE_IV
−0.75
−0.50
−0.25
0.00
β_IV

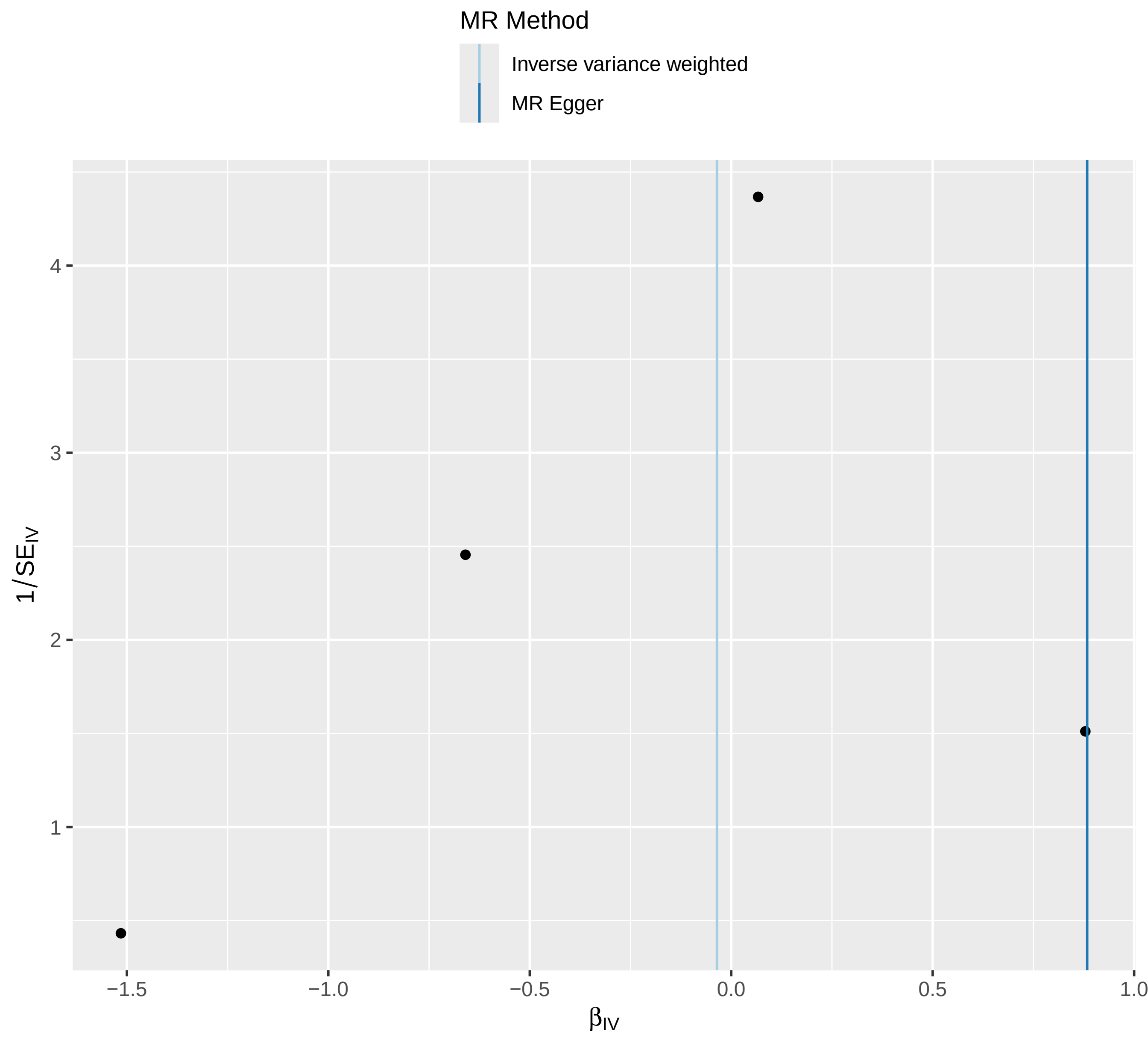

MR Method
Inverse variance weighted
MR Egger
4
3
2
1
1/SE$_{IV}$
−1.5
−1.0
−0.5
0.0
0.5
1.0
β$_{IV}$

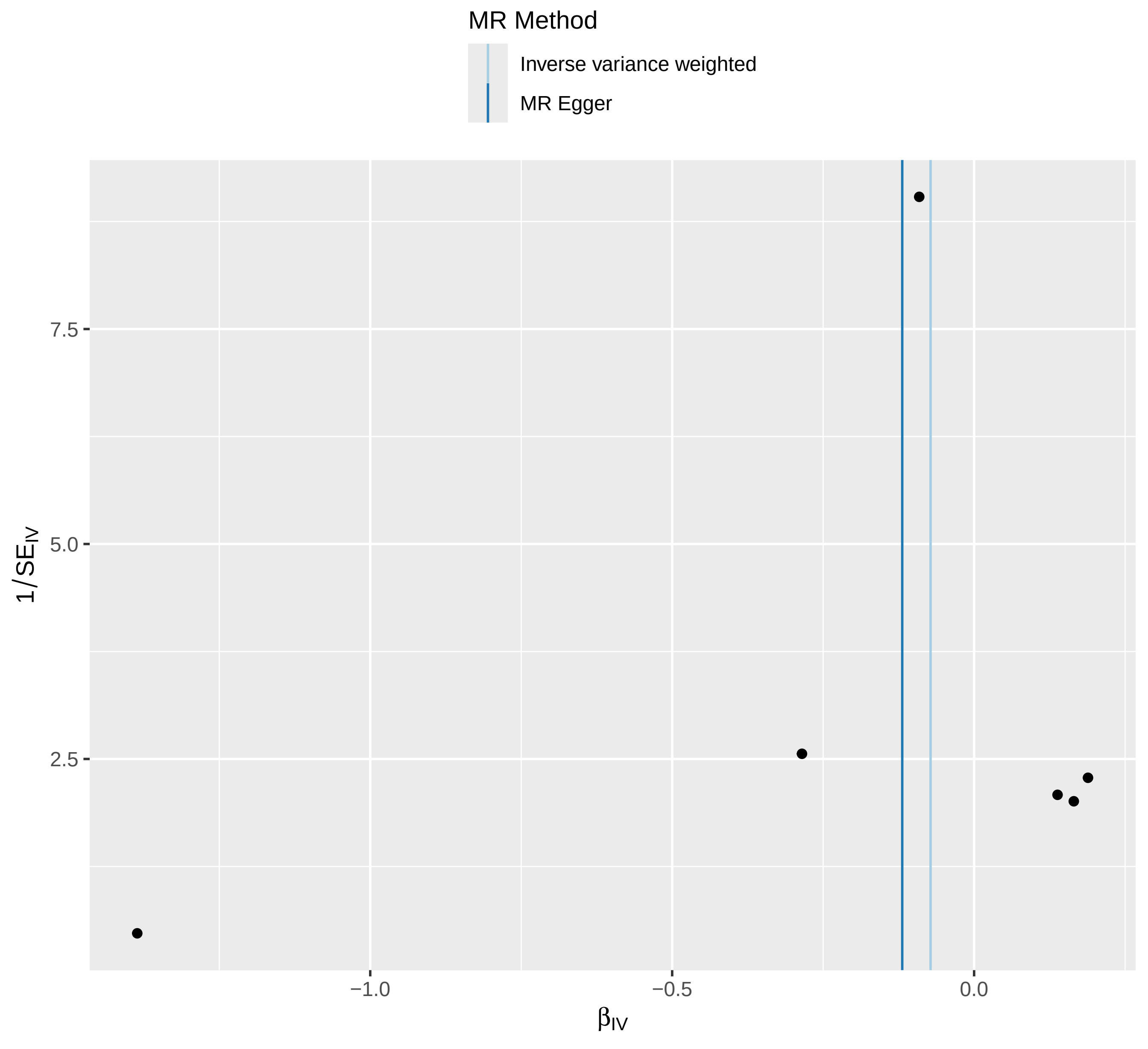
MR Method
Inverse variance weighted
MR Egger
7.5
5.0
2.5
1/SE_{IV}
−1.0
−0.5
0.0
β_{IV}

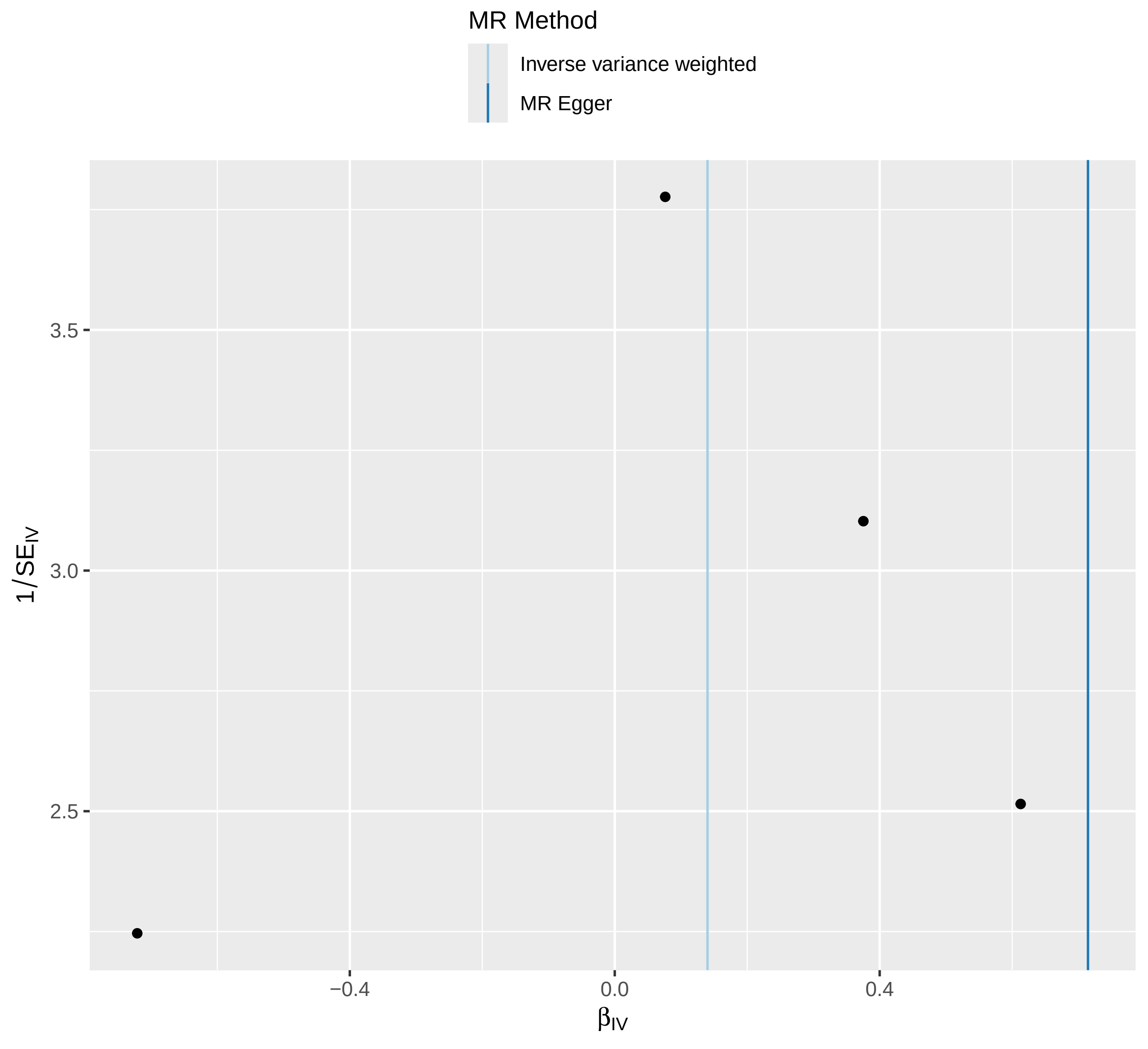

MR Method
Inverse variance weighted
MR Egger
3.5
3.0
2.5
1/SE_IV
−0.4
0.0
0.4
β_IV

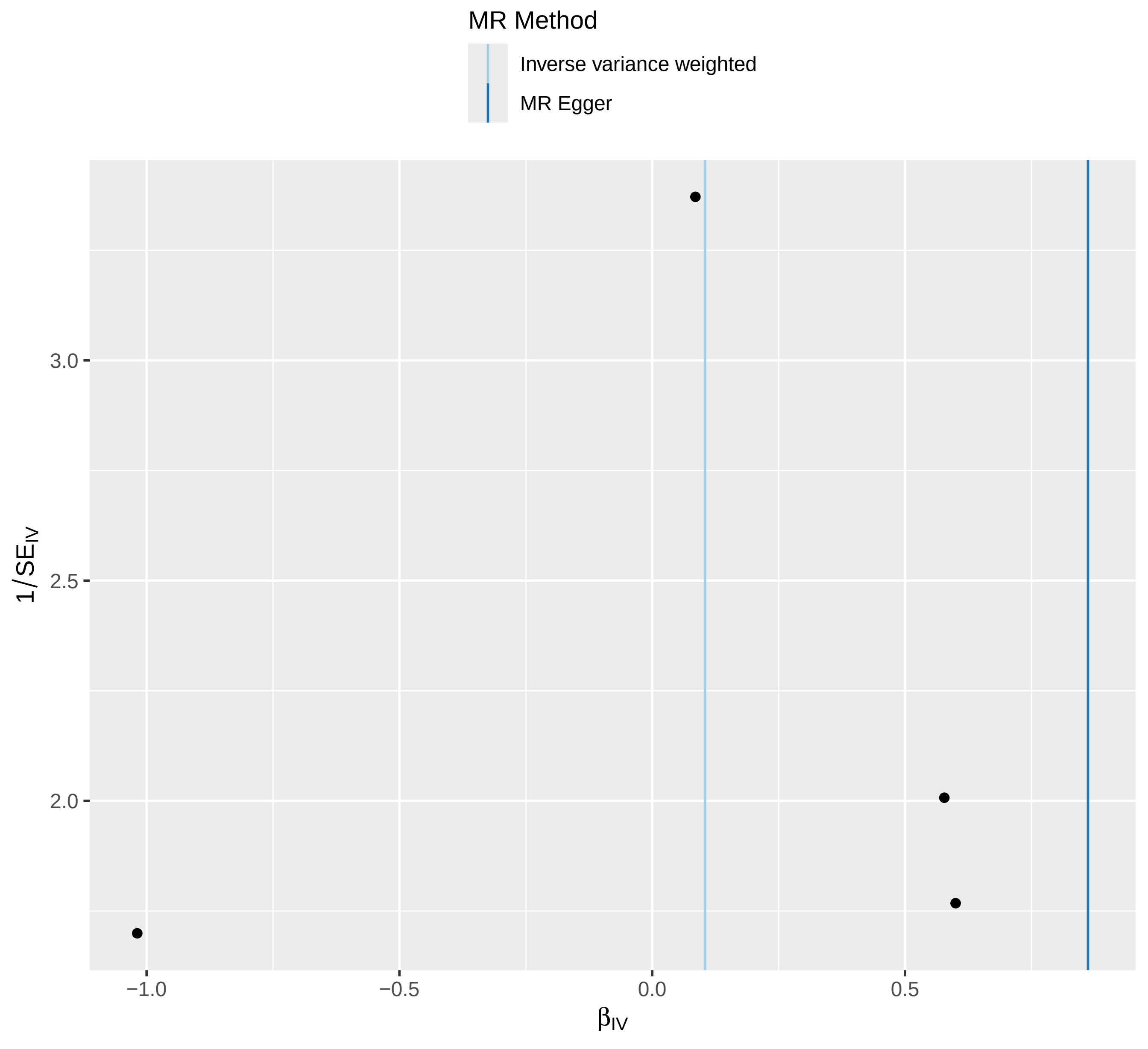

MR Method
Inverse variance weighted
MR Egger
3.0
2.5
2.0
1/SE_IV
−1.0
−0.5
0.0
0.5
β_IV

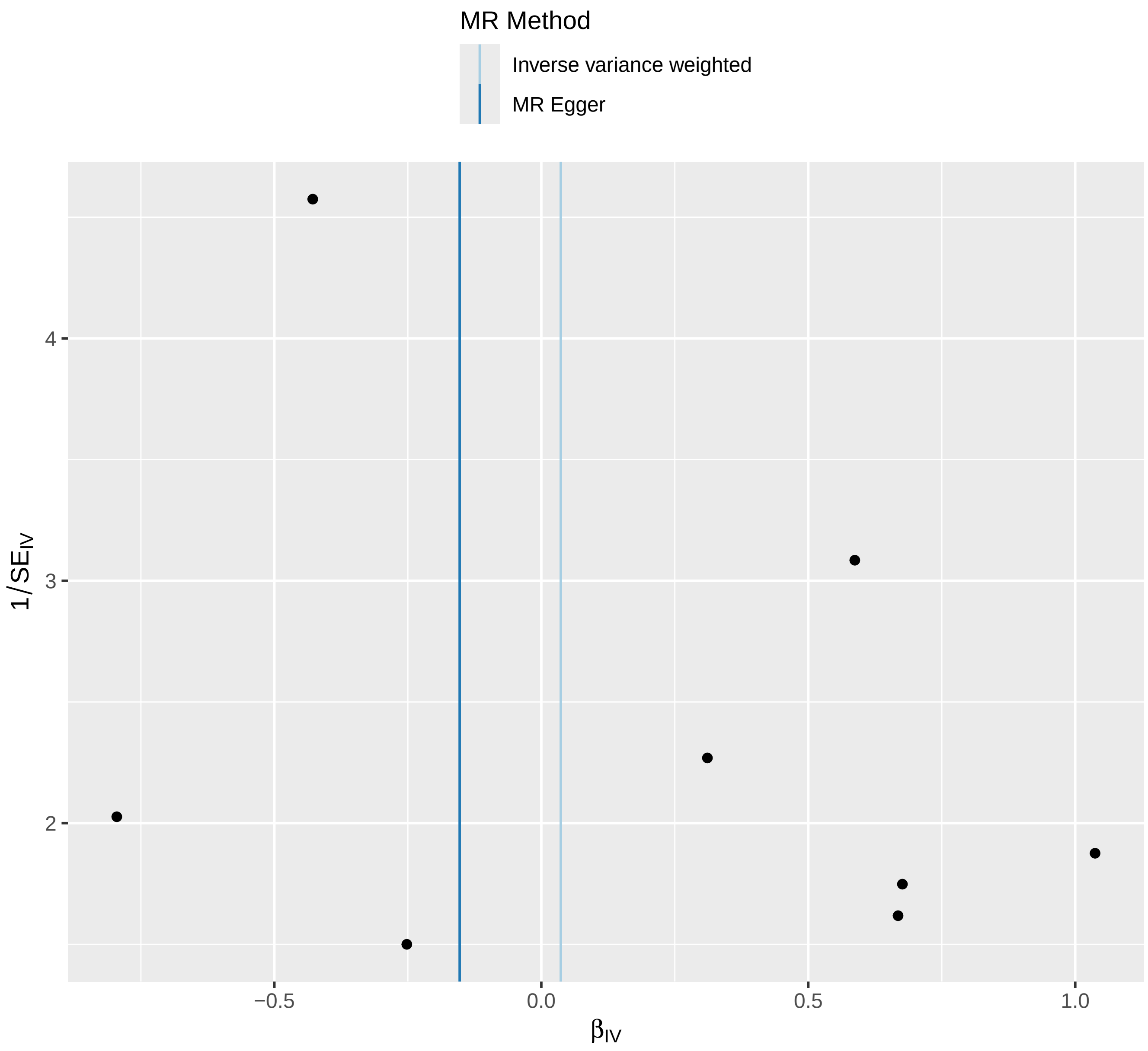
MR Method
Inverse variance weighted
MR Egger
4
3
2
1/SE_IV
−0.5
0.0
0.5
1.0
β_IV

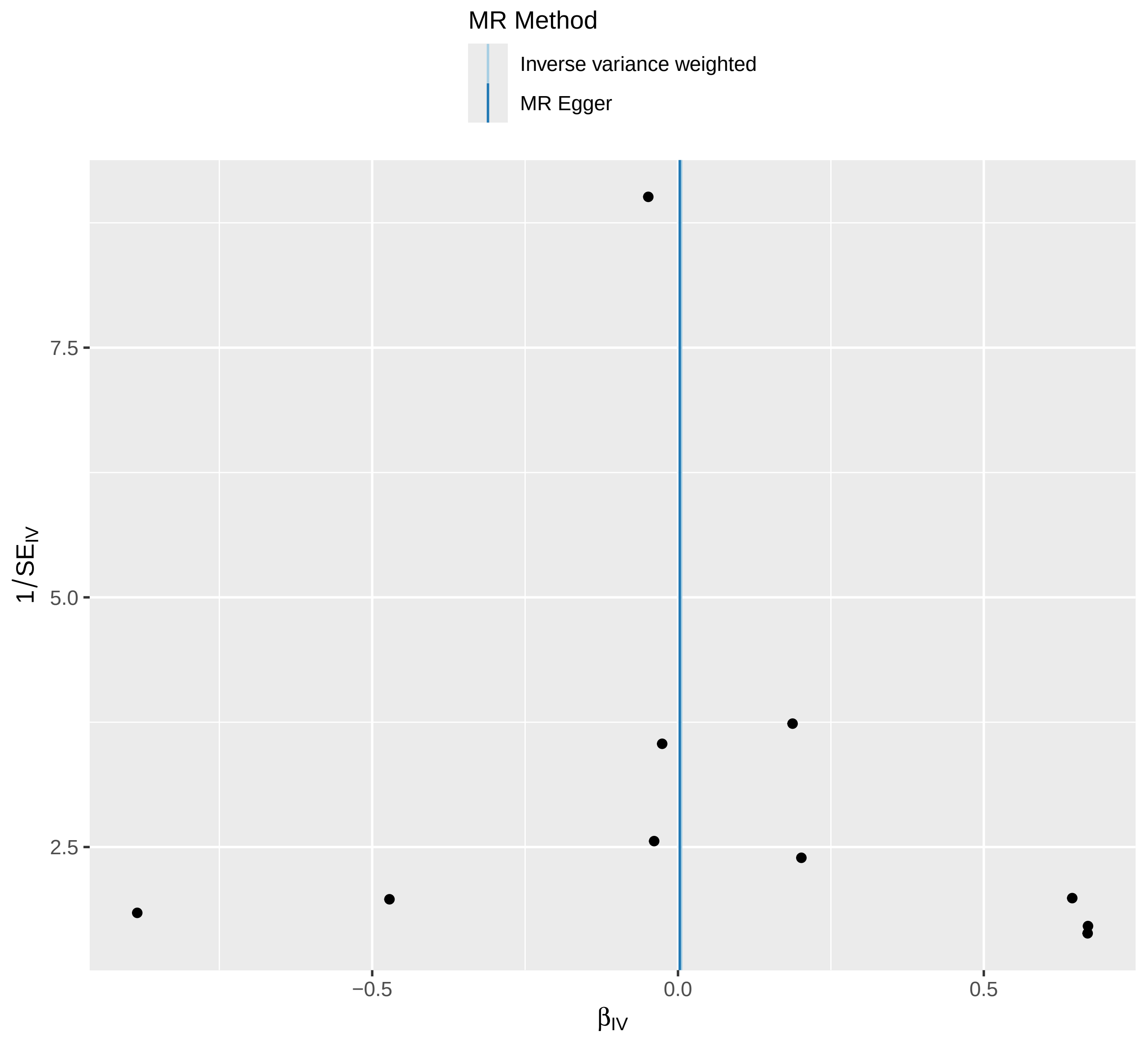
MR Method
Inverse variance weighted
MR Egger
7.5
5.0
2.5
1/SE_IV
−0.5
0.0
0.5
β_IV

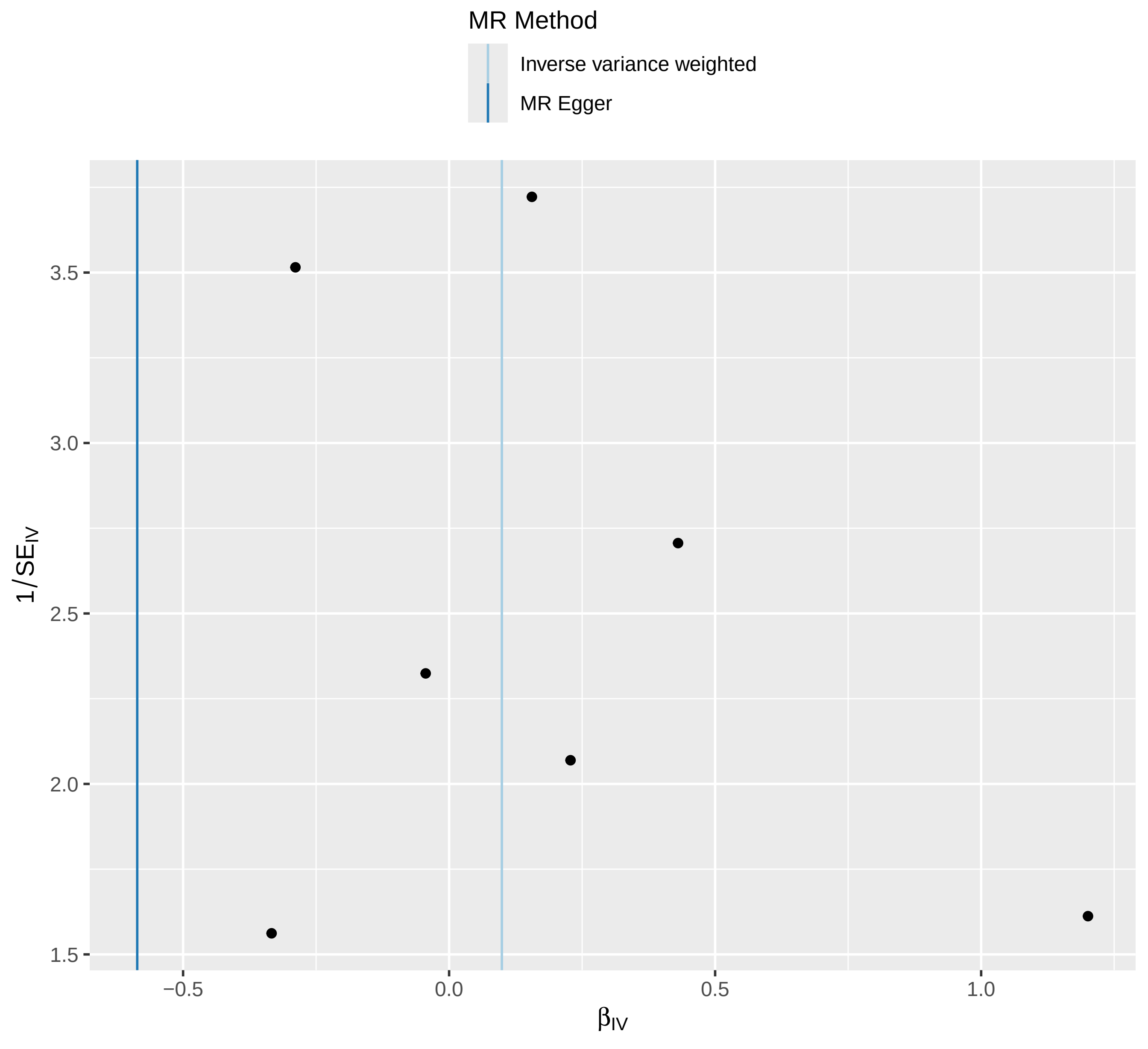

MR Method
Inverse variance weighted
MR Egger
1/SE$_{IV}$
3.5
3.0
2.5
2.0
1.5
−0.5
0.0
0.5
1.0
β$_{IV}$

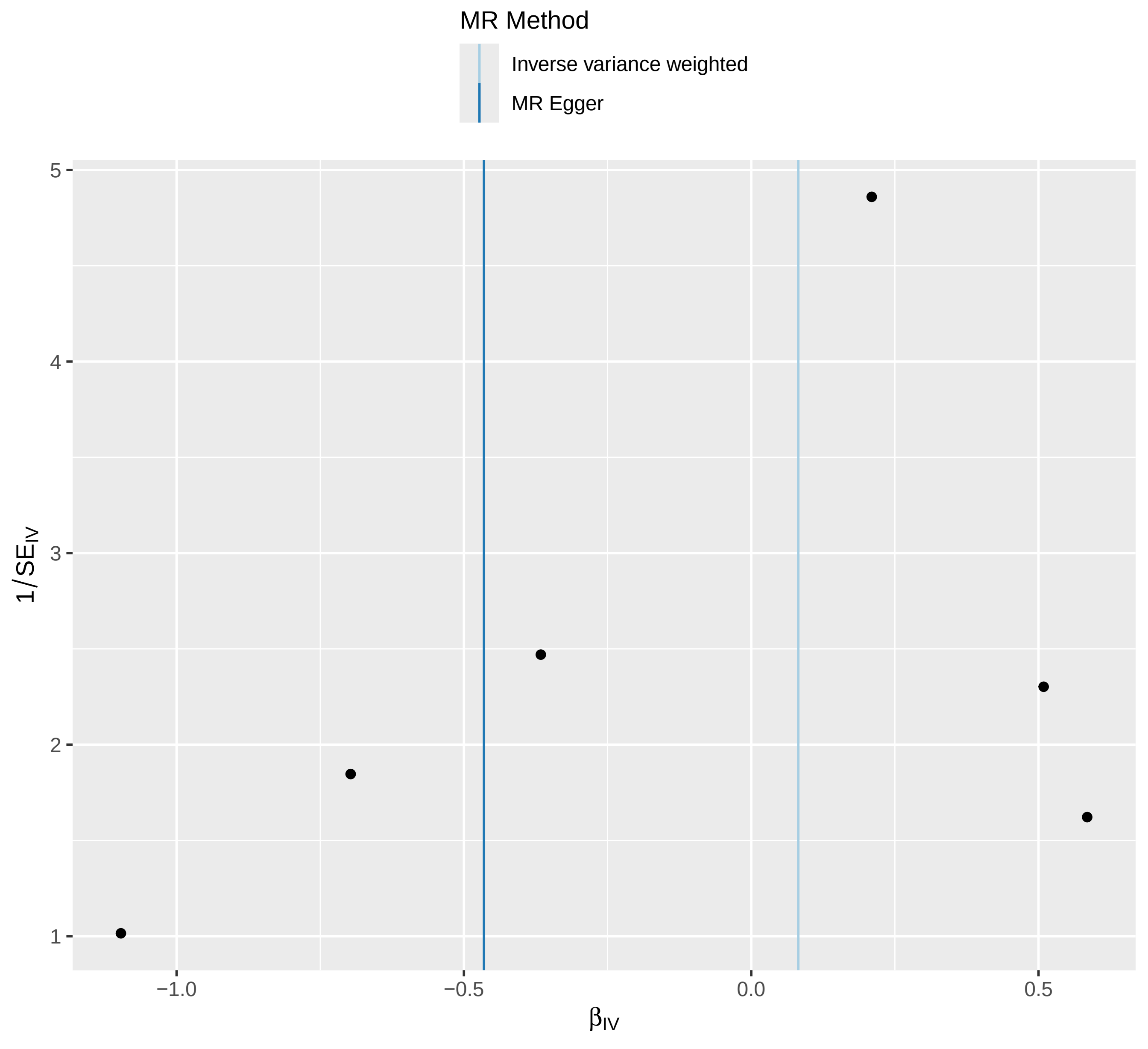

MR Method
Inverse variance weighted
MR Egger
5
4
3
2
1
1/SE$_{IV}$
−1.0
−0.5
0.0
0.5
β$_{IV}$

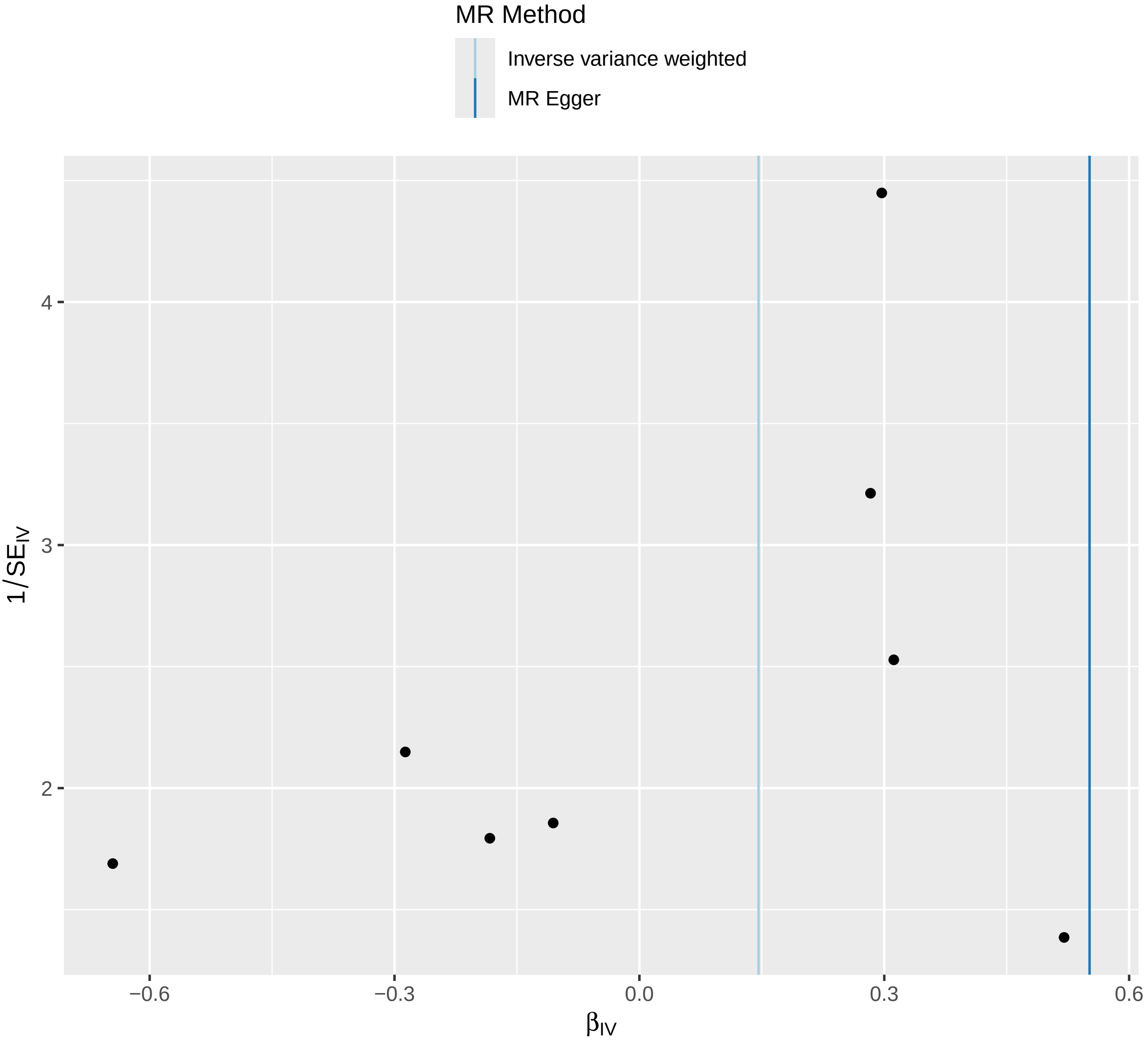

MR Method
Inverse variance weighted
MR Egger
4
3
2
1/SE_IV
−0.6
−0.3
0.0
0.3
0.6
β_IV

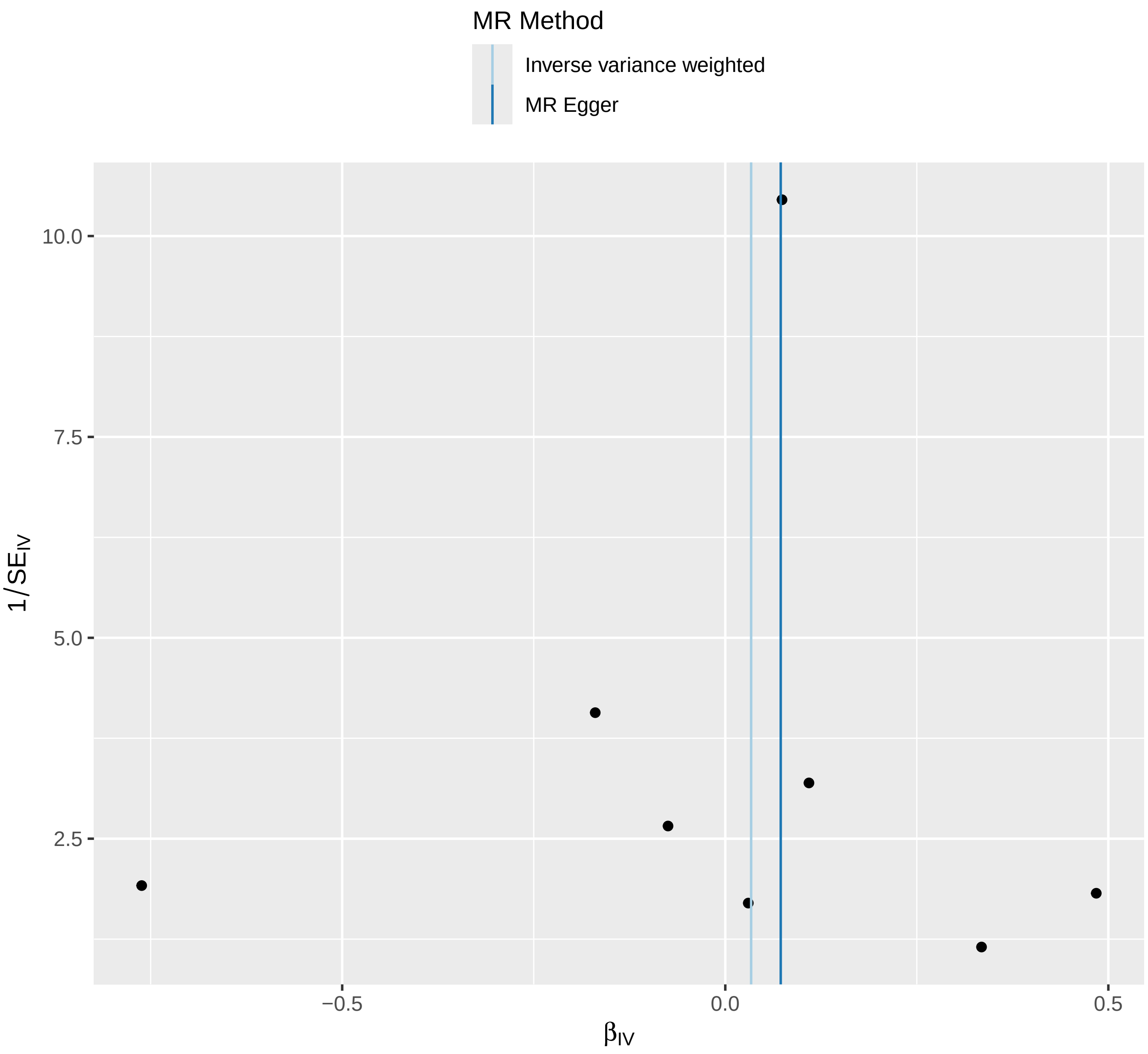

MR Method
Inverse variance weighted
MR Egger
10.0
7.5
5.0
2.5
1/SE$_{IV}$
−0.5
0.0
0.5
β$_{IV}$

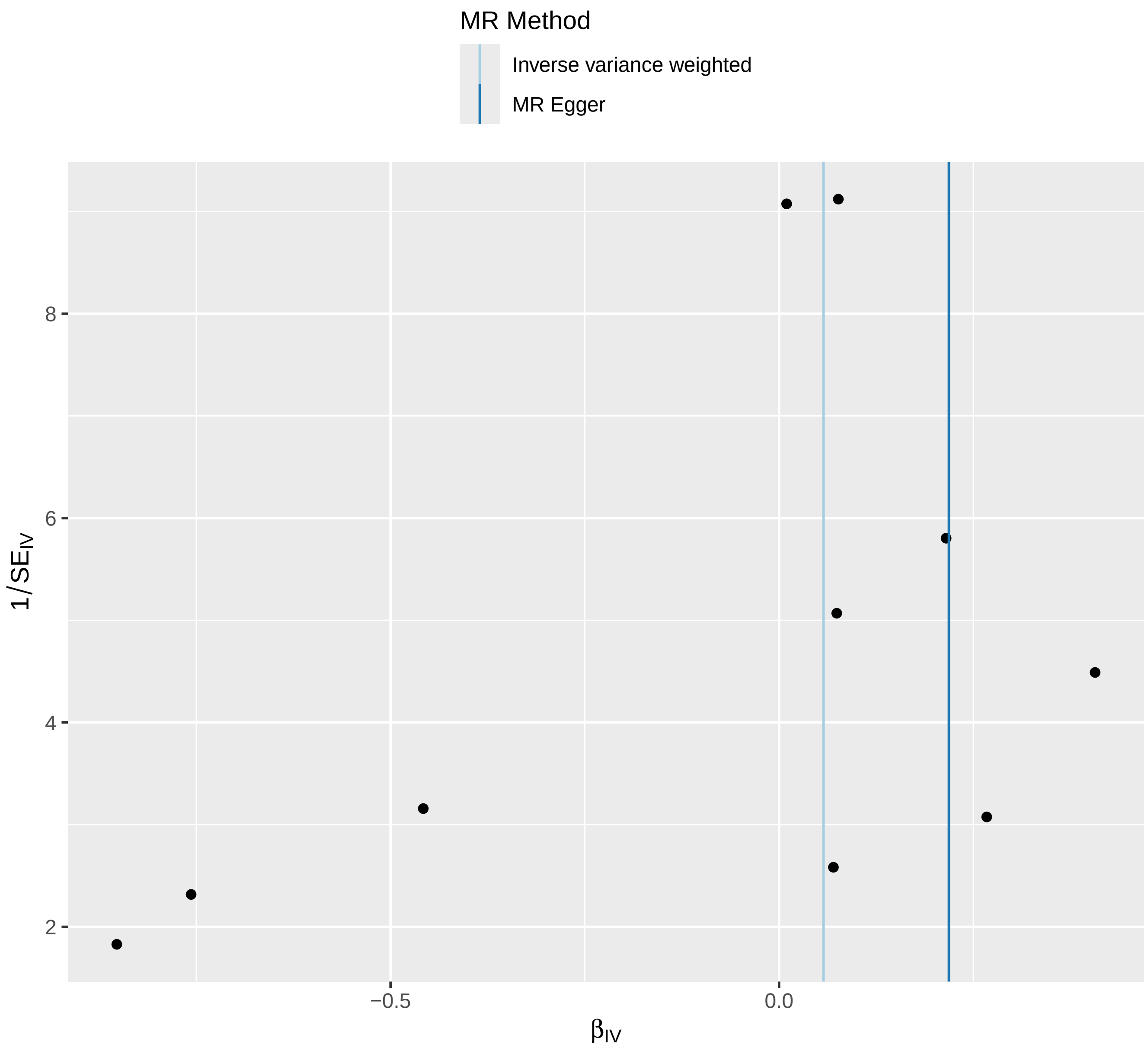

MR Method
Inverse variance weighted
MR Egger
8
6
4
2
1/SE$_{IV}$
−0.5
0.0
β$_{IV}$

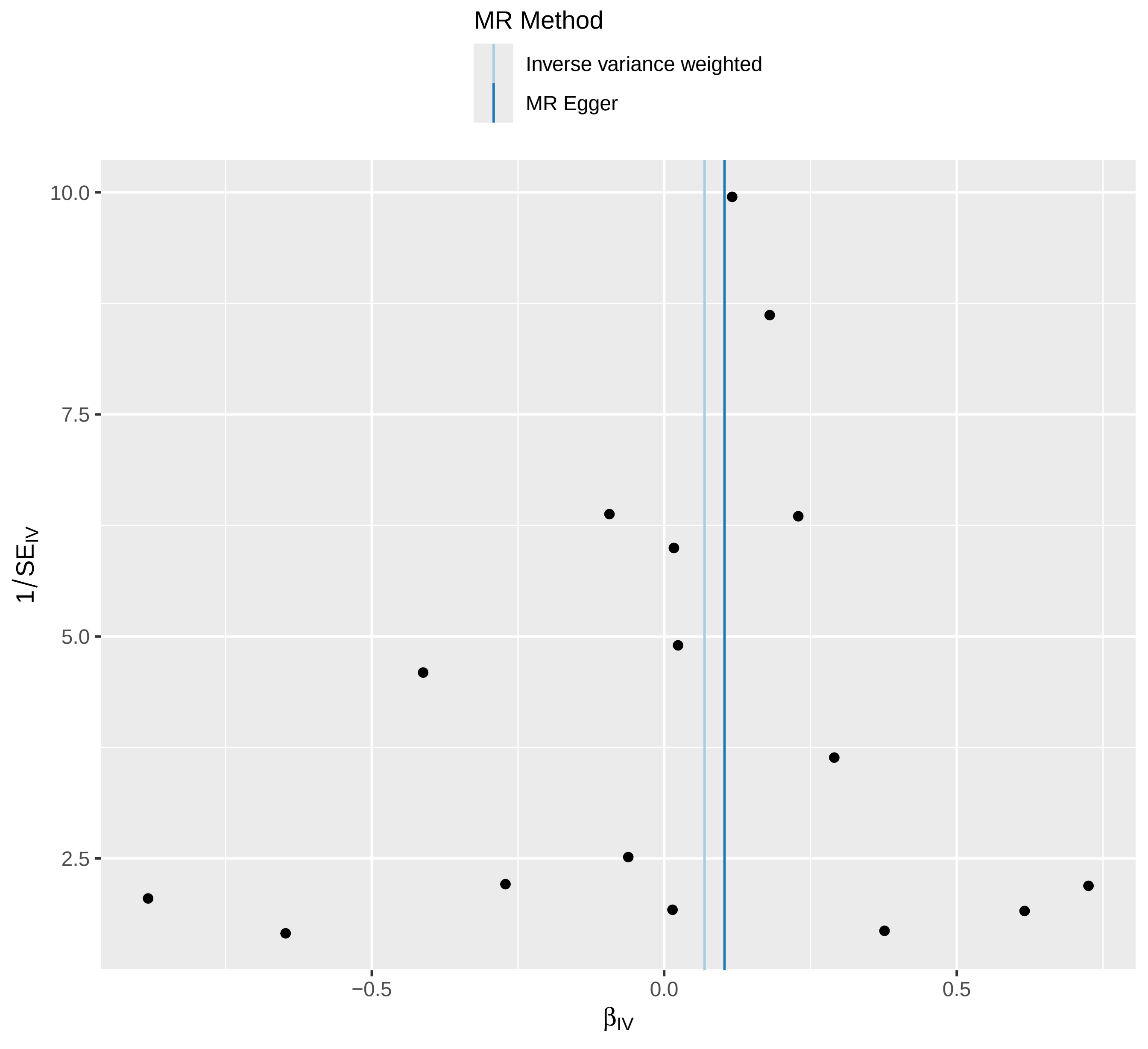
MR Method
Inverse variance weighted
MR Egger
10.0
7.5
5.0
2.5
1/SE_IV
−0.5
0.0
0.5
β_IV

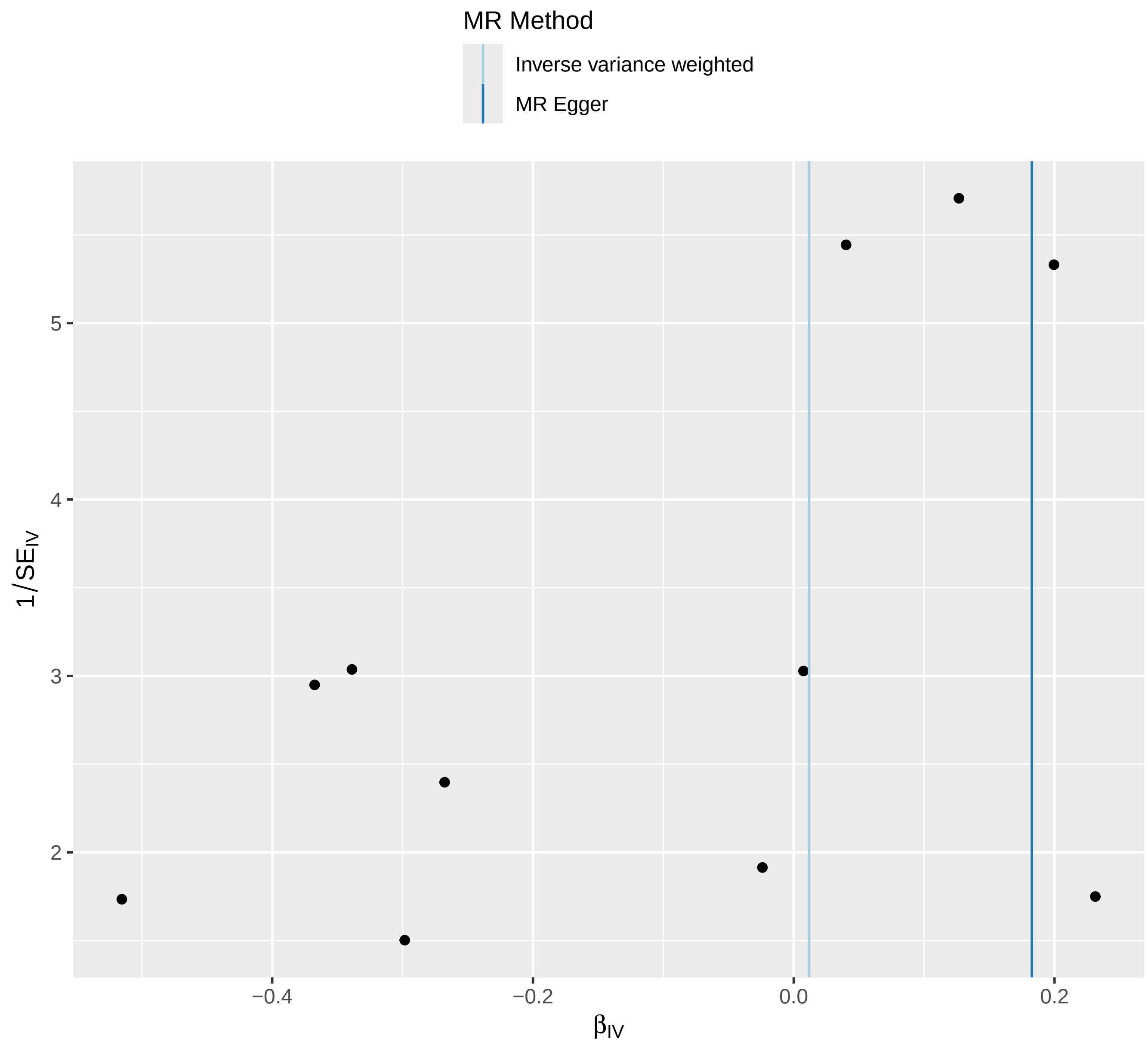

MR Method
Inverse variance weighted
MR Egger
1/SE_{IV}
5
4
3
2
−0.4
−0.2
0.0
0.2
β_{IV}

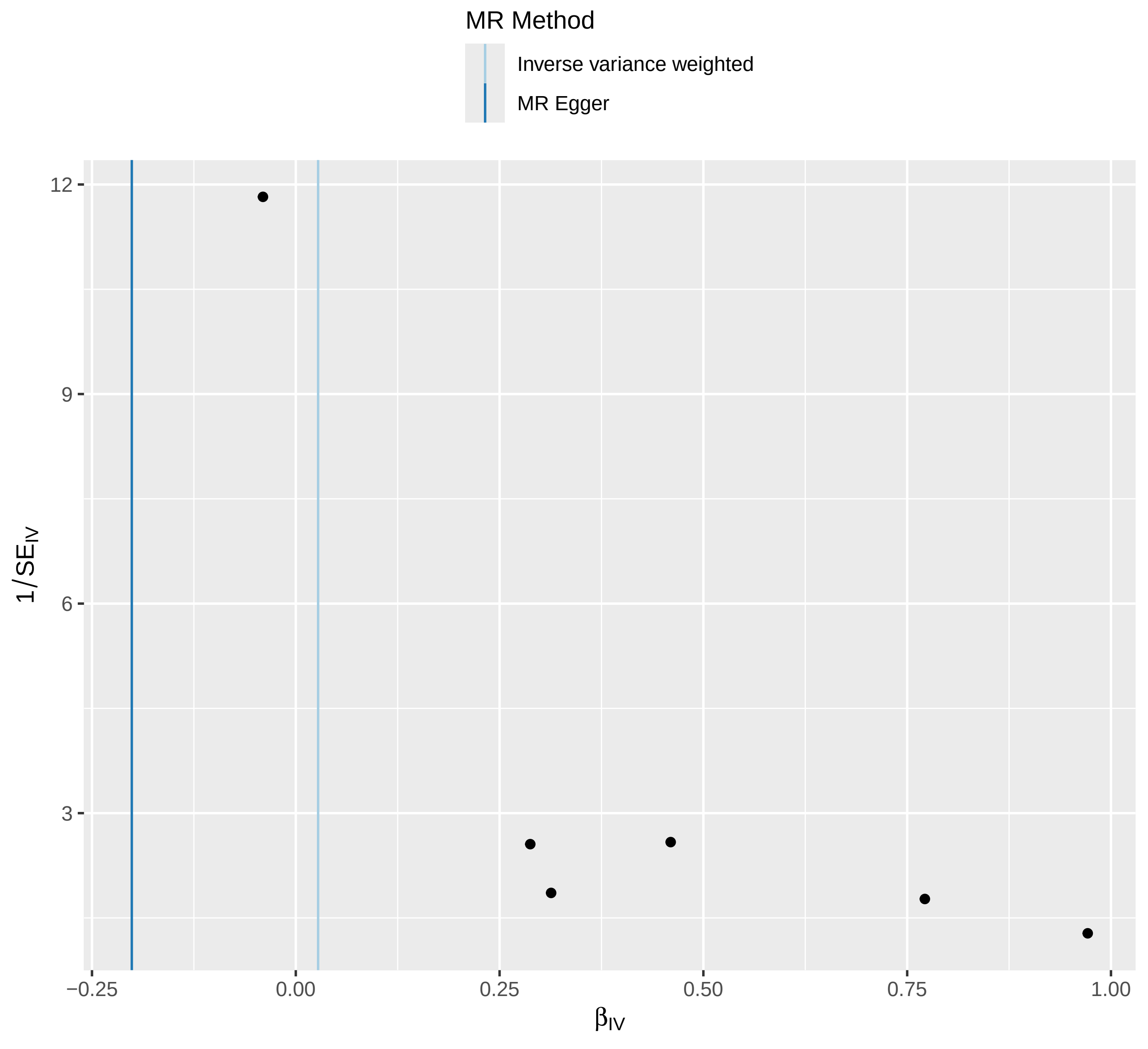
MR Method
Inverse variance weighted
MR Egger
$1/SE_{IV}$
12
9
6
3
−0.25
0.00
0.25
0.50
0.75
1.00
β_{IV}

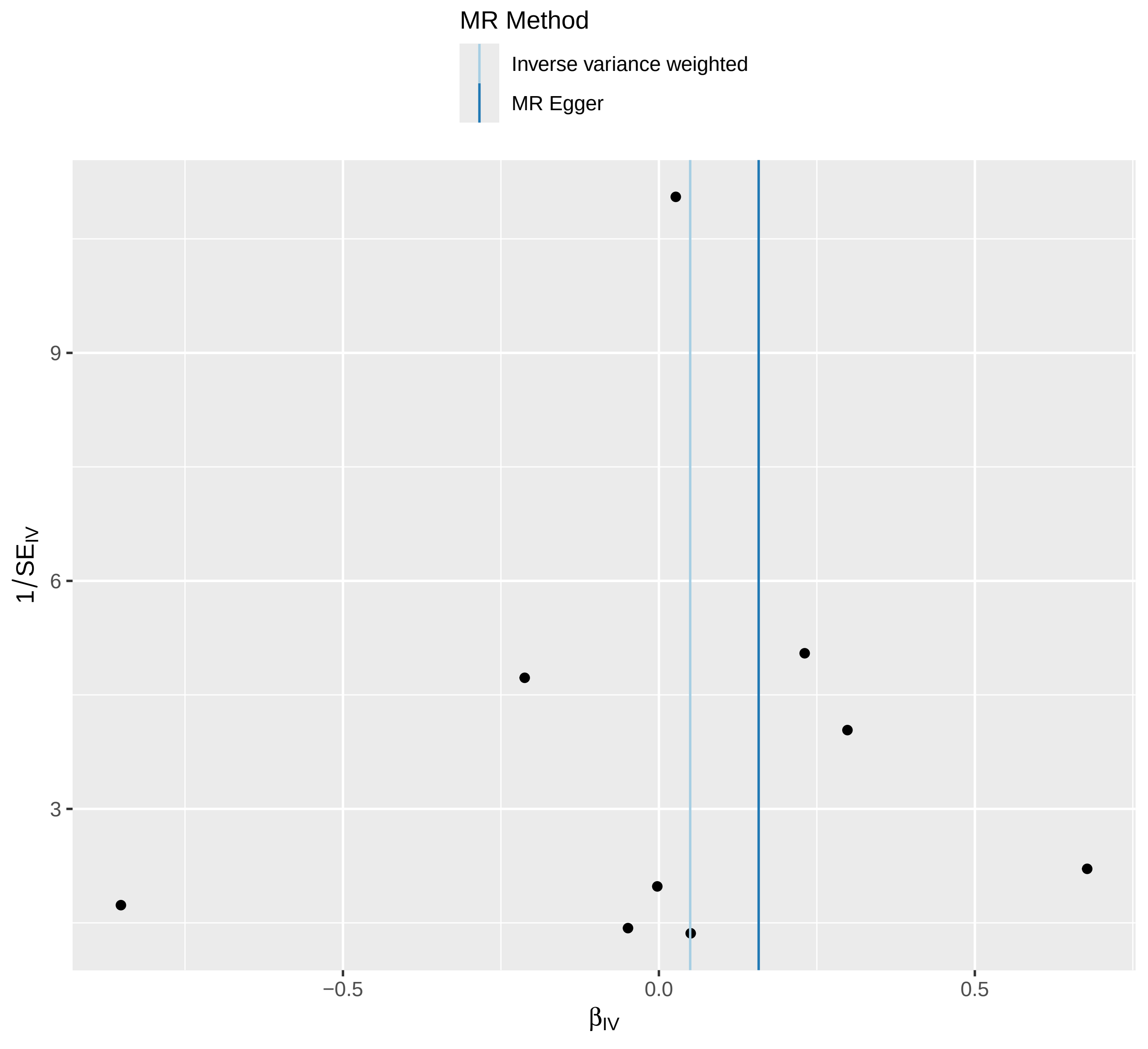

MR Method
Inverse variance weighted
MR Egger
9
6
3
1/SE_IV
−0.5
0.0
0.5
β_IV

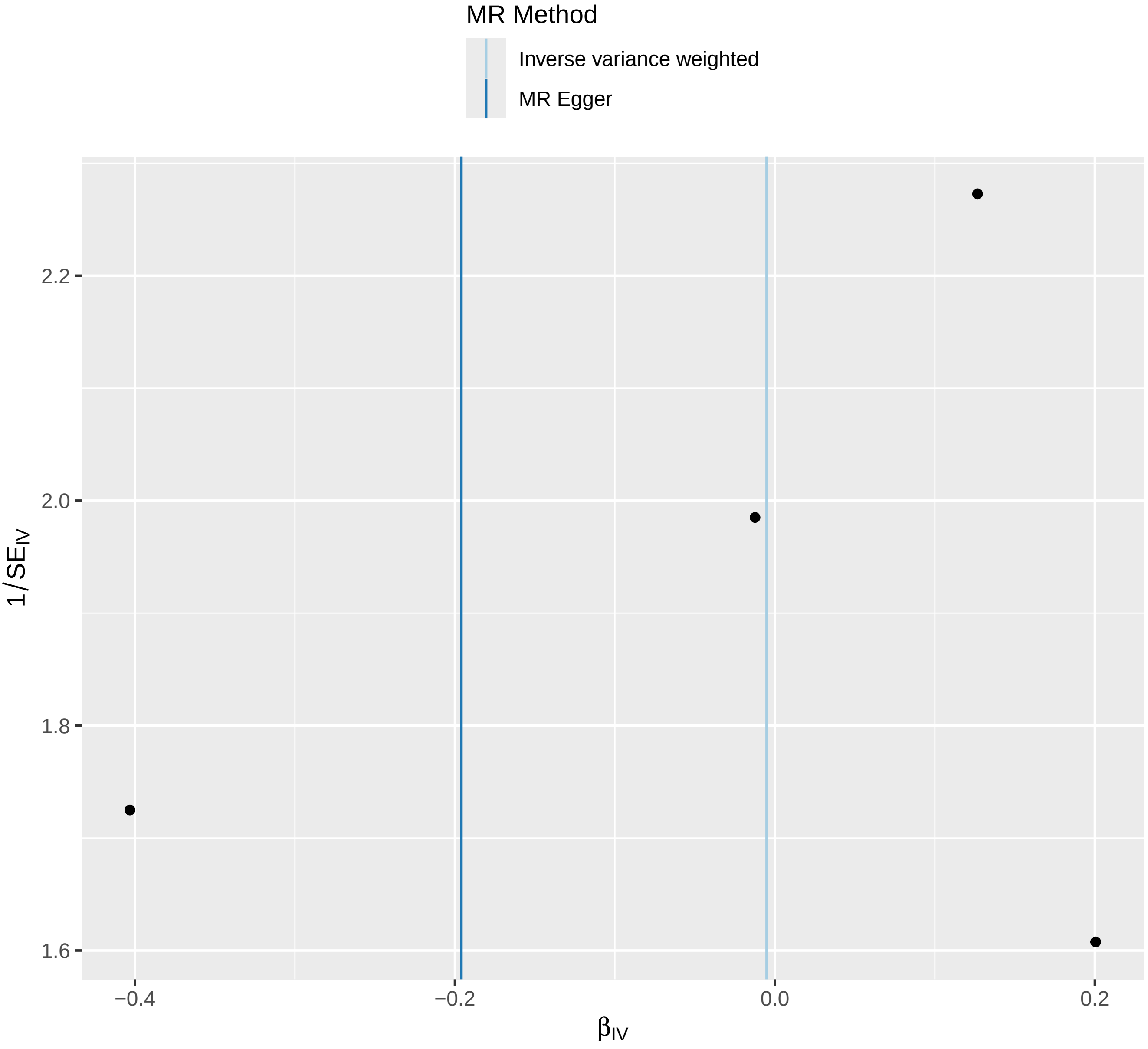

MR Method
Inverse variance weighted
MR Egger
2.2
2.0
1.8
1.6
1/SE$_{IV}$
−0.4
−0.2
0.0
0.2
β$_{IV}$

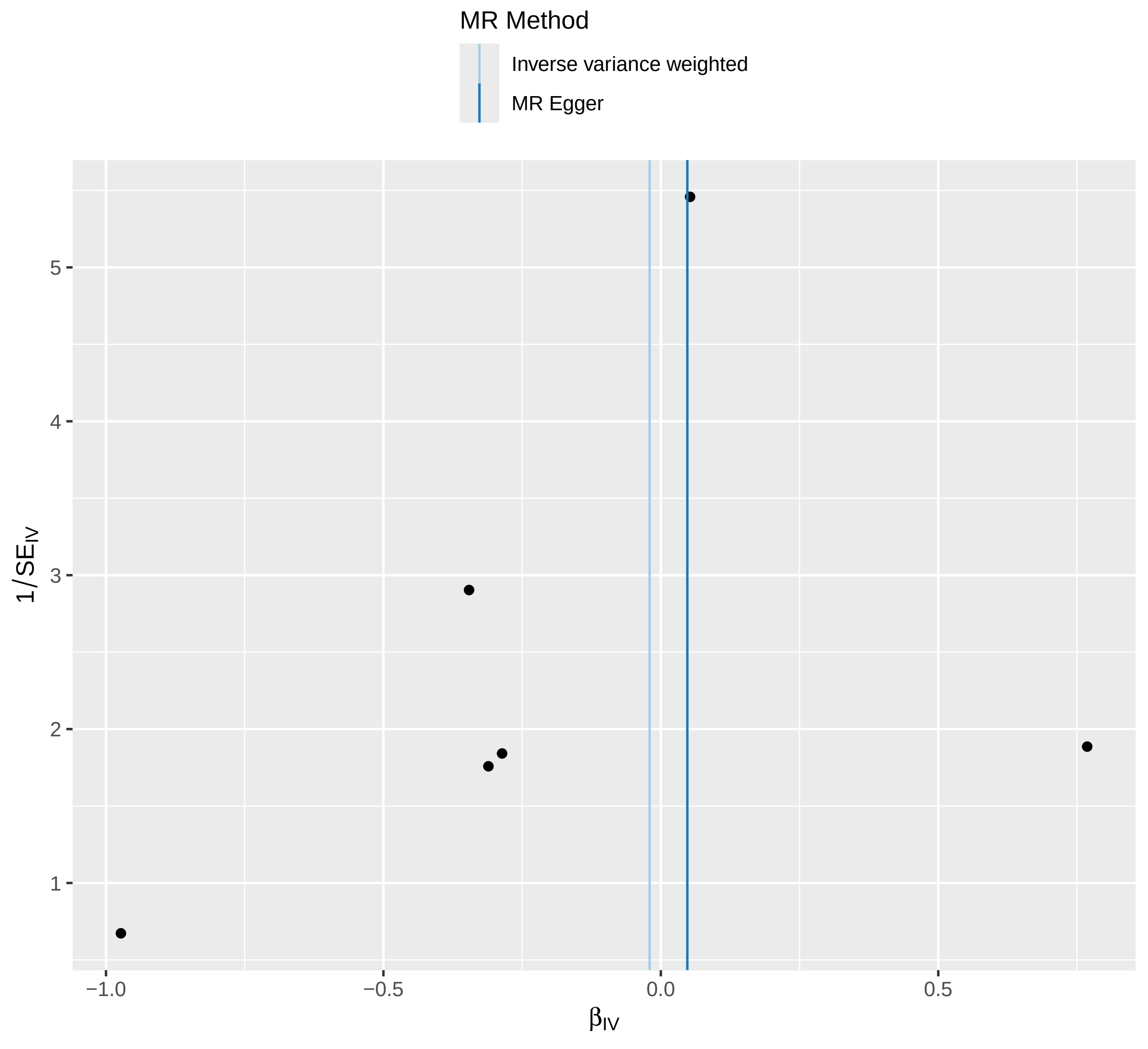
MR Method
Inverse variance weighted
MR Egger
1/SE_IV
5
4
3
2
1
−1.0
−0.5
0.0
0.5
β_IV

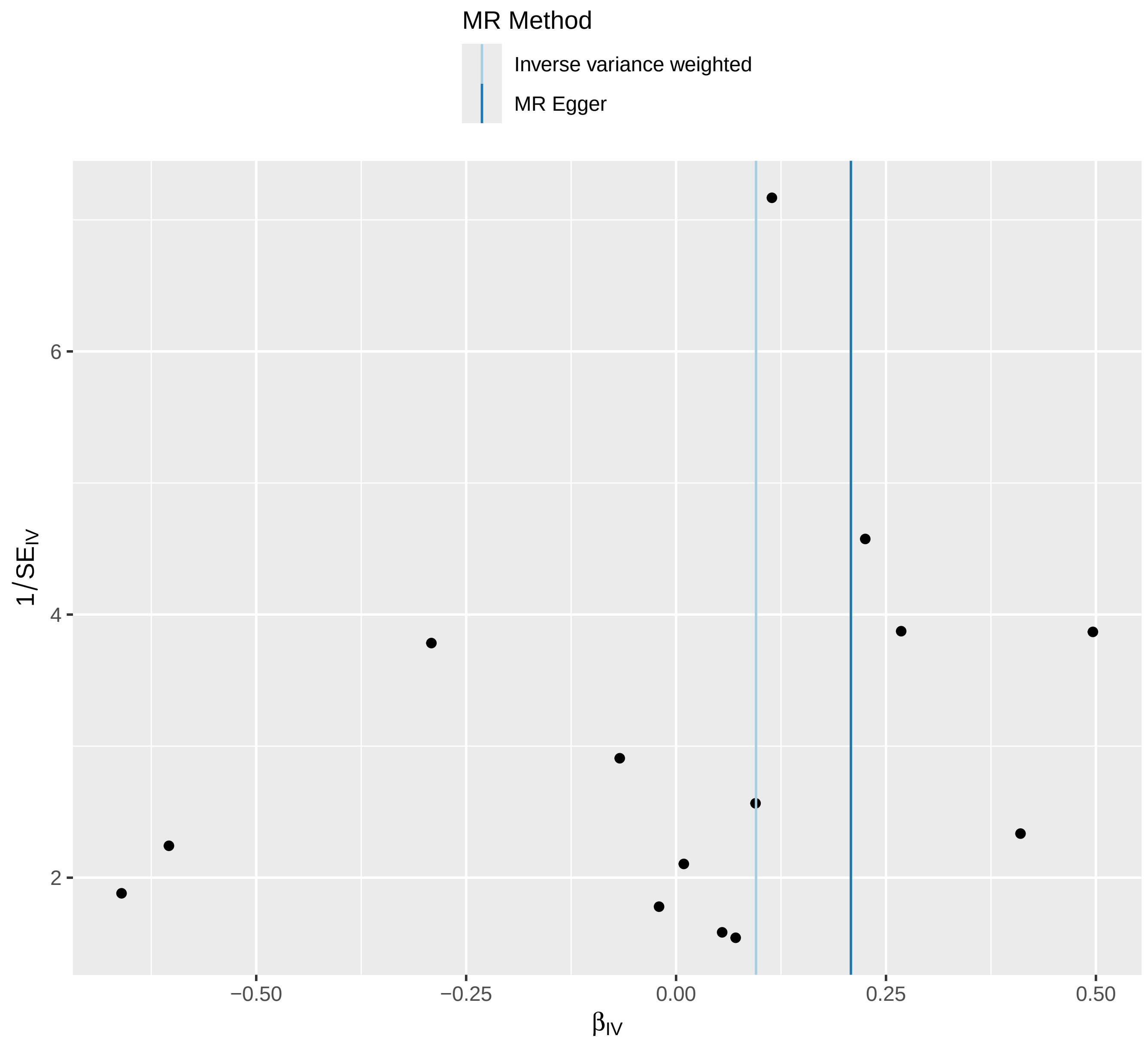
MR Method
Inverse variance weighted
MR Egger
6
4
2
1/SE$_{IV}$
−0.50
−0.25
0.00
0.25
0.50
β_{IV}

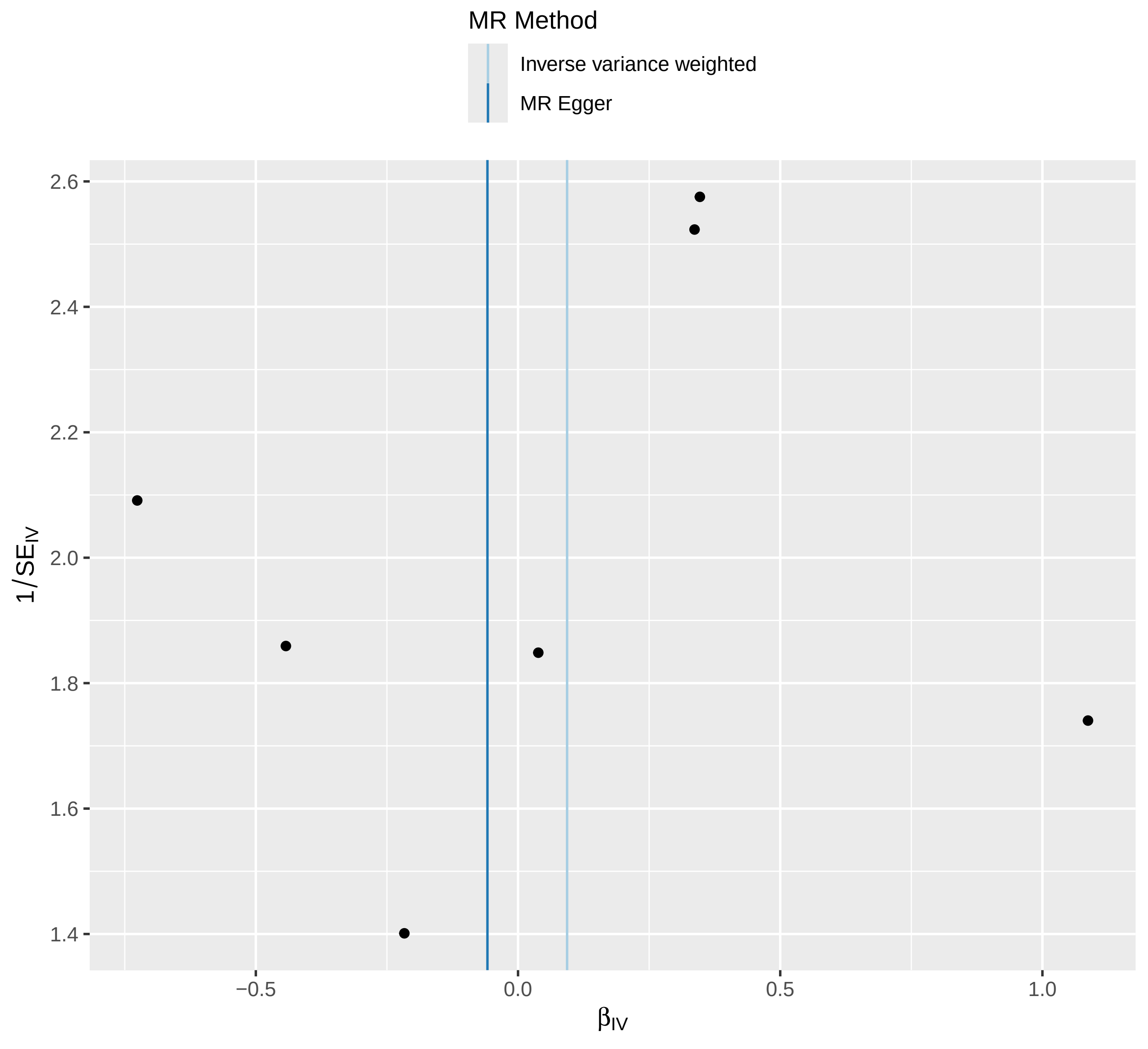
MR Method
Inverse variance weighted
MR Egger
2.6
2.4
2.2
2.0
1.8
1.6
1.4
1/SE_IV
−0.5
0.0
0.5
1.0
β_IV

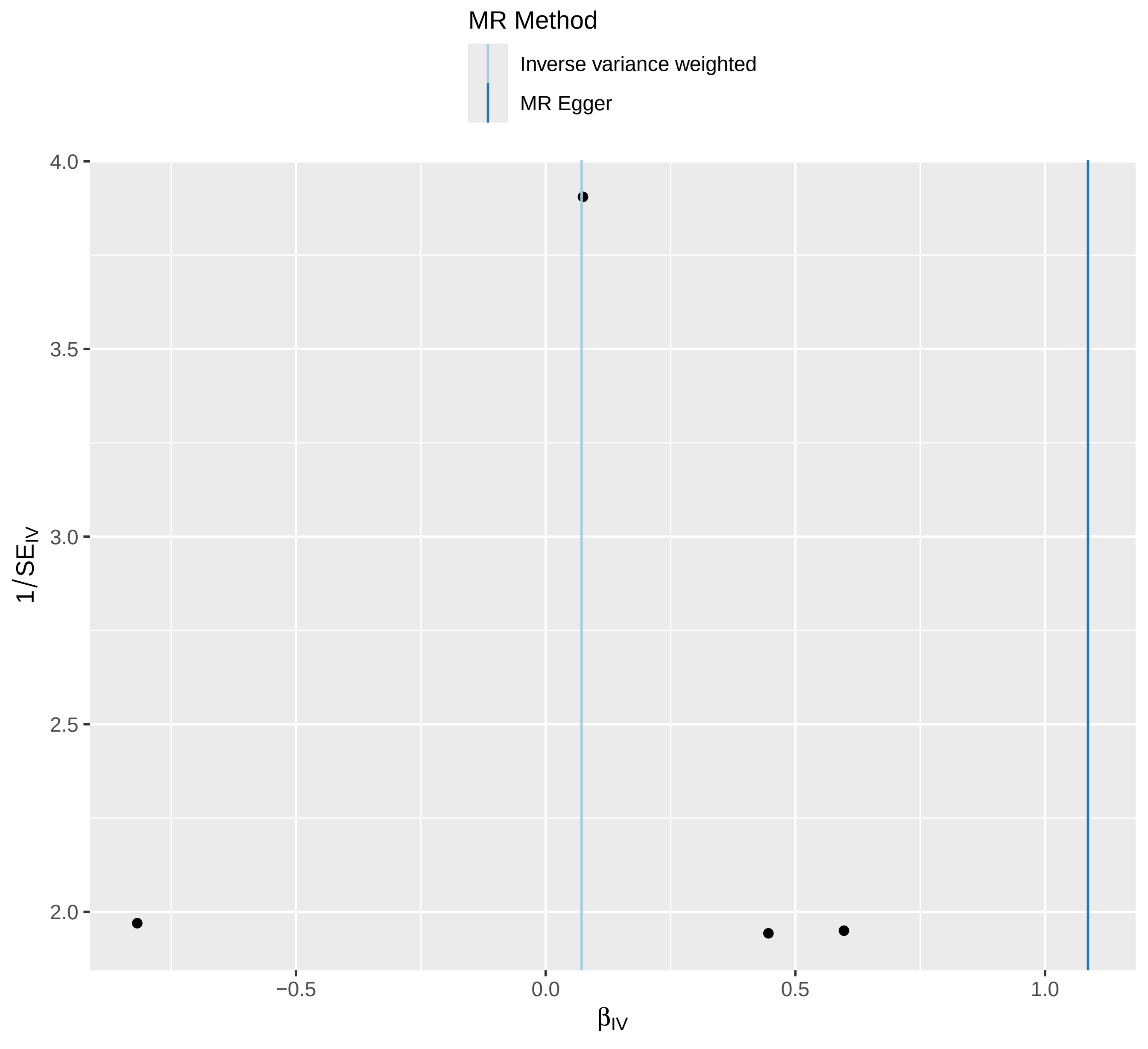
MR Method
Inverse variance weighted
MR Egger
4.0
3.5
3.0
2.5
2.0
1/SE$_{IV}$
−0.5
0.0
0.5
1.0
β$_{IV}$

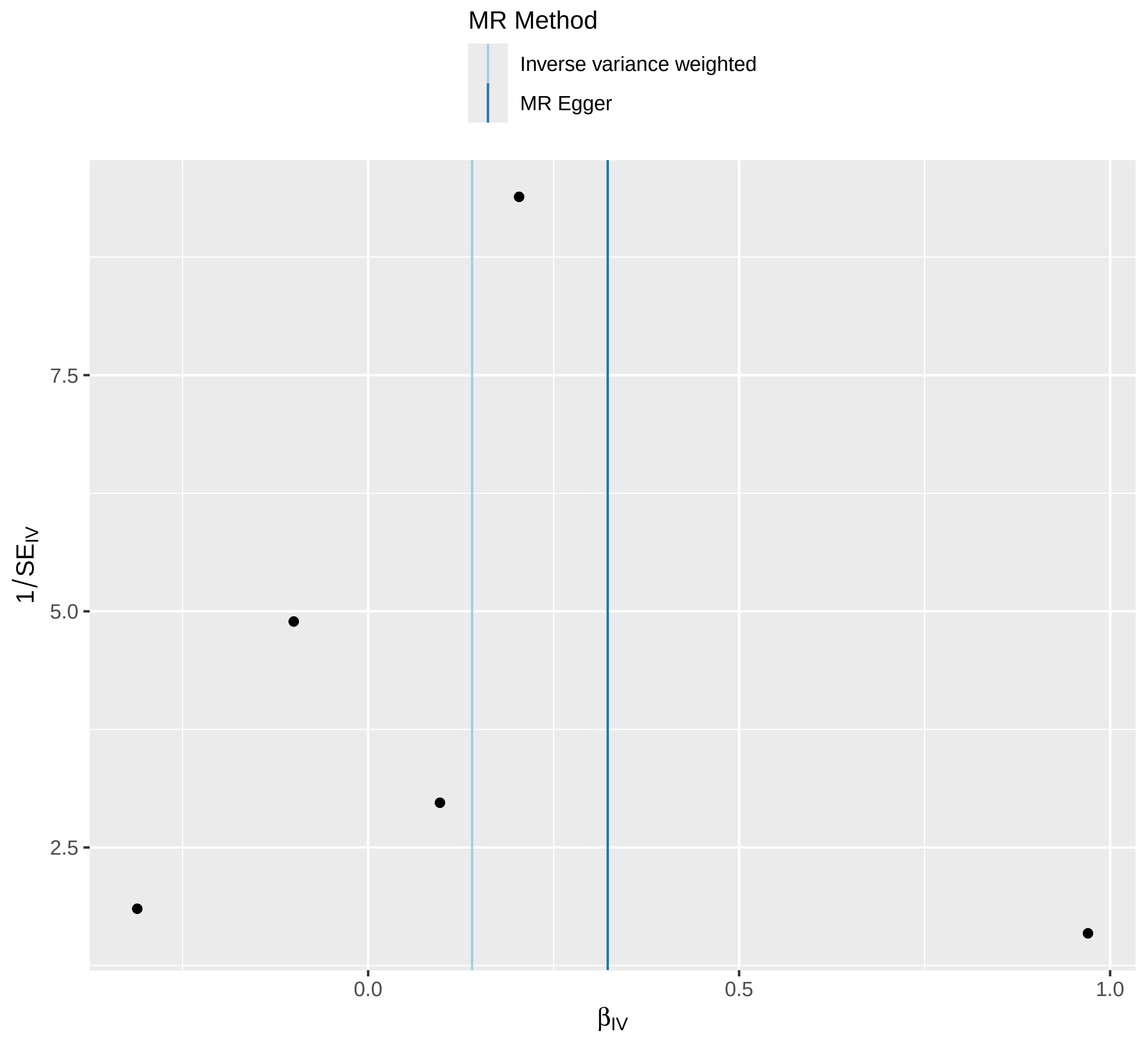
MR Method
Inverse variance weighted
MR Egger
7.5
5.0
2.5
1/SE_IV
0.0
0.5
1.0
β_IV

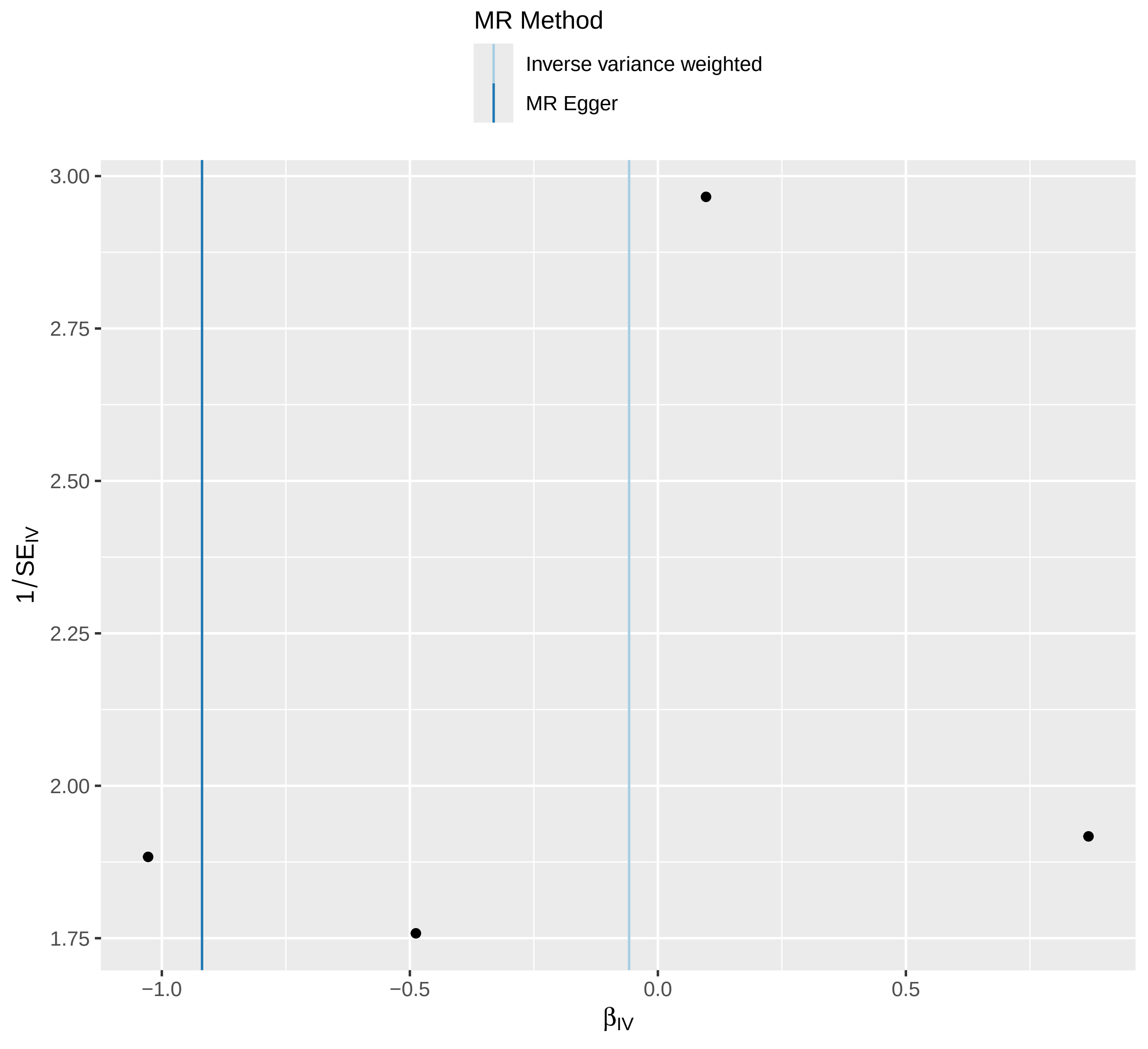

MR Method
Inverse variance weighted
MR Egger
3.00
2.75
2.50
2.25
2.00
1.75
$1/SE_{IV}$
−1.0
−0.5
0.0
0.5
β_{IV}

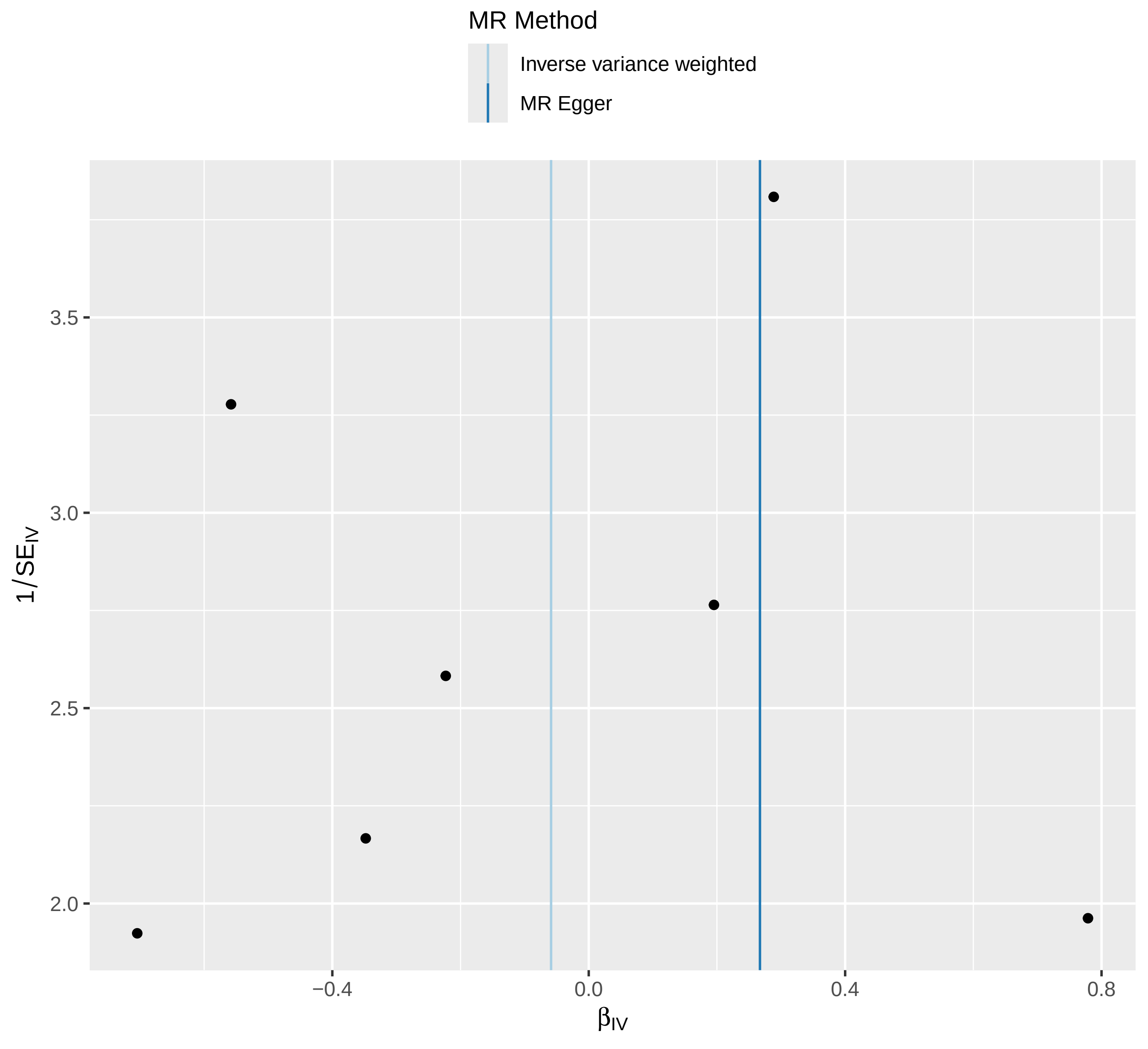
MR Method
Inverse variance weighted
MR Egger
1/SE$_{IV}$
3.5
3.0
2.5
2.0
−0.4
0.0
0.4
0.8
β$_{IV}$

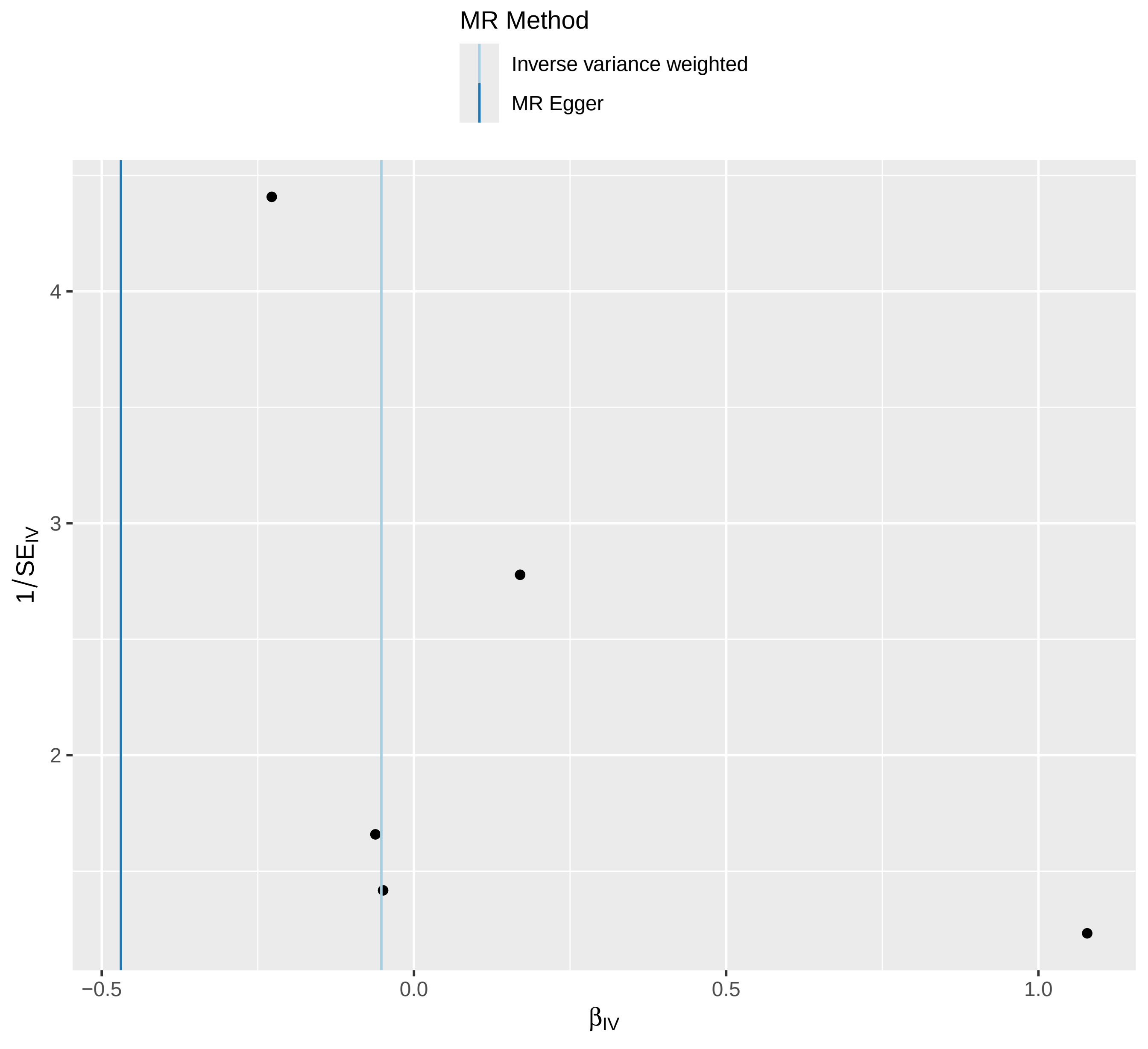
MR Method
Inverse variance weighted
MR Egger
4
3
2
1/SE_IV
−0.5
0.0
0.5
1.0
β_IV

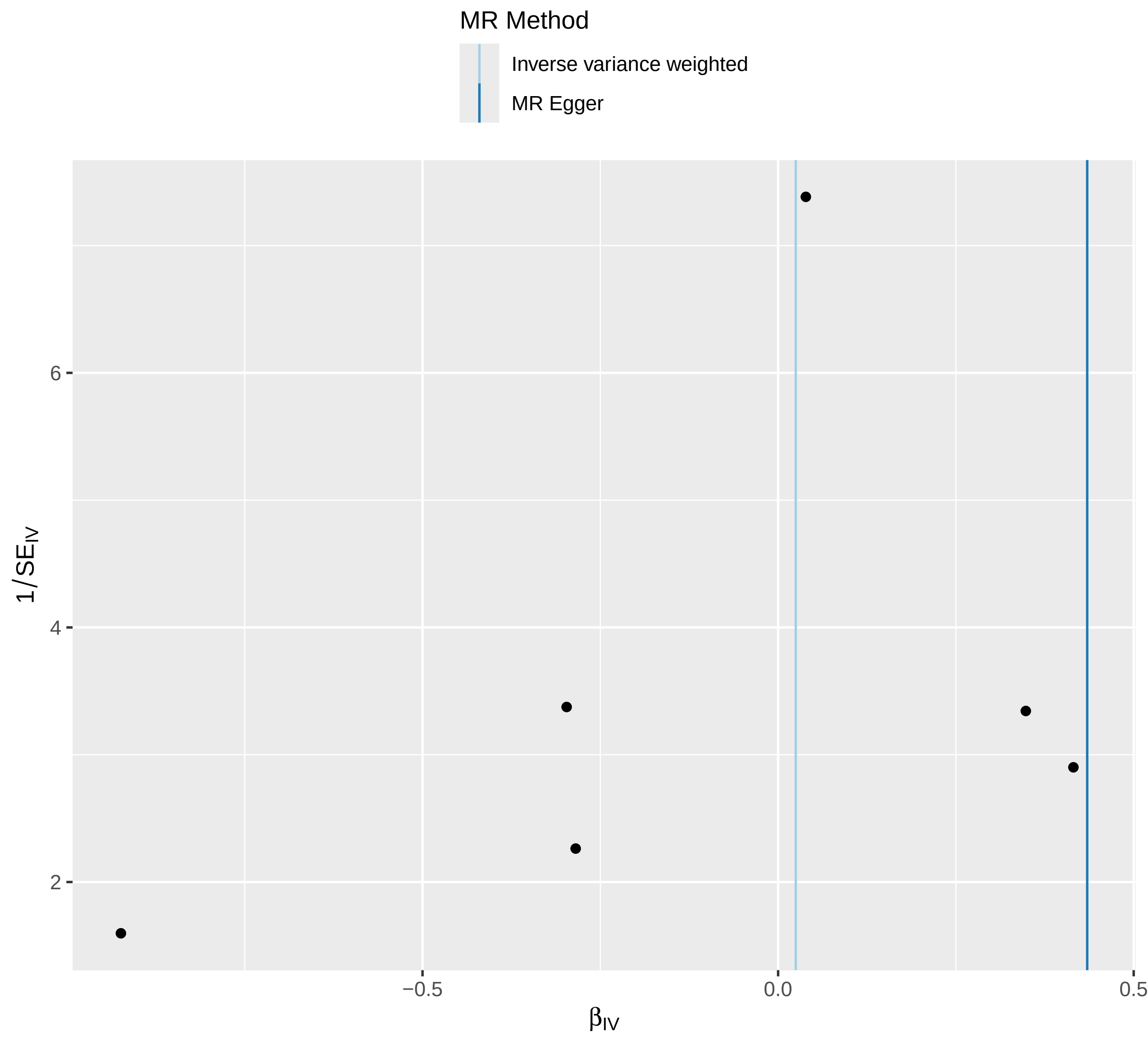
MR Method
Inverse variance weighted
MR Egger
6
4
2
1/SE$_{IV}$
−0.5
0.0
0.5
β_{IV}

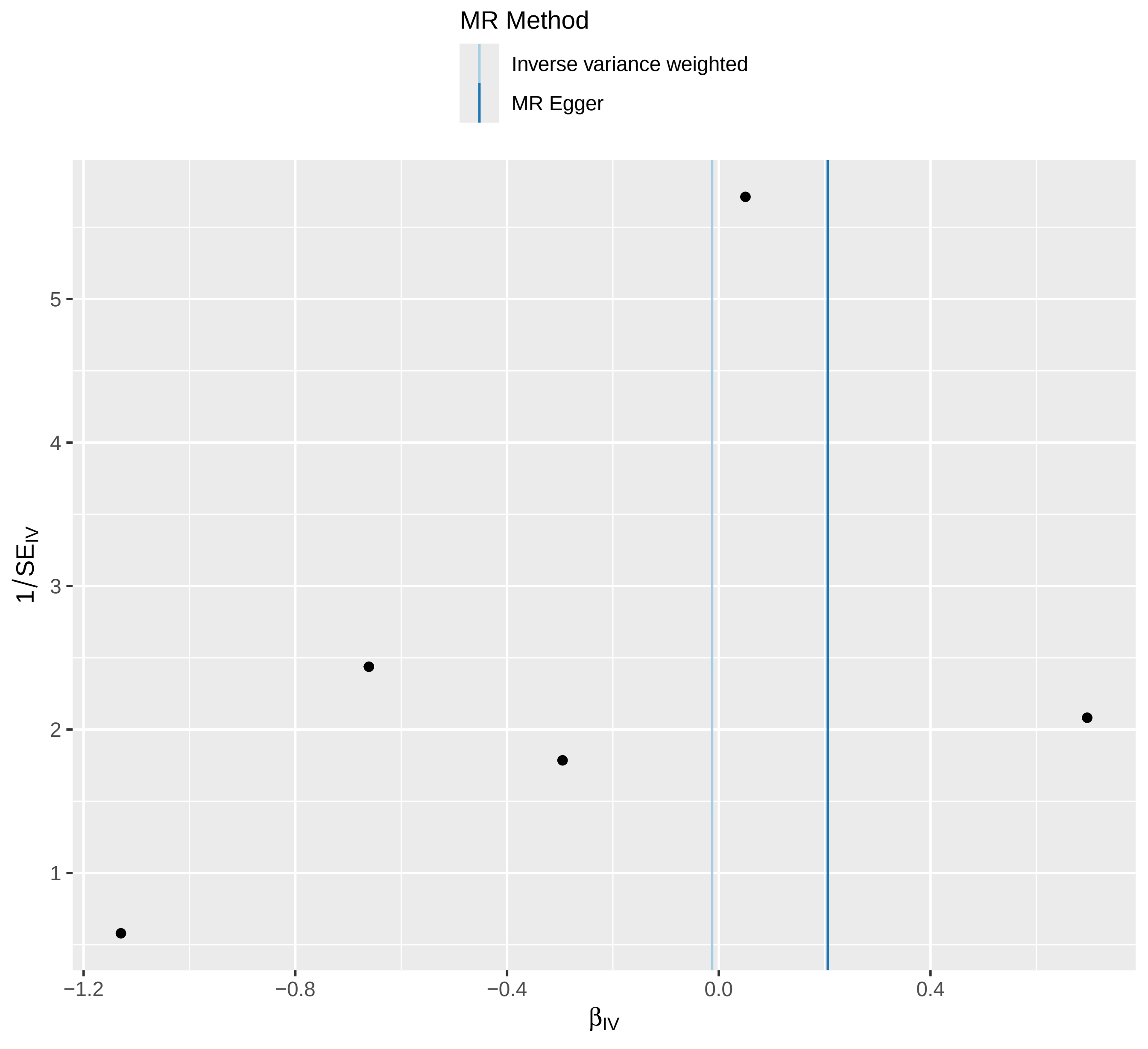

MR Method
Inverse variance weighted
MR Egger
5
4
3
2
1
1/SE_IV
−1.2
−0.8
−0.4
0.0
0.4
β_IV

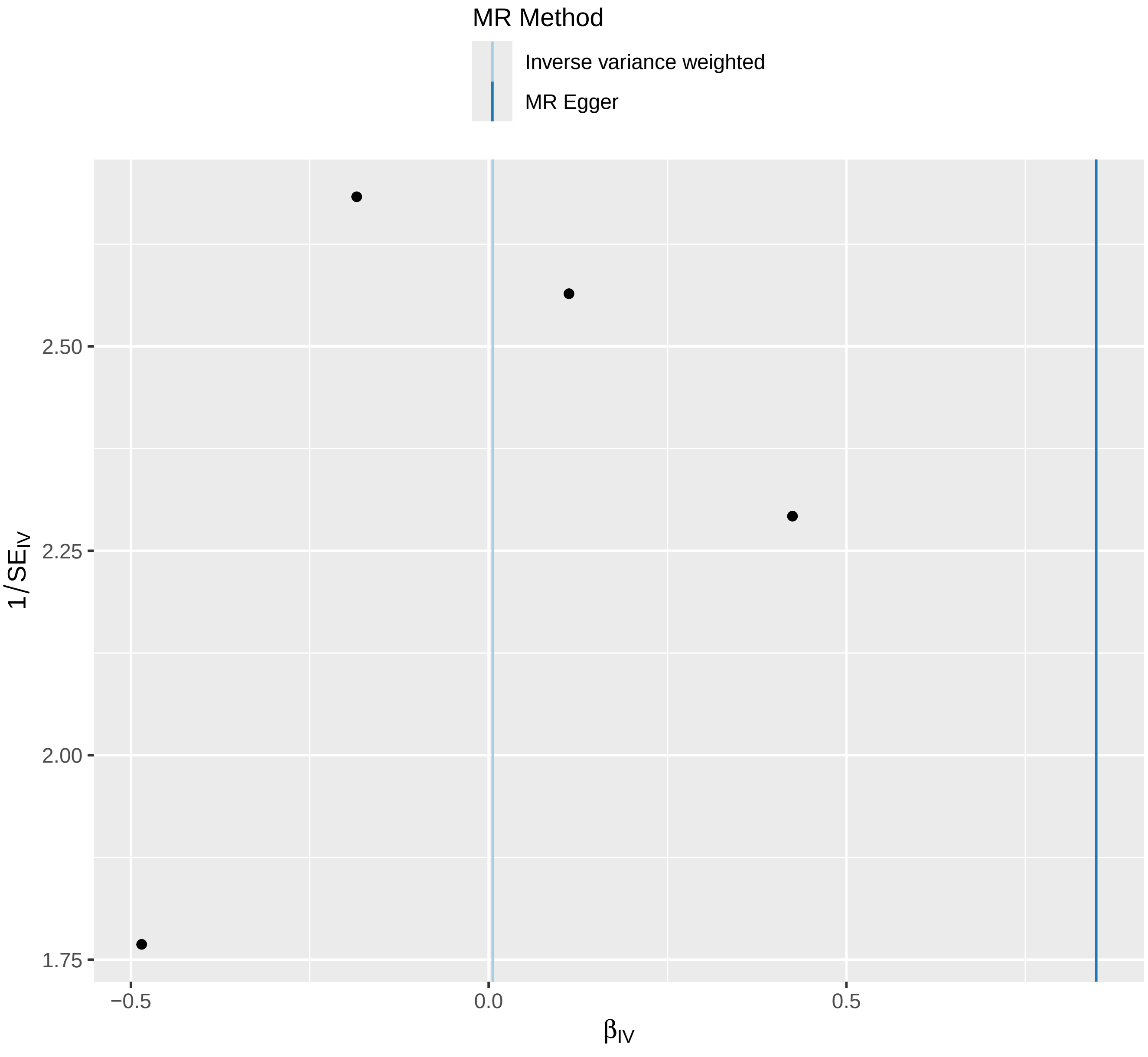
MR Method
Inverse variance weighted
MR Egger
2.50
2.25
2.00
1.75
$1/SE_{IV}$
−0.5
0.0
0.5
β_{IV}

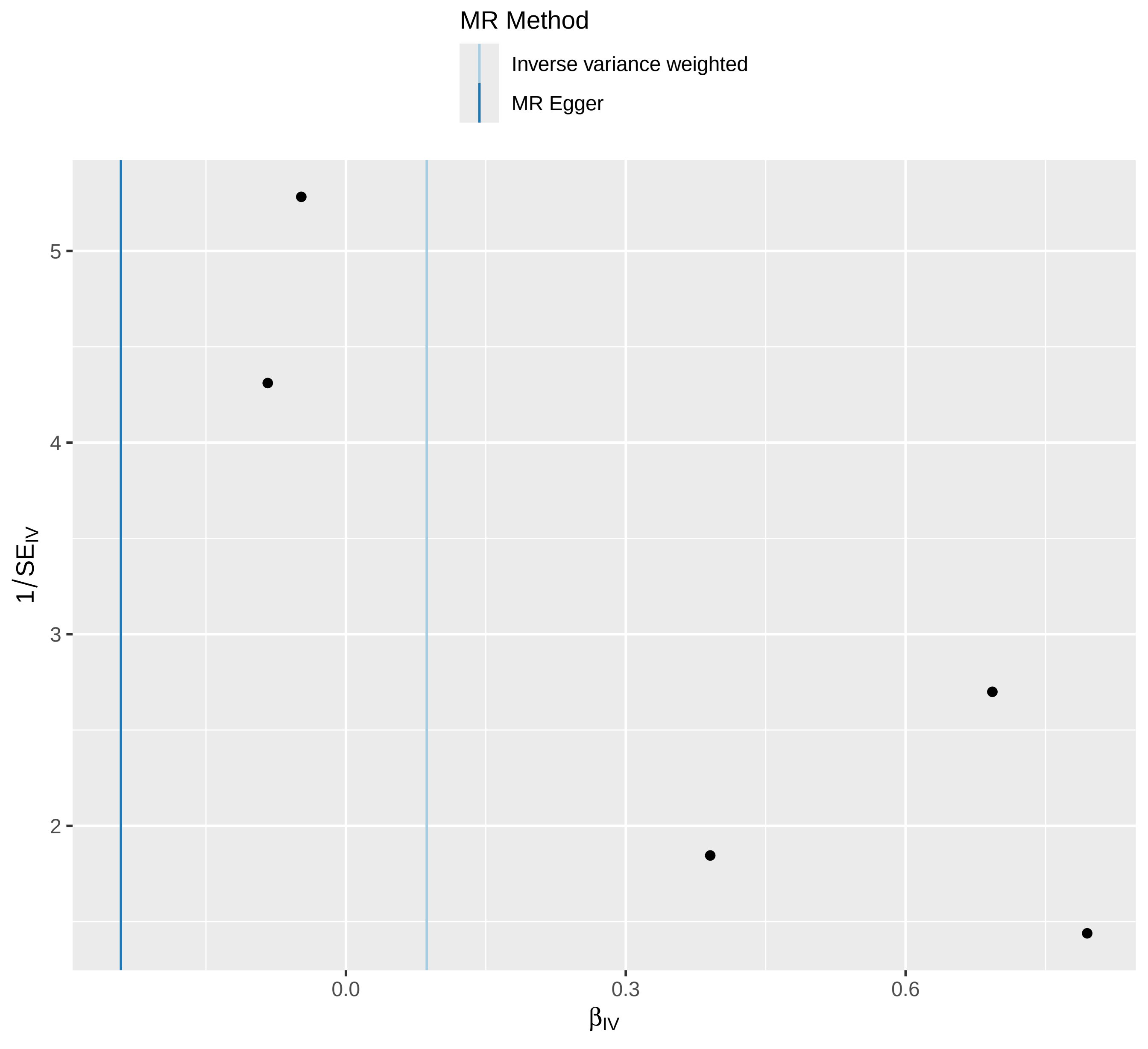
MR Method
Inverse variance weighted
MR Egger
5
4
3
2
1/SE$_{IV}$
0.0
0.3
0.6
β$_{IV}$

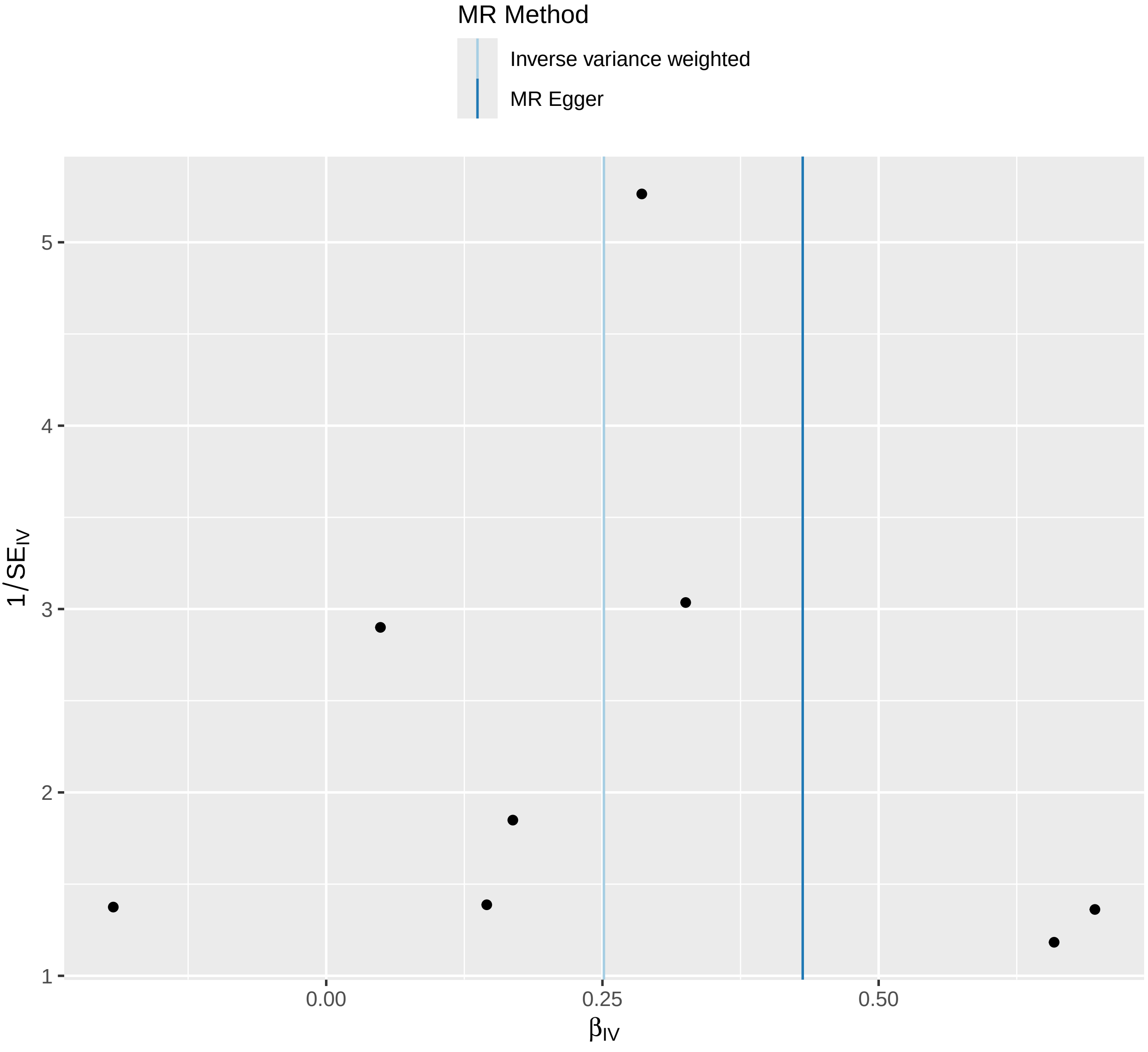

MR Method
Inverse variance weighted
MR Egger
5
4
3
2
1
$1/SE_{IV}$
0.00
0.25
0.50
β_{IV}

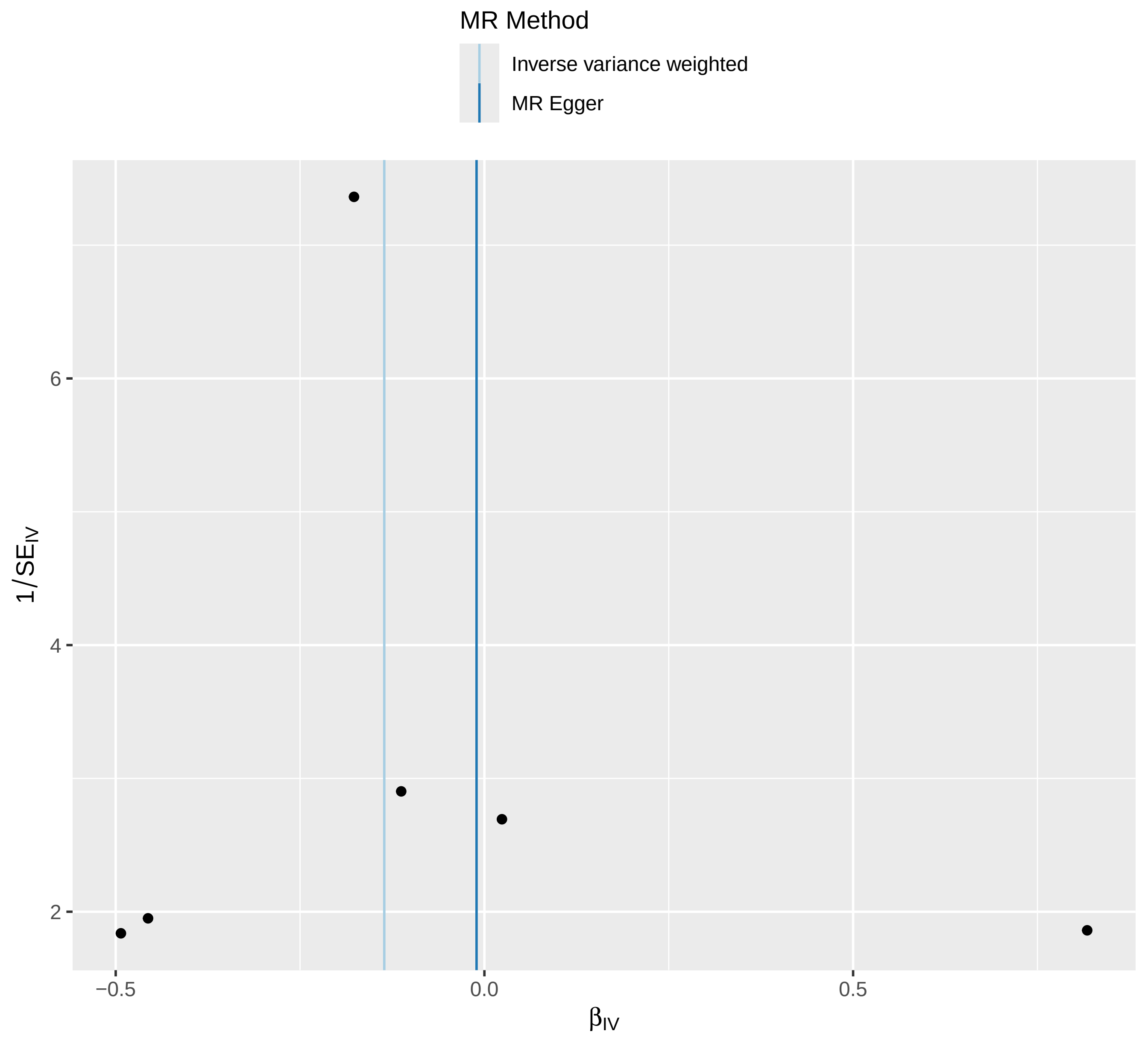

MR Method
Inverse variance weighted
MR Egger
6
4
2
1/SE_IV
−0.5
0.0
0.5
β_IV

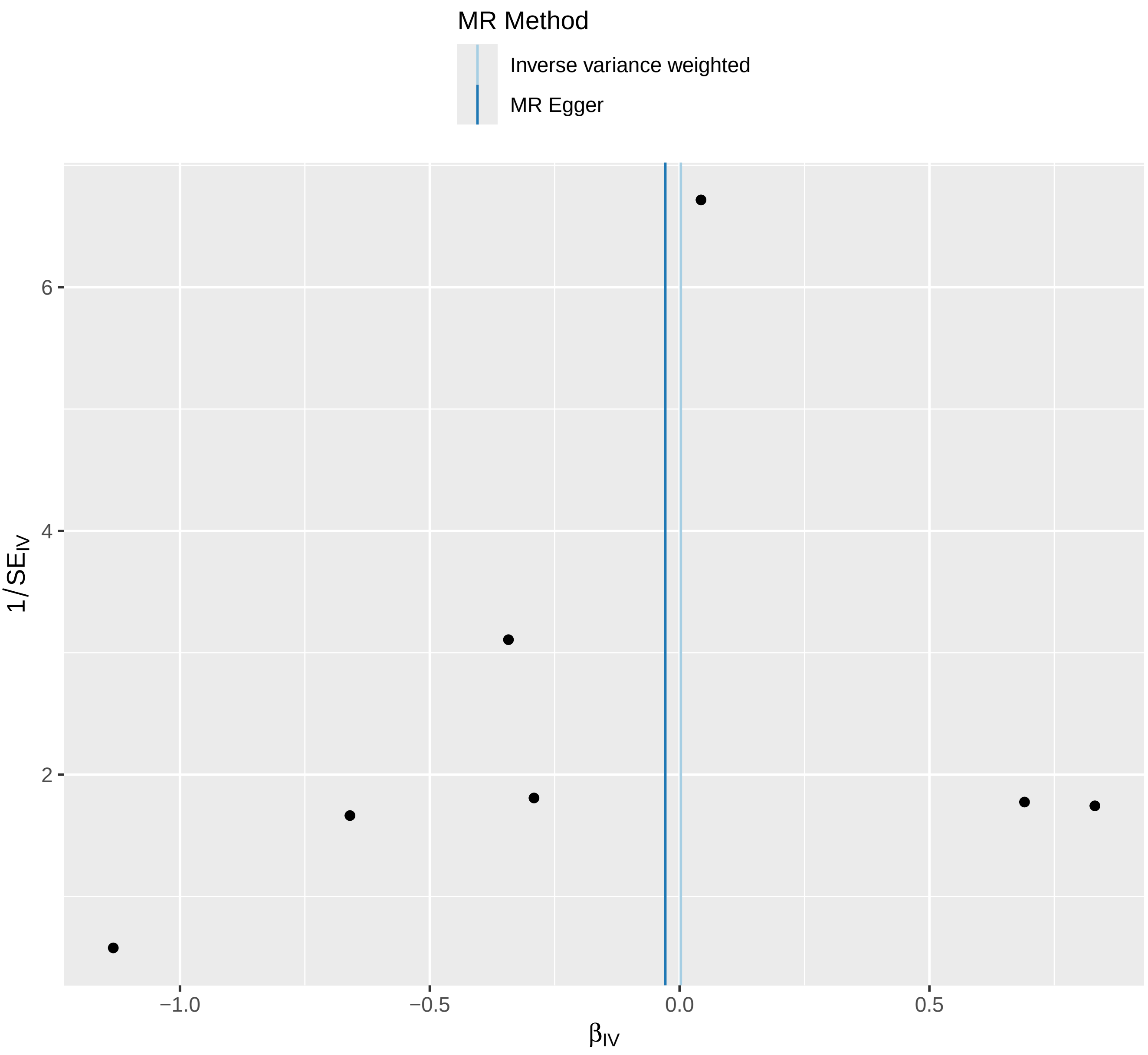
MR Method
Inverse variance weighted
MR Egger
6
4
2
1/SE$_{IV}$
−1.0
−0.5
0.0
0.5
β$_{IV}$

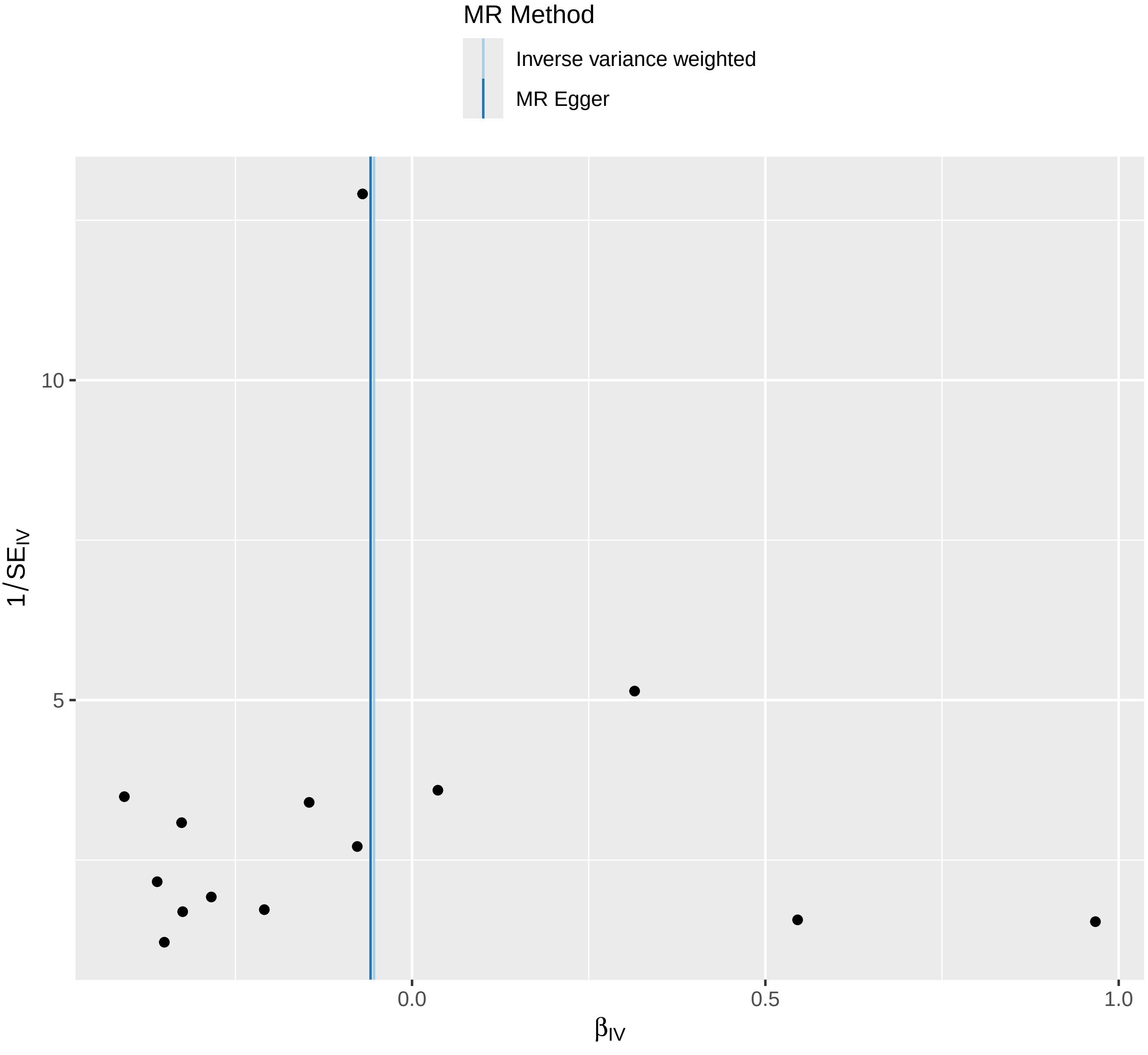
MR Method
Inverse variance weighted
MR Egger
10
5
0.0
0.5
1.0
1/SE$_{IV}$
β$_{IV}$

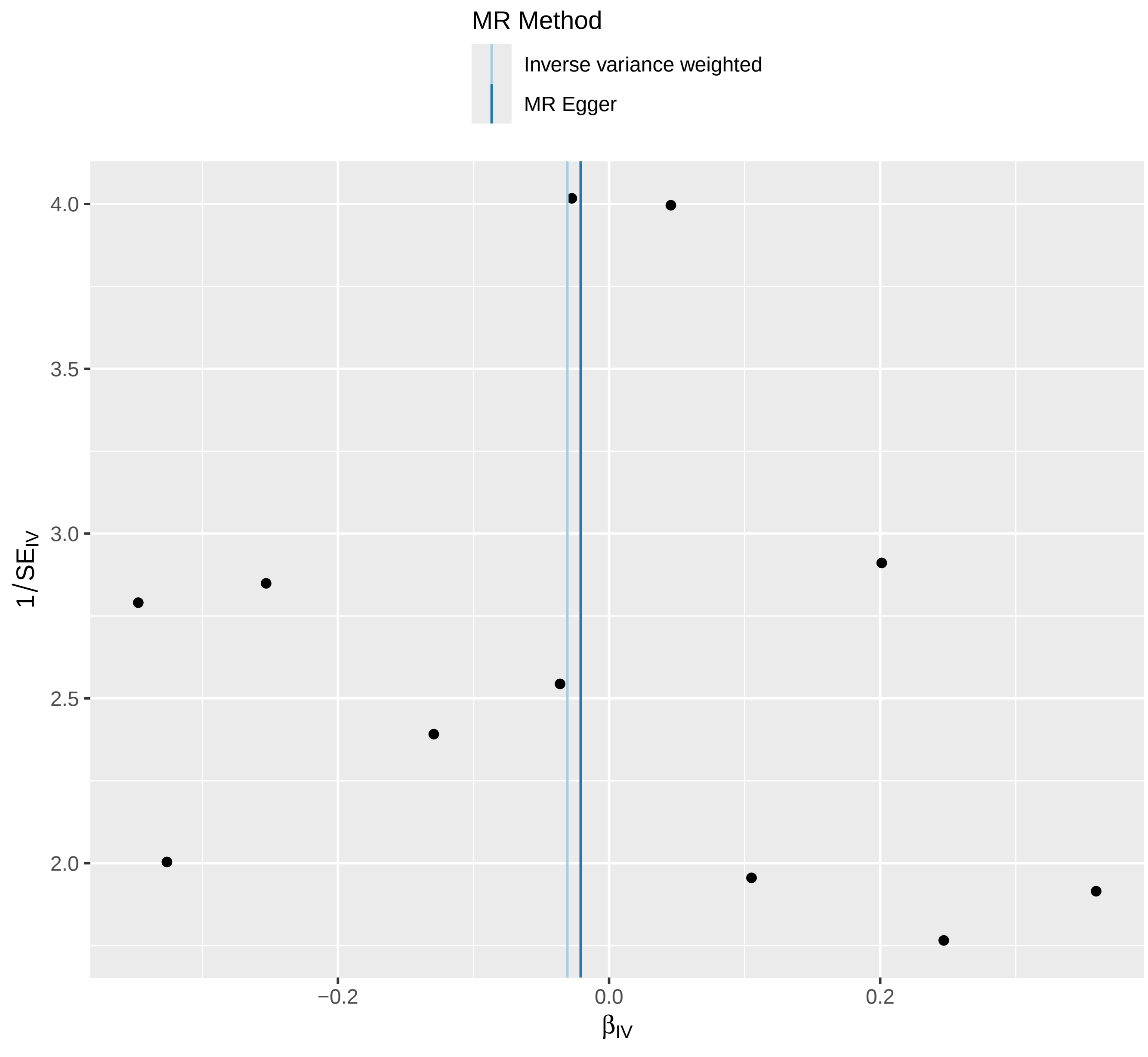

MR Method
Inverse variance weighted
MR Egger
4.0
3.5
3.0
2.5
2.0
1/SE_{IV}
−0.2
0.0
0.2
$β_{IV}$

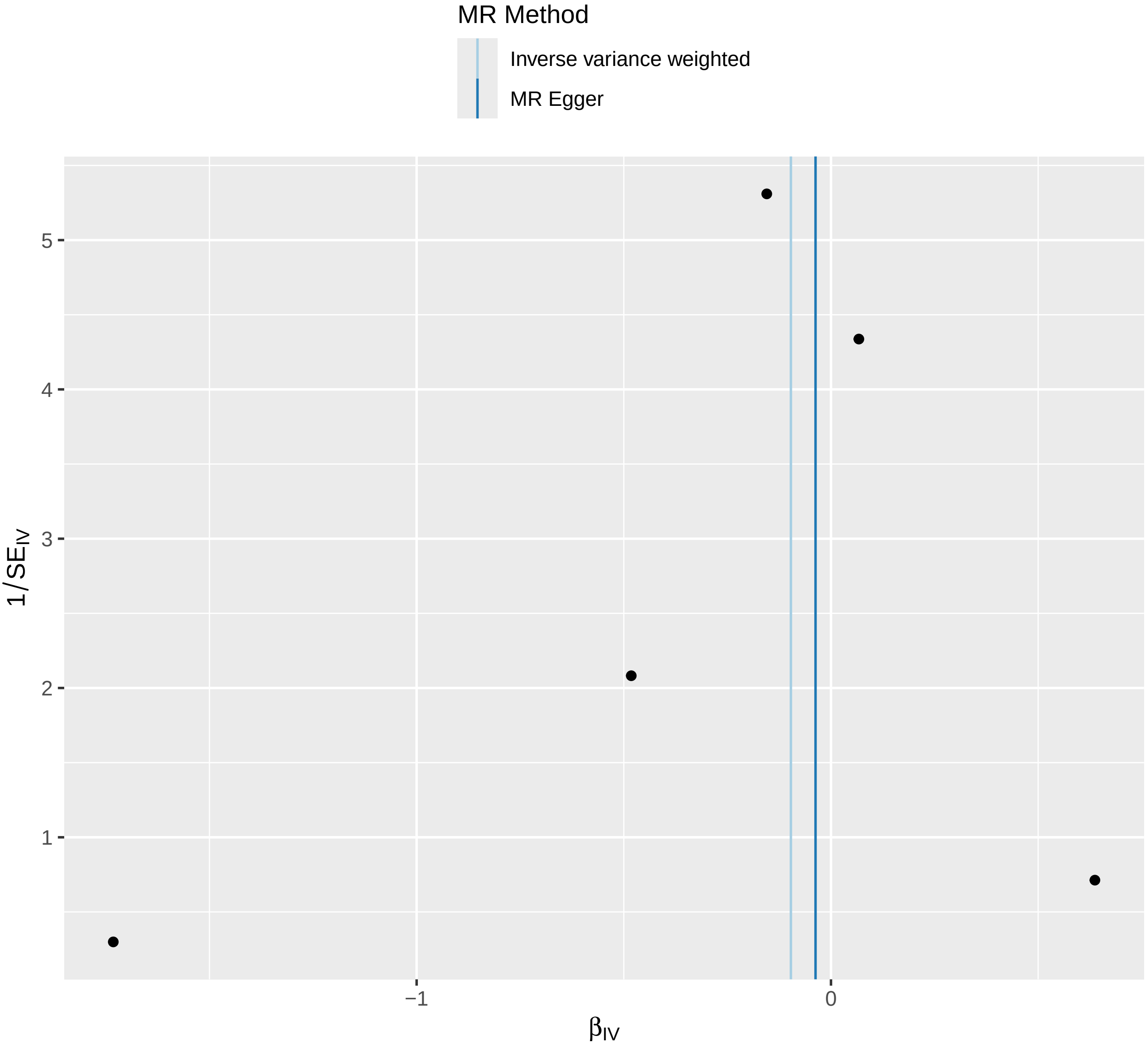
MR Method
Inverse variance weighted
MR Egger
5
4
3
2
1
1/SE$_{IV}$
−1
0
β$_{IV}$

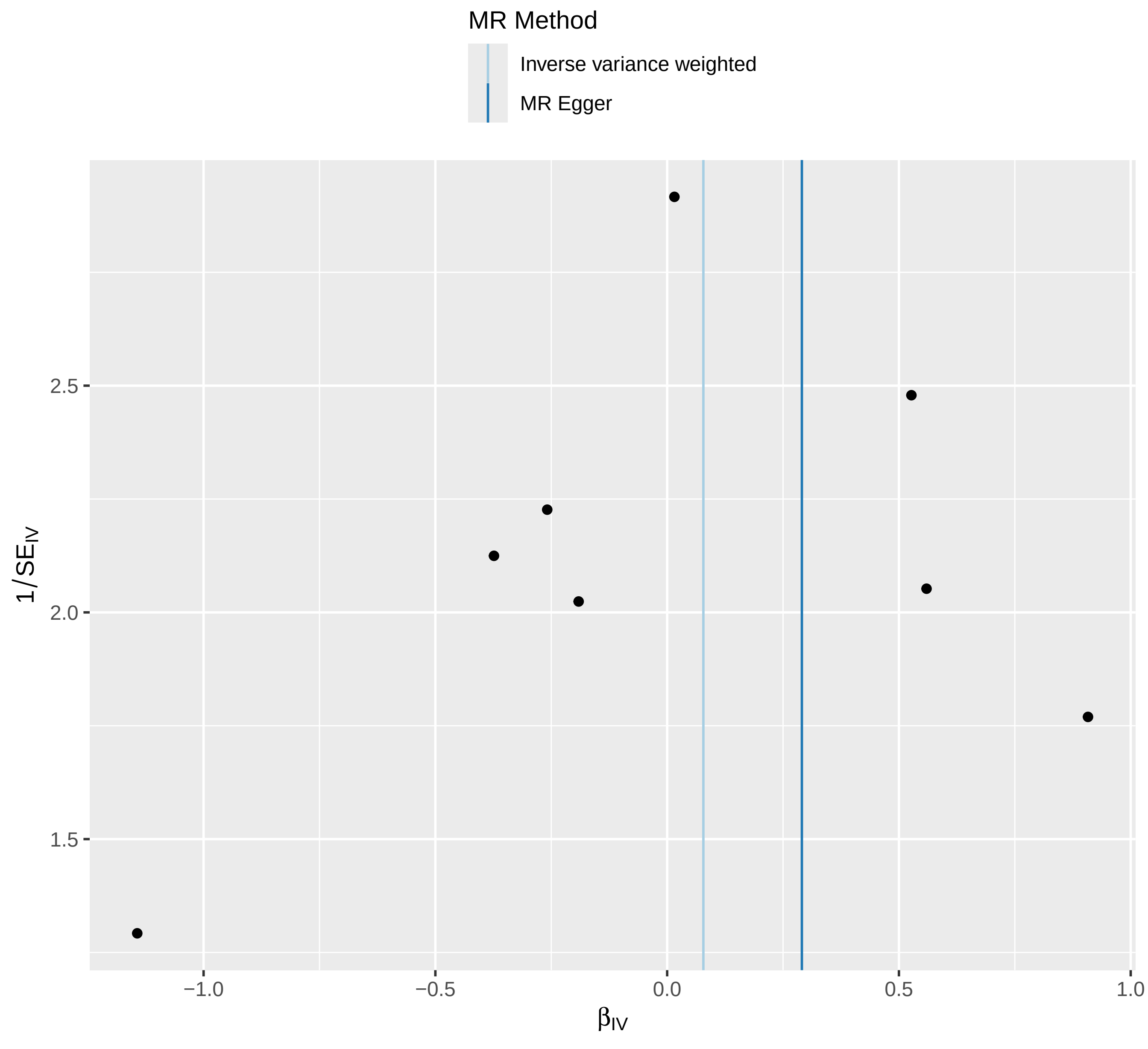

MR Method
Inverse variance weighted
MR Egger
2.5
2.0
1.5
1/SE$_{IV}$
−1.0
−0.5
0.0
0.5
1.0
β$_{IV}$

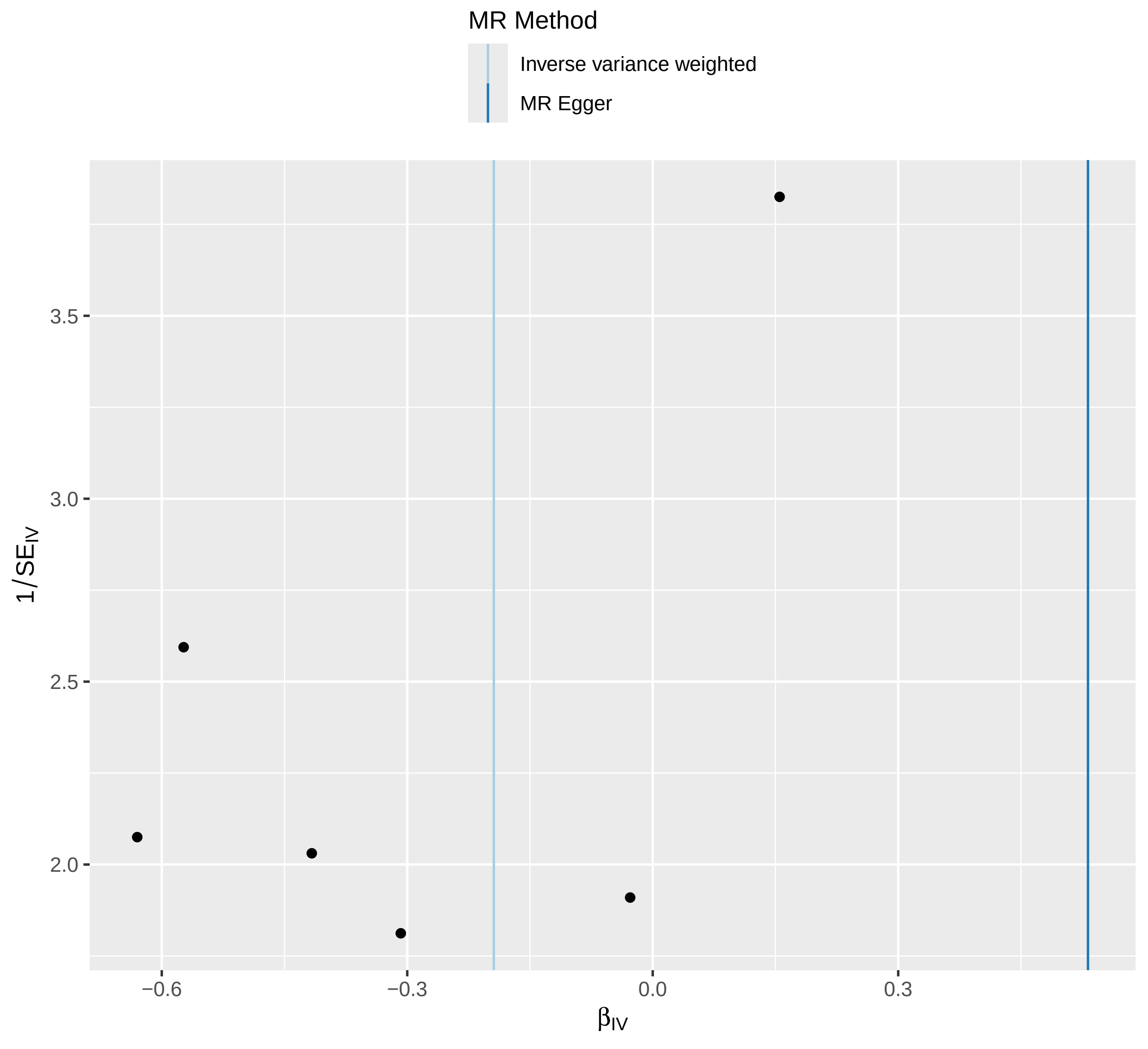

MR Method
Inverse variance weighted
MR Egger
3.5
3.0
2.5
2.0
1/SE$_{IV}$
−0.6
−0.3
0.0
0.3
β$_{IV}$

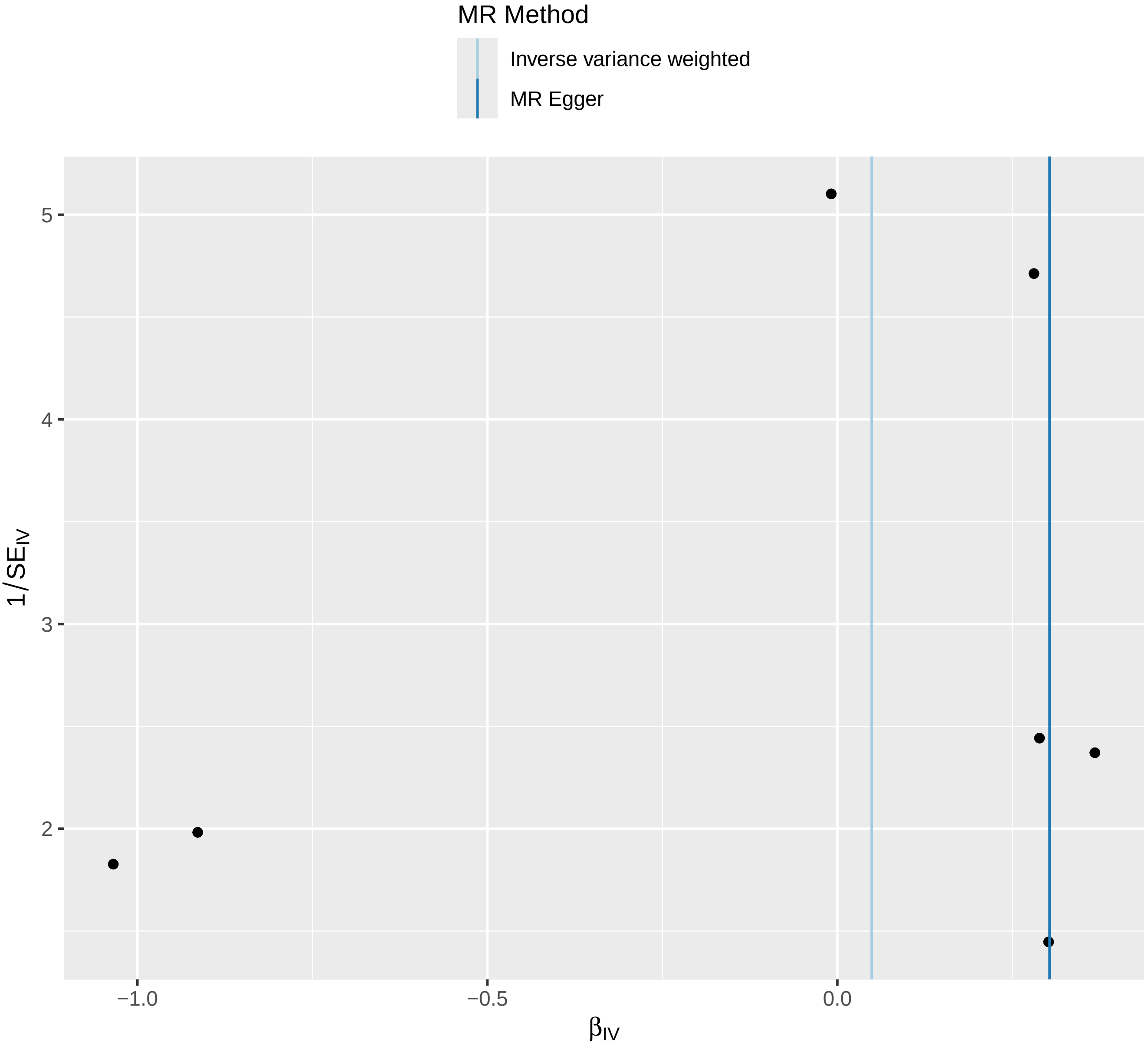

MR Method
Inverse variance weighted
MR Egger
5
4
3
2
−1.0
−0.5
0.0
$1/SE_{IV}$
β_{IV}

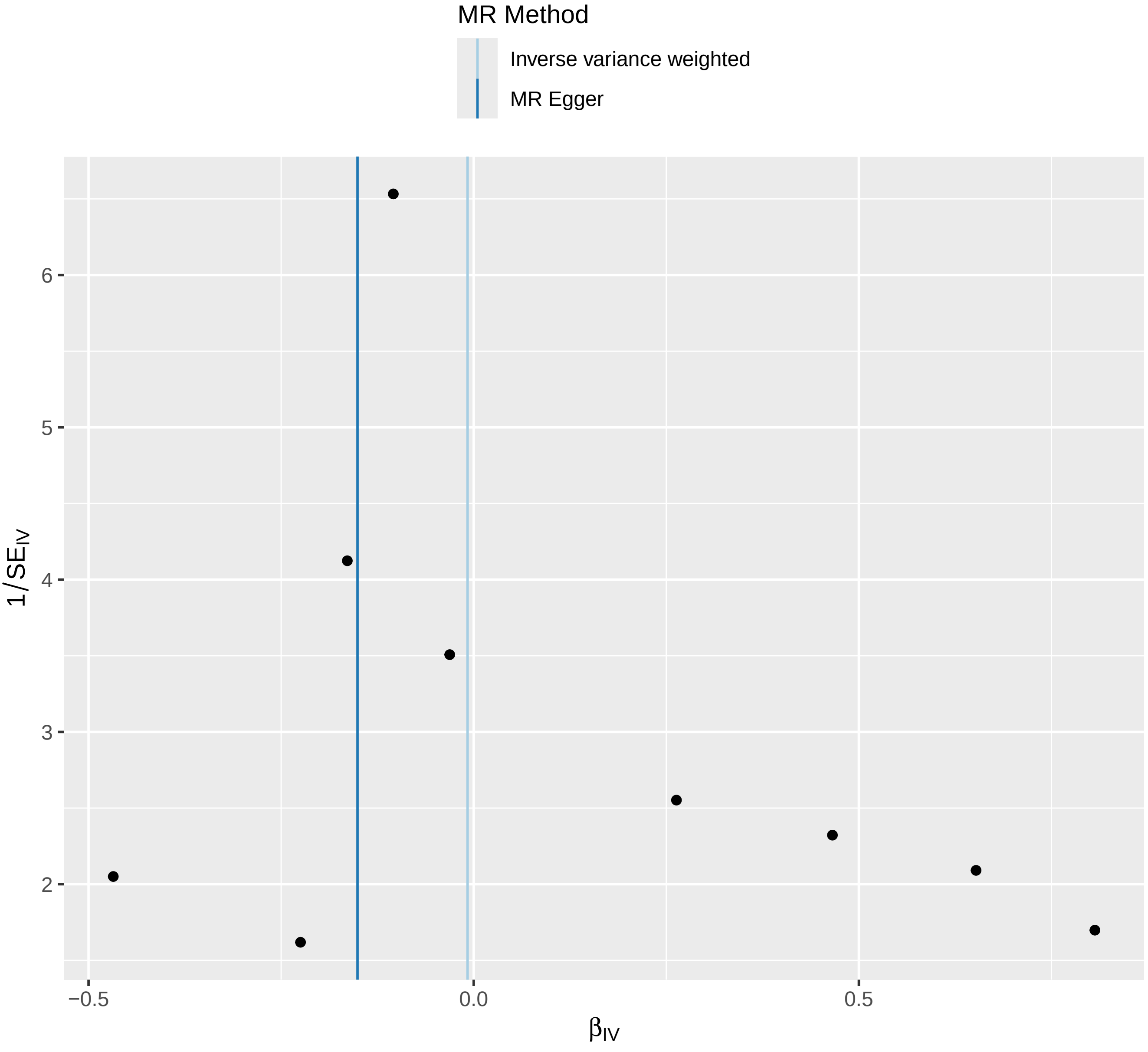
MR Method
Inverse variance weighted
MR Egger
1/SE$_{IV}$
β_{IV}
6
5
4
3
2
−0.5
0.0
0.5

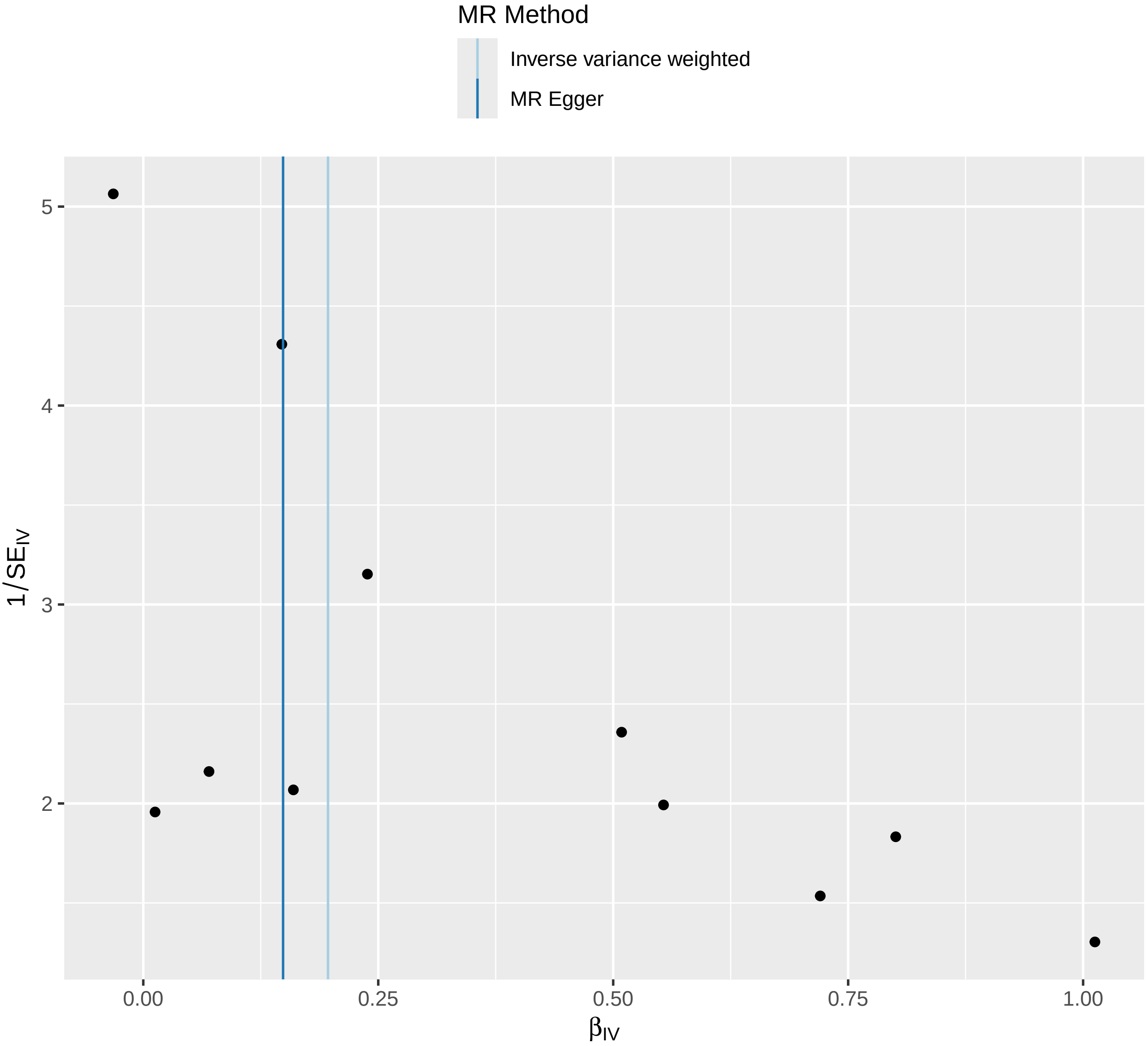

MR Method
Inverse variance weighted
MR Egger
1/SE_{IV}
5
4
3
2
0.00
0.25
0.50
0.75
1.00
β_{IV}

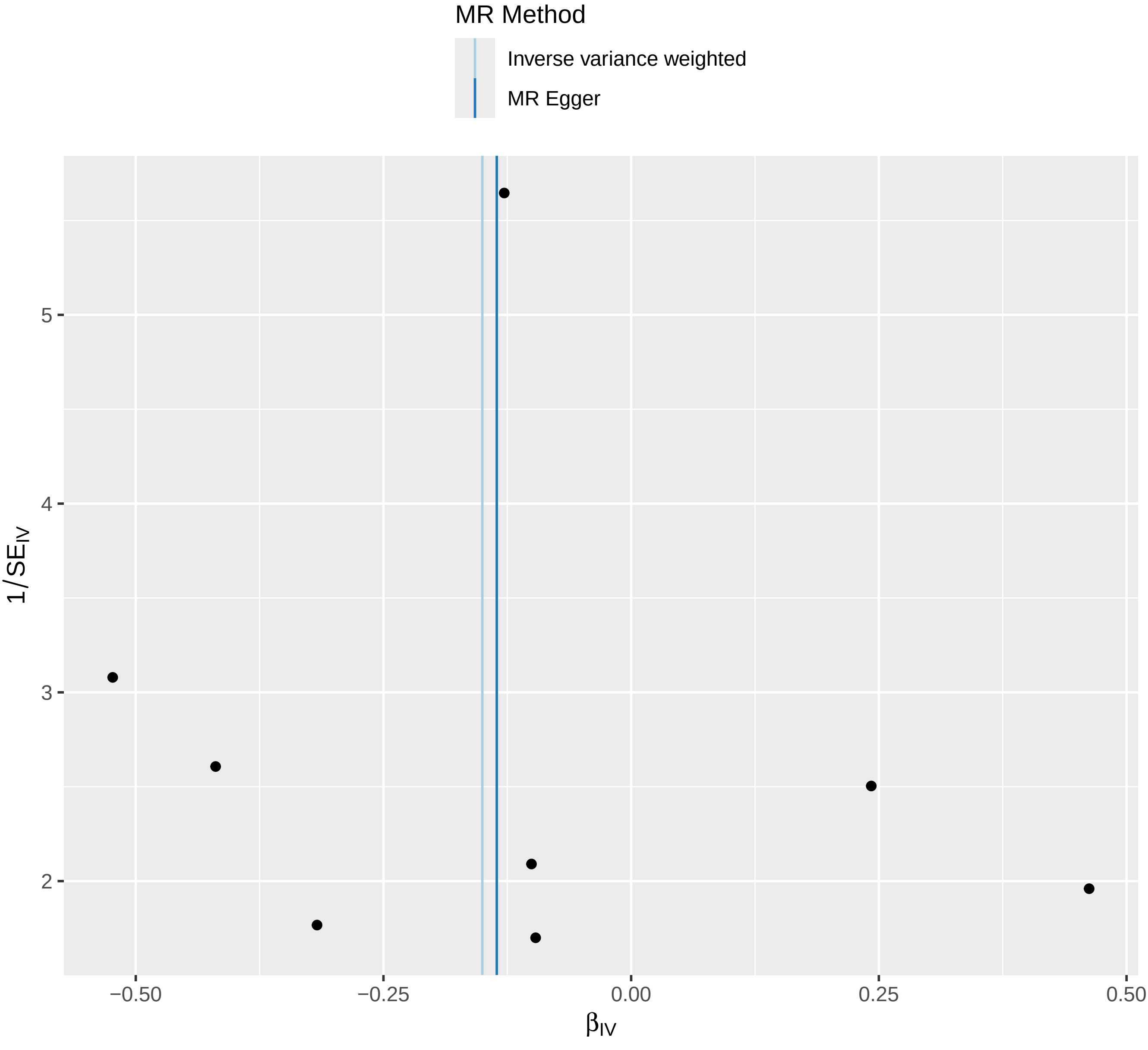

MR Method
Inverse variance weighted
MR Egger
5
4
3
2
1/SE$_{IV}$
−0.50
−0.25
0.00
0.25
0.50
β$_{IV}$

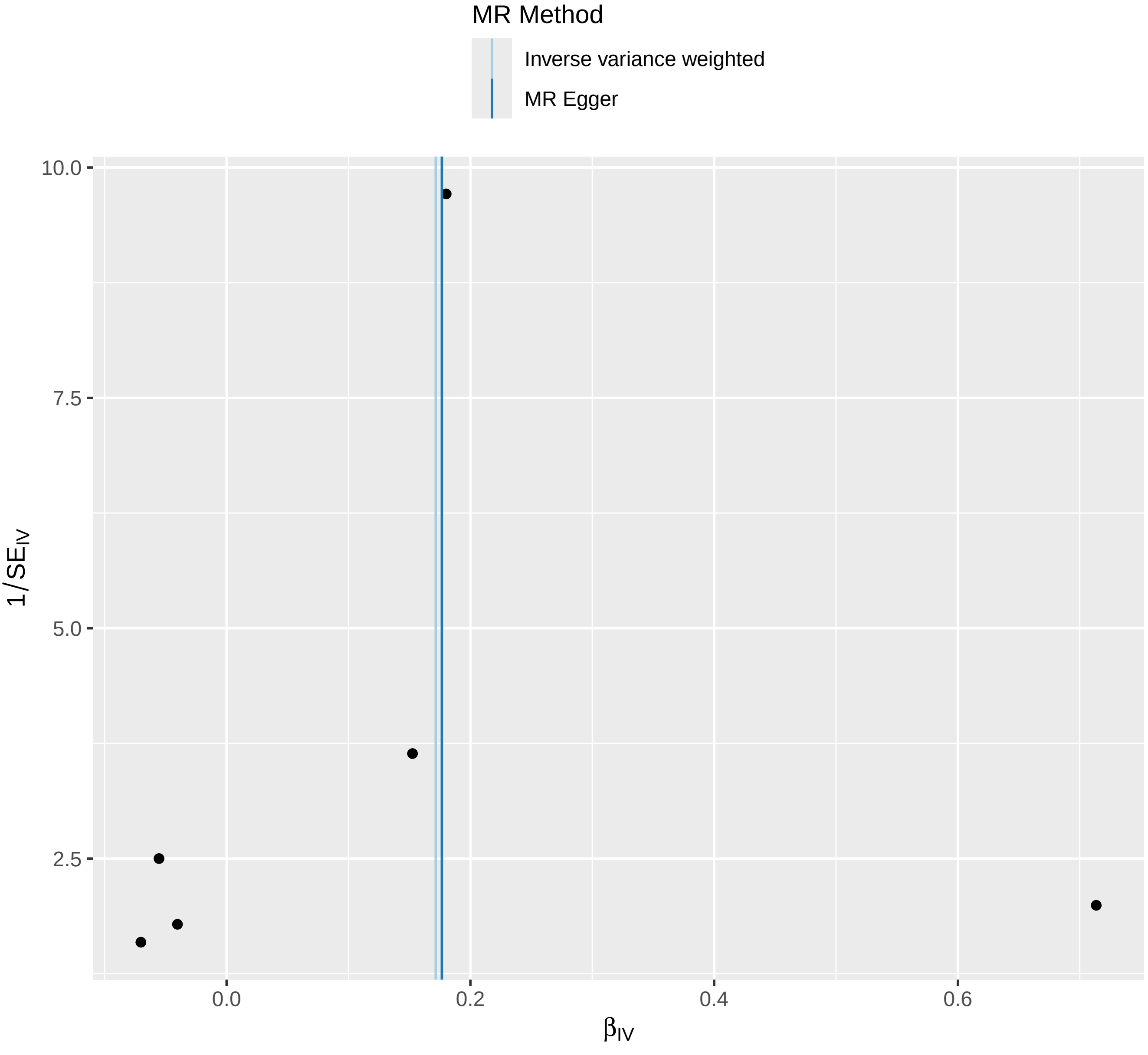
MR Method
Inverse variance weighted
MR Egger
10.0
7.5
5.0
2.5
$1/SE_{IV}$
0.0
0.2
0.4
0.6
β_{IV}

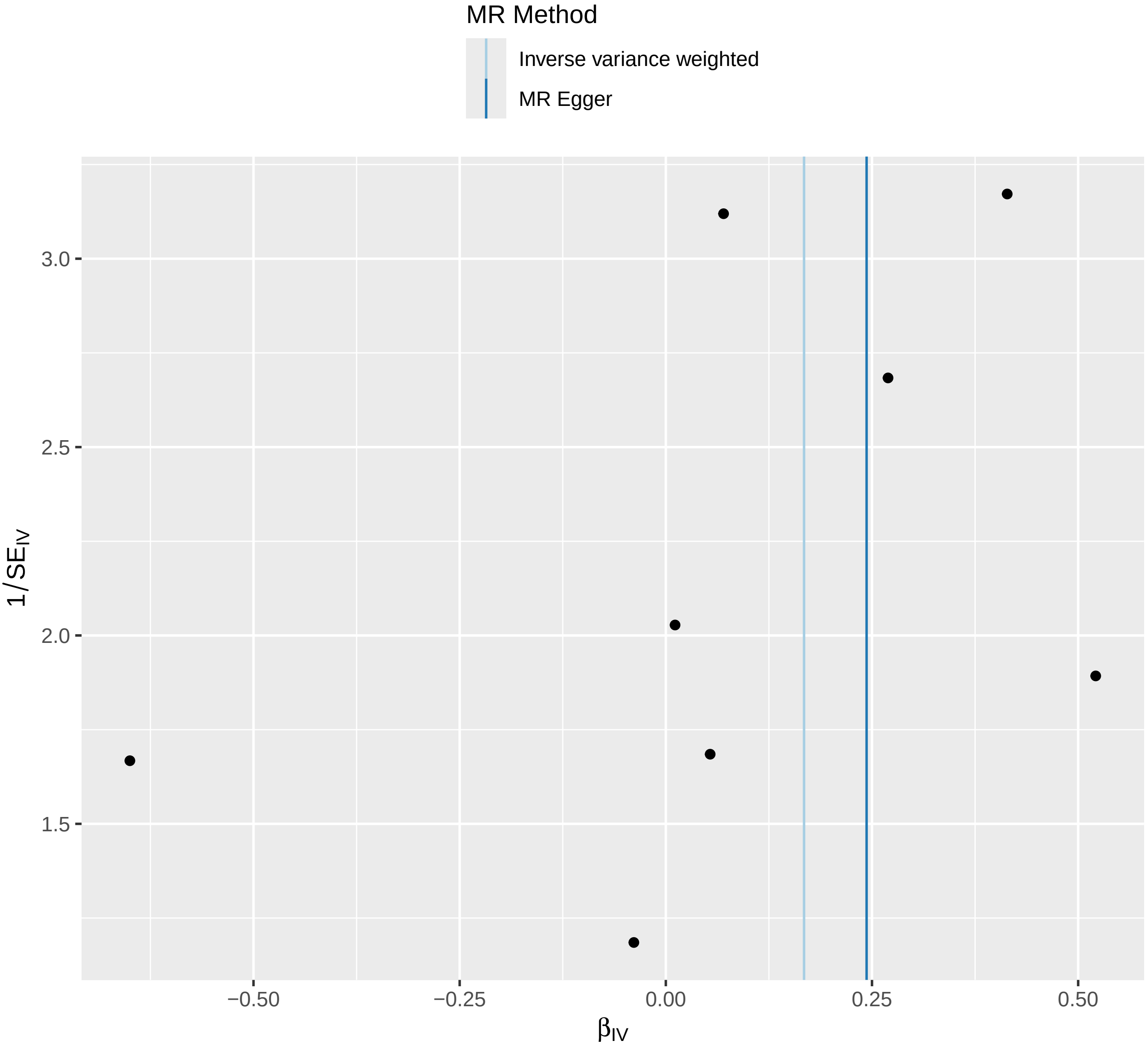

MR Method
Inverse variance weighted
MR Egger
3.0
2.5
2.0
1.5
$1/SE_{IV}$
−0.50
−0.25
0.00
0.25
0.50
β_{IV}

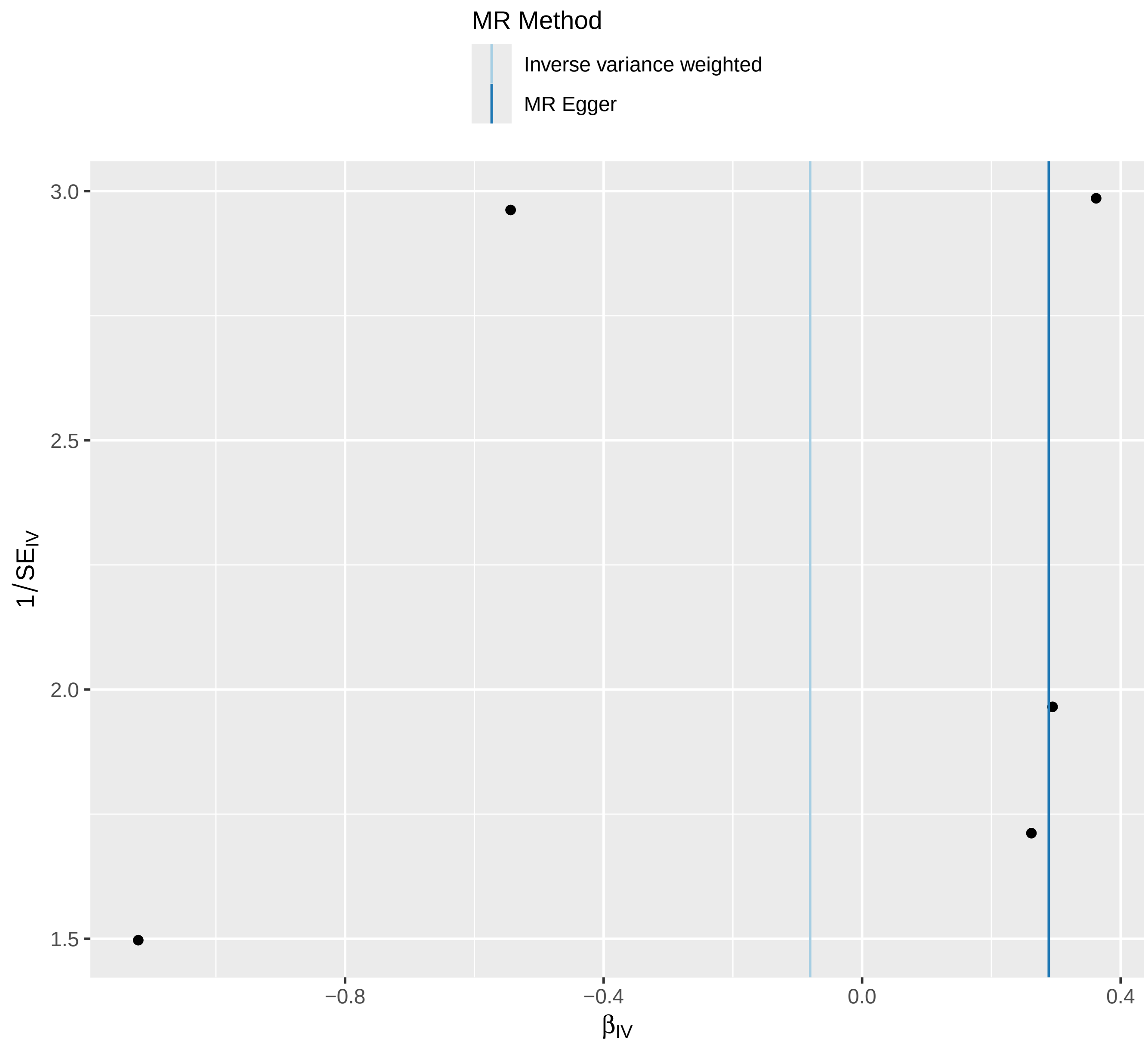

MR Method
Inverse variance weighted
MR Egger
3.0
2.5
2.0
1.5
1/SE$_{IV}$
−0.8
−0.4
0.0
0.4
β$_{IV}$

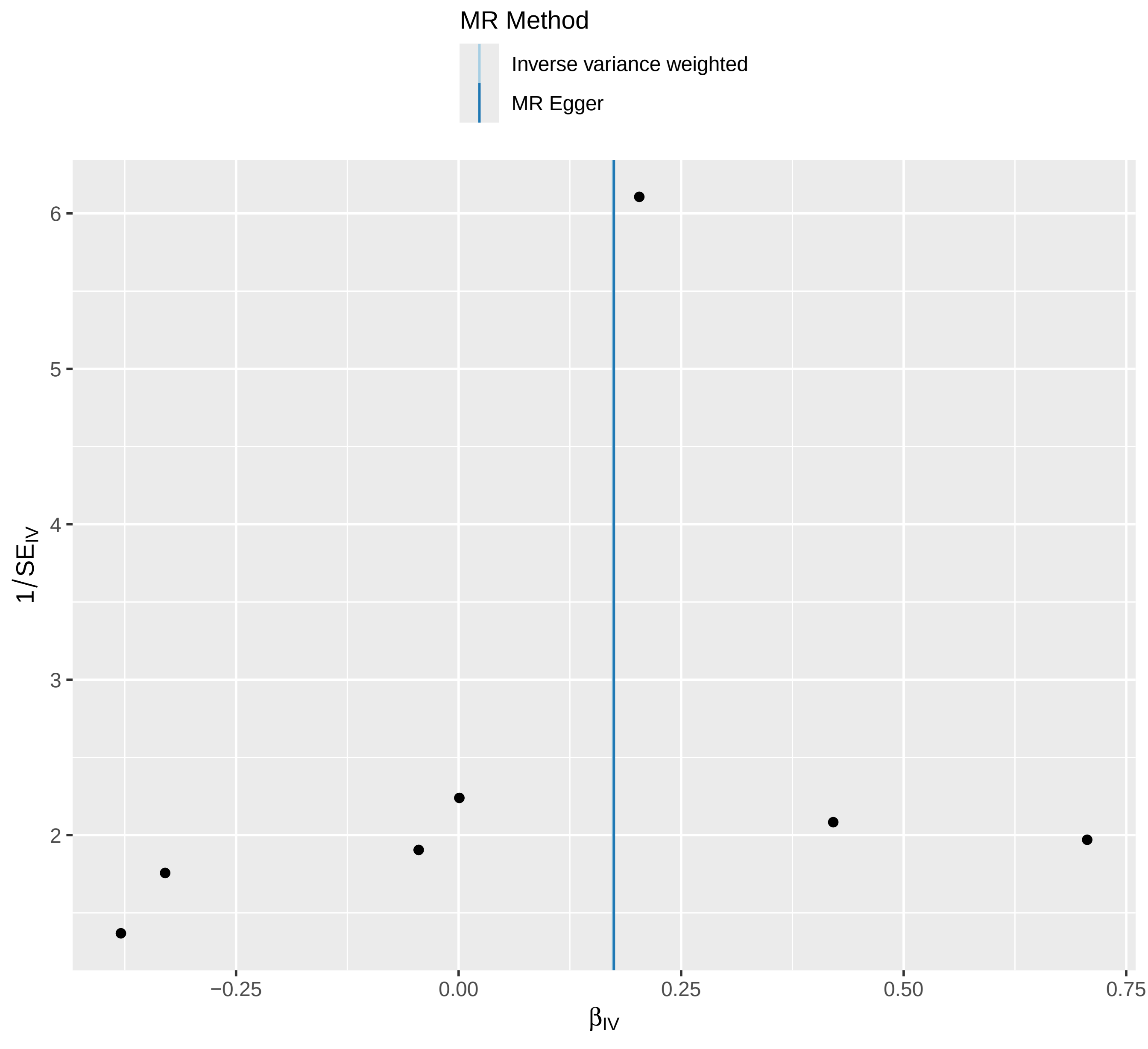
MR Method
Inverse variance weighted
MR Egger
1/SE_IV
β_IV
6
5
4
3
2
−0.25
0.00
0.25
0.50
0.75

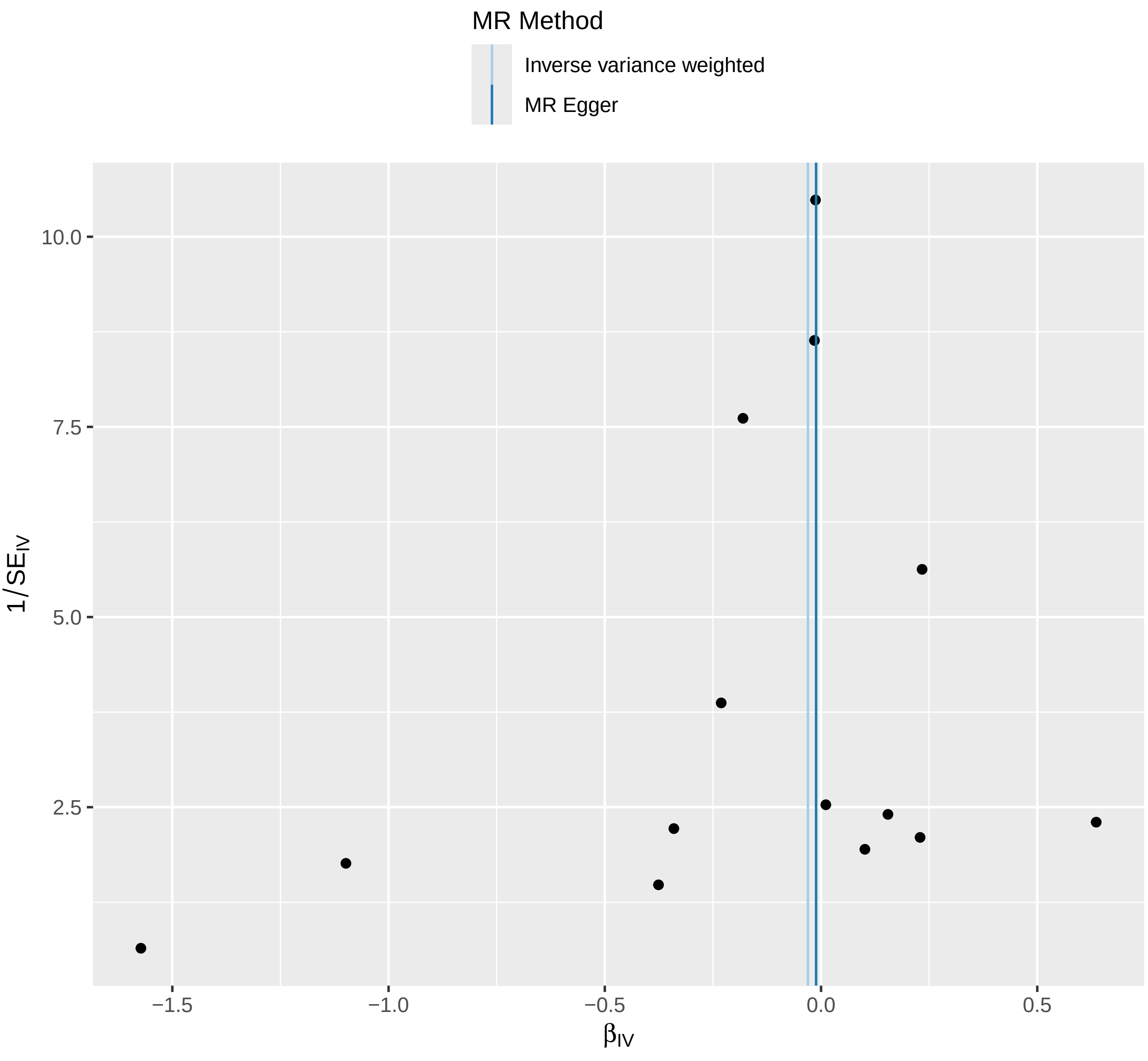

MR Method
Inverse variance weighted
MR Egger
10.0
7.5
5.0
2.5
1/SE_IV
−1.5
−1.0
−0.5
0.0
0.5
β_IV

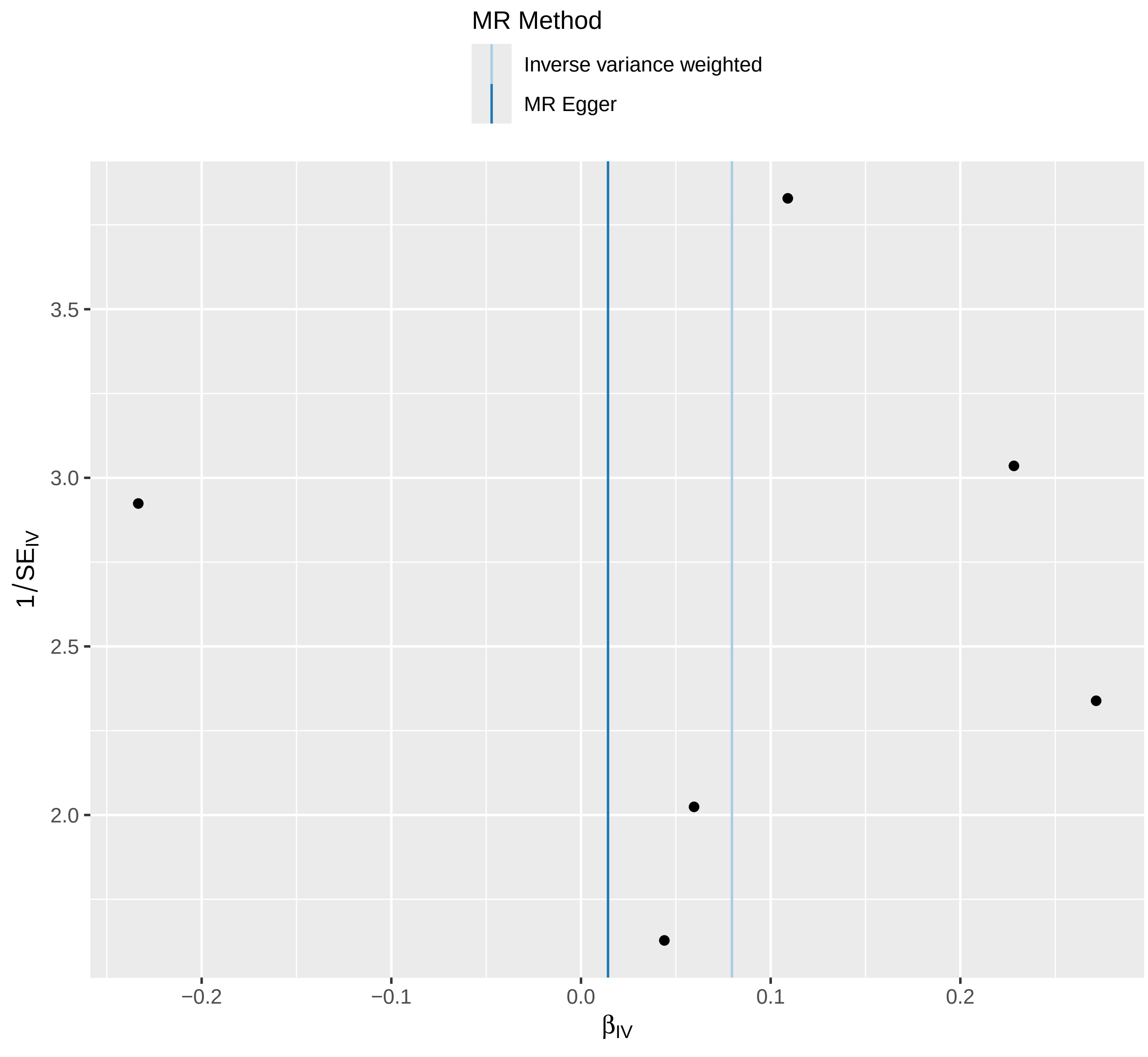
MR Method
Inverse variance weighted
MR Egger
3.5
3.0
2.5
2.0
1/SE$_{IV}$
−0.2
−0.1
0.0
0.1
0.2
β$_{IV}$

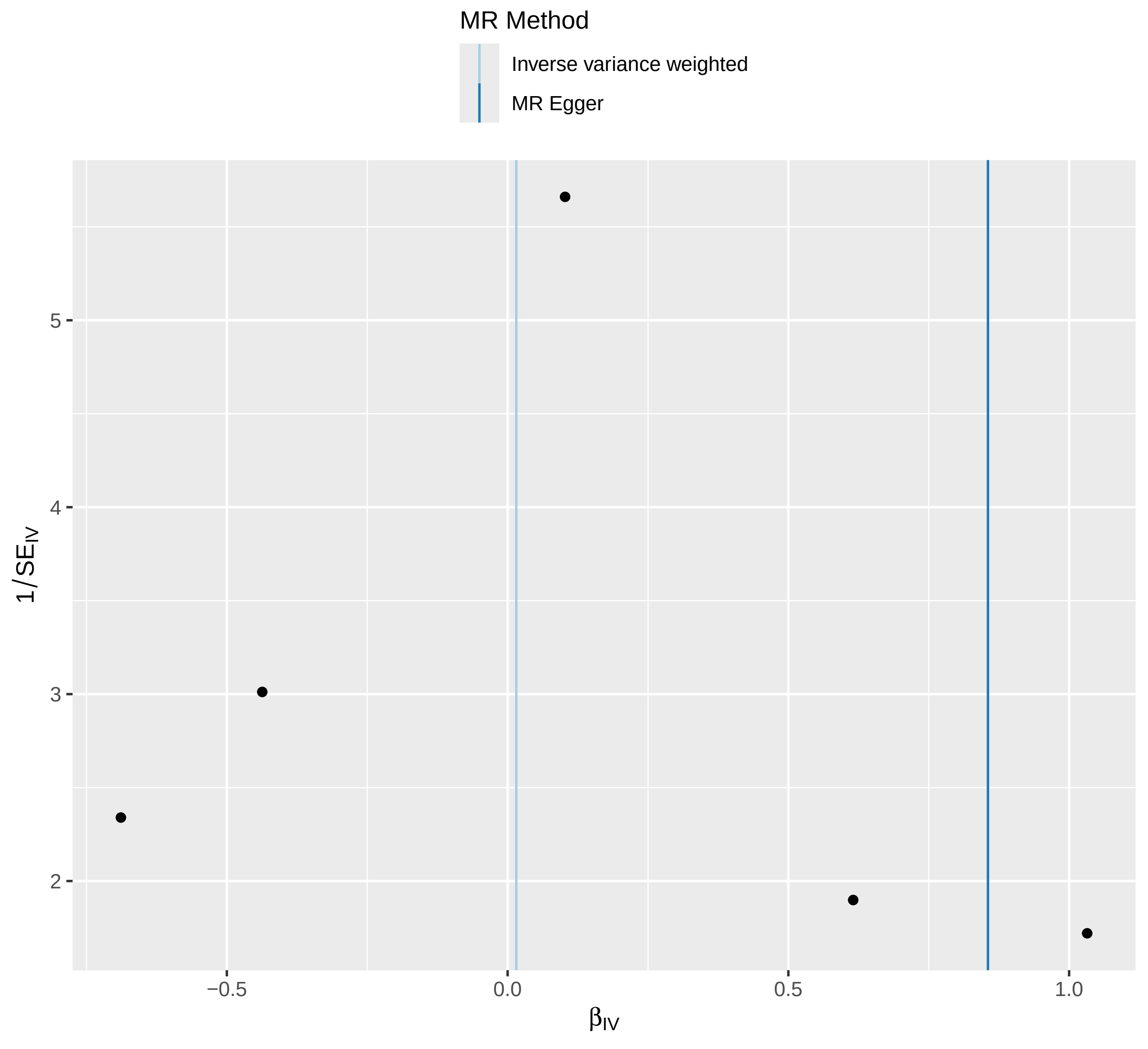
MR Method
Inverse variance weighted
MR Egger
1/SE_IV
5
4
3
2
β_IV
−0.5
0.0
0.5
1.0

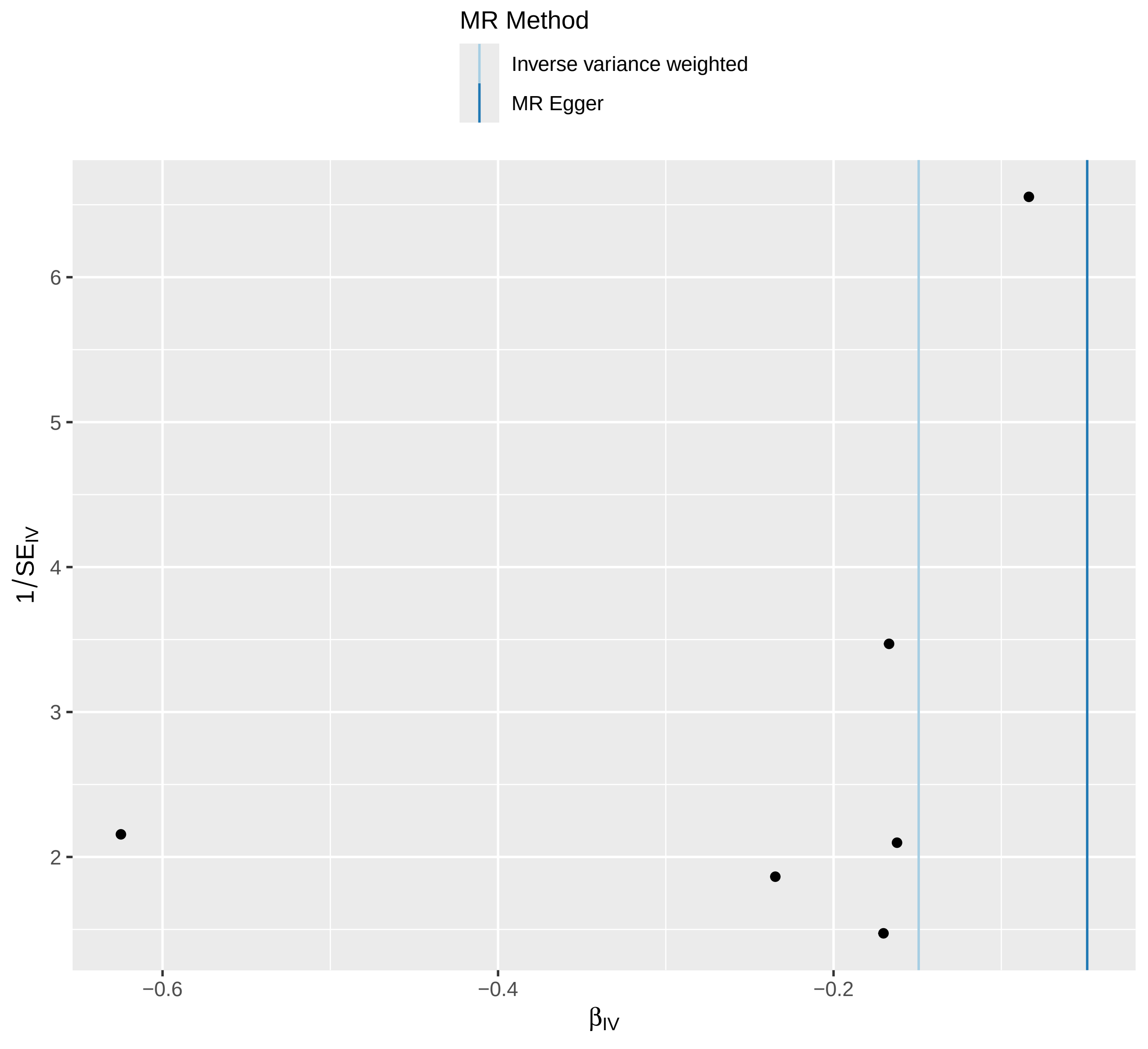

MR Method
Inverse variance weighted
MR Egger
6
5
4
3
2
1/SE_IV
−0.6
−0.4
−0.2
β_IV

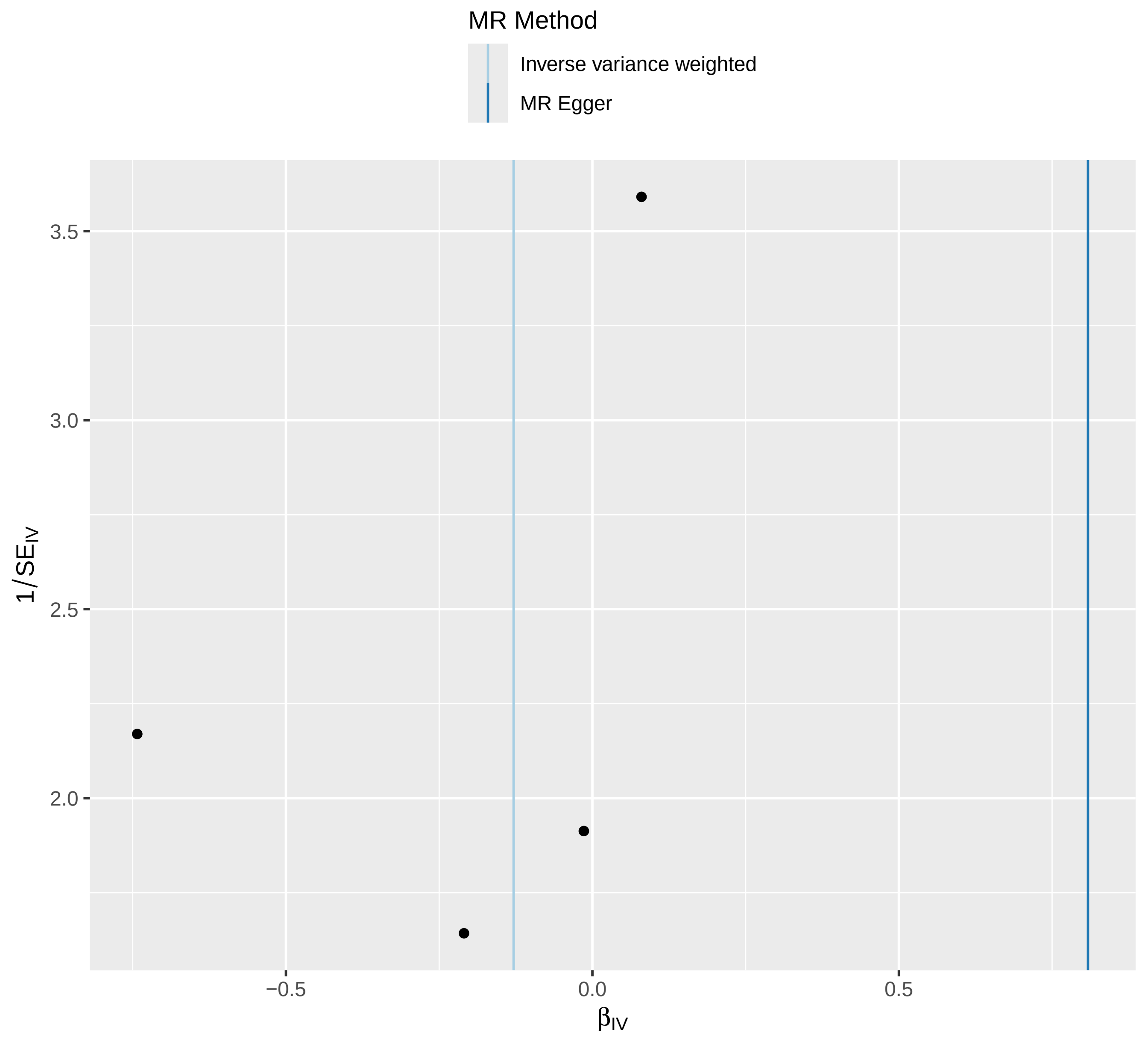
MR Method
Inverse variance weighted
MR Egger
3.5
3.0
2.5
2.0
1/SE_IV
−0.5
0.0
0.5
β_IV

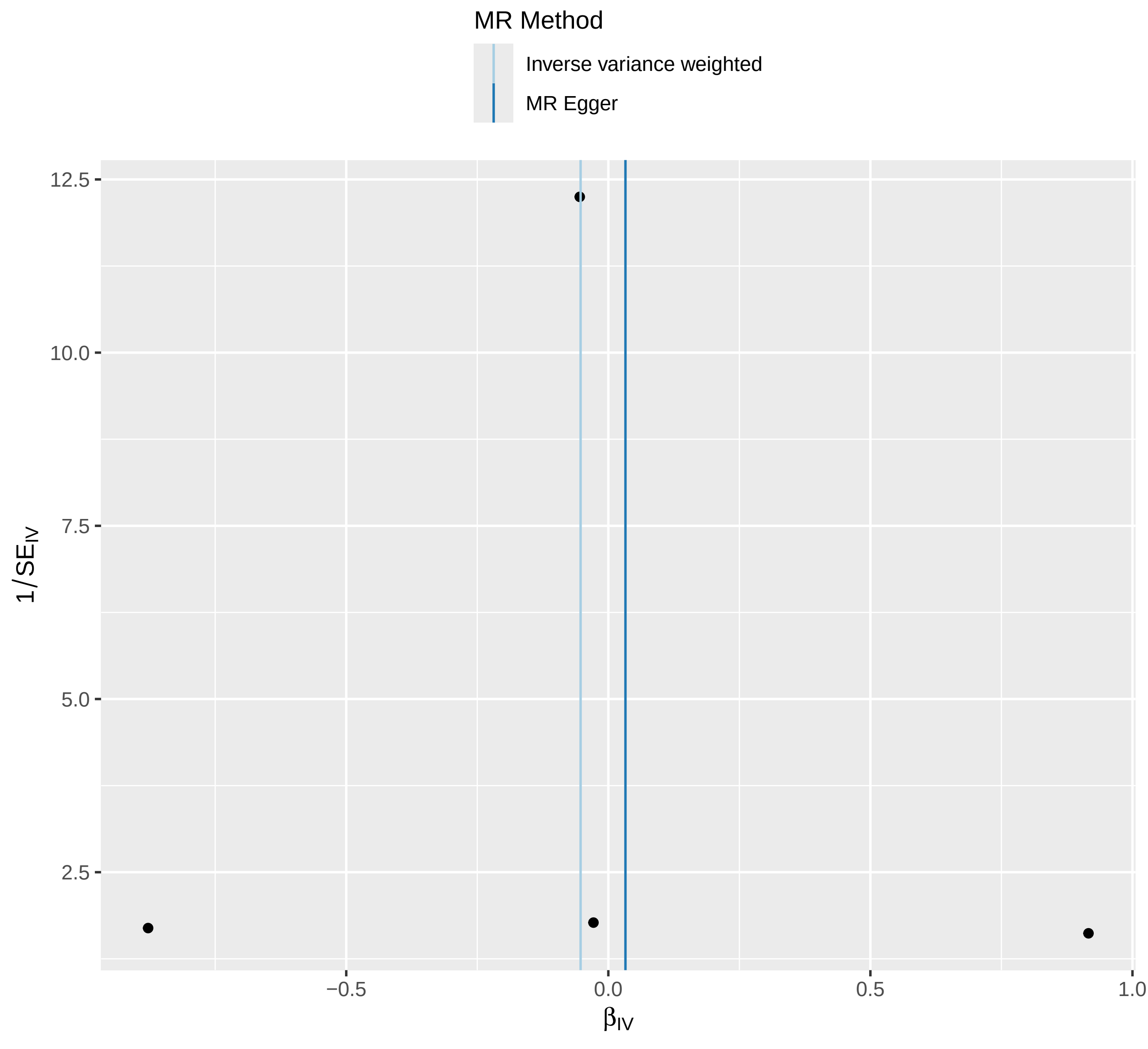
MR Method
Inverse variance weighted
MR Egger
12.5
10.0
7.5
5.0
2.5
1/SE_IV
−0.5
0.0
0.5
1.0
β_IV

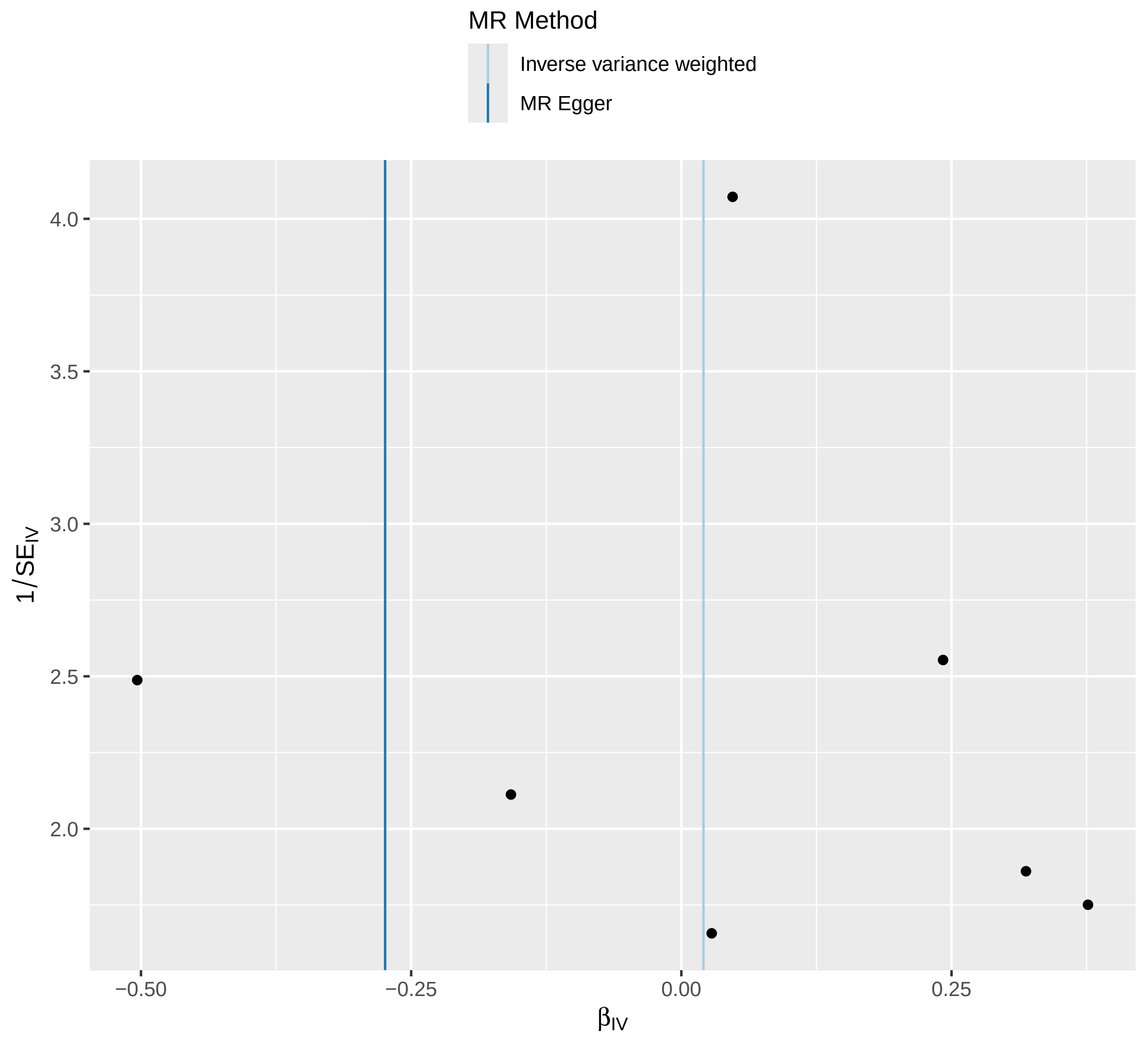
MR Method
Inverse variance weighted
MR Egger
4.0
3.5
3.0
2.5
2.0
1/SE$_{IV}$
−0.50
−0.25
0.00
0.25
β$_{IV}$

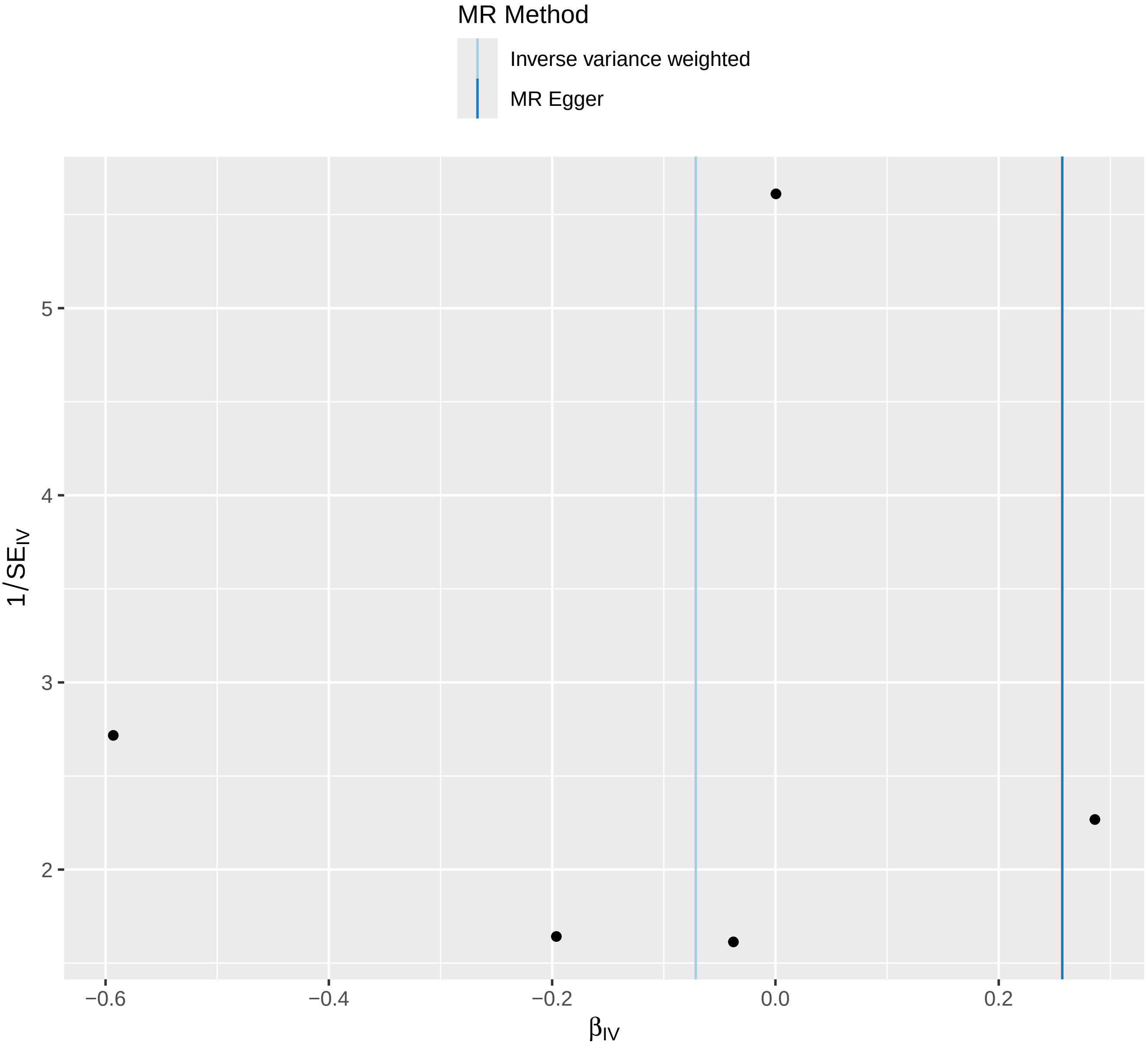

MR Method
Inverse variance weighted
MR Egger
5
4
3
2
1/SE$_{IV}$
−0.6
−0.4
−0.2
0.0
0.2
β$_{IV}$

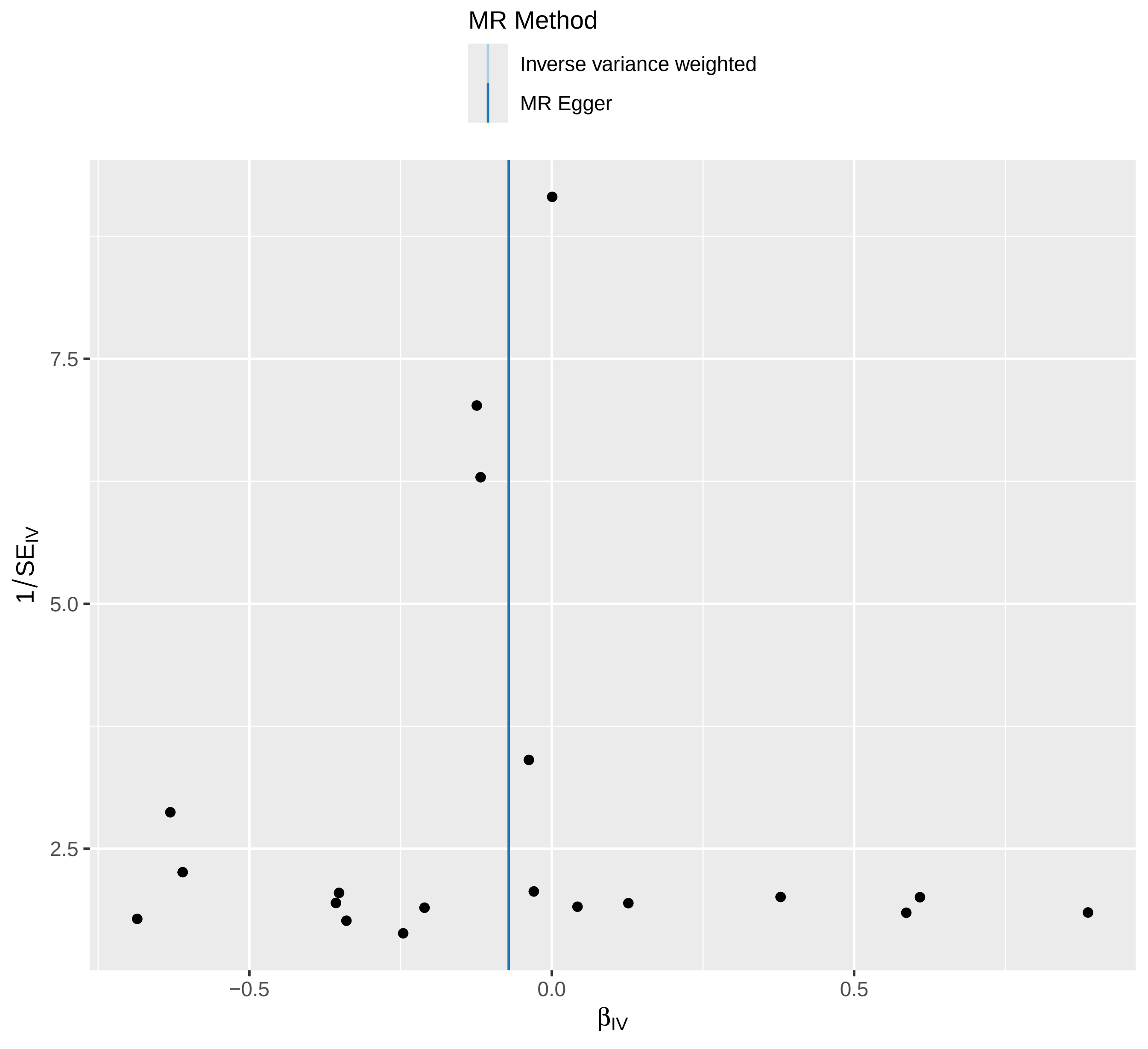

MR Method
Inverse variance weighted
MR Egger
7.5
5.0
2.5
1/SE$_{IV}$
−0.5
0.0
0.5
β$_{IV}$

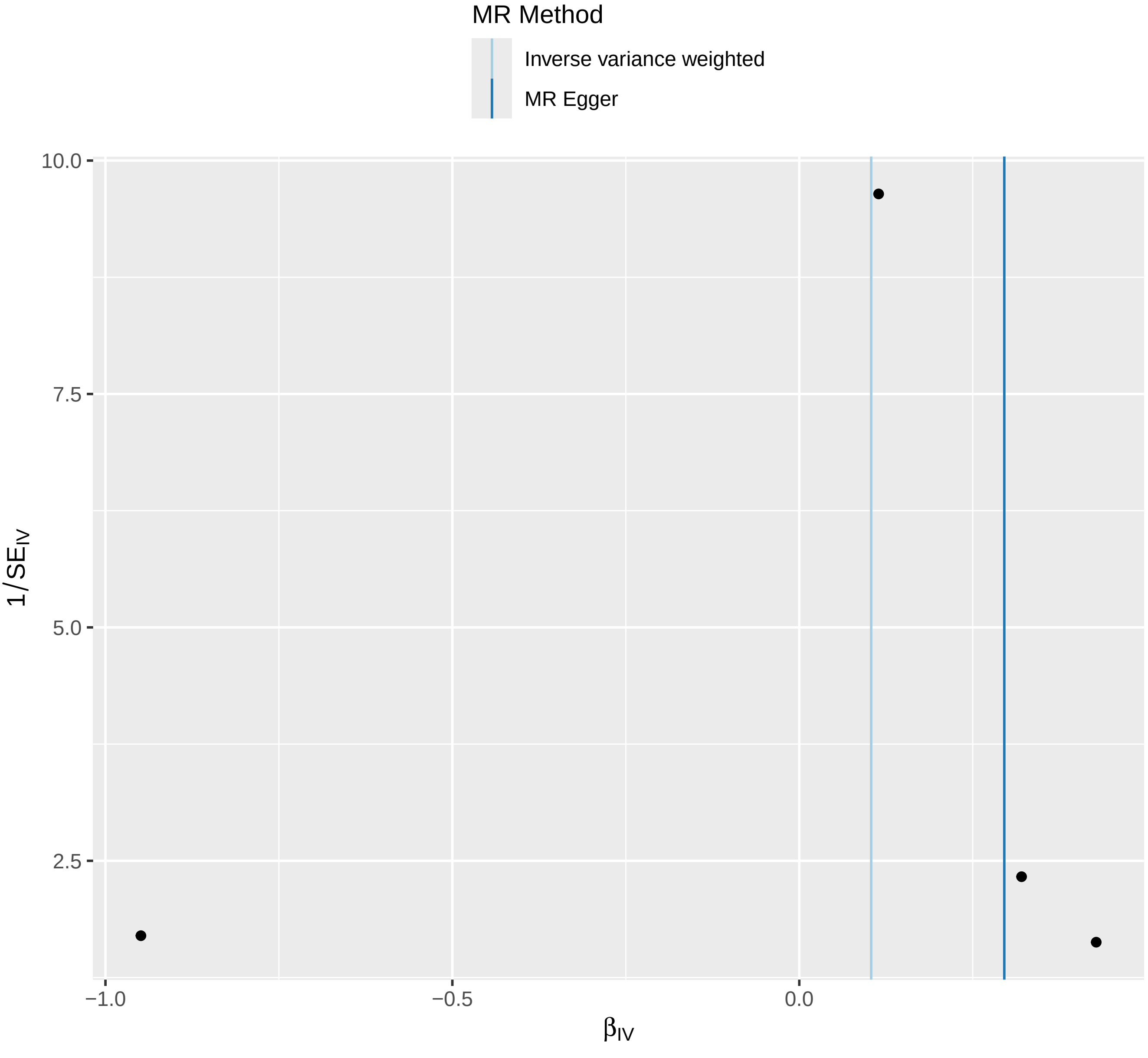

MR Method
Inverse variance weighted
MR Egger
10.0
7.5
5.0
2.5
$1/SE_{IV}$
−1.0
−0.5
0.0
β_{IV}

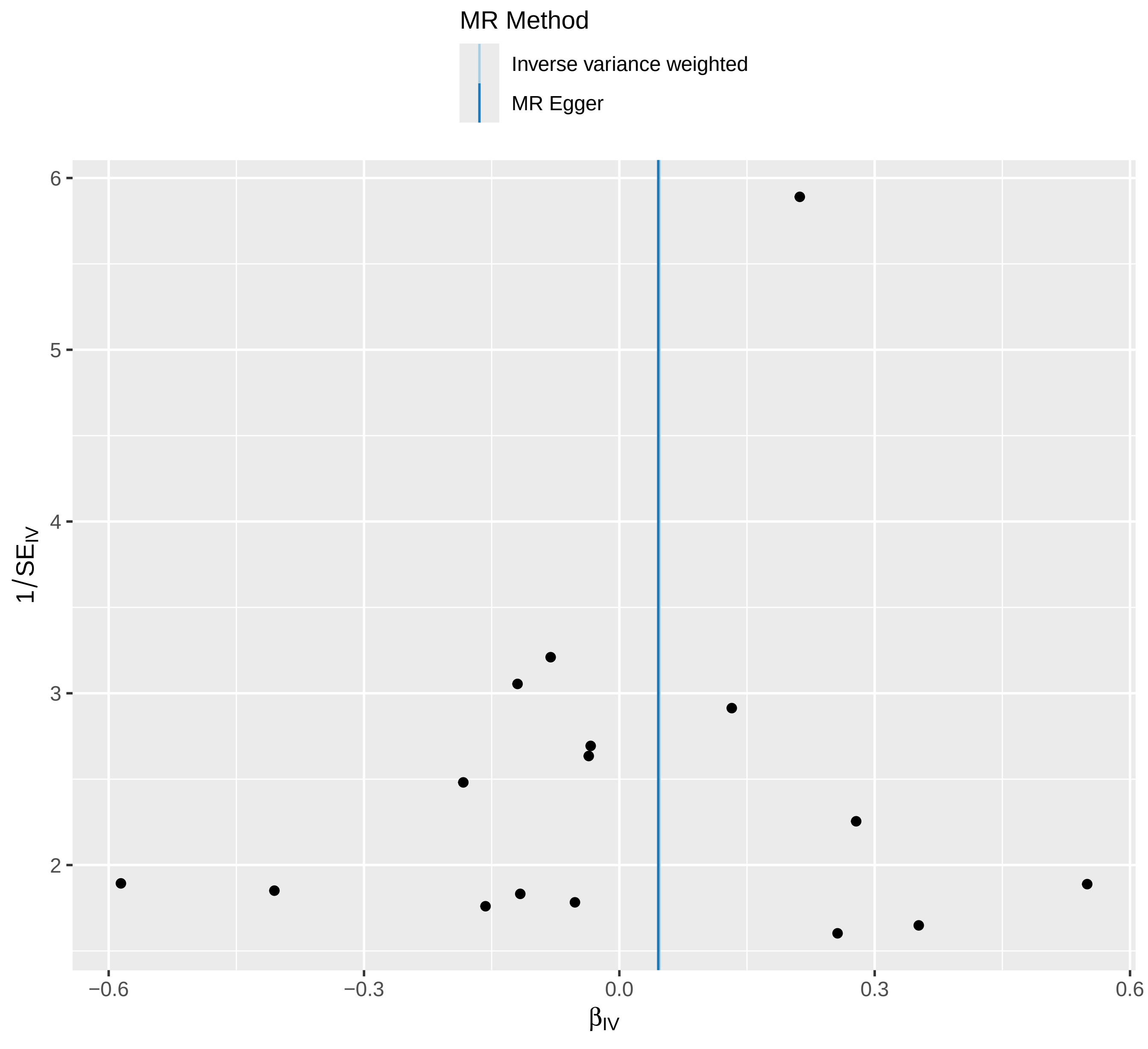

MR Method
Inverse variance weighted
MR Egger
6
5
4
3
2
1/SE$_{IV}$
−0.6
−0.3
0.0
0.3
0.6
β$_{IV}$

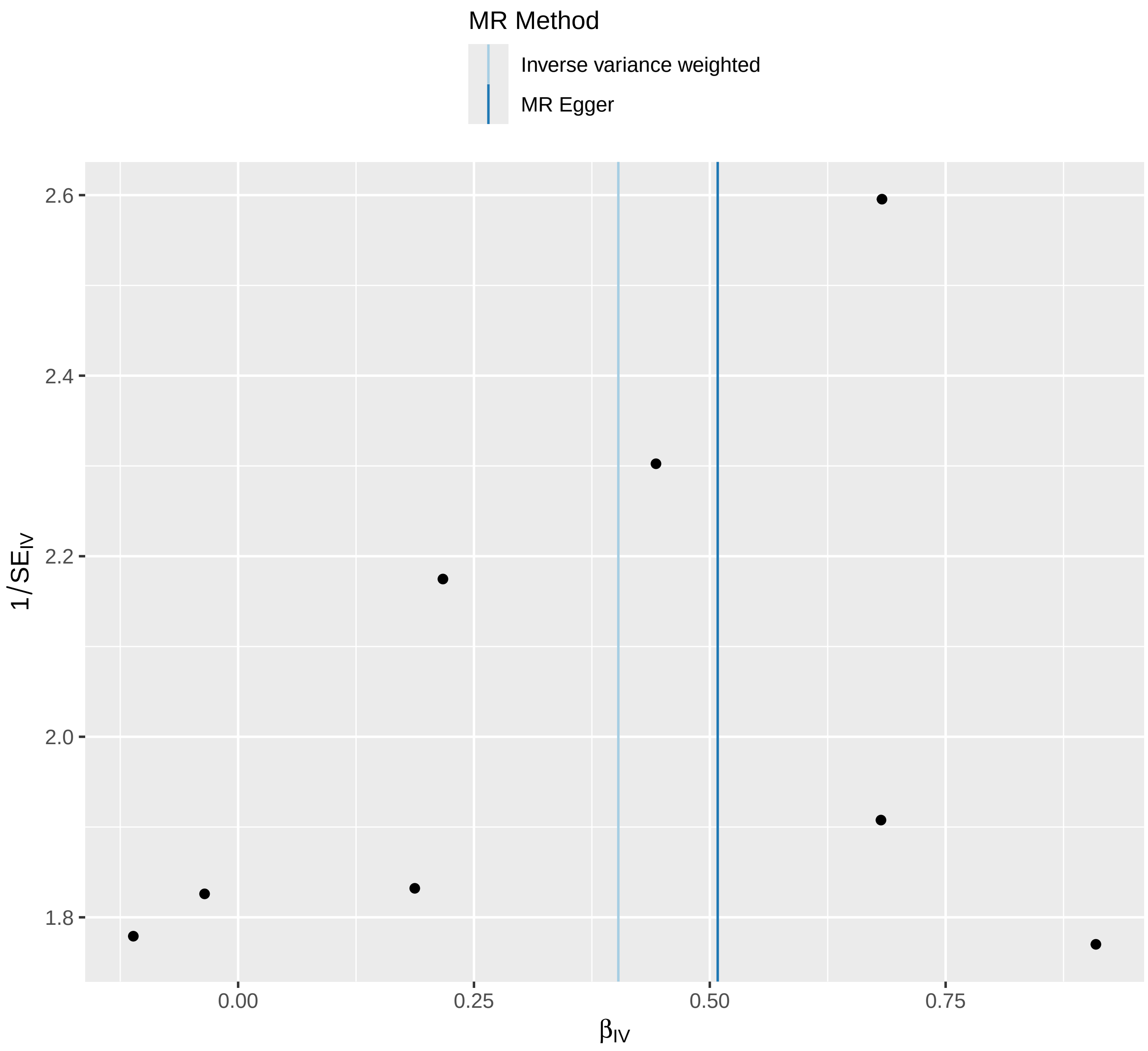

MR Method
Inverse variance weighted
MR Egger
2.6
2.4
2.2
2.0
1.8
$1/SE_{IV}$
0.00
0.25
0.50
0.75
β_{IV}

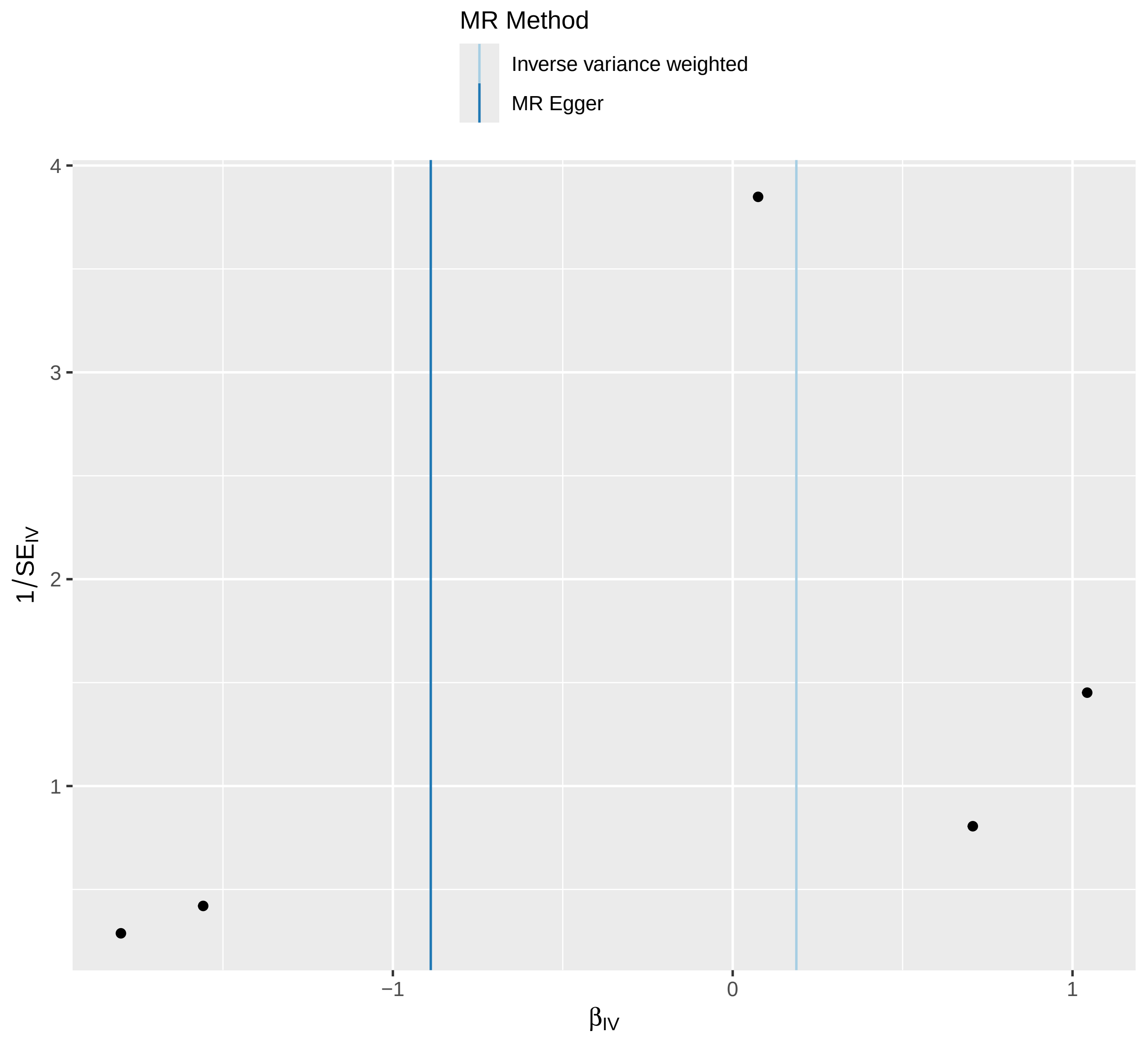

MR Method
Inverse variance weighted
MR Egger
4
3
2
1
1/SE_IV
−1
0
1
β_IV

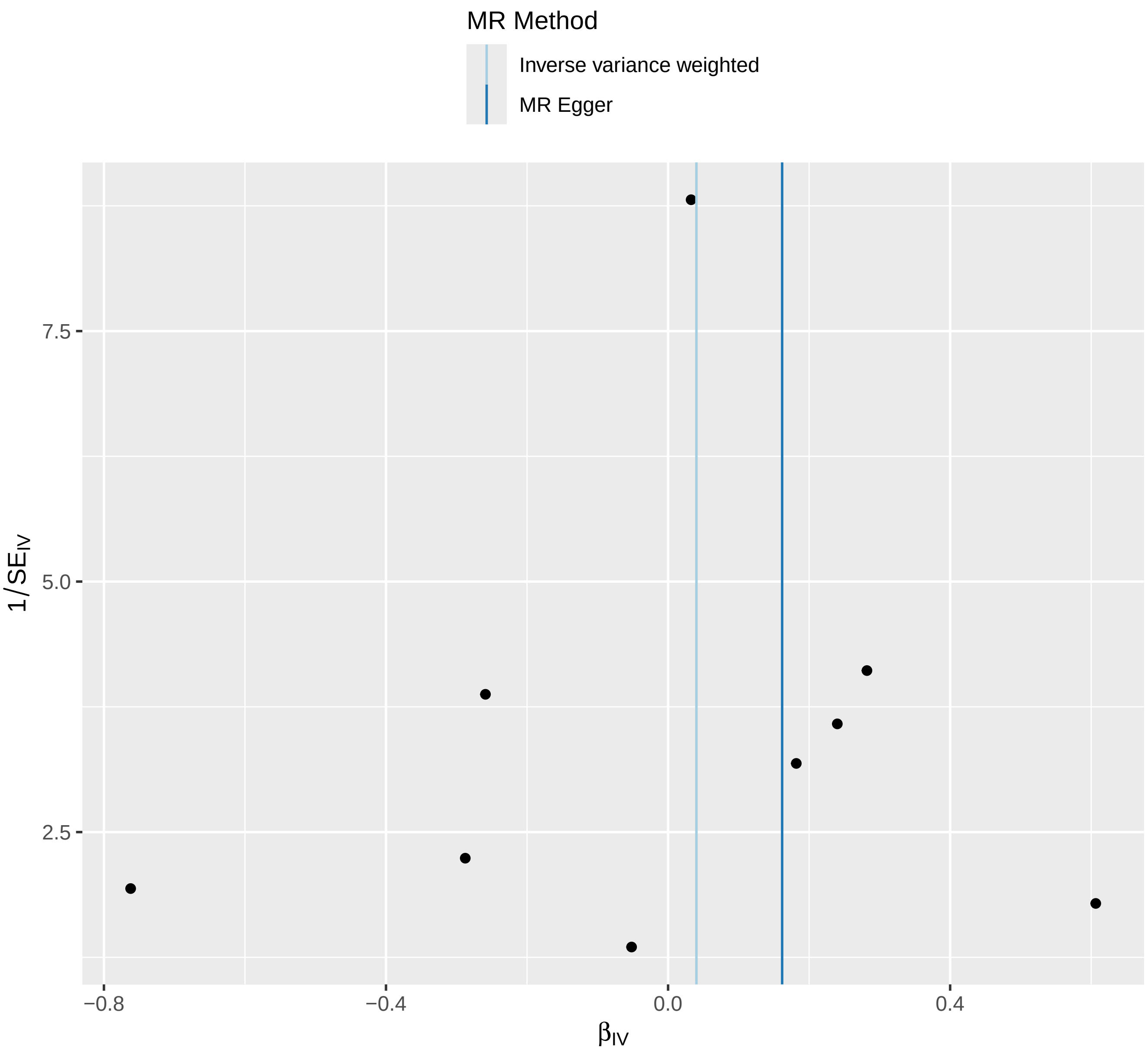

MR Method
Inverse variance weighted
MR Egger
7.5
5.0
2.5
1/SE_IV
−0.8
−0.4
0.0
0.4
β_IV

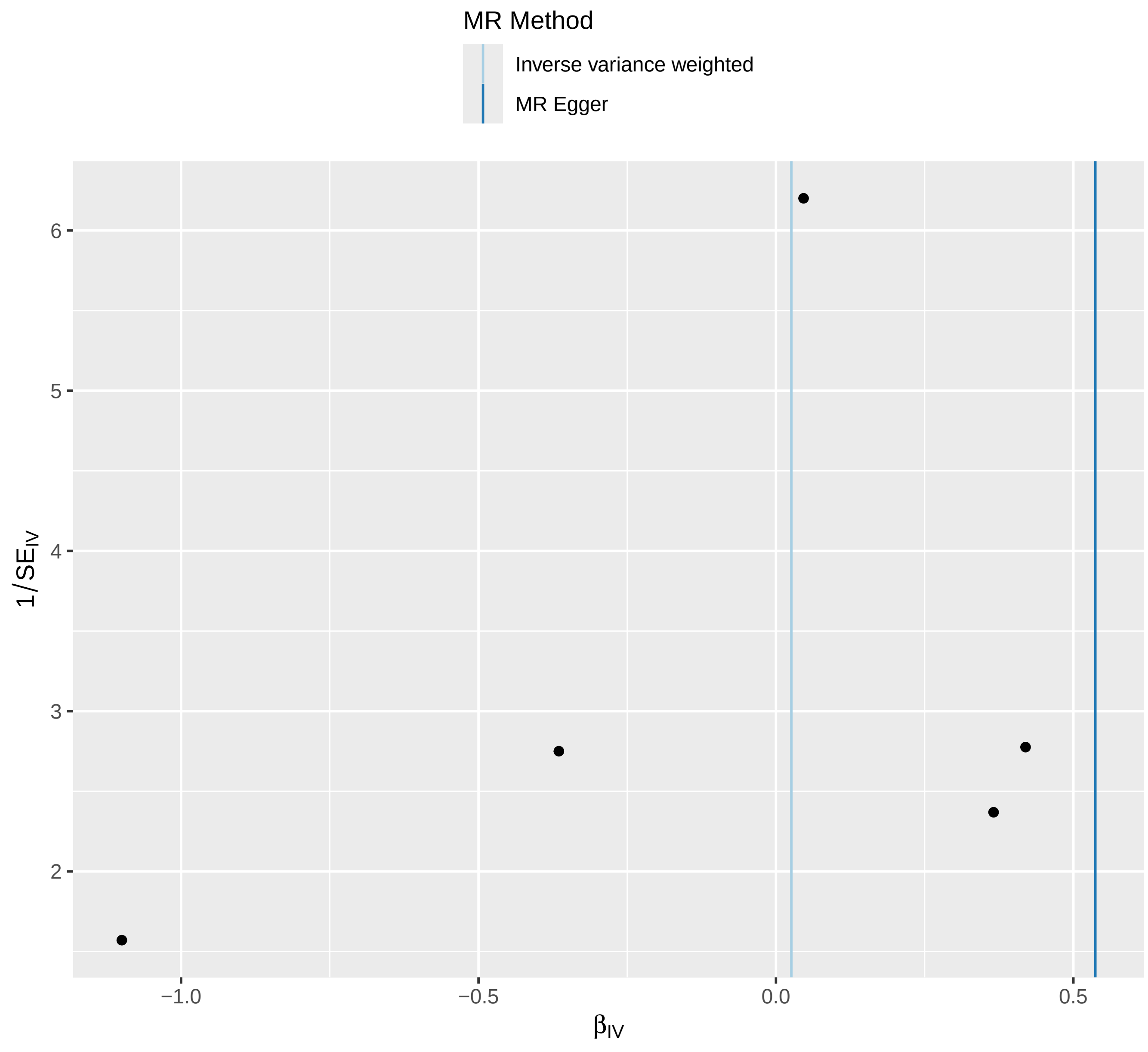

MR Method
Inverse variance weighted
MR Egger
6
5
4
3
2
$1/SE_{IV}$
−1.0
−0.5
0.0
0.5
β_{IV}

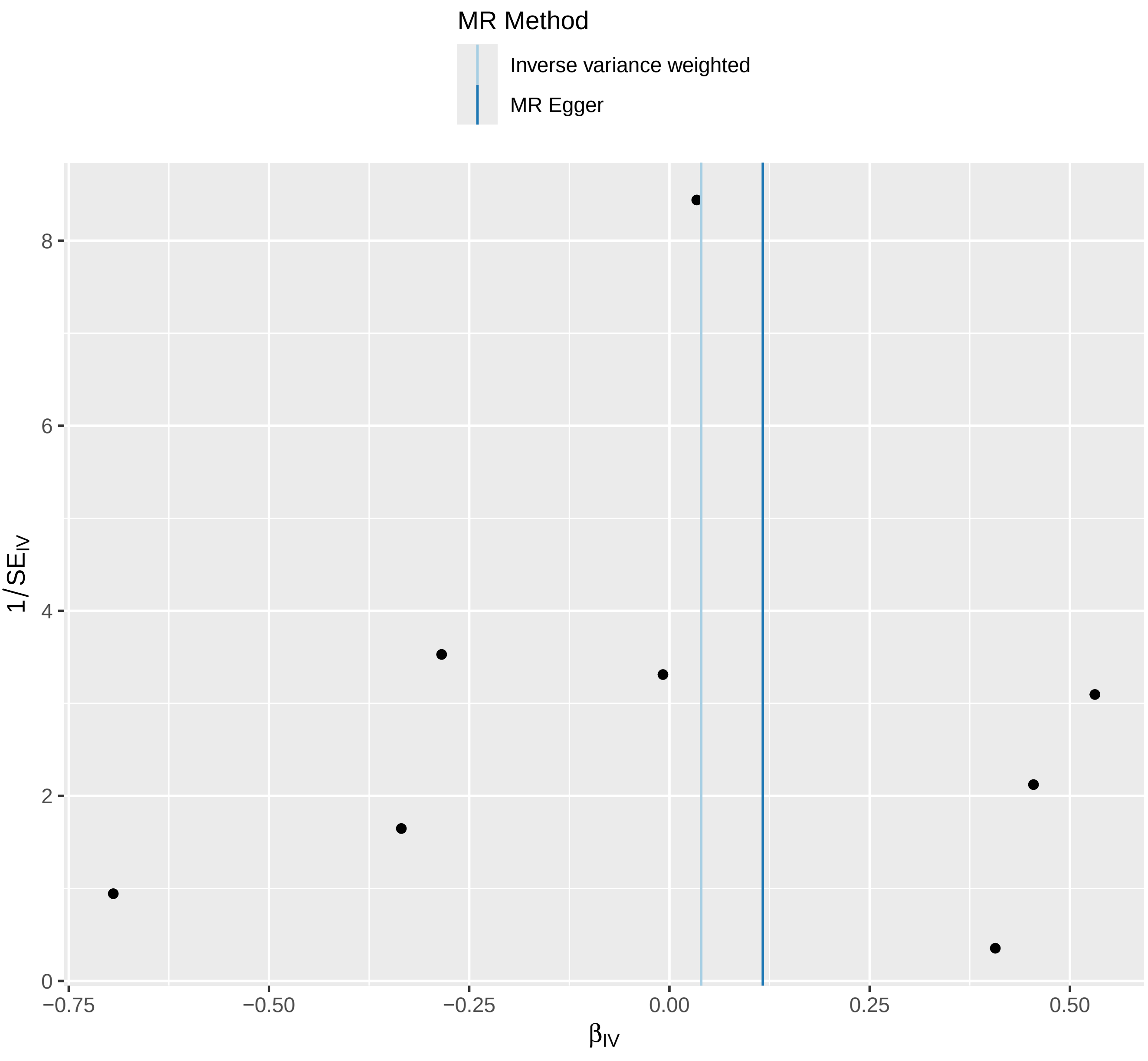
MR Method
Inverse variance weighted
MR Egger
8
6
4
2
0
1/SE_IV
−0.75
−0.50
−0.25
0.00
0.25
0.50
β_IV

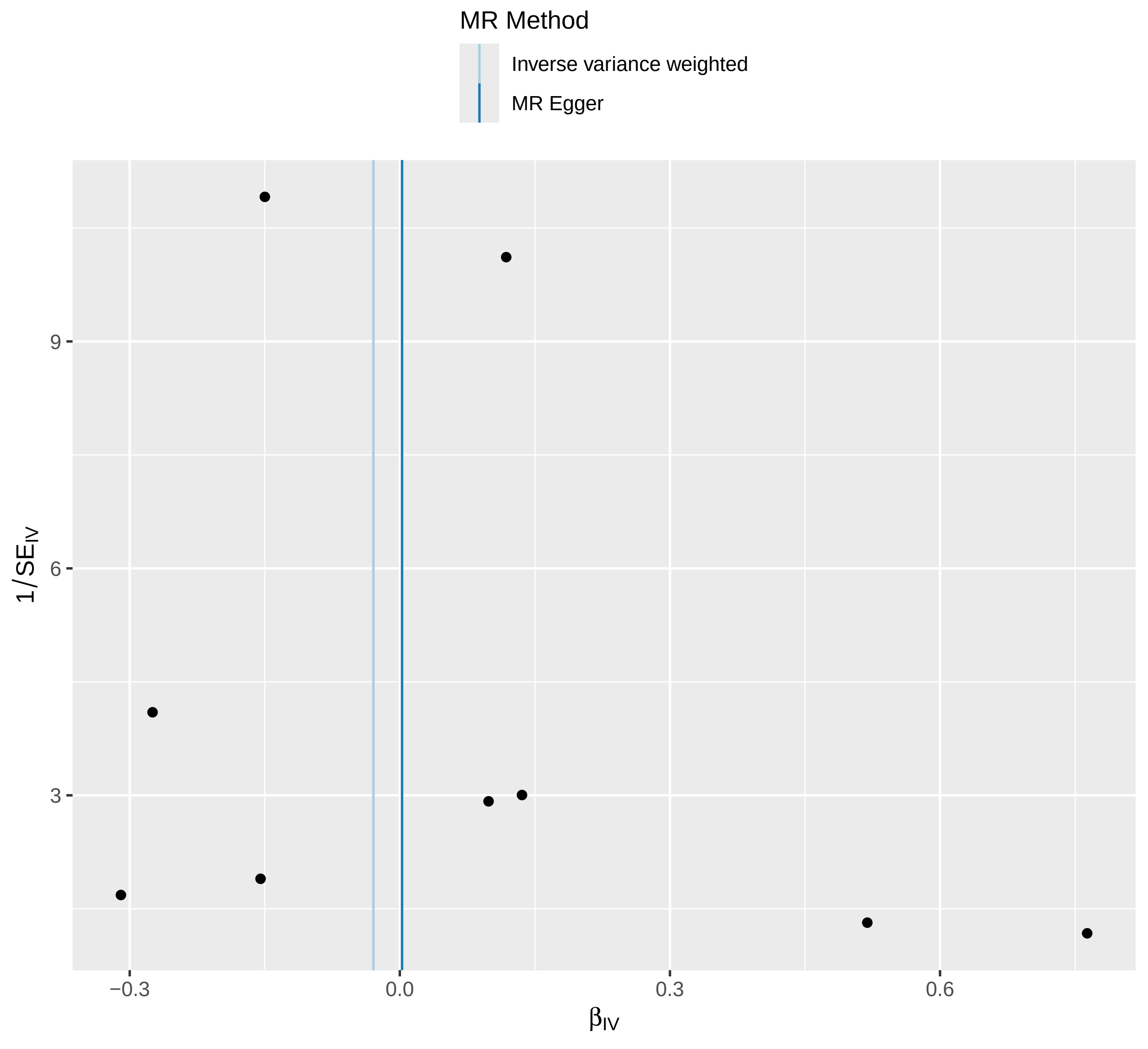
MR Method
Inverse variance weighted
MR Egger
9
6
3
−0.3
0.0
0.3
0.6
$1/SE_{IV}$
β_{IV}

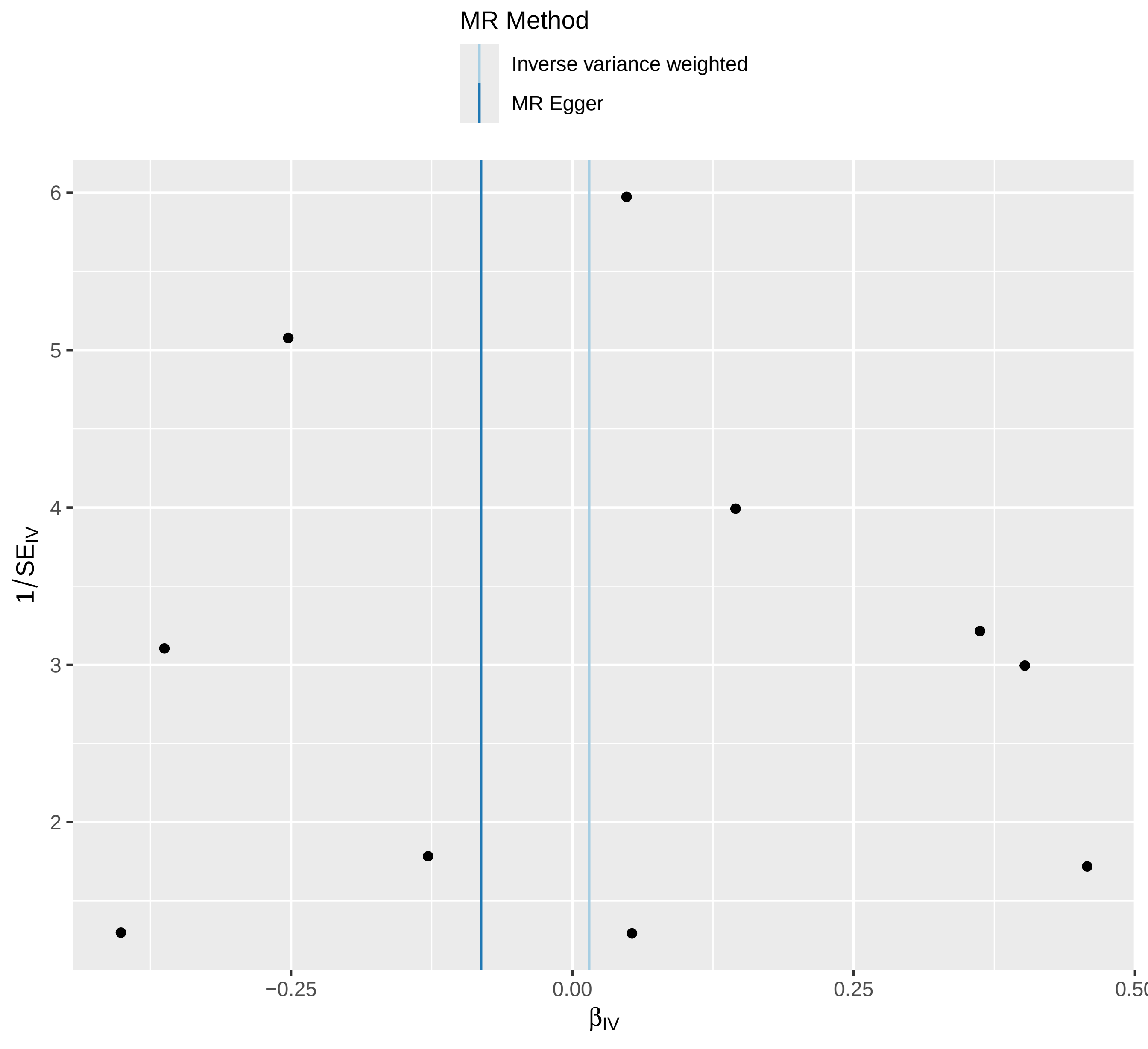

MR Method
Inverse variance weighted
MR Egger
6
5
4
3
2
1/SE_IV
−0.25
0.00
0.25
0.50
β_IV

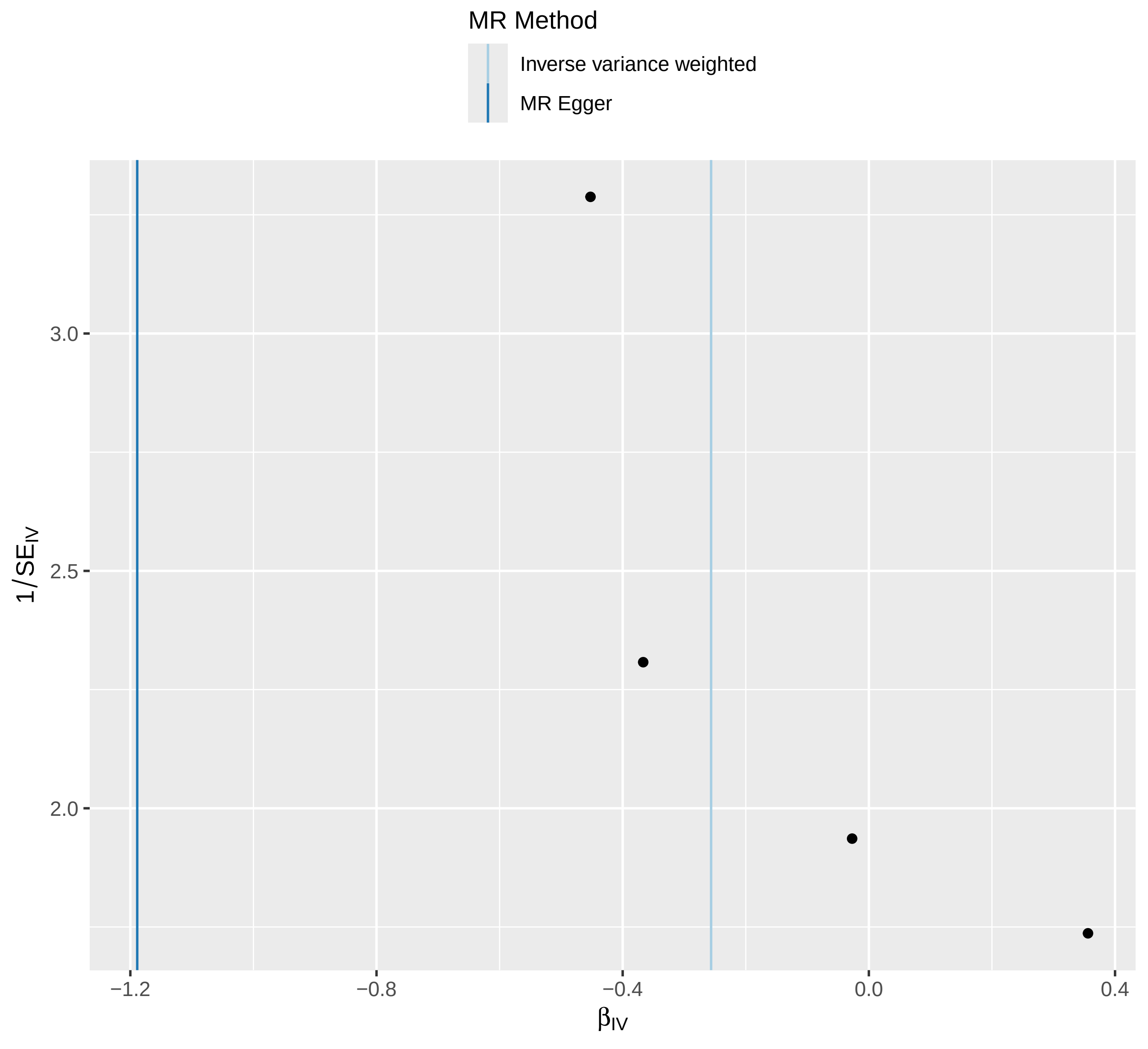
MR Method
Inverse variance weighted
MR Egger
3.0
2.5
2.0
1/SE$_{IV}$
−1.2
−0.8
−0.4
0.0
0.4
β$_{IV}$

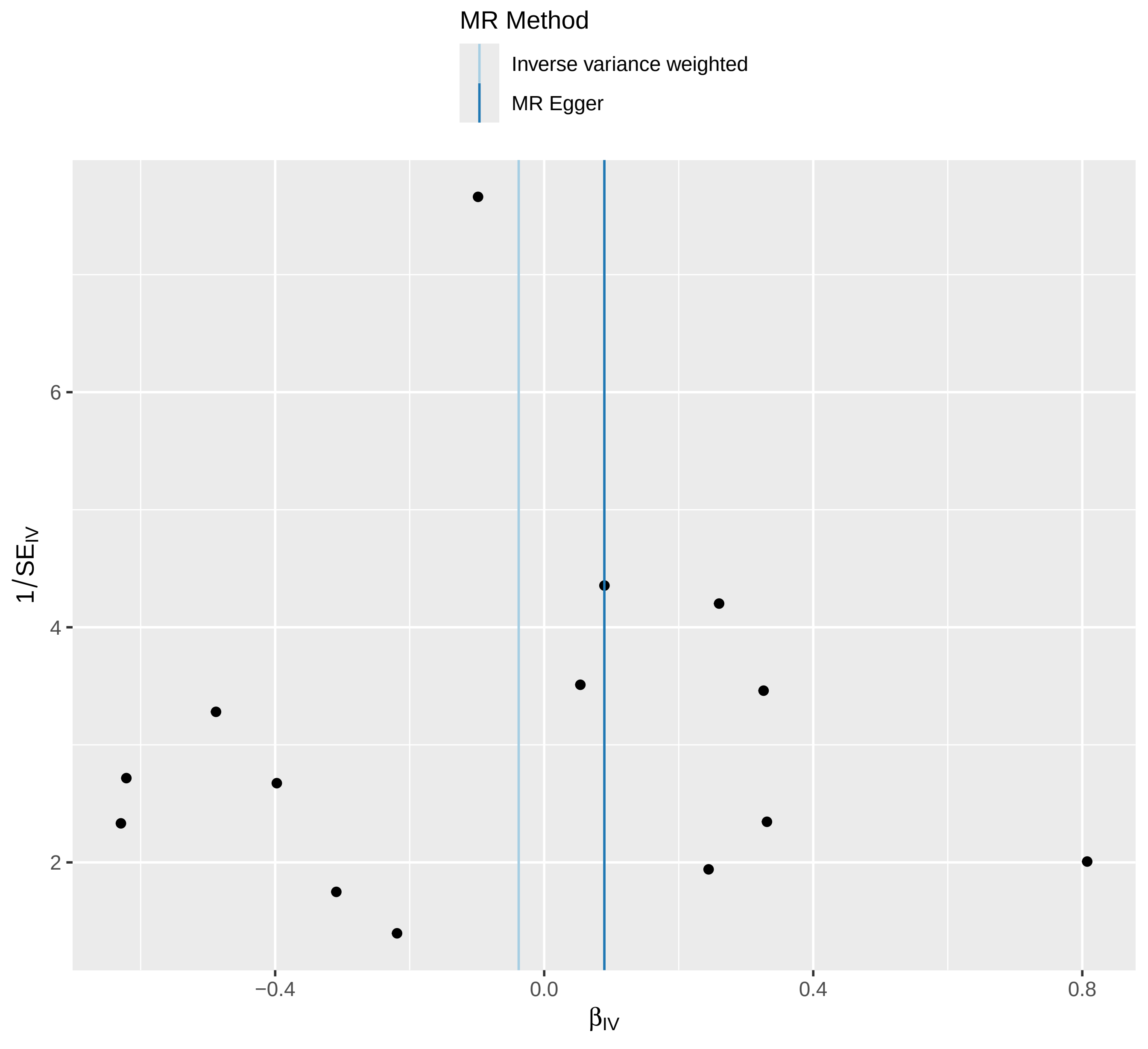

MR Method
Inverse variance weighted
MR Egger
6
4
2
1/SE$_{IV}$
−0.4
0.0
0.4
0.8
β$_{IV}$

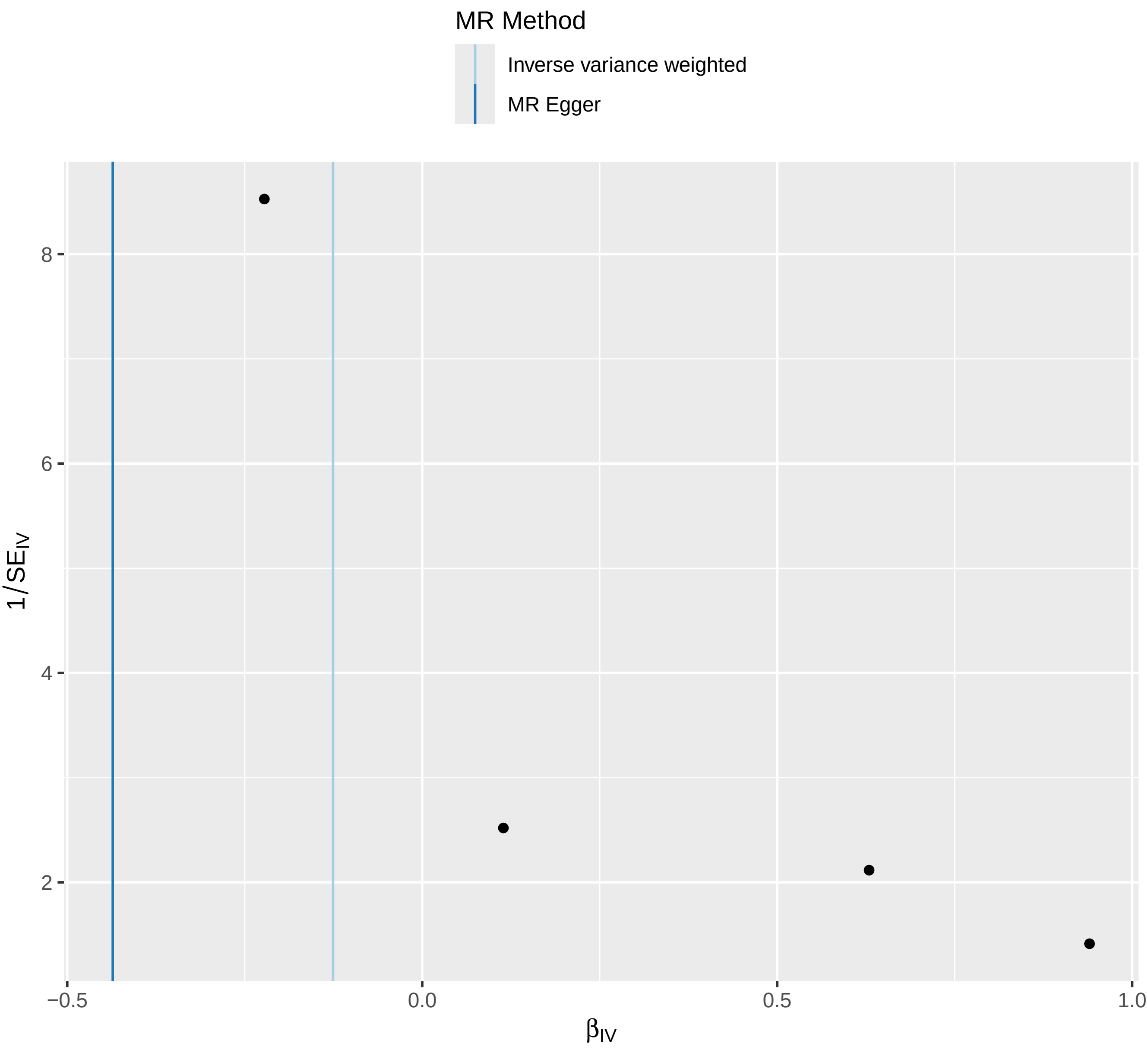

MR Method
Inverse variance weighted
MR Egger
8
6
4
2
1/SE_IV
−0.5
0.0
0.5
1.0
β_IV

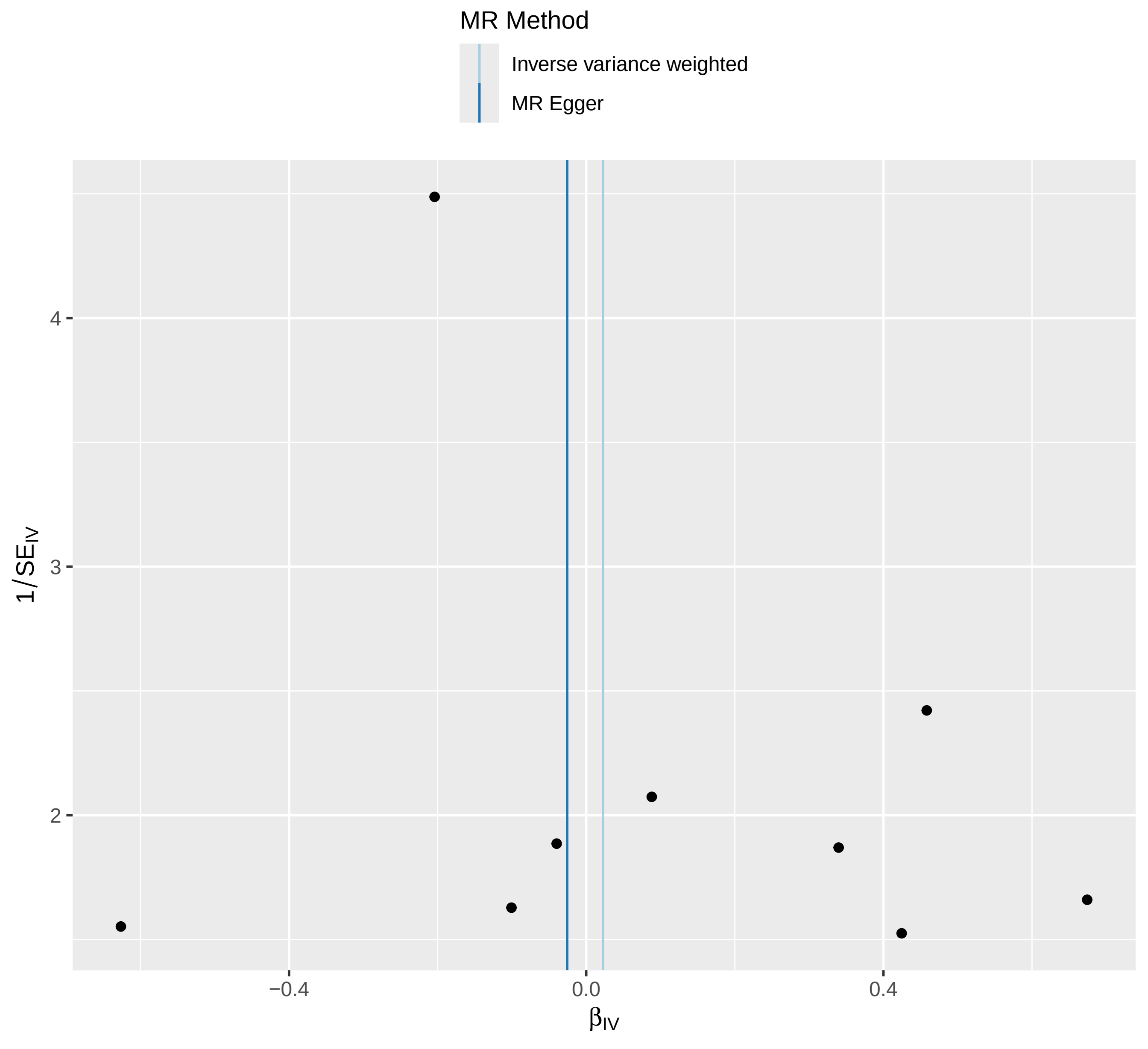
MR Method
Inverse variance weighted
MR Egger
4
3
2
1/SE$_{IV}$
−0.4
0.0
0.4
β$_{IV}$

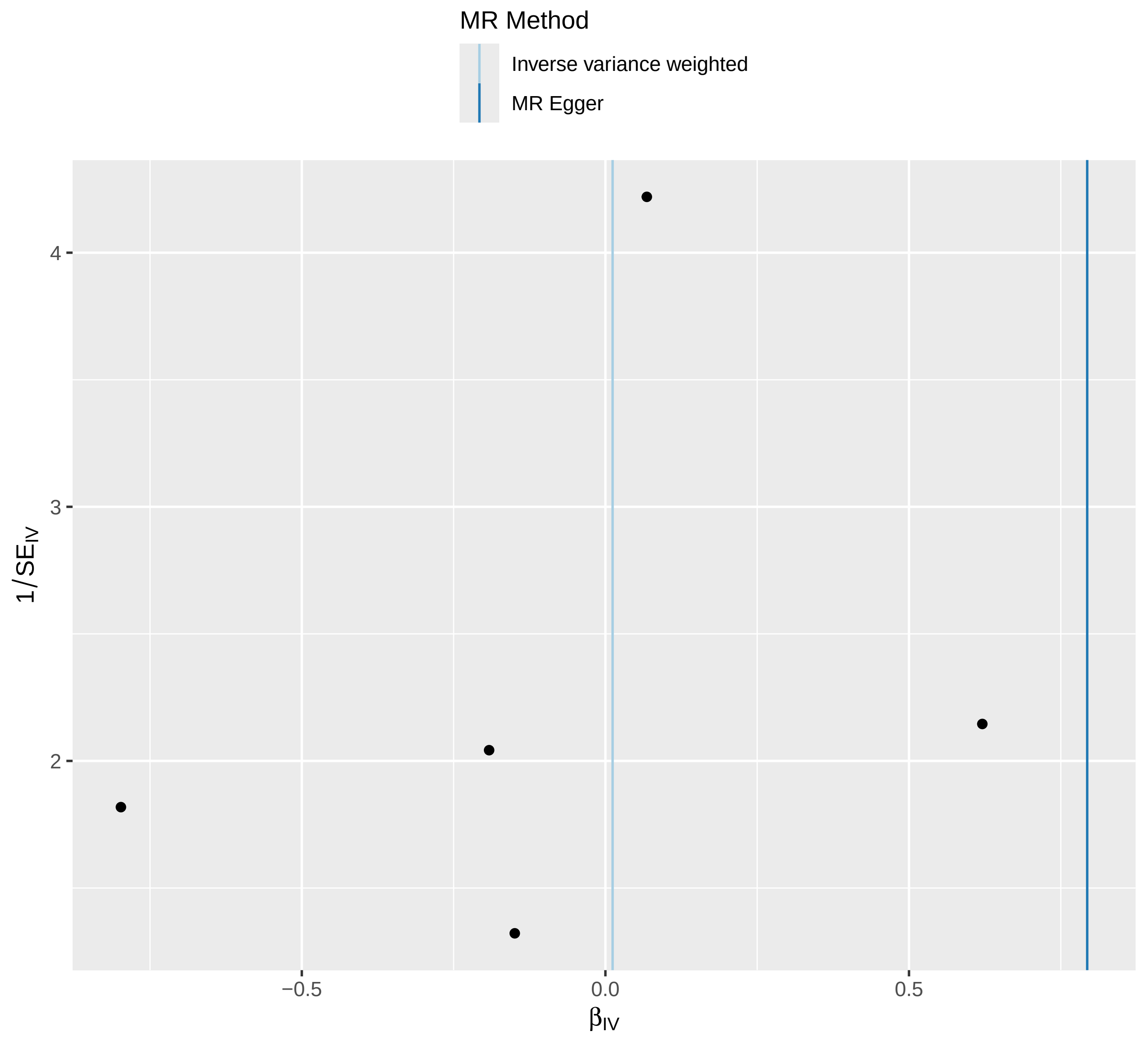
MR Method
Inverse variance weighted
MR Egger
4
3
2
1/SE_IV
−0.5
0.0
0.5
β_IV

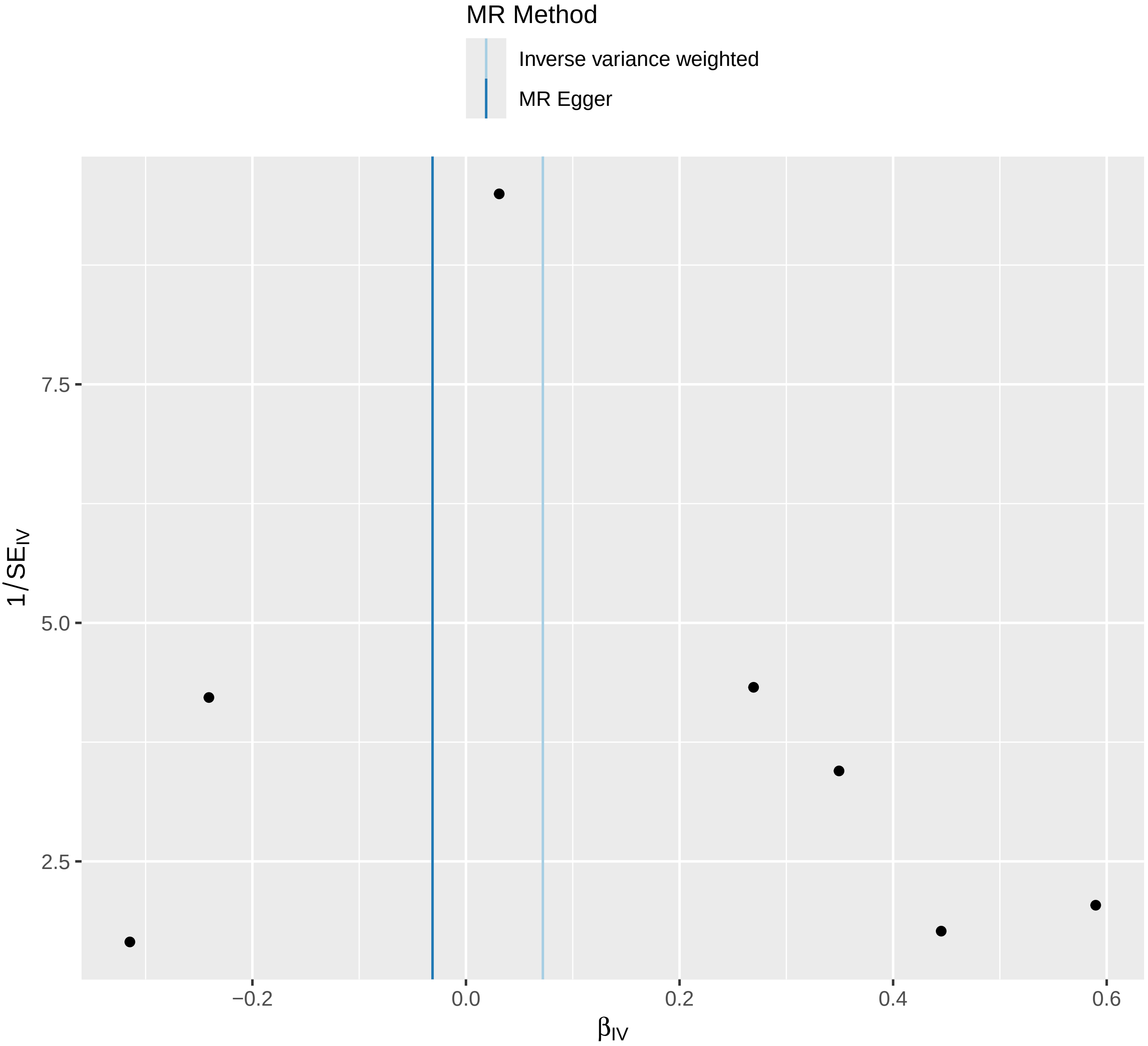

MR Method
Inverse variance weighted
MR Egger
7.5
5.0
2.5
$1/SE_{IV}$
−0.2
0.0
0.2
0.4
0.6
β_{IV}

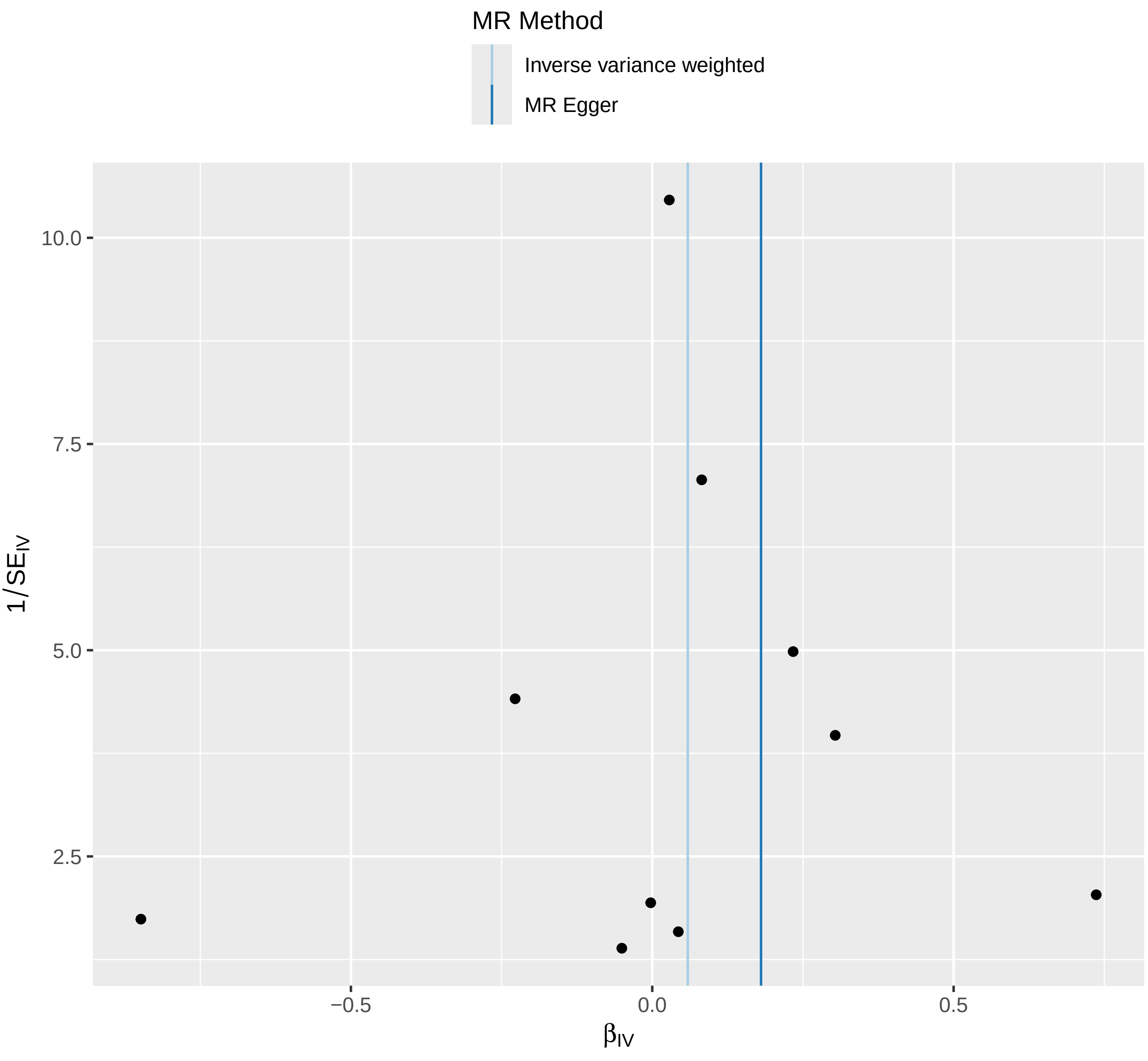
MR Method
Inverse variance weighted
MR Egger
10.0
7.5
5.0
2.5
1/SE_IV
−0.5
0.0
0.5
β_IV

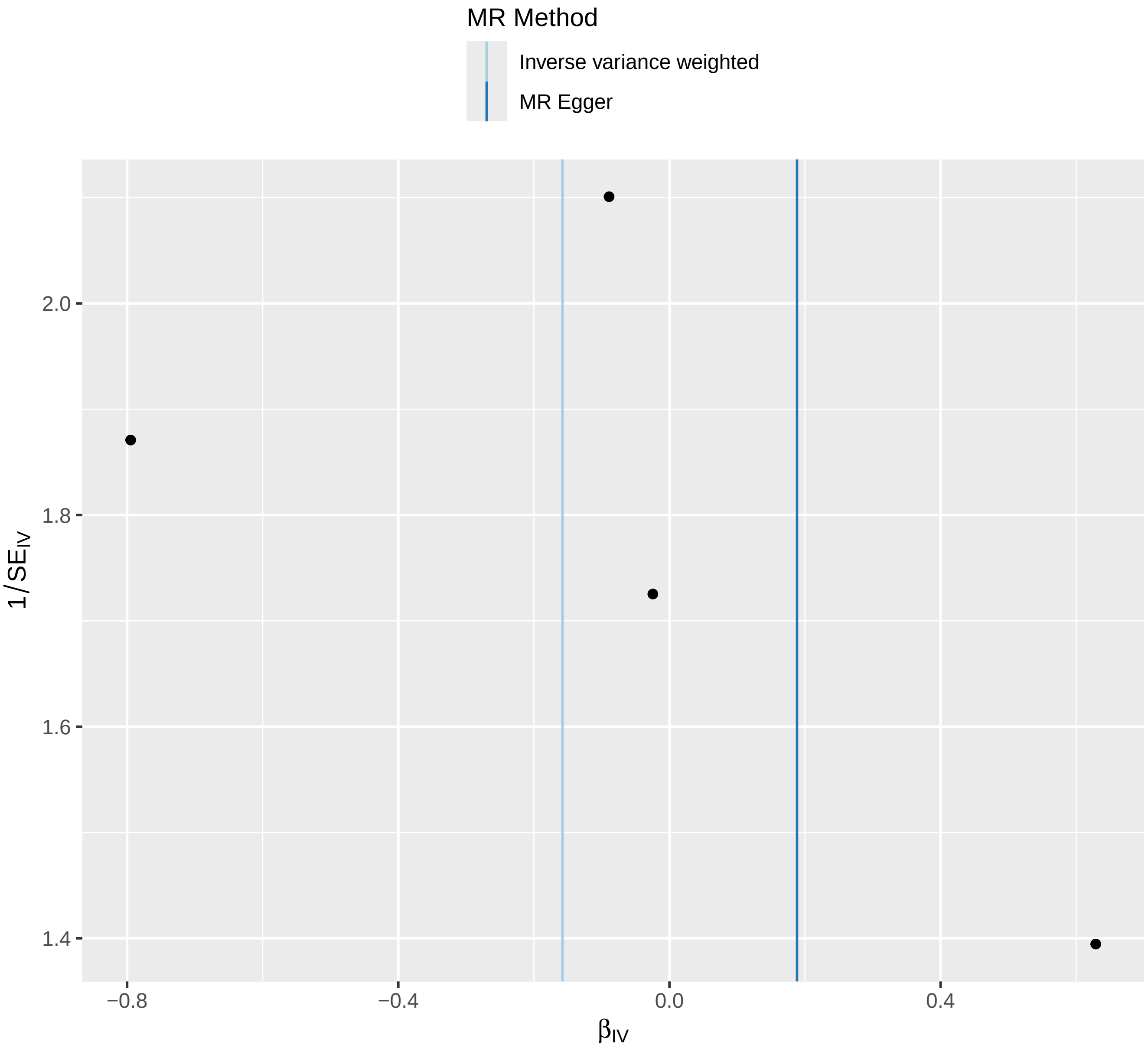

MR Method
Inverse variance weighted
MR Egger
2.0
1.8
1.6
1.4
1/SE_IV
−0.8
−0.4
0.0
0.4
β_IV

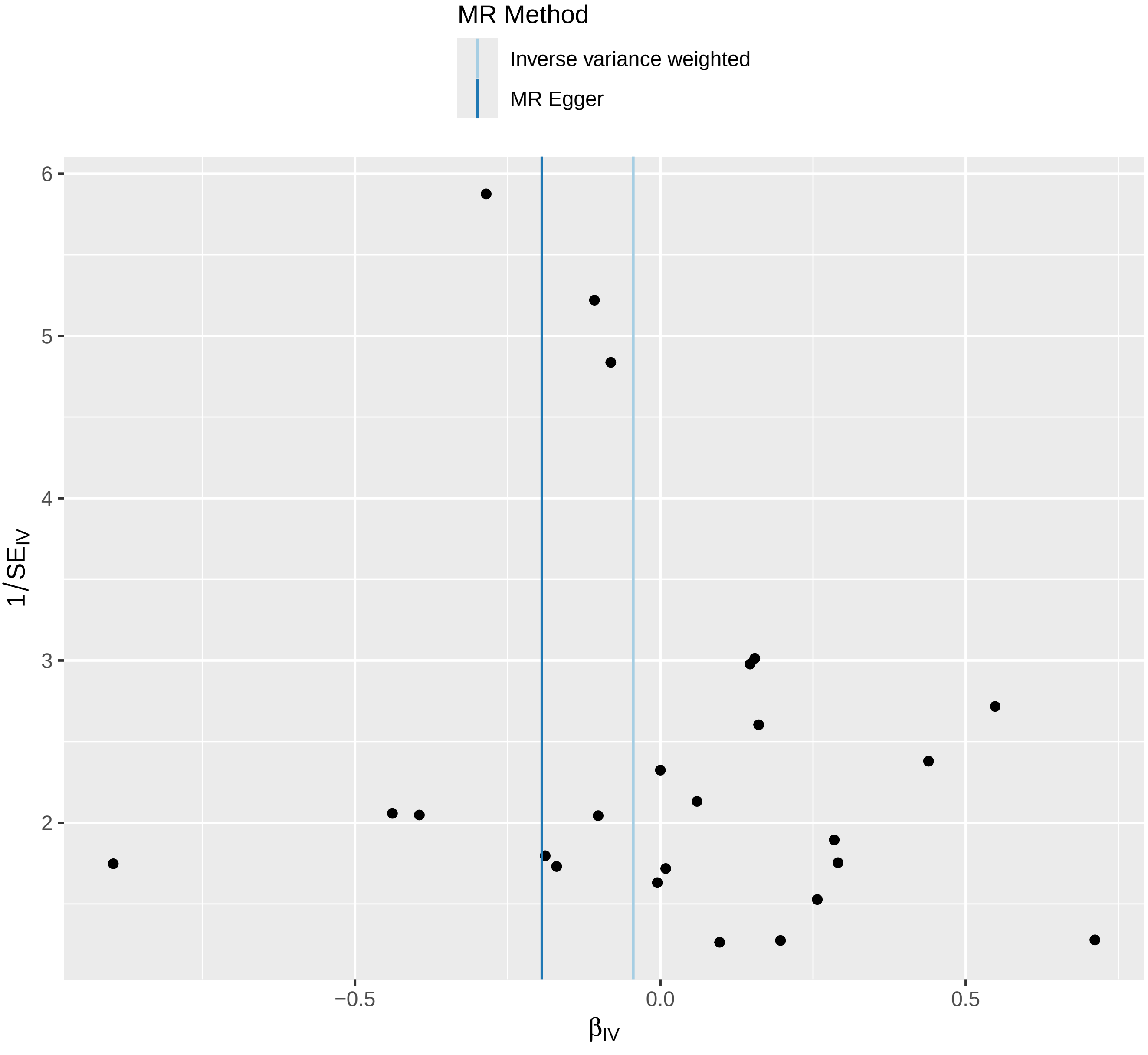

MR Method
Inverse variance weighted
MR Egger
1/SE_IV
β_IV
6
5
4
3
2
−0.5
0.0
0.5

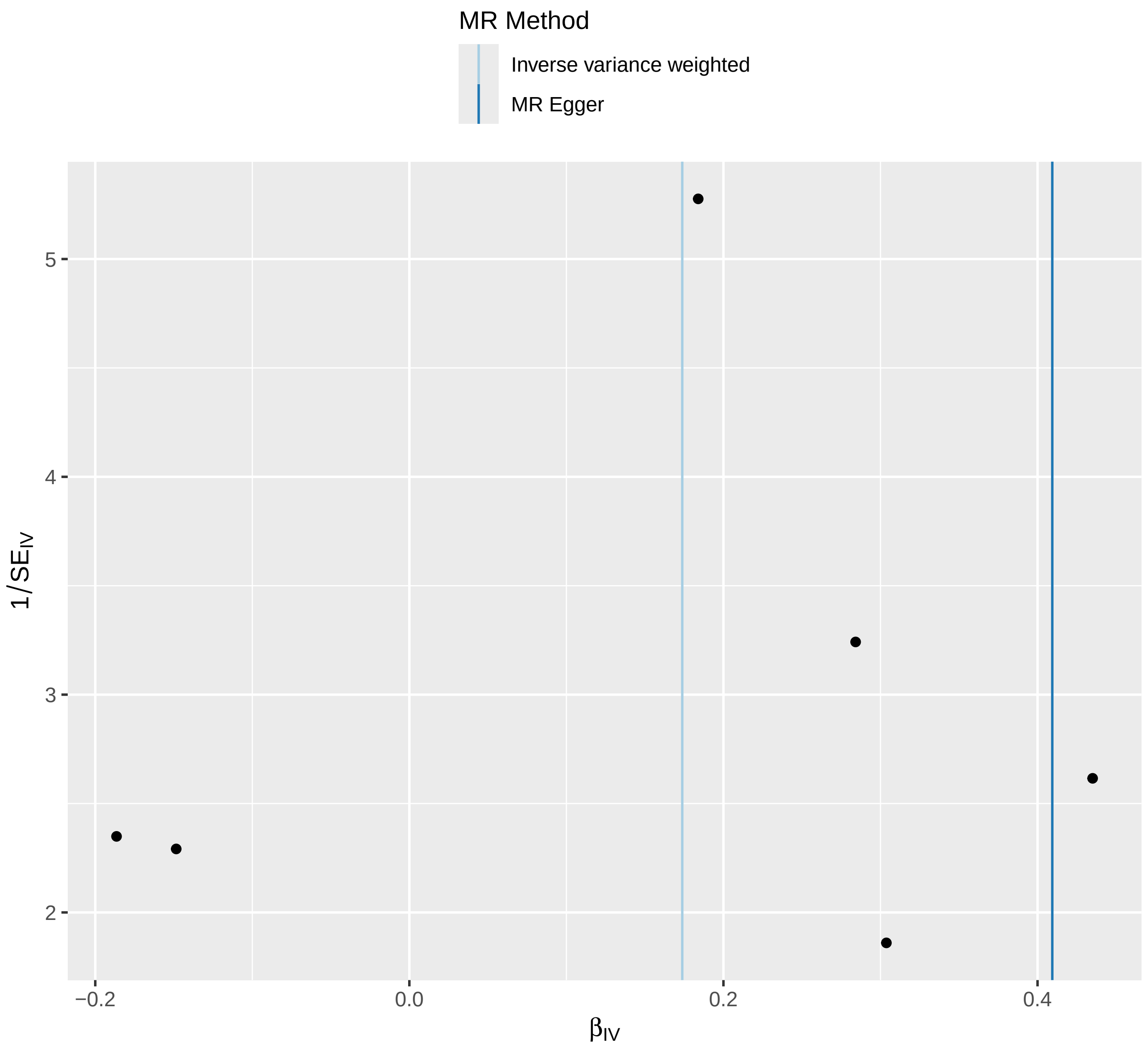

MR Method
Inverse variance weighted
MR Egger
5
4
3
2
1/SE_IV
−0.2
0.0
0.2
0.4
β_IV

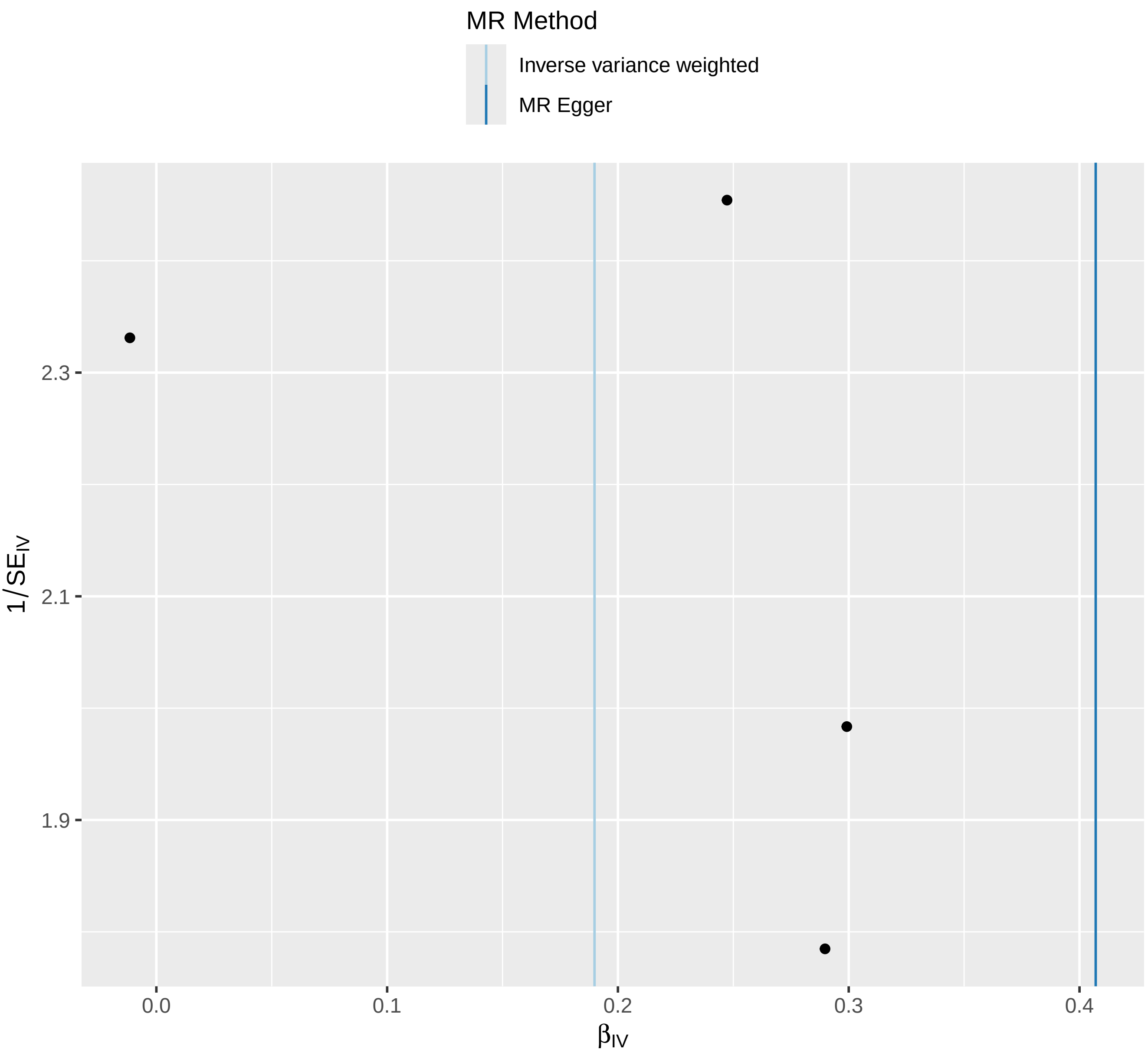

MR Method
Inverse variance weighted
MR Egger
2.3
2.1
1.9
1/SE$_{IV}$
0.0
0.1
0.2
0.3
0.4
β_{IV}

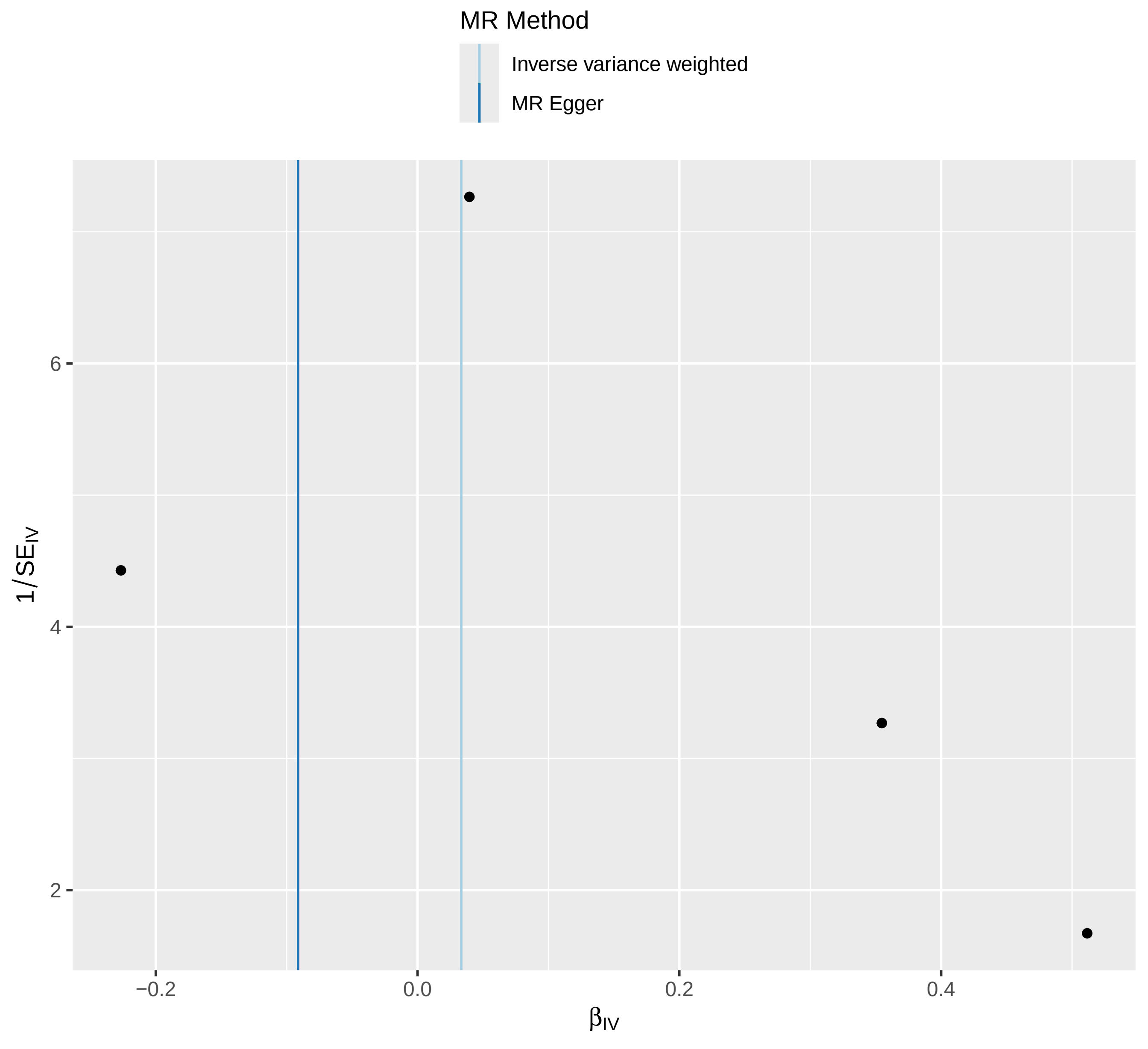

MR Method
Inverse variance weighted
MR Egger
6
4
2
−0.2
0.0
0.2
0.4
$1/SE_{IV}$
β_{IV}

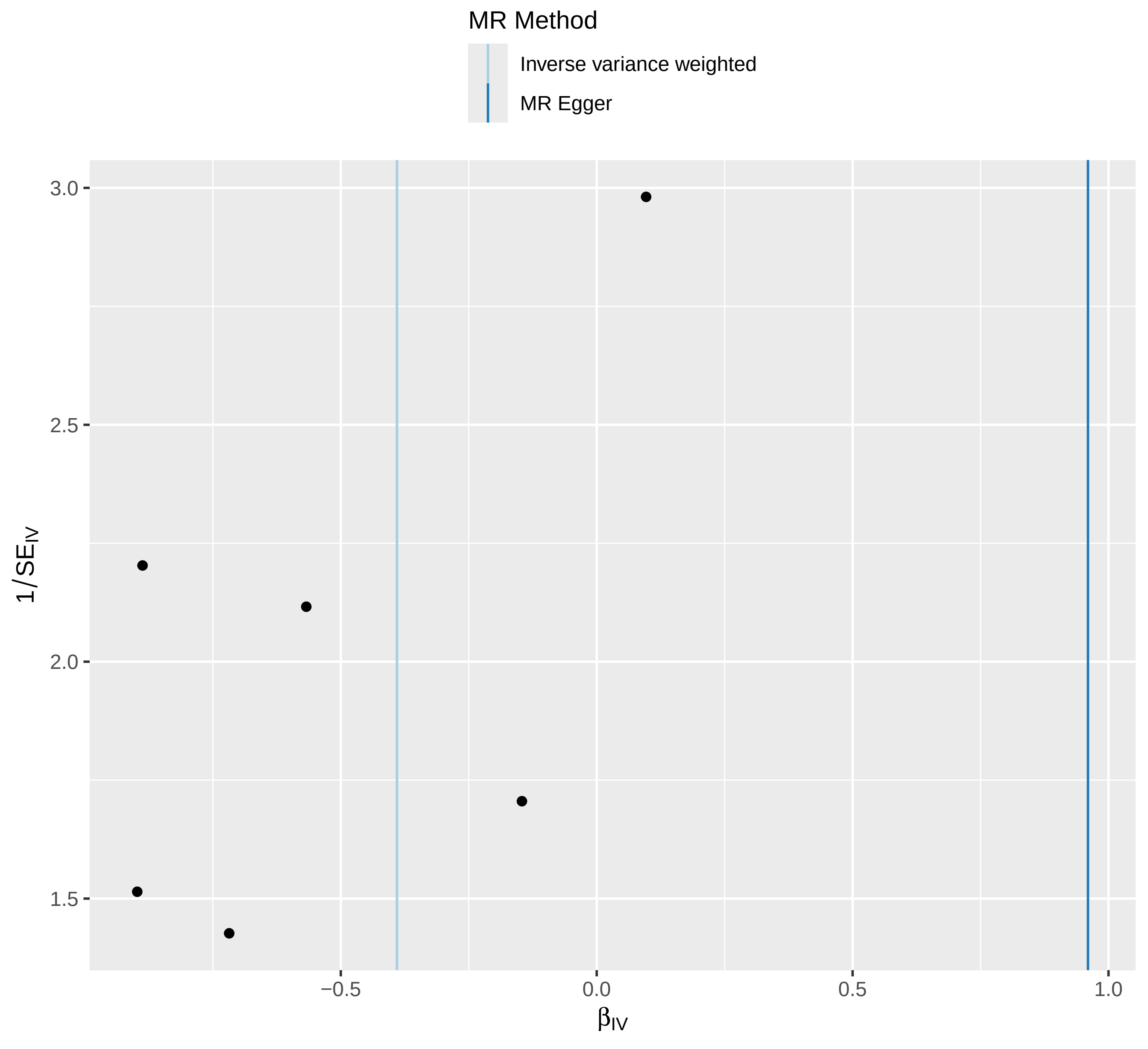
MR Method
Inverse variance weighted
MR Egger
3.0
2.5
2.0
1.5
1/SE_IV
−0.5
0.0
0.5
1.0
β_IV

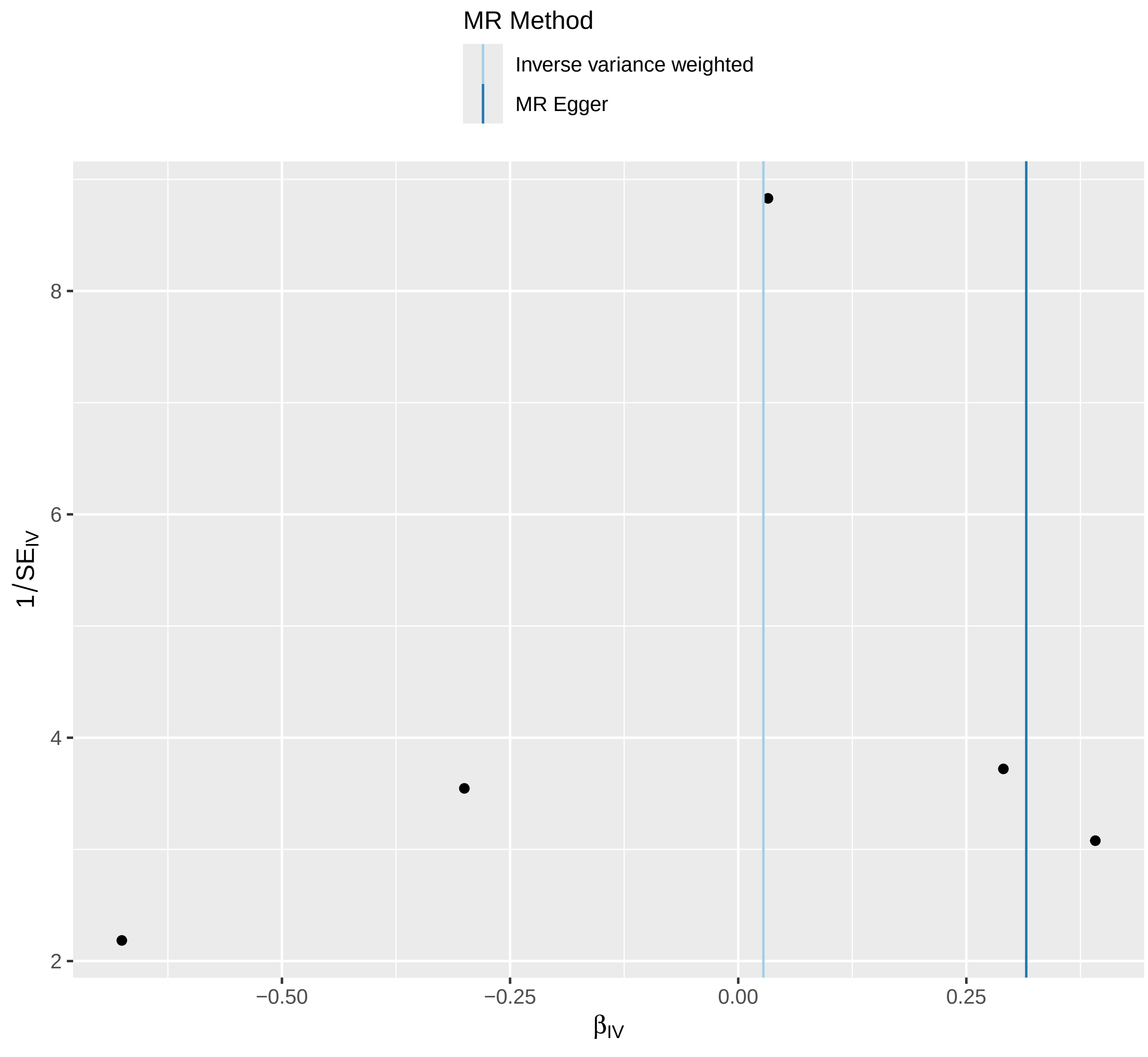
MR Method
Inverse variance weighted
MR Egger
8
6
4
2
1/SE$_{IV}$
−0.50
−0.25
0.00
0.25
β$_{IV}$

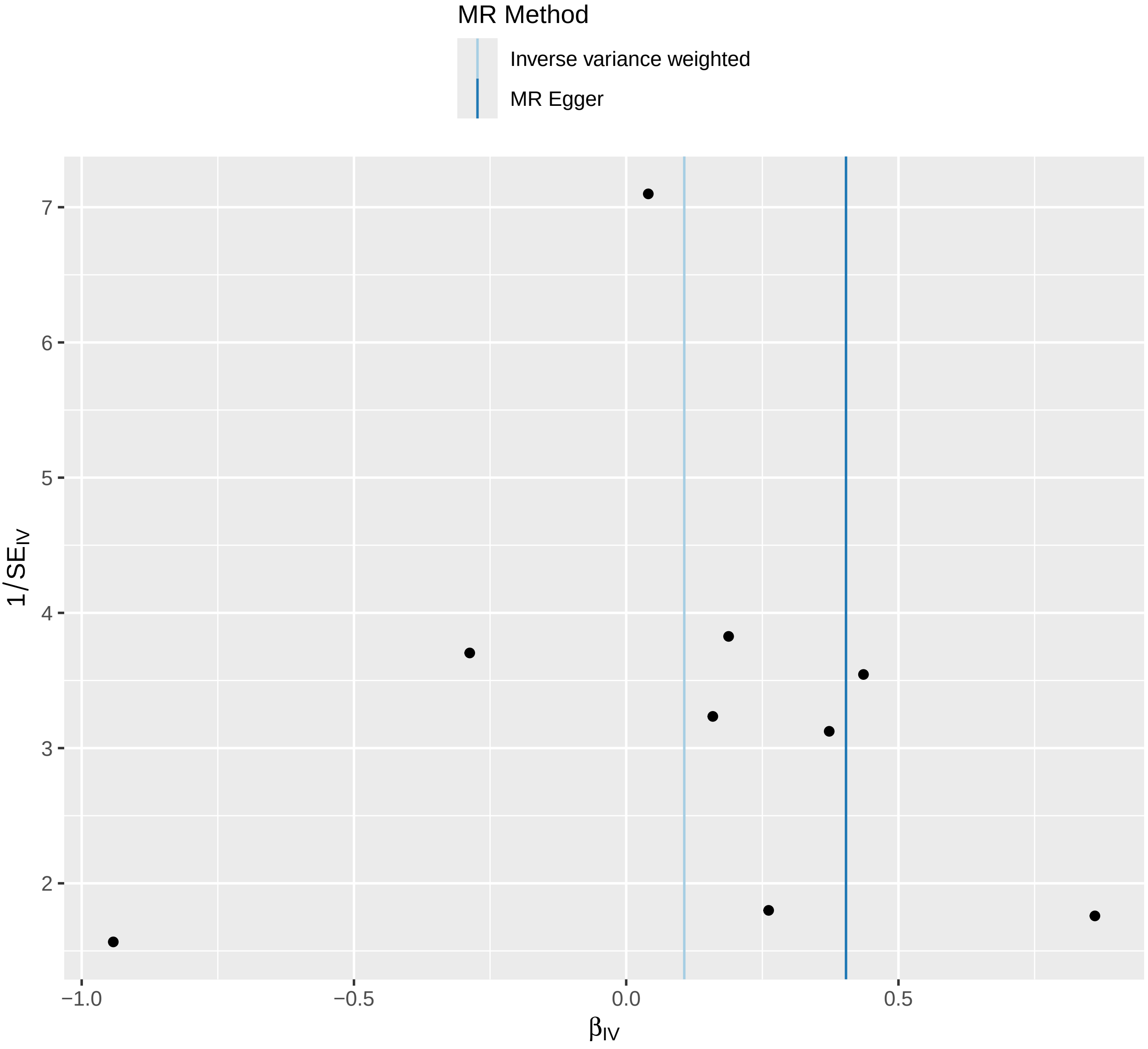

MR Method
Inverse variance weighted
MR Egger
1/SE_IV
7
6
5
4
3
2
−1.0
−0.5
0.0
0.5
β_IV

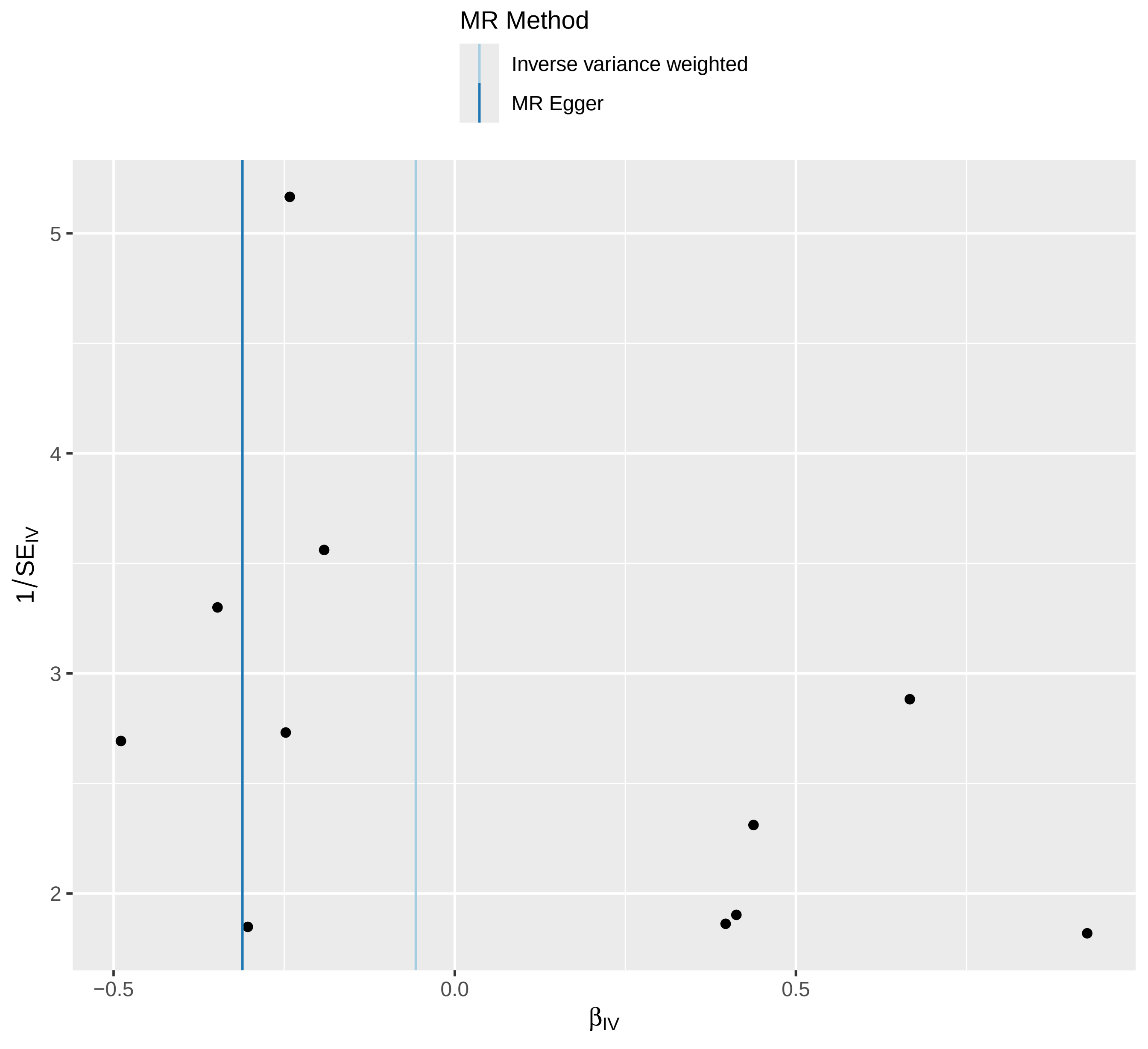
MR Method
Inverse variance weighted
MR Egger
5
4
3
2
−0.5
0.0
0.5
1/SE_IV
β_IV

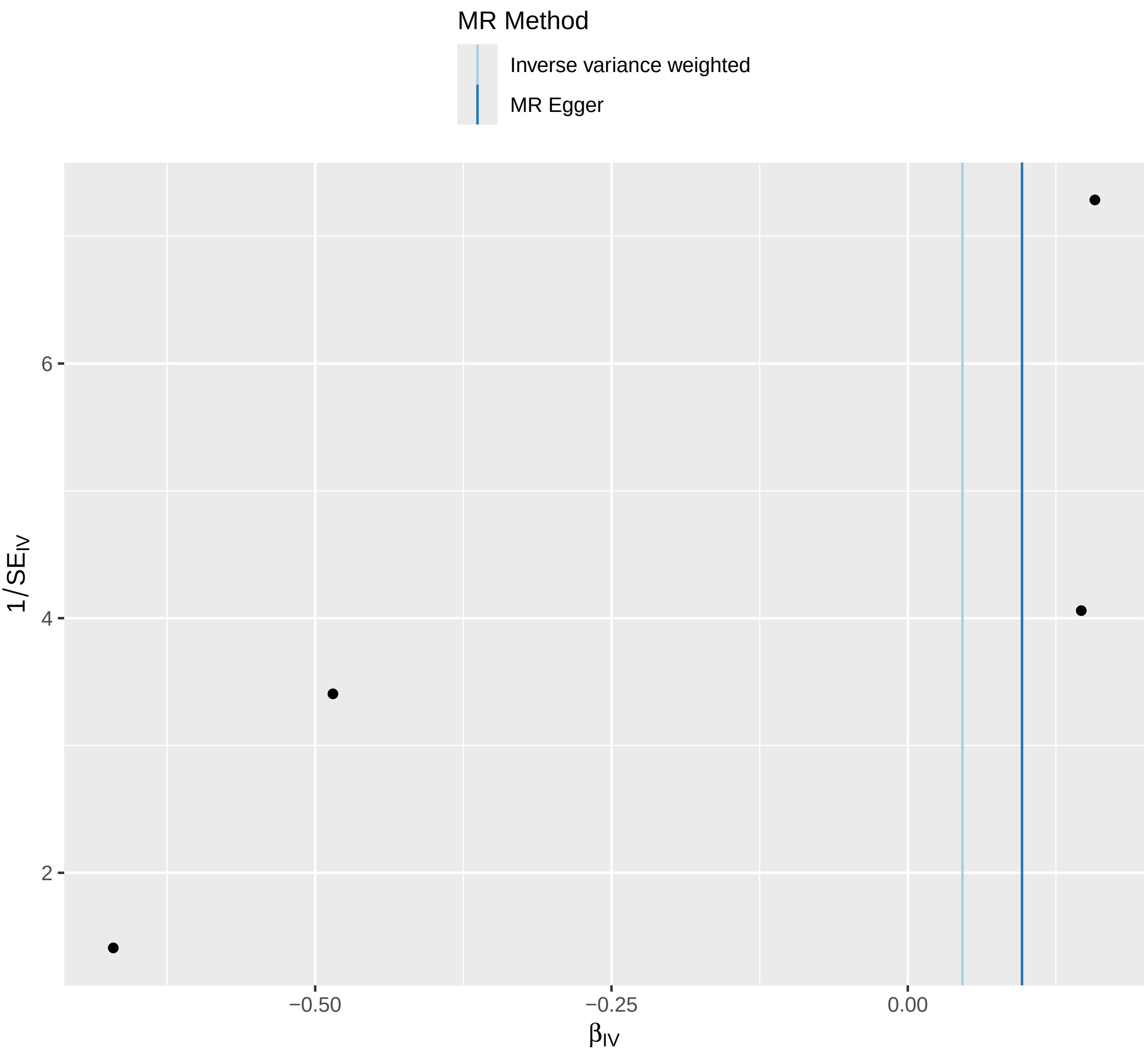

MR Method
Inverse variance weighted
MR Egger
6
4
2
1/SE$_{IV}$
−0.50
−0.25
0.00
β$_{IV}$

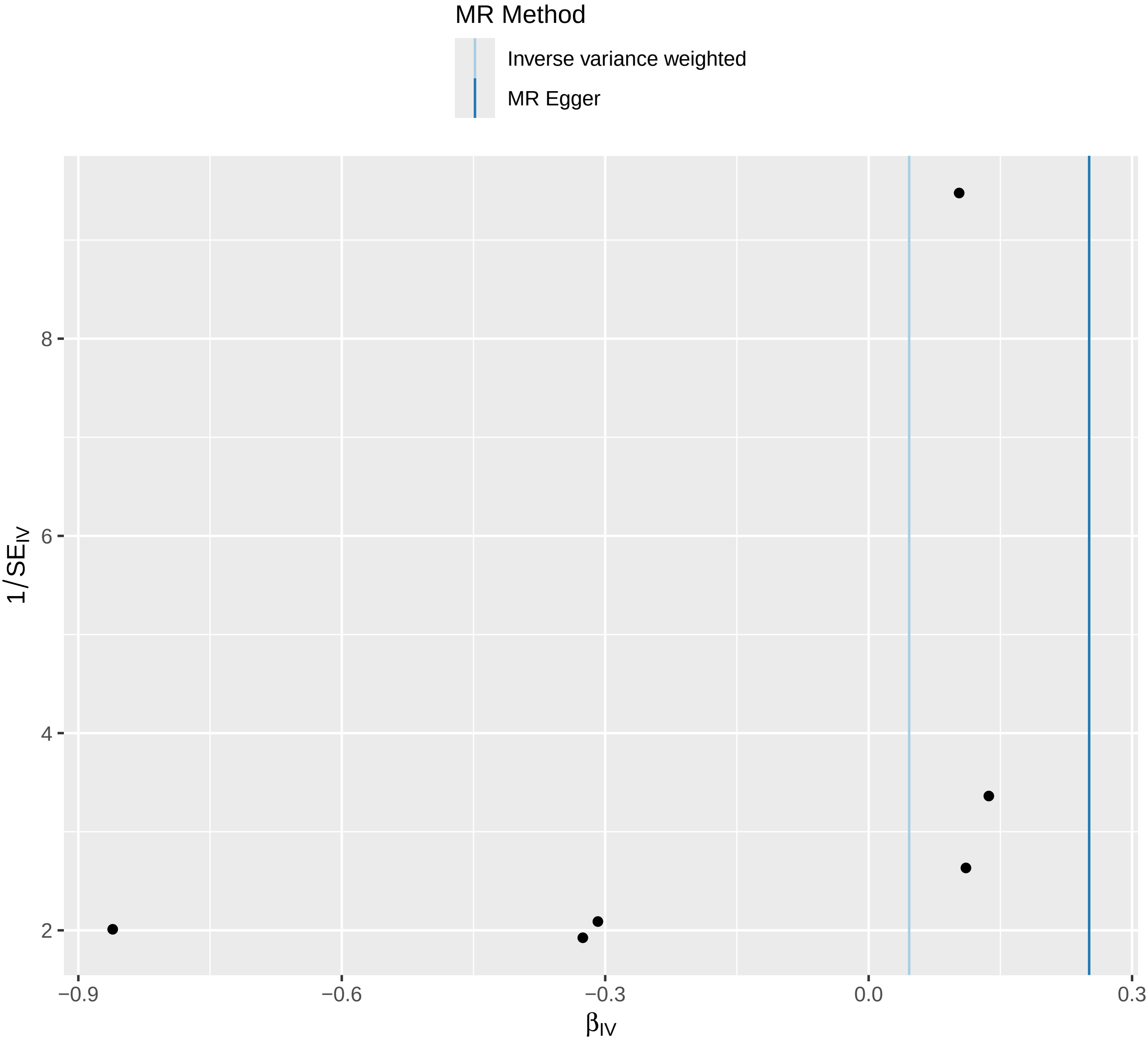
MR Method
Inverse variance weighted
MR Egger
1/SE$_{IV}$
β$_{IV}$
2
4
6
8
−0.9
−0.6
−0.3
0.0
0.3

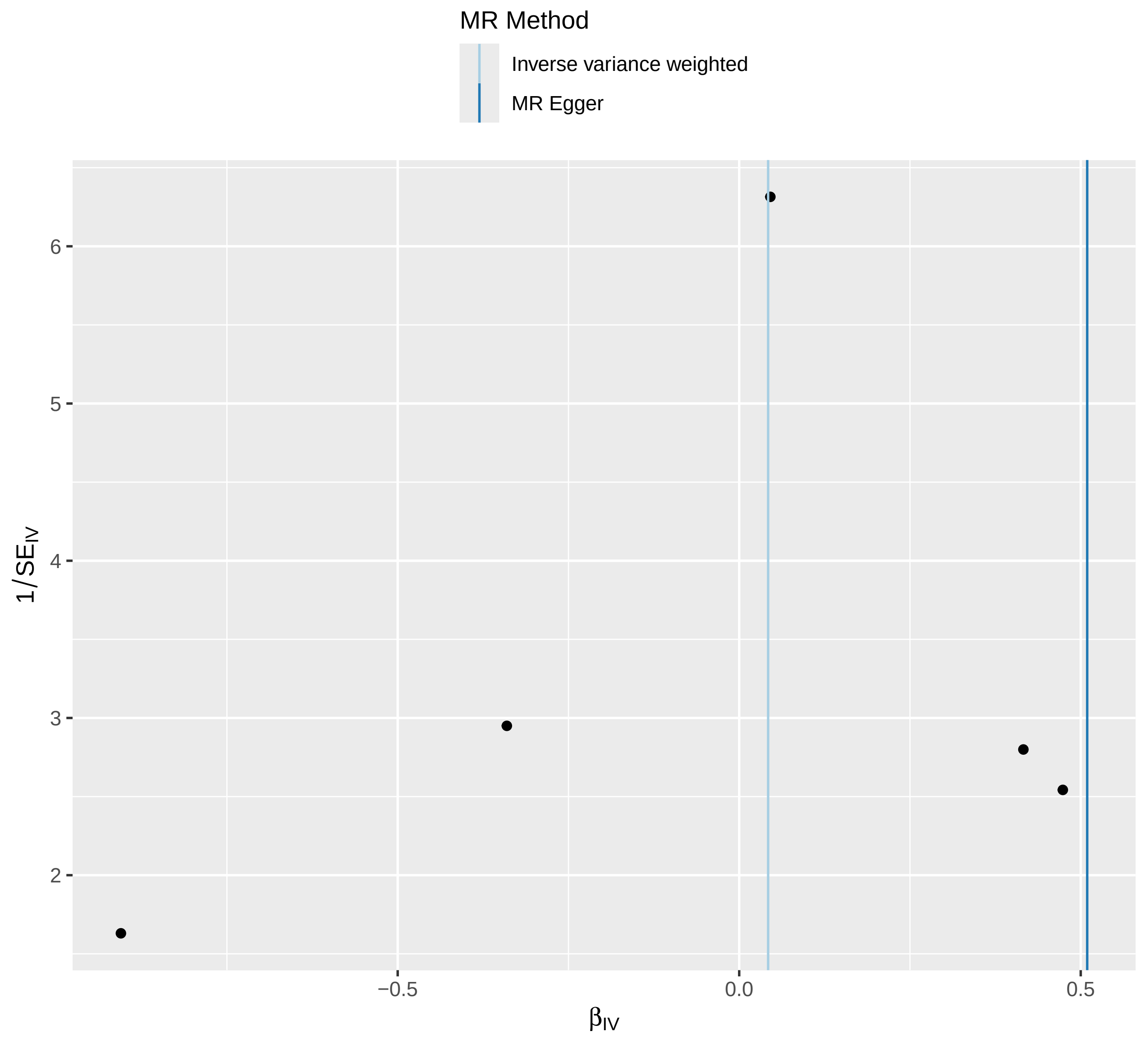
MR Method
Inverse variance weighted
MR Egger
1/SE$_{IV}$
2
3
4
5
6
β$_{IV}$
−0.5
0.0
0.5

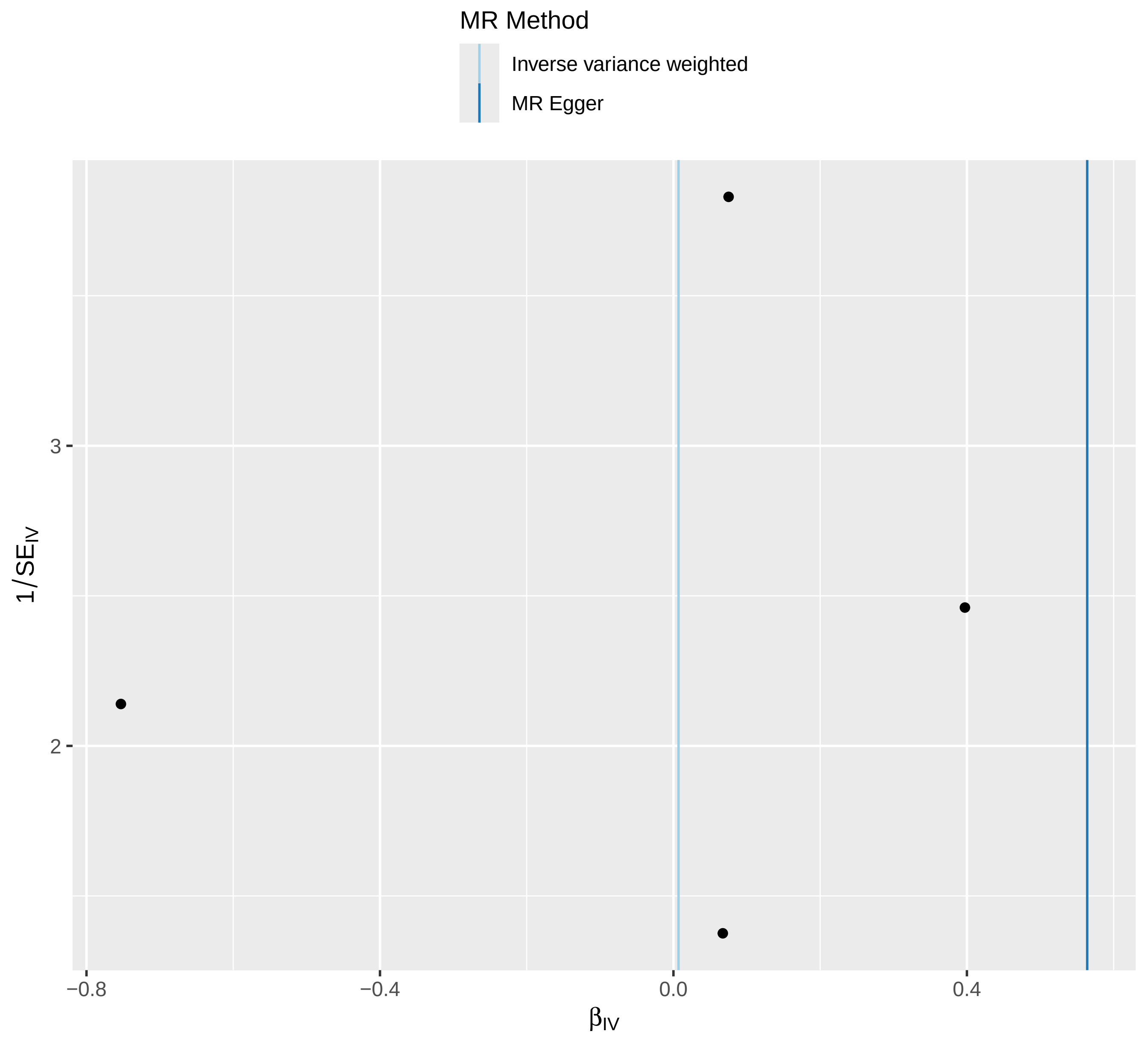
MR Method
Inverse variance weighted
MR Egger
1/SE$_{IV}$
3
2
−0.8
−0.4
0.0
0.4
β$_{IV}$

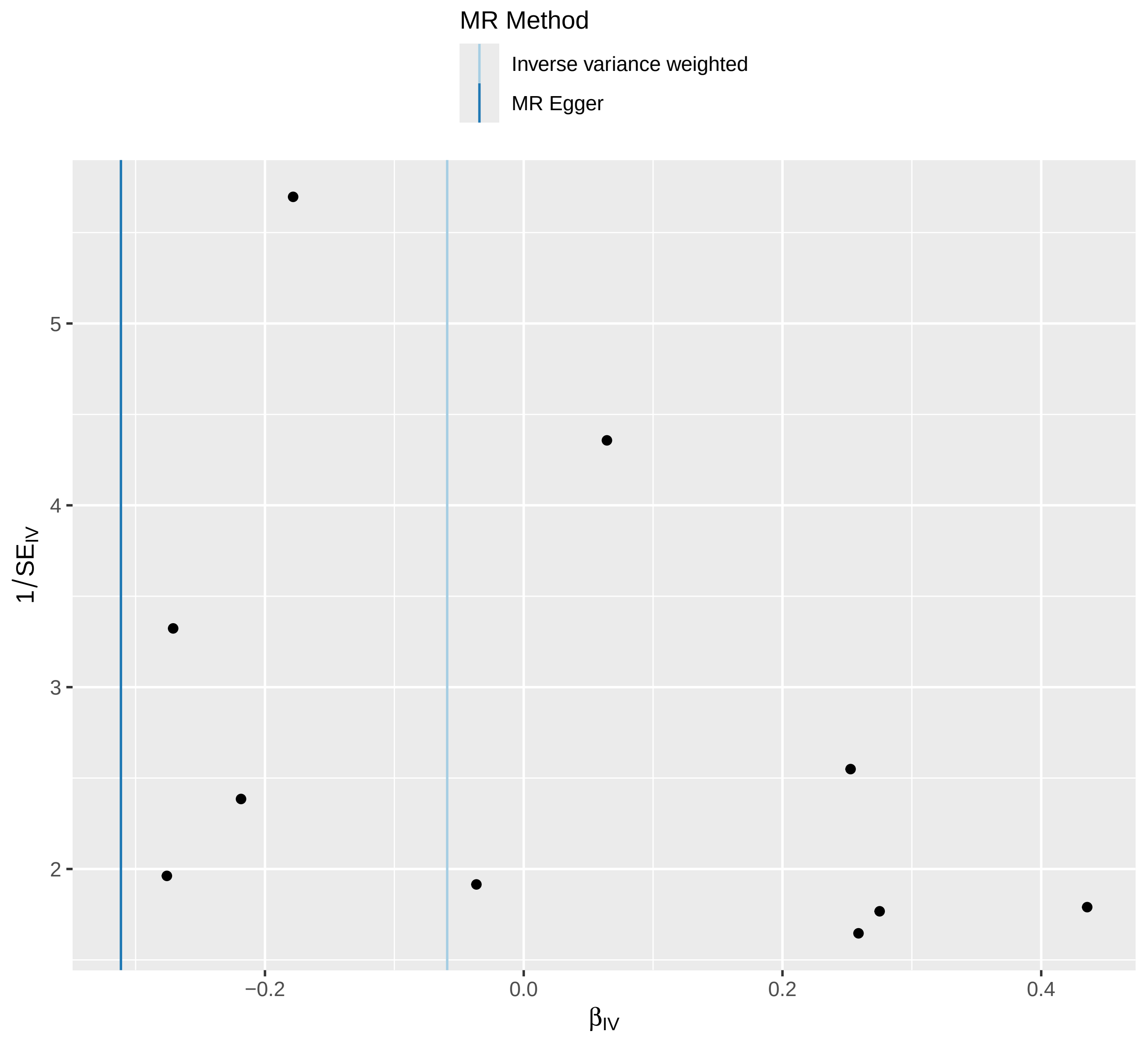
MR Method
Inverse variance weighted
MR Egger
1/SE_IV
β_IV
5
4
3
2
−0.2
0.0
0.2
0.4

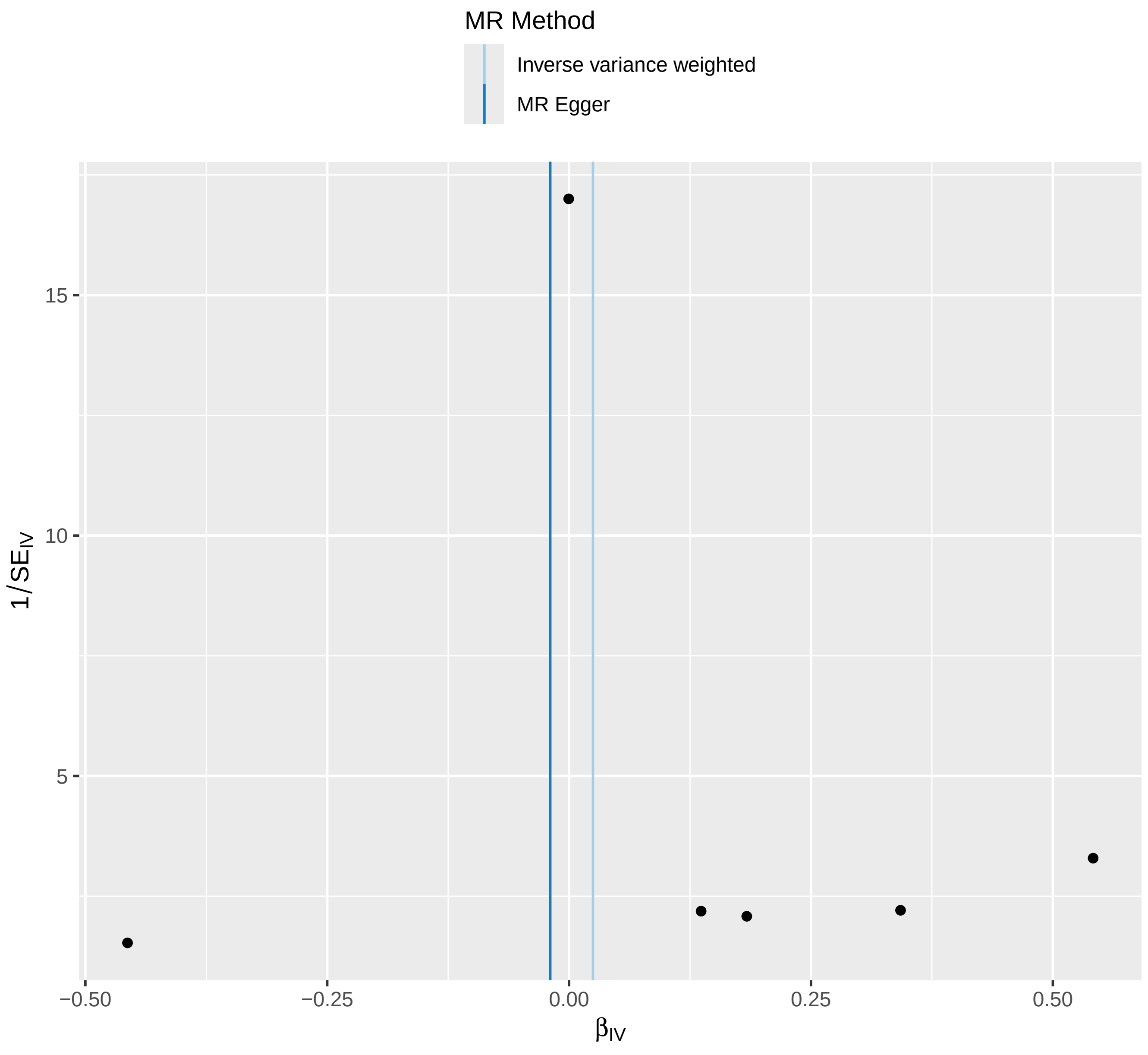
MR Method
Inverse variance weighted
MR Egger
15
10
5
$1/SE_{IV}$
−0.50
−0.25
0.00
0.25
0.50
β_{IV}

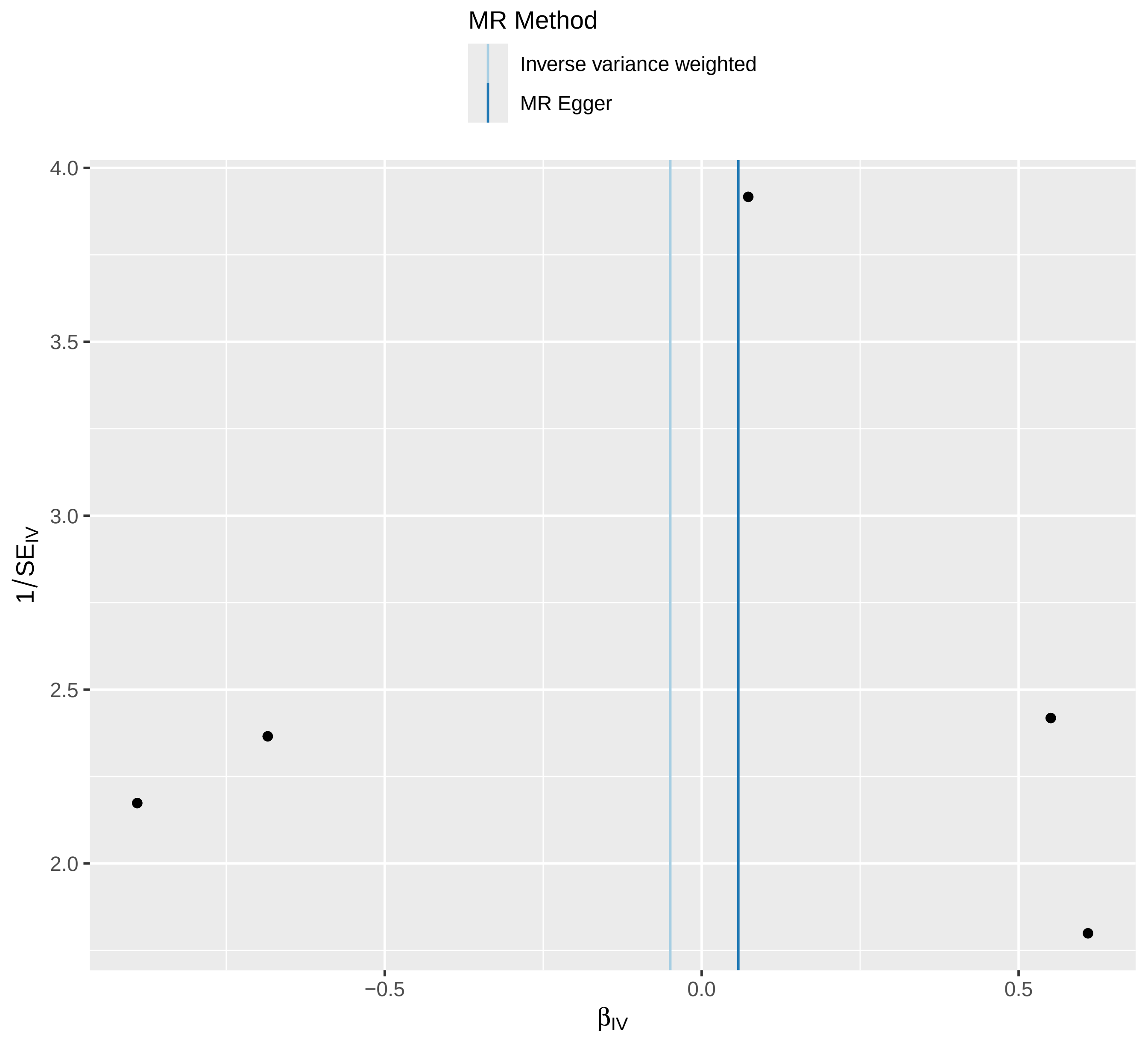
MR Method
Inverse variance weighted
MR Egger
4.0
3.5
3.0
2.5
2.0
$1/SE_{IV}$
−0.5
0.0
0.5
β_{IV}

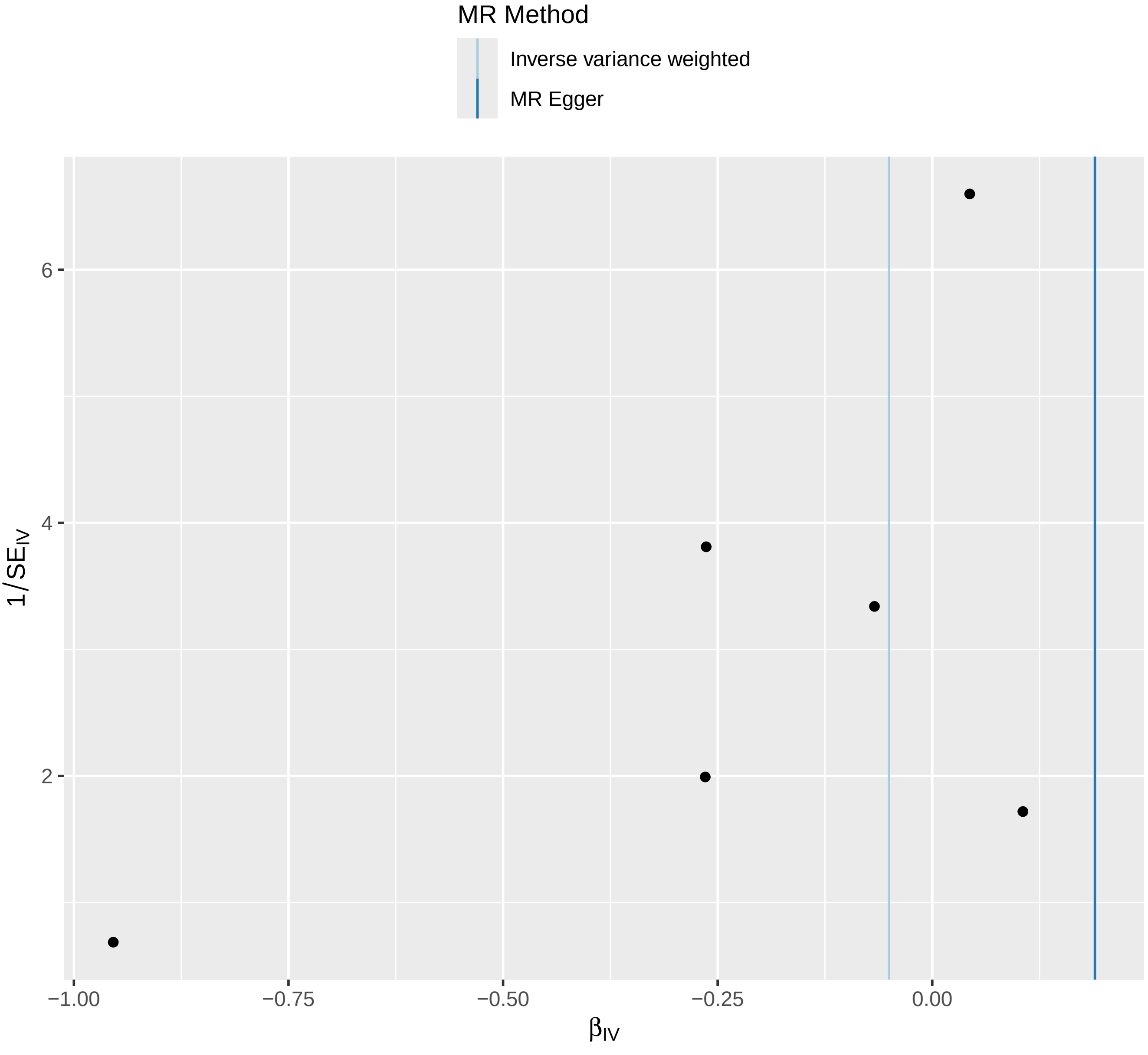

MR Method
Inverse variance weighted
MR Egger
6
4
2
$1/SE_{IV}$
−1.00
−0.75
−0.50
−0.25
0.00
β_{IV}

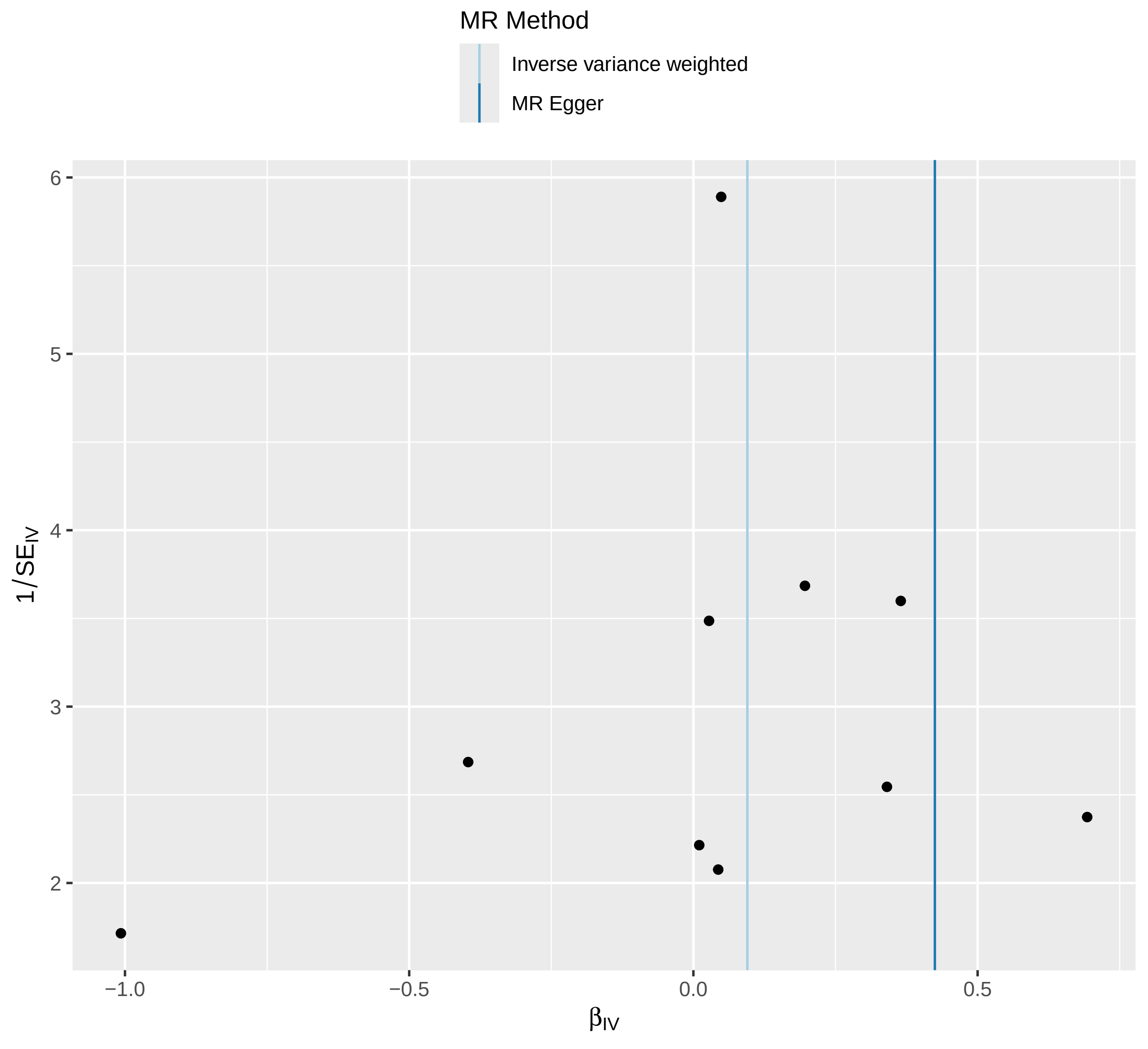

MR Method
Inverse variance weighted
MR Egger
6
5
4
3
2
1/SE_IV
−1.0
−0.5
0.0
0.5
β_IV

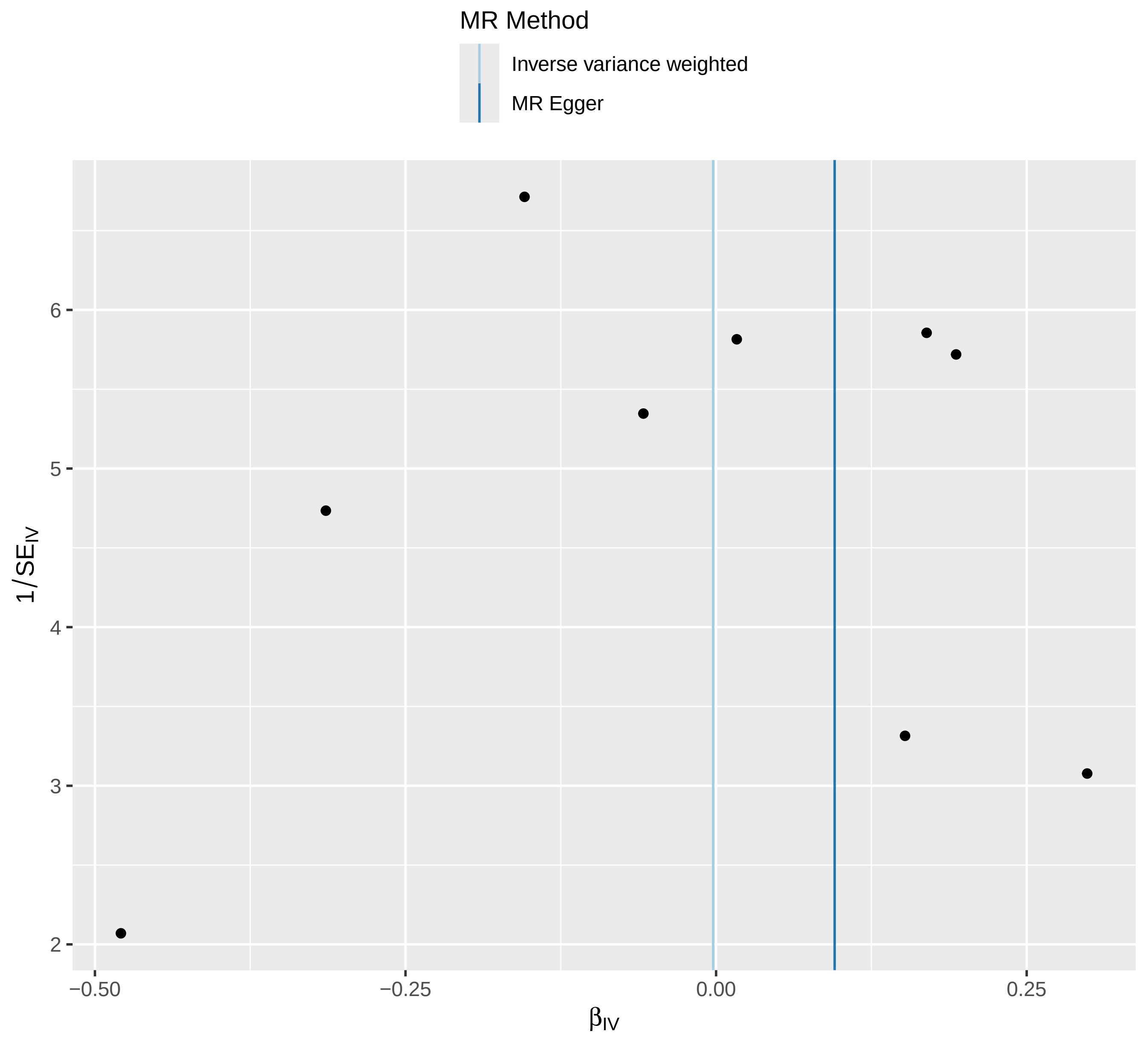
MR Method
Inverse variance weighted
MR Egger
1/SE_IV
β_IV
6
5
4
3
2
−0.50
−0.25
0.00
0.25

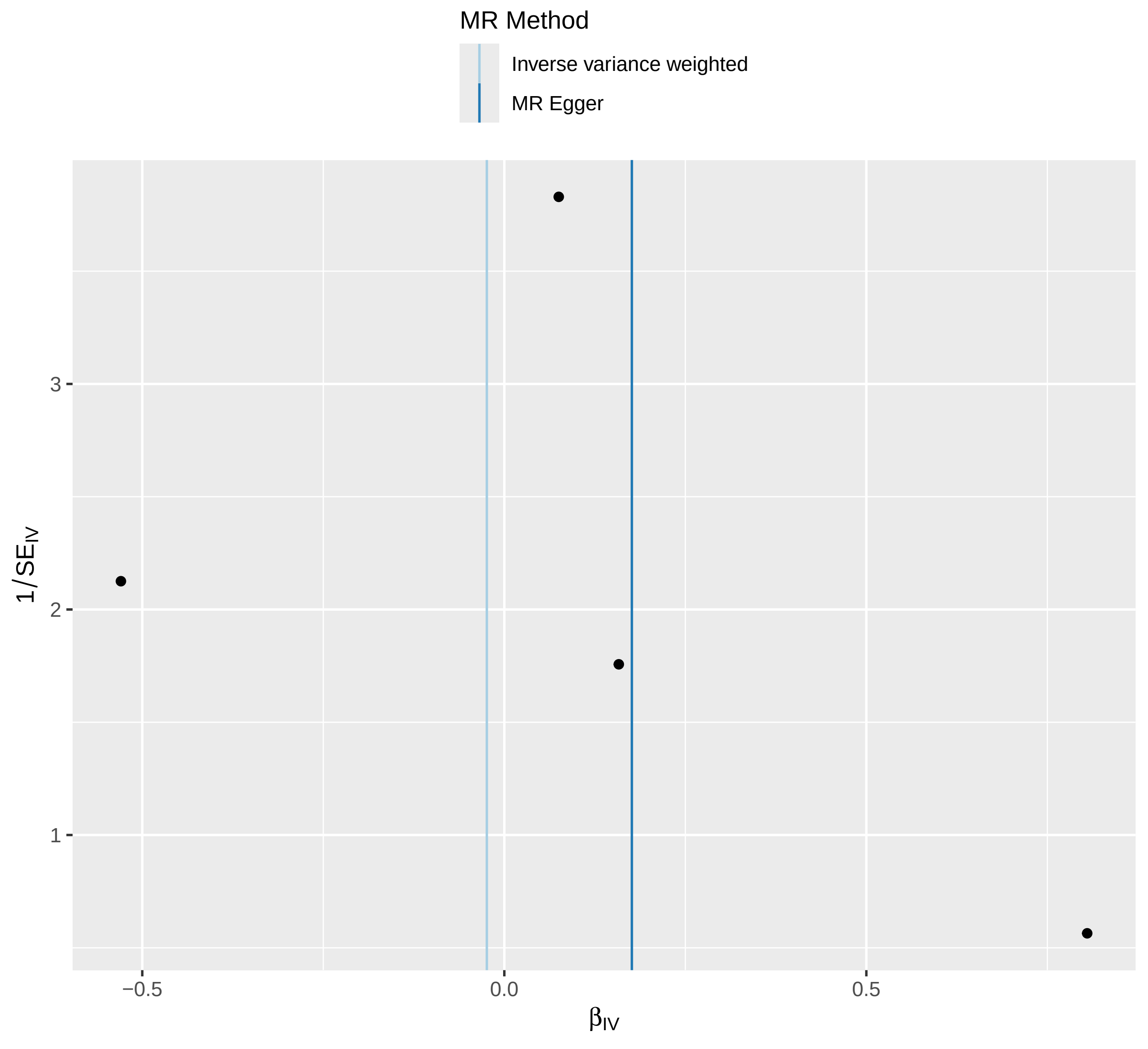
MR Method
Inverse variance weighted
MR Egger
1/SE_IV
3
2
1
−0.5
0.0
0.5
β_IV

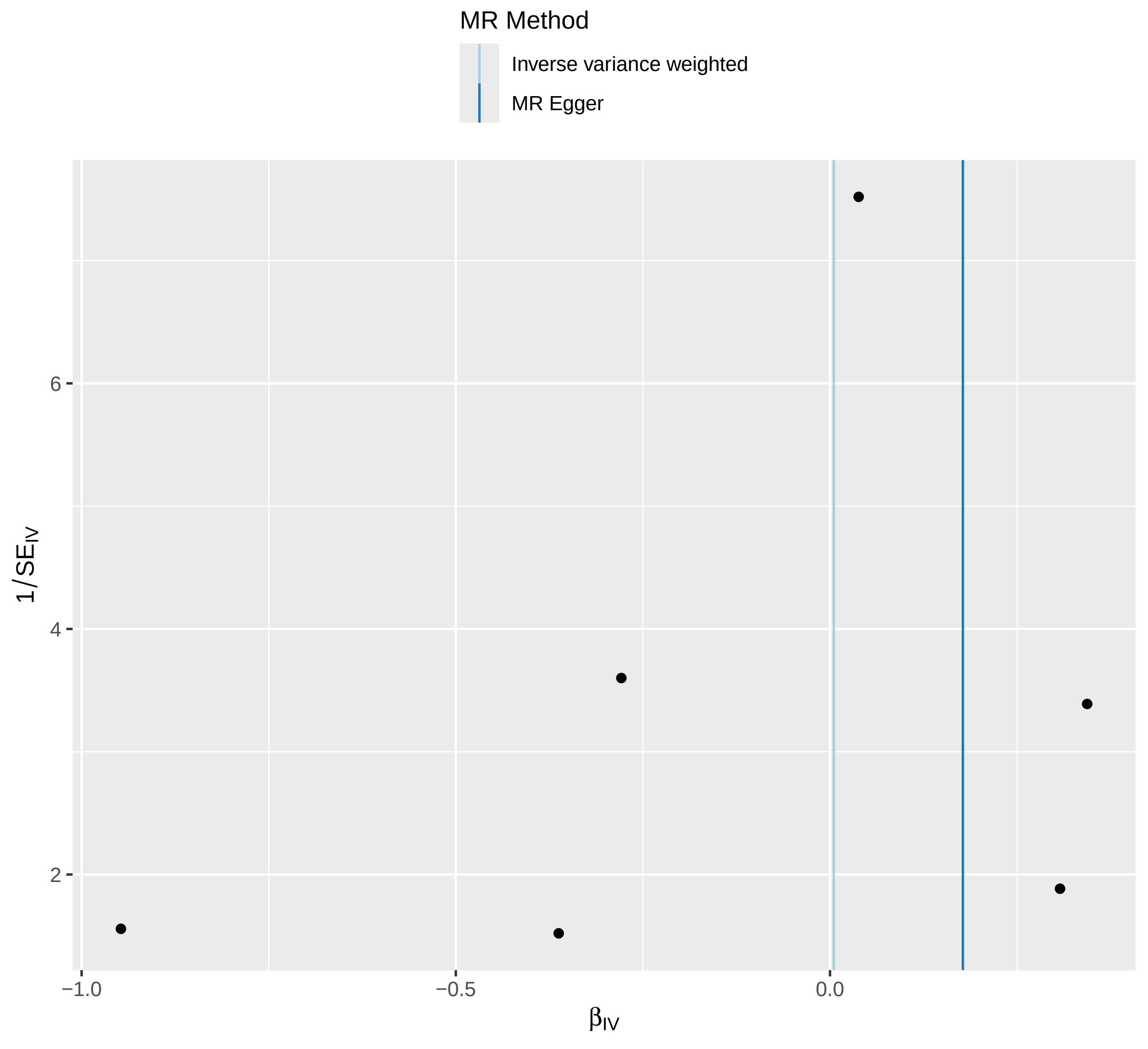
MR Method
Inverse variance weighted
MR Egger
1/SE_IV
6
4
2
−1.0
−0.5
0.0
β_IV

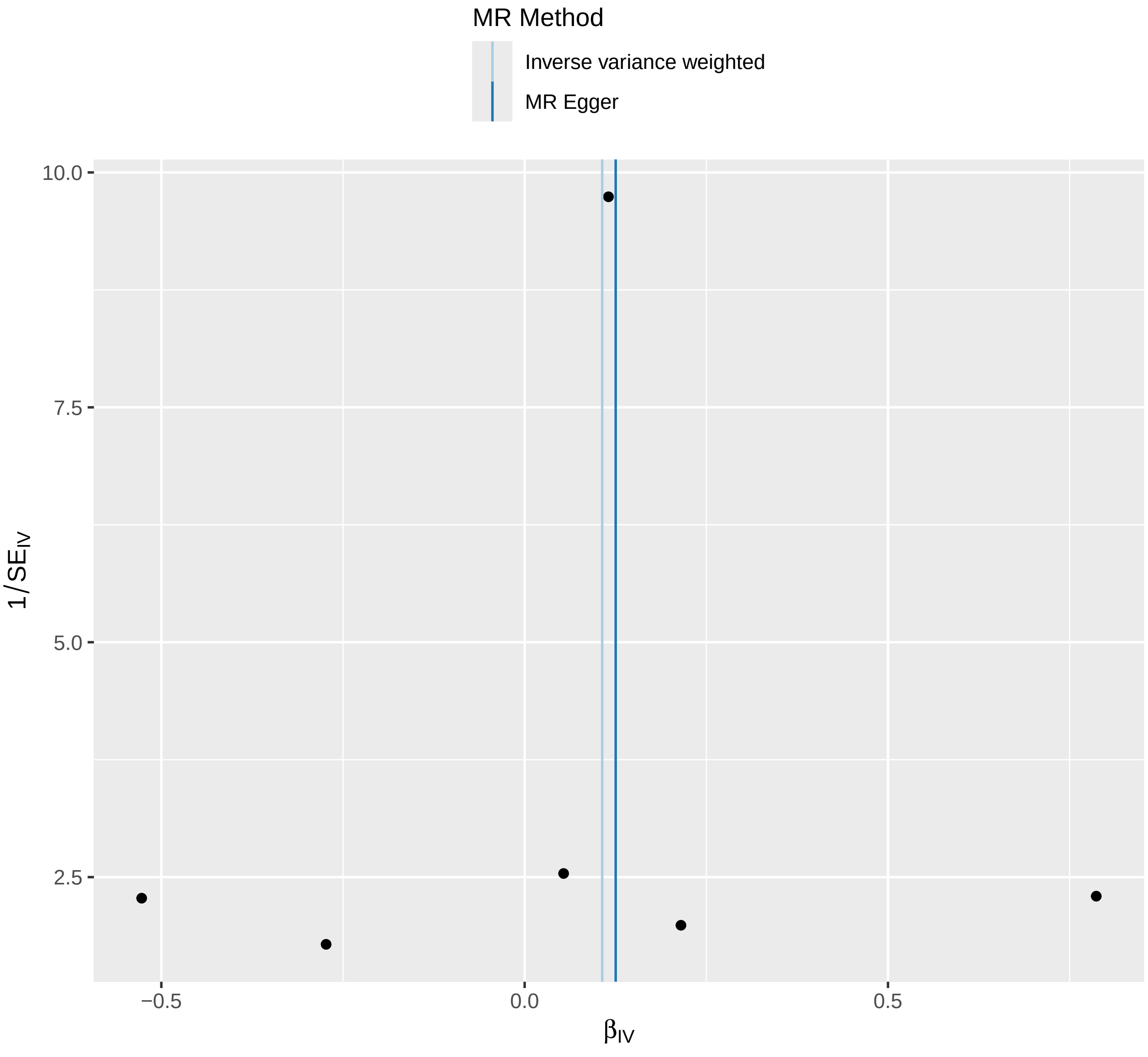

MR Method
Inverse variance weighted
MR Egger
10.0
7.5
5.0
2.5
1/SE$_{IV}$
−0.5
0.0
0.5
β$_{IV}$

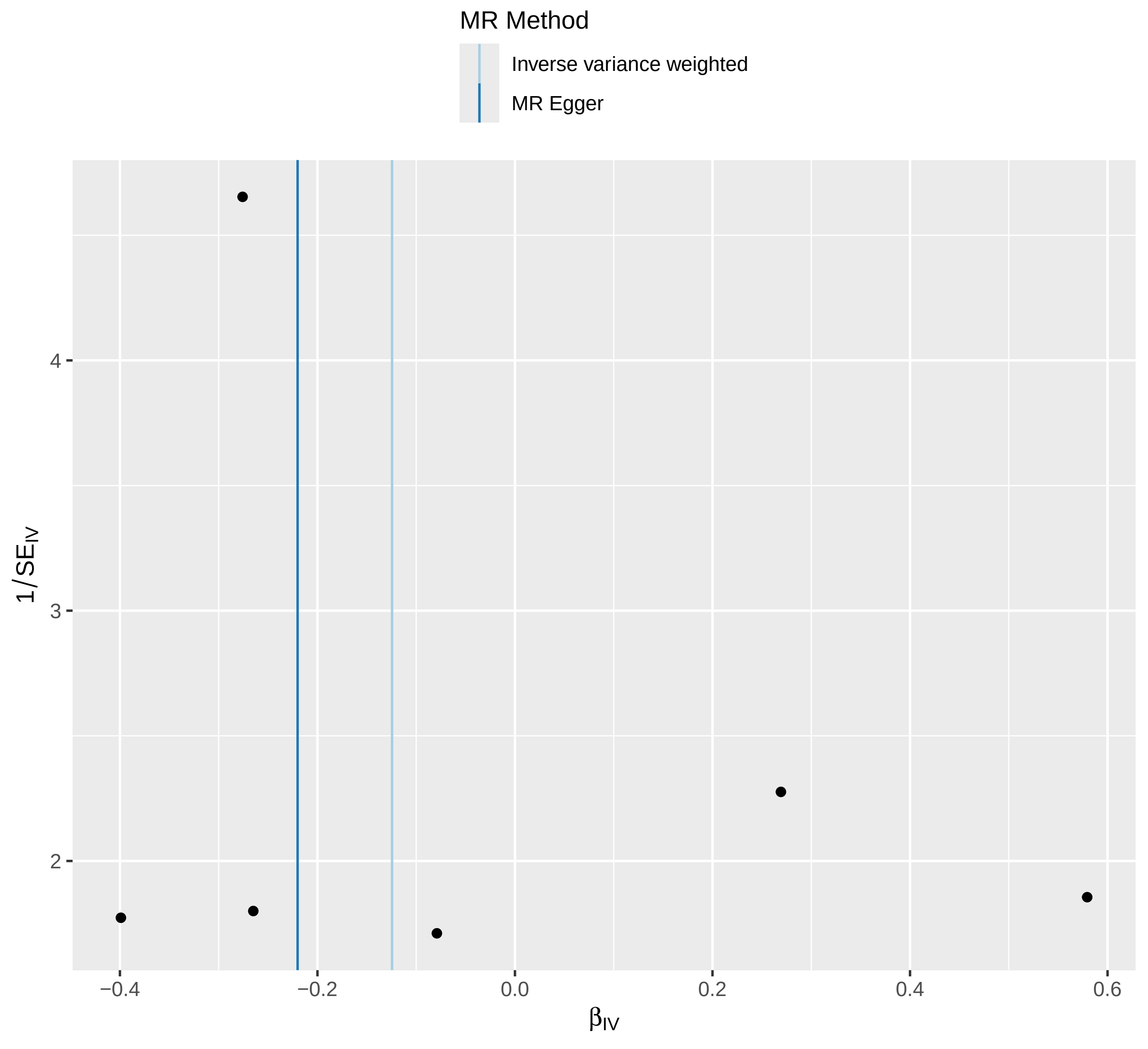
MR Method
Inverse variance weighted
MR Egger
1/SE_IV
4
3
2
−0.4
−0.2
0.0
0.2
0.4
0.6
β_IV

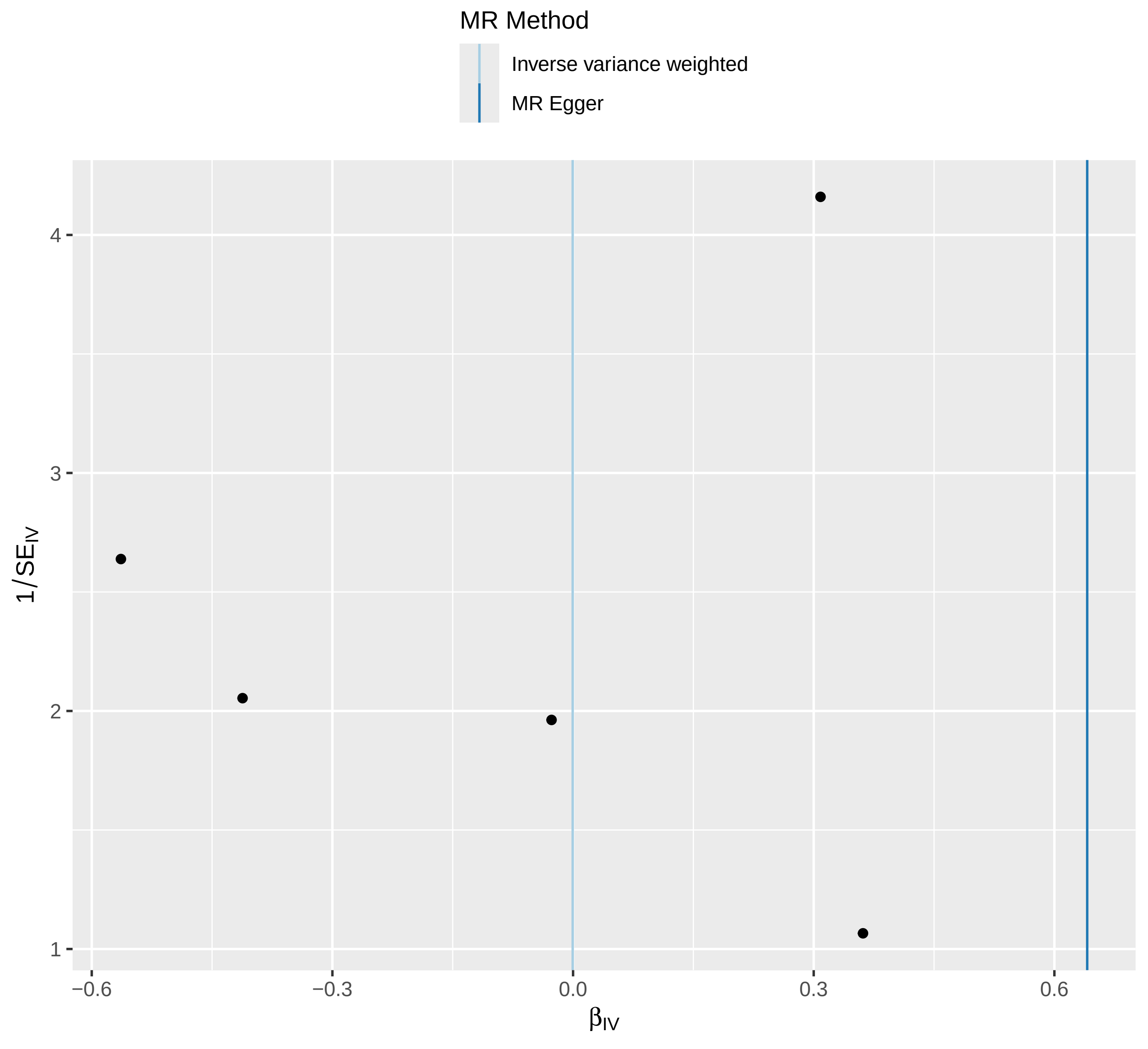
MR Method
Inverse variance weighted
MR Egger
4
3
2
1
$1/SE_{IV}$
−0.6
−0.3
0.0
0.3
0.6
β_{IV}

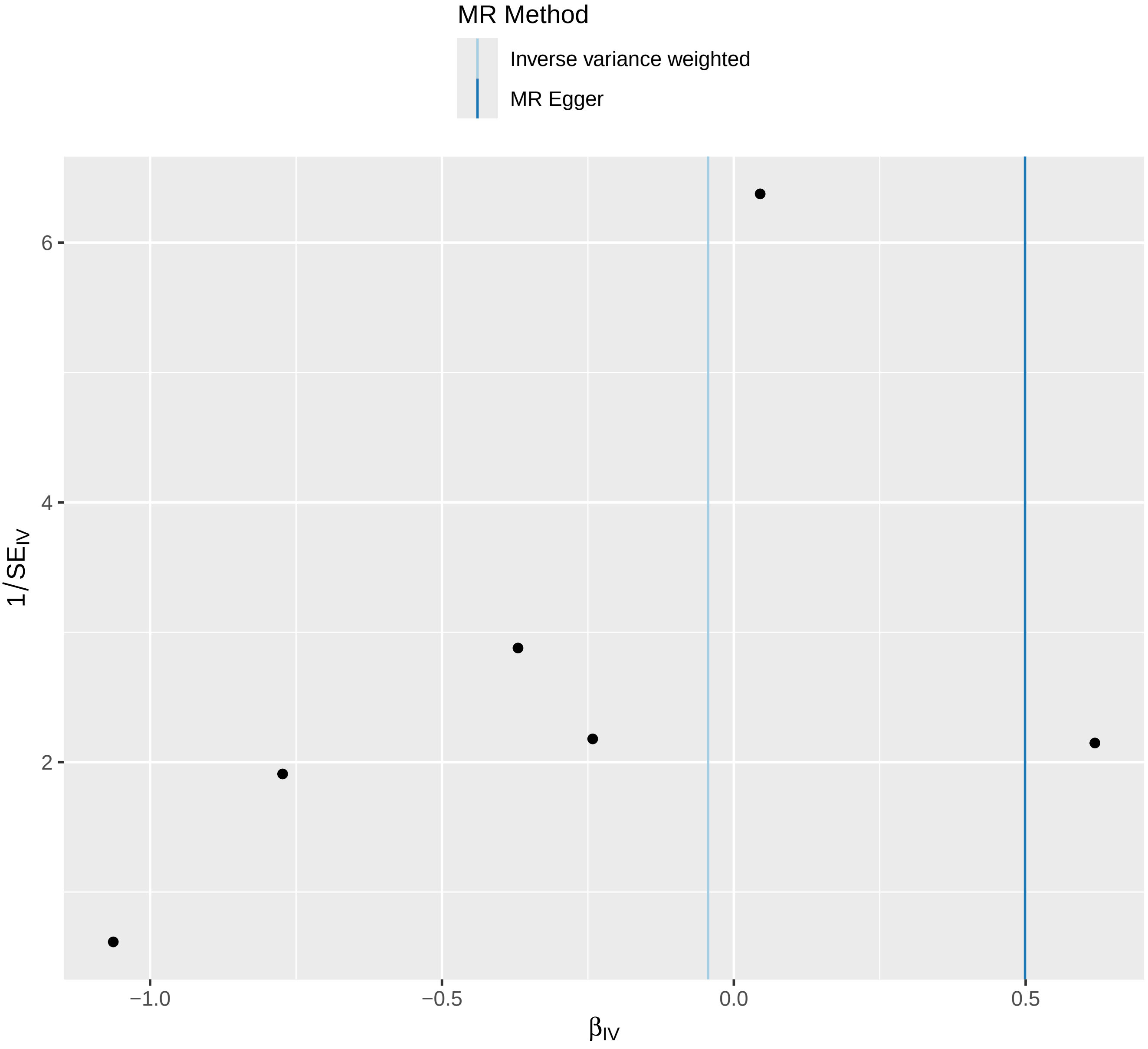

MR Method
Inverse variance weighted
MR Egger
6
4
2
1/SE$_{IV}$
−1.0
−0.5
0.0
0.5
β$_{IV}$

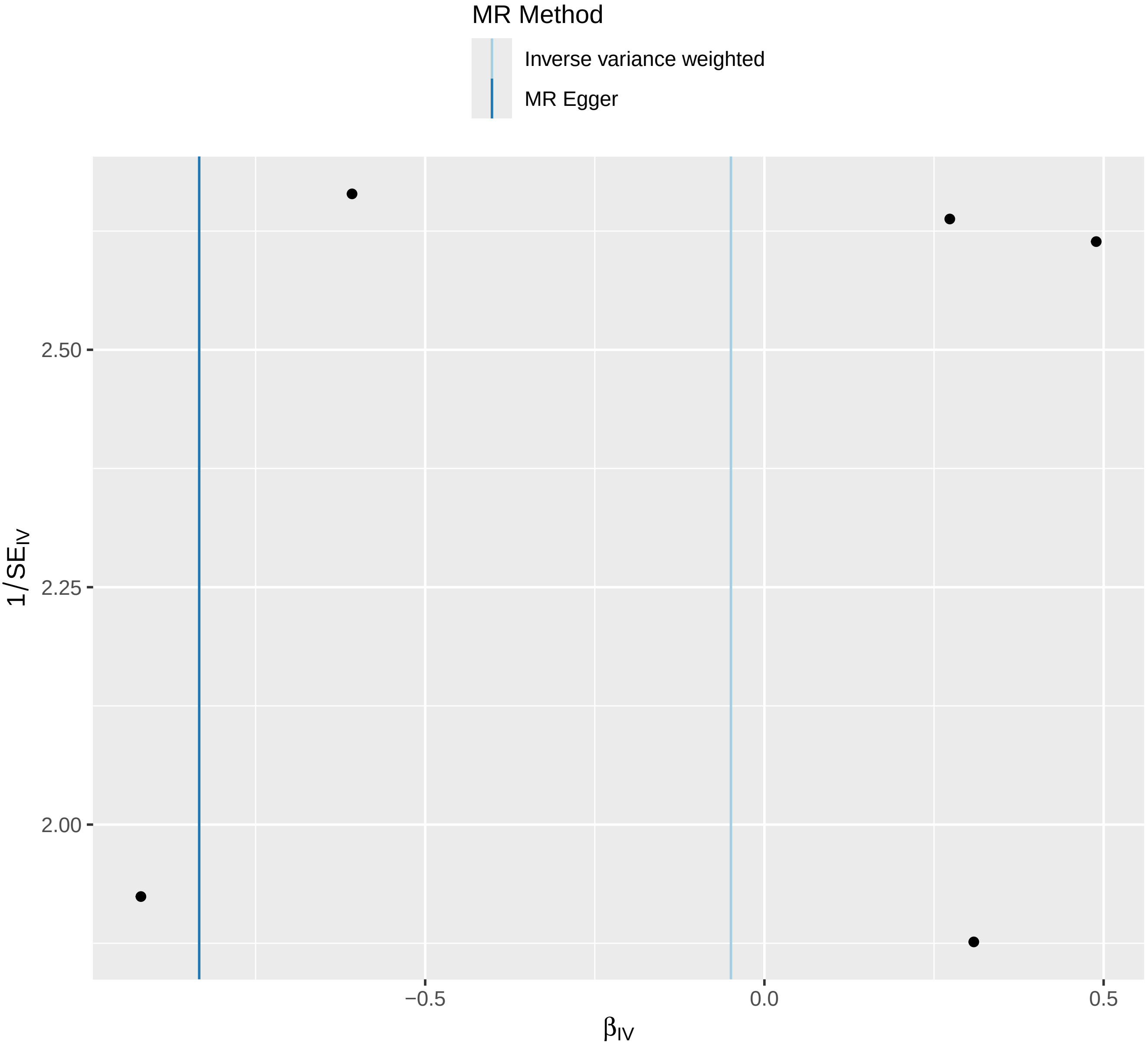
MR Method
Inverse variance weighted
MR Egger
2.50
2.25
2.00
1/SE_IV
−0.5
0.0
0.5
β_IV

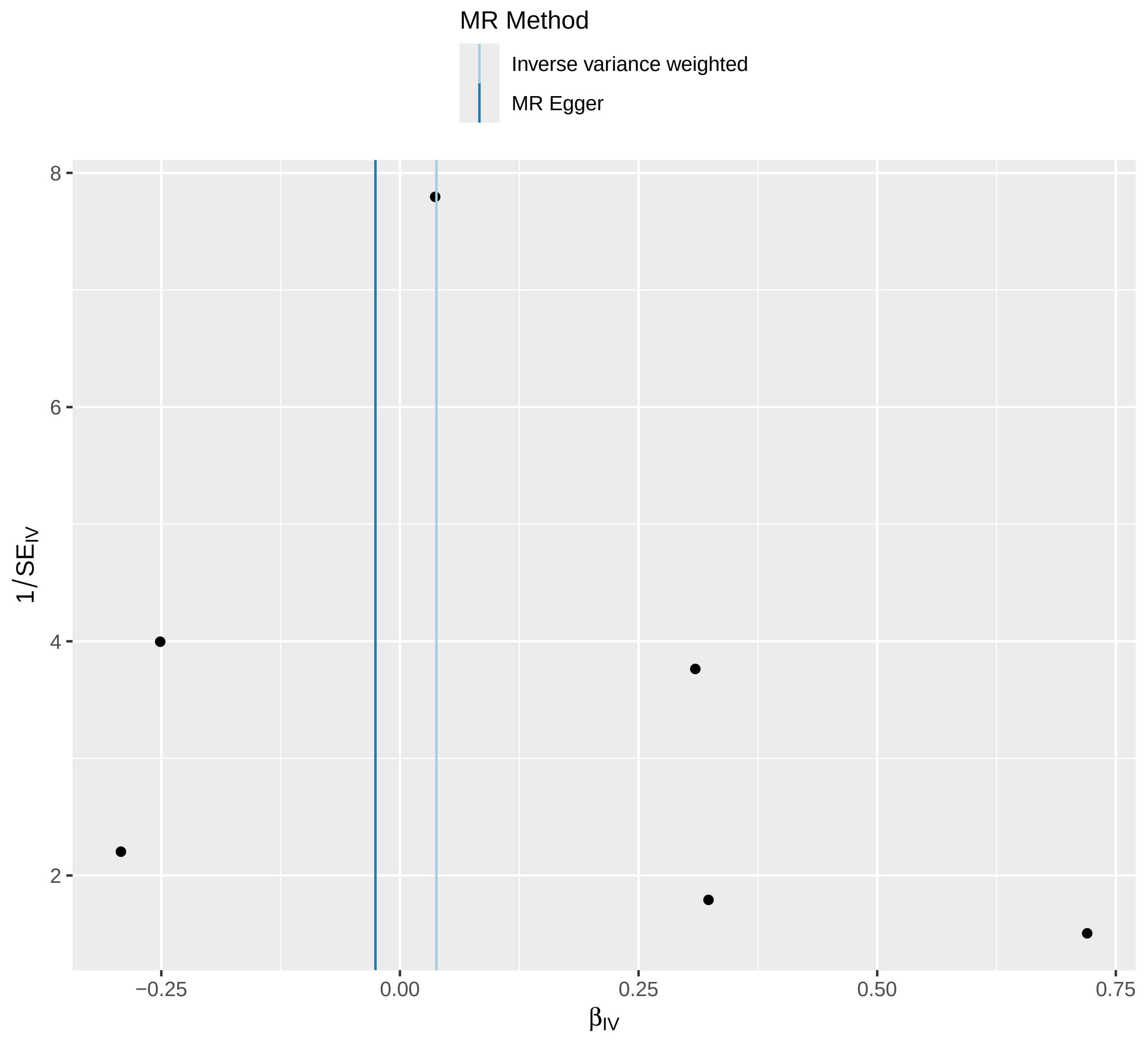

MR Method
Inverse variance weighted
MR Egger
8
6
4
2
1/SE$_{IV}$
−0.25
0.00
0.25
0.50
0.75
β$_{IV}$

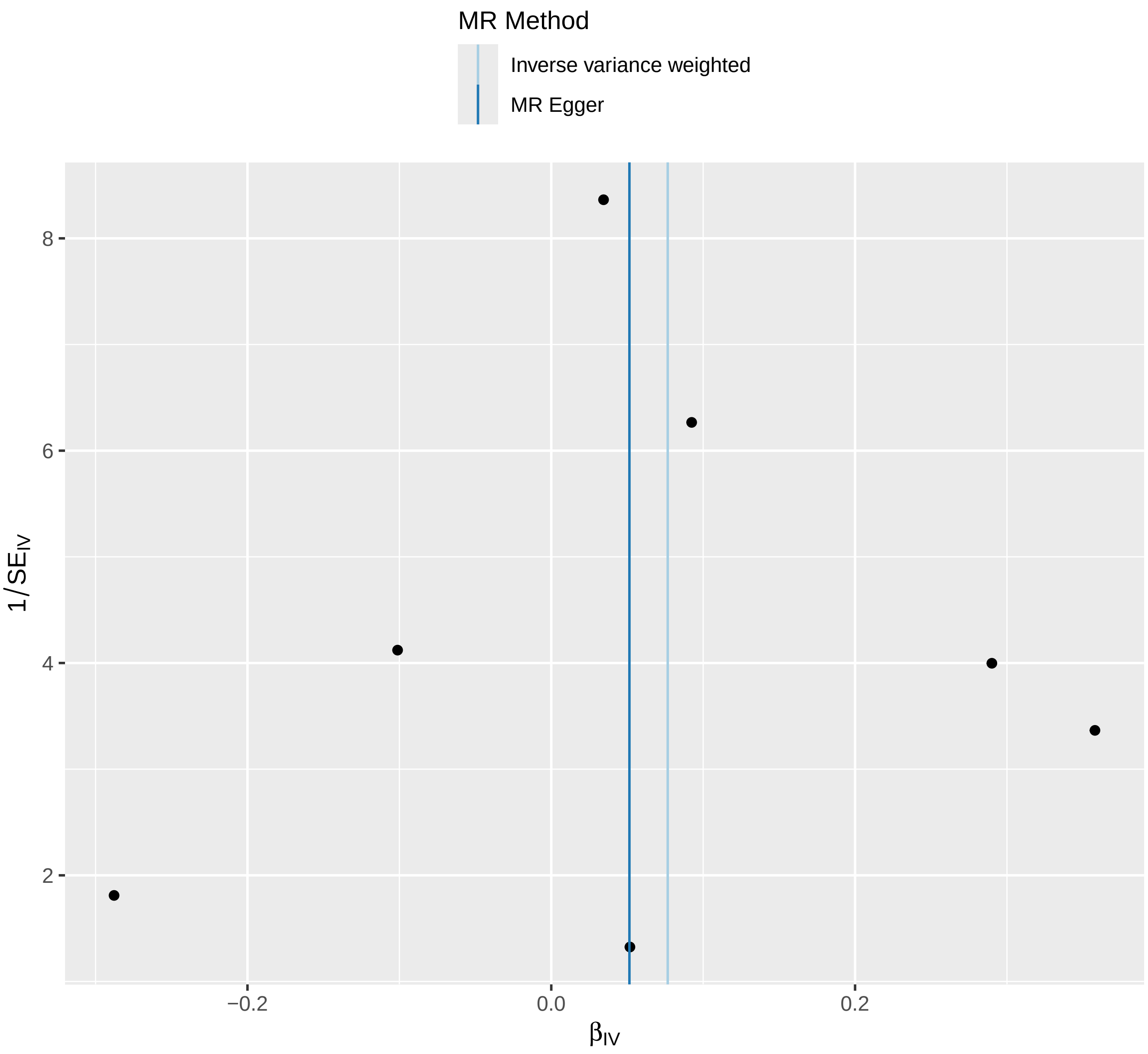

MR Method
Inverse variance weighted
MR Egger
1/SE$_{IV}$
8
6
4
2
−0.2
0.0
0.2
β$_{IV}$

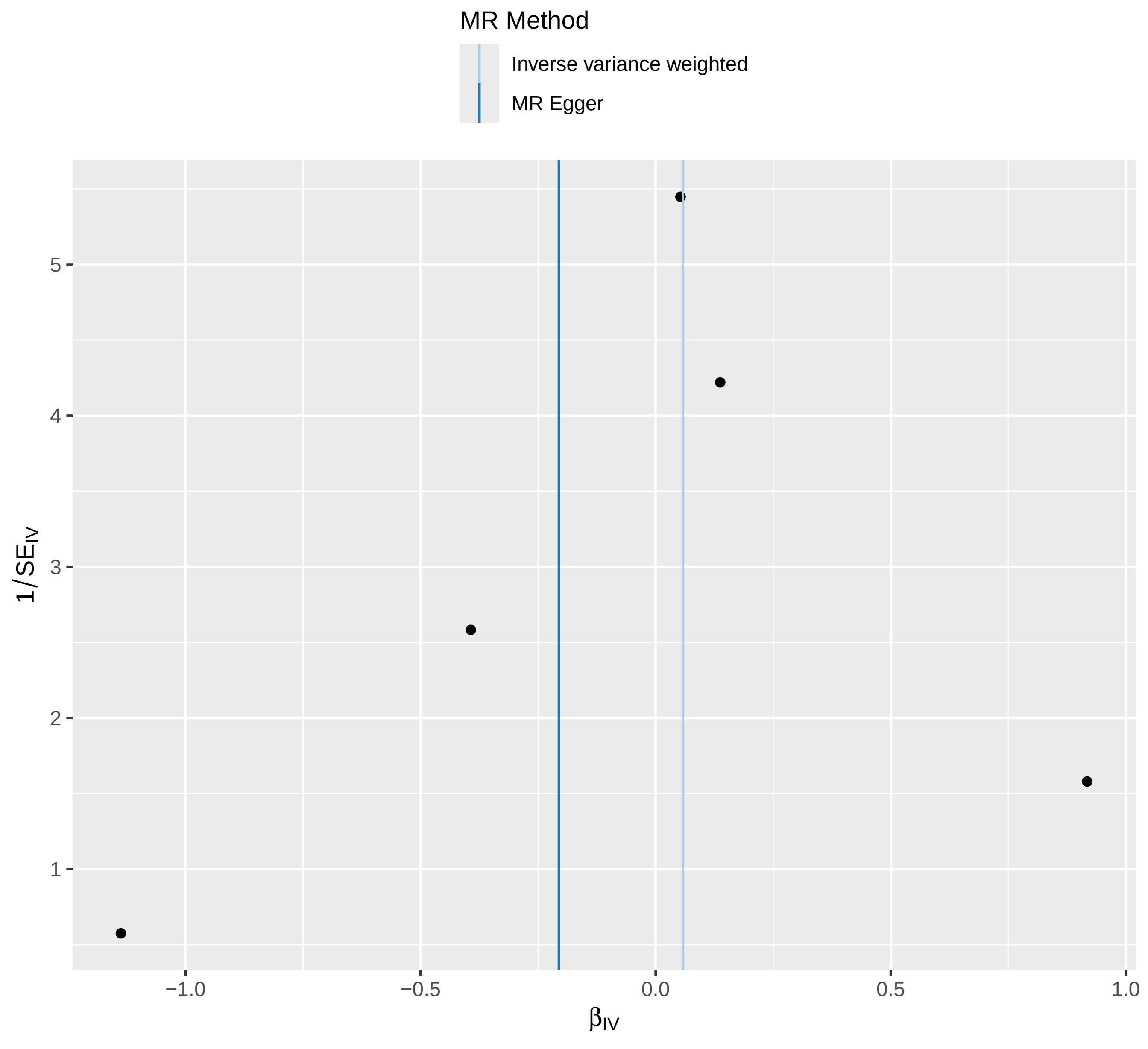

MR Method
Inverse variance weighted
MR Egger
5
4
3
2
1
1/SE_IV
−1.0
−0.5
0.0
0.5
1.0
β_IV

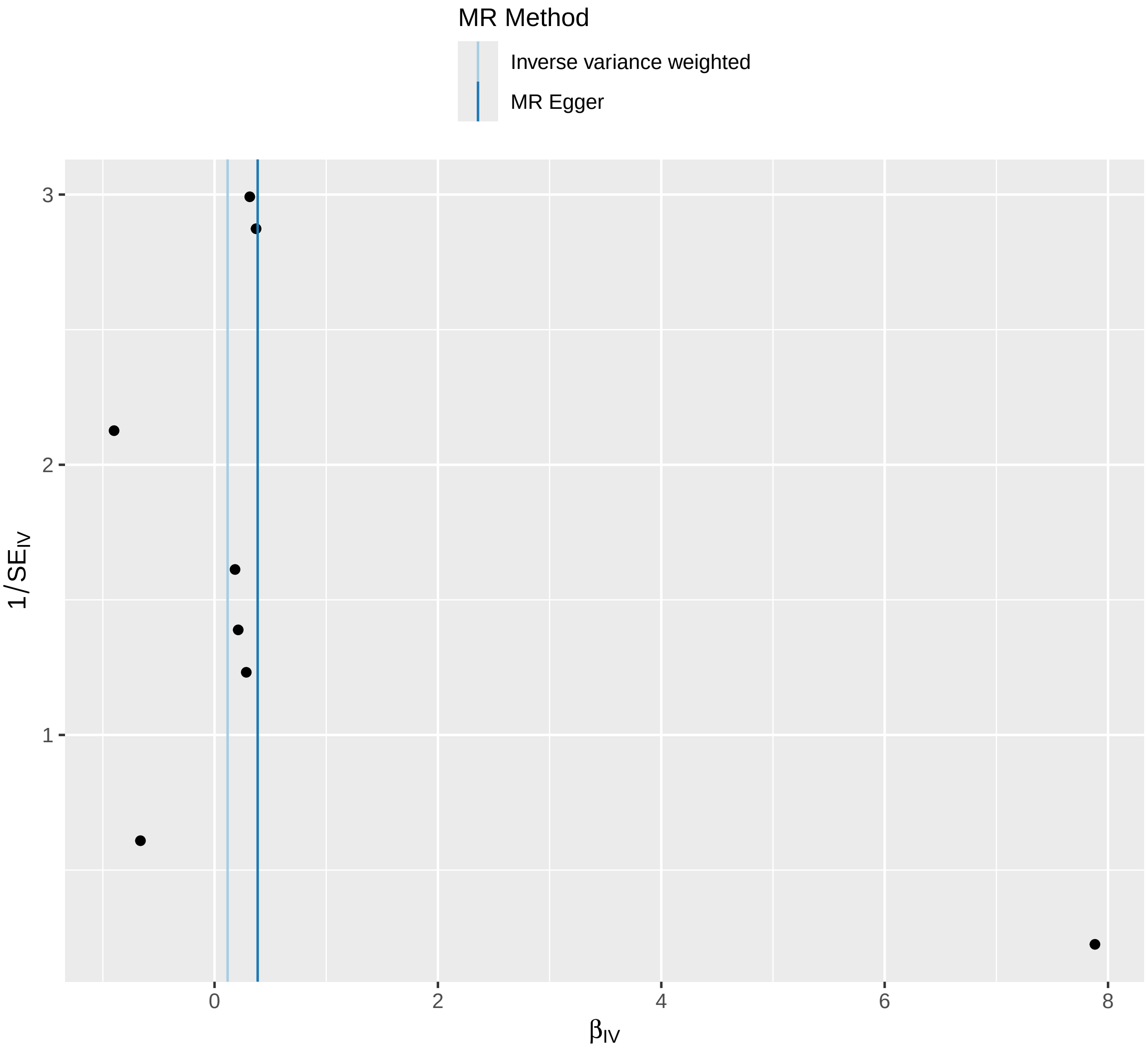
MR Method
Inverse variance weighted
MR Egger
3
2
1
$1/SE_{IV}$
0
2
4
6
8
β_{IV}

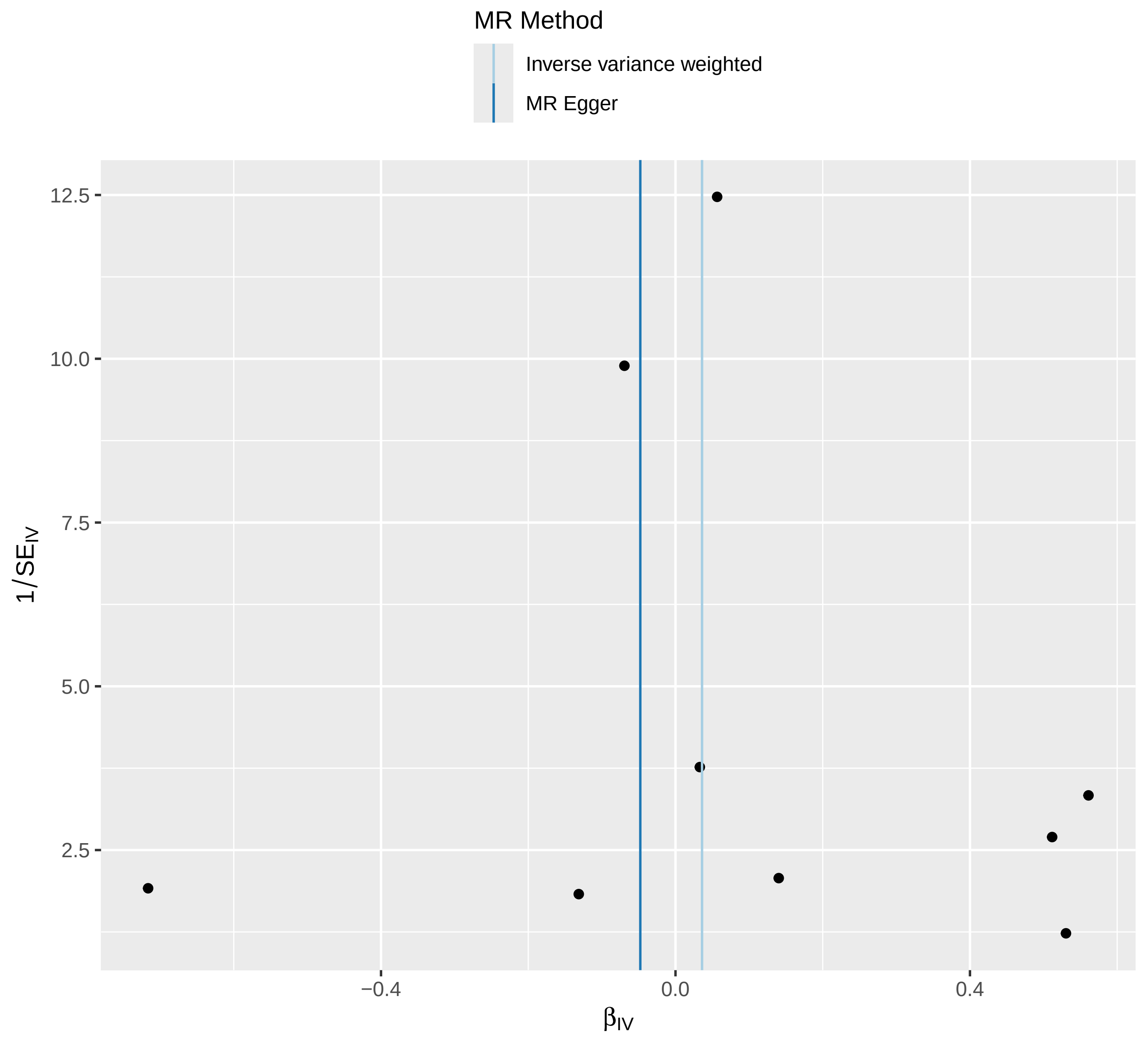
MR Method
Inverse variance weighted
MR Egger
12.5
10.0
7.5
5.0
2.5
1/SE_IV
−0.4
0.0
0.4
β_IV

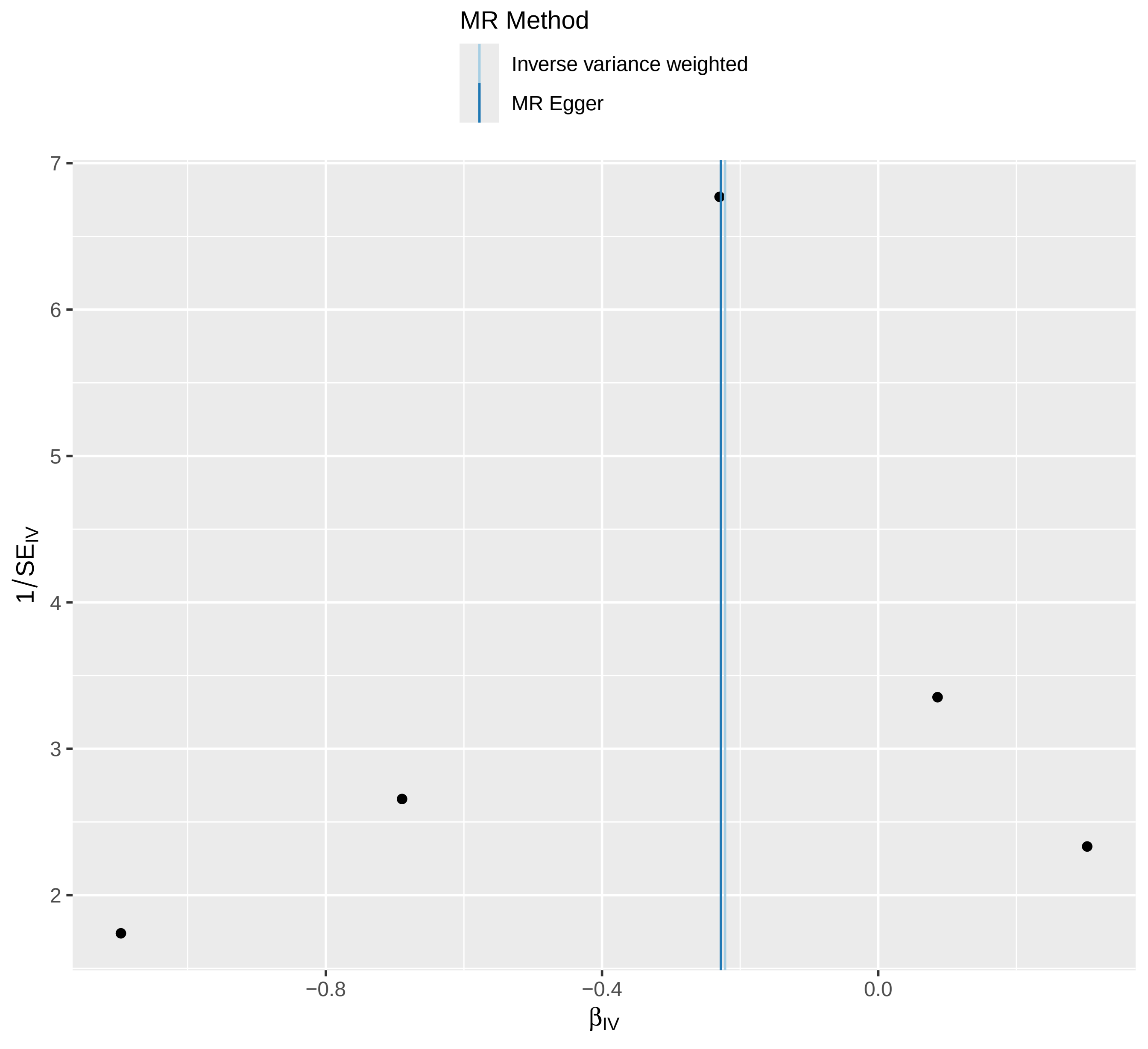

MR Method
Inverse variance weighted
MR Egger
7
6
5
4
3
2
1/SE$_{IV}$
−0.8
−0.4
0.0
β$_{IV}$

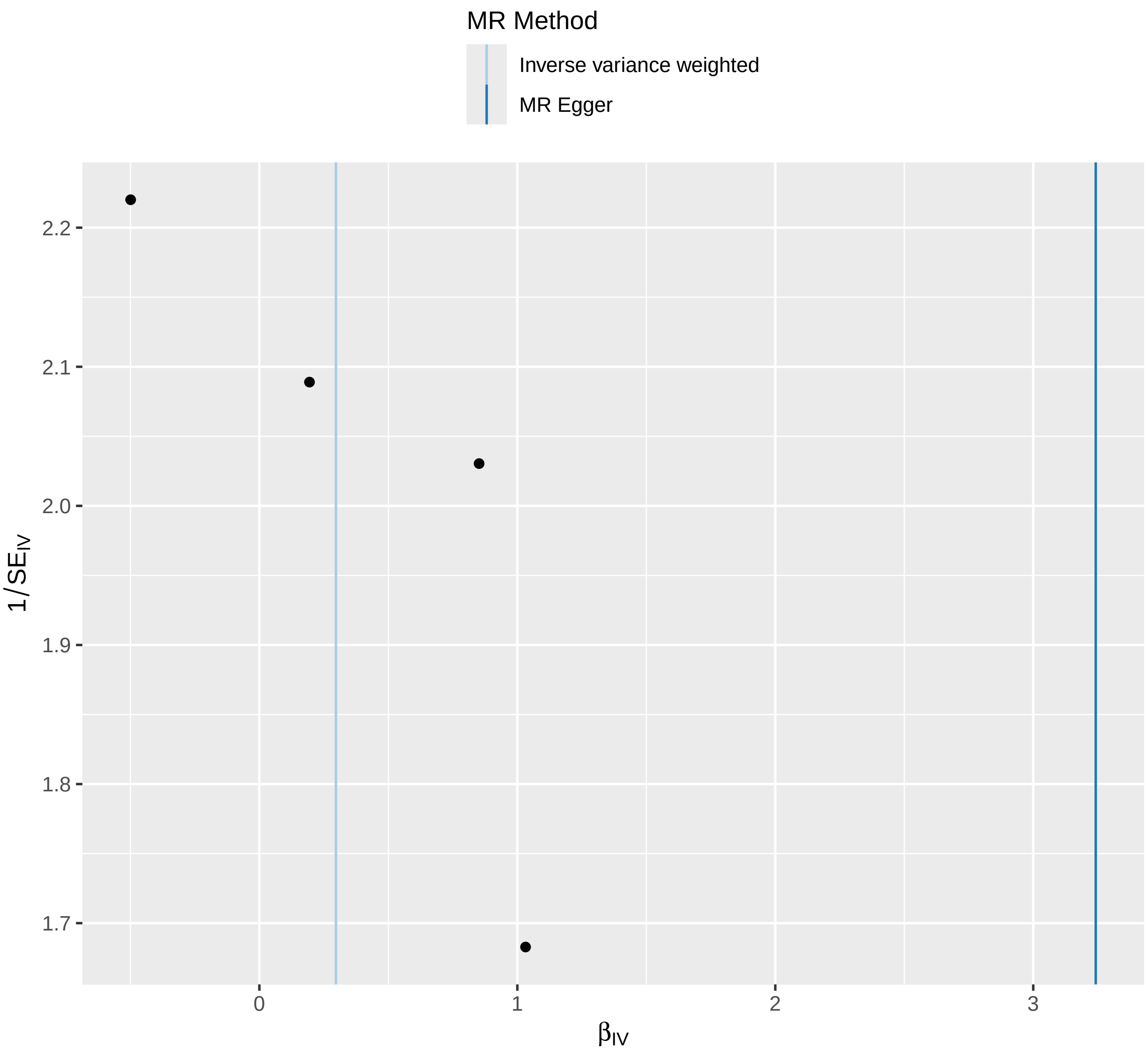
MR Method
Inverse variance weighted
MR Egger
2.2
2.1
2.0
1.9
1.8
1.7
1/SE_IV
0
1
2
3
β_IV

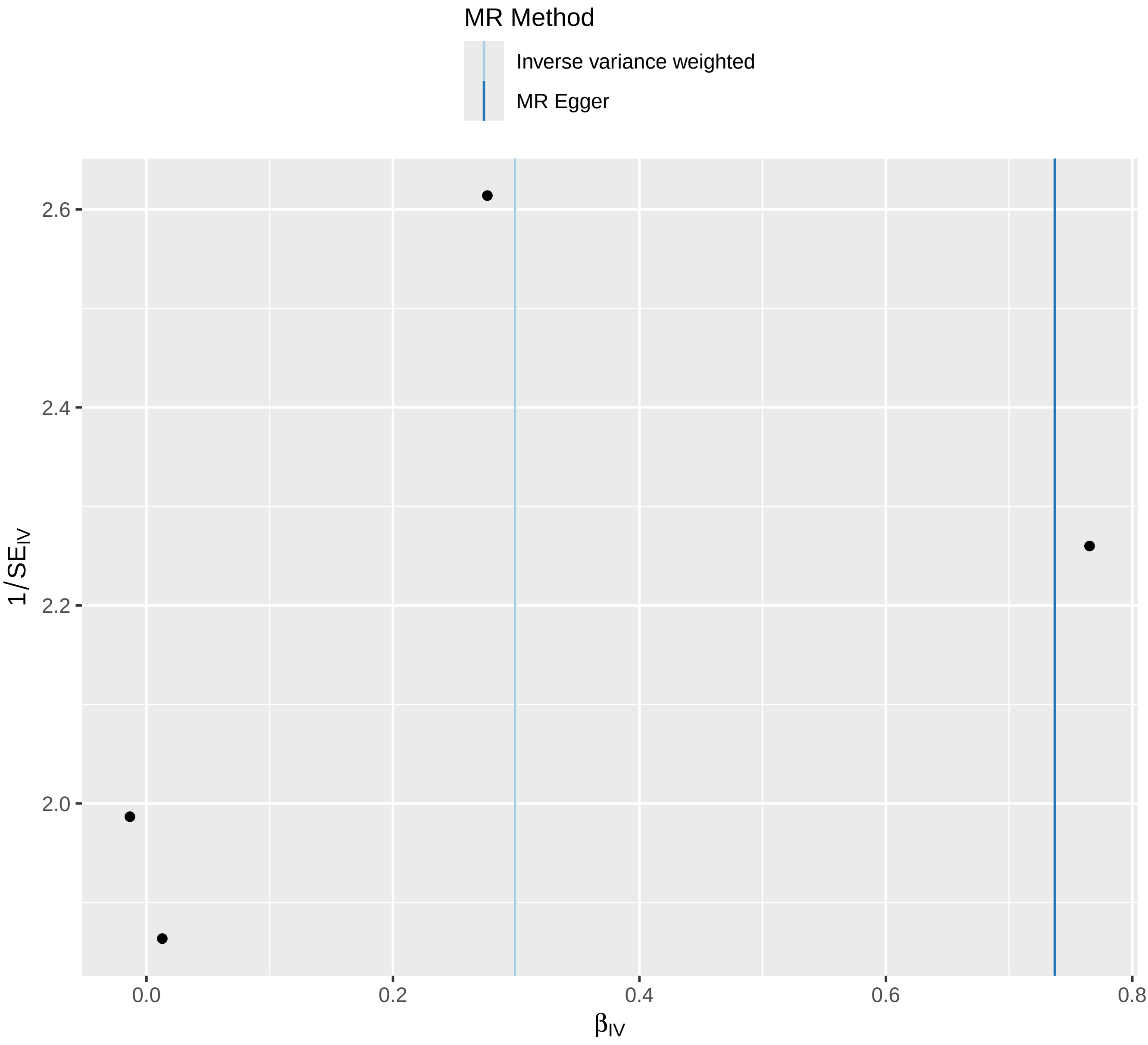

MR Method
Inverse variance weighted
MR Egger
2.6
2.4
2.2
2.0
$1/SE_{IV}$
0.0
0.2
0.4
0.6
0.8
β_{IV}

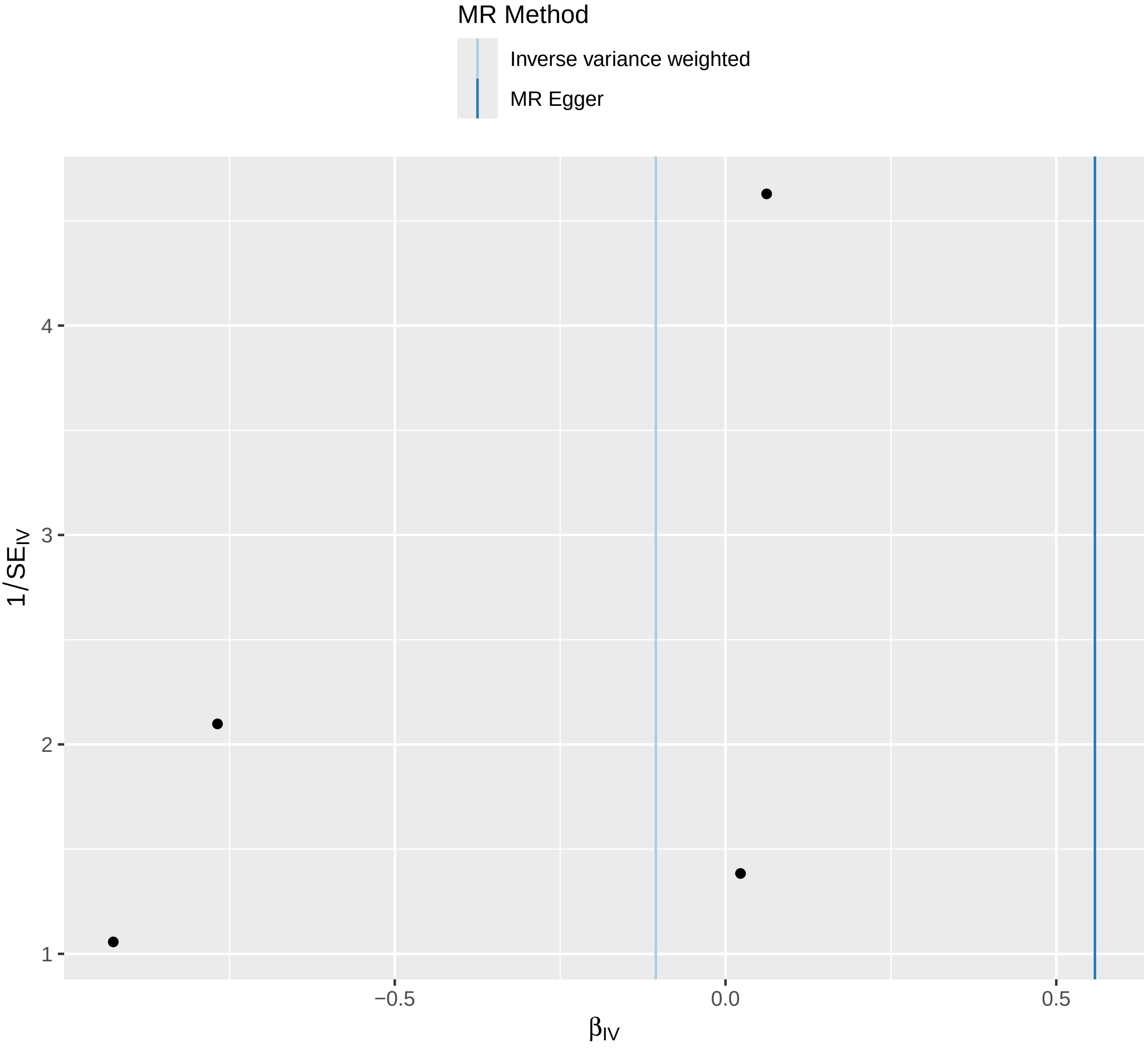

MR Method
Inverse variance weighted
MR Egger
1/SE$_{IV}$
4
3
2
1
−0.5
0.0
0.5
β$_{IV}$

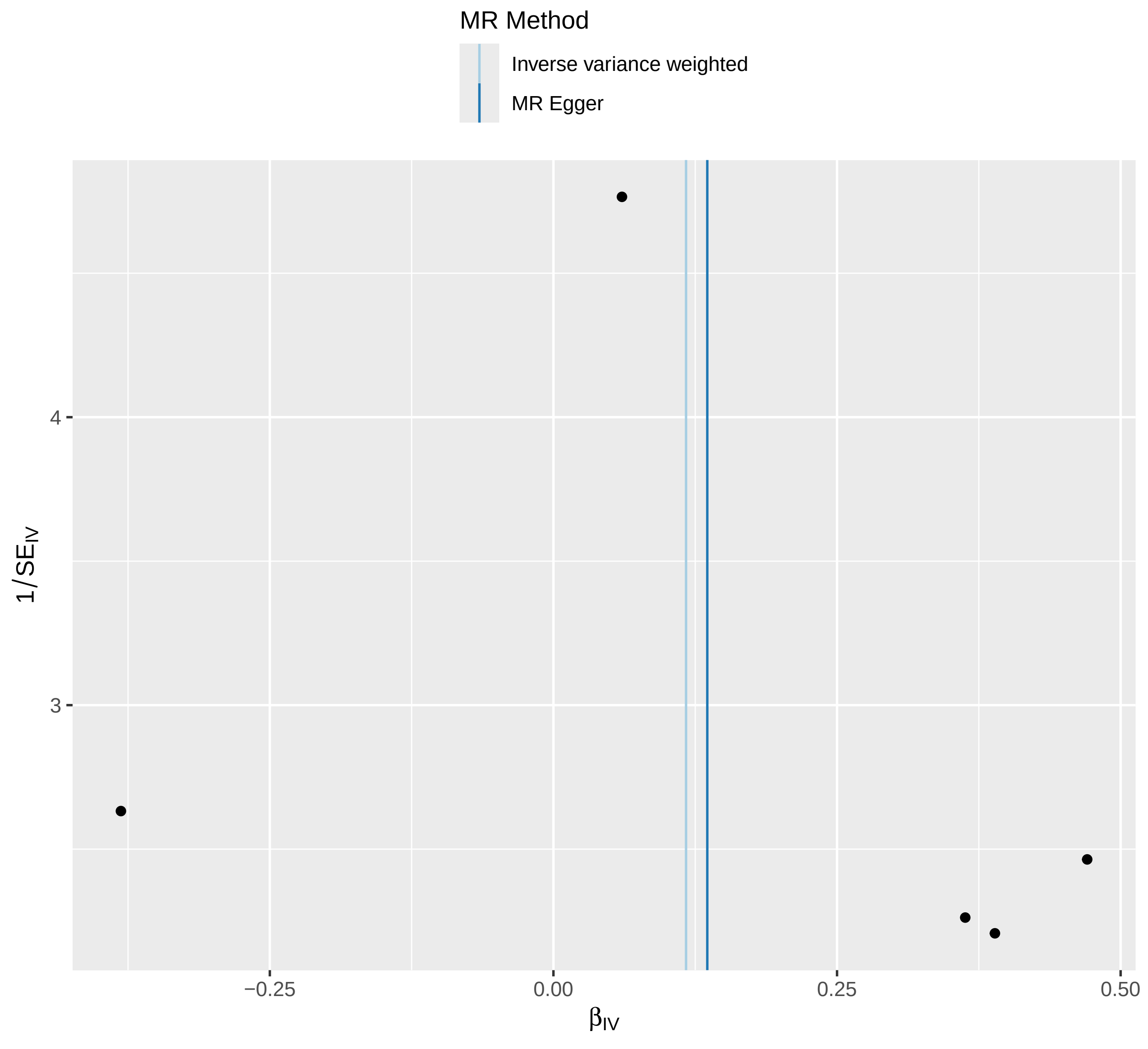

MR Method
Inverse variance weighted
MR Egger
4
3
1/SE_IV
−0.25
0.00
0.25
0.50
β_IV

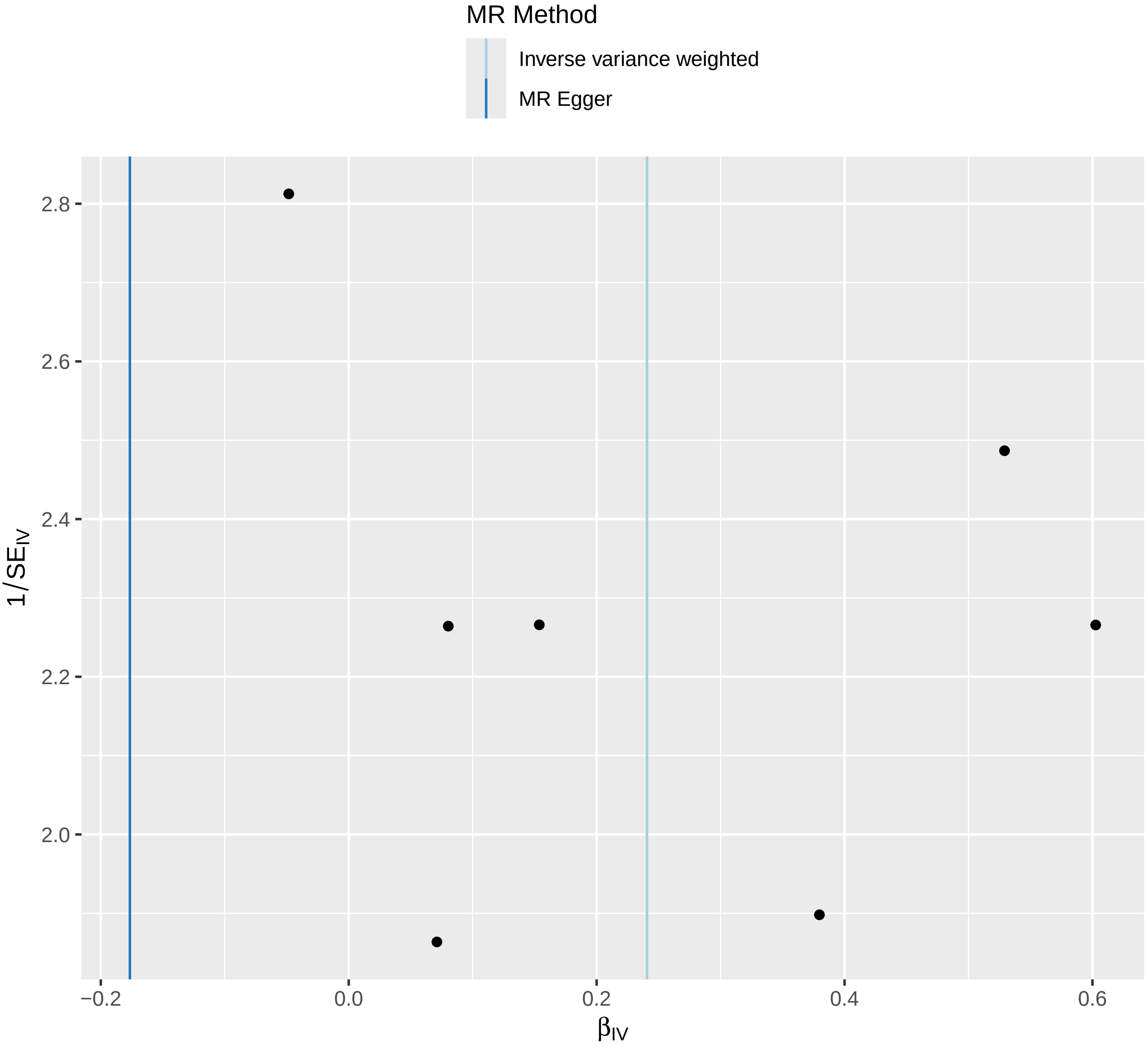

MR Method
Inverse variance weighted
MR Egger
2.8
2.6
2.4
2.2
2.0
$1/SE_{IV}$
−0.2
0.0
0.2
0.4
0.6
β_{IV}

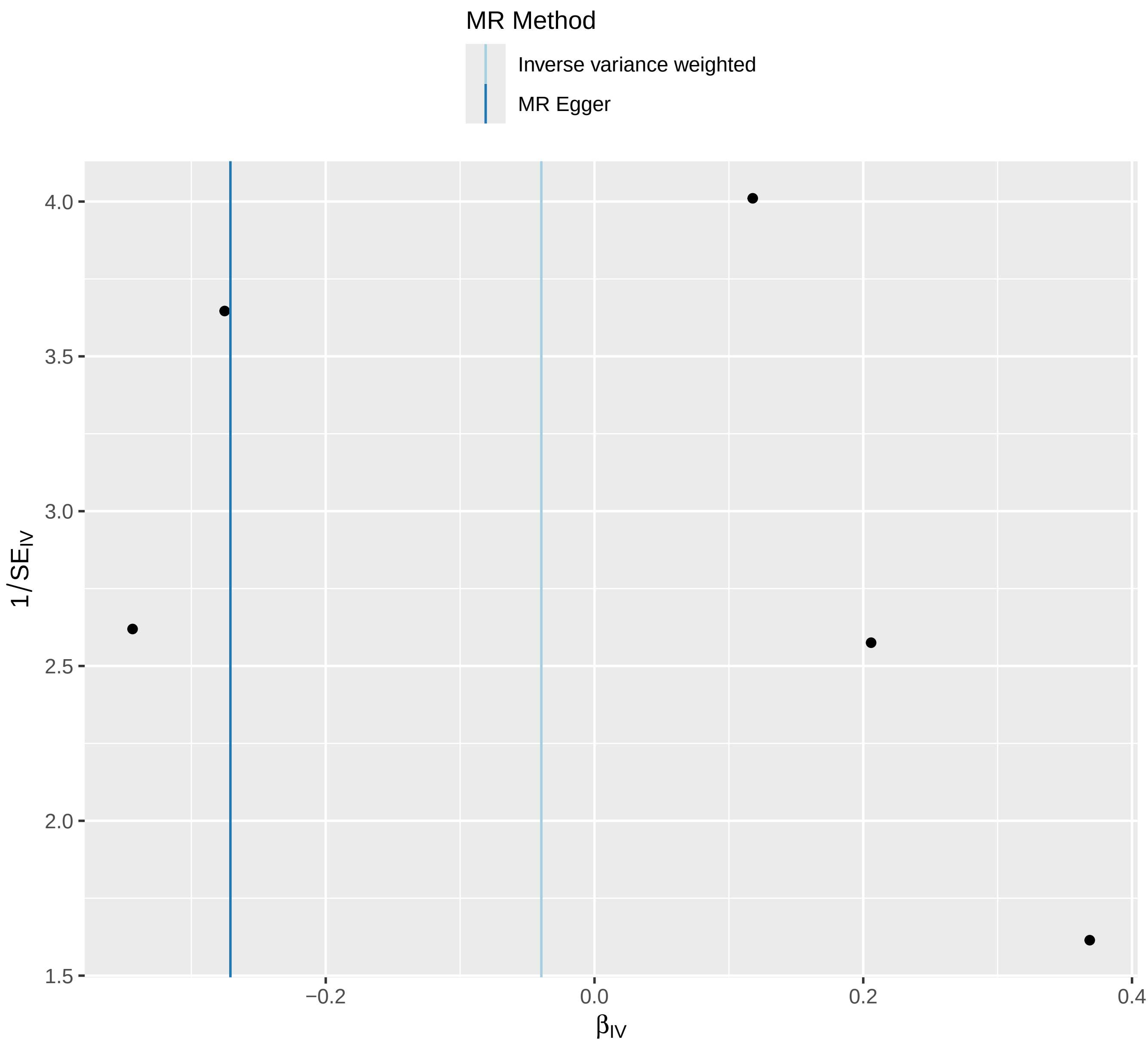
MR Method
Inverse variance weighted
MR Egger
4.0
3.5
3.0
2.5
2.0
1.5
$1/SE_{IV}$
−0.2
0.0
0.2
0.4
β_{IV}

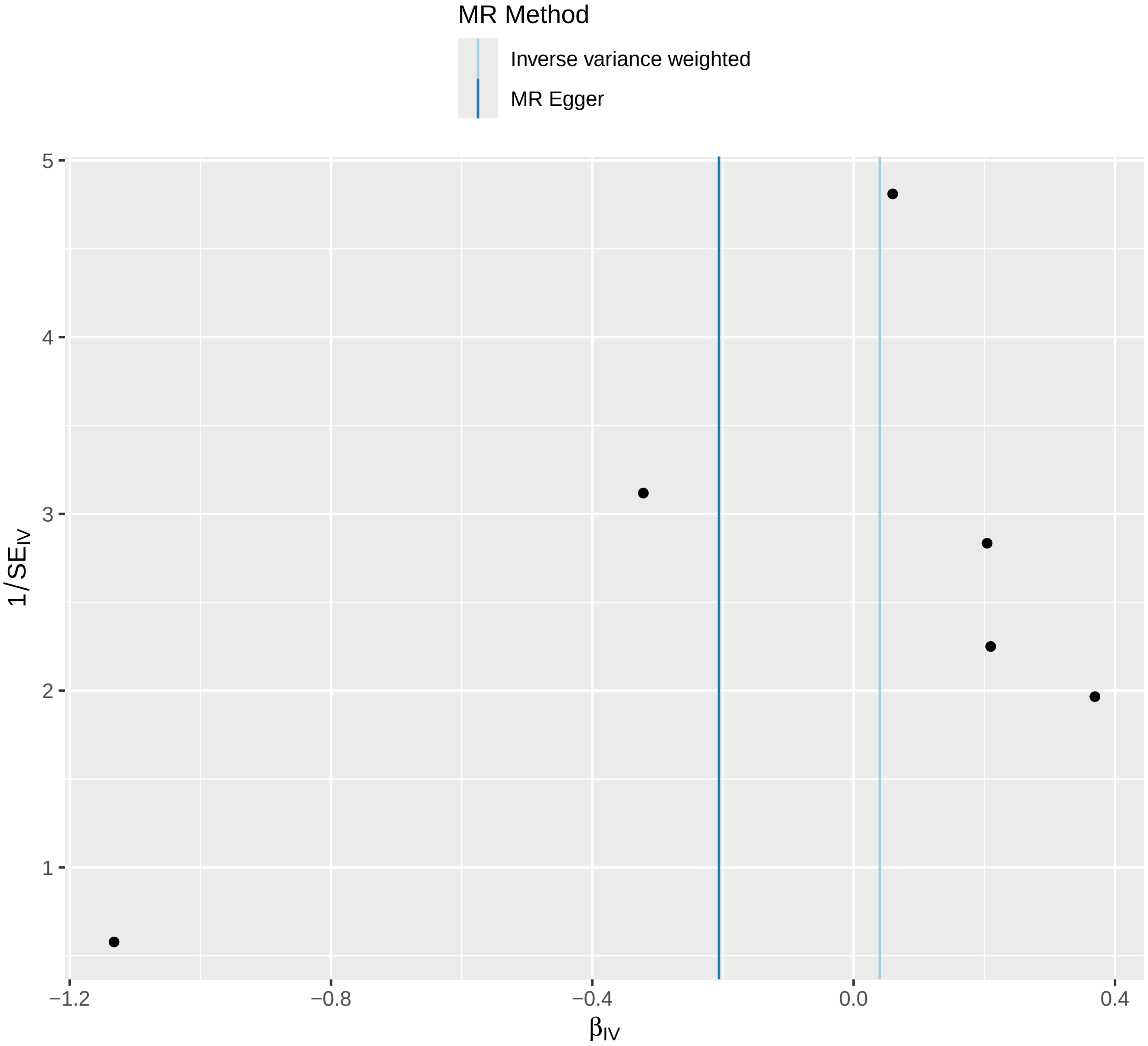

MR Method
Inverse variance weighted
MR Egger
1/SE$_{IV}$
β$_{IV}$
5
4
3
2
1
−1.2
−0.8
−0.4
0.0
0.4

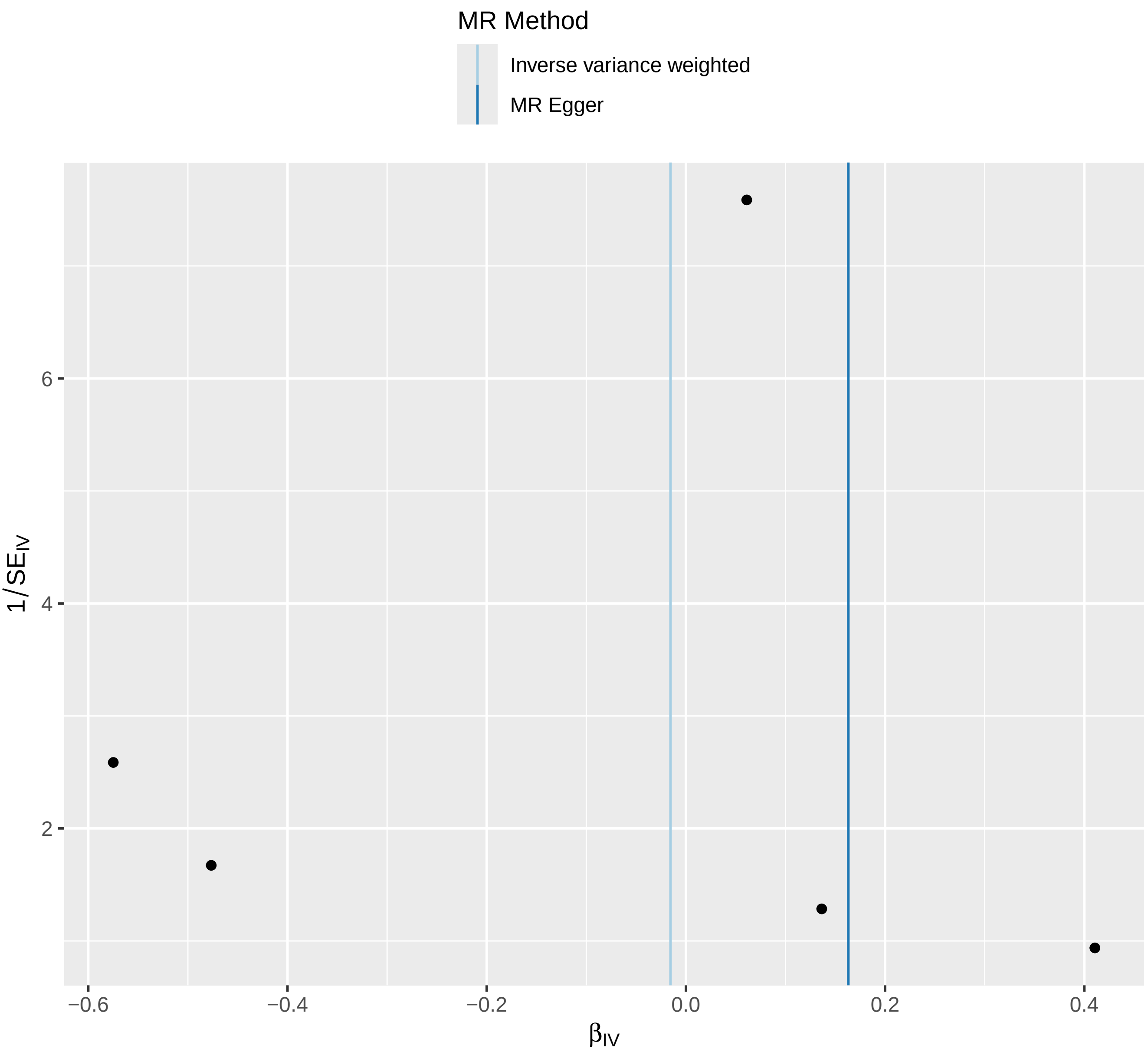
MR Method
Inverse variance weighted
MR Egger
1/SE$_{IV}$
6
4
2
−0.6
−0.4
−0.2
0.0
0.2
0.4
β$_{IV}$

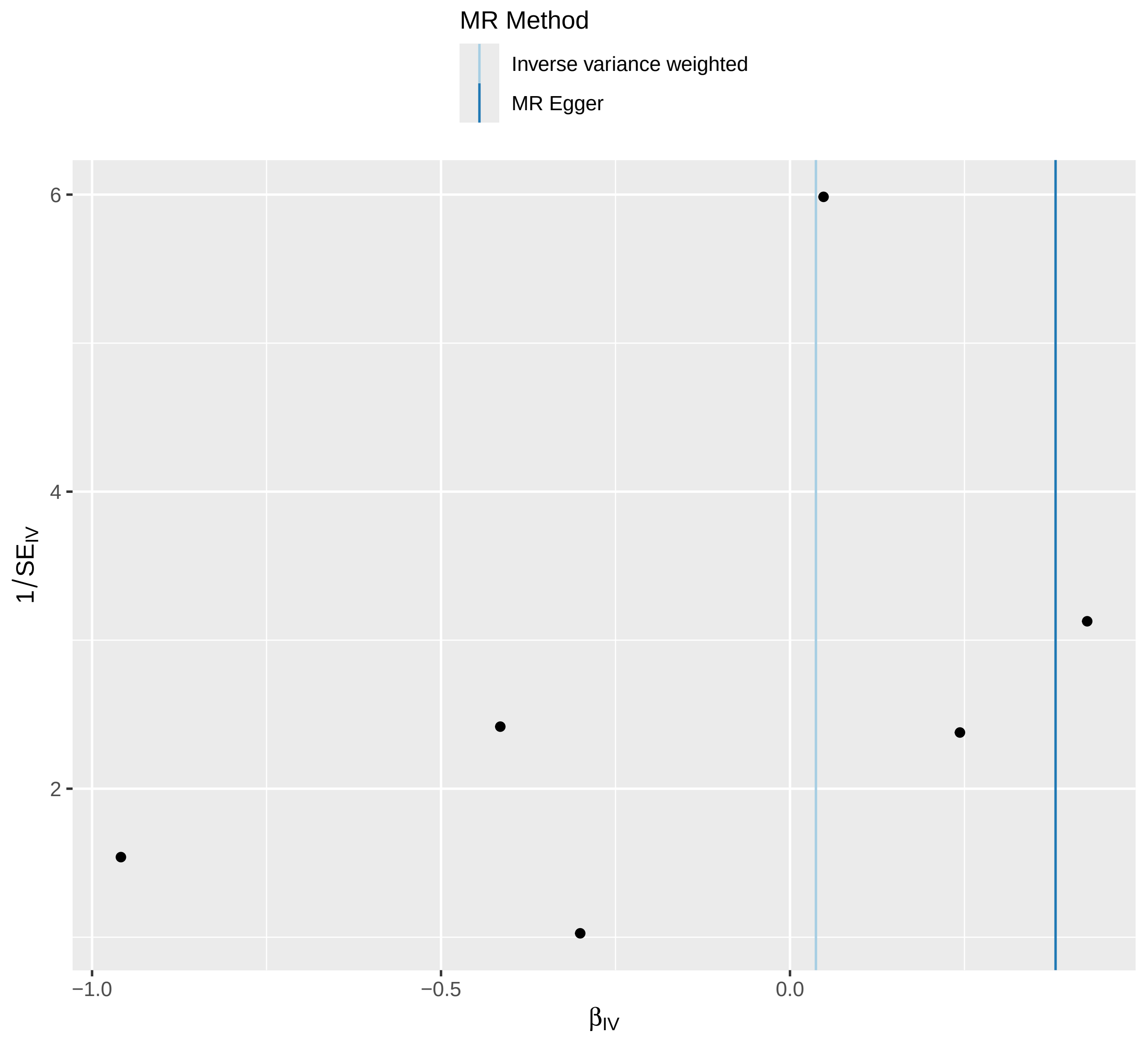
MR Method
Inverse variance weighted
MR Egger
6
4
2
1/SE$_{IV}$
−1.0
−0.5
0.0
β$_{IV}$

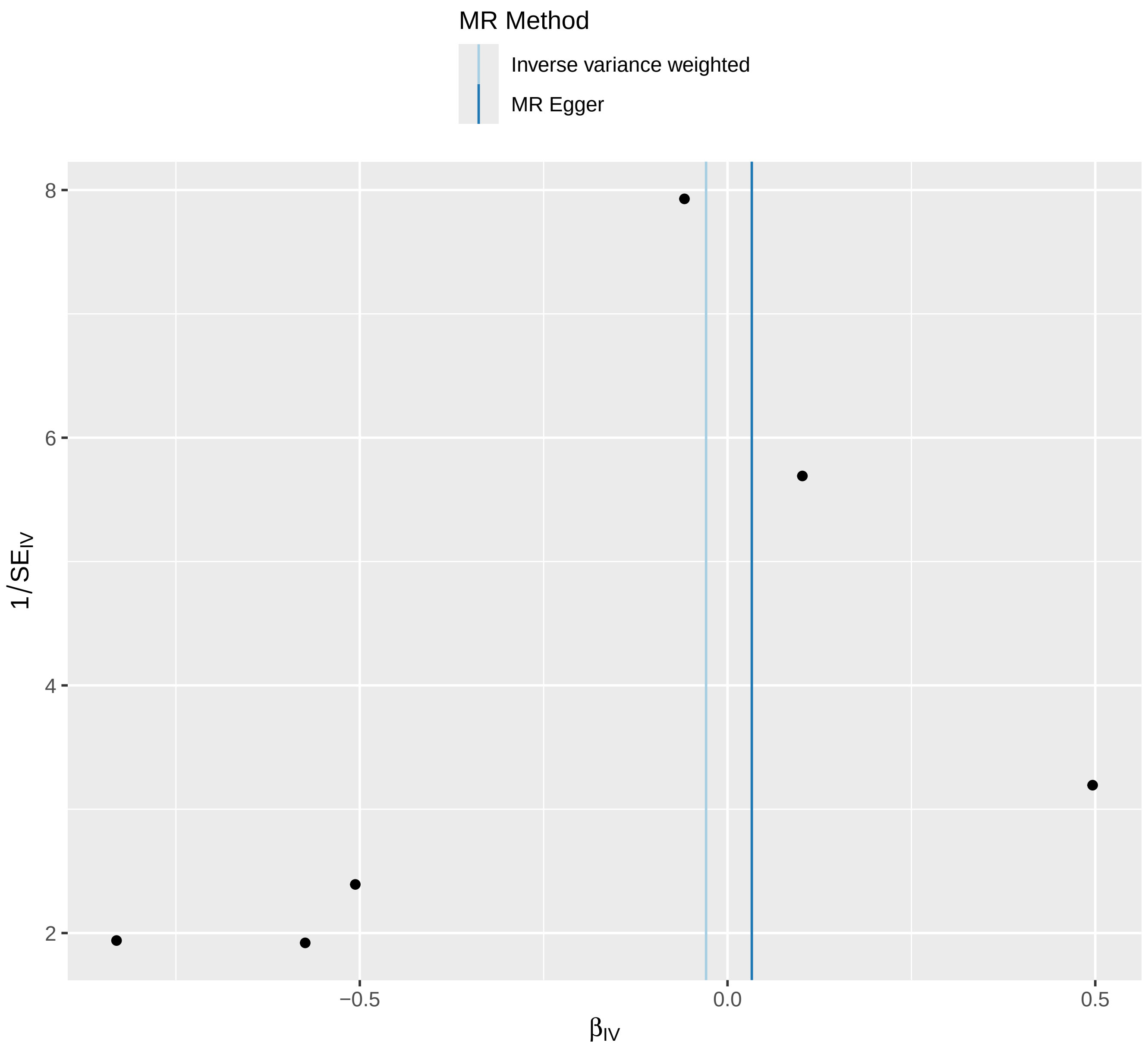

MR Method
Inverse variance weighted
MR Egger
8
6
4
2
−0.5
0.0
0.5
$1/SE_{IV}$
β_{IV}

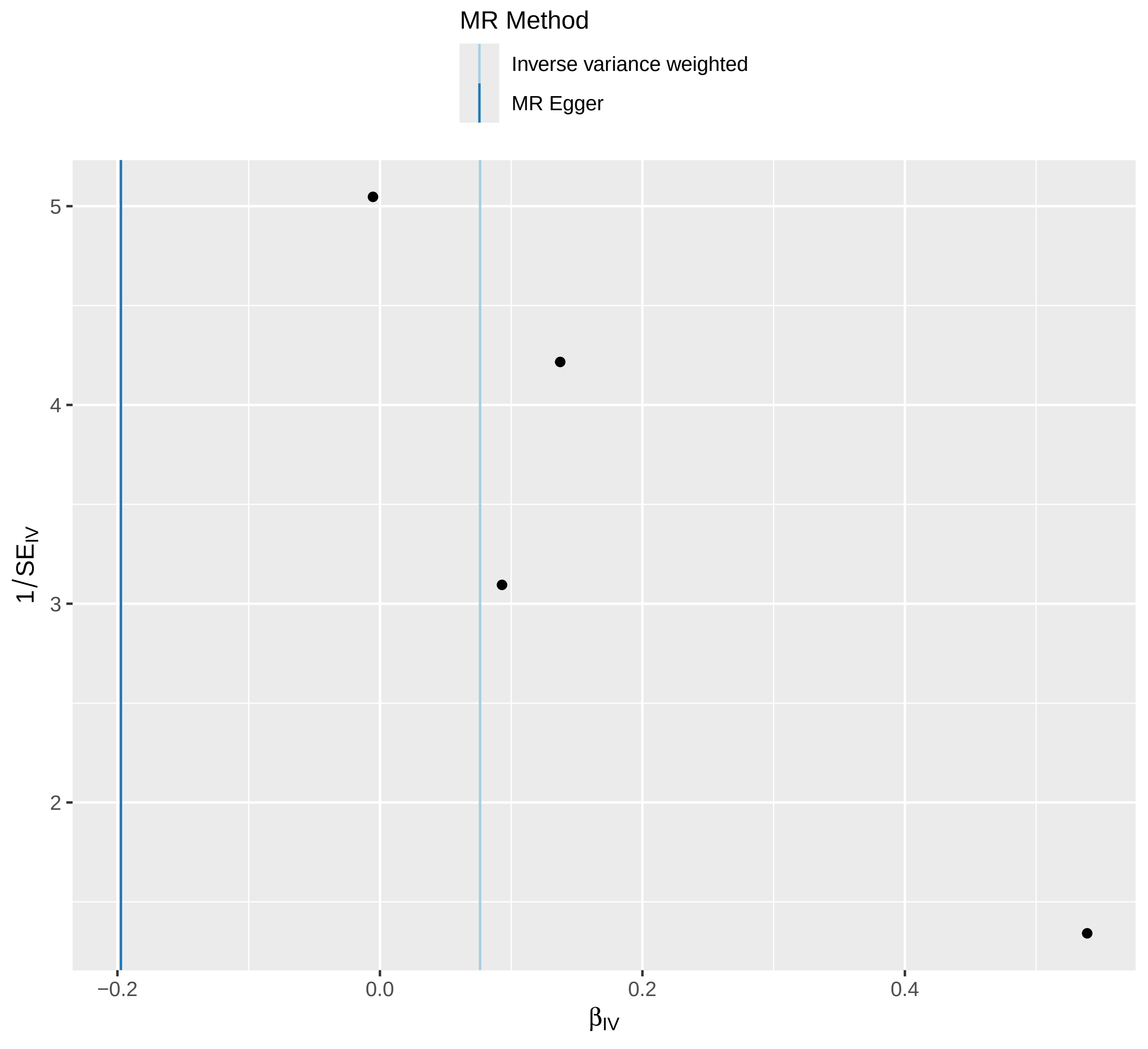

MR Method
Inverse variance weighted
MR Egger
5
4
3
2
1/SE_IV
−0.2
0.0
0.2
0.4
β_IV

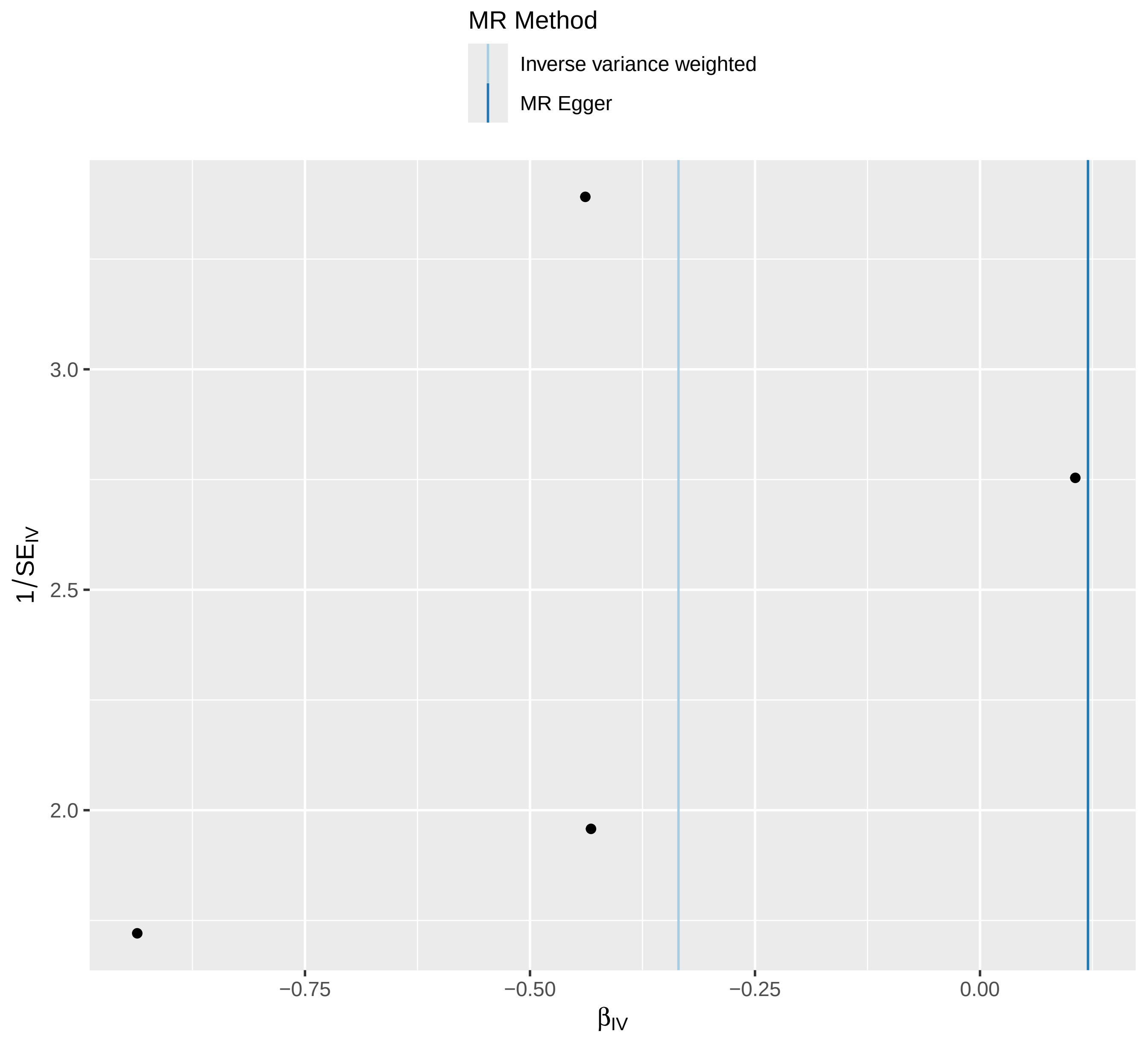
MR Method
Inverse variance weighted
MR Egger
3.0
2.5
2.0
1/SE_IV
−0.75
−0.50
−0.25
0.00
β_IV

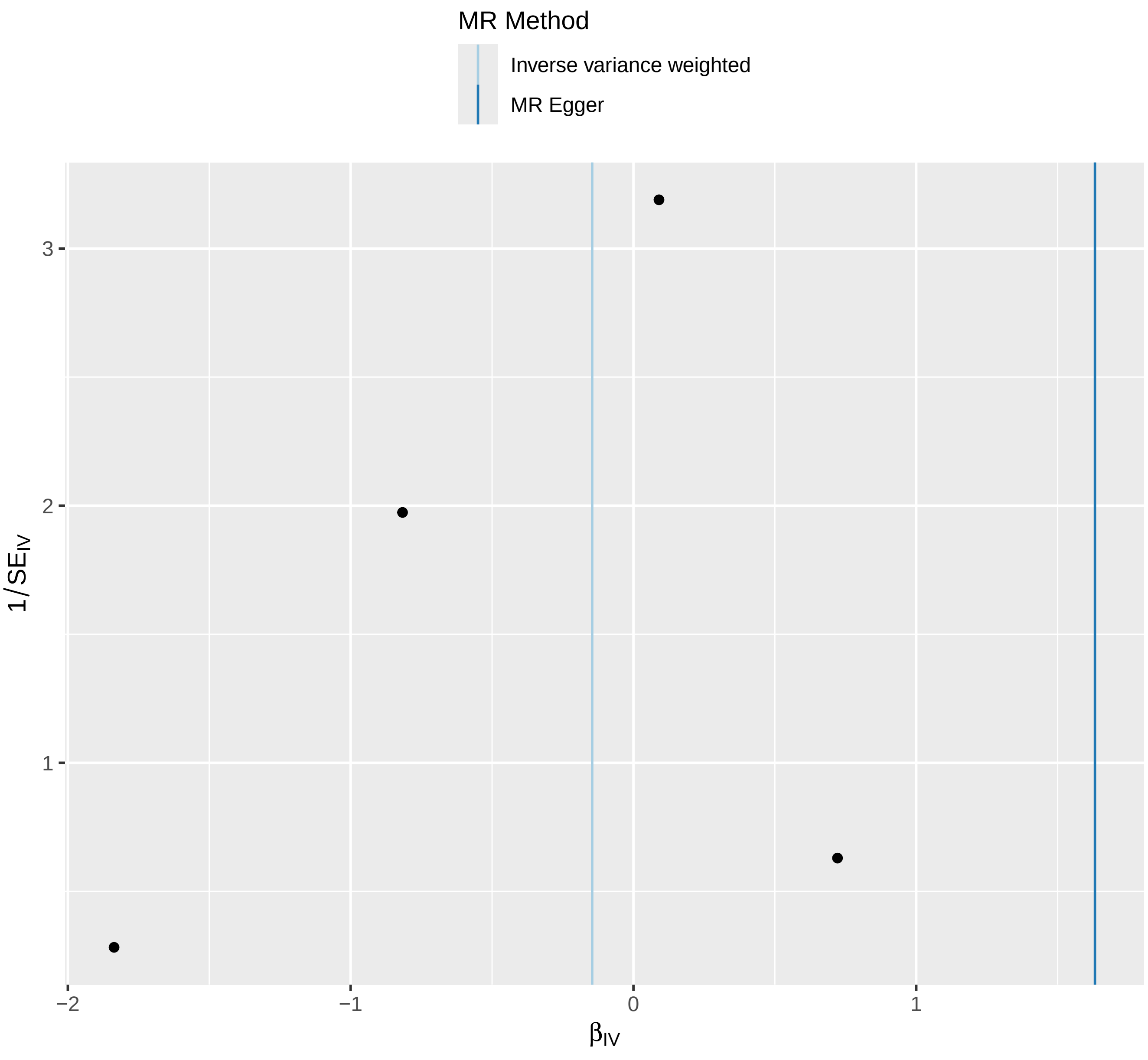
MR Method
Inverse variance weighted
MR Egger
3
2
1
1/SE_IV
−2
−1
0
1
β_IV

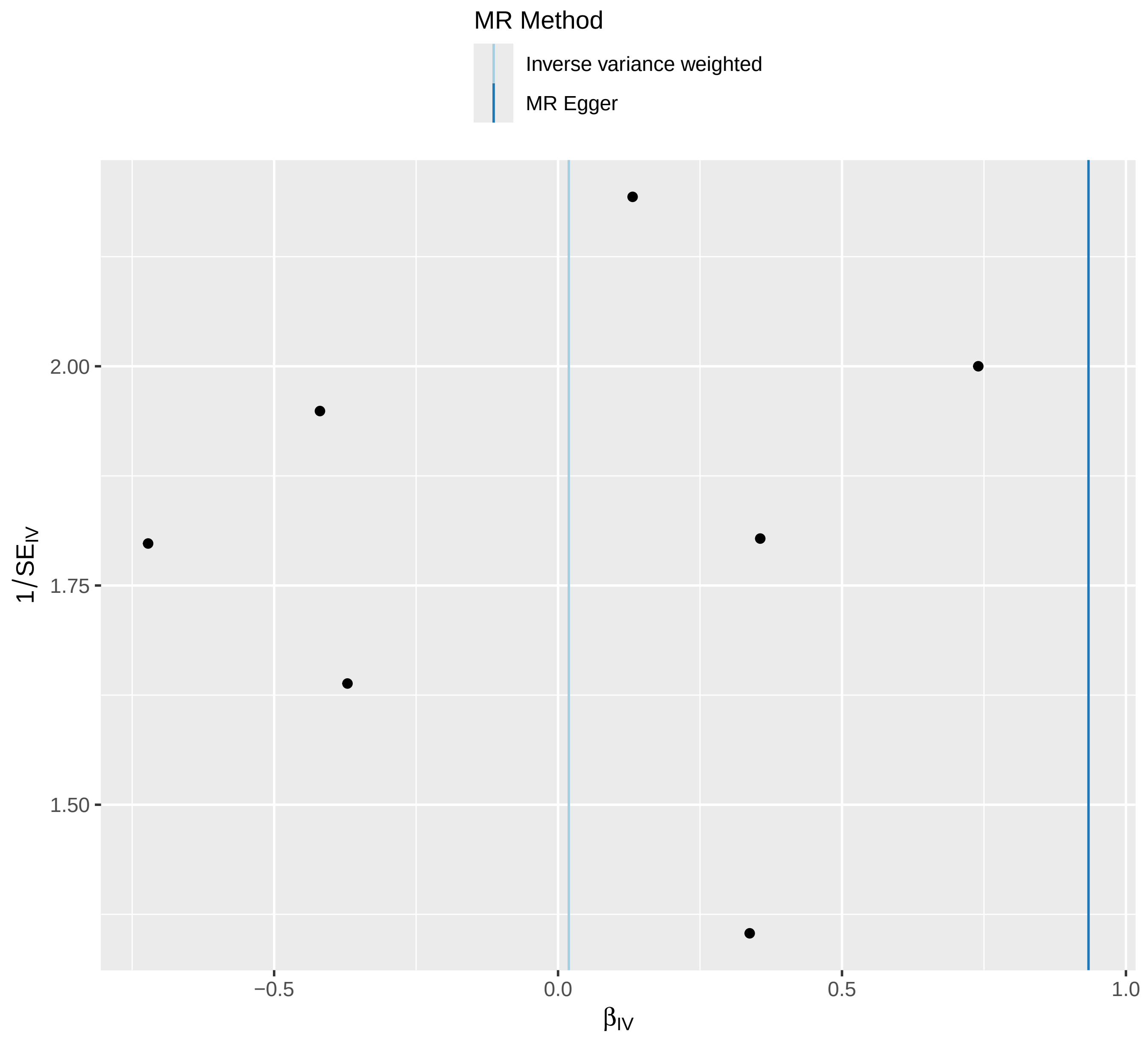

MR Method
Inverse variance weighted
MR Egger
2.00
1.75
1.50
1/SE_IV
−0.5
0.0
0.5
1.0
β_IV

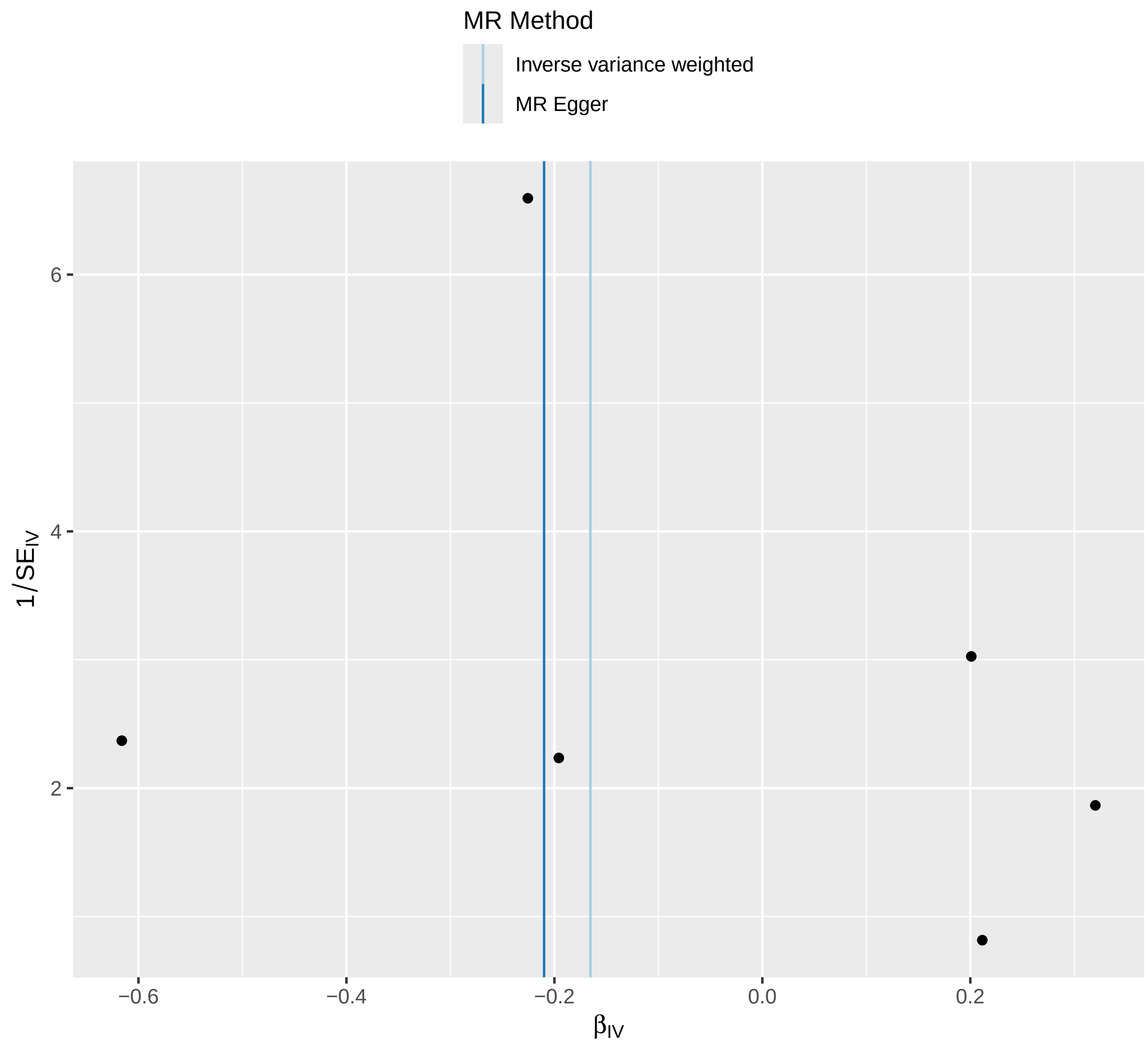
MR Method
Inverse variance weighted
MR Egger
6
4
2
1/SE$_{IV}$
−0.6
−0.4
−0.2
0.0
0.2
β$_{IV}$

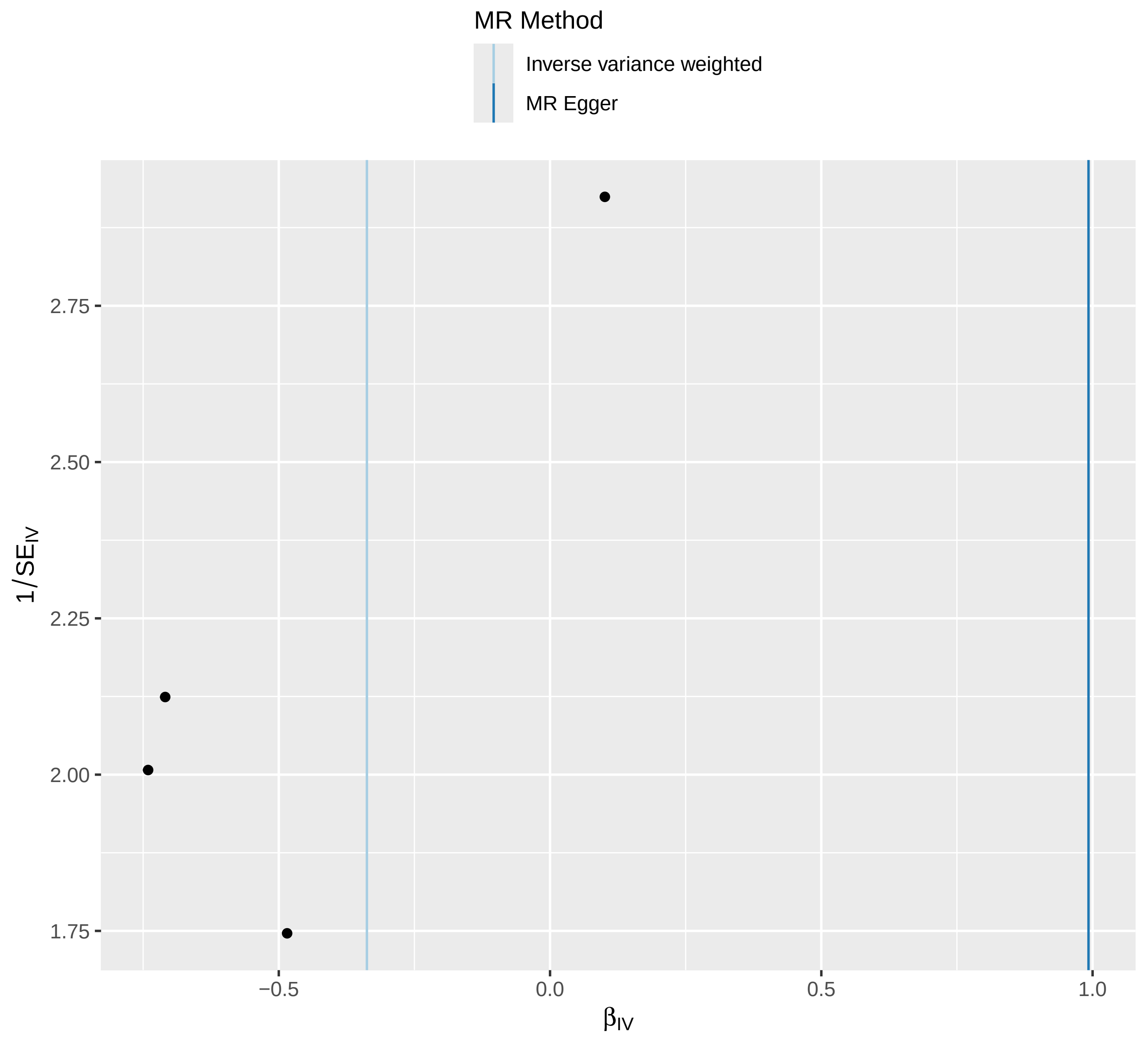

MR Method
Inverse variance weighted
MR Egger
2.75
2.50
2.25
2.00
1.75
1/SE$_{IV}$
−0.5
0.0
0.5
1.0
β$_{IV}$

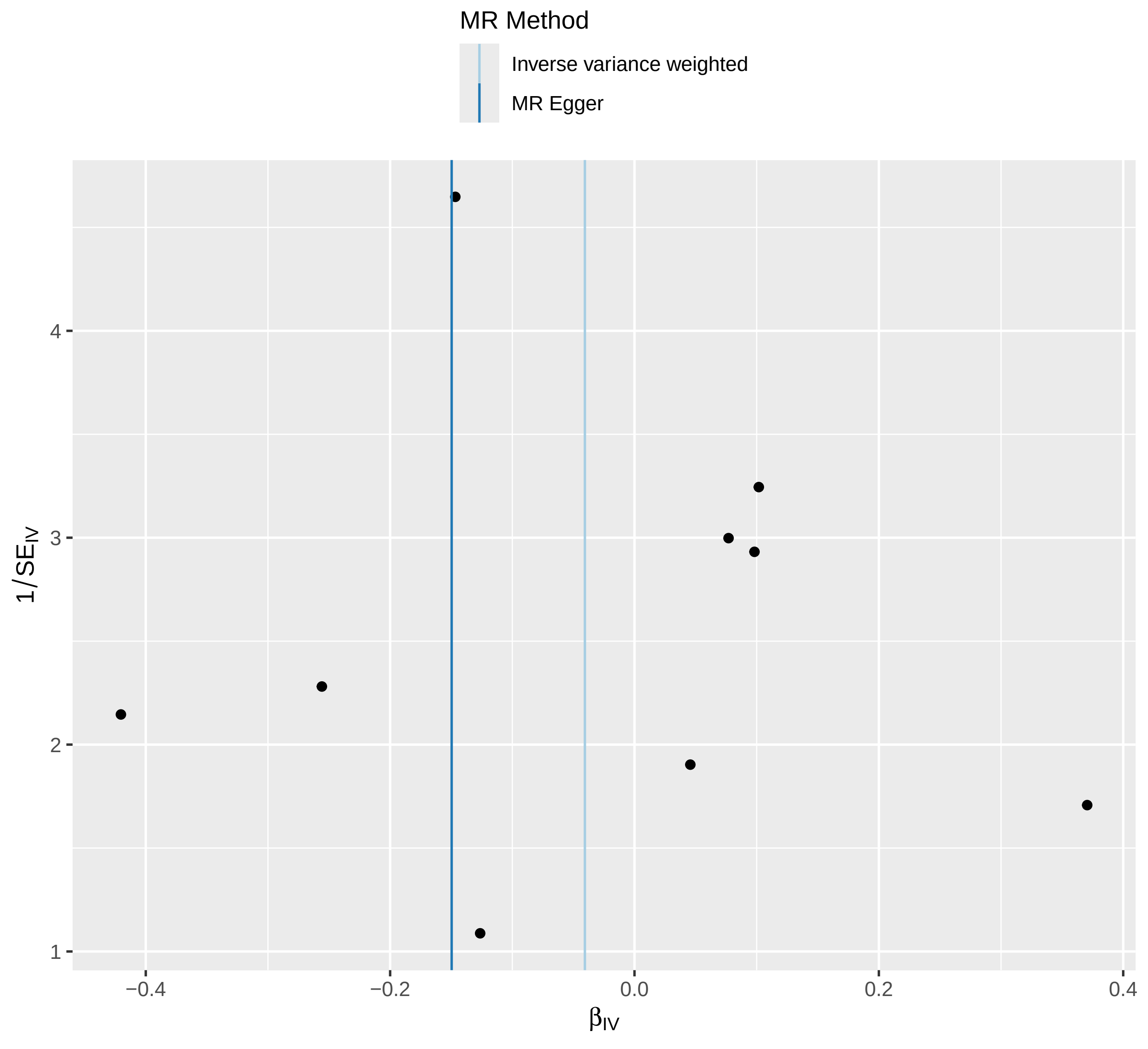
MR Method
Inverse variance weighted
MR Egger
$1/SE_{IV}$
β_{IV}
4
3
2
1
−0.4
−0.2
0.0
0.2
0.4

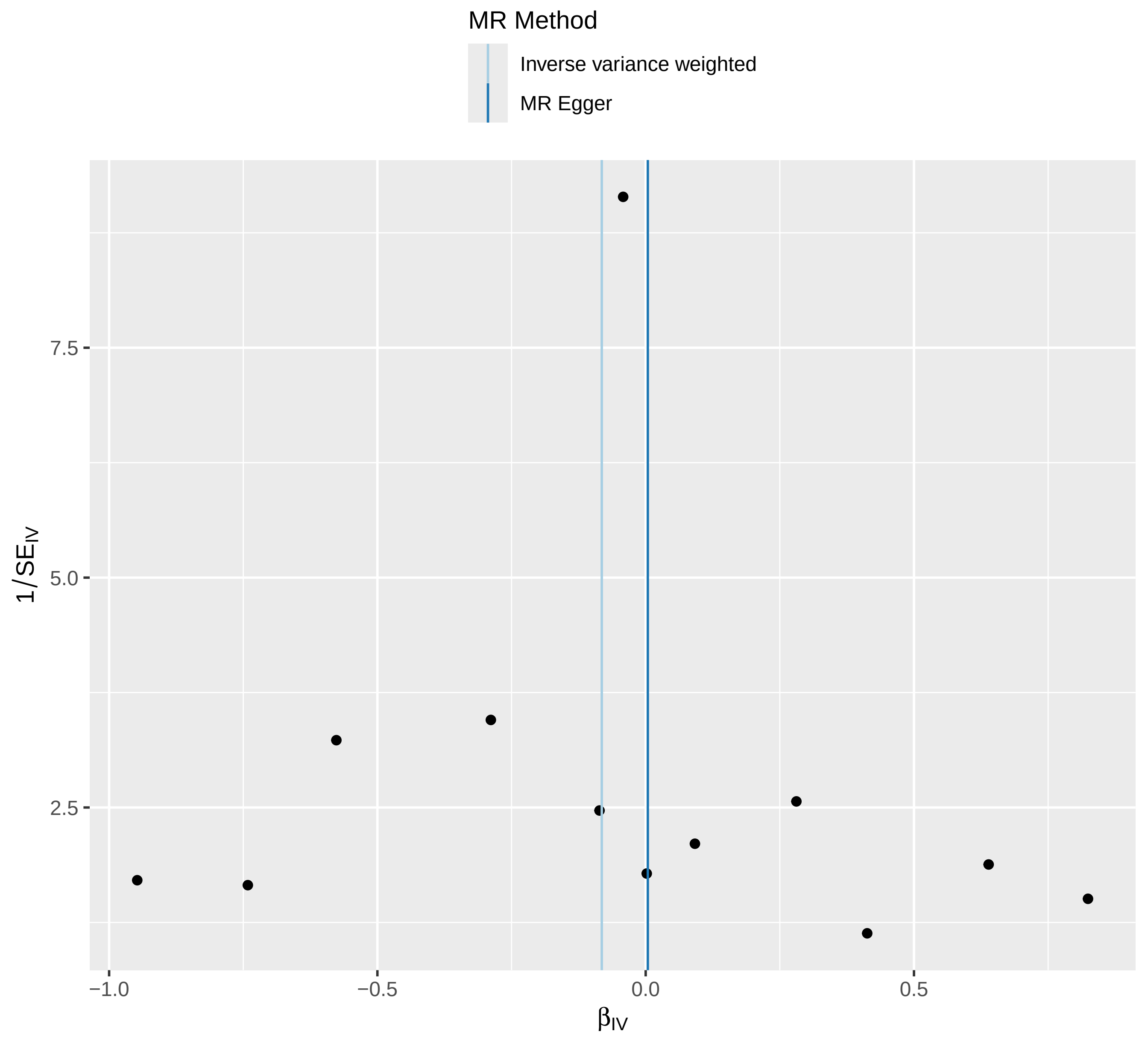
MR Method
Inverse variance weighted
MR Egger
7.5
5.0
2.5
1/SE_IV
−1.0
−0.5
0.0
0.5
β_IV

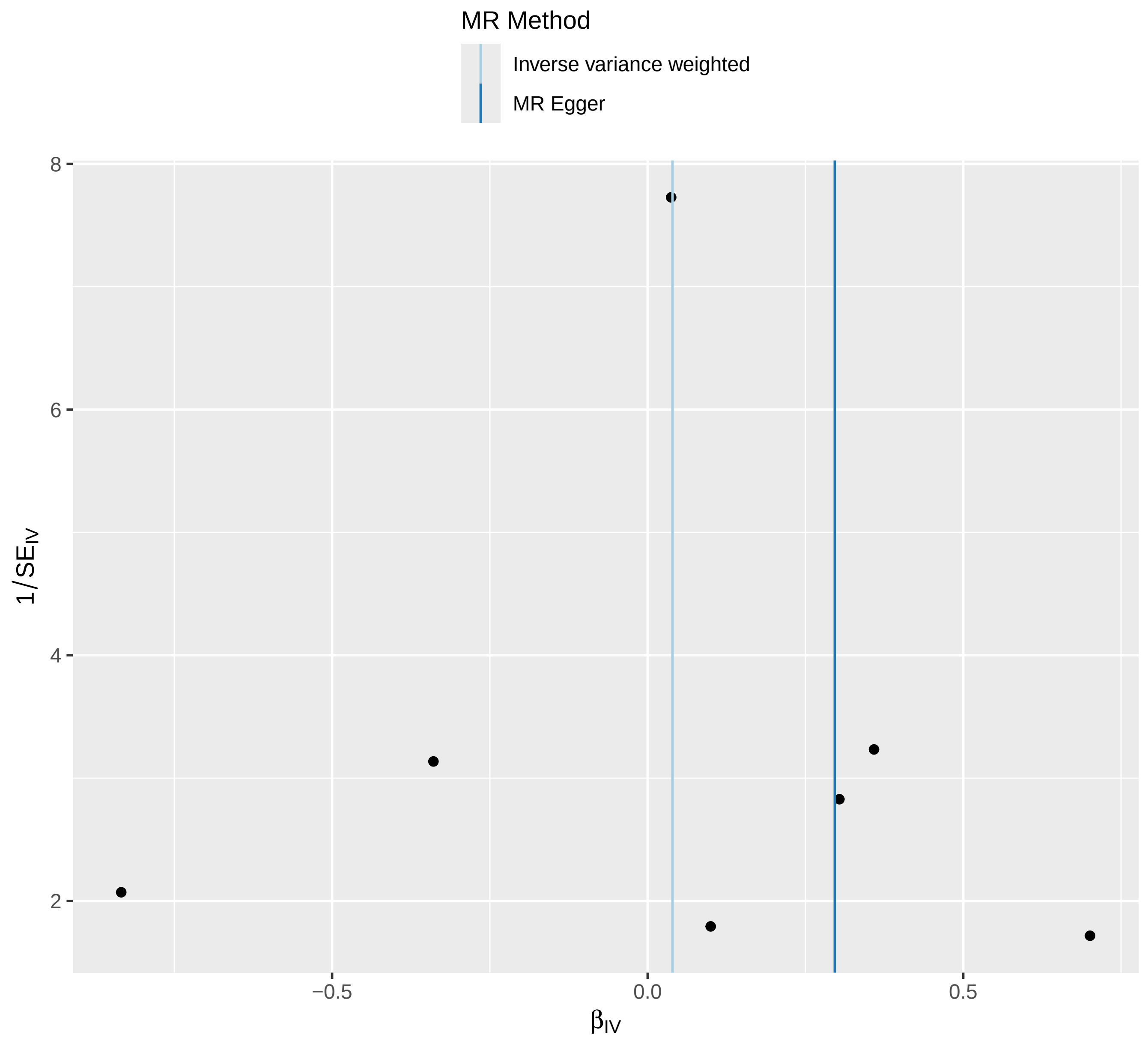

MR Method
Inverse variance weighted
MR Egger
8
6
4
2
1/SE$_{IV}$
−0.5
0.0
0.5
β$_{IV}$

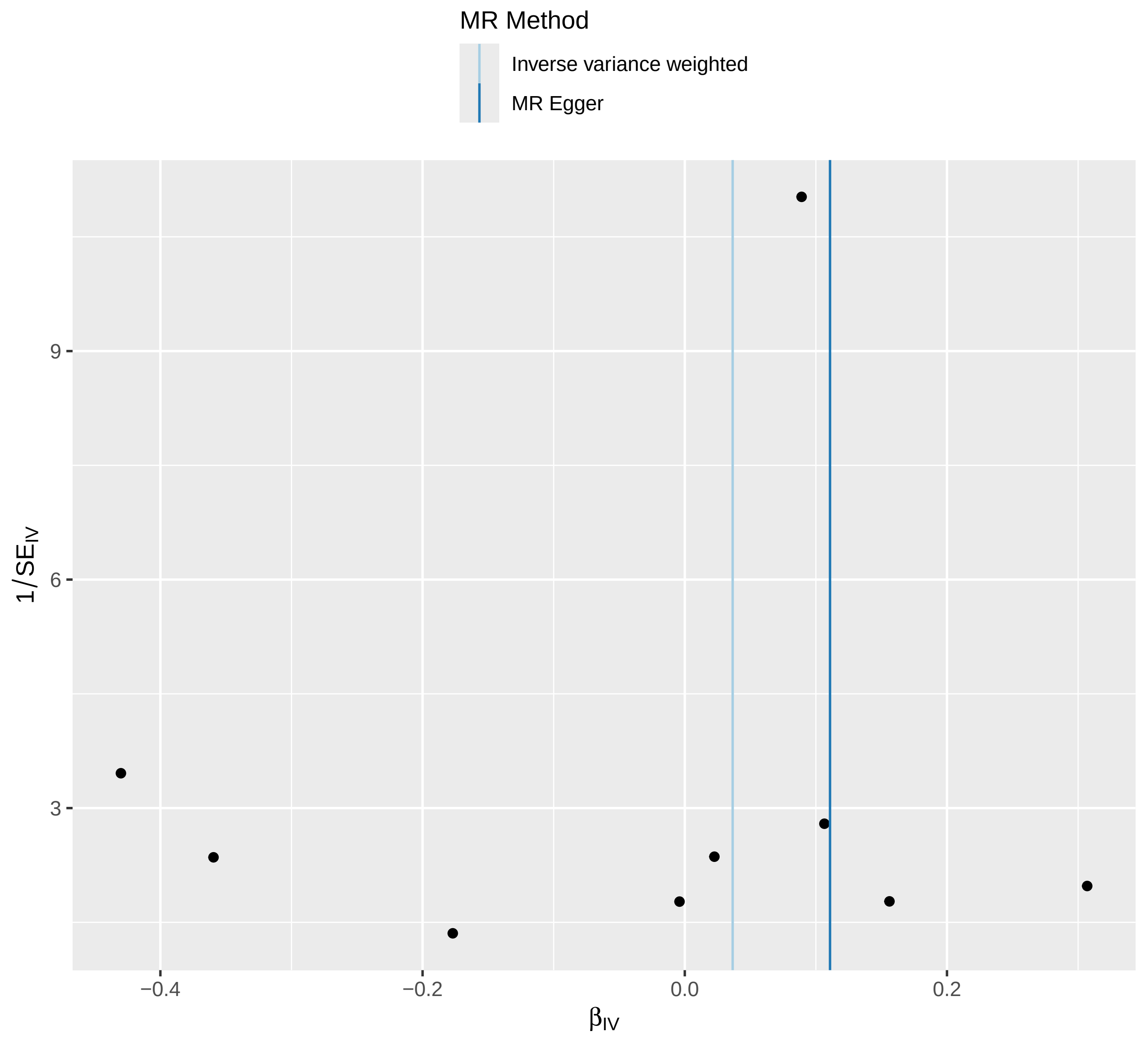

MR Method
Inverse variance weighted
MR Egger
9
6
3
1/SE$_{IV}$
−0.4
−0.2
0.0
0.2
β$_{IV}$

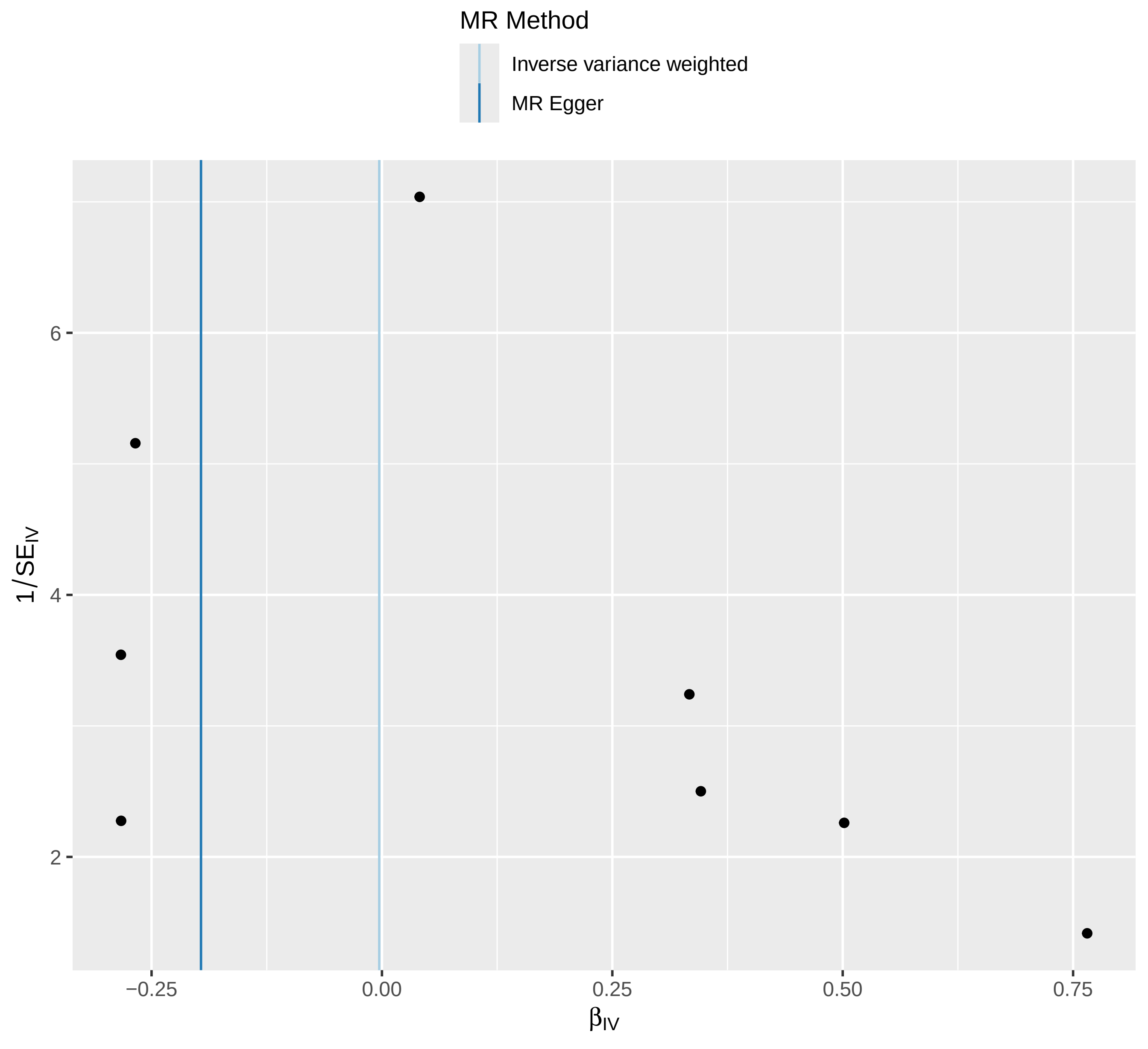
MR Method
Inverse variance weighted
MR Egger
6
4
2
1/SE_IV
−0.25
0.00
0.25
0.50
0.75
β_IV

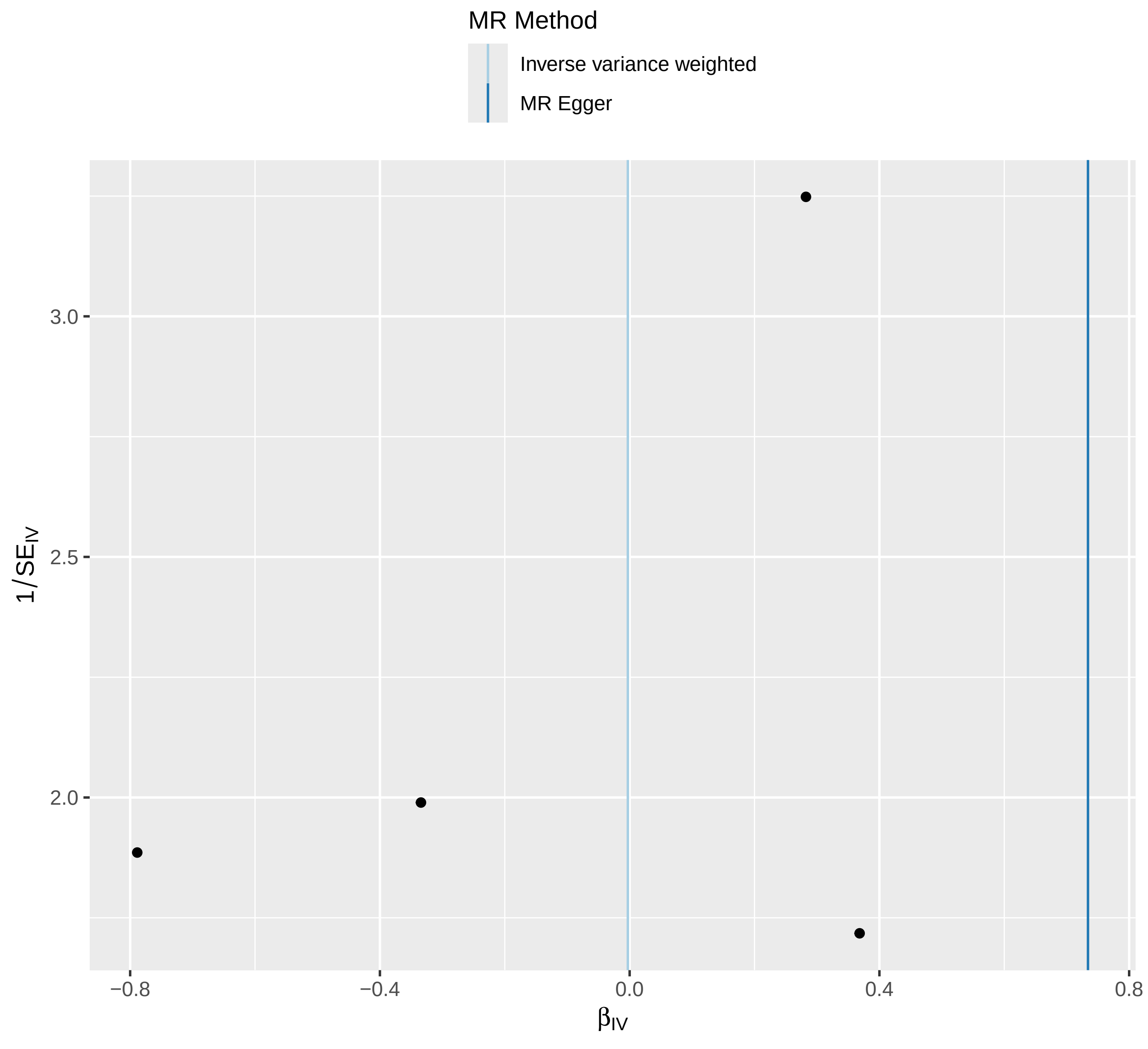
MR Method
Inverse variance weighted
MR Egger
3.0
2.5
2.0
1/SE$_{IV}$
−0.8
−0.4
0.0
0.4
0.8
β$_{IV}$

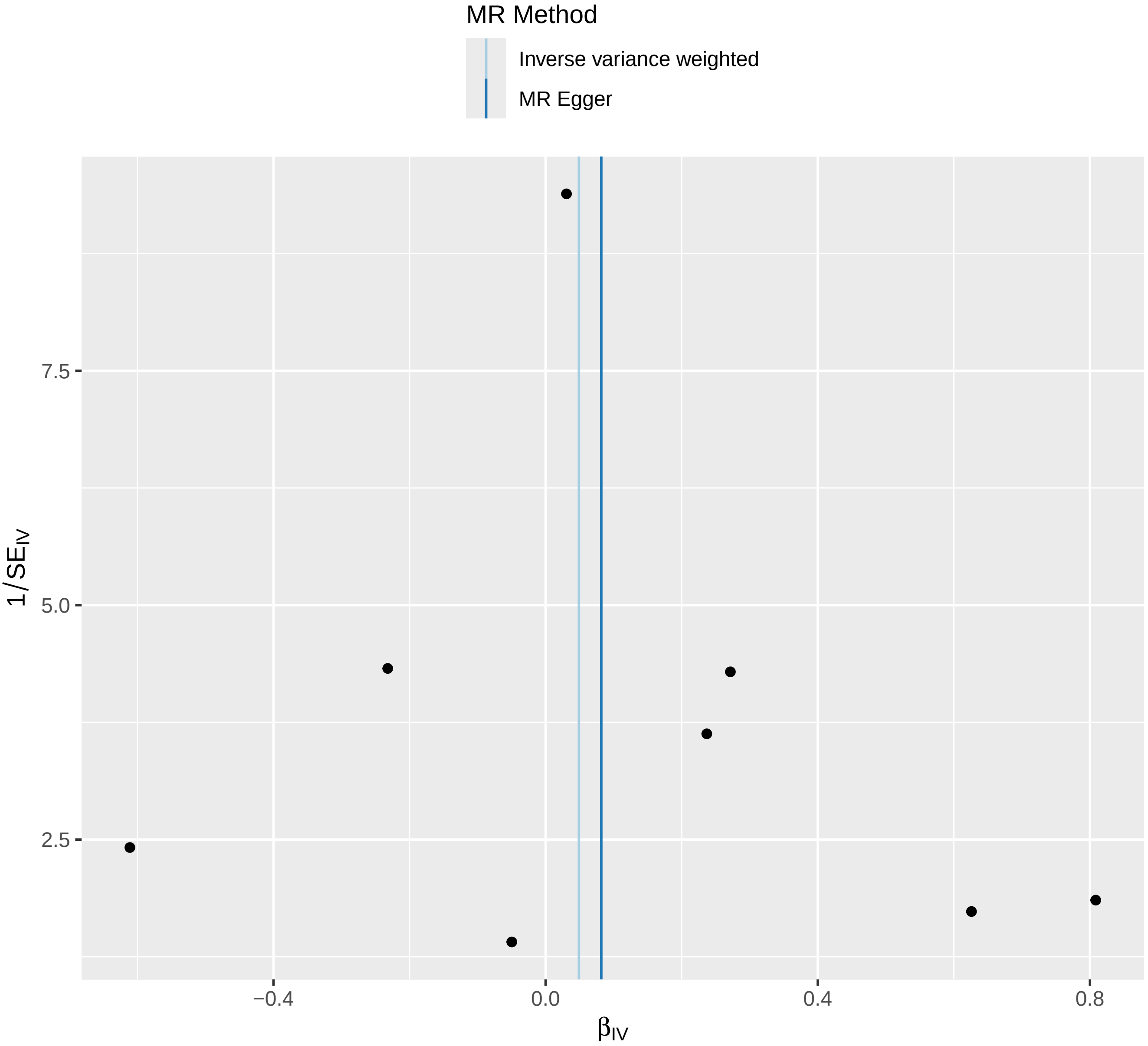
MR Method
Inverse variance weighted
MR Egger
1/SE$_{IV}$
7.5
5.0
2.5
−0.4
0.0
0.4
0.8
β$_{IV}$

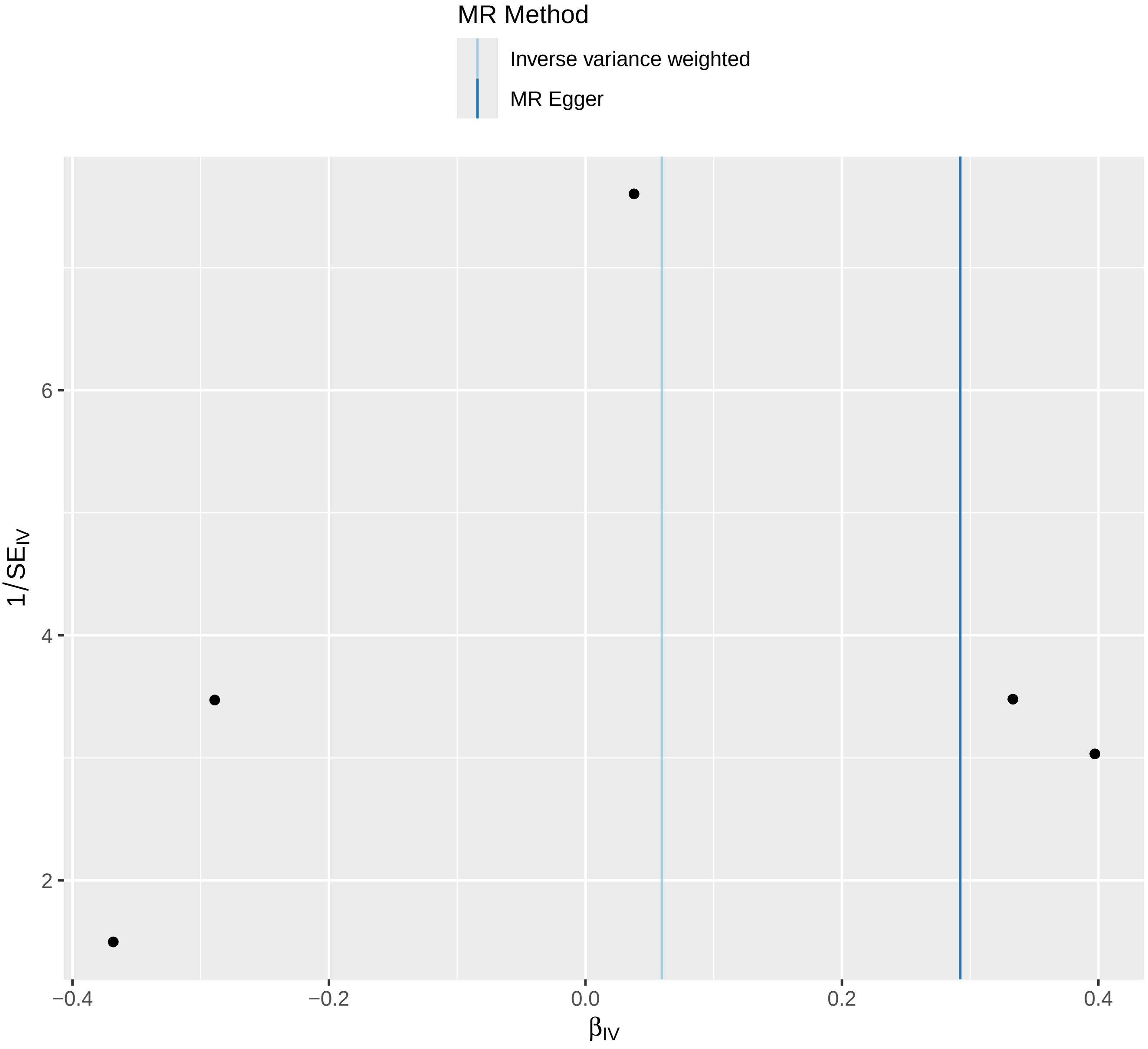

MR Method
Inverse variance weighted
MR Egger
6
4
2
1/SE_IV
−0.4
−0.2
0.0
0.2
0.4
β_IV

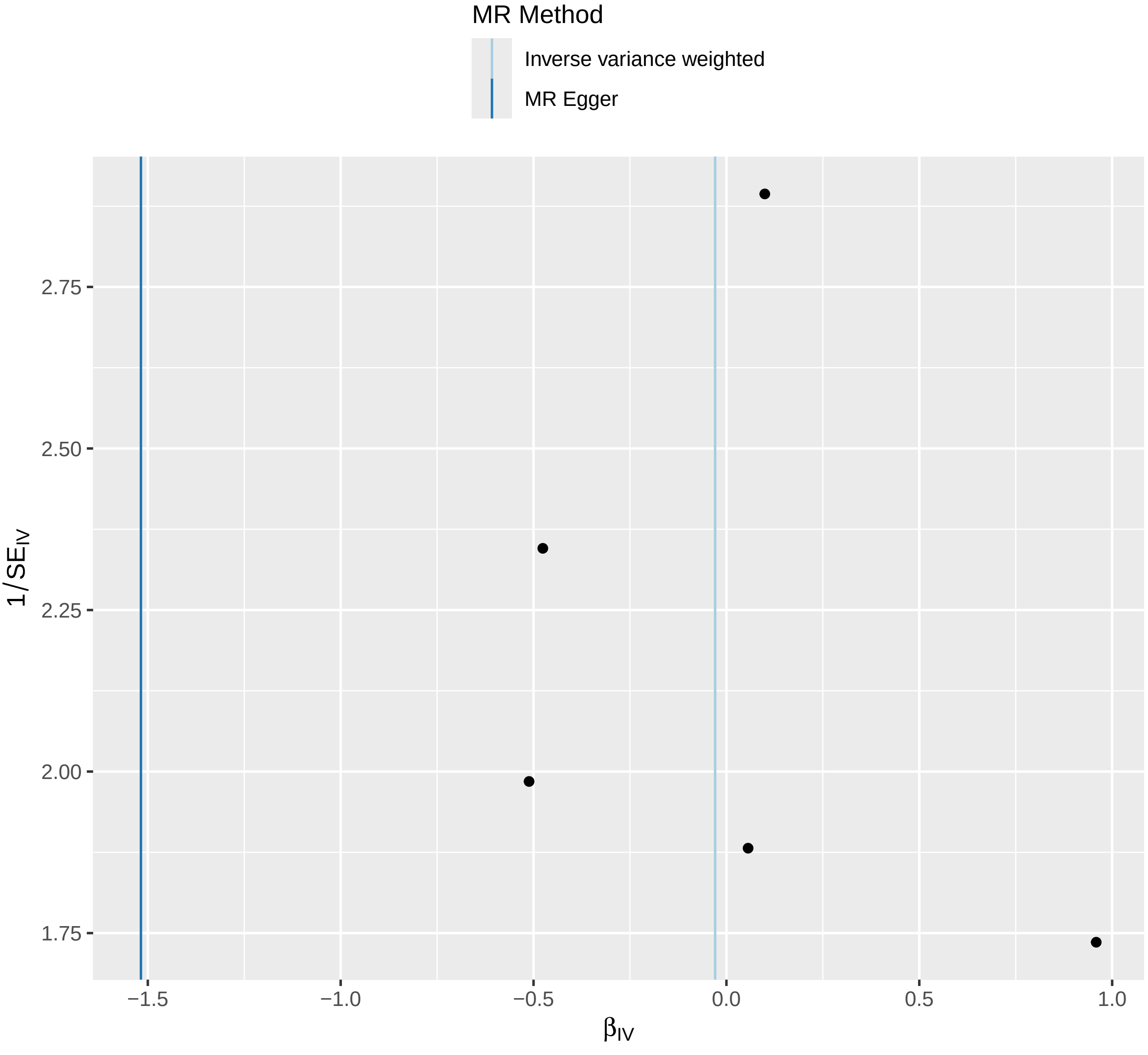
MR Method
Inverse variance weighted
MR Egger
1/SE$_{IV}$
2.75
2.50
2.25
2.00
1.75
−1.5
−1.0
−0.5
0.0
0.5
1.0
β$_{IV}$

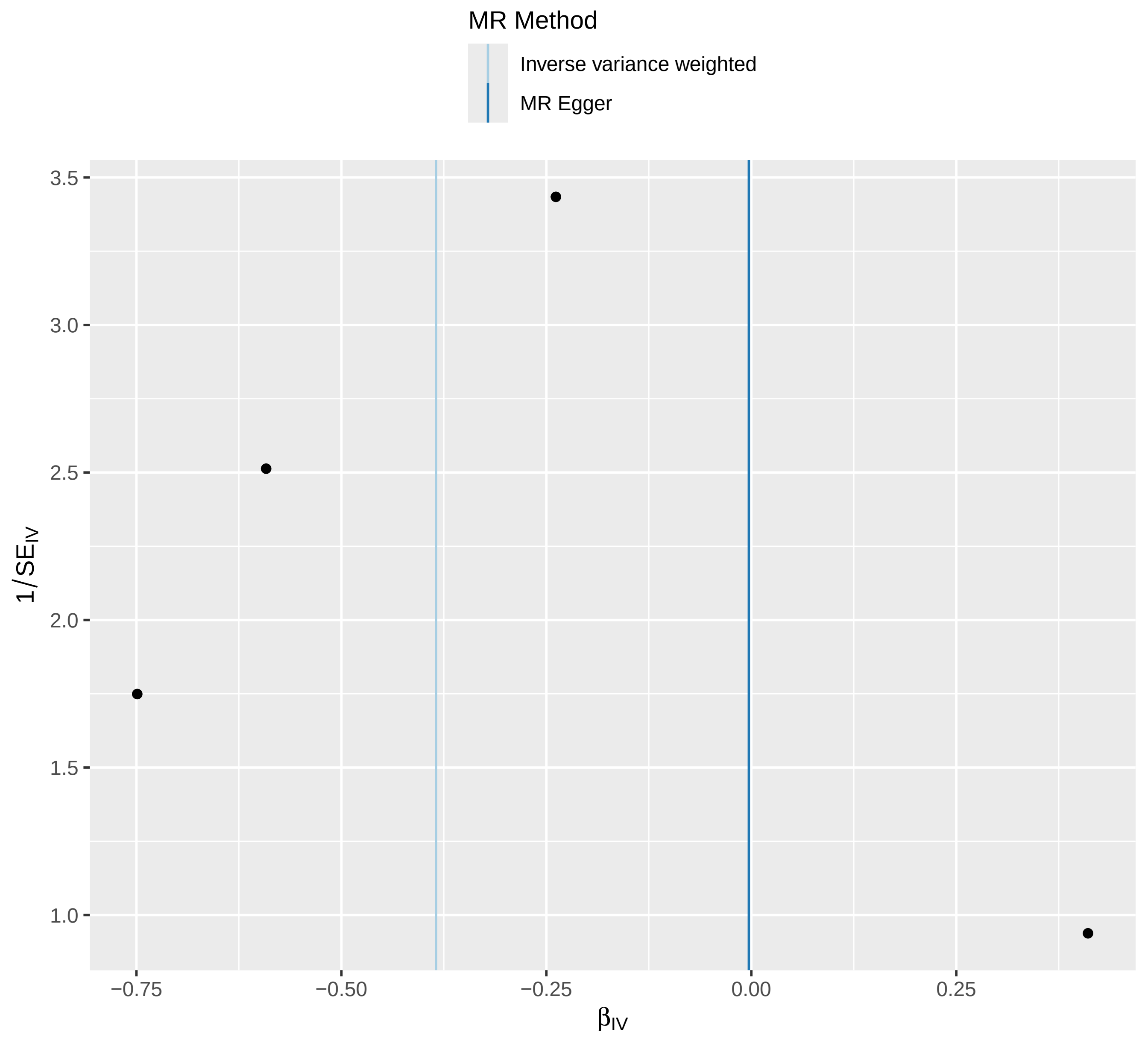

MR Method
Inverse variance weighted
MR Egger
1/SE_IV
β_IV
3.5
3.0
2.5
2.0
1.5
1.0
−0.75
−0.50
−0.25
0.00
0.25

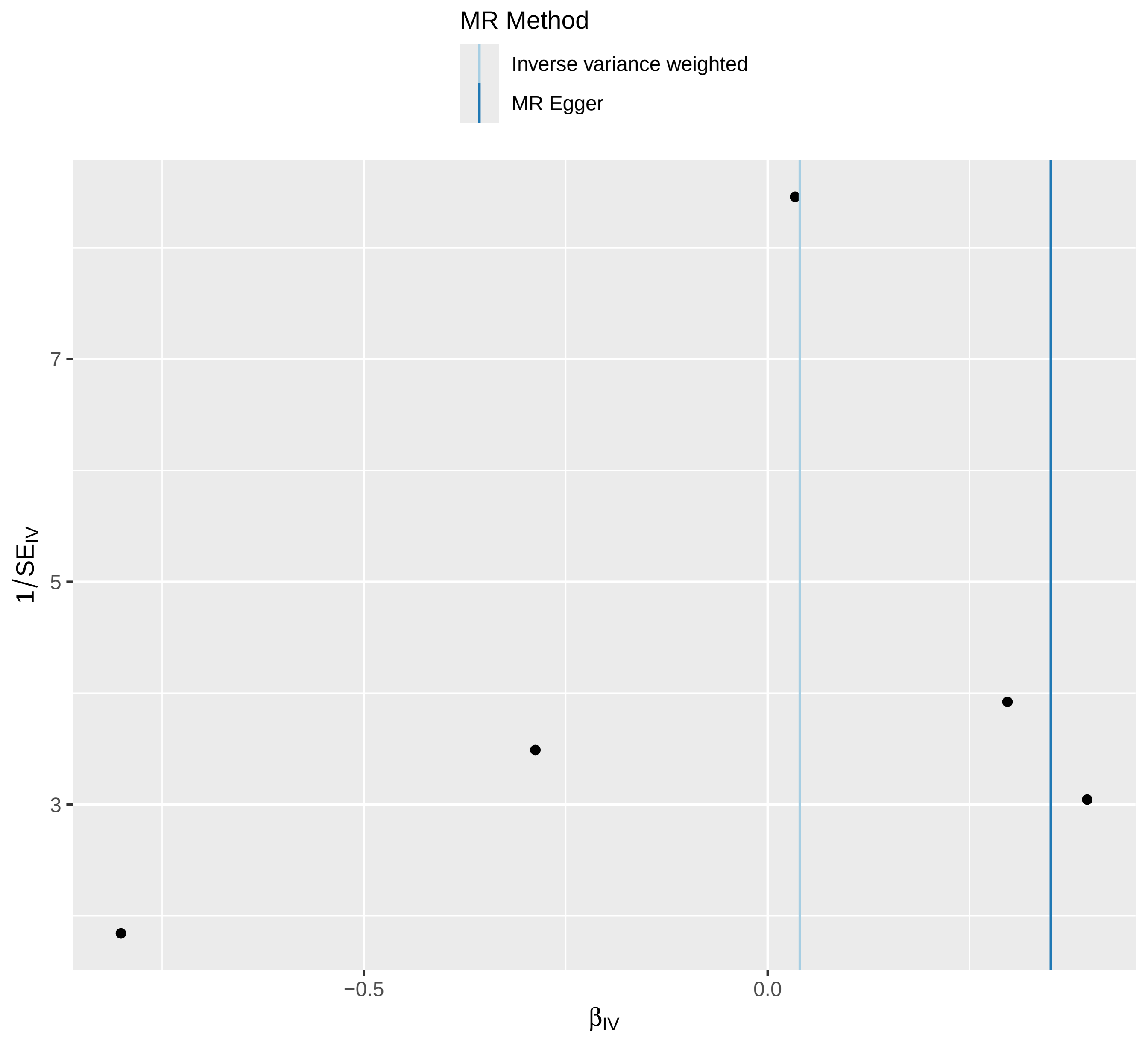
MR Method
Inverse variance weighted
MR Egger
7
5
3
1/SE$_{IV}$
−0.5
0.0
β$_{IV}$

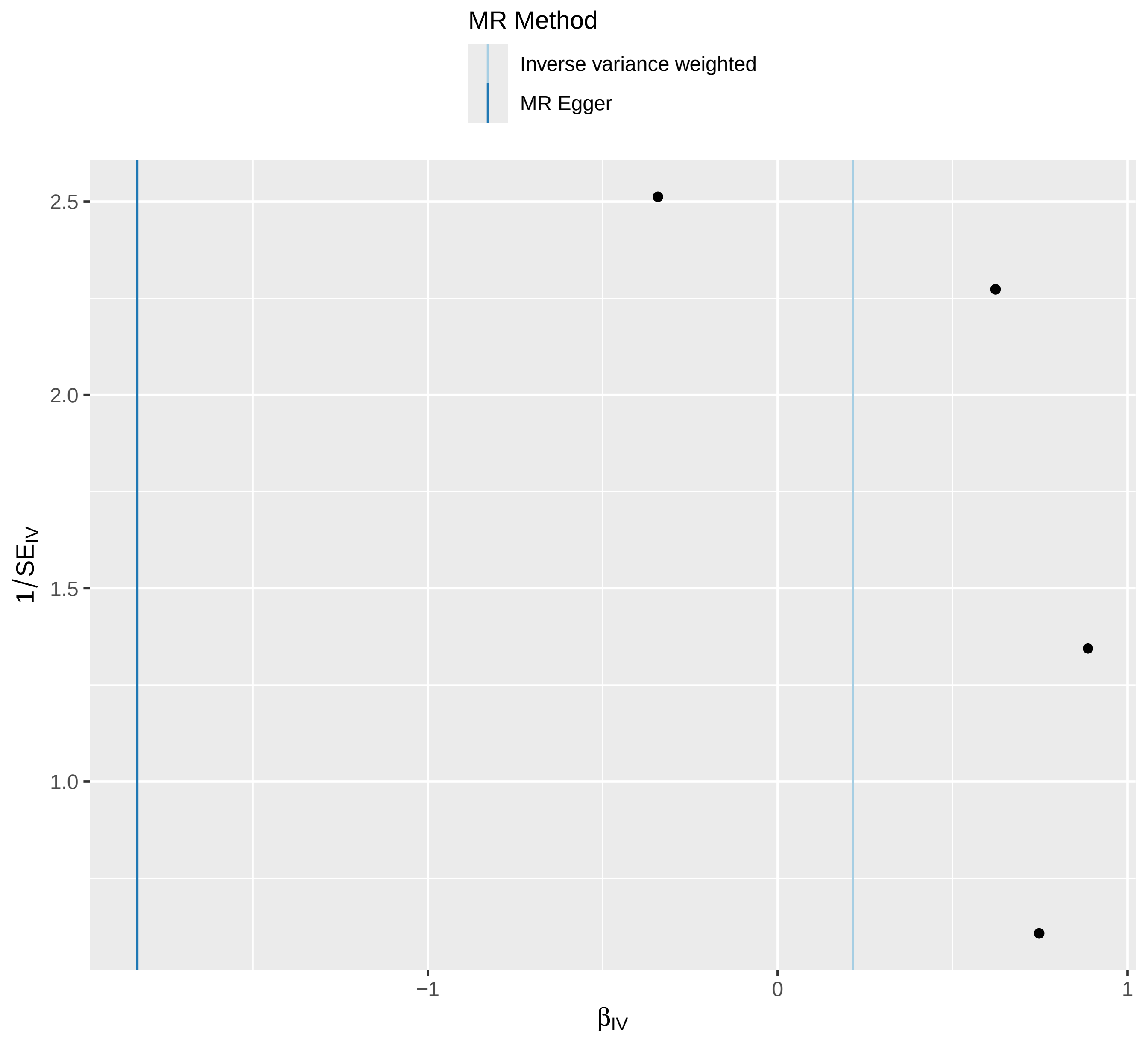

MR Method
Inverse variance weighted
MR Egger
2.5
2.0
1.5
1.0
1/SE_IV
−1
0
1
β_IV

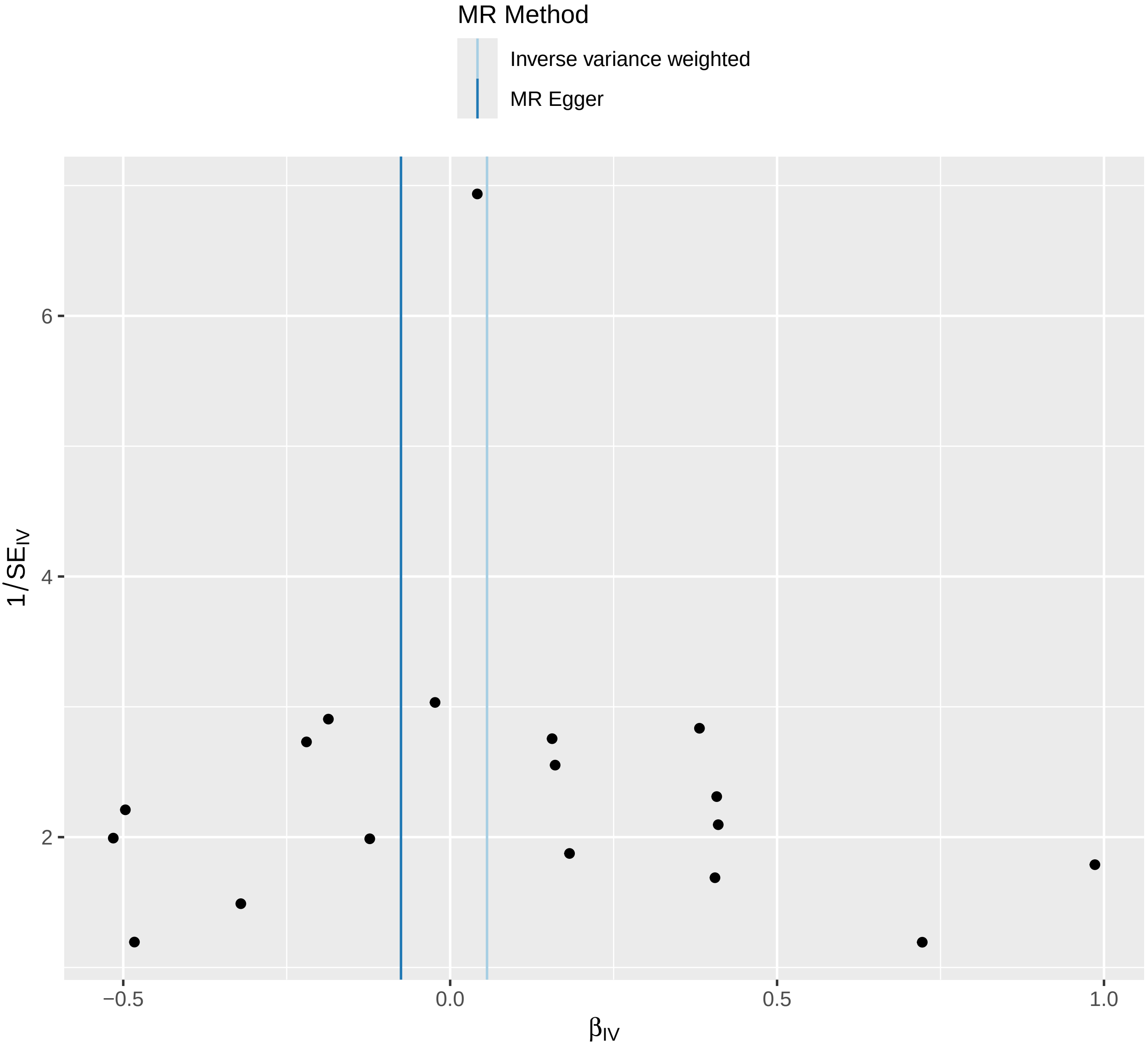

MR Method
Inverse variance weighted
MR Egger
6
4
2
1/SE$_{IV}$
−0.5
0.0
0.5
1.0
β$_{IV}$

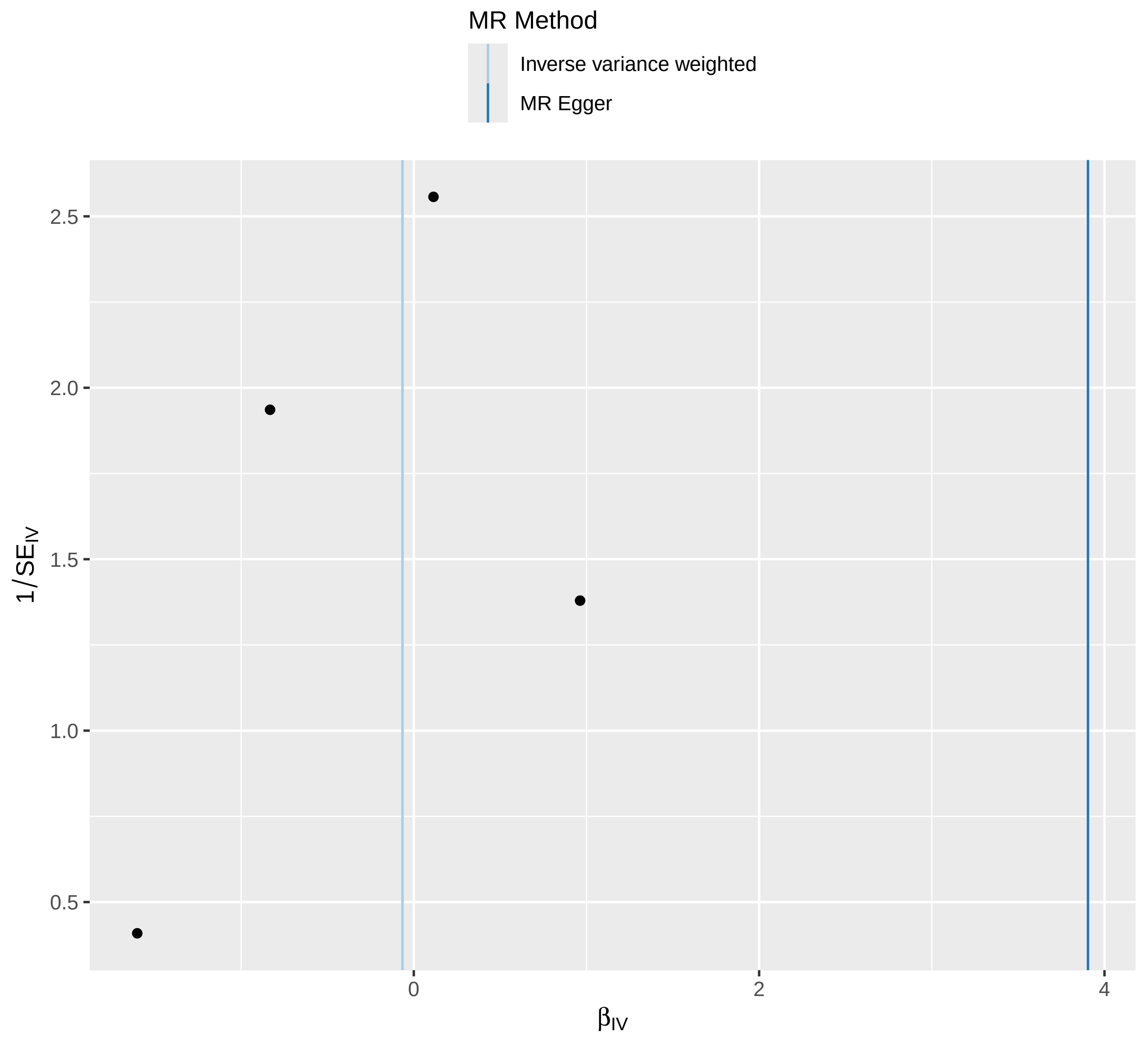
MR Method
Inverse variance weighted
MR Egger
2.5
2.0
1.5
1.0
0.5
$1/SE_{IV}$
0
2
4
β_{IV}

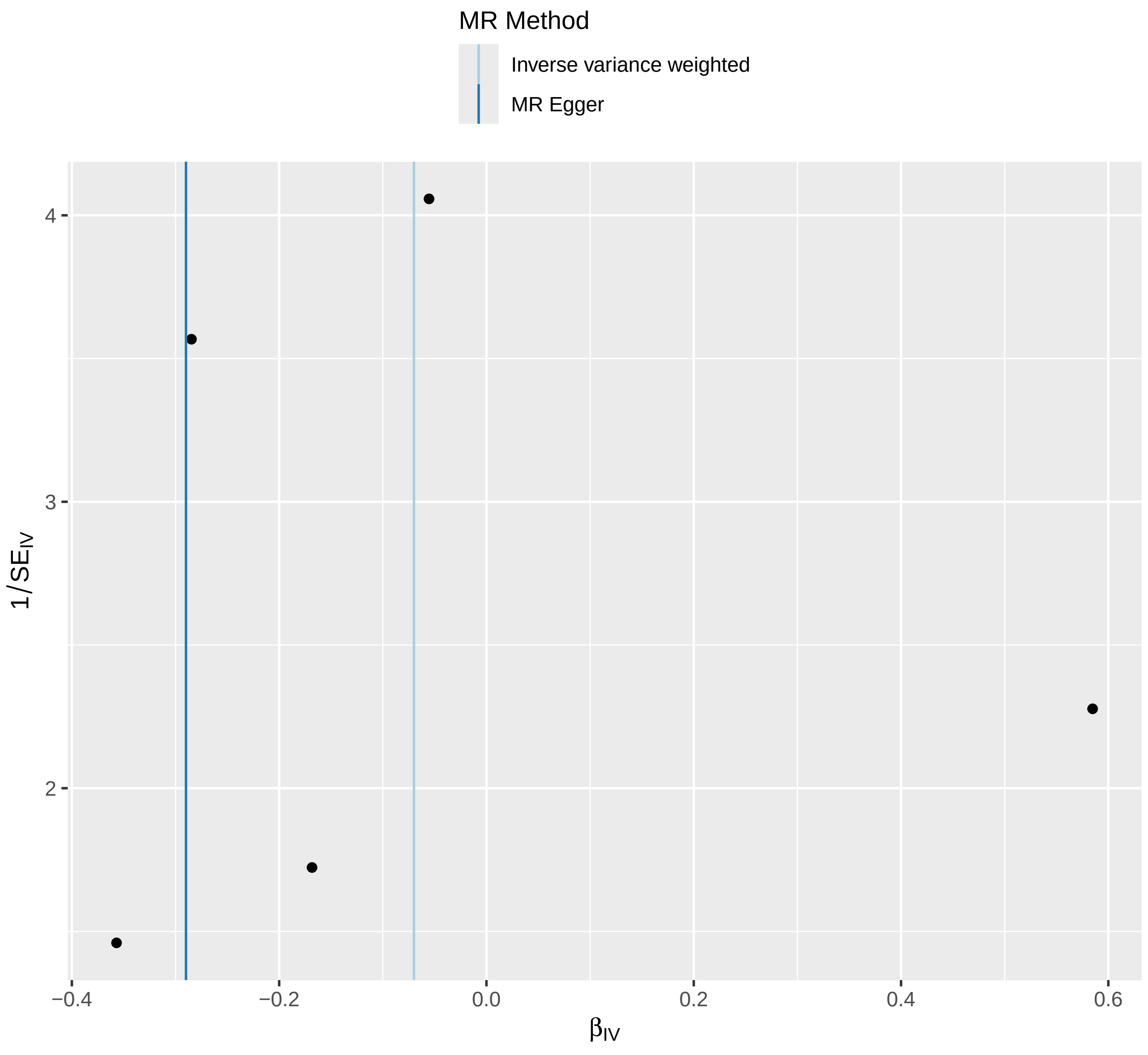
MR Method
Inverse variance weighted
MR Egger
4
3
2
1/SE_IV
−0.4
−0.2
0.0
0.2
0.4
0.6
β_IV

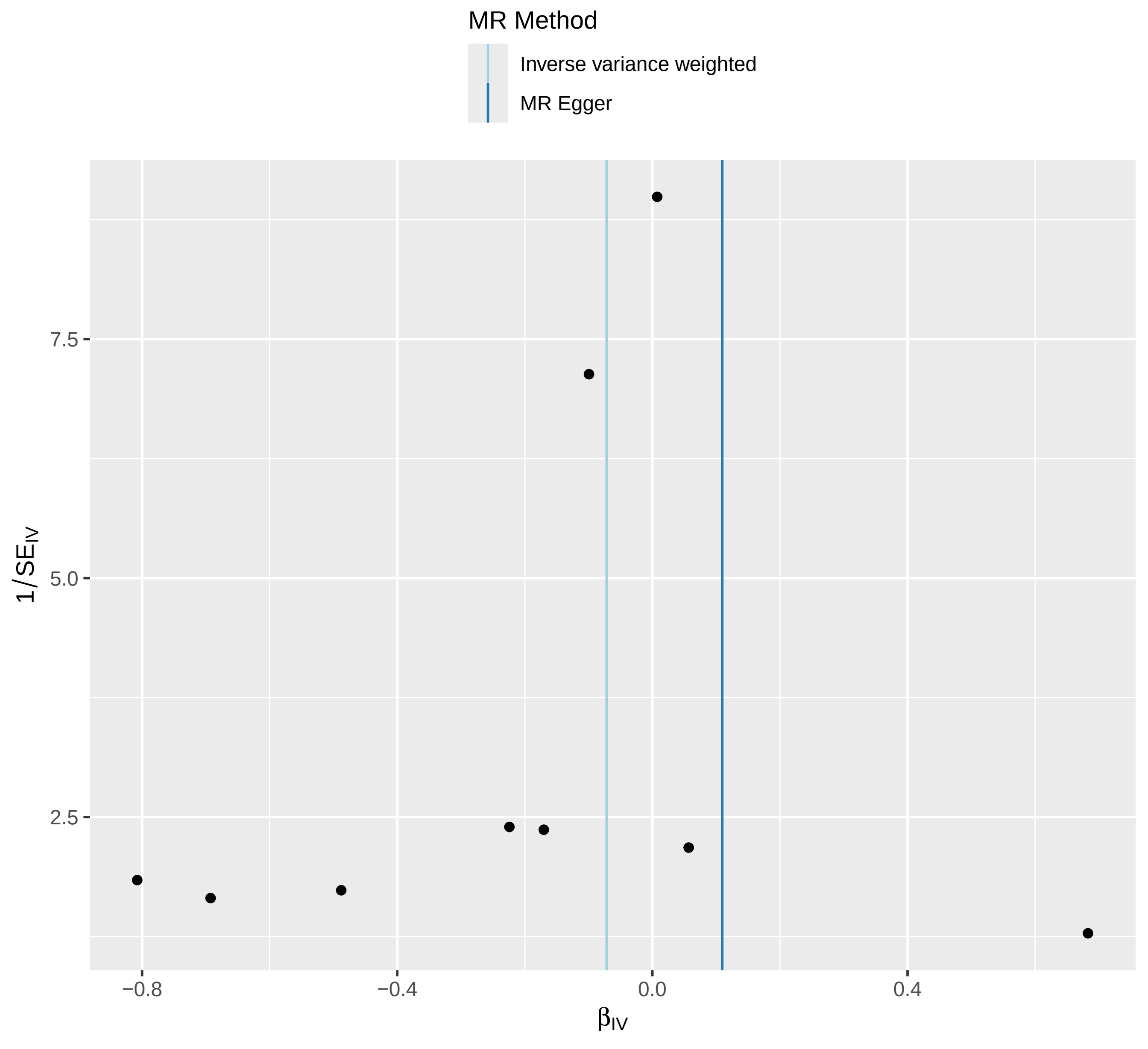
MR Method
Inverse variance weighted
MR Egger
7.5
5.0
2.5
−0.8
−0.4
0.0
0.4
$1/SE_{IV}$
β_{IV}

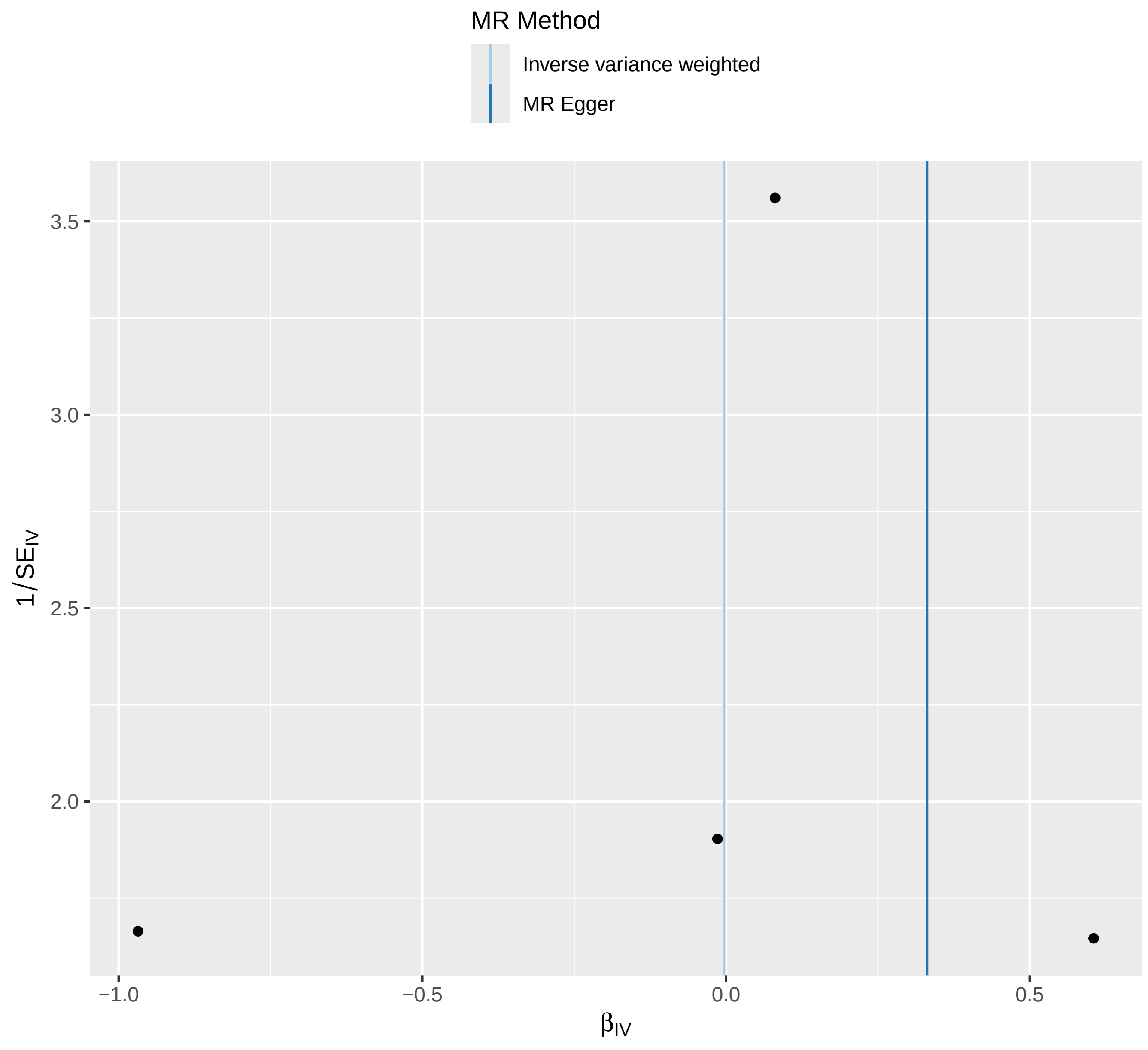
MR Method
Inverse variance weighted
MR Egger
3.5
3.0
2.5
2.0
1/SE_IV
−1.0
−0.5
0.0
0.5
β_IV

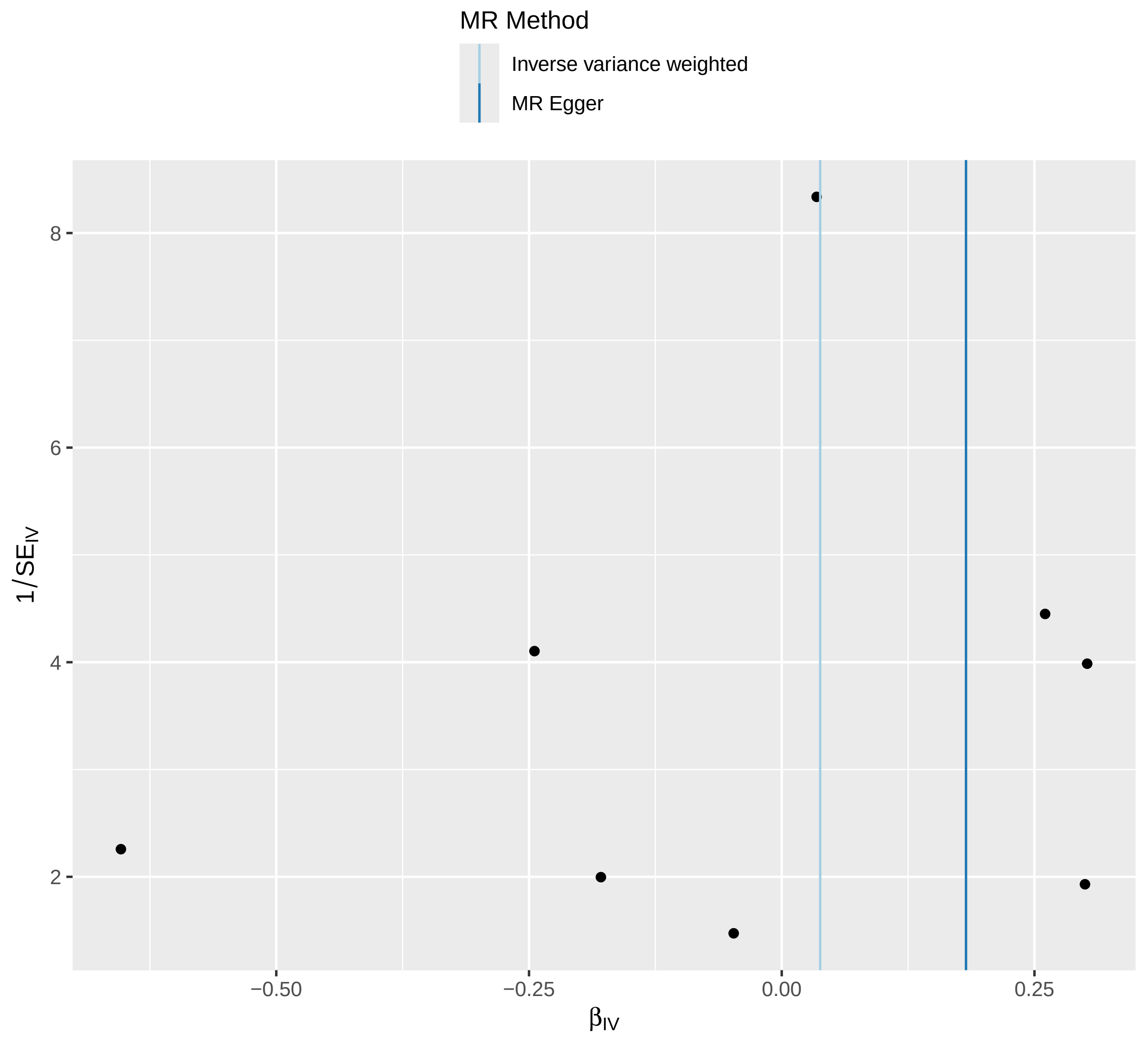

MR Method
Inverse variance weighted
MR Egger
1/SE$_{IV}$
8
6
4
2
−0.50
−0.25
0.00
0.25
β$_{IV}$

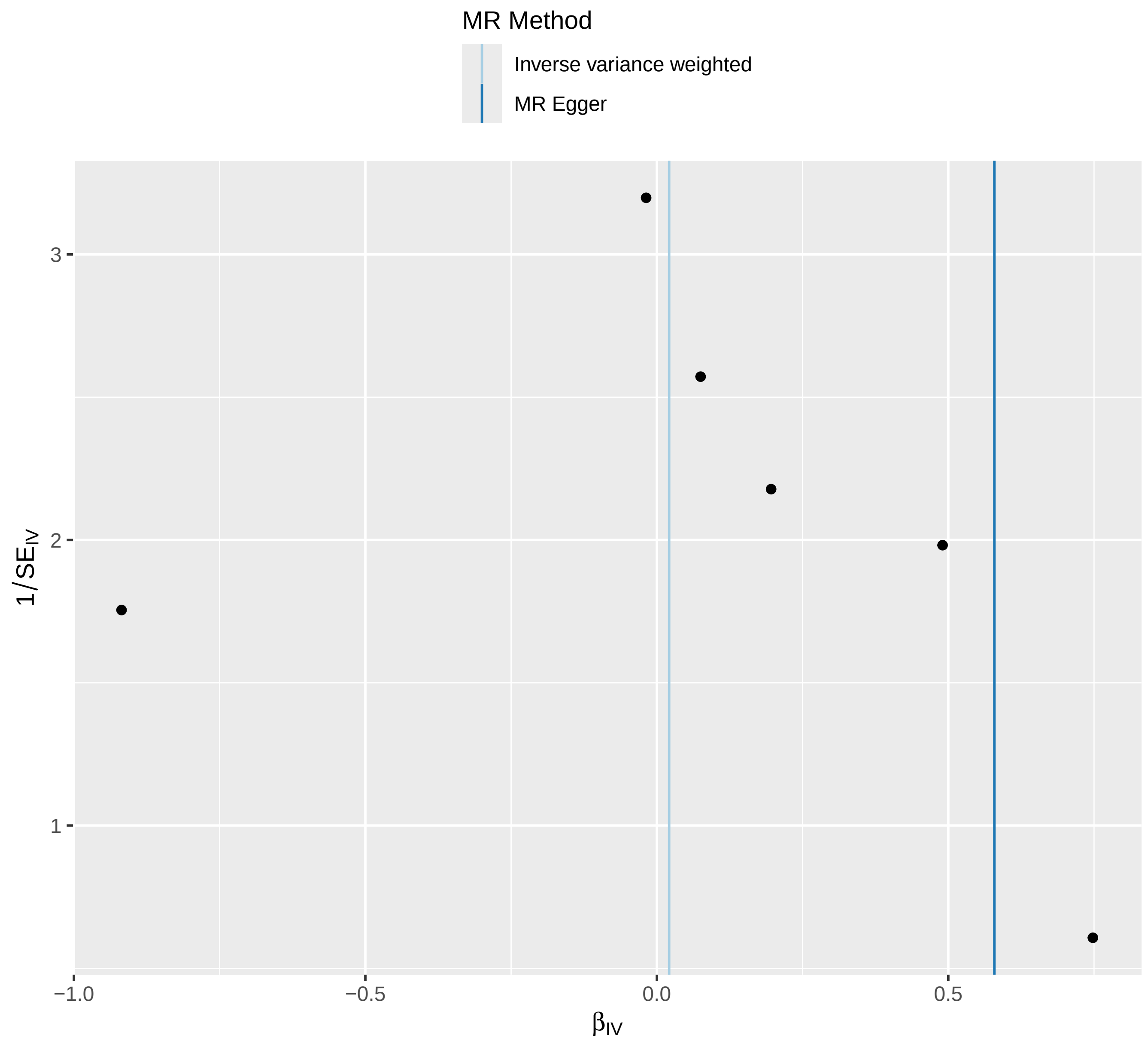

MR Method
Inverse variance weighted
MR Egger
3
2
1
1/SE$_{IV}$
−1.0
−0.5
0.0
0.5
β$_{IV}$

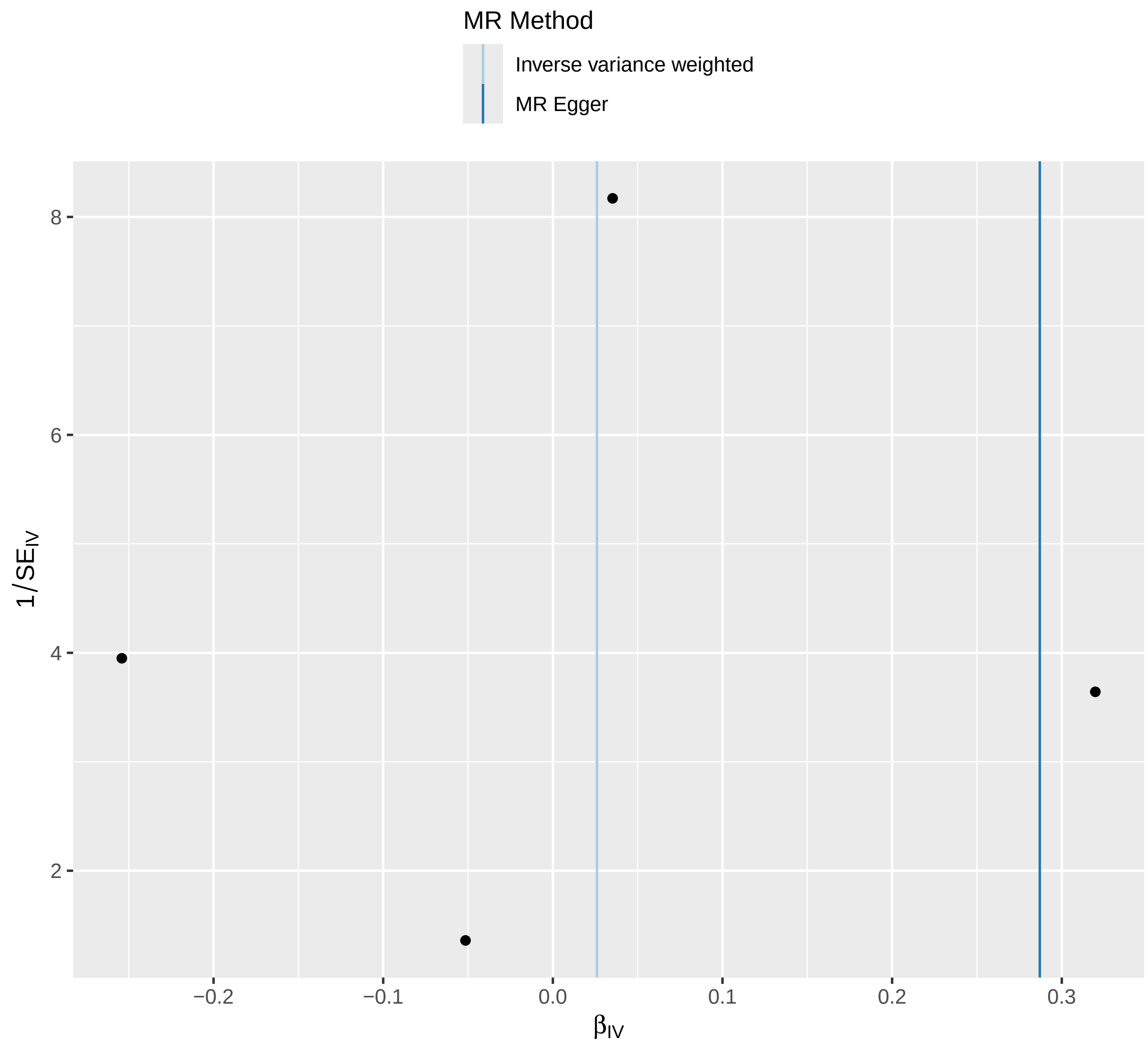

MR Method
Inverse variance weighted
MR Egger
8
6
4
2
1/SE_IV
−0.2
−0.1
0.0
0.1
0.2
0.3
β_IV

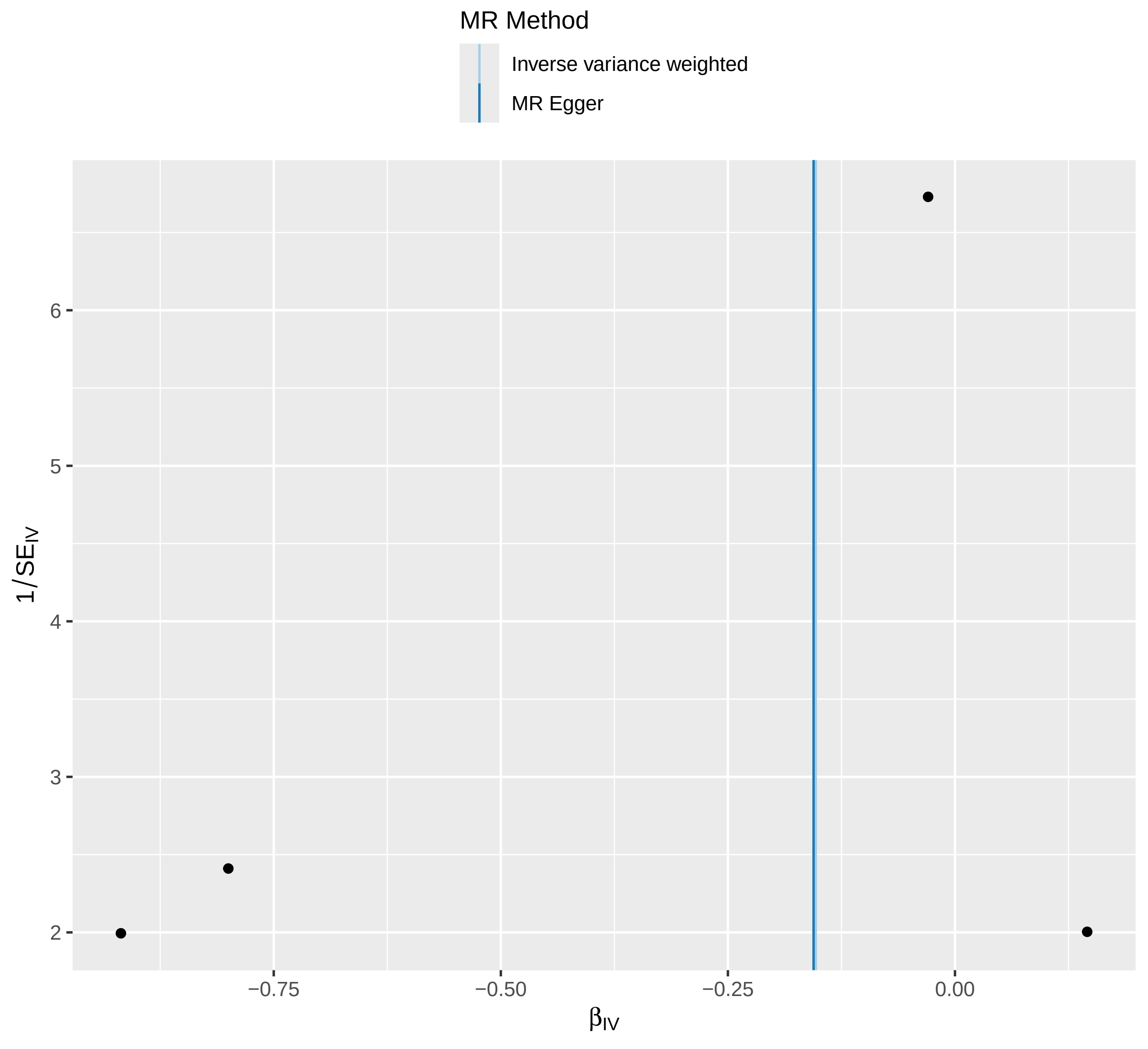
MR Method
Inverse variance weighted
MR Egger
1/SE_{IV}
6
5
4
3
2
−0.75
−0.50
−0.25
0.00
β_{IV}

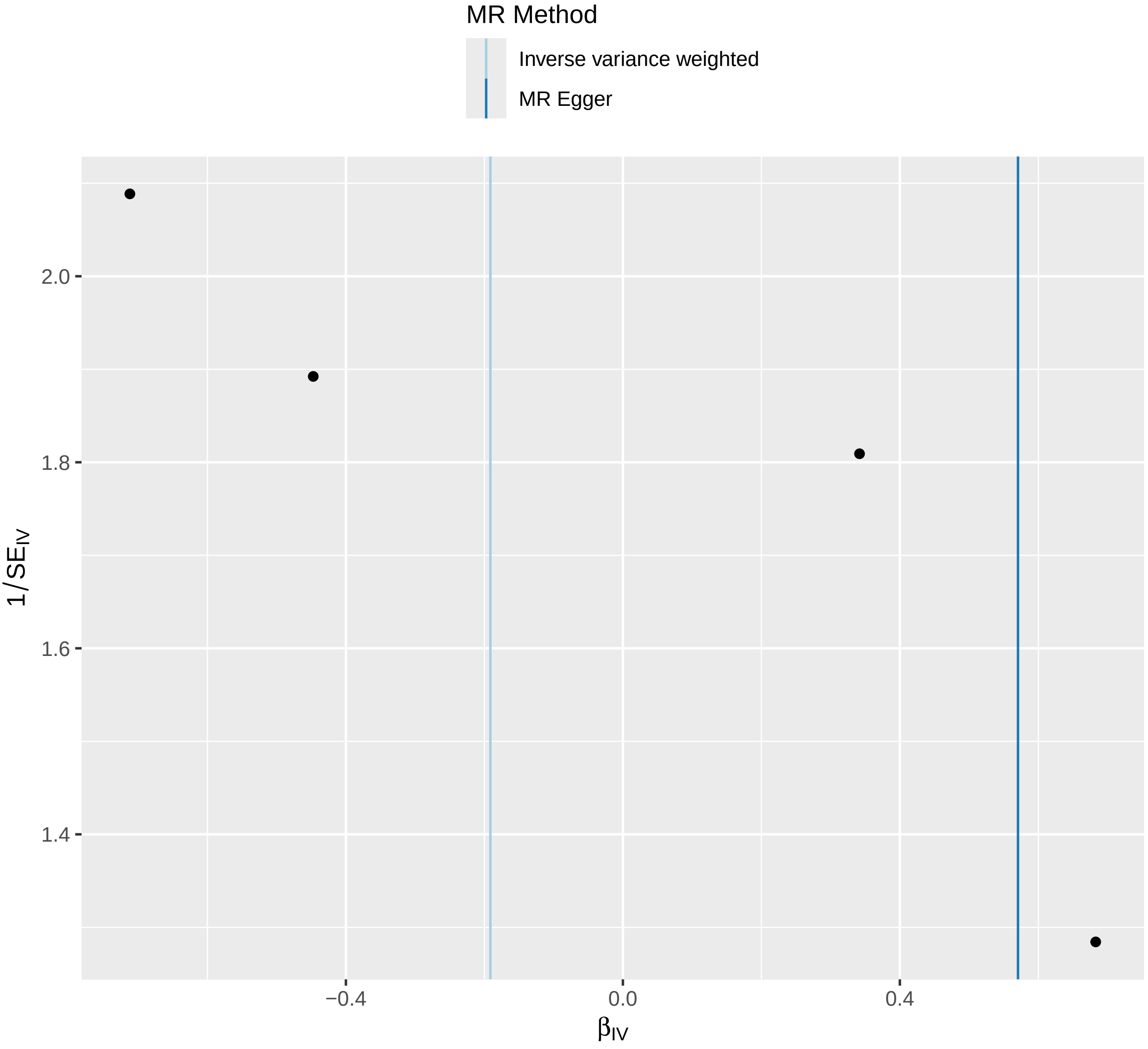

MR Method
Inverse variance weighted
MR Egger
2.0
1.8
1.6
1.4
1/SE_IV
−0.4
0.0
0.4
β_IV

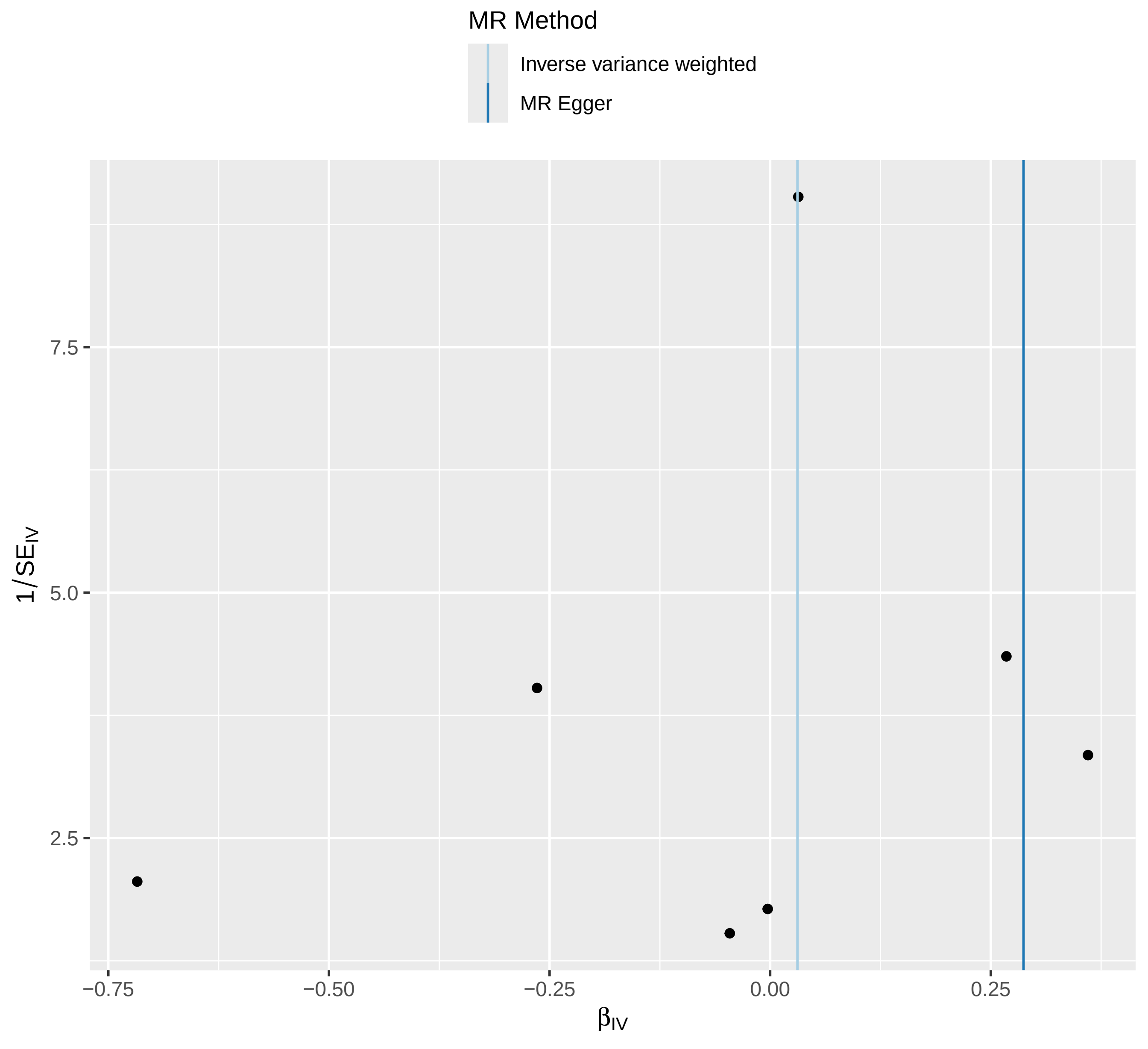

MR Method
Inverse variance weighted
MR Egger
7.5
5.0
2.5
1/SE$_{IV}$
−0.75
−0.50
−0.25
0.00
0.25
β$_{IV}$

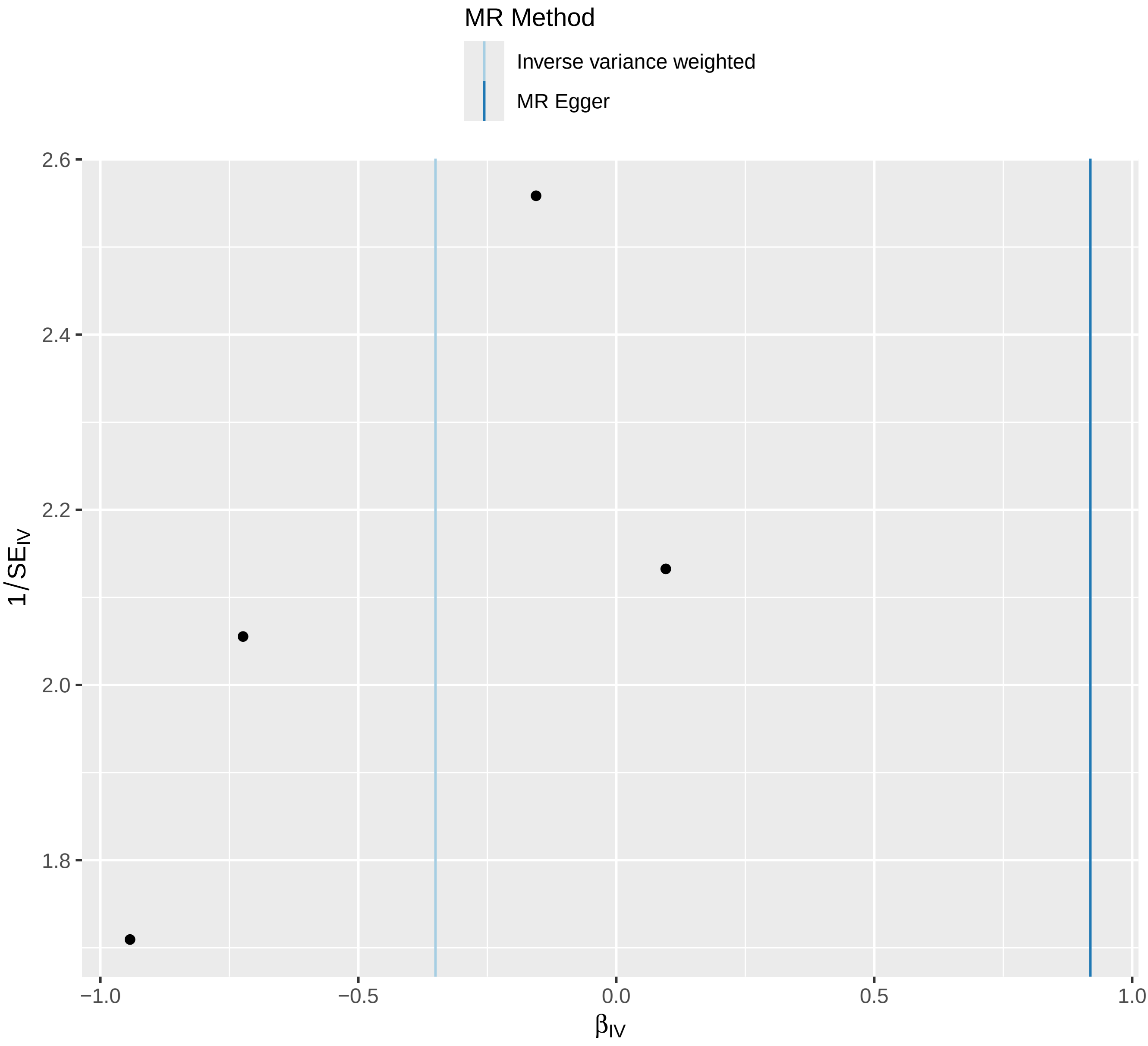

MR Method
Inverse variance weighted
MR Egger
2.6
2.4
2.2
2.0
1.8
1/SE_IV
−1.0
−0.5
0.0
0.5
1.0
β_IV

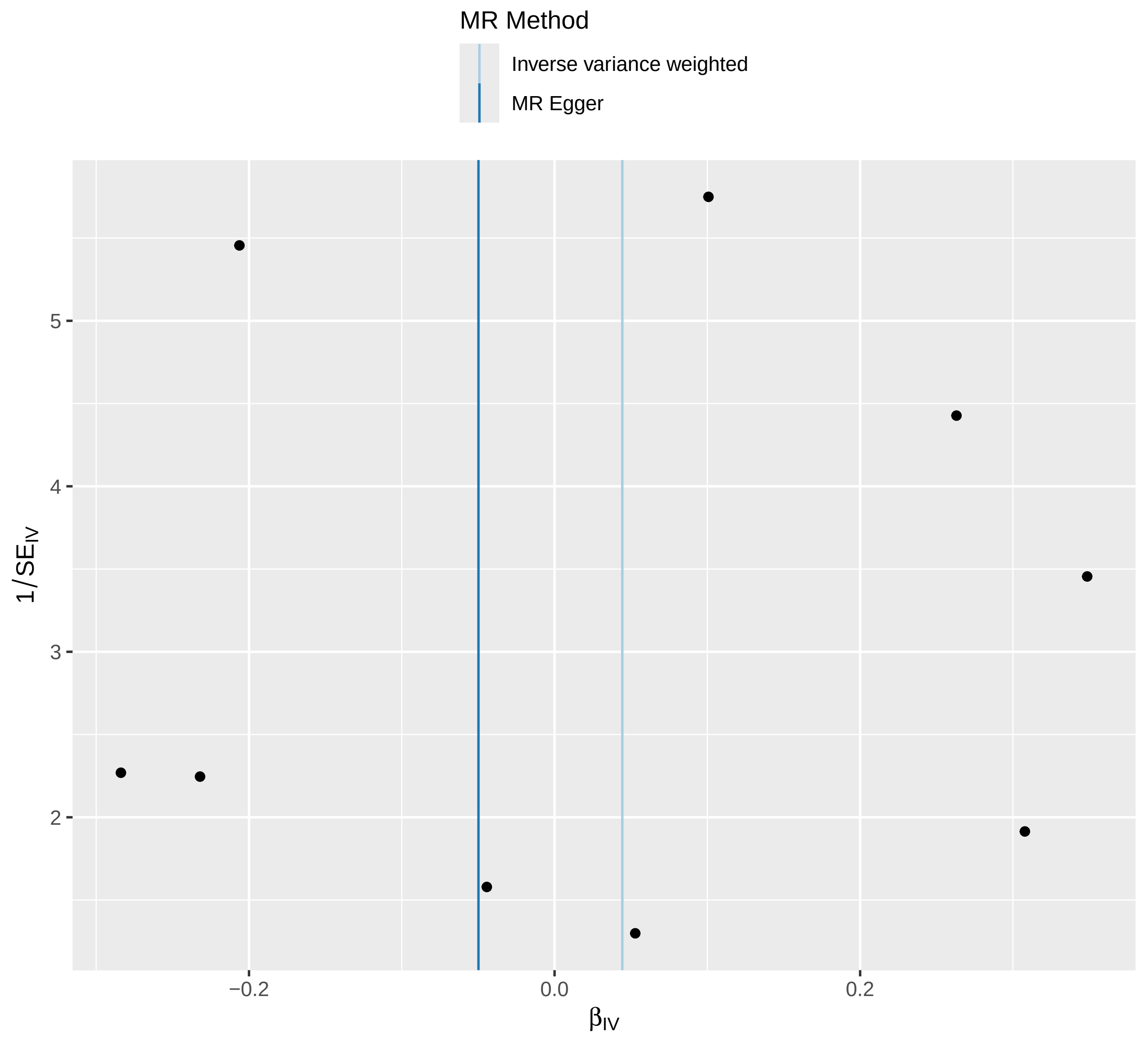

MR Method
Inverse variance weighted
MR Egger
5
4
3
2
1/SE$_{IV}$
−0.2
0.0
0.2
β$_{IV}$

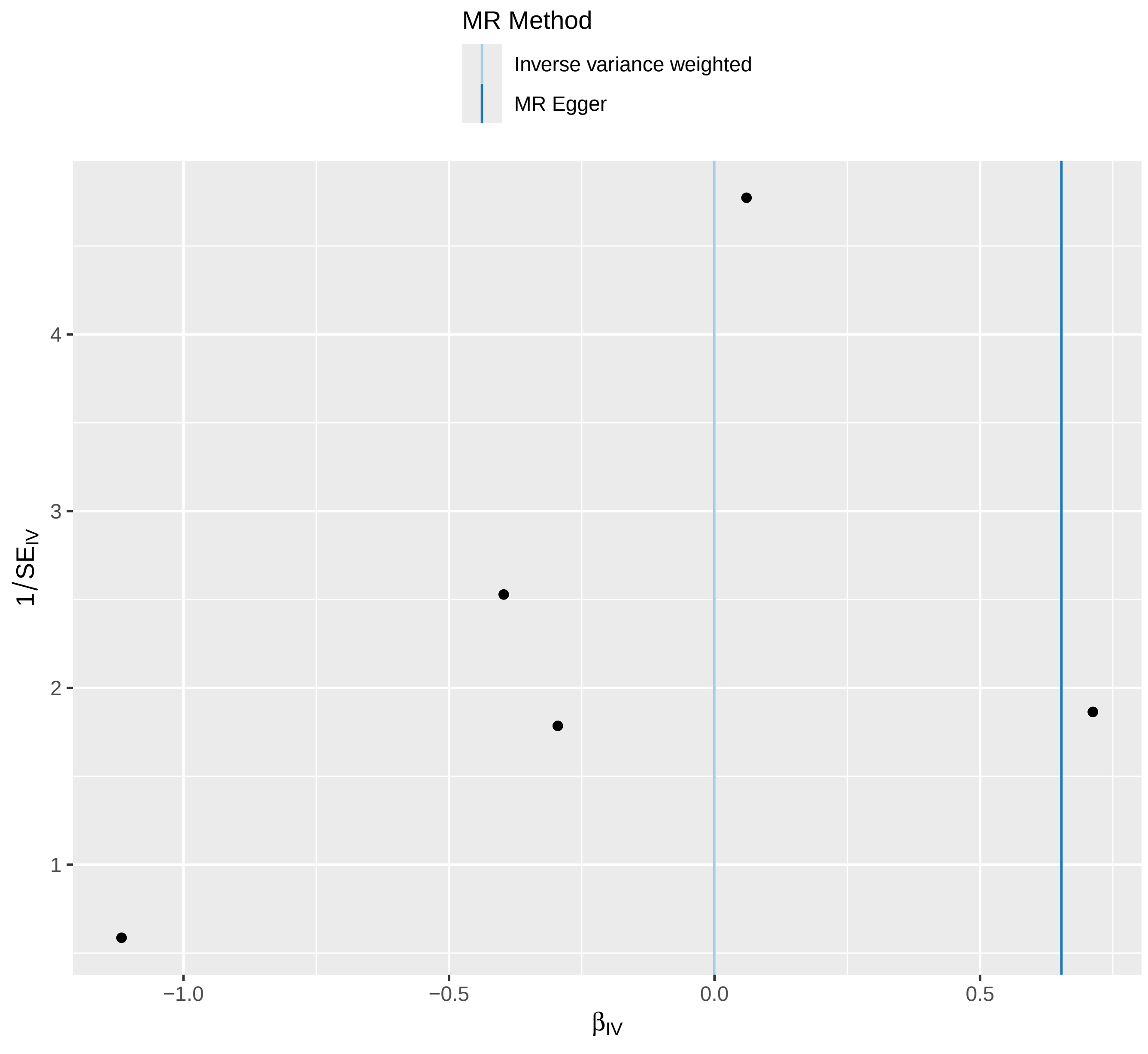
MR Method
Inverse variance weighted
MR Egger
1/SE_IV
4
3
2
1
−1.0
−0.5
0.0
0.5
β_IV

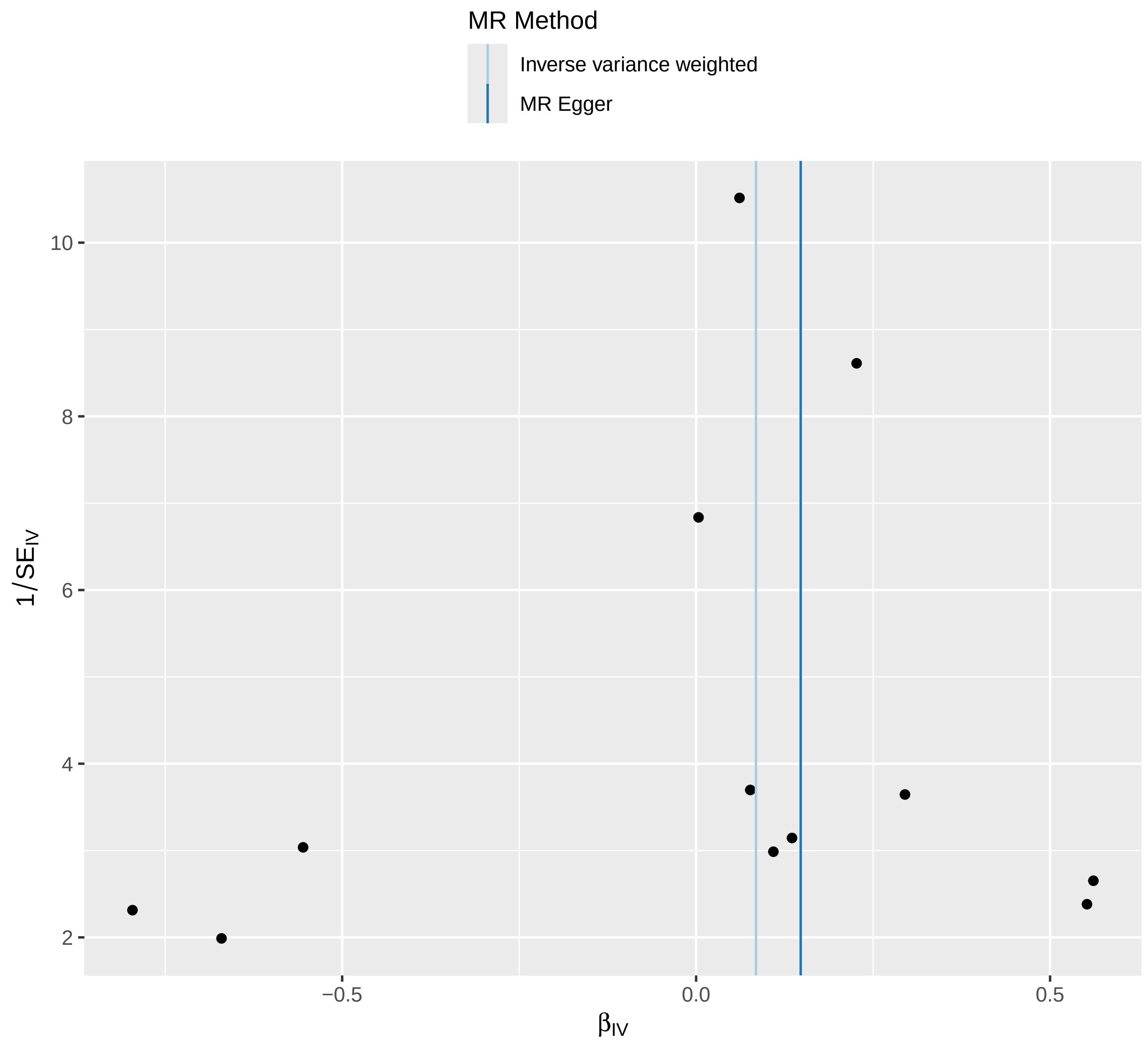
MR Method
Inverse variance weighted
MR Egger
10
8
6
4
2
1/SE$_{IV}$
−0.5
0.0
0.5
β$_{IV}$

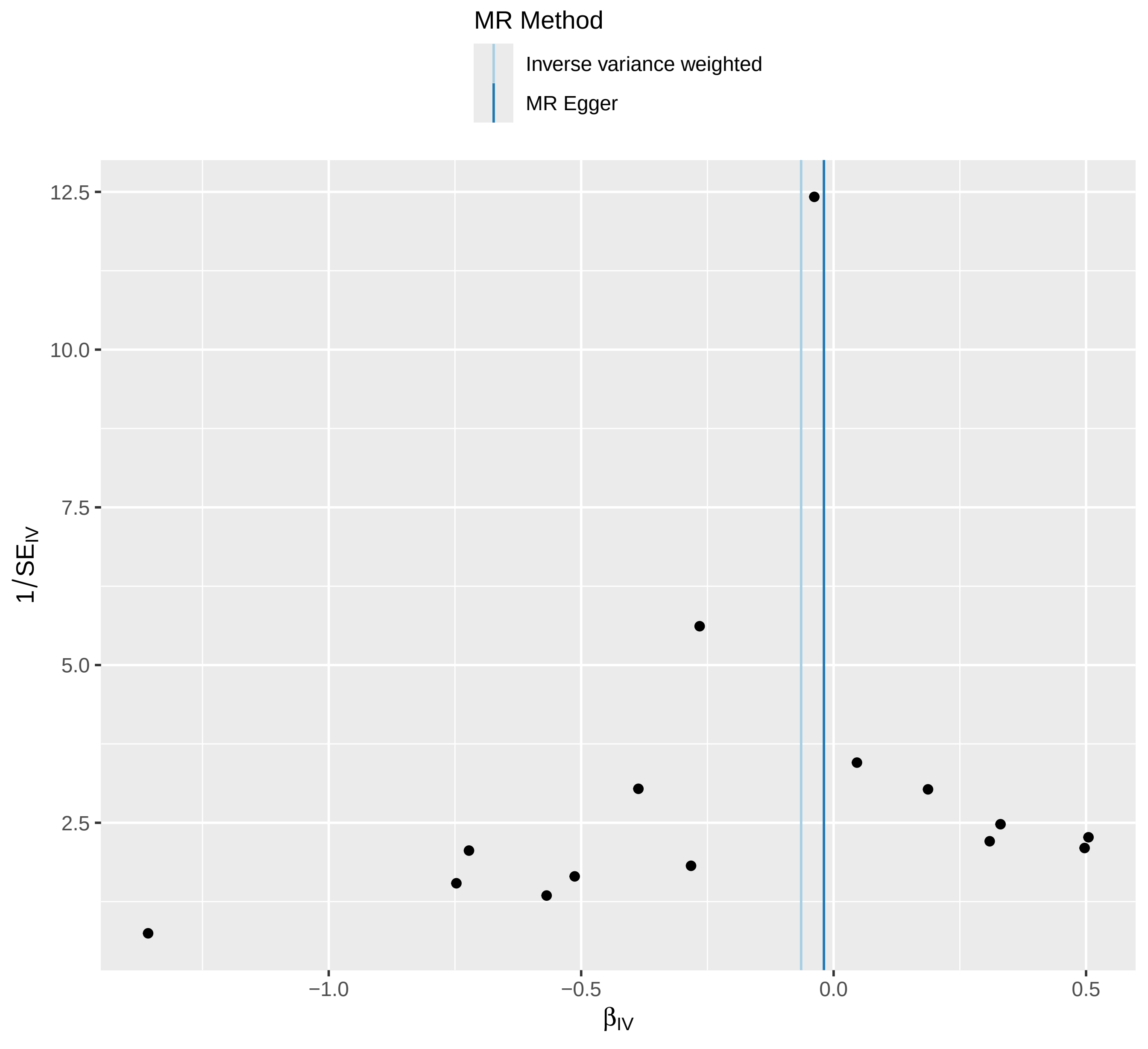

MR Method
Inverse variance weighted
MR Egger
12.5
10.0
7.5
5.0
2.5
1/SE_IV
−1.0
−0.5
0.0
0.5
β_IV

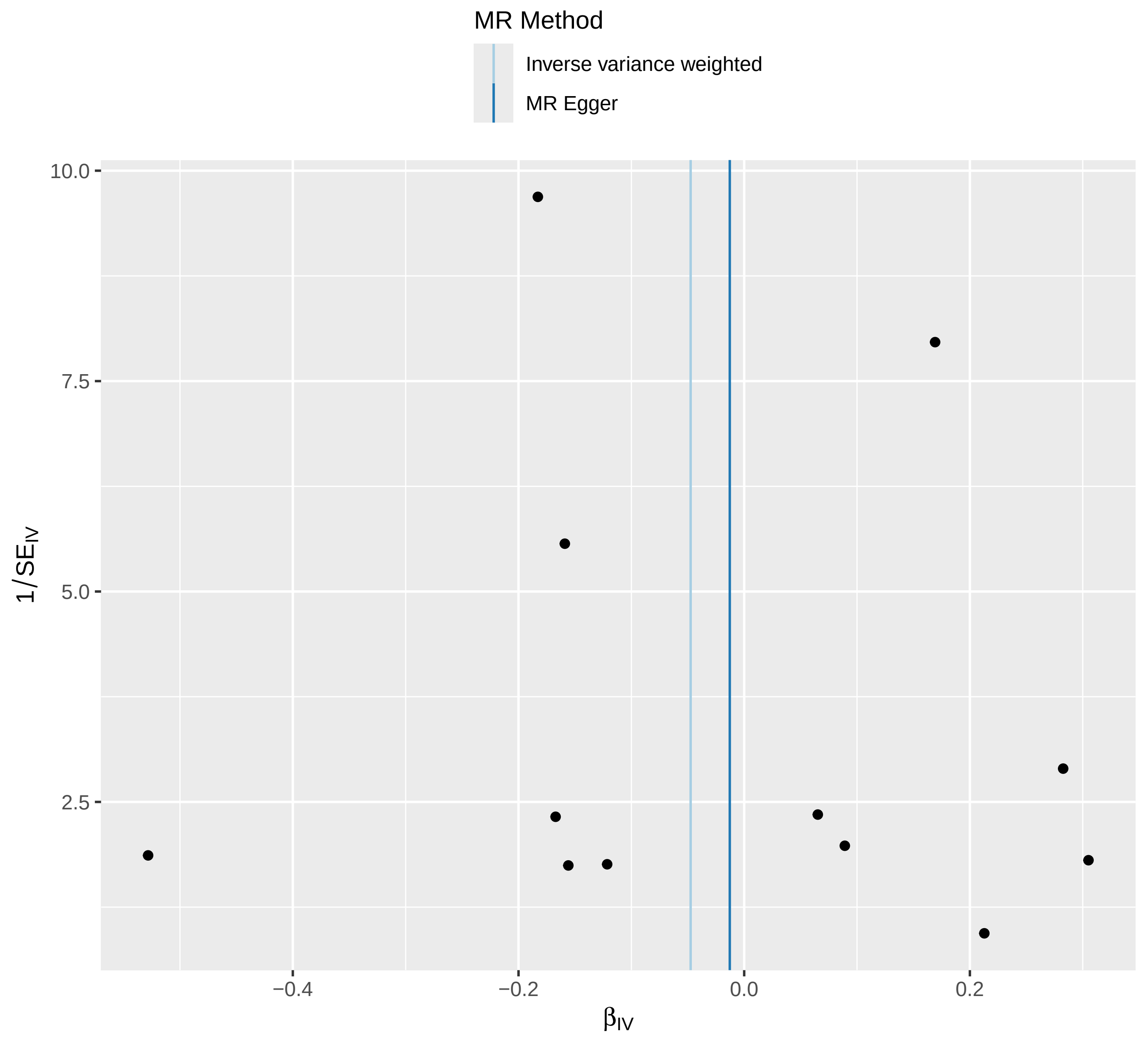
MR Method
Inverse variance weighted
MR Egger
10.0
7.5
5.0
2.5
1/SE_IV
−0.4
−0.2
0.0
0.2
β_IV

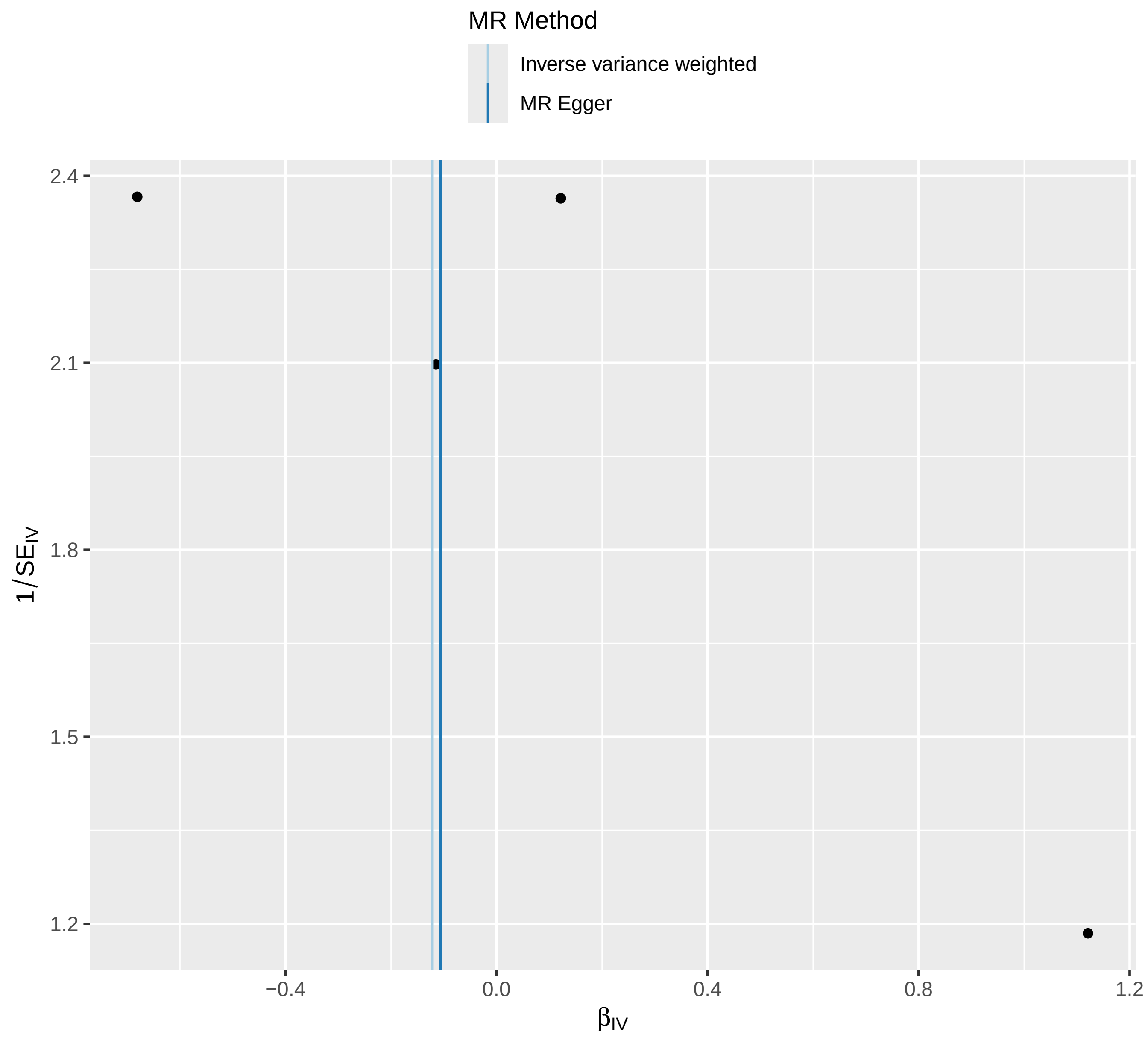
MR Method
Inverse variance weighted
MR Egger
2.4
2.1
1.8
1.5
1.2
1/SE_IV
−0.4
0.0
0.4
0.8
1.2
β_IV

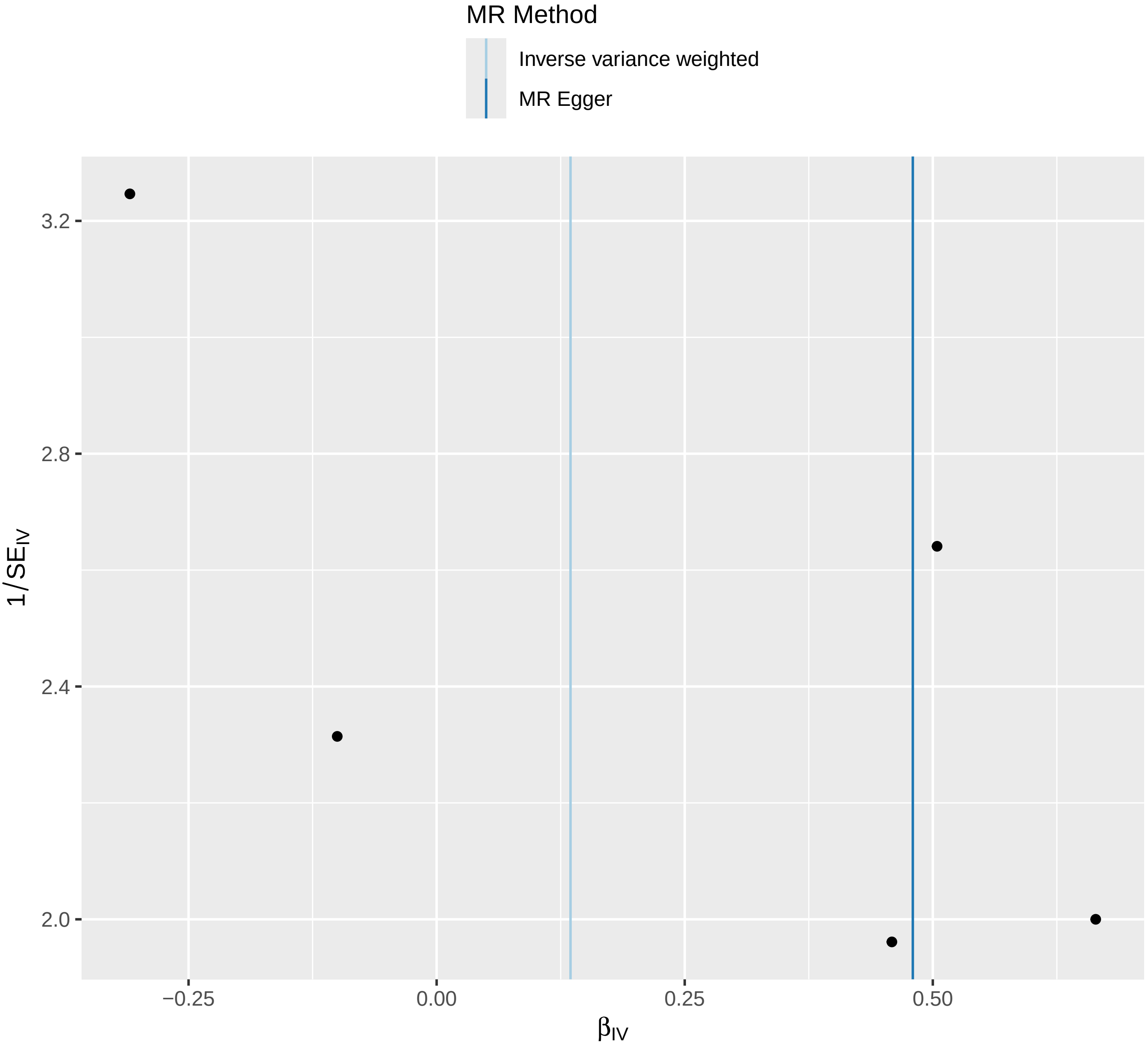

MR Method
Inverse variance weighted
MR Egger
3.2
2.8
2.4
2.0
1/SE_IV
−0.25
0.00
0.25
0.50
β_IV

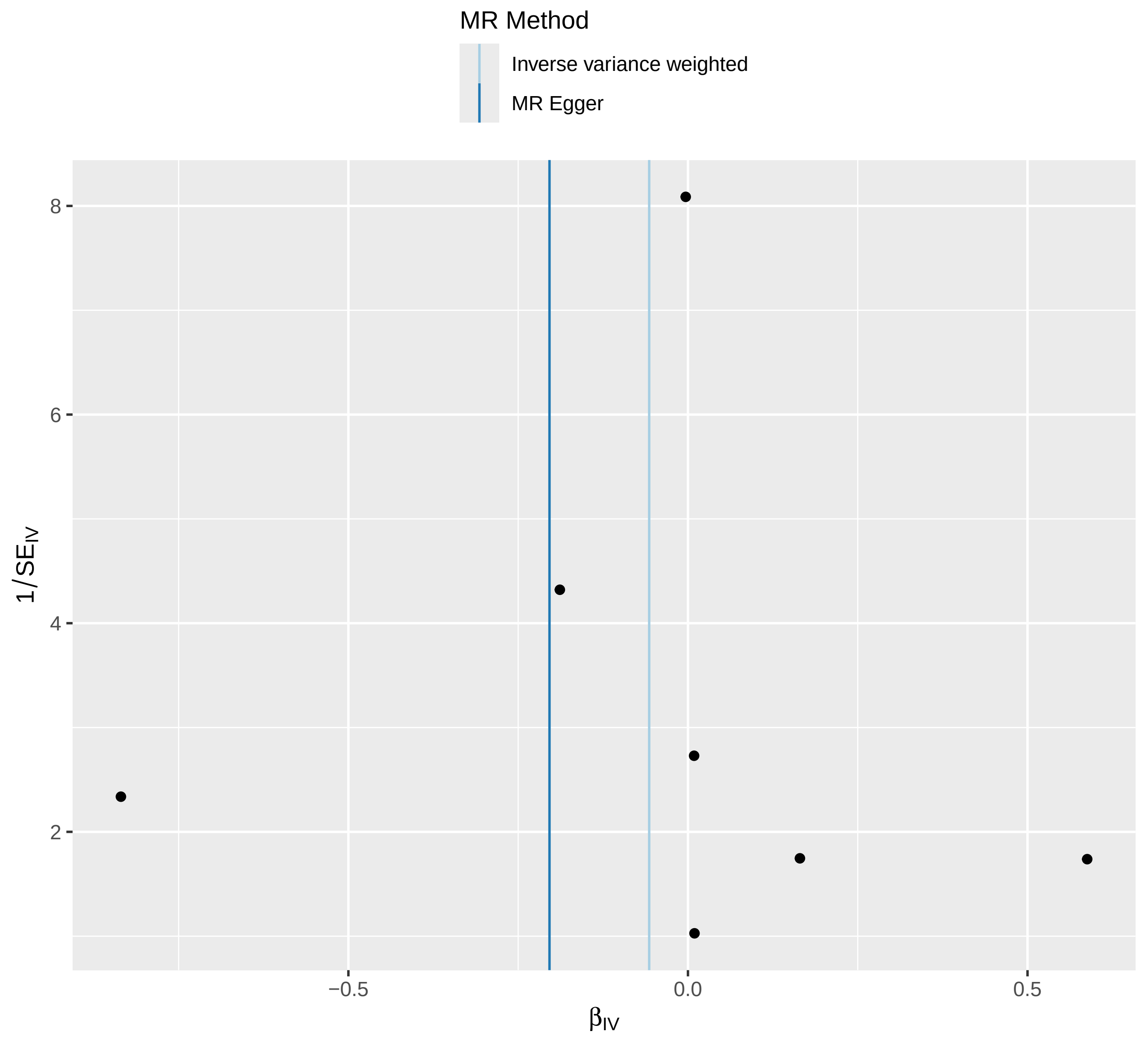

MR Method
Inverse variance weighted
MR Egger
8
6
4
2
1/SE$_{IV}$
−0.5
0.0
0.5
β$_{IV}$

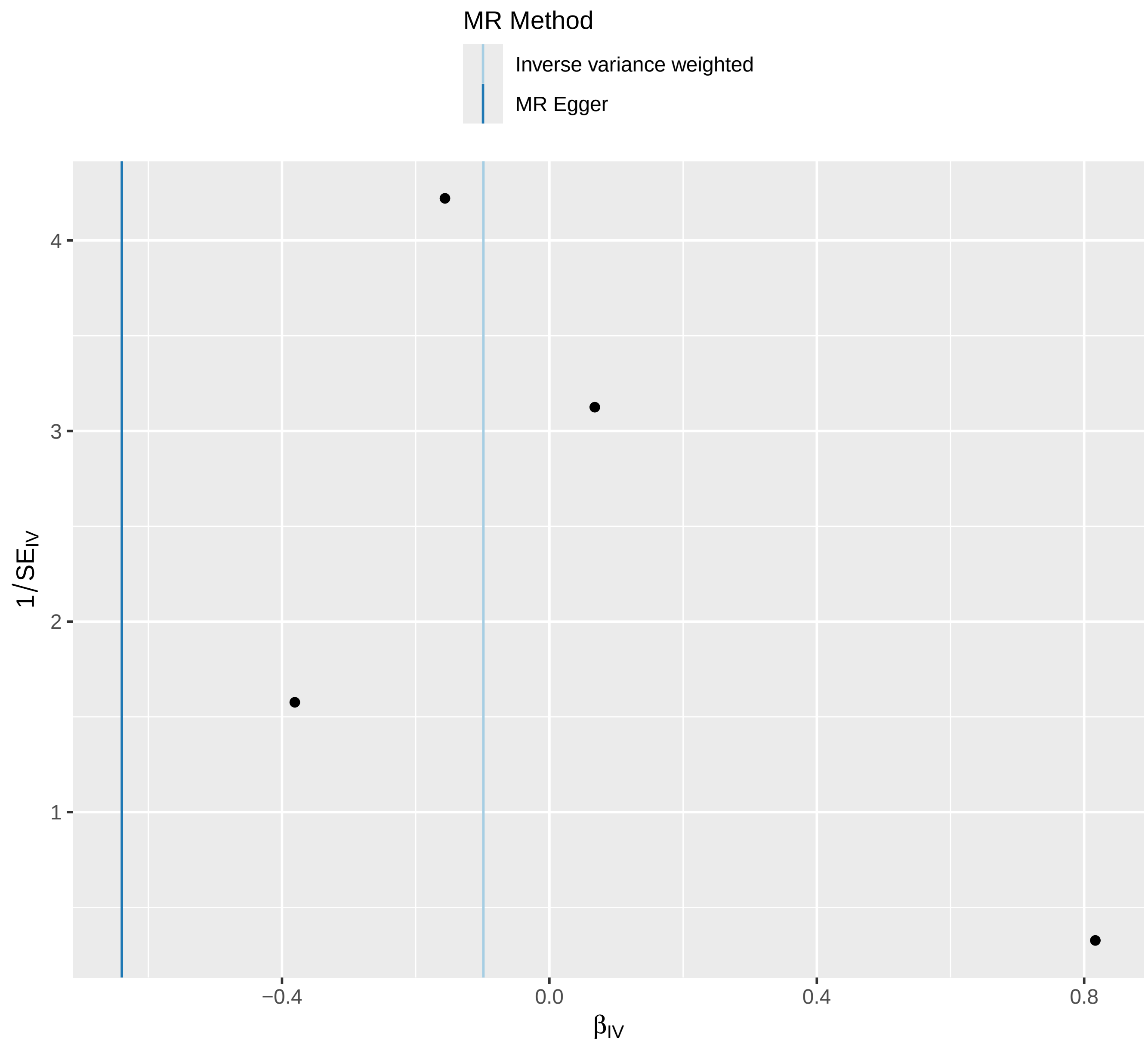
MR Method
Inverse variance weighted
MR Egger
1/SE_IV
β_IV
−0.4
0.0
0.4
0.8
1
2
3
4

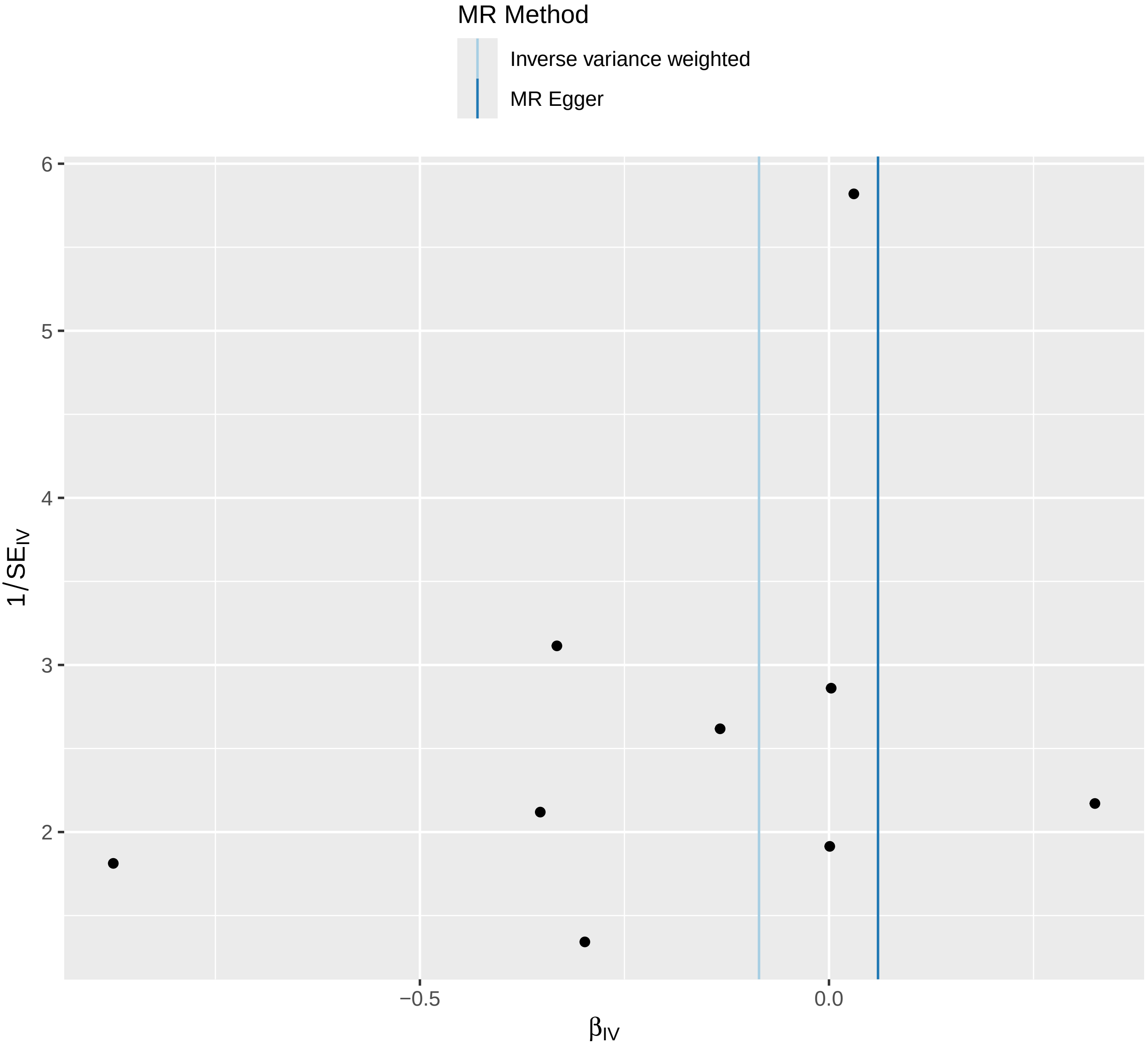
MR Method
Inverse variance weighted
MR Egger
1/SE_IV
β_IV
6
5
4
3
2
−0.5
0.0

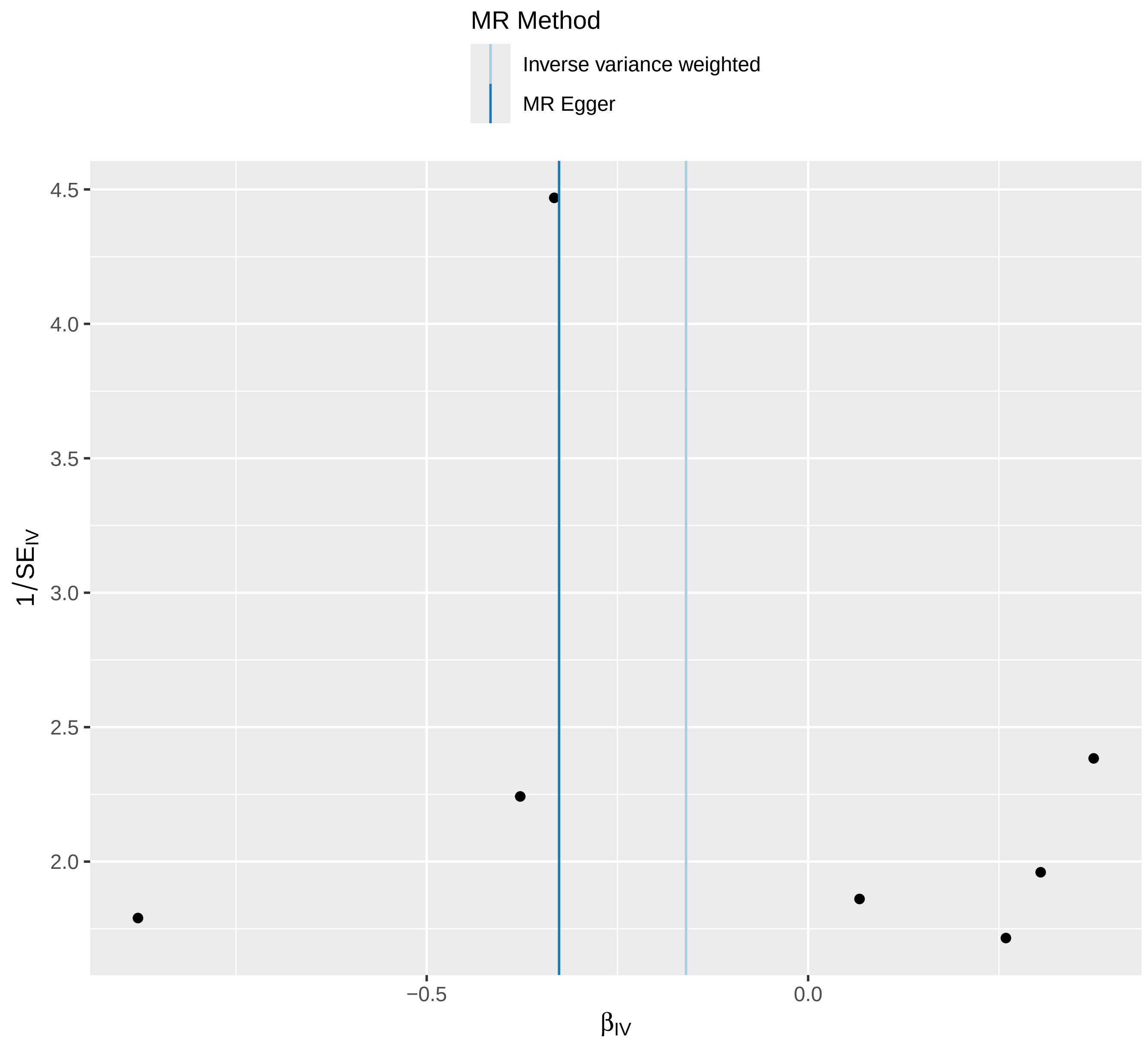
MR Method
Inverse variance weighted
MR Egger
4.5
4.0
3.5
3.0
2.5
2.0
1/SE_IV
−0.5
0.0
β_IV

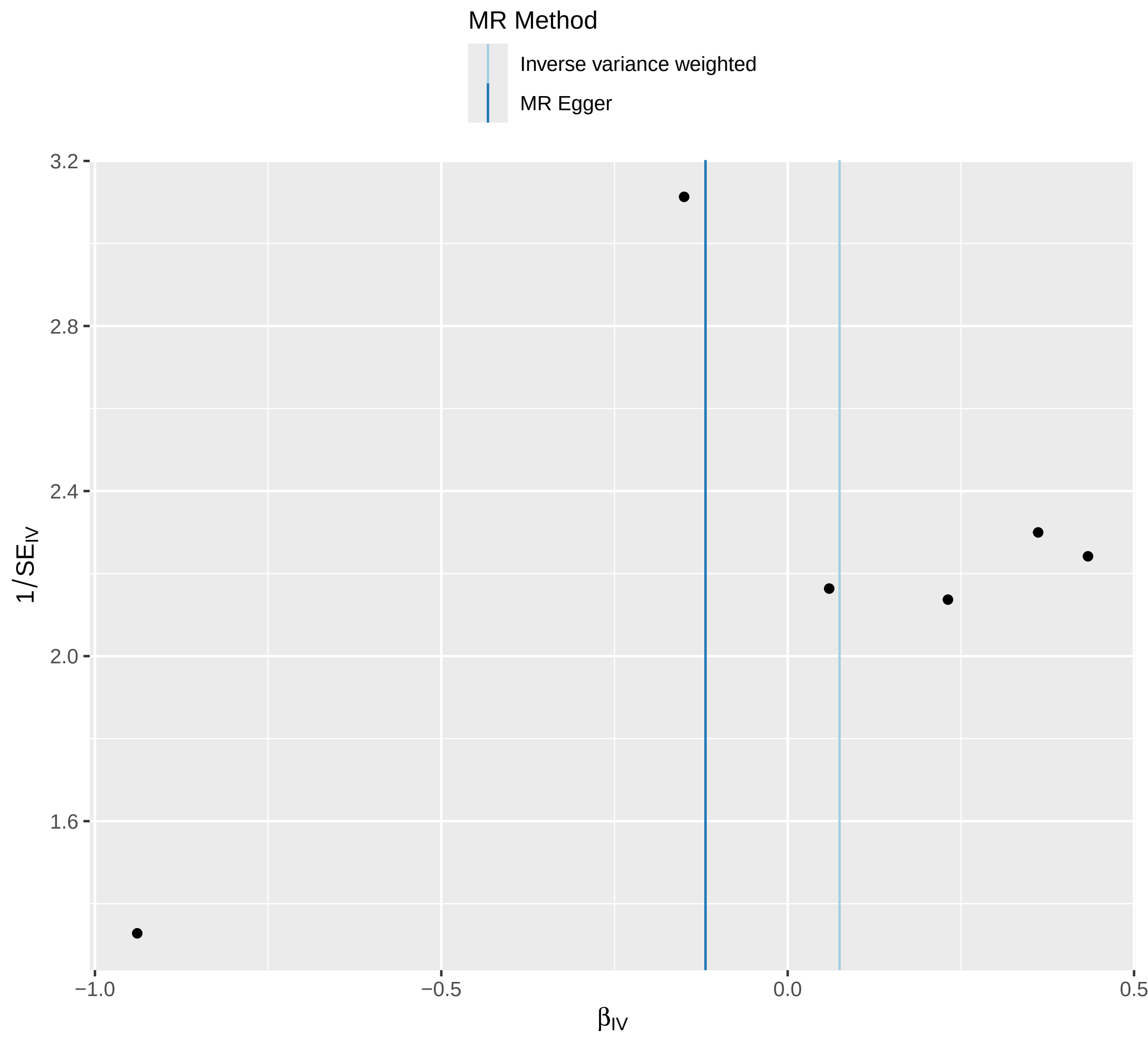
MR Method
Inverse variance weighted
MR Egger
3.2
2.8
2.4
2.0
1.6
1/SE_IV
−1.0
−0.5
0.0
0.5
β_IV

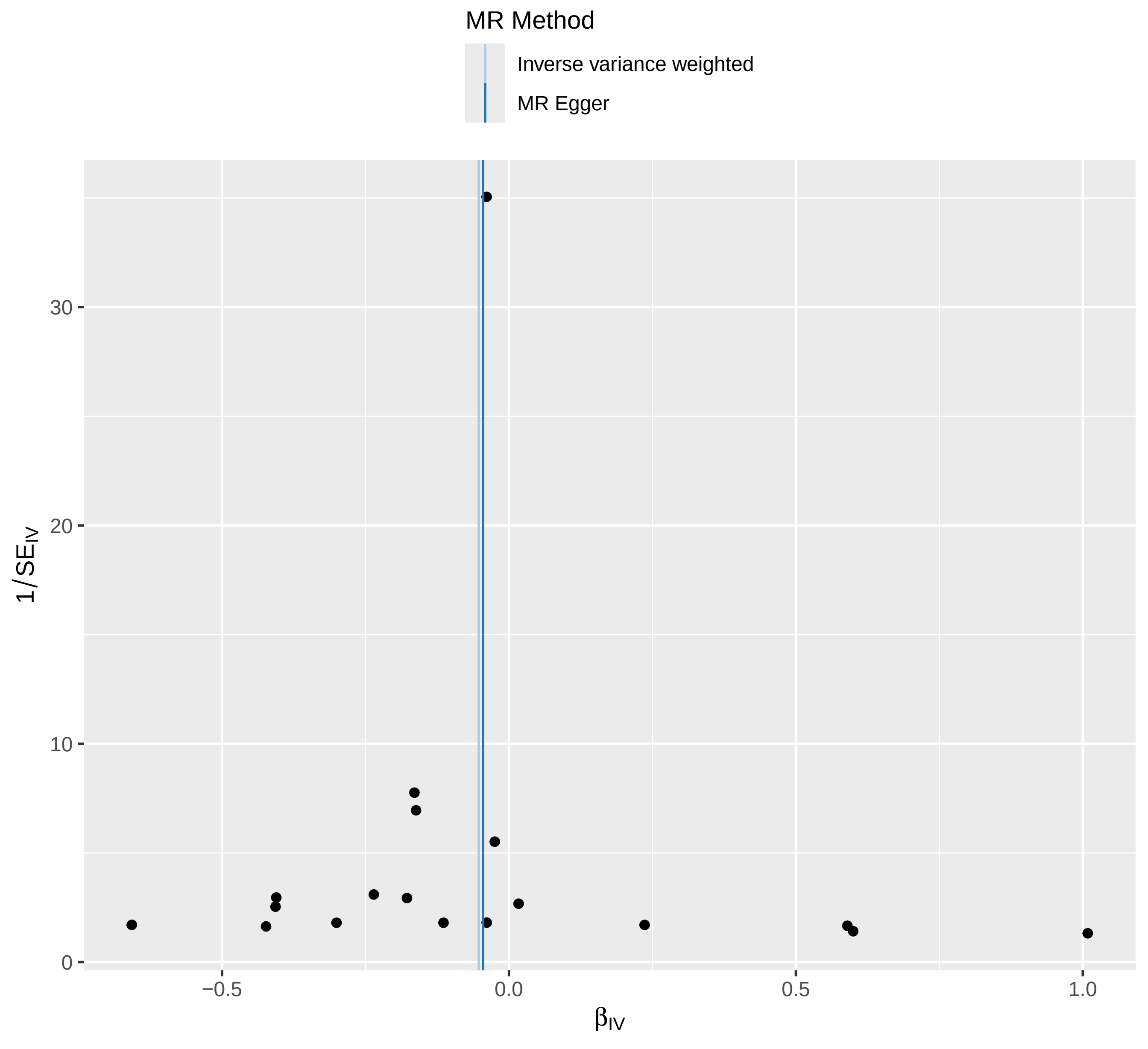

MR Method
Inverse variance weighted
MR Egger
30
20
10
0
$1/SE_{IV}$
−0.5
0.0
0.5
1.0
β_{IV}

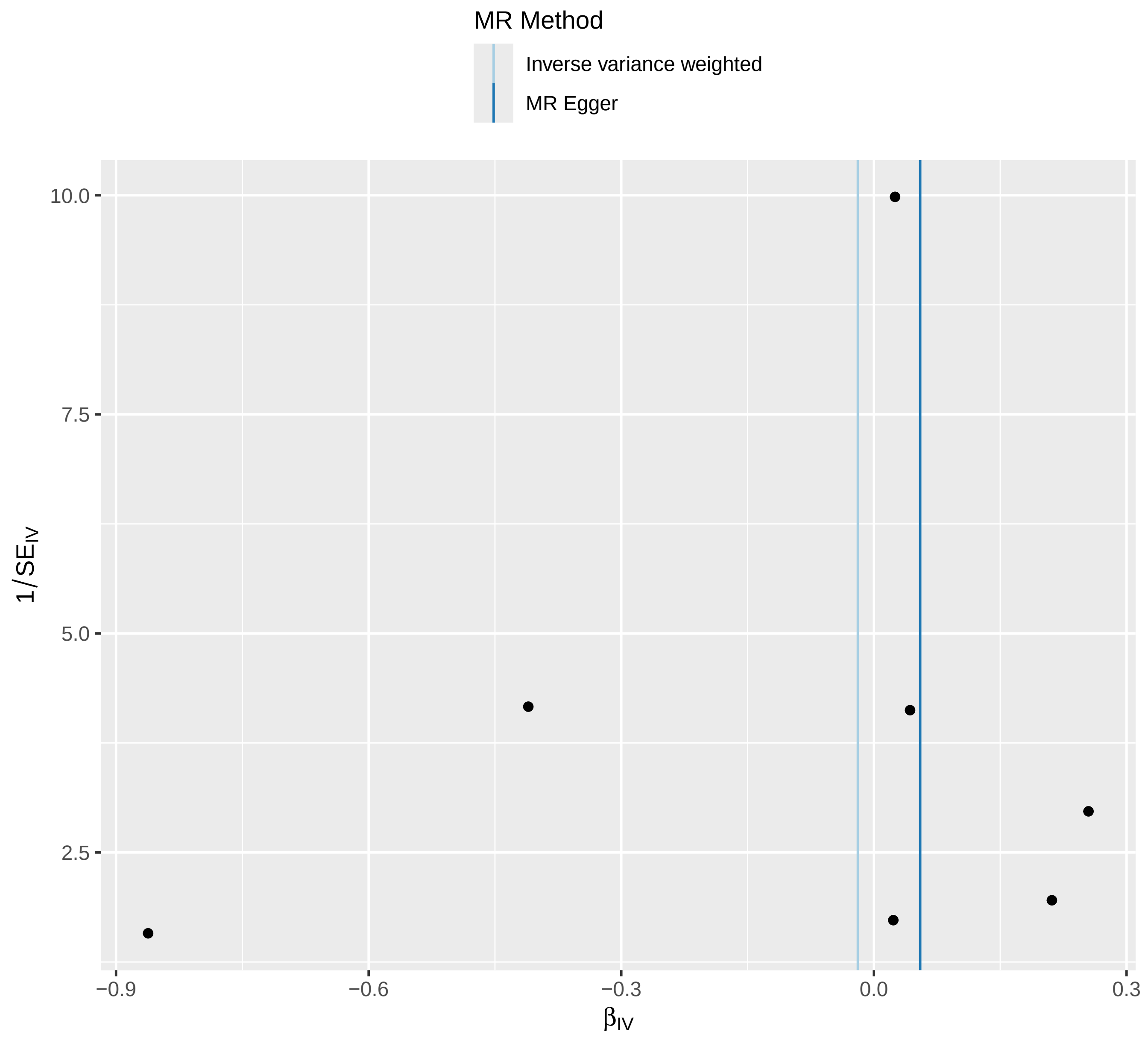

MR Method
Inverse variance weighted
MR Egger
10.0
7.5
5.0
2.5
$1/SE_{IV}$
−0.9
−0.6
−0.3
0.0
0.3
β_{IV}

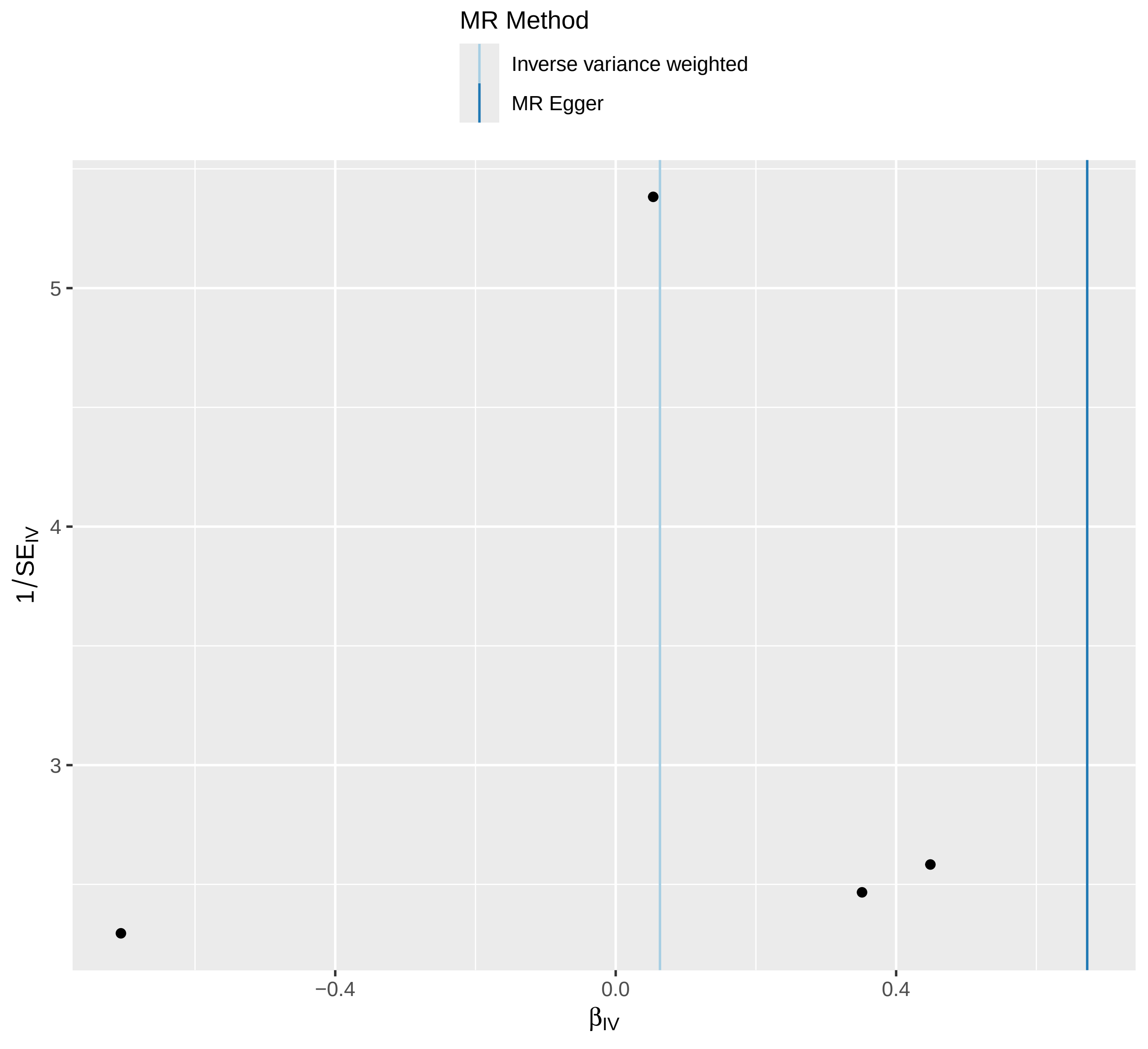

MR Method
Inverse variance weighted
MR Egger
5
4
3
$1/SE_{IV}$
−0.4
0.0
0.4
β_{IV}

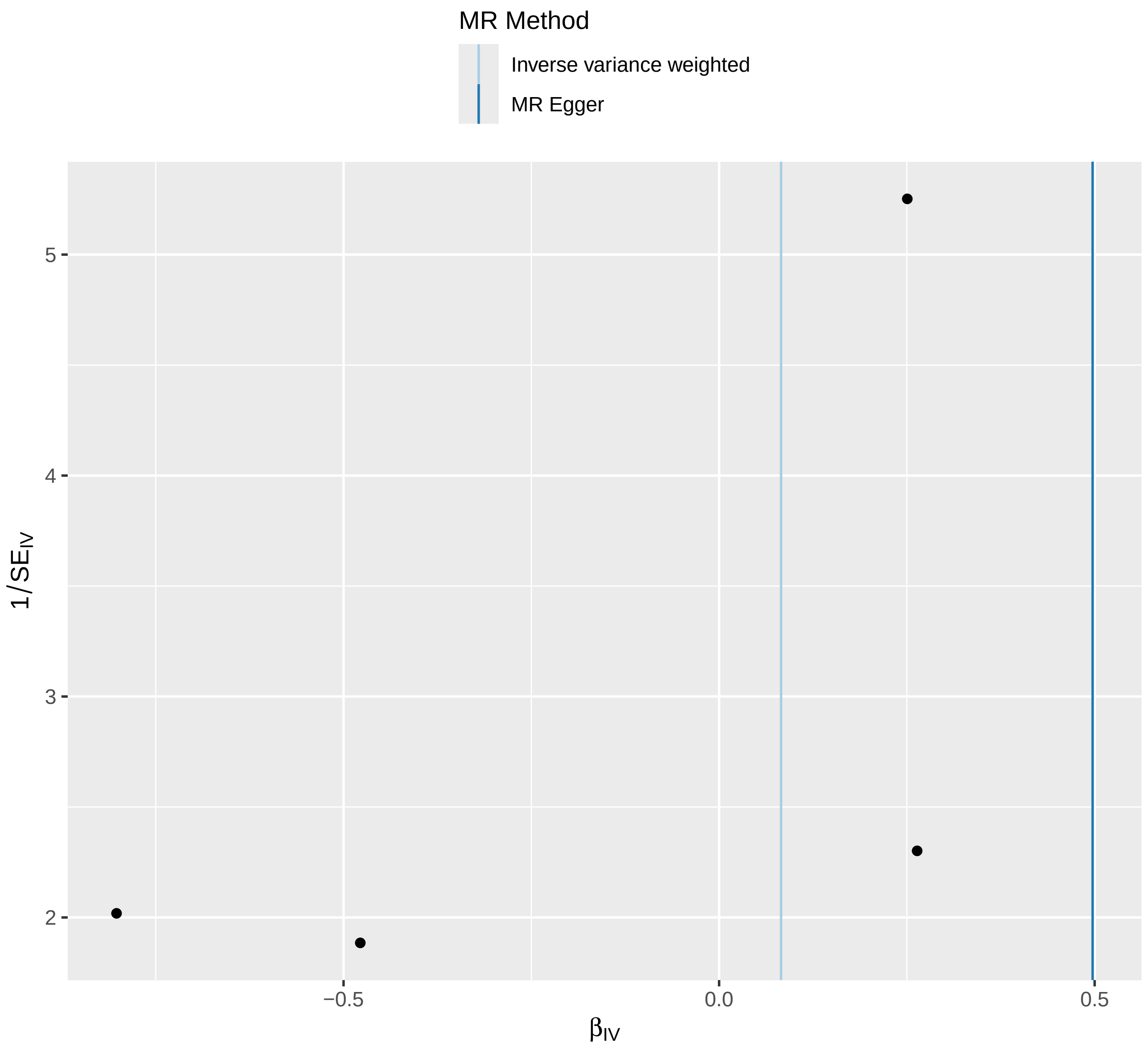
MR Method
Inverse variance weighted
MR Egger
5
4
3
2
1/SE$_{IV}$
−0.5
0.0
0.5
β$_{IV}$

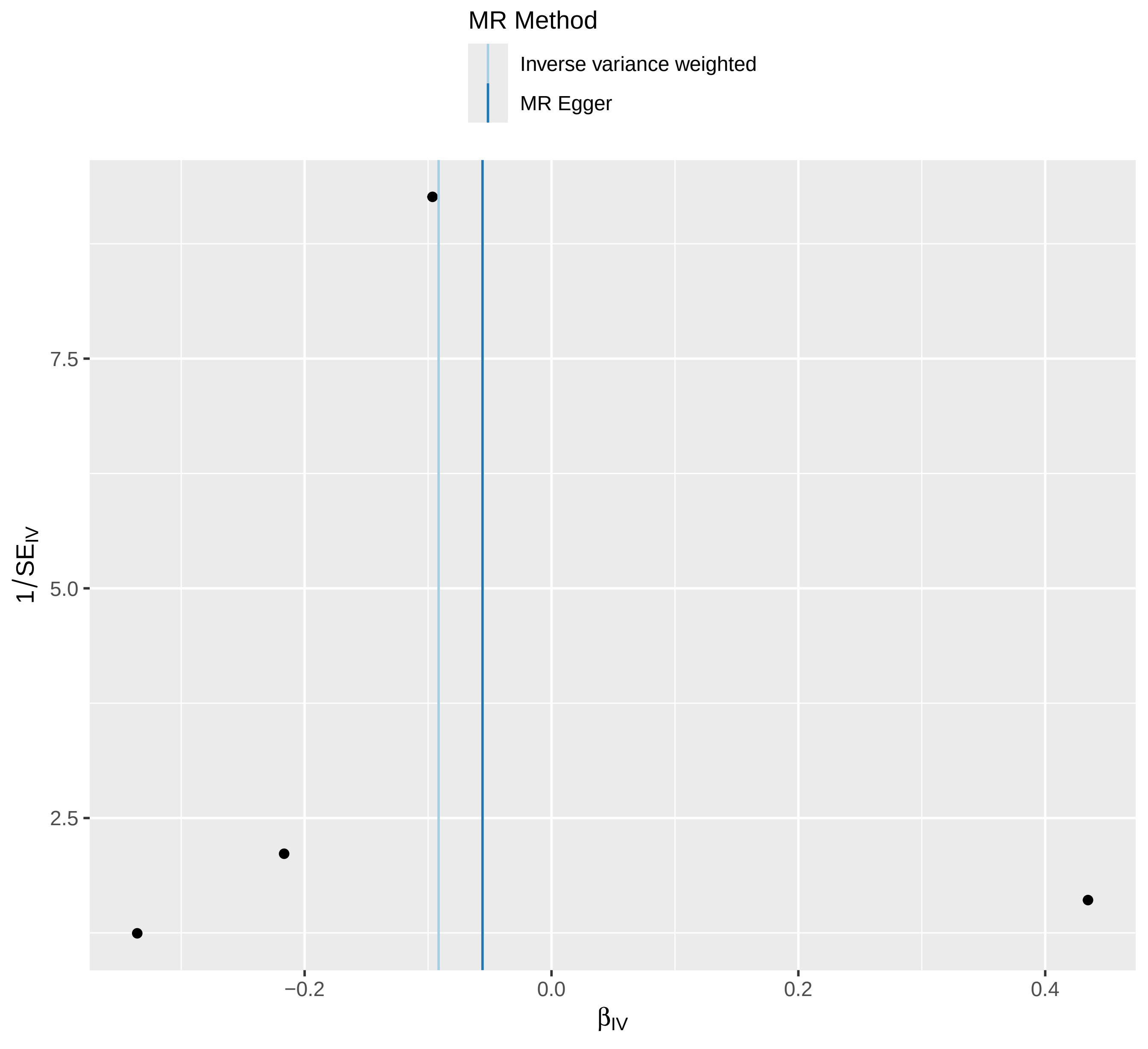
MR Method
Inverse variance weighted
MR Egger
7.5
5.0
2.5
−0.2
0.0
0.2
0.4
$1/SE_{IV}$
β_{IV}

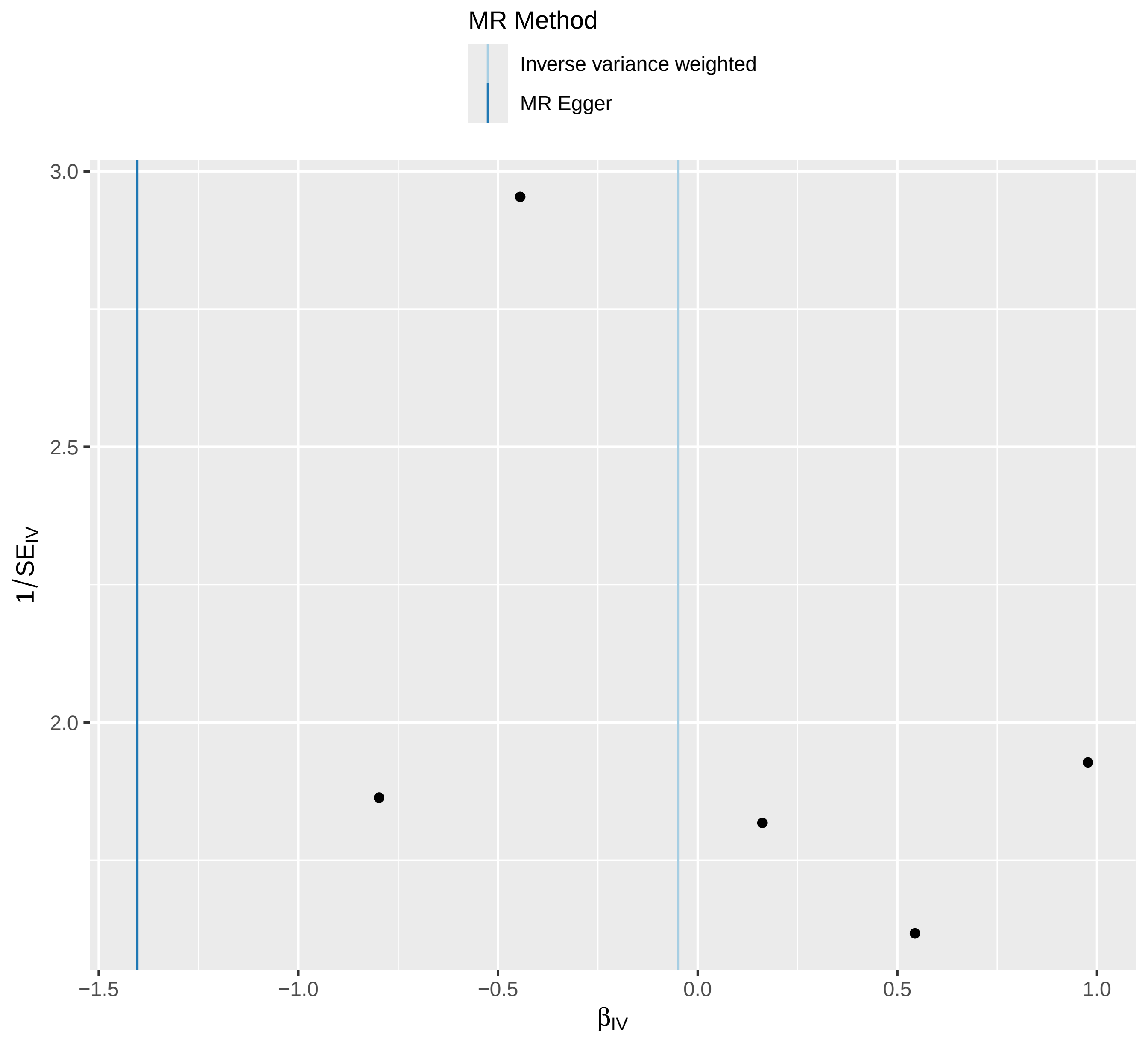
MR Method
Inverse variance weighted
MR Egger
3.0
2.5
2.0
1/SE$_{IV}$
−1.5
−1.0
−0.5
0.0
0.5
1.0
β$_{IV}$

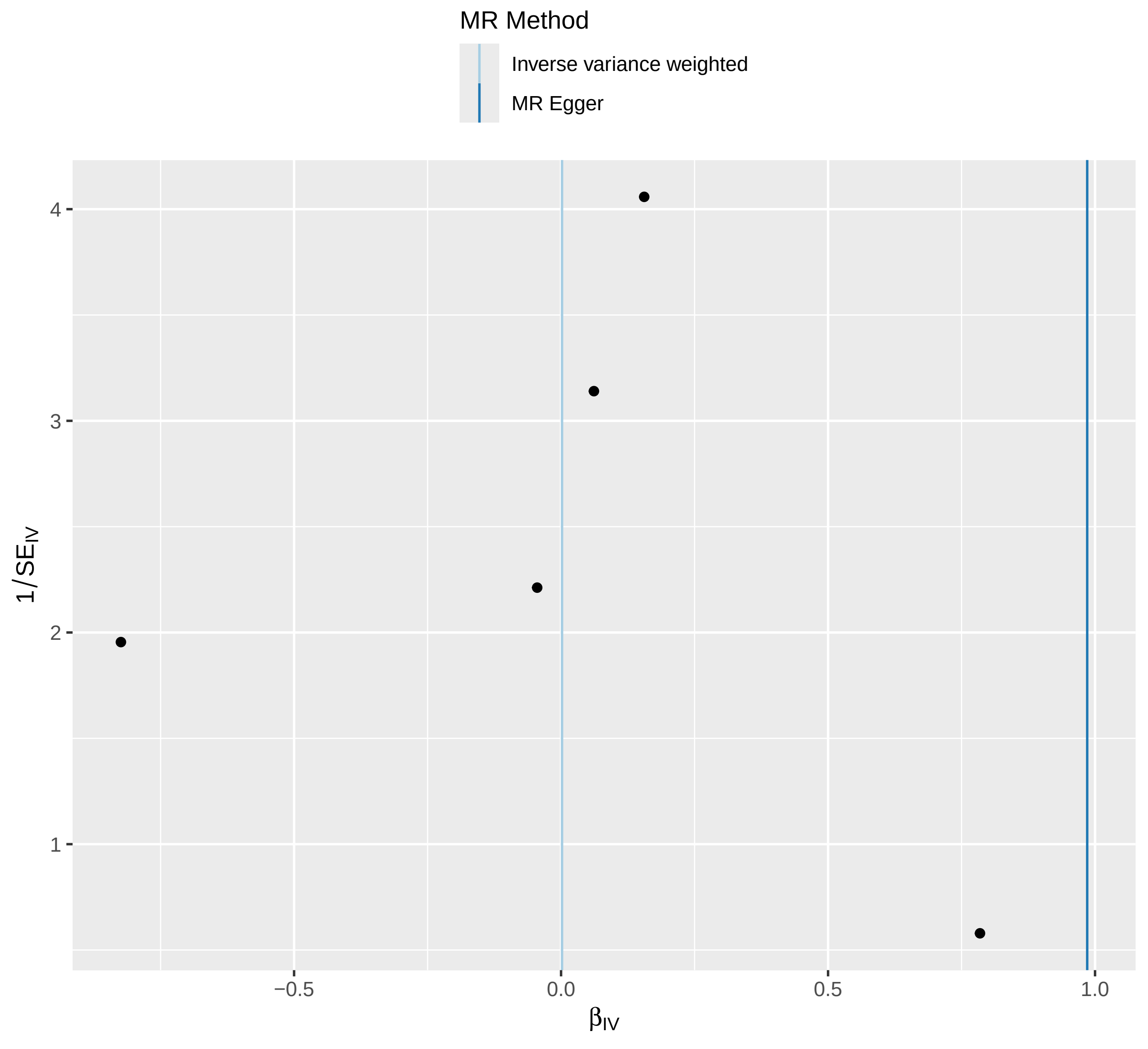
MR Method
Inverse variance weighted
MR Egger
1/SE_IV
4
3
2
1
−0.5
0.0
0.5
1.0
β_IV

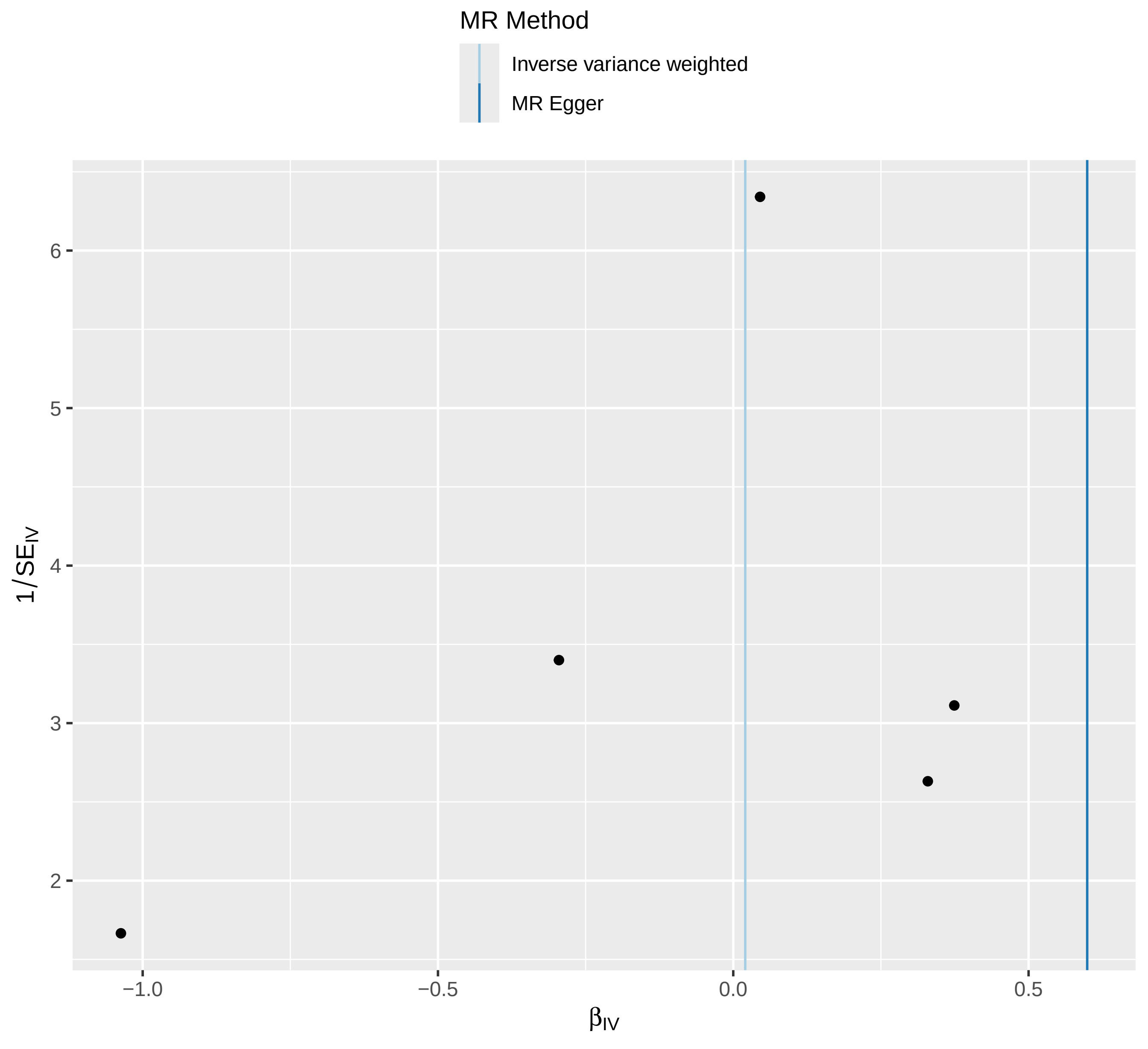

MR Method
Inverse variance weighted
MR Egger
6
5
4
3
2
1/SE_IV
−1.0
−0.5
0.0
0.5
β_IV

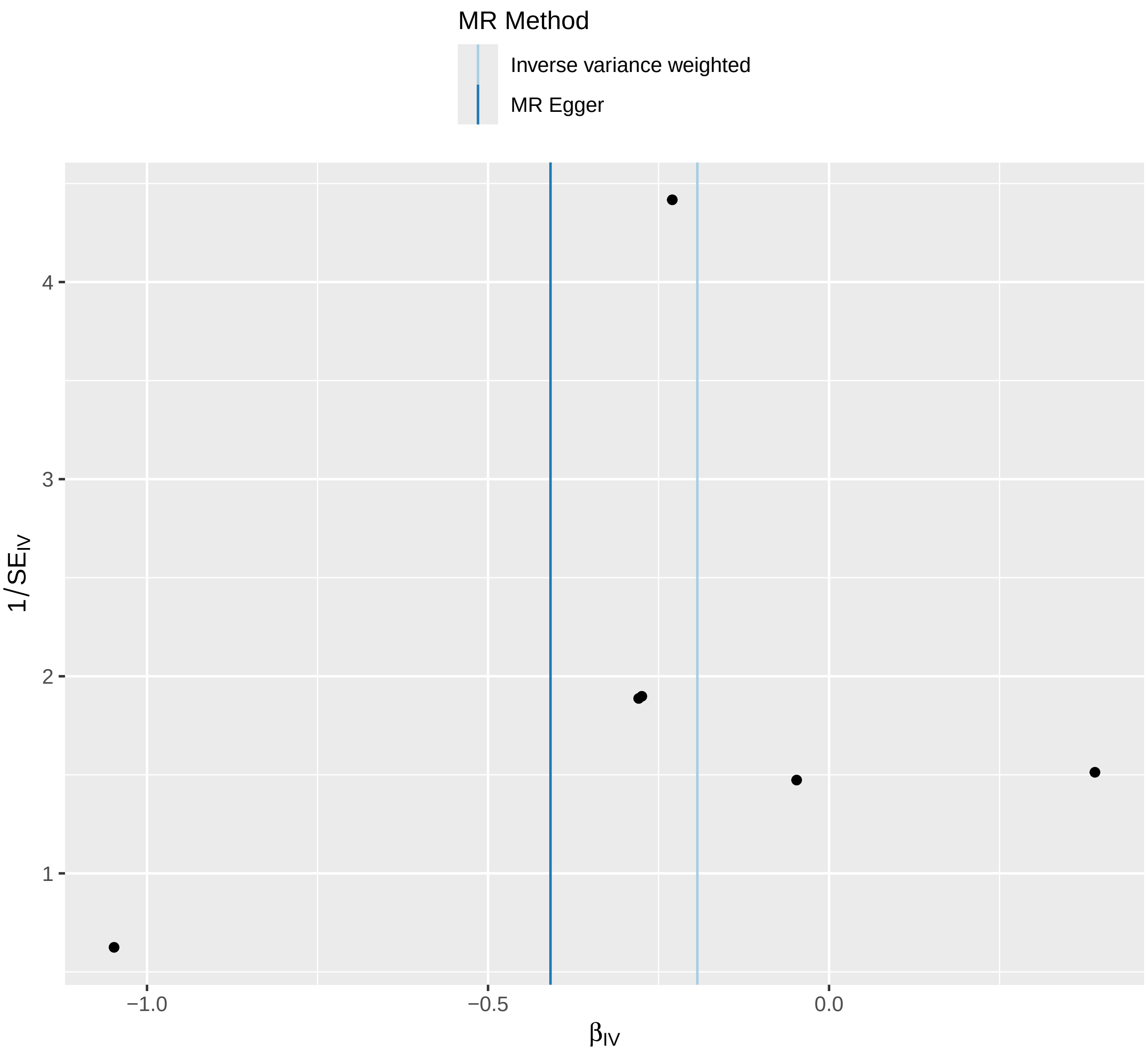

MR Method
Inverse variance weighted
MR Egger
4
3
2
1
1/SE_IV
−1.0
−0.5
0.0
β_IV

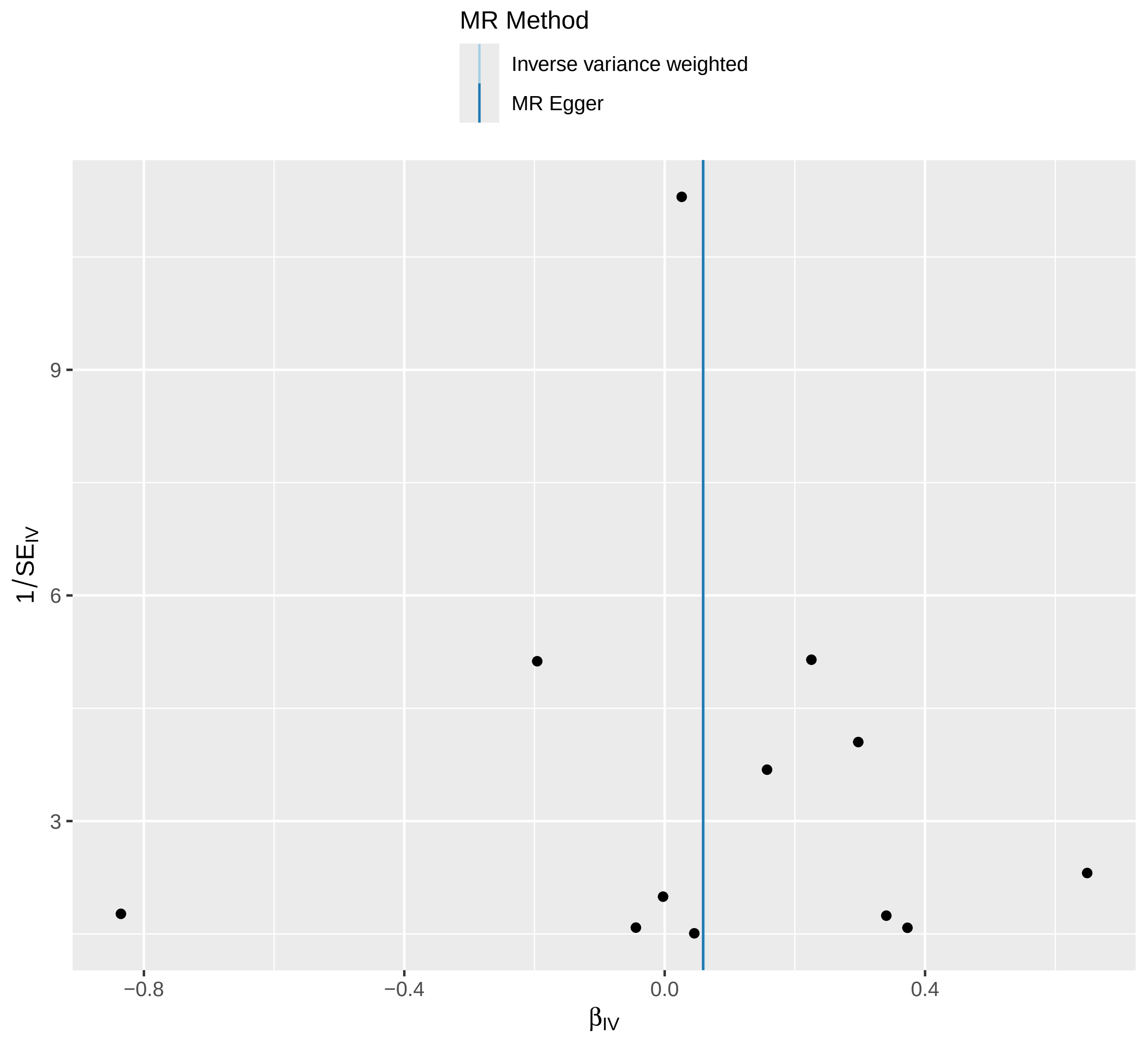

MR Method
Inverse variance weighted
MR Egger
9
6
3
1/SE$_{IV}$
−0.8
−0.4
0.0
0.4
β$_{IV}$

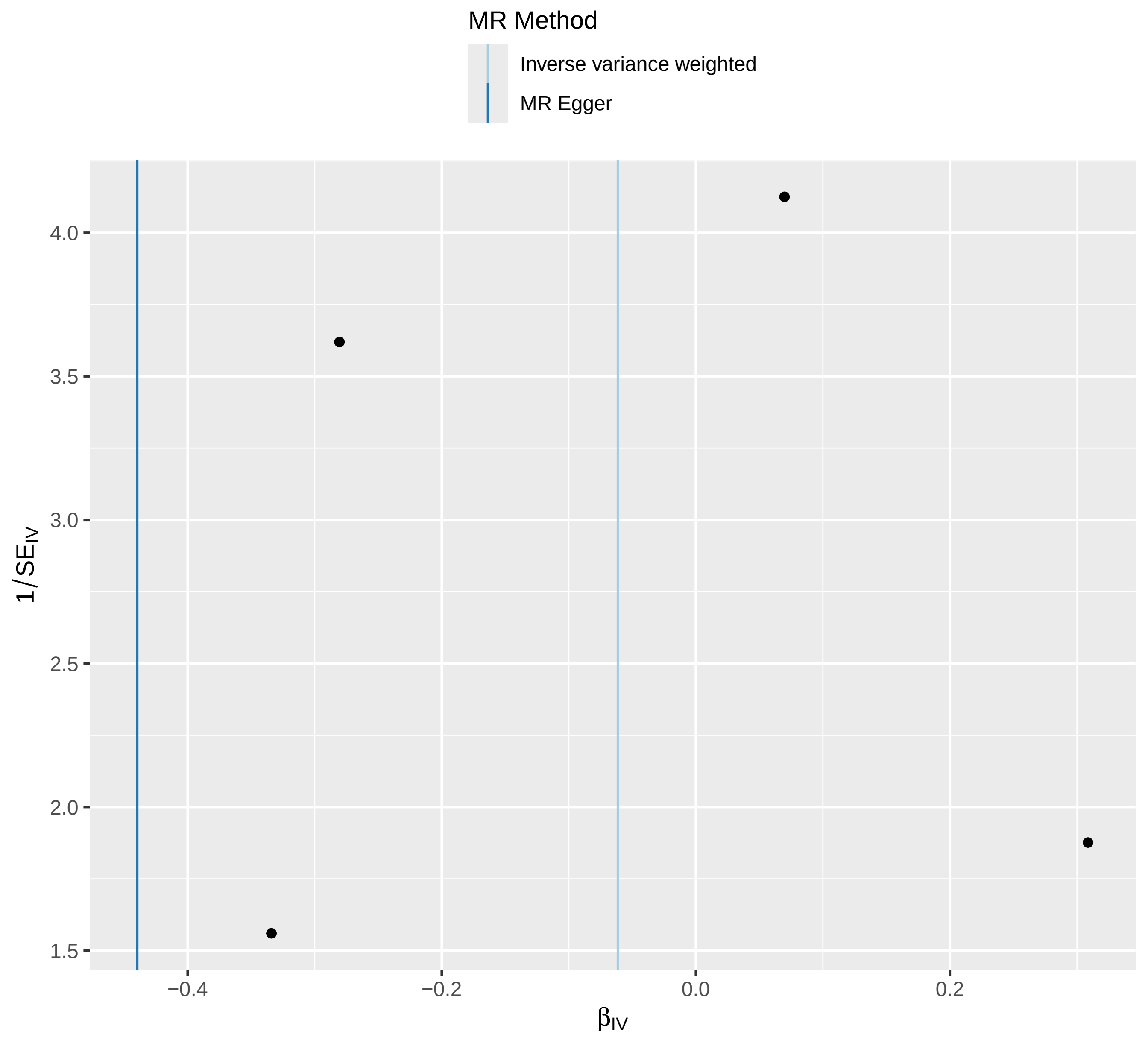

MR Method
Inverse variance weighted
MR Egger
4.0
3.5
3.0
2.5
2.0
1.5
$1/SE_{IV}$
−0.4
−0.2
0.0
0.2
β_{IV}

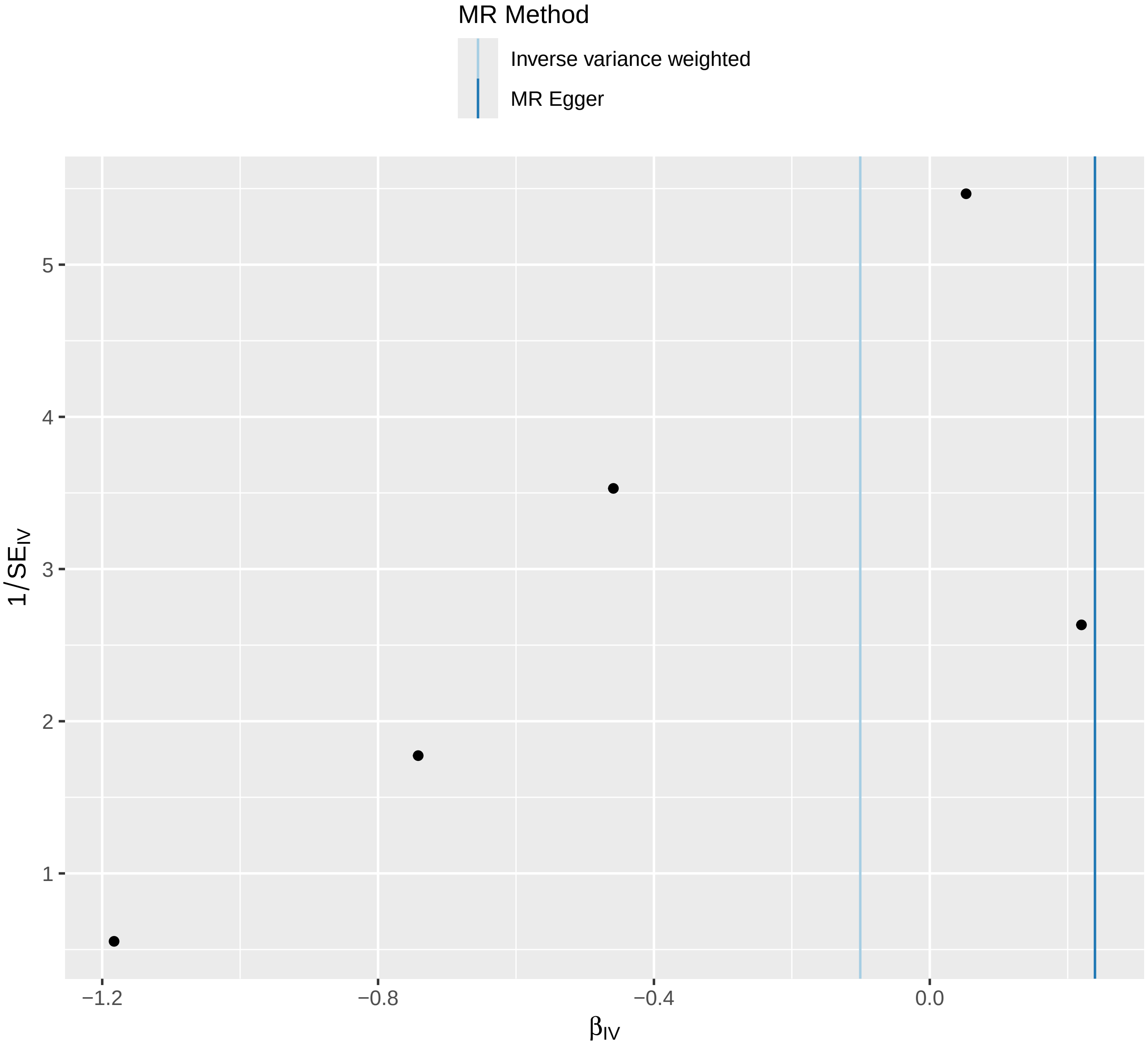
MR Method
Inverse variance weighted
MR Egger
1/SE$_{IV}$
β$_{IV}$
5
4
3
2
1
−1.2
−0.8
−0.4
0.0

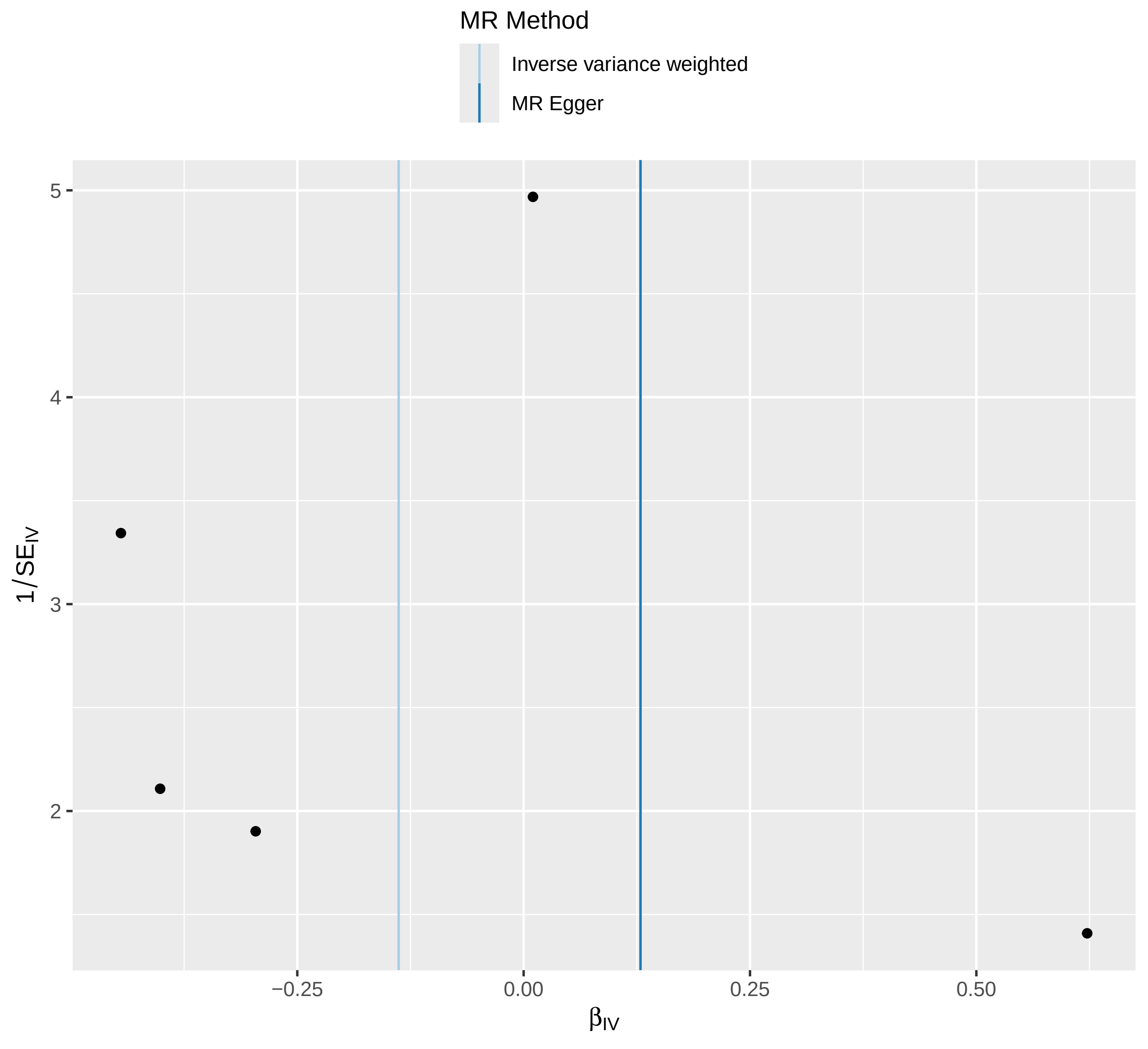
MR Method
Inverse variance weighted
MR Egger
5
4
3
2
1/SE_IV
−0.25
0.00
0.25
0.50
β_IV

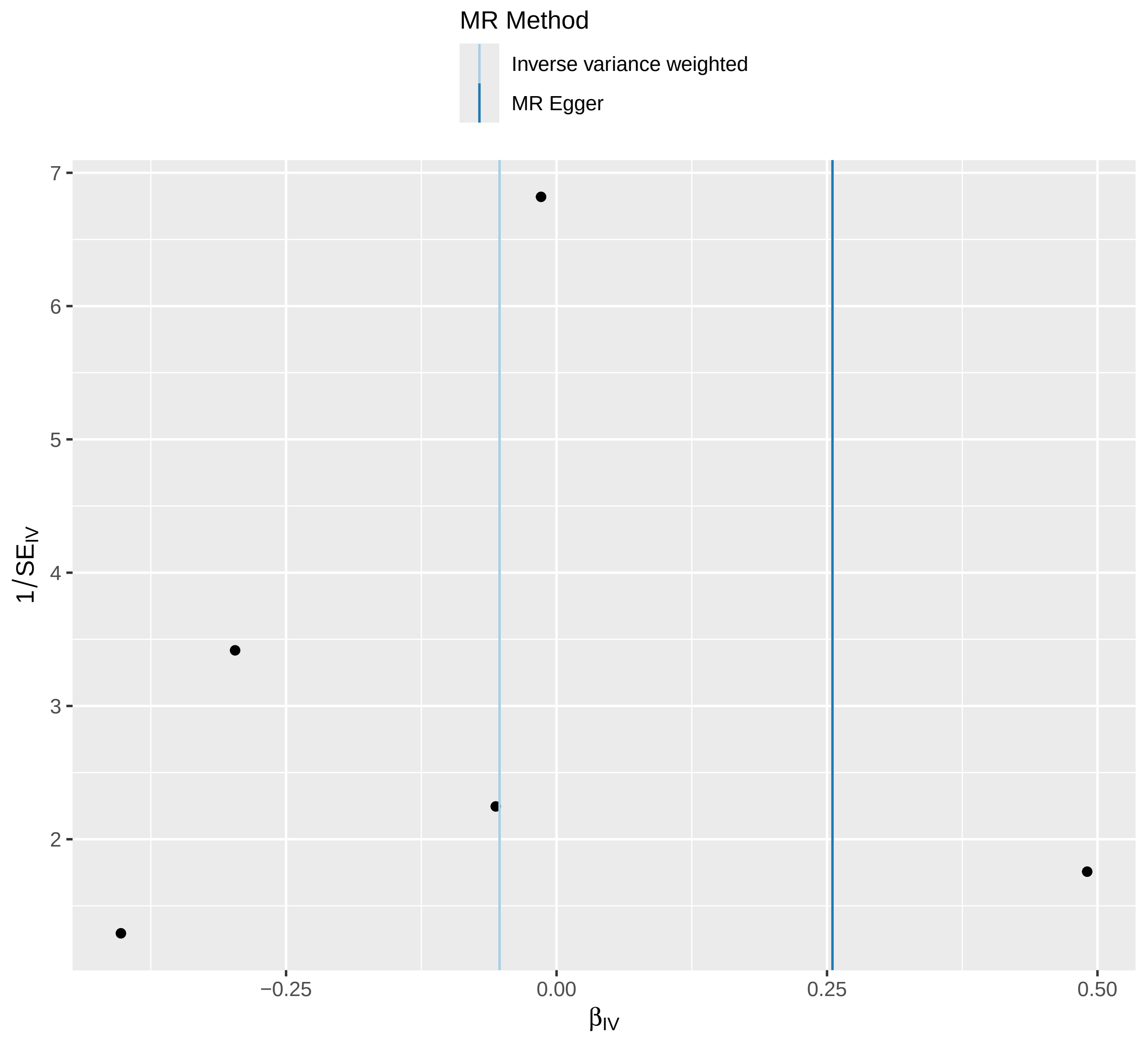

MR Method
Inverse variance weighted
MR Egger
1/SE_IV
β_IV
−0.25
0.00
0.25
0.50
2
3
4
5
6
7

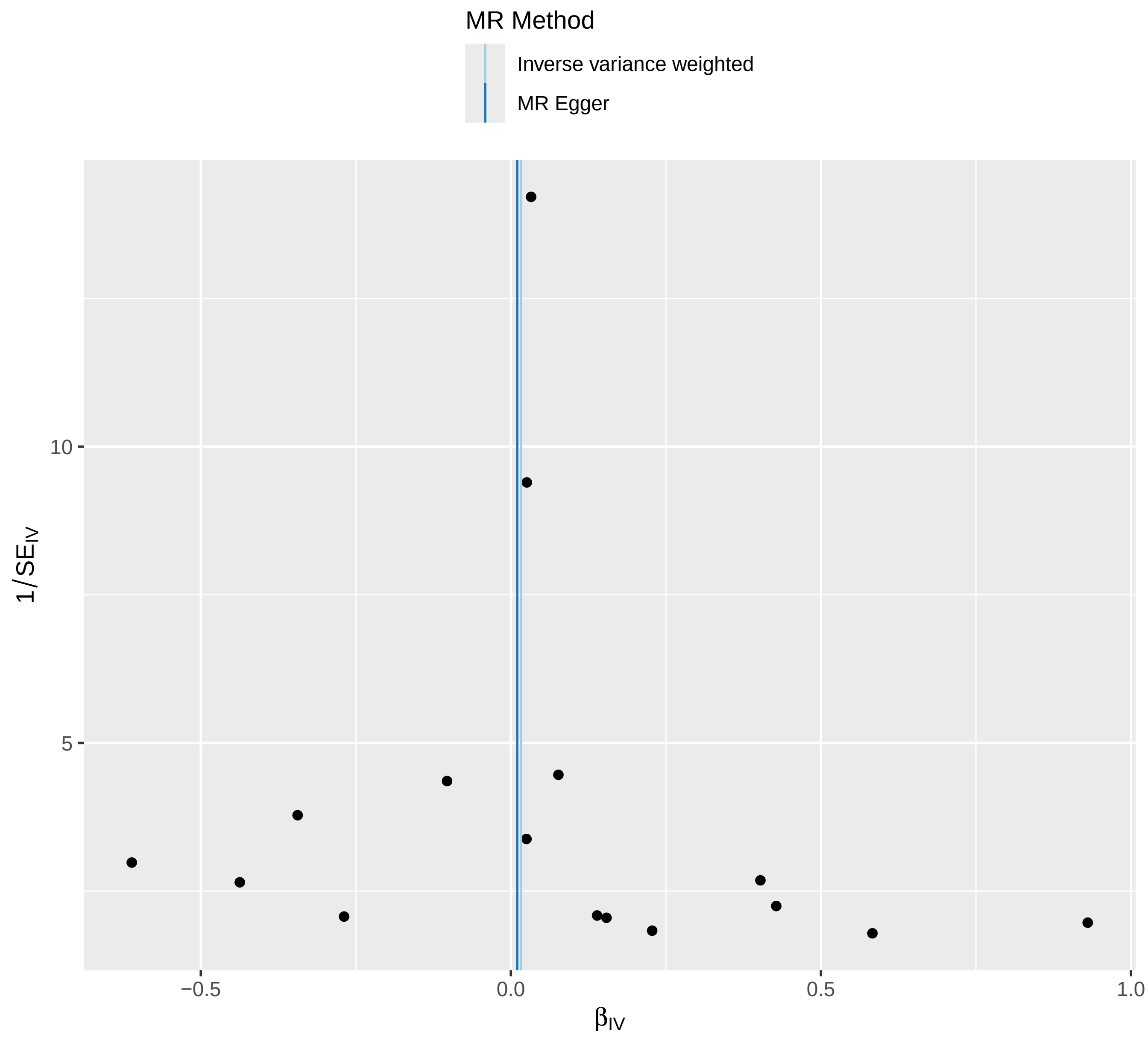
MR Method
Inverse variance weighted
MR Egger
1/SE$_{IV}$
10
5
β$_{IV}$
−0.5
0.0
0.5
1.0

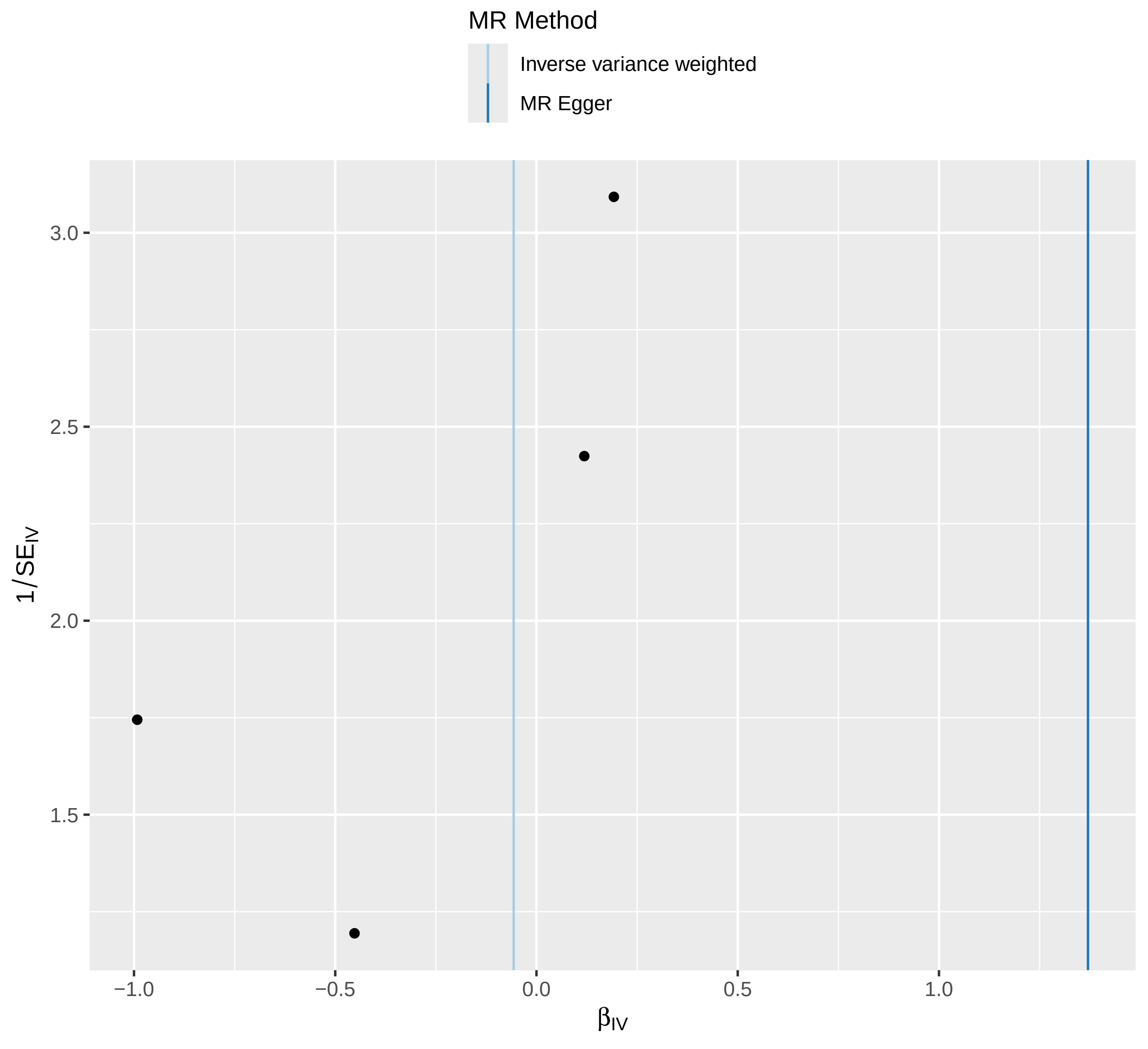

MR Method
Inverse variance weighted
MR Egger
3.0
2.5
2.0
1.5
$1/SE_{IV}$
−1.0
−0.5
0.0
0.5
1.0
β_{IV}

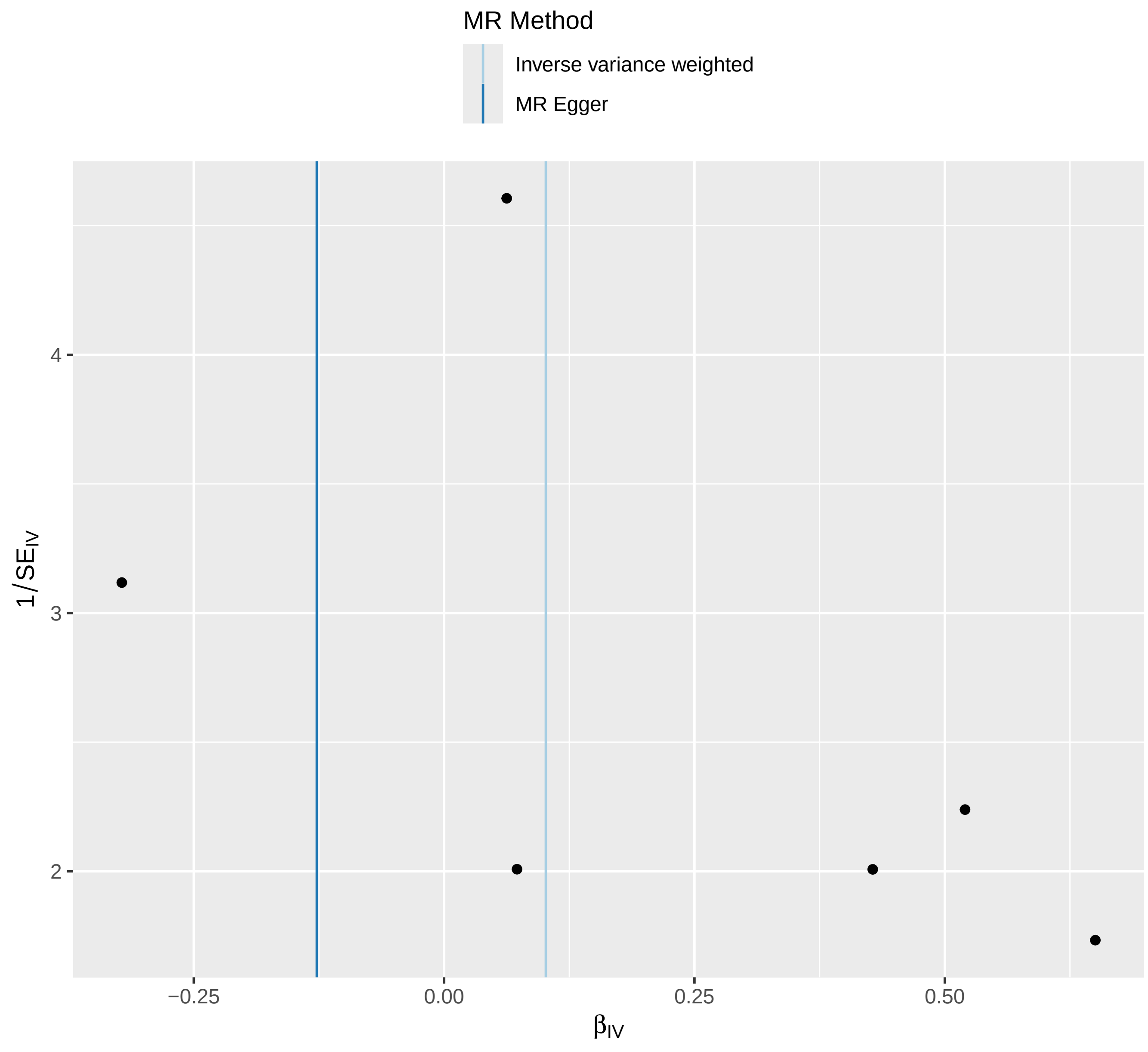
MR Method
Inverse variance weighted
MR Egger
4
3
2
1/SE$_{IV}$
−0.25
0.00
0.25
0.50
β$_{IV}$

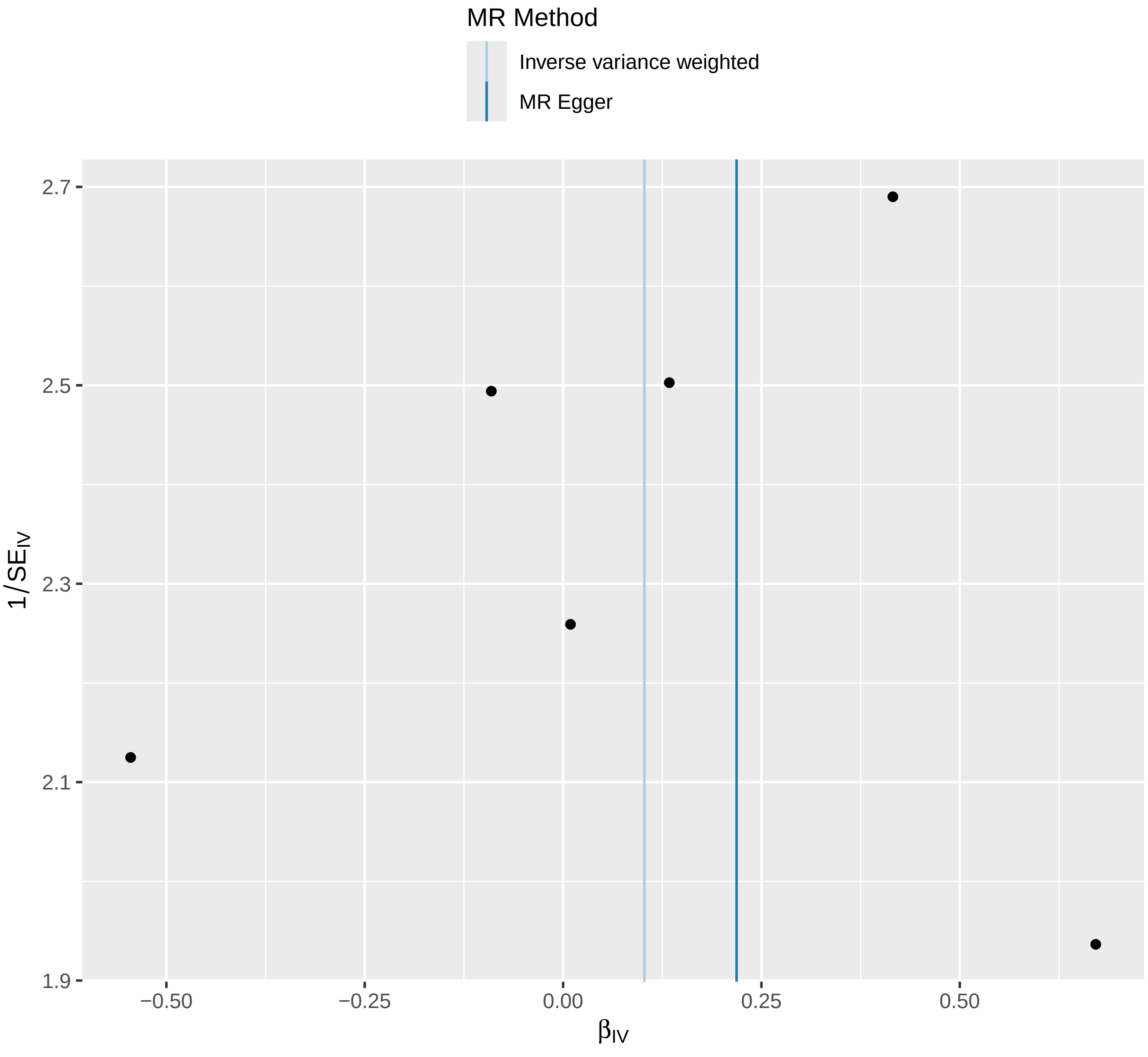

MR Method
Inverse variance weighted
MR Egger
2.7
2.5
2.3
2.1
1.9
1/SE_IV
−0.50
−0.25
0.00
0.25
0.50
β_IV

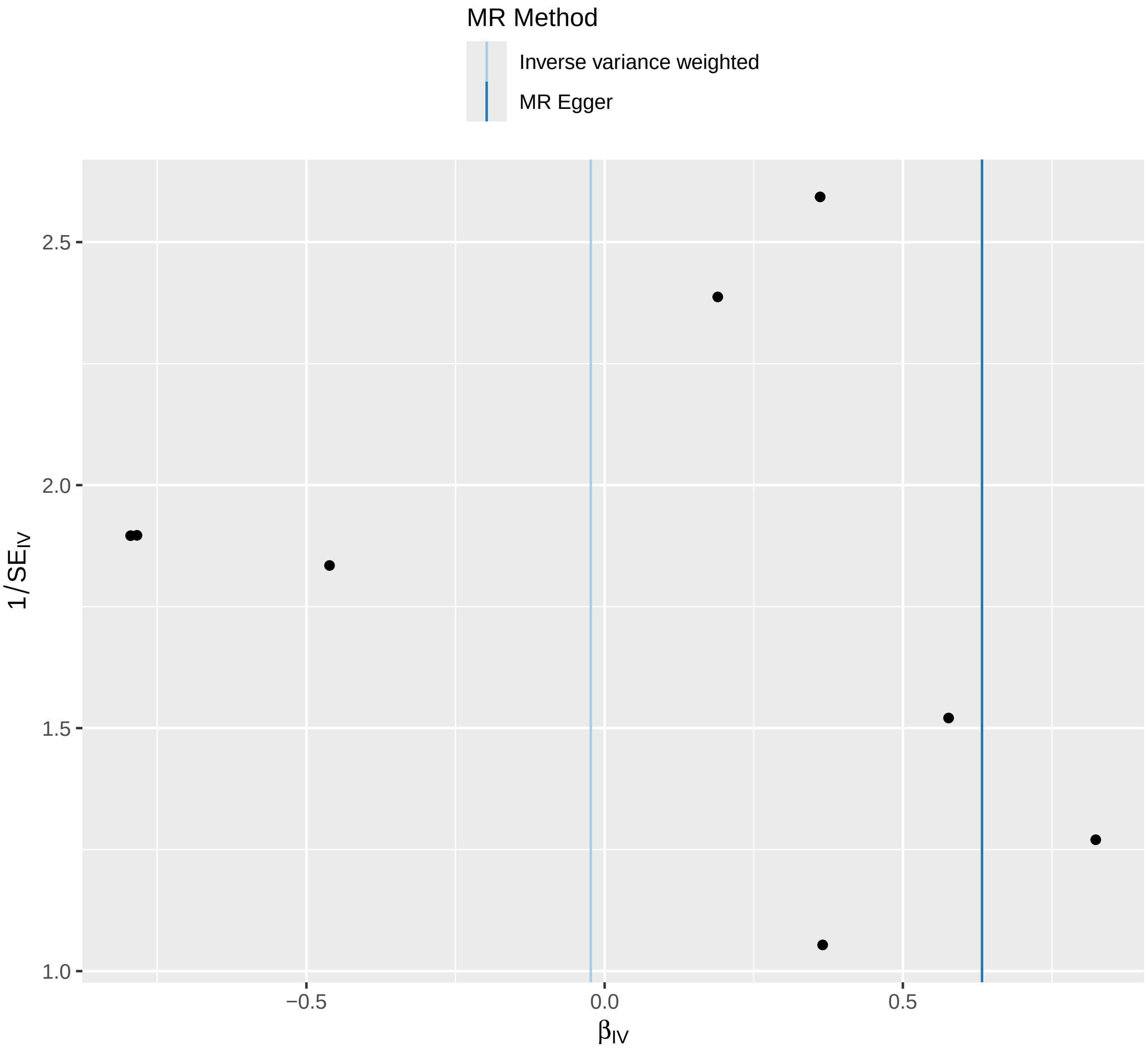

MR Method
Inverse variance weighted
MR Egger
2.5
2.0
1.5
1.0
1/SE_IV
−0.5
0.0
0.5
β_IV

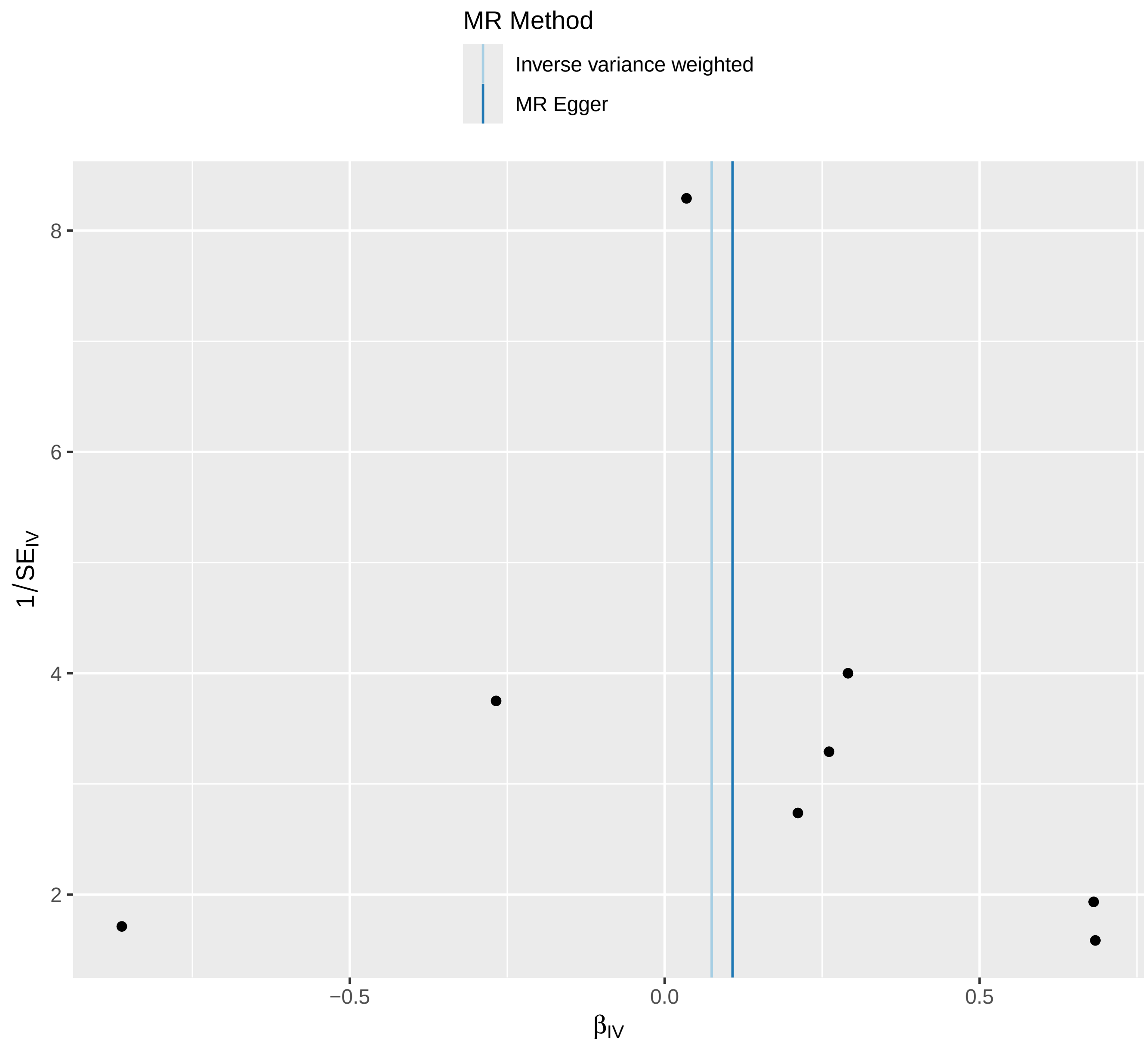

MR Method
Inverse variance weighted
MR Egger
1/SE_IV
8
6
4
2
−0.5
0.0
0.5
β_IV

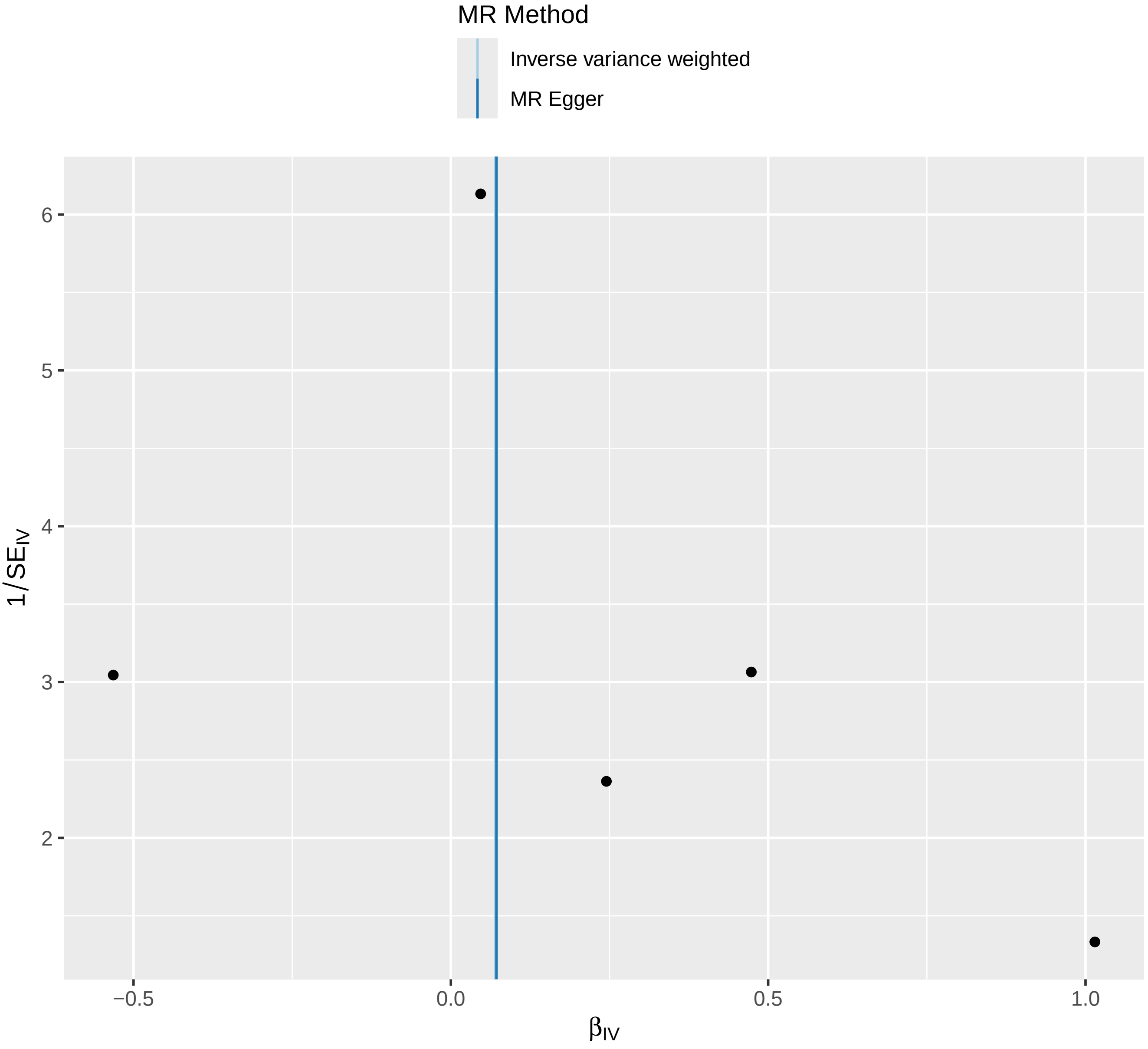

MR Method
Inverse variance weighted
MR Egger
6
5
4
3
2
1/SE_IV
−0.5
0.0
0.5
1.0
β_IV

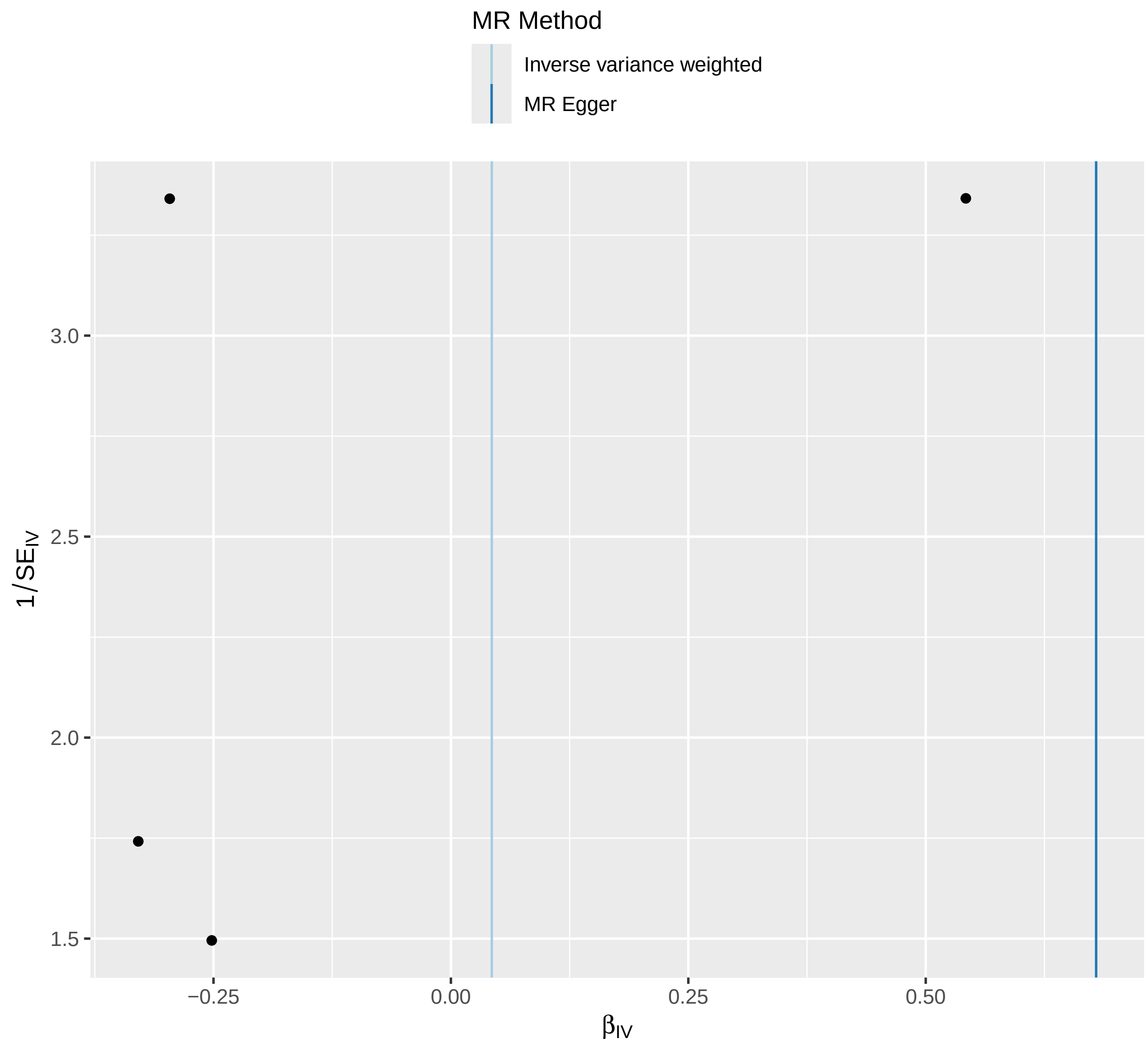

MR Method
Inverse variance weighted
MR Egger
3.0
2.5
2.0
1.5
$1/SE_{IV}$
−0.25
0.00
0.25
0.50
β_{IV}

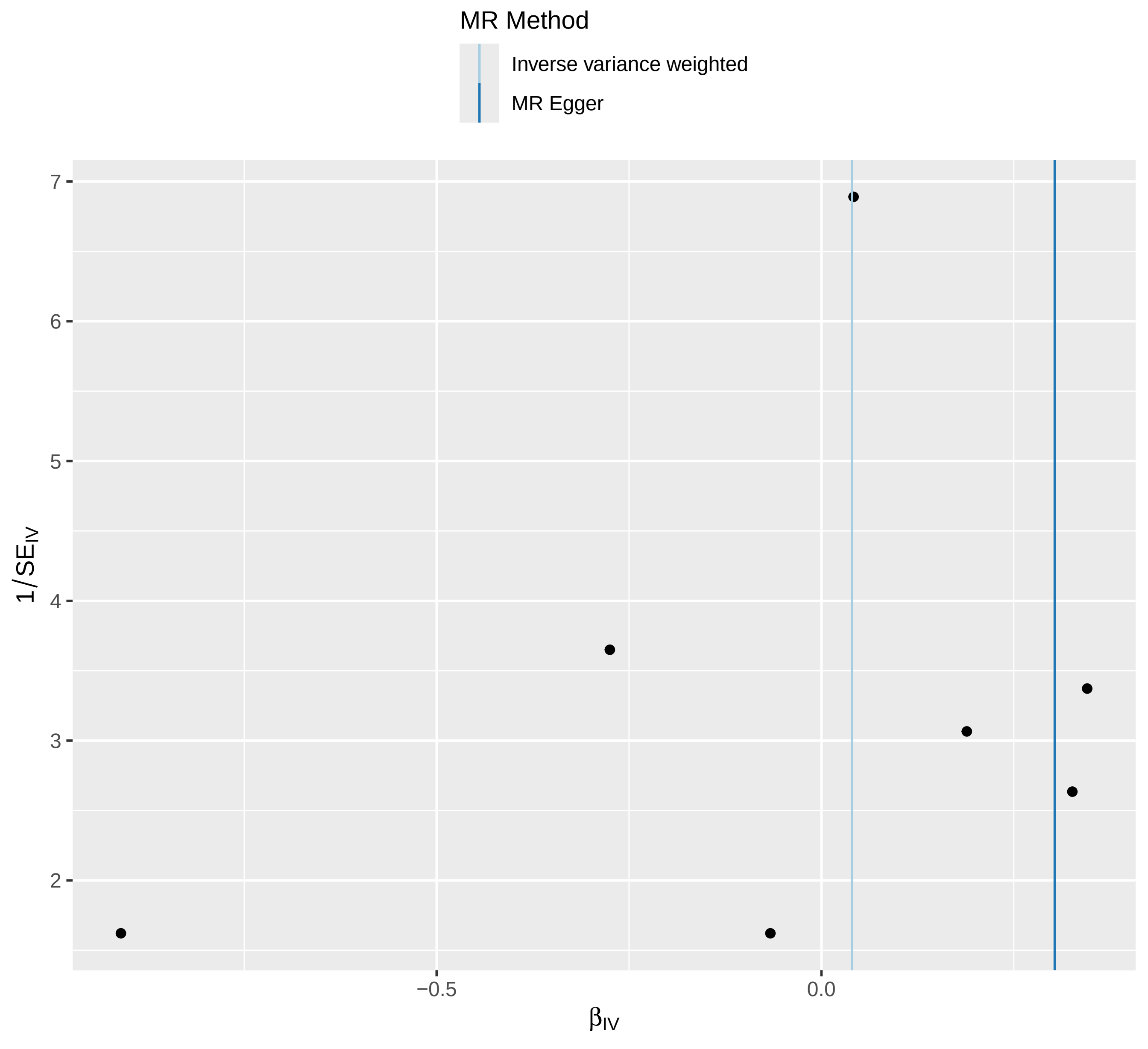
MR Method
Inverse variance weighted
MR Egger
7
6
5
4
3
2
$1/SE_{IV}$
−0.5
0.0
β_{IV}

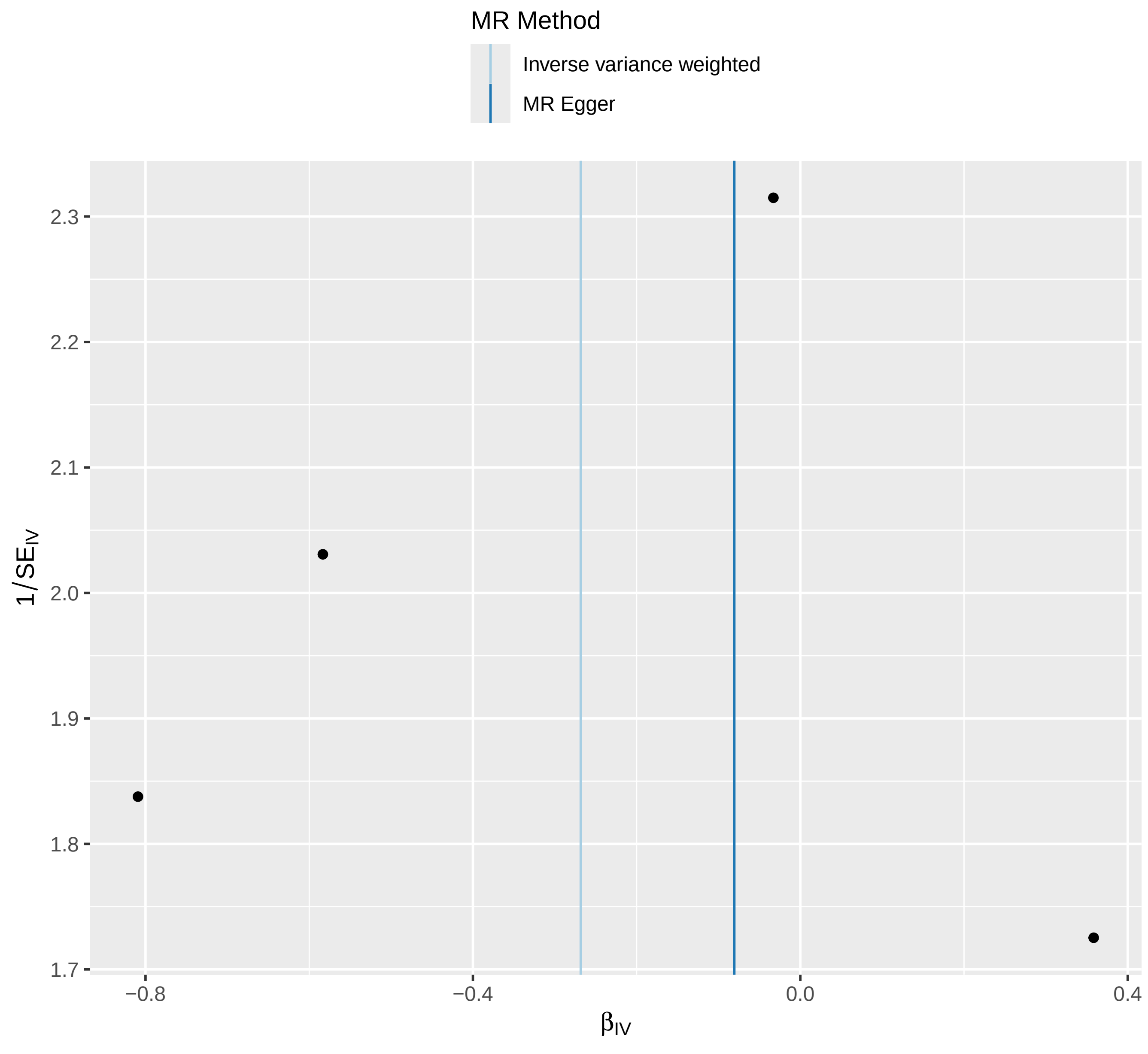

MR Method
Inverse variance weighted
MR Egger
2.3
2.2
2.1
2.0
1.9
1.8
1.7
1/SE$_{IV}$
−0.8
−0.4
0.0
0.4
β$_{IV}$

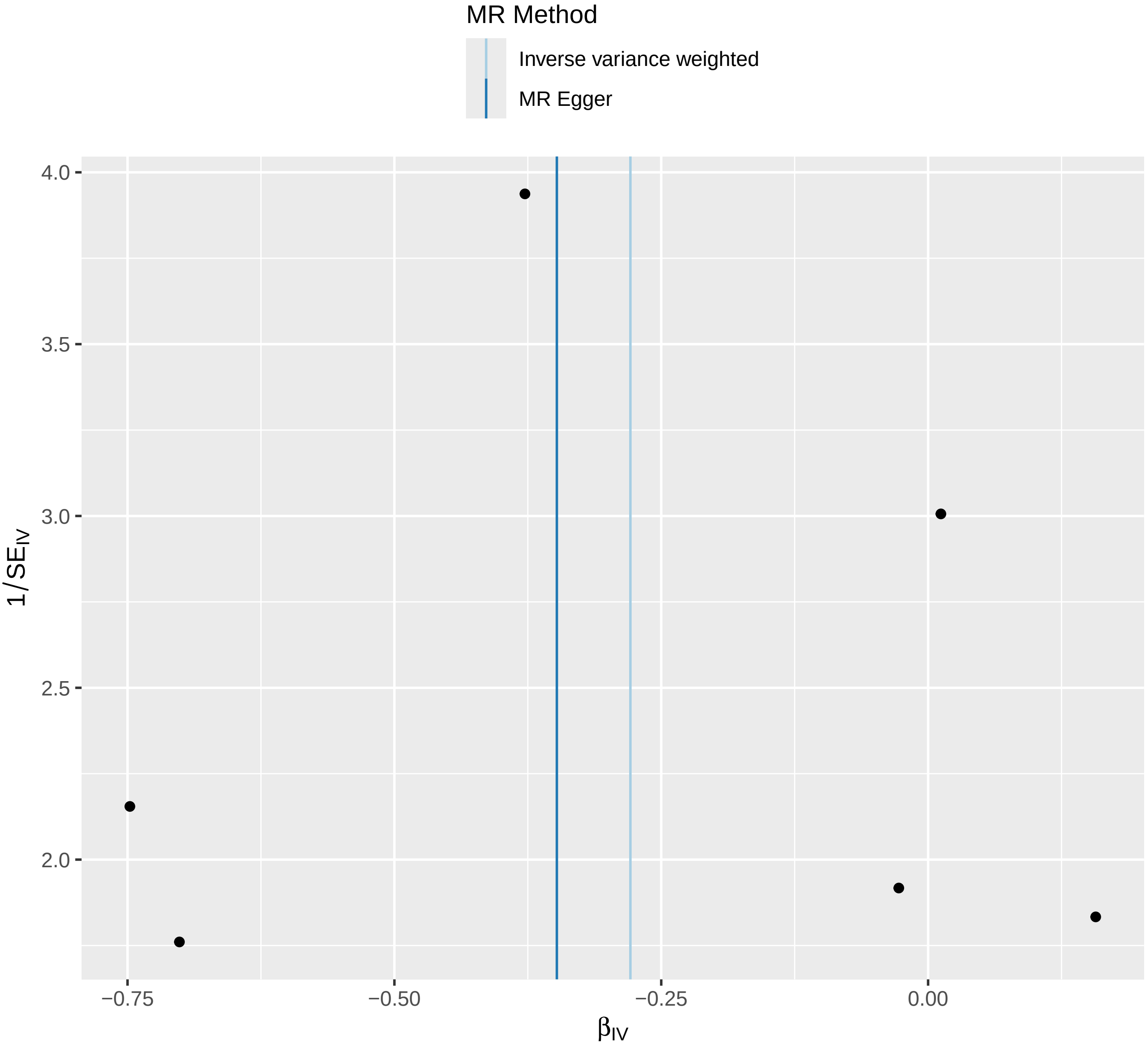
MR Method
Inverse variance weighted
MR Egger
4.0
3.5
3.0
2.5
2.0
1/SE$_{IV}$
−0.75
−0.50
−0.25
0.00
β$_{IV}$

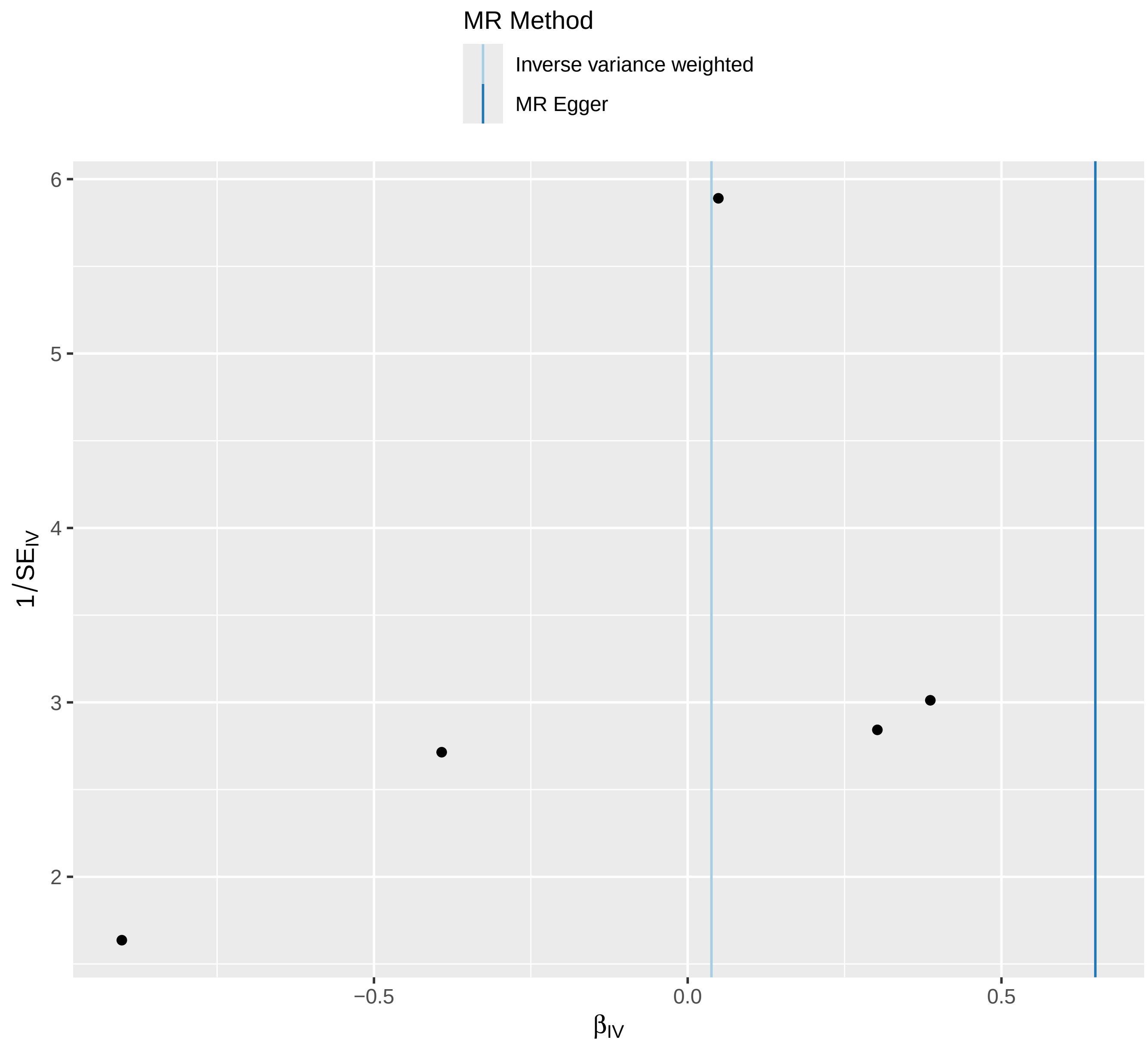
MR Method
Inverse variance weighted
MR Egger
6
5
4
3
2
1/SE_IV
−0.5
0.0
0.5
β_IV

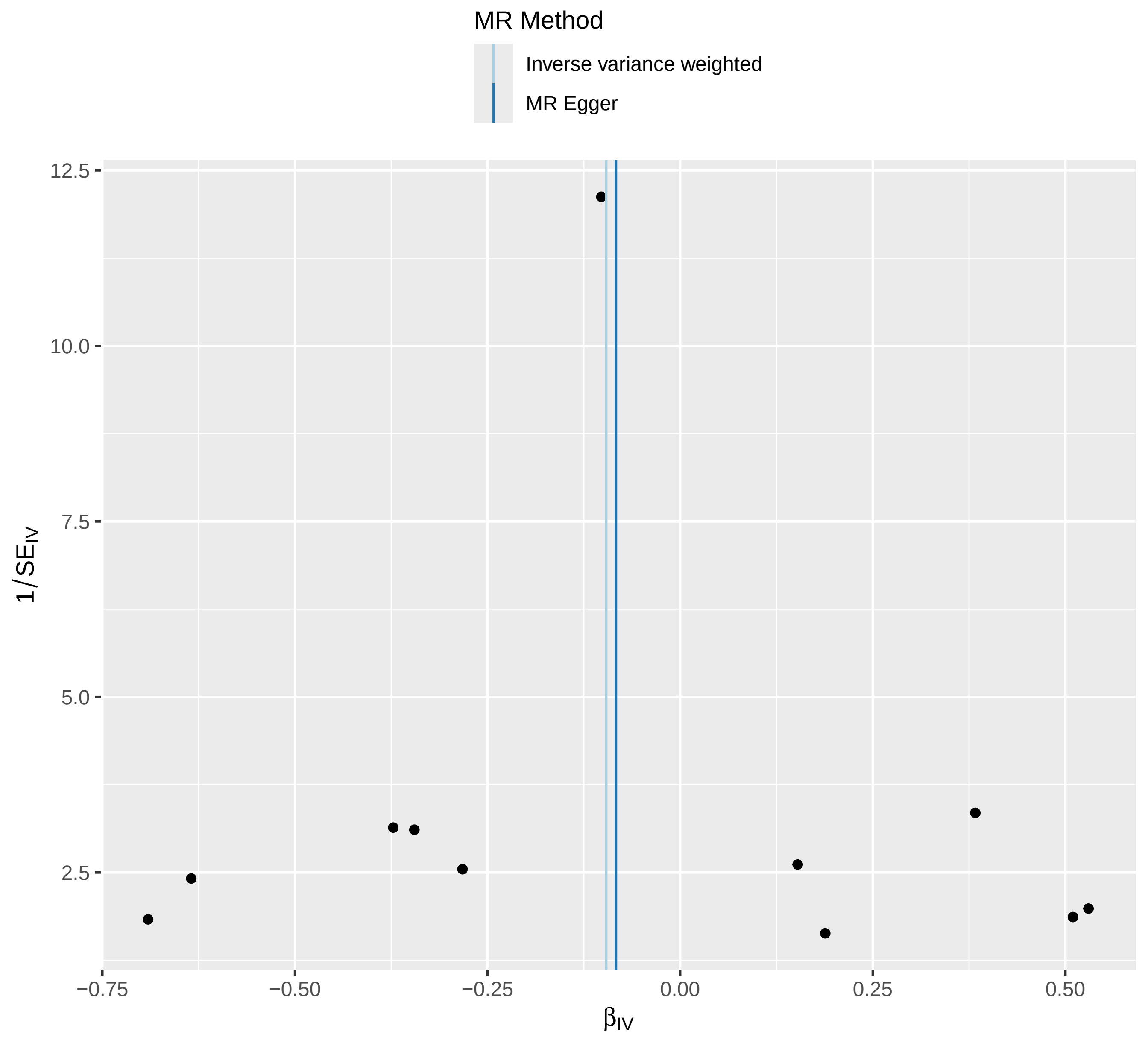
MR Method
Inverse variance weighted
MR Egger
12.5
10.0
7.5
5.0
2.5
1/SE_IV
−0.75
−0.50
−0.25
0.00
0.25
0.50
β_IV

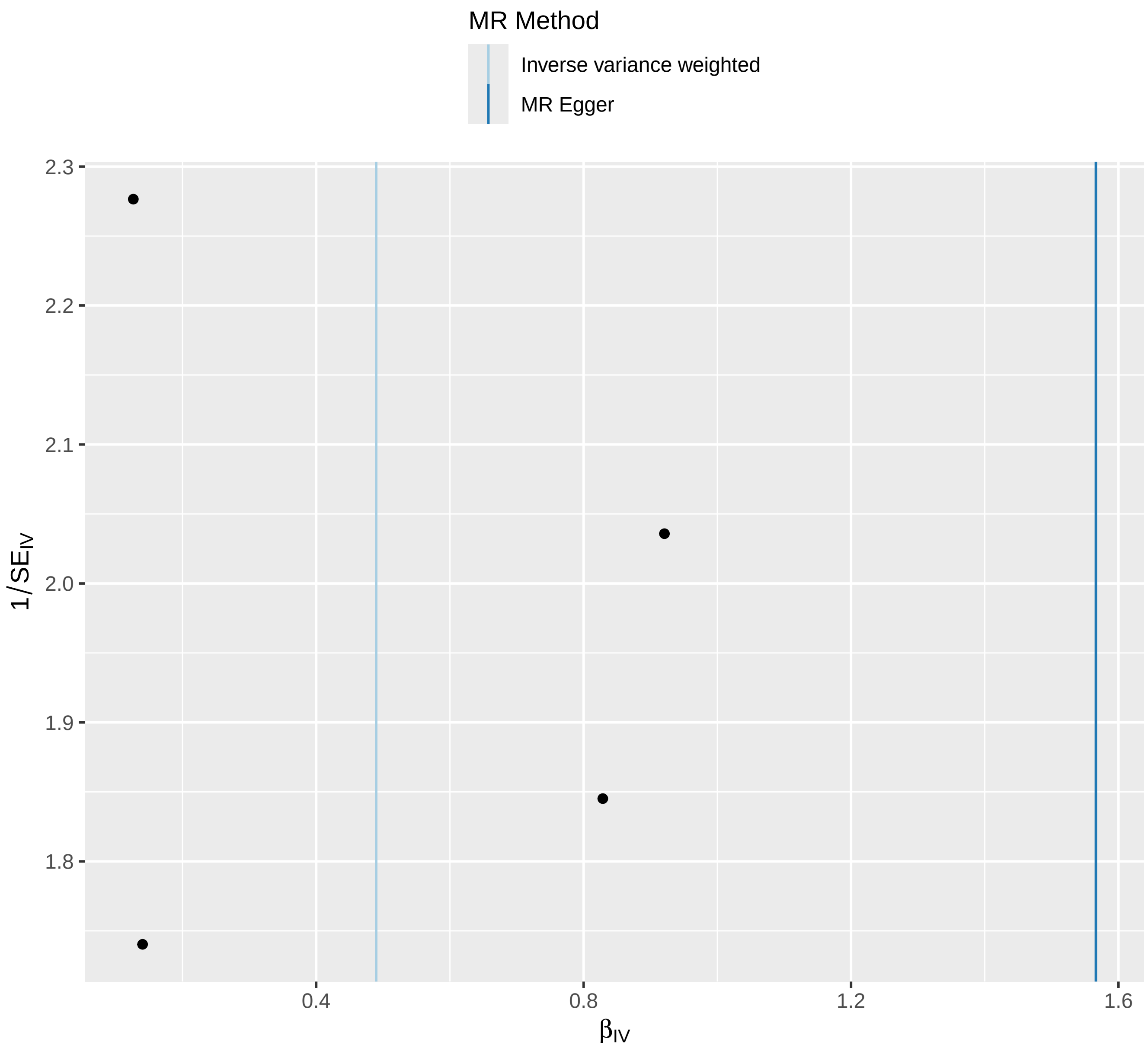

MR Method
Inverse variance weighted
MR Egger
2.3
2.2
2.1
2.0
1.9
1.8
1/SE_IV
0.4
0.8
1.2
1.6
β_IV

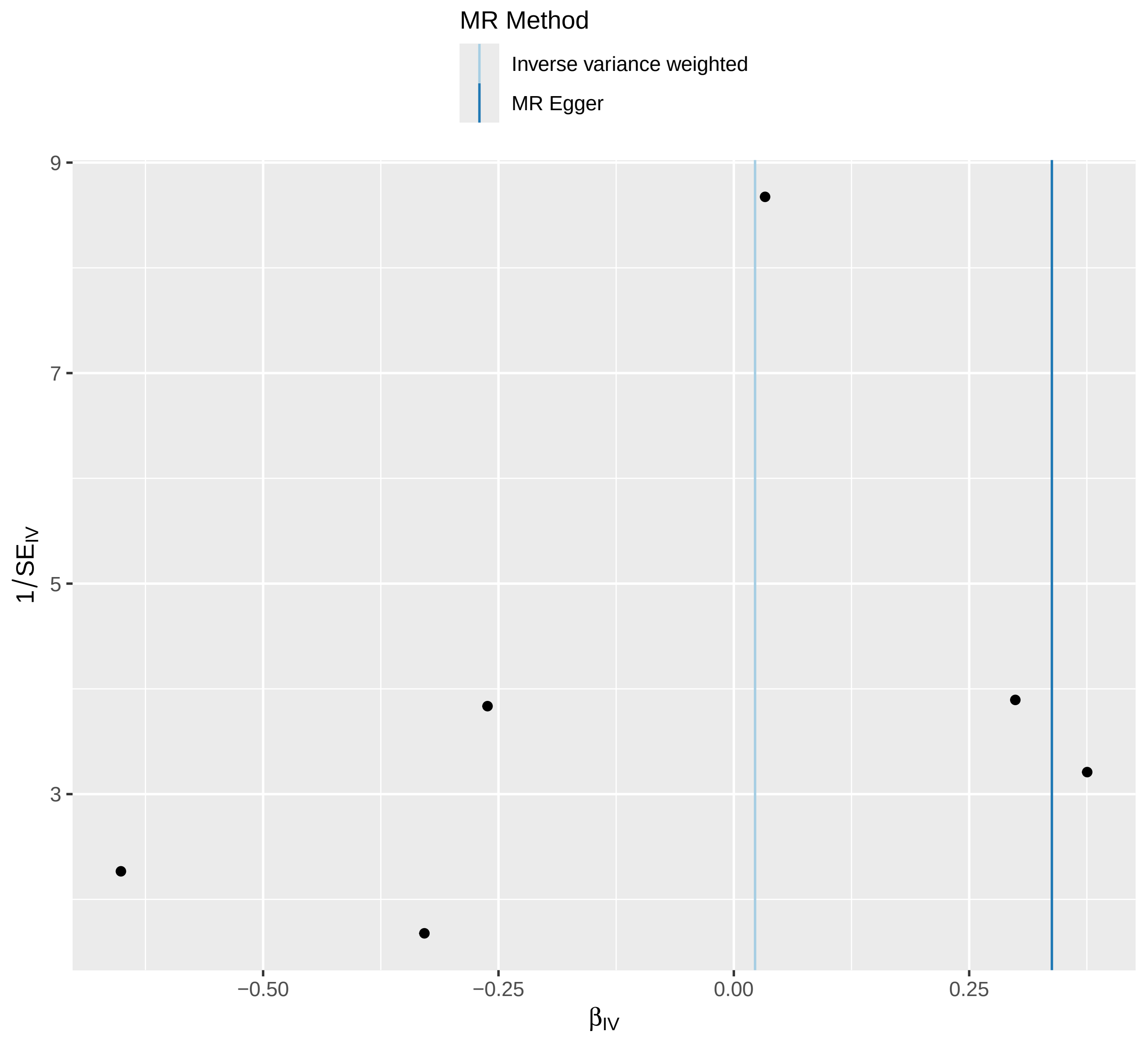

MR Method
Inverse variance weighted
MR Egger
9
7
5
3
1/SE_IV
−0.50
−0.25
0.00
0.25
β_IV

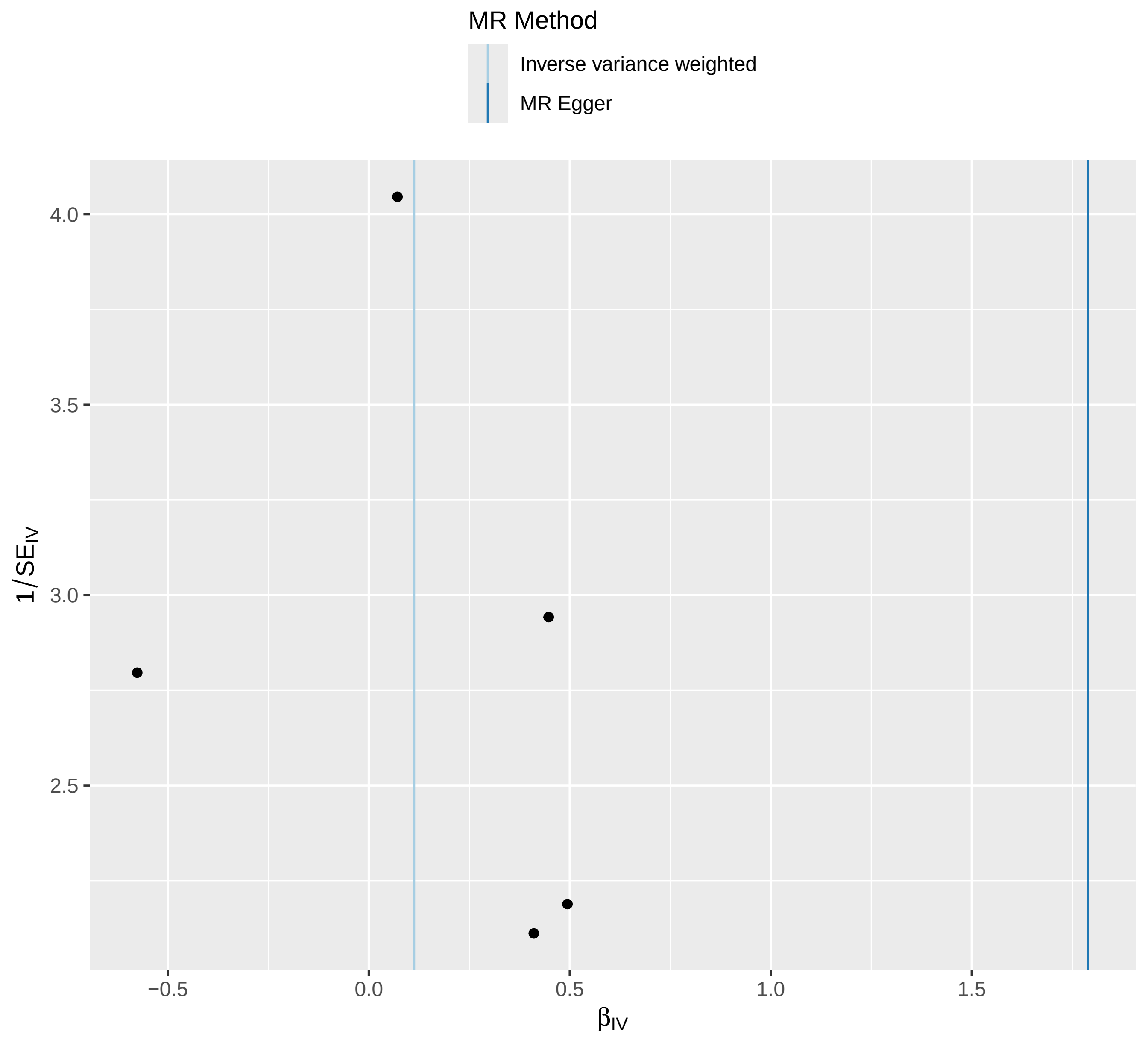
MR Method
Inverse variance weighted
MR Egger
1/SE_IV
4.0
3.5
3.0
2.5
−0.5
0.0
0.5
1.0
1.5
β_IV

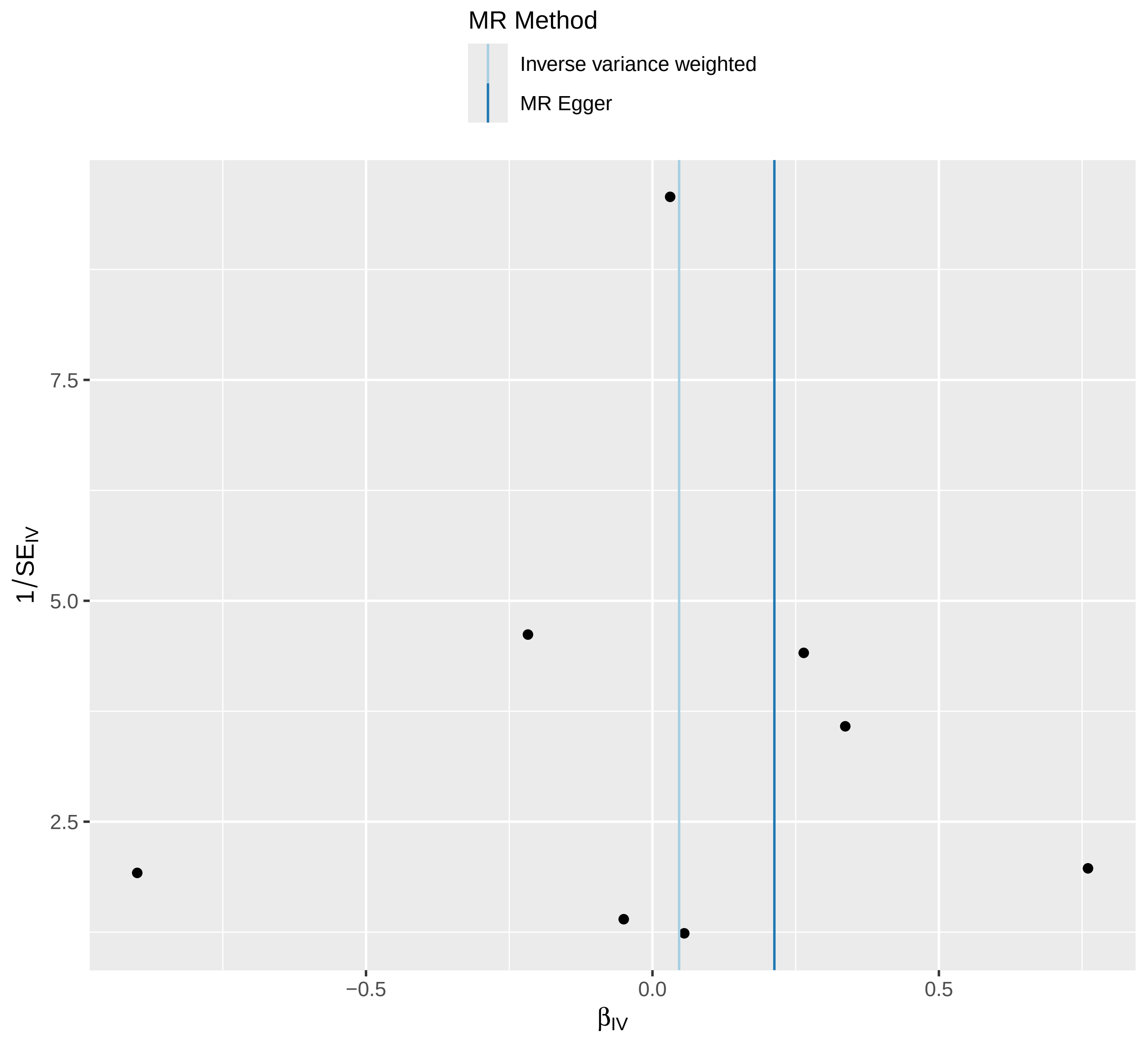
MR Method
Inverse variance weighted
MR Egger
7.5
5.0
2.5
1/SE$_{IV}$
−0.5
0.0
0.5
β$_{IV}$

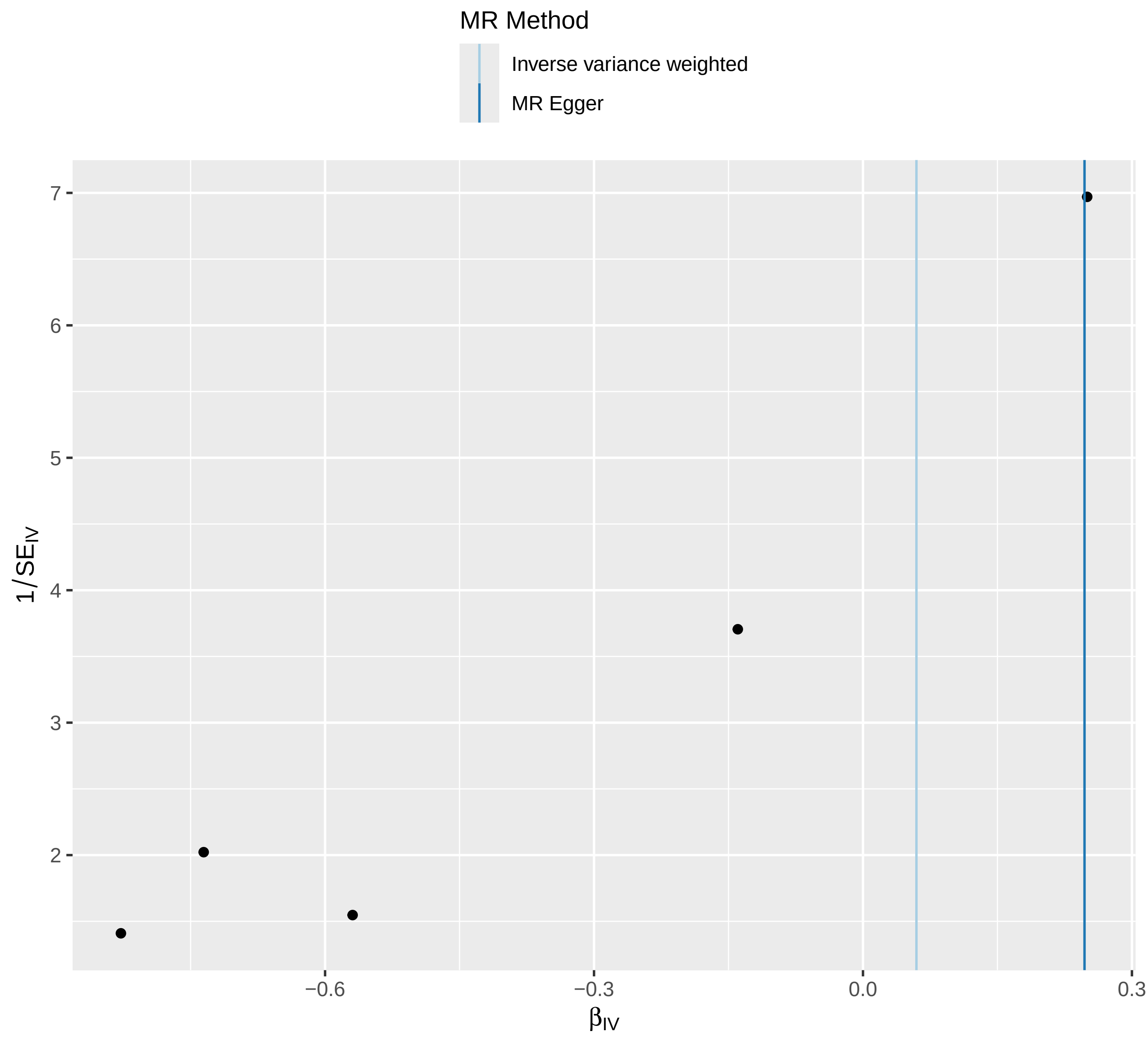
MR Method
Inverse variance weighted
MR Egger
1/SE$_{IV}$
7
6
5
4
3
2
−0.6
−0.3
0.0
0.3
β$_{IV}$

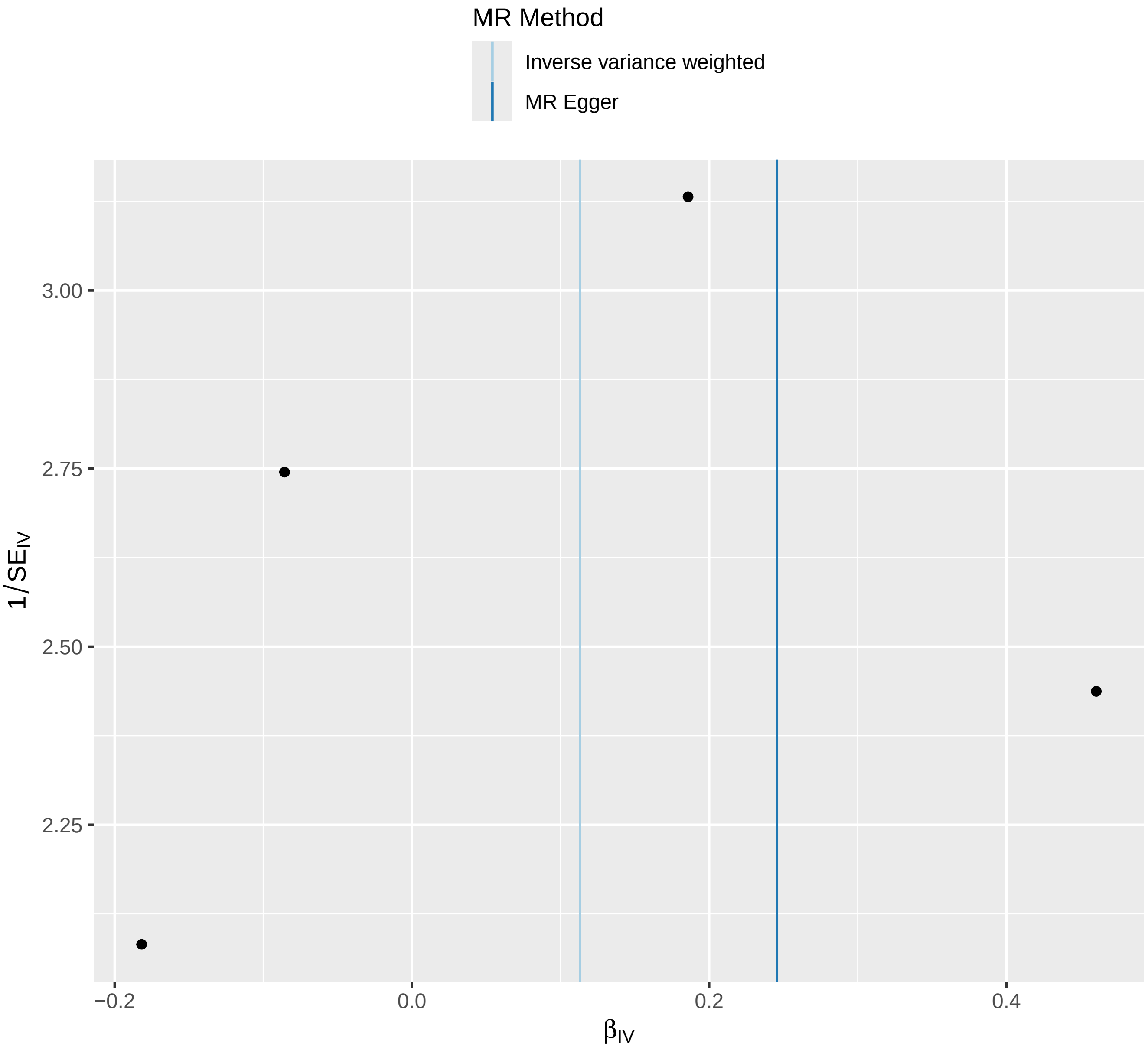
MR Method
Inverse variance weighted
MR Egger
3.00
2.75
2.50
2.25
1/SE$_{IV}$
−0.2
0.0
0.2
0.4
β$_{IV}$

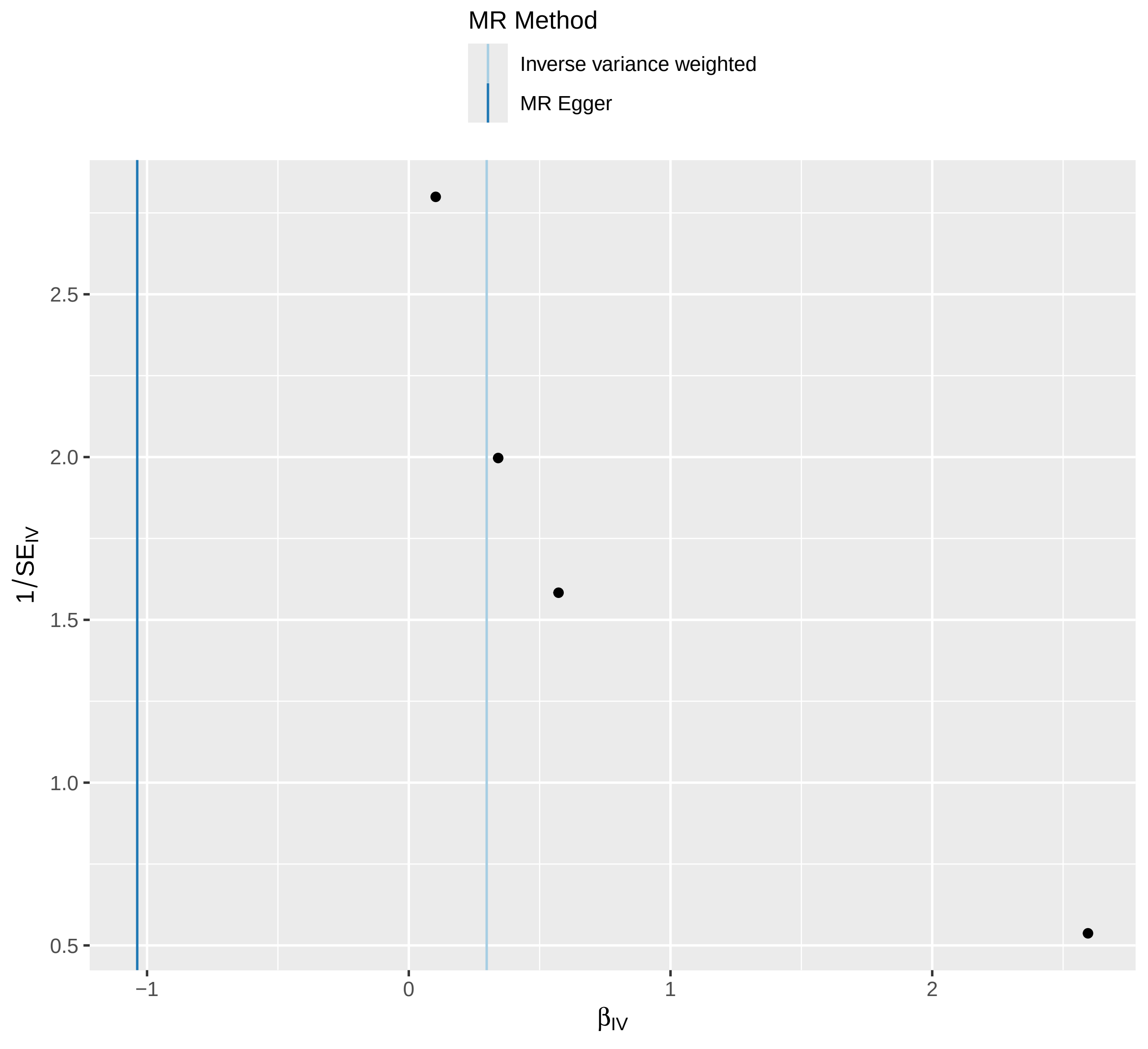
MR Method
Inverse variance weighted
MR Egger
2.5
2.0
1.5
1.0
0.5
$1/SE_{IV}$
−1
0
1
2
β_{IV}

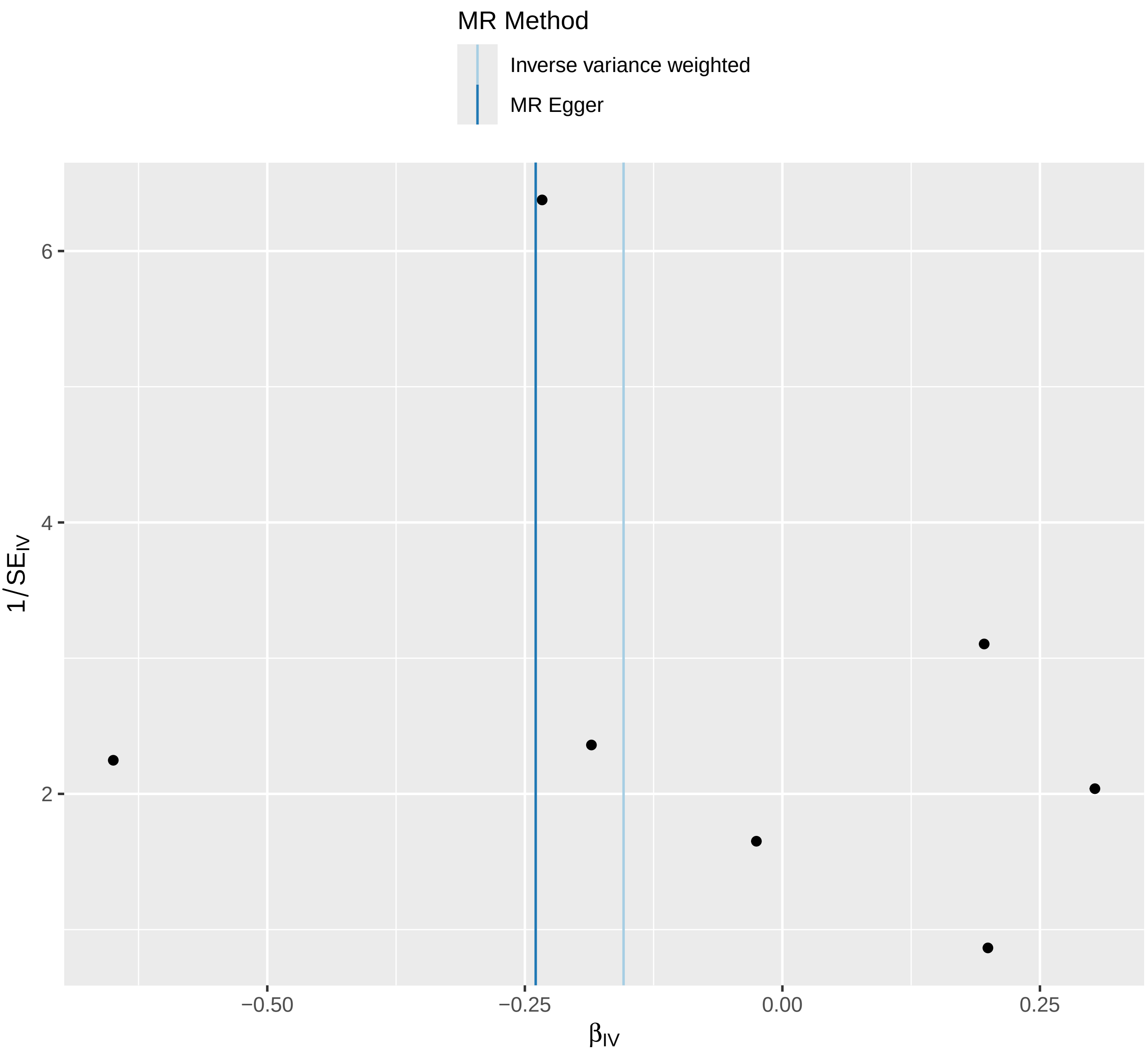

MR Method
Inverse variance weighted
MR Egger
6
4
2
1/SE$_{IV}$
−0.50
−0.25
0.00
0.25
β$_{IV}$

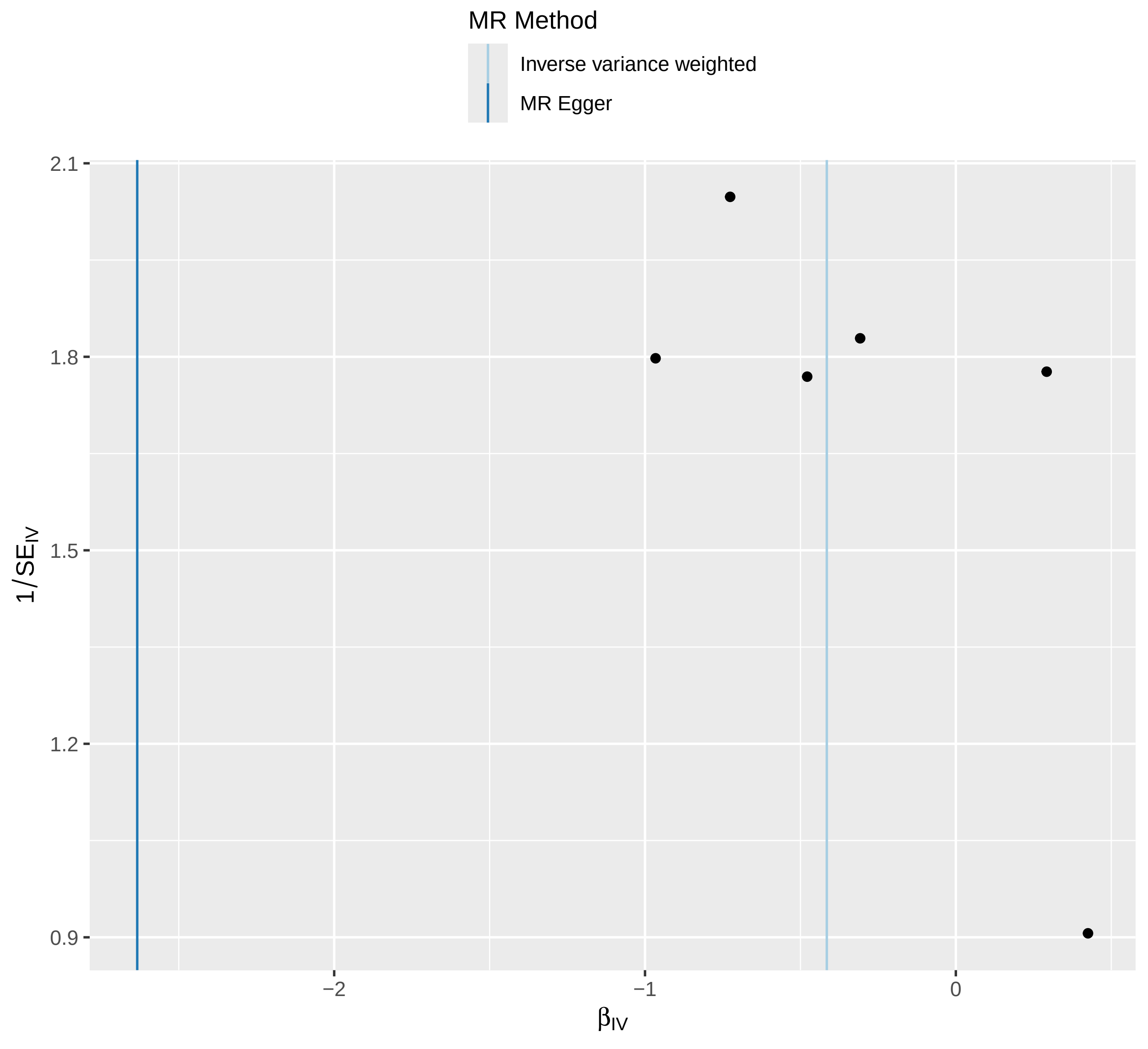

MR Method
Inverse variance weighted
MR Egger
2.1
1.8
1.5
1.2
0.9
1/SE_IV
−2
−1
0
β_IV

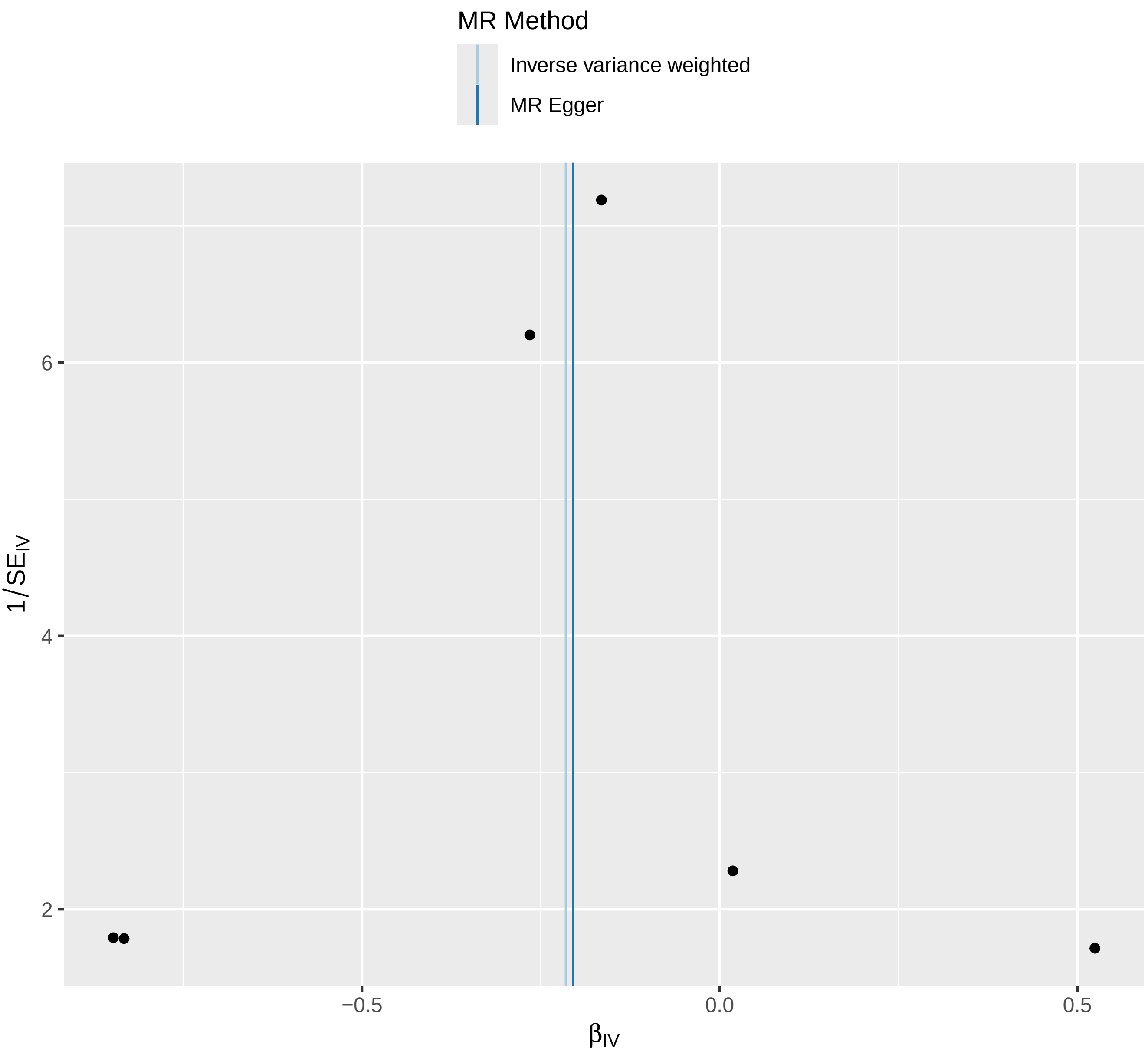
MR Method
Inverse variance weighted
MR Egger
6
4
2
1/SE$_{IV}$
−0.5
0.0
0.5
β$_{IV}$

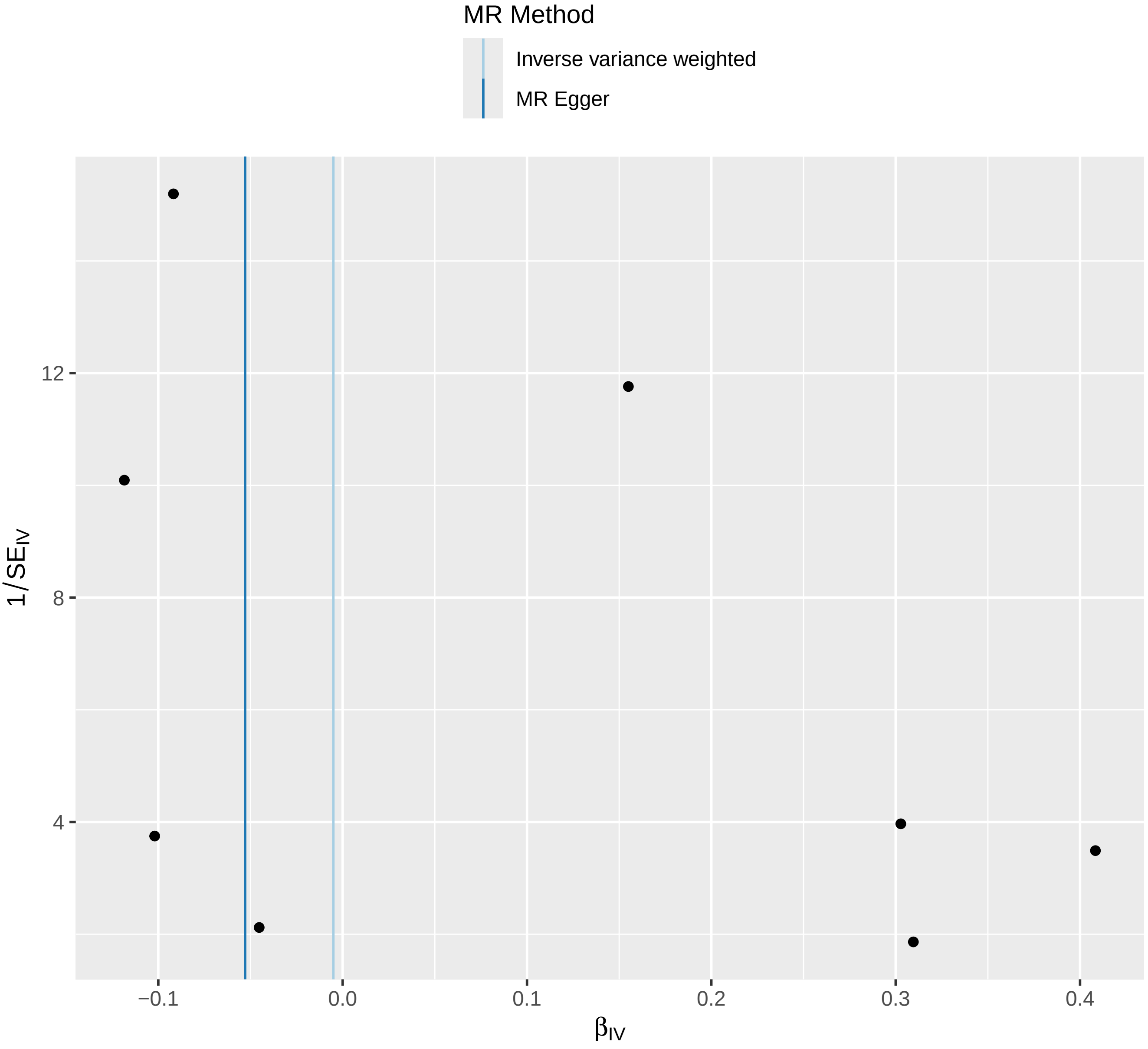

MR Method
Inverse variance weighted
MR Egger
1/SE$_{IV}$
12
8
4
−0.1
0.0
0.1
0.2
0.3
0.4
β$_{IV}$

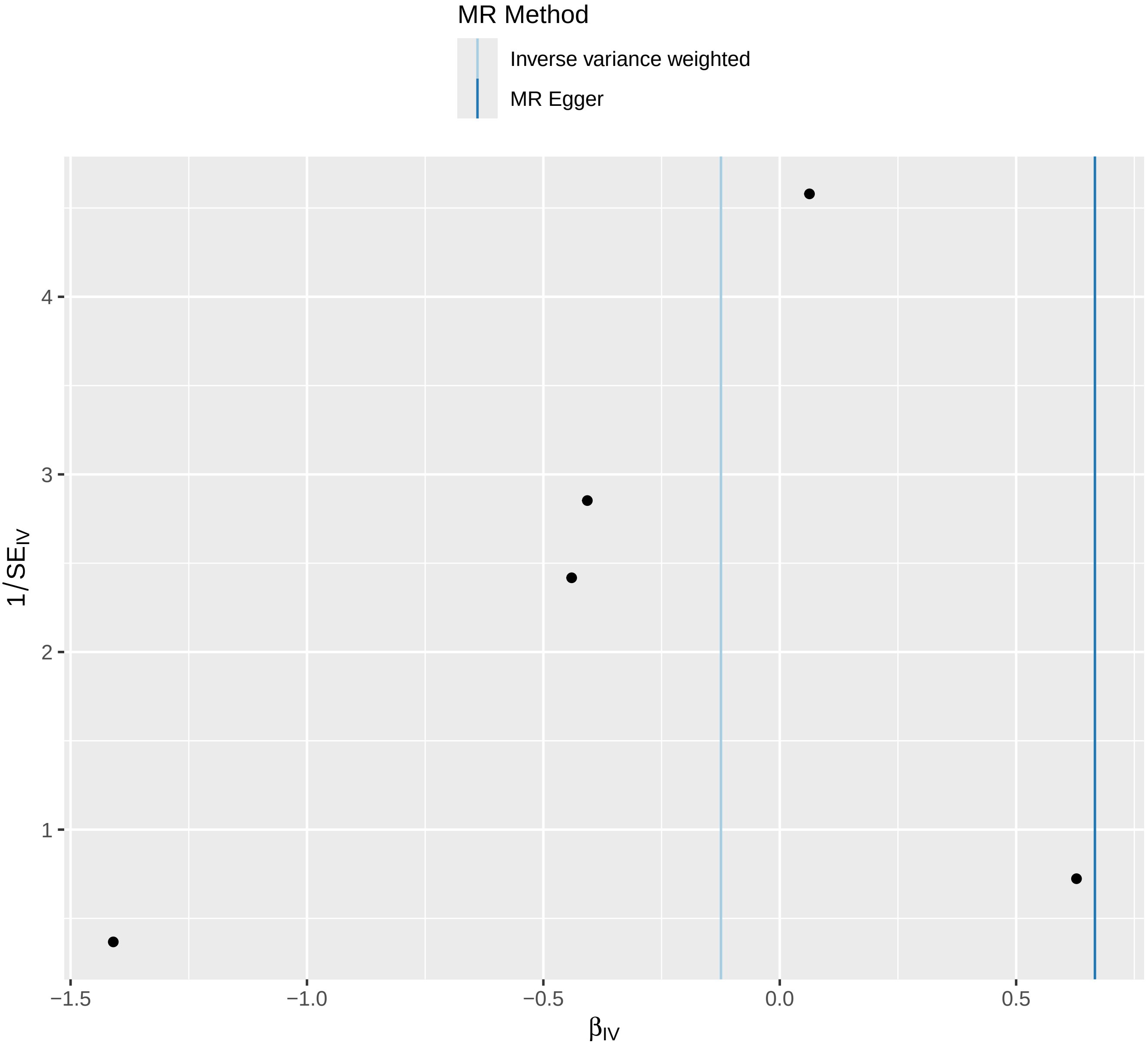

MR Method
Inverse variance weighted
MR Egger
4
3
2
1
1/SE$_{IV}$
−1.5
−1.0
−0.5
0.0
0.5
β$_{IV}$

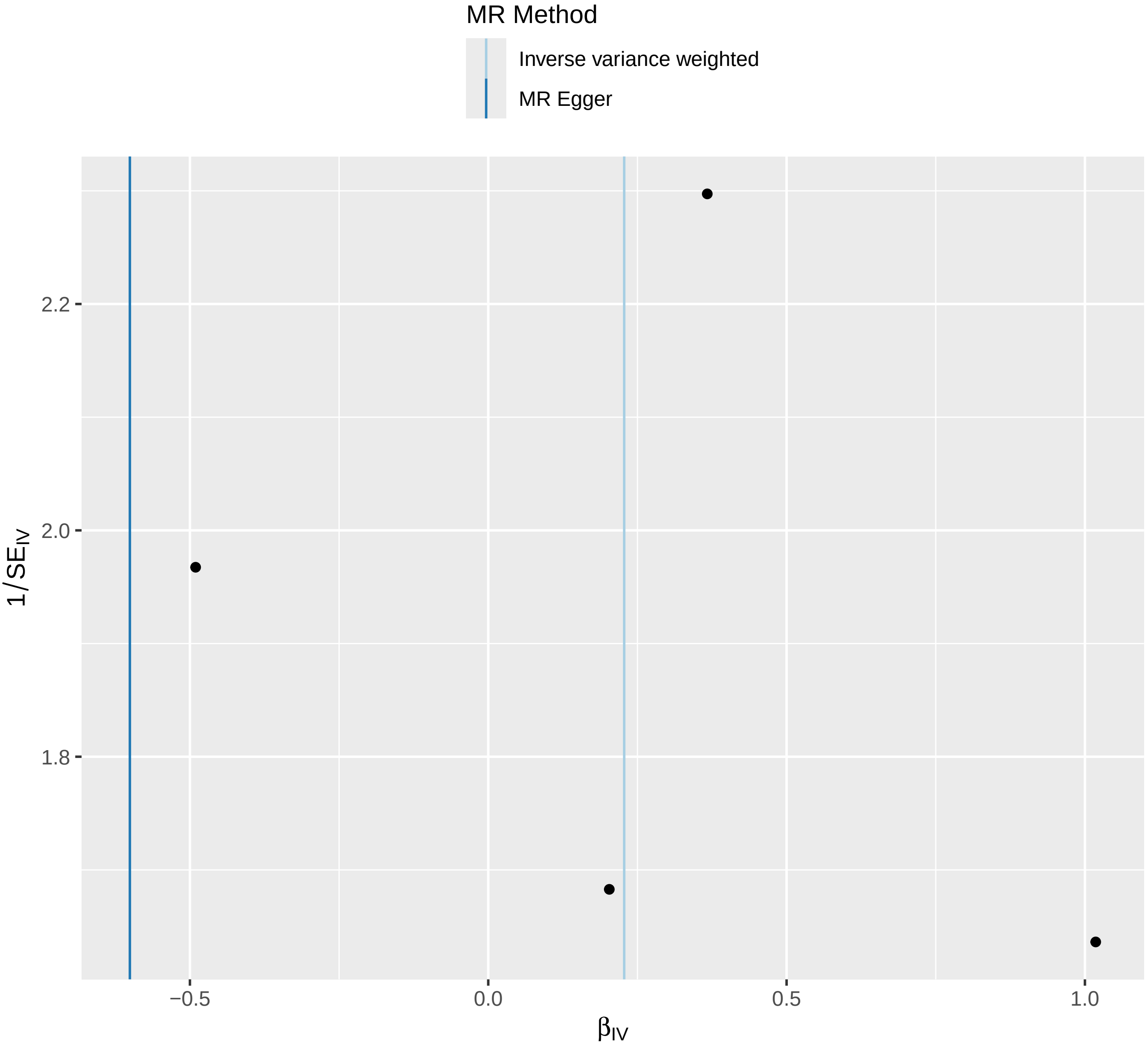

MR Method
Inverse variance weighted
MR Egger
2.2
2.0
1.8
1/SE_IV
−0.5
0.0
0.5
1.0
β_IV

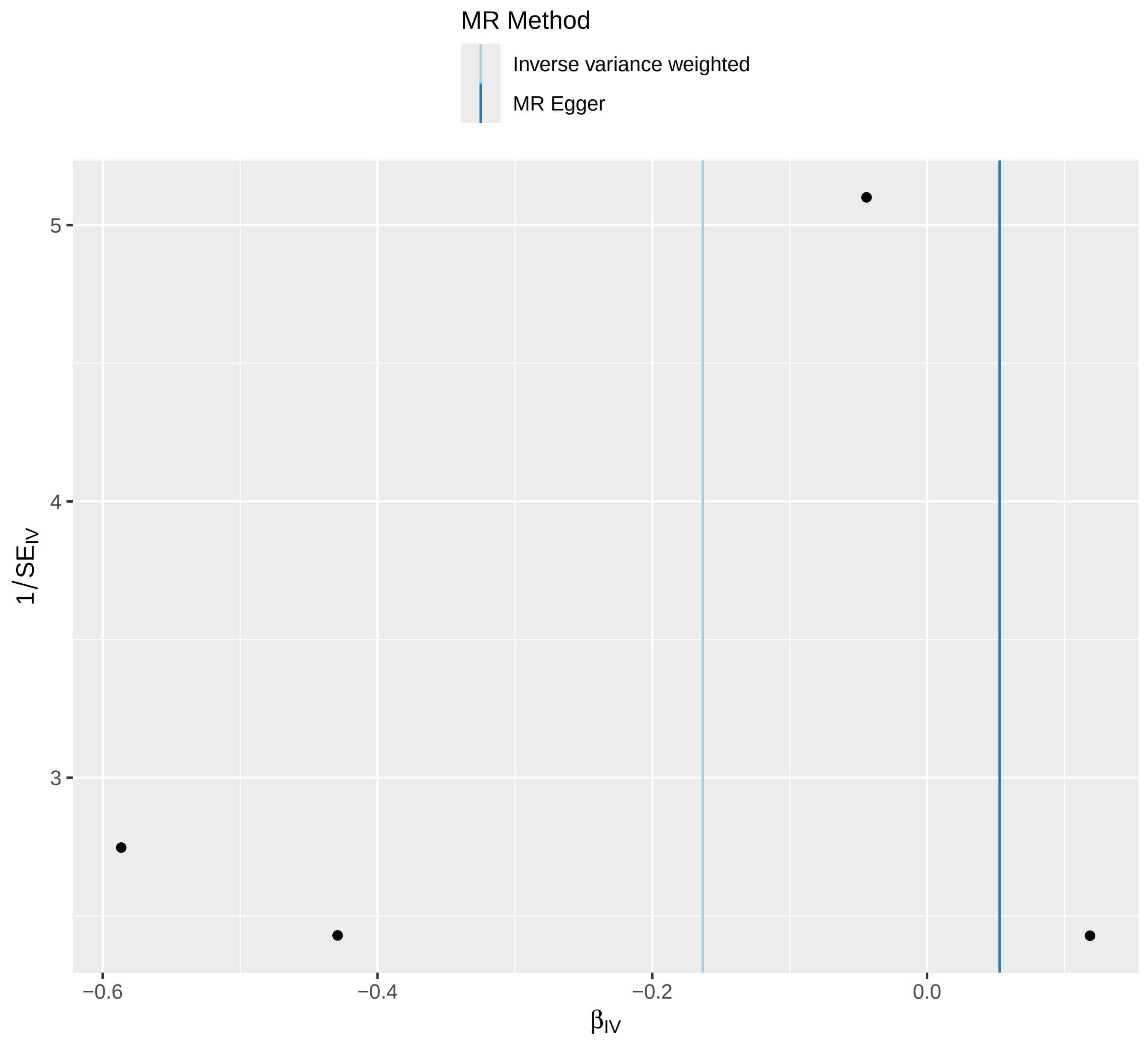

MR Method
Inverse variance weighted
MR Egger
5
4
3
1/SE_IV
−0.6
−0.4
−0.2
0.0
β_IV

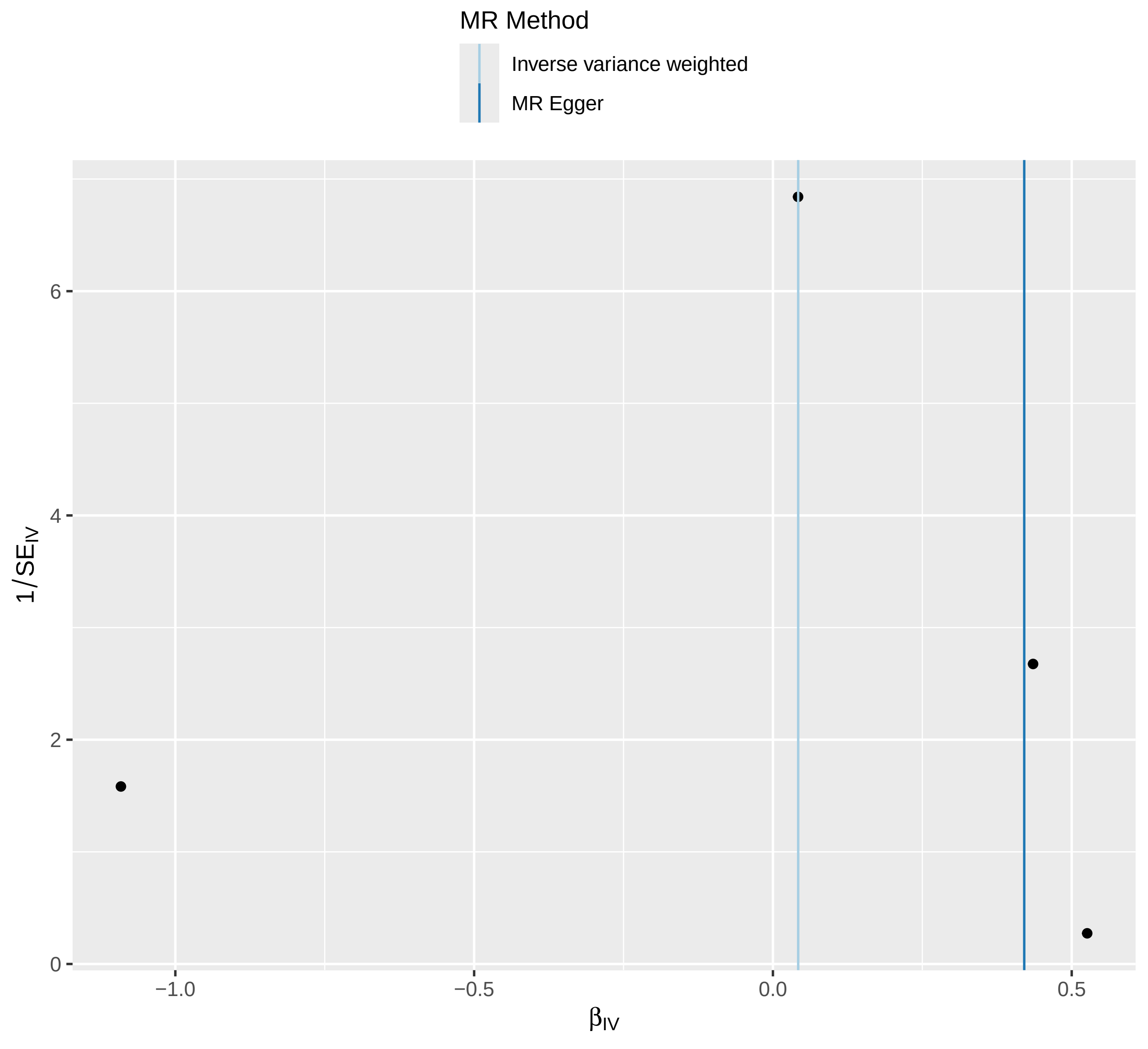
MR Method
Inverse variance weighted
MR Egger
6
4
2
0
−1.0
−0.5
0.0
0.5
1/SE_IV
β_IV

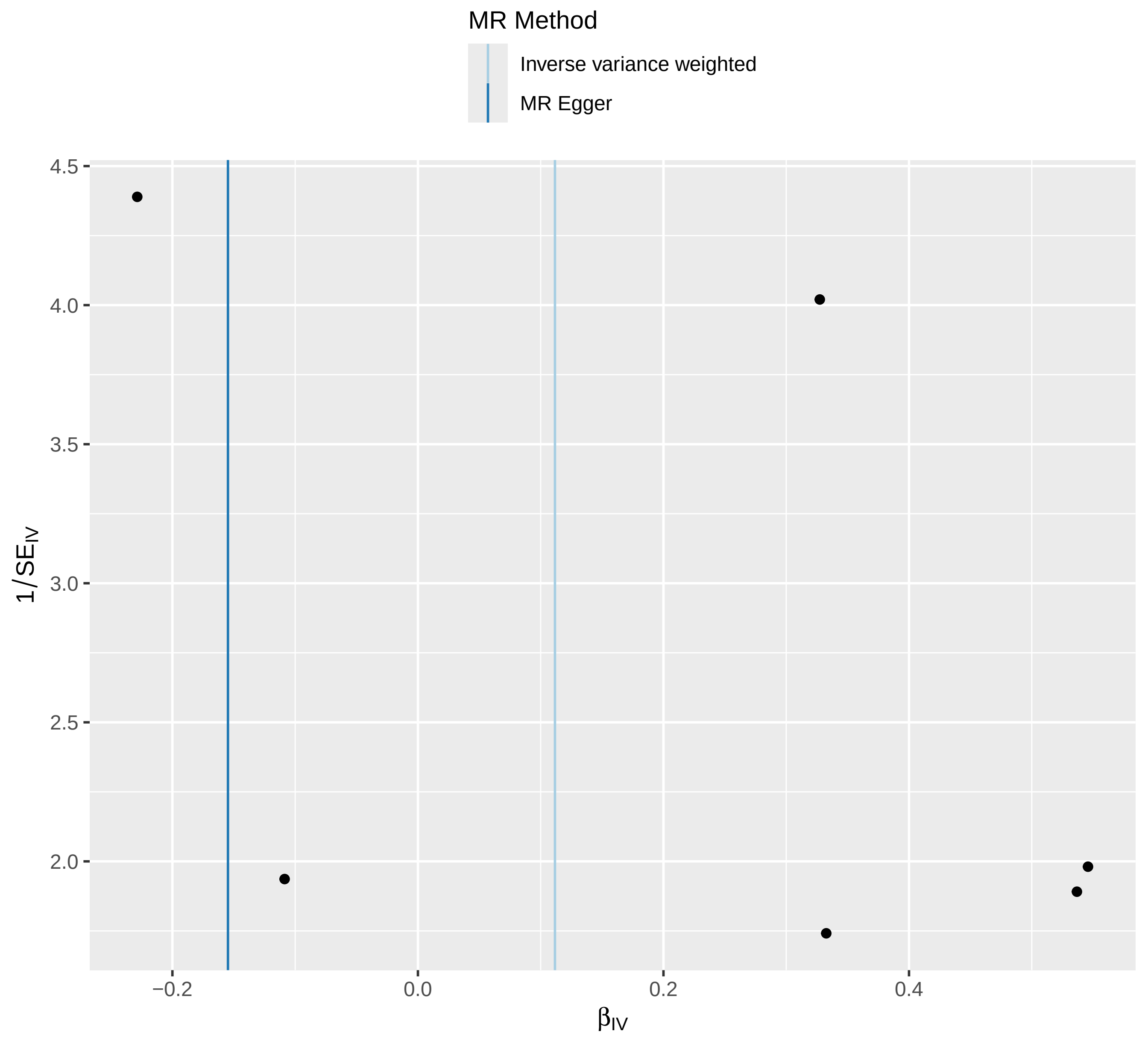

MR Method
Inverse variance weighted
MR Egger
4.5
4.0
3.5
3.0
2.5
2.0
1/SE_IV
−0.2
0.0
0.2
0.4
β_IV

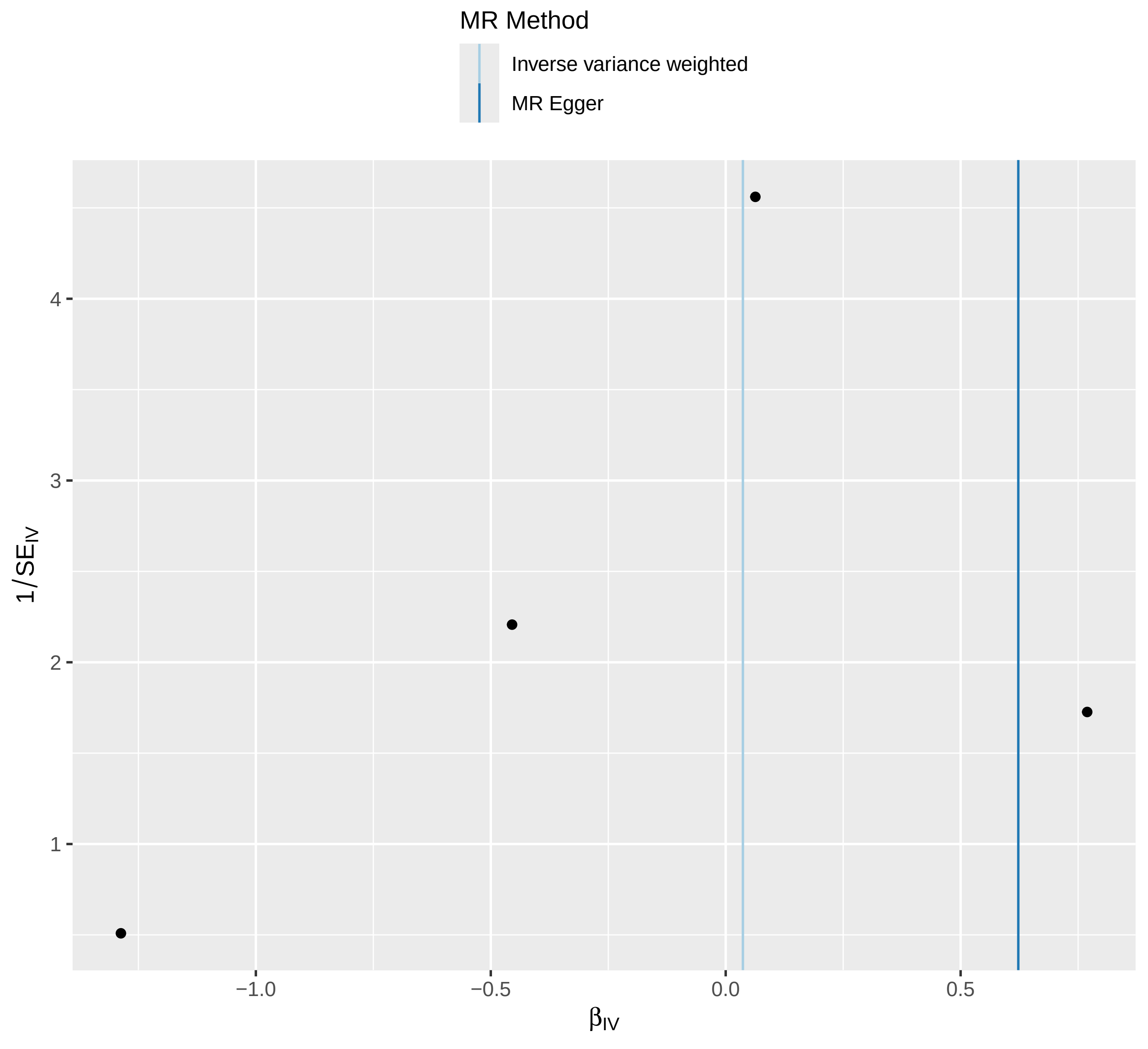
MR Method
Inverse variance weighted
MR Egger
1/SE_IV
4
3
2
1
−1.0
−0.5
0.0
0.5
β_IV

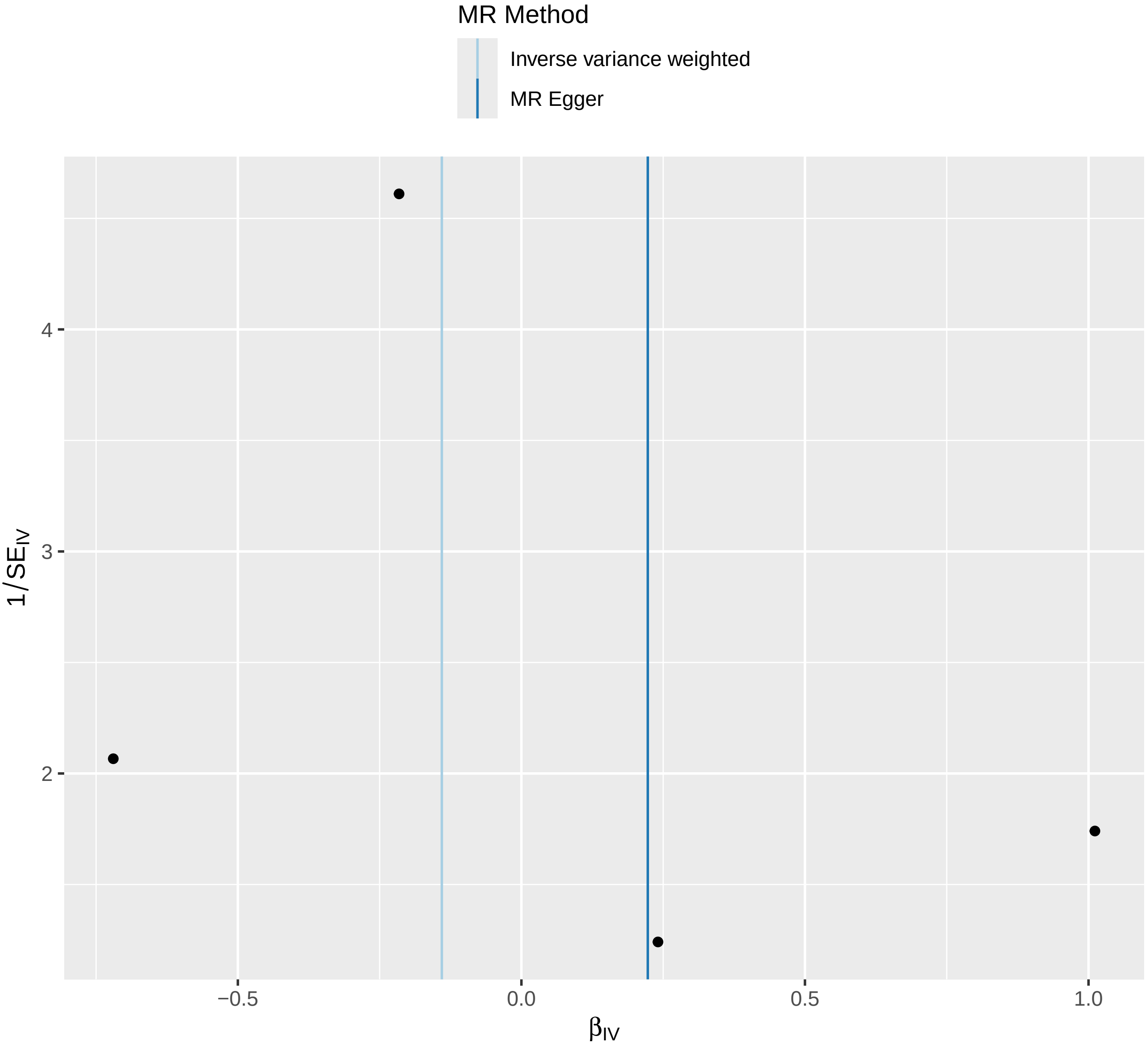
MR Method
Inverse variance weighted
MR Egger
1/SE_IV
4
3
2
−0.5
0.0
0.5
1.0
β_IV

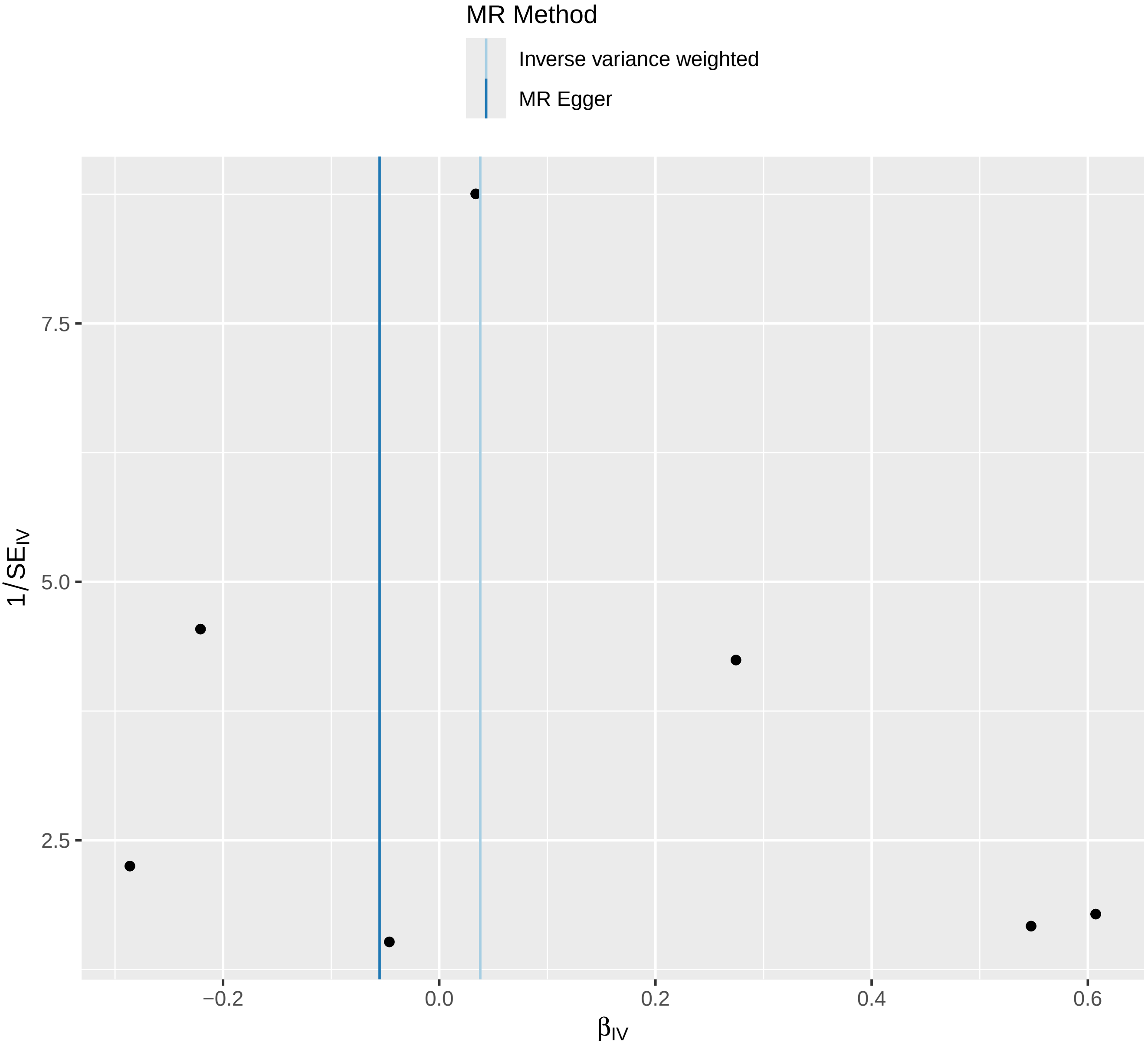

MR Method
Inverse variance weighted
MR Egger
7.5
5.0
2.5
1/SE$_{IV}$
−0.2
0.0
0.2
0.4
0.6
β$_{IV}$

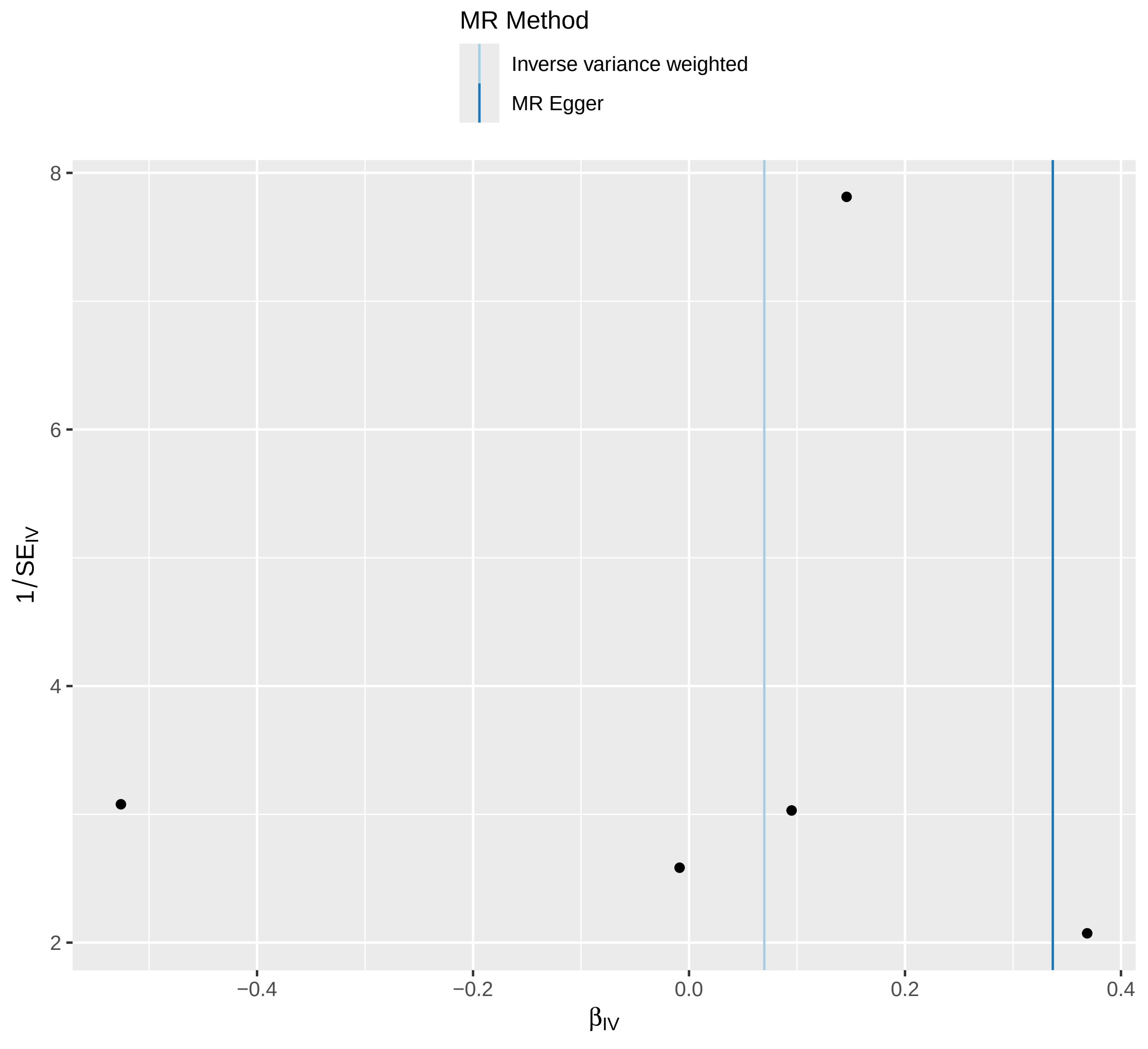
MR Method
Inverse variance weighted
MR Egger
8
6
4
2
−0.4
−0.2
0.0
0.2
0.4
$1/SE_{IV}$
β_{IV}

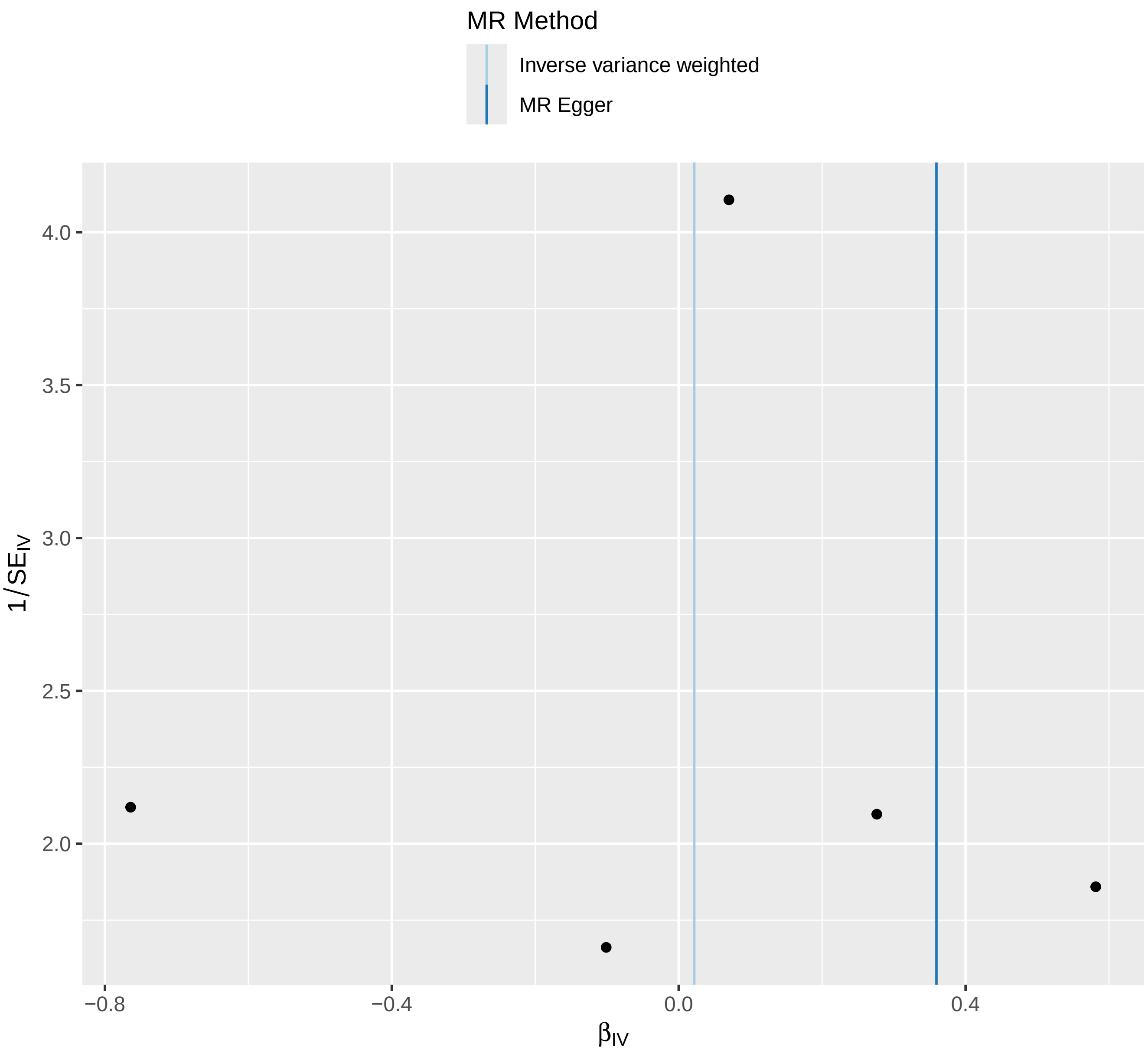

MR Method
Inverse variance weighted
MR Egger
4.0
3.5
3.0
2.5
2.0
1/SE$_{IV}$
−0.8
−0.4
0.0
0.4
β$_{IV}$

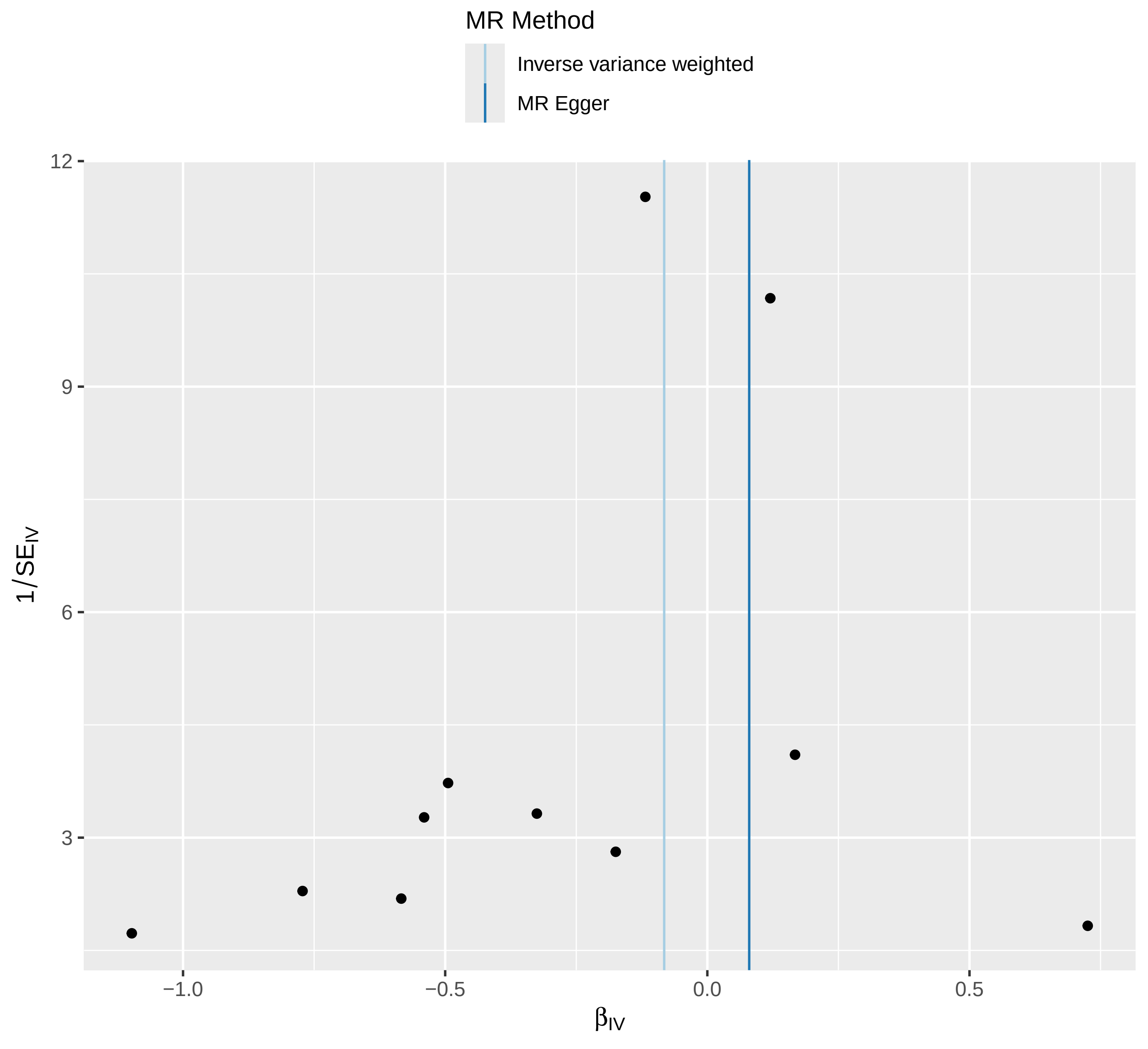

MR Method
Inverse variance weighted
MR Egger
12
9
6
3
1/SE_IV
−1.0
−0.5
0.0
0.5
β_IV

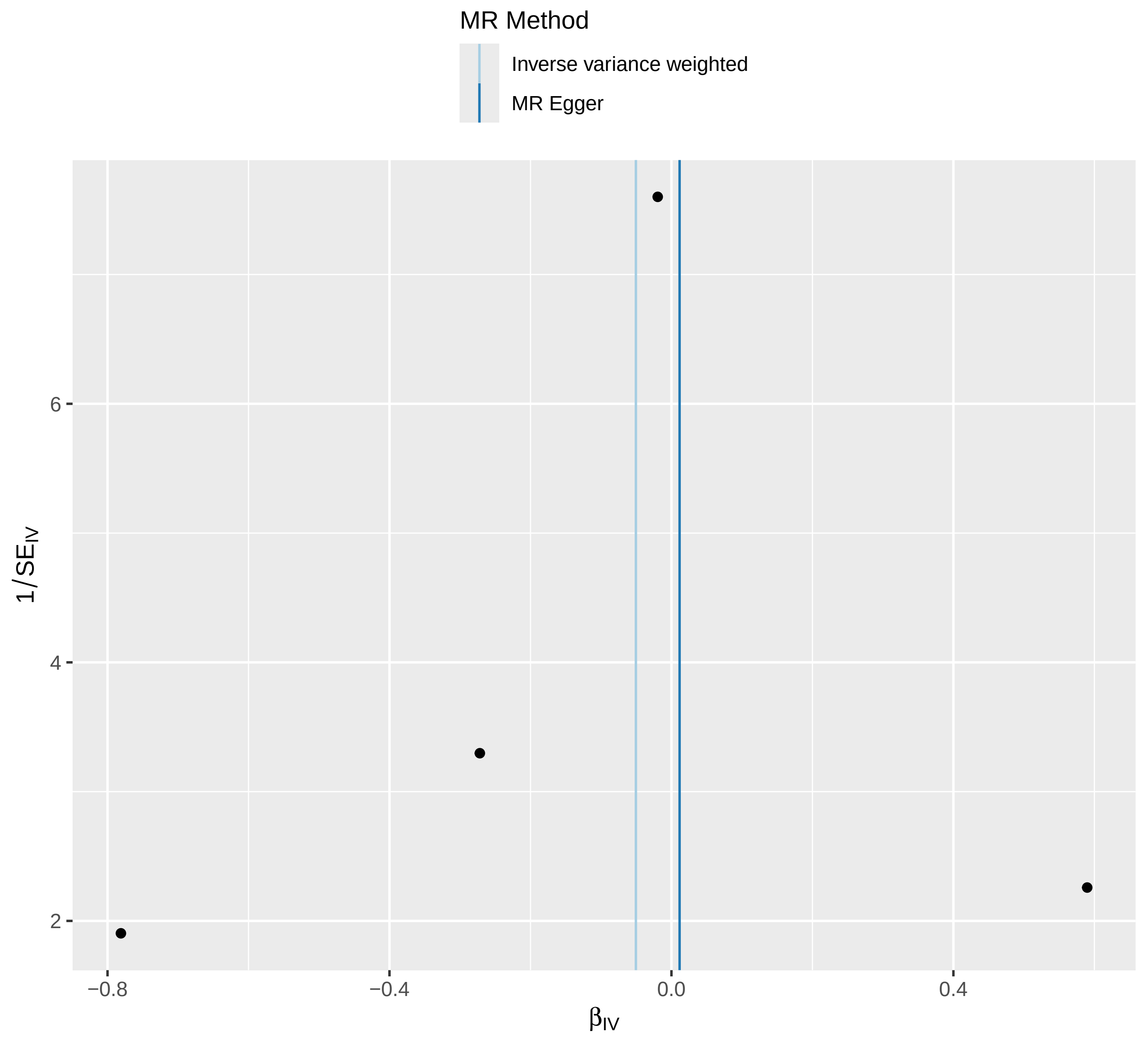

MR Method
Inverse variance weighted
MR Egger
6
4
2
−0.8
−0.4
0.0
0.4
$1/SE_{IV}$
β_{IV}

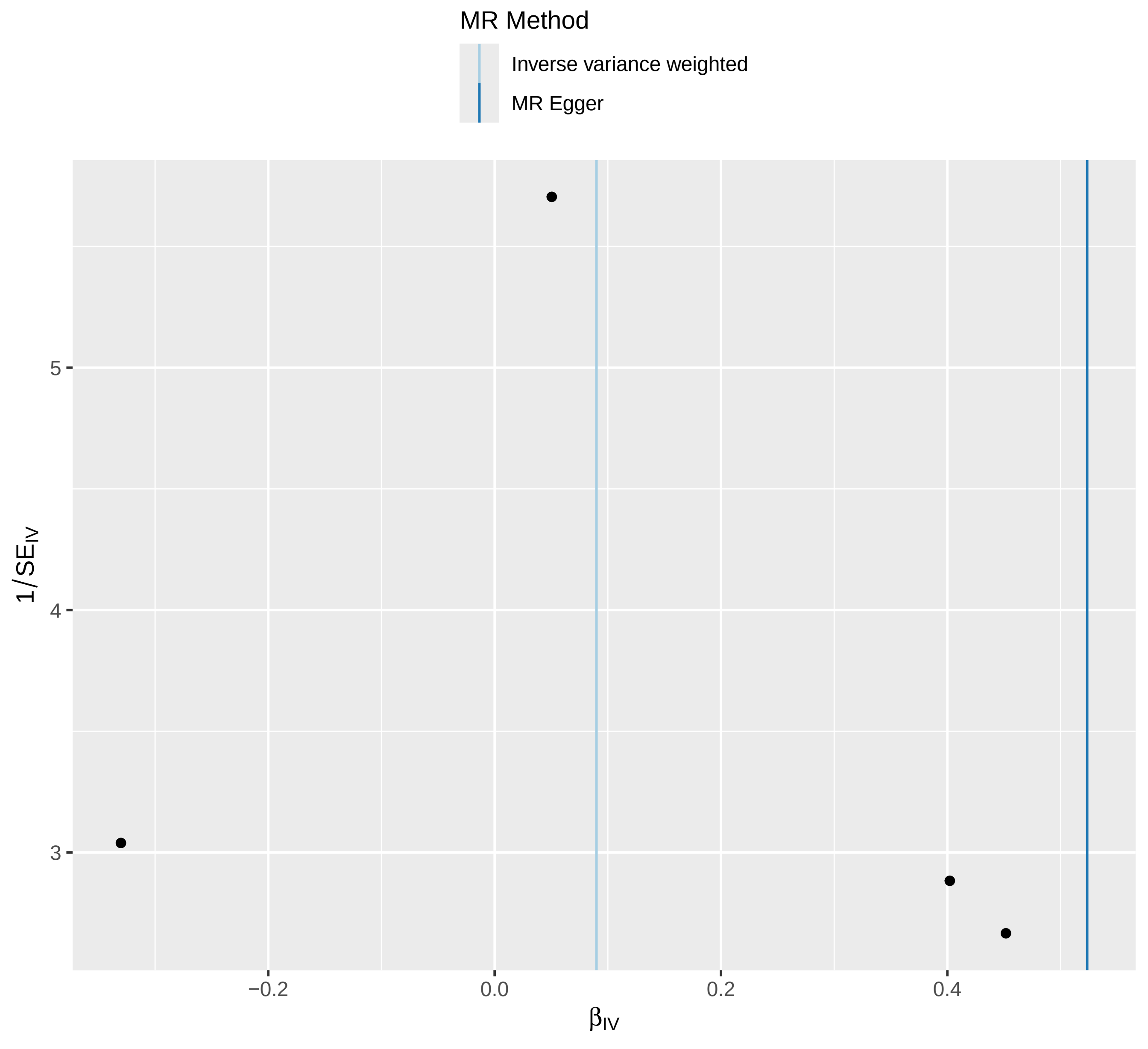

MR Method
Inverse variance weighted
MR Egger
1/SE_IV
5
4
3
−0.2
0.0
0.2
0.4
β_IV

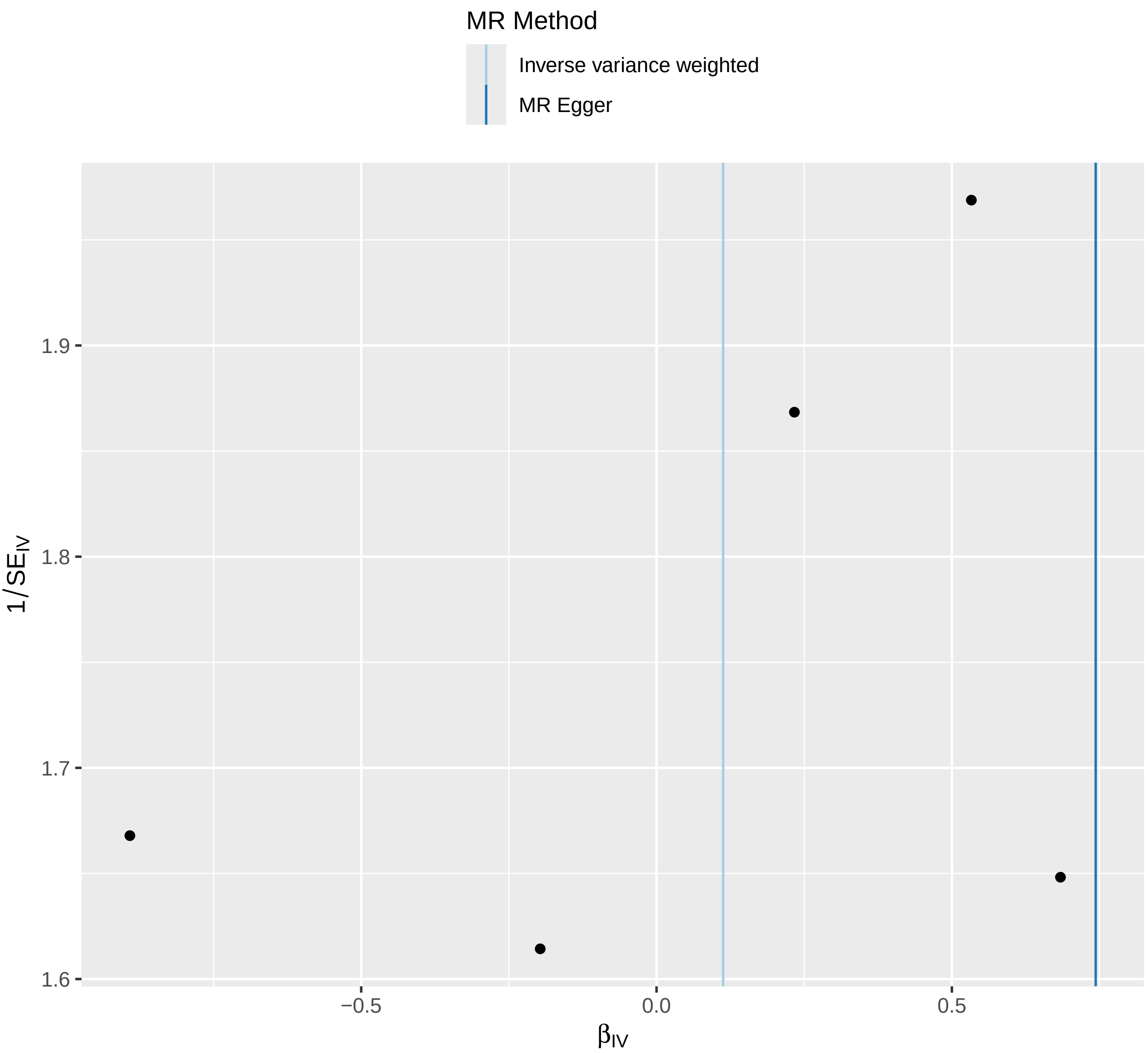

MR Method
Inverse variance weighted
MR Egger
1.9
1.8
1.7
1.6
−0.5
0.0
0.5
$1/SE_{IV}$
β_{IV}

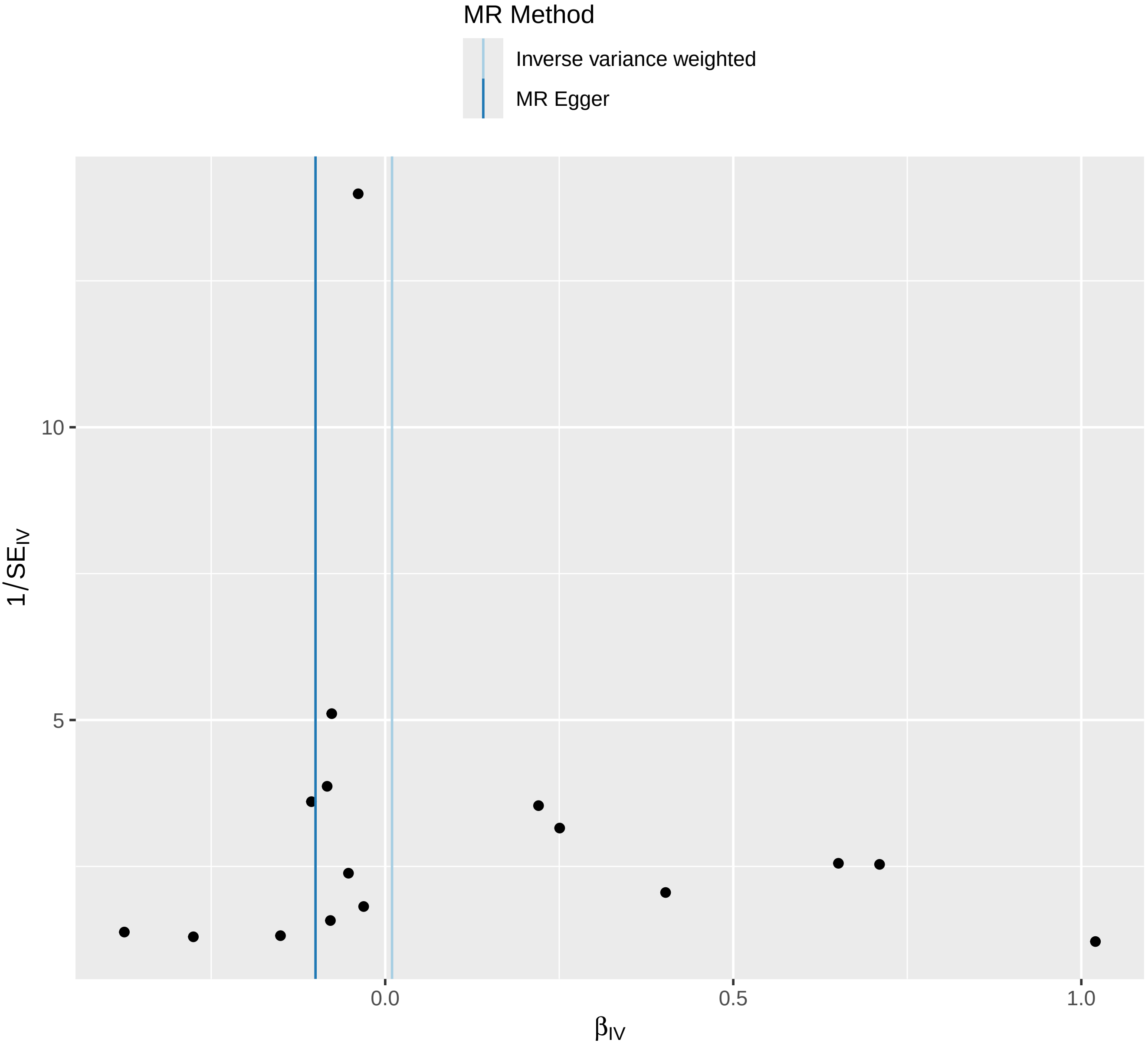

MR Method
Inverse variance weighted
MR Egger
10
5
1/SE_IV
0.0
0.5
1.0
β_IV

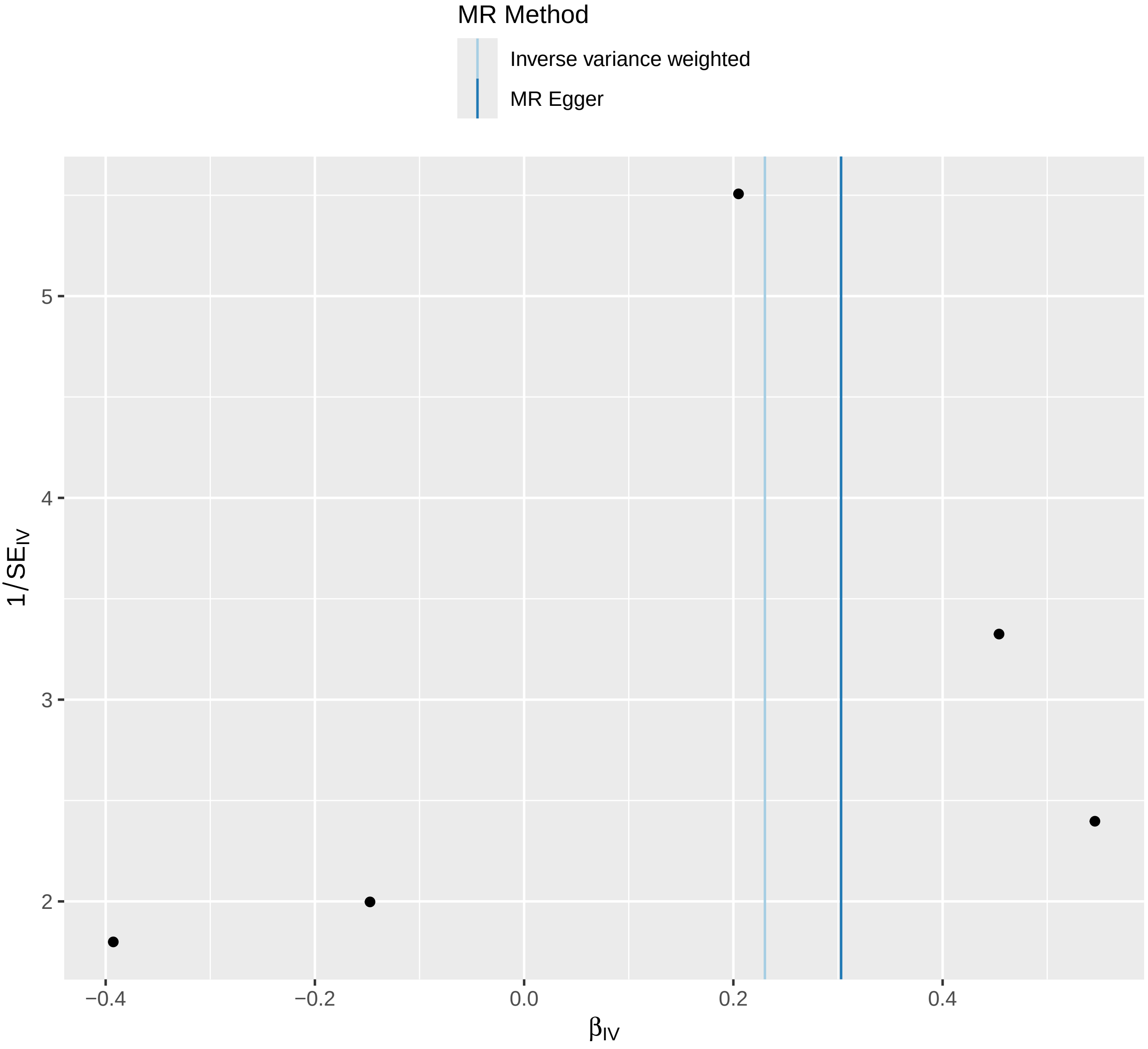
MR Method
Inverse variance weighted
MR Egger
5
4
3
2
1/SE_IV
−0.4
−0.2
0.0
0.2
0.4
β_IV

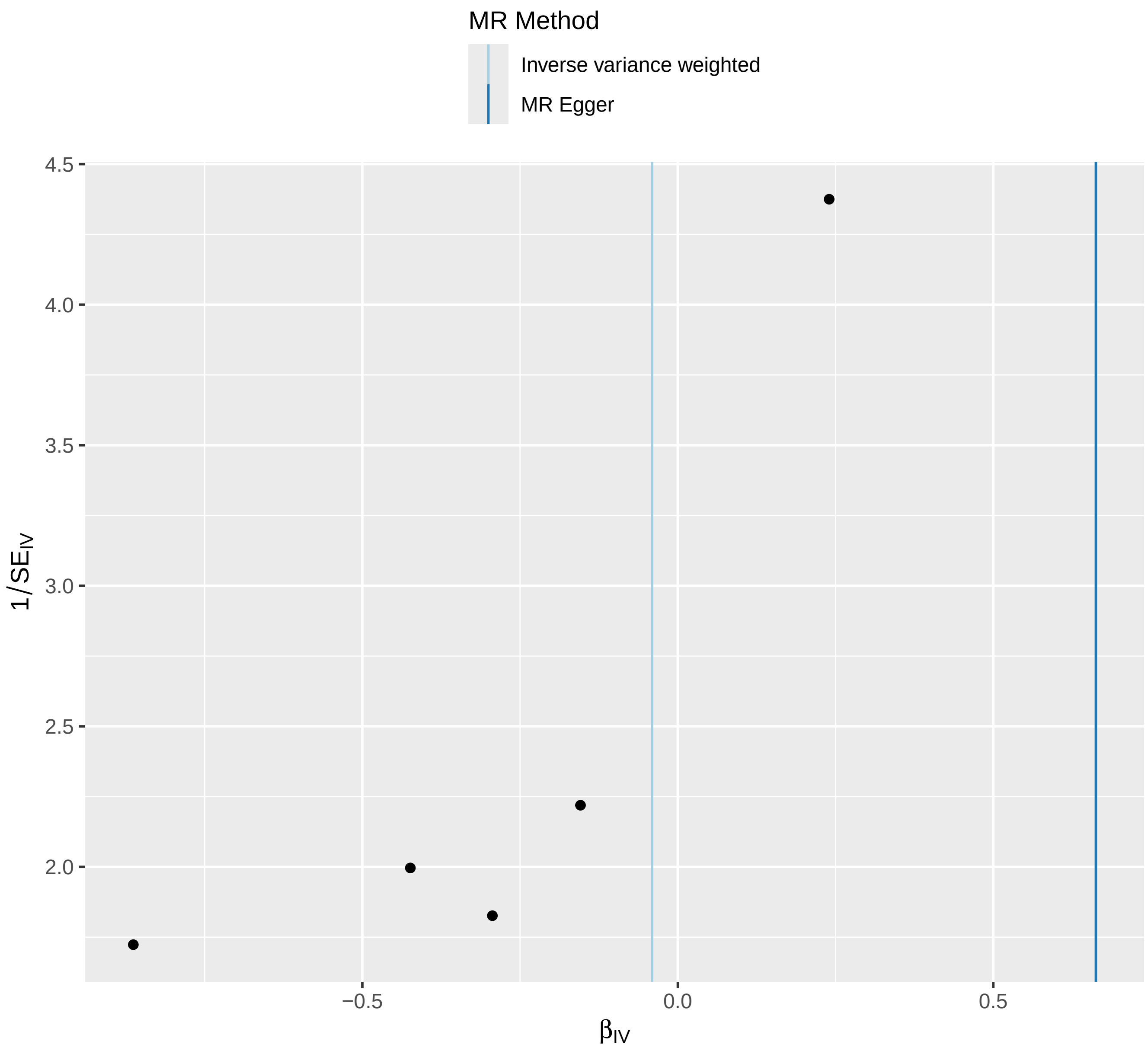

MR Method
Inverse variance weighted
MR Egger
4.5
4.0
3.5
3.0
2.5
2.0
1/SE_IV
−0.5
0.0
0.5
β_IV

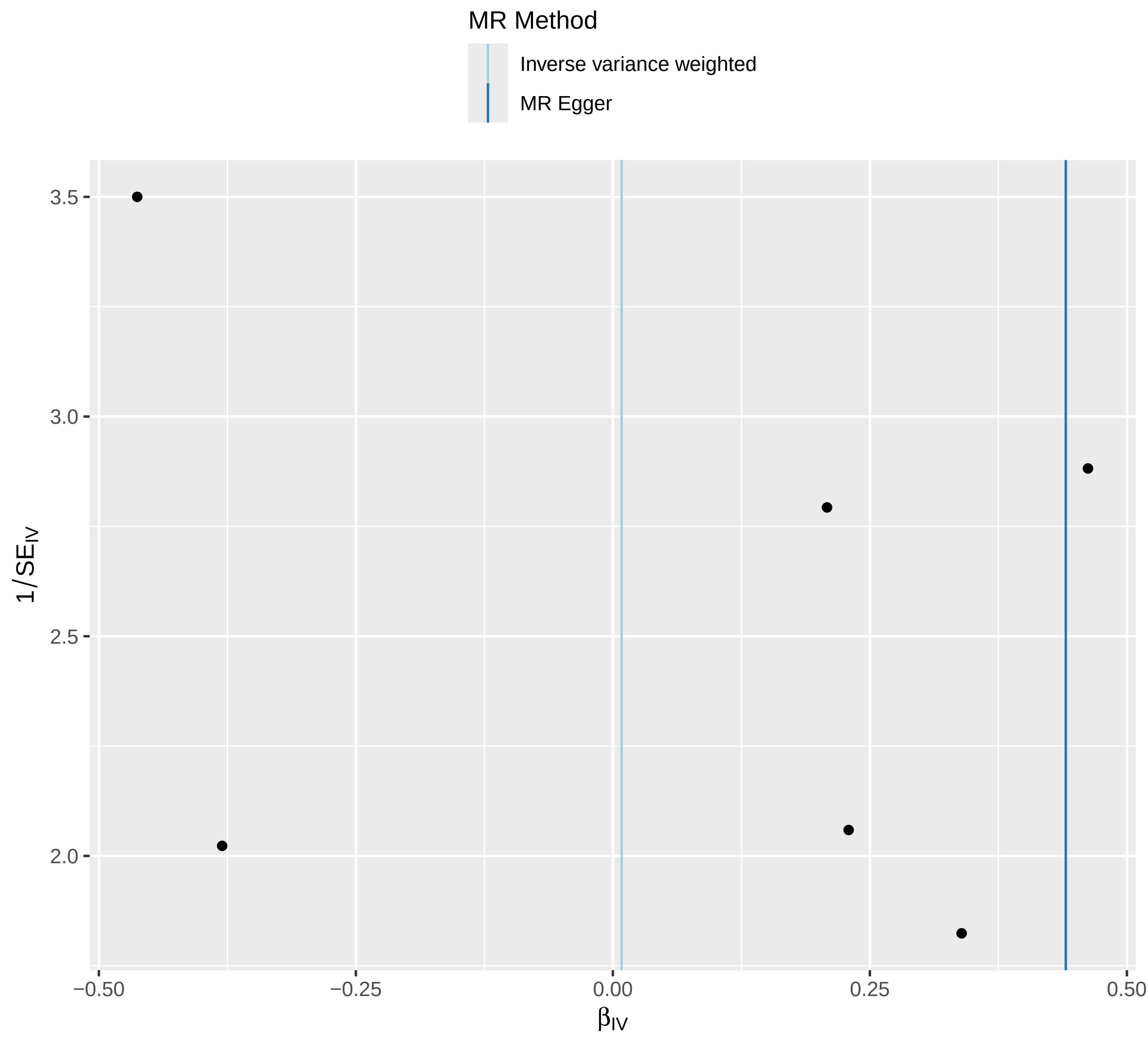
MR Method
Inverse variance weighted
MR Egger
3.5
3.0
2.5
2.0
1/SE_IV
−0.50
−0.25
0.00
0.25
0.50
β_IV

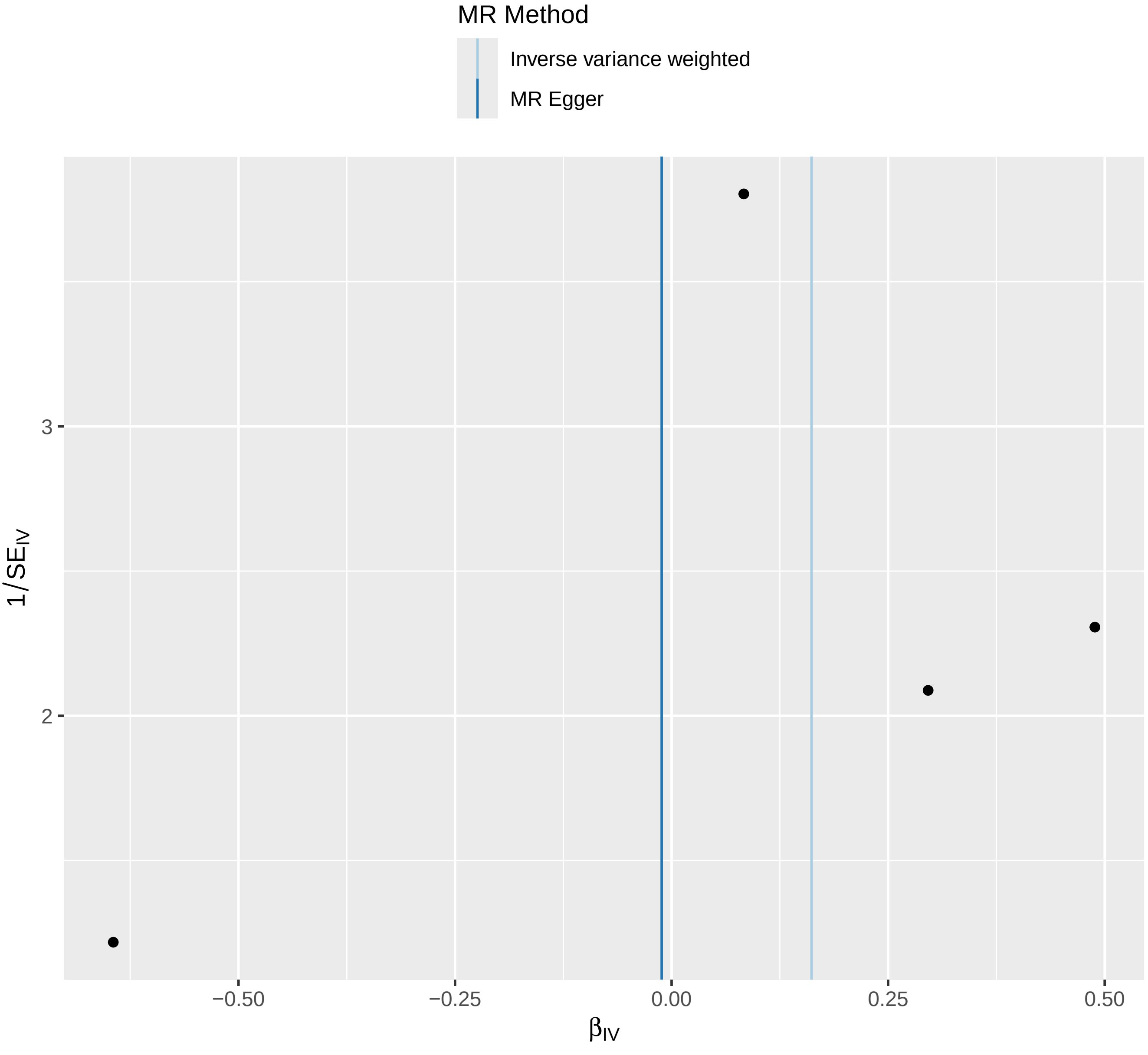

MR Method
Inverse variance weighted
MR Egger
3
2
1/SE_IV
−0.50
−0.25
0.00
0.25
0.50
β_IV

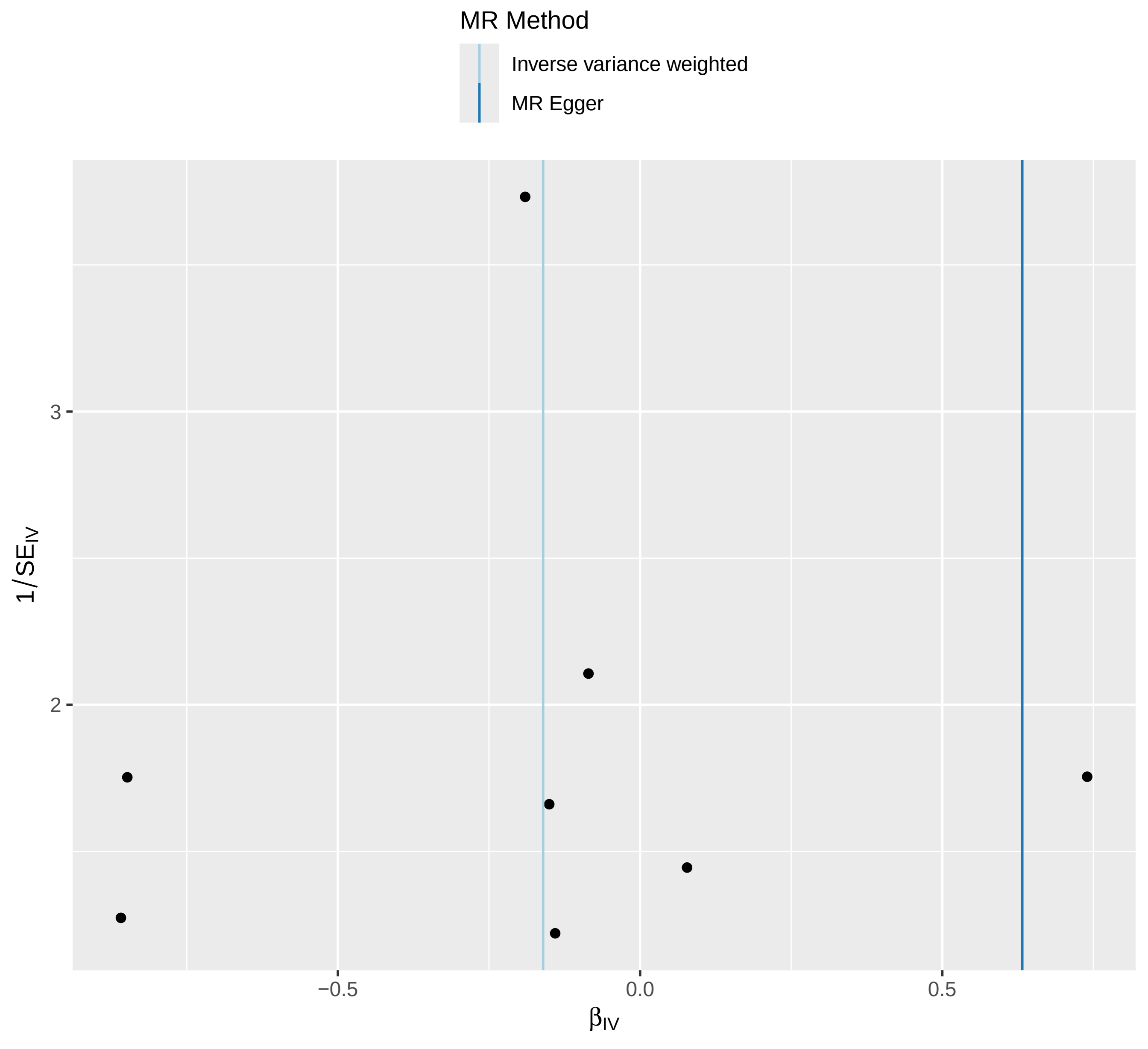

MR Method
Inverse variance weighted
MR Egger
3
2
1/SE_IV
−0.5
0.0
0.5
β_IV

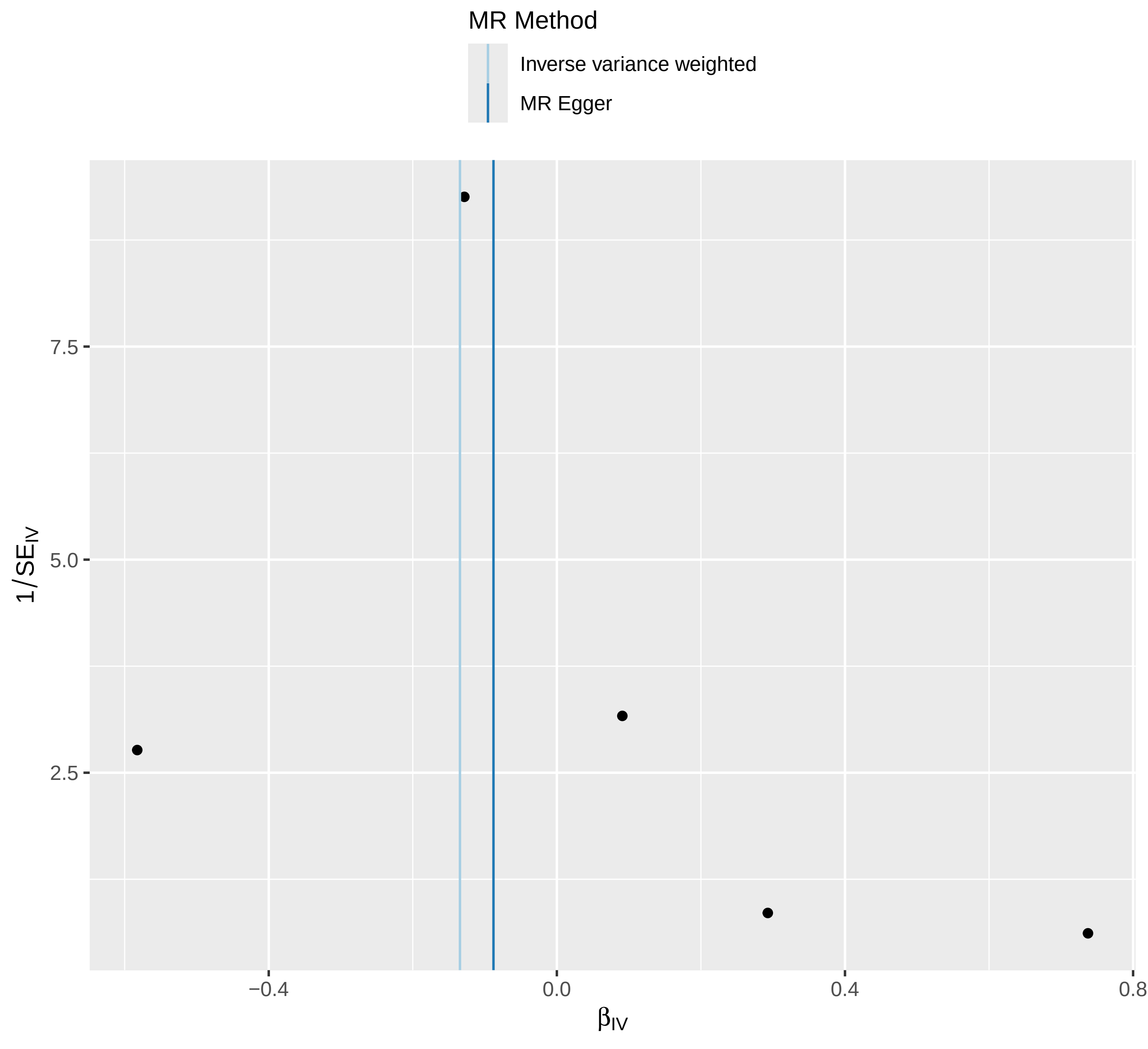

MR Method
Inverse variance weighted
MR Egger
7.5
5.0
2.5
−0.4
0.0
0.4
0.8
1/SE_IV
β_IV

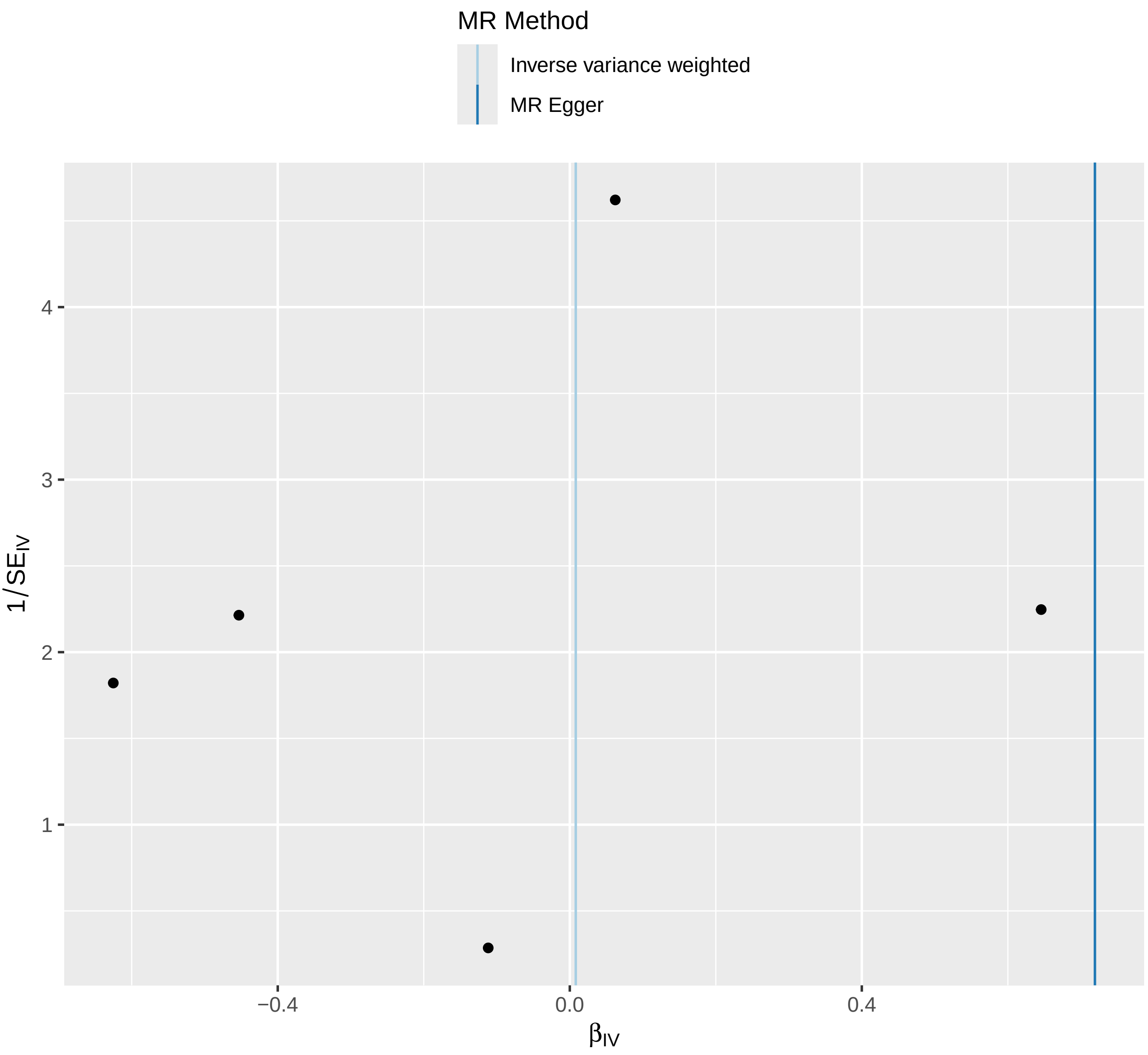
MR Method
Inverse variance weighted
MR Egger
4
3
2
1
1/SE_IV
−0.4
0.0
0.4
β_IV

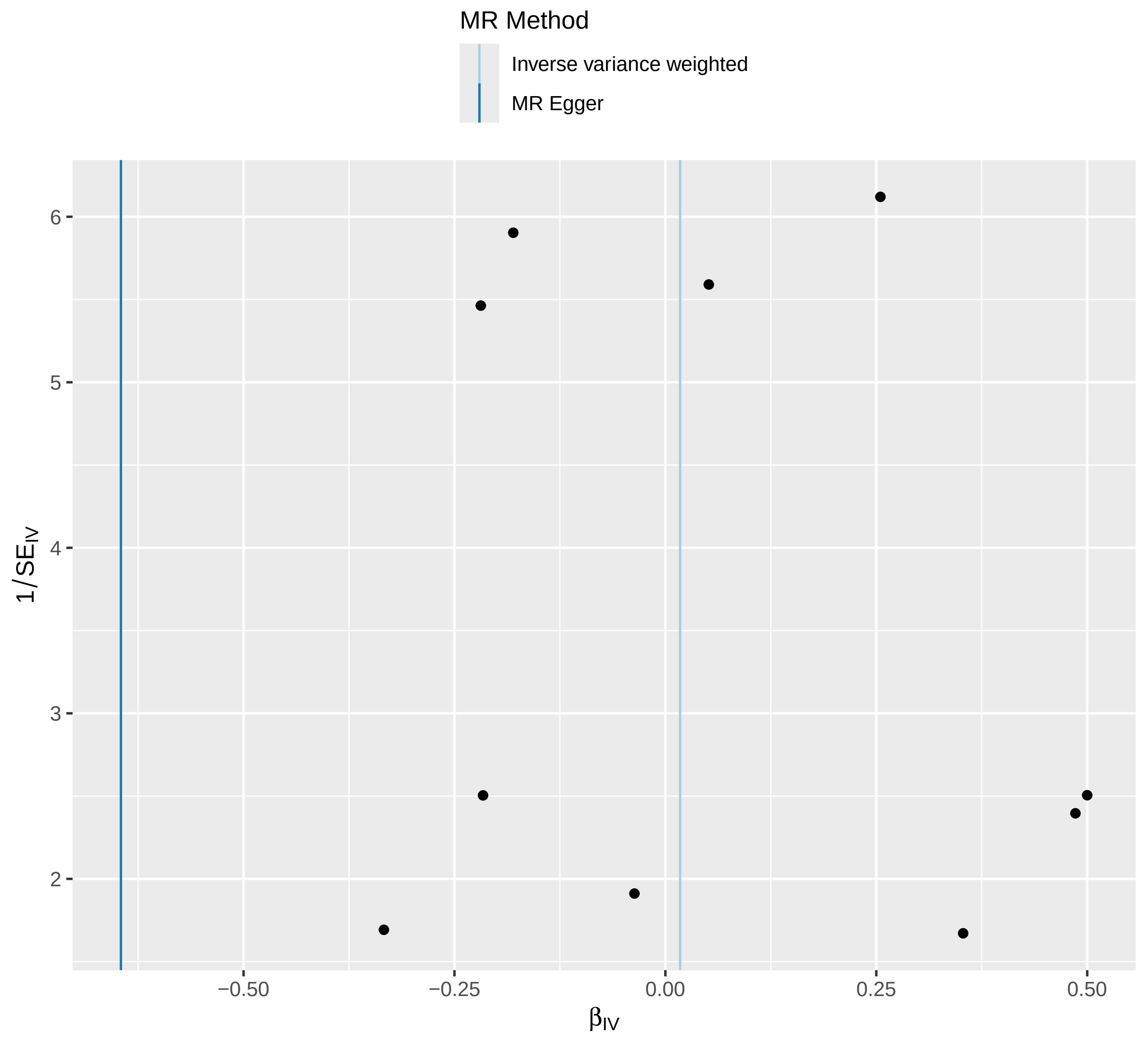

MR Method
Inverse variance weighted
MR Egger
6
5
4
3
2
1/SE_IV
−0.50
−0.25
0.00
0.25
0.50
β_IV

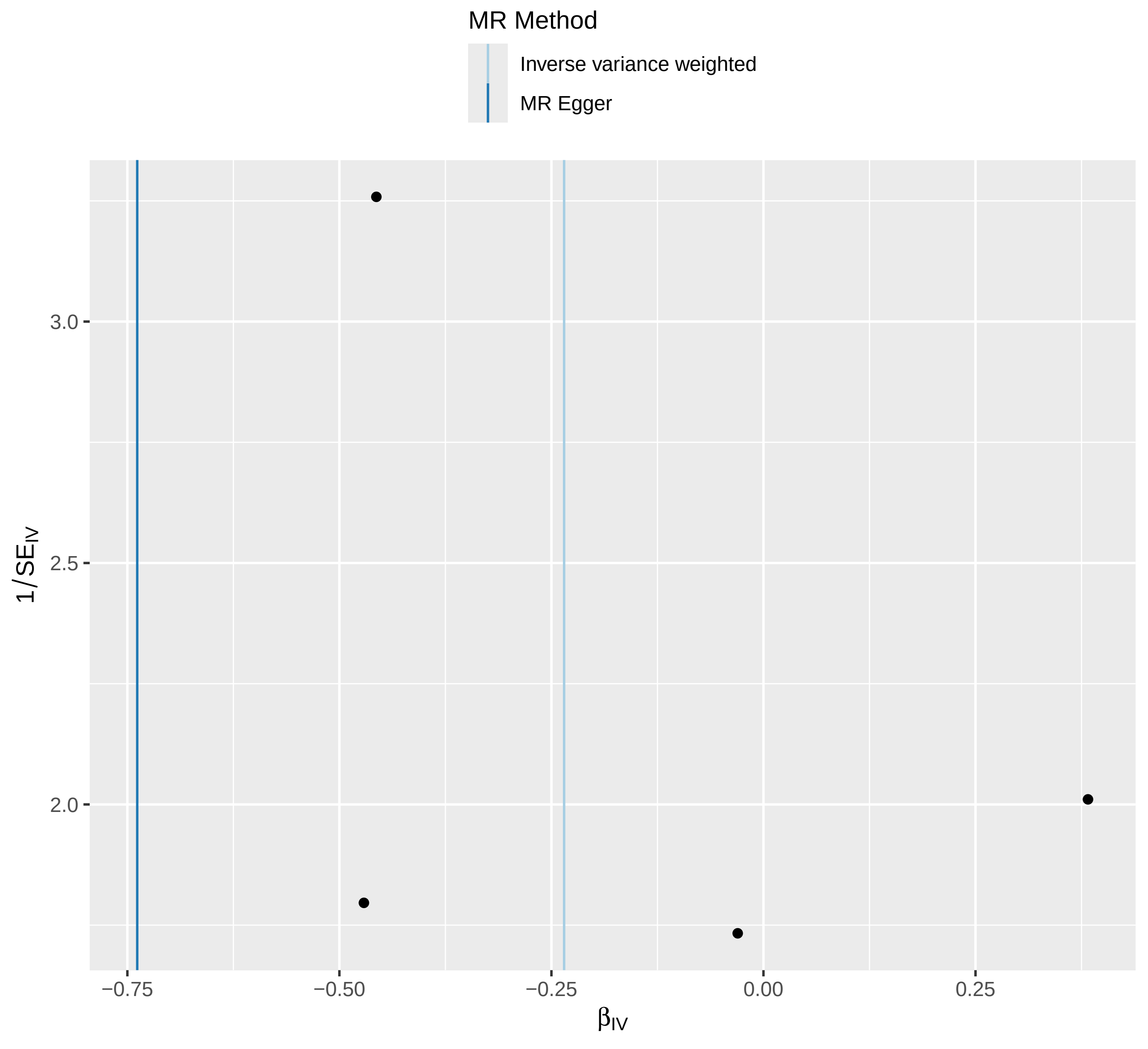
MR Method
Inverse variance weighted
MR Egger
3.0
2.5
2.0
1/SE_IV
−0.75
−0.50
−0.25
0.00
0.25
β_IV

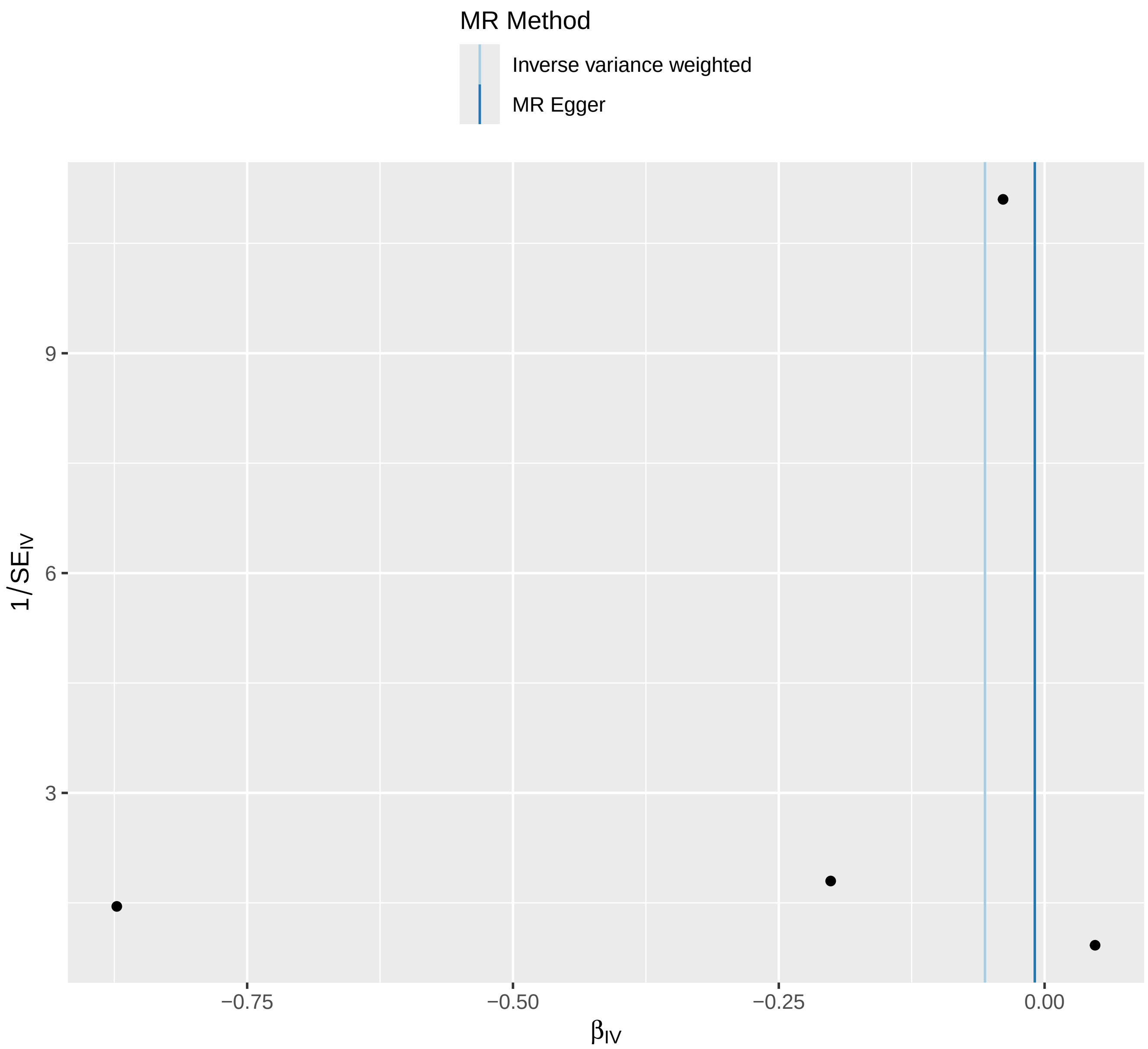
MR Method
Inverse variance weighted
MR Egger
9
6
3
1/SE$_{IV}$
−0.75
−0.50
−0.25
0.00
β$_{IV}$

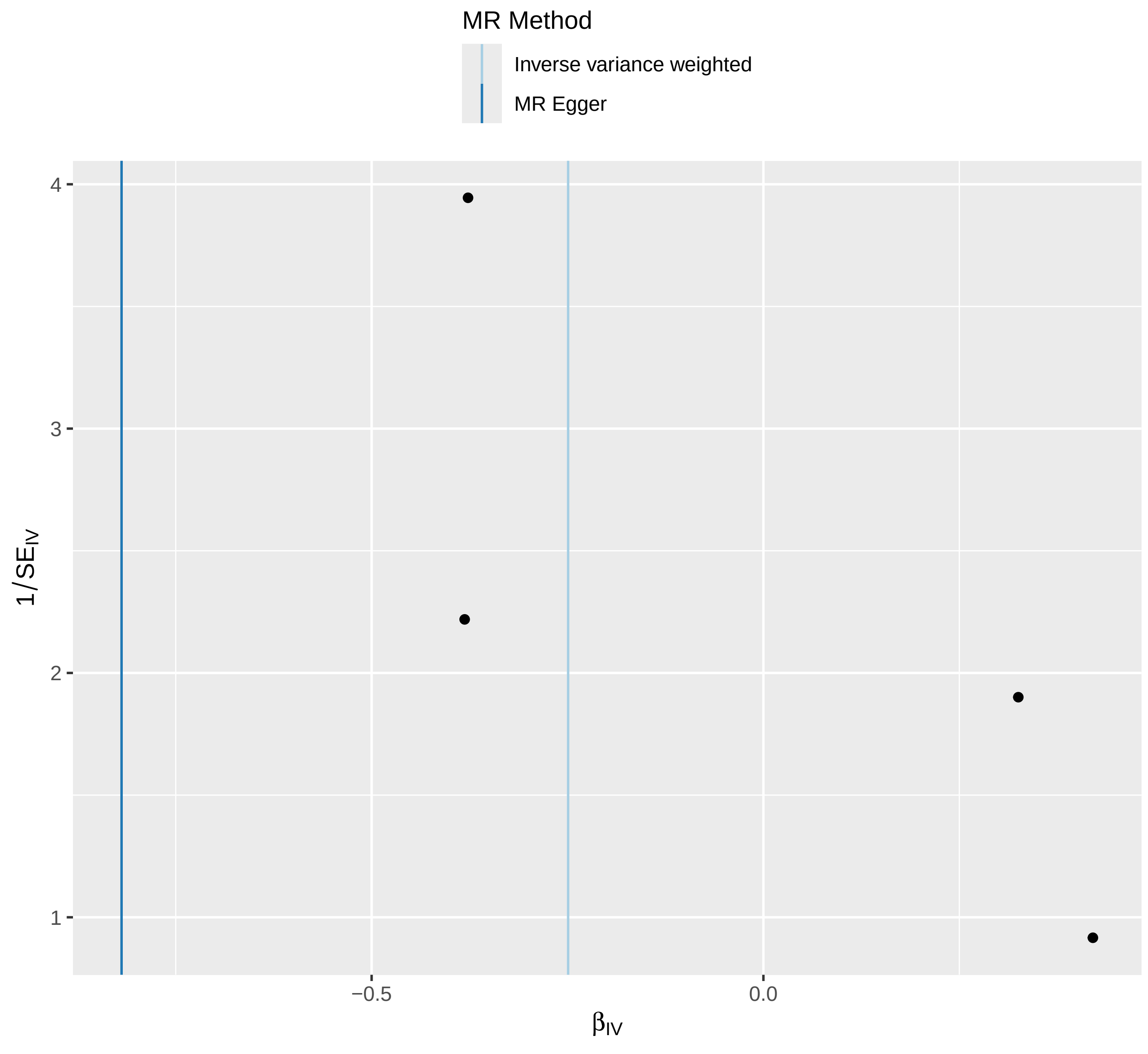
MR Method
Inverse variance weighted
MR Egger
4
3
2
1
1/SE$_{IV}$
−0.5
0.0
β$_{IV}$

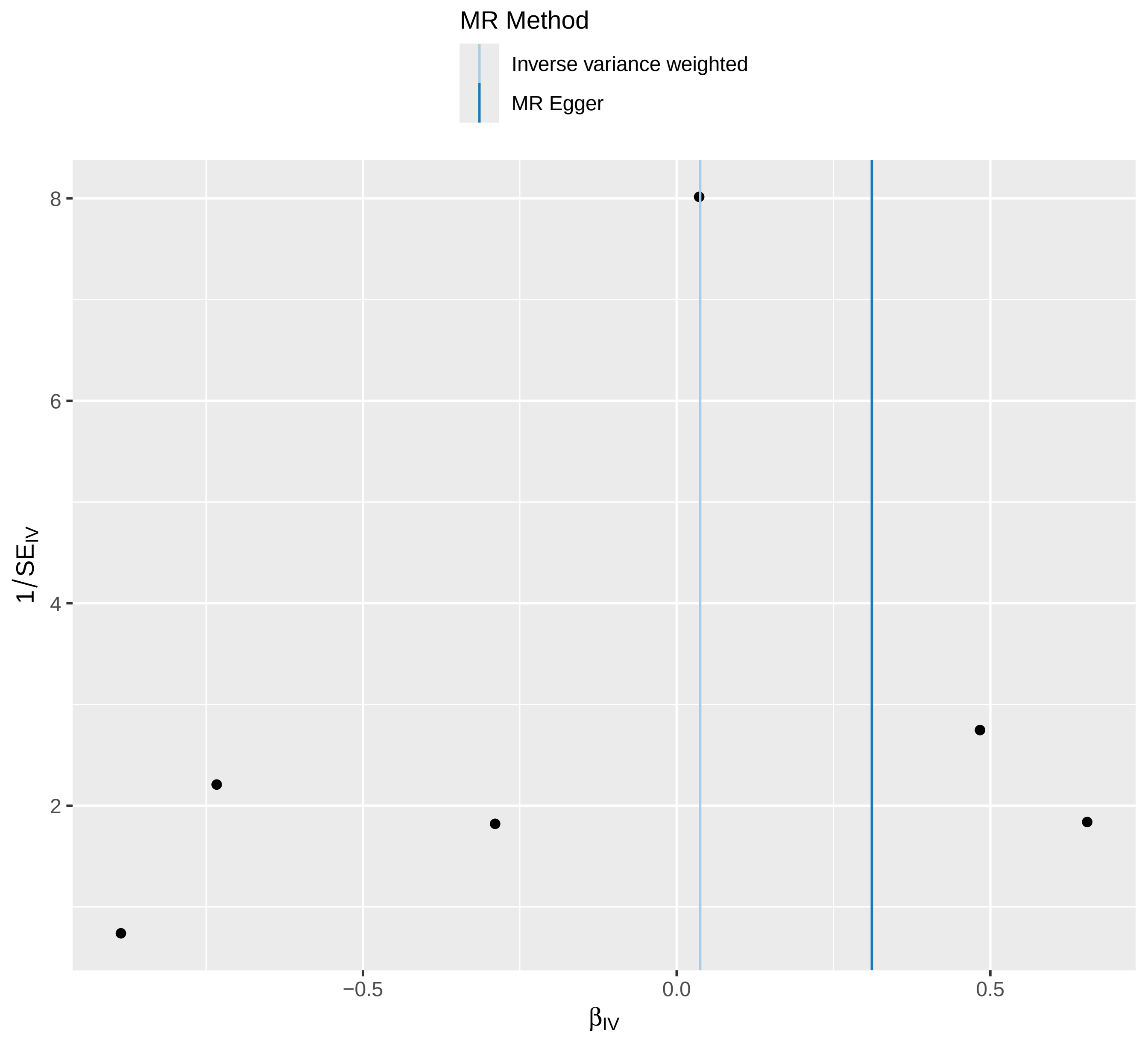

MR Method
Inverse variance weighted
MR Egger
8
6
4
2
1/SE$_{IV}$
−0.5
0.0
0.5
β$_{IV}$

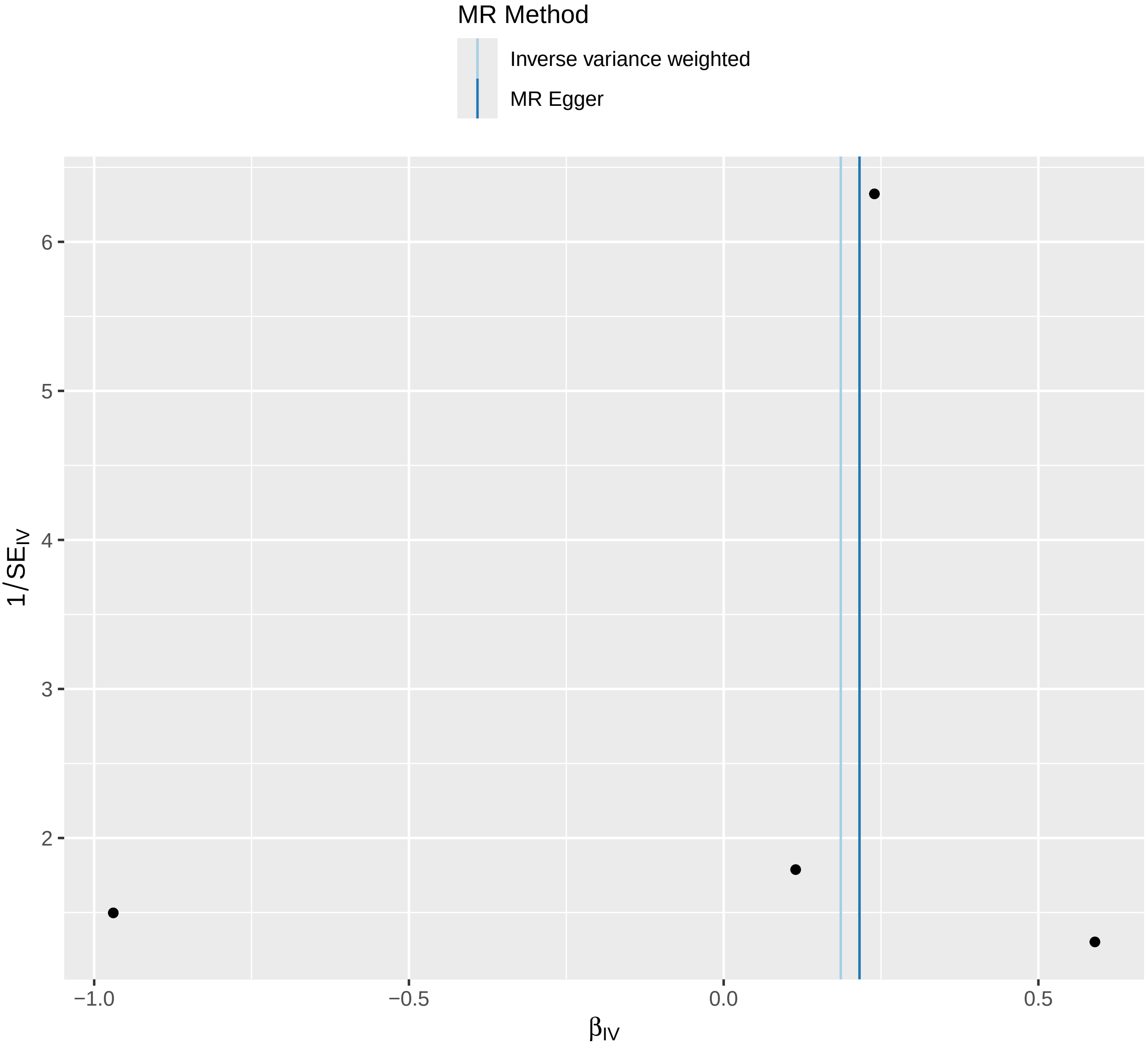

MR Method
Inverse variance weighted
MR Egger
1/SE_IV
6
5
4
3
2
−1.0
−0.5
0.0
0.5
β_IV

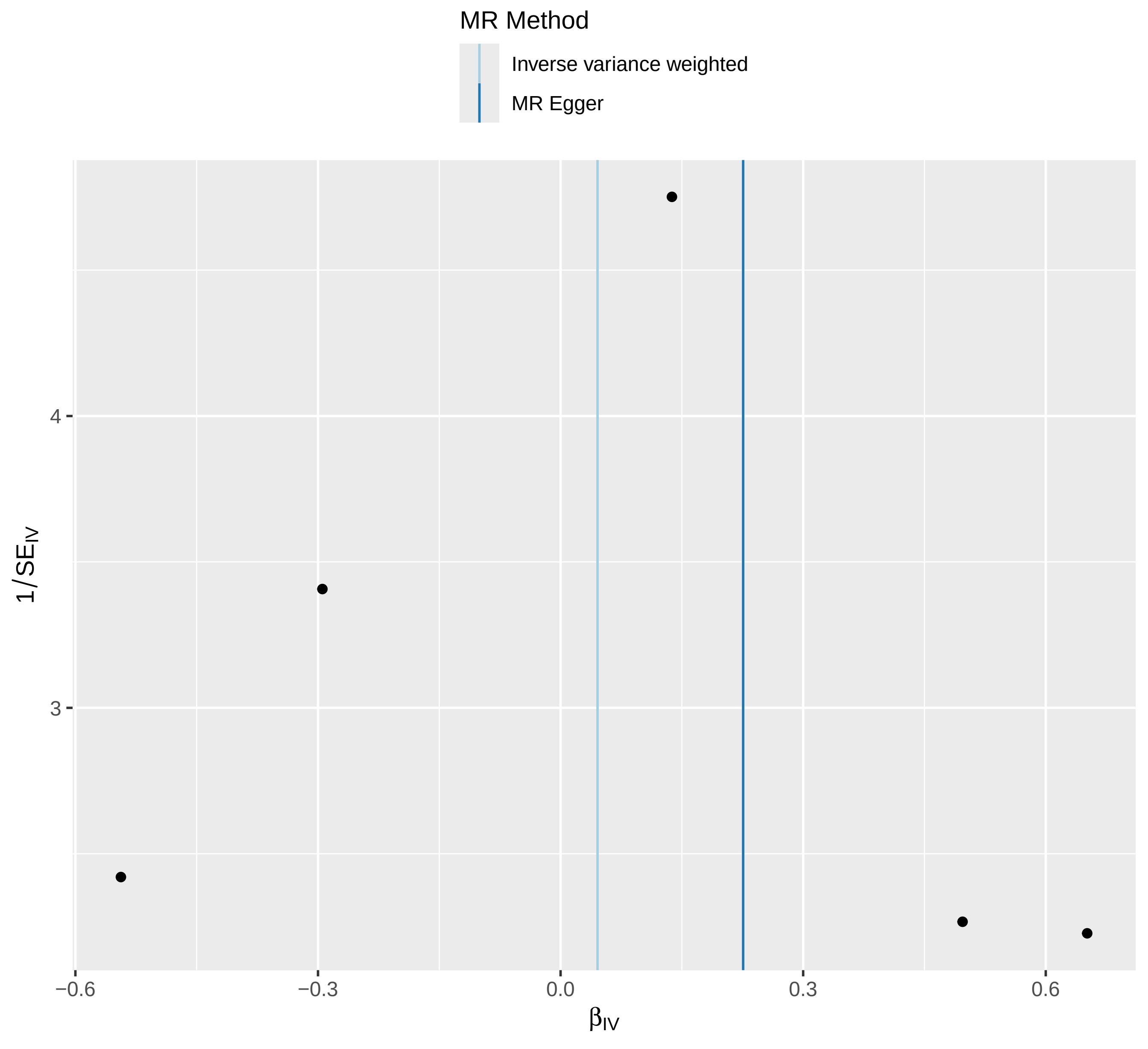
MR Method
Inverse variance weighted
MR Egger
4
3
1/SE_IV
−0.6
−0.3
0.0
0.3
0.6
β_IV

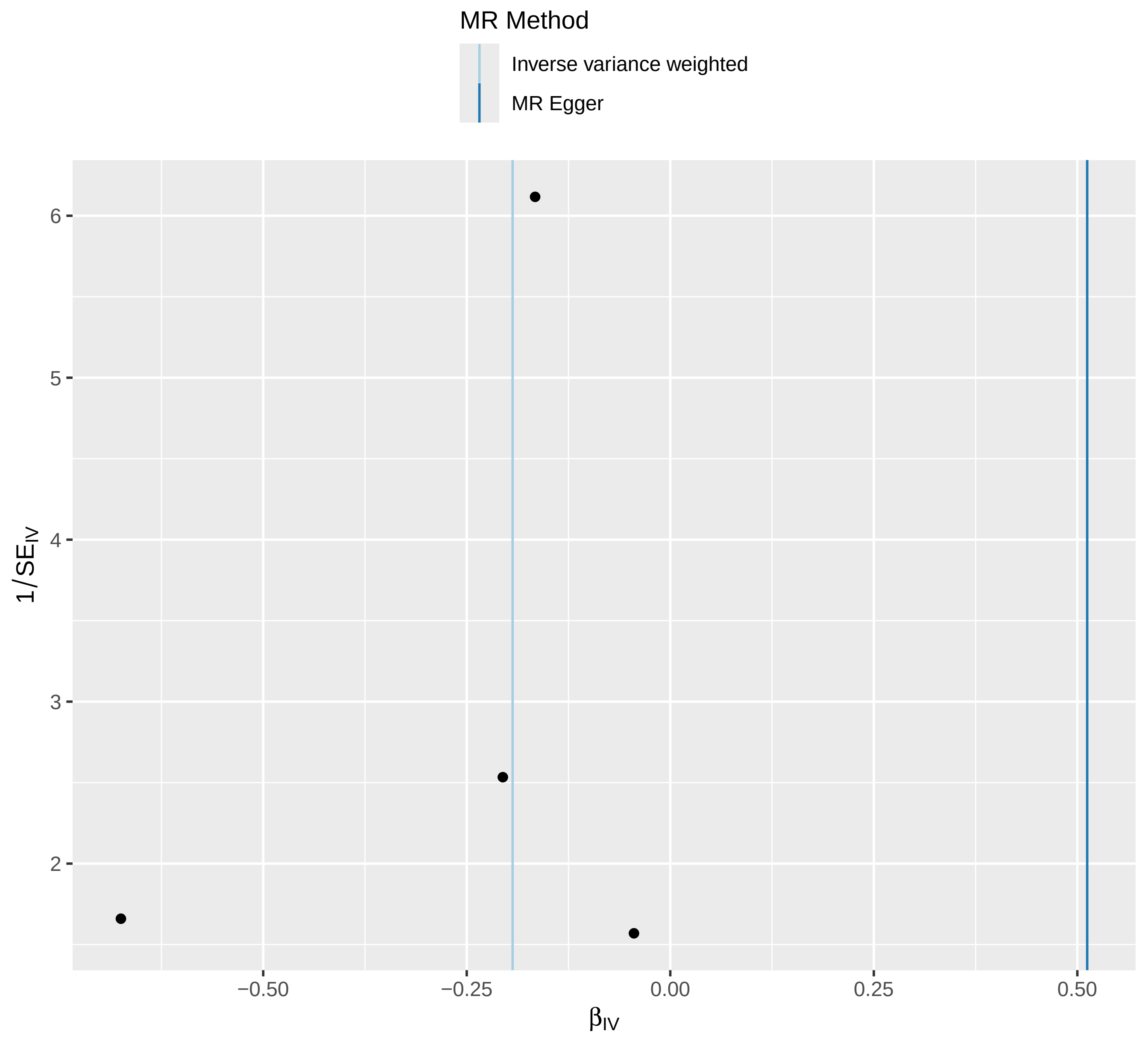
MR Method
Inverse variance weighted
MR Egger
6
5
4
3
2
1/SE_IV
−0.50
−0.25
0.00
0.25
0.50
β_IV

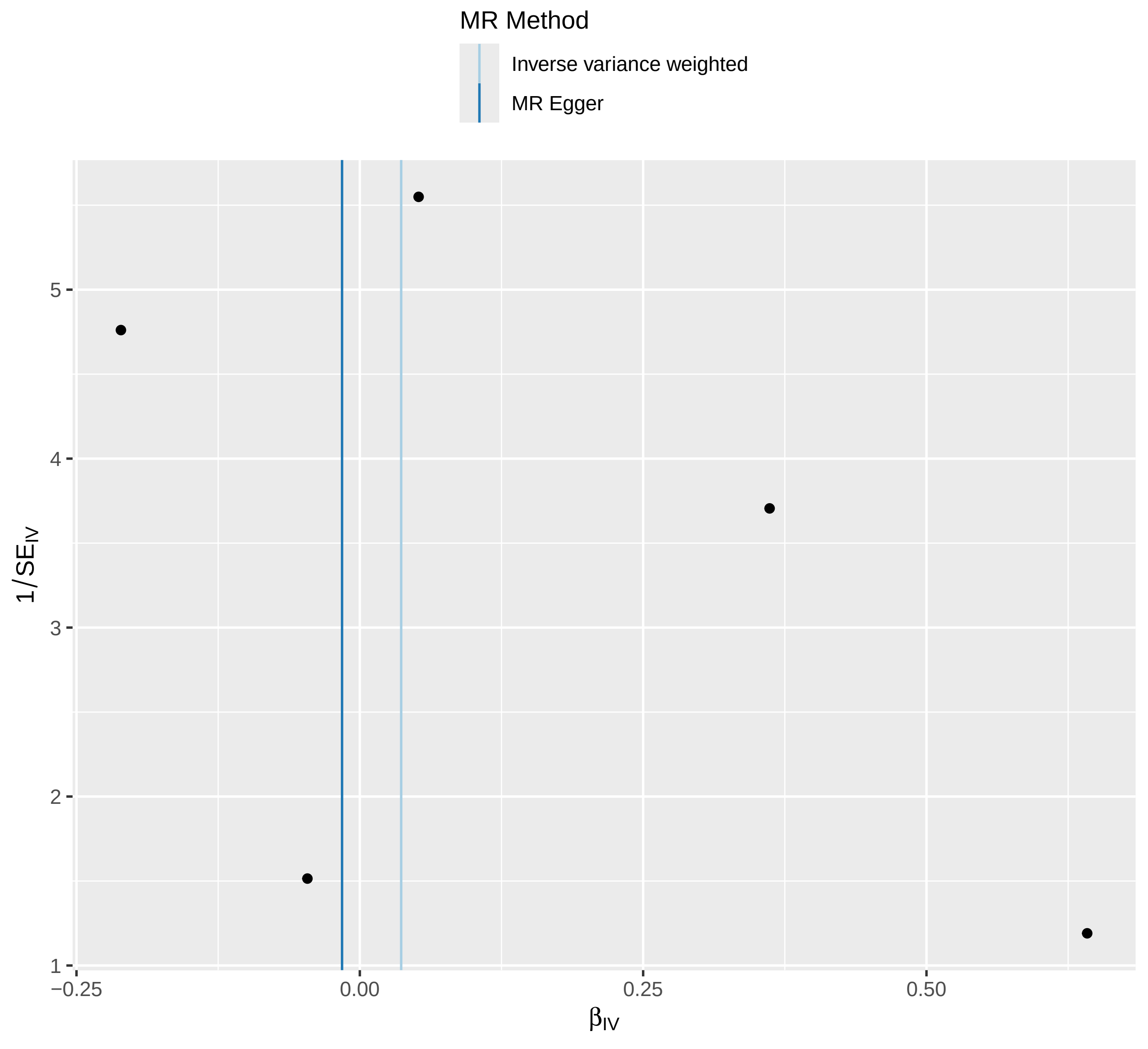

MR Method
Inverse variance weighted
MR Egger
1
2
3
4
5
1/SE$_{IV}$
−0.25
0.00
0.25
0.50
β$_{IV}$

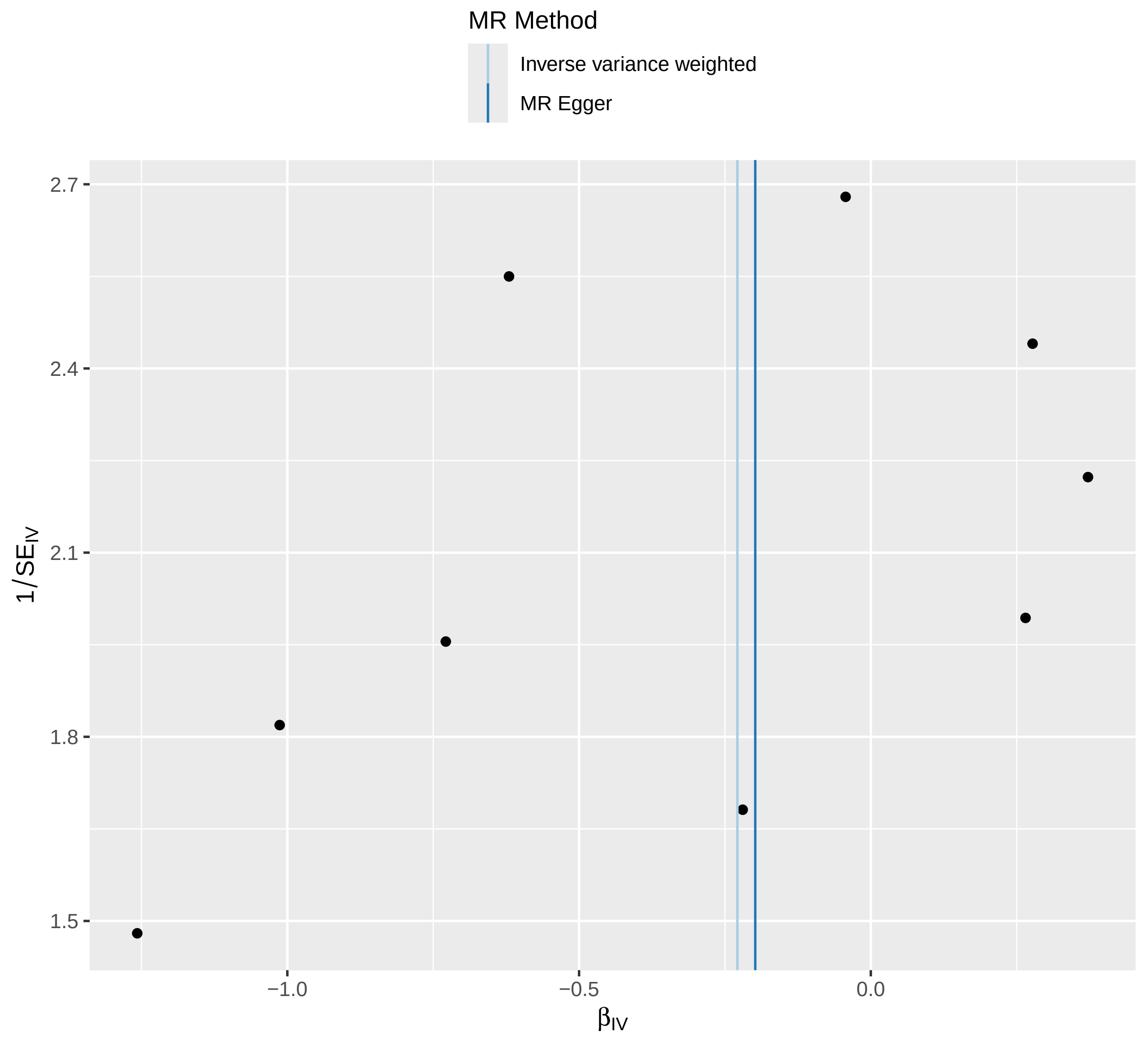

MR Method
Inverse variance weighted
MR Egger
2.7
2.4
2.1
1.8
1.5
$1/SE_{IV}$
−1.0
−0.5
0.0
β_{IV}

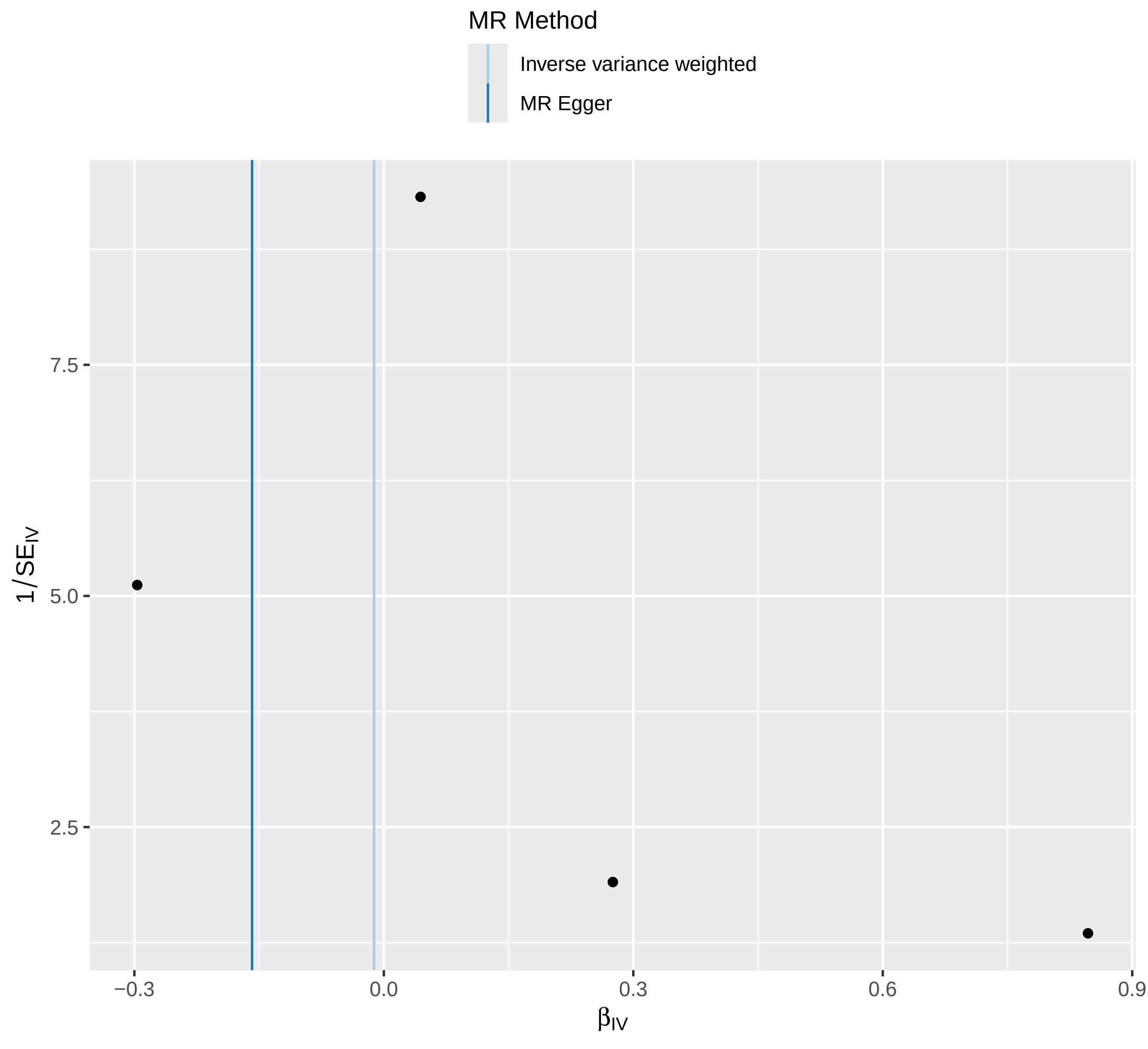

MR Method
Inverse variance weighted
MR Egger
7.5
5.0
2.5
1/SE$_{IV}$
−0.3
0.0
0.3
0.6
0.9
β$_{IV}$

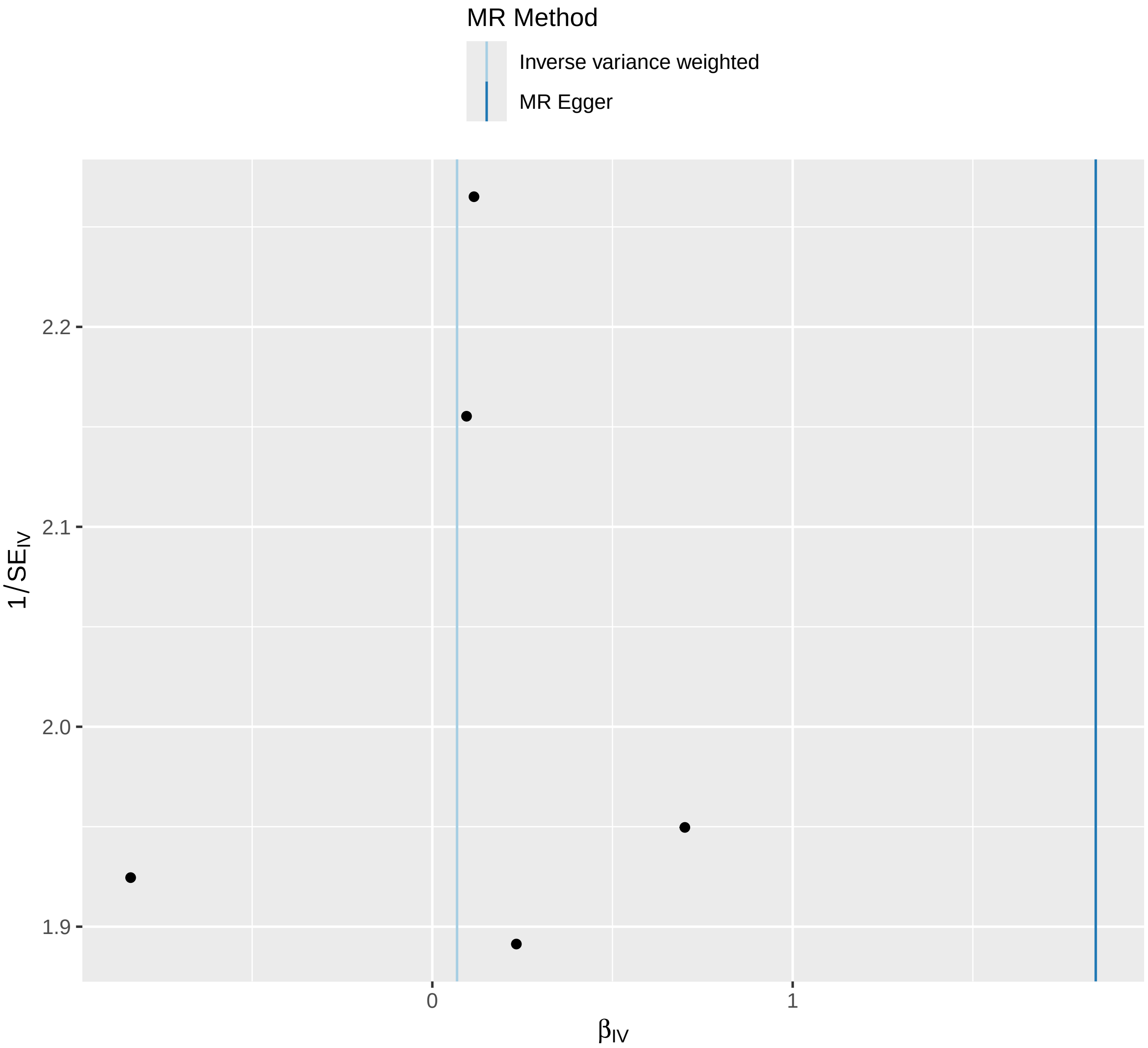
MR Method
Inverse variance weighted
MR Egger
2.2
2.1
2.0
1.9
1/SE_IV
0
1
β_IV

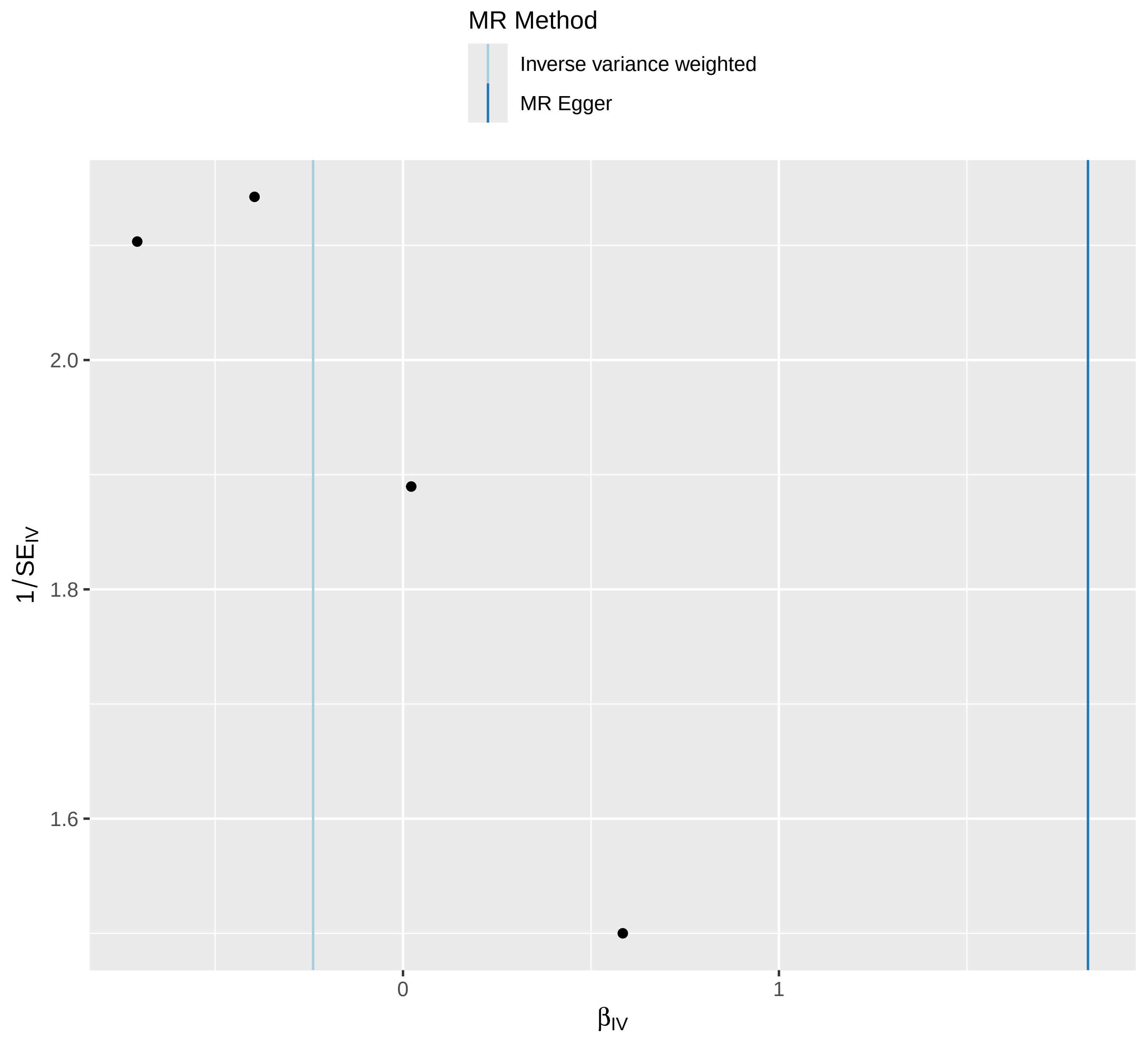

MR Method
Inverse variance weighted
MR Egger
2.0
1.8
1.6
1/SE$_{IV}$
0
1
β$_{IV}$

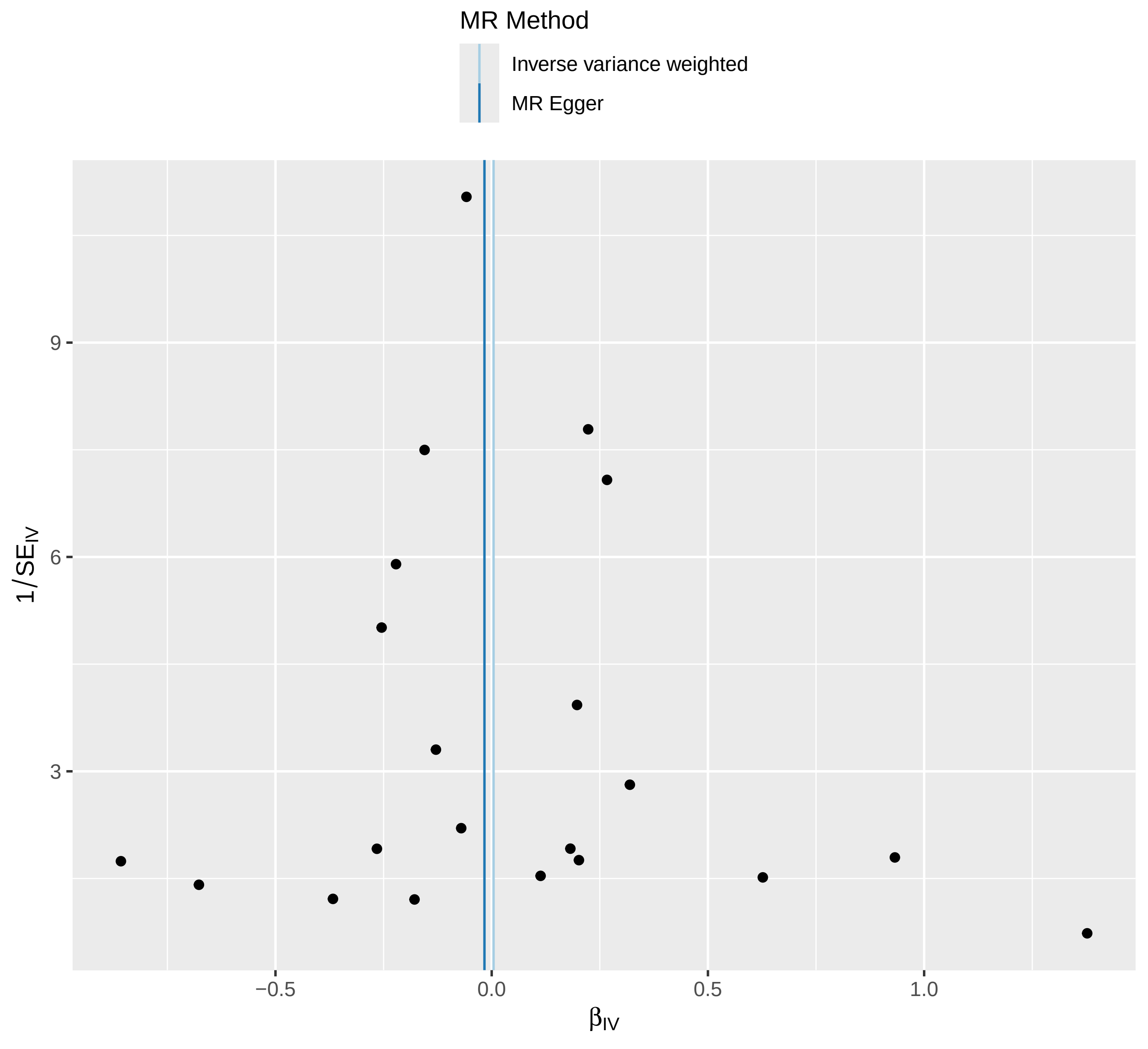

MR Method
Inverse variance weighted
MR Egger
9
6
3
1/SE$_{IV}$
−0.5
0.0
0.5
1.0
β$_{IV}$

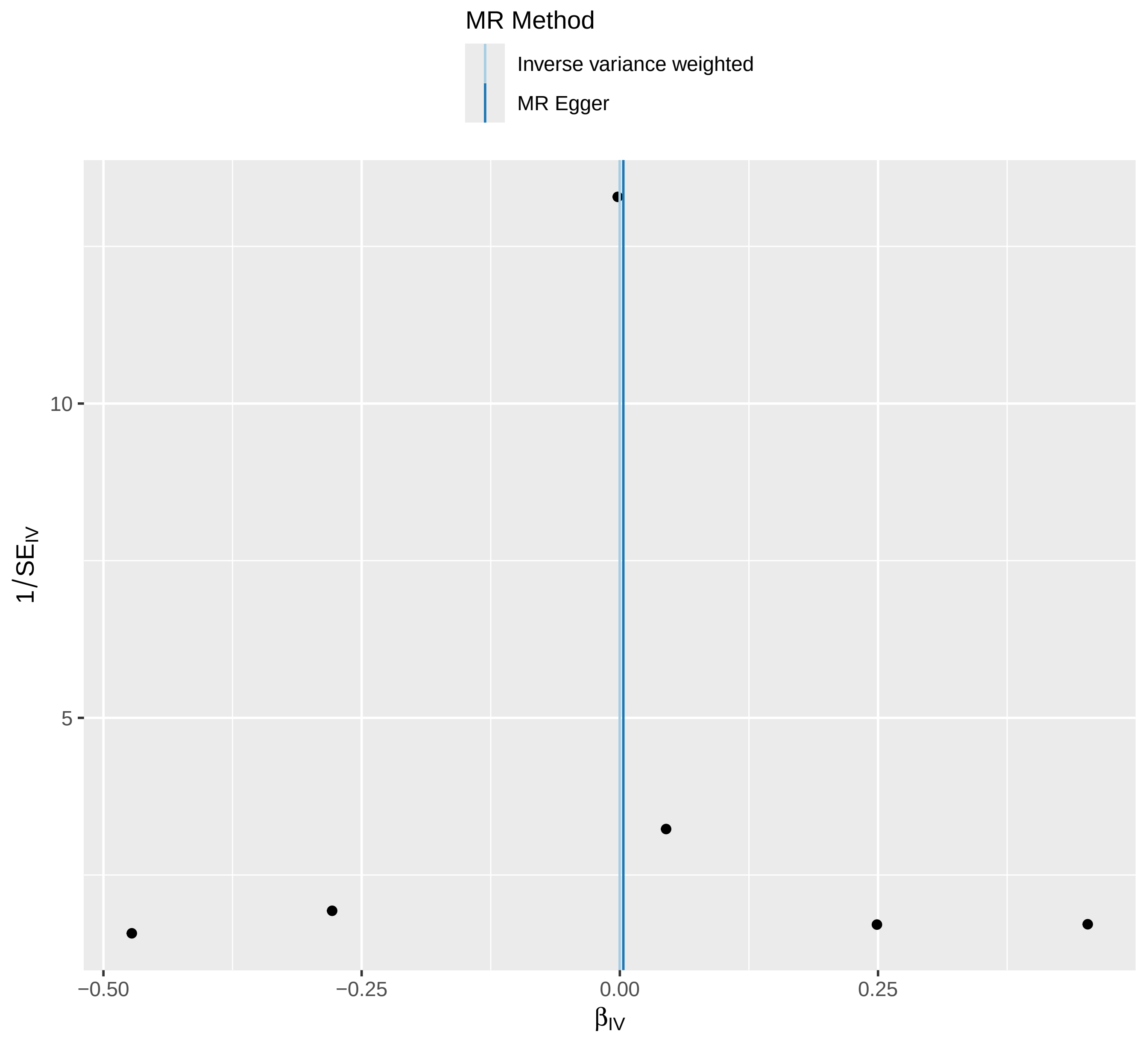

MR Method
Inverse variance weighted
MR Egger
10
5
1/SE$_{IV}$
−0.50
−0.25
0.00
0.25
β$_{IV}$

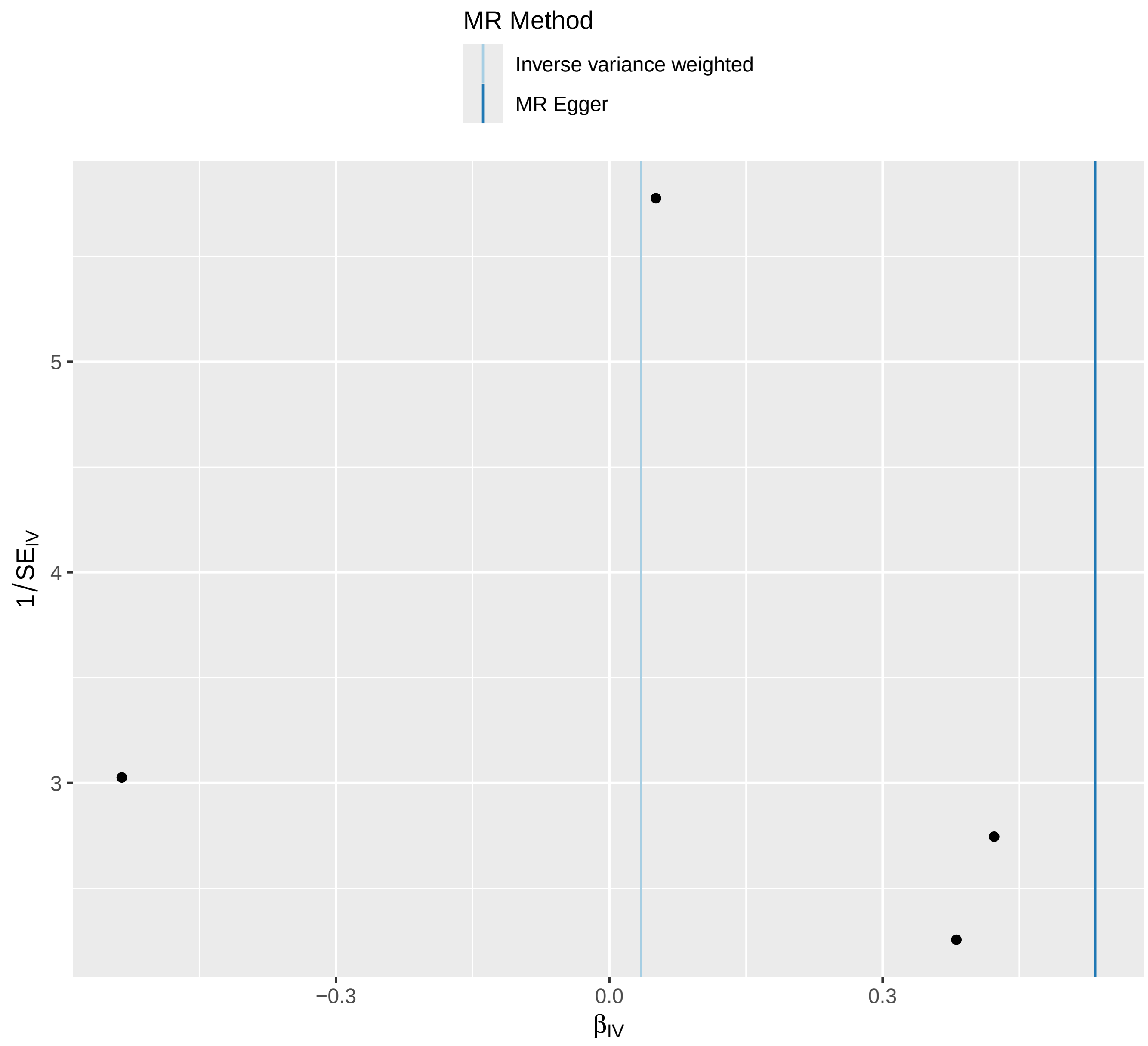
MR Method
Inverse variance weighted
MR Egger
5
4
3
1/SE_IV
−0.3
0.0
0.3
β_IV

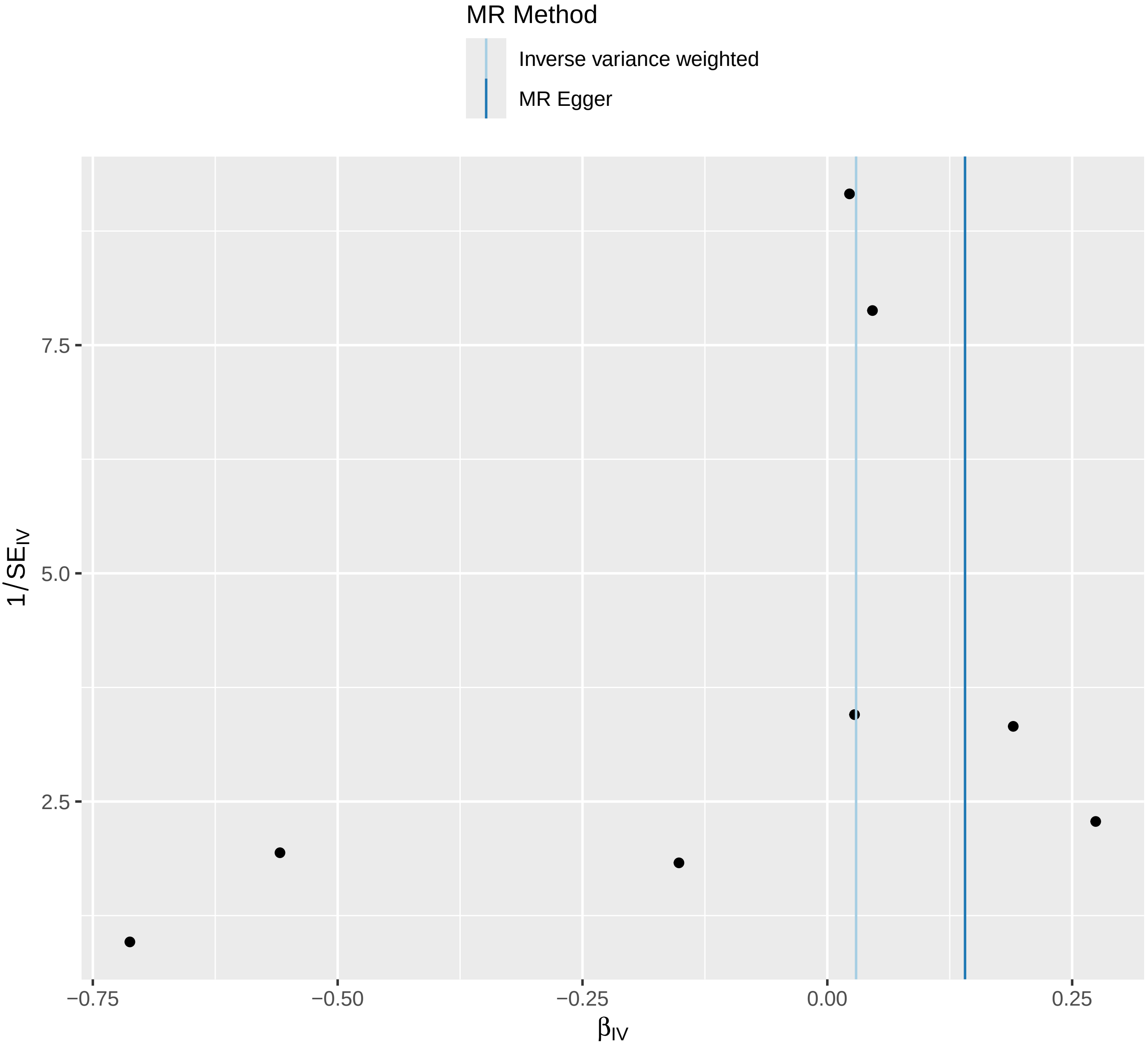

MR Method
Inverse variance weighted
MR Egger
7.5
5.0
2.5
1/SE_IV
−0.75
−0.50
−0.25
0.00
0.25
β_IV

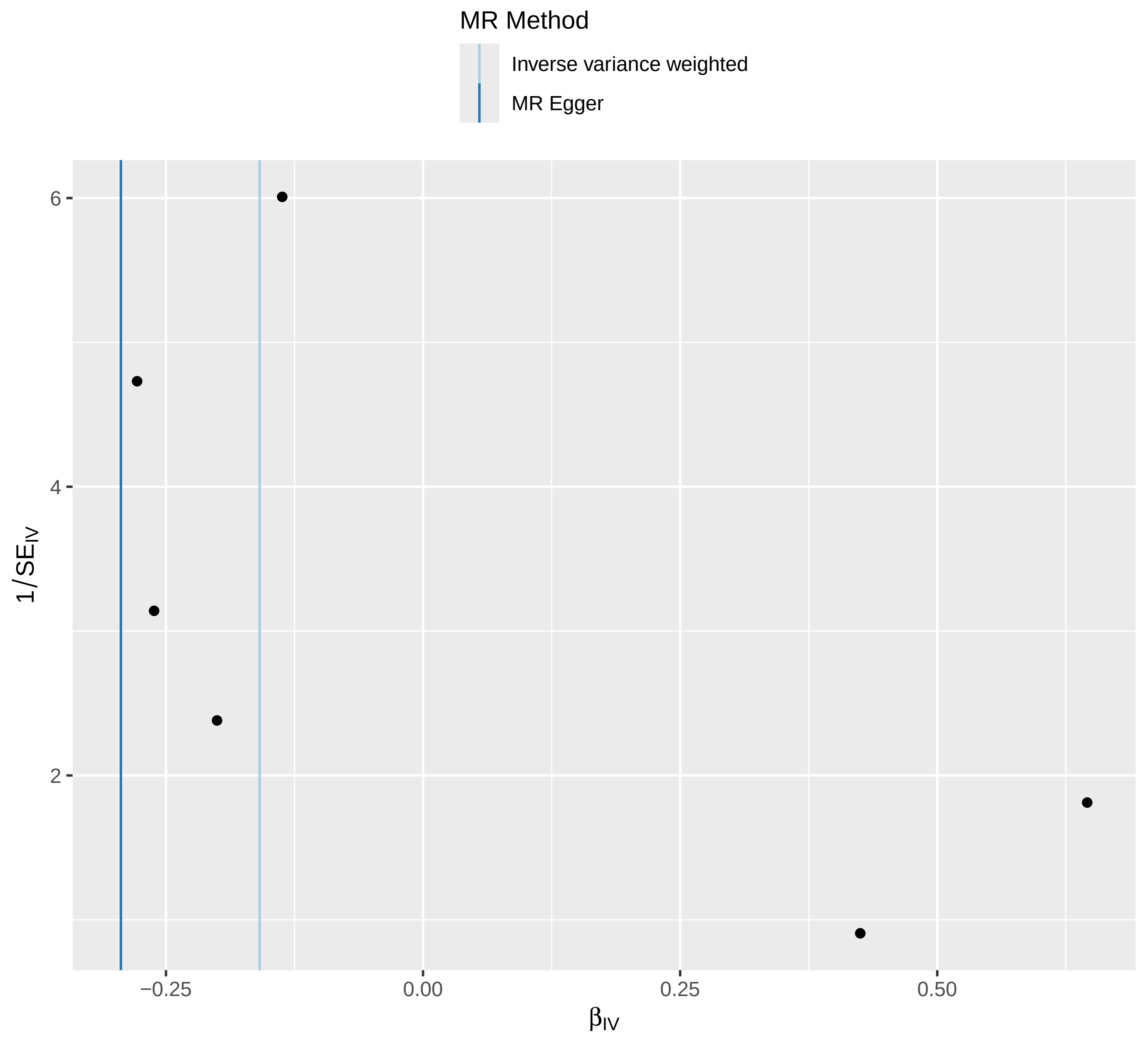

MR Method
Inverse variance weighted
MR Egger
6
4
2
1/SE$_{IV}$
−0.25
0.00
0.25
0.50
β$_{IV}$

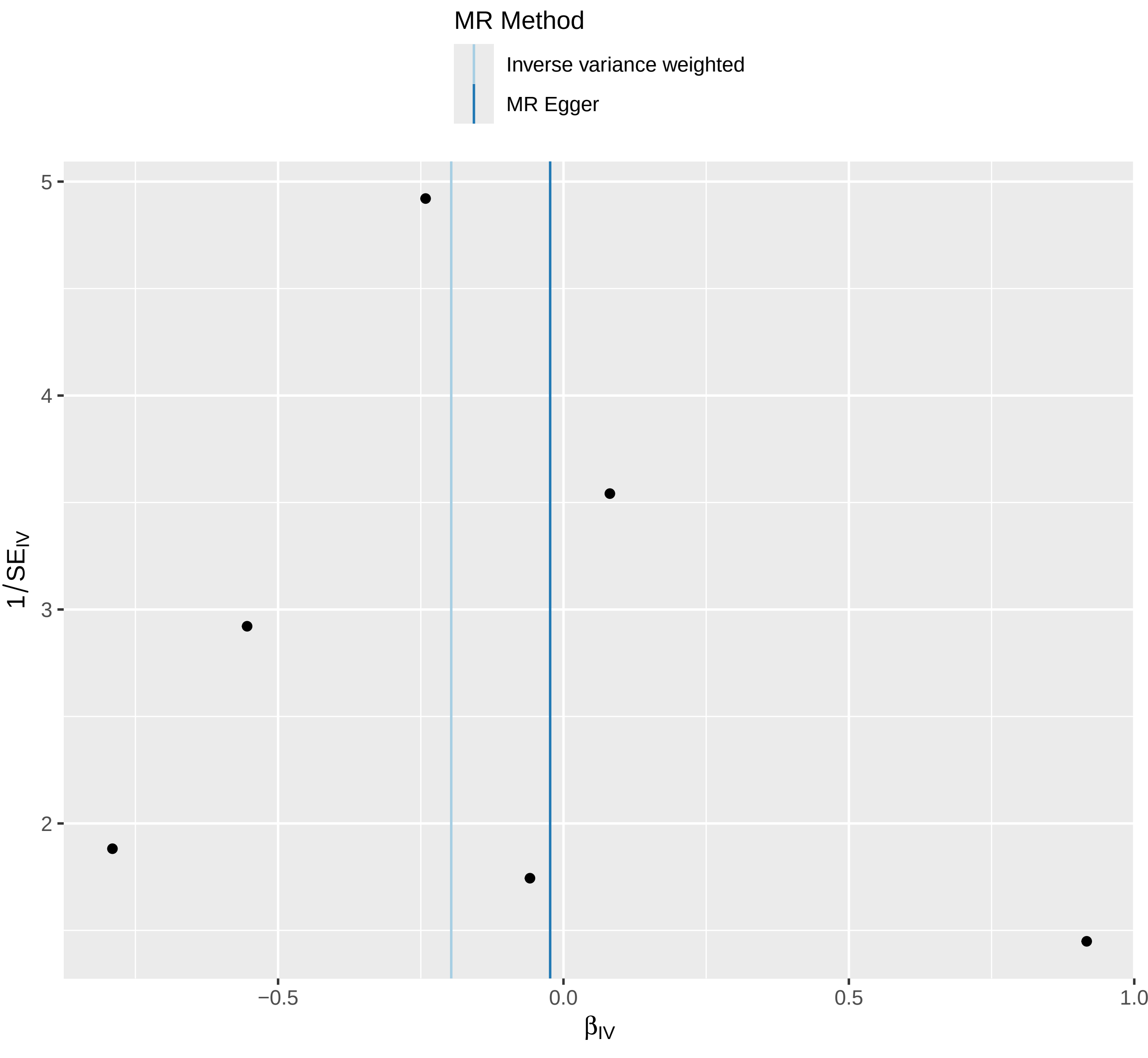

MR Method
Inverse variance weighted
MR Egger
5
4
3
2
$1/SE_{IV}$
−0.5
0.0
0.5
1.0
β_{IV}

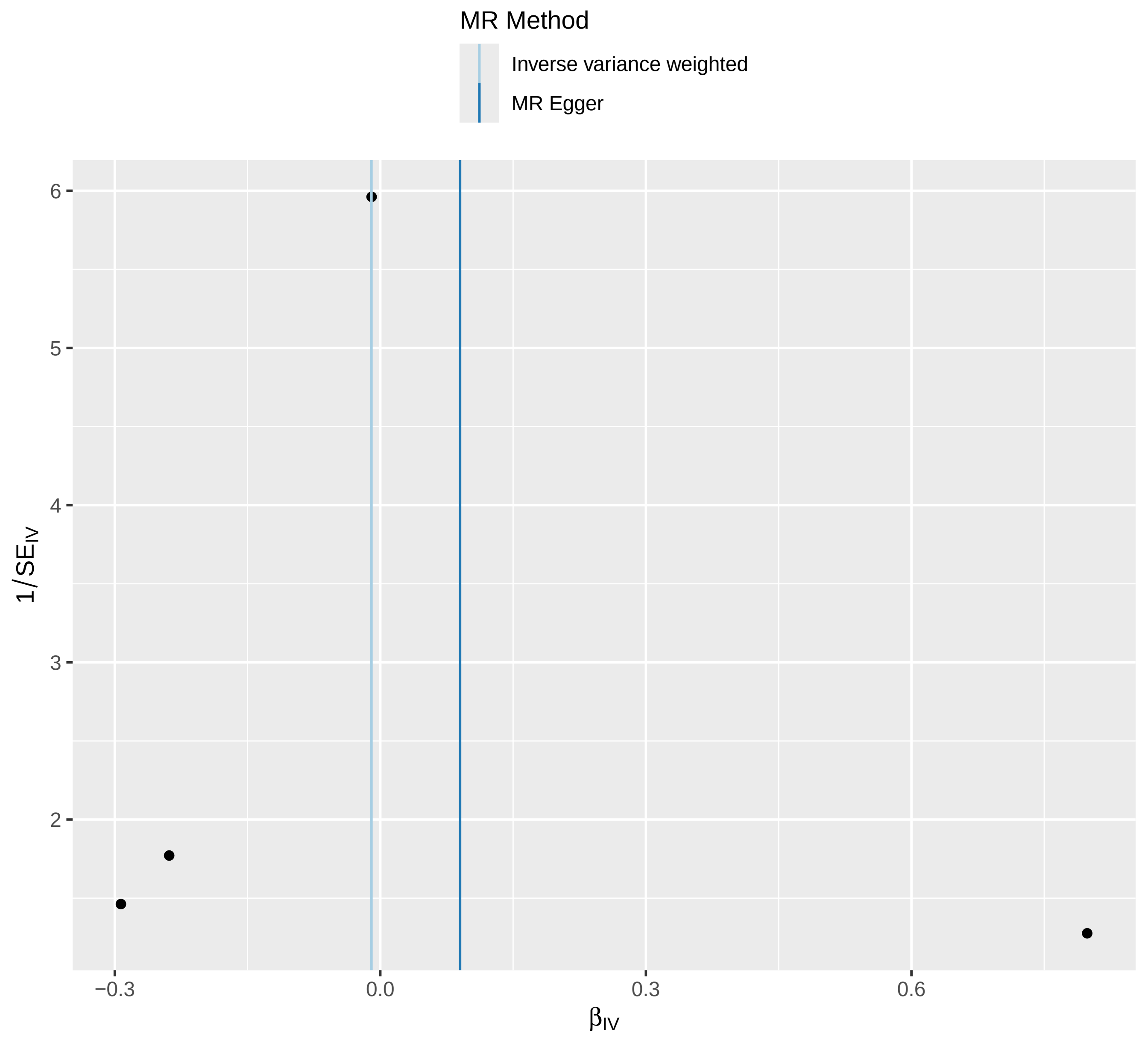
MR Method
Inverse variance weighted
MR Egger
6
5
4
3
2
1/SE_IV
−0.3
0.0
0.3
0.6
β_IV

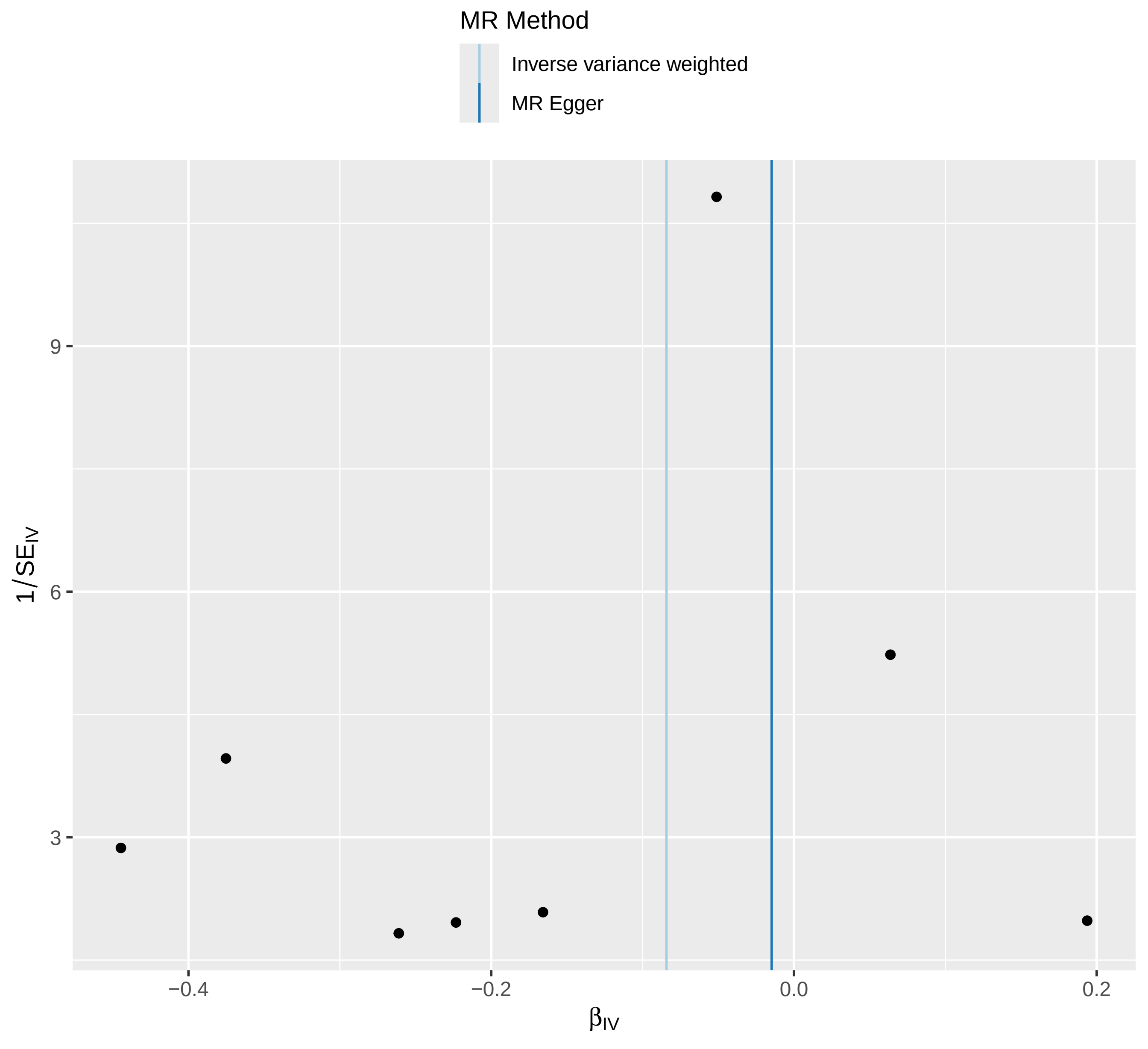

MR Method
Inverse variance weighted
MR Egger
1/SE_IV
9
6
3
−0.4
−0.2
0.0
0.2
β_IV

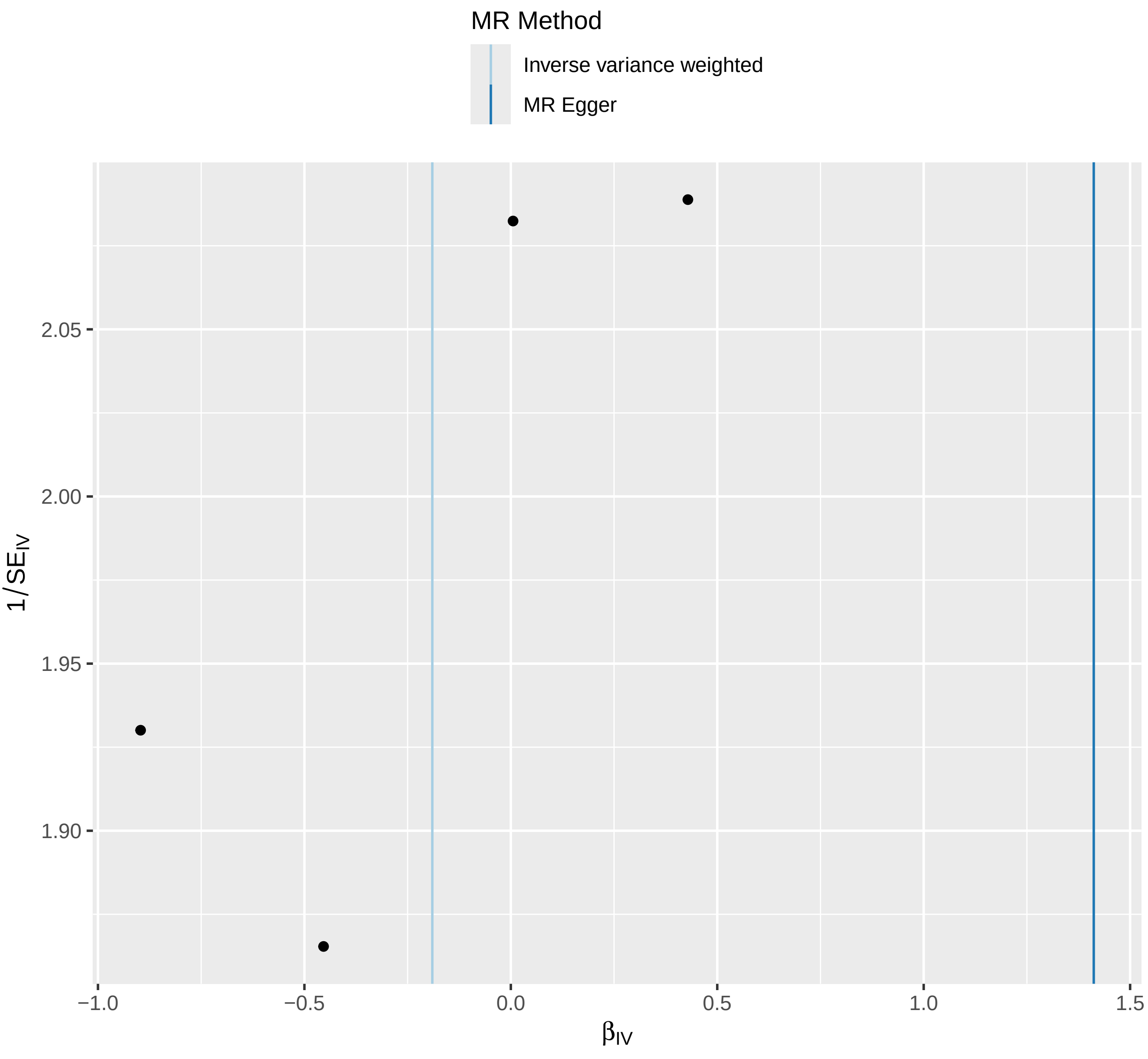

MR Method
Inverse variance weighted
MR Egger
2.05
2.00
1.95
1.90
1/SE_IV
−1.0
−0.5
0.0
0.5
1.0
1.5
β_IV

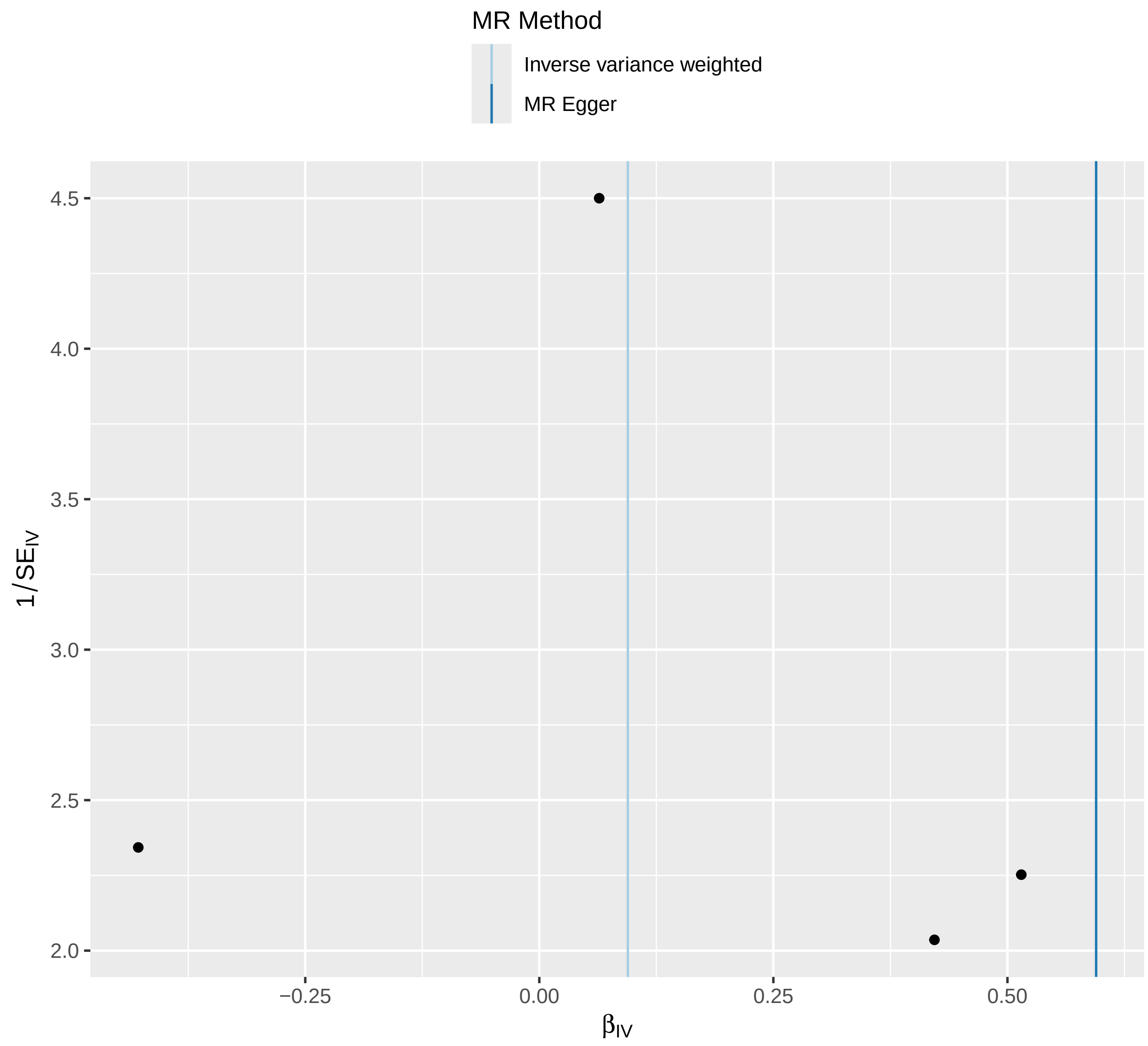

MR Method
Inverse variance weighted
MR Egger
4.5
4.0
3.5
3.0
2.5
2.0
1/SE_IV
−0.25
0.00
0.25
0.50
β_IV

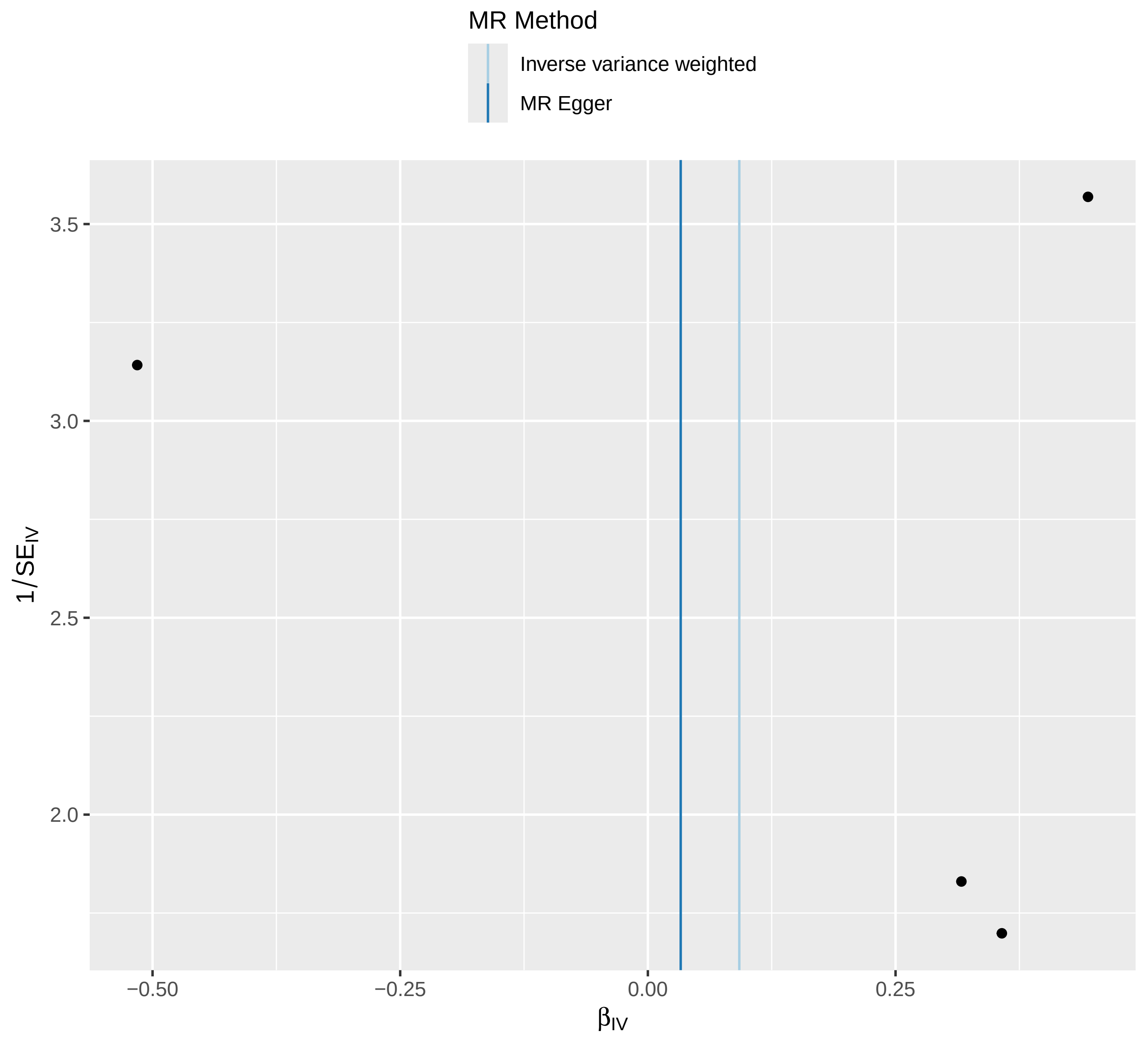

MR Method
Inverse variance weighted
MR Egger
3.5
3.0
2.5
2.0
1/SE$_{IV}$
−0.50
−0.25
0.00
0.25
β$_{IV}$

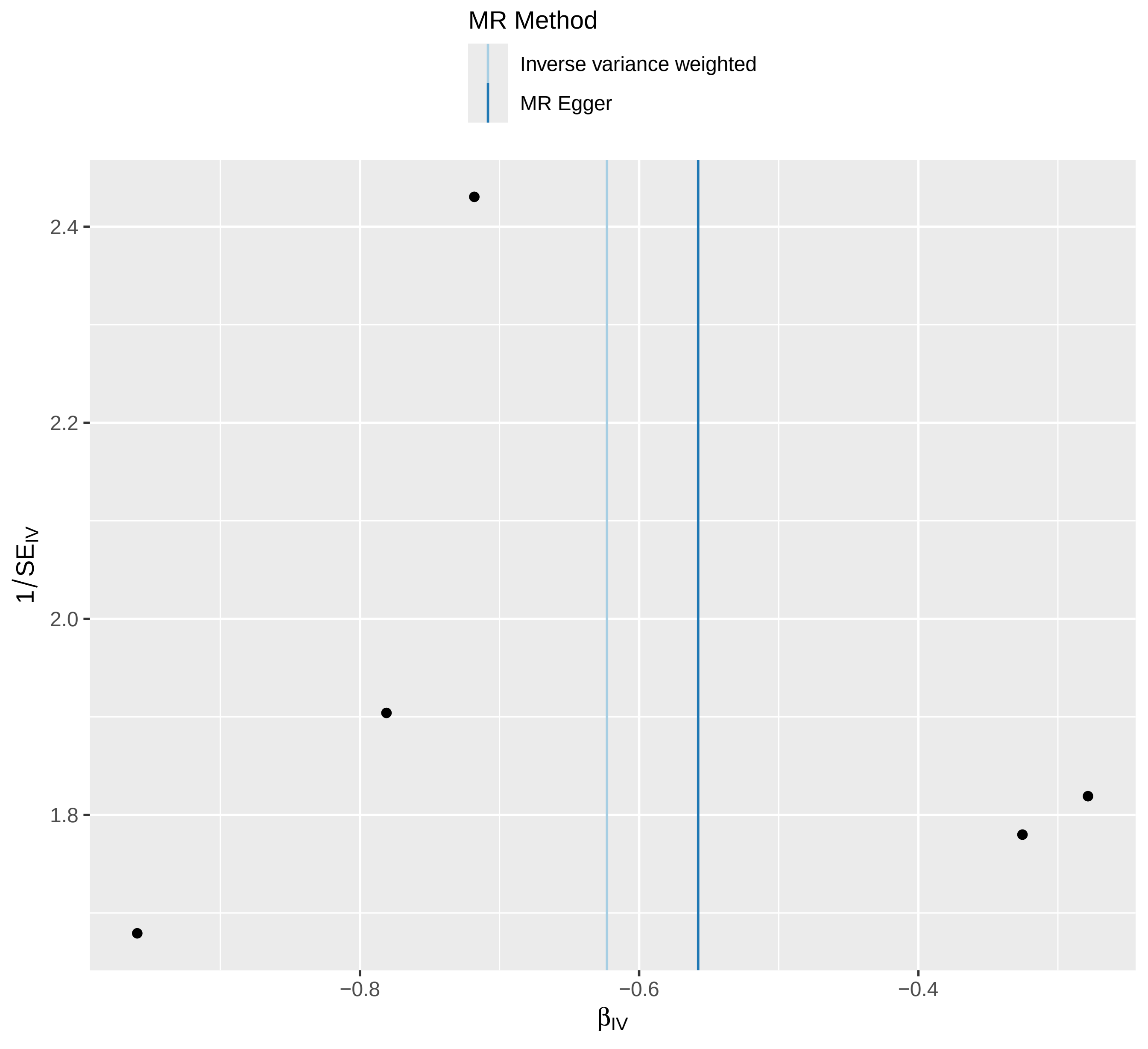
MR Method
Inverse variance weighted
MR Egger
1/SE_IV
2.4
2.2
2.0
1.8
β_IV
−0.8
−0.6
−0.4

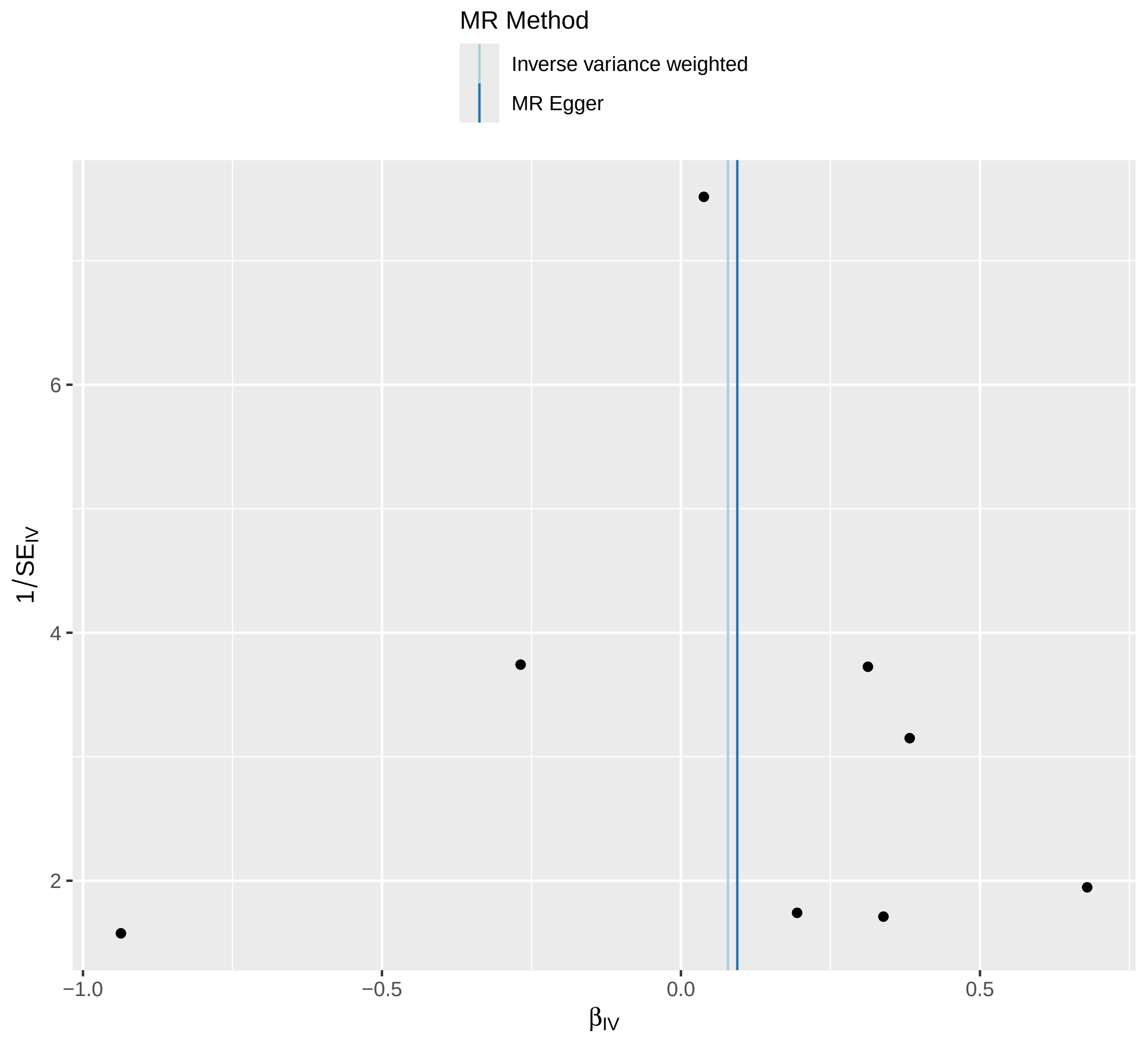

MR Method
Inverse variance weighted
MR Egger
6
4
2
1/SE$_{IV}$
−1.0
−0.5
0.0
0.5
β$_{IV}$

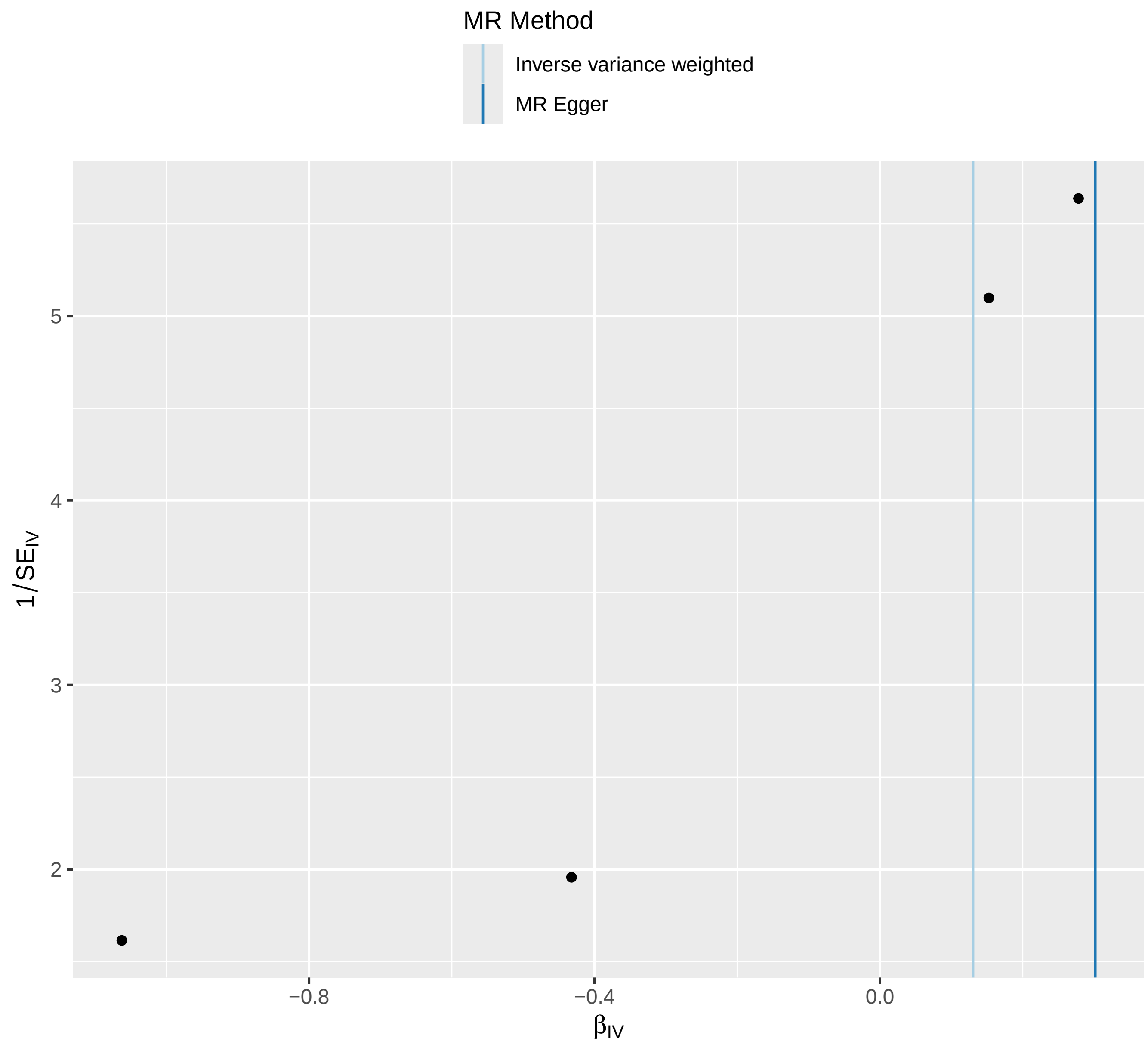
MR Method
Inverse variance weighted
MR Egger
5
4
3
2
1/SE$_{IV}$
−0.8
−0.4
0.0
β$_{IV}$

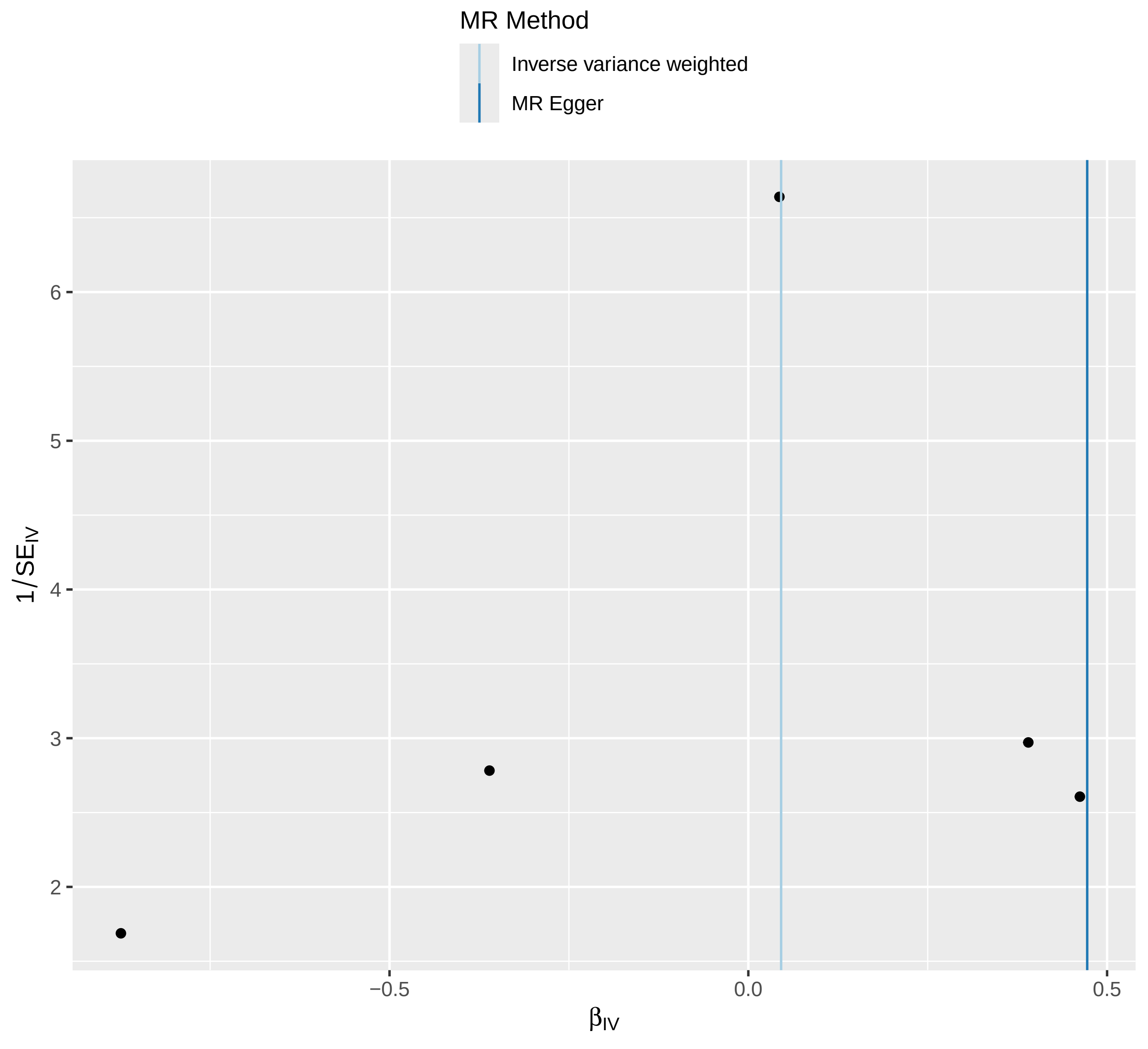
MR Method
Inverse variance weighted
MR Egger
1/SE_IV
6
5
4
3
2
−0.5
0.0
0.5
β_IV

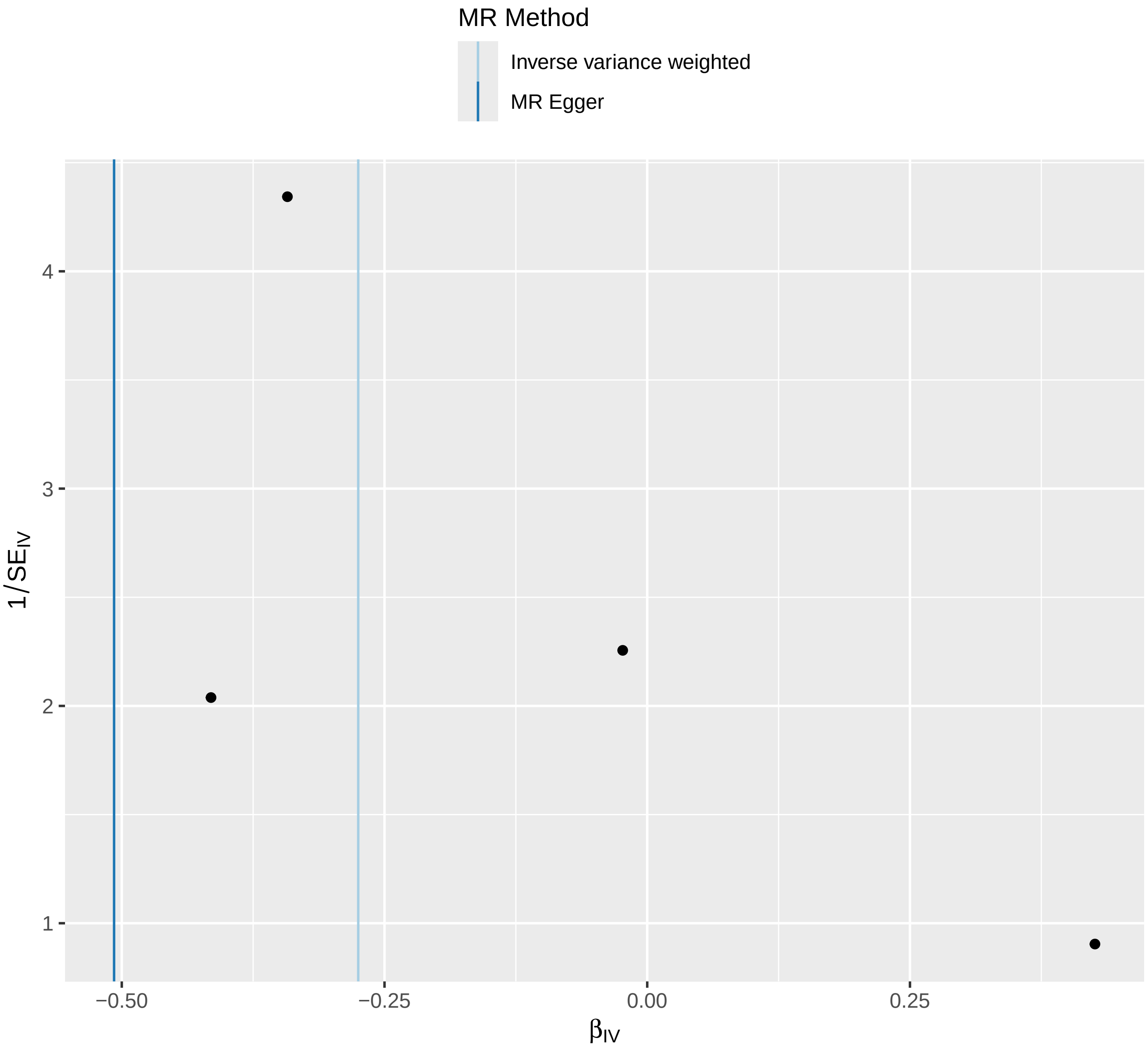

MR Method
Inverse variance weighted
MR Egger
4
3
2
1
1/SE_IV
−0.50
−0.25
0.00
0.25
β_IV

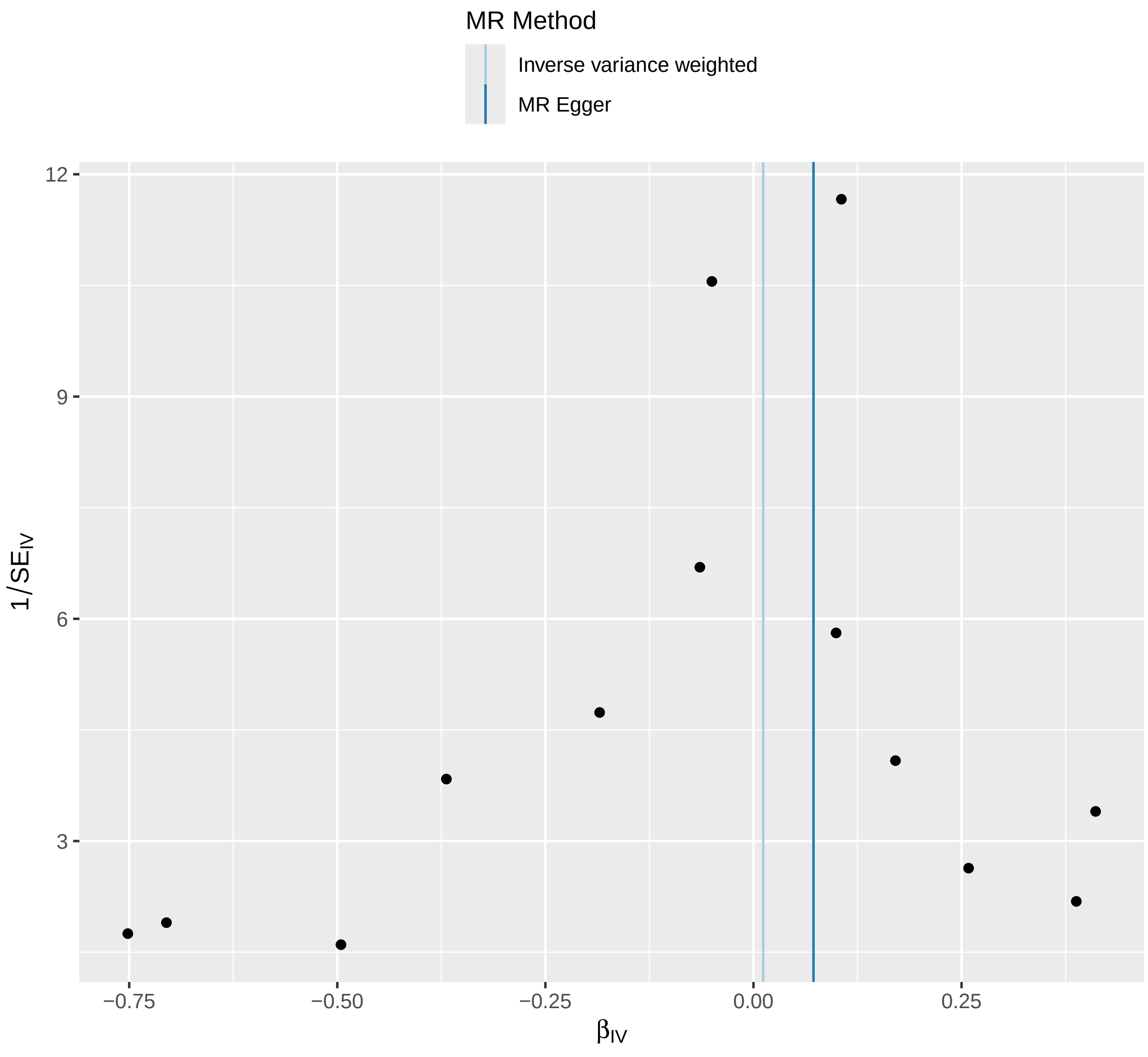
MR Method
Inverse variance weighted
MR Egger
12
9
6
3
1/SE_{IV}
−0.75
−0.50
−0.25
0.00
0.25
β_{IV}

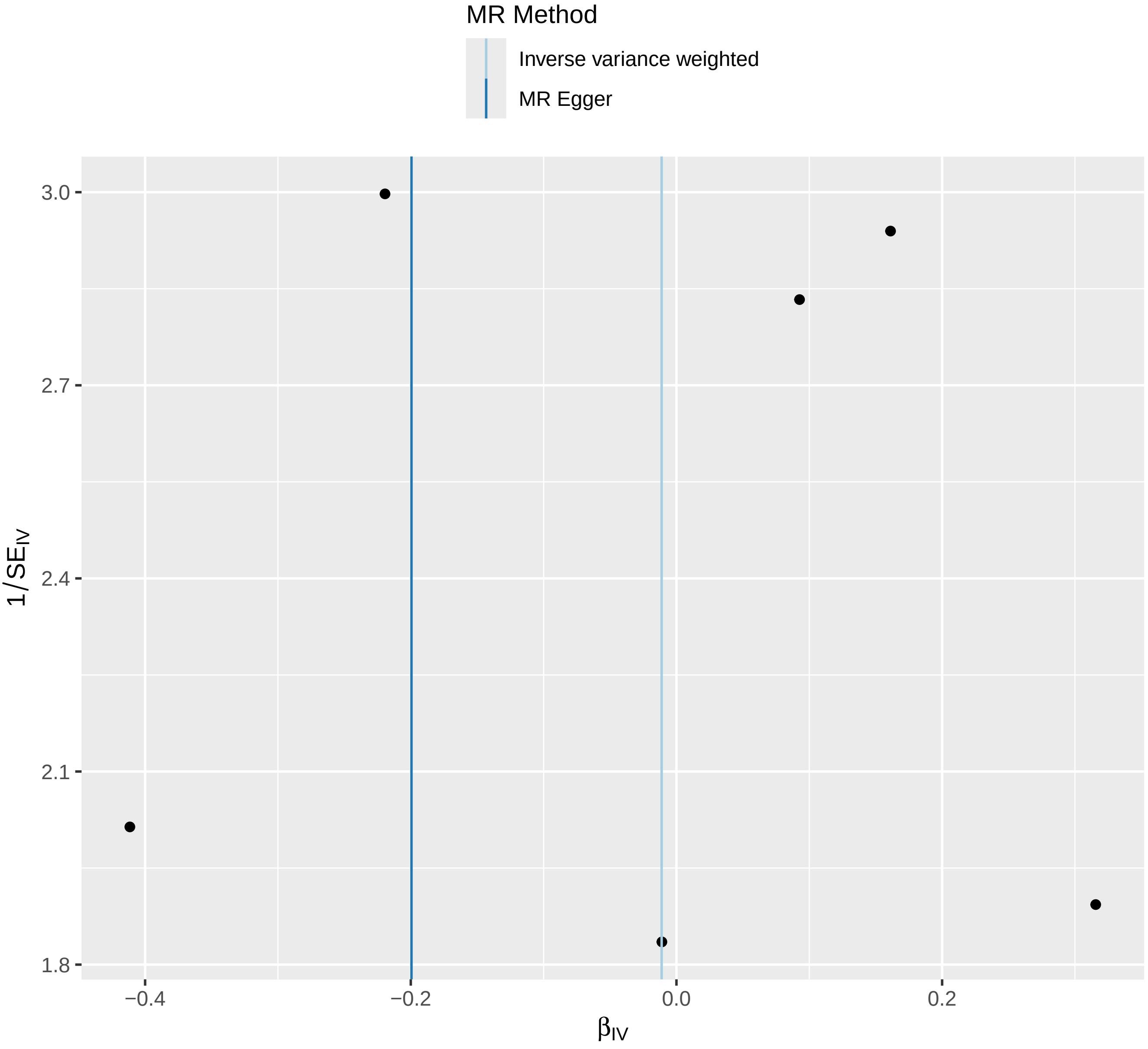

MR Method
Inverse variance weighted
MR Egger
3.0
2.7
2.4
2.1
1.8
1/SE$_{IV}$
−0.4
−0.2
0.0
0.2
β$_{IV}$

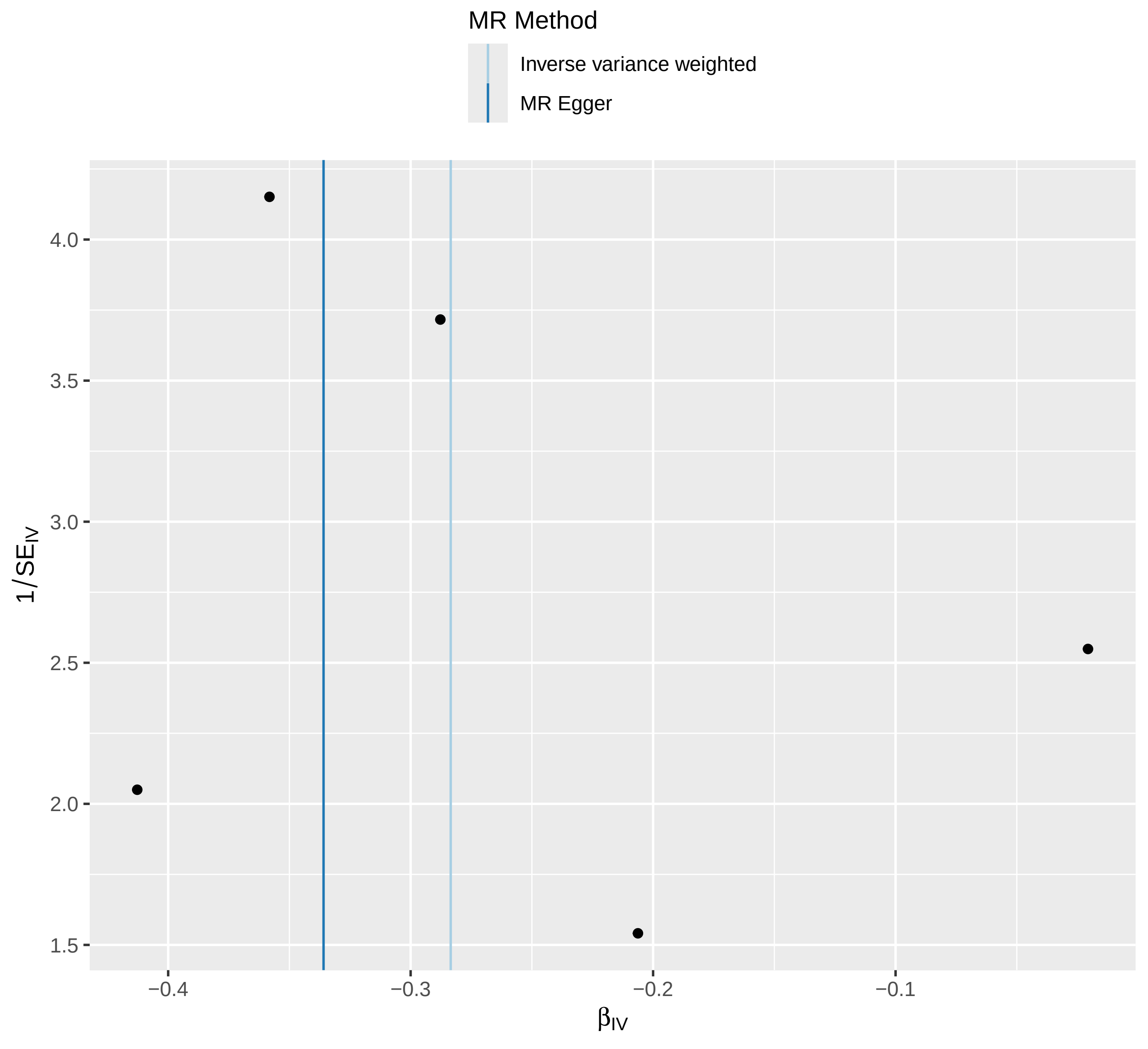

MR Method
Inverse variance weighted
MR Egger
4.0
3.5
3.0
2.5
2.0
1.5
1/SE_IV
−0.4
−0.3
−0.2
−0.1
β_IV

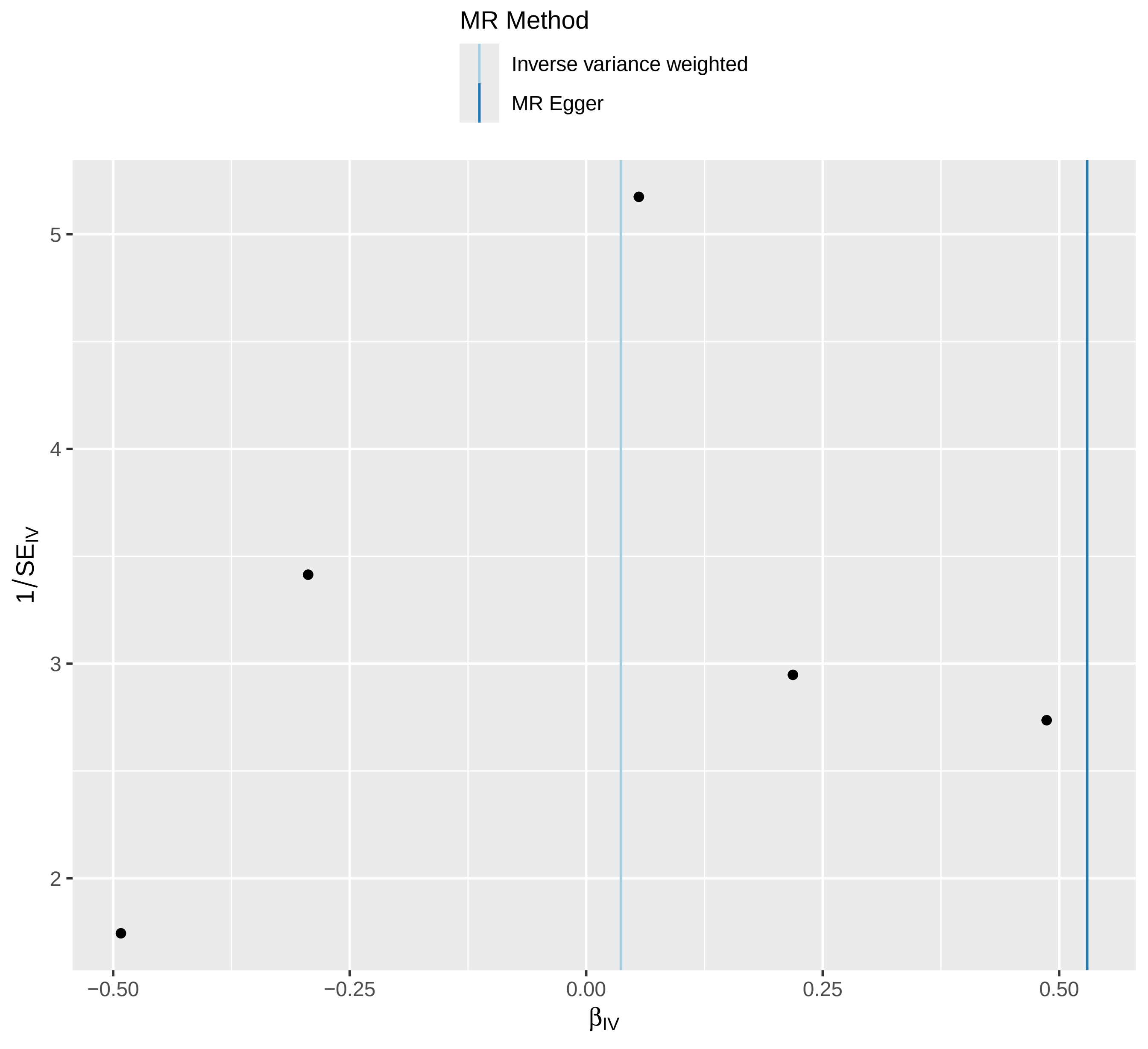
MR Method
Inverse variance weighted
MR Egger
5
4
3
2
1/SE_IV
−0.50
−0.25
0.00
0.25
0.50
β_IV

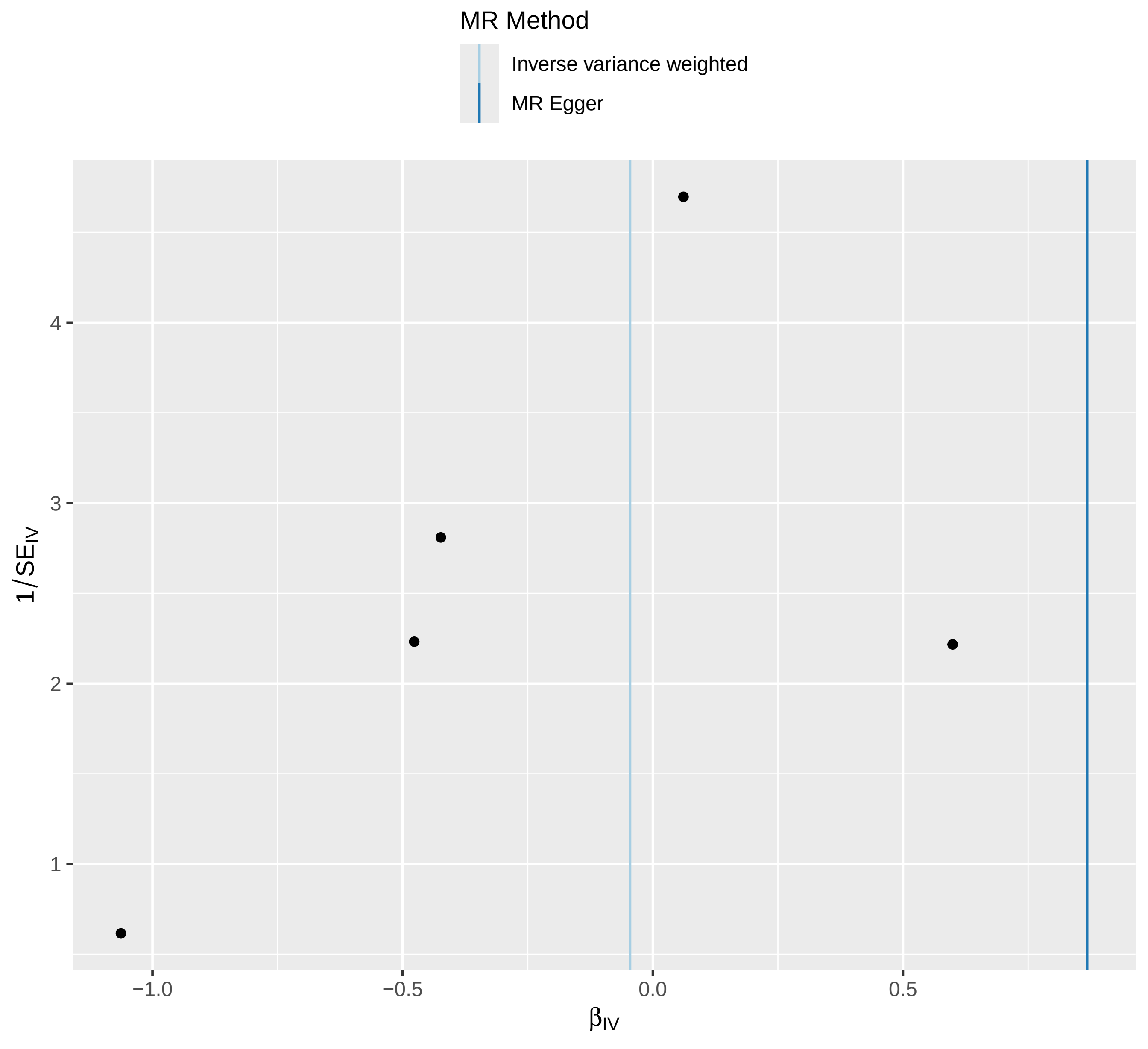

MR Method
Inverse variance weighted
MR Egger
4
3
2
1
1/SE_IV
−1.0
−0.5
0.0
0.5
β_IV

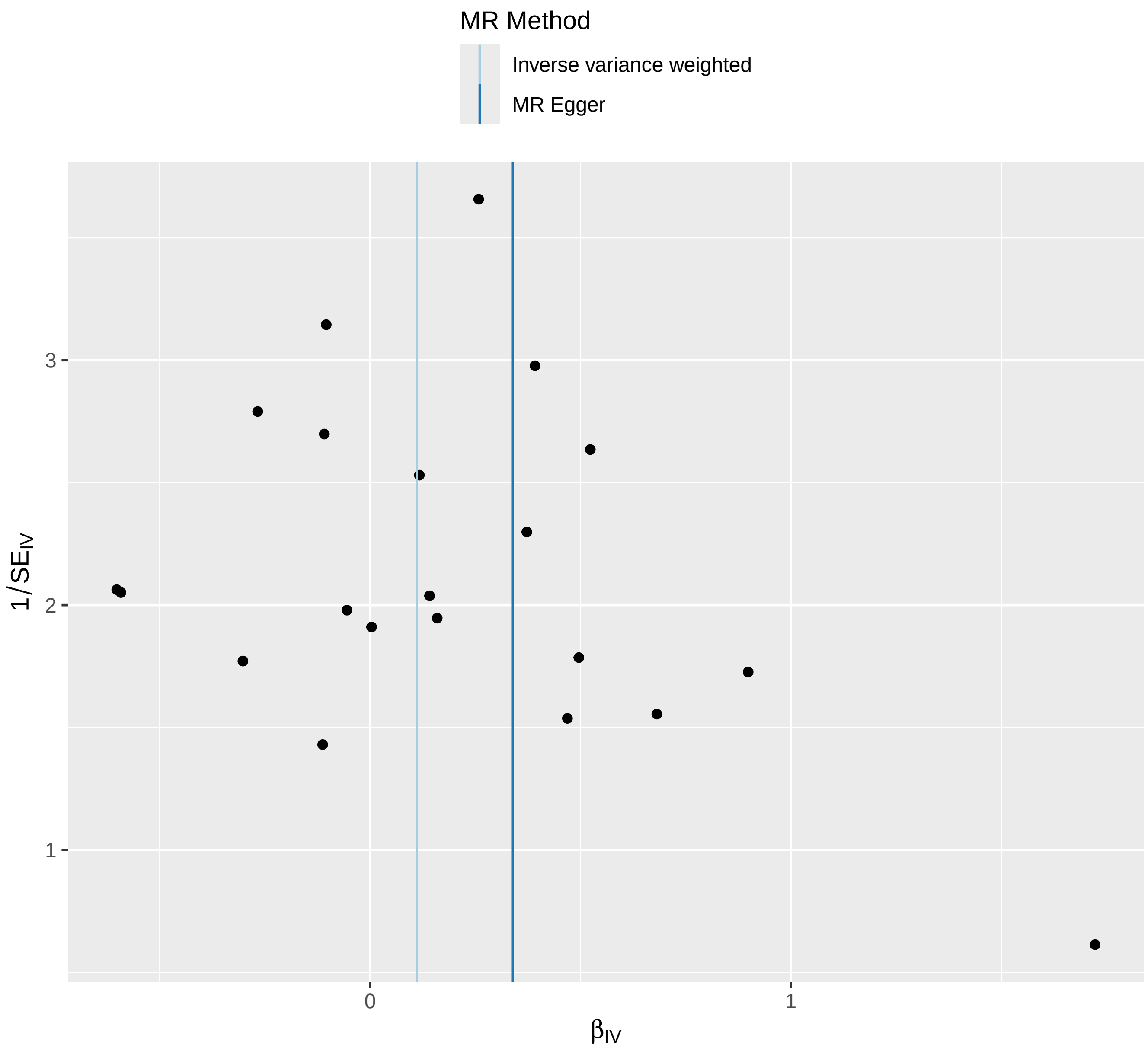

MR Method
Inverse variance weighted
MR Egger
3
2
1
1/SE_IV
0
1
β_IV

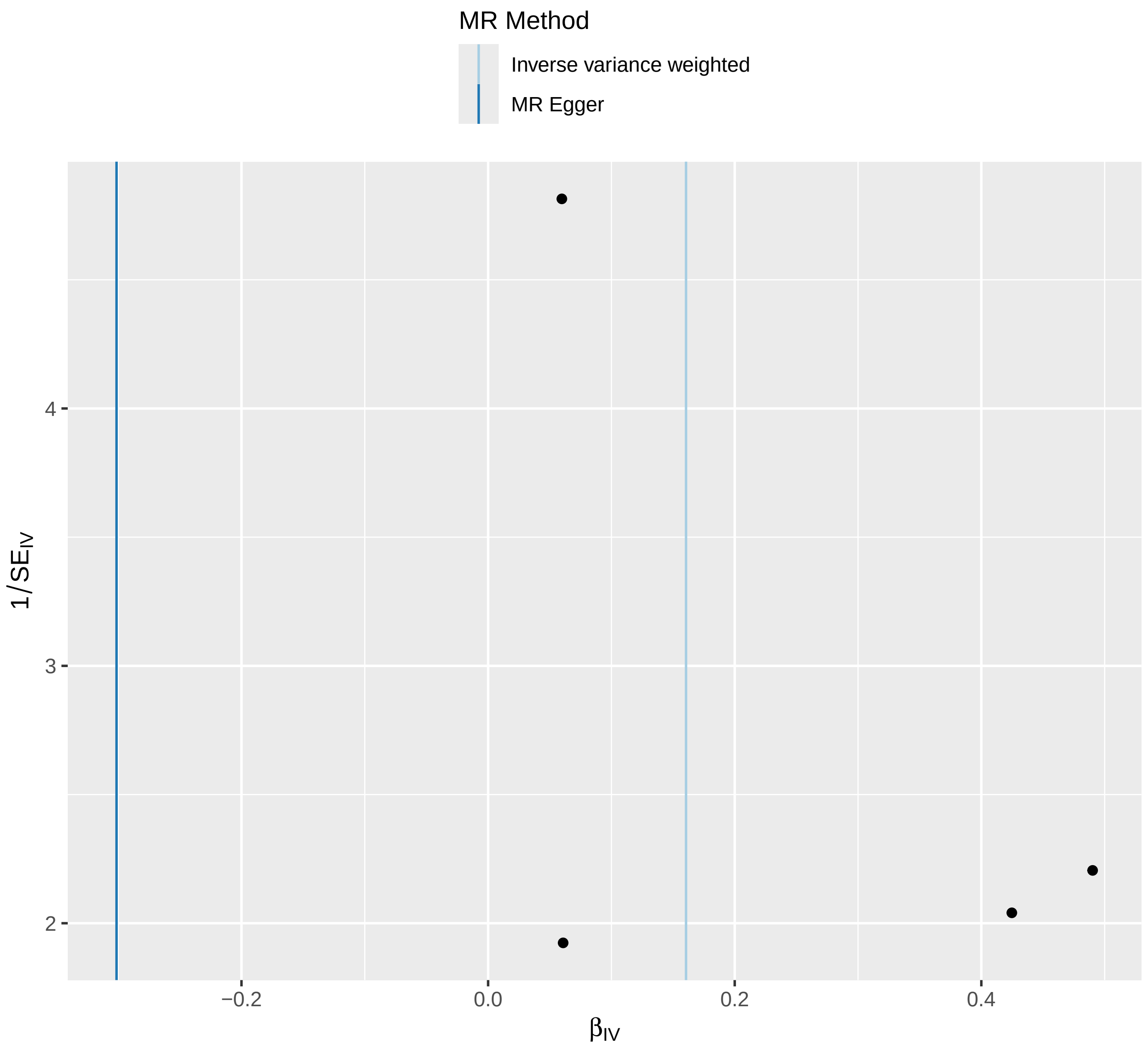
MR Method
Inverse variance weighted
MR Egger
4
3
2
1/SE_{IV}
−0.2
0.0
0.2
0.4
β_{IV}

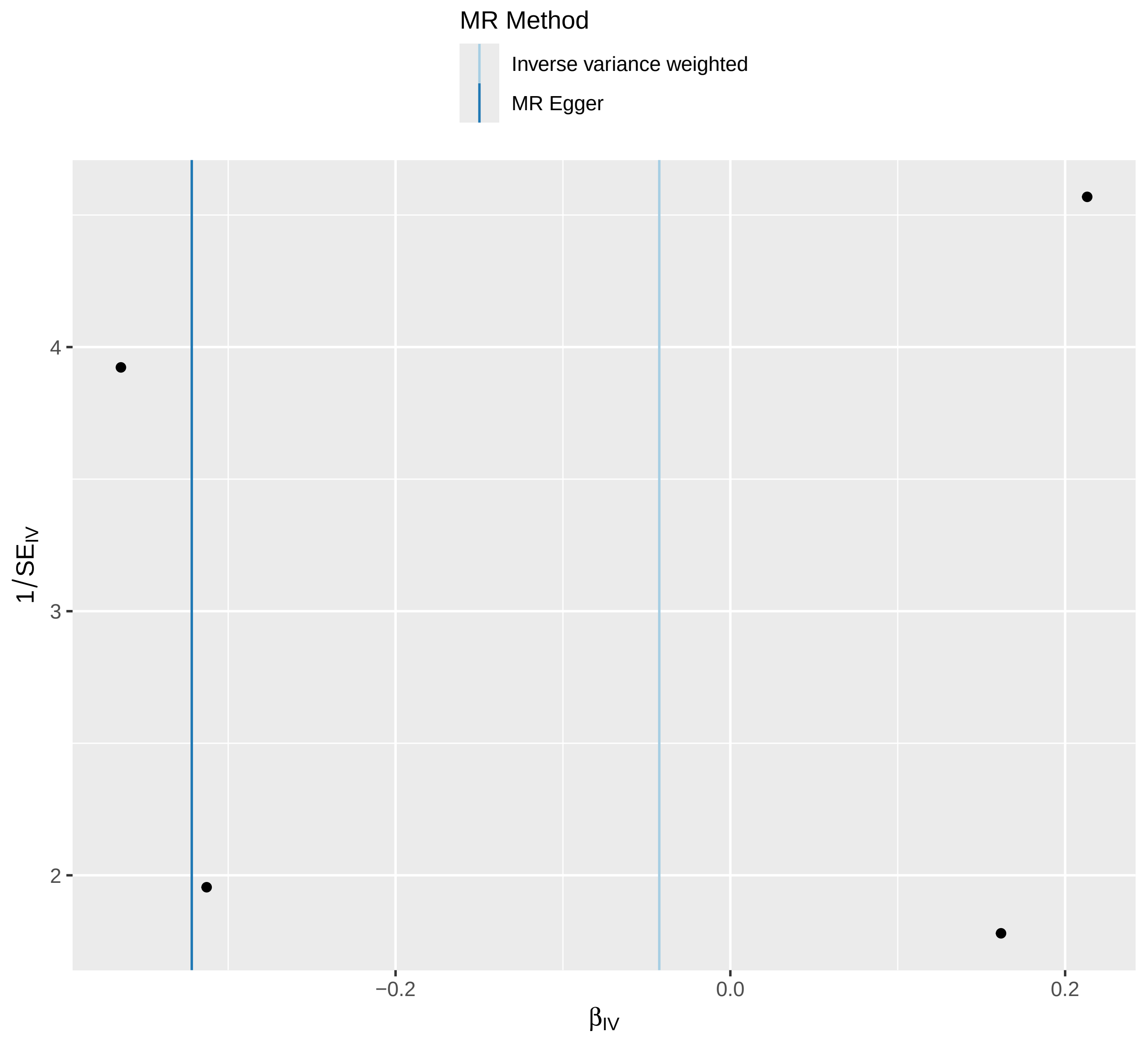
MR Method
Inverse variance weighted
MR Egger
4
3
2
−0.2
0.0
0.2
β_{IV}
$1/SE_{IV}$

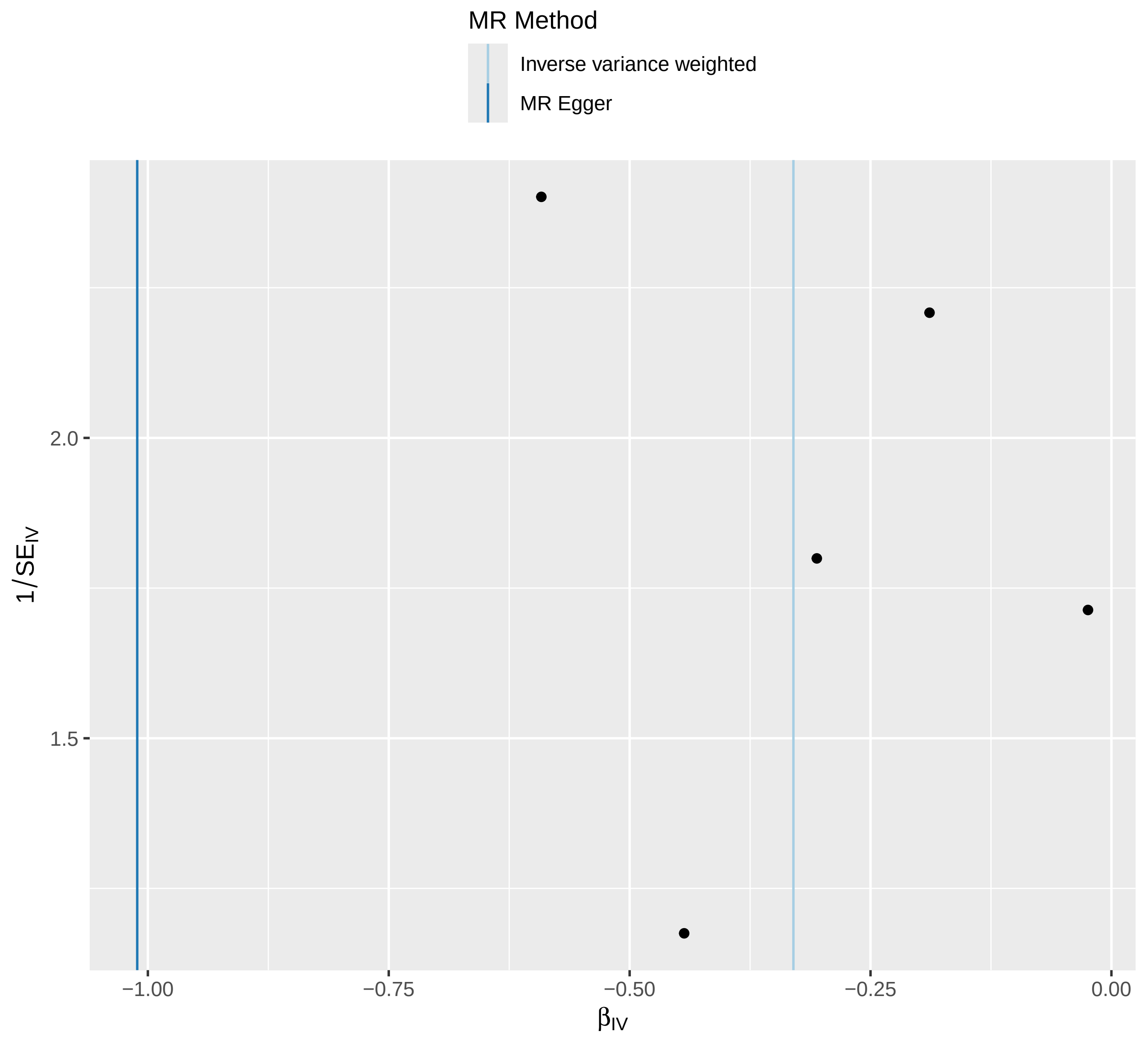
MR Method
Inverse variance weighted
MR Egger
2.0
1.5
1/SE$_{IV}$
−1.00
−0.75
−0.50
−0.25
0.00
β$_{IV}$

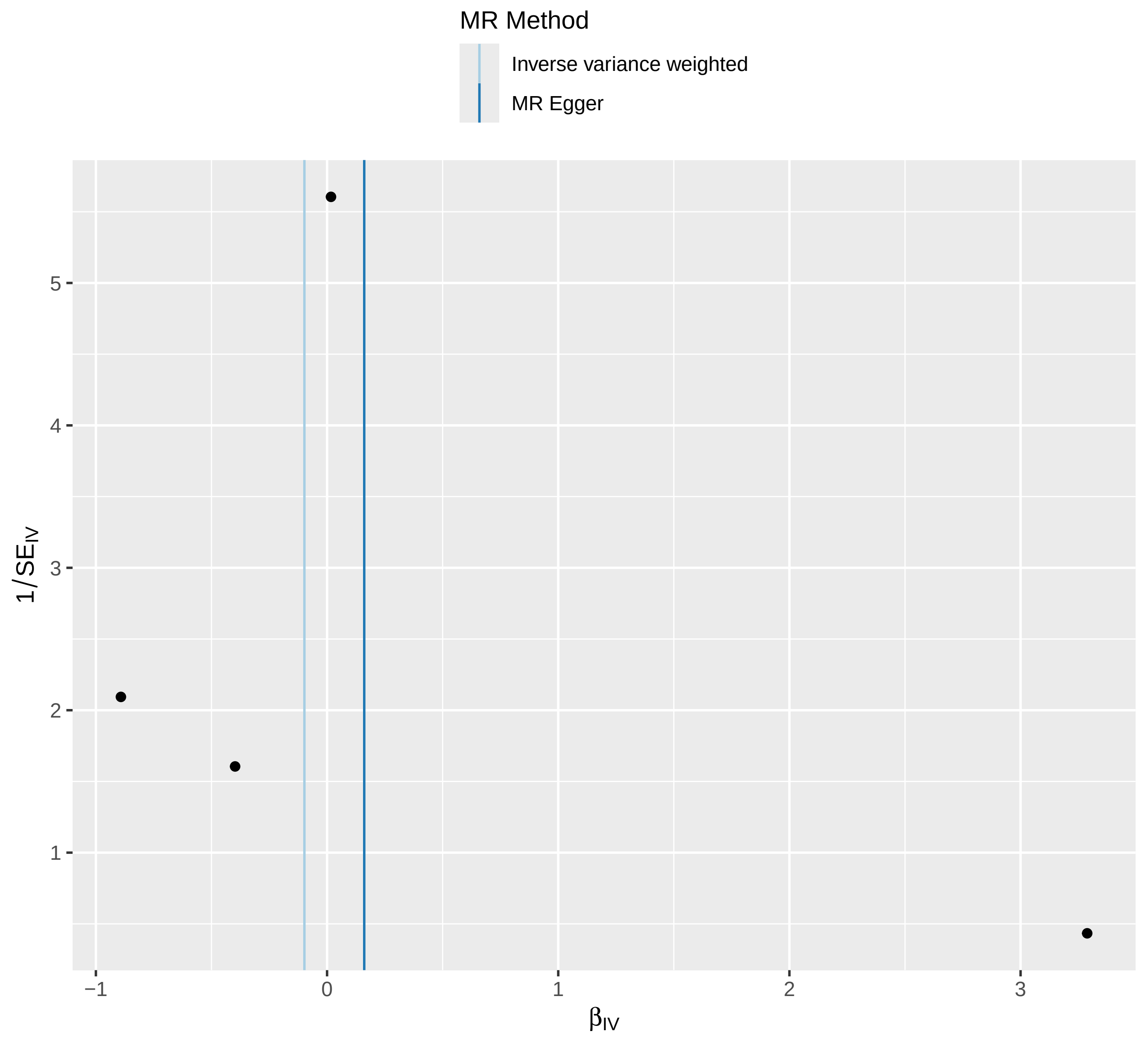
MR Method
Inverse variance weighted
MR Egger
1/SE$_{IV}$
β$_{IV}$
5
4
3
2
1
−1
0
1
2
3

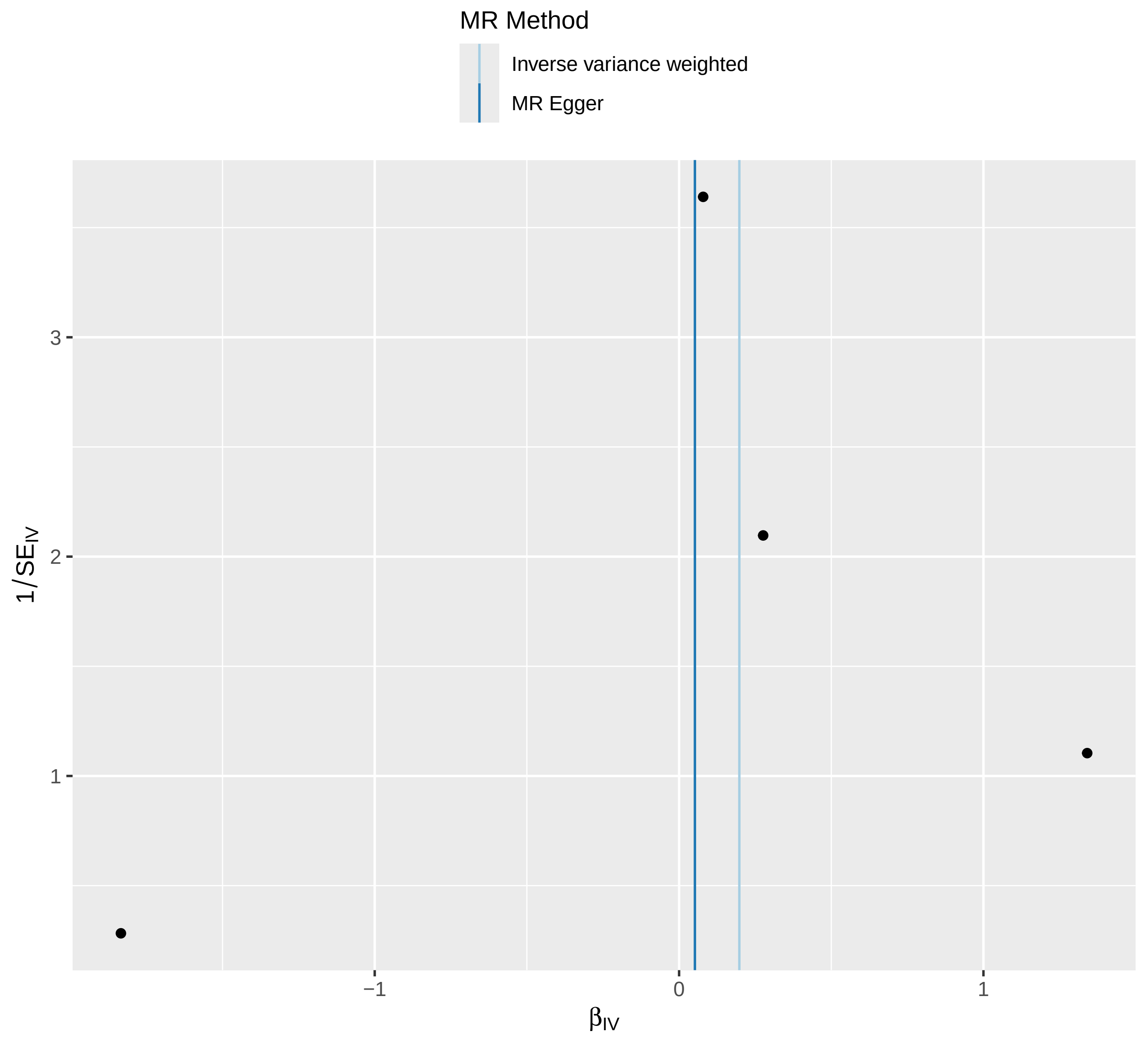

MR Method
Inverse variance weighted
MR Egger
1/SE$_{IV}$
3
2
1
−1
0
1
β$_{IV}$

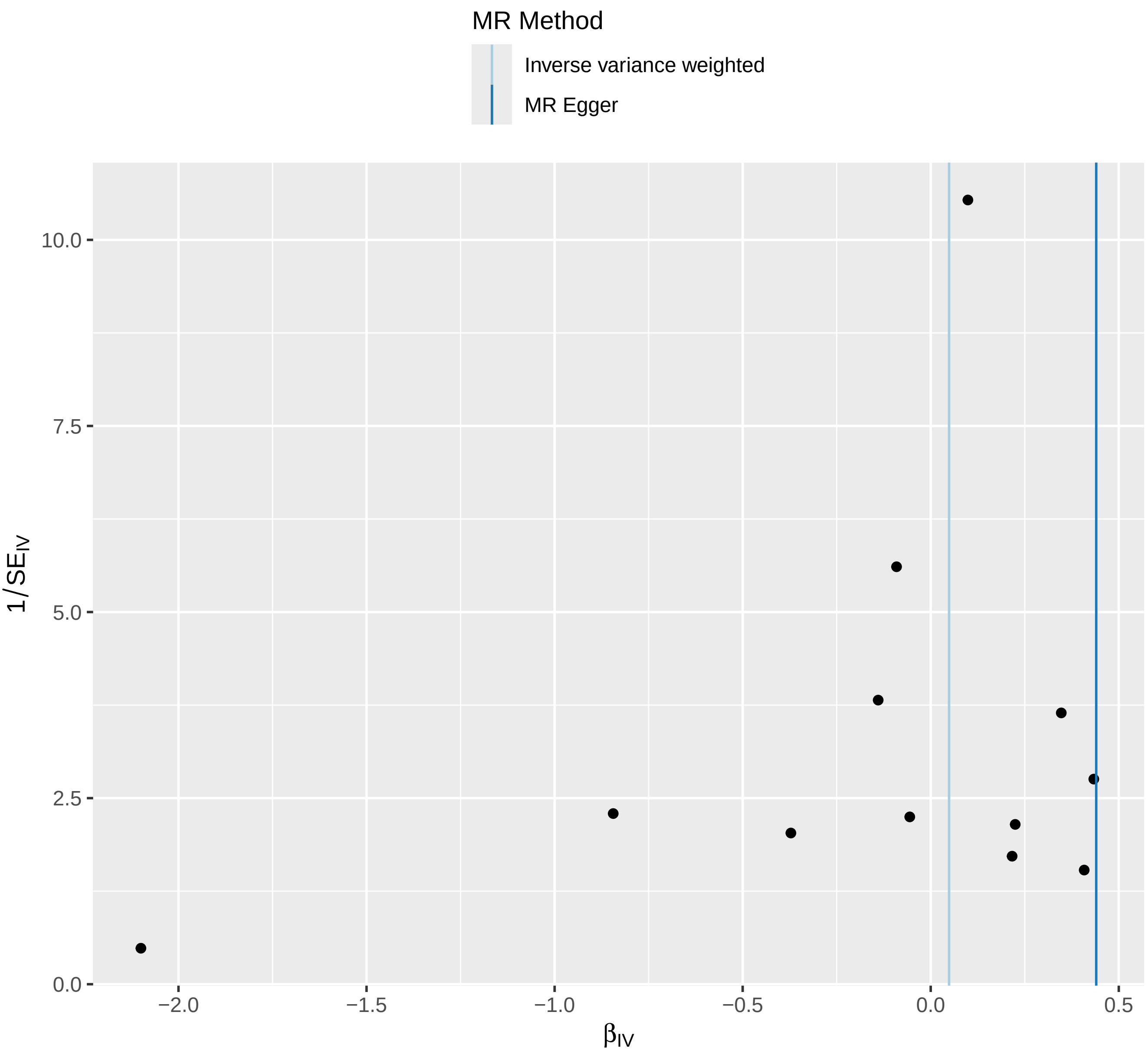

MR Method
Inverse variance weighted
MR Egger
10.0
7.5
5.0
2.5
0.0
1/SE$_{IV}$
−2.0
−1.5
−1.0
−0.5
0.0
0.5
β$_{IV}$

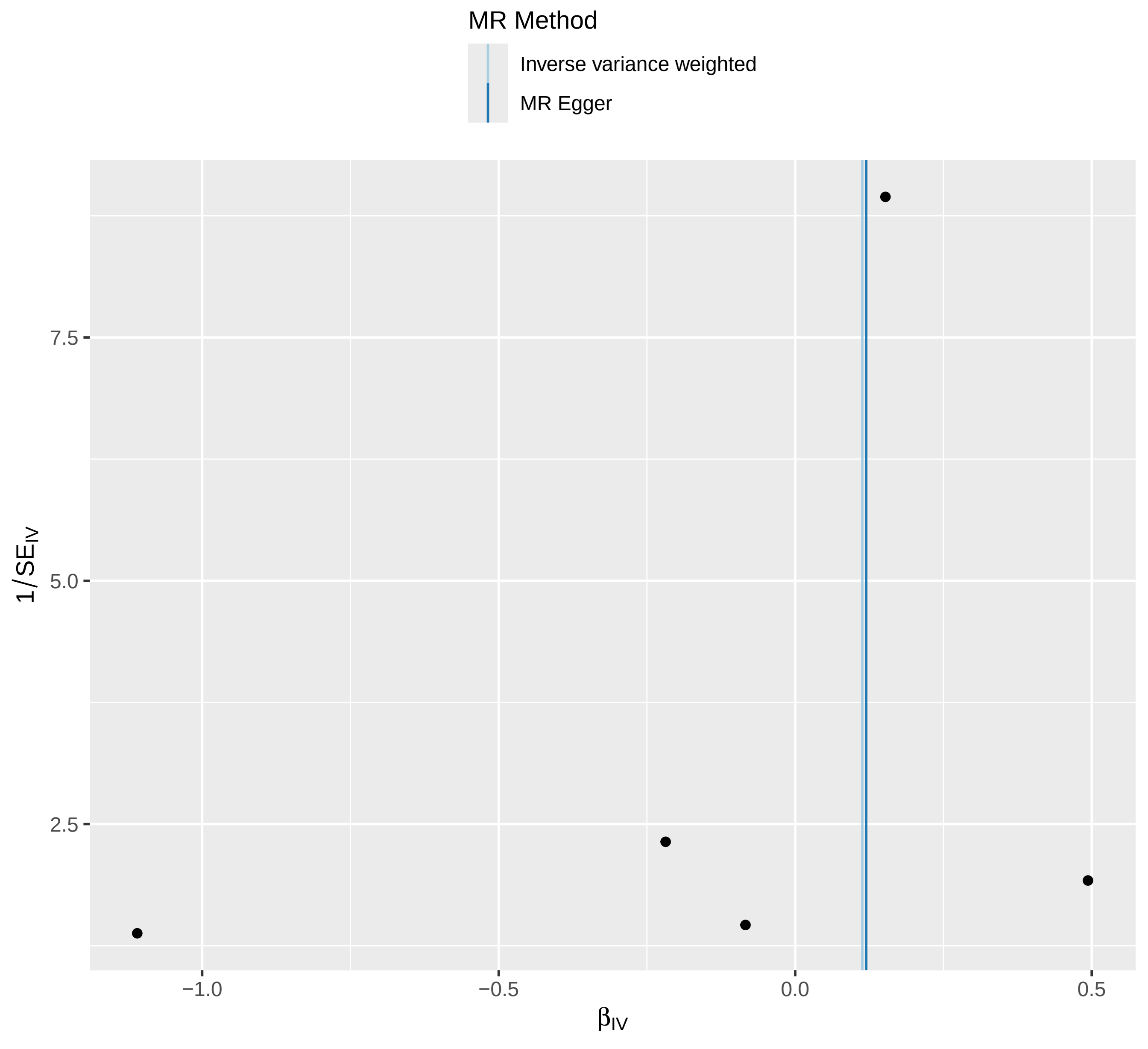
MR Method
Inverse variance weighted
MR Egger
7.5
5.0
2.5
1/SE$_{IV}$
−1.0
−0.5
0.0
0.5
β$_{IV}$

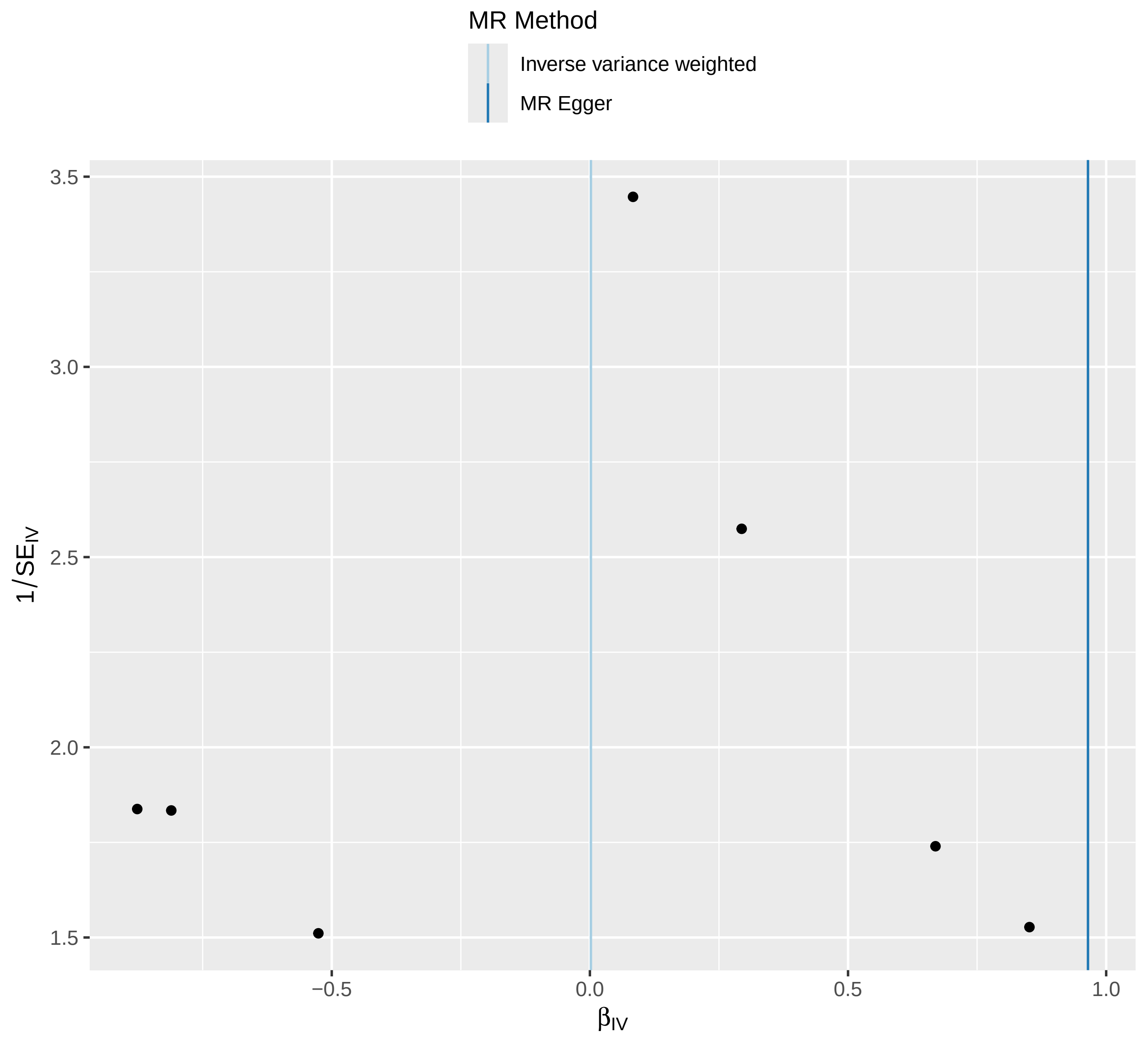
MR Method
Inverse variance weighted
MR Egger
3.5
3.0
2.5
2.0
1.5
1/SE_IV
−0.5
0.0
0.5
1.0
β_IV

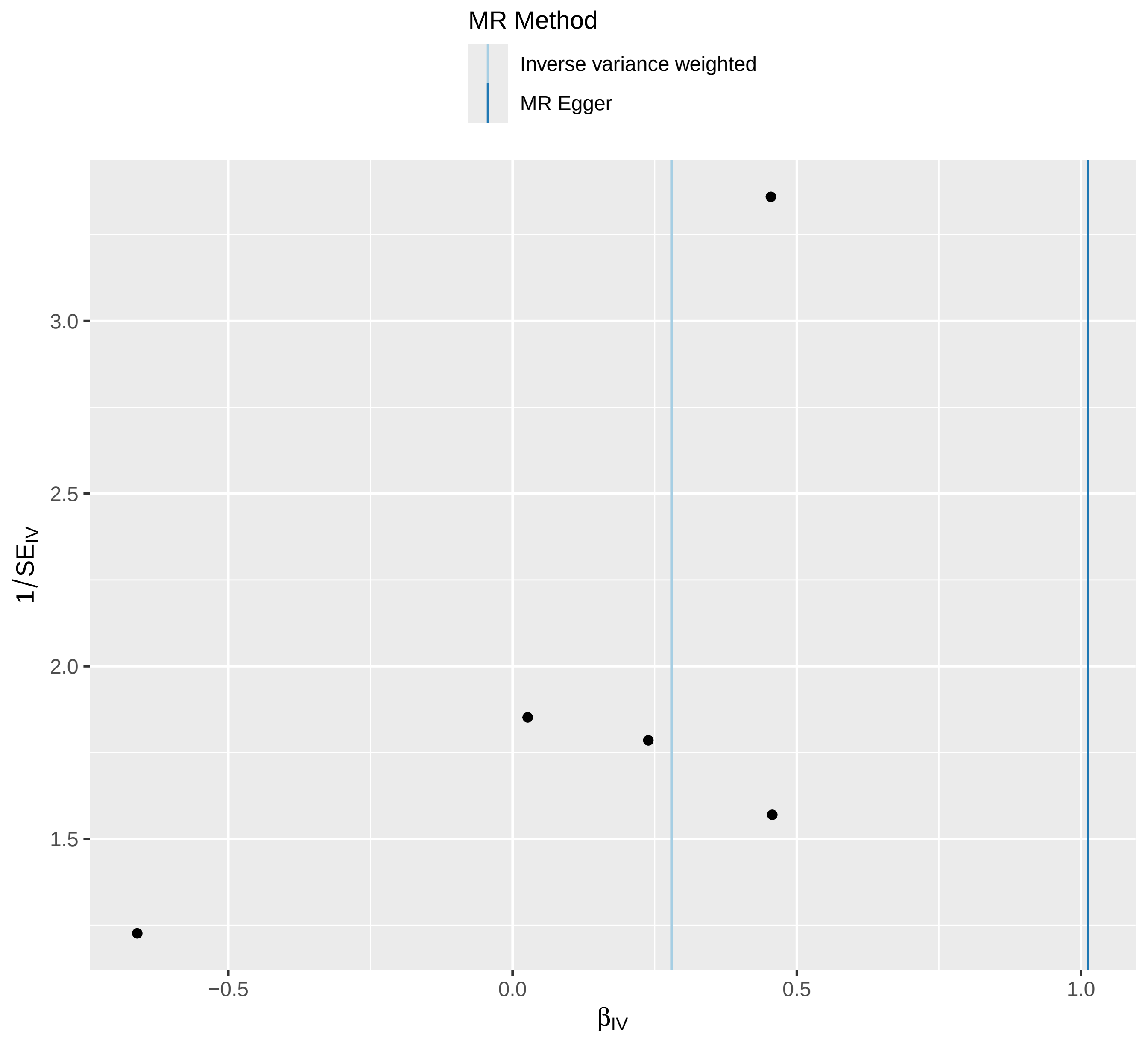
MR Method
Inverse variance weighted
MR Egger
3.0
2.5
2.0
1.5
−0.5
0.0
0.5
1.0
1/SE_IV
β_IV

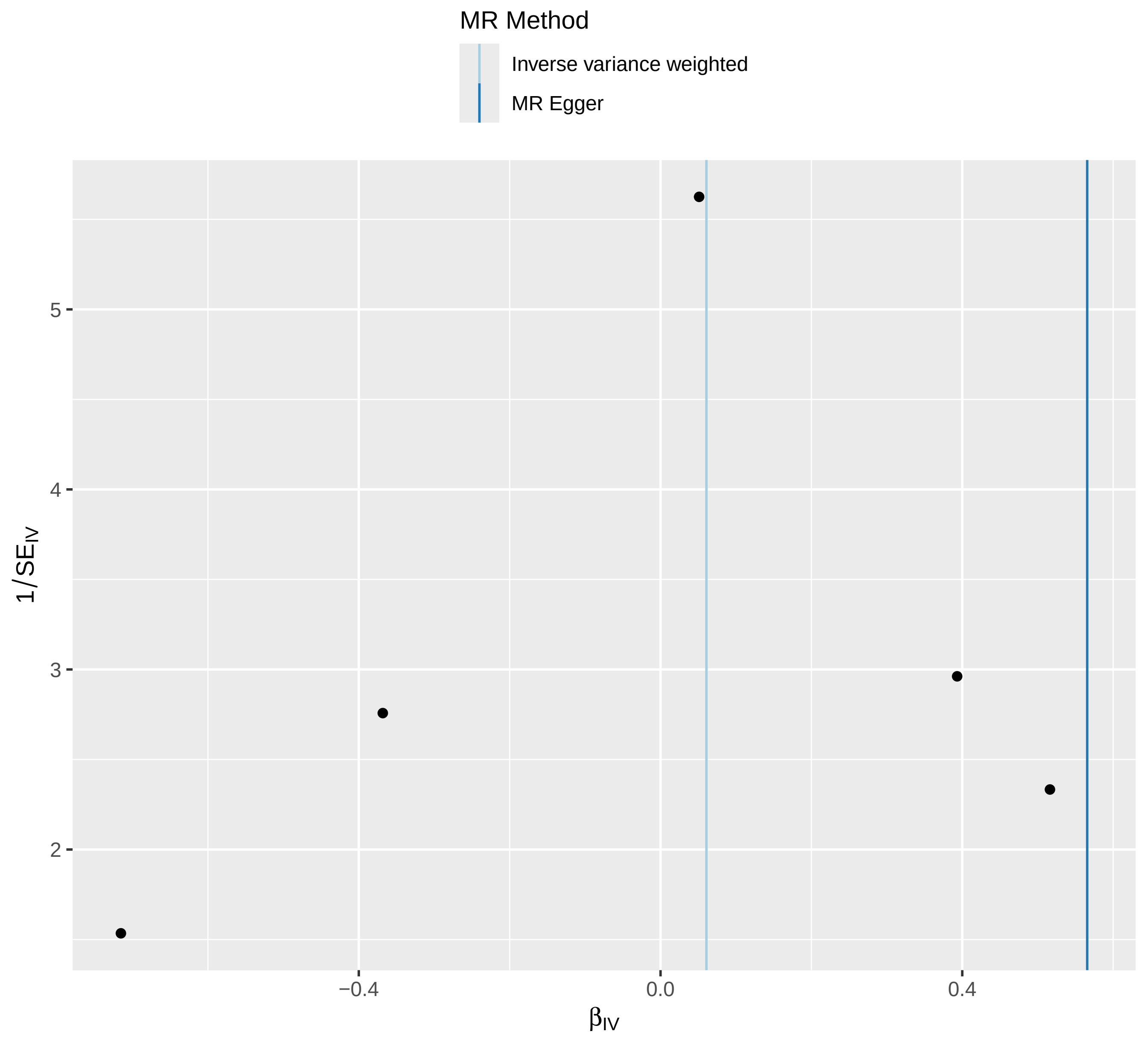
MR Method
Inverse variance weighted
MR Egger
1/SE_IV
β_IV
5
4
3
2
−0.4
0.0
0.4

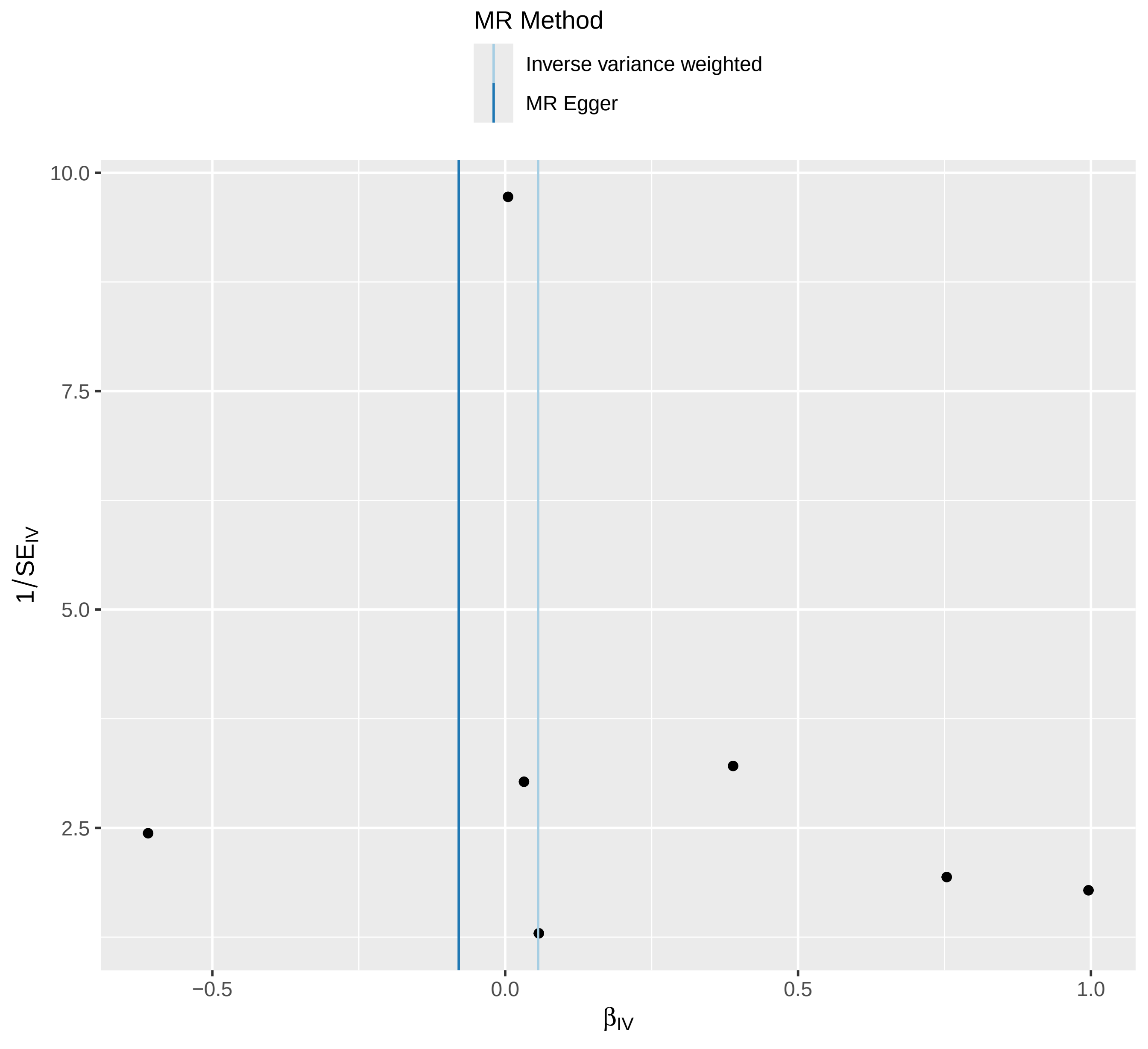
MR Method
Inverse variance weighted
MR Egger
10.0
7.5
5.0
2.5
1/SE_IV
−0.5
0.0
0.5
1.0
β_IV

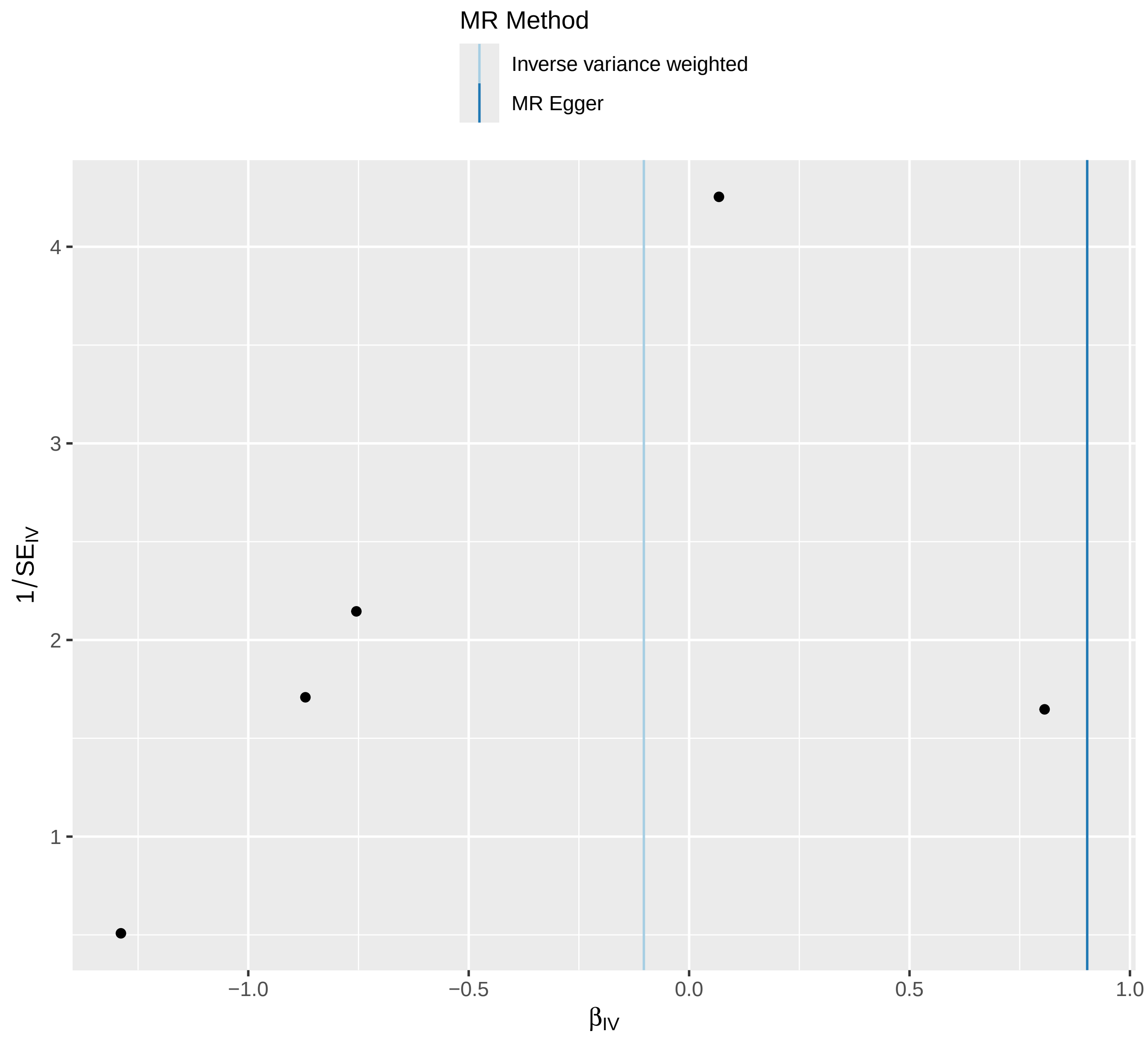

MR Method
Inverse variance weighted
MR Egger
4
3
2
1
1/SE$_{IV}$
−1.0
−0.5
0.0
0.5
1.0
β$_{IV}$

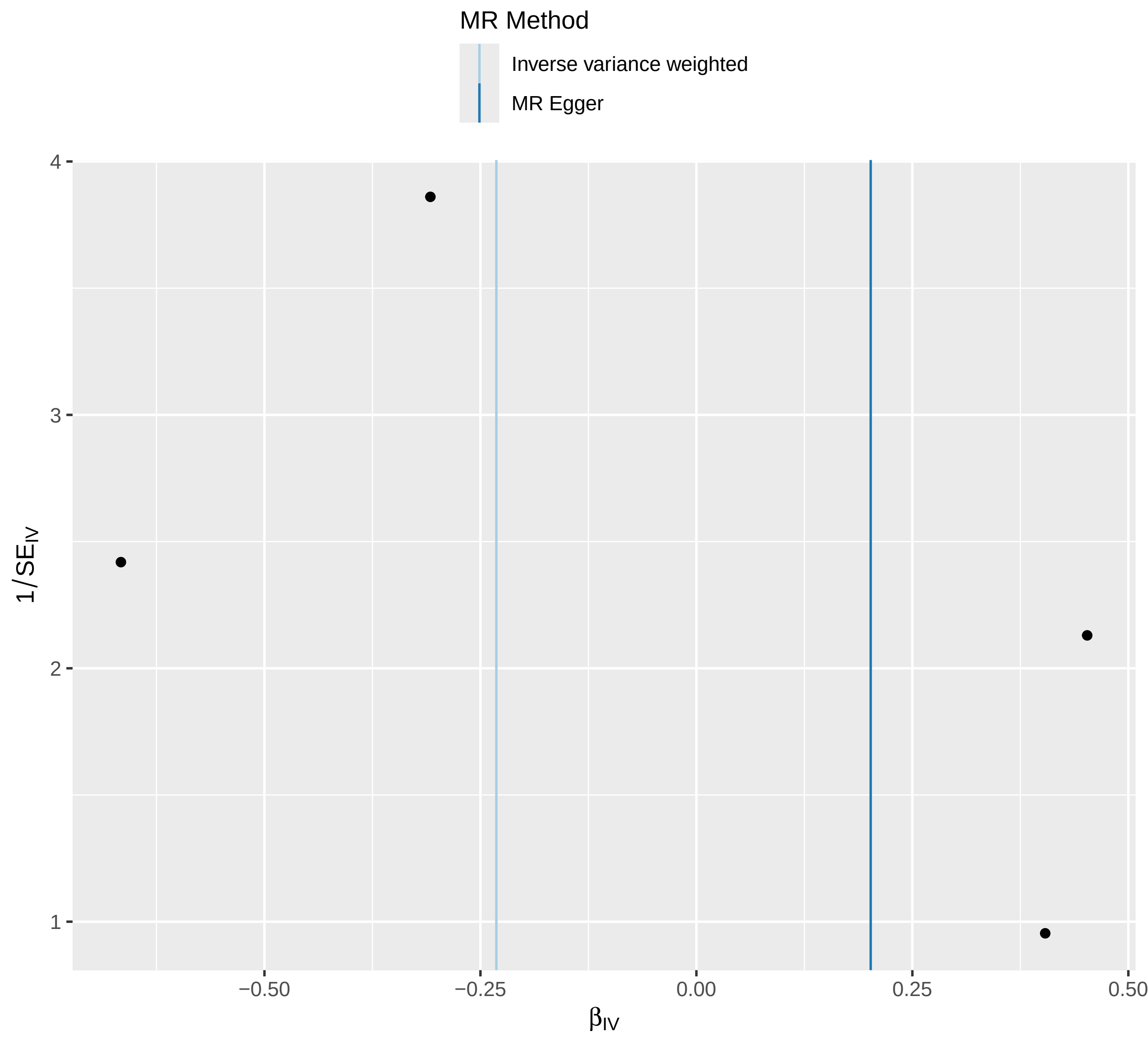

MR Method
Inverse variance weighted
MR Egger
4
3
2
1
−0.50
−0.25
0.00
0.25
0.50
$1/SE_{IV}$
β_{IV}

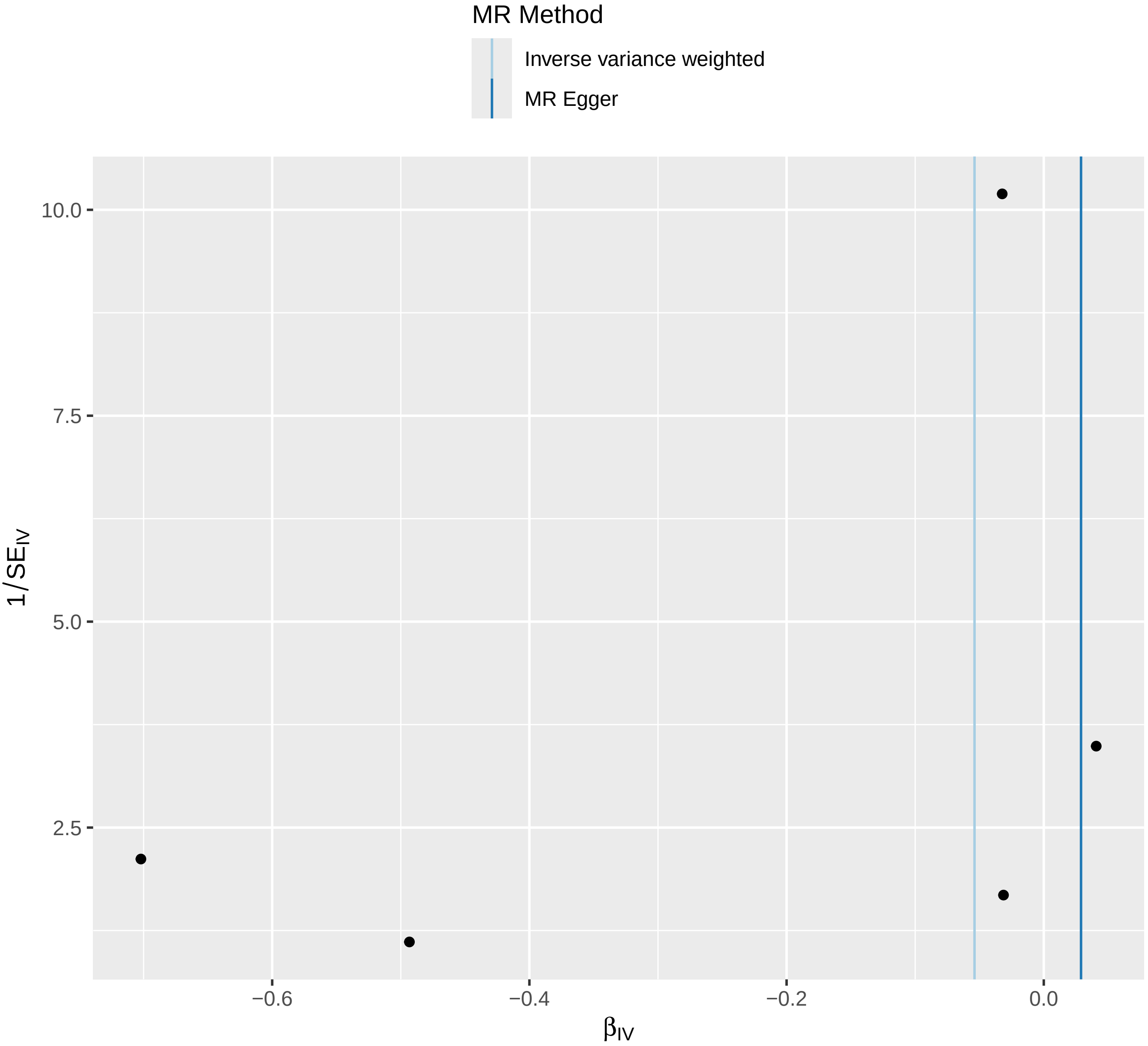

MR Method
Inverse variance weighted
MR Egger
10.0
7.5
5.0
2.5
1/SE_IV
−0.6
−0.4
−0.2
0.0
β_IV

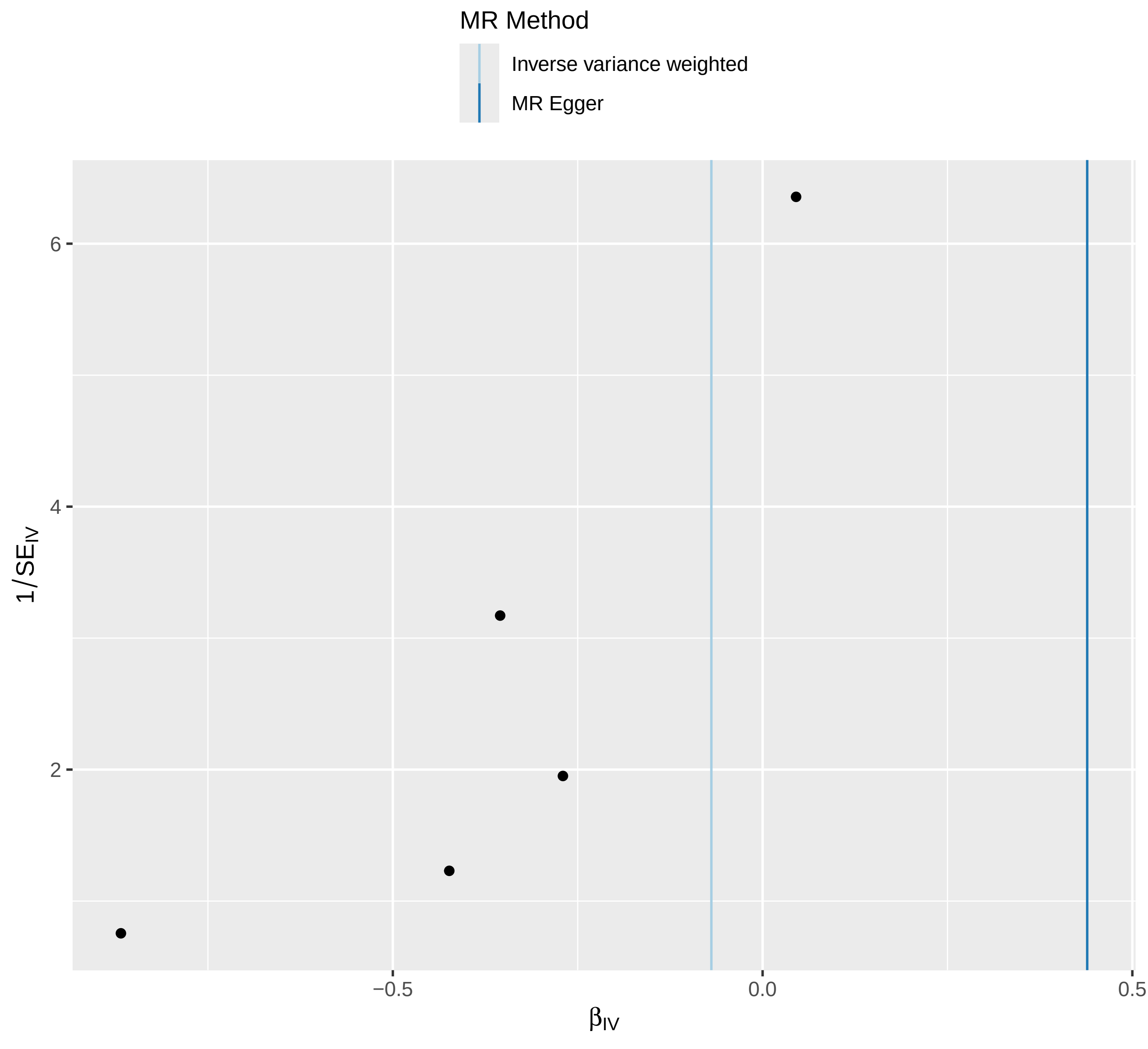

MR Method
Inverse variance weighted
MR Egger
6
4
2
1/SE_IV
−0.5
0.0
0.5
β_IV

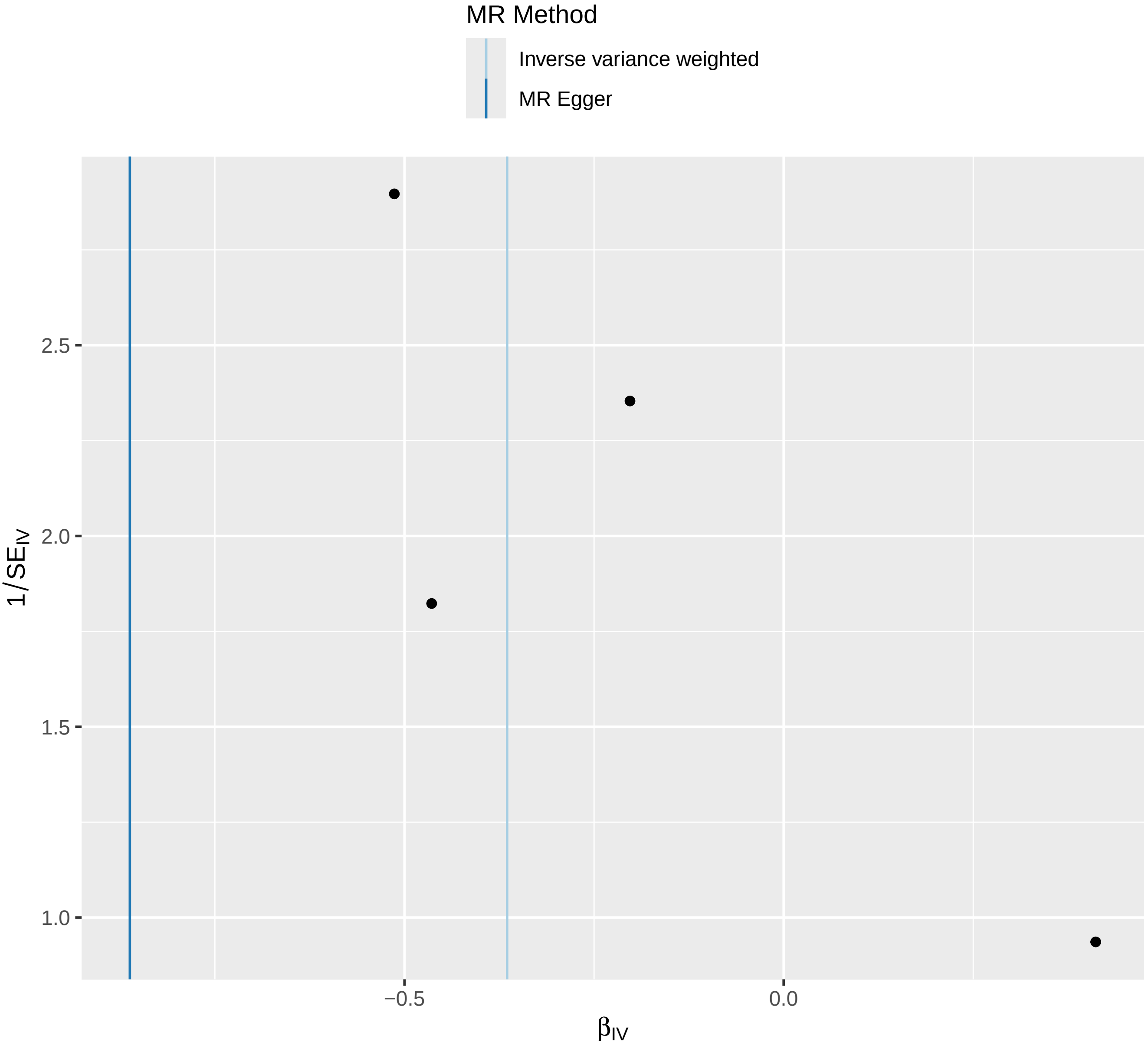

MR Method
Inverse variance weighted
MR Egger
2.5
2.0
1.5
1.0
−0.5
0.0
$1/SE_{IV}$
β_{IV}

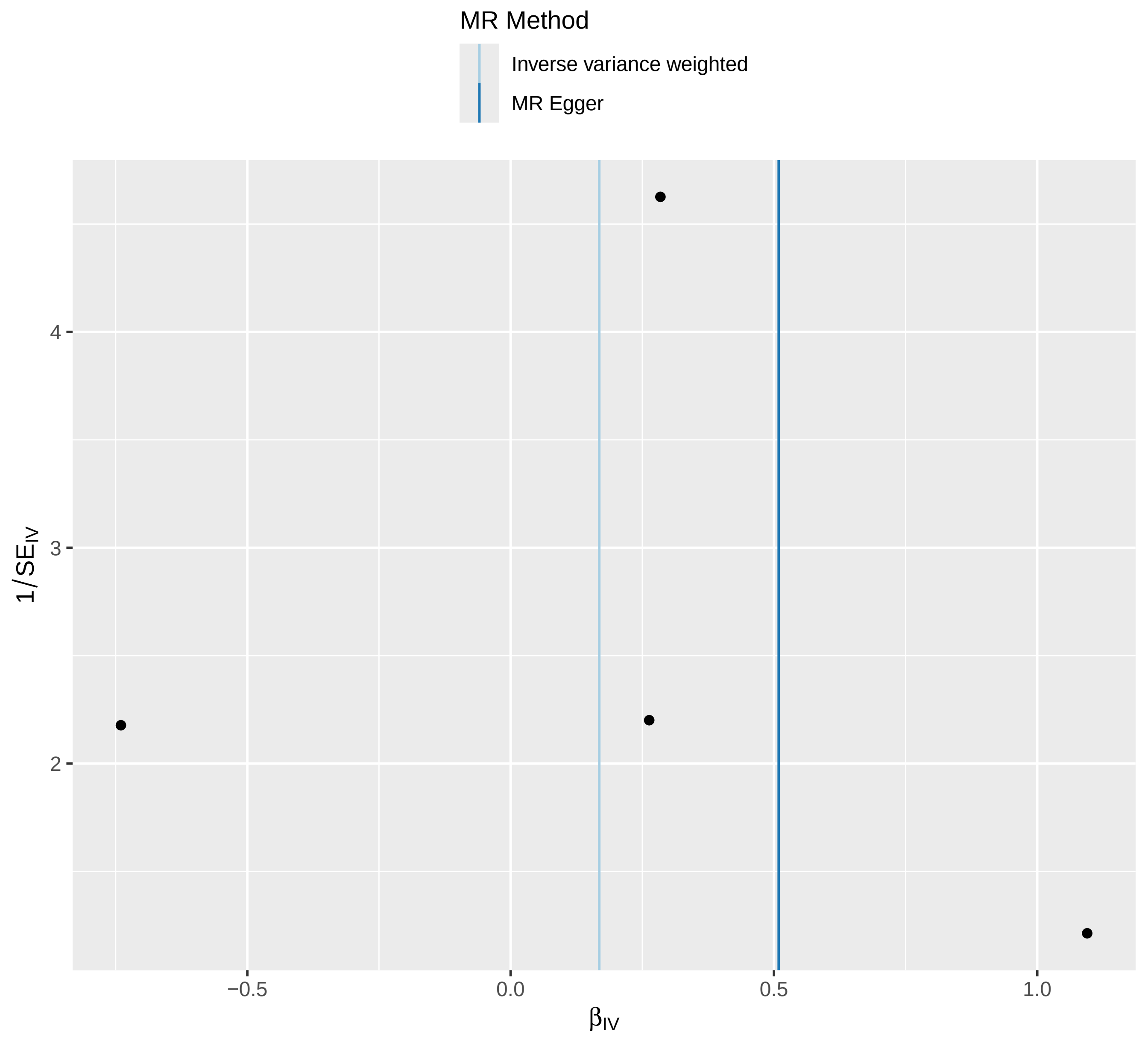
MR Method
Inverse variance weighted
MR Egger
4
3
2
1/SE$_{IV}$
−0.5
0.0
0.5
1.0
β$_{IV}$

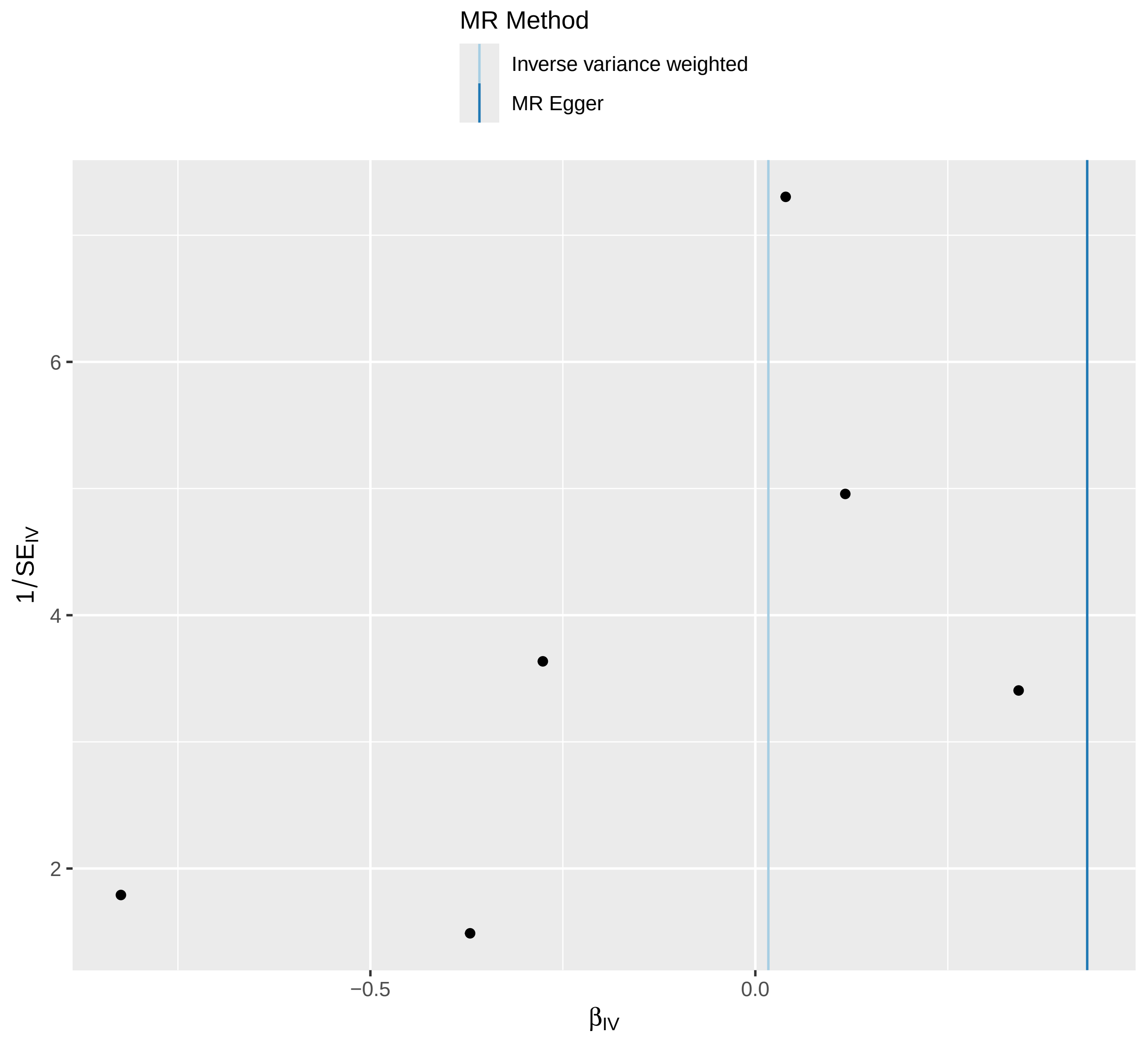
MR Method
Inverse variance weighted
MR Egger
6
4
2
1/SE_{IV}
−0.5
0.0
β_{IV}

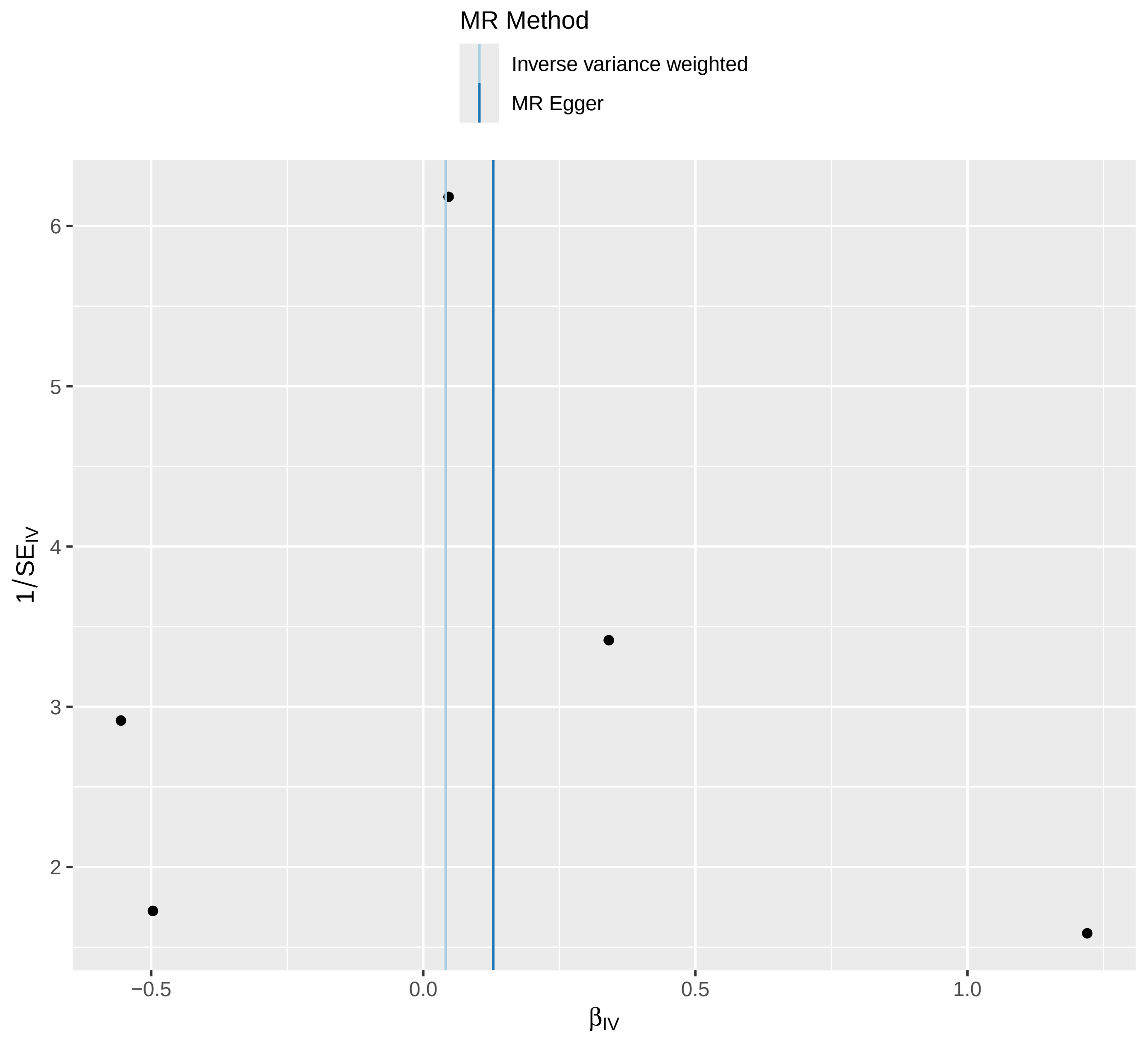

MR Method
Inverse variance weighted
MR Egger
6
5
4
3
2
1/SE_IV
−0.5
0.0
0.5
1.0
β_IV

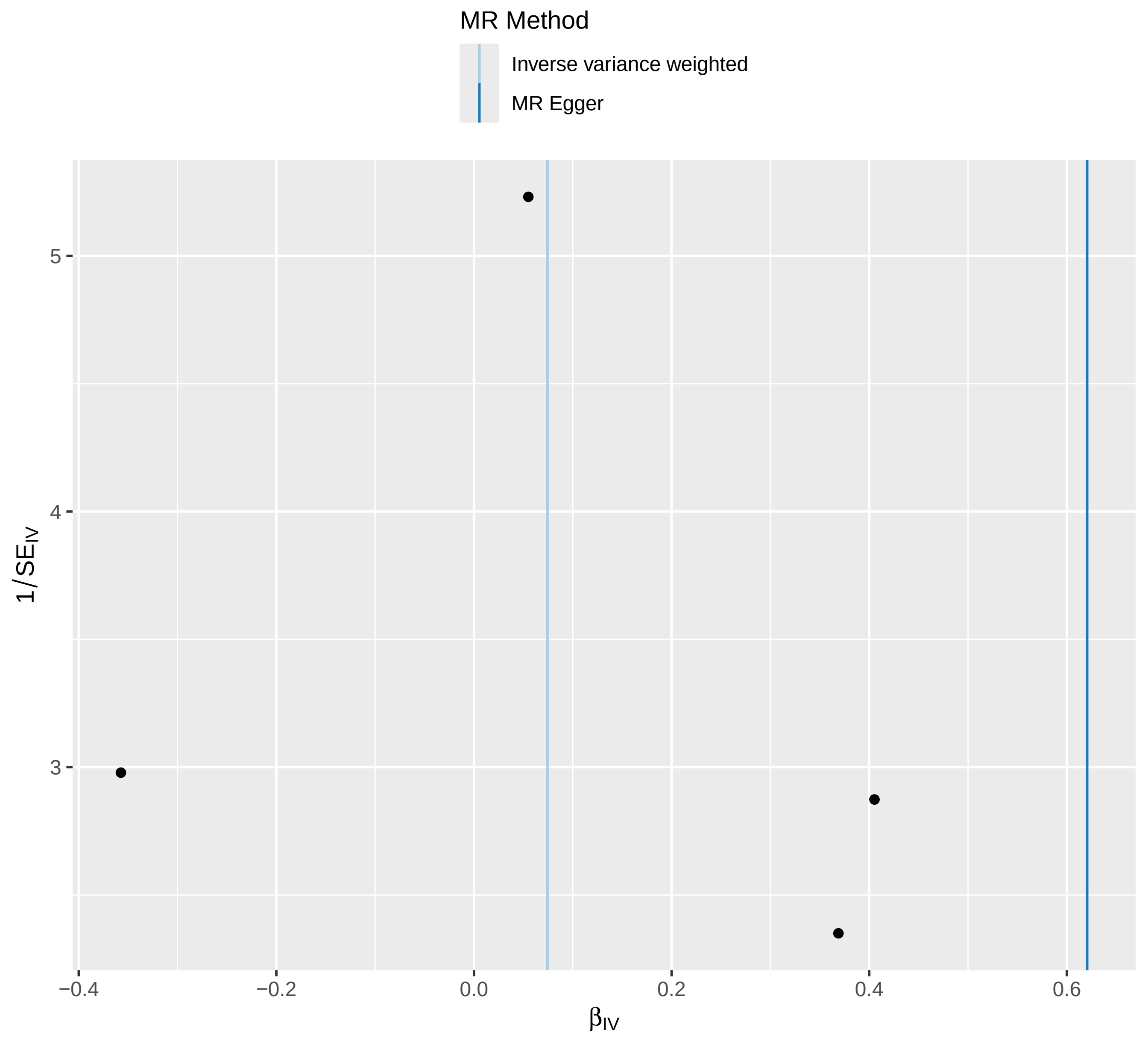
MR Method
Inverse variance weighted
MR Egger
5
4
3
$1/SE_{IV}$
−0.4
−0.2
0.0
0.2
0.4
0.6
β_{IV}

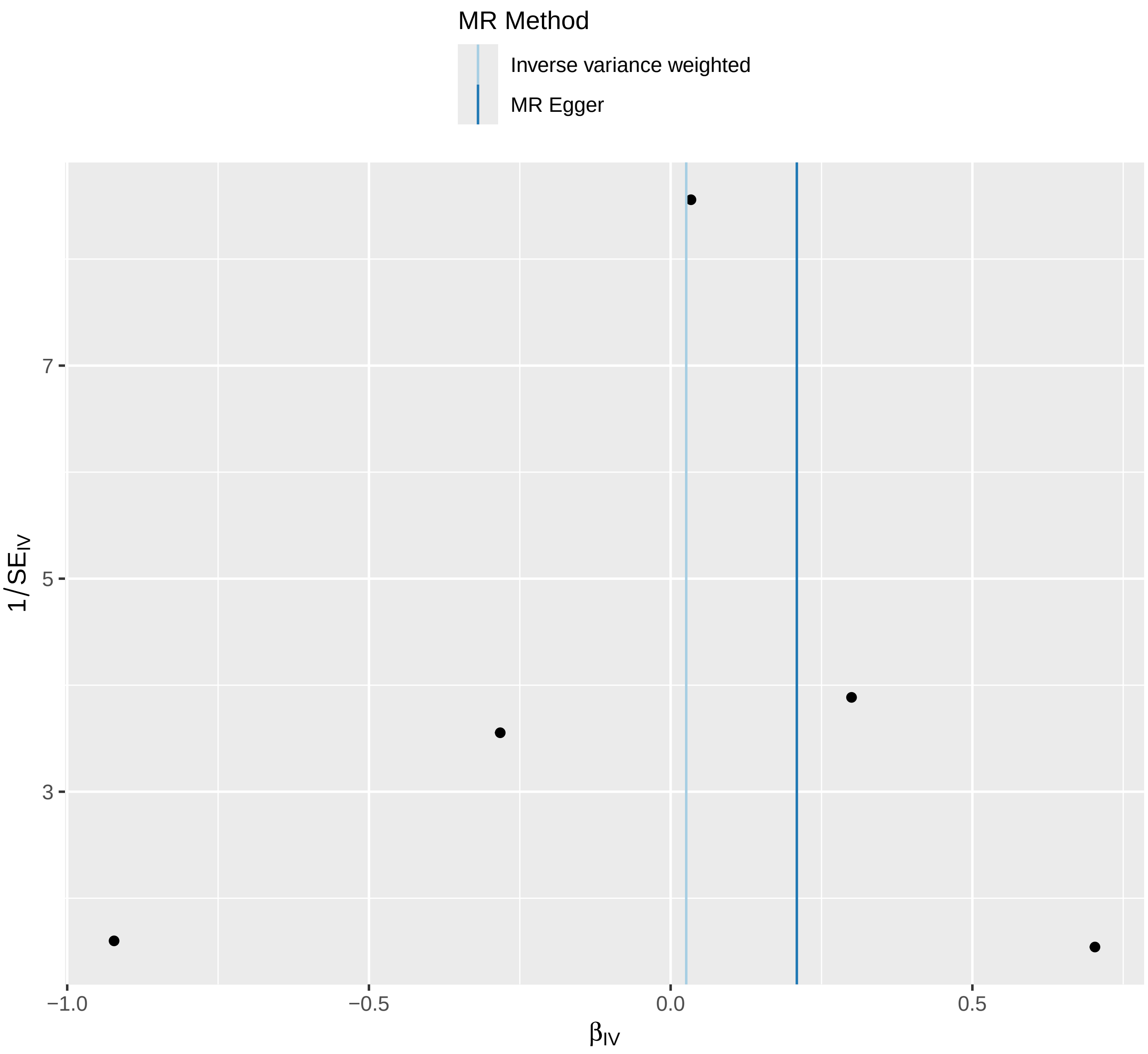
MR Method
Inverse variance weighted
MR Egger
7
5
3
$1/SE_{IV}$
−1.0
−0.5
0.0
0.5
β_{IV}

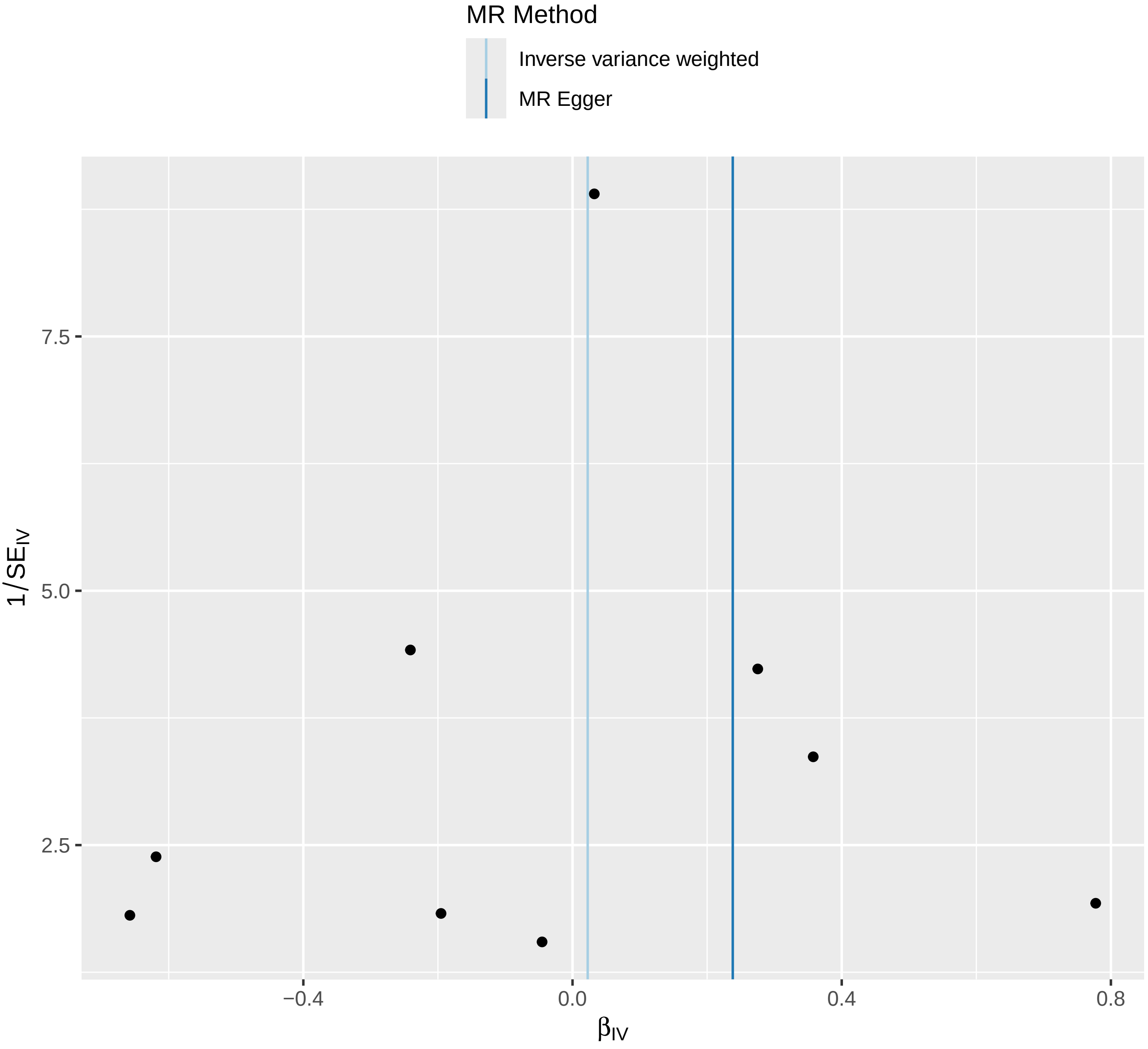
MR Method
Inverse variance weighted
MR Egger
7.5
5.0
2.5
1/SE$_{IV}$
−0.4
0.0
0.4
0.8
β$_{IV}$

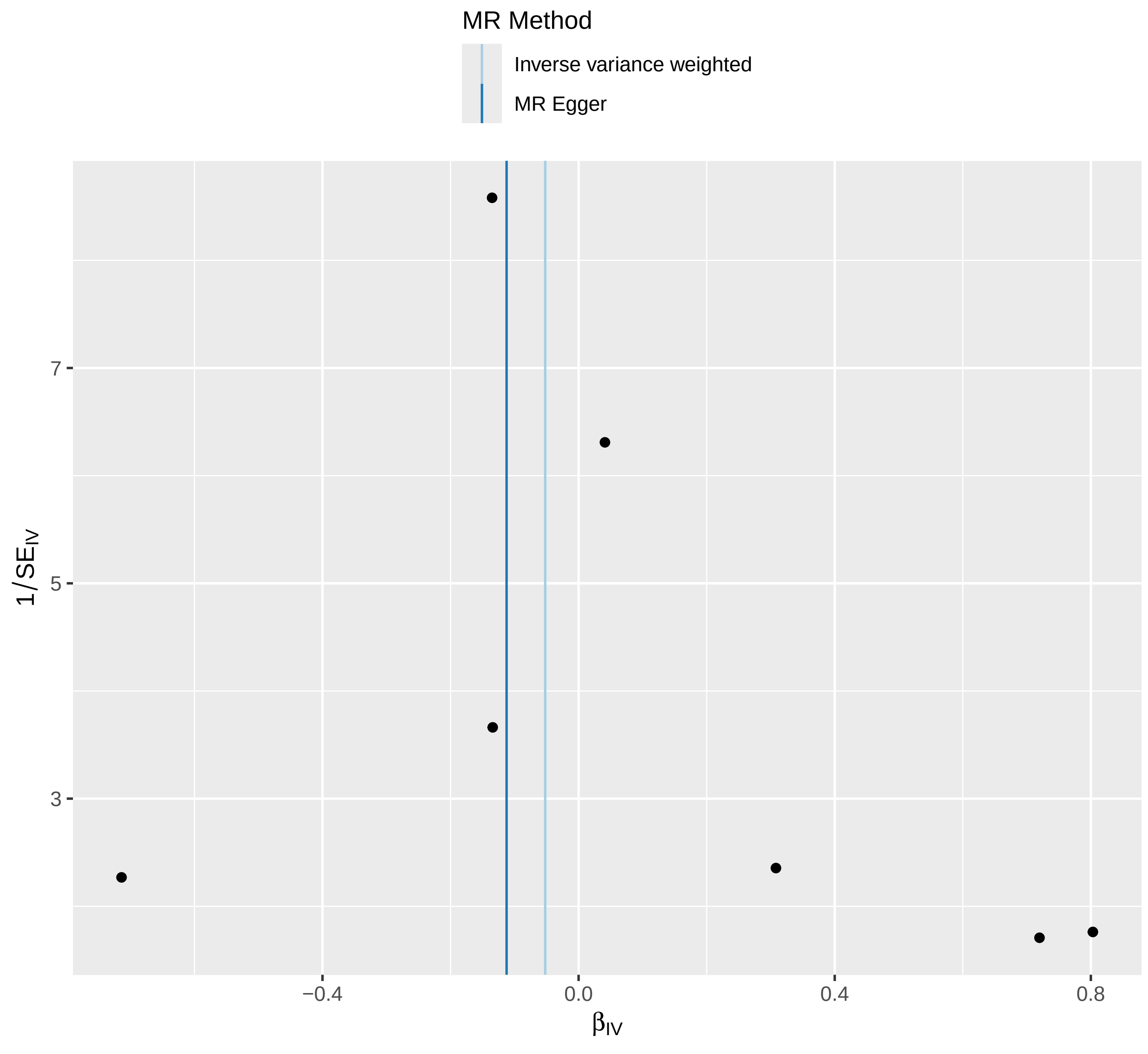
MR Method
Inverse variance weighted
MR Egger
7
5
3
1/SE_IV
−0.4
0.0
0.4
0.8
β_IV

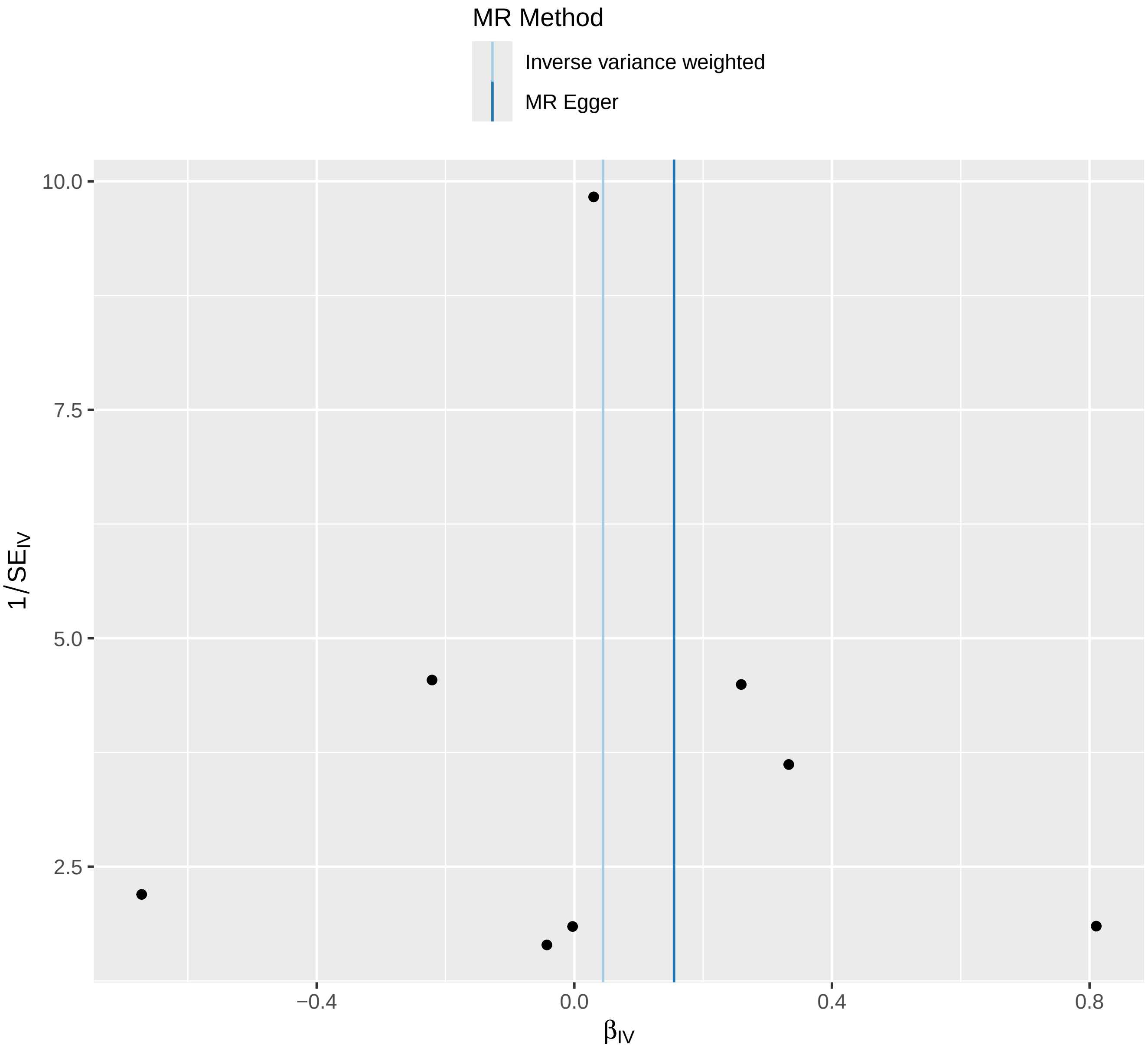
MR Method
Inverse variance weighted
MR Egger
10.0
7.5
5.0
2.5
1/SE$_{IV}$
−0.4
0.0
0.4
0.8
β$_{IV}$

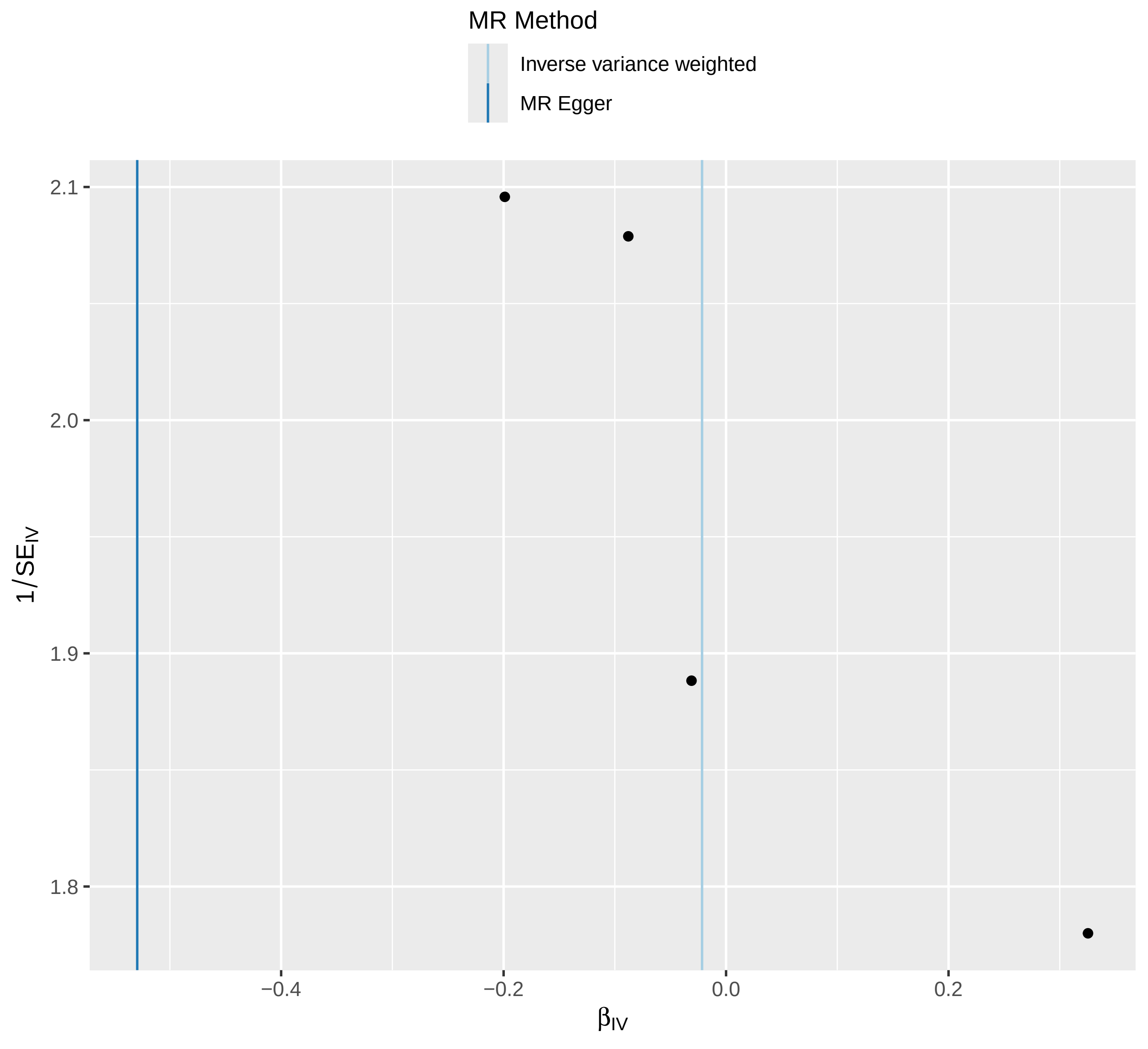

MR Method
Inverse variance weighted
MR Egger
2.1
2.0
1.9
1.8
$1/SE_{IV}$
−0.4
−0.2
0.0
0.2
β_{IV}

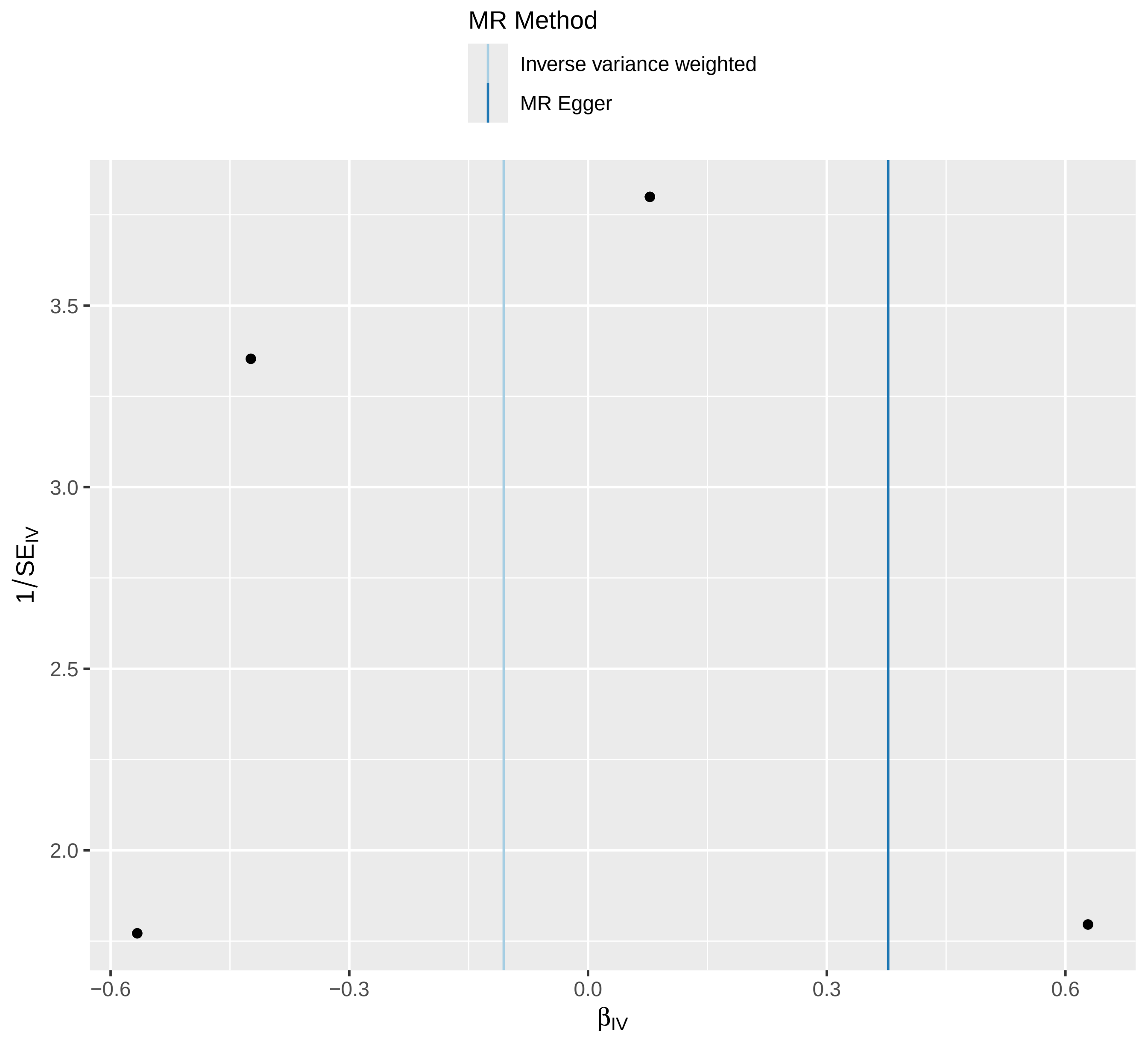
MR Method
Inverse variance weighted
MR Egger
3.5
3.0
2.5
2.0
1/SE$_{IV}$
−0.6
−0.3
0.0
0.3
0.6
β$_{IV}$

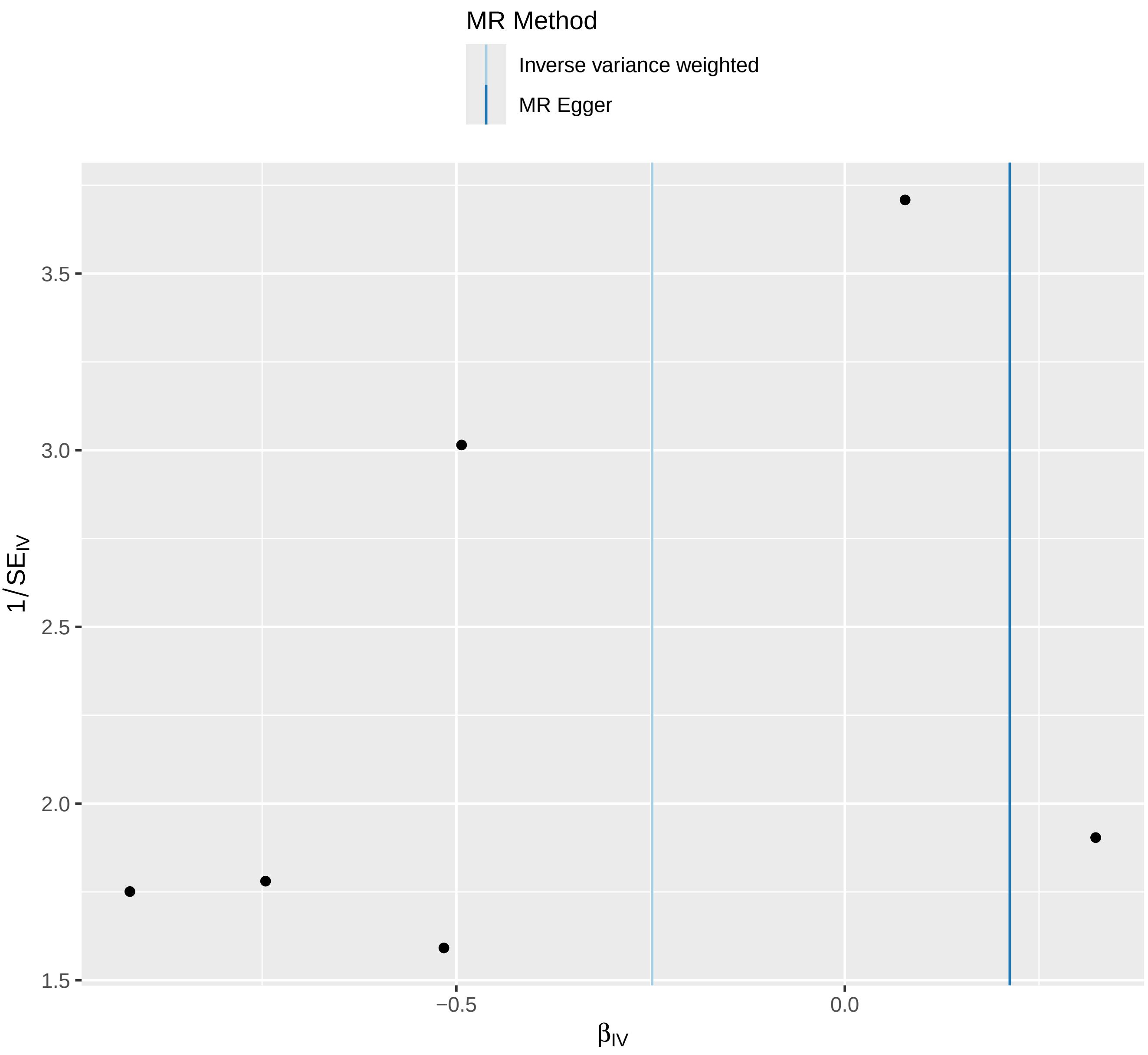
MR Method
Inverse variance weighted
MR Egger
3.5
3.0
2.5
2.0
1.5
$1/SE_{IV}$
−0.5
0.0
β_{IV}

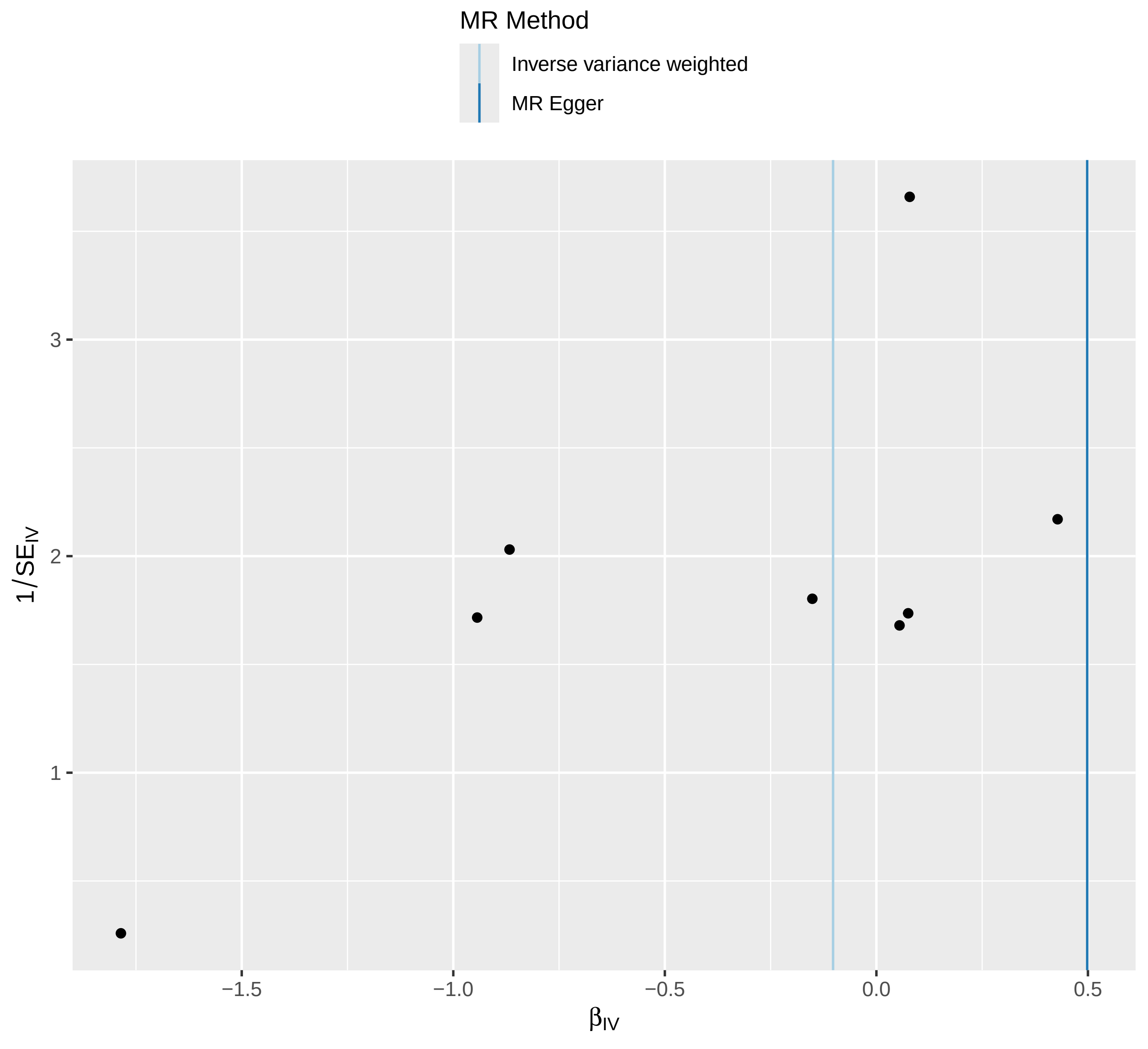

MR Method
Inverse variance weighted
MR Egger
3
2
1
$1/SE_{IV}$
−1.5
−1.0
−0.5
0.0
0.5
β_{IV}

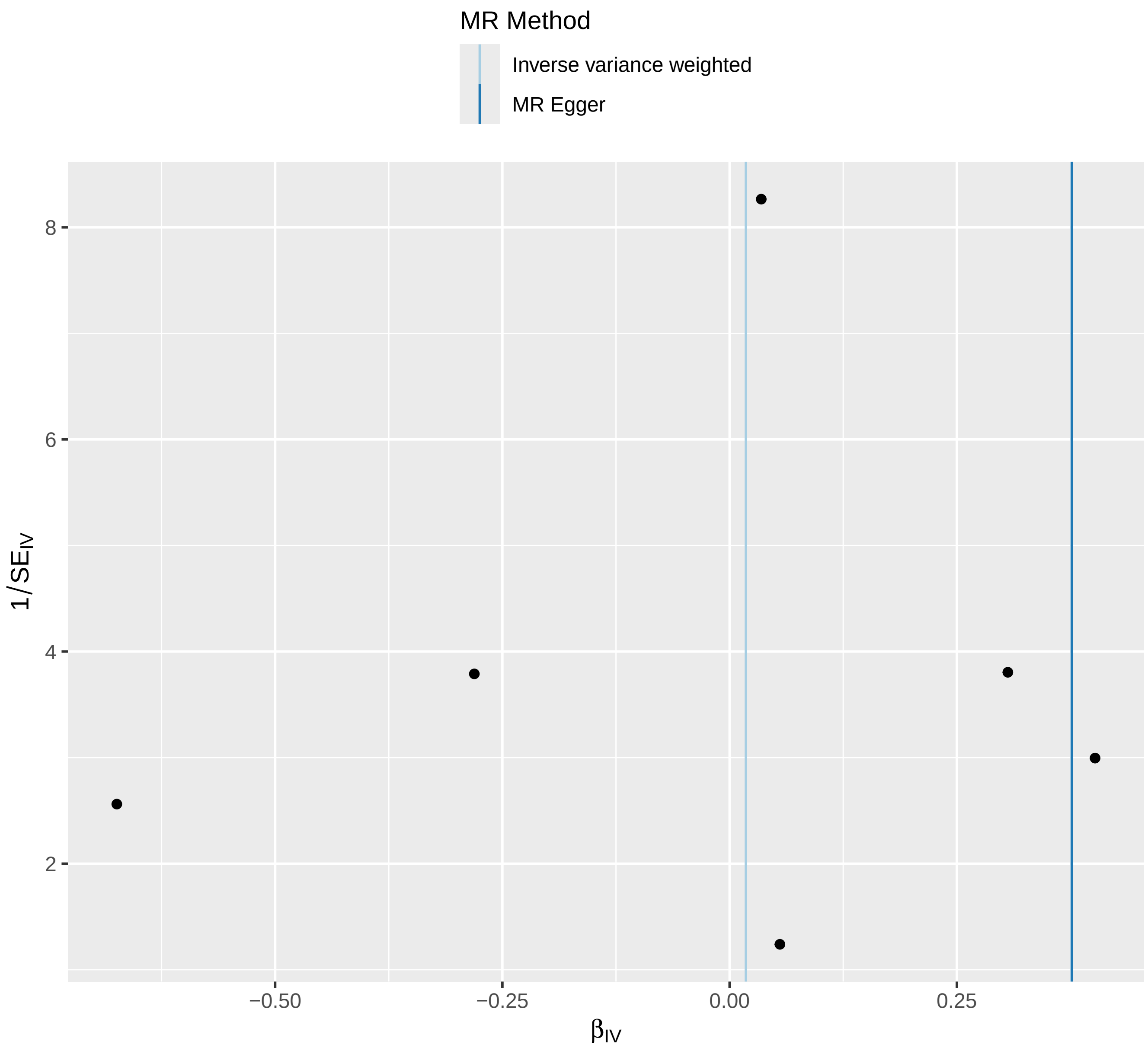

MR Method
Inverse variance weighted
MR Egger
8
6
4
2
$1/SE_{IV}$
−0.50
−0.25
0.00
0.25
β_{IV}

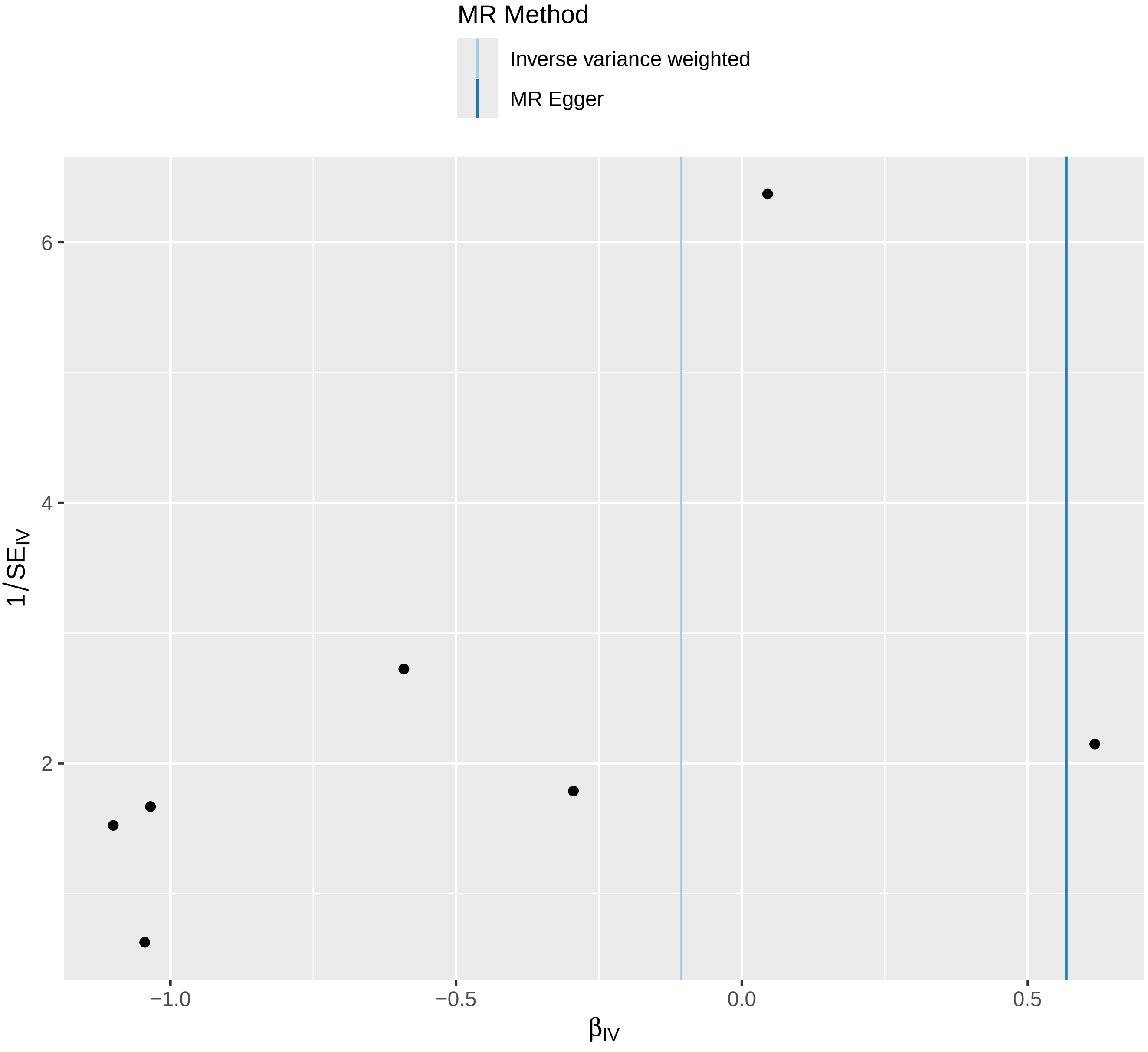

MR Method
Inverse variance weighted
MR Egger
6
4
2
1/SE_IV
−1.0
−0.5
0.0
0.5
β_IV

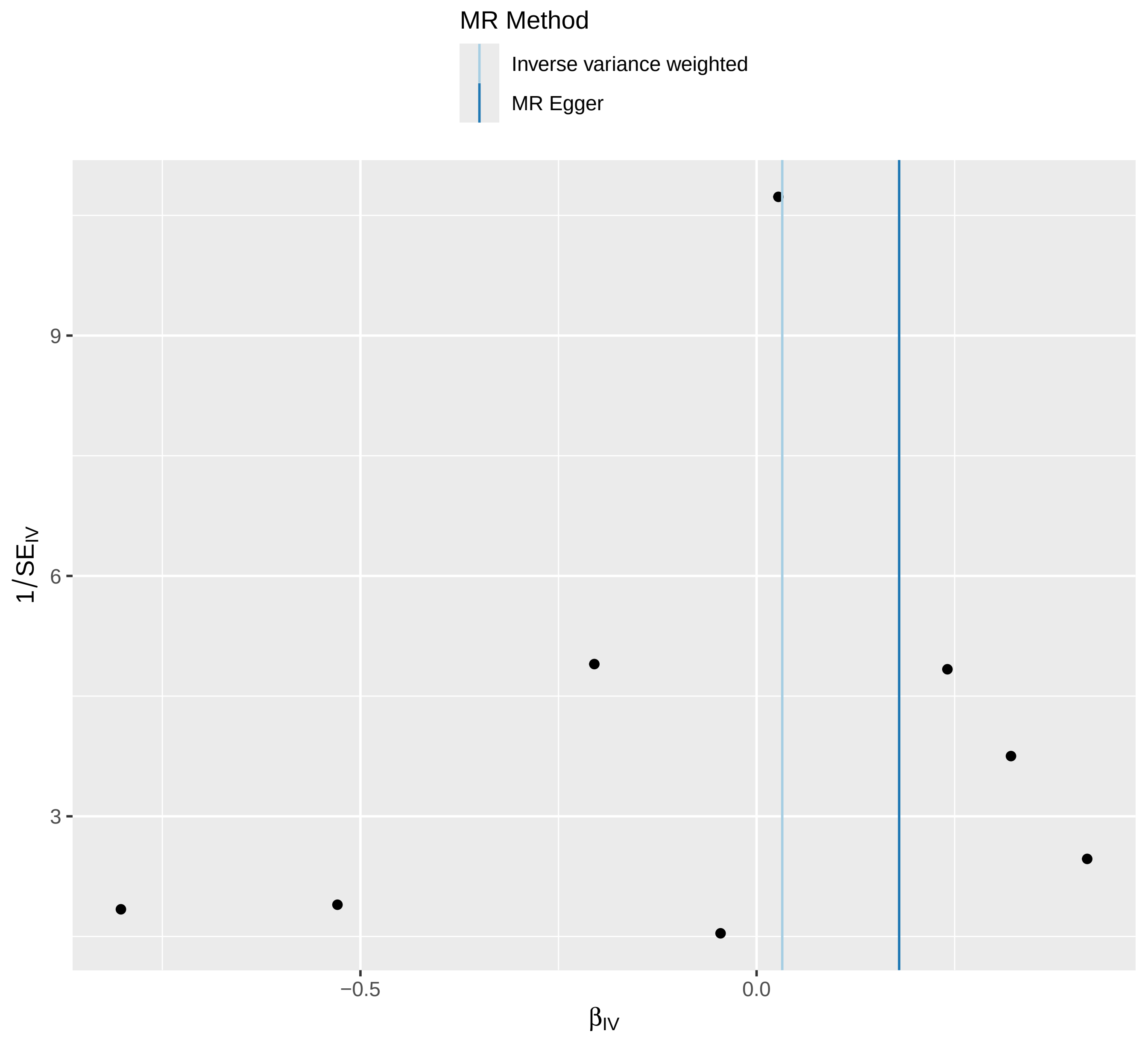
MR Method
Inverse variance weighted
MR Egger
9
6
3
1/SE$_{IV}$
−0.5
0.0
β$_{IV}$

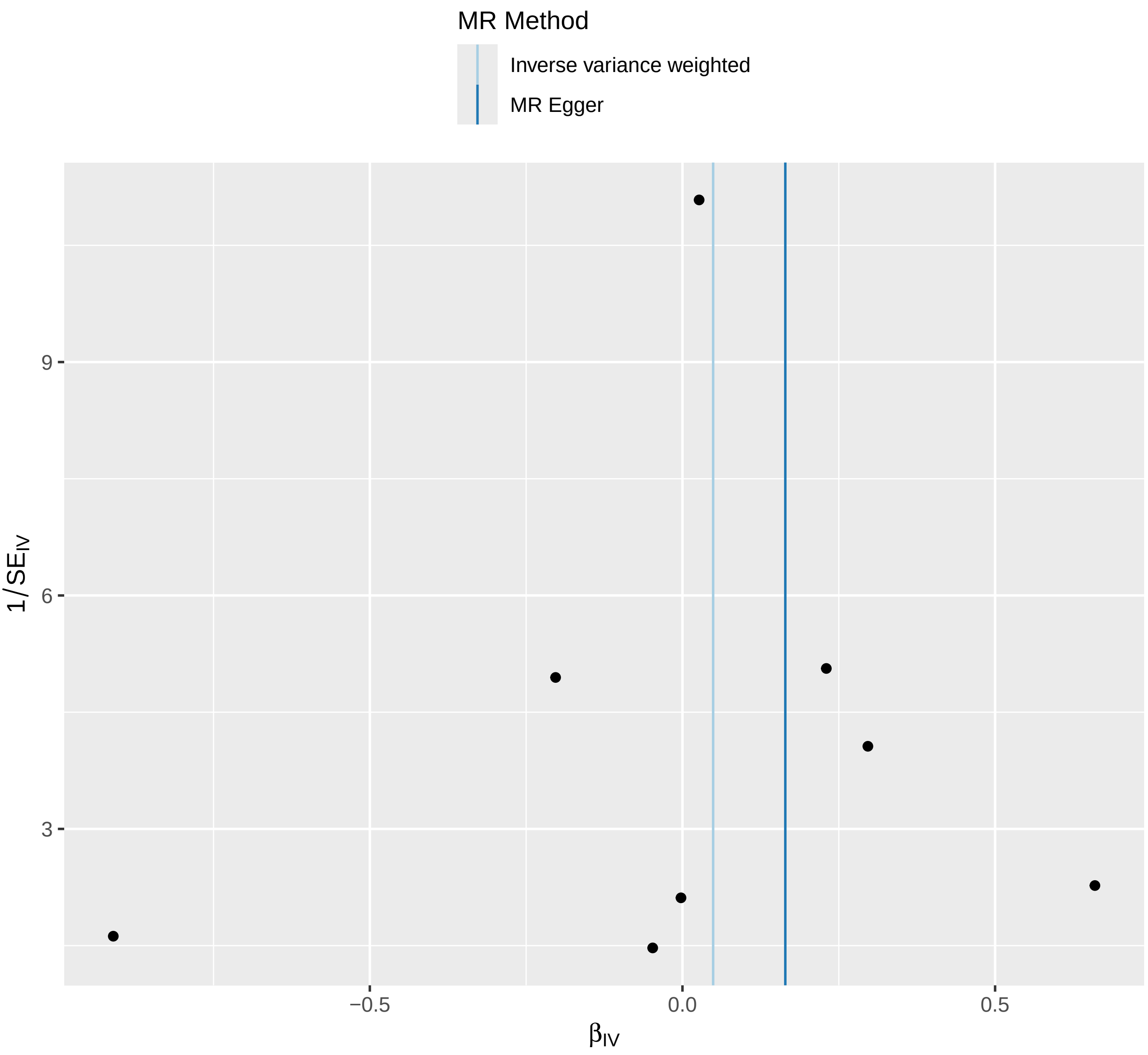
MR Method
Inverse variance weighted
MR Egger
9
6
3
1/SE$_{IV}$
−0.5
0.0
0.5
β$_{IV}$

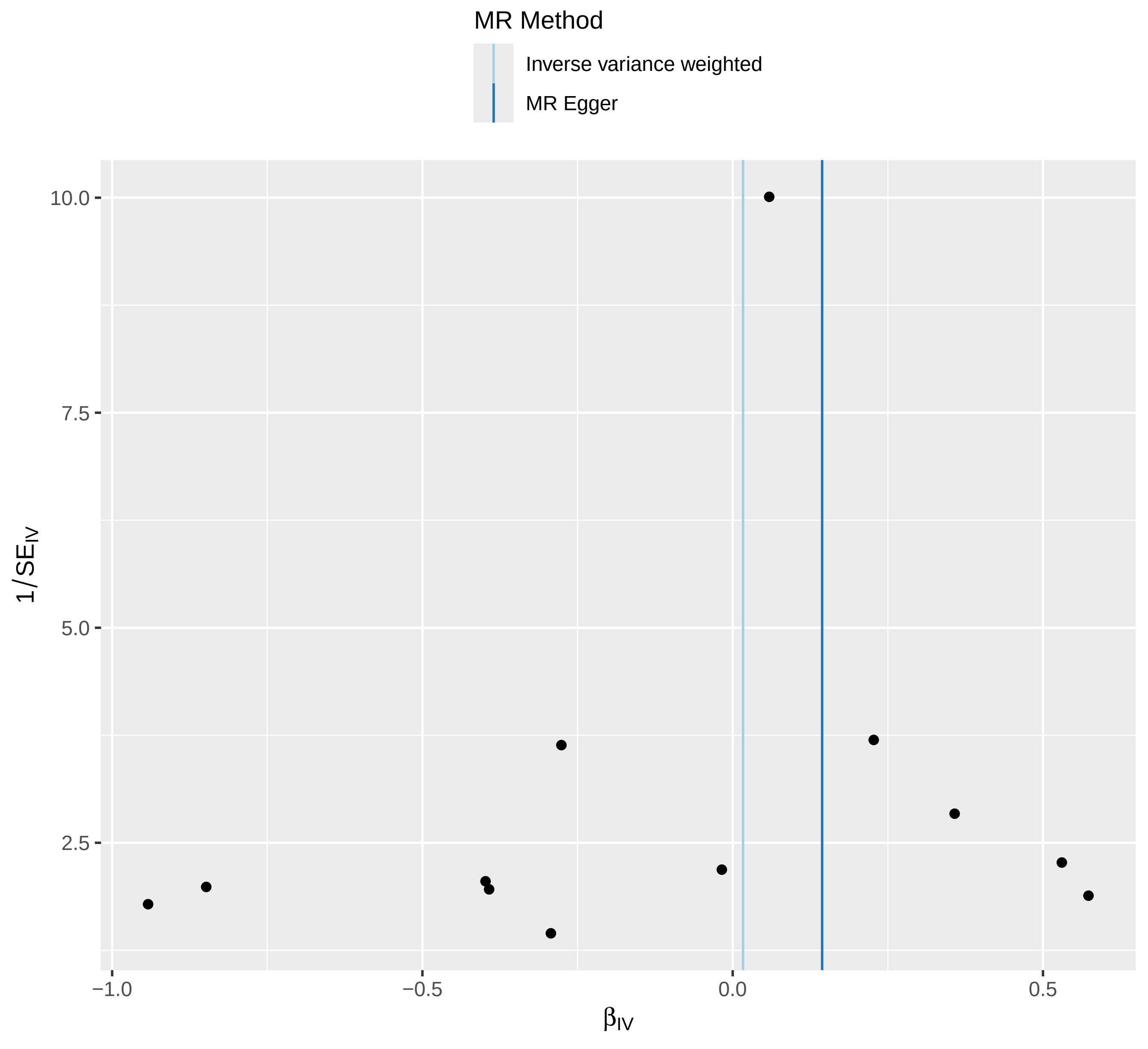
MR Method
Inverse variance weighted
MR Egger
10.0
7.5
5.0
2.5
1/SE_IV
−1.0
−0.5
0.0
0.5
β_IV

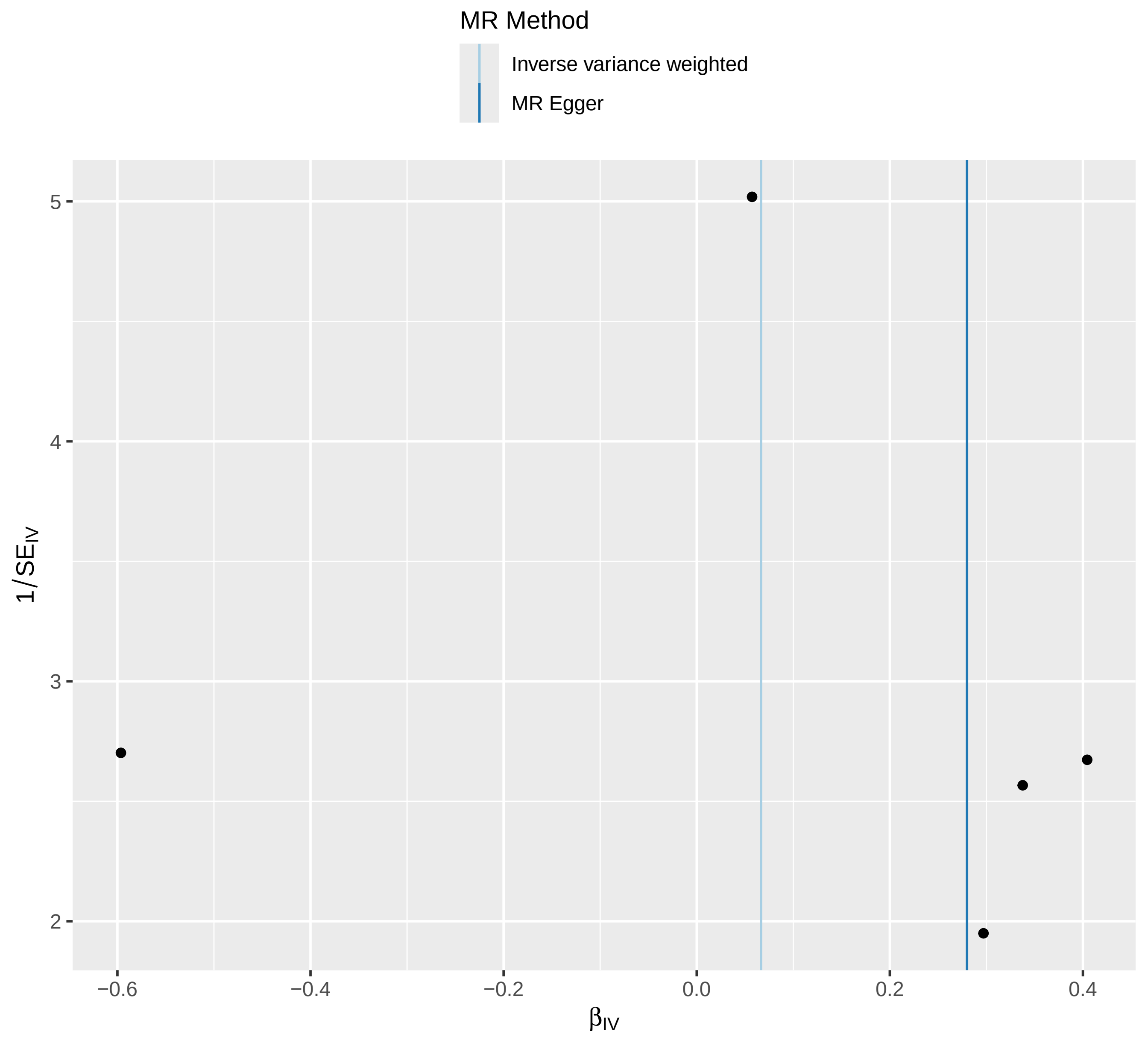

MR Method
Inverse variance weighted
MR Egger
5
4
3
2
1/SE$_{IV}$
−0.6
−0.4
−0.2
0.0
0.2
0.4
β$_{IV}$

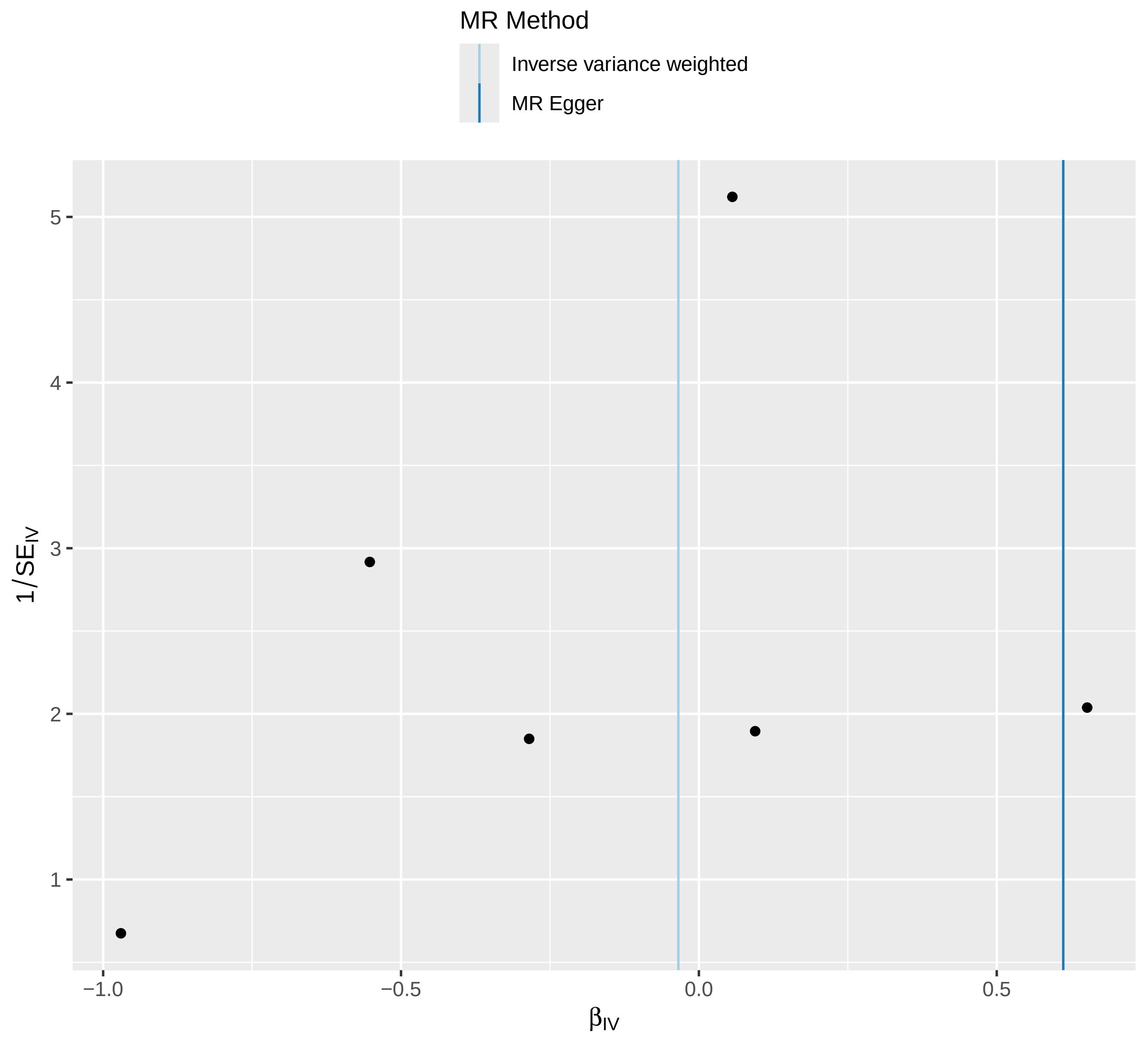

MR Method
Inverse variance weighted
MR Egger
5
4
3
2
1
1/SE_IV
−1.0
−0.5
0.0
0.5
β_IV

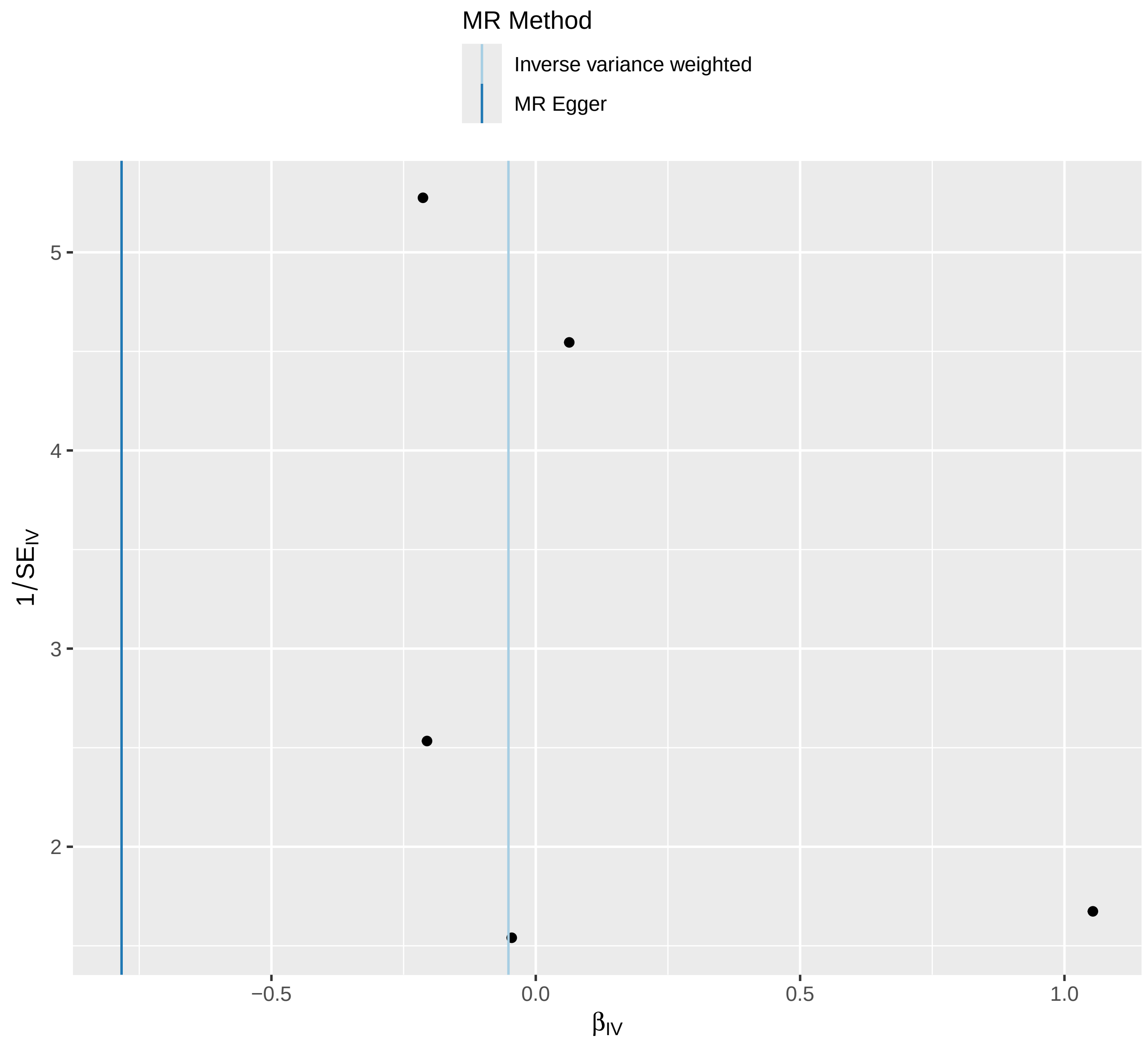
MR Method
Inverse variance weighted
MR Egger
5
4
3
2
1/SE_IV
−0.5
0.0
0.5
1.0
β_IV

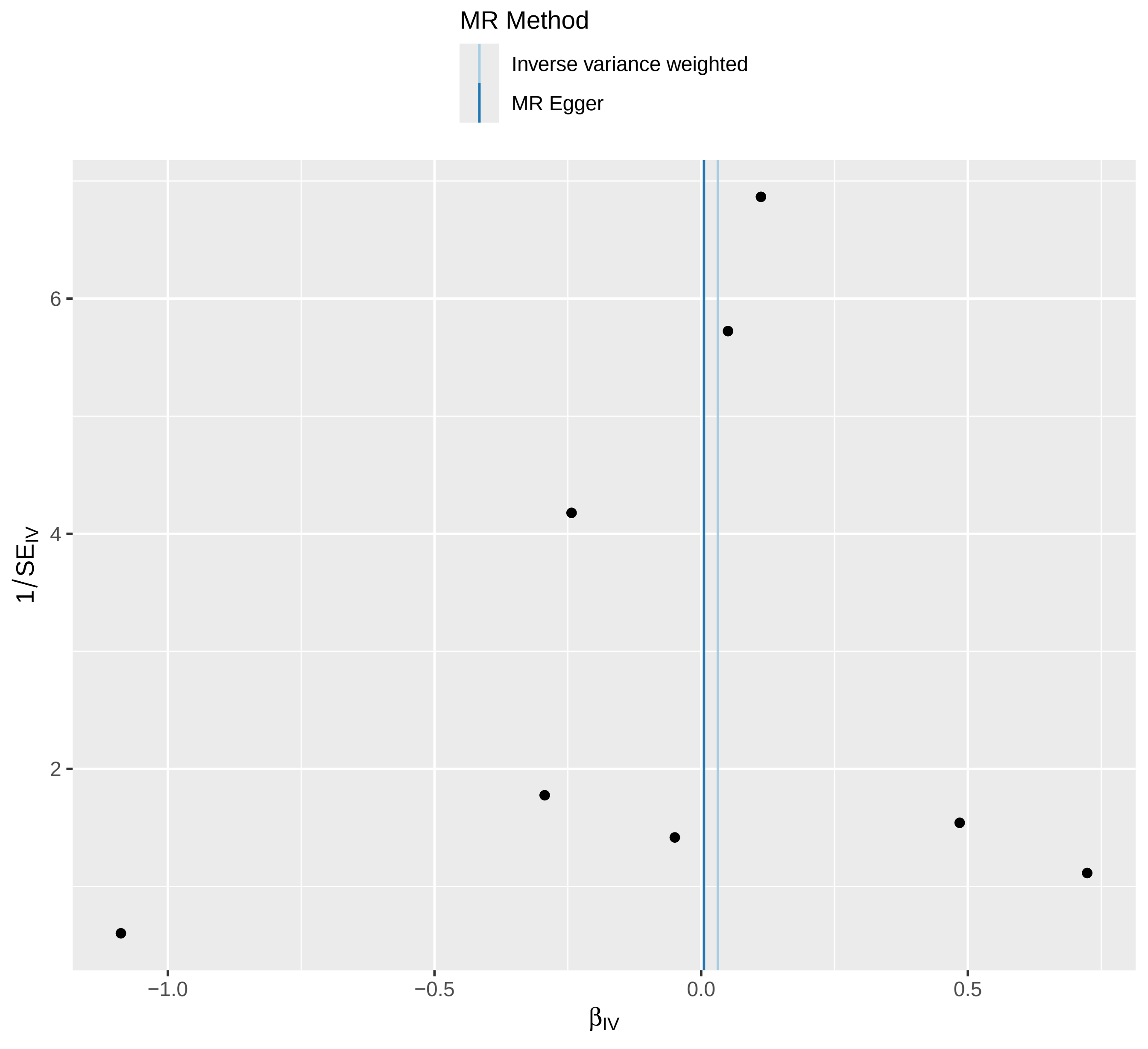

MR Method
Inverse variance weighted
MR Egger
6
4
2
1/SE_IV
−1.0
−0.5
0.0
0.5
β_IV

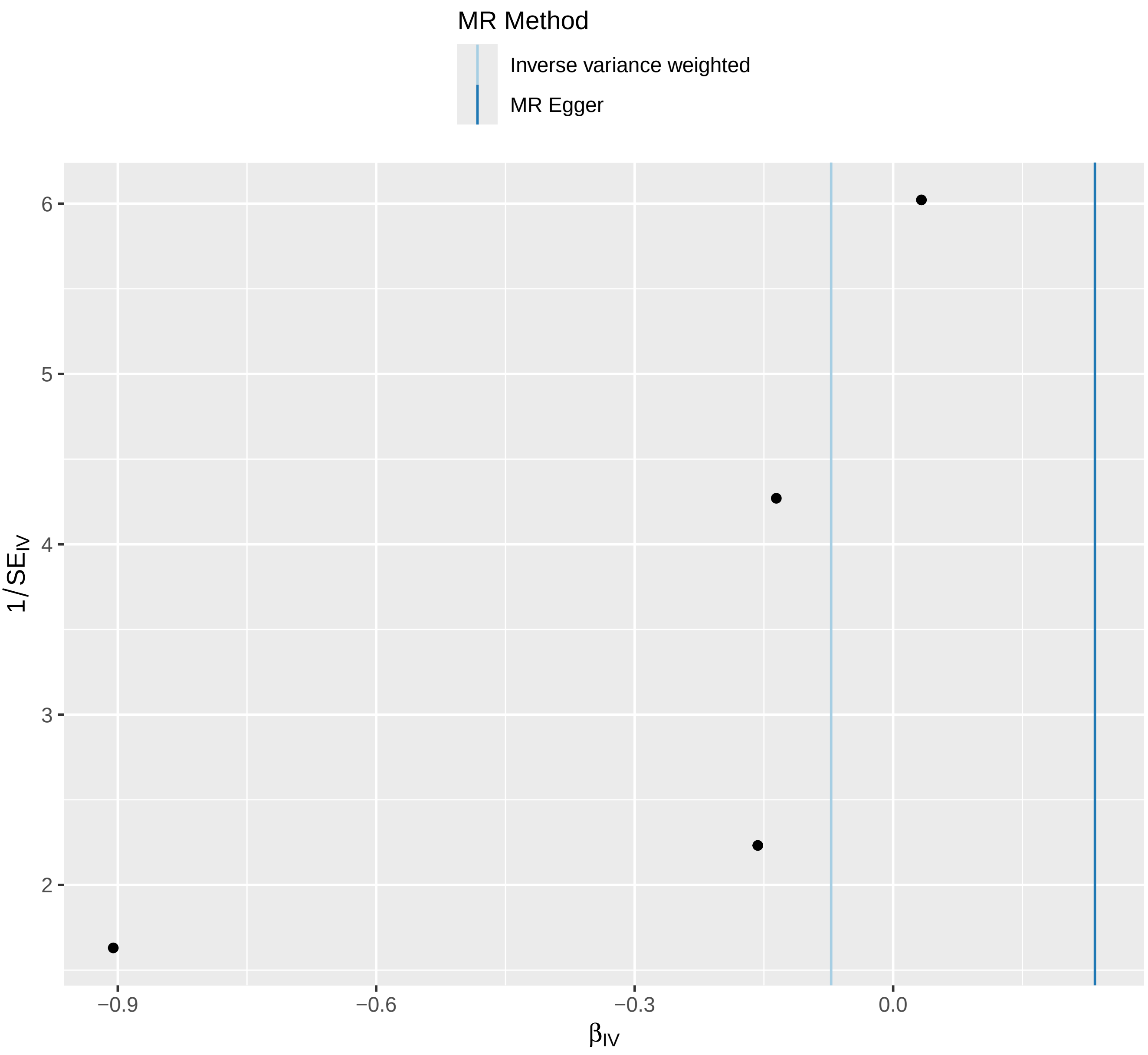
MR Method
Inverse variance weighted
MR Egger
6
5
4
3
2
1/SE$_{IV}$
−0.9
−0.6
−0.3
0.0
β$_{IV}$

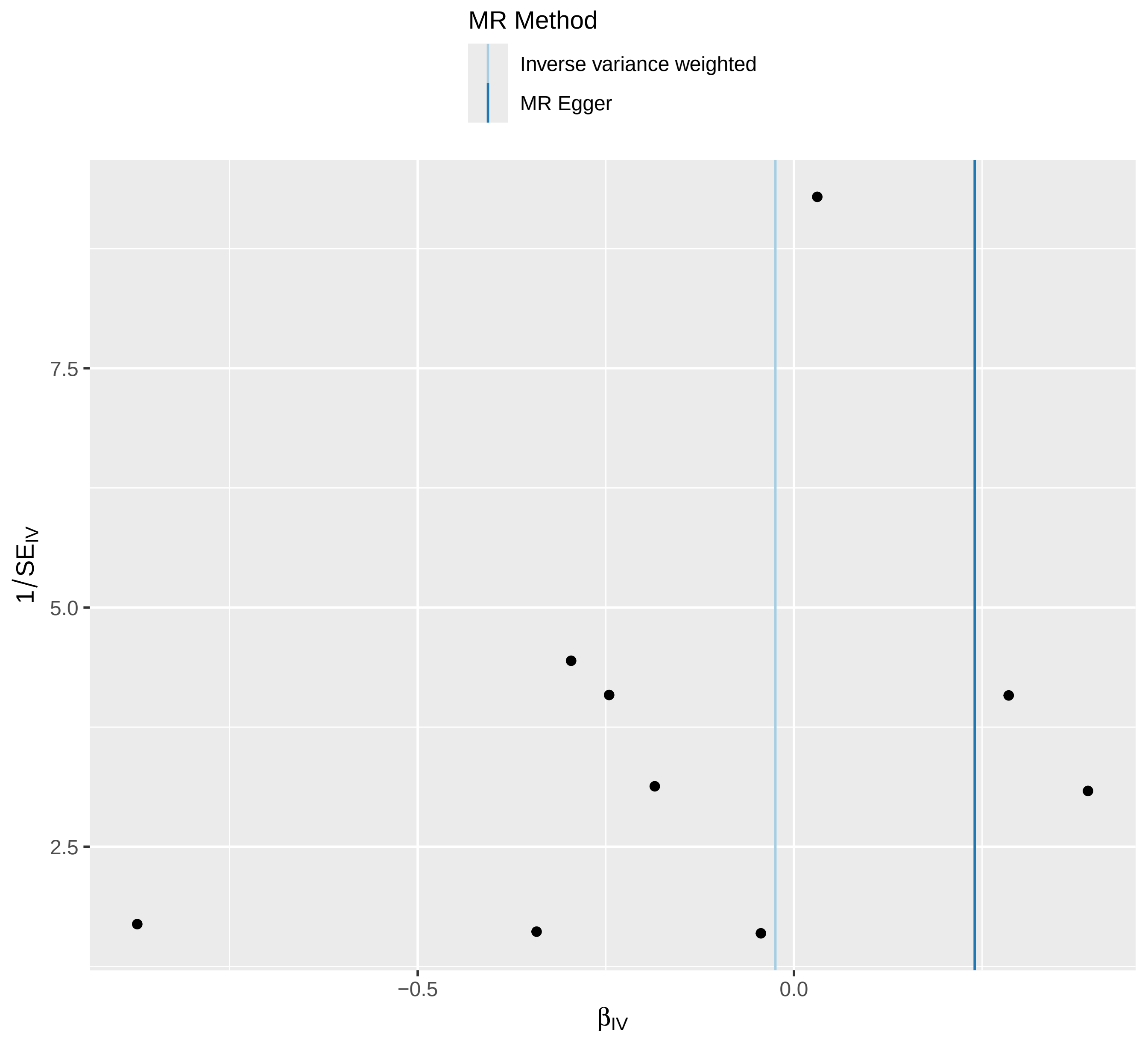

MR Method
Inverse variance weighted
MR Egger
7.5
5.0
2.5
−0.5
0.0
1/SE_IV
β_IV

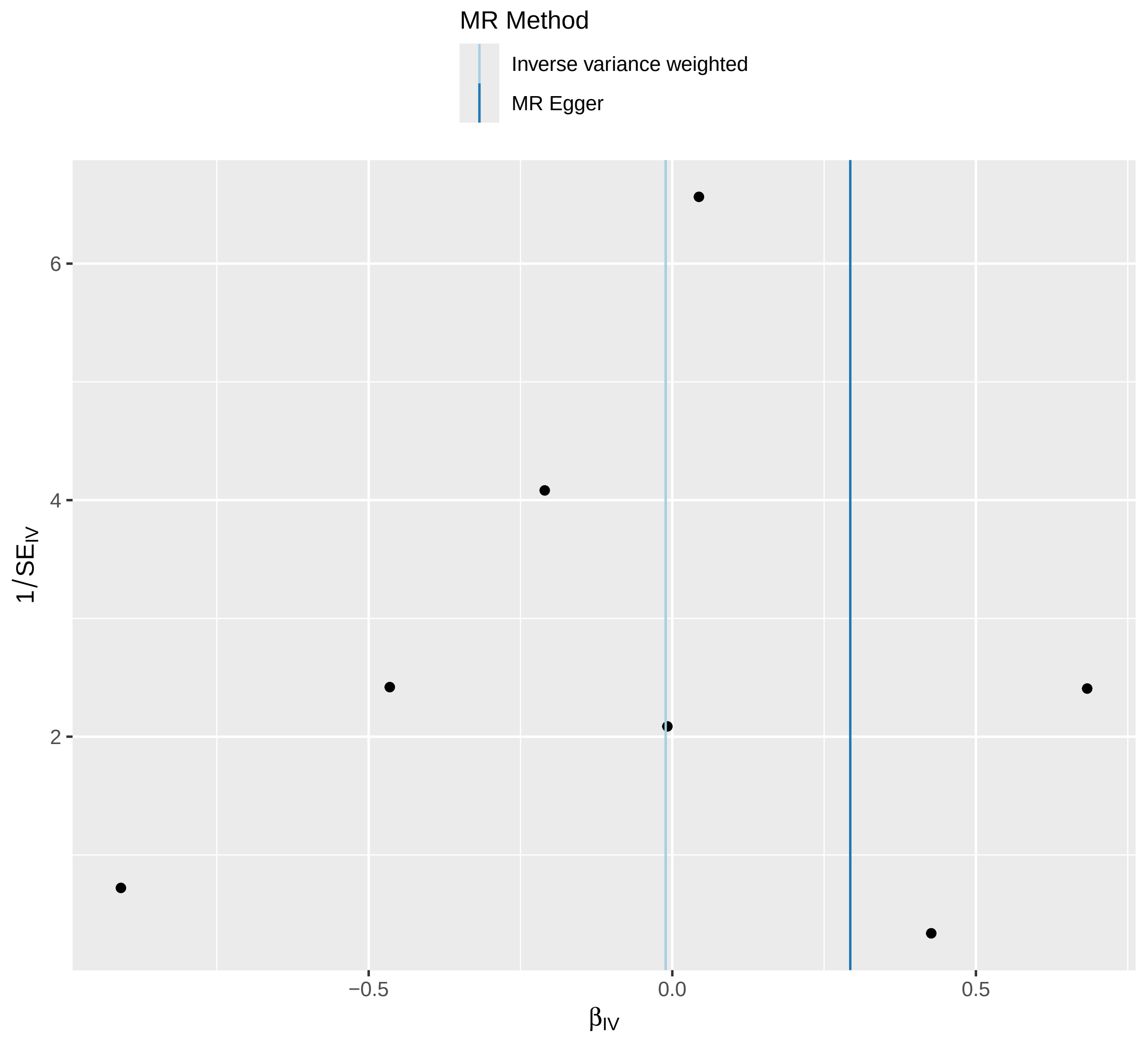

MR Method
Inverse variance weighted
MR Egger
6
4
2
1/SE_IV
−0.5
0.0
0.5
β_IV

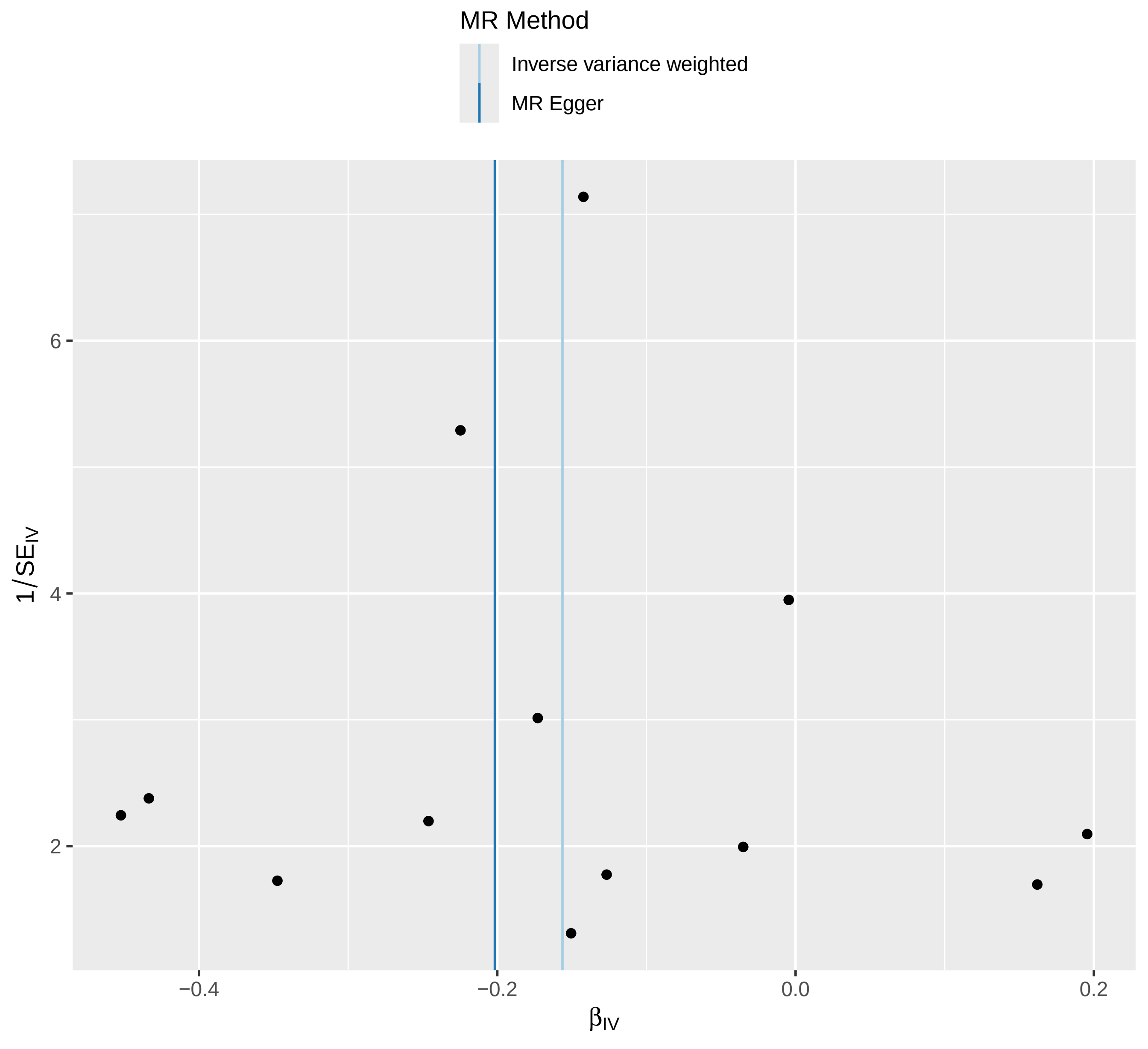

MR Method
Inverse variance weighted
MR Egger
6
4
2
1/SE_IV
−0.4
−0.2
0.0
0.2
β_IV

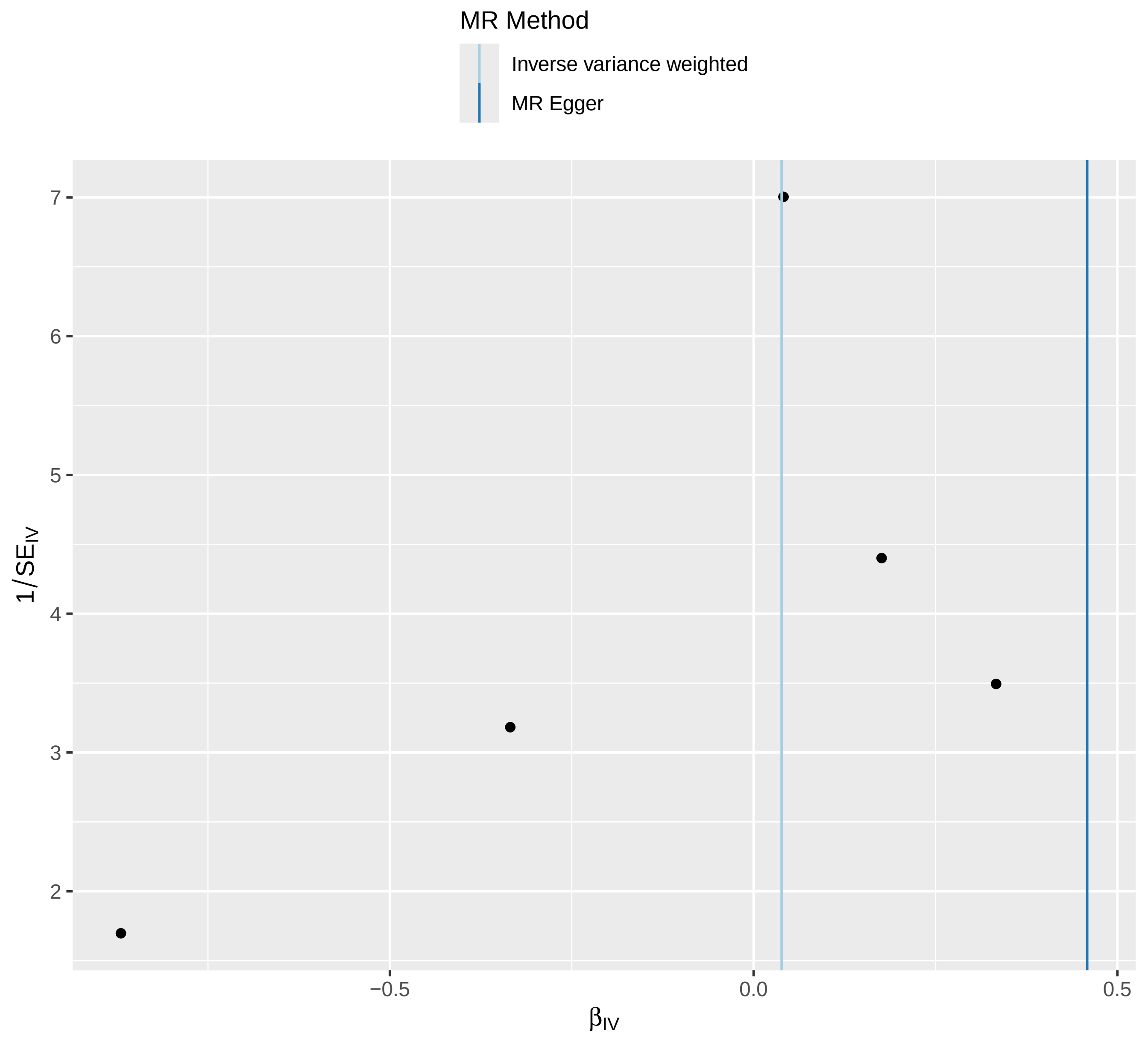

MR Method
Inverse variance weighted
MR Egger
7
6
5
4
3
2
1/SE_IV
−0.5
0.0
0.5
β_IV

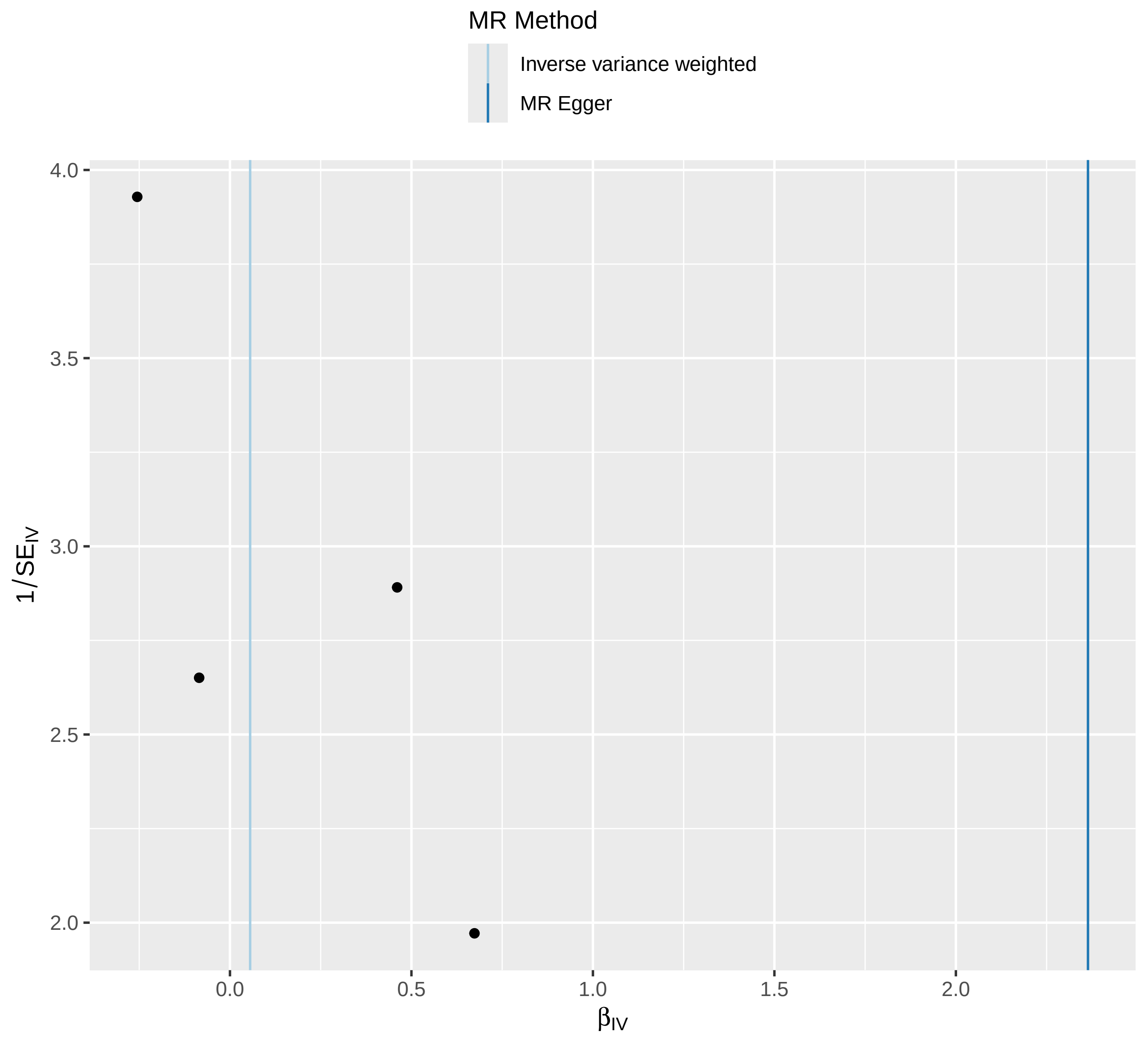

MR Method
Inverse variance weighted
MR Egger
4.0
3.5
3.0
2.5
2.0
$1/SE_{IV}$
0.0
0.5
1.0
1.5
2.0
β_{IV}

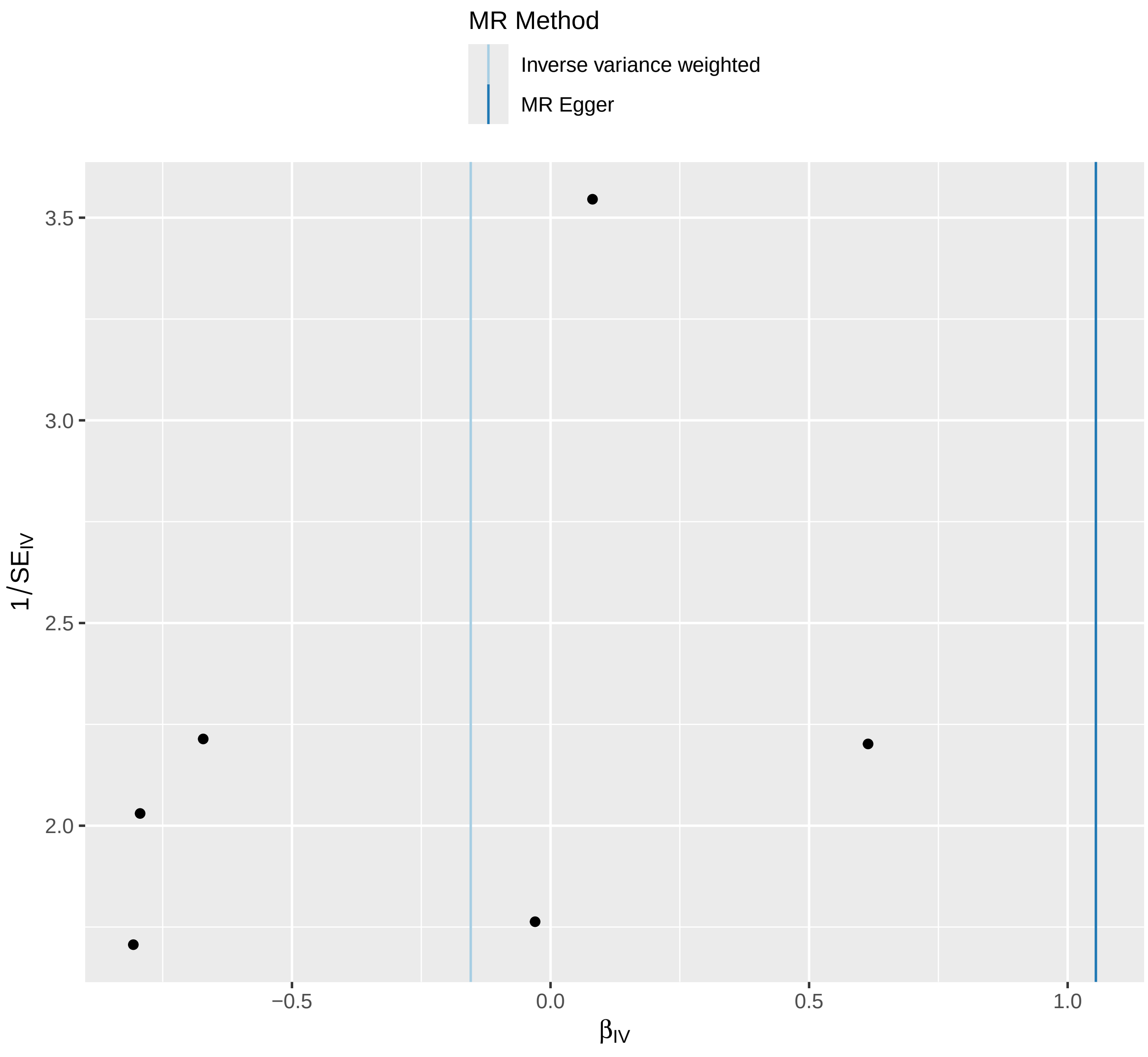
MR Method
Inverse variance weighted
MR Egger
3.5
3.0
2.5
2.0
$1/SE_{IV}$
−0.5
0.0
0.5
1.0
β_{IV}

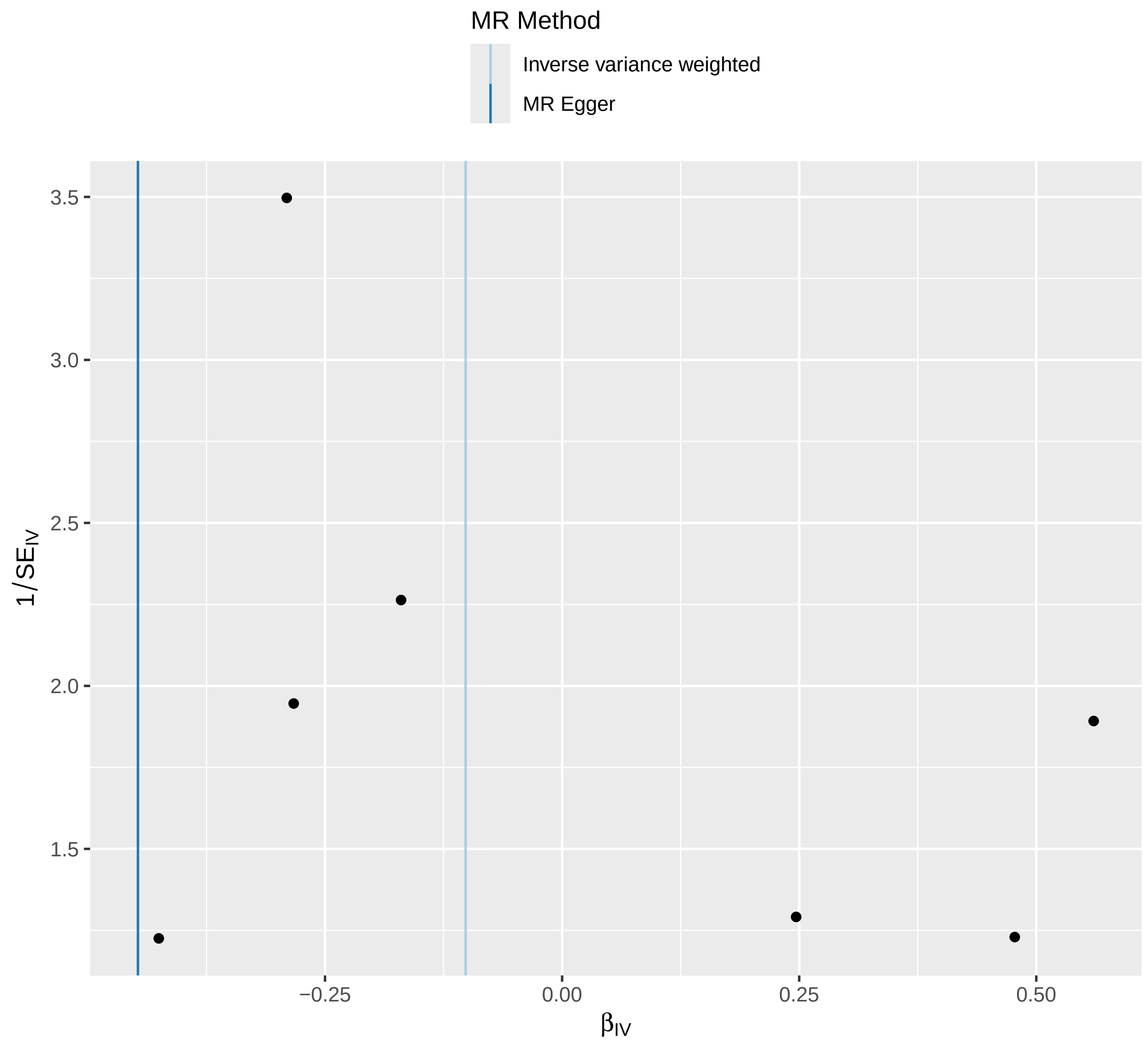

MR Method
Inverse variance weighted
MR Egger
3.5
3.0
2.5
2.0
1.5
1/SE_IV
−0.25
0.00
0.25
0.50
β_IV

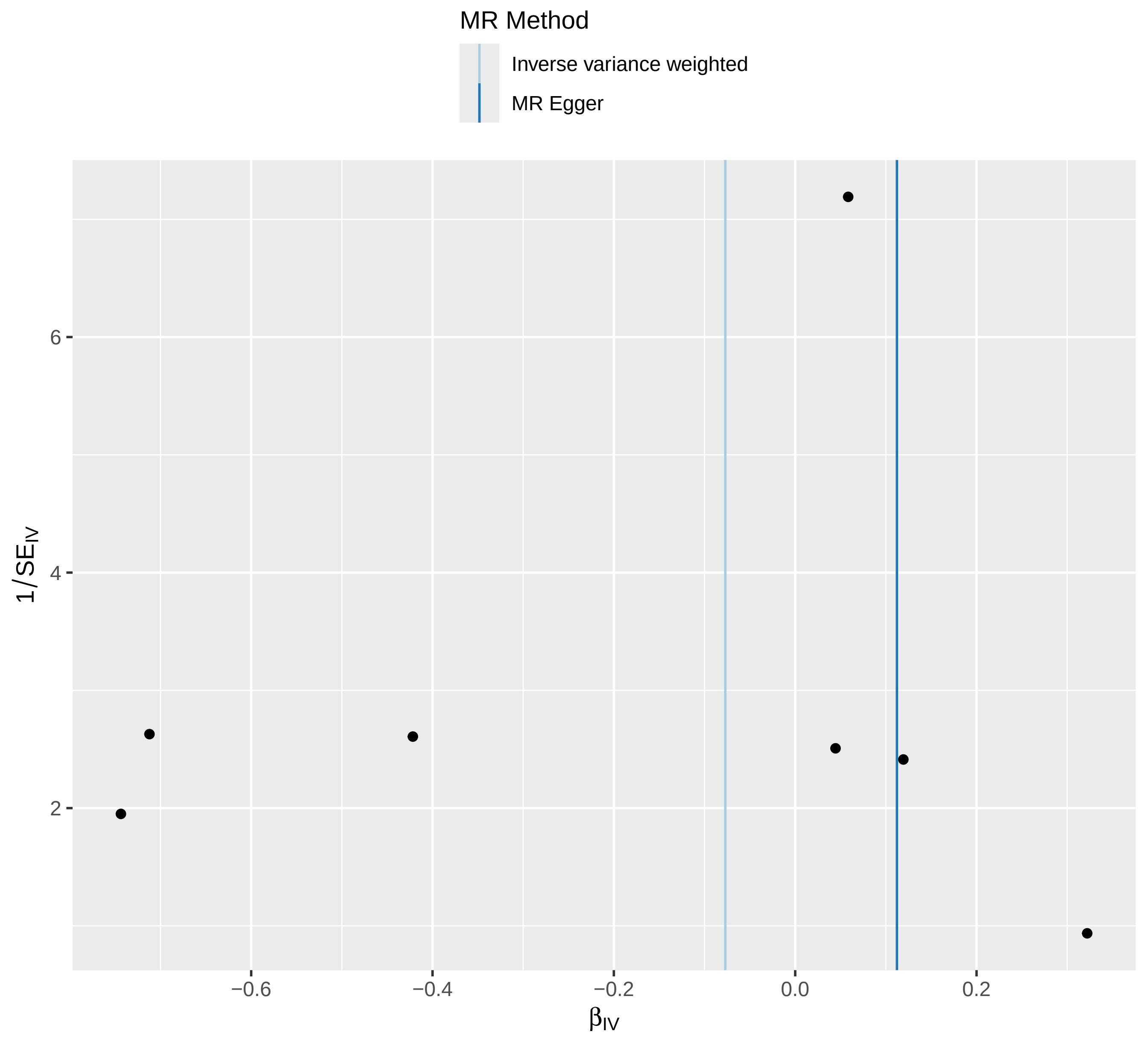

MR Method
Inverse variance weighted
MR Egger
6
4
2
1/SE$_{IV}$
−0.6
−0.4
−0.2
0.0
0.2
β$_{IV}$

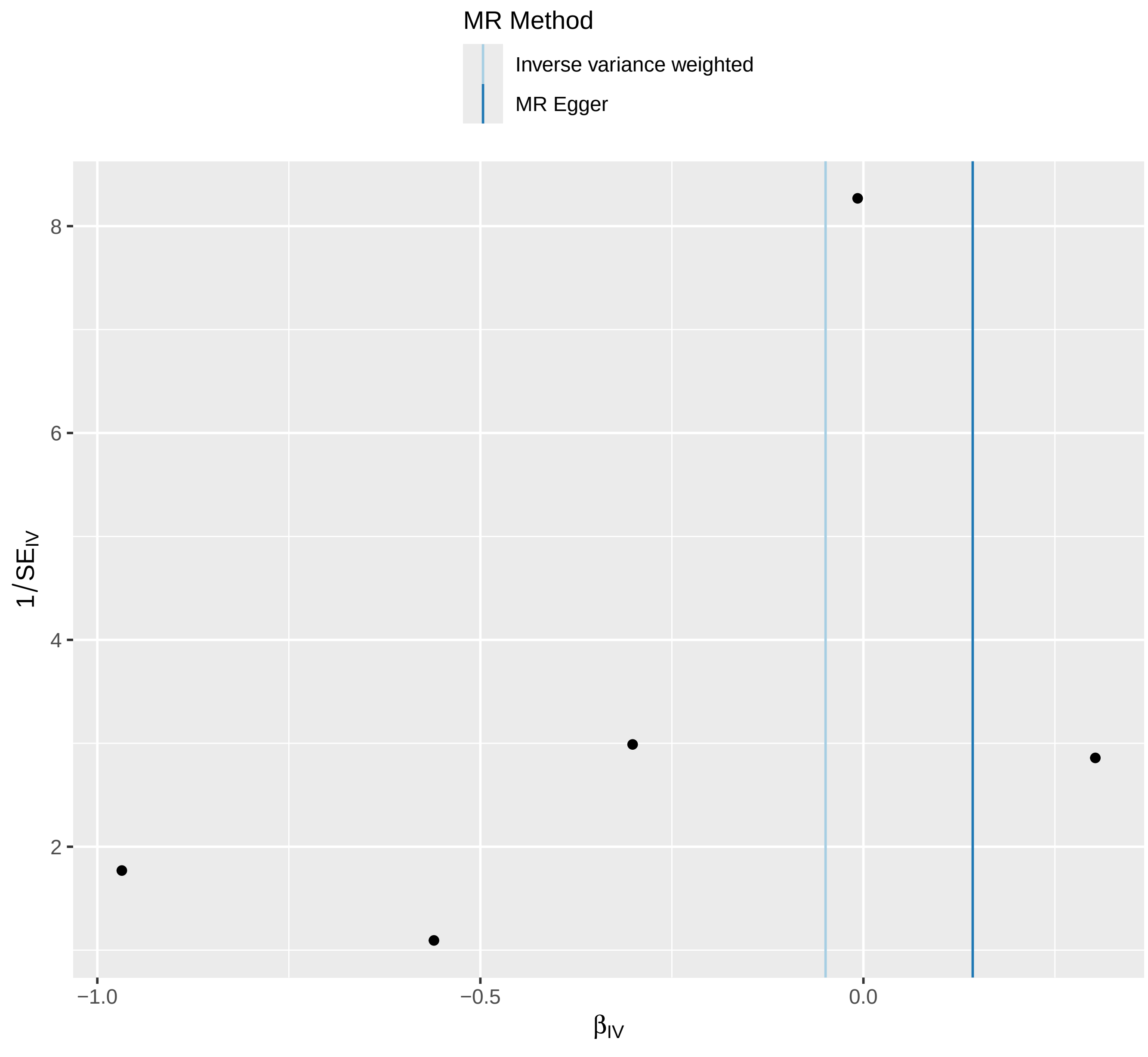

MR Method
Inverse variance weighted
MR Egger
1/SE_IV
8
6
4
2
−1.0
−0.5
0.0
β_IV

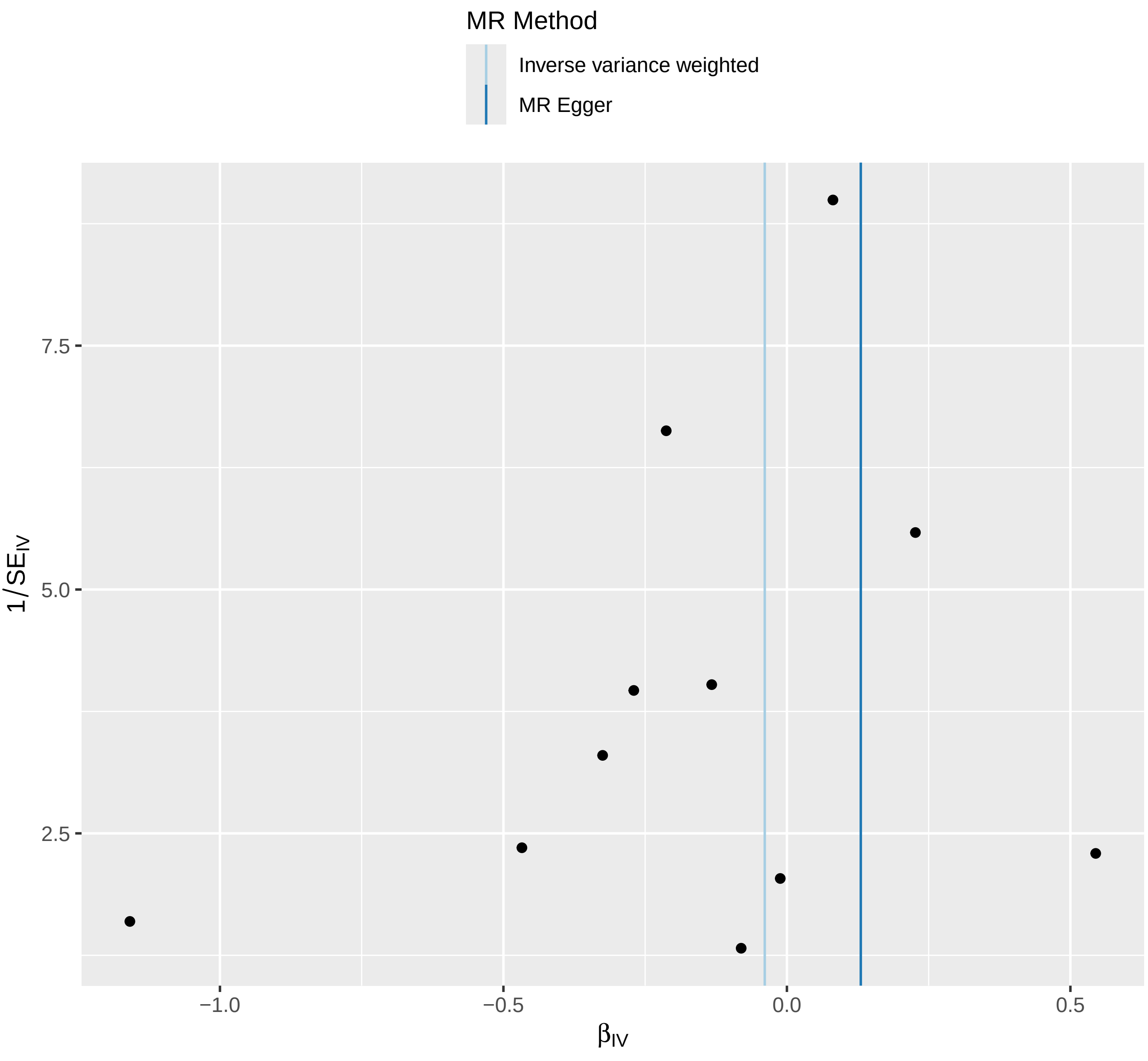

MR Method
Inverse variance weighted
MR Egger
7.5
5.0
2.5
1/SE$_{IV}$
−1.0
−0.5
0.0
0.5
β$_{IV}$

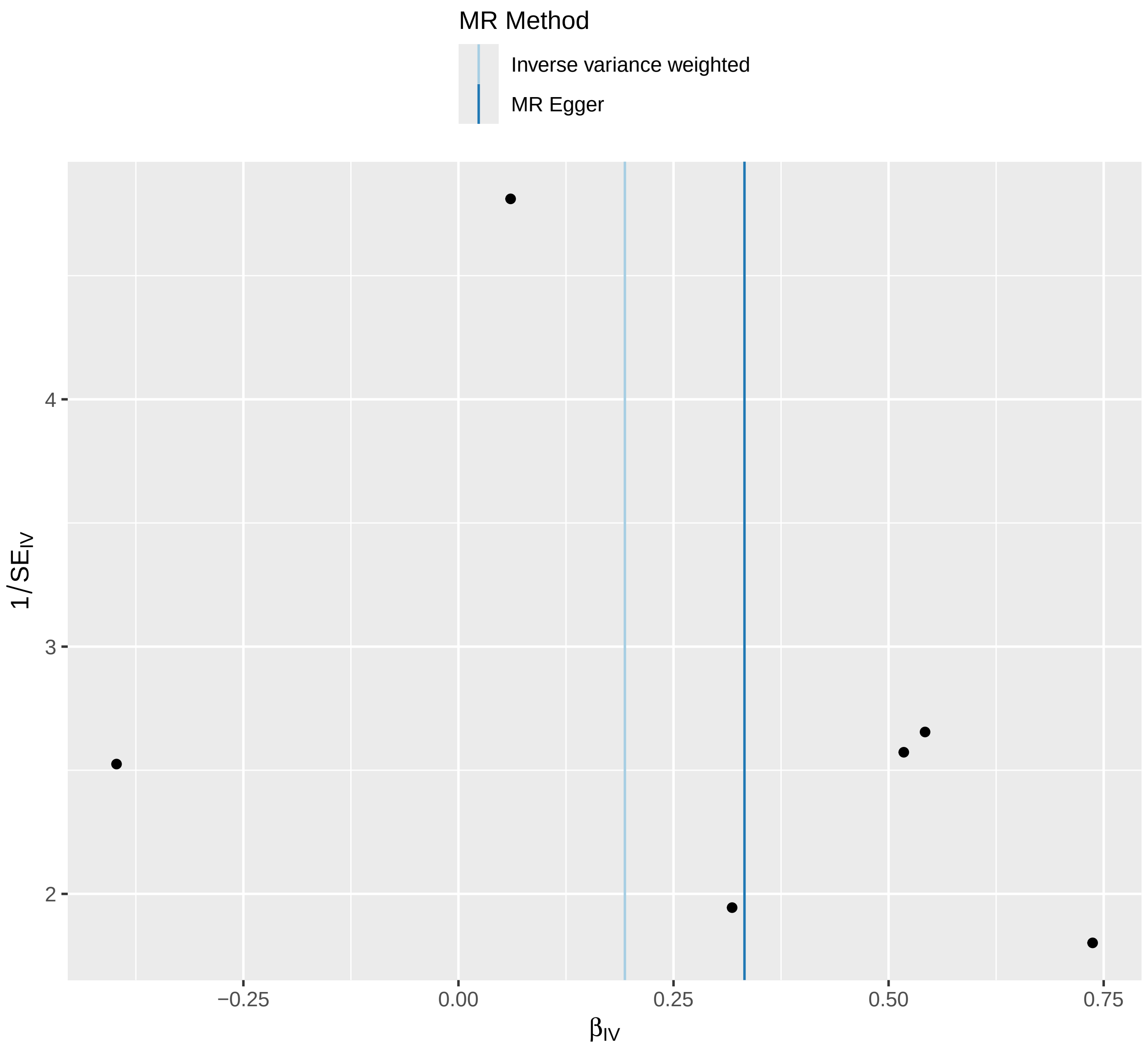
MR Method
Inverse variance weighted
MR Egger
1/SE$_{IV}$
4
3
2
−0.25
0.00
0.25
0.50
0.75
β$_{IV}$

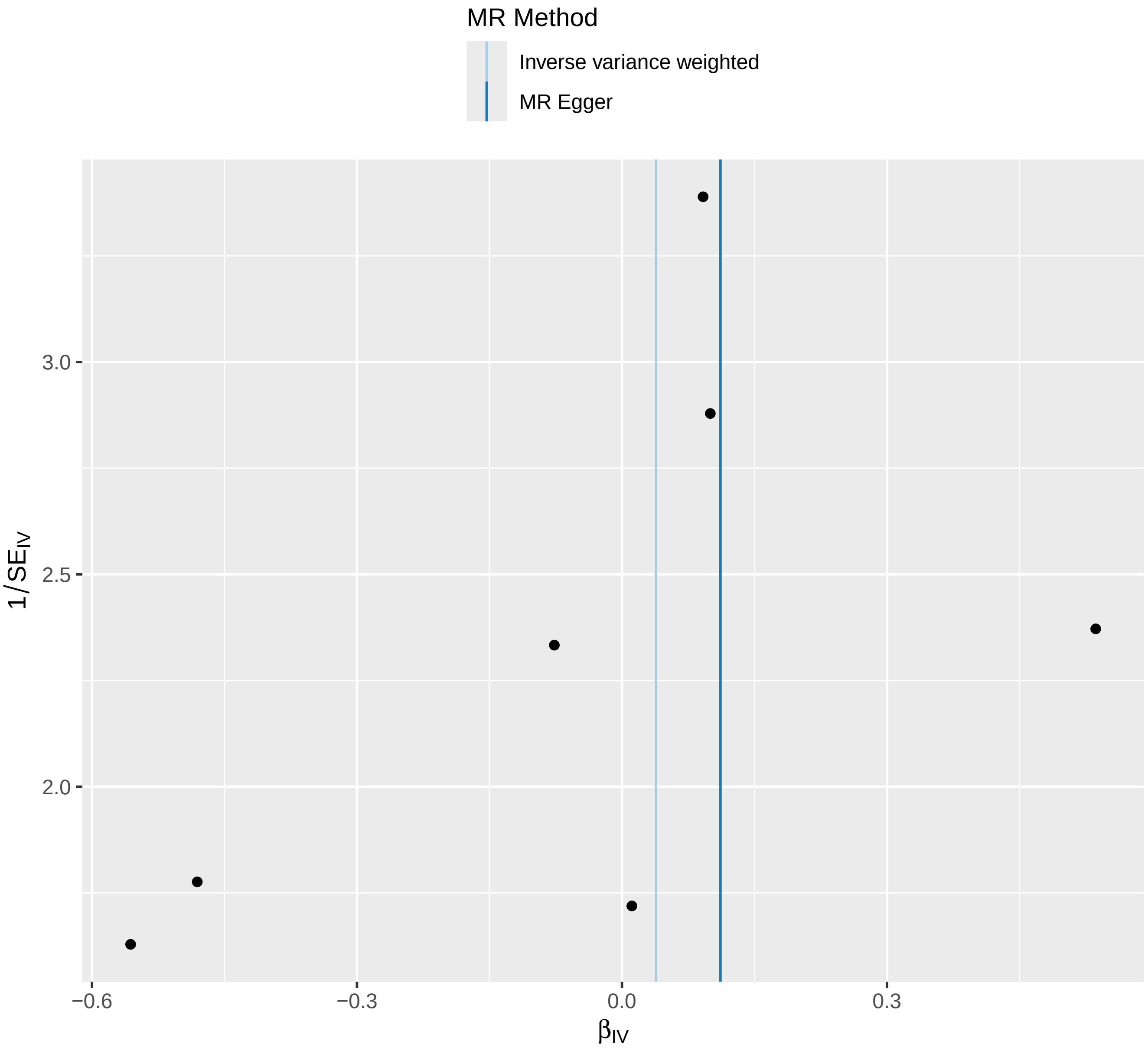
MR Method
Inverse variance weighted
MR Egger
3.0
2.5
2.0
1/SE$_{IV}$
−0.6
−0.3
0.0
0.3
β$_{IV}$

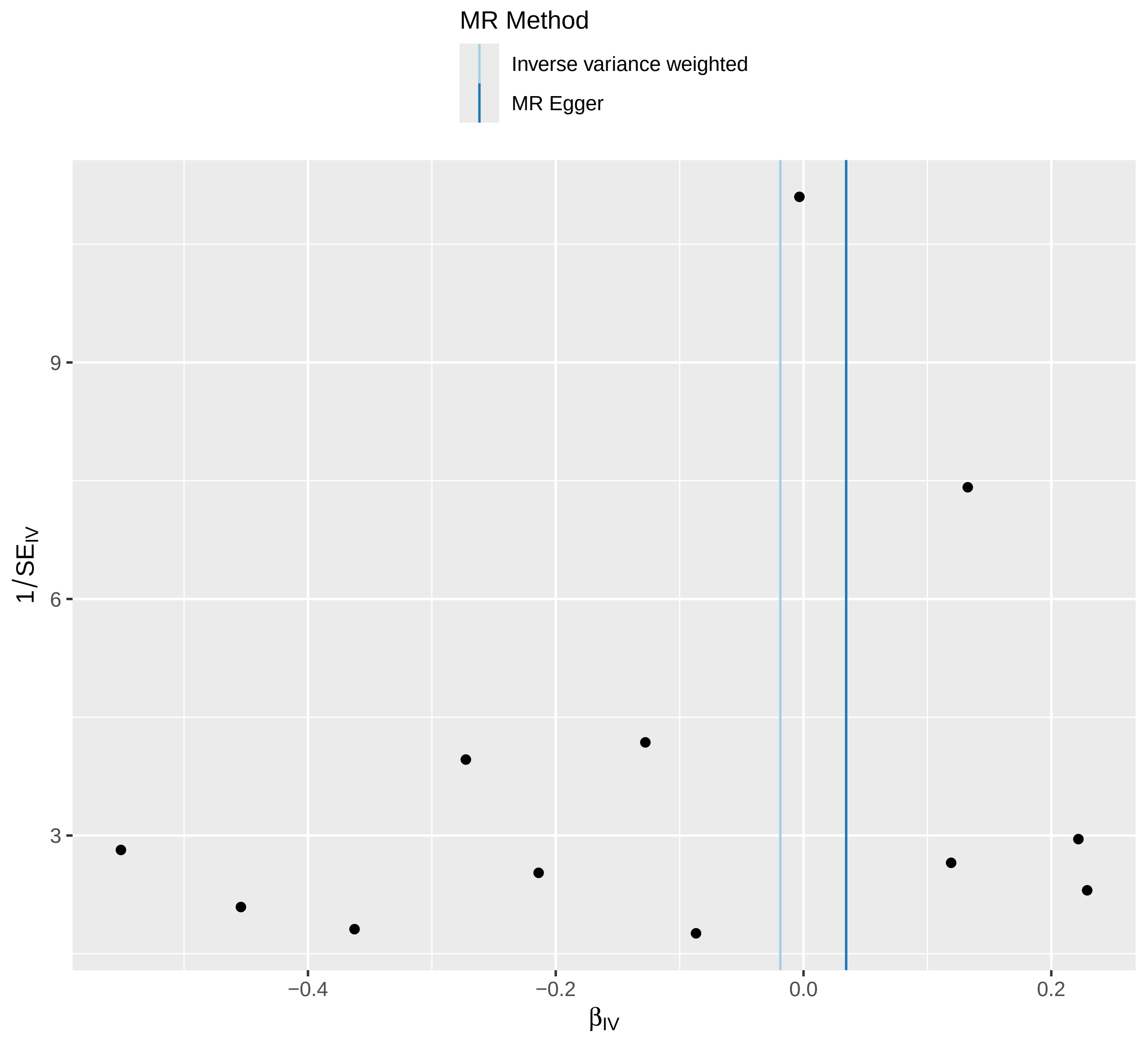

MR Method
Inverse variance weighted
MR Egger
9
6
3
1/SE_IV
−0.4
−0.2
0.0
0.2
β_IV

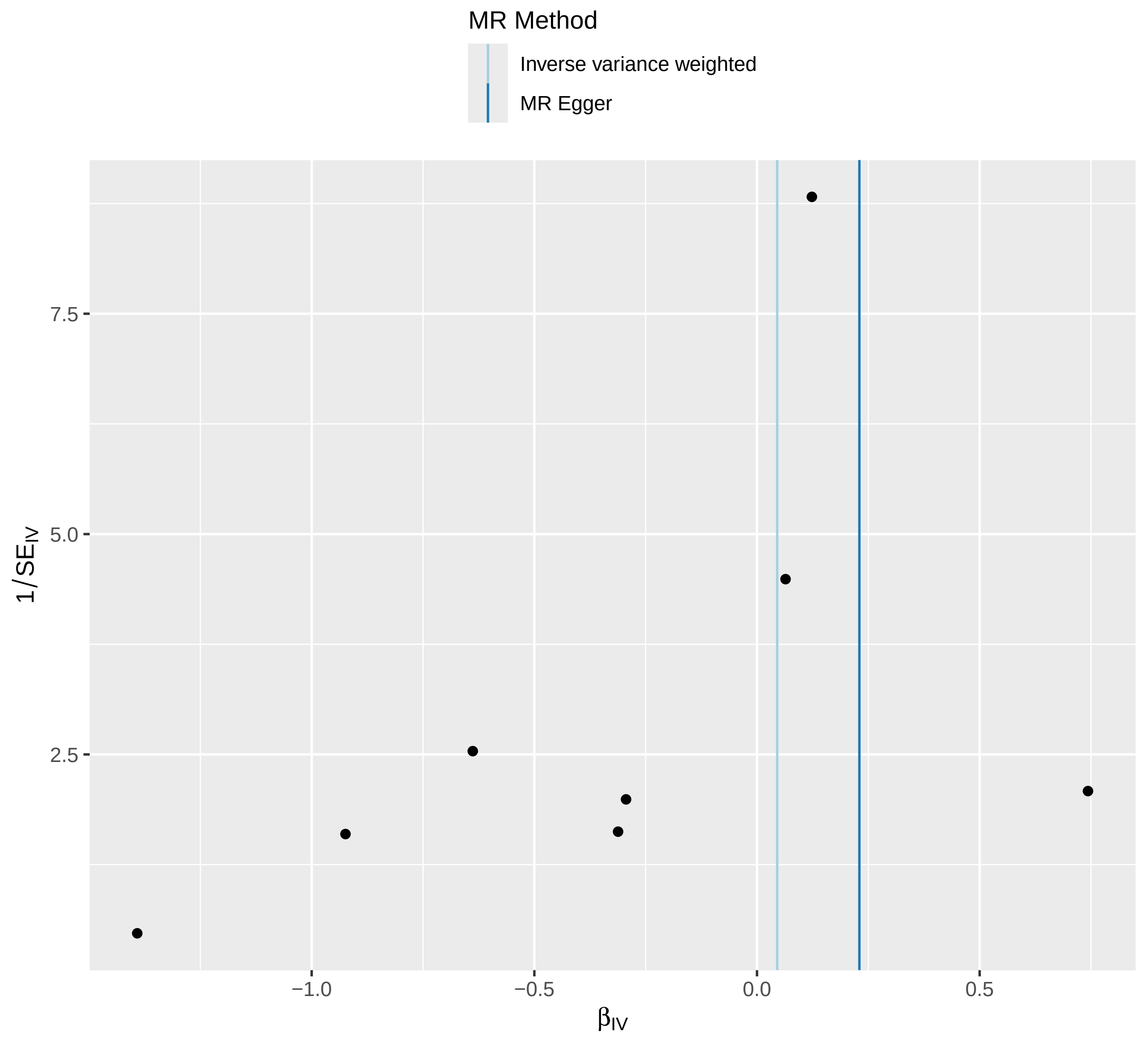
MR Method
Inverse variance weighted
MR Egger
7.5
5.0
2.5
1/SE_IV
−1.0
−0.5
0.0
0.5
β_IV

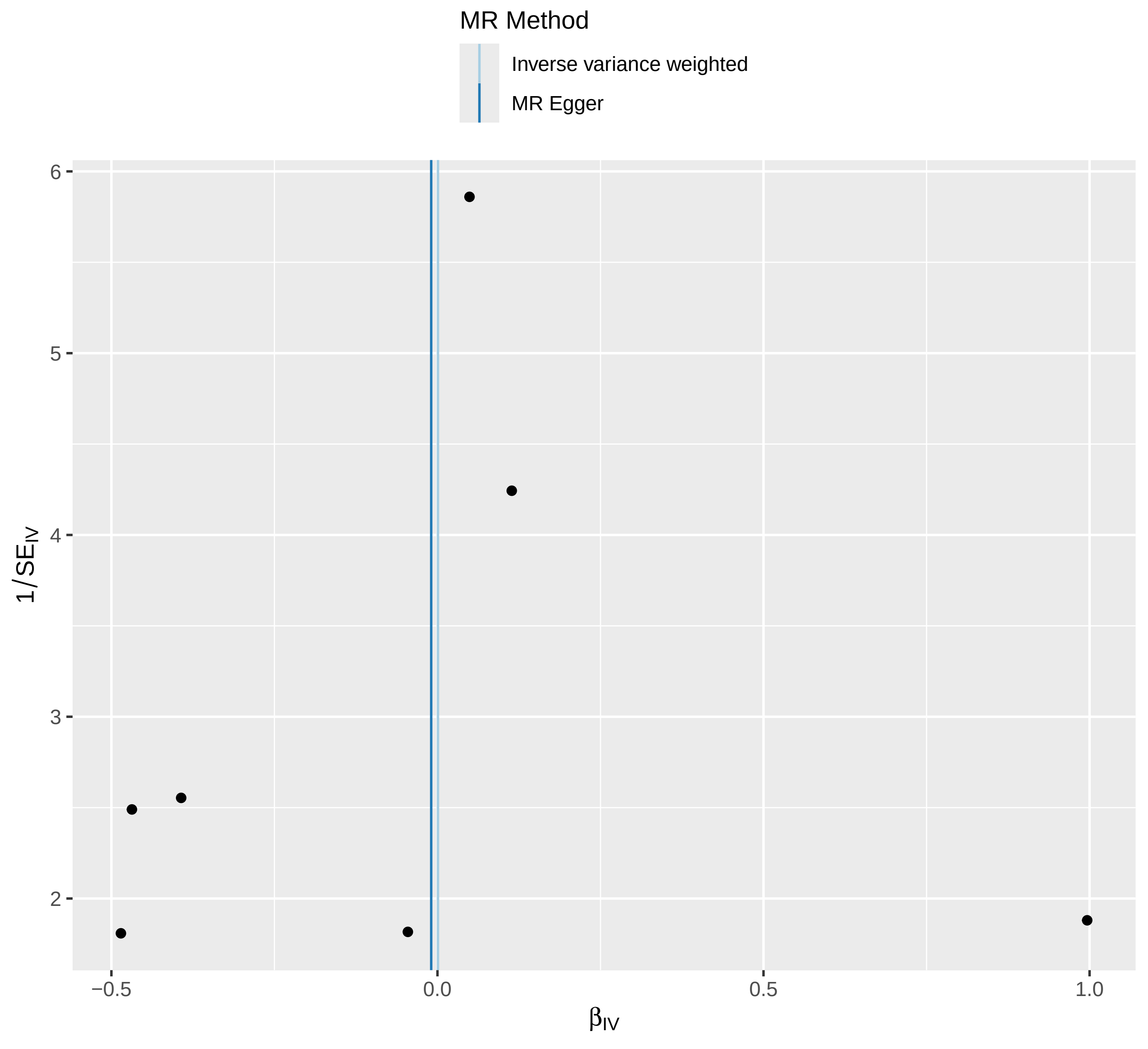

MR Method
Inverse variance weighted
MR Egger
6
5
4
3
2
1/SE_IV
−0.5
0.0
0.5
1.0
β_IV

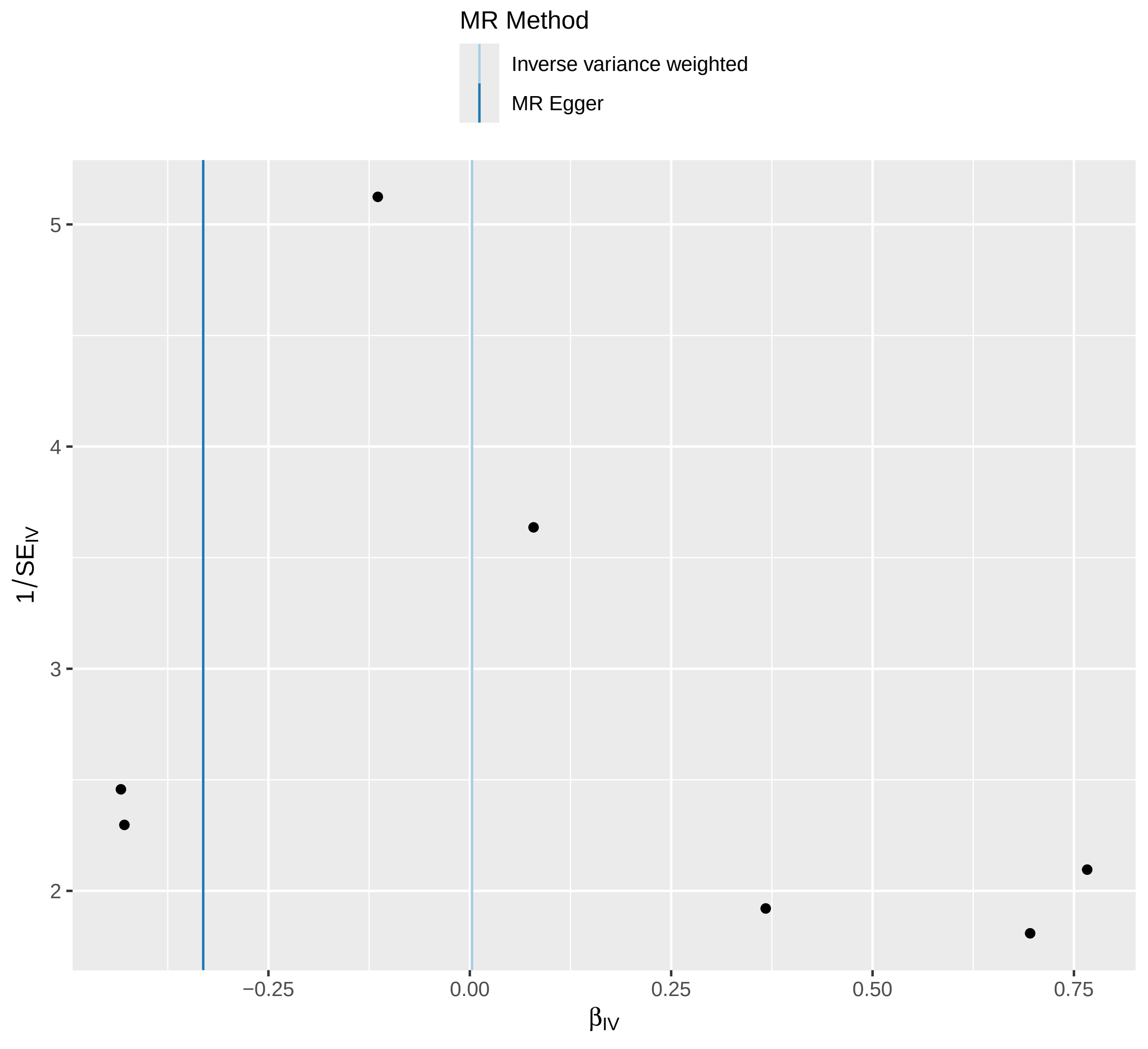

MR Method
Inverse variance weighted
MR Egger
5
4
3
2
1/SE_IV
−0.25
0.00
0.25
0.50
0.75
β_IV

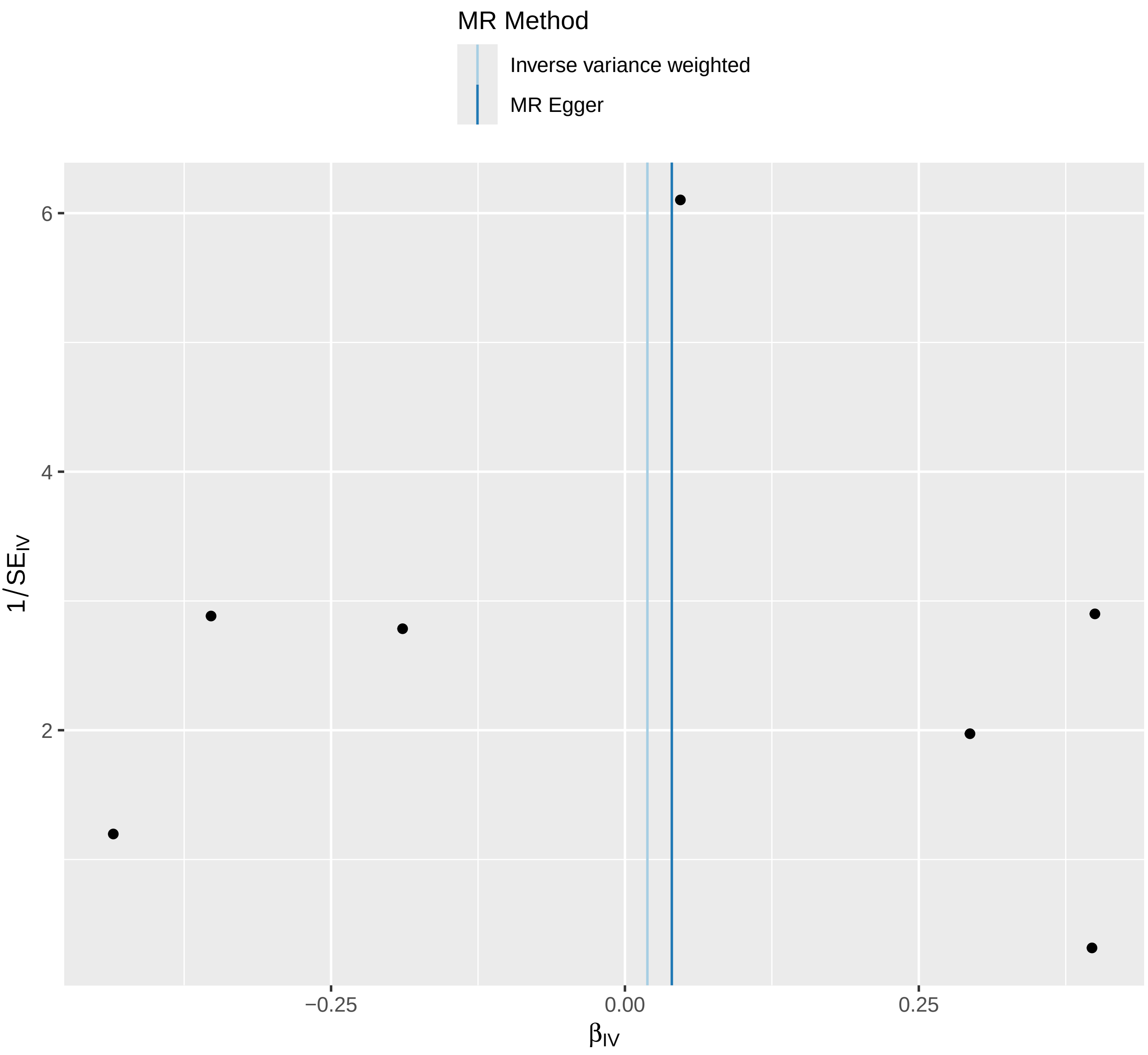

MR Method
Inverse variance weighted
MR Egger
6
4
2
$1/SE_{IV}$
−0.25
0.00
0.25
β_{IV}

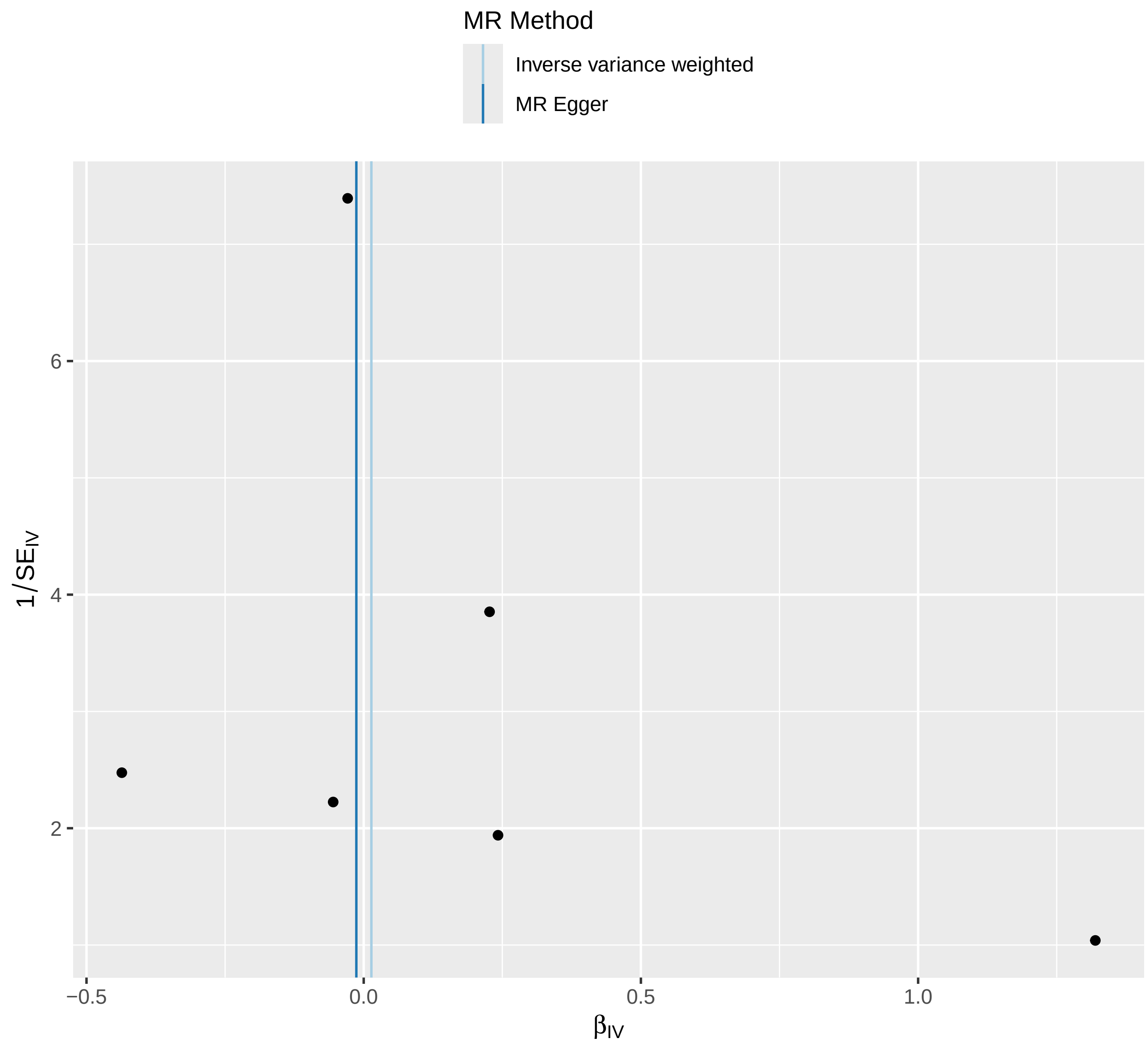
MR Method
Inverse variance weighted
MR Egger
6
4
2
1/SE_IV
−0.5
0.0
0.5
1.0
β_IV

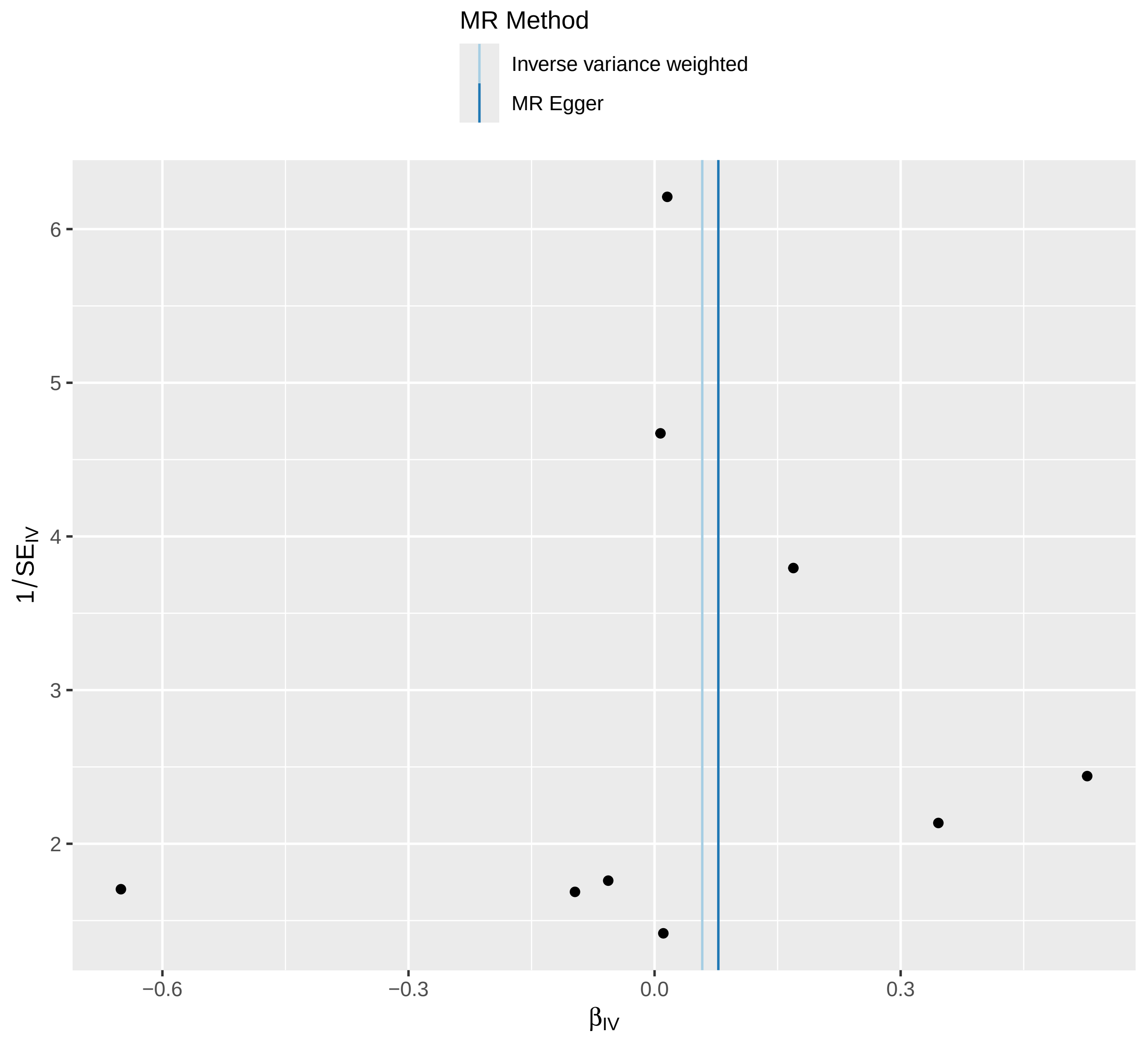
MR Method
Inverse variance weighted
MR Egger
6
5
4
3
2
1/SE_IV
−0.6
−0.3
0.0
0.3
β_IV

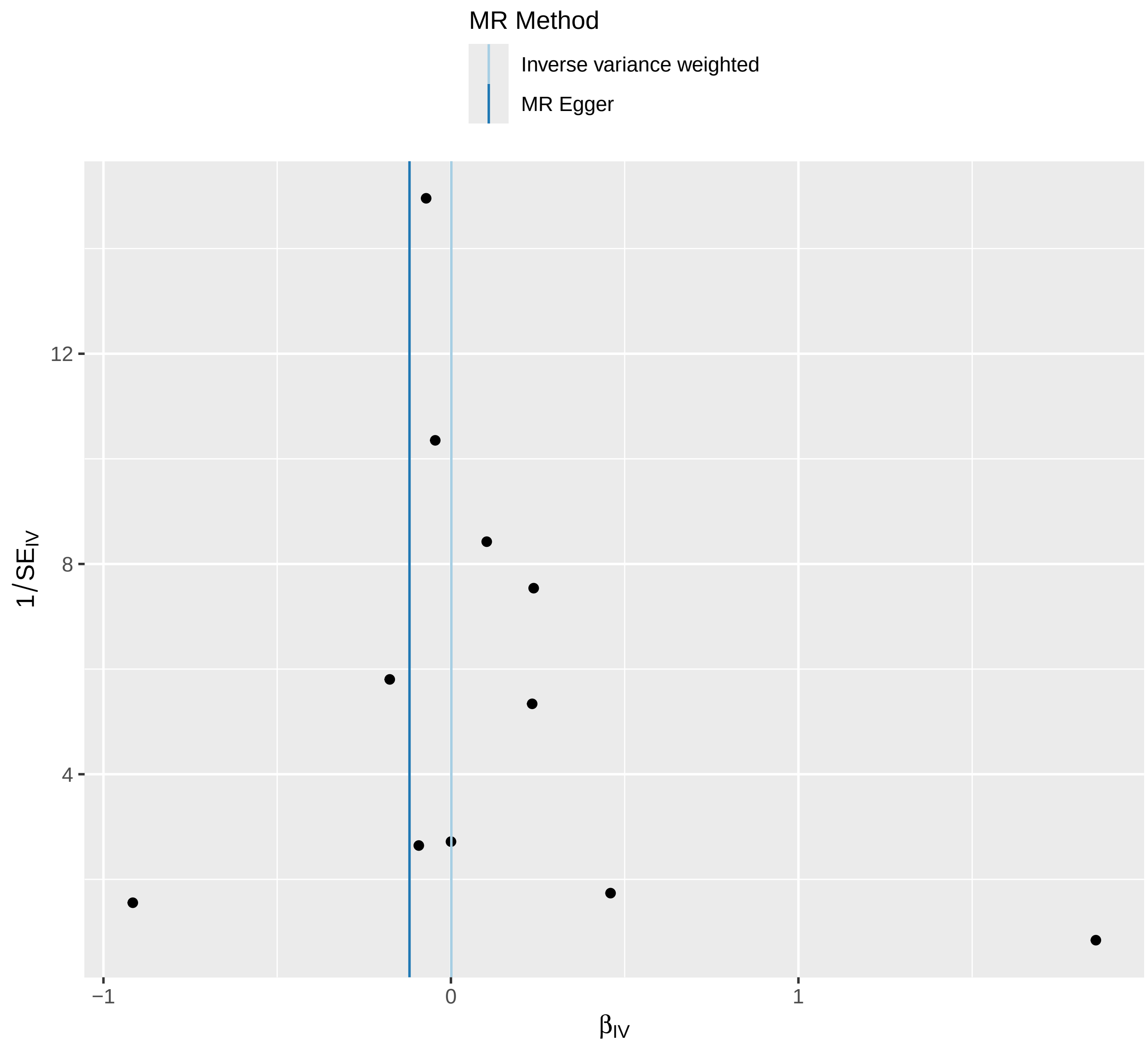
MR Method
Inverse variance weighted
MR Egger
12
8
4
1/SE_IV
−1
0
1
β_IV

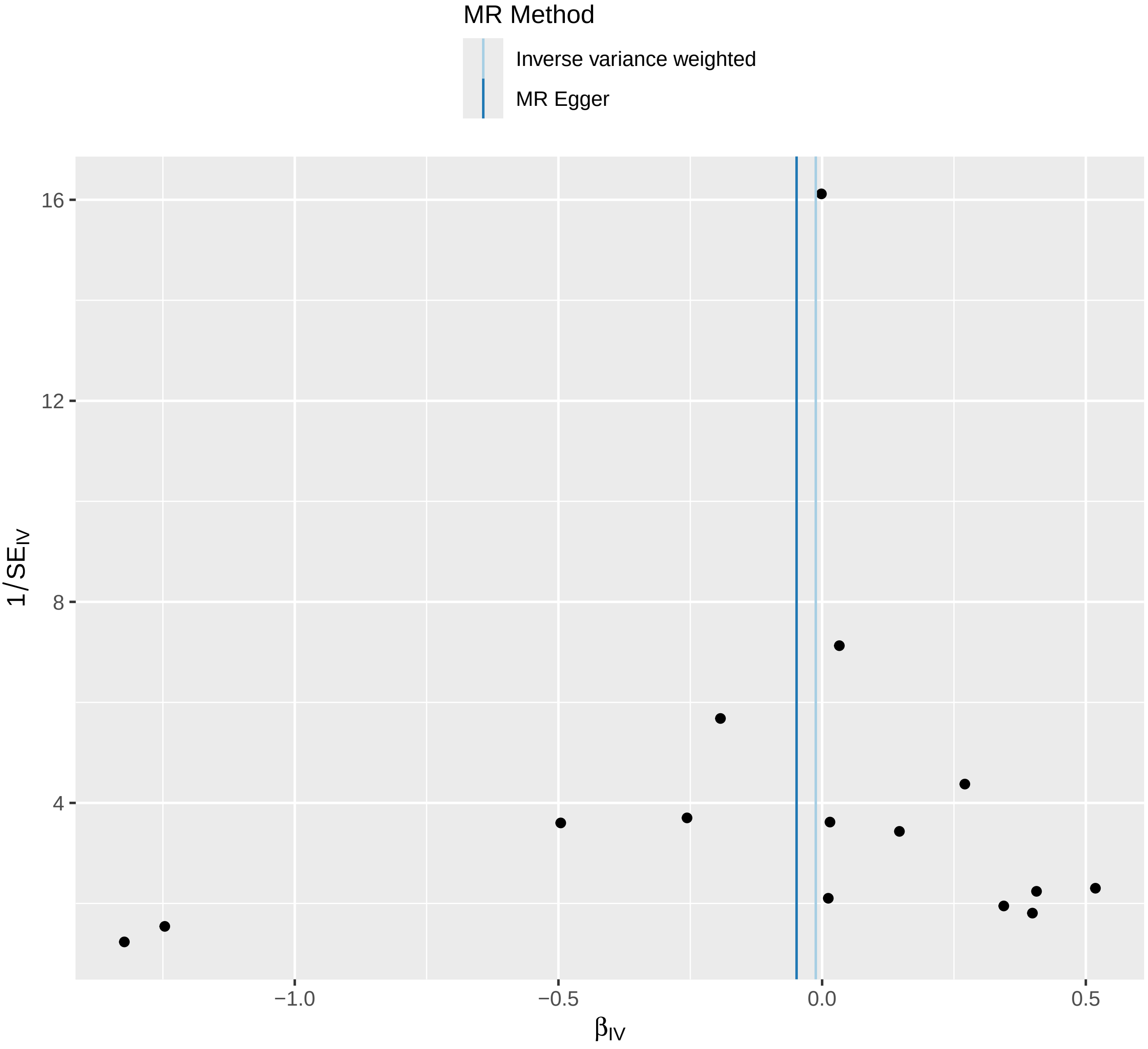
MR Method
Inverse variance weighted
MR Egger
16
12
8
4
$1/SE_{IV}$
−1.0
−0.5
0.0
0.5
β_{IV}

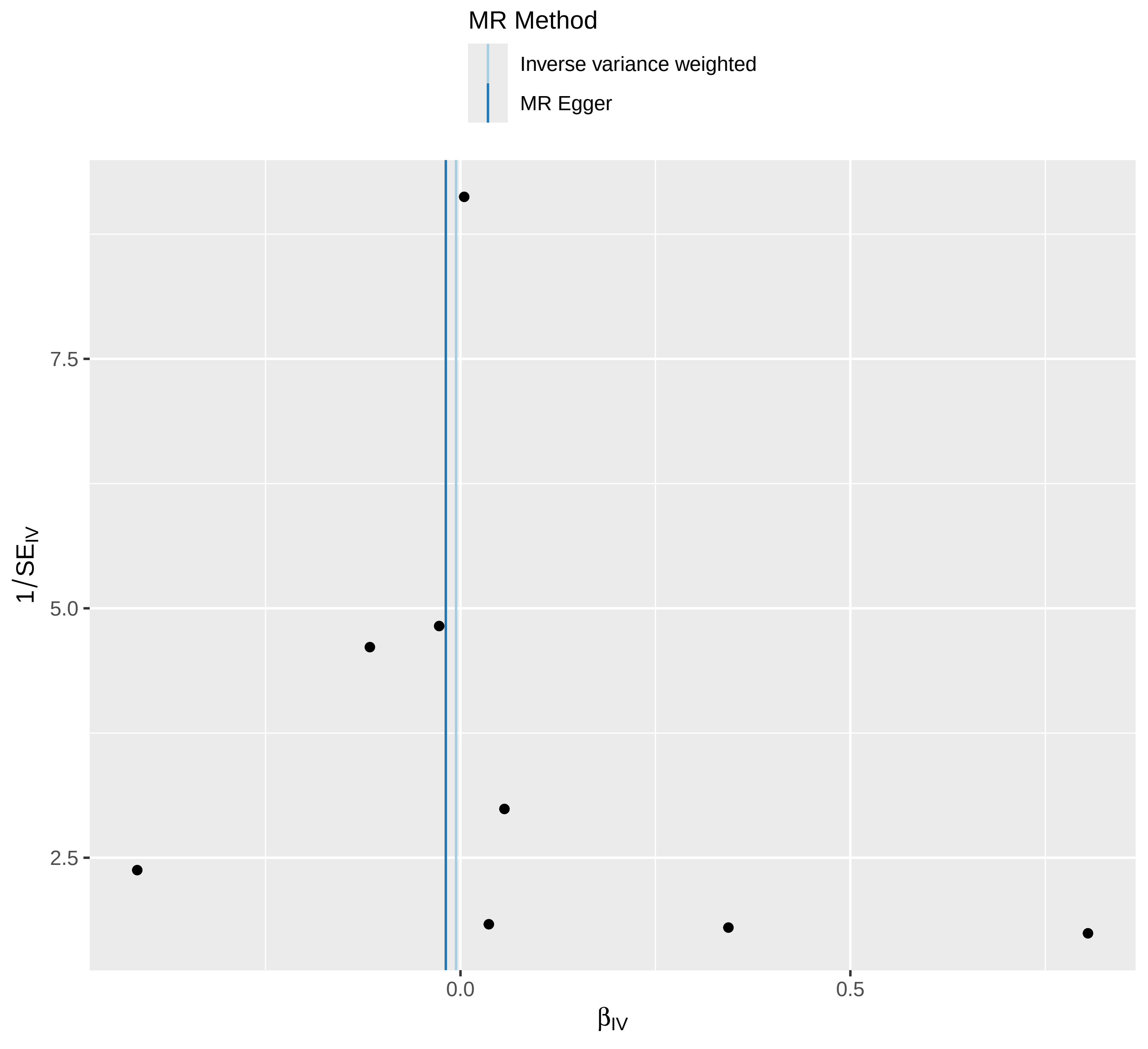

MR Method
Inverse variance weighted
MR Egger
7.5
5.0
2.5
$1/SE_{IV}$
0.0
0.5
β_{IV}

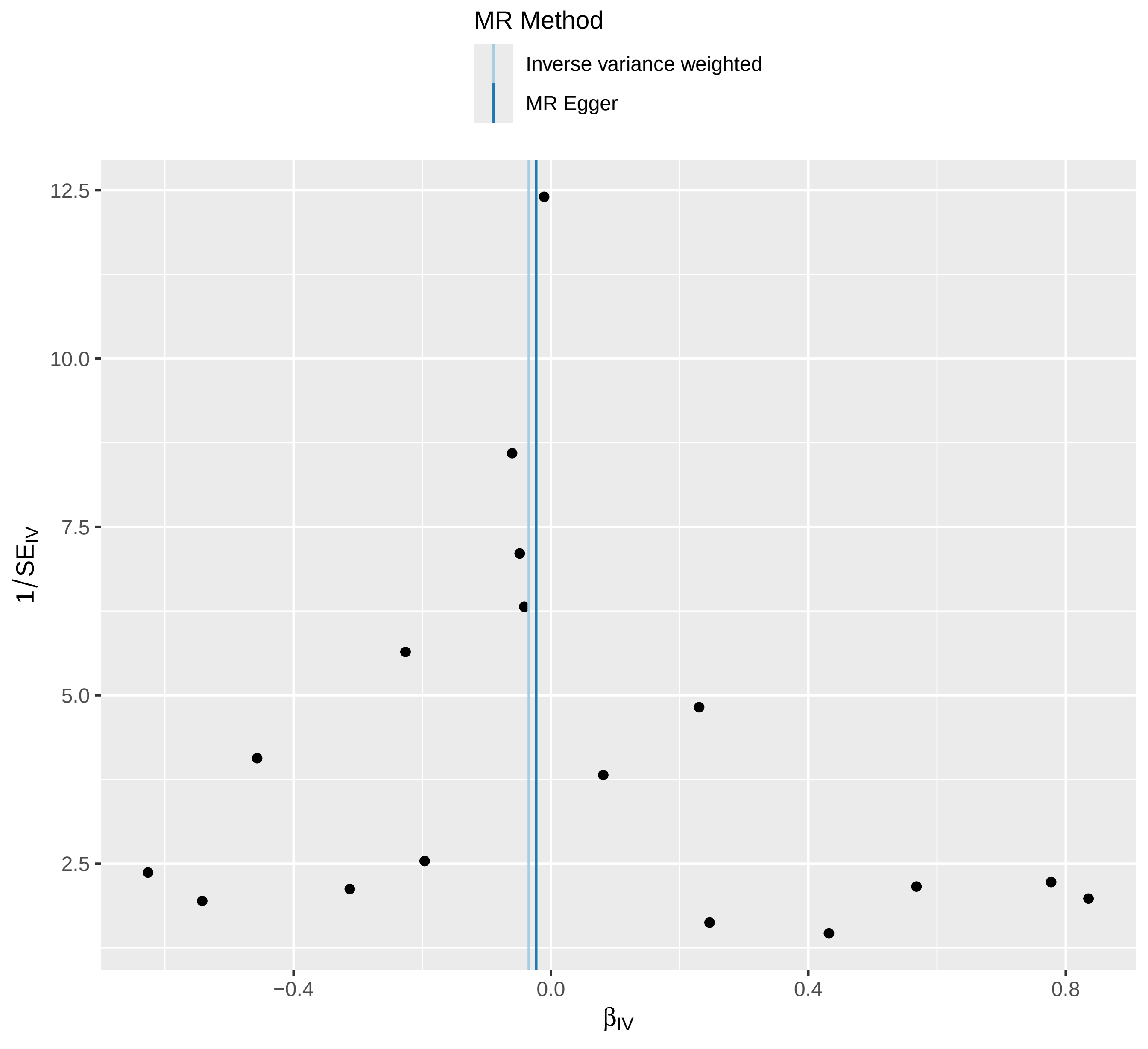
MR Method
Inverse variance weighted
MR Egger
12.5
10.0
7.5
5.0
2.5
1/SE_IV
−0.4
0.0
0.4
0.8
β_IV

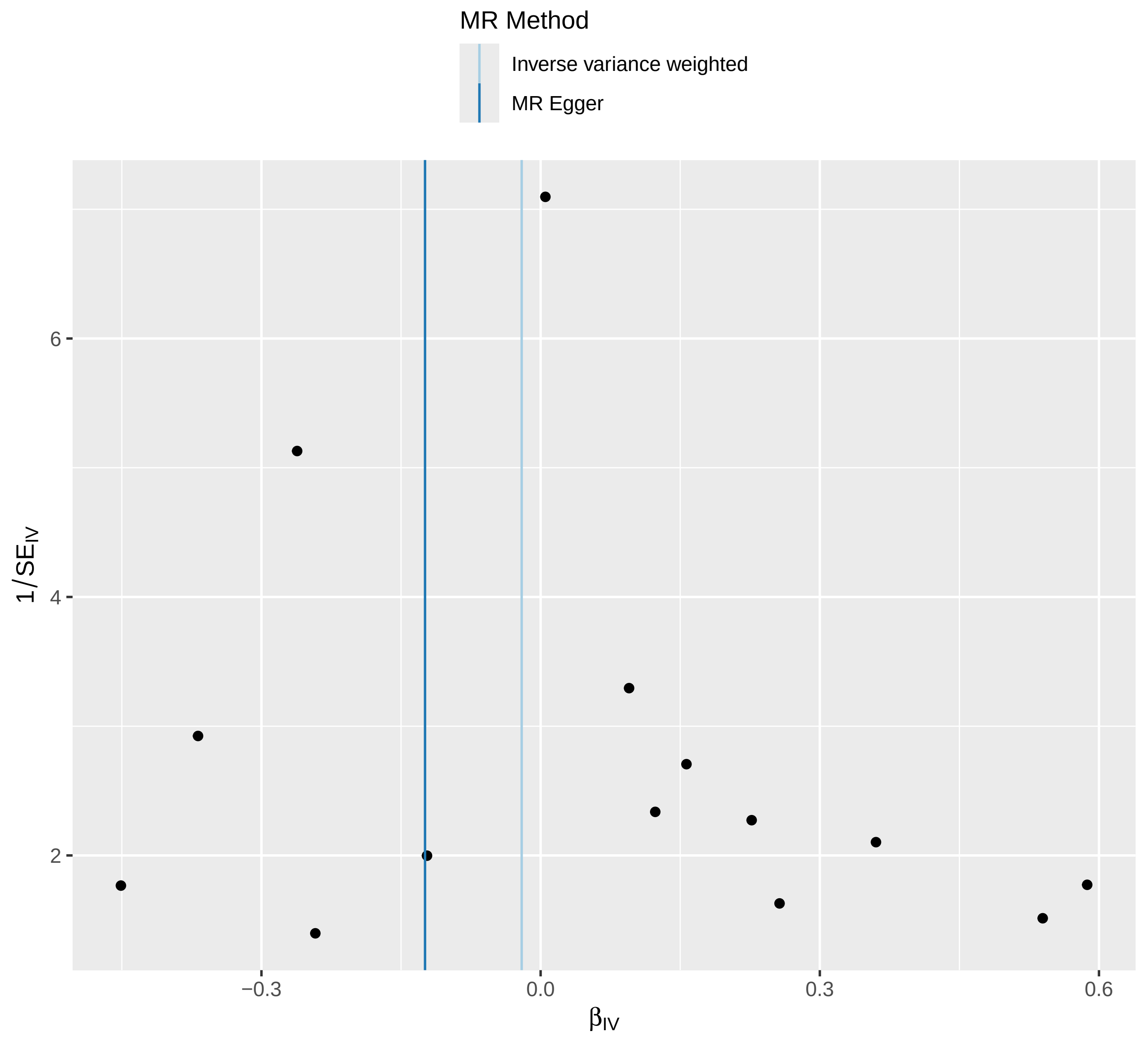
MR Method
Inverse variance weighted
MR Egger
1/SE_IV
6
4
2
−0.3
0.0
0.3
0.6
β_IV

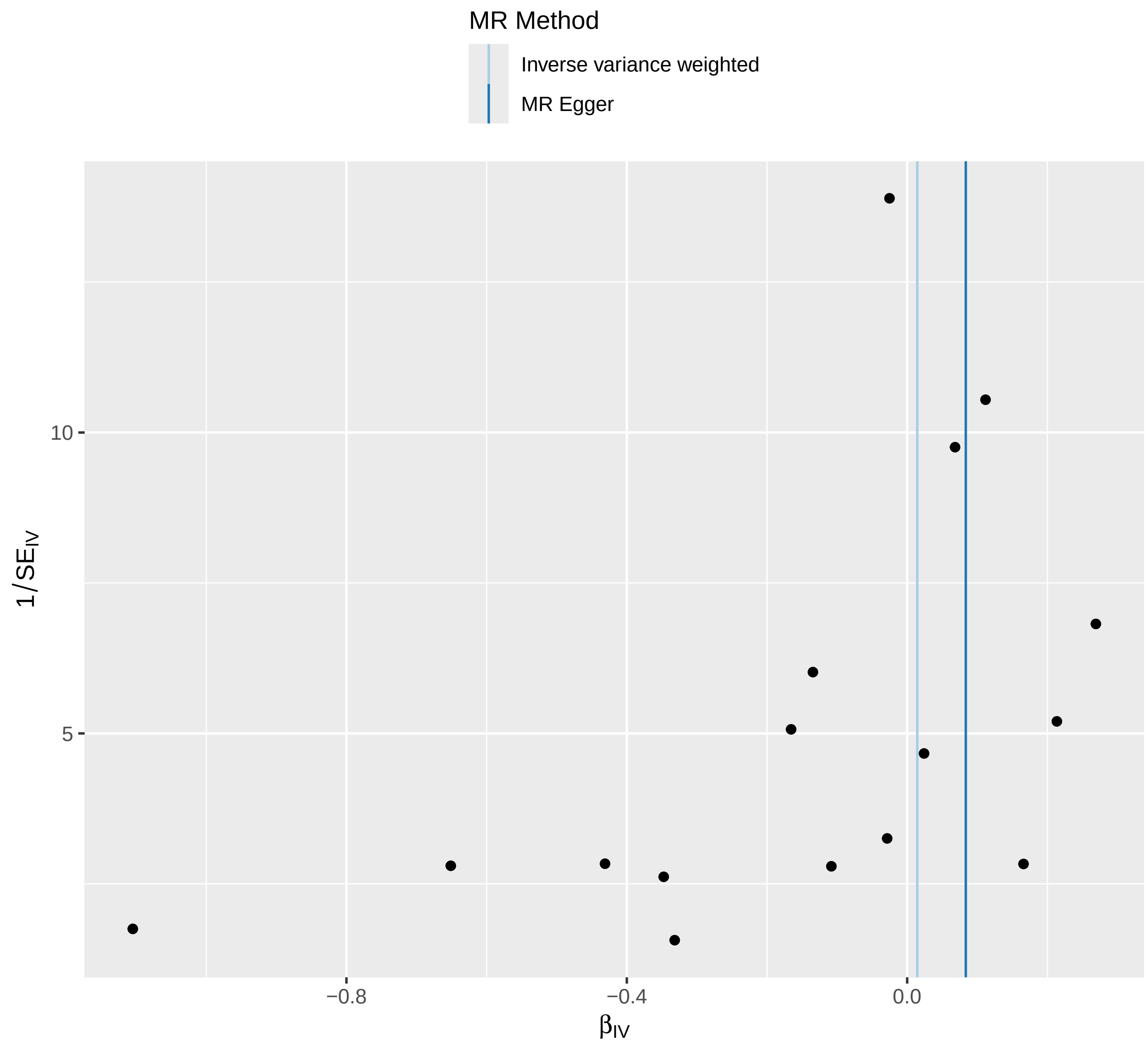

MR Method
Inverse variance weighted
MR Egger
10
5
1/SE$_{IV}$
−0.8
−0.4
0.0
β$_{IV}$

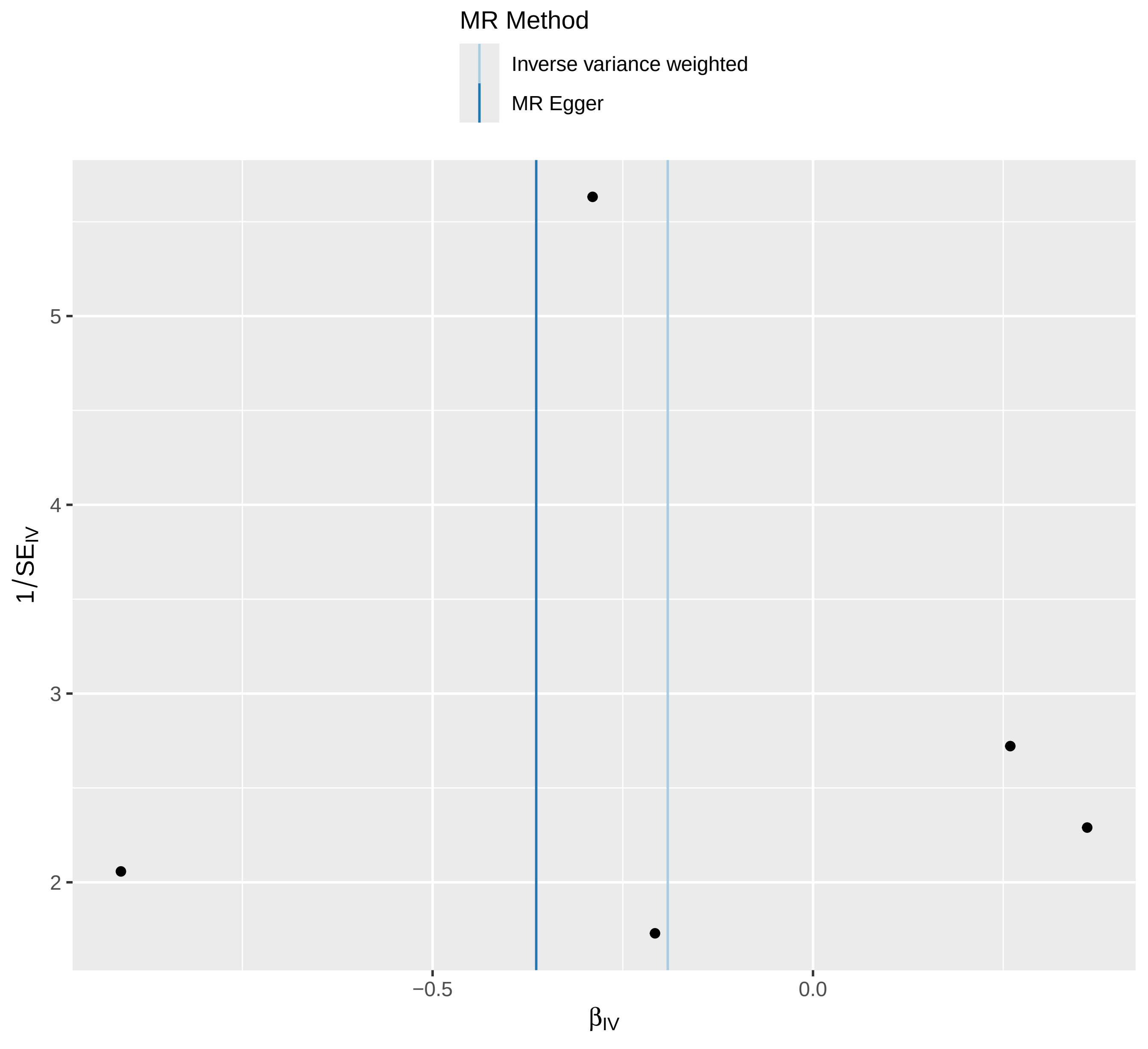

MR Method
Inverse variance weighted
MR Egger
5
4
3
2
1/SE_{IV}
−0.5
0.0
β_{IV}

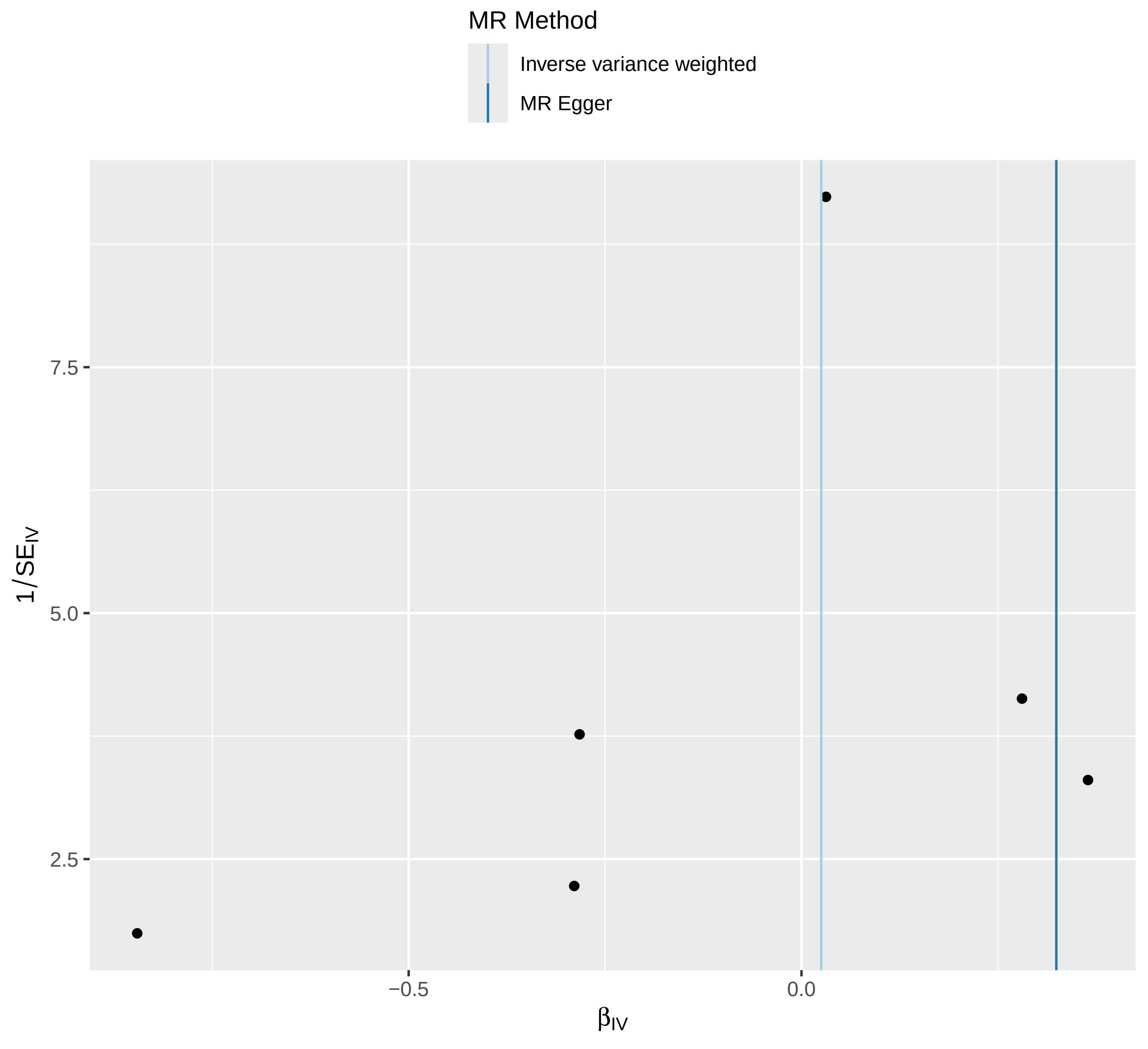

MR Method
Inverse variance weighted
MR Egger
7.5
5.0
2.5
$1/SE_{IV}$
−0.5
0.0
β_{IV}

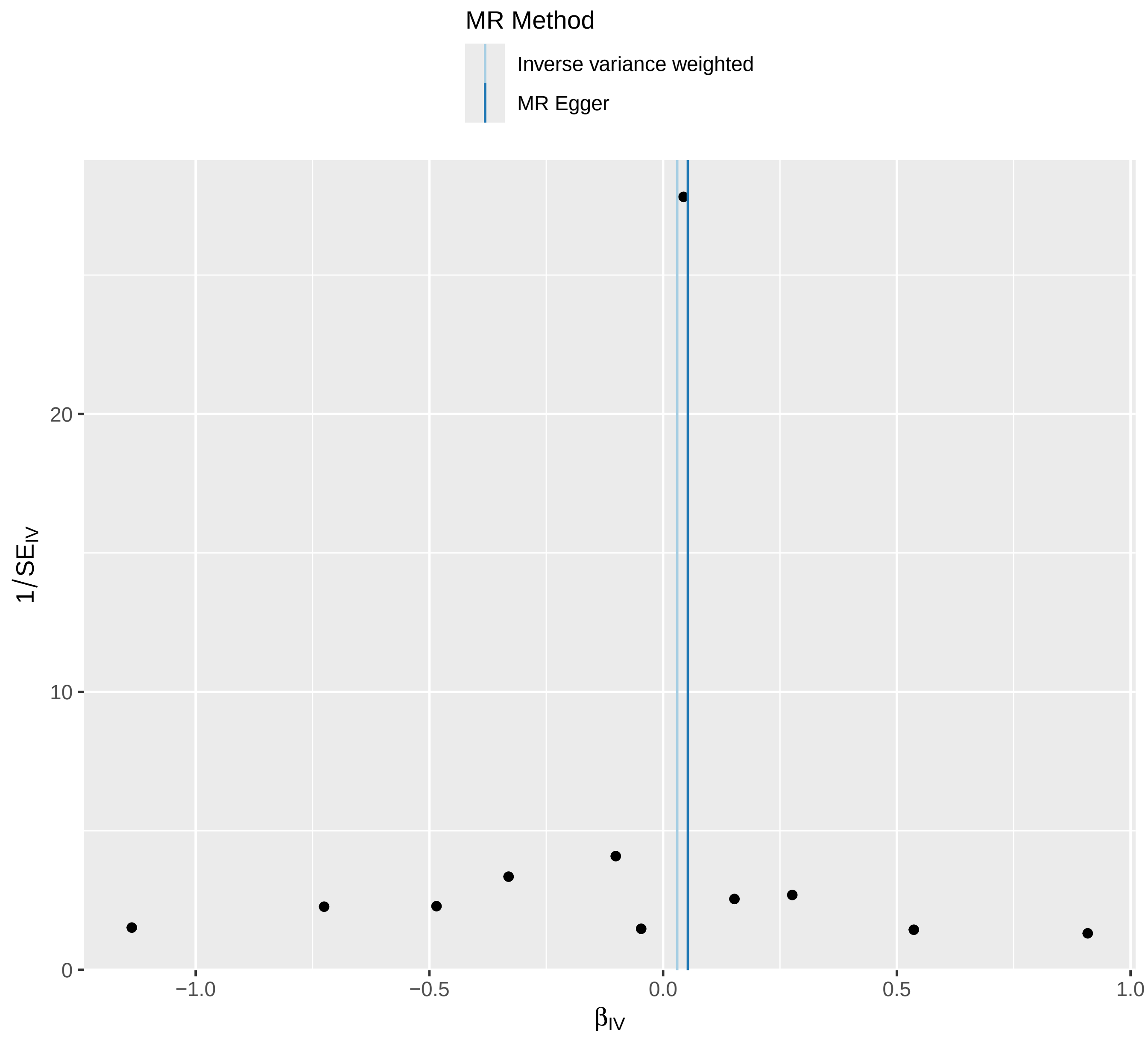

MR Method
Inverse variance weighted
MR Egger
1/SE$_{IV}$
β_{IV}
0
10
20
−1.0
−0.5
0.0
0.5
1.0

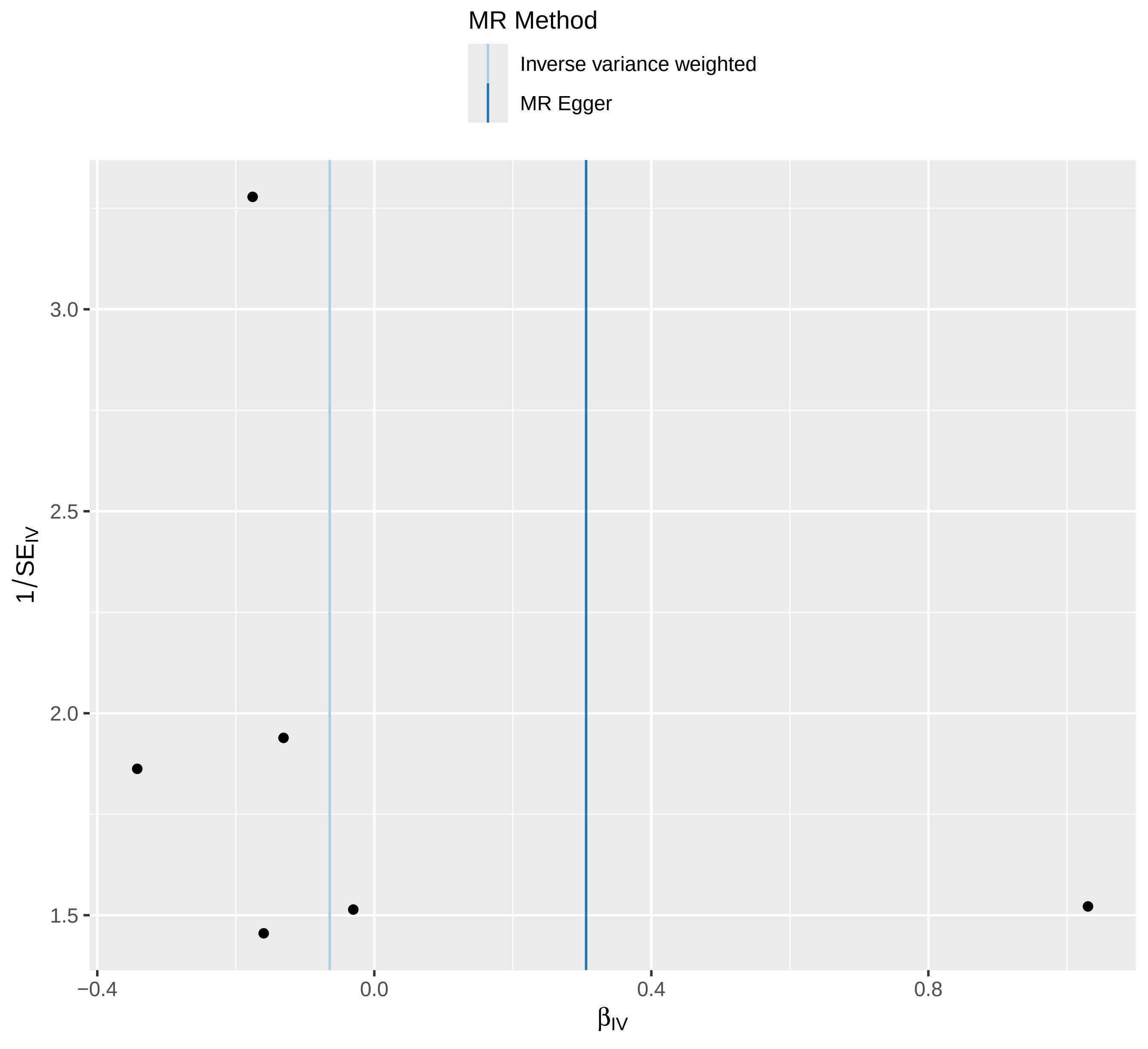

MR Method
Inverse variance weighted
MR Egger
3.0
2.5
2.0
1.5
1/SE$_{IV}$
−0.4
0.0
0.4
0.8
β$_{IV}$

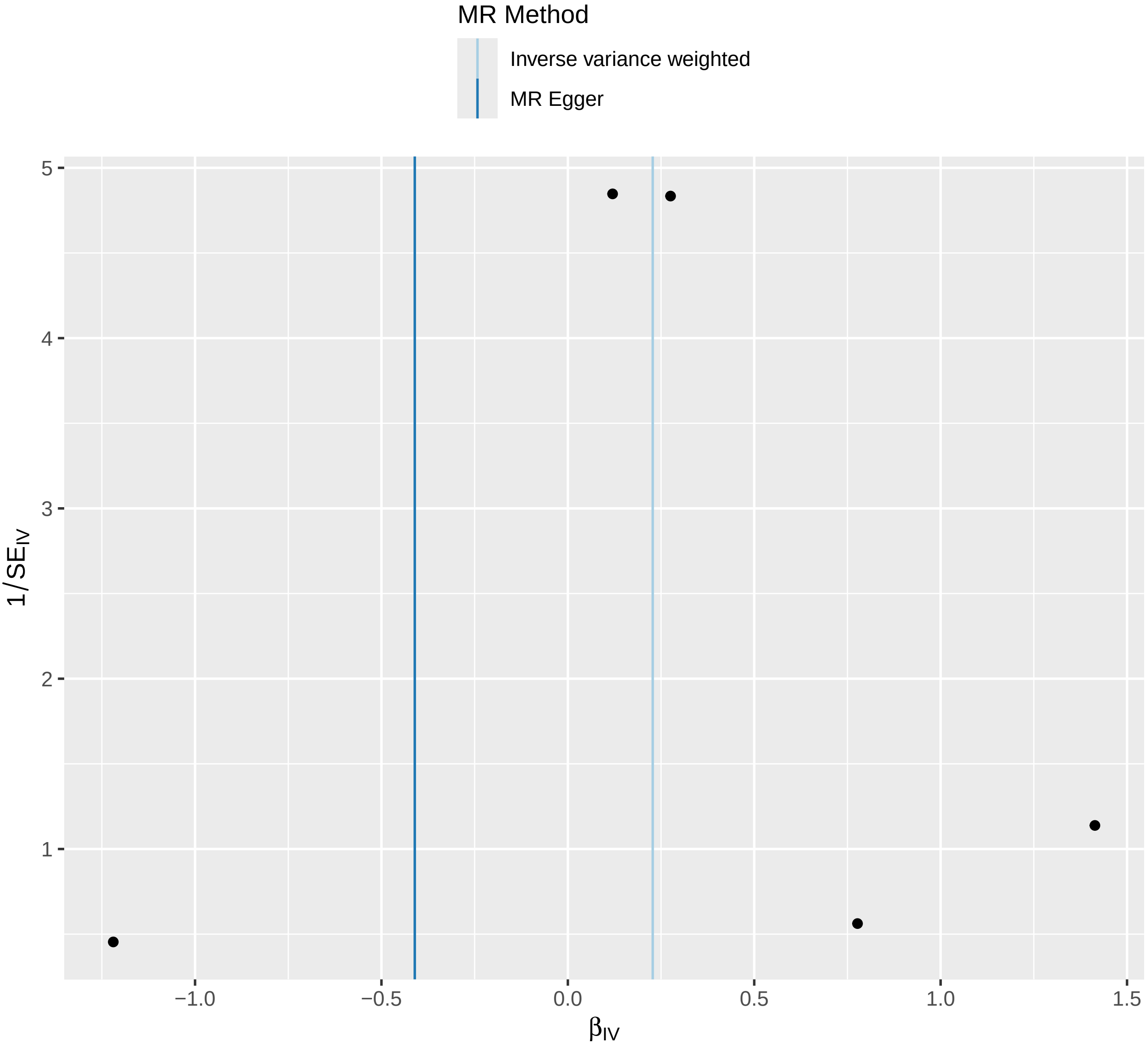
MR Method
Inverse variance weighted
MR Egger
5
4
3
2
1
$1/SE_{IV}$
−1.0
−0.5
0.0
0.5
1.0
1.5
β_{IV}

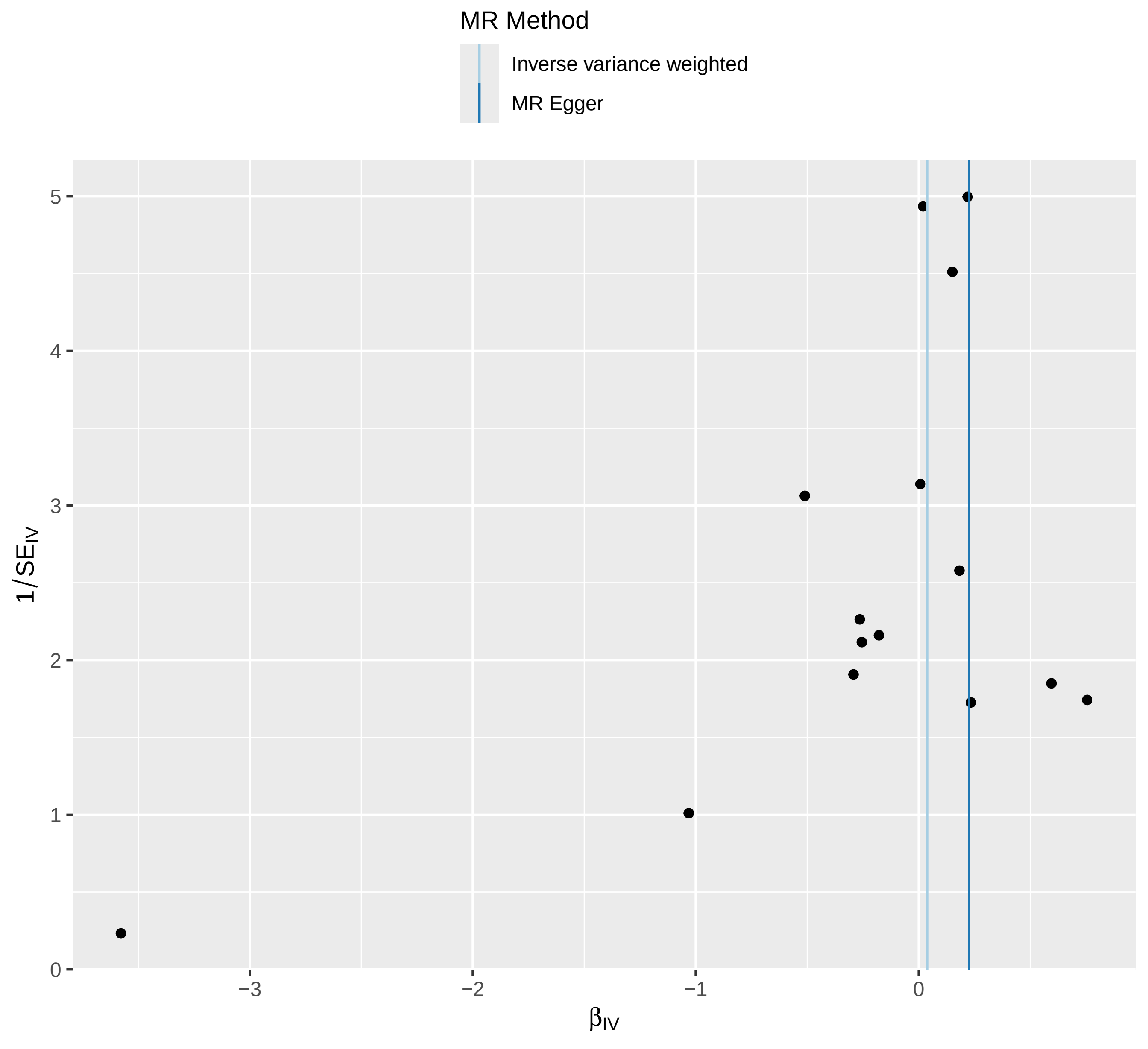

MR Method
Inverse variance weighted
MR Egger
1/SE_IV
β_IV
5
4
3
2
1
0
−3
−2
−1
0

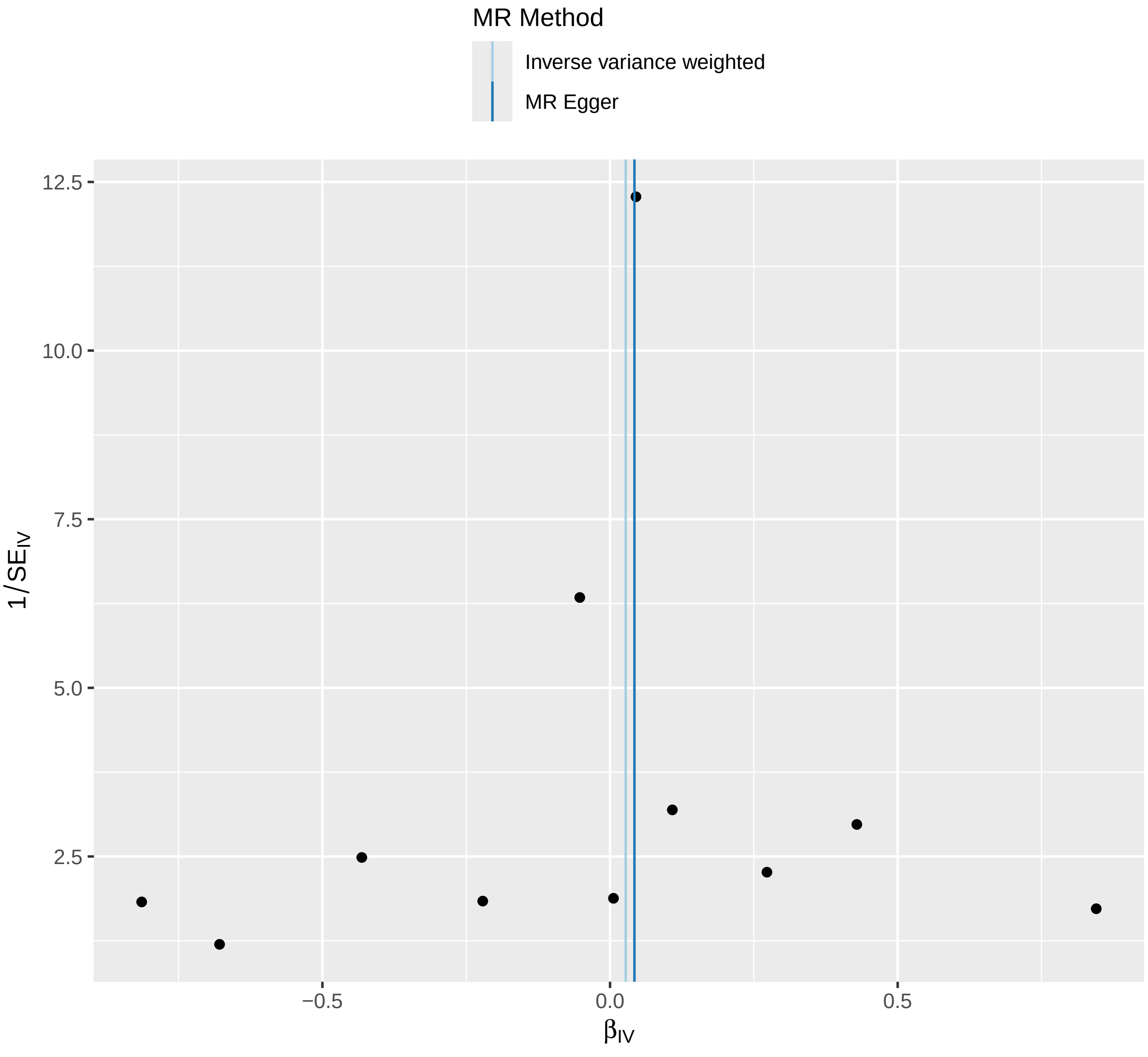

MR Method
Inverse variance weighted
MR Egger
12.5
10.0
7.5
5.0
2.5
1/SE_IV
−0.5
0.0
0.5
β_IV

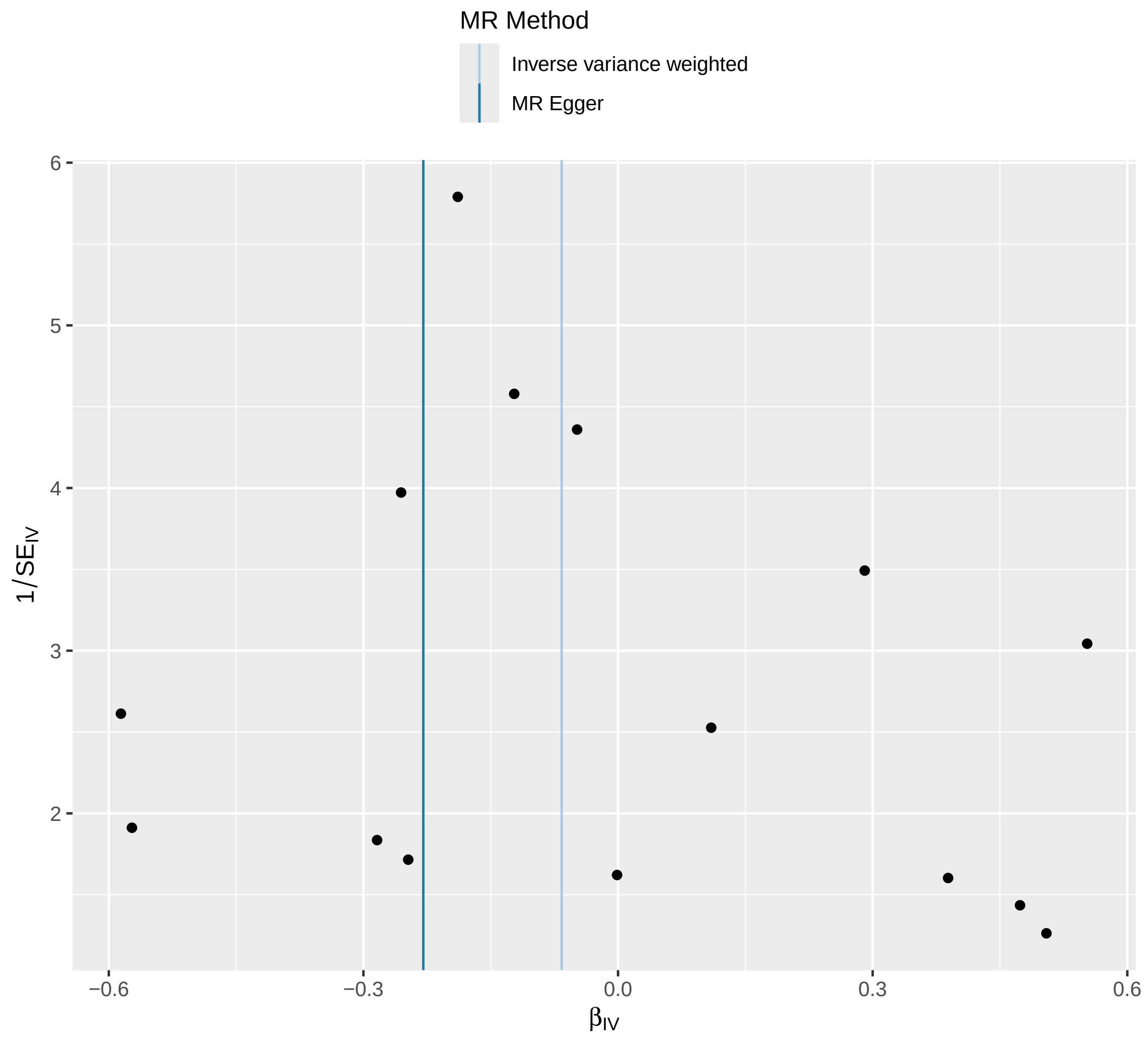
MR Method
Inverse variance weighted
MR Egger
1/SE_IV
β_IV
6
5
4
3
2
−0.6
−0.3
0.0
0.3
0.6

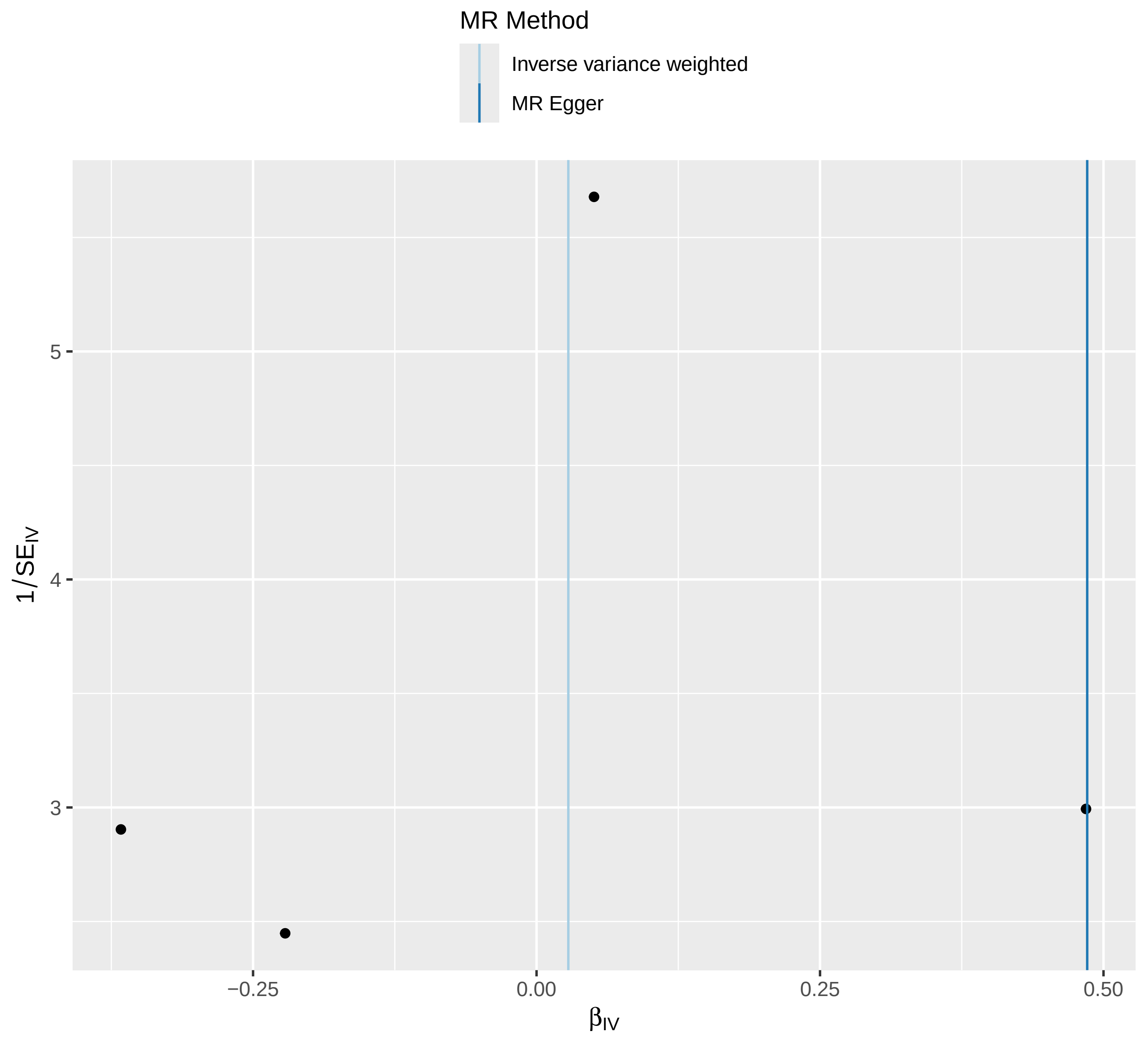

MR Method
Inverse variance weighted
MR Egger
5
4
3
1/SE$_{IV}$
−0.25
0.00
0.25
0.50
β$_{IV}$

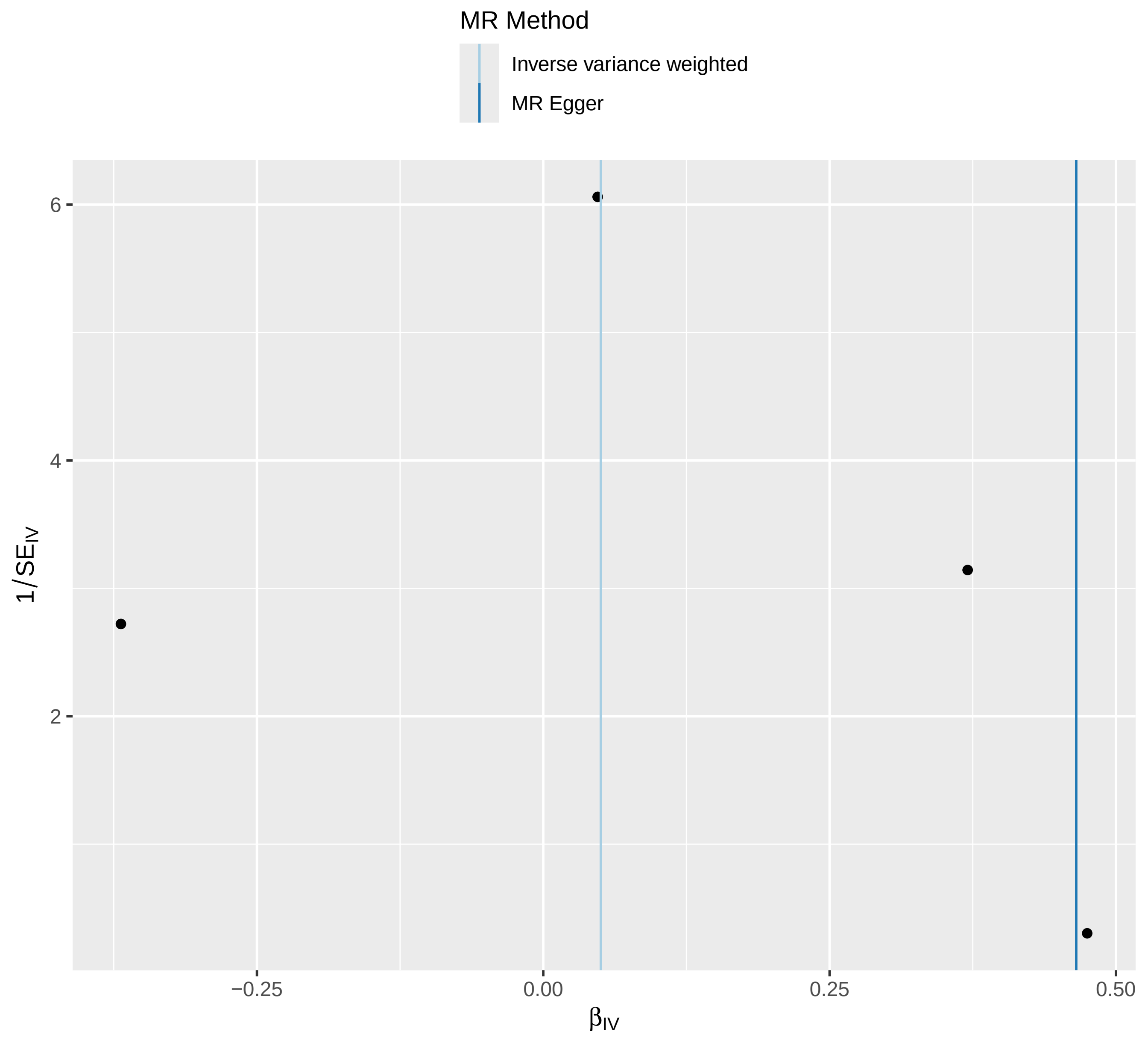

MR Method
Inverse variance weighted
MR Egger
6
4
2
1/SE$_{IV}$
−0.25
0.00
0.25
0.50
β$_{IV}$

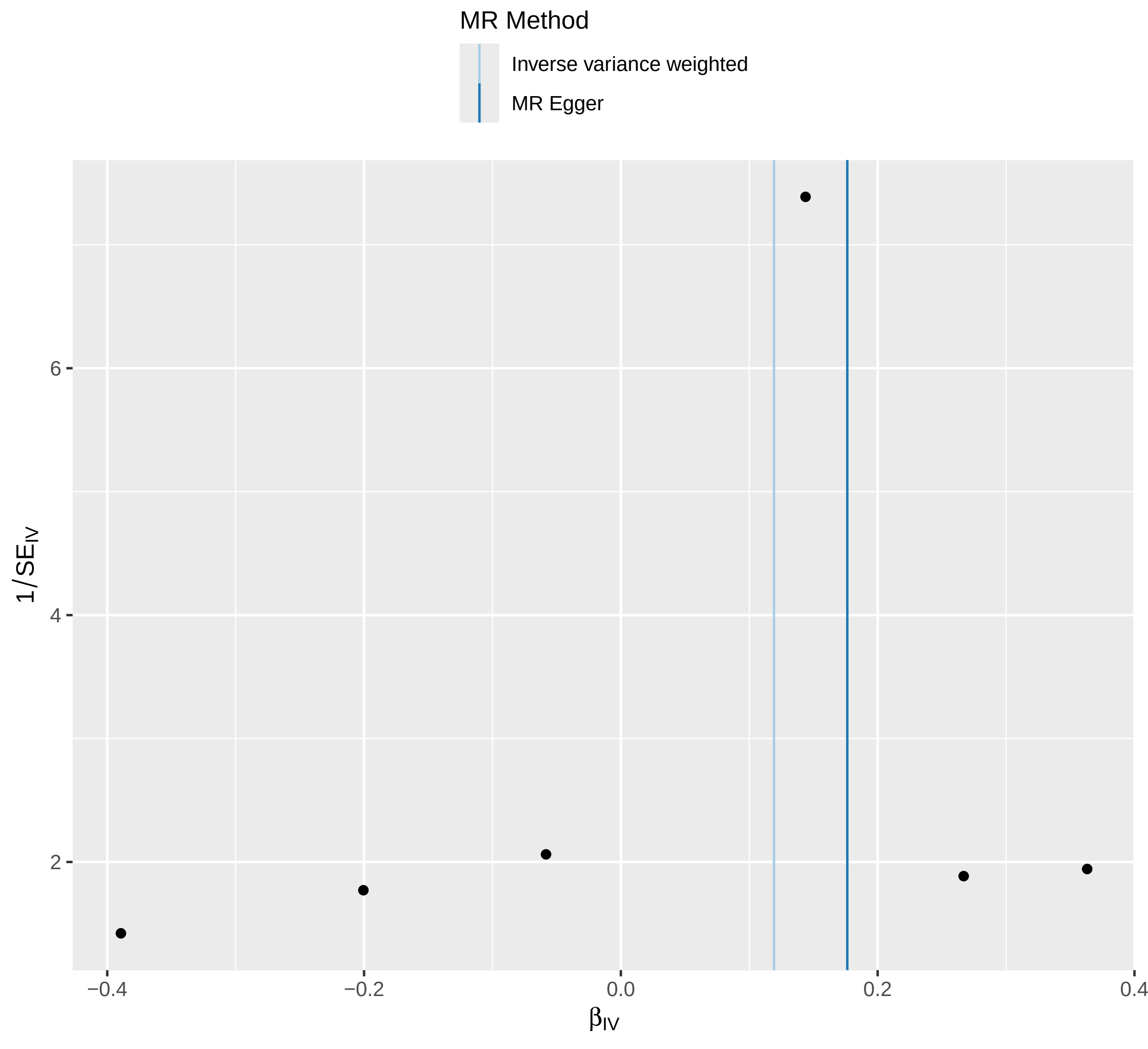

MR Method
Inverse variance weighted
MR Egger
6
4
2
$1/SE_{IV}$
−0.4
−0.2
0.0
0.2
0.4
β_{IV}

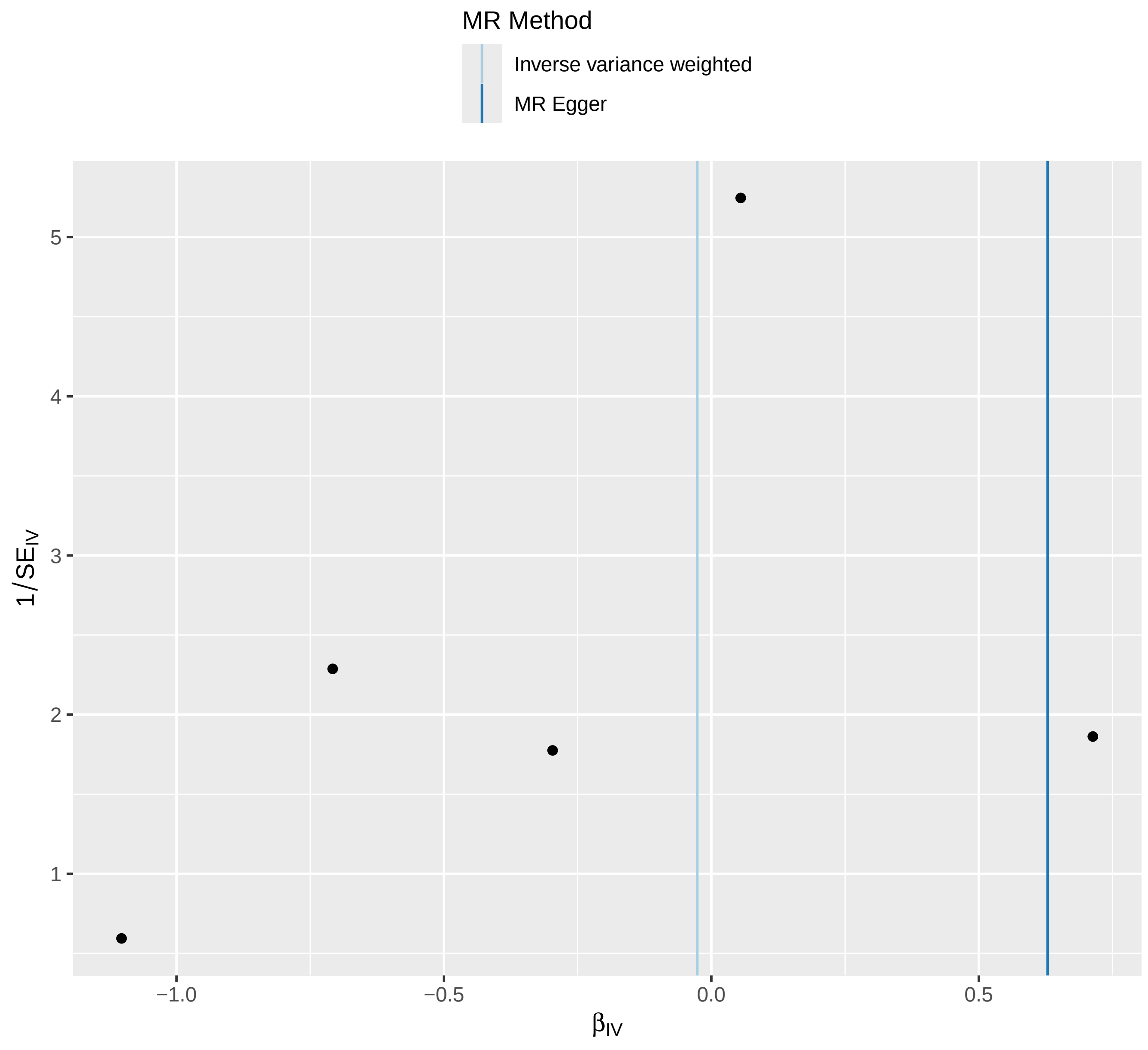

MR Method
Inverse variance weighted
MR Egger
5
4
3
2
1
1/SE_IV
−1.0
−0.5
0.0
0.5
β_IV

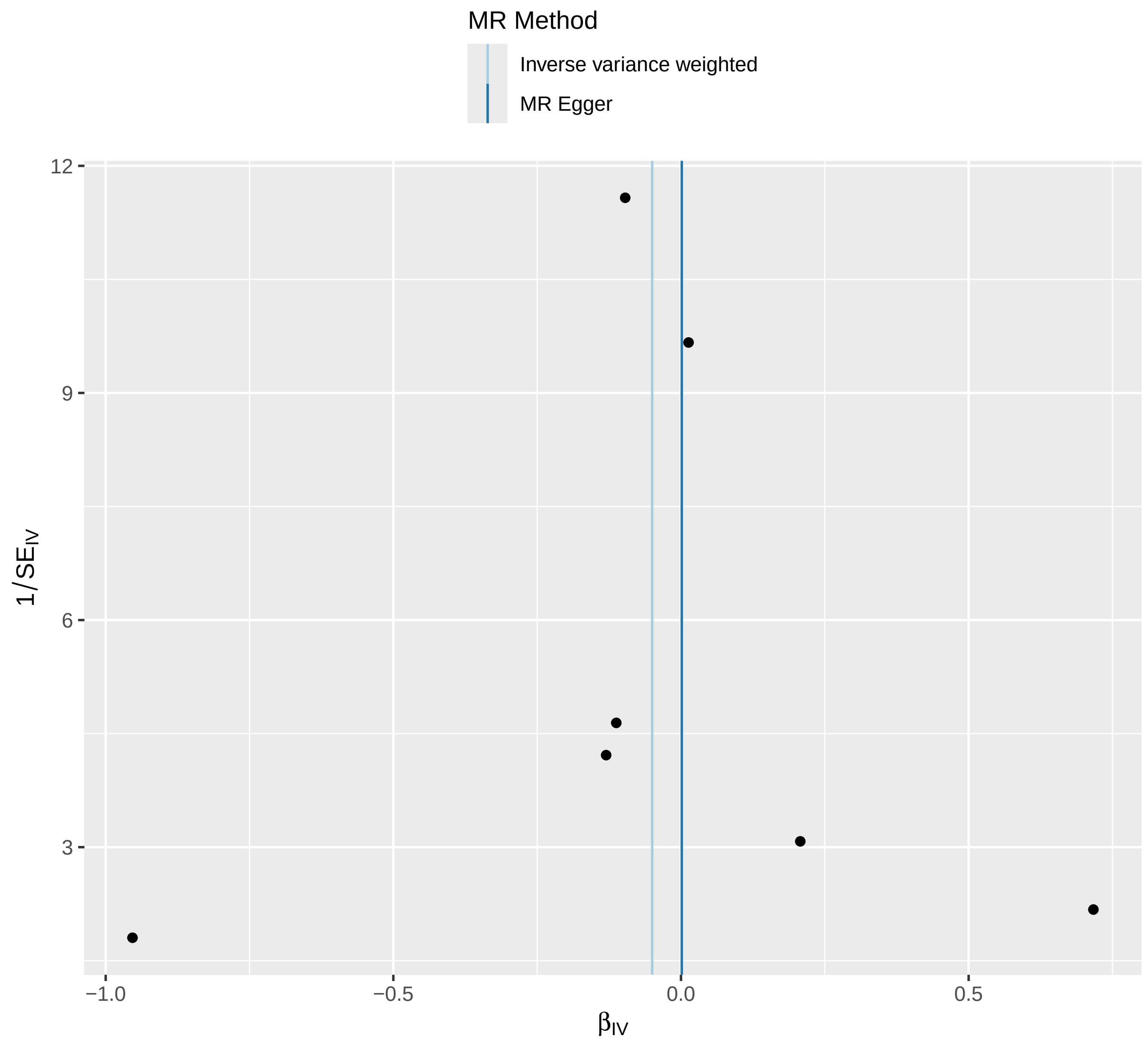
MR Method
Inverse variance weighted
MR Egger
12
9
6
3
1/SE$_{IV}$
−1.0
−0.5
0.0
0.5
β$_{IV}$

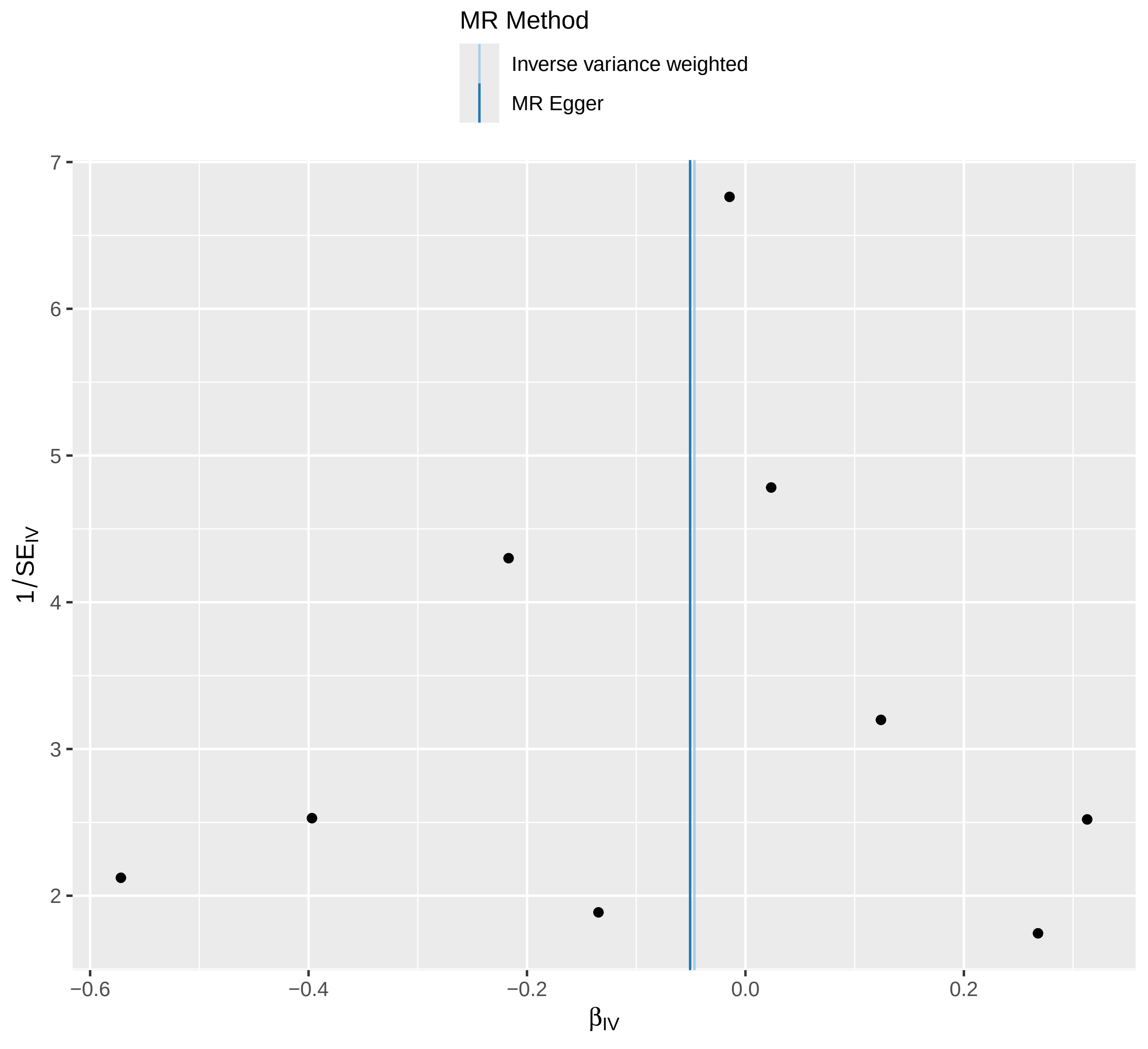

MR Method
Inverse variance weighted
MR Egger
7
6
5
4
3
2
1/SE_IV
−0.6
−0.4
−0.2
0.0
0.2
β_IV

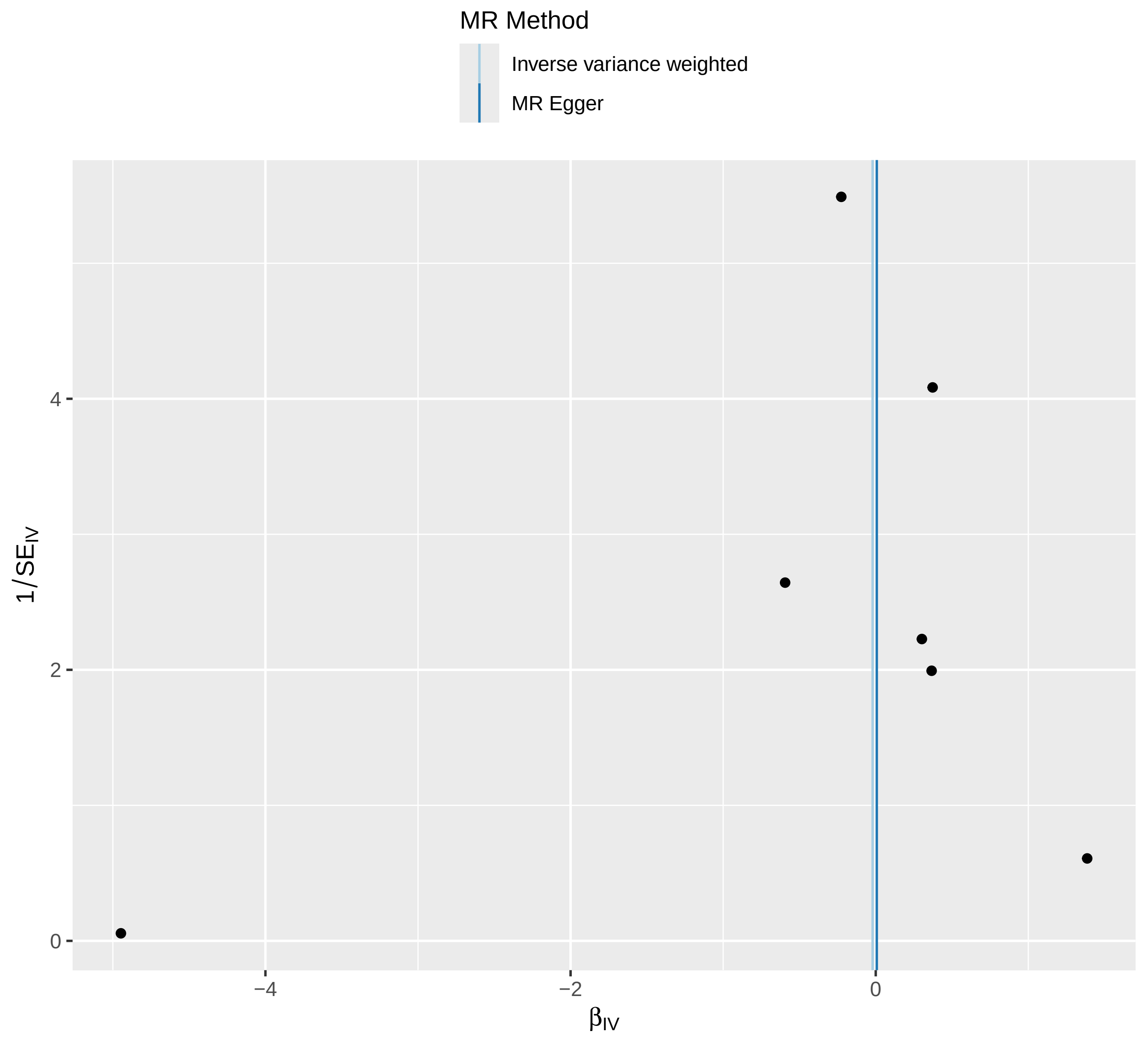
MR Method
Inverse variance weighted
MR Egger
4
2
0
−4
−2
0
β_IV
1/SE_IV

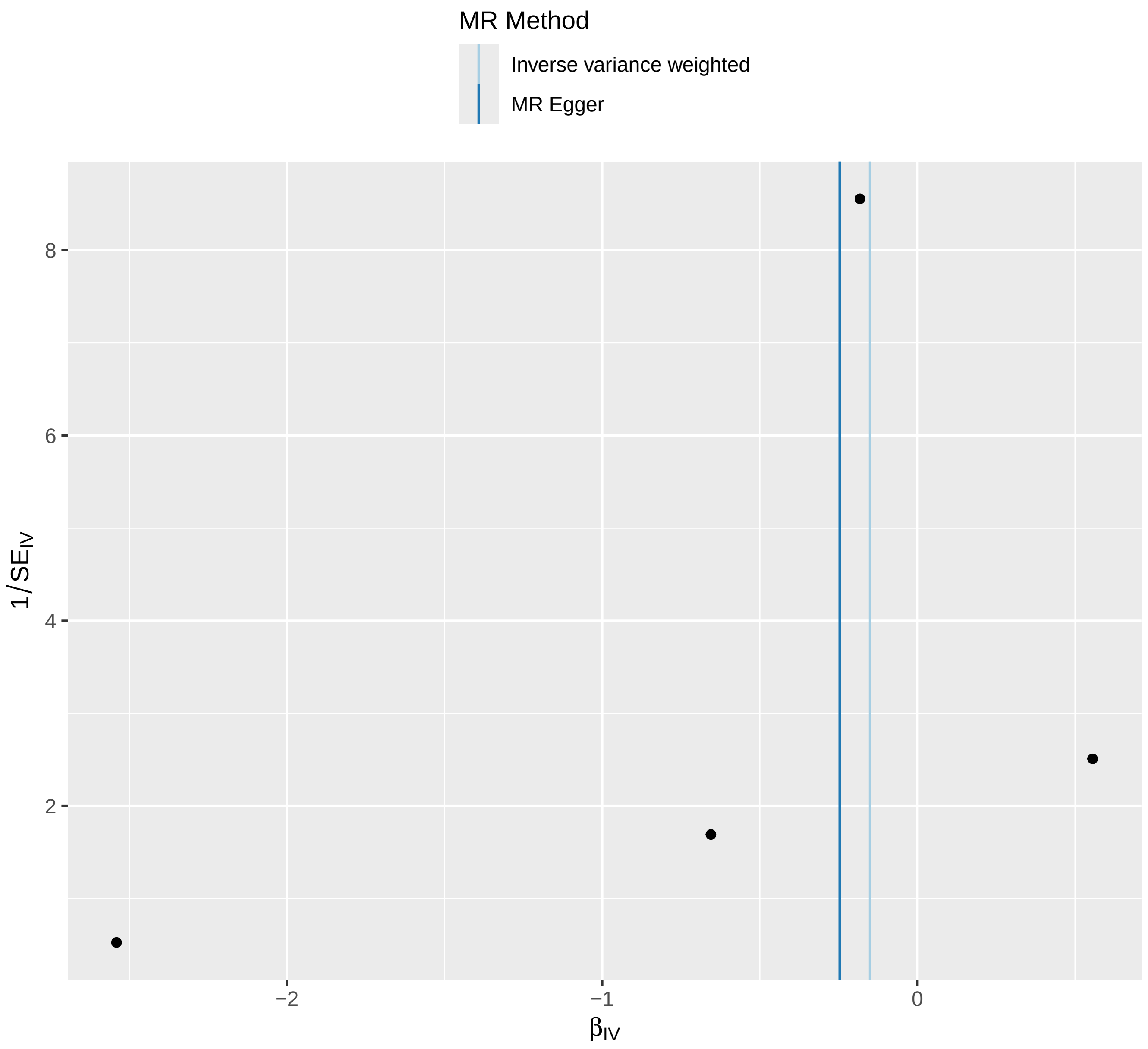
MR Method
Inverse variance weighted
MR Egger
8
6
4
2
1/SE$_{IV}$
−2
−1
0
β_{IV}

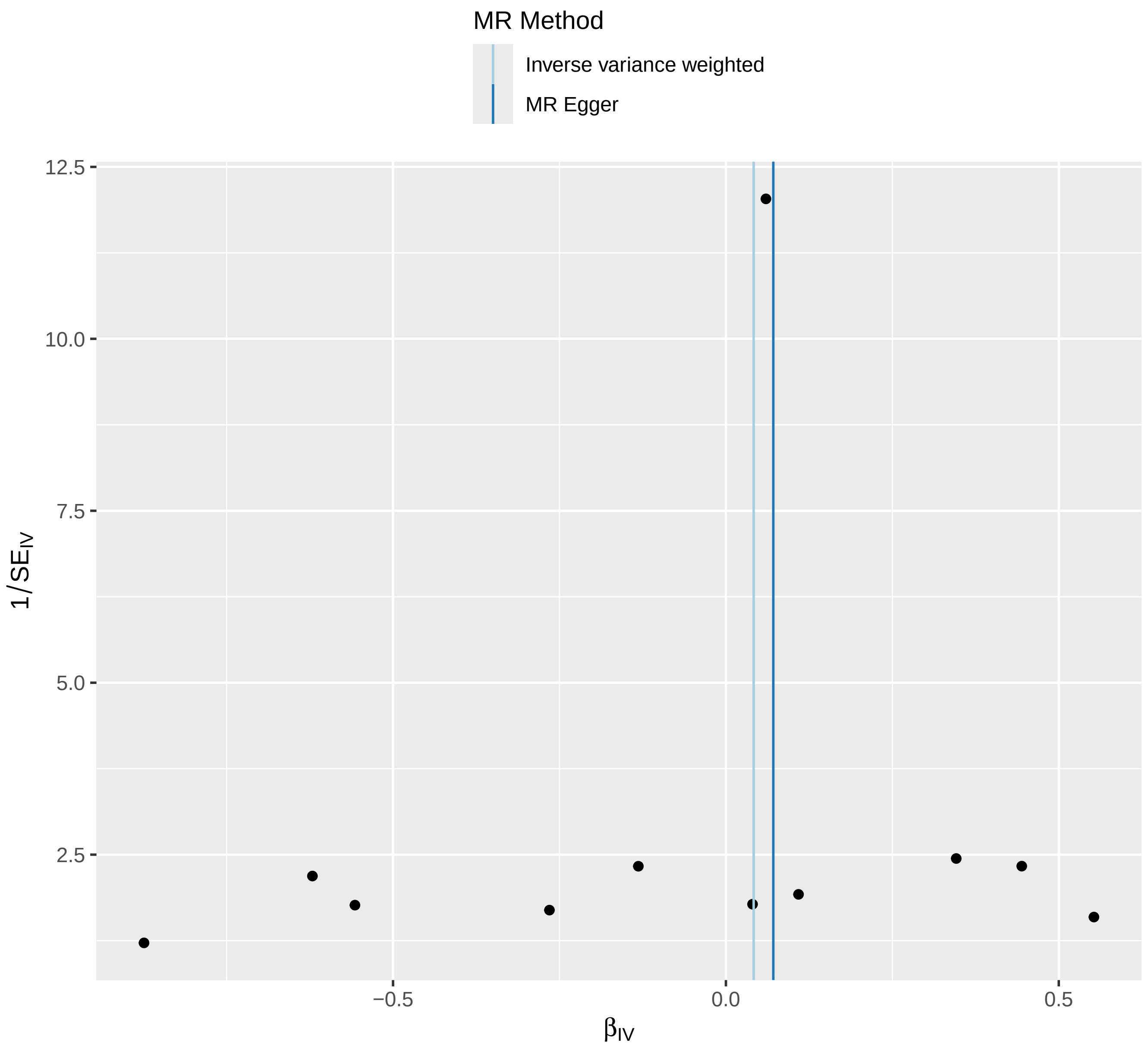
MR Method
Inverse variance weighted
MR Egger
12.5
10.0
7.5
5.0
2.5
1/SE_IV
−0.5
0.0
0.5
β_IV

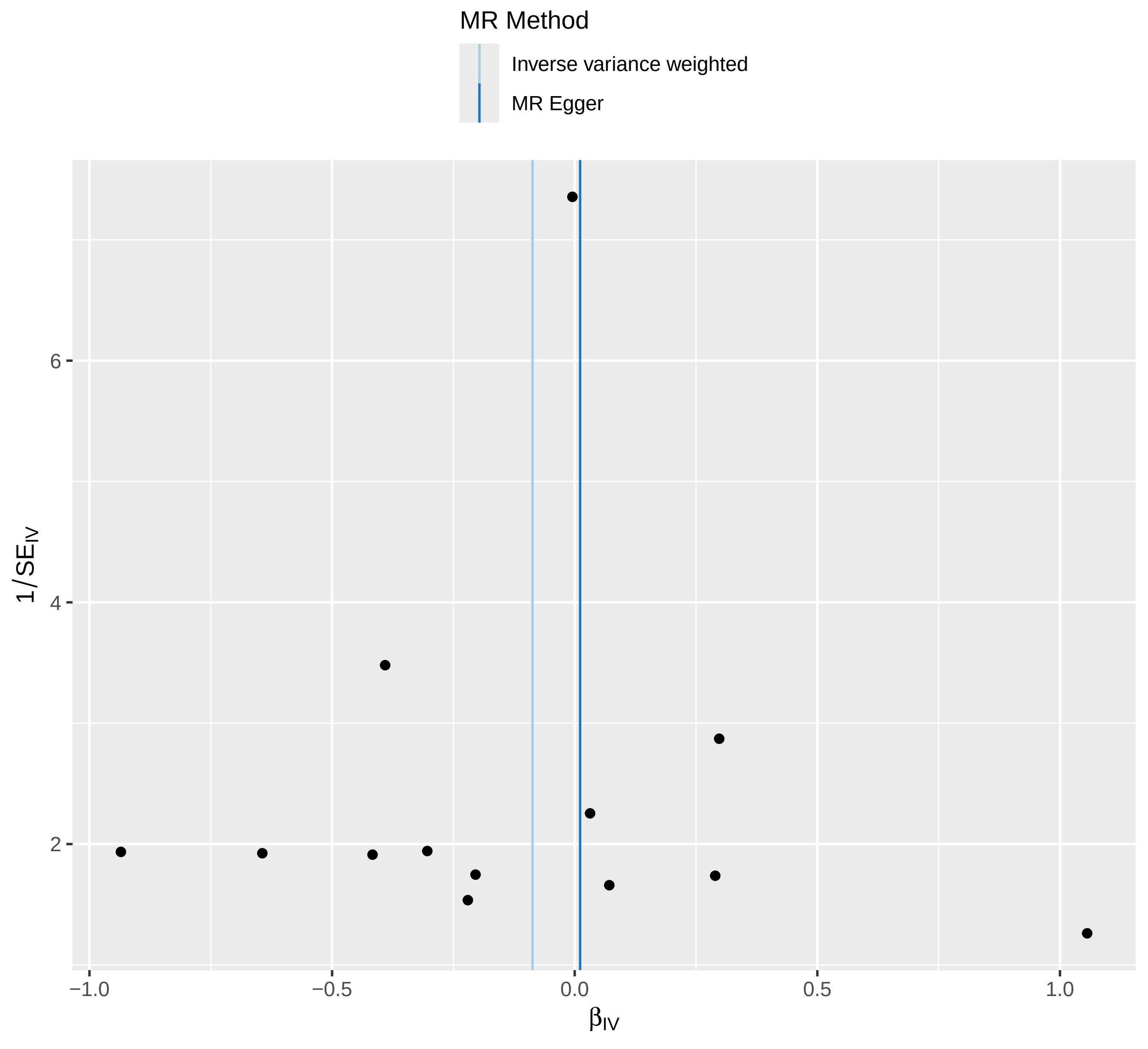

MR Method
Inverse variance weighted
MR Egger
6
4
2
1/SE_IV
−1.0
−0.5
0.0
0.5
1.0
β_IV

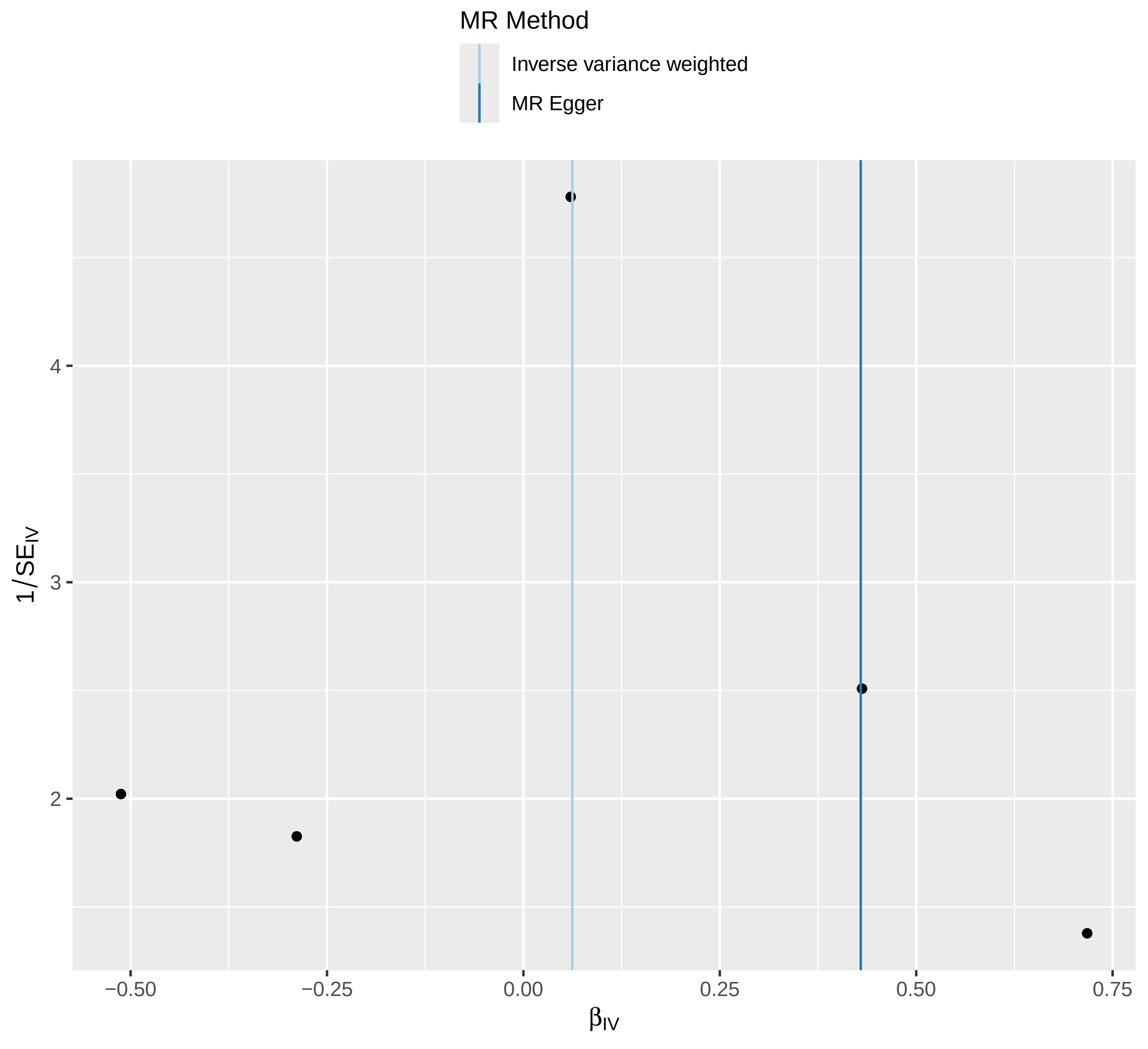
MR Method
Inverse variance weighted
MR Egger
4
3
2
1/SE_IV
−0.50
−0.25
0.00
0.25
0.50
0.75
β_IV

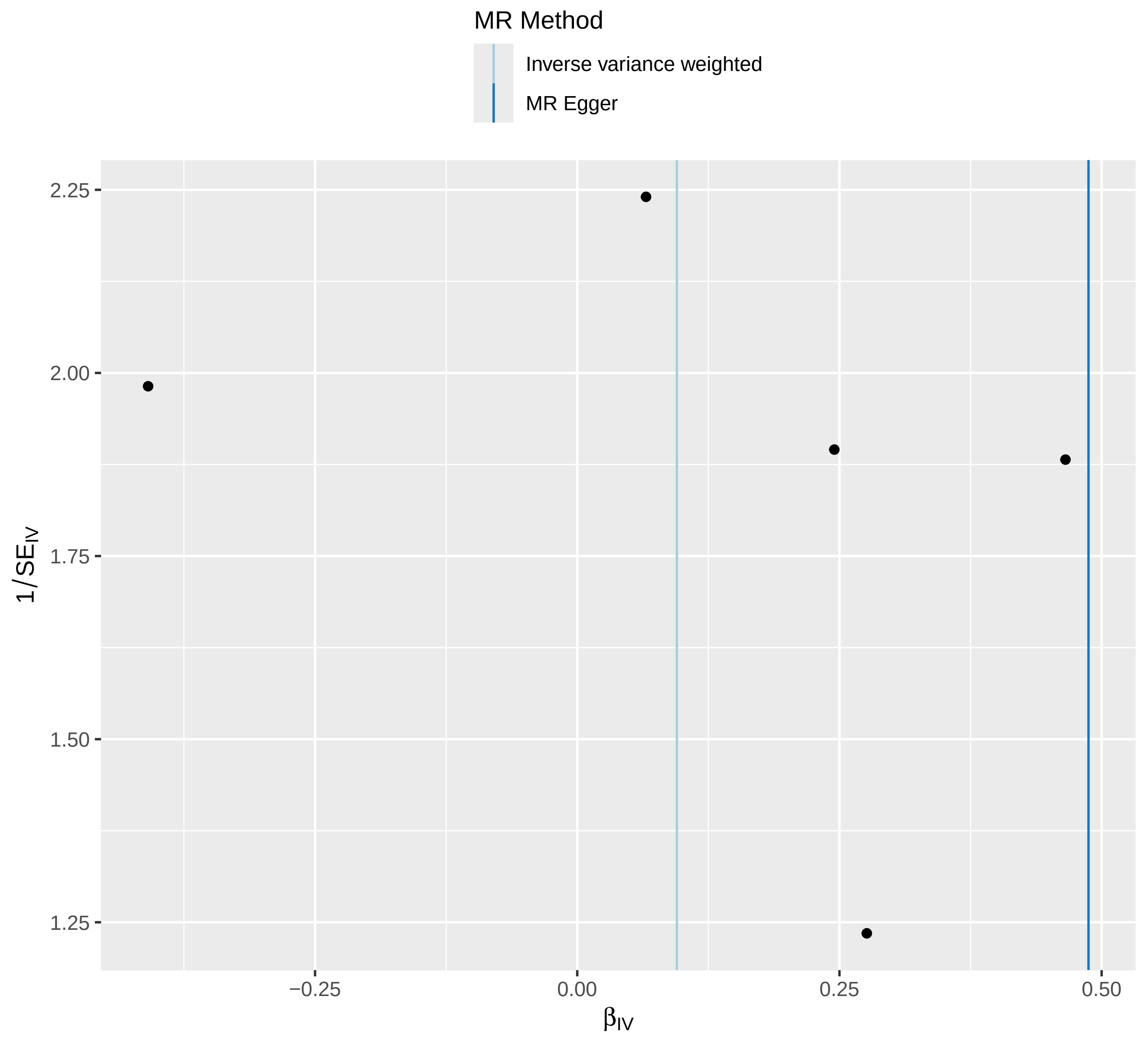

MR Method
Inverse variance weighted
MR Egger
2.25
2.00
1.75
1.50
1.25
1/SE_IV
−0.25
0.00
0.25
0.50
β_IV

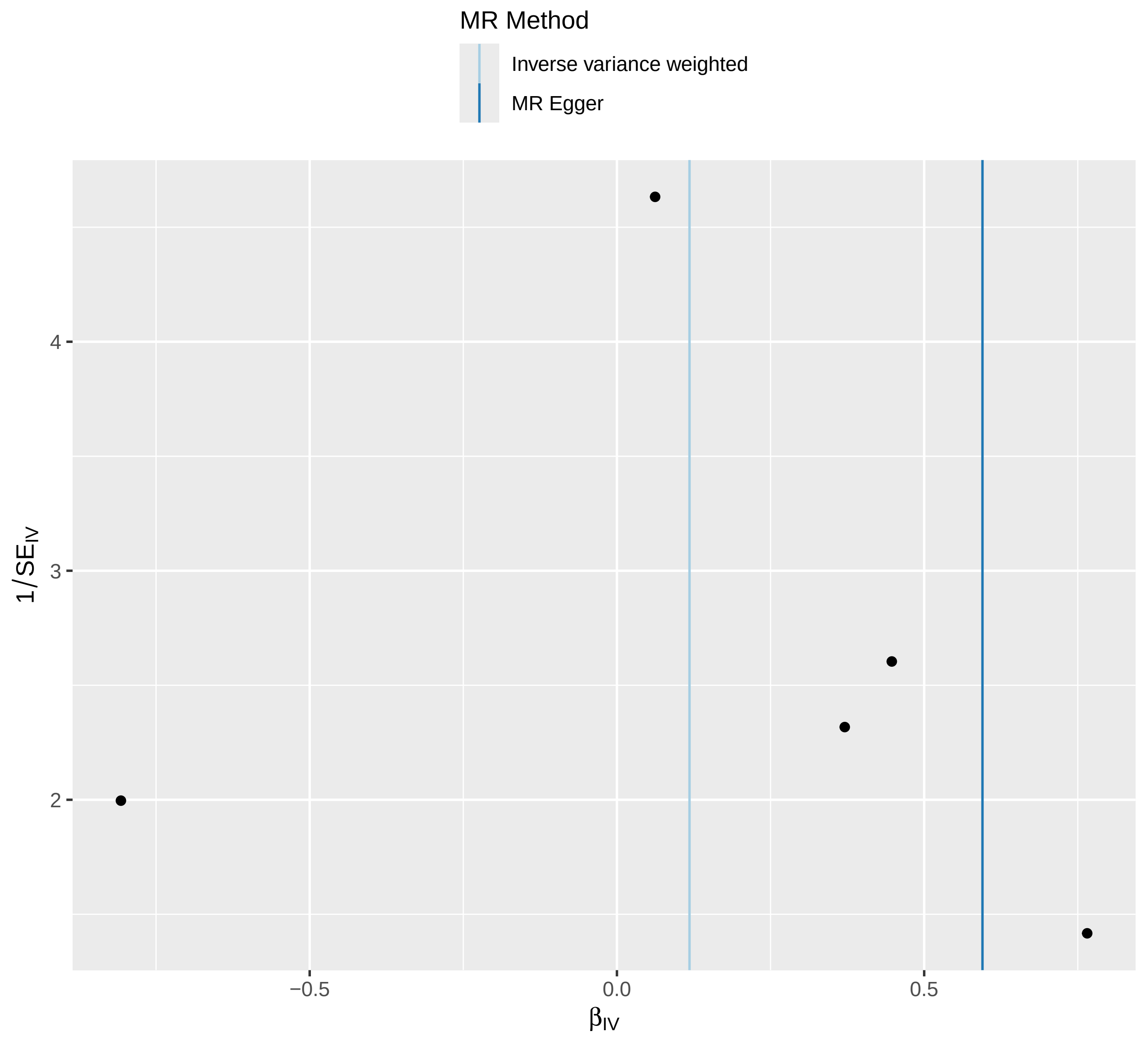
MR Method
Inverse variance weighted
MR Egger
4
3
2
1/SE$_{IV}$
−0.5
0.0
0.5
β$_{IV}$

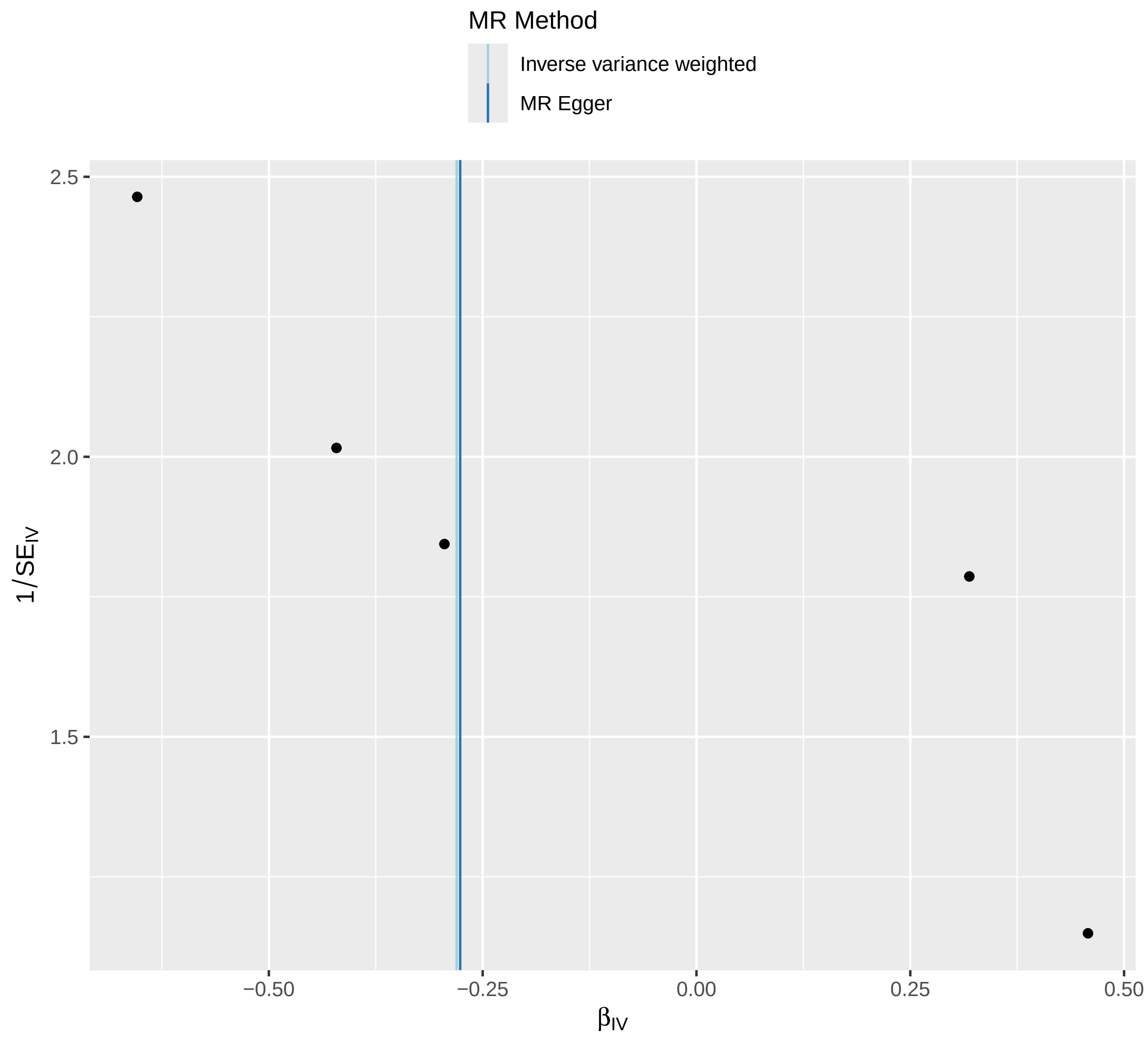

MR Method
Inverse variance weighted
MR Egger
2.5
2.0
1.5
1/SE_IV
−0.50
−0.25
0.00
0.25
0.50
β_IV

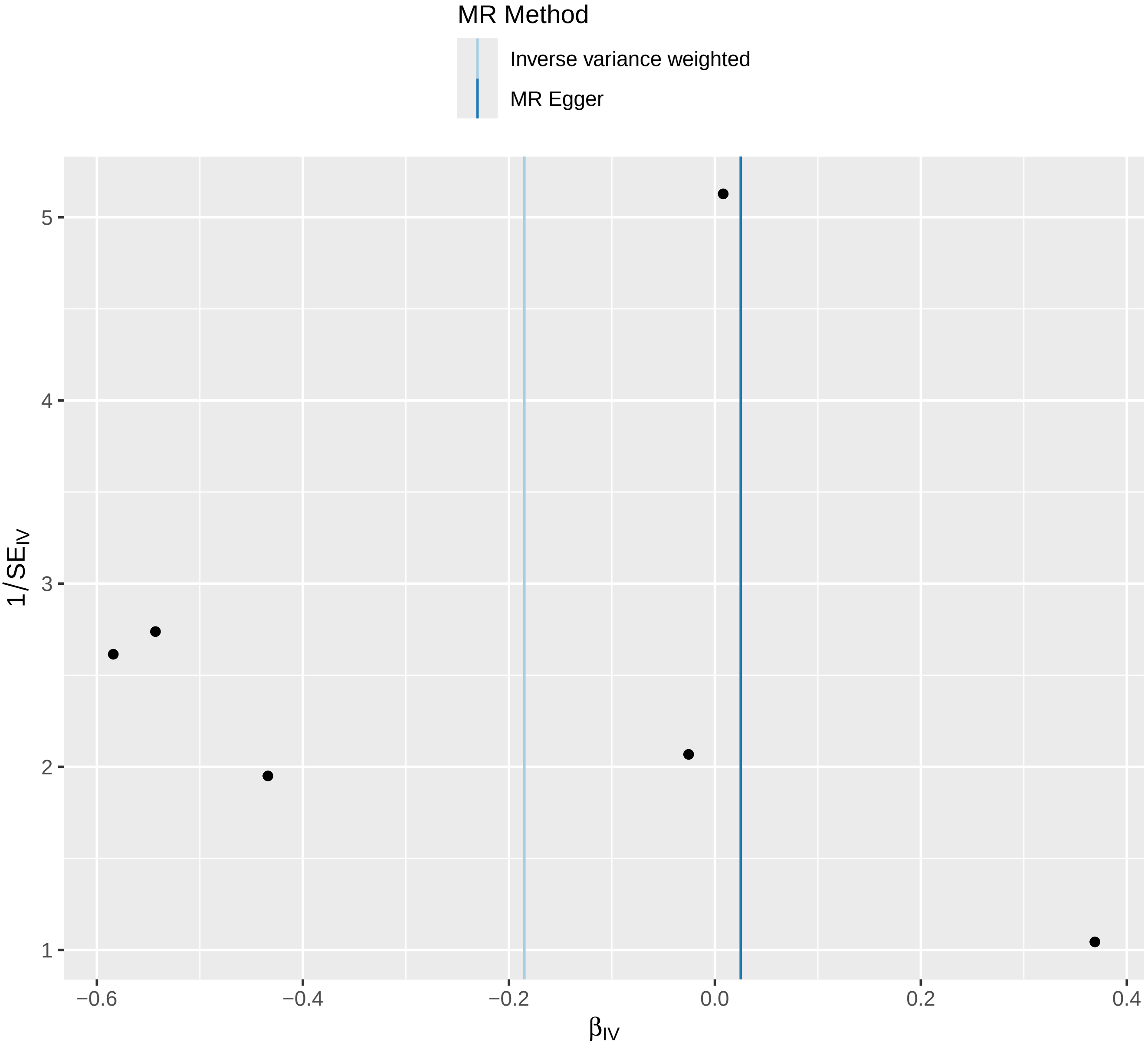

MR Method
Inverse variance weighted
MR Egger
1/SE_IV
5
4
3
2
1
−0.6
−0.4
−0.2
0.0
0.2
0.4
β_IV

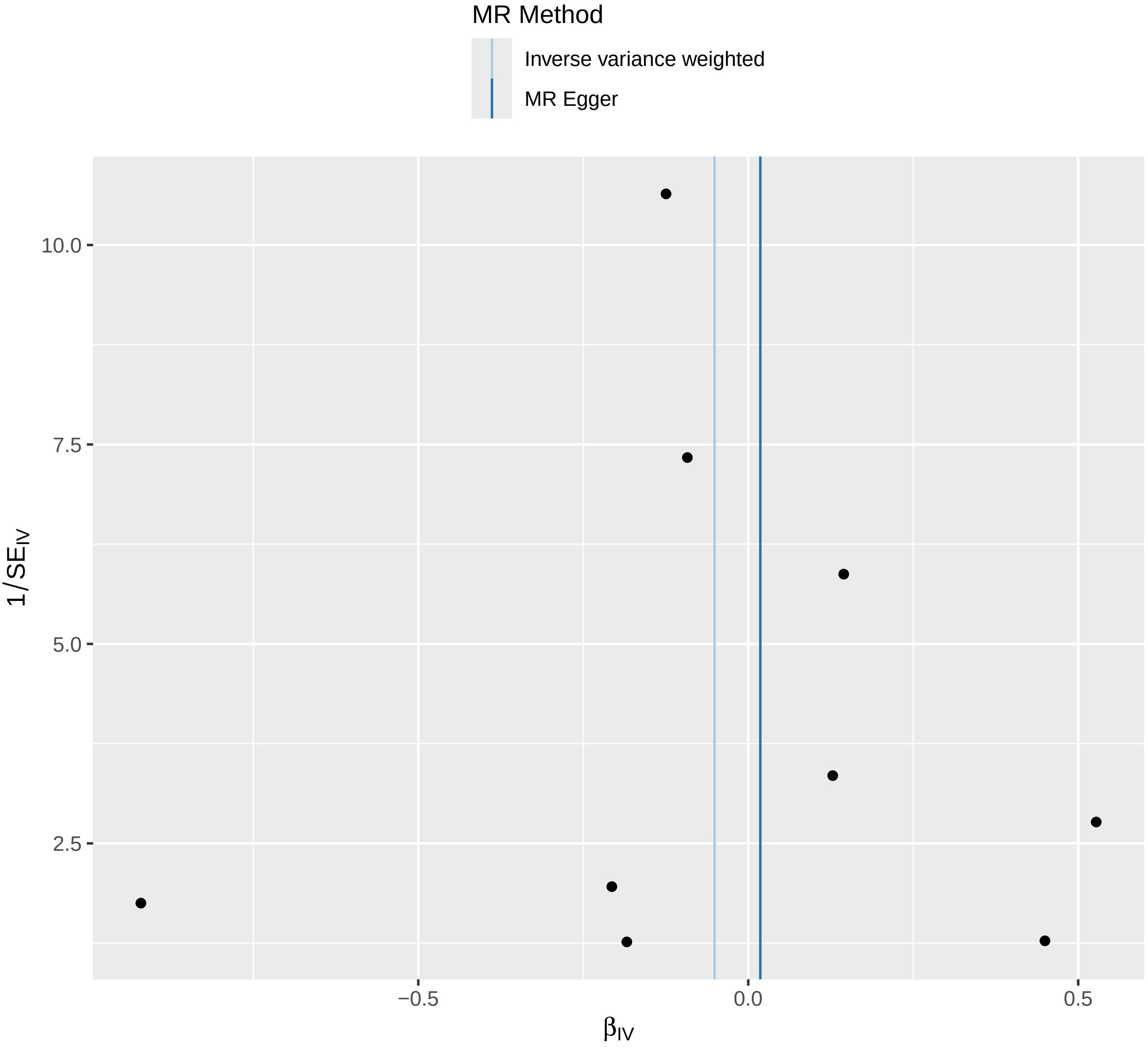

MR Method
Inverse variance weighted
MR Egger
10.0
7.5
5.0
2.5
1/SE_IV
−0.5
0.0
0.5
β_IV

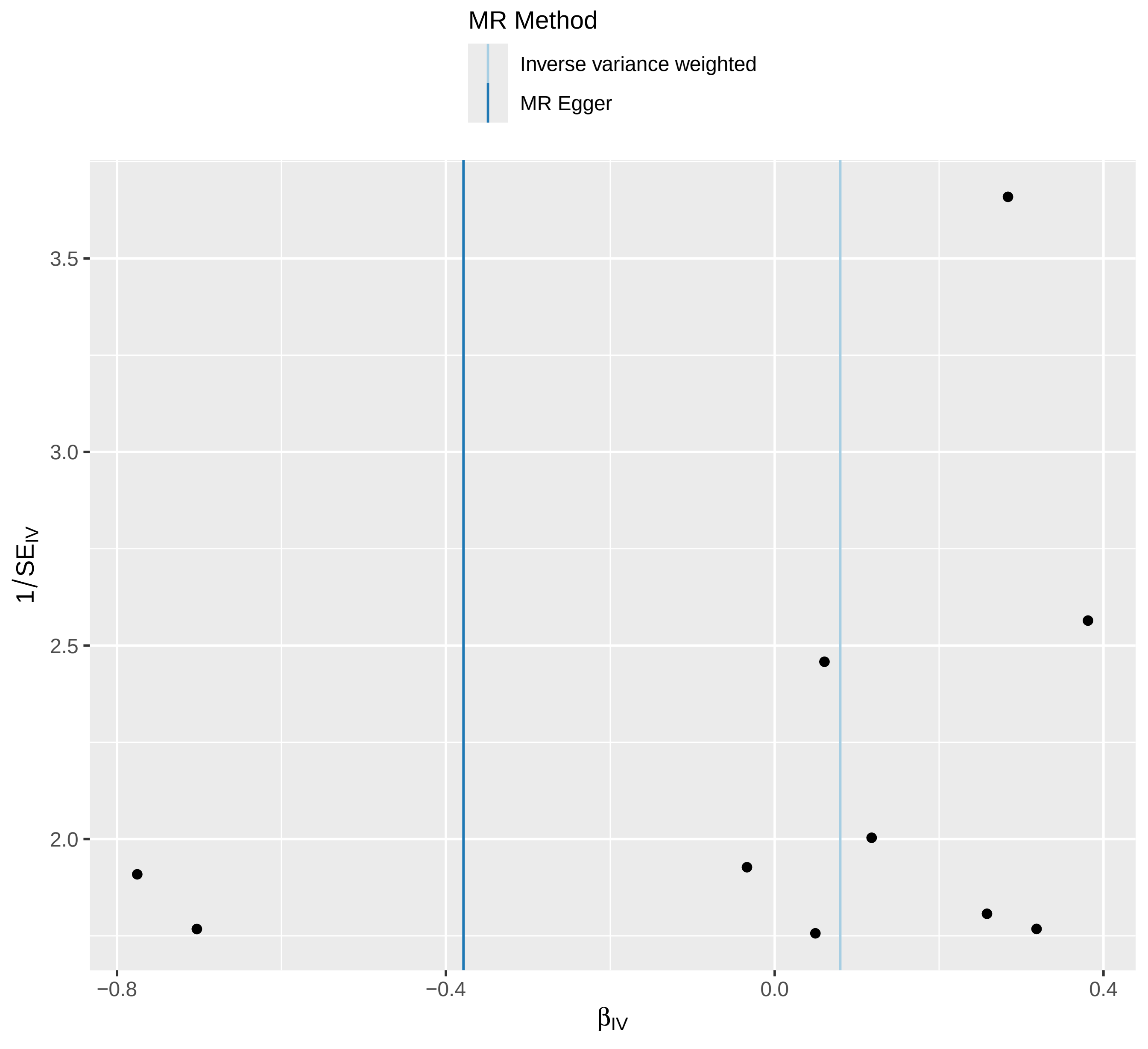
MR Method
Inverse variance weighted
MR Egger
3.5
3.0
2.5
2.0
1/SE$_{IV}$
−0.8
−0.4
0.0
0.4
β$_{IV}$

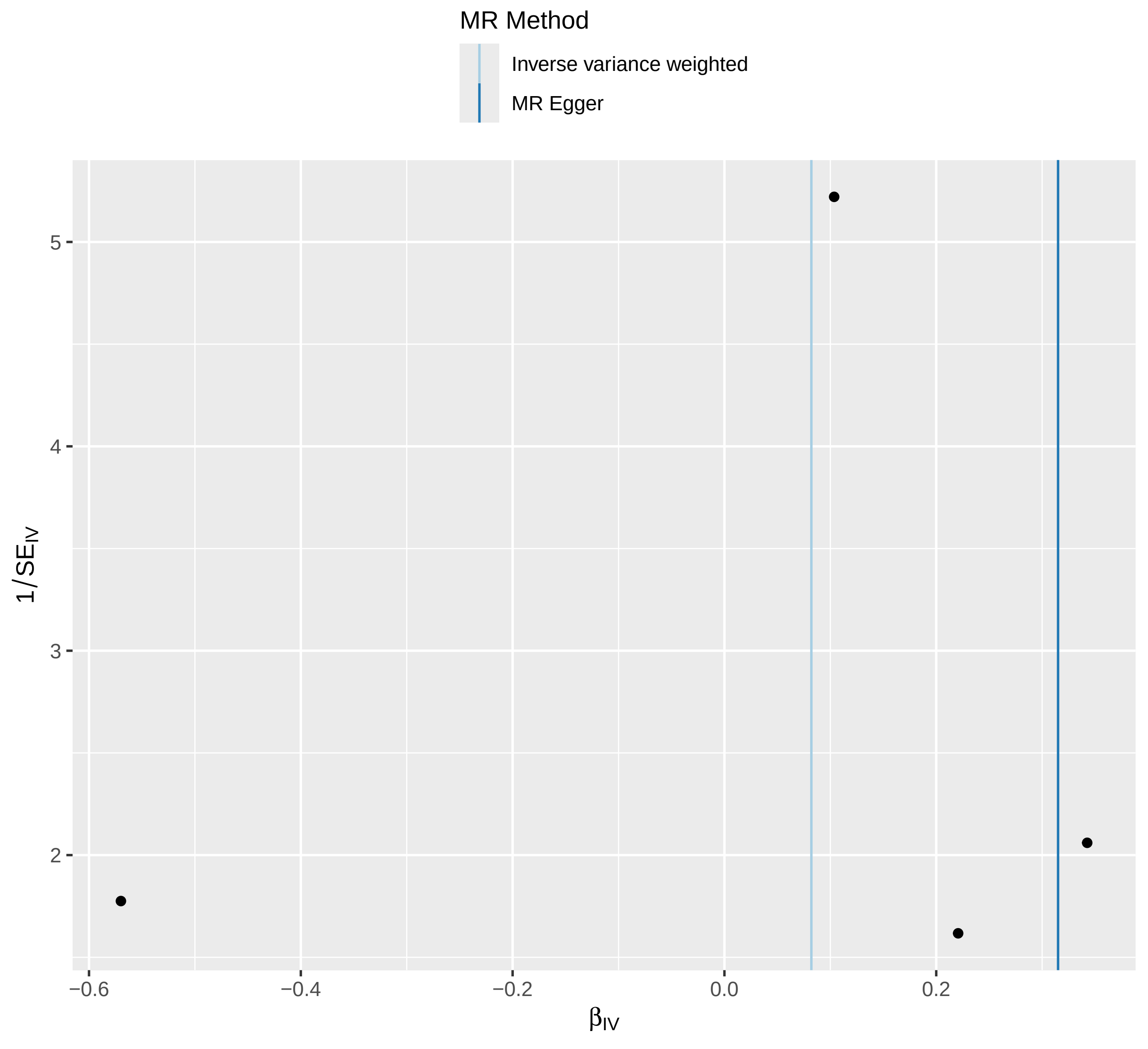

MR Method
Inverse variance weighted
MR Egger
1/SE_IV
5
4
3
2
−0.6
−0.4
−0.2
0.0
0.2
β_IV

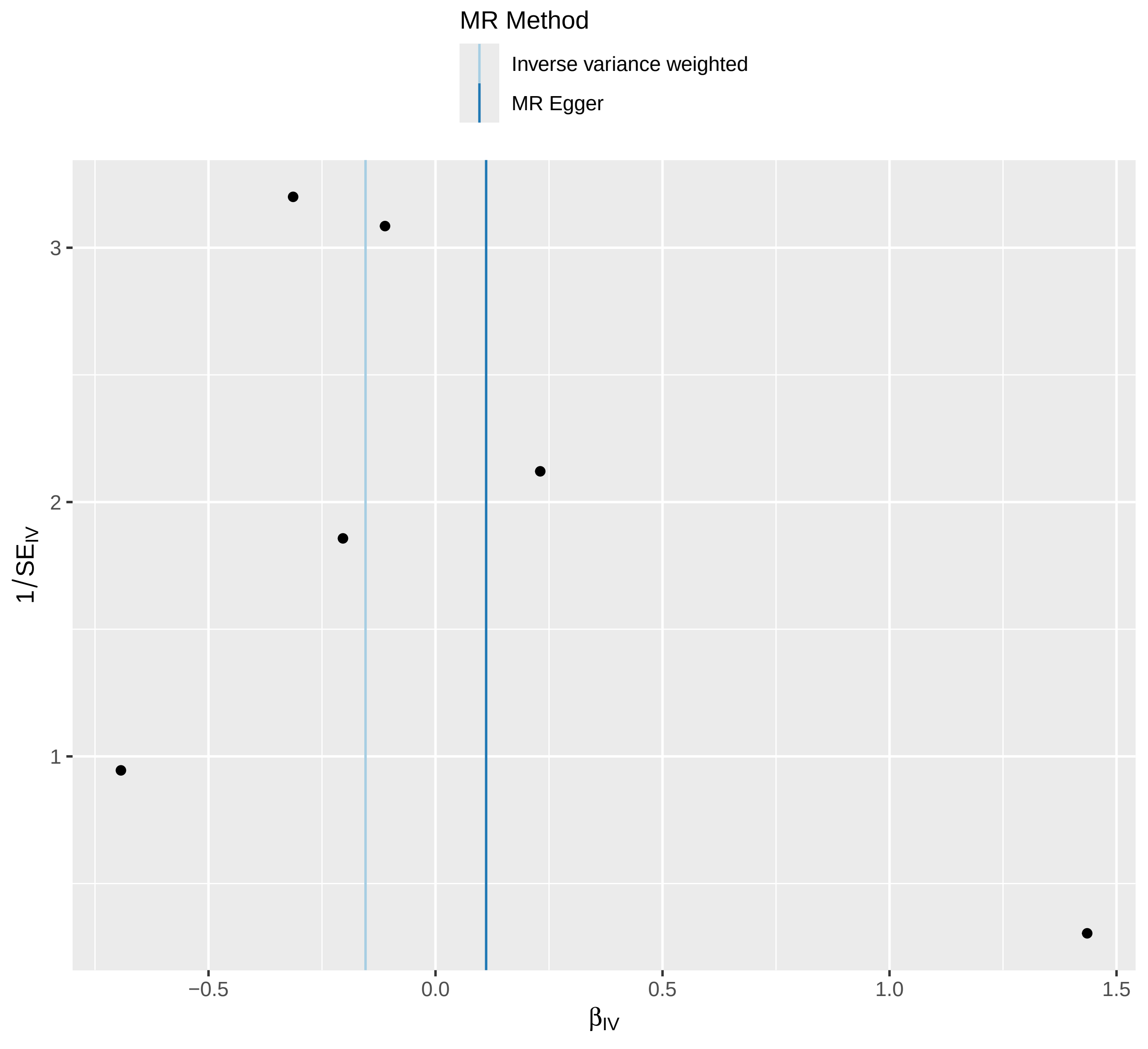

MR Method
Inverse variance weighted
MR Egger
3
2
1
1/SE_IV
−0.5
0.0
0.5
1.0
1.5
β_IV

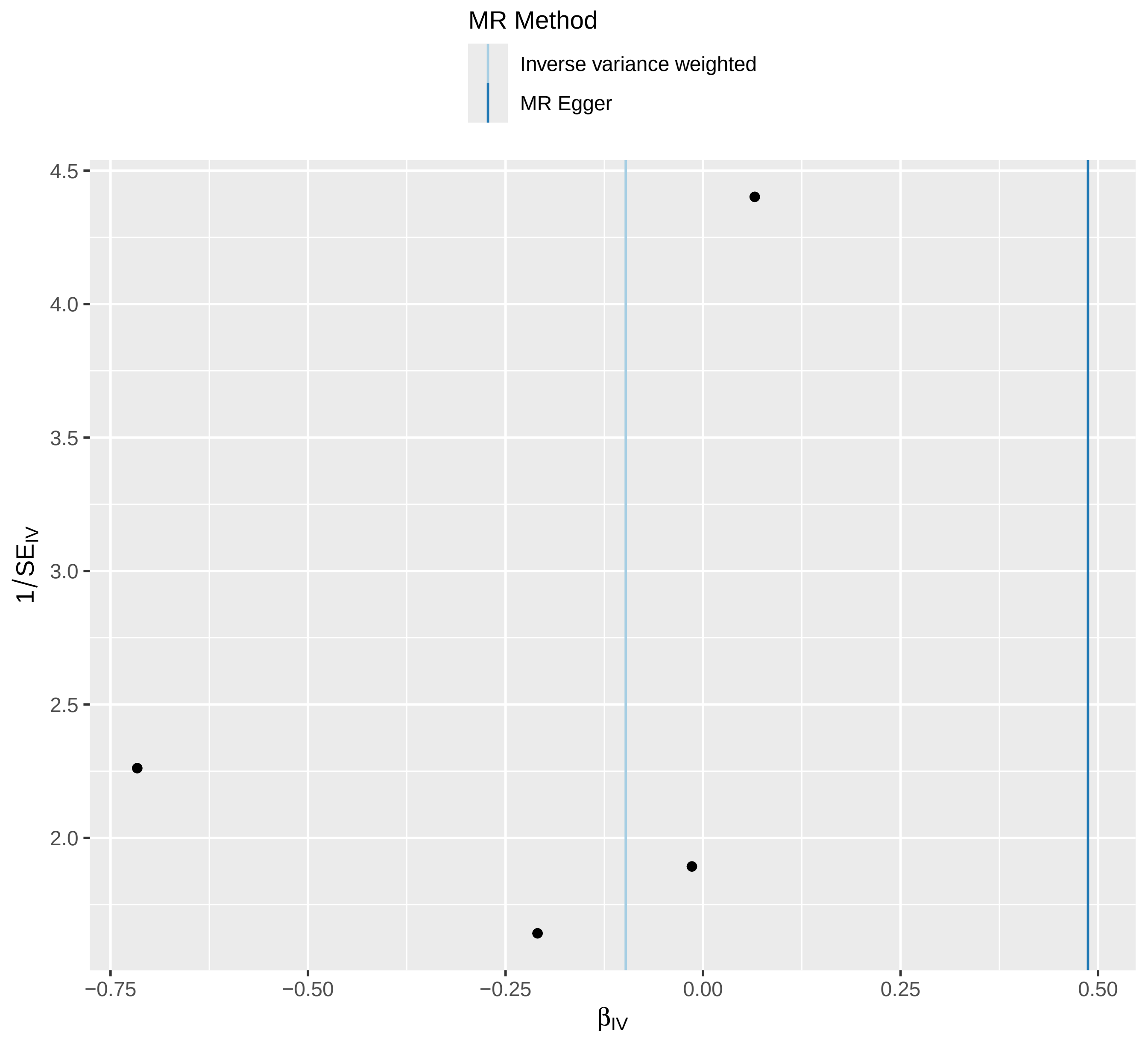

MR Method
Inverse variance weighted
MR Egger
4.5
4.0
3.5
3.0
2.5
2.0
1/SE_IV
−0.75
−0.50
−0.25
0.00
0.25
0.50
β_IV

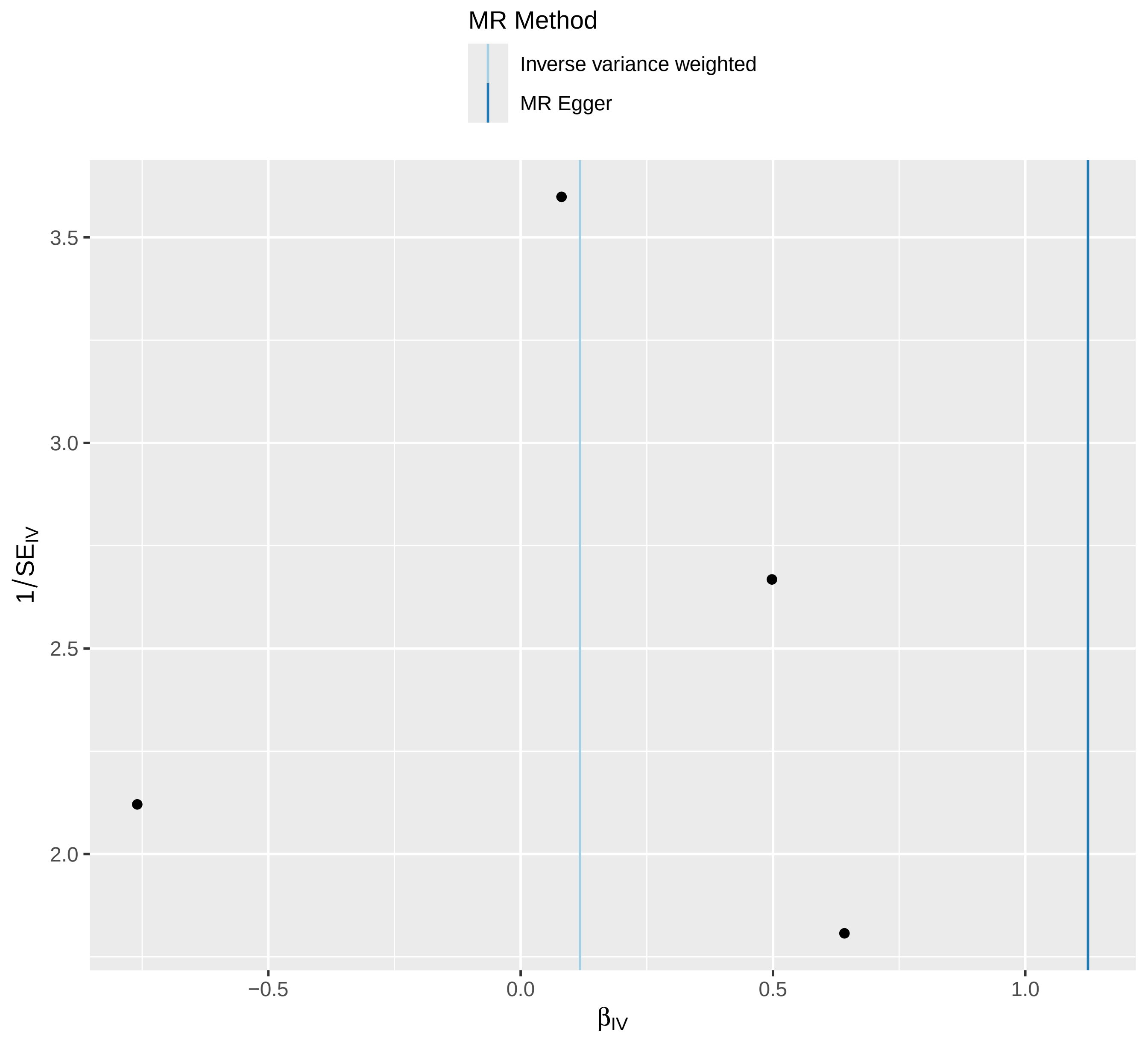
MR Method
Inverse variance weighted
MR Egger
3.5
3.0
2.5
2.0
1/SE_IV
−0.5
0.0
0.5
1.0
β_IV

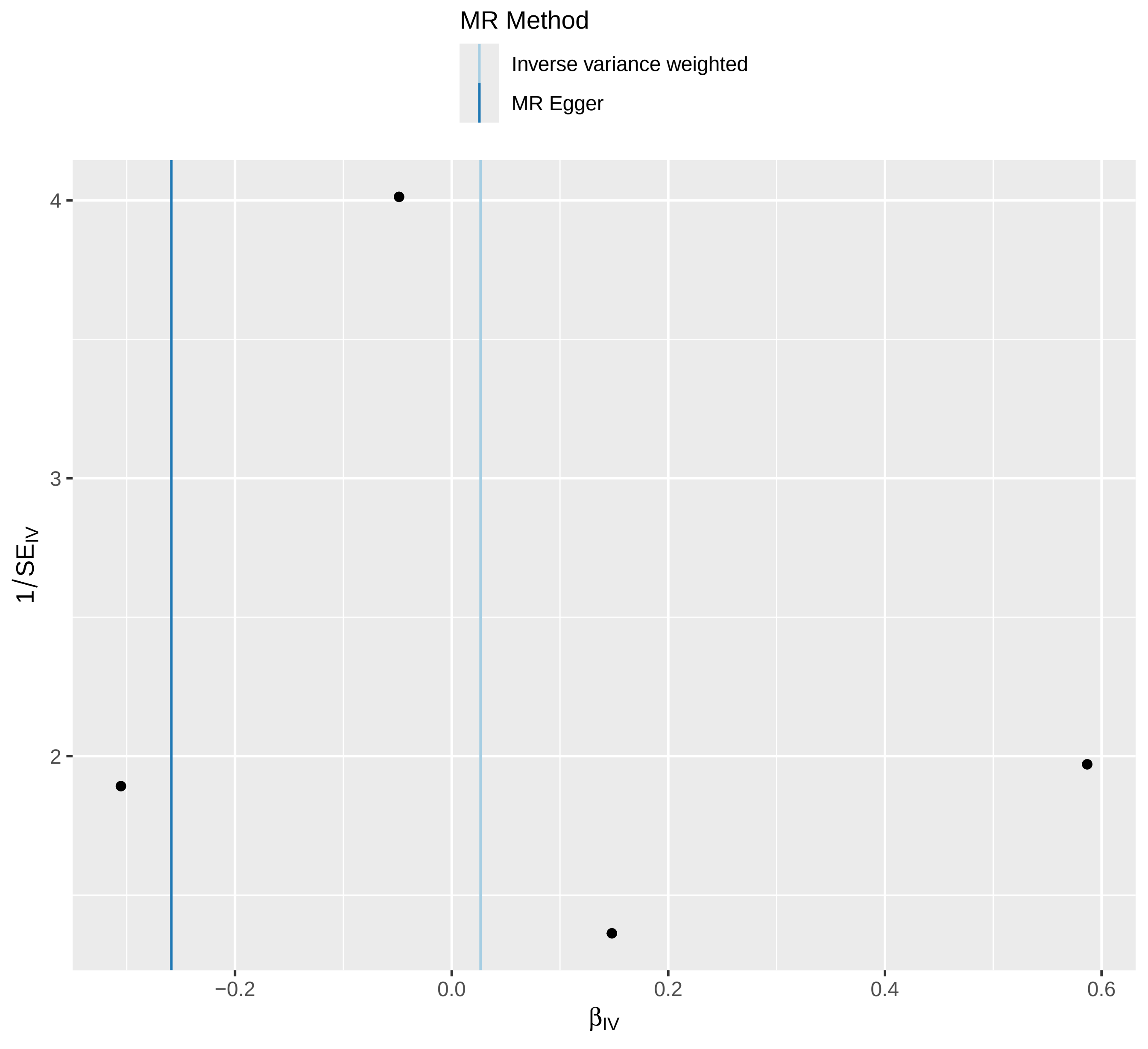

MR Method
Inverse variance weighted
MR Egger
4
3
2
1/SE_IV
−0.2
0.0
0.2
0.4
0.6
β_IV

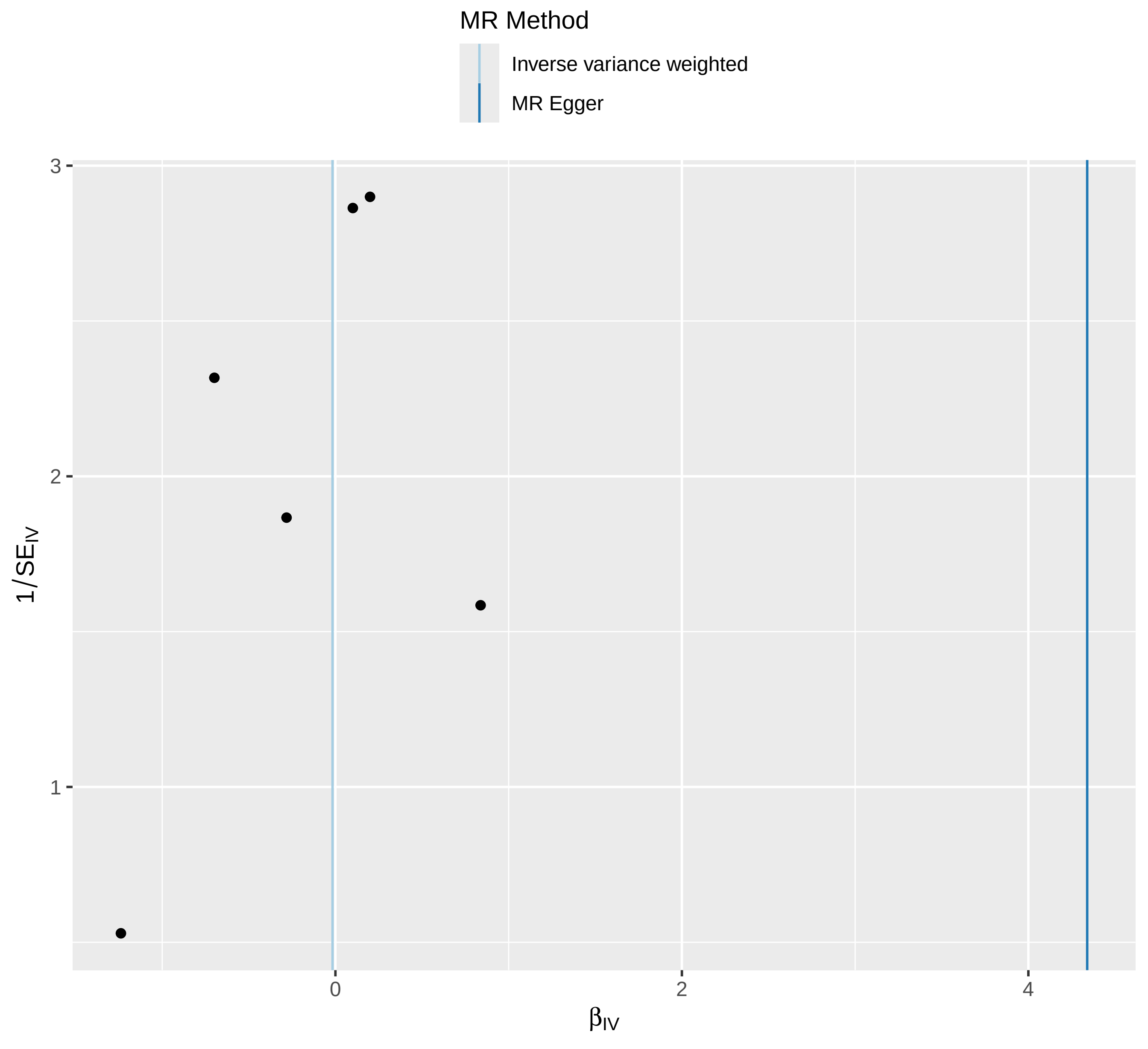

MR Method
Inverse variance weighted
MR Egger
3
2
1
0
2
4
$1/SE_{IV}$
β_{IV}

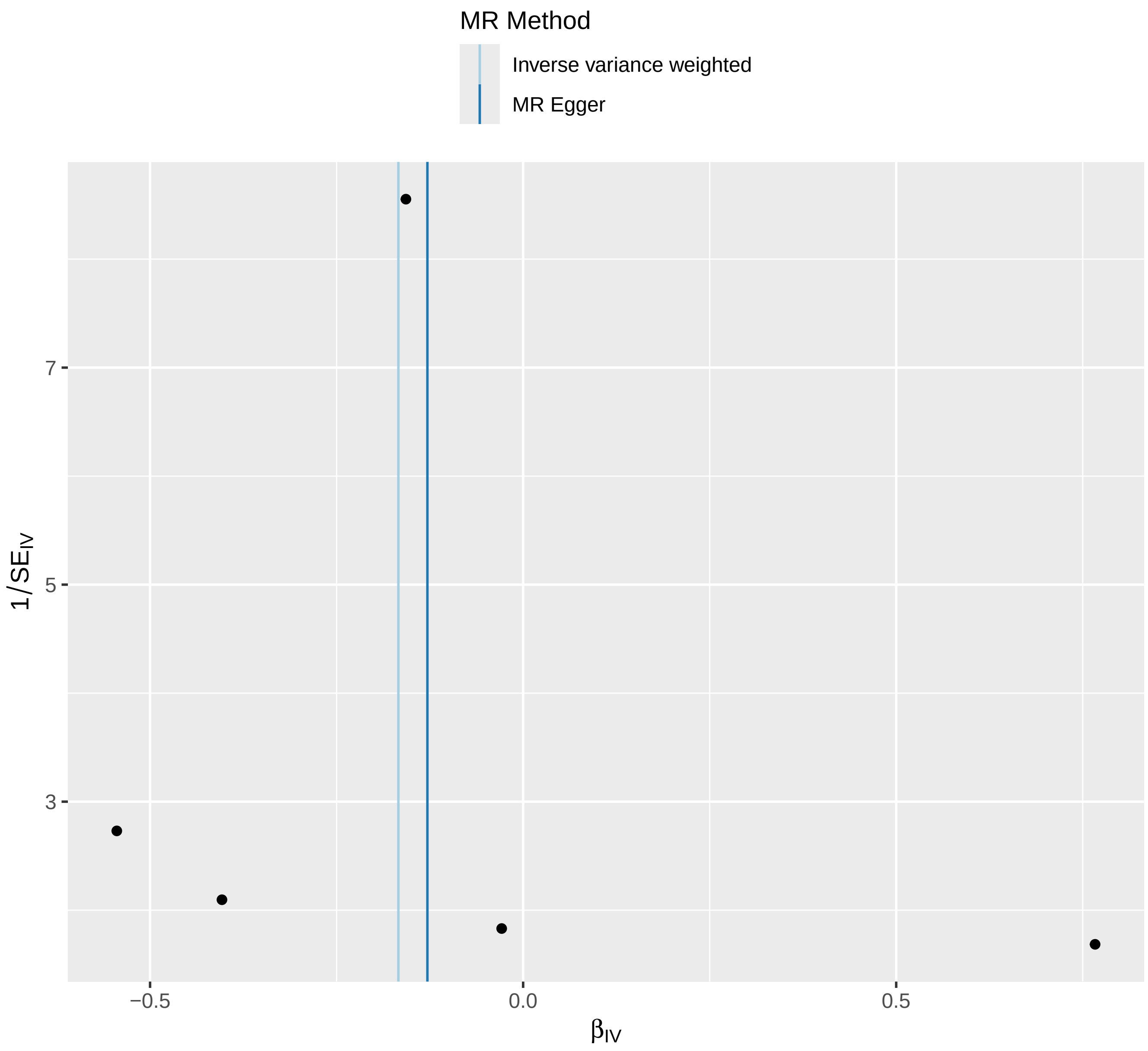
MR Method
Inverse variance weighted
MR Egger
7
5
3
1/SE_IV
−0.5
0.0
0.5
β_IV

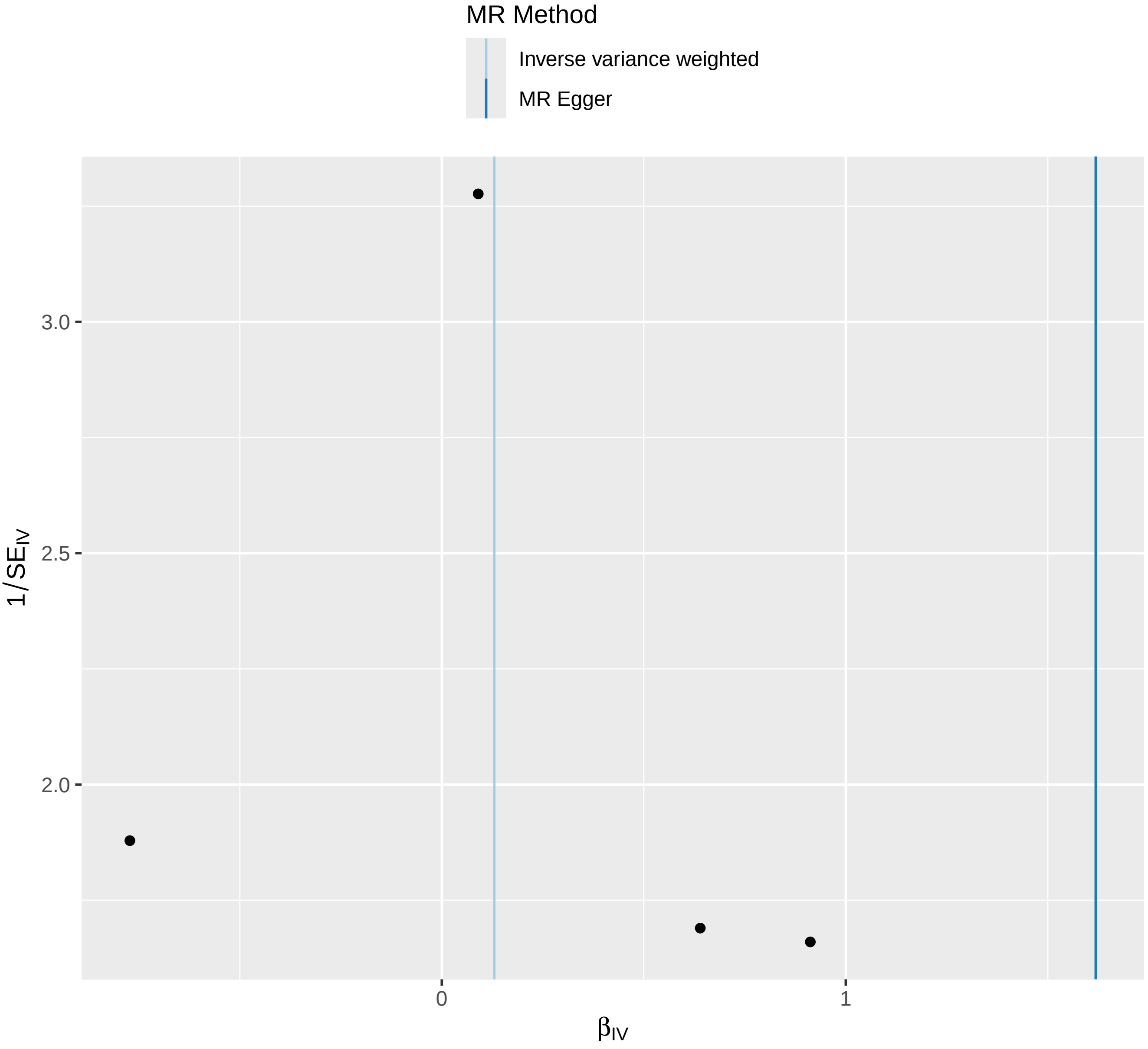

MR Method
Inverse variance weighted
MR Egger
3.0
2.5
2.0
$1/SE_{IV}$
0
1
β_{IV}

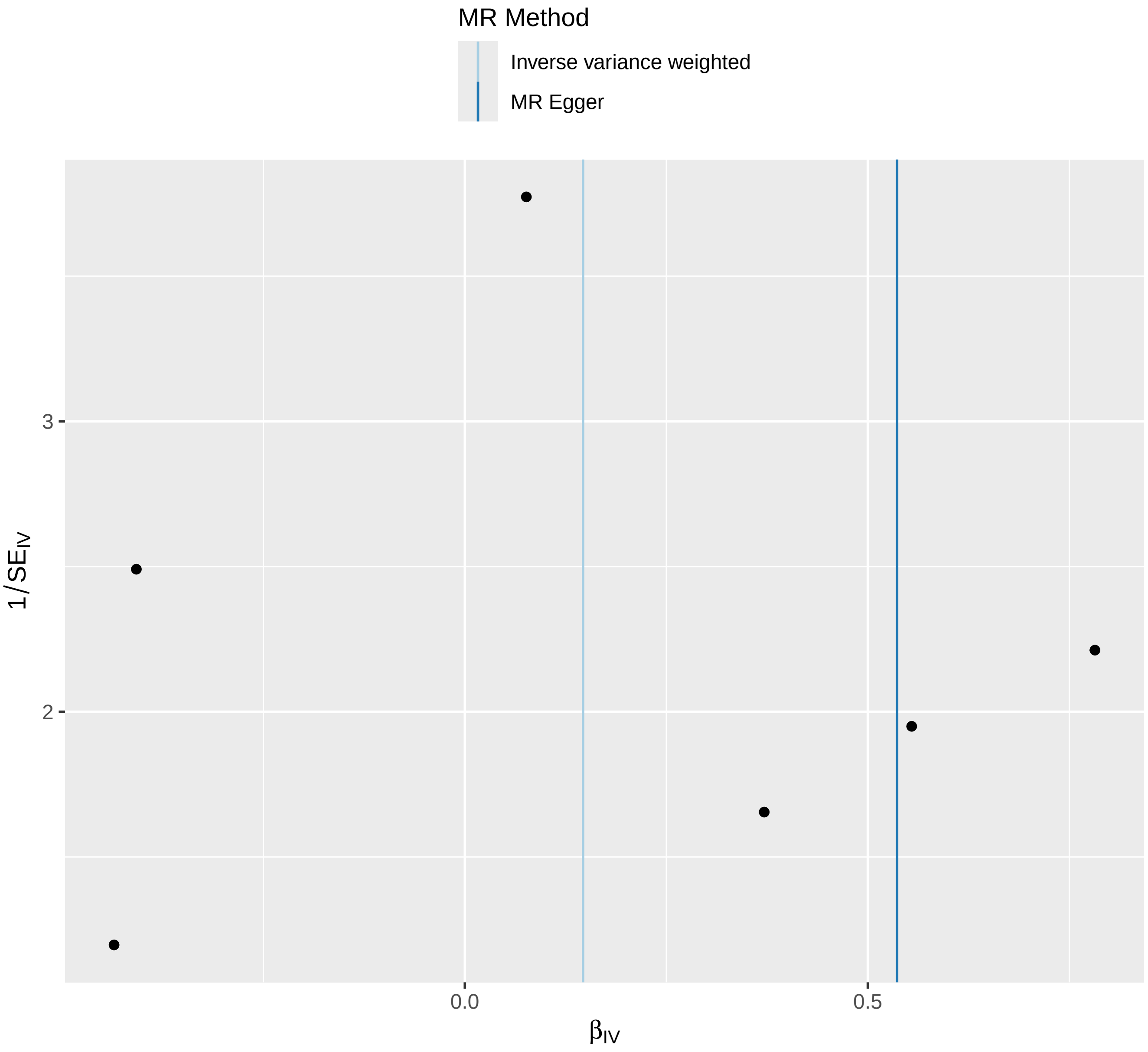

MR Method
Inverse variance weighted
MR Egger
3
2
1/SE_IV
0.0
0.5
β_IV

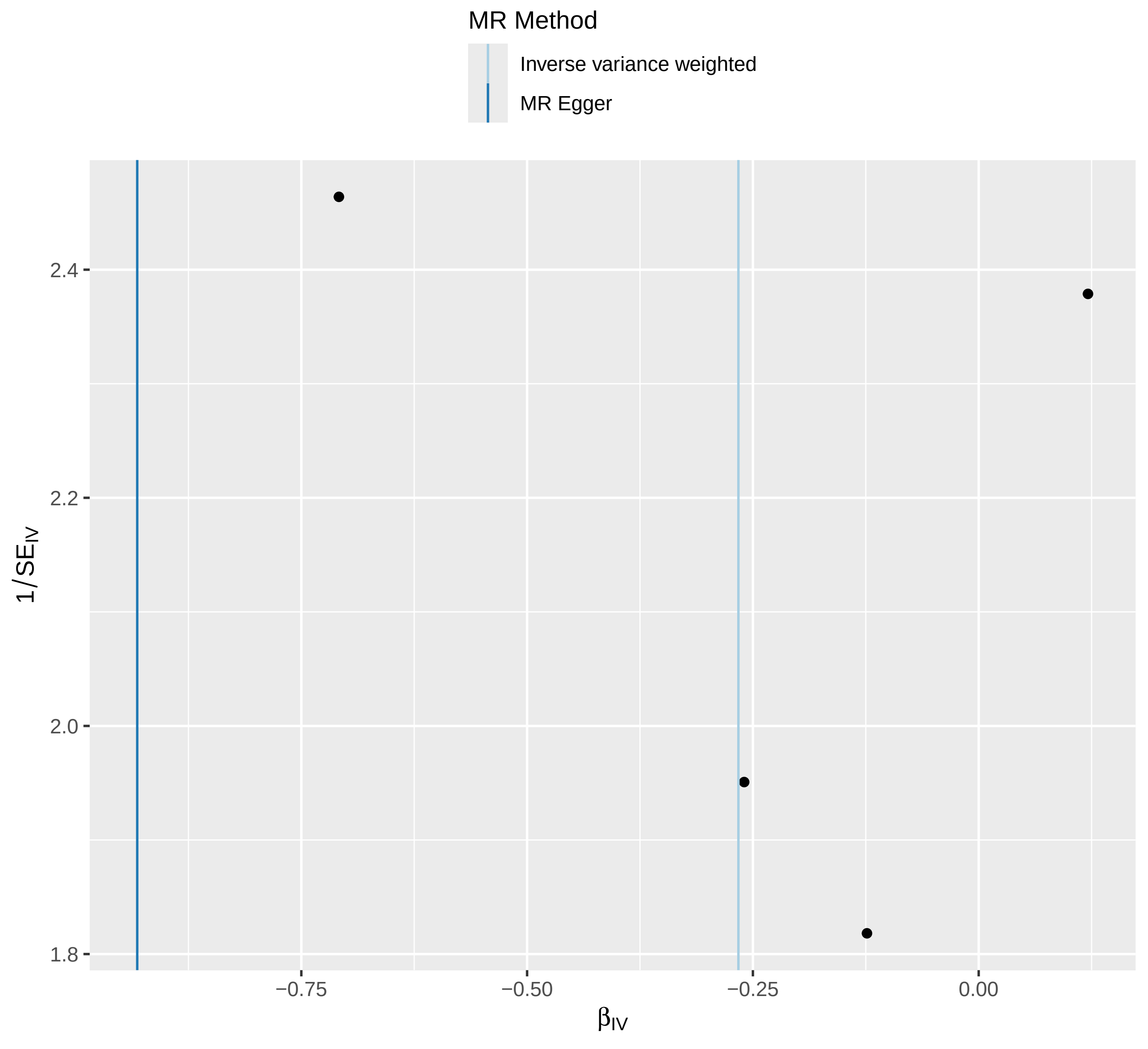

MR Method
Inverse variance weighted
MR Egger
2.4
2.2
2.0
1.8
1/SE_IV
−0.75
−0.50
−0.25
0.00
β_IV

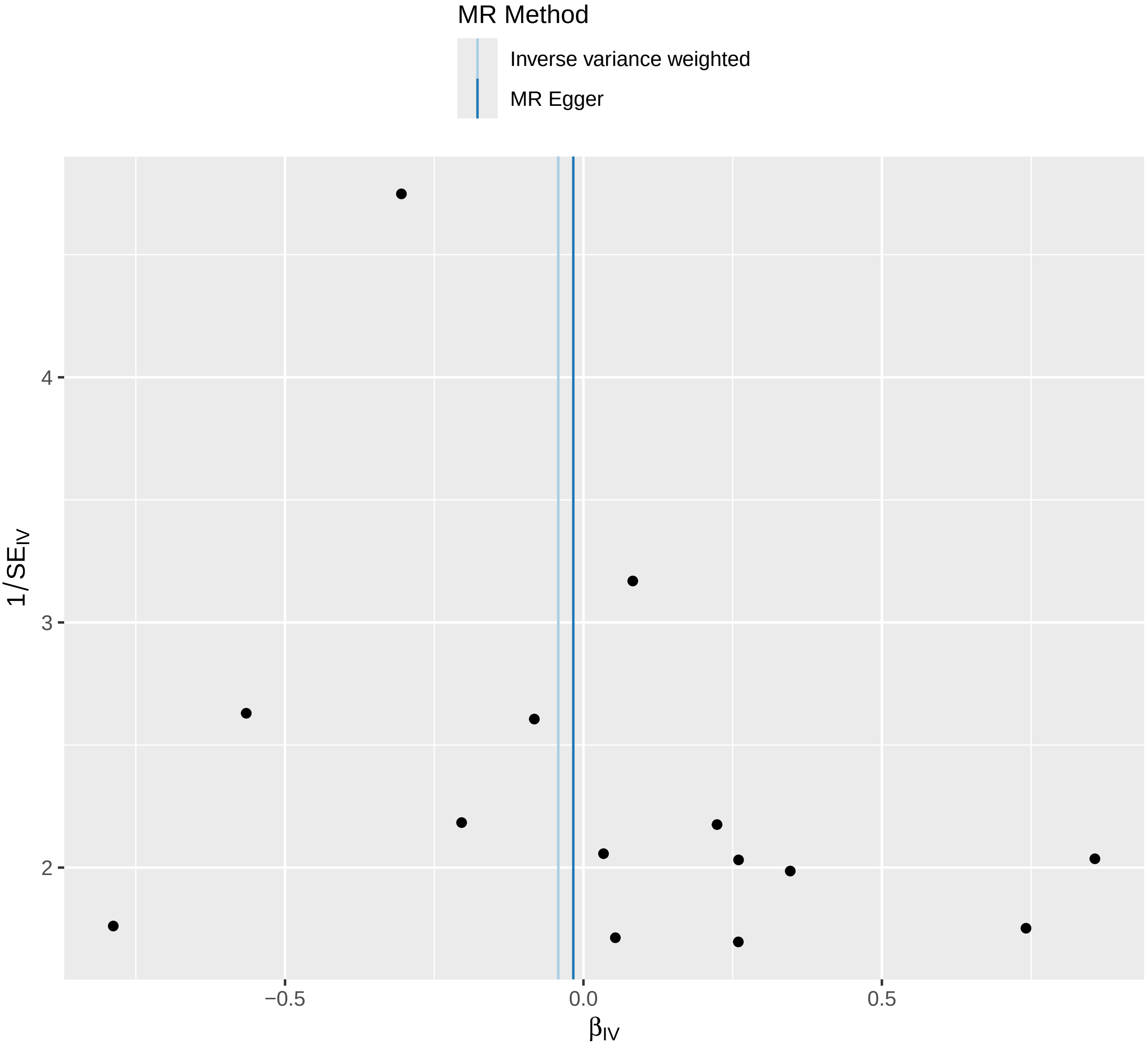
MR Method
Inverse variance weighted
MR Egger
1/SE$_{IV}$
4
3
2
−0.5
0.0
0.5
β$_{IV}$

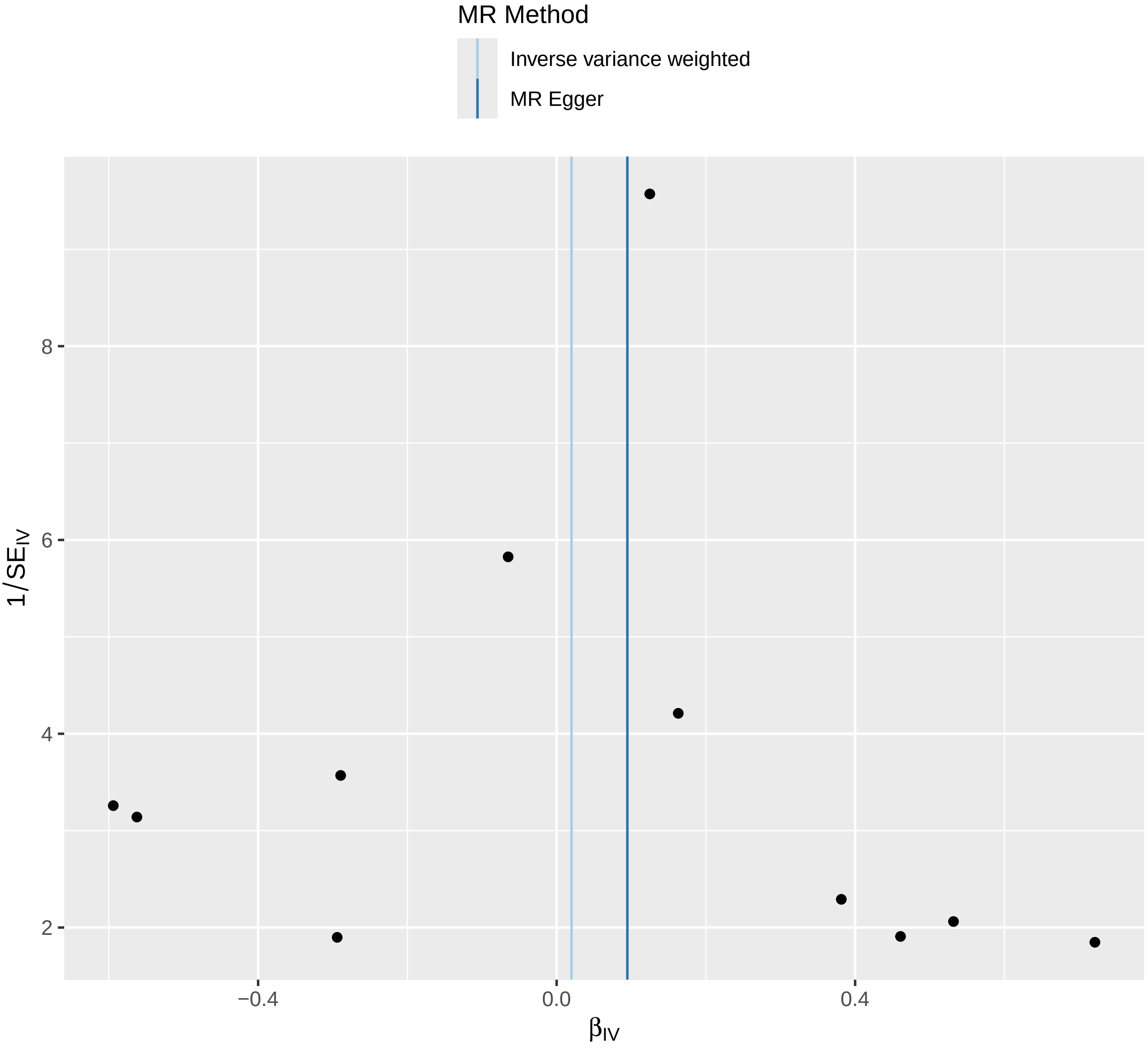
MR Method
Inverse variance weighted
MR Egger
8
6
4
2
−0.4
0.0
0.4
$1/SE_{IV}$
β_{IV}

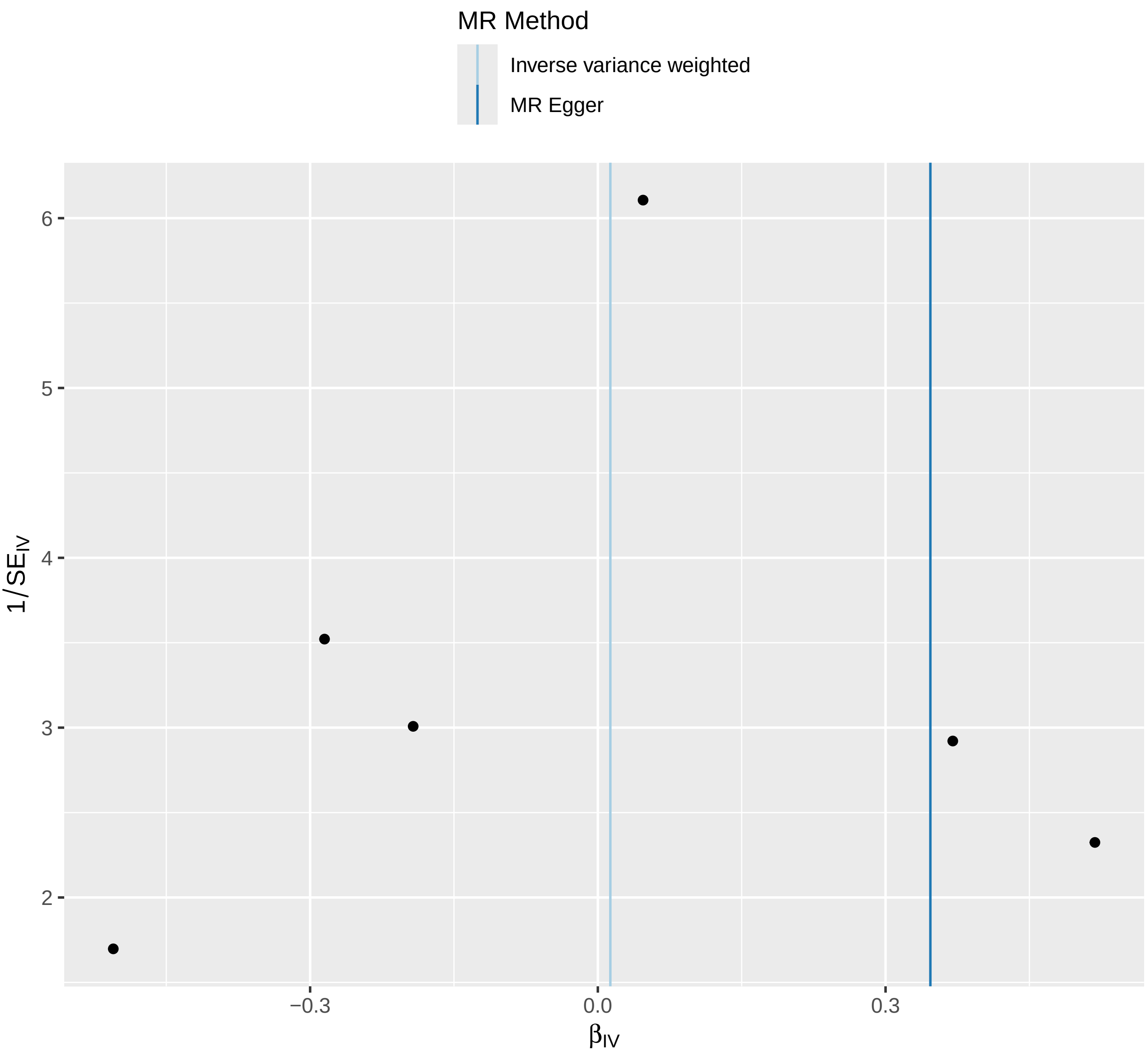
MR Method
Inverse variance weighted
MR Egger
6
5
4
3
2
1/SE_IV
−0.3
0.0
0.3
β_IV

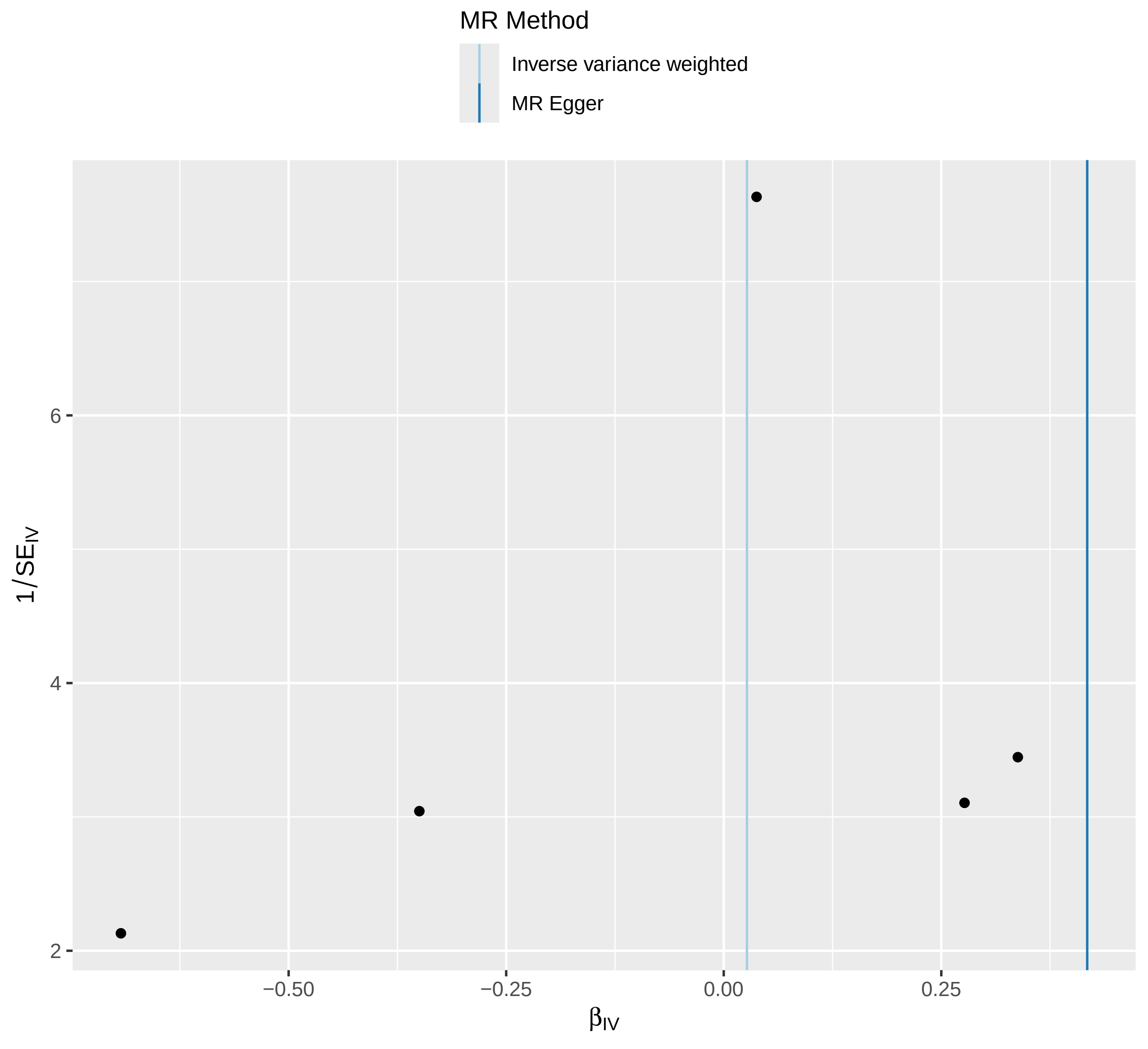
MR Method
Inverse variance weighted
MR Egger
6
4
2
1/SE$_{IV}$
−0.50
−0.25
0.00
0.25
β_{IV}

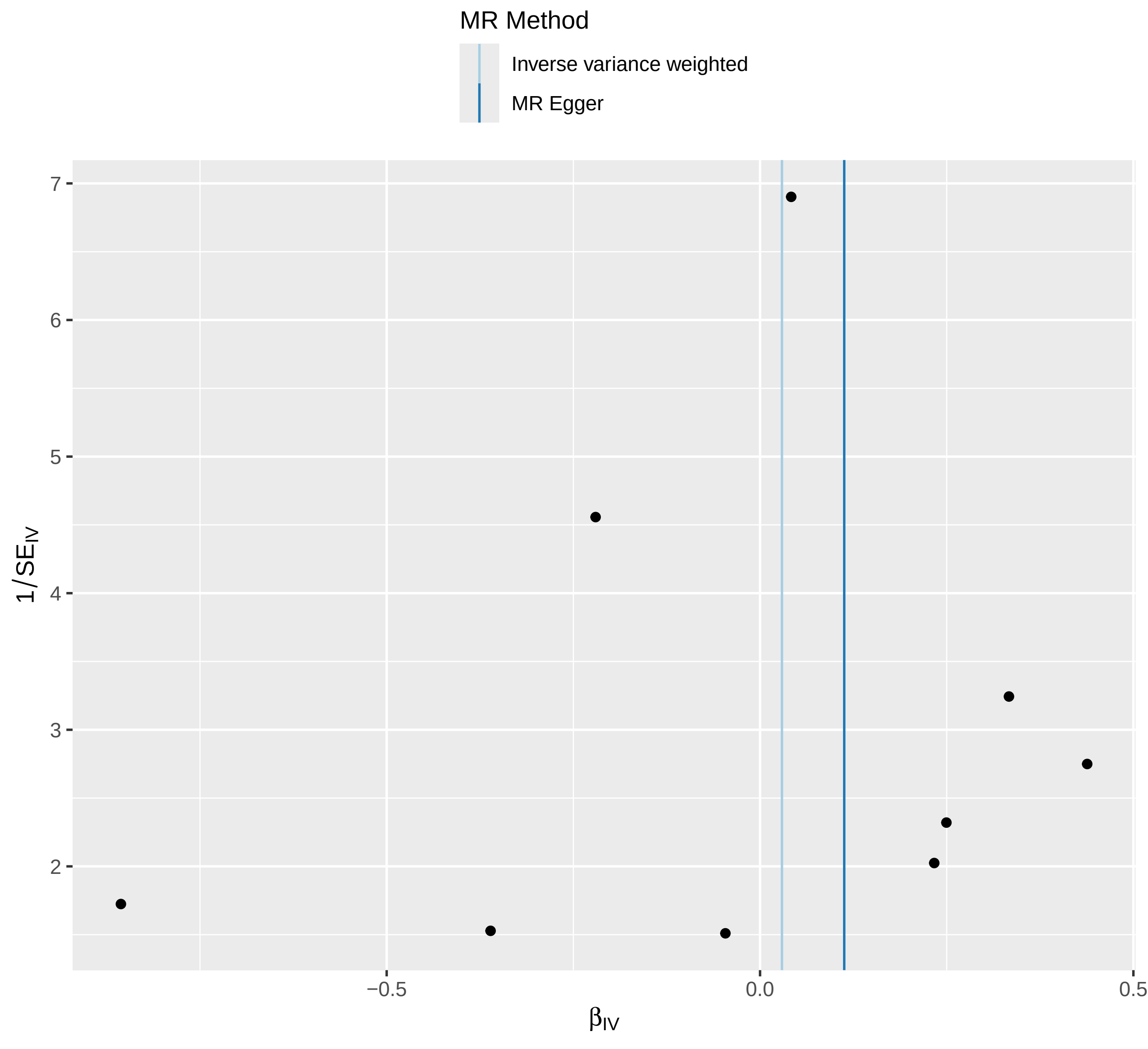

MR Method
Inverse variance weighted
MR Egger
7
6
5
4
3
2
$1/SE_{IV}$
−0.5
0.0
0.5
β_{IV}

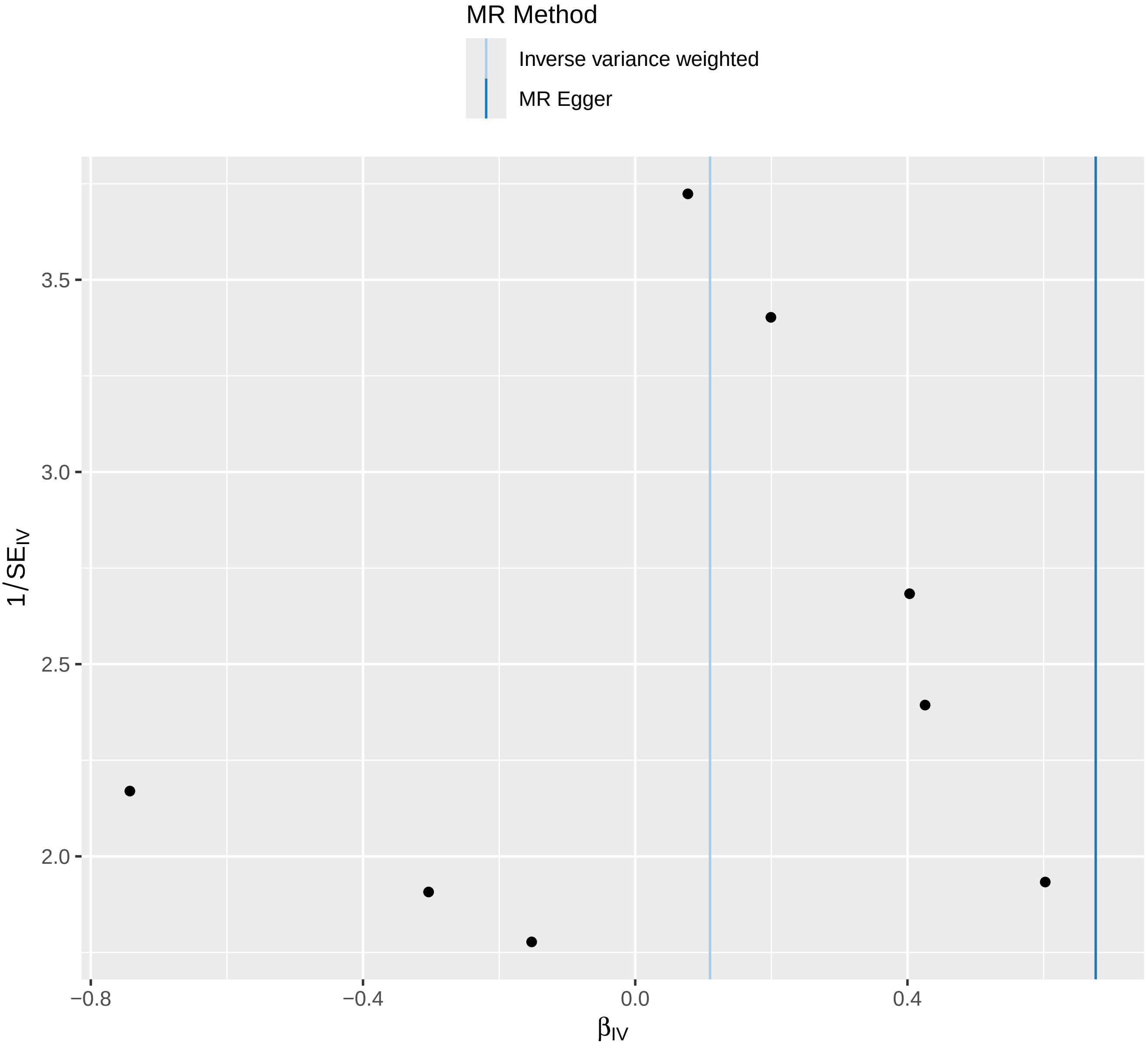

MR Method
Inverse variance weighted
MR Egger
3.5
3.0
2.5
2.0
$1/SE_{IV}$
−0.8
−0.4
0.0
0.4
β_{IV}

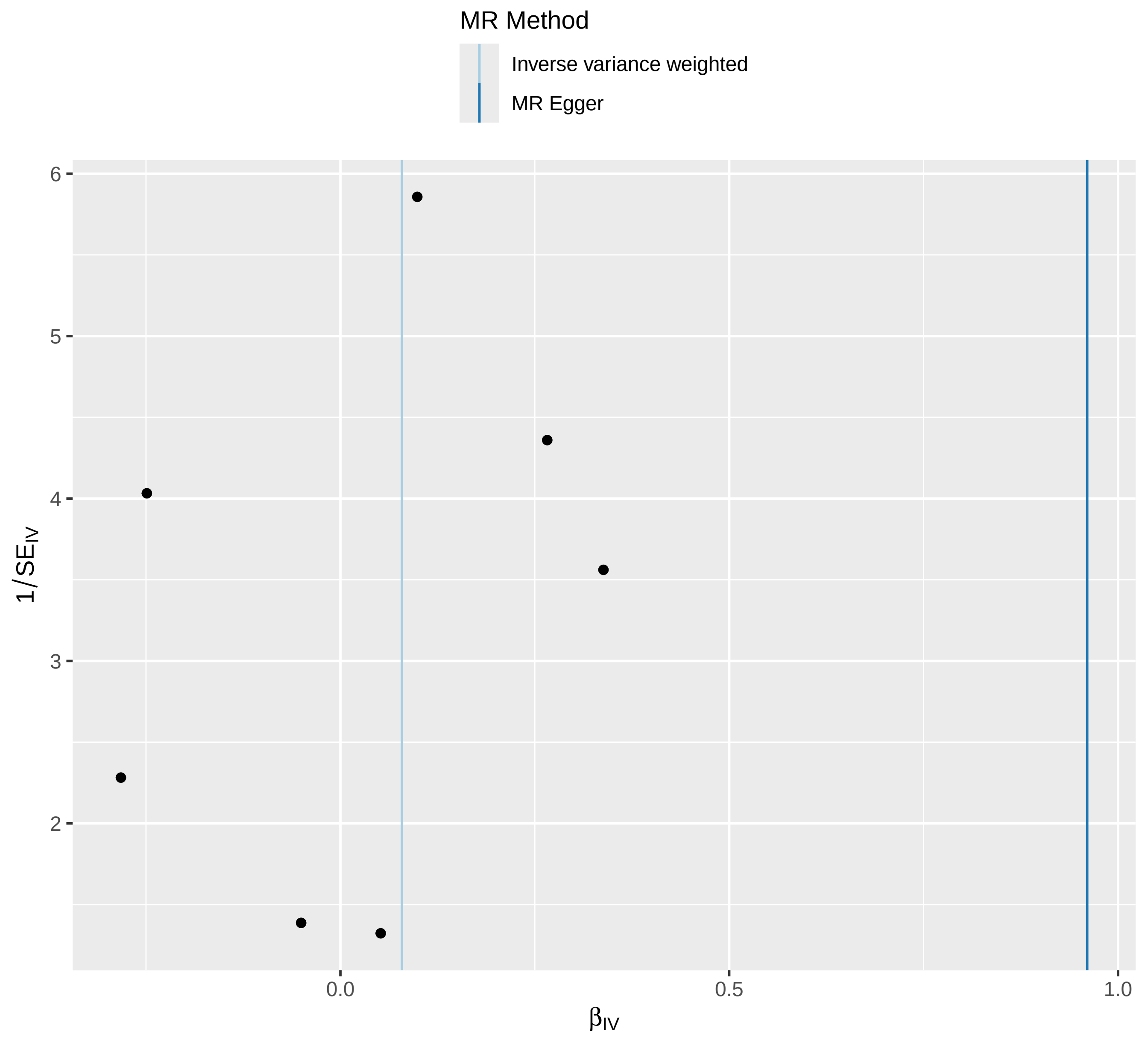

MR Method
Inverse variance weighted
MR Egger
6
5
4
3
2
1/SE_IV
0.0
0.5
1.0
β_IV

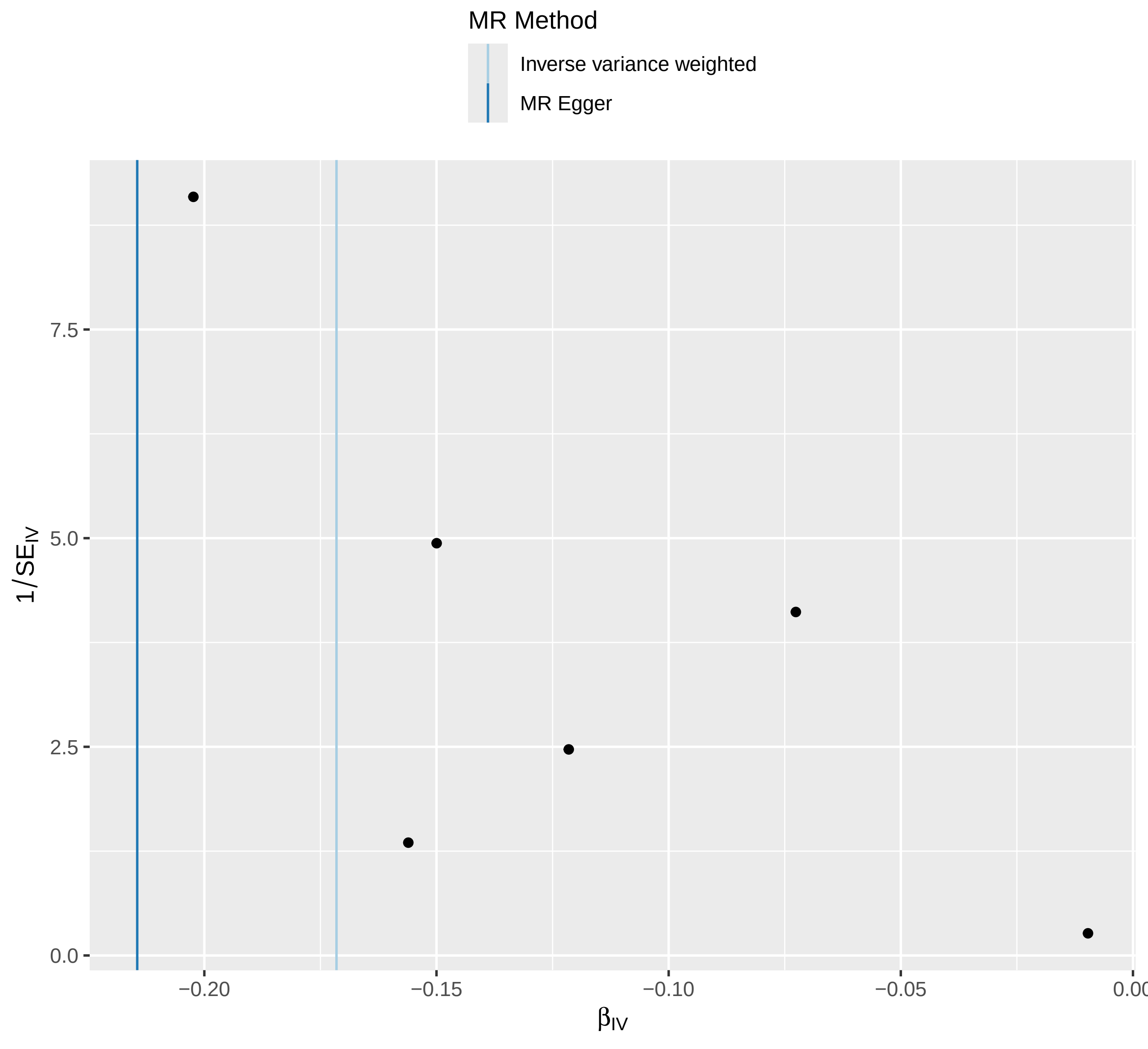
MR Method
Inverse variance weighted
MR Egger
7.5
5.0
2.5
0.0
$1/SE_{IV}$
−0.20
−0.15
−0.10
−0.05
0.00
β_{IV}

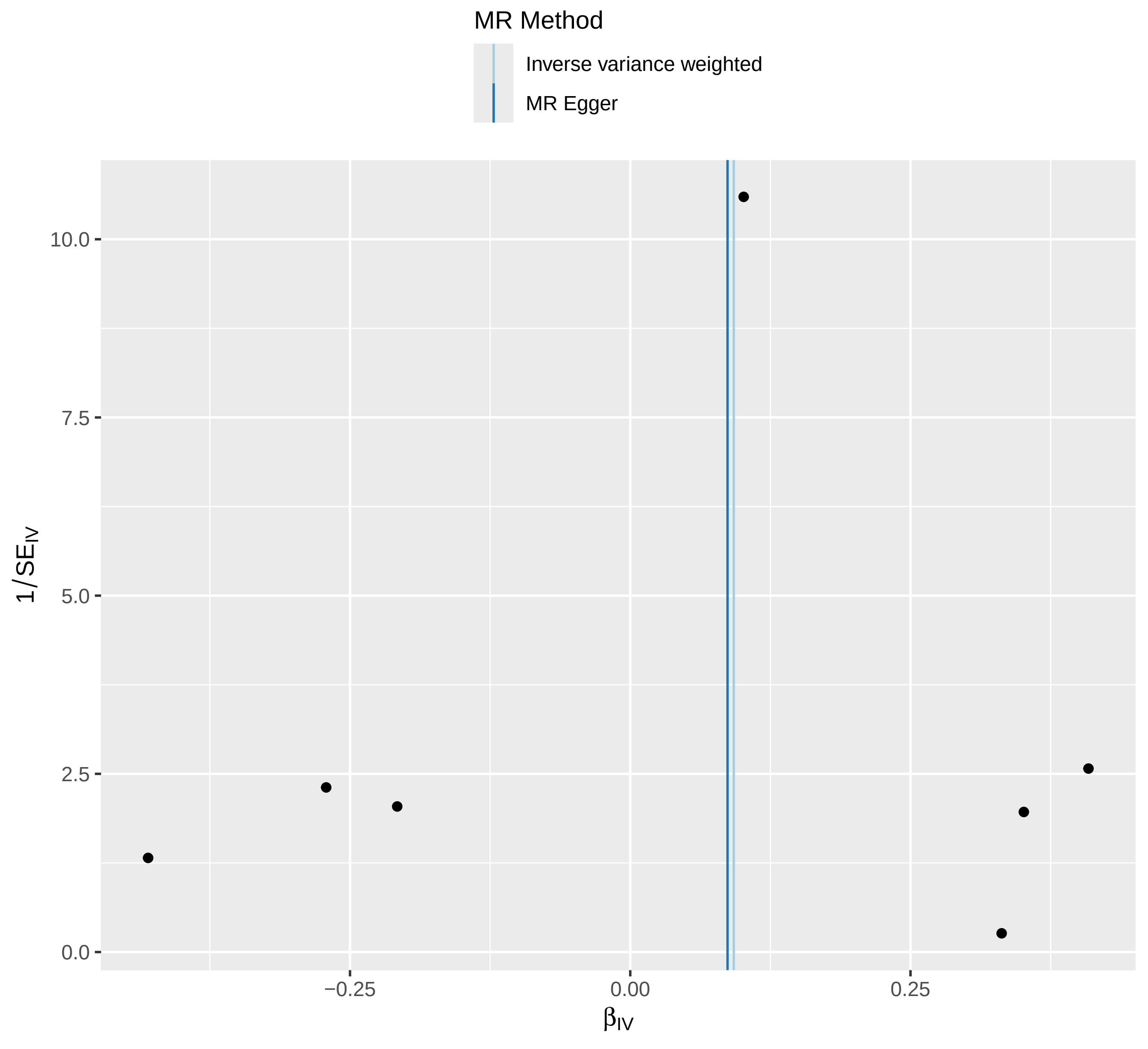
MR Method
Inverse variance weighted
MR Egger
10.0
7.5
5.0
2.5
0.0
1/SE$_{IV}$
−0.25
0.00
0.25
β$_{IV}$

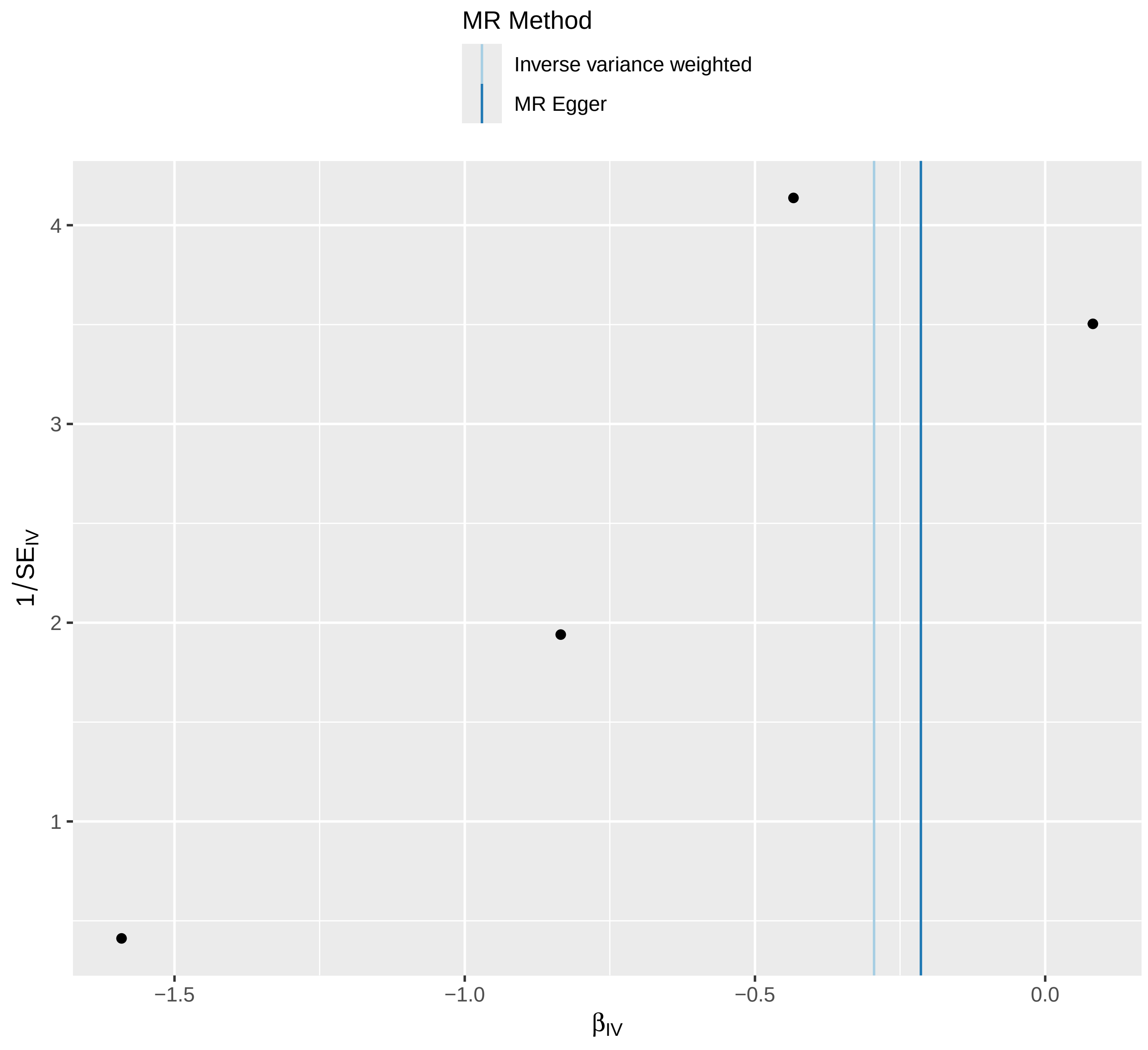
MR Method
Inverse variance weighted
MR Egger
4
3
2
1
1/SE_IV
−1.5
−1.0
−0.5
0.0
β_IV

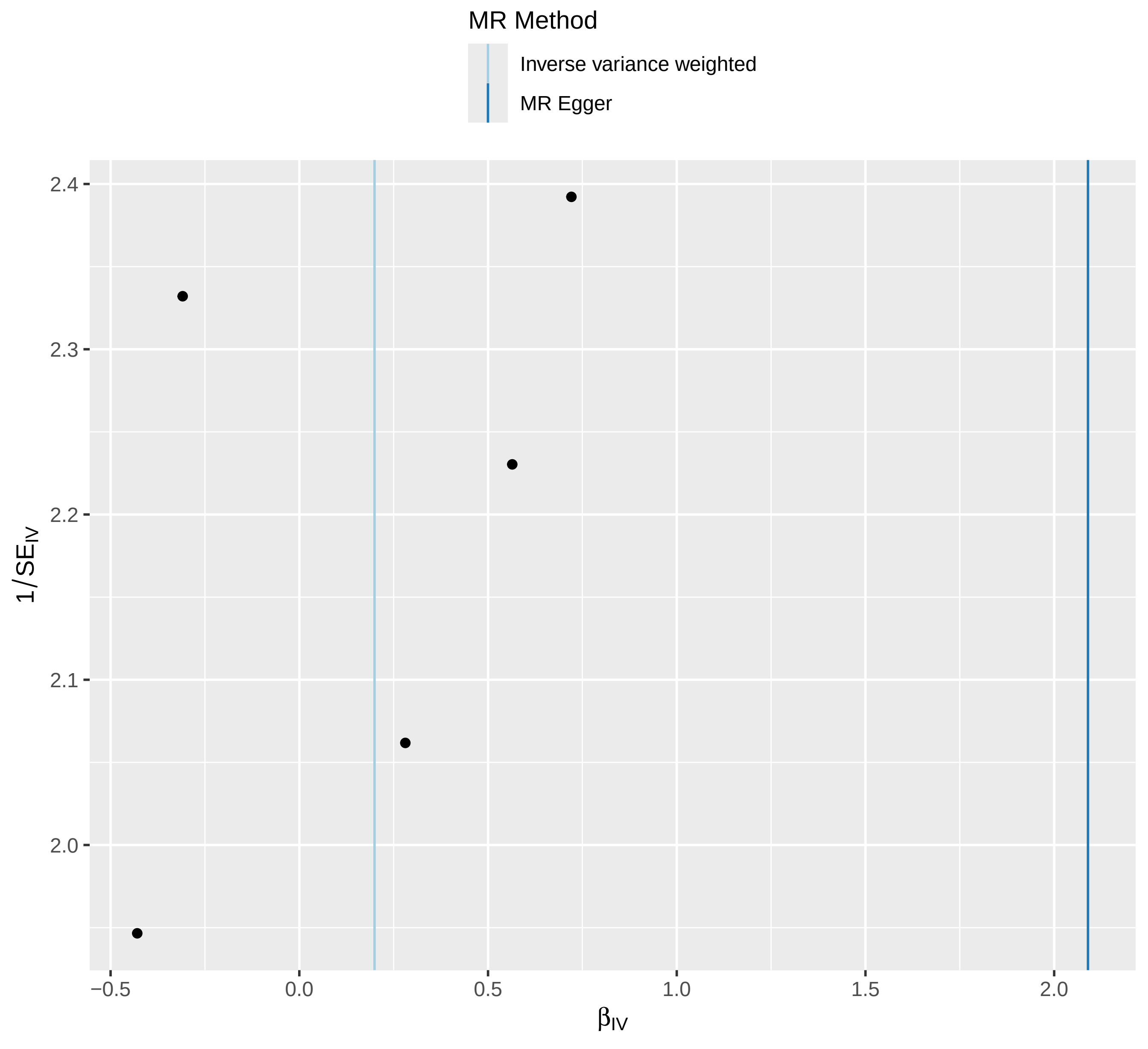
MR Method
Inverse variance weighted
MR Egger
2.4
2.3
2.2
2.1
2.0
$1/SE_{IV}$
−0.5
0.0
0.5
1.0
1.5
2.0
β_{IV}

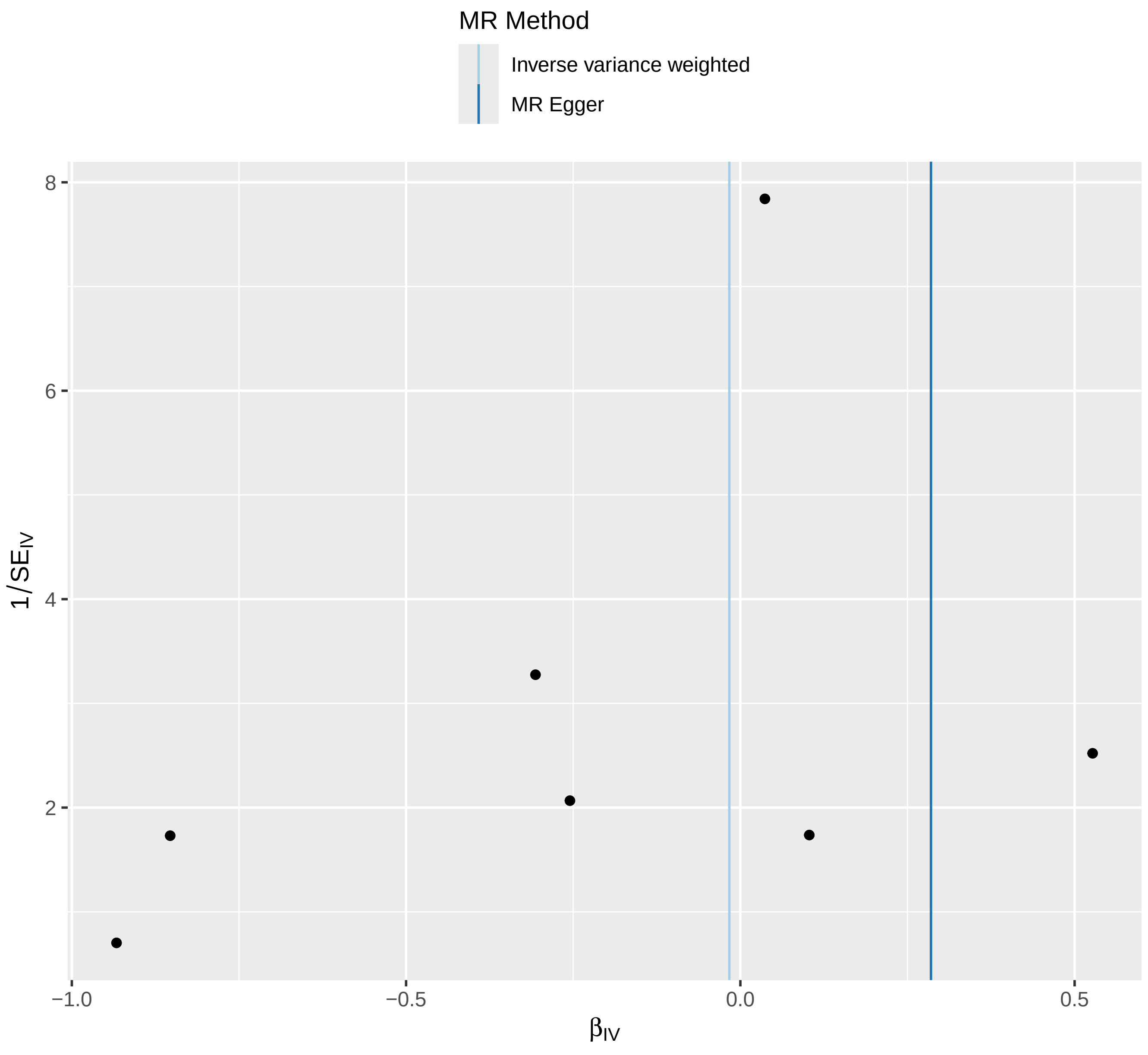

MR Method
Inverse variance weighted
MR Egger
8
6
4
2
1/SE$_{IV}$
−1.0
−0.5
0.0
0.5
β$_{IV}$

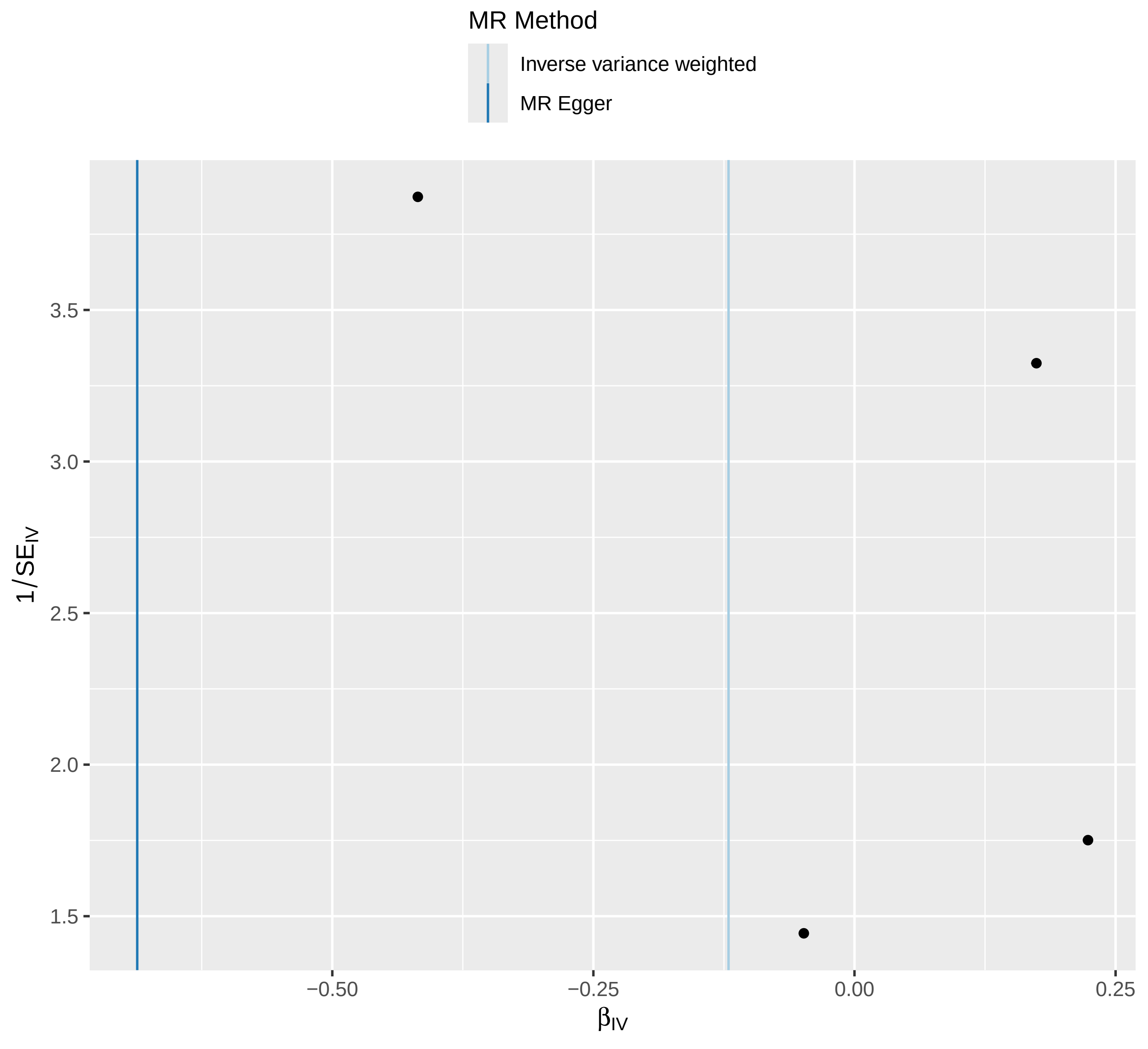

MR Method
Inverse variance weighted
MR Egger
1/SE_IV
3.5
3.0
2.5
2.0
1.5
−0.50
−0.25
0.00
0.25
β_IV

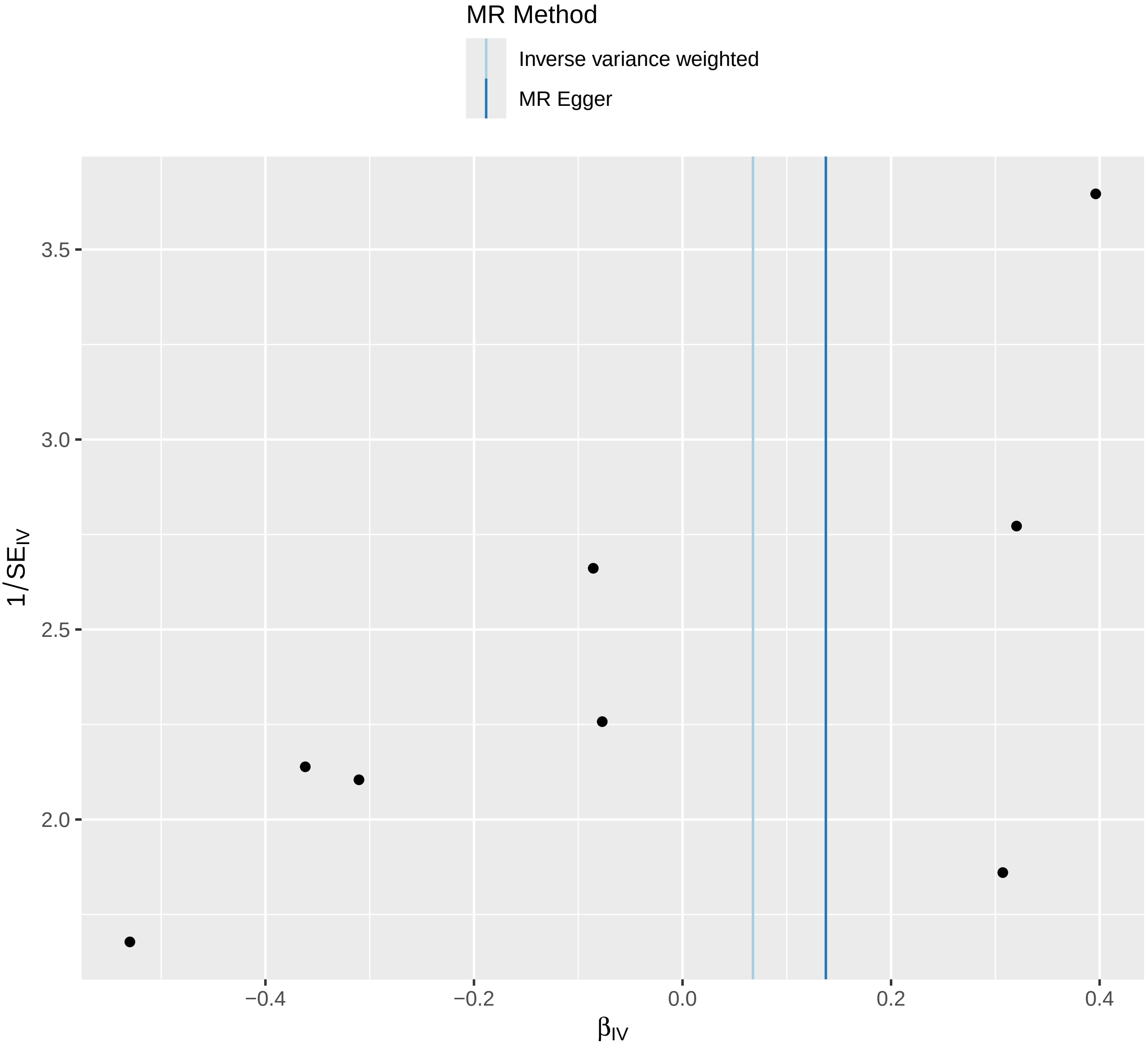

MR Method
Inverse variance weighted
MR Egger
3.5
3.0
2.5
2.0
1/SE_IV
−0.4
−0.2
0.0
0.2
0.4
β_IV

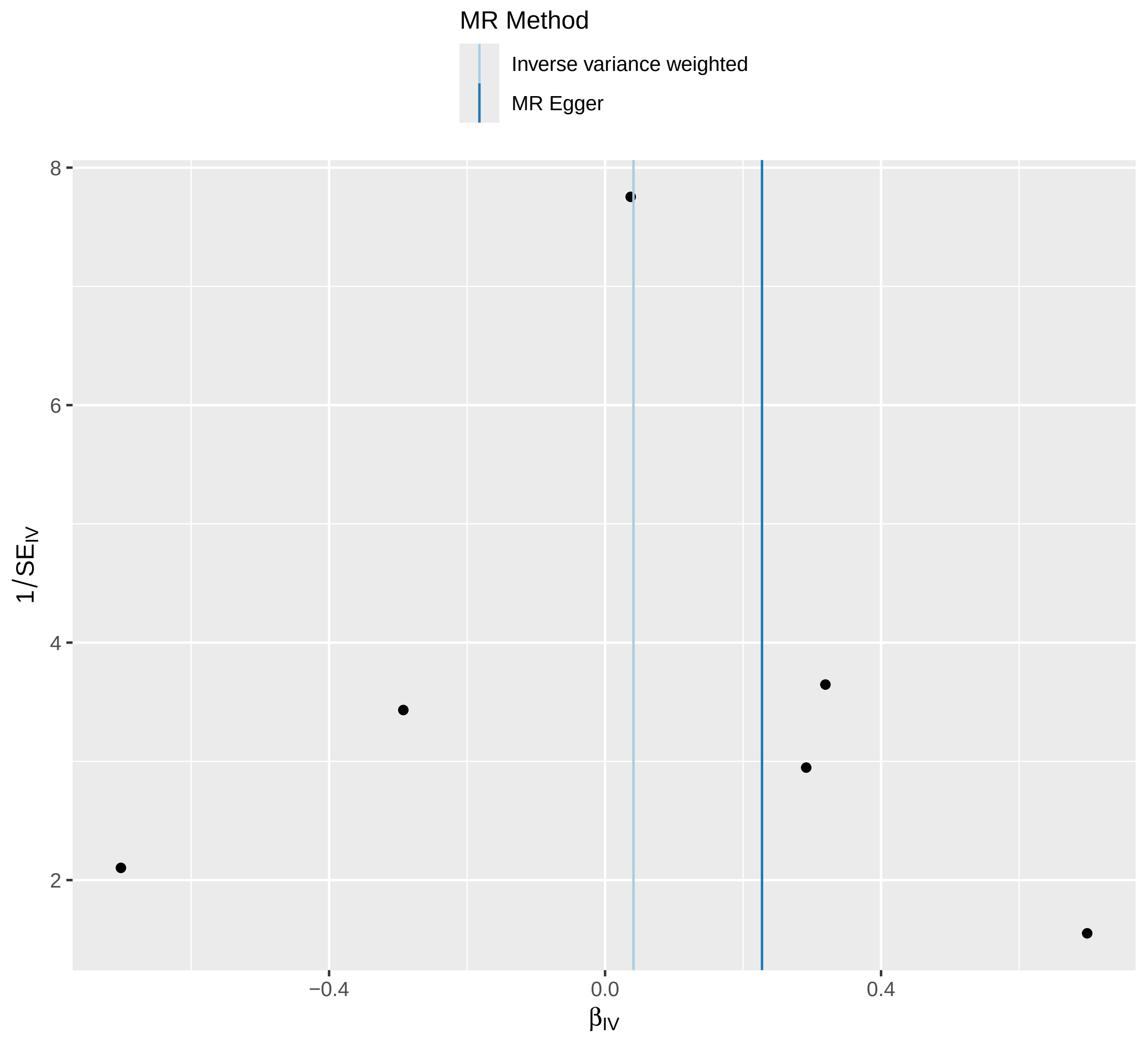
MR Method
Inverse variance weighted
MR Egger
1/SE_IV
β_IV
8
6
4
2
−0.4
0.0
0.4

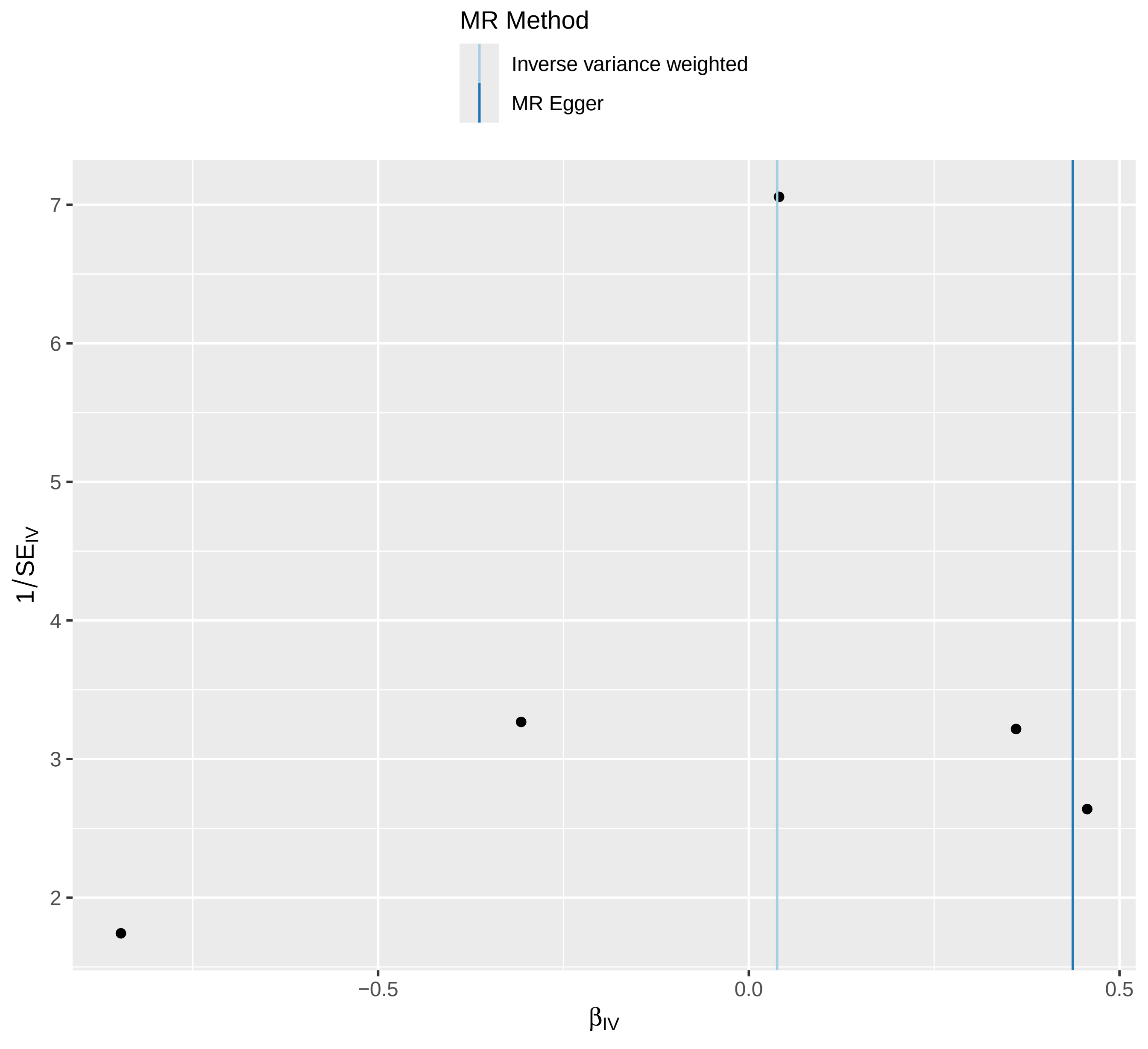
MR Method
Inverse variance weighted
MR Egger
1/SE$_{IV}$
7
6
5
4
3
2
−0.5
0.0
0.5
β$_{IV}$

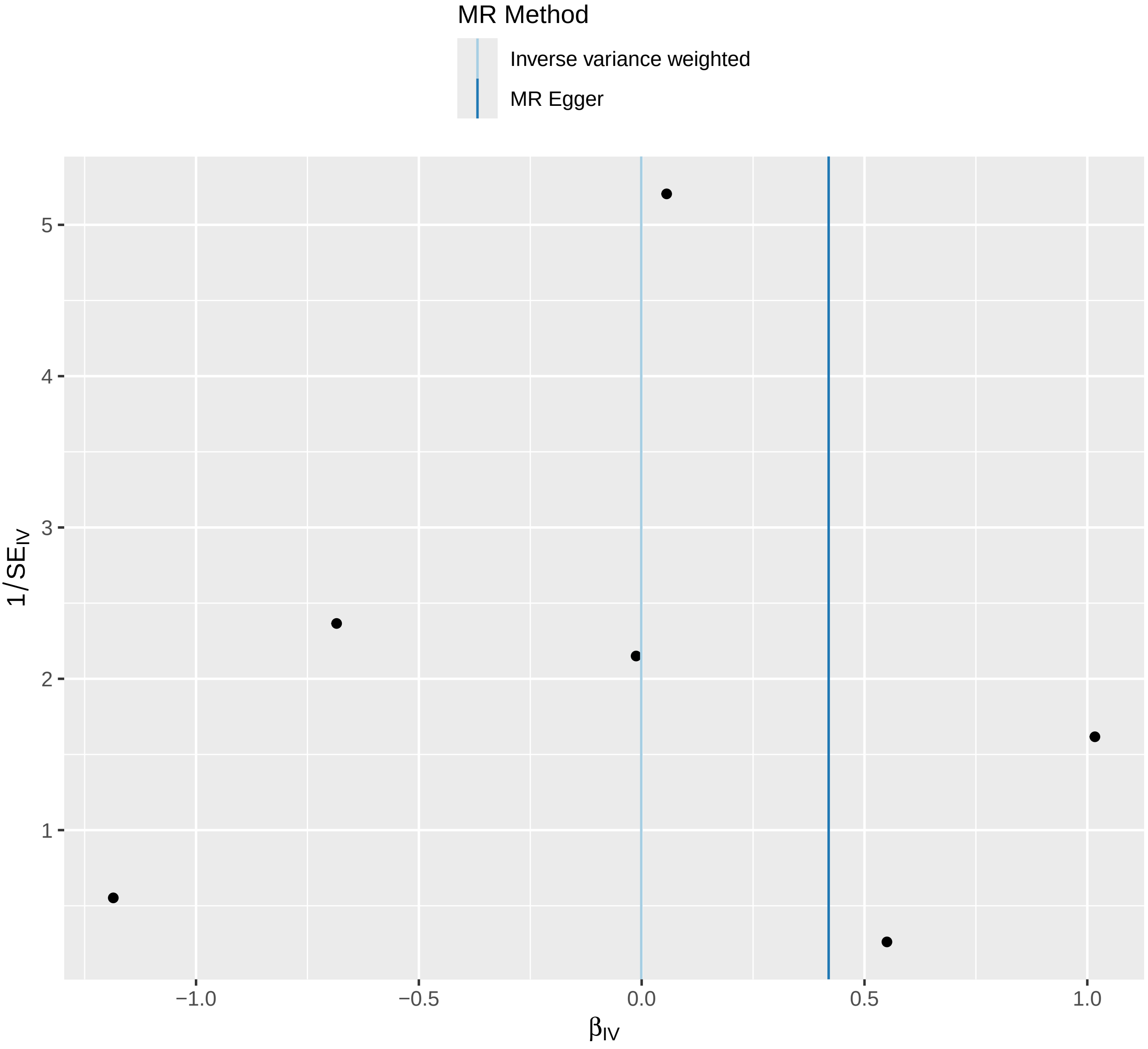

MR Method
Inverse variance weighted
MR Egger
5
4
3
2
1
1/SE_IV
−1.0
−0.5
0.0
0.5
1.0
β_IV

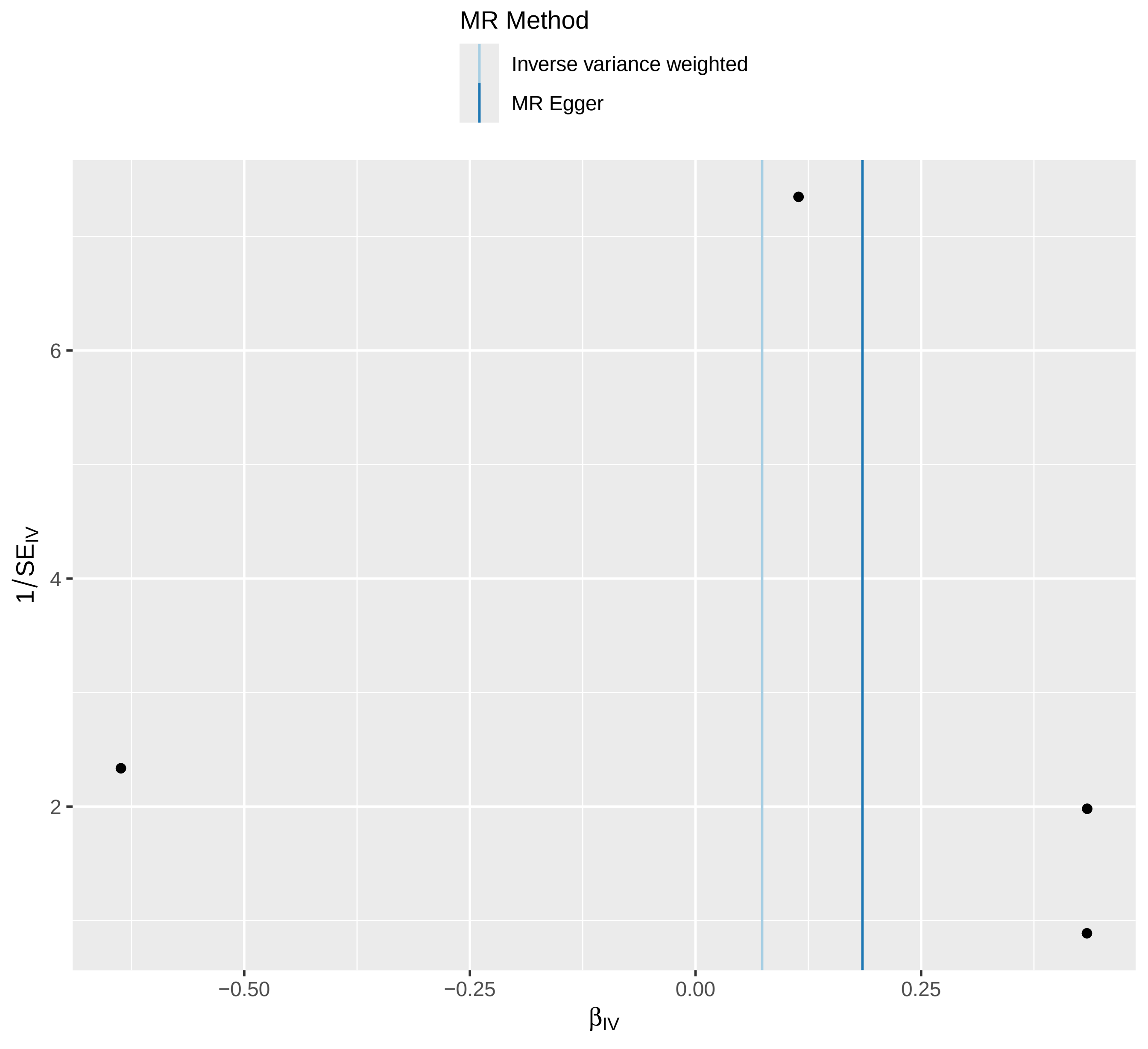

MR Method
Inverse variance weighted
MR Egger
1/SE_IV
6
4
2
−0.50
−0.25
0.00
0.25
β_IV

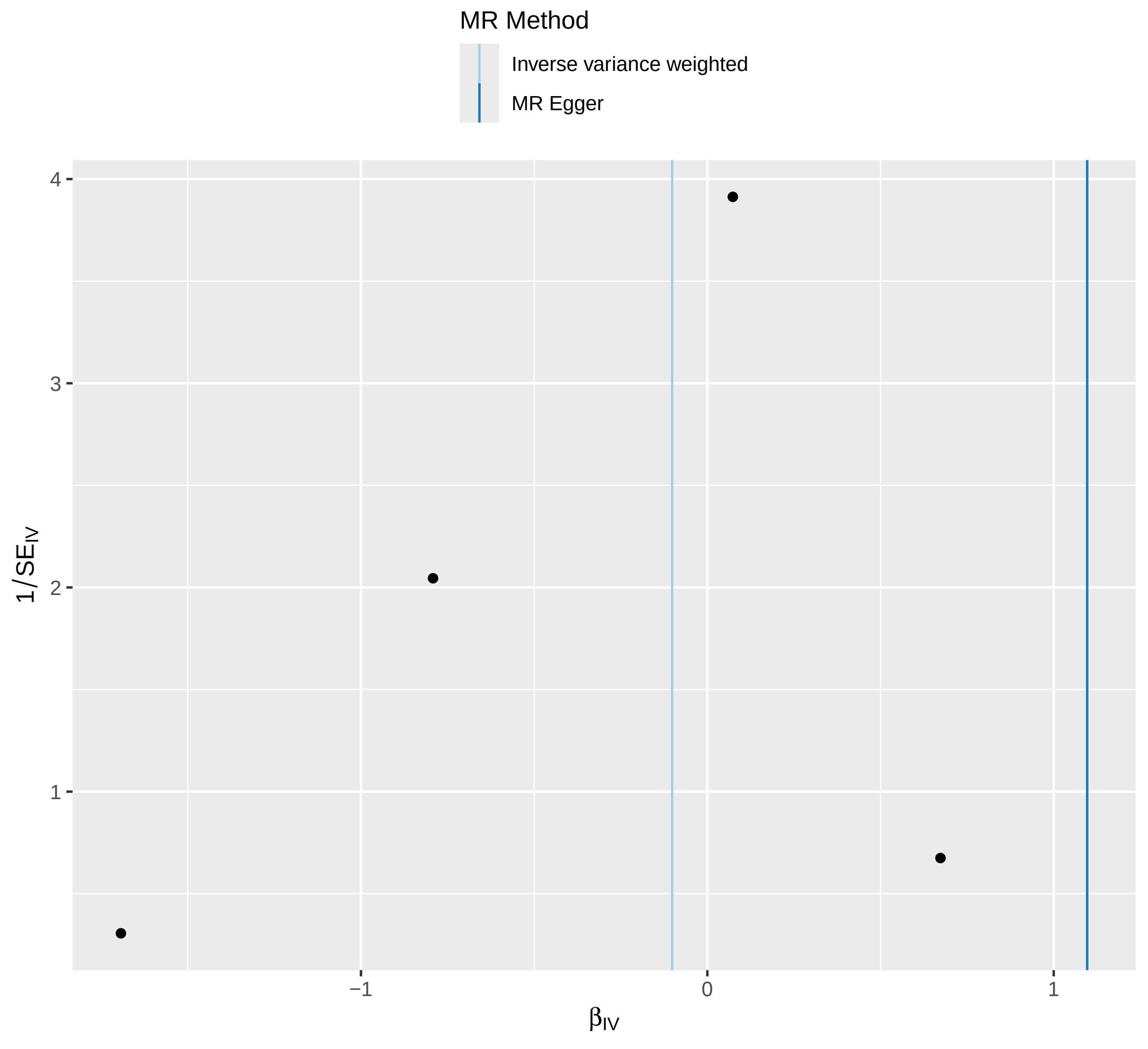

MR Method
Inverse variance weighted
MR Egger
4
3
2
1
1/SE_IV
−1
0
1
β_IV

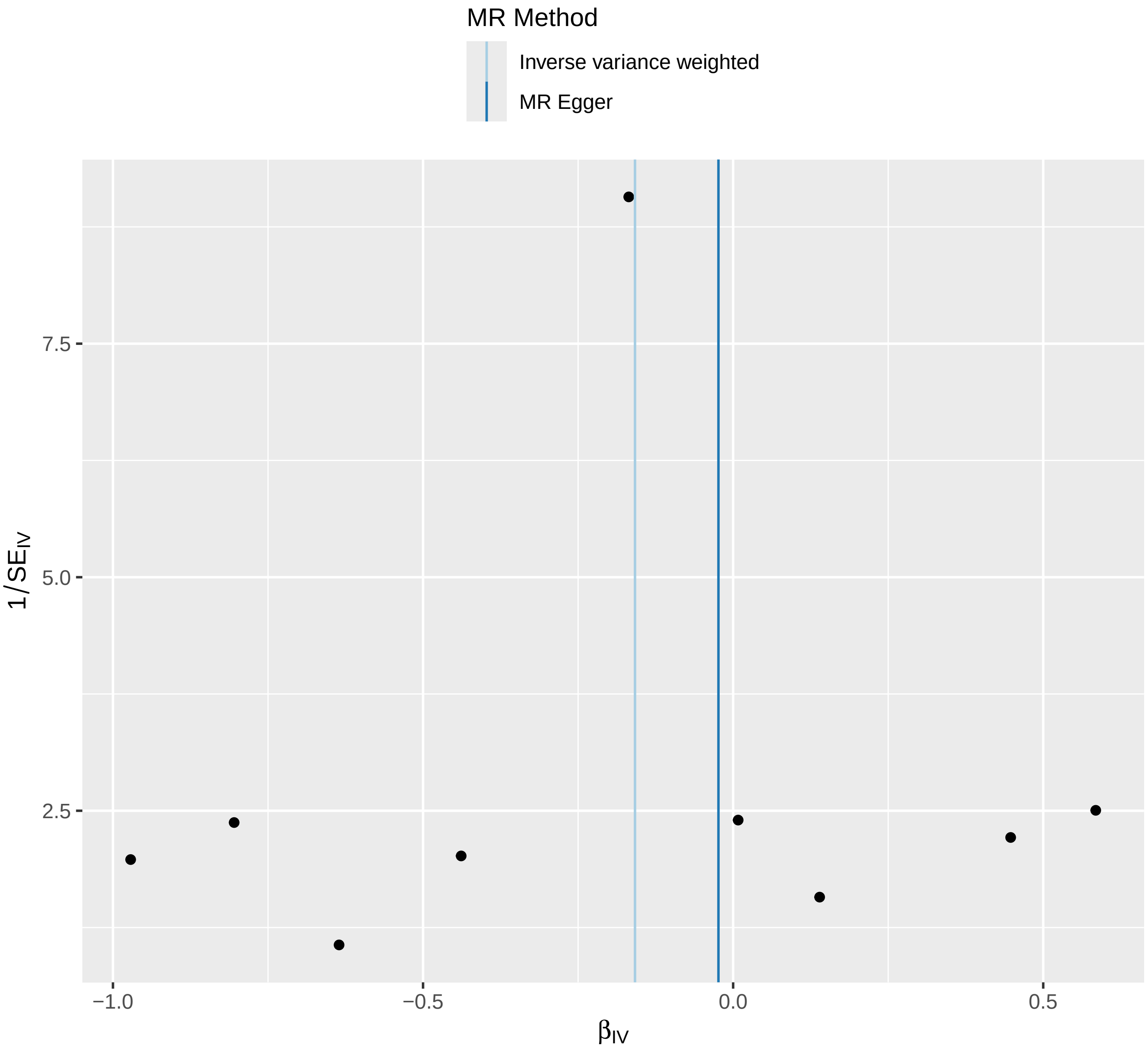
MR Method
Inverse variance weighted
MR Egger
7.5
5.0
2.5
1/SE$_{IV}$
−1.0
−0.5
0.0
0.5
β$_{IV}$

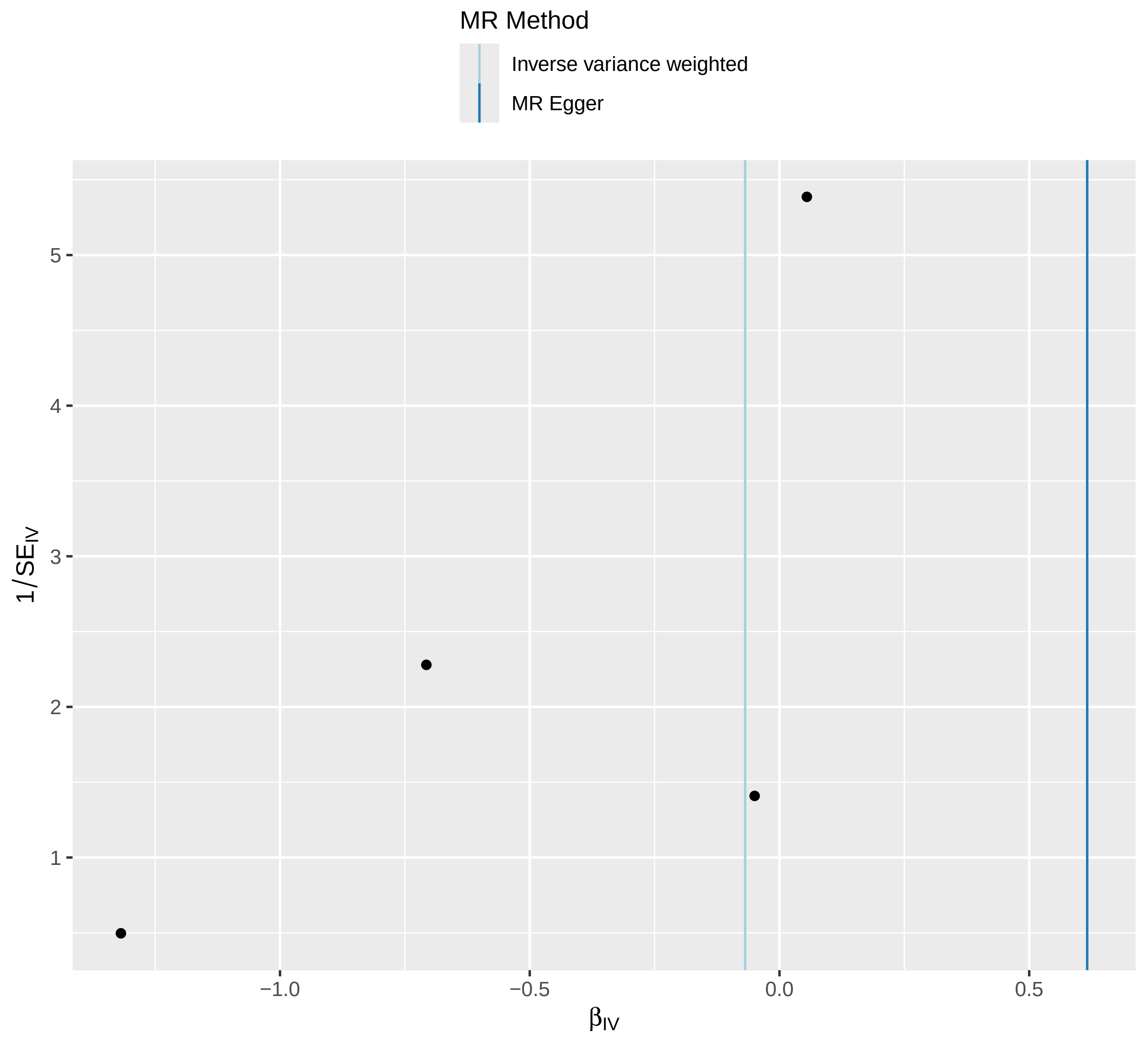
MR Method
Inverse variance weighted
MR Egger
1/SE$_{IV}$
β_{IV}
−1.0
−0.5
0.0
0.5
1
2
3
4
5

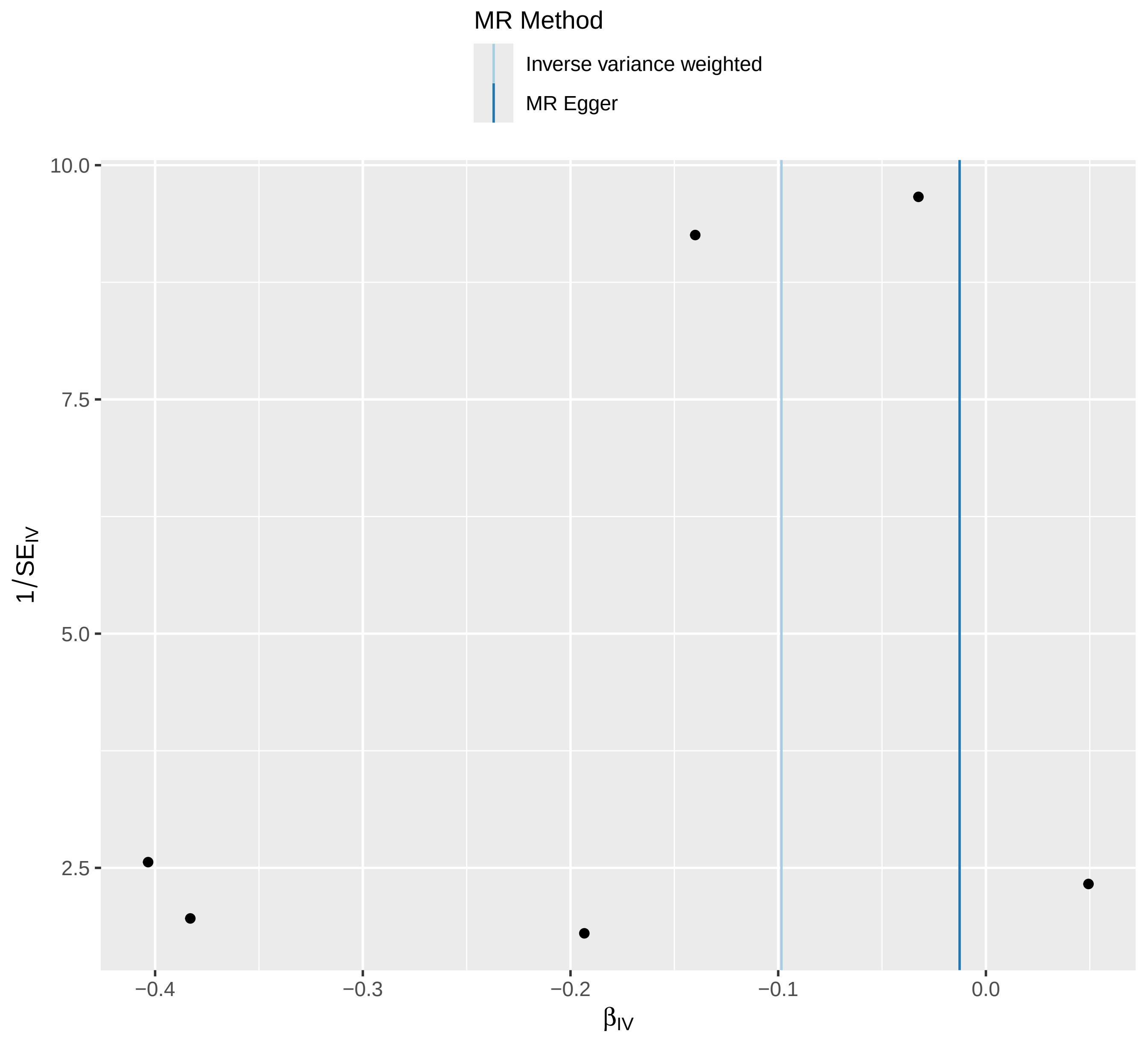
MR Method
Inverse variance weighted
MR Egger
10.0
7.5
5.0
2.5
1/SE_IV
−0.4
−0.3
−0.2
−0.1
0.0
β_IV

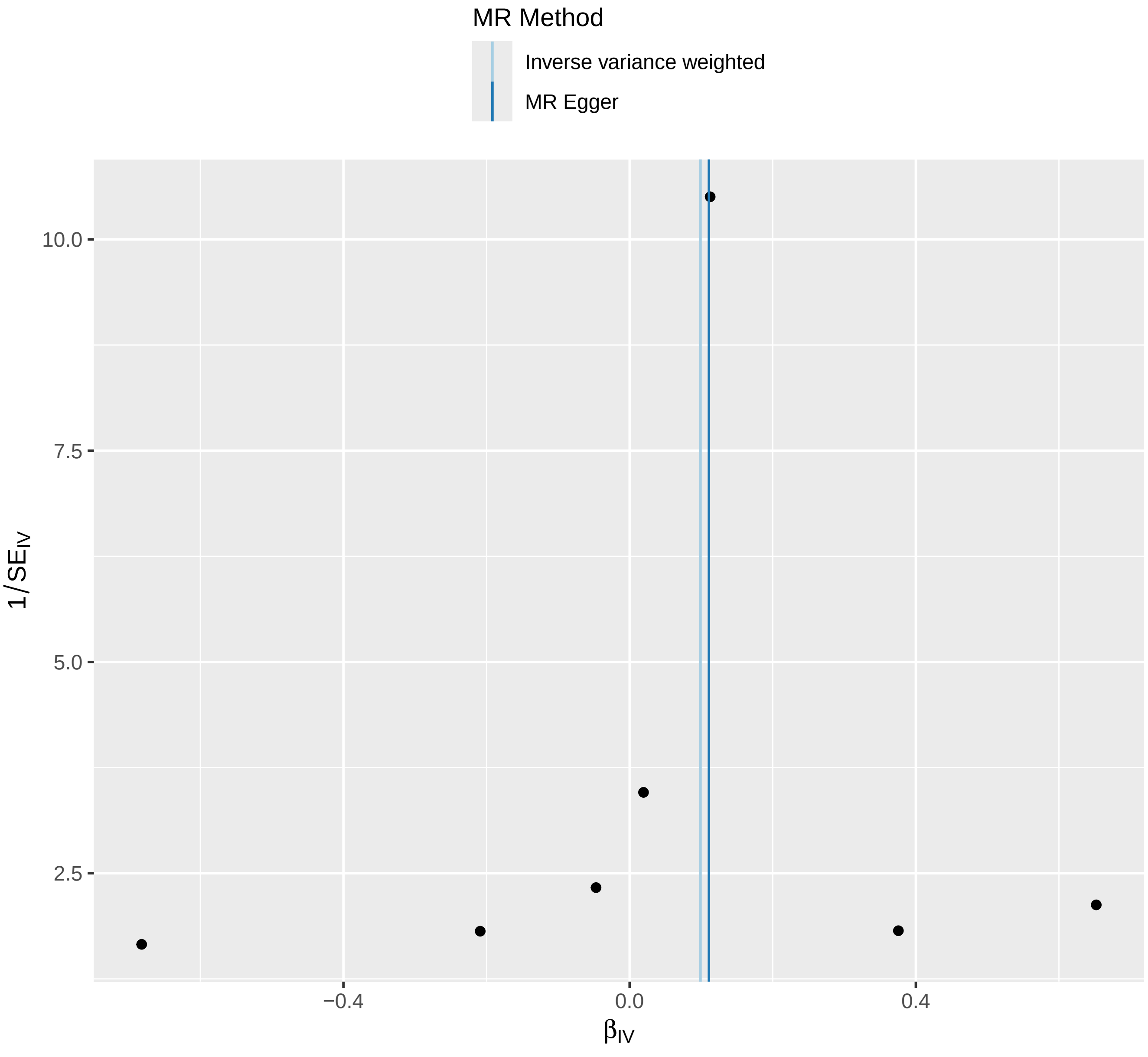

MR Method
Inverse variance weighted
MR Egger
10.0
7.5
5.0
2.5
1/SE$_{IV}$
−0.4
0.0
0.4
β$_{IV}$

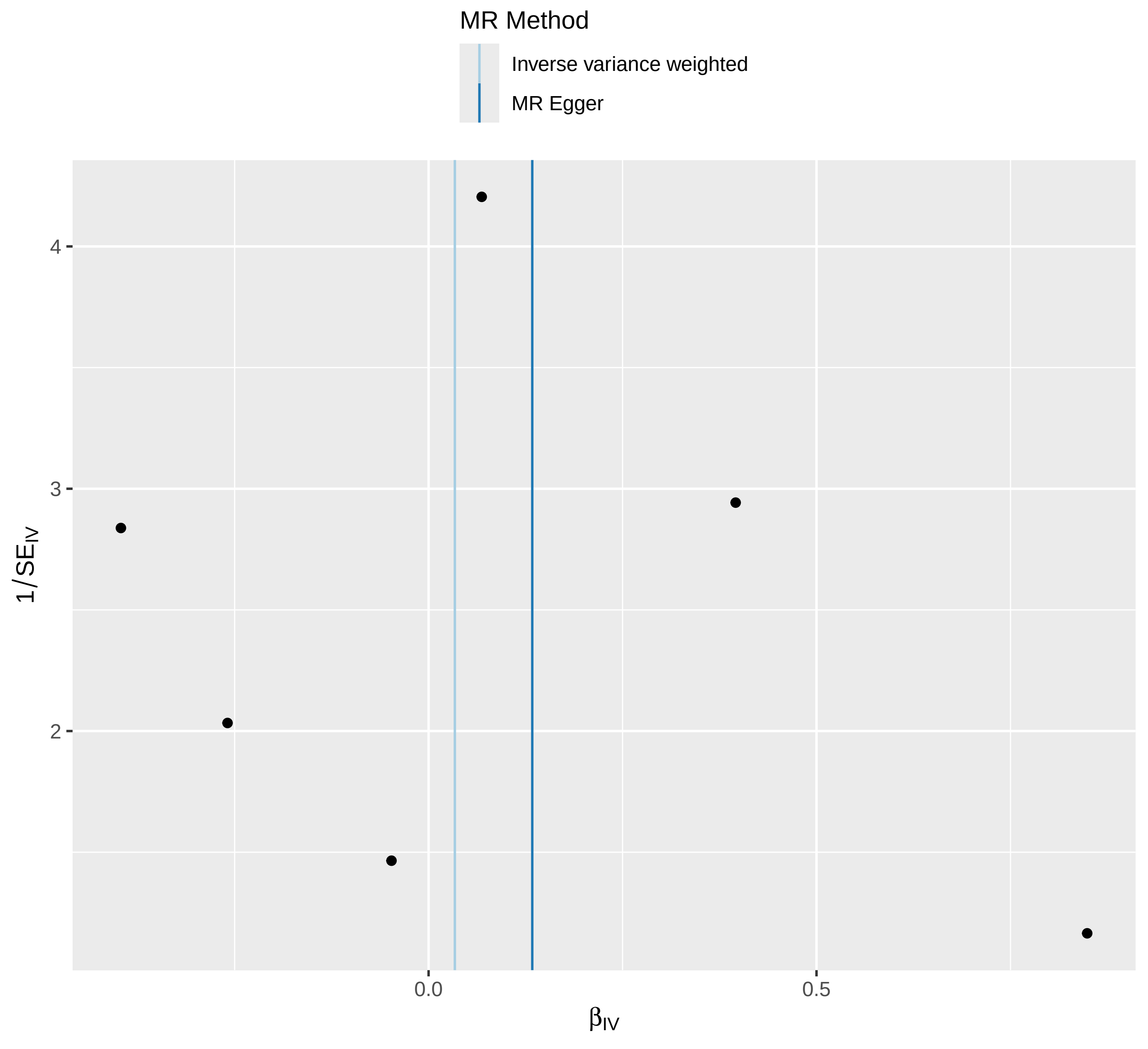
MR Method
Inverse variance weighted
MR Egger
4
3
2
$1/SE_{IV}$
0.0
0.5
β_{IV}

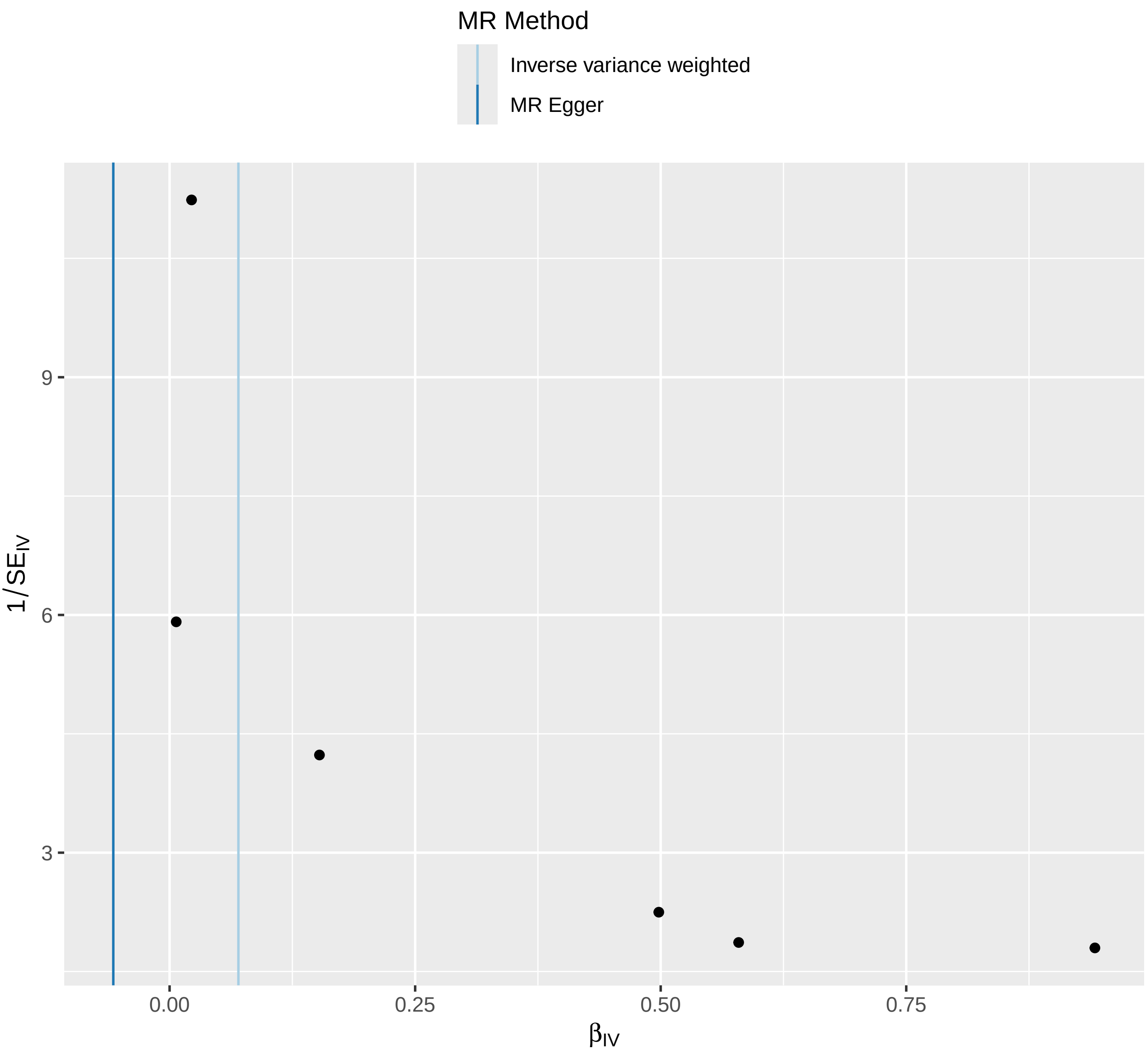

MR Method
Inverse variance weighted
MR Egger
9
6
3
$1/SE_{IV}$
0.00
0.25
0.50
0.75
β_{IV}

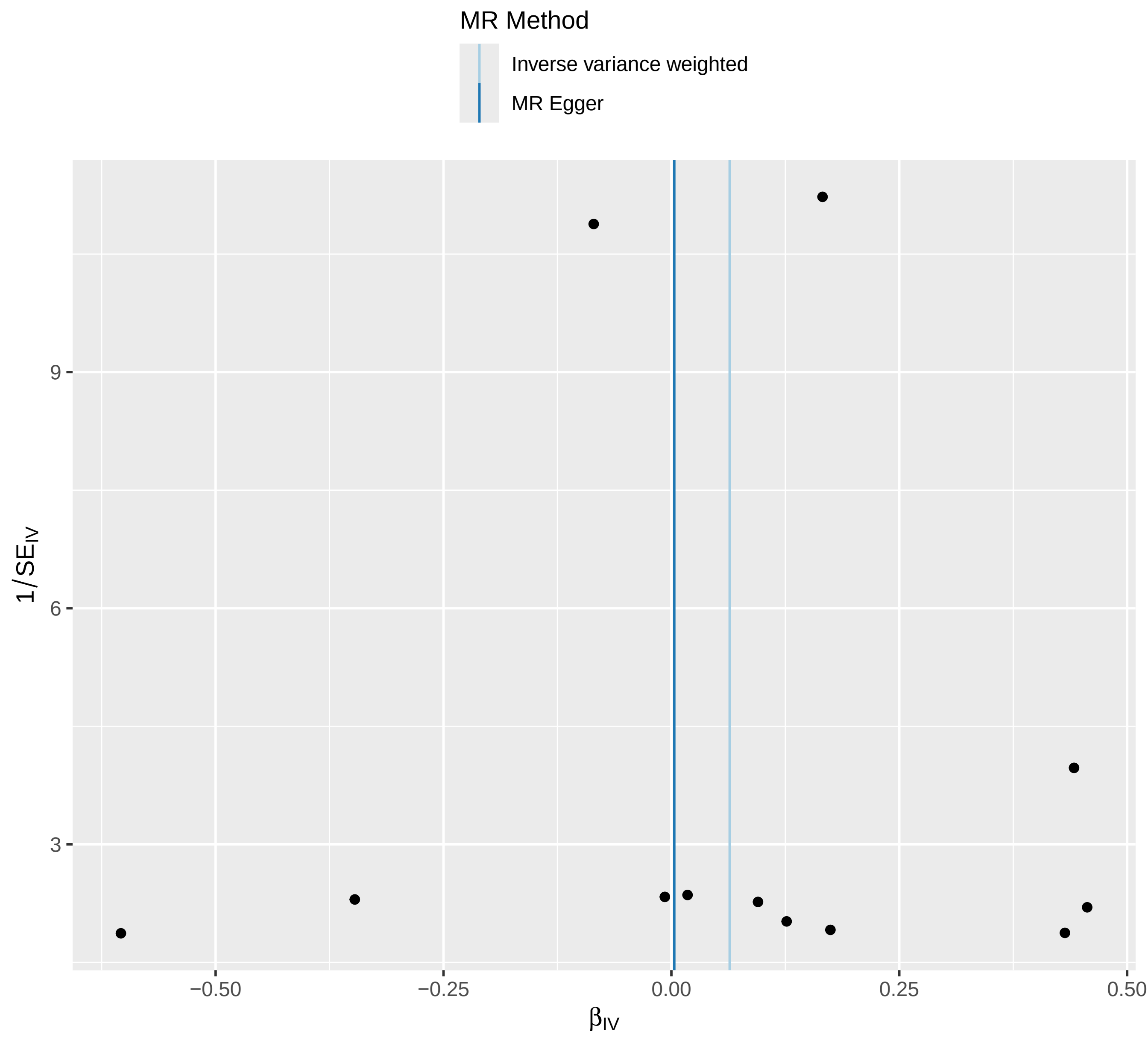

MR Method
Inverse variance weighted
MR Egger
9
6
3
$1/SE_{IV}$
−0.50
−0.25
0.00
0.25
0.50
β_{IV}

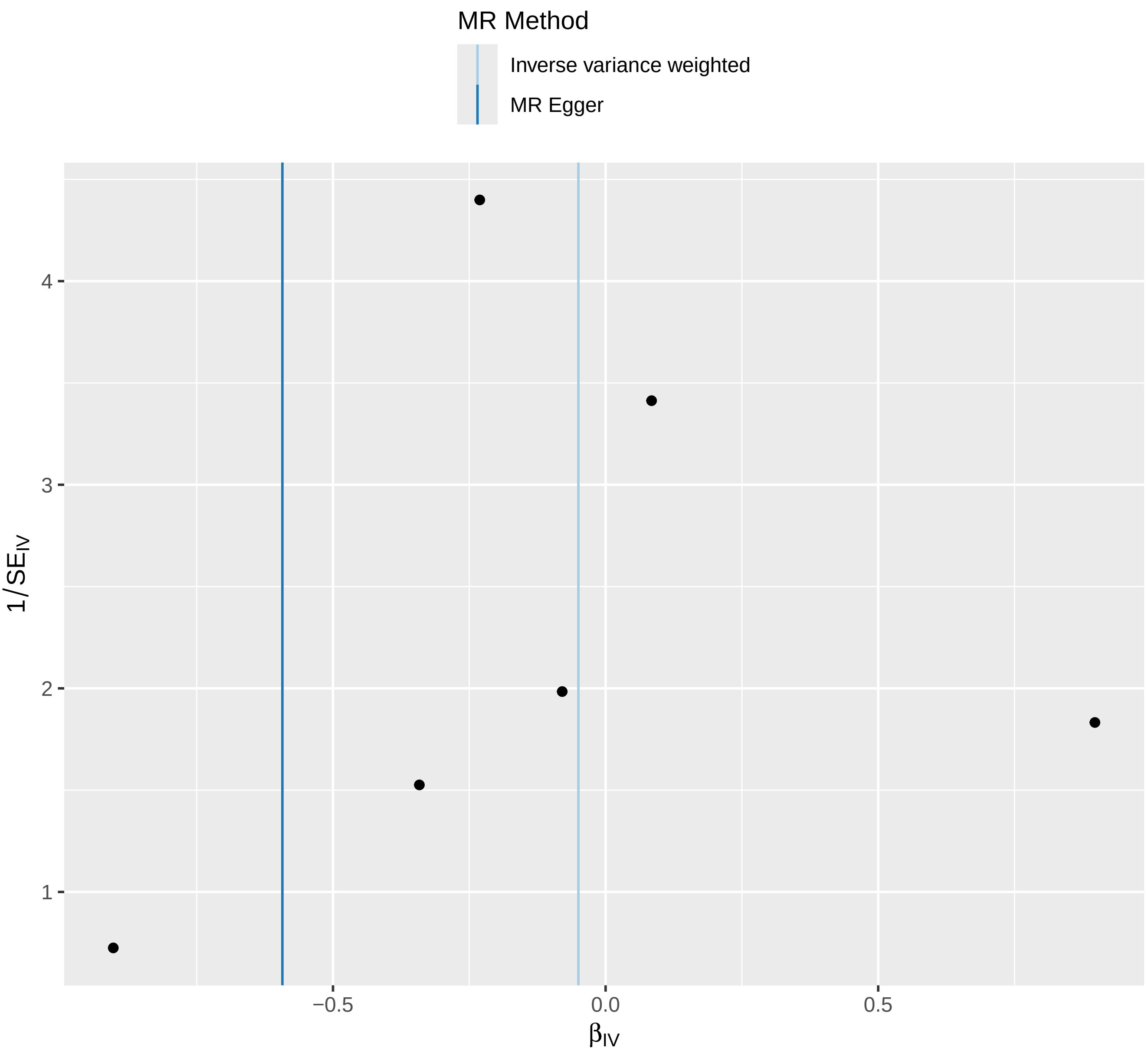

MR Method
Inverse variance weighted
MR Egger
4
3
2
1
1/SE_IV
−0.5
0.0
0.5
β_IV

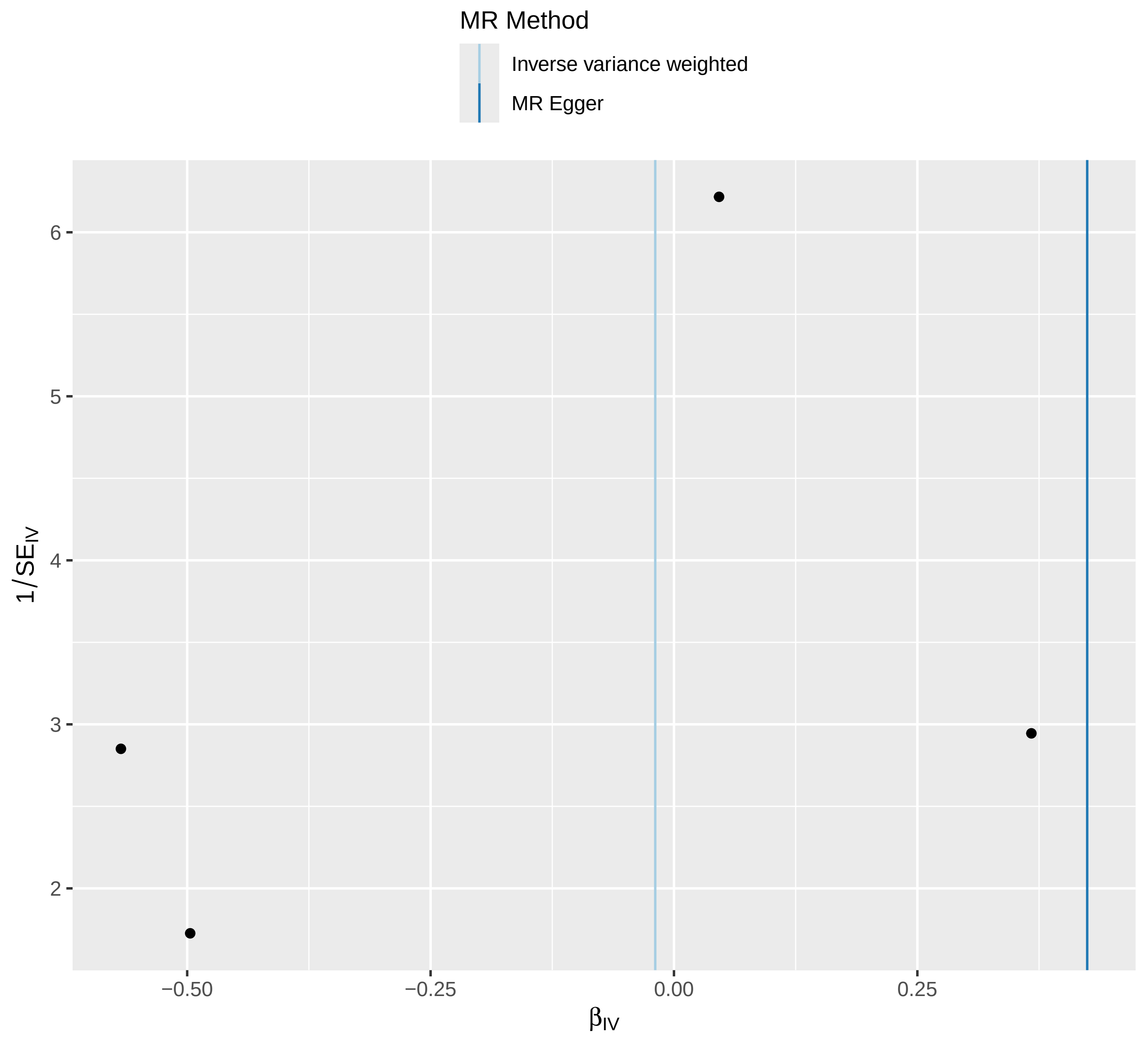
MR Method
Inverse variance weighted
MR Egger
1/SE_IV
6
5
4
3
2
β_IV
−0.50
−0.25
0.00
0.25

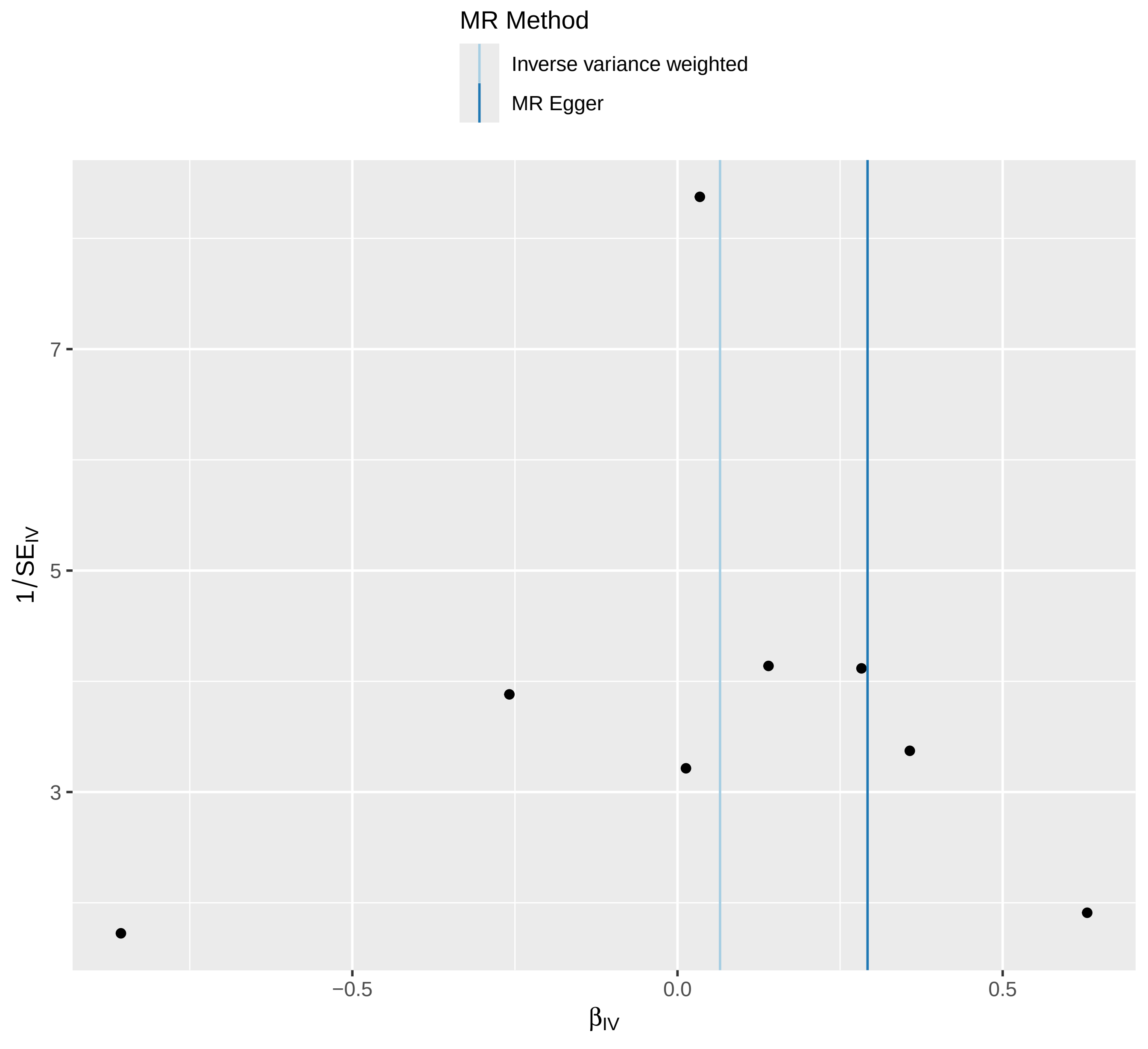
MR Method
Inverse variance weighted
MR Egger
7
5
3
1/SE_IV
−0.5
0.0
0.5
β_IV

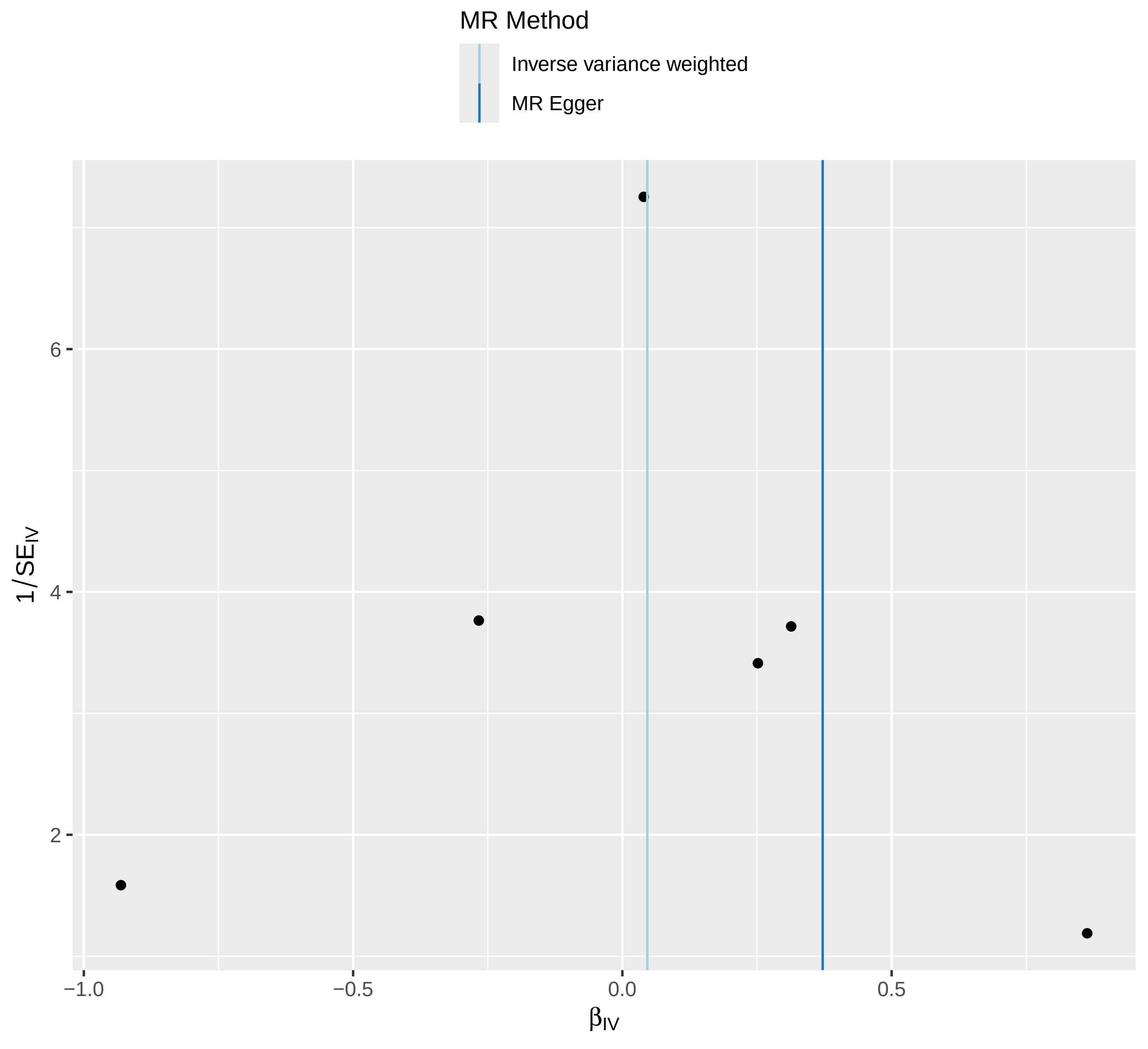
MR Method
Inverse variance weighted
MR Egger
6
4
2
$1/SE_{IV}$
−1.0
−0.5
0.0
0.5
β_{IV}

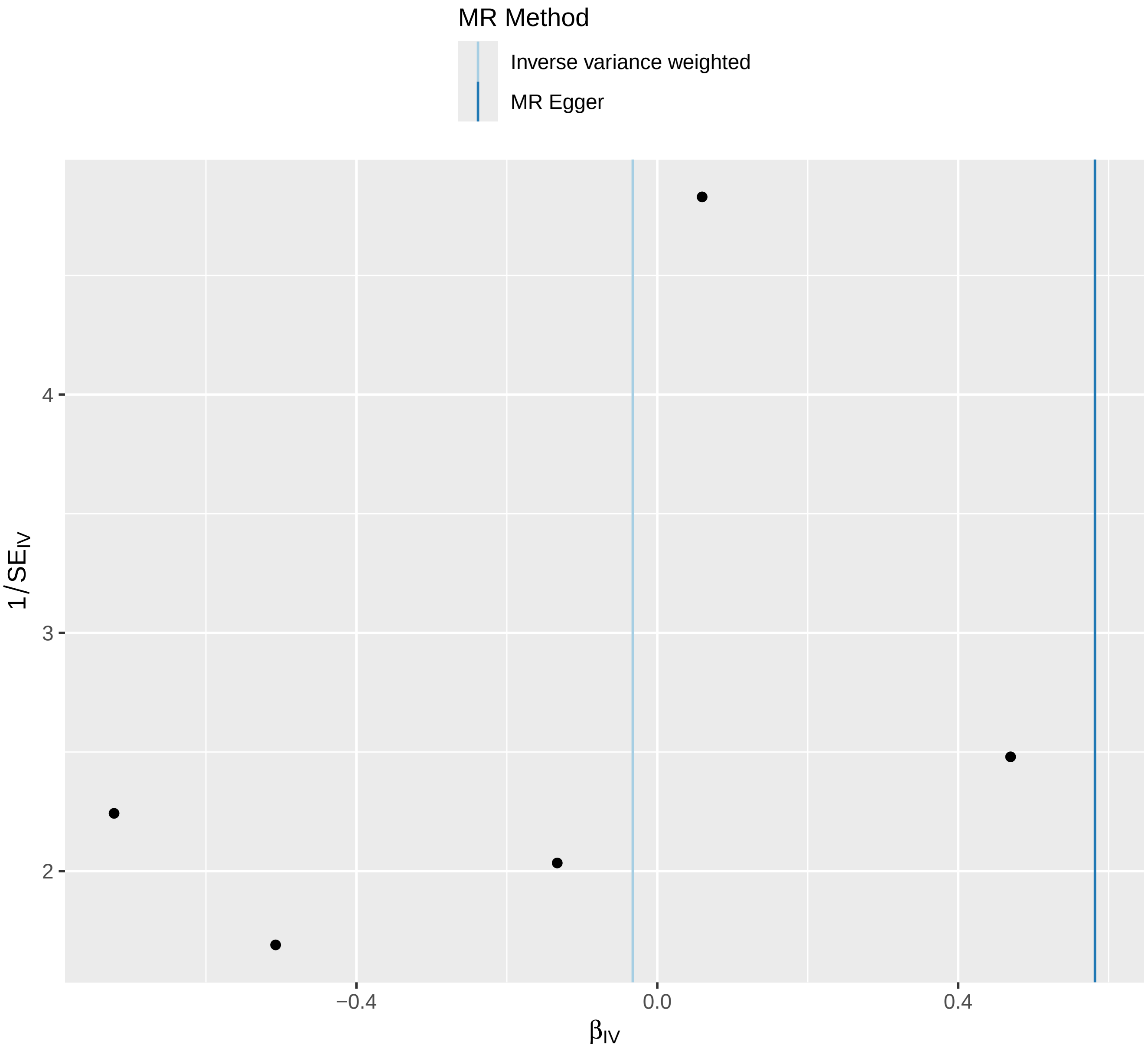

MR Method
Inverse variance weighted
MR Egger
4
3
2
1/SE$_{IV}$
−0.4
0.0
0.4
β$_{IV}$

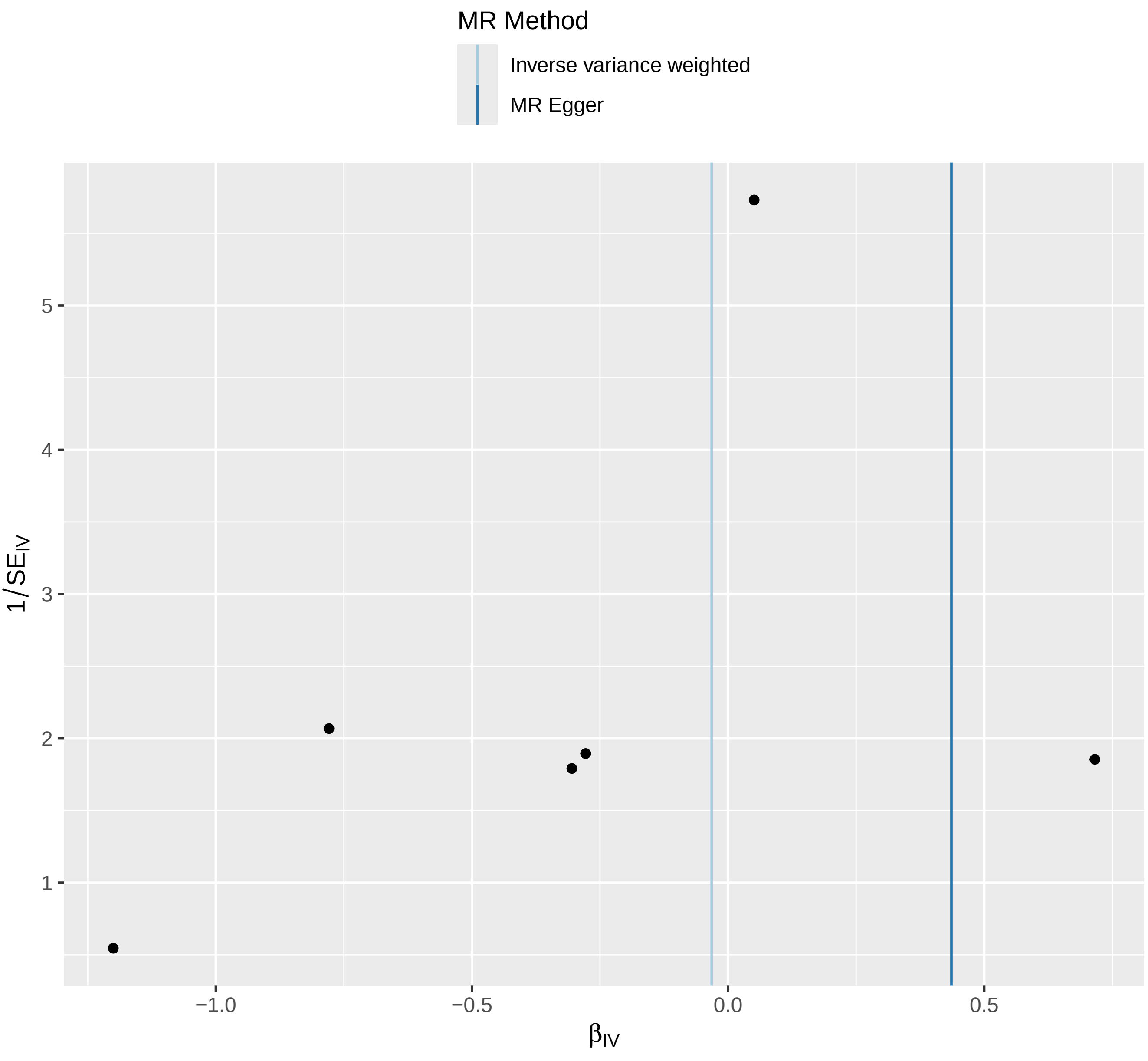

MR Method
Inverse variance weighted
MR Egger
5
4
3
2
1
1/SE$_{IV}$
−1.0
−0.5
0.0
0.5
β$_{IV}$

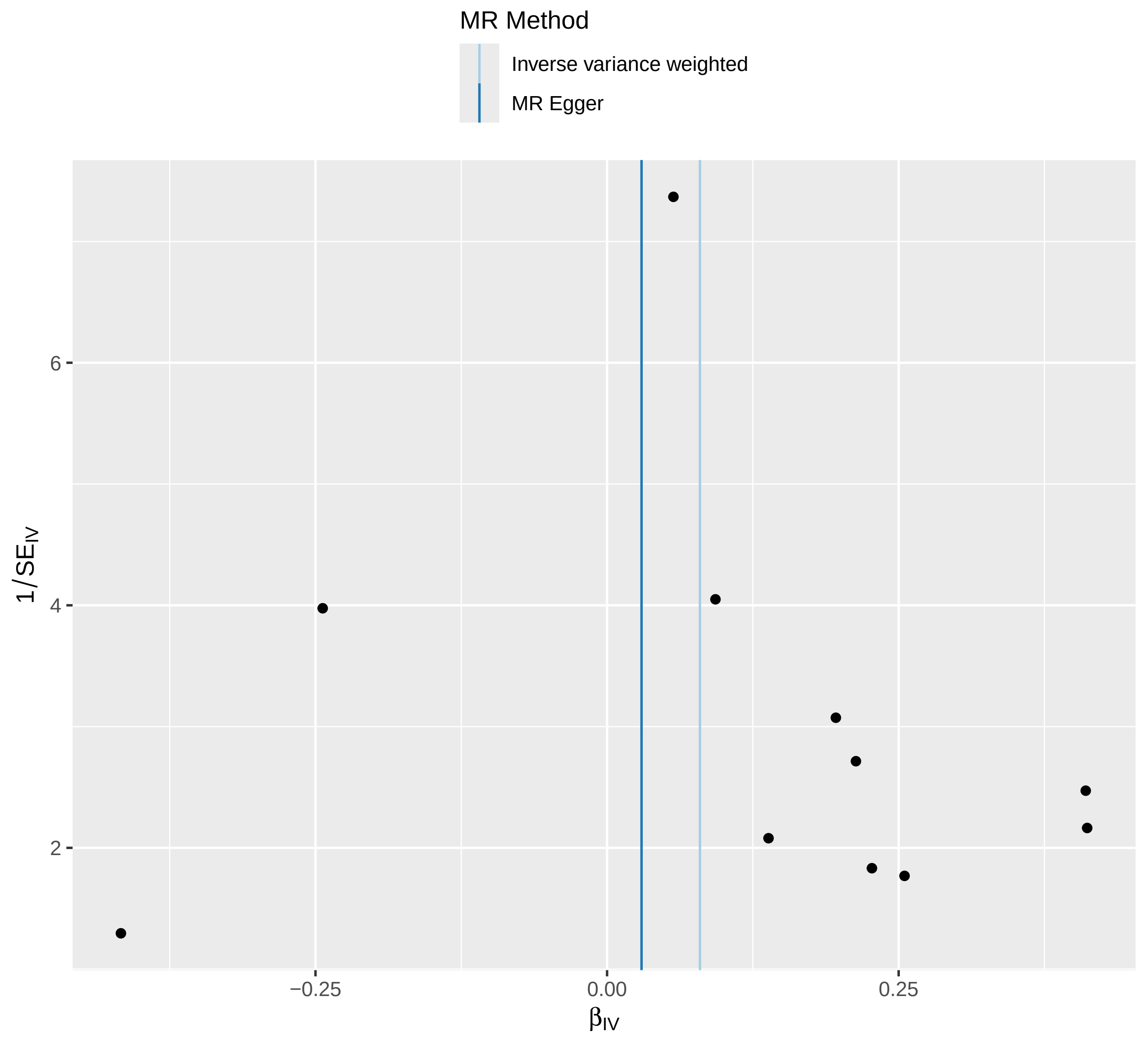
MR Method
Inverse variance weighted
MR Egger
1/SE_IV
6
4
2
−0.25
0.00
0.25
β_IV

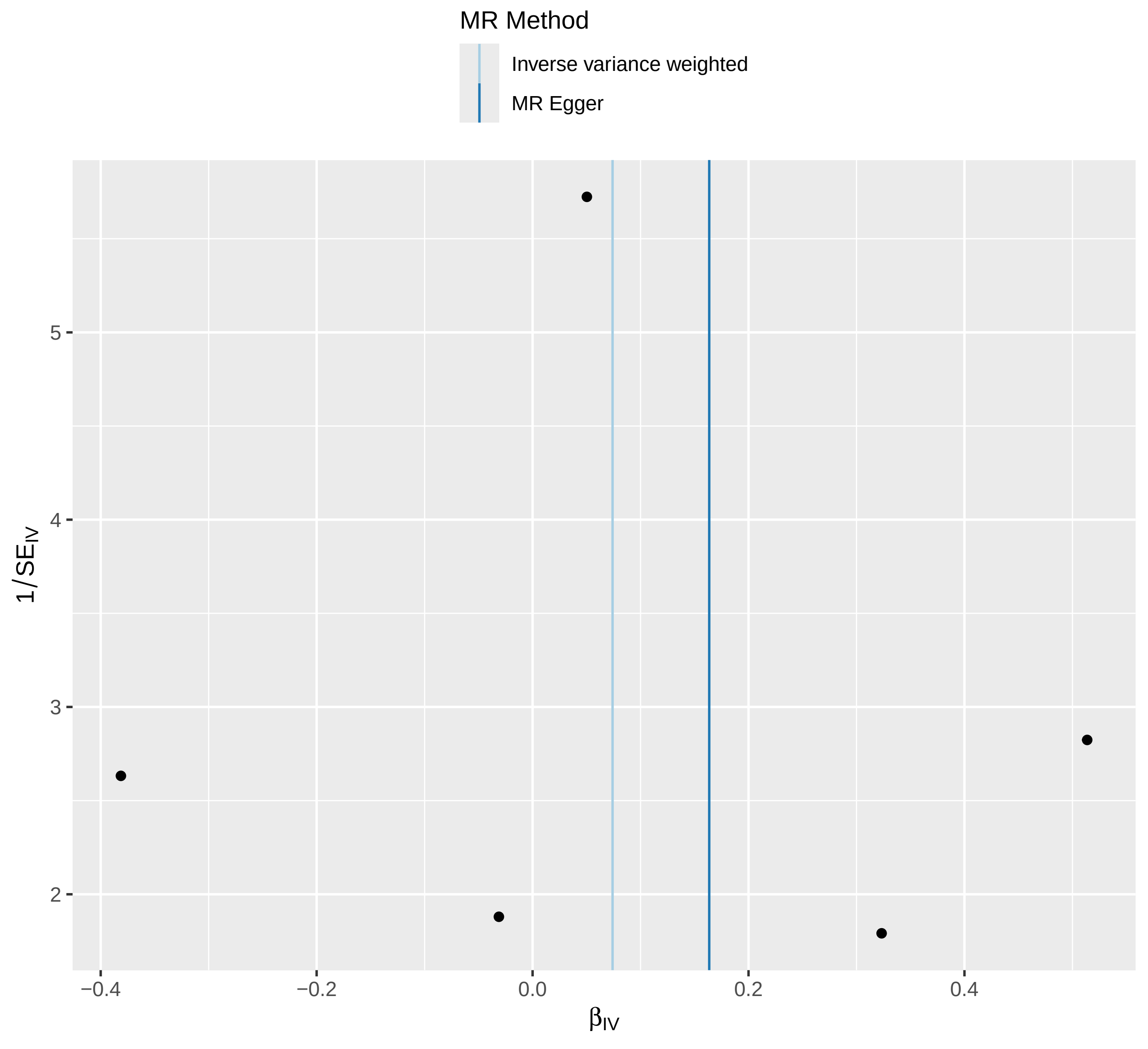
MR Method
Inverse variance weighted
MR Egger
1/SE$_{IV}$
5
4
3
2
−0.4
−0.2
0.0
0.2
0.4
β$_{IV}$

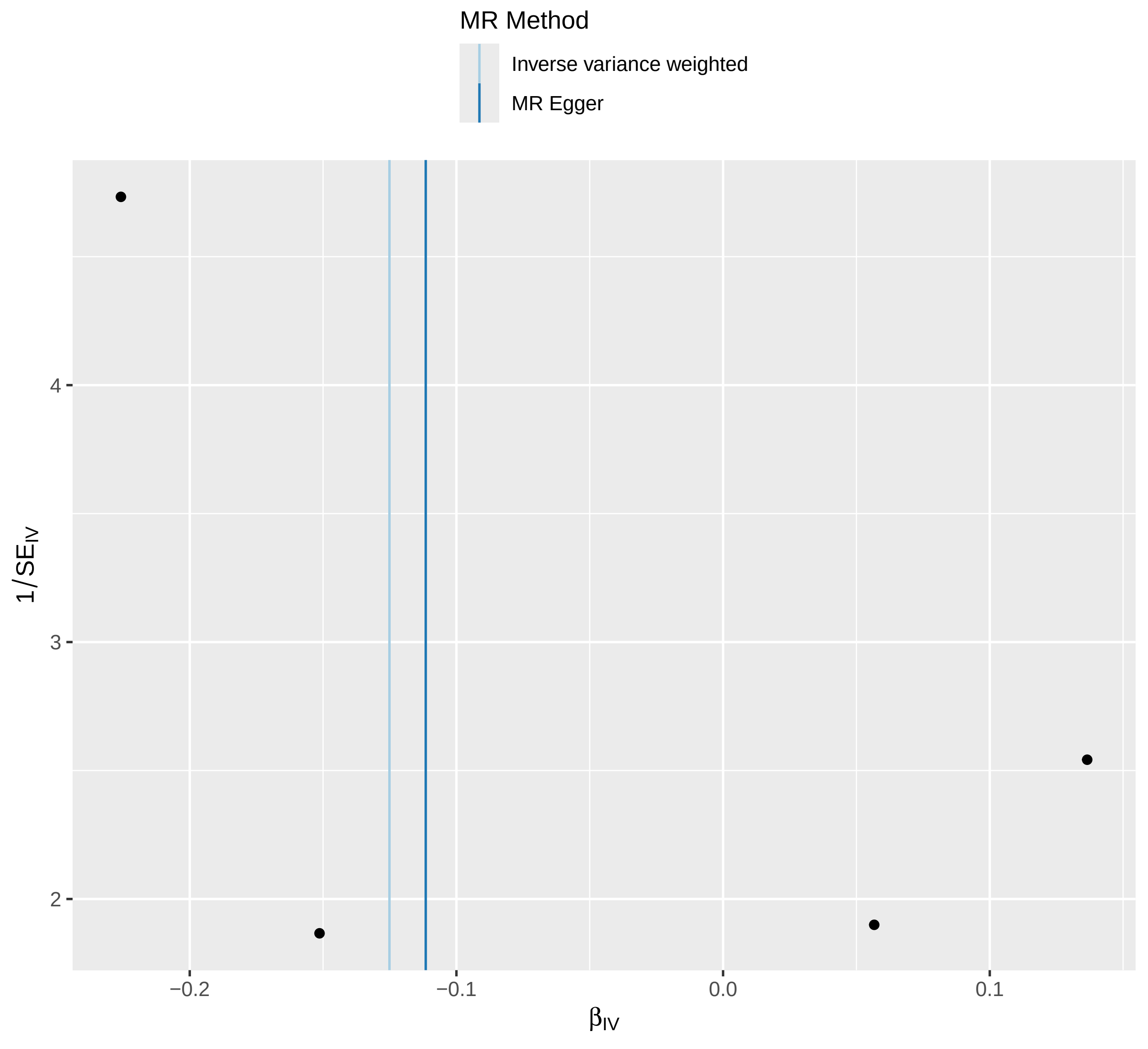

MR Method
Inverse variance weighted
MR Egger
4
3
2
1/SE$_{IV}$
−0.2
−0.1
0.0
0.1
β$_{IV}$

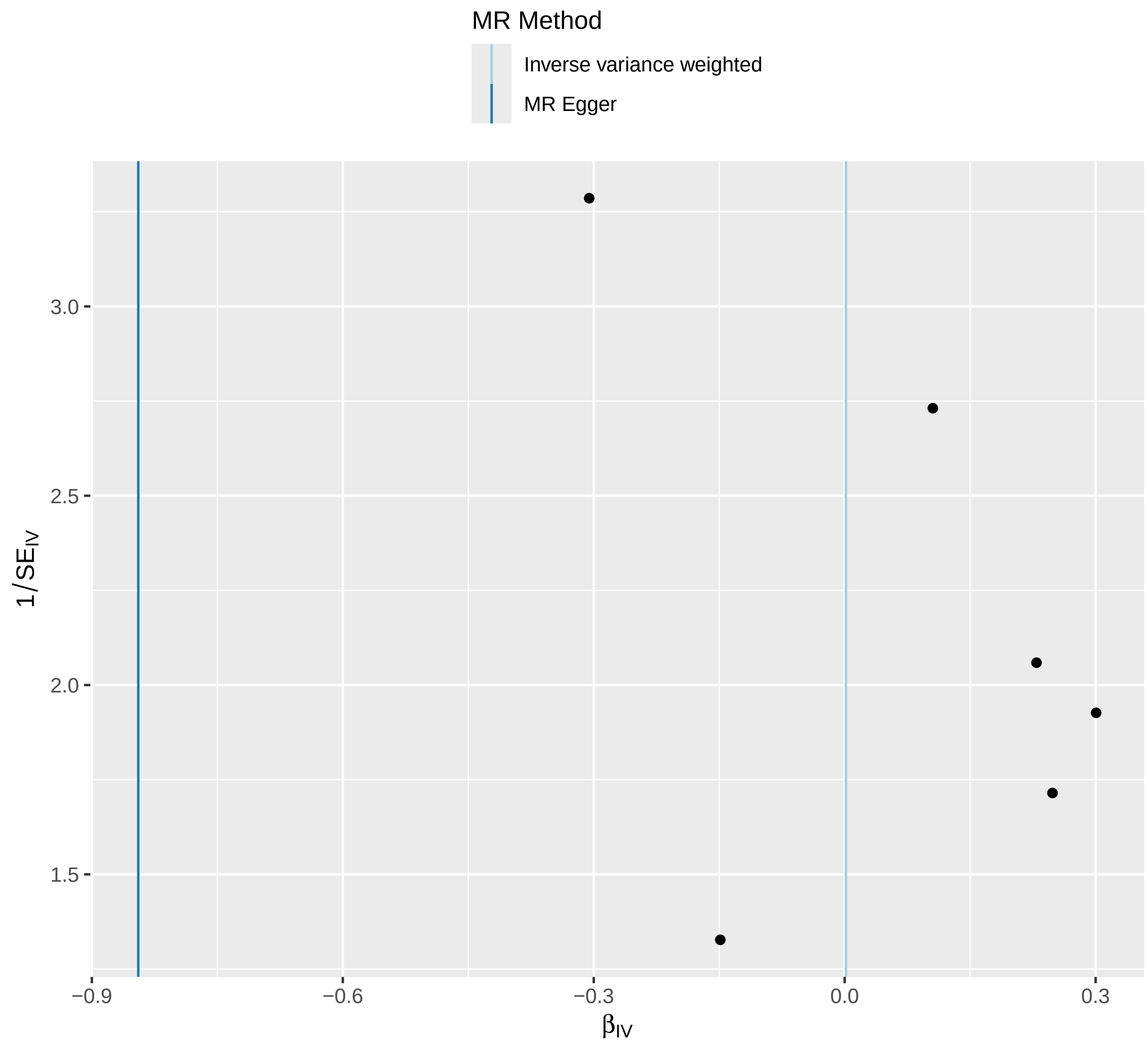

MR Method
Inverse variance weighted
MR Egger
3.0
2.5
2.0
1.5
$1/SE_{IV}$
−0.9
−0.6
−0.3
0.0
0.3
β_{IV}

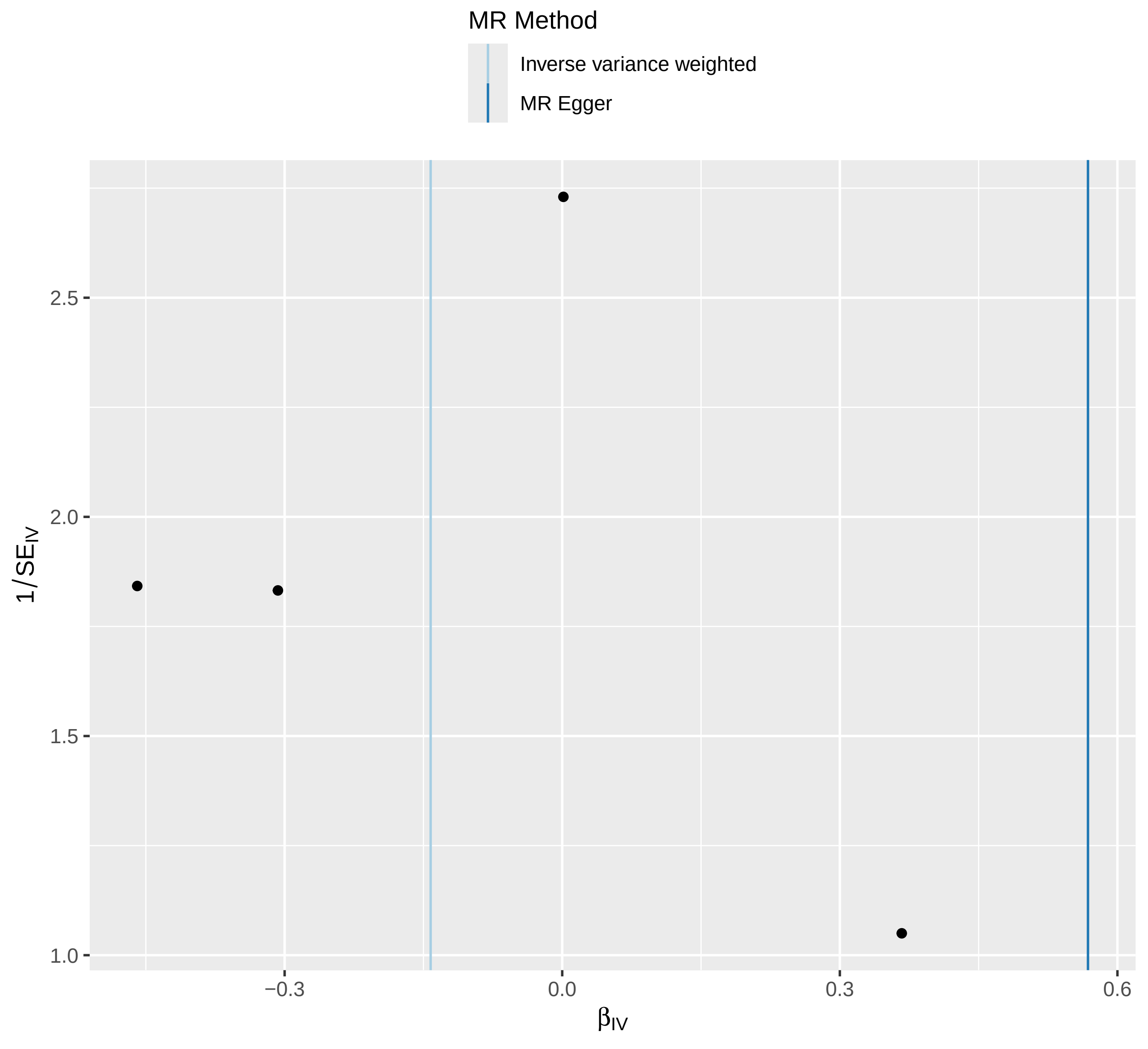
MR Method
Inverse variance weighted
MR Egger
2.5
2.0
1.5
1.0
1/SE$_{IV}$
−0.3
0.0
0.3
0.6
β$_{IV}$

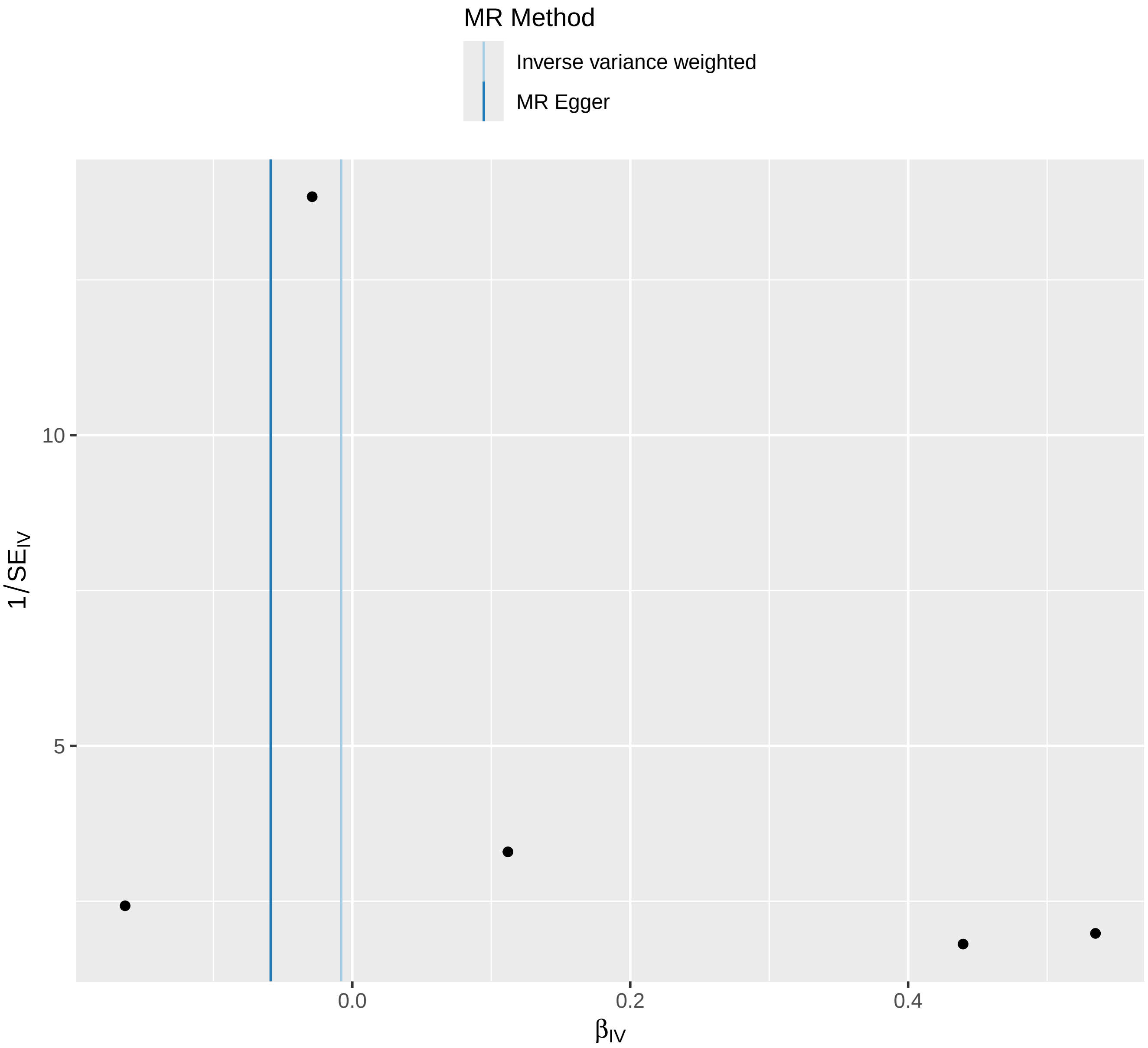
MR Method
Inverse variance weighted
MR Egger
10
5
$1/SE_{IV}$
0.0
0.2
0.4
β_{IV}

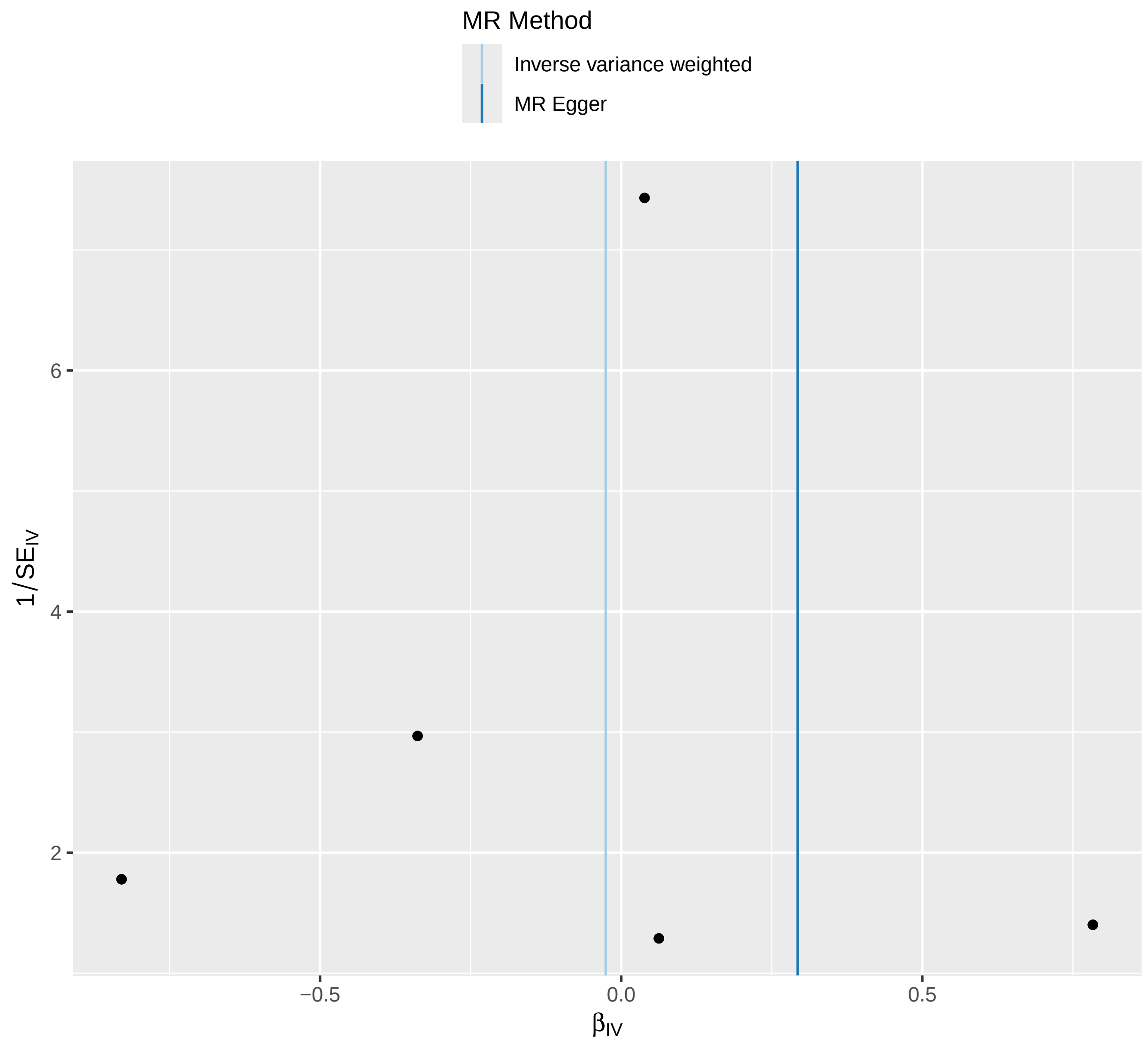

MR Method
Inverse variance weighted
MR Egger
6
4
2
−0.5
0.0
0.5
1/SE_IV
β_IV

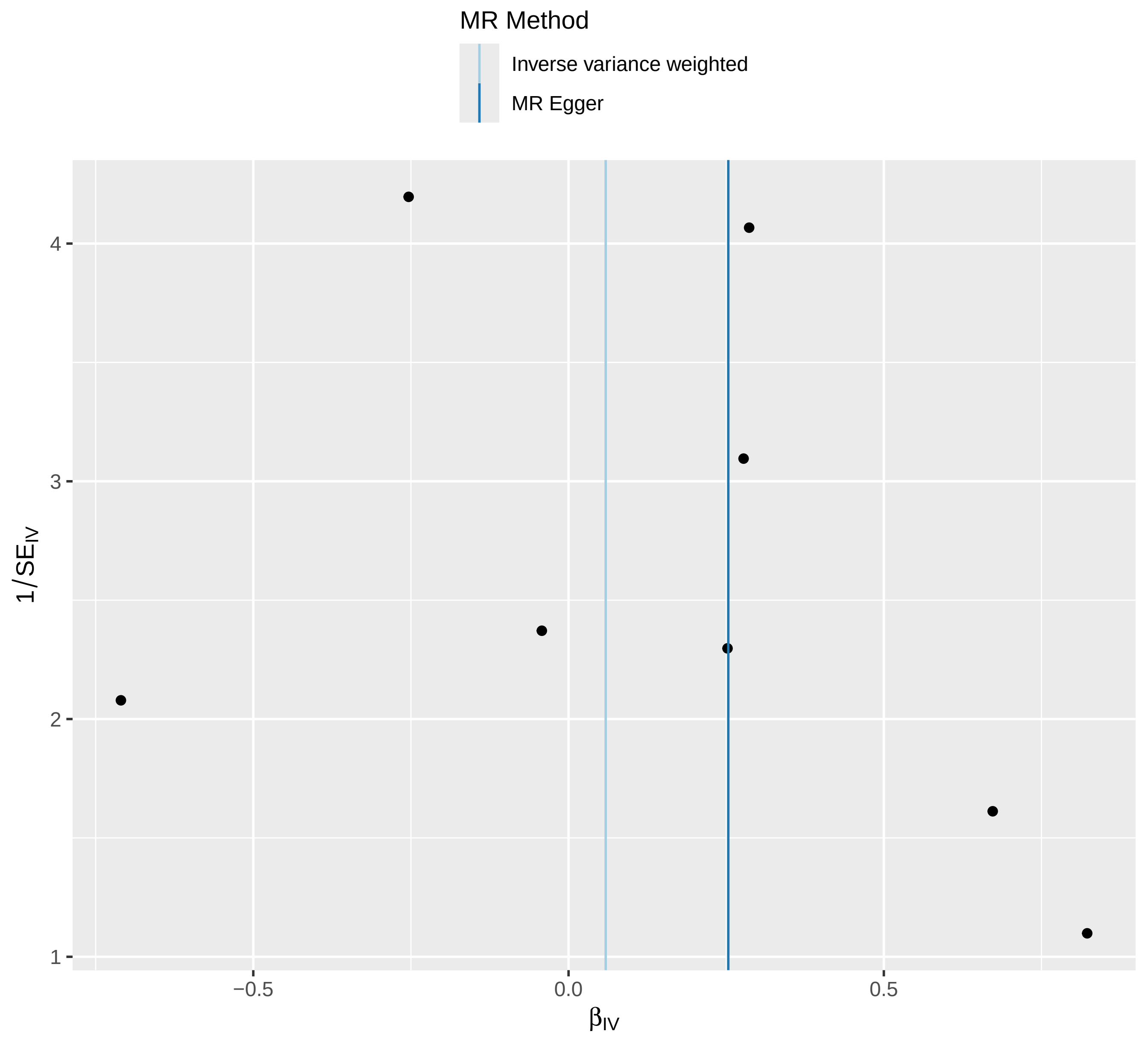
MR Method
Inverse variance weighted
MR Egger
1/SE$_{IV}$
β_{IV}
−0.5
0.0
0.5
1
2
3
4

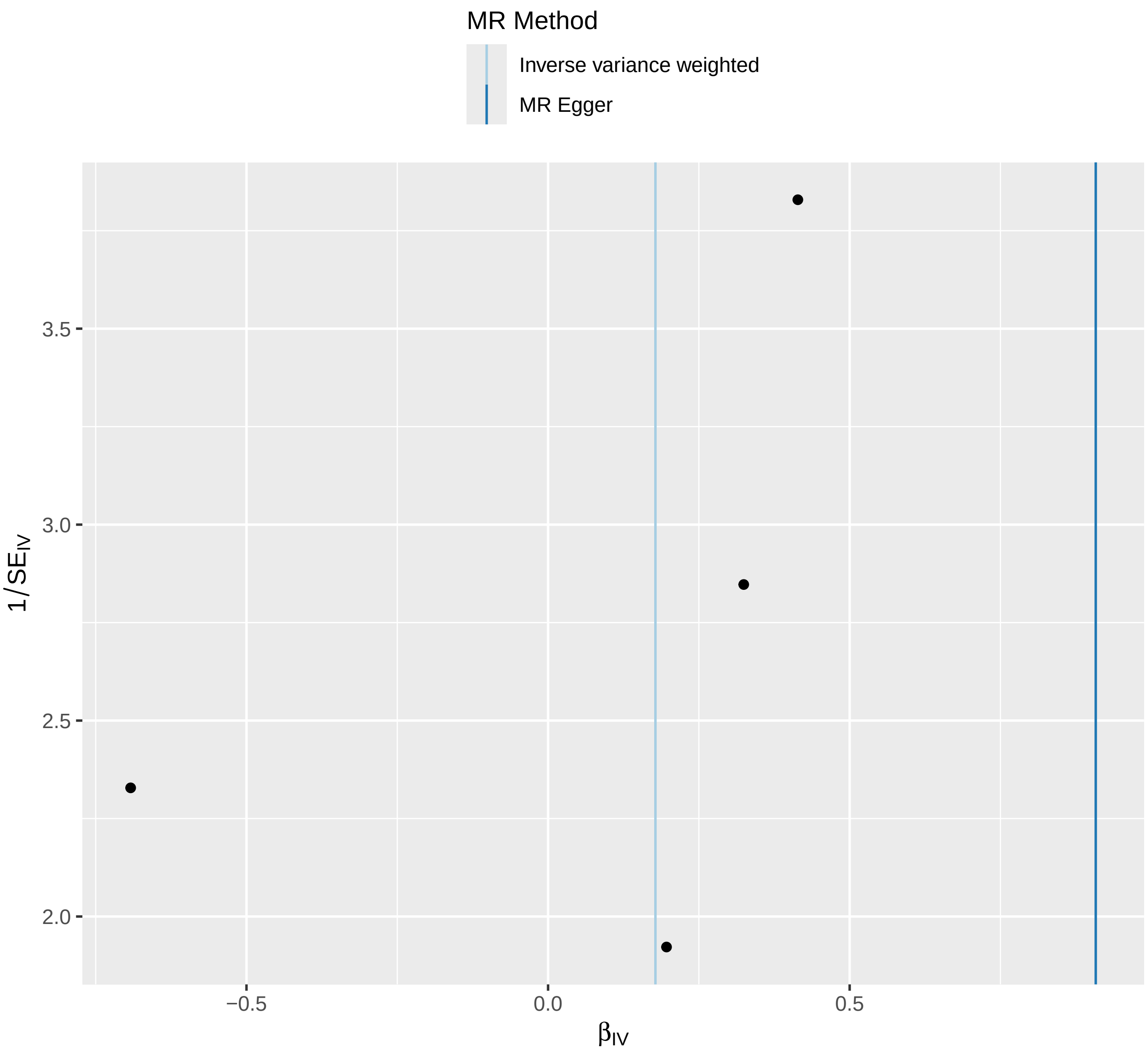
MR Method
Inverse variance weighted
MR Egger
3.5
3.0
2.5
2.0
1/SE$_{IV}$
−0.5
0.0
0.5
β$_{IV}$

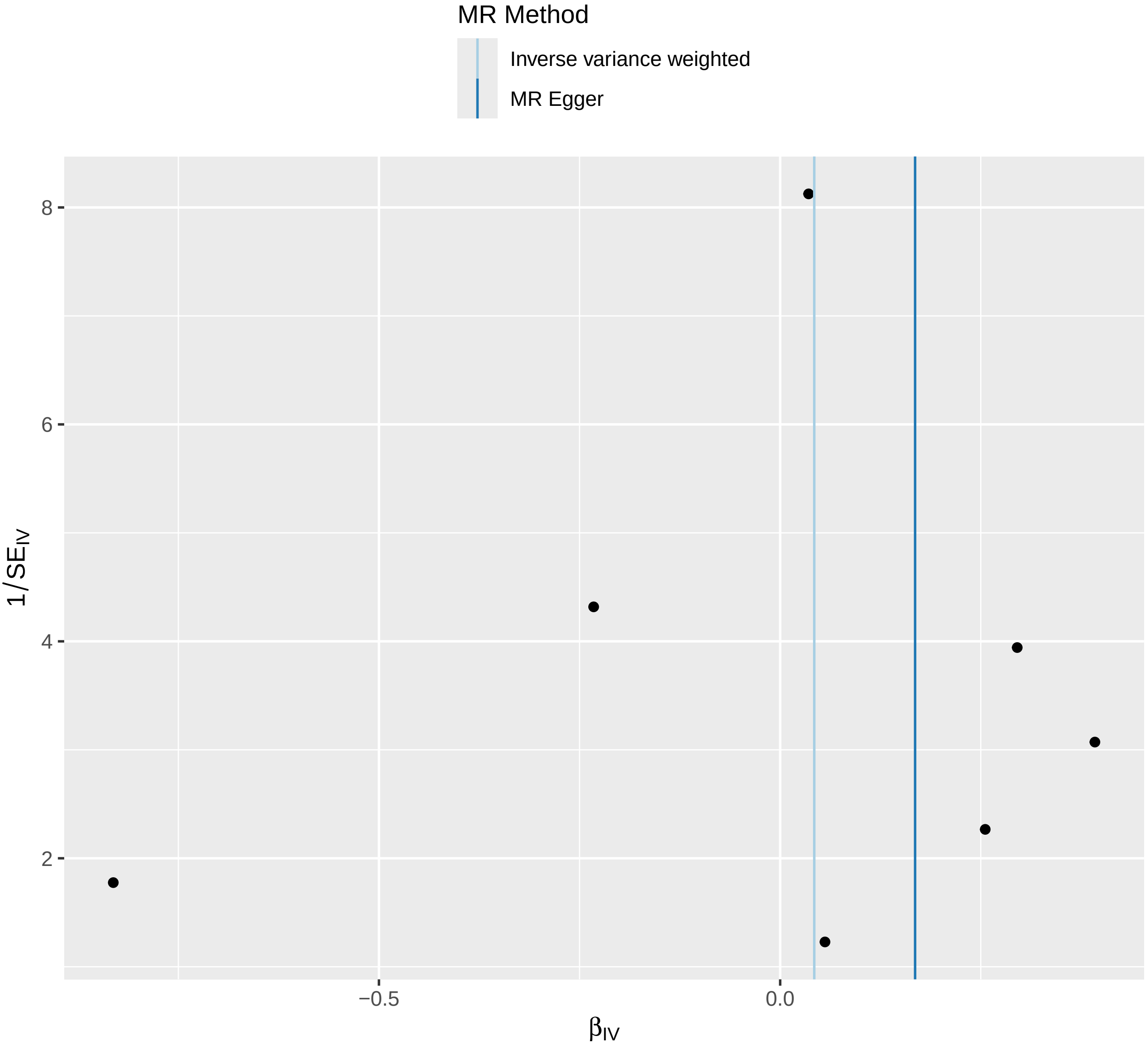

MR Method
Inverse variance weighted
MR Egger
8
6
4
2
1/SE_IV
−0.5
0.0
β_IV

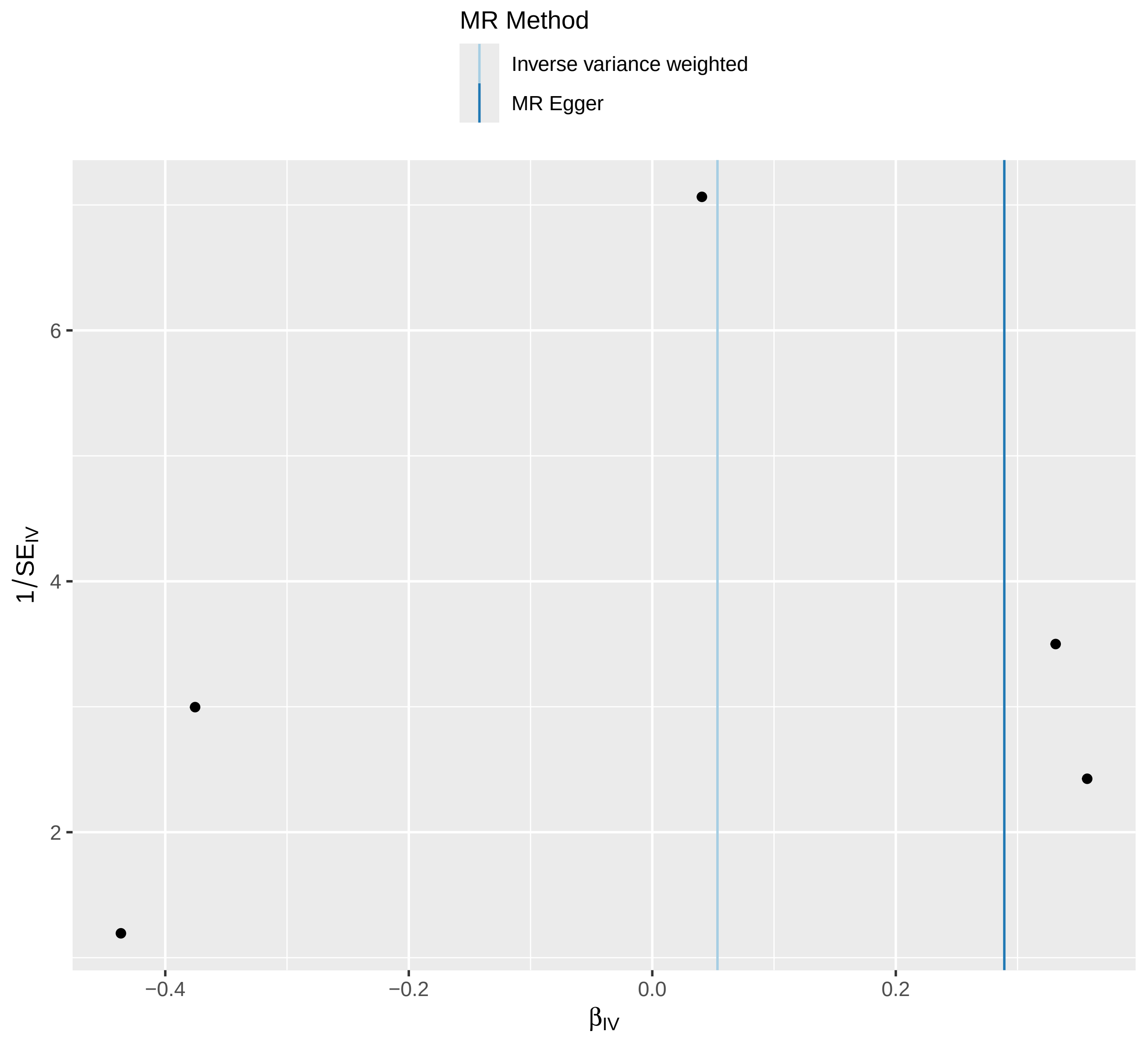

MR Method
Inverse variance weighted
MR Egger
6
4
2
−0.4
−0.2
0.0
0.2
$1/SE_{IV}$
β_{IV}

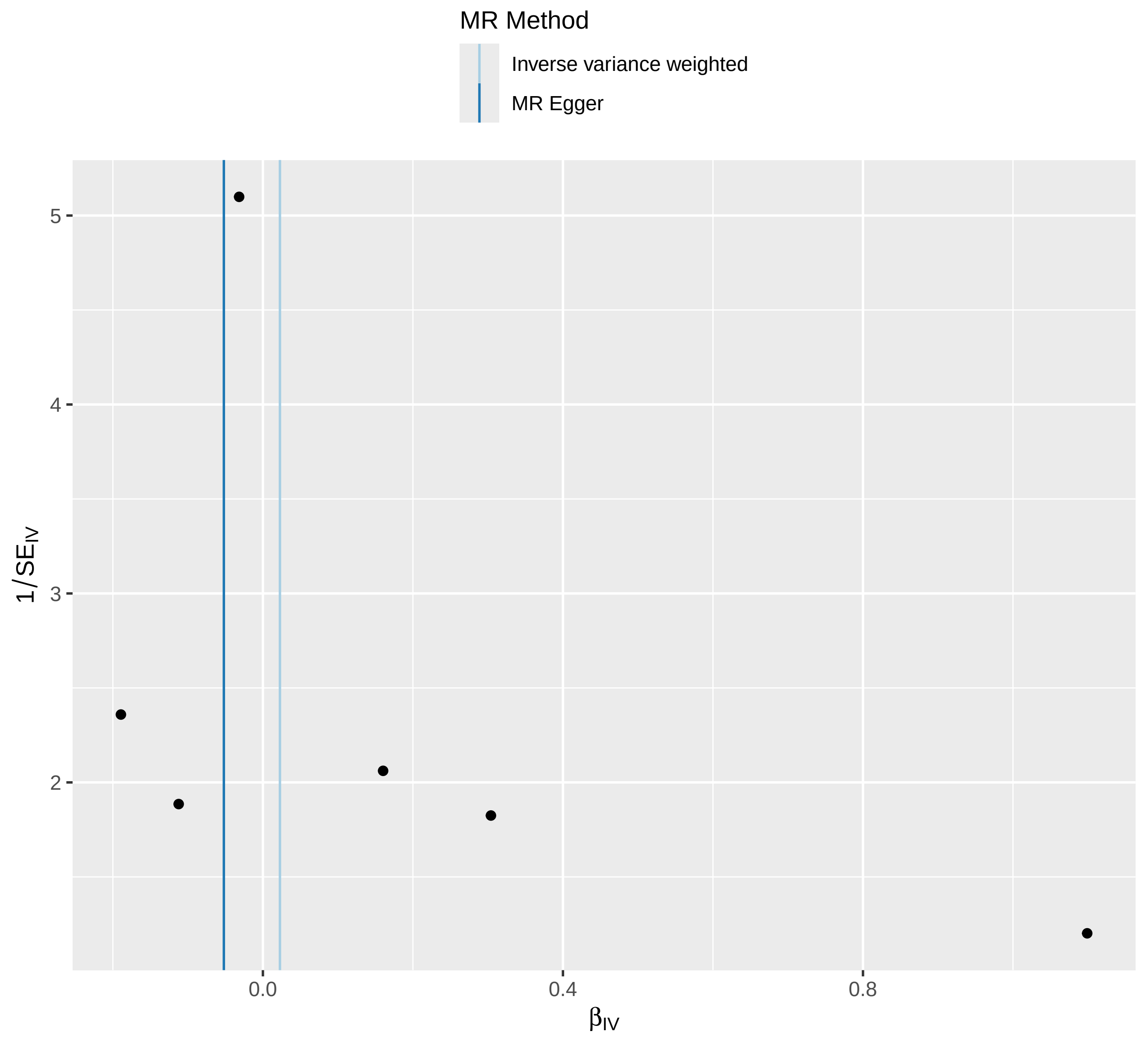

MR Method
Inverse variance weighted
MR Egger
5
4
3
2
1/SE$_{IV}$
0.0
0.4
0.8
β$_{IV}$

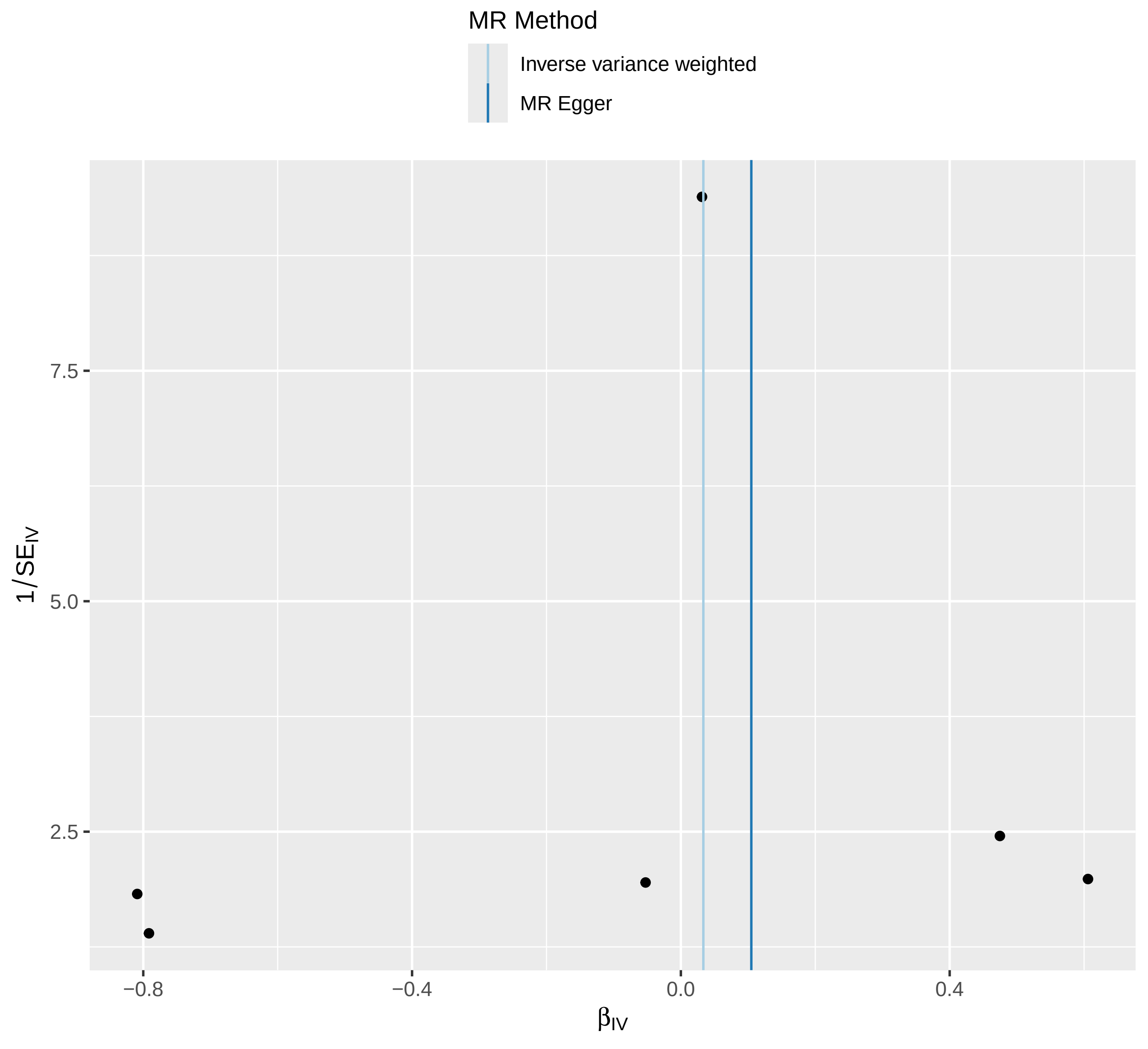
MR Method
Inverse variance weighted
MR Egger
7.5
5.0
2.5
1/SE_IV
−0.8
−0.4
0.0
0.4
β_IV

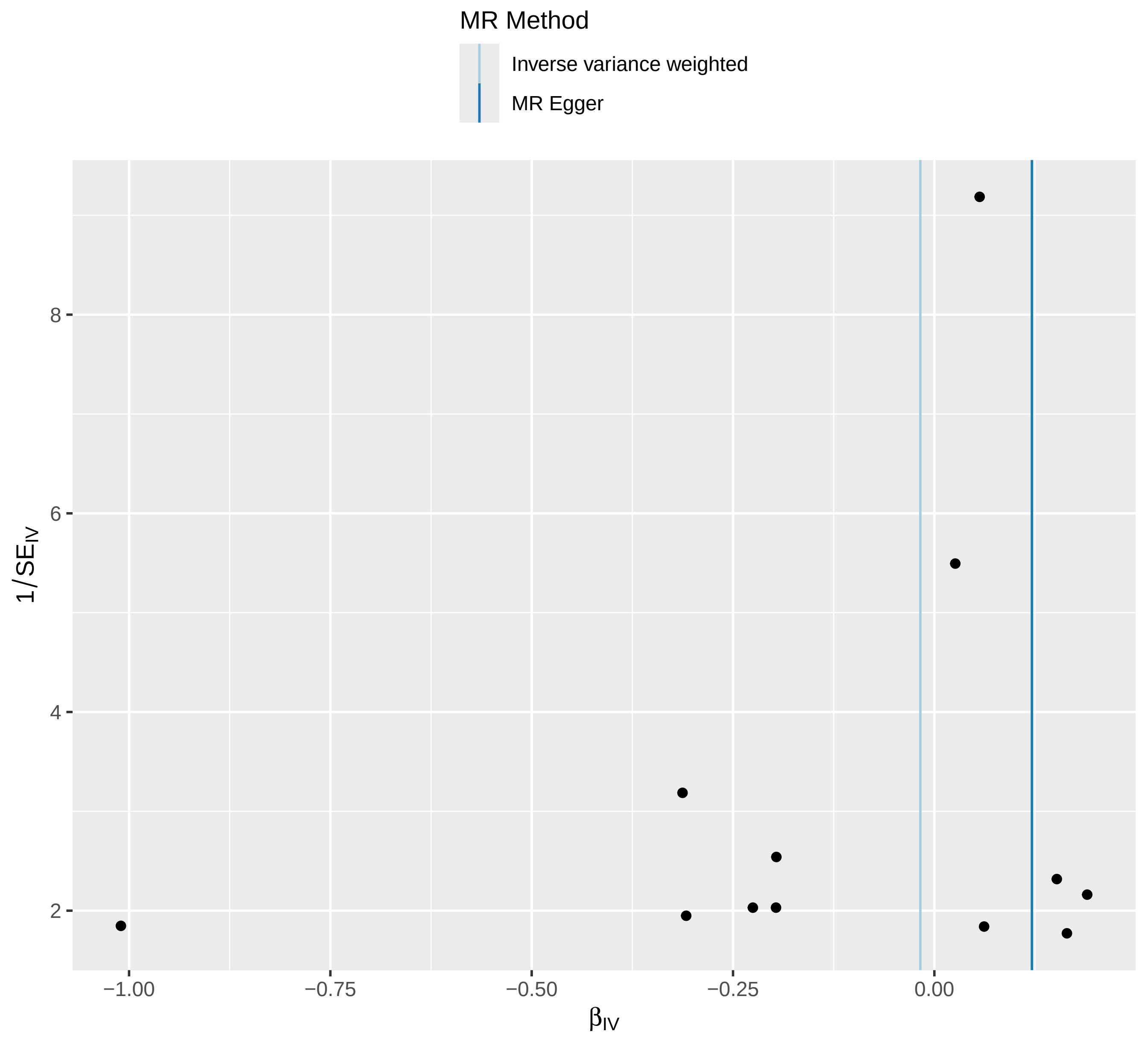

MR Method
Inverse variance weighted
MR Egger
8
6
4
2
1/SE_IV
−1.00
−0.75
−0.50
−0.25
0.00
β_IV

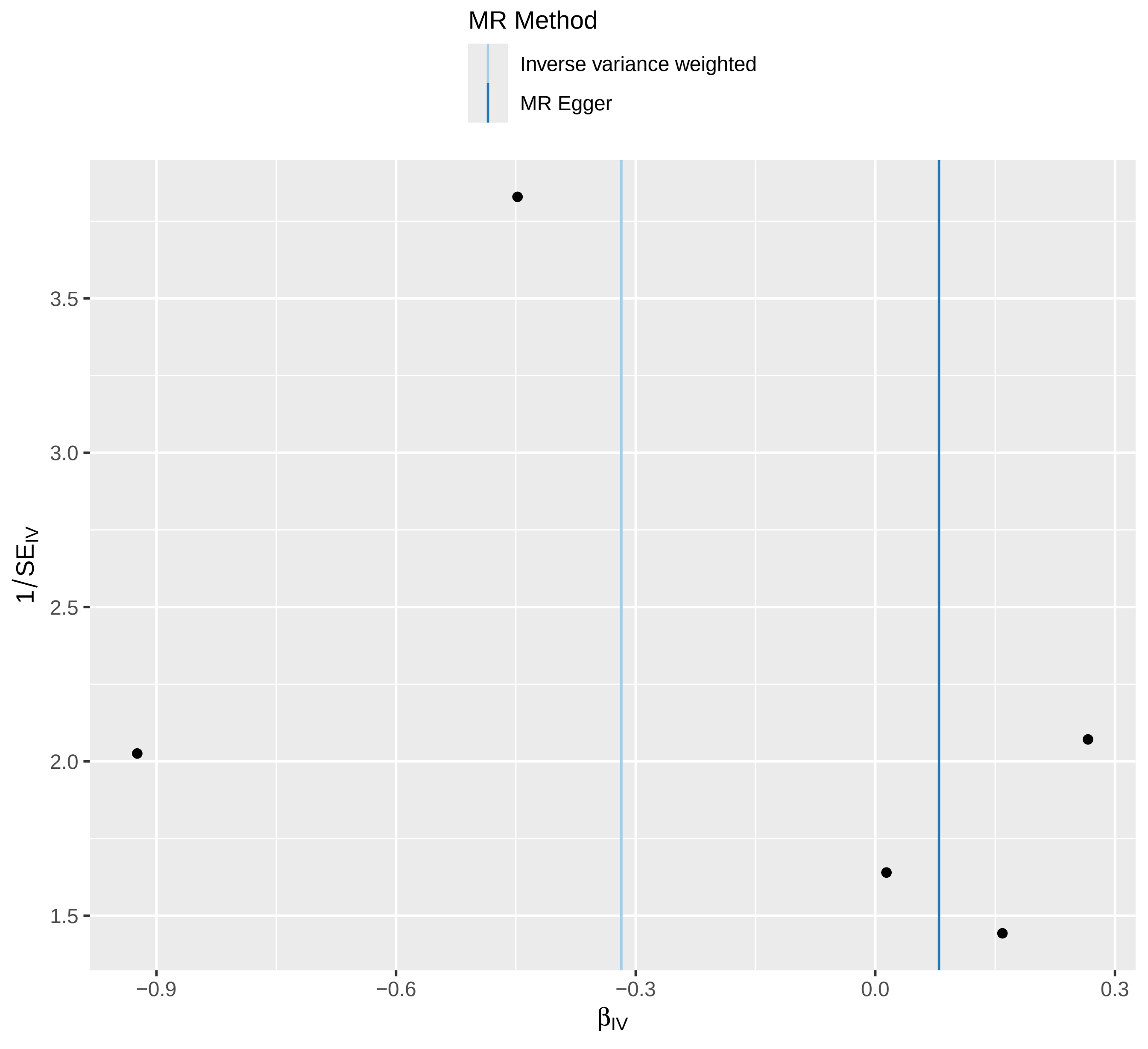

MR Method
Inverse variance weighted
MR Egger
1/SE_IV
3.5
3.0
2.5
2.0
1.5
−0.9
−0.6
−0.3
0.0
0.3
β_IV

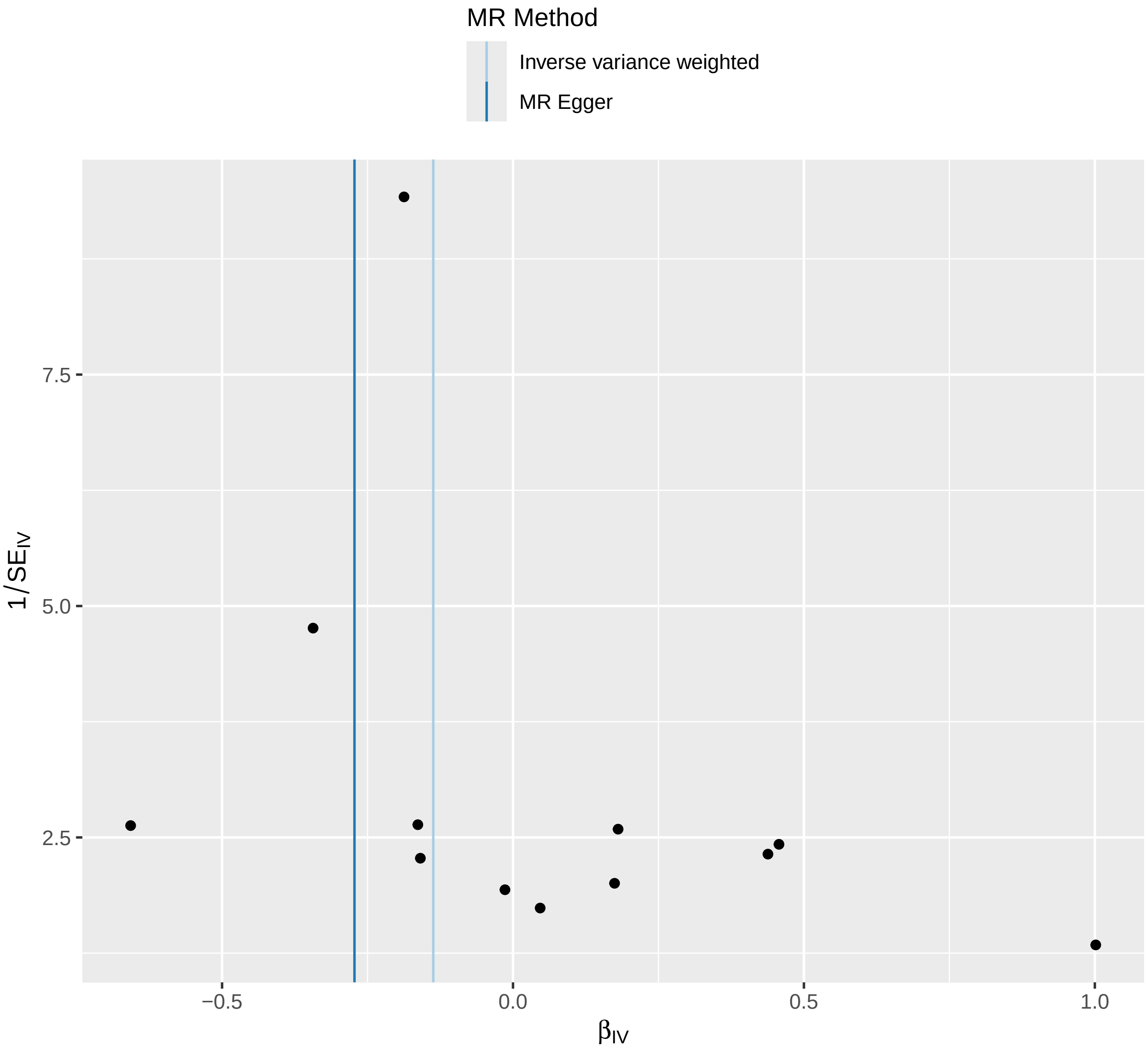

MR Method
Inverse variance weighted
MR Egger
7.5
5.0
2.5
1/SE$_{IV}$
−0.5
0.0
0.5
1.0
β$_{IV}$

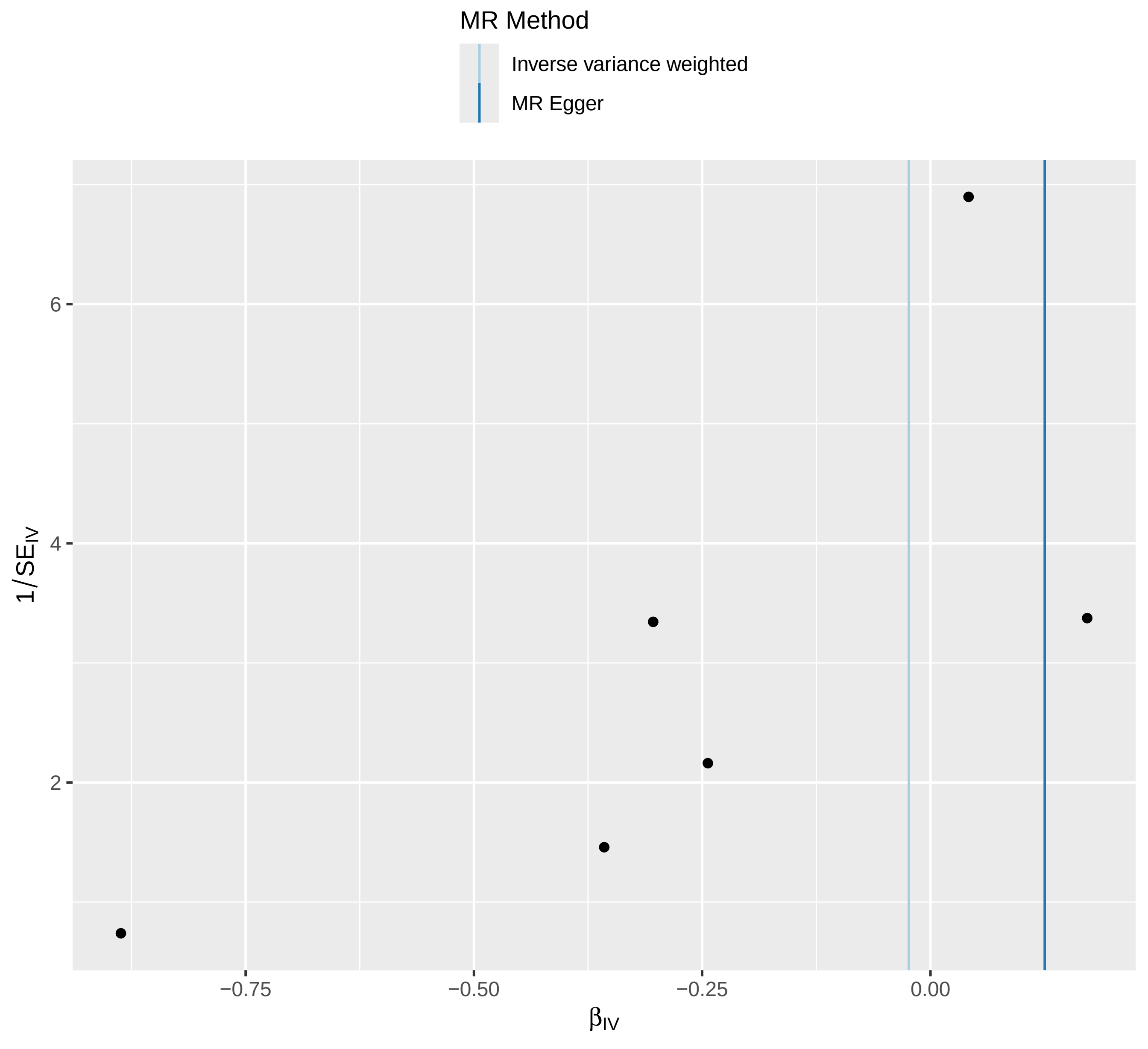

MR Method
Inverse variance weighted
MR Egger
6
4
2
1/SE$_{IV}$
−0.75
−0.50
−0.25
0.00
β$_{IV}$

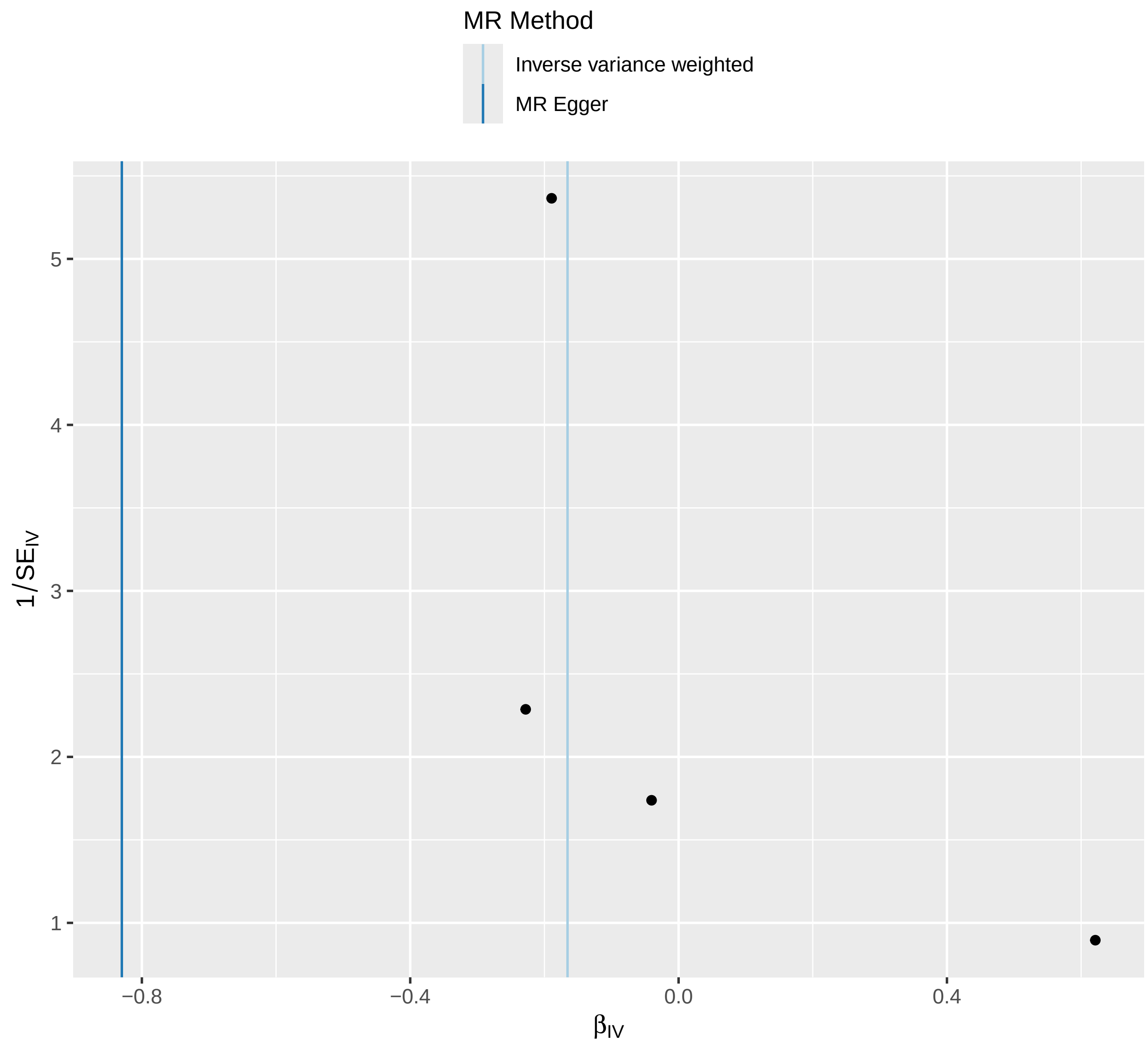
MR Method
Inverse variance weighted
MR Egger
1/SE_IV
5
4
3
2
1
−0.8
−0.4
0.0
0.4
β_IV

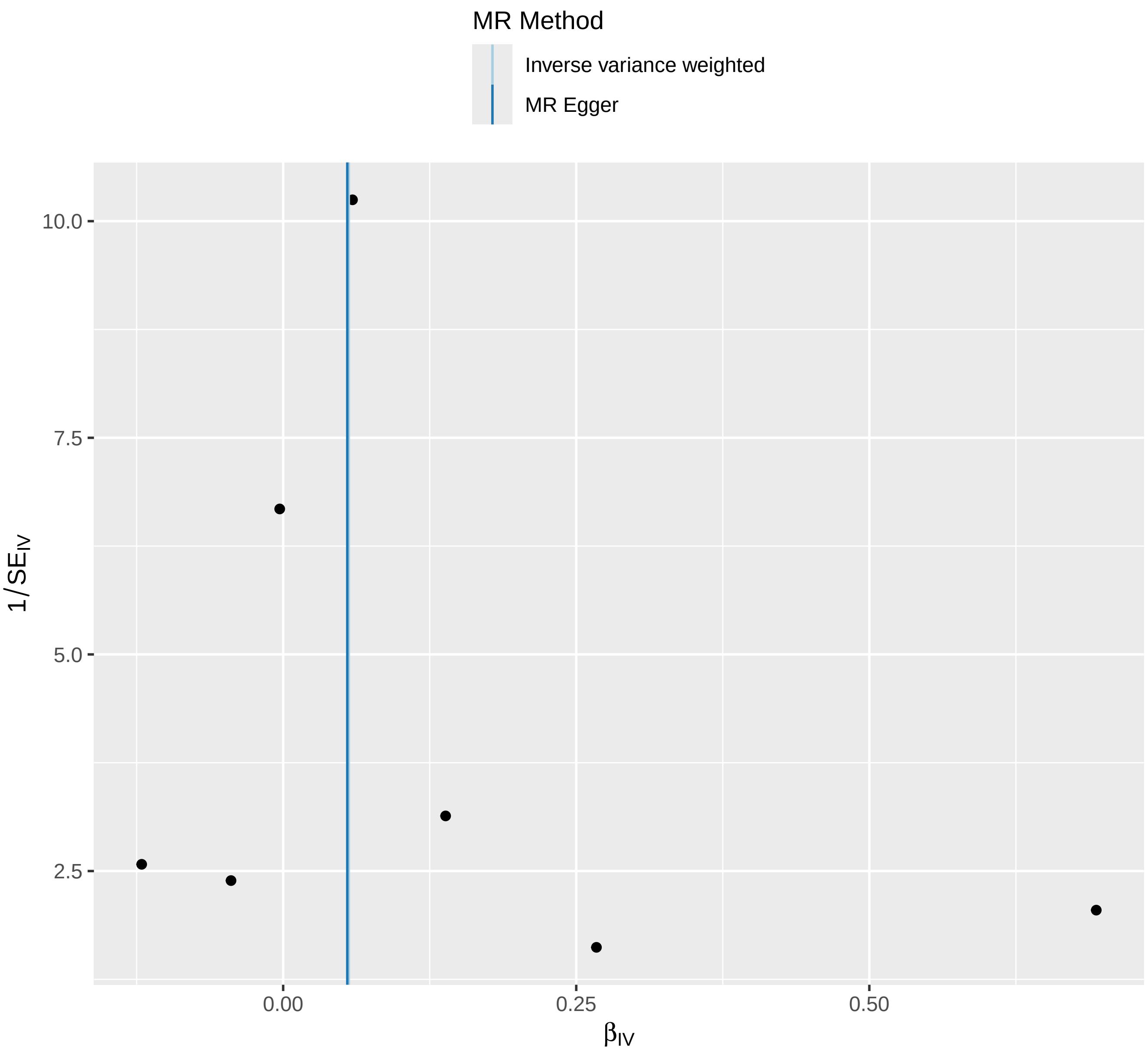
MR Method
Inverse variance weighted
MR Egger
10.0
7.5
5.0
2.5
0.00
0.25
0.50
$1/SE_{IV}$
β_{IV}

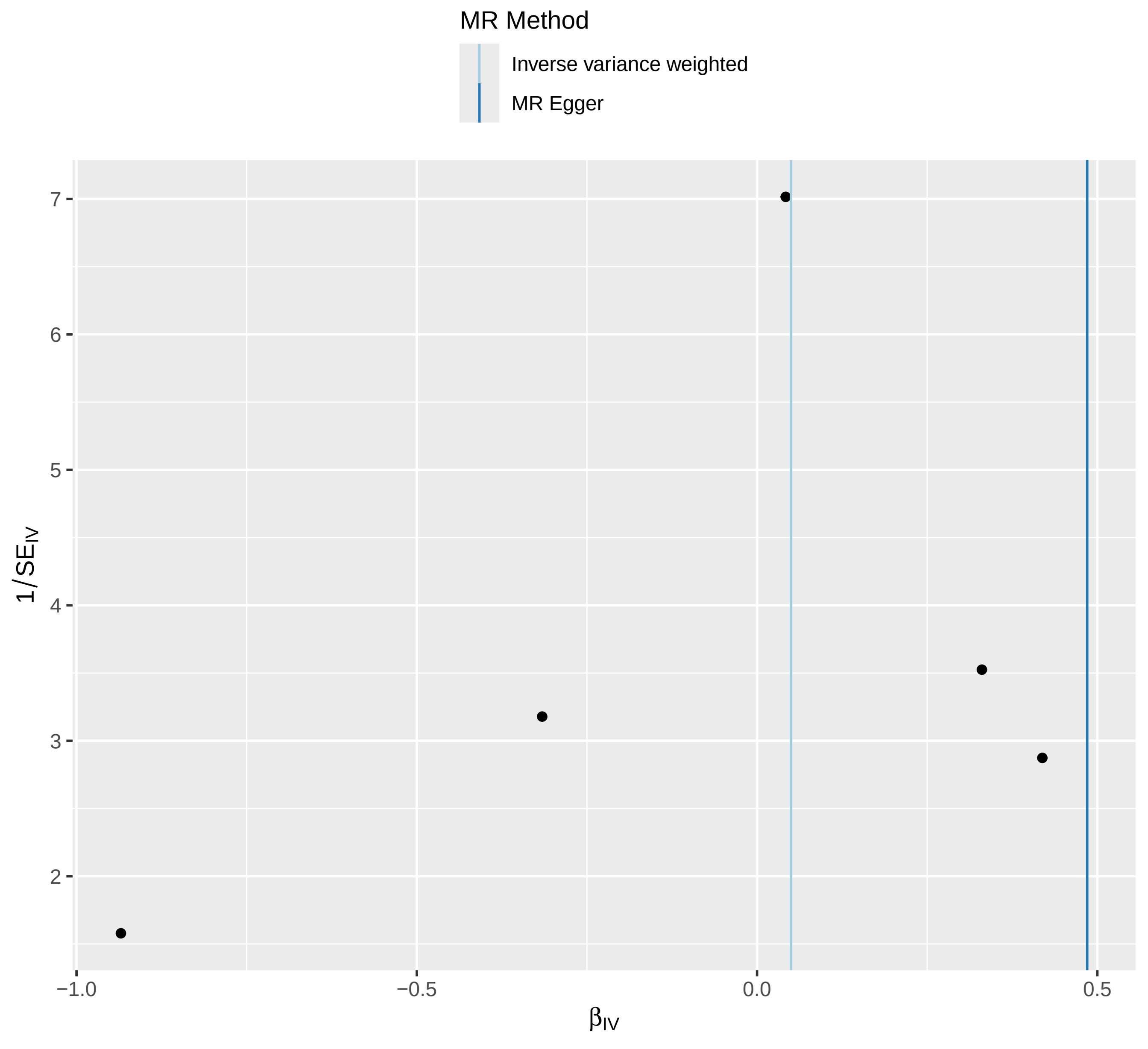
MR Method
Inverse variance weighted
MR Egger
1/SE_IV
β_IV
7
6
5
4
3
2
−1.0
−0.5
0.0
0.5

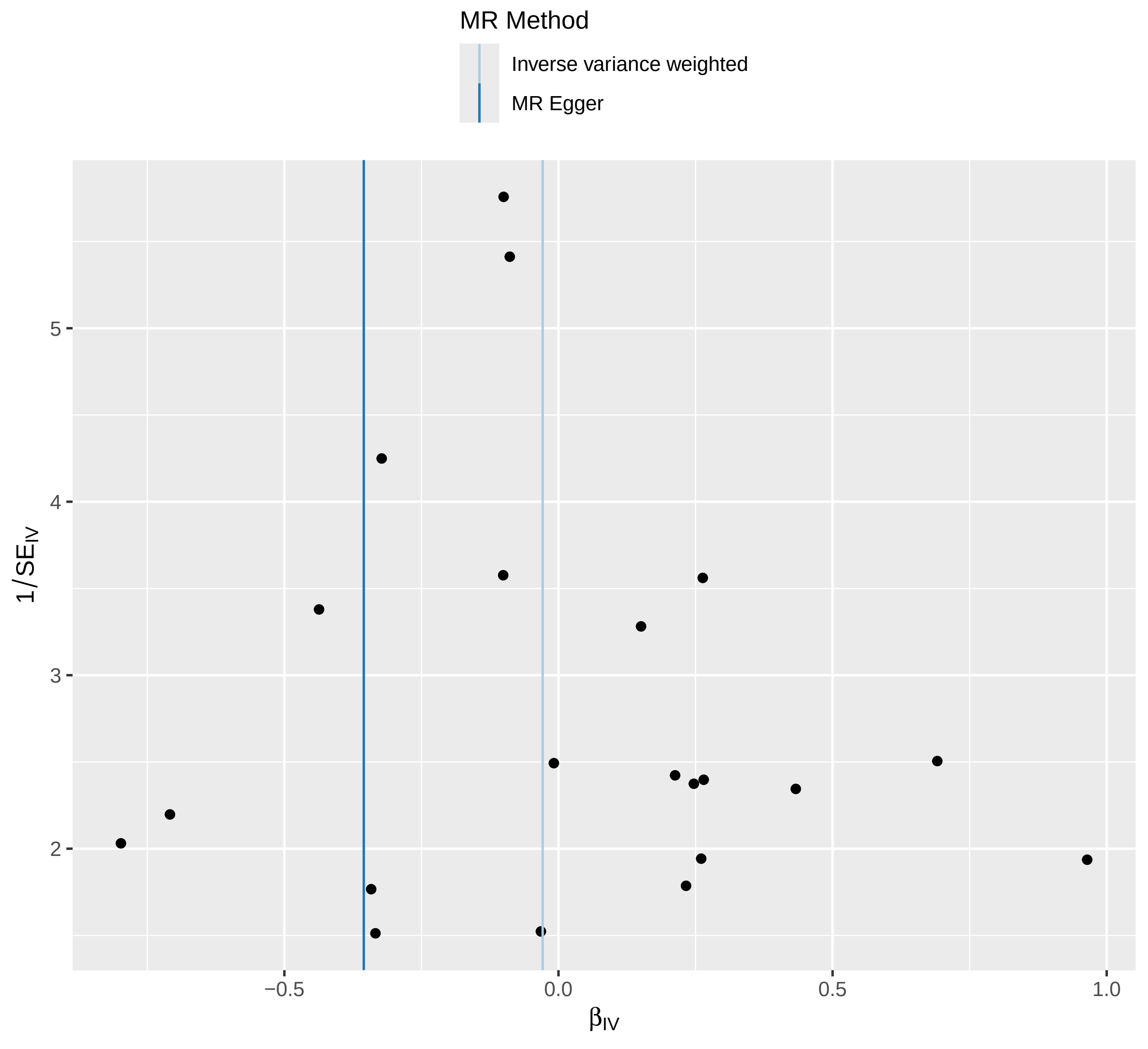

MR Method
Inverse variance weighted
MR Egger
5
4
3
2
1/SE_IV
−0.5
0.0
0.5
1.0
β_IV

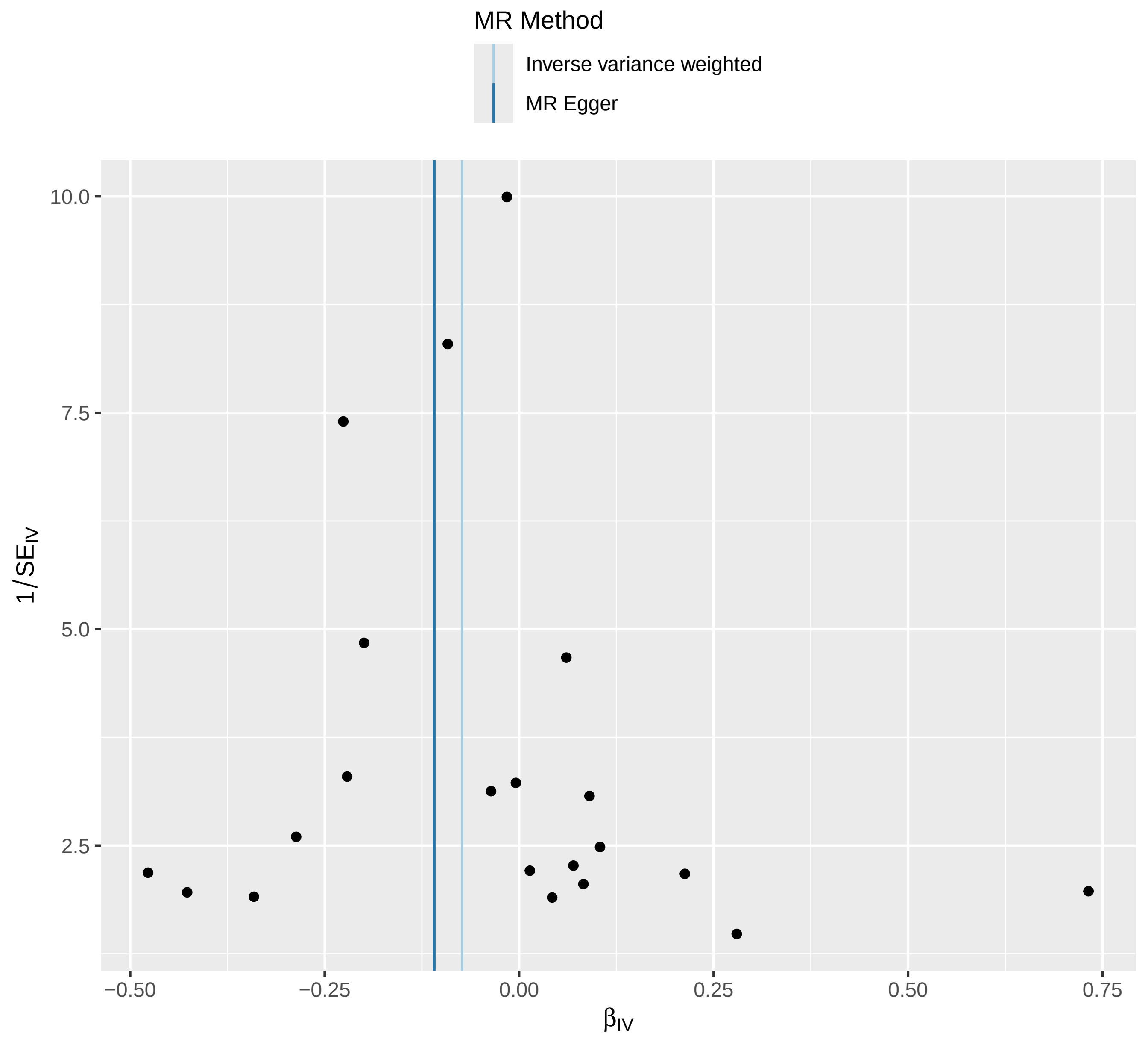

MR Method
Inverse variance weighted
MR Egger
10.0
7.5
5.0
2.5
1/SE$_{IV}$
−0.50
−0.25
0.00
0.25
0.50
0.75
β$_{IV}$

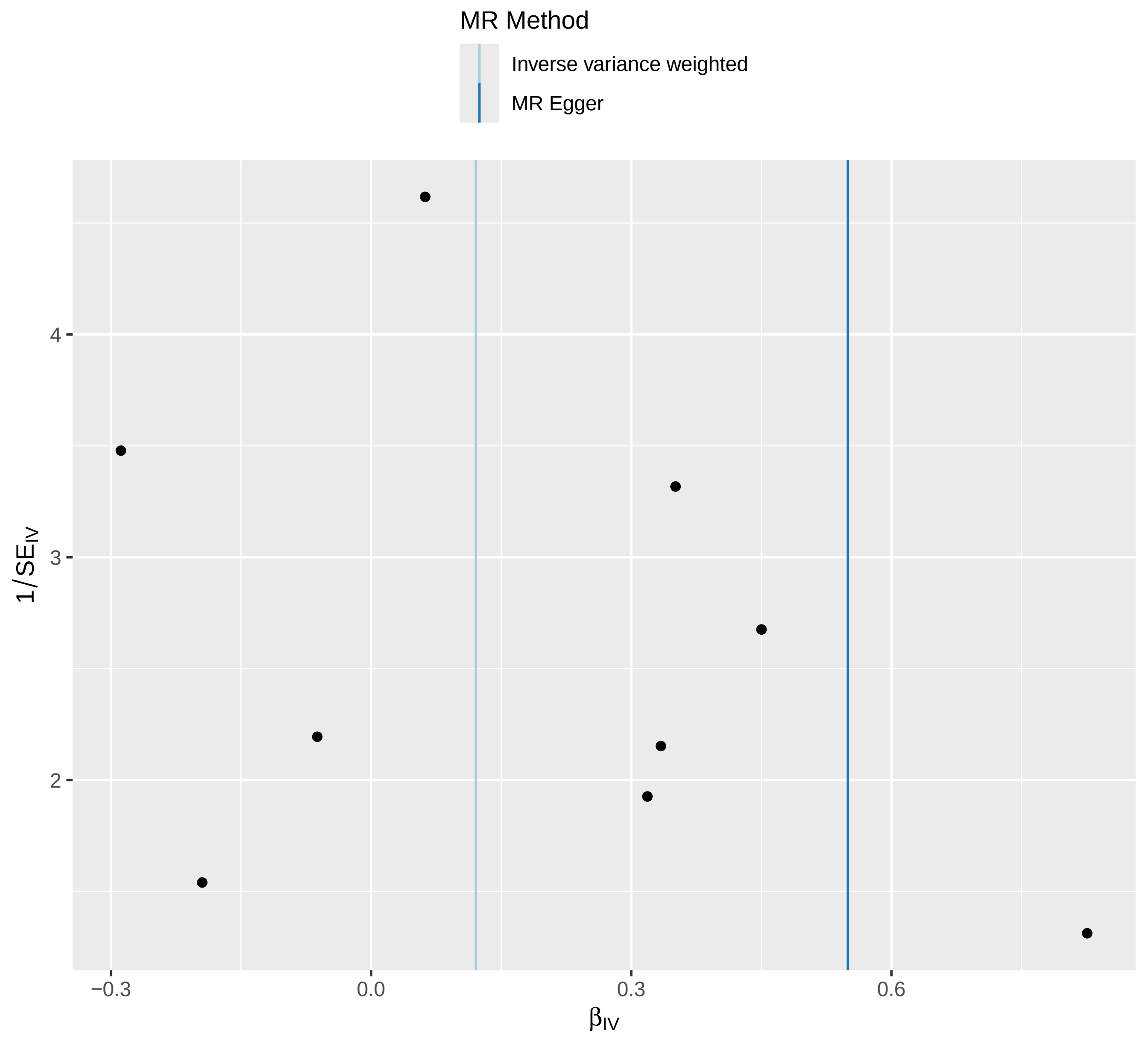
MR Method
Inverse variance weighted
MR Egger
1/SE_IV
4
3
2
−0.3
0.0
0.3
0.6
β_IV

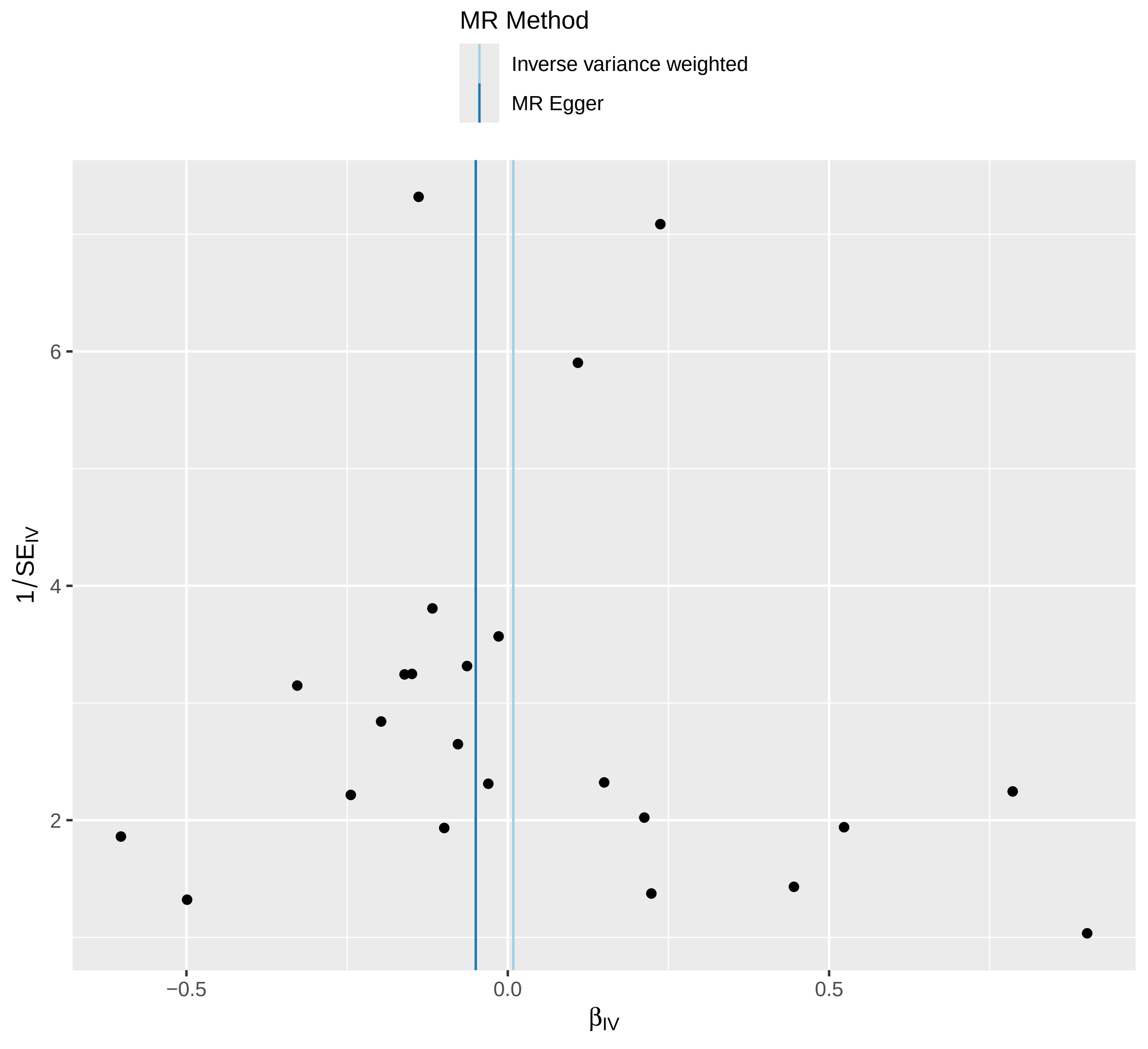
MR Method
Inverse variance weighted
MR Egger
6
4
2
$1/SE_{IV}$
−0.5
0.0
0.5
β_{IV}

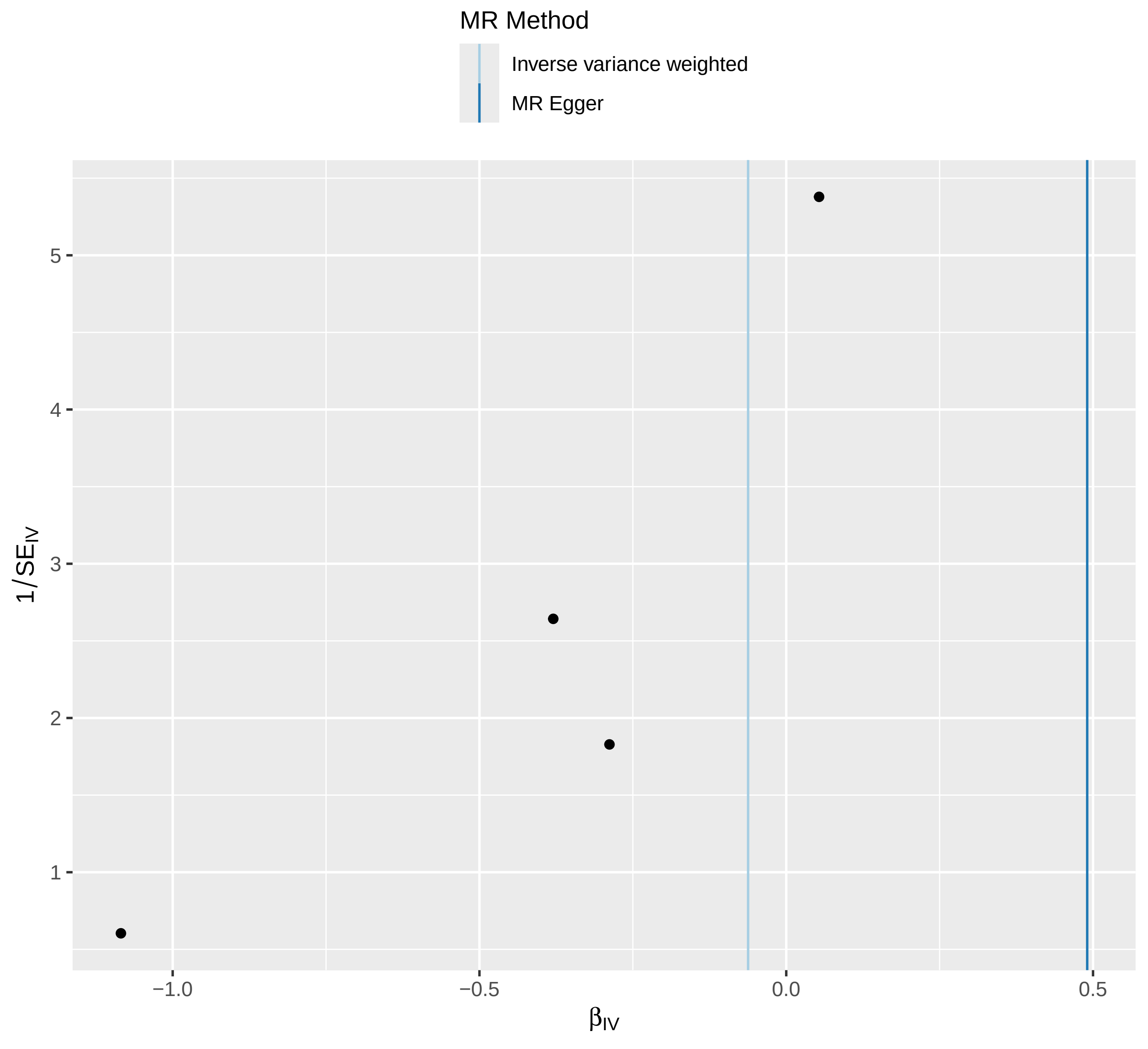

MR Method
Inverse variance weighted
MR Egger
1/SE_{IV}
5
4
3
2
1
−1.0
−0.5
0.0
0.5
β_{IV}

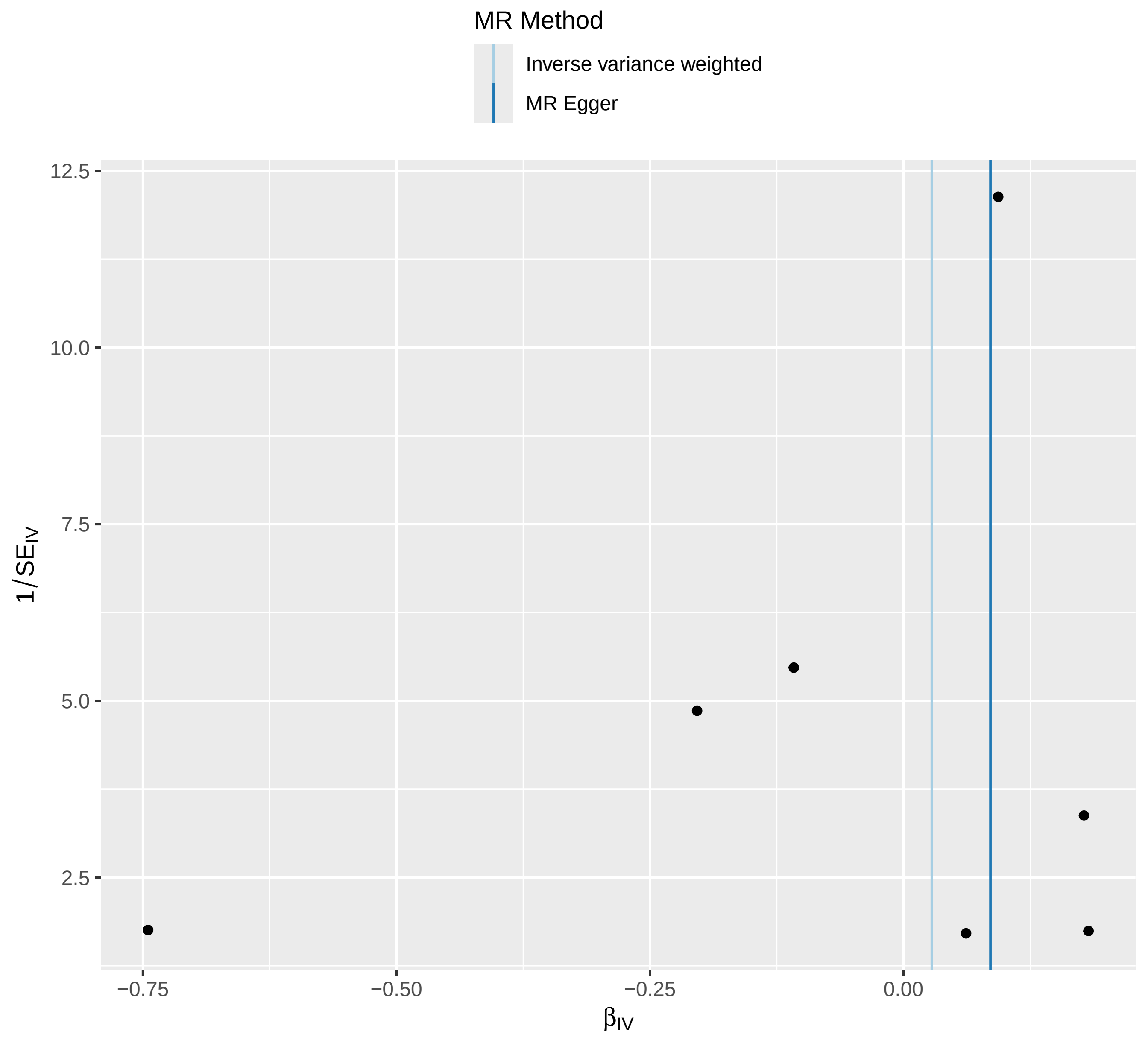

MR Method
Inverse variance weighted
MR Egger
12.5
10.0
7.5
5.0
2.5
1/SE_IV
−0.75
−0.50
−0.25
0.00
β_IV

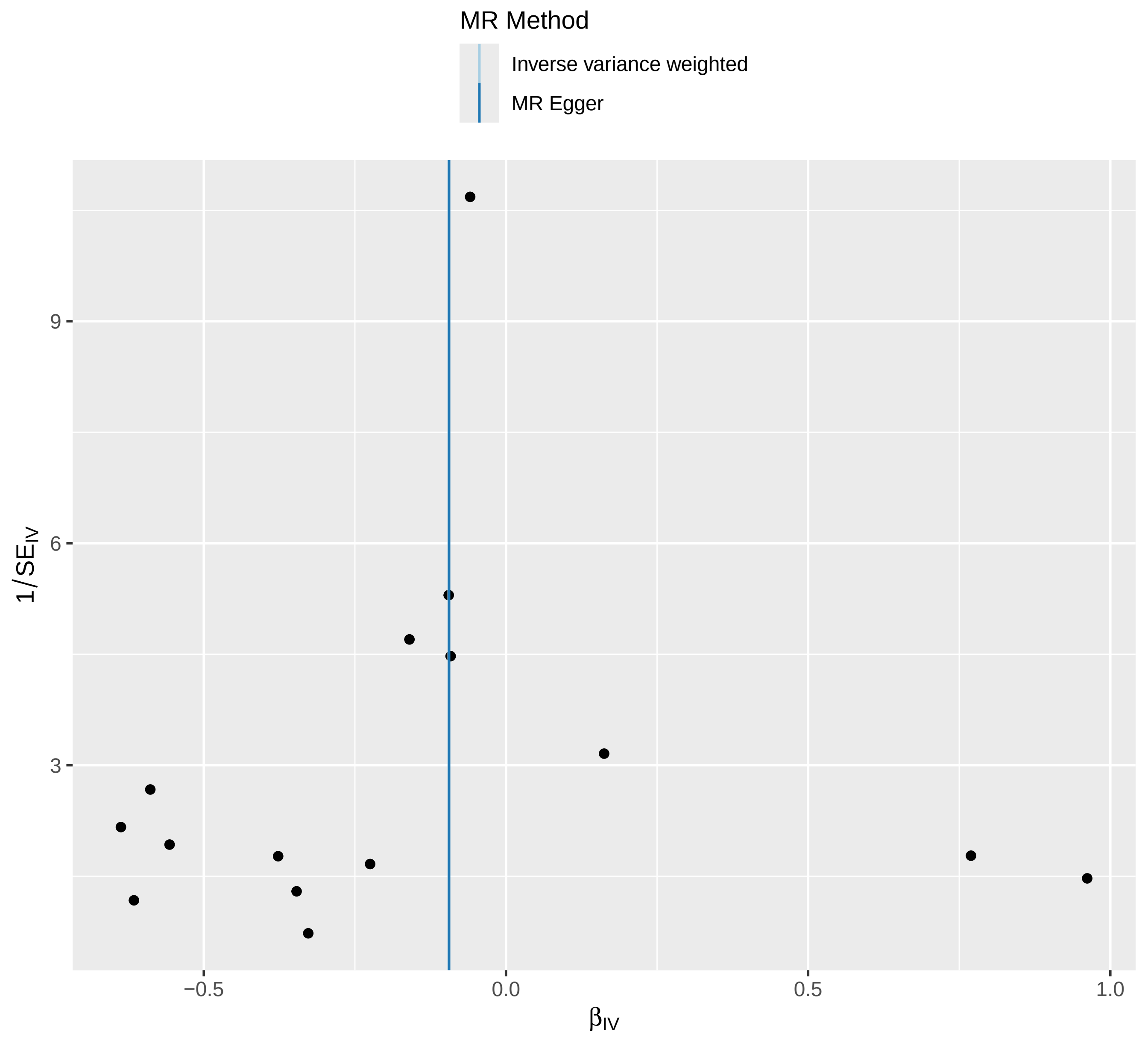

MR Method
Inverse variance weighted
MR Egger
9
6
3
1/SE_IV
−0.5
0.0
0.5
1.0
β_IV

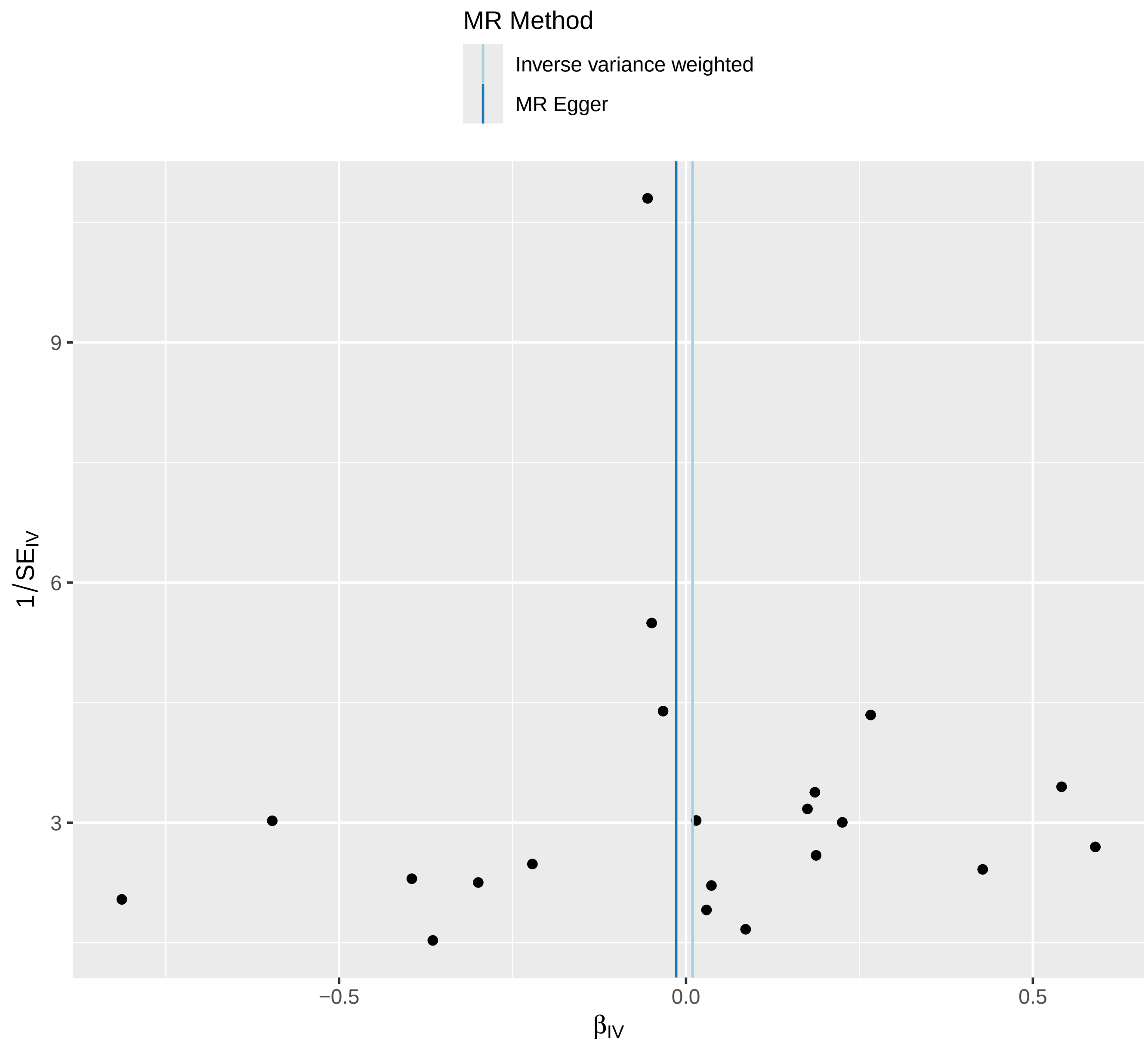

MR Method
Inverse variance weighted
MR Egger
9
6
3
1/SE_IV
−0.5
0.0
0.5
β_IV

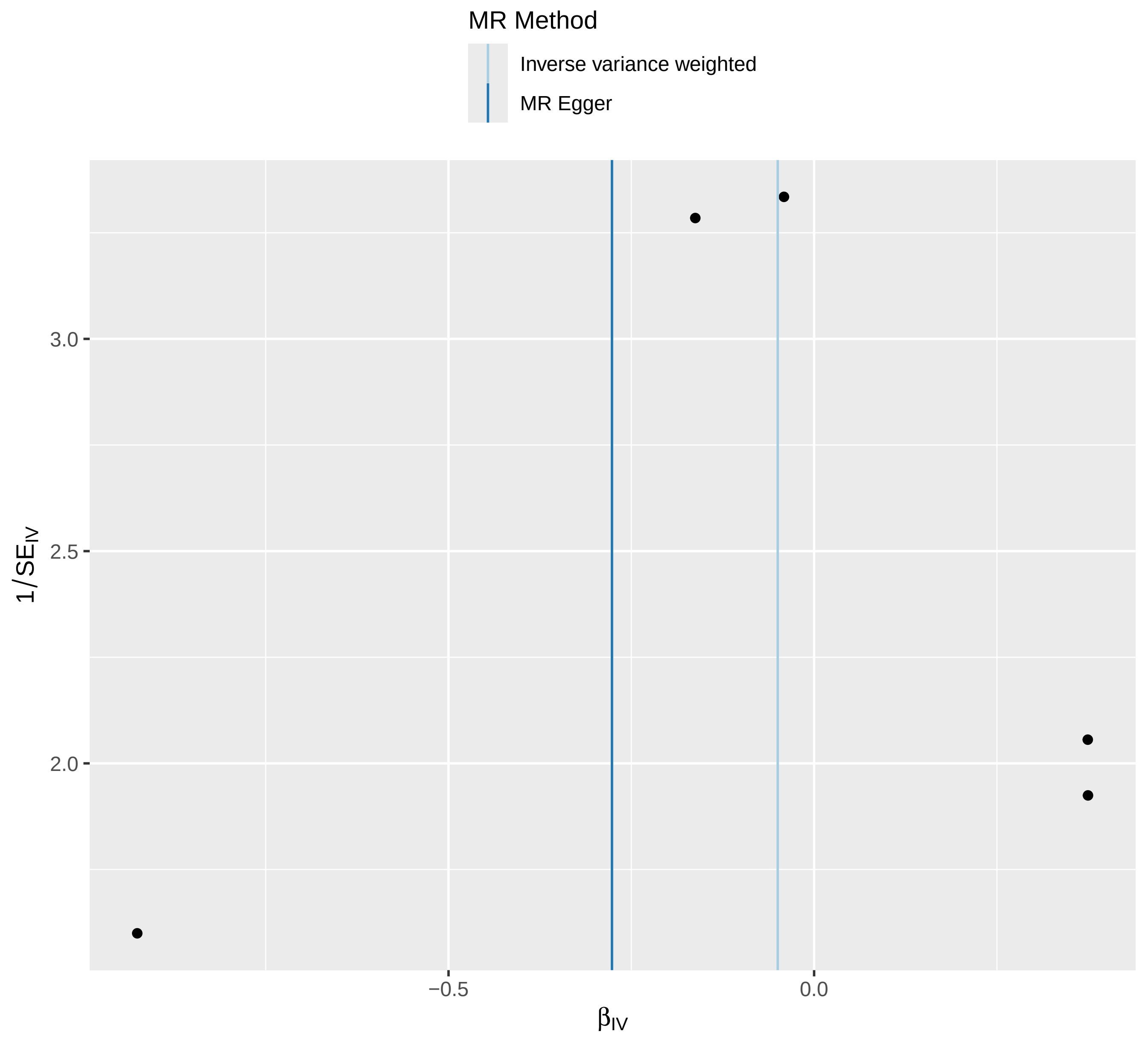

MR Method
Inverse variance weighted
MR Egger
3.0
2.5
2.0
1/SE_IV
−0.5
0.0
β_IV

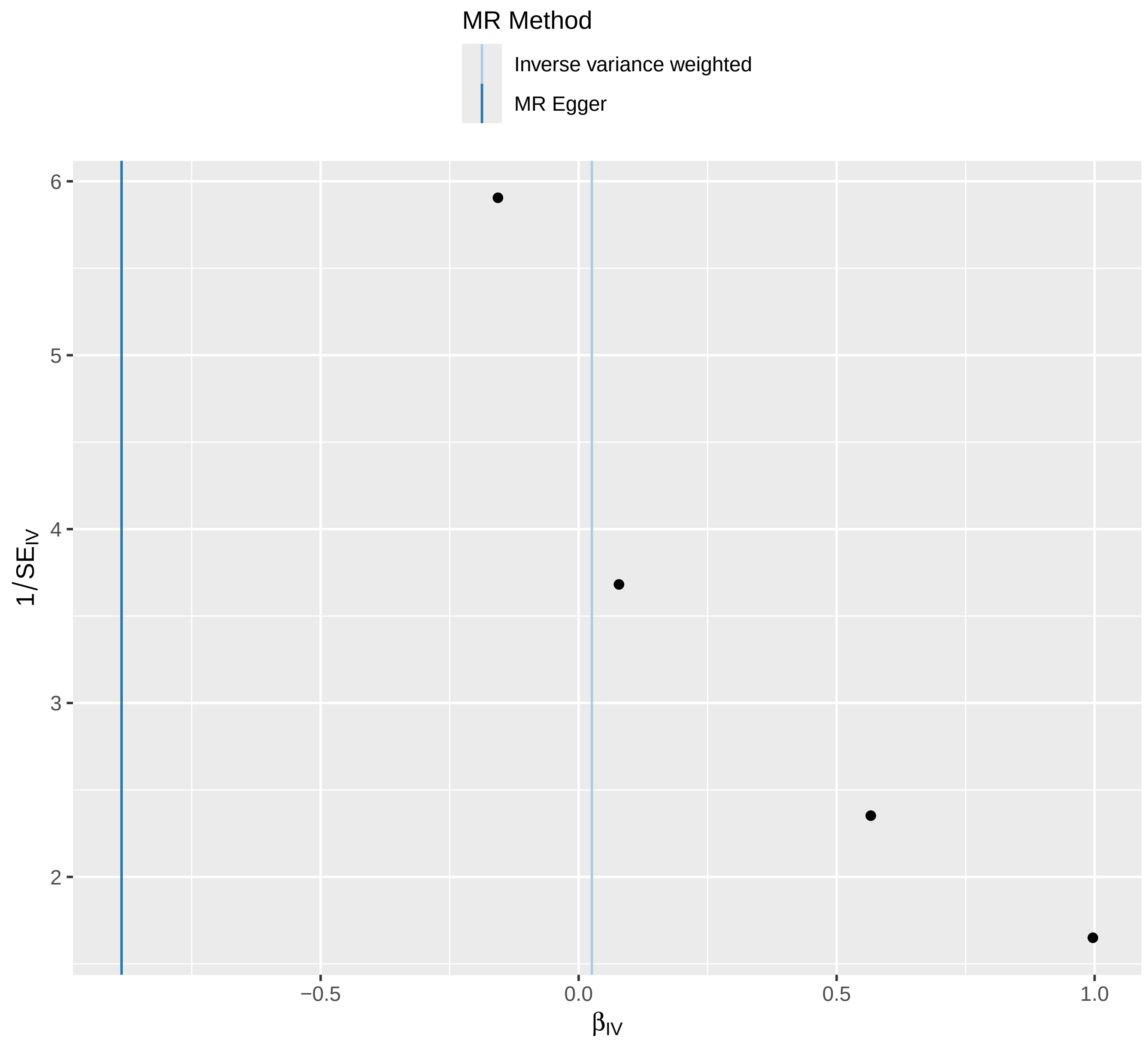

MR Method
Inverse variance weighted
MR Egger
1/SE_IV
6
5
4
3
2
−0.5
0.0
0.5
1.0
β_IV

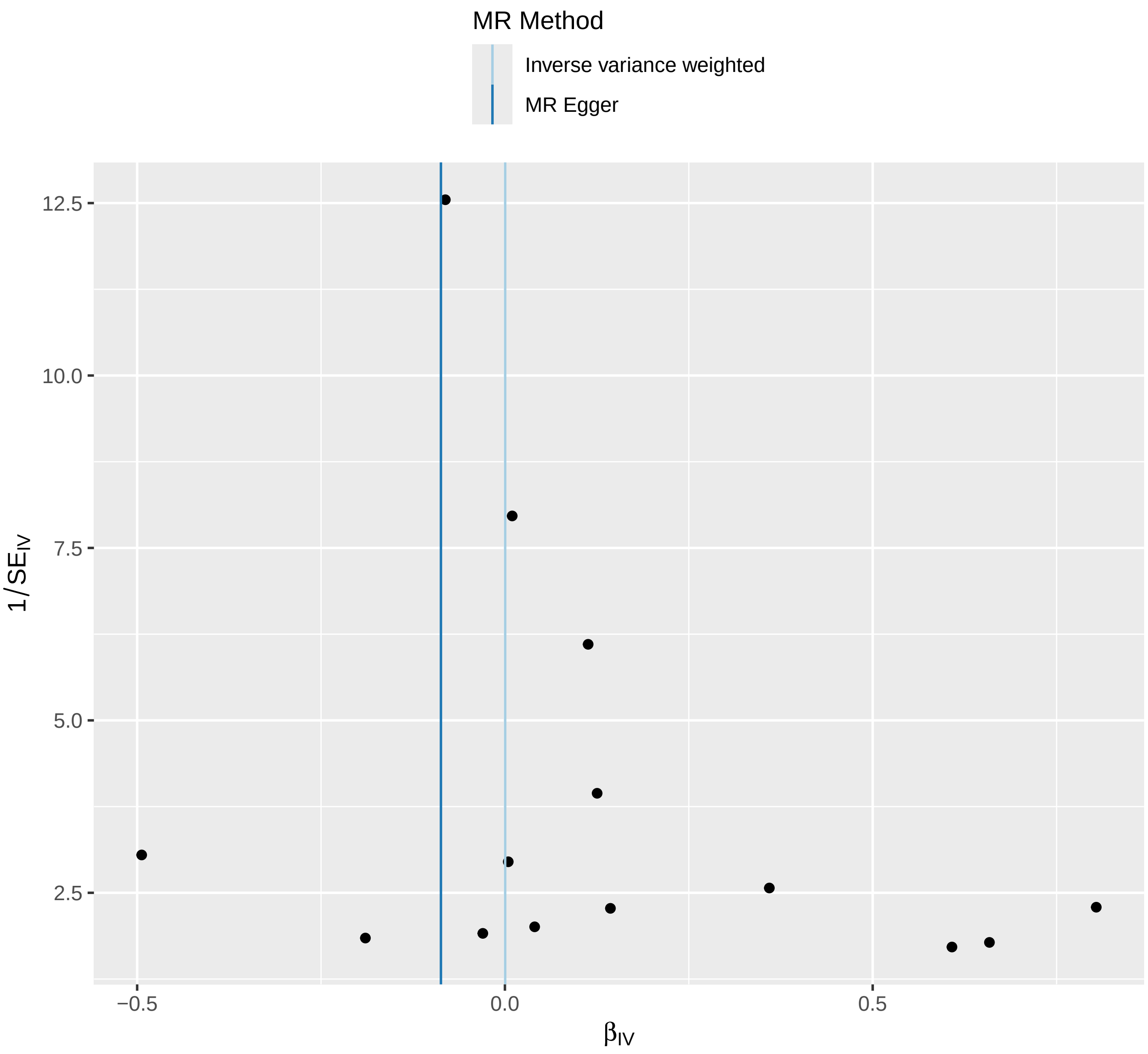
MR Method
Inverse variance weighted
MR Egger
12.5
10.0
7.5
5.0
2.5
$1/SE_{IV}$
−0.5
0.0
0.5
β_{IV}

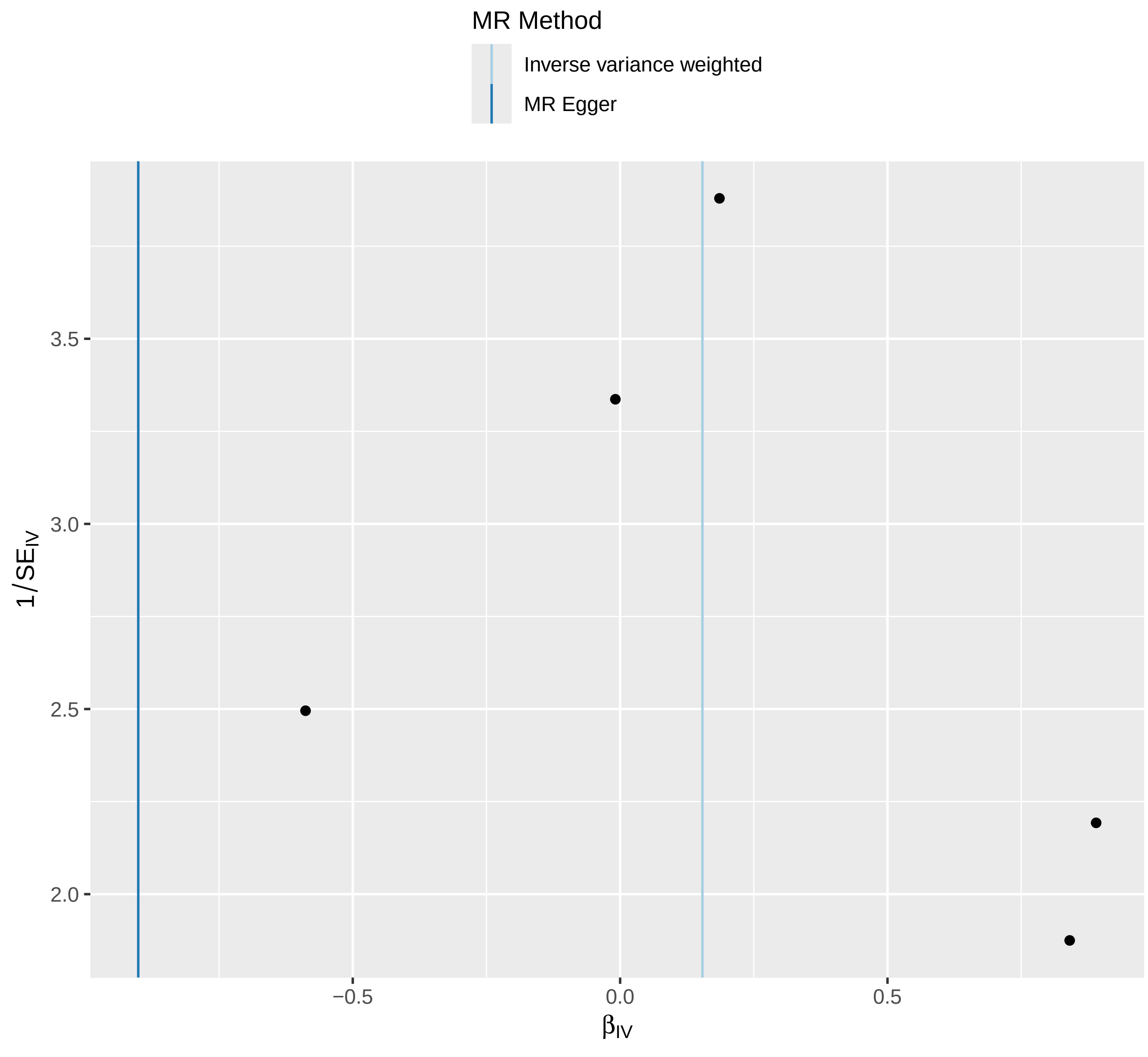

MR Method
Inverse variance weighted
MR Egger
3.5
3.0
2.5
2.0
1/SE$_{IV}$
−0.5
0.0
0.5
β$_{IV}$

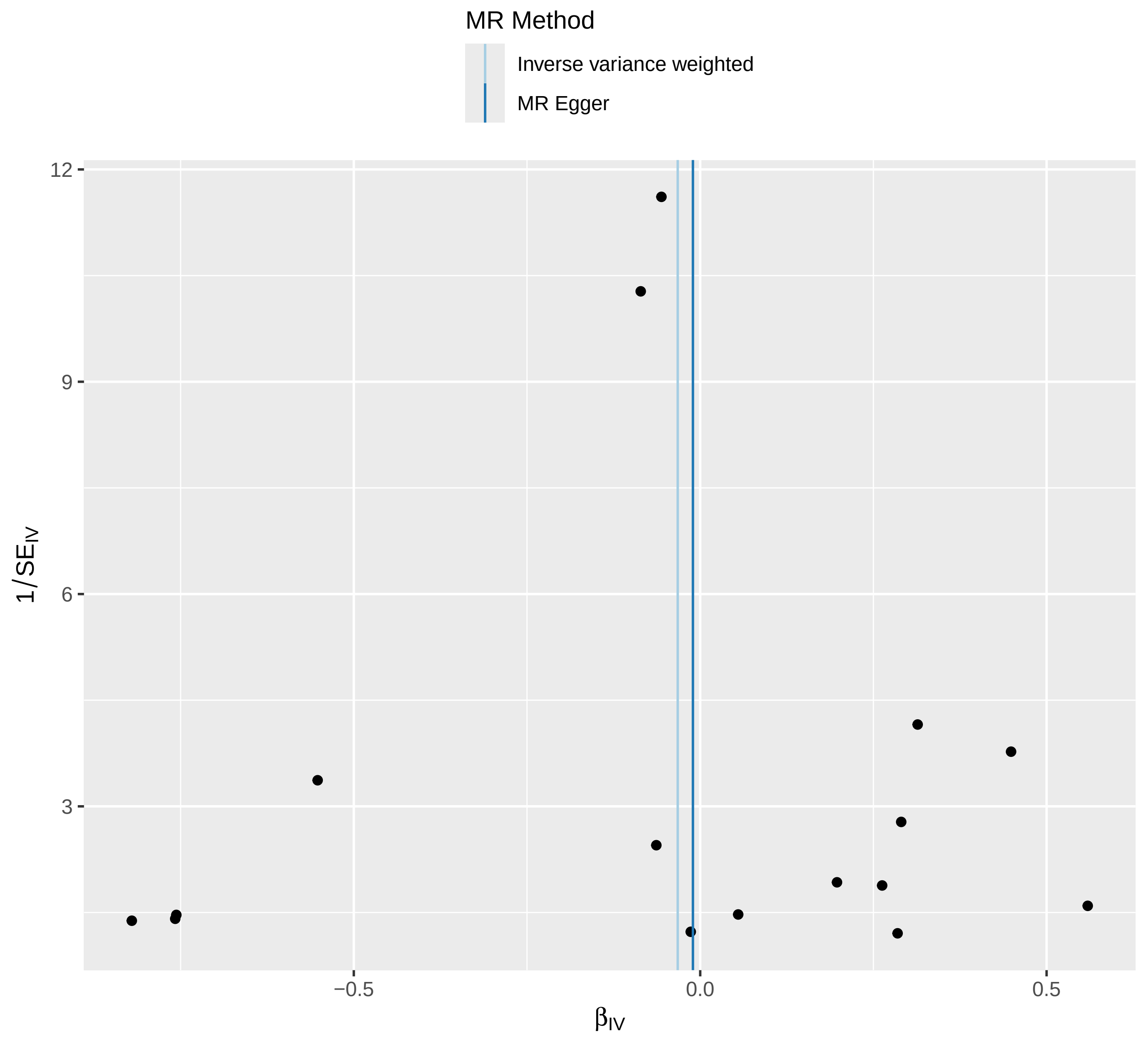

MR Method
Inverse variance weighted
MR Egger
12
9
6
3
1/SE_IV
−0.5
0.0
0.5
β_IV

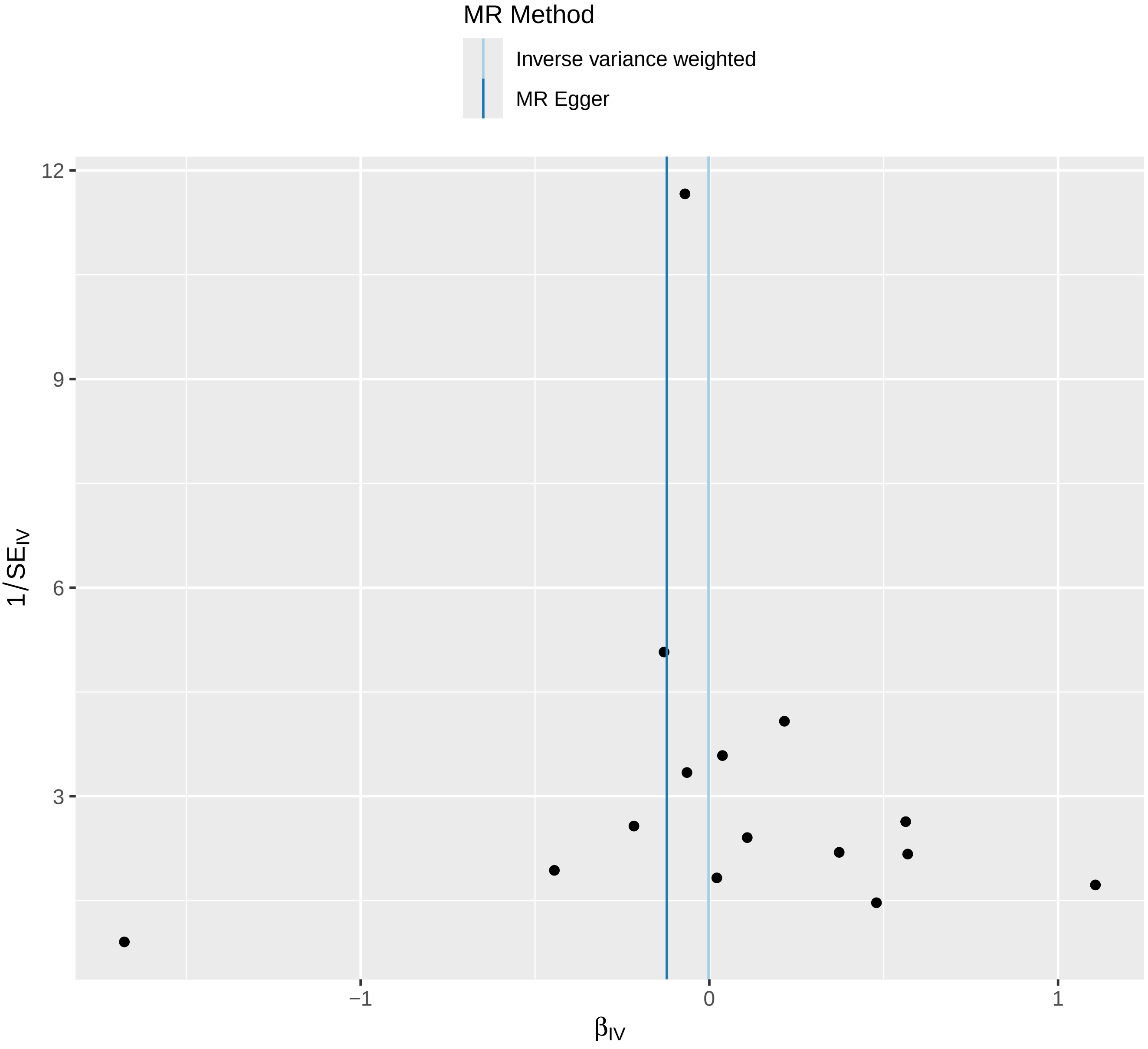

MR Method
Inverse variance weighted
MR Egger
12
9
6
3
−1
0
1
$1/SE_{IV}$
β_{IV}

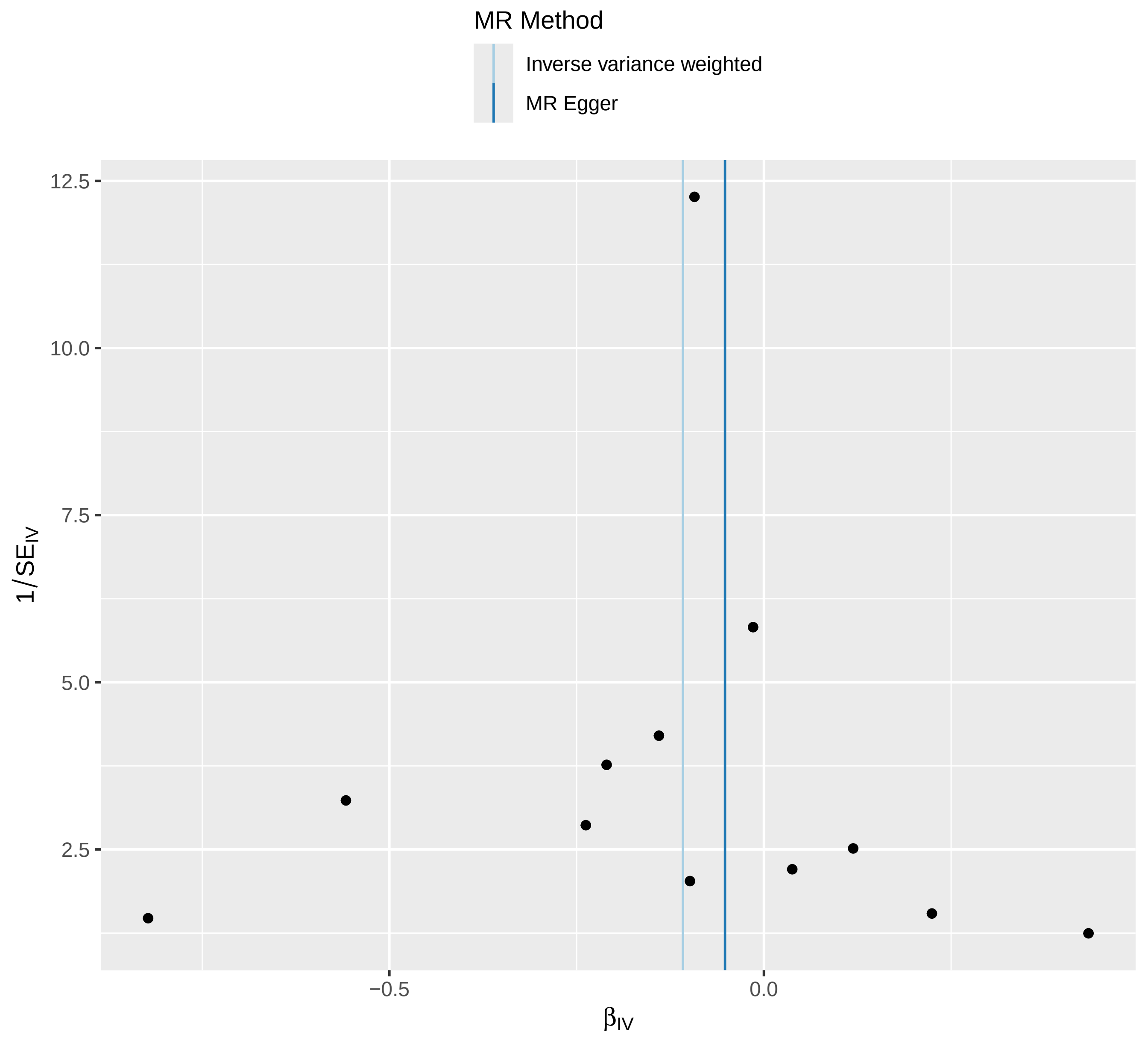
MR Method
Inverse variance weighted
MR Egger
12.5
10.0
7.5
5.0
2.5
1/SE_IV
−0.5
0.0
β_IV

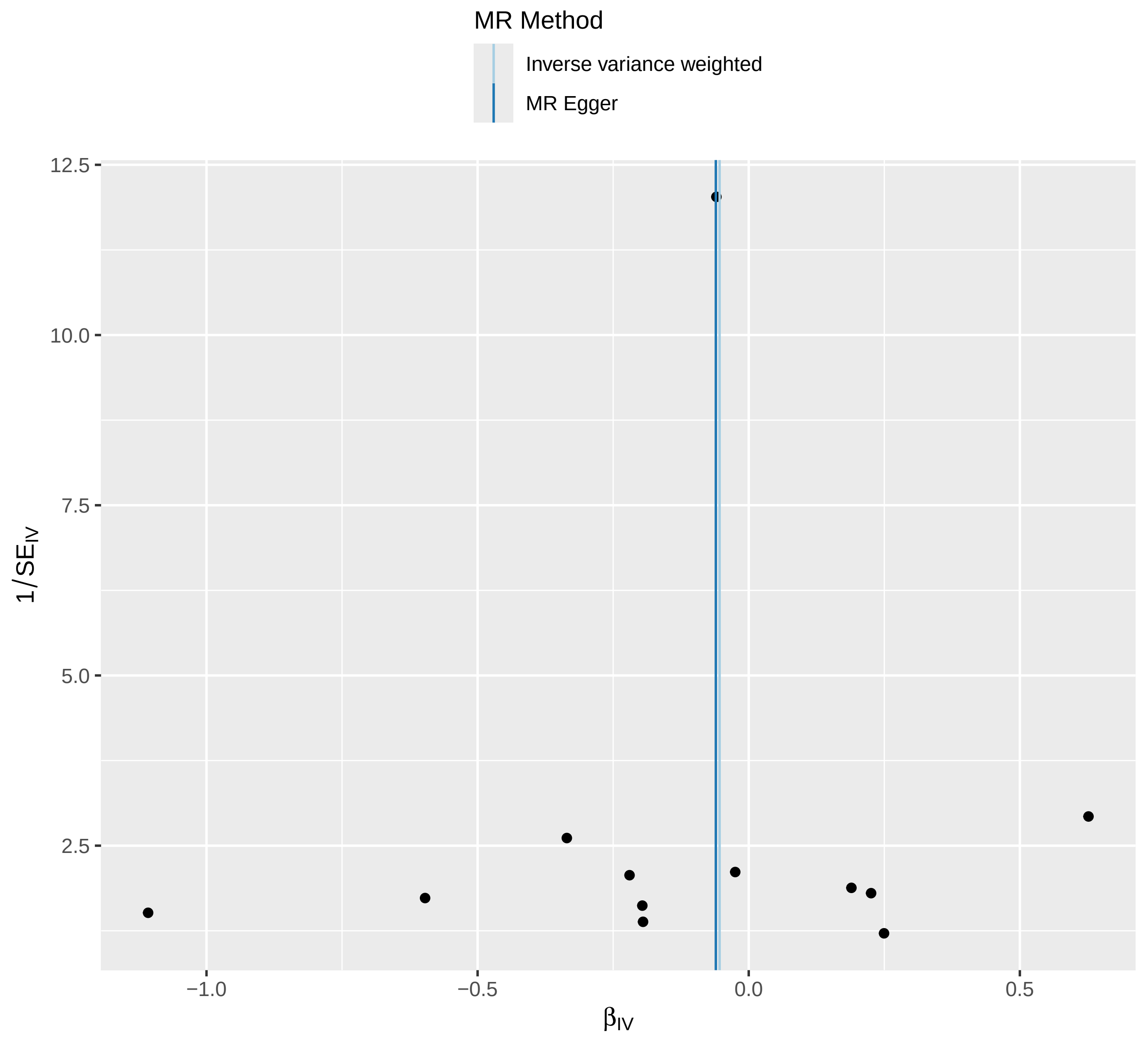

MR Method
Inverse variance weighted
MR Egger
12.5
10.0
7.5
5.0
2.5
1/SE$_{IV}$
−1.0
−0.5
0.0
0.5
β$_{IV}$

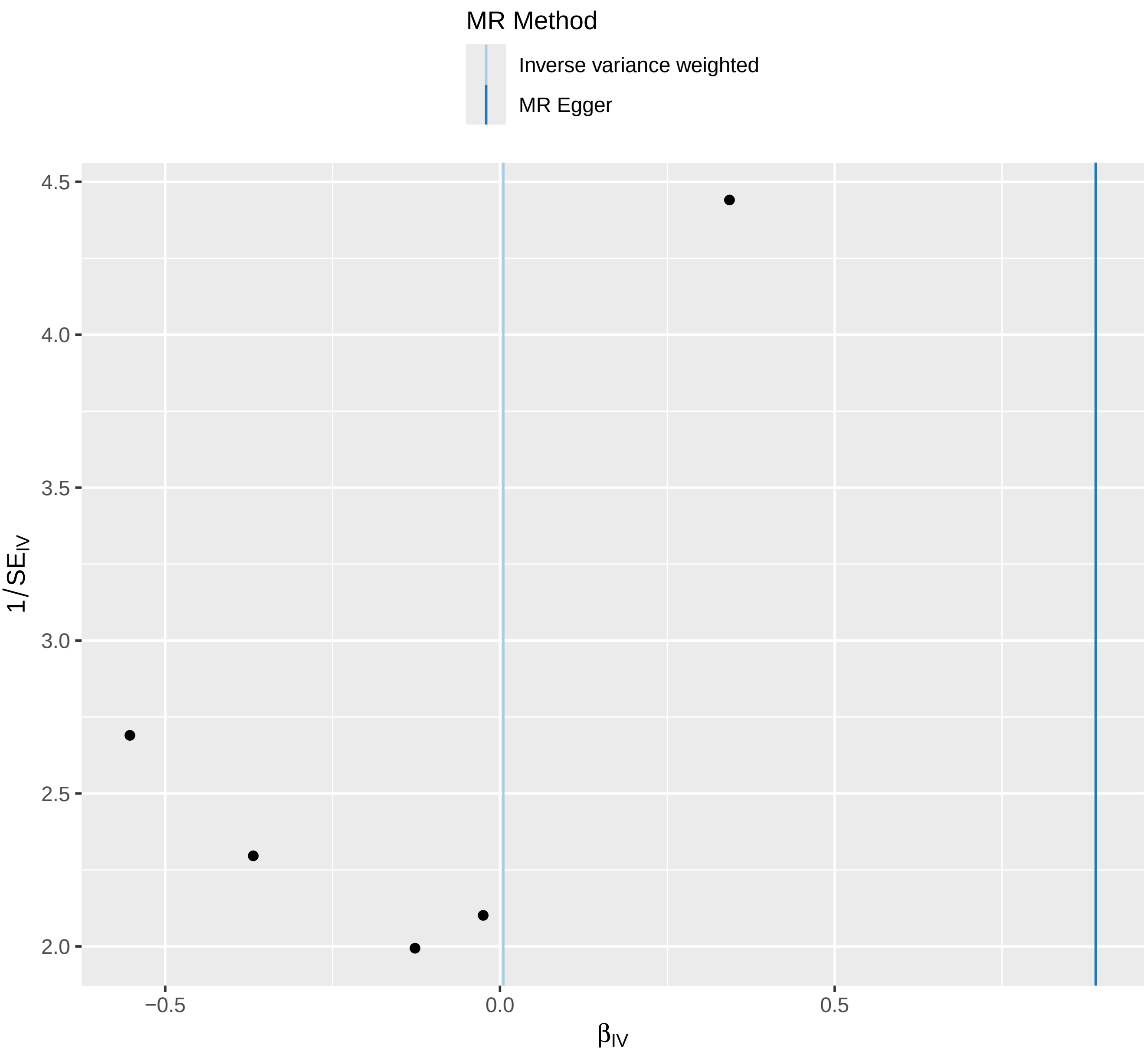
MR Method
Inverse variance weighted
MR Egger
4.5
4.0
3.5
3.0
2.5
2.0
1/SE_IV
−0.5
0.0
0.5
β_IV

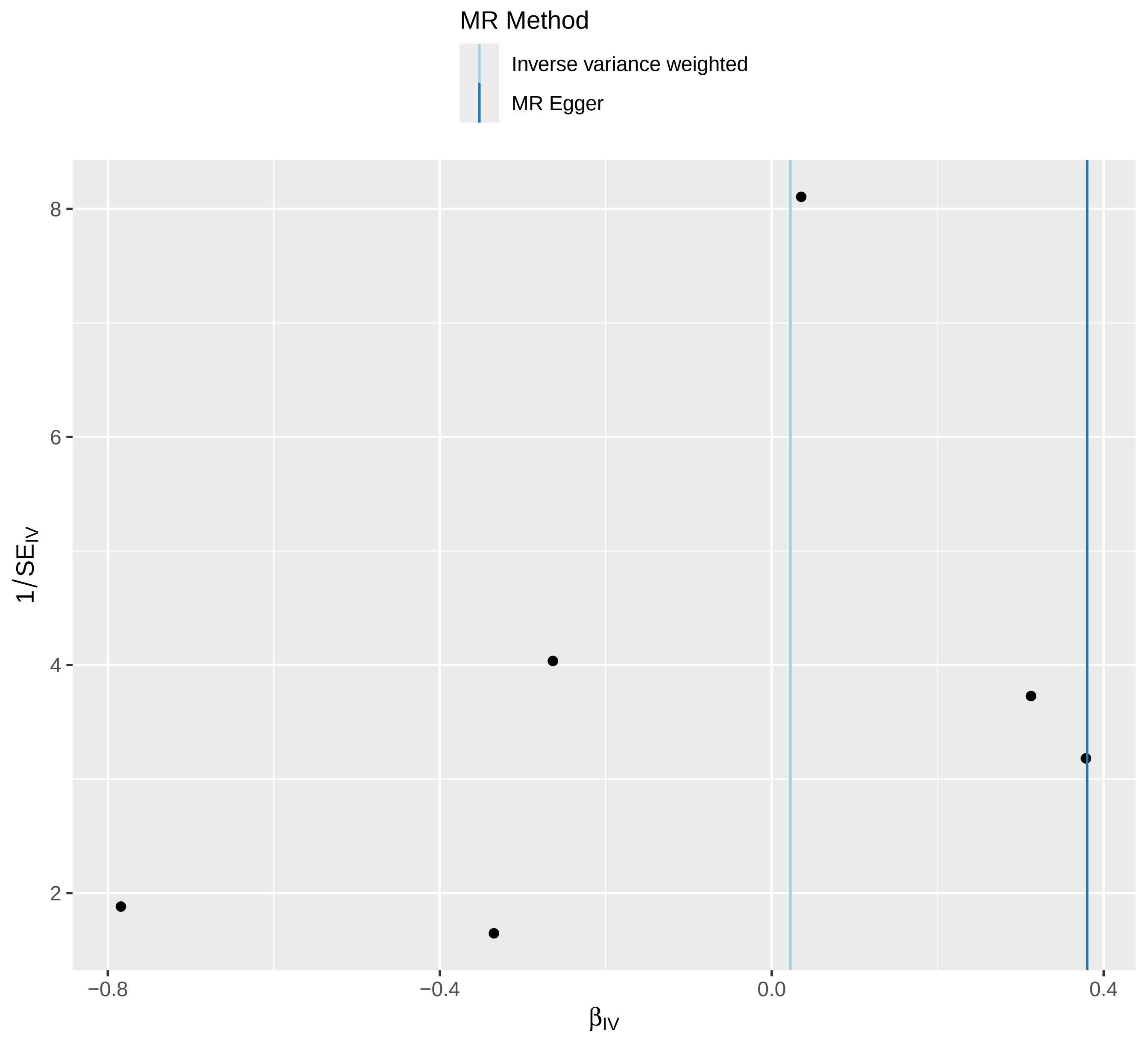

MR Method
Inverse variance weighted
MR Egger
8
6
4
2
1/SE$_{IV}$
−0.8
−0.4
0.0
0.4
β$_{IV}$

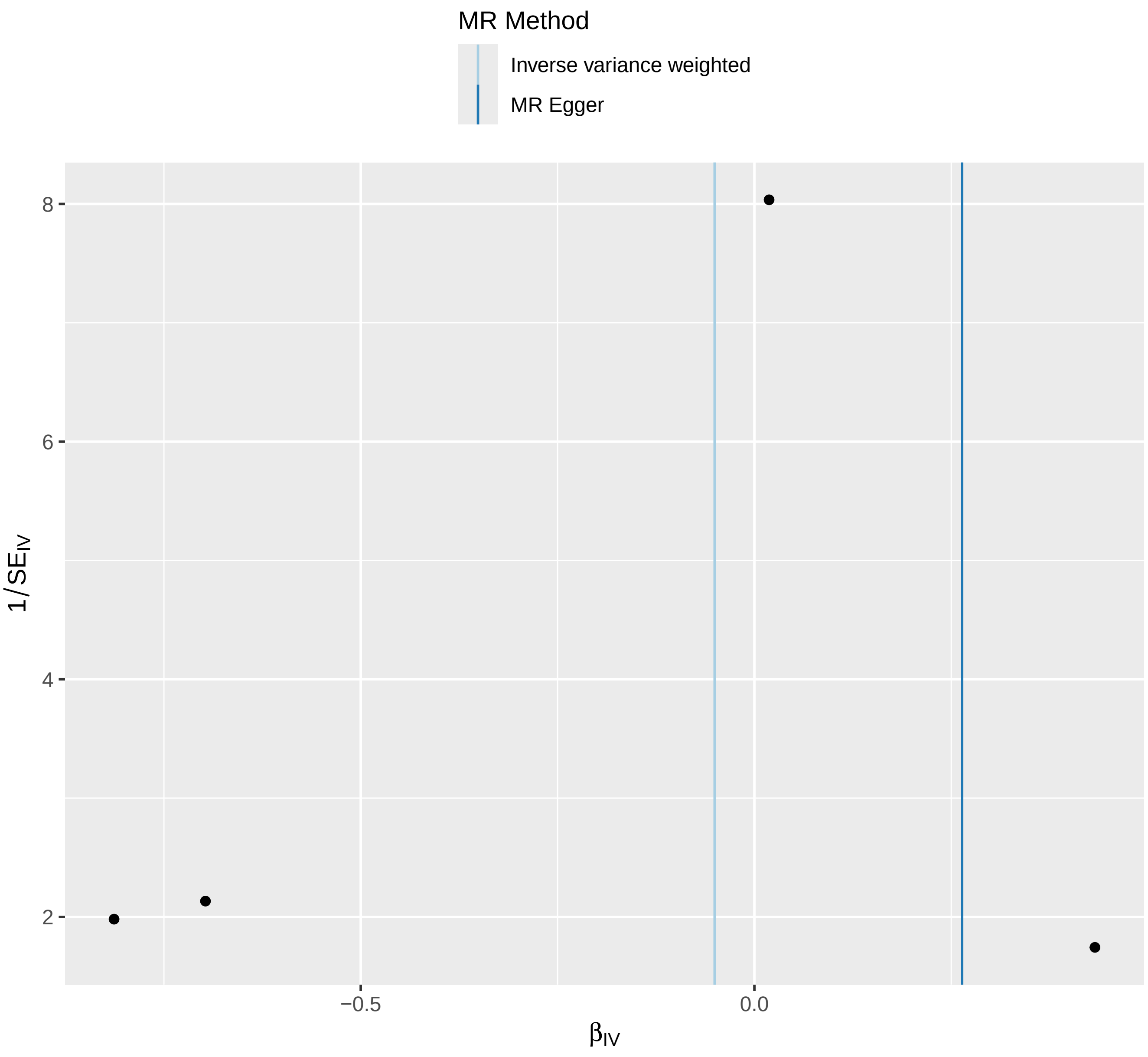

MR Method
Inverse variance weighted
MR Egger
8
6
4
2
1/SE$_{IV}$
−0.5
0.0
β$_{IV}$

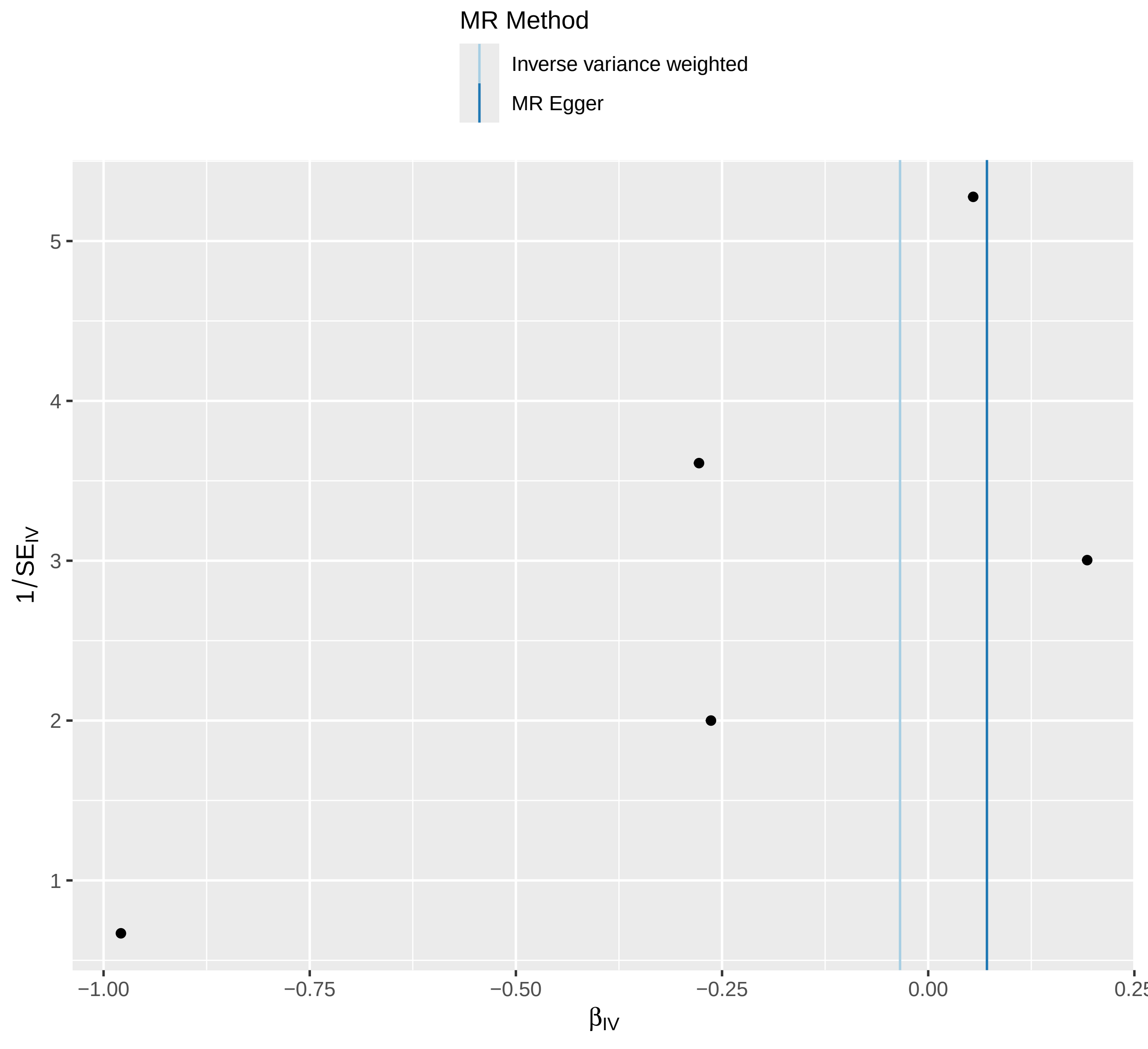
MR Method
Inverse variance weighted
MR Egger
1/SE_{IV}
5
4
3
2
1
−1.00
−0.75
−0.50
−0.25
0.00
0.25
β_{IV}

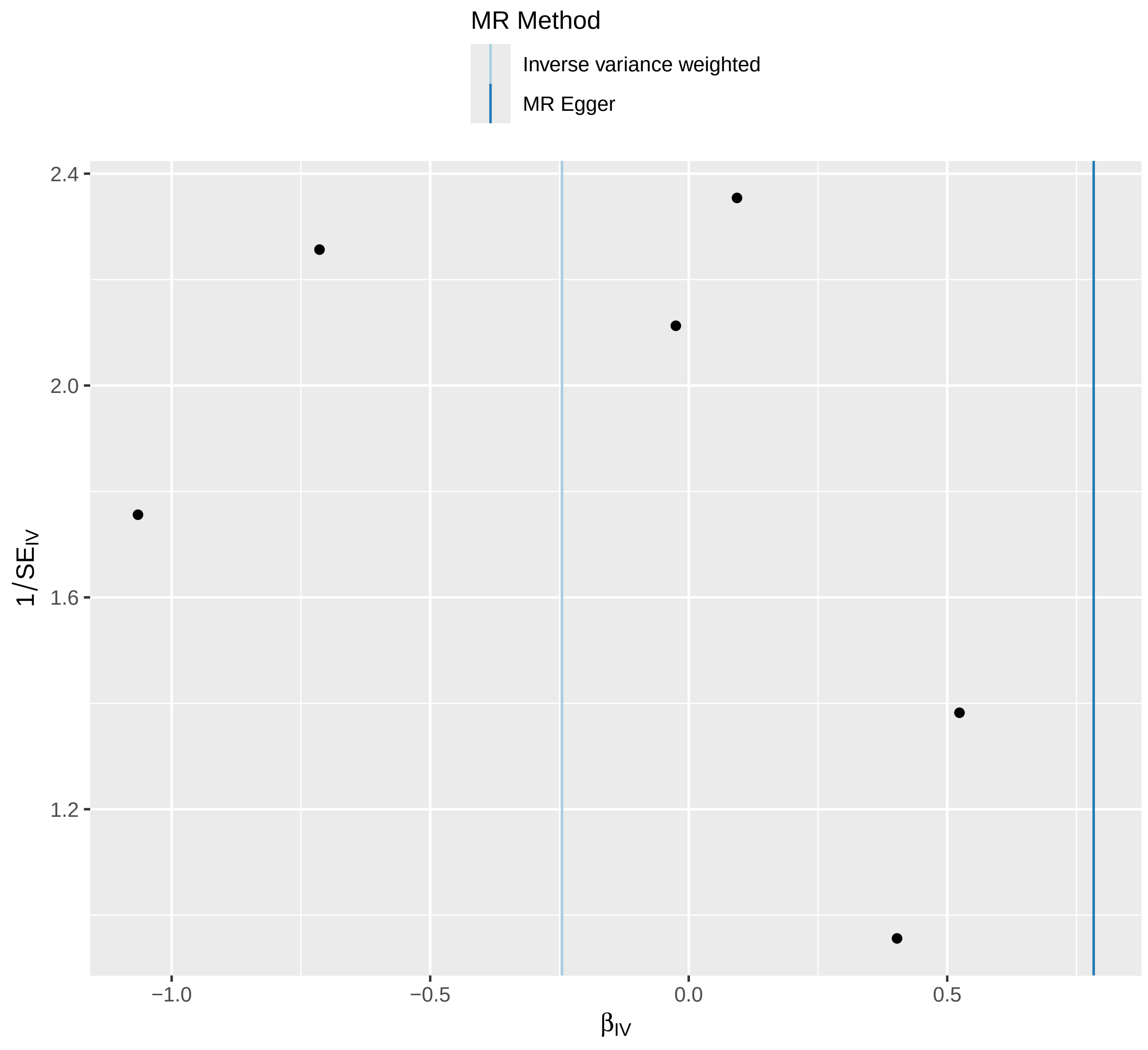
MR Method
Inverse variance weighted
MR Egger
1/SE_IV
β_IV
2.4
2.0
1.6
1.2
−1.0
−0.5
0.0
0.5

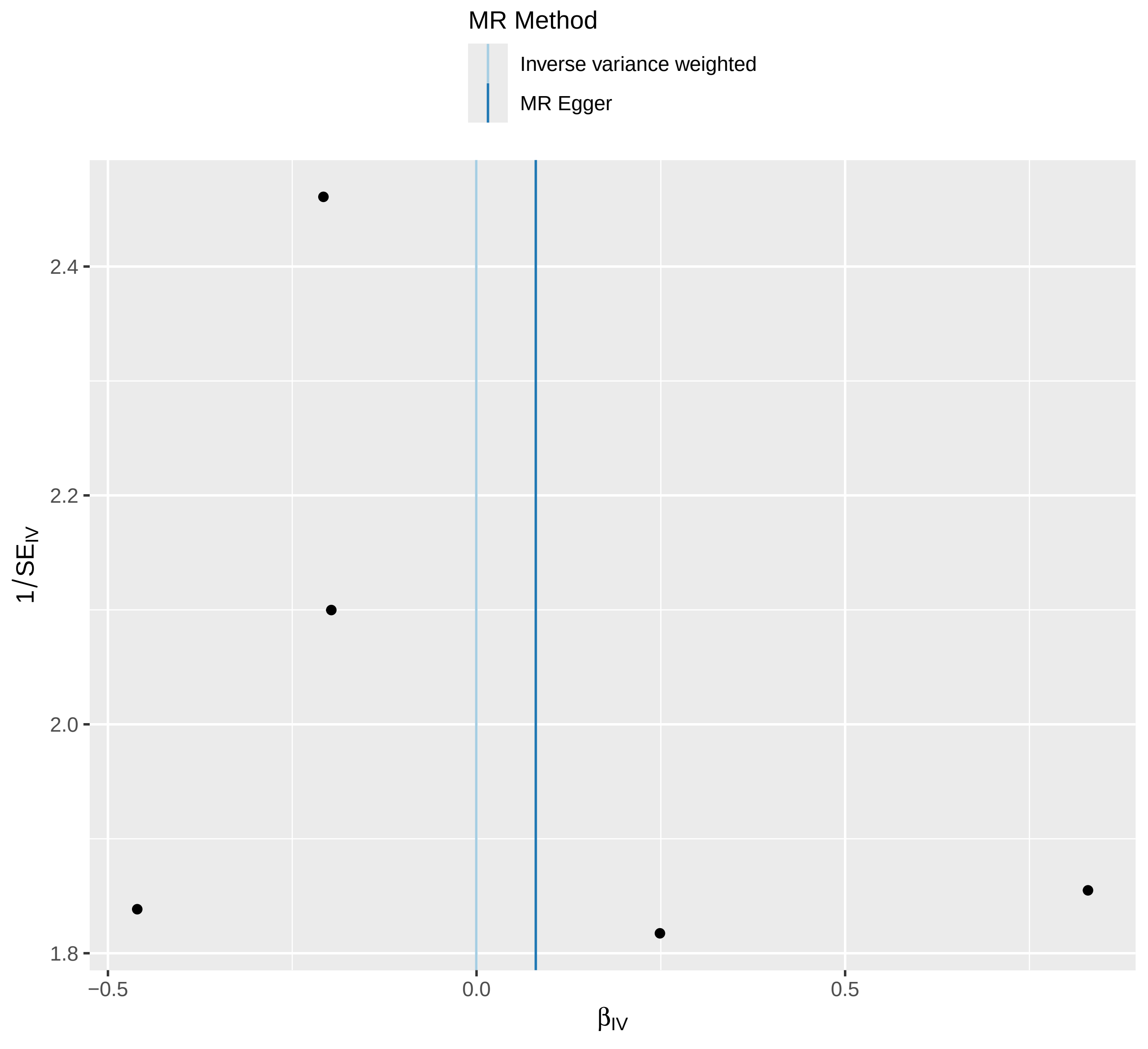

MR Method
Inverse variance weighted
MR Egger
2.4
2.2
2.0
1.8
1/SE_IV
−0.5
0.0
0.5
β_IV

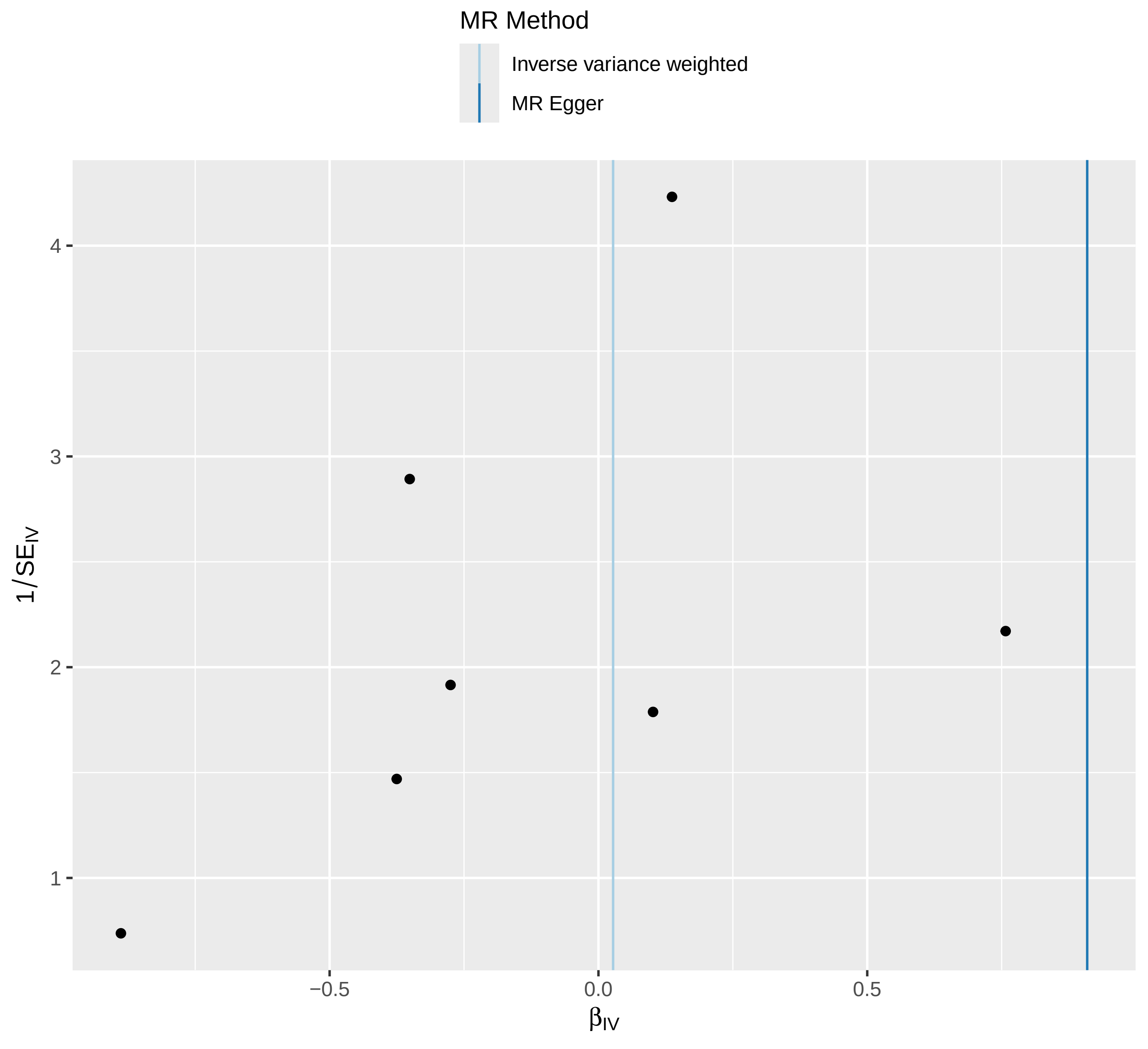

MR Method
Inverse variance weighted
MR Egger
4
3
2
1
1/SE$_{IV}$
−0.5
0.0
0.5
β$_{IV}$

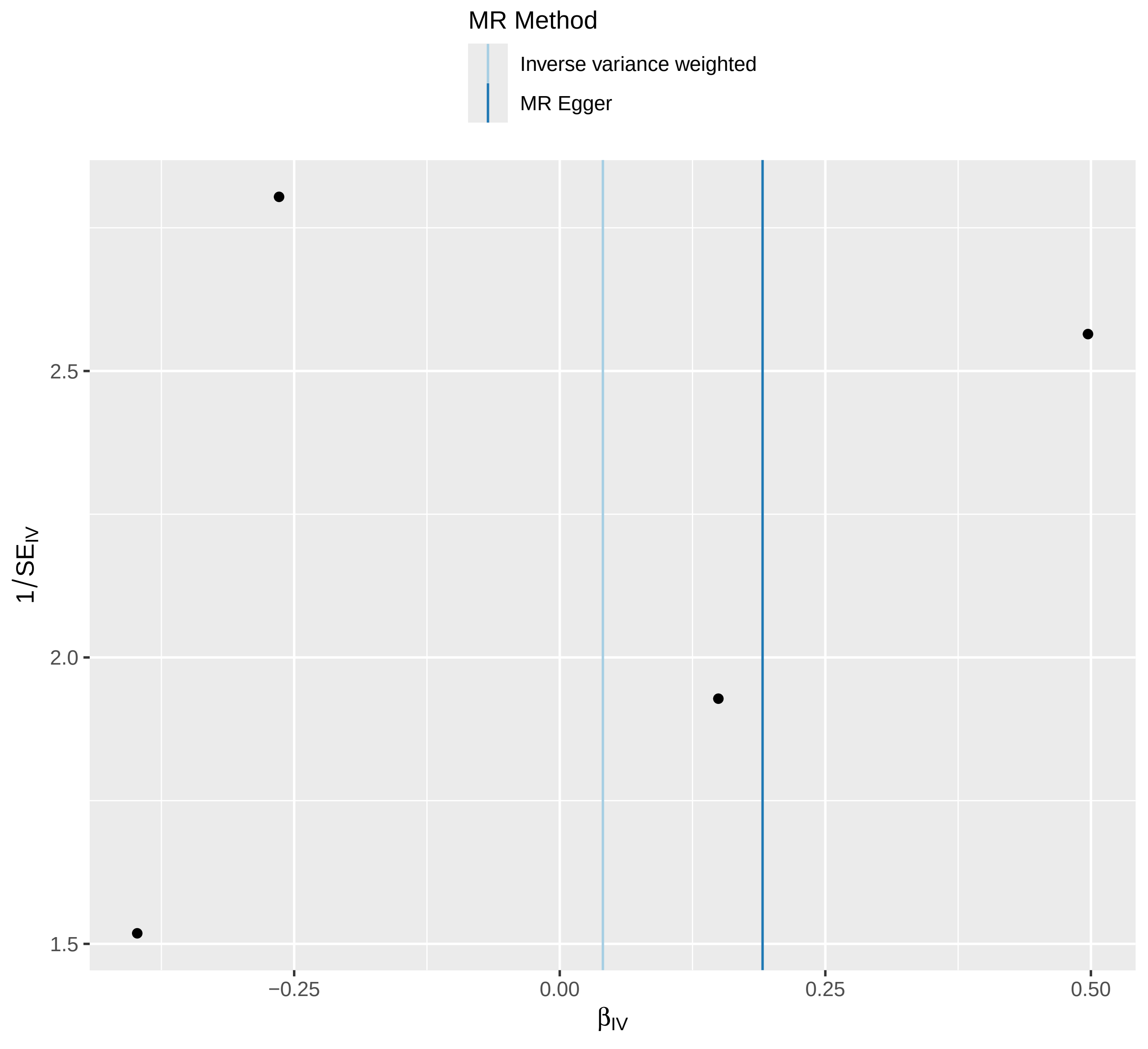
MR Method
Inverse variance weighted
MR Egger
2.5
2.0
1.5
−0.25
0.00
0.25
0.50
$1/SE_{IV}$
β_{IV}

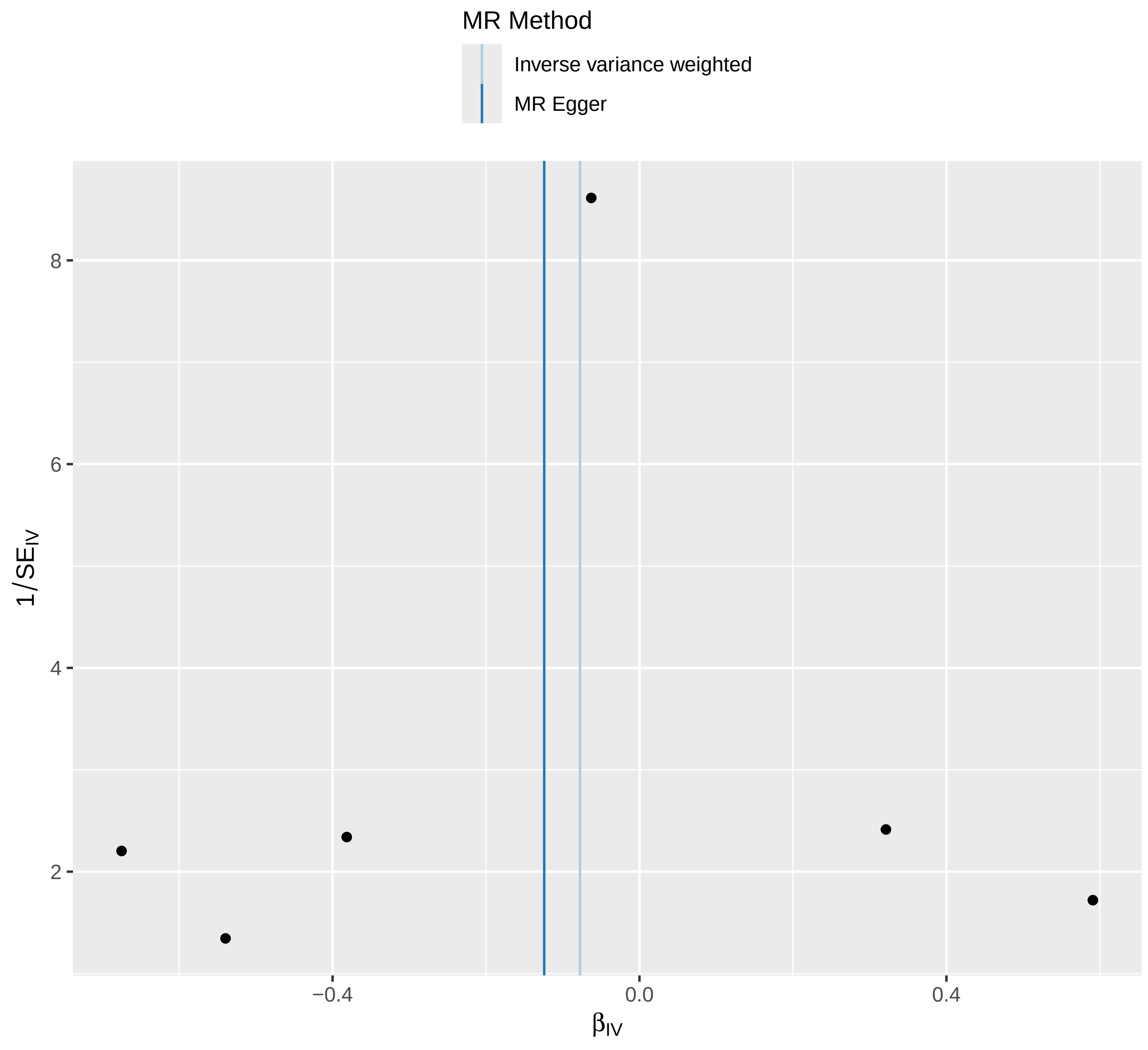
MR Method
Inverse variance weighted
MR Egger
8
6
4
2
1/SE$_{IV}$
−0.4
0.0
0.4
β$_{IV}$

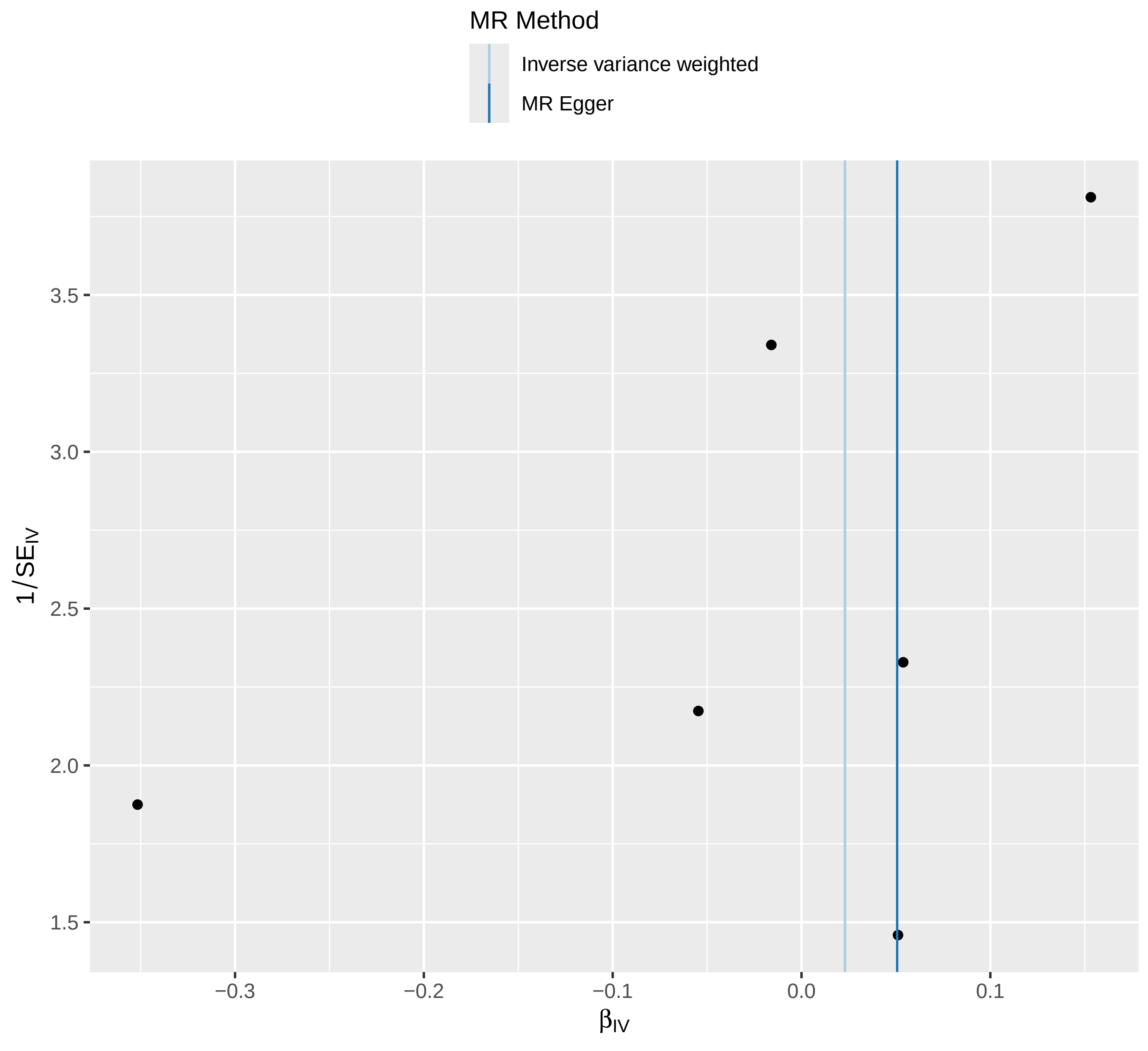

MR Method
Inverse variance weighted
MR Egger
3.5
3.0
2.5
2.0
1.5
1/SE_IV
−0.3
−0.2
−0.1
0.0
0.1
β_IV

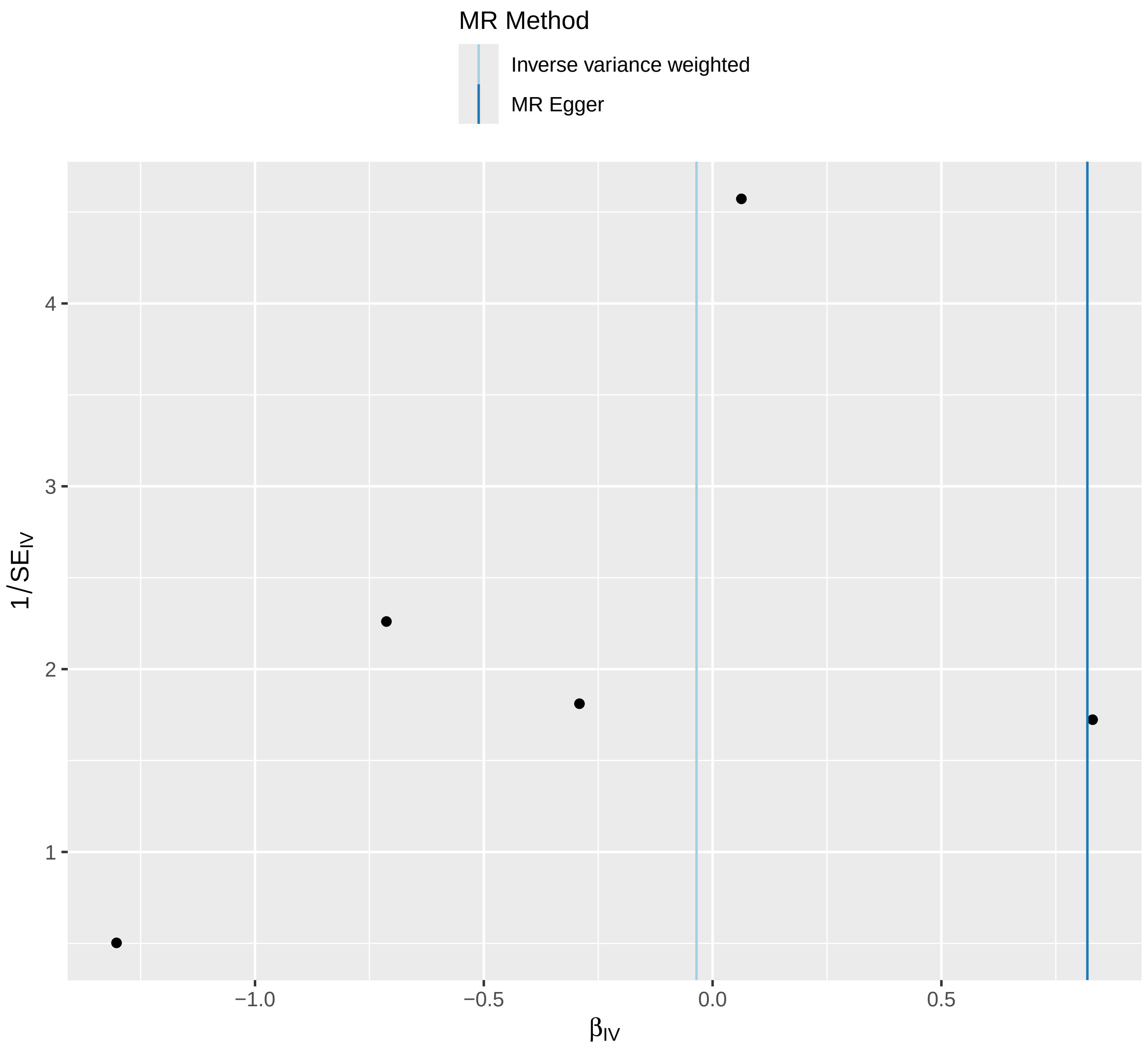

MR Method
Inverse variance weighted
MR Egger
4
3
2
1
1/SE_IV
−1.0
−0.5
0.0
0.5
β_IV

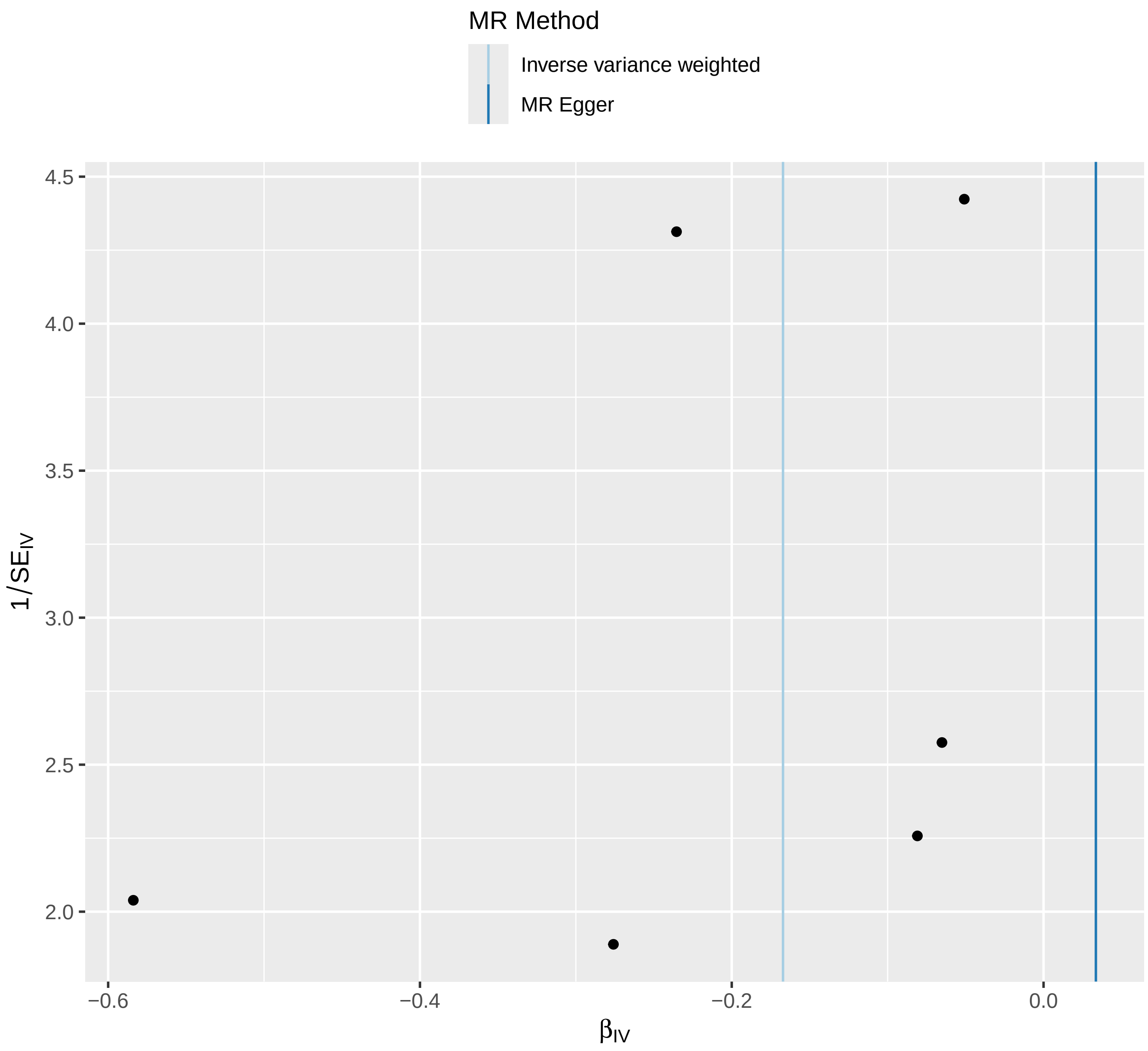
MR Method
Inverse variance weighted
MR Egger
4.5
4.0
3.5
3.0
2.5
2.0
1/SE$_{IV}$
−0.6
−0.4
−0.2
0.0
β$_{IV}$

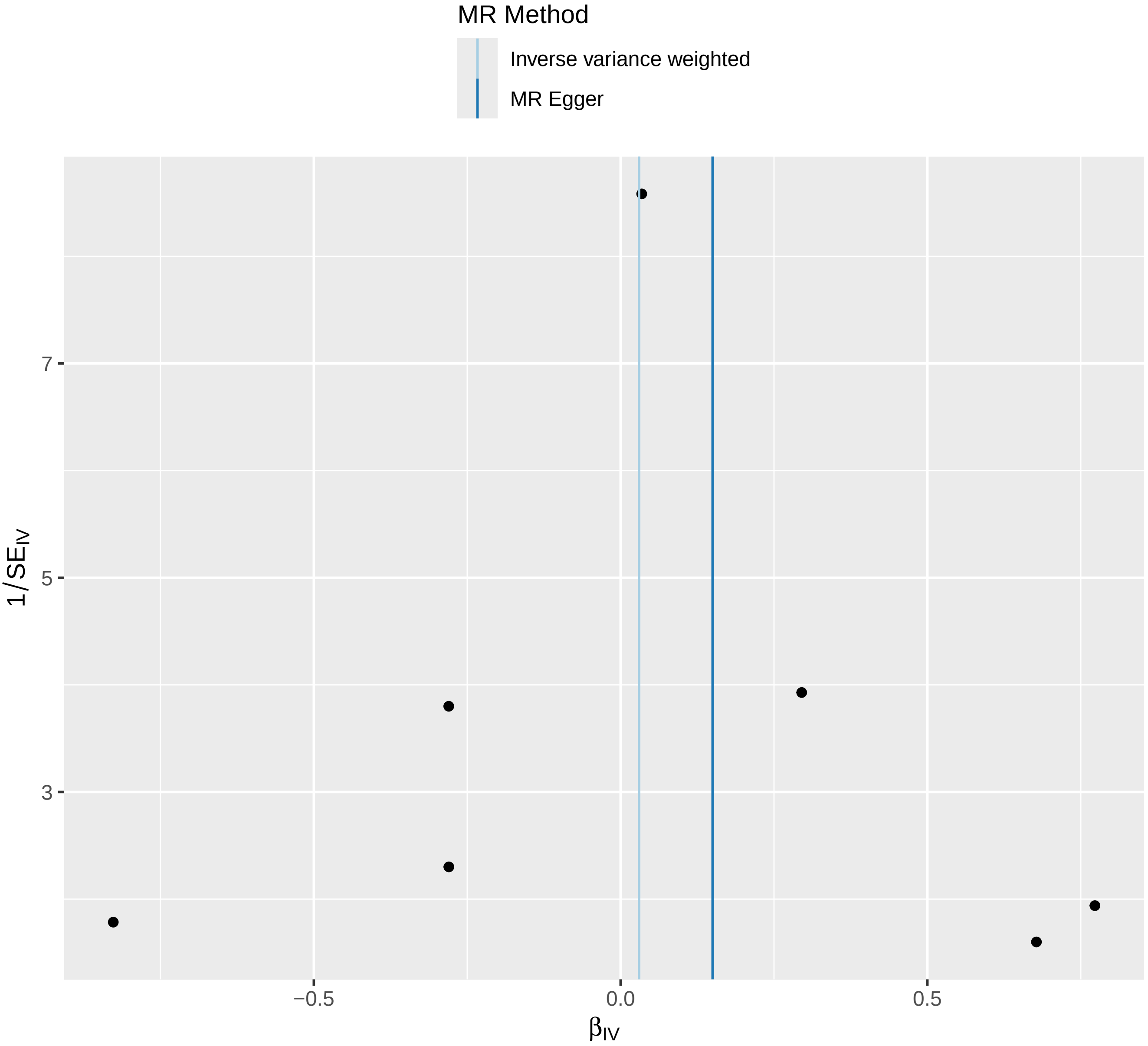

MR Method
Inverse variance weighted
MR Egger
1/SE_IV
7
5
3
−0.5
0.0
0.5
β_IV

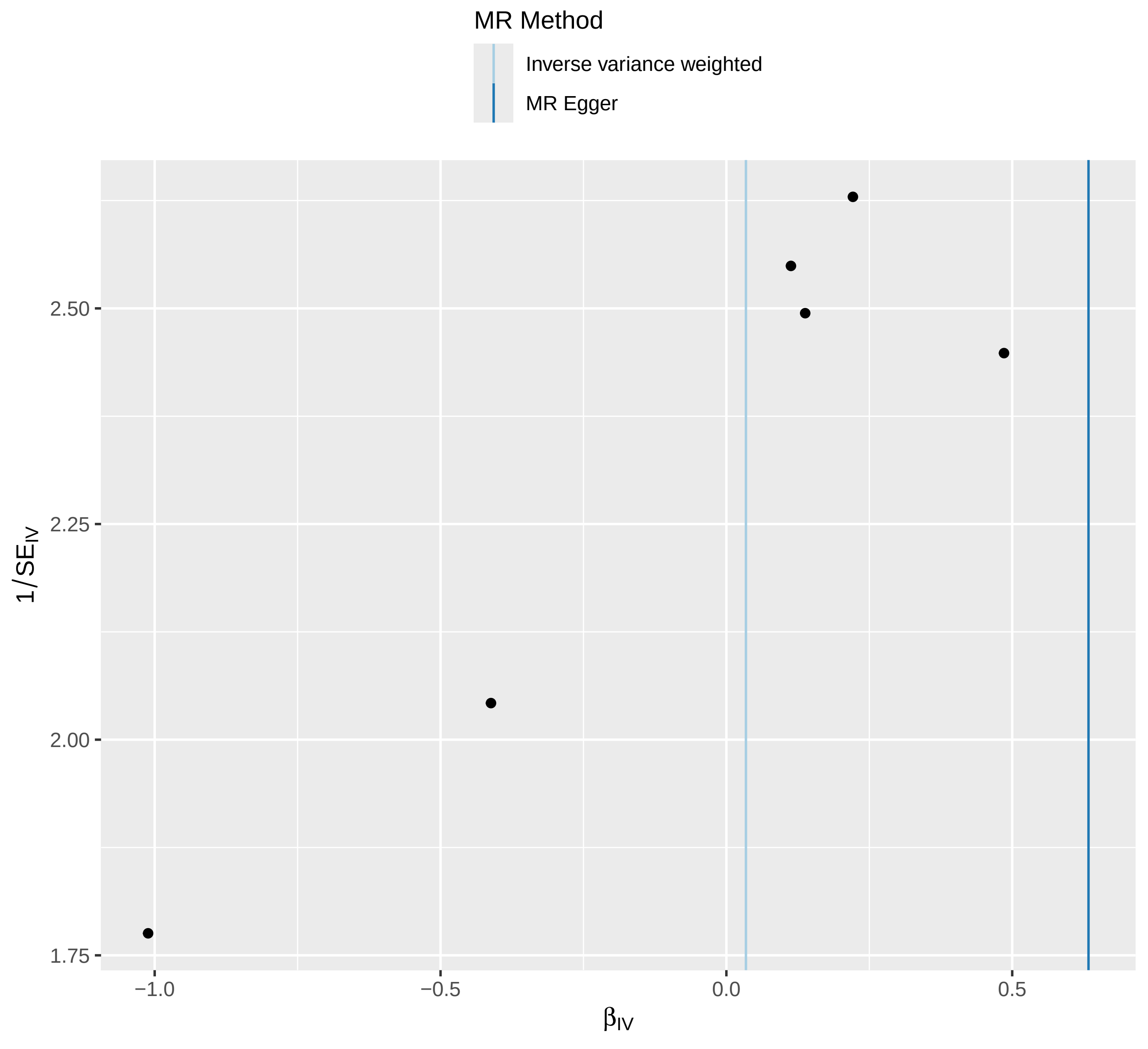

MR Method
Inverse variance weighted
MR Egger
2.50
2.25
2.00
1.75
1/SE_IV
−1.0
−0.5
0.0
0.5
β_IV

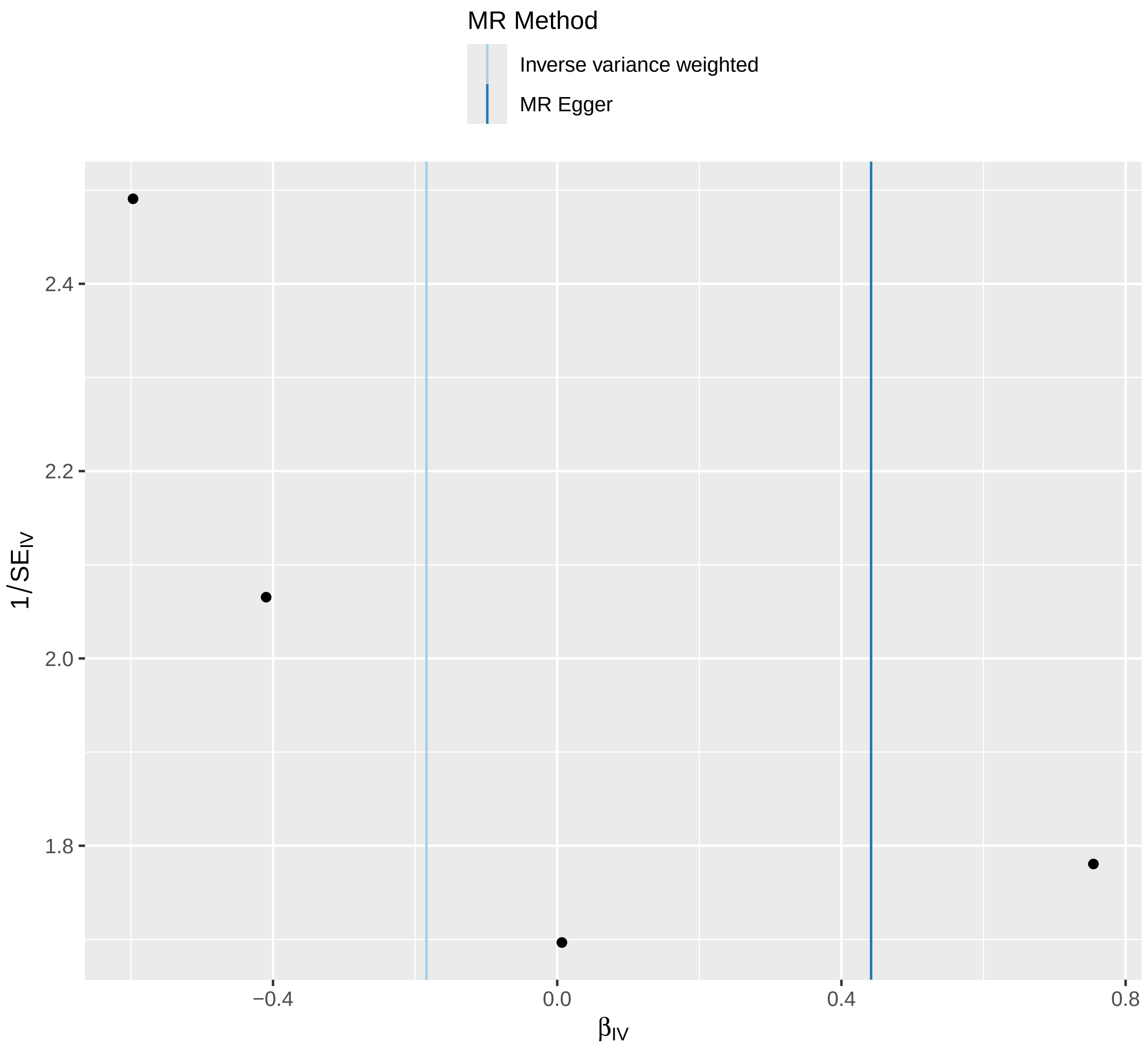
MR Method
Inverse variance weighted
MR Egger
2.4
2.2
2.0
1.8
1/SE_IV
−0.4
0.0
0.4
0.8
β_IV

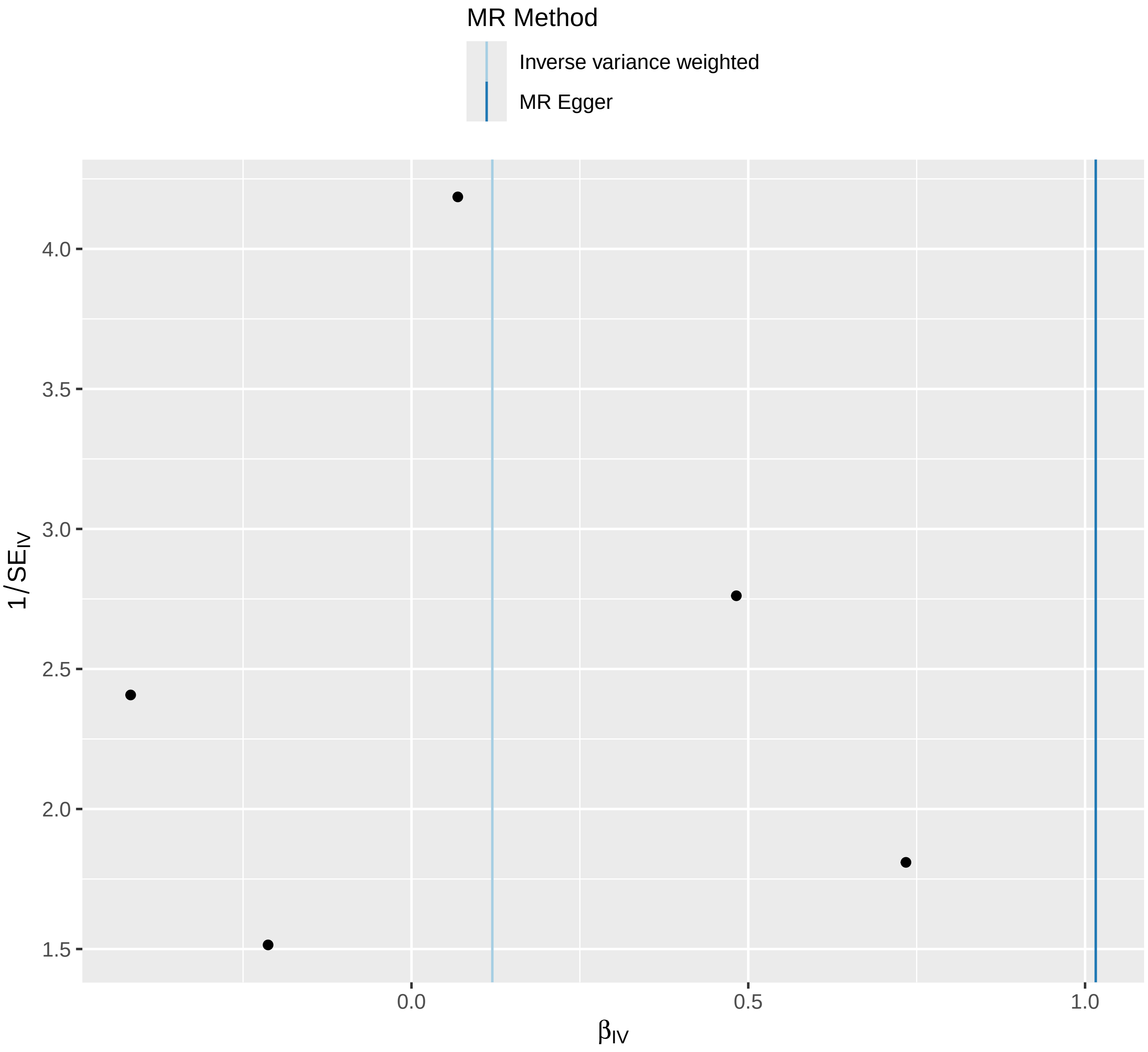

MR Method
Inverse variance weighted
MR Egger
4.0
3.5
3.0
2.5
2.0
1.5
1/SE_IV
0.0
0.5
1.0
β_IV

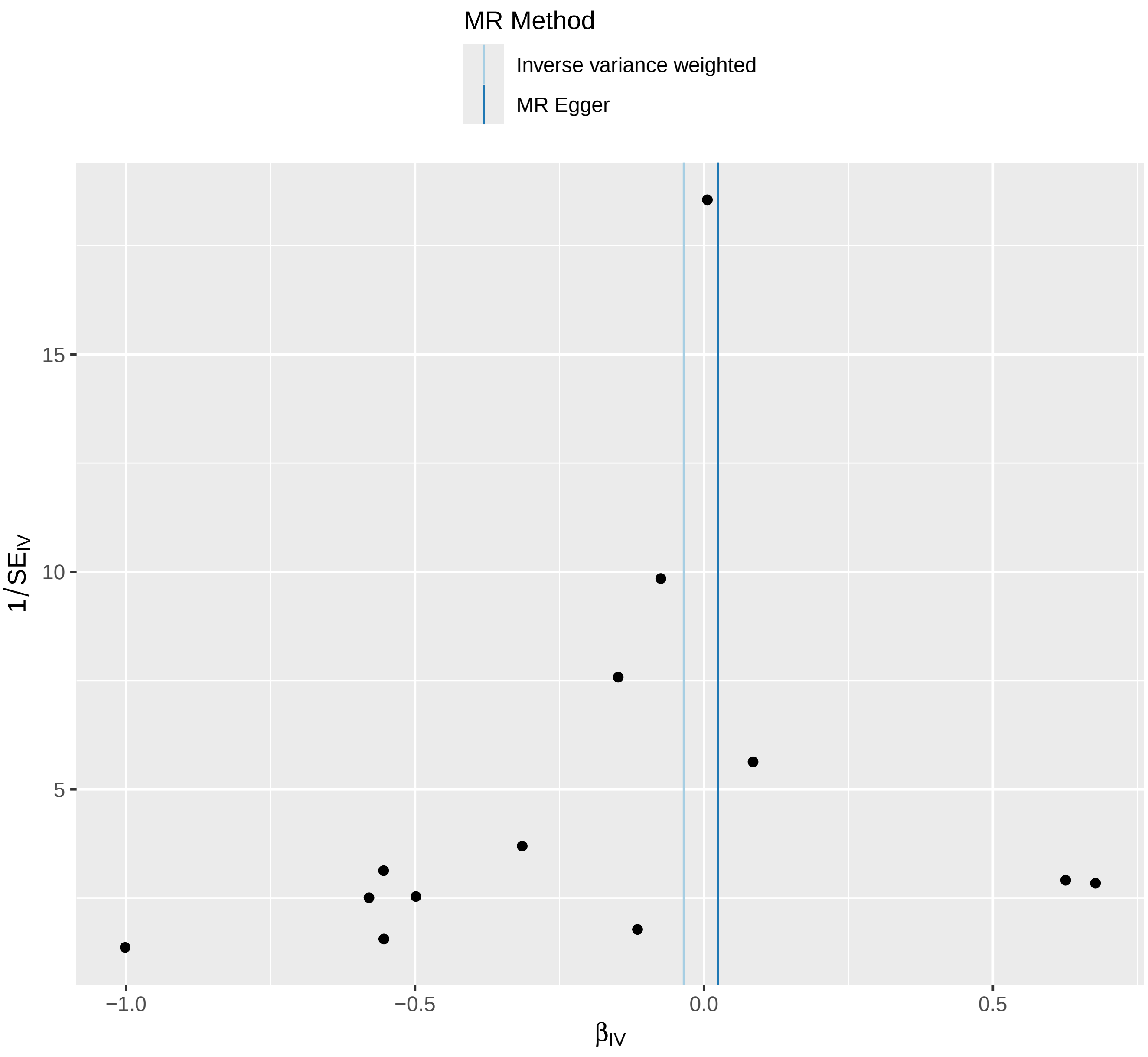
MR Method
Inverse variance weighted
MR Egger
15
10
5
$1/SE_{IV}$
−1.0
−0.5
0.0
0.5
β_{IV}

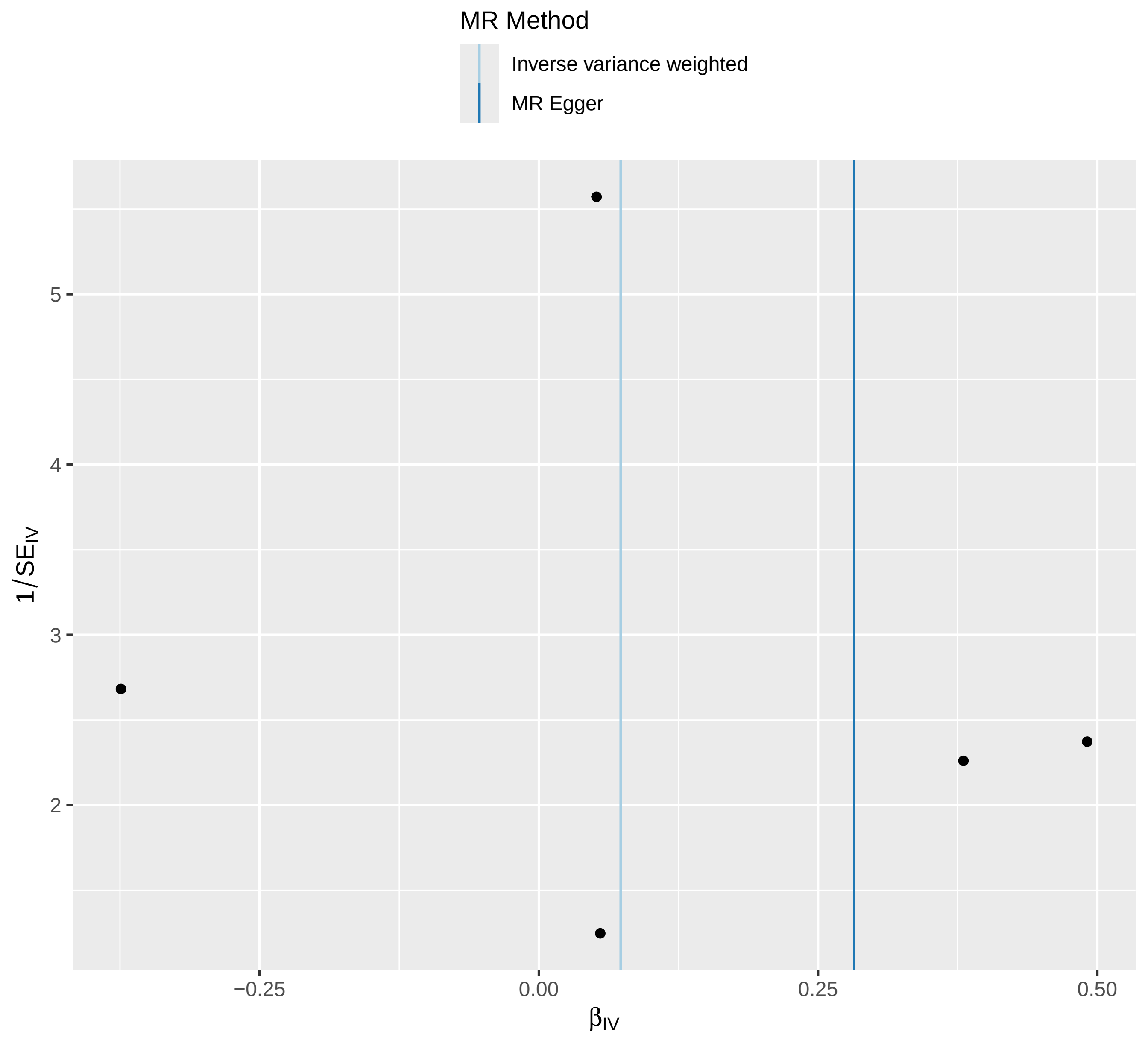
MR Method
Inverse variance weighted
MR Egger
1/SE_{IV}
β_{IV}
2
3
4
5
−0.25
0.00
0.25
0.50

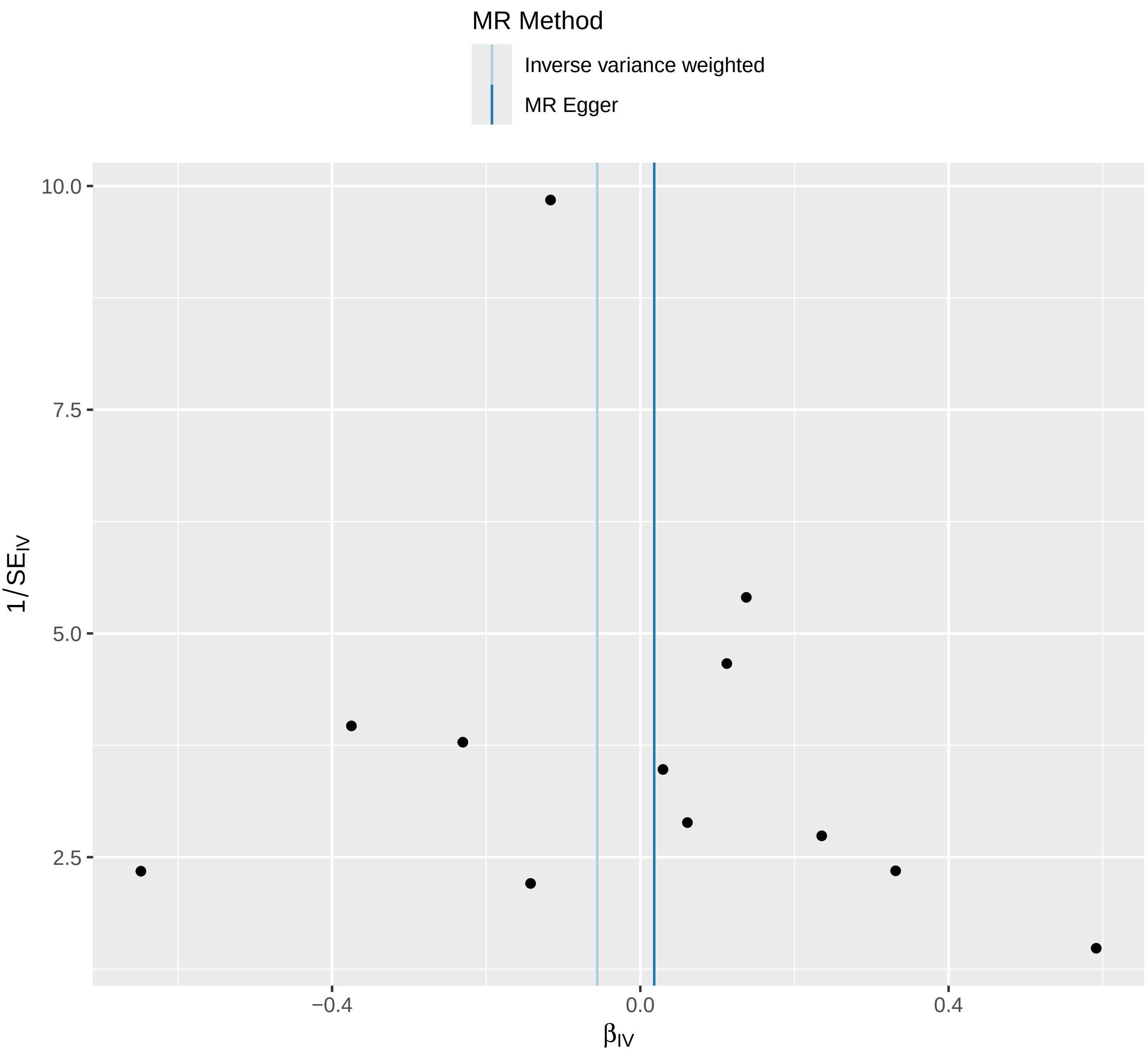
MR Method
Inverse variance weighted
MR Egger
10.0
7.5
5.0
2.5
1/SE_IV
−0.4
0.0
0.4
β_IV

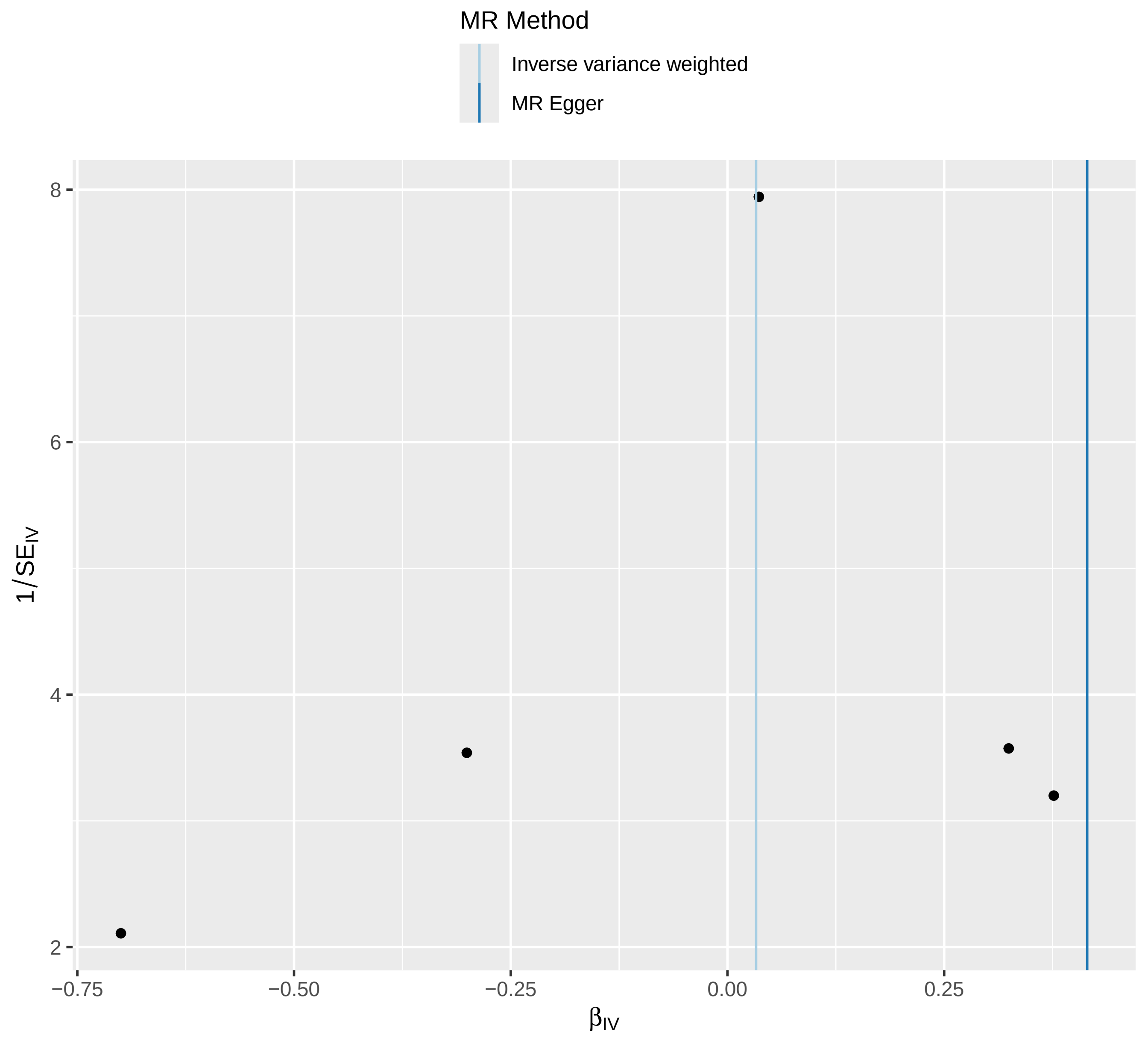

MR Method
Inverse variance weighted
MR Egger
8
6
4
2
1/SE$_{IV}$
−0.75
−0.50
−0.25
0.00
0.25
β$_{IV}$

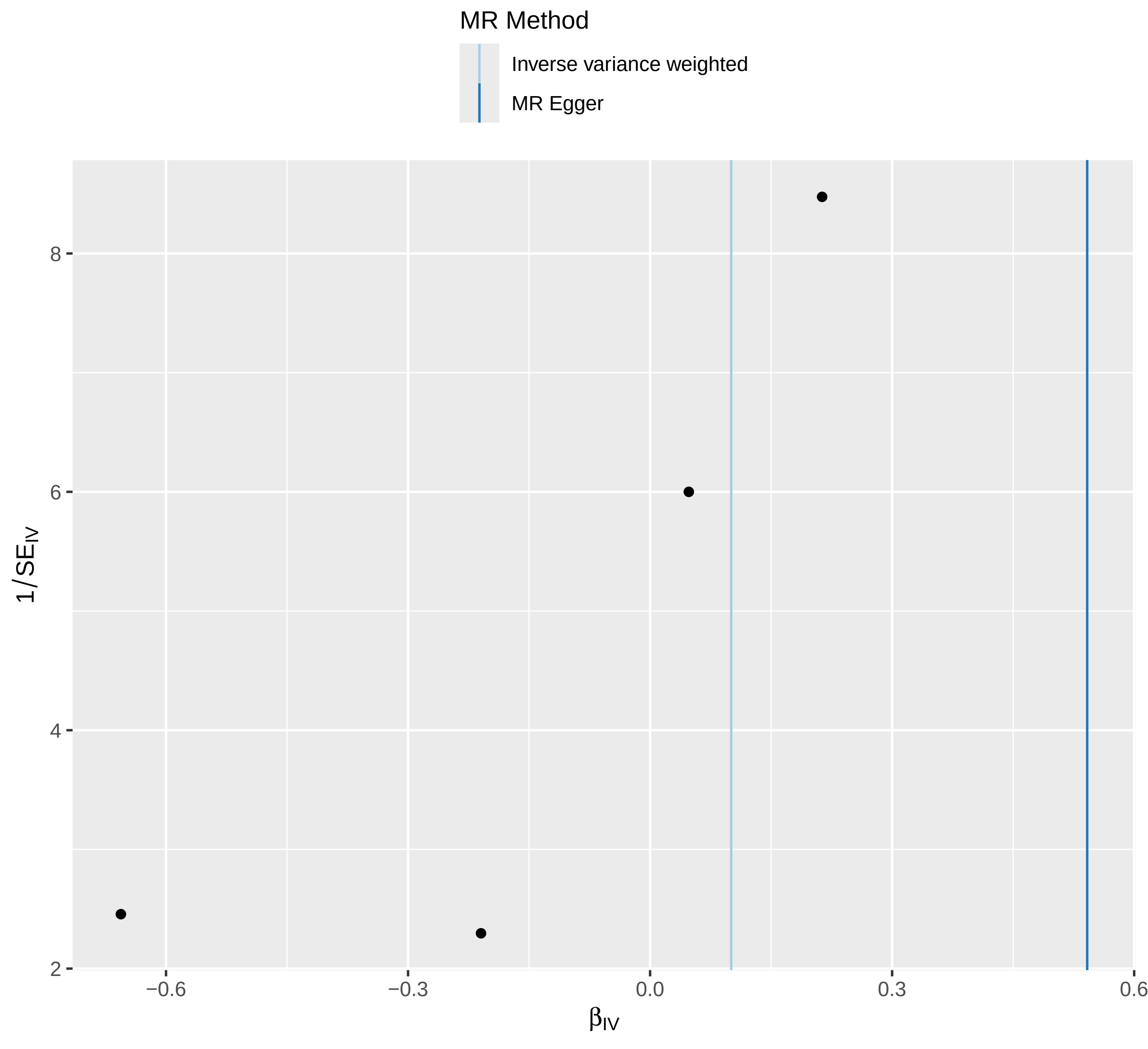

MR Method
Inverse variance weighted
MR Egger
1/SE$_{IV}$
β$_{IV}$
8
6
4
2
−0.6
−0.3
0.0
0.3
0.6

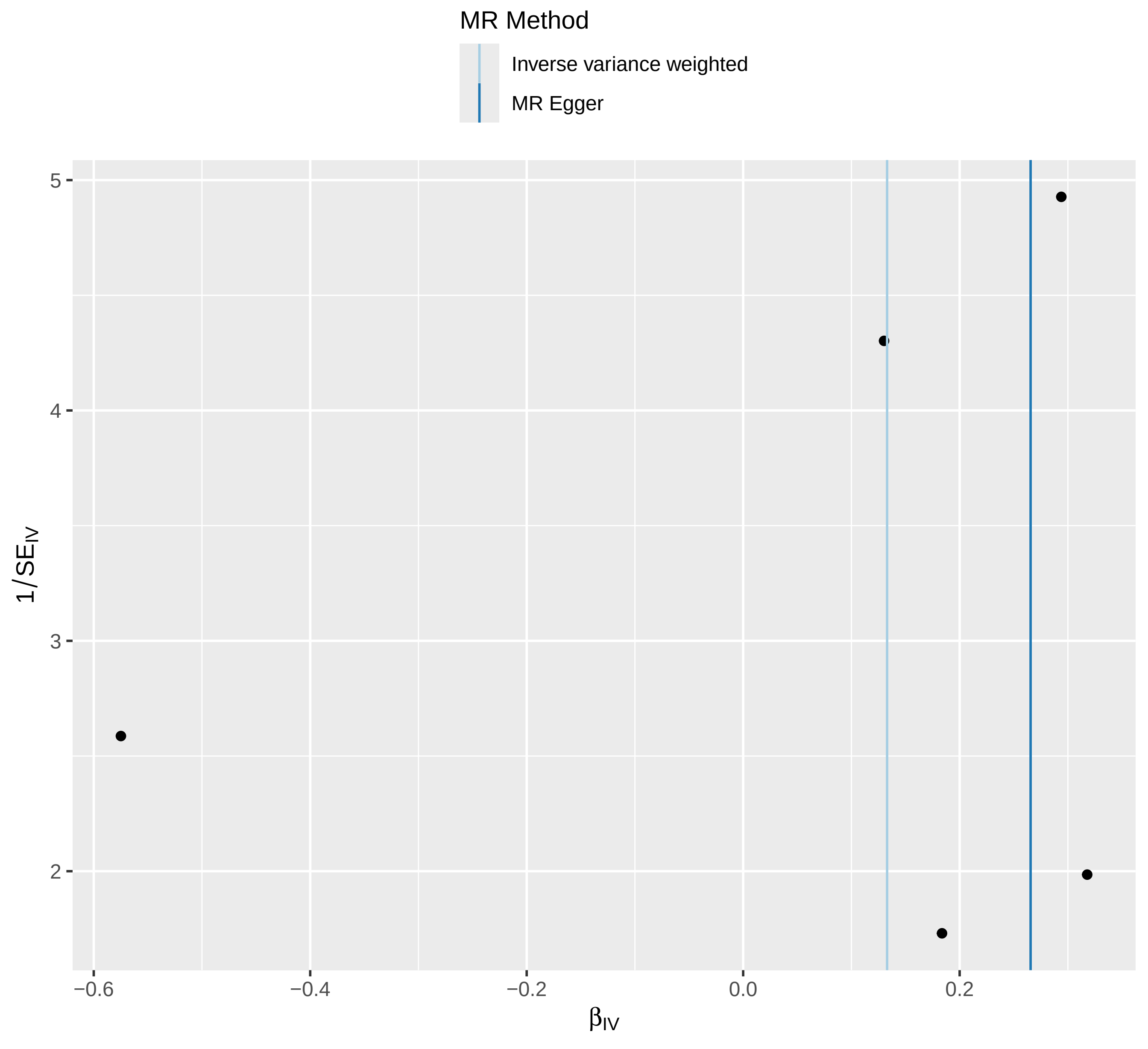

MR Method
Inverse variance weighted
MR Egger
5
4
3
2
−0.6
−0.4
−0.2
0.0
0.2
1/SE_IV
β_IV

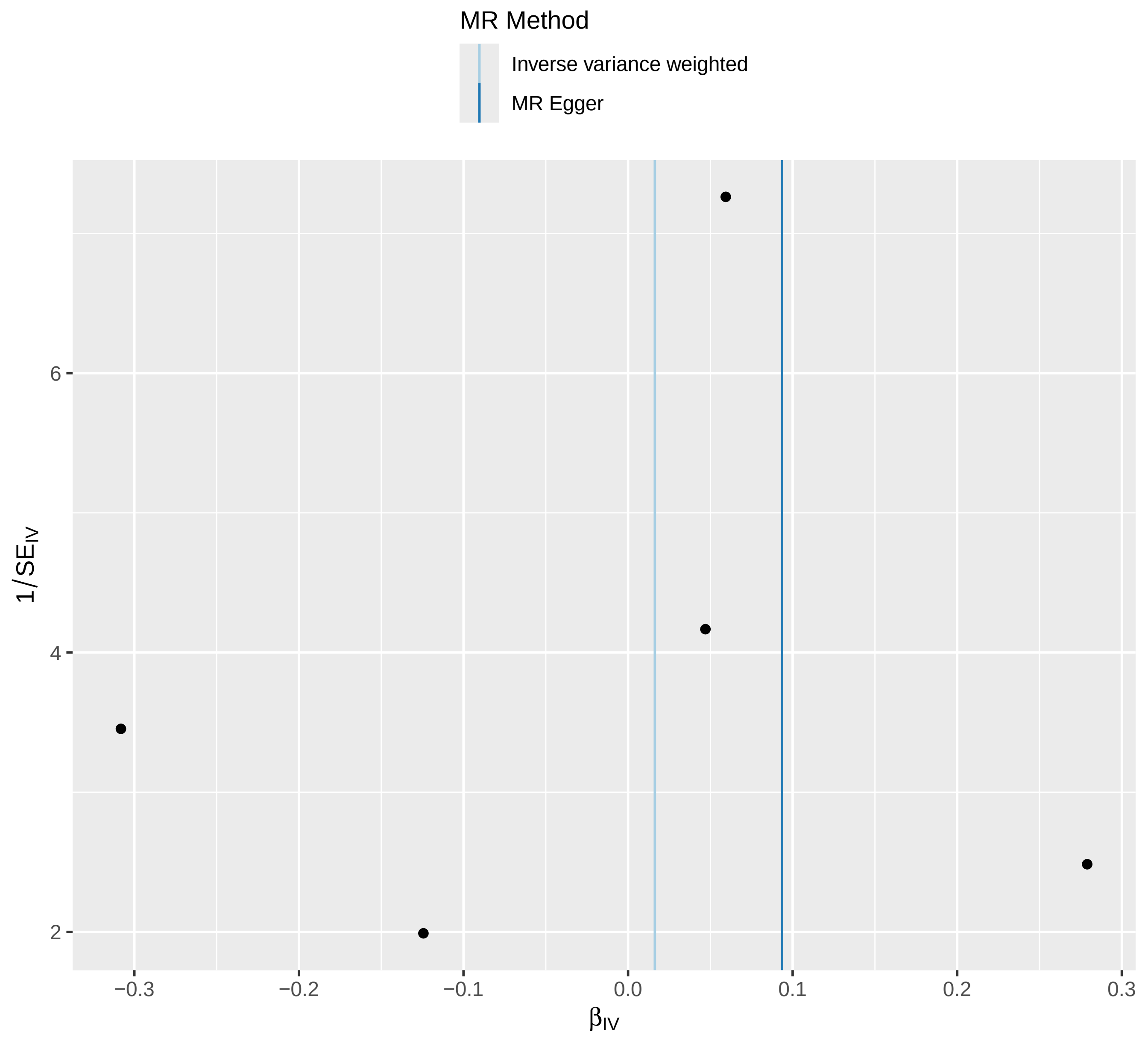
MR Method
Inverse variance weighted
MR Egger
1/SE_IV
6
4
2
−0.3
−0.2
−0.1
0.0
0.1
0.2
0.3
β_IV

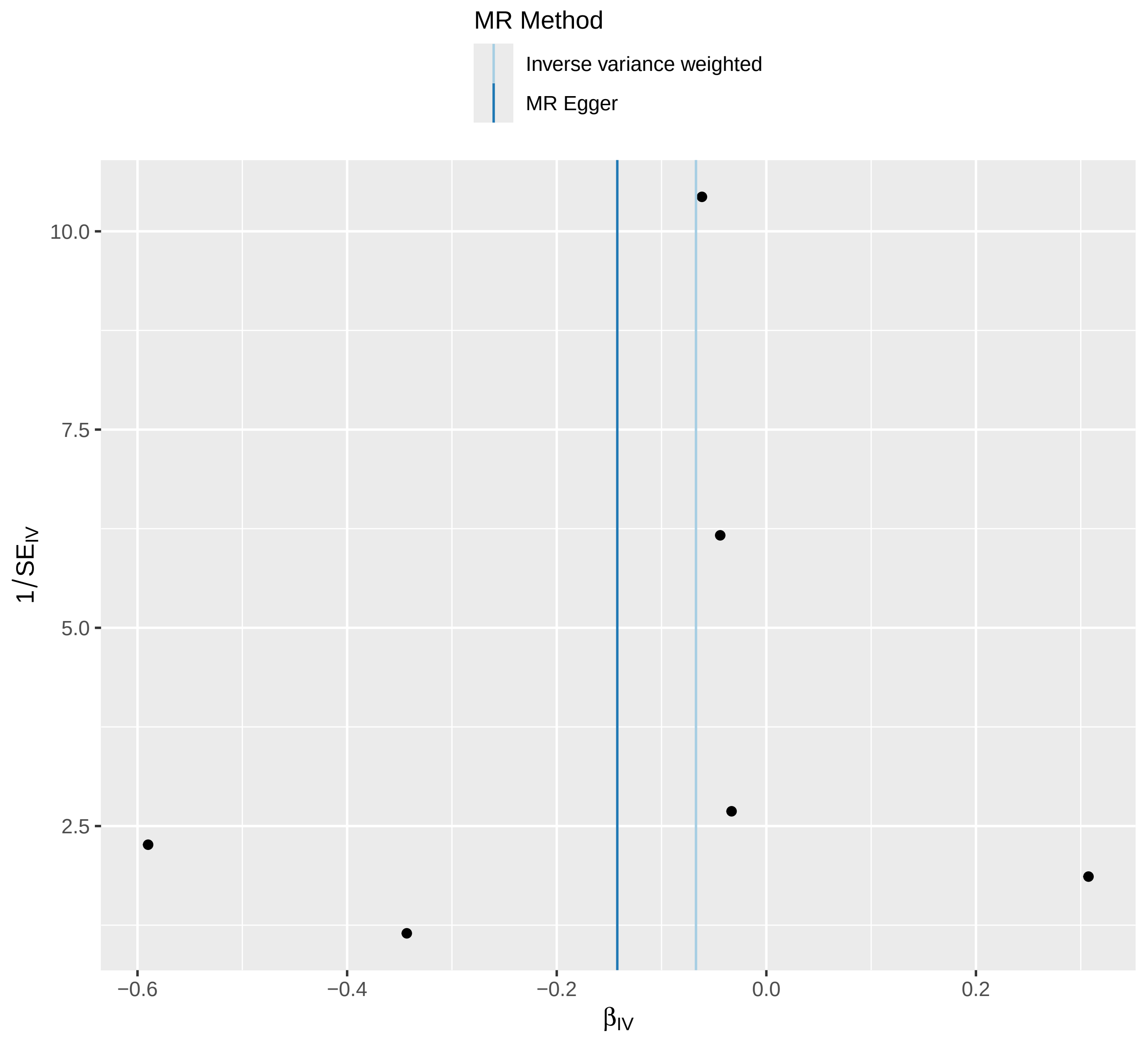

MR Method
Inverse variance weighted
MR Egger
10.0
7.5
5.0
2.5
$1/SE_{IV}$
−0.6
−0.4
−0.2
0.0
0.2
β_{IV}

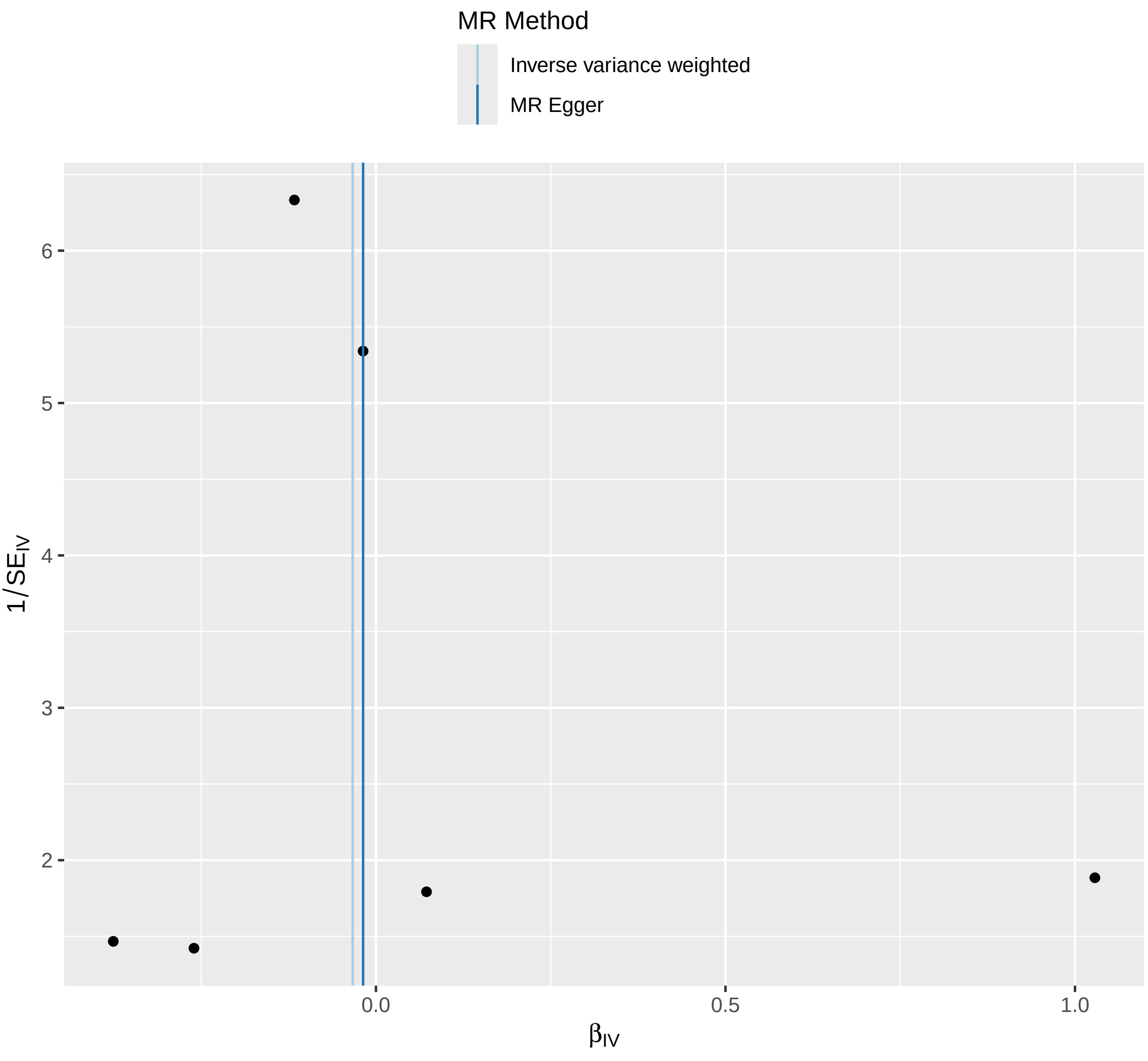

MR Method
Inverse variance weighted
MR Egger
6
5
4
3
2
1/SE_IV
0.0
0.5
1.0
β_IV

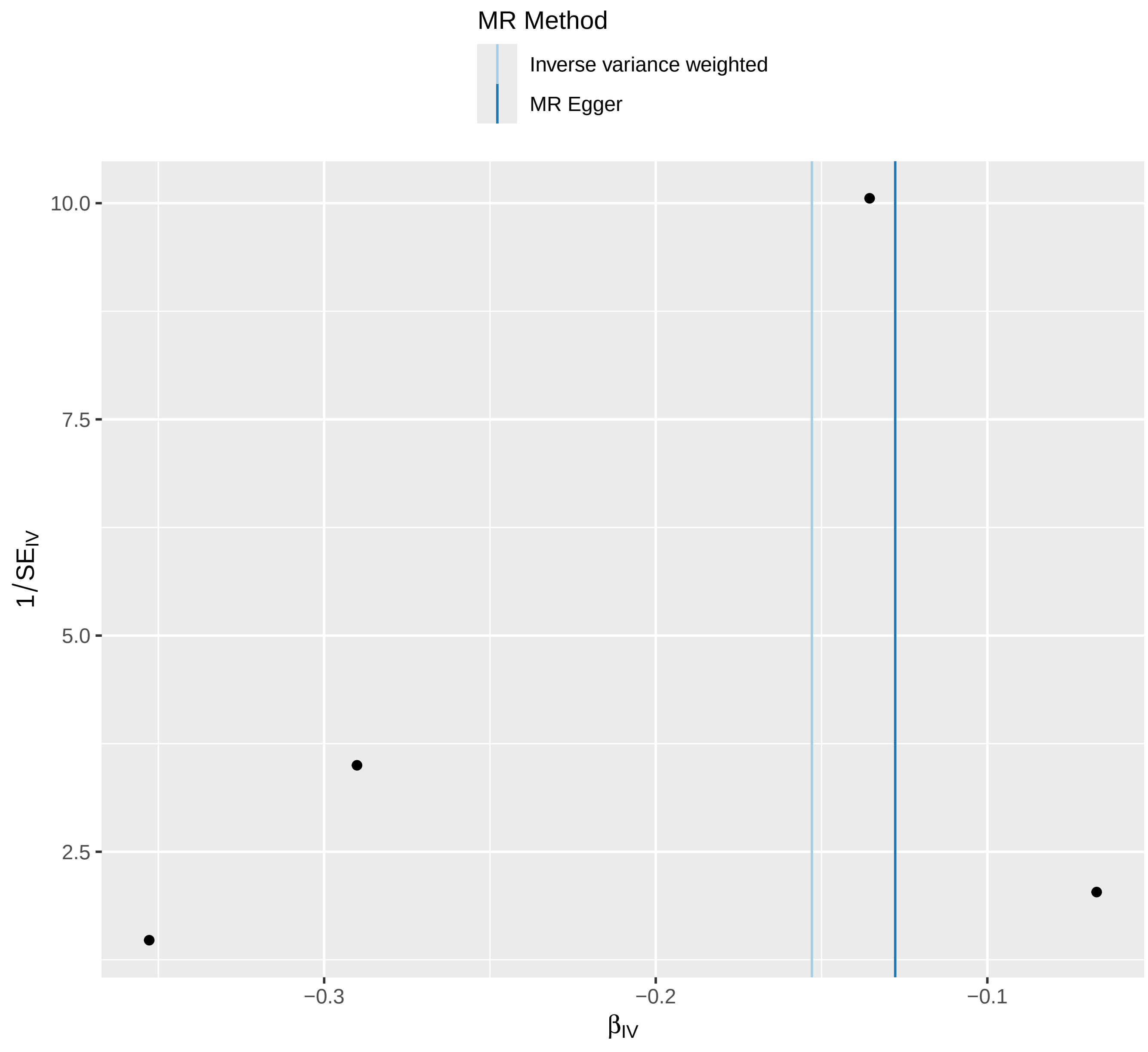

MR Method
Inverse variance weighted
MR Egger
10.0
7.5
5.0
2.5
1/SE_IV
−0.3
−0.2
−0.1
β_IV

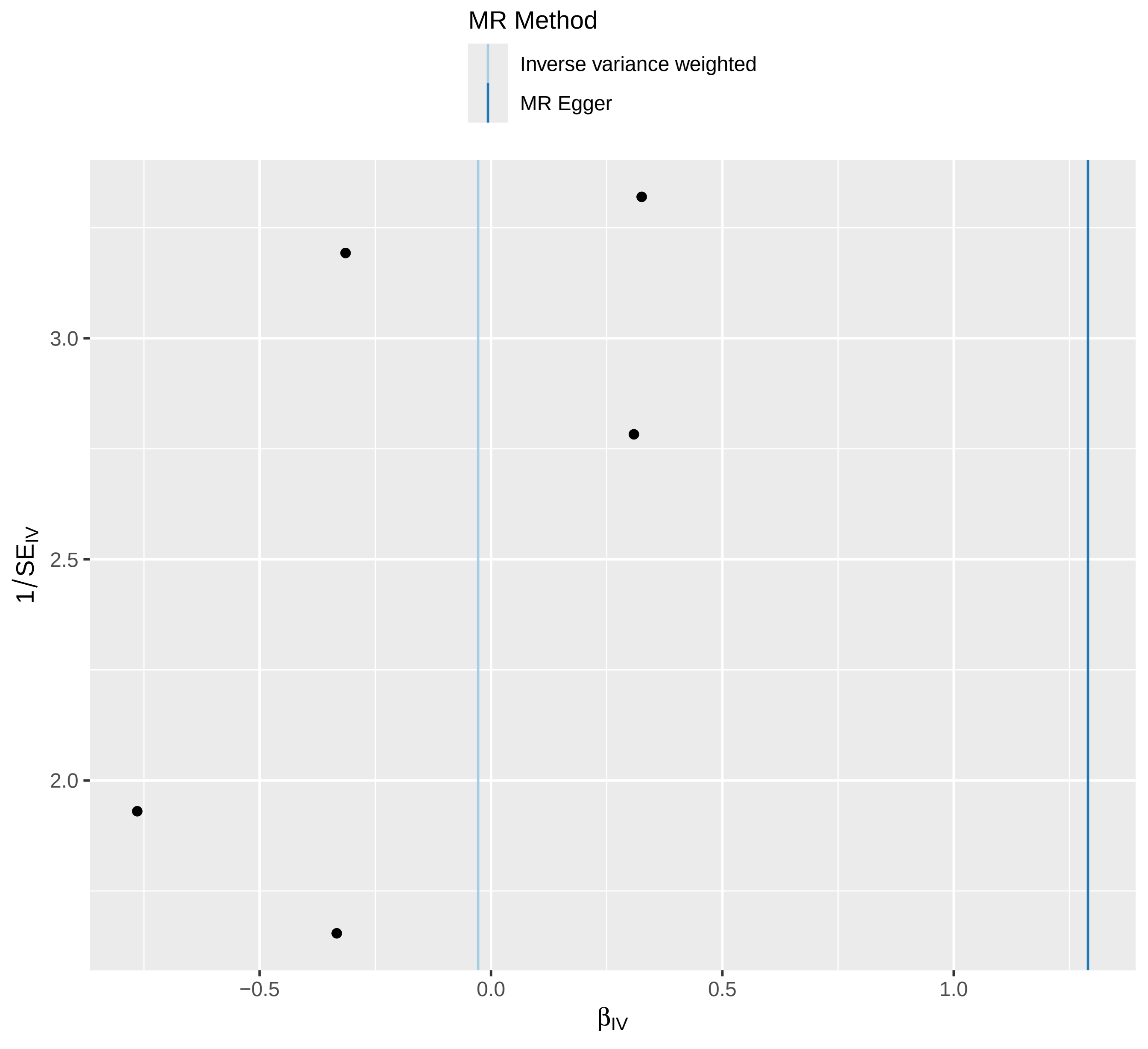
MR Method
Inverse variance weighted
MR Egger
3.0
2.5
2.0
$1/SE_{IV}$
−0.5
0.0
0.5
1.0
β_{IV}

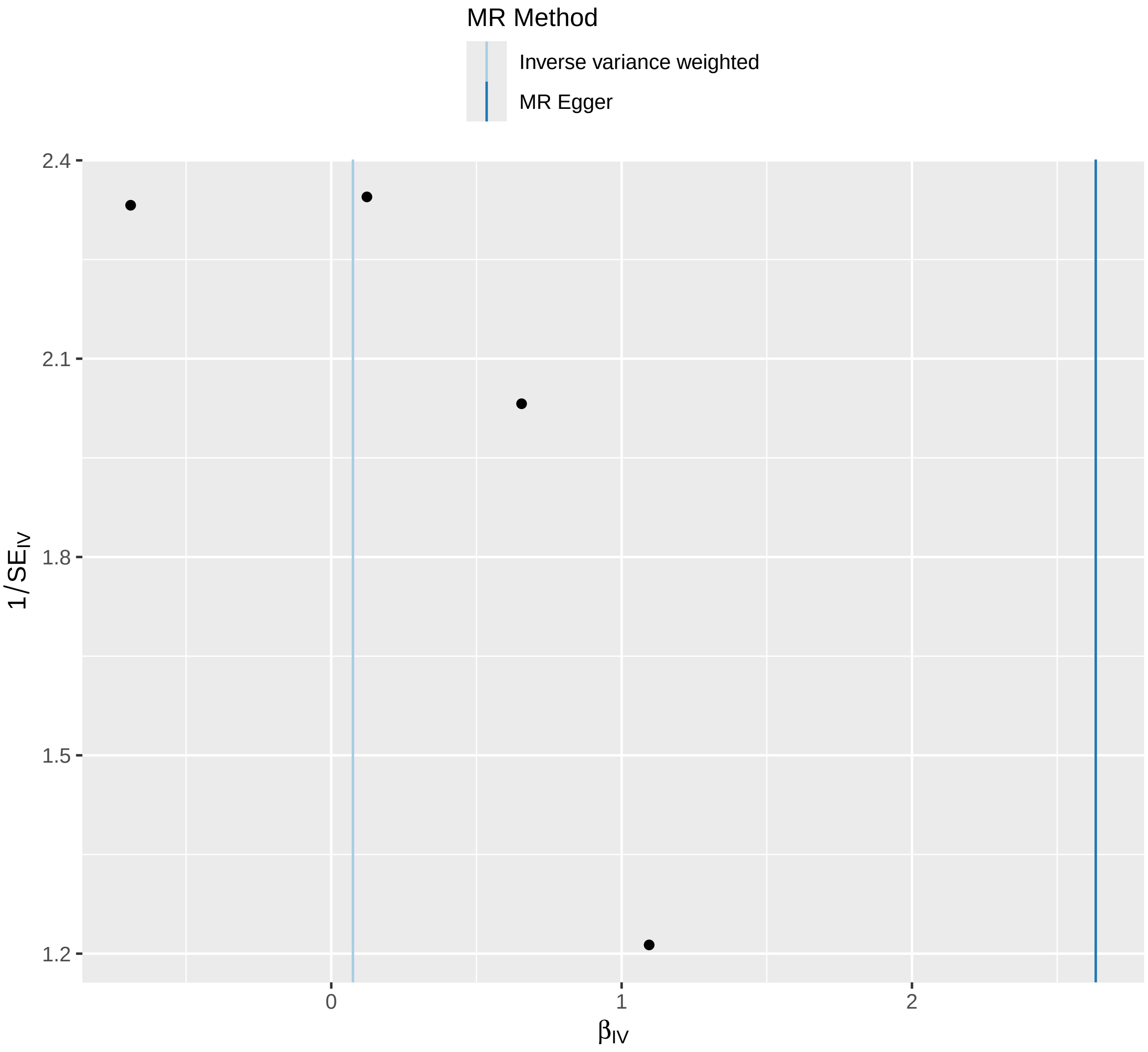

MR Method
Inverse variance weighted
MR Egger
2.4
2.1
1.8
1.5
1.2
1/SE$_{IV}$
0
1
2
β$_{IV}$

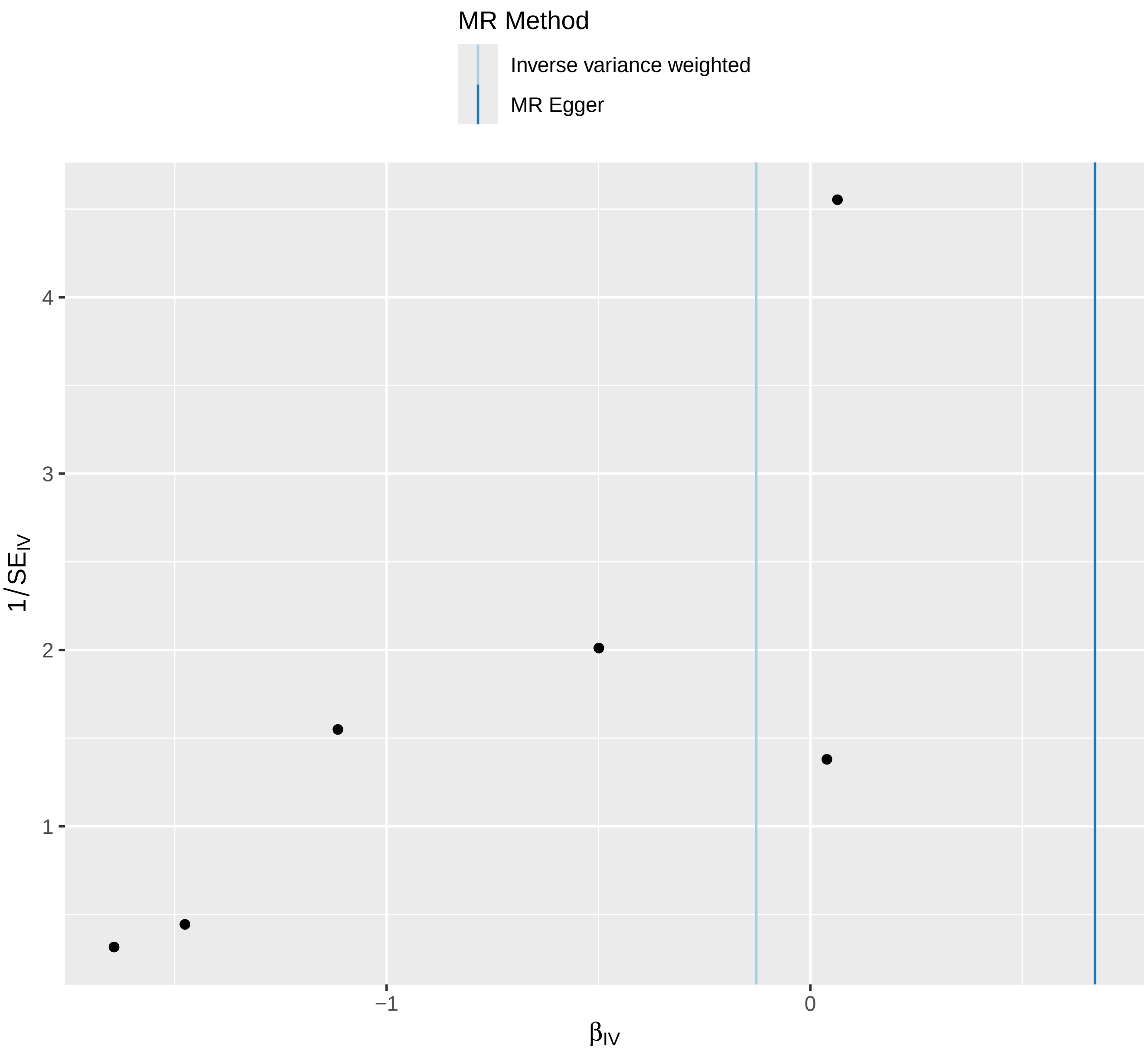
MR Method
Inverse variance weighted
MR Egger
4
3
2
1
$1/SE_{IV}$
−1
0
β_{IV}

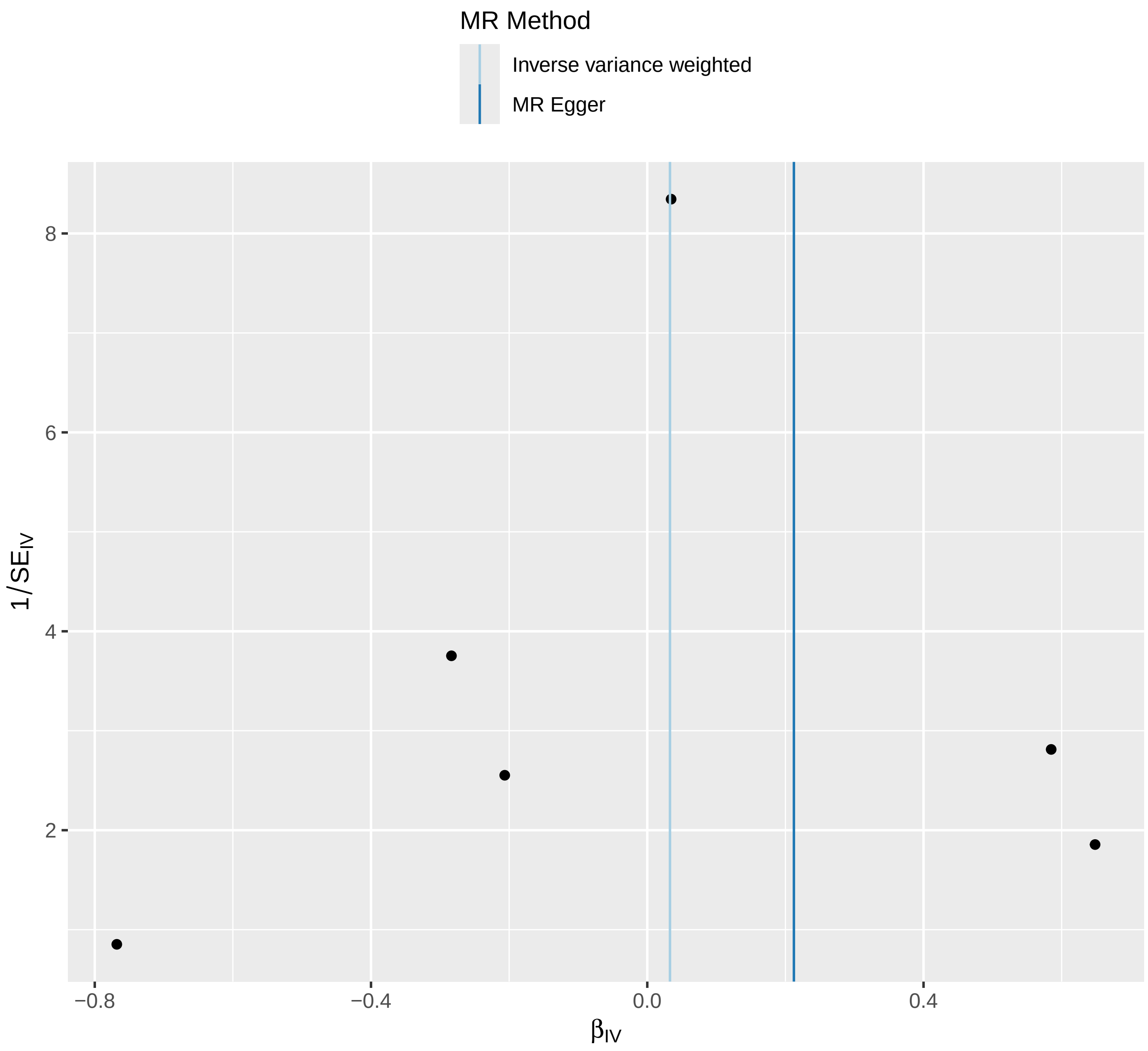

MR Method
Inverse variance weighted
MR Egger
8
6
4
2
1/SE$_{IV}$
−0.8
−0.4
0.0
0.4
β$_{IV}$

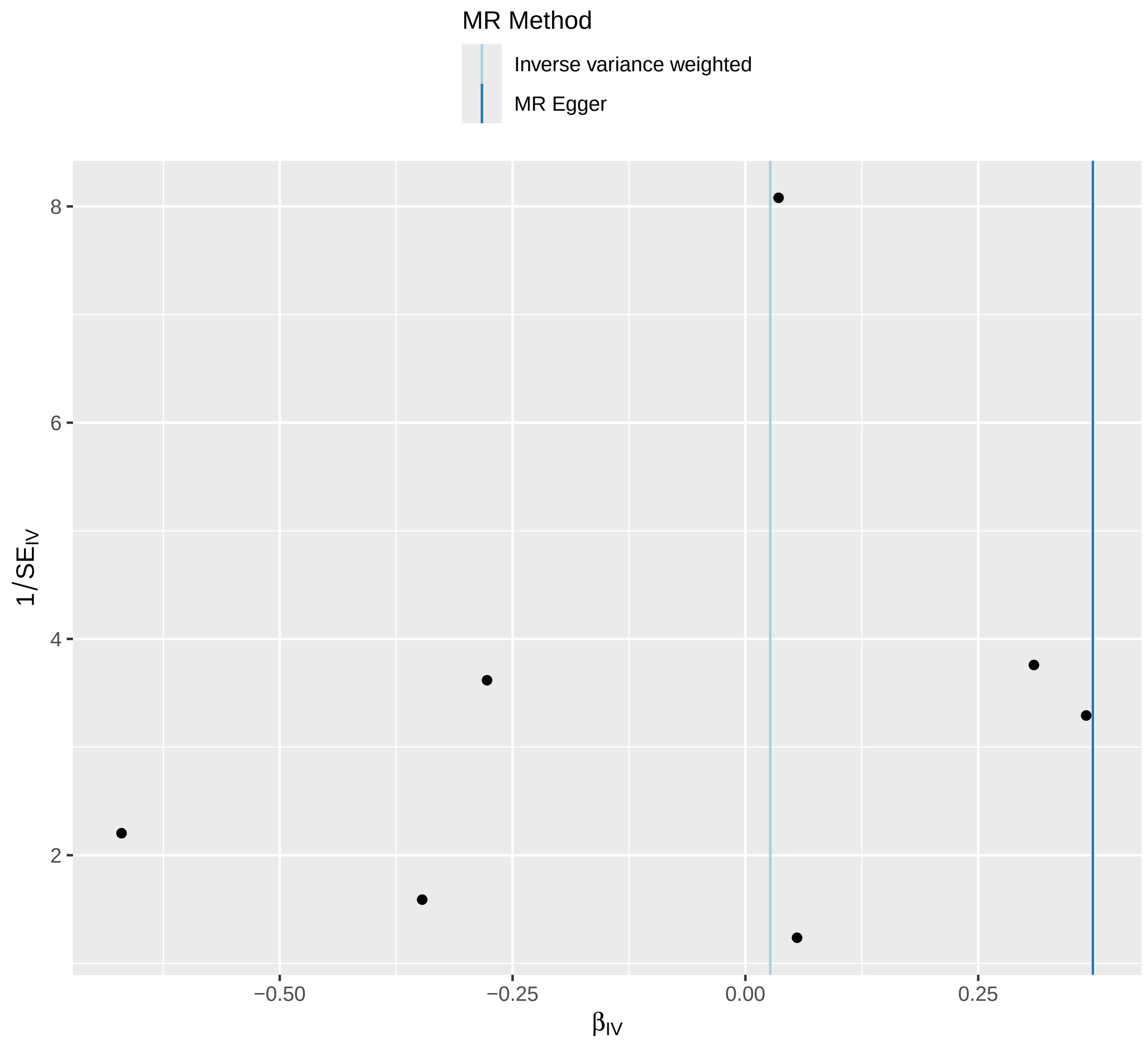

MR Method
Inverse variance weighted
MR Egger
8
6
4
2
1/SE_IV
−0.50
−0.25
0.00
0.25
β_IV

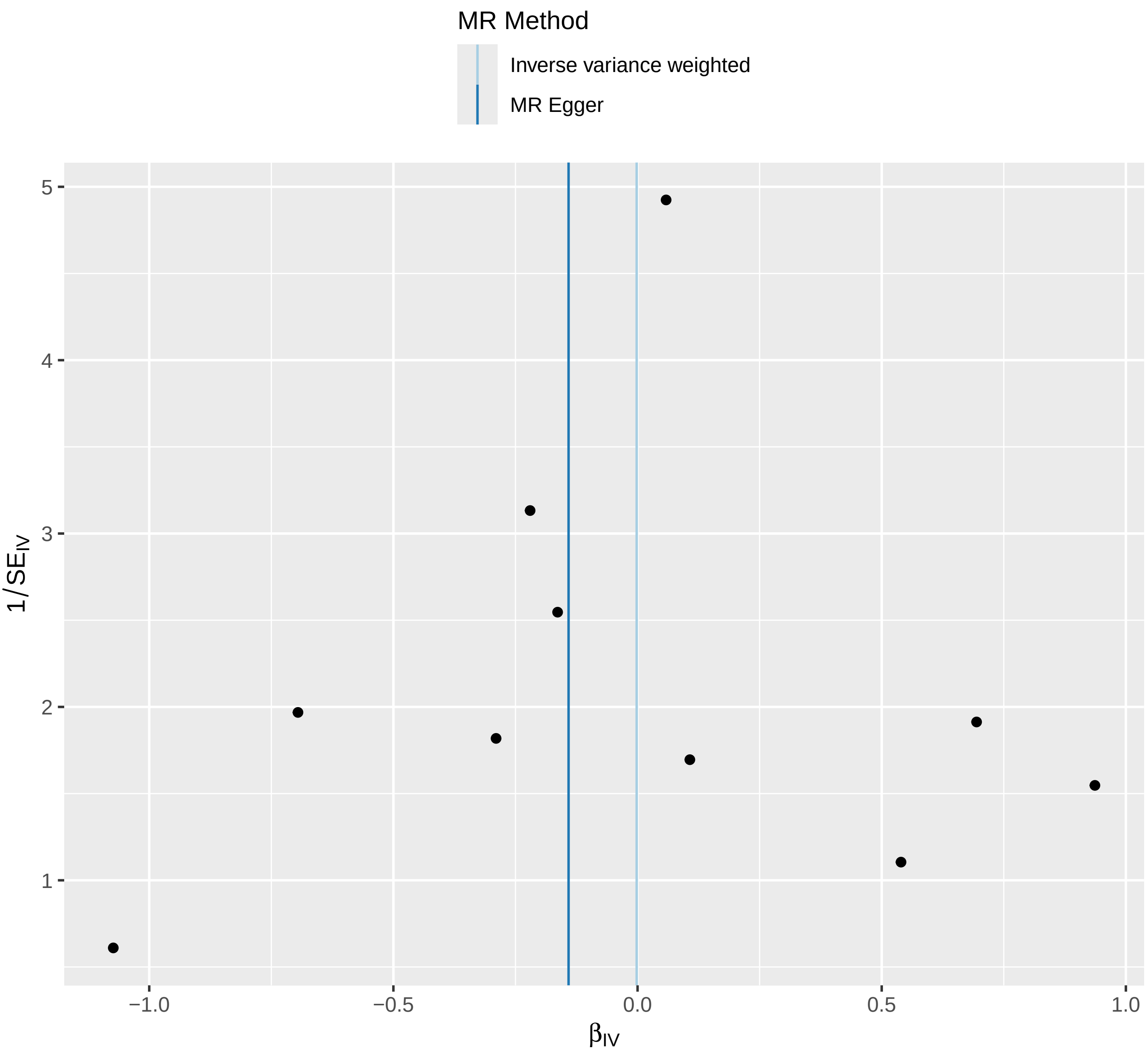
MR Method
Inverse variance weighted
MR Egger
5
4
3
2
1
1/SE$_{IV}$
−1.0
−0.5
0.0
0.5
1.0
β$_{IV}$

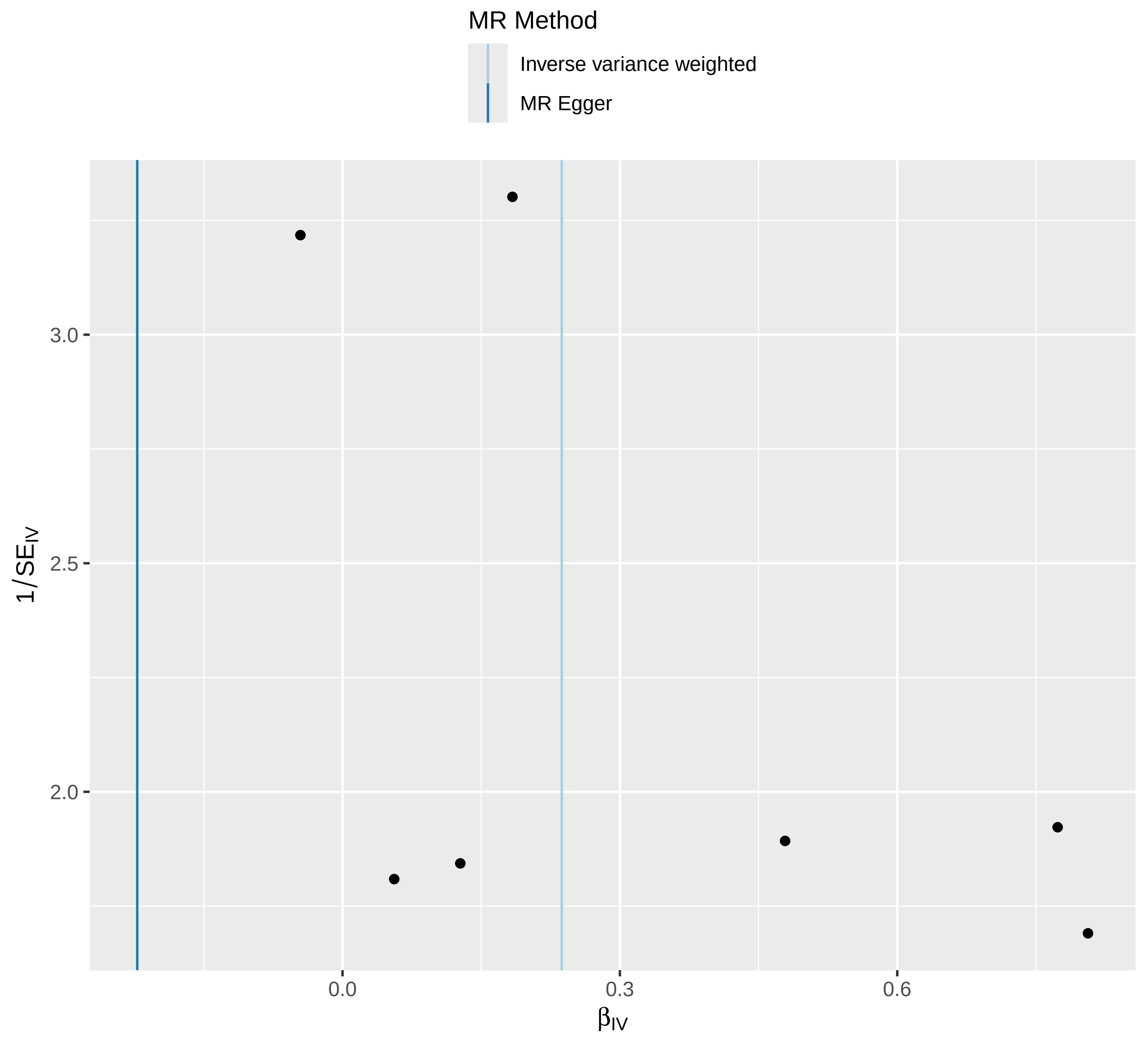

MR Method
Inverse variance weighted
MR Egger
3.0
2.5
2.0
0.0
0.3
0.6
1/SE_IV
β_IV

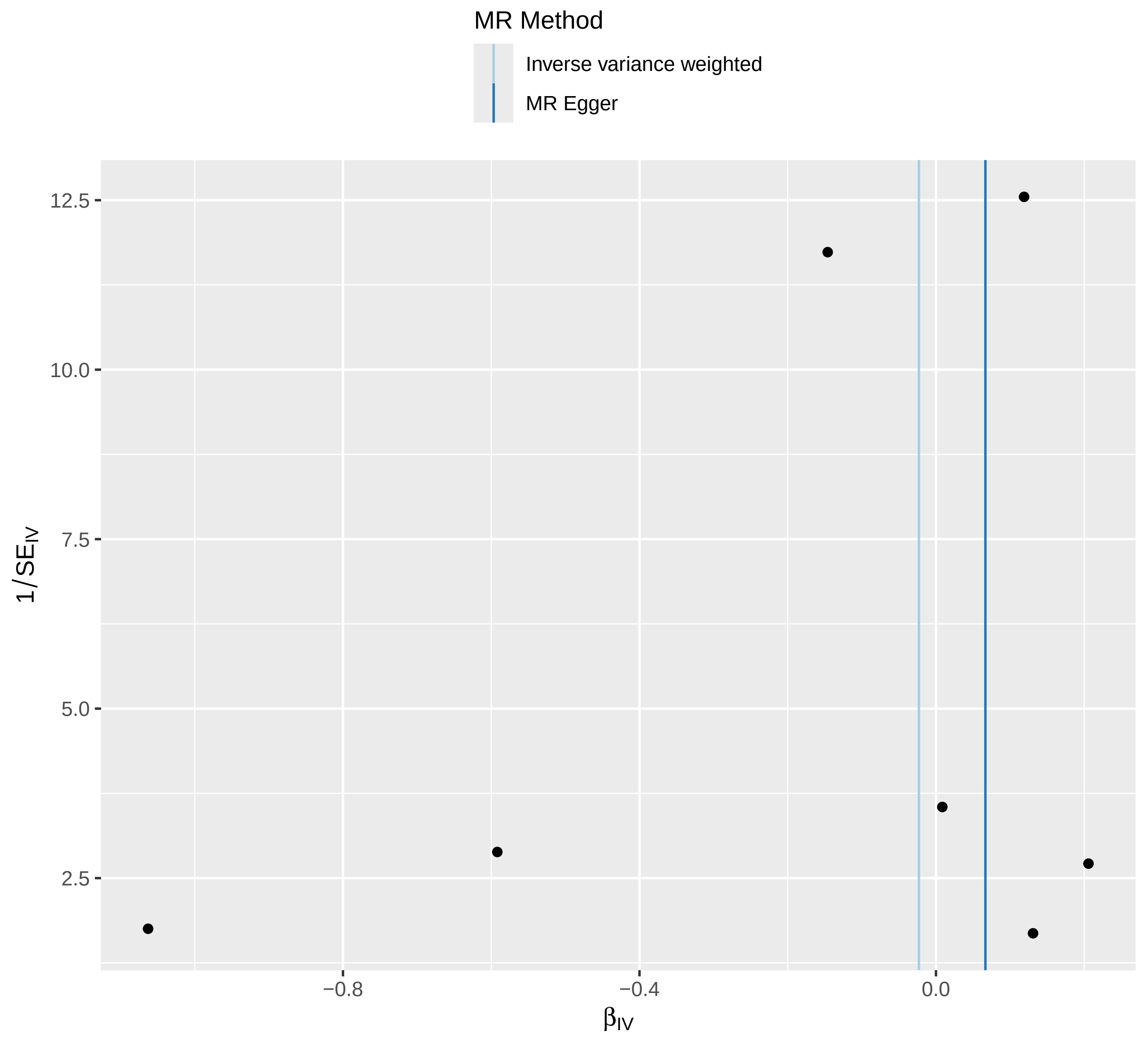
MR Method
Inverse variance weighted
MR Egger
12.5
10.0
7.5
5.0
2.5
$1/SE_{IV}$
−0.8
−0.4
0.0
β_{IV}

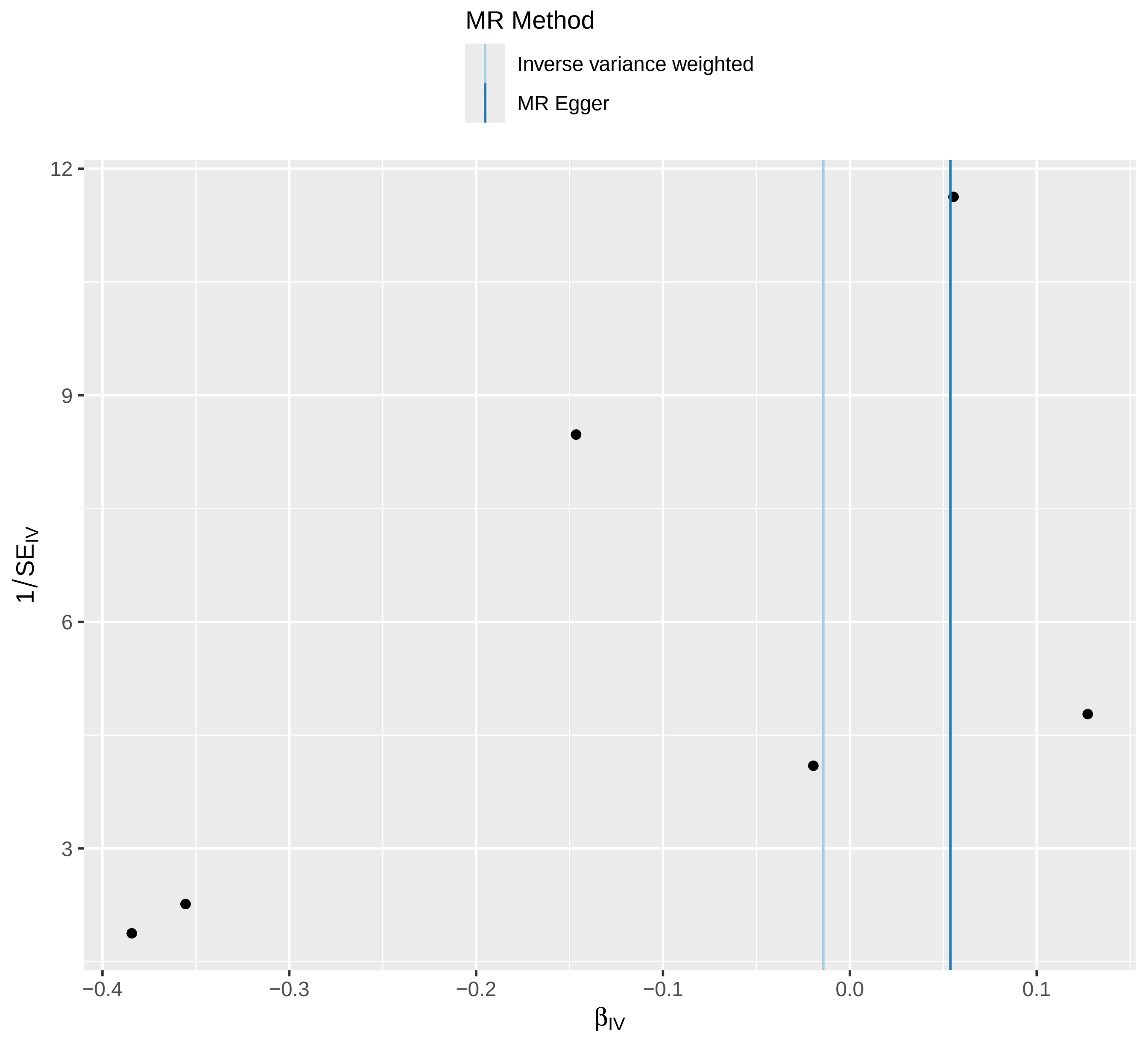

MR Method
Inverse variance weighted
MR Egger
12
9
6
3
1/SE_IV
−0.4
−0.3
−0.2
−0.1
0.0
0.1
β_IV

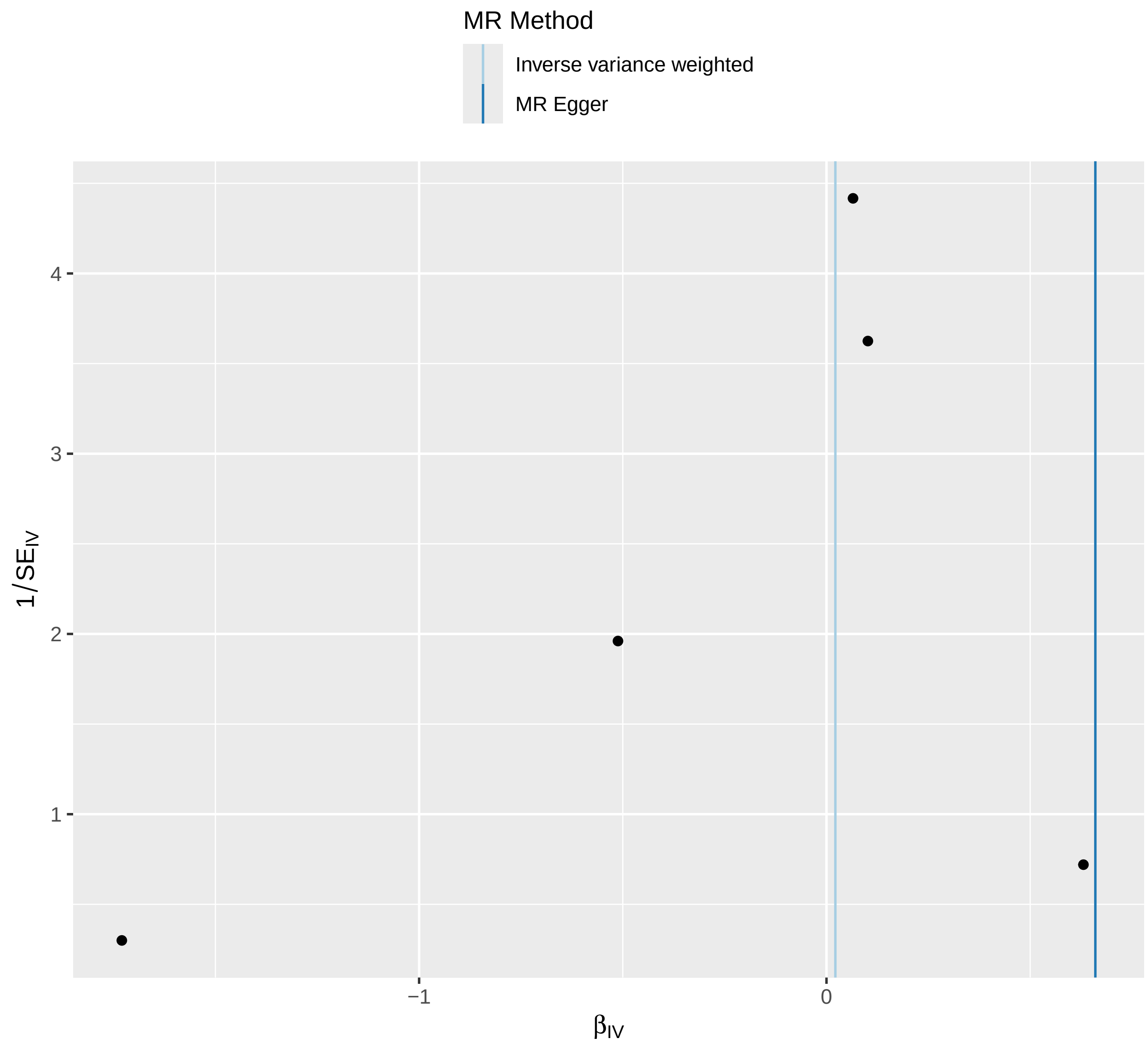
MR Method
Inverse variance weighted
MR Egger
1/SE_{IV}
4
3
2
1
−1
0
β_{IV}

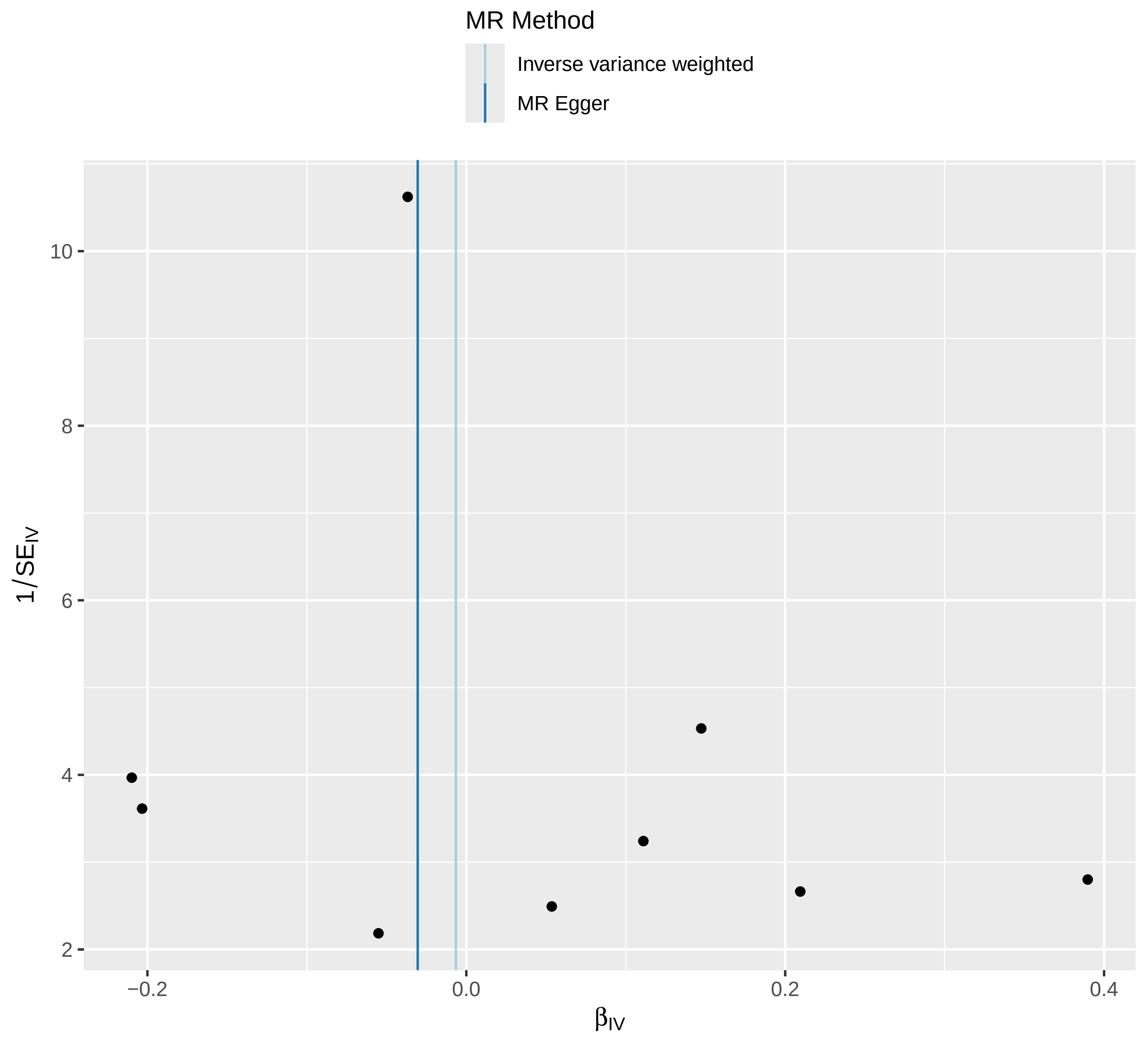

MR Method
Inverse variance weighted
MR Egger
10
8
6
4
2
1/SE_IV
−0.2
0.0
0.2
0.4
β_IV

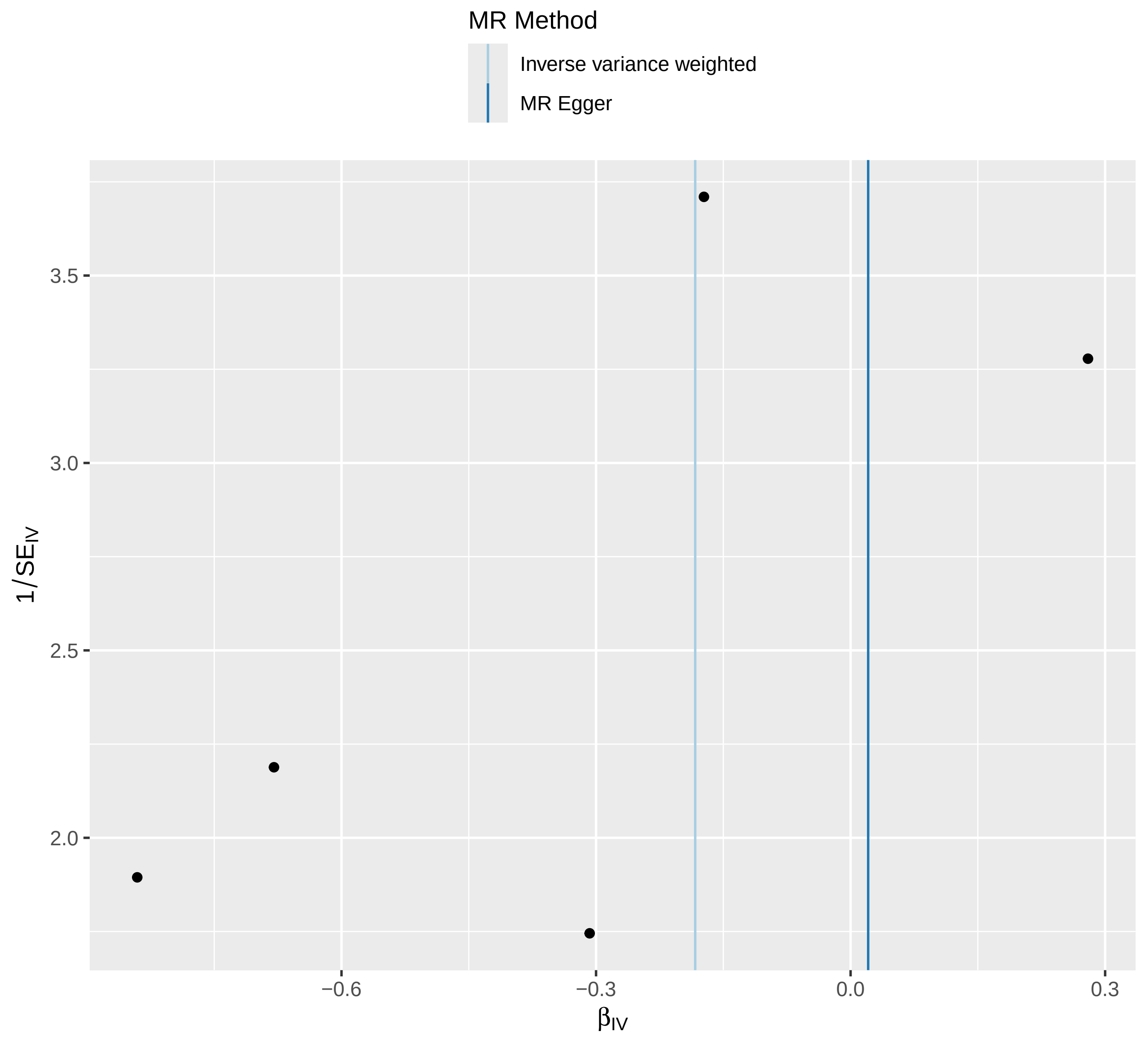
MR Method
Inverse variance weighted
MR Egger
1/SE_{IV}
3.5
3.0
2.5
2.0
−0.6
−0.3
0.0
0.3
β_{IV}

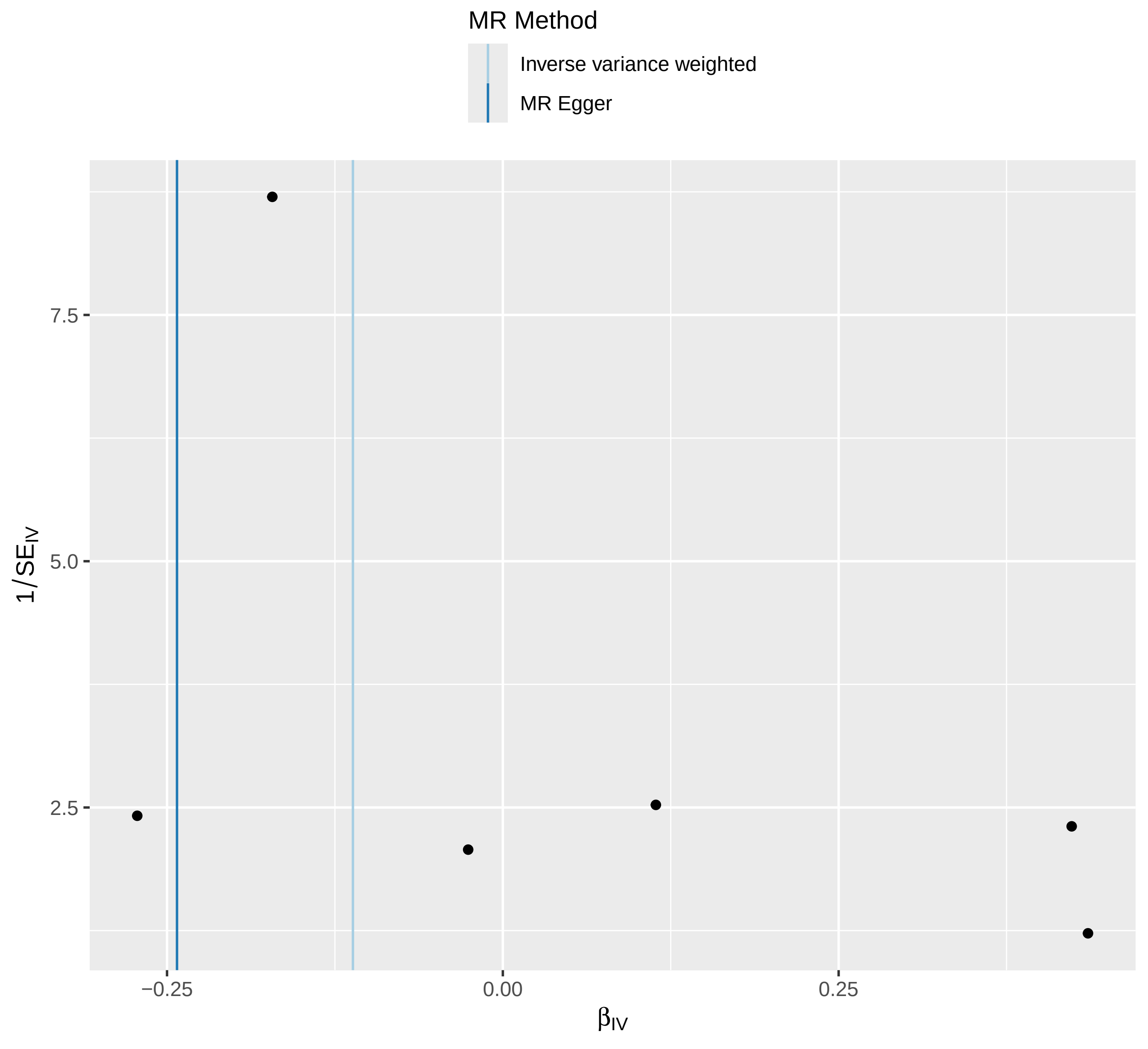
MR Method
Inverse variance weighted
MR Egger
7.5
5.0
2.5
1/SE_IV
−0.25
0.00
0.25
β_IV

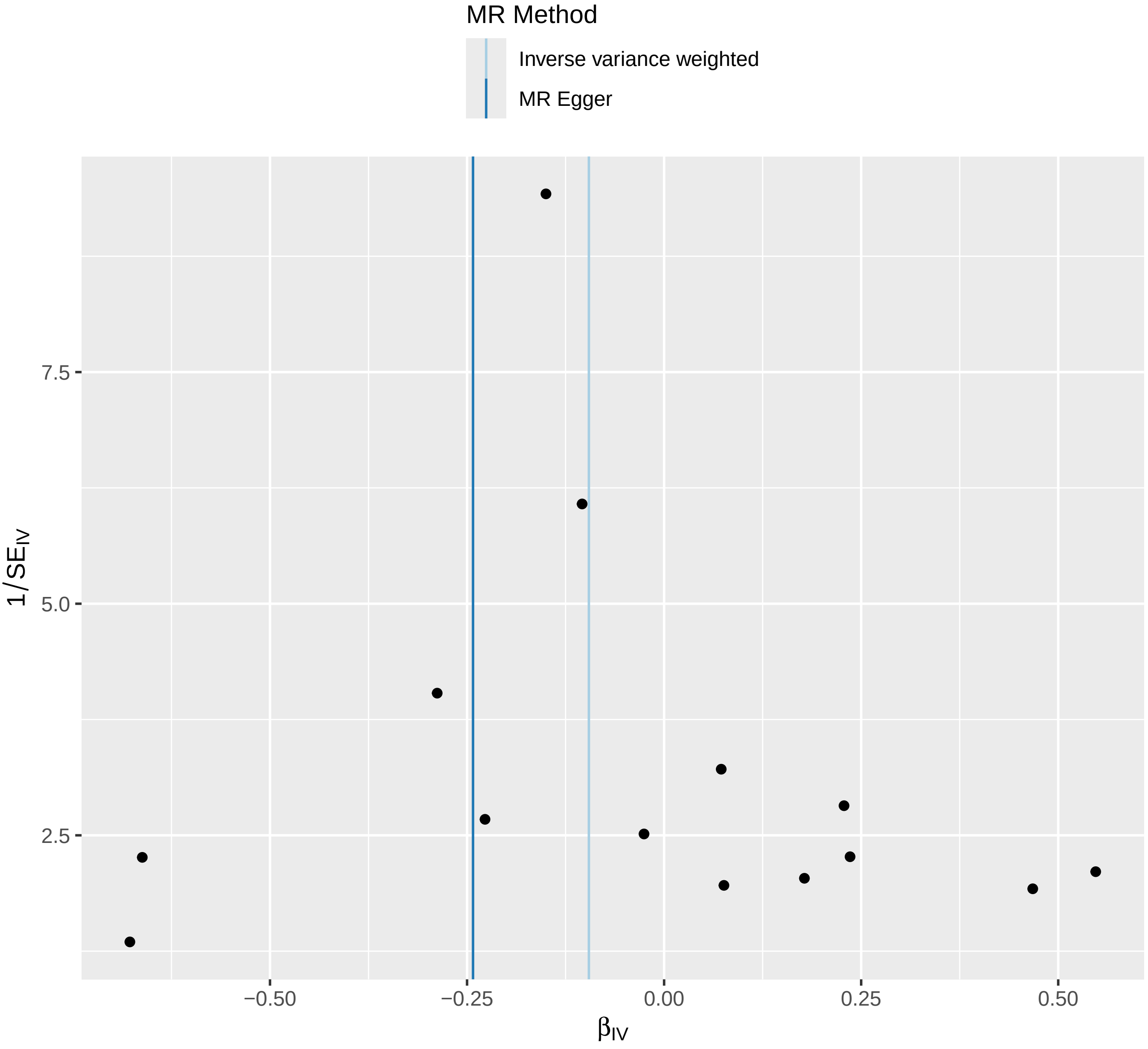

MR Method
Inverse variance weighted
MR Egger
7.5
5.0
2.5
−0.50
−0.25
0.00
0.25
0.50
$1/SE_{IV}$
β_{IV}

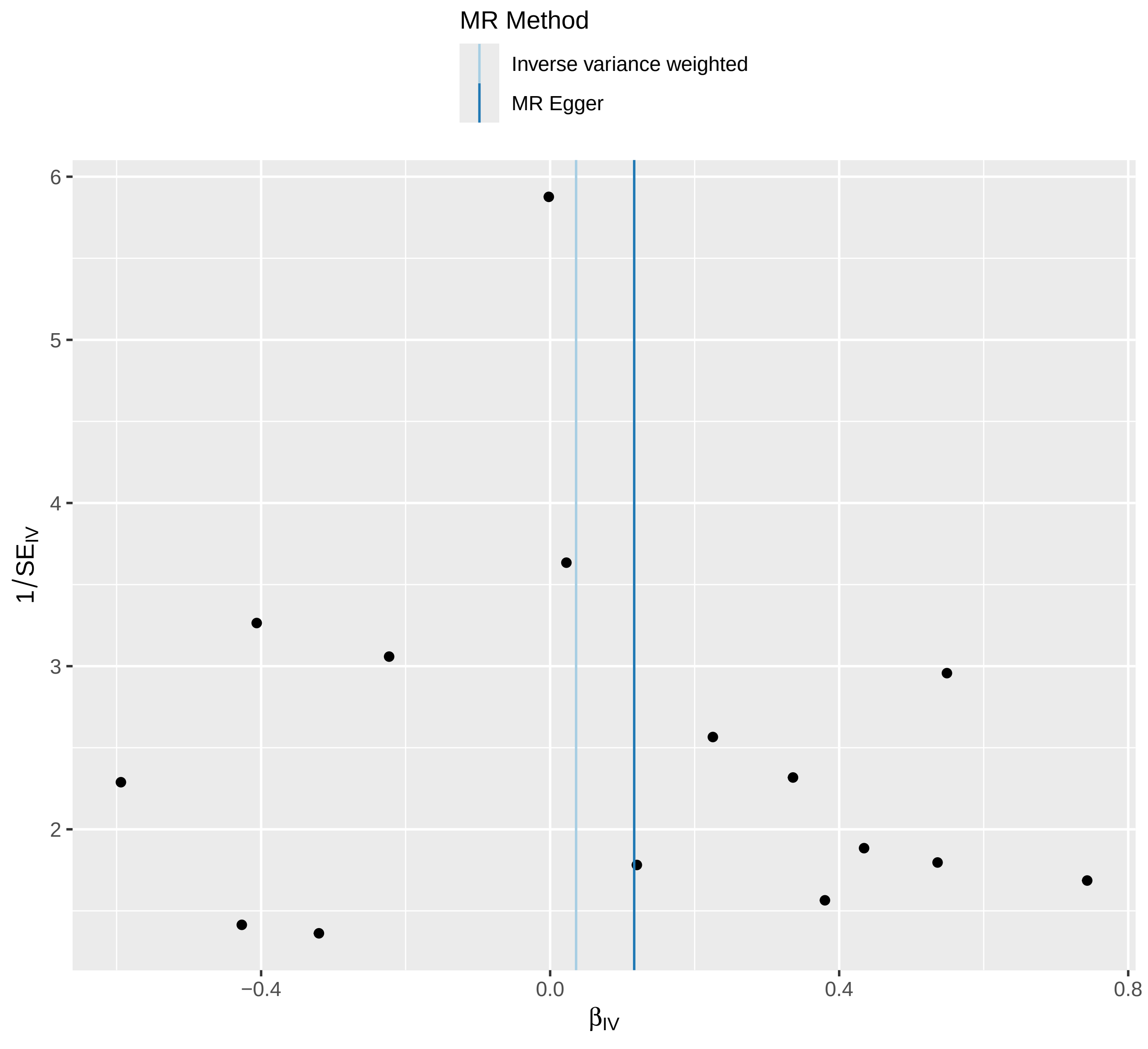
MR Method
Inverse variance weighted
MR Egger
6
5
4
3
2
1/SE$_{IV}$
−0.4
0.0
0.4
0.8
β$_{IV}$

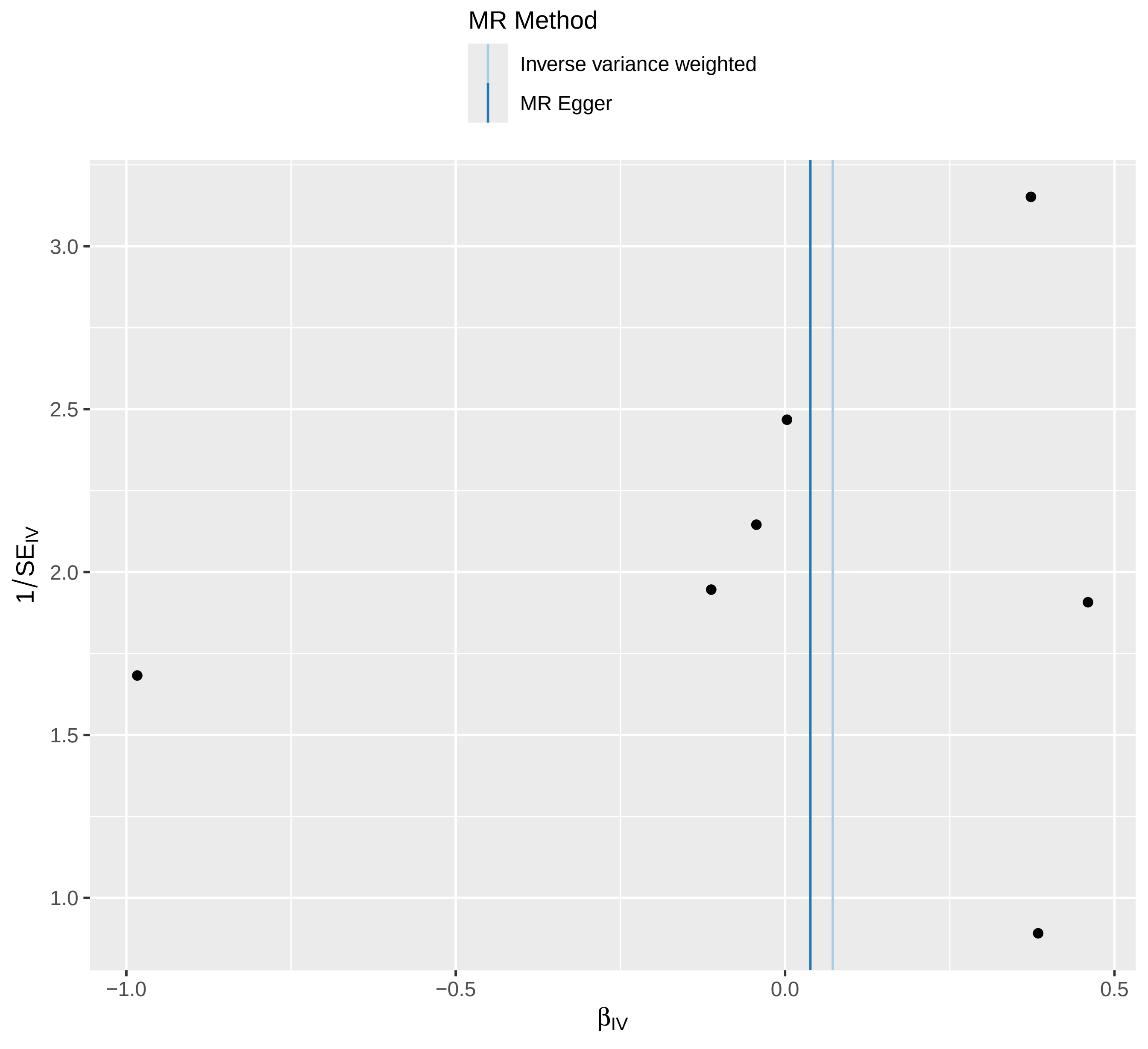
MR Method
Inverse variance weighted
MR Egger
3.0
2.5
2.0
1.5
1.0
1/SE_IV
−1.0
−0.5
0.0
0.5
β_IV

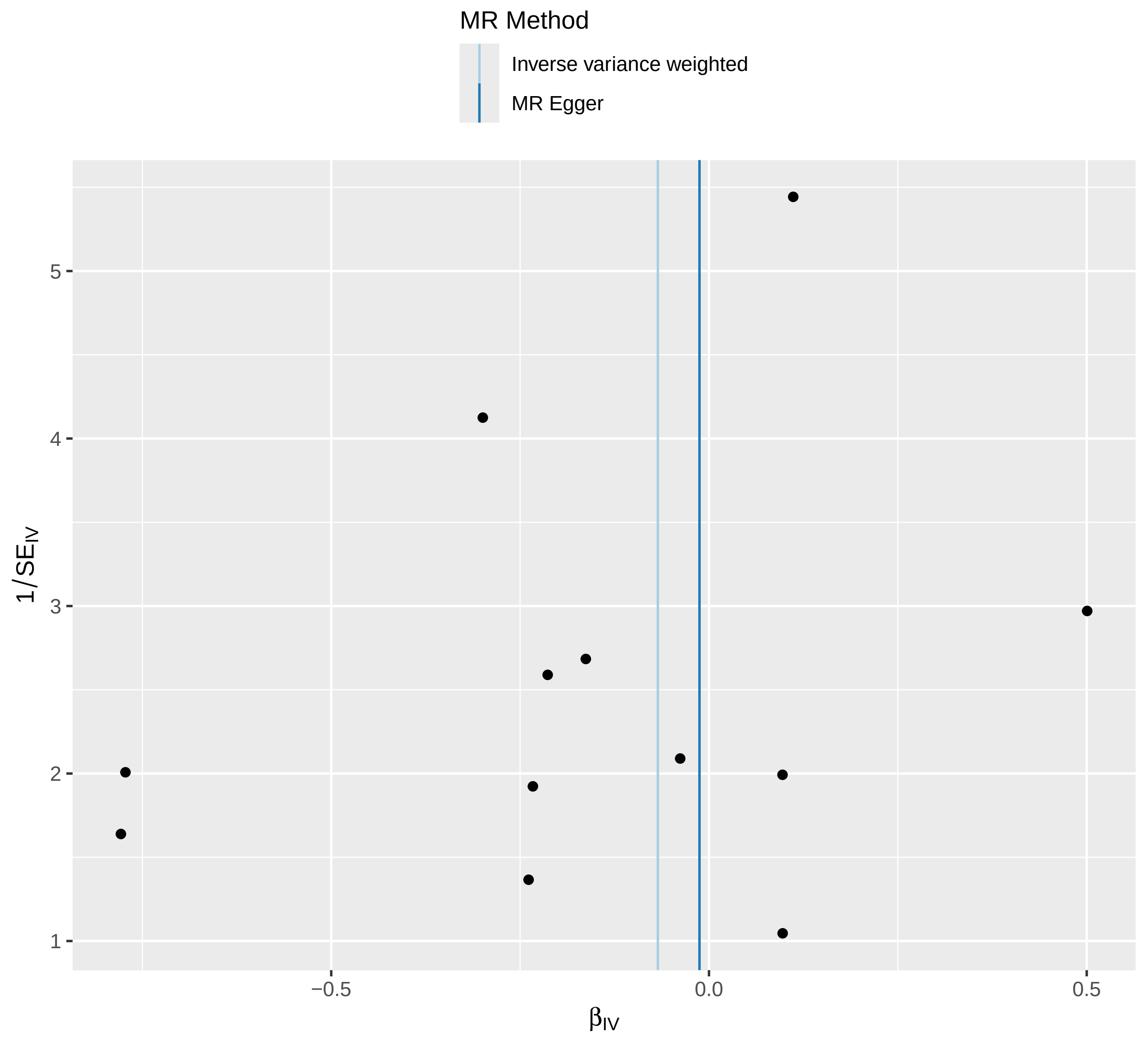

MR Method
Inverse variance weighted
MR Egger
1/SE$_{IV}$
5
4
3
2
1
−0.5
0.0
0.5
β$_{IV}$

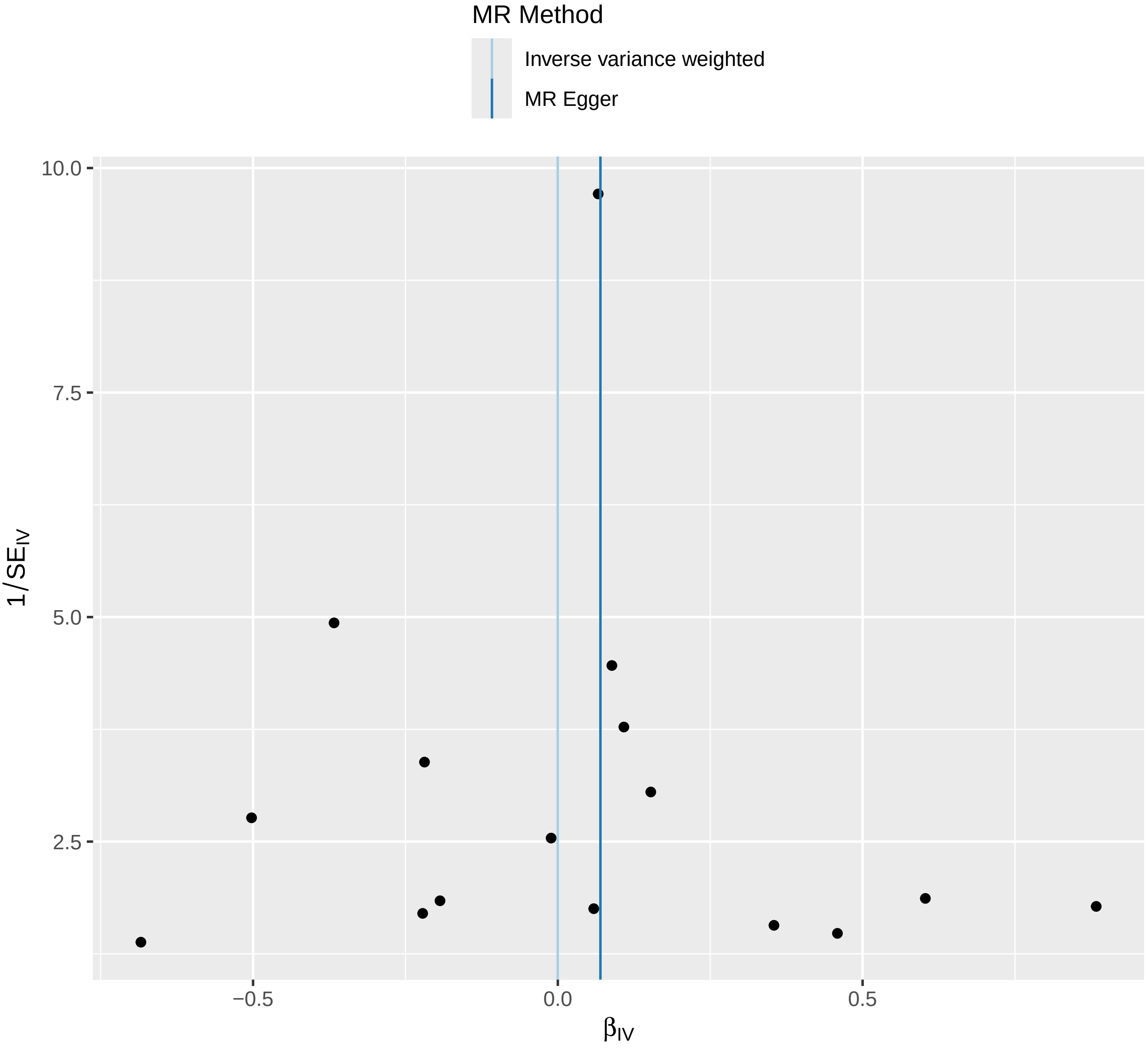

MR Method
Inverse variance weighted
MR Egger
10.0
7.5
5.0
2.5
$1/SE_{IV}$
−0.5
0.0
0.5
β_{IV}

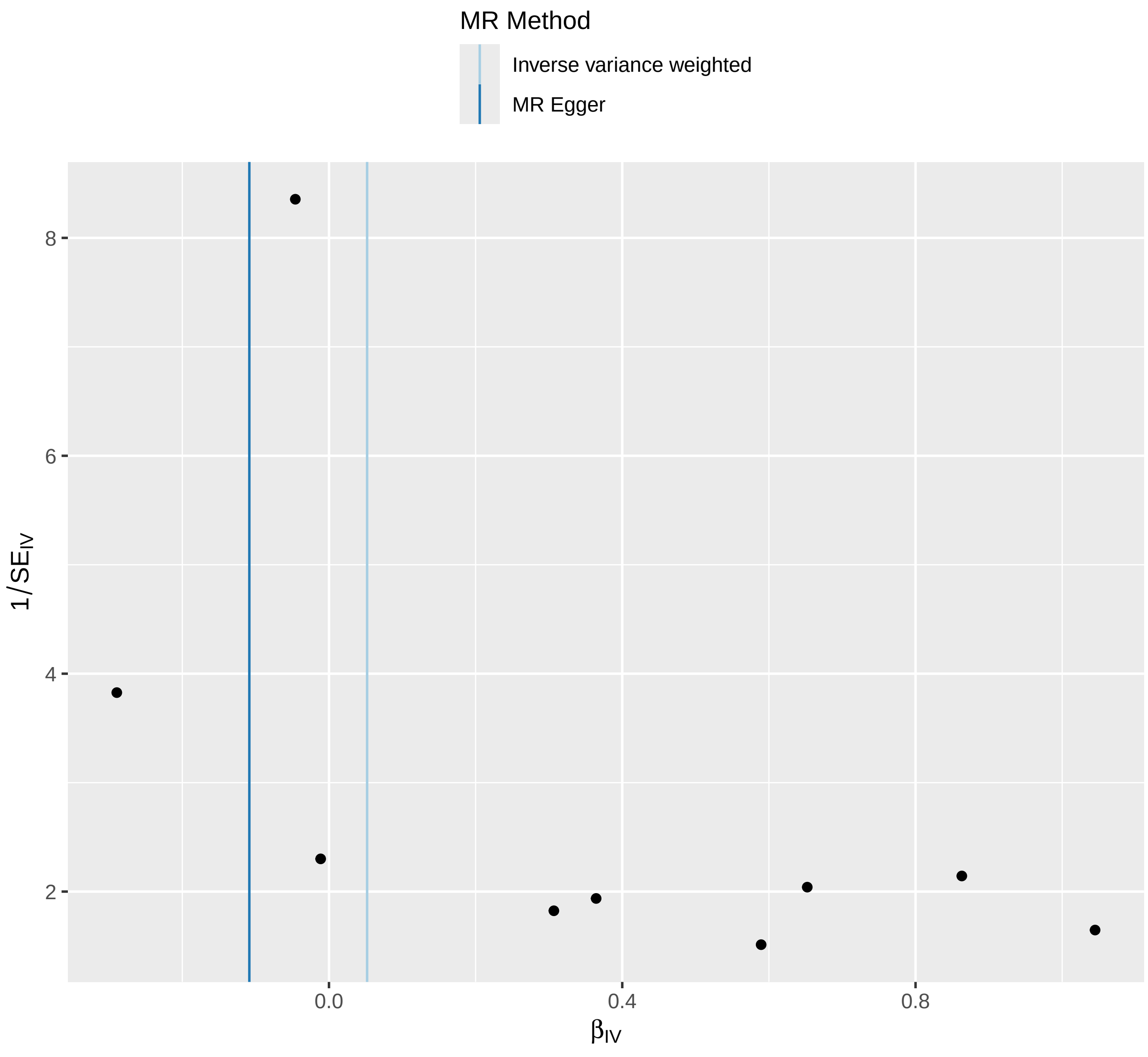

MR Method
Inverse variance weighted
MR Egger
8
6
4
2
$1/SE_{IV}$
0.0
0.4
0.8
β_{IV}

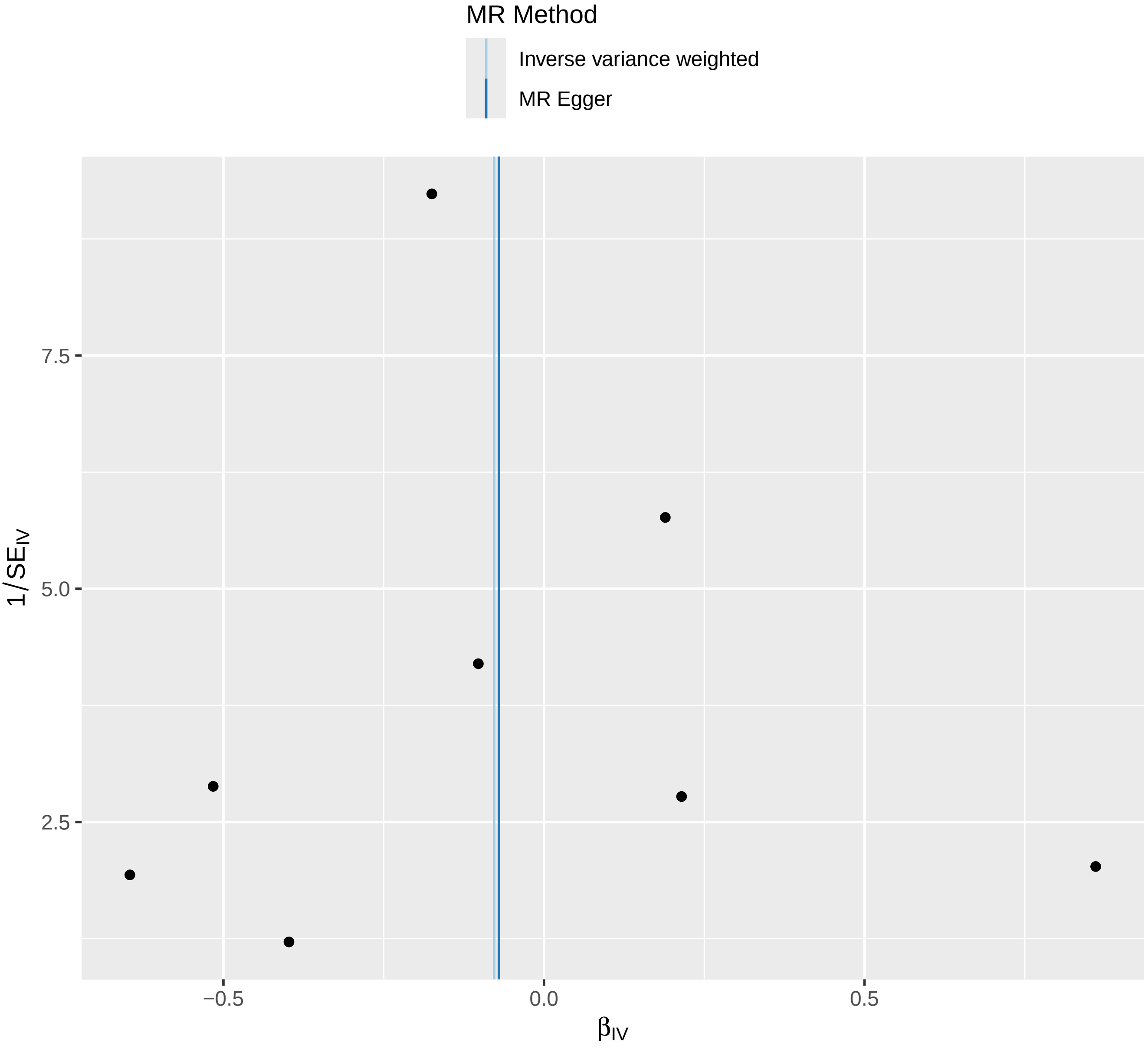
MR Method
Inverse variance weighted
MR Egger
7.5
5.0
2.5
1/SE$_{IV}$
−0.5
0.0
0.5
β$_{IV}$

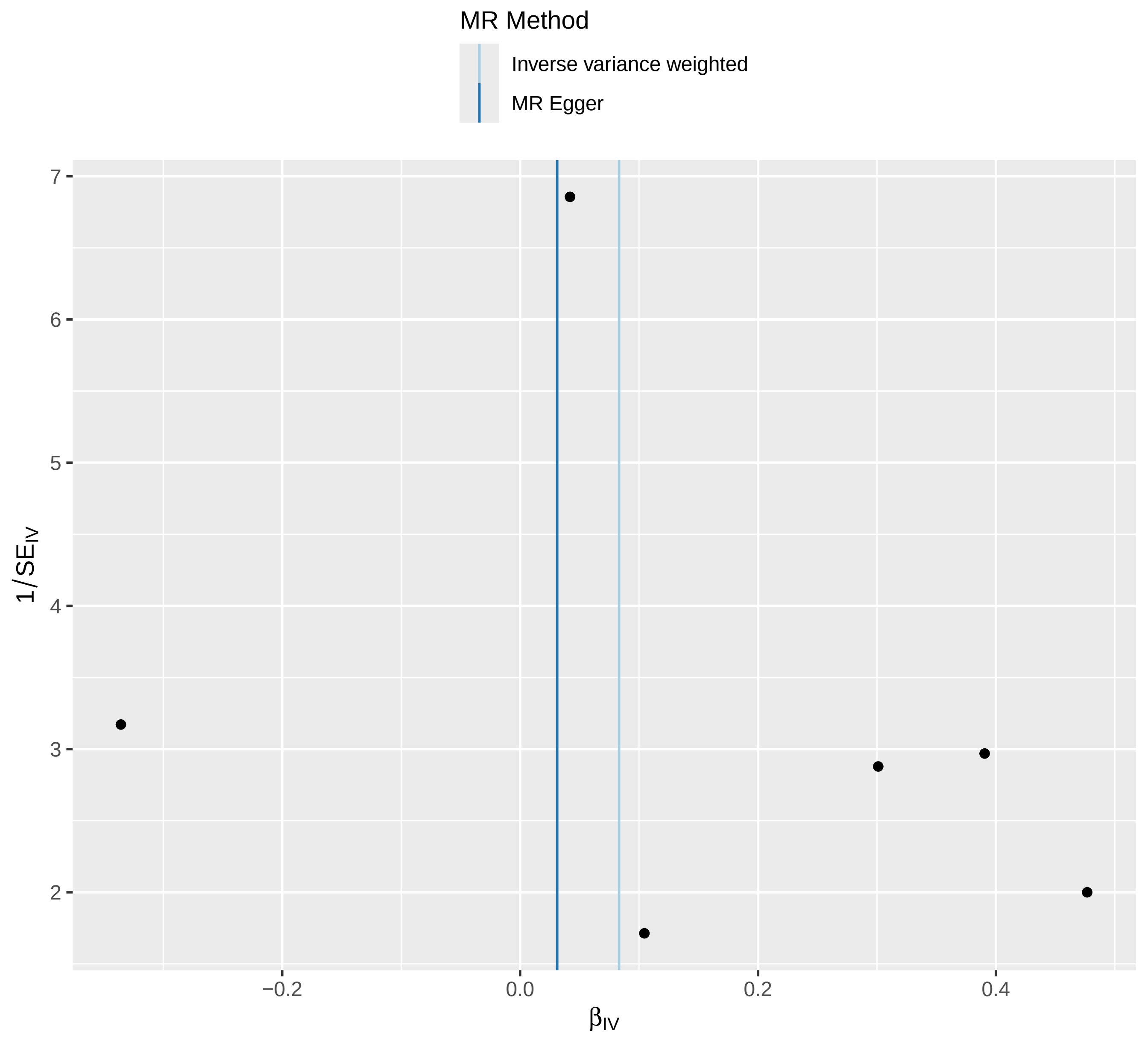
MR Method
Inverse variance weighted
MR Egger
1/SE_IV
β_IV
7
6
5
4
3
2
−0.2
0.0
0.2
0.4

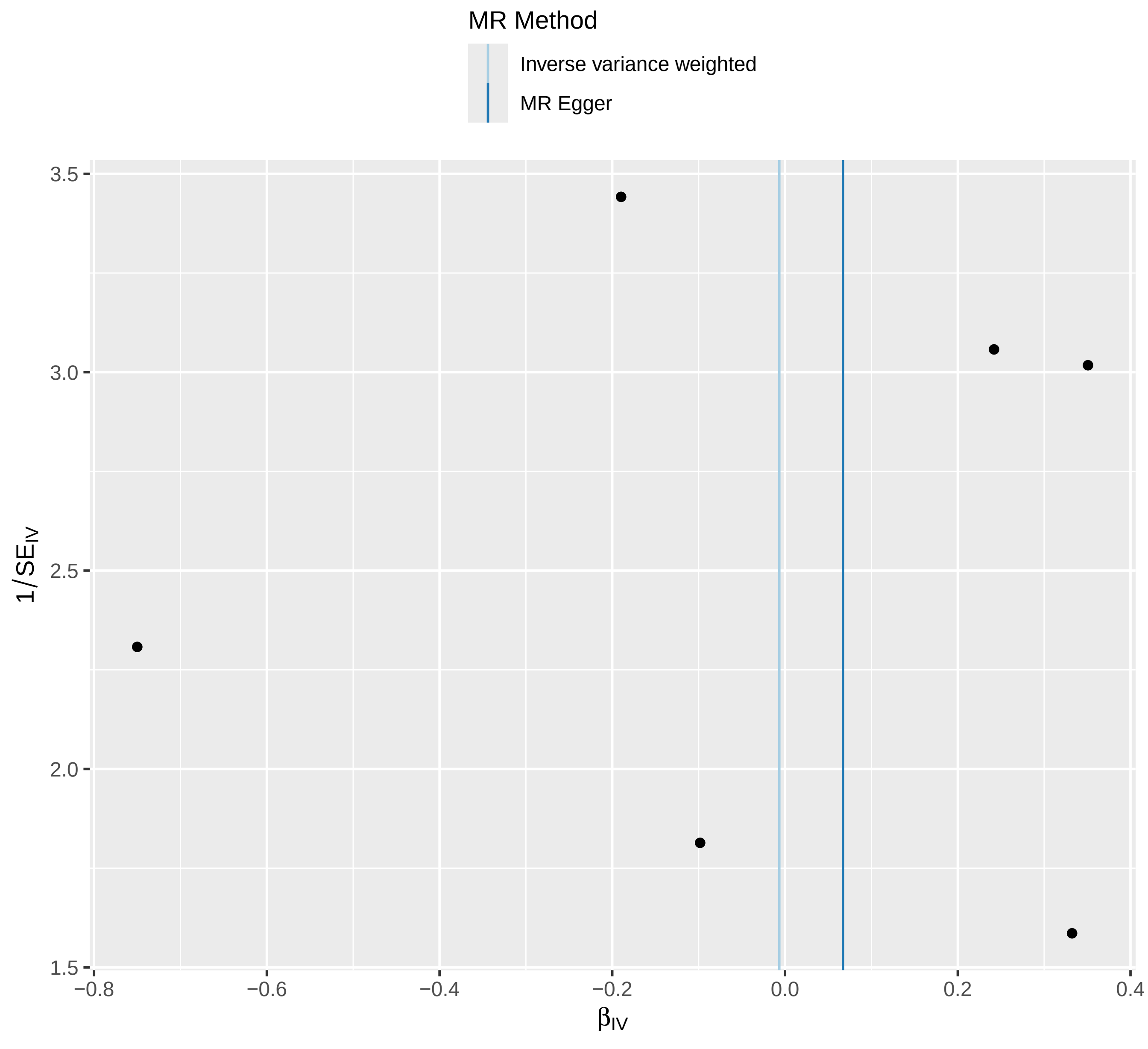

MR Method
Inverse variance weighted
MR Egger
1/SE_IV
3.5
3.0
2.5
2.0
1.5
−0.8
−0.6
−0.4
−0.2
0.0
0.2
0.4
β_IV

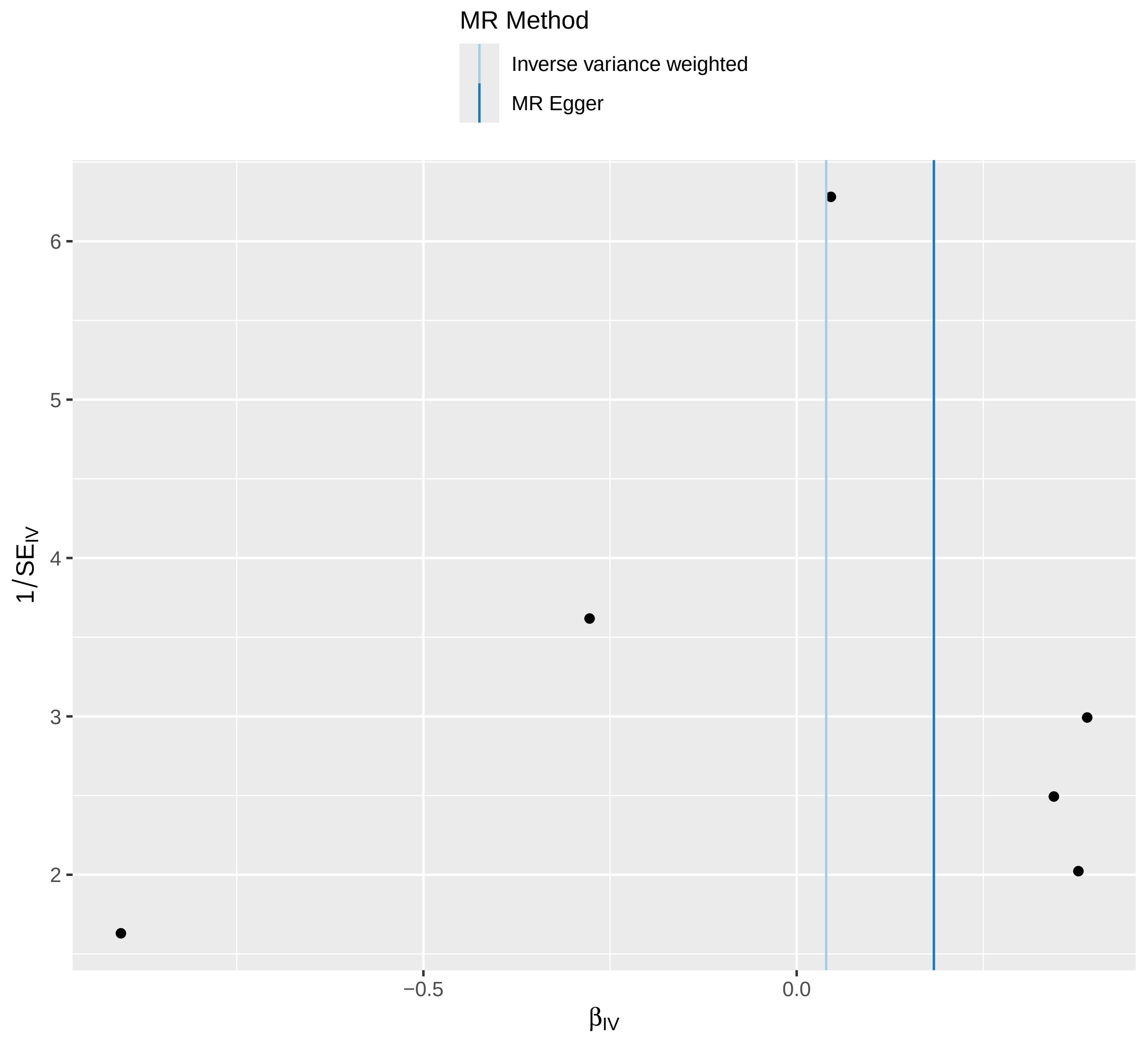

MR Method
Inverse variance weighted
MR Egger
1/SE_IV
6
5
4
3
2
−0.5
0.0
β_IV

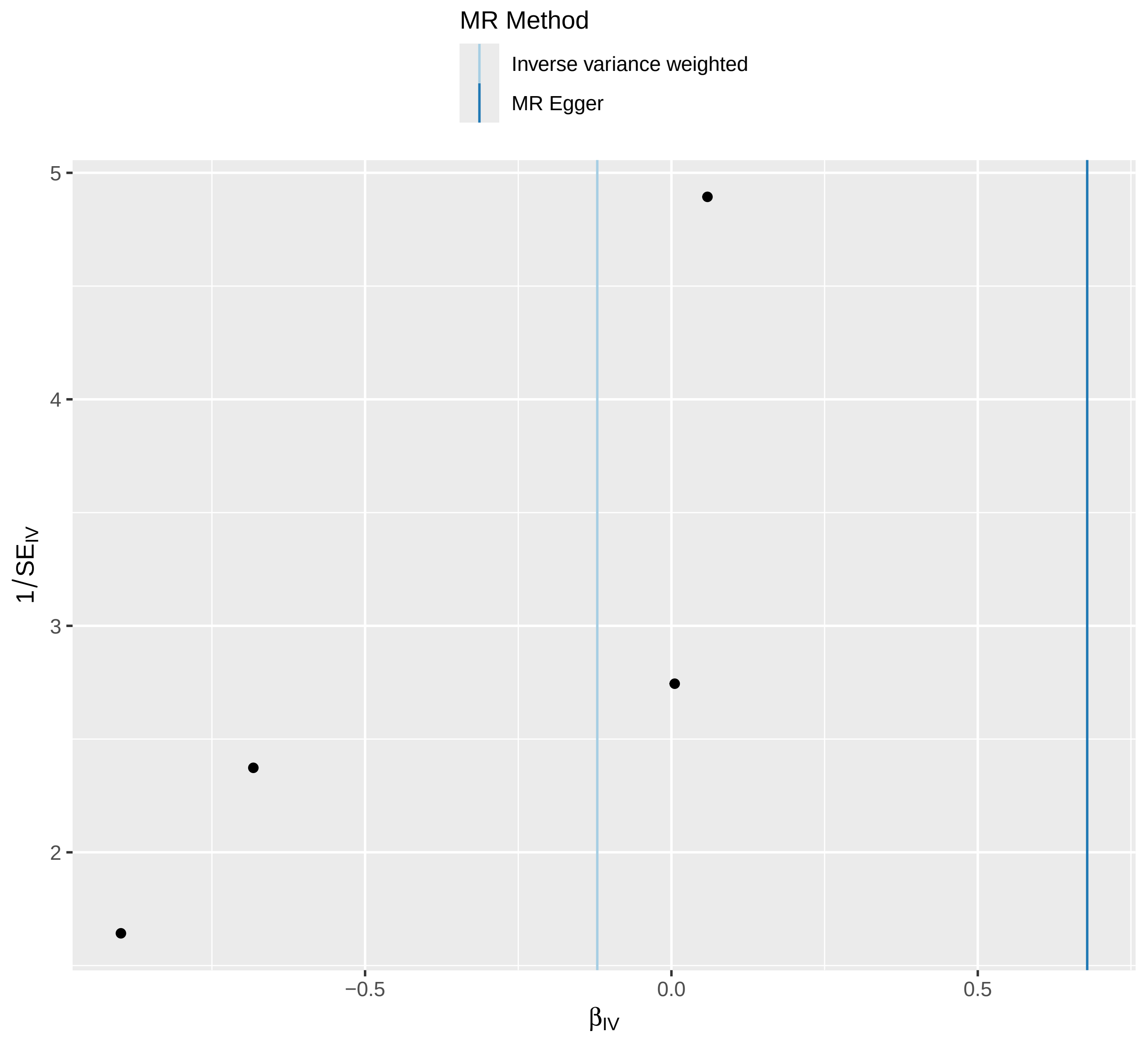
MR Method
Inverse variance weighted
MR Egger
1/SE$_{IV}$
5
4
3
2
−0.5
0.0
0.5
β$_{IV}$

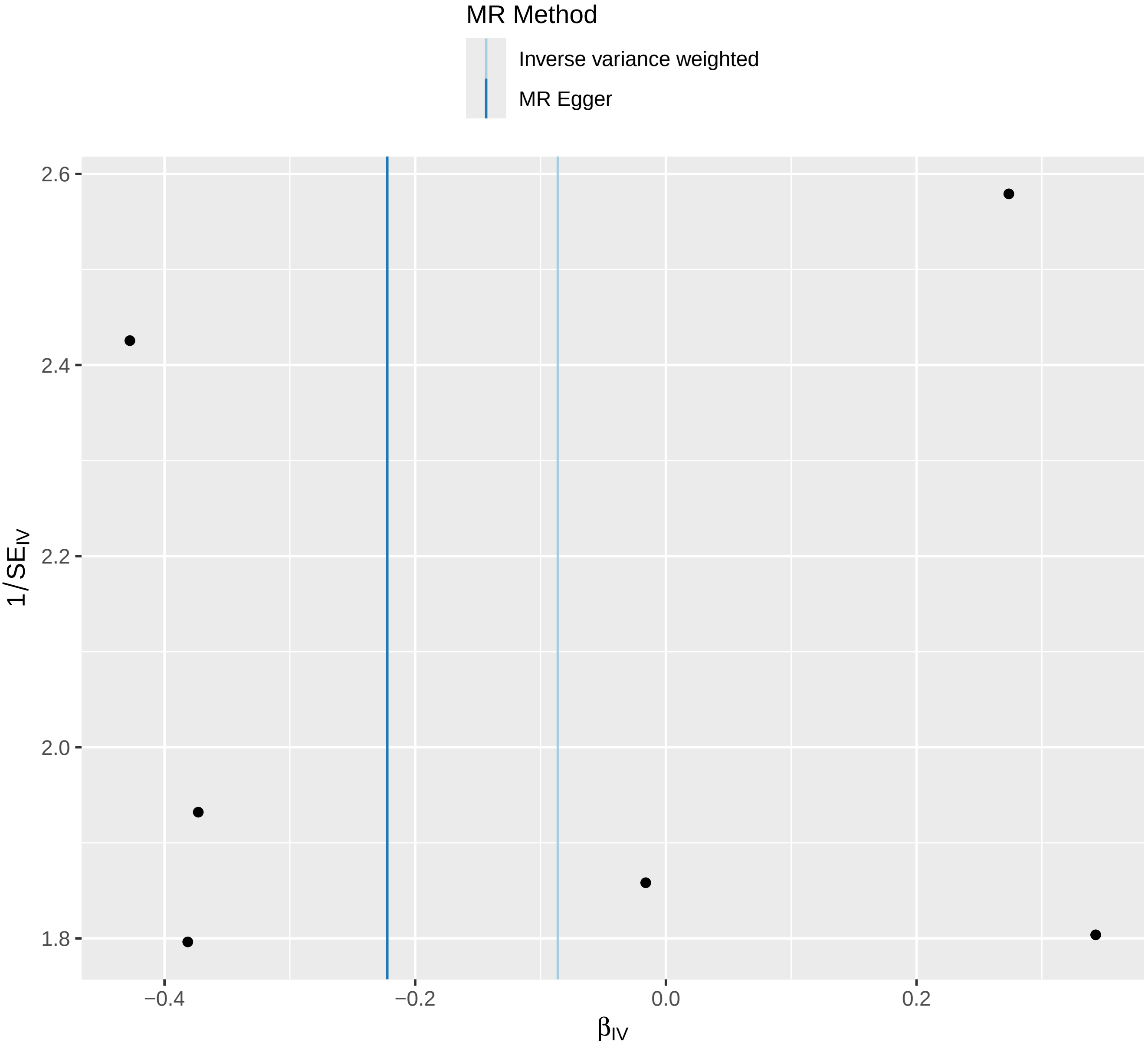

MR Method
Inverse variance weighted
MR Egger
2.6
2.4
2.2
2.0
1.8
$1/SE_{IV}$
−0.4
−0.2
0.0
0.2
β_{IV}

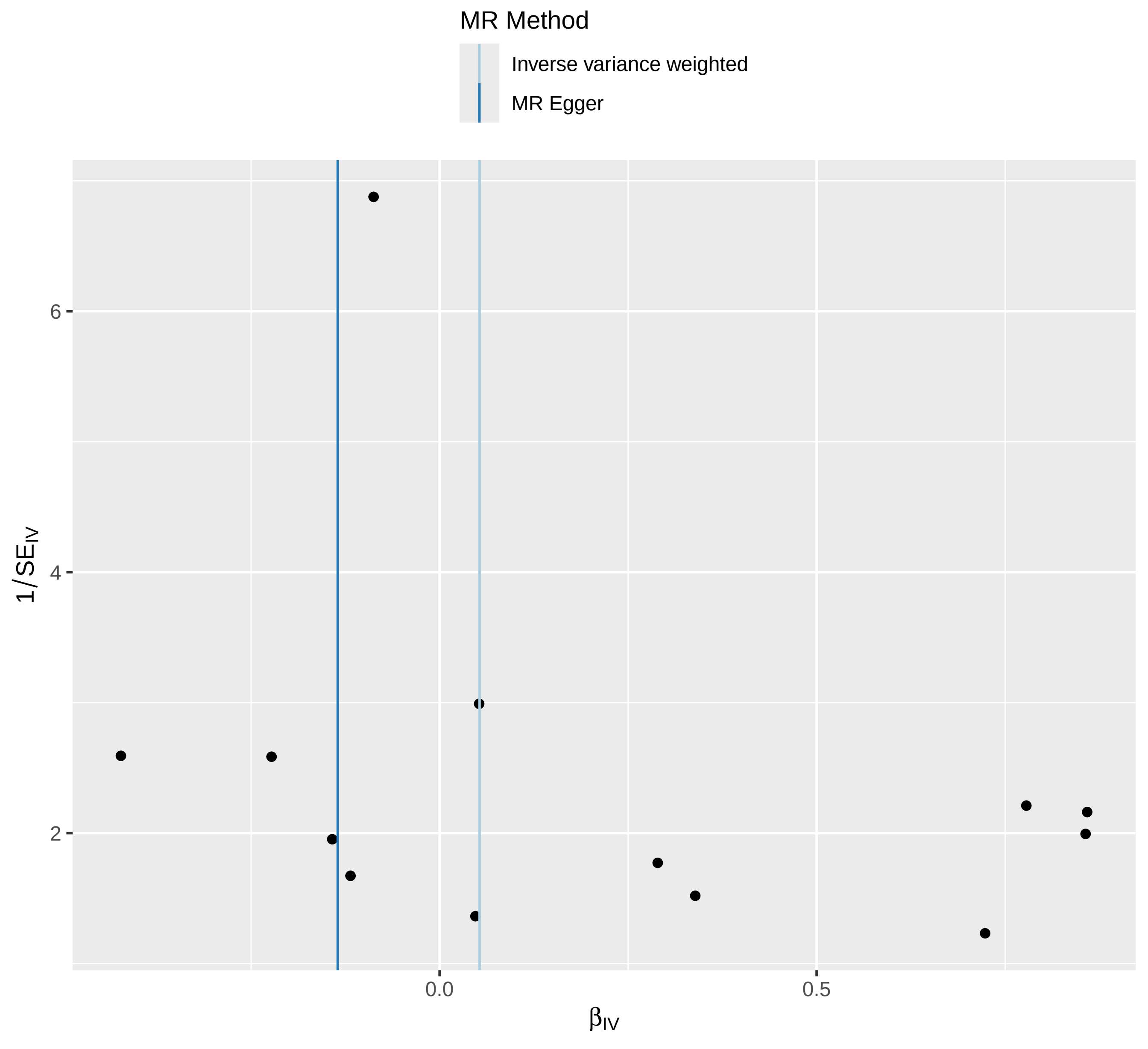
MR Method
Inverse variance weighted
MR Egger
6
4
2
1/SE$_{IV}$
0.0
0.5
β$_{IV}$

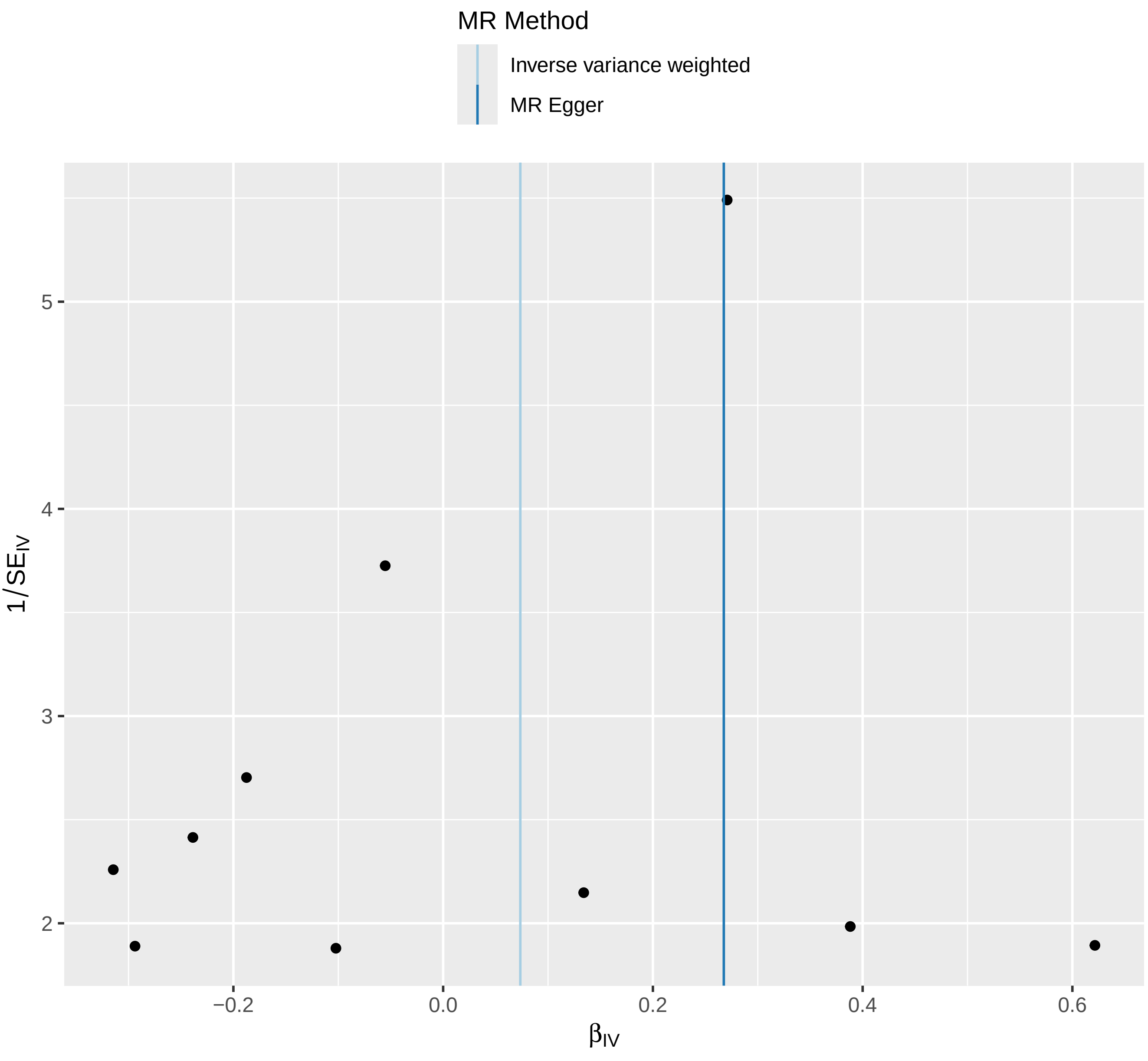

MR Method
Inverse variance weighted
MR Egger
5
4
3
2
1/SE$_{IV}$
−0.2
0.0
0.2
0.4
0.6
β$_{IV}$

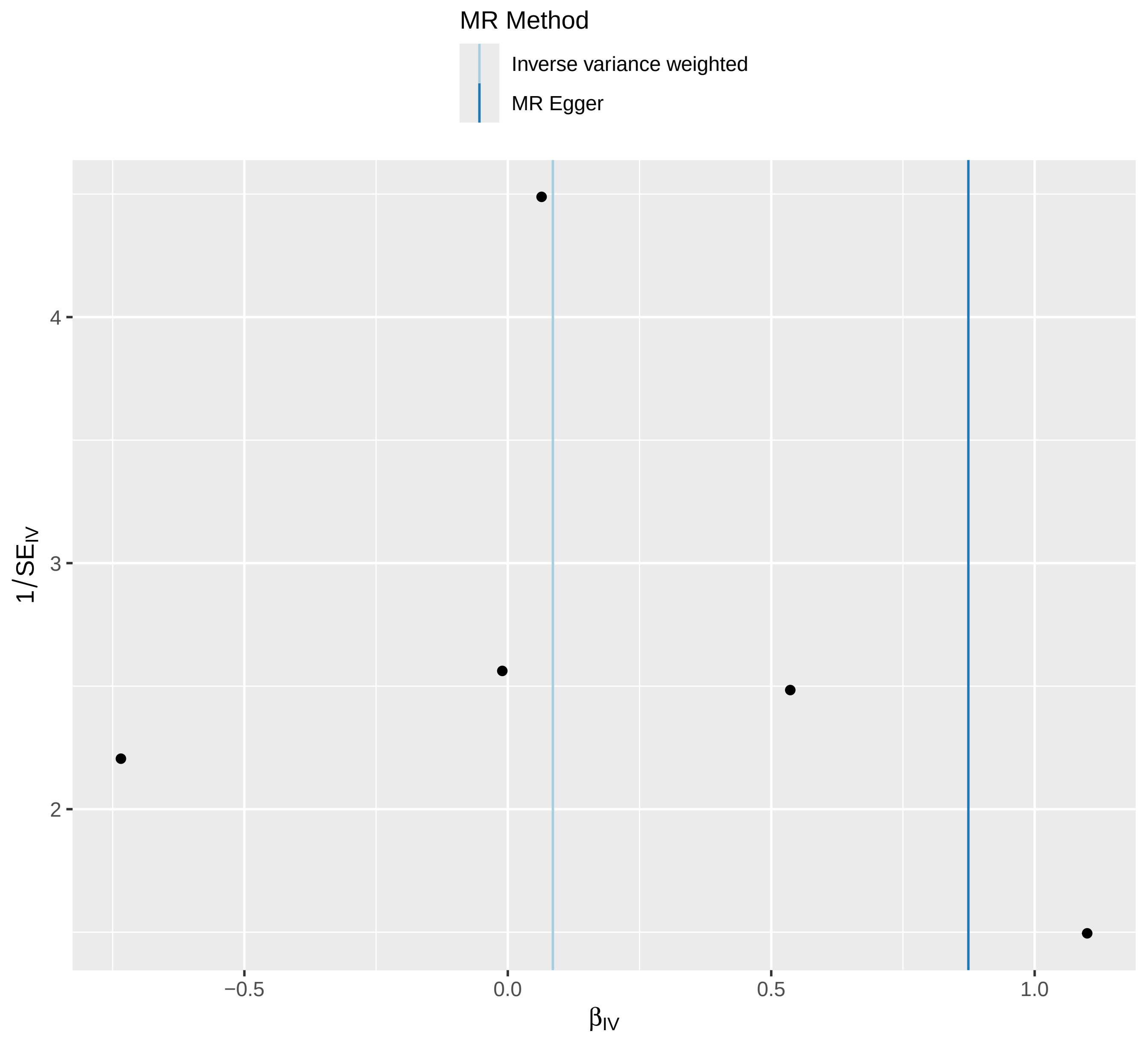
MR Method
Inverse variance weighted
MR Egger
4
3
2
1/SE$_{IV}$
−0.5
0.0
0.5
1.0
β$_{IV}$

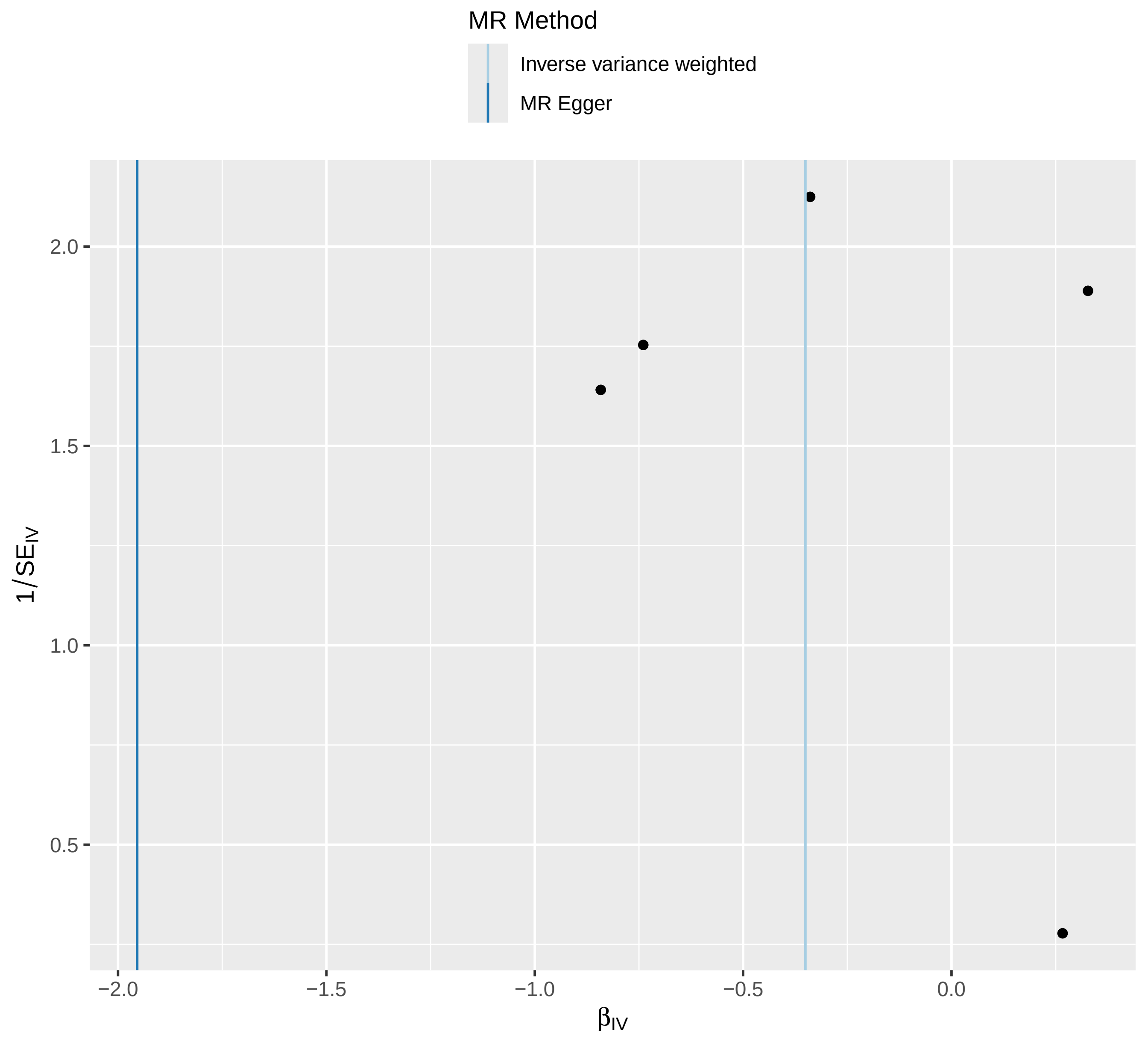

MR Method
Inverse variance weighted
MR Egger
2.0
1.5
1.0
0.5
−2.0
−1.5
−1.0
−0.5
0.0
$1/SE_{IV}$
β_{IV}

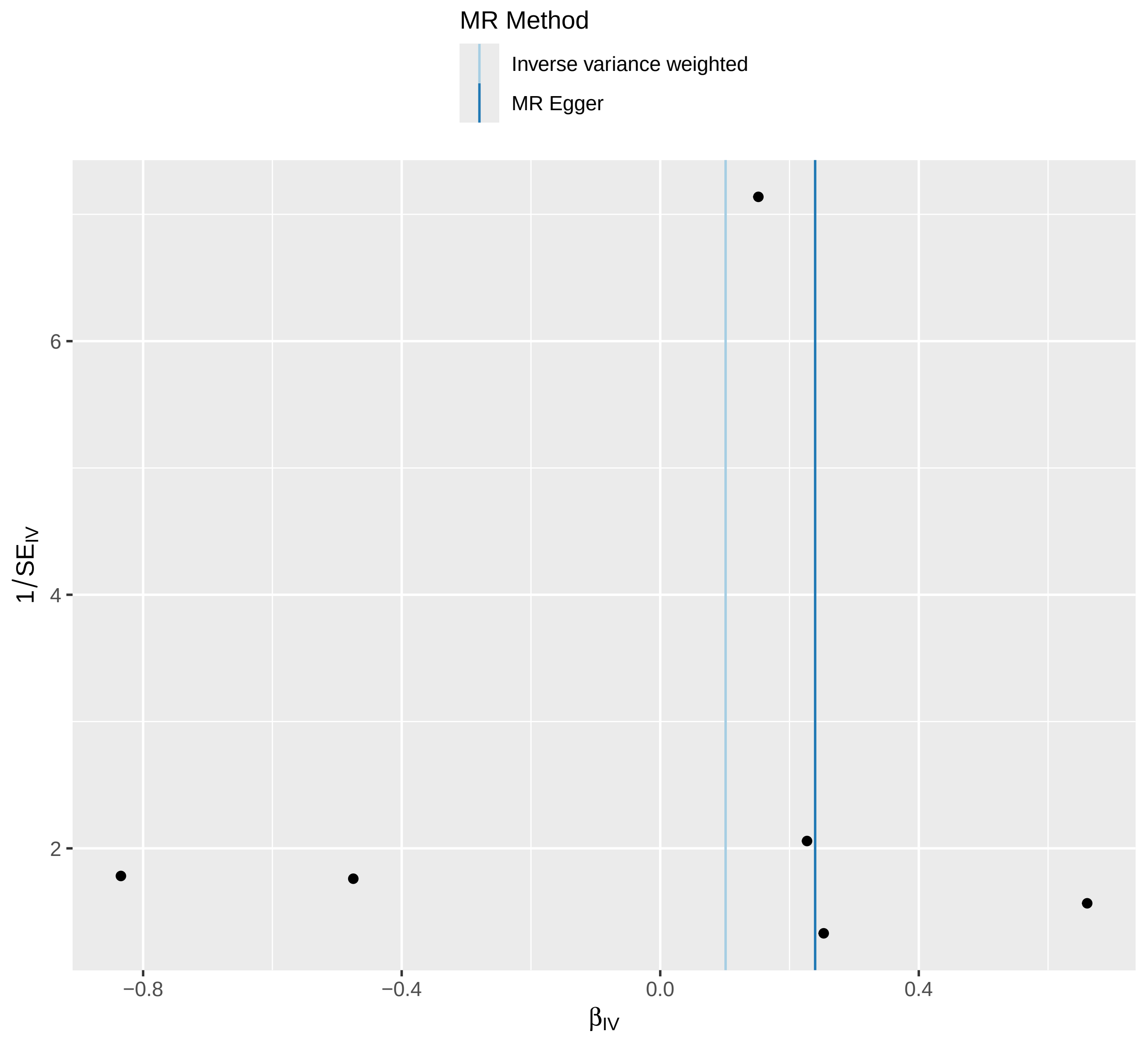

MR Method
Inverse variance weighted
MR Egger
1/SE_IV
6
4
2
−0.8
−0.4
0.0
0.4
β_IV

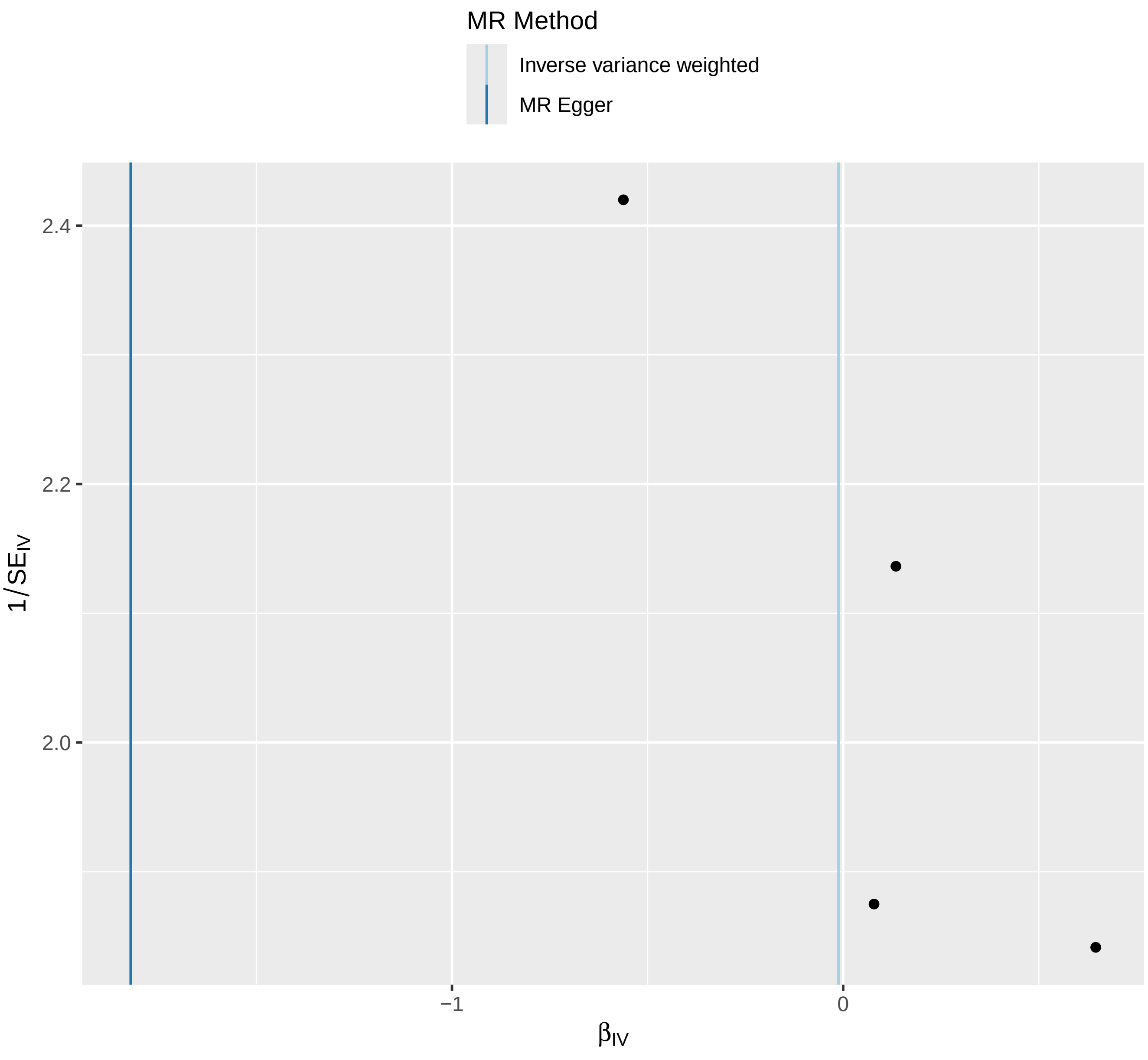

MR Method
Inverse variance weighted
MR Egger
2.4
2.2
2.0
$1/SE_{IV}$
−1
0
β_{IV}

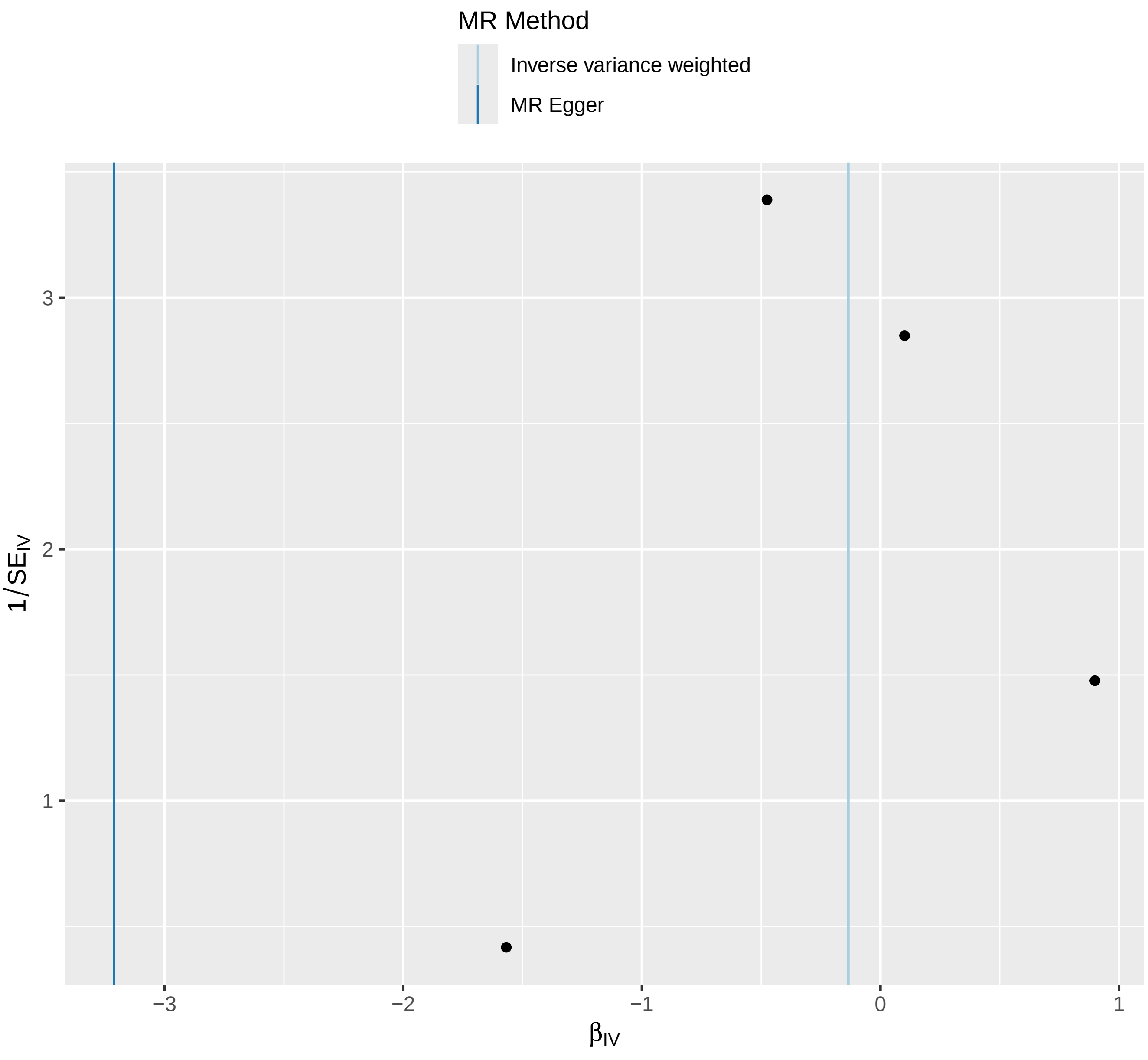
MR Method
Inverse variance weighted
MR Egger
1/SE$_{IV}$
3
2
1
−3
−2
−1
0
1
β$_{IV}$

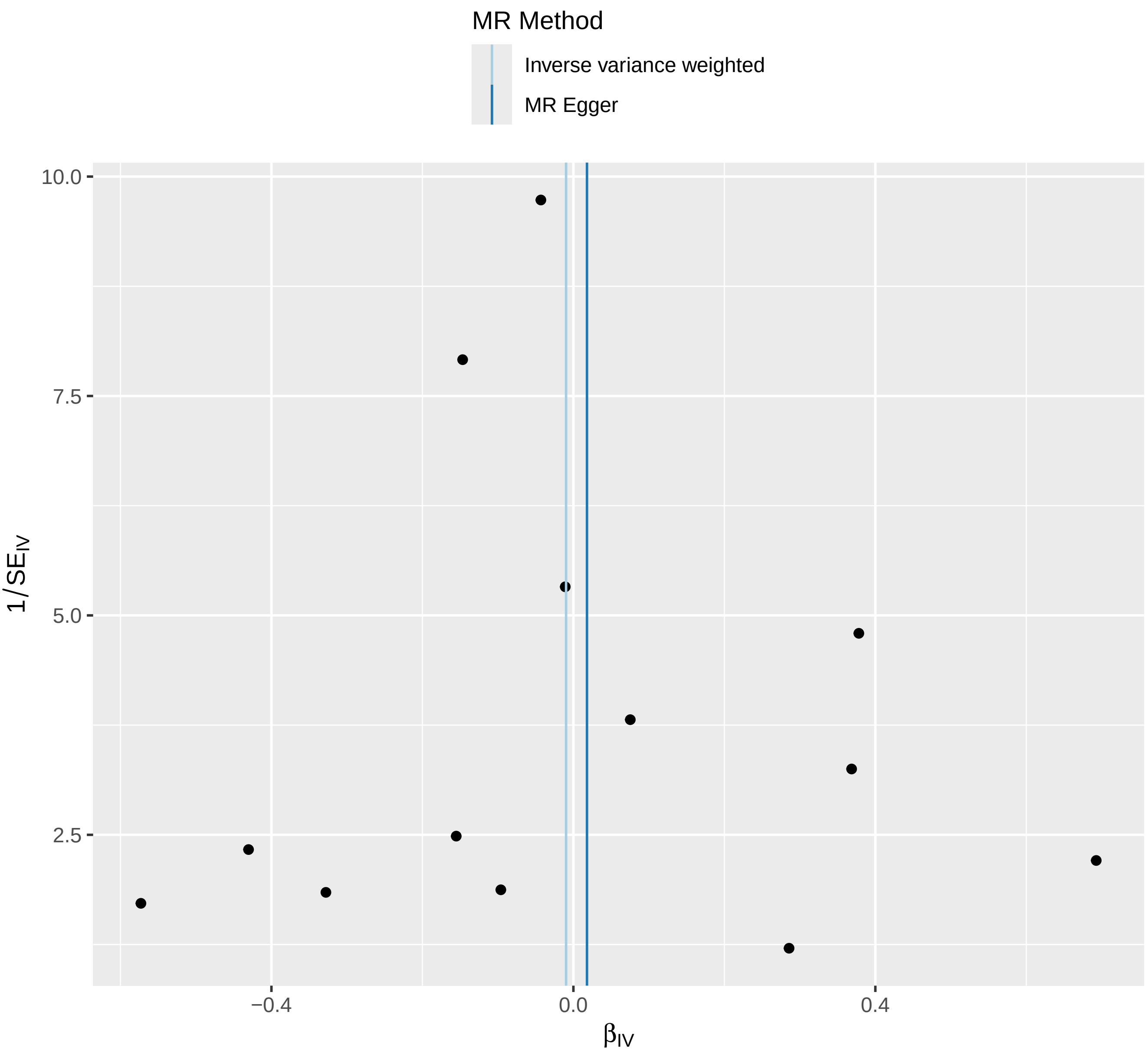
MR Method
Inverse variance weighted
MR Egger
10.0
7.5
5.0
2.5
1/SE_IV
−0.4
0.0
0.4
β_IV

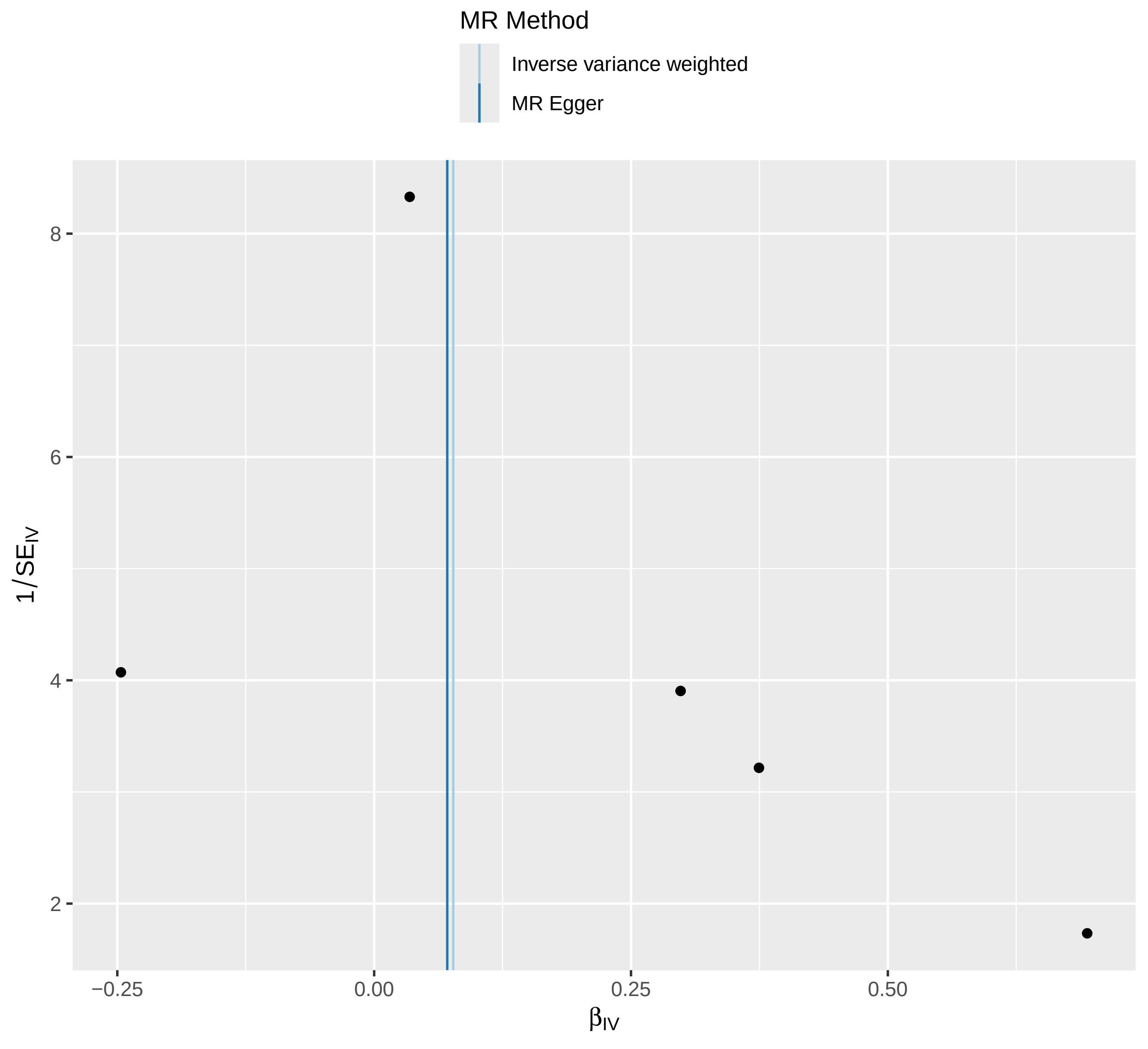
MR Method
Inverse variance weighted
MR Egger
8
6
4
2
1/SE$_{IV}$
−0.25
0.00
0.25
0.50
β$_{IV}$

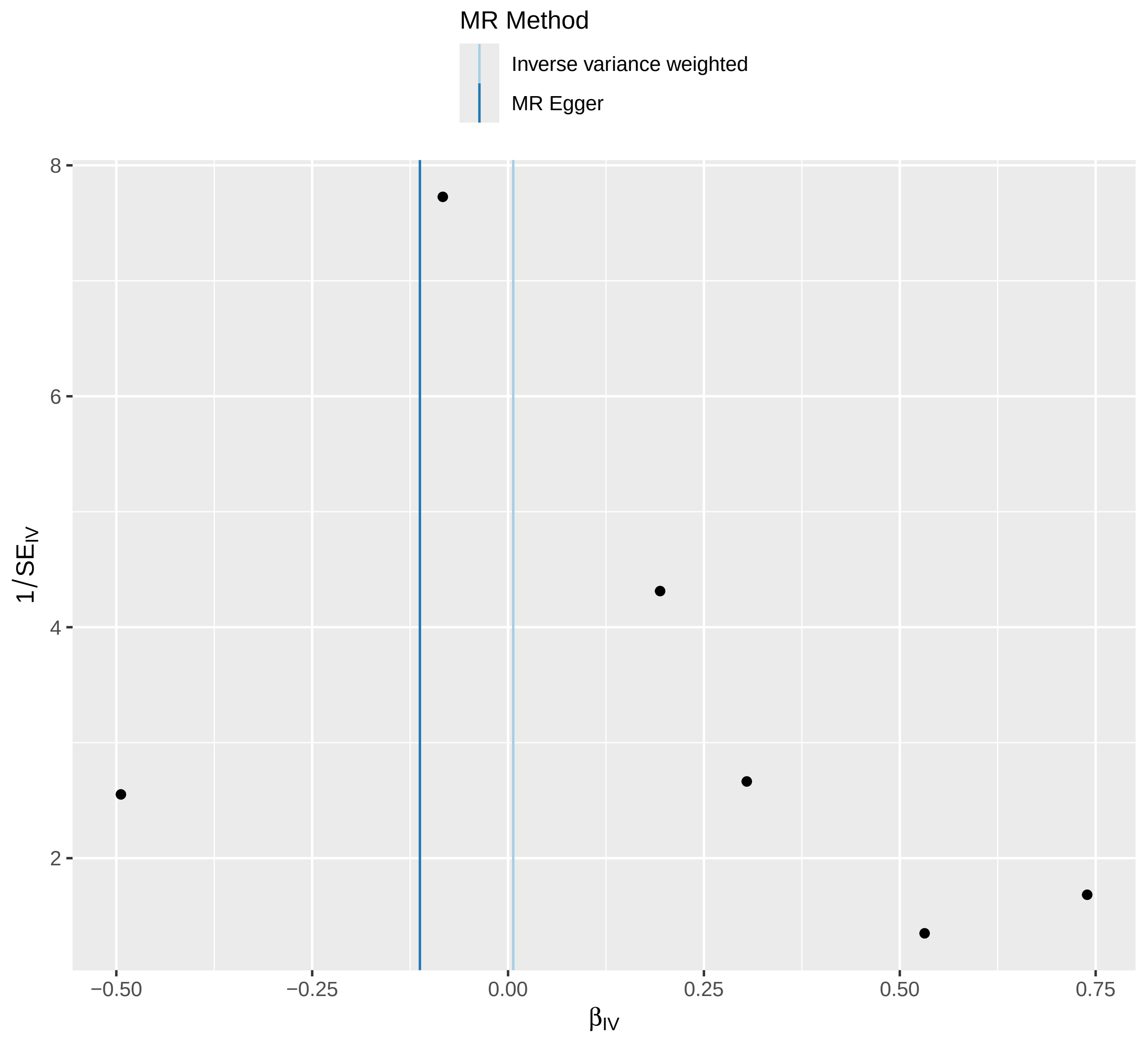
MR Method
Inverse variance weighted
MR Egger
8
6
4
2
1/SE_IV
−0.50
−0.25
0.00
0.25
0.50
0.75
β_IV

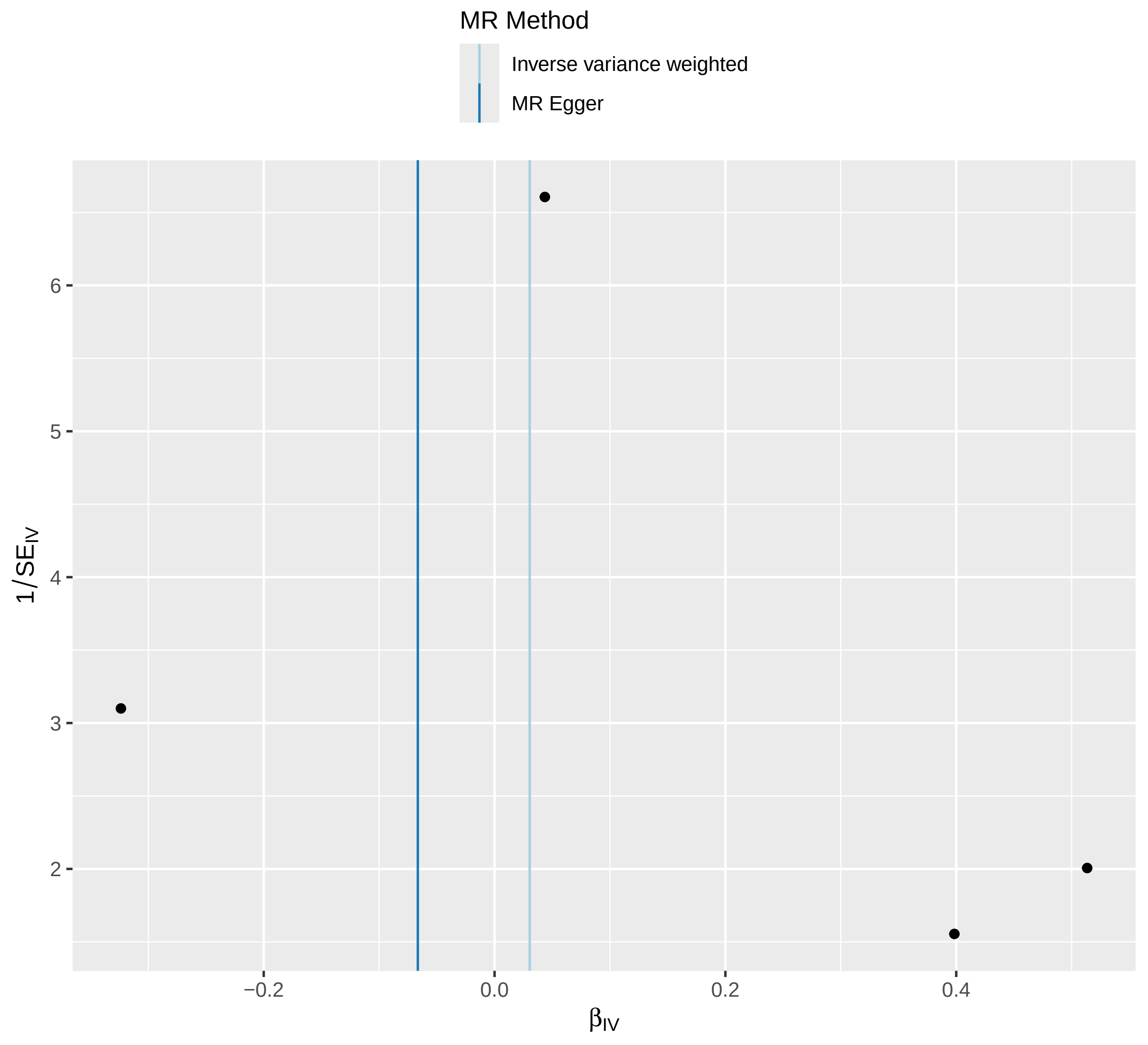
MR Method
Inverse variance weighted
MR Egger
6
5
4
3
2
1/SE$_{IV}$
−0.2
0.0
0.2
0.4
β$_{IV}$

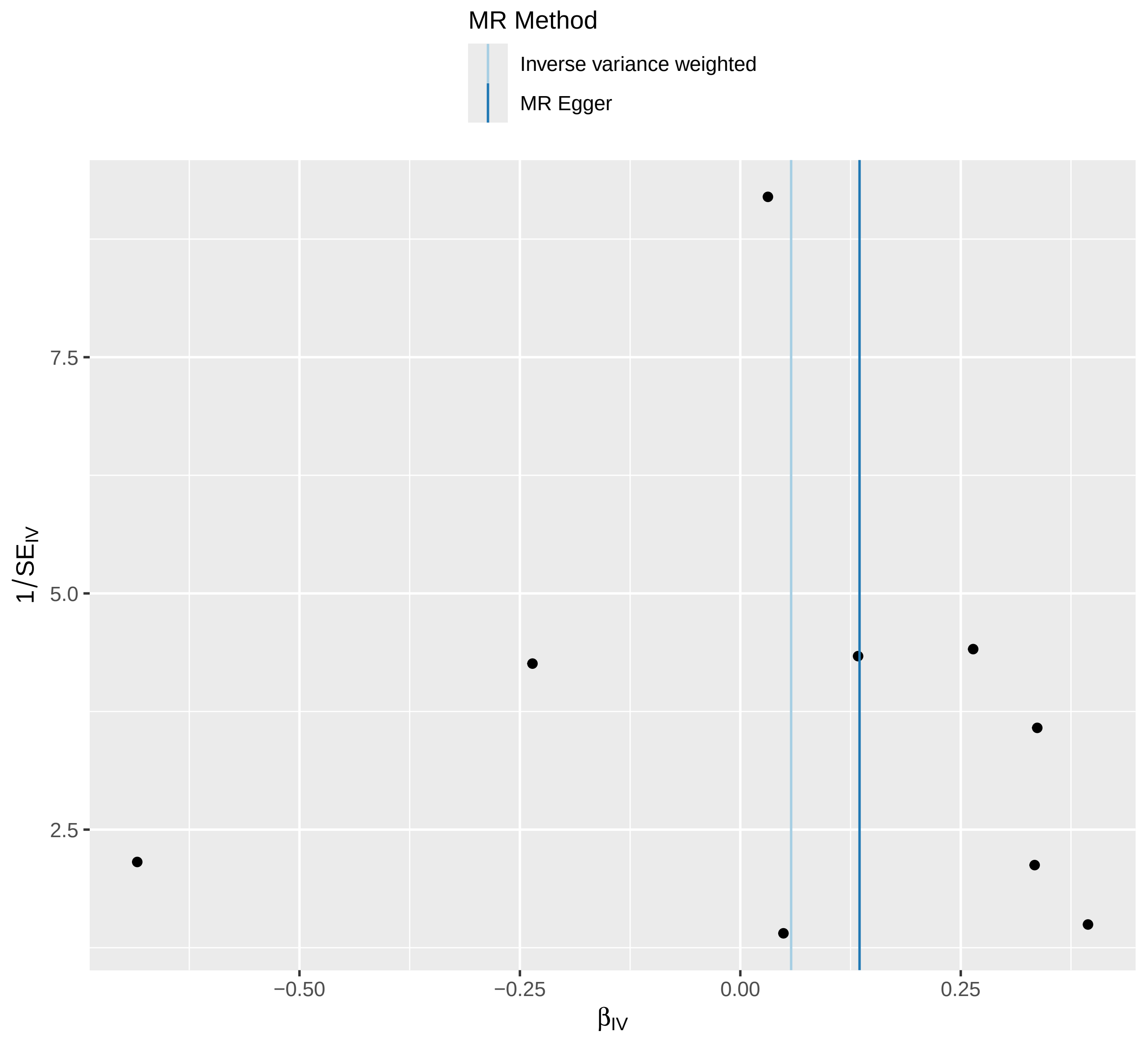
MR Method
Inverse variance weighted
MR Egger
7.5
5.0
2.5
1/SE_IV
−0.50
−0.25
0.00
0.25
β_IV

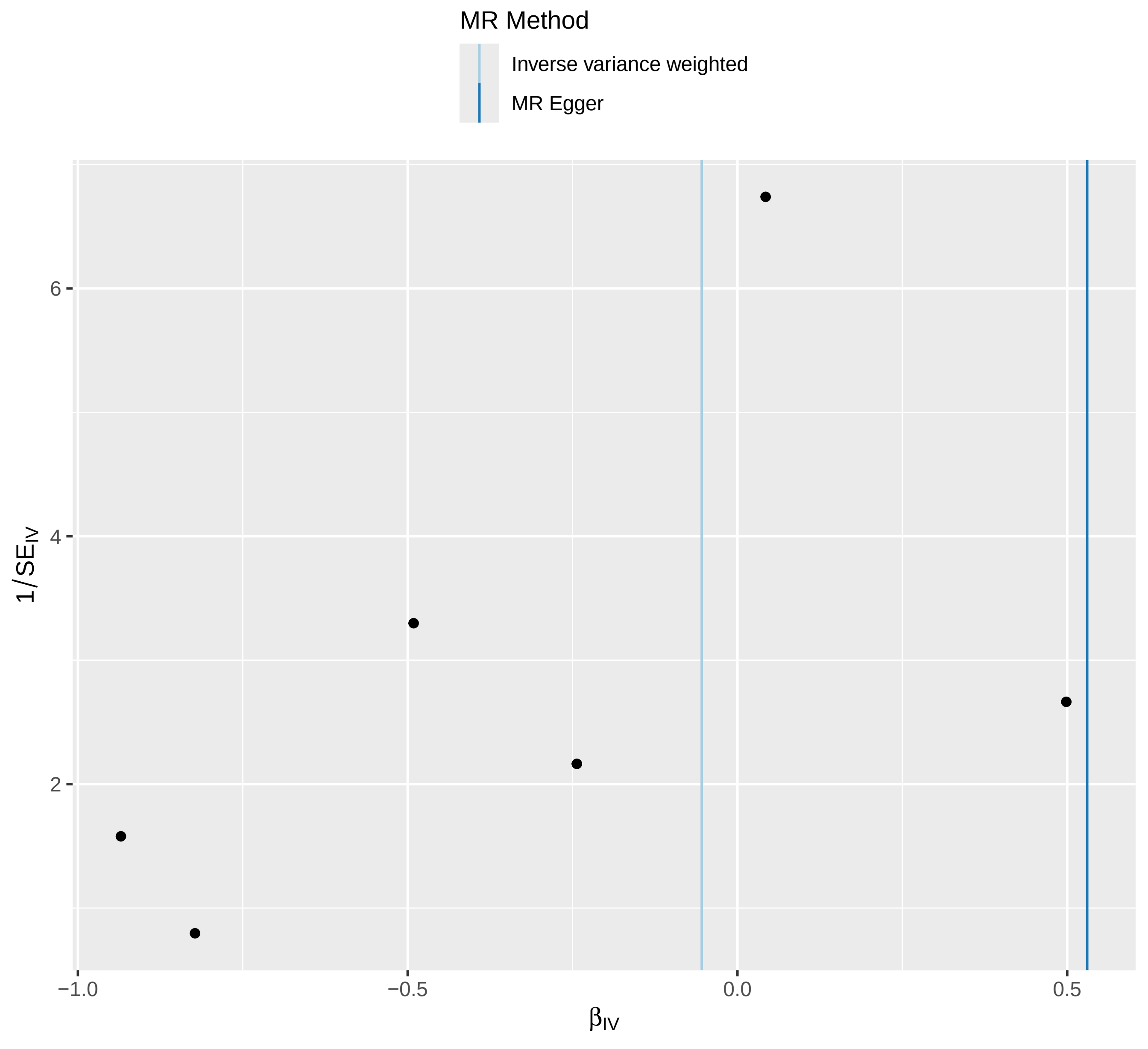

MR Method
Inverse variance weighted
MR Egger
6
4
2
1/SE$_{IV}$
−1.0
−0.5
0.0
0.5
β$_{IV}$

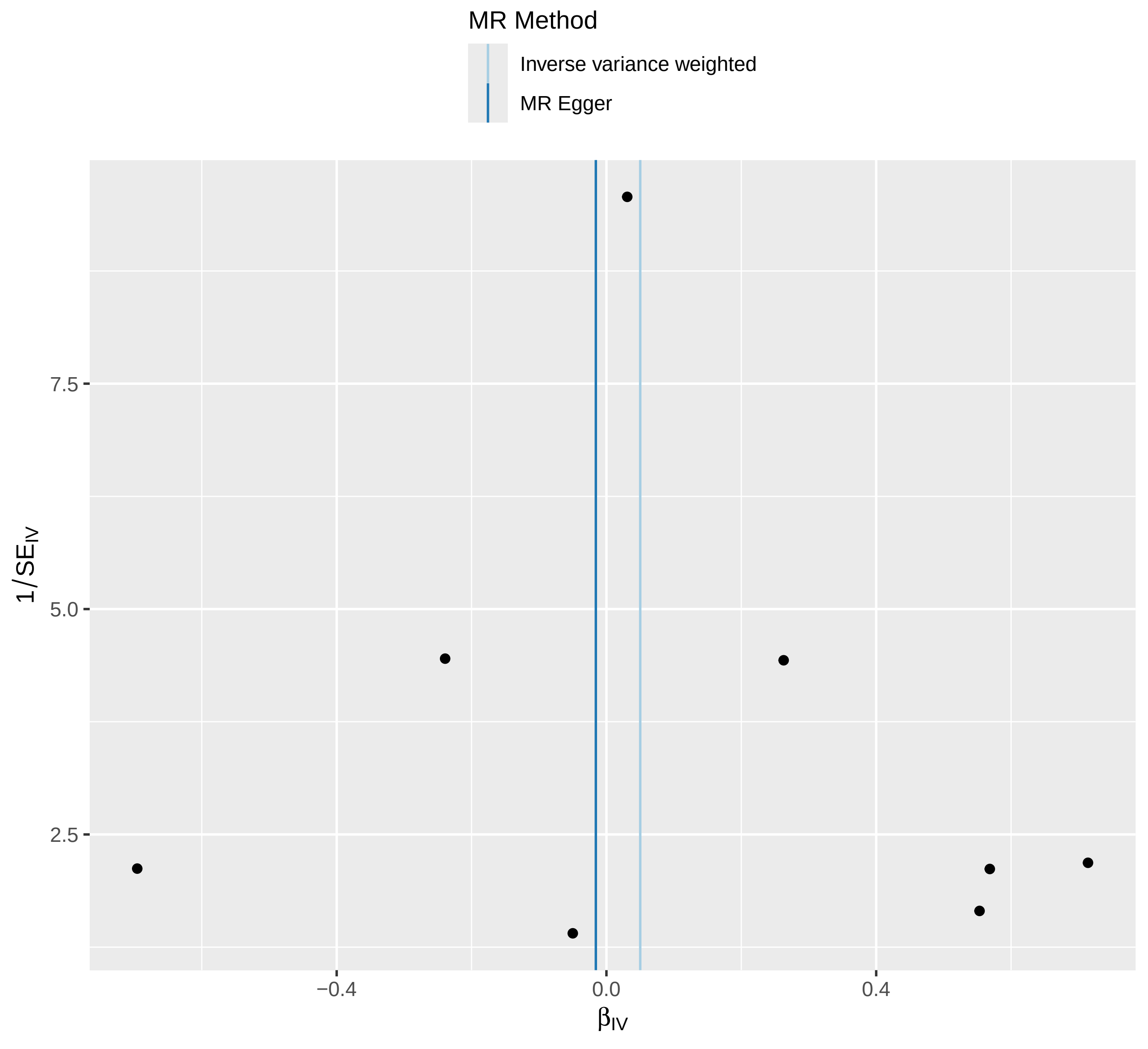

MR Method
Inverse variance weighted
MR Egger
7.5
5.0
2.5
$1/SE_{IV}$
−0.4
0.0
0.4
β_{IV}

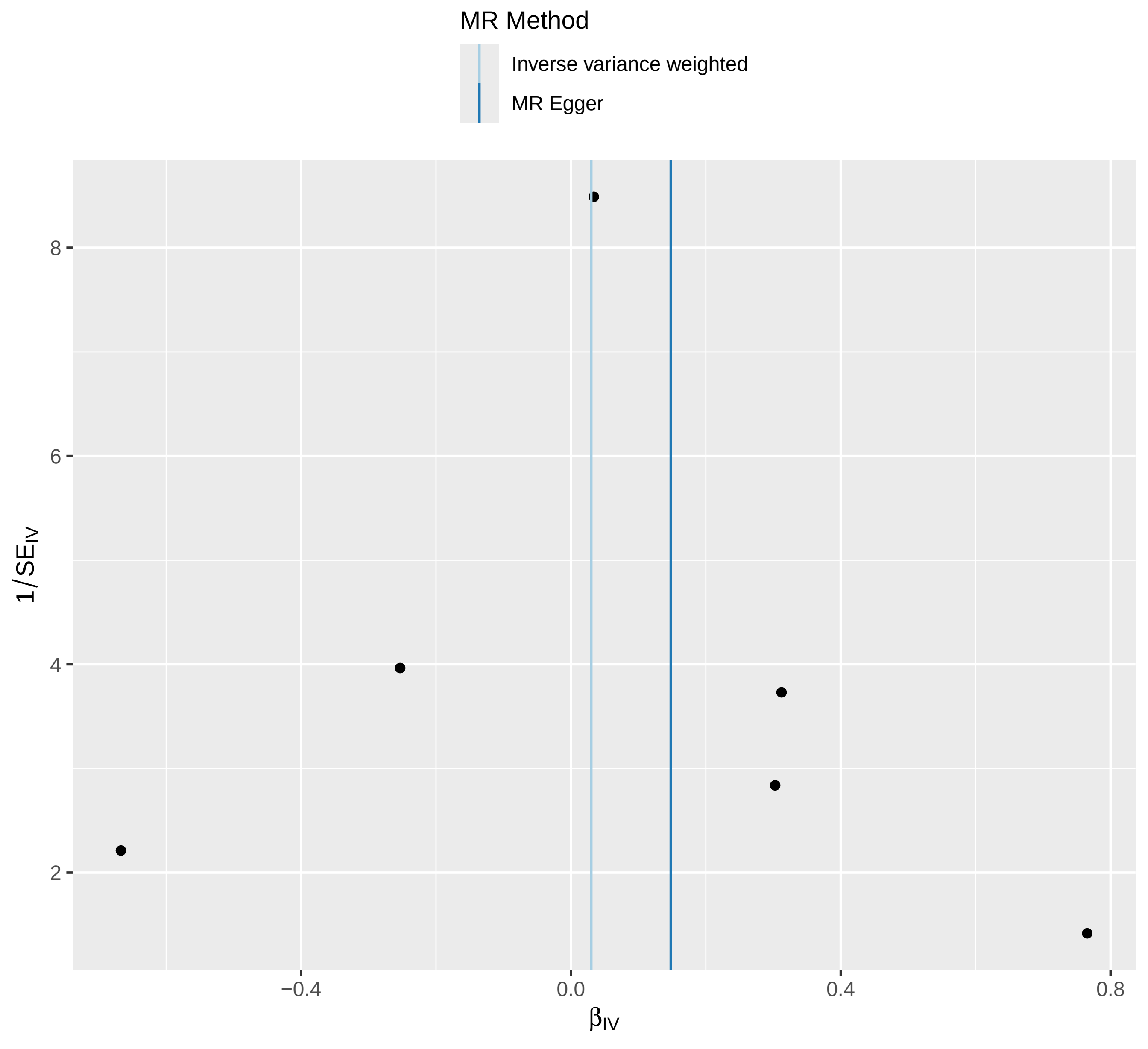

MR Method
Inverse variance weighted
MR Egger
8
6
4
2
1/SE_IV
−0.4
0.0
0.4
0.8
β_IV

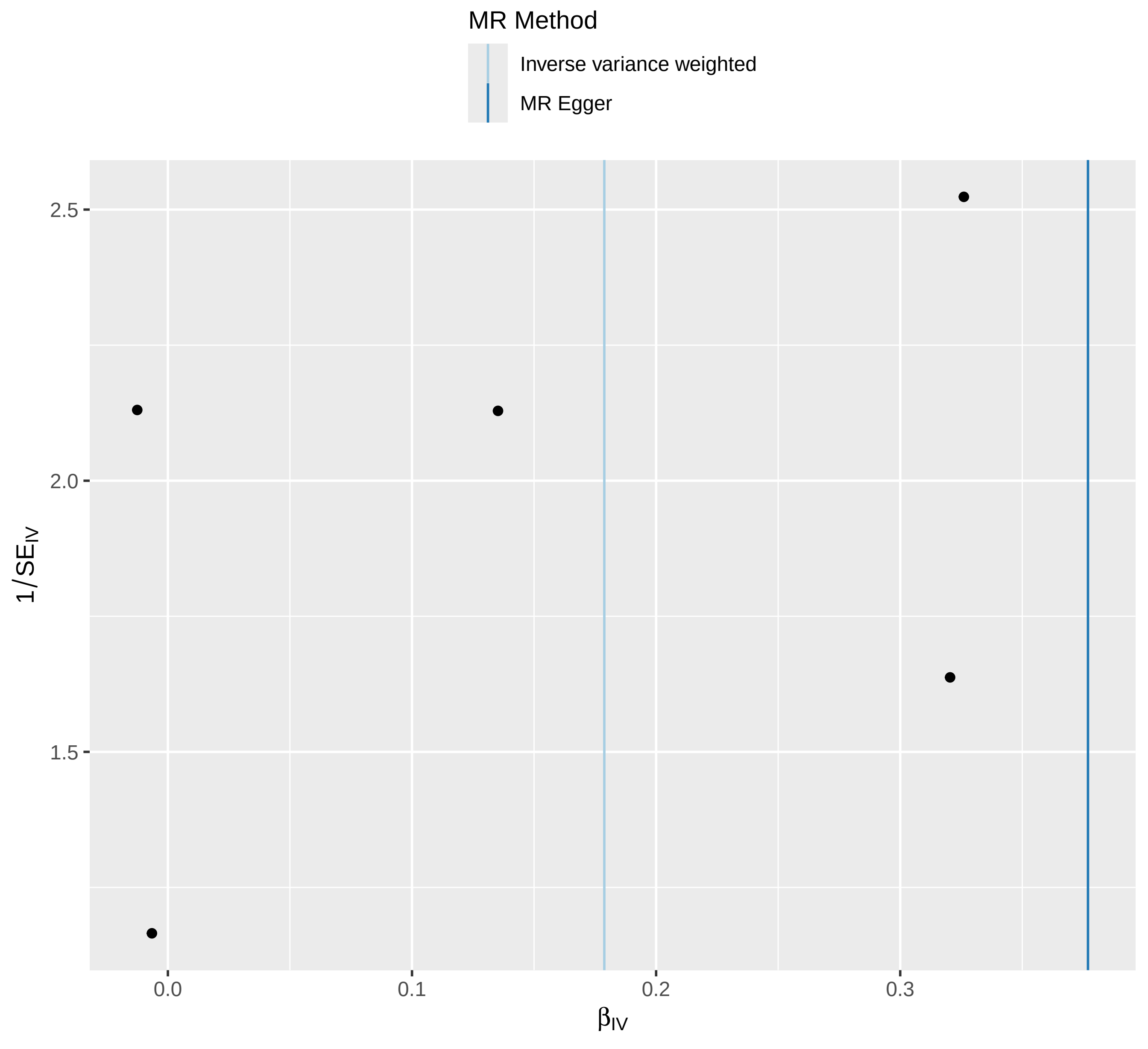

MR Method
Inverse variance weighted
MR Egger
2.5
2.0
1.5
1/SE_IV
0.0
0.1
0.2
0.3
β_IV

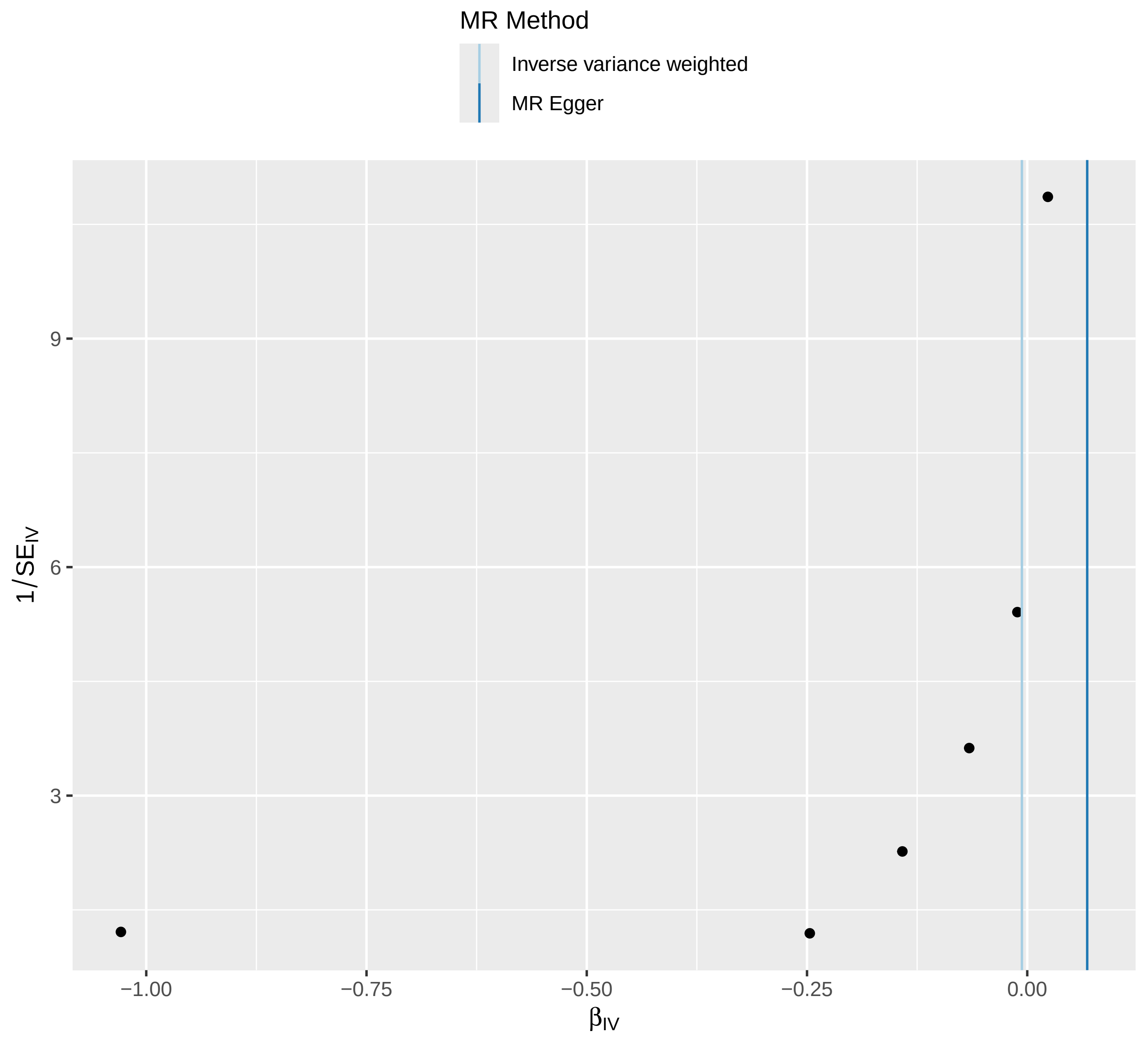

MR Method
Inverse variance weighted
MR Egger
1/SE_{IV}
9
6
3
−1.00
−0.75
−0.50
−0.25
0.00
$β_{IV}$

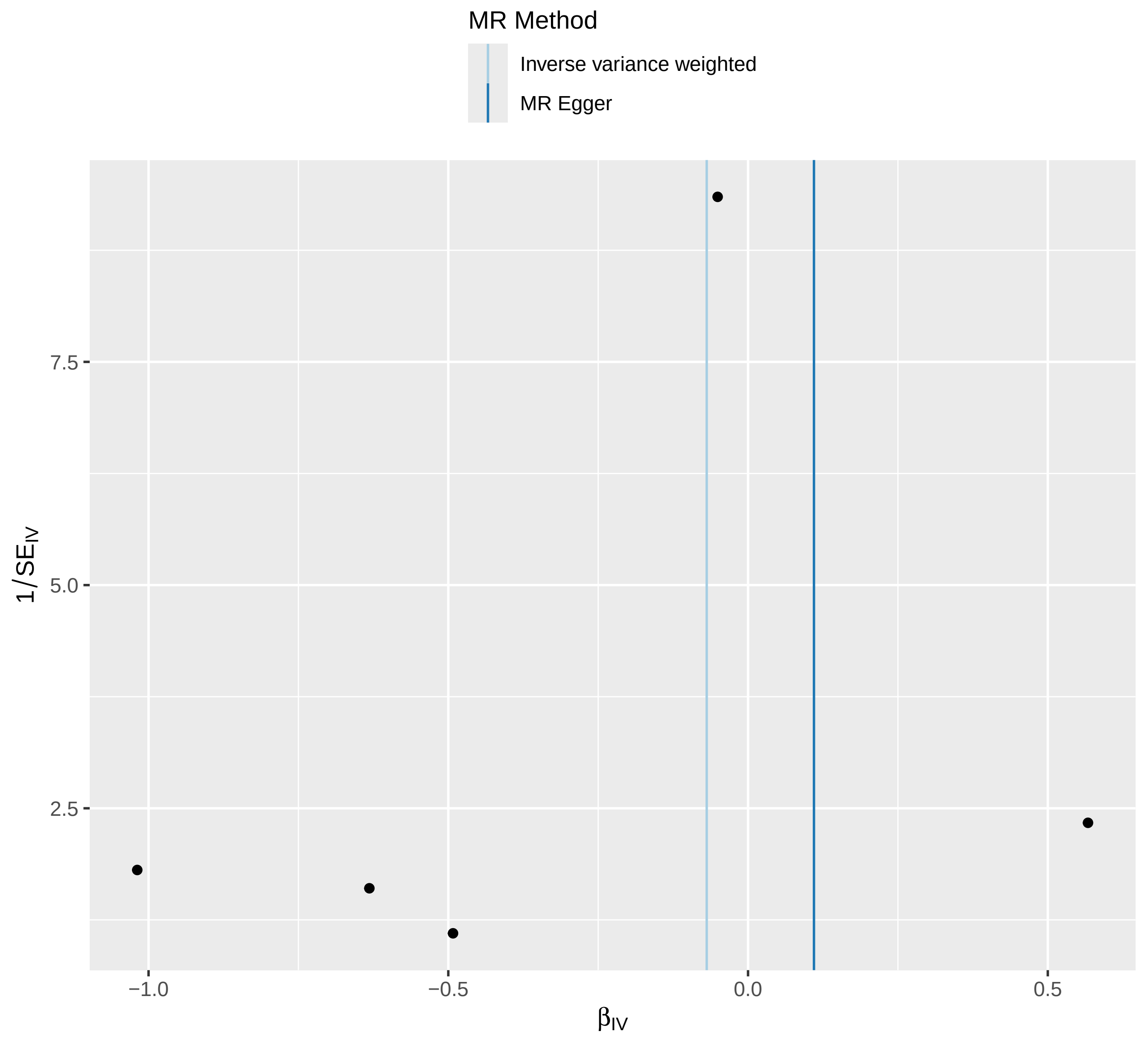
MR Method
Inverse variance weighted
MR Egger
7.5
5.0
2.5
1/SE$_{IV}$
−1.0
−0.5
0.0
0.5
β$_{IV}$

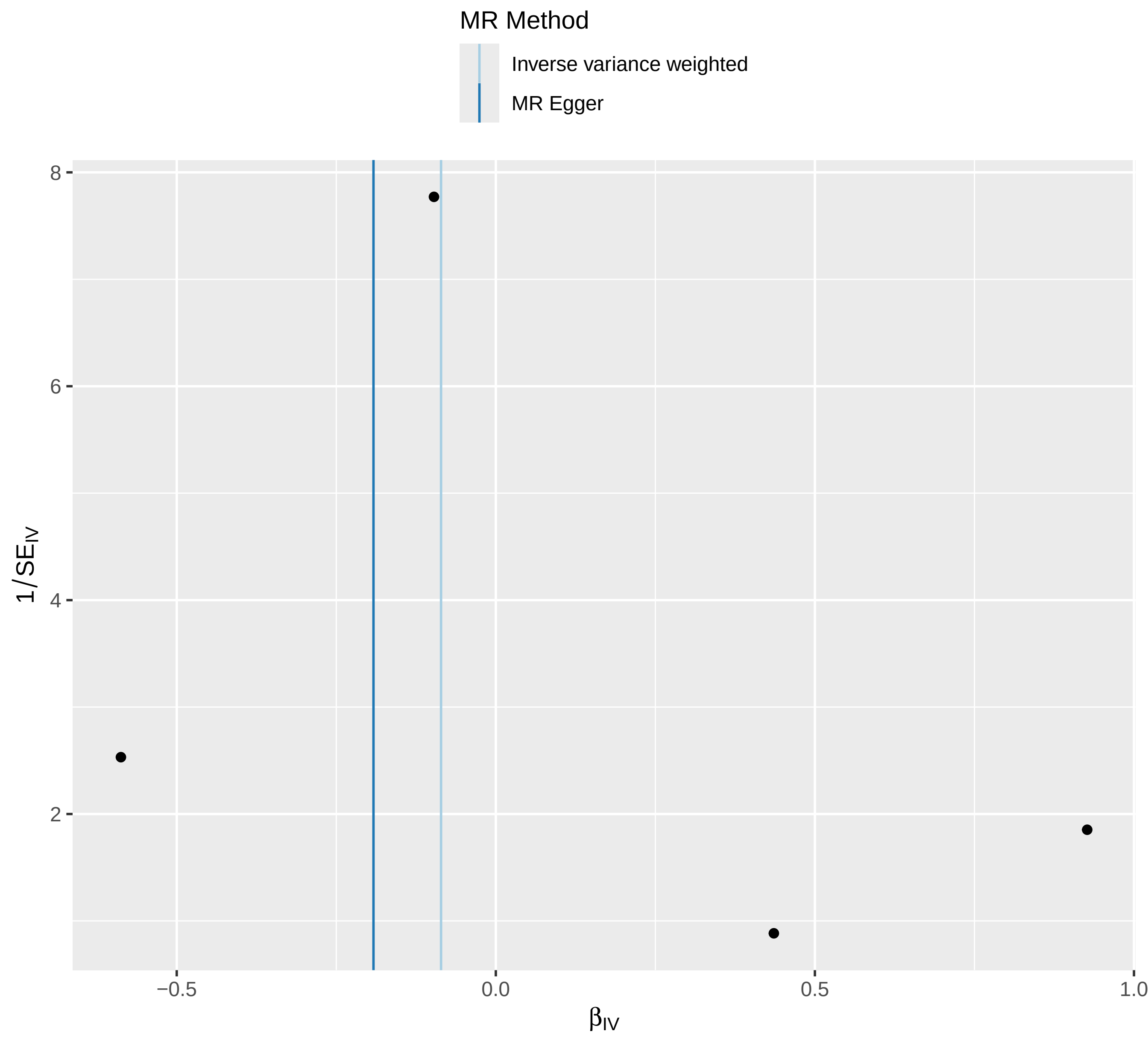

MR Method
Inverse variance weighted
MR Egger
8
6
4
2
1/SE_IV
−0.5
0.0
0.5
1.0
β_IV

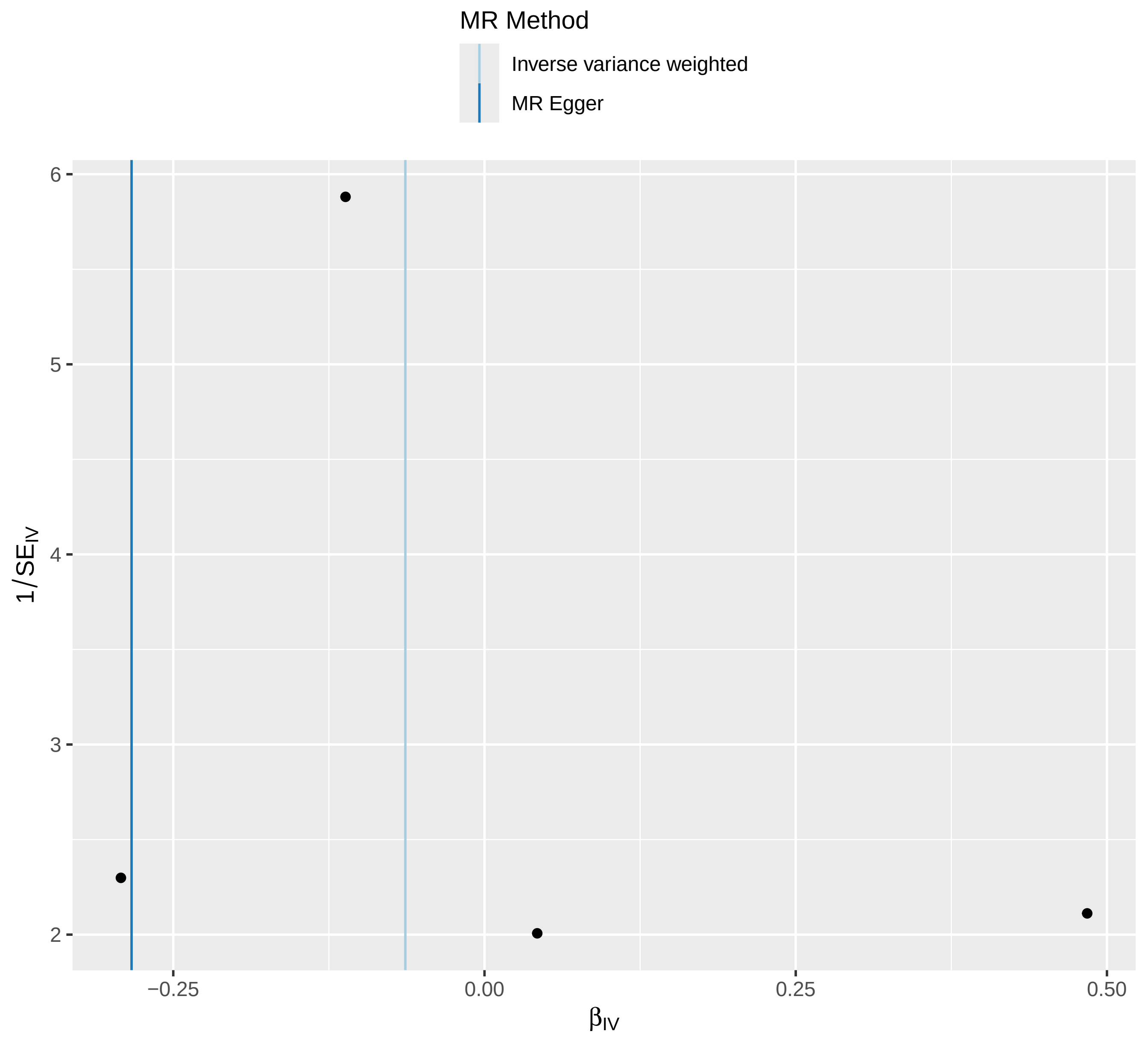
MR Method
Inverse variance weighted
MR Egger
6
5
4
3
2
$1/SE_{IV}$
−0.25
0.00
0.25
0.50
β_{IV}

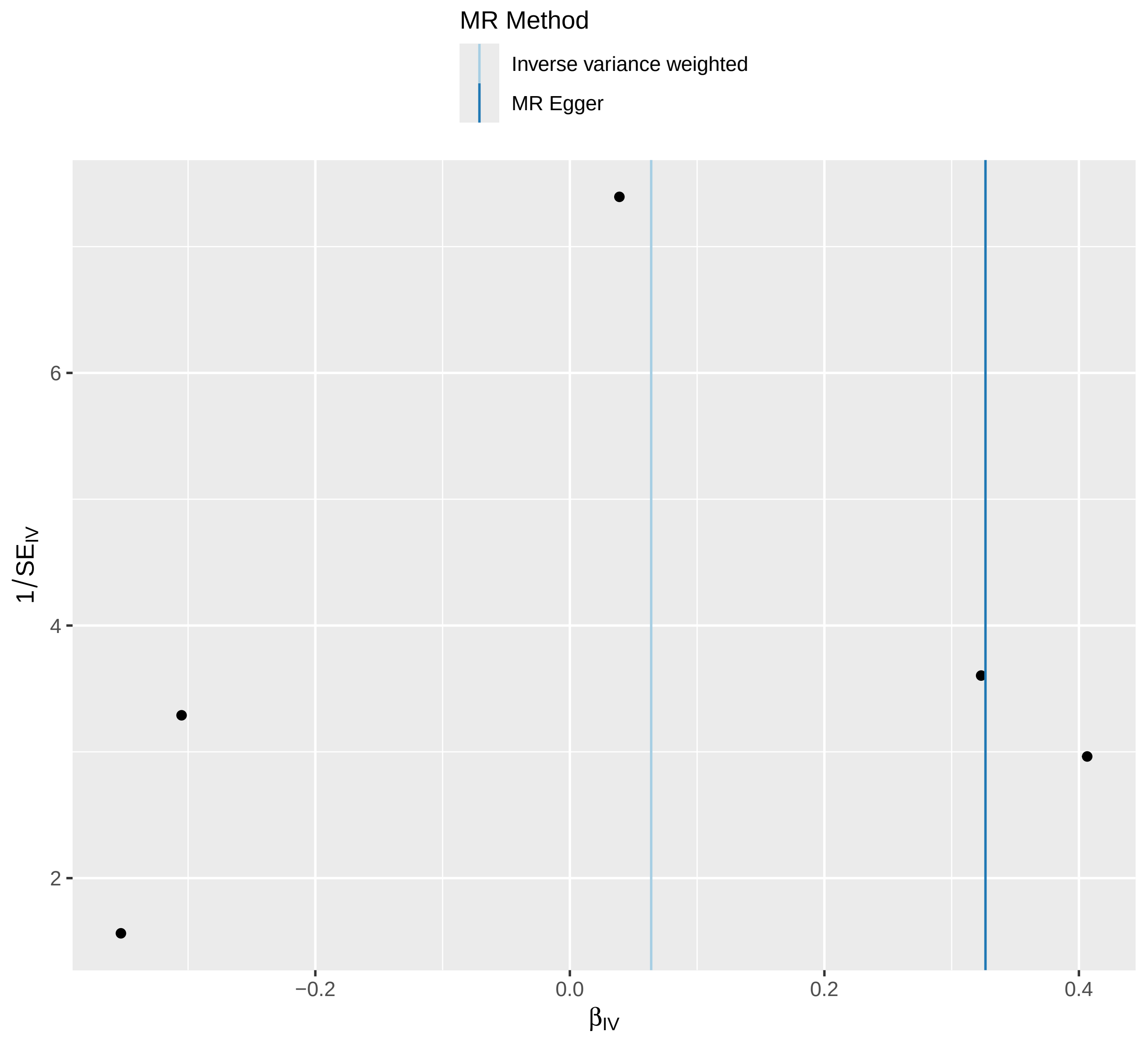

MR Method
Inverse variance weighted
MR Egger
6
4
2
1/SE_IV
−0.2
0.0
0.2
0.4
β_IV

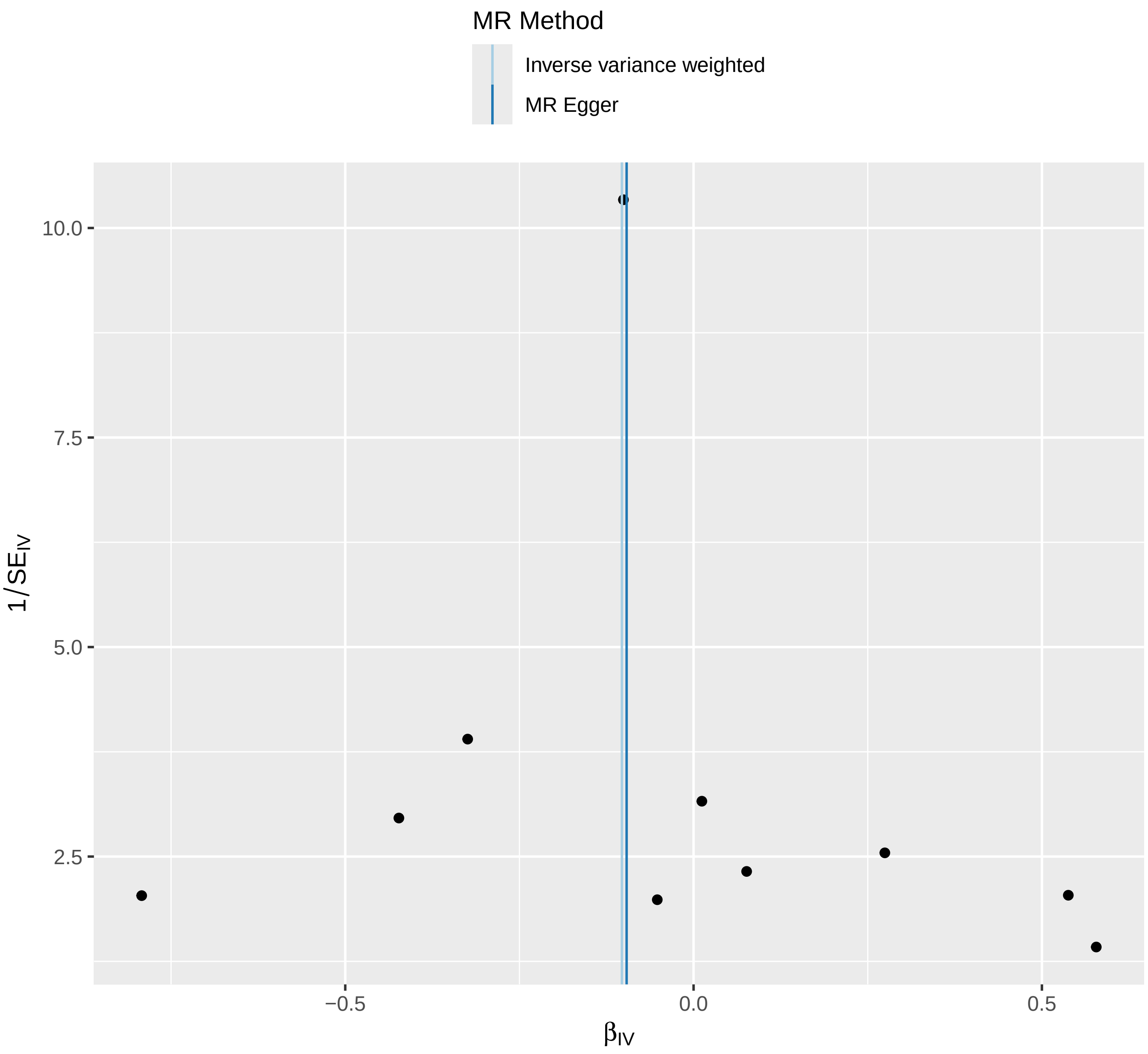

MR Method
Inverse variance weighted
MR Egger
10.0
7.5
5.0
2.5
$1/SE_{IV}$
−0.5
0.0
0.5
β_{IV}

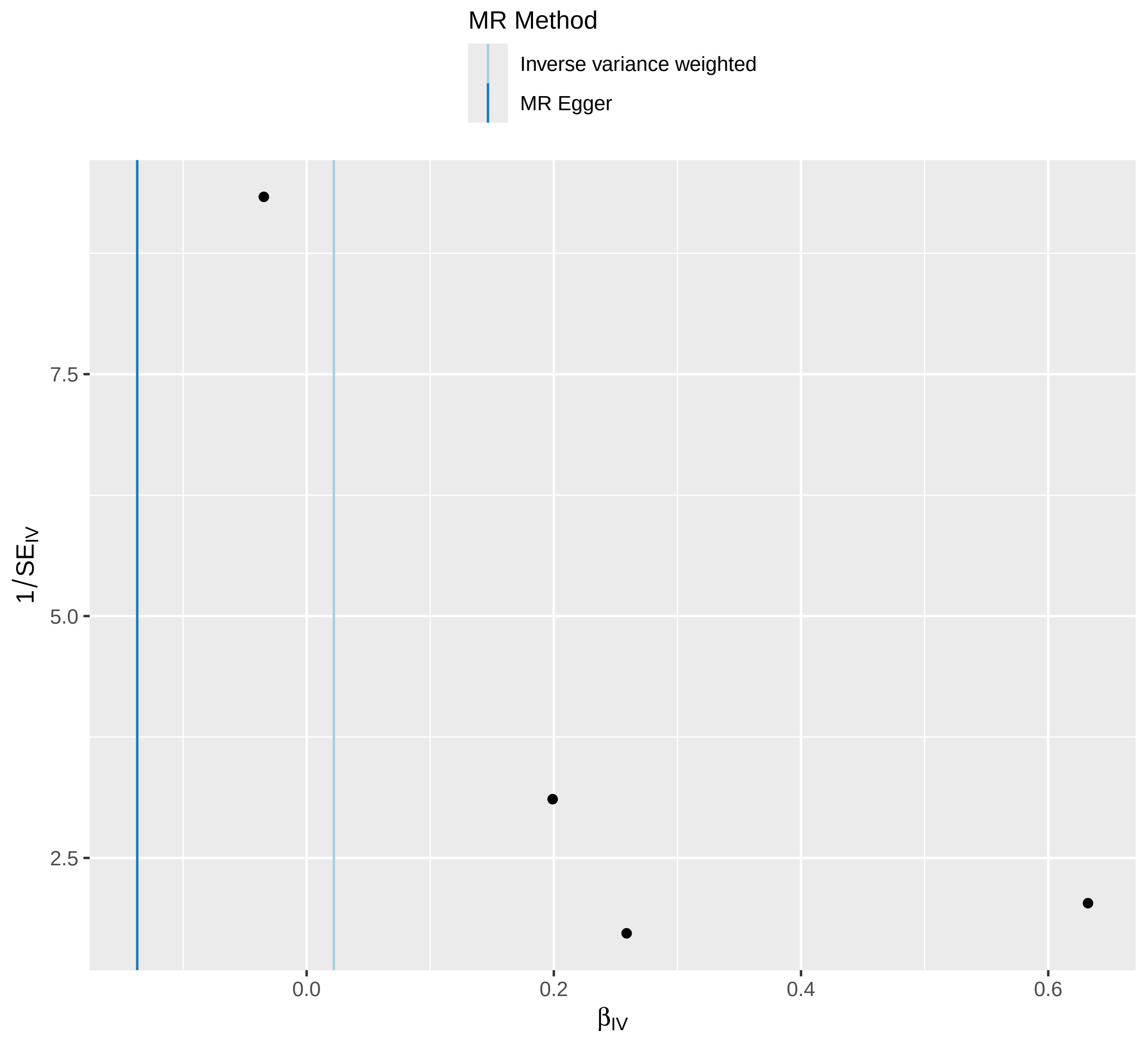
MR Method
Inverse variance weighted
MR Egger
7.5
5.0
2.5
1/SE_IV
0.0
0.2
0.4
0.6
β_IV

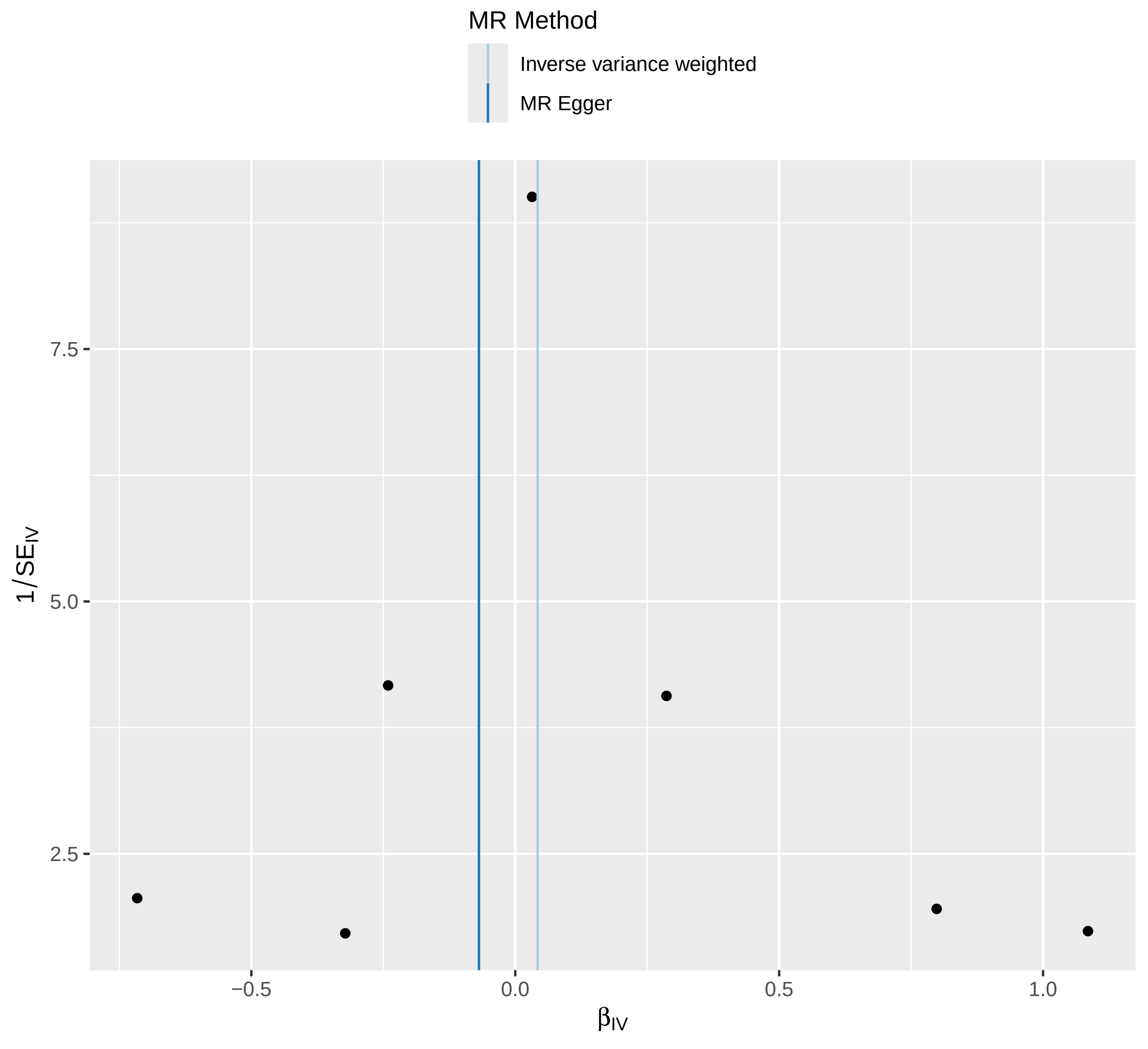
MR Method
Inverse variance weighted
MR Egger
7.5
5.0
2.5
1/SE_IV
−0.5
0.0
0.5
1.0
β_IV

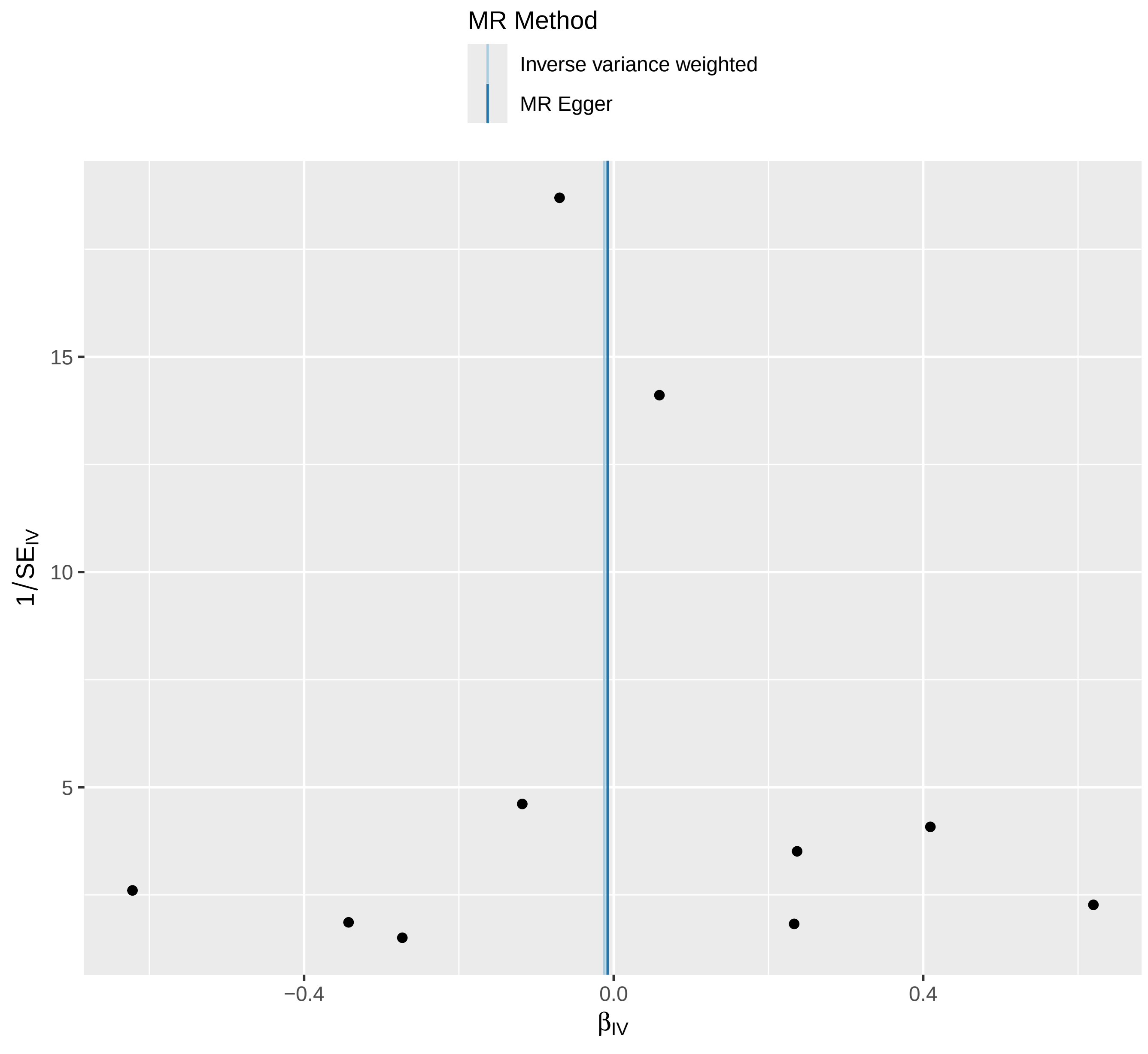

MR Method
Inverse variance weighted
MR Egger
15
10
5
−0.4
0.0
0.4
1/SE_IV
β_IV

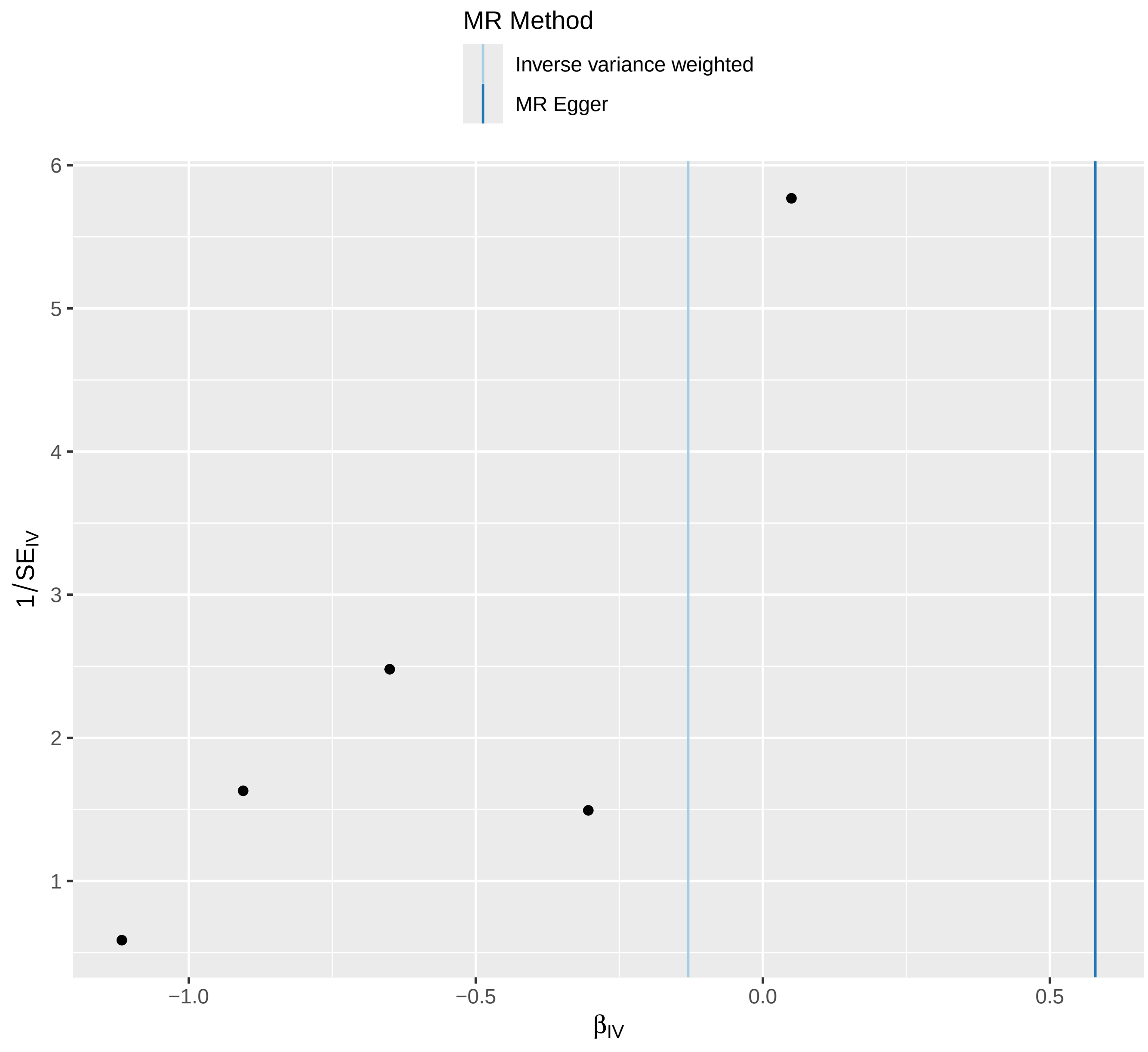

MR Method
Inverse variance weighted
MR Egger
6
5
4
3
2
1
$1/SE_{IV}$
−1.0
−0.5
0.0
0.5
β_{IV}

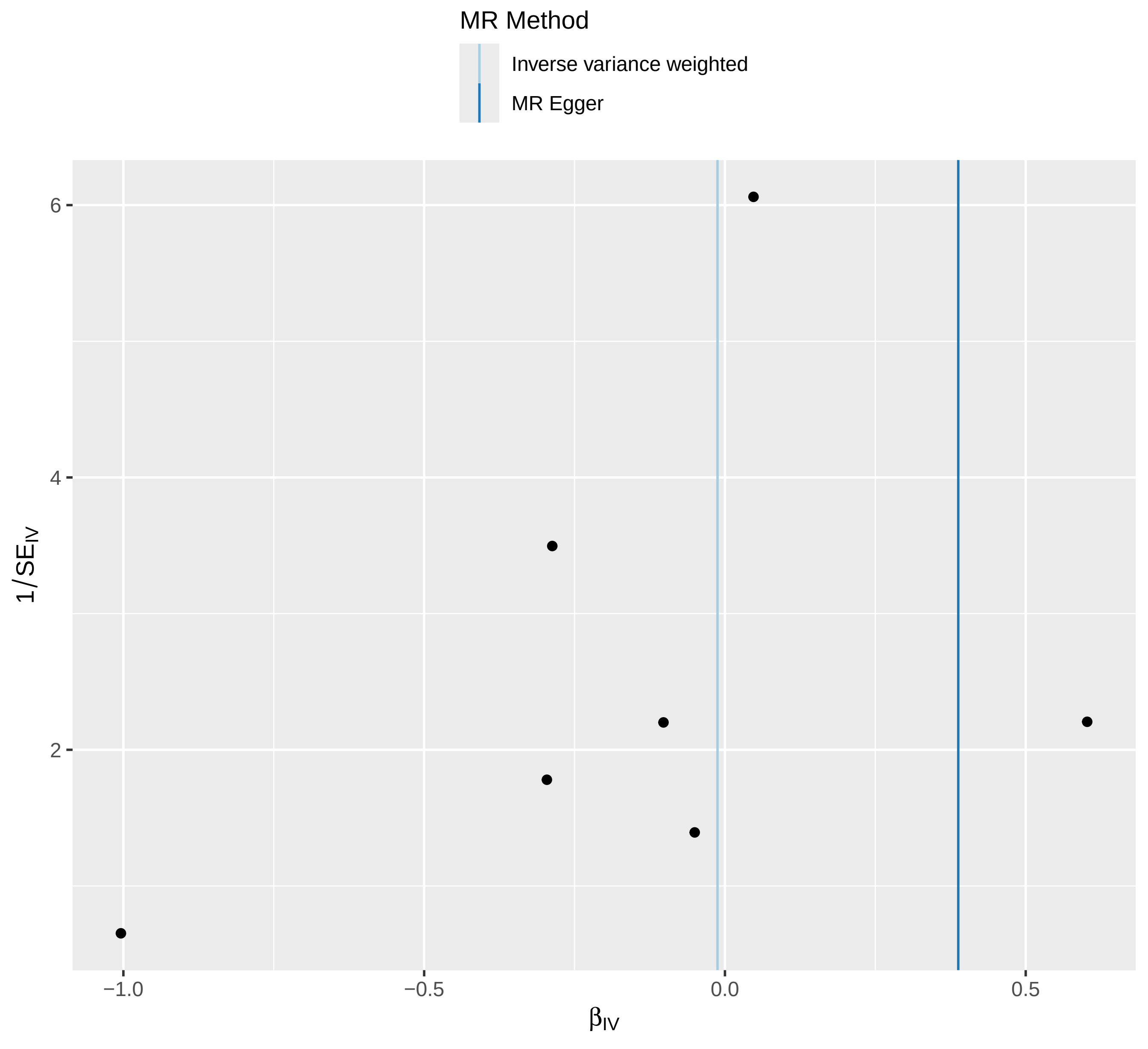
MR Method
Inverse variance weighted
MR Egger
6
4
2
1/SE$_{IV}$
−1.0
−0.5
0.0
0.5
β$_{IV}$

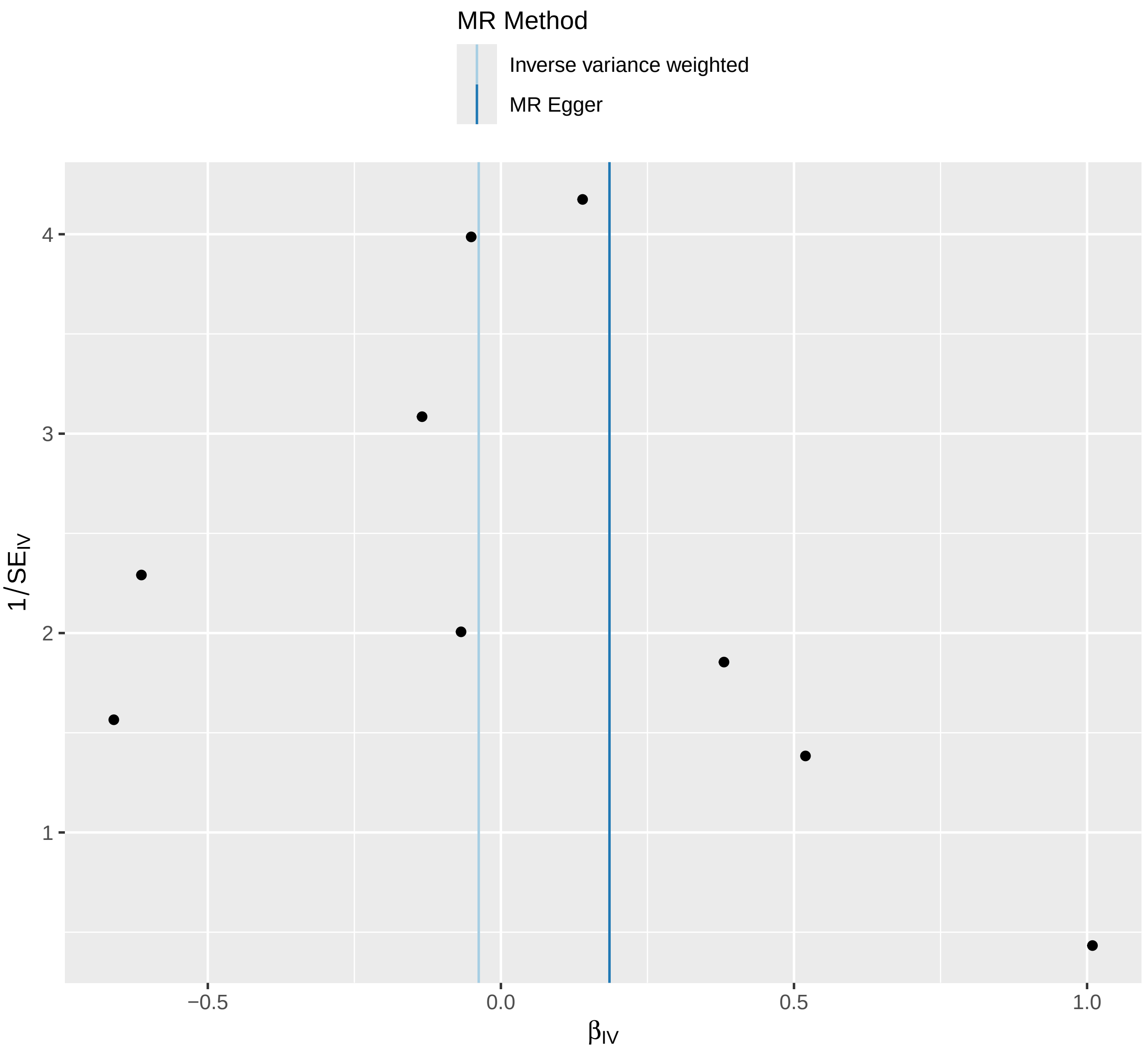

MR Method
Inverse variance weighted
MR Egger
4
3
2
1
$1/SE_{IV}$
−0.5
0.0
0.5
1.0
β_{IV}

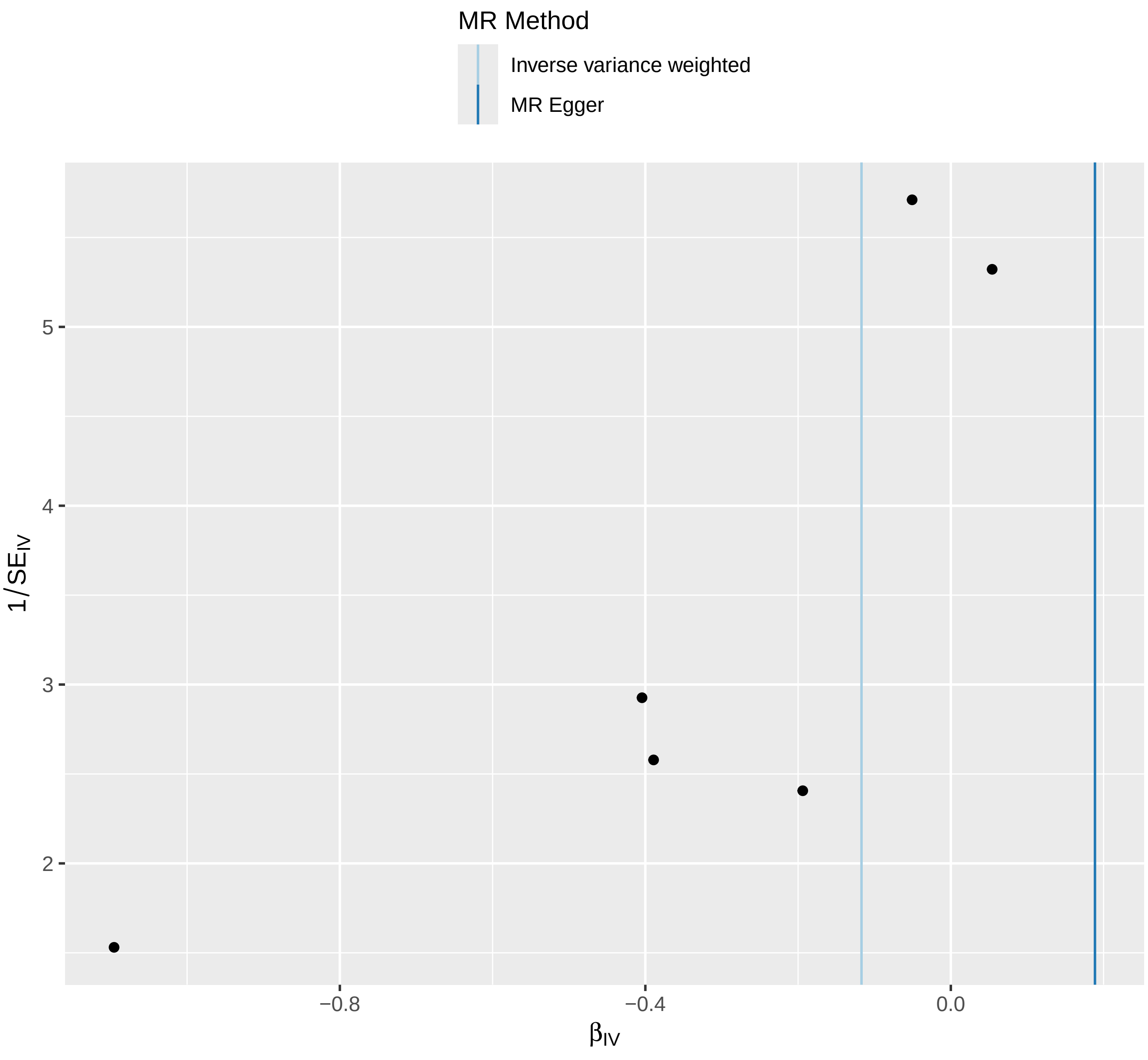
MR Method
Inverse variance weighted
MR Egger
5
4
3
2
1/SE$_{IV}$
−0.8
−0.4
0.0
β$_{IV}$

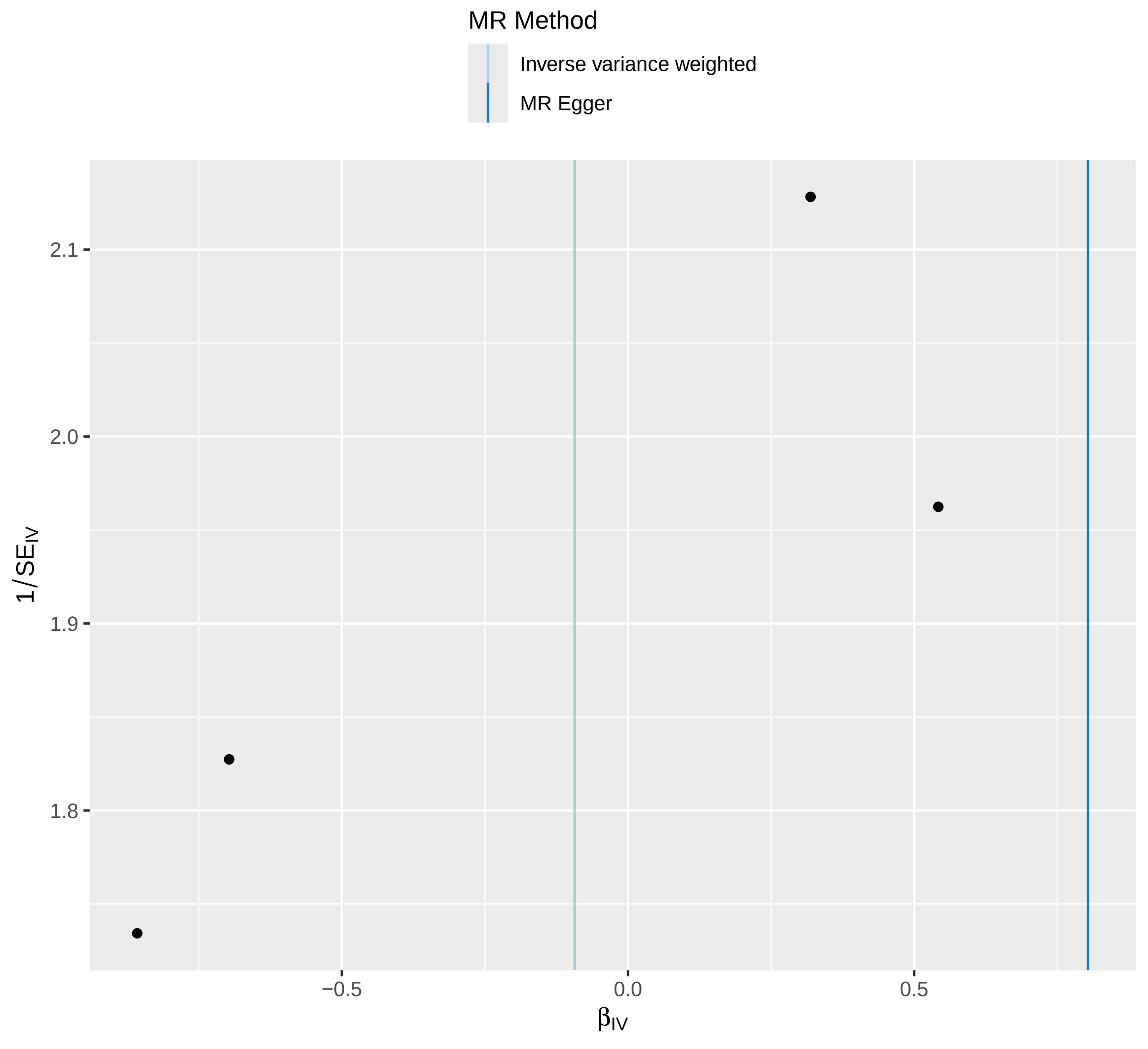
MR Method
Inverse variance weighted
MR Egger
2.1
2.0
1.9
1.8
1/SE$_{IV}$
−0.5
0.0
0.5
β$_{IV}$

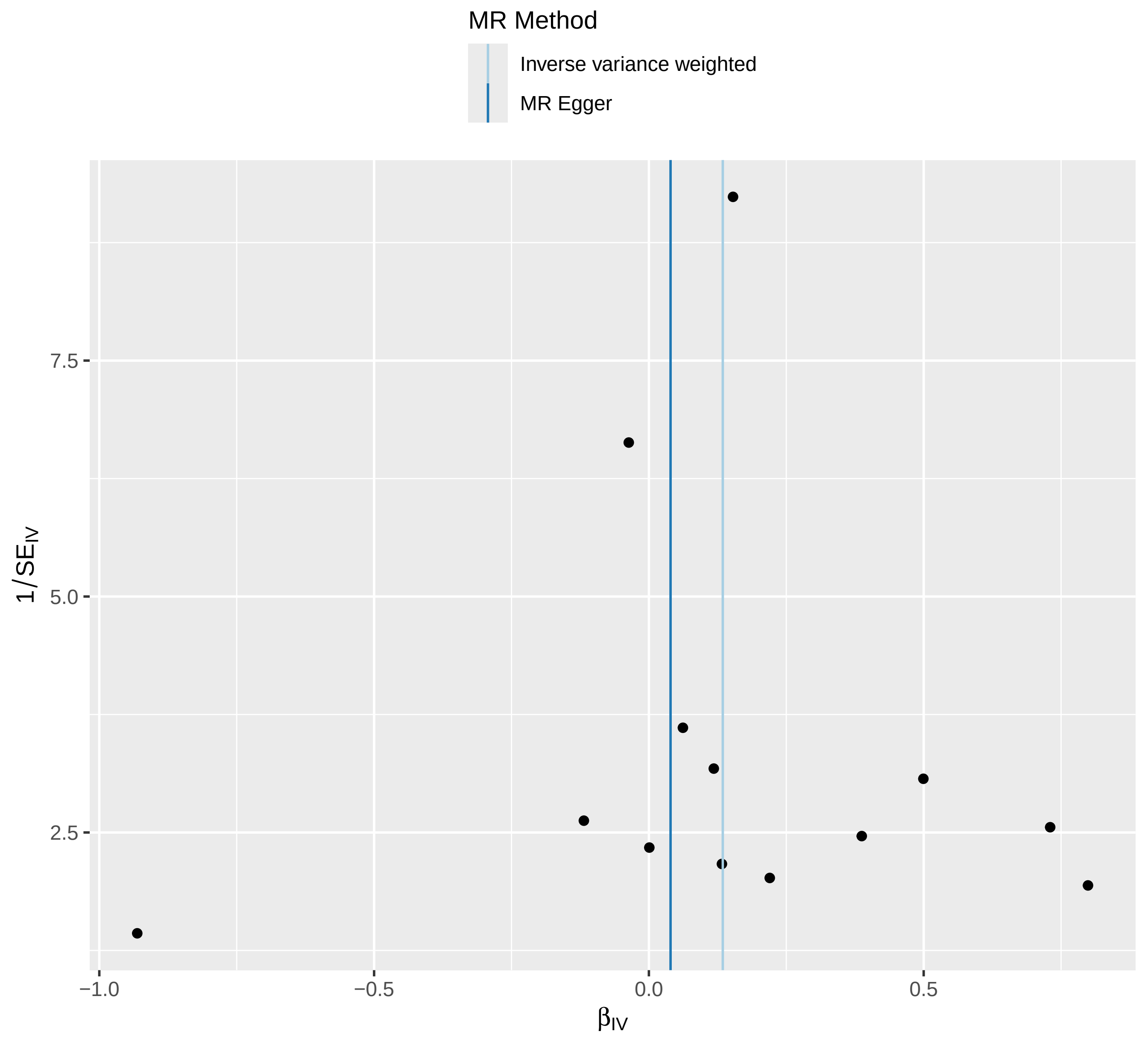

MR Method
Inverse variance weighted
MR Egger
7.5
5.0
2.5
1/SE$_{IV}$
−1.0
−0.5
0.0
0.5
β$_{IV}$

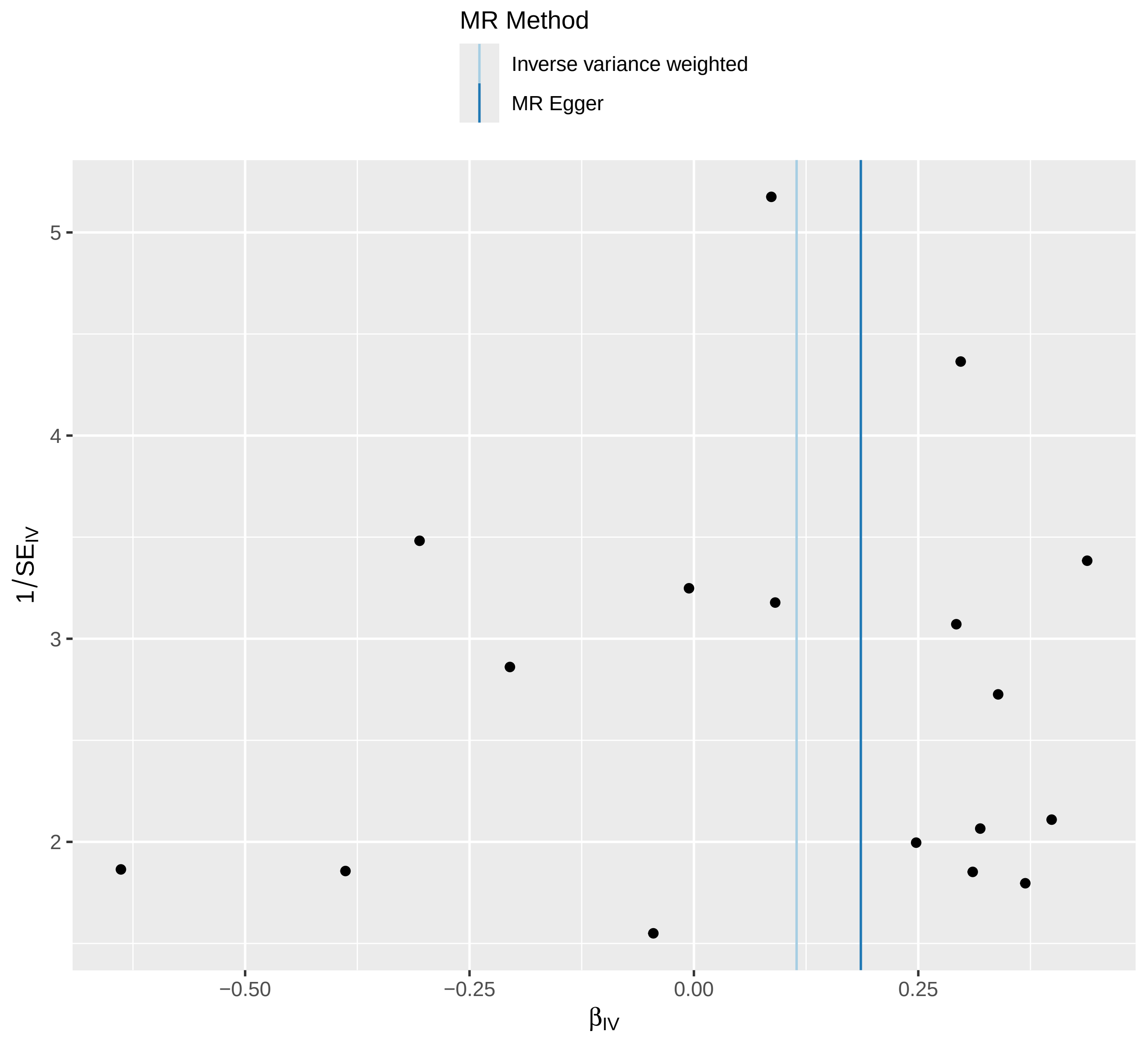
MR Method
Inverse variance weighted
MR Egger
5
4
3
2
$1/SE_{IV}$
−0.50
−0.25
0.00
0.25
β_{IV}

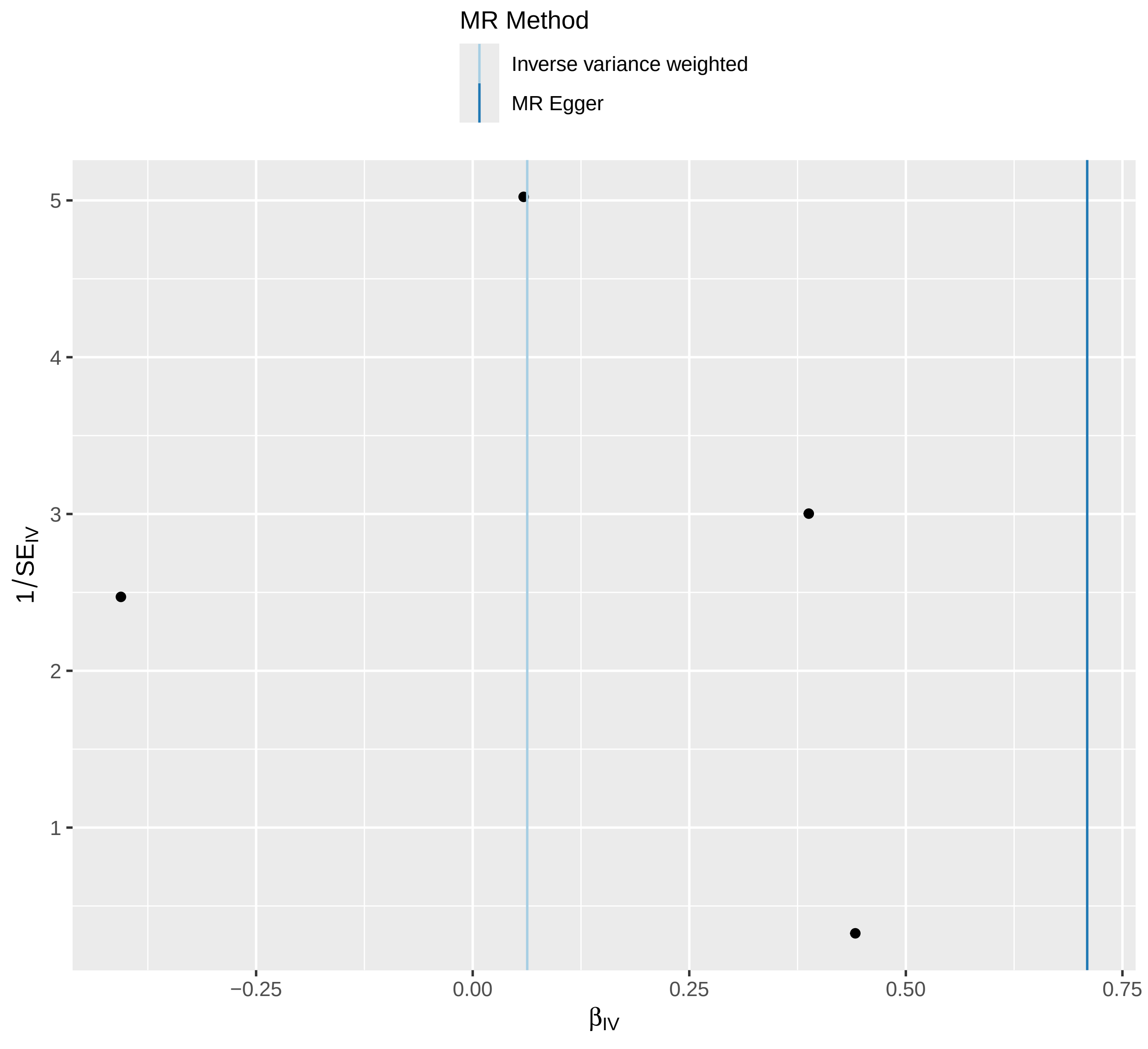
MR Method
Inverse variance weighted
MR Egger
1/SE$_{IV}$
5
4
3
2
1
−0.25
0.00
0.25
0.50
0.75
β$_{IV}$

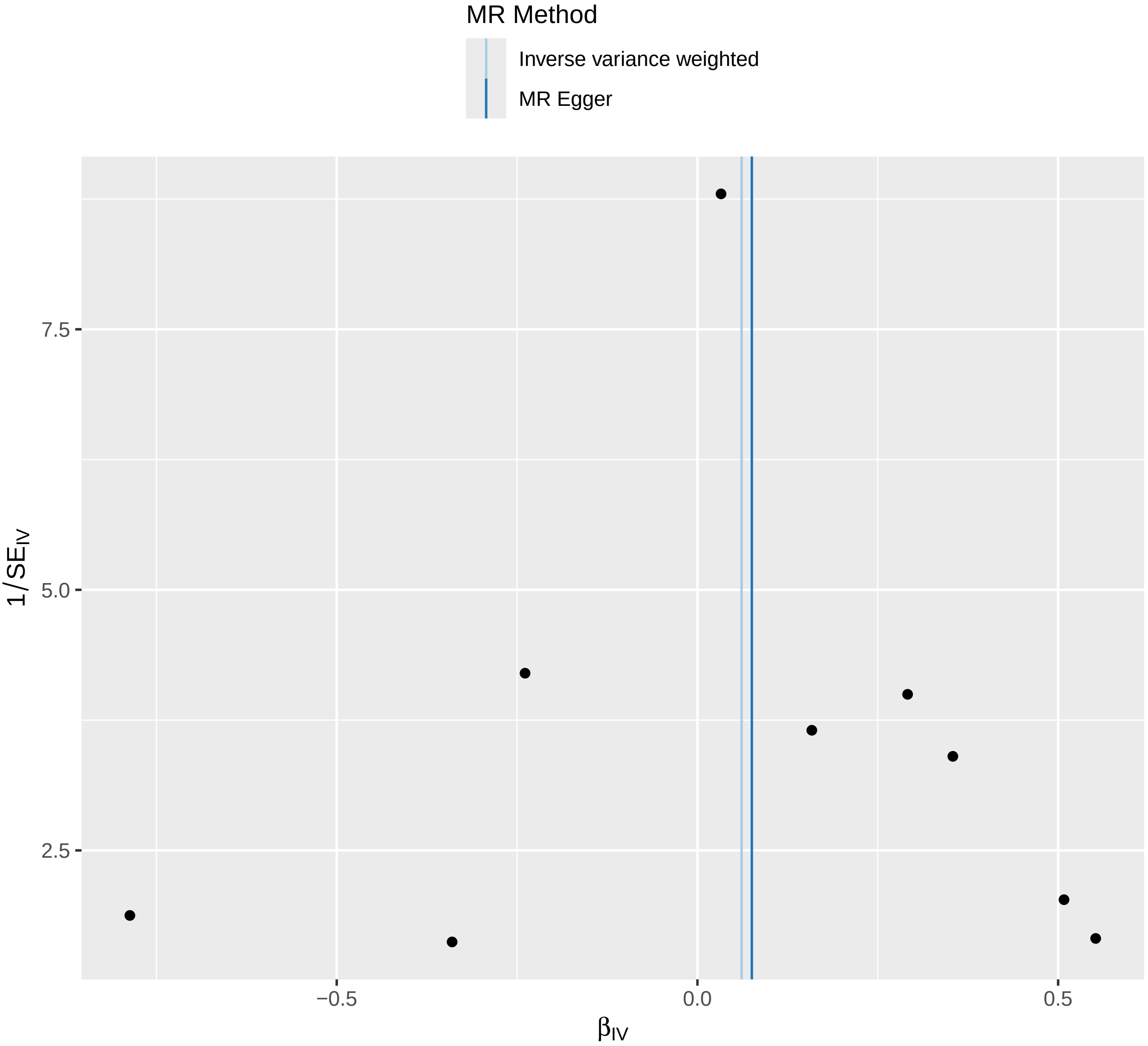

MR Method
Inverse variance weighted
MR Egger
7.5
5.0
2.5
1/SE$_{IV}$
−0.5
0.0
0.5
β_{IV}

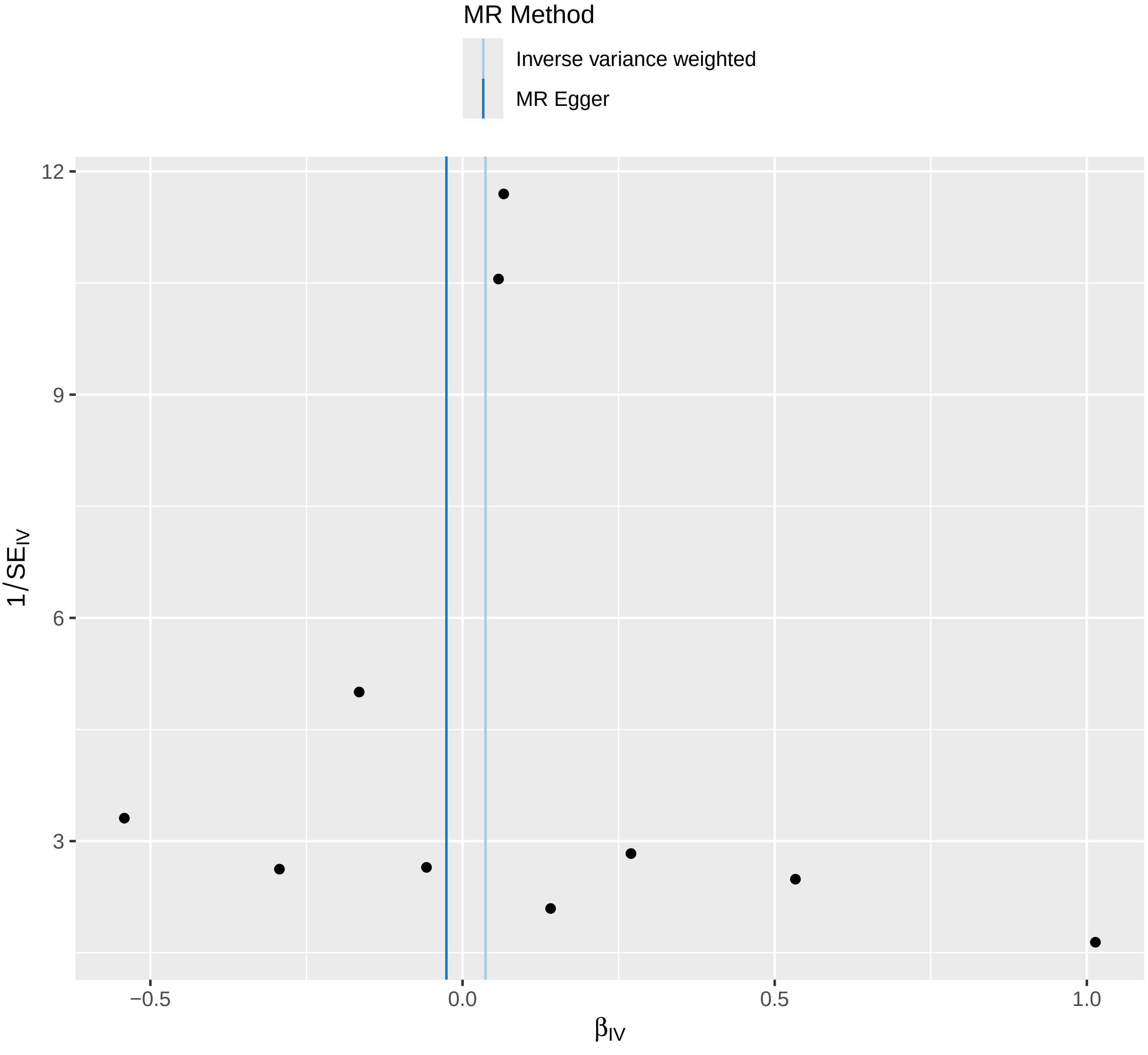

MR Method
Inverse variance weighted
MR Egger
12
9
6
3
$1/SE_{IV}$
−0.5
0.0
0.5
1.0
β_{IV}

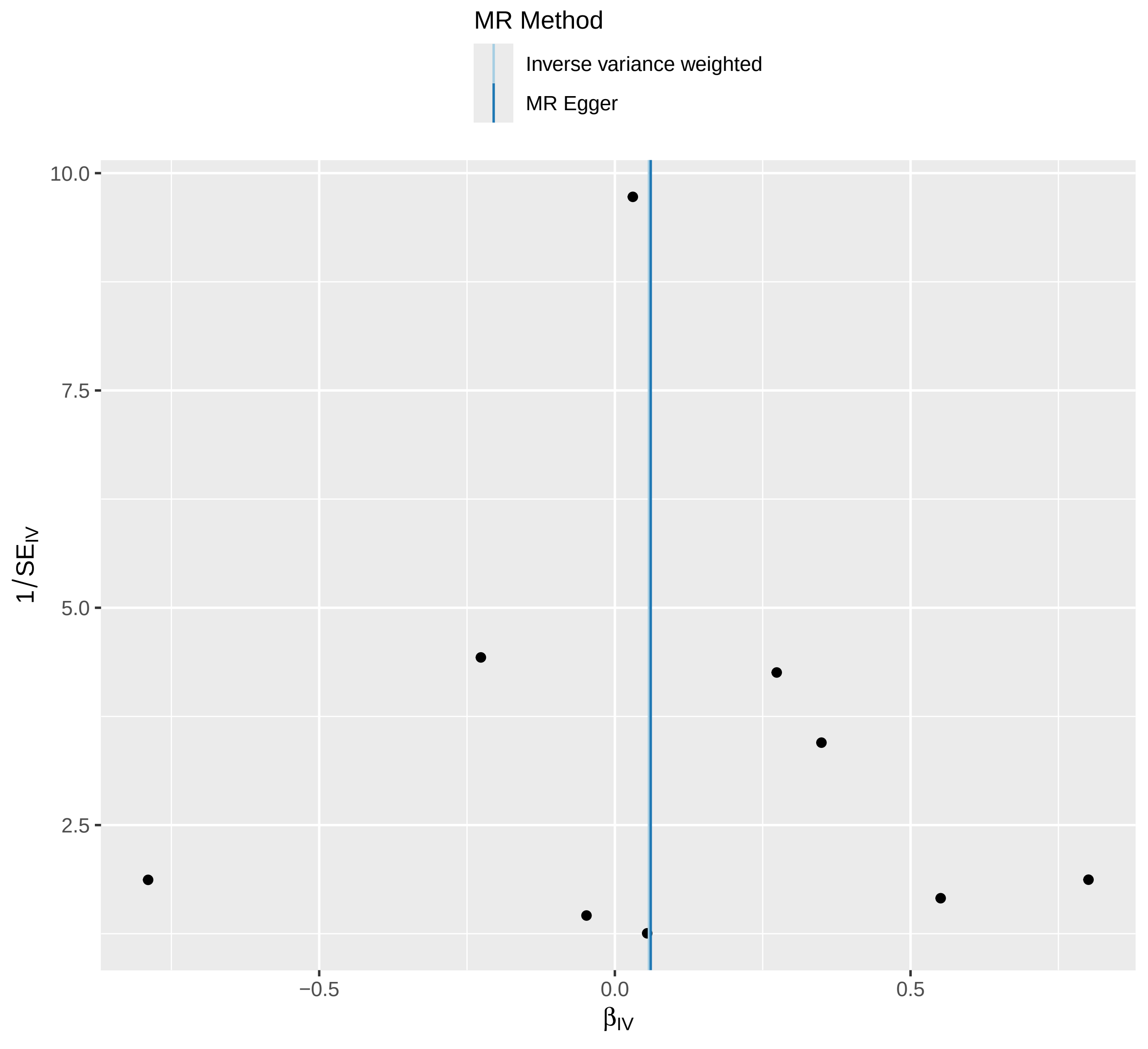
MR Method
Inverse variance weighted
MR Egger
10.0
7.5
5.0
2.5
1/SE$_{IV}$
−0.5
0.0
0.5
β$_{IV}$

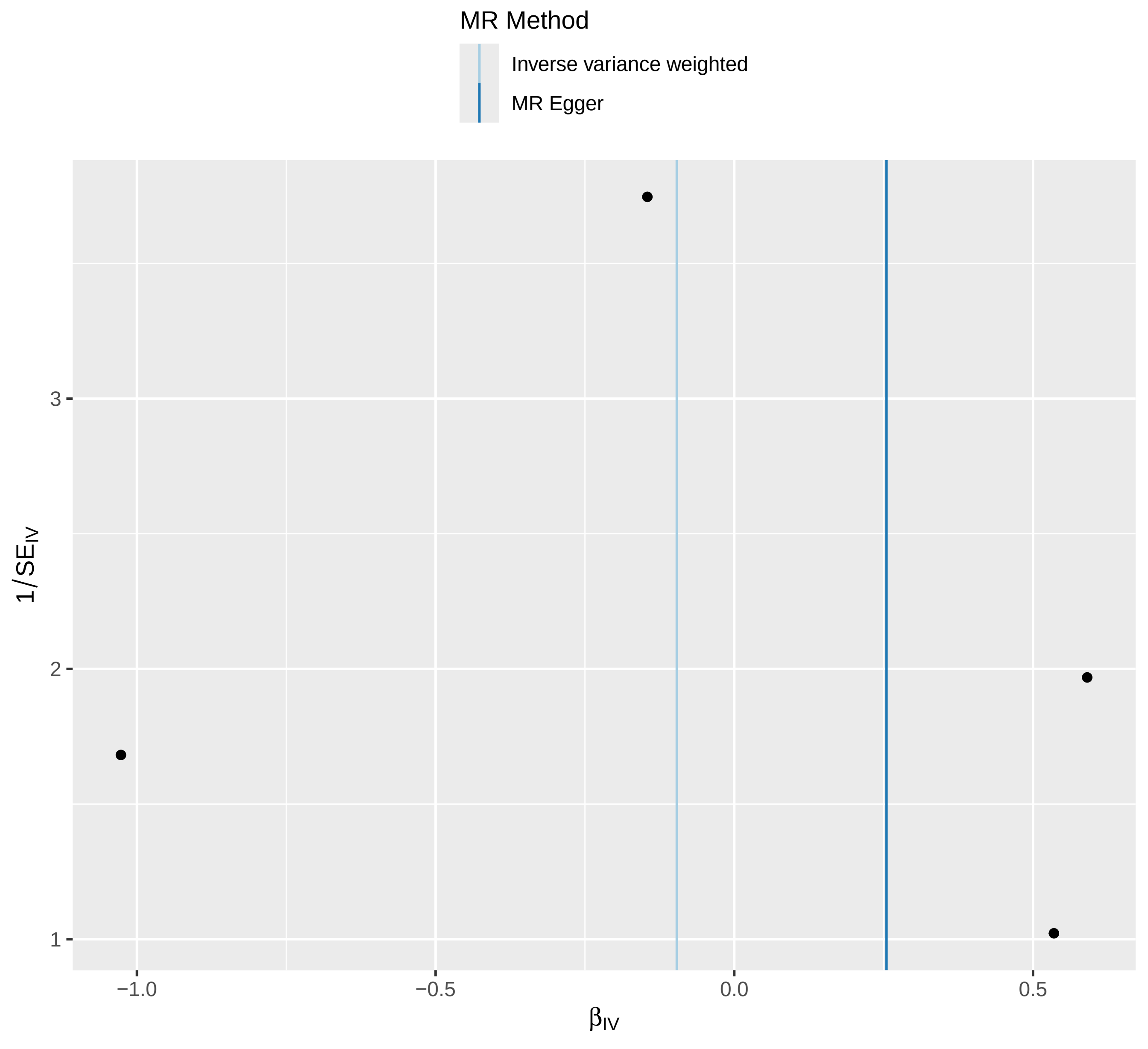
MR Method
Inverse variance weighted
MR Egger
1/SE_IV
3
2
1
−1.0
−0.5
0.0
0.5
β_IV

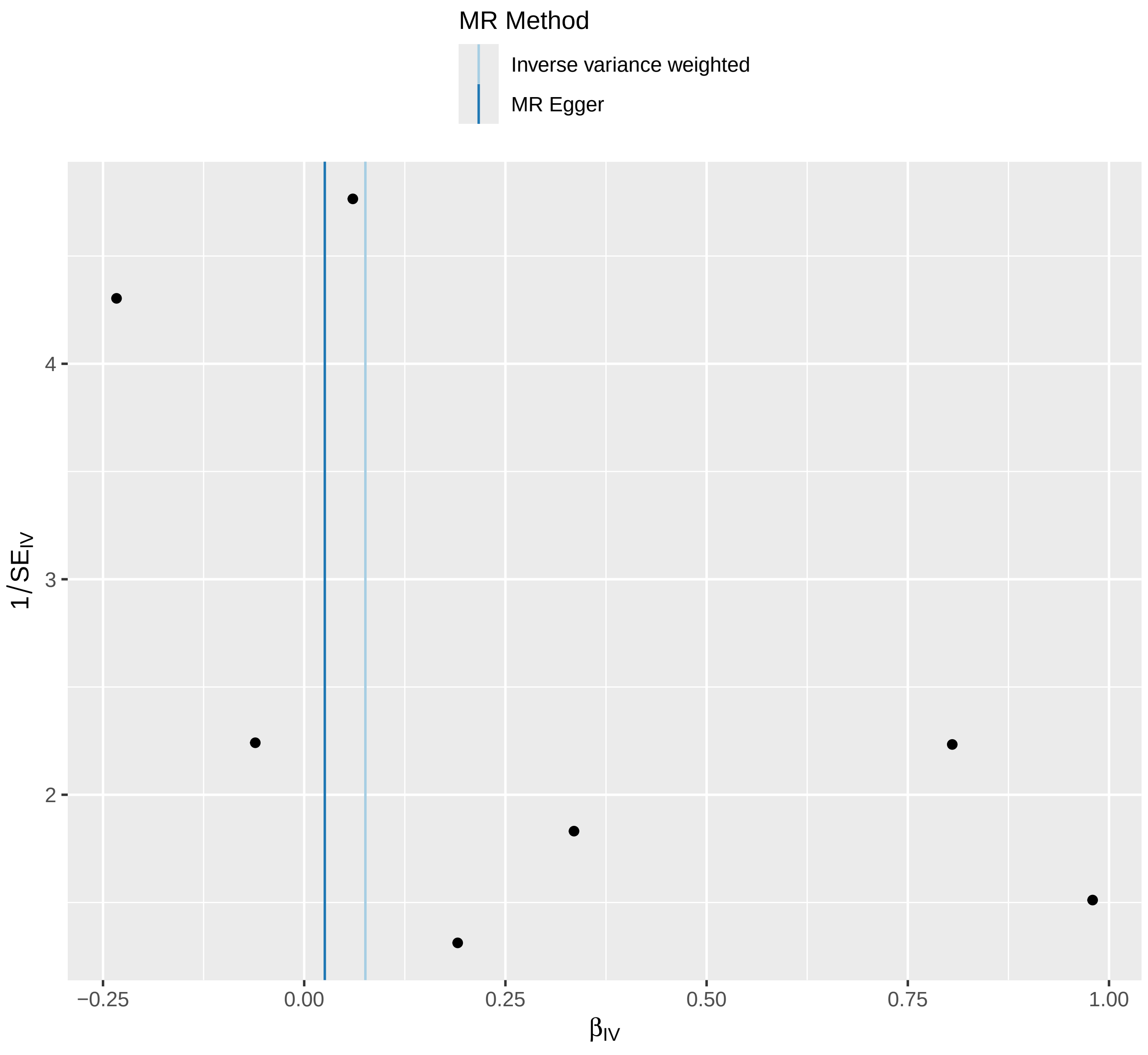
MR Method
Inverse variance weighted
MR Egger
1/SE$_{IV}$
4
3
2
−0.25
0.00
0.25
0.50
0.75
1.00
β$_{IV}$

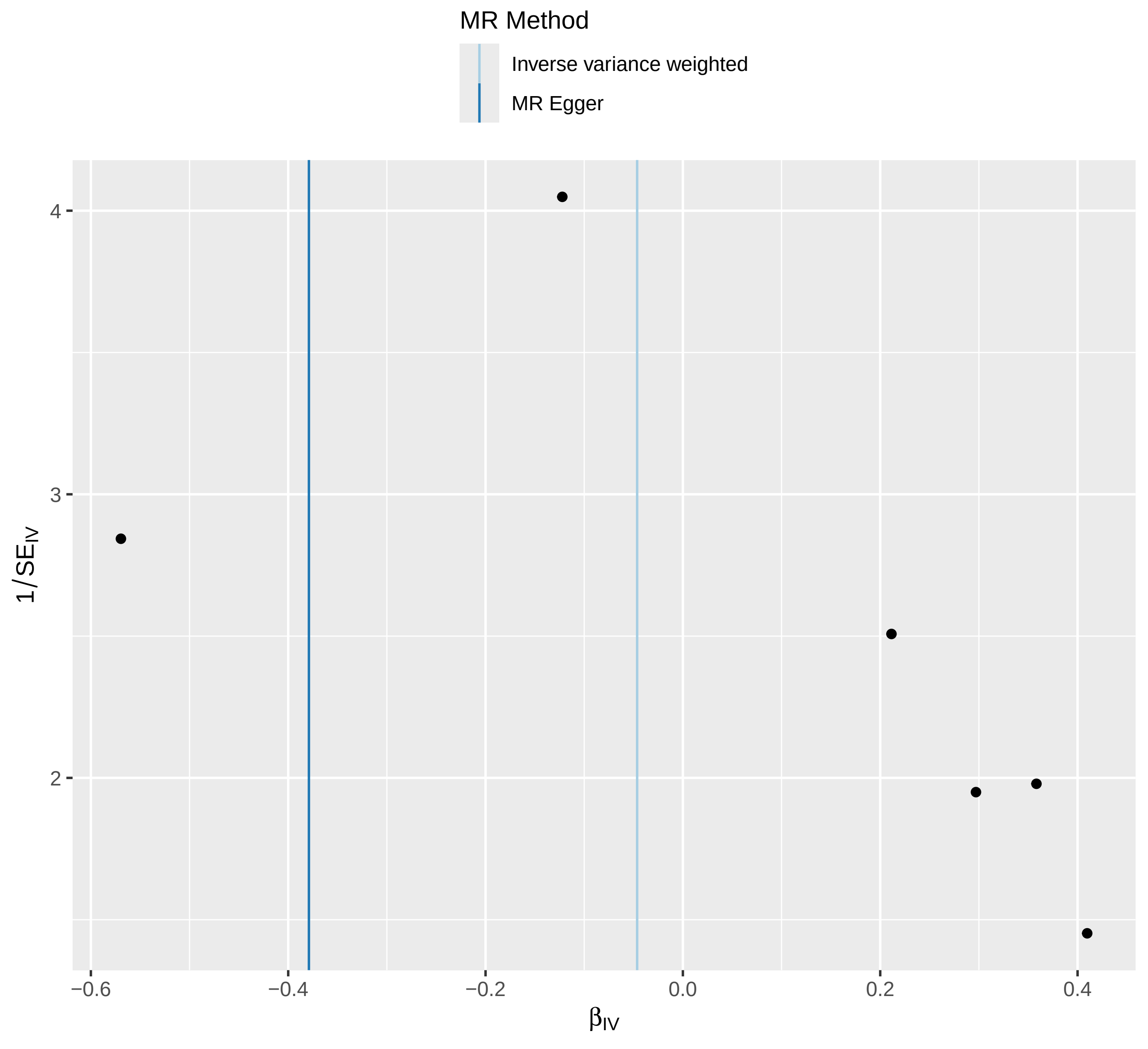
MR Method
Inverse variance weighted
MR Egger
4
3
2
1/SE_IV
−0.6
−0.4
−0.2
0.0
0.2
0.4
β_IV

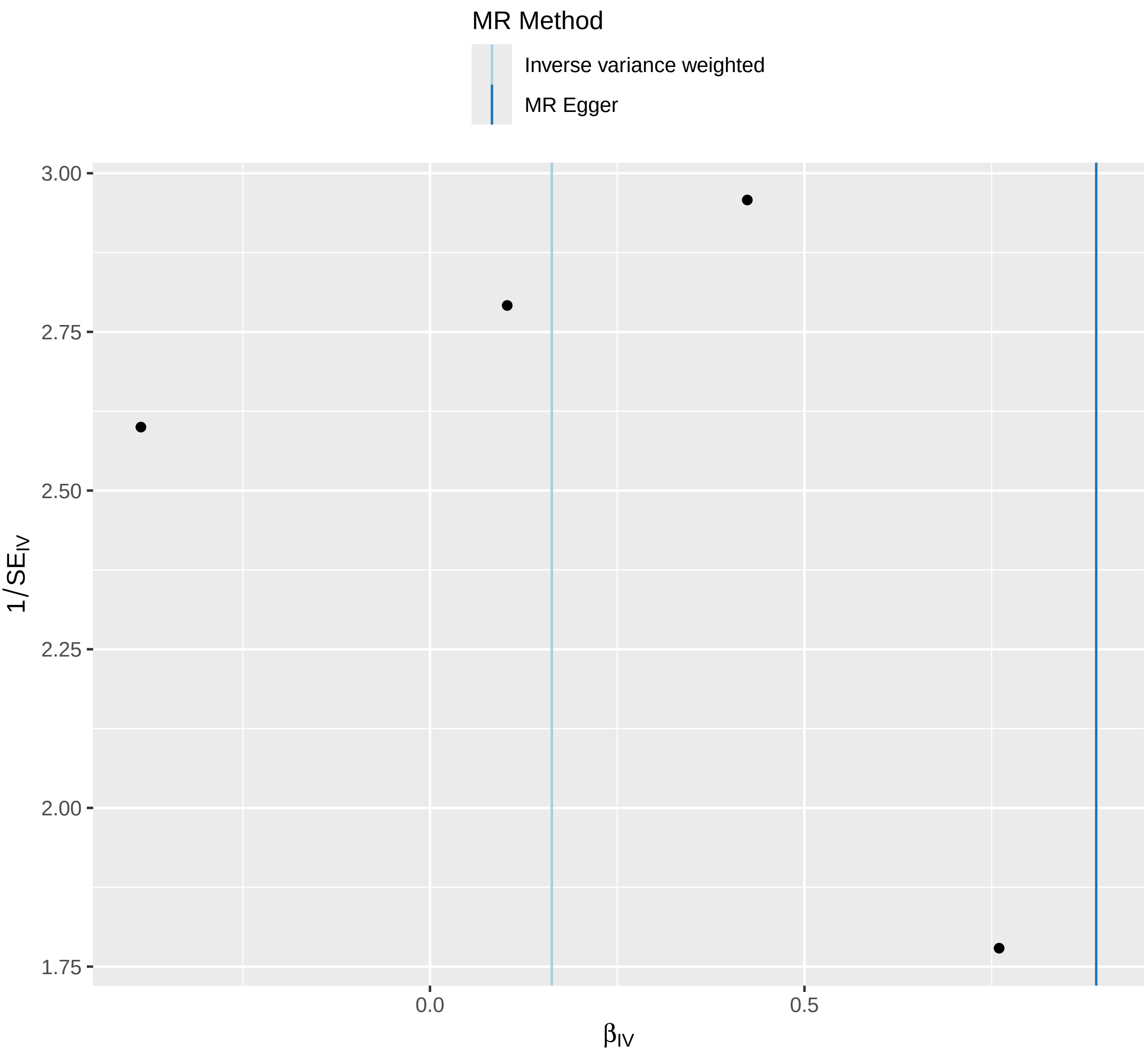

MR Method
Inverse variance weighted
MR Egger
3.00
2.75
2.50
2.25
2.00
1.75
1/SE$_{IV}$
0.0
0.5
β$_{IV}$

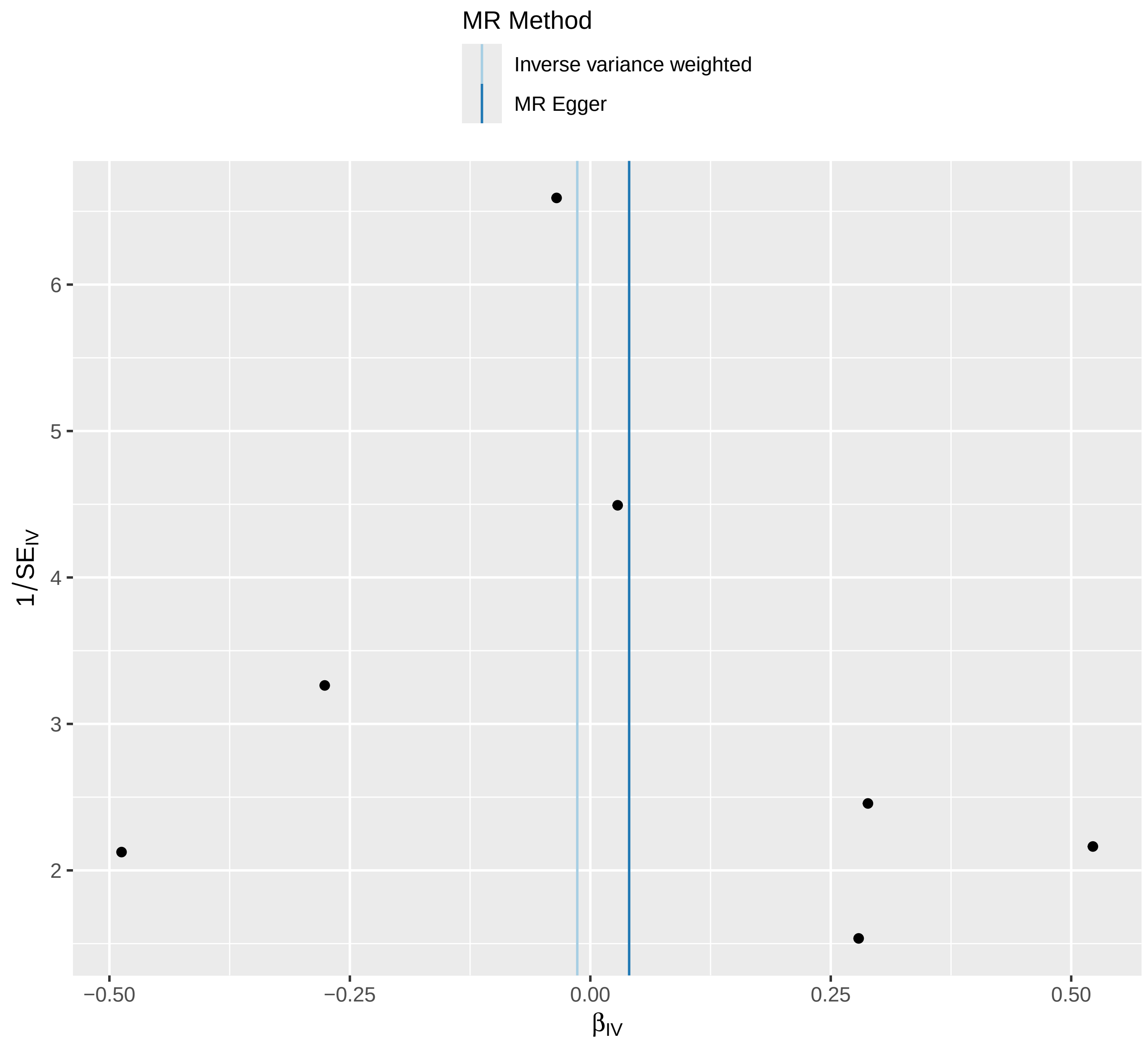
MR Method
Inverse variance weighted
MR Egger
1/SE_IV
β_IV
6
5
4
3
2
−0.50
−0.25
0.00
0.25
0.50

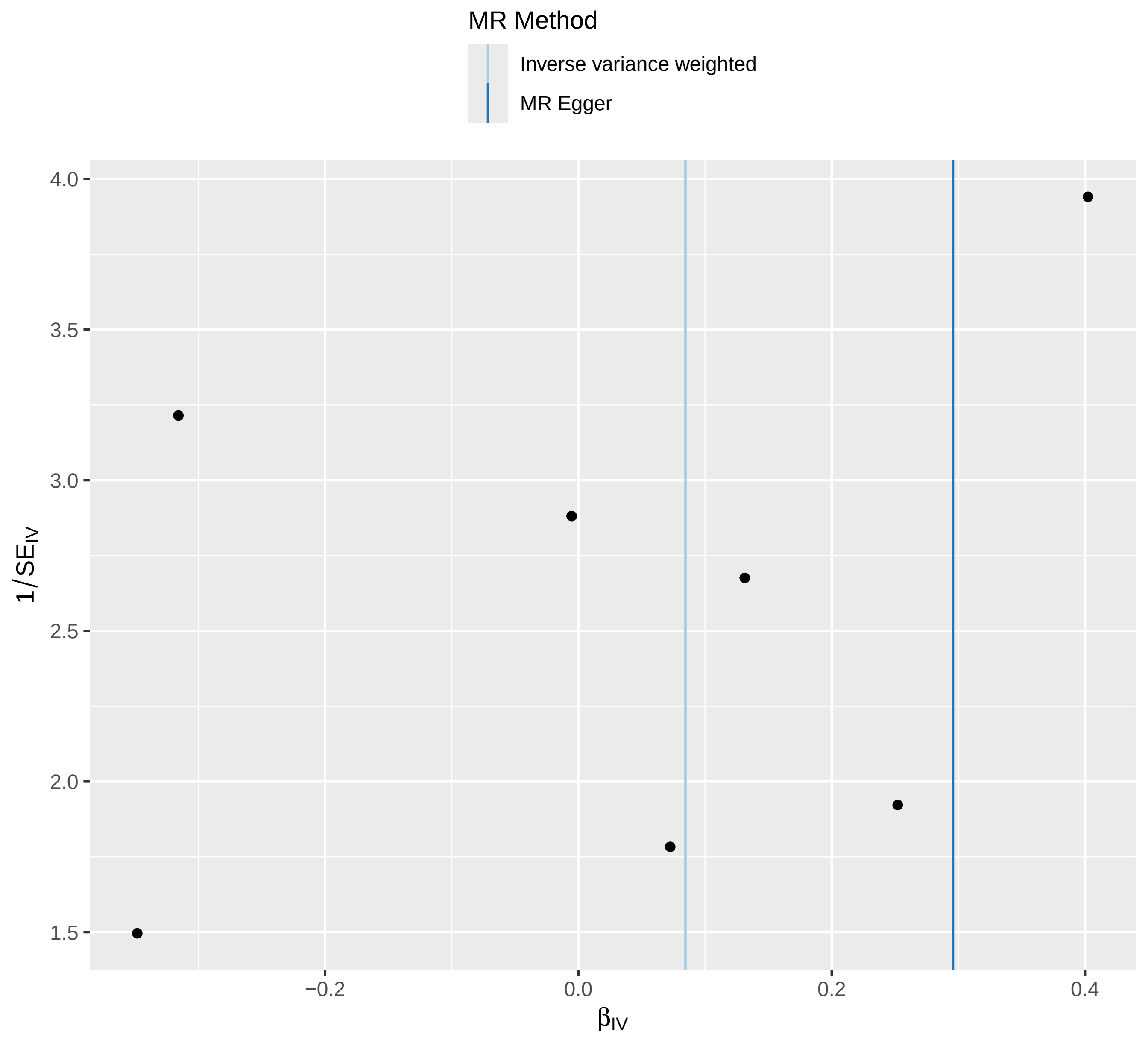
MR Method
Inverse variance weighted
MR Egger
4.0
3.5
3.0
2.5
2.0
1.5
$1/SE_{IV}$
−0.2
0.0
0.2
0.4
β_{IV}

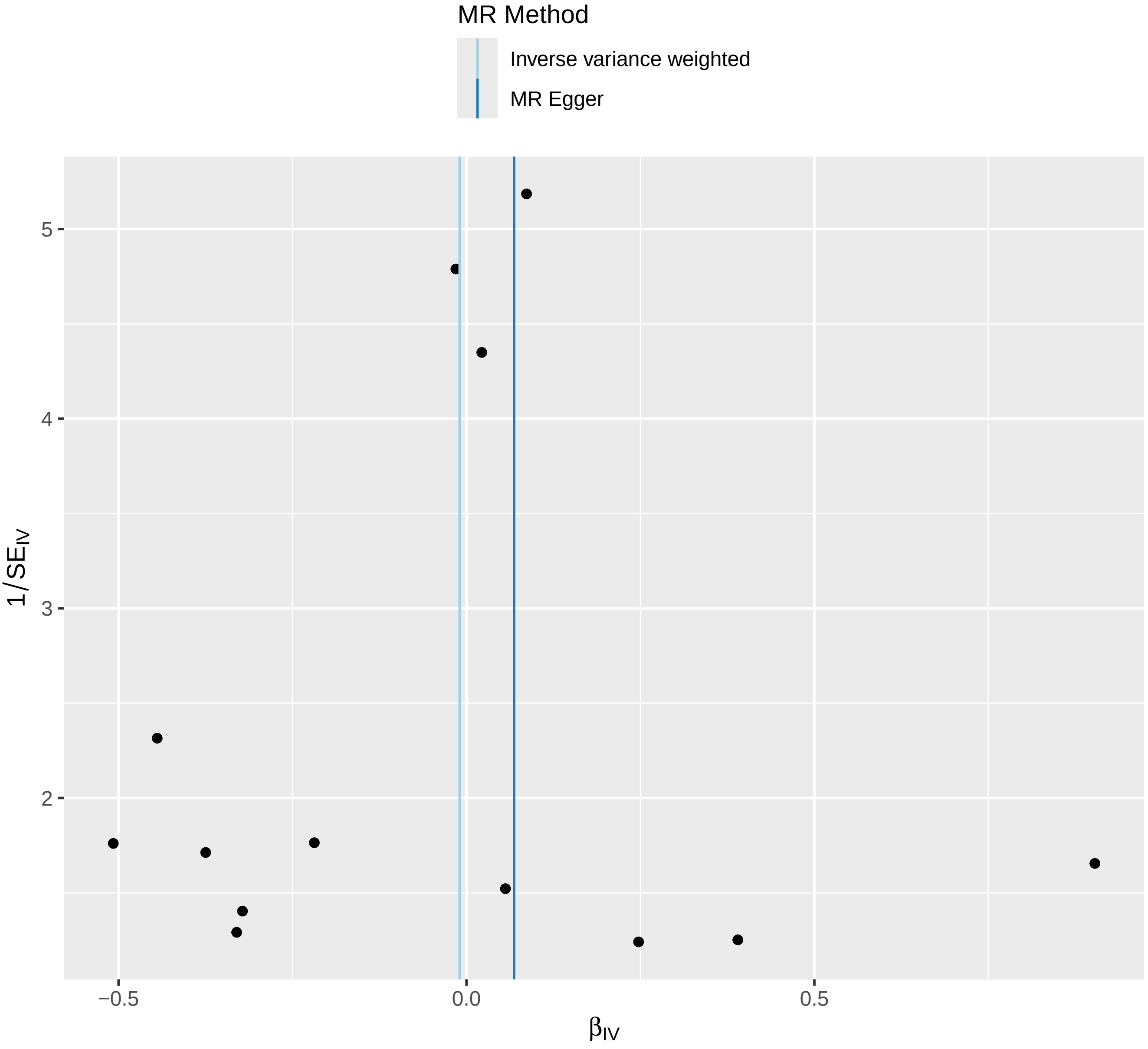

MR Method
Inverse variance weighted
MR Egger
5
4
3
2
$1/SE_{IV}$
−0.5
0.0
0.5
β_{IV}

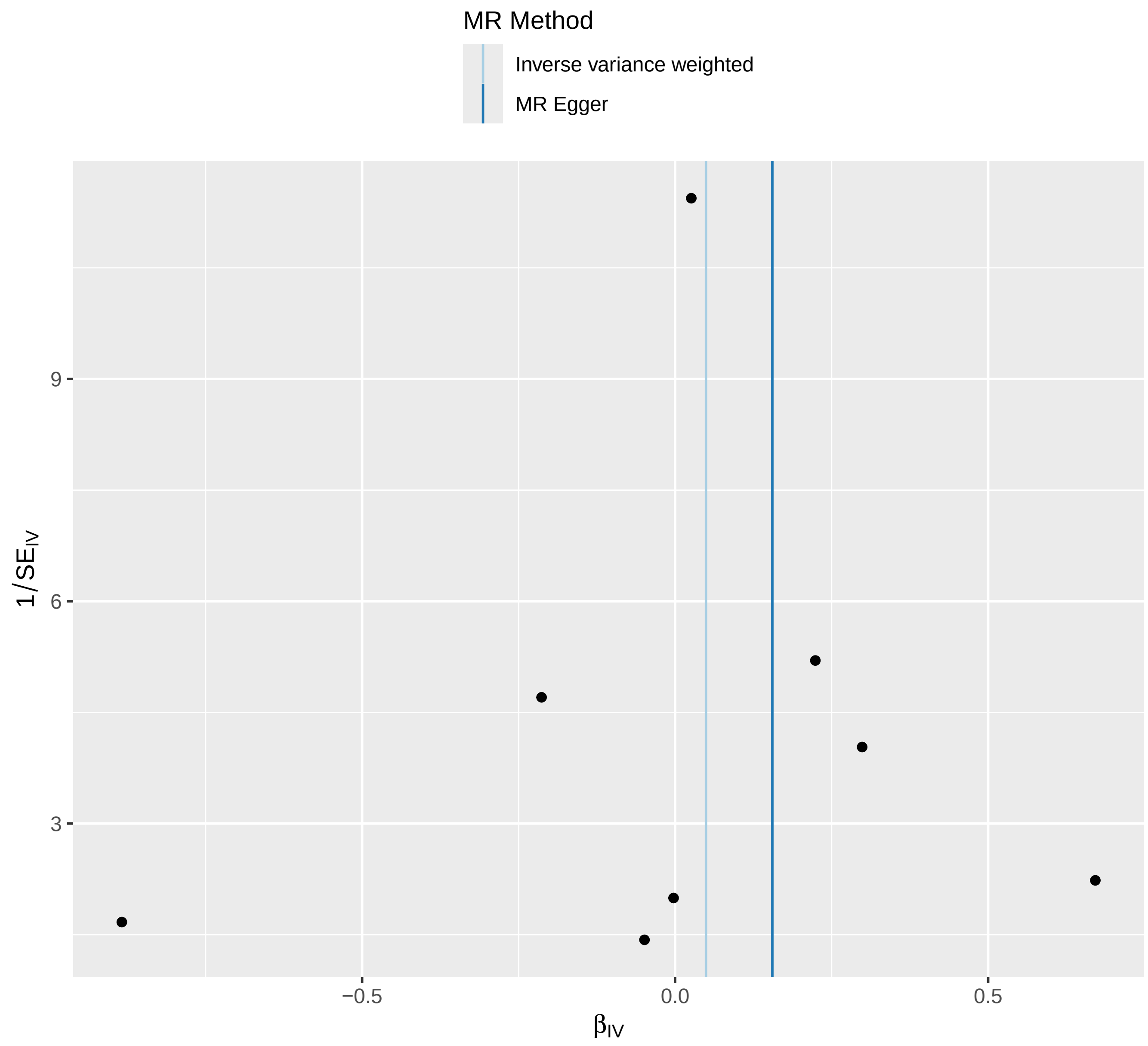
MR Method
Inverse variance weighted
MR Egger
1/SE_IV
9
6
3
−0.5
0.0
0.5
β_IV

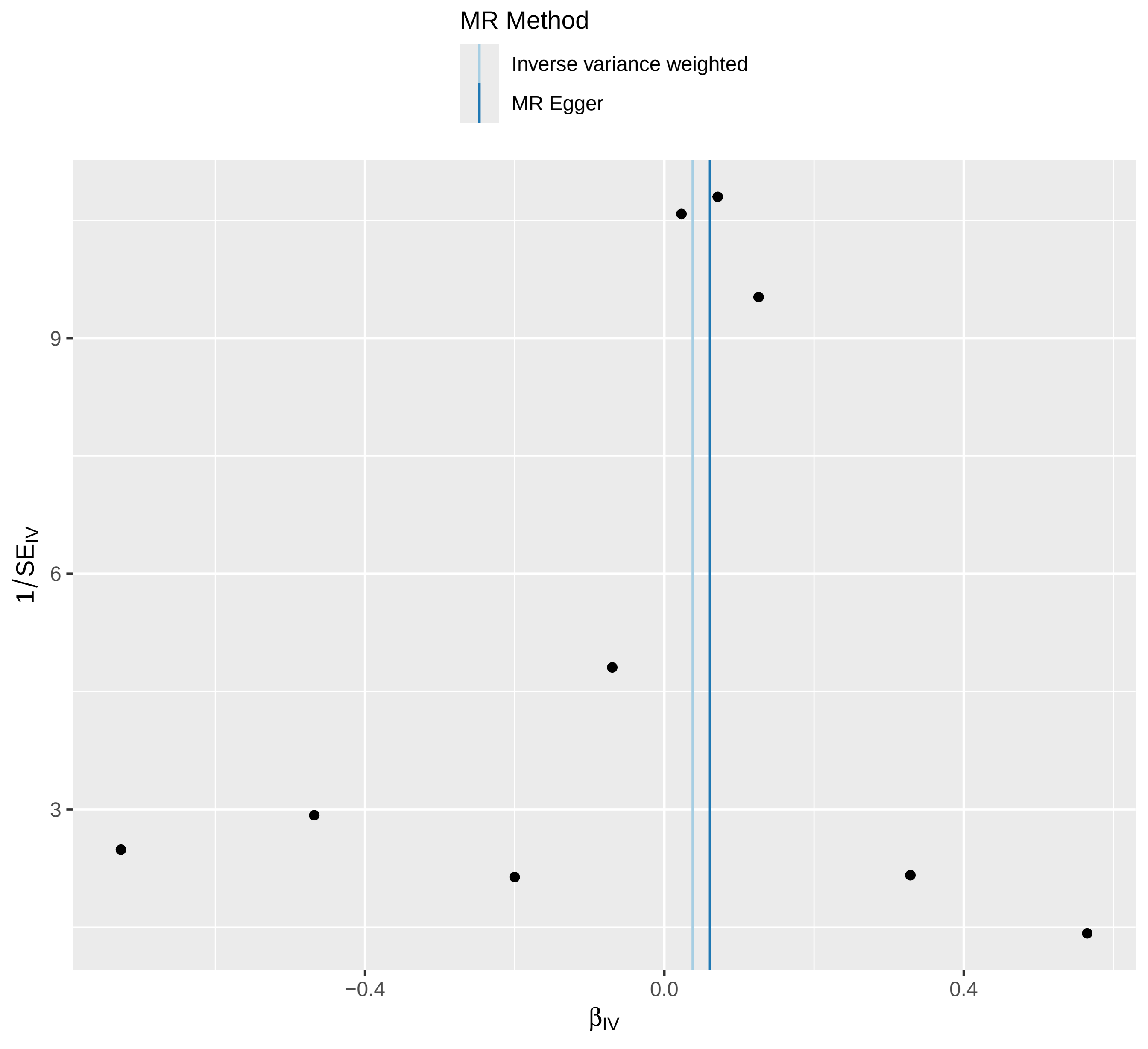

MR Method
Inverse variance weighted
MR Egger
9
6
3
1/SE$_{IV}$
−0.4
0.0
0.4
β_{IV}

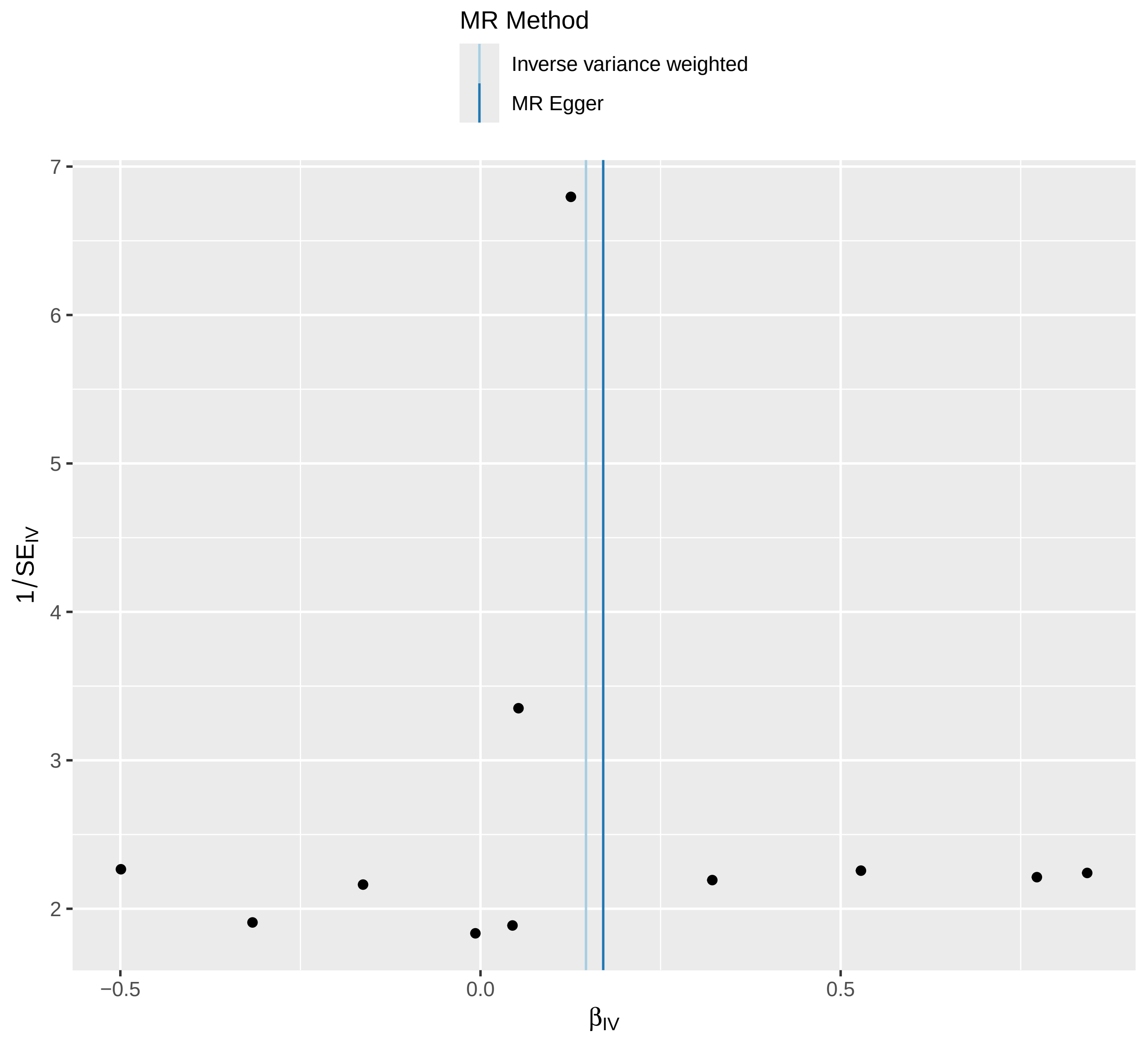
MR Method
Inverse variance weighted
MR Egger
1/SE_{IV}
β_{IV}
7
6
5
4
3
2
−0.5
0.0
0.5

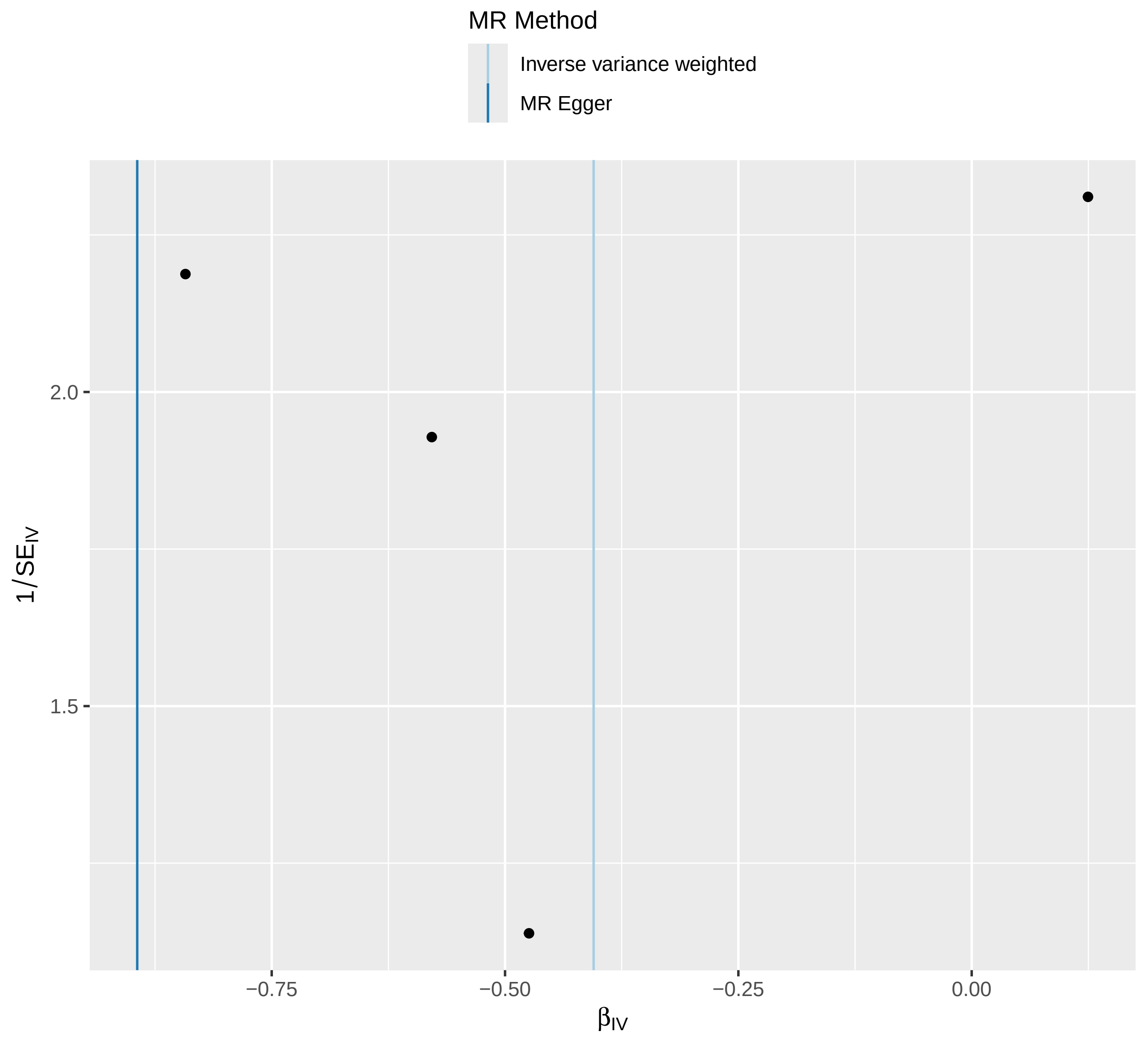

MR Method
Inverse variance weighted
MR Egger
2.0
1.5
1/SE$_{IV}$
−0.75
−0.50
−0.25
0.00
β$_{IV}$

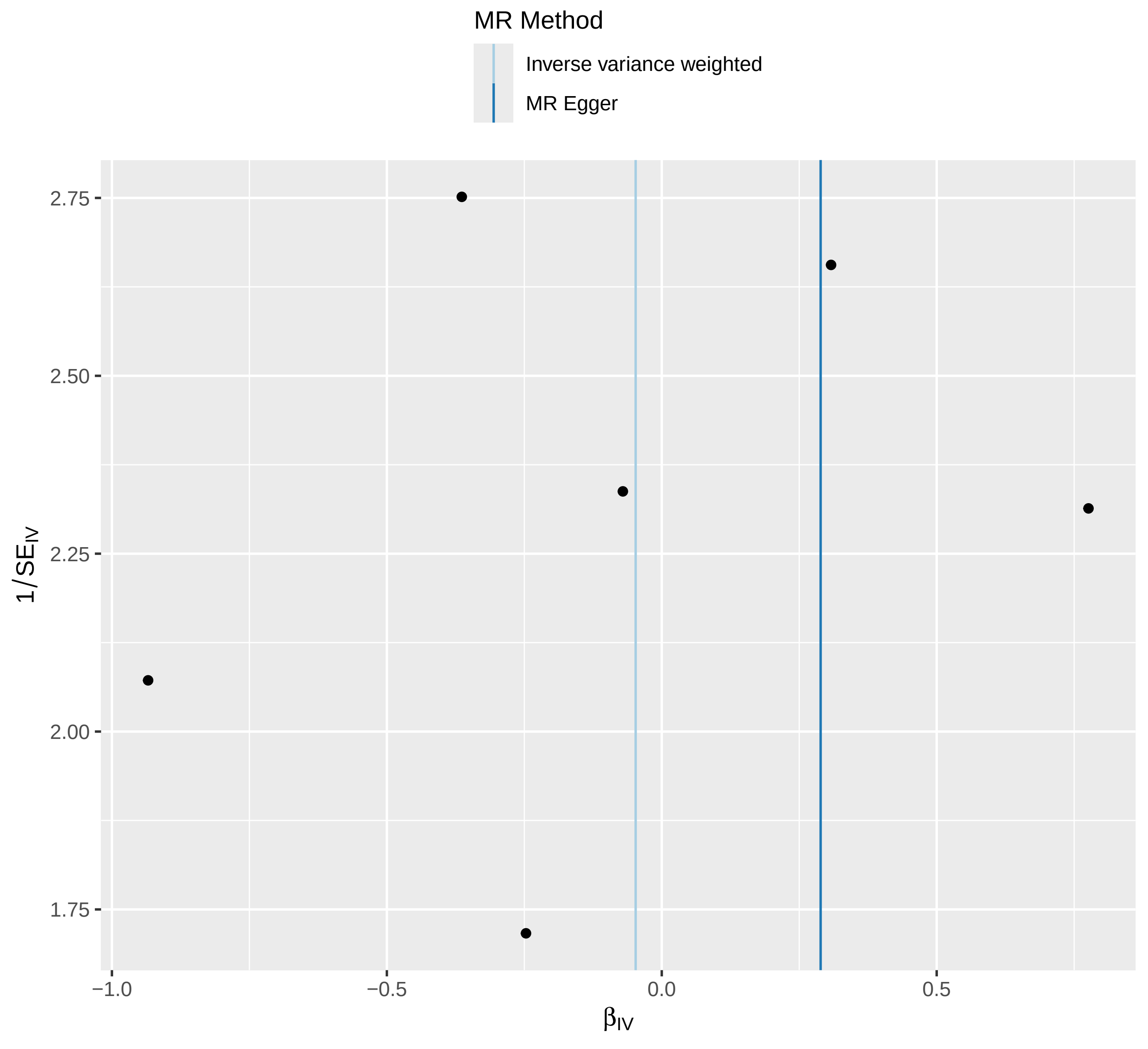
MR Method
Inverse variance weighted
MR Egger
2.75
2.50
2.25
2.00
1.75
$1/SE_{IV}$
−1.0
−0.5
0.0
0.5
β_{IV}

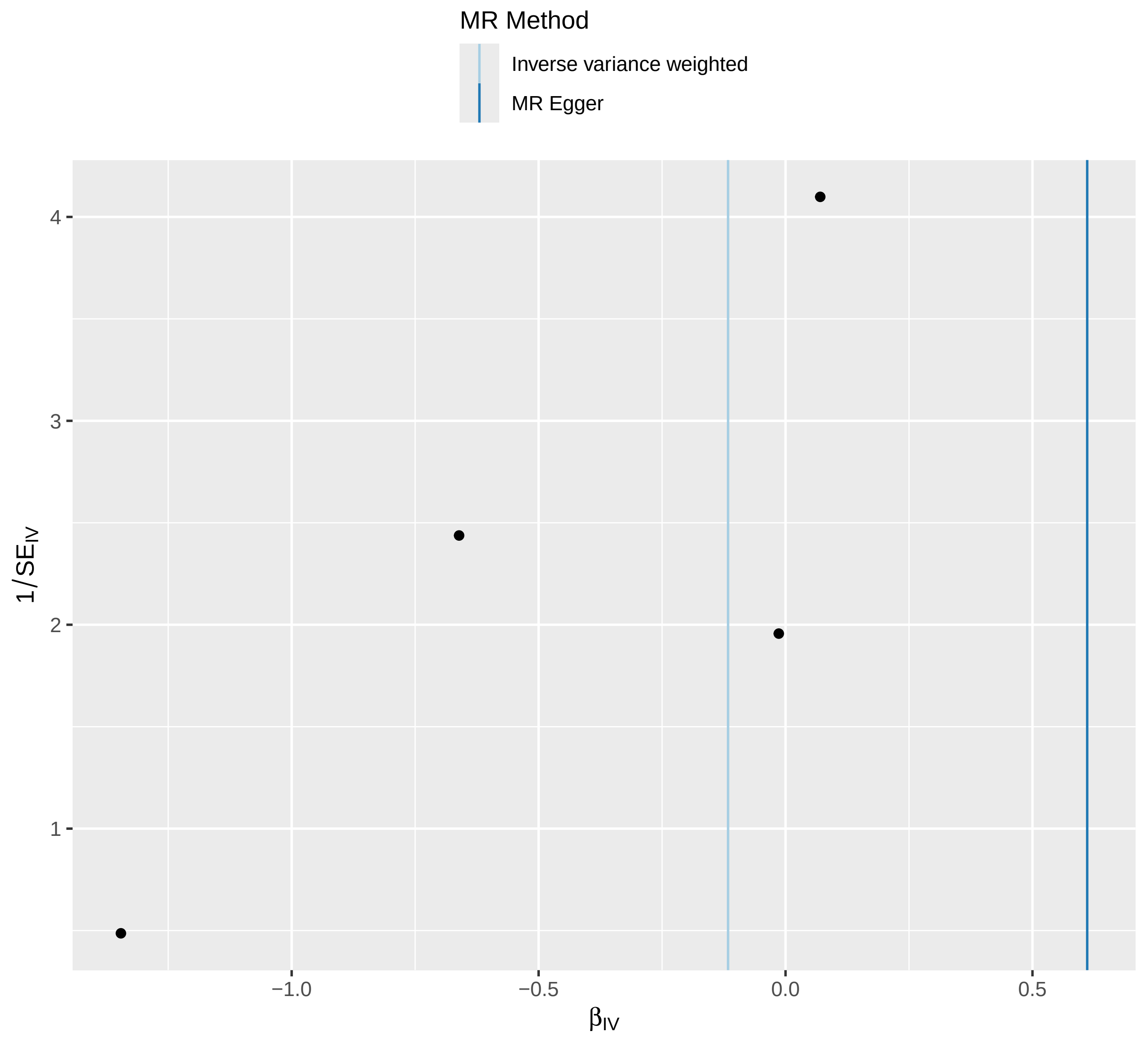
MR Method
Inverse variance weighted
MR Egger
4
3
2
1
$1/SE_{IV}$
−1.0
−0.5
0.0
0.5
β_{IV}

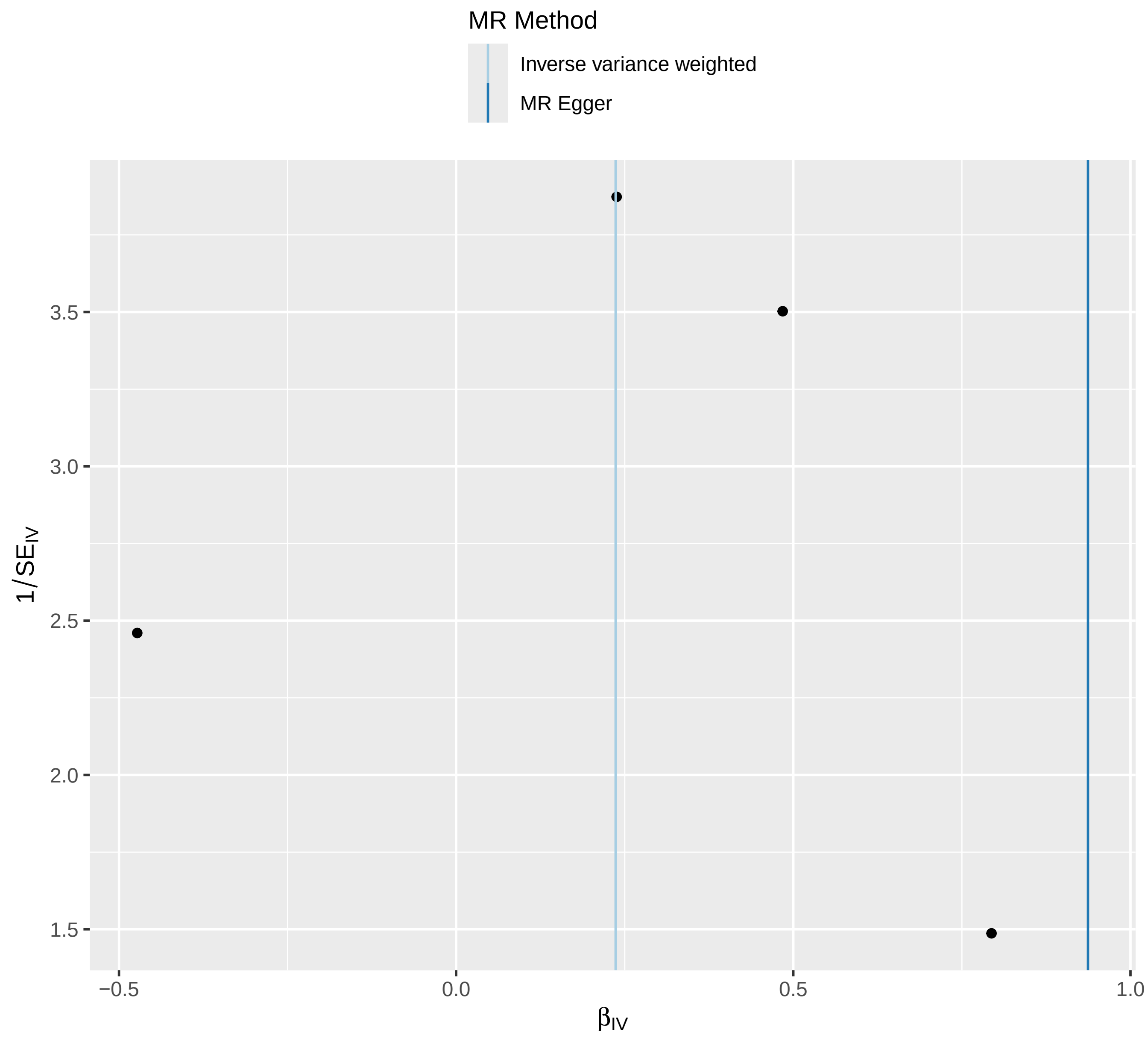

MR Method
Inverse variance weighted
MR Egger
3.5
3.0
2.5
2.0
1.5
$1/SE_{IV}$
−0.5
0.0
0.5
1.0
β_{IV}

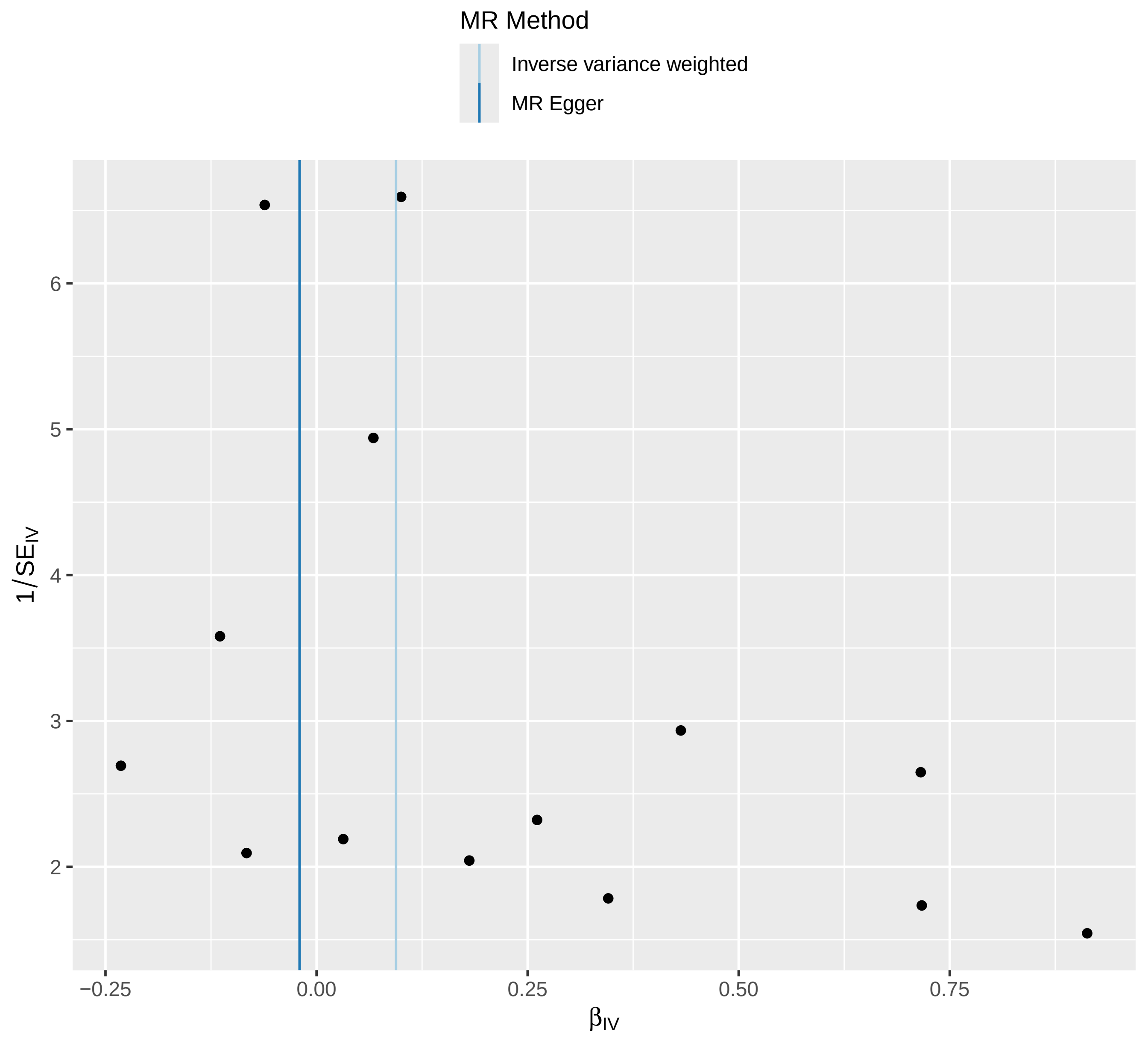

MR Method
Inverse variance weighted
MR Egger
1/SE_IV
6
5
4
3
2
−0.25
0.00
0.25
0.50
0.75
β_IV

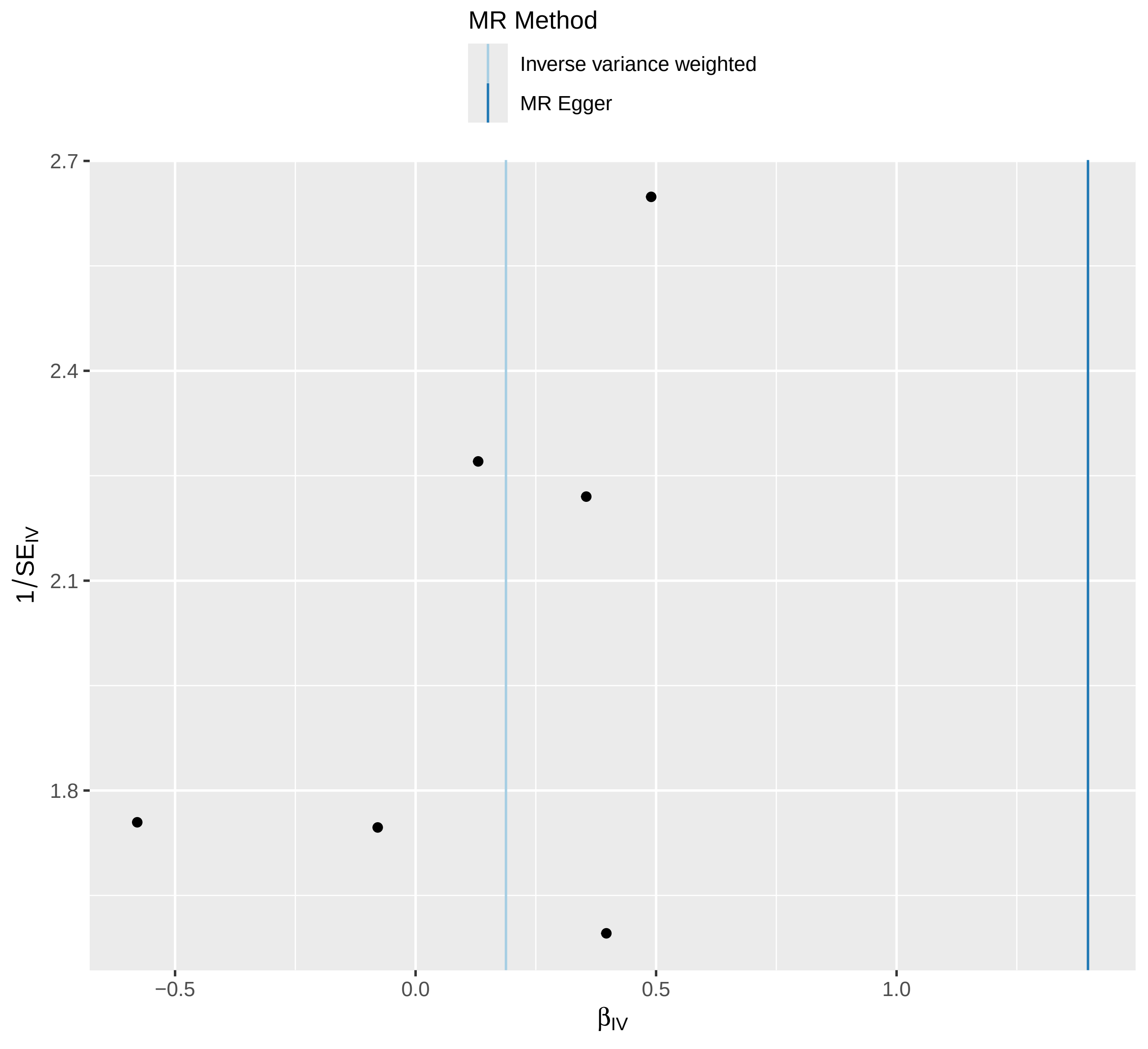
MR Method
Inverse variance weighted
MR Egger
2.7
2.4
2.1
1.8
1/SE_IV
−0.5
0.0
0.5
1.0
β_IV

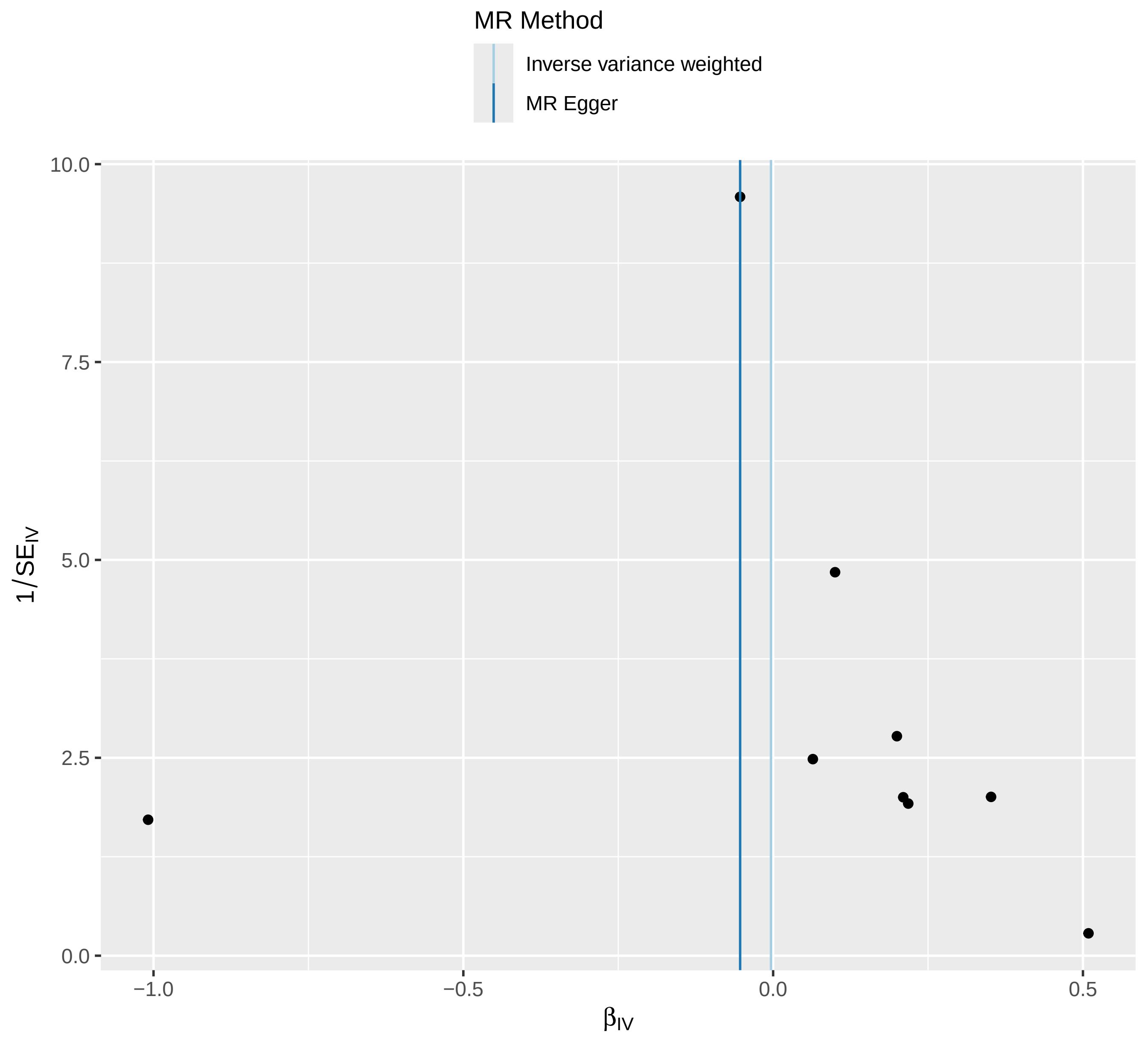

MR Method
Inverse variance weighted
MR Egger
10.0
7.5
5.0
2.5
0.0
−1.0
−0.5
0.0
0.5
$1/SE_{IV}$
β_{IV}

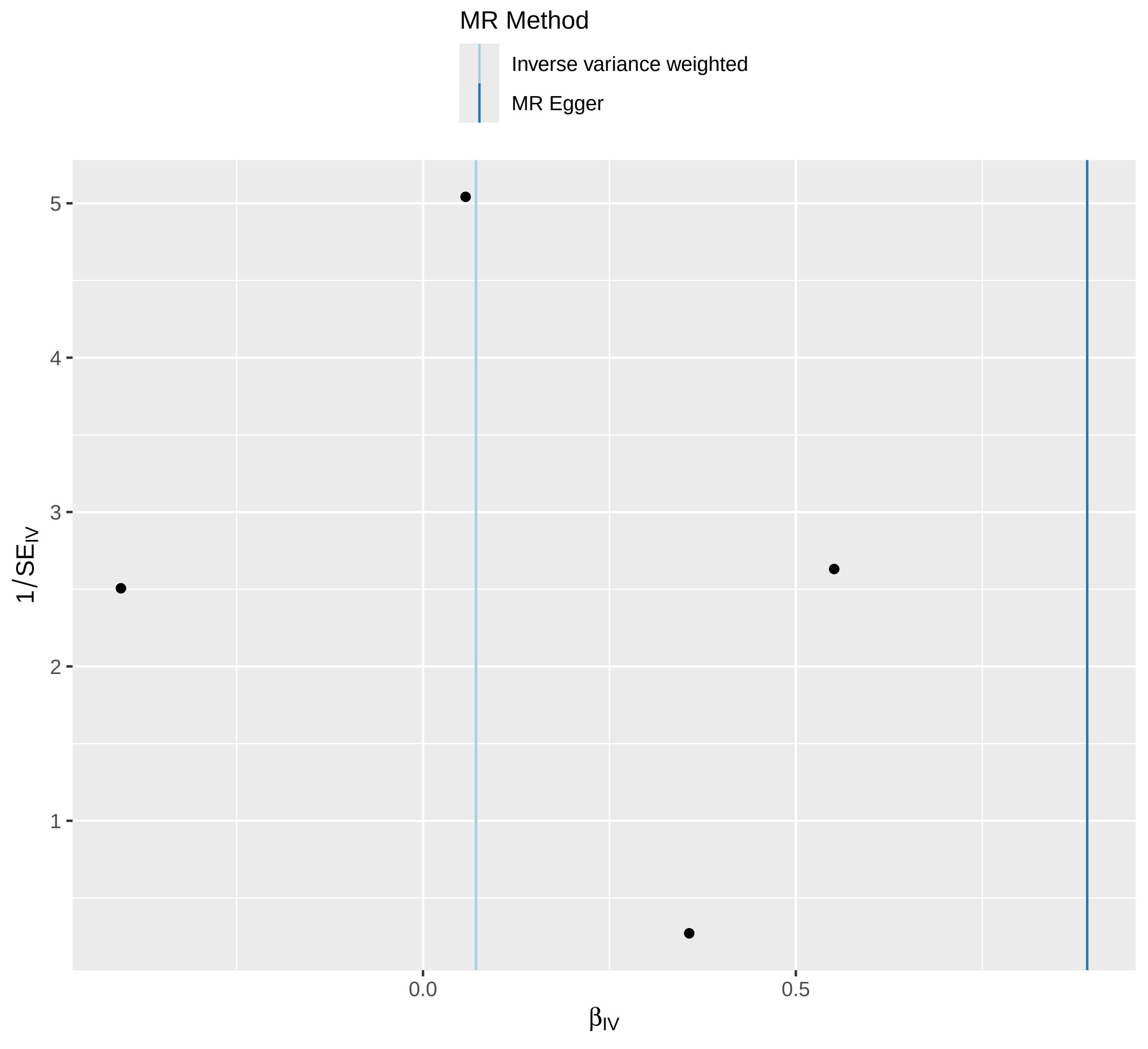

MR Method
Inverse variance weighted
MR Egger
5
4
3
2
1
1/SE_IV
0.0
0.5
β_IV

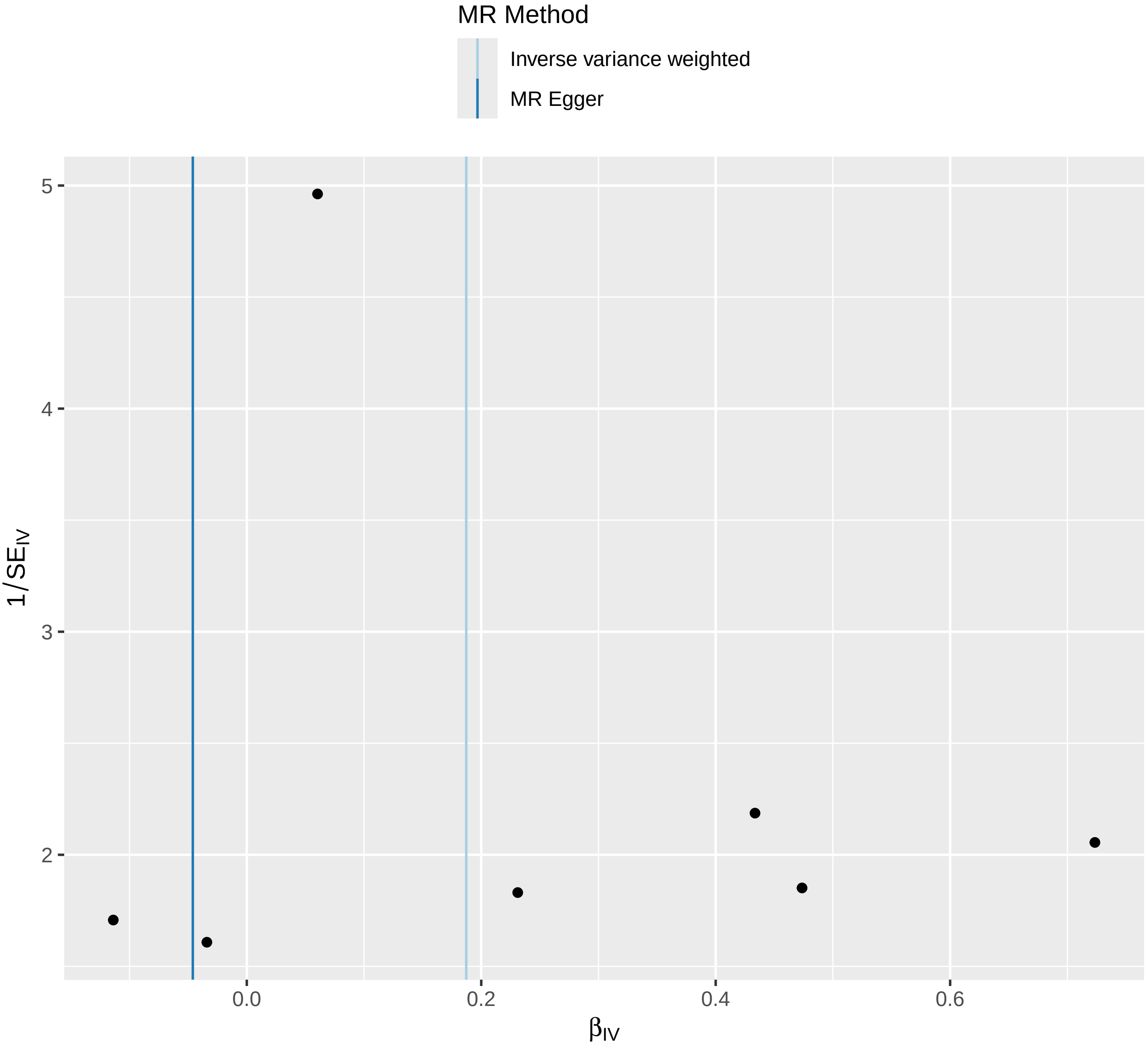

MR Method
Inverse variance weighted
MR Egger
5
4
3
2
0.0
0.2
0.4
0.6
1/SE_IV
β_IV

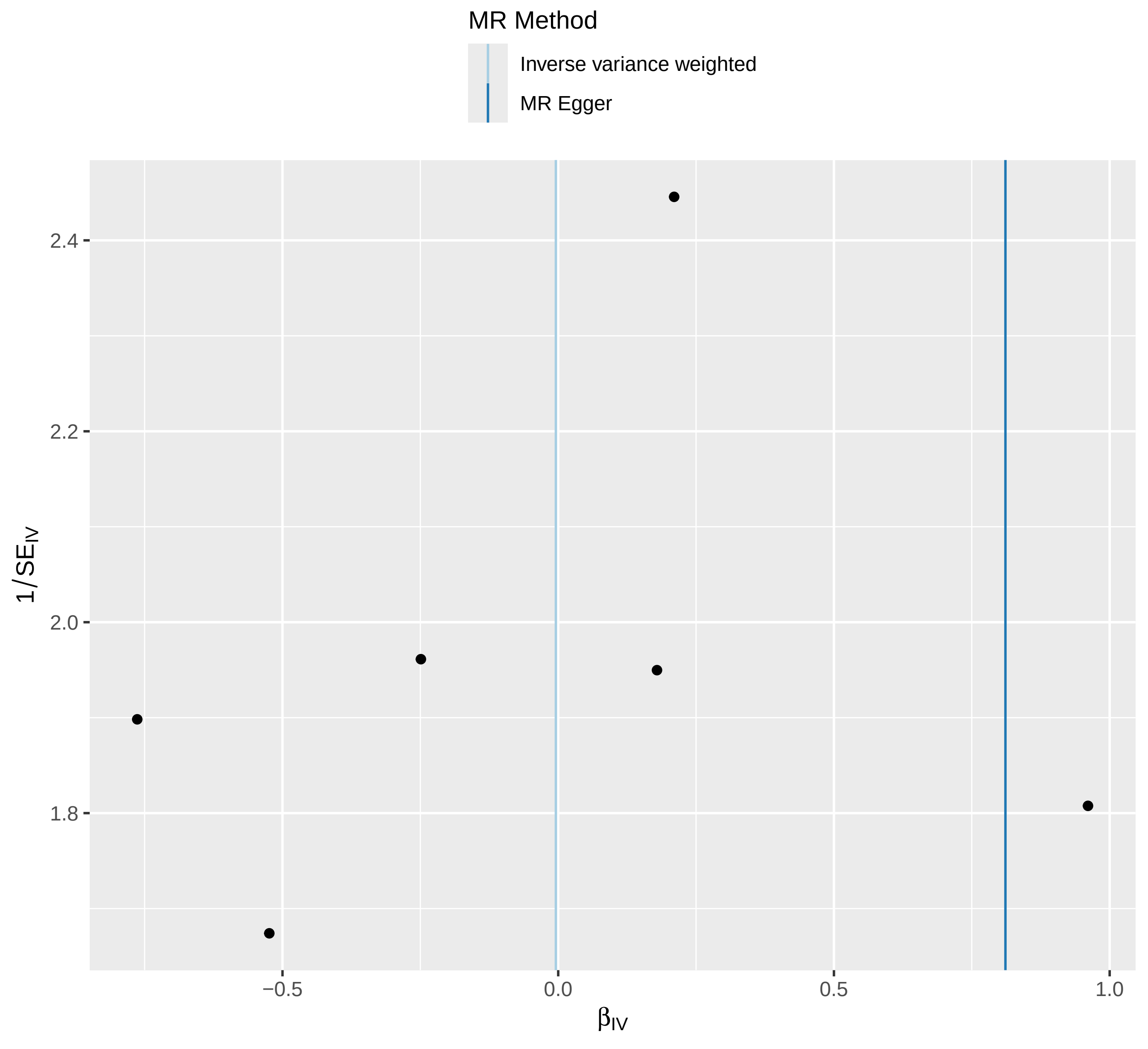

MR Method
Inverse variance weighted
MR Egger
2.4
2.2
2.0
1.8
1/SE_IV
−0.5
0.0
0.5
1.0
β_IV

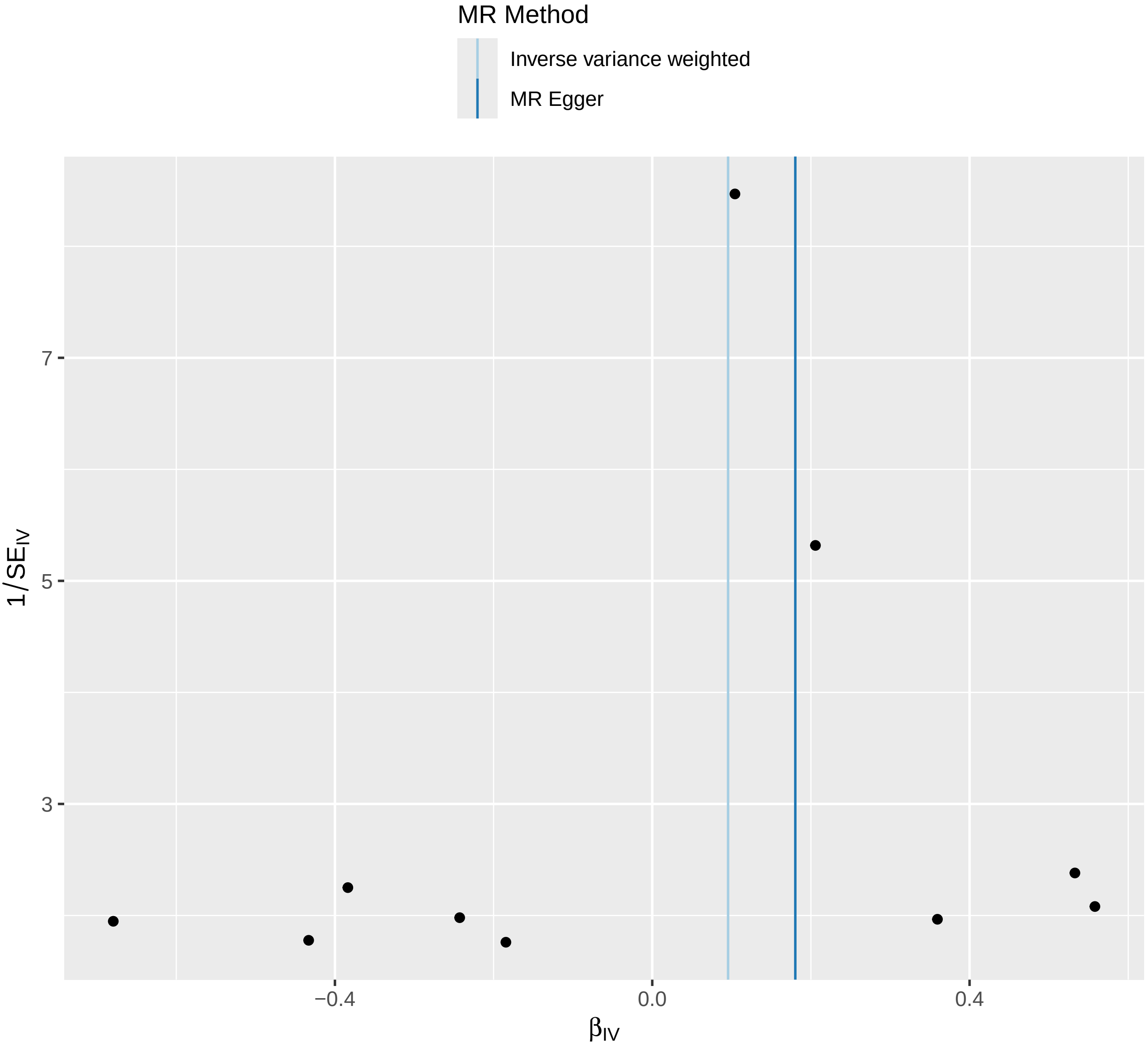
MR Method
Inverse variance weighted
MR Egger
7
5
3
1/SE_IV
−0.4
0.0
0.4
β_IV

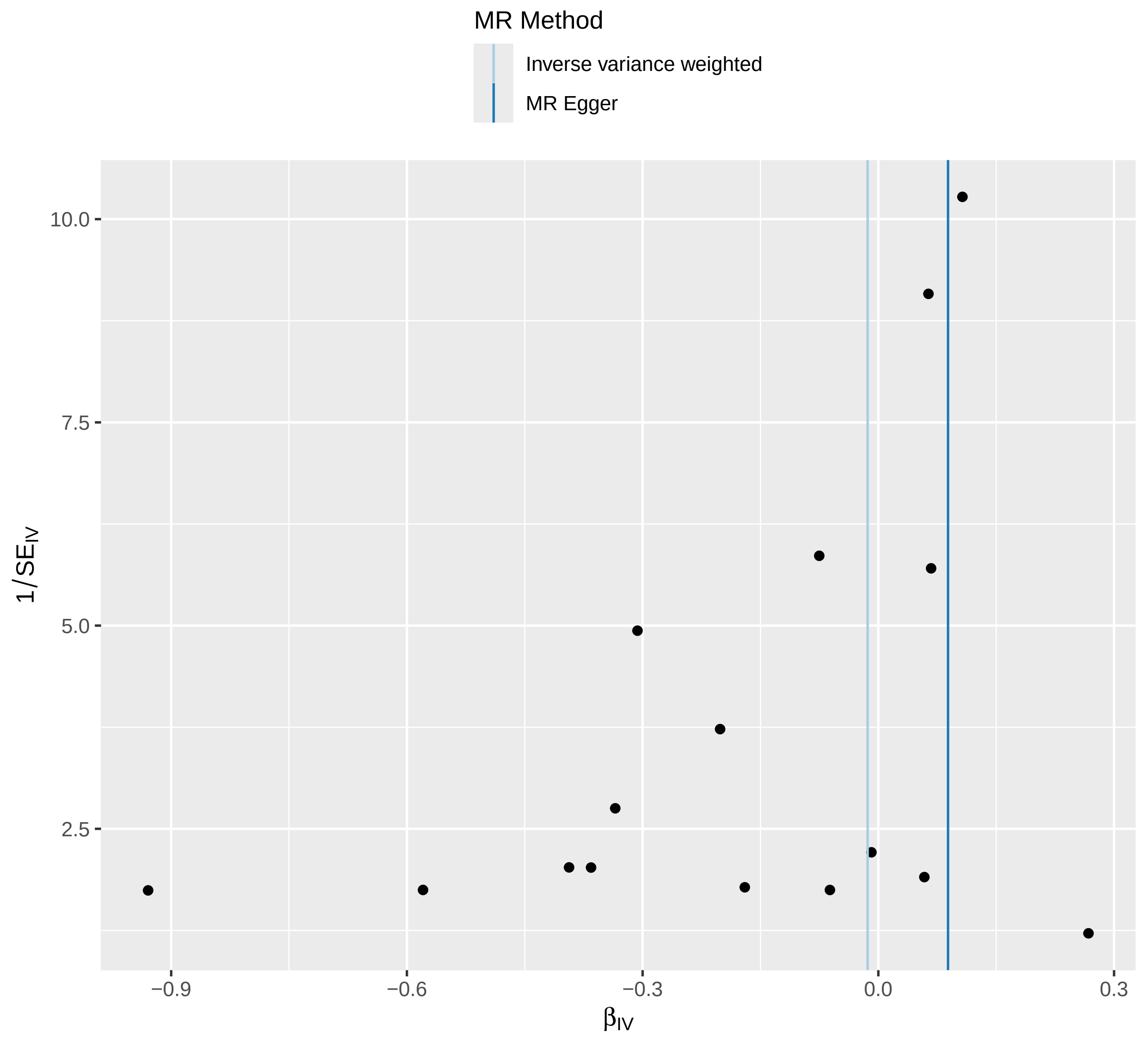

MR Method
Inverse variance weighted
MR Egger
10.0
7.5
5.0
2.5
1/SE_IV
−0.9
−0.6
−0.3
0.0
0.3
β_IV

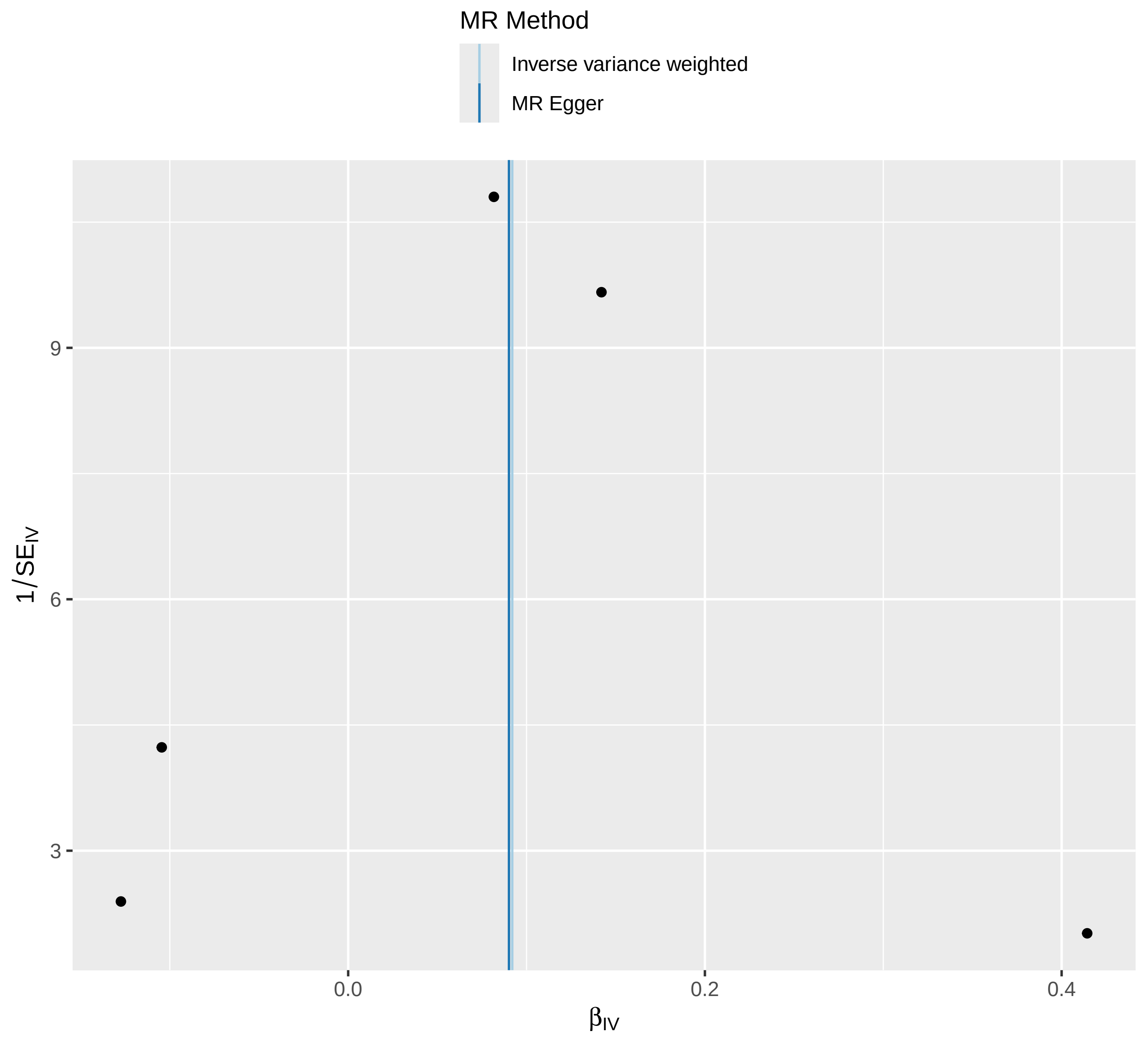

MR Method
Inverse variance weighted
MR Egger
9
6
3
1/SE_IV
0.0
0.2
0.4
β_IV

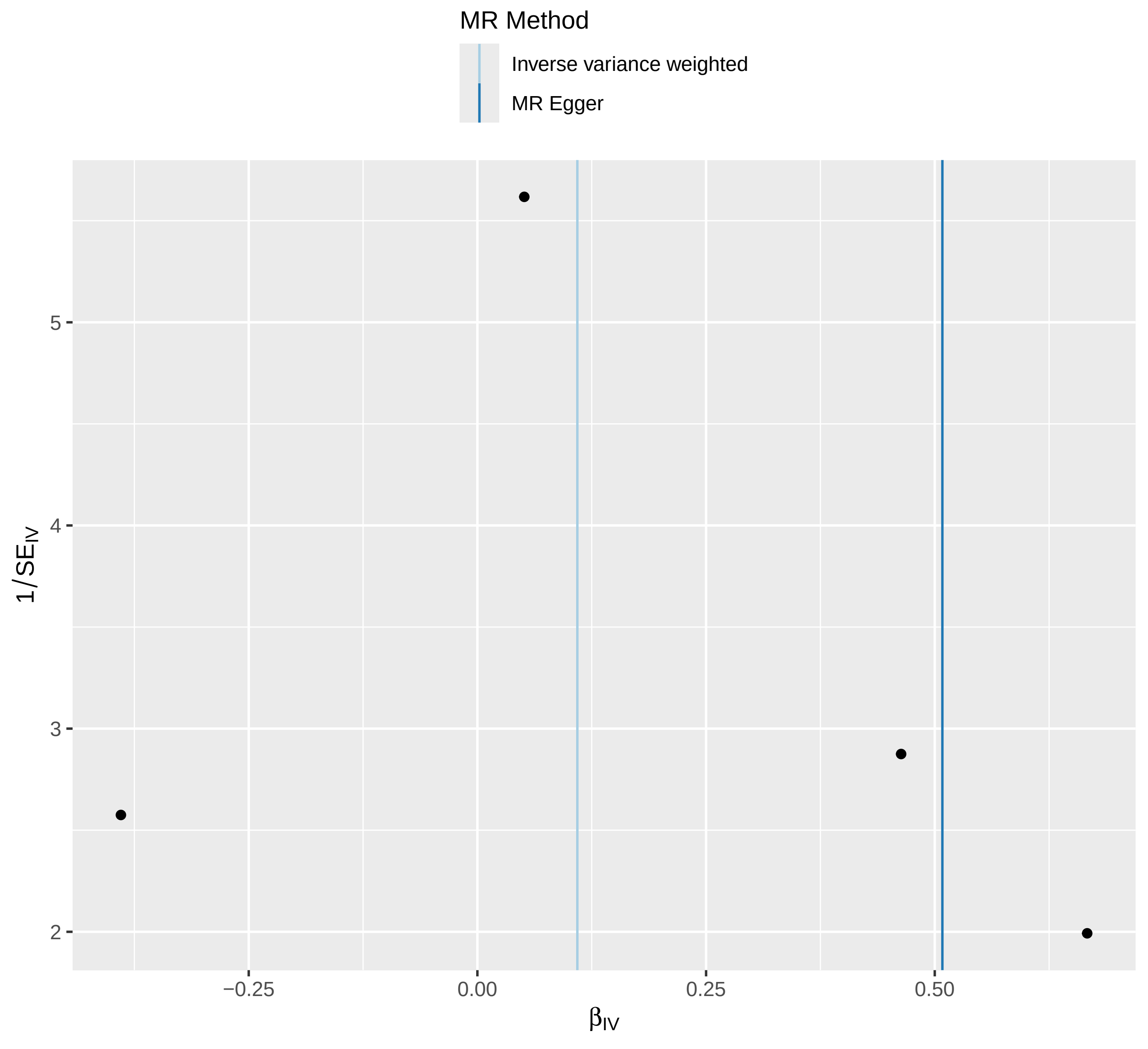

MR Method
Inverse variance weighted
MR Egger
1/SE_IV
5
4
3
2
−0.25
0.00
0.25
0.50
β_IV

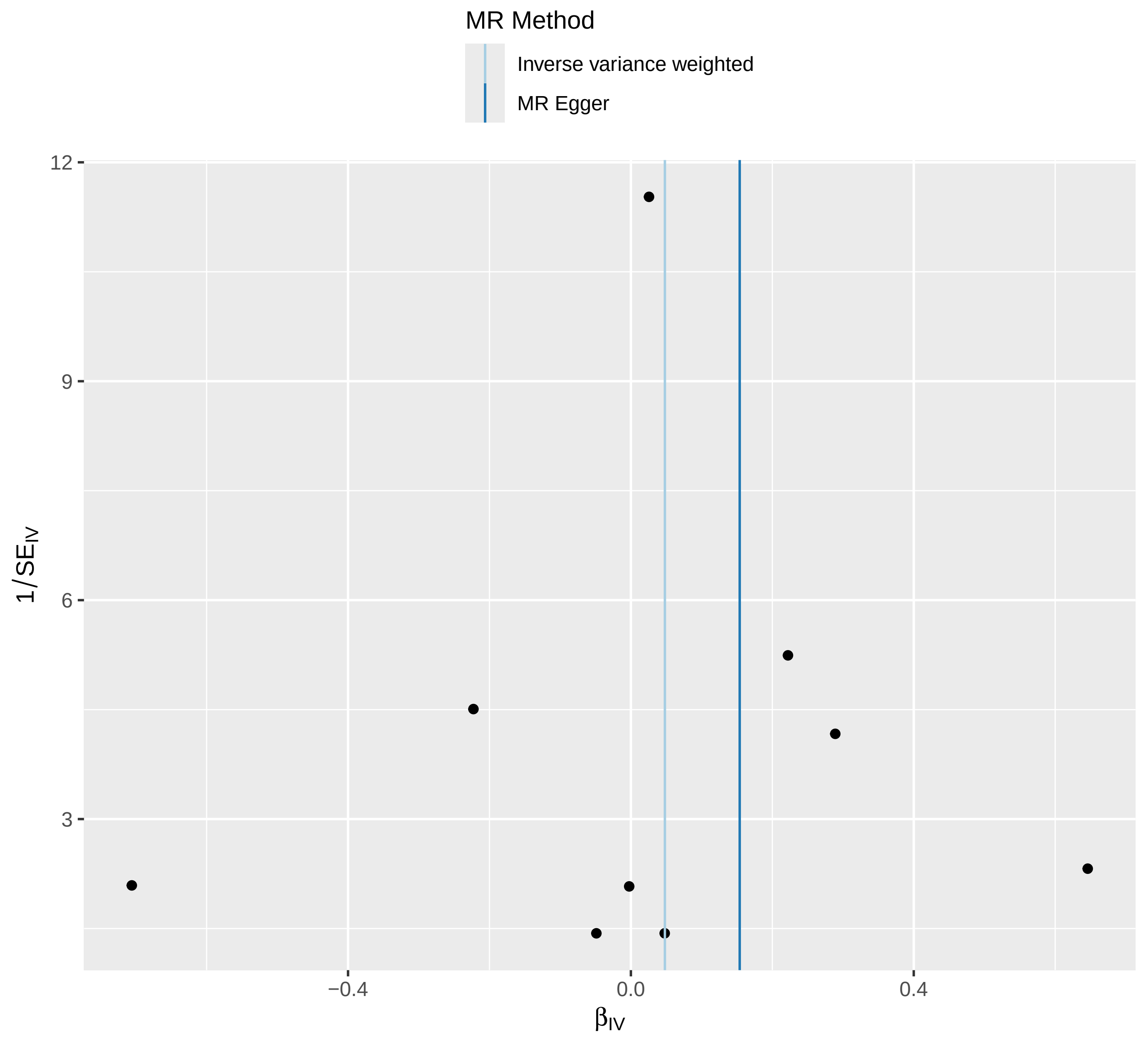
MR Method
Inverse variance weighted
MR Egger
12
9
6
3
$1/SE_{IV}$
−0.4
0.0
0.4
β_{IV}

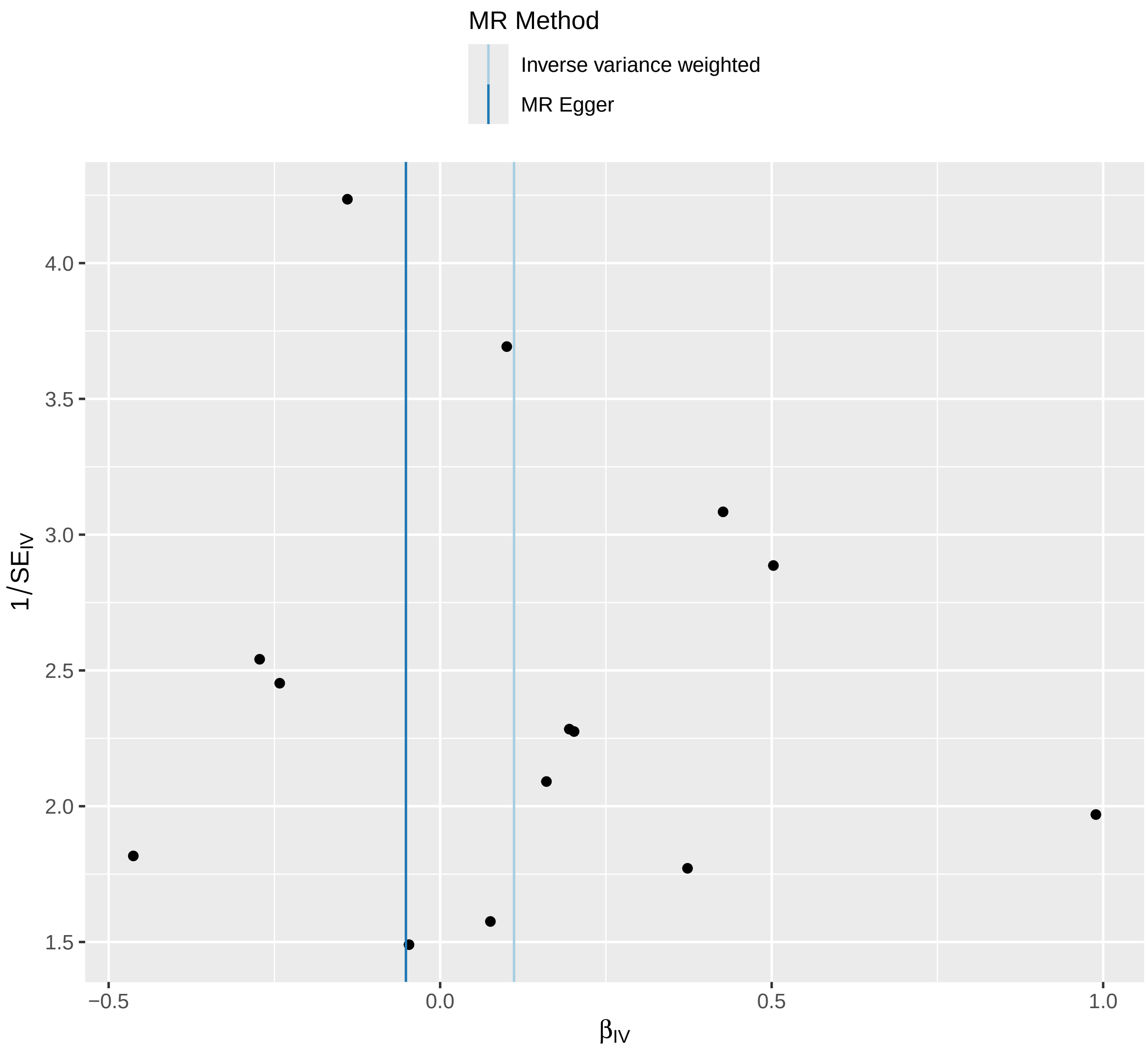

MR Method
Inverse variance weighted
MR Egger
4.0
3.5
3.0
2.5
2.0
1.5
1/SE_IV
−0.5
0.0
0.5
1.0
β_IV

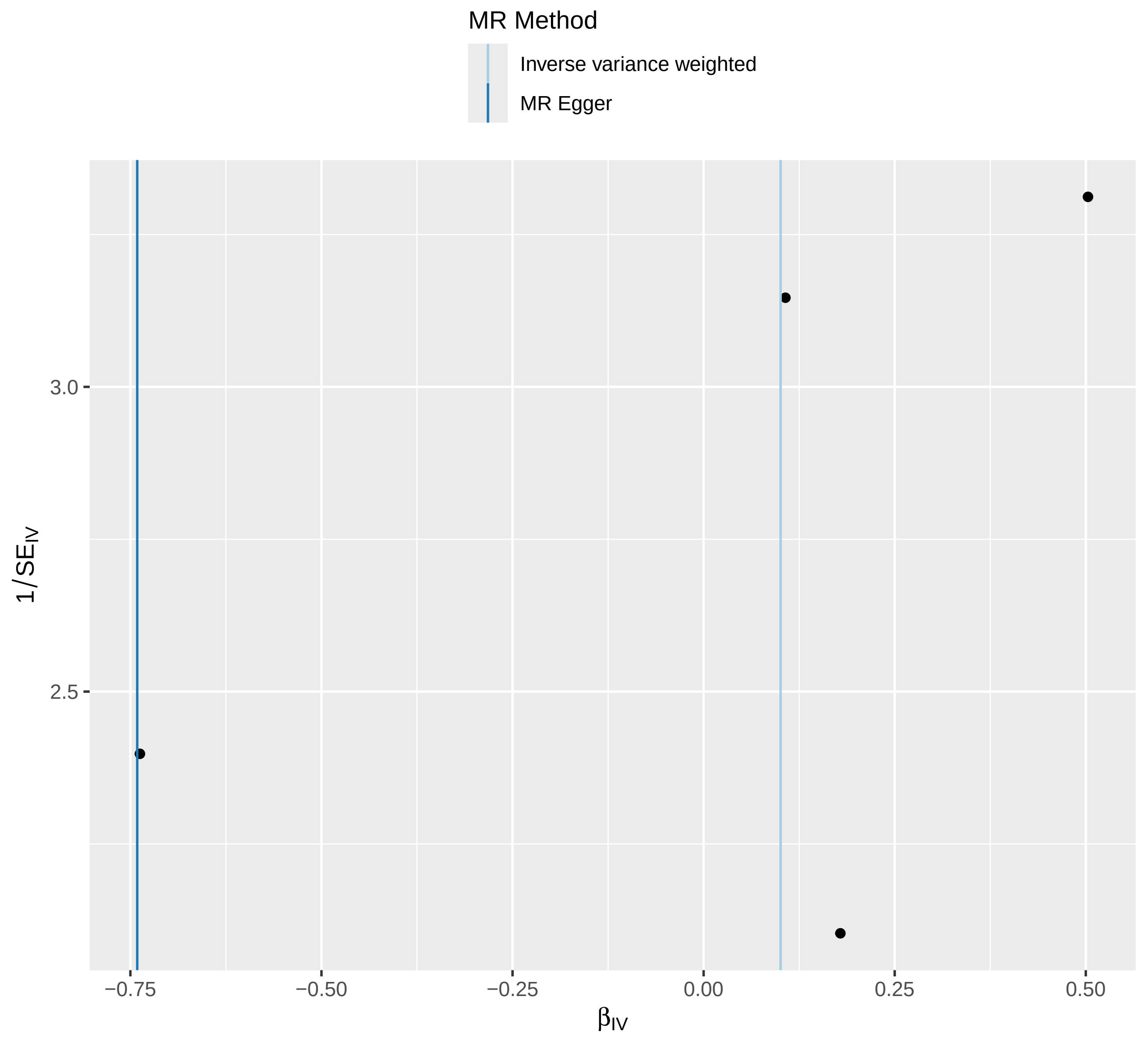

MR Method
Inverse variance weighted
MR Egger
3.0
2.5
1/SE$_{IV}$
−0.75
−0.50
−0.25
0.00
0.25
0.50
β$_{IV}$

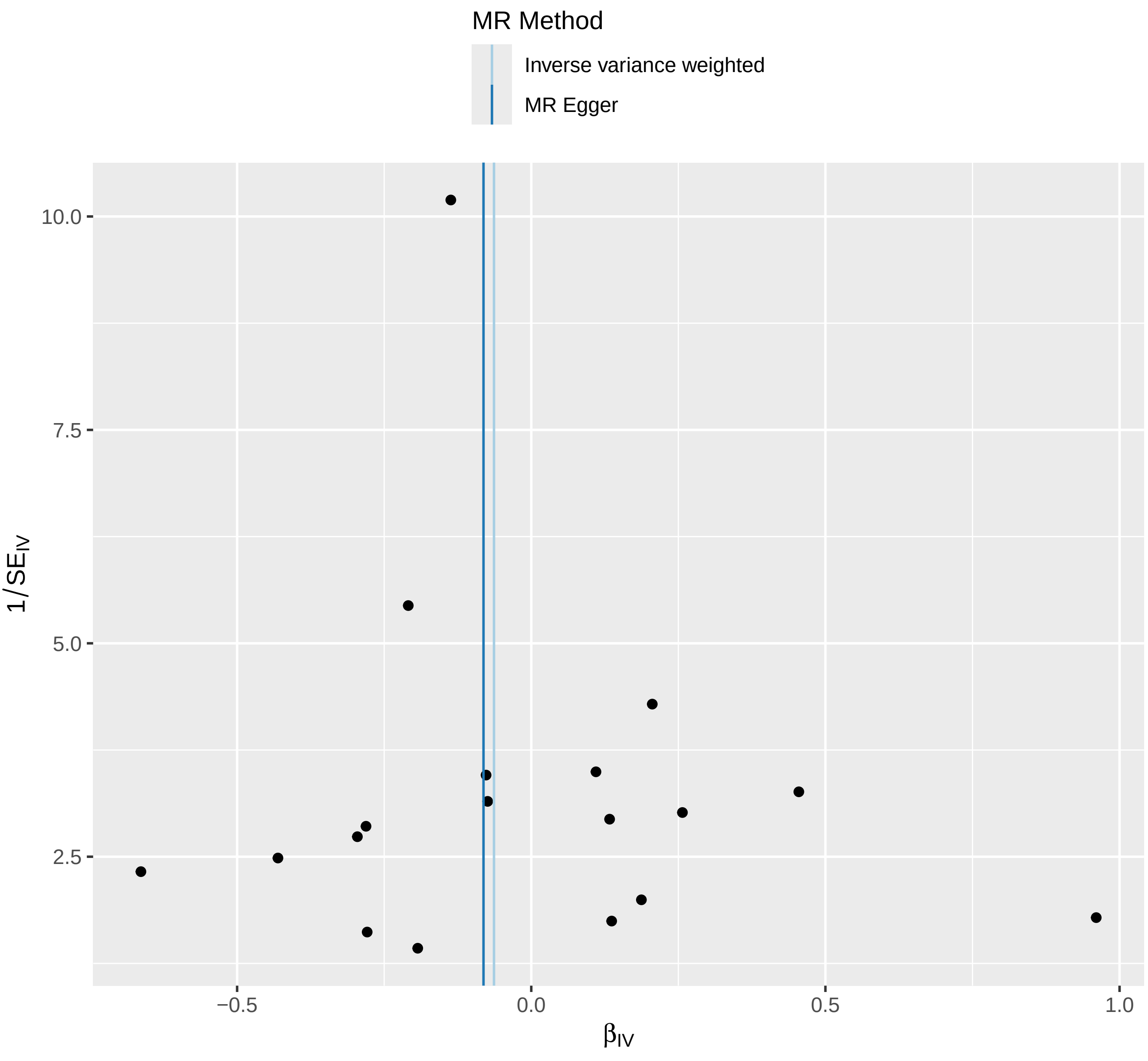
MR Method
Inverse variance weighted
MR Egger
10.0
7.5
5.0
2.5
1/SE_IV
−0.5
0.0
0.5
1.0
β_IV

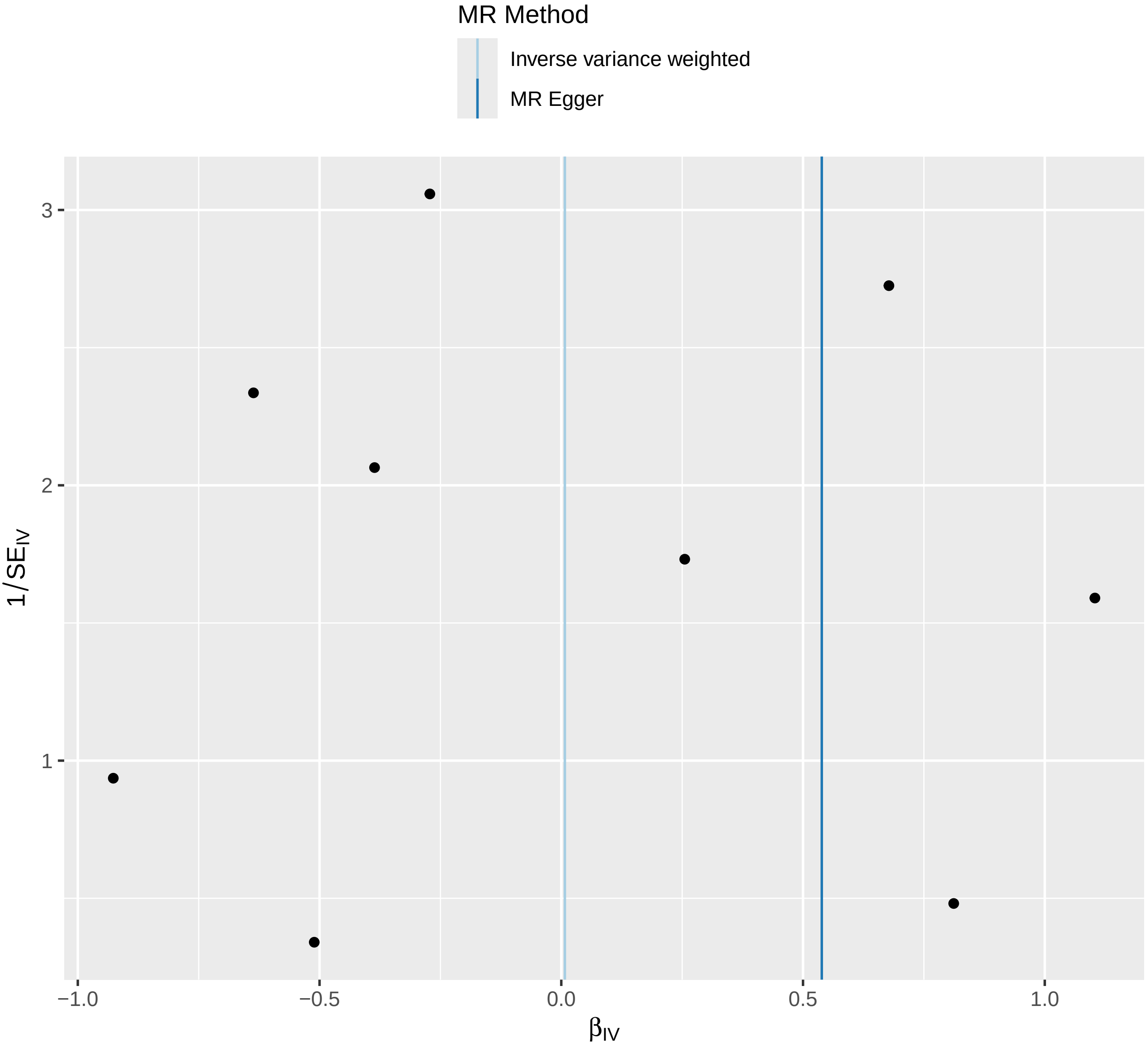

MR Method
Inverse variance weighted
MR Egger
3
2
1
1/SE_{IV}
−1.0
−0.5
0.0
0.5
1.0
β_{IV}

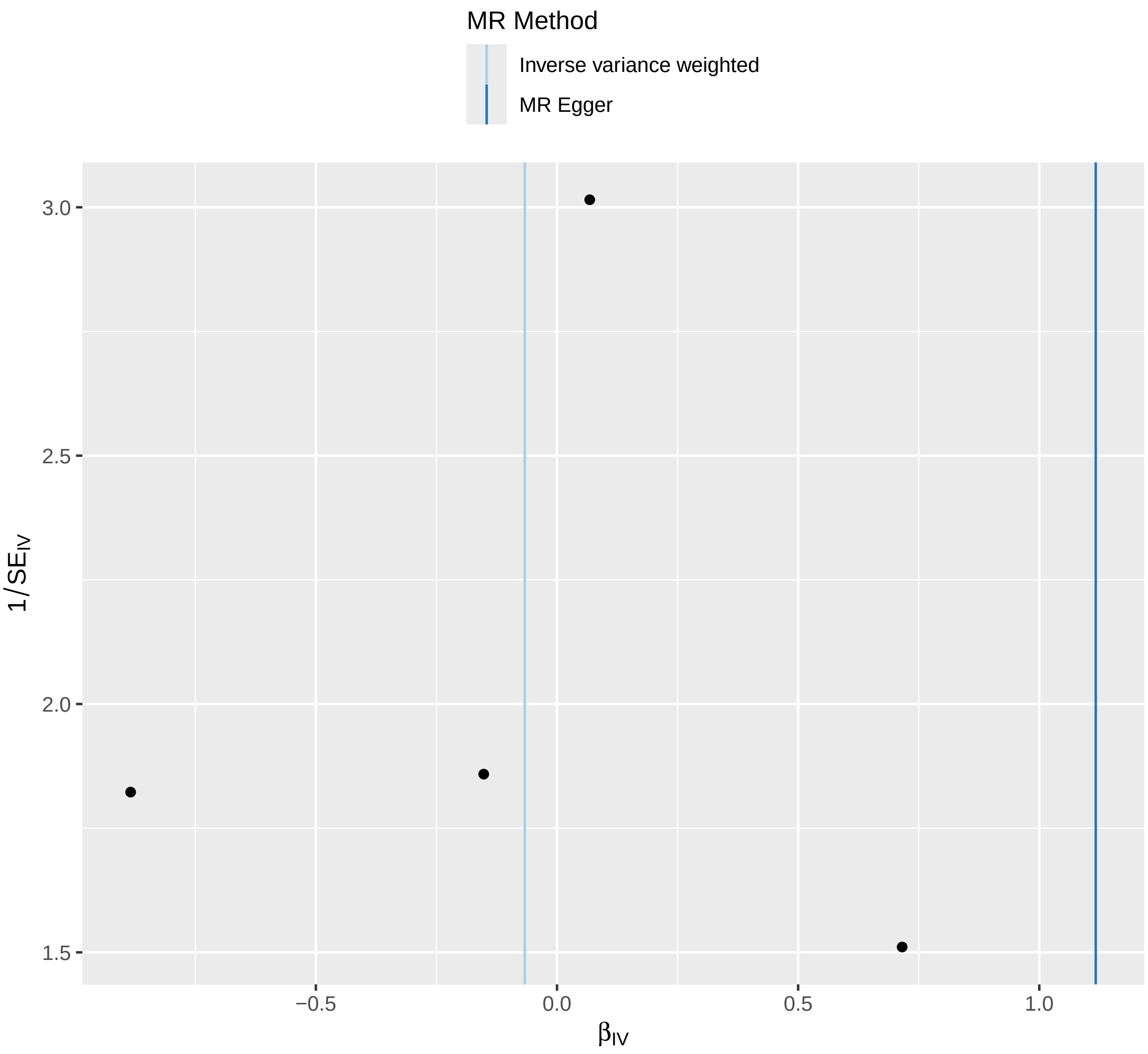
MR Method
Inverse variance weighted
MR Egger
3.0
2.5
2.0
1.5
1/SE_IV
−0.5
0.0
0.5
1.0
β_IV

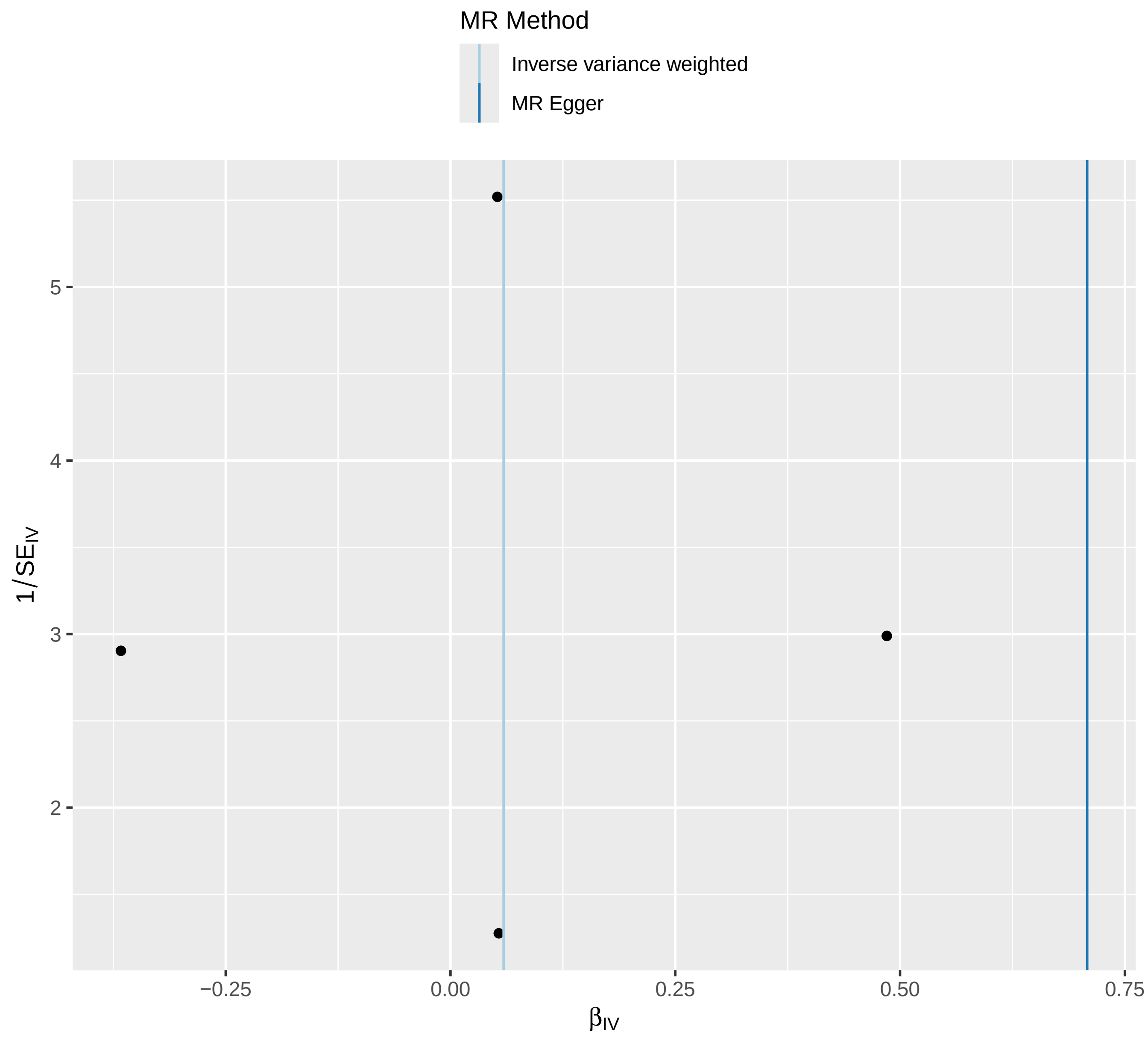
MR Method
Inverse variance weighted
MR Egger
1/SE_IV
β_IV
5
4
3
2
−0.25
0.00
0.25
0.50
0.75

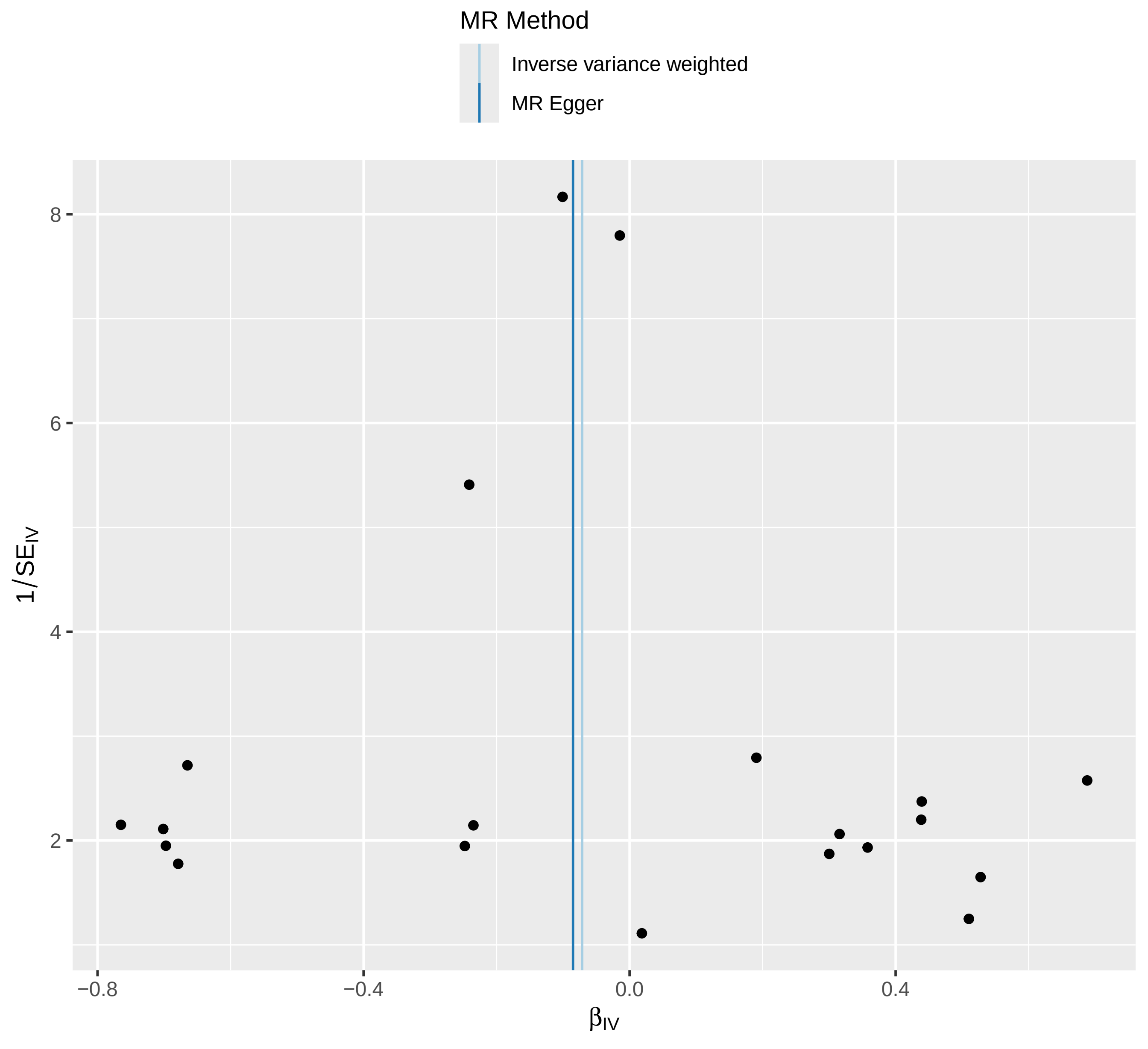
MR Method
Inverse variance weighted
MR Egger
1/SE$_{IV}$
8
6
4
2
−0.8
−0.4
0.0
0.4
β$_{IV}$

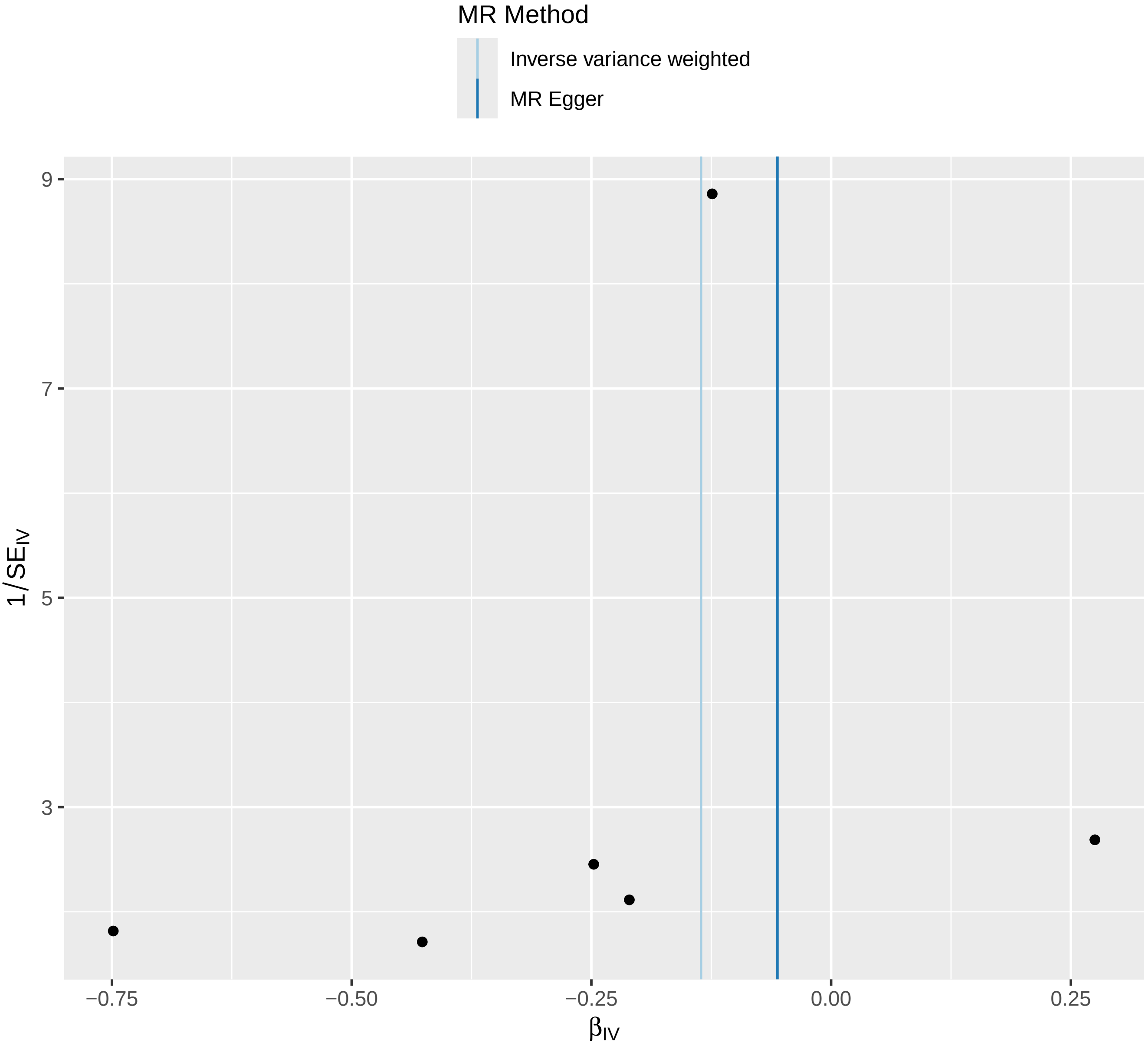

MR Method
Inverse variance weighted
MR Egger
9
7
5
3
1/SE$_{IV}$
−0.75
−0.50
−0.25
0.00
0.25
β$_{IV}$

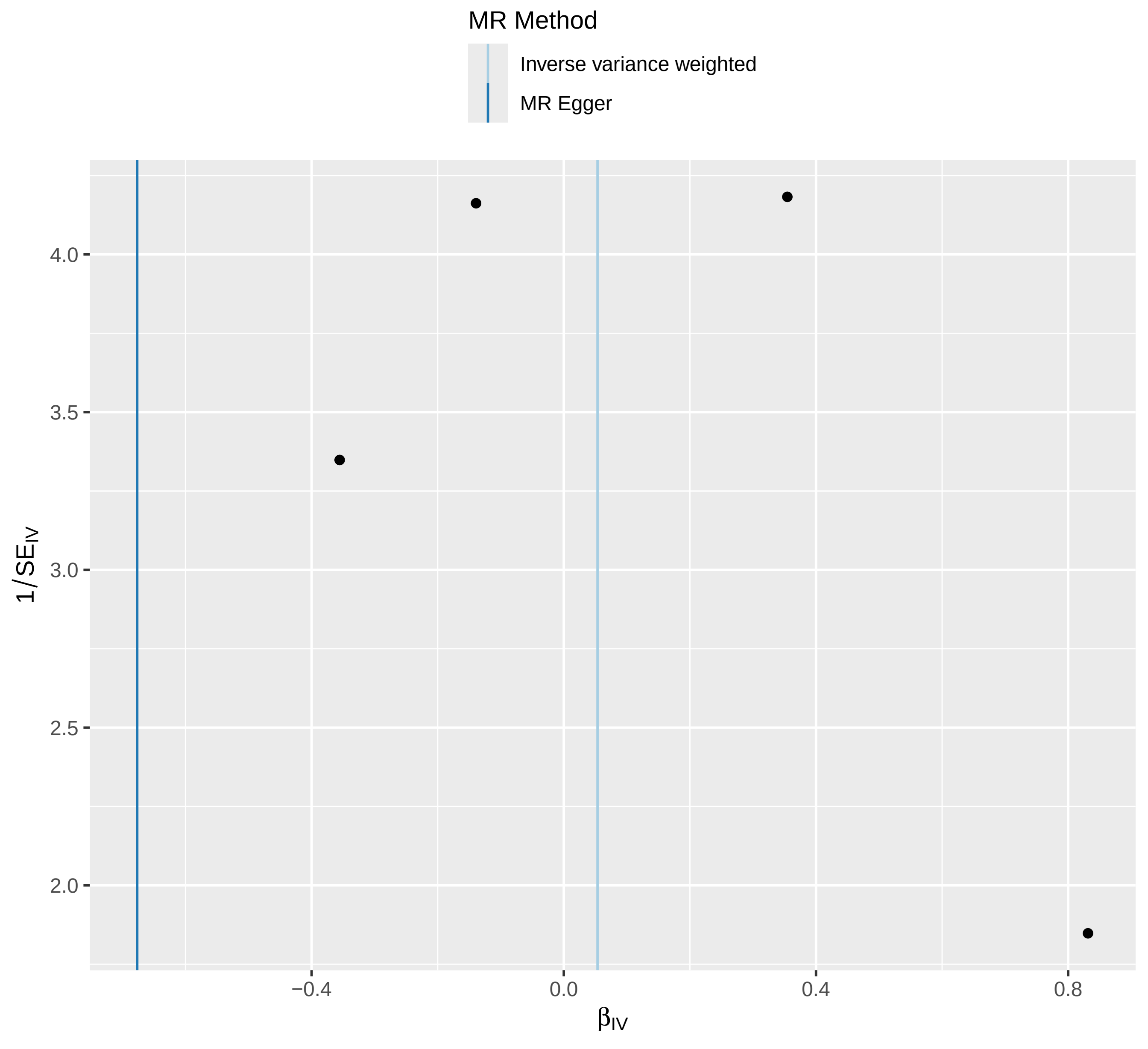

MR Method
Inverse variance weighted
MR Egger
4.0
3.5
3.0
2.5
2.0
1/SE$_{IV}$
−0.4
0.0
0.4
0.8
β$_{IV}$

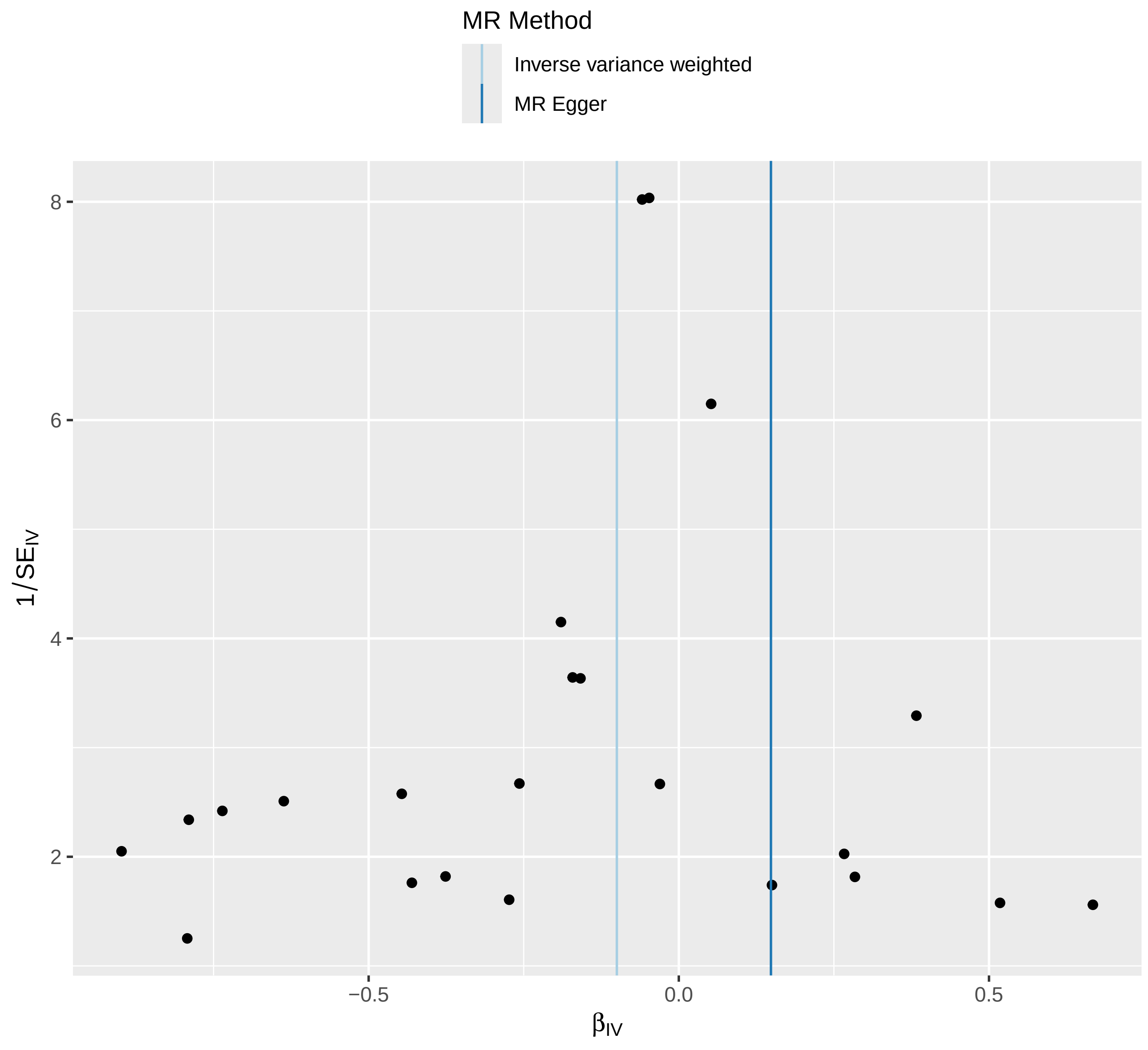

MR Method
Inverse variance weighted
MR Egger
8
6
4
2
1/SE$_{IV}$
−0.5
0.0
0.5
β$_{IV}$

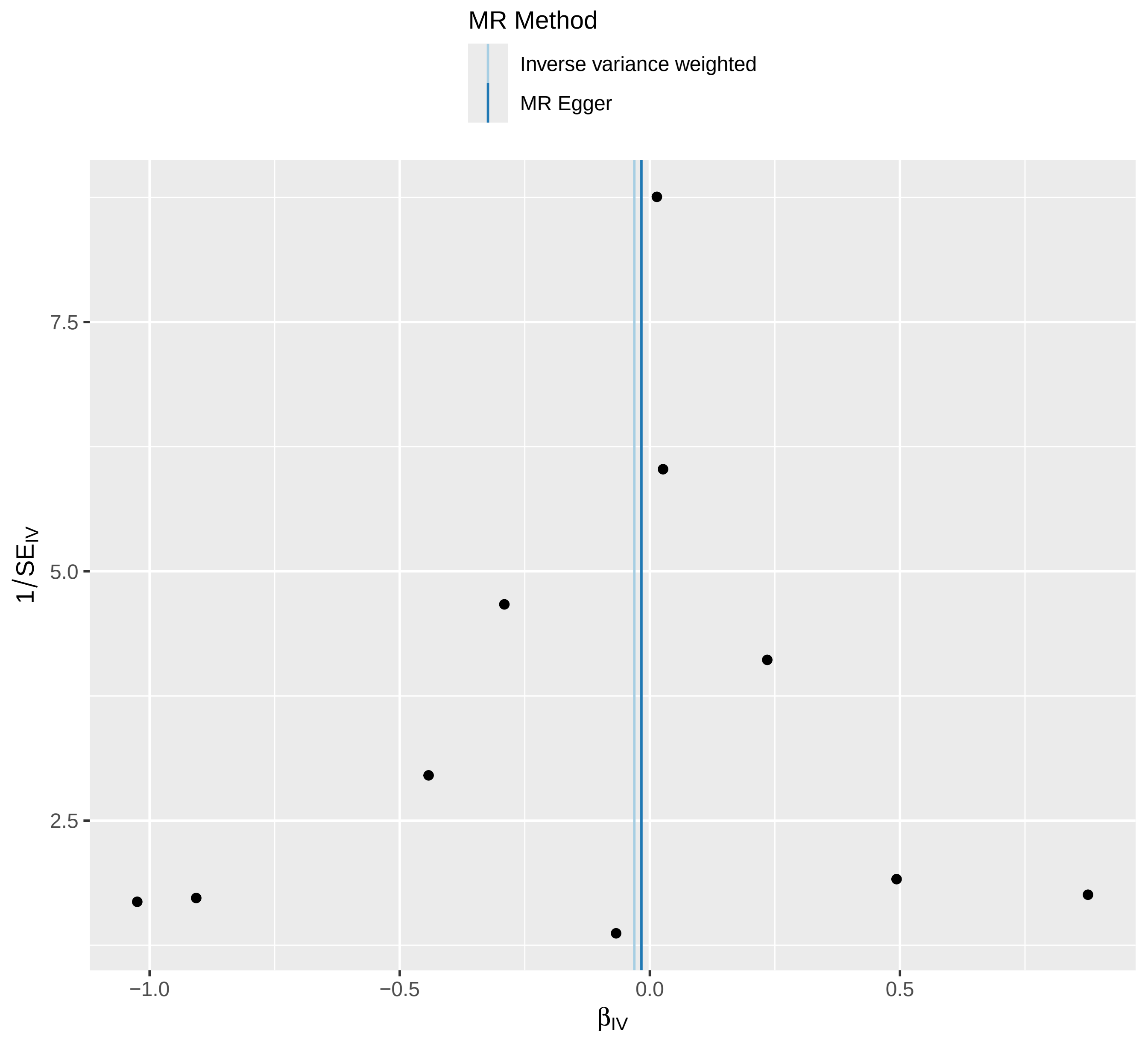
MR Method
Inverse variance weighted
MR Egger
7.5
5.0
2.5
1/SE_IV
−1.0
−0.5
0.0
0.5
β_IV

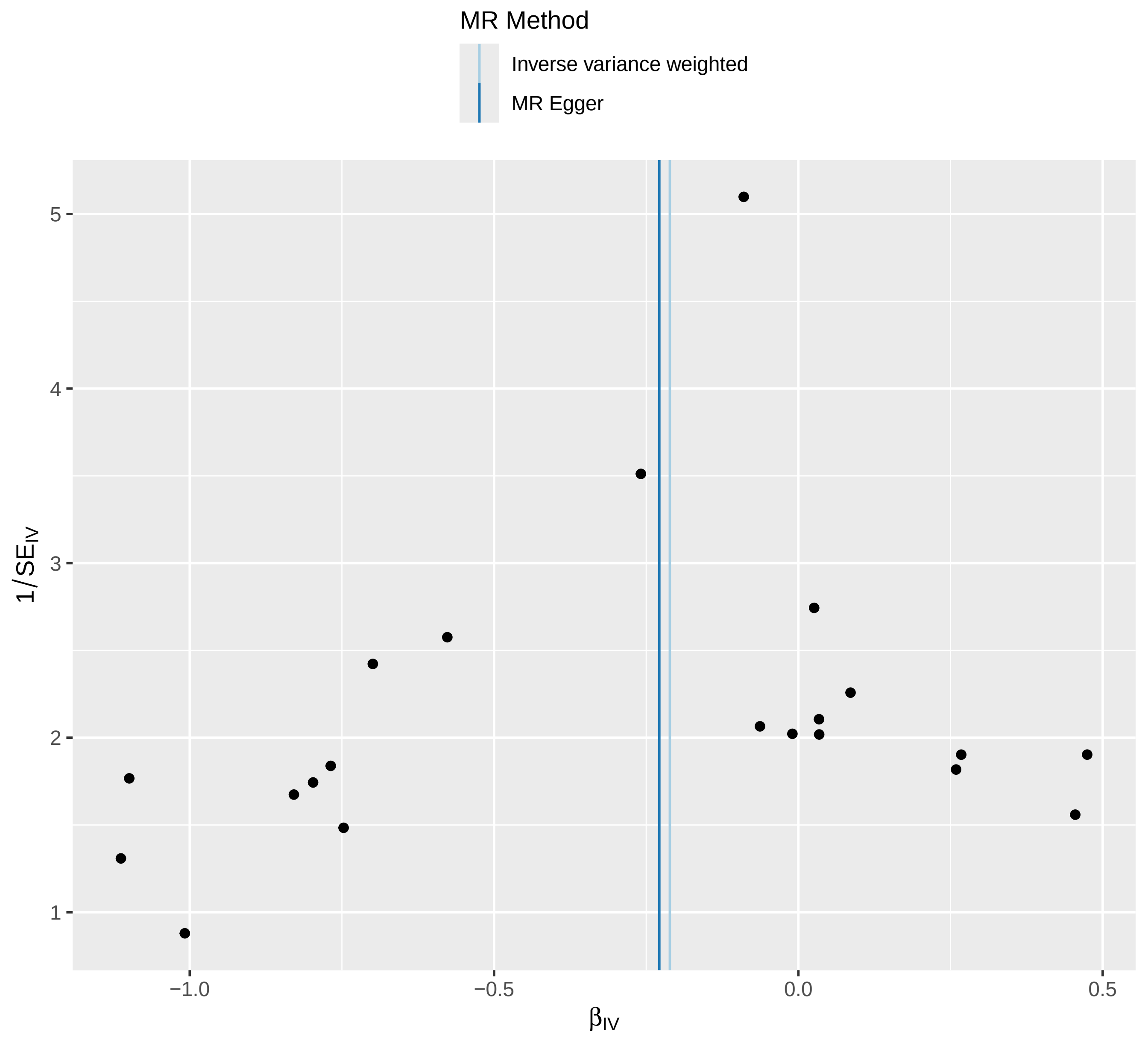
MR Method
Inverse variance weighted
MR Egger
5
4
3
2
1
1/SE$_{IV}$
−1.0
−0.5
0.0
0.5
β$_{IV}$

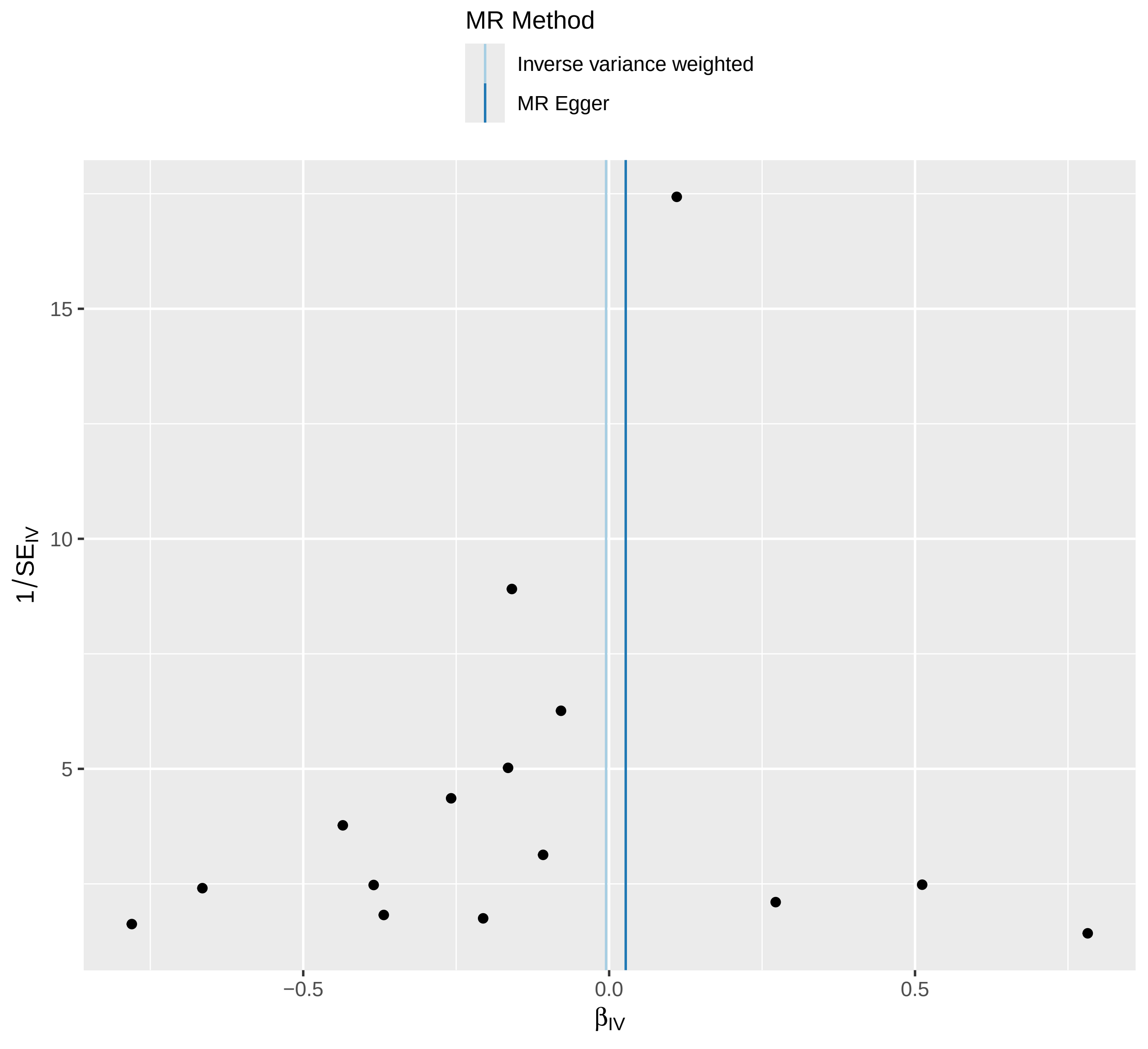

MR Method
Inverse variance weighted
MR Egger
15
10
5
1/SE$_{IV}$
−0.5
0.0
0.5
β$_{IV}$

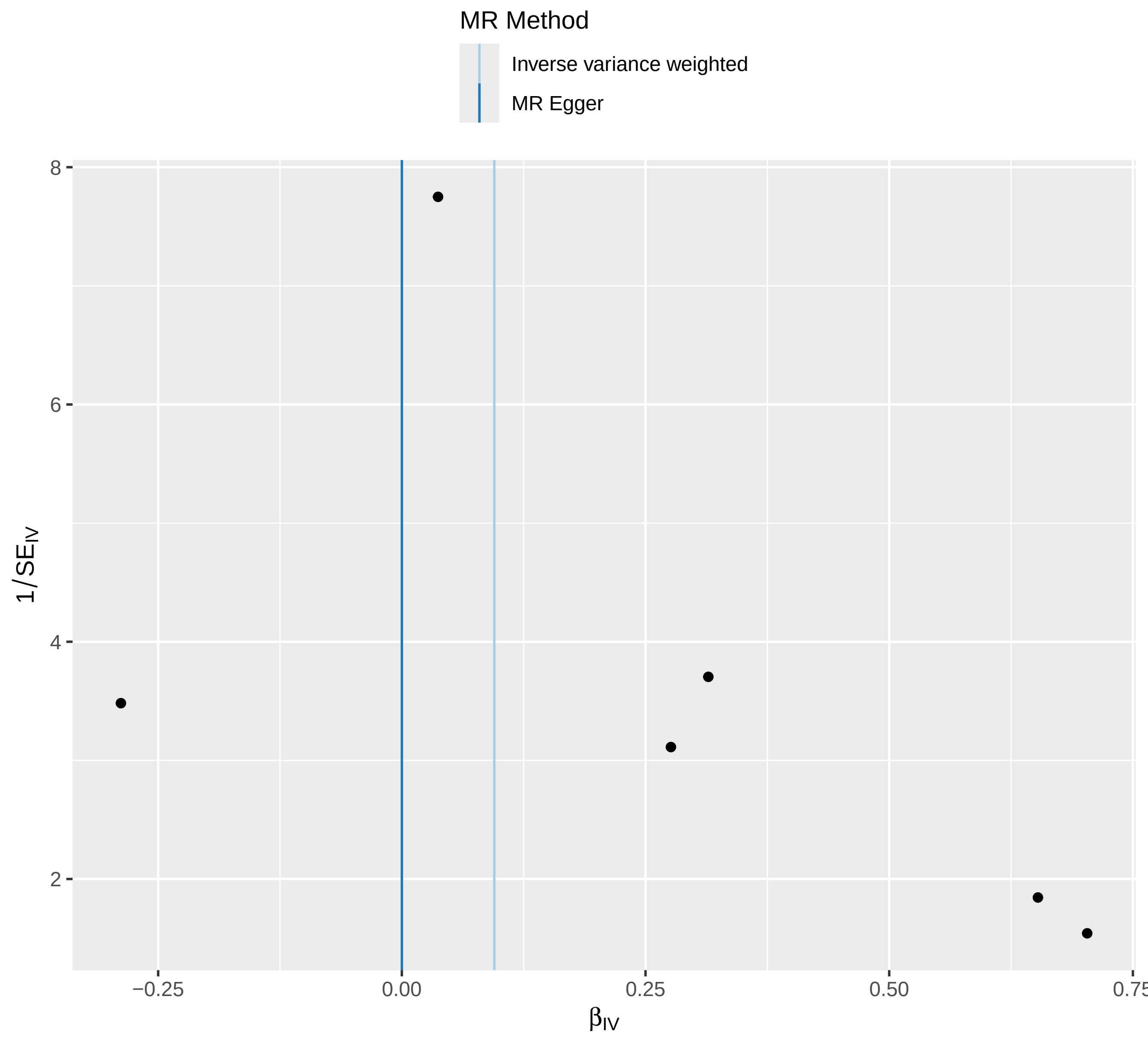
MR Method
Inverse variance weighted
MR Egger
8
6
4
2
1/SE$_{IV}$
−0.25
0.00
0.25
0.50
0.75
β$_{IV}$

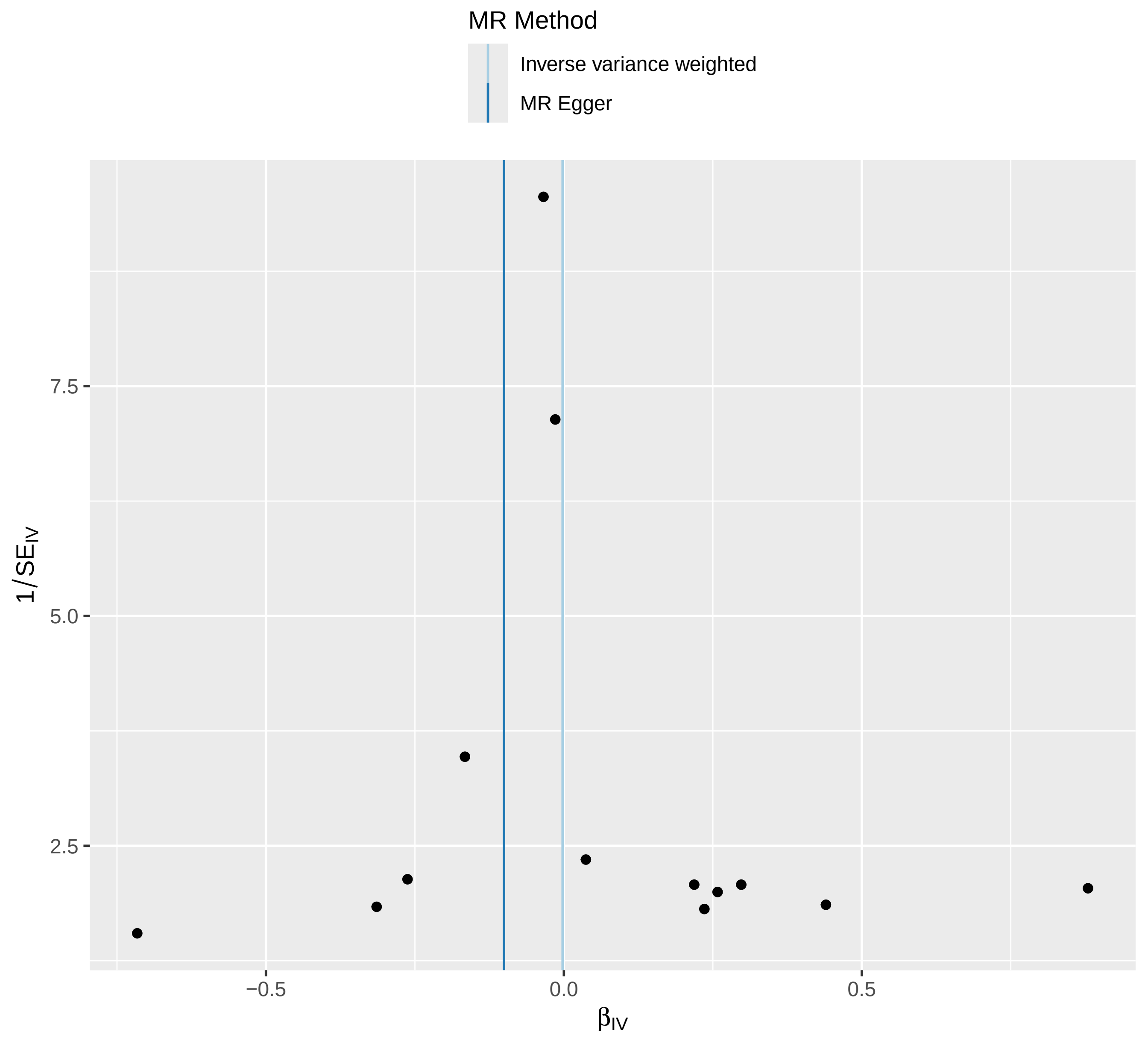
MR Method
Inverse variance weighted
MR Egger
7.5
5.0
2.5
1/SE_IV
−0.5
0.0
0.5
β_IV

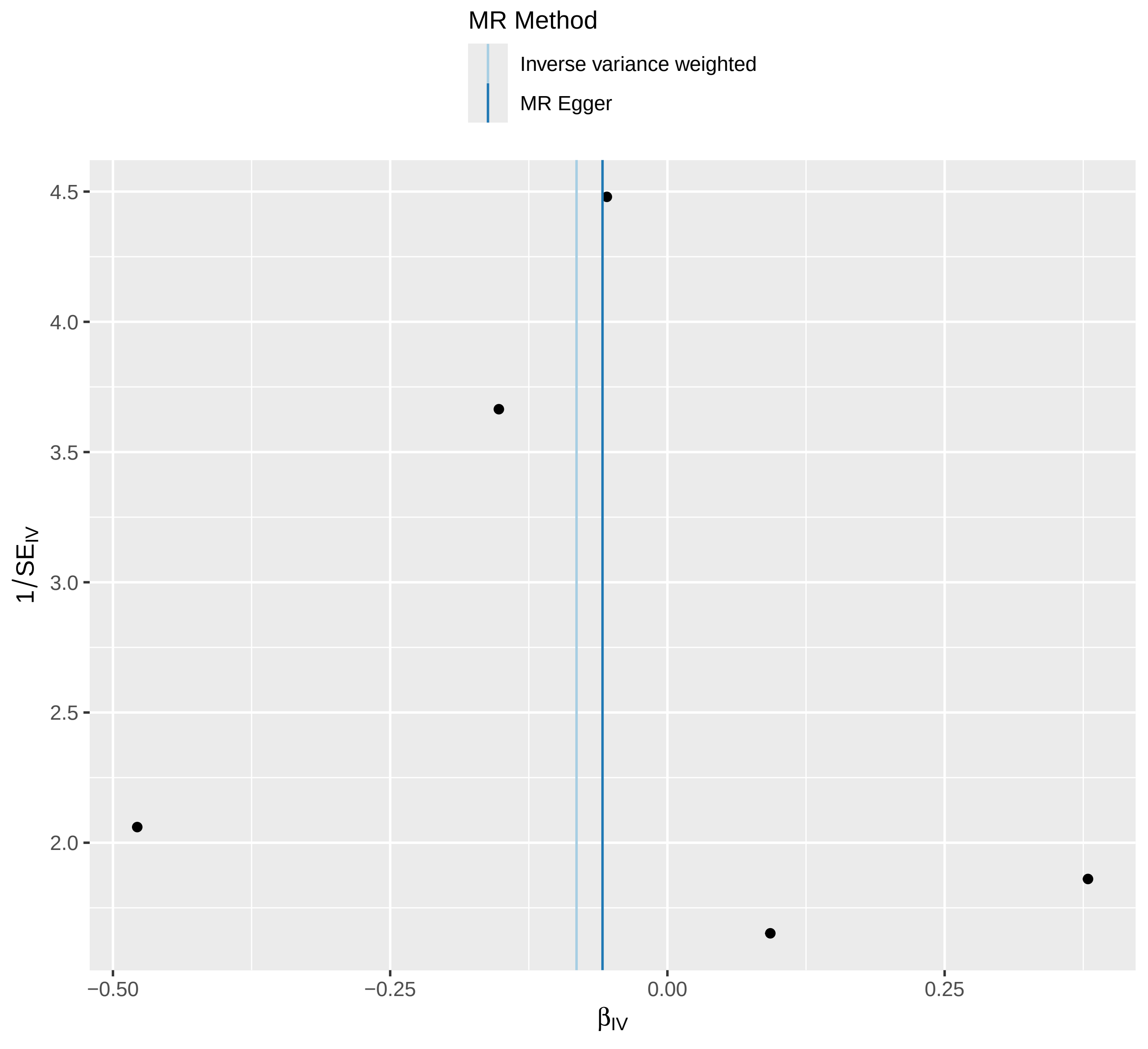
MR Method
Inverse variance weighted
MR Egger
4.5
4.0
3.5
3.0
2.5
2.0
1/SE$_{IV}$
−0.50
−0.25
0.00
0.25
β$_{IV}$

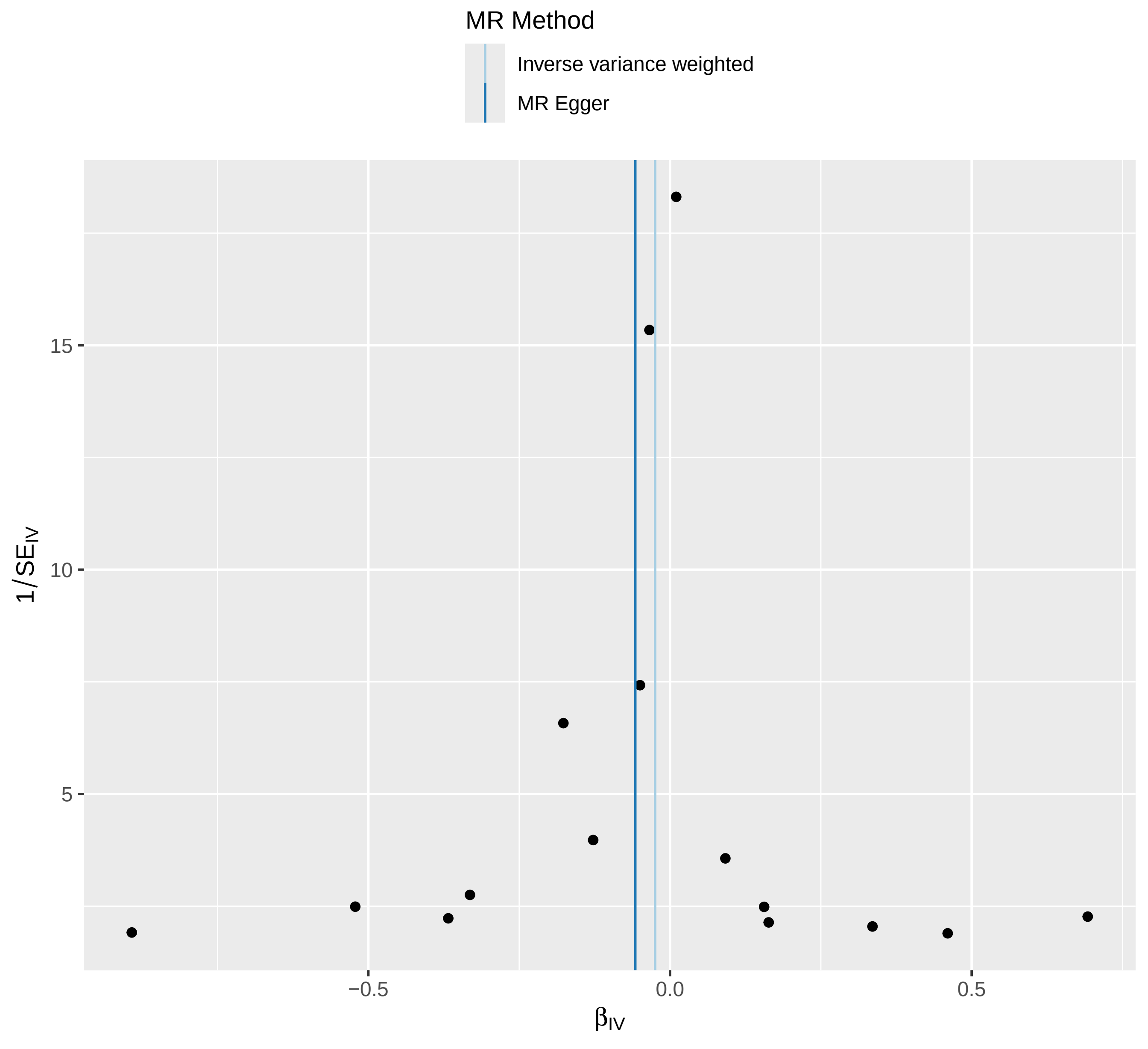
MR Method
Inverse variance weighted
MR Egger
1/SE_IV
15
10
5
−0.5
0.0
0.5
β_IV

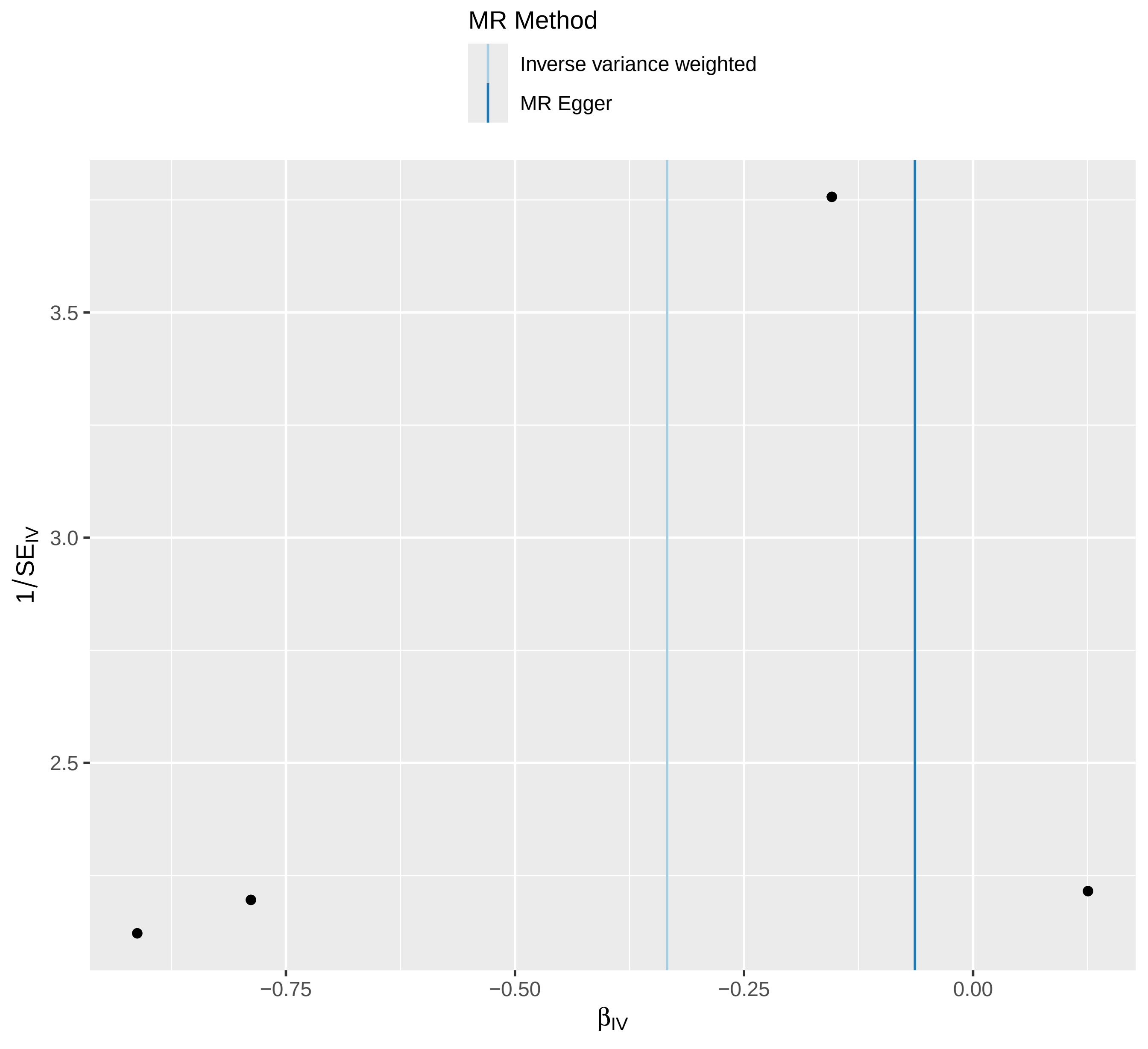

MR Method
Inverse variance weighted
MR Egger
3.5
3.0
2.5
1/SE_IV
−0.75
−0.50
−0.25
0.00
β_IV

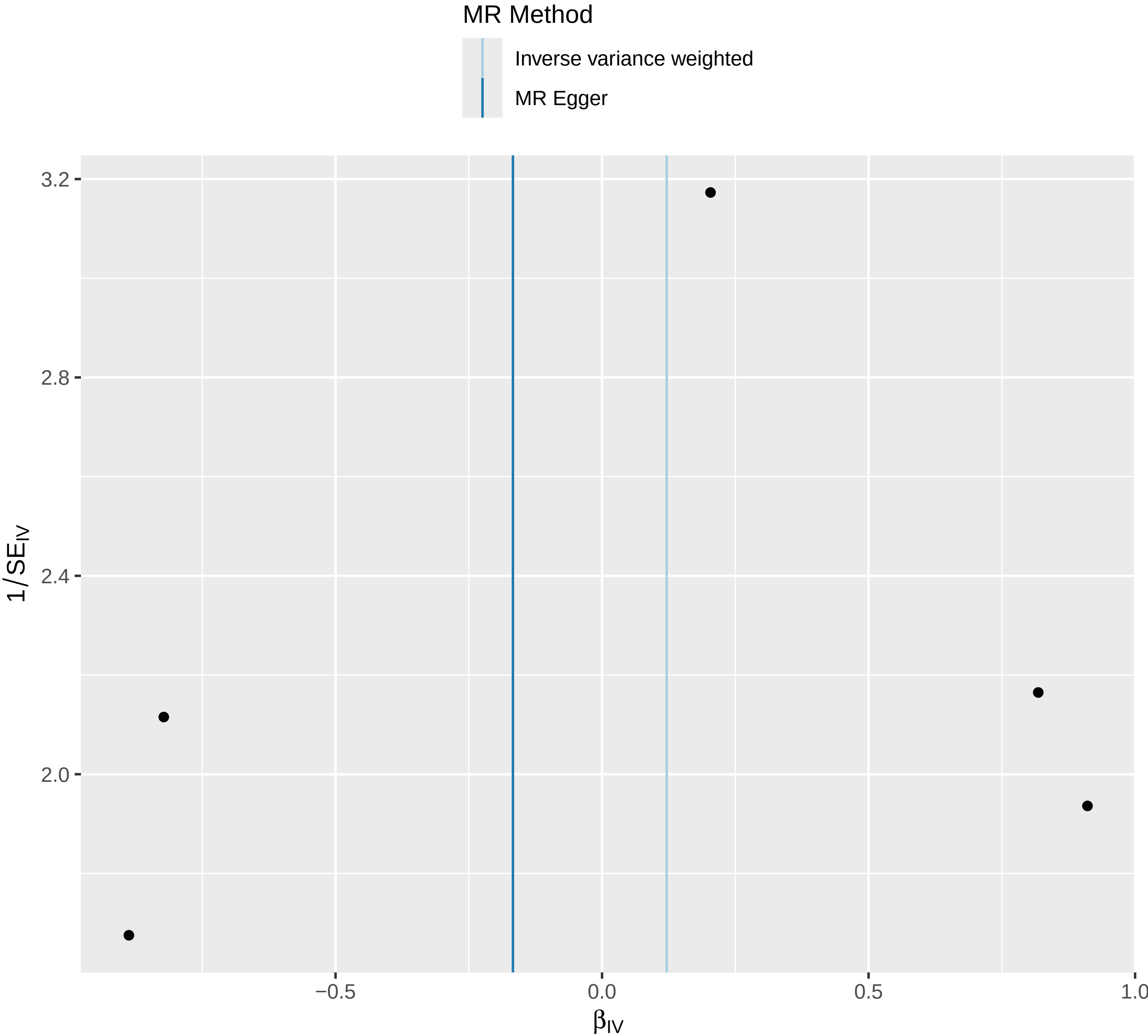

MR Method
Inverse variance weighted
MR Egger
3.2
2.8
2.4
2.0
1/SE_IV
−0.5
0.0
0.5
1.0
β_IV

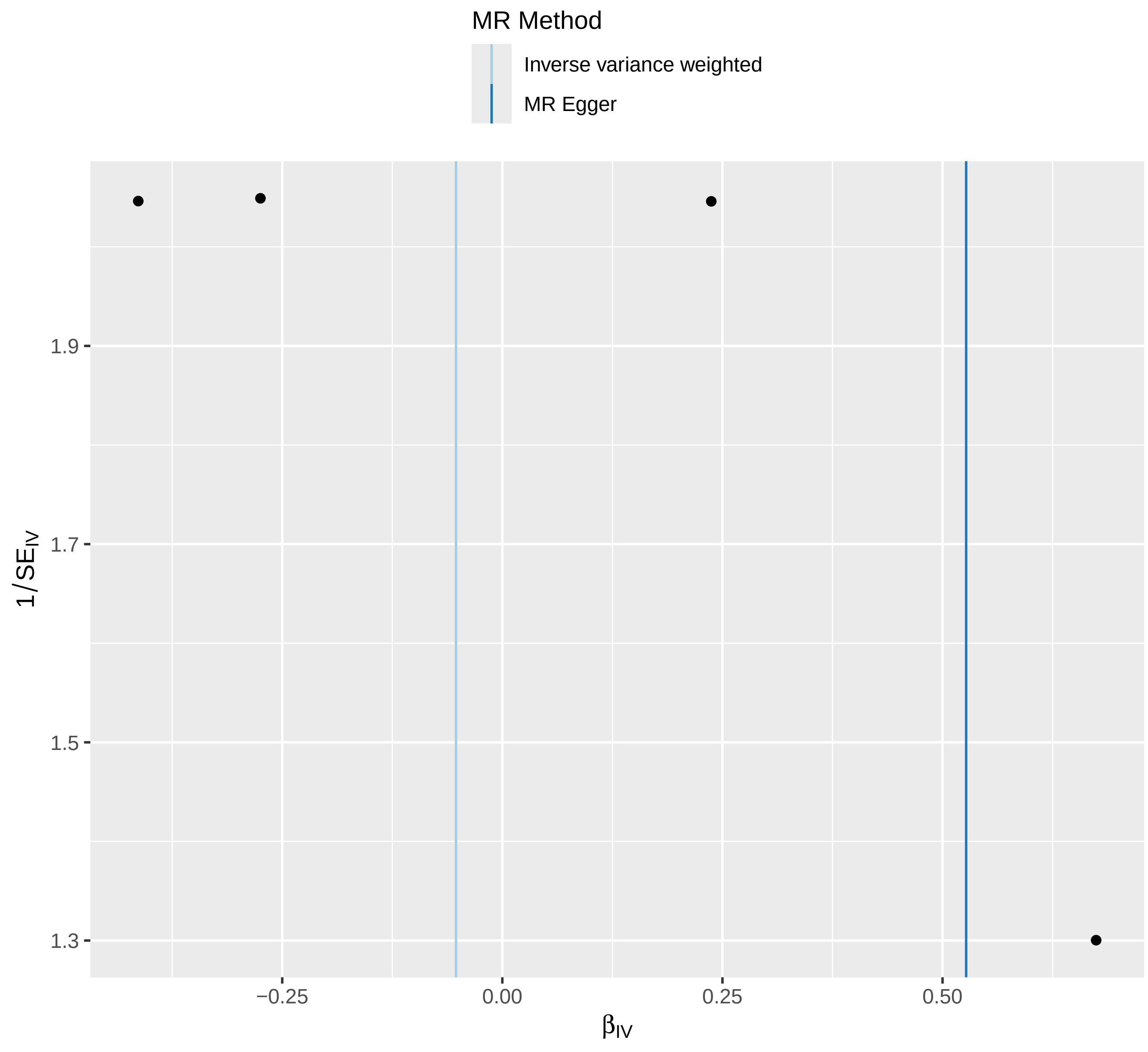
MR Method
Inverse variance weighted
MR Egger
1.9
1.7
1.5
1.3
1/SE_IV
−0.25
0.00
0.25
0.50
β_IV

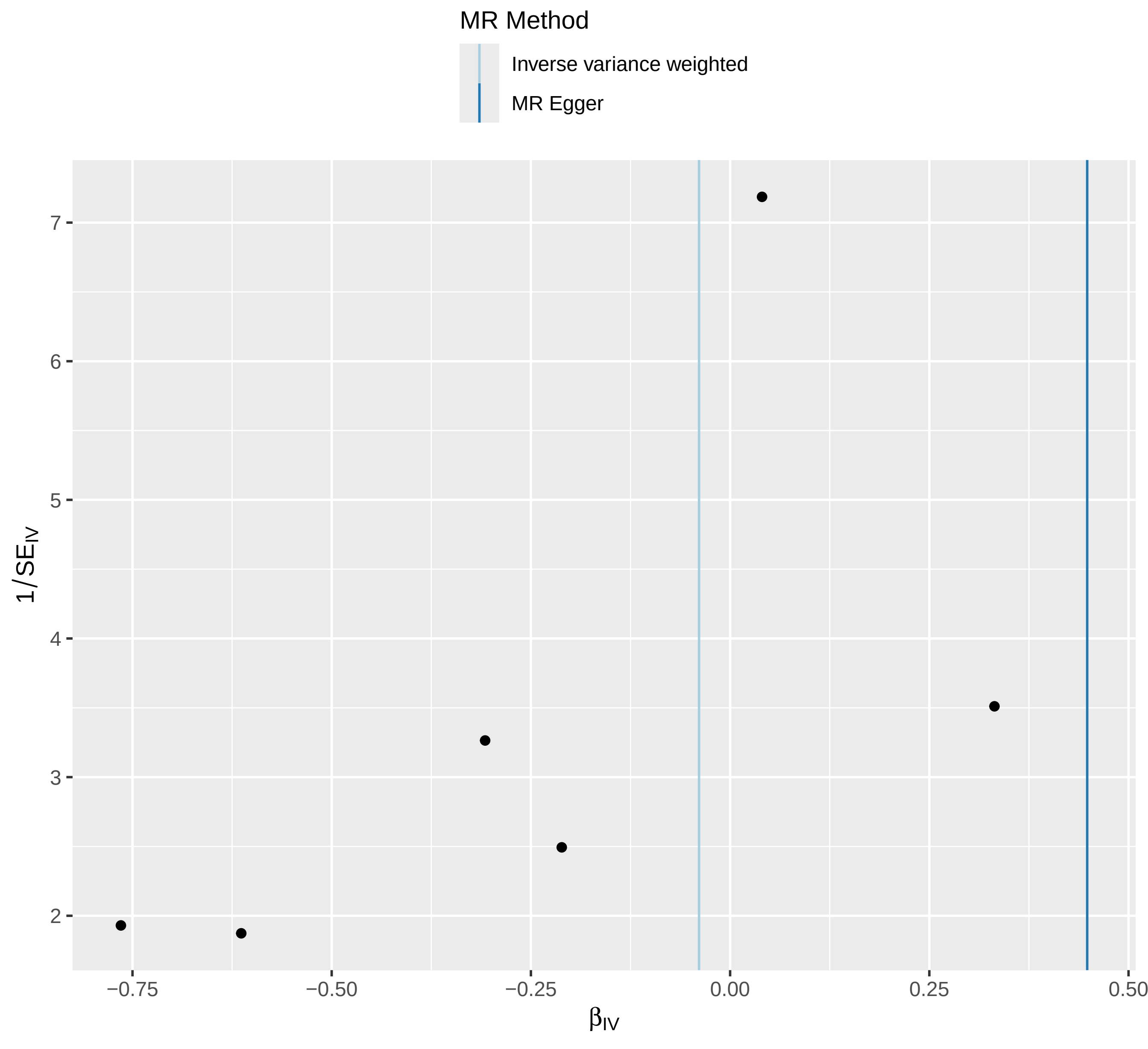
MR Method
Inverse variance weighted
MR Egger
$1/SE_{IV}$
β_{IV}
7
6
5
4
3
2
−0.75
−0.50
−0.25
0.00
0.25
0.50

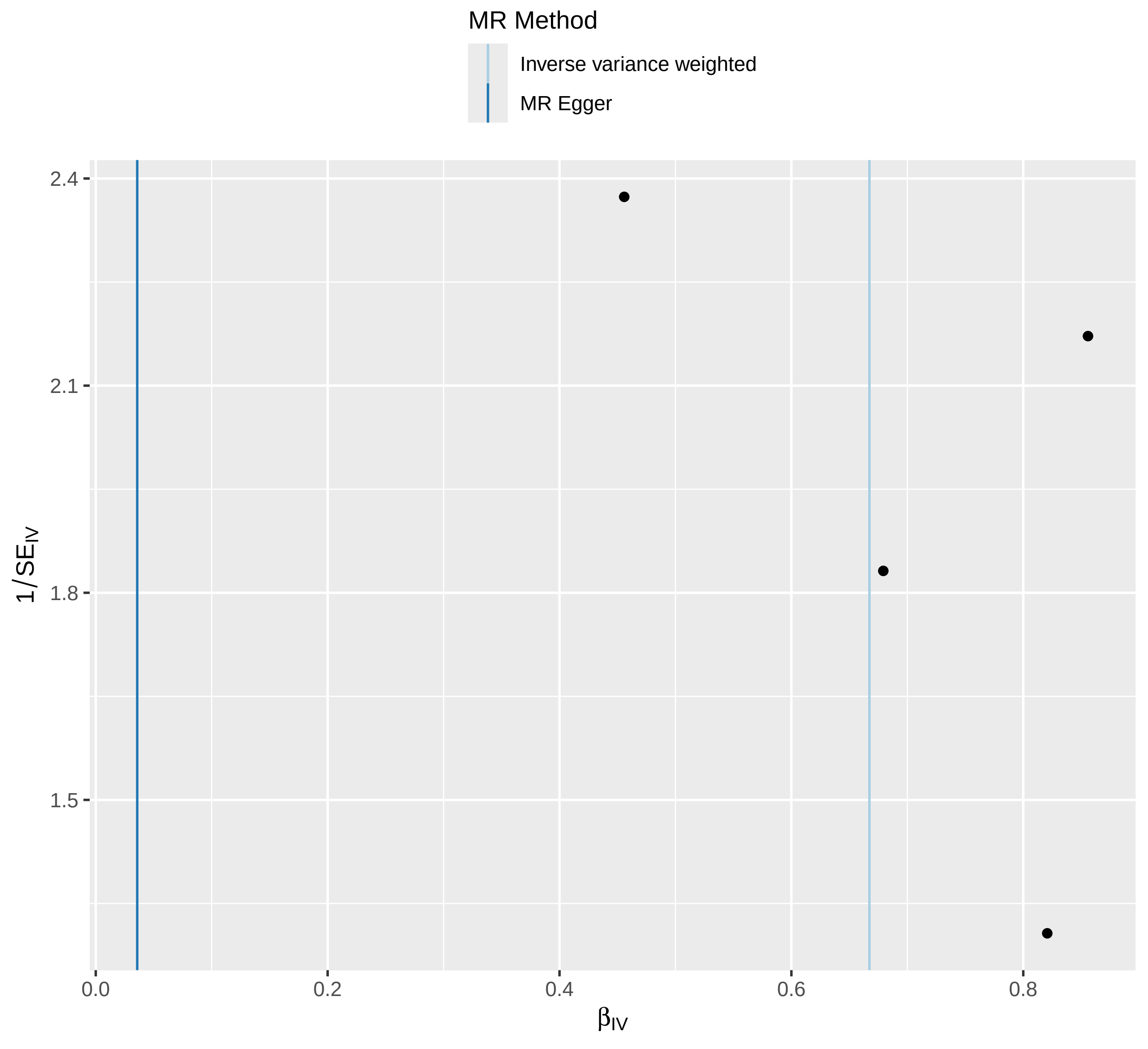
MR Method
Inverse variance weighted
MR Egger
2.4
2.1
1.8
1.5
$1/SE_{IV}$
0.0
0.2
0.4
0.6
0.8
β_{IV}

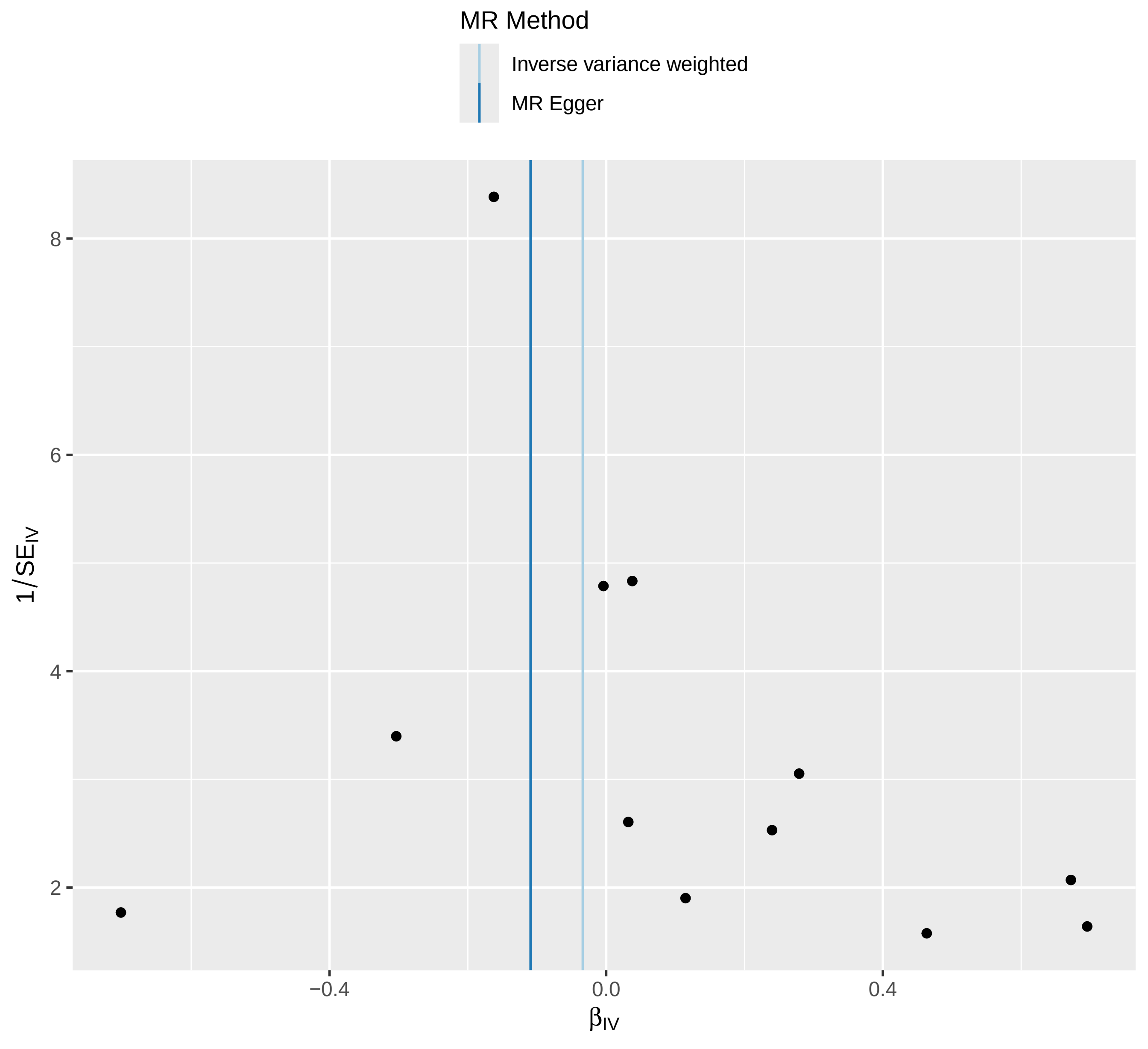
MR Method
Inverse variance weighted
MR Egger
8
6
4
2
1/SE_IV
−0.4
0.0
0.4
β_IV

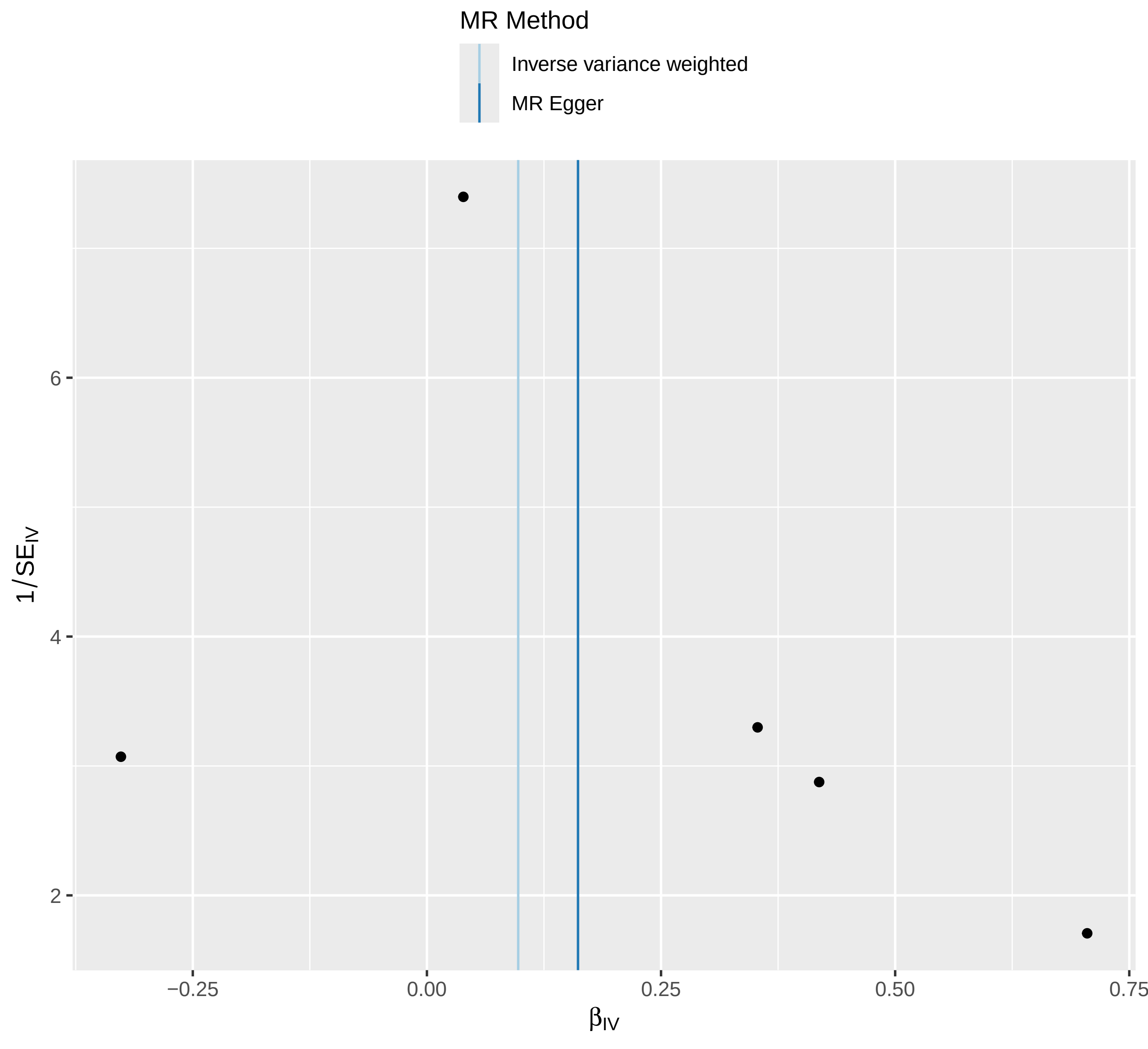

MR Method
Inverse variance weighted
MR Egger
1/SE_IV
6
4
2
−0.25
0.00
0.25
0.50
0.75
β_IV

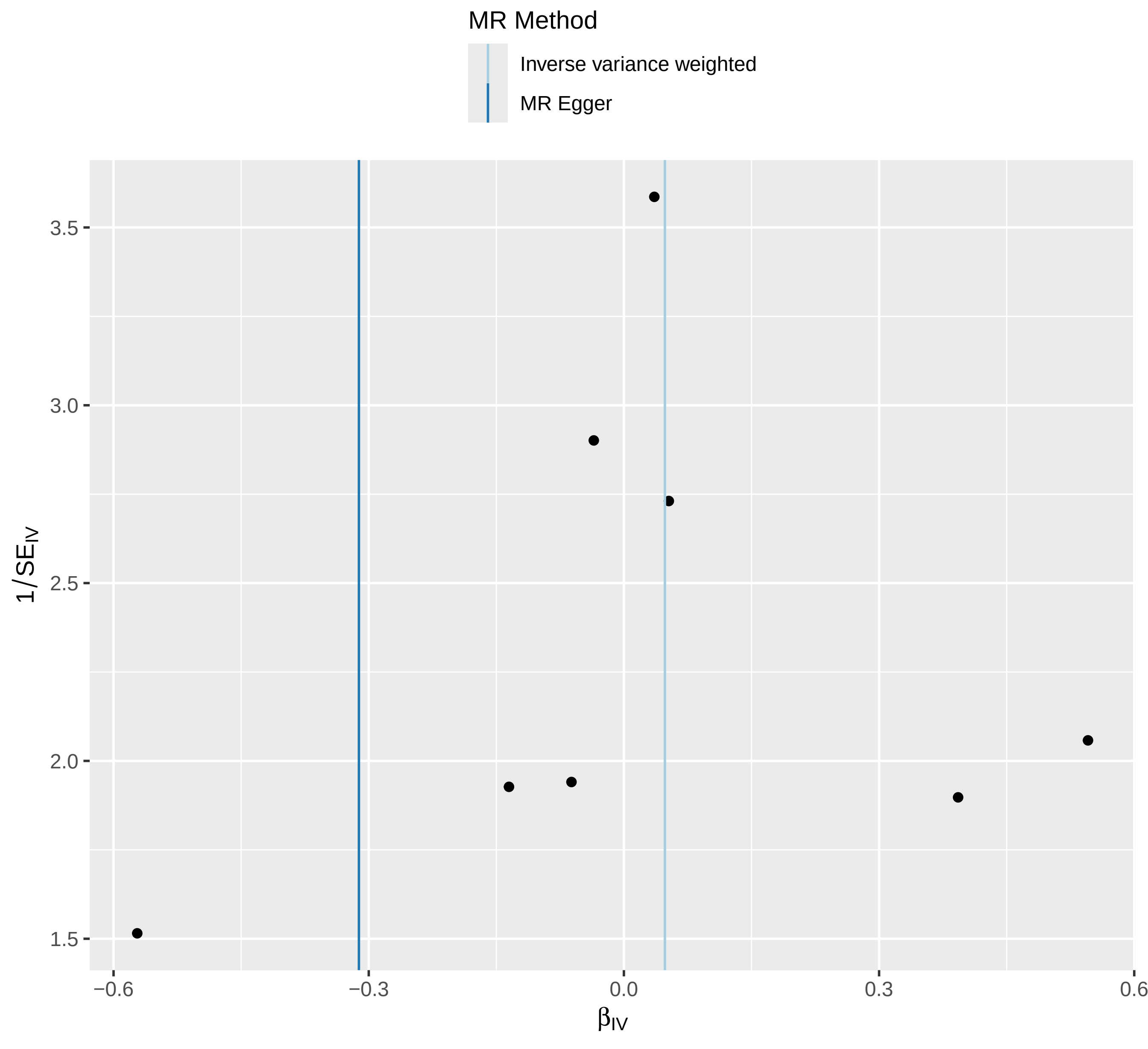
MR Method
Inverse variance weighted
MR Egger
3.5
3.0
2.5
2.0
1.5
$1/SE_{IV}$
−0.6
−0.3
0.0
0.3
0.6
β_{IV}

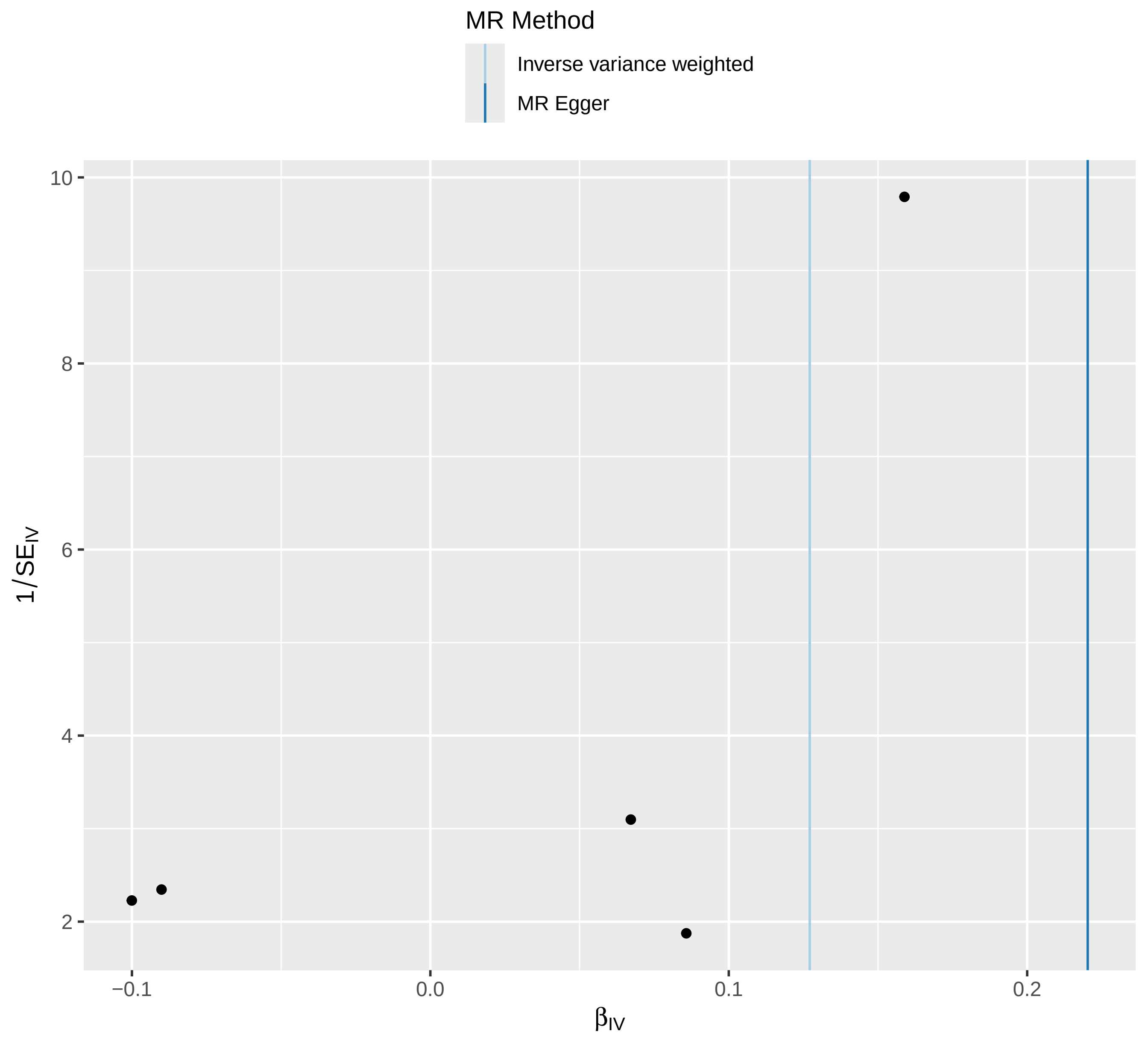
MR Method
Inverse variance weighted
MR Egger
10
8
6
4
2
1/SE_IV
−0.1
0.0
0.1
0.2
β_IV

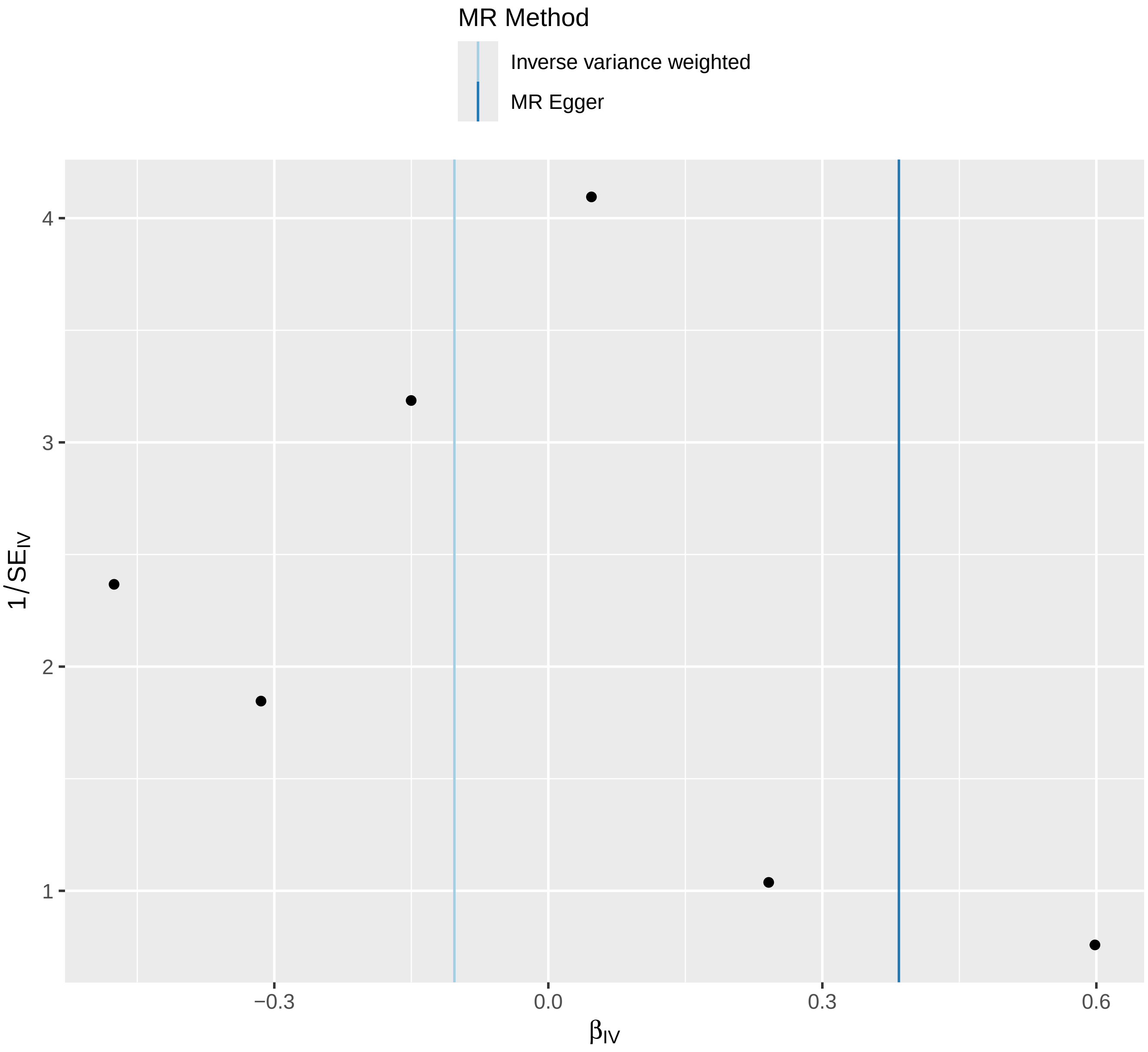
MR Method
Inverse variance weighted
MR Egger
$1/SE_{IV}$
4
3
2
1
−0.3
0.0
0.3
0.6
β_{IV}

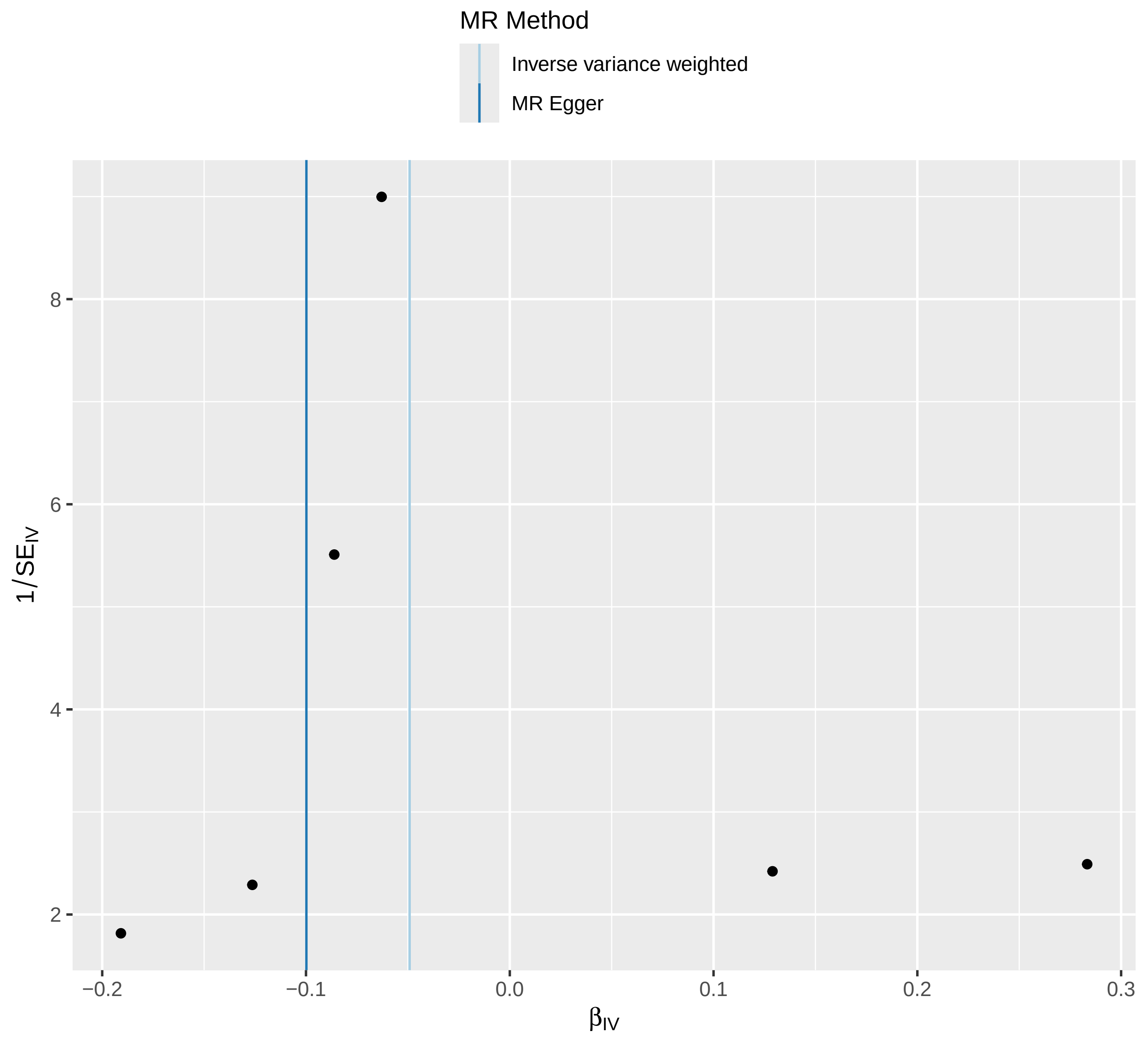

MR Method
Inverse variance weighted
MR Egger
8
6
4
2
1/SE_IV
−0.2
−0.1
0.0
0.1
0.2
0.3
β_IV

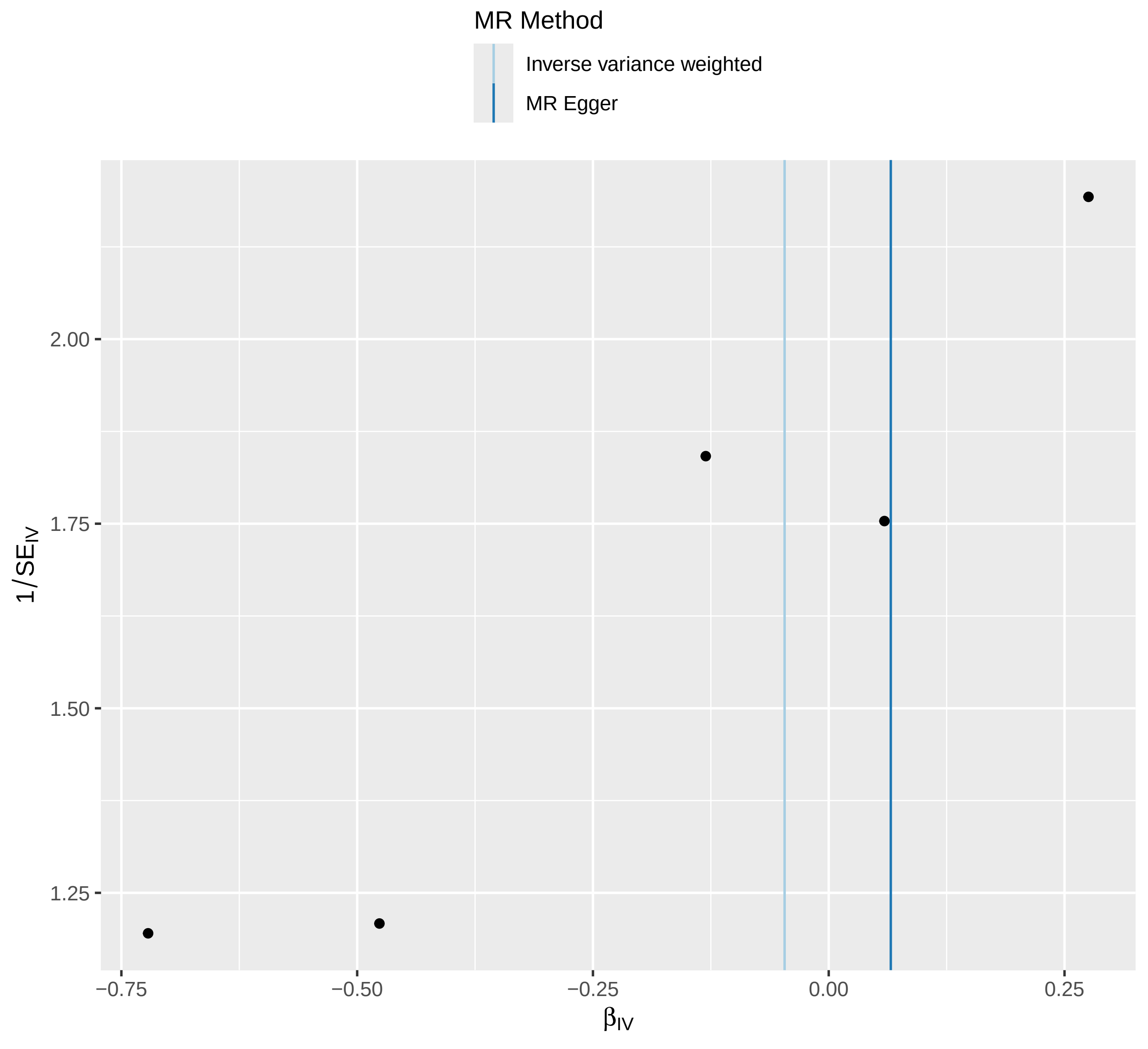
MR Method
Inverse variance weighted
MR Egger
2.00
1.75
1.50
1.25
$1/SE_{IV}$
−0.75
−0.50
−0.25
0.00
0.25
β_{IV}

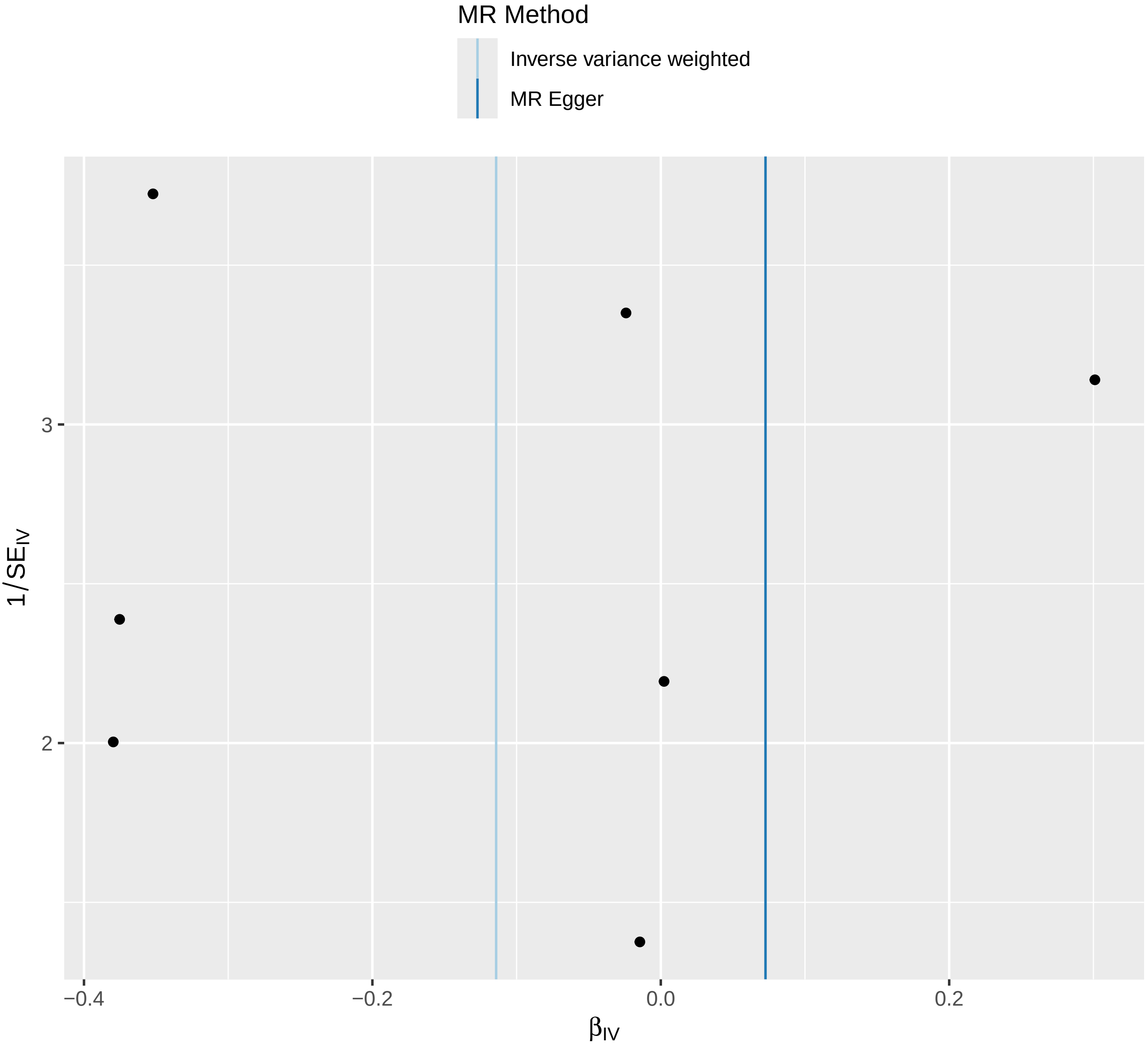
MR Method
Inverse variance weighted
MR Egger
3
2
1/SE_IV
−0.4
−0.2
0.0
0.2
β_IV

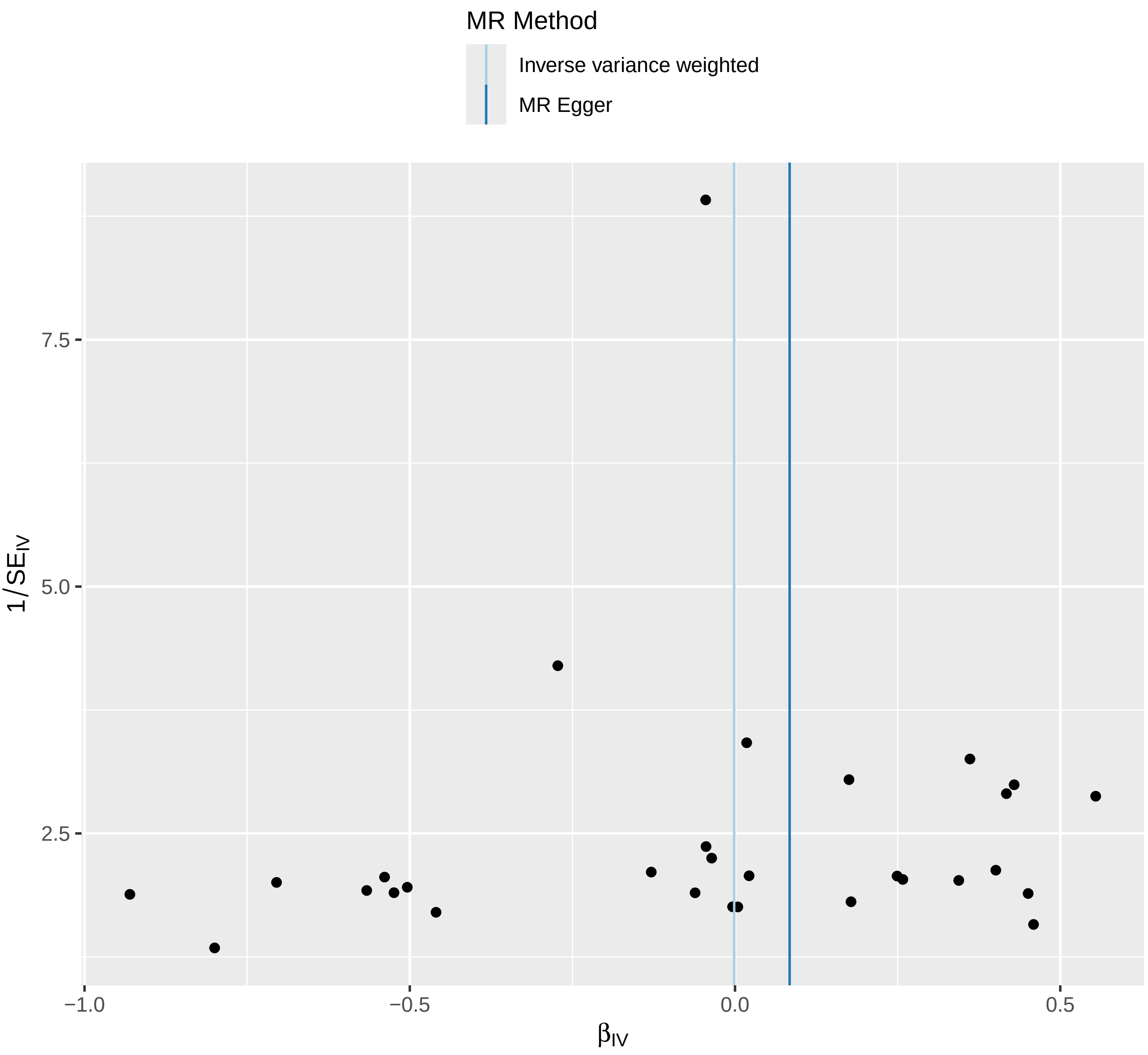
MR Method
Inverse variance weighted
MR Egger
7.5
5.0
2.5
1/SE_IV
−1.0
−0.5
0.0
0.5
β_IV

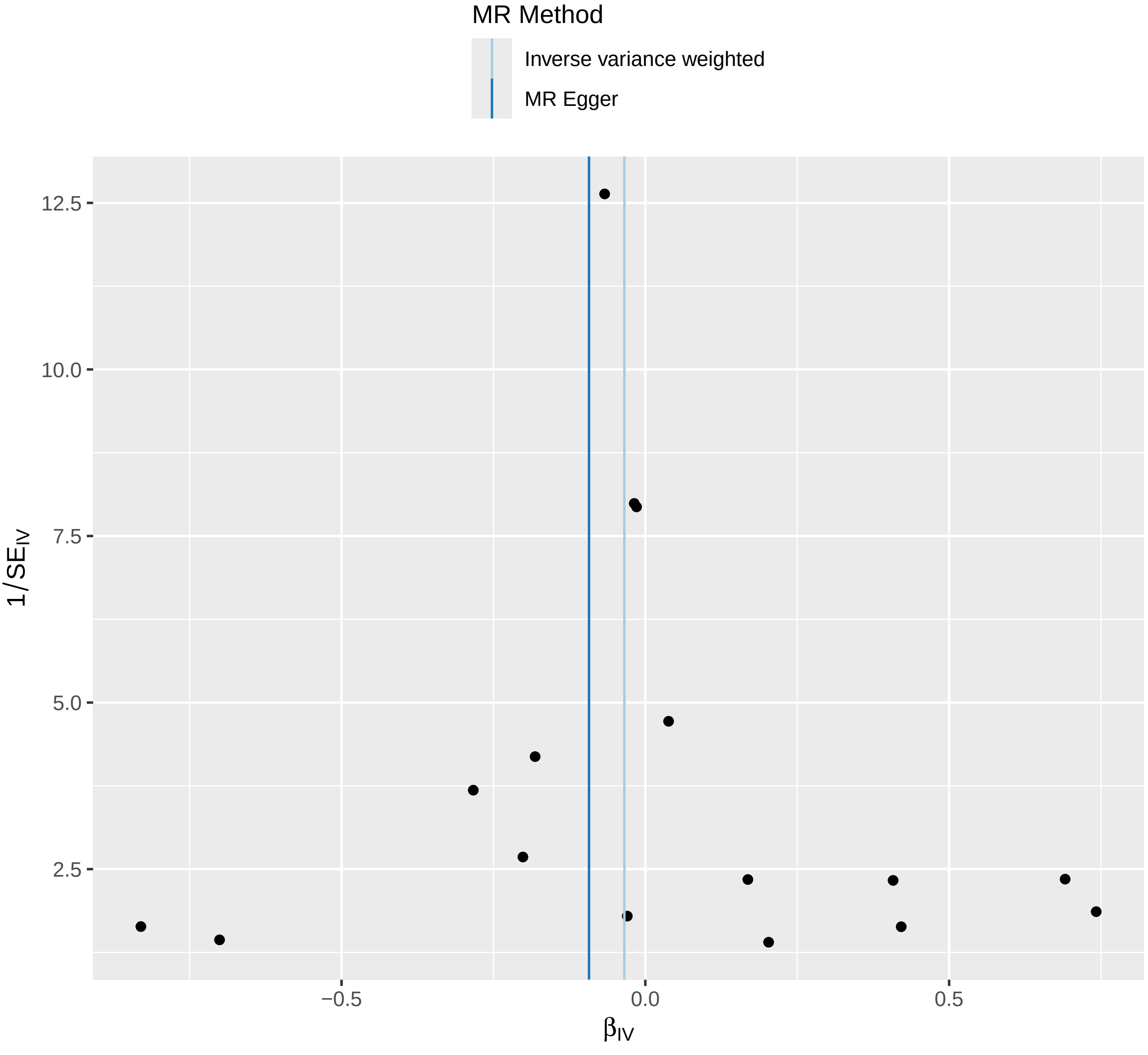
MR Method
Inverse variance weighted
MR Egger
12.5
10.0
7.5
5.0
2.5
−0.5
0.0
0.5
$1/SE_{IV}$
β_{IV}

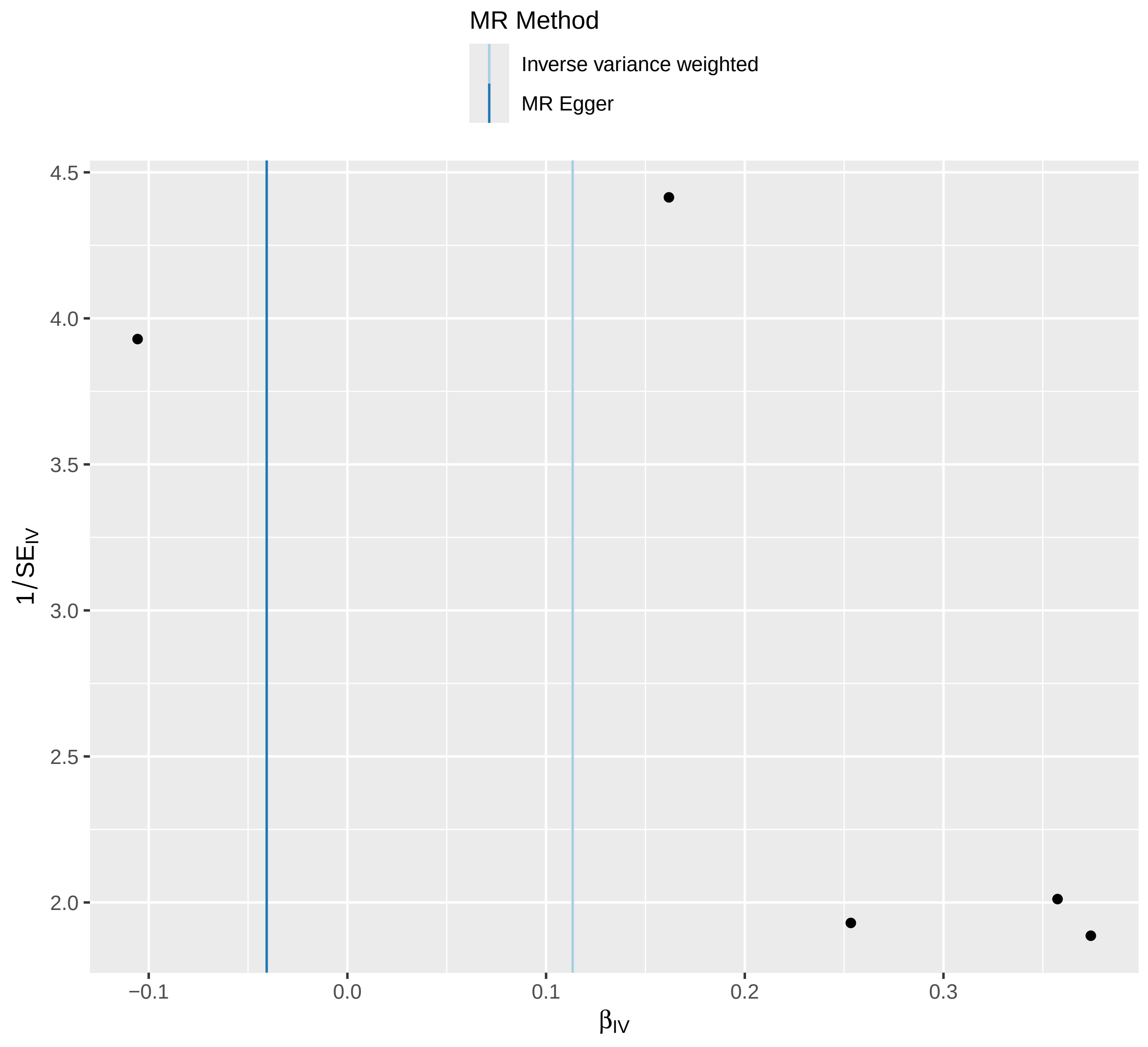

MR Method
Inverse variance weighted
MR Egger
4.5
4.0
3.5
3.0
2.5
2.0
1/SE_IV
−0.1
0.0
0.1
0.2
0.3
β_IV

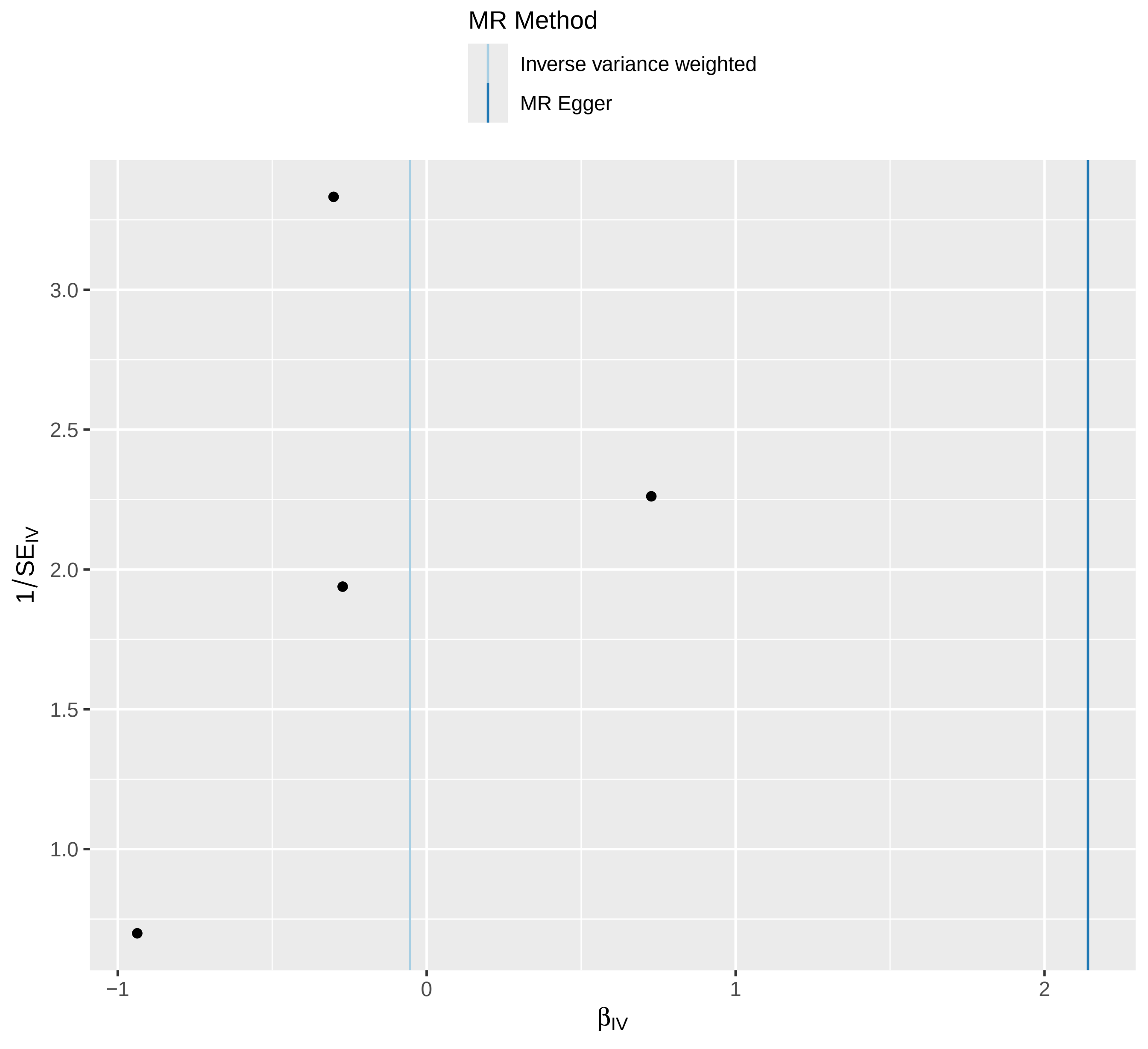
MR Method
Inverse variance weighted
MR Egger
1/SE$_{IV}$
3.0
2.5
2.0
1.5
1.0
−1
0
1
2
β$_{IV}$

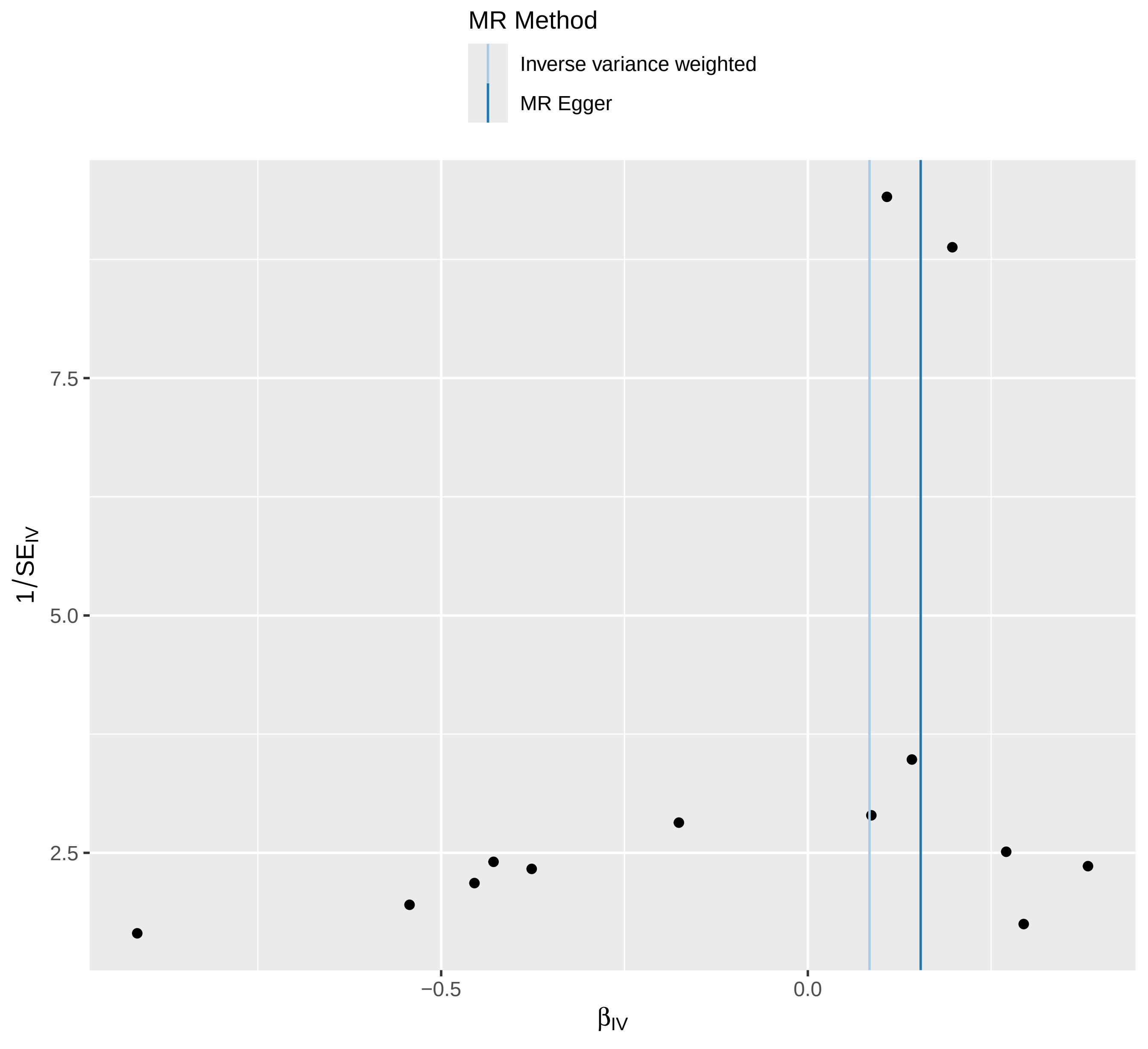

MR Method
Inverse variance weighted
MR Egger
7.5
5.0
2.5
1/SE_IV
−0.5
0.0
β_IV

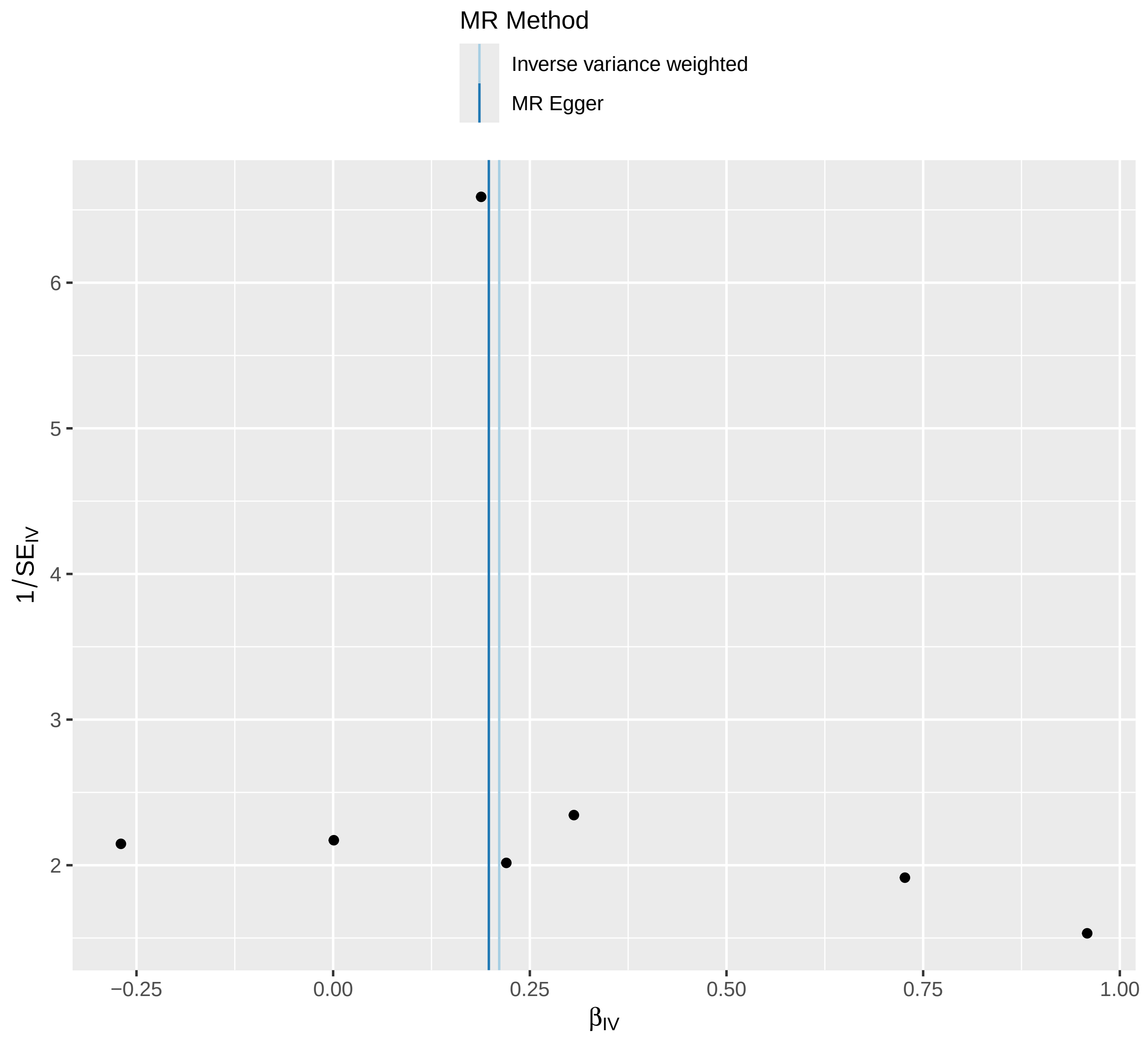

MR Method
Inverse variance weighted
MR Egger
6
5
4
3
2
1/SE_IV
−0.25
0.00
0.25
0.50
0.75
1.00
β_IV

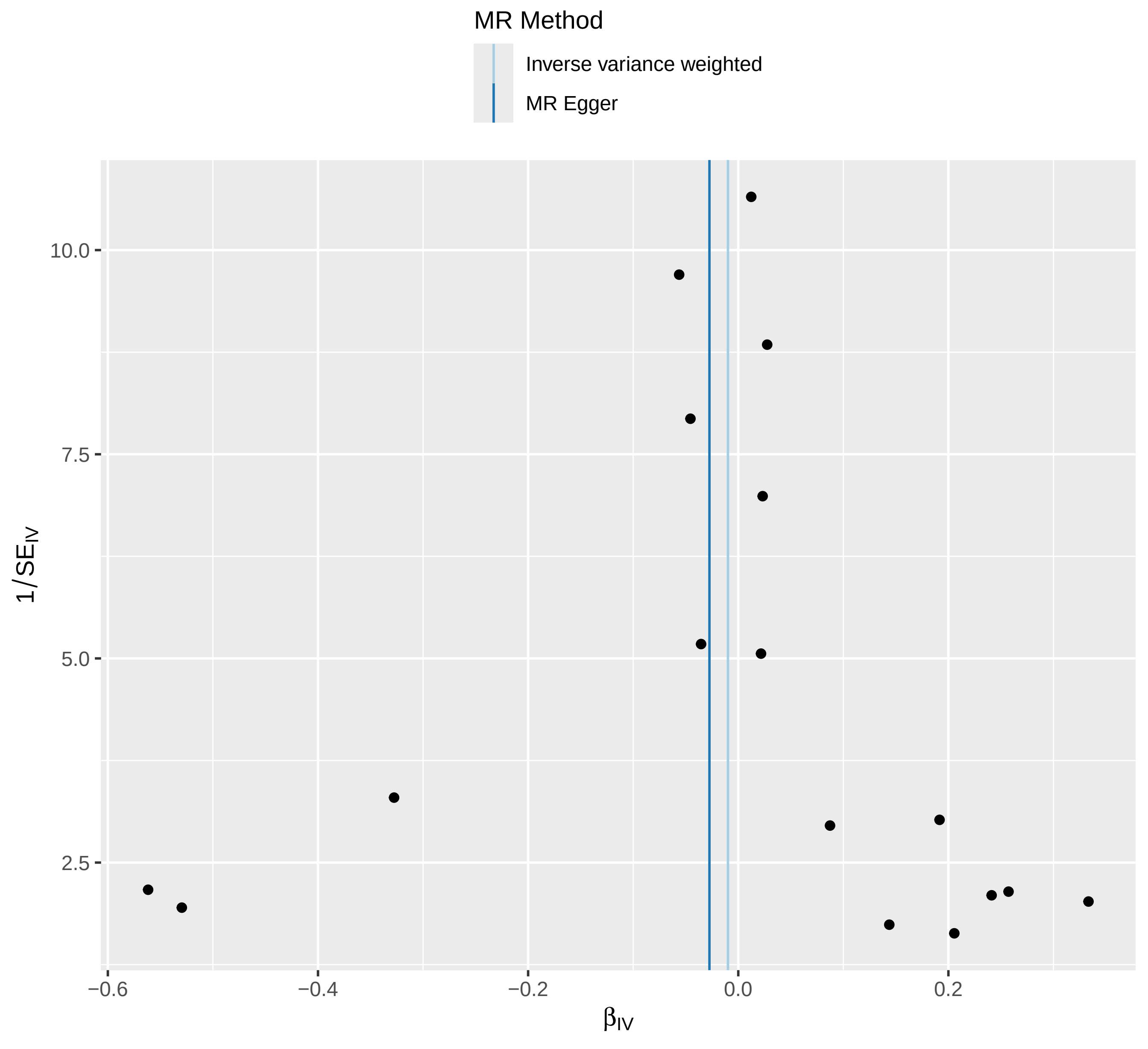
MR Method
Inverse variance weighted
MR Egger
10.0
7.5
5.0
2.5
1/SE_IV
−0.6
−0.4
−0.2
0.0
0.2
β_IV

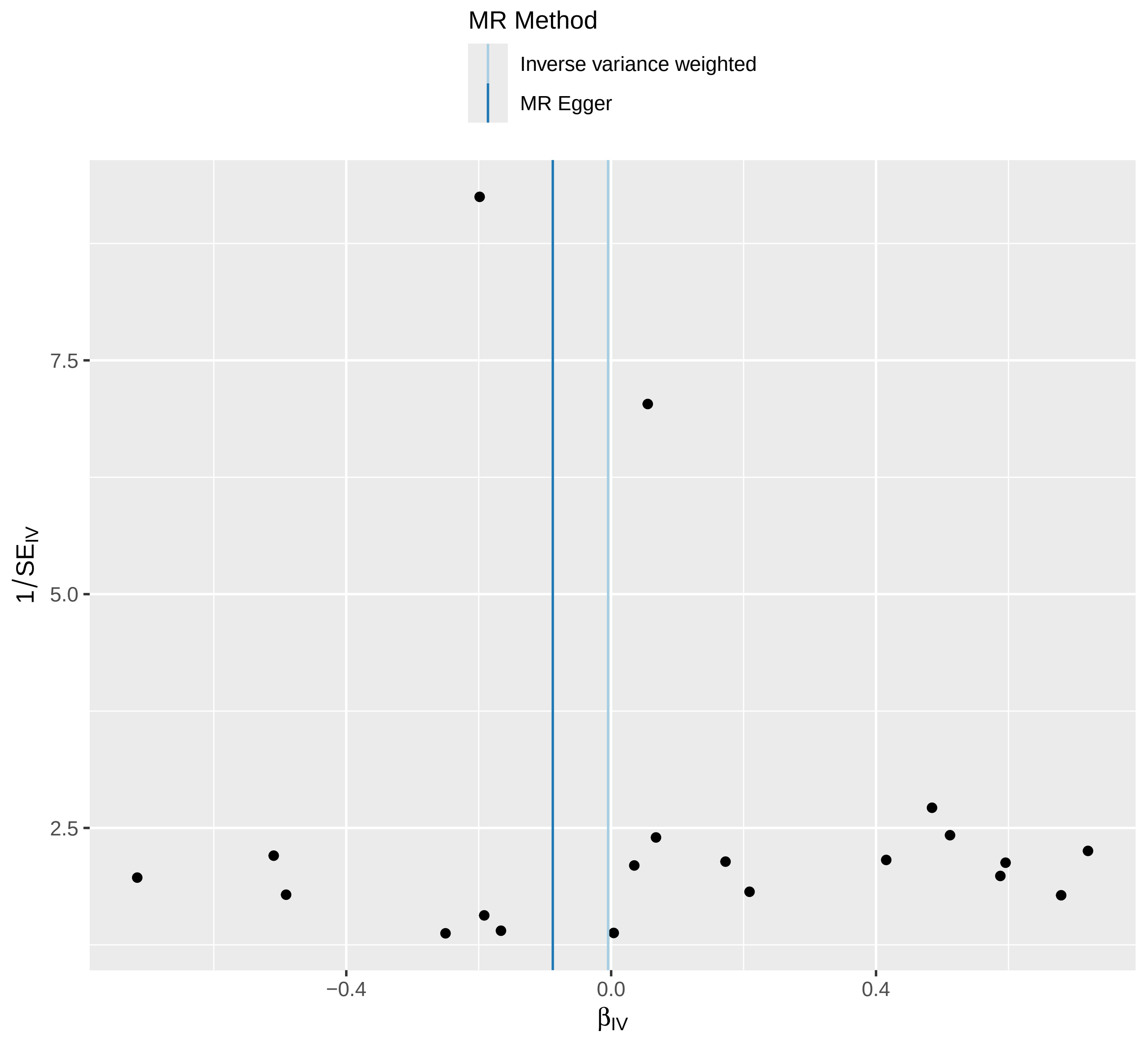
MR Method
Inverse variance weighted
MR Egger
7.5
5.0
2.5
1/SE_IV
−0.4
0.0
0.4
β_IV

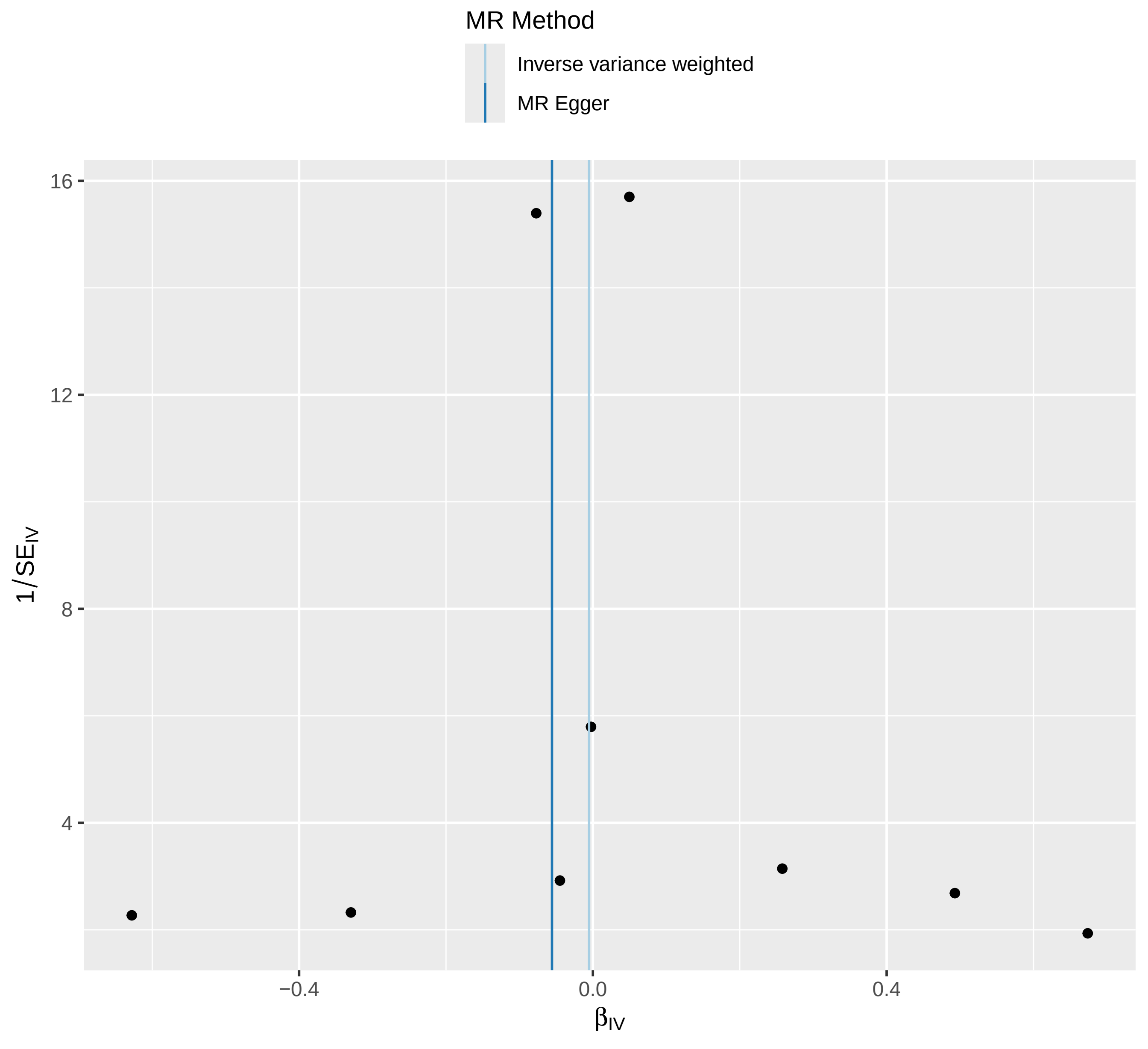

MR Method
Inverse variance weighted
MR Egger
16
12
8
4
1/SE_{IV}
−0.4
0.0
0.4
β_{IV}

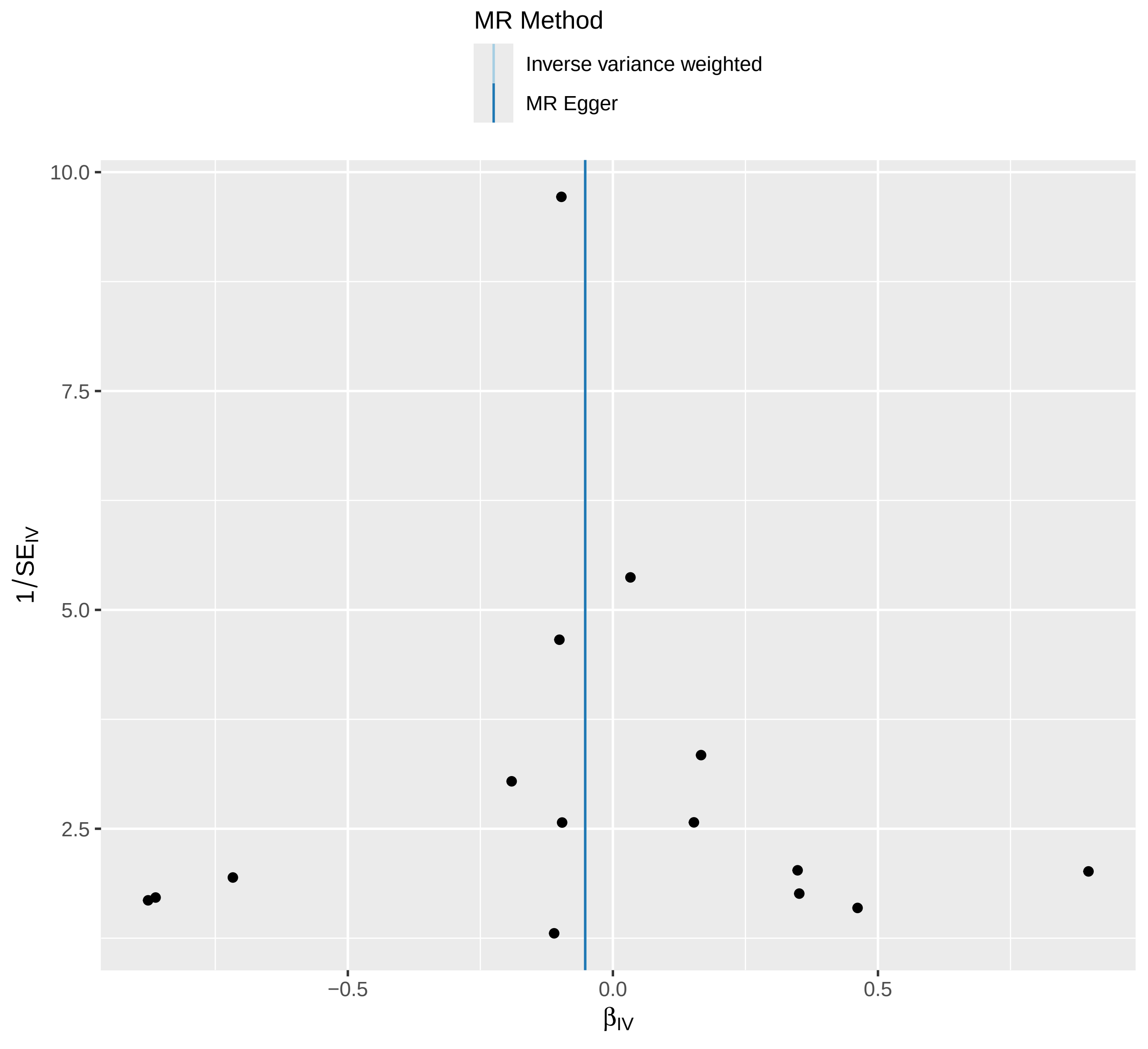
MR Method
Inverse variance weighted
MR Egger
10.0
7.5
5.0
2.5
1/SE$_{IV}$
−0.5
0.0
0.5
β$_{IV}$

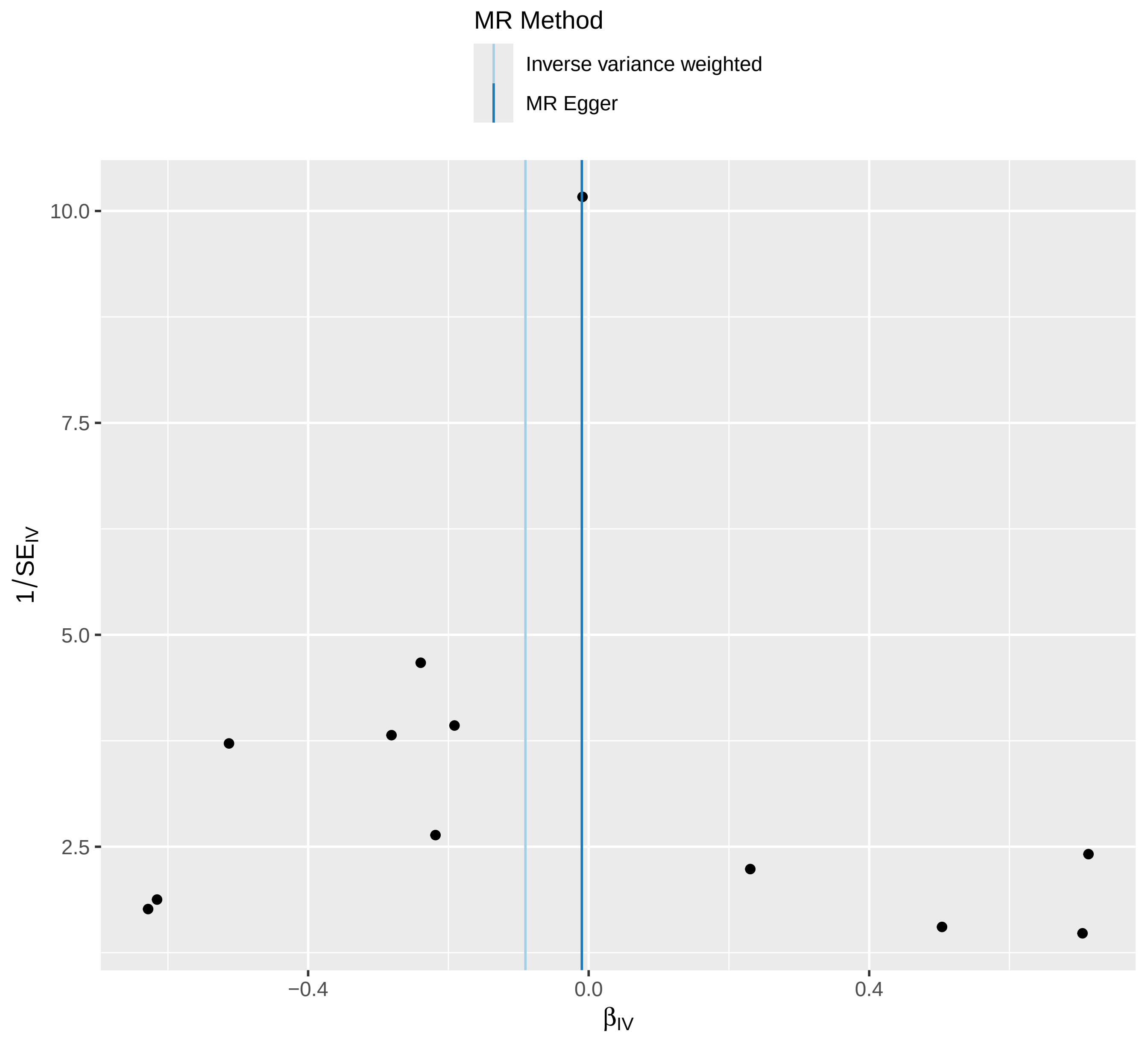

MR Method
Inverse variance weighted
MR Egger
10.0
7.5
5.0
2.5
1/SE$_{IV}$
−0.4
0.0
0.4
β$_{IV}$

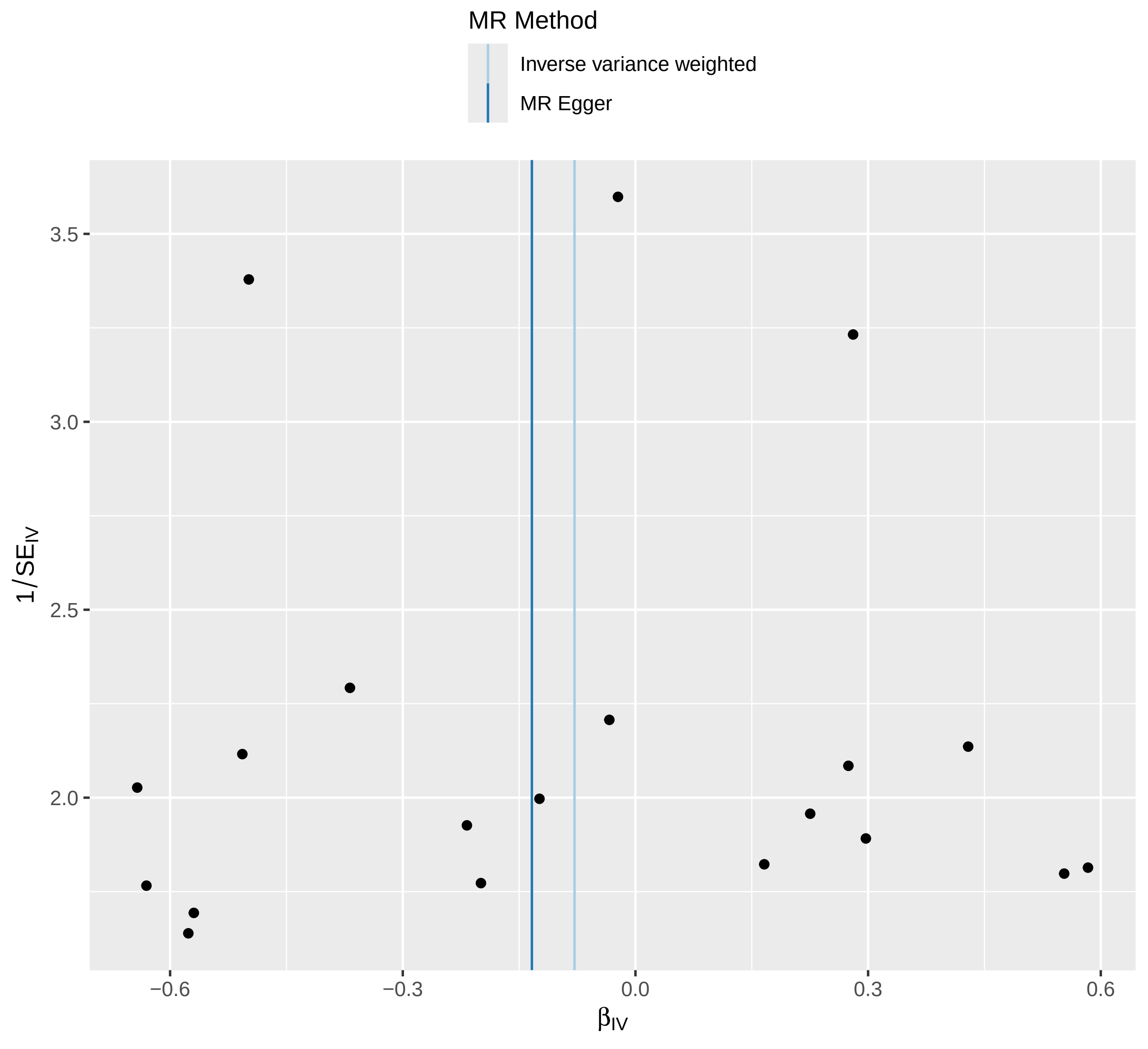
MR Method
Inverse variance weighted
MR Egger
3.5
3.0
2.5
2.0
1/SE$_{IV}$
−0.6
−0.3
0.0
0.3
0.6
β$_{IV}$

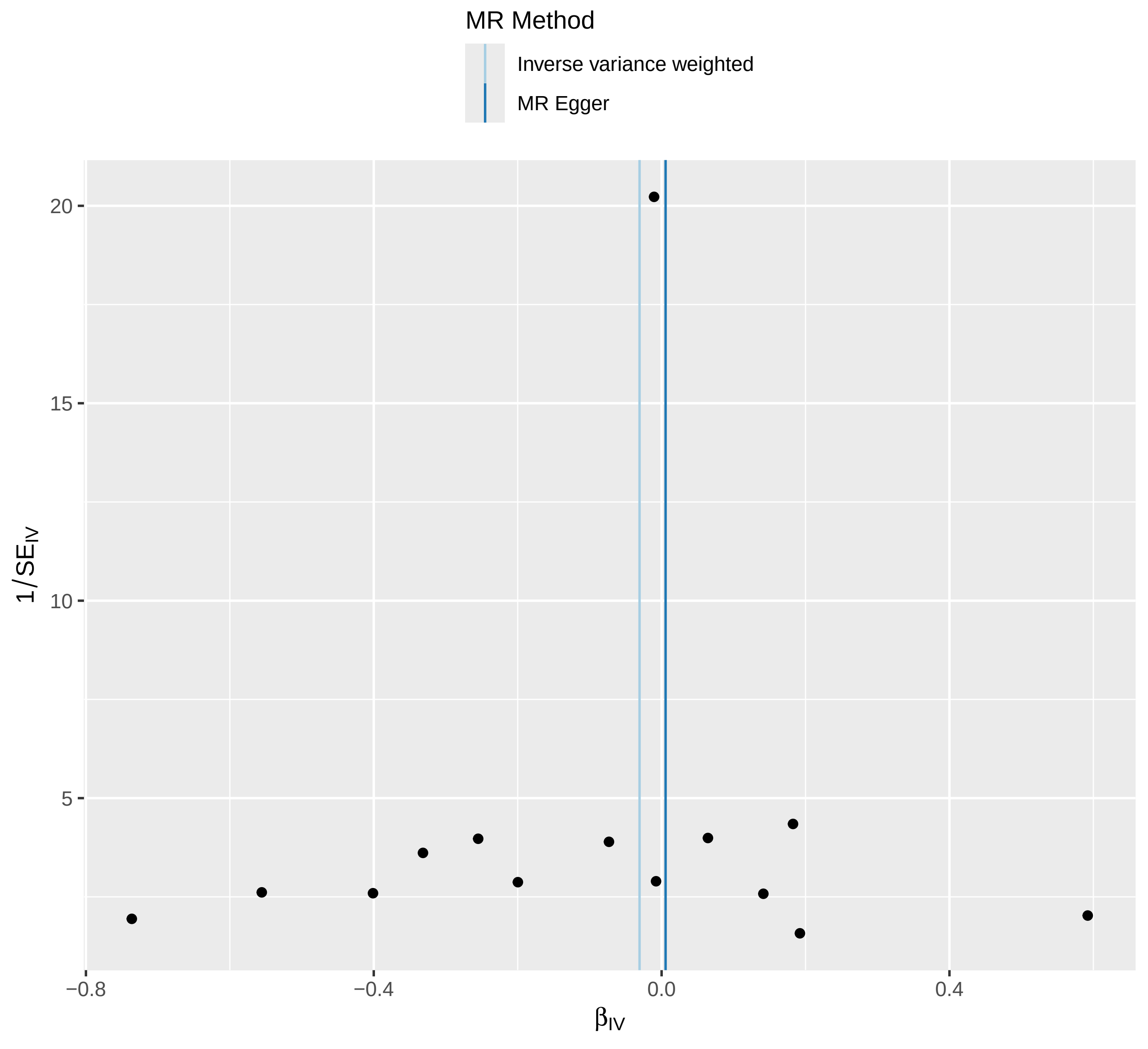

MR Method
Inverse variance weighted
MR Egger
1/SE_IV
20
15
10
5
−0.8
−0.4
0.0
0.4
β_IV

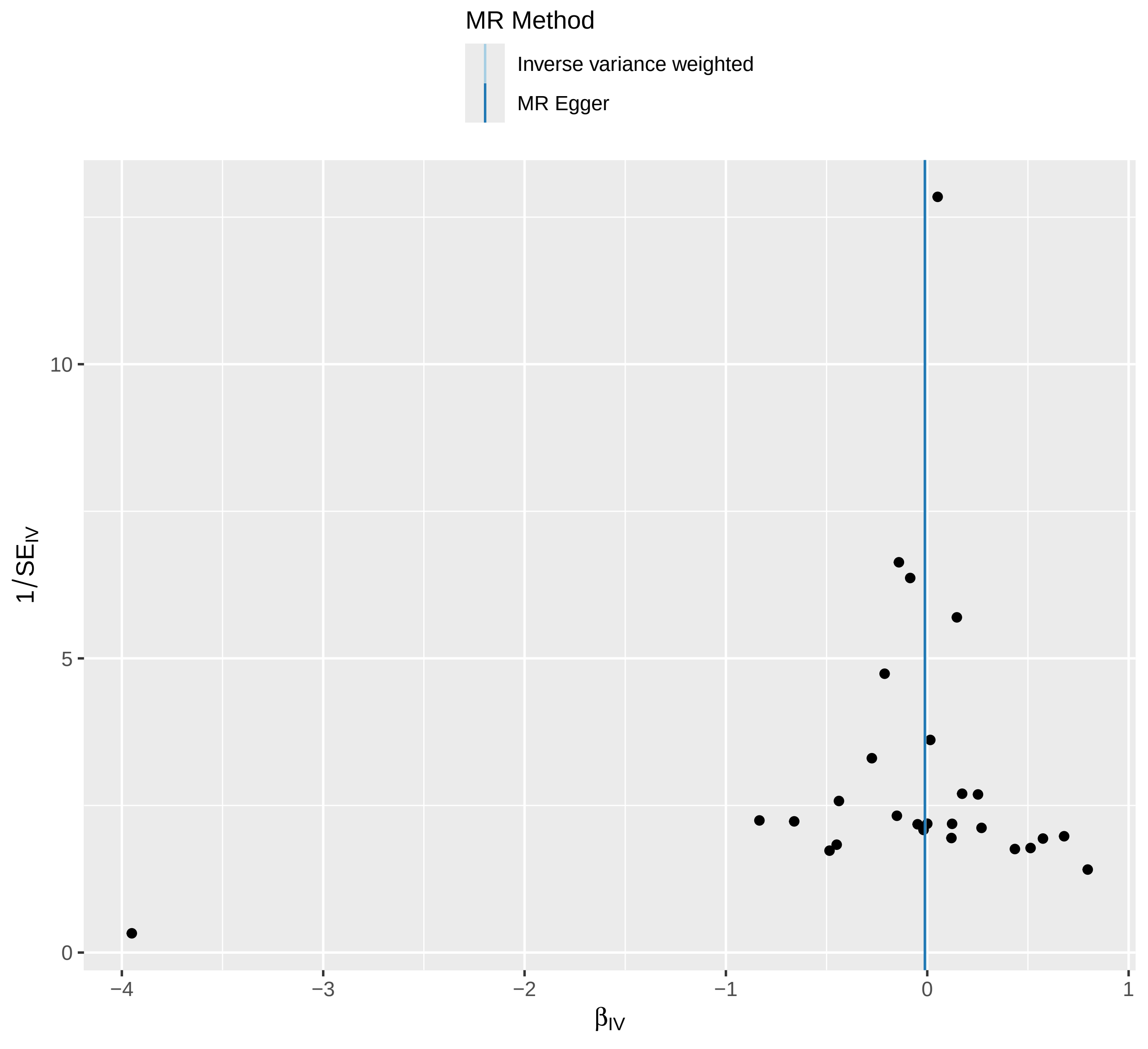

MR Method
Inverse variance weighted
MR Egger
10
5
0
−4
−3
−2
−1
0
1
$1/SE_{IV}$
β_{IV}

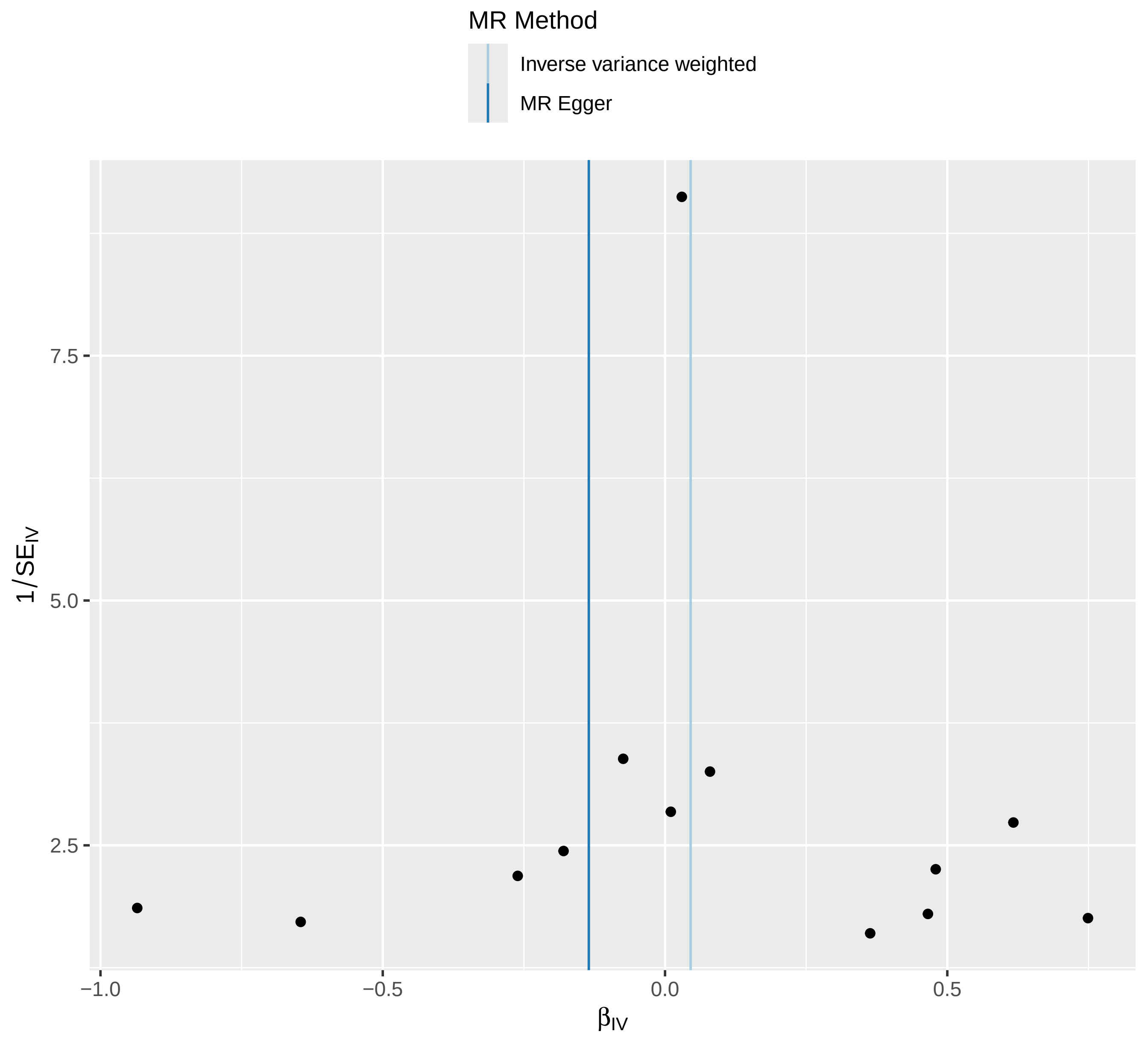
MR Method
Inverse variance weighted
MR Egger
7.5
5.0
2.5
1/SE$_{IV}$
−1.0
−0.5
0.0
0.5
β$_{IV}$

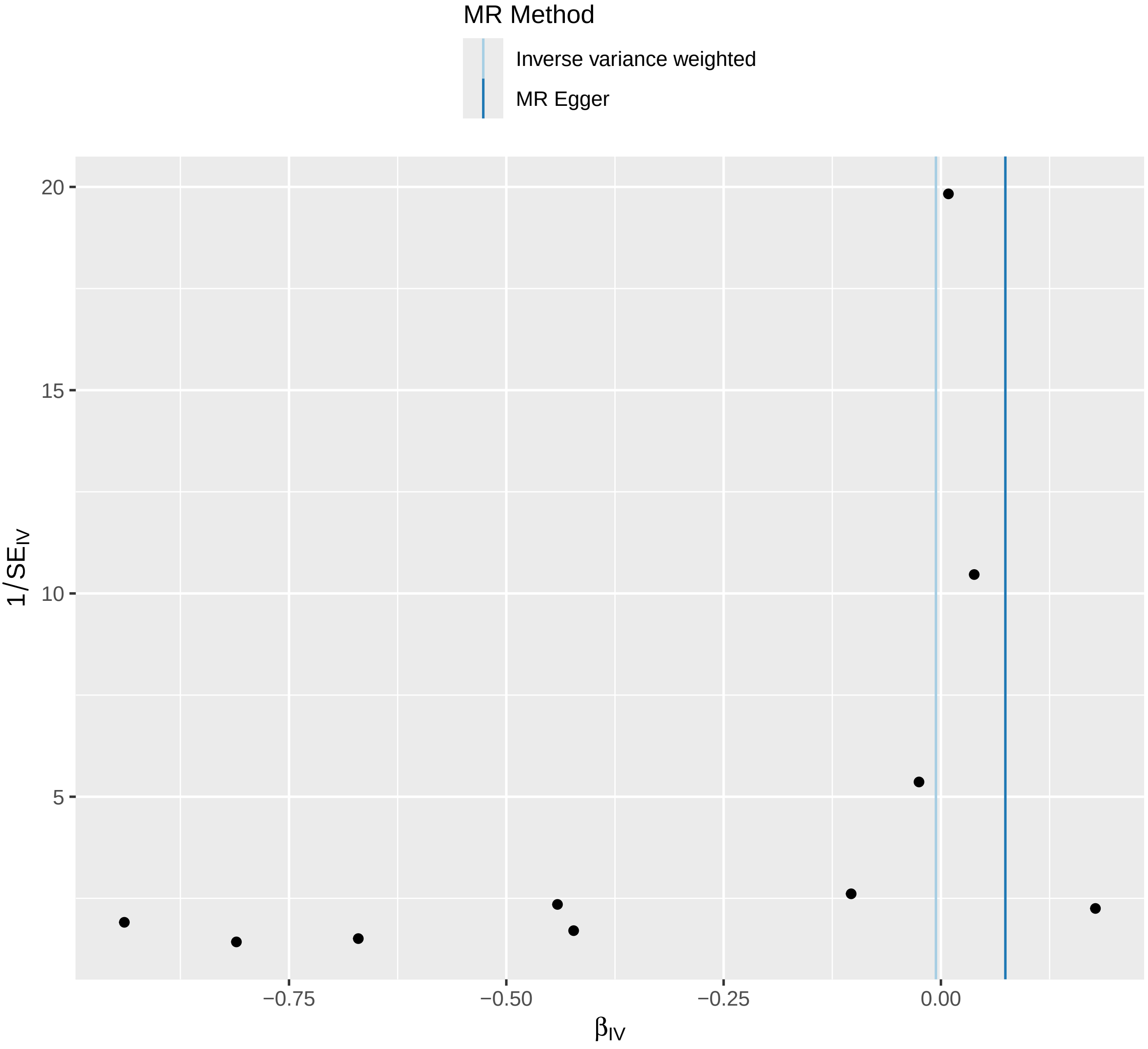
MR Method
Inverse variance weighted
MR Egger
20
15
10
5
1/SE_IV
−0.75
−0.50
−0.25
0.00
β_IV

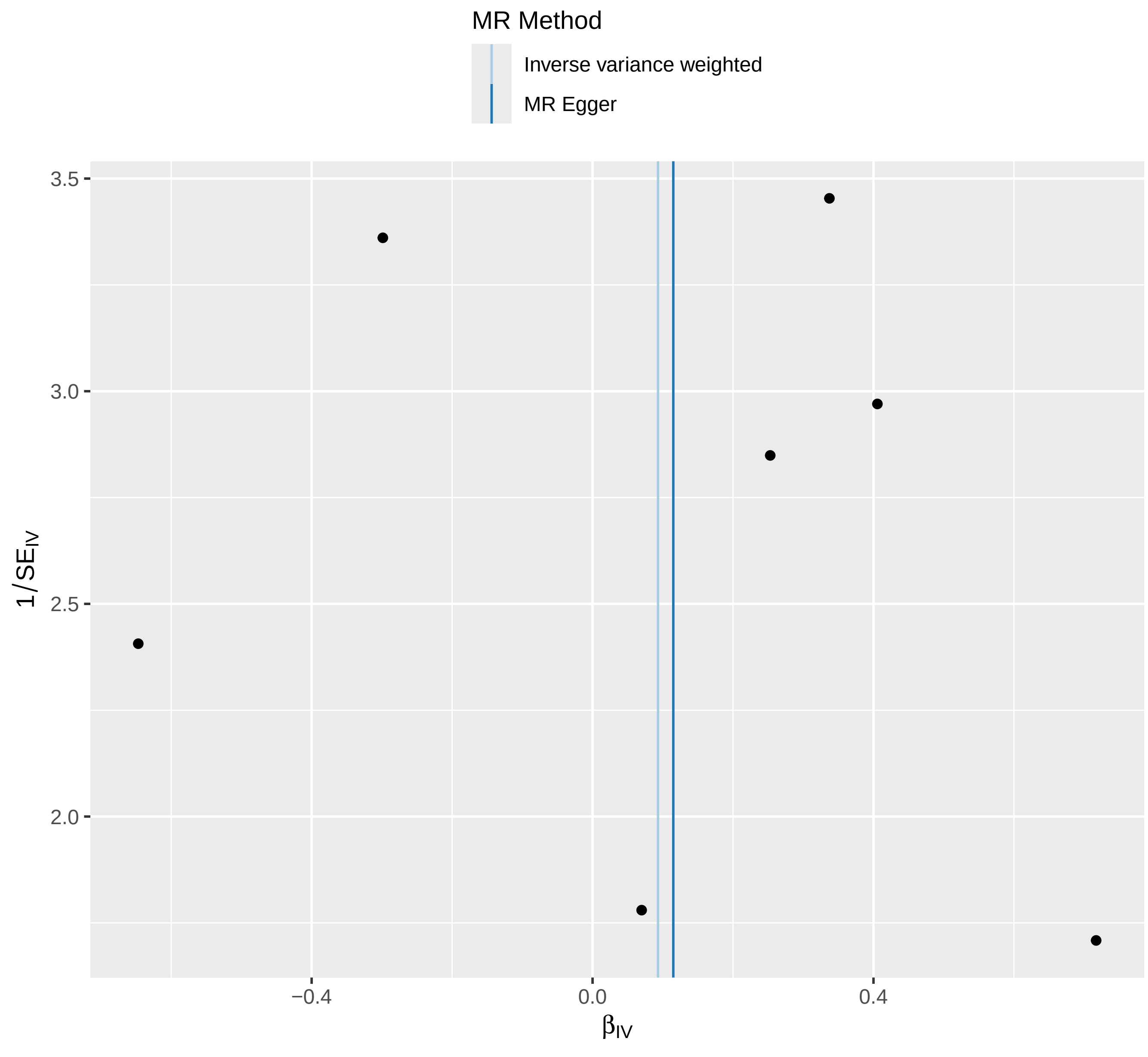
MR Method
Inverse variance weighted
MR Egger
3.5
3.0
2.5
2.0
1/SE$_{IV}$
−0.4
0.0
0.4
β$_{IV}$

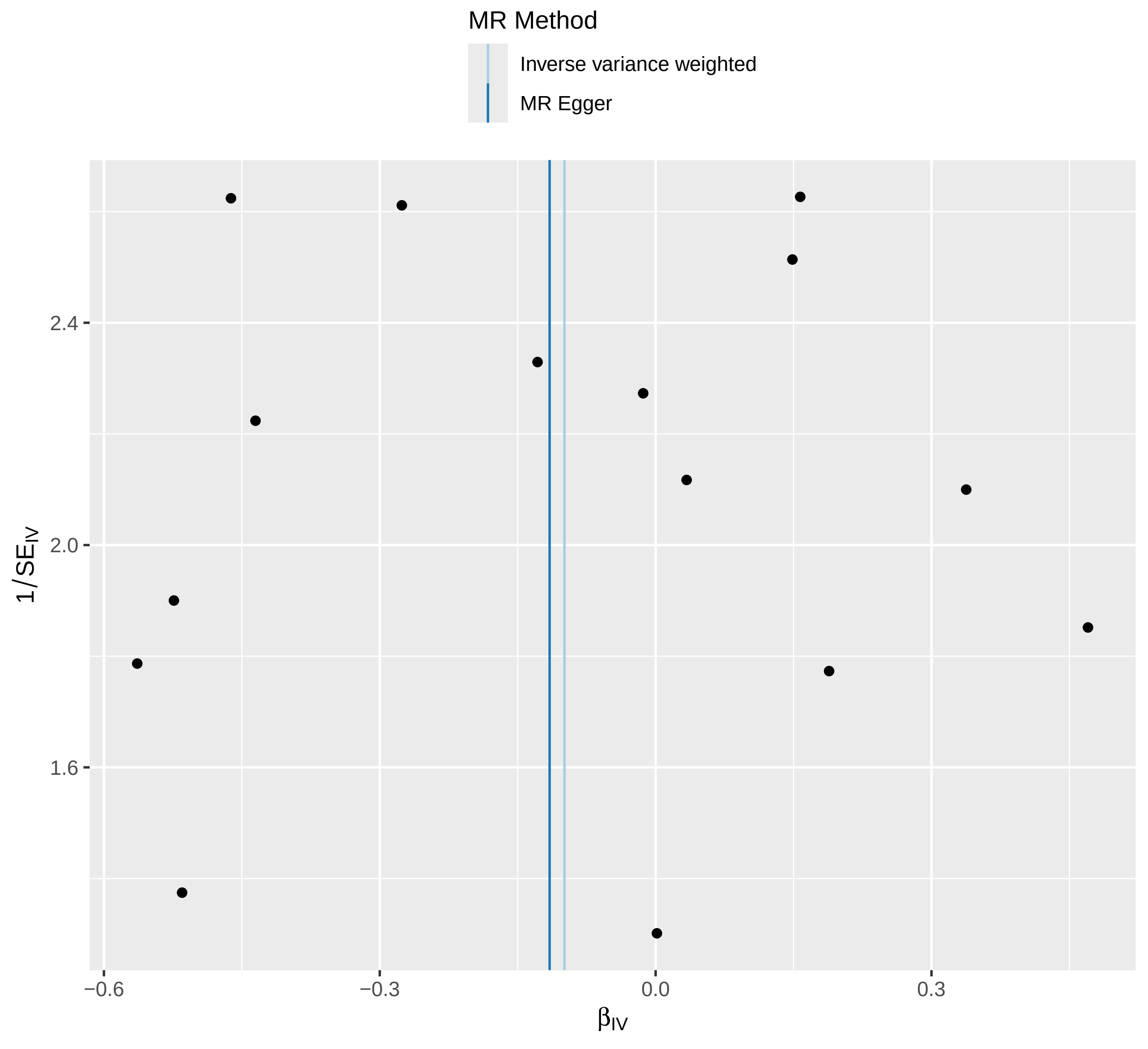

MR Method
Inverse variance weighted
MR Egger
2.4
2.0
1.6
1/SE_IV
−0.6
−0.3
0.0
0.3
β_IV

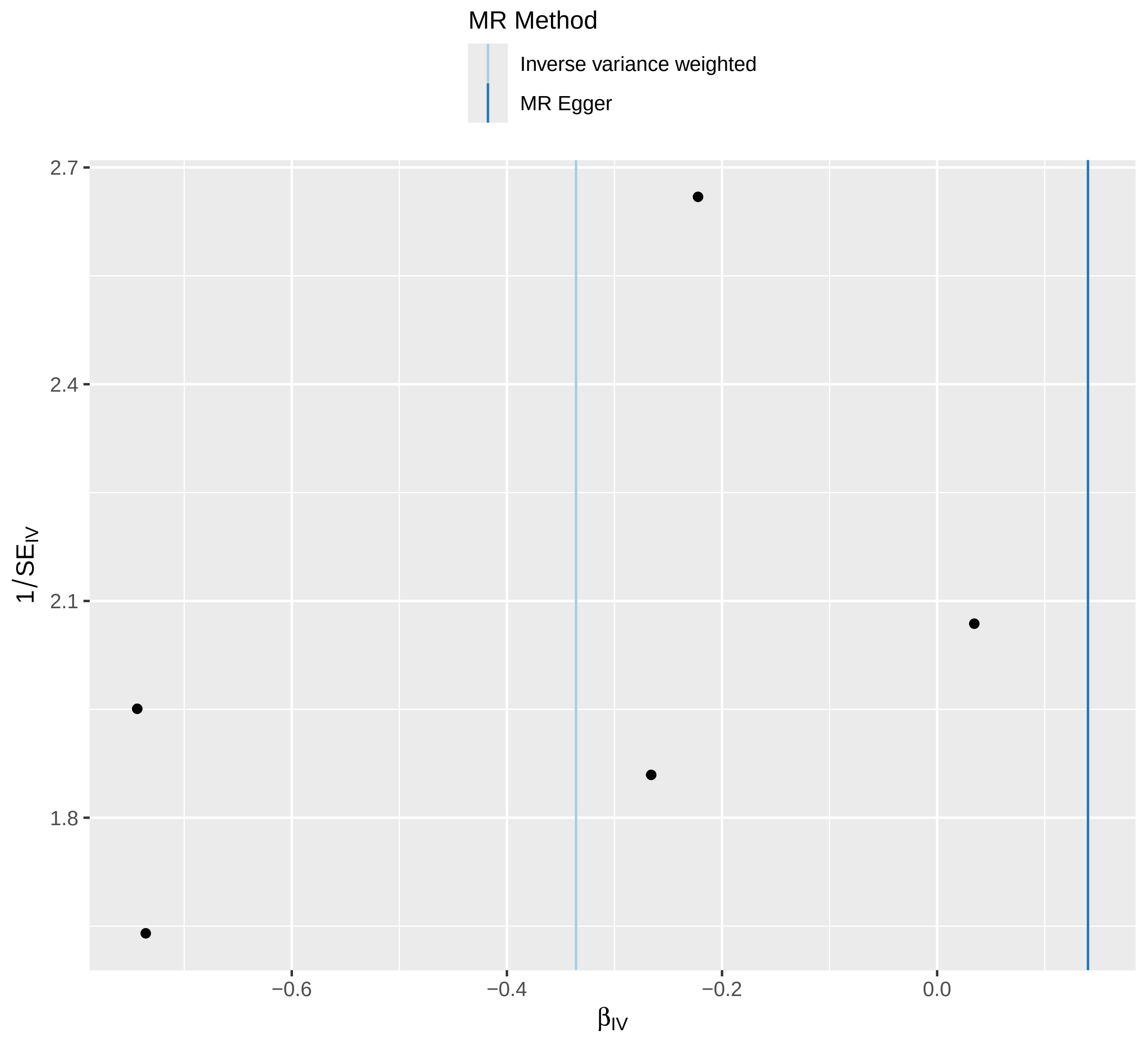
MR Method
Inverse variance weighted
MR Egger
2.7
2.4
2.1
1.8
1/SE_IV
−0.6
−0.4
−0.2
0.0
β_IV

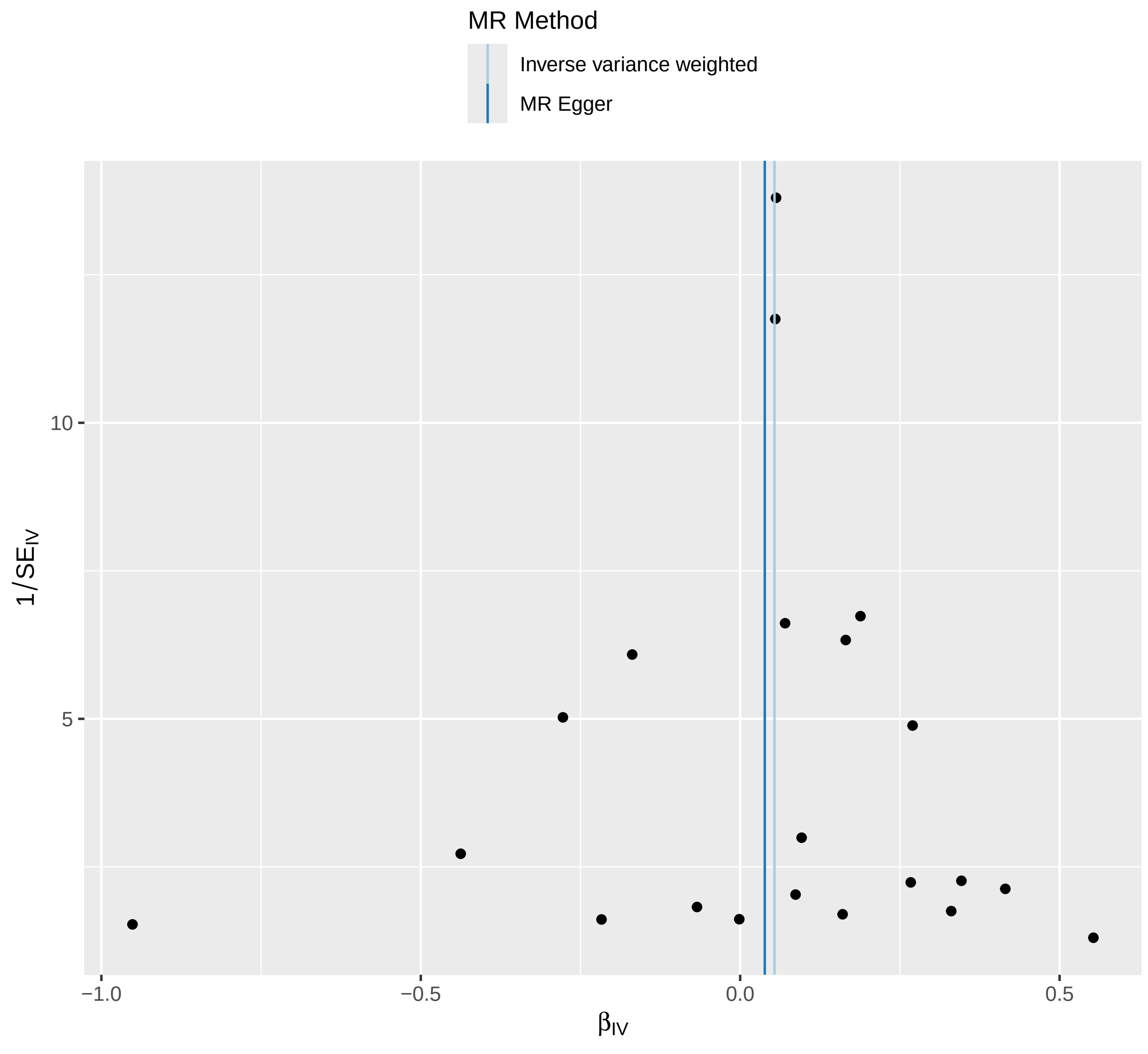
MR Method
Inverse variance weighted
MR Egger
10
5
1/SE_IV
−1.0
−0.5
0.0
0.5
β_IV

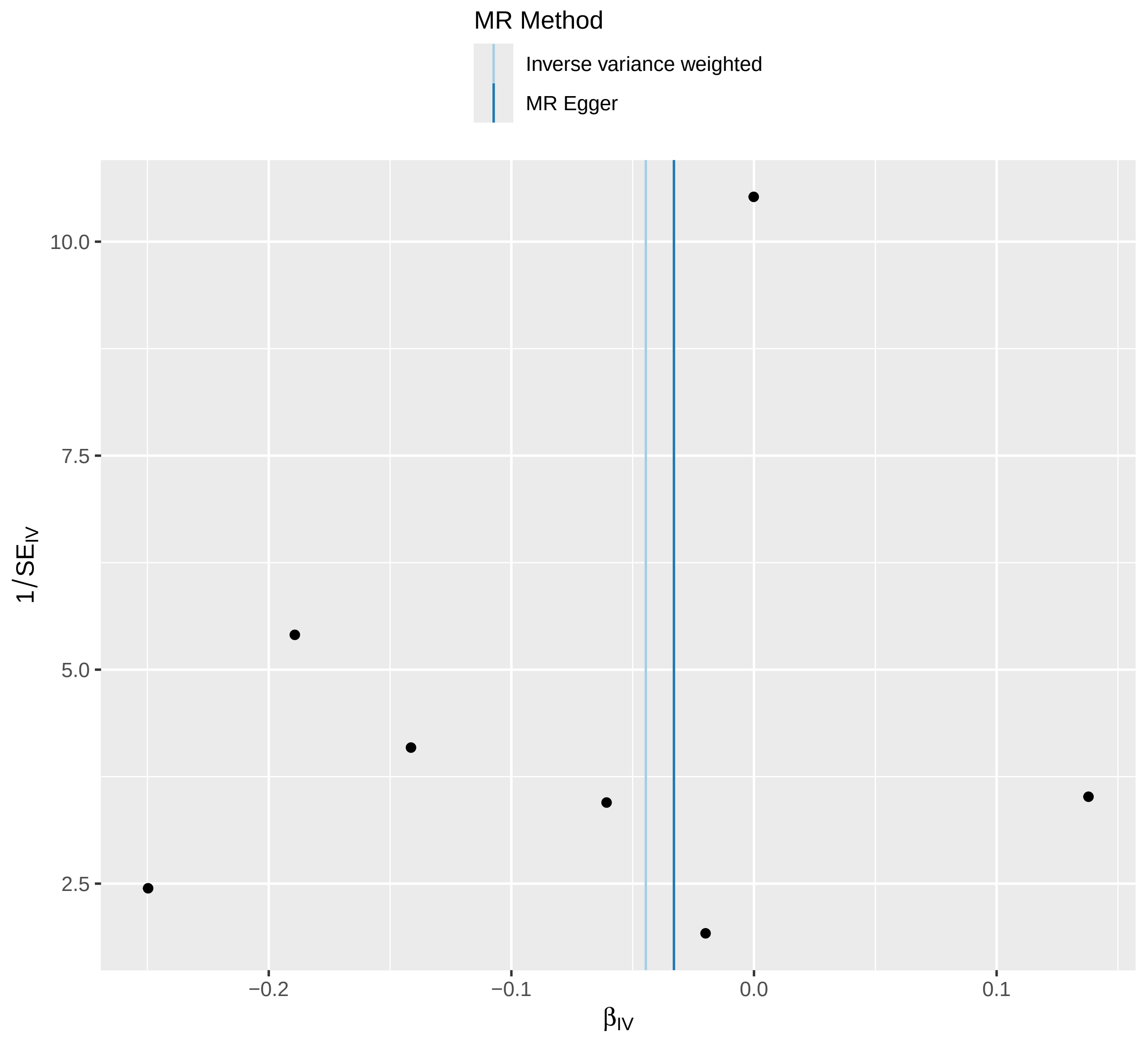
MR Method
Inverse variance weighted
MR Egger
1/SE_IV
10.0
7.5
5.0
2.5
−0.2
−0.1
0.0
0.1
β_IV

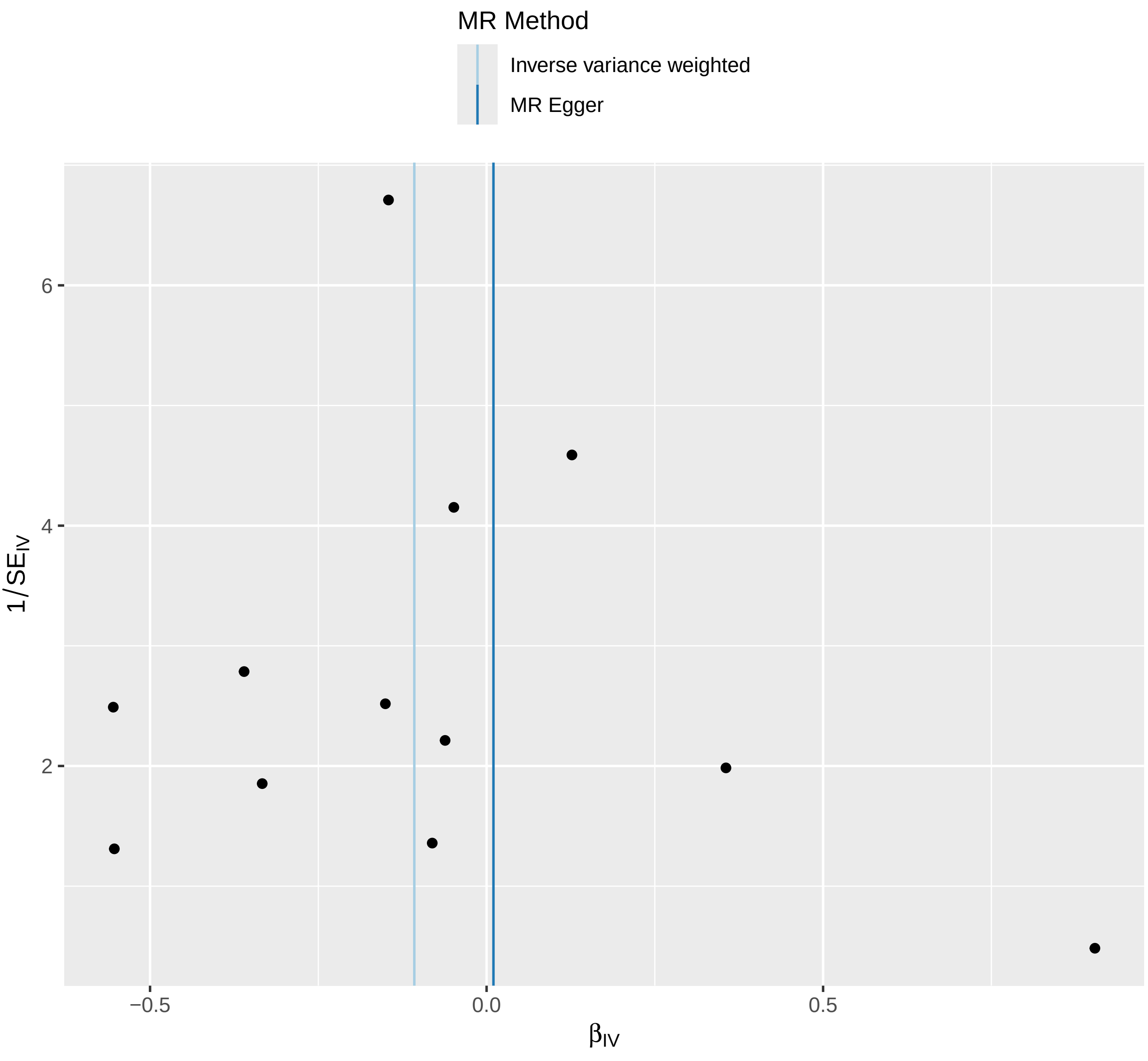

MR Method
Inverse variance weighted
MR Egger
6
4
2
1/SE$_{IV}$
−0.5
0.0
0.5
β_{IV}

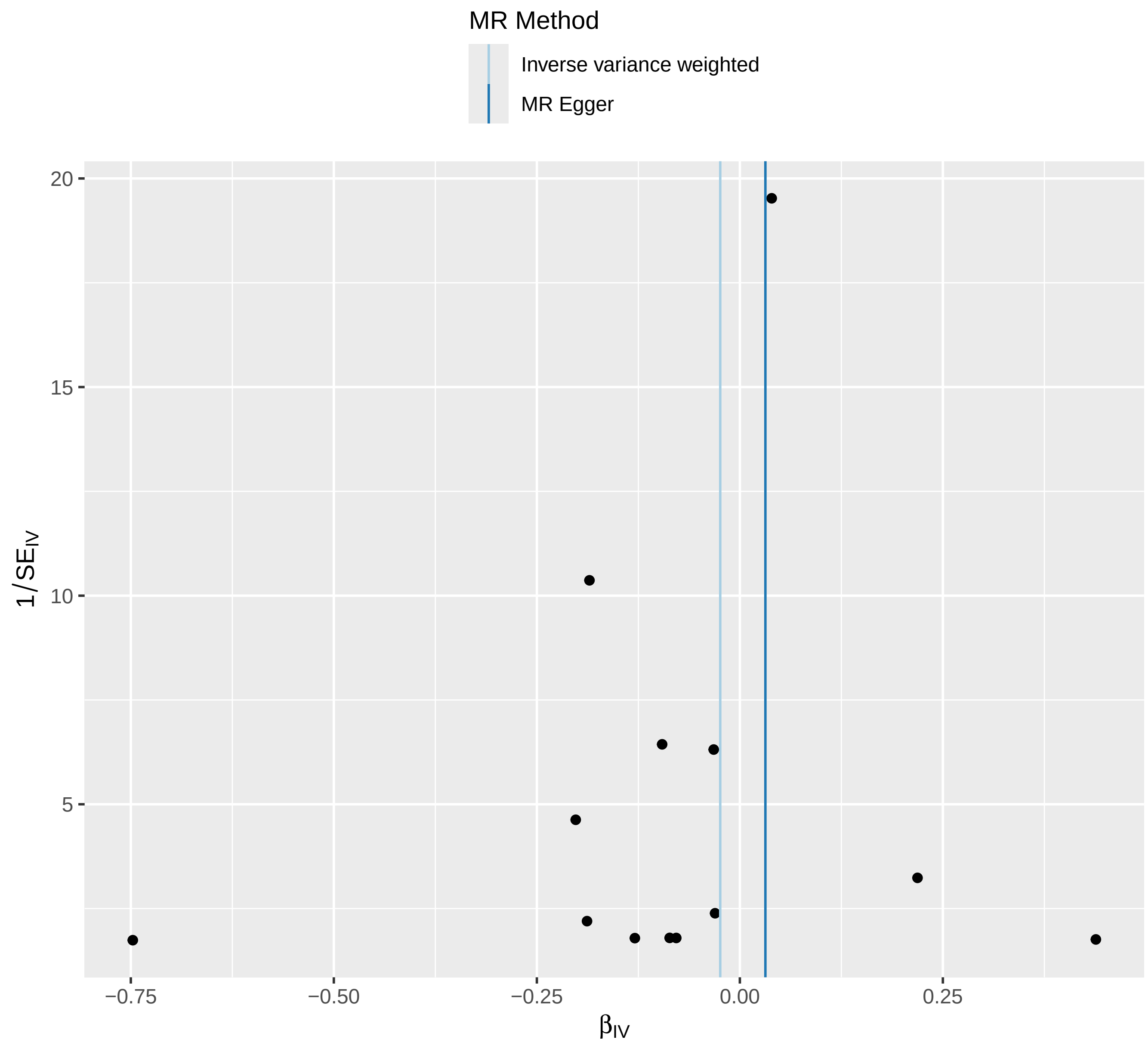
MR Method
Inverse variance weighted
MR Egger
20
15
10
5
1/SE$_{IV}$
−0.75
−0.50
−0.25
0.00
0.25
β$_{IV}$

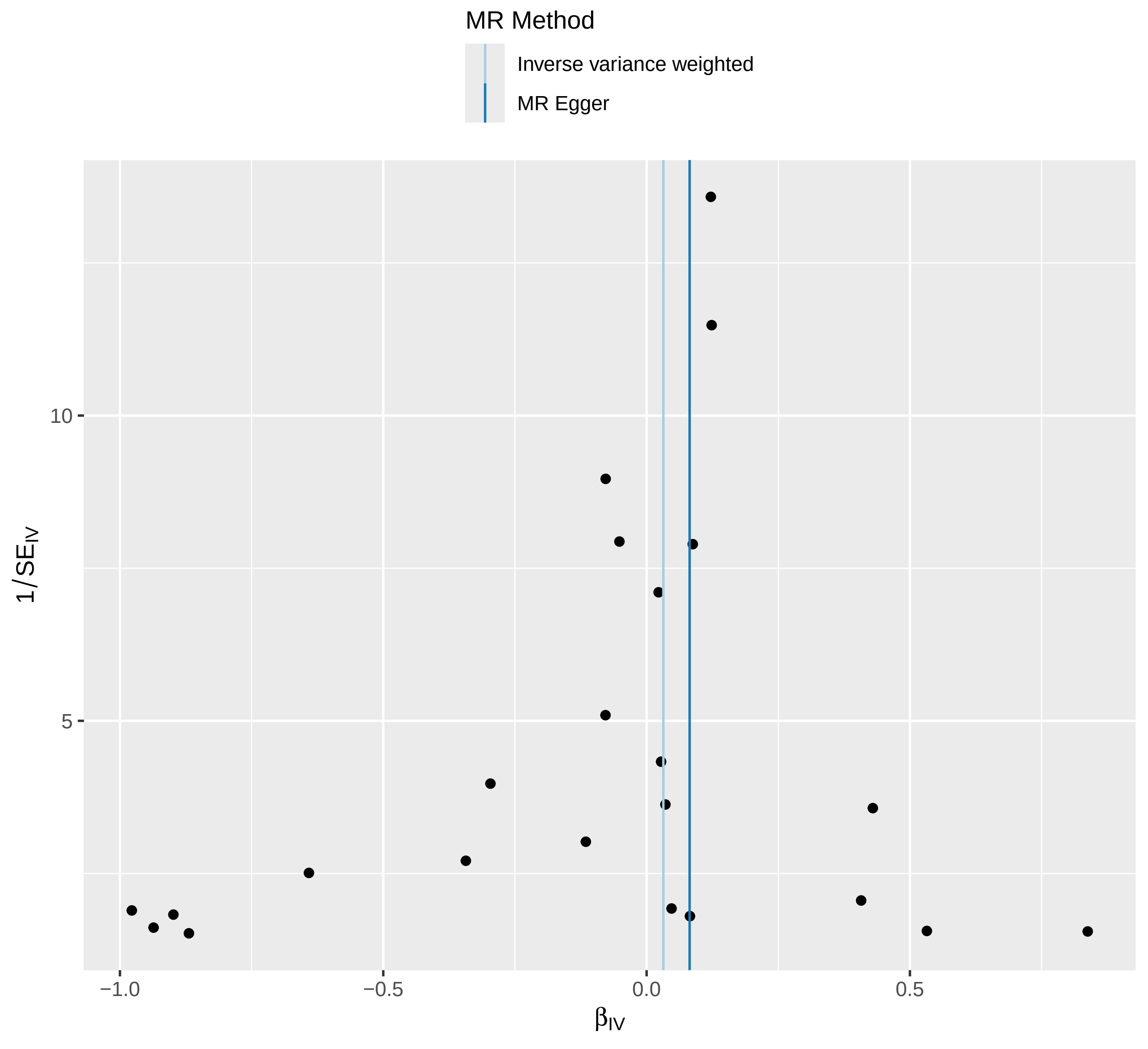

MR Method
Inverse variance weighted
MR Egger
10
5
1/SE_IV
−1.0
−0.5
0.0
0.5
β_IV

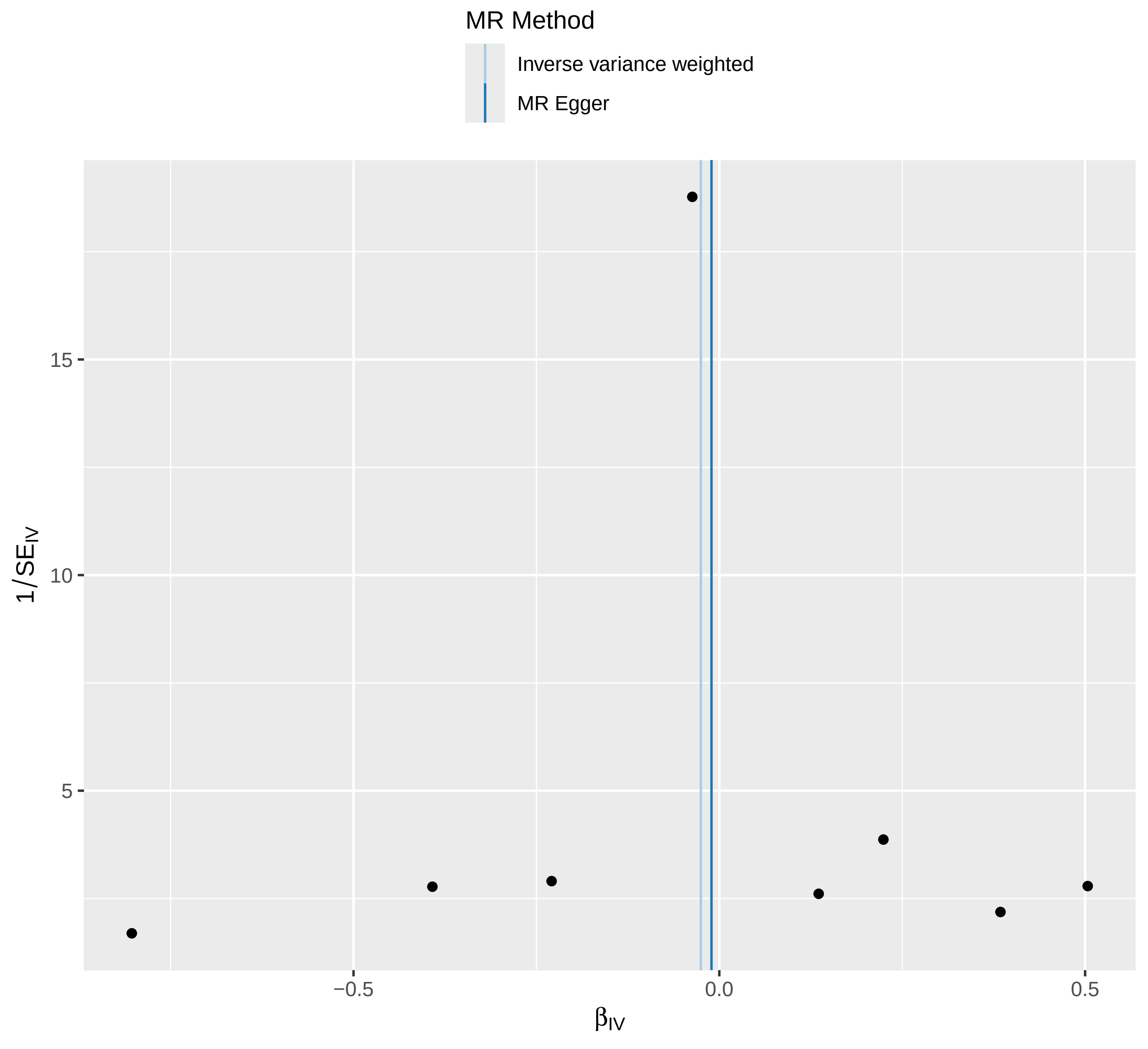
MR Method
Inverse variance weighted
MR Egger
15
10
5
1/SE$_{IV}$
−0.5
0.0
0.5
β$_{IV}$

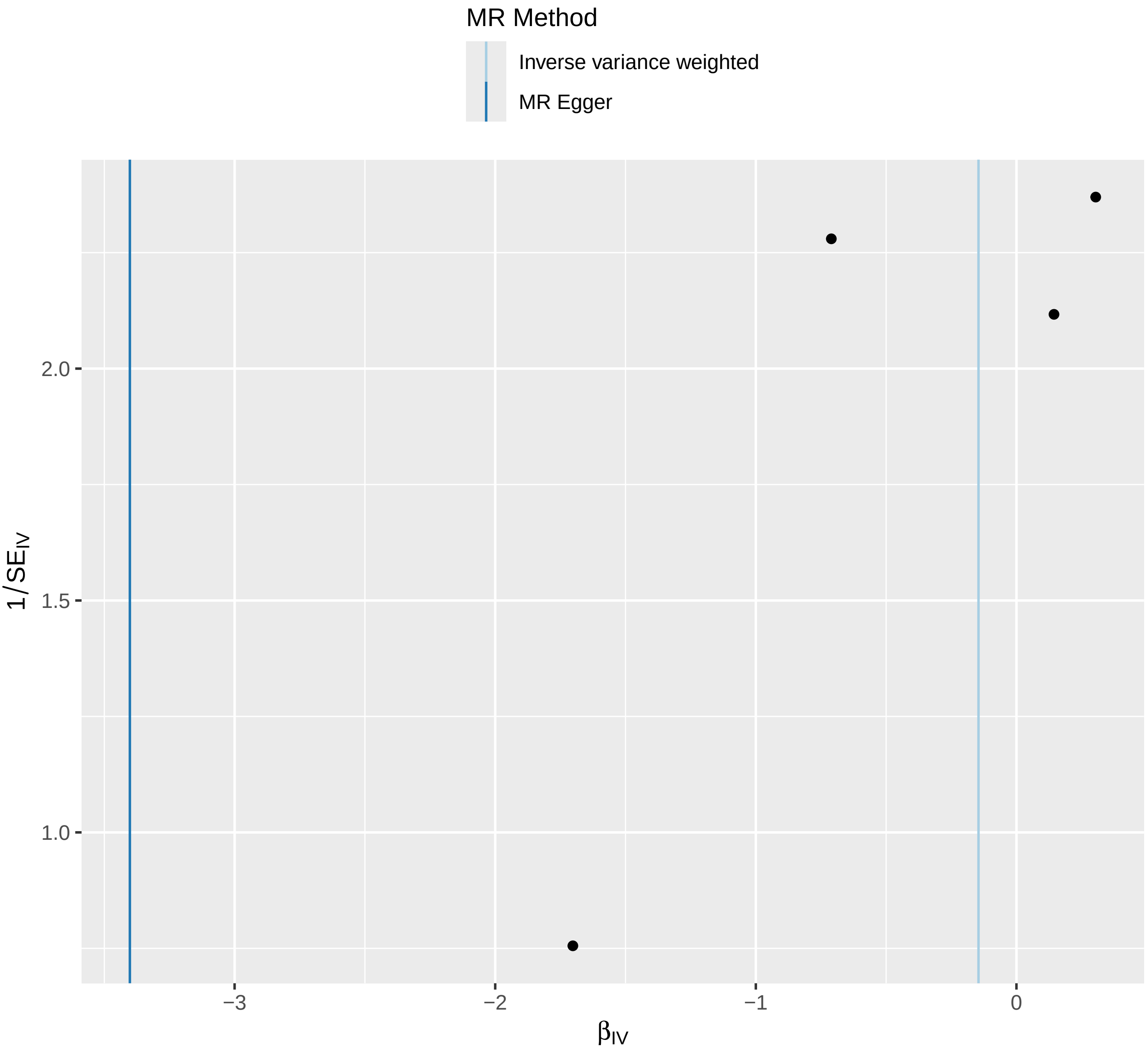
MR Method
Inverse variance weighted
MR Egger
1/SE$_{IV}$
2.0
1.5
1.0
−3
−2
−1
0
β$_{IV}$

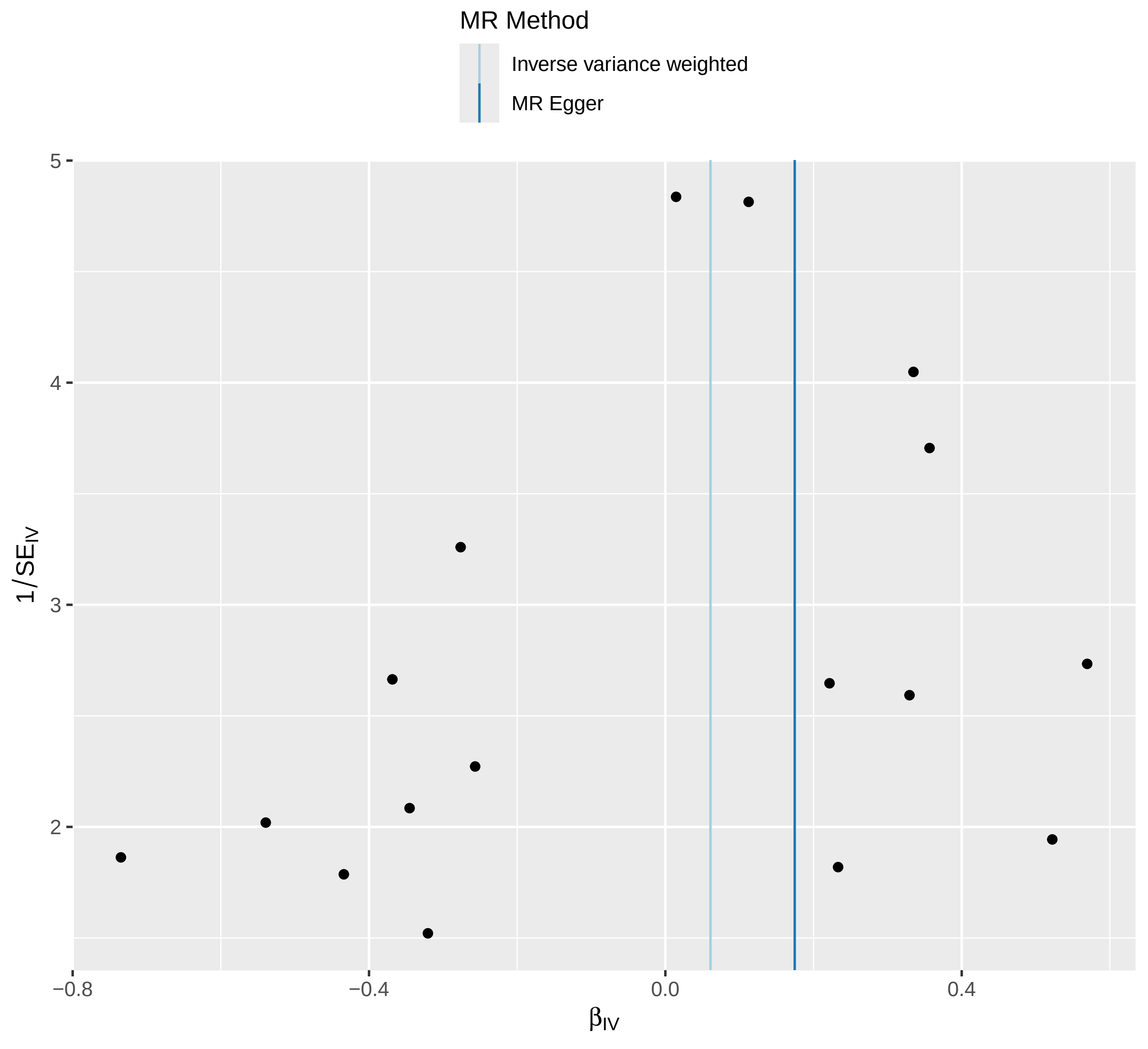

MR Method
Inverse variance weighted
MR Egger
5
4
3
2
1/SE$_{IV}$
−0.8
−0.4
0.0
0.4
β$_{IV}$

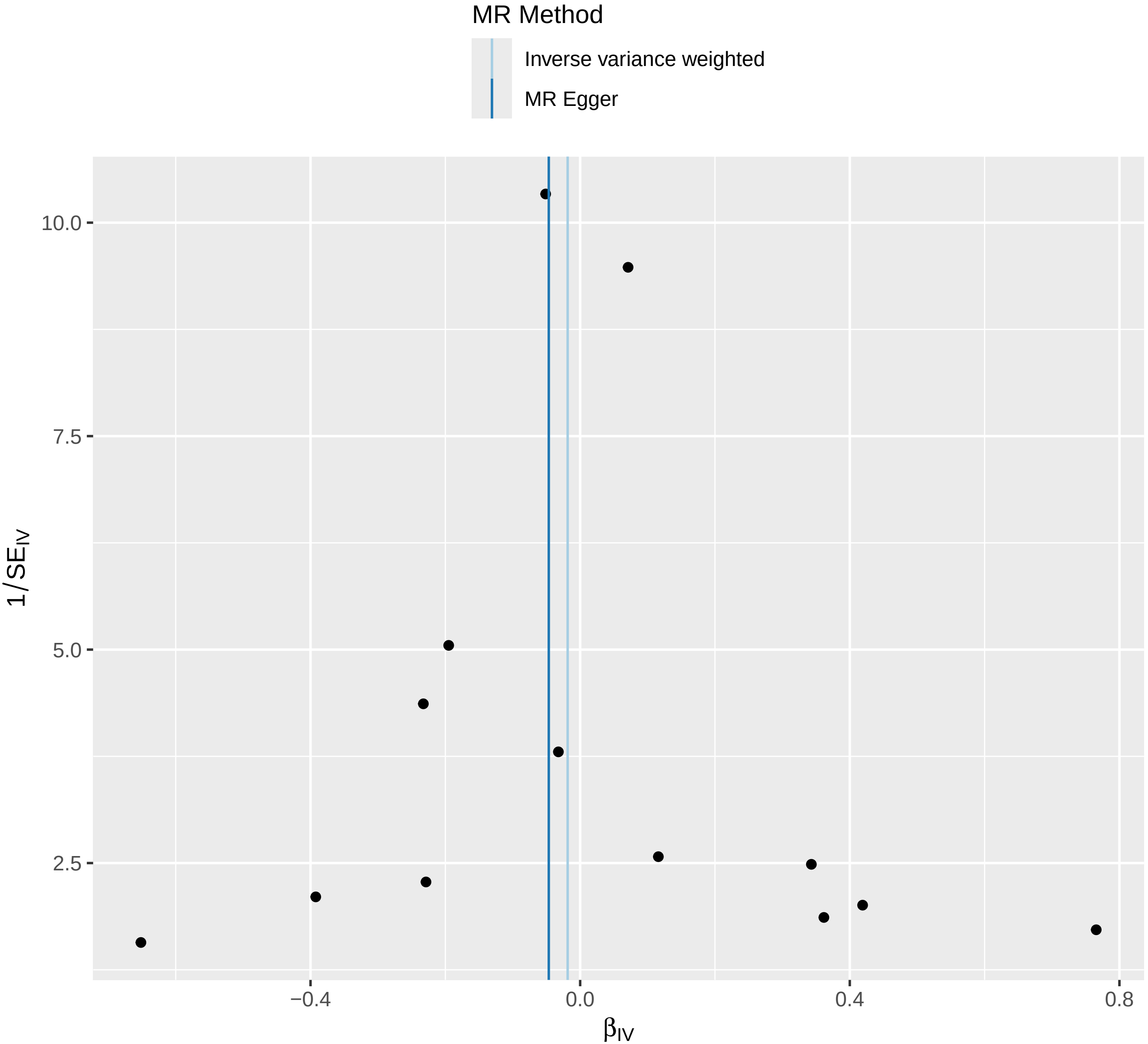

MR Method
Inverse variance weighted
MR Egger
10.0
7.5
5.0
2.5
1/SE_IV
−0.4
0.0
0.4
0.8
β_IV

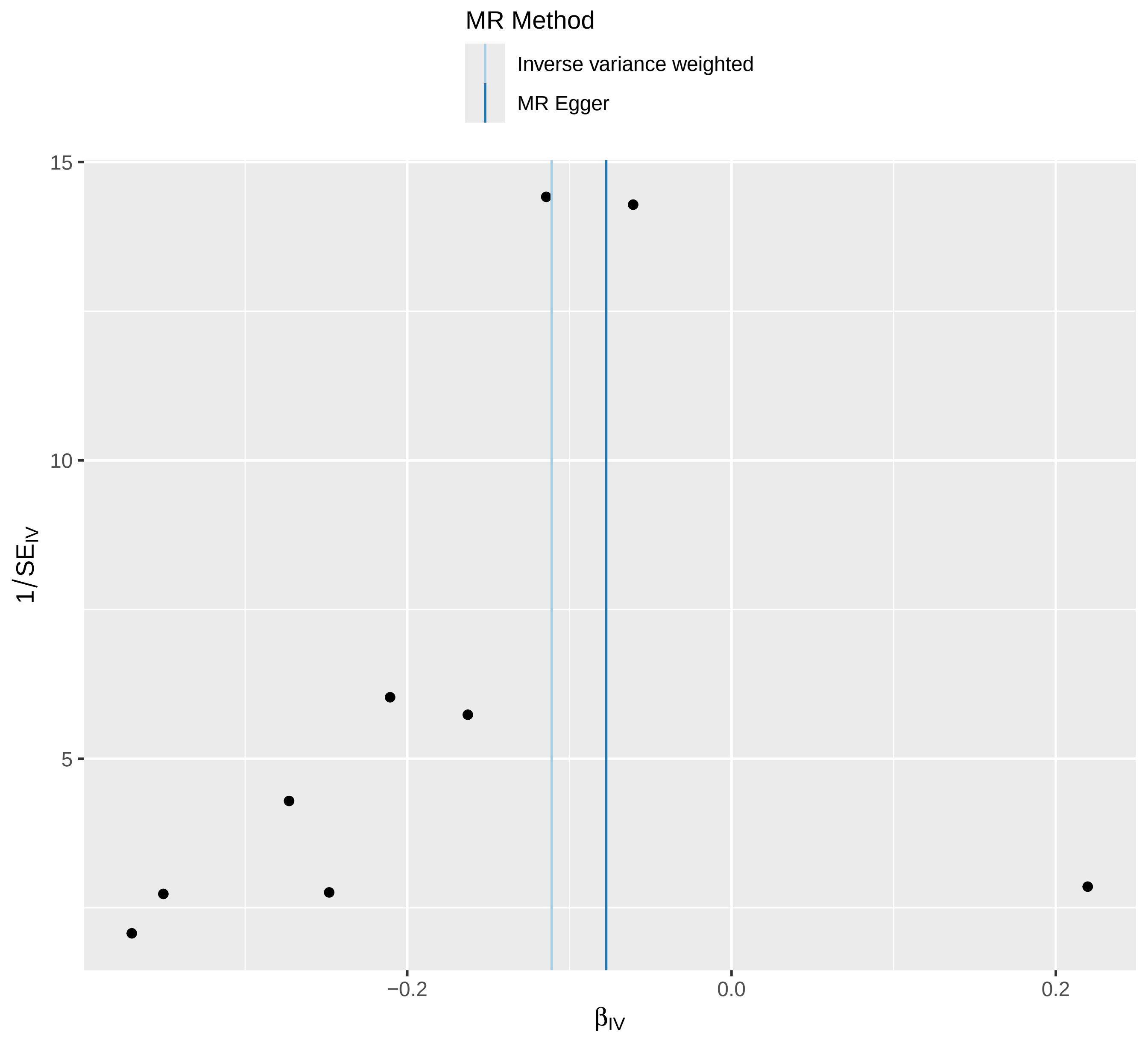

MR Method
Inverse variance weighted
MR Egger
15
10
5
−0.2
0.0
0.2
1/SE_IV
β_IV

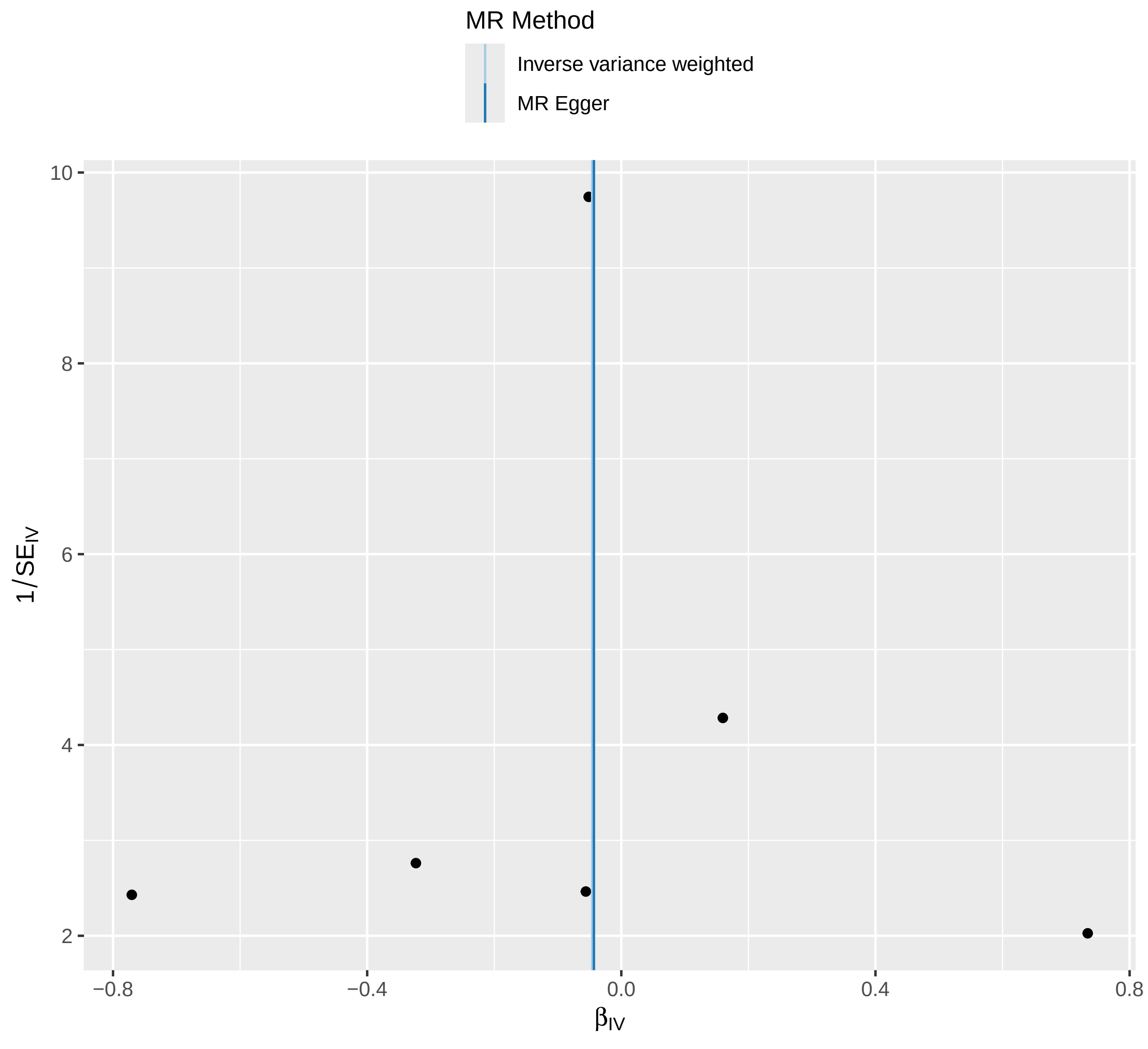

MR Method
Inverse variance weighted
MR Egger
10
8
6
4
2
1/SE$_{IV}$
−0.8
−0.4
0.0
0.4
0.8
β$_{IV}$

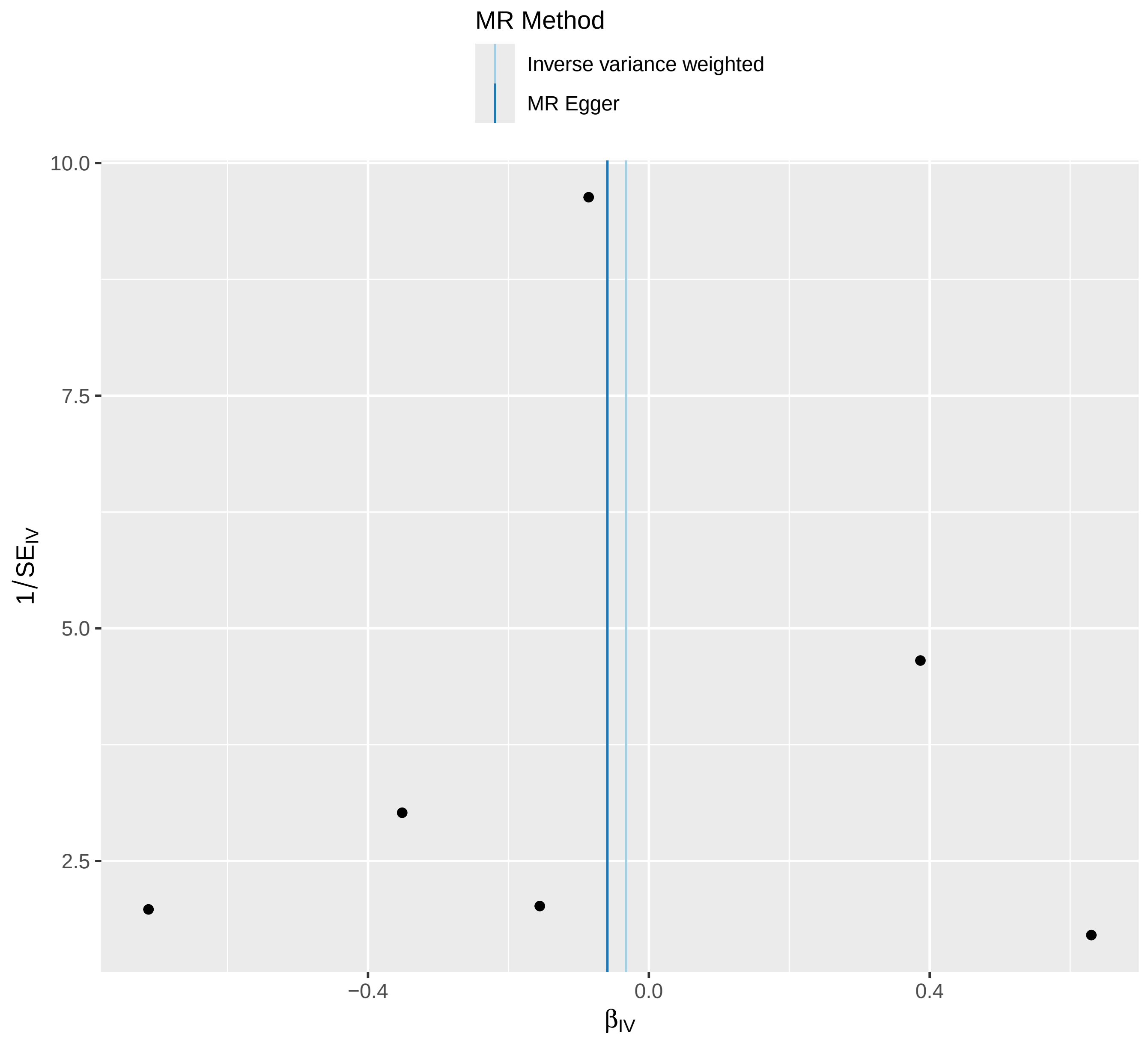

MR Method
Inverse variance weighted
MR Egger
10.0
7.5
5.0
2.5
1/SE_IV
−0.4
0.0
0.4
β_IV

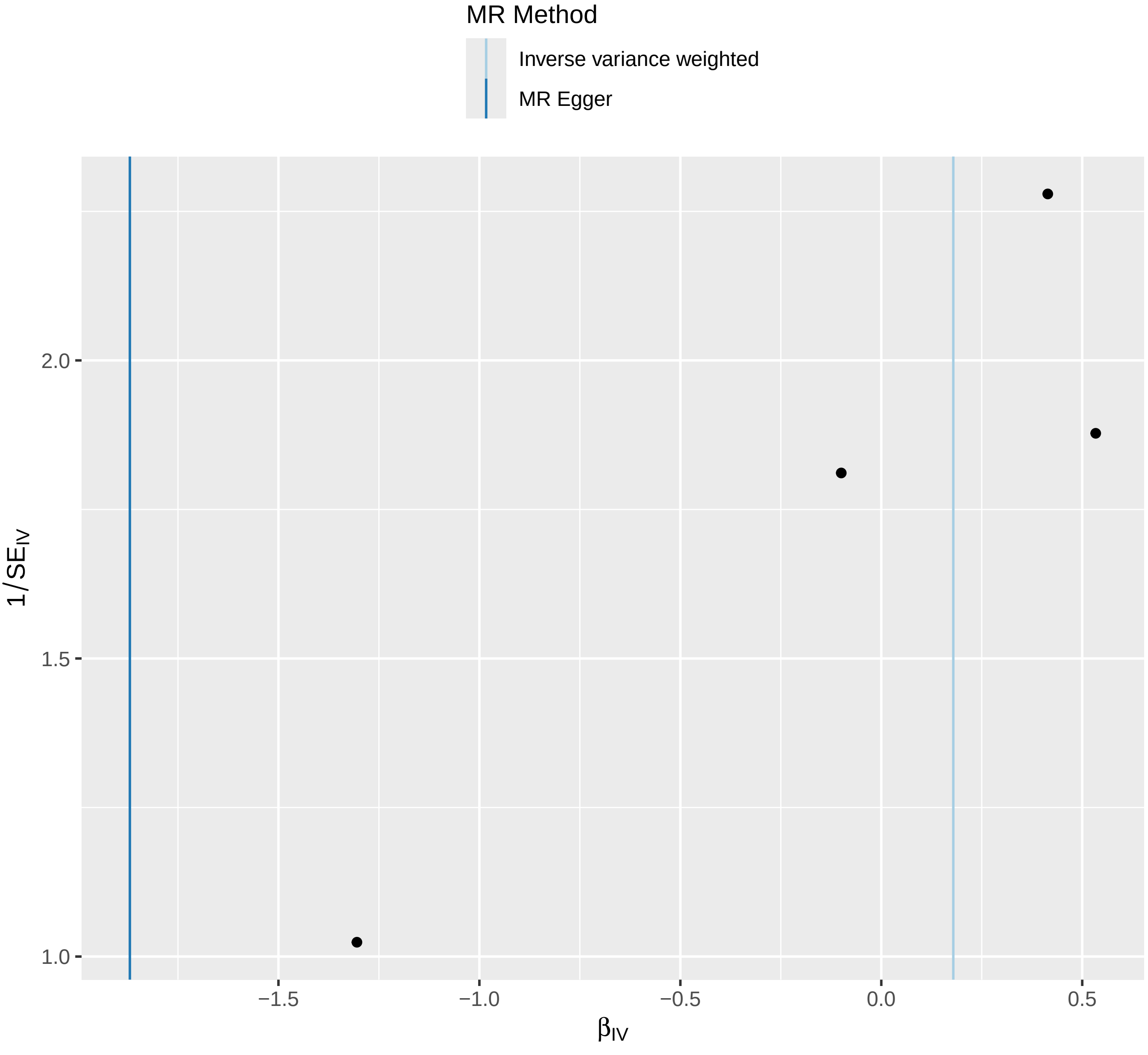

MR Method
Inverse variance weighted
MR Egger
2.0
1.5
1.0
1/SE$_{IV}$
−1.5
−1.0
−0.5
0.0
0.5
β$_{IV}$

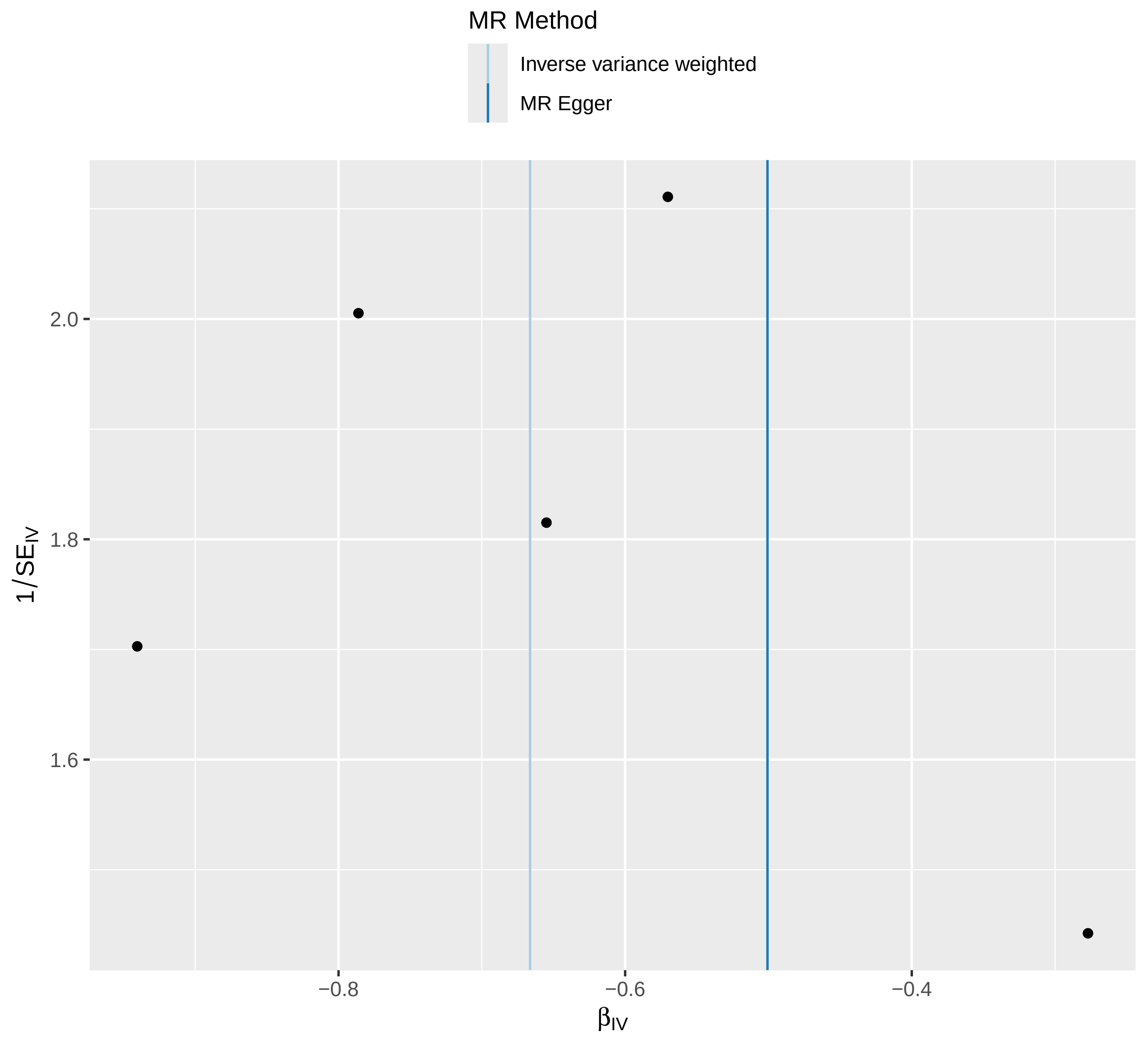
MR Method
Inverse variance weighted
MR Egger
2.0
1.8
1.6
$1/SE_{IV}$
−0.8
−0.6
−0.4
β_{IV}

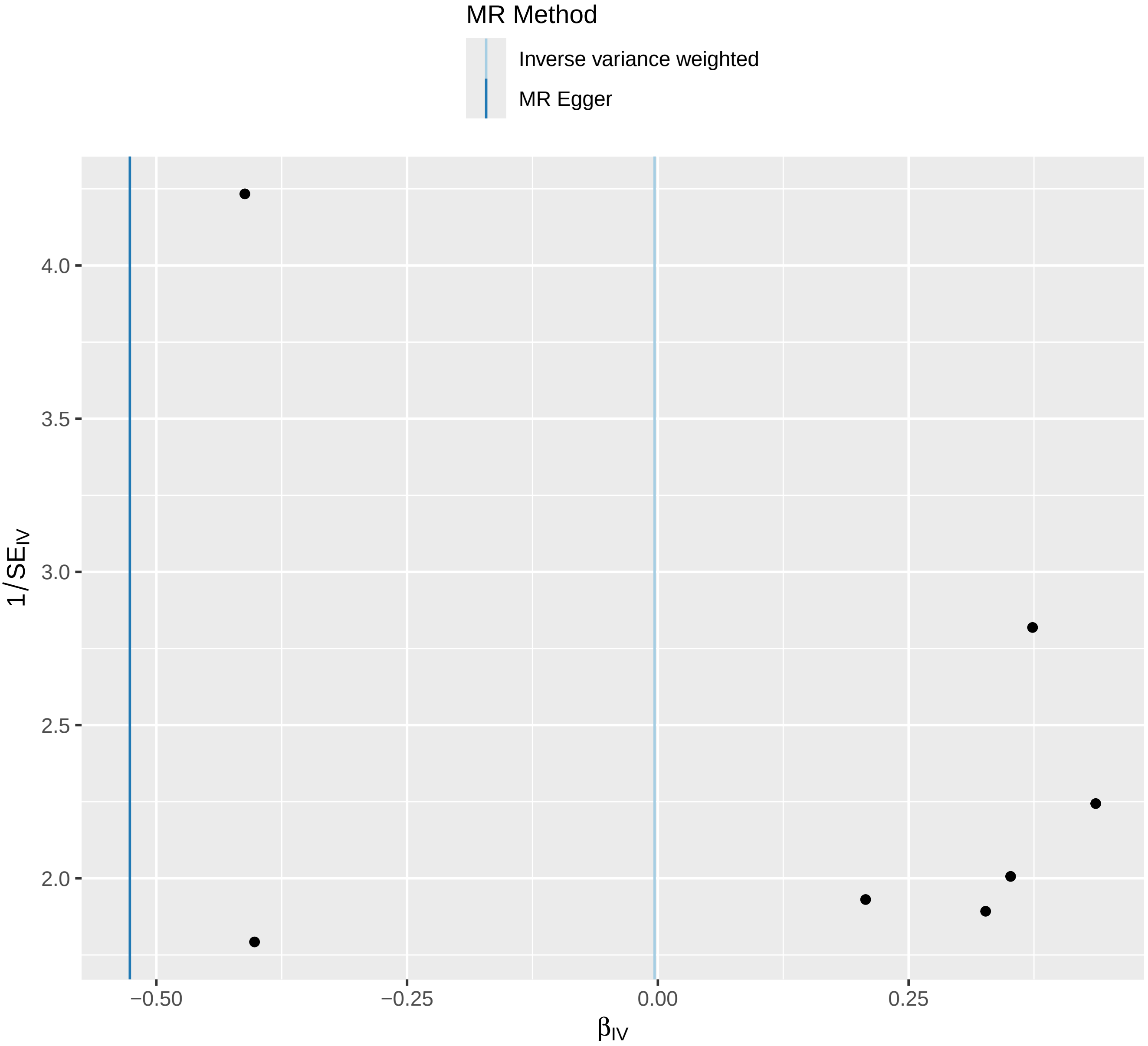

MR Method
Inverse variance weighted
MR Egger
4.0
3.5
3.0
2.5
2.0
$1/SE_{IV}$
−0.50
−0.25
0.00
0.25
β_{IV}

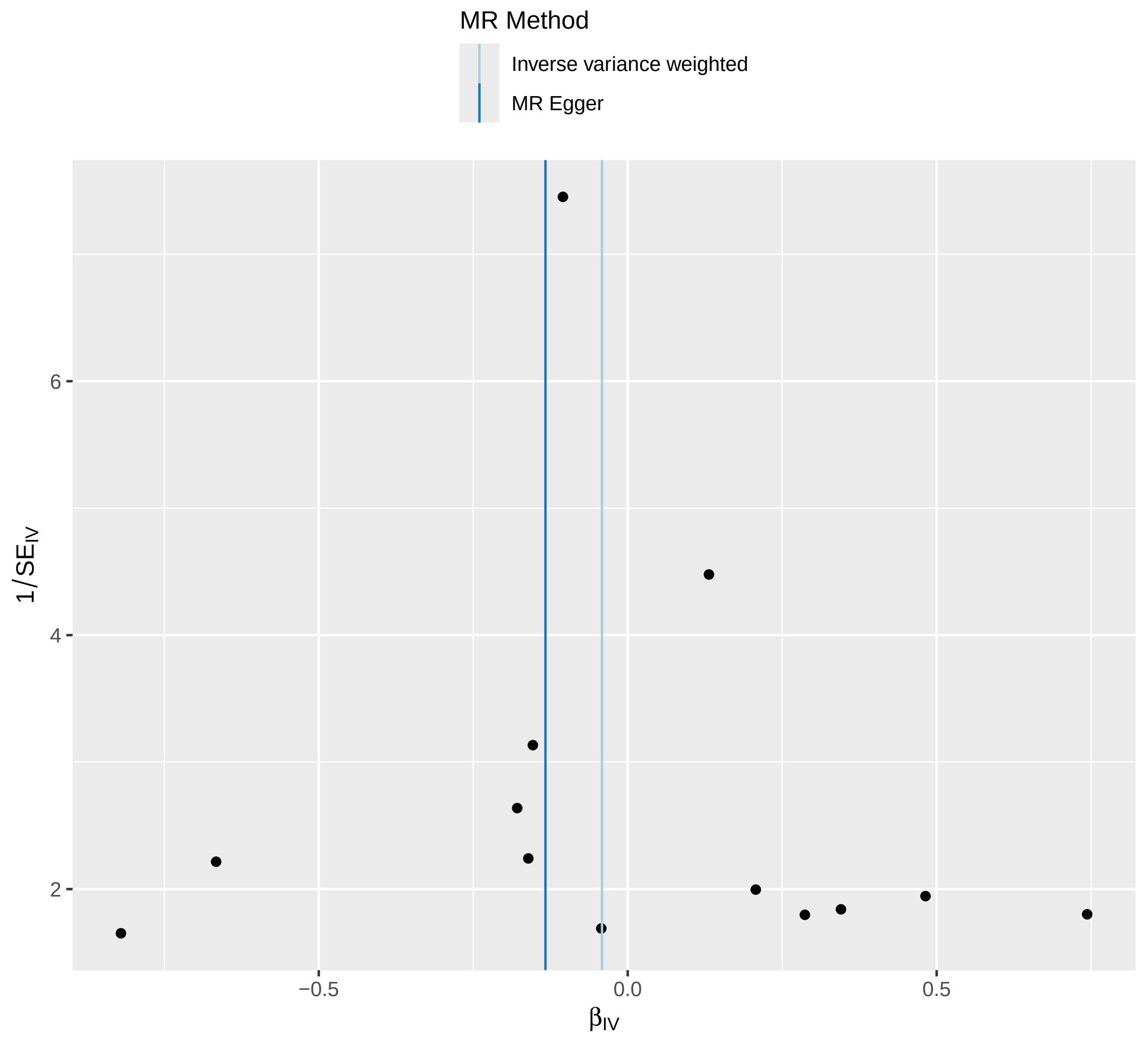

MR Method
Inverse variance weighted
MR Egger
6
4
2
1/SE$_{IV}$
−0.5
0.0
0.5
β$_{IV}$

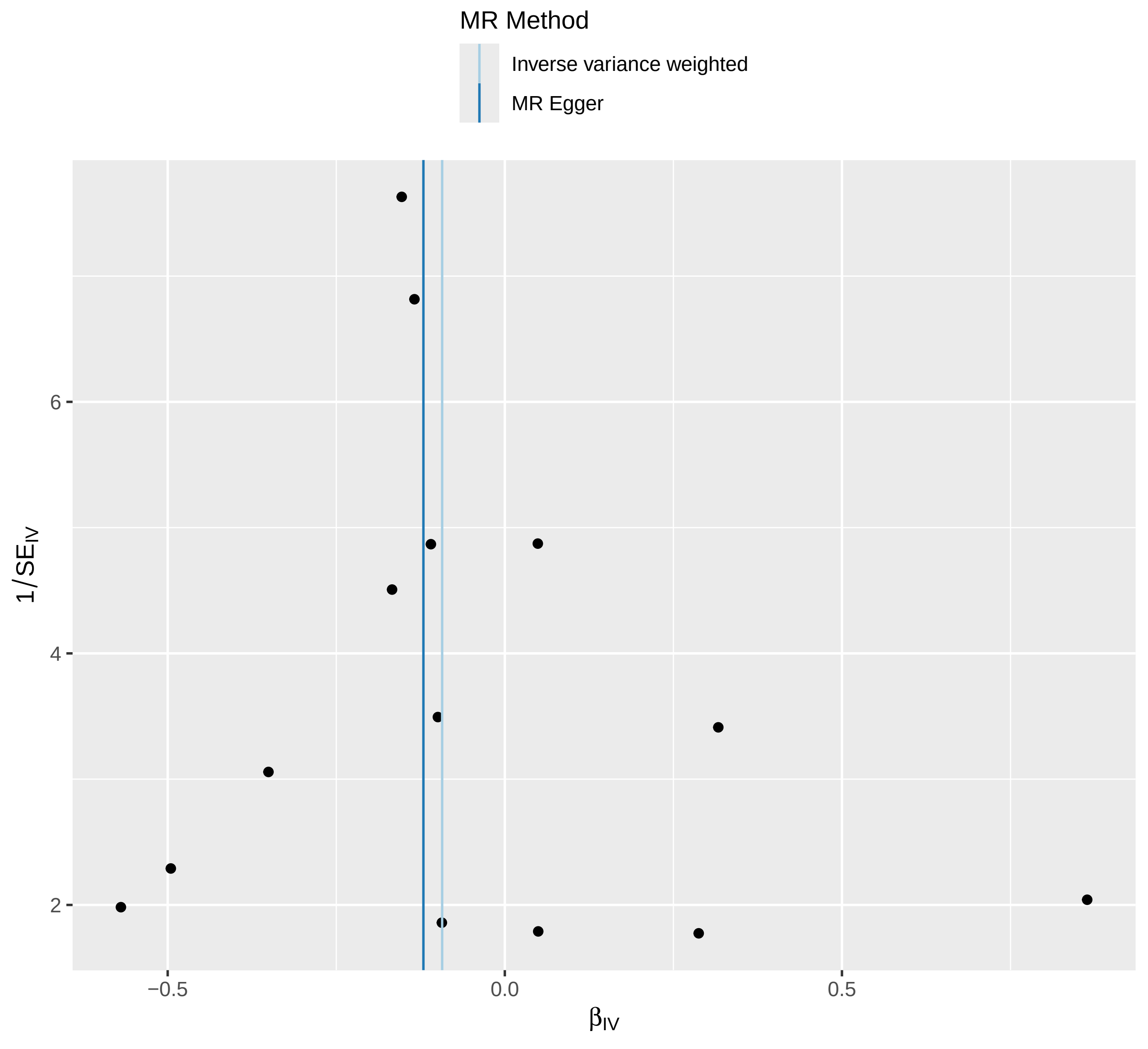
MR Method
Inverse variance weighted
MR Egger
6
4
2
−0.5
0.0
0.5
$1/SE_{IV}$
β_{IV}

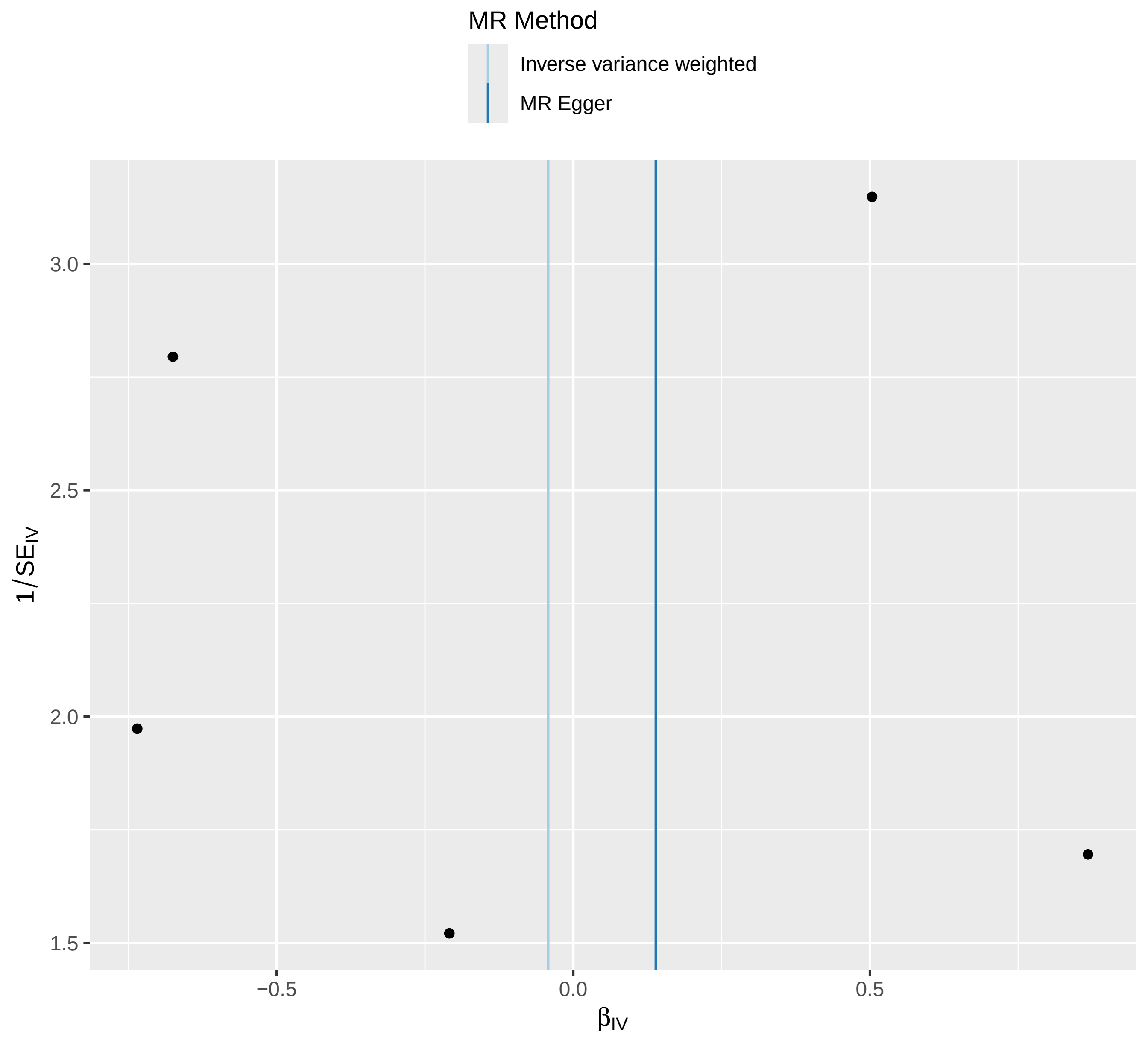
MR Method
Inverse variance weighted
MR Egger
3.0
2.5
2.0
1.5
$1/SE_{IV}$
−0.5
0.0
0.5
β_{IV}

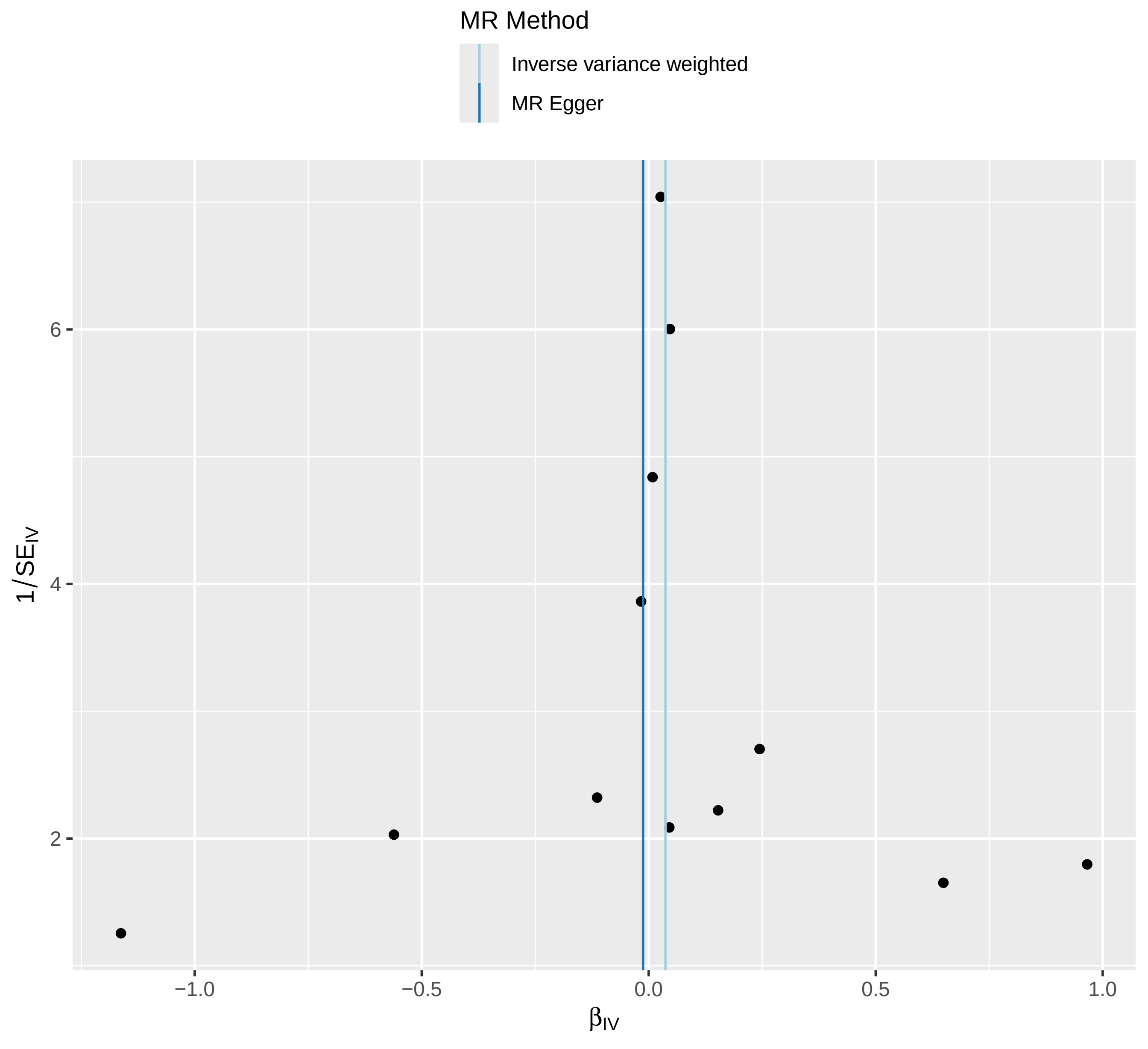
MR Method
Inverse variance weighted
MR Egger
1/SE_IV
6
4
2
−1.0
−0.5
0.0
0.5
1.0
β_IV

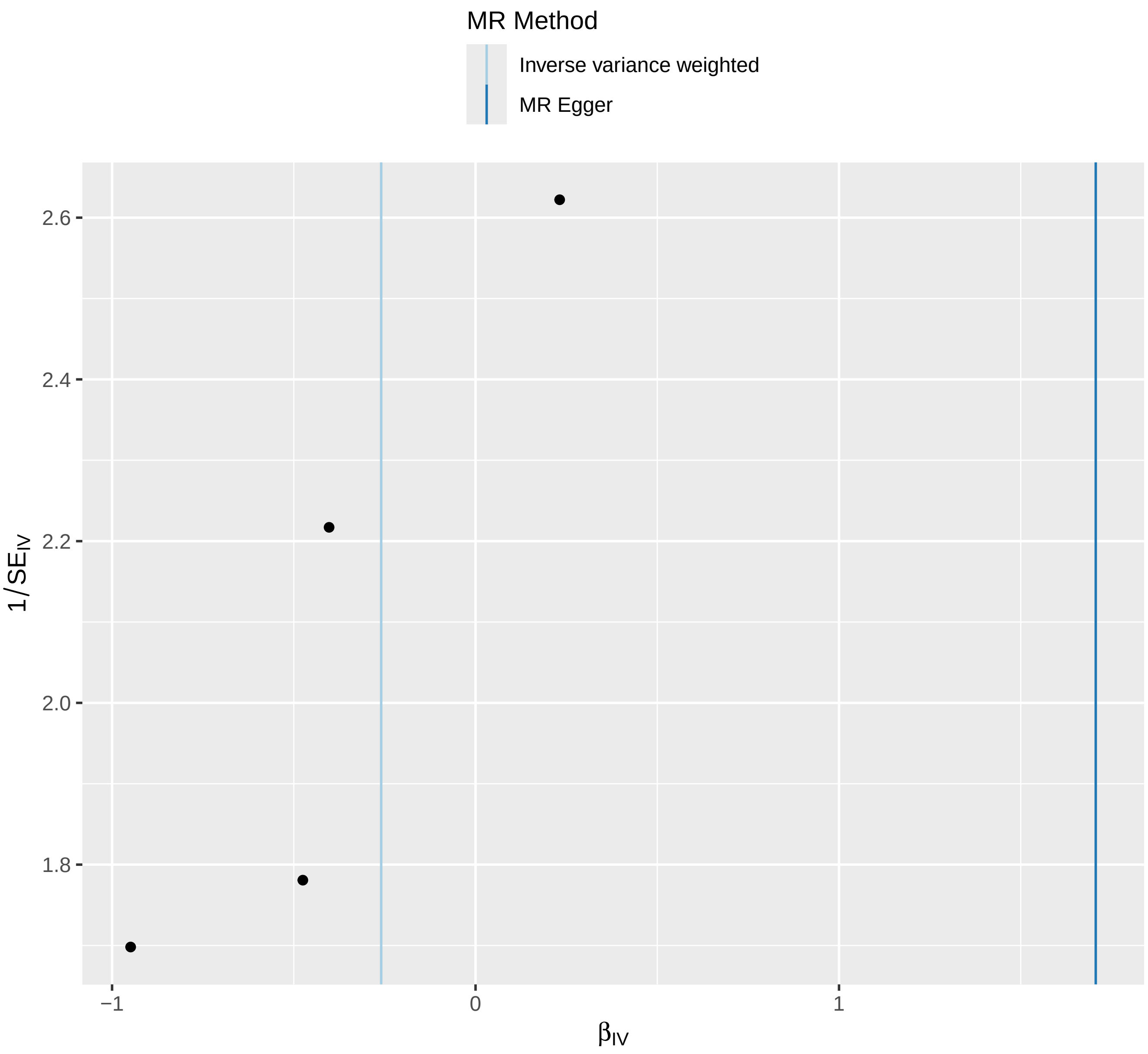
MR Method
Inverse variance weighted
MR Egger
2.6
2.4
2.2
2.0
1.8
$1/SE_{IV}$
−1
0
1
β_{IV}

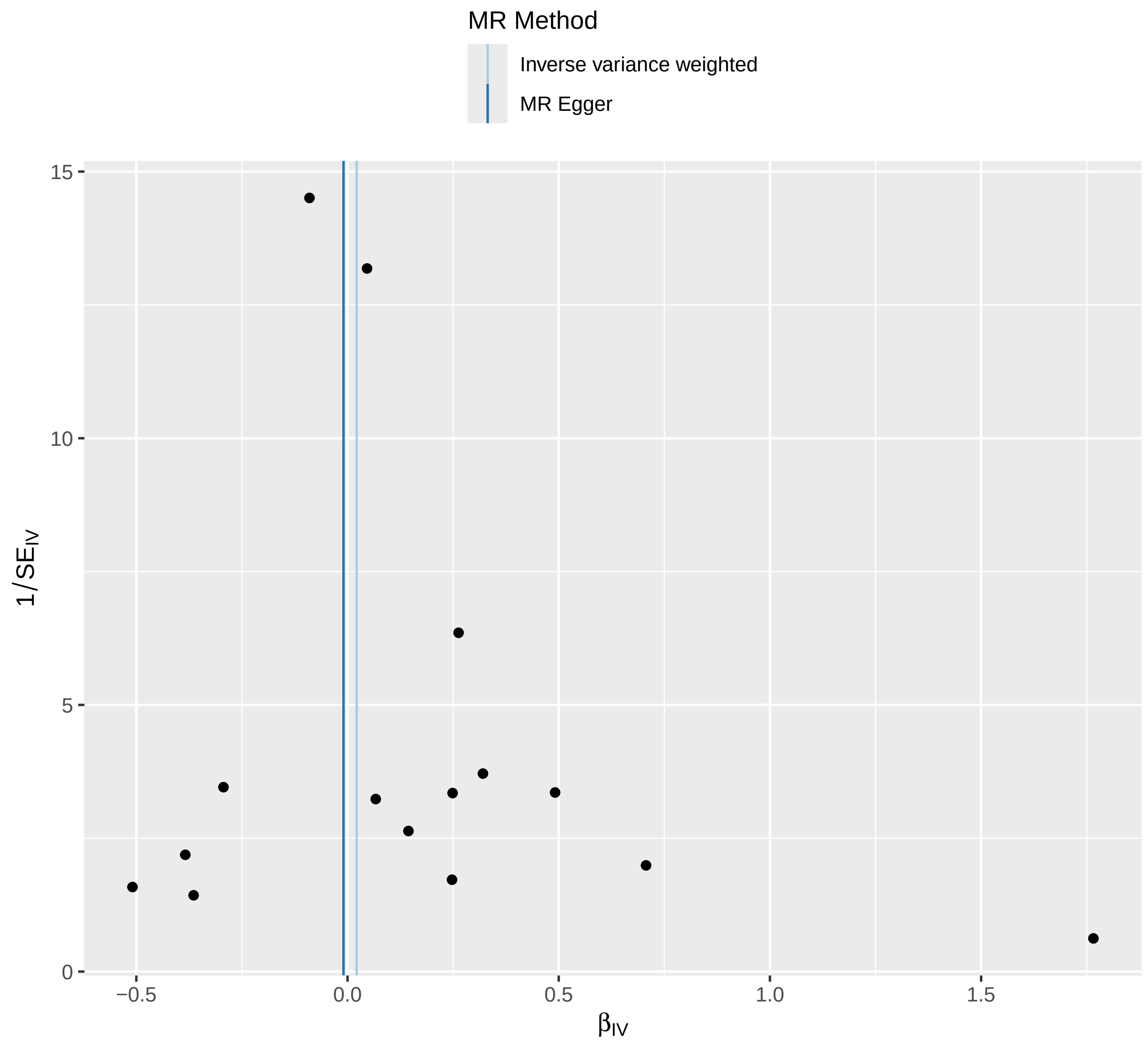
MR Method
Inverse variance weighted
MR Egger
15
10
5
0
1/SE$_{IV}$
−0.5
0.0
0.5
1.0
1.5
β$_{IV}$

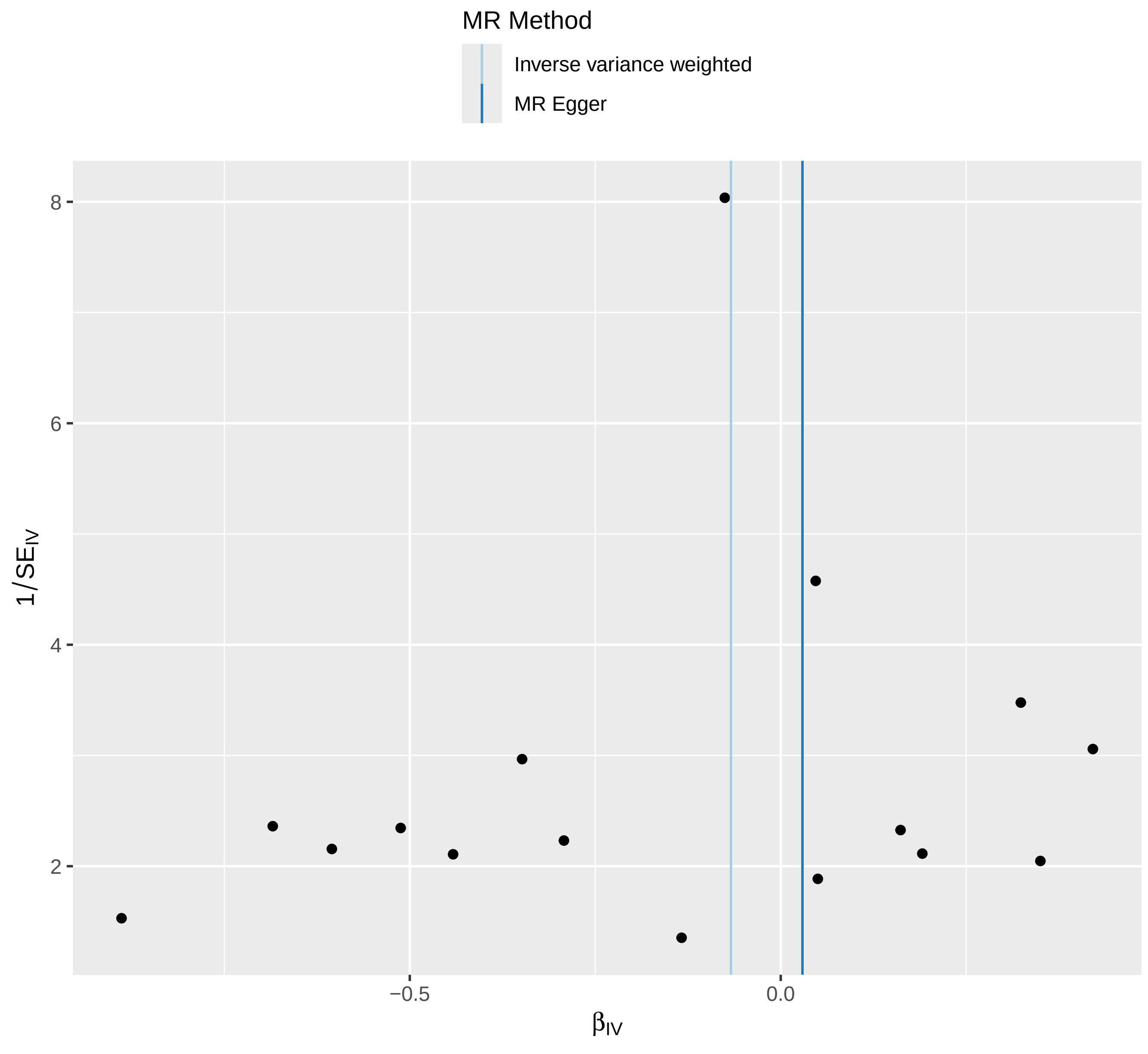

MR Method
Inverse variance weighted
MR Egger
8
6
4
2
1/SE$_{IV}$
−0.5
0.0
β$_{IV}$

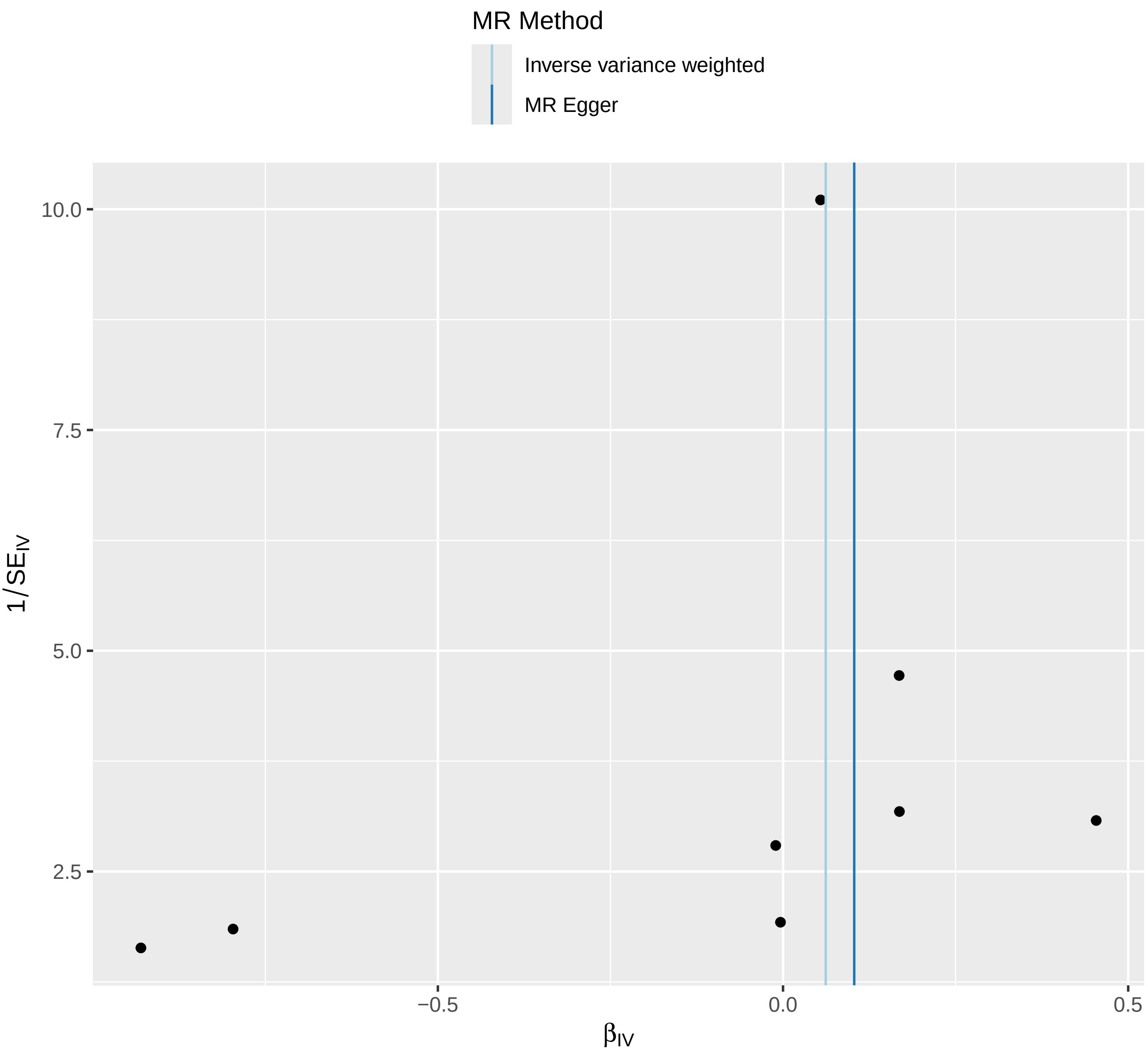

MR Method
Inverse variance weighted
MR Egger
10.0
7.5
5.0
2.5
1/SE$_{IV}$
−0.5
0.0
0.5
β$_{IV}$

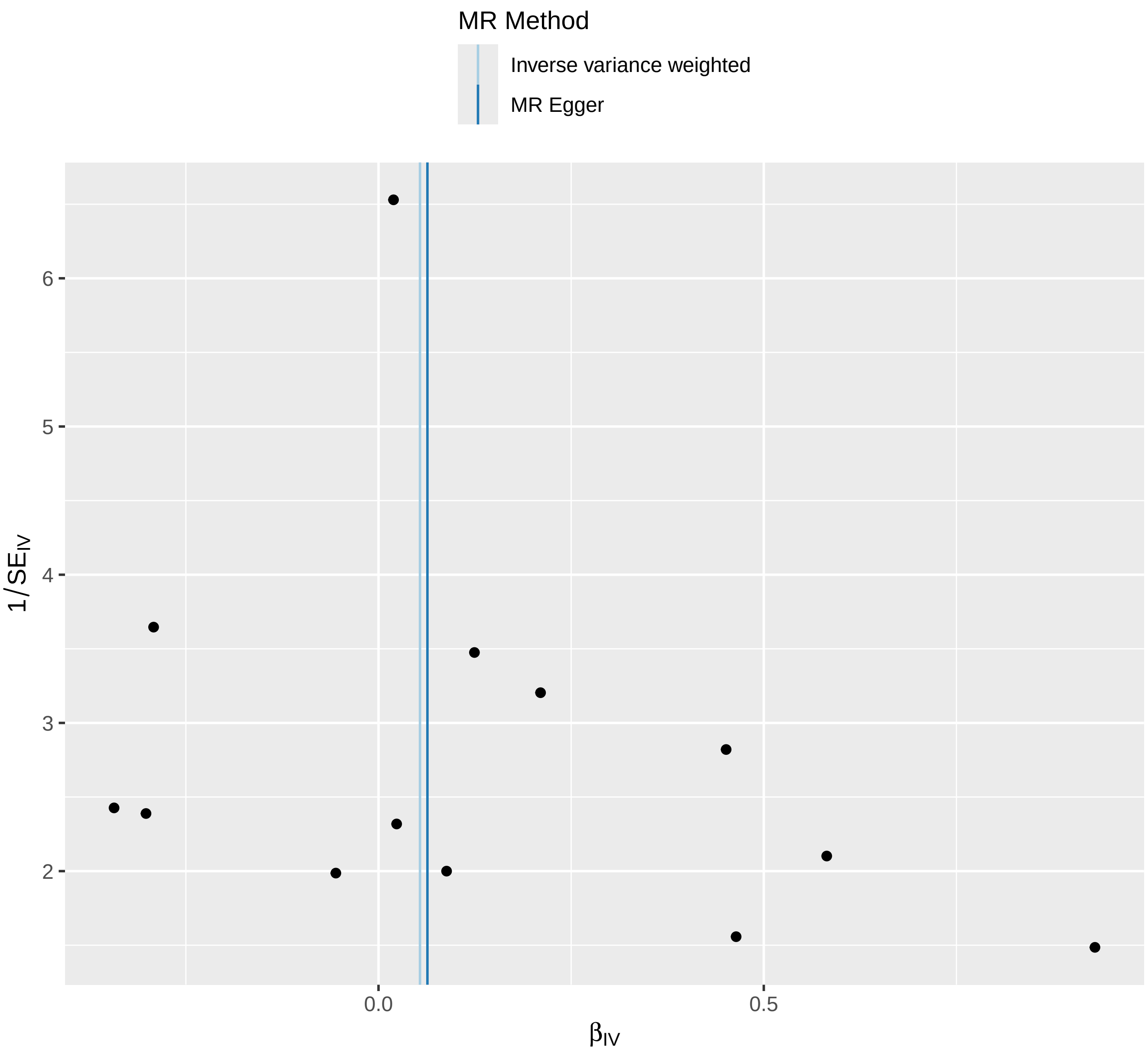

MR Method
Inverse variance weighted
MR Egger
6
5
4
3
2
$1/SE_{IV}$
0.0
0.5
β_{IV}

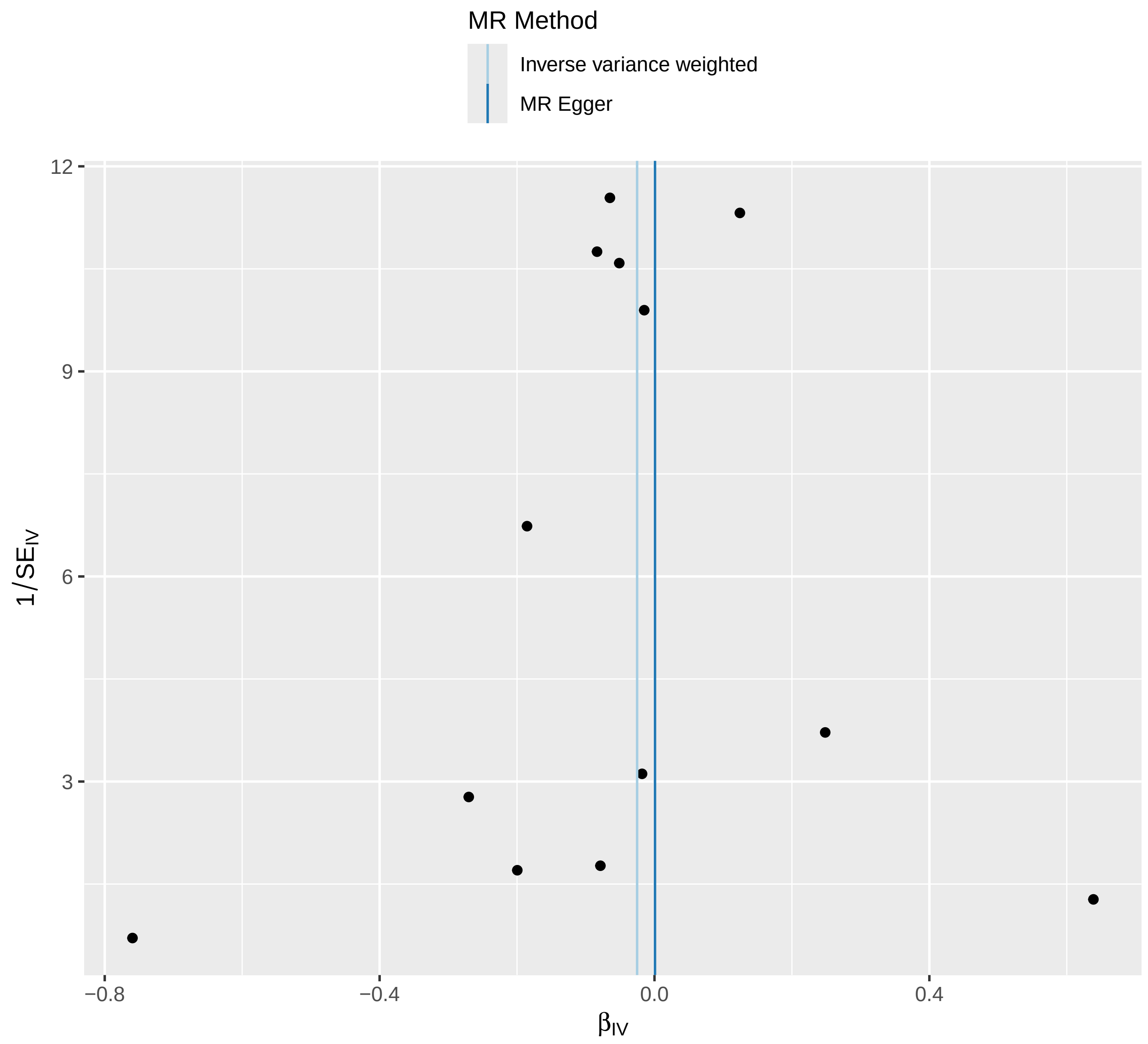

MR Method
Inverse variance weighted
MR Egger
12
9
6
3
1/SE_IV
−0.8
−0.4
0.0
0.4
β_IV

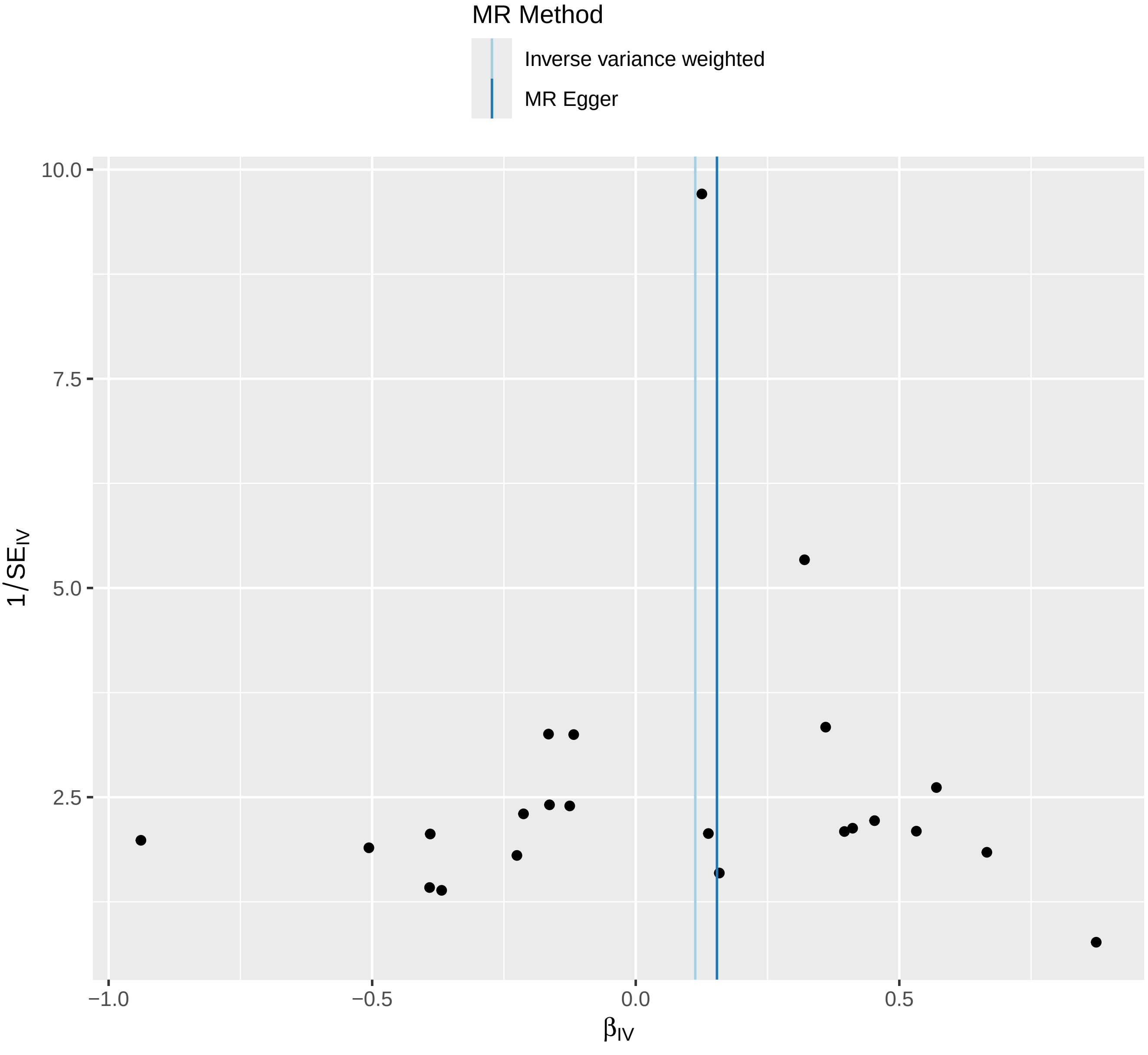

MR Method
Inverse variance weighted
MR Egger
10.0
7.5
5.0
2.5
$1/SE_{IV}$
−1.0
−0.5
0.0
0.5
β_{IV}

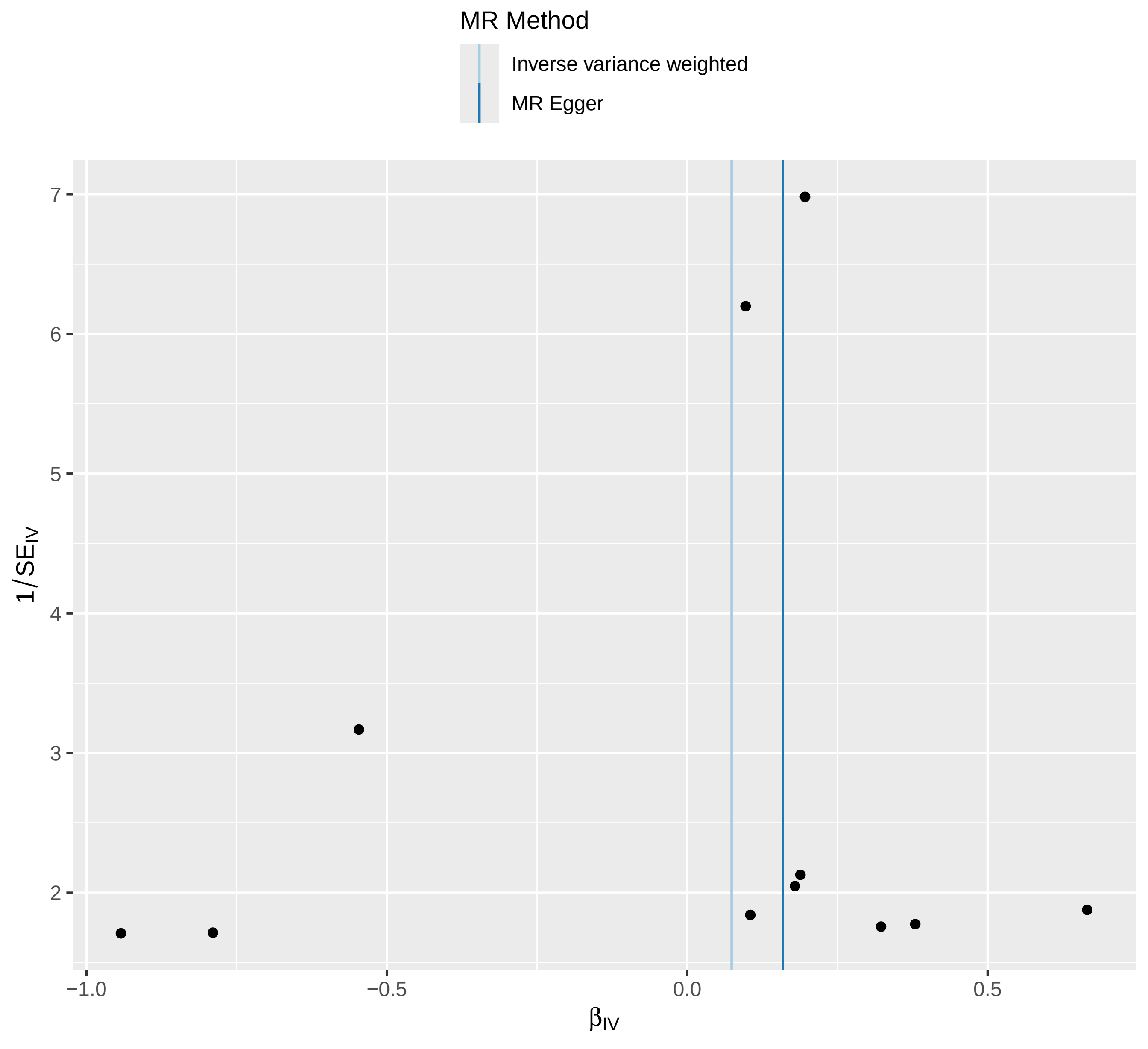
MR Method
Inverse variance weighted
MR Egger
7
6
5
4
3
2
1/SE_IV
−1.0
−0.5
0.0
0.5
β_IV

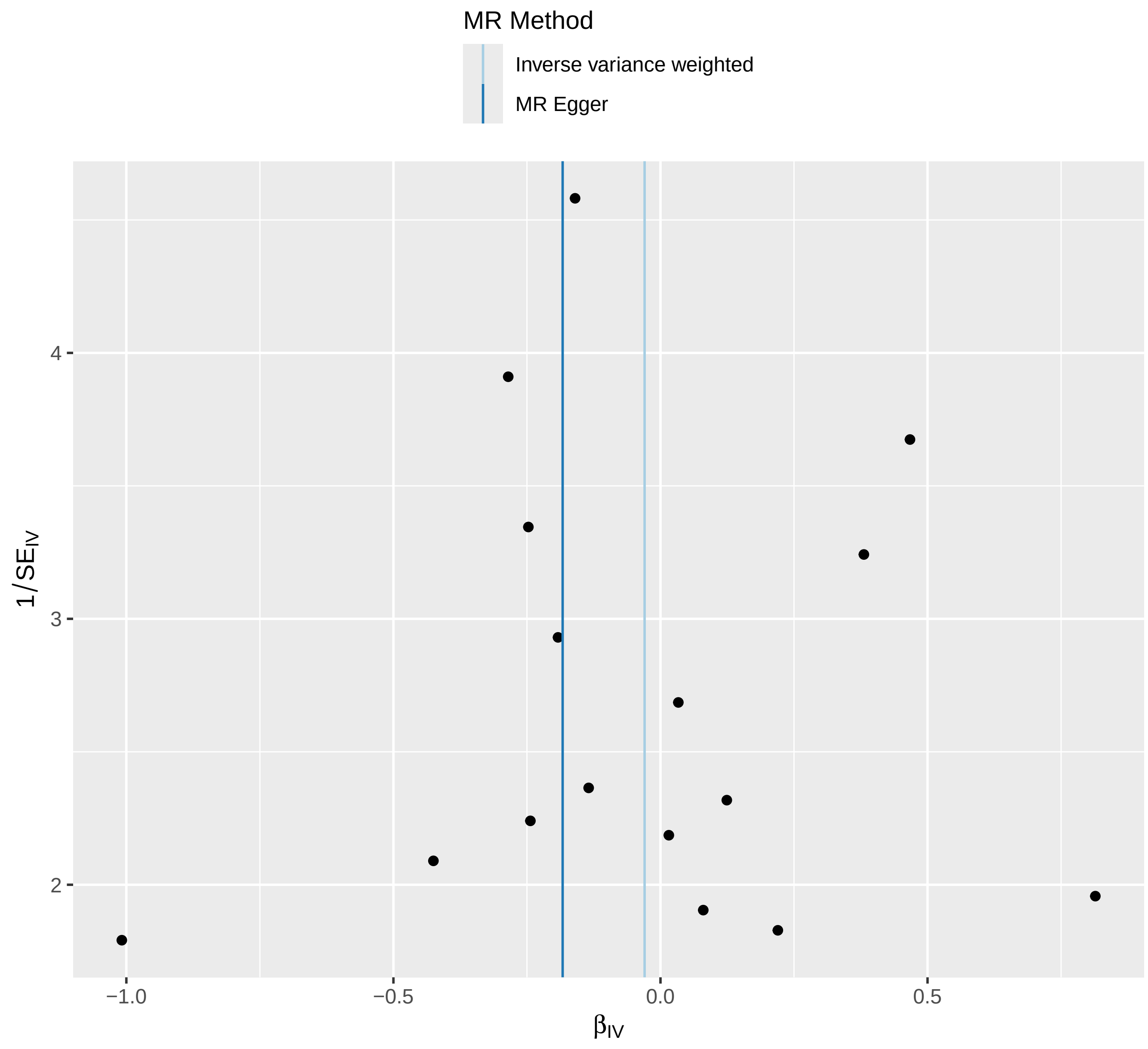

MR Method
Inverse variance weighted
MR Egger
4
3
2
1/SE_IV
−1.0
−0.5
0.0
0.5
β_IV

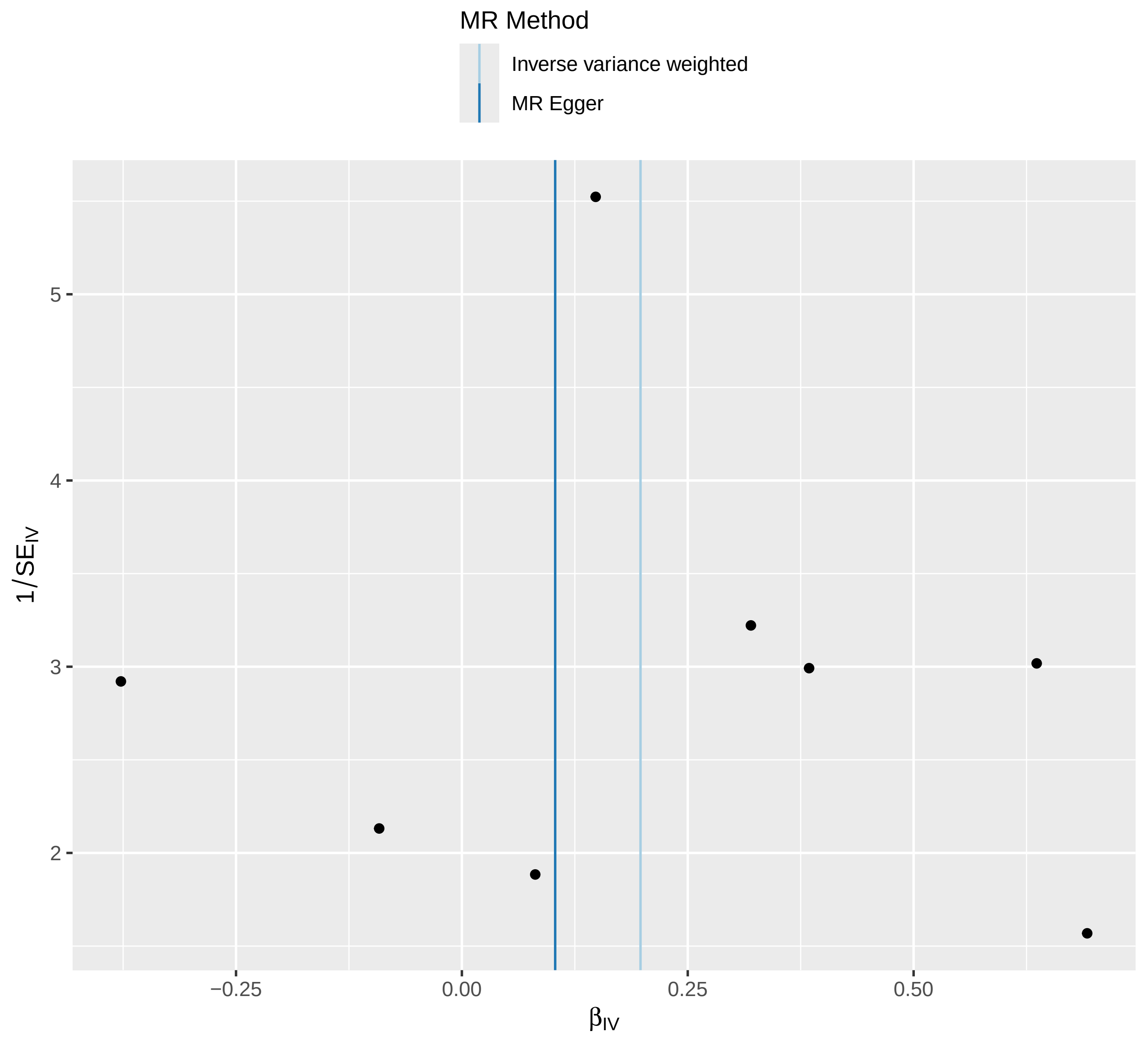

MR Method
Inverse variance weighted
MR Egger
5
4
3
2
1/SE_IV
−0.25
0.00
0.25
0.50
β_IV

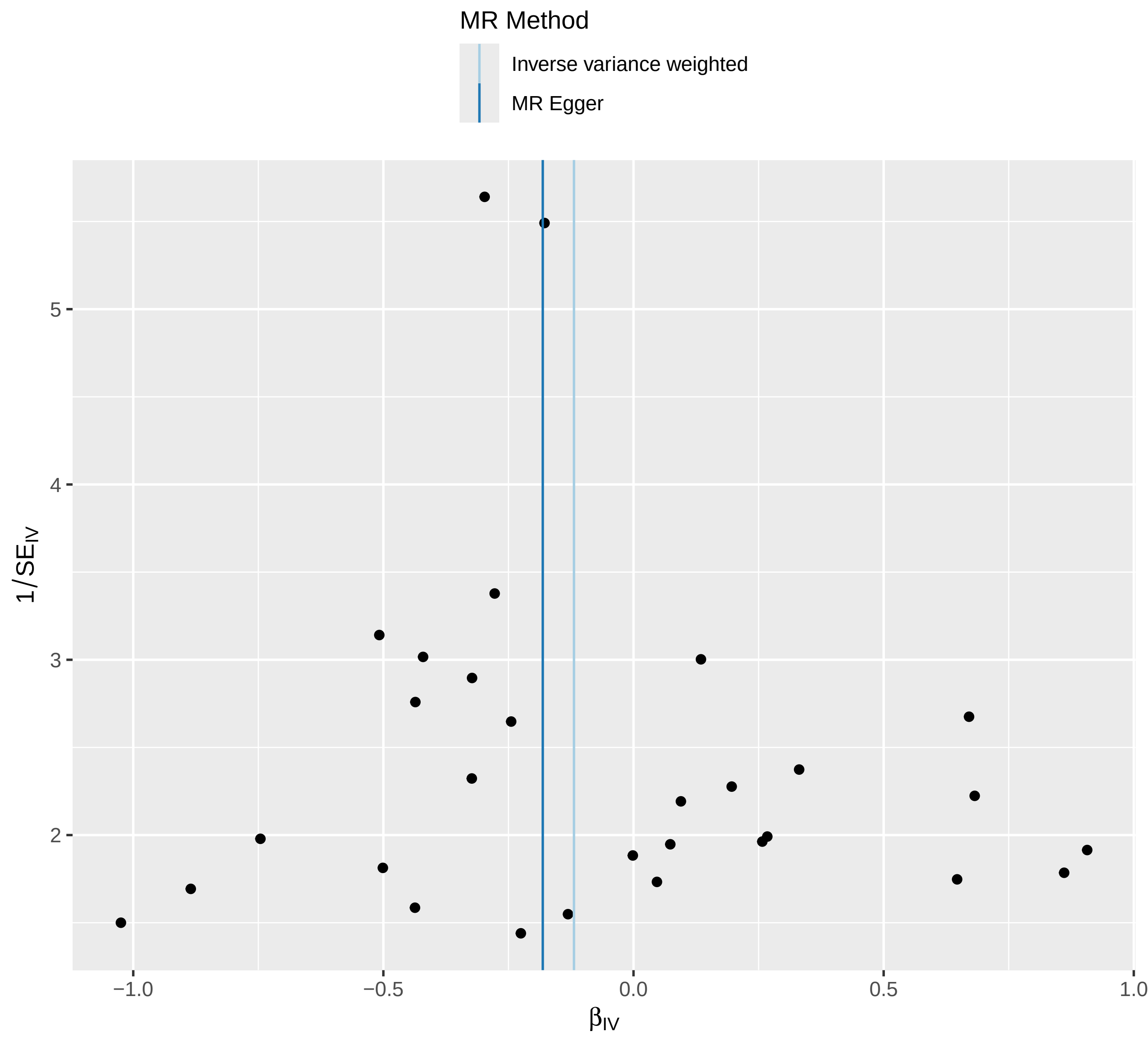

MR Method
Inverse variance weighted
MR Egger
5
4
3
2
1/SE_IV
−1.0
−0.5
0.0
0.5
1.0
β_IV

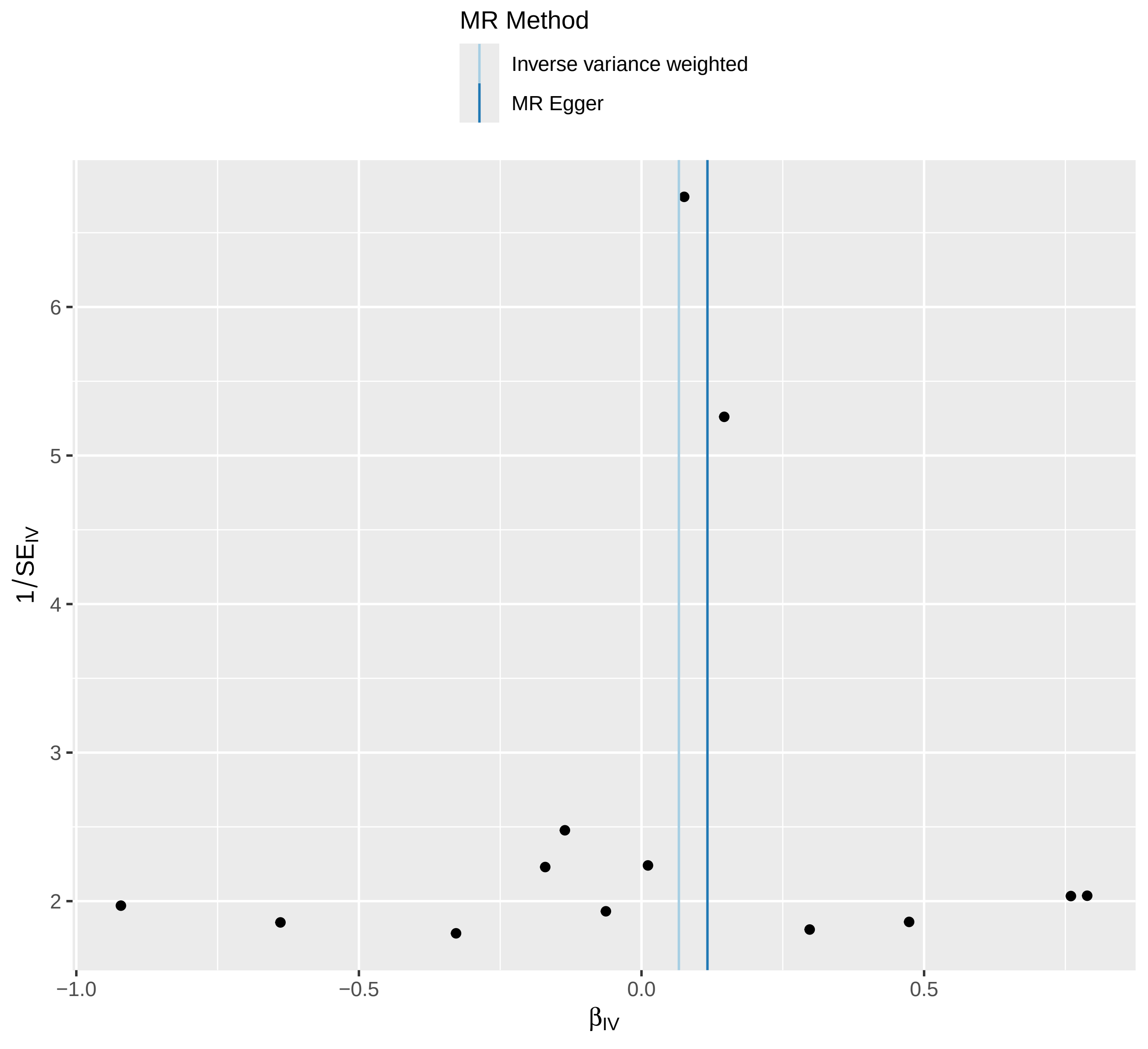

MR Method
Inverse variance weighted
MR Egger
1/SE_IV
6
5
4
3
2
−1.0
−0.5
0.0
0.5
β_IV

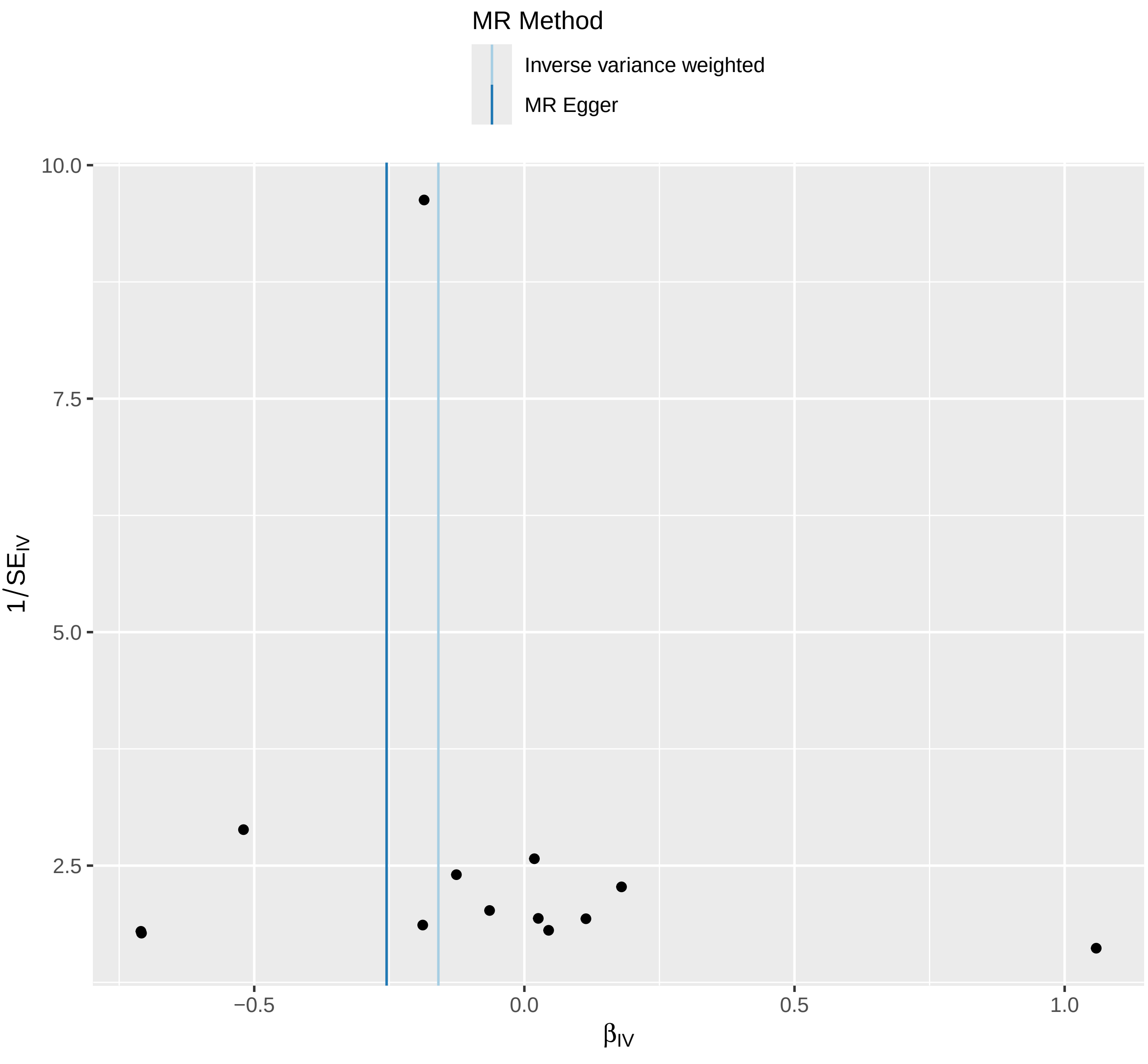

MR Method
Inverse variance weighted
MR Egger
10.0
7.5
5.0
2.5
1/SE$_{IV}$
−0.5
0.0
0.5
1.0
β$_{IV}$

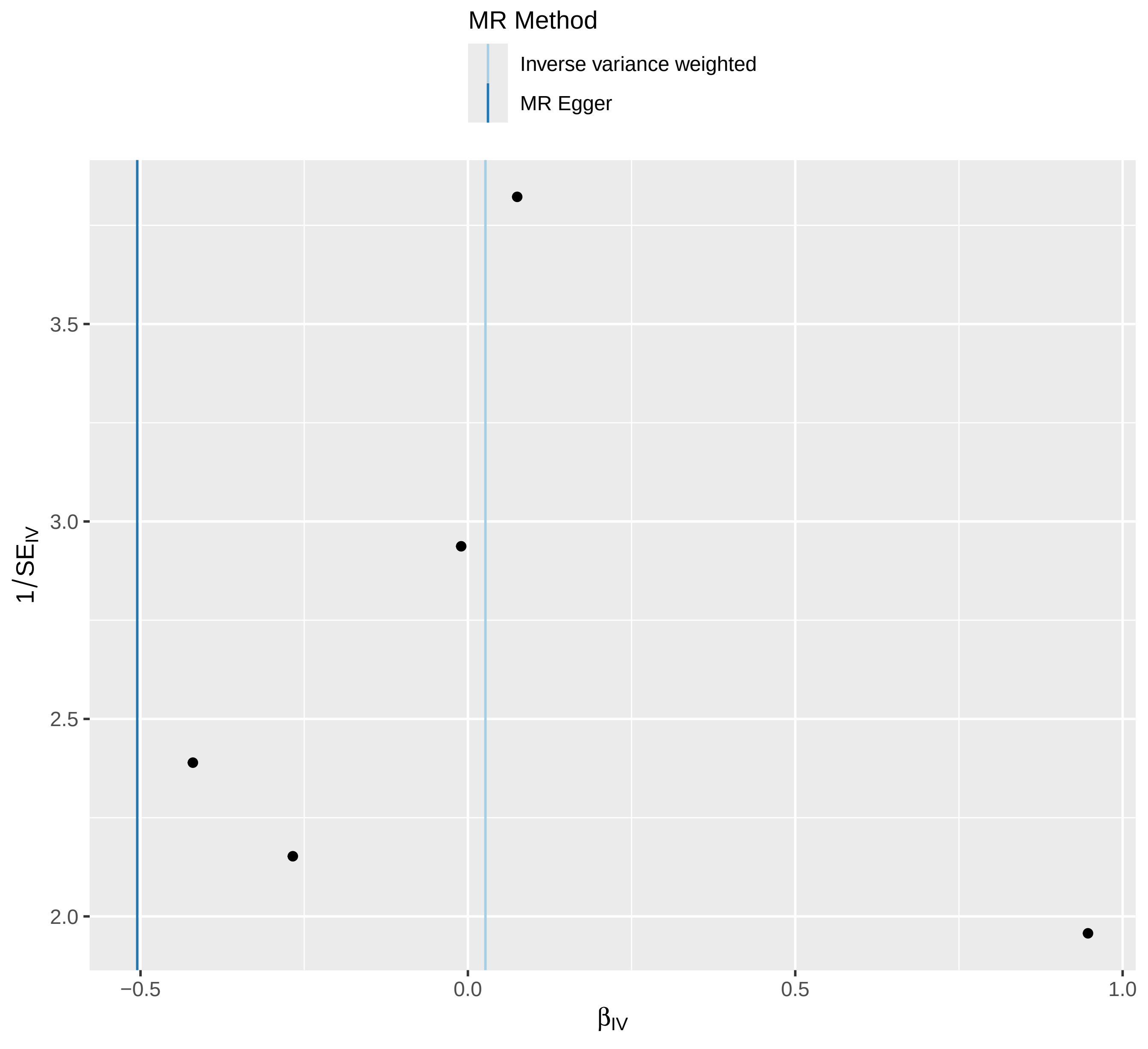
MR Method
Inverse variance weighted
MR Egger
3.5
3.0
2.5
2.0
1/SE_IV
−0.5
0.0
0.5
1.0
β_IV

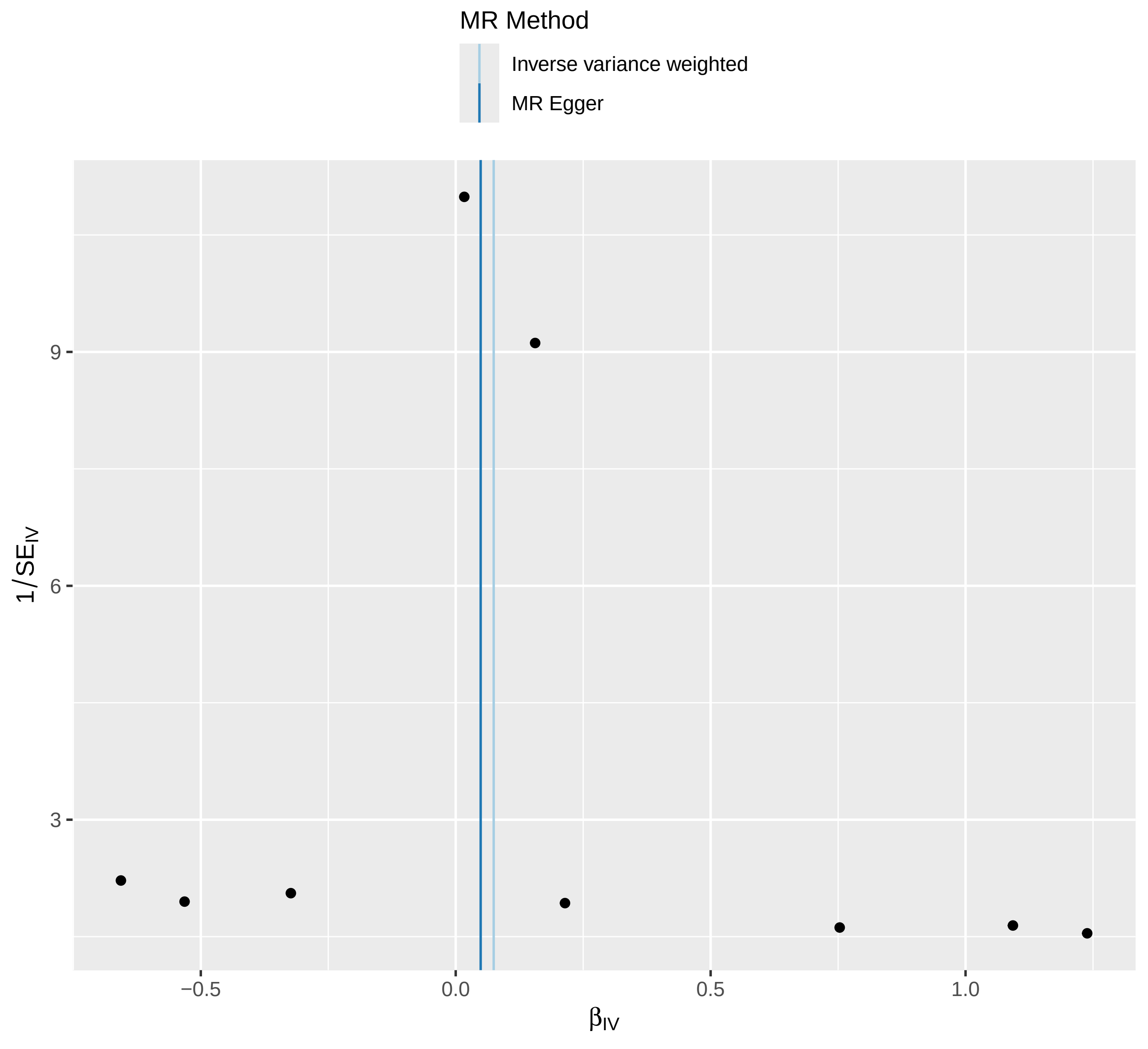

MR Method
Inverse variance weighted
MR Egger
9
6
3
1/SE_{IV}
−0.5
0.0
0.5
1.0
β_{IV}

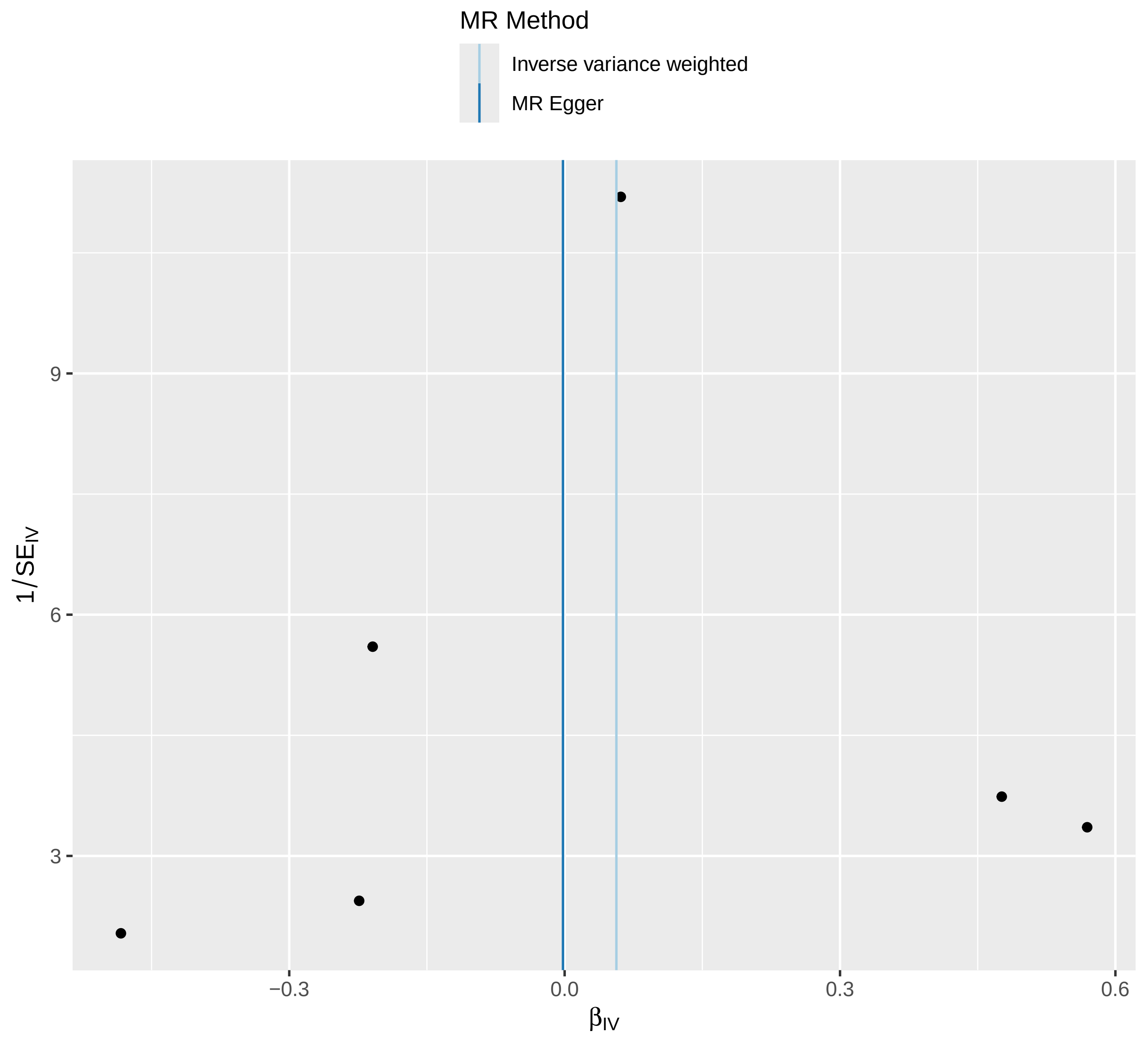

MR Method
Inverse variance weighted
MR Egger
9
6
3
1/SE_IV
−0.3
0.0
0.3
0.6
β_IV

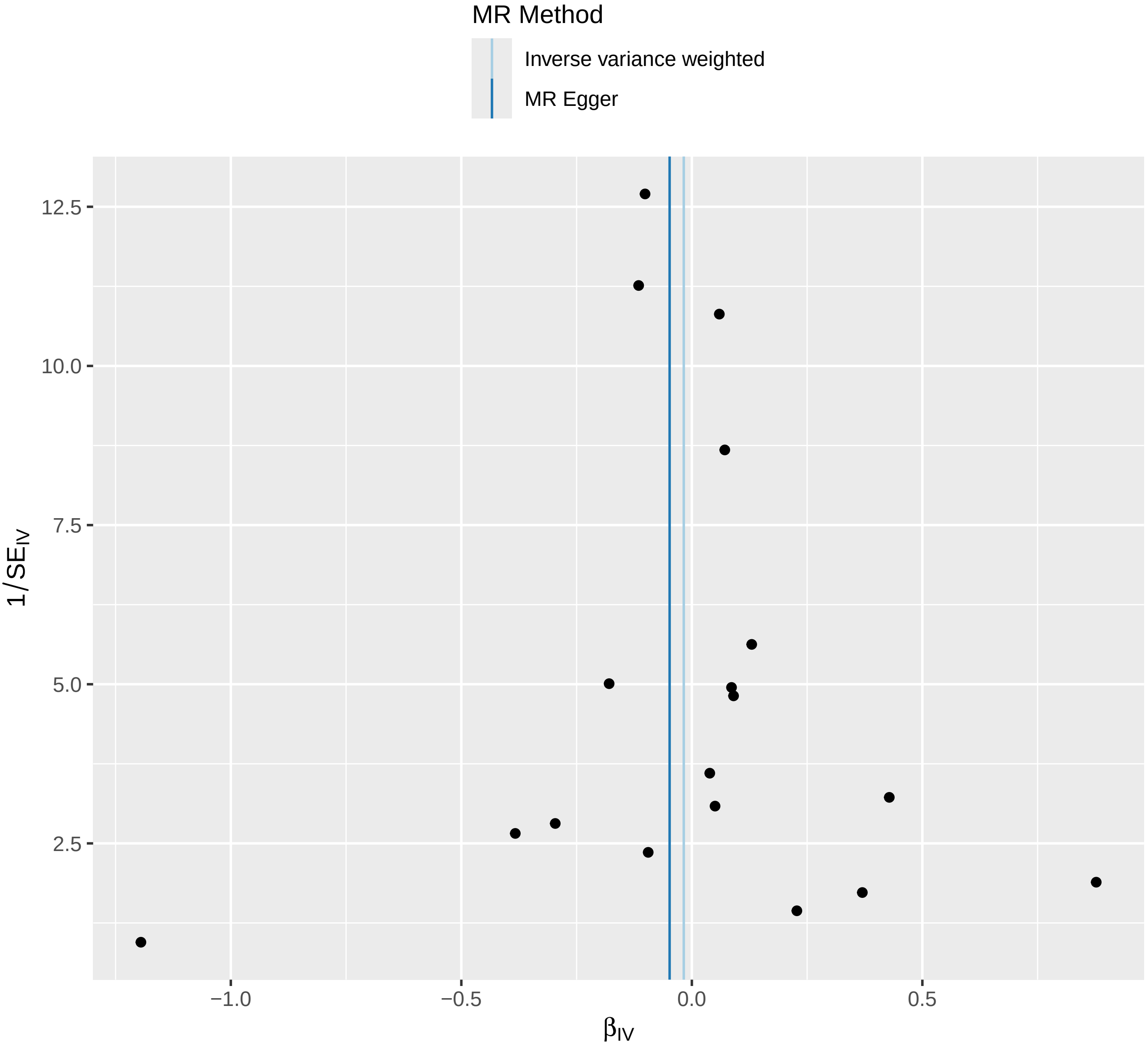

MR Method
Inverse variance weighted
MR Egger
12.5
10.0
7.5
5.0
2.5
$1/SE_{IV}$
−1.0
−0.5
0.0
0.5
β_{IV}

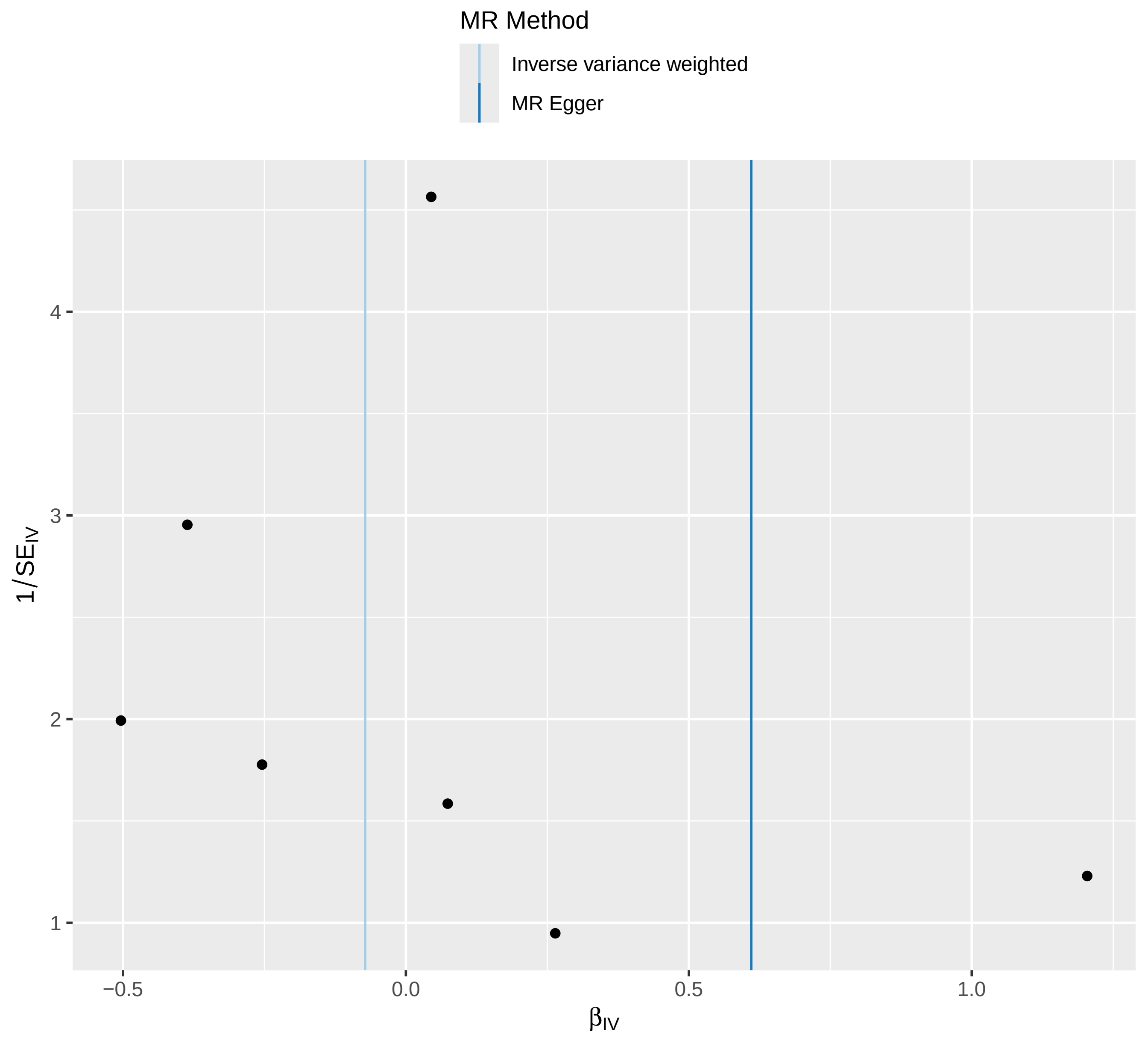

MR Method
Inverse variance weighted
MR Egger
1/SE$_{IV}$
4
3
2
1
−0.5
0.0
0.5
1.0
β$_{IV}$

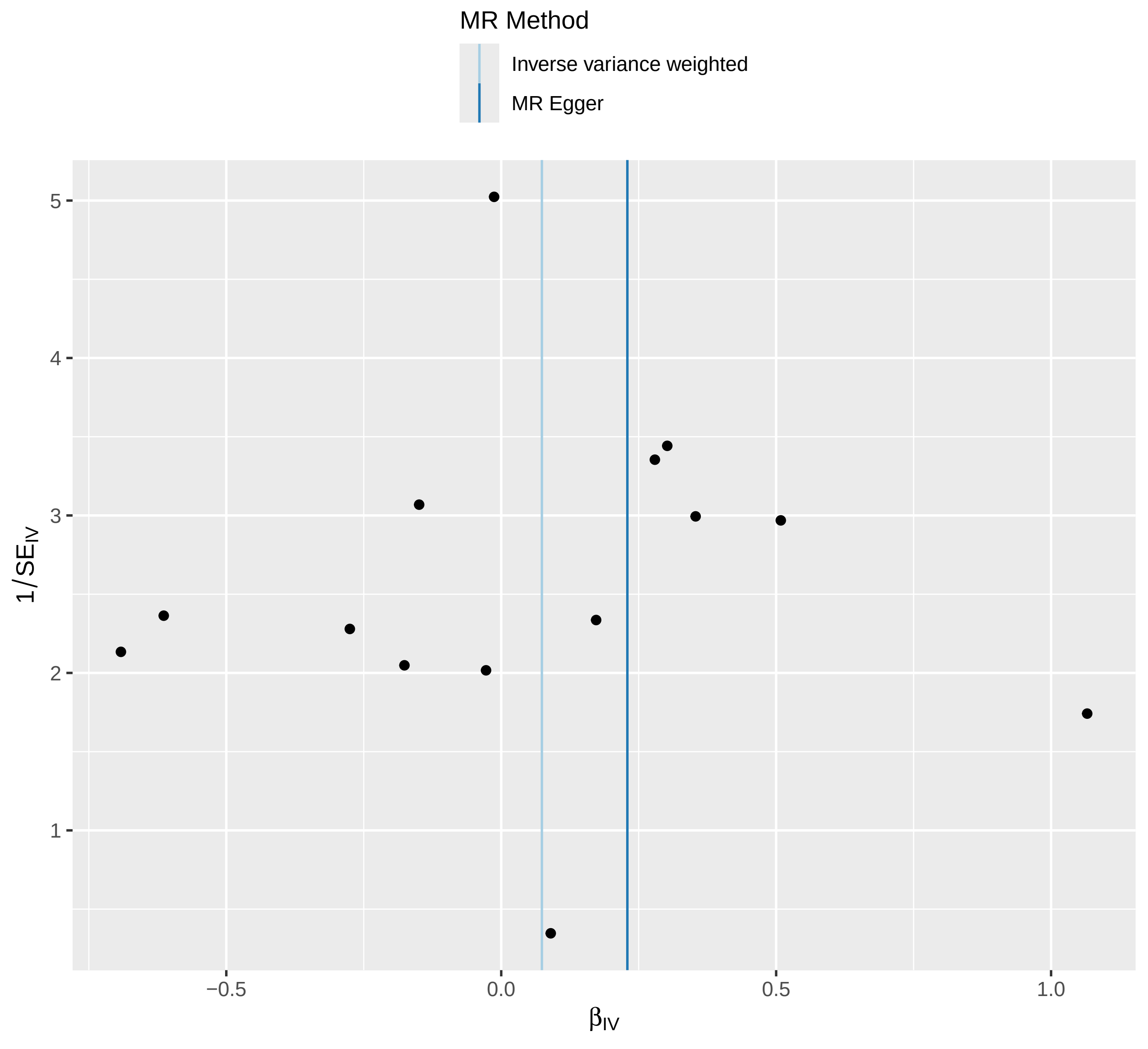

MR Method
Inverse variance weighted
MR Egger
5
4
3
2
1
1/SE_IV
−0.5
0.0
0.5
1.0
β_IV

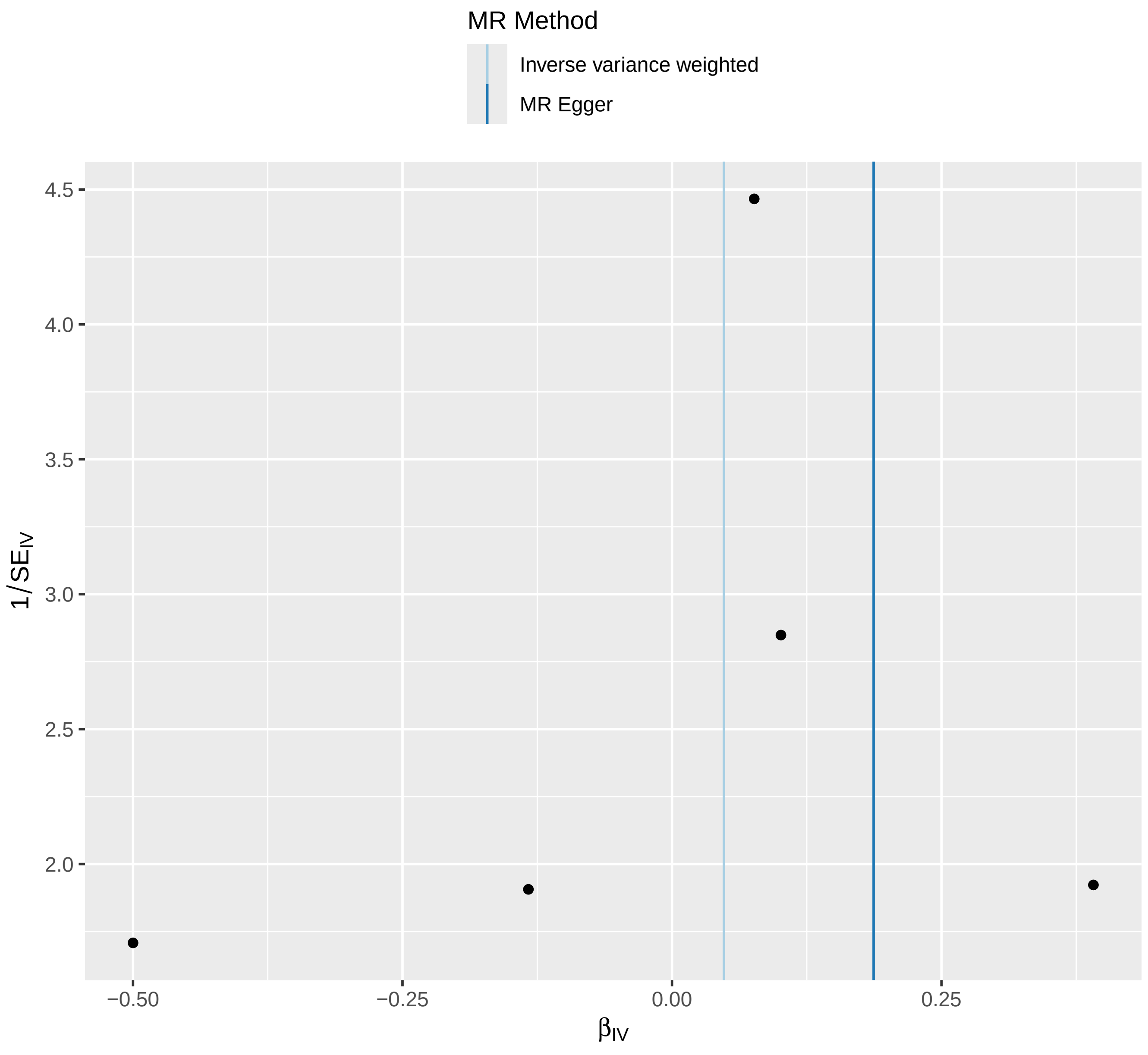

MR Method
Inverse variance weighted
MR Egger
4.5
4.0
3.5
3.0
2.5
2.0
1/SE$_{IV}$
−0.50
−0.25
0.00
0.25
β$_{IV}$

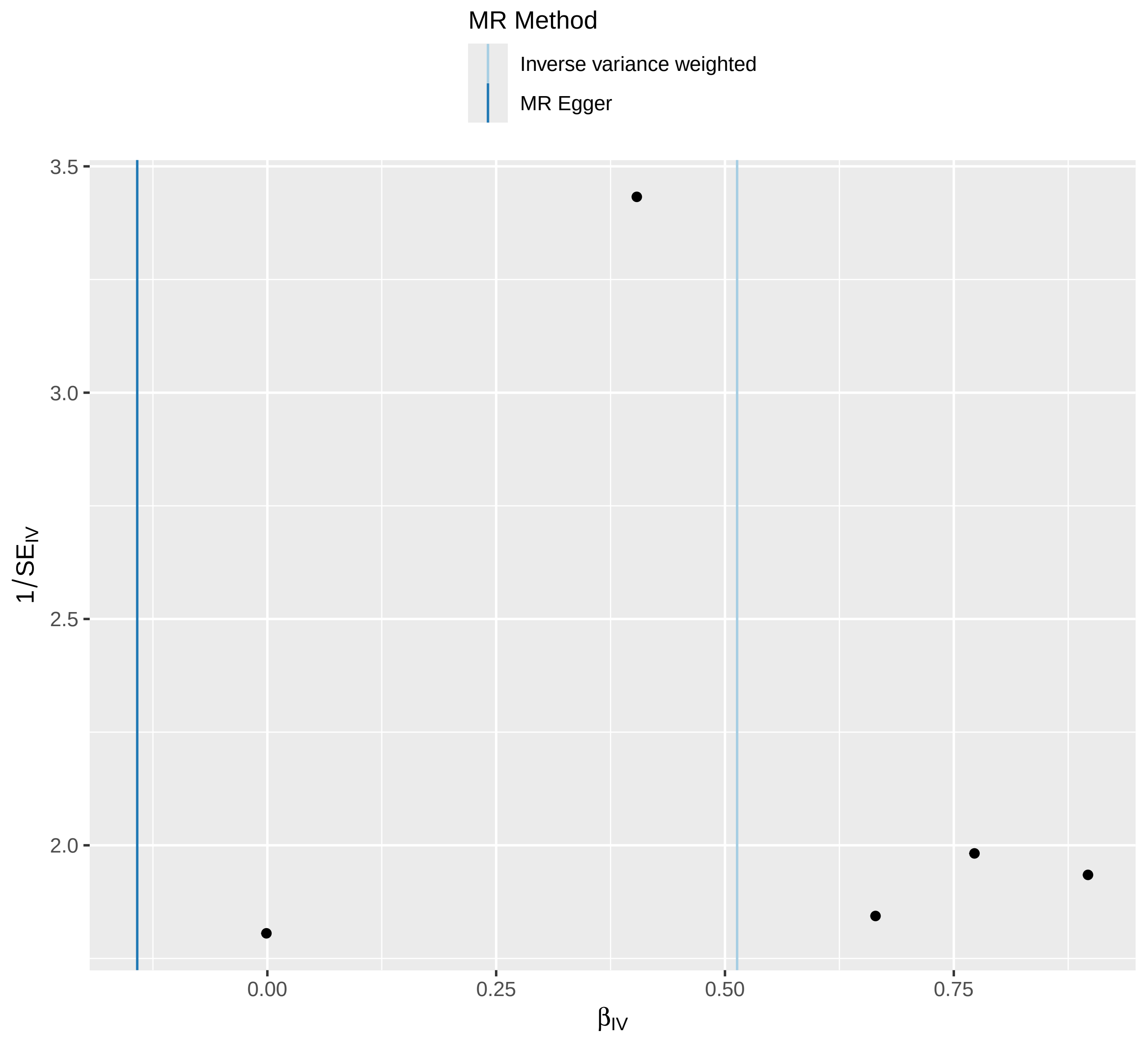
MR Method
Inverse variance weighted
MR Egger
3.5
3.0
2.5
2.0
1/SE_IV
0.00
0.25
0.50
0.75
β_IV

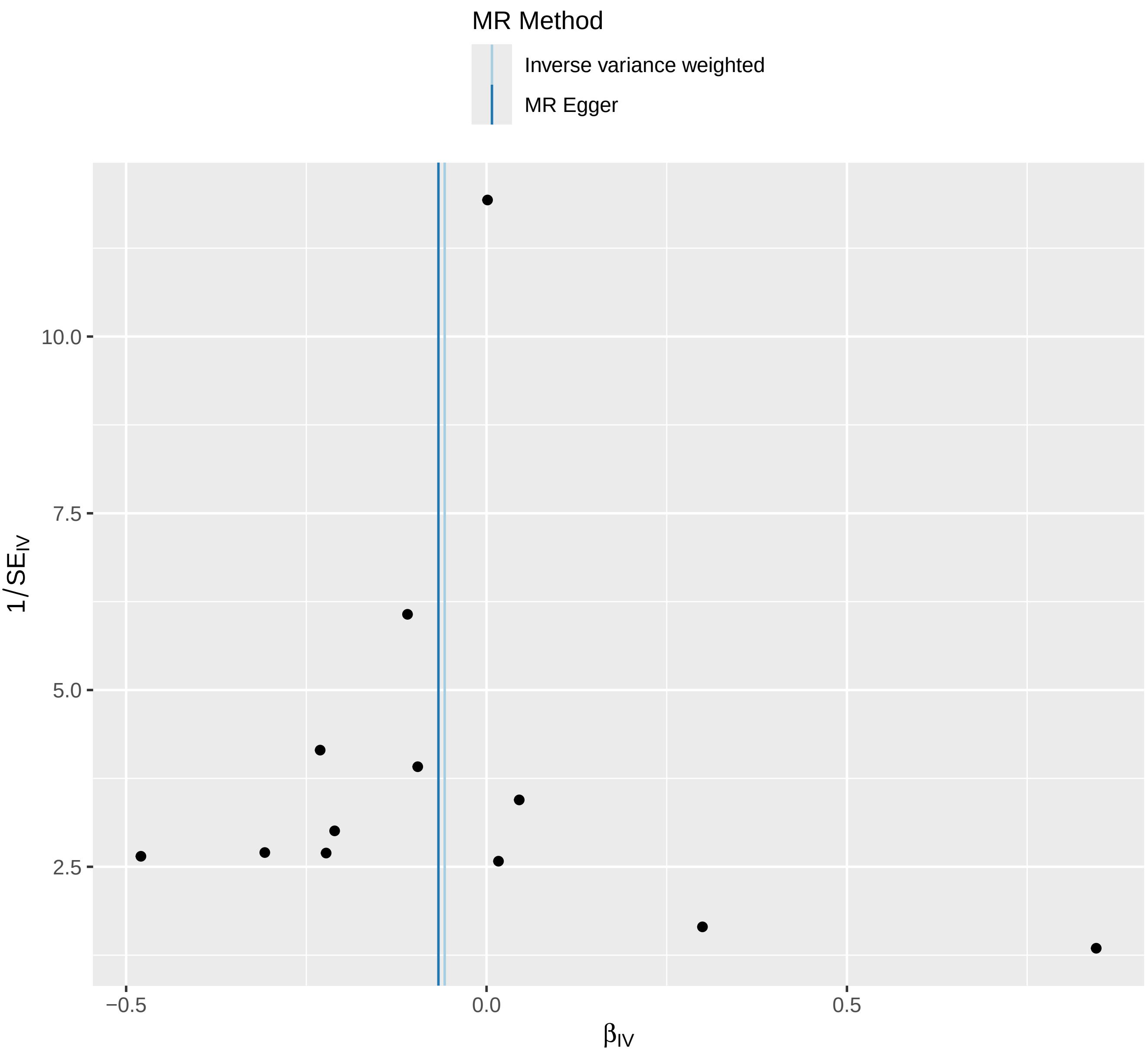

MR Method
Inverse variance weighted
MR Egger
10.0
7.5
5.0
2.5
1/SE$_{IV}$
−0.5
0.0
0.5
β$_{IV}$

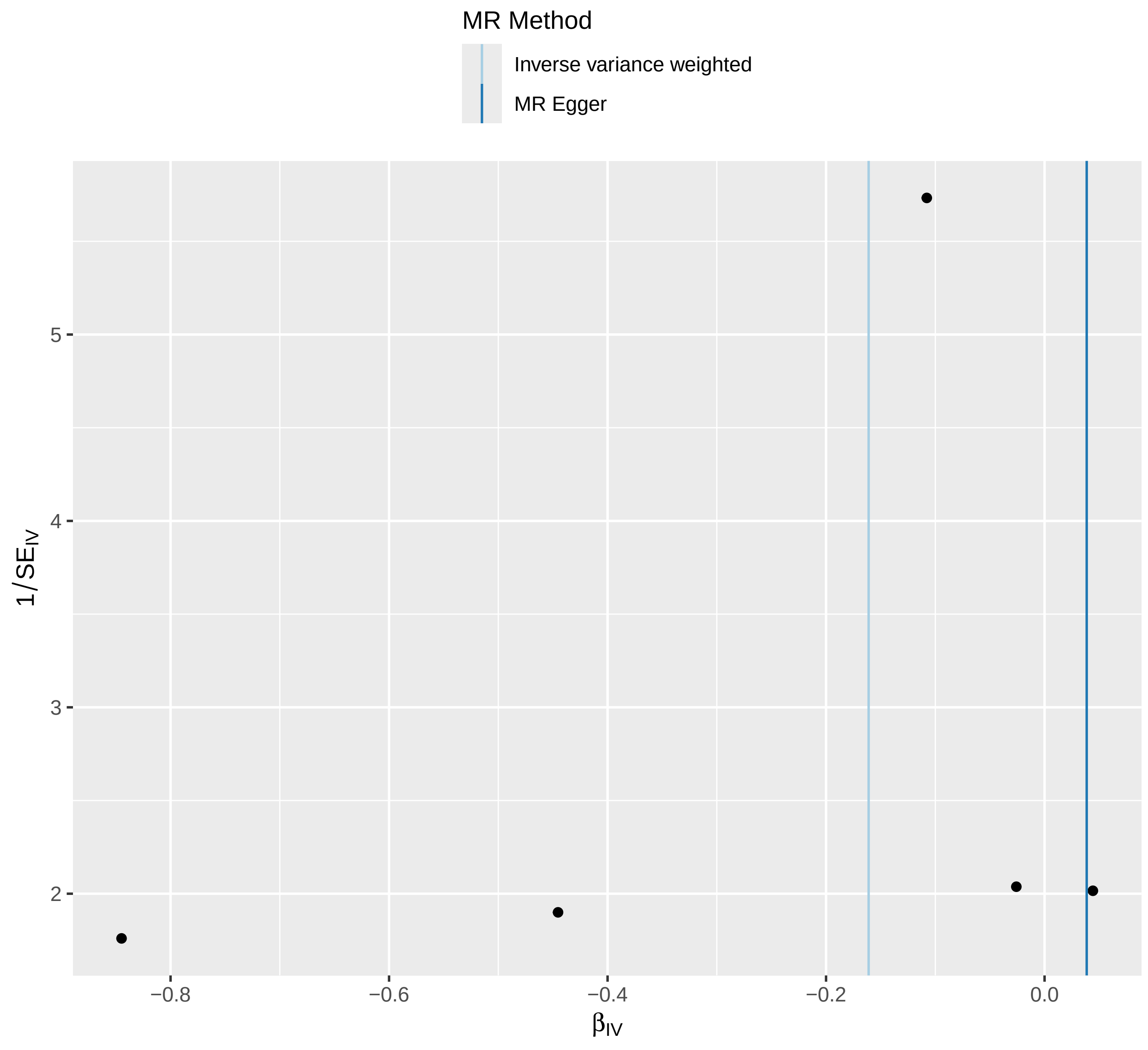

MR Method
Inverse variance weighted
MR Egger
5
4
3
2
1/SE$_{IV}$
−0.8
−0.6
−0.4
−0.2
0.0
β$_{IV}$

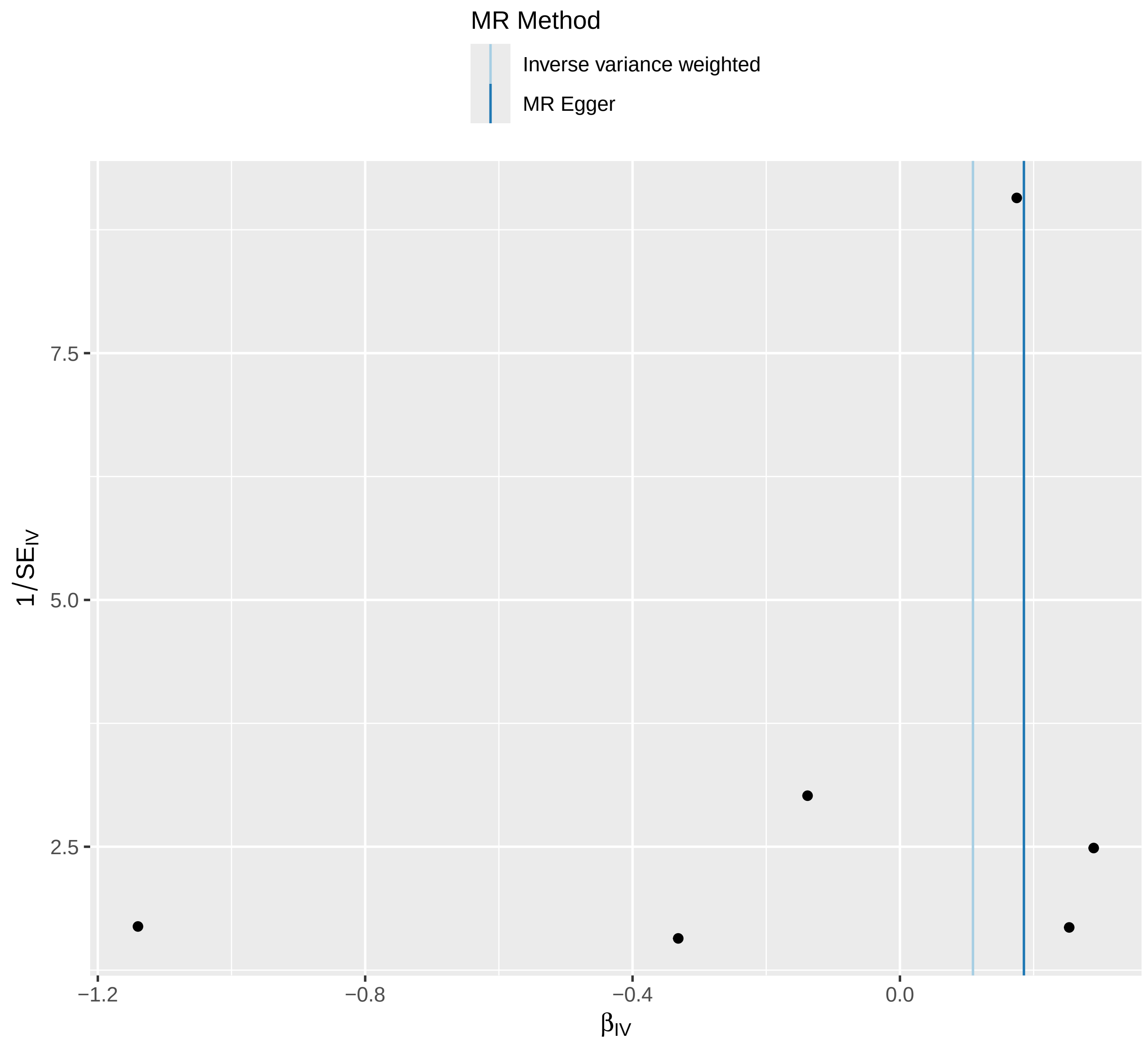

MR Method
Inverse variance weighted
MR Egger
1/SE_IV
7.5
5.0
2.5
−1.2
−0.8
−0.4
0.0
β_IV

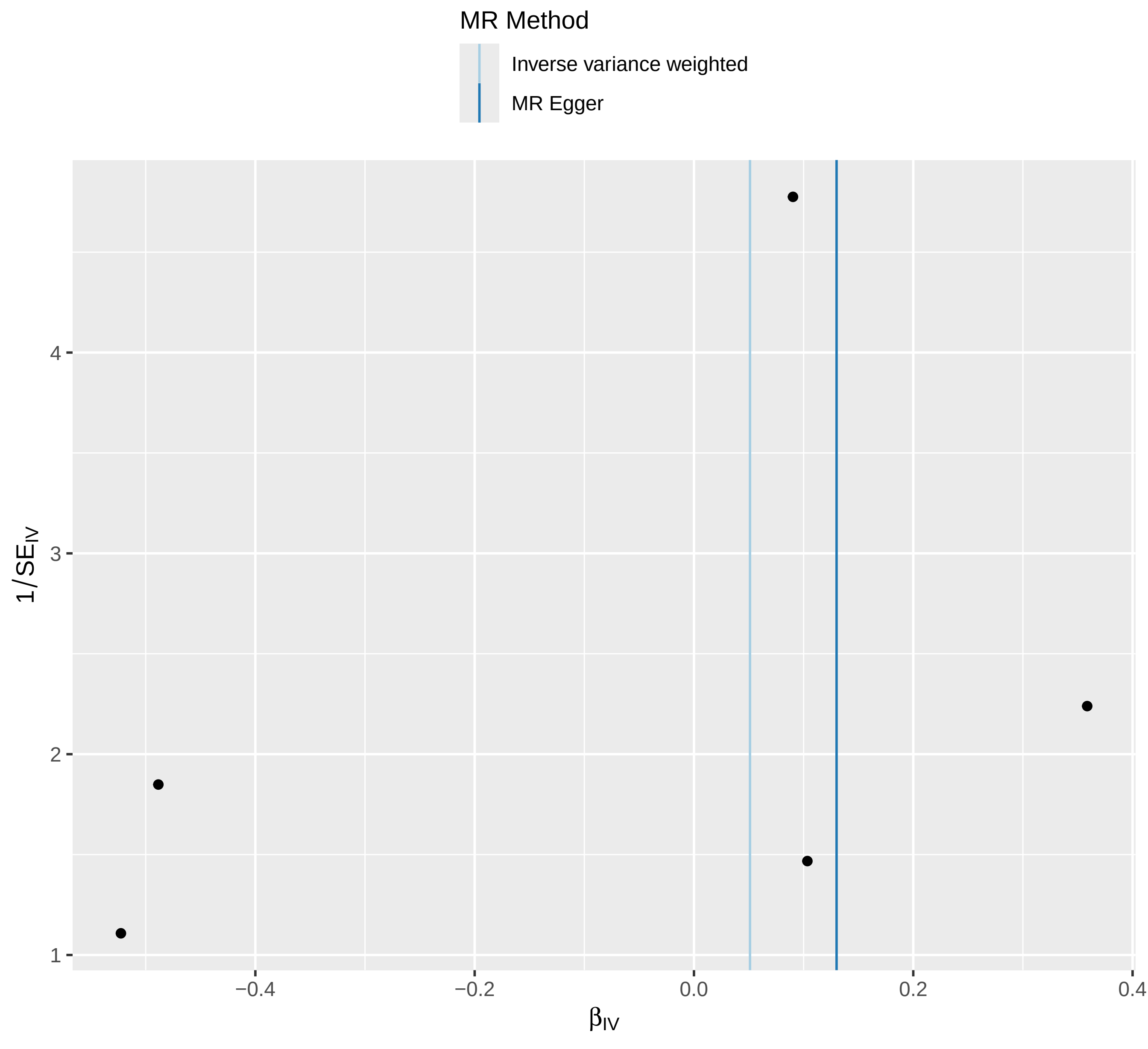

MR Method
Inverse variance weighted
MR Egger
1/SE_IV
4
3
2
1
−0.4
−0.2
0.0
0.2
0.4
β_IV

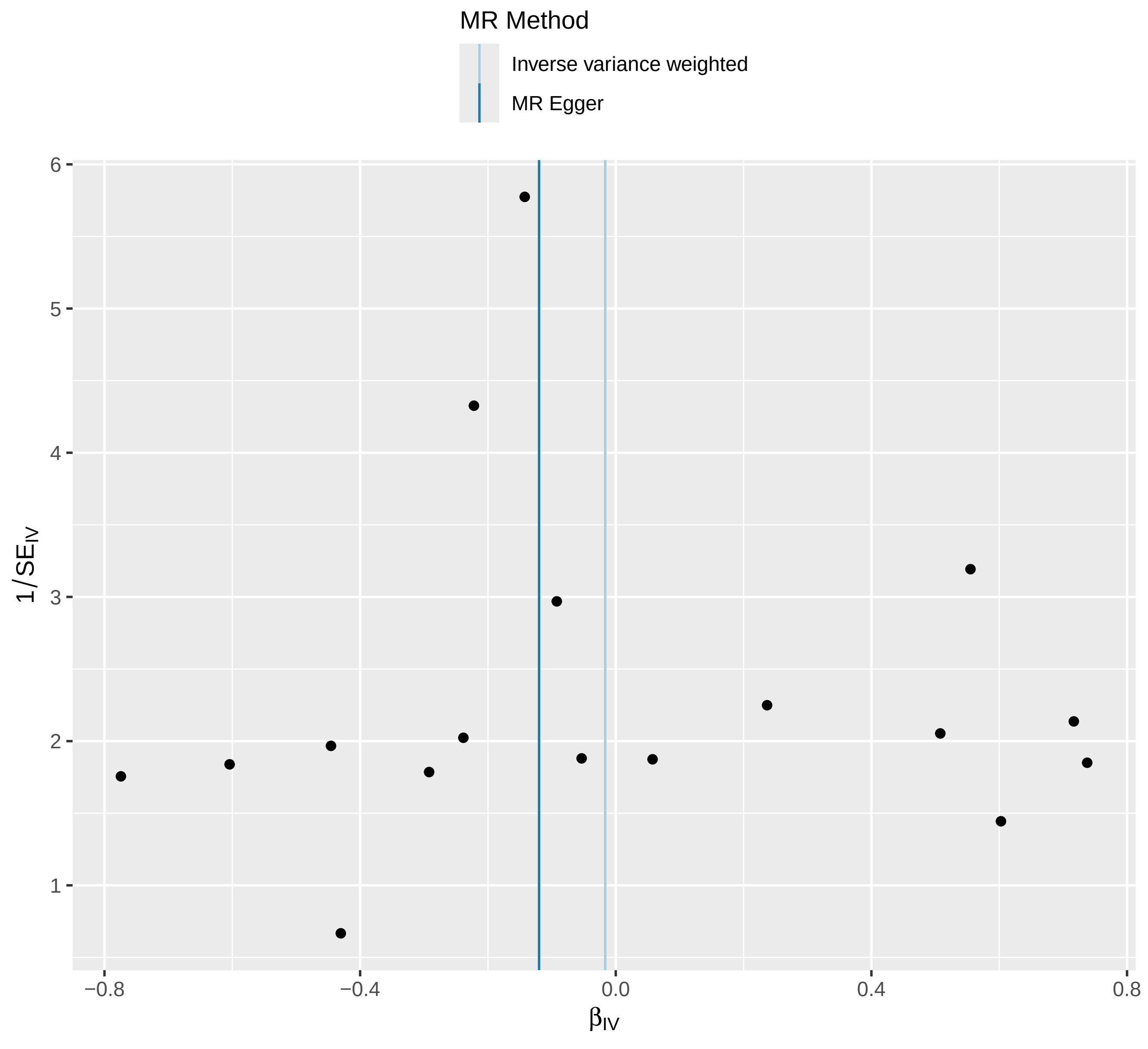
MR Method
Inverse variance weighted
MR Egger
1/SE_IV
β_IV
−0.8
−0.4
0.0
0.4
0.8
1
2
3
4
5
6

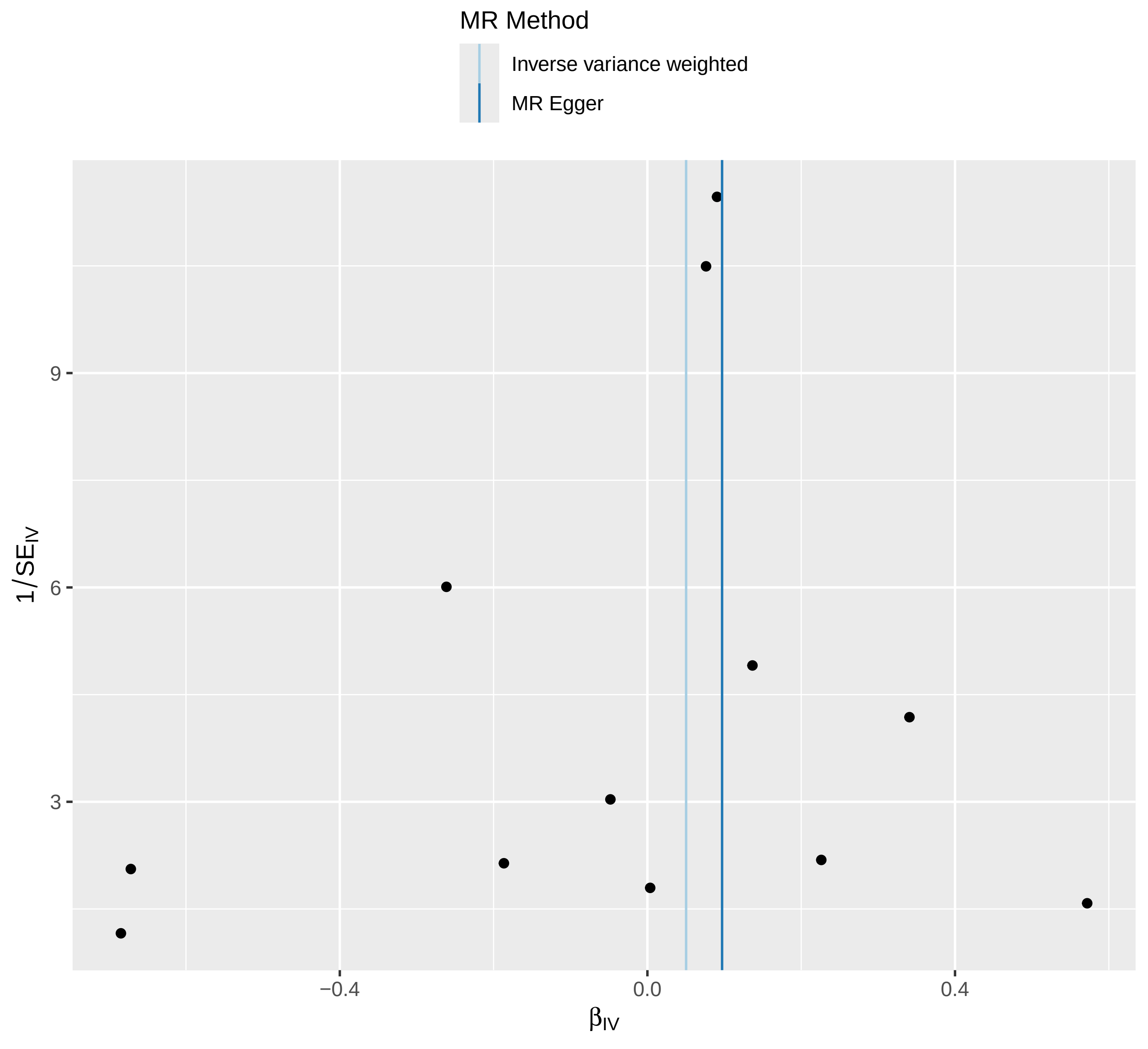

MR Method
Inverse variance weighted
MR Egger
9
6
3
1/SE$_{IV}$
−0.4
0.0
0.4
β$_{IV}$

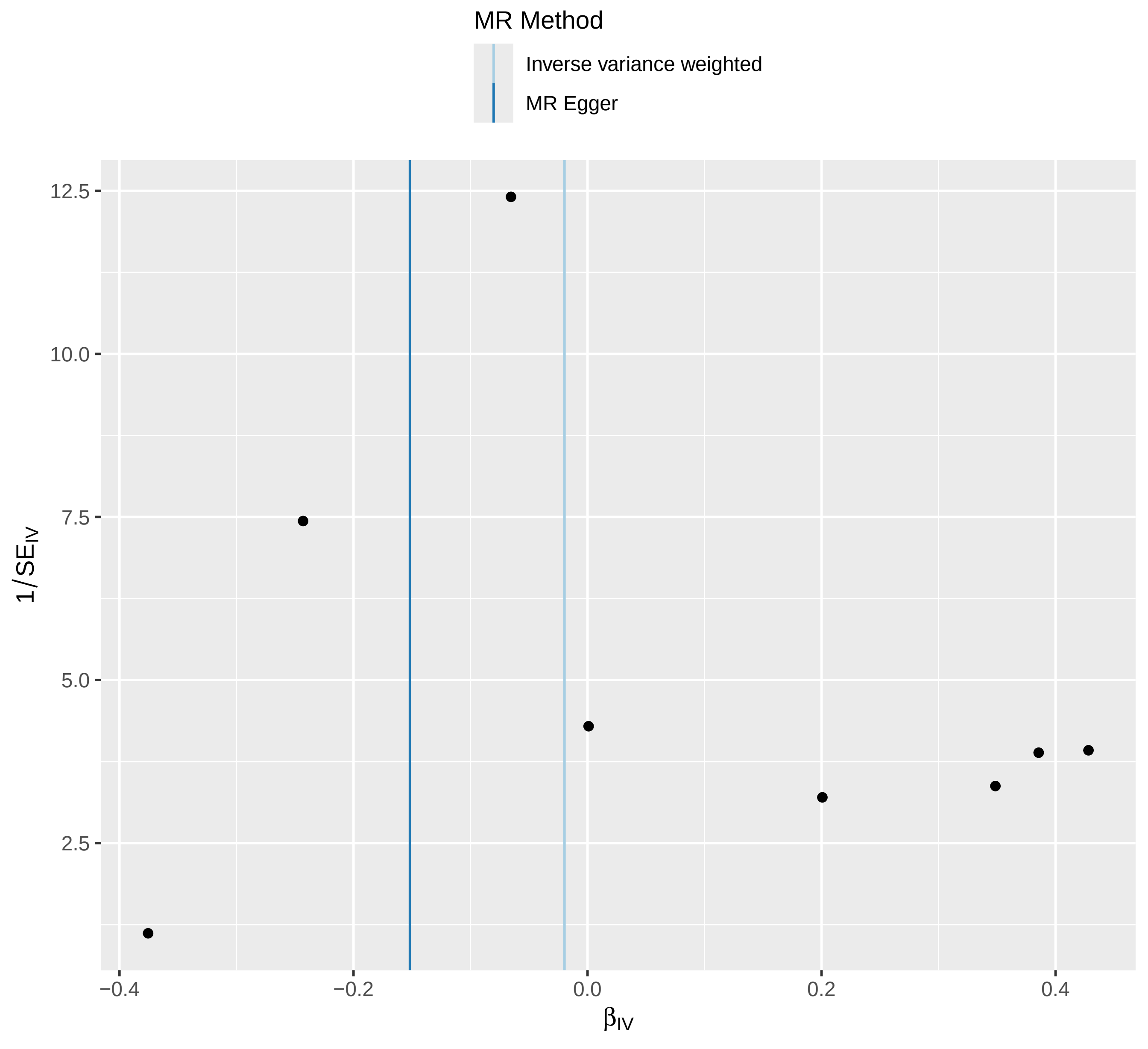

MR Method
Inverse variance weighted
MR Egger
12.5
10.0
7.5
5.0
2.5
1/SE_IV
−0.4
−0.2
0.0
0.2
0.4
β_IV

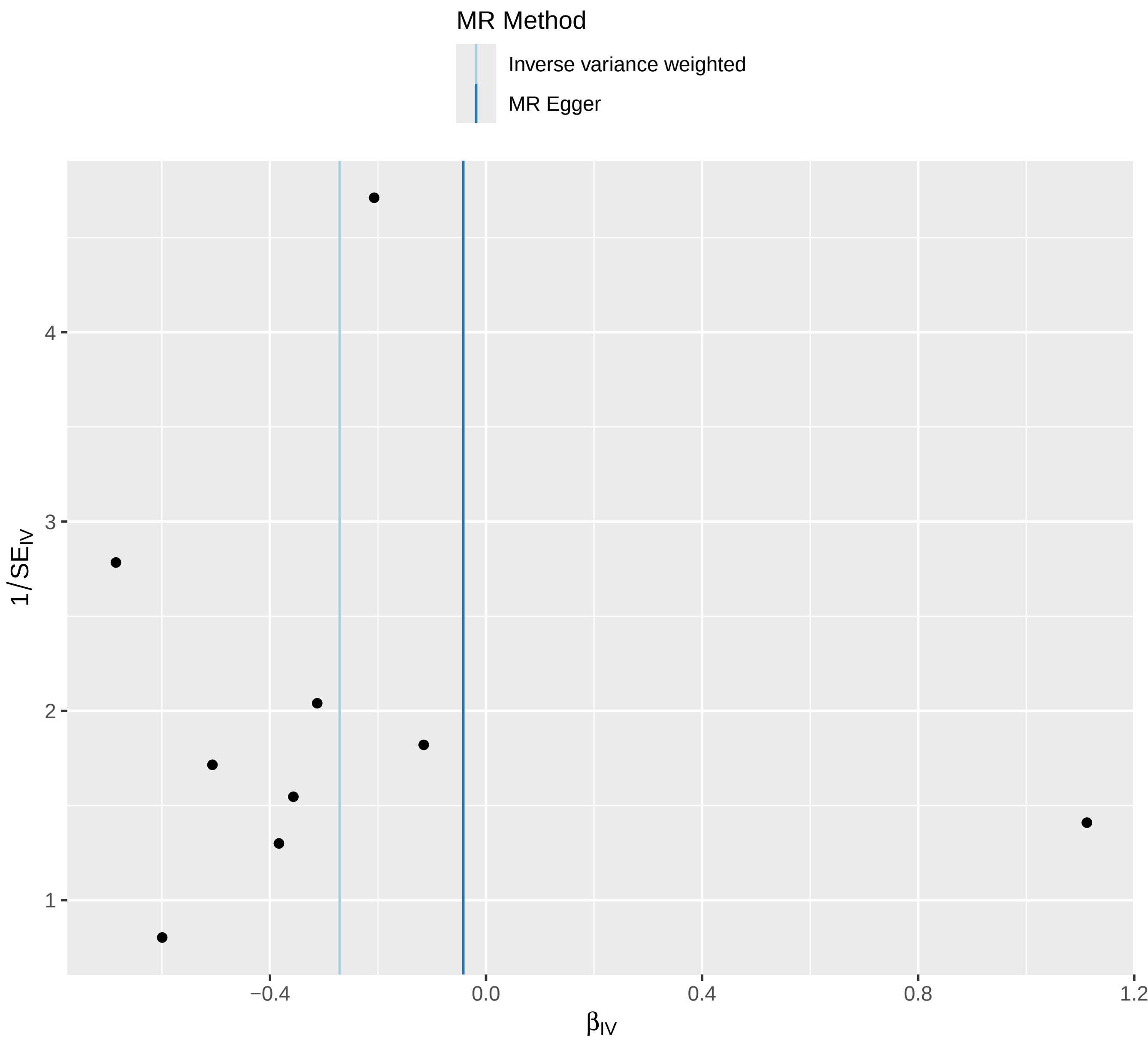

MR Method
Inverse variance weighted
MR Egger
4
3
2
1
$1/SE_{IV}$
−0.4
0.0
0.4
0.8
1.2
β_{IV}

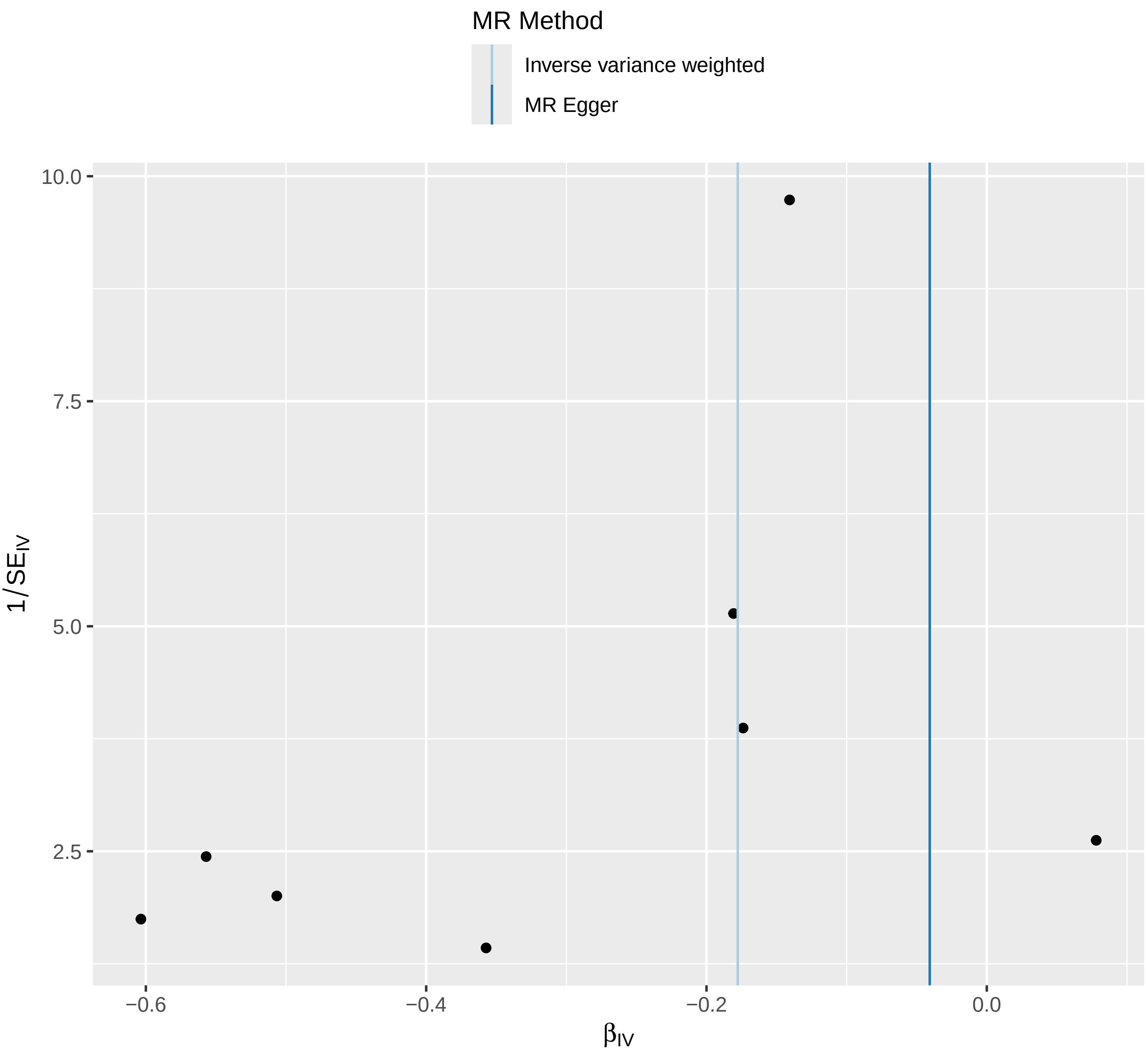
MR Method
Inverse variance weighted
MR Egger
10.0
7.5
5.0
2.5
1/SE_IV
−0.6
−0.4
−0.2
0.0
β_IV

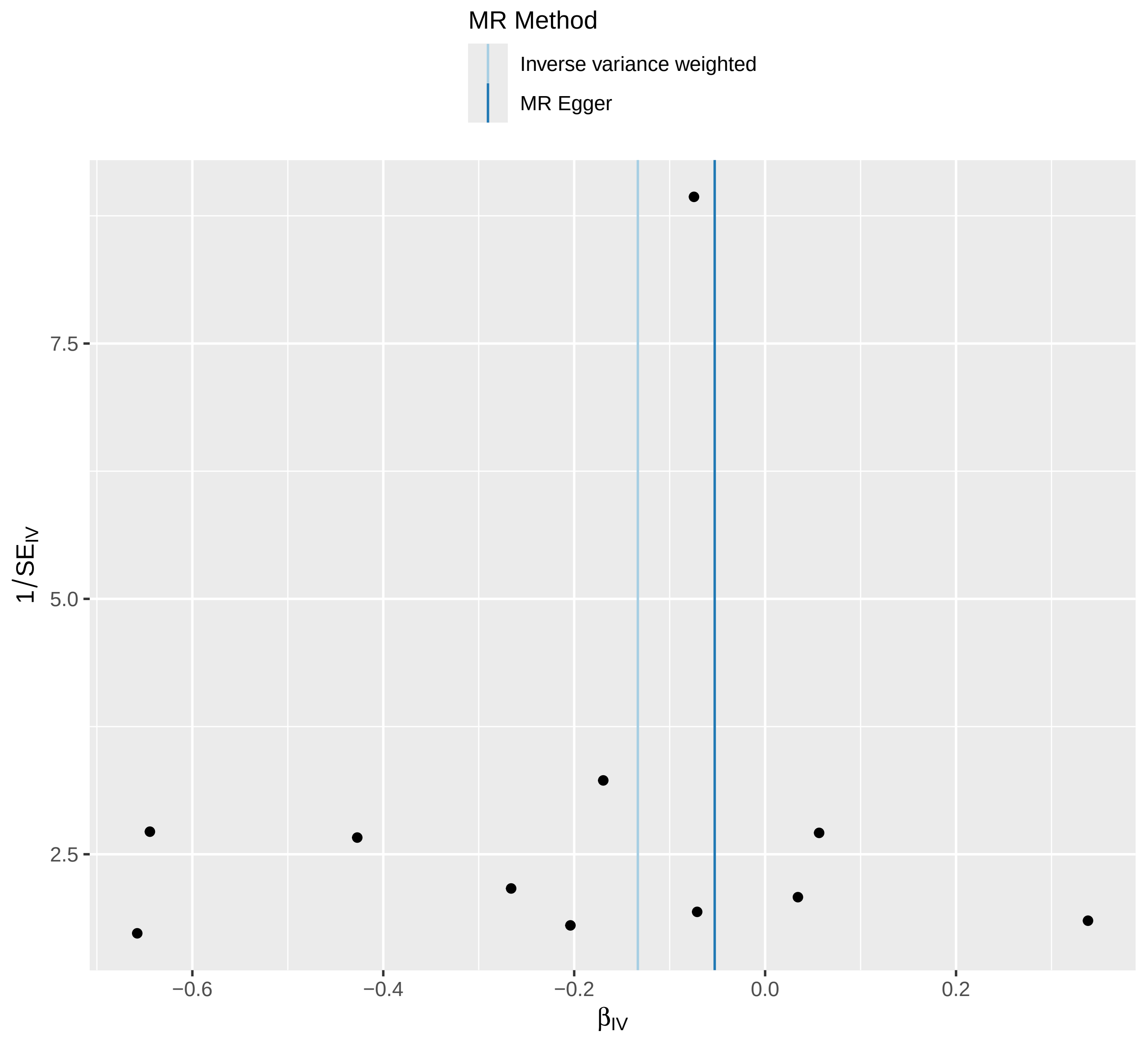

MR Method
Inverse variance weighted
MR Egger
7.5
5.0
2.5
$1/SE_{IV}$
−0.6
−0.4
−0.2
0.0
0.2
β_{IV}

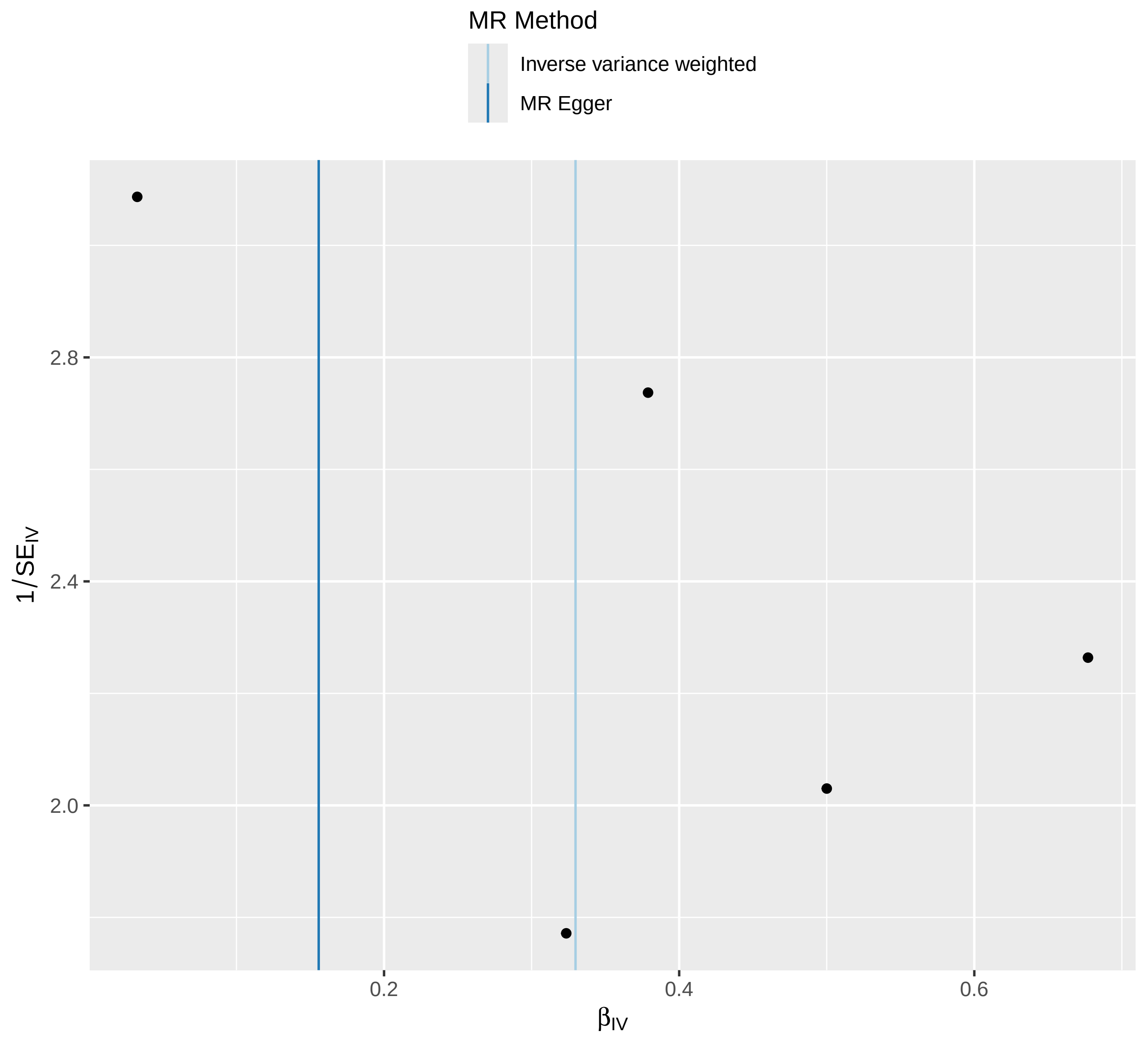

MR Method
Inverse variance weighted
MR Egger
2.8
2.4
2.0
1/SE$_{IV}$
0.2
0.4
0.6
β$_{IV}$

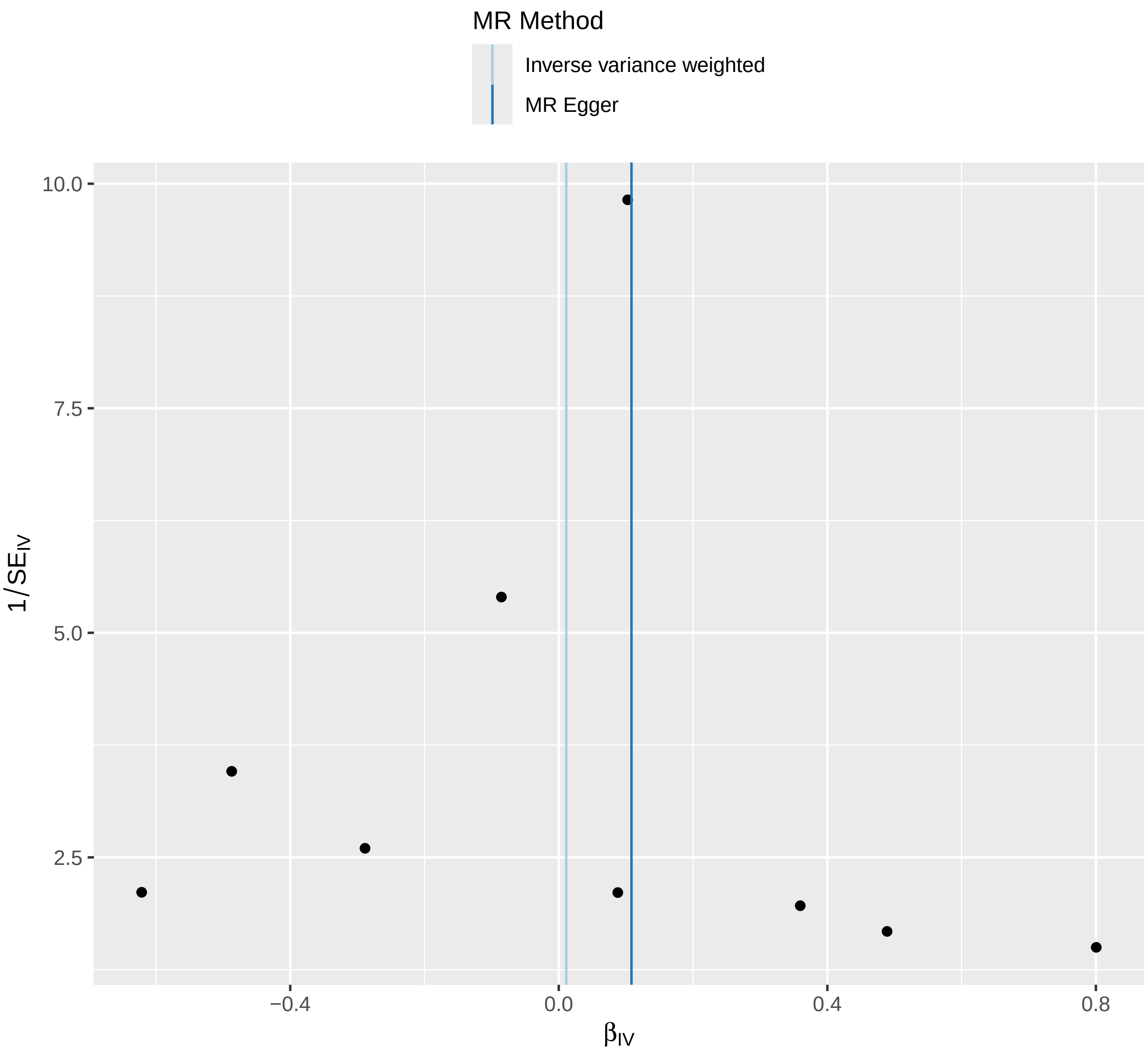
MR Method
Inverse variance weighted
MR Egger
10.0
7.5
5.0
2.5
1/SE$_{IV}$
−0.4
0.0
0.4
0.8
β$_{IV}$

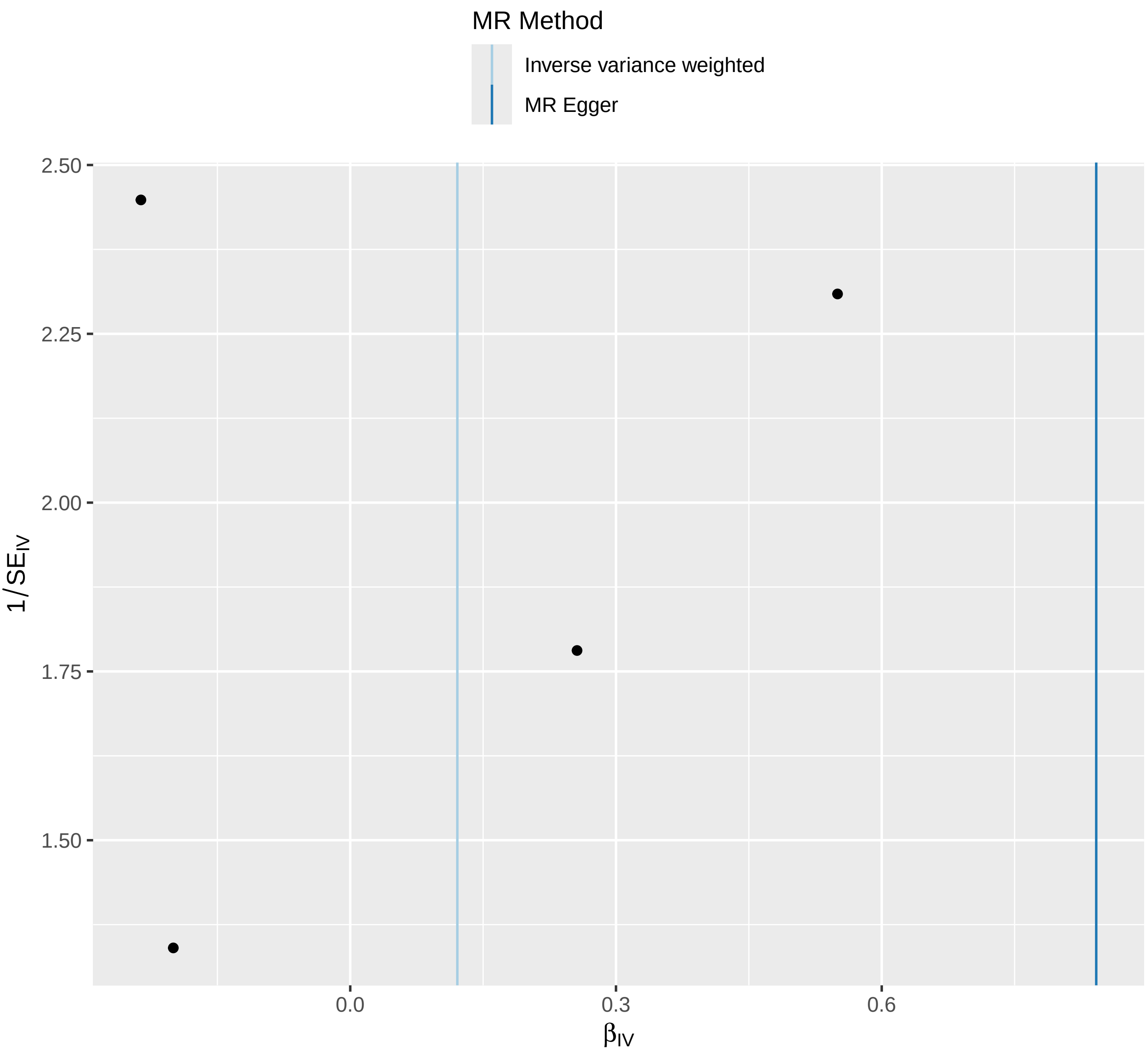
MR Method
Inverse variance weighted
MR Egger
2.50
2.25
2.00
1.75
1.50
$1/SE_{IV}$
0.0
0.3
0.6
β_{IV}

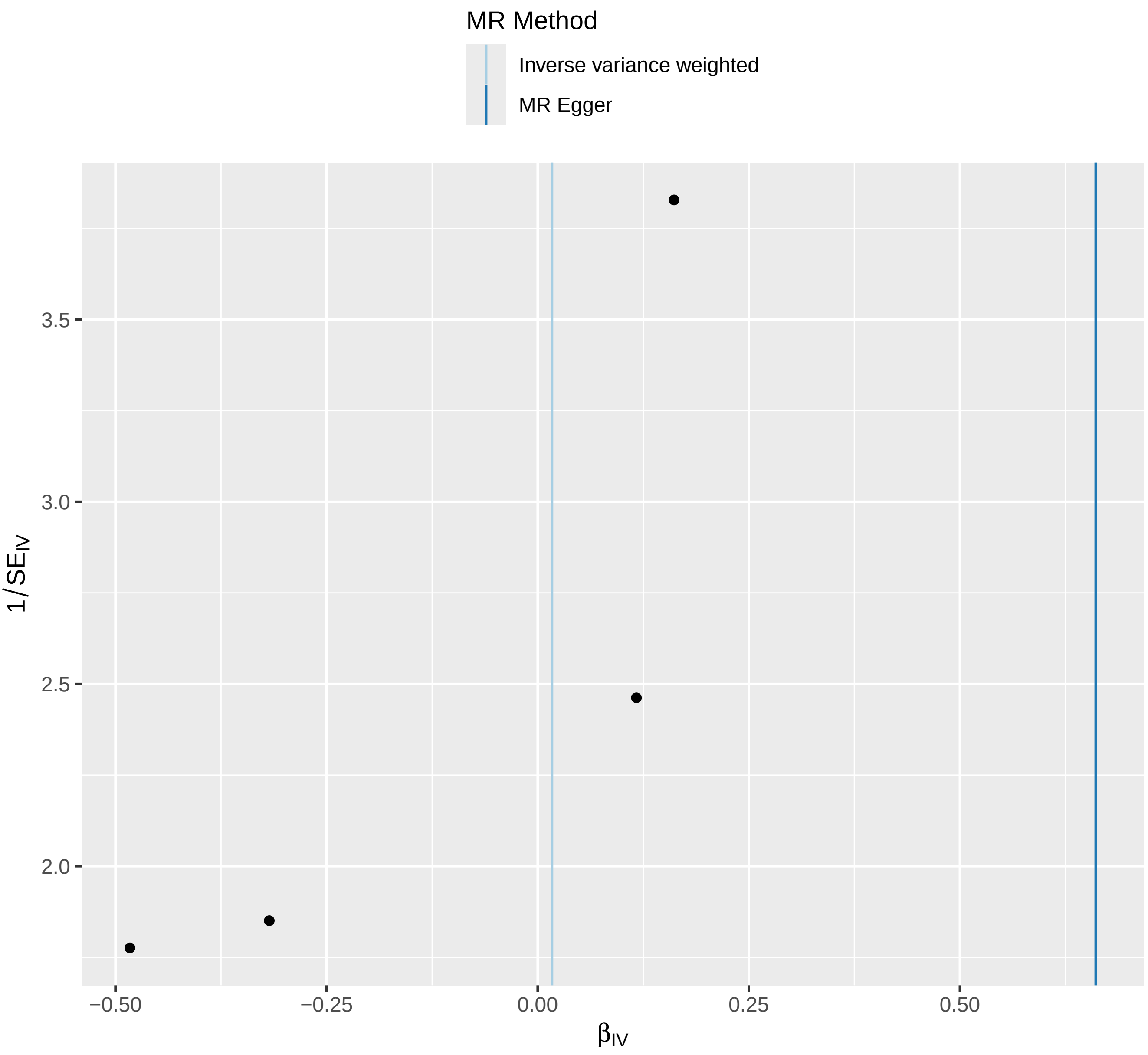
MR Method
Inverse variance weighted
MR Egger
3.5
3.0
2.5
2.0
1/SE$_{IV}$
−0.50
−0.25
0.00
0.25
0.50
β$_{IV}$

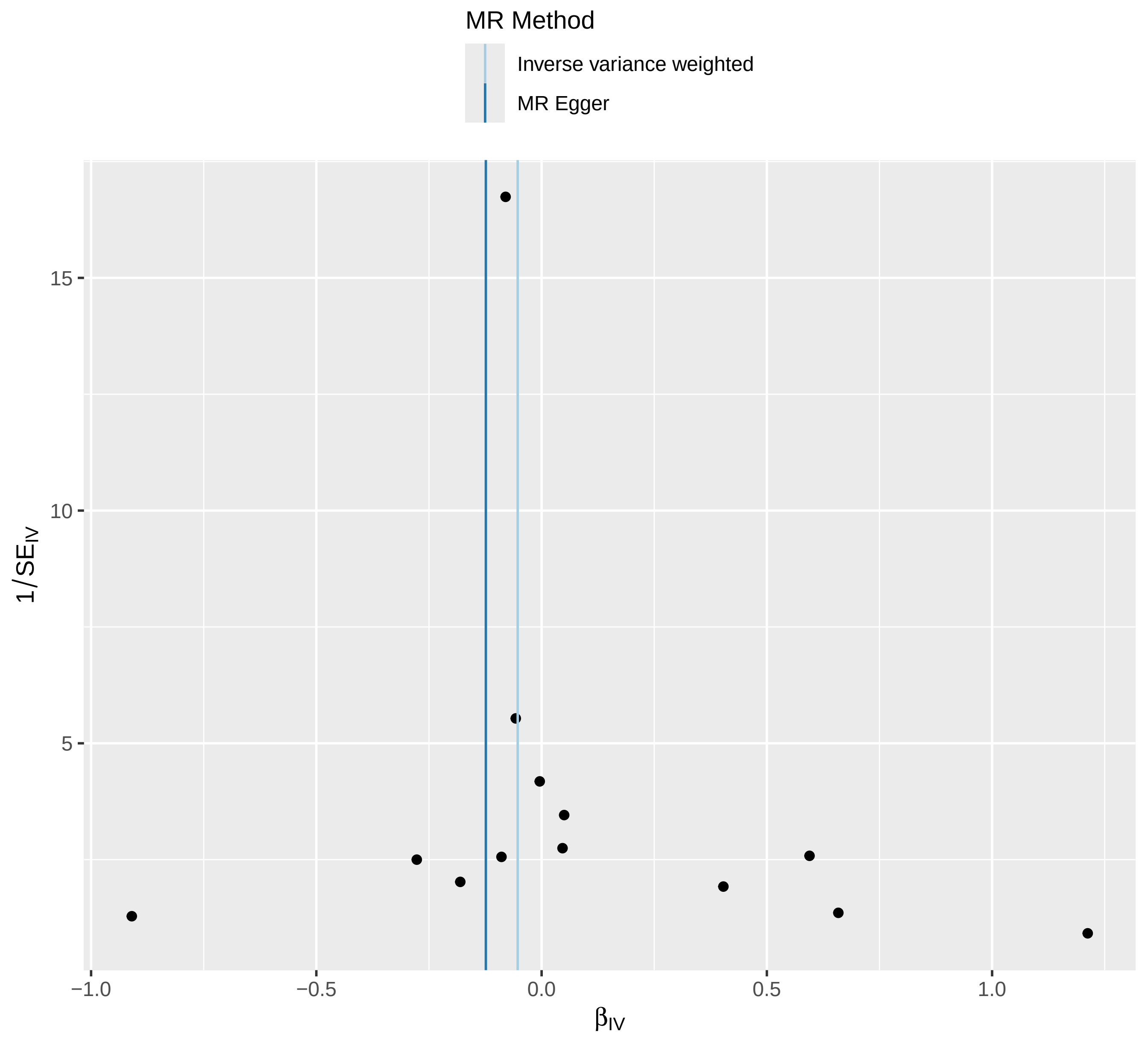
MR Method
Inverse variance weighted
MR Egger
15
10
5
1/SE$_{IV}$
−1.0
−0.5
0.0
0.5
1.0
β$_{IV}$

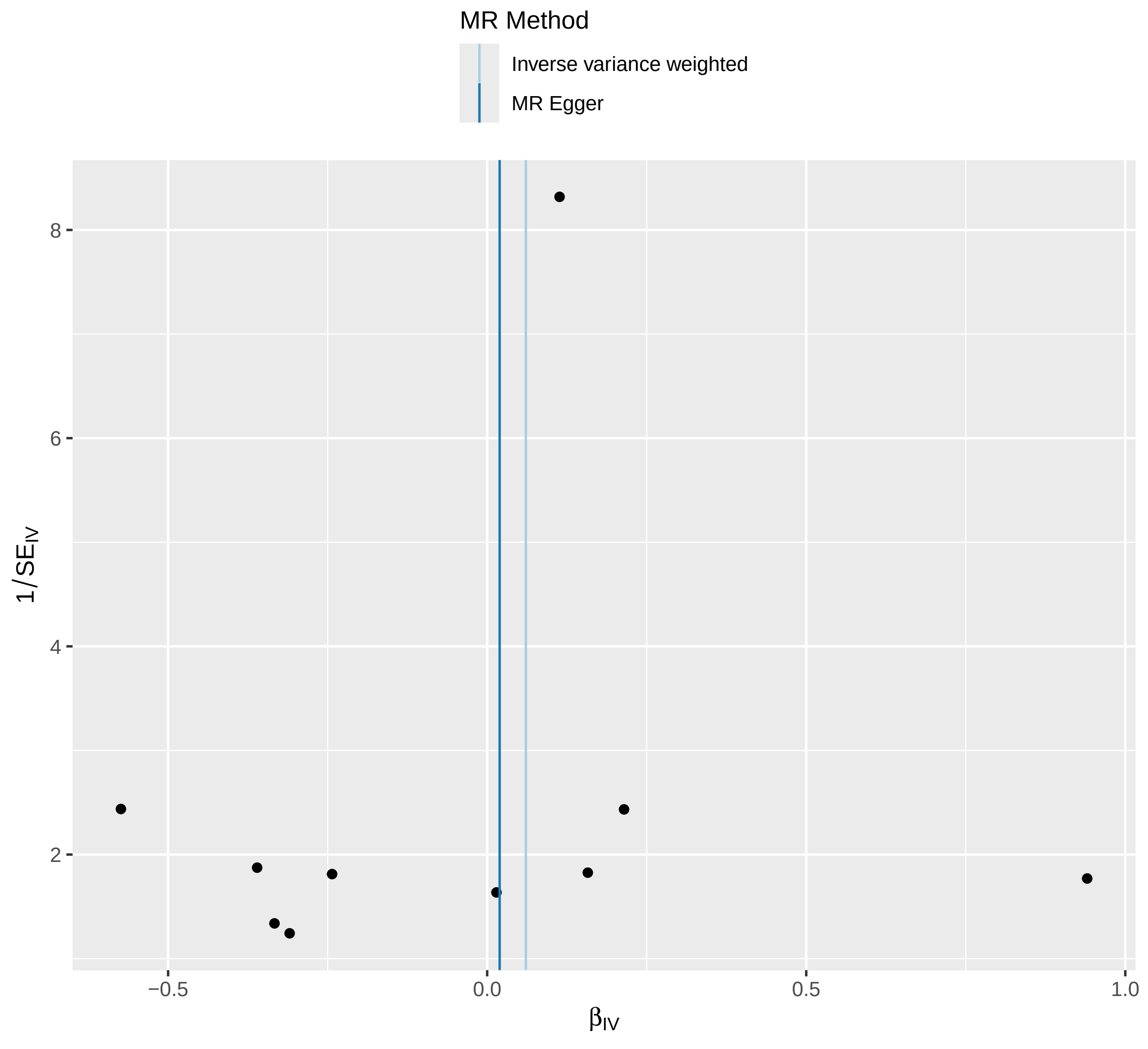
MR Method
Inverse variance weighted
MR Egger
8
6
4
2
1/SE$_{IV}$
−0.5
0.0
0.5
1.0
β_{IV}

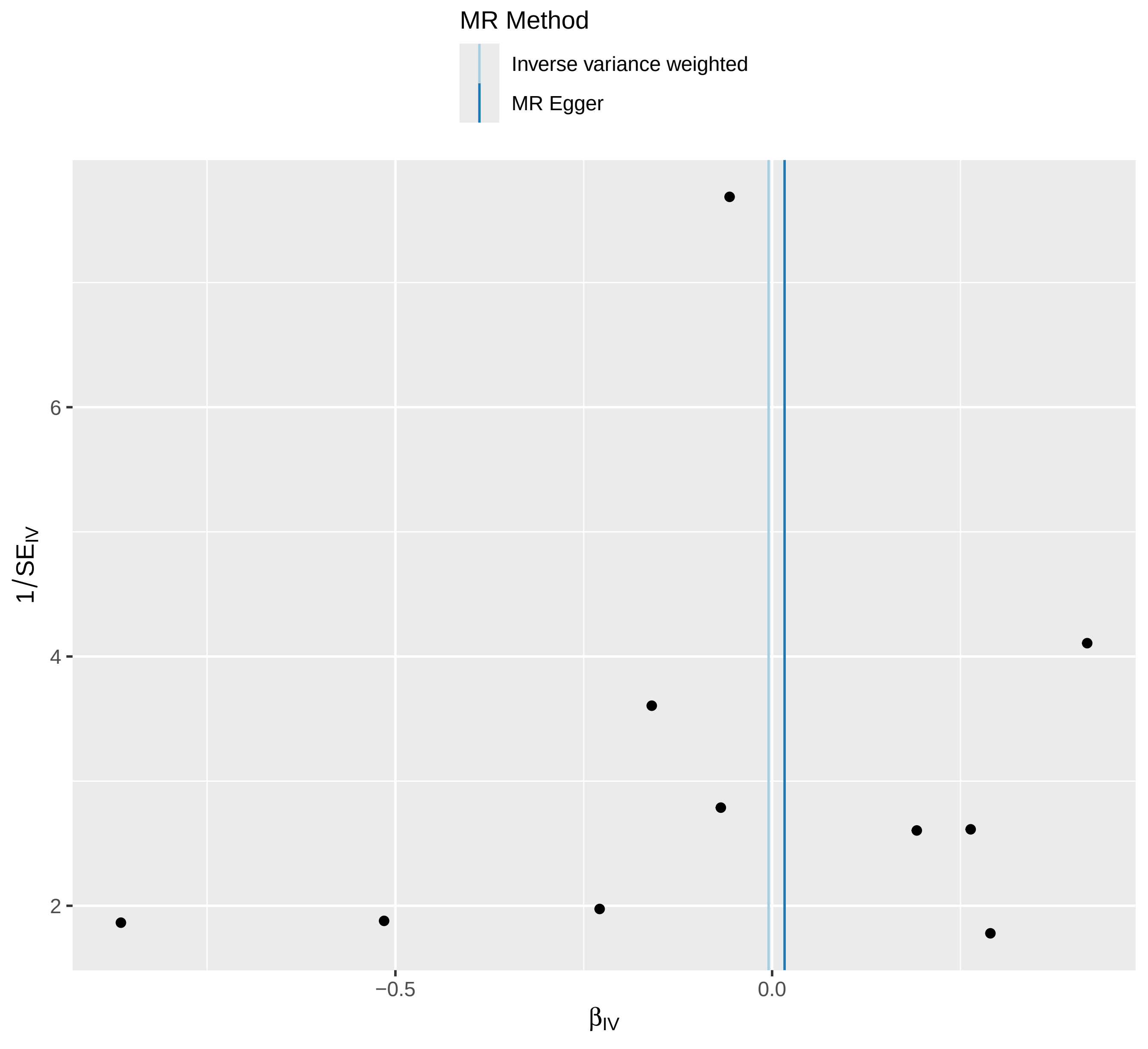
MR Method
Inverse variance weighted
MR Egger
$1/SE_{IV}$
6
4
2
−0.5
0.0
β_{IV}

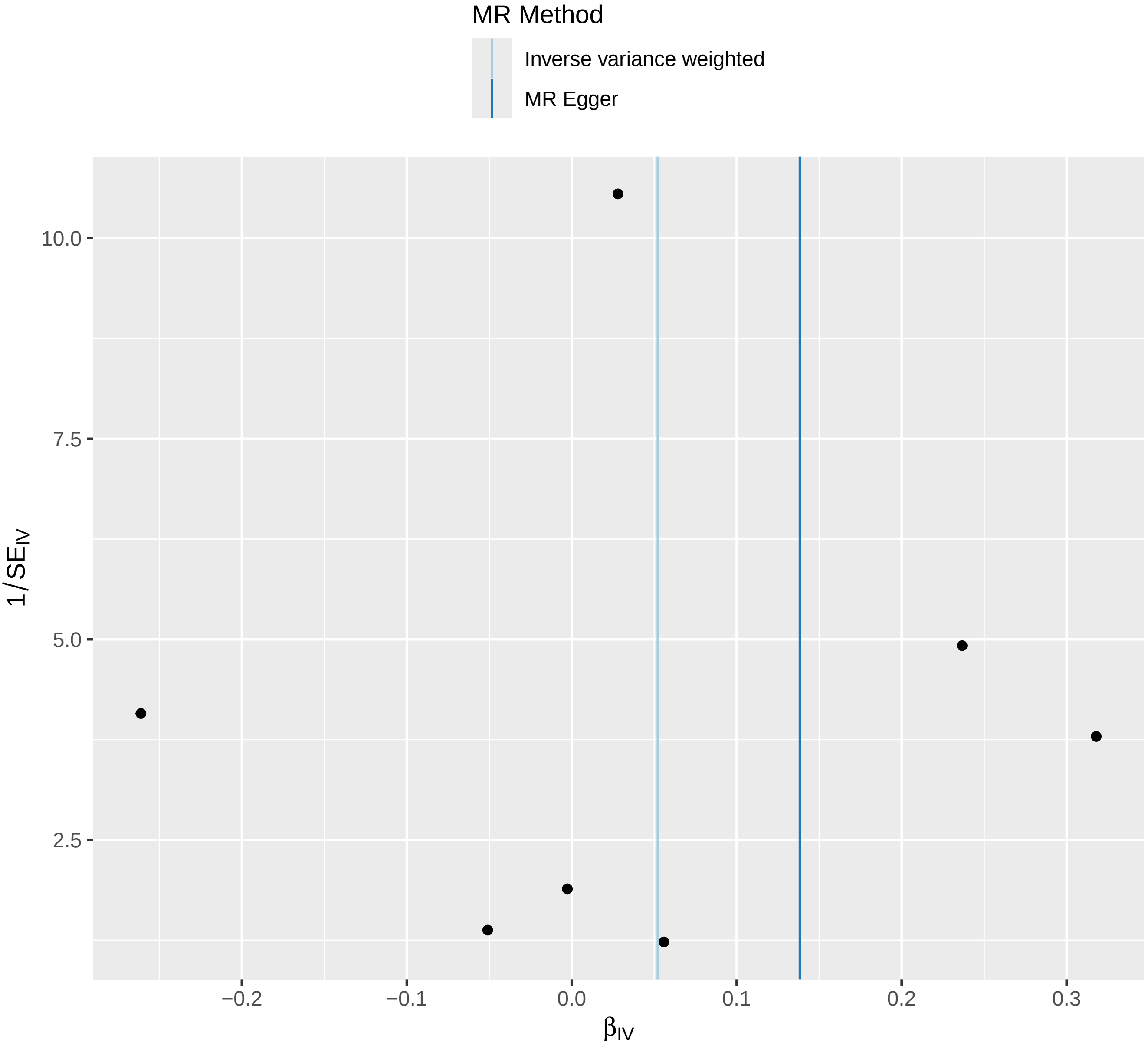

MR Method
Inverse variance weighted
MR Egger
10.0
7.5
5.0
2.5
$1/SE_{IV}$
−0.2
−0.1
0.0
0.1
0.2
0.3
β_{IV}

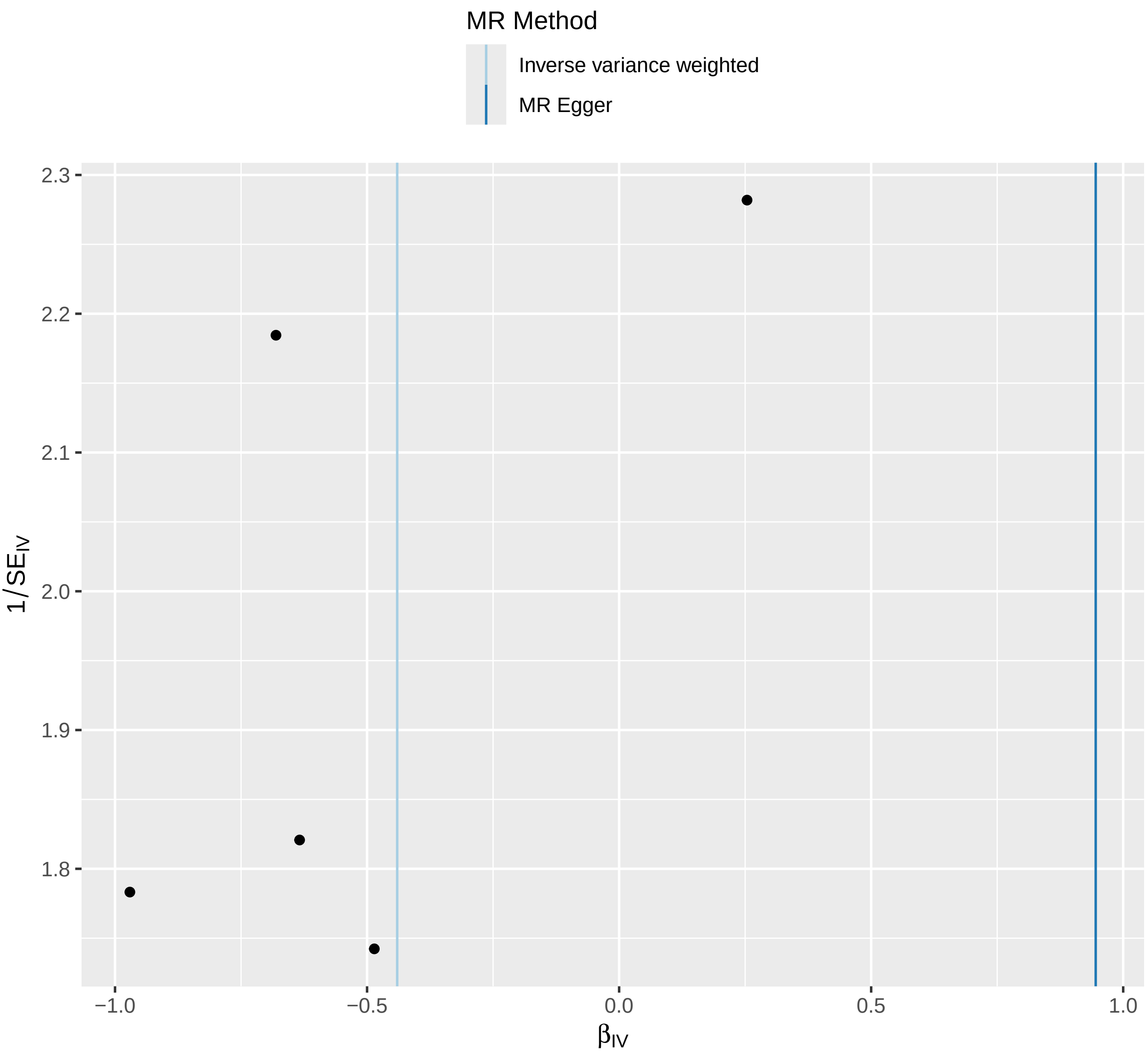
MR Method
Inverse variance weighted
MR Egger
2.3
2.2
2.1
2.0
1.9
1.8
1/SE_IV
−1.0
−0.5
0.0
0.5
1.0
β_IV

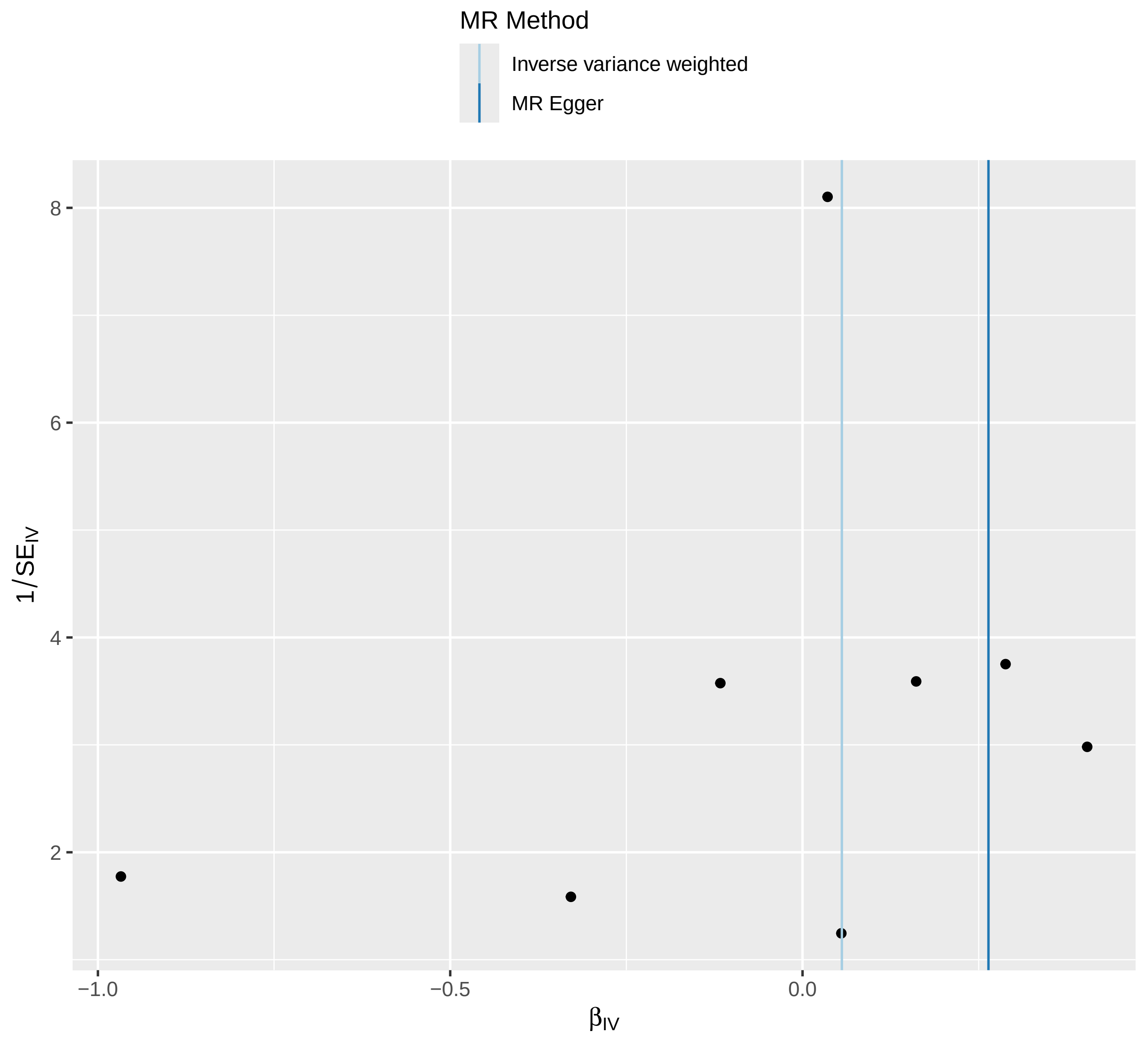
MR Method
Inverse variance weighted
MR Egger
8
6
4
2
1/SE_IV
−1.0
−0.5
0.0
β_IV

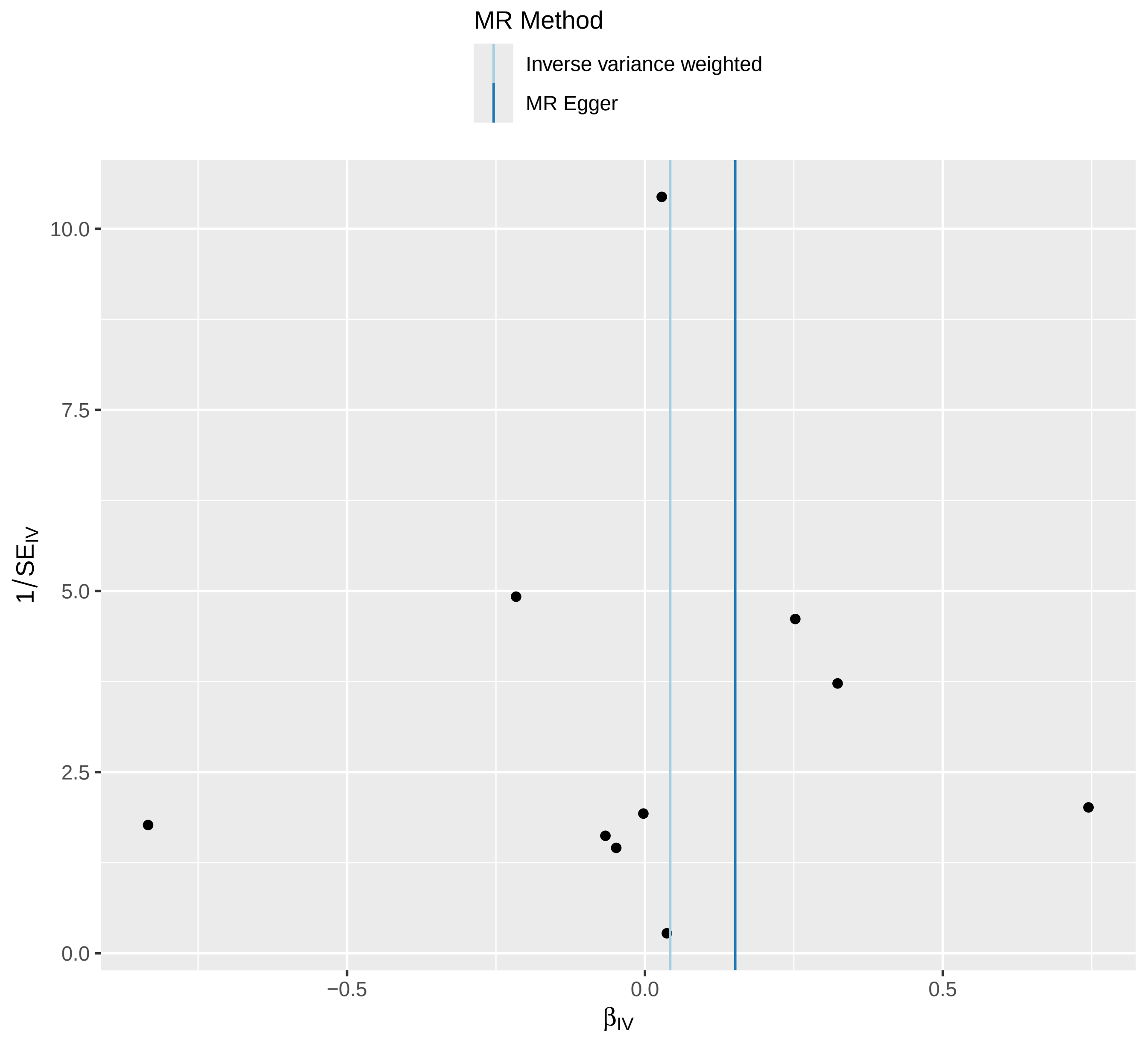

MR Method
Inverse variance weighted
MR Egger
10.0
7.5
5.0
2.5
0.0
1/SE_{IV}
−0.5
0.0
0.5
β_{IV}

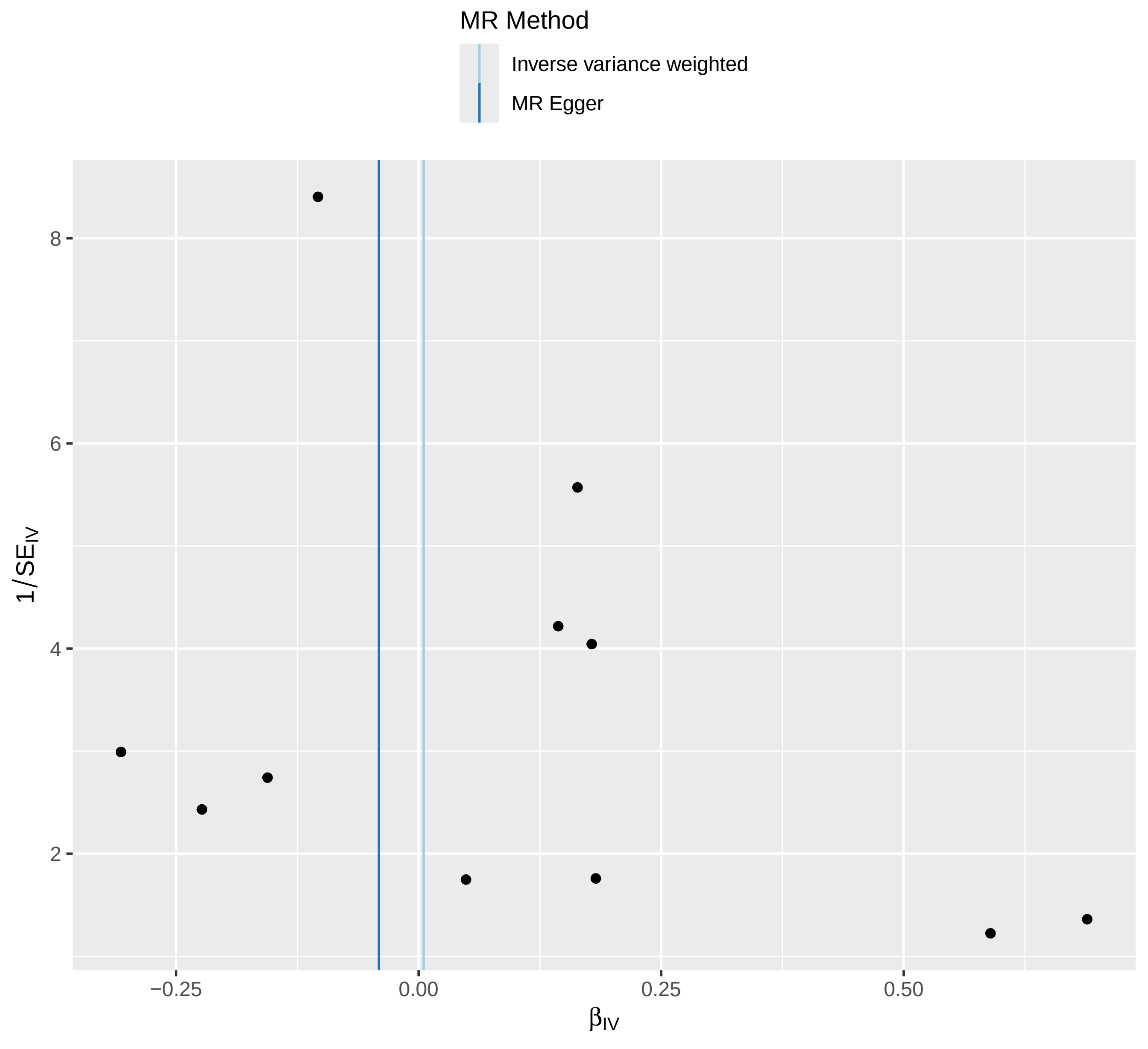
MR Method
Inverse variance weighted
MR Egger
8
6
4
2
1/SE_{IV}
−0.25
0.00
0.25
0.50
β_{IV}

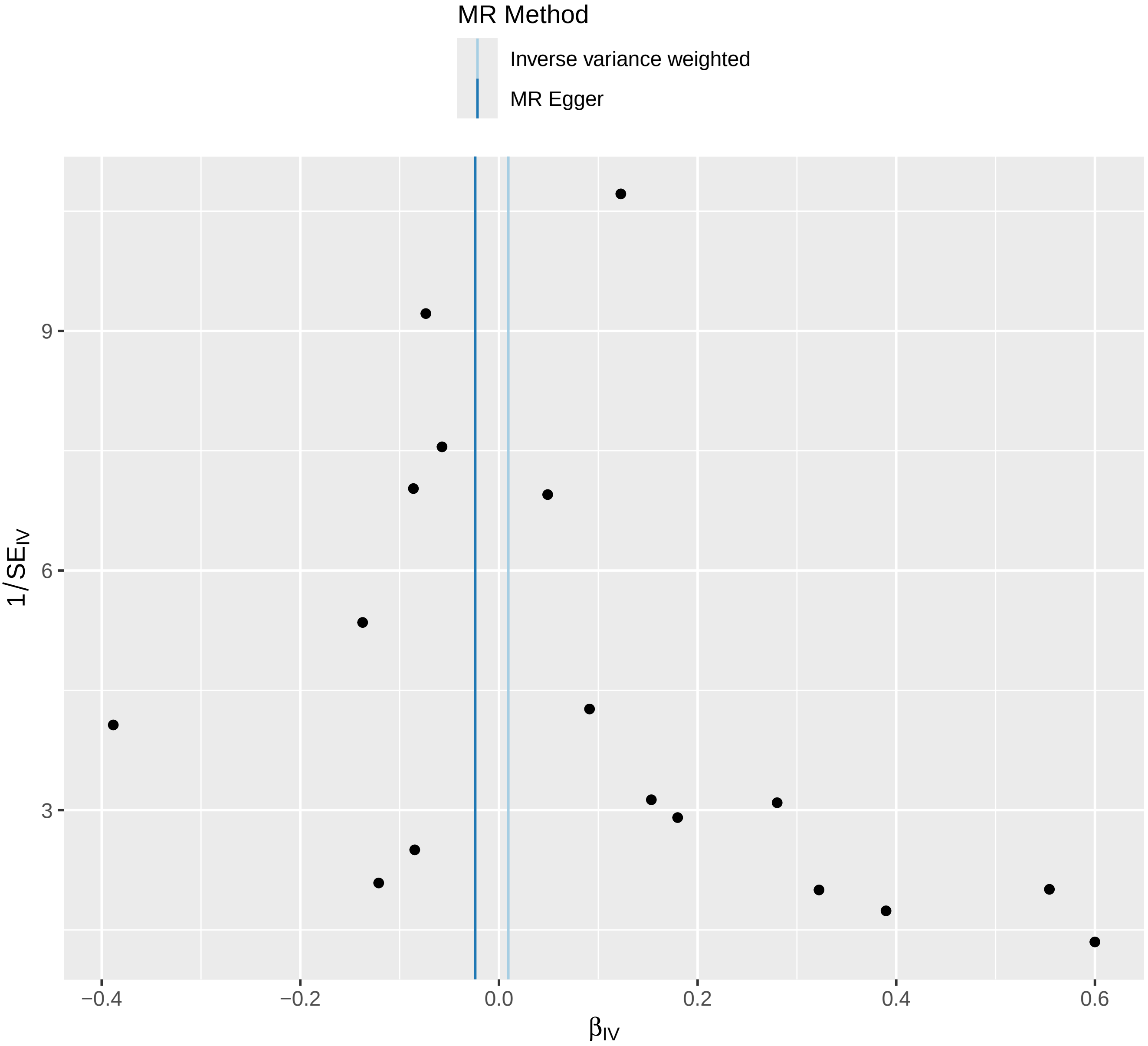

MR Method
Inverse variance weighted
MR Egger
1/SE$_{IV}$
9
6
3
−0.4
−0.2
0.0
0.2
0.4
0.6
β$_{IV}$

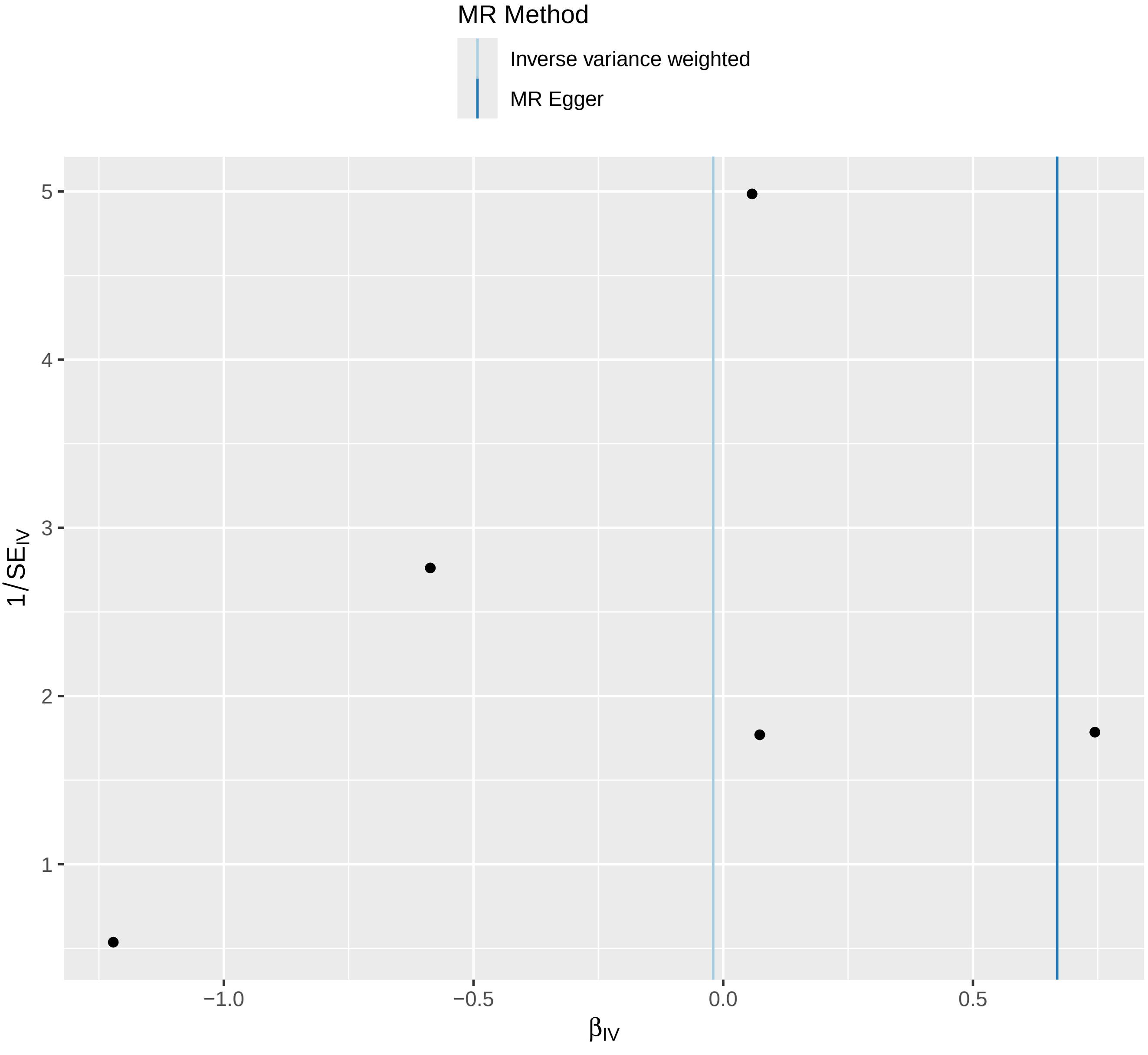
MR Method
Inverse variance weighted
MR Egger
1/SE_IV
β_IV
−1.0
−0.5
0.0
0.5
1
2
3
4
5

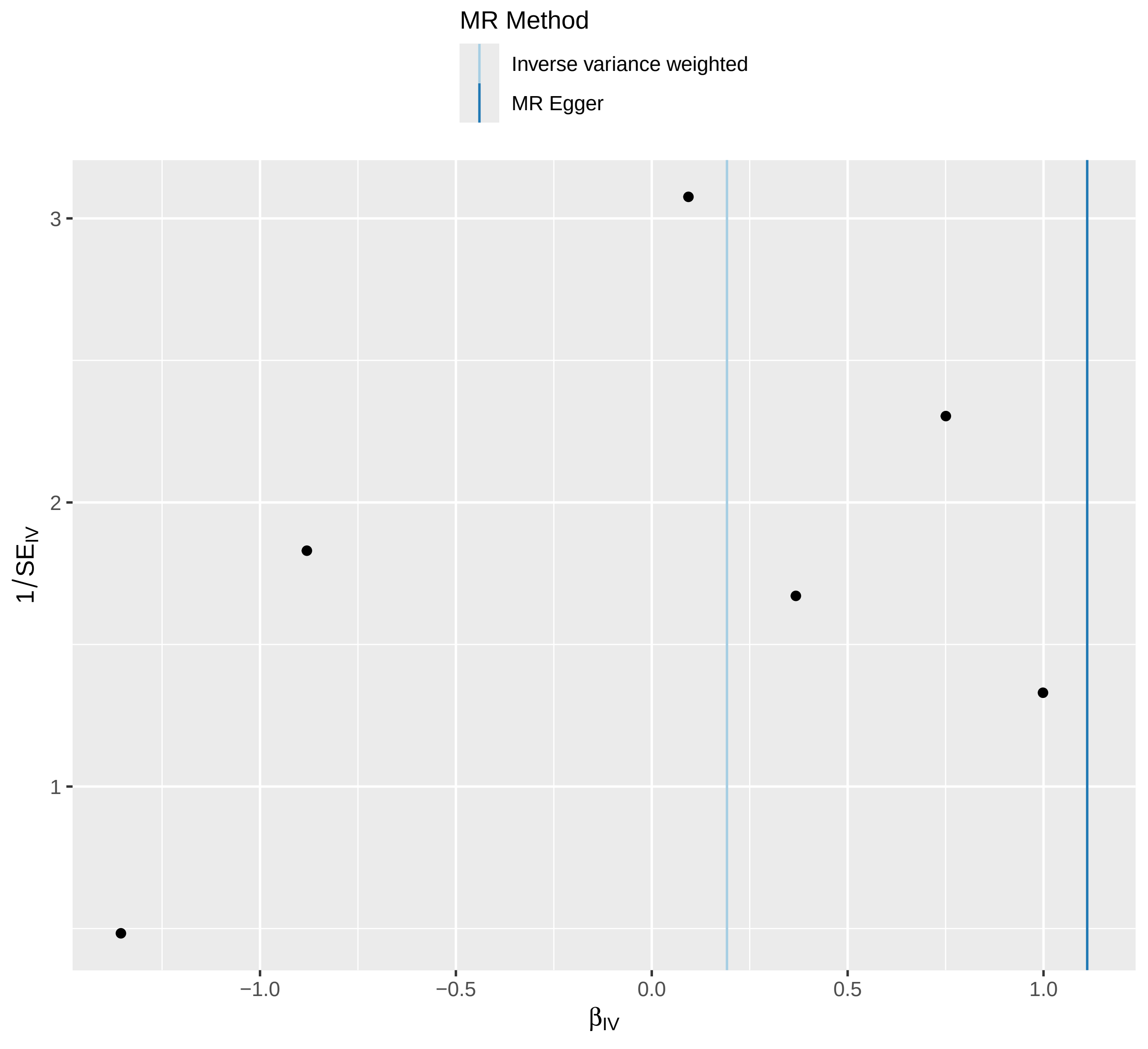

MR Method
Inverse variance weighted
MR Egger
3
2
1
$1/SE_{IV}$
−1.0
−0.5
0.0
0.5
1.0
β_{IV}

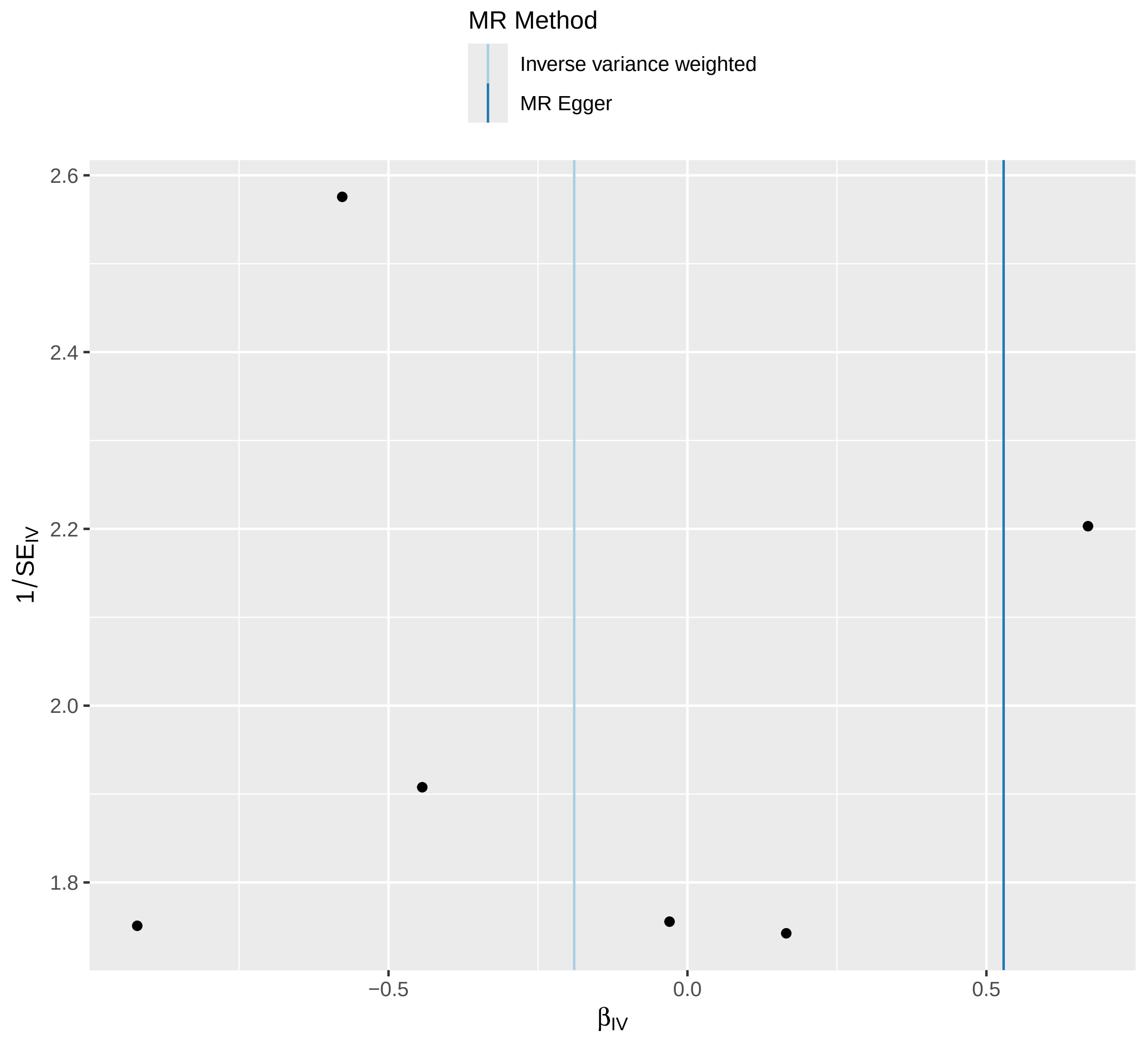

MR Method
Inverse variance weighted
MR Egger
2.6
2.4
2.2
2.0
1.8
1/SE$_{IV}$
−0.5
0.0
0.5
β_{IV}

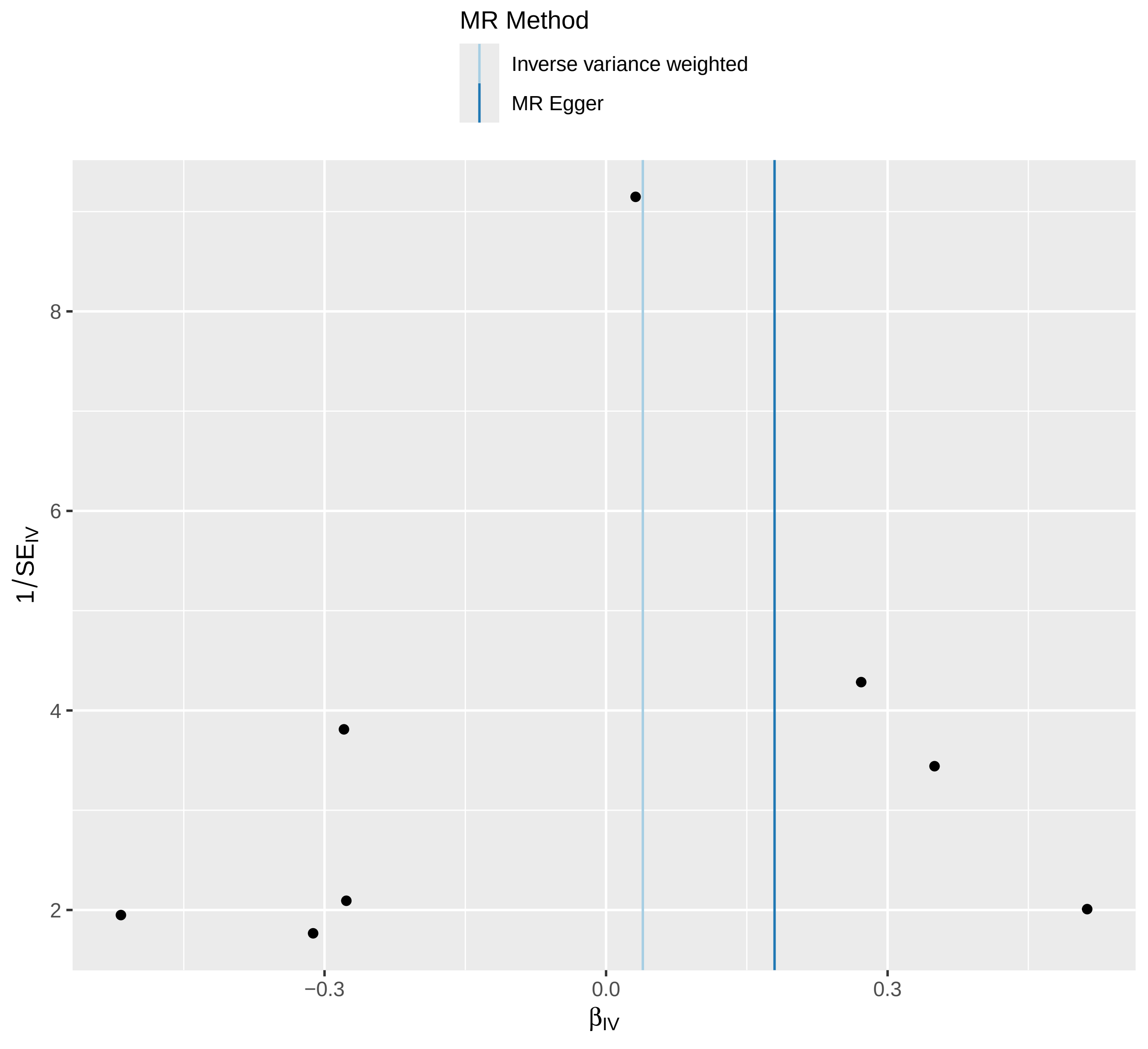
MR Method
Inverse variance weighted
MR Egger
8
6
4
2
1/SE_IV
−0.3
0.0
0.3
β_IV

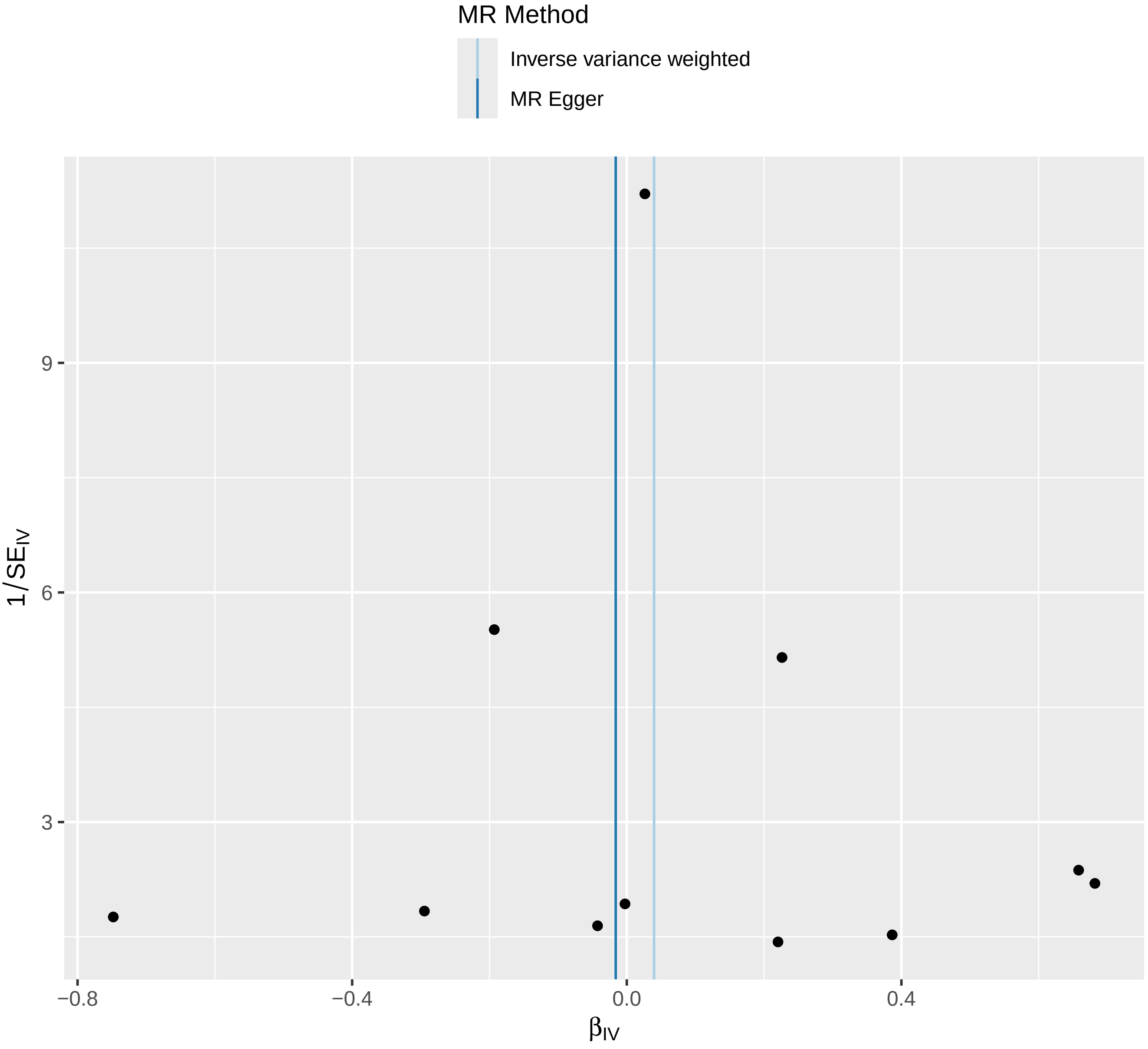

MR Method
Inverse variance weighted
MR Egger
9
6
3
1/SE_IV
−0.8
−0.4
0.0
0.4
β_IV

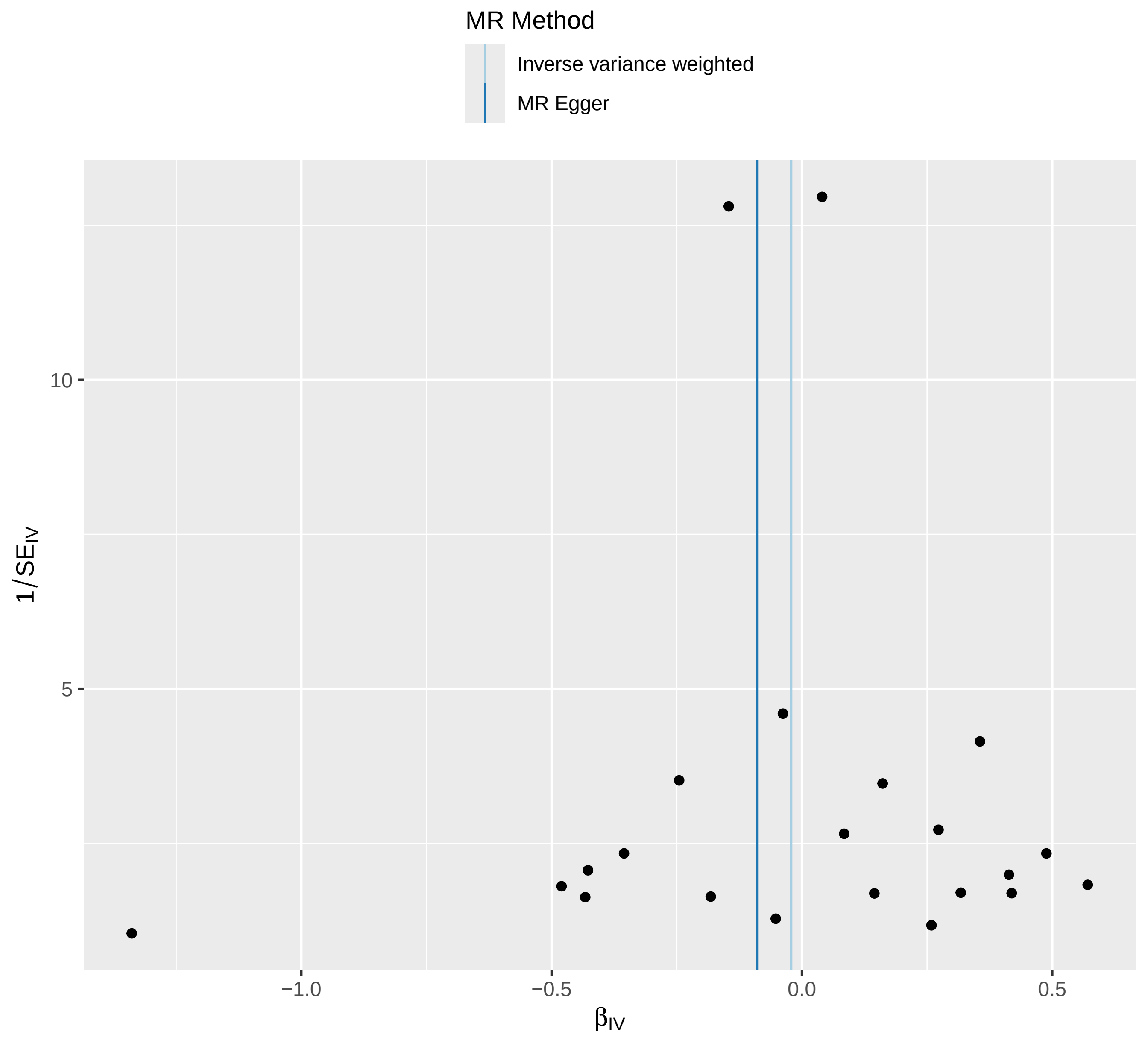

MR Method
Inverse variance weighted
MR Egger
10
5
1/SE_IV
−1.0
−0.5
0.0
0.5
β_IV

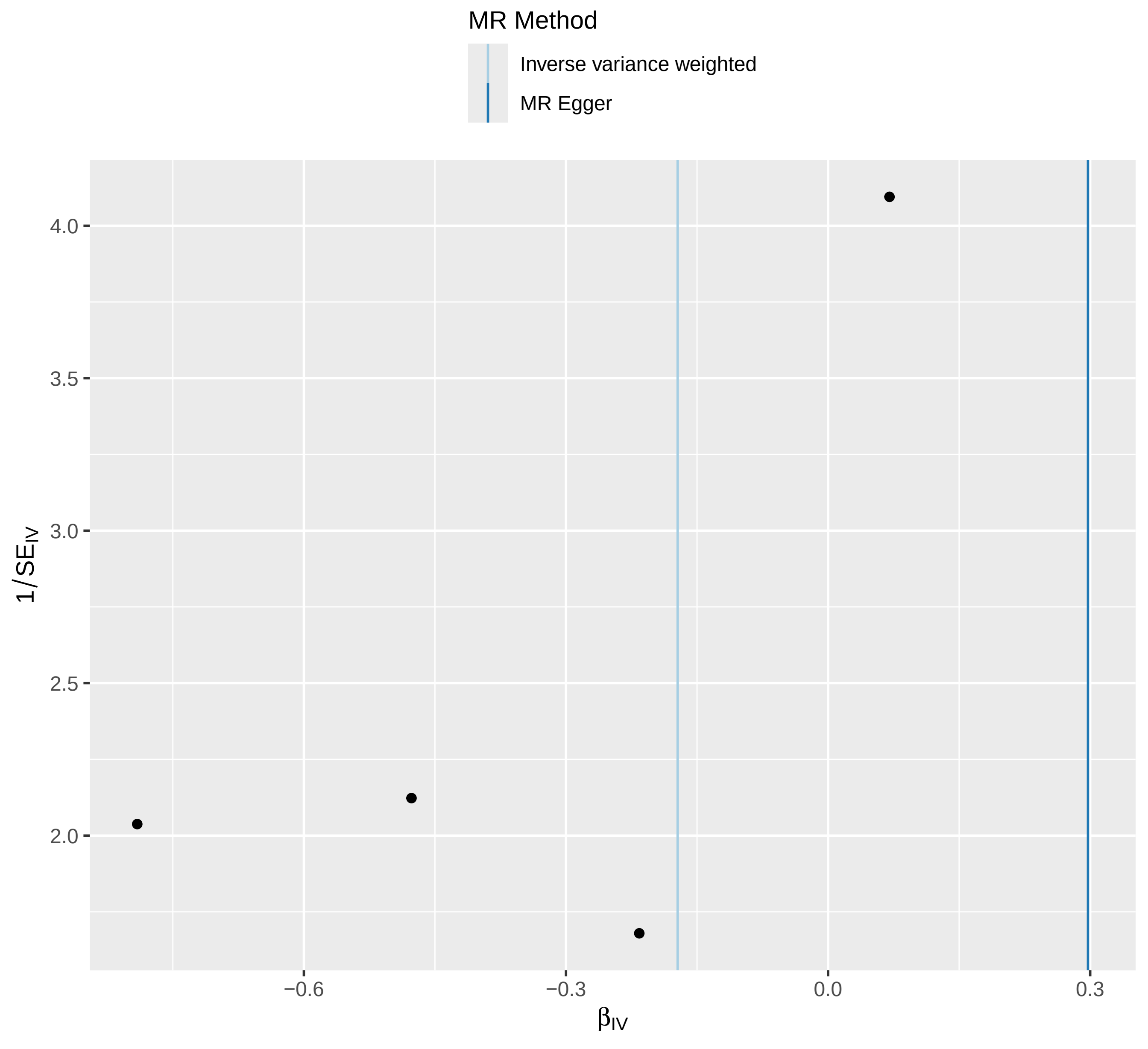
MR Method
Inverse variance weighted
MR Egger
4.0
3.5
3.0
2.5
2.0
1/SE$_{IV}$
−0.6
−0.3
0.0
0.3
β$_{IV}$

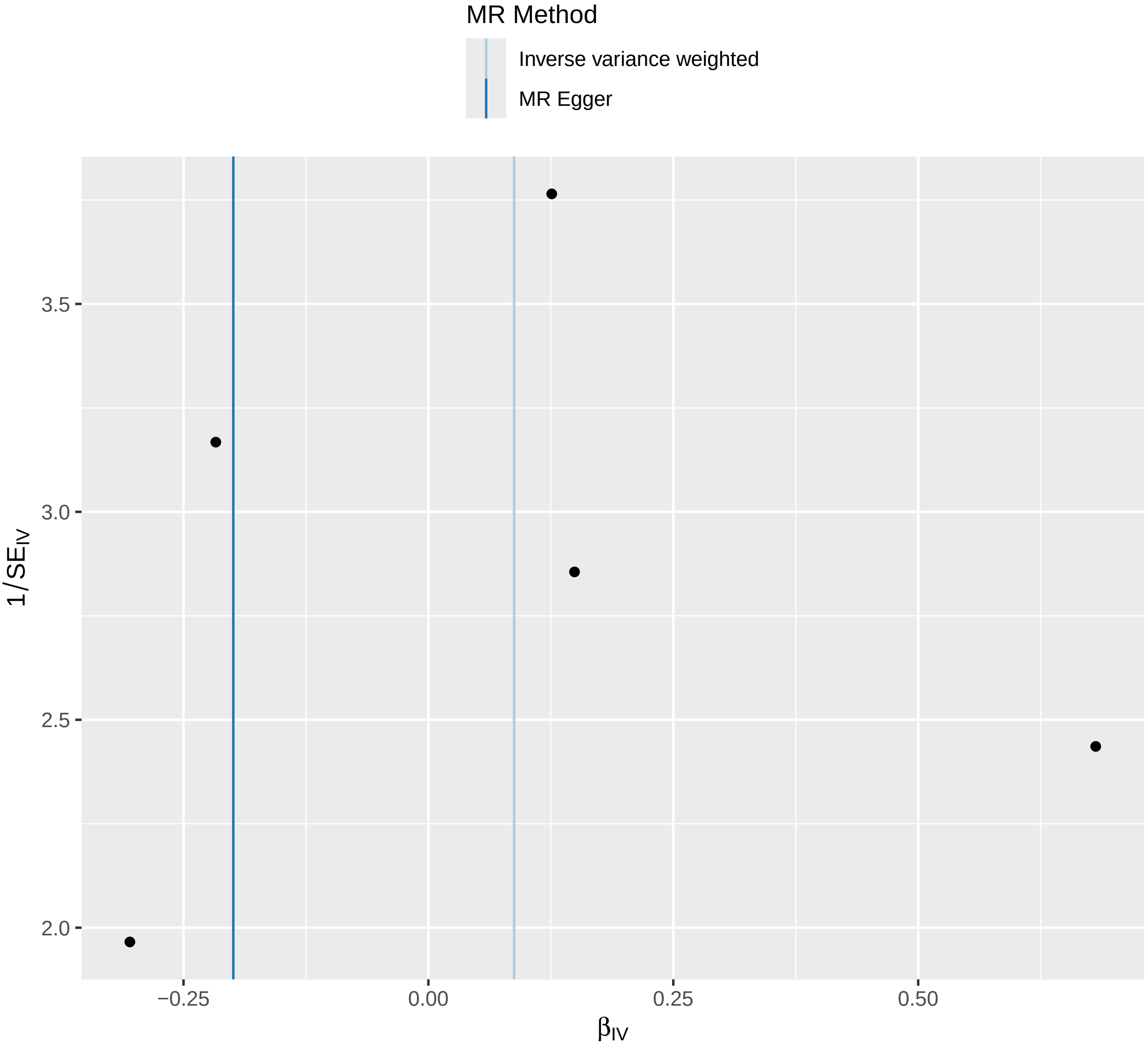
MR Method
Inverse variance weighted
MR Egger
3.5
3.0
2.5
2.0
1/SE$_{IV}$
−0.25
0.00
0.25
0.50
β$_{IV}$

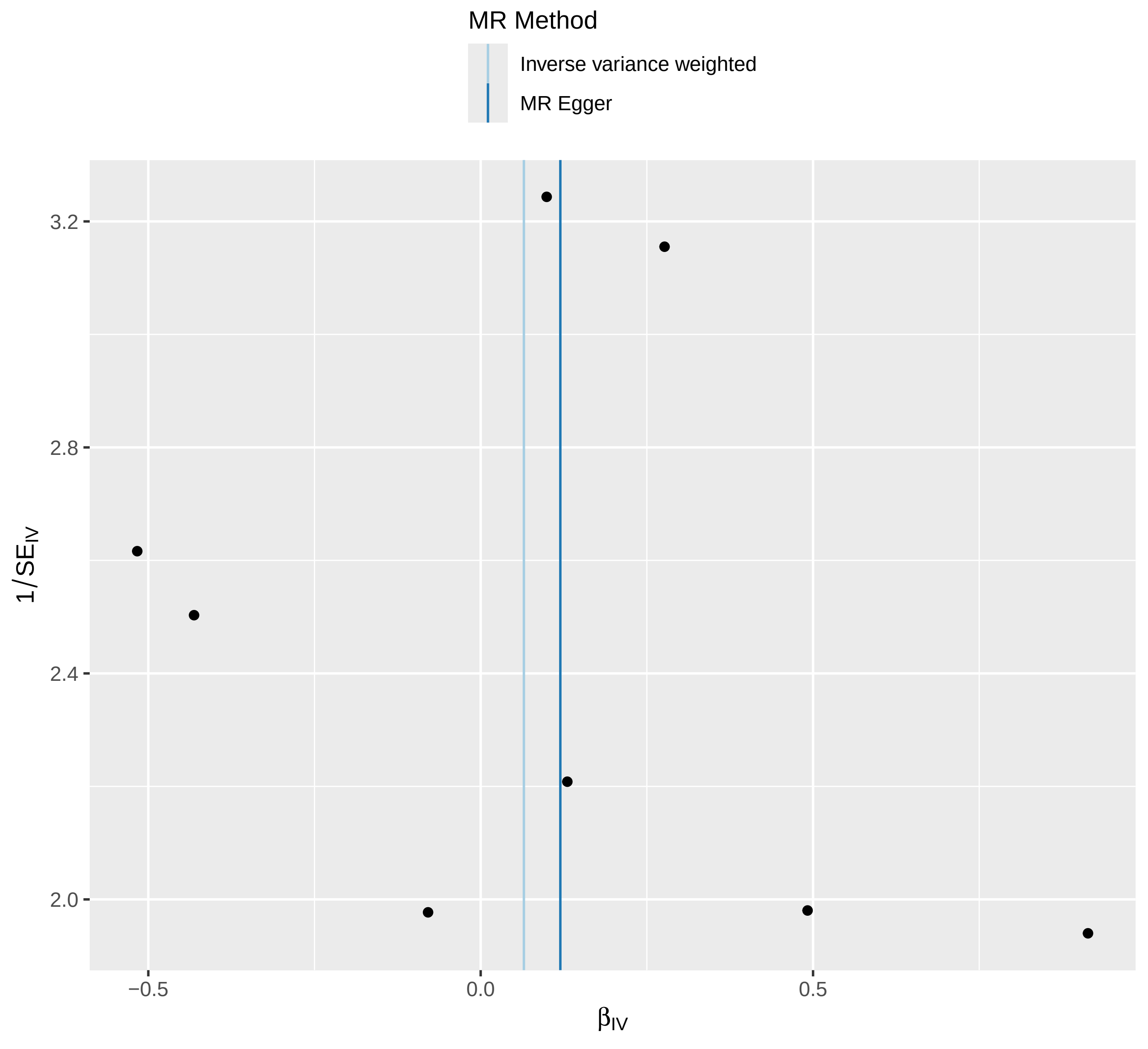
MR Method
Inverse variance weighted
MR Egger
3.2
2.8
2.4
2.0
1/SE_IV
−0.5
0.0
0.5
β_IV

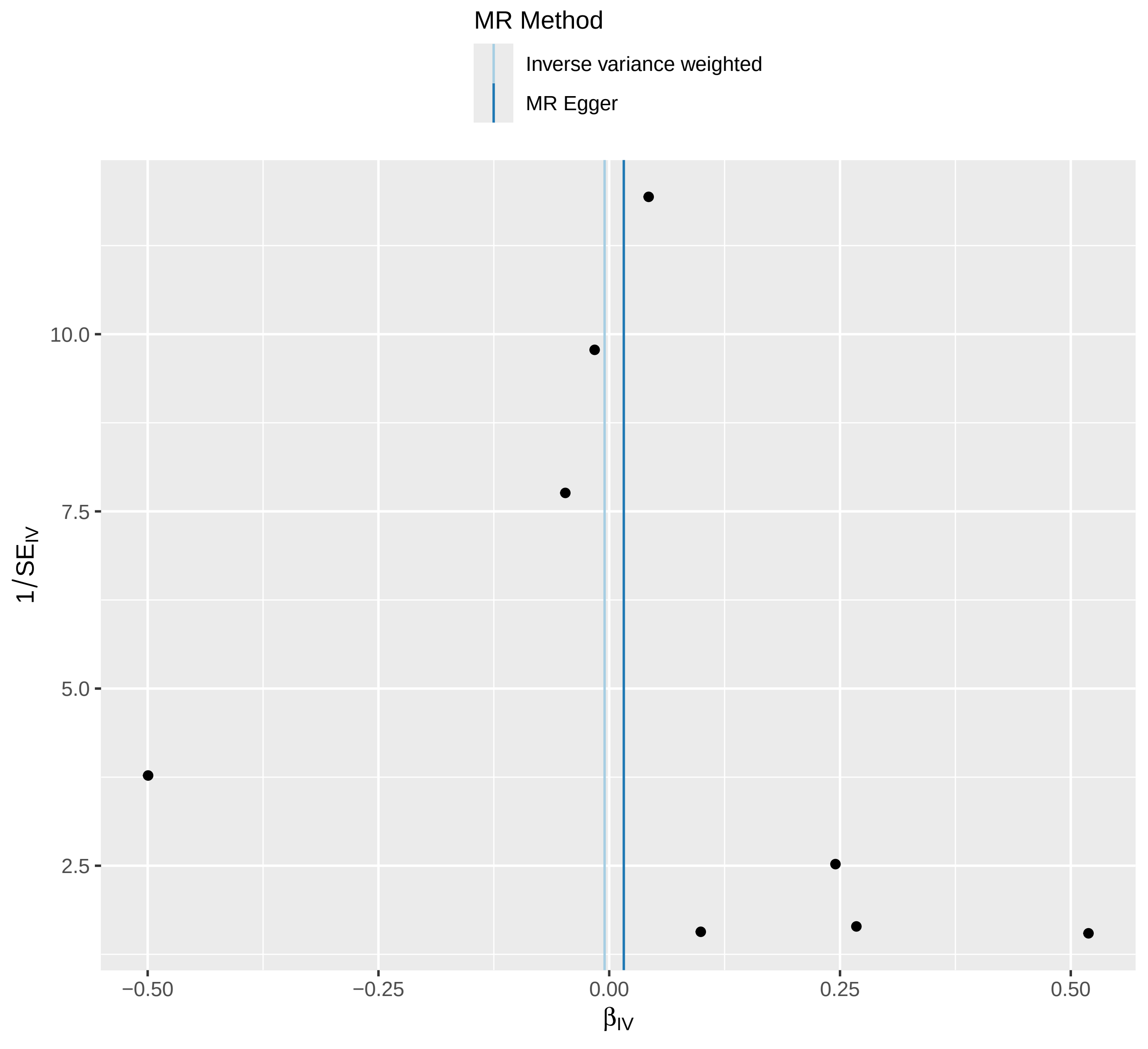

MR Method
Inverse variance weighted
MR Egger
10.0
7.5
5.0
2.5
1/SE$_{IV}$
−0.50
−0.25
0.00
0.25
0.50
β$_{IV}$

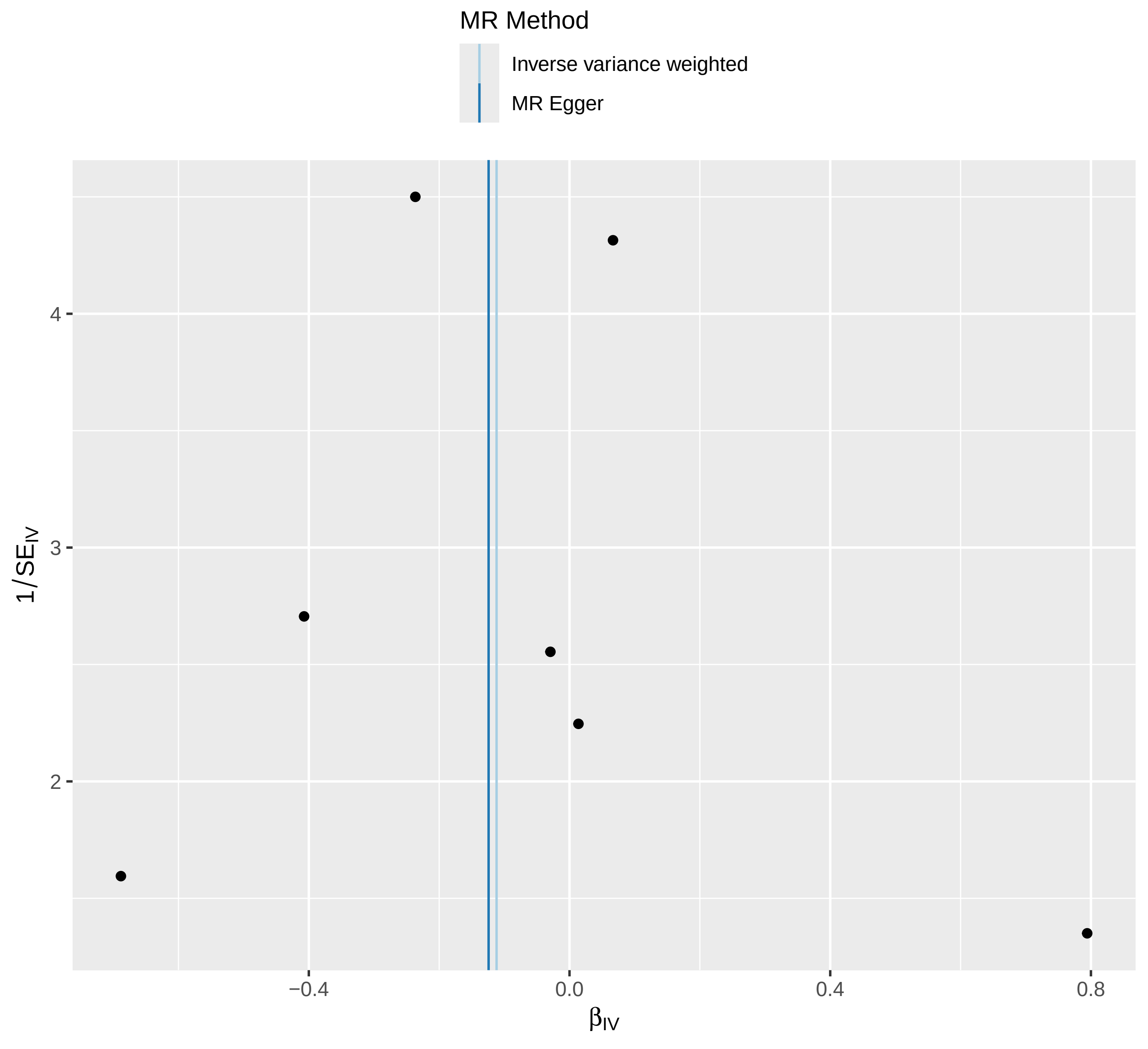

MR Method
Inverse variance weighted
MR Egger
4
3
2
$1/SE_{IV}$
−0.4
0.0
0.4
0.8
β_{IV}

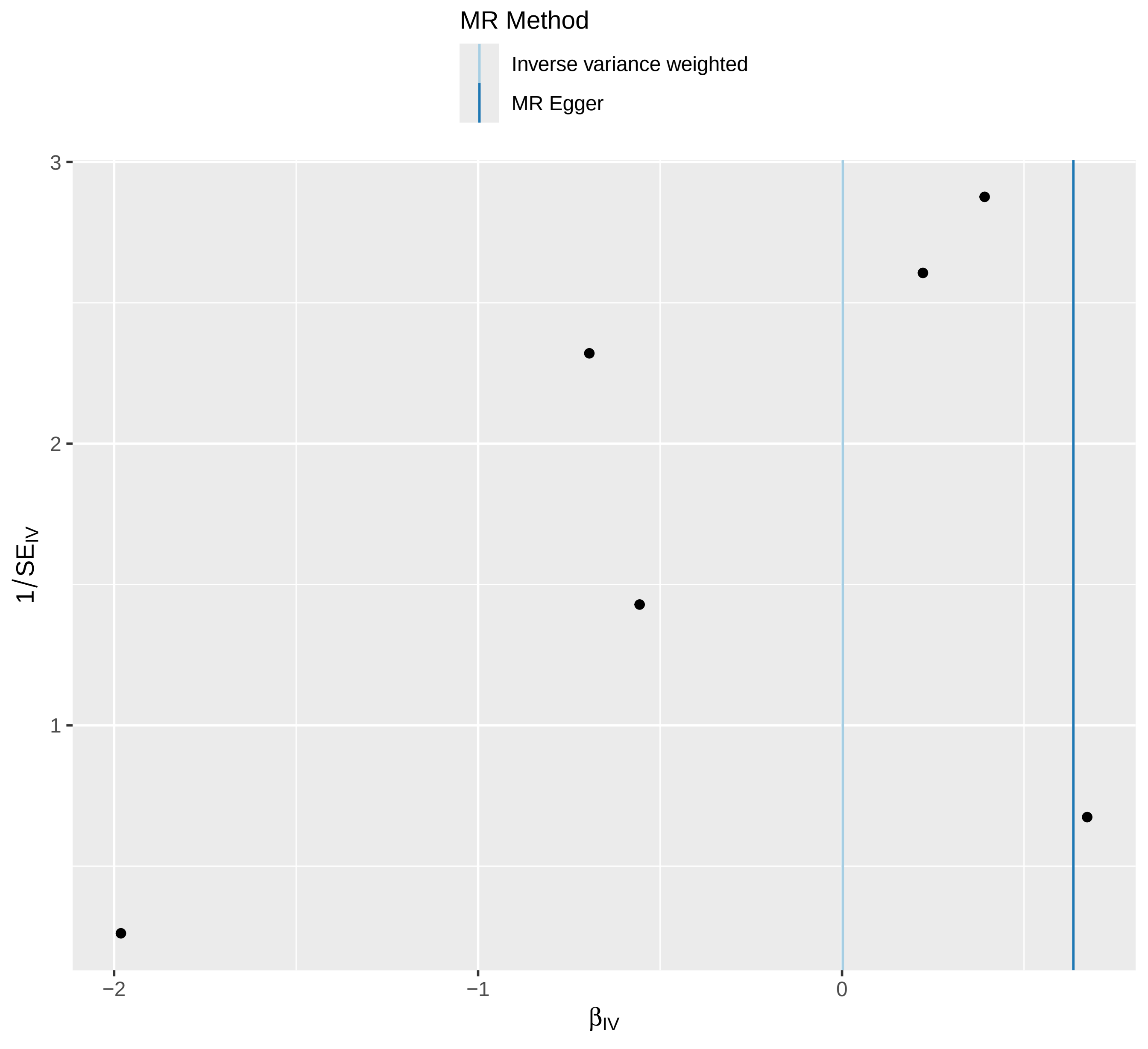
MR Method
Inverse variance weighted
MR Egger
3
2
1
1/SE$_{IV}$
−2
−1
0
β$_{IV}$

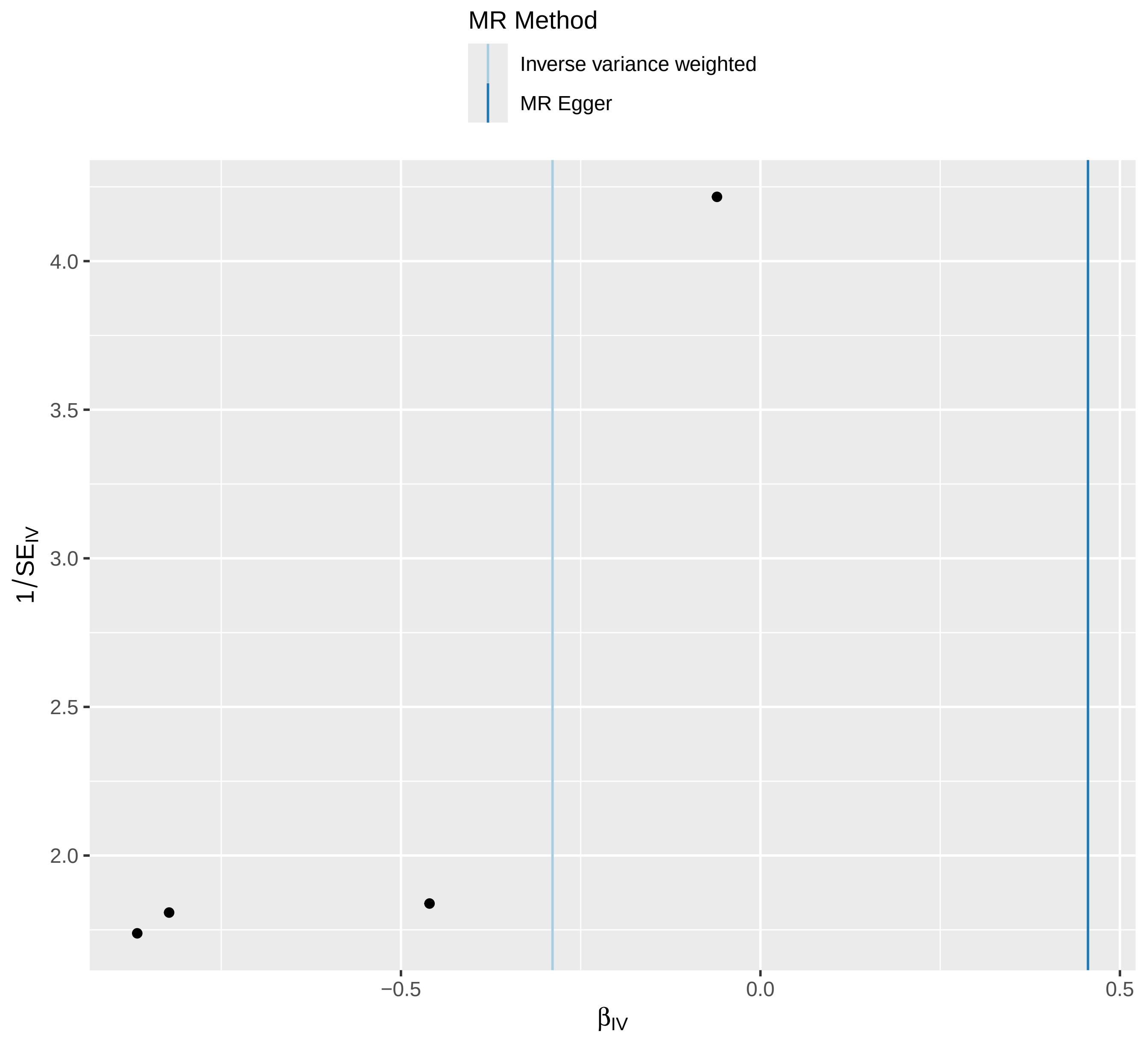
MR Method
Inverse variance weighted
MR Egger
1/SE_IV
4.0
3.5
3.0
2.5
2.0
−0.5
0.0
0.5
β_IV

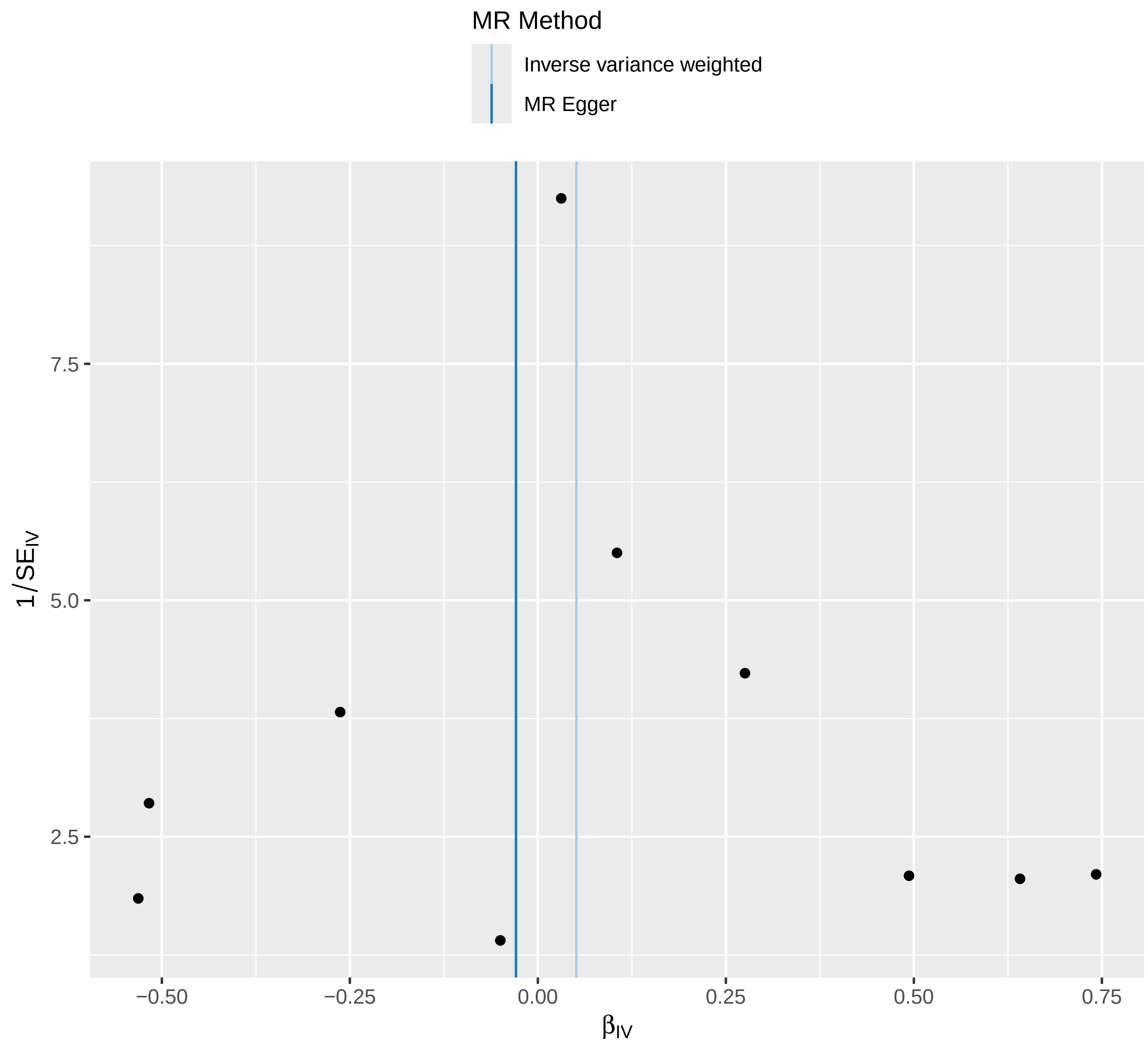
MR Method
Inverse variance weighted
MR Egger
7.5
5.0
2.5
1/SE_IV
−0.50
−0.25
0.00
0.25
0.50
0.75
β_IV

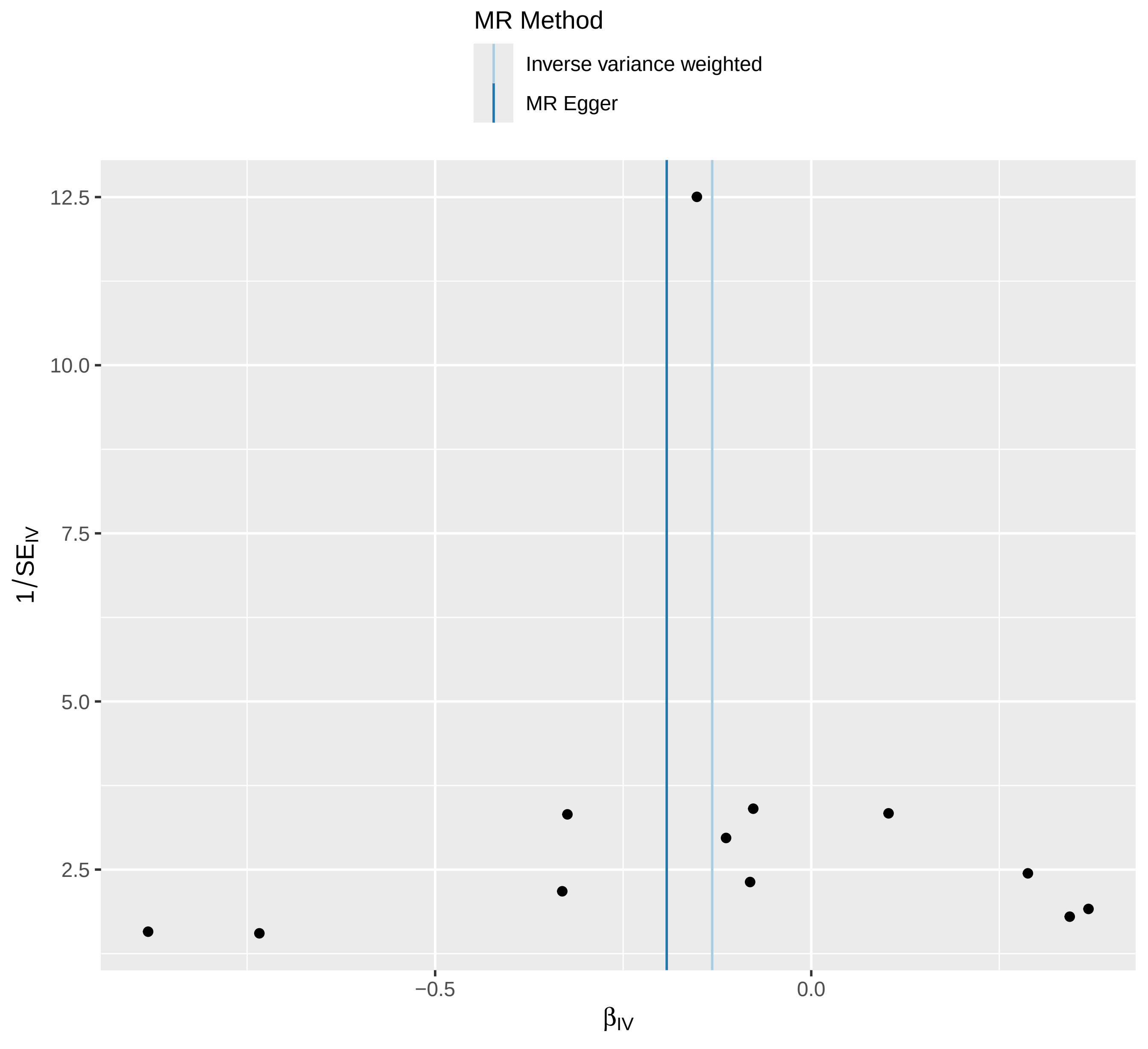
MR Method
Inverse variance weighted
MR Egger
12.5
10.0
7.5
5.0
2.5
1/SE_IV
−0.5
0.0
β_IV

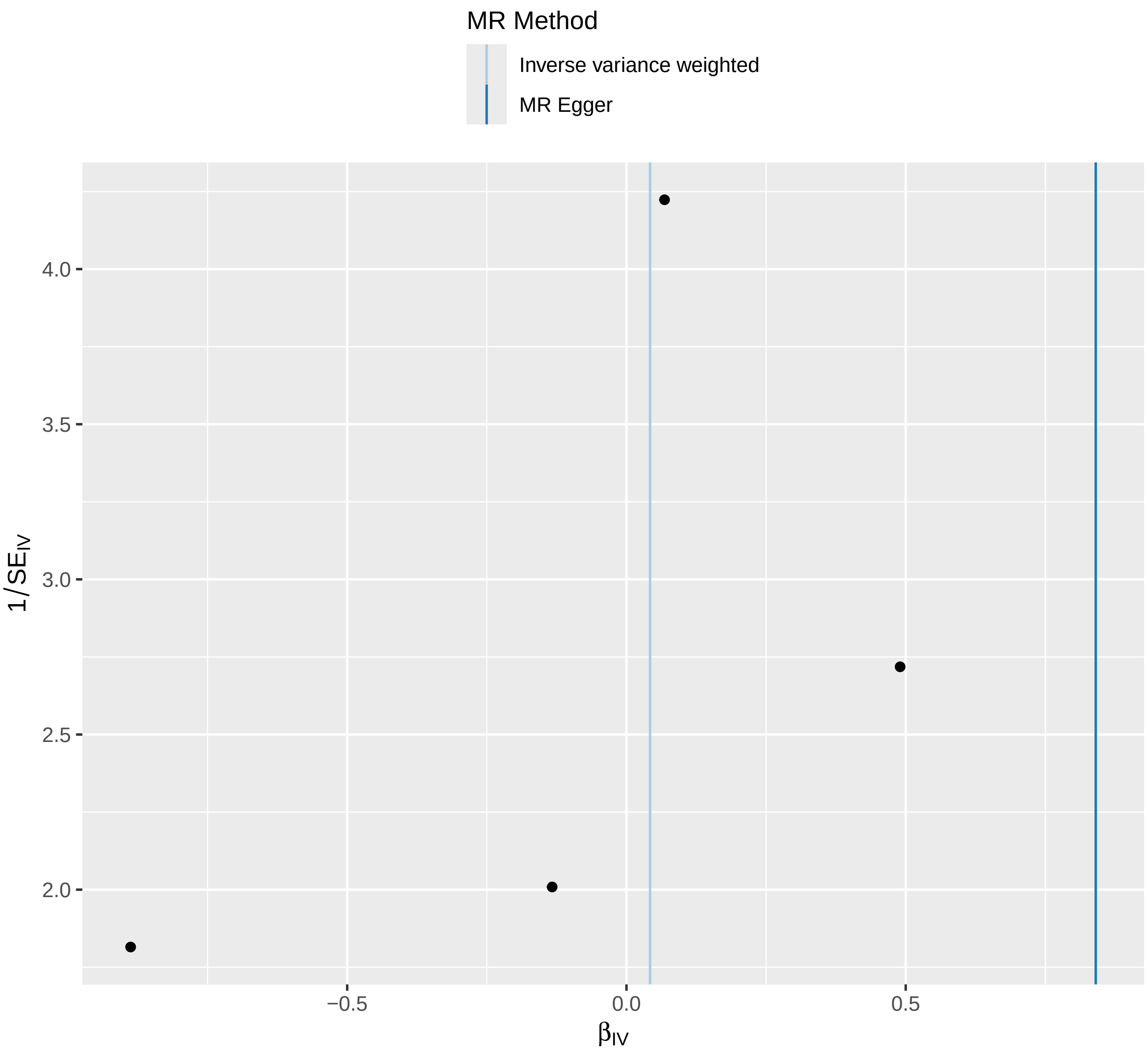
MR Method
Inverse variance weighted
MR Egger
4.0
3.5
3.0
2.5
2.0
1/SE_IV
−0.5
0.0
0.5
β_IV

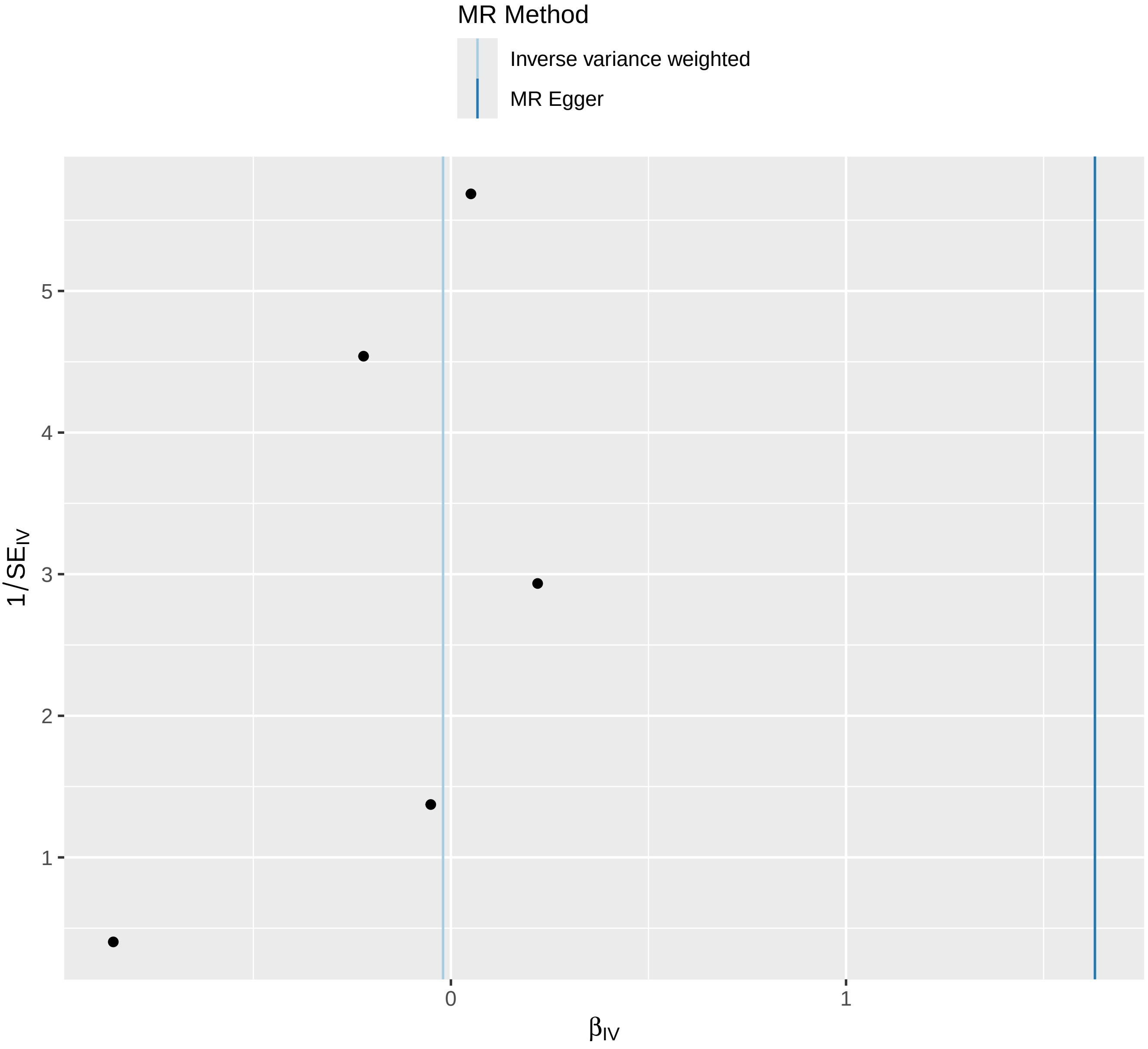

MR Method
Inverse variance weighted
MR Egger
1/SE_IV
5
4
3
2
1
0
1
β_IV

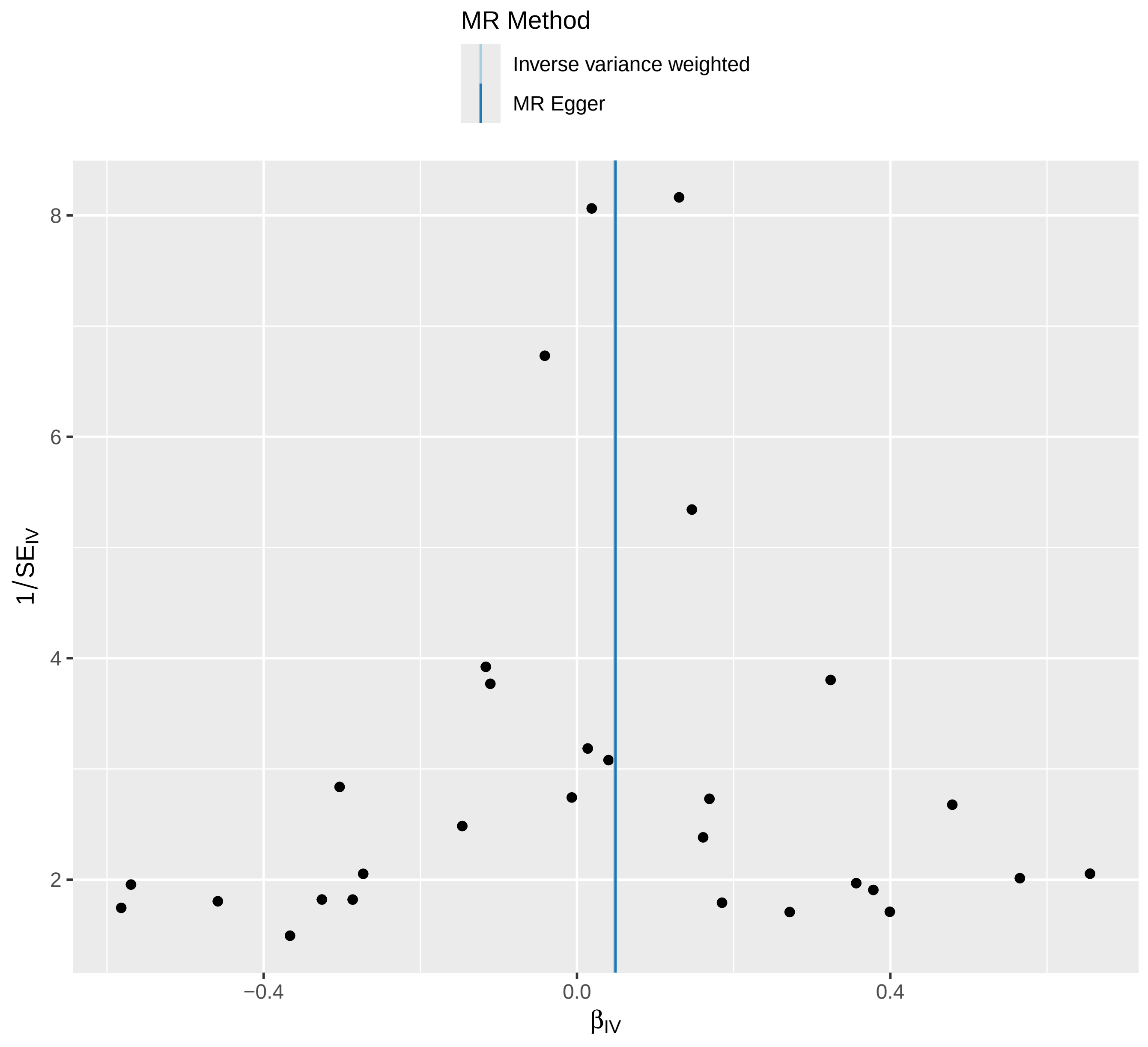
MR Method
Inverse variance weighted
MR Egger
1/SE$_{IV}$
β$_{IV}$
8
6
4
2
−0.4
0.0
0.4

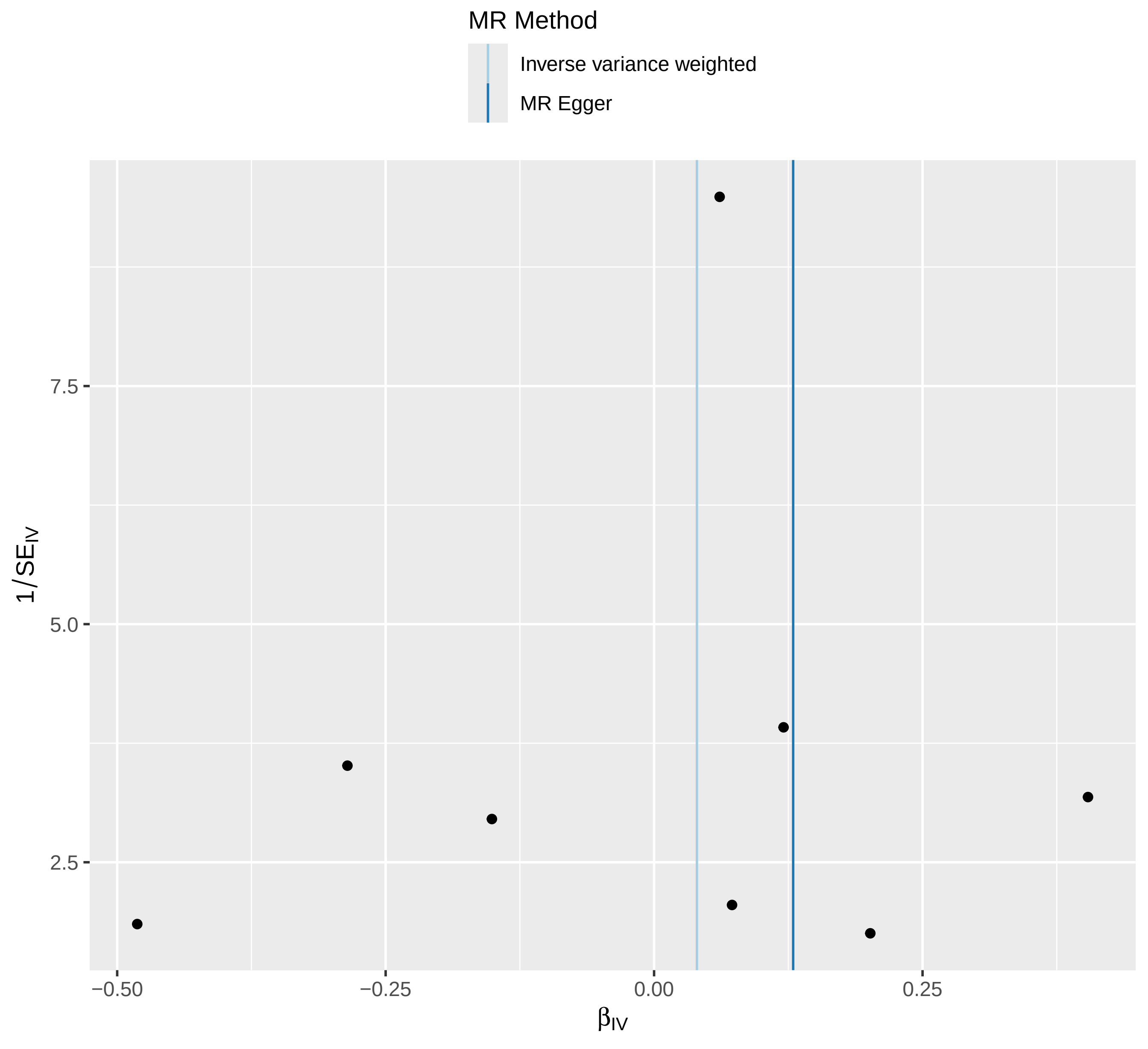

MR Method
Inverse variance weighted
MR Egger
7.5
5.0
2.5
$1/SE_{IV}$
−0.50
−0.25
0.00
0.25
β_{IV}

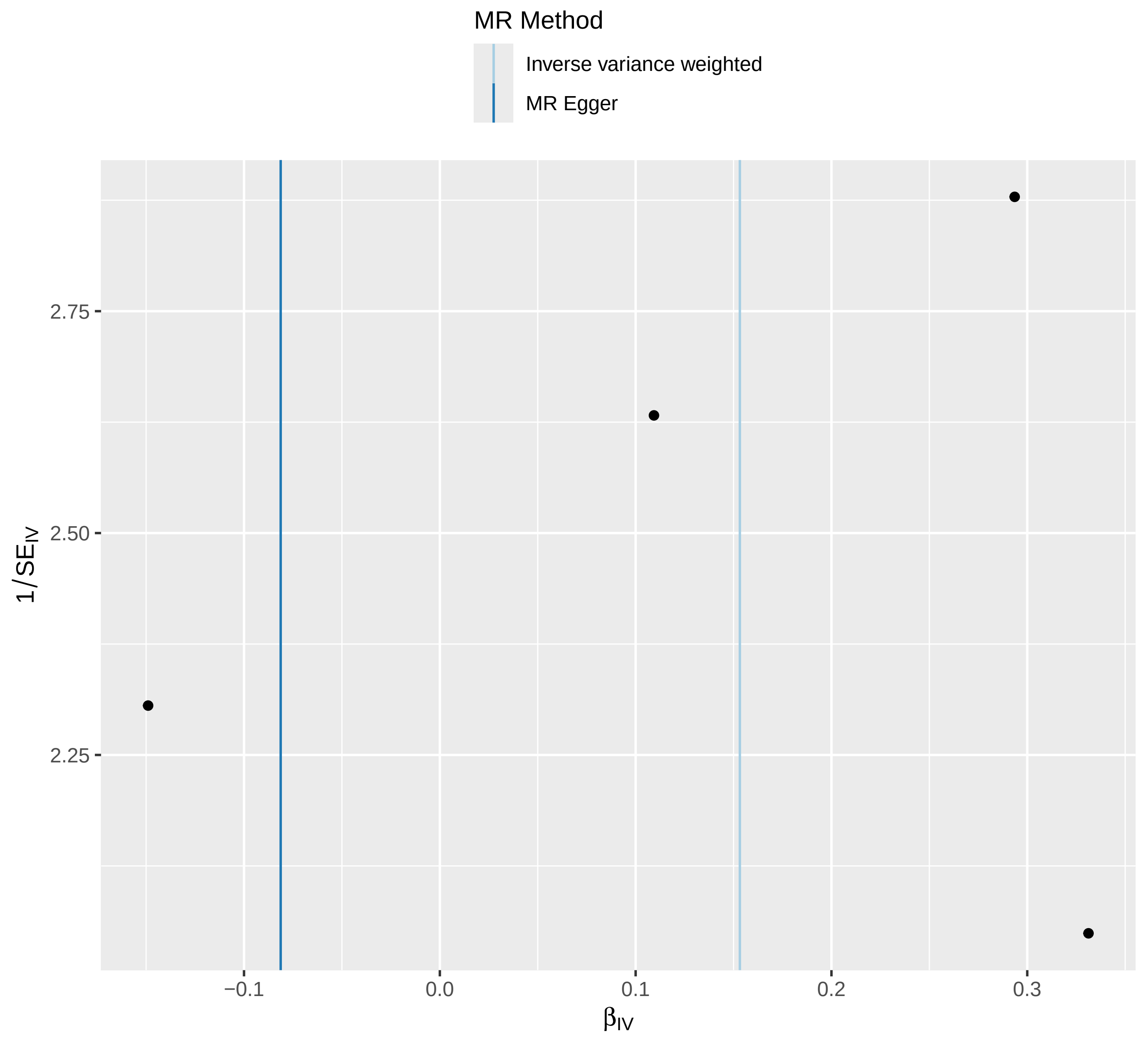

MR Method
Inverse variance weighted
MR Egger
2.75
2.50
2.25
1/SE$_{IV}$
−0.1
0.0
0.1
0.2
0.3
β$_{IV}$

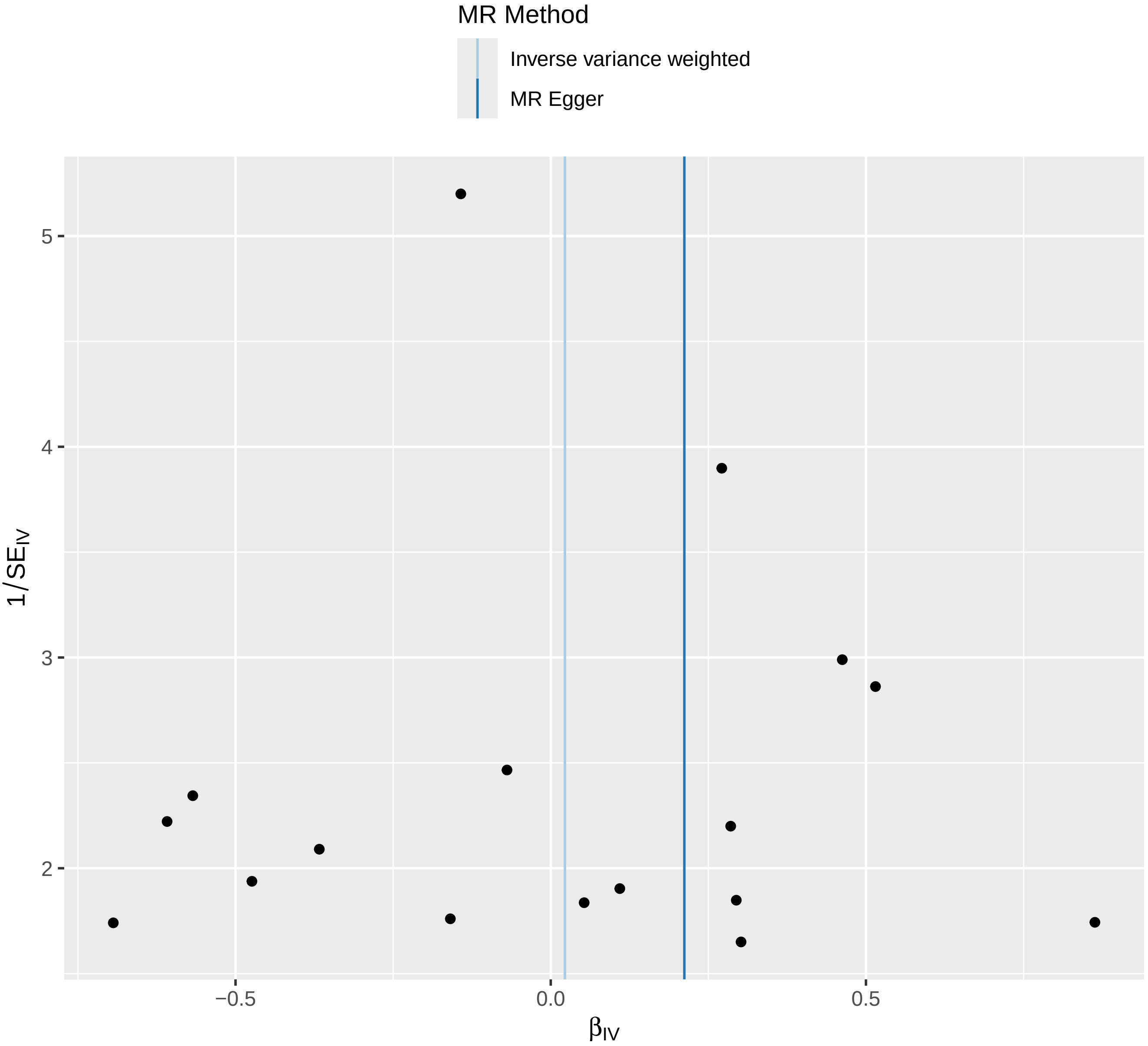

MR Method
Inverse variance weighted
MR Egger
5
4
3
2
1/SE_IV
−0.5
0.0
0.5
β_IV

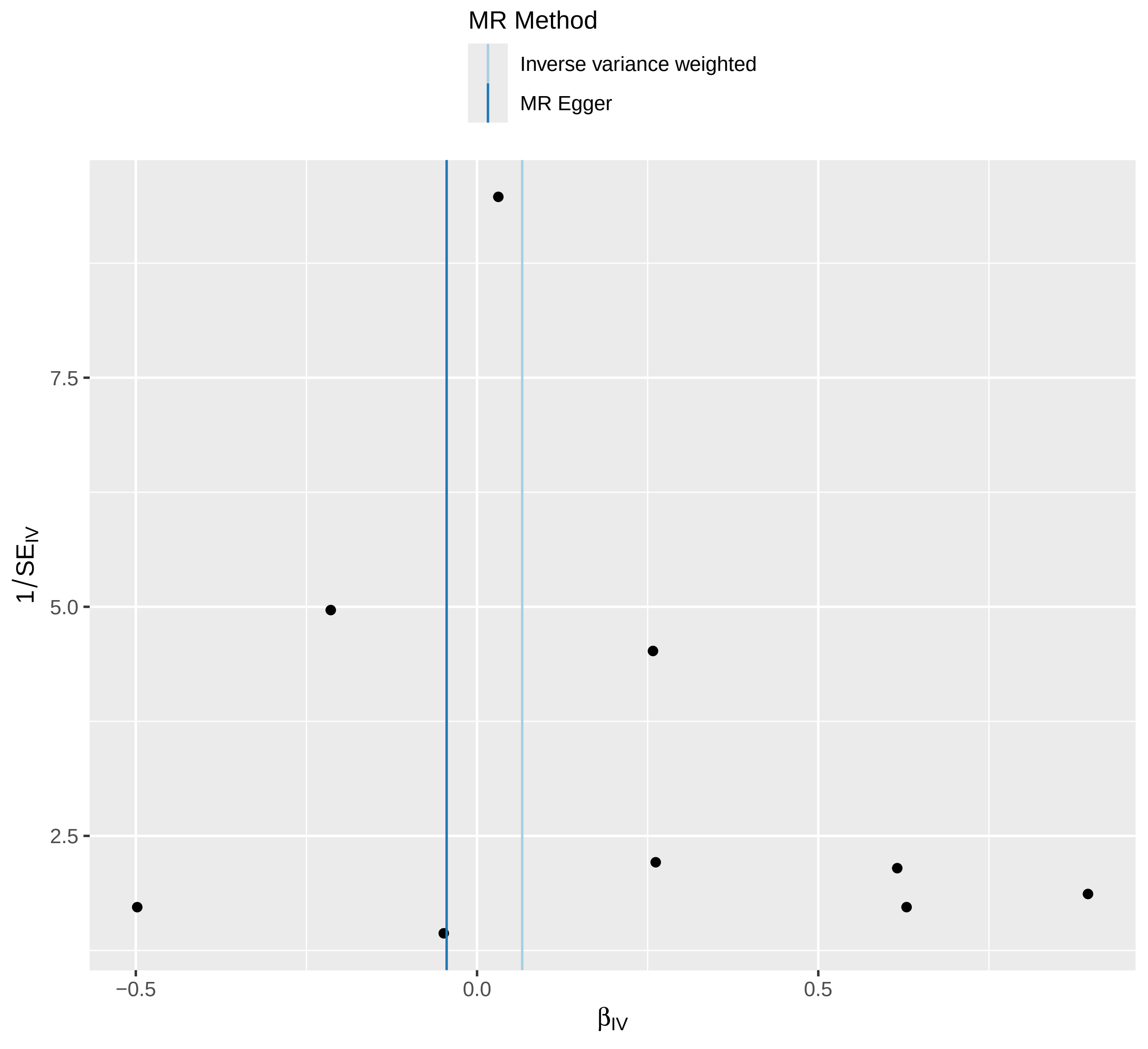

MR Method
Inverse variance weighted
MR Egger
7.5
5.0
2.5
1/SE_IV
−0.5
0.0
0.5
β_IV

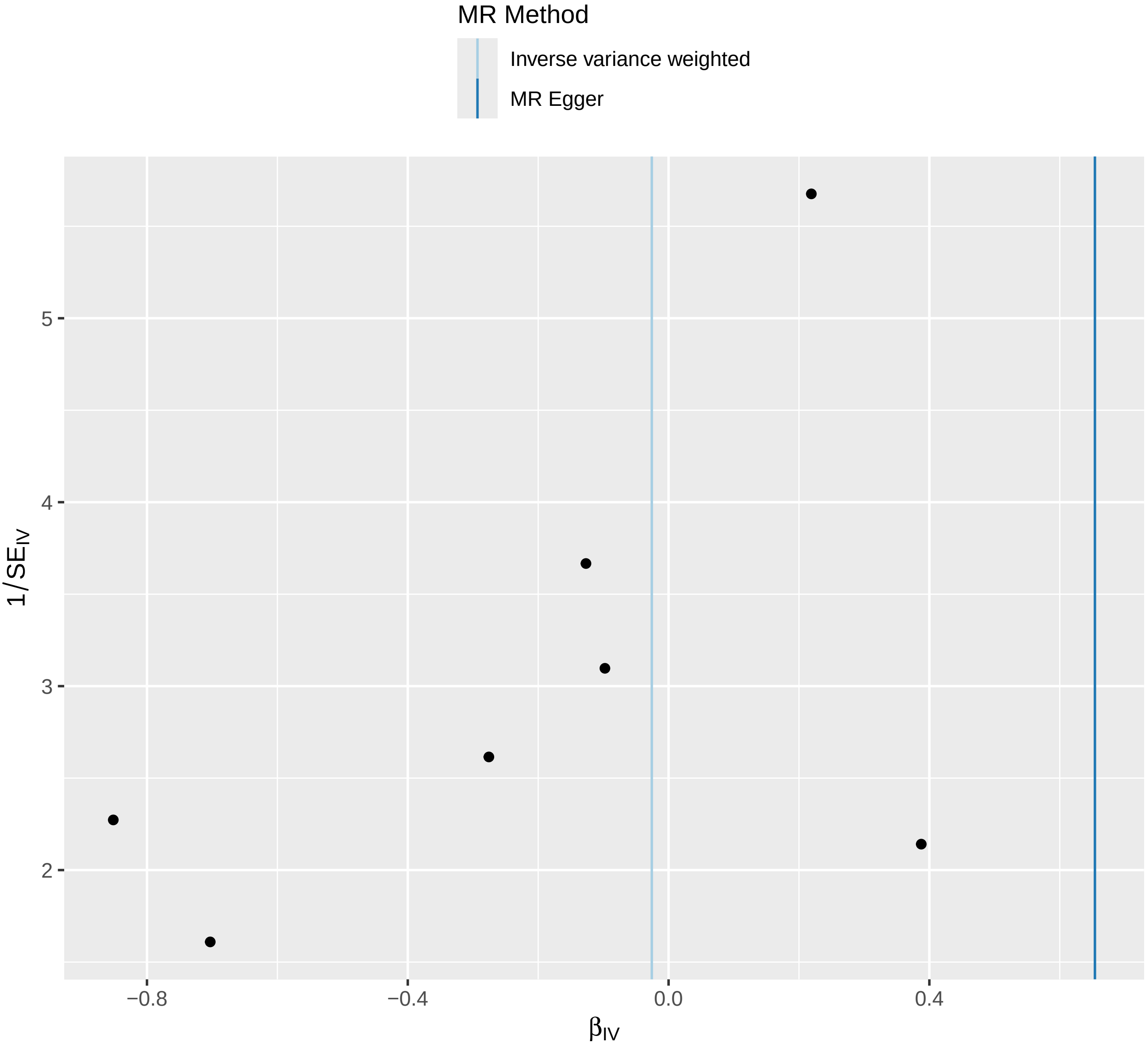
MR Method
Inverse variance weighted
MR Egger
1/SE_IV
β_IV
−0.8
−0.4
0.0
0.4
2
3
4
5

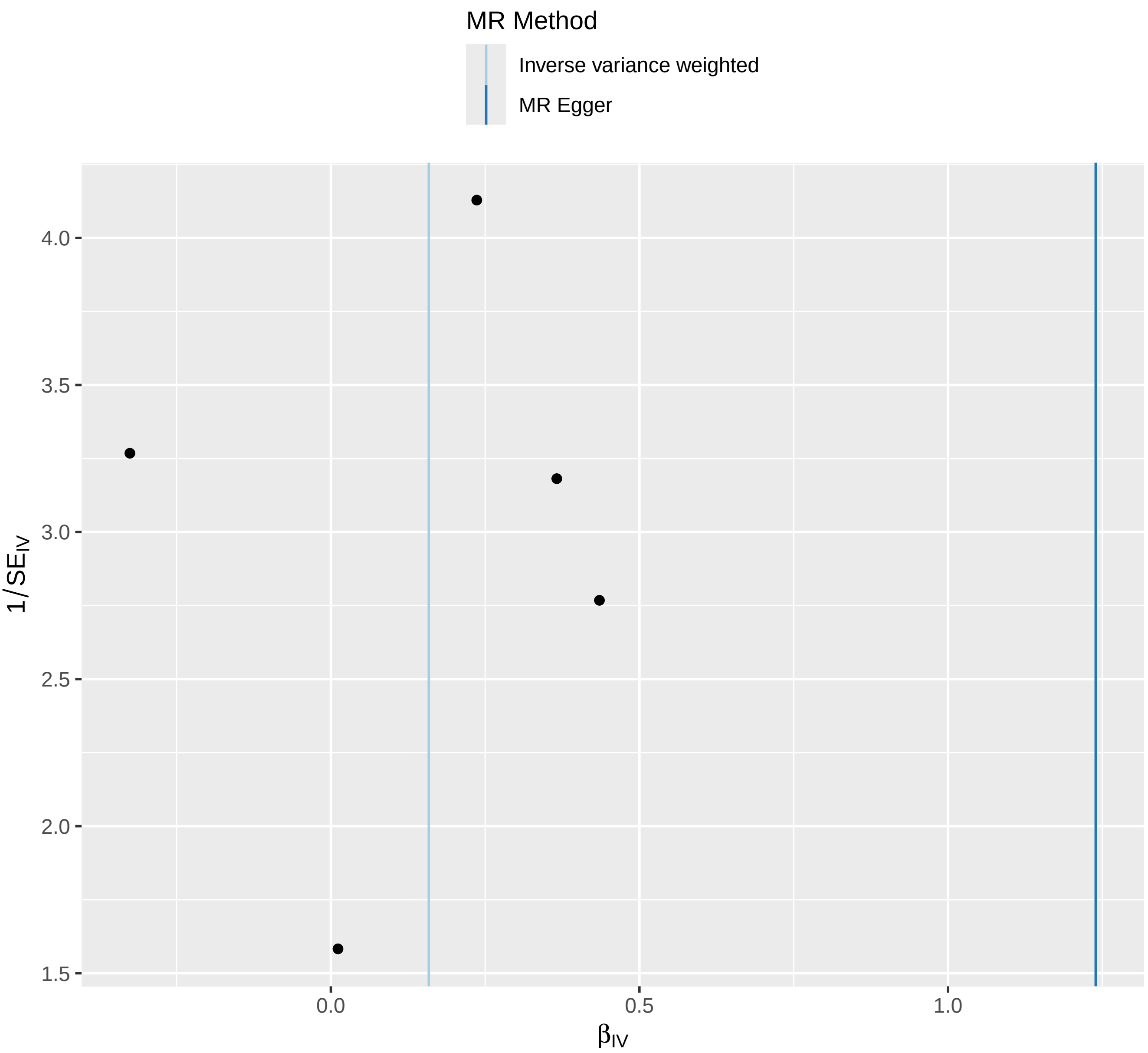
MR Method
Inverse variance weighted
MR Egger
1/SE_IV
4.0
3.5
3.0
2.5
2.0
1.5
0.0
0.5
1.0
β_IV

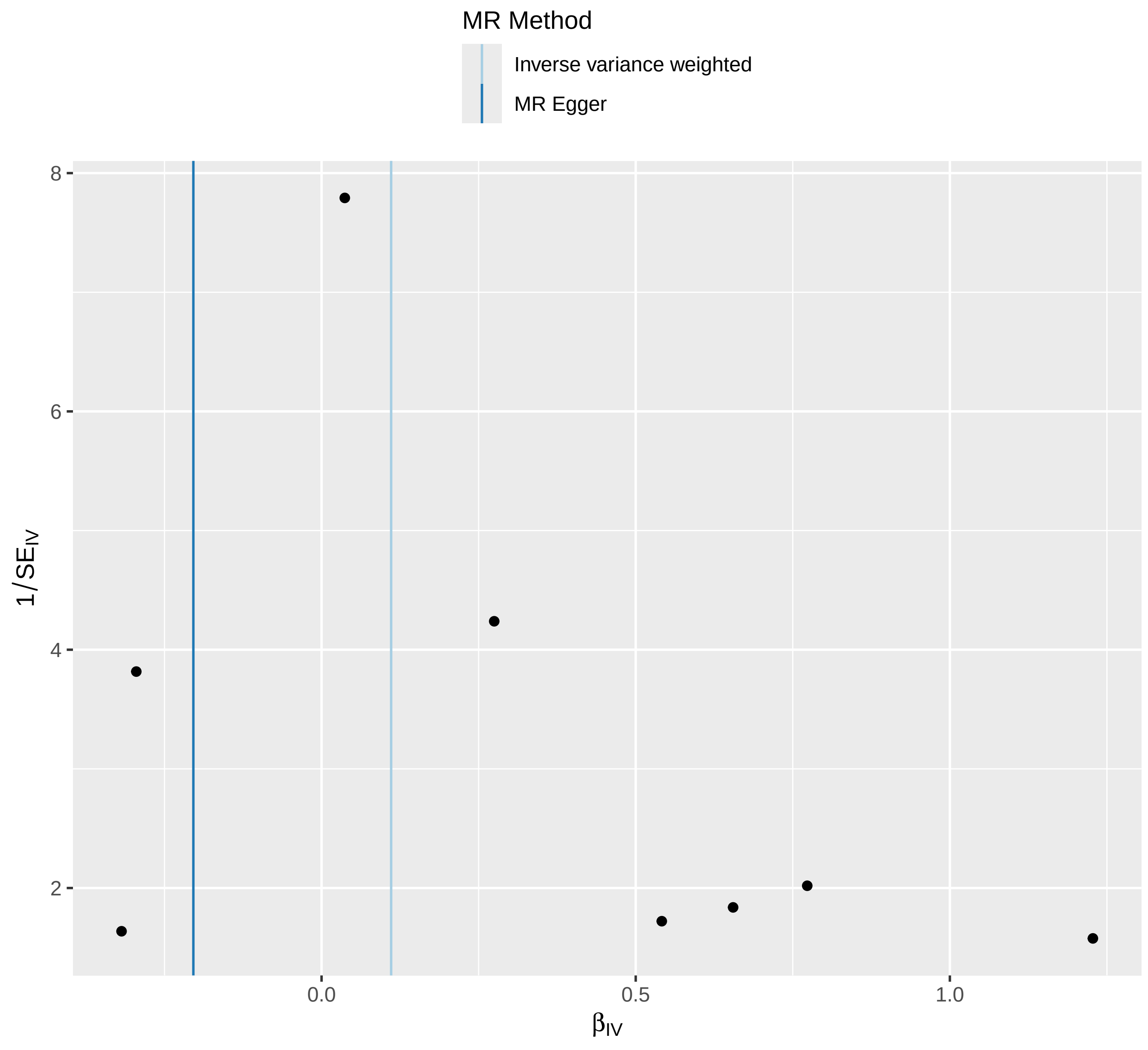

MR Method
Inverse variance weighted
MR Egger
8
6
4
2
1/SE_IV
0.0
0.5
1.0
β_IV

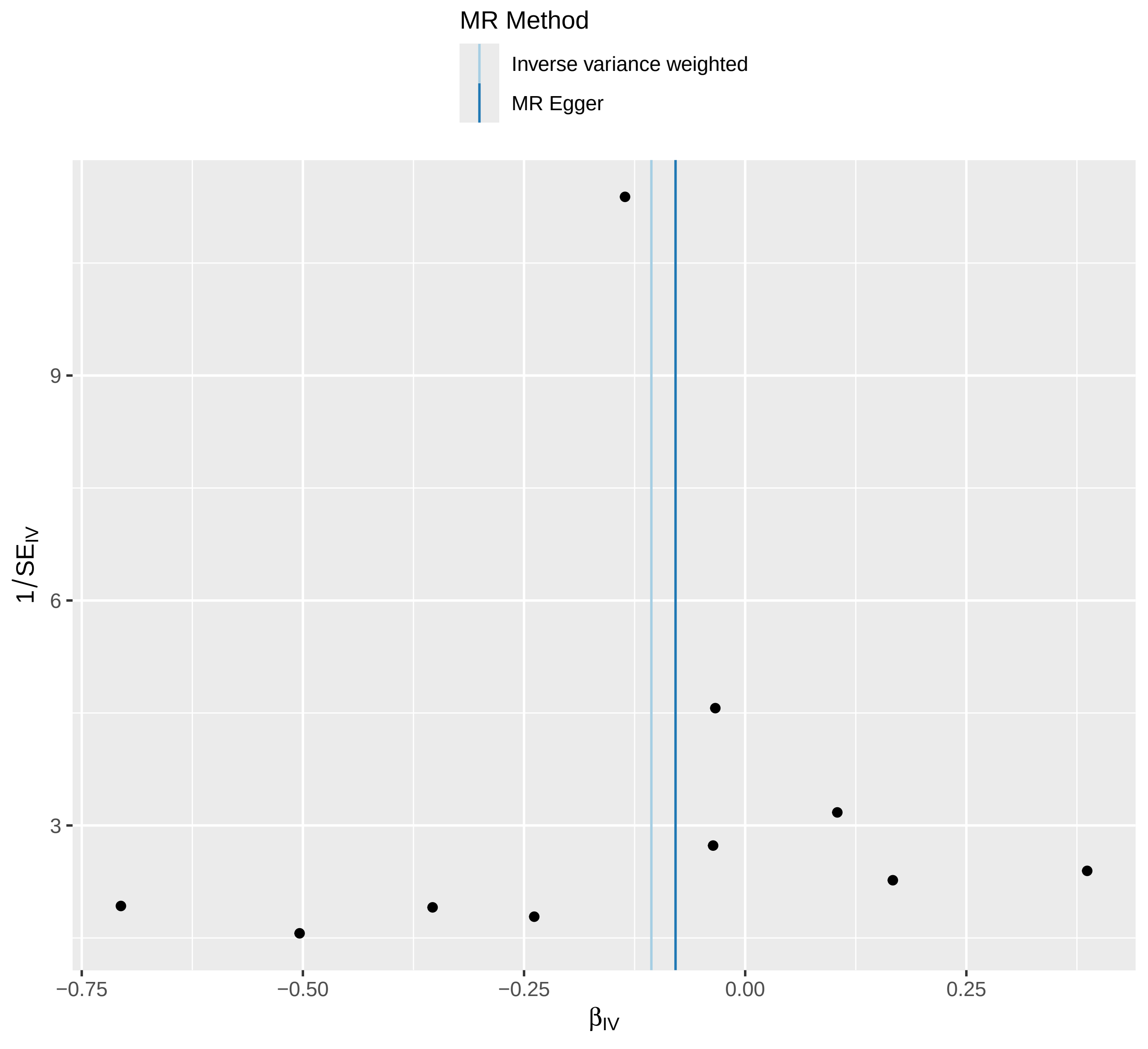

MR Method
Inverse variance weighted
MR Egger
1/SE_IV
9
6
3
−0.75
−0.50
−0.25
0.00
0.25
β_IV

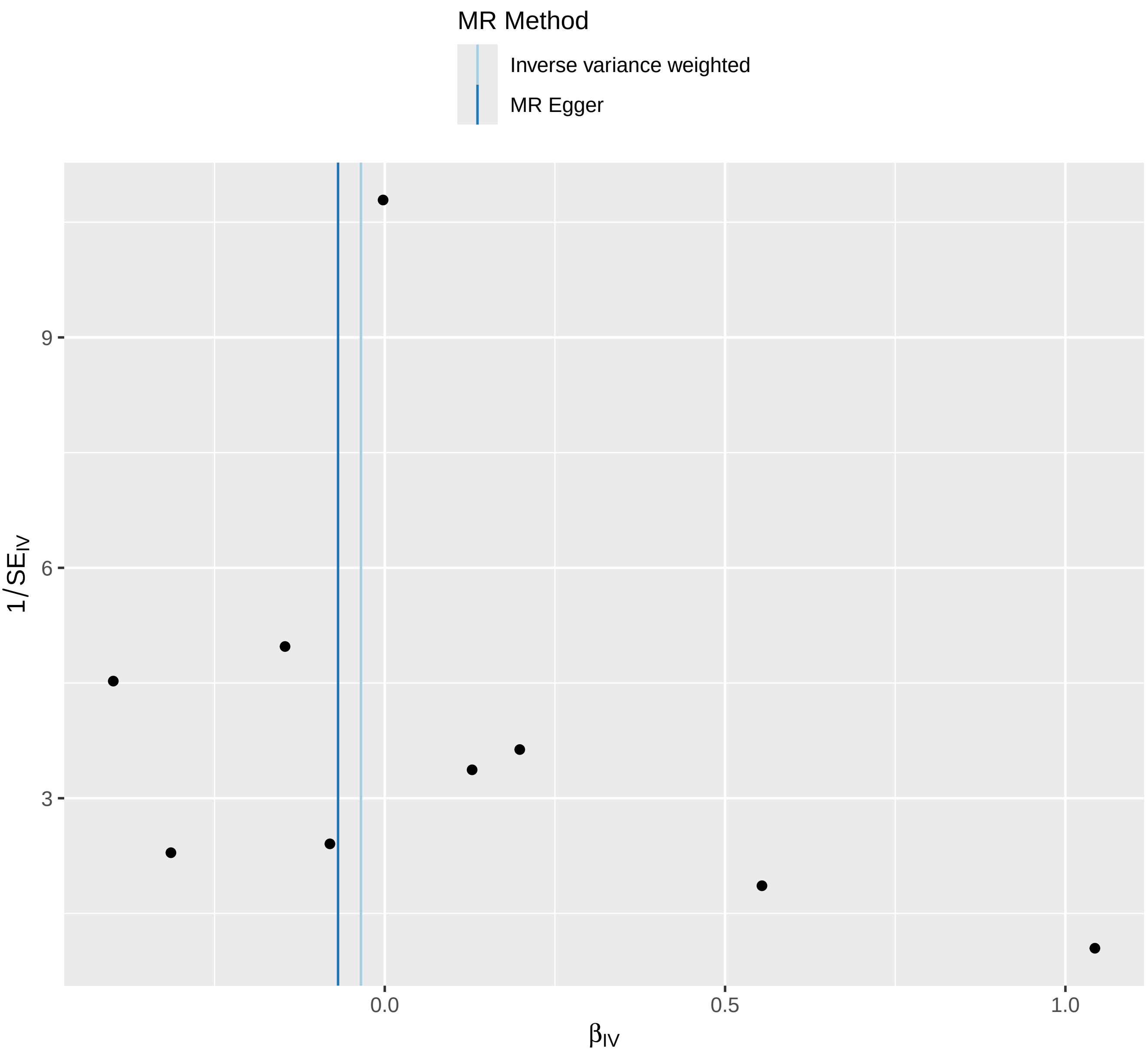
MR Method
Inverse variance weighted
MR Egger
9
6
3
1/SE_IV
0.0
0.5
1.0
β_IV

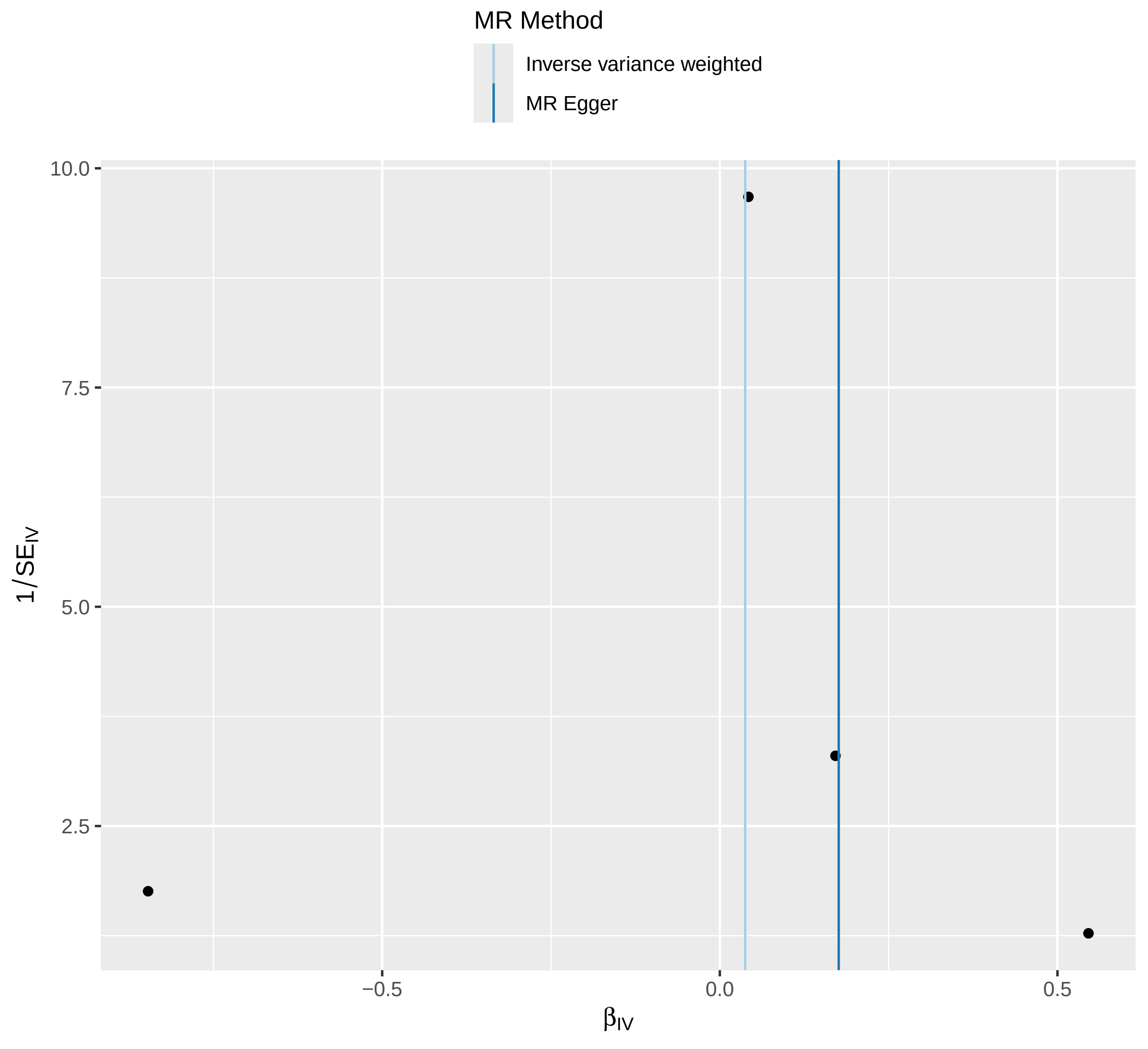

MR Method
Inverse variance weighted
MR Egger
10.0
7.5
5.0
2.5
1/SE$_{IV}$
−0.5
0.0
0.5
β$_{IV}$

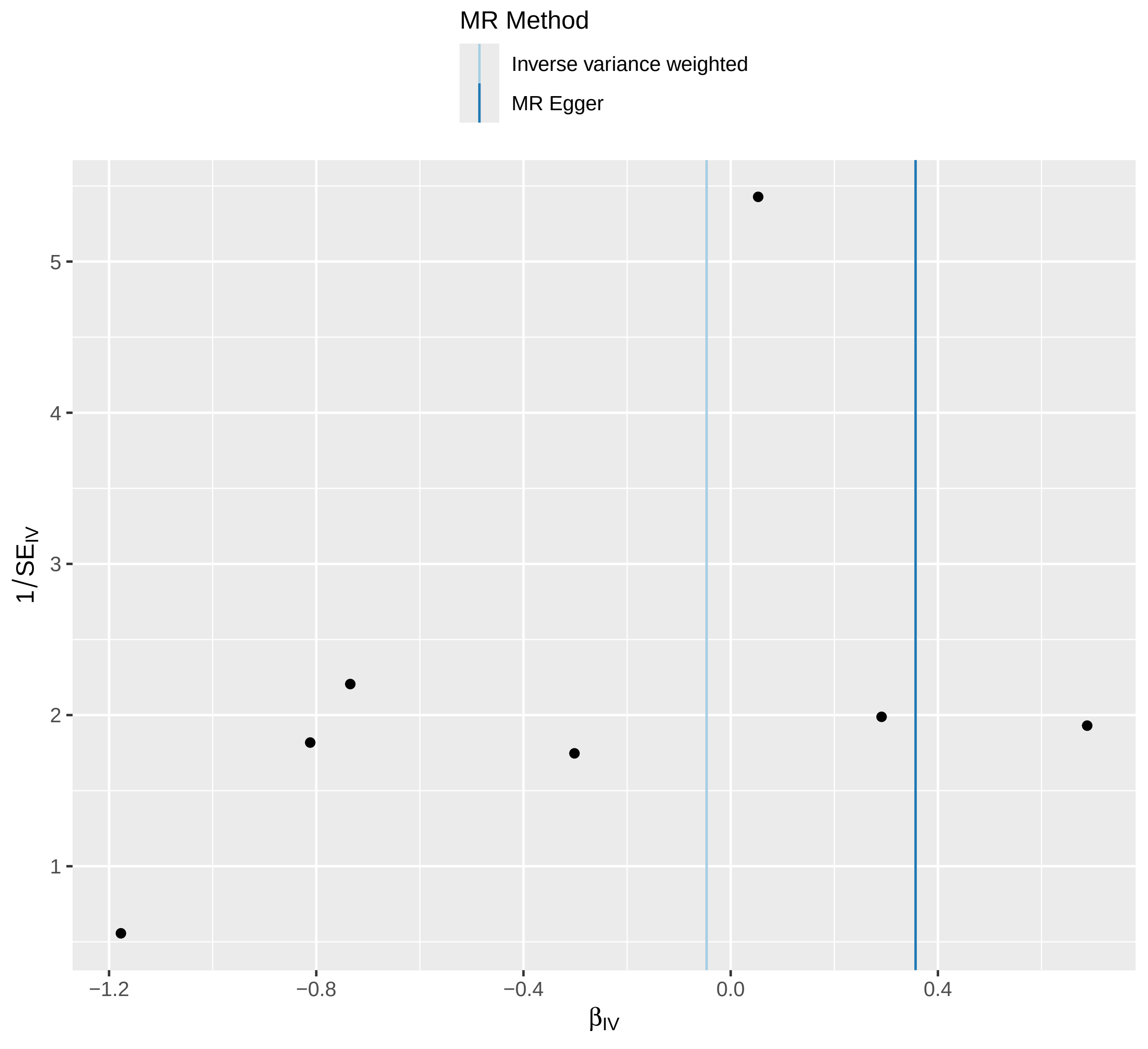

MR Method
Inverse variance weighted
MR Egger
1/SE$_{IV}$
β$_{IV}$
5
4
3
2
1
−1.2
−0.8
−0.4
0.0
0.4

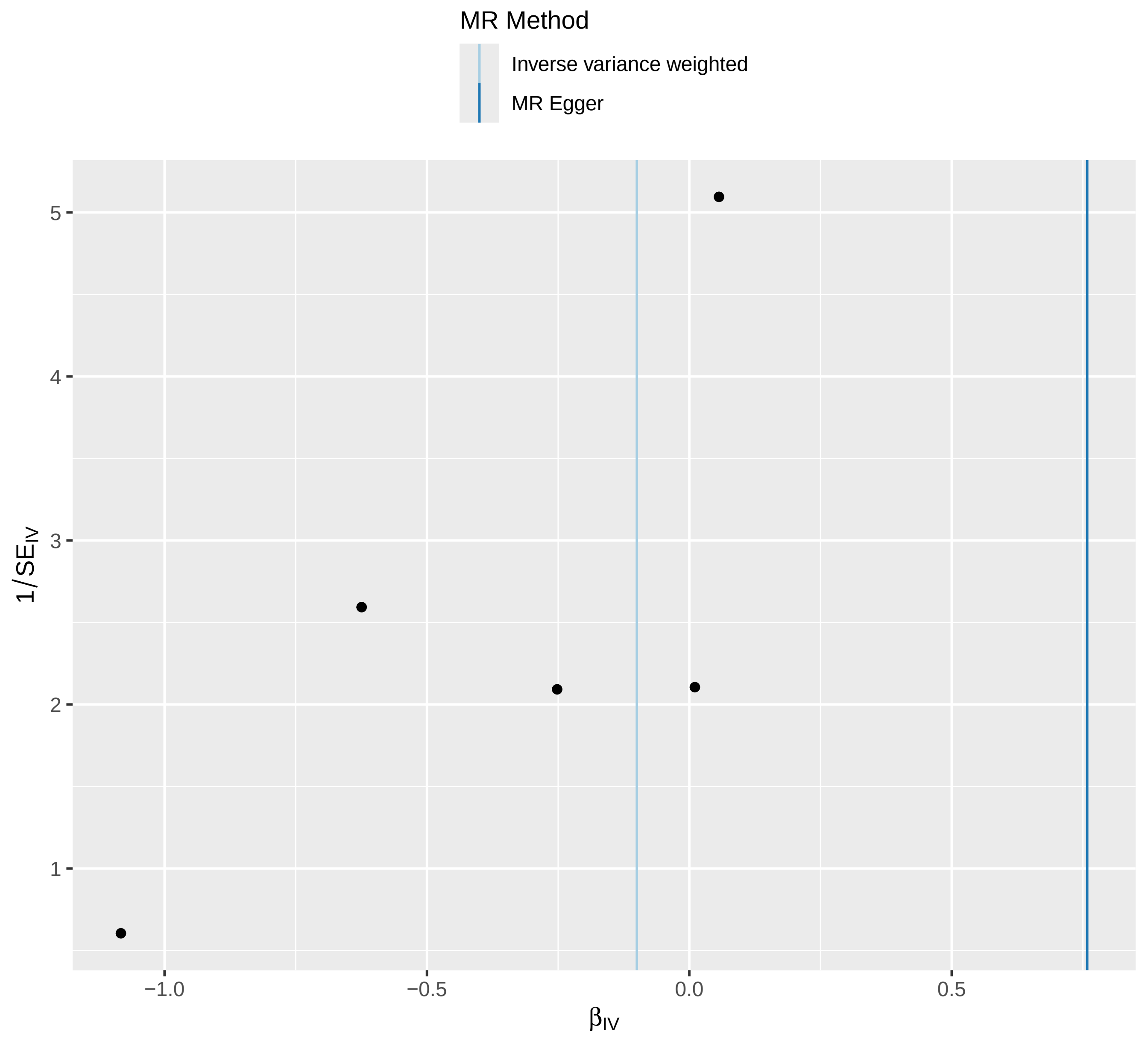
MR Method
Inverse variance weighted
MR Egger
1/SE_{IV}
β_{IV}
5
4
3
2
1
−1.0
−0.5
0.0
0.5

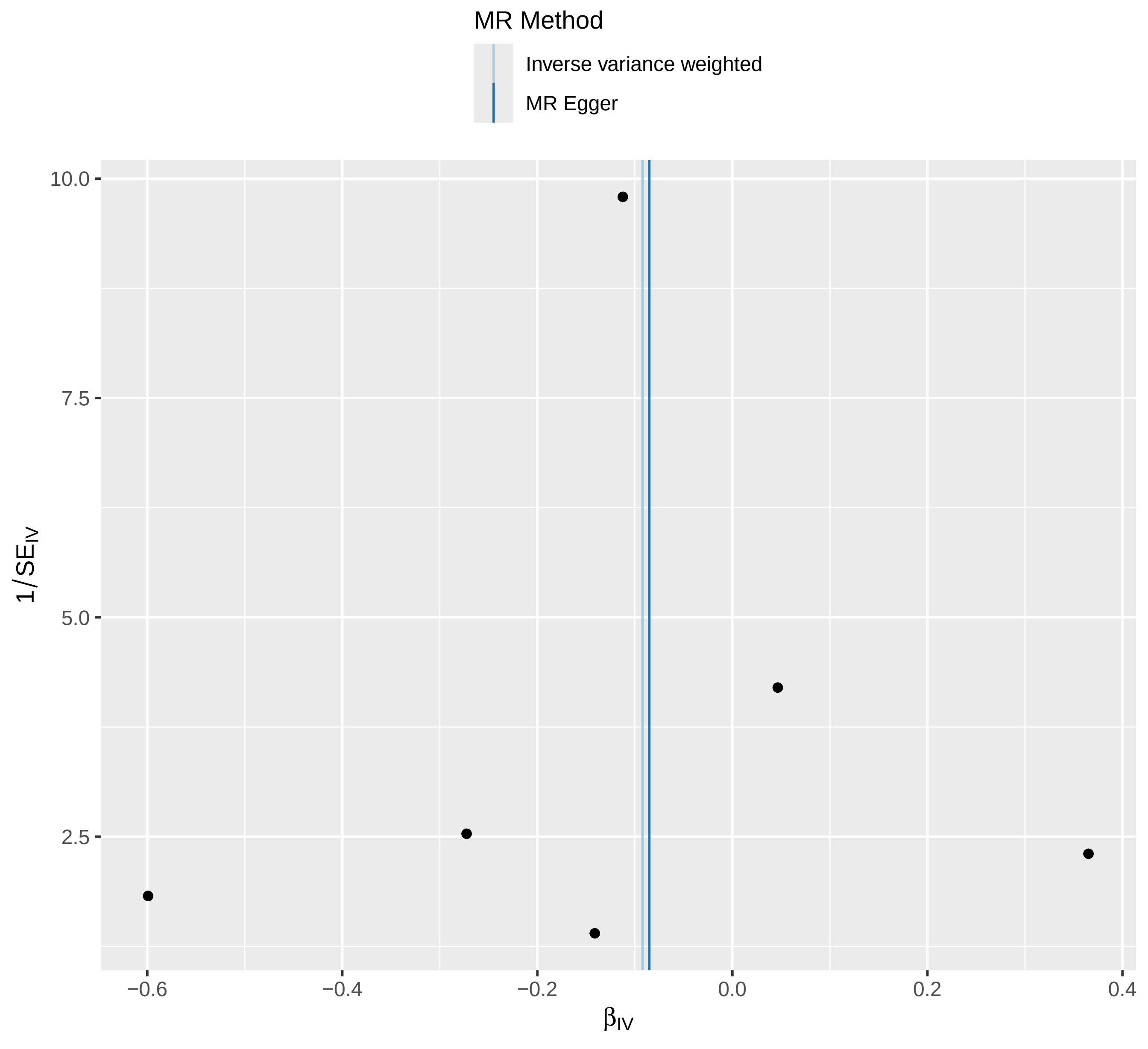
MR Method
Inverse variance weighted
MR Egger
1/SE_IV
10.0
7.5
5.0
2.5
−0.6
−0.4
−0.2
0.0
0.2
0.4
β_IV

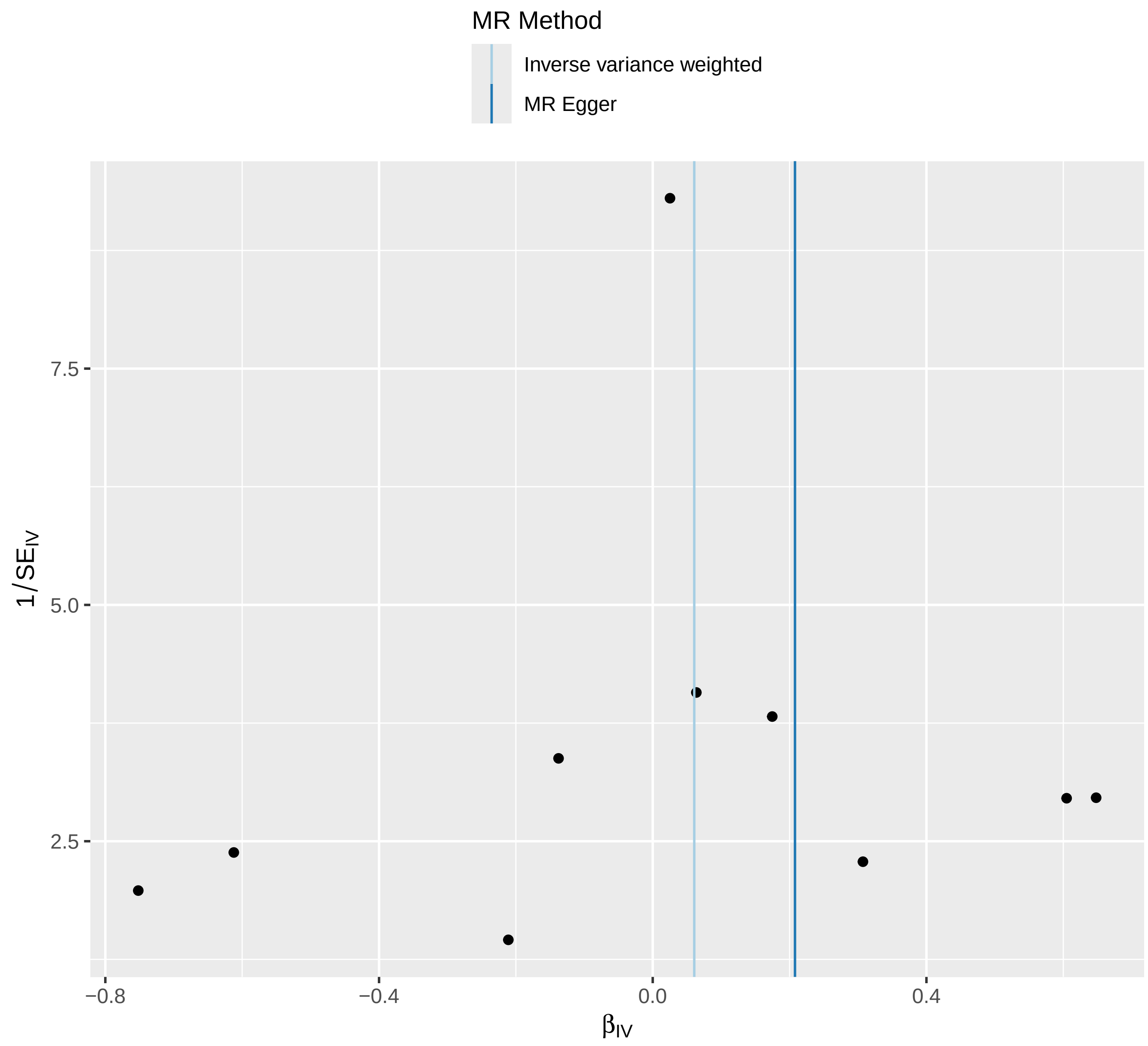

MR Method
Inverse variance weighted
MR Egger
7.5
5.0
2.5
$1/SE_{IV}$
−0.8
−0.4
0.0
0.4
β_{IV}

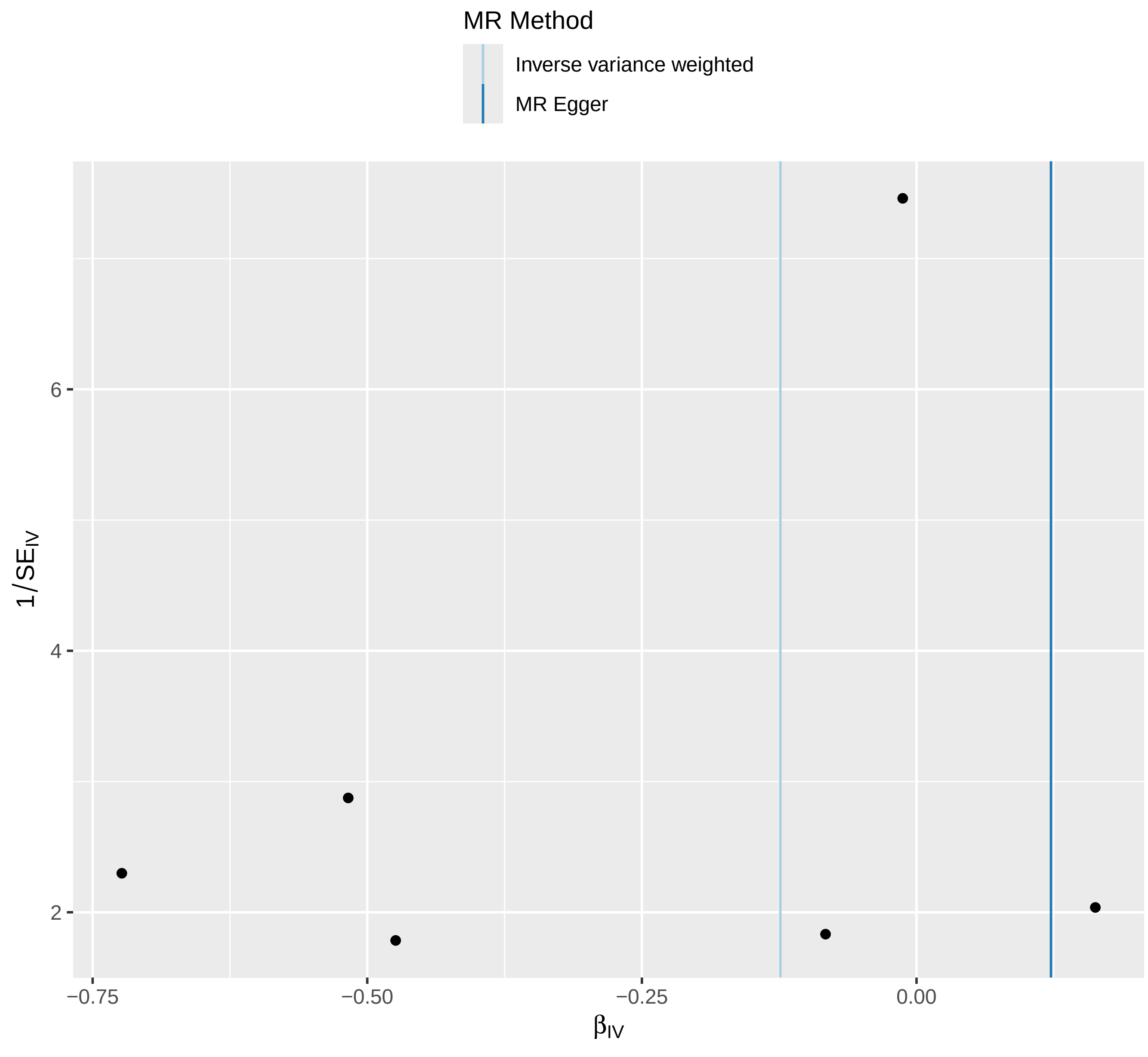

MR Method
Inverse variance weighted
MR Egger
6
4
2
1/SE$_{IV}$
−0.75
−0.50
−0.25
0.00
β$_{IV}$

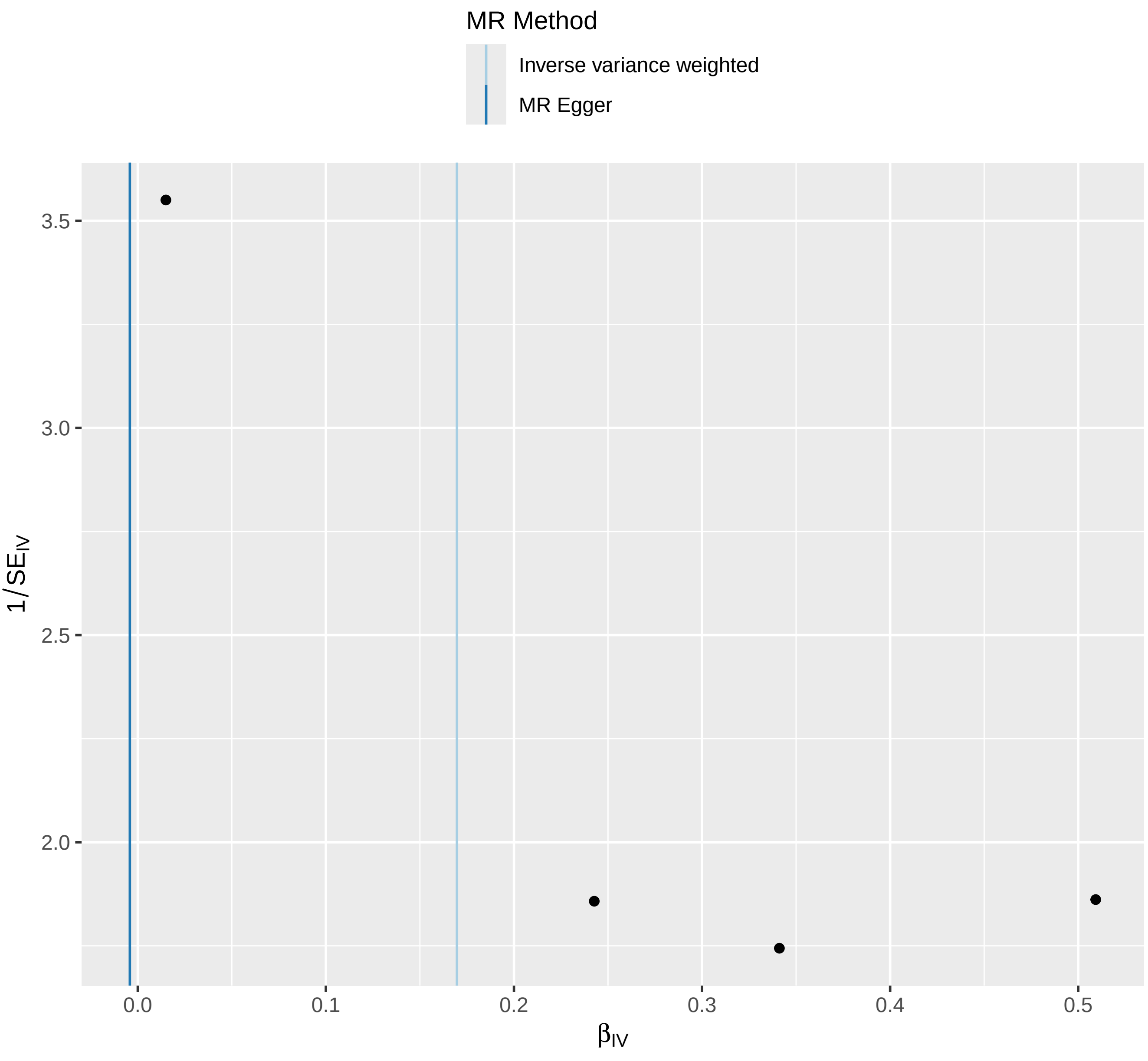
MR Method
Inverse variance weighted
MR Egger
3.5
3.0
2.5
2.0
1/SE_IV
0.0
0.1
0.2
0.3
0.4
0.5
β_IV

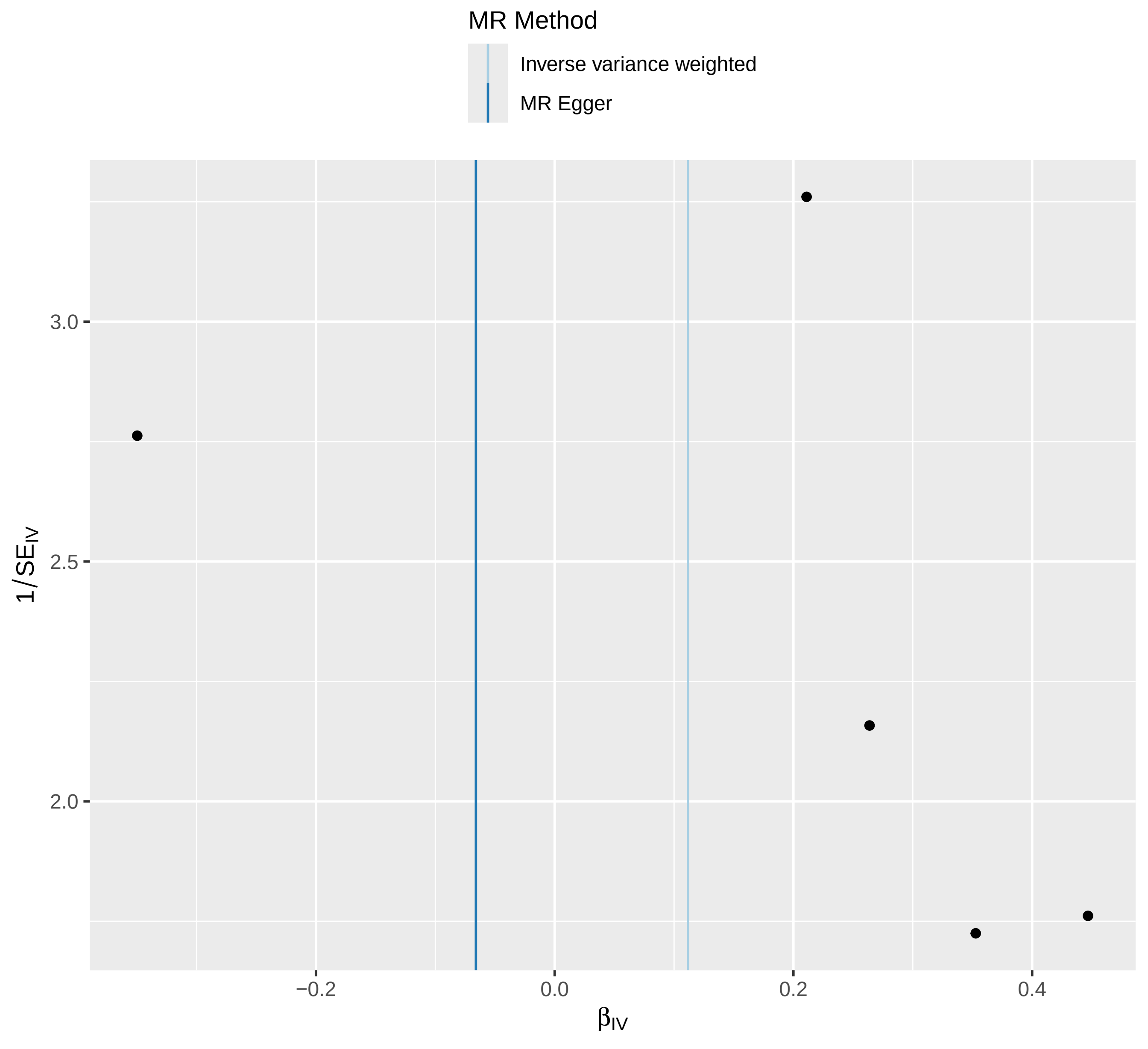
MR Method
Inverse variance weighted
MR Egger
3.0
2.5
2.0
1/SE$_{IV}$
−0.2
0.0
0.2
0.4
β_{IV}

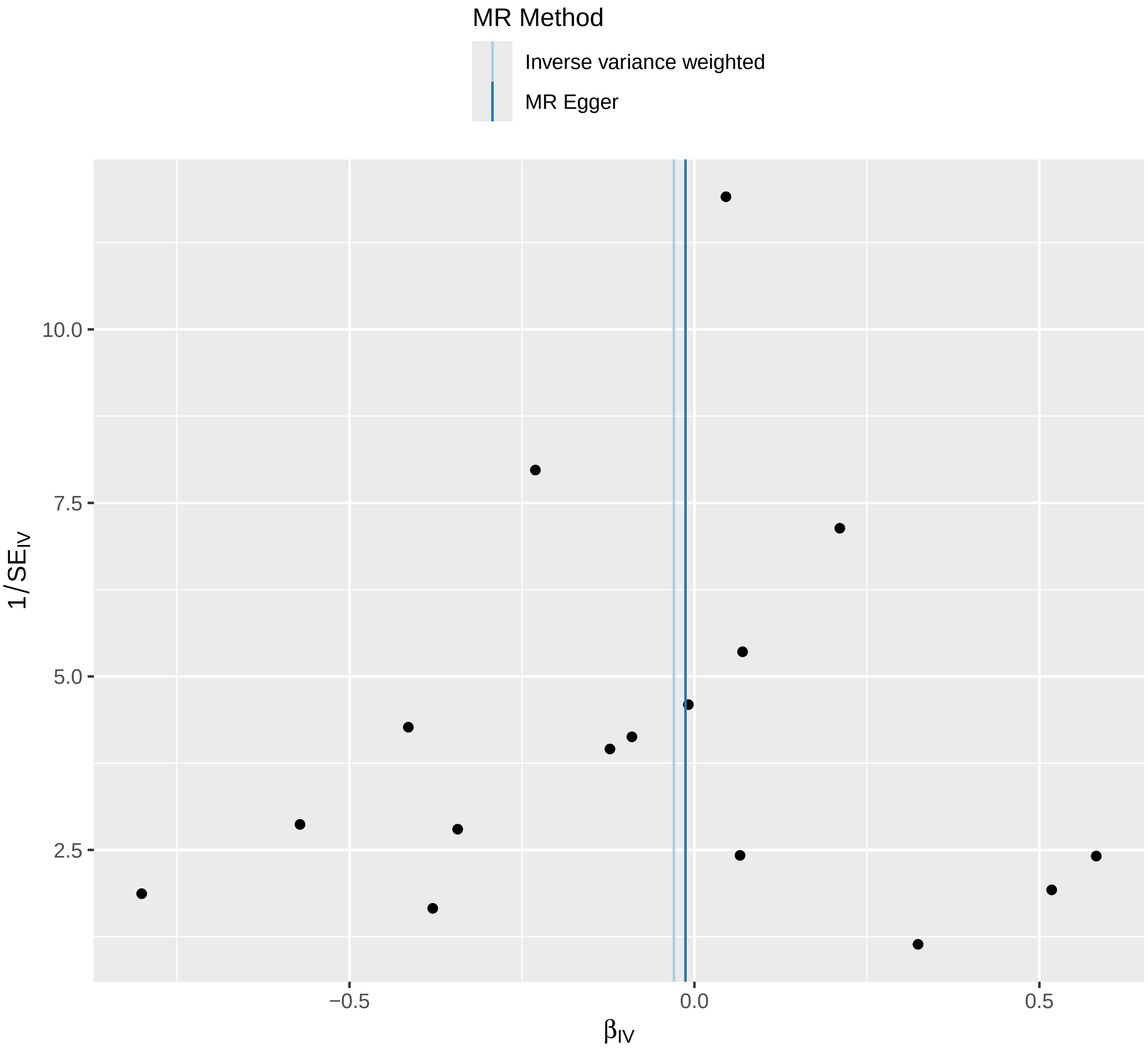

MR Method
Inverse variance weighted
MR Egger
10.0
7.5
5.0
2.5
1/SE$_{IV}$
−0.5
0.0
0.5
β$_{IV}$

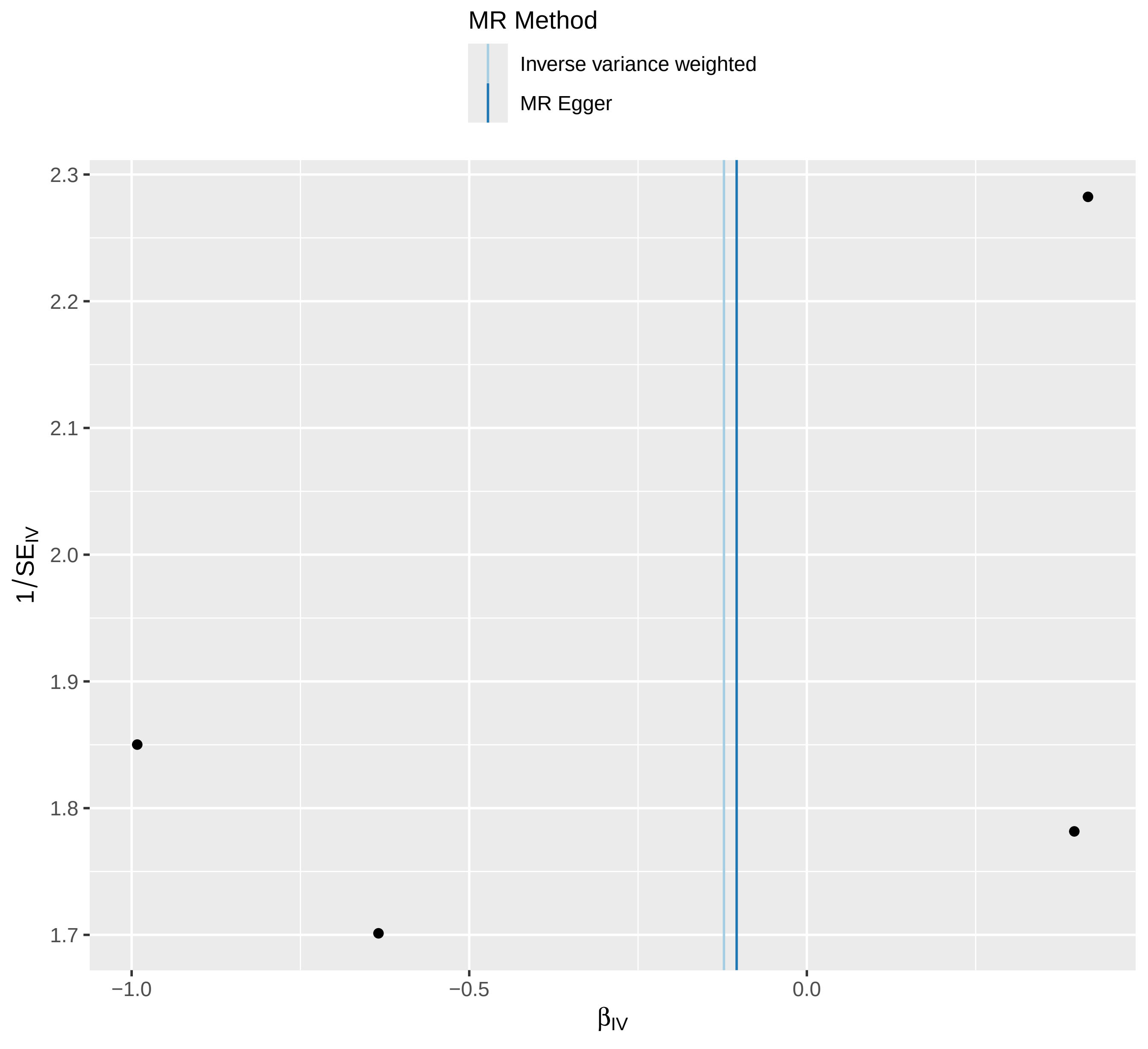
MR Method
Inverse variance weighted
MR Egger
2.3
2.2
2.1
2.0
1.9
1.8
1.7
1/SE_IV
−1.0
−0.5
0.0
β_IV

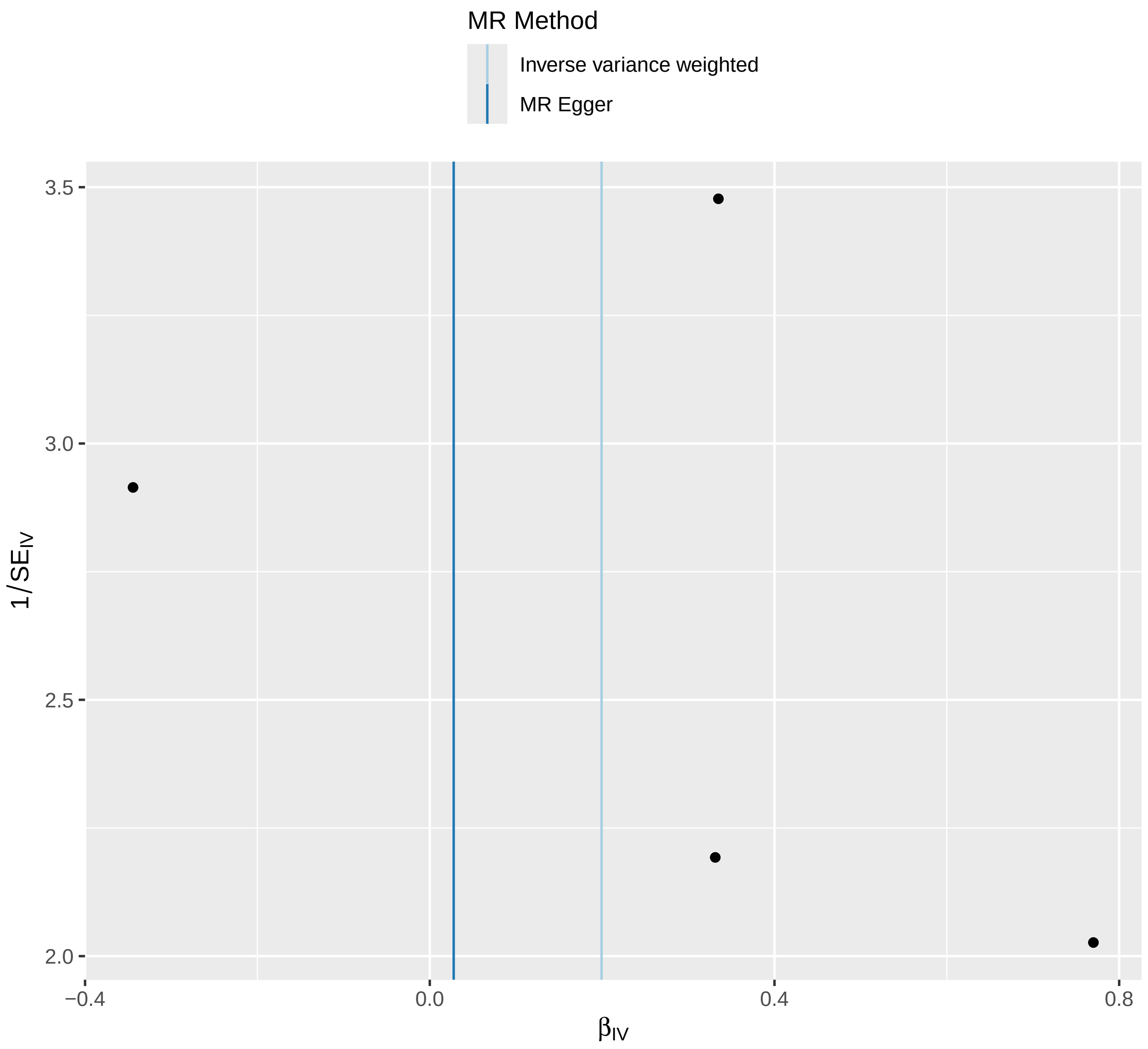

MR Method
Inverse variance weighted
MR Egger
3.5
3.0
2.5
2.0
1/SE_IV
−0.4
0.0
0.4
0.8
β_IV

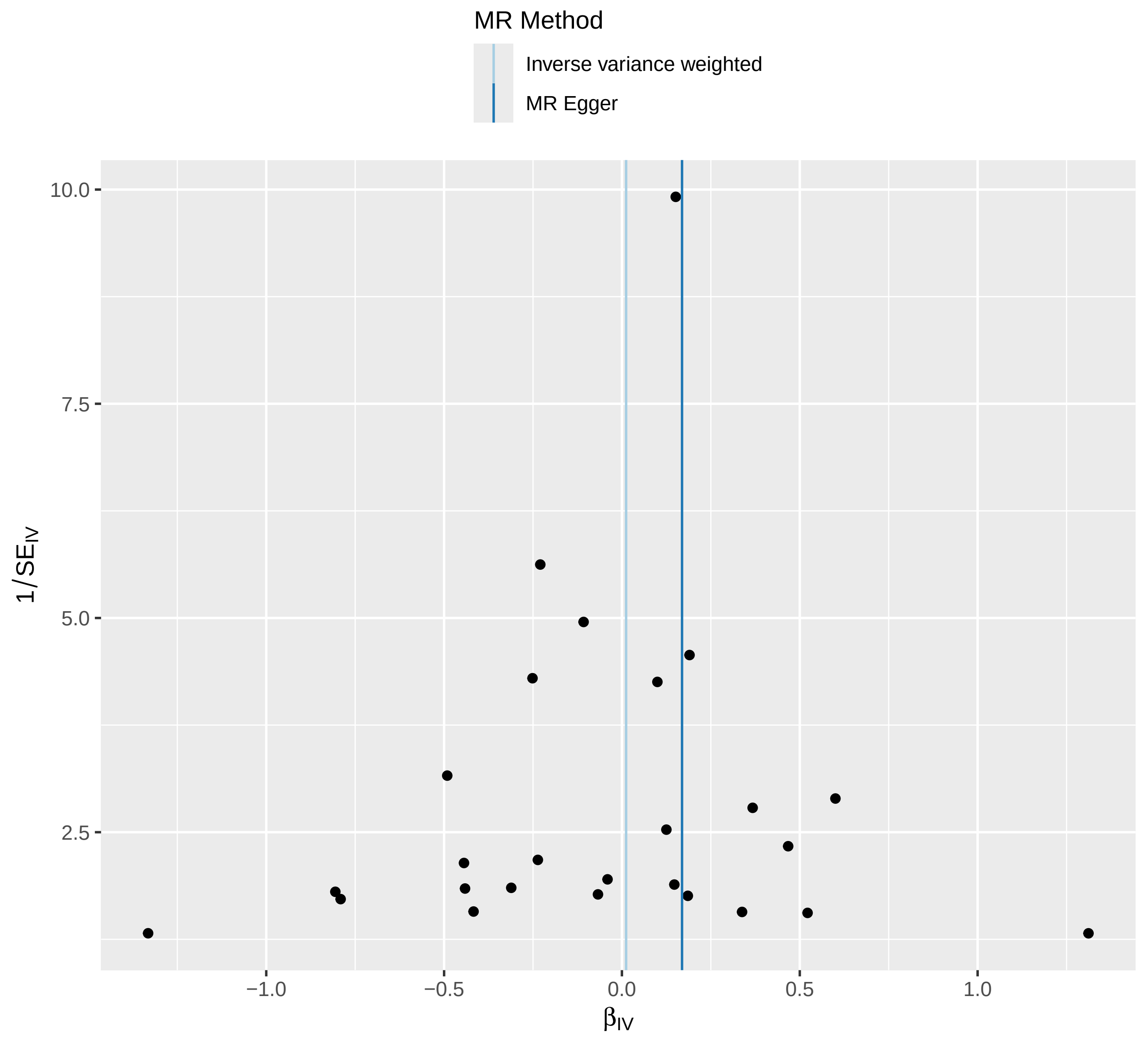

MR Method
Inverse variance weighted
MR Egger
1/SE_IV
10.0
7.5
5.0
2.5
−1.0
−0.5
0.0
0.5
1.0
β_IV

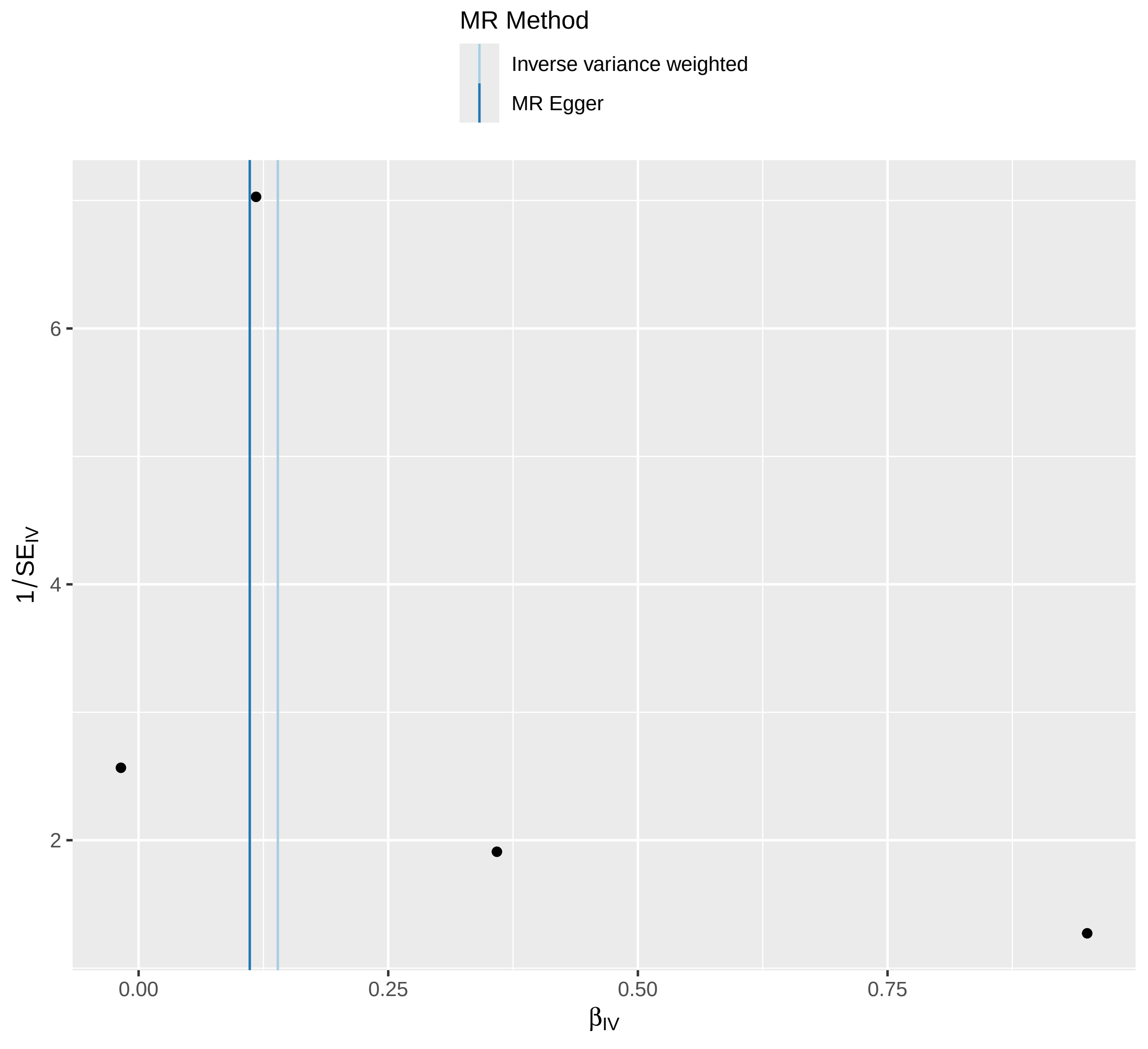
MR Method
Inverse variance weighted
MR Egger
6
4
2
1/SE_IV
0.00
0.25
0.50
0.75
β_IV

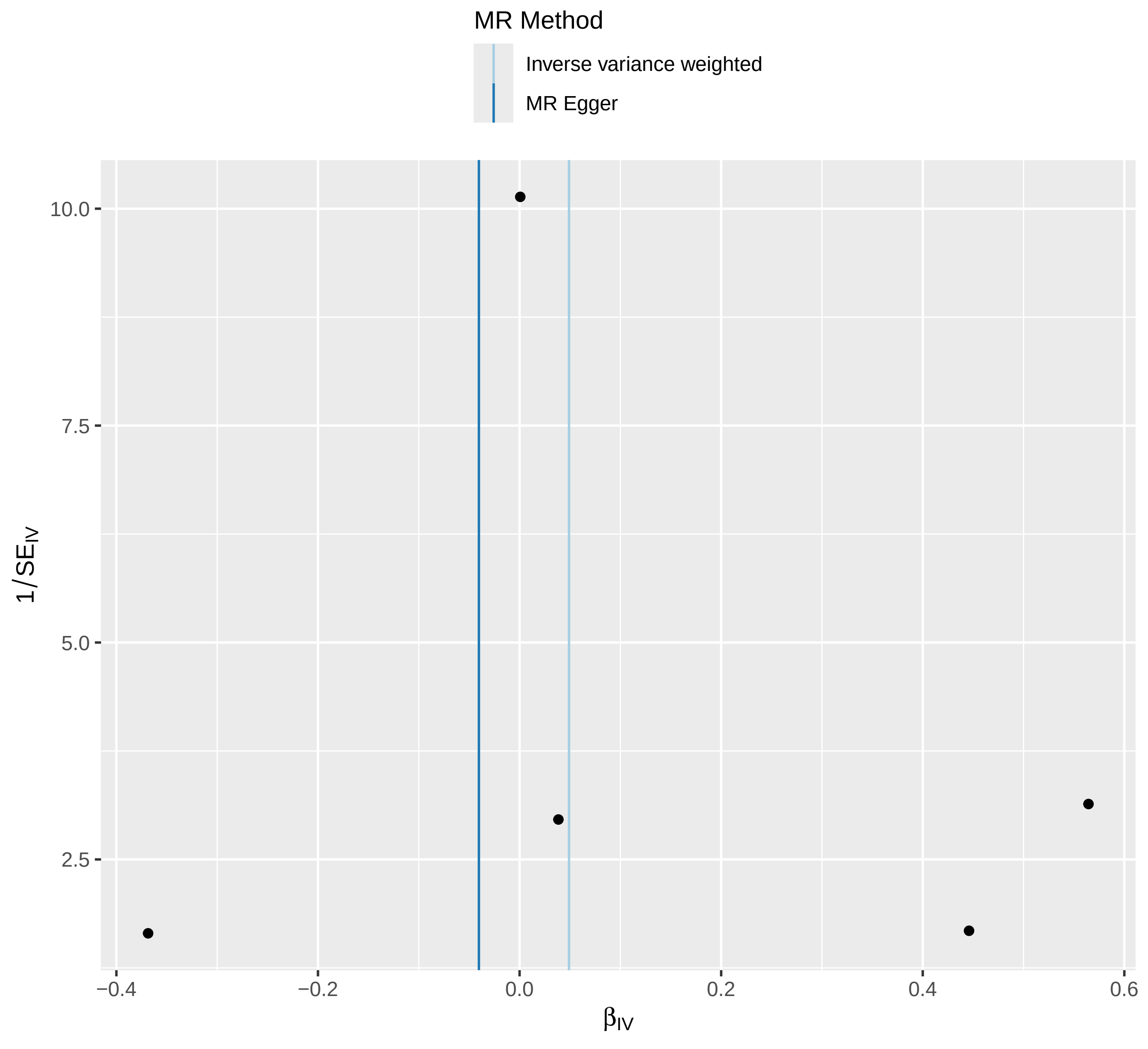

MR Method
Inverse variance weighted
MR Egger
10.0
7.5
5.0
2.5
1/SE_IV
−0.4
−0.2
0.0
0.2
0.4
0.6
β_IV

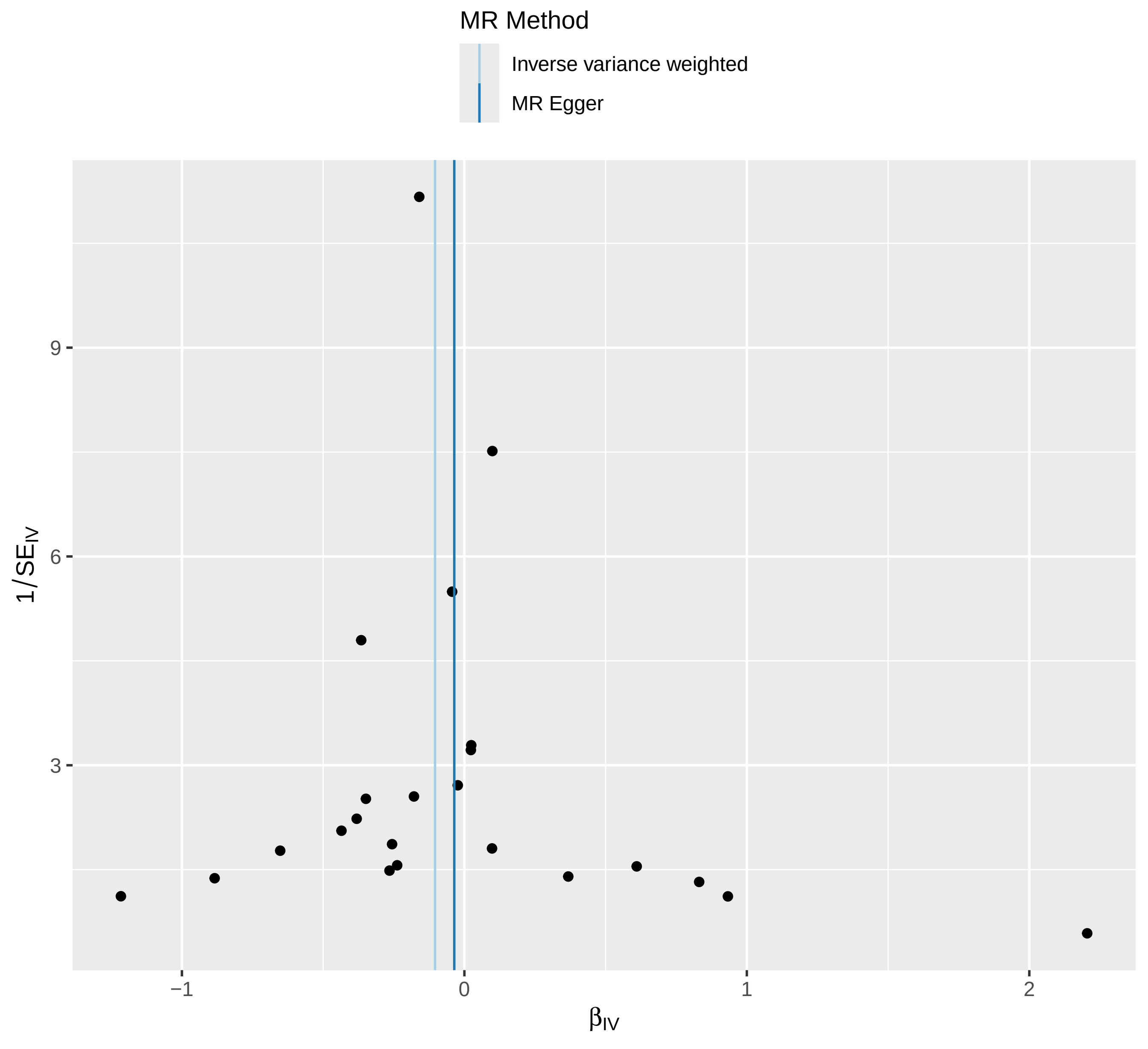

MR Method
Inverse variance weighted
MR Egger
1/SE_{IV}
9
6
3
−1
0
1
2
β_{IV}

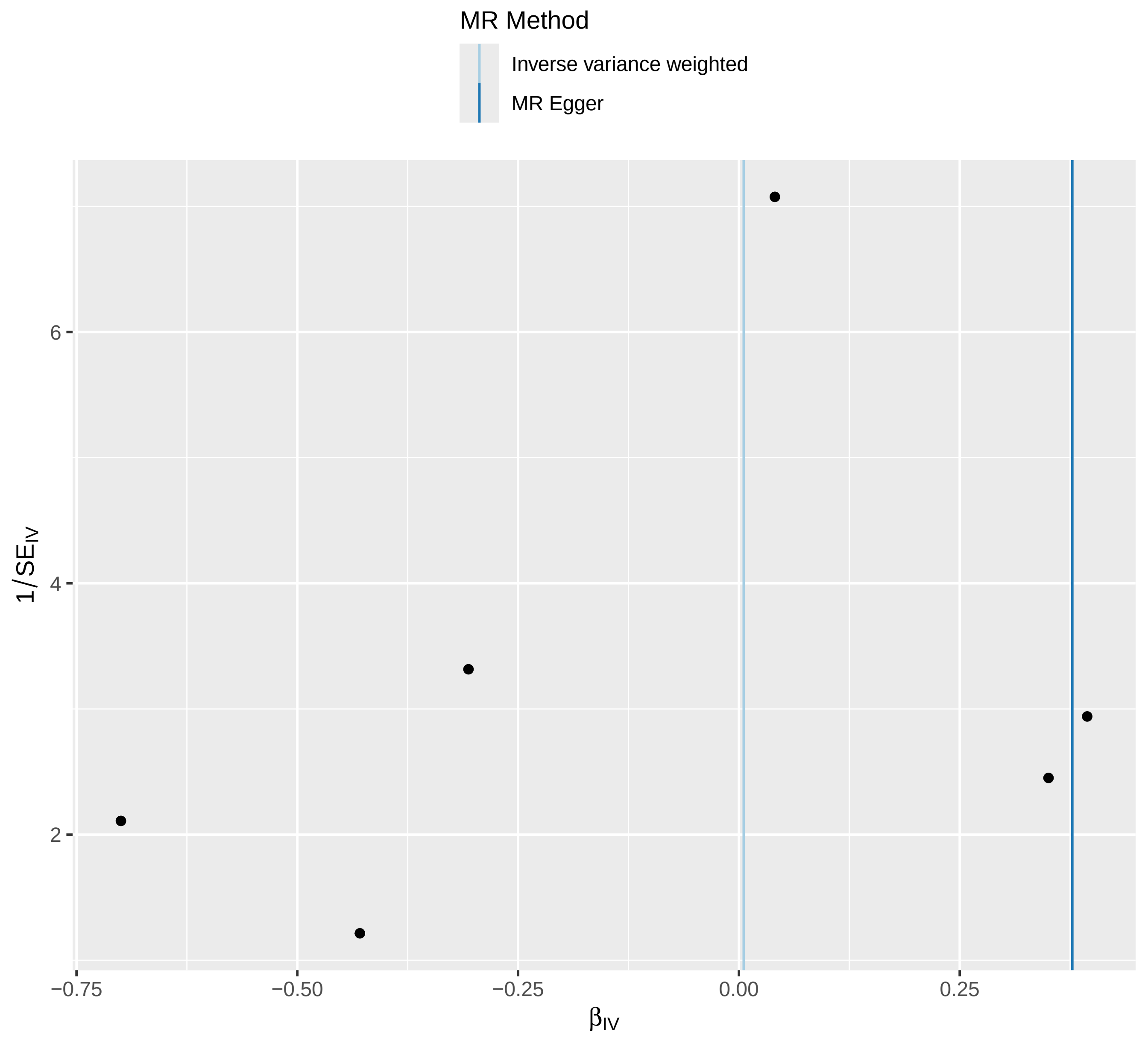

MR Method
Inverse variance weighted
MR Egger
6
4
2
1/SE_IV
−0.75
−0.50
−0.25
0.00
0.25
β_IV

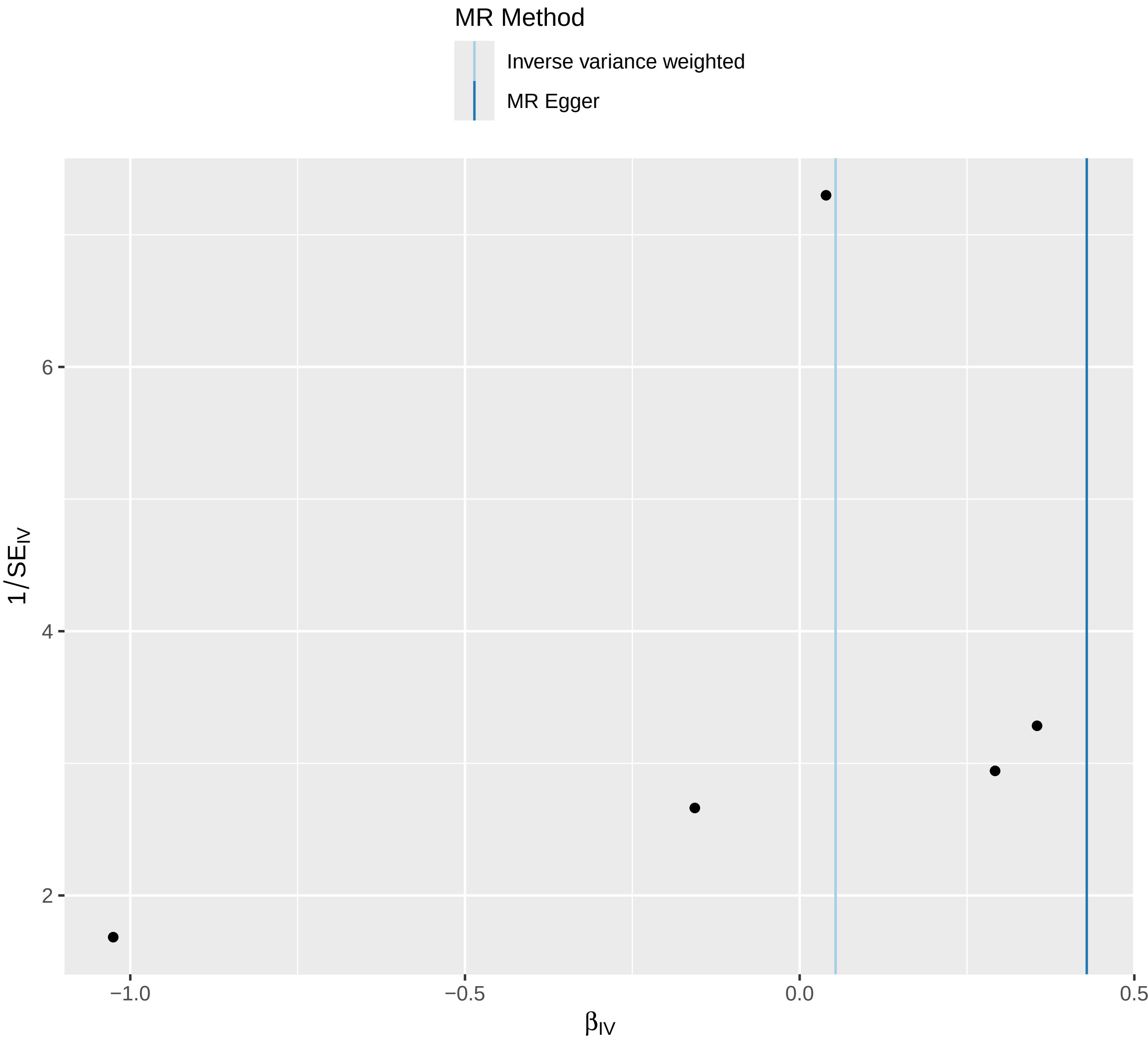

MR Method
Inverse variance weighted
MR Egger
6
4
2
1/SE_IV
−1.0
−0.5
0.0
0.5
β_IV

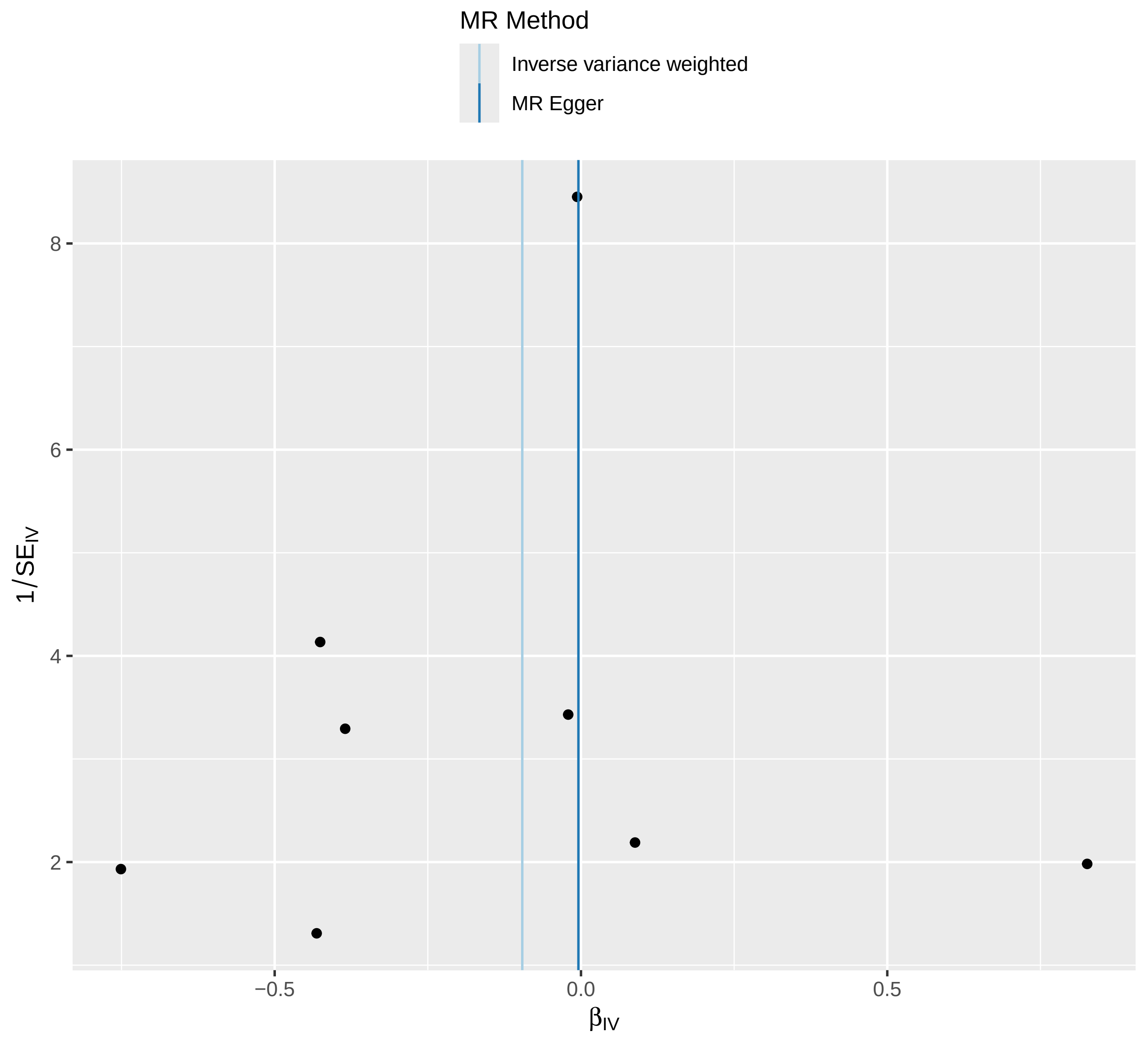
MR Method
Inverse variance weighted
MR Egger
8
6
4
2
1/SE_IV
−0.5
0.0
0.5
β_IV

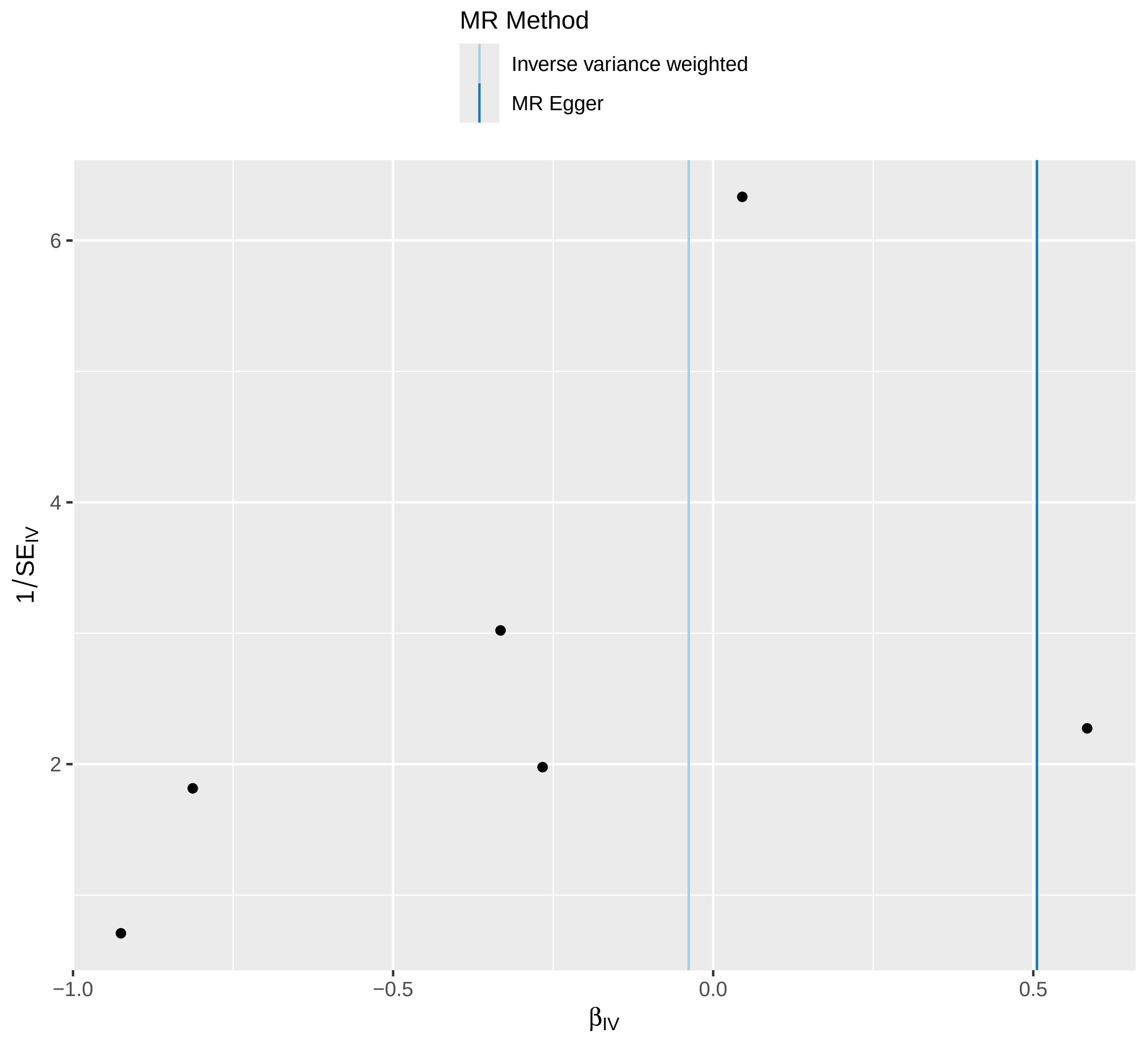

MR Method
Inverse variance weighted
MR Egger
6
4
2
1/SE$_{IV}$
−1.0
−0.5
0.0
0.5
β$_{IV}$

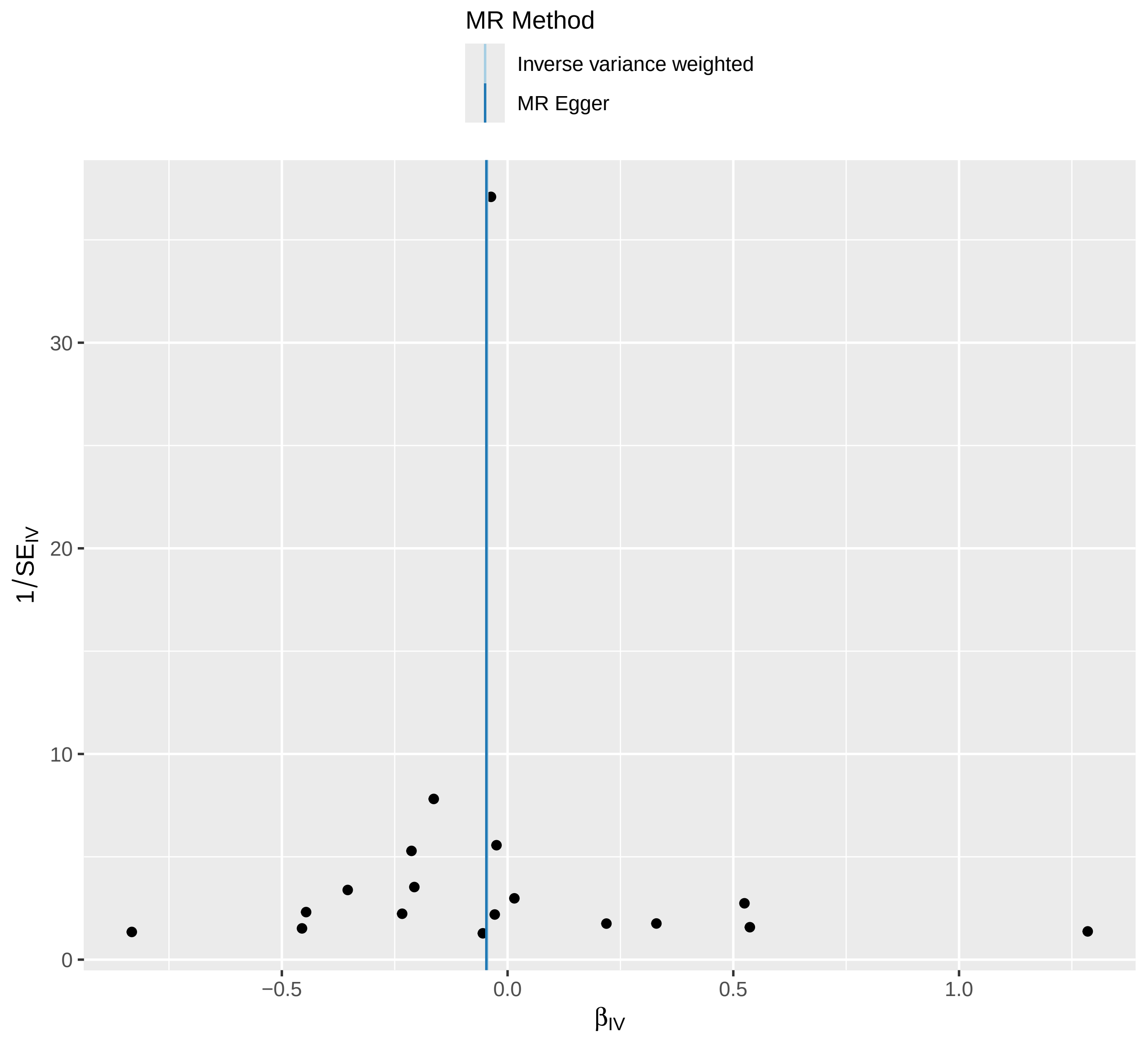
MR Method
Inverse variance weighted
MR Egger
30
20
10
0
1/SE$_{IV}$
−0.5
0.0
0.5
1.0
β$_{IV}$

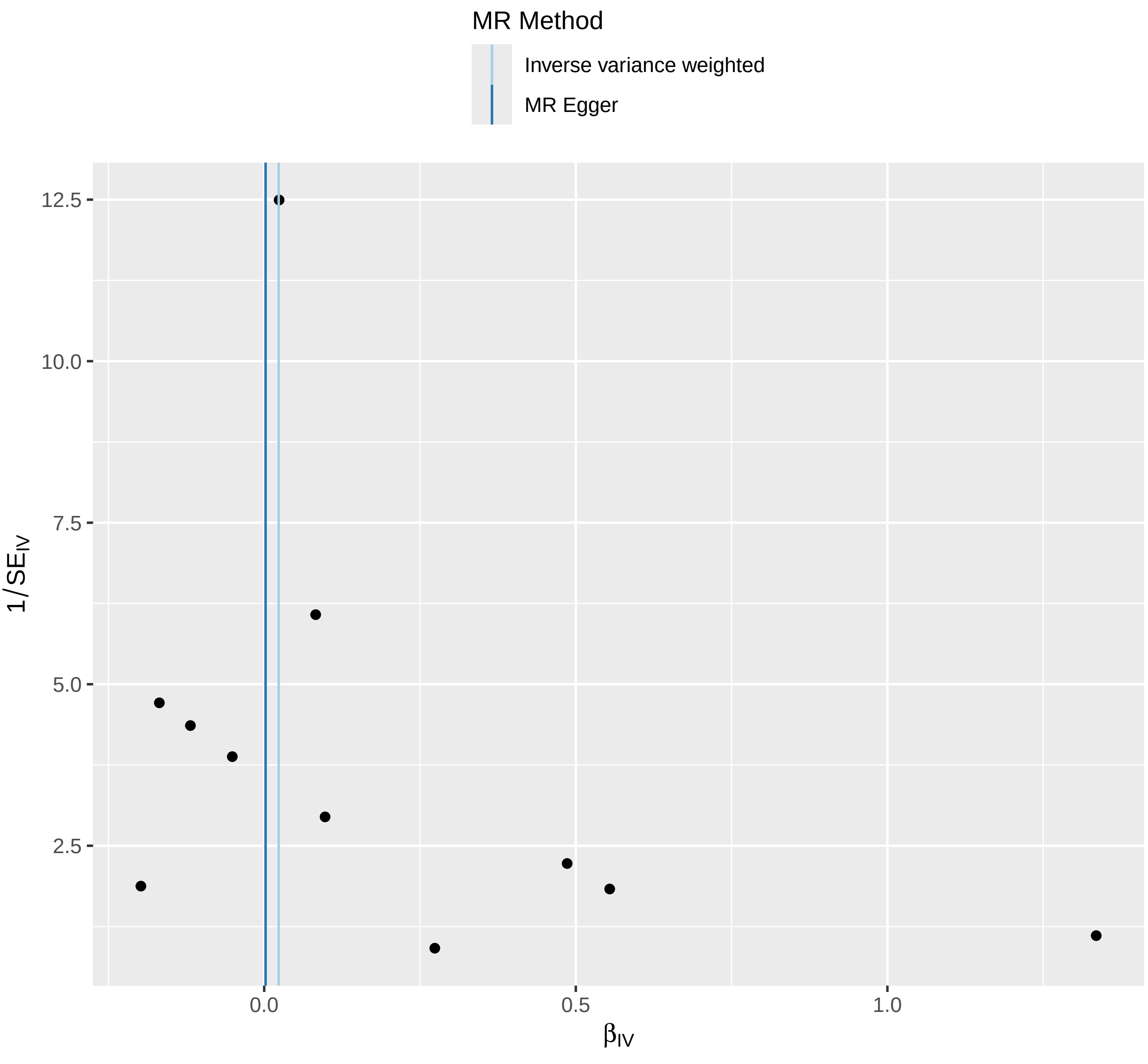

MR Method
Inverse variance weighted
MR Egger
12.5
10.0
7.5
5.0
2.5
$1/SE_{IV}$
0.0
0.5
1.0
β_{IV}

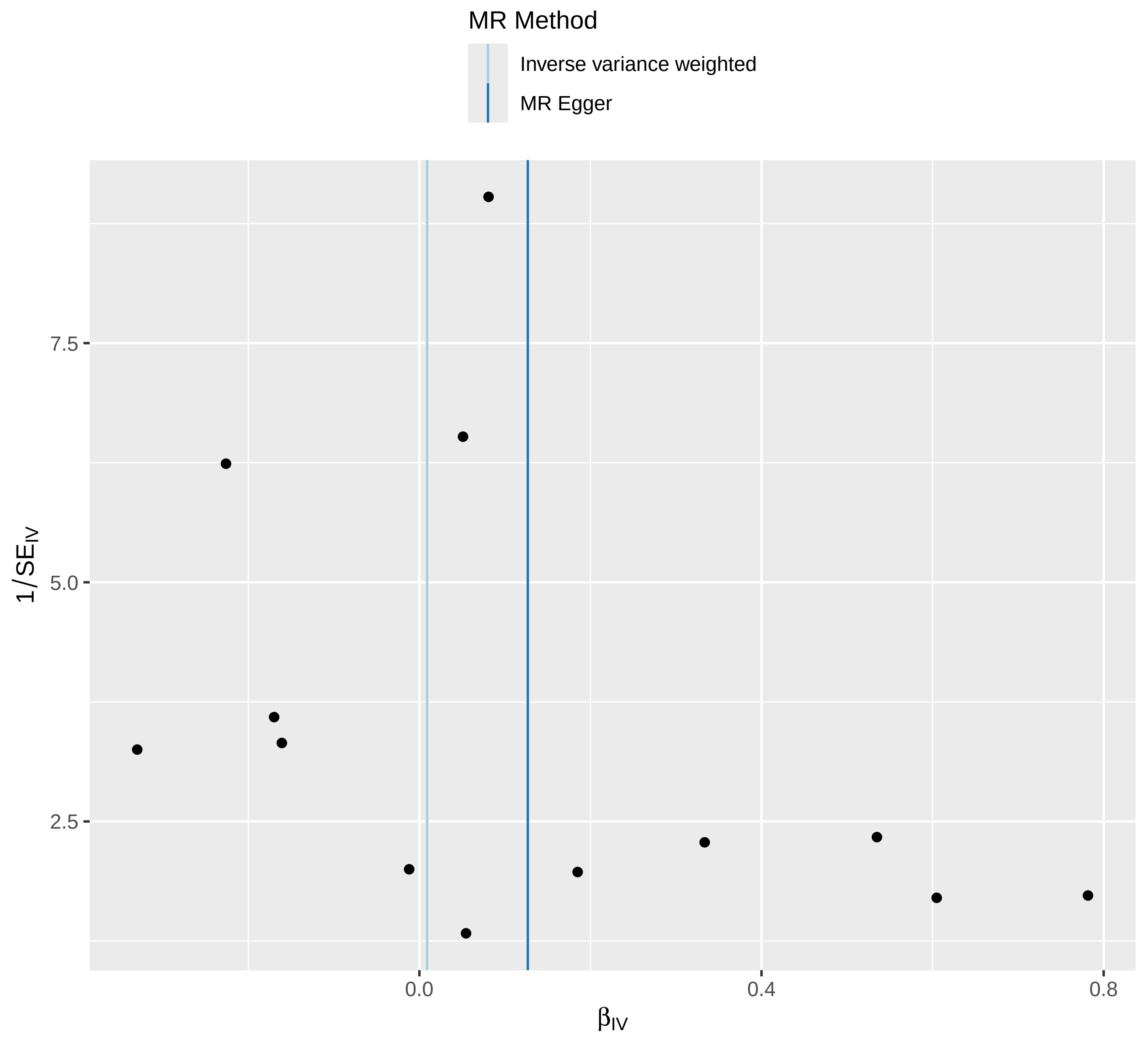

MR Method
Inverse variance weighted
MR Egger
7.5
5.0
2.5
1/SE$_{IV}$
0.0
0.4
0.8
β$_{IV}$

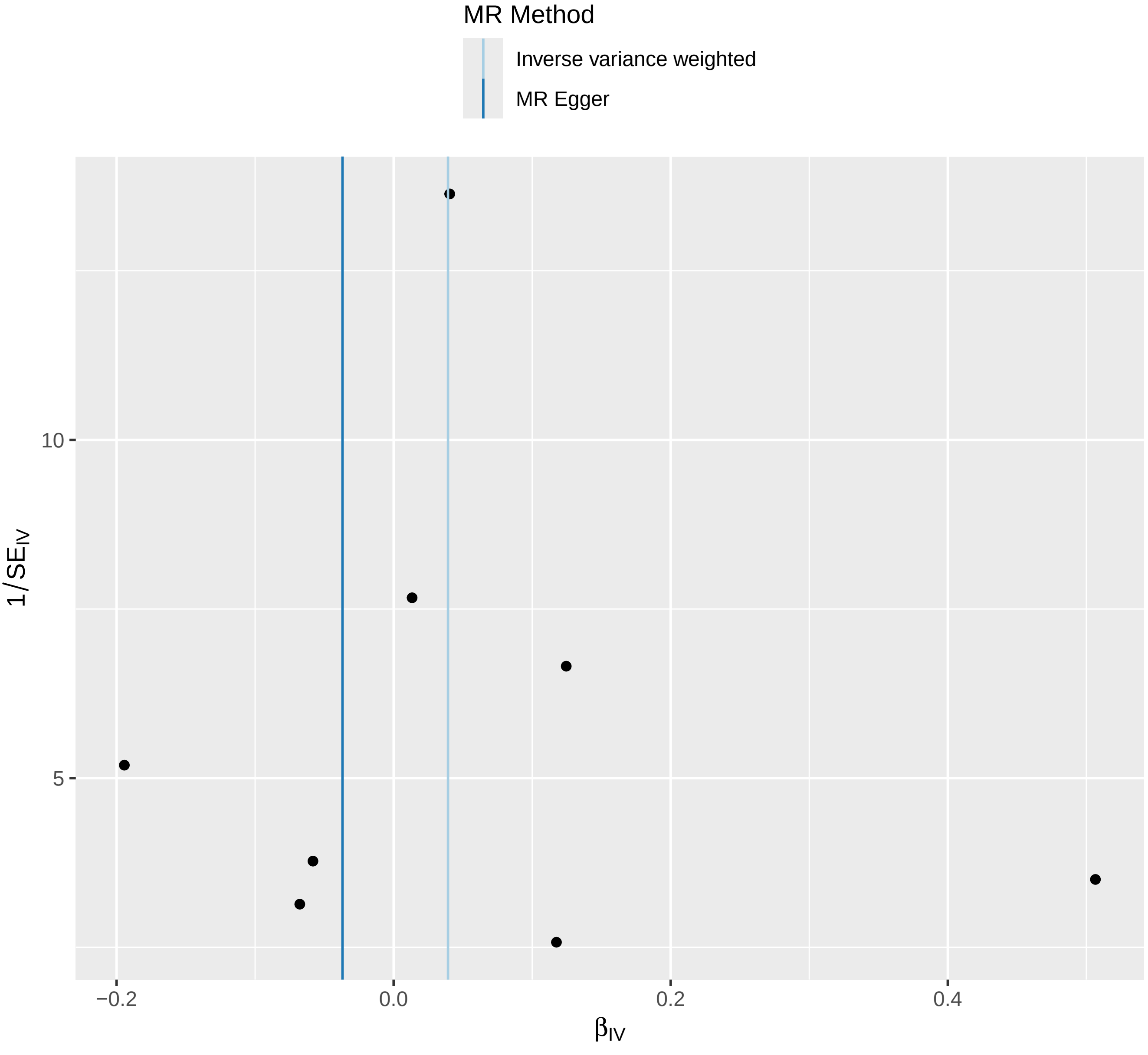

MR Method
Inverse variance weighted
MR Egger
10
5
1/SE_IV
−0.2
0.0
0.2
0.4
β_IV

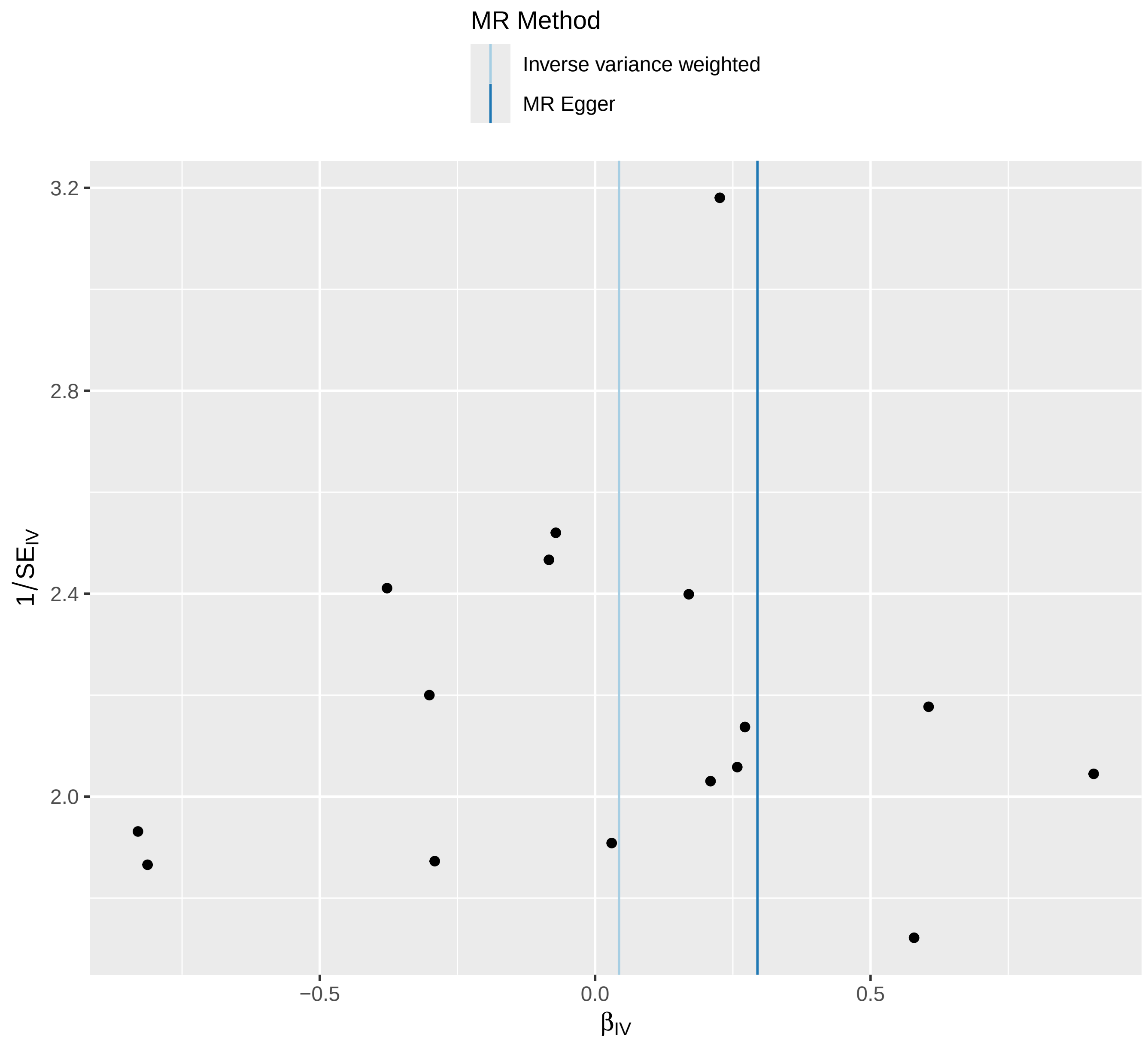
MR Method
Inverse variance weighted
MR Egger
3.2
2.8
2.4
2.0
1/SE$_{IV}$
−0.5
0.0
0.5
β$_{IV}$

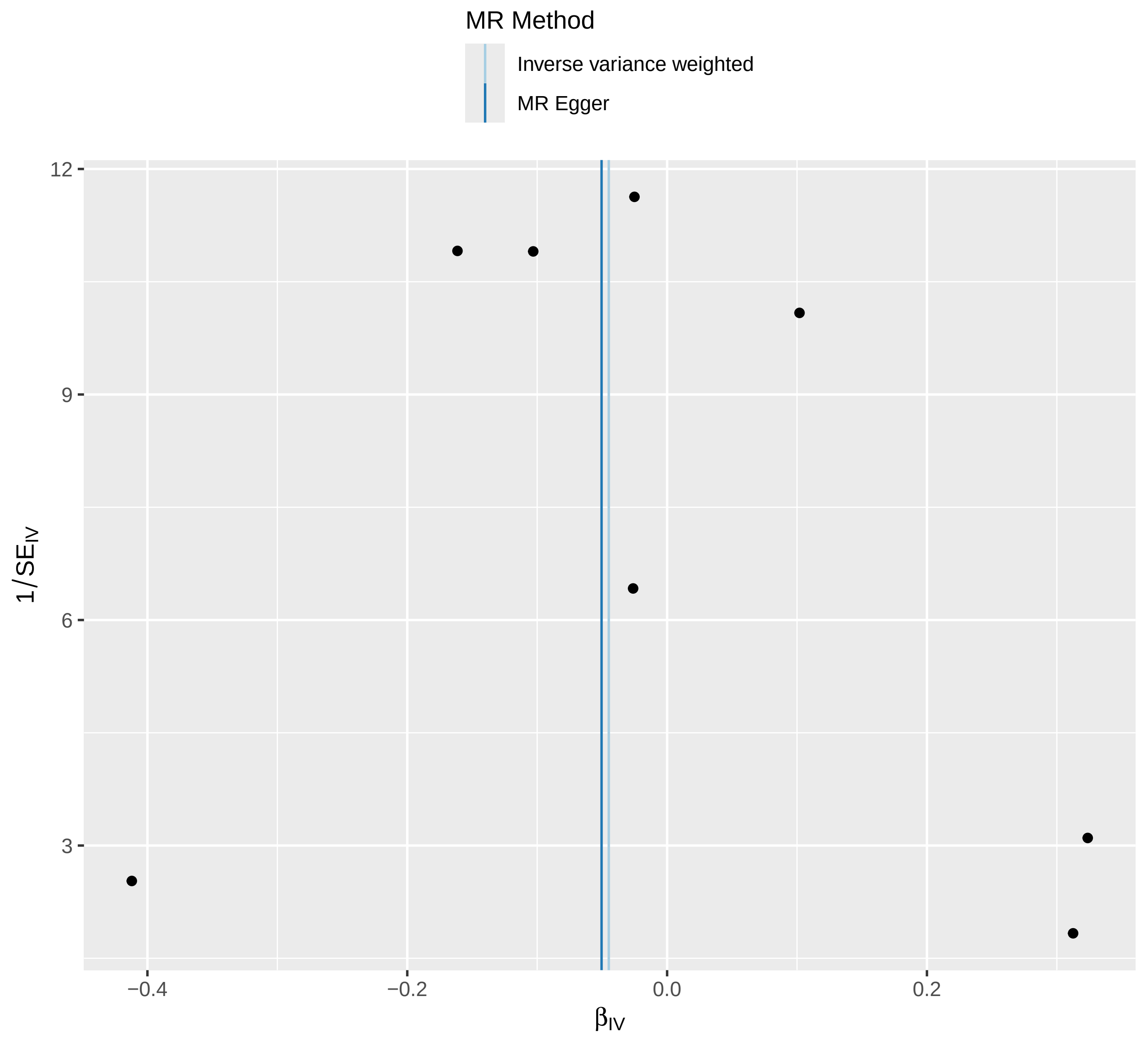

MR Method
Inverse variance weighted
MR Egger
1/SE$_{IV}$
12
9
6
3
−0.4
−0.2
0.0
0.2
β$_{IV}$

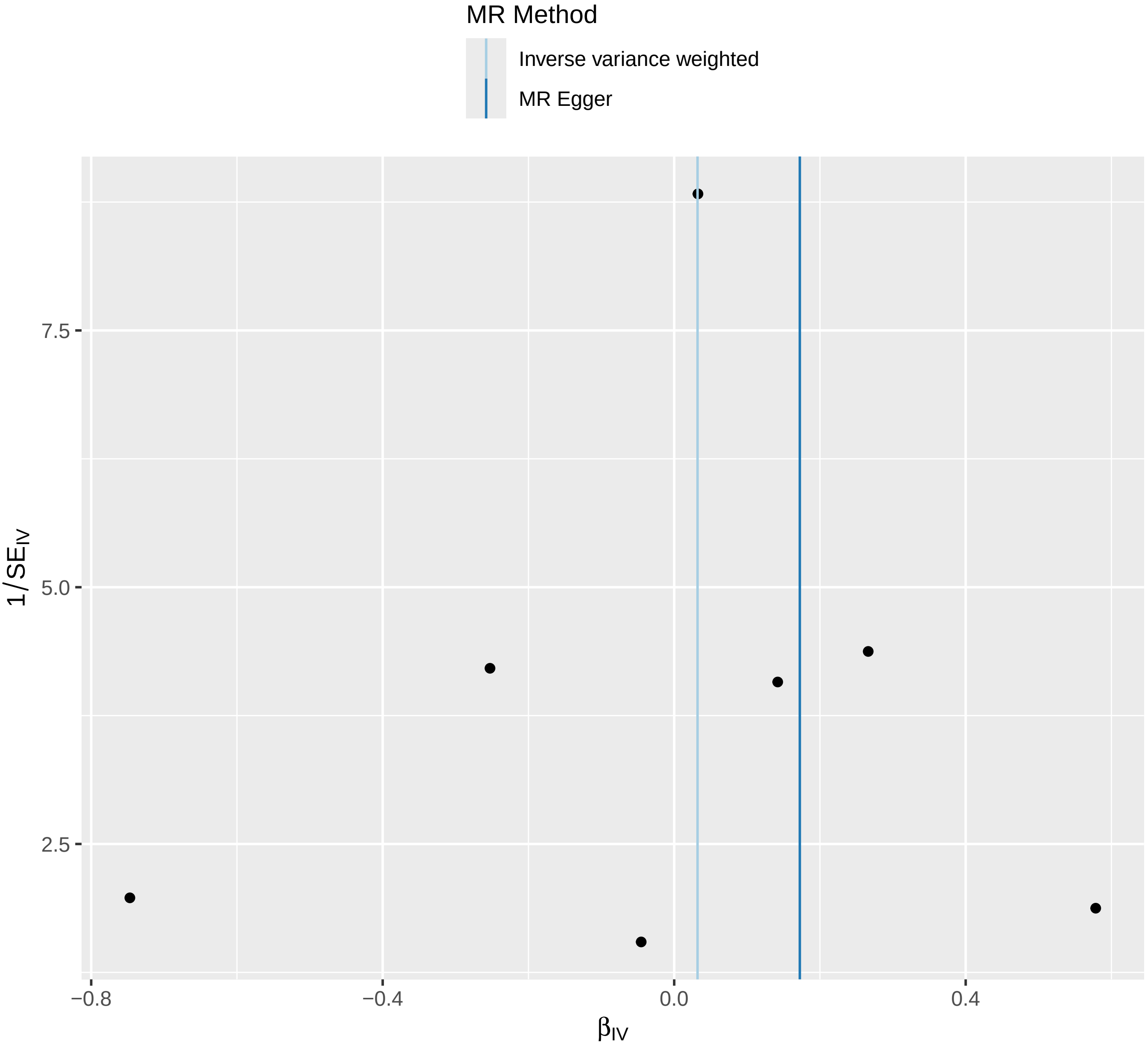
MR Method
Inverse variance weighted
MR Egger
7.5
5.0
2.5
1/SE_IV
−0.8
−0.4
0.0
0.4
β_IV

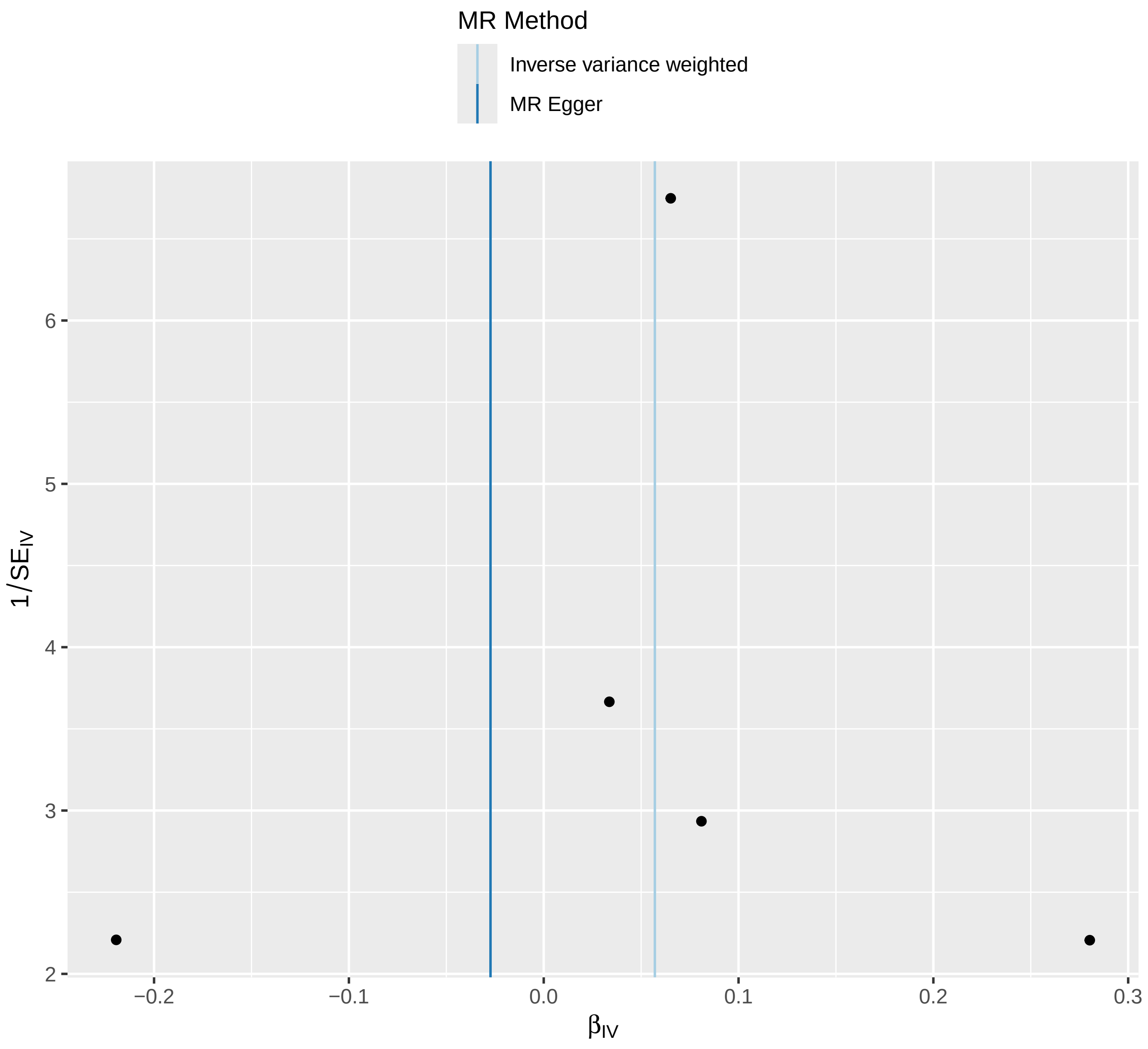

MR Method
Inverse variance weighted
MR Egger
6
5
4
3
2
1/SE_IV
−0.2
−0.1
0.0
0.1
0.2
0.3
β_IV

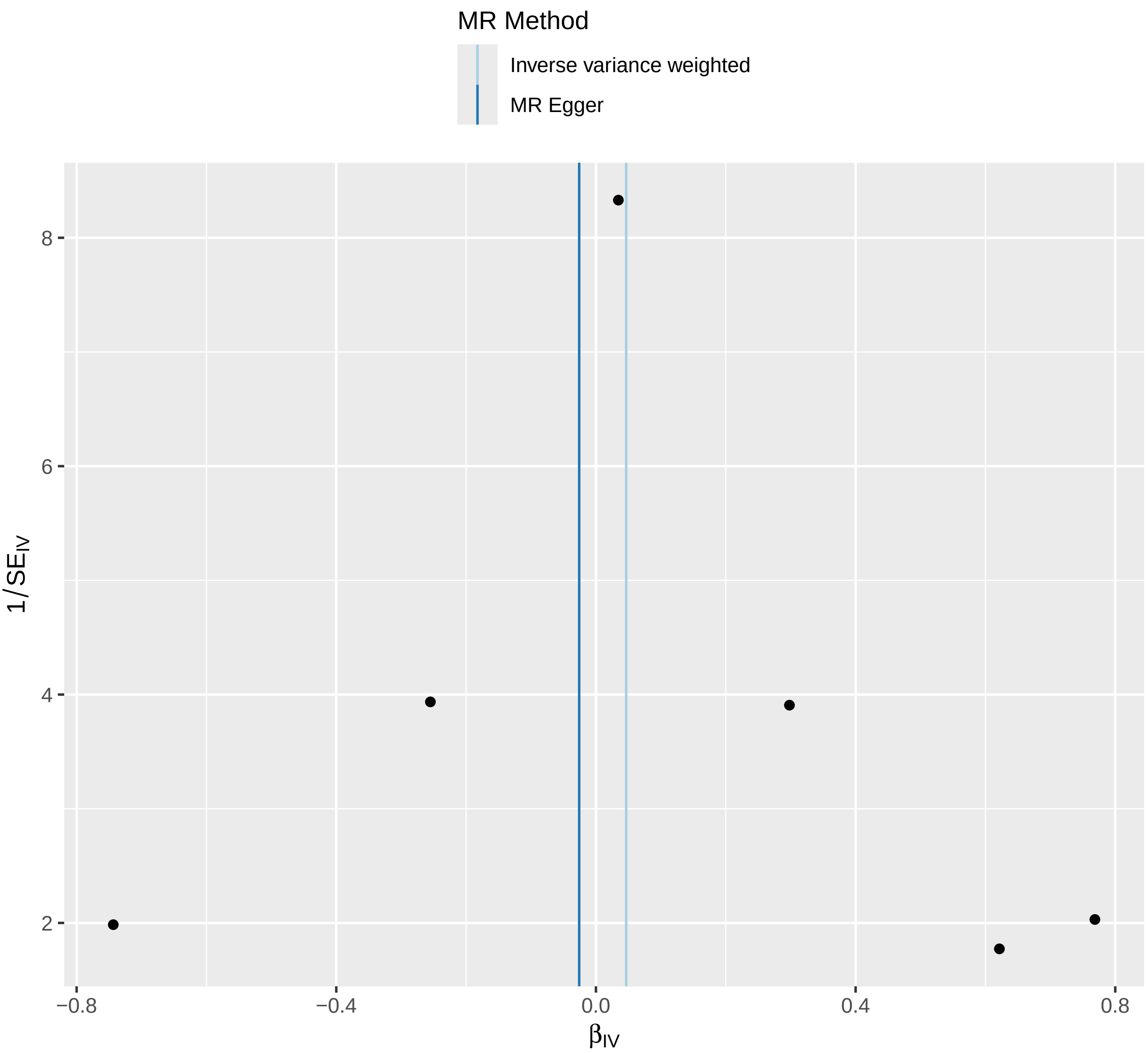
MR Method
Inverse variance weighted
MR Egger
8
6
4
2
1/SE_IV
−0.8
−0.4
0.0
0.4
0.8
β_IV

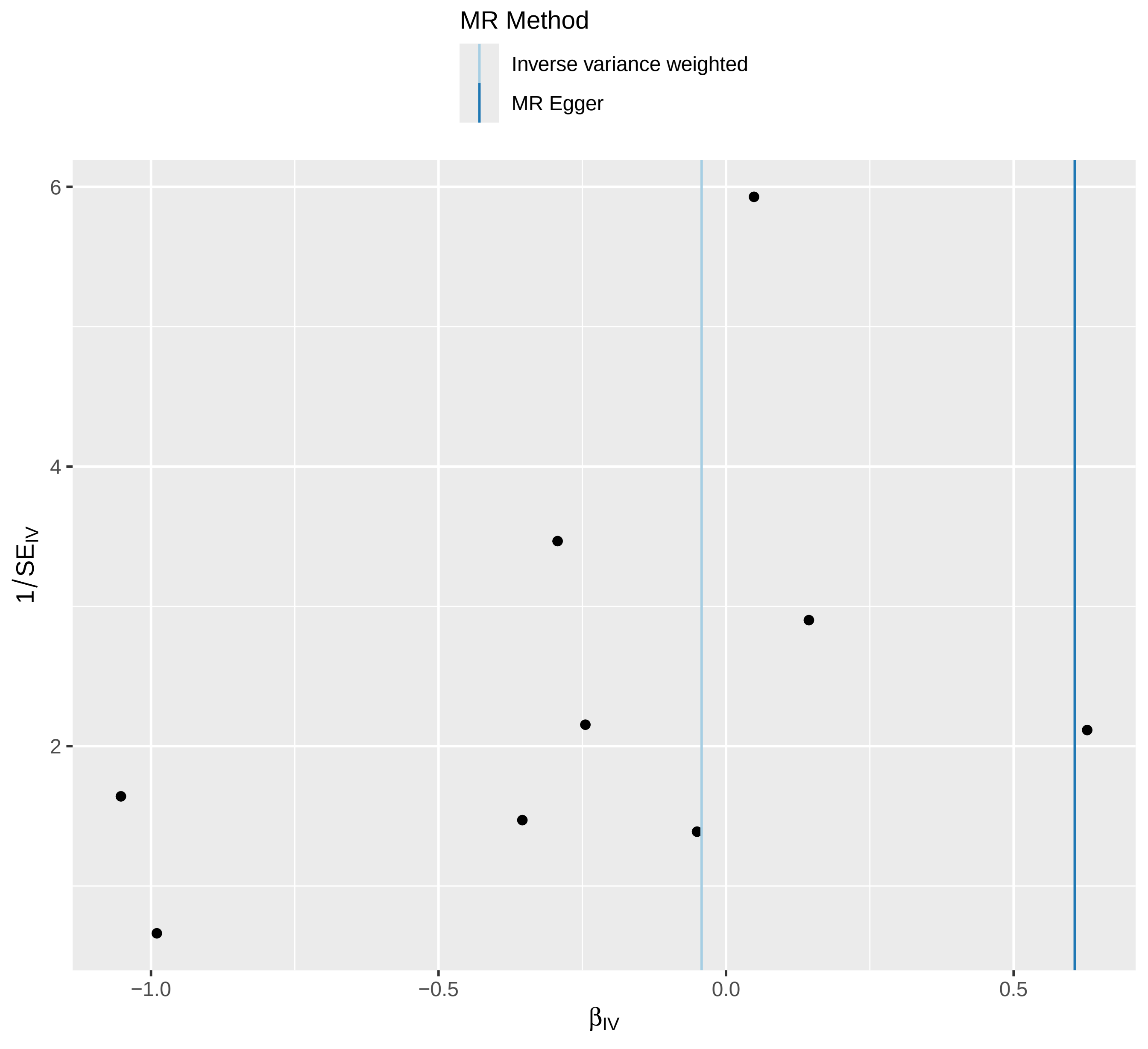
MR Method
Inverse variance weighted
MR Egger
6
4
2
1/SE$_{IV}$
−1.0
−0.5
0.0
0.5
β$_{IV}$

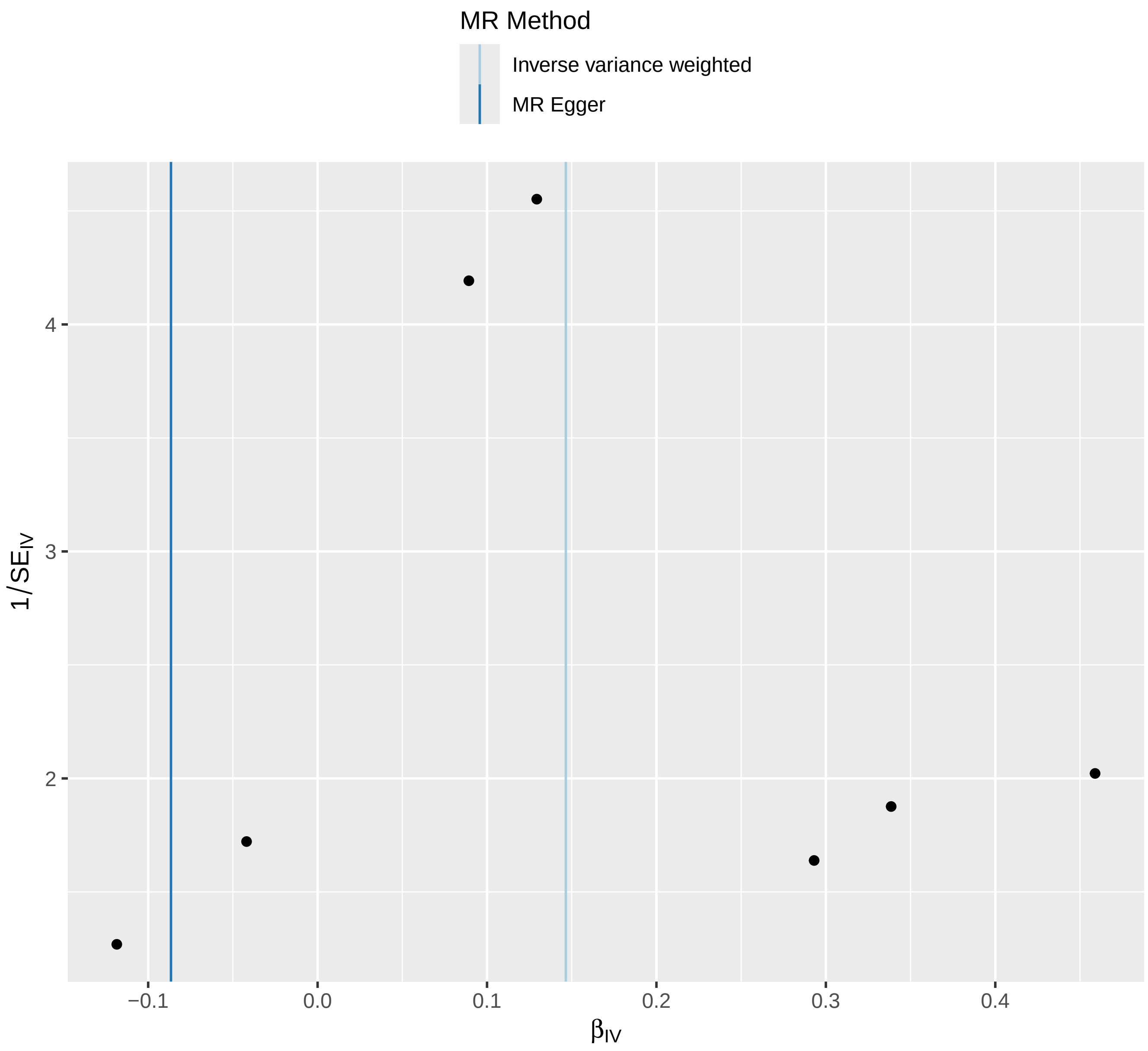

MR Method
Inverse variance weighted
MR Egger
4
3
2
1/SE$_{IV}$
−0.1
0.0
0.1
0.2
0.3
0.4
β$_{IV}$

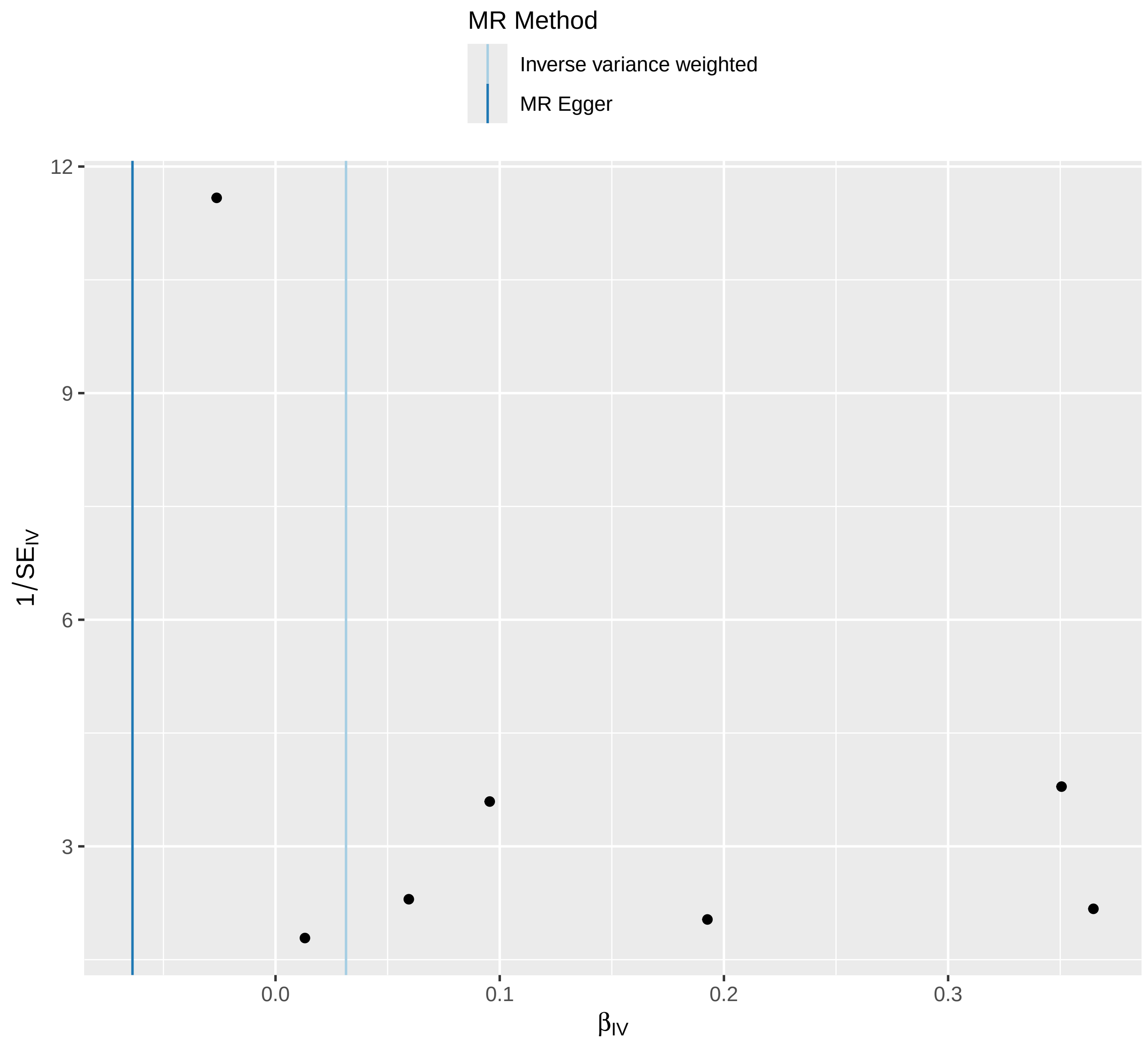
MR Method
Inverse variance weighted
MR Egger
12
9
6
3
1/SE_IV
0.0
0.1
0.2
0.3
β_IV

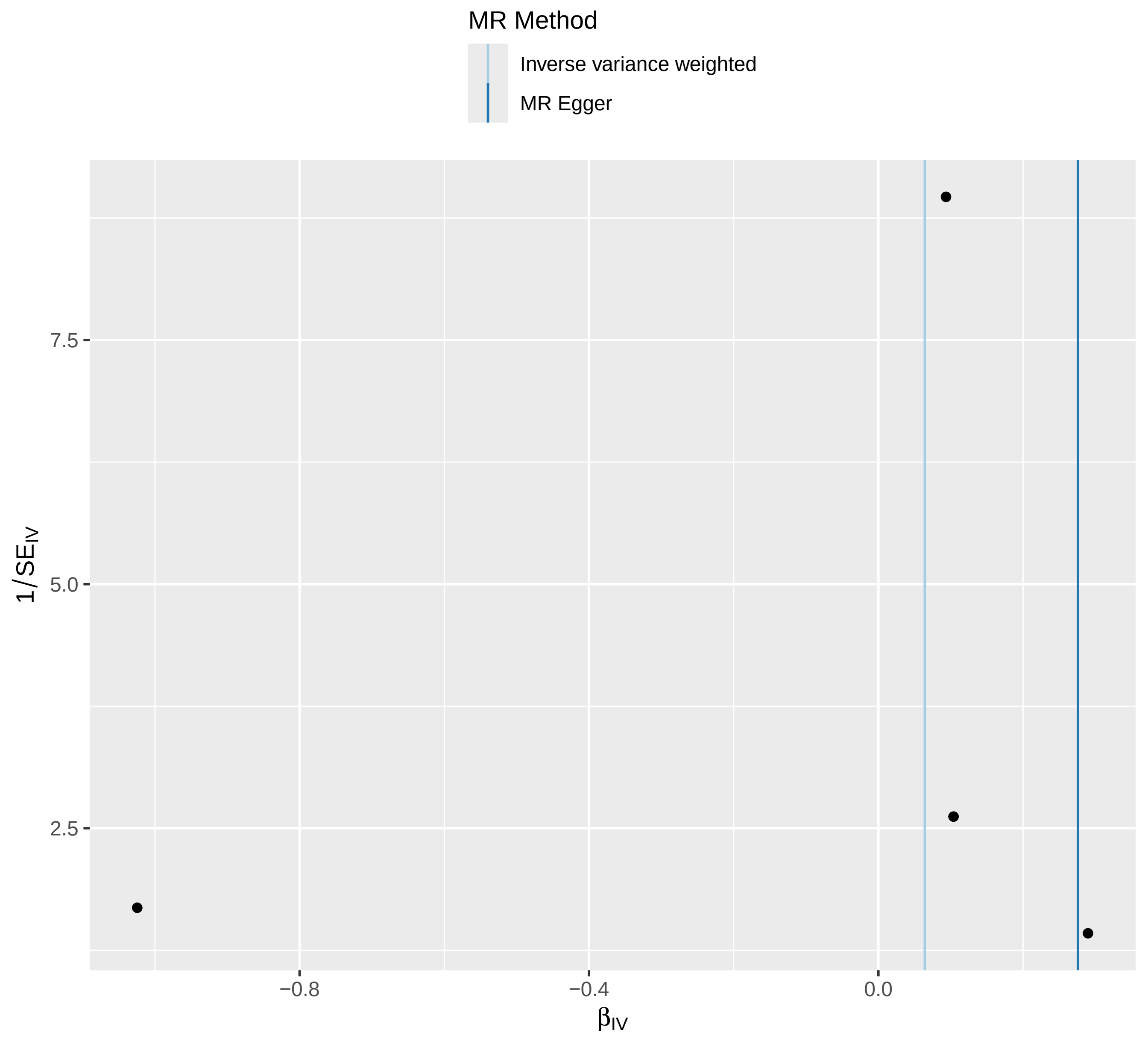
MR Method
Inverse variance weighted
MR Egger
7.5
5.0
2.5
1/SE$_{IV}$
−0.8
−0.4
0.0
β$_{IV}$

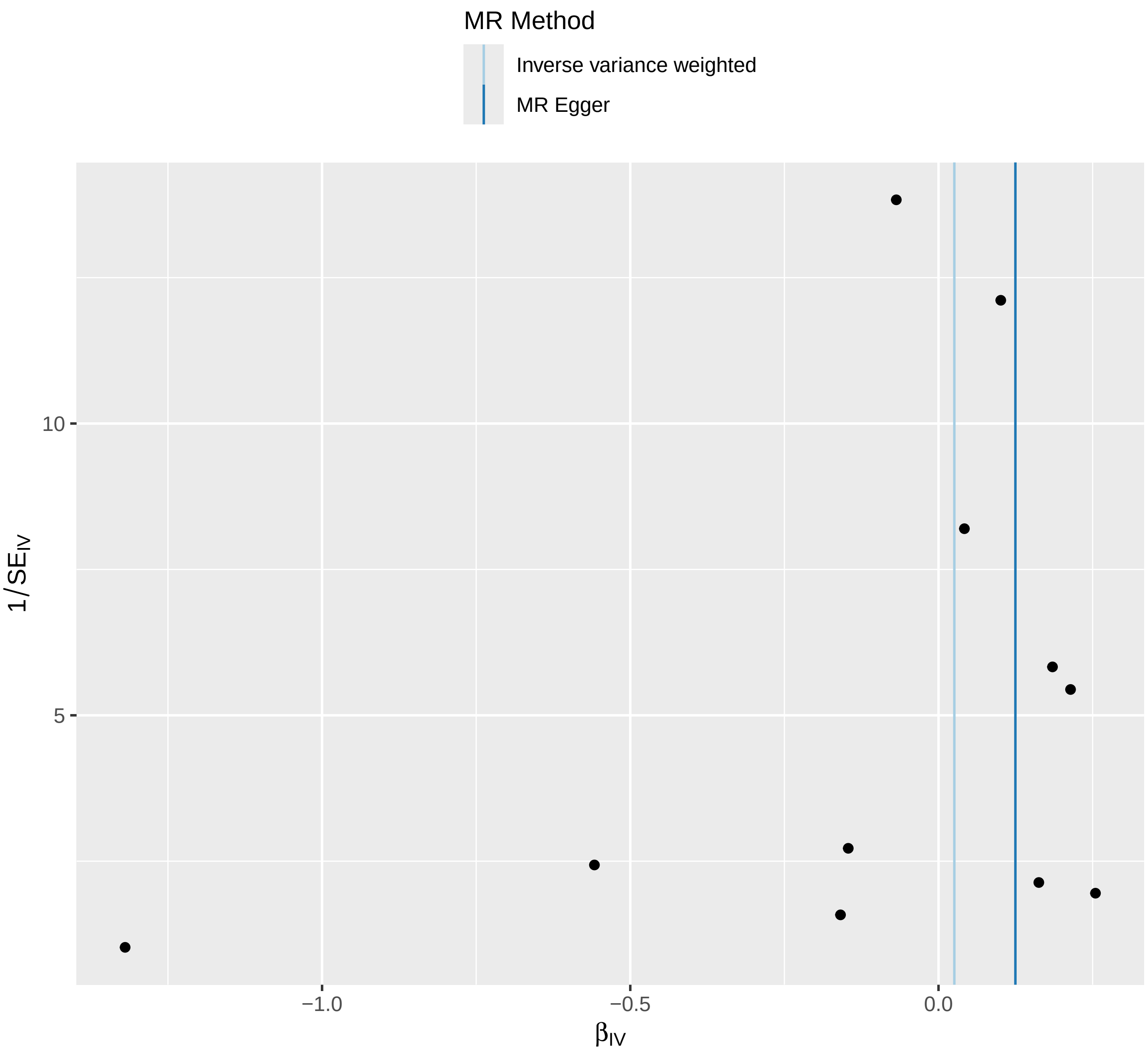

MR Method
Inverse variance weighted
MR Egger
10
5
−1.0
−0.5
0.0
β_{IV}
$1/SE_{IV}$

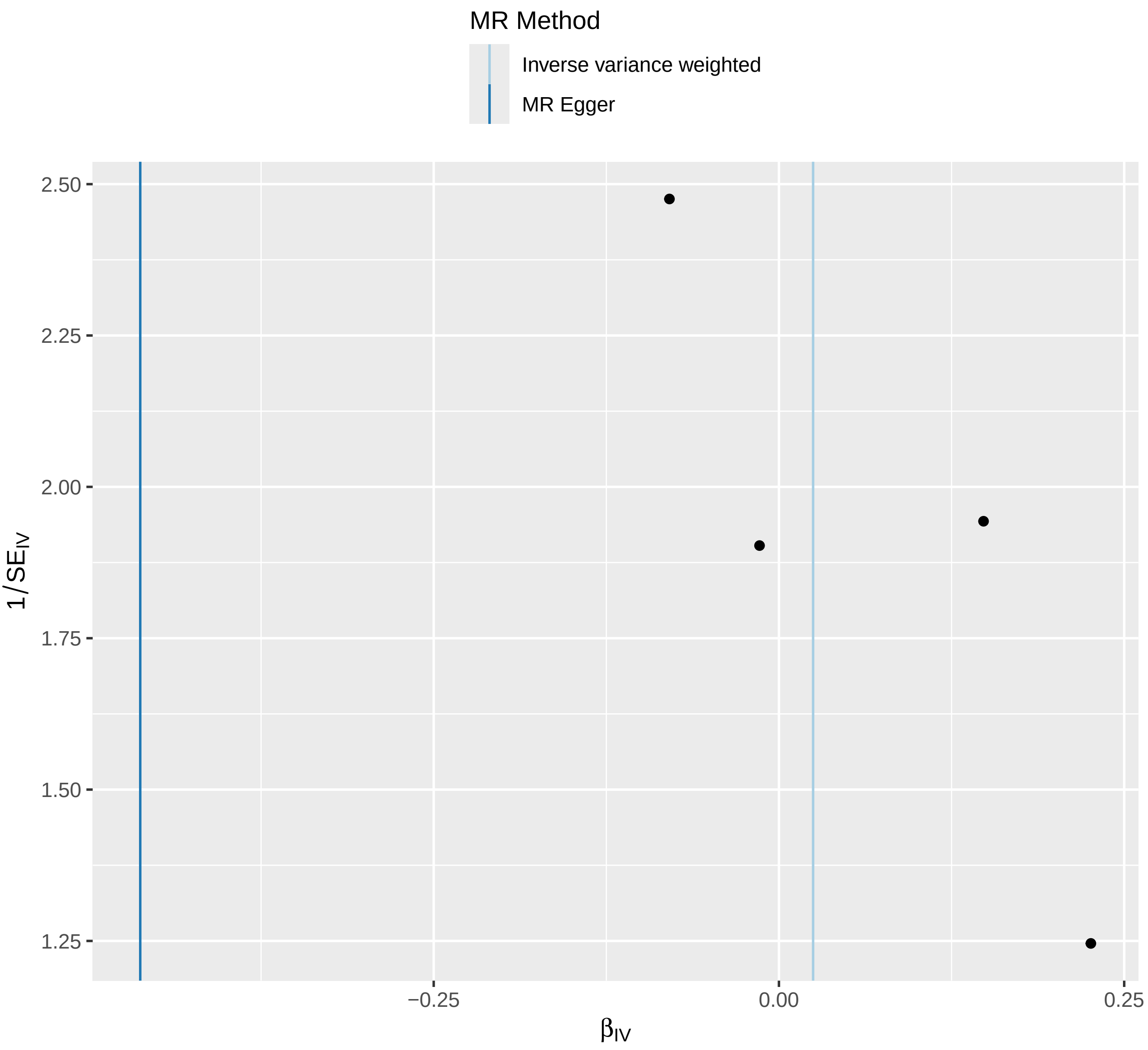
MR Method
Inverse variance weighted
MR Egger
2.50
2.25
2.00
1.75
1.50
1.25
$1/SE_{IV}$
−0.25
0.00
0.25
β_{IV}

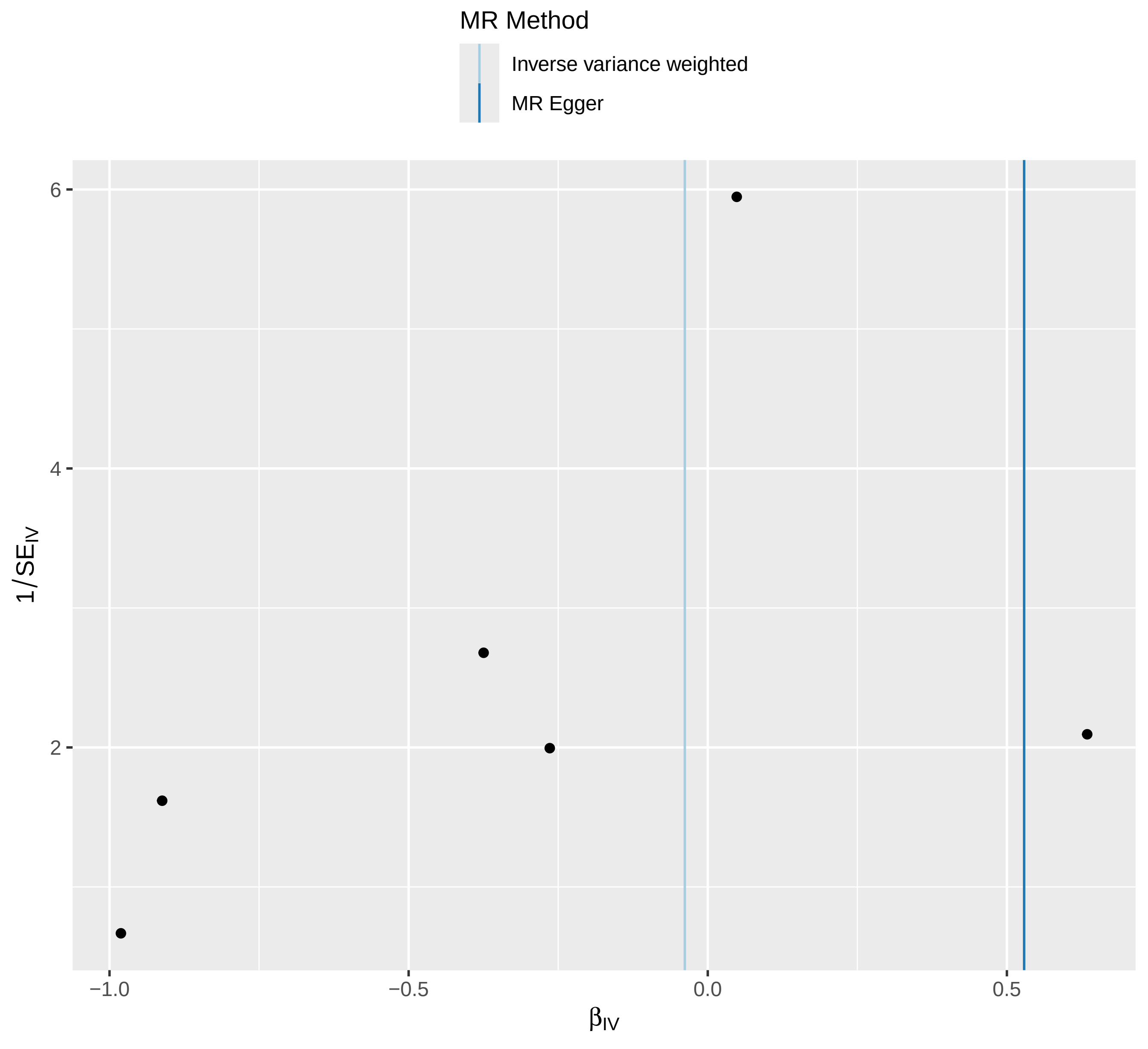
MR Method
Inverse variance weighted
MR Egger
6
4
2
1/SE$_{IV}$
−1.0
−0.5
0.0
0.5
β$_{IV}$

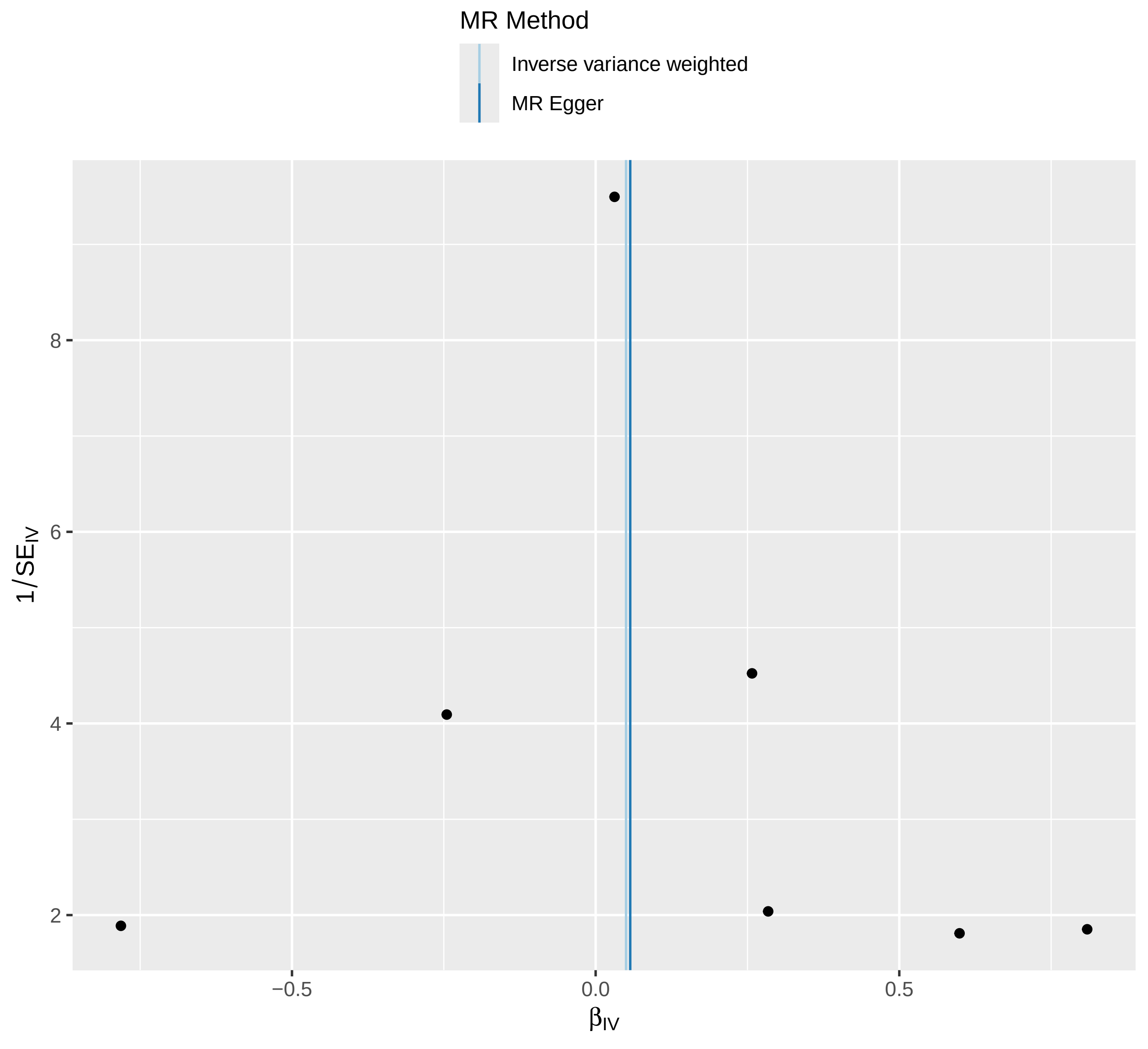
MR Method
Inverse variance weighted
MR Egger
1/SE$_{IV}$
8
6
4
2
−0.5
0.0
0.5
β$_{IV}$

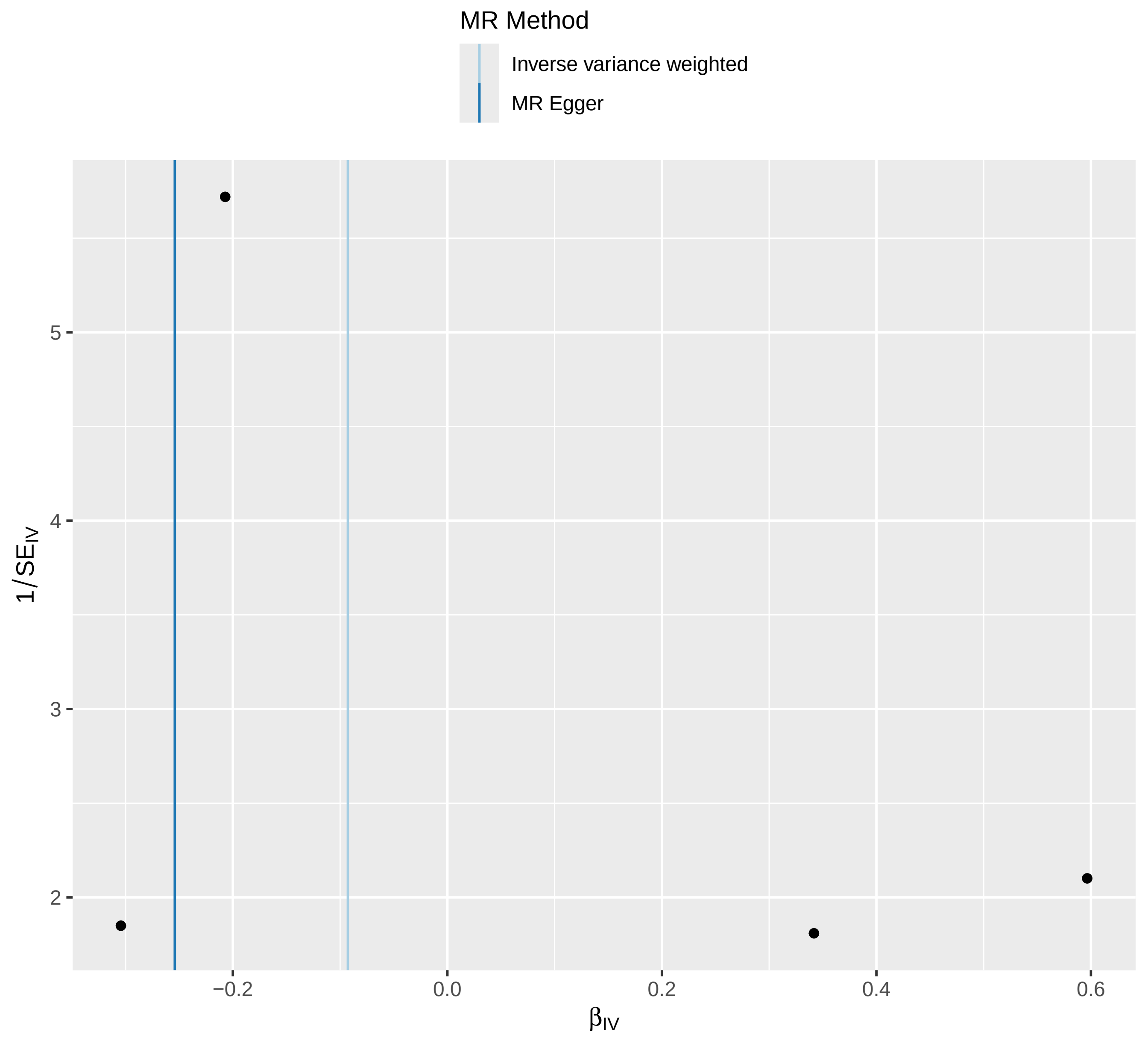
MR Method
Inverse variance weighted
MR Egger
1/SE$_{IV}$
5
4
3
2
−0.2
0.0
0.2
0.4
0.6
β_{IV}

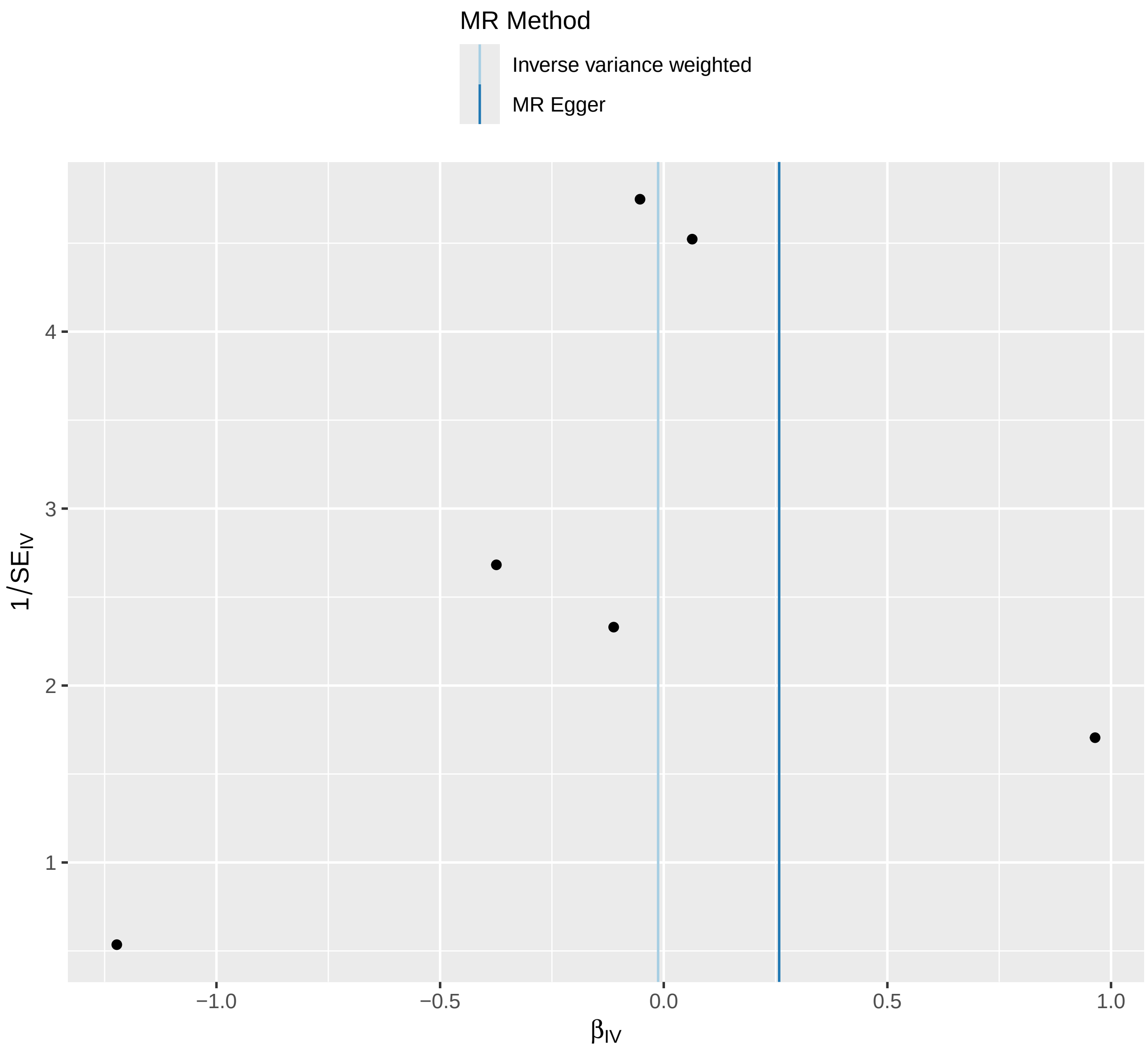

MR Method
Inverse variance weighted
MR Egger
4
3
2
1
$1/SE_{IV}$
−1.0
−0.5
0.0
0.5
1.0
β_{IV}

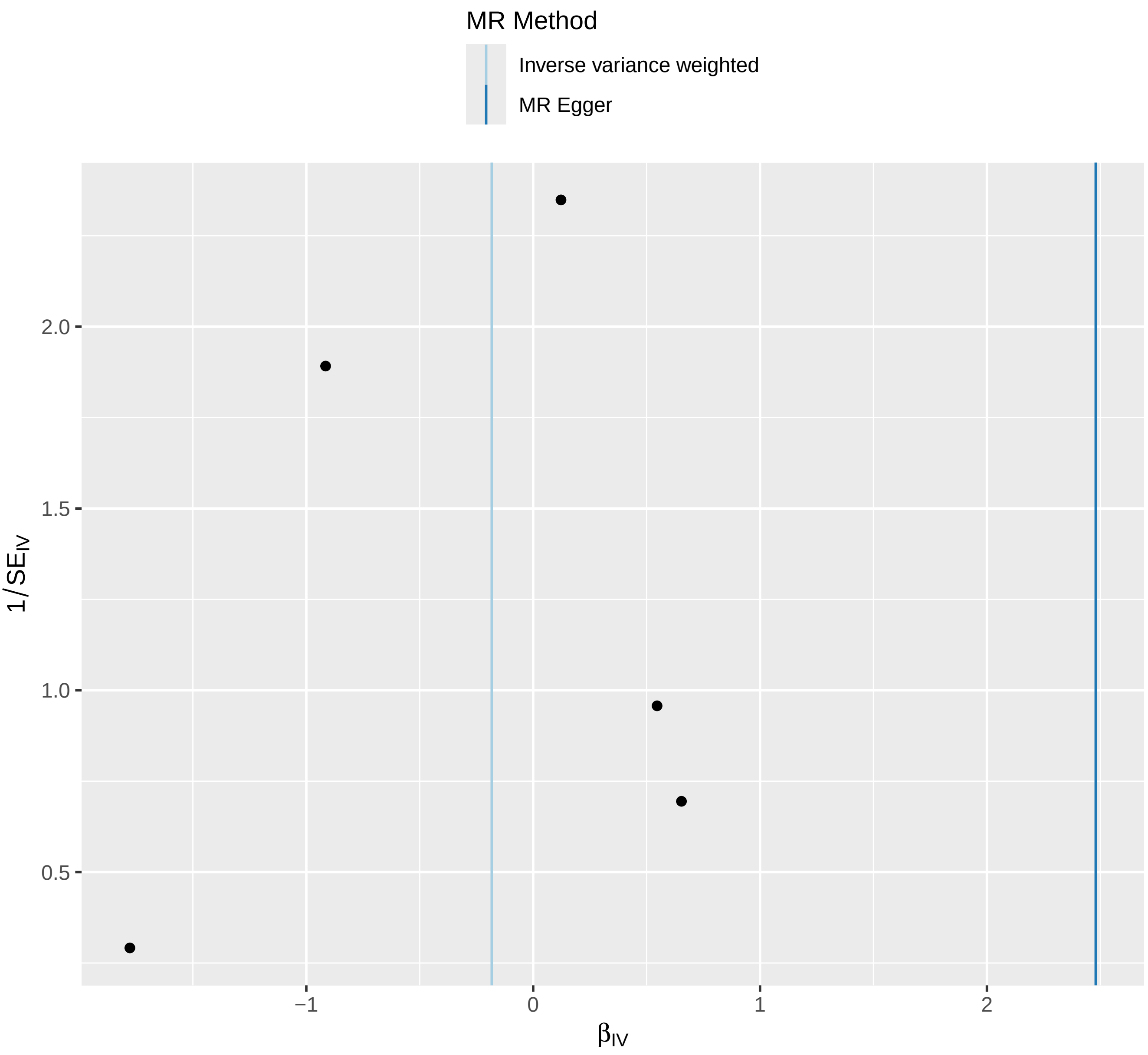
MR Method
Inverse variance weighted
MR Egger
2.0
1.5
1.0
0.5
−1
0
1
2
$1/SE_{IV}$
β_{IV}

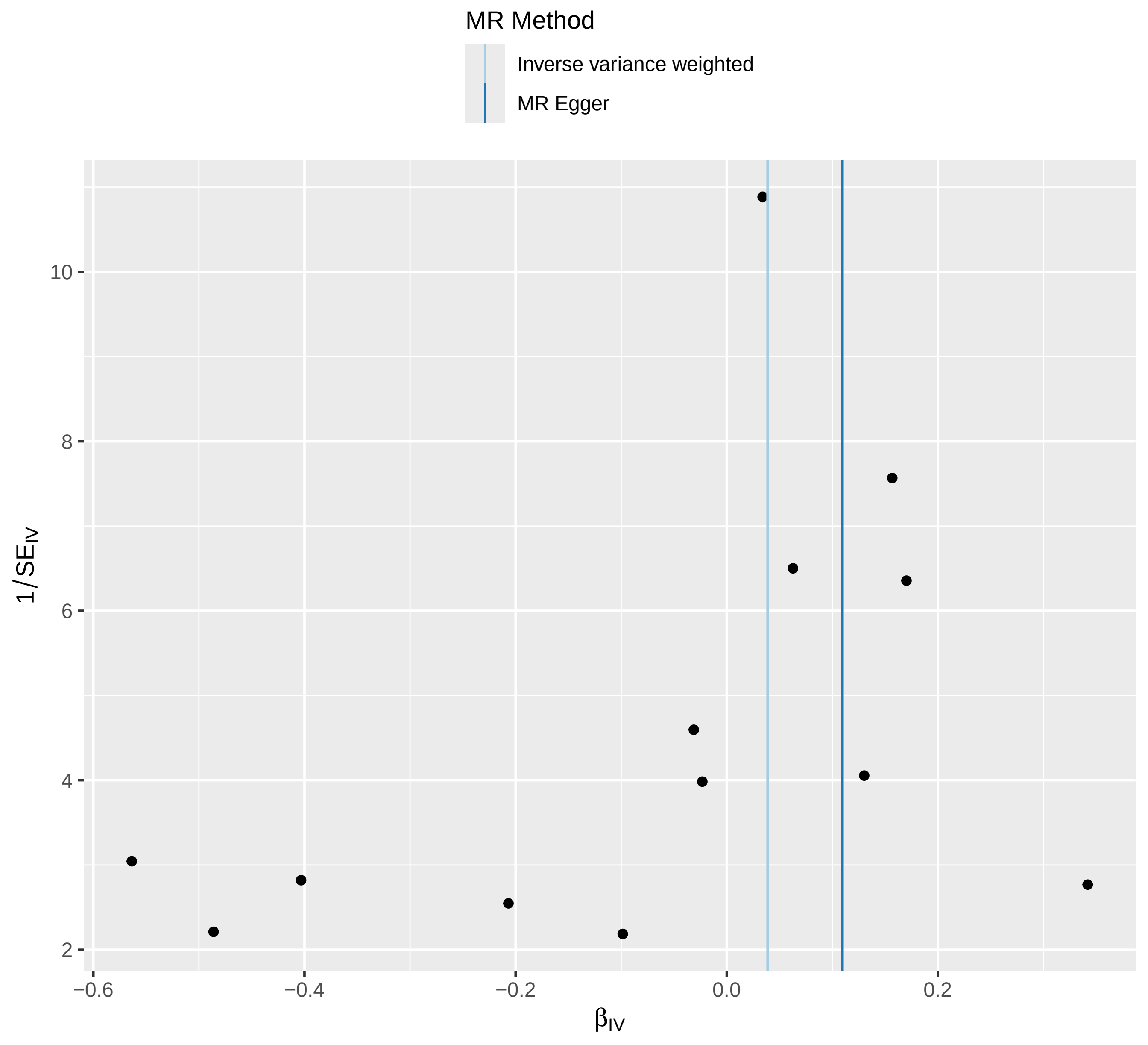

MR Method
Inverse variance weighted
MR Egger
1/SE_IV
10
8
6
4
2
−0.6
−0.4
−0.2
0.0
0.2
β_IV

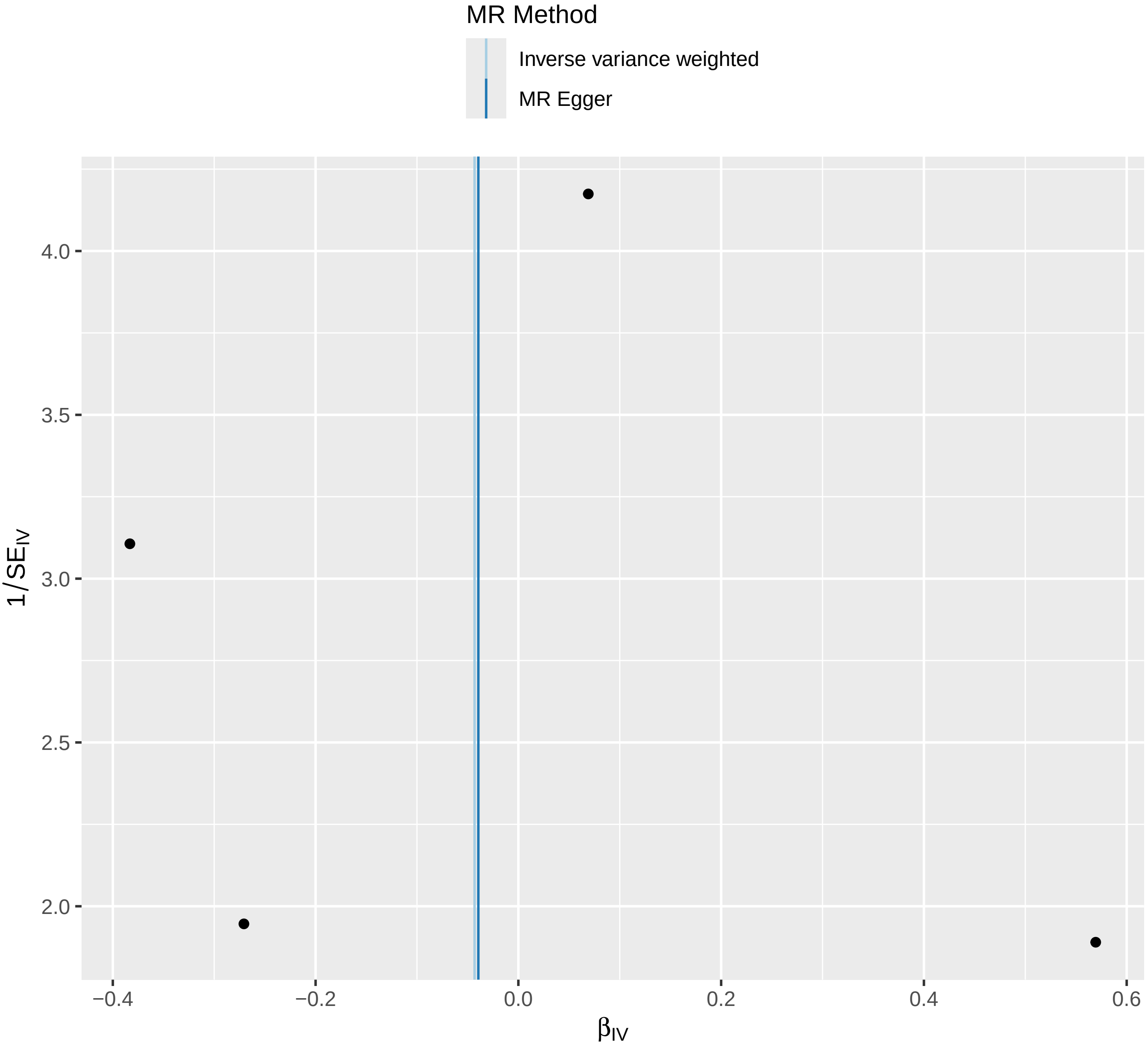
MR Method
Inverse variance weighted
MR Egger
1/SE_IV
β_IV
4.0
3.5
3.0
2.5
2.0
−0.4
−0.2
0.0
0.2
0.4
0.6

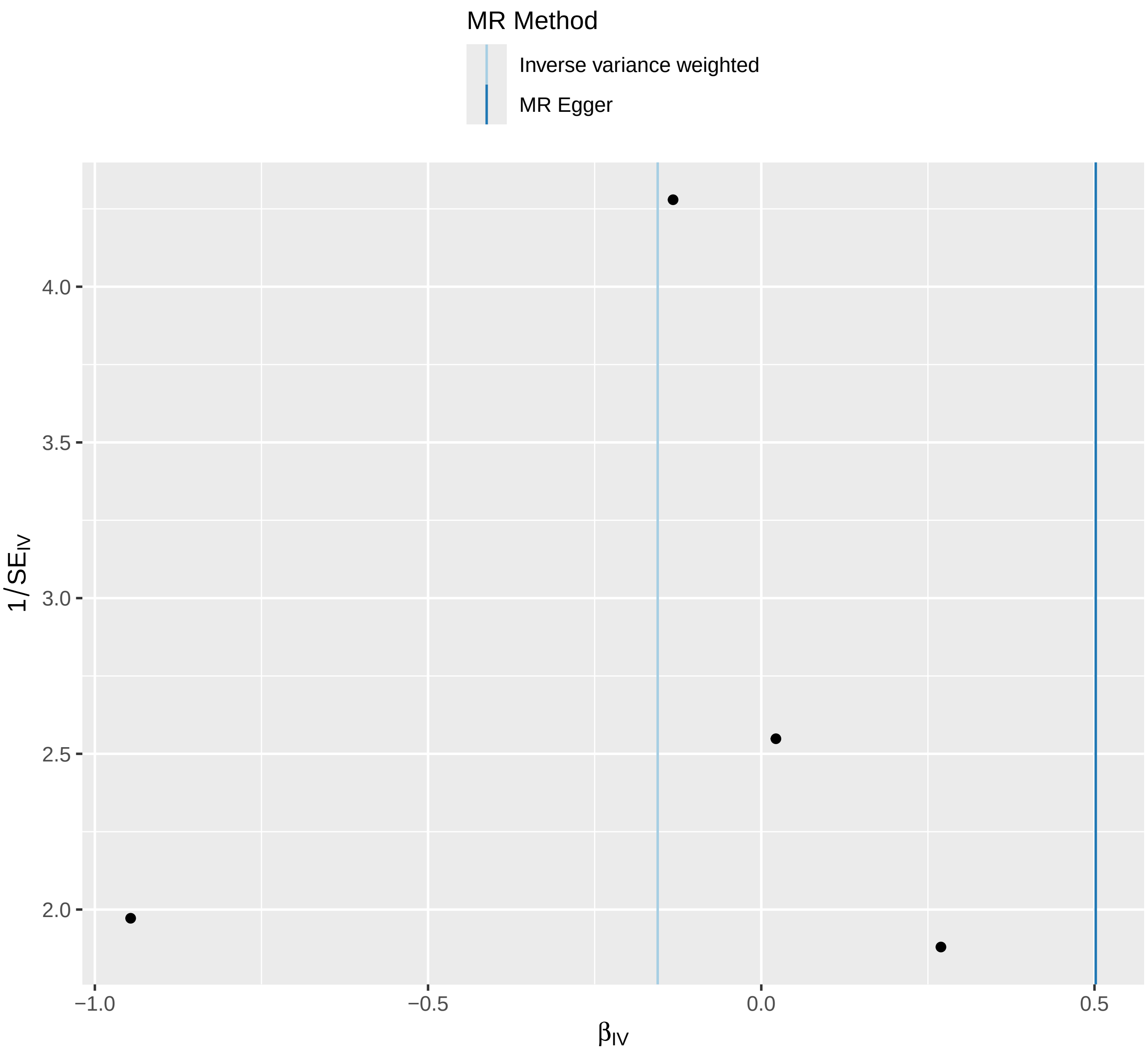
MR Method
Inverse variance weighted
MR Egger
4.0
3.5
3.0
2.5
2.0
$1/SE_{IV}$
−1.0
−0.5
0.0
0.5
β_{IV}

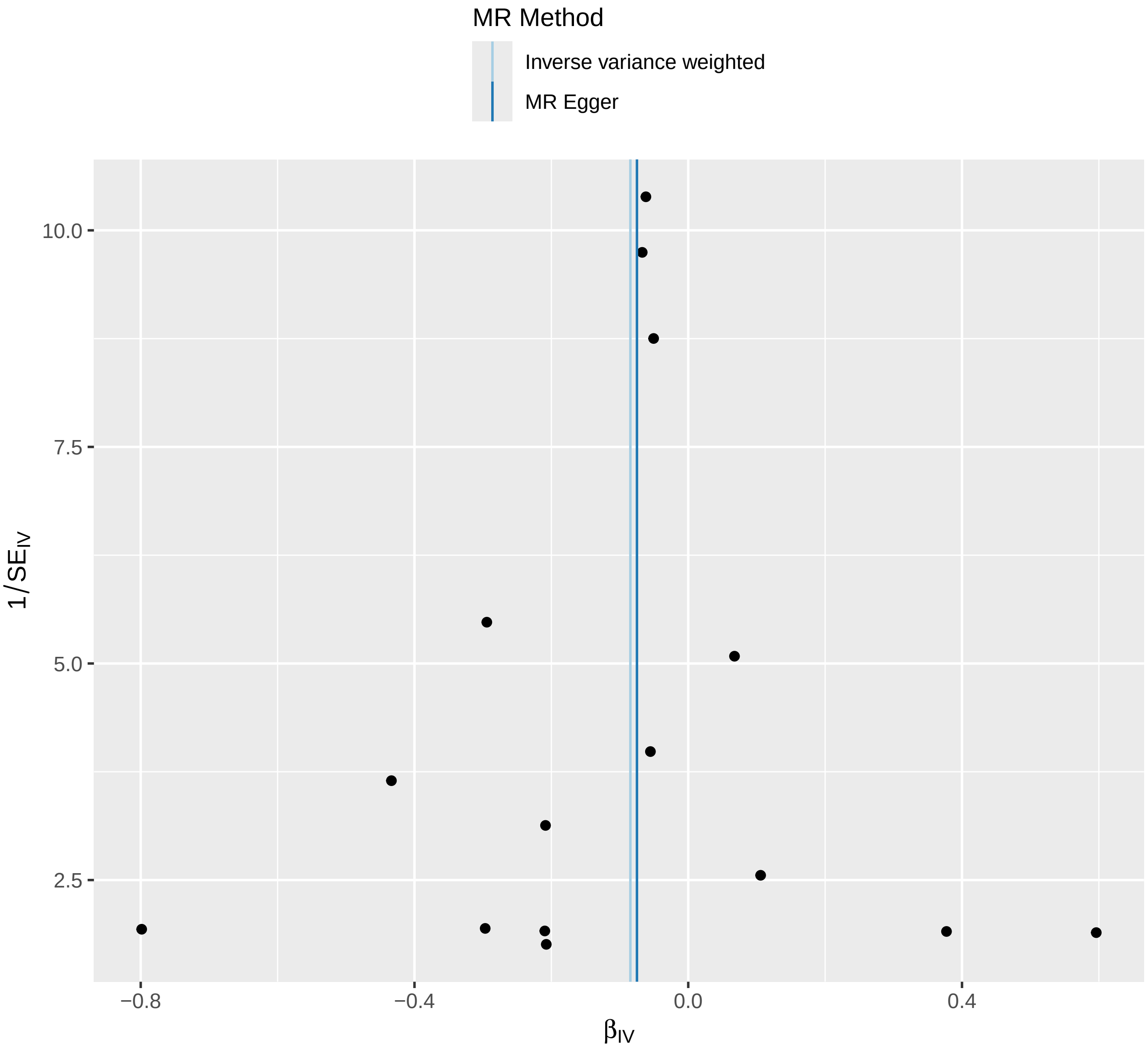

MR Method
Inverse variance weighted
MR Egger
10.0
7.5
5.0
2.5
1/SE_IV
−0.8
−0.4
0.0
0.4
β_IV

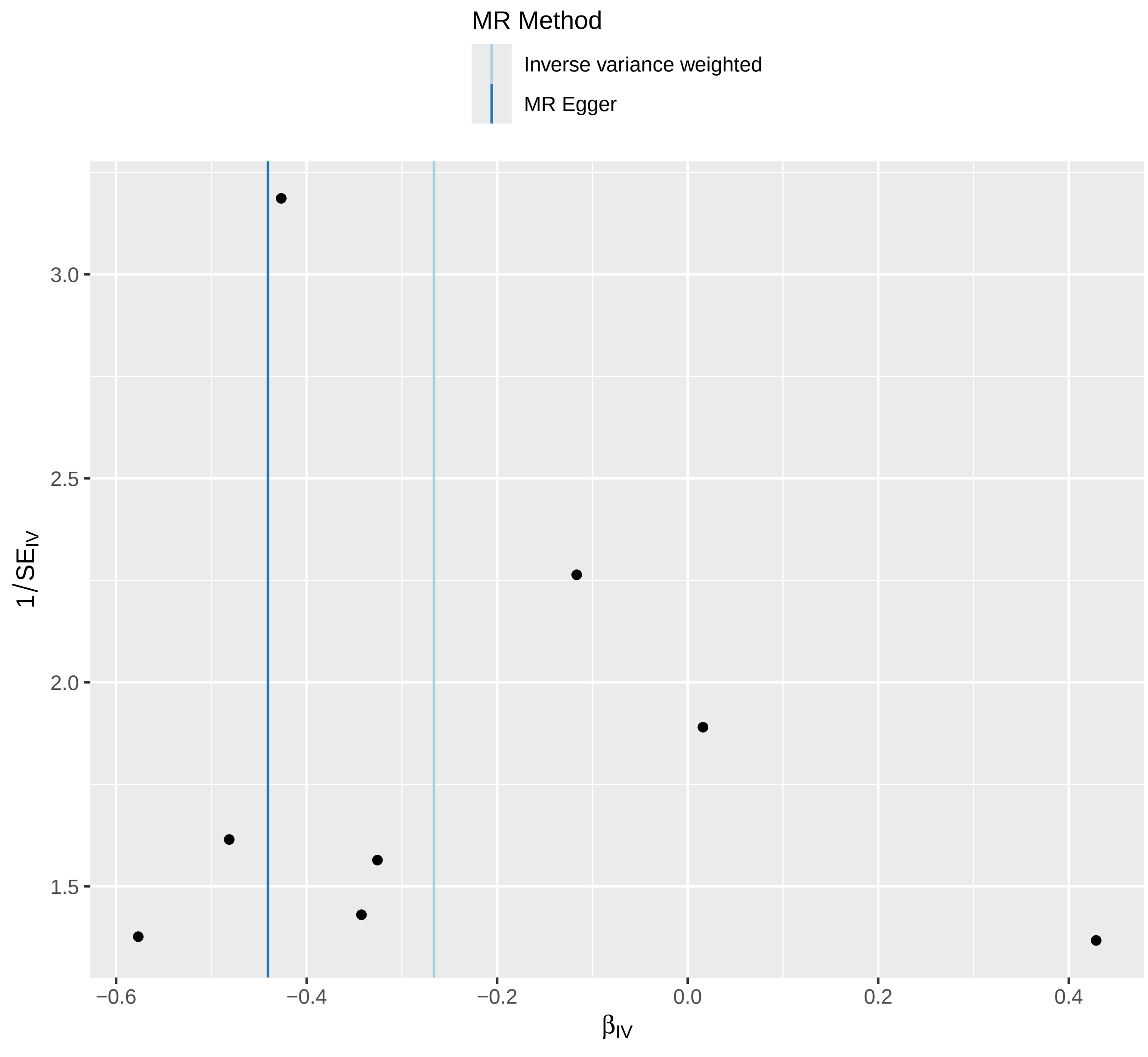
MR Method
Inverse variance weighted
MR Egger
1/SE_{IV}
3.0
2.5
2.0
1.5
−0.6
−0.4
−0.2
0.0
0.2
0.4
β_{IV}

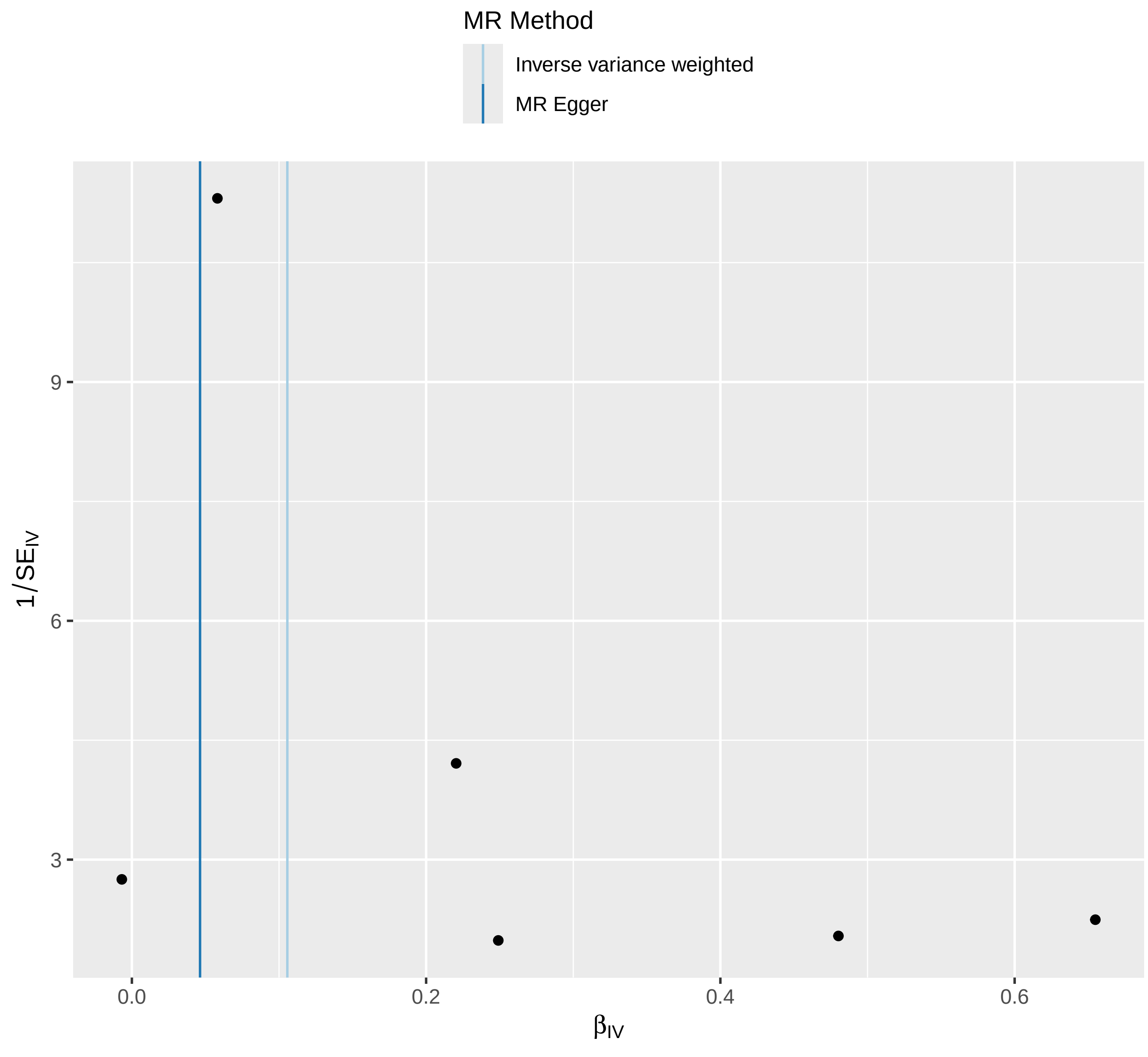

MR Method
Inverse variance weighted
MR Egger
9
6
3
1/SE_IV
0.0
0.2
0.4
0.6
β_IV

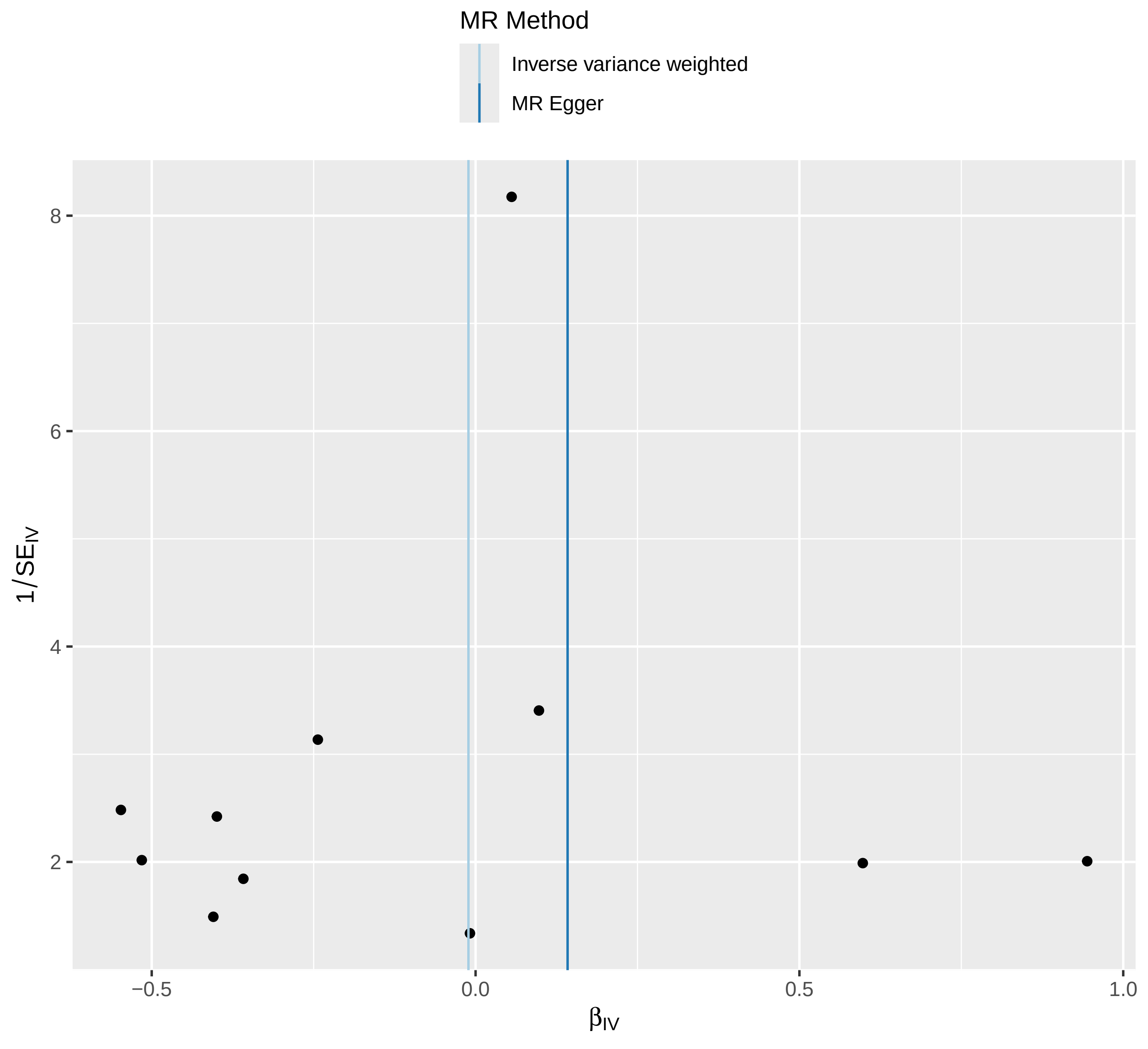

MR Method
Inverse variance weighted
MR Egger
8
6
4
2
$1/SE_{IV}$
−0.5
0.0
0.5
1.0
β_{IV}

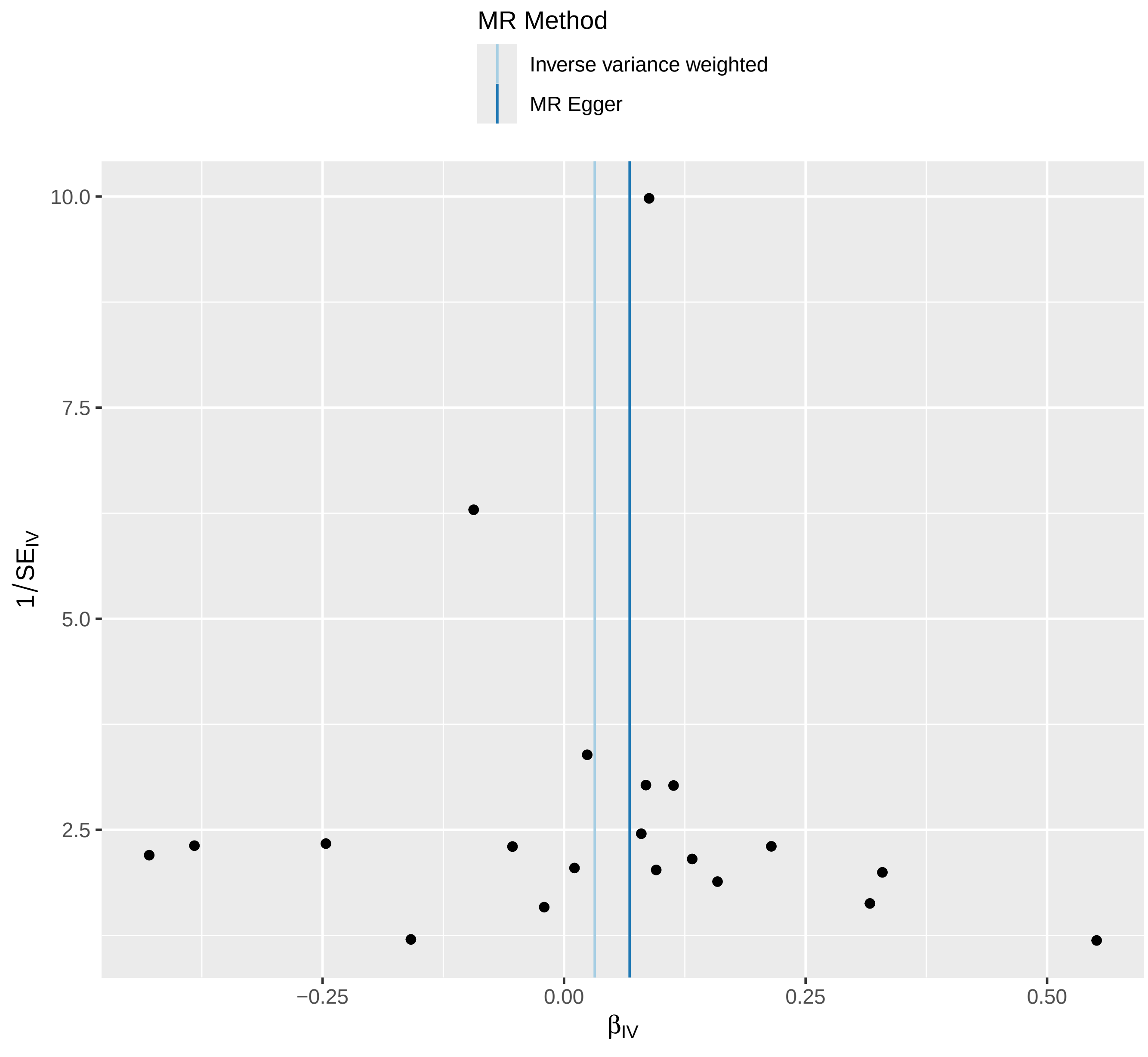

MR Method
Inverse variance weighted
MR Egger
10.0
7.5
5.0
2.5
−0.25
0.00
0.25
0.50
$1/SE_{IV}$
β_{IV}

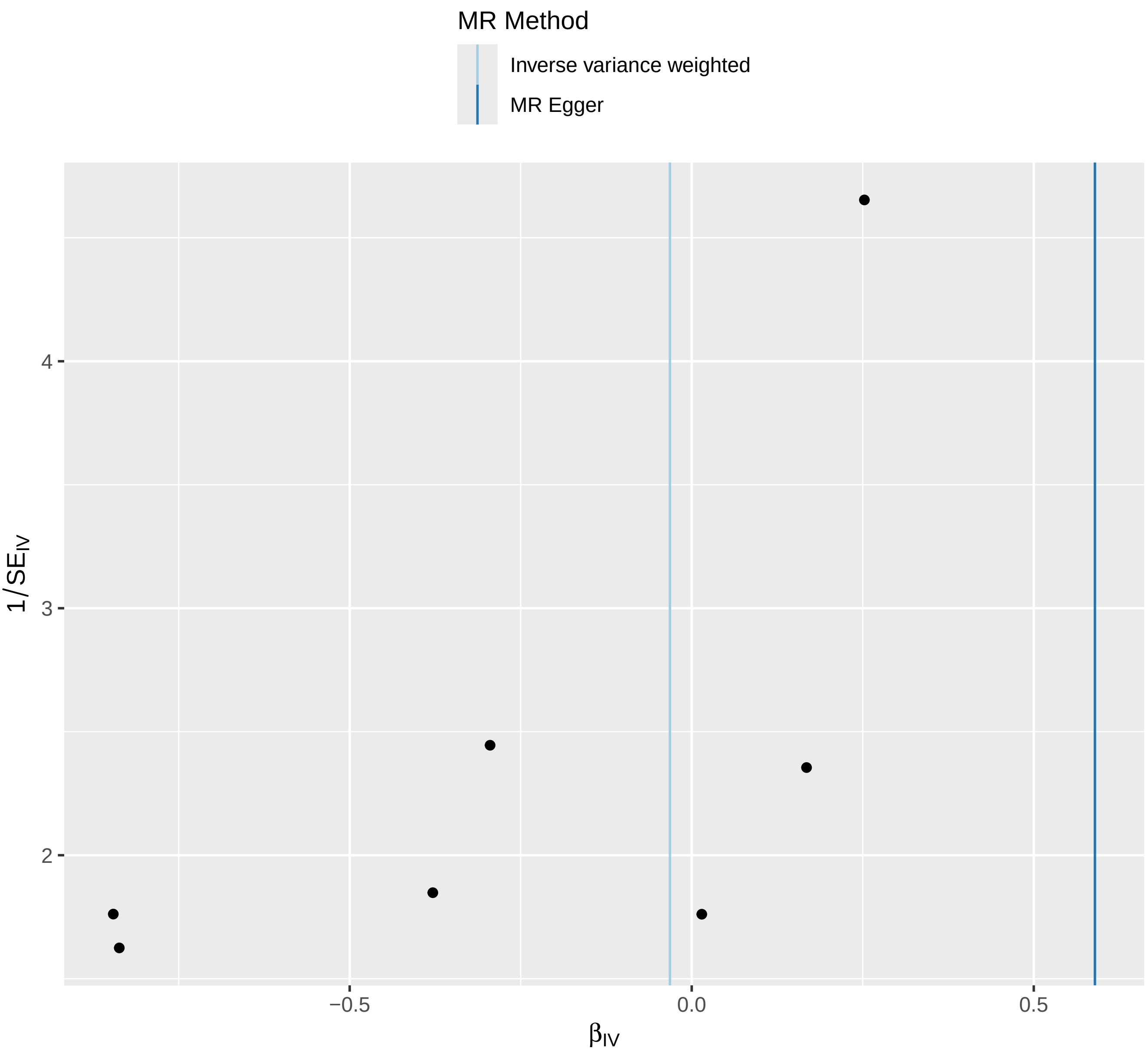

MR Method
Inverse variance weighted
MR Egger
4
3
2
1/SE$_{IV}$
−0.5
0.0
0.5
β$_{IV}$

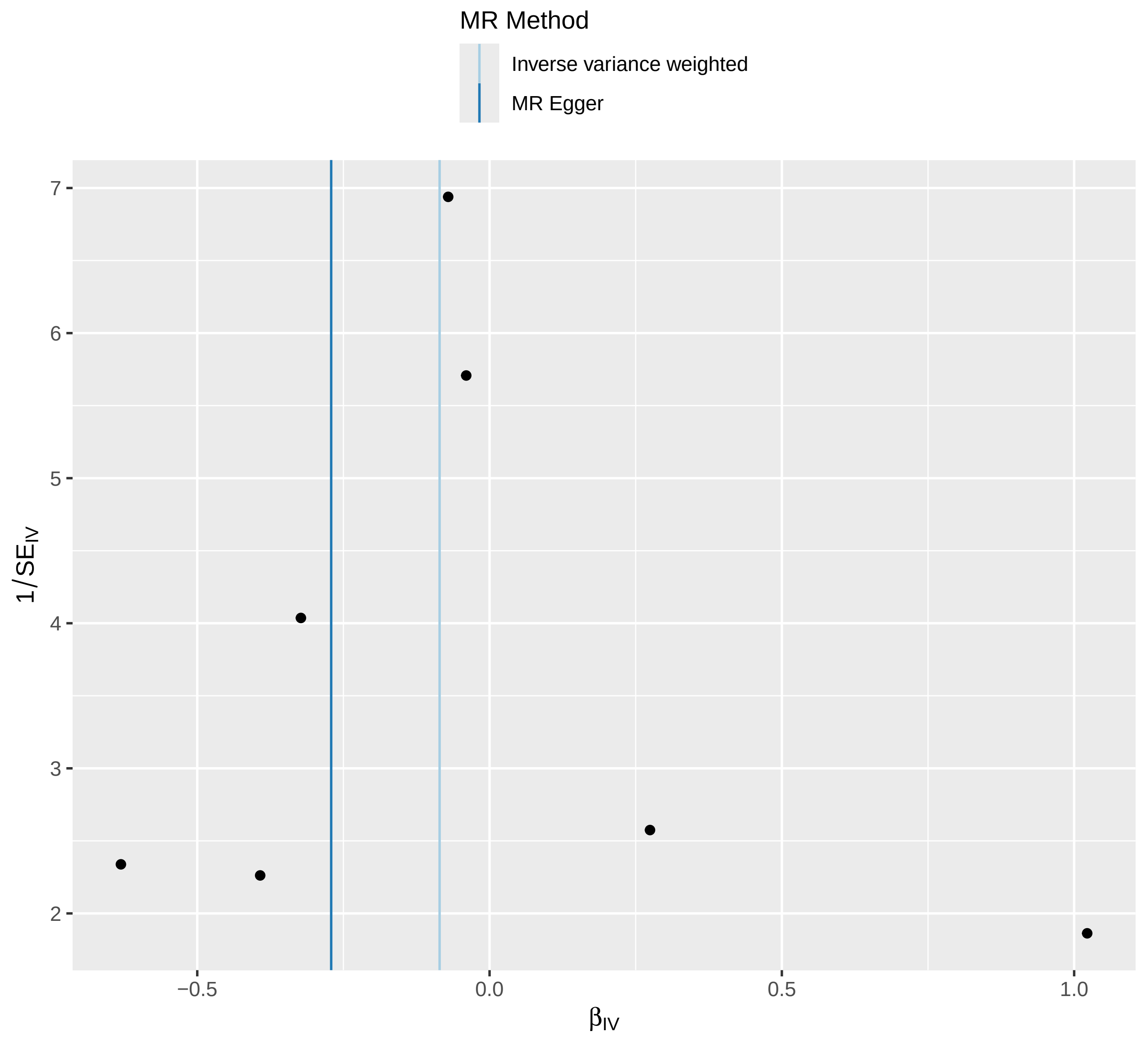

MR Method
Inverse variance weighted
MR Egger
7
6
5
4
3
2
1/SE$_{IV}$
−0.5
0.0
0.5
1.0
β$_{IV}$

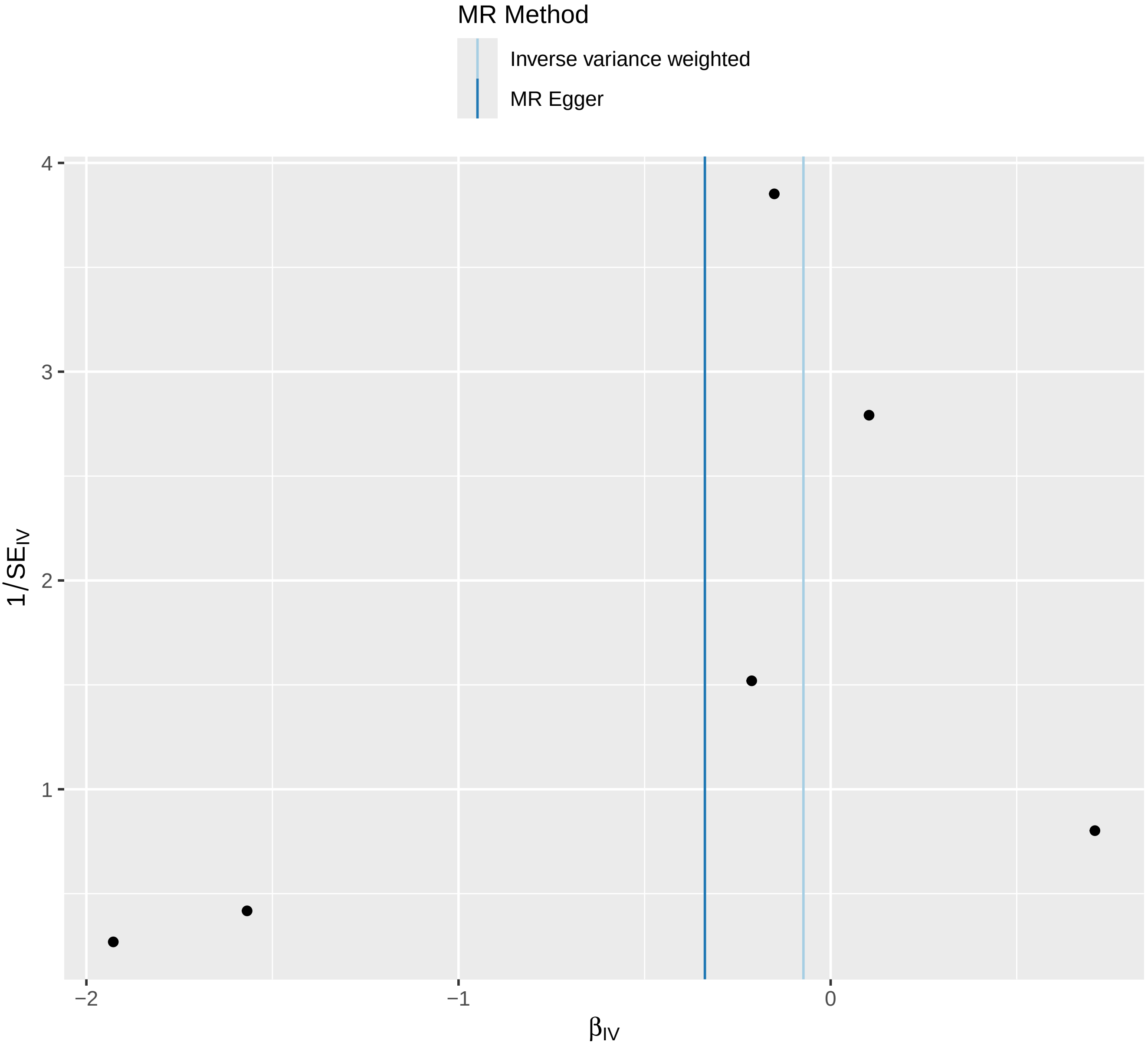
MR Method
Inverse variance weighted
MR Egger
4
3
2
1
1/SE_IV
−2
−1
0
β_IV

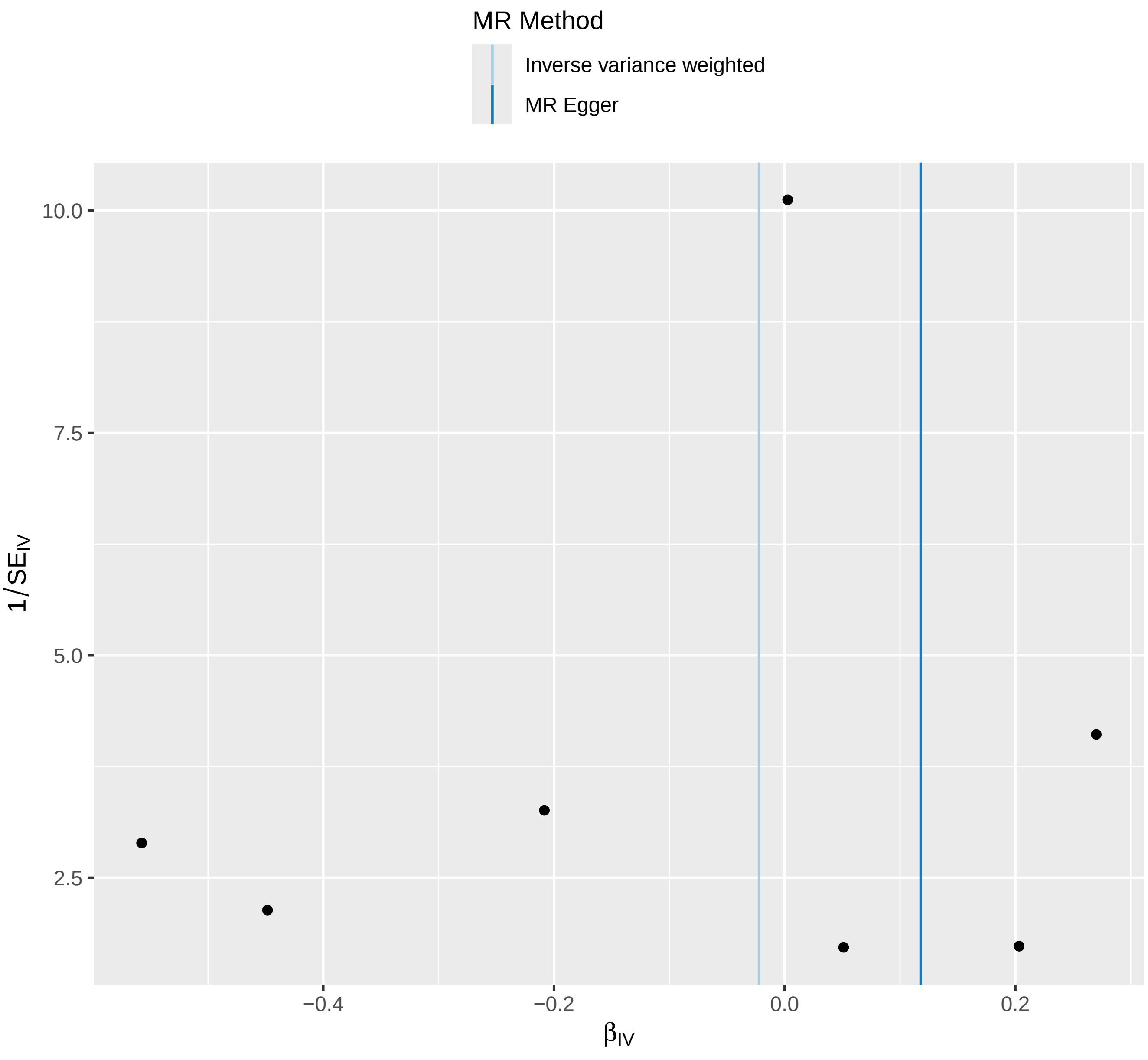
MR Method
Inverse variance weighted
MR Egger
10.0
7.5
5.0
2.5
1/SE_IV
−0.4
−0.2
0.0
0.2
β_IV

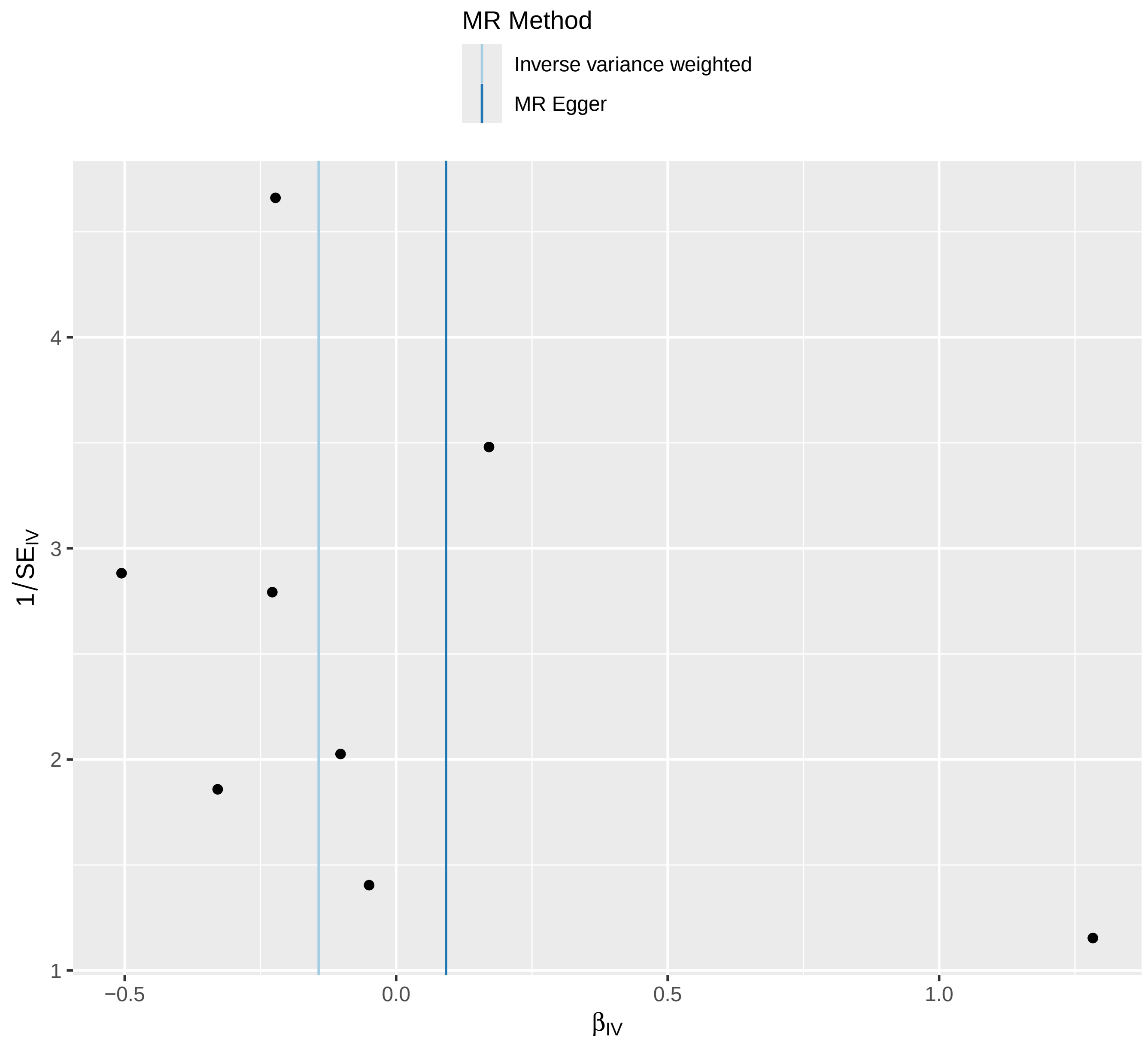

MR Method
Inverse variance weighted
MR Egger
1/SE$_{IV}$
4
3
2
1
−0.5
0.0
0.5
1.0
β$_{IV}$

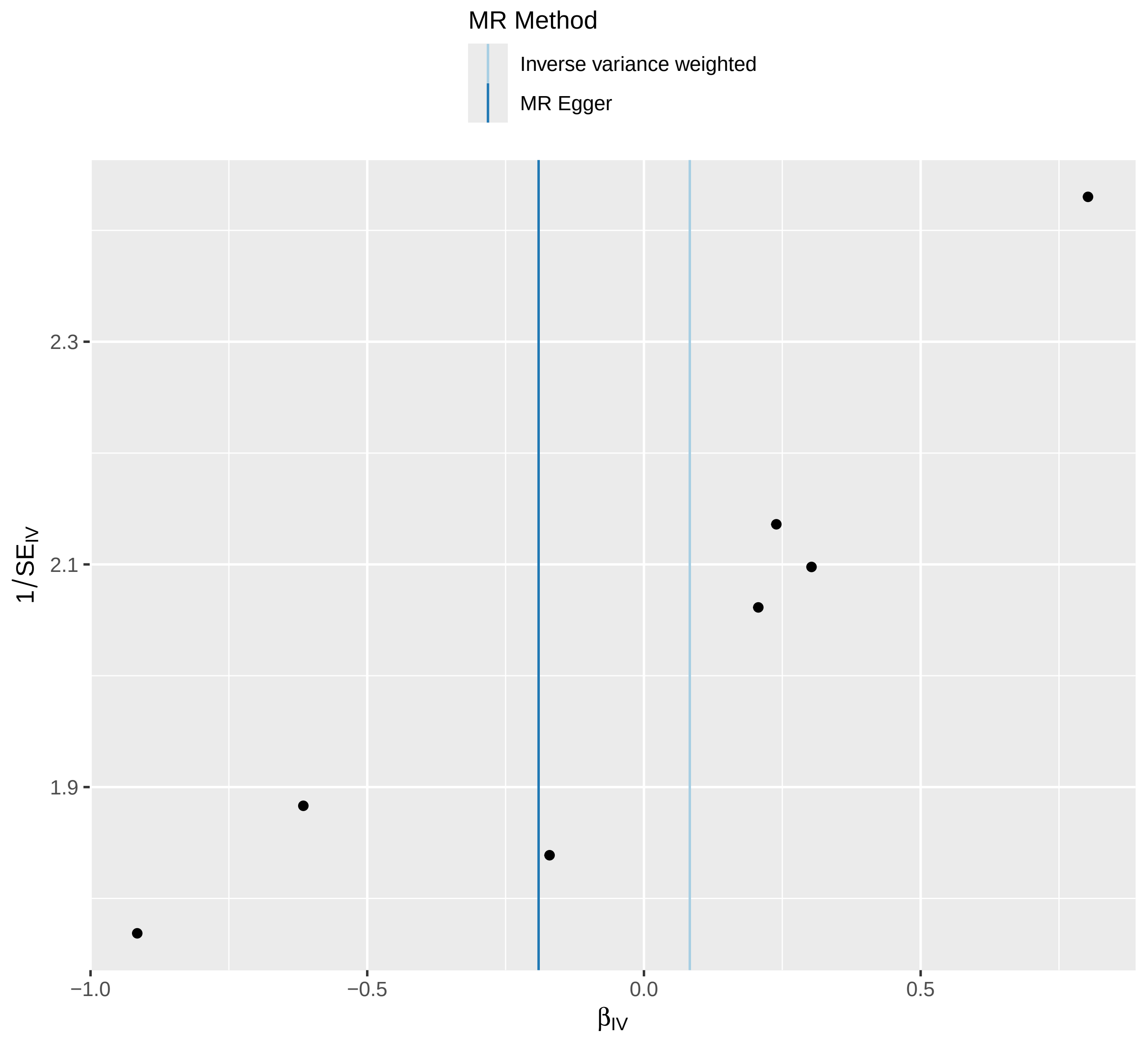

MR Method
Inverse variance weighted
MR Egger
2.3
2.1
1.9
1/SE$_{IV}$
−1.0
−0.5
0.0
0.5
β$_{IV}$

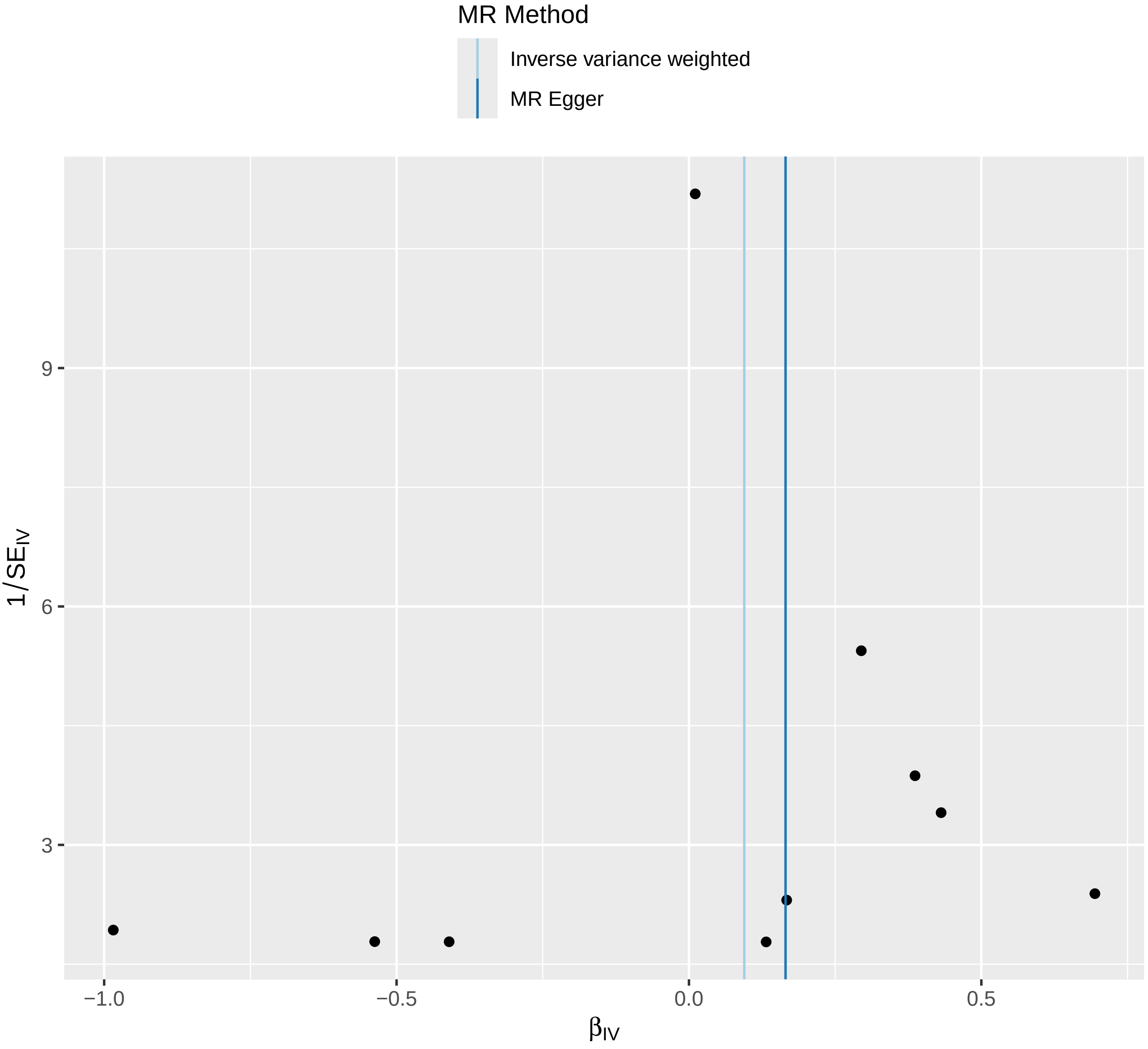

MR Method
Inverse variance weighted
MR Egger
9
6
3
1/SE_IV
−1.0
−0.5
0.0
0.5
β_IV

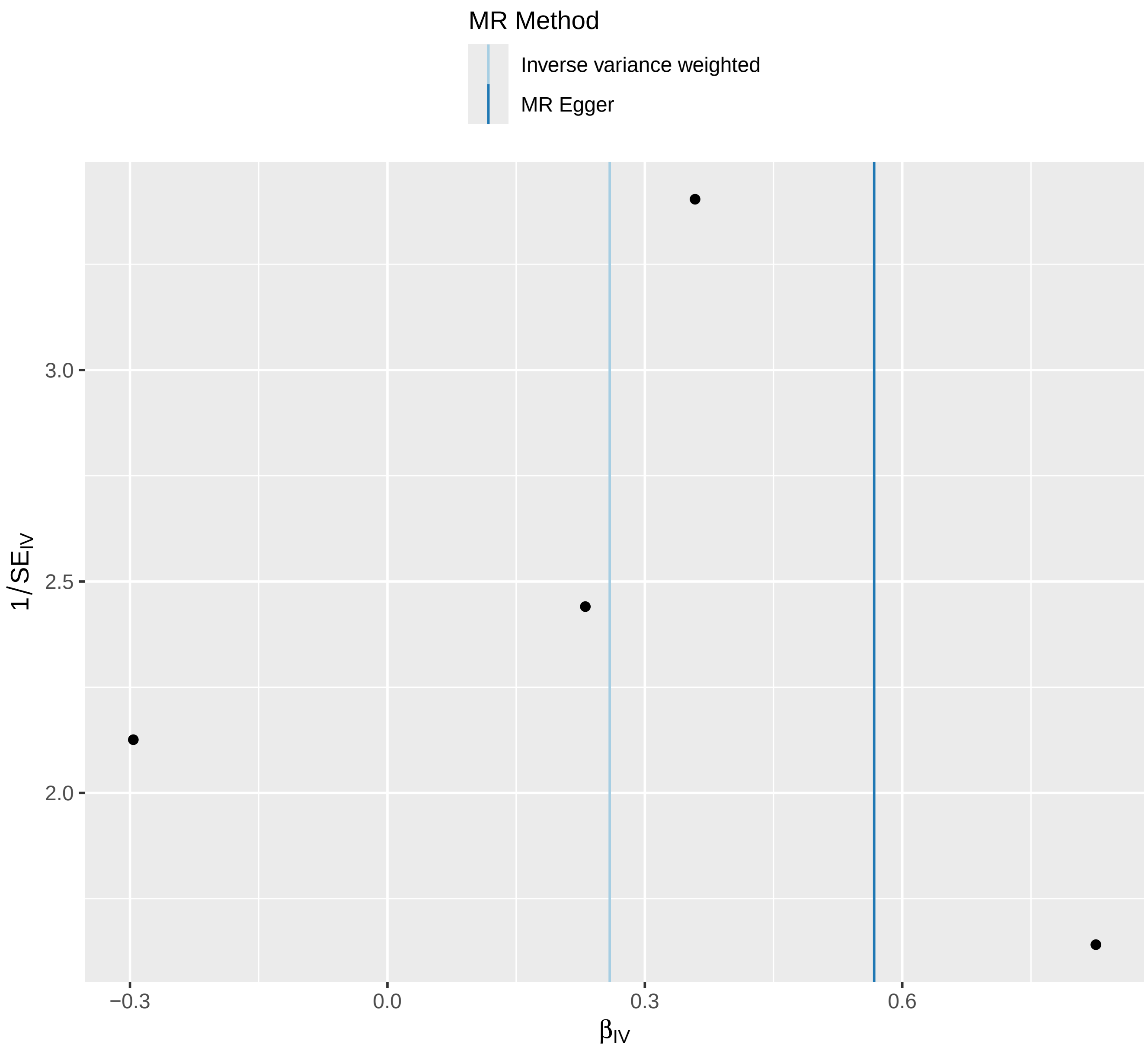

MR Method
Inverse variance weighted
MR Egger
3.0
2.5
2.0
1/SE$_{IV}$
−0.3
0.0
0.3
0.6
β$_{IV}$

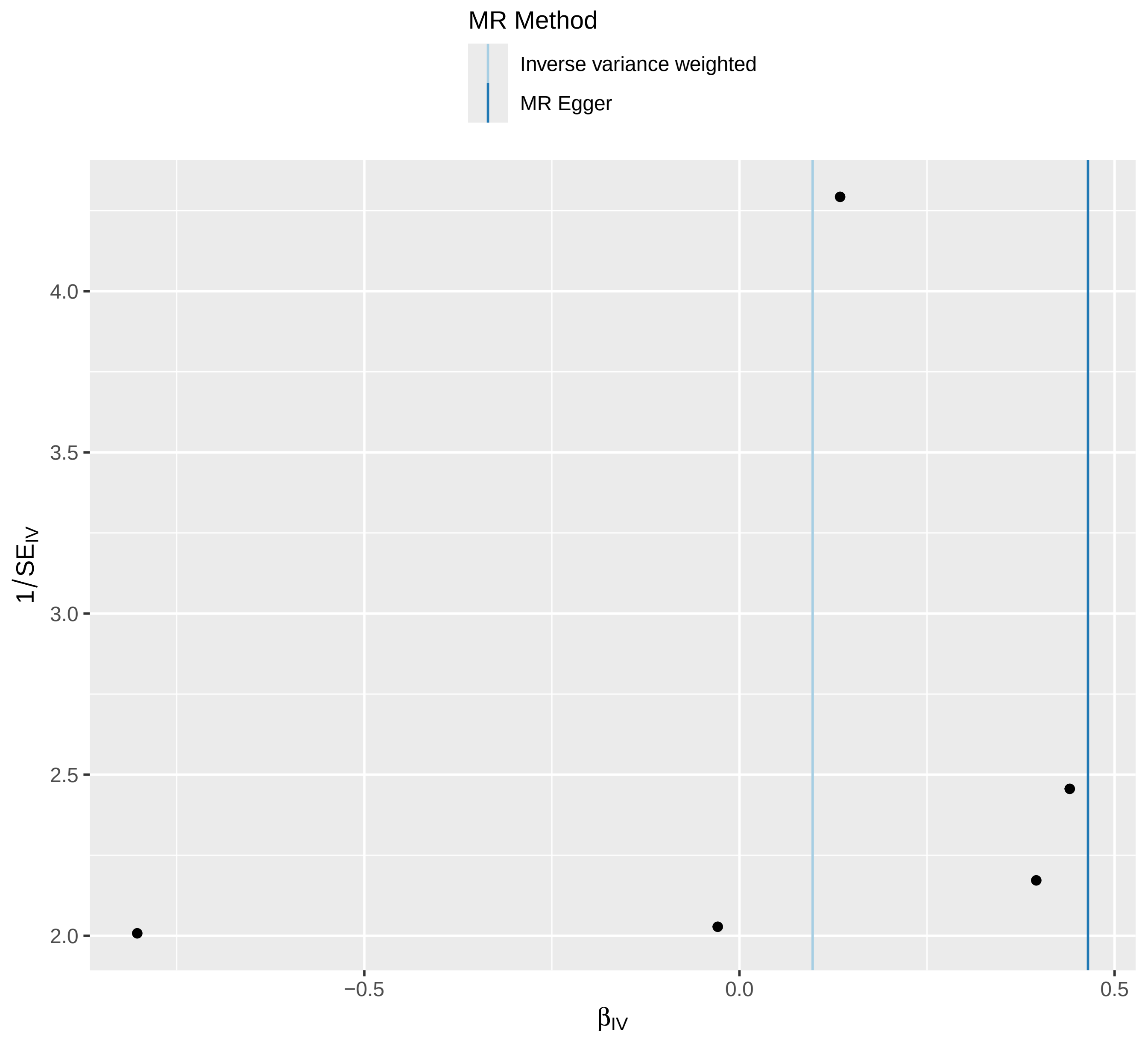

MR Method
Inverse variance weighted
MR Egger
4.0
3.5
3.0
2.5
2.0
1/SE$_{IV}$
−0.5
0.0
0.5
β_{IV}

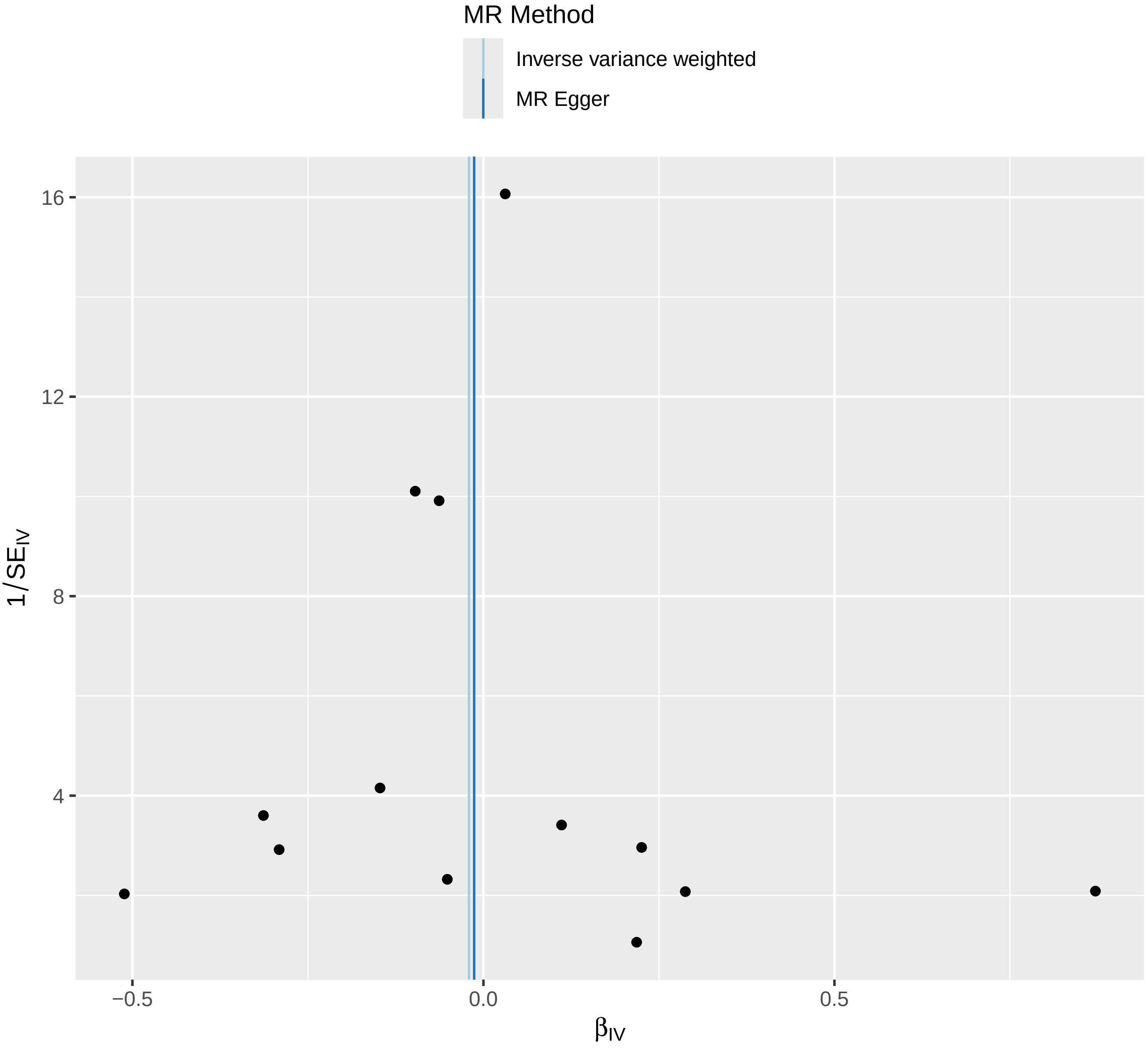
MR Method
Inverse variance weighted
MR Egger
16
12
8
4
1/SE_IV
−0.5
0.0
0.5
β_IV

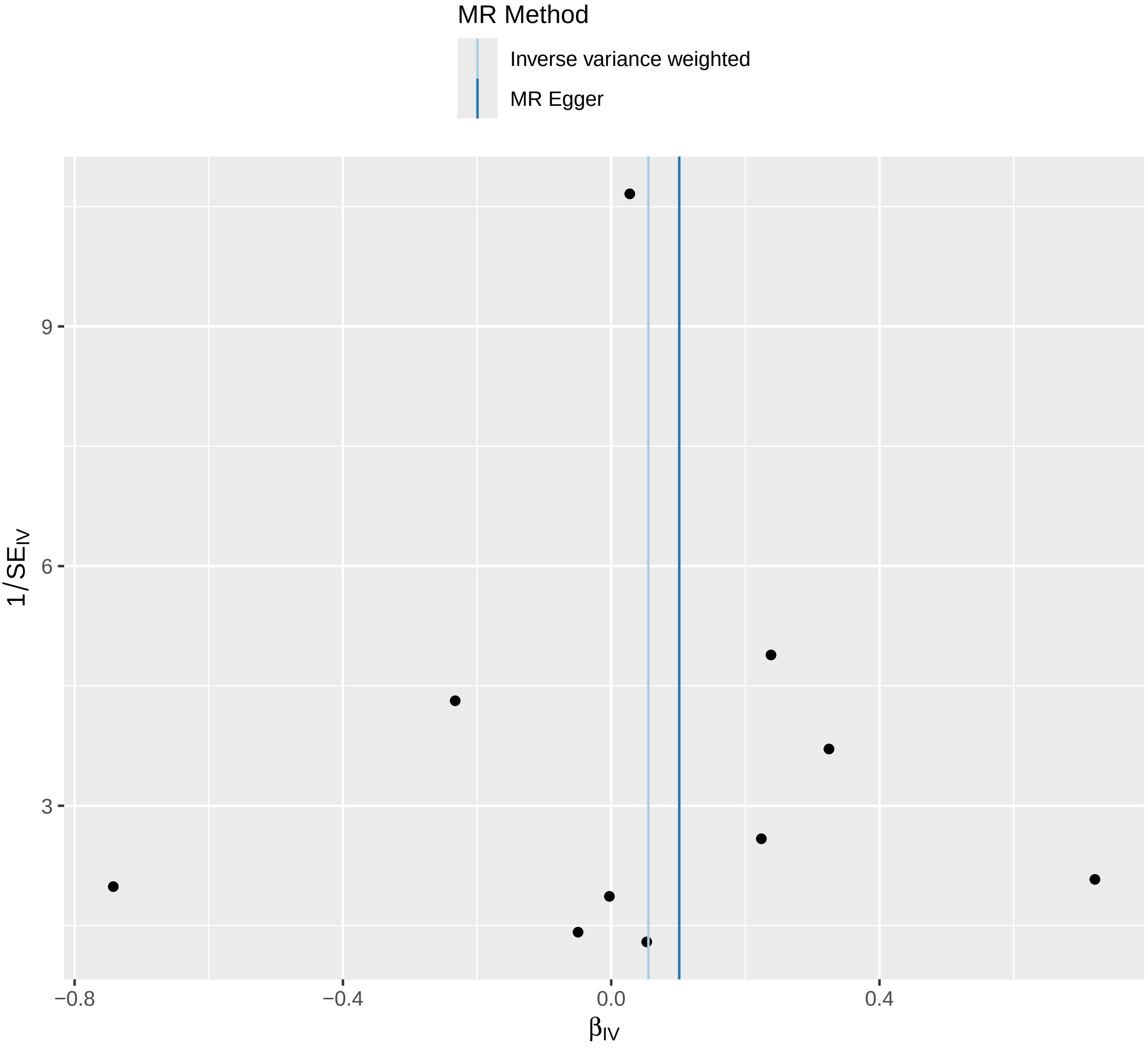
MR Method
Inverse variance weighted
MR Egger
9
6
3
1/SE_IV
−0.8
−0.4
0.0
0.4
β_IV

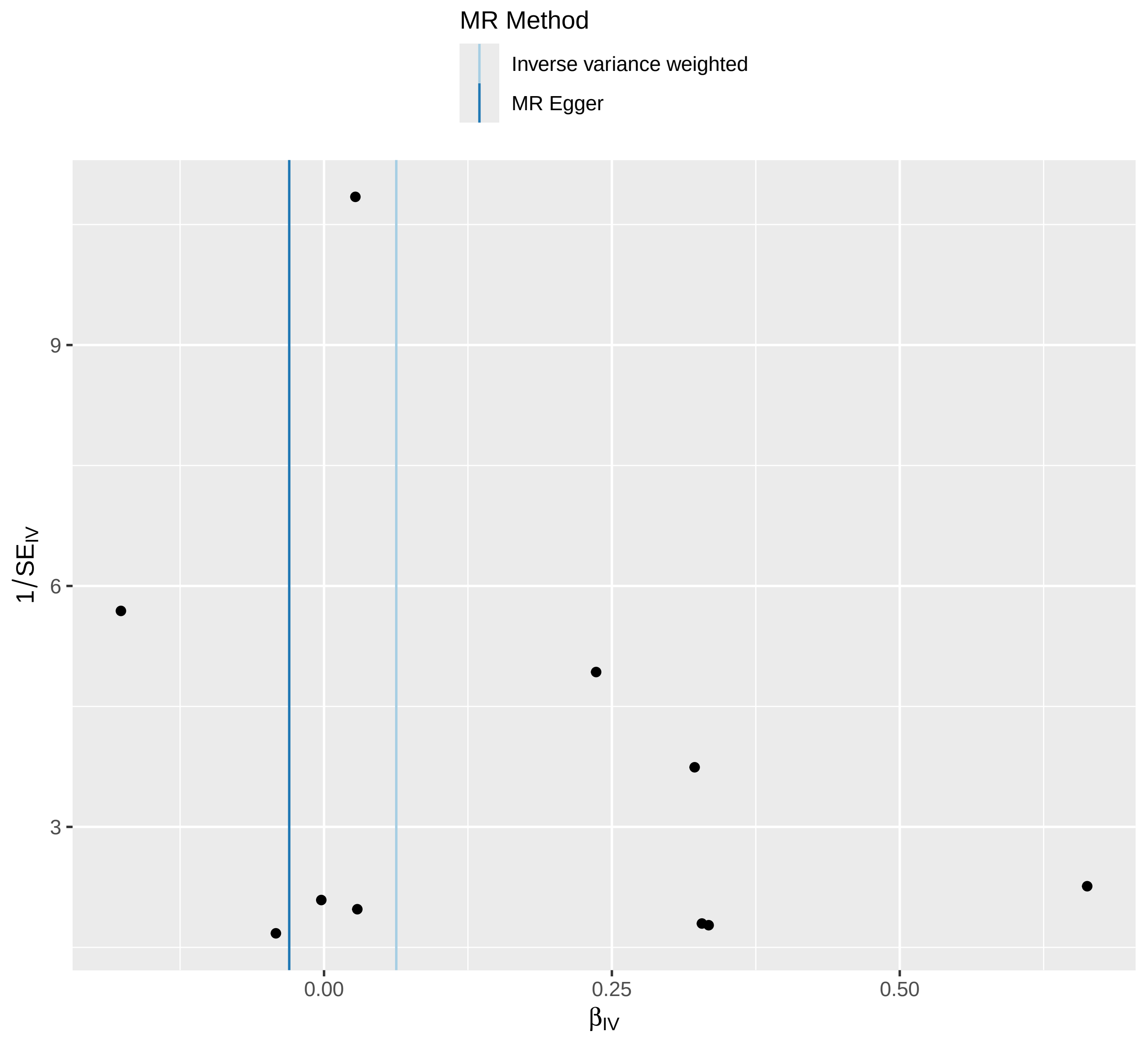

MR Method
Inverse variance weighted
MR Egger
9
6
3
0.00
0.25
0.50
$1/SE_{IV}$
β_{IV}

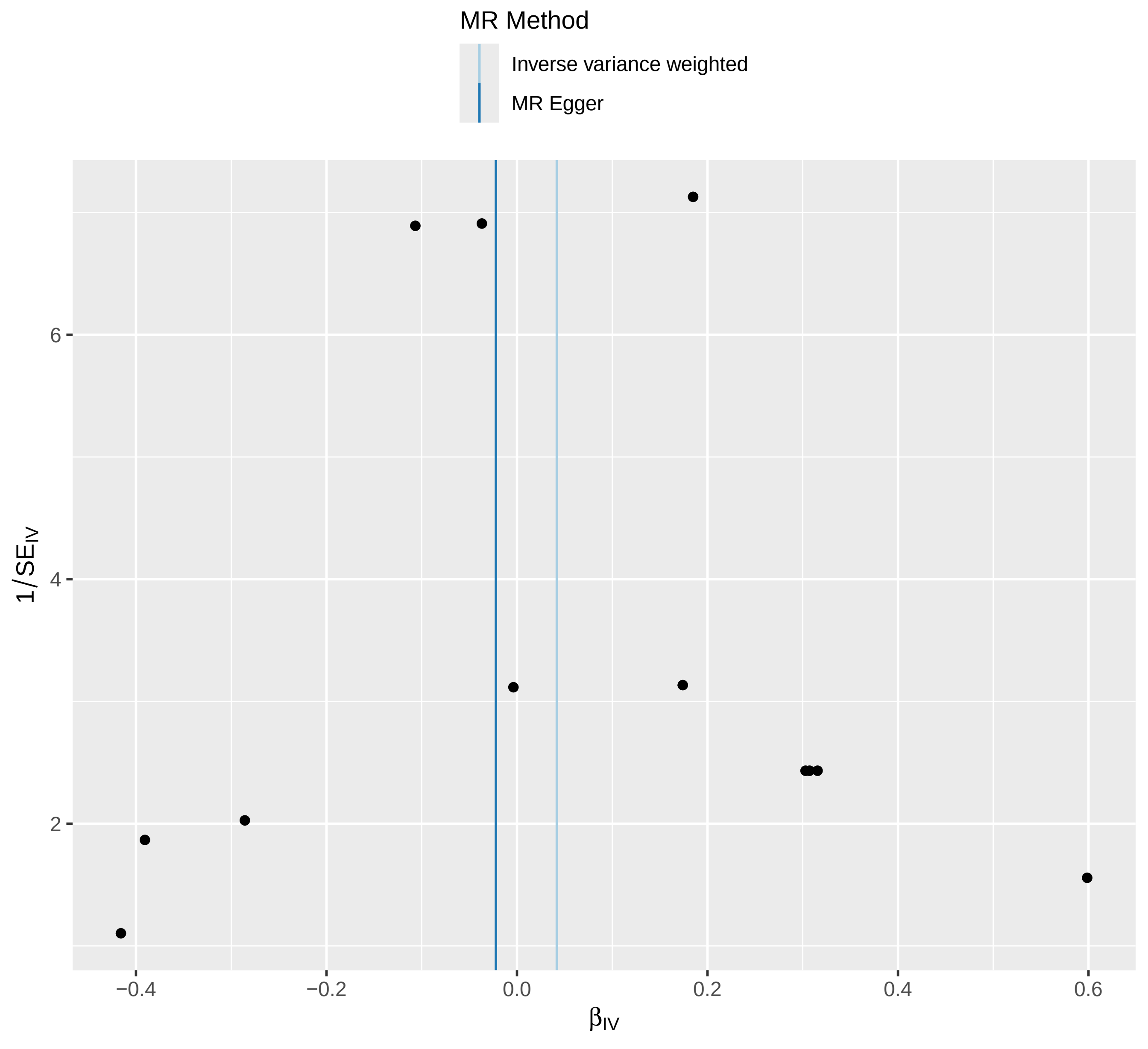
MR Method
Inverse variance weighted
MR Egger
1/SE$_{IV}$
6
4
2
−0.4
−0.2
0.0
0.2
0.4
0.6
β$_{IV}$

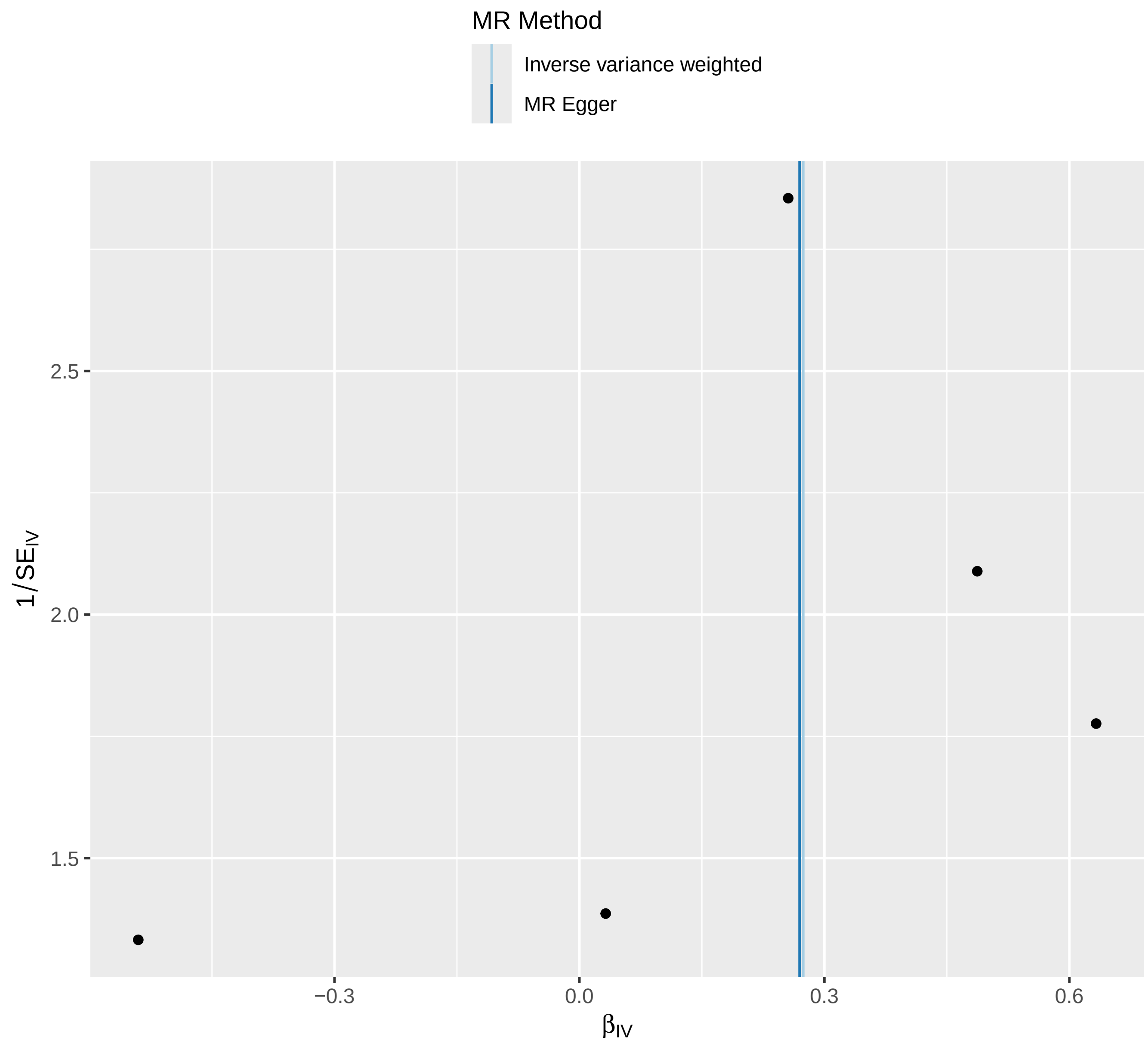
MR Method
Inverse variance weighted
MR Egger
2.5
2.0
1.5
1/SE_IV
−0.3
0.0
0.3
0.6
β_IV

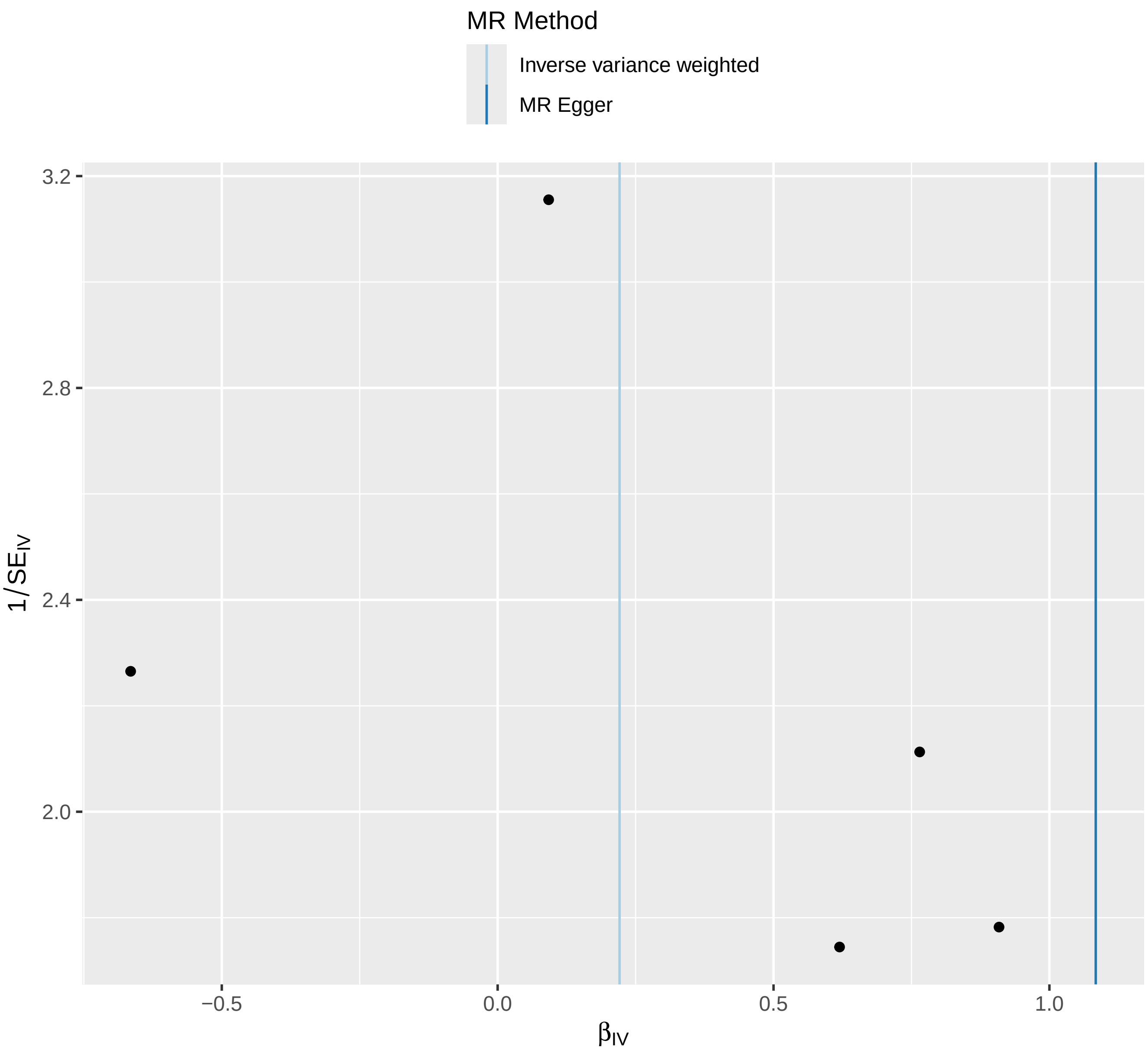
MR Method
Inverse variance weighted
MR Egger
3.2
2.8
2.4
2.0
1/SE$_{IV}$
−0.5
0.0
0.5
1.0
β$_{IV}$

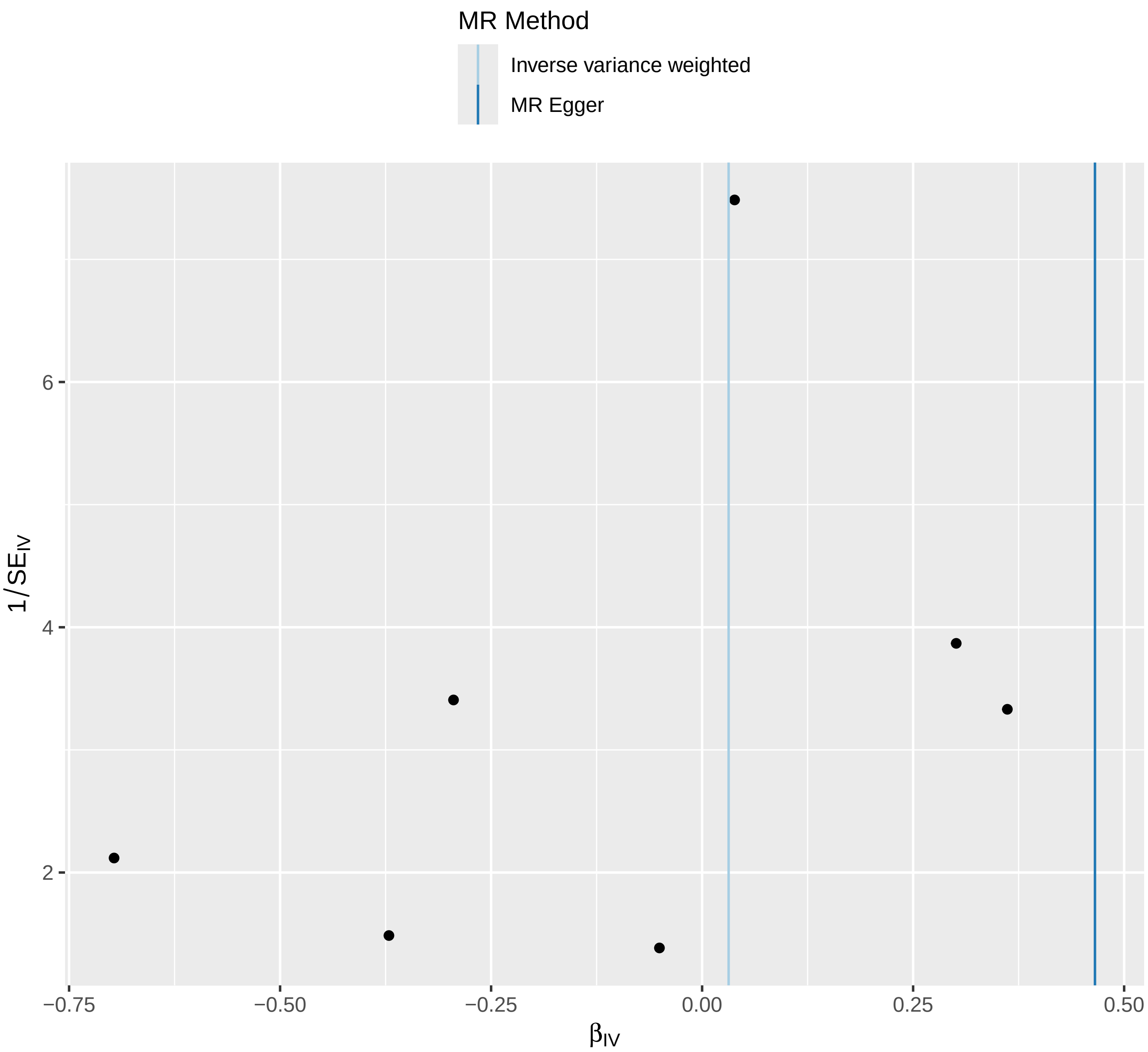
MR Method
Inverse variance weighted
MR Egger
1/SE_IV
β_IV
6
4
2
−0.75
−0.50
−0.25
0.00
0.25
0.50

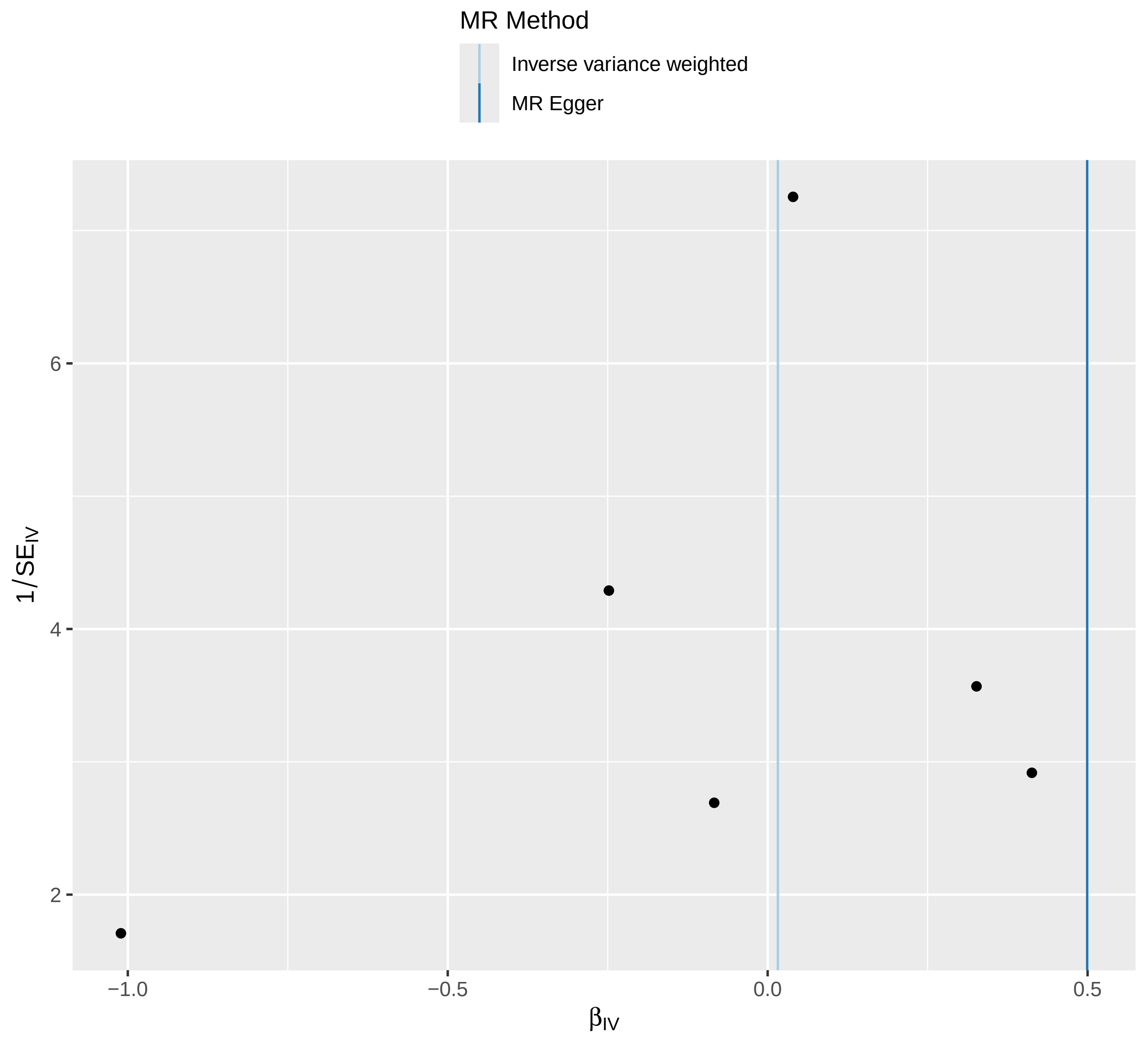
MR Method
Inverse variance weighted
MR Egger
1/SE_IV
6
4
2
−1.0
−0.5
0.0
0.5
β_IV

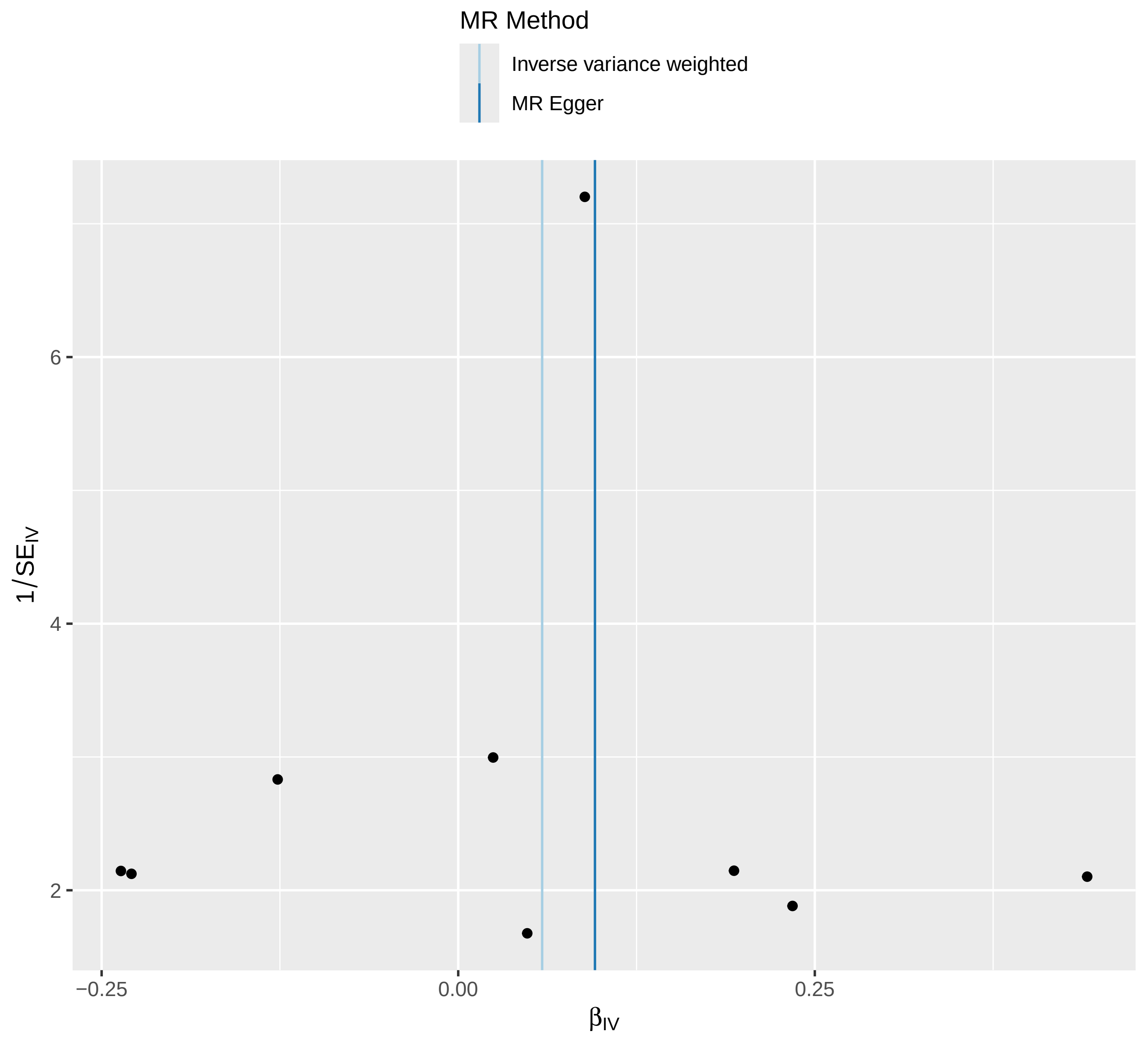
MR Method
Inverse variance weighted
MR Egger
6
4
2
1/SE_IV
−0.25
0.00
0.25
β_IV

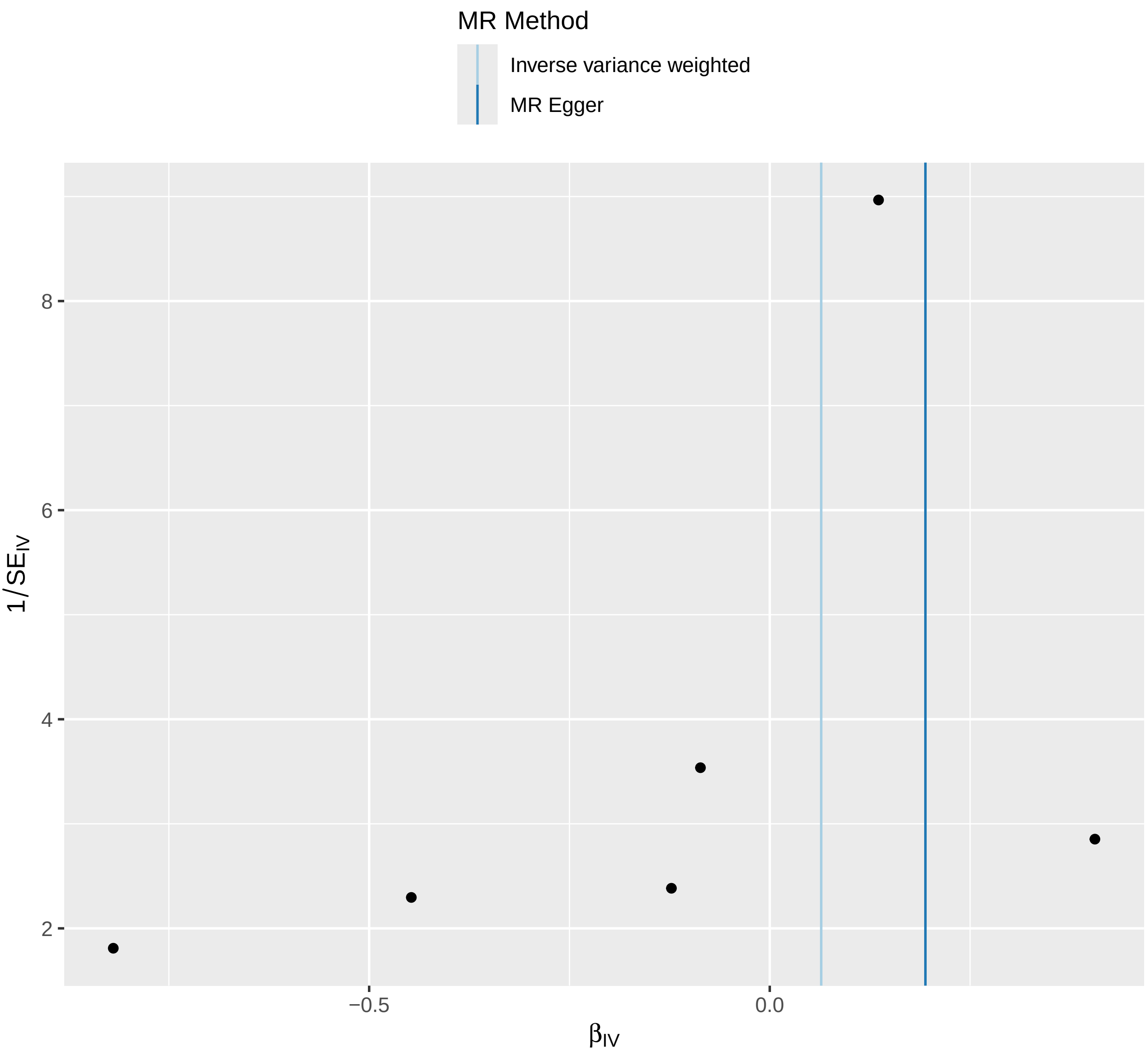
MR Method
Inverse variance weighted
MR Egger
8
6
4
2
1/SE_IV
−0.5
0.0
β_IV

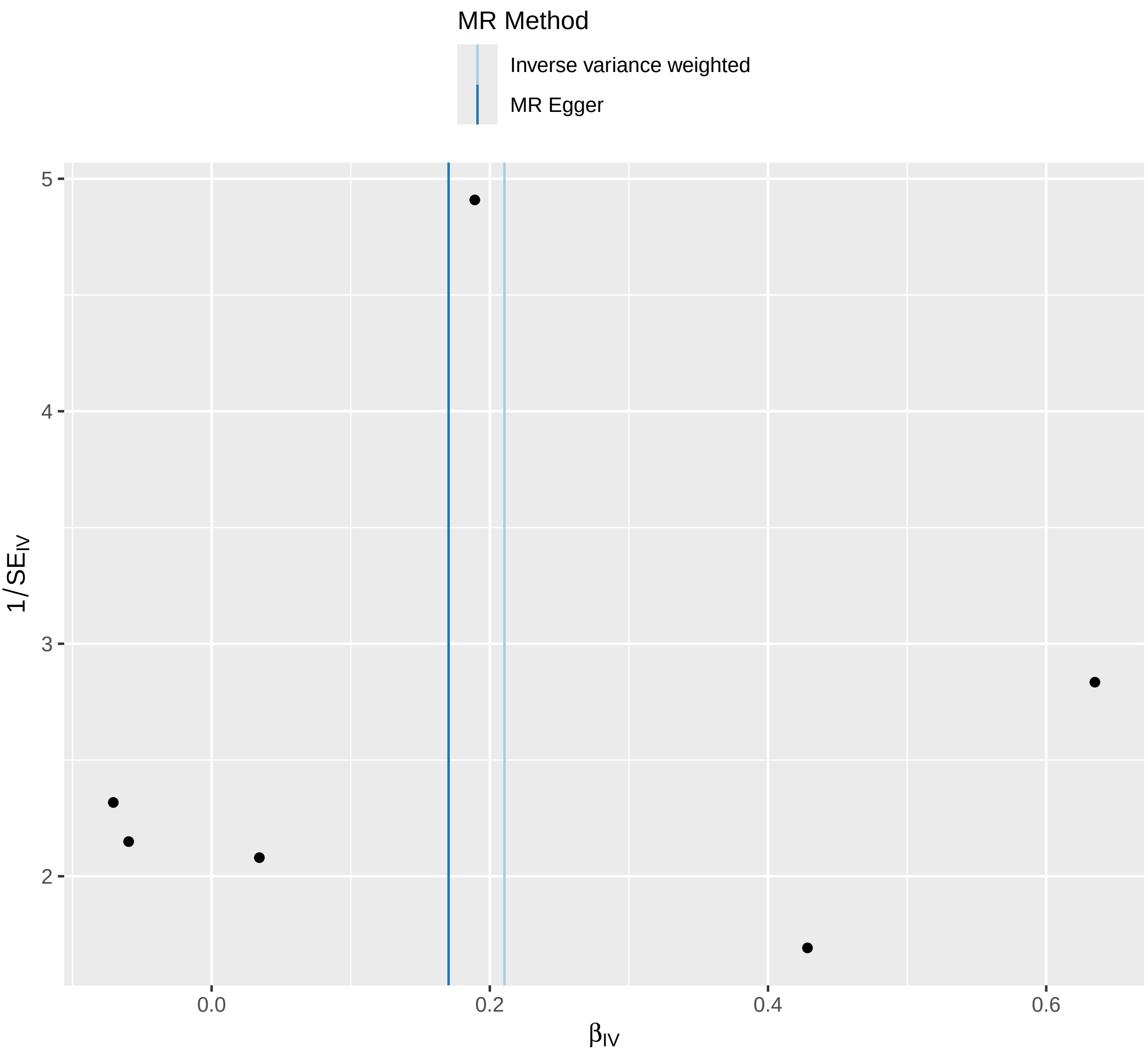

MR Method
Inverse variance weighted
MR Egger
5
4
3
2
1/SE_IV
0.0
0.2
0.4
0.6
β_IV

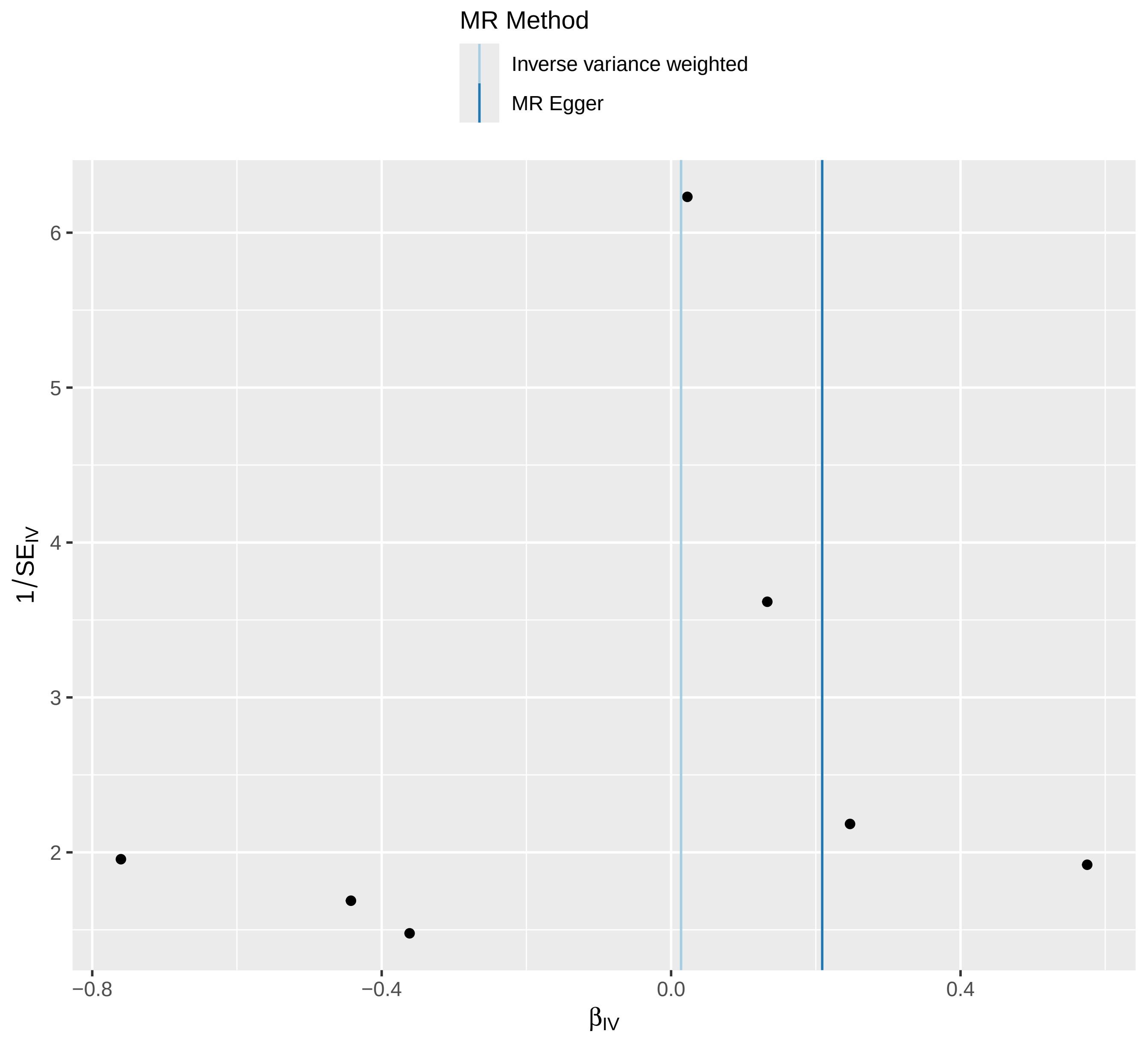
MR Method
Inverse variance weighted
MR Egger
6
5
4
3
2
1/SE_IV
−0.8
−0.4
0.0
0.4
β_IV

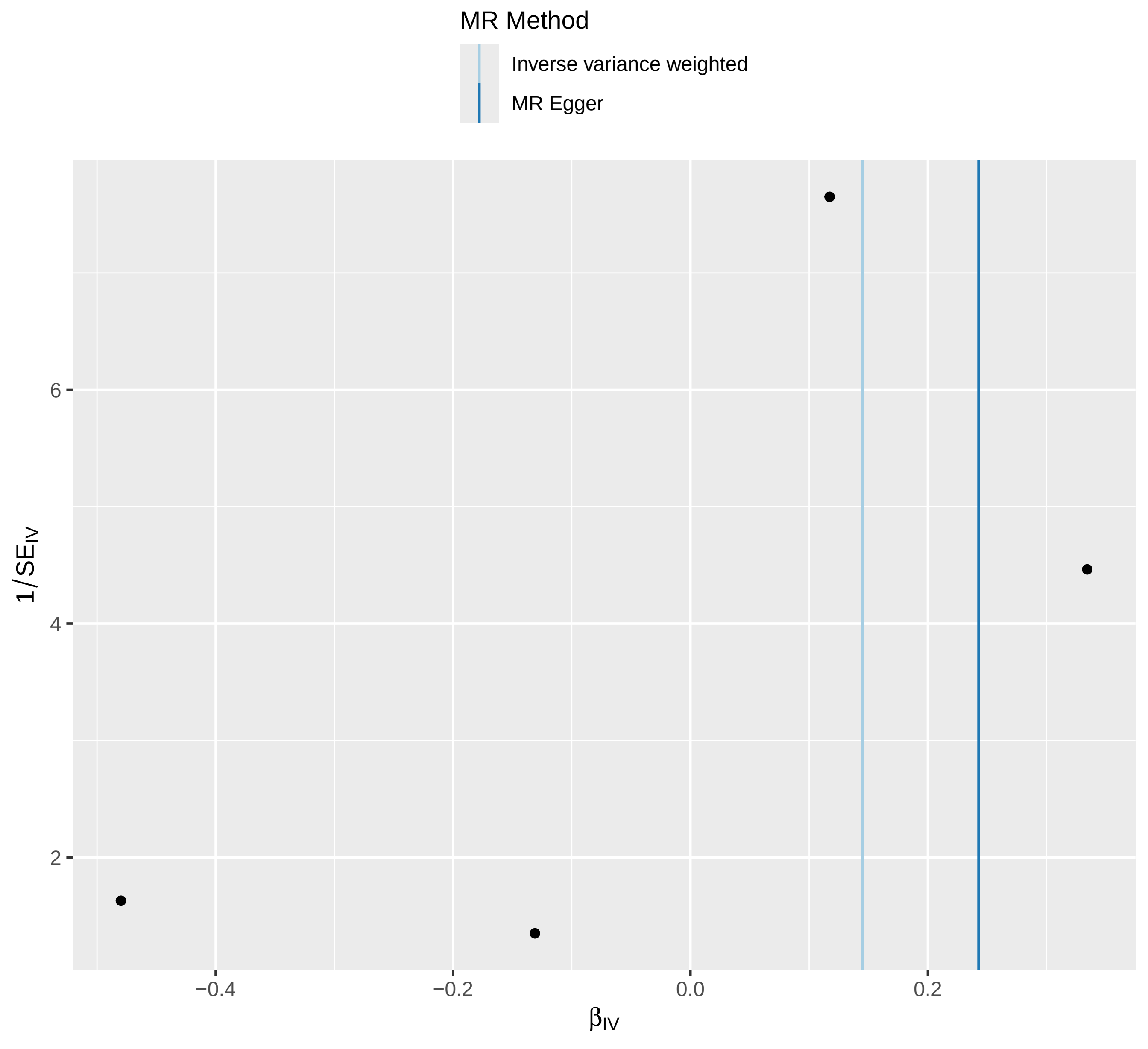

MR Method
Inverse variance weighted
MR Egger
1/SE_IV
6
4
2
−0.4
−0.2
0.0
0.2
β_IV

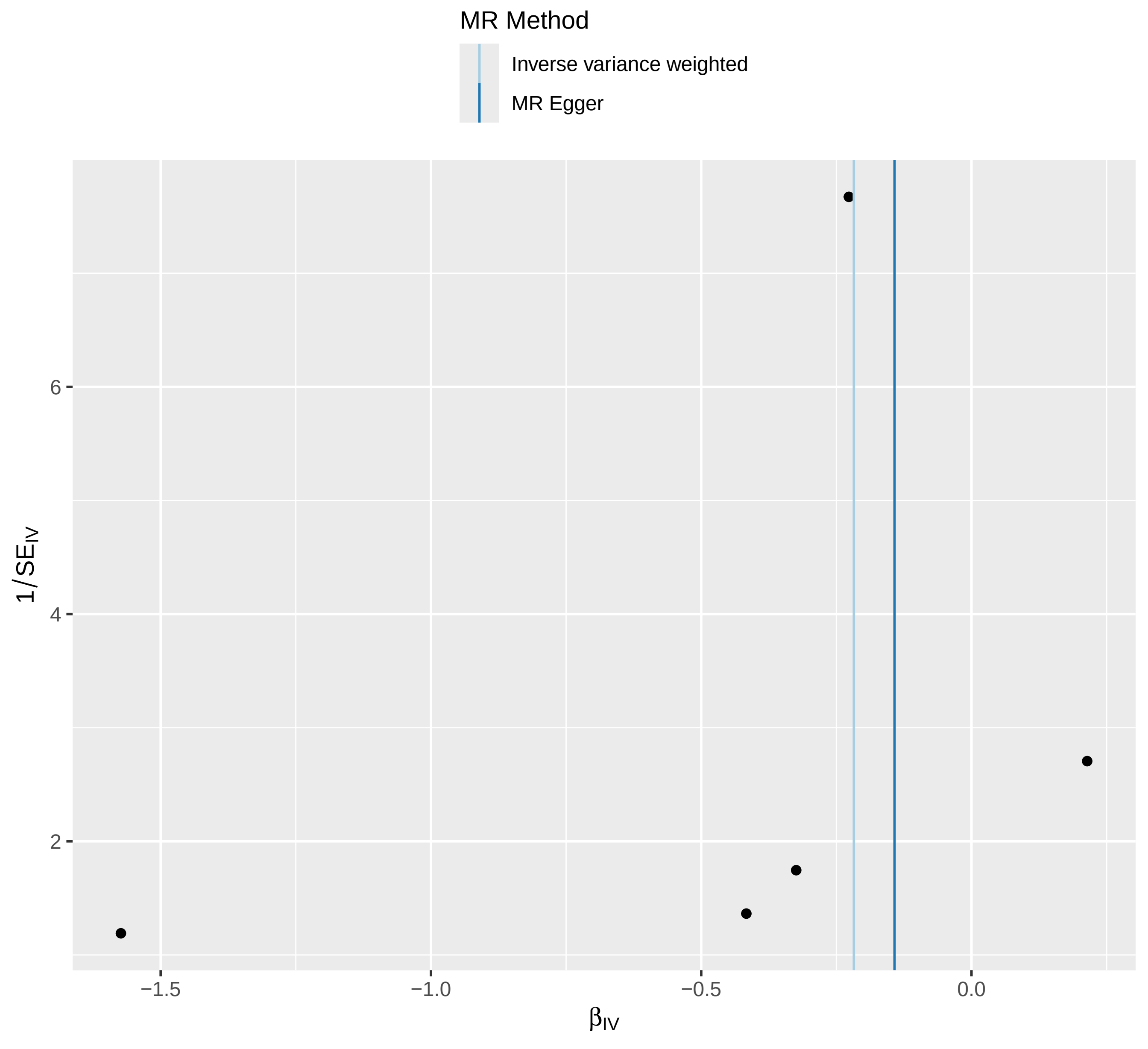
MR Method
Inverse variance weighted
MR Egger
6
4
2
1/SE$_{IV}$
−1.5
−1.0
−0.5
0.0
β_{IV}

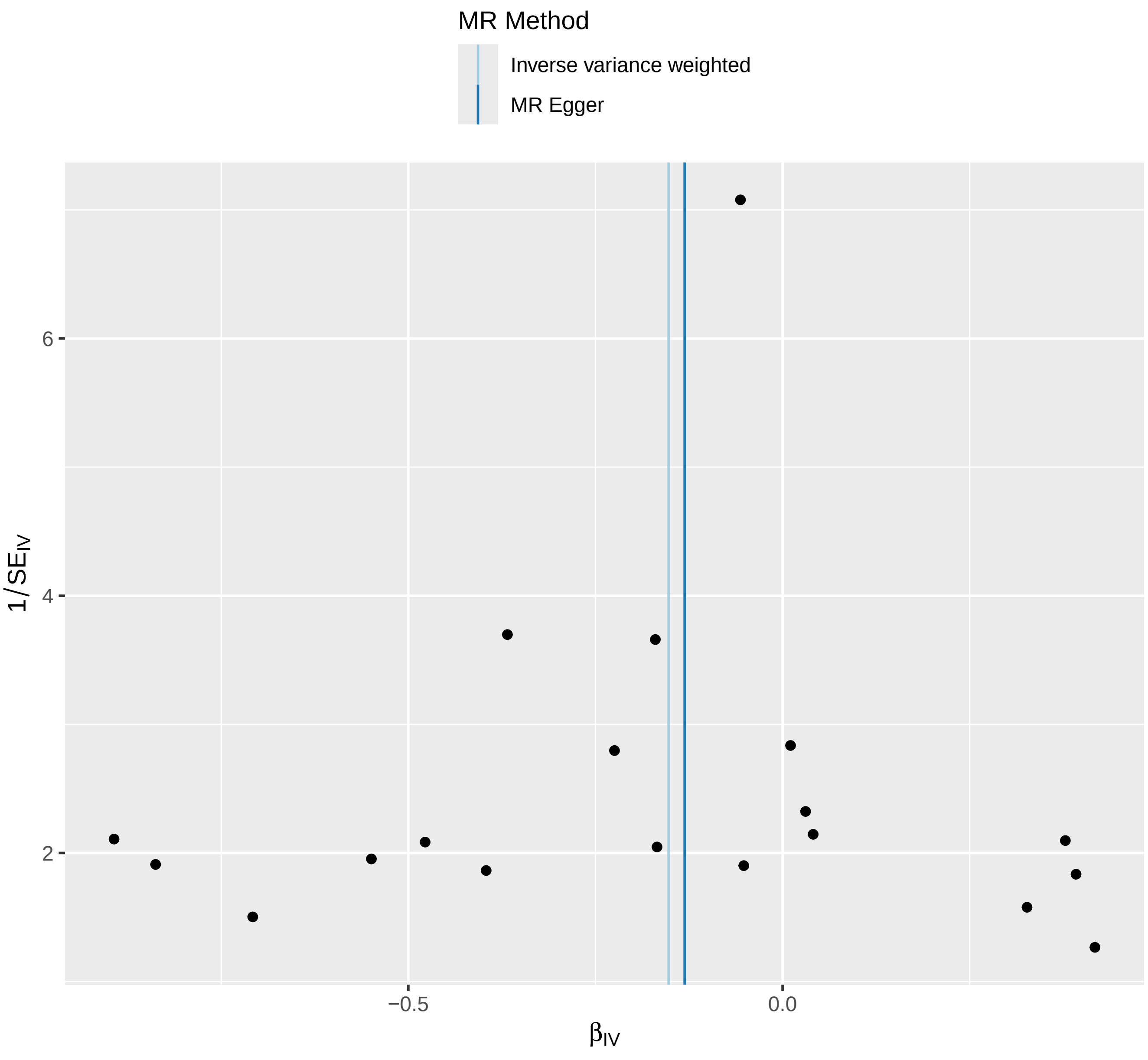
MR Method
Inverse variance weighted
MR Egger
1/SE_IV
6
4
2
−0.5
0.0
β_IV

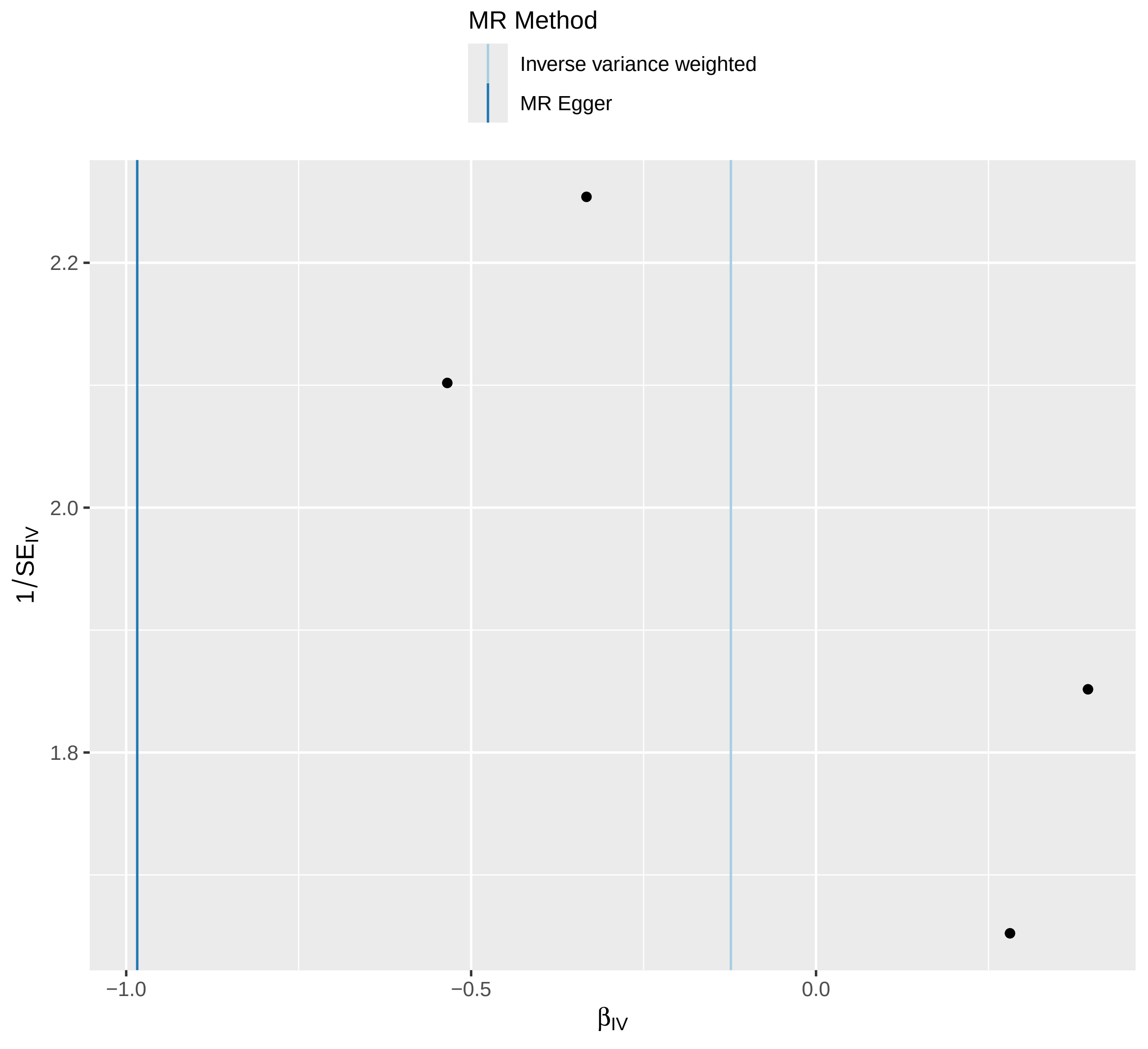
MR Method
Inverse variance weighted
MR Egger
2.2
2.0
1.8
1/SE_IV
−1.0
−0.5
0.0
β_IV

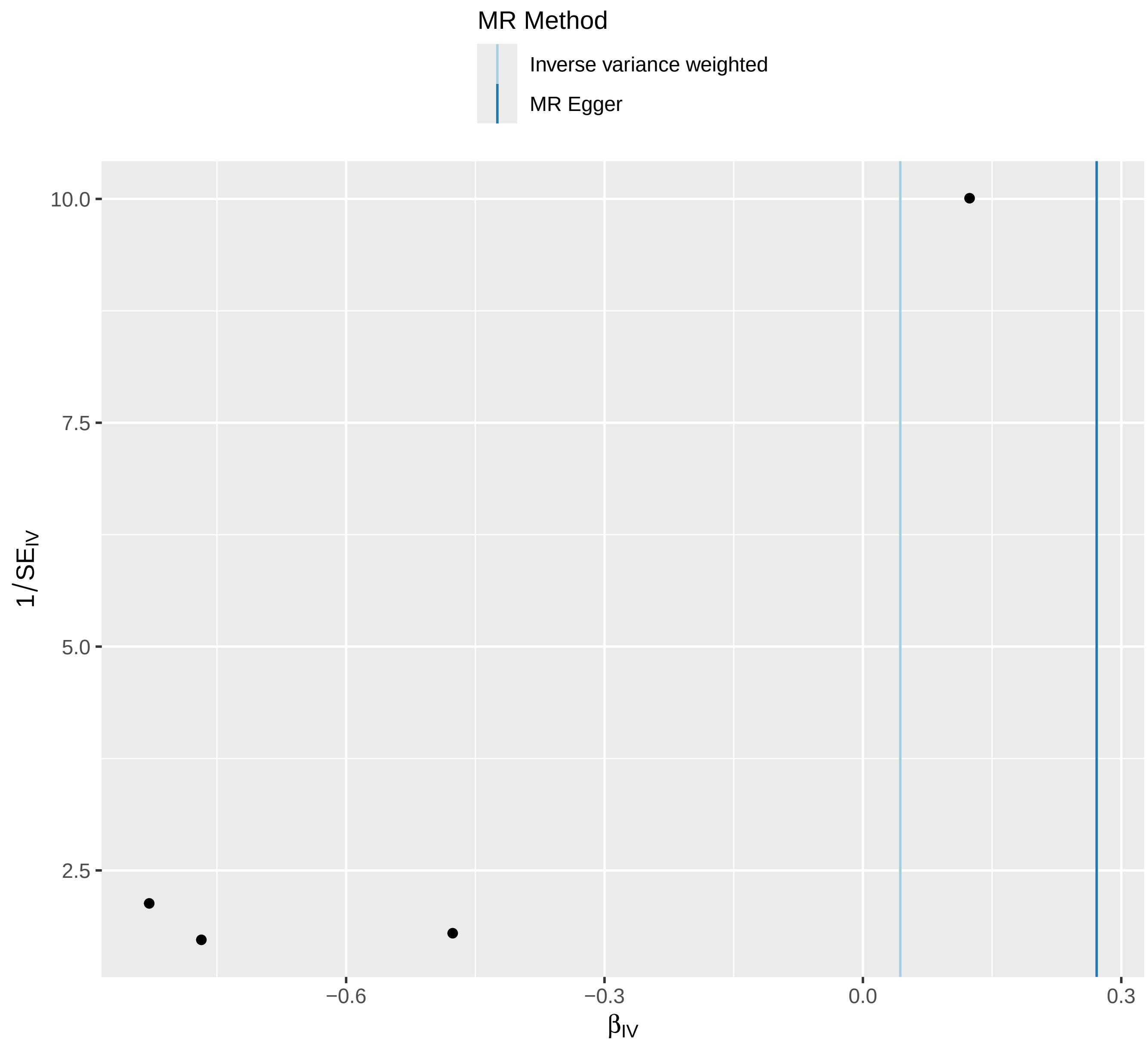
MR Method
Inverse variance weighted
MR Egger
10.0
7.5
5.0
2.5
1/SE$_{IV}$
−0.6
−0.3
0.0
0.3
β$_{IV}$

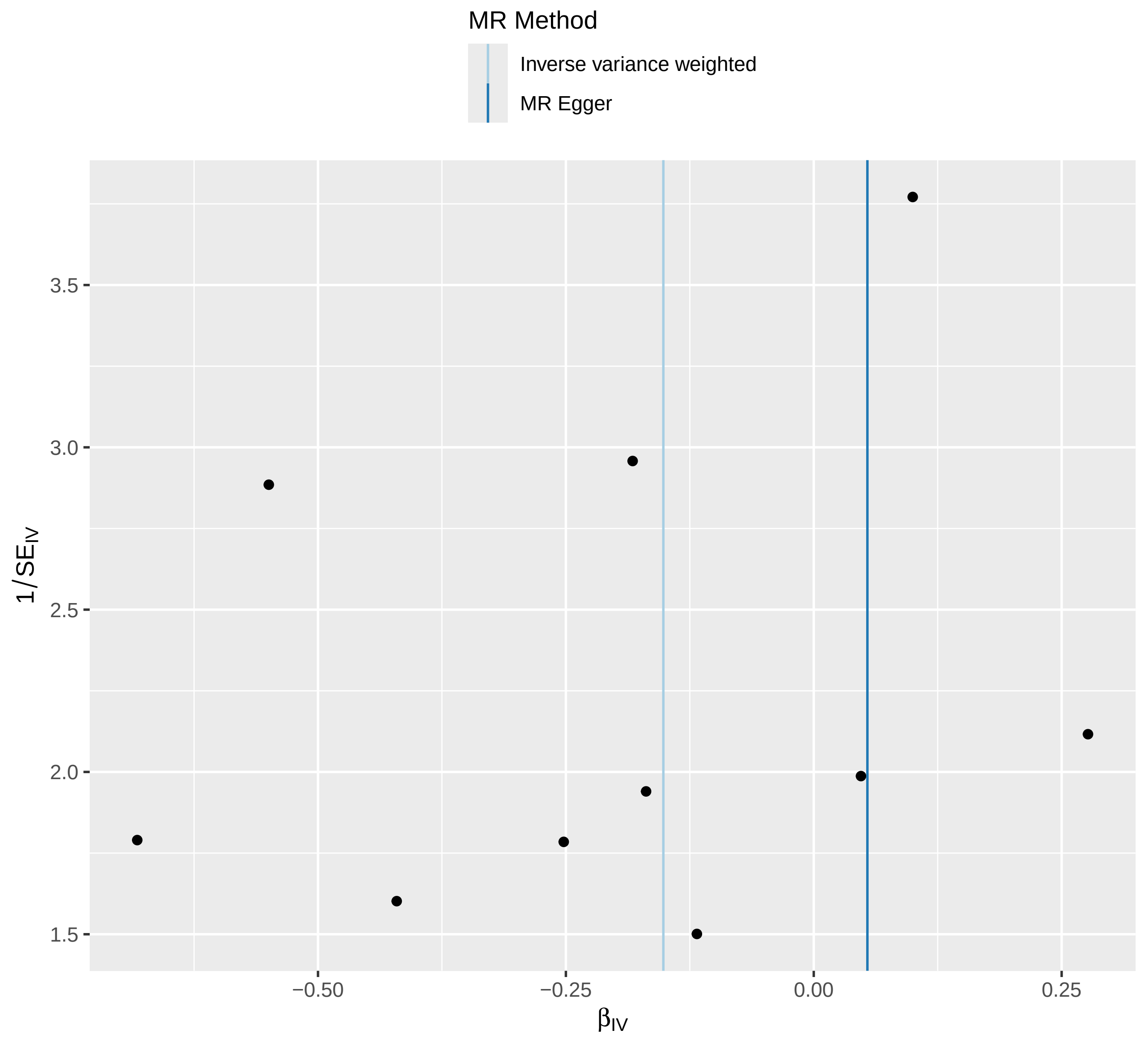
MR Method
Inverse variance weighted
MR Egger
3.5
3.0
2.5
2.0
1.5
1/SE_IV
−0.50
−0.25
0.00
0.25
β_IV

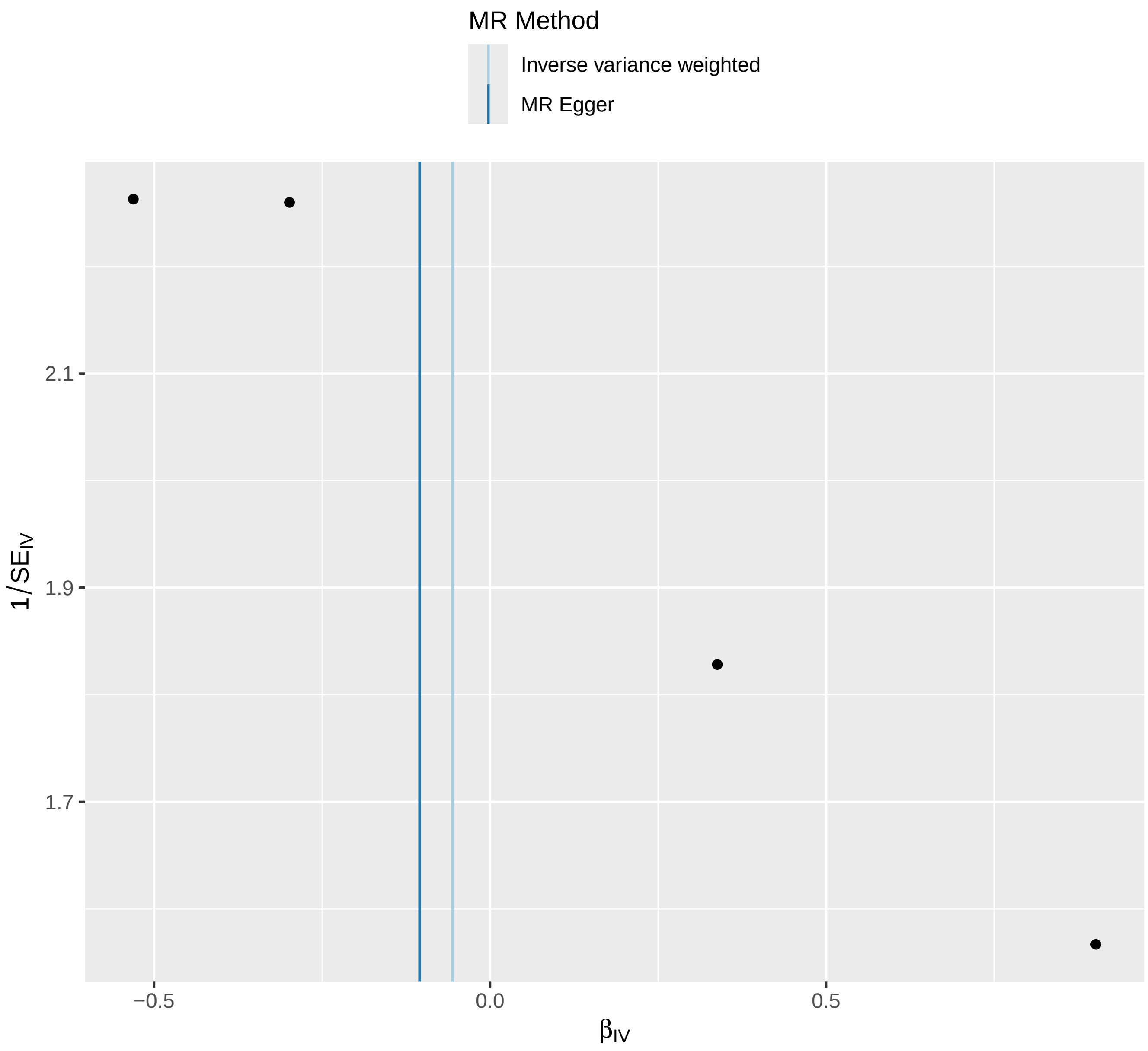

MR Method
Inverse variance weighted
MR Egger
2.1
1.9
1.7
$1/SE_{IV}$
−0.5
0.0
0.5
β_{IV}

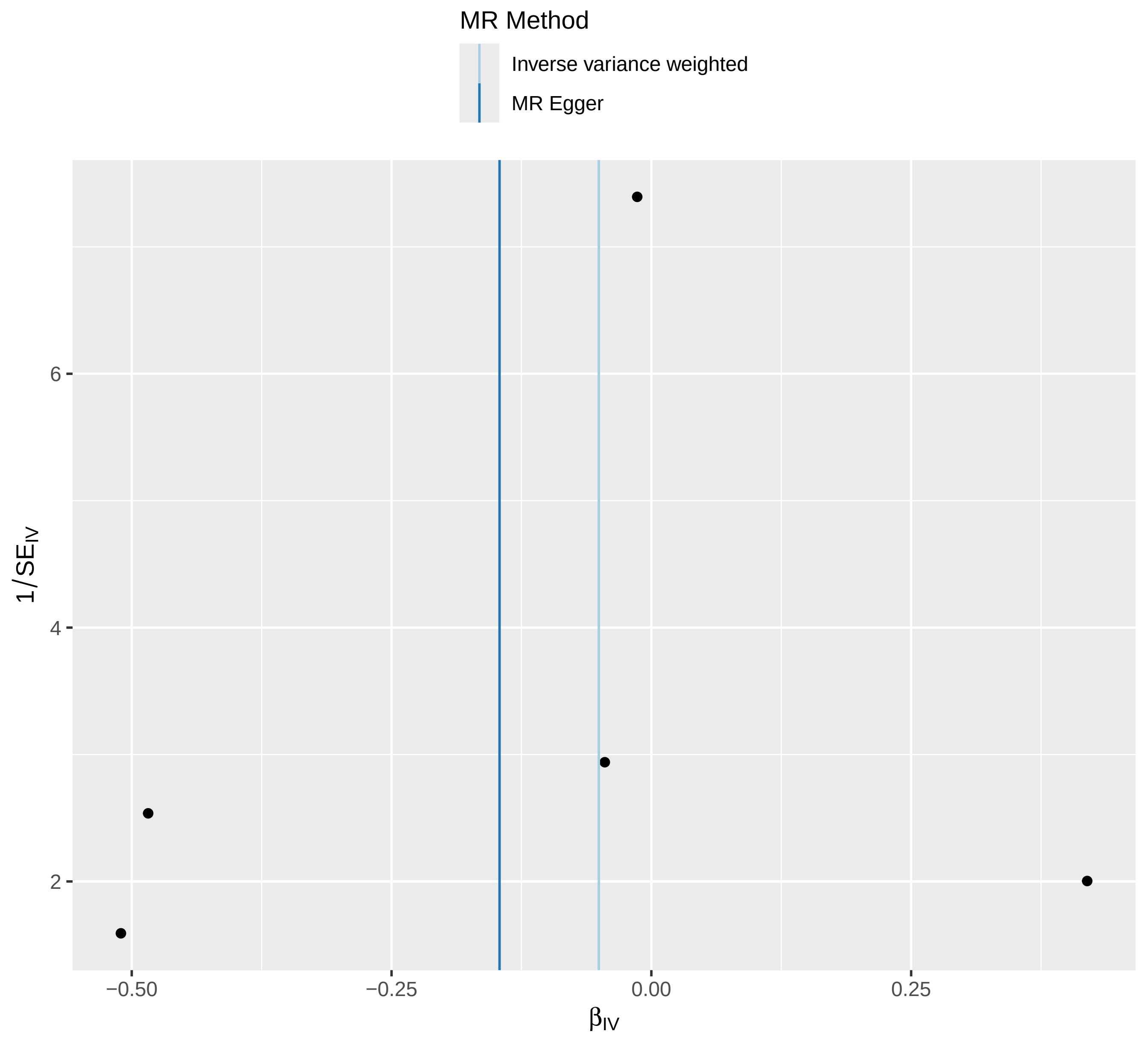

MR Method
Inverse variance weighted
MR Egger
1/SE_IV
6
4
2
−0.50
−0.25
0.00
0.25
β_IV

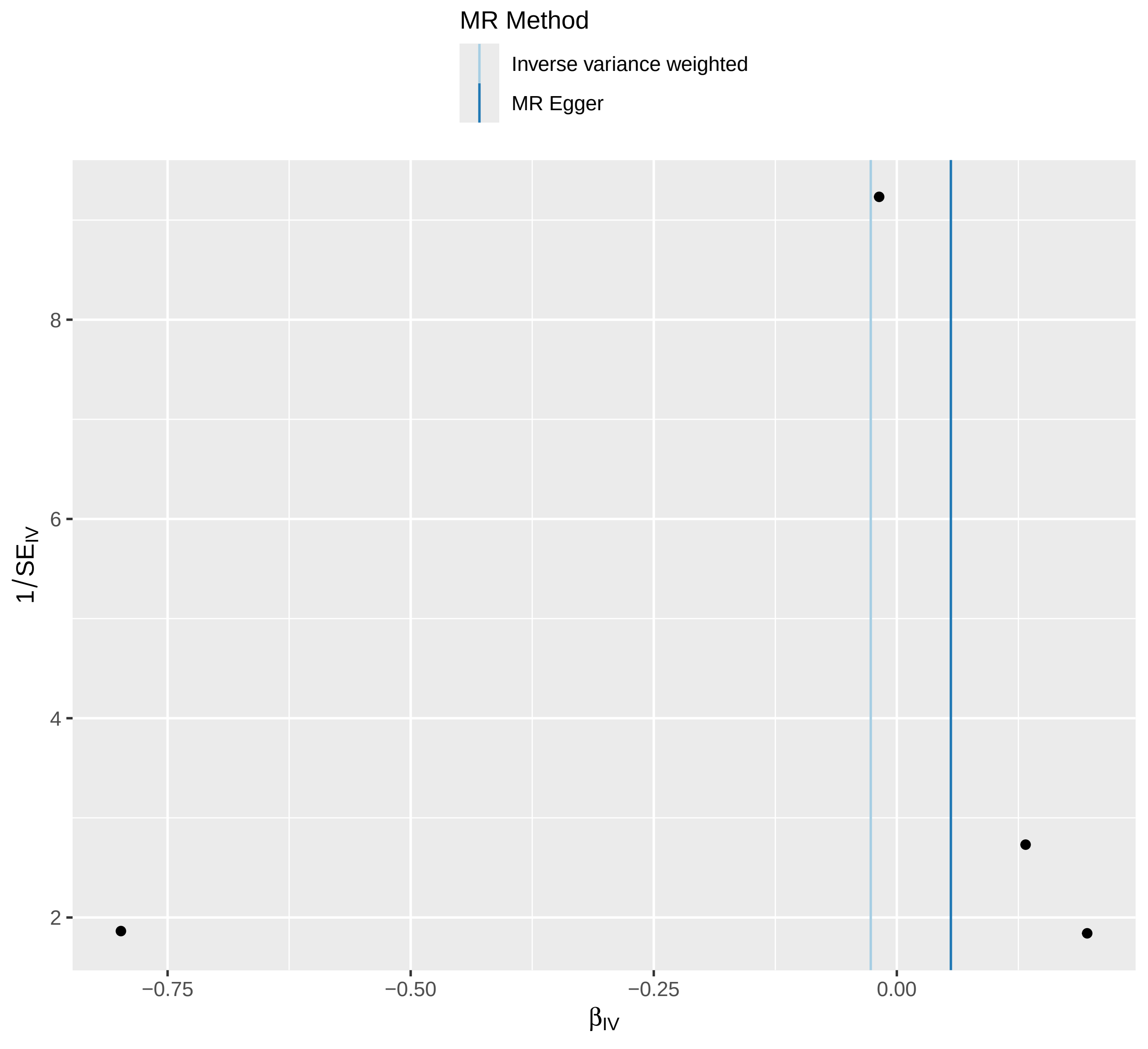
MR Method
Inverse variance weighted
MR Egger
8
6
4
2
1/SE_IV
−0.75
−0.50
−0.25
0.00
β_IV

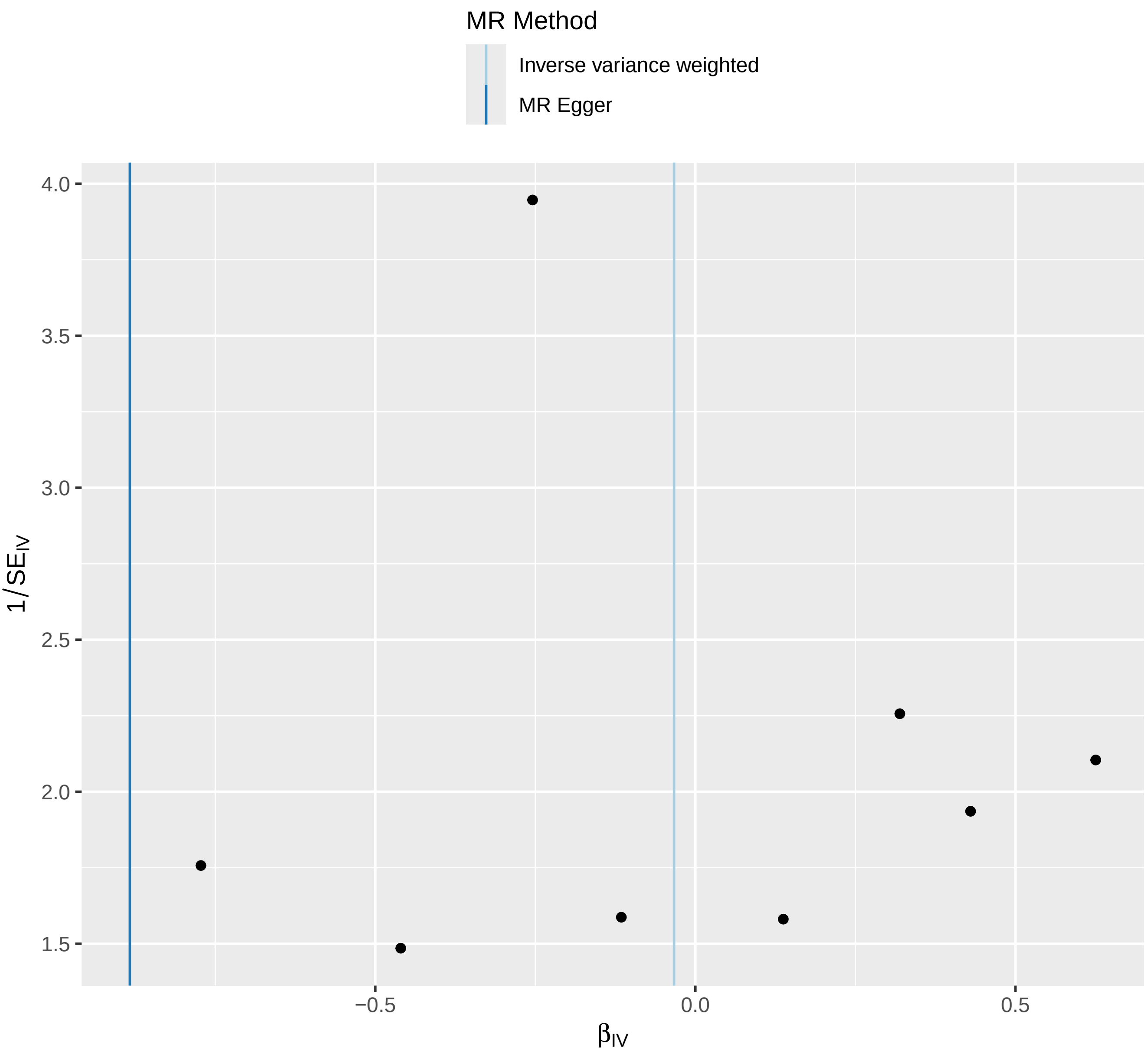
MR Method
Inverse variance weighted
MR Egger
4.0
3.5
3.0
2.5
2.0
1.5
1/SE_IV
−0.5
0.0
0.5
β_IV

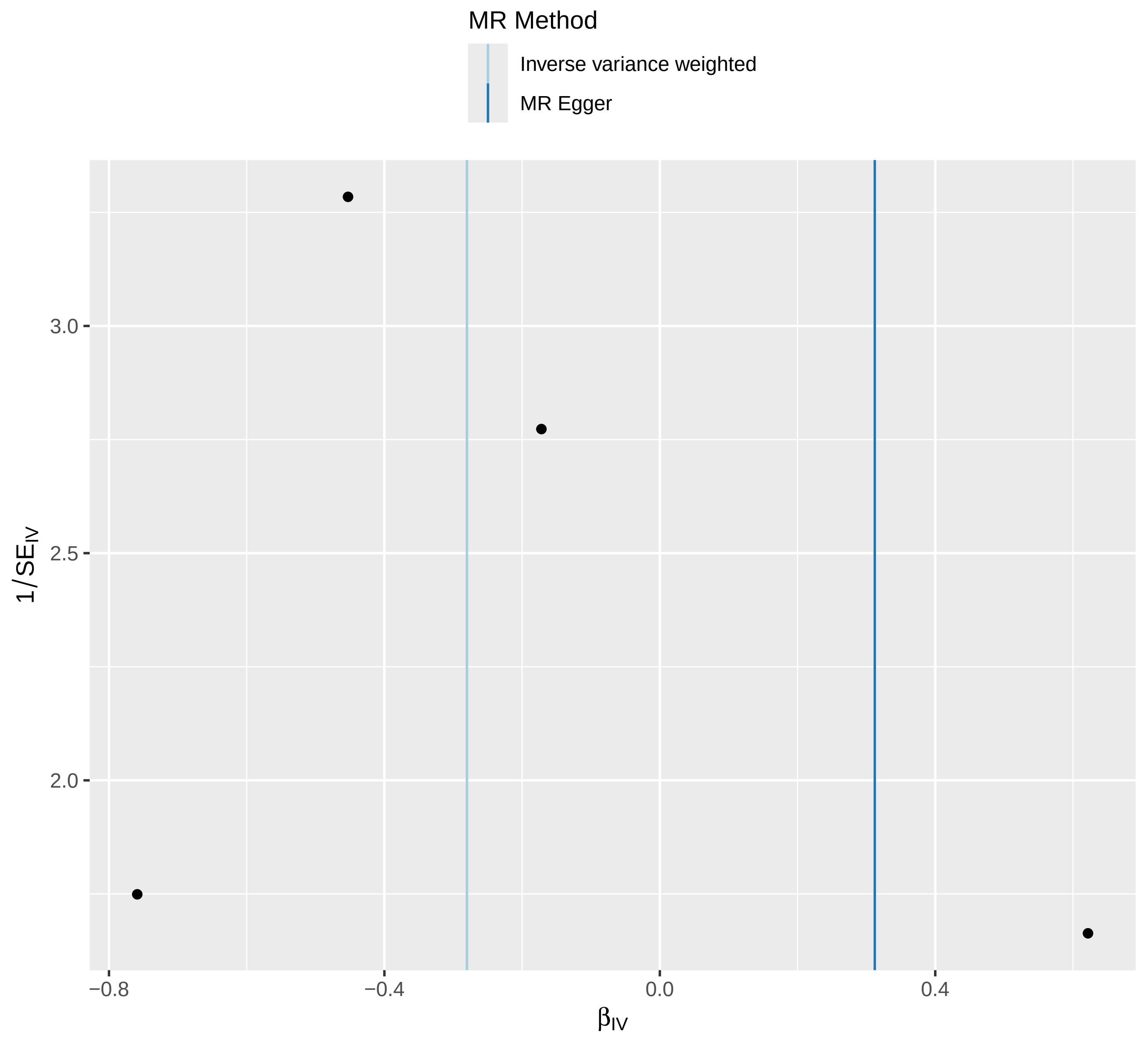

MR Method
Inverse variance weighted
MR Egger
3.0
2.5
2.0
1/SE$_{IV}$
−0.8
−0.4
0.0
0.4
β$_{IV}$

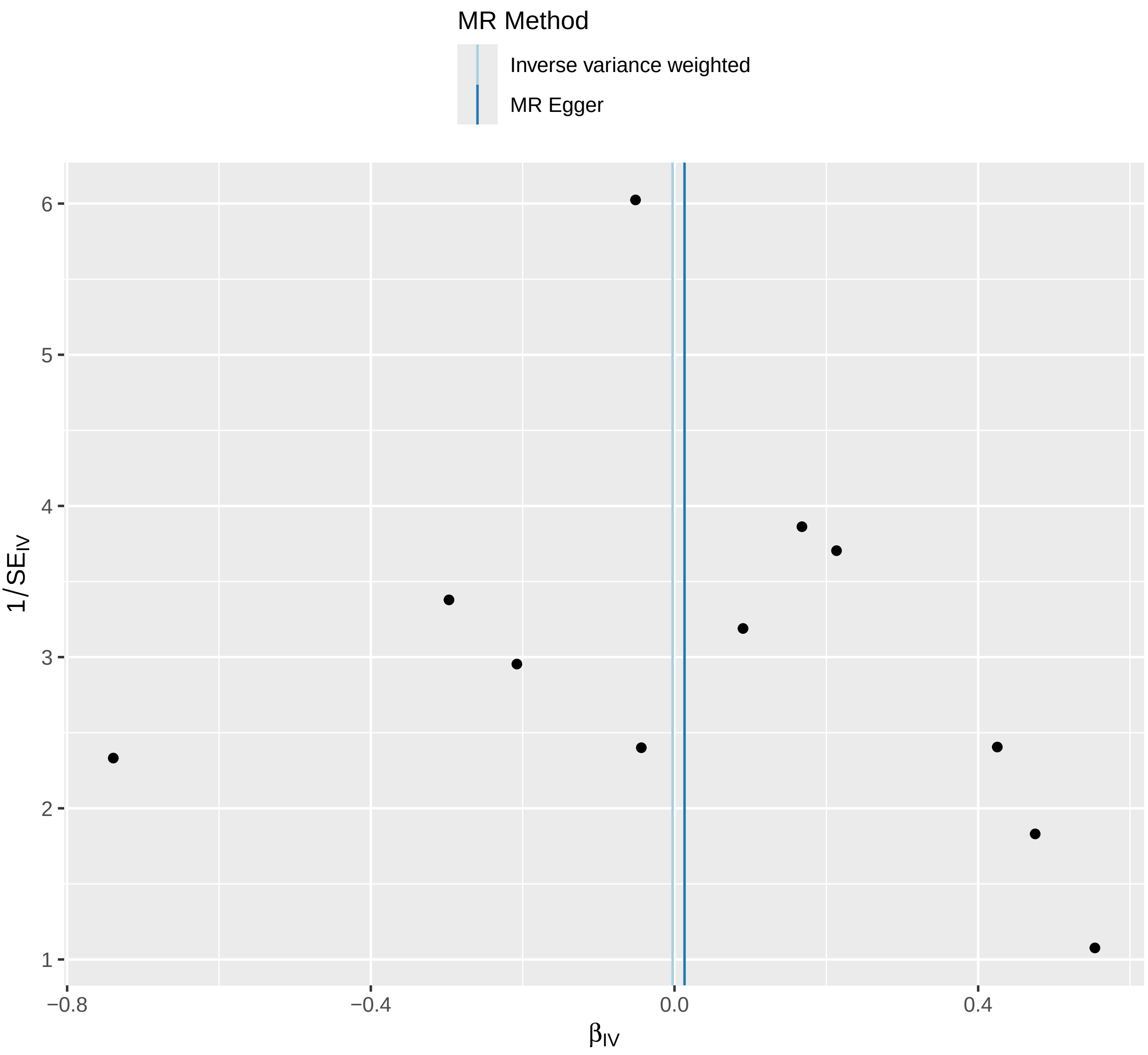

MR Method
Inverse variance weighted
MR Egger
6
5
4
3
2
1
$1/SE_{IV}$
−0.8
−0.4
0.0
0.4
β_{IV}

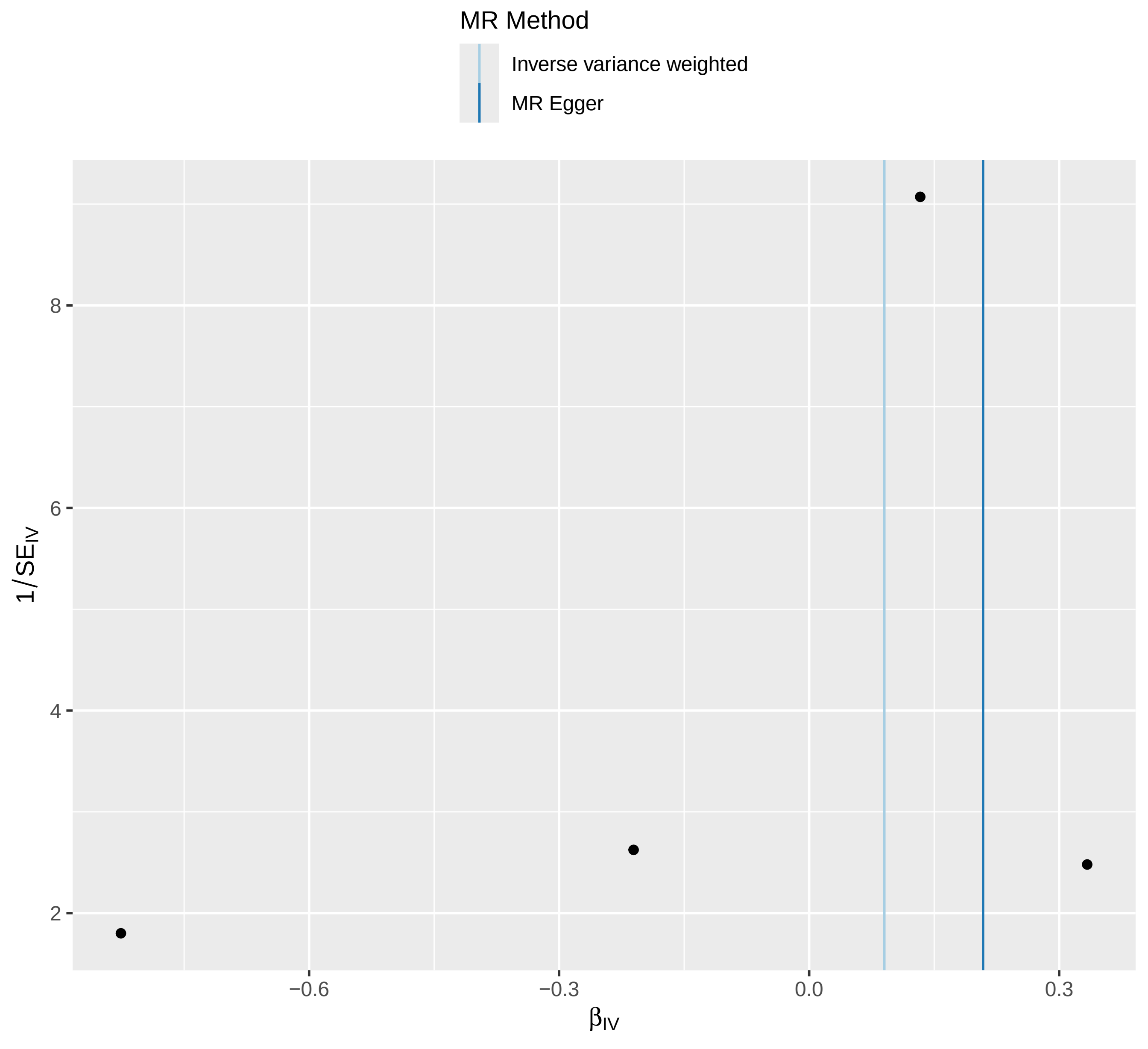
MR Method
Inverse variance weighted
MR Egger
8
6
4
2
1/SE_IV
−0.6
−0.3
0.0
0.3
β_IV

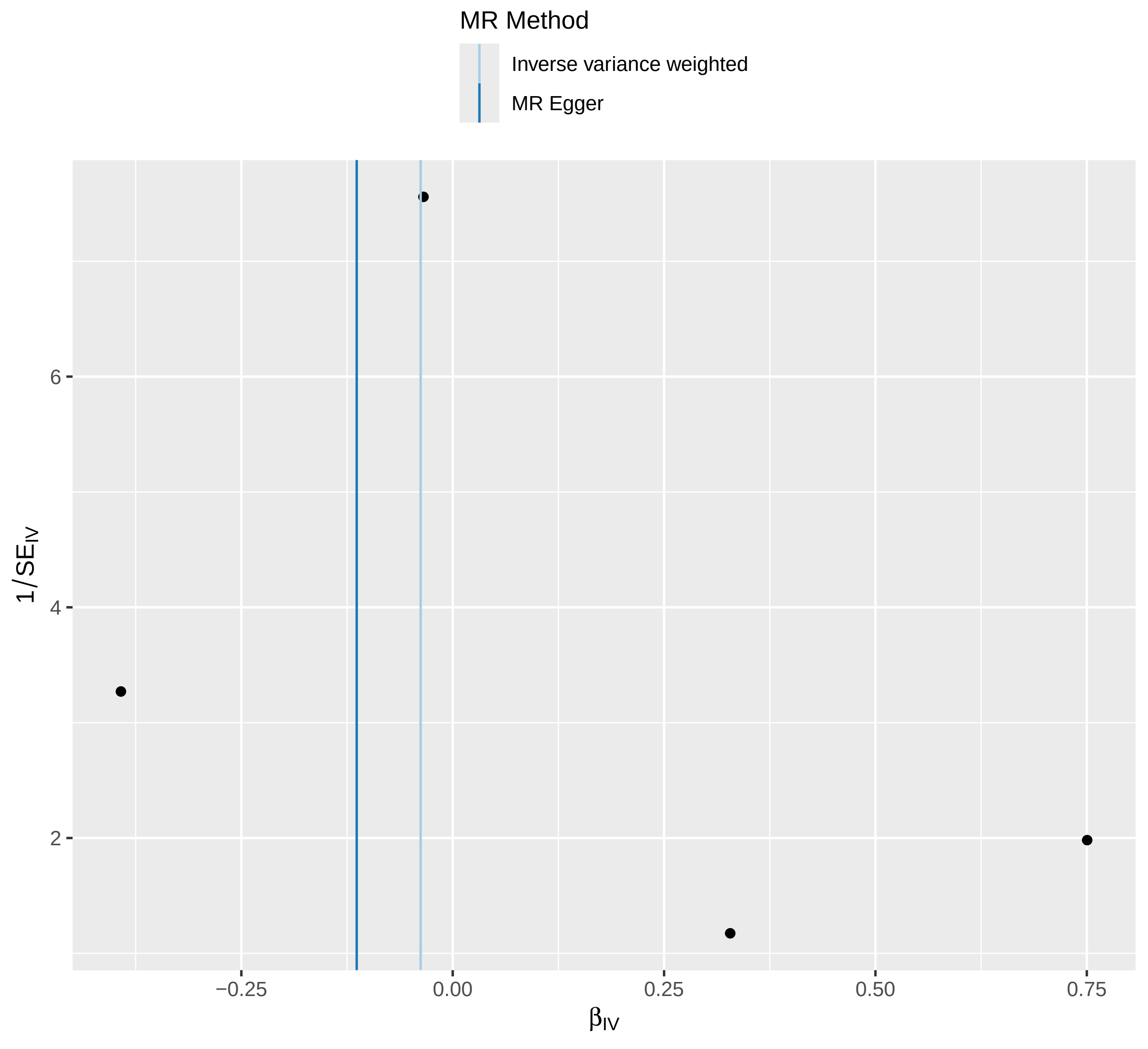

MR Method
Inverse variance weighted
MR Egger
6
4
2
1/SE_IV
−0.25
0.00
0.25
0.50
0.75
β_IV

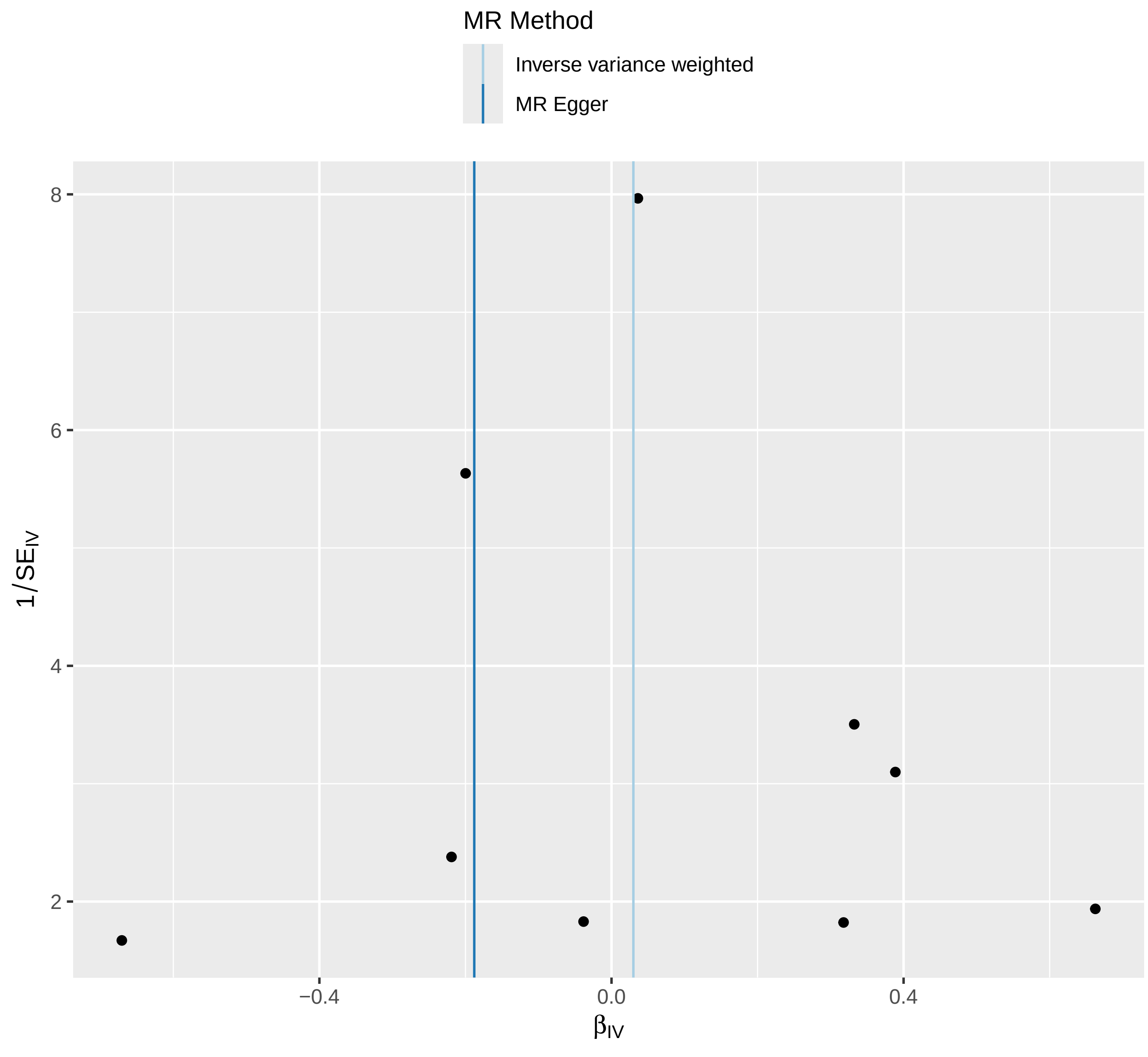
MR Method
Inverse variance weighted
MR Egger
8
6
4
2
1/SE_IV
−0.4
0.0
0.4
β_IV

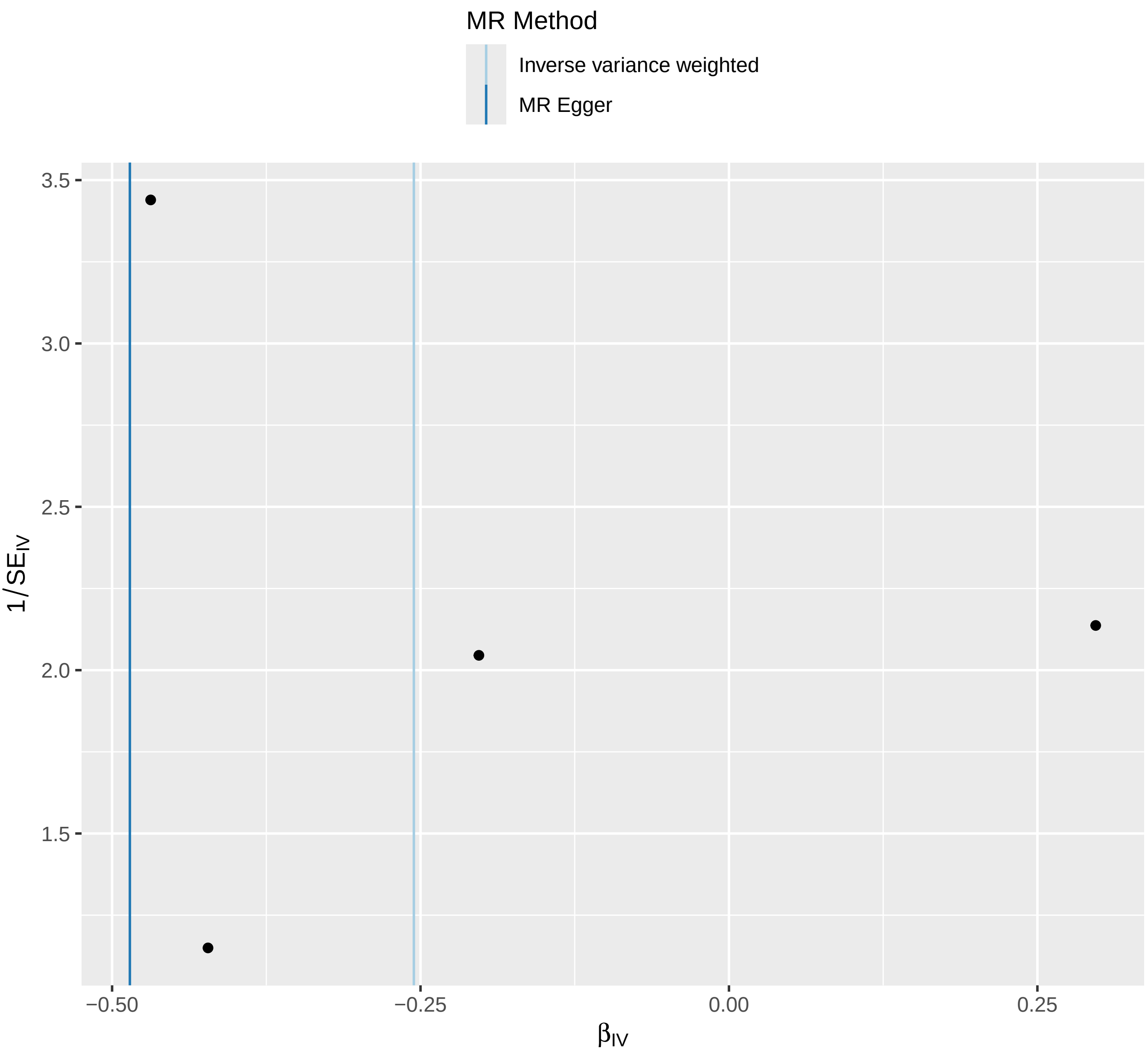
MR Method
Inverse variance weighted
MR Egger
3.5
3.0
2.5
2.0
1.5
1/SE_IV
−0.50
−0.25
0.00
0.25
β_IV

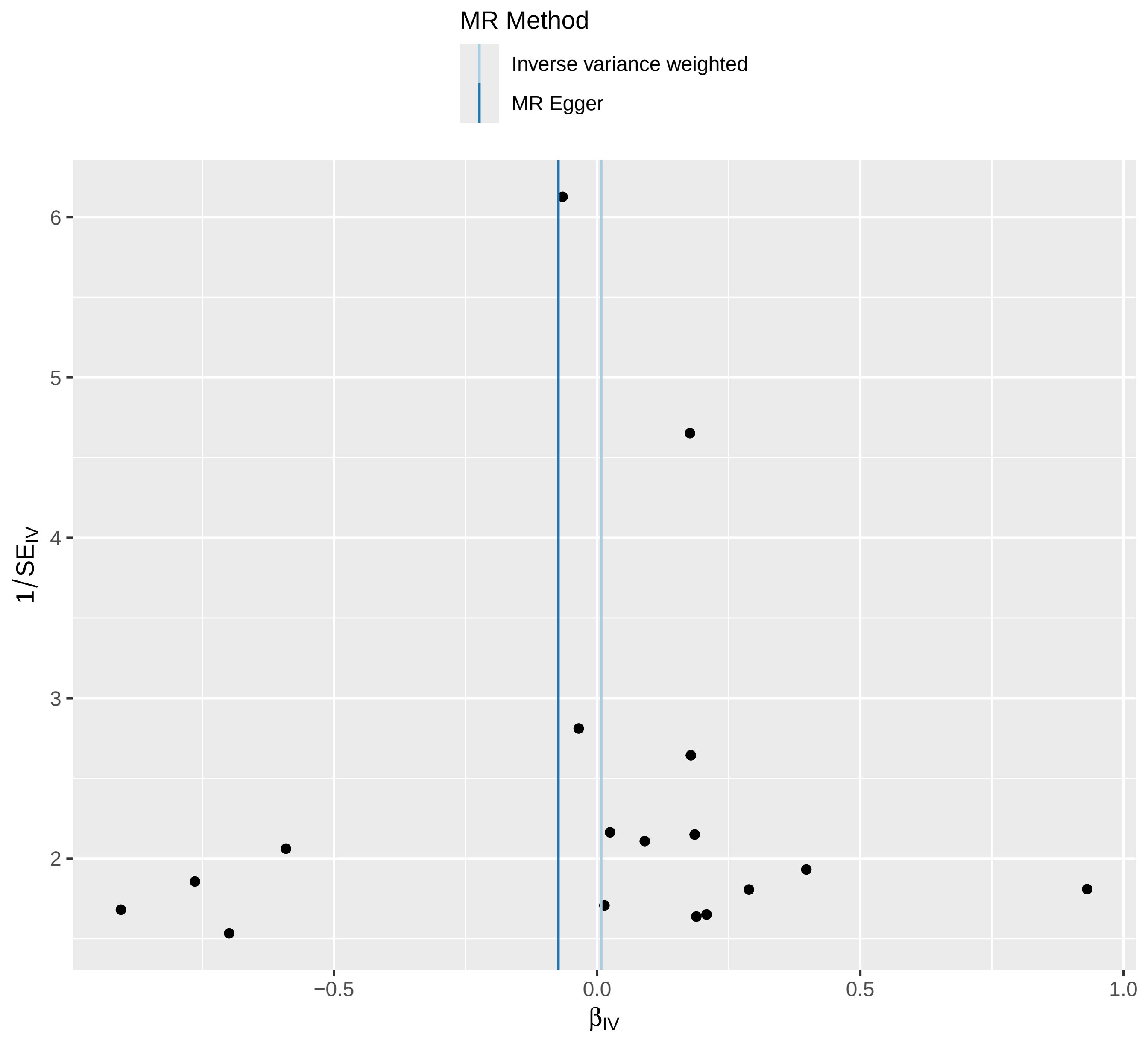

MR Method
Inverse variance weighted
MR Egger
6
5
4
3
2
1/SE$_{IV}$
−0.5
0.0
0.5
1.0
β$_{IV}$

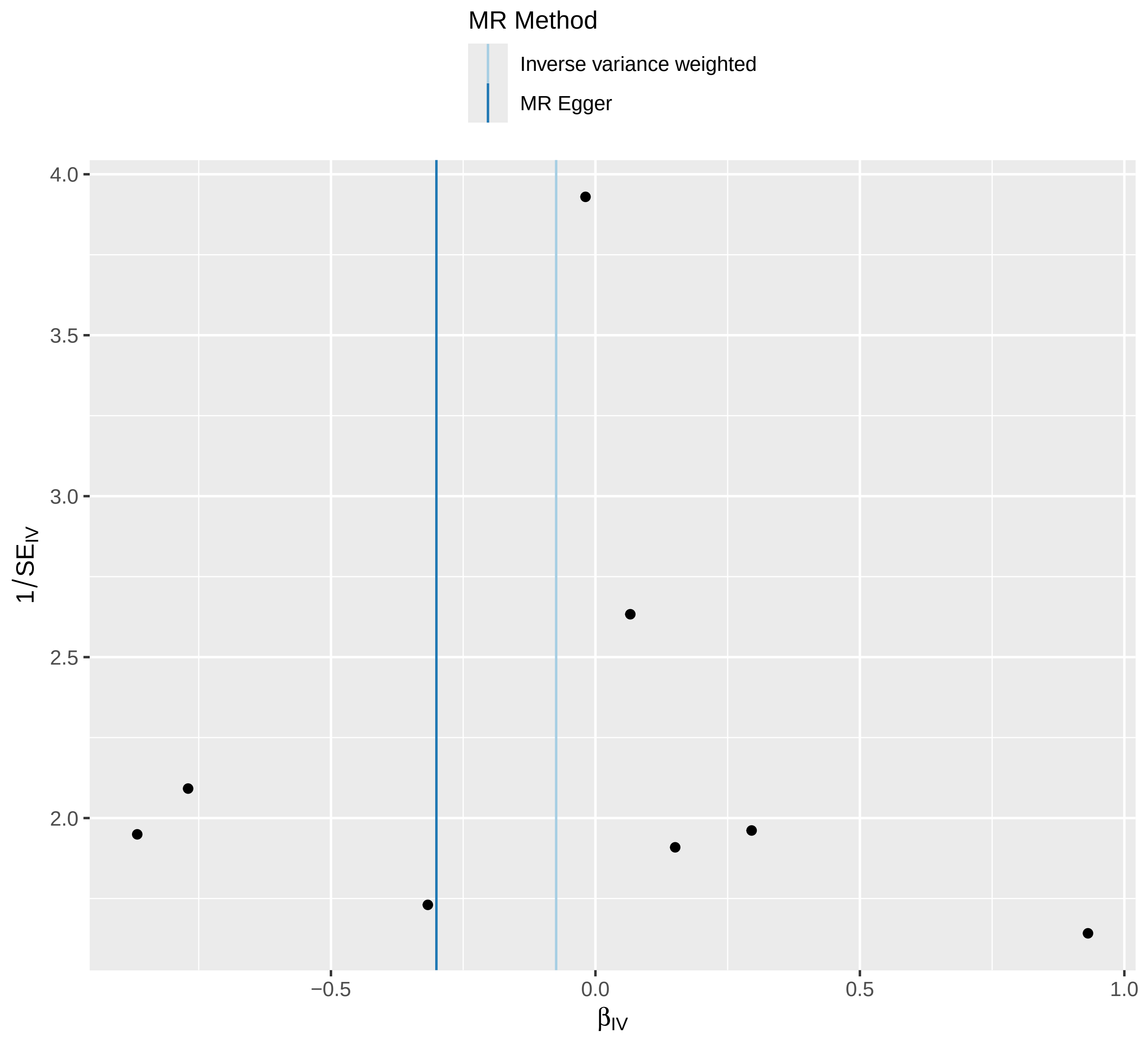

MR Method
Inverse variance weighted
MR Egger
4.0
3.5
3.0
2.5
2.0
1/SE$_{IV}$
−0.5
0.0
0.5
1.0
β$_{IV}$

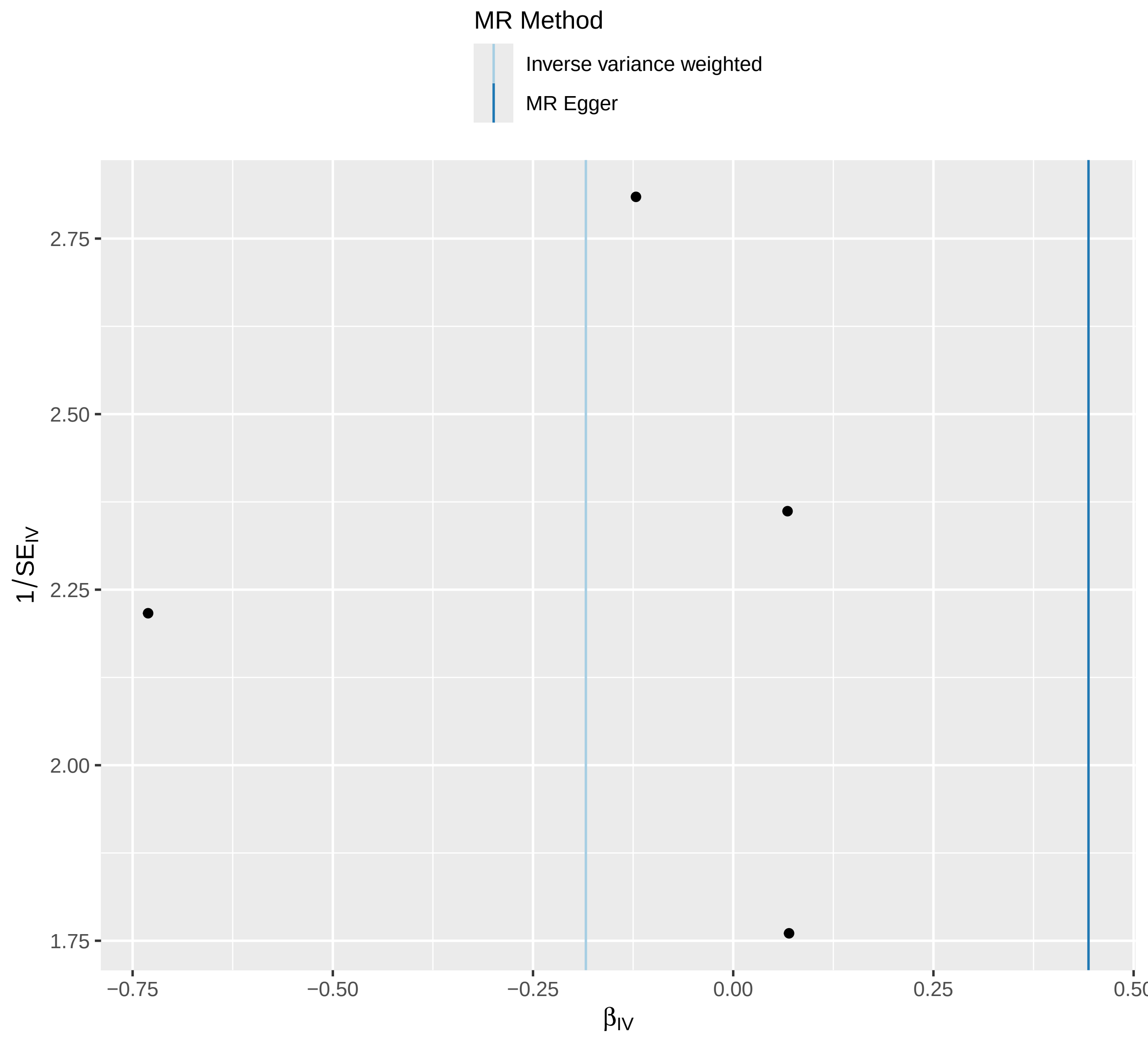

MR Method
Inverse variance weighted
MR Egger
2.75
2.50
2.25
2.00
1.75
1/SE_IV
−0.75
−0.50
−0.25
0.00
0.25
0.50
β_IV

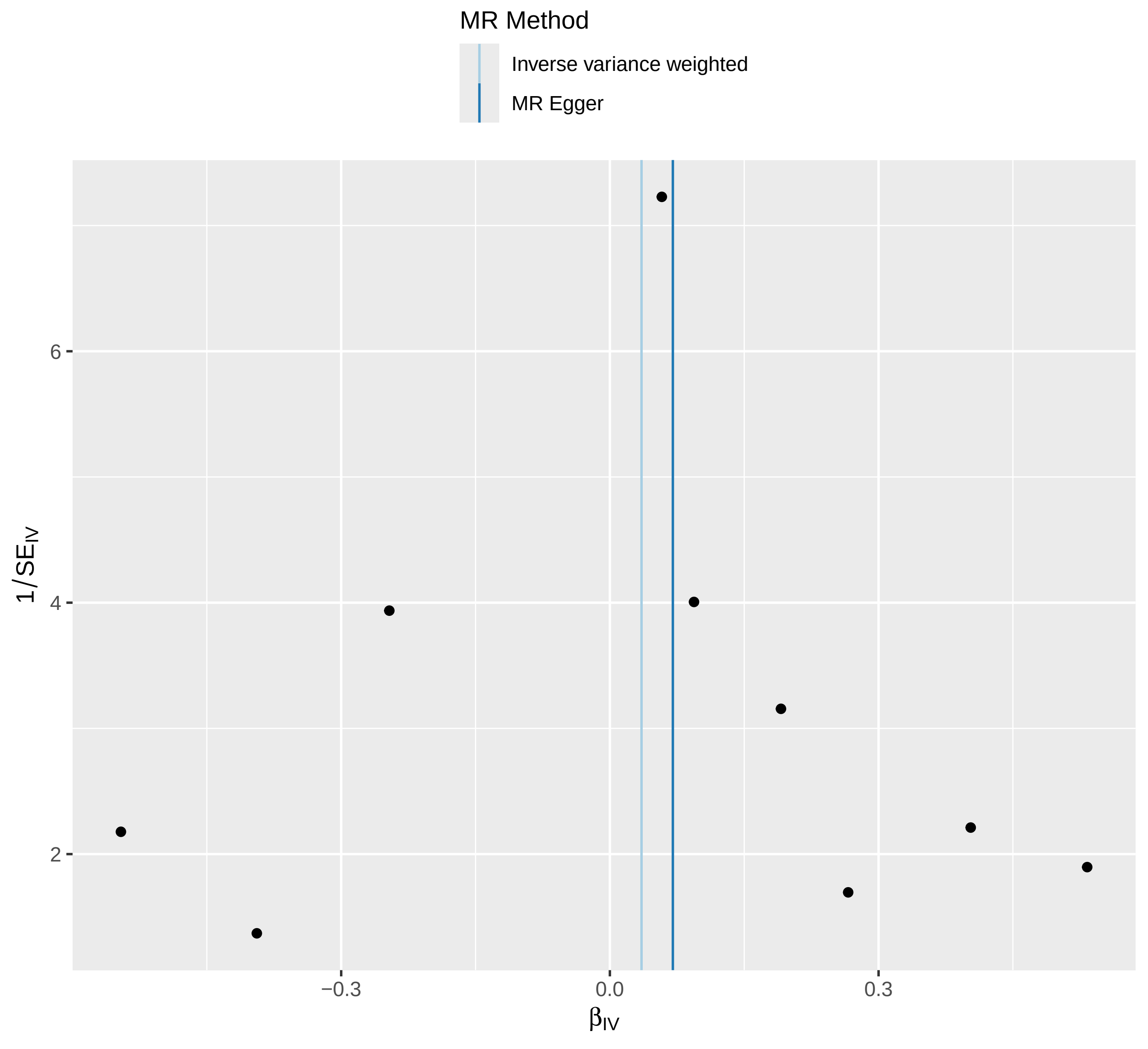

MR Method
Inverse variance weighted
MR Egger
1/SE_IV
6
4
2
−0.3
0.0
0.3
β_IV

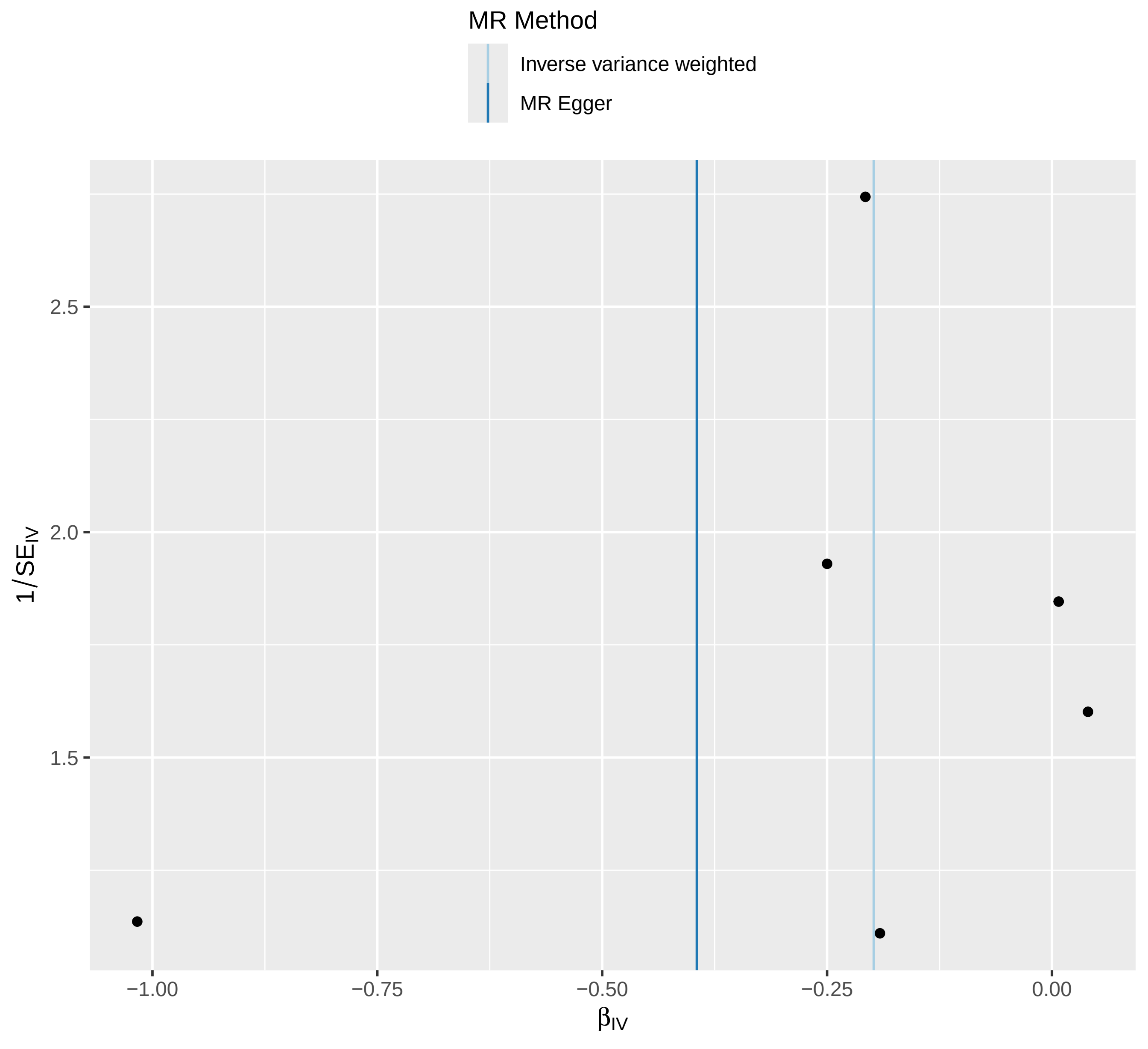

MR Method
Inverse variance weighted
MR Egger
2.5
2.0
1.5
1/SE$_{IV}$
−1.00
−0.75
−0.50
−0.25
0.00
β$_{IV}$

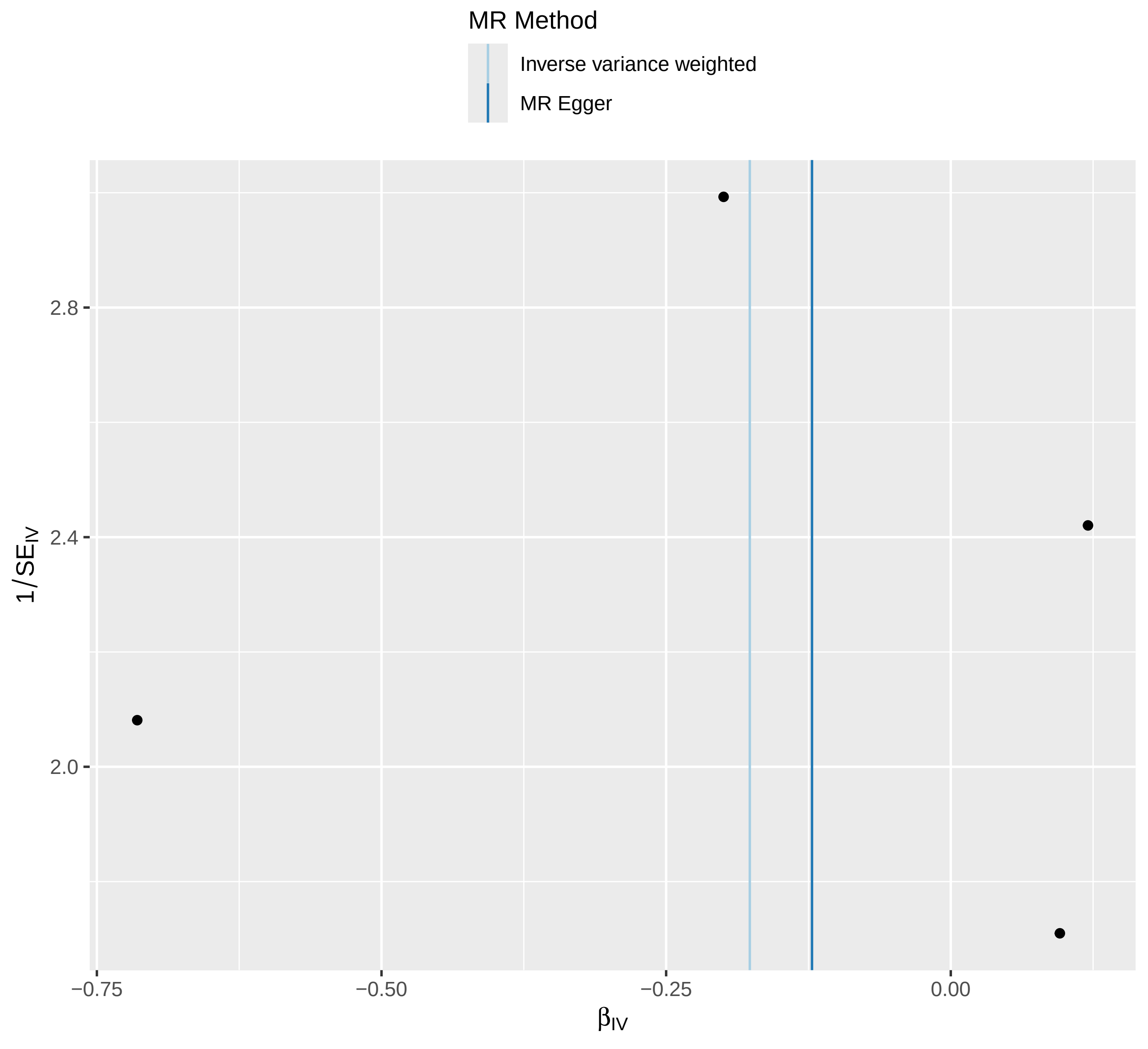

MR Method
Inverse variance weighted
MR Egger
2.8
2.4
2.0
1/SE_IV
−0.75
−0.50
−0.25
0.00
β_IV

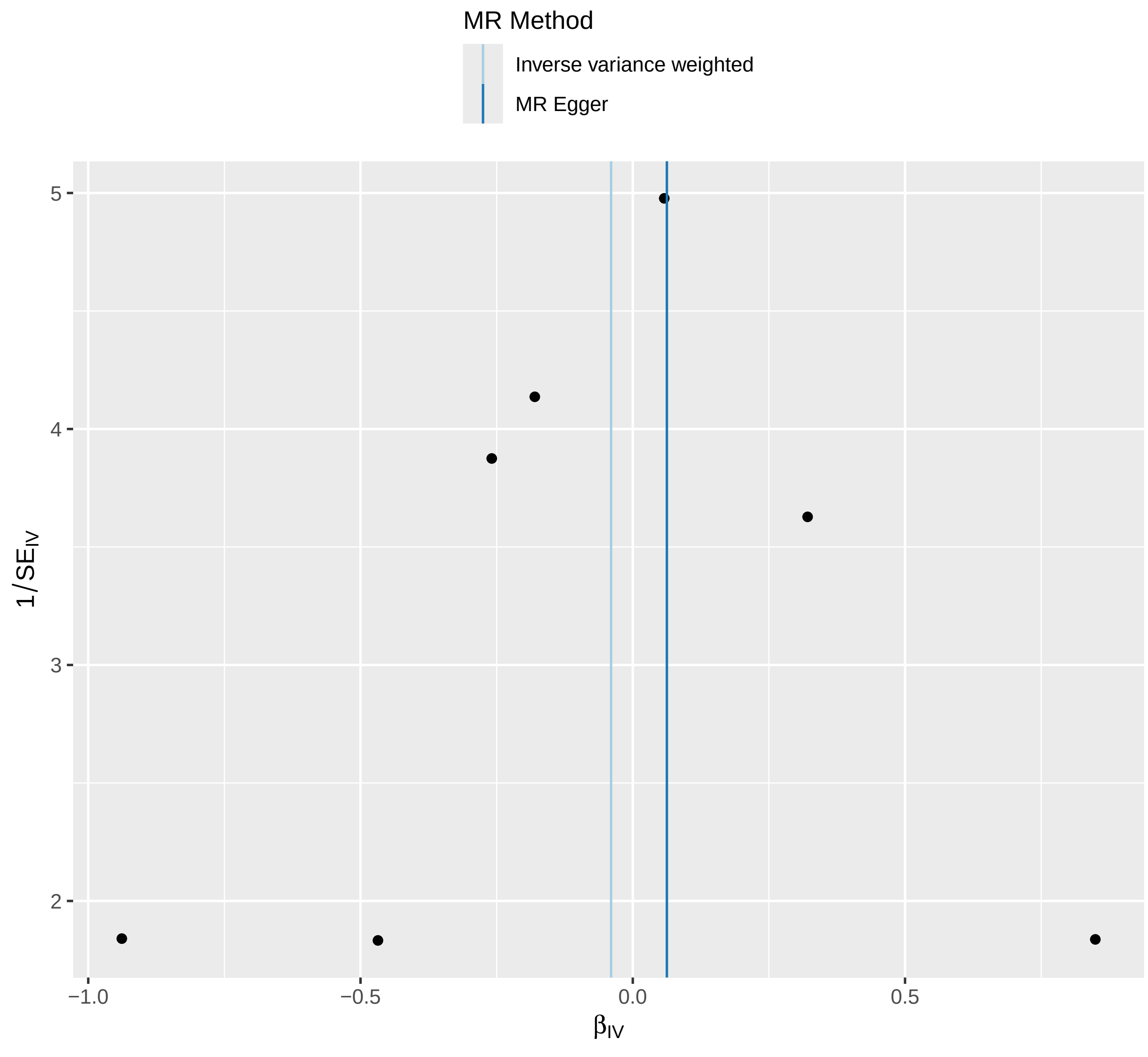

MR Method
Inverse variance weighted
MR Egger
5
4
3
2
1/SE$_{IV}$
−1.0
−0.5
0.0
0.5
β$_{IV}$

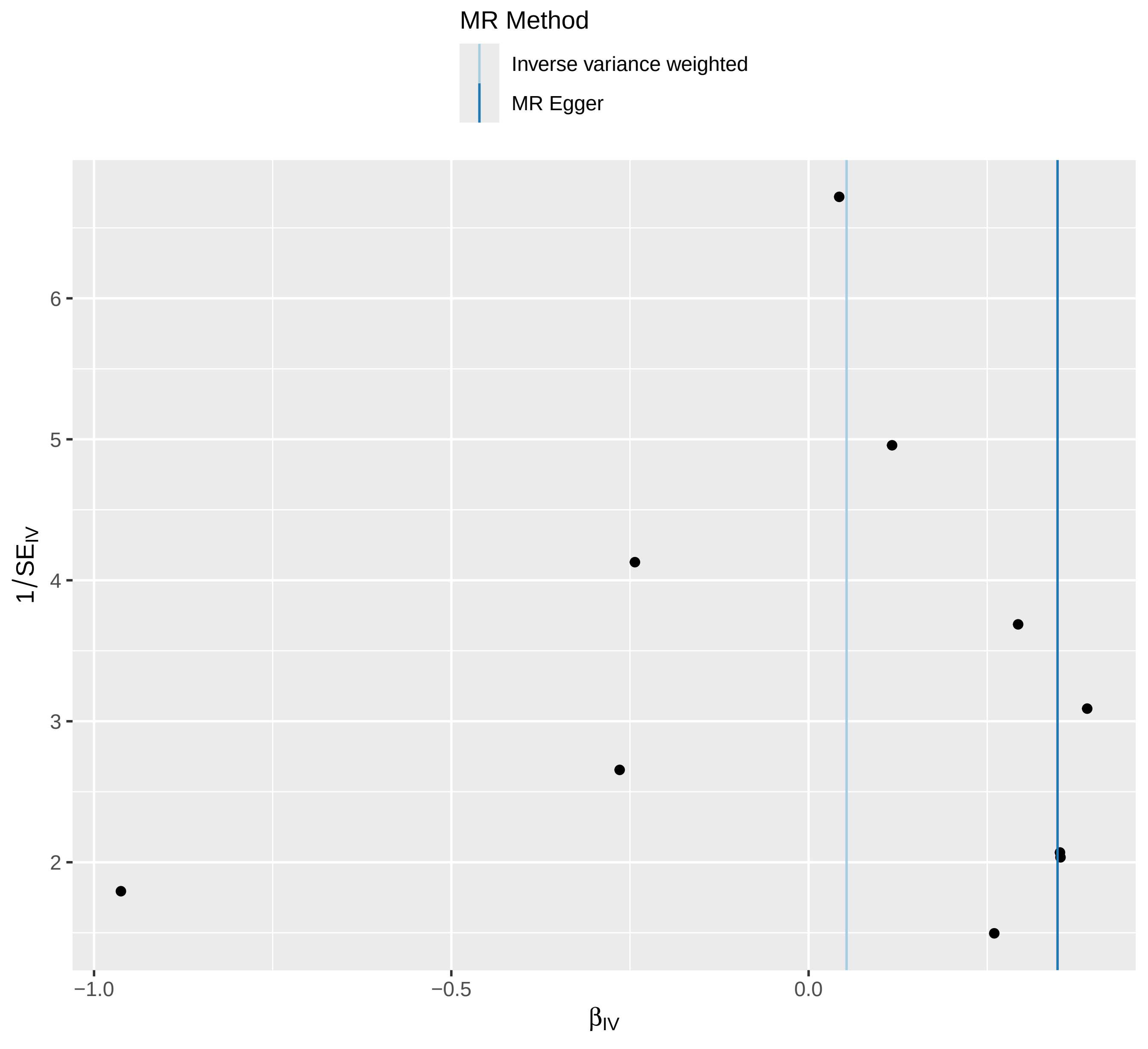

MR Method
Inverse variance weighted
MR Egger
1/SE_IV
β_IV
6
5
4
3
2
−1.0
−0.5
0.0

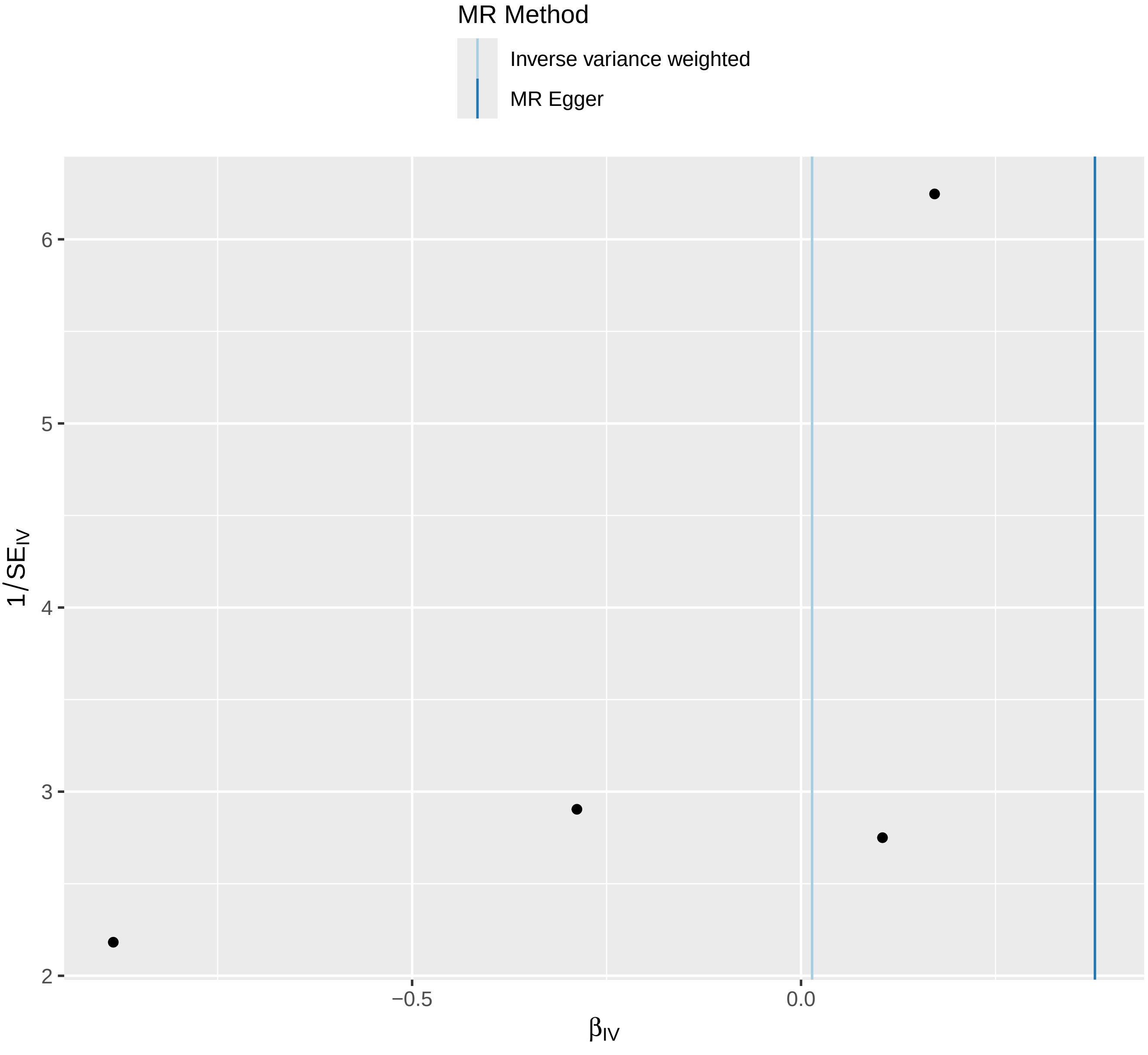

MR Method
Inverse variance weighted
MR Egger
6
5
4
3
2
1/SE_IV
−0.5
0.0
β_IV

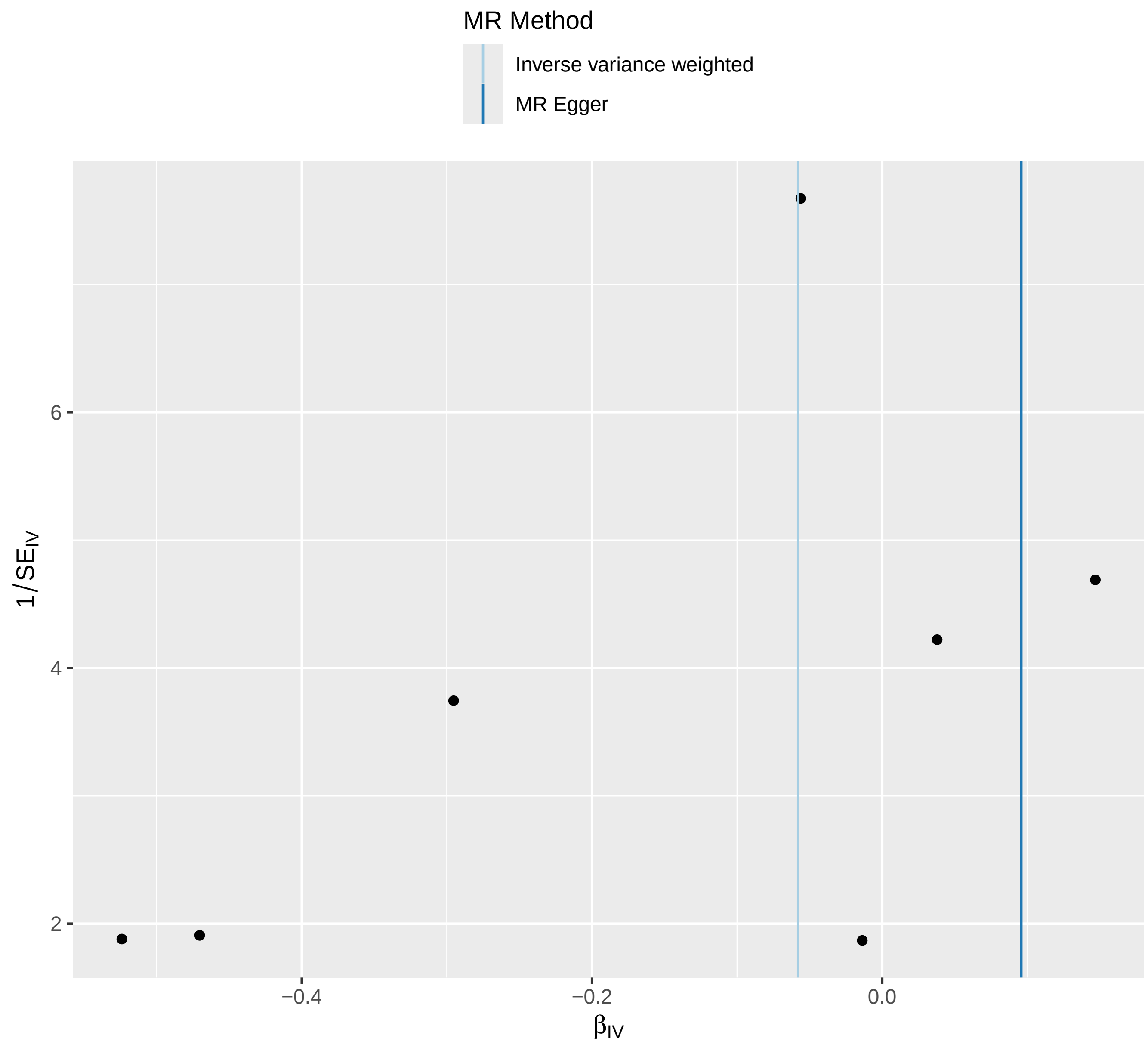

MR Method
Inverse variance weighted
MR Egger
6
4
2
1/SE$_{IV}$
−0.4
−0.2
0.0
β$_{IV}$

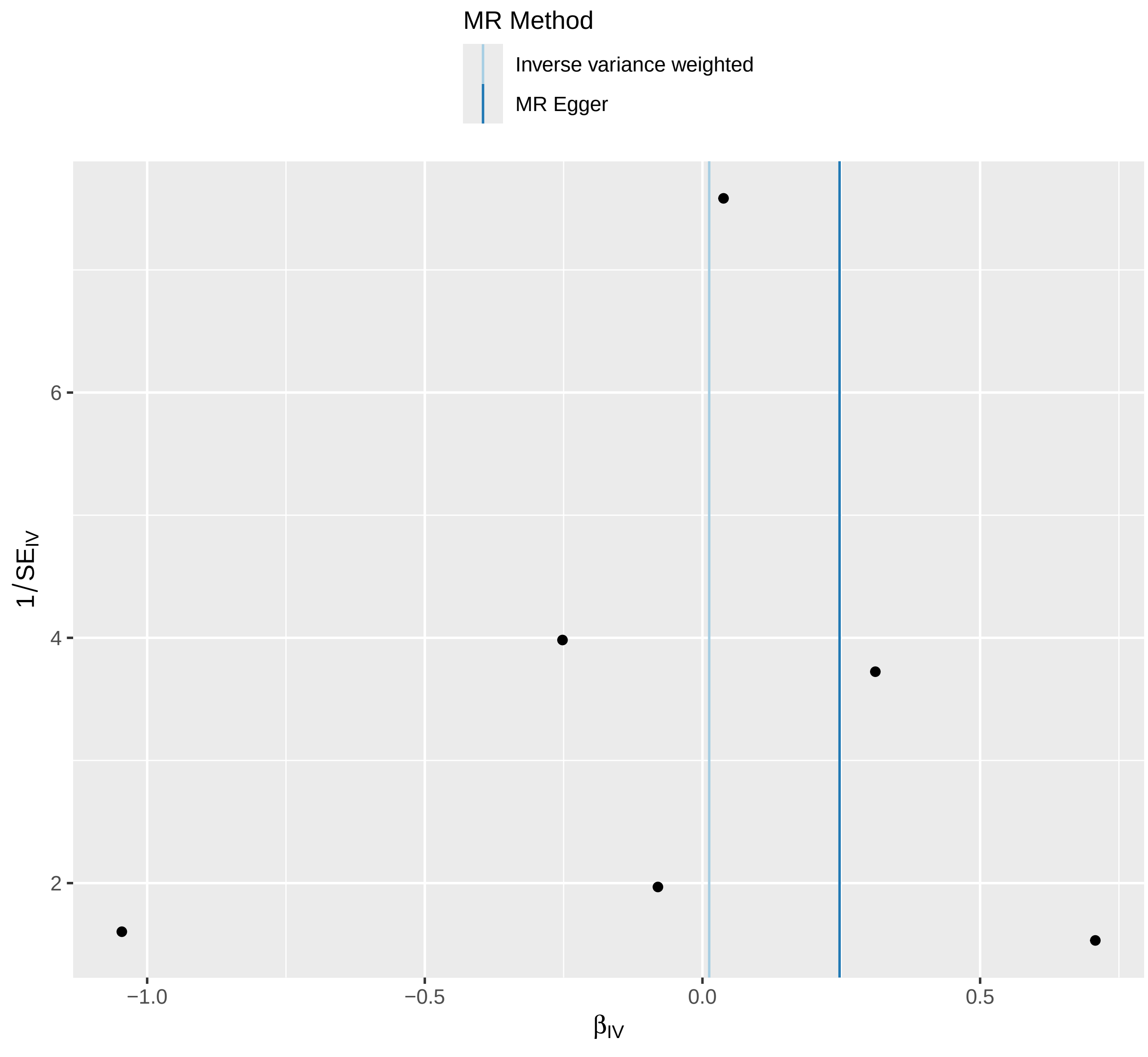

MR Method
Inverse variance weighted
MR Egger
6
4
2
$1/SE_{IV}$
−1.0
−0.5
0.0
0.5
β_{IV}

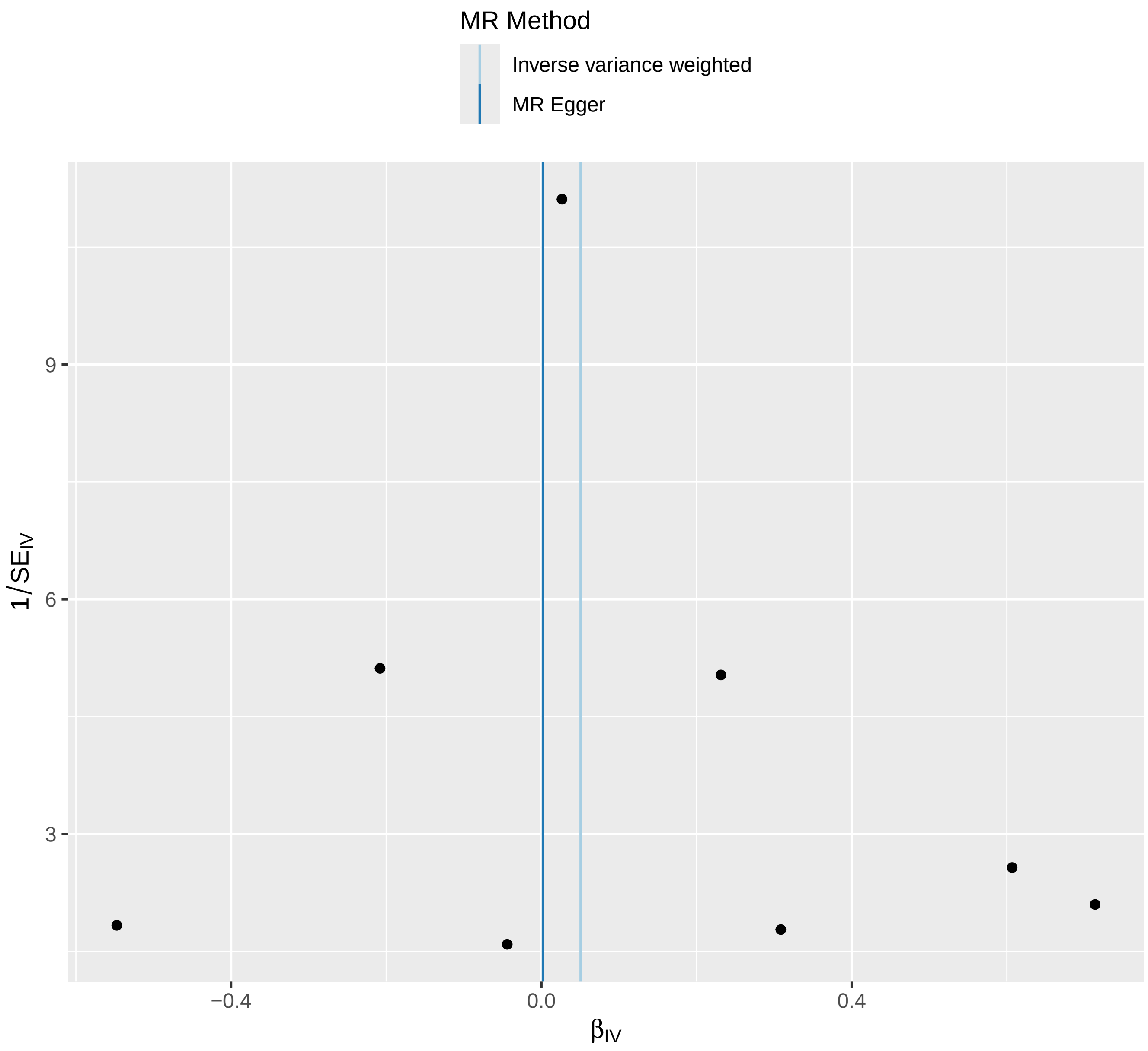

MR Method
Inverse variance weighted
MR Egger
9
6
3
1/SE_IV
−0.4
0.0
0.4
β_IV

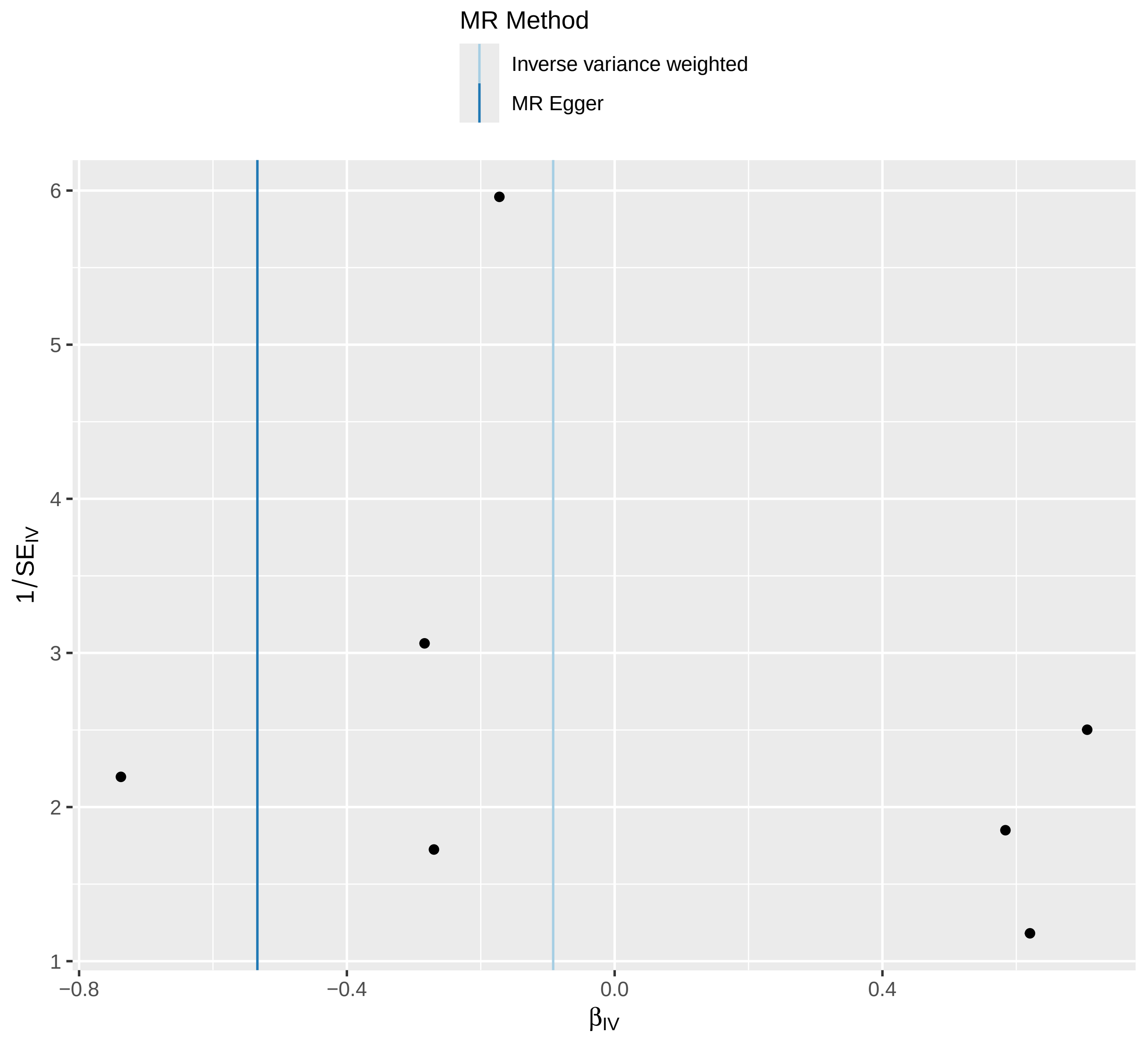

MR Method
Inverse variance weighted
MR Egger
6
5
4
3
2
1
1/SE_IV
−0.8
−0.4
0.0
0.4
β_IV

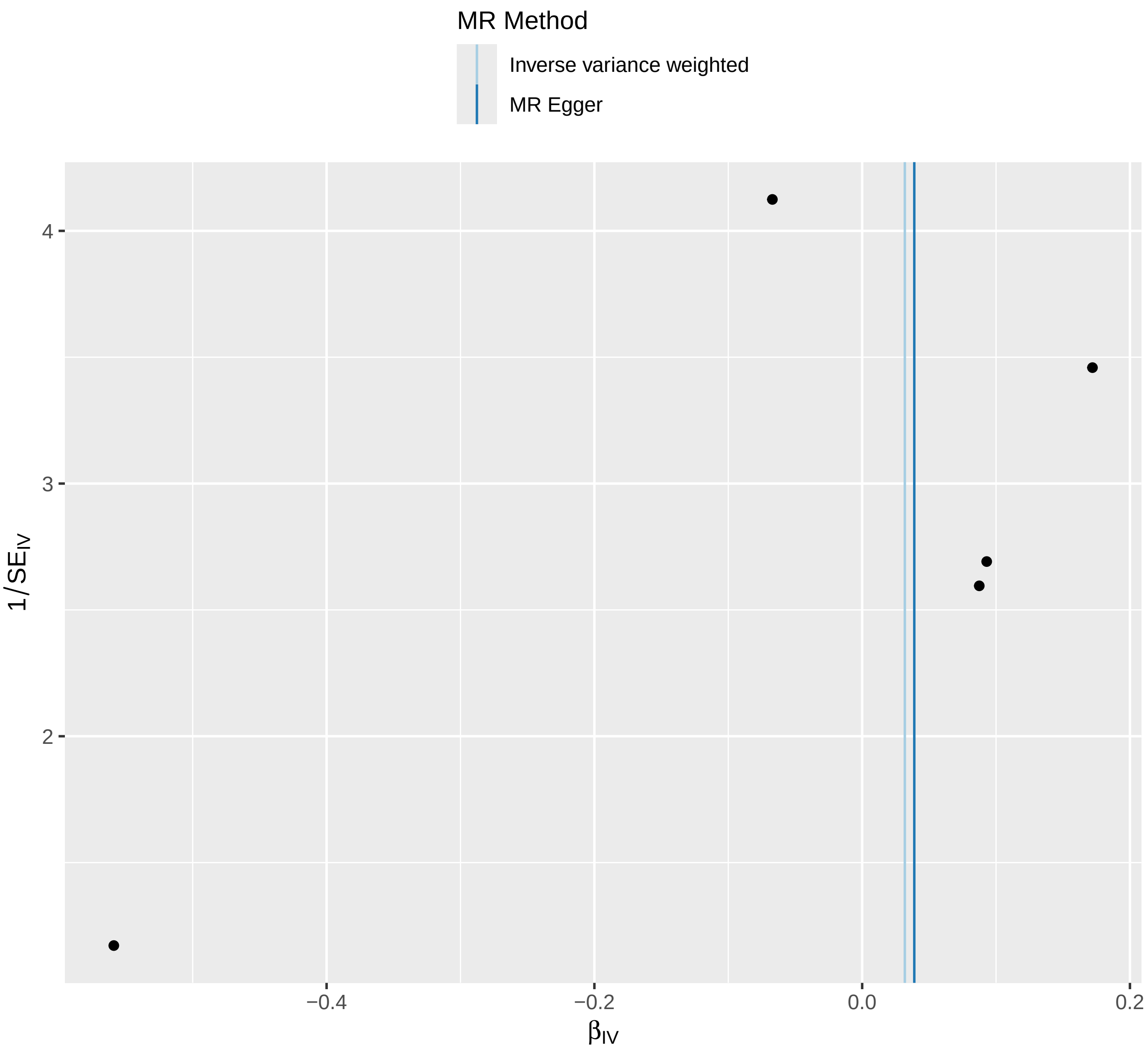
MR Method
Inverse variance weighted
MR Egger
4
3
2
1/SE_IV
−0.4
−0.2
0.0
0.2
β_IV

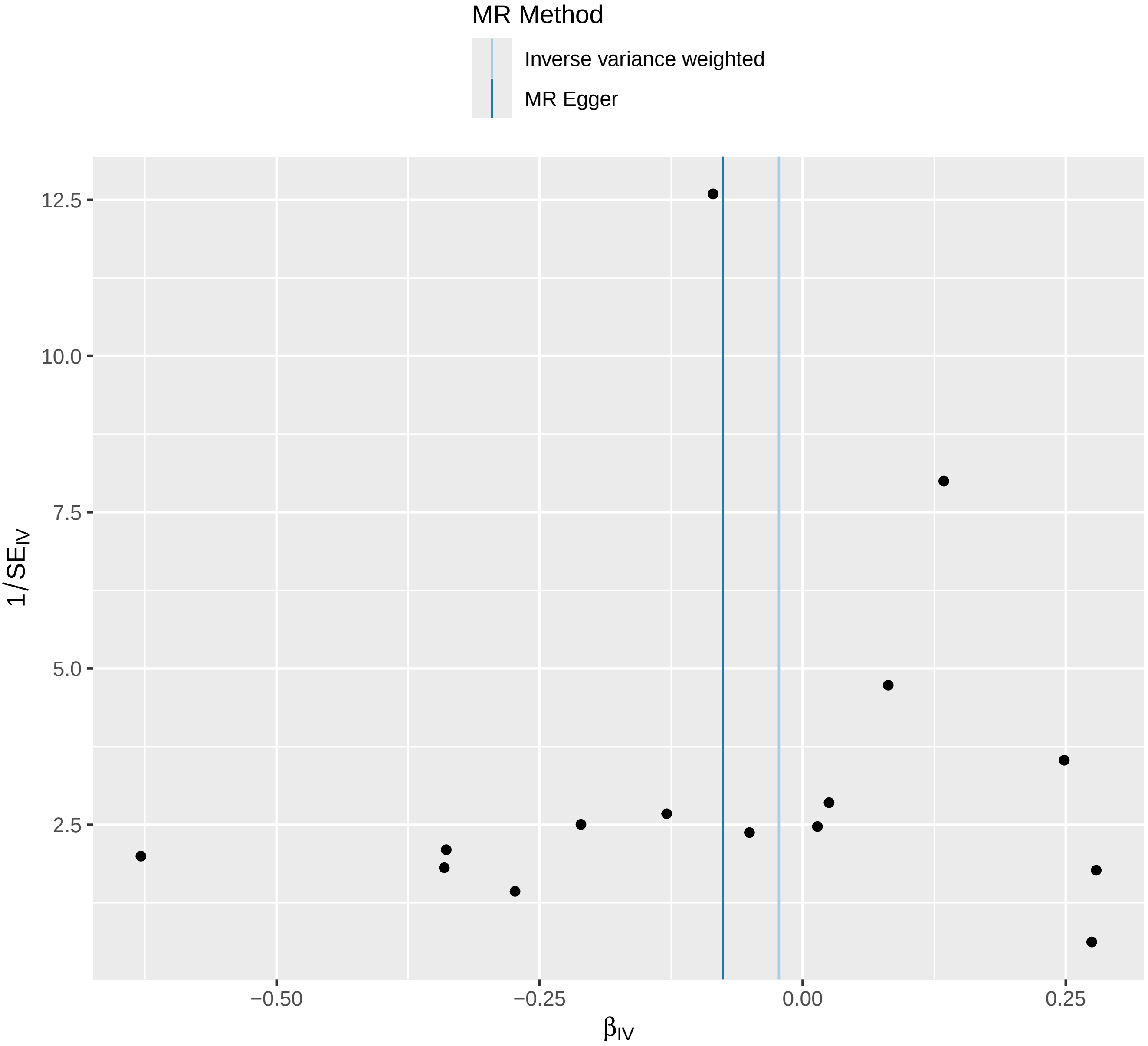

MR Method
Inverse variance weighted
MR Egger
12.5
10.0
7.5
5.0
2.5
1/SE_IV
−0.50
−0.25
0.00
0.25
β_IV

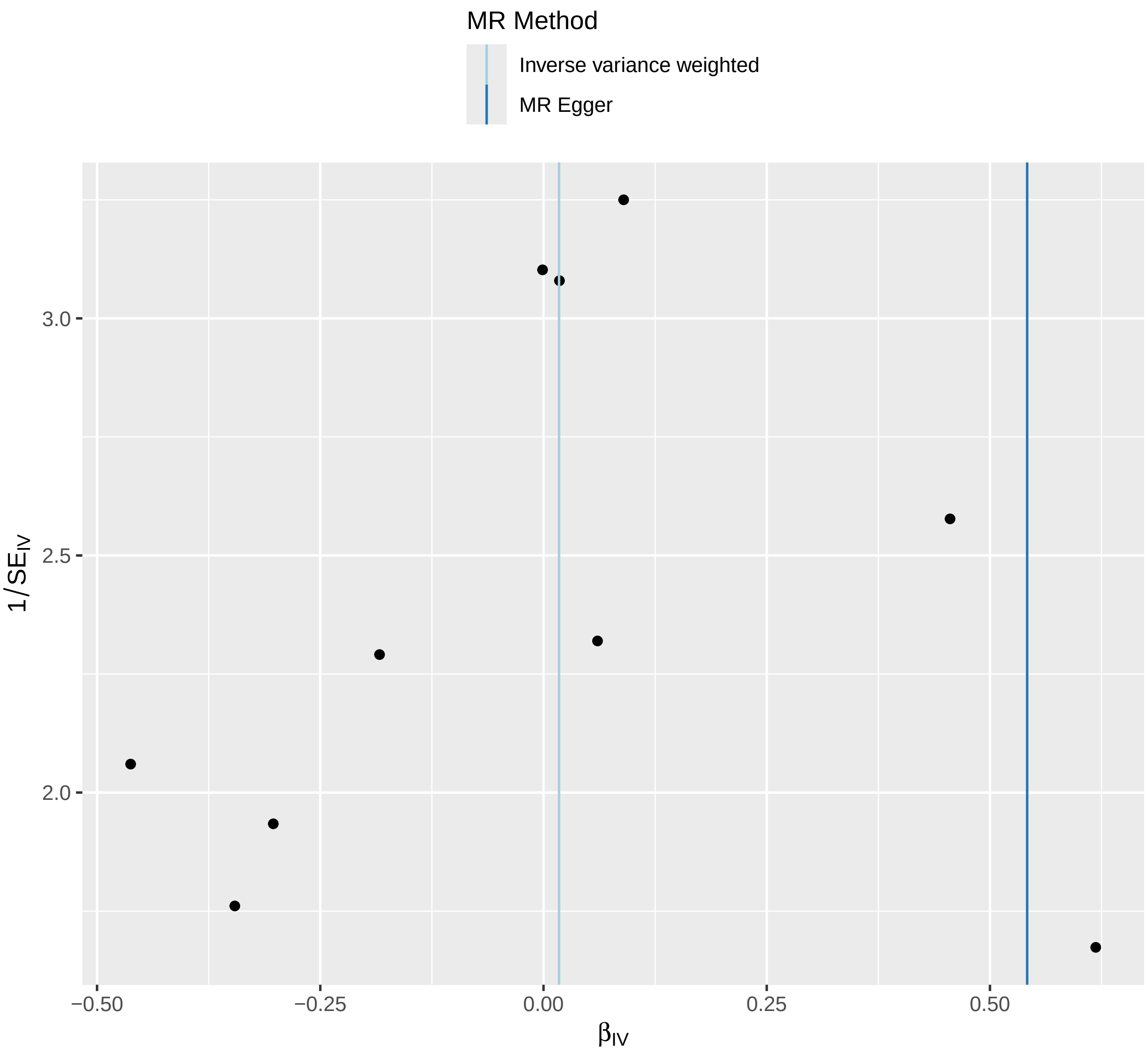

MR Method
Inverse variance weighted
MR Egger
3.0
2.5
2.0
1/SE_IV
−0.50
−0.25
0.00
0.25
0.50
β_IV

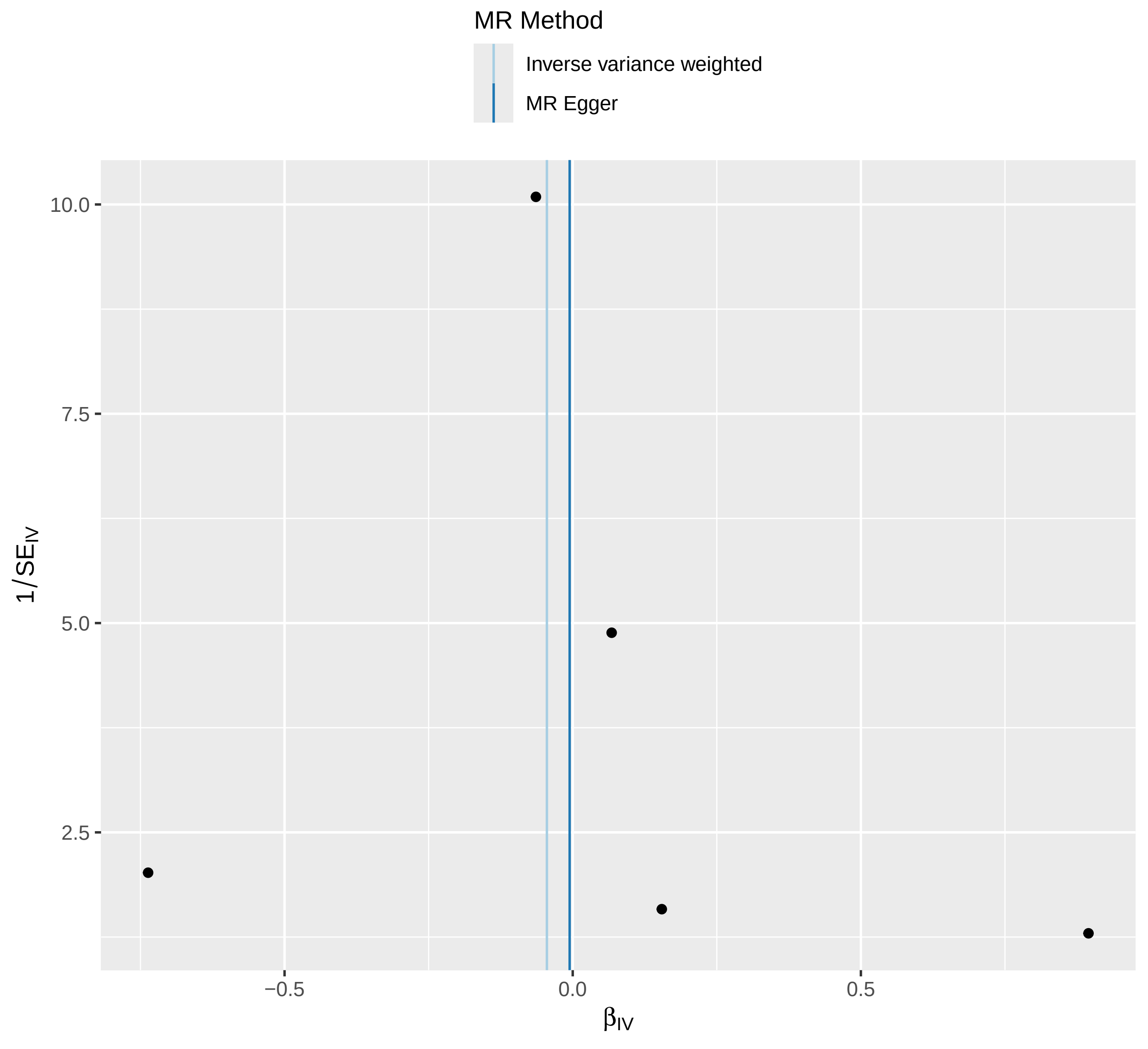
MR Method
Inverse variance weighted
MR Egger
10.0
7.5
5.0
2.5
−0.5
0.0
0.5
$1/SE_{IV}$
β_{IV}

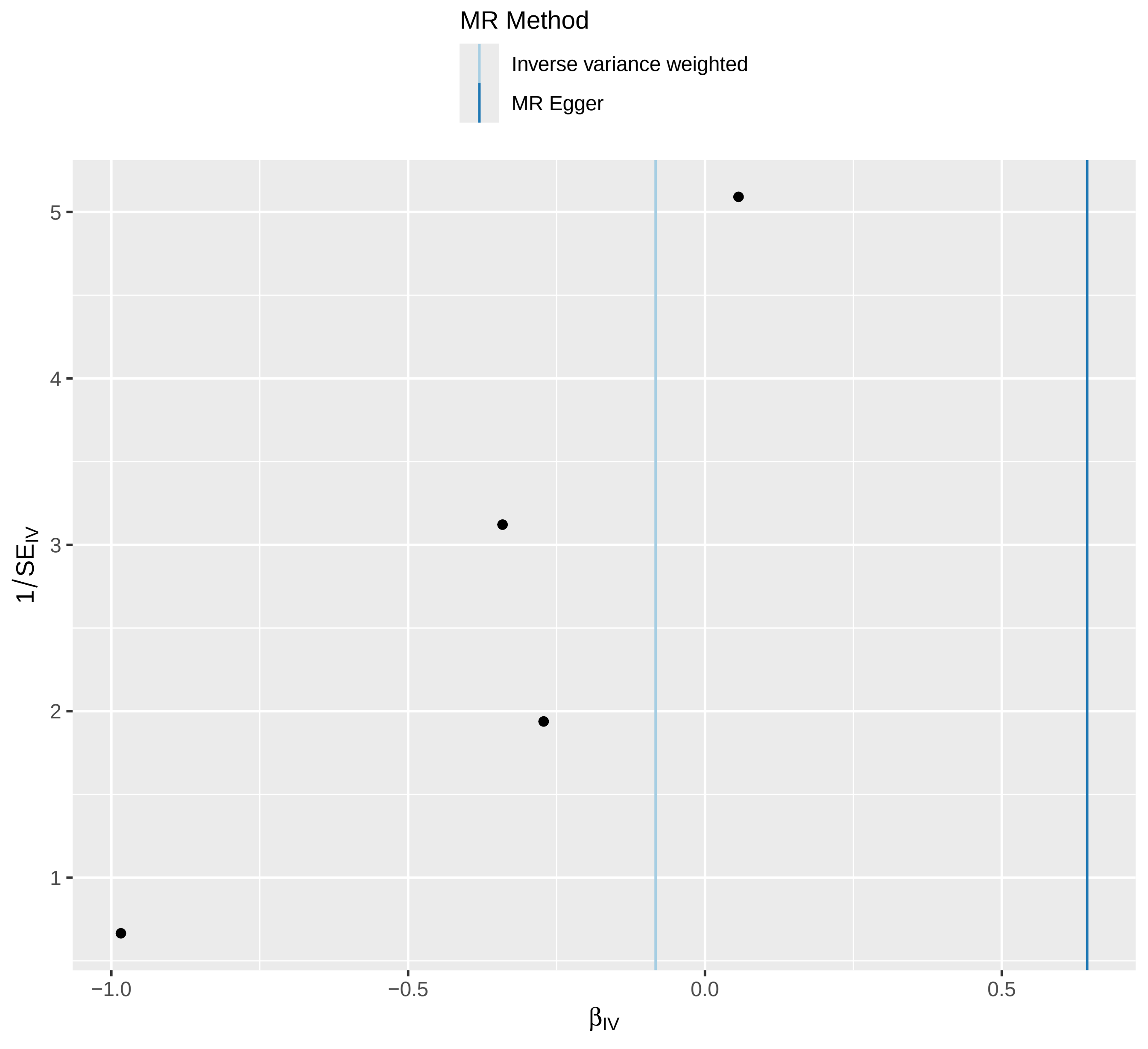

MR Method
Inverse variance weighted
MR Egger
5
4
3
2
1
1/SE_IV
−1.0
−0.5
0.0
0.5
β_IV

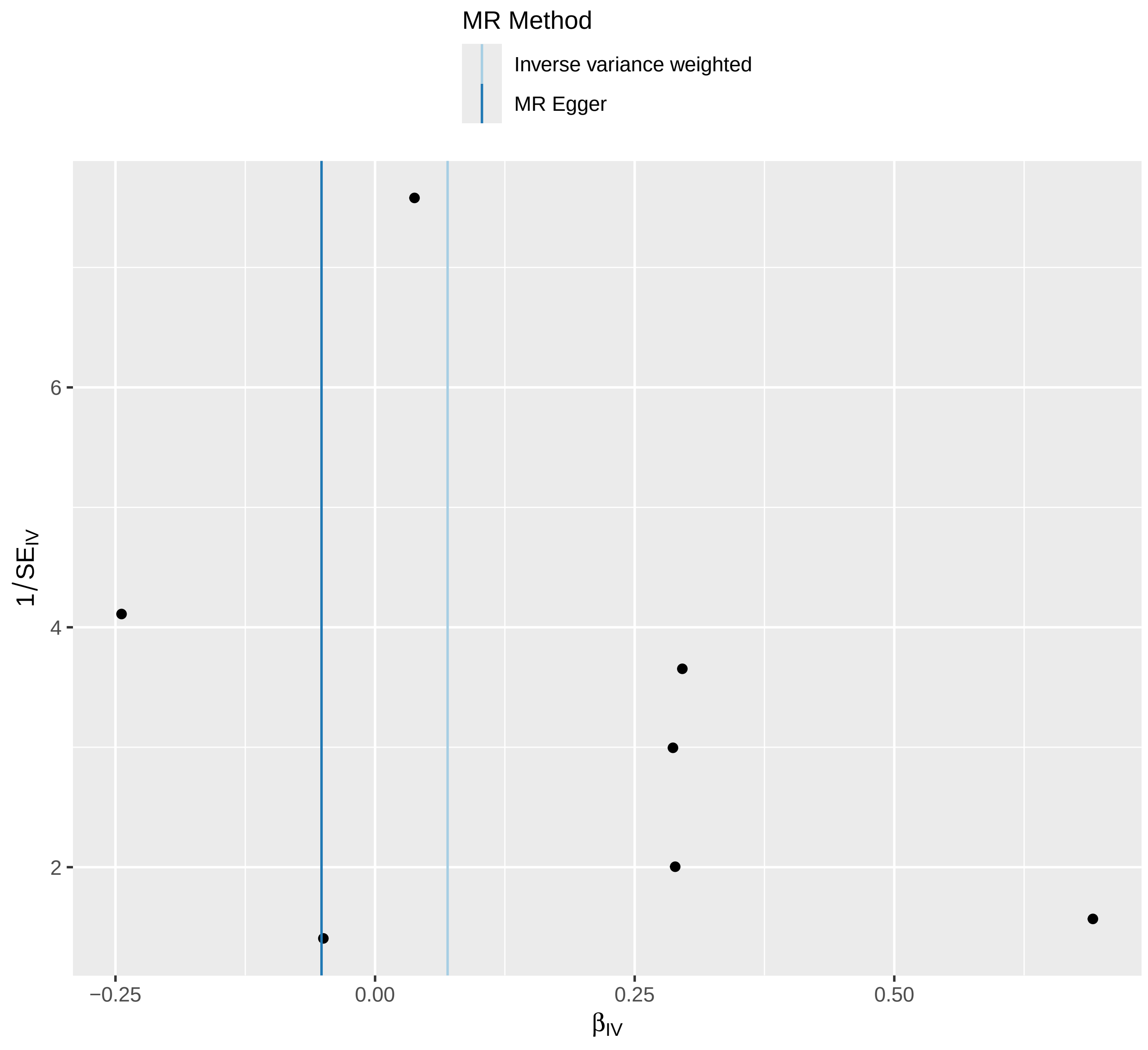
MR Method
Inverse variance weighted
MR Egger
$1/SE_{IV}$
6
4
2
−0.25
0.00
0.25
0.50
β_{IV}

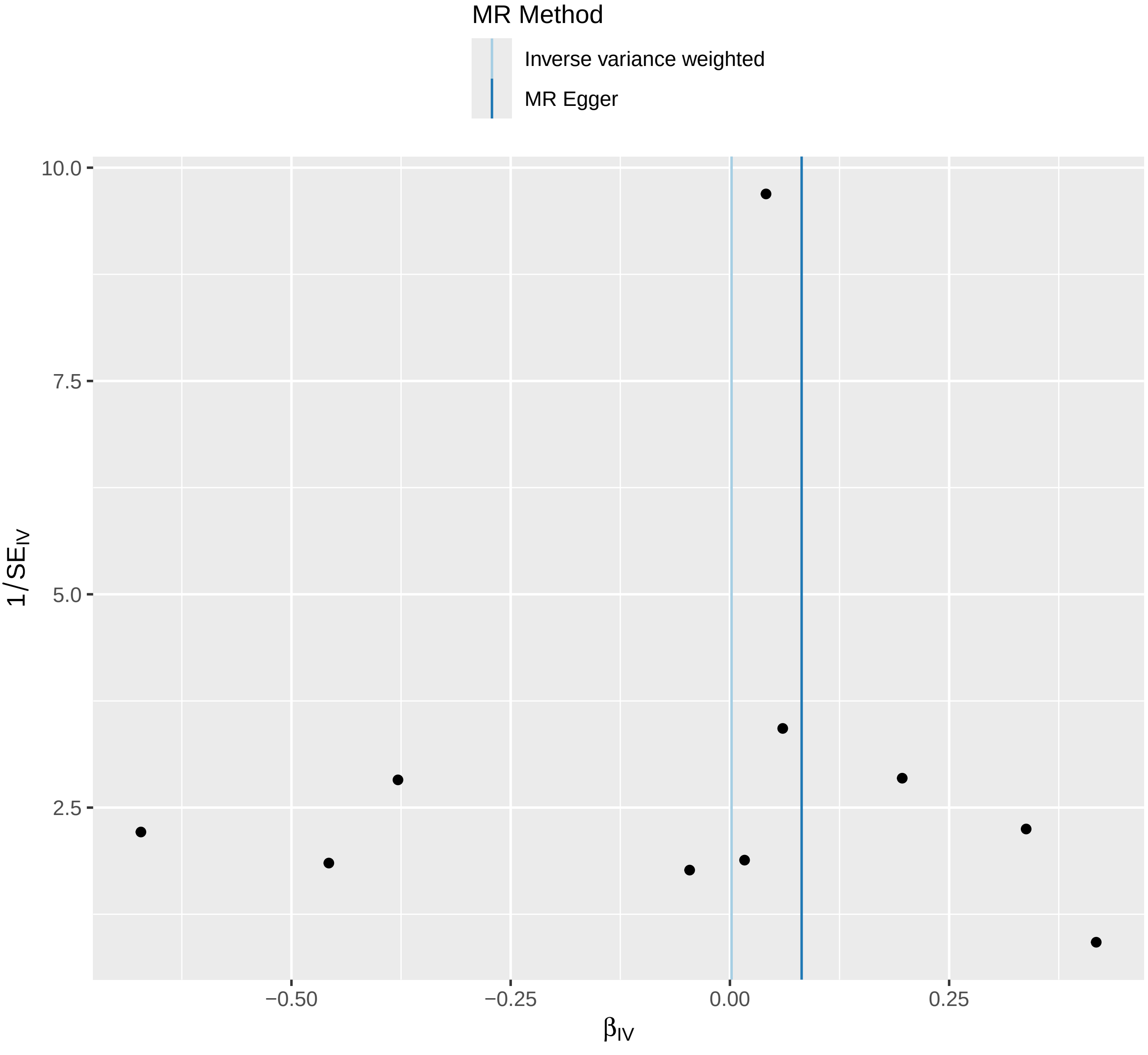
MR Method
Inverse variance weighted
MR Egger
10.0
7.5
5.0
2.5
1/SE$_{IV}$
−0.50
−0.25
0.00
0.25
β$_{IV}$

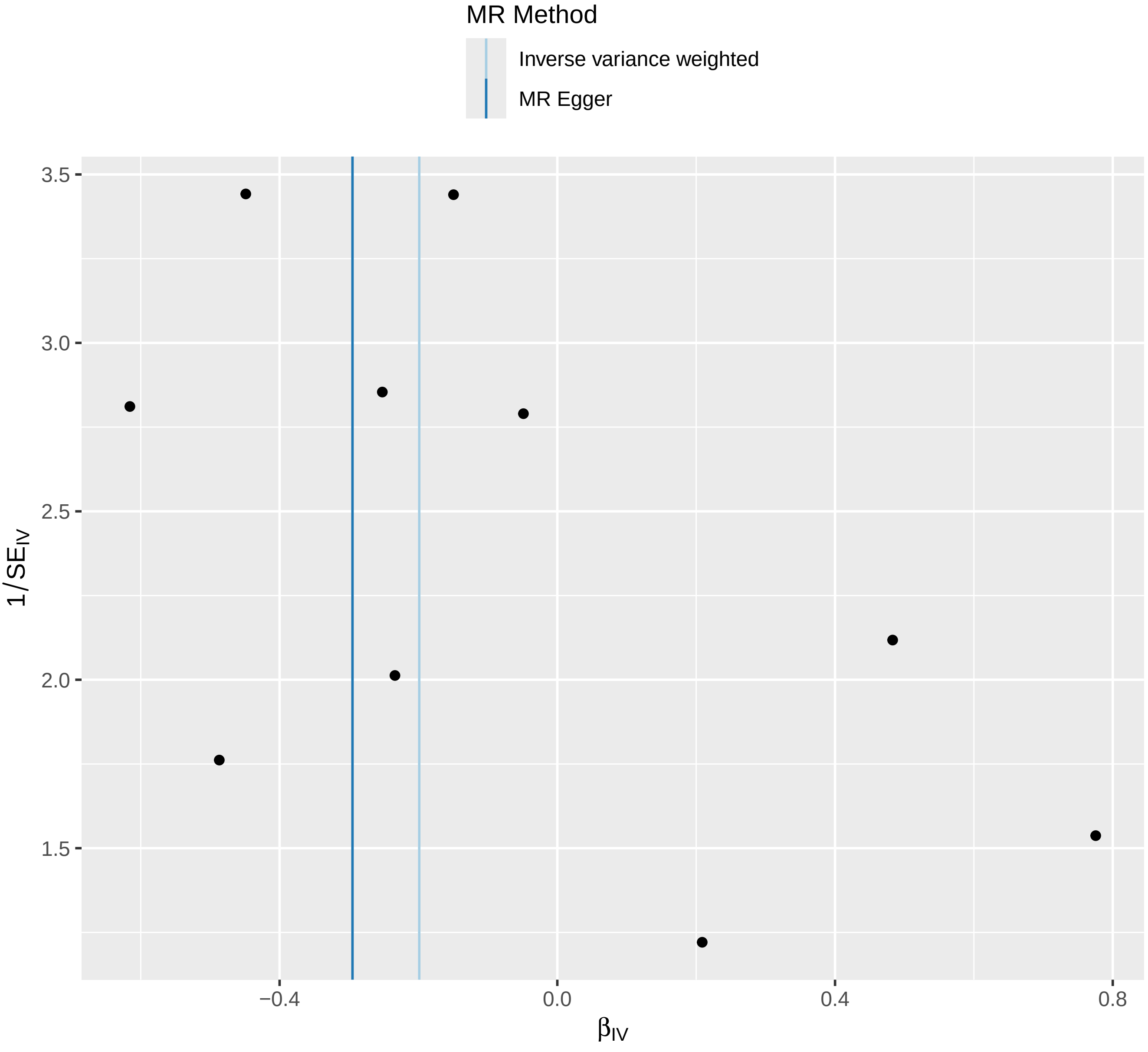

MR Method
Inverse variance weighted
MR Egger
3.5
3.0
2.5
2.0
1.5
$1/SE_{IV}$
−0.4
0.0
0.4
0.8
β_{IV}

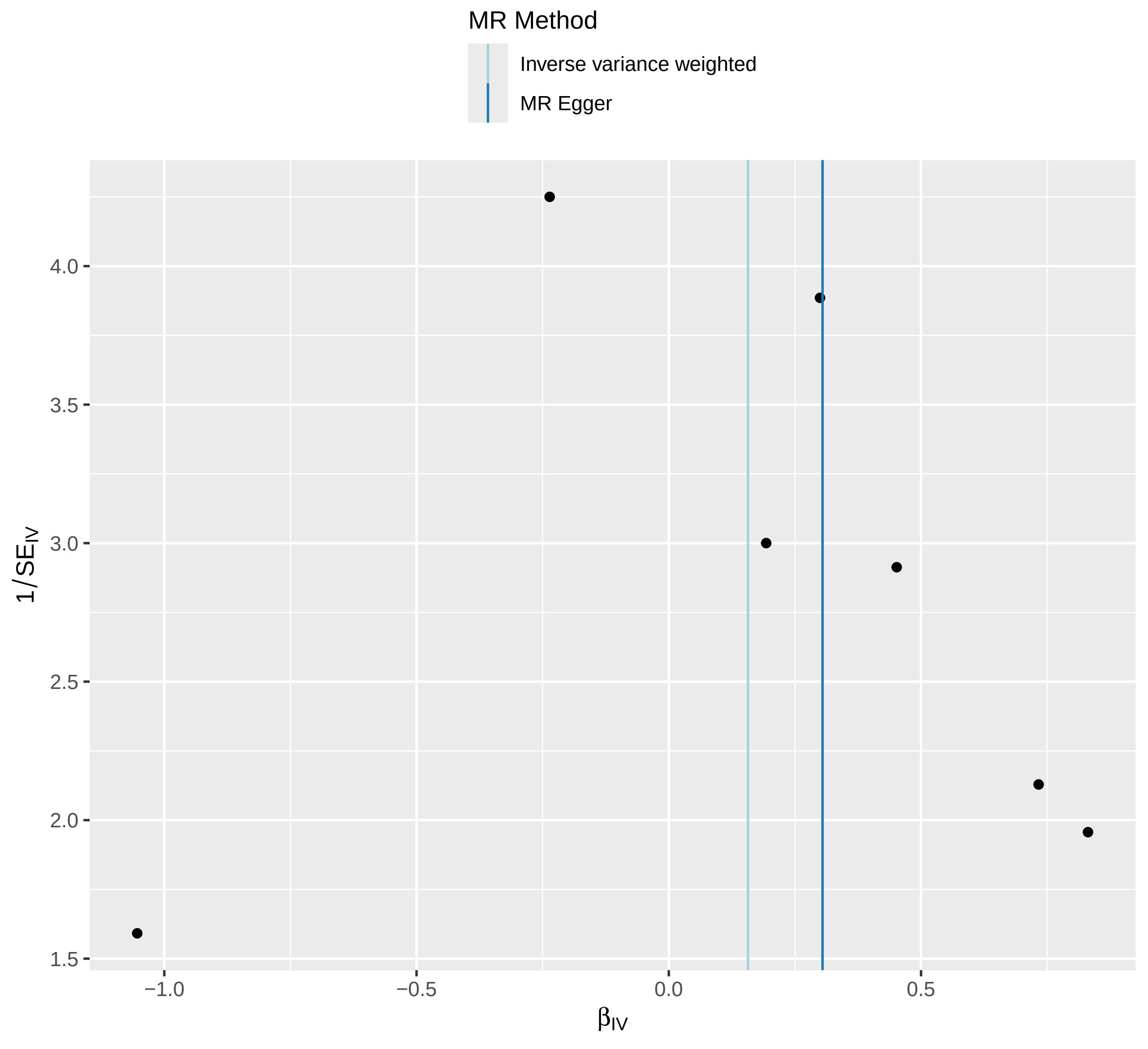
MR Method
Inverse variance weighted
MR Egger
1/SE_IV
4.0
3.5
3.0
2.5
2.0
1.5
−1.0
−0.5
0.0
0.5
β_IV

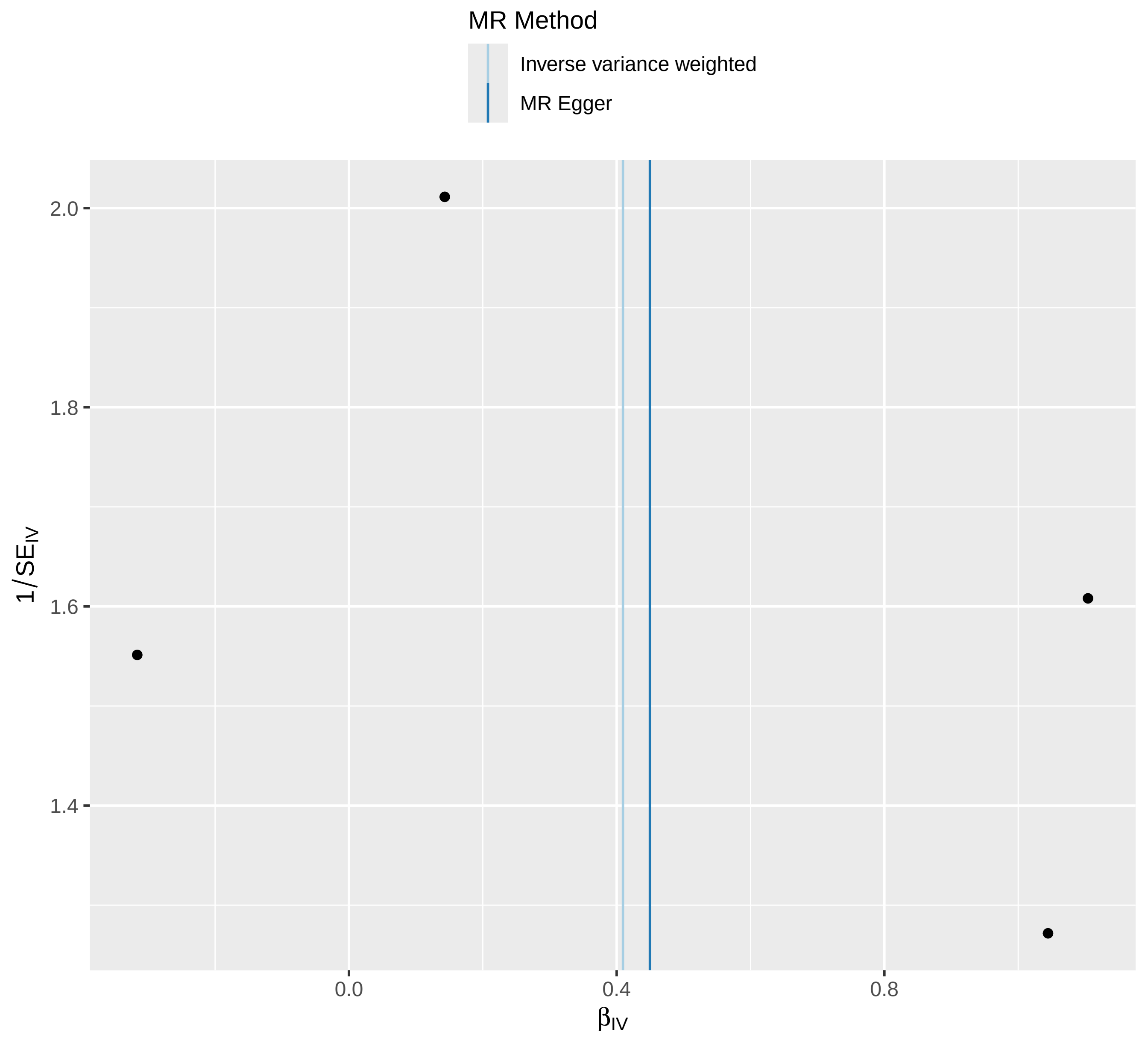

MR Method
Inverse variance weighted
MR Egger
2.0
1.8
1.6
1.4
0.0
0.4
0.8
$1/SE_{IV}$
β_{IV}

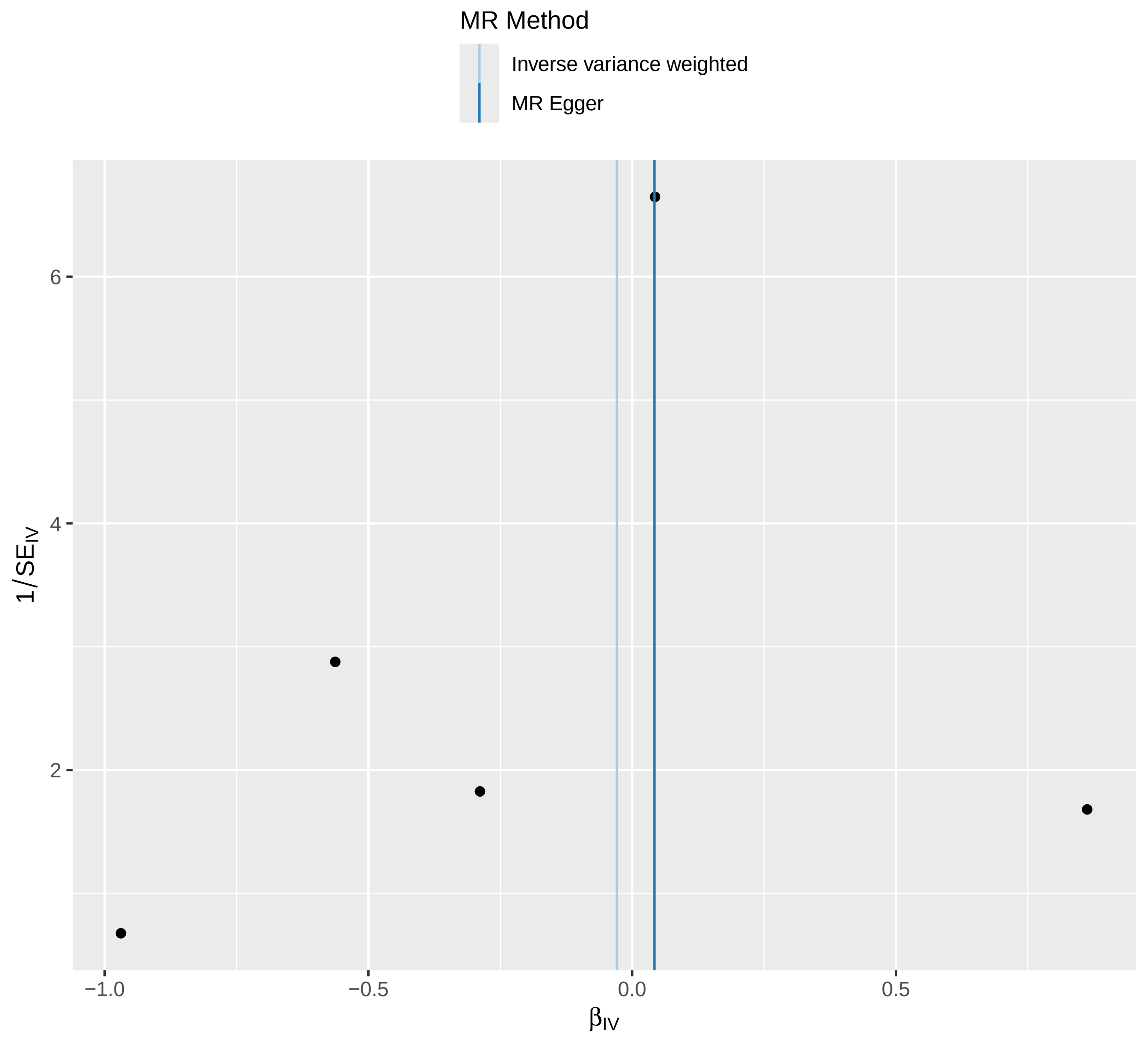

MR Method
Inverse variance weighted
MR Egger
6
4
2
1/SE_IV
−1.0
−0.5
0.0
0.5
β_IV

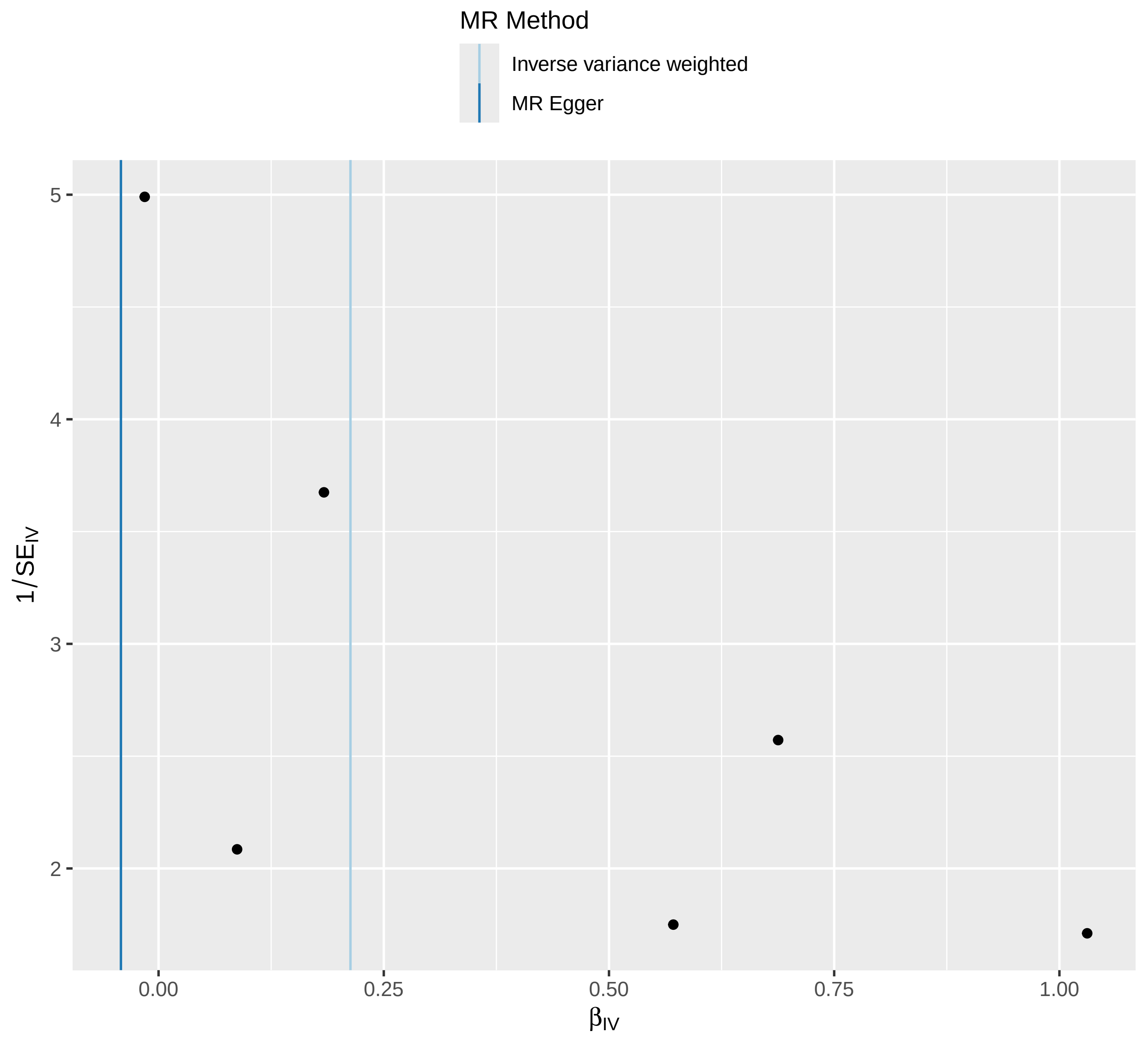
MR Method
Inverse variance weighted
MR Egger
5
4
3
2
1/SE_IV
0.00
0.25
0.50
0.75
1.00
β_IV

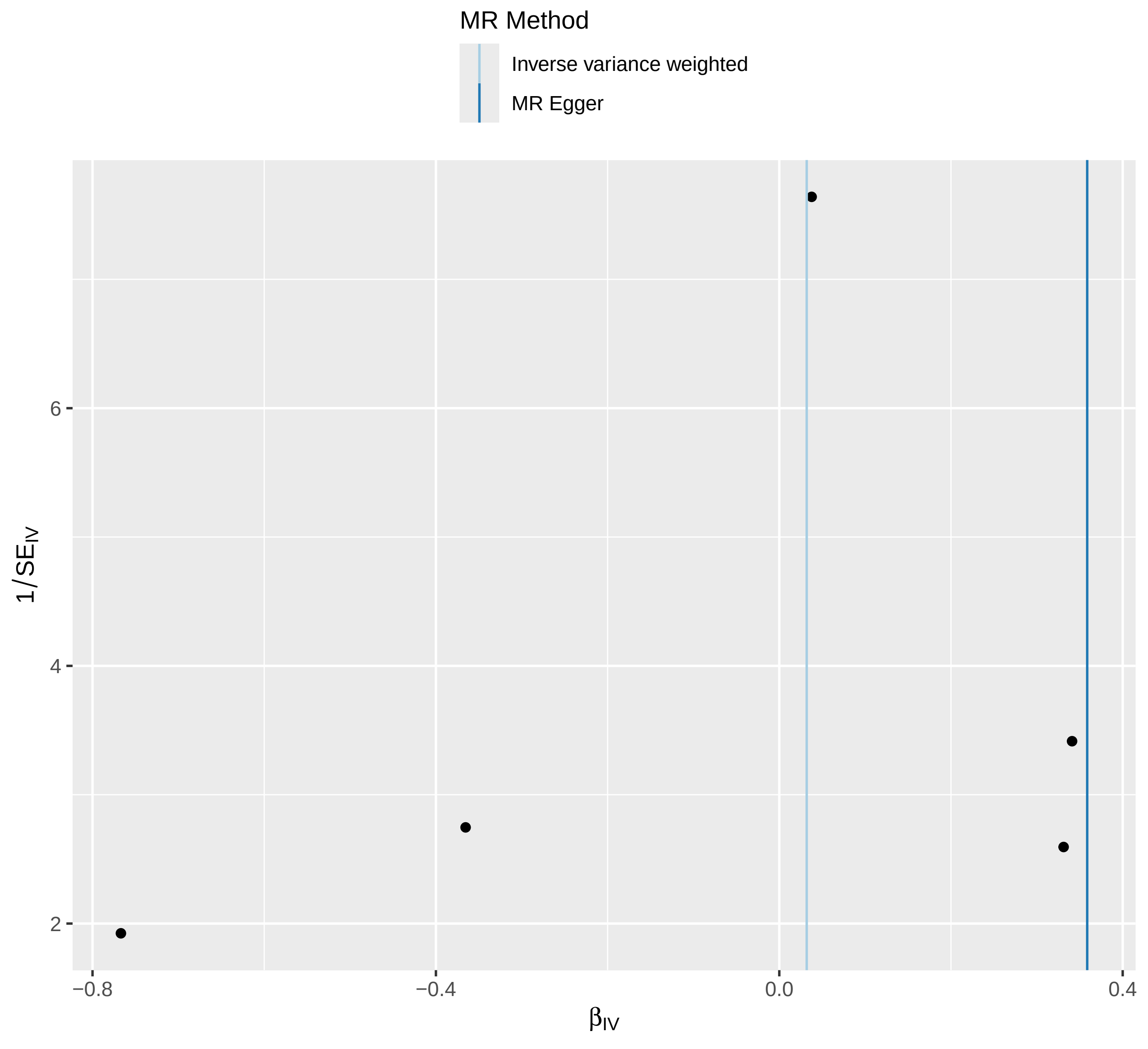

MR Method
Inverse variance weighted
MR Egger
6
4
2
1/SE$_{IV}$
−0.8
−0.4
0.0
0.4
β$_{IV}$

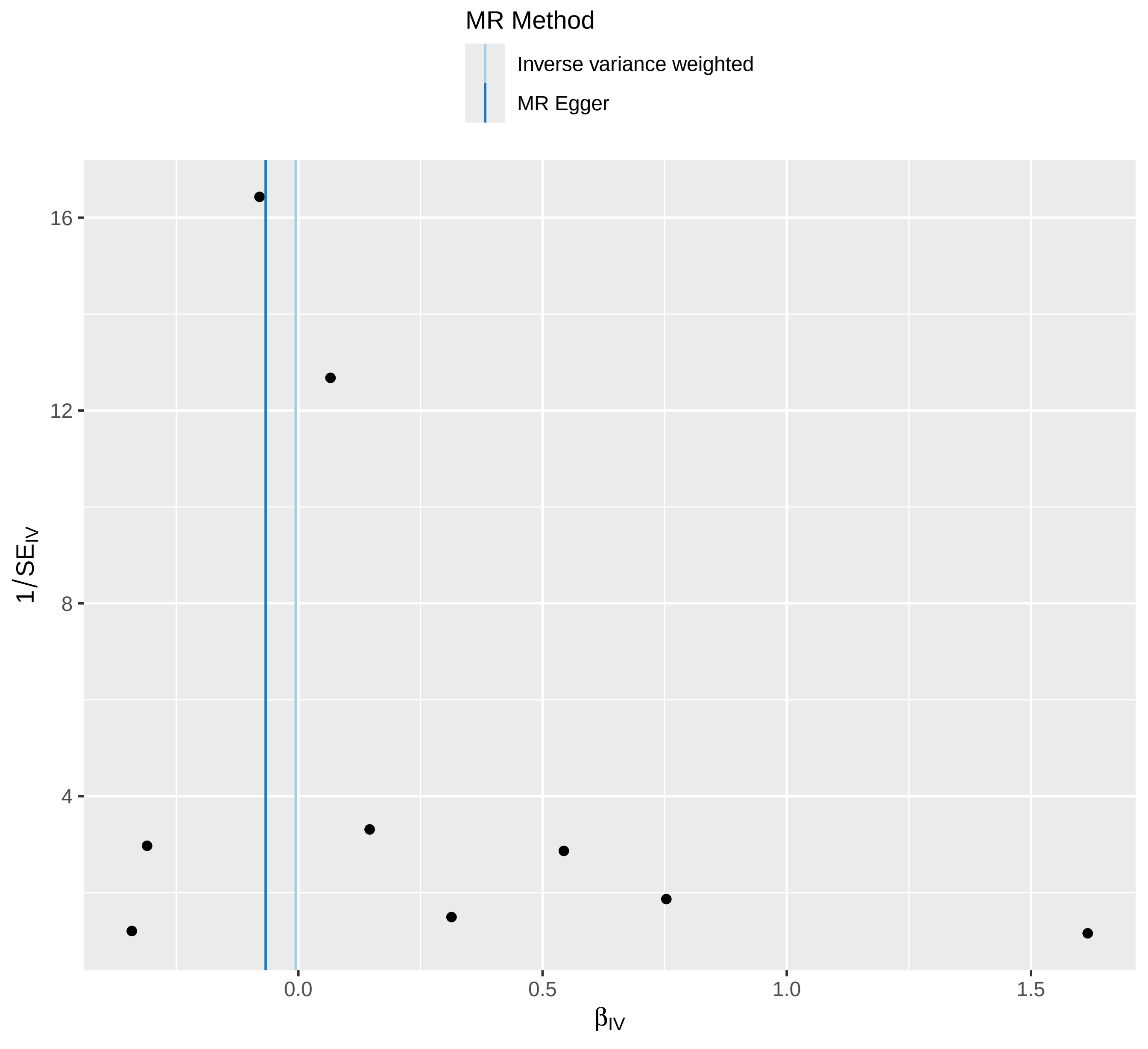
MR Method
Inverse variance weighted
MR Egger
16
12
8
4
1/SE$_{IV}$
0.0
0.5
1.0
1.5
β$_{IV}$

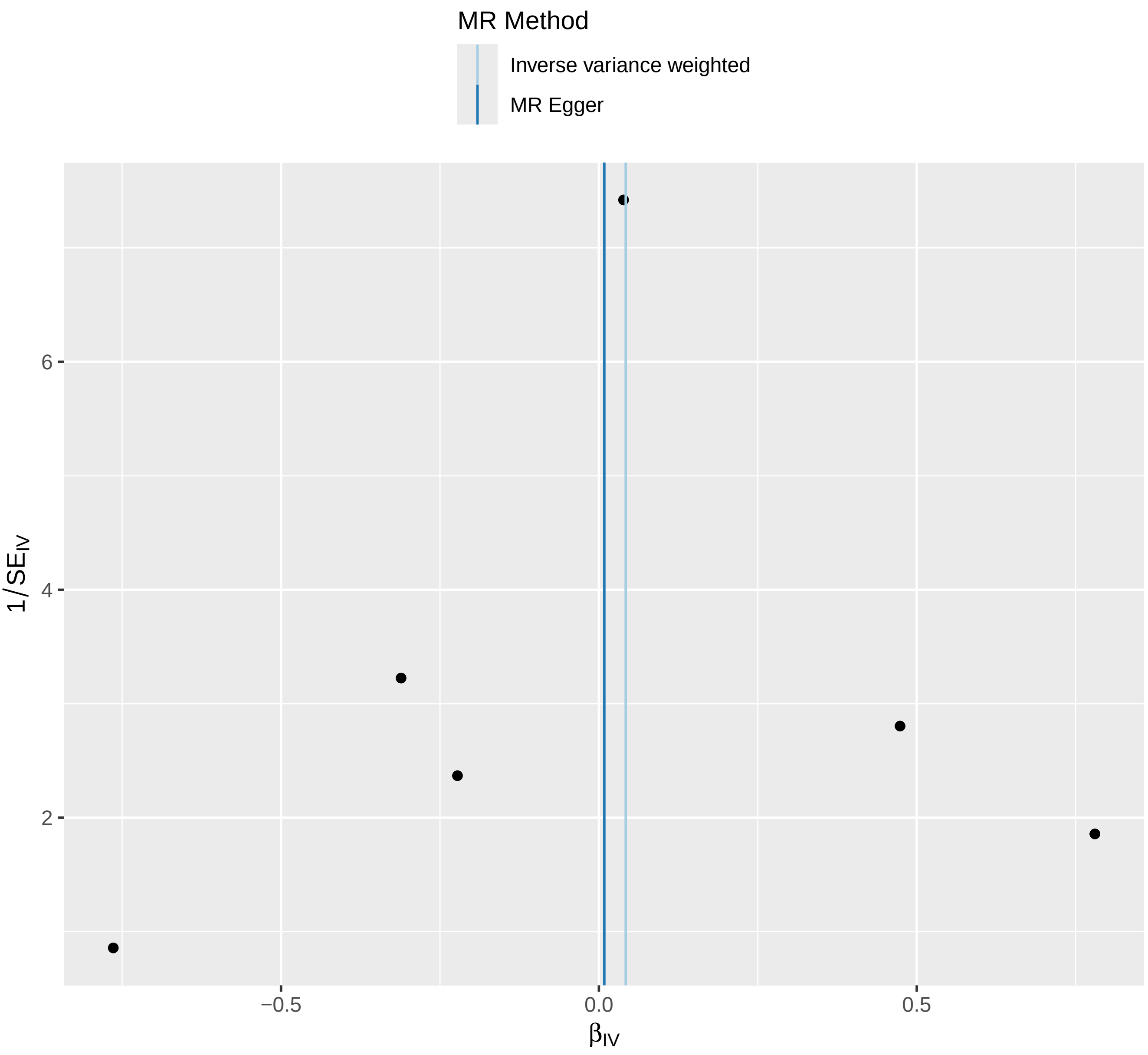
MR Method
Inverse variance weighted
MR Egger
6
4
2
1/SE$_{IV}$
−0.5
0.0
0.5
β$_{IV}$

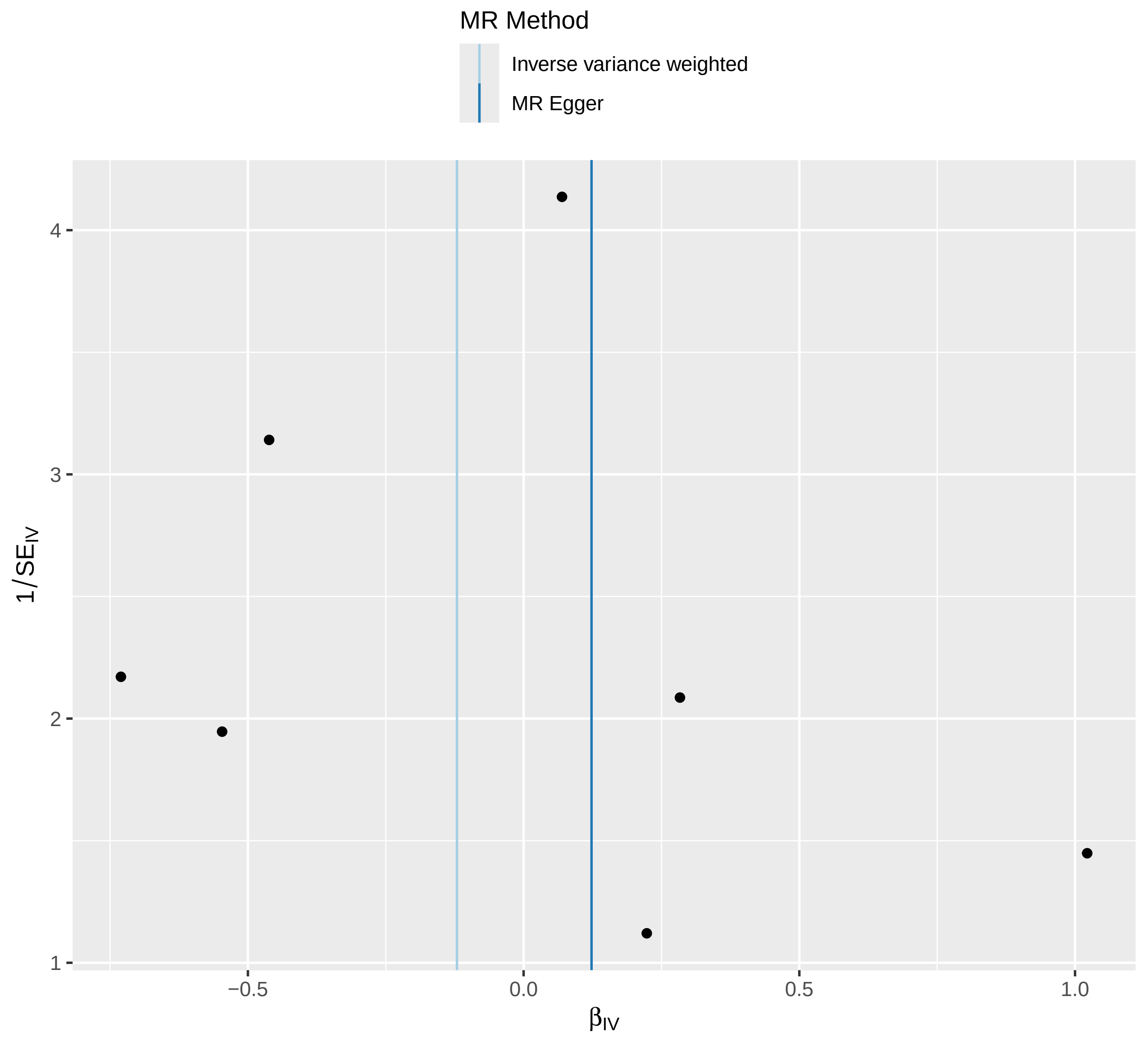

MR Method
Inverse variance weighted
MR Egger
4
3
2
1
1/SE_IV
−0.5
0.0
0.5
1.0
β_IV

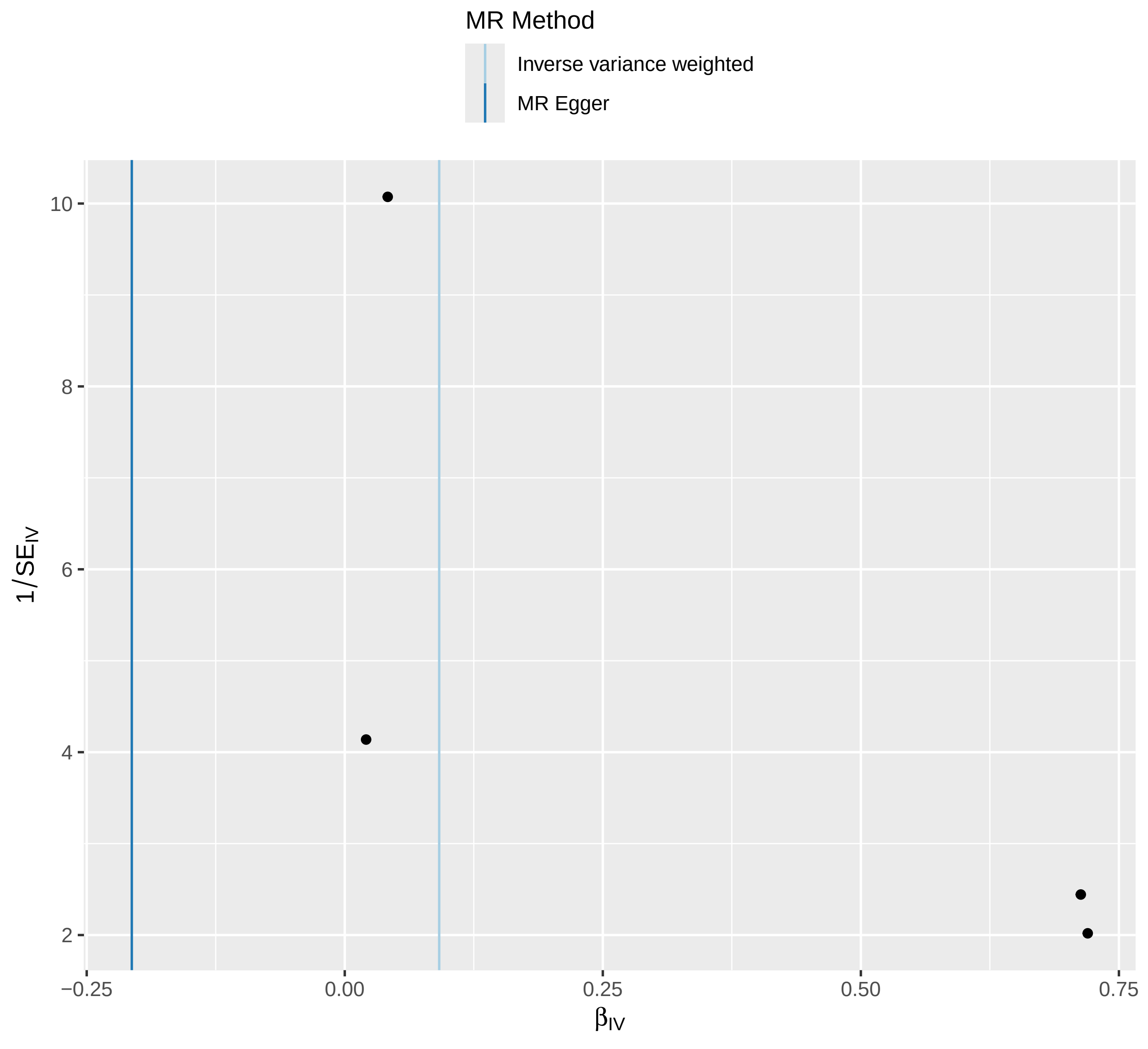

MR Method
Inverse variance weighted
MR Egger
10
8
6
4
2
1/SE$_{IV}$
−0.25
0.00
0.25
0.50
0.75
β$_{IV}$

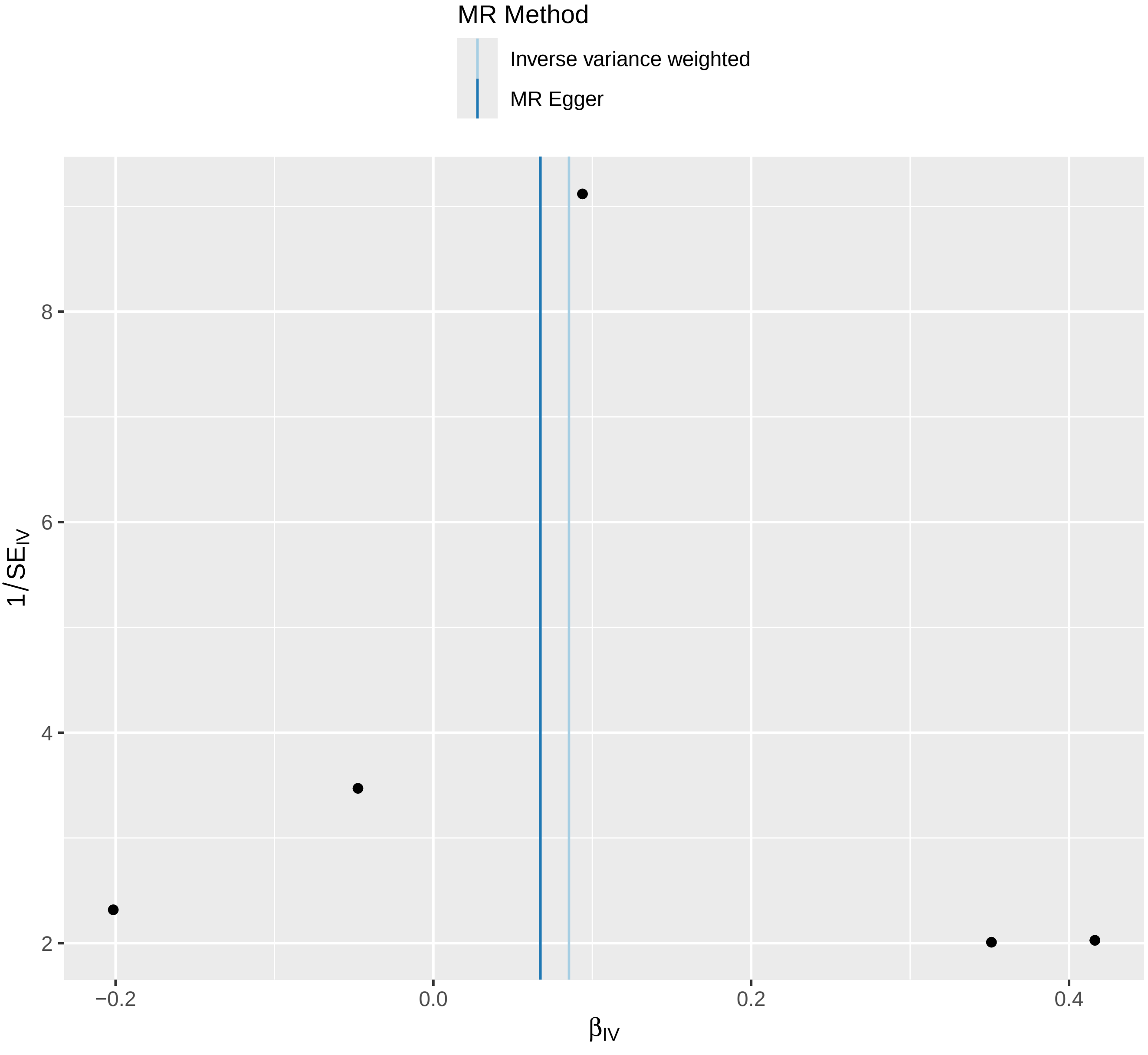
MR Method
Inverse variance weighted
MR Egger
8
6
4
2
1/SE_IV
−0.2
0.0
0.2
0.4
β_IV

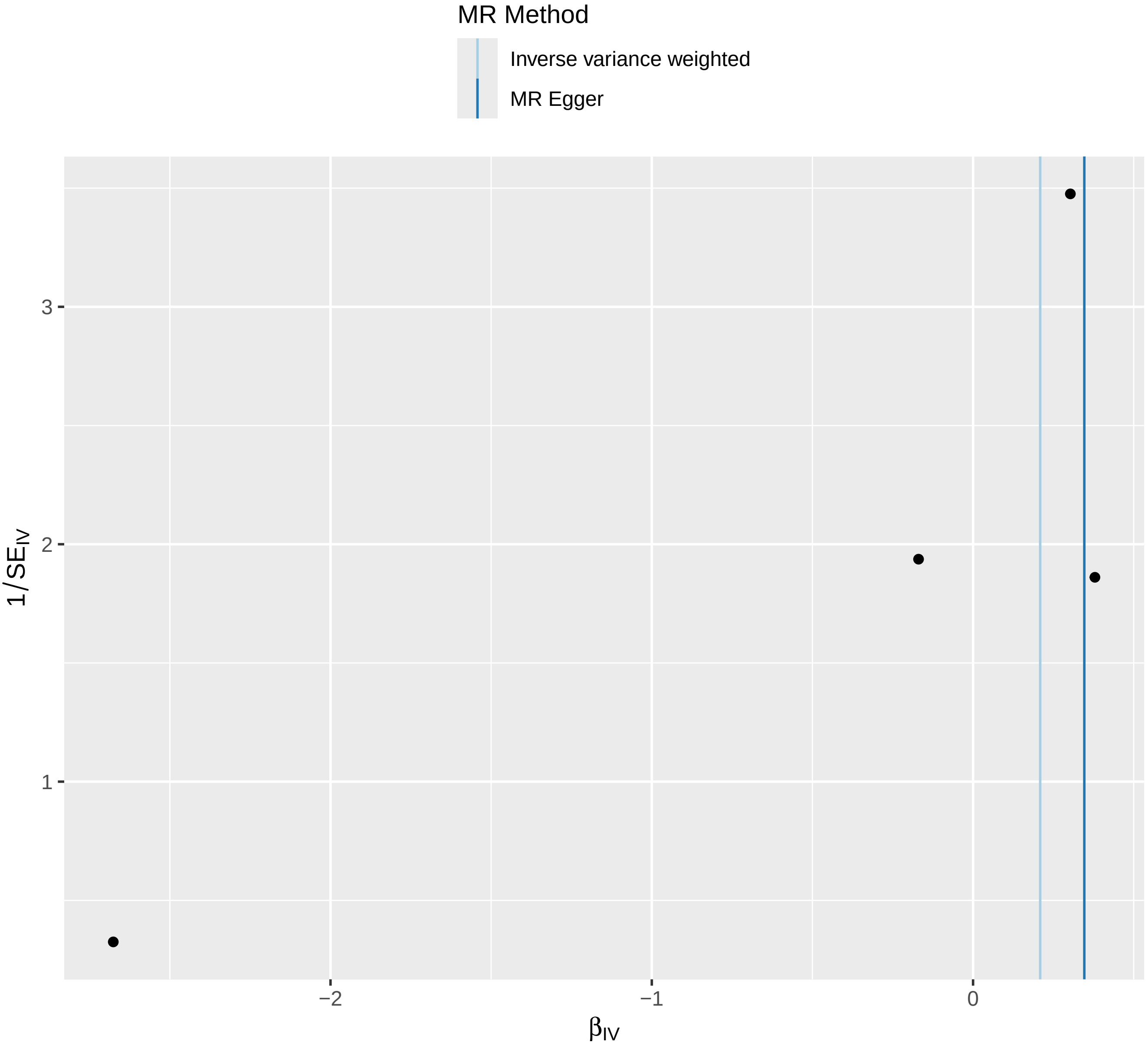

MR Method
Inverse variance weighted
MR Egger
1/SE$_{IV}$
3
2
1
−2
−1
0
β$_{IV}$

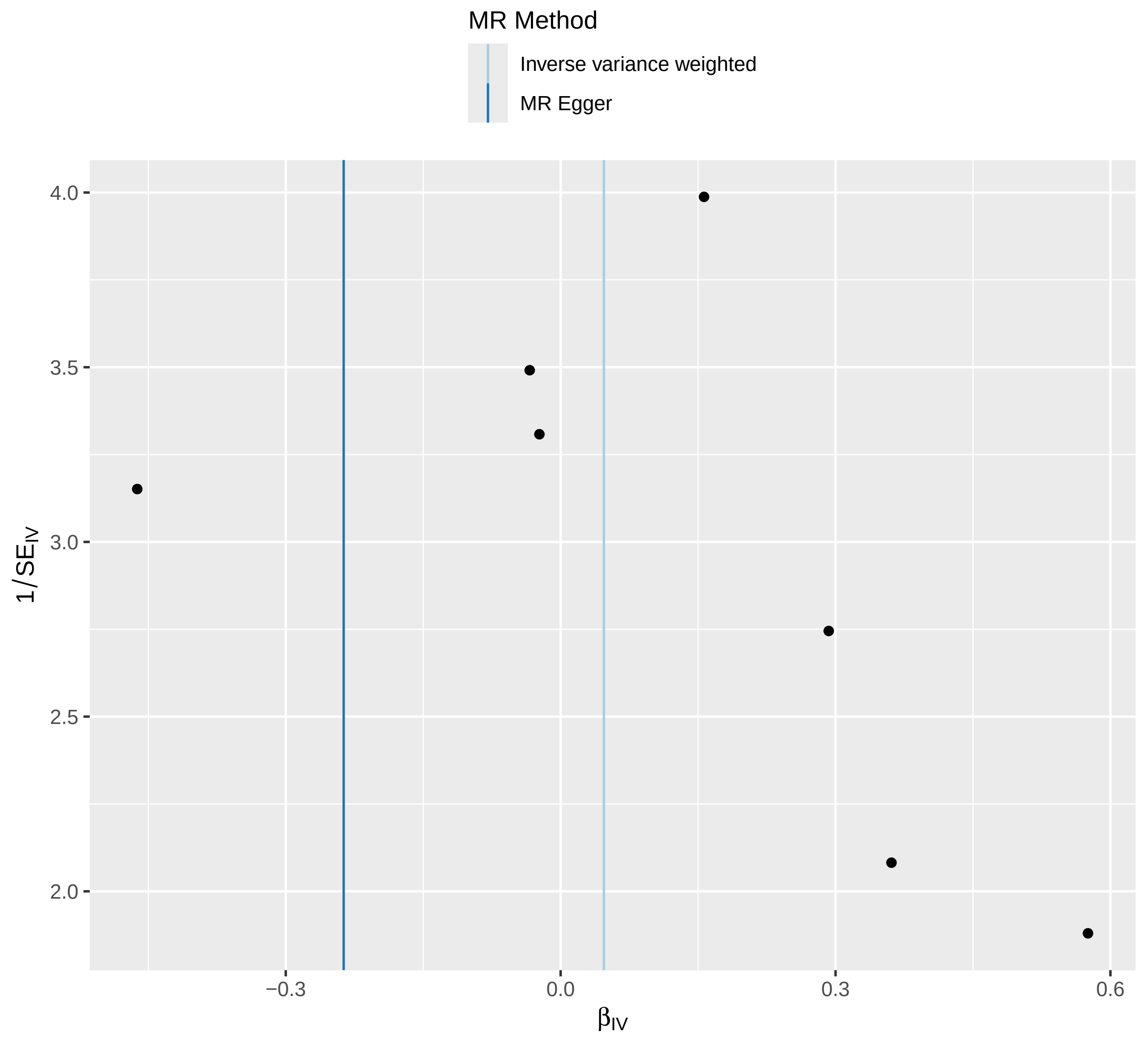

MR Method
Inverse variance weighted
MR Egger
4.0
3.5
3.0
2.5
2.0
1/SE$_{IV}$
−0.3
0.0
0.3
0.6
β$_{IV}$

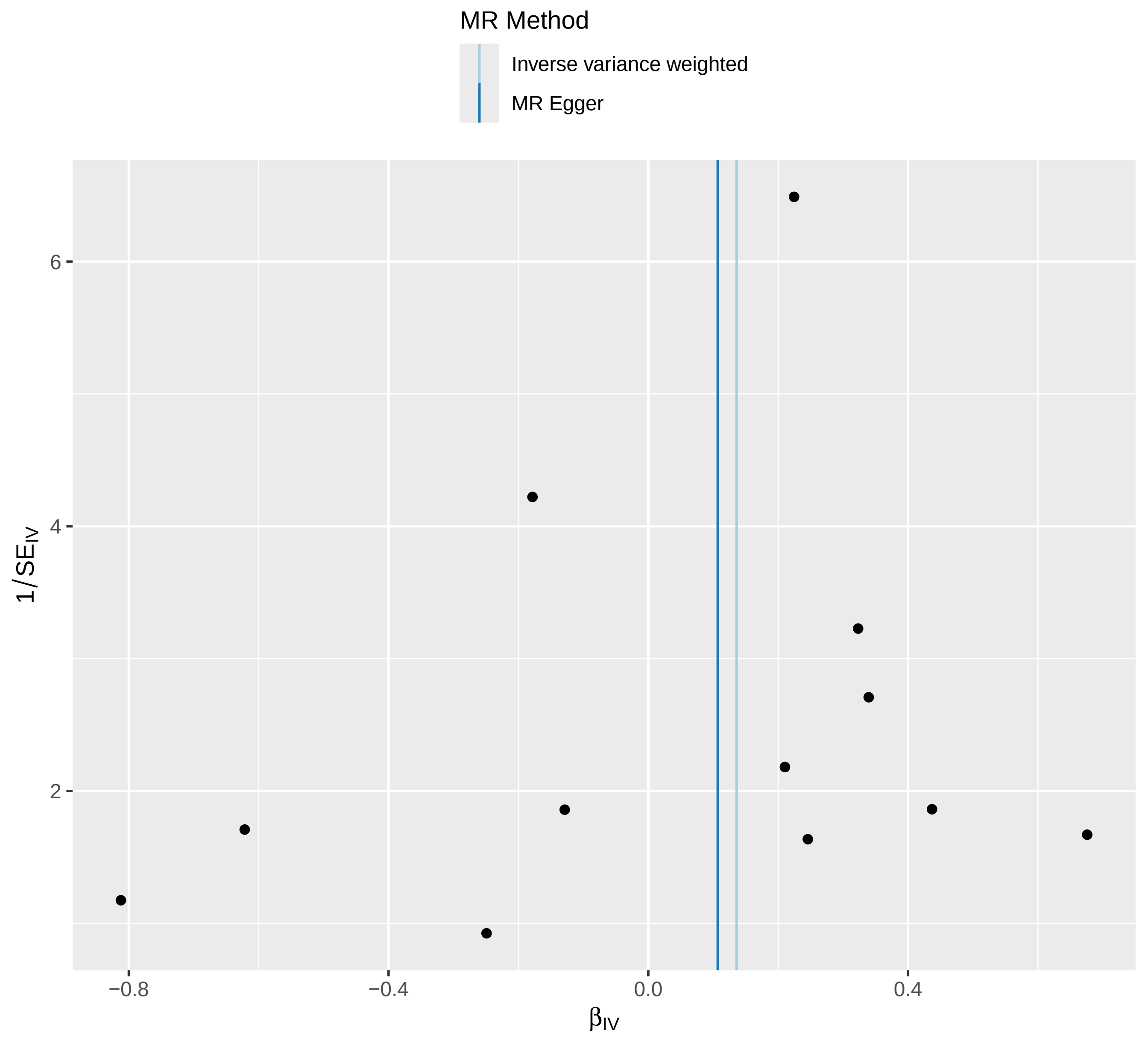
MR Method
Inverse variance weighted
MR Egger
6
4
2
$1/SE_{IV}$
−0.8
−0.4
0.0
0.4
β_{IV}

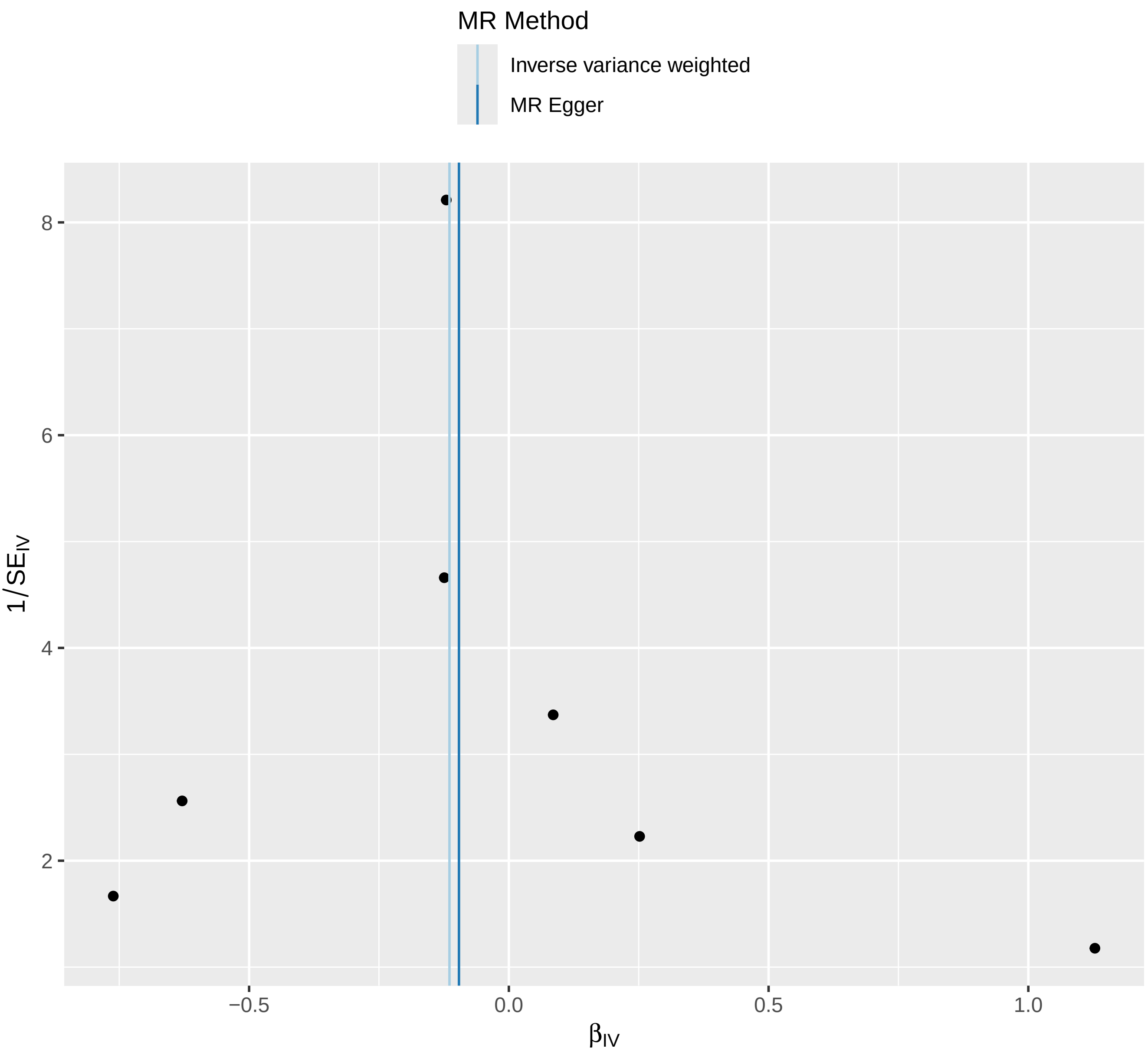

MR Method
Inverse variance weighted
MR Egger
8
6
4
2
1/SE_IV
−0.5
0.0
0.5
1.0
β_IV

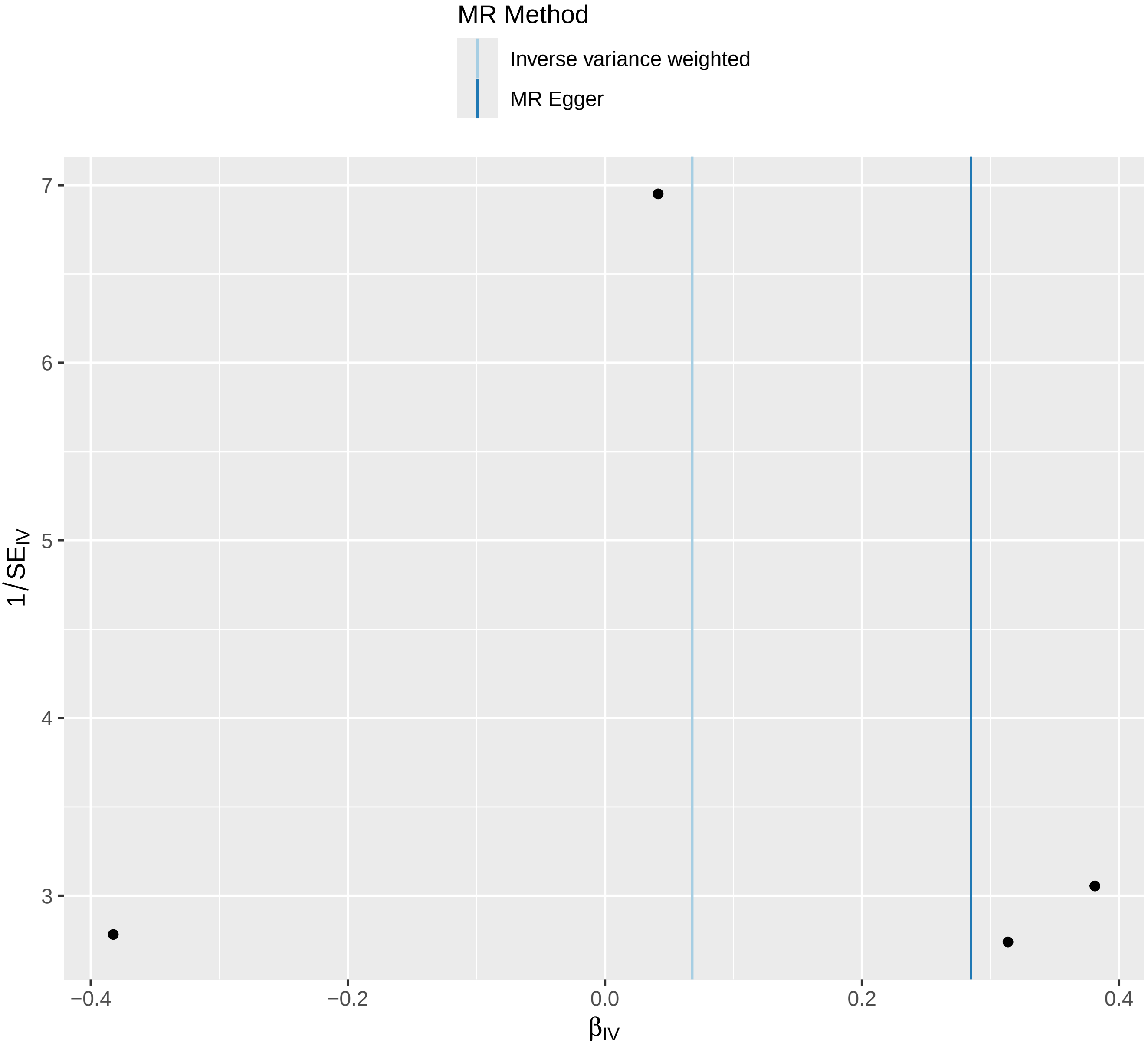

MR Method
Inverse variance weighted
MR Egger
7
6
5
4
3
$1/SE_{IV}$
−0.4
−0.2
0.0
0.2
0.4
β_{IV}

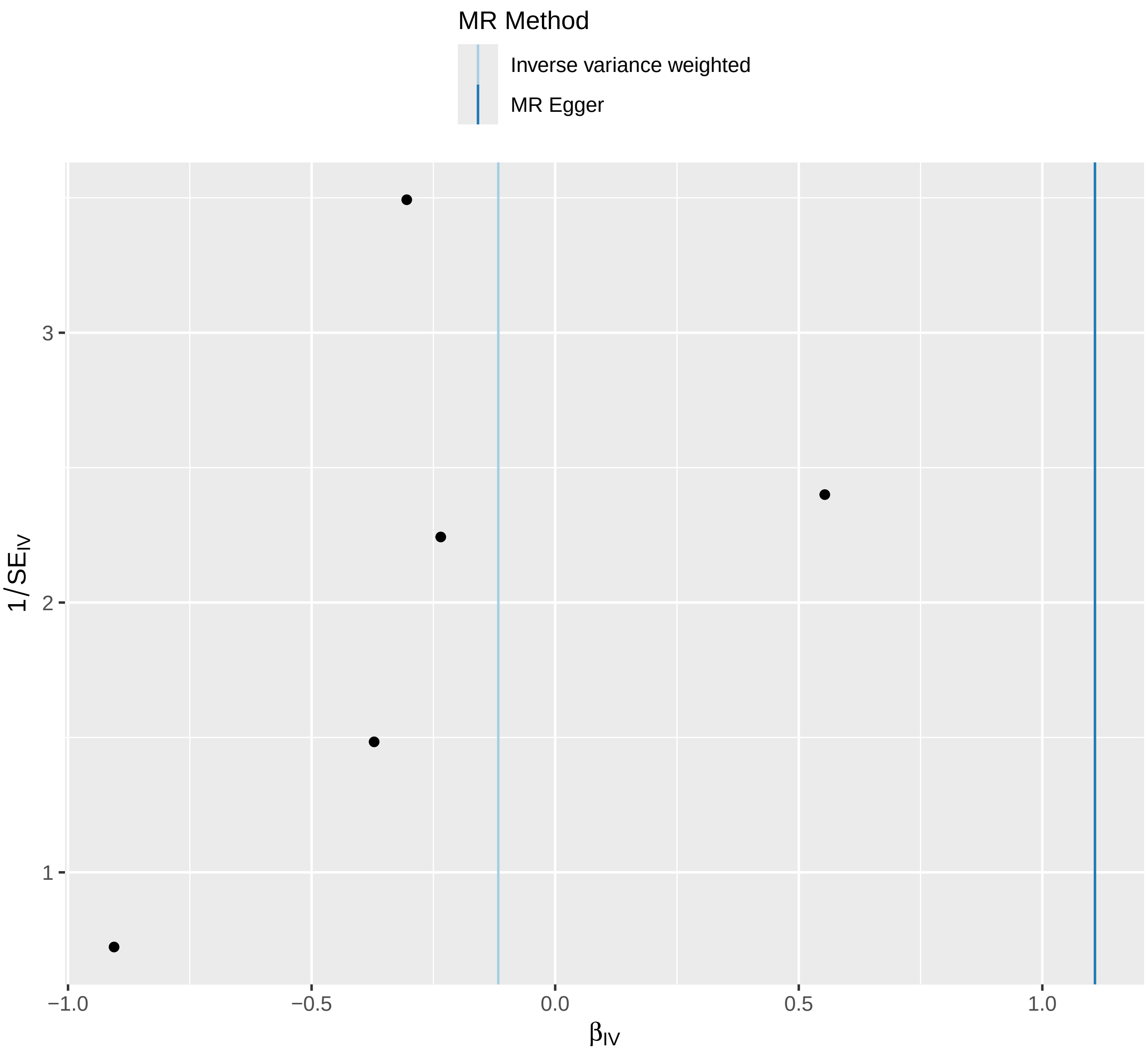
MR Method
Inverse variance weighted
MR Egger
3
2
1
1/SE_IV
−1.0
−0.5
0.0
0.5
1.0
β_IV

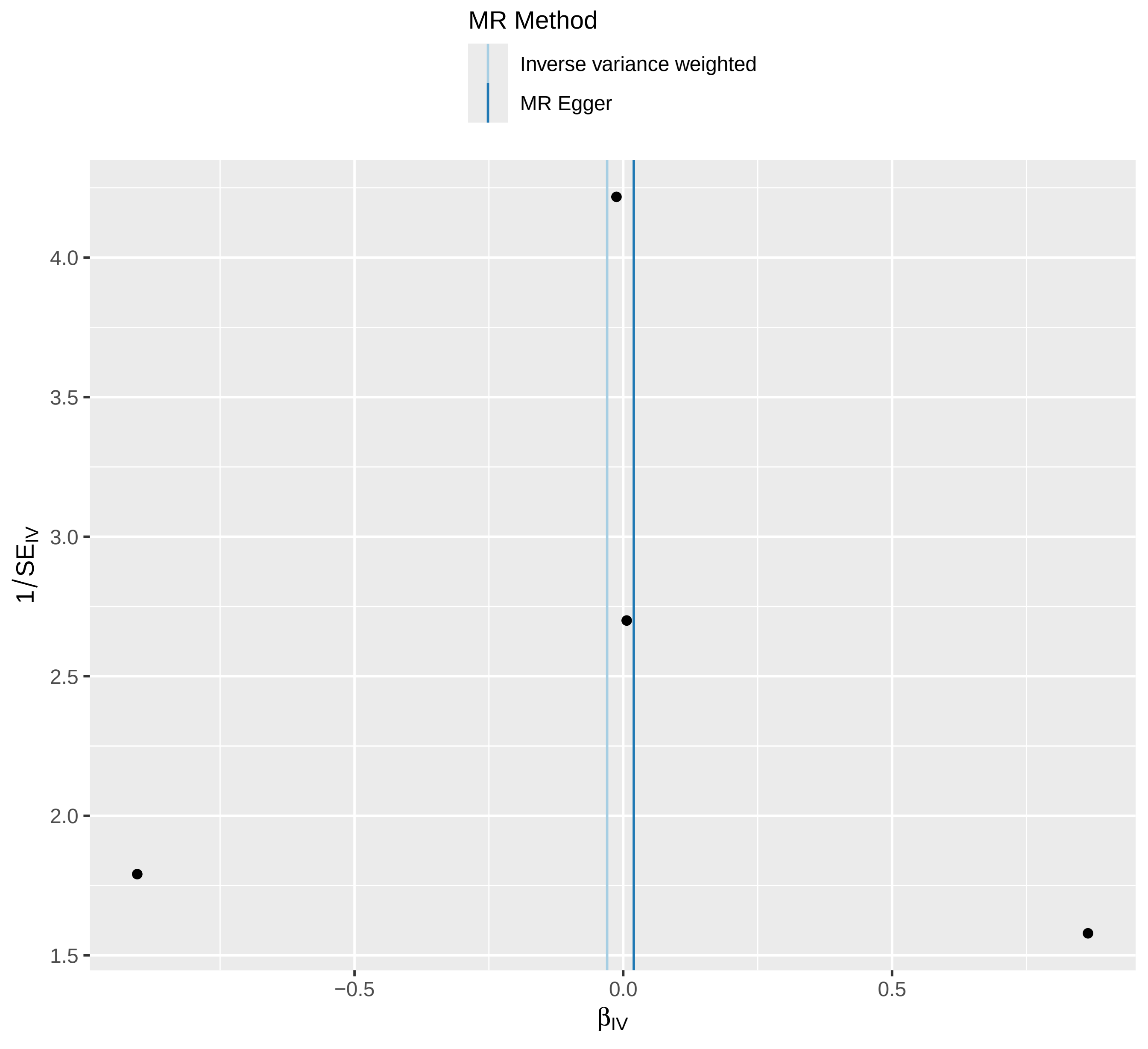
MR Method
Inverse variance weighted
MR Egger
1/SE_IV
β_IV
4.0
3.5
3.0
2.5
2.0
1.5
−0.5
0.0
0.5

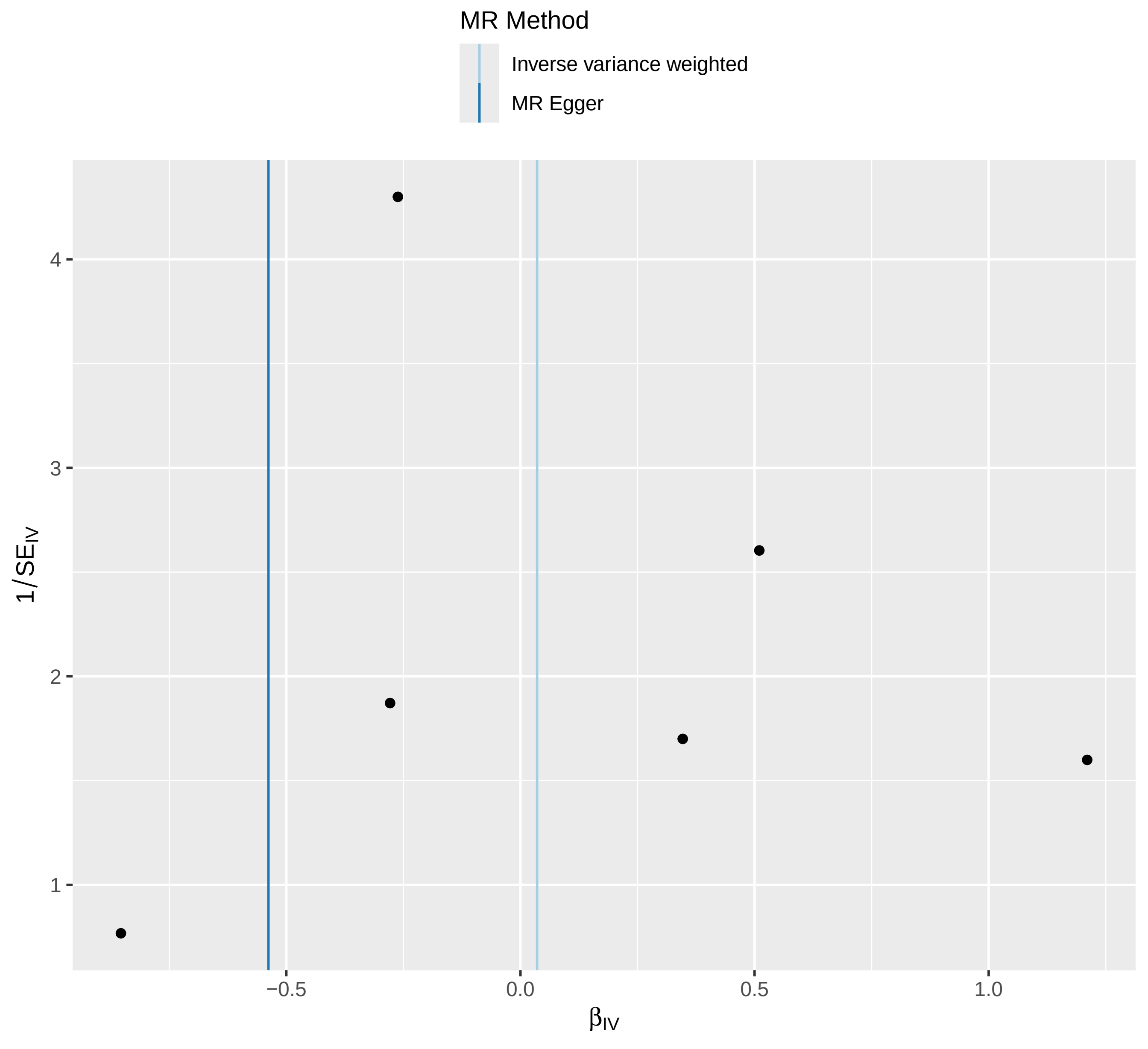
MR Method
Inverse variance weighted
MR Egger
1/SE_IV
4
3
2
1
−0.5
0.0
0.5
1.0
β_IV

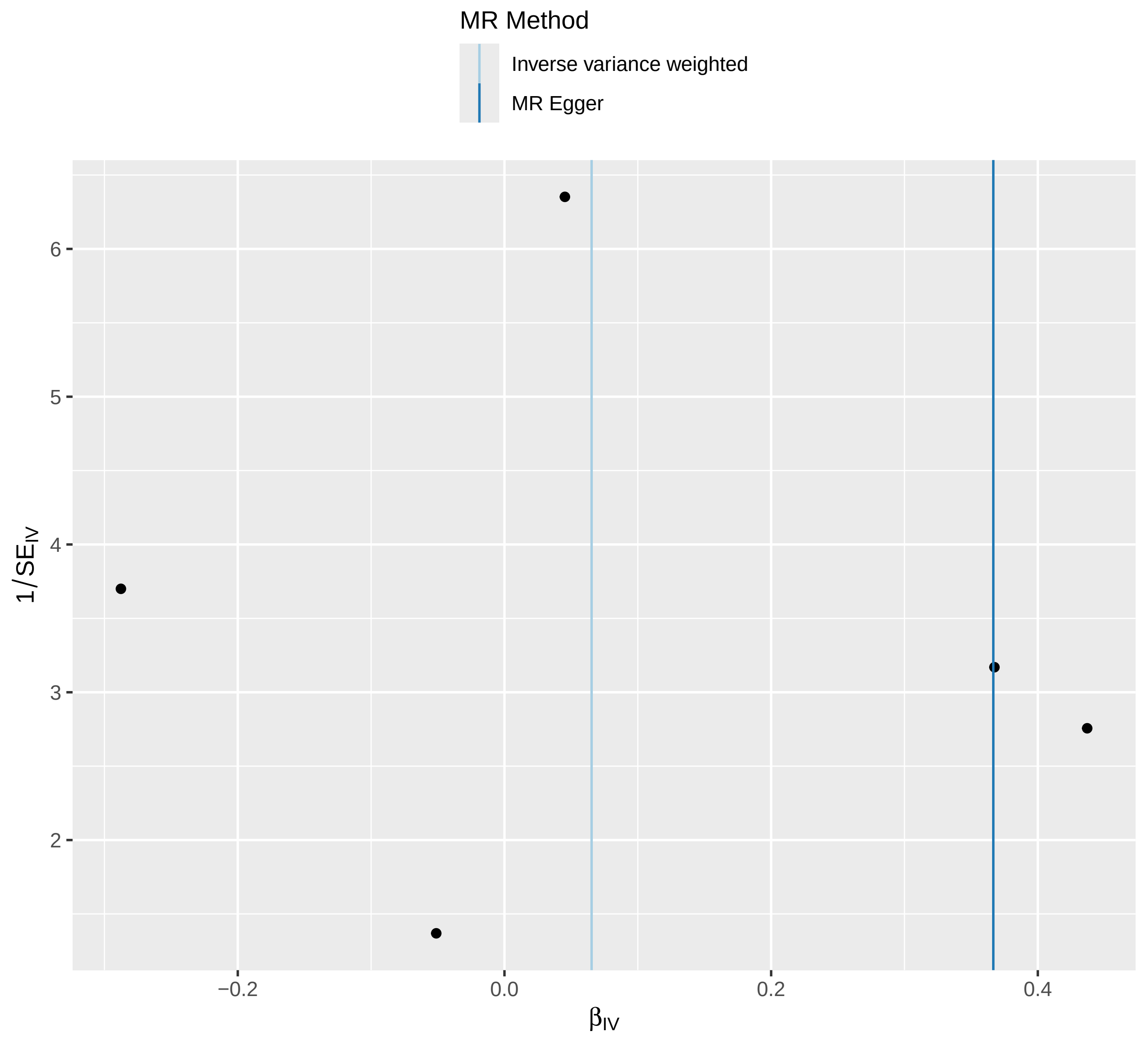

MR Method
Inverse variance weighted
MR Egger
6
5
4
3
2
$1/SE_{IV}$
−0.2
0.0
0.2
0.4
β_{IV}

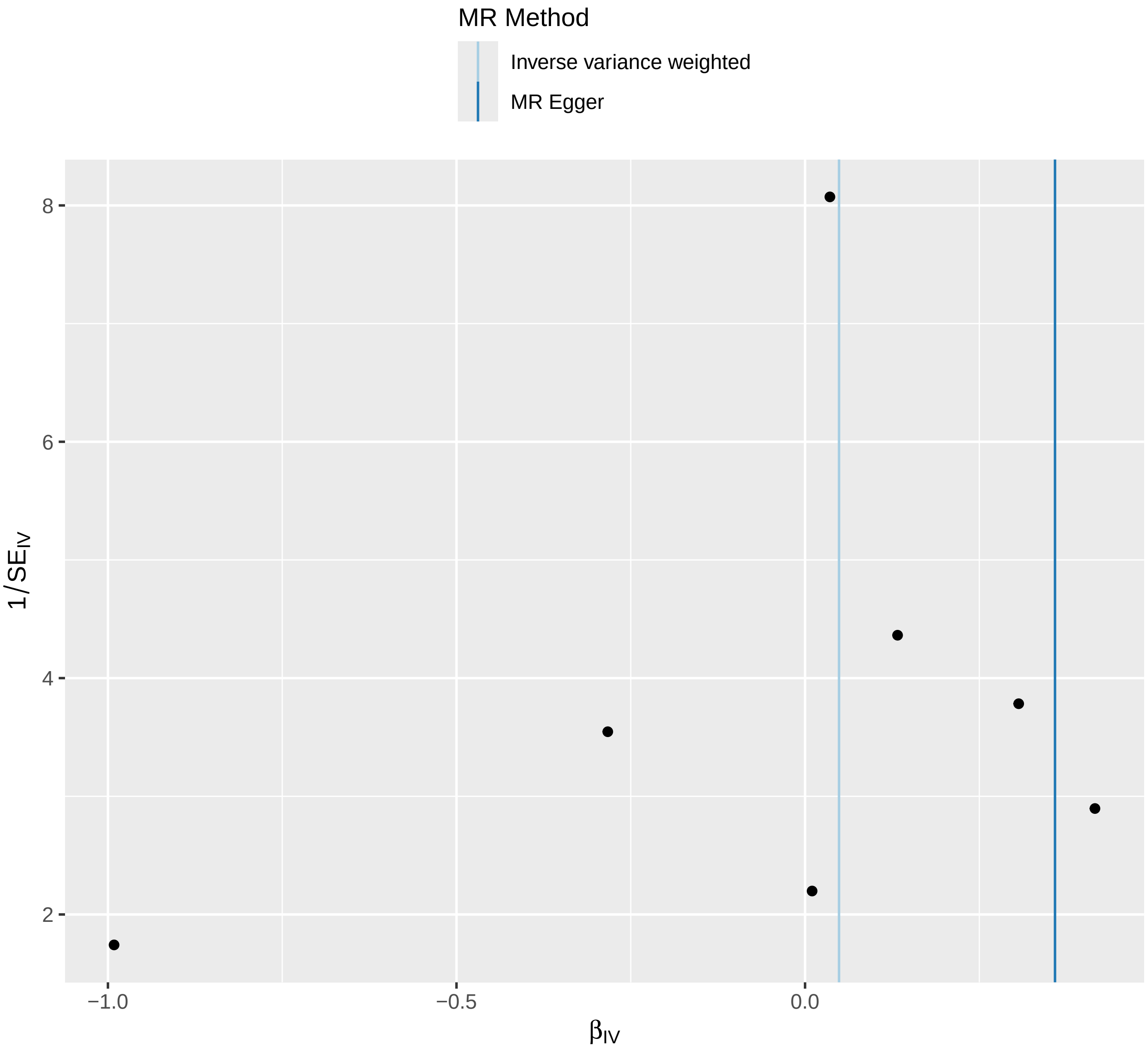

MR Method
Inverse variance weighted
MR Egger
8
6
4
2
1/SE_IV
−1.0
−0.5
0.0
β_IV

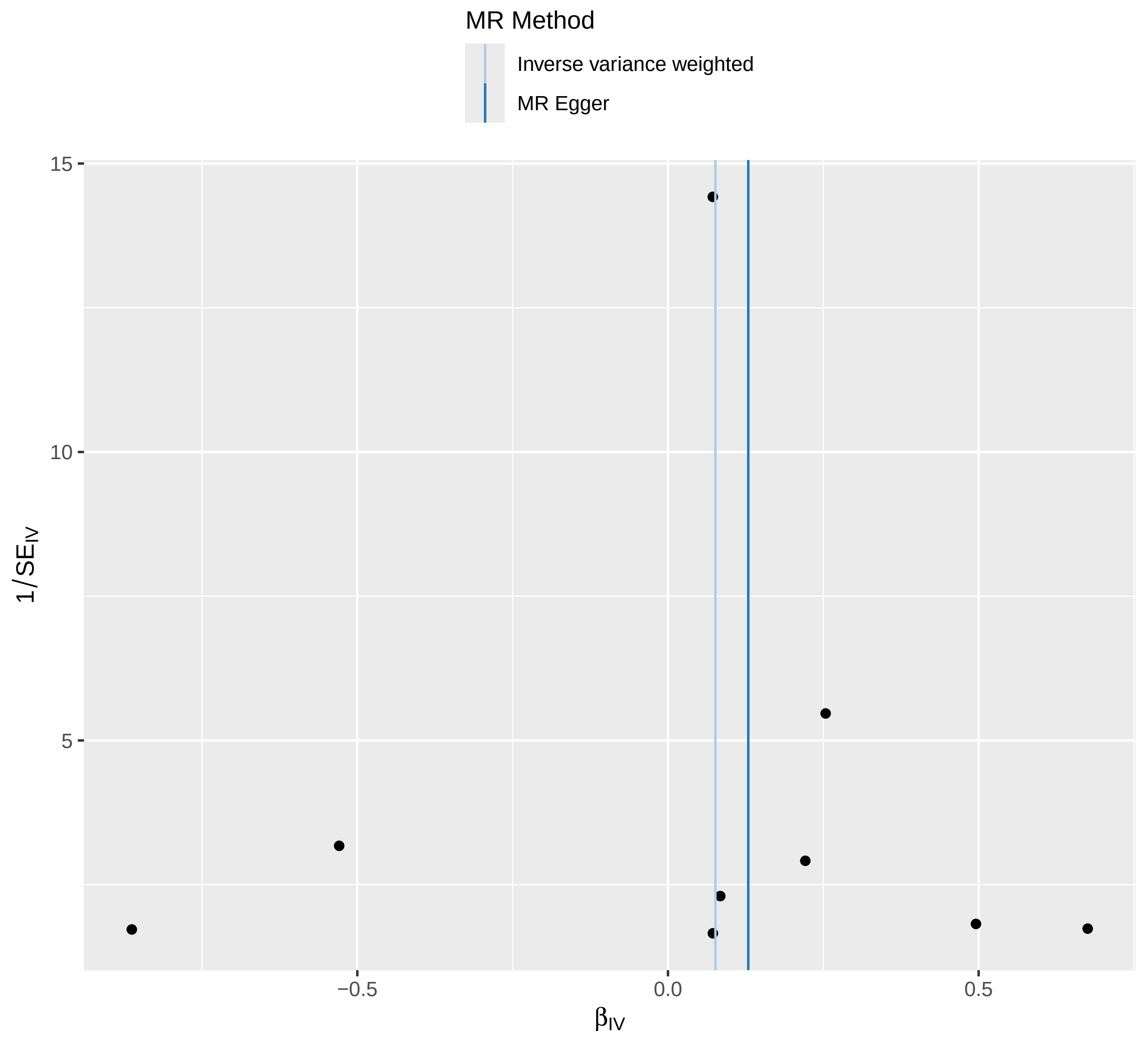

MR Method
Inverse variance weighted
MR Egger
15
10
5
1/SE_IV
−0.5
0.0
0.5
β_IV

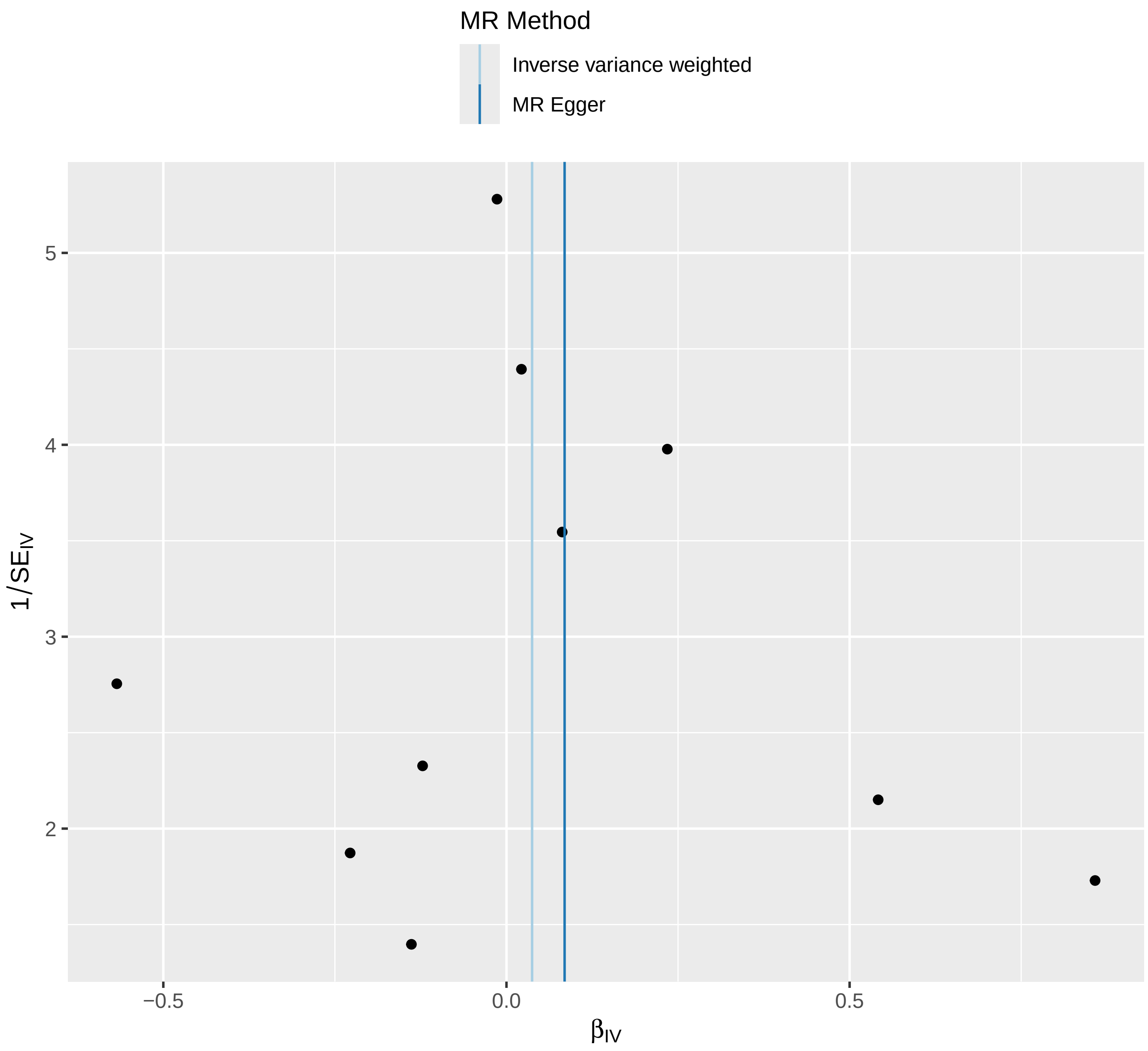
MR Method
Inverse variance weighted
MR Egger
5
4
3
2
$1/SE_{IV}$
−0.5
0.0
0.5
β_{IV}

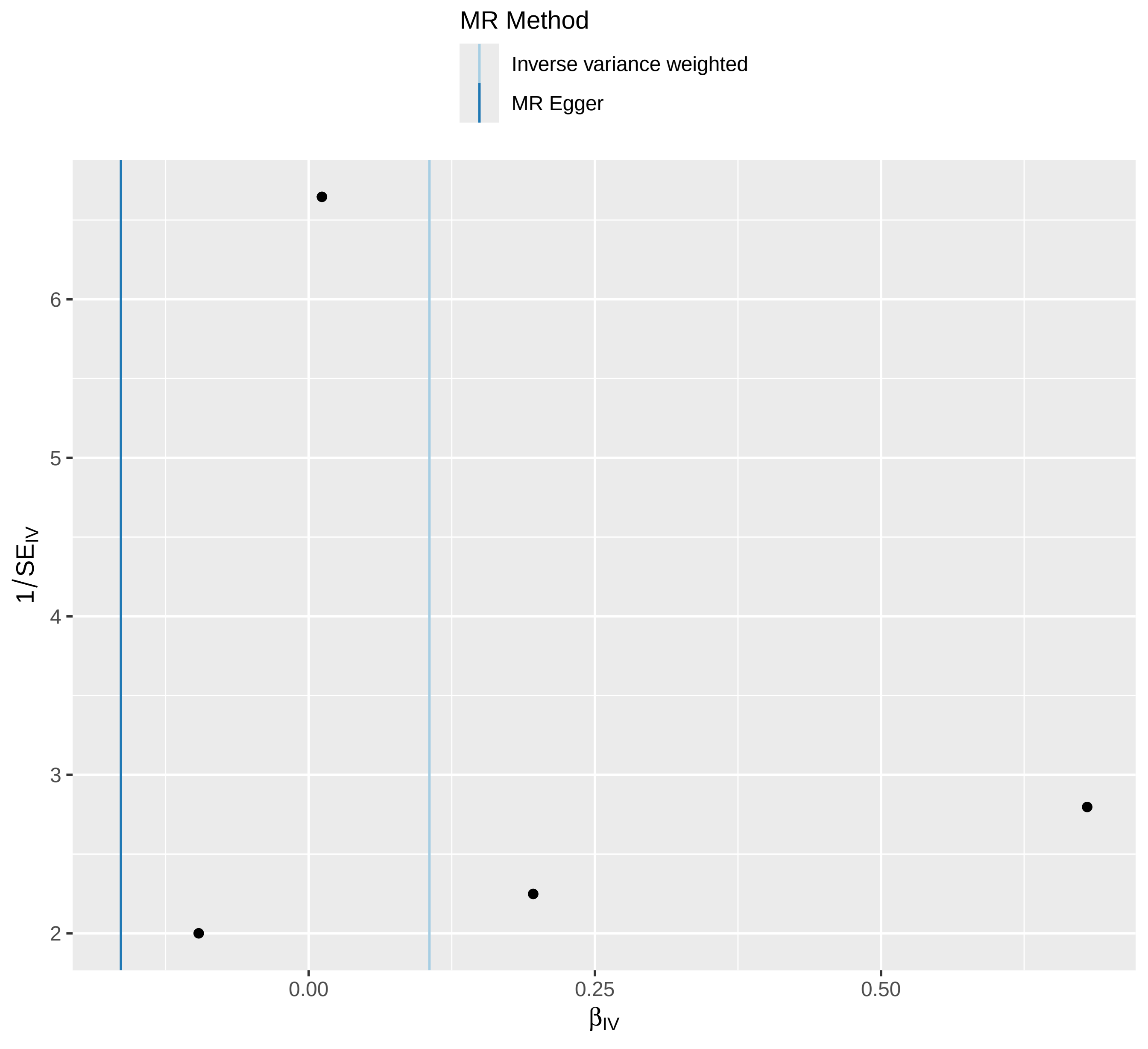

MR Method
Inverse variance weighted
MR Egger
6
5
4
3
2
1/SE$_{IV}$
0.00
0.25
0.50
β$_{IV}$

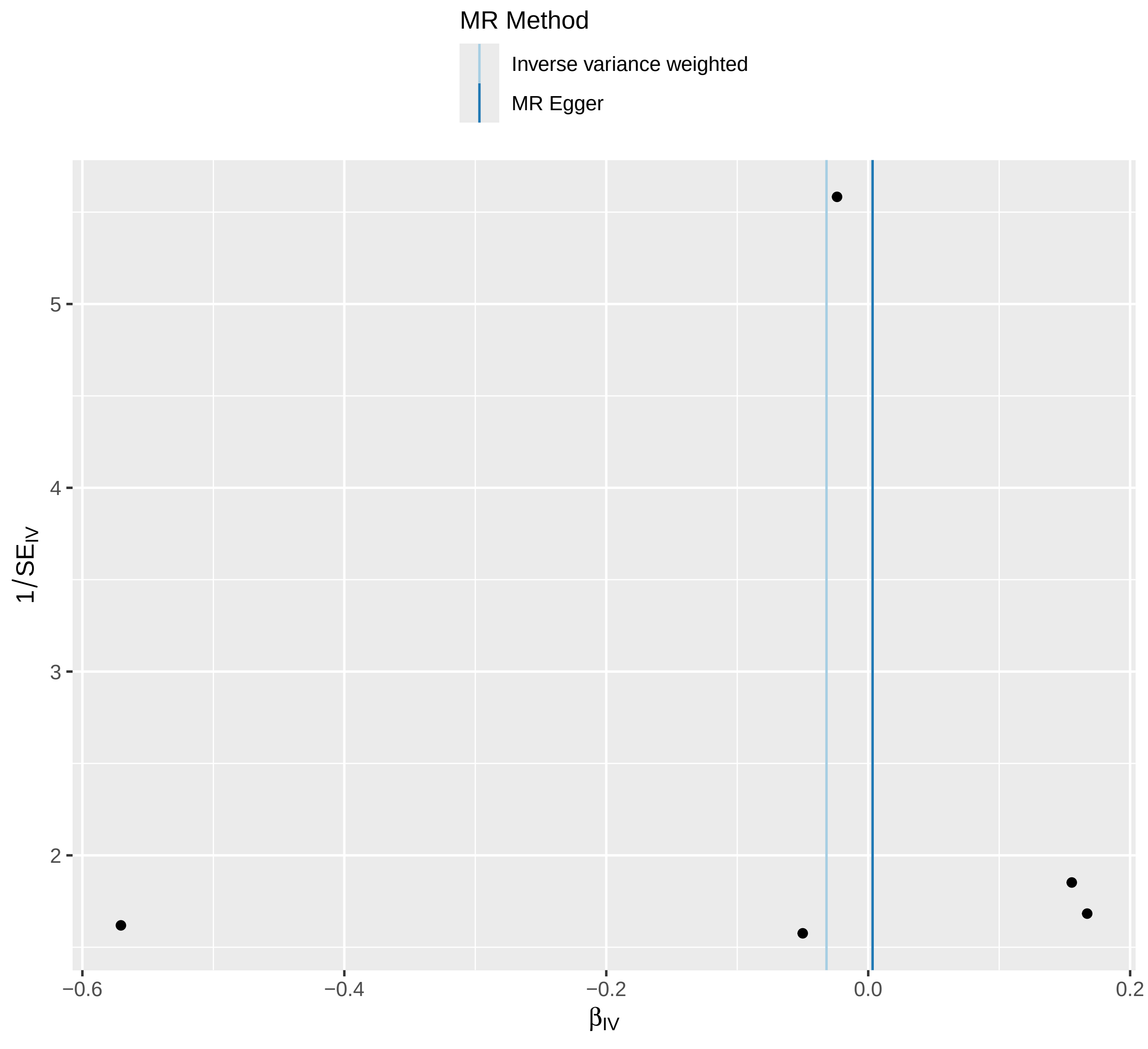

MR Method
Inverse variance weighted
MR Egger
5
4
3
2
1/SE$_{IV}$
−0.6
−0.4
−0.2
0.0
0.2
β$_{IV}$

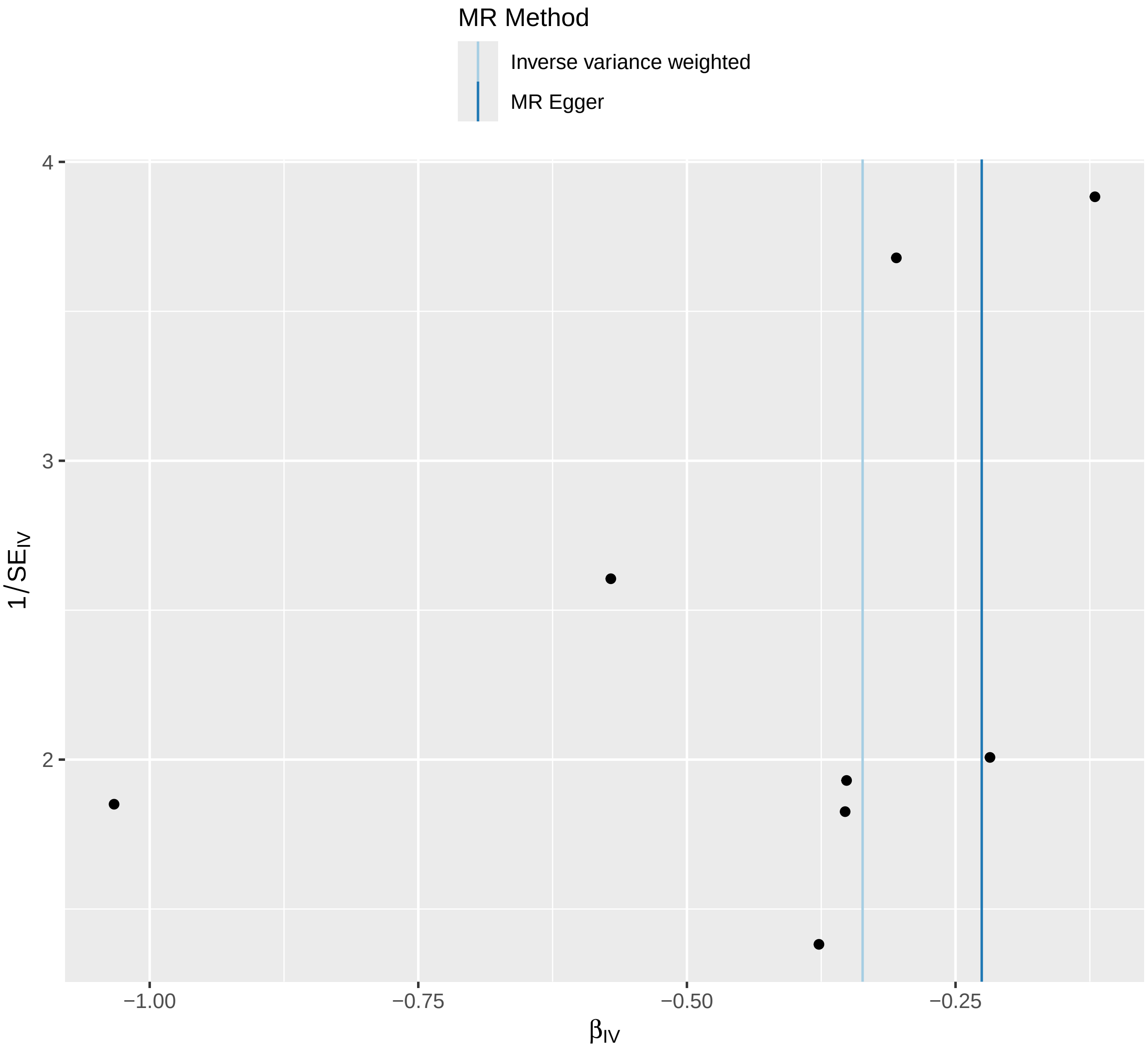
MR Method
Inverse variance weighted
MR Egger
4
3
2
1/SE_IV
−1.00
−0.75
−0.50
−0.25
β_IV

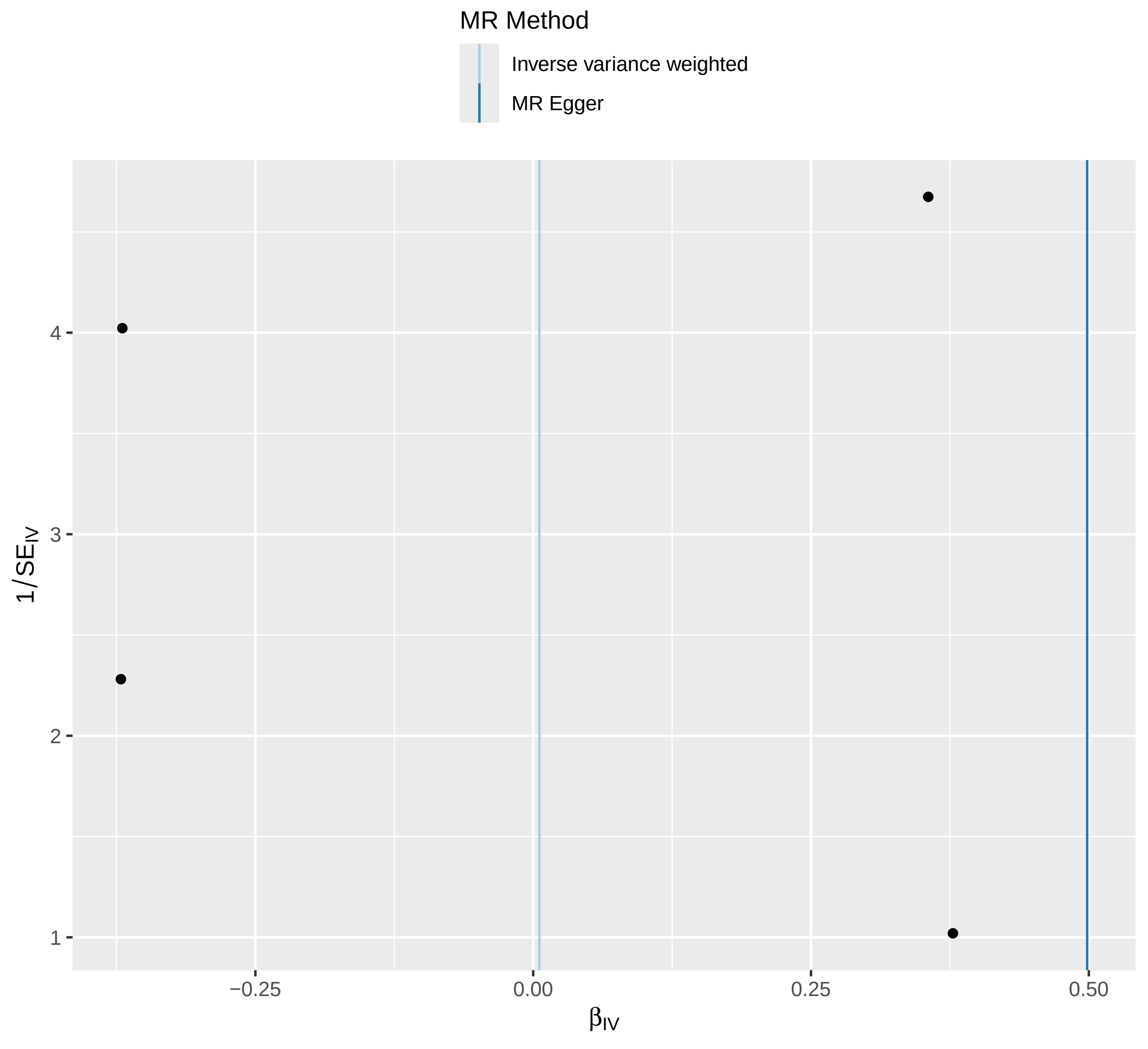

MR Method
Inverse variance weighted
MR Egger
4
3
2
1
1/SE_IV
−0.25
0.00
0.25
0.50
β_IV

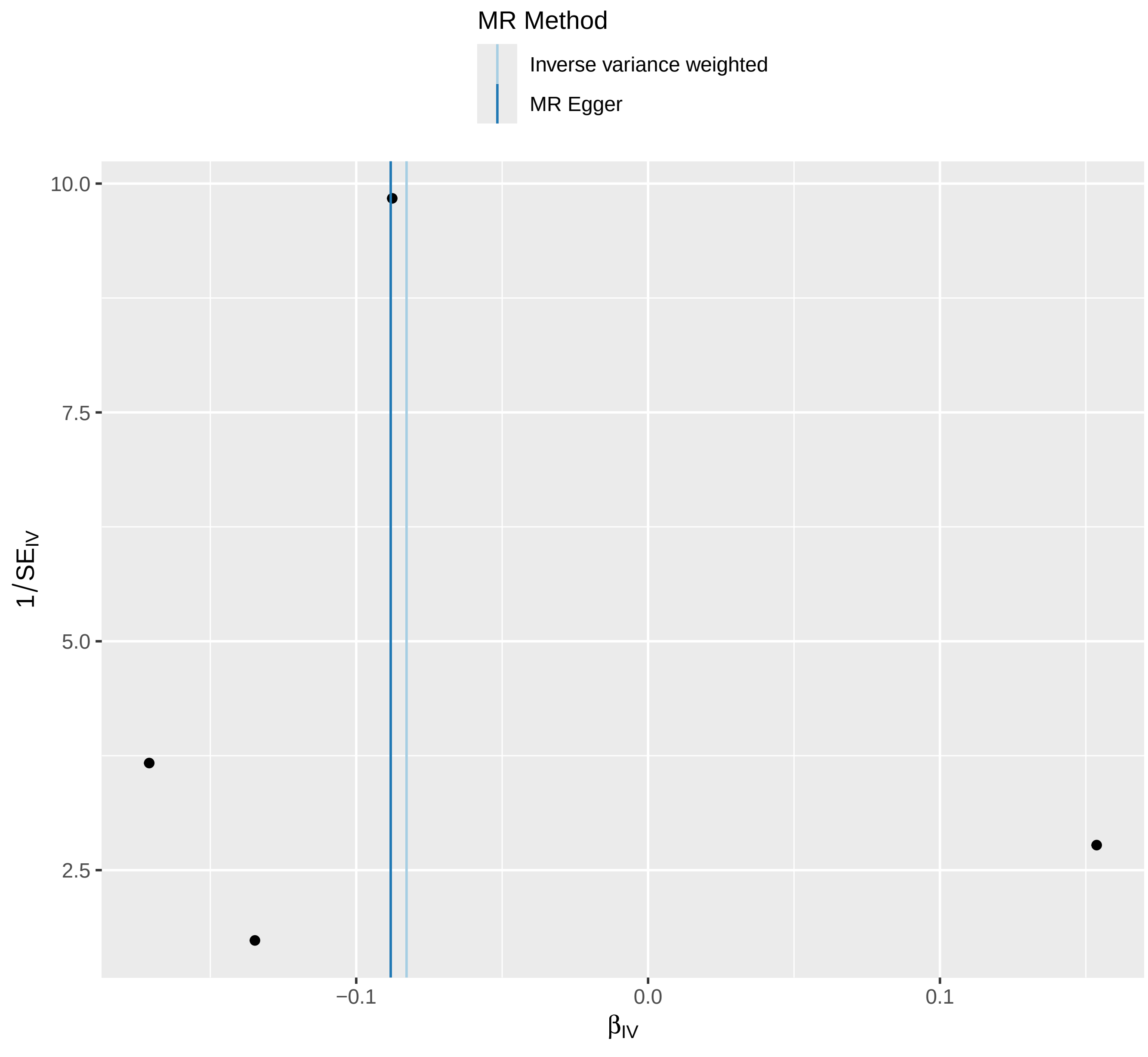
MR Method
Inverse variance weighted
MR Egger
10.0
7.5
5.0
2.5
1/SE$_{IV}$
−0.1
0.0
0.1
β$_{IV}$

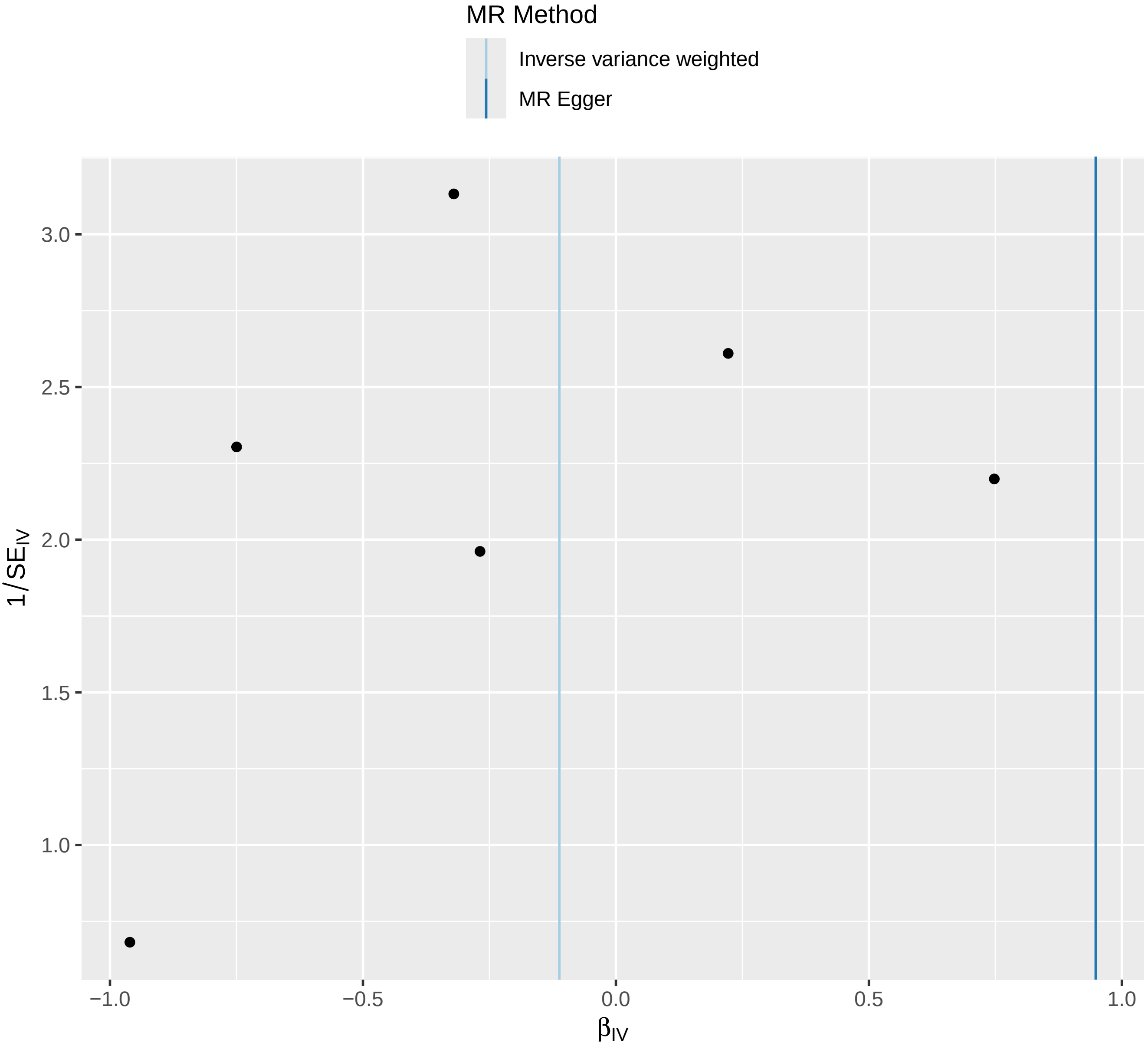

MR Method
Inverse variance weighted
MR Egger
3.0
2.5
2.0
1.5
1.0
$1/SE_{IV}$
−1.0
−0.5
0.0
0.5
1.0
β_{IV}

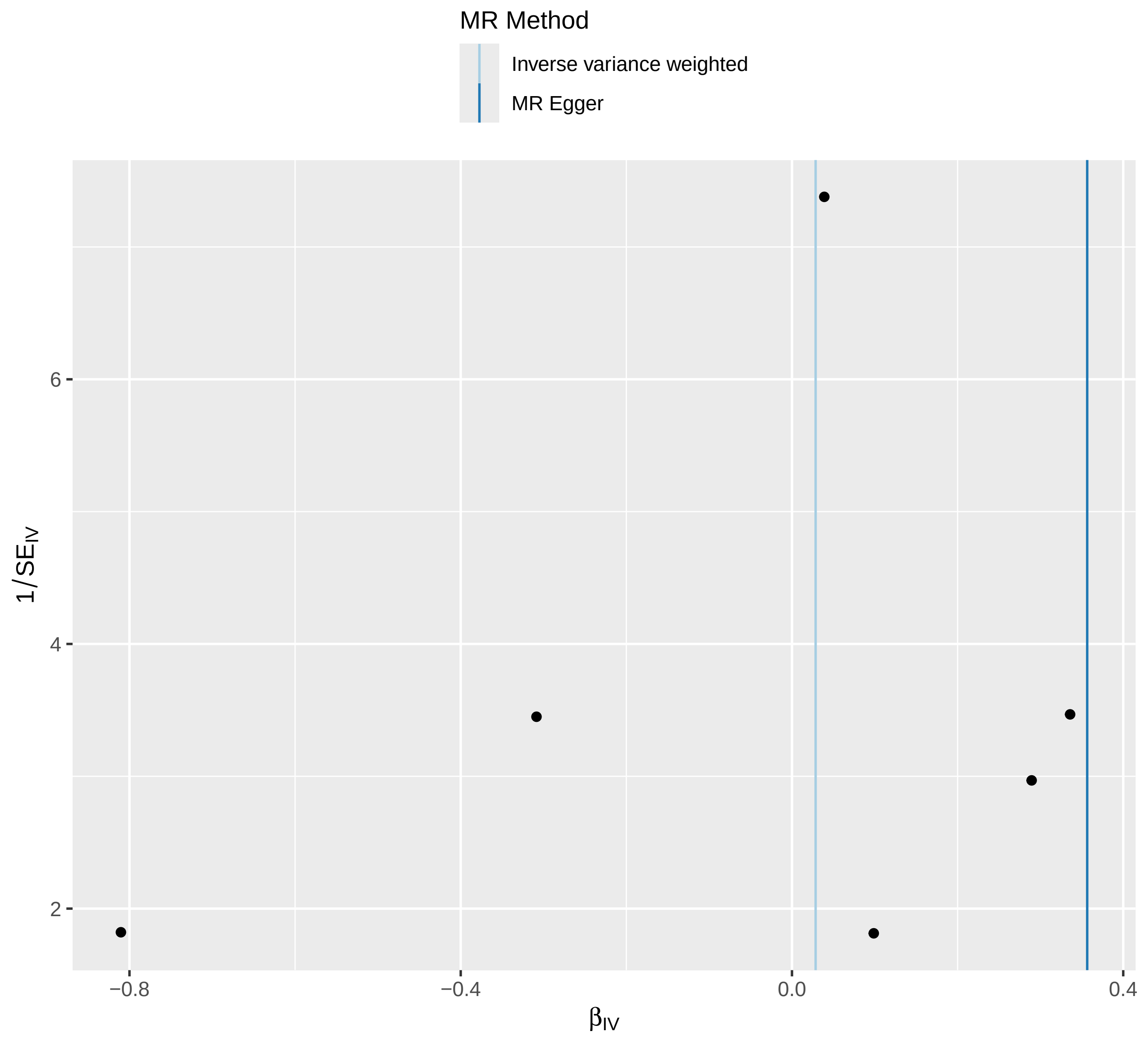
MR Method
Inverse variance weighted
MR Egger
1/SE$_{IV}$
6
4
2
−0.8
−0.4
0.0
0.4
β$_{IV}$

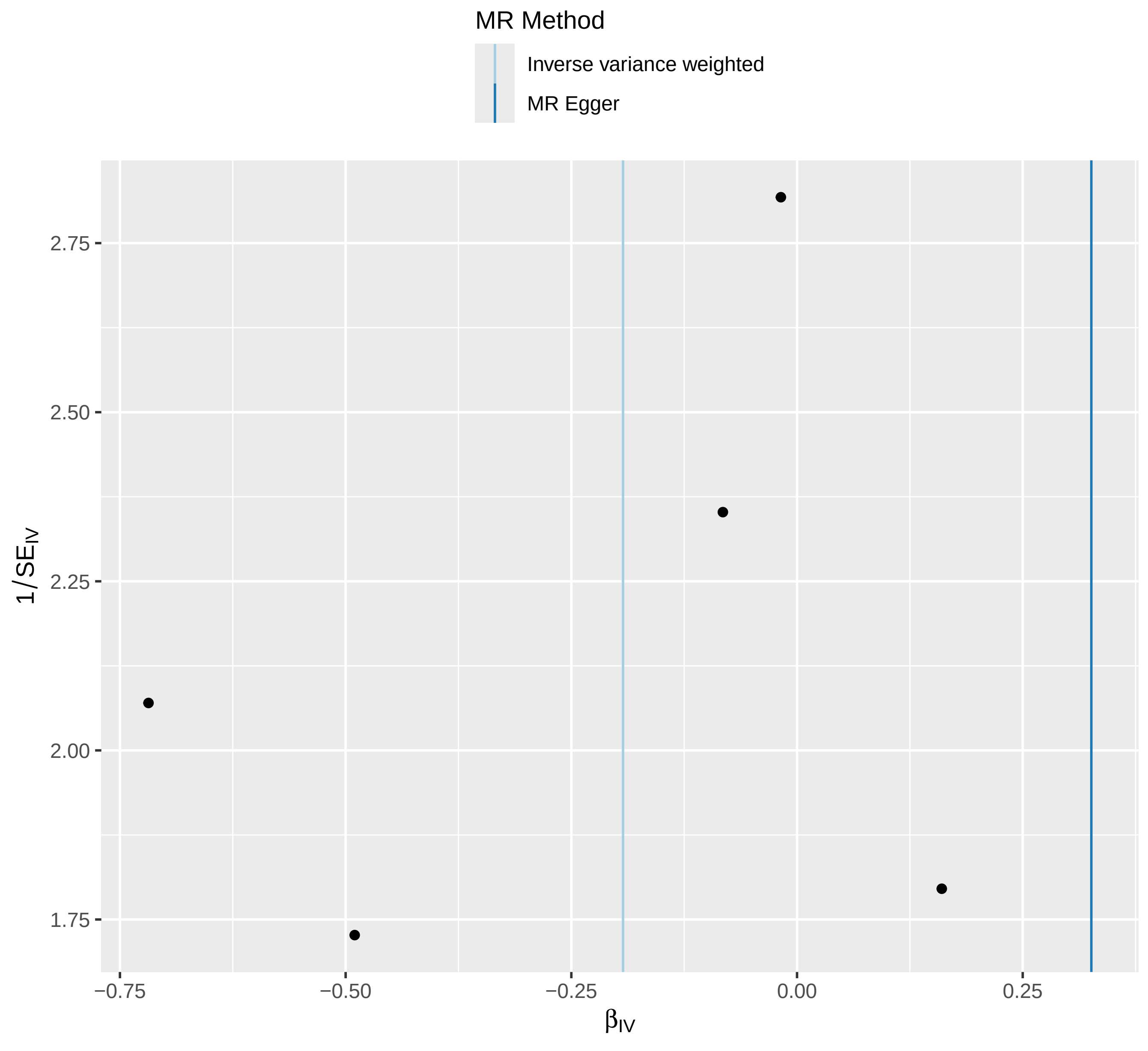
MR Method
Inverse variance weighted
MR Egger
2.75
2.50
2.25
2.00
1.75
$1/SE_{IV}$
−0.75
−0.50
−0.25
0.00
0.25
β_{IV}

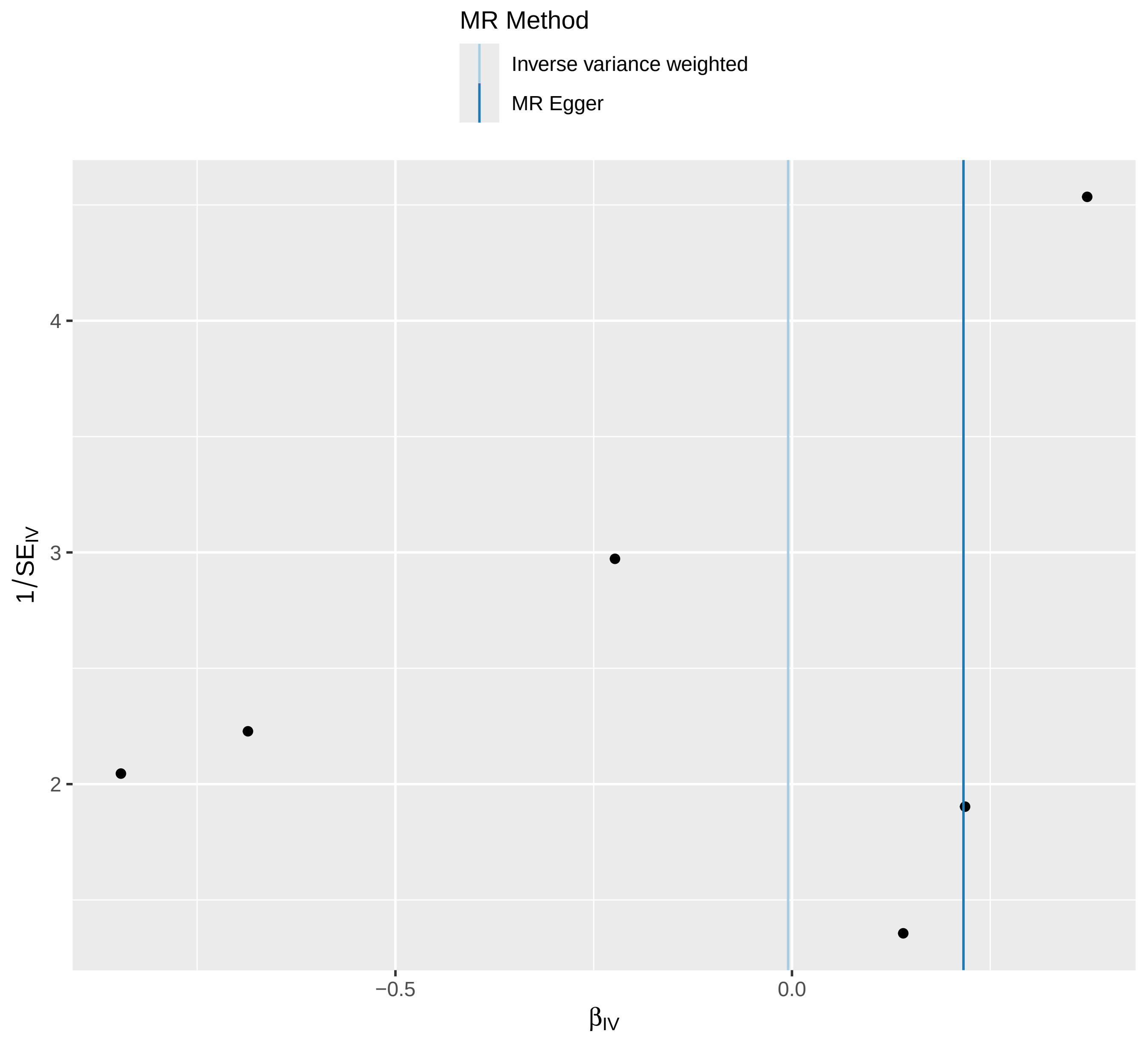
MR Method
Inverse variance weighted
MR Egger
4
3
2
1/SE_IV
−0.5
0.0
β_IV

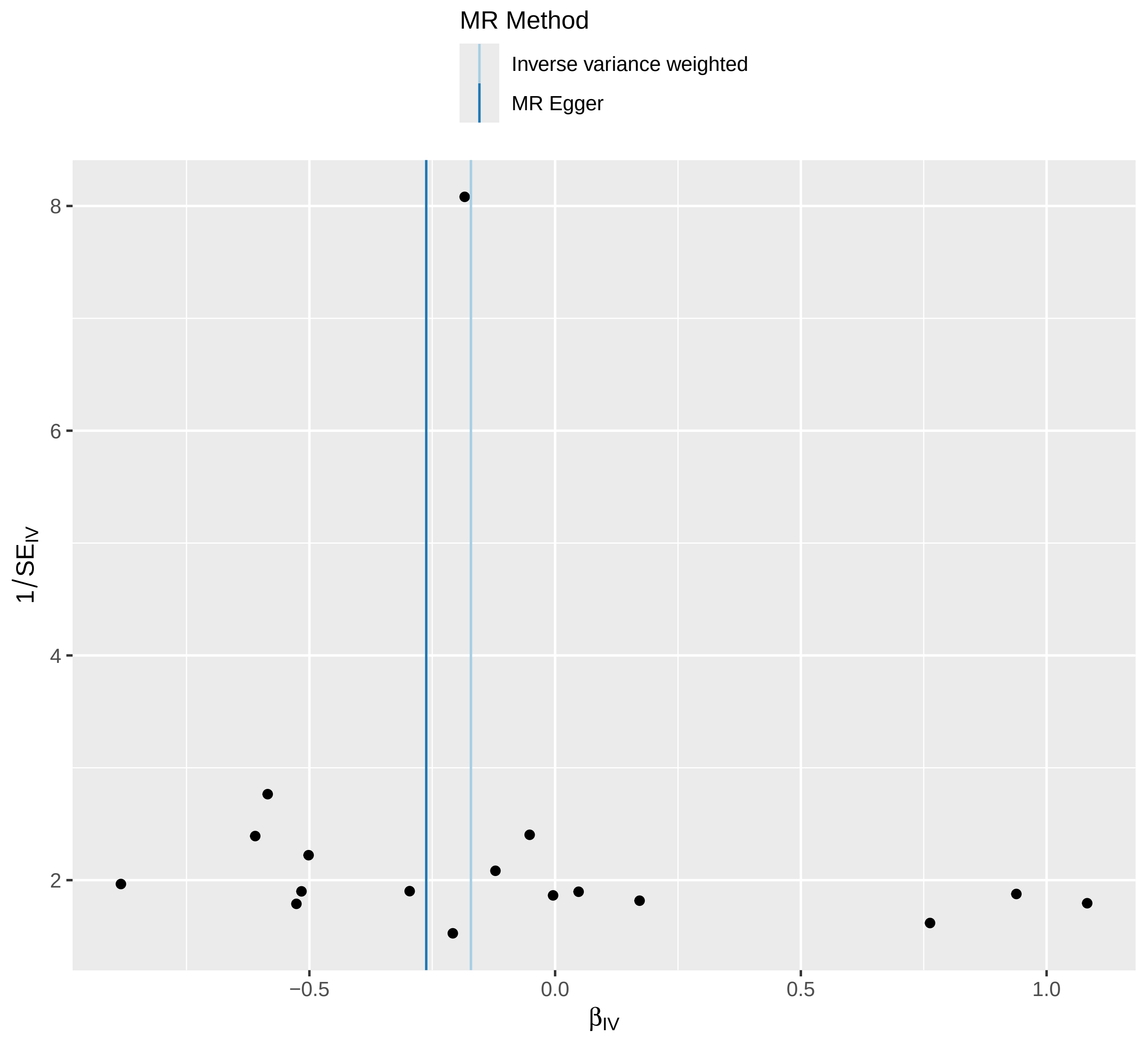

MR Method
Inverse variance weighted
MR Egger
8
6
4
2
1/SE_IV
−0.5
0.0
0.5
1.0
β_IV

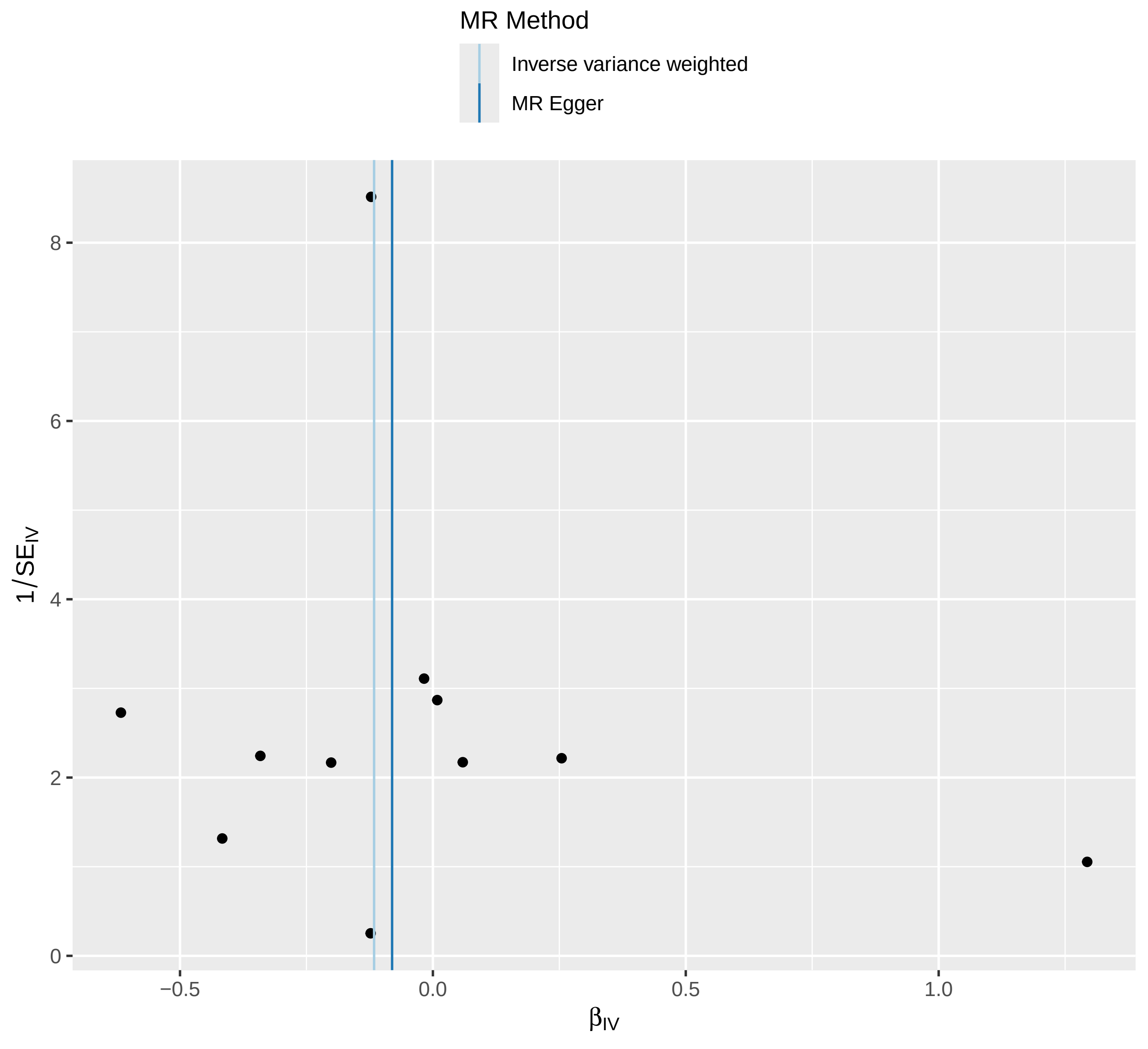

MR Method
Inverse variance weighted
MR Egger
8
6
4
2
0
−0.5
0.0
0.5
1.0
1/SE_IV
β_IV

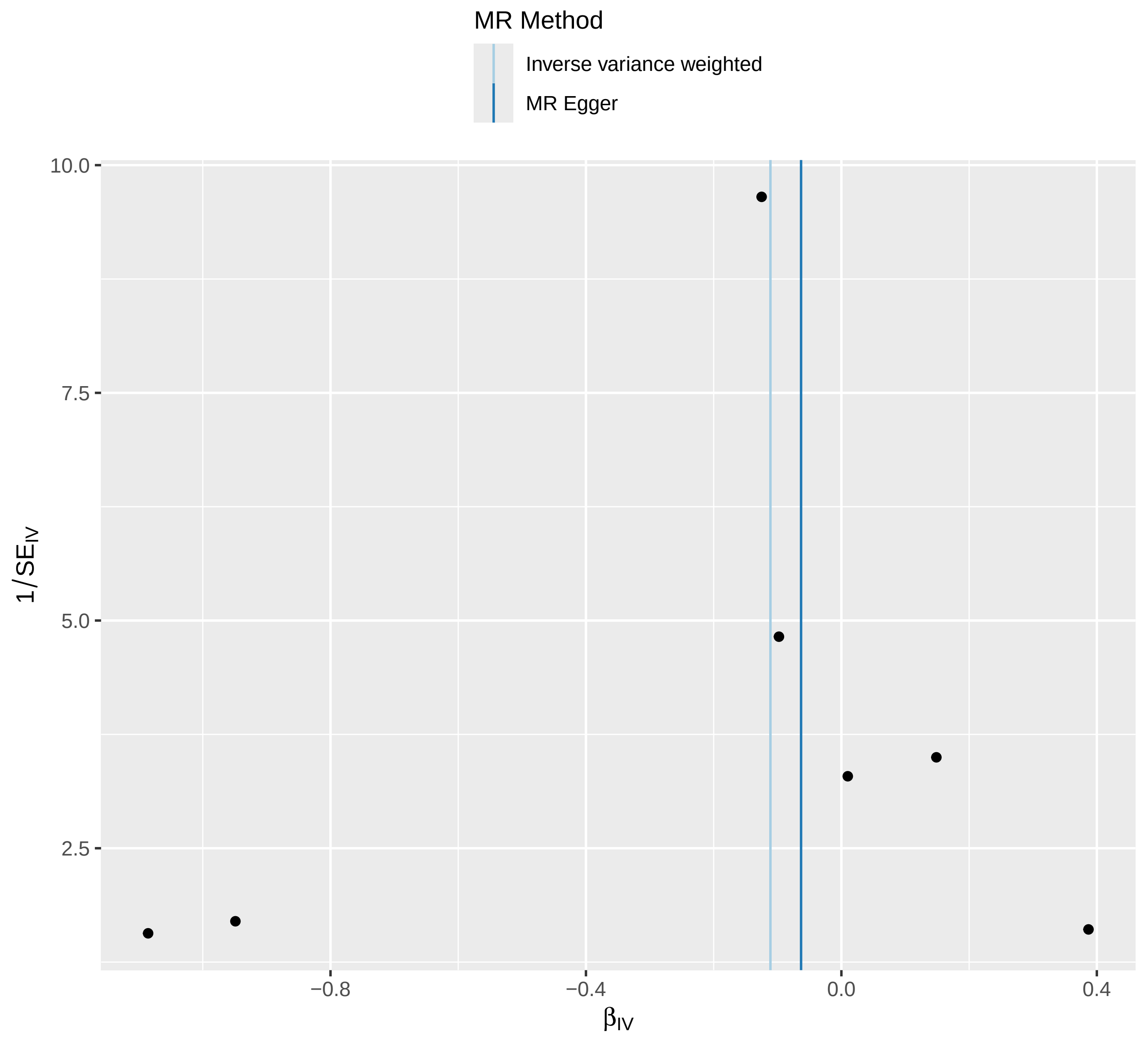
MR Method
Inverse variance weighted
MR Egger
10.0
7.5
5.0
2.5
$1/SE_{IV}$
−0.8
−0.4
0.0
0.4
β_{IV}

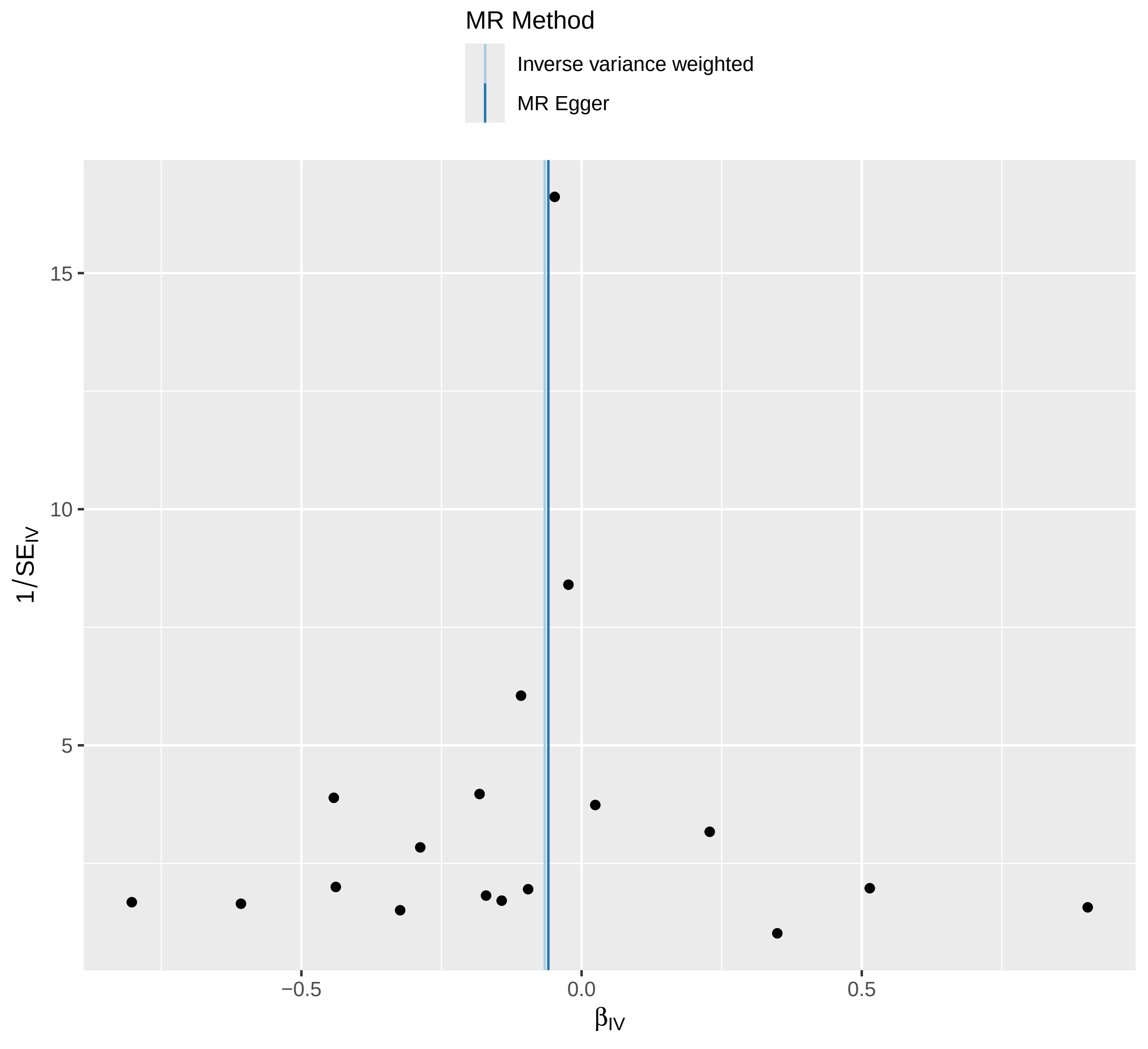
MR Method
Inverse variance weighted
MR Egger
15
10
5
$1/SE_{IV}$
−0.5
0.0
0.5
β_{IV}

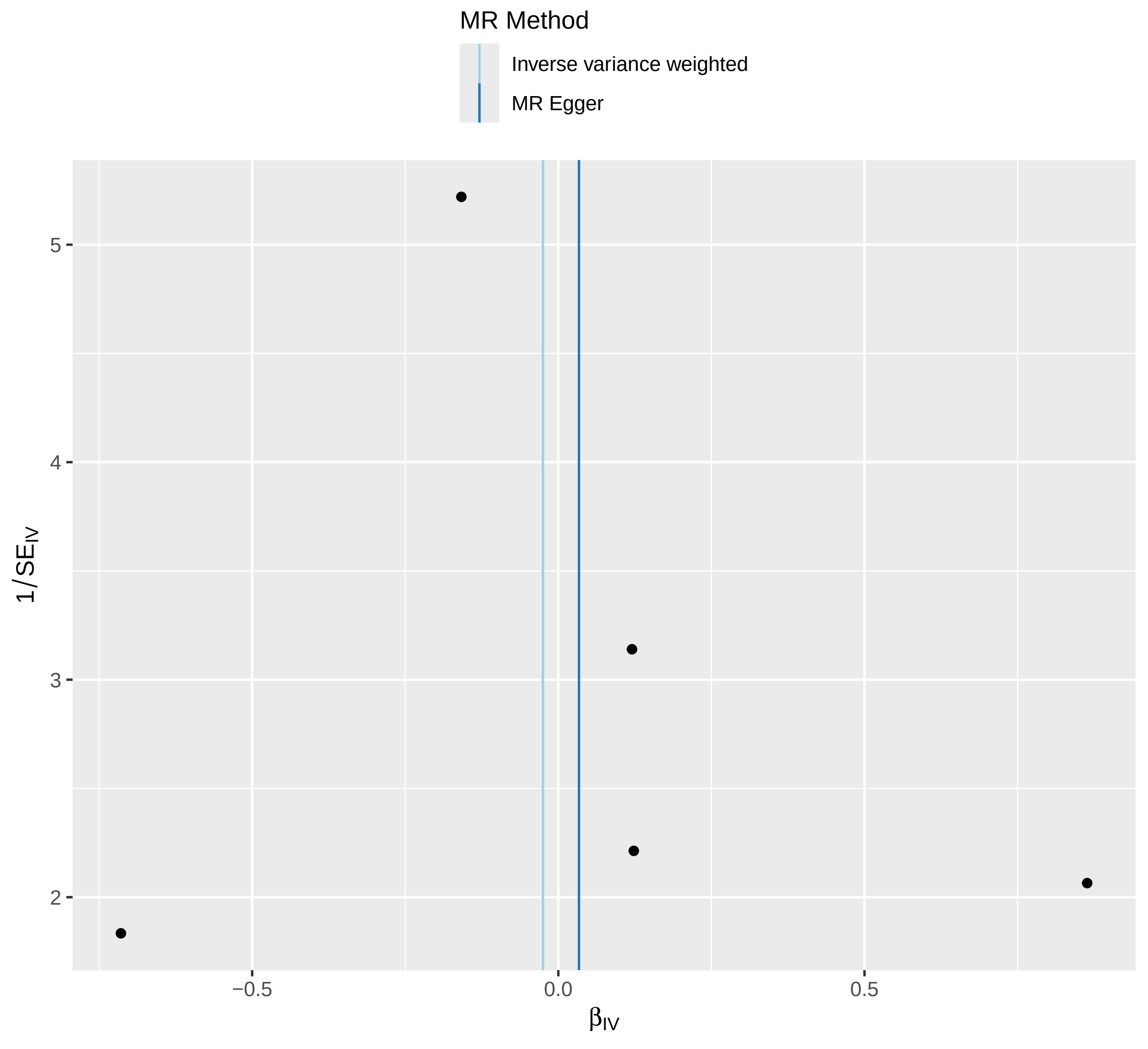
MR Method
Inverse variance weighted
MR Egger
5
4
3
2
1/SE$_{IV}$
−0.5
0.0
0.5
β$_{IV}$

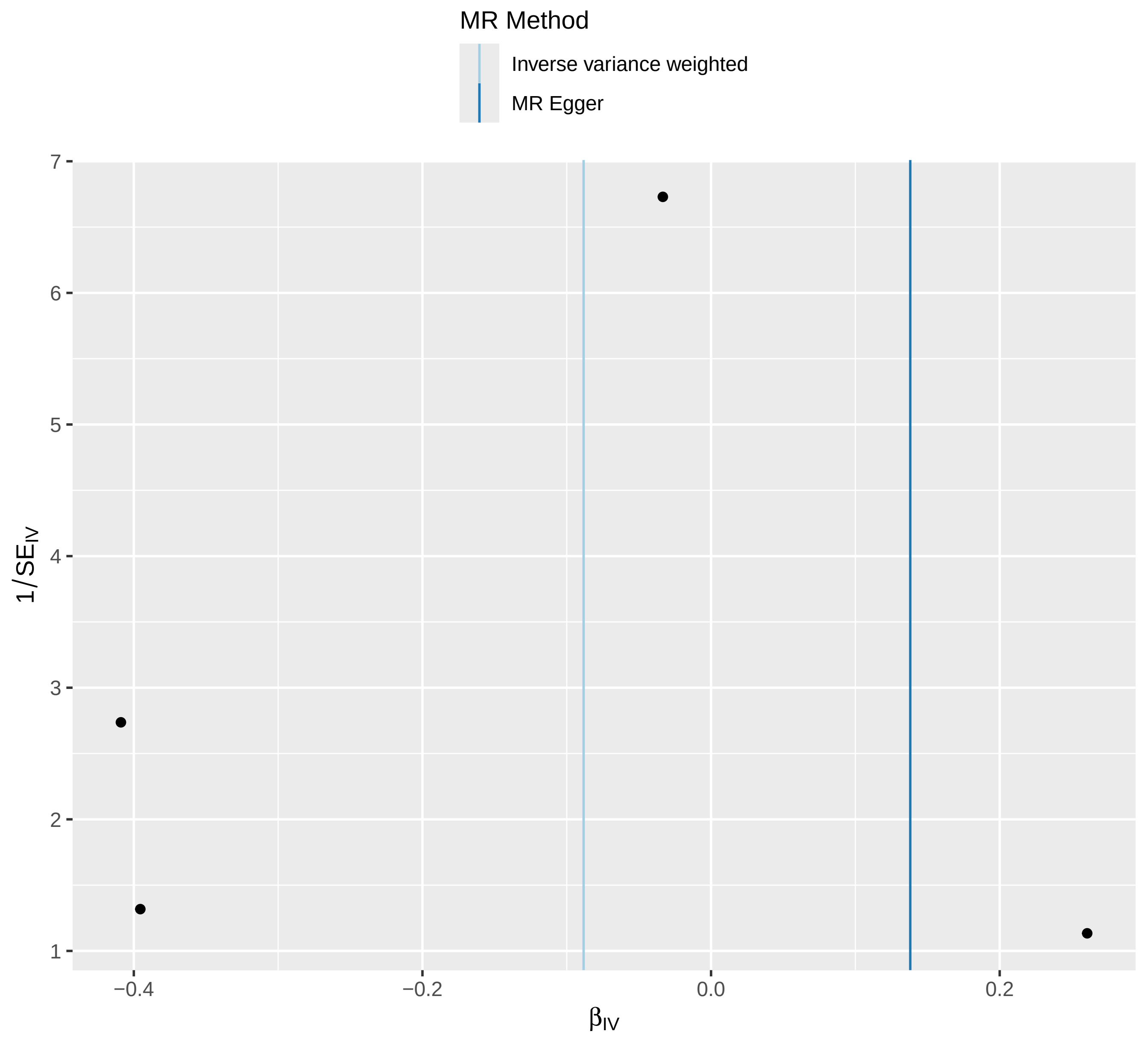

MR Method
Inverse variance weighted
MR Egger
1/SE$_{IV}$
β_{IV}
7
6
5
4
3
2
1
−0.4
−0.2
0.0
0.2

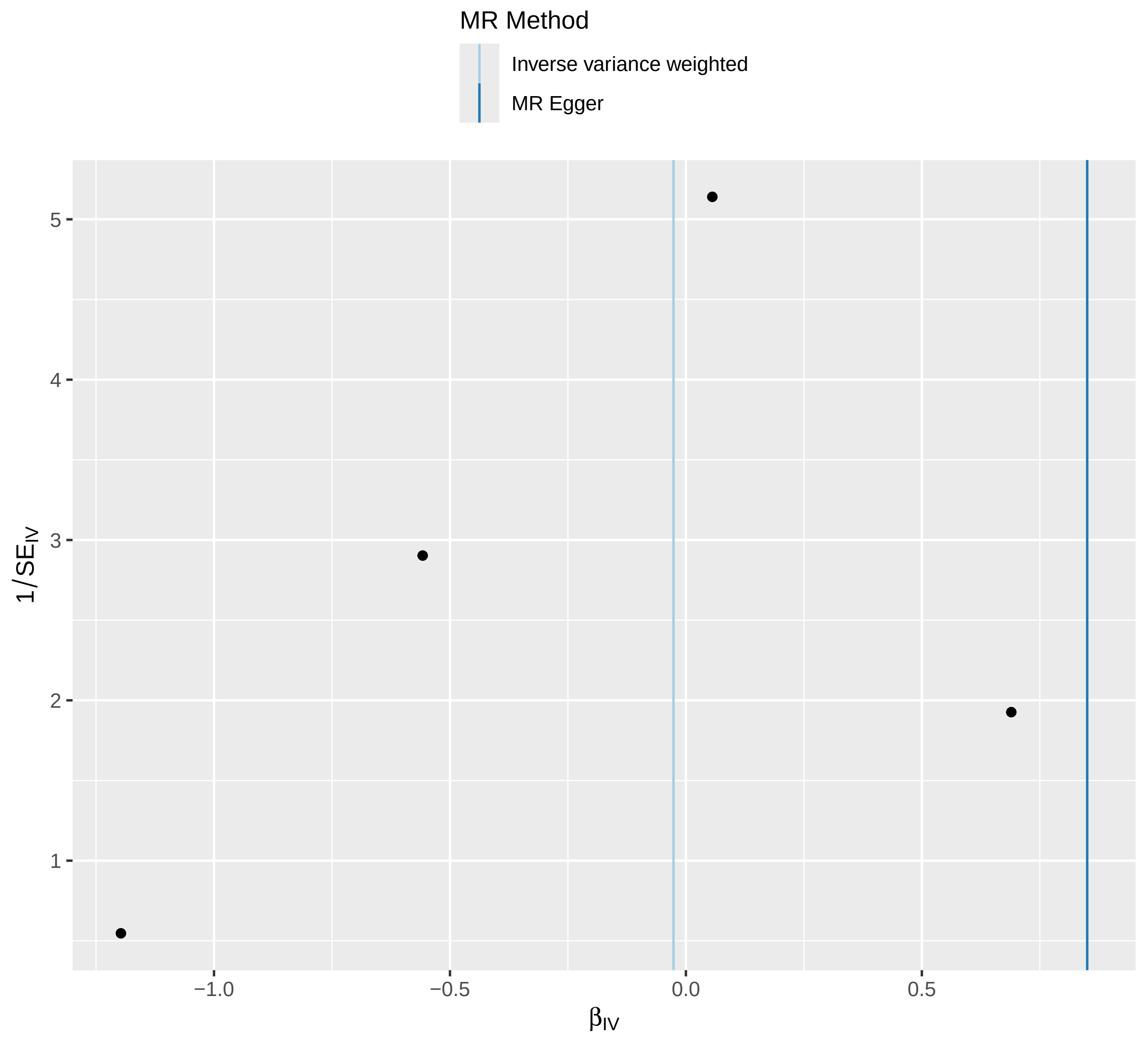
MR Method
Inverse variance weighted
MR Egger
1/SE_IV
5
4
3
2
1
−1.0
−0.5
0.0
0.5
β_IV

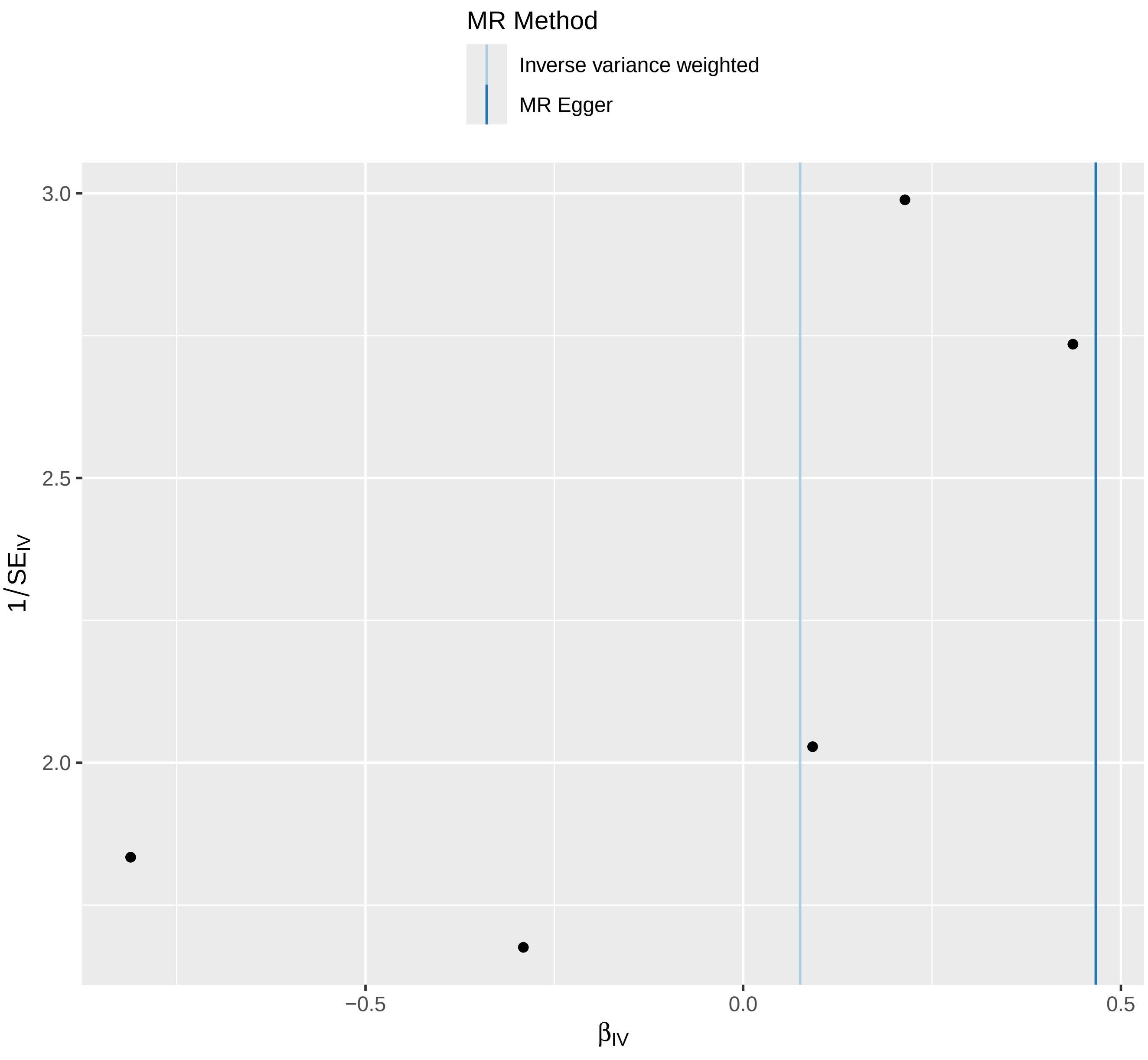
MR Method
Inverse variance weighted
MR Egger
3.0
2.5
2.0
1/SE$_{IV}$
−0.5
0.0
0.5
β$_{IV}$

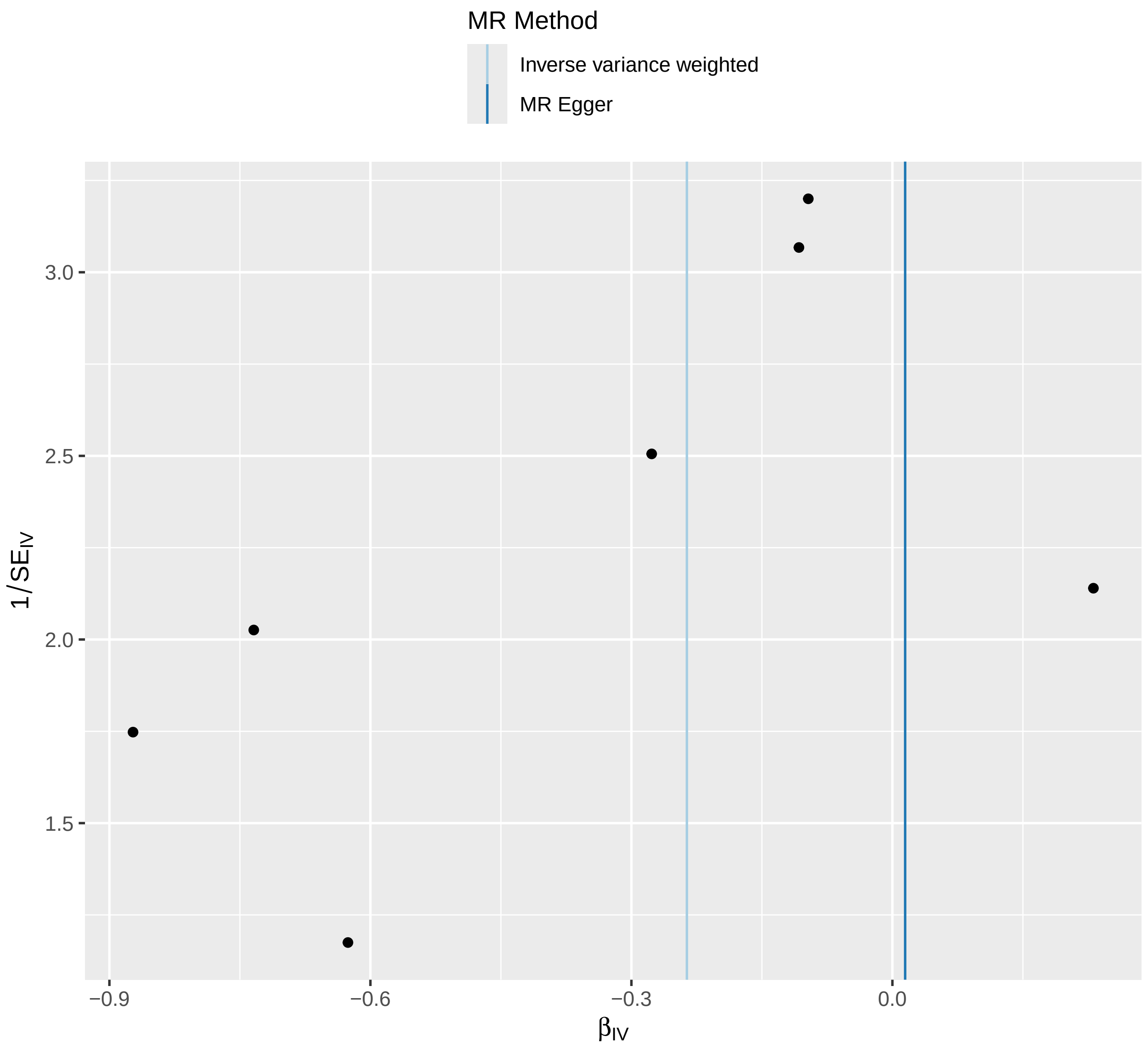

MR Method
Inverse variance weighted
MR Egger
1/SE_IV
3.0
2.5
2.0
1.5
−0.9
−0.6
−0.3
0.0
β_IV

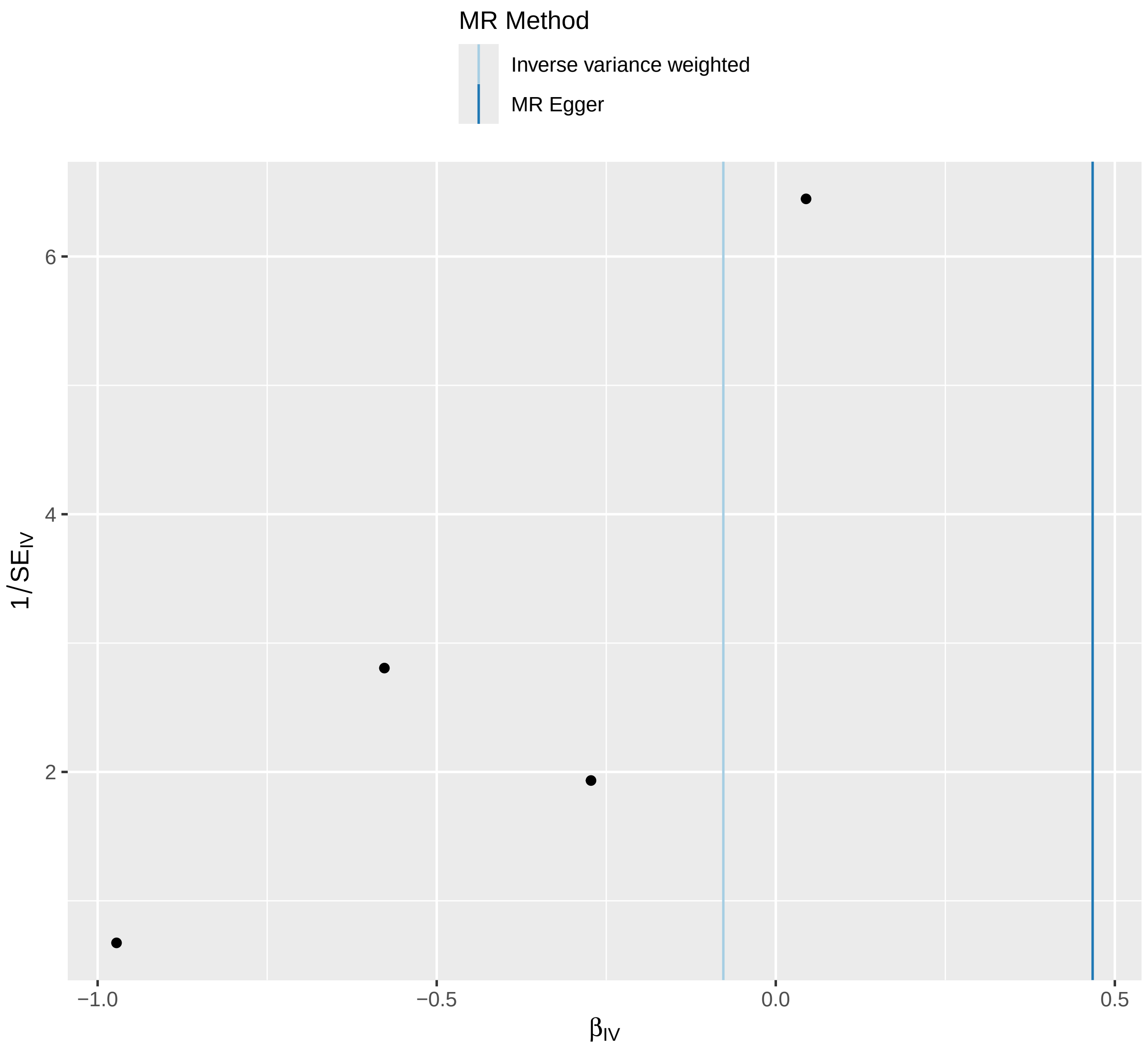
MR Method
Inverse variance weighted
MR Egger
1/SE$_{IV}$
6
4
2
−1.0
−0.5
0.0
0.5
β$_{IV}$

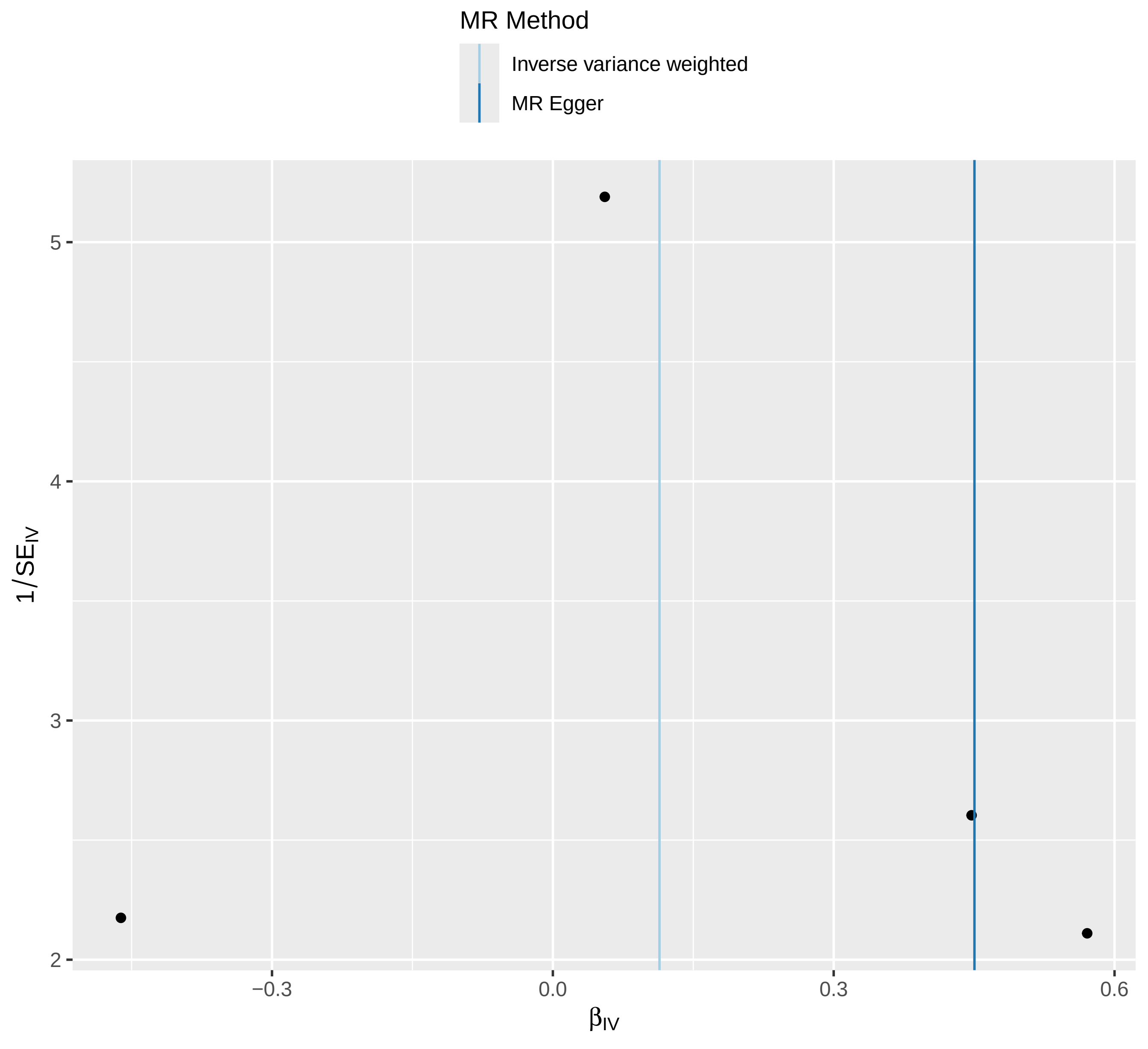

MR Method
Inverse variance weighted
MR Egger
5
4
3
2
1/SE$_{IV}$
−0.3
0.0
0.3
0.6
β$_{IV}$

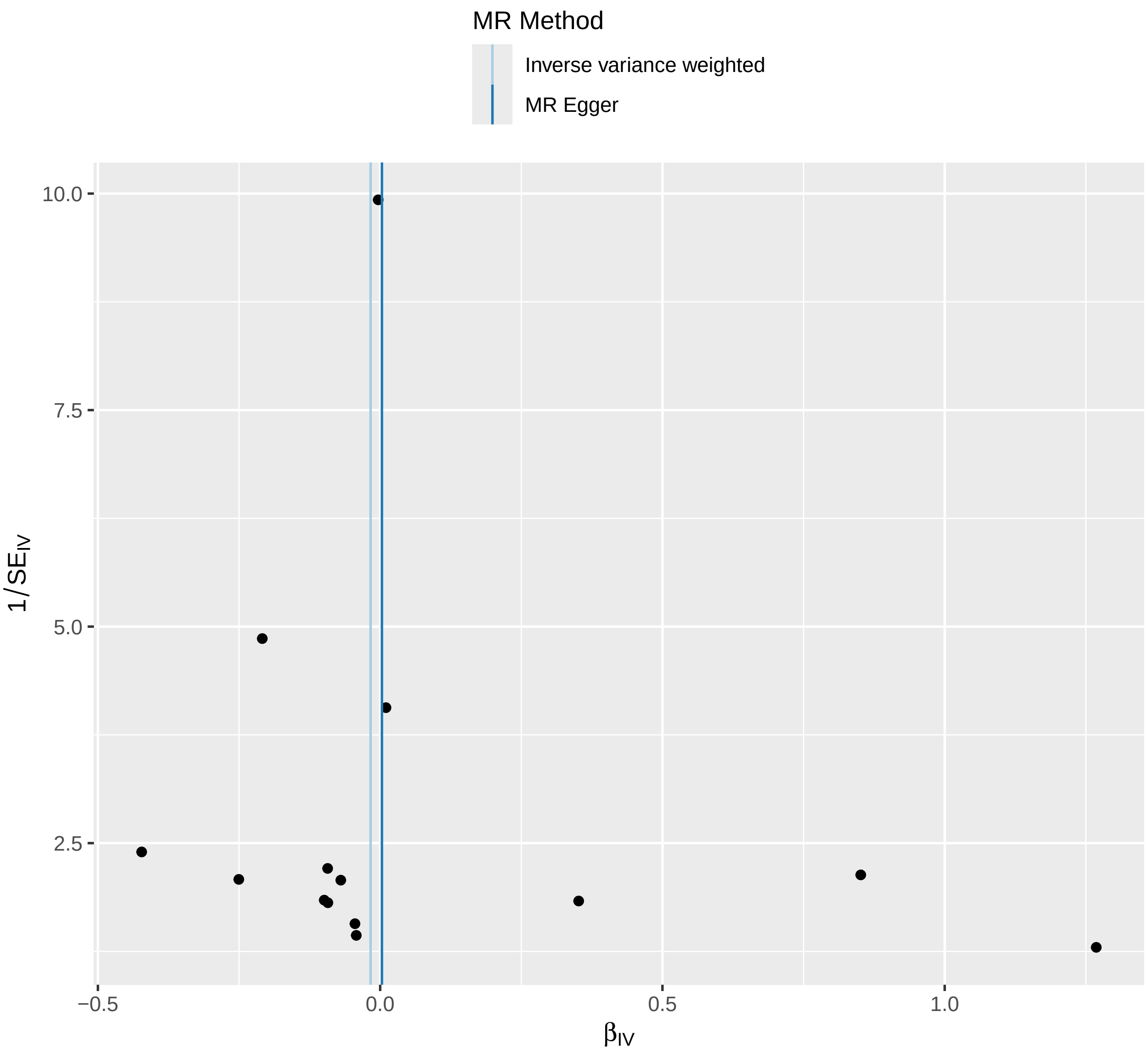
MR Method
Inverse variance weighted
MR Egger
10.0
7.5
5.0
2.5
$1/SE_{IV}$
−0.5
0.0
0.5
1.0
β_{IV}

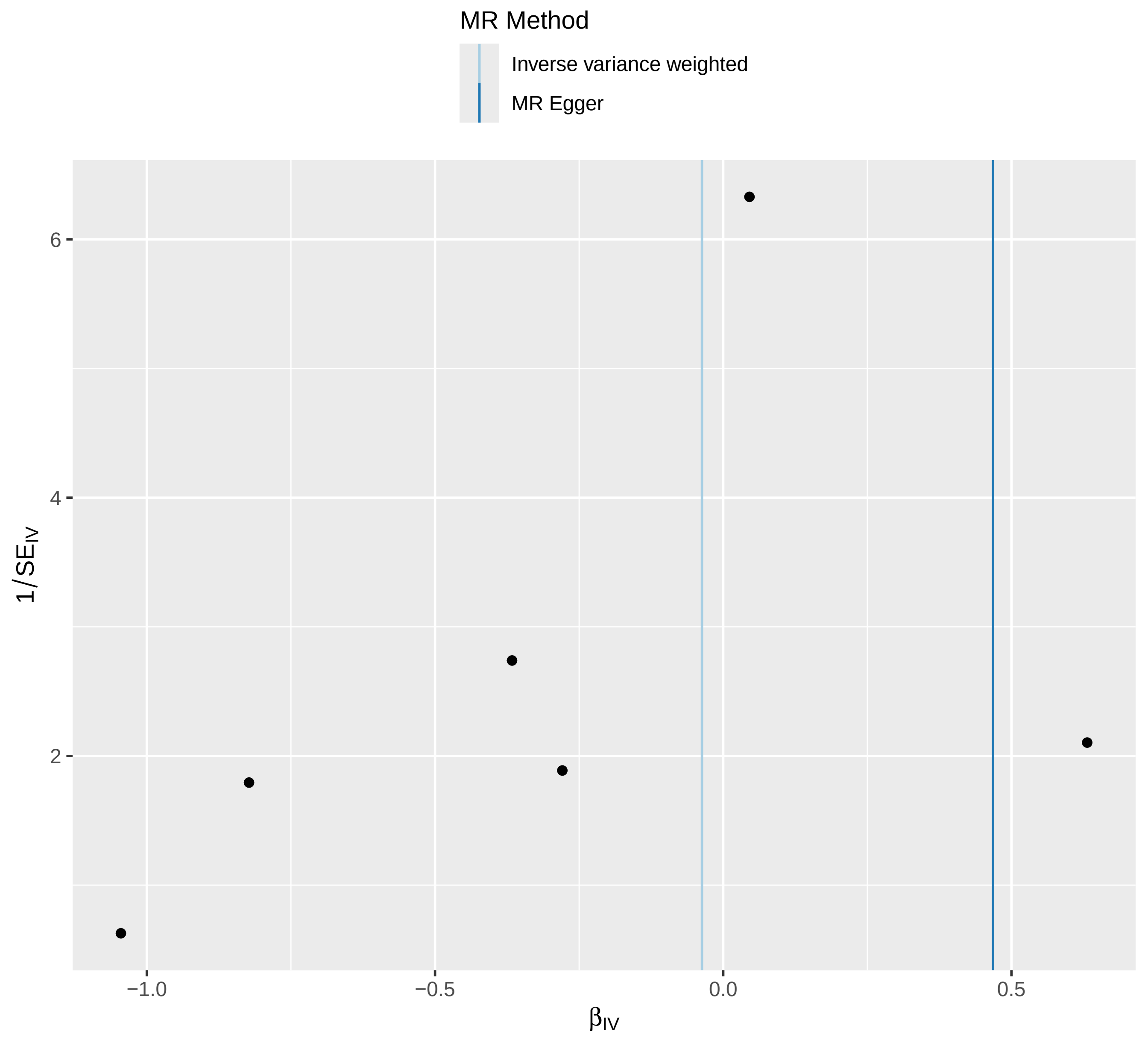

MR Method
Inverse variance weighted
MR Egger
6
4
2
1/SE$_{IV}$
−1.0
−0.5
0.0
0.5
β$_{IV}$

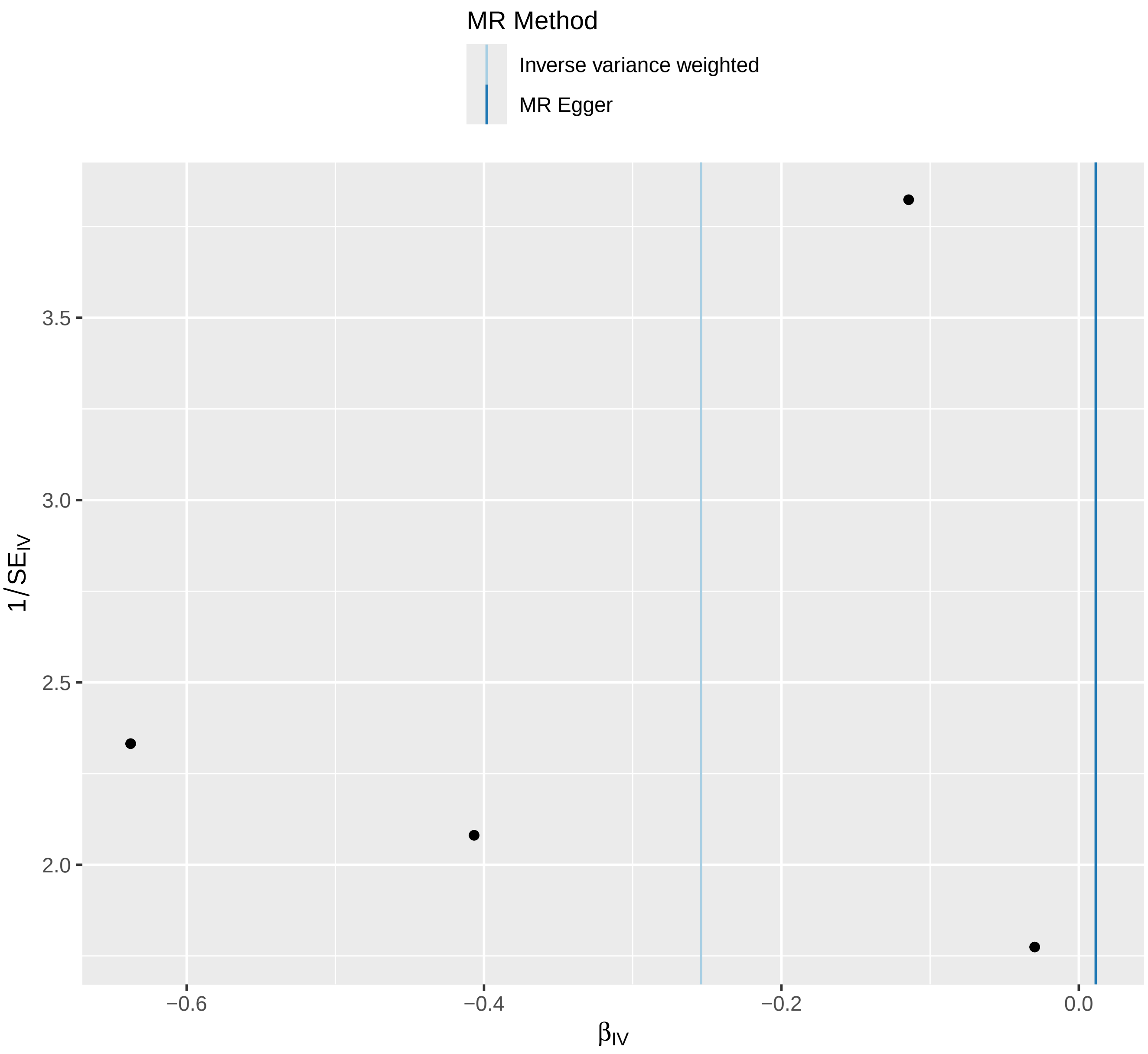
MR Method
Inverse variance weighted
MR Egger
3.5
3.0
2.5
2.0
$1/SE_{IV}$
−0.6
−0.4
−0.2
0.0
β_{IV}

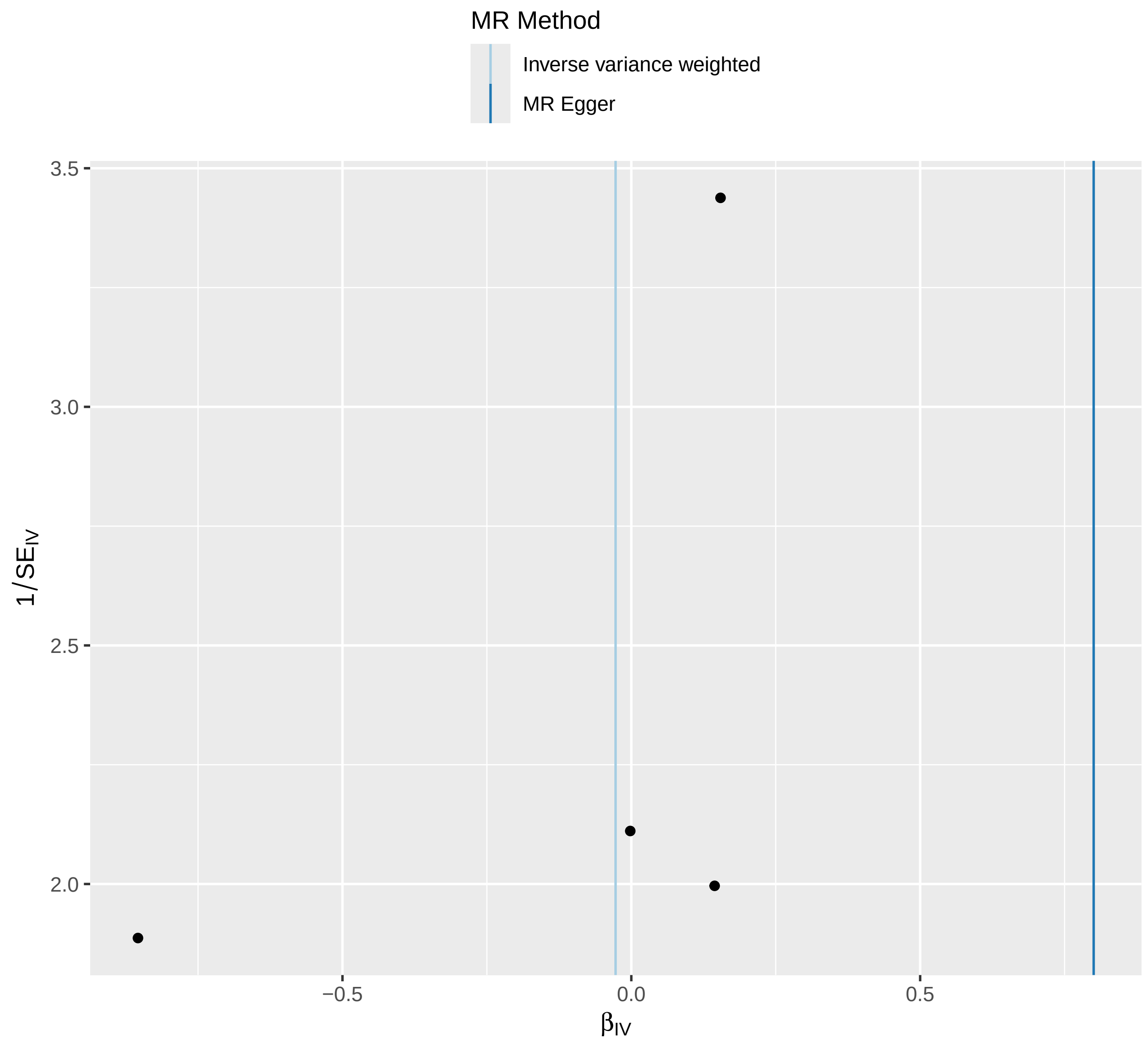
MR Method
Inverse variance weighted
MR Egger
3.5
3.0
2.5
2.0
1/SE_IV
−0.5
0.0
0.5
β_IV

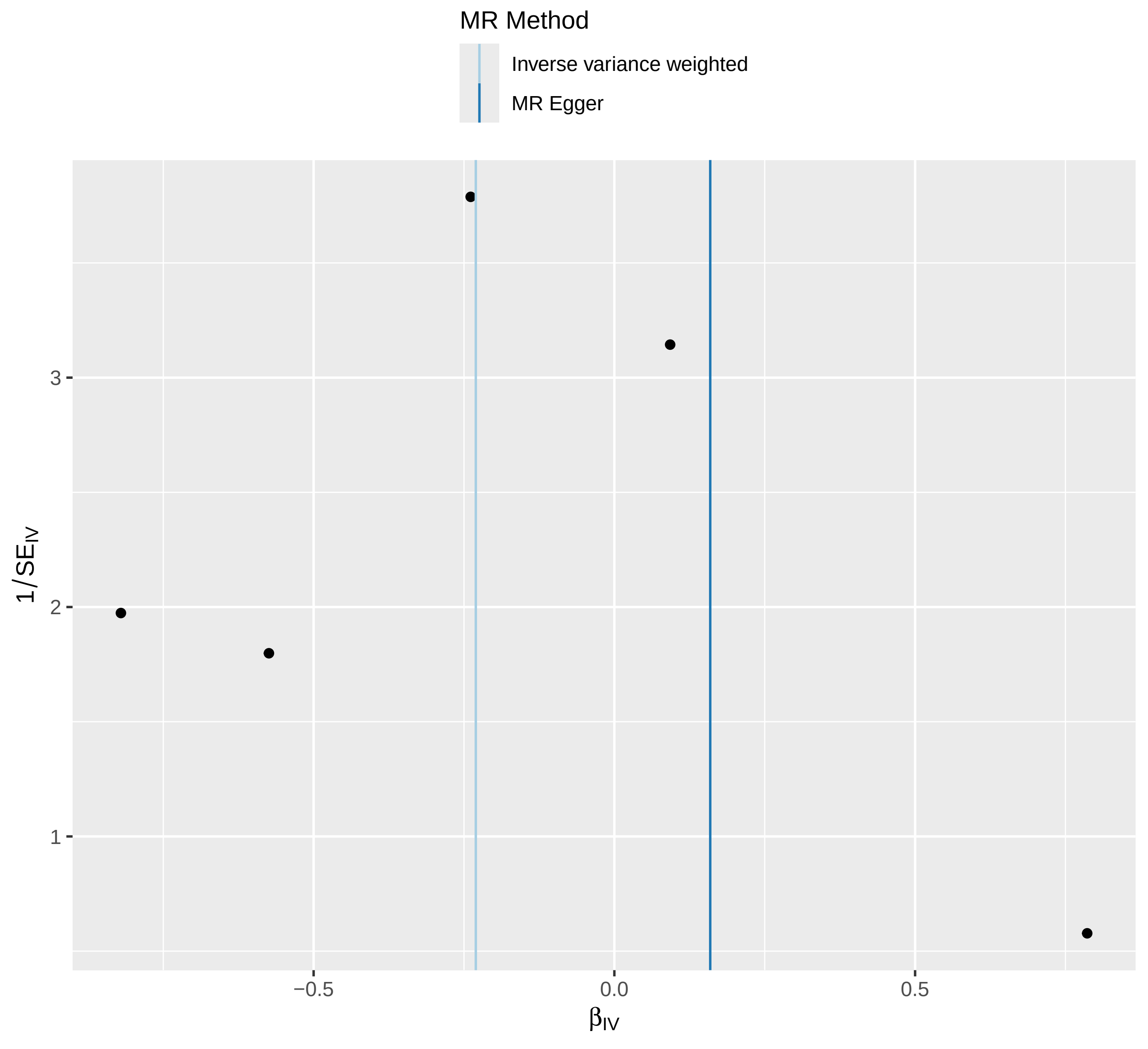
MR Method
Inverse variance weighted
MR Egger
1/SE_IV
3
2
1
−0.5
0.0
0.5
β_IV

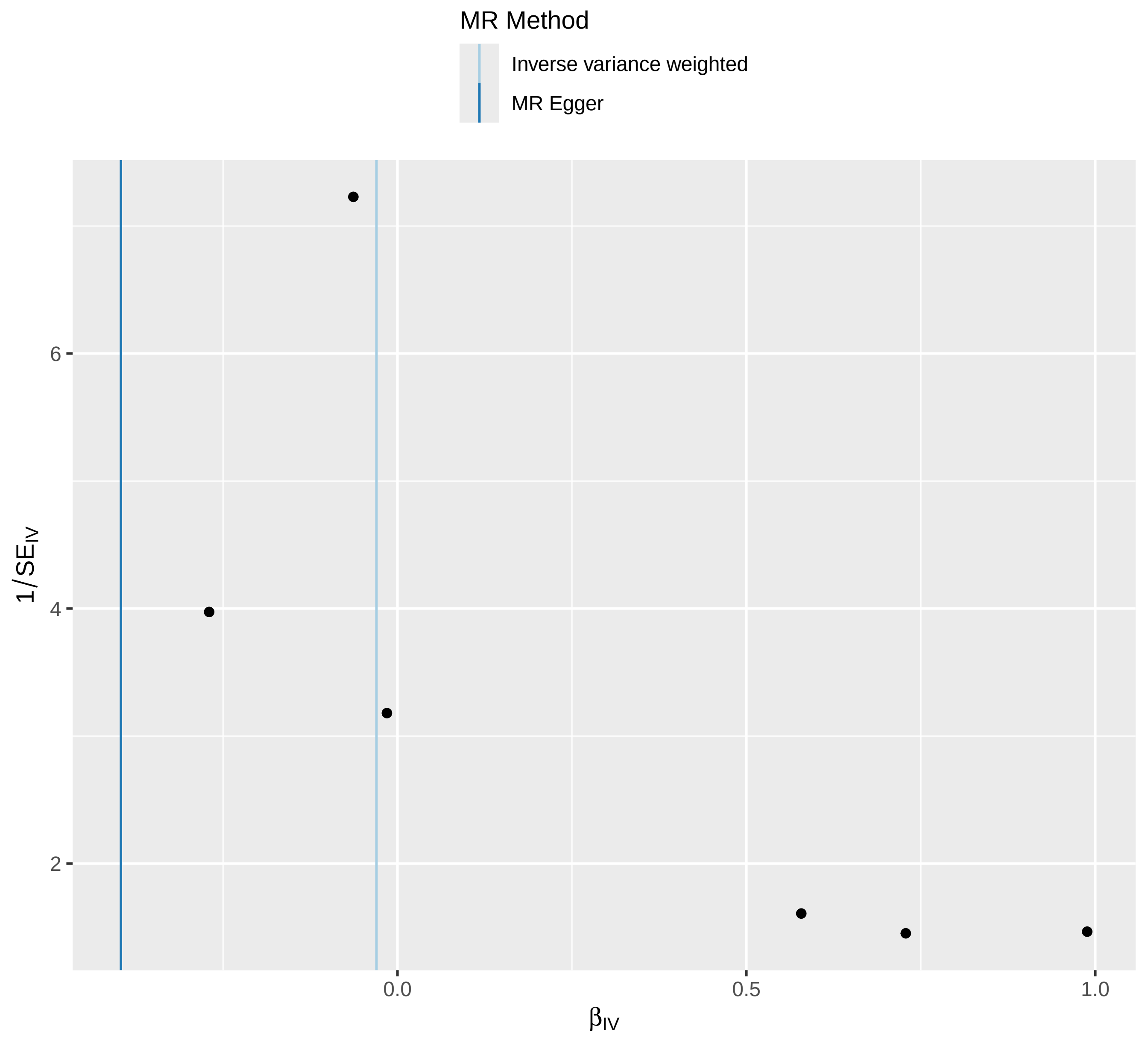

MR Method
Inverse variance weighted
MR Egger
6
4
2
1/SE$_{IV}$
0.0
0.5
1.0
β$_{IV}$

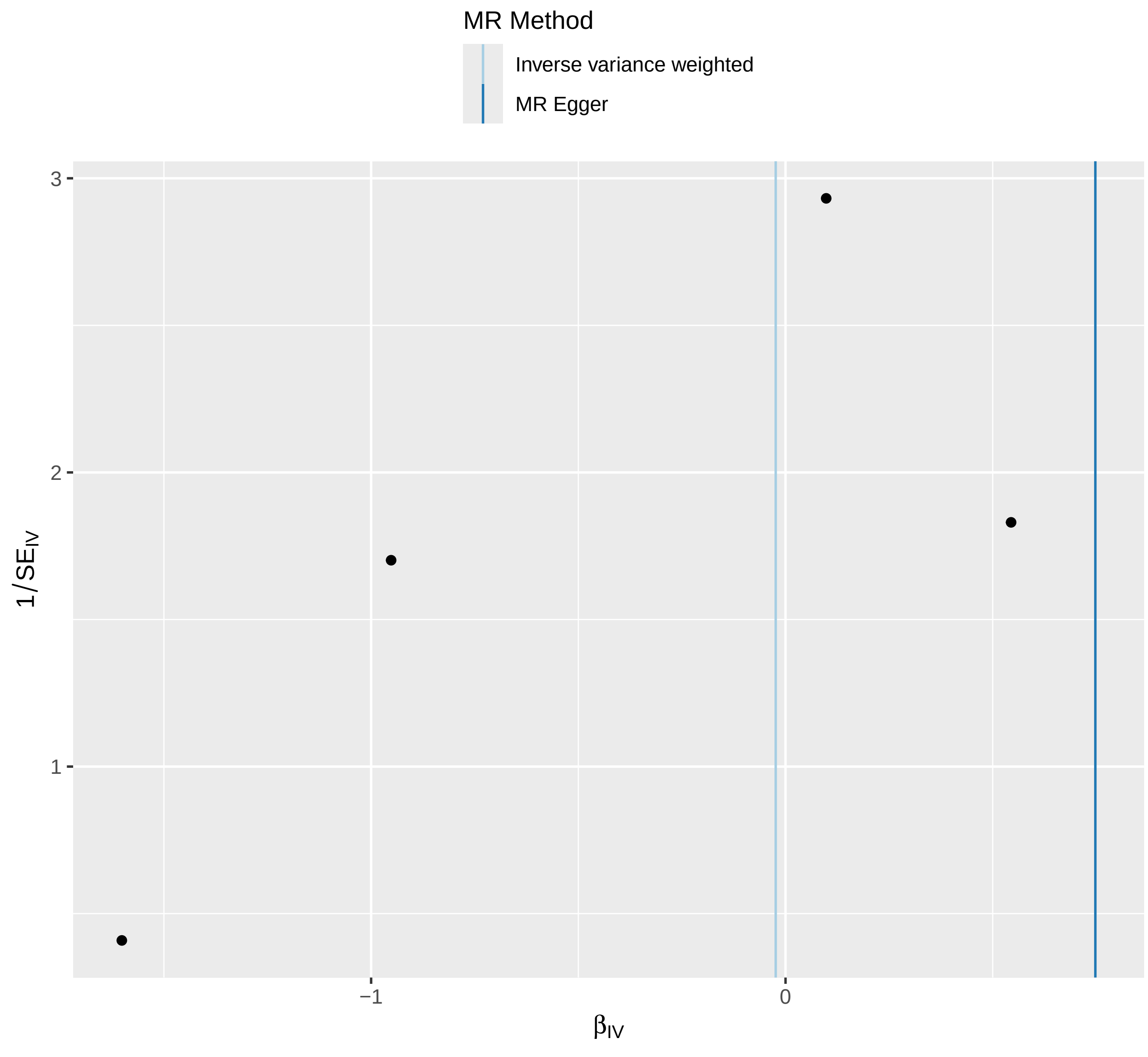

MR Method
Inverse variance weighted
MR Egger
3
2
1
1/SE$_{IV}$
−1
0
β$_{IV}$

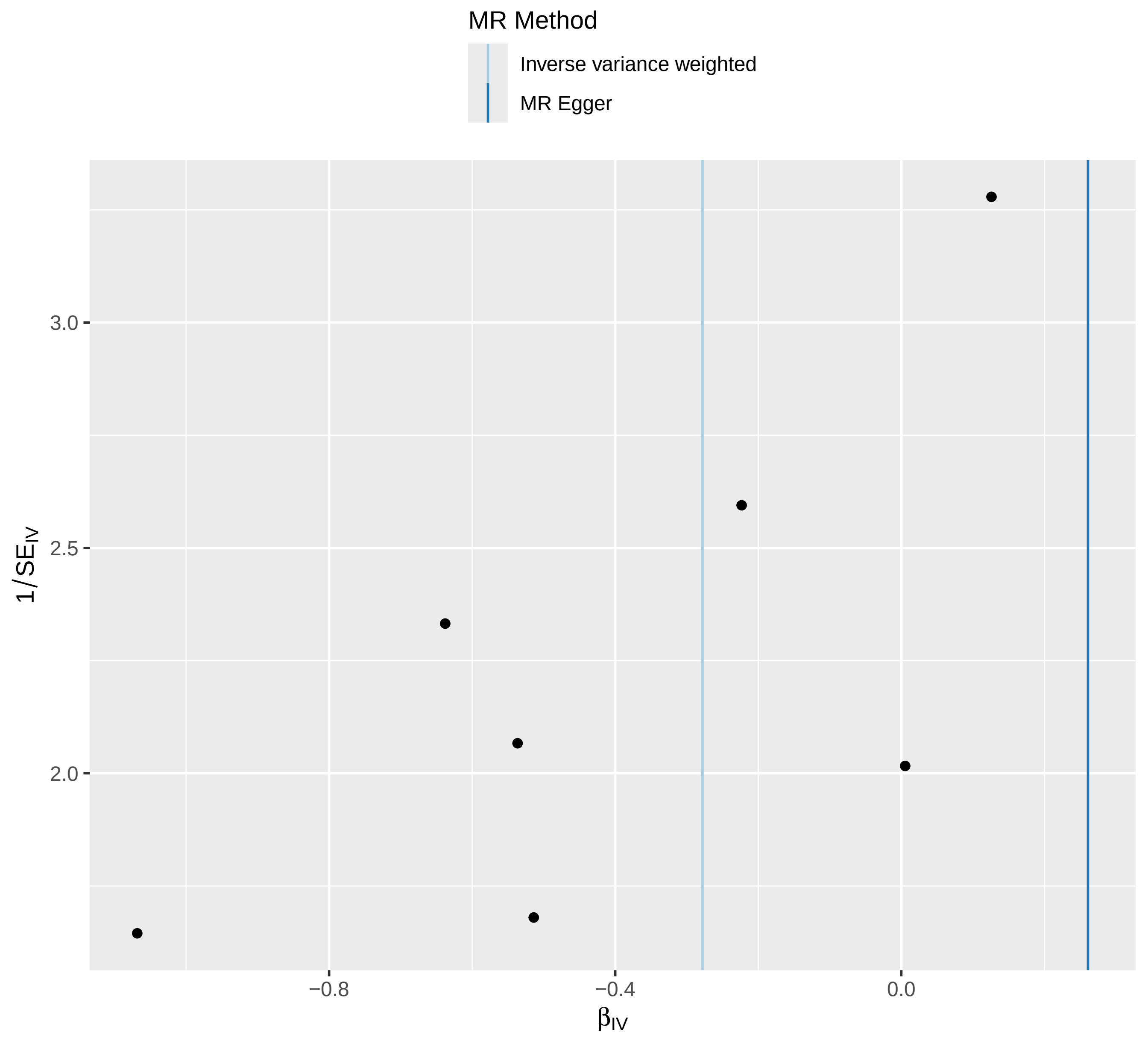

MR Method
Inverse variance weighted
MR Egger
3.0
2.5
2.0
1/SE$_{IV}$
−0.8
−0.4
0.0
β$_{IV}$

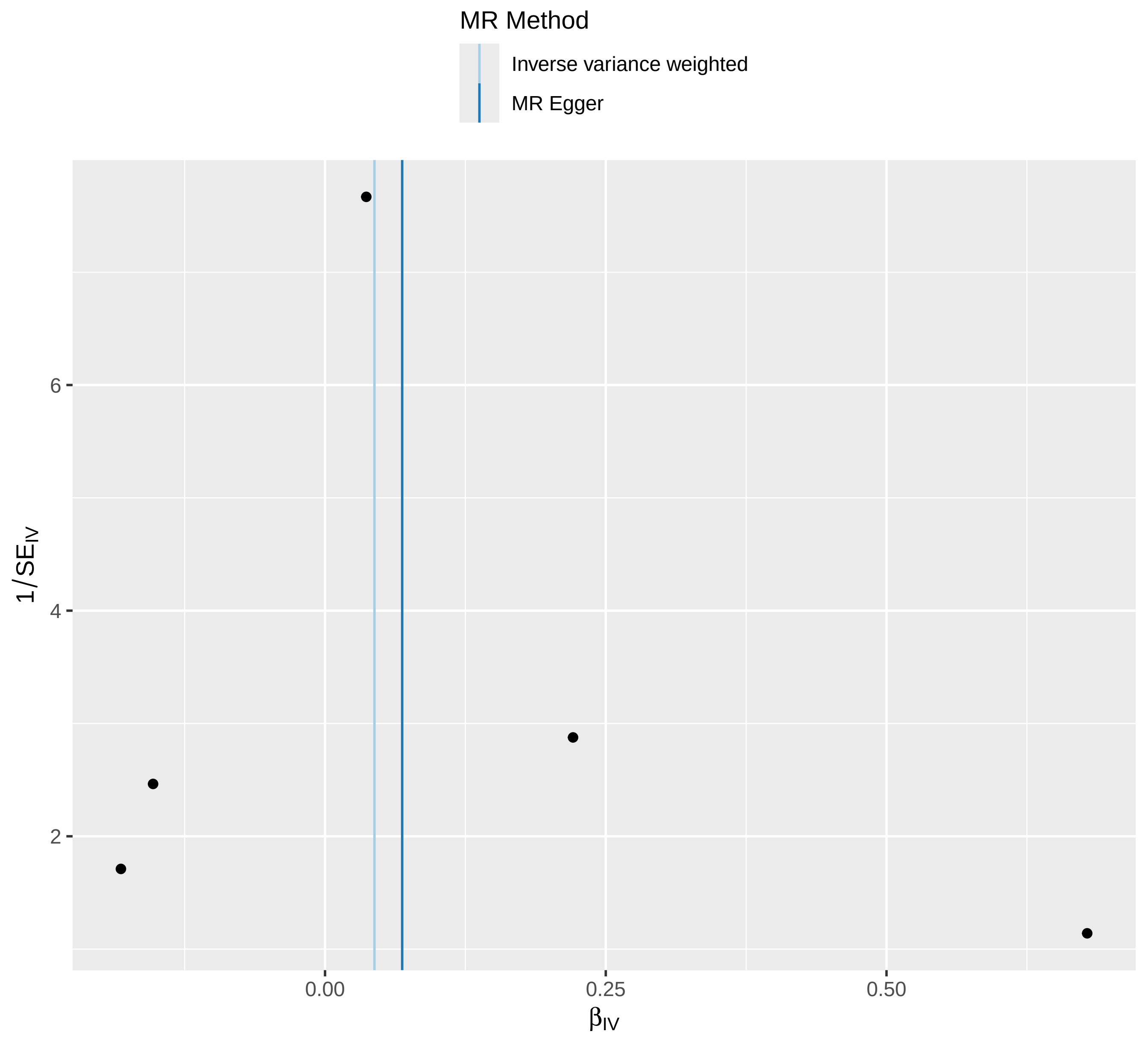

MR Method
Inverse variance weighted
MR Egger
6
4
2
1/SE_IV
0.00
0.25
0.50
β_IV

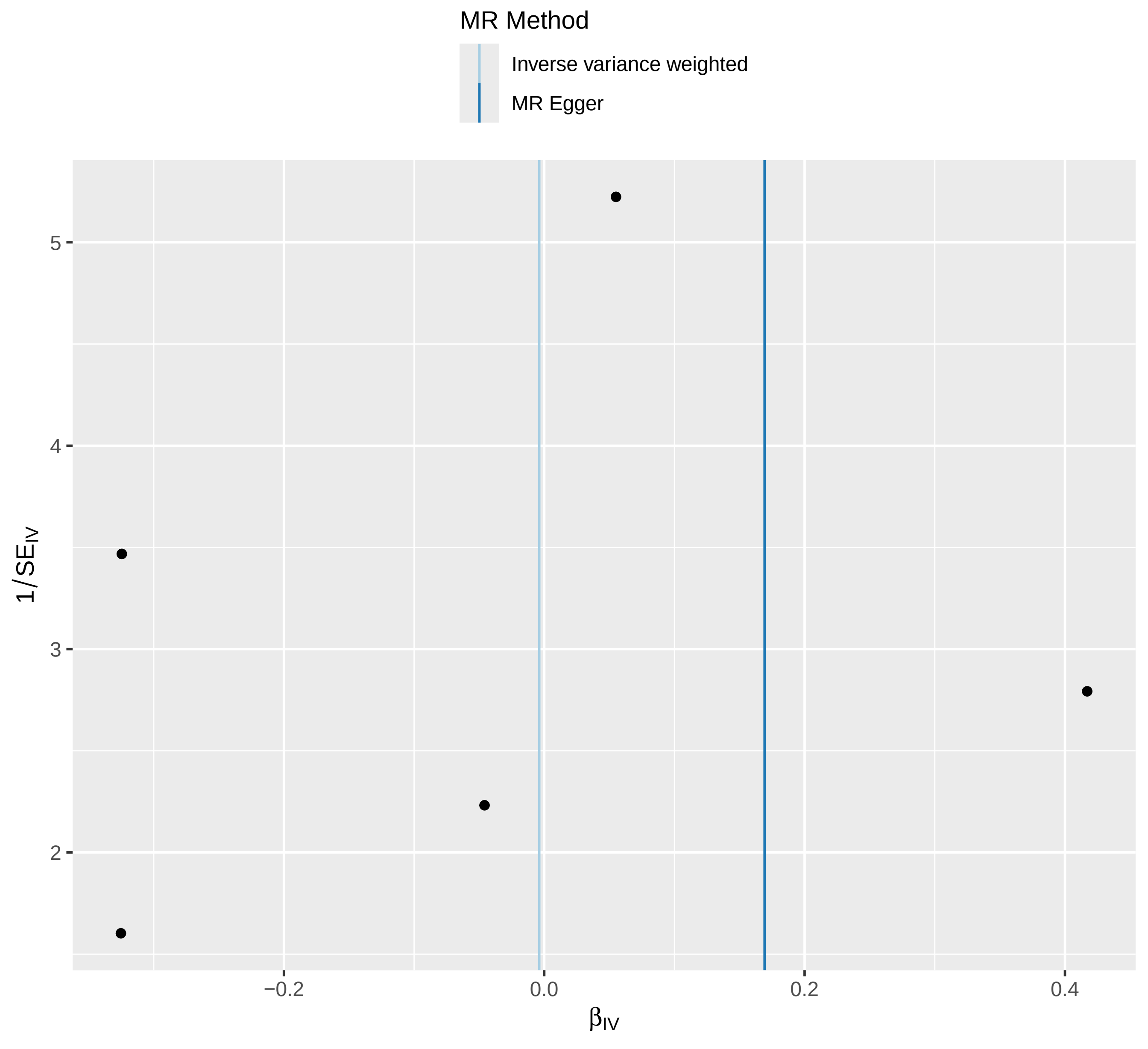

MR Method
Inverse variance weighted
MR Egger
5
4
3
2
1/SE_IV
−0.2
0.0
0.2
0.4
β_IV

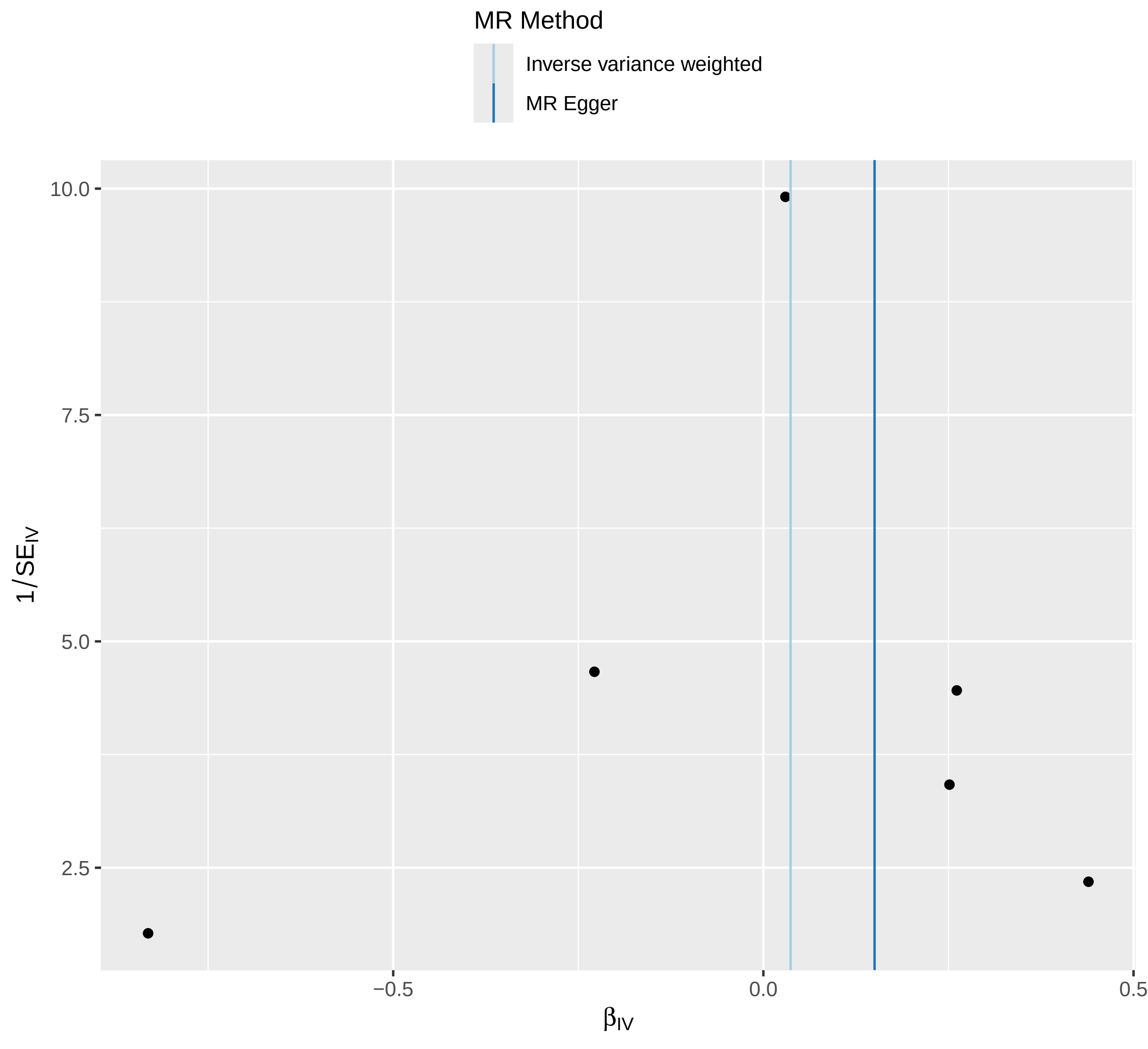

MR Method
Inverse variance weighted
MR Egger
10.0
7.5
5.0
2.5
1/SE_IV
−0.5
0.0
0.5
β_IV

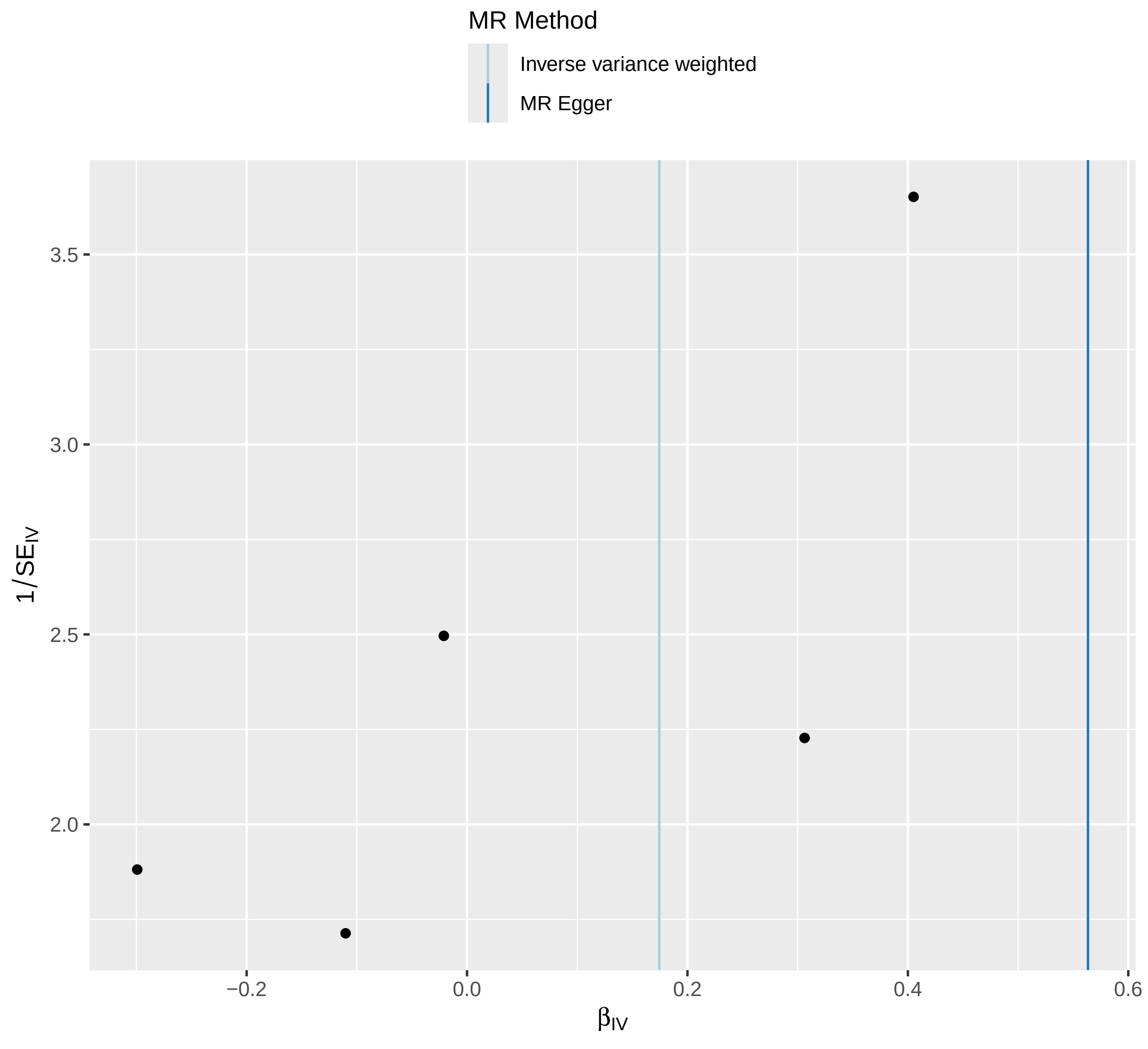

MR Method
Inverse variance weighted
MR Egger
3.5
3.0
2.5
2.0
1/SE_IV
−0.2
0.0
0.2
0.4
0.6
β_IV

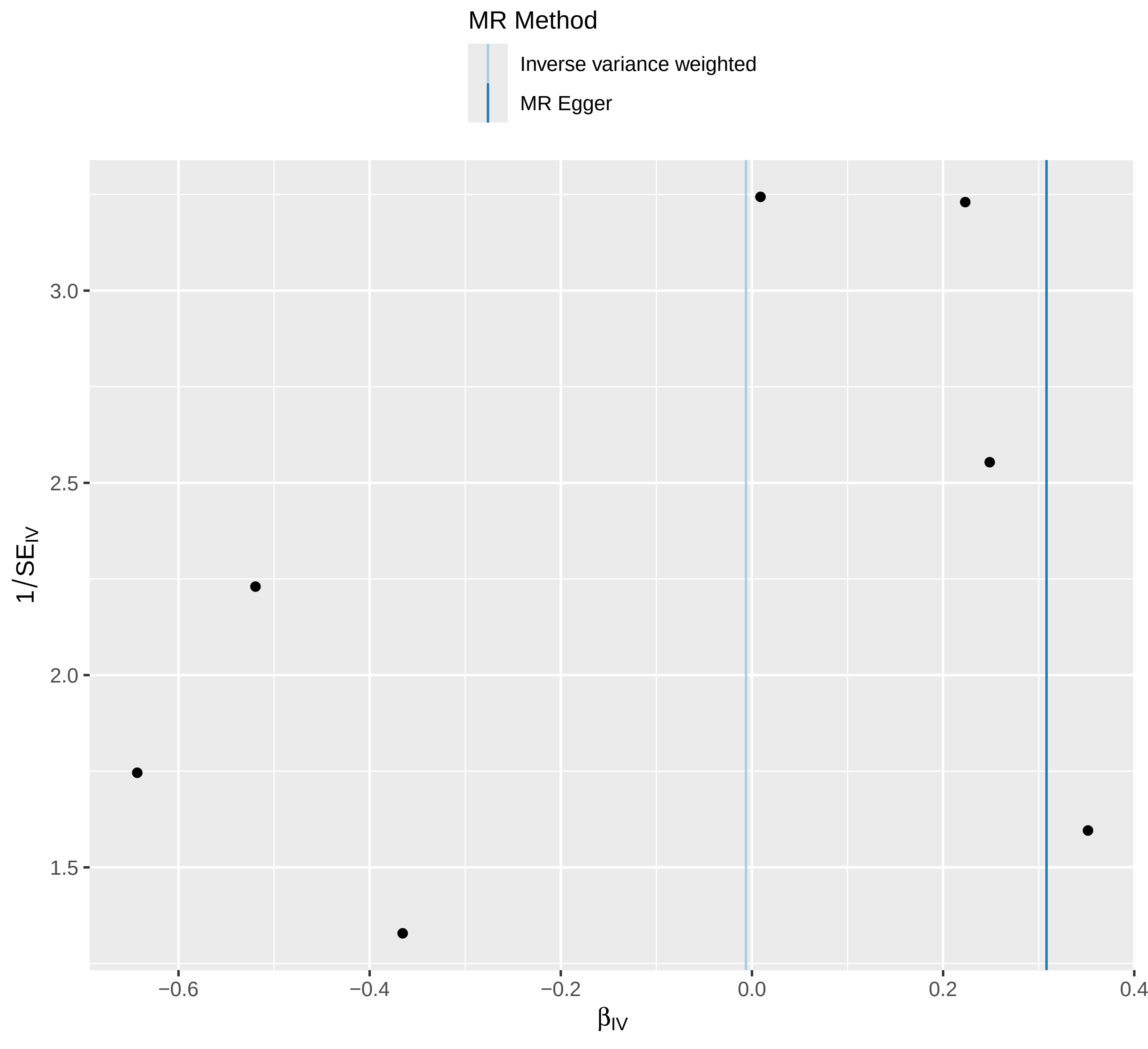
MR Method
Inverse variance weighted
MR Egger
3.0
2.5
2.0
1.5
1/SE_IV
−0.6
−0.4
−0.2
0.0
0.2
0.4
β_IV

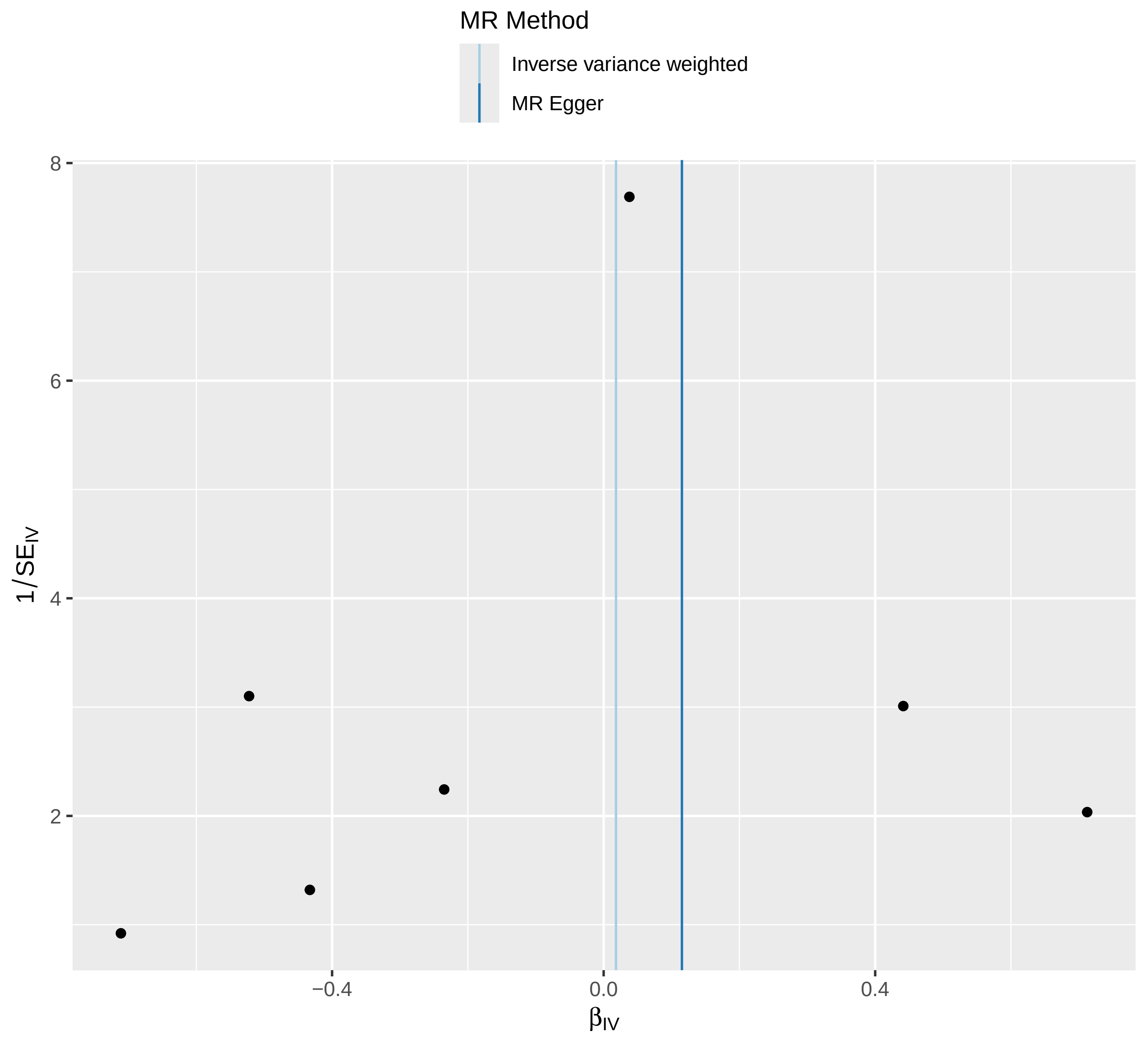

MR Method
Inverse variance weighted
MR Egger
8
6
4
2
1/SE$_{IV}$
−0.4
0.0
0.4
β$_{IV}$

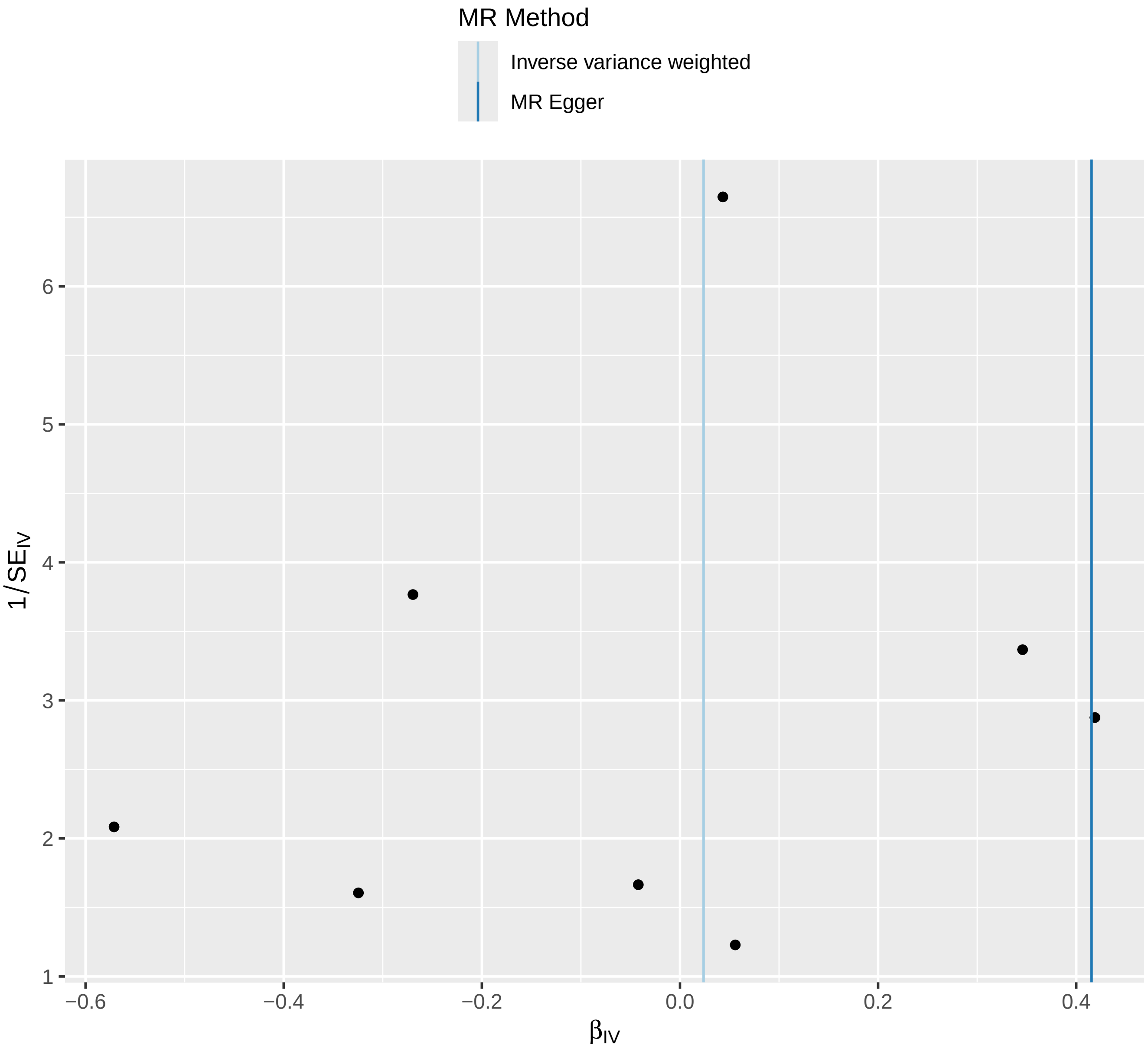

MR Method
Inverse variance weighted
MR Egger
6
5
4
3
2
1
1/SE$_{IV}$
−0.6
−0.4
−0.2
0.0
0.2
0.4
β$_{IV}$

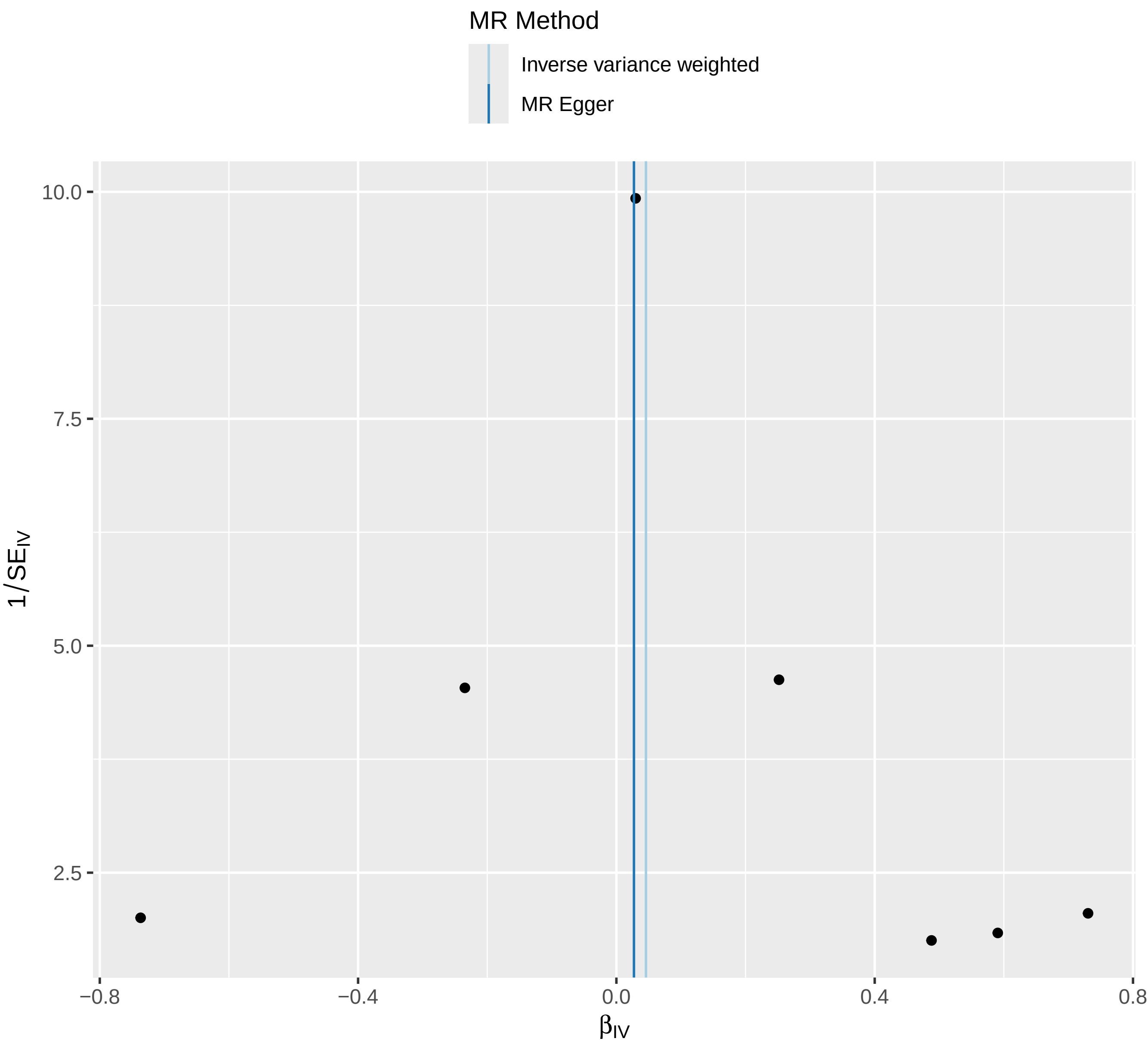
MR Method
Inverse variance weighted
MR Egger
10.0
7.5
5.0
2.5
1/SE_IV
−0.8
−0.4
0.0
0.4
0.8
β_IV

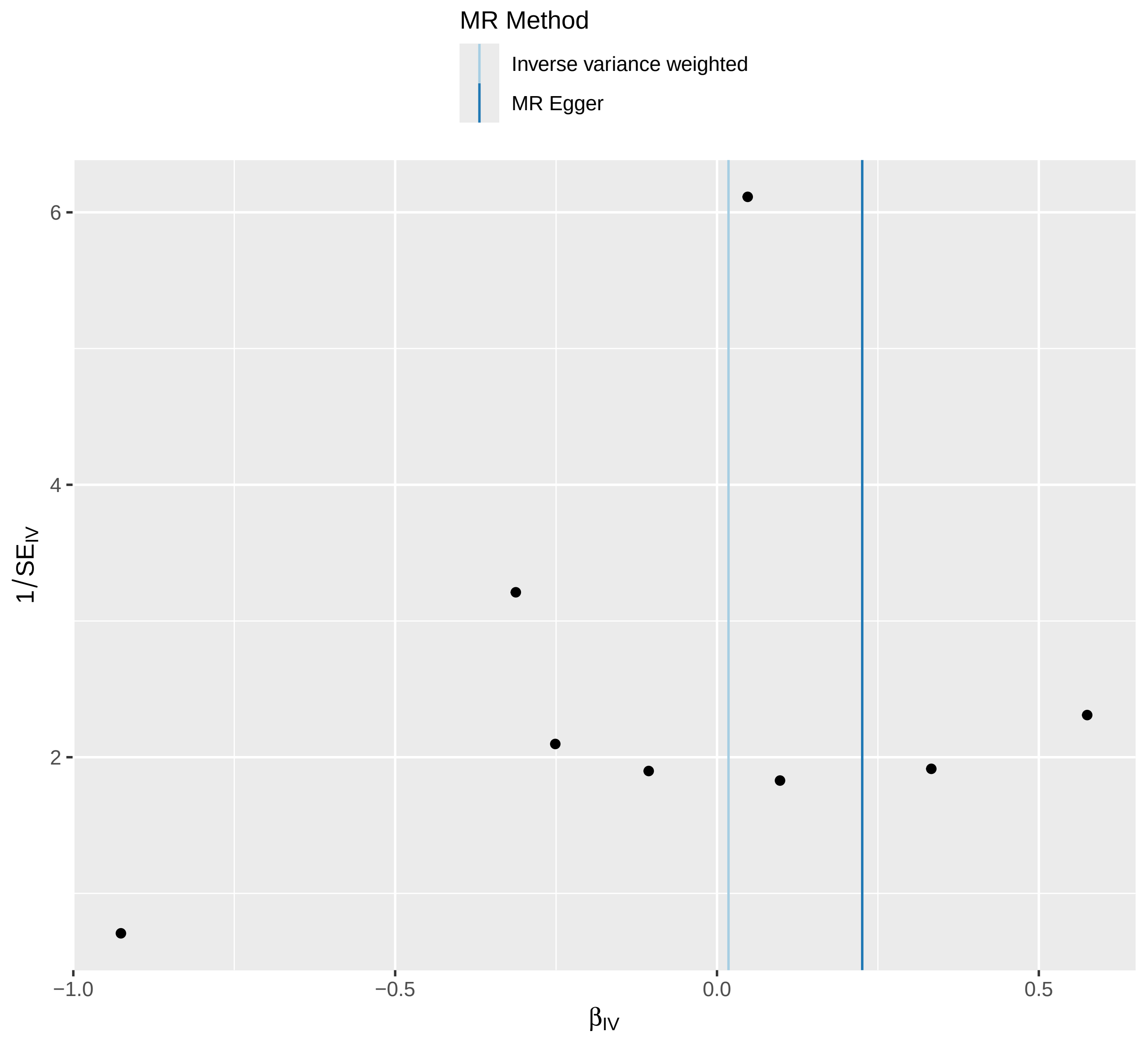

MR Method
Inverse variance weighted
MR Egger
6
4
2
1/SE_IV
−1.0
−0.5
0.0
0.5
β_IV

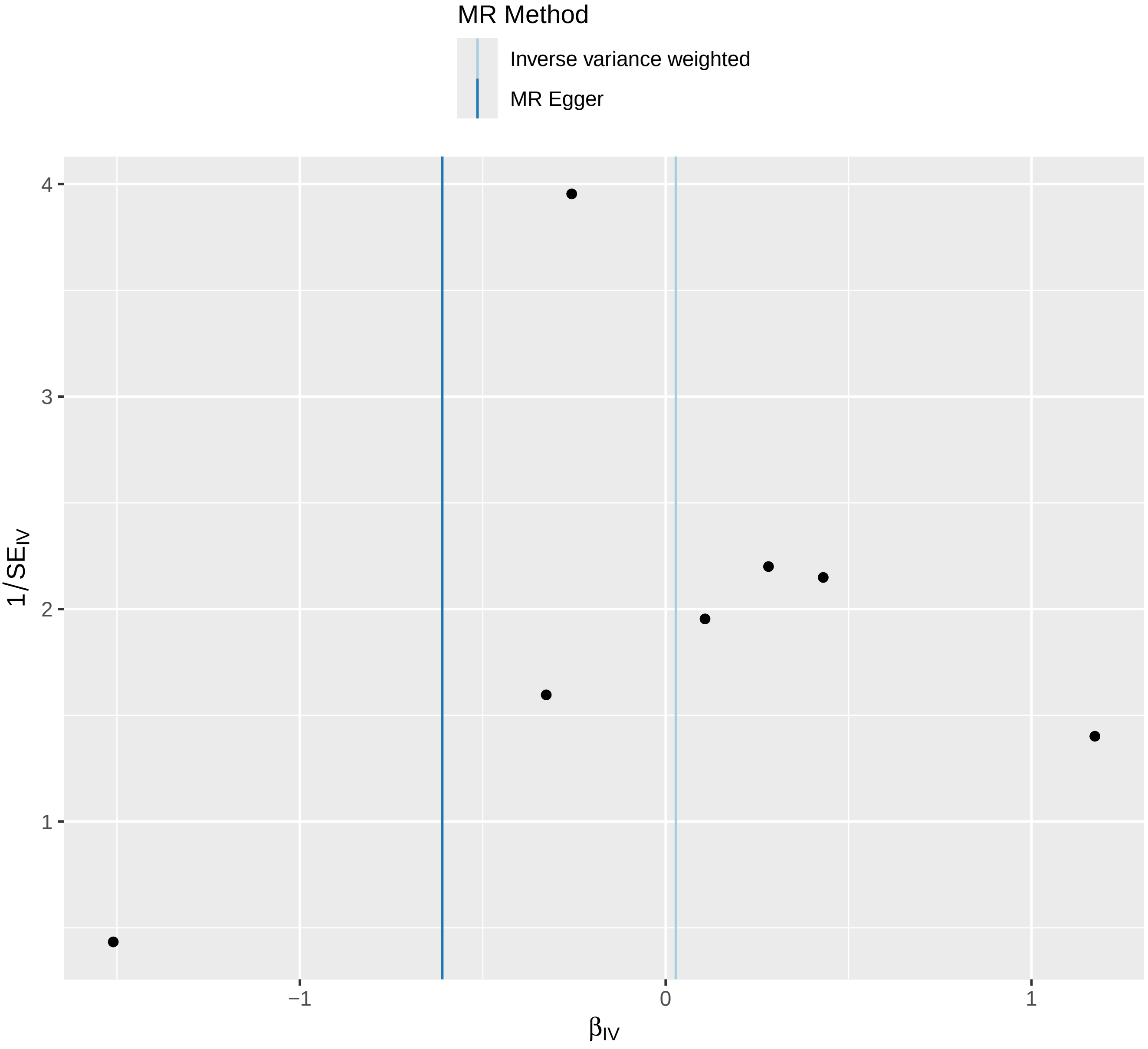
MR Method
Inverse variance weighted
MR Egger
4
3
2
1
1/SE_IV
−1
0
1
β_IV

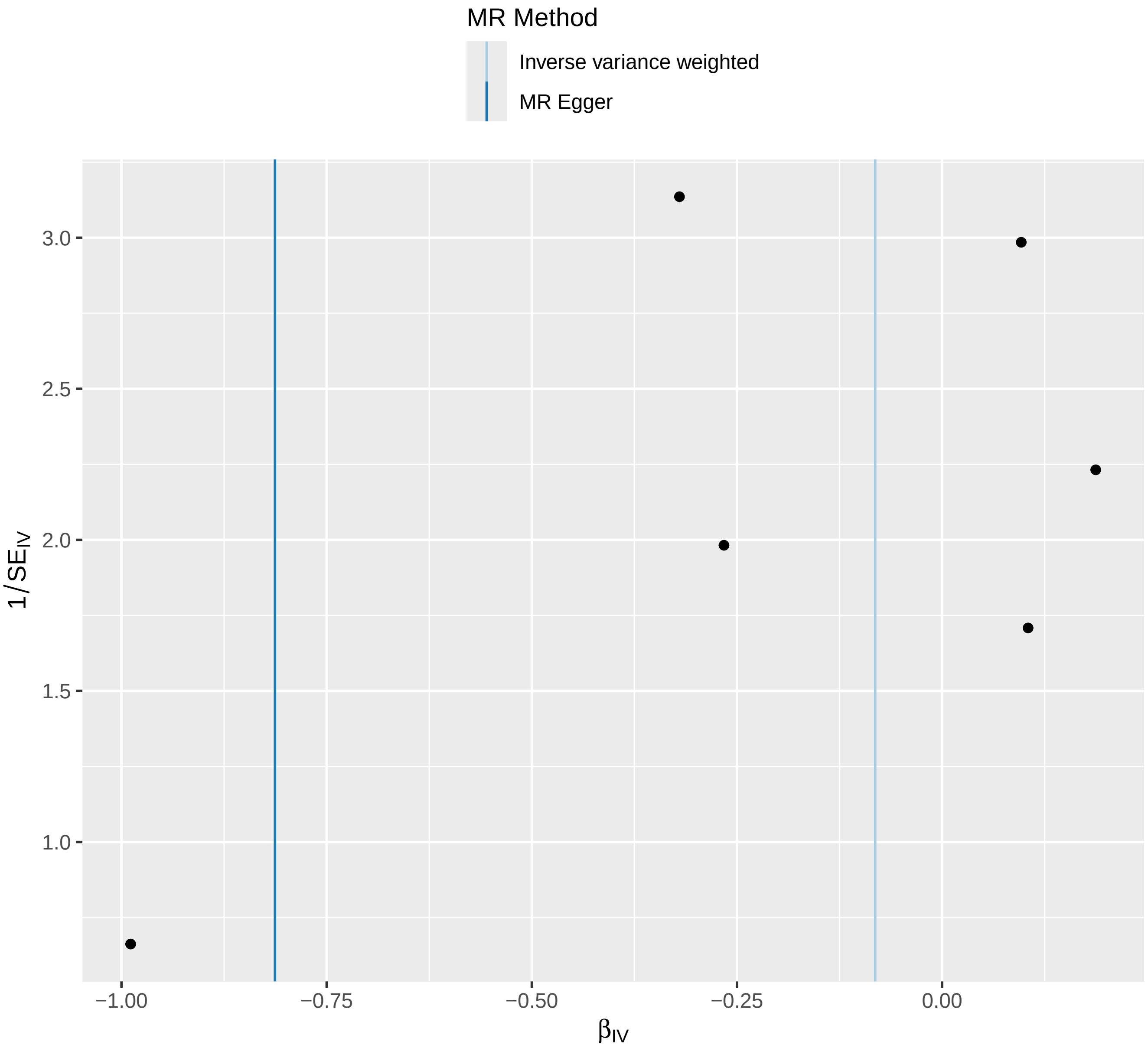

MR Method
Inverse variance weighted
MR Egger
3.0
2.5
2.0
1.5
1.0
1/SE_IV
−1.00
−0.75
−0.50
−0.25
0.00
β_IV

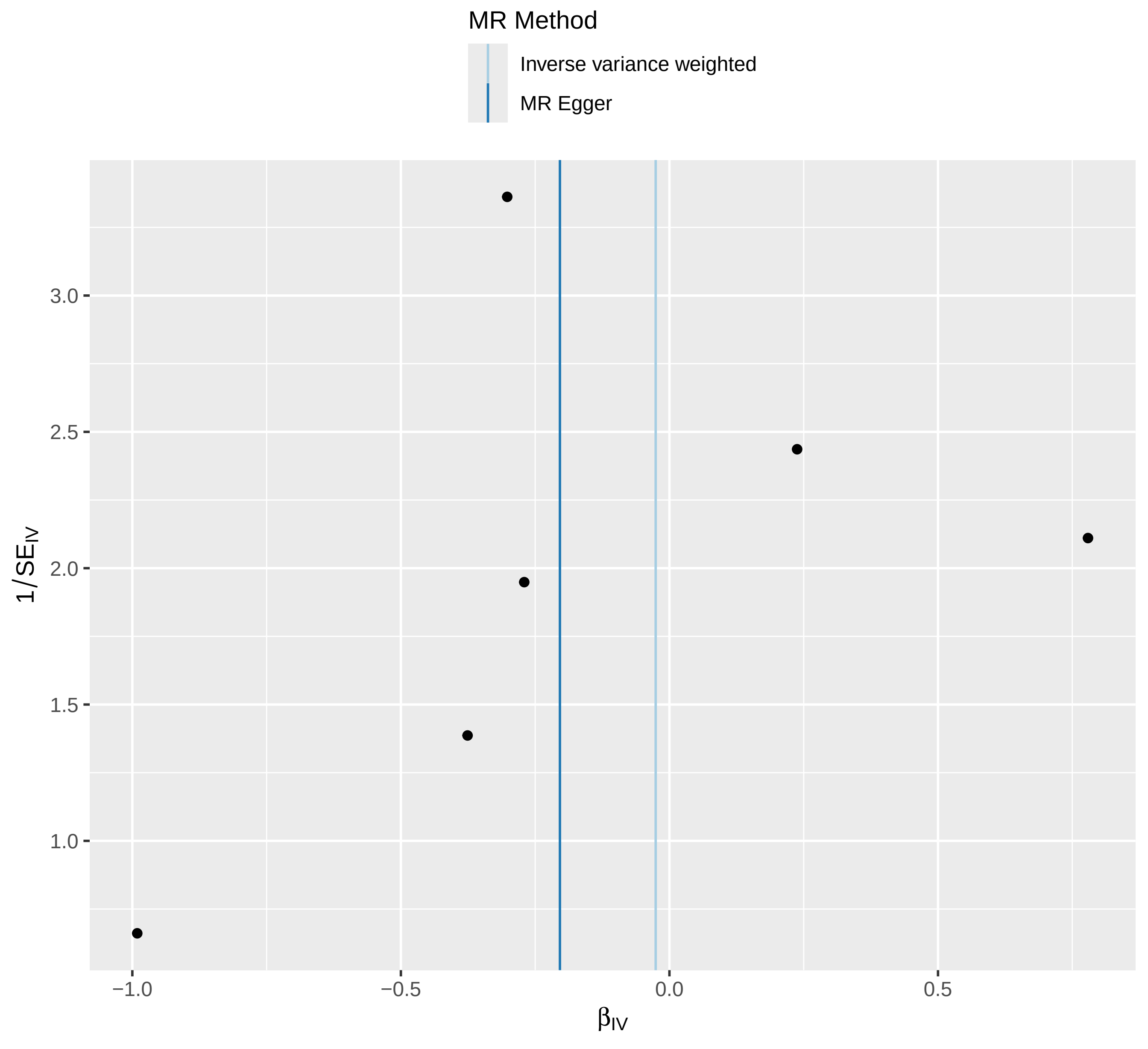
MR Method
Inverse variance weighted
MR Egger
3.0
2.5
2.0
1.5
1.0
−1.0
−0.5
0.0
0.5
$1/SE_{IV}$
β_{IV}

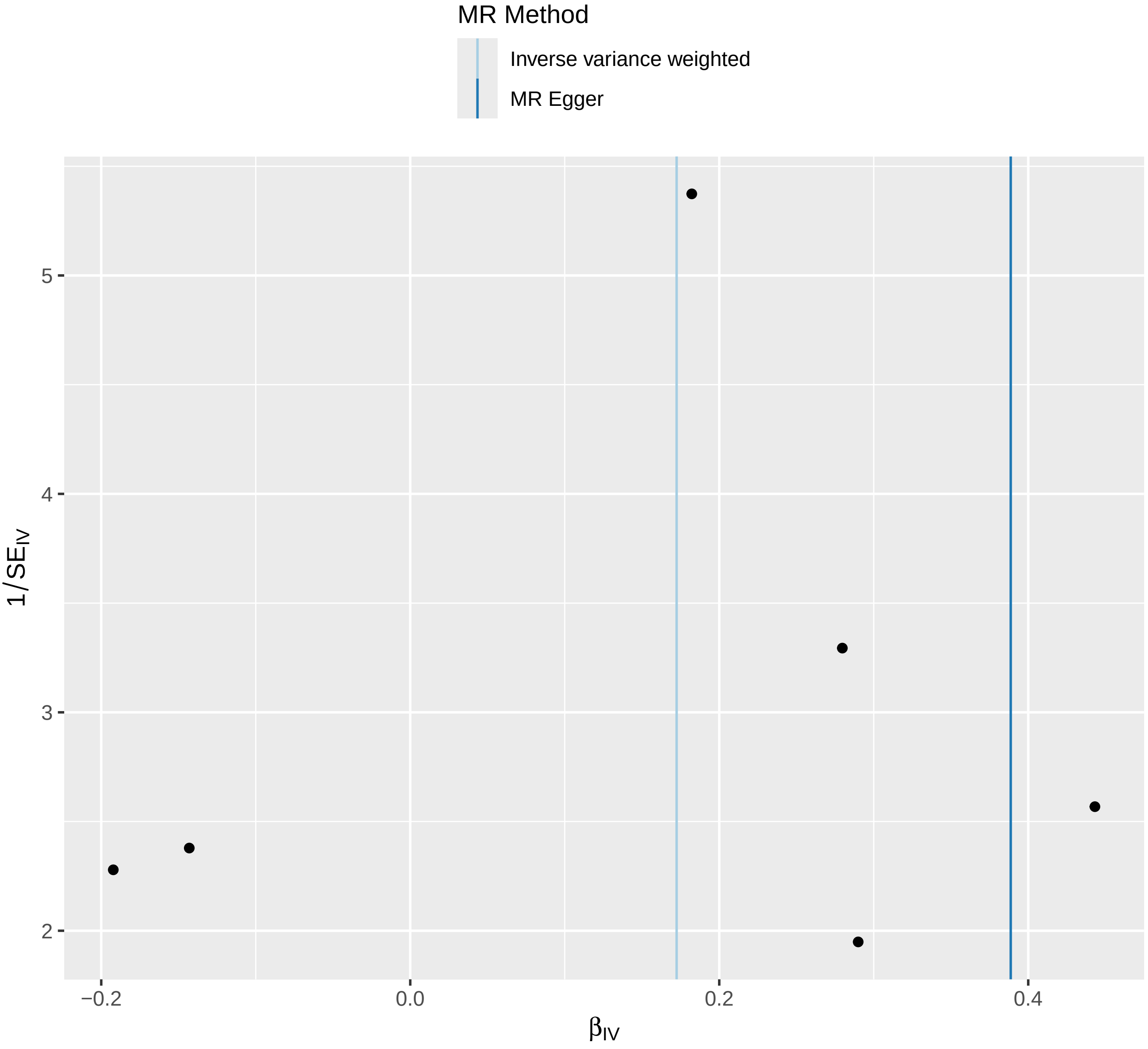

MR Method
Inverse variance weighted
MR Egger
5
4
3
2
1/SE_IV
−0.2
0.0
0.2
0.4
β_IV

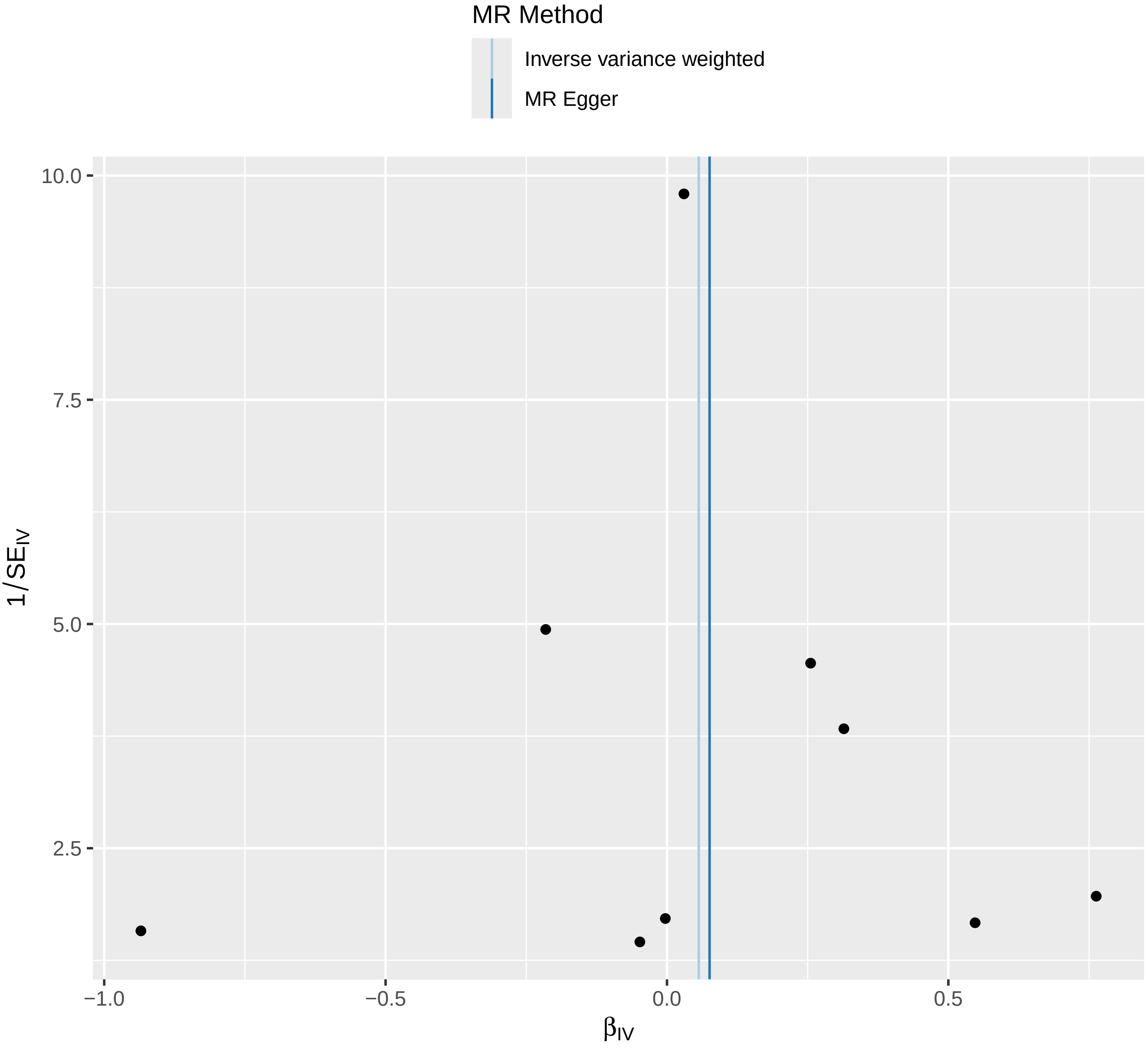
MR Method
Inverse variance weighted
MR Egger
10.0
7.5
5.0
2.5
$1/SE_{IV}$
−1.0
−0.5
0.0
0.5
β_{IV}

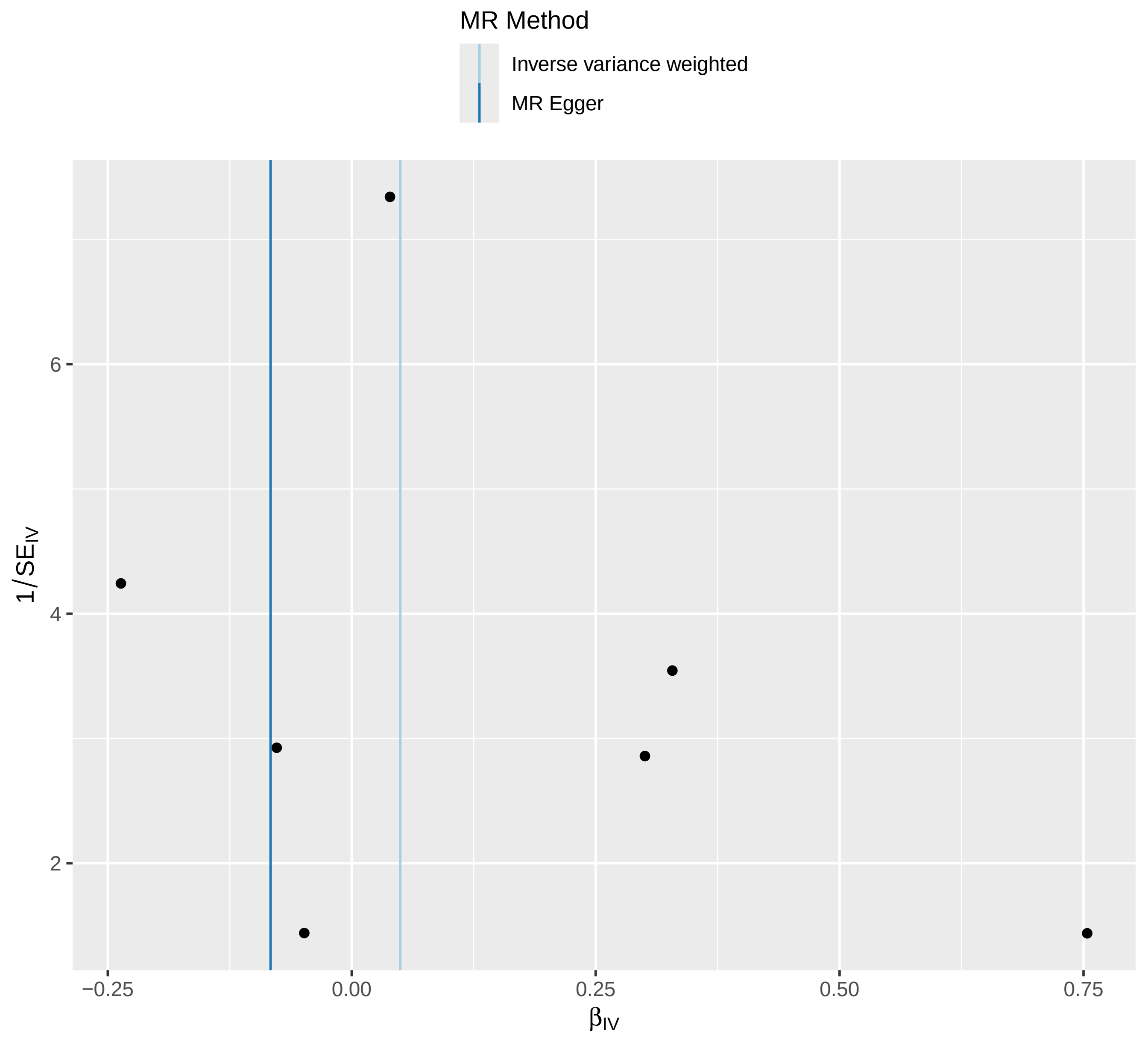

MR Method
Inverse variance weighted
MR Egger
1/SE_IV
β_IV
6
4
2
−0.25
0.00
0.25
0.50
0.75

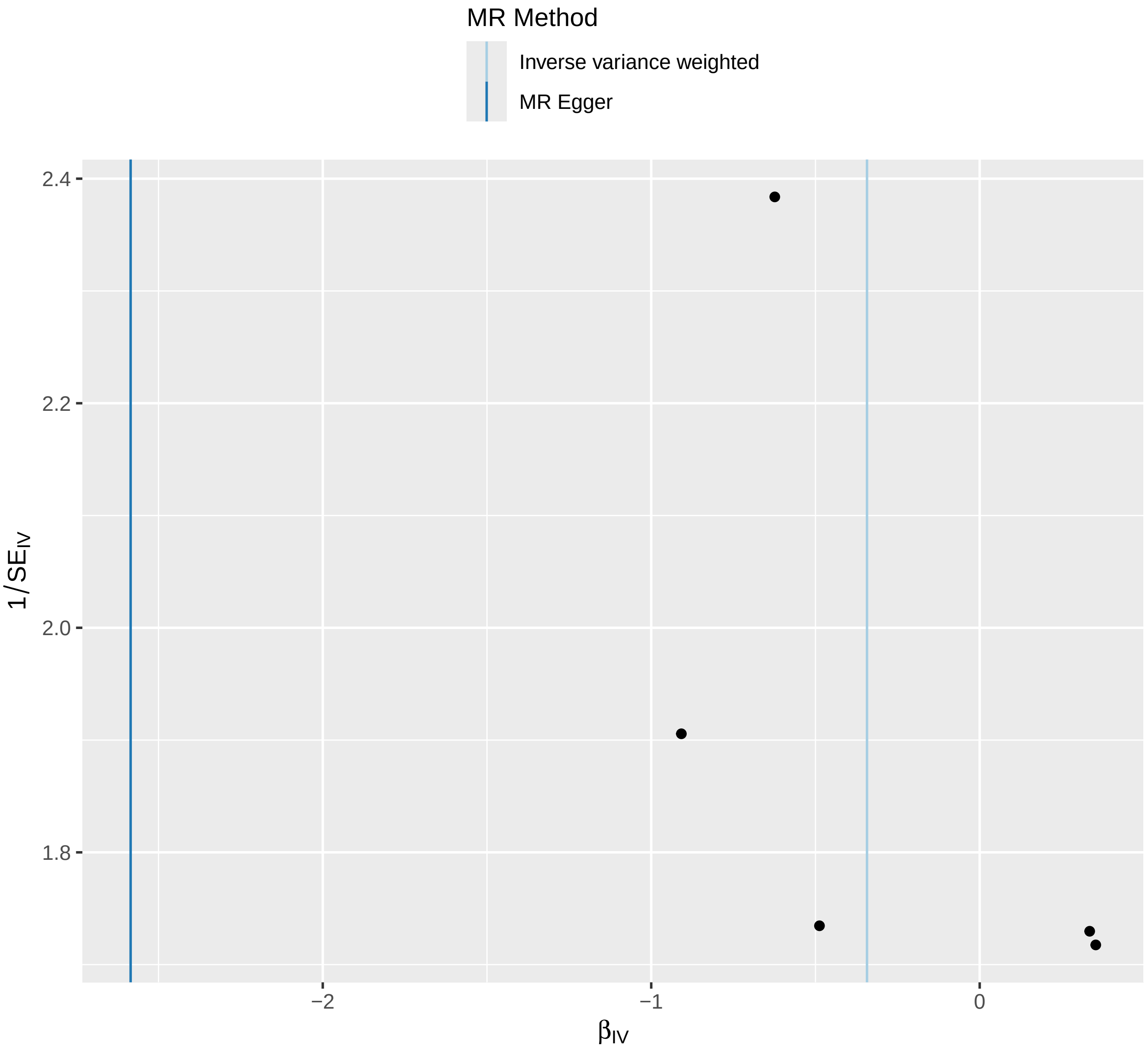
MR Method
Inverse variance weighted
MR Egger
2.4
2.2
2.0
1.8
1/SE$_{IV}$
−2
−1
0
β$_{IV}$

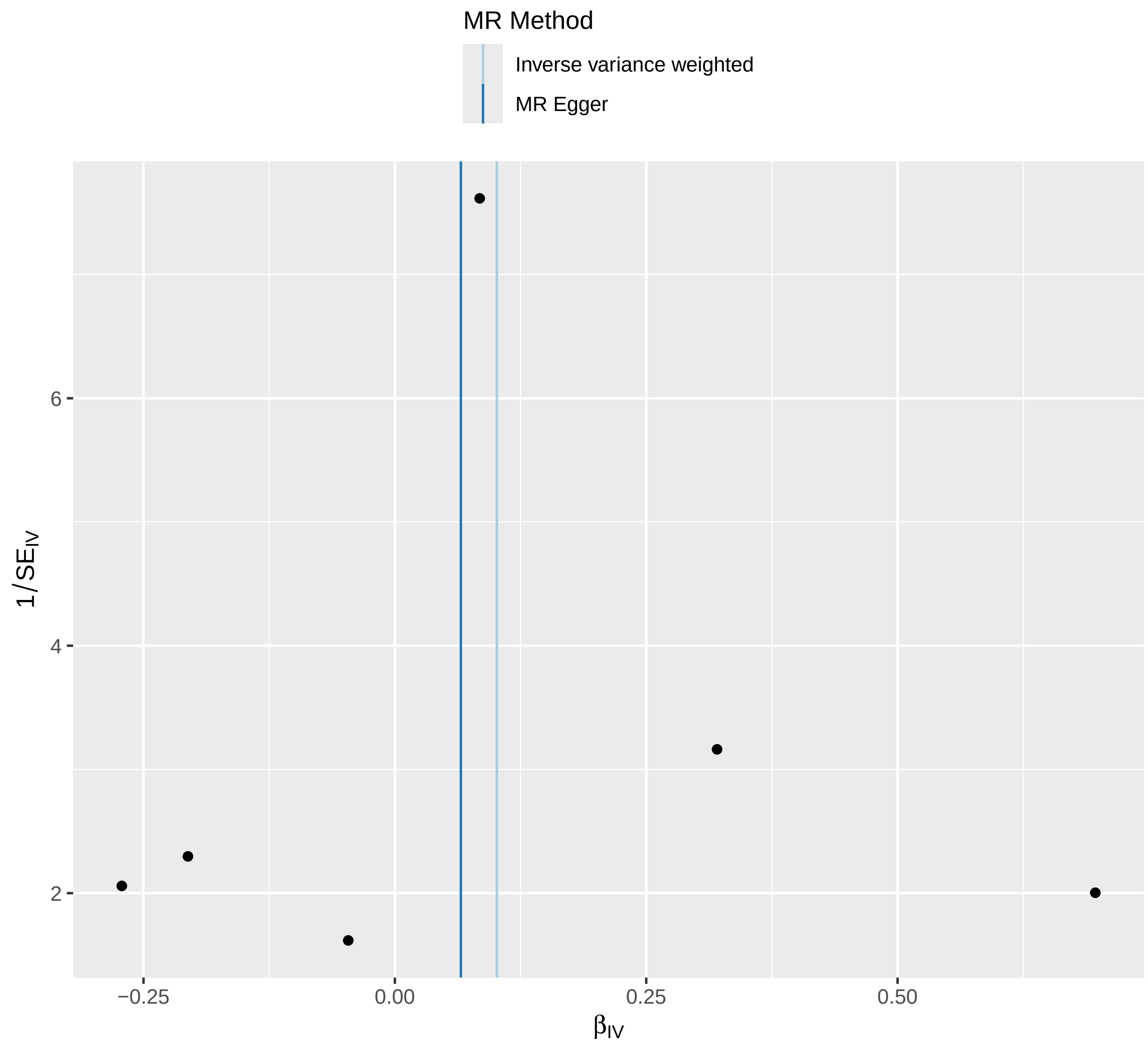
MR Method
Inverse variance weighted
MR Egger
1/SE_IV
6
4
2
−0.25
0.00
0.25
0.50
β_IV

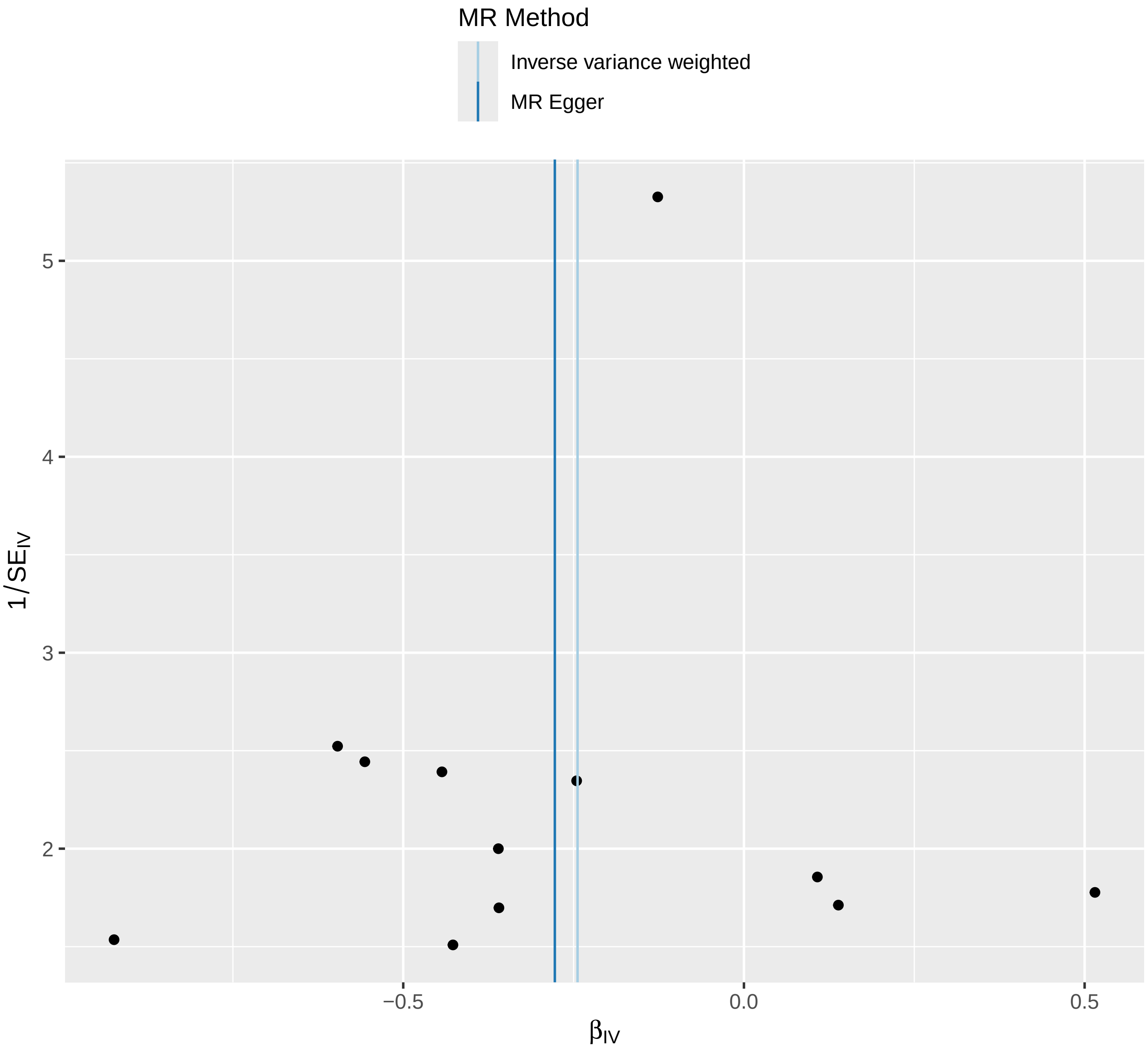
MR Method
Inverse variance weighted
MR Egger
5
4
3
2
1/SE_IV
−0.5
0.0
0.5
β_IV

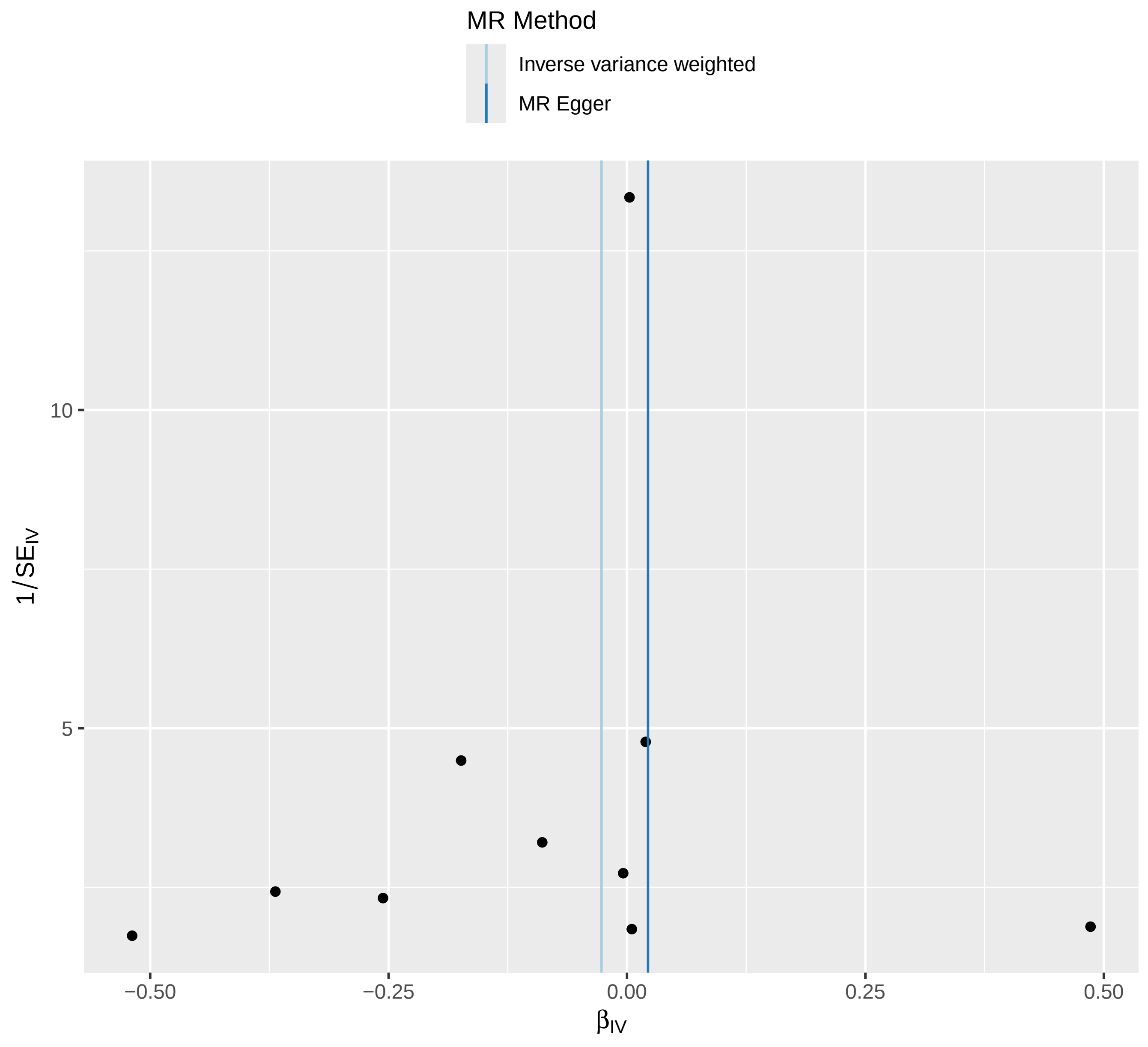
MR Method
Inverse variance weighted
MR Egger
10
5
$1/SE_{IV}$
−0.50
−0.25
0.00
0.25
0.50
β_{IV}

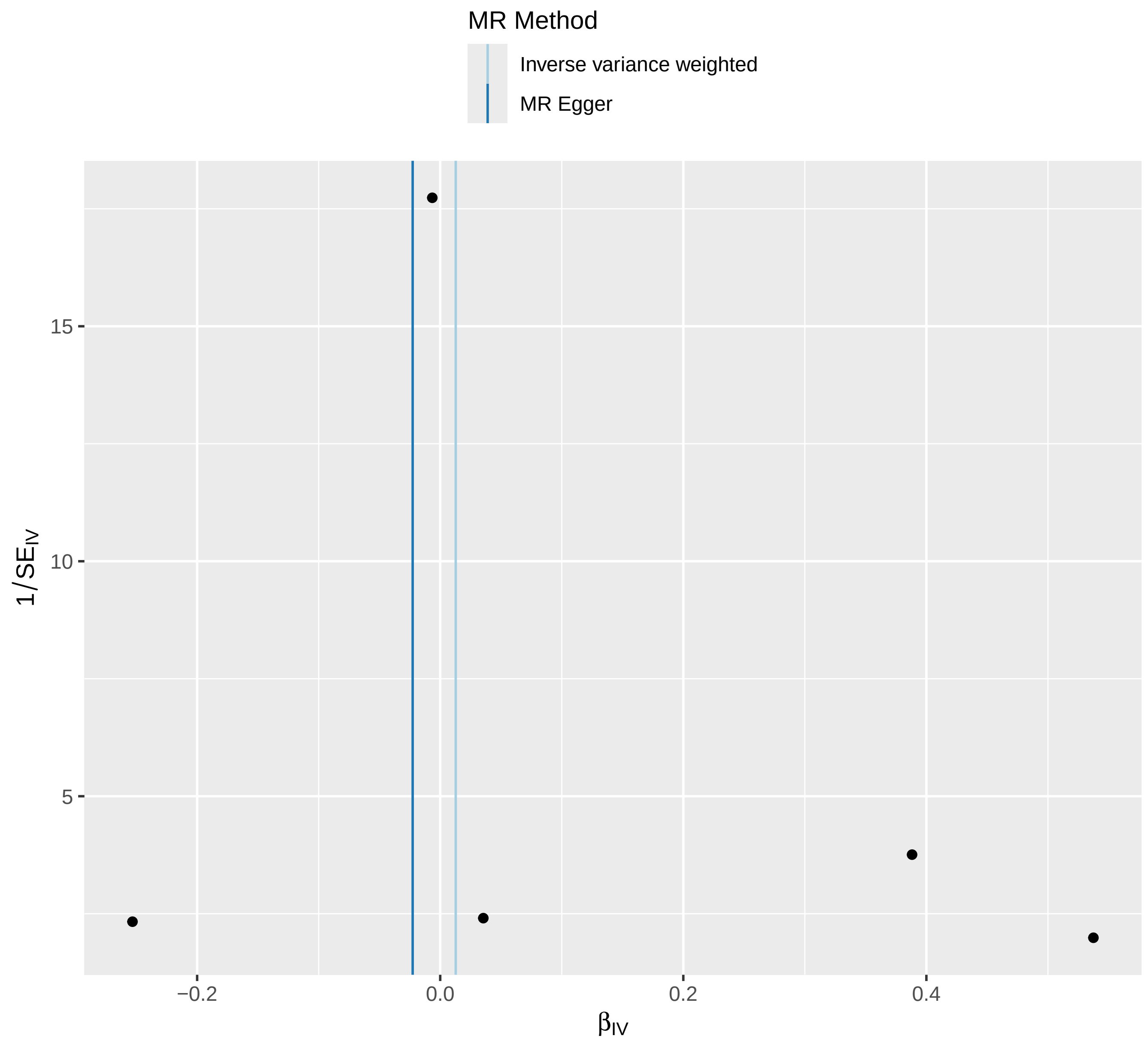

MR Method
Inverse variance weighted
MR Egger
15
10
5
−0.2
0.0
0.2
0.4
β_{IV}
$1/SE_{IV}$

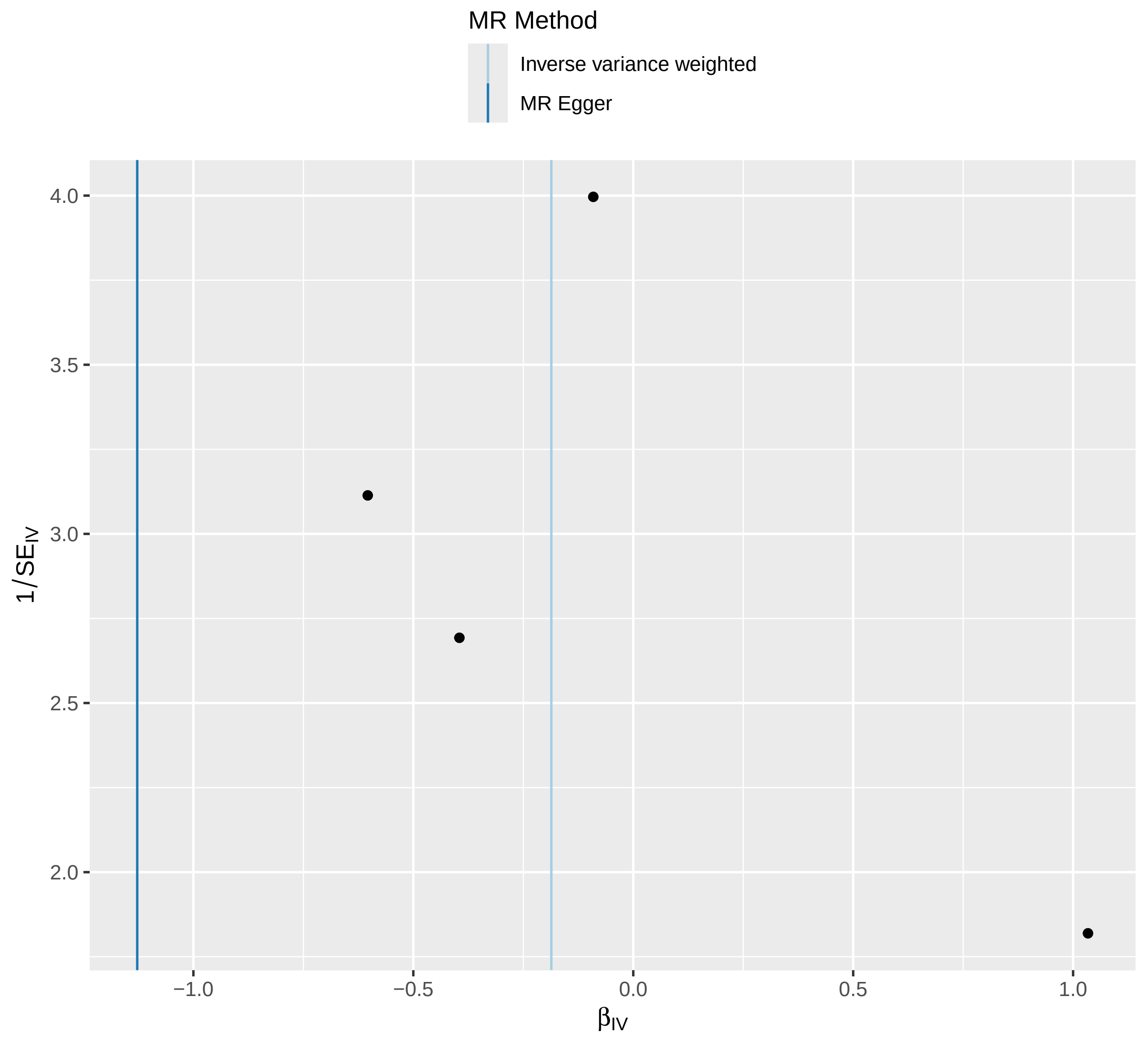

MR Method
Inverse variance weighted
MR Egger
4.0
3.5
3.0
2.5
2.0
1/SE_IV
−1.0
−0.5
0.0
0.5
1.0
β_IV

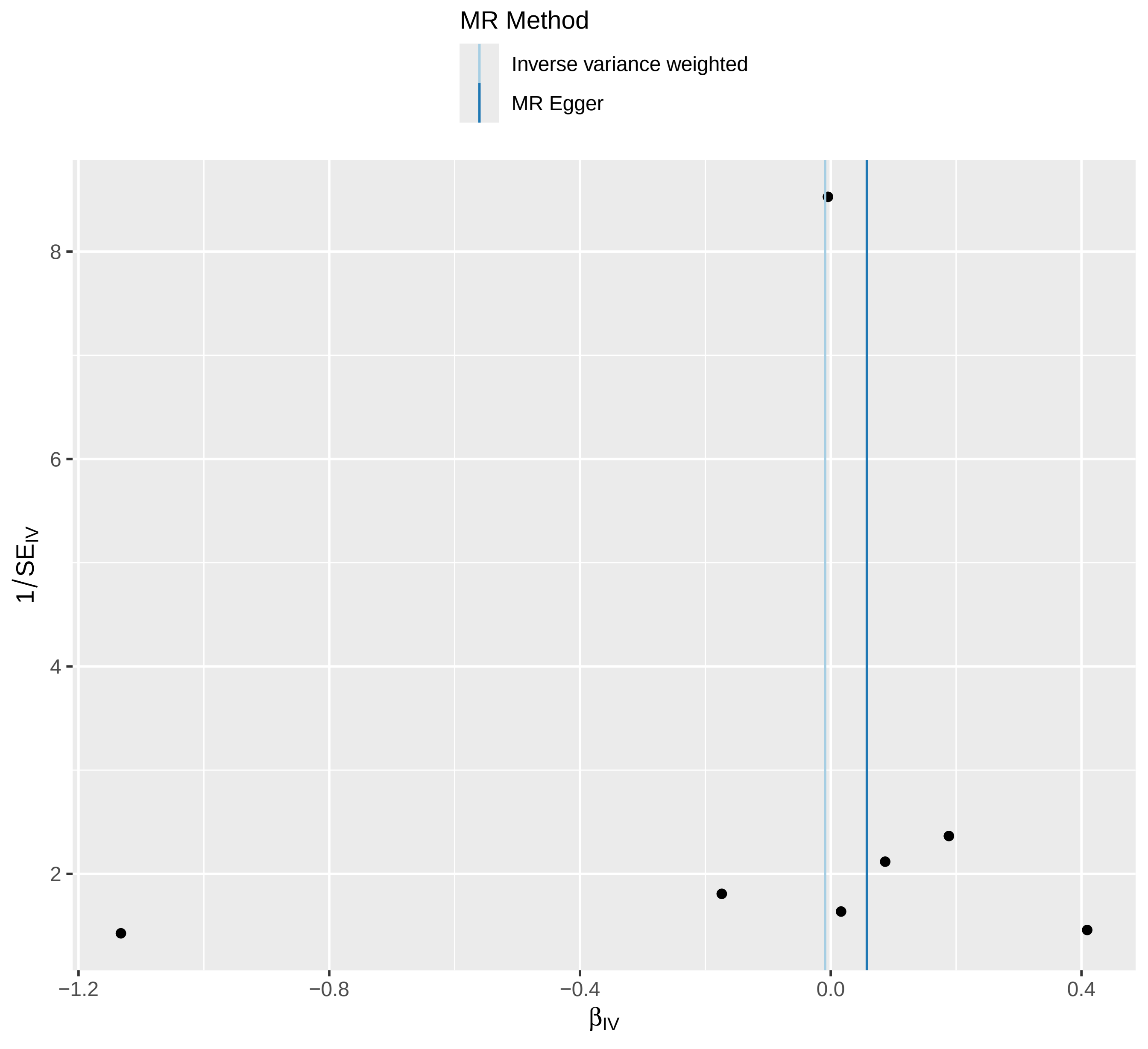
MR Method
Inverse variance weighted
MR Egger
1/SE_IV
8
6
4
2
−1.2
−0.8
−0.4
0.0
0.4
β_IV

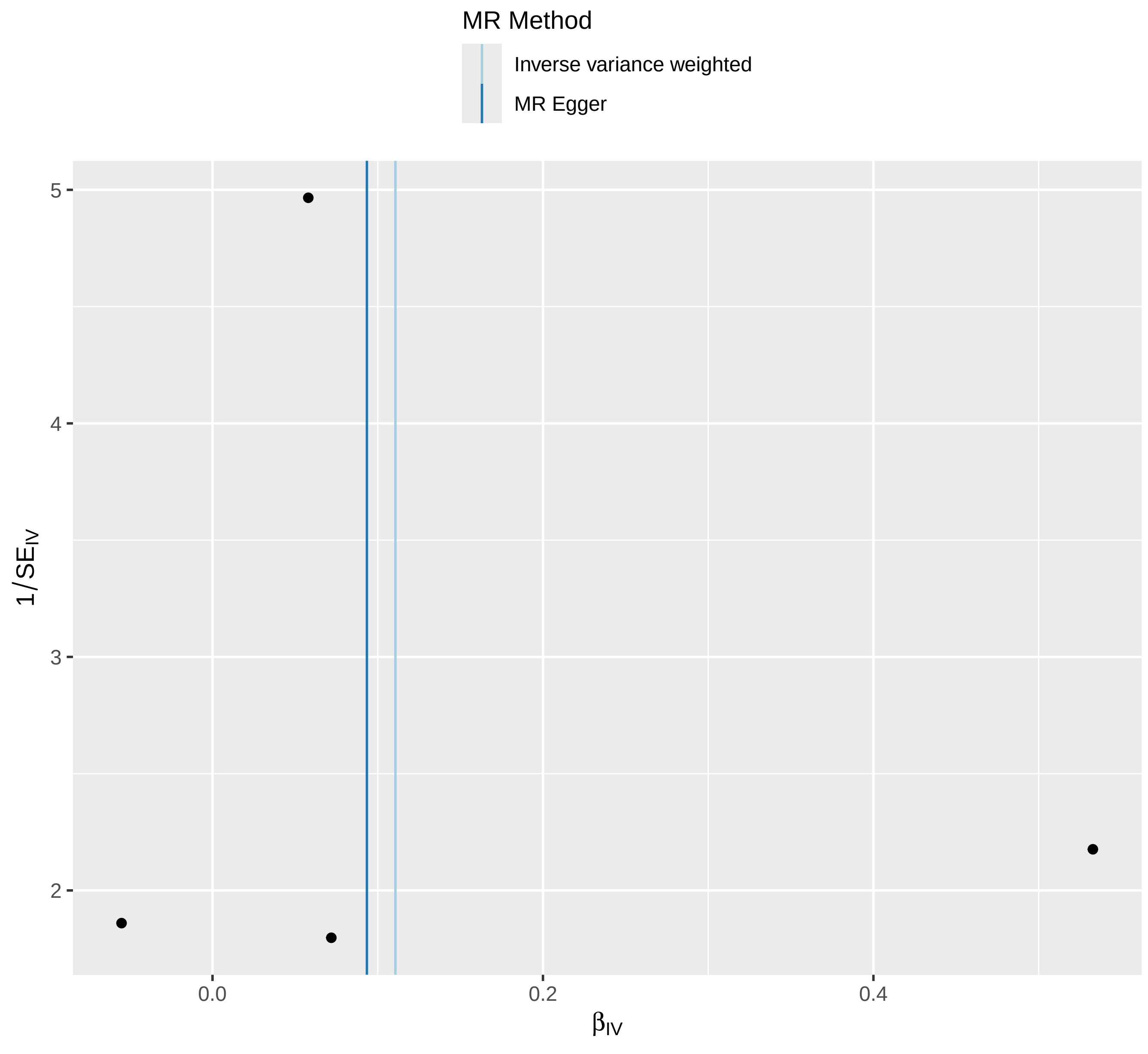

MR Method
Inverse variance weighted
MR Egger
5
4
3
2
0.0
0.2
0.4
$1/SE_{IV}$
β_{IV}

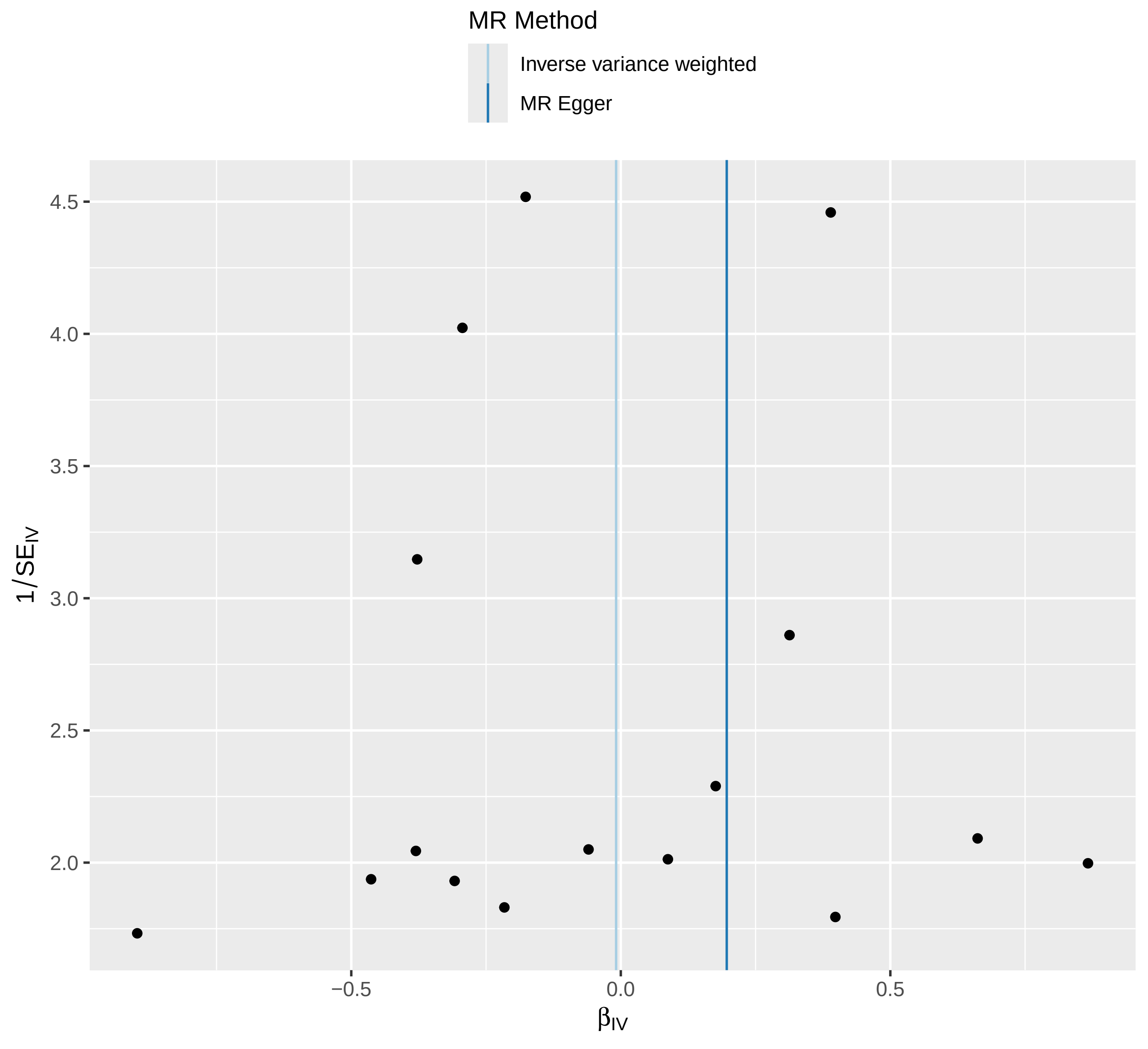

MR Method
Inverse variance weighted
MR Egger
4.5
4.0
3.5
3.0
2.5
2.0
1/SE_IV
−0.5
0.0
0.5
β_IV

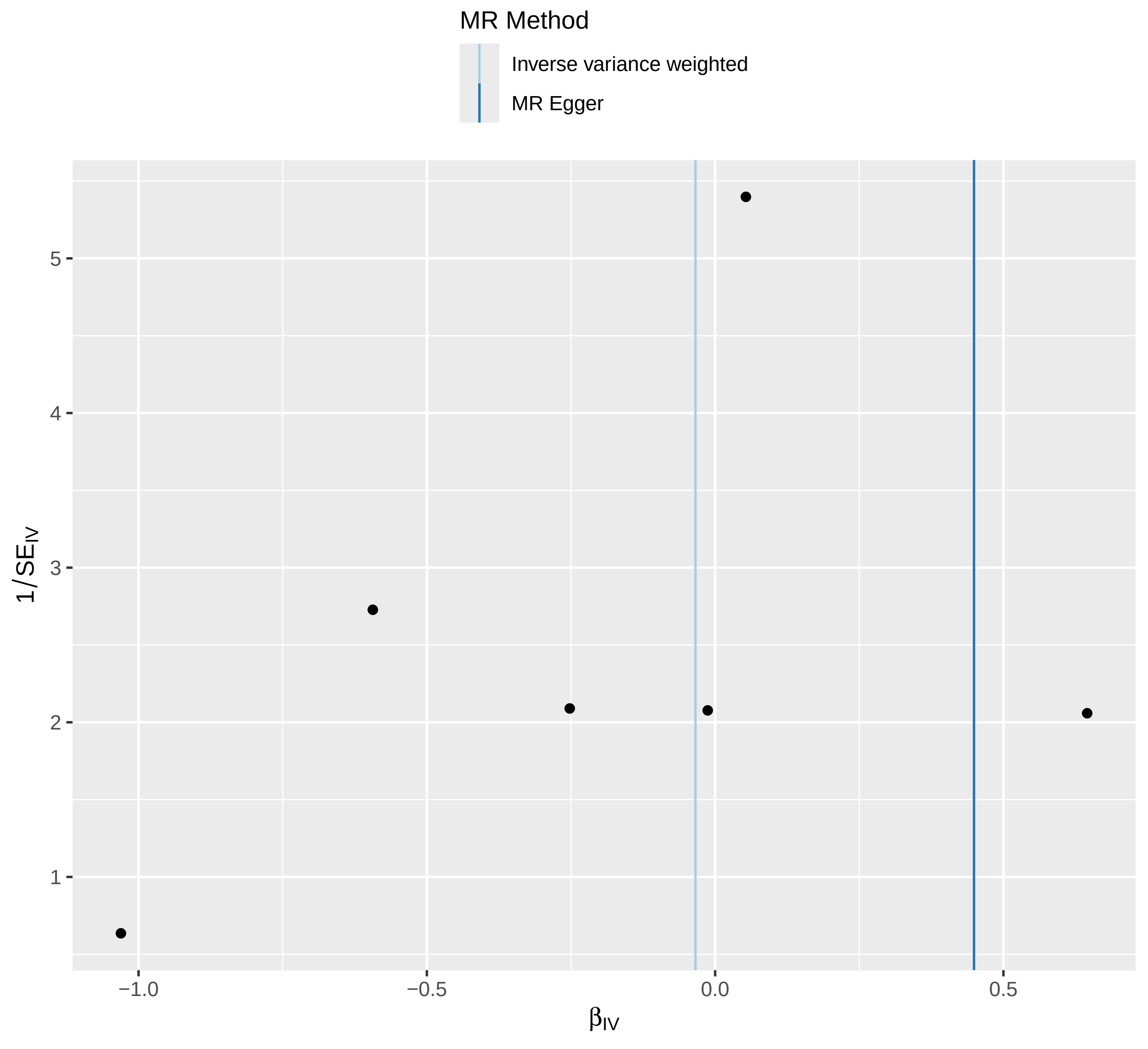

MR Method
Inverse variance weighted
MR Egger
1/SE_IV
β_IV
5
4
3
2
1
−1.0
−0.5
0.0
0.5

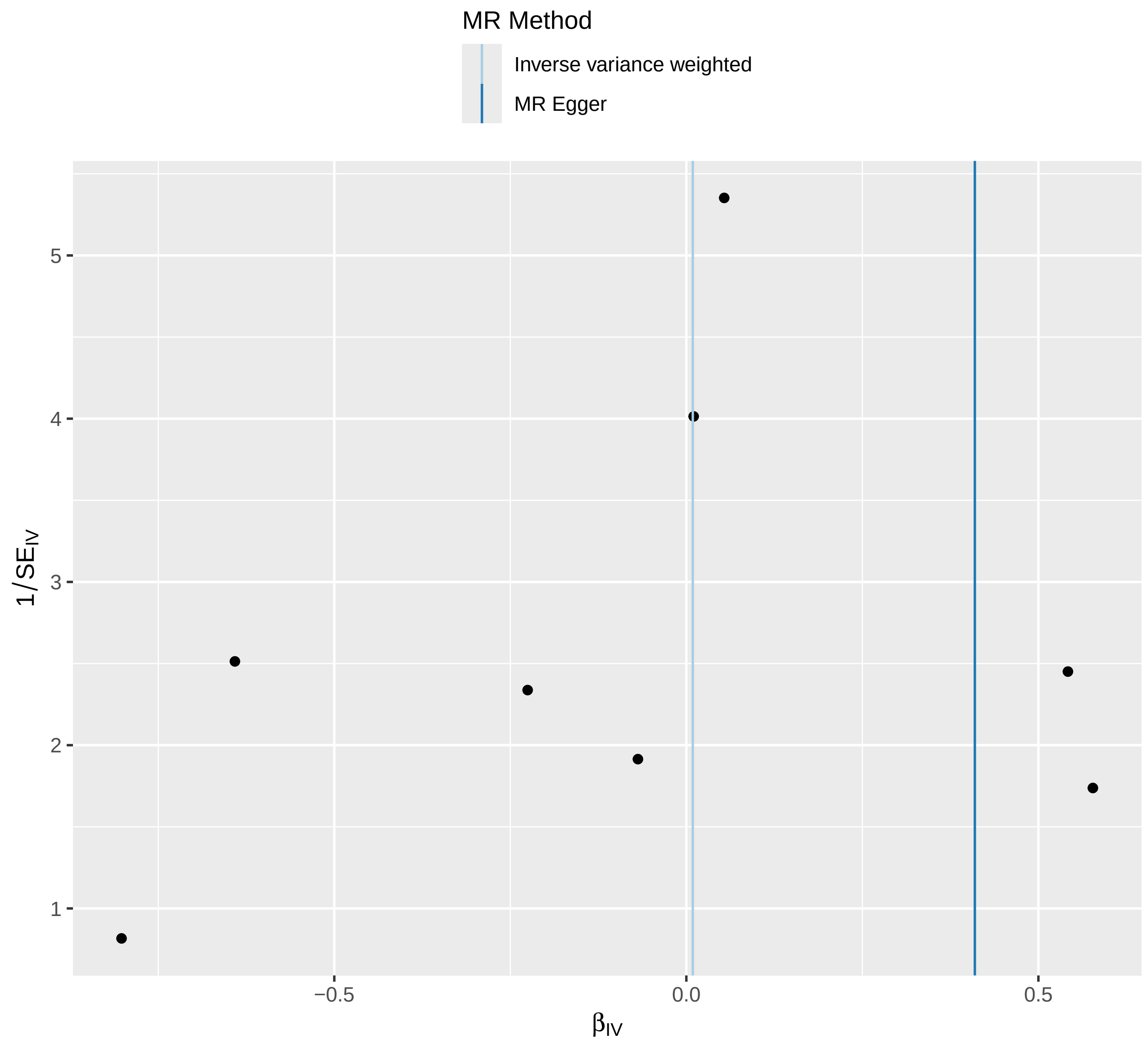

MR Method
Inverse variance weighted
MR Egger
1/SE_IV
5
4
3
2
1
−0.5
0.0
0.5
β_IV

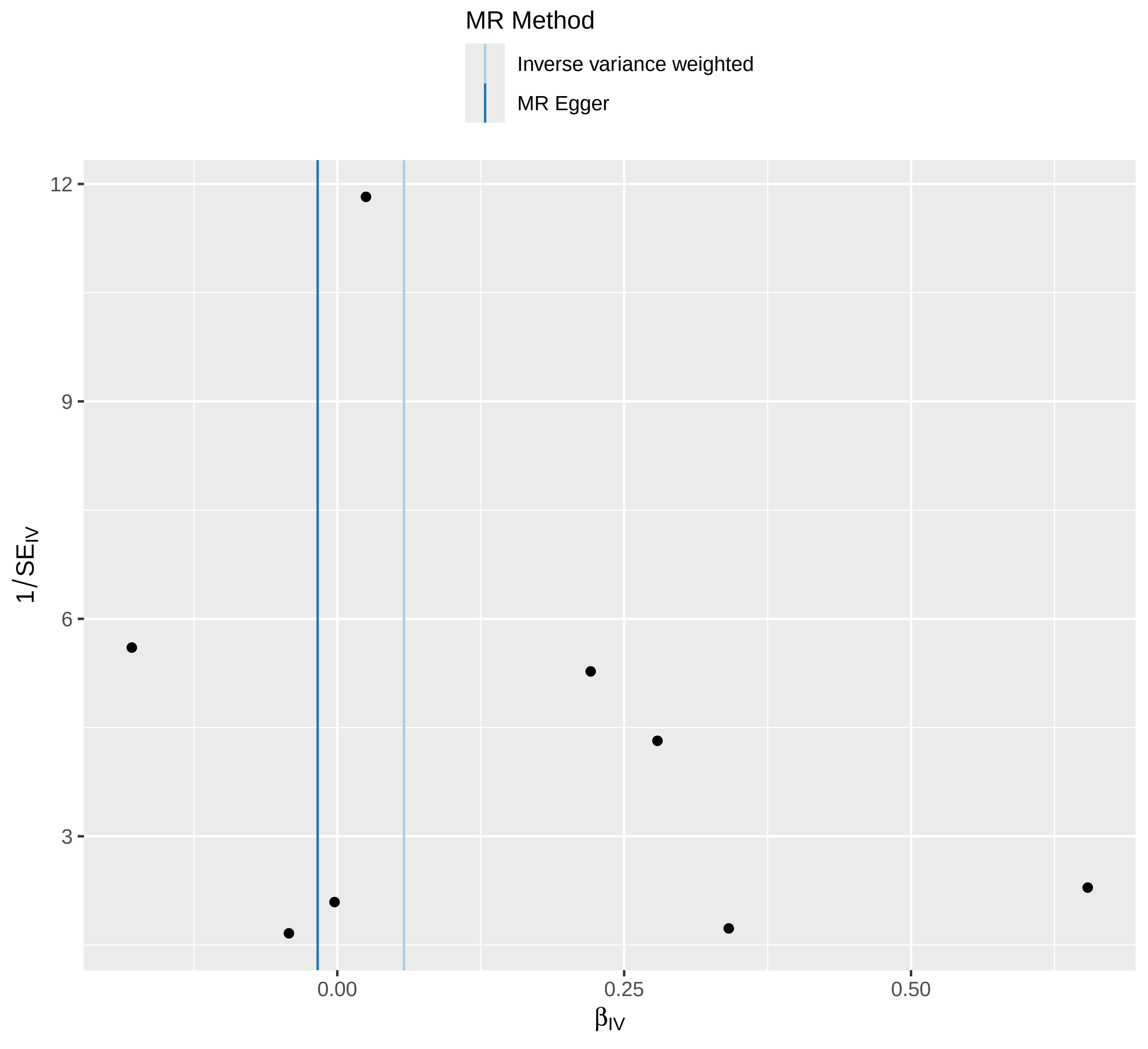
MR Method
Inverse variance weighted
MR Egger
12
9
6
3
1/SE_IV
0.00
0.25
0.50
β_IV

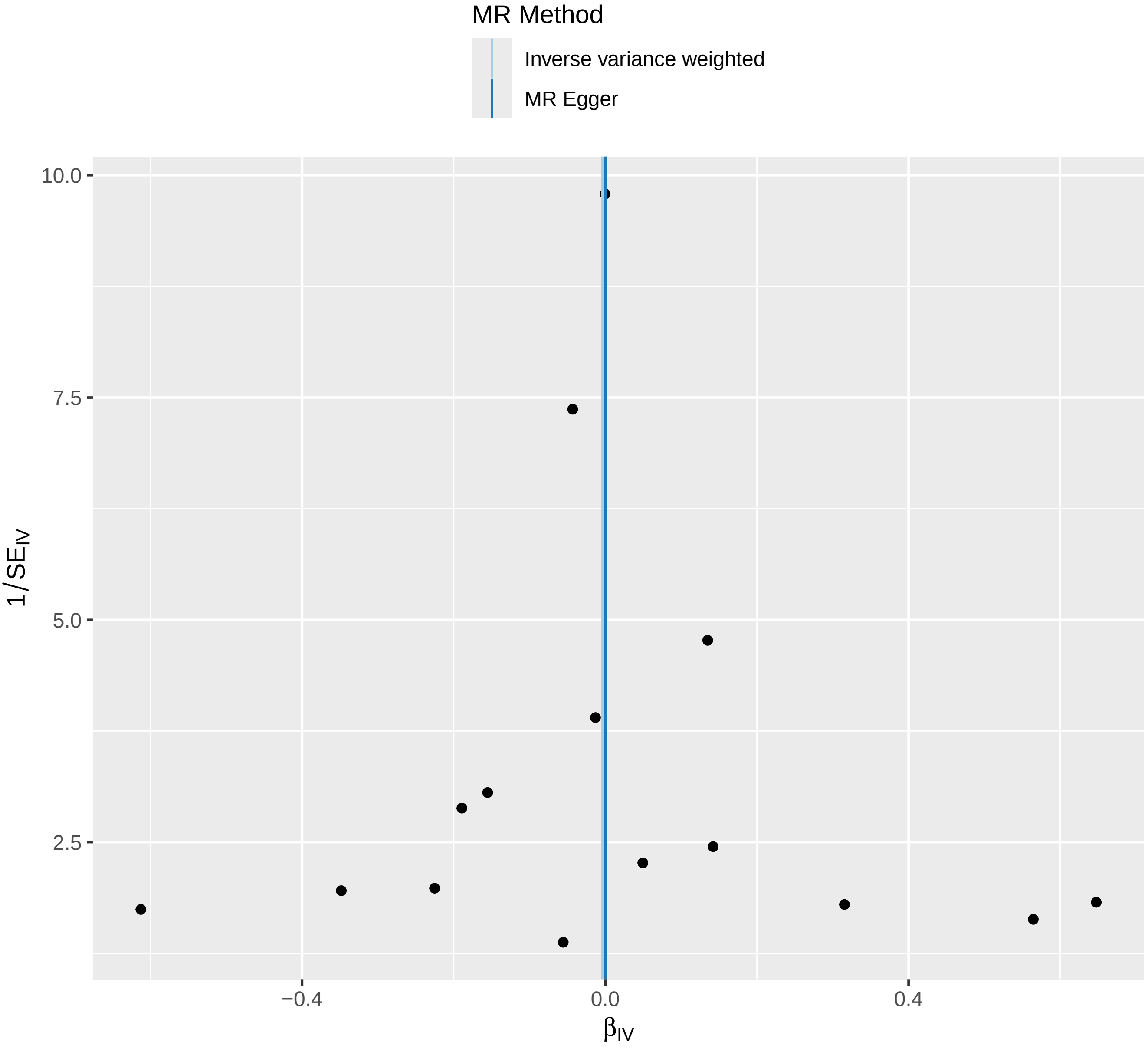
MR Method
Inverse variance weighted
MR Egger
10.0
7.5
5.0
2.5
$1/SE_{IV}$
−0.4
0.0
0.4
β_{IV}

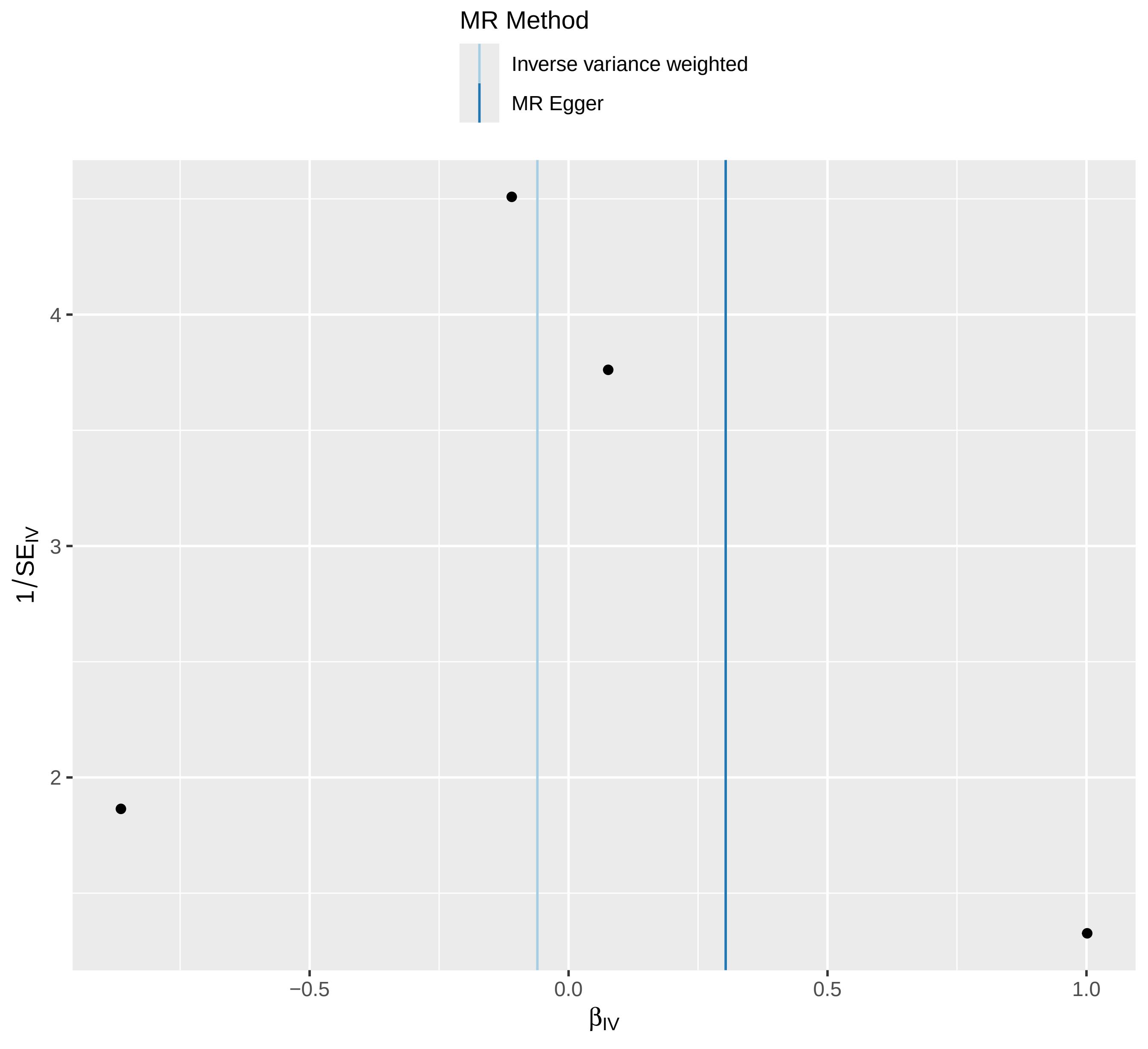
MR Method
Inverse variance weighted
MR Egger
1/SE_IV
4
3
2
−0.5
0.0
0.5
1.0
β_IV

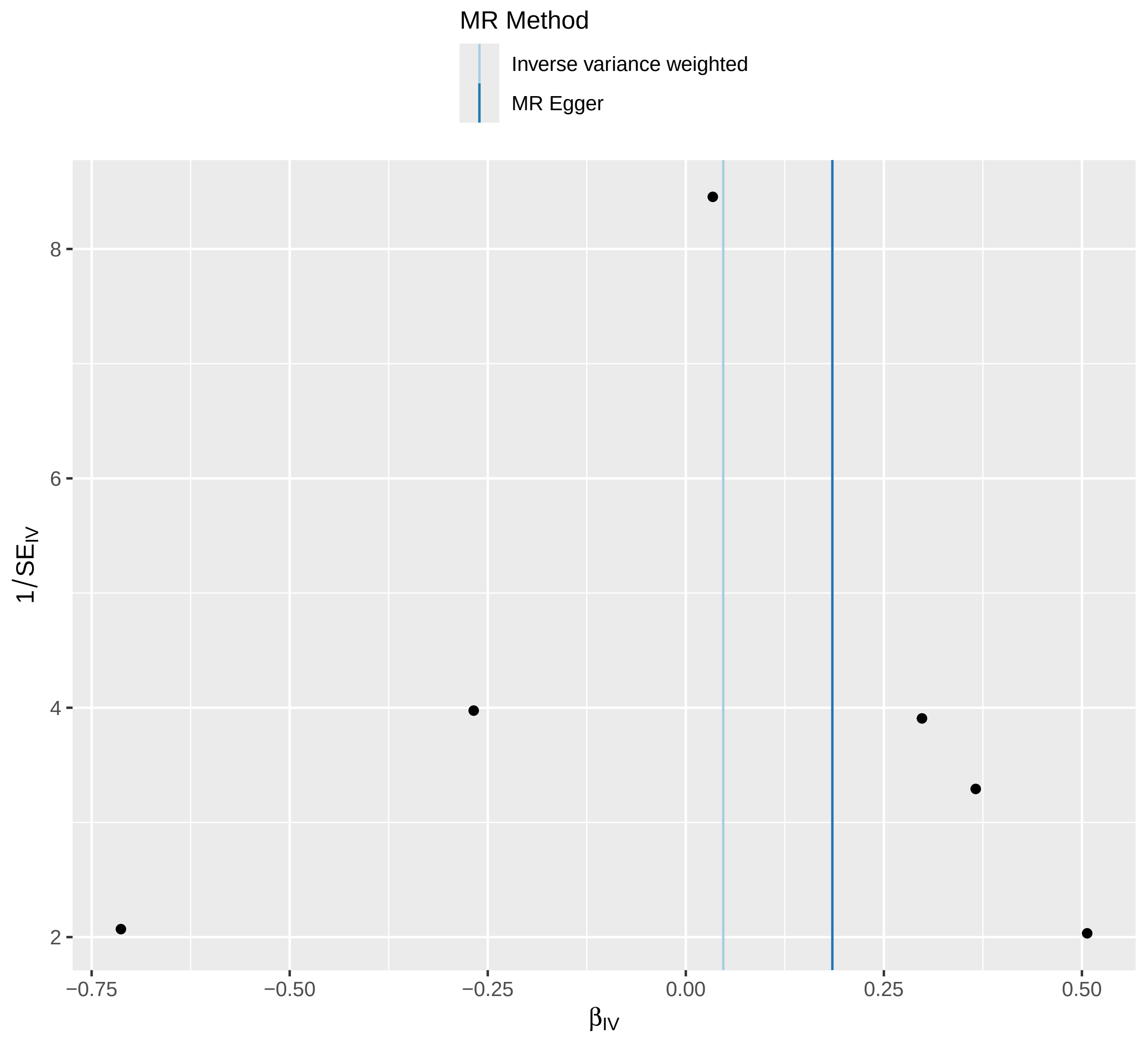
MR Method
Inverse variance weighted
MR Egger
1/SE$_{IV}$
8
6
4
2
−0.75
−0.50
−0.25
0.00
0.25
0.50
β$_{IV}$

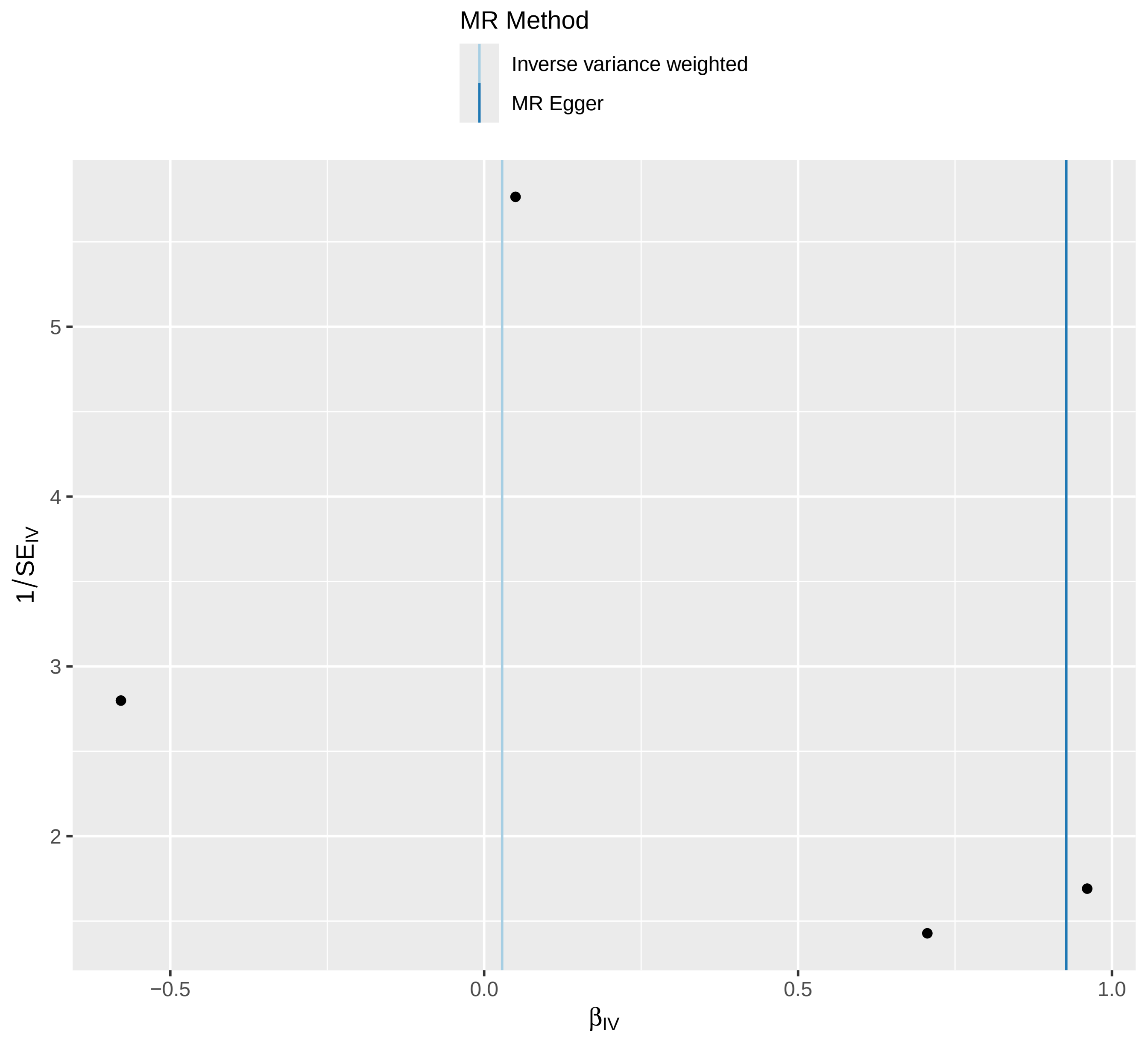
MR Method
Inverse variance weighted
MR Egger
1/SE$_{IV}$
2
3
4
5
−0.5
0.0
0.5
1.0
β$_{IV}$

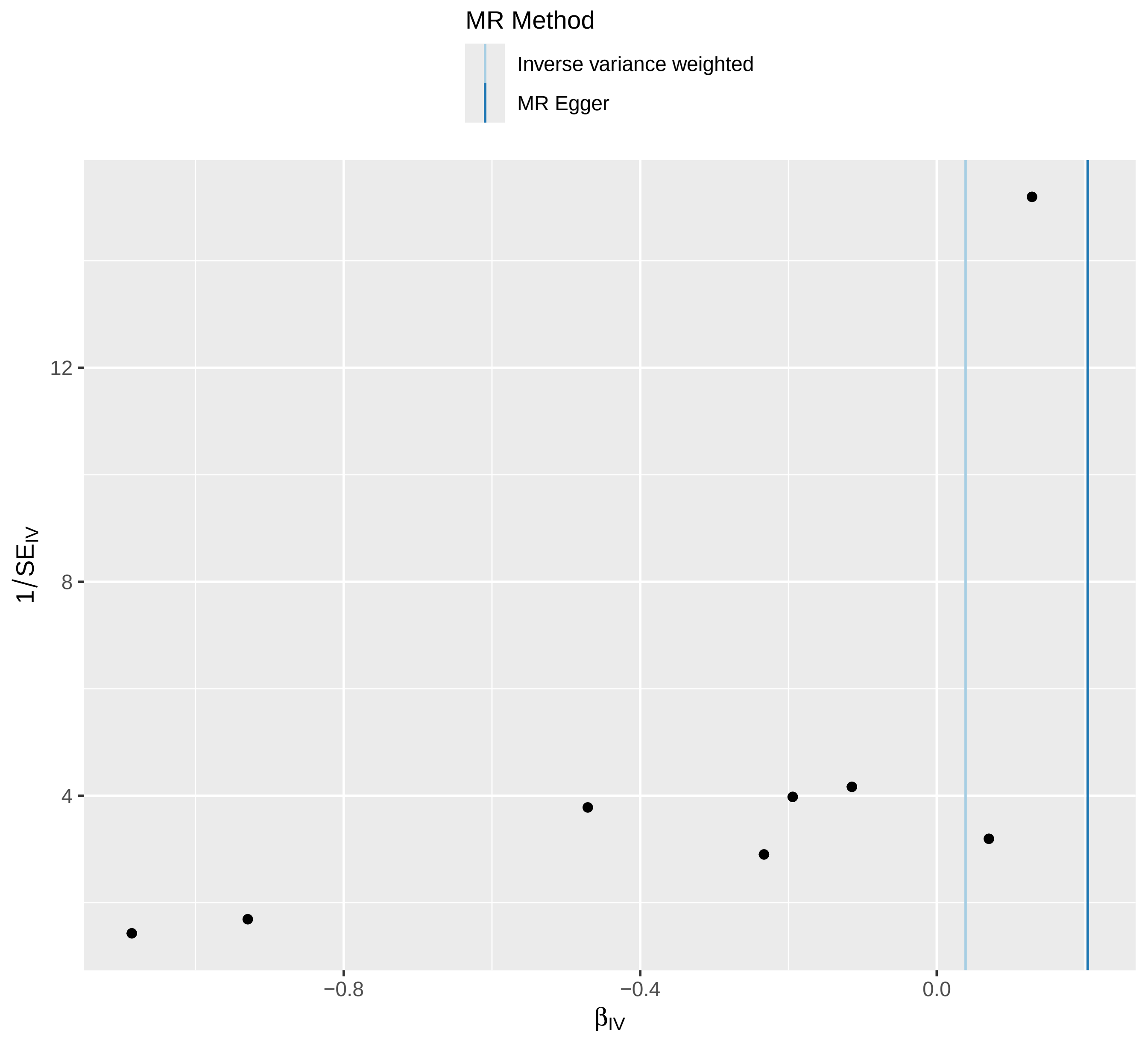
MR Method
Inverse variance weighted
MR Egger
12
8
4
−0.8
−0.4
0.0
1/SE_IV
β_IV

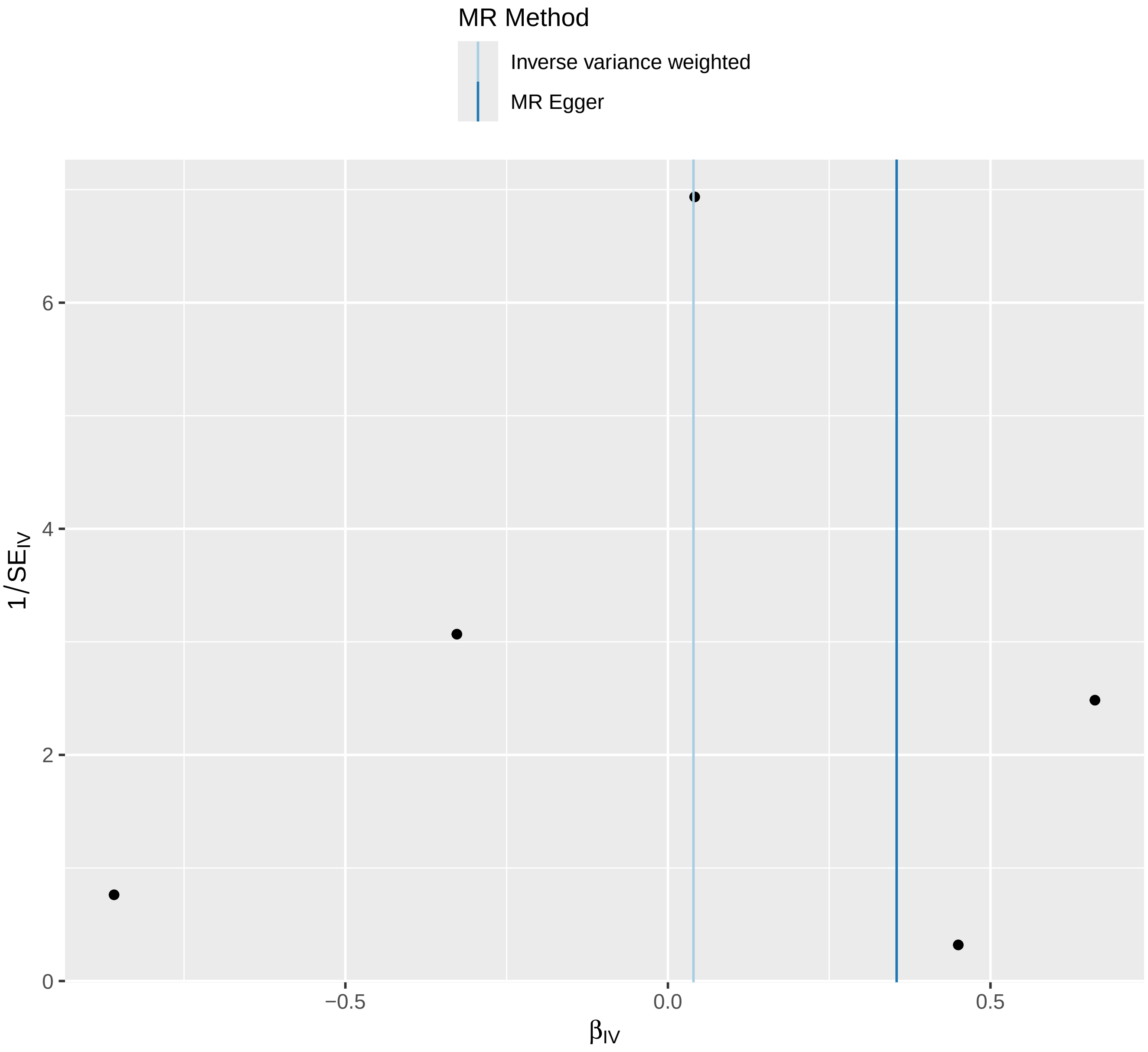

MR Method
Inverse variance weighted
MR Egger
6
4
2
0
1/SE$_{IV}$
−0.5
0.0
0.5
β$_{IV}$

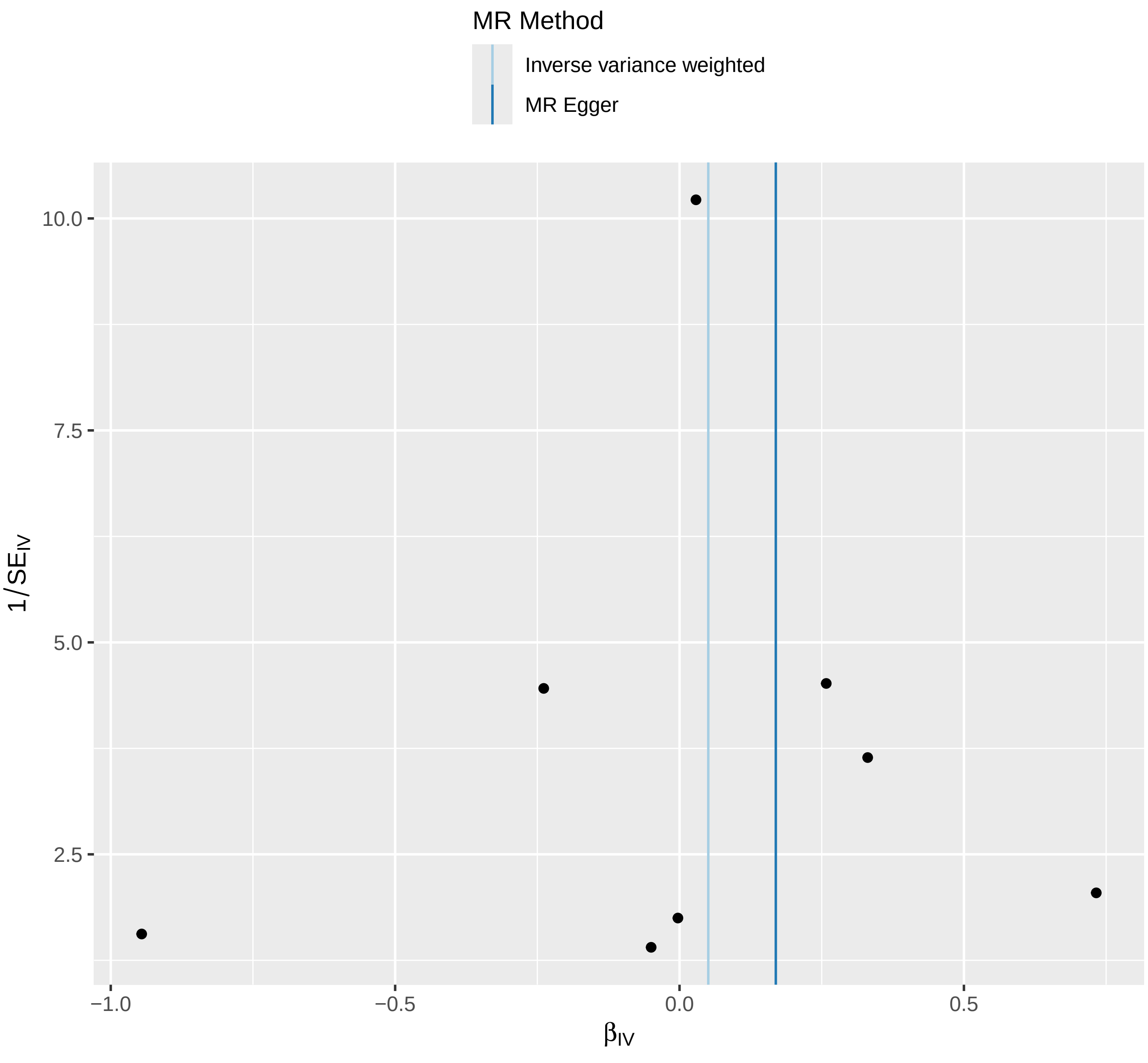
MR Method
Inverse variance weighted
MR Egger
10.0
7.5
5.0
2.5
1/SE$_{IV}$
−1.0
−0.5
0.0
0.5
β$_{IV}$

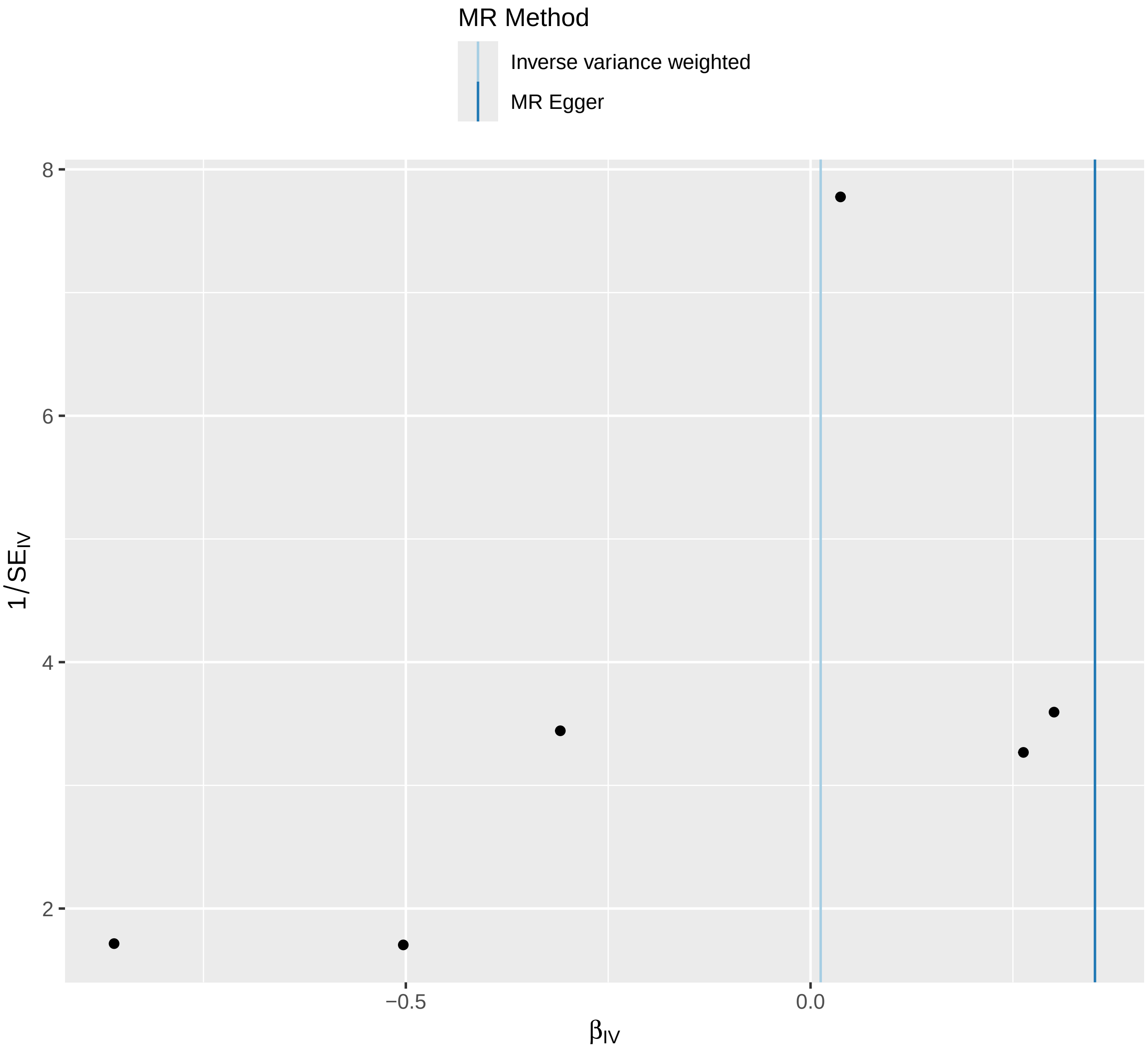
MR Method
Inverse variance weighted
MR Egger
8
6
4
2
1/SE_{IV}
−0.5
0.0
β_{IV}

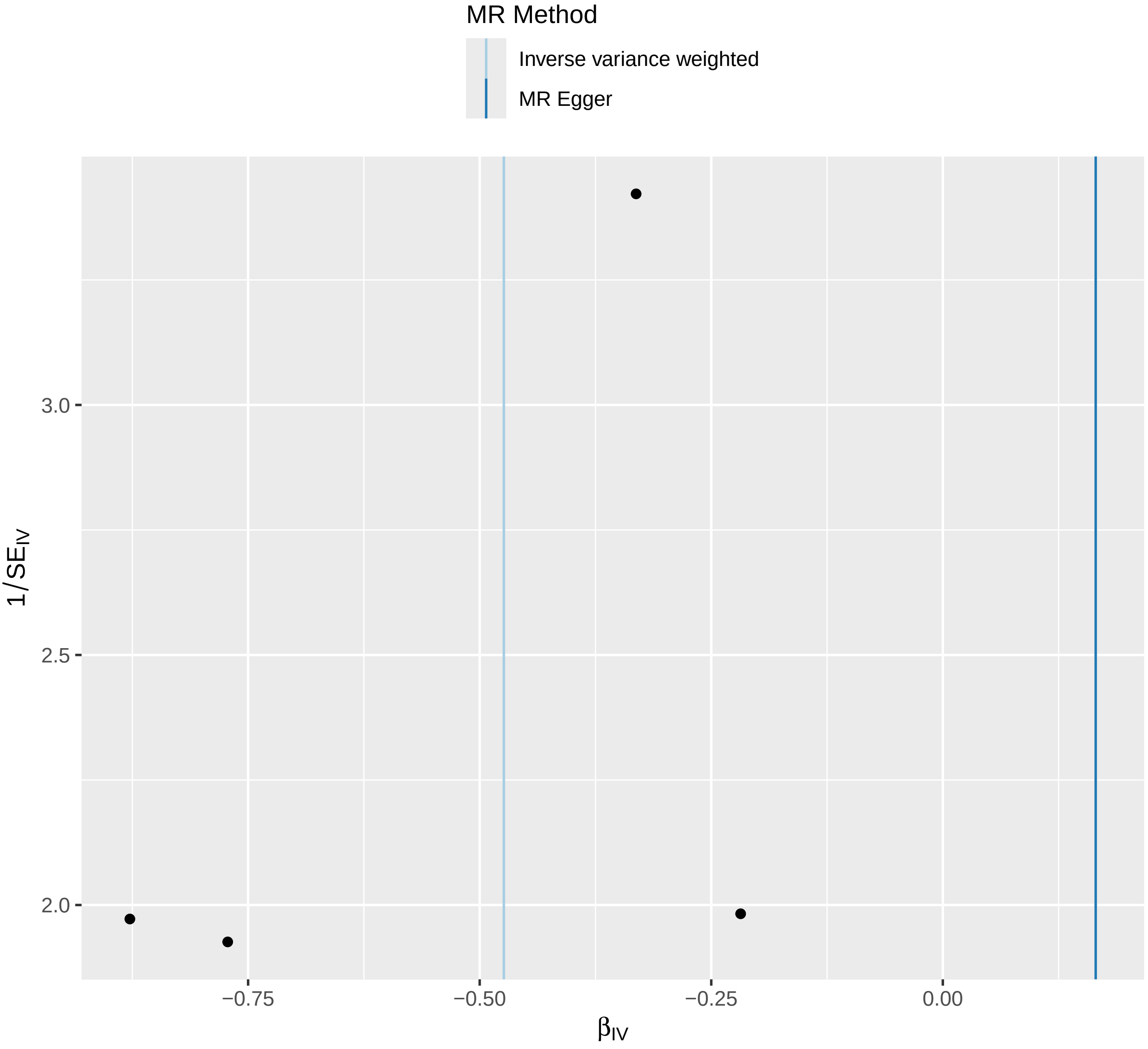

MR Method
Inverse variance weighted
MR Egger
3.0
2.5
2.0
1/SE_IV
−0.75
−0.50
−0.25
0.00
β_IV

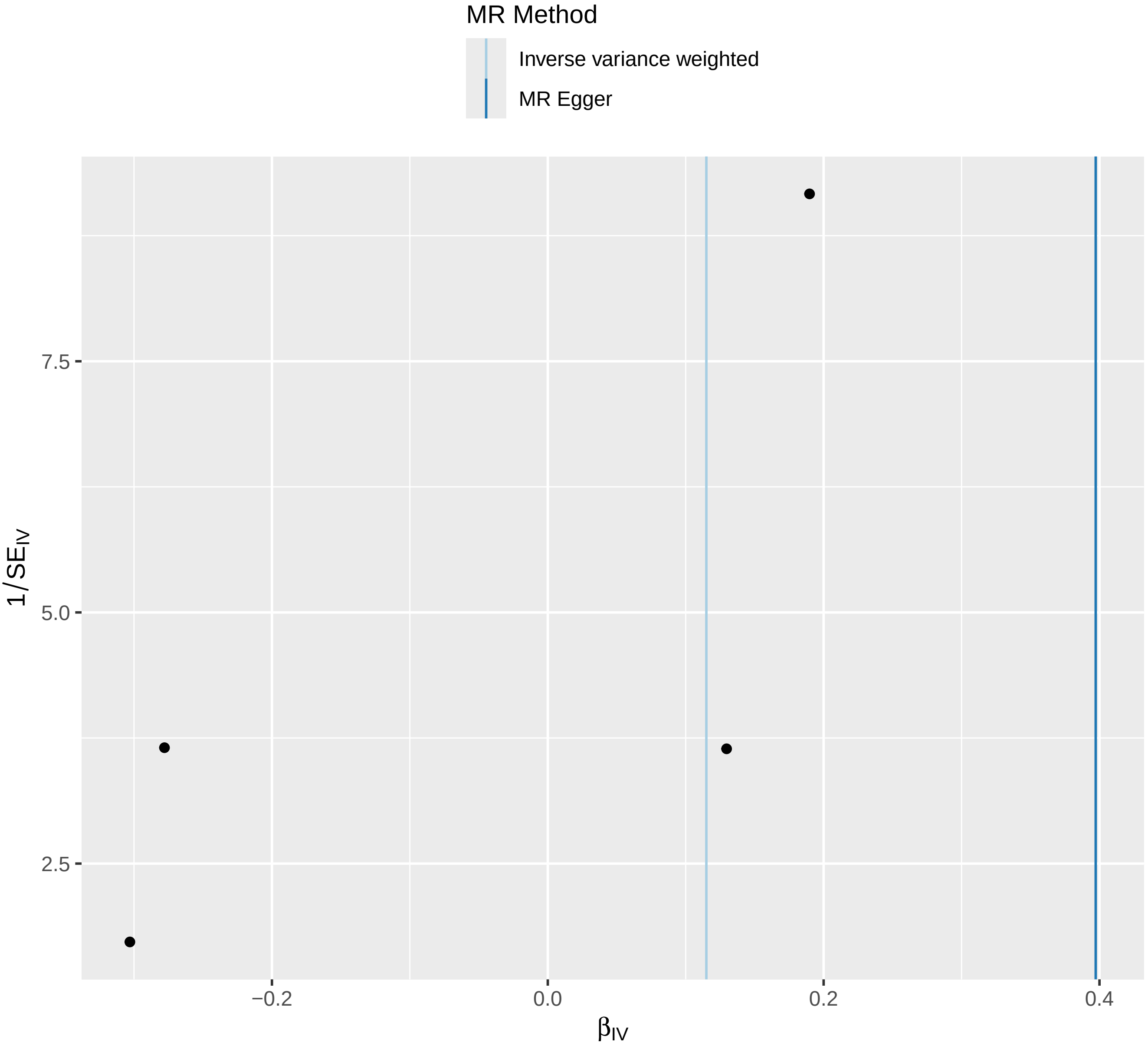

MR Method
Inverse variance weighted
MR Egger
7.5
5.0
2.5
$1/SE_{IV}$
−0.2
0.0
0.2
0.4
β_{IV}

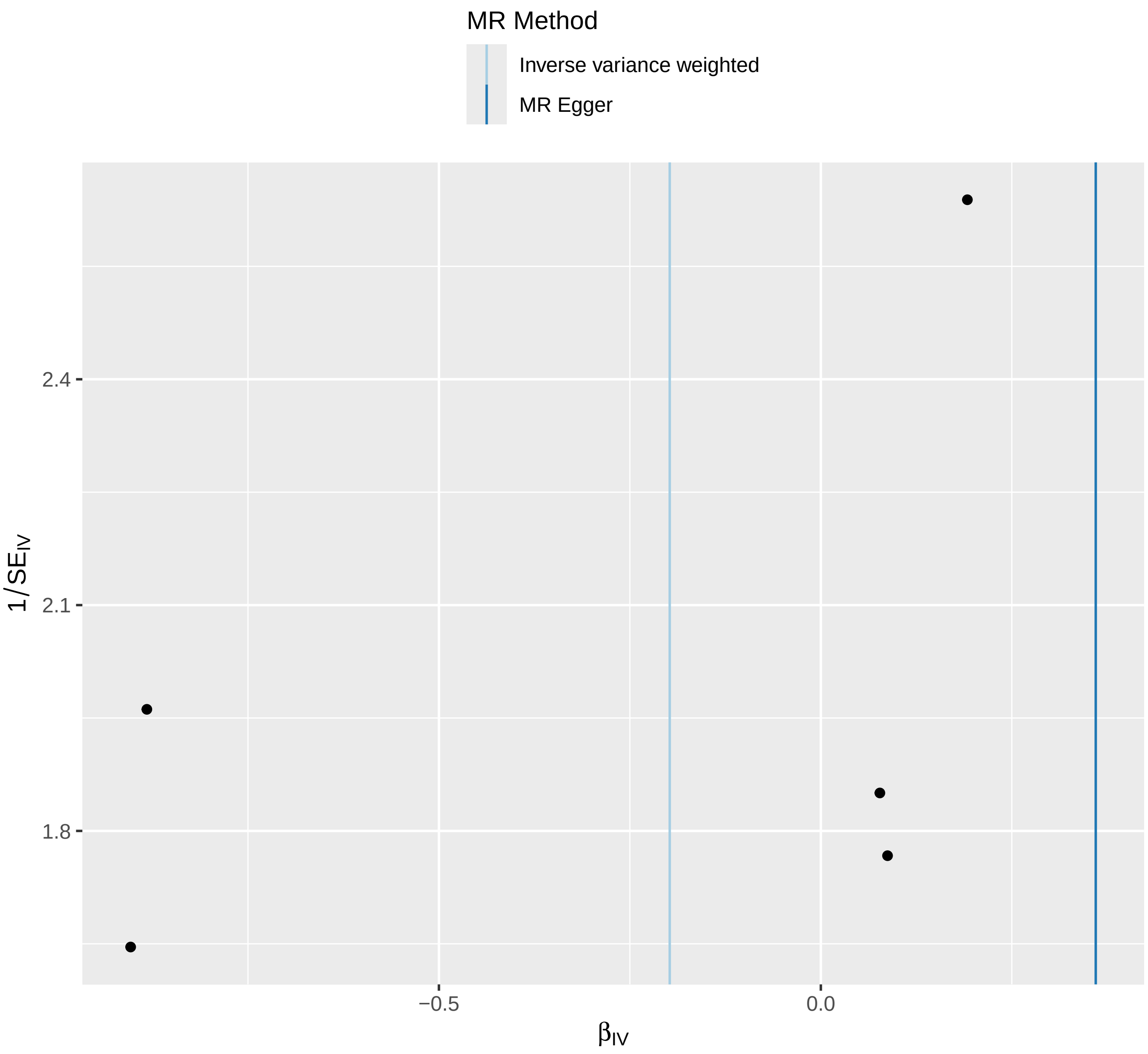

MR Method
Inverse variance weighted
MR Egger
2.4
2.1
1.8
1/SE$_{IV}$
−0.5
0.0
β$_{IV}$

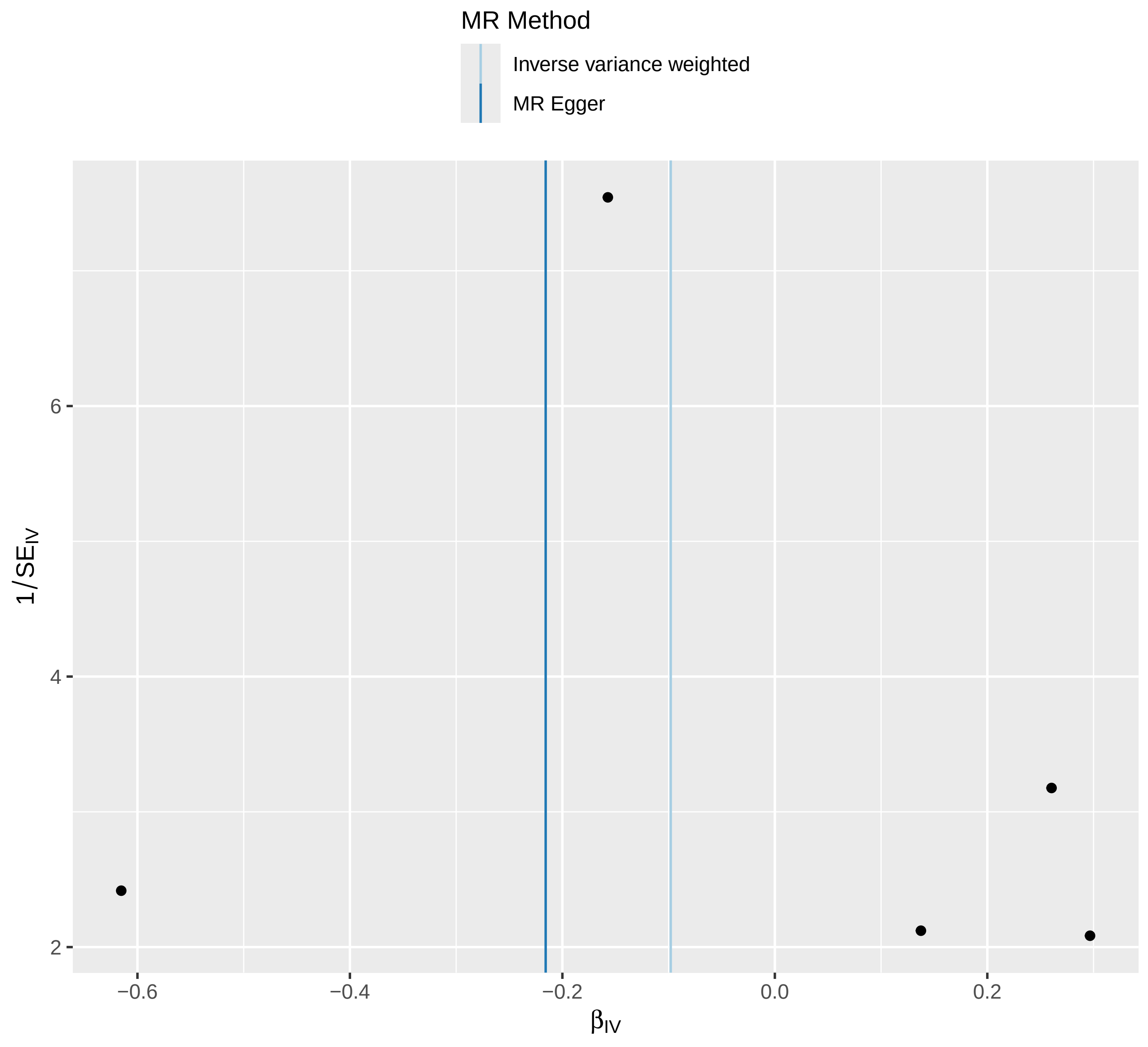
MR Method
Inverse variance weighted
MR Egger
1/SE$_{IV}$
6
4
2
−0.6
−0.4
−0.2
0.0
0.2
β$_{IV}$

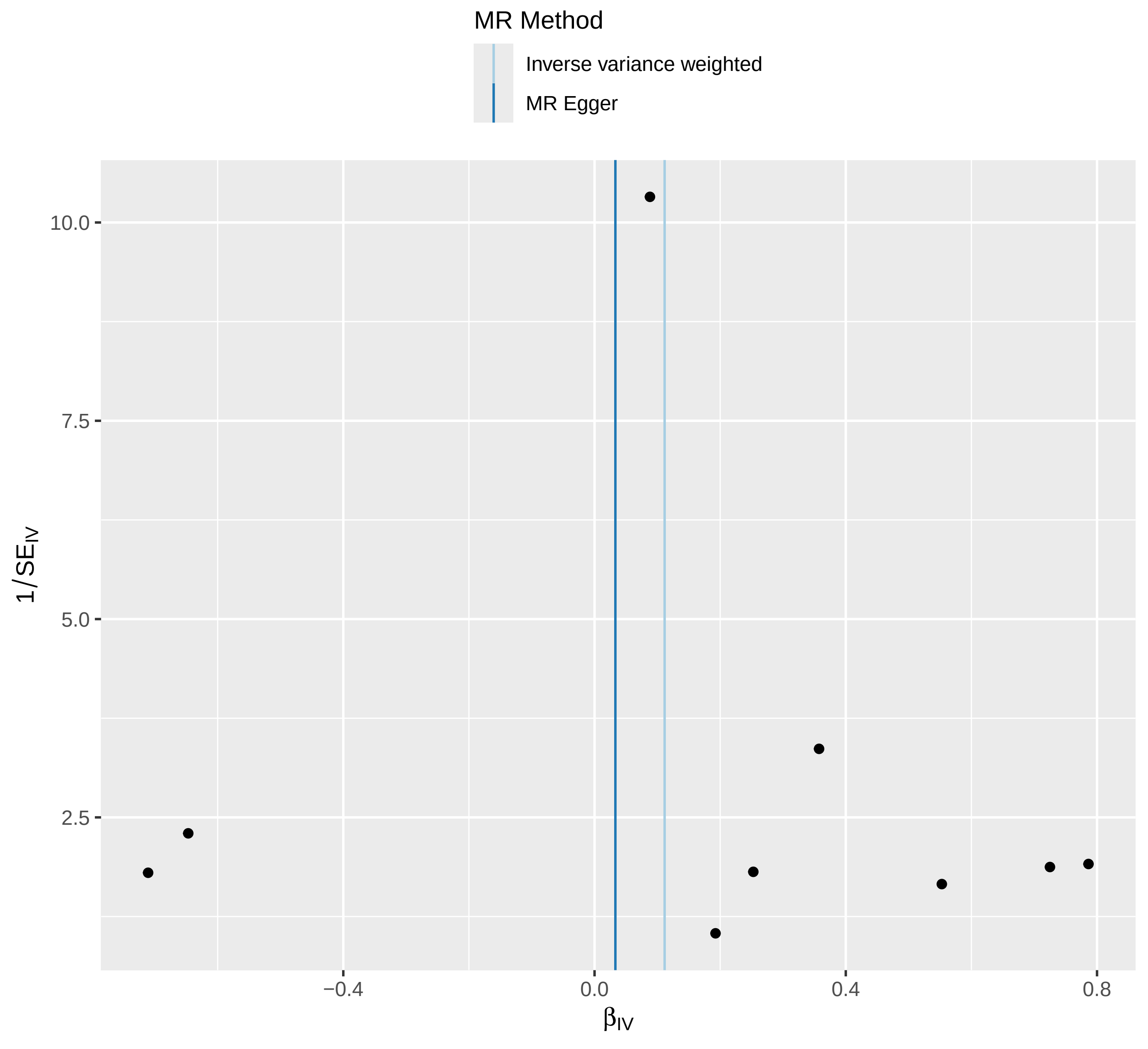
MR Method
Inverse variance weighted
MR Egger
10.0
7.5
5.0
2.5
$1/SE_{IV}$
−0.4
0.0
0.4
0.8
β_{IV}

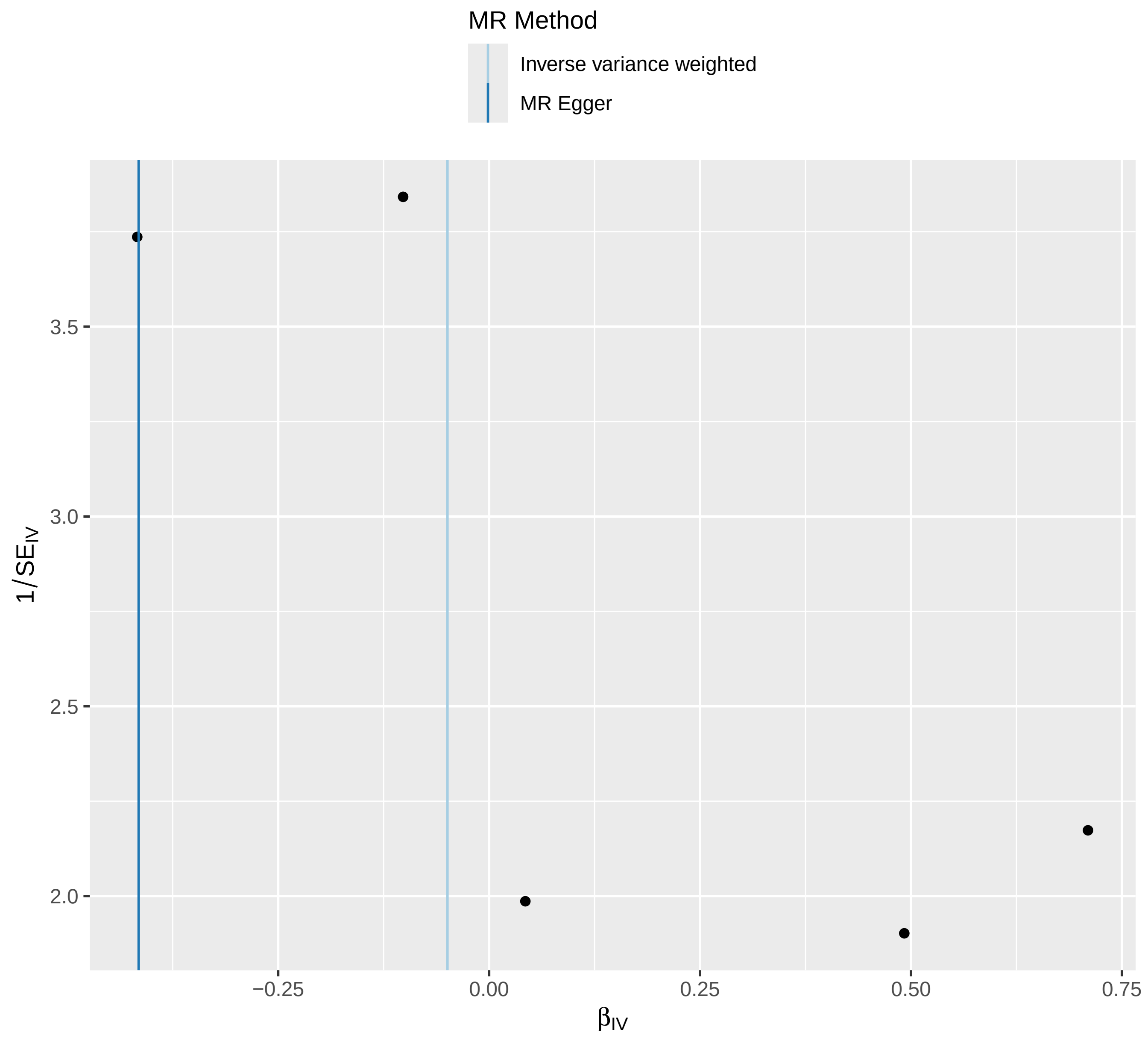
MR Method
Inverse variance weighted
MR Egger
3.5
3.0
2.5
2.0
1/SE_IV
−0.25
0.00
0.25
0.50
0.75
β_IV

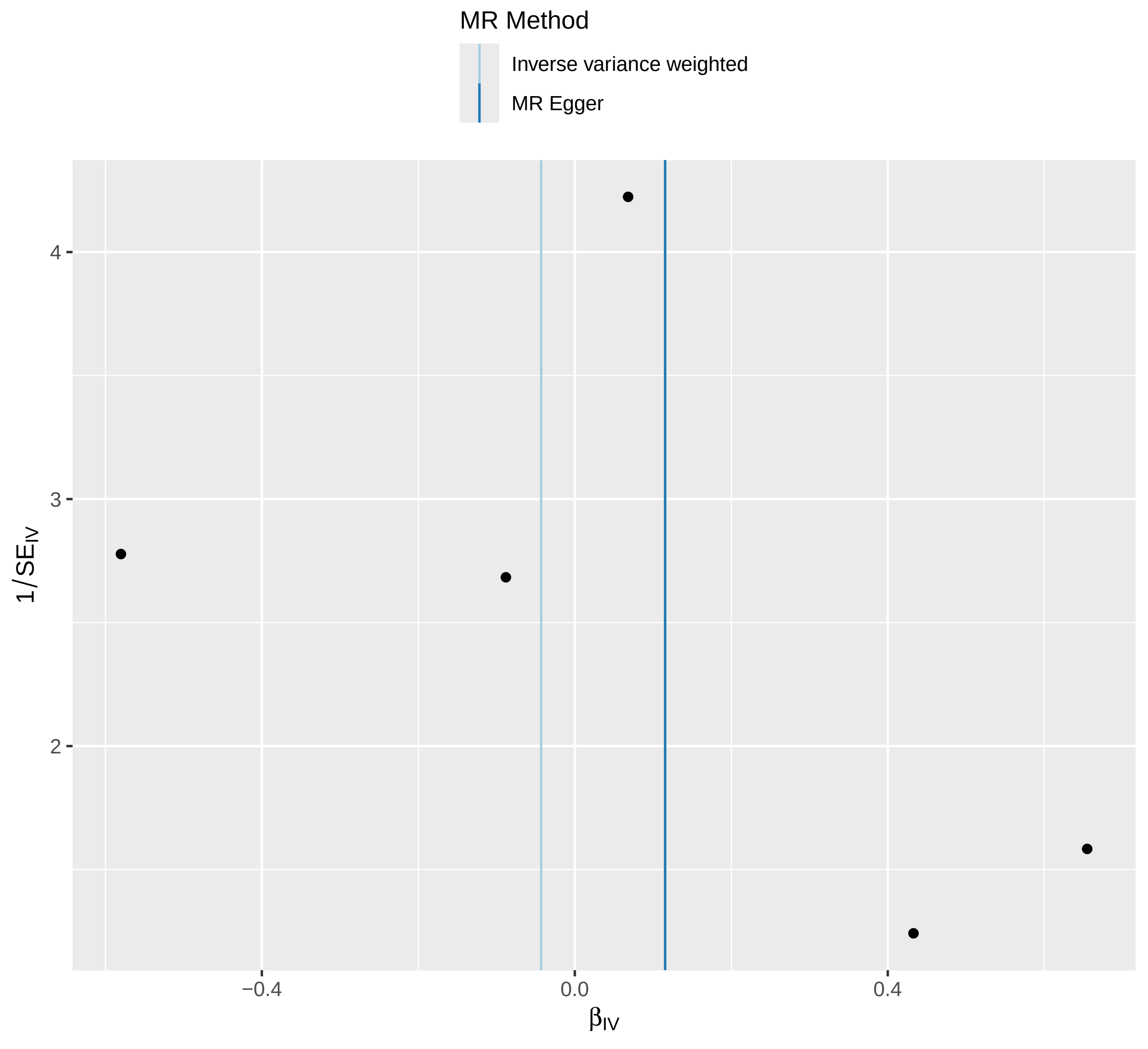
MR Method
Inverse variance weighted
MR Egger
4
3
2
1/SE_IV
−0.4
0.0
0.4
β_IV

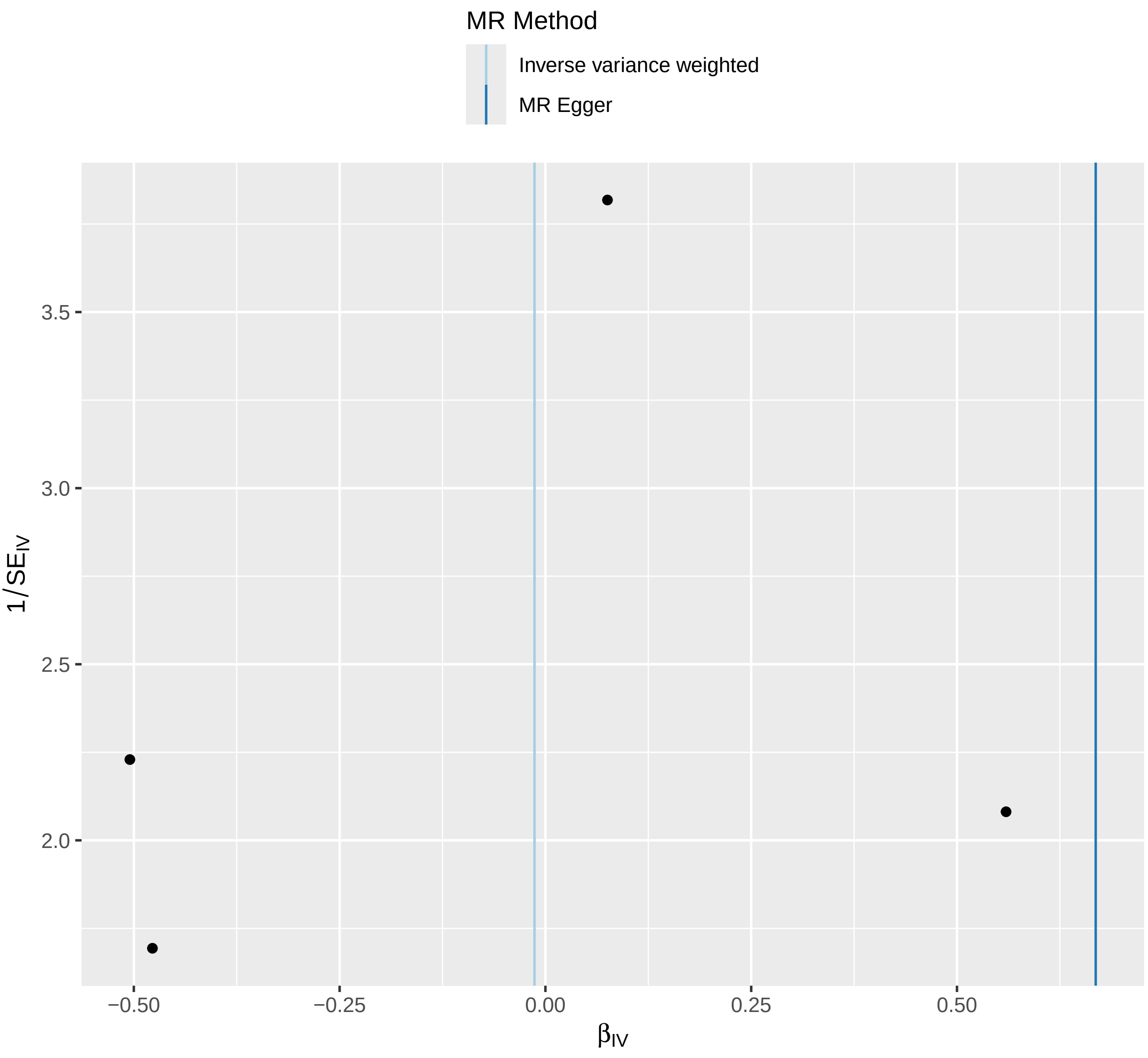
MR Method
Inverse variance weighted
MR Egger
3.5
3.0
2.5
2.0
1/SE_IV
−0.50
−0.25
0.00
0.25
0.50
β_IV

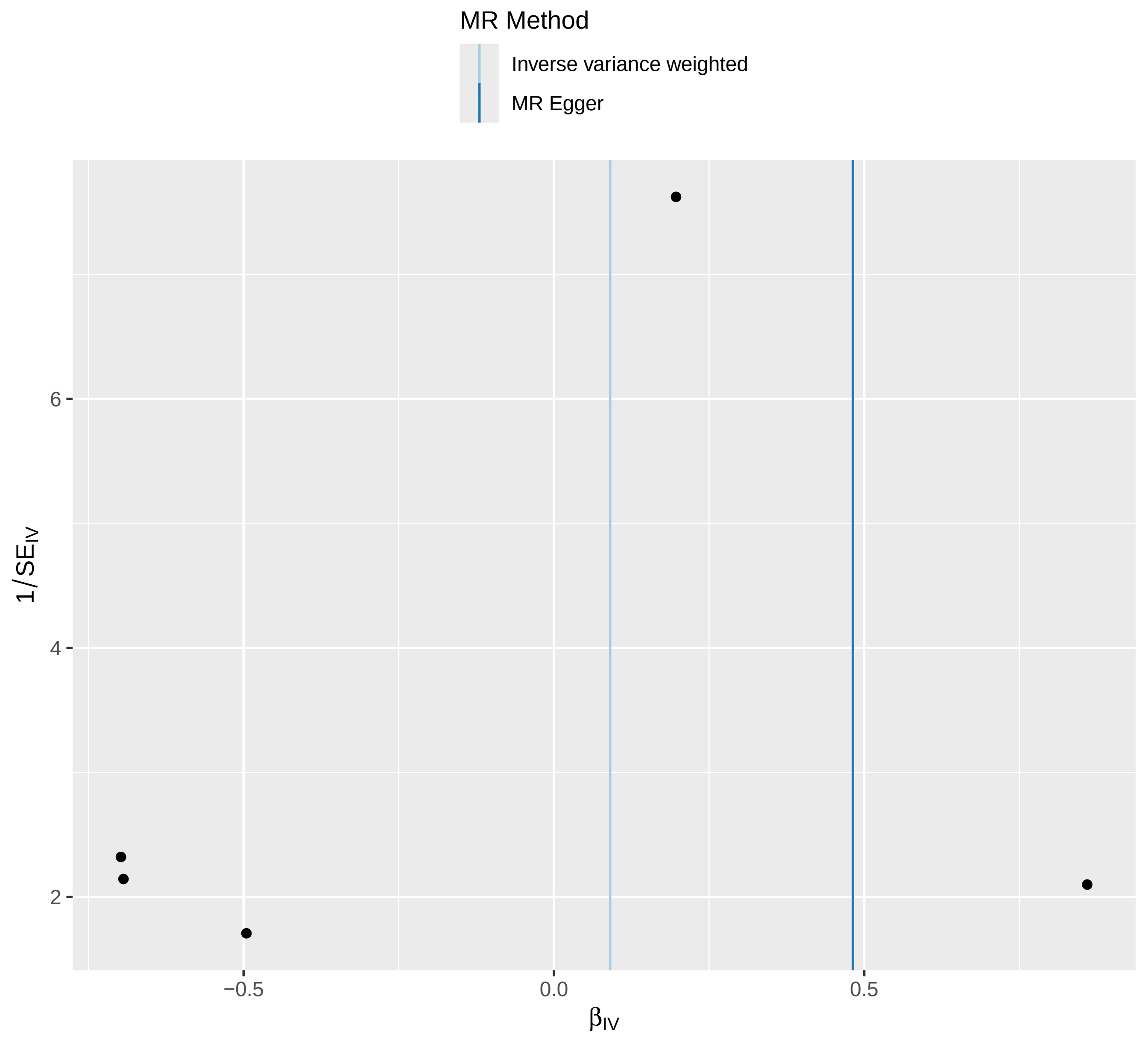

MR Method
Inverse variance weighted
MR Egger
6
4
2
1/SE_IV
−0.5
0.0
0.5
β_IV

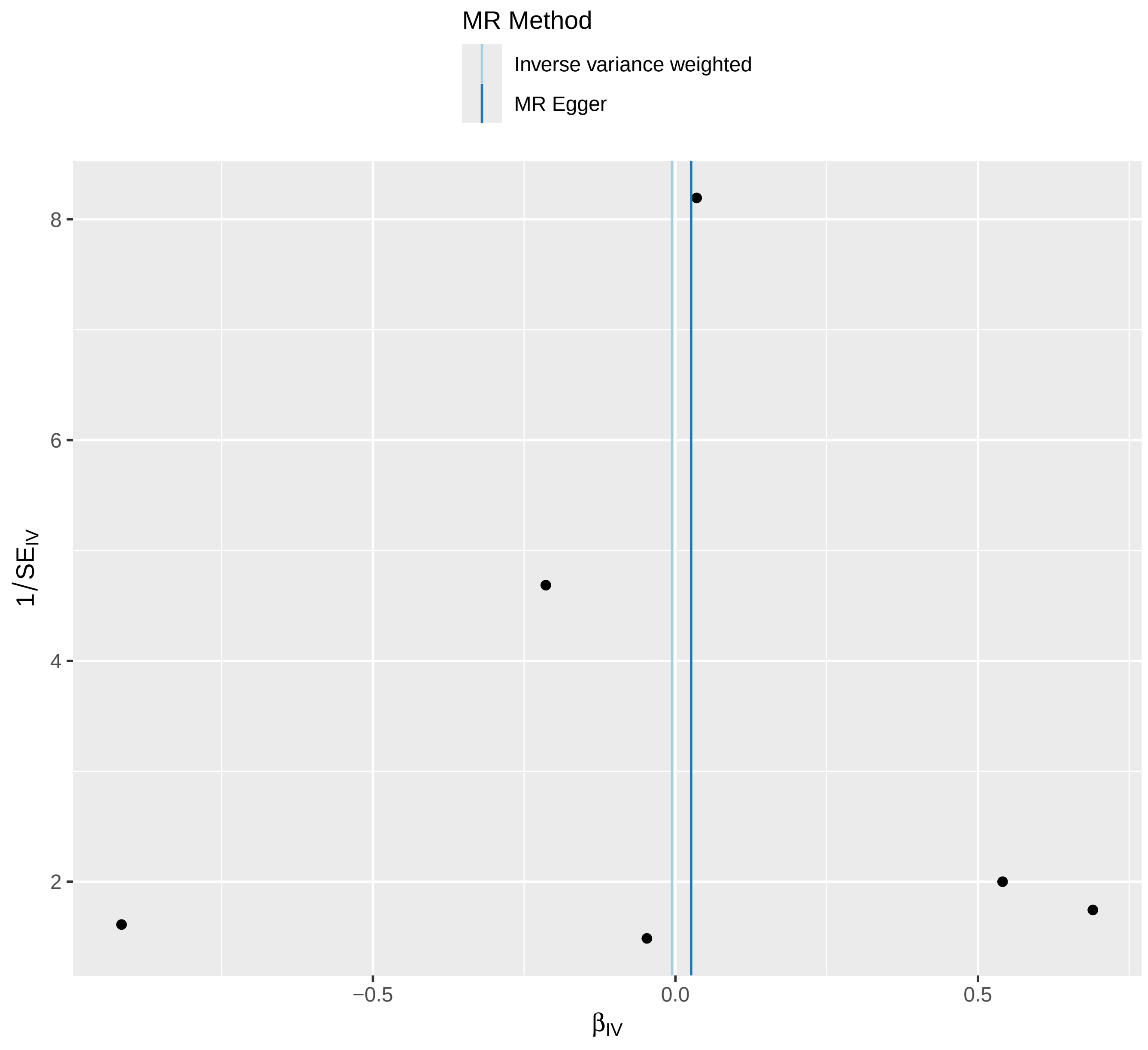

MR Method
Inverse variance weighted
MR Egger
8
6
4
2
1/SE$_{IV}$
−0.5
0.0
0.5
β$_{IV}$

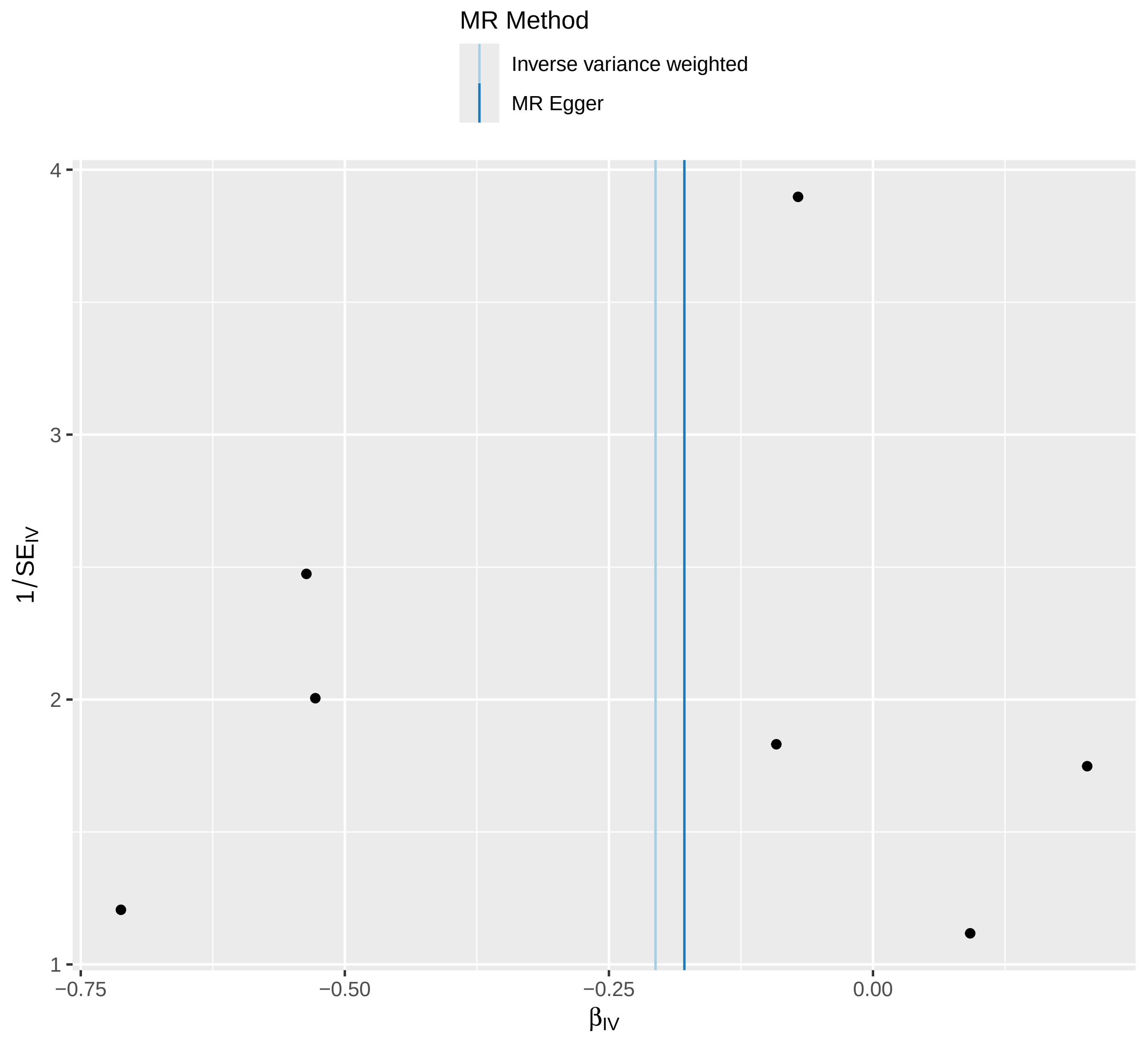

MR Method
Inverse variance weighted
MR Egger
4
3
2
1
1/SE_IV
−0.75
−0.50
−0.25
0.00
β_IV

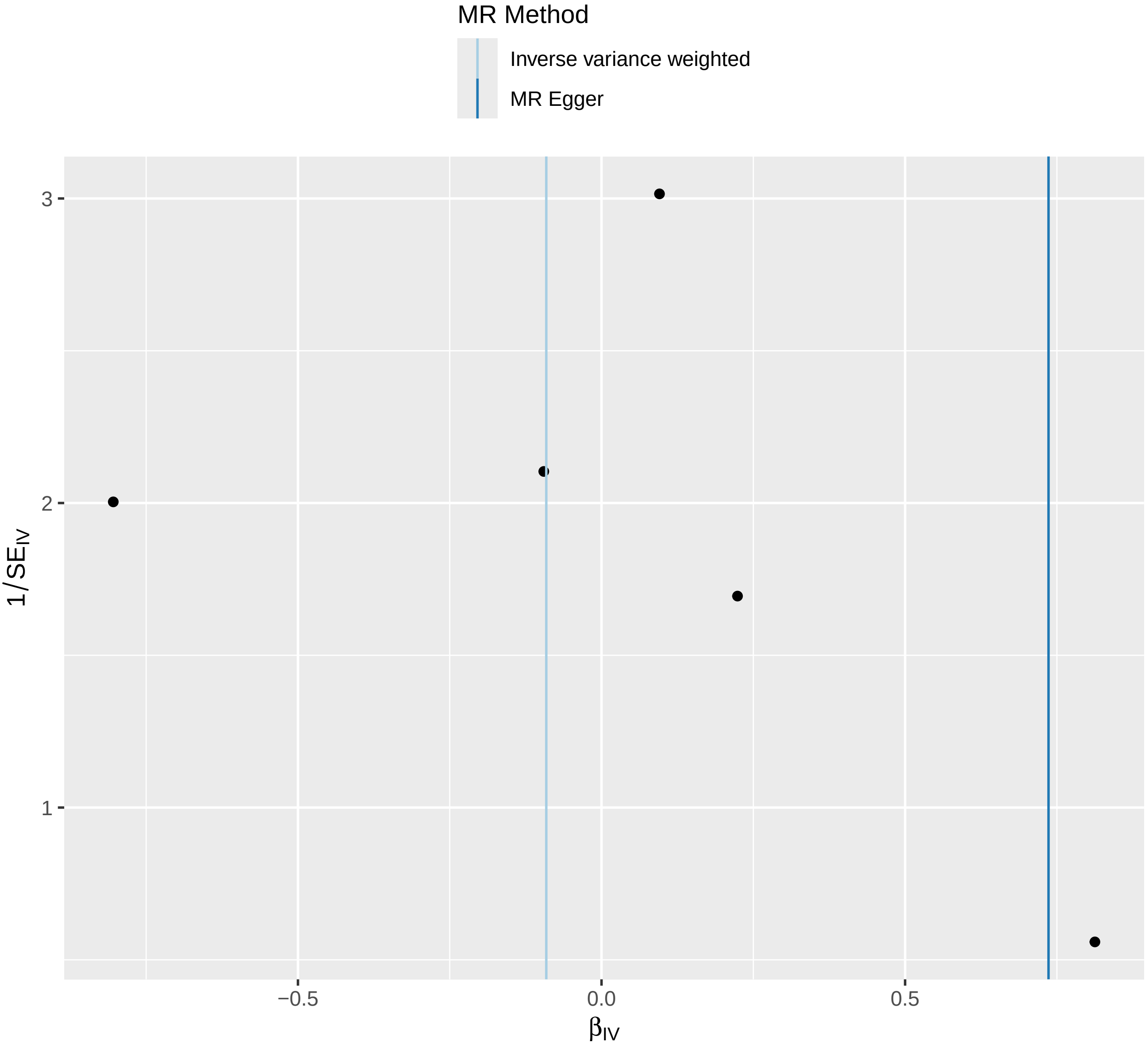

MR Method
Inverse variance weighted
MR Egger
3
2
1
1/SE_IV
−0.5
0.0
0.5
β_IV

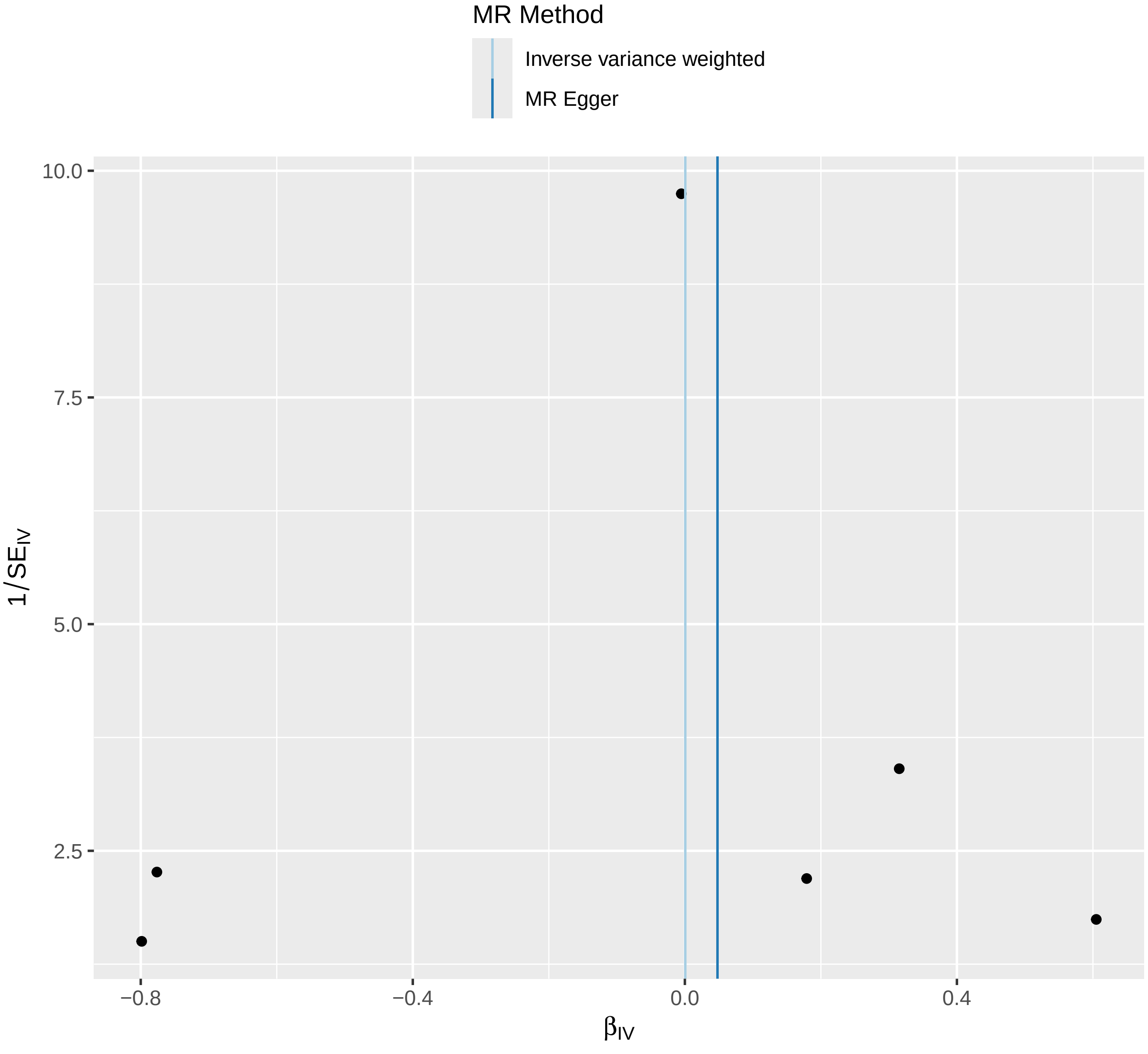

MR Method
Inverse variance weighted
MR Egger
10.0
7.5
5.0
2.5
1/SE_IV
−0.8
−0.4
0.0
0.4
β_IV

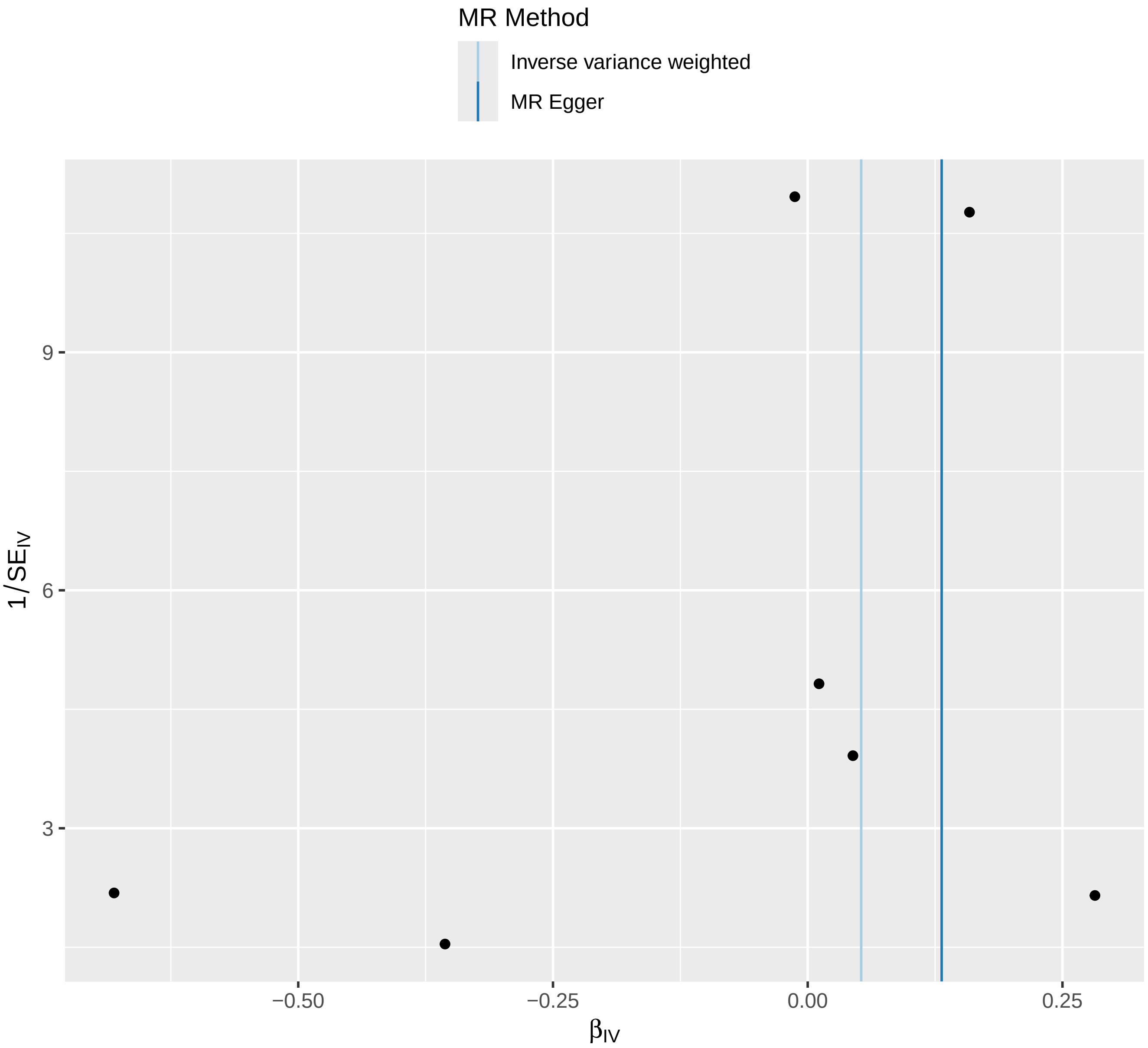

MR Method
Inverse variance weighted
MR Egger
9
6
3
1/SE_IV
−0.50
−0.25
0.00
0.25
β_IV

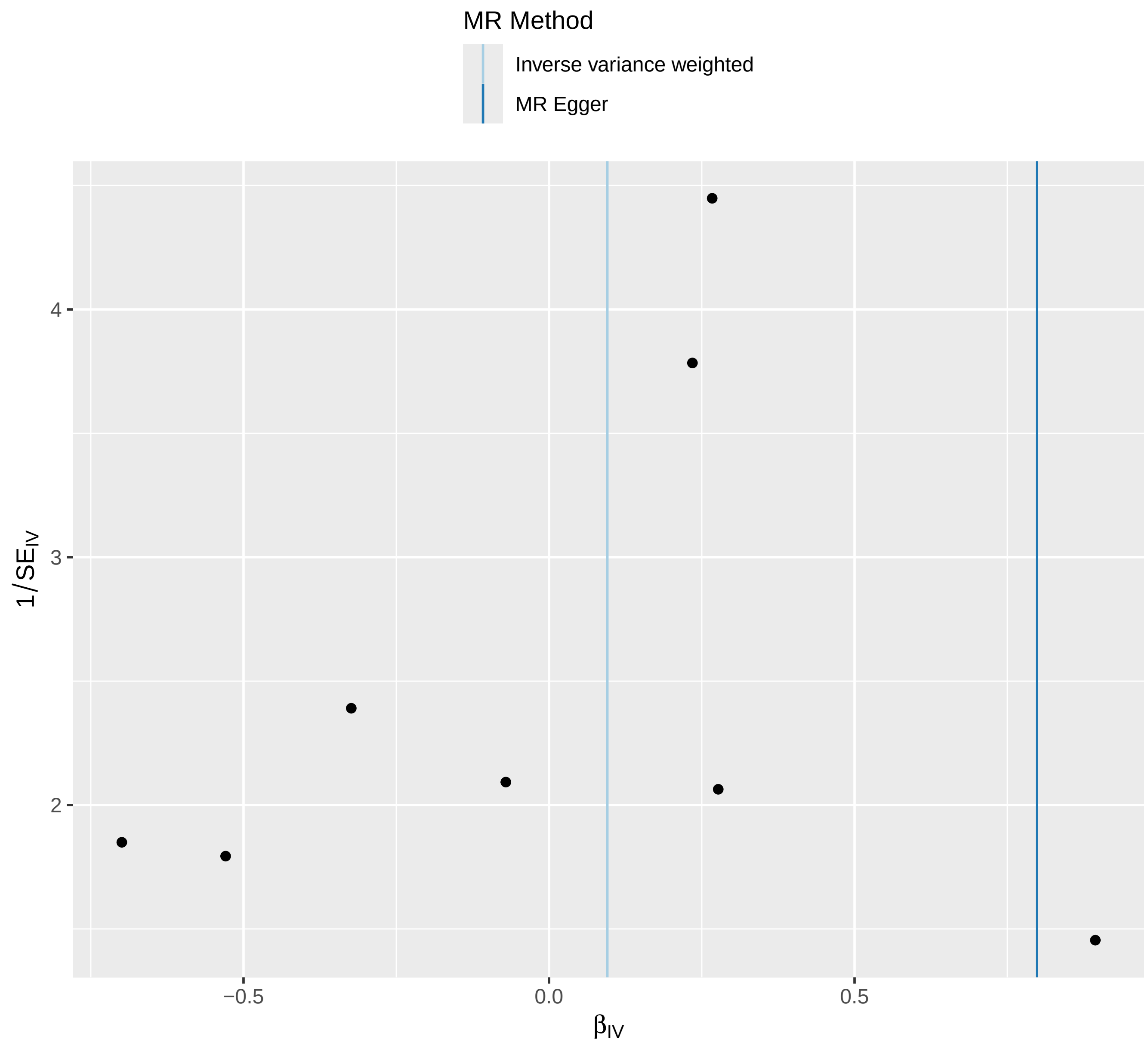
MR Method
Inverse variance weighted
MR Egger
4
3
2
1/SE_IV
−0.5
0.0
0.5
β_IV

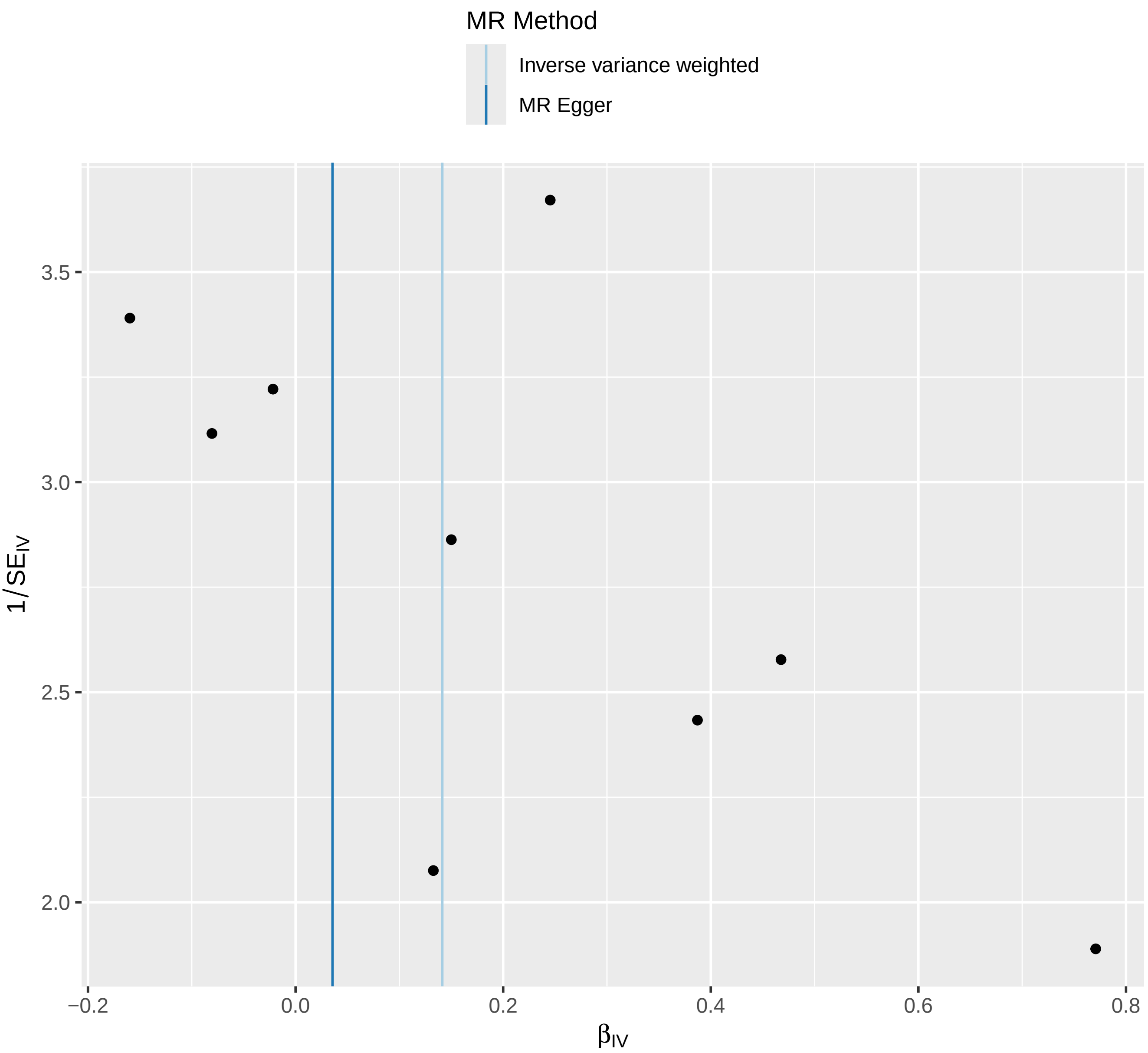
MR Method
Inverse variance weighted
MR Egger
3.5
3.0
2.5
2.0
$1/SE_{IV}$
−0.2
0.0
0.2
0.4
0.6
0.8
β_{IV}

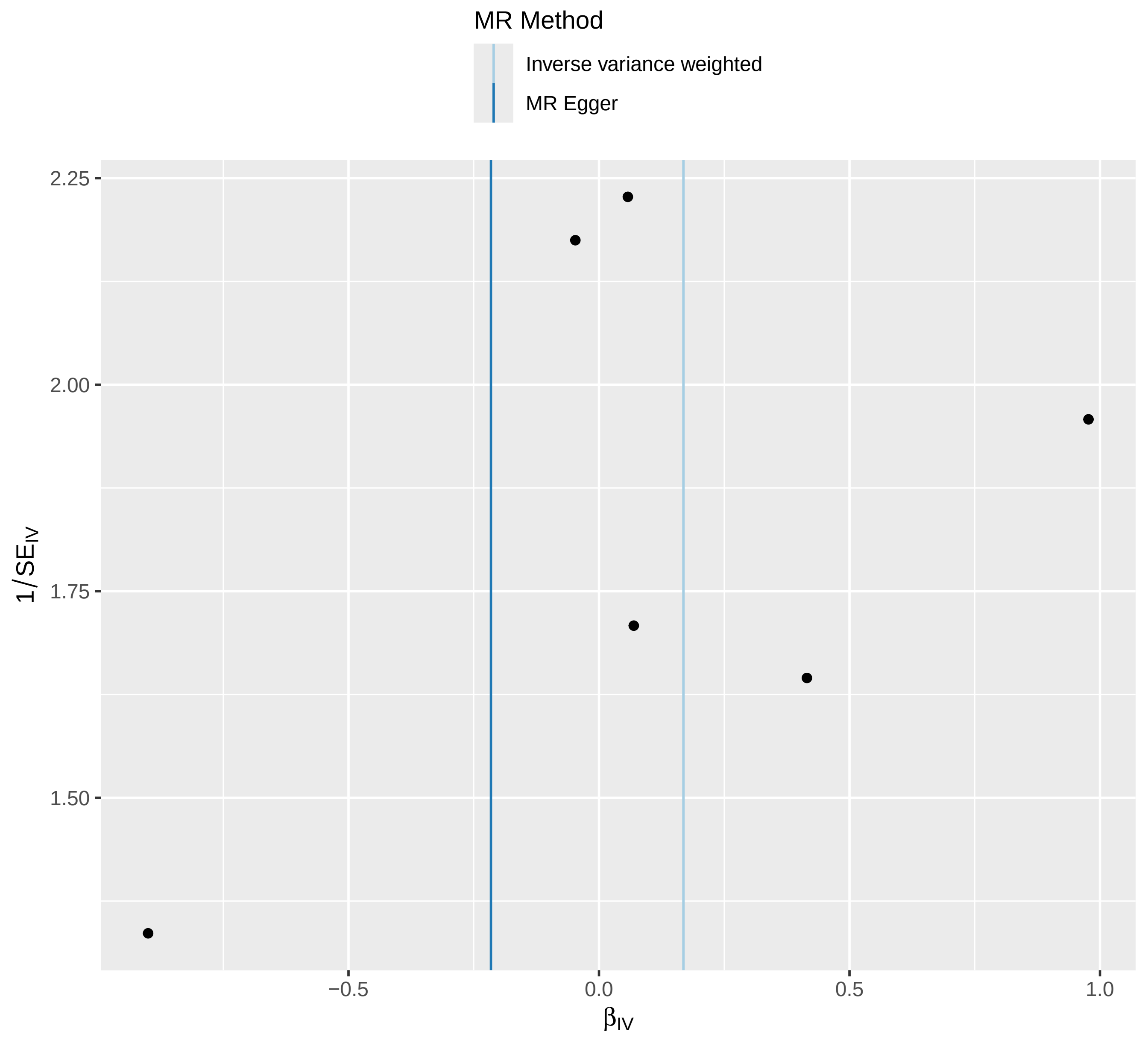
MR Method
Inverse variance weighted
MR Egger
2.25
2.00
1.75
1.50
1/SE_IV
−0.5
0.0
0.5
1.0
β_IV

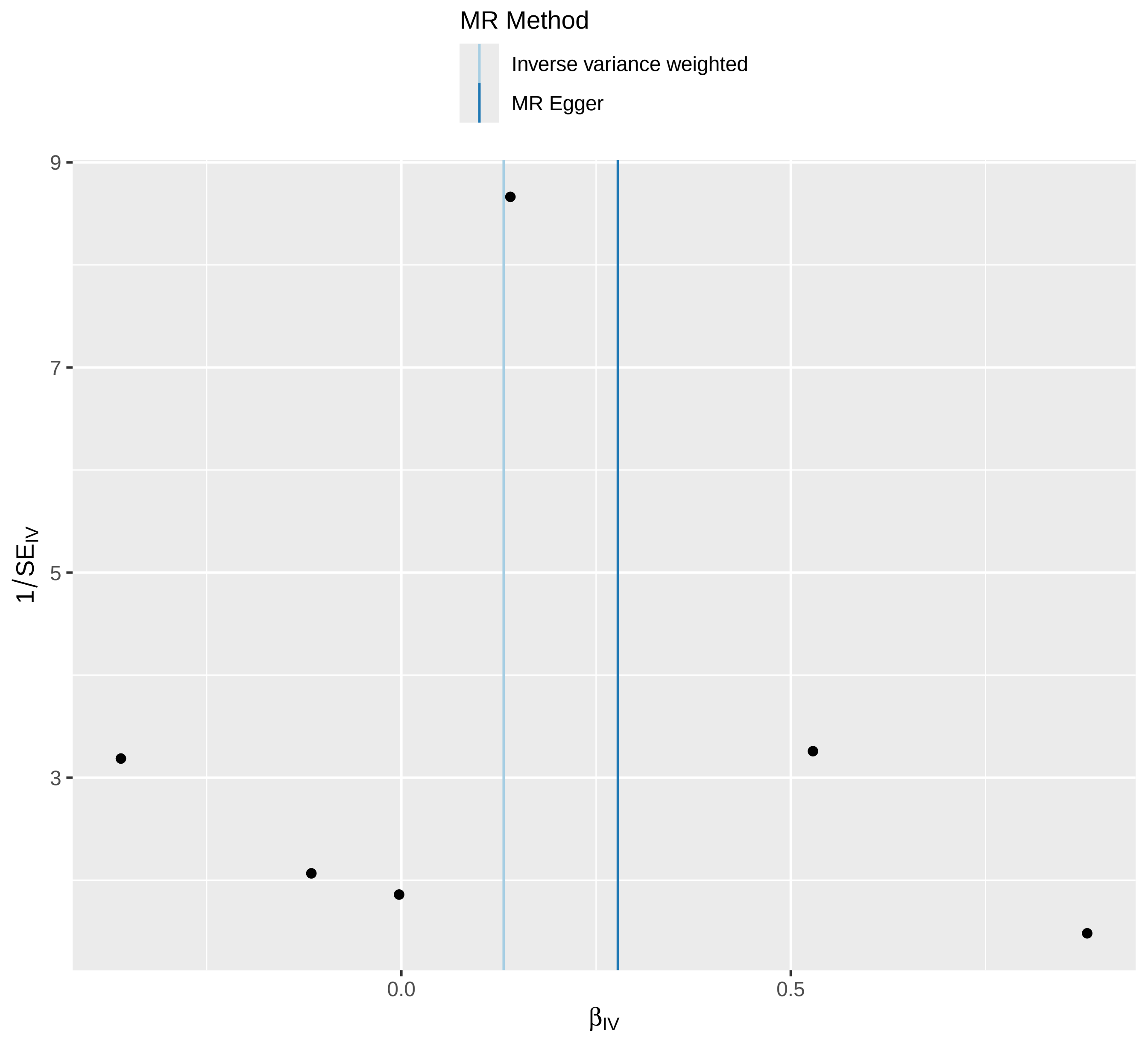
MR Method
Inverse variance weighted
MR Egger
9
7
5
3
1/SE_IV
0.0
0.5
β_IV

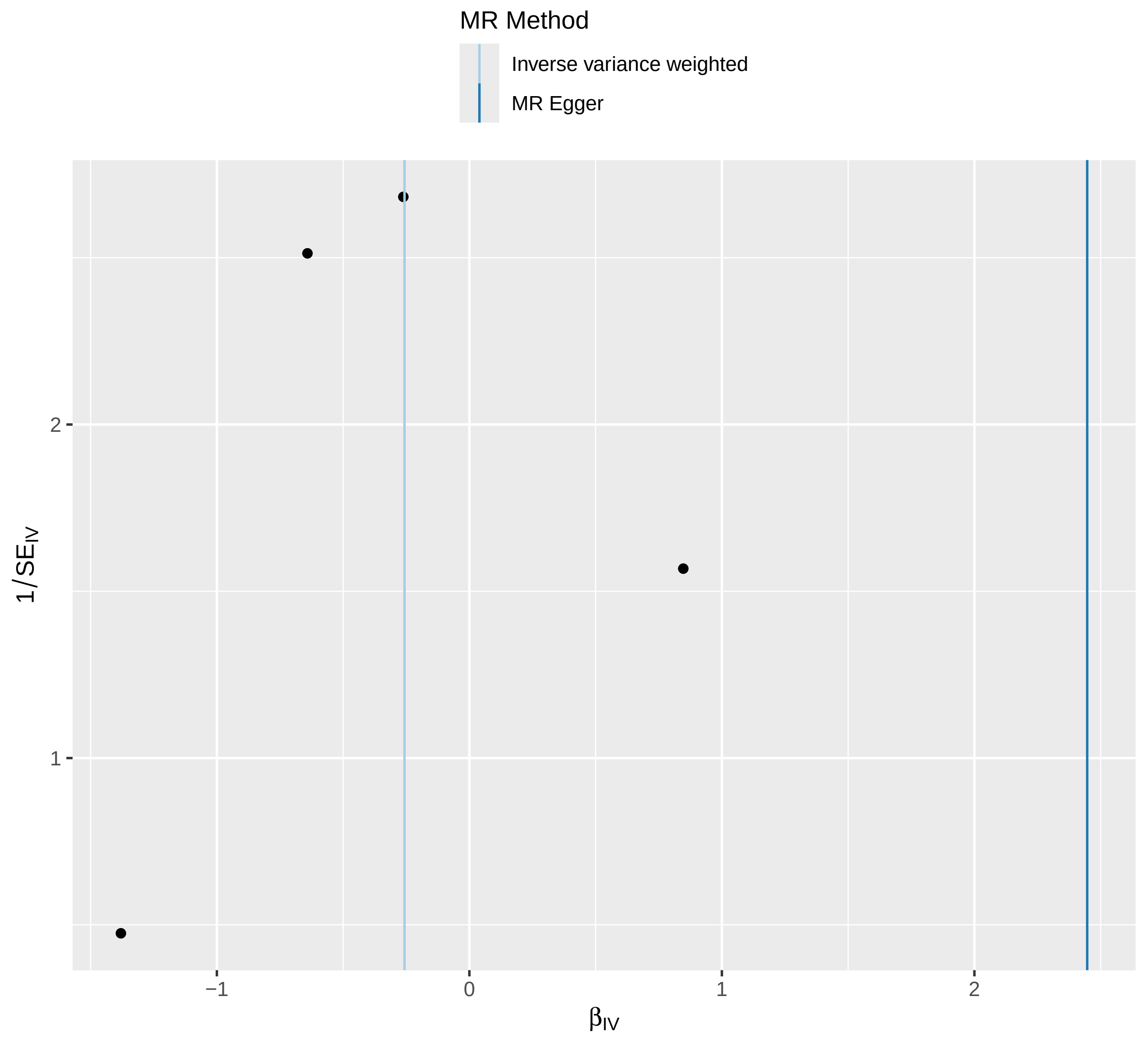
MR Method
Inverse variance weighted
MR Egger
2
1
1/SE$_{IV}$
−1
0
1
2
β$_{IV}$

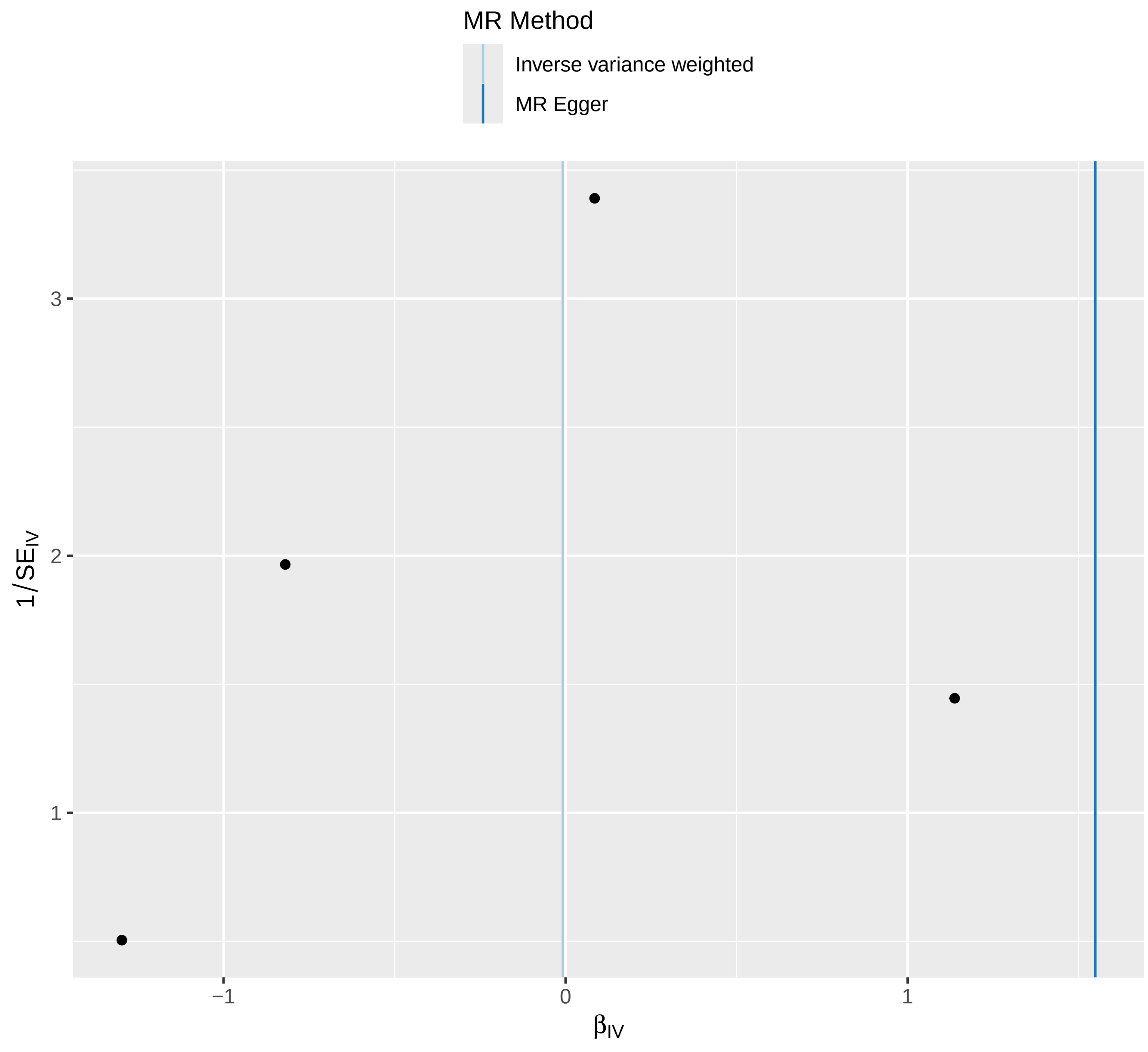

MR Method
Inverse variance weighted
MR Egger
3
2
1
1/SE$_{IV}$
−1
0
1
β$_{IV}$

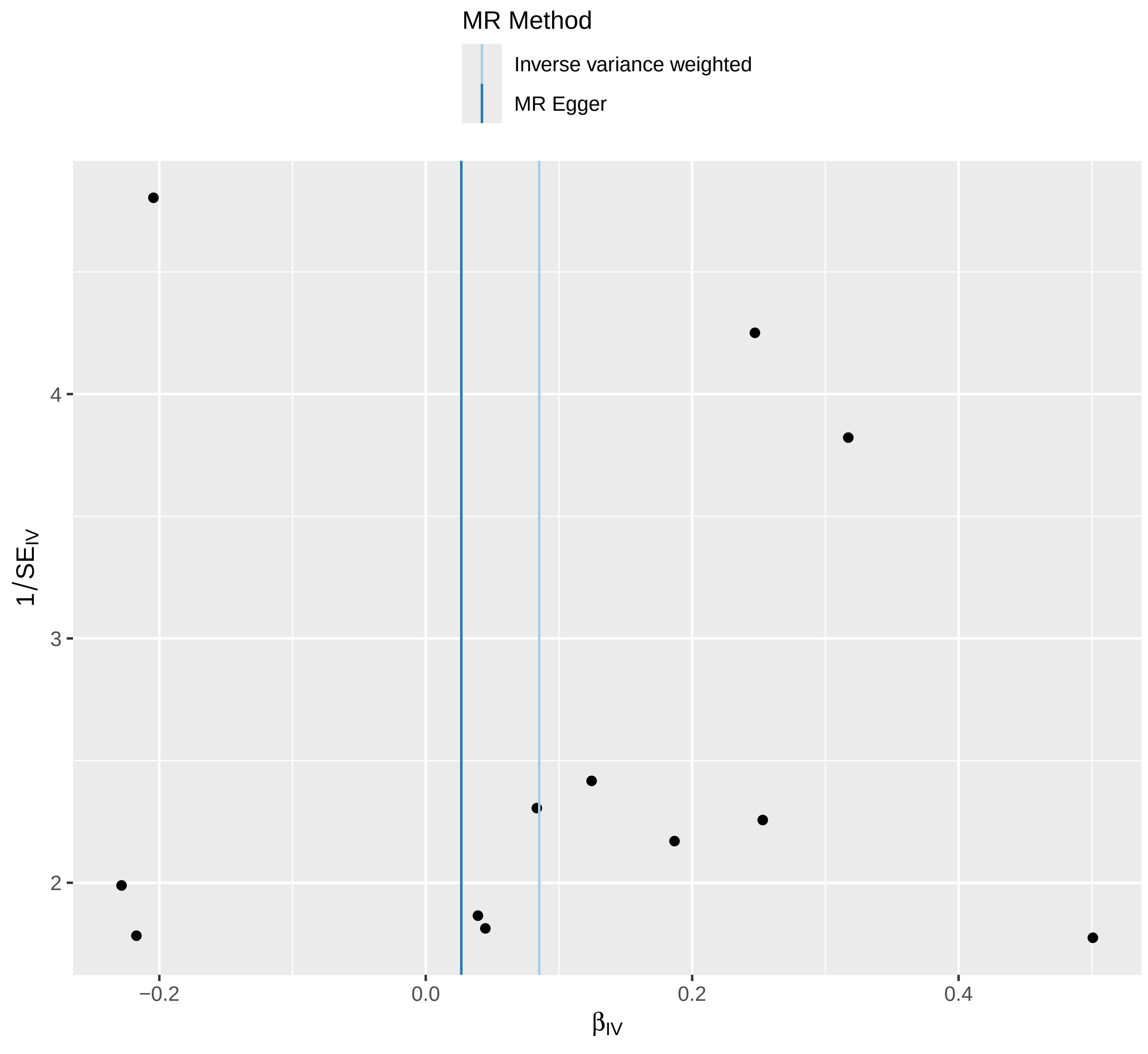
MR Method
Inverse variance weighted
MR Egger
4
3
2
1/SE$_{IV}$
−0.2
0.0
0.2
0.4
β$_{IV}$

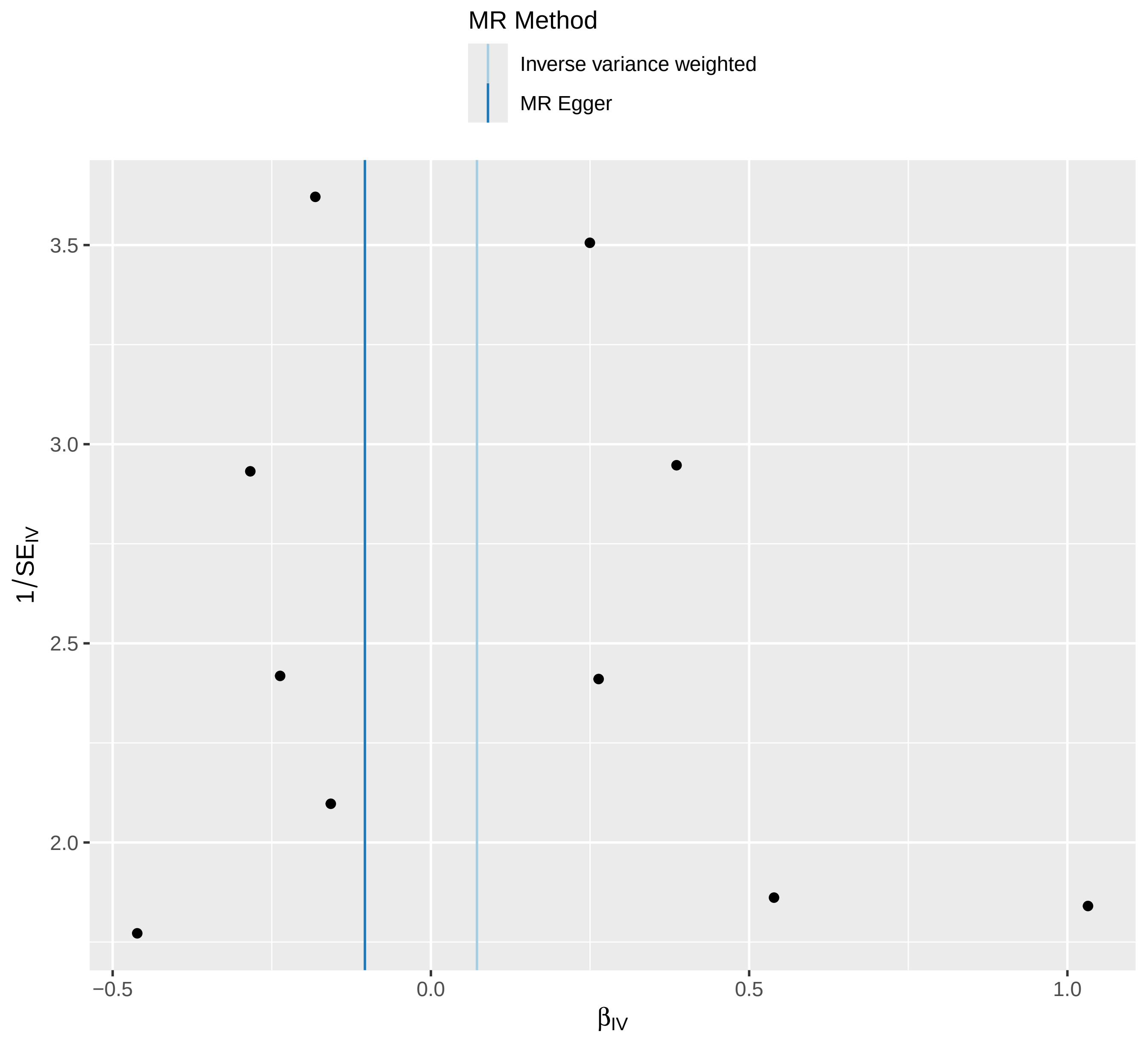
MR Method
Inverse variance weighted
MR Egger
3.5
3.0
2.5
2.0
1/SE_IV
−0.5
0.0
0.5
1.0
β_IV

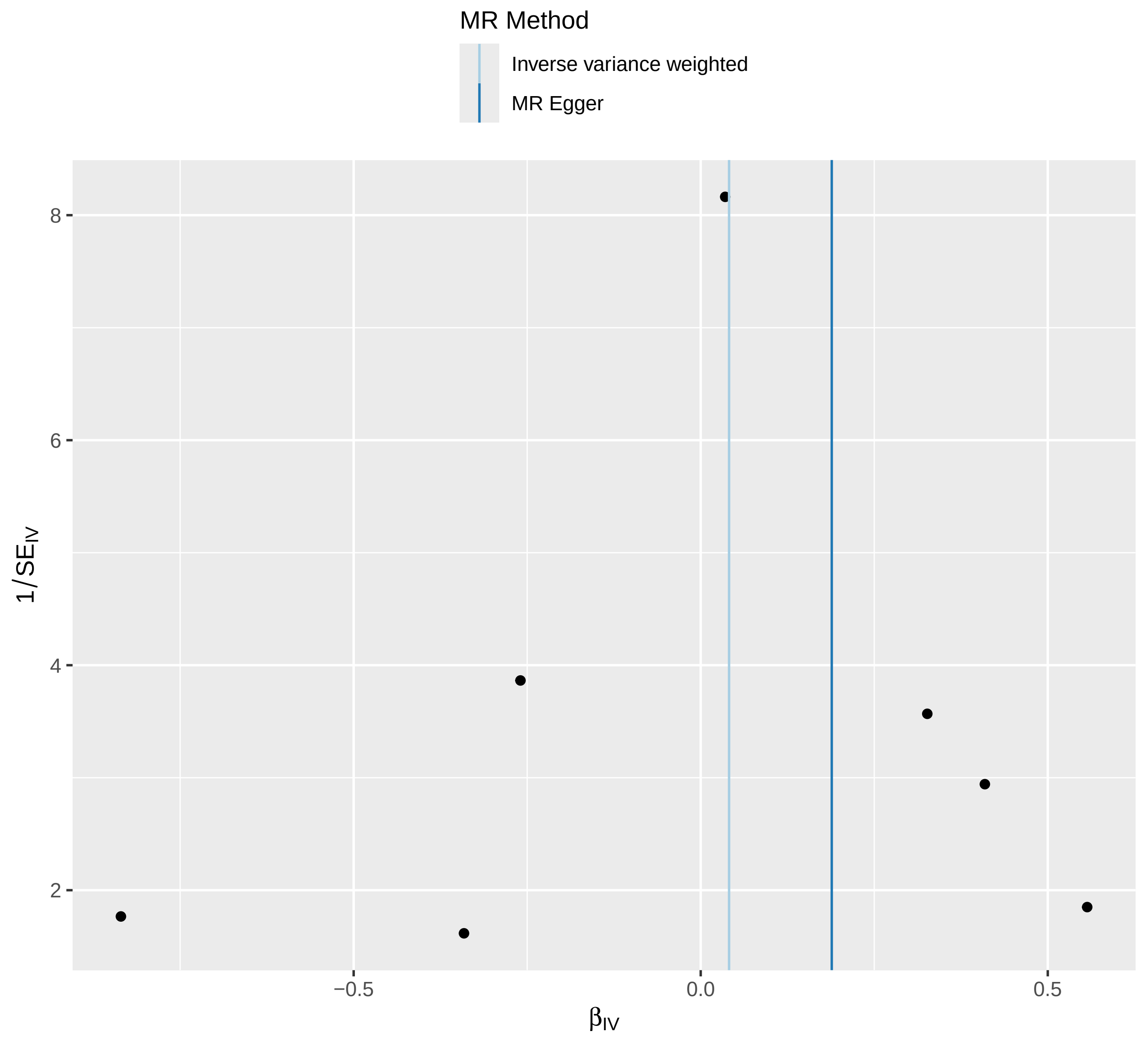
MR Method
Inverse variance weighted
MR Egger
8
6
4
2
1/SE$_{IV}$
−0.5
0.0
0.5
β$_{IV}$

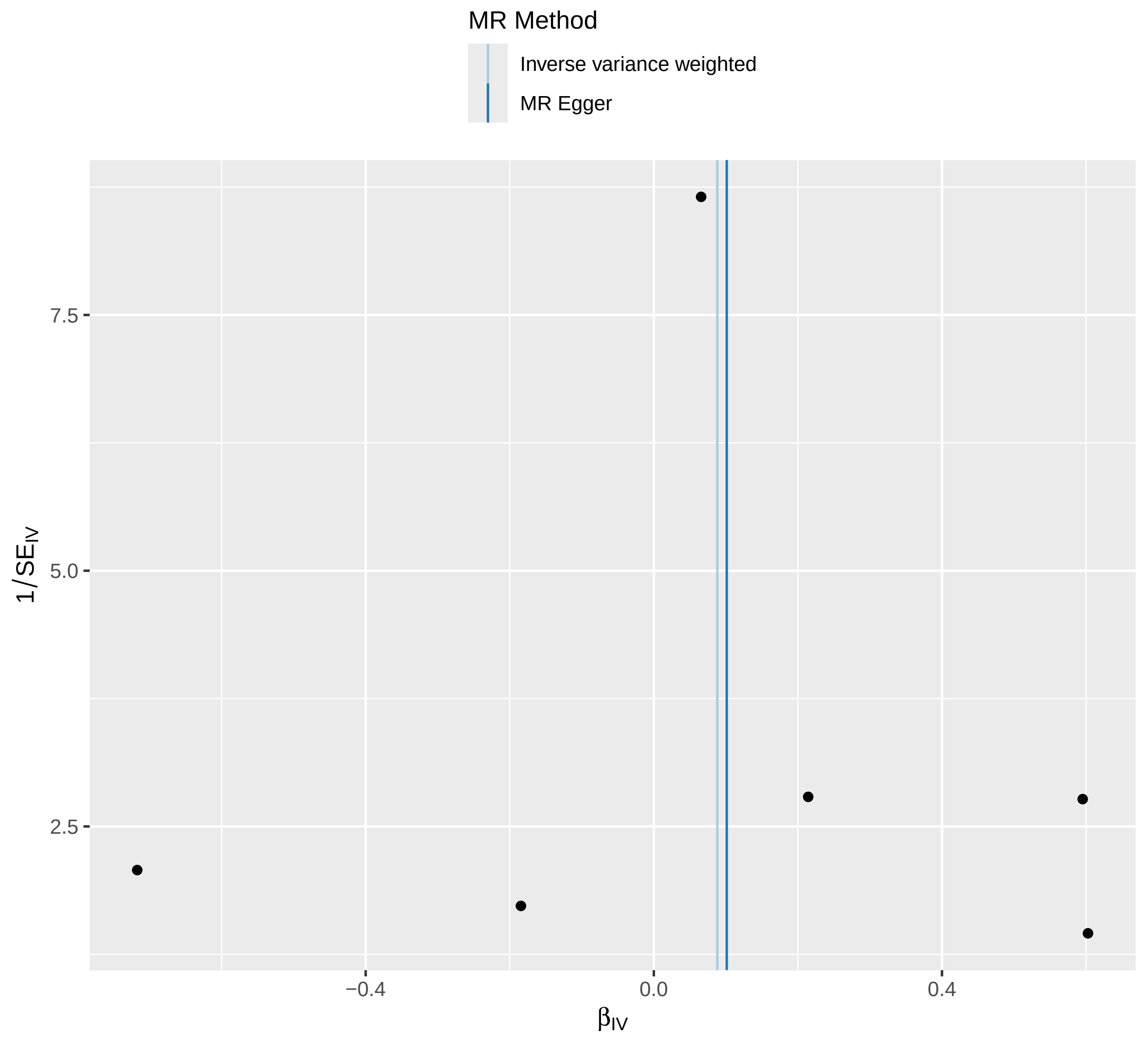

MR Method
Inverse variance weighted
MR Egger
7.5
5.0
2.5
1/SE_IV
−0.4
0.0
0.4
β_IV

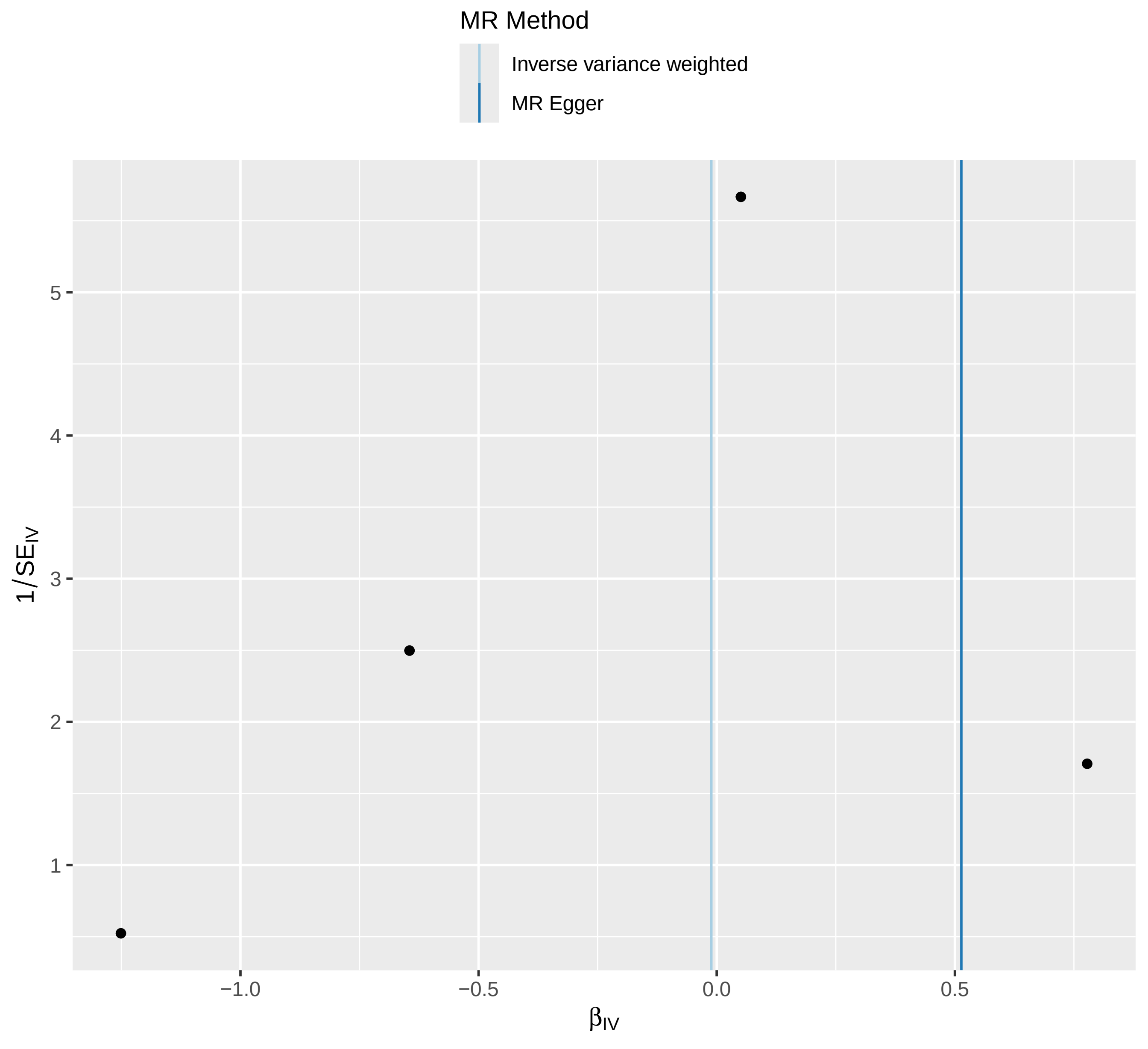
MR Method
Inverse variance weighted
MR Egger
5
4
3
2
1
$1/SE_{IV}$
−1.0
−0.5
0.0
0.5
β_{IV}

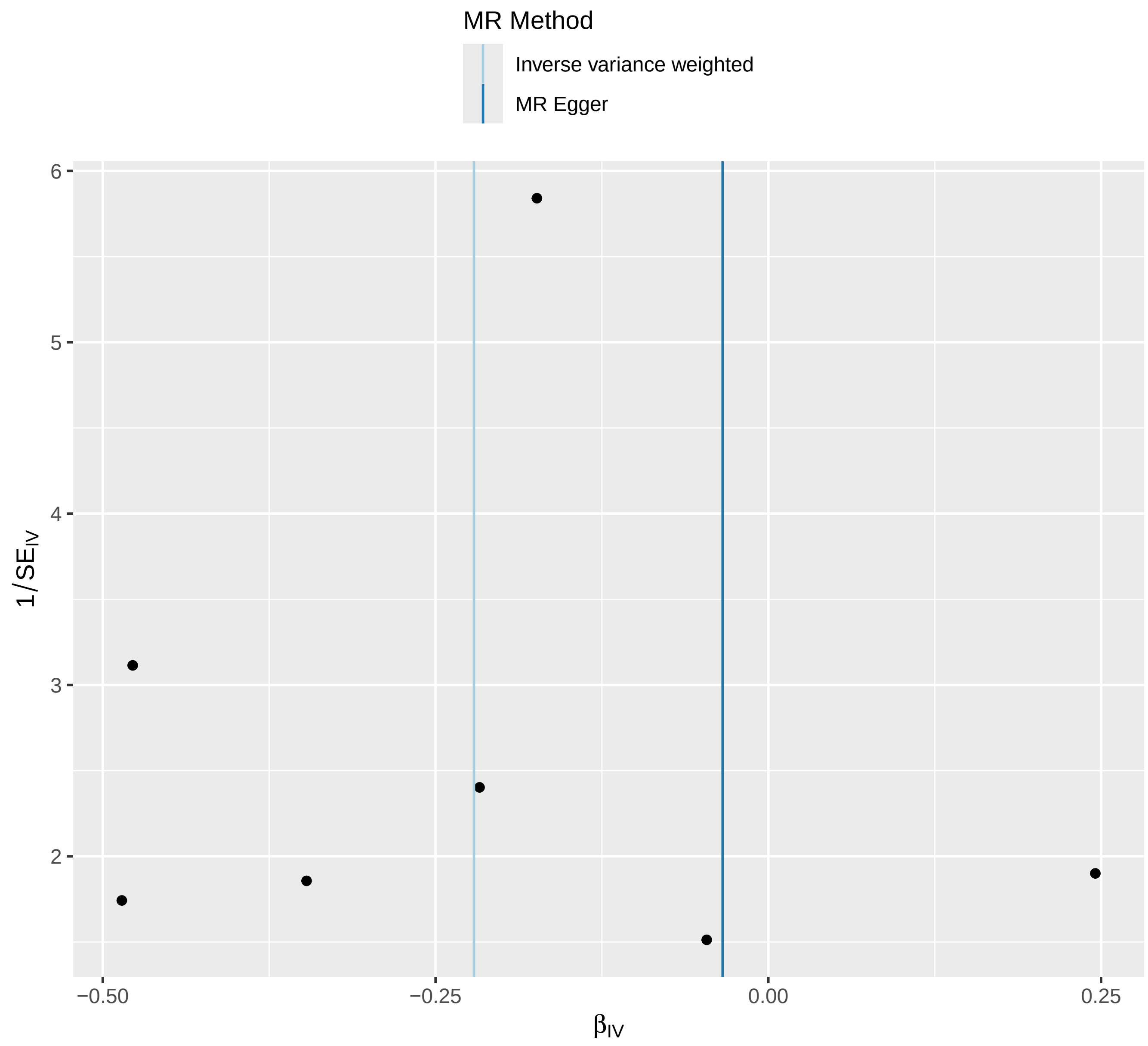
MR Method
Inverse variance weighted
MR Egger
1/SE$_{IV}$
6
5
4
3
2
−0.50
−0.25
0.00
0.25
β$_{IV}$

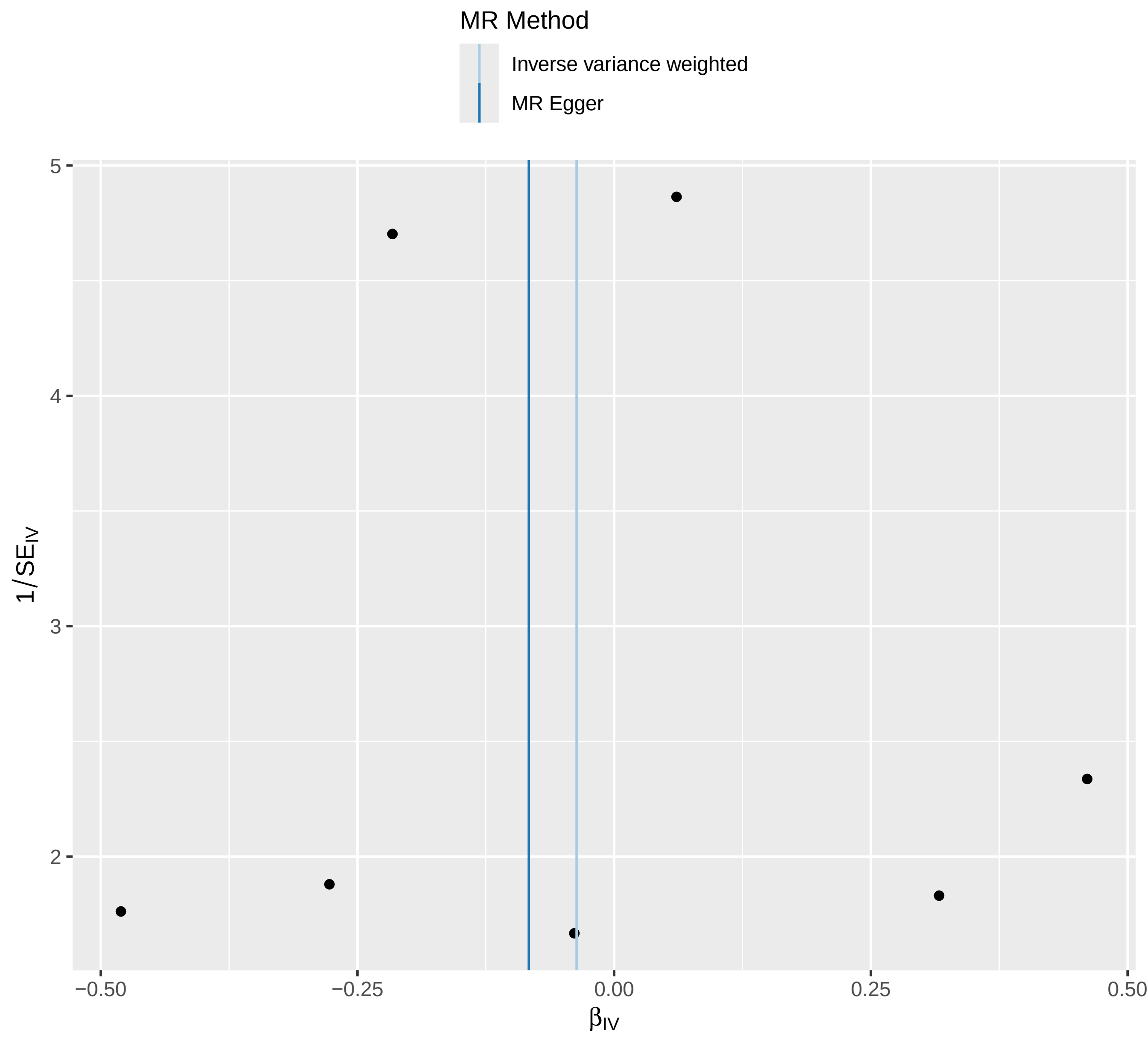
MR Method
Inverse variance weighted
MR Egger
5
4
3
2
1/SE_IV
−0.50
−0.25
0.00
0.25
0.50
β_IV

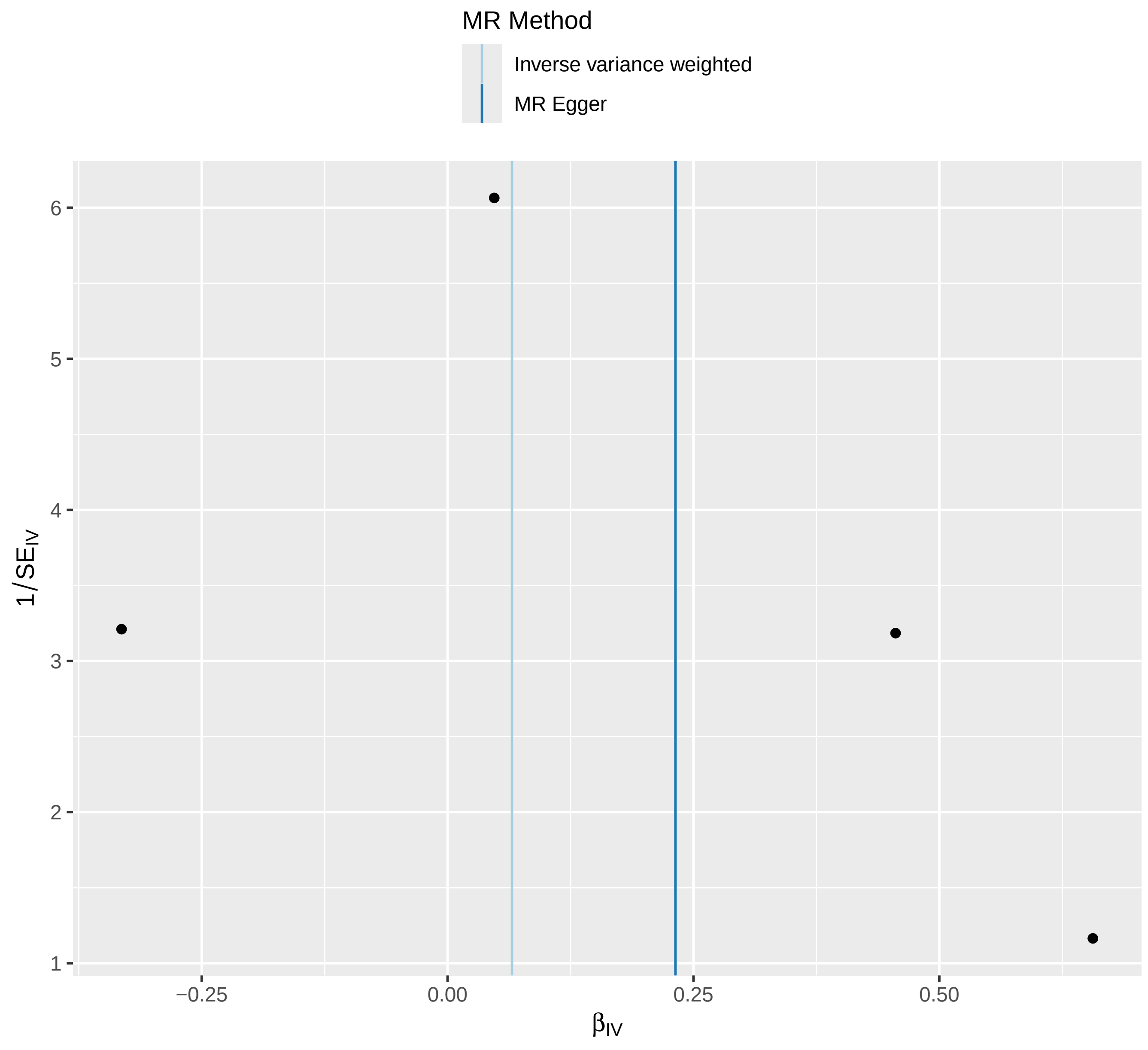

MR Method
Inverse variance weighted
MR Egger
6
5
4
3
2
1
1/SE_IV
−0.25
0.00
0.25
0.50
β_IV

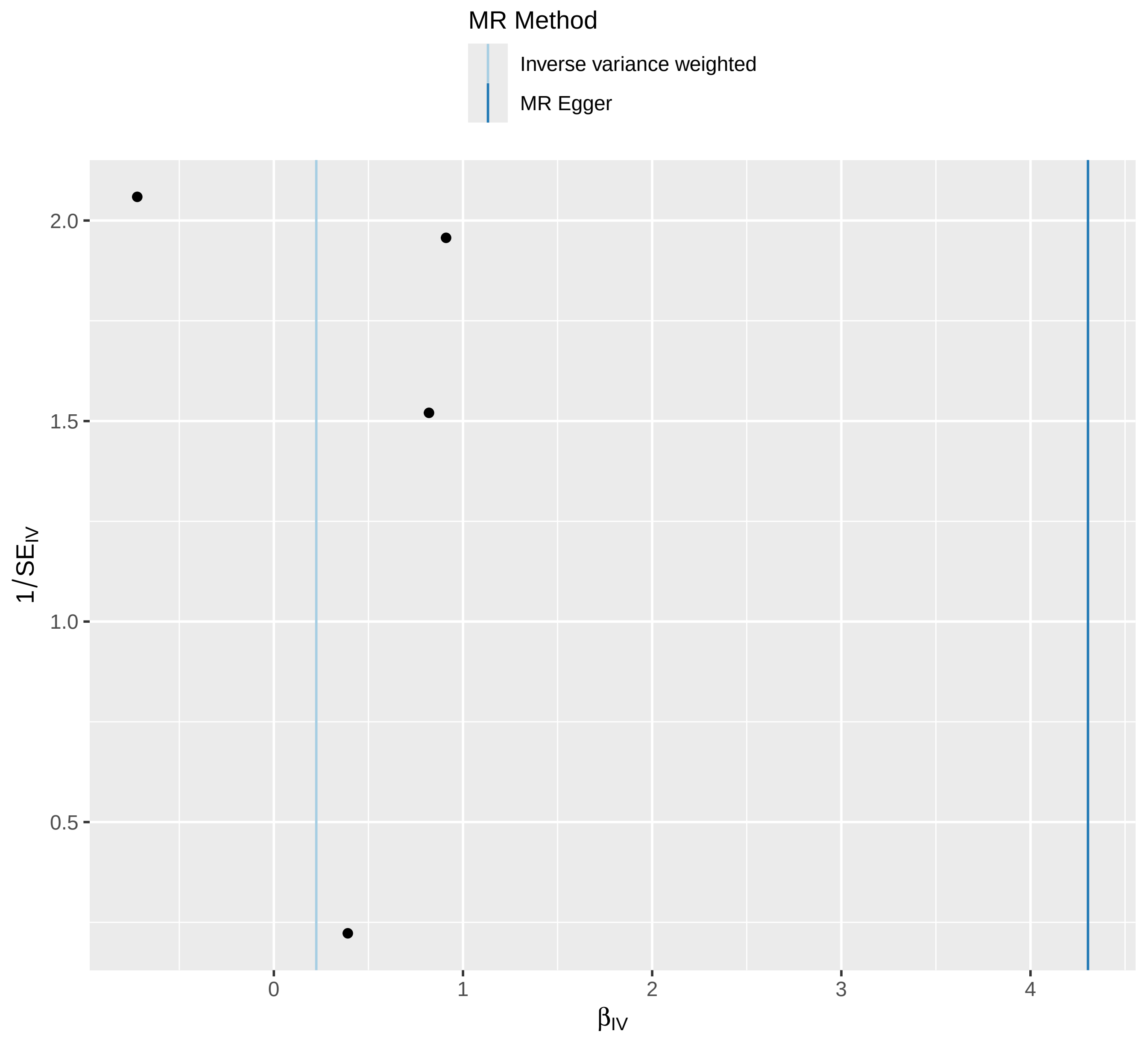
MR Method
Inverse variance weighted
MR Egger
1/SE$_{IV}$
2.0
1.5
1.0
0.5
0
1
2
3
4
β$_{IV}$

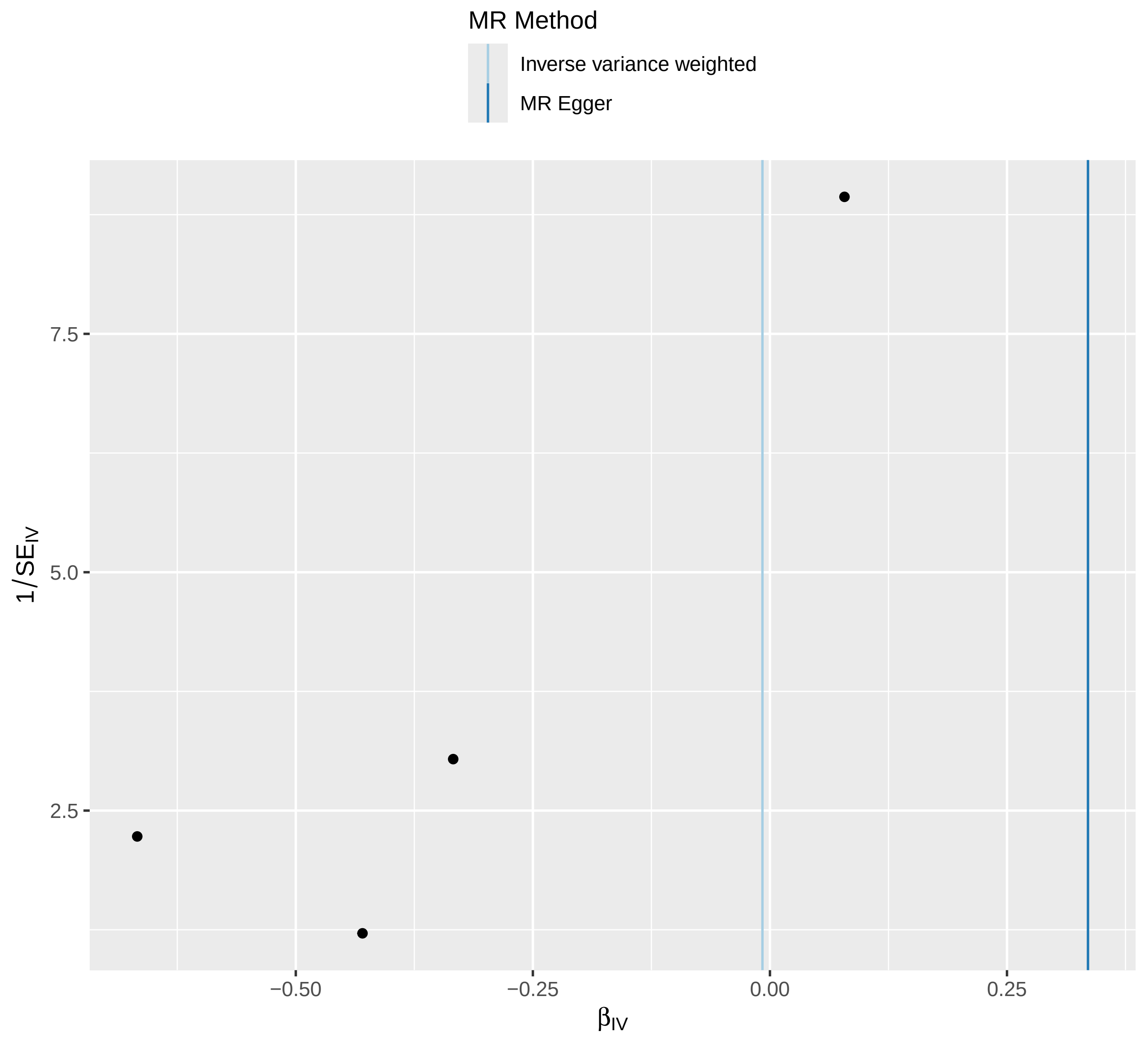
MR Method
Inverse variance weighted
MR Egger
7.5
5.0
2.5
$1/SE_{IV}$
−0.50
−0.25
0.00
0.25
β_{IV}

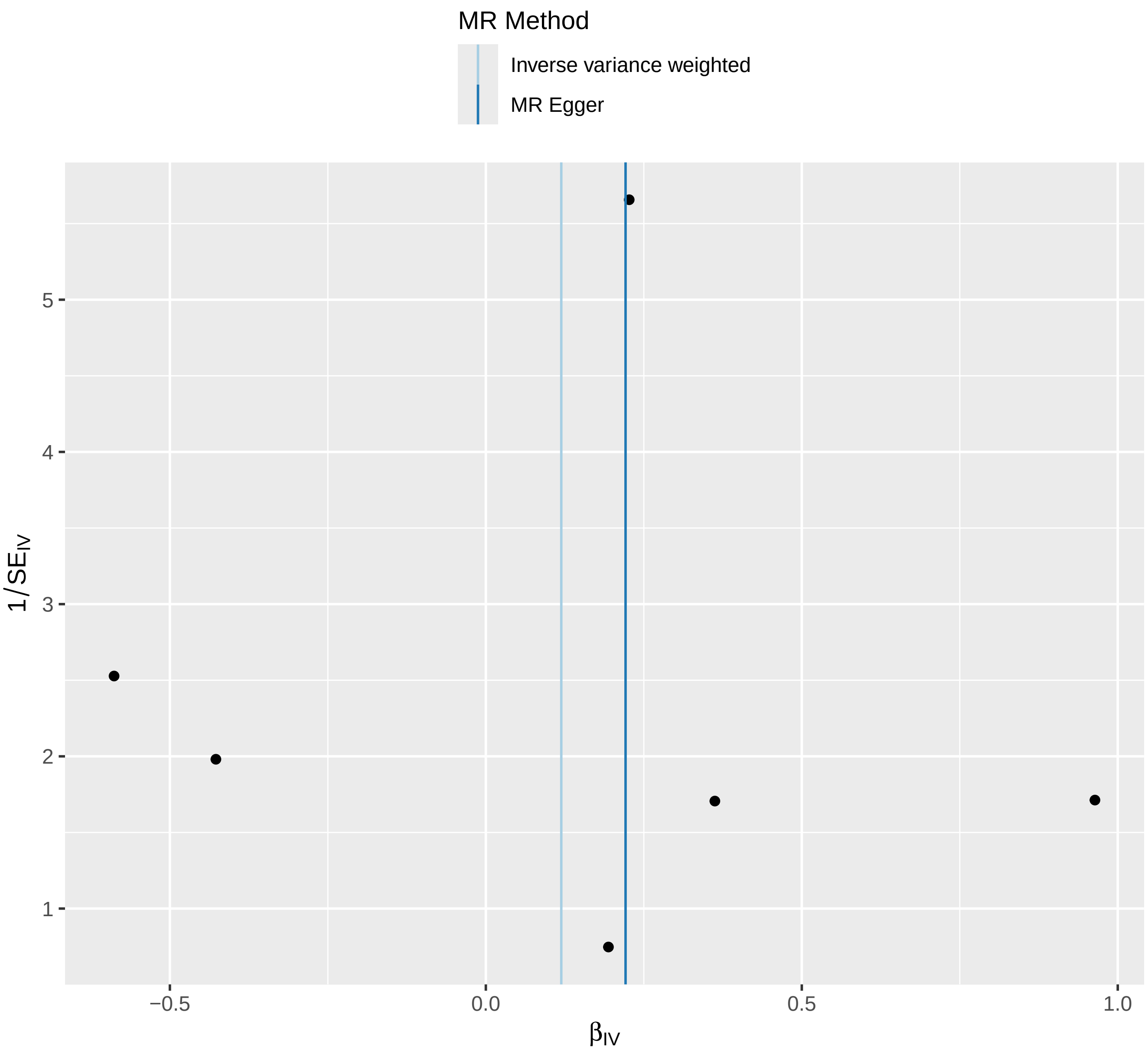

MR Method
Inverse variance weighted
MR Egger
1/SE_IV
β_IV
−0.5
0.0
0.5
1.0
1
2
3
4
5

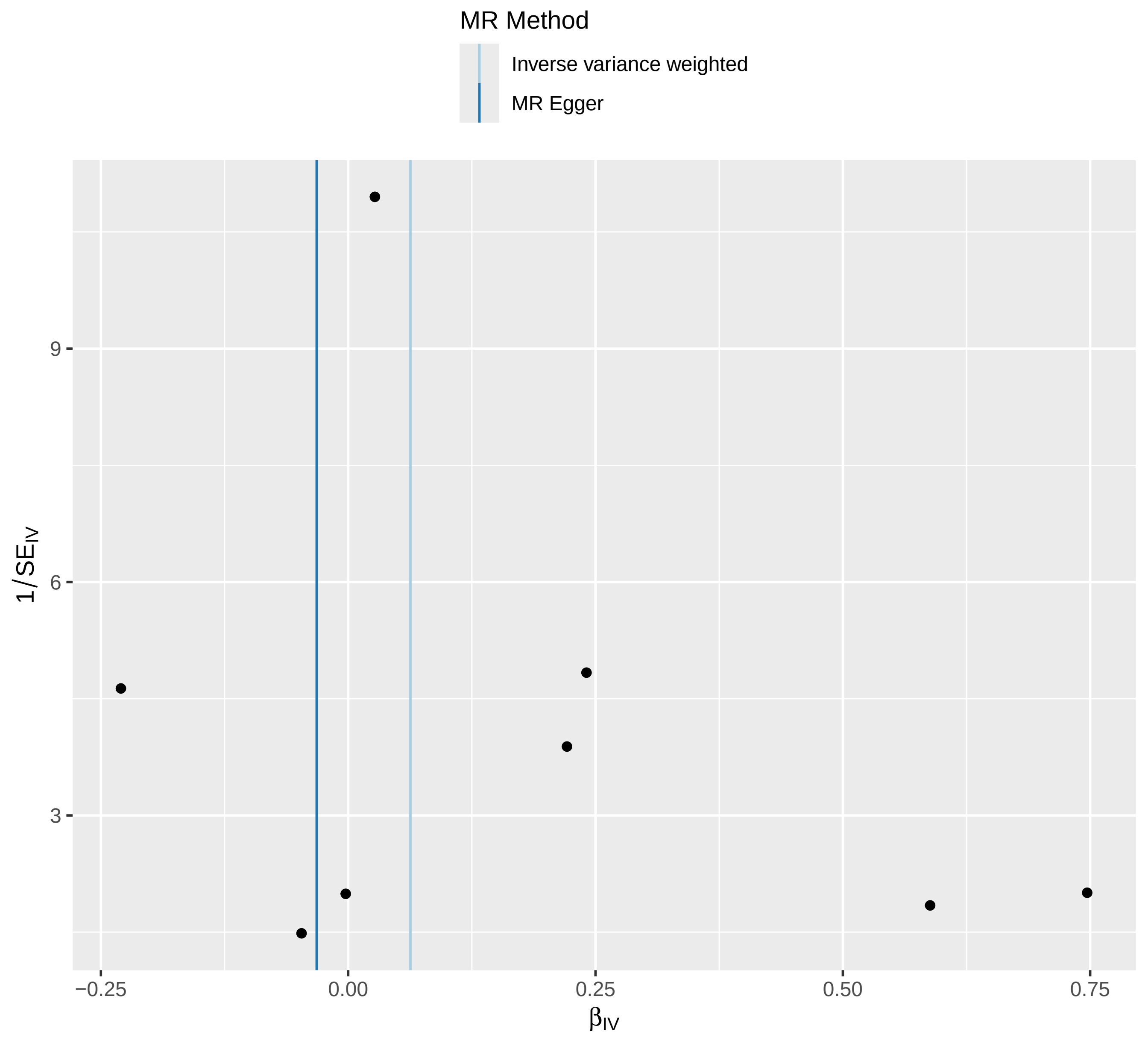

MR Method
Inverse variance weighted
MR Egger
9
6
3
1/SE_IV
−0.25
0.00
0.25
0.50
0.75
β_IV
